# Molecular investigations on a chimeric strain of *Staphylococcus aureus* sequence type 80

**DOI:** 10.1371/journal.pone.0232071

**Published:** 2020-10-14

**Authors:** Darius Gawlik, Antje Ruppelt-Lorz, Elke Müller, Annett Reißig, Helmut Hotzel, Sascha D. Braun, Bo Söderquist, Albrecht Ziegler-Cordts, Claudia Stein, Mathias W. Pletz, Ralf Ehricht, Stefan Monecke

**Affiliations:** 1 Institute of Infectious Diseases and Infection Control, University Hospital, Jena, Germany; 2 PTC—Phage Technology Center GmbH, Bönen, Germany; 3 Institute for Medical Microbiology and Hygiene, Technical University of Dresden, Dresden, Germany; 4 Leibniz Institute of Photonic Technology (IPHT), Jena, Germany; 5 InfectoGnostics Research Campus, Jena, Germany; 6 Friedrich-Loeffler-Institut, Institute of Bacterial Infections and Zoonoses, Jena, Germany; 7 School of Medical Sciences, Department of Laboratory Medicine, Clinical Microbiology, Faculty of Medicine and Health, Örebro University, Örebro, Sweden; 8 T-Systems Multimedia Solutions GmbH, Dresden, Germany; 9 Institute of Physical Chemistry, Jena University, Jena, Germany; University of Mississippi Medical Center, UNITED STATES

## Abstract

A PVL-positive, methicillin-susceptible *Staphylococcus aureus* was cultured from pus from cervical lymphadenitis of a patient of East-African origin. Microarray hybridisation assigned the isolate to clonal complex (CC) 80 but revealed unusual features, including the presence of the ORF-CM14 enterotoxin homologue and of an ACME-III element as well as the absence of *etD* and *edinB*. The isolate was subjected to both, Illumina and Nanopore sequencing allowing characterisation of deviating regions within the strain´s genome. Atypical features of this strain were attributable to the presence of two genomic regions that originated from other *S*. *aureus* lineages and that comprised, respectively, 3% and 1.4% of the genome. One deviating region extended from *walJ* to *sirB*. It comprised ORF-CM14 and the ACME-III element. A homologous but larger fragment was also found in an atypical *S*. *aureus* CC1/ST567 strain whose lineage might have served as donor of this genomic region. This region itself is a chimera comprising fragments from CC1 as well as fragments of unknown origin. The other deviating region comprised the region from *htsB* to *ecfA2*, *i*.*e*., another 3% of the genome. It was very similar to CC1 sequences. Either this suggests an incorporation of CC1 DNA into the study strain, or alternatively a recombination event affecting “canonical” CC80. Thus, the study strain bears witness of several recombination events affecting supposedly core genomic genes. Although the exact mechanism is not yet clear, such chimerism seems to be an additional pathway in the evolution of *S*. *aureus*. This could facilitate also a transmission of virulence and resistance factors and therefore offer an additional evolutionary advantage.

## Introduction

*Staphylococcus aureus* (*S*. *aureus*) is a versatile pathogen that colonises or infects a large fraction of the world´s human population as well as several species of animals. Thus, it can asymptomatically colonise its carriers, or alternatively cause various infections ranging from superficial skin and soft tissue infections to severe bloodstream infections. Many of its virulence factors are variably present and their genes are localized on mobile genetic elements such as plasmids, phages, transposons or on pathogenicity islands. In recent decades, some strains of *S*. *aureus* acquired resistance to many or most antibiotics. Again, resistance genes are localized on mobile, or potentially mobile, genetic elements such as staphylococcal chromosomal cassette *mec* (SCC*mec*) cassettes. Despite a vast variety of variable, mobile elements, and despite some incremental, mutation-driven variation, the overall structure of the *S*. *aureus* genome is conservative with all core genomic elements being present in all strains in one uniform sequential arrangement. Multilocus sequence typing (MLST) enables the assignment of isolates to taxonomic units, sequence types (ST) and clonal complexes (CC), based on numbered alleles of seven housekeeping genes assuming that these genes cannot be lost or truncated because of their crucial function and that the accumulation of mutations in their sequences is purely a function of time. This lead to a model of a clonal evolution of the *S*. *aureus* core genome that is driven by a time-dependent accumulation of single point mutations allowing classification based on a few marker genes into a limited number of clonal complexes comprising a number of sequence types that differ only in random mutations in these marker genes as well as of others. However, there are observations that certain non-mobile markers (among them, alleles of *agr* genes, genes determining capsule types or MLST markers) occur in non-related lineages [1–4]. This suggests that multiple recombination events affected at least most lineages that subsequently evolved and expanded clonally [4] and that large-scale recombination events played a role in driving the evolution of *S*. *aureus* along a frequent exchange of mobile genetic elements [3].

Some *S*. *aureus* strains show evidence of large-scale recombination events, with large fragments of their genomes clearly originating from other lineages and being inserted at the appropriate position of the recipient strain. Such a phenomenon was first postulated for ST239 where a CC30 DNA fragment of approximately 635,000 base pairs (ca. 20% of the genome) is integrated into a CC8 recipient with the integration site being localised around *oriC* [5, 6]. Another sequence type, ST2249, harbours ST239 DNA comprising fragments of both, CC8 and CC30 that in turn are integrated into a CC45 genome [7]. Further examples for chimeric strains are ST34 and ST42 (where CC10 fragments are integrated into CC30 genomes) [5] or CC398 strains that harbour fragments of CC9 origin [8, 9] as well as ST71 that carries a large insert of unknown provenance in a CC97 backbone genome [10, 11].

In the present paper, we describe another, chimeric strain that comprises a backbone of CC80 genomic DNA and two separate large inserts. *S*. *aureus* CC80 is a lineage that is commonly associated with recurrent and/or severe skin and soft tissue infections (SSTI) since a majority of isolates carries the phage-borne virulence factor Panton-Valentine leukocidin (PVL). One PVL-positive, methicillin-resistant CC80-MRSA-IVc strain is sporadically found in Western Europe [12–20] while it is widespread in Mediterranean countries including Greece [21, 22], Turkey [23], Lebanon [24], Malta [25], Tunisia [26, 27] and Algeria [28–30] as well as in the Middle East/Arabian Gulf regions [31–34]. Methicillin-susceptible CC80 are uncommon but geographically widespread in Africa [35–37] from where this lineage originated [20].

The isolate described herein was initially subjected to microarray hybridisation, primarily for typing and detection of resistance and toxin genes. The procedure revealed unusual features for a CC80 isolate (presence of ORF-CM14 and absence of *edinB* and *etD*) that could be explained by a large-scale horizontal gene transfer. This observation prompted further investigations including Illumina and Nanopore sequencing of its entire genome and a search for the donor strain of regions assumed to be introduced by horizontal gene transfer.

## Material and methods

### Clinical background and isolates

A patient of East African background was admitted to the Dresden University Hospital (Dresden, Saxony, Germany) with a cervical SSTI that initially was suspected to be suppurative tuberculous lymphadenitis. While no mycobacteria were detected (neither immediately by microscopy after staining for acid-fast bacilli nor subsequently in MGIT and Ogawa cultures), culture of pus yielded a PVL-positive, methicillin-susceptible *S*. *aureus* (isolate Dresden-275757).

A second isolate (Oerebro-086360) was further characterised because of certain similarities with the study isolate (see below). It was isolated in Oerebro, Sweden, originating from an approximately 50 years old female patient with lobar pneumonia probably secondary to an influenza B infection. She was a Swedish citizen and denied any travel outside Sweden.

### Microarray-based typing

The *S*. *aureus* isolates were initially characterized using different DNA microarray-based assays. Probes, primers as well as amplification and hybridization protocols have previously been described in detail [38–40].

### Ilumina sequencing

Sequencing of the two strains was performed at two geographically distant facilities and at different dates (Jena, Germany, and Örebro, Sweden, in spring and autumn 2018, respectively), ruling out any possibility of carry-over contaminations.

Genomic DNA of Dresden-275757 was prepared using a Qiagen kit (Qiagen, Hilden, Germany) after an enzymatic lysis step with lysostaphin, lysozyme and RNAse as previously described [38–40]. Afterwards, whole-genome sequencing was carried out using the Illumina HiSeq2500 genome analyser and the Illumina Experiment Manager 1.13.1. (Illumina, Essex, UK). The Nextera XT DNA Library Prep Kit (FC-131-1096; Illumina, San Diego, CA, U.S.A.) and the Nextera XT Index Kit v2, Set A (FC-131-2001; Illumina) were used for preparation of the library according to the manufacturer (Document #15031942 v 02, 2017). An average coverage of 139 was achieved. The reads were assembled to contigs using SPAdes. Sequencing of Oerebro-086360 was performed as previously described [41]. DNA was automatically extracted using the QIAsymphony DSP Virus/Pathogen kit (Qiagen, Hilden, Germany) following manufacturer’s instructions. Sequencing was done with the Nextera XT kit (Illumina Inc, San Diego, CA, USA) on an Illumina MiSeq. The reads with a coverage of 120 were *de novo* assembled with version 1.1.04 of Velvet26.

### Nanopore sequencing

The Nanopore Oxford MinION platform was used for whole-genome sequencing of Dresden-275757. A detailed protocol is given in the S1 File. Briefly, no size selection was performed using 0.5 v/v AMPure XP beads (Beckman Coulter GmbH, Krefeld, Germany) to avoid DNA fragments smaller than 1.000 bp. The DNA library was generated using the native barcoding expansion kit EXP-NBD103 and the sequencing kit SQK-LSK109 (Oxford Nanopore Technologies, Oxford, UK) according to manufacturer’s instructions. The flow cell FLO-MIN106 (R9-Version) was primed by the flow cell priming kit EXP-FLP001 (Oxford Nanopore). The protocol “1D Native barcoding genomic DNA” was used in version NBE_9065_v109_revB_23May2018 (Last update: 03/09/2018). The albacore basecaller (Oxford Nanopore) translated the minion raw data (FAST5) into short long quality tagged sequence reads (FASTQ). After barcode trimming using Porechop (https://github.com/rrwick/Porechop, release v0.2.4), canu in version v1.7.1 (https://github.com/marbl/canu, release v1.7.1) was used to assemble the short all reads with a minimum coverage of 50. After nano-polishing (https://github.com/jts/nanopolish, release v0.11.3), the corrected sequence data were used for a direct comparison to the Illumina sequence data (see below).

### Bioinformatic analysis

Iterated BLAST searches were used for analysis of individual contigs in this genome (https://blast.ncbi.nlm.nih.gov/Blast.cgi). This analysis was conducted using automated scripts for full text comparison and BLAST analysis and an in-house database of known, annotated and previously identified *S*. *aureus* genomes, genes and fragments to the query sequence. This enables the determination of identity, gene content, clonal parentage and of position within the genome of each contig given the constant order of core genomic genes in *S*. *aureus*. Finally, Nanopore and Ilumina sequences were aligned manually for reasons discussed below.

The sequence was compared to the representative CC80 strain 11819–97, GenBank CP003194.1/SAMN02603886. This is a PVL-positive CC80-MRSA-IVc with–as essentially all canonical CC80 strains–with an *etD/edinB* pathogenicity island. Its genome has a size of 2,846,546 nt and an average G/C content of 32.9%. Other sequences analysed included the CC8 strain COL, GenBank CP000046, and to MW2, BA000033, as reference sequence for CC1.

For comparison, two genomes where provisionally assembled from raw sequencing reads from the Short Read Archive (https://www.ncbi.nlm.nih.gov/sra/) and analysed using the program blastn (Camacho et al., 2009) from the NCBI blast+ suite. All sites were identified that matched the probe sequences. A probe without mismatches was assigned signal intensity of 0.9; with one mismatch, a signal of 0.6; with two mismatches, a signal of 0.3; with three mismatches, a signal of 0.1. The results were analysed in the same way as real array hybridisation experiments allowing identification of similar/related strains among published genome data [42].

## Results

### Comparison of sequencing methods

A total of 36 Illumina contigs was considered to be chromosomal. Another one contained typically plasmid-borne sequences (including *blaZ* and *cadX*; see below). The average fragment size of the library was 220 nt. Visual inspection and comparison of these contigs to the Nanopore sequences revealed faulty assemblies of four contigs that needed to be split into two “sub-contigs” each in order to allow alignment to the Nanopore sequence. Most significantly, Illumina failed to resolve a ca. 5,000 nt region within the ACME-III element that consisted of repetitive sequences. On the other hand, Nanopore showed a poor resolution of poly-A and poly-T sequence fragments resulting in the loss of approximately 15,800 nucleotides across the entire chromosome.

### Characterisation of the clinical isolate

Array hybridization revealed the presence of the enterotoxin homologue ORF-CM14 and of an ACME-III element, as well as the absence of *edinB* and *etD*. Otherwise, the isolate matched previously characterized CC80 strains (see S2 File). In order to explain these discrepancies, it was sequenced using both, Illumina and Nanopore methods and resulting sequences were aligned resulting in a continuous chromosome with a total length of 2,789,663 nt and an overall G/C content of 32.98%. MLST was performed based on the consensus genome sequence and it yielded ST-80 (*arcC*-1, *aroE*-3, *glpF*-1, *gmk*-14, *pta*-11, *tpi*-51, *yqiL*-10).

**Fig 1:**
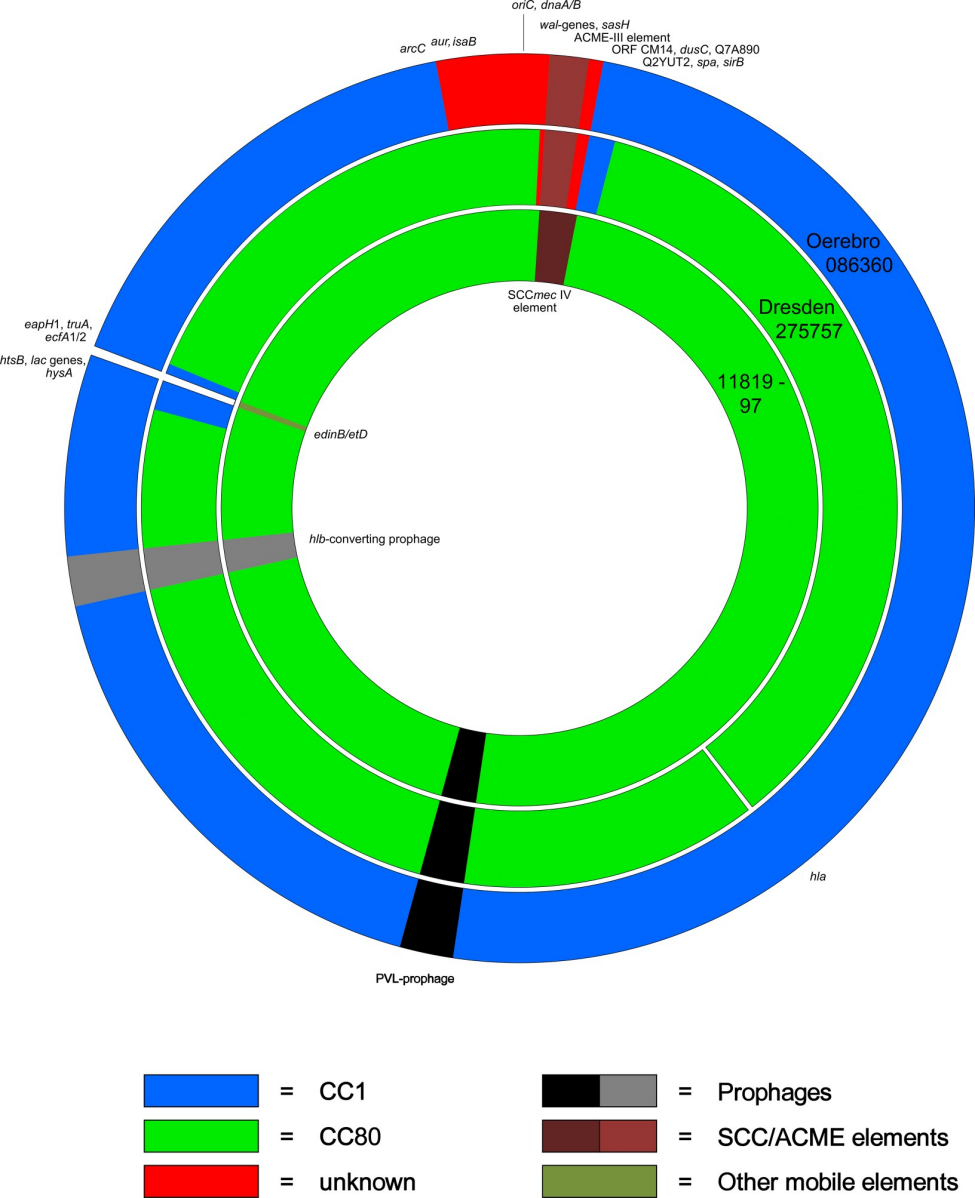
Schematic diagram of the genomes of Oerebro-086360 (outer circle), Dresden-275757 (middle circle) and the reference genome CC80, 11819–97, GenBank CP003194.1 (inner circle). Genomic fragments are colour-coded depending on their origin.

A comparison of core chromosomal genes revealed that two separate regions in Dresden-275757 differed from CP003194 confirming and explaining the differences to canonical CC80 as observed in the array experiment (Fig 1).

### Deviating Region 1

Deviating Region 1 extends from *walJ* (locus tag MS7_0024 in CP003194, SACOL0023 in CP000046) with a putative recombination sites located in the intergenic region between *walL* and *walJ*. It extends to certainly include *sirB* (MS7_0106, SACOL0098), possibly even to *sbnE* (MS7_0112, SACOL0104) although the differences to canonical CC80 sequences are not large enough to clearly determine a recombination site. It can be estimated at 84,363 nt (based on a consensus of the Illumina and the Nanopore sequences, and including *walJ* to *sirB*). This corresponds to roughly 3% of the genome and includes *ca*. 34,000 nt belonging to the ACME-element.

The gene content of Deviating Region 1 is described in S5 File/Table 1 where it is also compared to a CC80 reference sequence CP003194 as well as to Oerebro-086360.

Deviating Region 1 consists of four different fragments. The first comprises the genes between *walJ* (MS7_0024; SACOL0023) and *orfX*.

The second one is an ACME-III element including the *opp* operon. This is a potentially motile element and thus it is not necessarily connected to the genomic replacement in this strain. It will be discussed below.

A third fragment includes, among other genes, the enterotoxin homologue ORF-CM14. It extends to Q7A890/Q2YUT2 (MS7_0086/MS7_0087; SACOL0076/SACOL0077). This fragment does not contain the enterotoxin H gene *seh* or a *seh*-derived pseudogene (MS7_0080), which are characteristic for CC1 or CC80, respectively.

A forth fragment of Deviating Region 1 includes the gene encoding staphylococcal protein A. It can be assigned to RIDOM *spa* type t1849; 07-23-34-33-13. This *spa* type is related, but not identical, to both, those of CC1 (such as t127; 07-23-21-16-34-33-13) and CC80 (such as t044; 07-23-12-34-34-33-34). The RIDOM database shows 10 matches (https://spa.ridom.de/spa-t1849.shtml; as of 2020, April 3rd), including three belonging to a German project on characterisation of African *S*. *aureus* isolates [35, 43, 44] but without disclosing their MLST types or further details. Several other genes in this fragment match CC1 sequences (see S5 File).

### The SCC element as part of Deviating Region 1

Deviating Region 1 also comprises *orfX*, *i*.*e*., the integration site of SCC elements. While most published isolates and sequences of CC80 harbour SCC*mec* IVa or IVc elements, Dresden-275757 carries an SCC element without *mecA/C* genes.

The gene content of the SCC element is summarized in S5 File/Table 2. Sequences are provided in S6 File. In short, the element consists of

- a type II restriction-modification system,
- *ccrA/B1* recombinase genes and adjacent genes showing some similarity or relationship to SCC*mec* IX sequences (strain JCSC6690, GenBank AB705452.1),
- a large gene with repetitive sequences that is very similar to the gene encoding a hypothetical protein DLJ55_14705 in the chromosomal DNA of strain MOK042 (GenBank CP029627.1) as well as on a plasmid of a ST508/CC45 strain, AR_0471 (chromosome CP029652.1, plasmid CP029650.1)
- and an oligopeptide permease operon, *i*.*e*., *opp* genes or ACME-III as well as some genes for “putative proteins” as known from the ST42 strain C427, GenBank ACSQ.

A search of the short read archive of GenBank revealed two near-identical sequences of deviant CC80 strains. One of them (SAMEA3671725) harbours ACME-III while it is absent from the other one (SAMEA48342418). When performing a BLAST search with the NCBI GenBank, no significant hits over the entire length of the SCC element were obtained indicating that this element has not yet been observed, although most of its genes have already been found in other SCC elements.

### Identification and characterisation of the ST567 isolate Oerebro-086360 as a potential donor of Deviating Region 1

The observation of the enterotoxin homologue ORF-CM14 rather than of the enterotoxin H gene *seh* normally present in canonical CC1 strains, followed by a set of CC1-like genes strongly suggests that Deviating Region 1 is of chimeric origin itself. Our database of ca. 25,000 microarray hybridization profiles was searched for potential donors of Deviating Region 1, *i*.*e*., for strains that harbour ORF-CM14 in a CC1-like core genomic backbone. Only one isolate, Oerebro-086360 a deviant strain CC1 (ST567, MLST profile 10-1-1-1-1-1-1, *spa* type, t1242; 07-23-12-34-34-16-34-33-13) matched these criteria. Thus it was also sequenced using Illumina Miseq.

Oerebro-086360 is a PVL-positive CC1 MSSA that differs from canonical CC1 in several features including a presence of the ORF-CM14 enterotoxin homologue and an absence of *seh*. Other differences compared to canonical CC1 strains are the presence of deviant alleles of *aur* and *isaB* as well as an absence of *cna* and Q2G1R6/*cstB* (BA000033.2: 66419–67753). It also harboured an ACME-III element (*opp* genes and *ccrAB1*). The MLST marker *arcC* was different compared to ST1 (*arcC* 10 instead of *arcC* 1) but this difference is due to a single nucleotide polymorphism indicating mutation rather than recombination.

These observations are consistent with integration of a large alien insert around *oriC*. Excluding the ACME-III element, this insert can therefore be estimated to comprise roughly 150,000 nt, ranging from between *arcC* and *aur* across *oriC* and *orfX* to Q7A890/Q2YUT2 (see Fig 1).

Deviating Region 1 of Dresden-275757 and the corresponding region in the ST567 isolate Oerebro-086360 can be considered identical. This includes the gene content and the gene sequences, the presence and sequence of an ACME-III element and the fault line between Q7A890 and Q2YUT2 separating a region of unknown origin from CC1-like sequences.

The ACME-III elements of Dresden-275757 and Oerebro-086360 were largely identical to each other in both, gene content and gene sequences (see S6 File).

Therefore, we assume the lineage or strain represented by isolate Oerebro-086360 to be the donor of Deviating Region 1 in the lineage of Dresden-275757. However, the lineage of Oerebro-086360 is itself of chimeric nature comprising a large insert of DNA from a yet unidentified donor into a CC1 genome.

### Deviating Region 2

Deviating Region 2 (S5 File/Table 3, Fig 1) extends from *htsB* (MS7_2199, SACOL2166) to Q8NVB9 (MS7_2323, SACOL2297) or to *ecfA2* (MS7_2242; SACOL2211) having a size of 33,939 to 38,645 nt (1.2 to 1.4% of the genome, which is smaller than the corresponding fragment of the CC80 reference sequence which encompasses 115,604 nt). The reason is that it spans the integration site that in canonical CC80 harbours a motile genomic element comprising of *hsdS*, *hsdM*, *etD*, F3TKB7, *edinB* and F5W4X2 (MS7_2226 to MS7_2231). This element is absent from Dresden-275757. It is also absent from all CC1 sequences.

Deviating Region 2 also comprises a gene cluster from *rplQ* (MS7_2243; SACOL2212) to *rpsJ* (MS7_2271; SACOL2240) encoding several ribosomal proteins. These genes are highly conserved among all *S*. *aureus* sequences. However, when BLASTing (https://blast.ncbi.nlm.nih.gov/Blast.cgi) contig 16 (that entirely is a part of Deviating Region 2; the others are on 21 and 19), the five highest scoring matches over the entire length of the query sequence (68,165 nt) are CC1 genomes (with, e.g., 23 nt mismatches and 2 nt gaps for BX571857.1). In general, this region in Dresden-275757 is more related to CC1 than to canonical CC80 sequences. It also appears to be closer to Oerebro-086360 than to MW2 but given the overall similarity of all sequences concerned, this is hard to assess. The adjacent regions, up- and downstream of Deviating Region 2, are very similar in Dresden-275757, Oerebro-086360 as well as reference CC1 and CC80 sequences.

### The *hla* gene and its neighbouring genes

When comparing the sequence as well as the hybridization profile of Dresden-275757 to the CC80 reference sequence, the absence of the *hla* gene and its neighbouring genes (A5IS45, Q6GHS5, A5IS47, A6U0Y3, Q2FZB4, *i*.*e*., MS7_1116 to MS7_1120 or SACOL1171 to SACOL1175) can be detected. The presence of *hla* appears to be variable in the deviant CC80 lineage; SAMEA48342418 also lacks *hla* while it is present in SAMEA3671725.

### Prophages

When excising the phage sequence (Contig-0007:RC, positions 133,678 to end and Contig-0012 positions 1 to 42,938) and performing a NCBI BLAST search, the four best matches, with identities of 99.97%, are all PVL phages from CC80 strains, phiSa2wa_st80 (MG029515.1), NCTC13435 (LN831036.1), GR2 (CP010402.1) and 11819–97 (CP003194.1). The PVL prophage in Dresden-275757 is integrated into the same site of the chromosome as the one in CP003194.1. The prophage sequences from both strains are co-linear and they comprise the same set of genes.

The *hlb*-converting phage in Dresden-275757 differed from CP003194.1, although it also carried virulence-associated genes *scn* and *sak*. A NCBI Blast of Contig-0009RC positions 81,223 to 123,488 yielded as best matches (with more than 99.6% identity) the *hlb*-converting phages from BB155 (LN854556.1), 55-99-44 (CP024998.1) and SA17_S6 (CP010941.1). These strains belong to ST152 (https://pubmlst.org/bigsdb?db=pubmlst_saureus_seqdef&page=sequenceQuery), a rather common lineage in Africa [38–44].

### Resistance genes

Dresden-275757 carried the *blaZ/I/R* operon and a cadmium resistance operon (*cadD/cadX*) together on one contig without any known chromosomal markers, thus presumably on a plasmid.

Other resistance genes that frequently can be encountered in canonical CC80, namely *aphA3*, *aadE* and *sat* (neomycin, streptomycin and streptothricin resistance) as well as *far1/fusB* and *tet*(K) (fusidic acid and tetracycline resistance), were absent.

## Discussion

We identified a virulent, PVL-positive CC80 MSSA that differed in key features from canonical CC80 strains. Analysis was performed using array hybridisation, Illumina and Nanopore sequencing. While array hybridisation yields less information than sequencing, it can be routinely performed fast, automatically and economically on high numbers of clinical isolates that, in the present case, allowed the identification of the initial isolate Dresden-275757 as being of special interest as well as of Oerebro-086360 as putative donor. Illumina sequencing provided short reads of high quality sequences, but it has difficulties with repetitive sequences that, as the most relevant problem in the current project, led to a virtual miss of DLJ55_14705 within the ACME-III element. Nanopore sequencing proved unreliable with regard especially to poly-T and poly-A sequences, but it can handle repetitive sequences much better which in *S*. *aureus* also include MSCRAMM genes such as *spa*. With two large core genomic replacements being present in one single isolate, we assume that such-large scale horizontal gene transfers might be more common in *S*. *aureus* than previously perceived, and that the resolution of MLST with seven markers is not high enough to identify all chimeric strains. However, the combination and interaction of microarray-based assays as screening tool and NGS allows reliable identification and detailed analysis of such strains [45].

Deviating Region 1 is located close to *oriC* which appears to be a hotspot for chromosomal replacements (see Introduction). It comprises sequences identical to the ones from the atypical CC1/ST567 strain Oerebro-086360. This includes an ACME-III element. It also includes a stretch of DNA upstream and downstream of ACME-III with the latter part including ORF-CM14. Theoretically, this might give a hint on the putative donor of Deviating Region 1.

Possible donors for ORF-CM14 to both isolates obviously must include strains form ORF-CM14 positive lineages that are ST12, ST71, ST93, ST121, ST509, ST567, CC772, CC705, ST707, ST760, ST816, ST848, ST1094, ST1643, ST2272, ST2425, ST2616 and ST2972 (based on published sequences and author´s own microarray data). Unfortunately, genome sequences of several of these STs are not available and those that are available do not match fully the sequence of Deviating Region 1. When comparing ORF-CM14 sequences, those of JKD6159; CP002114.2 [76914–77693] (ST93) and SS-015; FQIU01000002 [597790–598569] (ST2972) are the most closely related ones. When performing BLAST on the non-CC1-region of Oerebro-086360, the highest scoring hits are two ST2272 sequences (AP019712.1 and AP019713.1). When directly comparing sequences in question, the differences indicate that ST2272 was not likely to be the direct donor (with an average difference of 1.8% for *dnaA*, *dnaN*, *yaaA*, *recF*, *gyrB*, *gyrA*, *nnrD*, *hutH*, *serS*, *azlC*, *sam-L1*, *metX*, *yybS*, *gdpP*, *rplI*, *dnaC*, *purA*, *walR*, *walK*, *walH*, *walI*, *walJ*, *sasH*, Q6GKL1, Q6GKL6, ORF-CM14, *dusC*, A6TXM6, A6QD71, Q6GKK6 and Q2YUT2 from Tokyo12482, GenBank AP019713.1, to Oerebro-086360).

In both isolates, a fault-line can be observed between Q7A890 and Q2YUT2 separating downstream sequences of unknown origin from those upstream that are related to CC1 (*i*.*e*., the right border between “red” and “blue” sectors in Fig 1 and the last two columns of S5 File). This means Deviating Region 1 of Dresden-275757 includes the fault line separating the alien insert in Oerebro-086360 from the canonical CC1 core genome of that strain. This makes it very likely that an Oerebro-086360-like strain was indeed the donor of Deviating Region 1, and that this region itself is of chimeric nature, spanning CC1 and non-CC1 sequences (see Fig 1). The upstream fault line in Dresden-275757 separating CC1 from CC80 sequences (between *sirB* and *spa* or *sbnE*) cannot exactly been determined because of the general relatedness of CC1 and CC80 sequences.

Deviating Region 1 of Dresden-275757 and Oerebro-086360 also comprise an ACME-III element. The presence of *opp* genes and *ccrA/B-1* recombinase genes are reminiscent of the CC34 strain 21342 (GenBank AHKU) although the sequence of *ccrB*-1 appears to be more related to the one from SCC*mec* IX. It also includes, as revealed primarily by Nanopore sequencing, a gene with repetitive sequences that is very similar to the gene encoding a hypothetical protein DLJ55_14705 in strain MOK042. This strain belongs to ST71, a lineage that comprises a large insert of unknown origin in a CC97 genome [10, 11]. In strain MOK042, the gene encoding DLJ55_14705 is localised on that insert but it is not a part of a SCC element.

In addition, there is a second Deviant Region elsewhere in the genome of Dresden-275757. It is localised at a position distant from known “recombination hotspots” around *oriC* and the conjugative transposon ICE6013 [3] (with the latter being identical to canonical CC80). Gene content and sequences of Deviant Region 2 are highly similar to CC1 sequences including Oerebro-086360 but clearly differ from canonical CC80. Differences include, but are not limited to, an absence of *edinB* and *etD*. The region in question includes genes whose origin cannot be determined because of their high degree of conservation. For this reason, the exact boundaries of the Deviating Region cannot be identified. Interestingly, the regions adjacent to Deviant Region 2 are very similar in all sequences analysed, *i*.*e*., Dresden-275757, Oerebro-086360 as well as the reference CC1 and CC80 sequences (with differences being less than 0.5%). This could suggest that the region corresponding to Deviant Region 2 was “deviant” not in Dresden-275757 but, compared to the other three sequences, in the CC80 reference sequence. This might indicate that Deviant Region 2 in Dresden-275757 was not an alien insert of CC1 origin but that its sequence represents shared, ancestral CC1/CC80 stock and that the corresponding region in canonical CC80 (including *edinB* and *etD*) itself was an insert from another, yet unidentified, lineage.

In conclusion, the core genome of Dresden-275757 bears evidence of at least two, possibly three large-scale recombination events. First, ORF-CM14, among other genes, was introduced into a CC1 strain and, second, the resulting ORF-CM14/CC1 composite fragment was introduced into CC80. In addition, another recombination event introduced either Deviating Region 2 from CC1 into the ancestor of Dresden-275757 or the corresponding region, possibly together with *edinB* and *etD*, from an unknown donor into canonical CC80.

Thus, such complex and large-scale recombination events are unlikely to be rare and exceptional, despite a distinct clonal nature of *S*. *aureus* [46]. Although the exact mechanism is not clear, chimerism, or horizontal gene transfer of core genomic fragments not associated with mobile genetic elements, seems to be an additional pathway in the evolution of *S*. *aureus*, possibly being responsible for a transmission of virulence factors (such as ORF-CM14 in the case described herein) or of resistance genes [7]. From a more theoretical point of view, large-scale genomic substitutions, chimerism or hybridisation facilitate evolutionary leaps that cannot be achieved by accumulation of single point mutations or that would require immeasurably much more time to be achieved by mutations. If one considers the ability to evolve and adapt as an evolutionary advantage, an organism that can shuffle, swap or exchange major parts of its genome by whatever unknown mechanism should be in a better position than a strictly clonal organism.

## Supporting information

S1 File Protocol for nanopore sequencing. (PDF)

S2 File Array hybridization patterns of strains discussed. (PDF)

S3 File Annotated sequence of Dresden-275757. (PDF)

S4 File Annotated sequence of Oerebro-086360. (PDF)

S5 File S5 Table/1, Genes in Deviating Region 1 in comparison to canonical CC80, to Oerebro-086360 and to canonical CC1, S5 Table/2, The ACME-III element in Dresden-275757 and Oerebro-086360. S5 Table/3, Genes in Deviating Region 2 in comparison to canonical CC80, to Oerebro-086360 and to canonical CC1.
(PDF)

S6 File Fasta-file with the ACME III sequences and markers. (FAS)

## References

[1] Robinson DA, Monk AB, Cooper JE, Feil EJ, Enright MC. Evolutionary Genetics of the Accessory Gene Regulator (agr) Locus in *Staphylococcus aureus*. J Bacteriol. 2005;187(24):8312–21. 10.1128/JB.187.24.8312-8321.2005 16321935 PMC1317016

[2] Watanabe S, Ito T, Sasaki T, Li S, Uchiyama I, Kishii K, et al Genetic diversity of staphylocoagulase genes (*coa*): insight into the evolution of variable chromosomal virulence factors in *Staphylococcus aureus*. PLoS ONE. 2009;4(5):e5714 10.1371/journal.pone.0005714 . 19492076 PMC2683563

[3] Everitt RG, Didelot X, Batty EM, Miller RR, Knox K, Young BC, et al Mobile elements drive recombination hotspots in the core genome of *Staphylococcus aureus*. Nature communications. 2014;5:3956–. 10.1038/ncomms4956 . 24853639 PMC4036114

[4] Driebe EM, Sahl JW, Roe C, Bowers JR, Schupp JM, Gillece JD, et al Using Whole Genome Analysis to Examine Recombination across Diverse Sequence Types of *Staphylococcus aureus*. PLOS ONE. 2015;10(7):e0130955 10.1371/journal.pone.0130955 26161978 PMC4498916

[5] Robinson DA, Enright MC. Evolution of *Staphylococcus aureus* by Large Chromosomal Replacements. J Bacteriol. 2004;186(4):1060–4. 10.1128/jb.186.4.1060-1064.2004 14762000 PMC344219

[6] Holden MTG, Lindsay JA, Corton C, Quail MA, Cockfield JD, Pathak S, et al Genome Sequence of a Recently Emerged, Highly Transmissible, Multi-Antibiotic- and Antiseptic-Resistant Variant of Methicillin-Resistant *Staphylococcus aureus*, Sequence Type 239 (TW). Journal of Bacteriology. 2010;192(3):888–92. 10.1128/JB.01255-09 19948800 PMC2812470

[7] Nimmo GR, Steen JA, Monecke S, Ehricht R, Slickers P, Thomas JC, et al ST2249-MRSA-III: a second major recombinant methicillin-resistant *Staphylococcus aureus* clone causing healthcare infection in the 1970s. Clin Microbiol Infect. 2015;21(5):444–50. Epub 2015/02/25. 10.1016/j.cmi.2014.12.018 25708549 PMC4564996

[8] Fetsch A, Kraushaar B, Kasbohrer A, Hammerl JA. Turkey Meat as Source of CC9/CC398 Methicillin-Resistant *Staphylococcus aureus* in Humans? Clin Infect Dis. 2017;64(1):102–3. Epub 2016/10/30. 10.1093/cid/ciw687 27794020 PMC5159606

[9] Monecke S, Slickers P, Gawlik D, Müller E, Reissig A, Ruppelt-Lorz A, et al Variability of SCC*mec* elements in livestock-associated CC398 MRSA. Veterinary Microbiology. 2018;217:36–46. 10.1016/j.vetmic.2018.02.024 29615254

[10] Spoor LE, Richardson E, Richards AC, Wilson GJ, Mendonca C, Gupta RK, et al Recombination-mediated remodelling of host–pathogen interactions during *Staphylococcus aureus* niche adaptation. Microbial Genomics. 2015;1(4). 10.1099/mgen.0.000036. PMC5320625 28348819

[11] Budd KE, McCoy F, Monecke S, Cormican P, Mitchell J, Keane OM. Extensive Genomic Diversity among Bovine-Adapted *Staphylococcus aureus*: Evidence for a Genomic Rearrangement within CC97. PLoS ONE. 2015;10(8):e0134592 10.1371/journal.pone.0134592 26317849 PMC4552844

[12] Faria NA, Oliveira DC, Westh H, Monnet DL, Larsen AR, Skov R, et al Epidemiology of emerging methicillin-resistant *Staphylococcus aureus* (MRSA) in Denmark: a nationwide study in a country with low prevalence of MRSA infection. J Clin Microbiol. 2005;43(4):1836–42. 10.1128/JCM.43.4.1836-1842.2005 . 15815005 PMC1081382

[13] Holmes A, Ganner M, McGuane S, Pitt TL, Cookson BD, Kearns AM. *Staphylococcus aureus* isolates carrying Panton-Valentine leucocidin genes in England and Wales: frequency, characterization, and association with clinical disease. J Clin Microbiol. 2005;43(5):2384–90. 10.1128/JCM.43.5.2384-2390.2005 . 15872271 PMC1153723

[14] Krziwanek K, Luger C, Sammer B, Stumvoll S, Stammler M, Metz-Gercek S, et al PVL-positive MRSA in Austria. Eur J Clin Microbiol Infect Dis. 2007;26(12):931–5. 10.1007/s10096-007-0391-4 . 17891548

[15] Monecke S, Aamot HV, Stieber B, Ruppelt A, Ehricht R. Characterization of PVL-positive MRSA from Norway. Apmis. 2014;122(7):580–4. Epub 2013/10/11. 10.1111/apm.12181 . 24106794

[16] Monecke S, Slickers P, Hotzel H, Richter-Huhn G, Pohle M, Weber S, et al Microarray-based characterisation of a Panton-Valentine leukocidin-positive community-acquired strain of methicillin-resistant *Staphylococcus aureus*. Clin Microbiol Infect. 2006;12(8):718–28. 10.1111/j.1469-0691.2006.01420.x 16842566

[17] Monecke S, Jatzwauk L, Weber S, Slickers P, Ehricht R. DNA microarray-based genotyping of methicillin-resistant *Staphylococcus aureus* strains from Eastern Saxony. Clin Microbiol Infect. 2008;14(6):534–45. Epub 2008/04/01. 10.1111/j.1469-0691.2008.01986.x . 18373691

[18] Otter JA, French GL. The emergence of community-associated methicillin-resistant *Staphylococcus aureus* at a London teaching hospital, 2000–2006. Clin Microbiol Infect. 2008;14(7):670–6. 10.1111/j.1469-0691.2008.02017.x . 18558939

[19] Rossney AS, Shore AC, Morgan PM, Fitzgibbon MM, O'Connell B, Coleman DC. The emergence and importation of diverse genotypes of methicillin-resistant *Staphylococcus aureus* (MRSA) harboring the Panton-Valentine leukocidin gene (pvl) reveal that pvl is a poor marker for community-acquired MRSA strains in Ireland. J Clin Microbiol. 2007;45(8):2554–63. 10.1128/JCM.00245-07 . 17581935 PMC1951240

[20] Stegger M, Wirth T, Andersen PS, Skov RL, De Grassi A, Simoes PM, et al Origin and evolution of European community-acquired methicillin-resistant *Staphylococcus aureus*. mbio. 2014;5(5):e01044–14. Epub 2014/08/28. 10.1128/mBio.01044-14 25161186 PMC4173770

[21] Aires de Sousa M, Bartzavali C, Spiliopoulou I, Sanches IS, Crisostomo MI, de Lencastre H. Two international methicillin-resistant *Staphylococcus aureus* clones endemic in a university hospital in Patras, Greece. J Clin Microbiol. 2003;41(5):2027–32. 10.1128/jcm.41.5.2027-2032.2003 . 12734244 PMC154747

[22] Vourli S, Perimeni D, Makri A, Polemis M, Voyiatzi A, Vatopoulos A. Community acquired MRSA infections in a paediatric population in Greece. Euro Surveill. 2005;10(5):78–9. . 16077207

[23] Maier J, Melzl H, Reischl U, Drubel I, Witte W, Lehn N, et al Panton-Valentine leukocidin-positive methicillin-resistant *Staphylococcus aureus* in Germany associated with travel or foreign family origin. Eur J Clin Microbiol Infect Dis. 2005;24(9):637–9. 10.1007/s10096-005-0008-8 16167136

[24] Tokajian ST, Khalil PA, Jabbour D, Rizk M, Farah MJ, Hashwa FA, et al Molecular characterization of *Staphylococcus aureus* in Lebanon. Epidemiol Infect. 2010;138(5):707–12. 10.1017/S0950268810000440 . 20202283

[25] Scicluna E, Shore A, Thuermer A, Ehricht R, Slickers P, Borg M, et al Characterisation of MRSA from Malta and the description of a Maltese epidemic MRSA strain. Eur J Clin Microbiol Infect Dis. 2010;29(2):163–70. 10.1007/s10096-009-0834-1 19911206

[26] Ben Nejma M, Mastouri M, Bel Hadj Jrad B, Nour M. Characterization of ST80 Panton-Valentine leukocidin-positive community-acquired methicillin-resistant *Staphylococcus aureus* clone in Tunisia. Diagn Microbiol Infect Dis. 2013;77(1):20–4. Epub 2008/04/09. 10.1016/j.diagmicrobio.2008.02.010 . 18394845

[27] Ben Slama K, Gharsa H, Klibi N, Jouini A, Lozano C, Gomez-Sanz E, et al Nasal carriage of *Staphylococcus aureus* in healthy humans with different levels of contact with animals in Tunisia: genetic lineages, methicillin resistance, and virulence factors. Eur J Clin Microbiol Infect Dis. 2011;30(4):499–508. Epub 2010/11/16. 10.1007/s10096-010-1109-6 . 21076928

[28] Antri K, Rouzic N, Dauwalder O, Boubekri I, Bes M, Lina G, et al High prevalence of methicillin-resistant *Staphylococcus aureus* clone ST80-IV in hospital and community settings in Algiers. Clin Microbiol Infect. 2011;17(4):526–32. 10.1111/j.1469-0691.2010.03273.x 20518793

[29] Basset P, Amhis W, Blanc DS. Changing molecular epidemiology of methicillin-resistant *Staphylococcus aureus* in an Algerian hospital. J Infect Dev Ctries. 2015;9(2):206–9. Epub 2015/02/24. 10.3855/jidc.4620 . 25699496

[30] Djoudi F, Bonura C, Benallaoua S, Touati A, Touati D, Aleo A, et al Panton-Valentine leukocidin positive sequence type 80 methicillin-resistant *Staphylococcus aureus* carrying a staphylococcal cassette chromosome *mec* type IVc is dominant in neonates and children in an Algiers hospital. New Microbiol. 2013;36(1):49–55. Epub 2013/02/26. . 23435815

[31] Boswihi SS, Udo EE, Al-Sweih N. Shifts in the Clonal Distribution of Methicillin-Resistant *Staphylococcus aureus* in Kuwait Hospitals: 1992–2010. PLoS ONE. 2016;11(9):e0162744 10.1371/journal.pone.0162744 27631623 PMC5025013

[32] Monecke S, Skakni L, Hasan R, Ruppelt A, Ghazal SS, Hakawi A, et al Characterisation of MRSA strains isolated from patients in a hospital in Riyadh, Kingdom of Saudi Arabia. BMC Microbiol. 2012;12(1):146 Epub 2012/07/25. 10.1186/1471-2180-12-146 . 22823982 PMC3464608

[33] Udo EE, Al-Lawati BAH, Al-Muharmi Z, Thukral SS. Genotyping of methicillin-resistant *Staphylococcus aureus* in the Sultan Qaboos University Hospital, Oman reveals the dominance of Panton–Valentine leucocidin-negative ST6-IV/t304 clone. New Microbes and New Infections. 2014;2(4):100–5. 10.1002/nmi2.47 PMC4184578. 25356354 PMC4184578

[34] Udo EE, Sarkhoo E. Genetic analysis of high-level mupirocin resistance in the ST80 clone of community-associated meticillin-resistant *Staphylococcus aureus*. J Med Microbiol. 2010;59(Pt 2):193–9. 10.1099/jmm.0.013268-0 . 19833783

[35] Shittu AO, Oyedara O, Kenneth OO, Raji A, Peters G, von Müller L, et al An assessment on DNA microarray and sequence-based methods for the characterization of methicillin-susceptible *Staphylococcus aureus* from Nigeria. Frontiers in Microbiology. 2015;6 10.3389/fmicb.2015.01160 26539185 PMC4612102

[36] Ghebremedhin B, Olugbosi MO, Raji AM, Layer F, Bakare RA, Konig B, et al Emergence of a community-associated methicillin-resistant *Staphylococcus aureus* strain with a unique resistance profile in Southwest Nigeria. J Clin Microbiol. 2009;47(9):2975–80. 10.1128/JCM.00648-09 . 19571020 PMC2738091

[37] Schaumburg F, Kock R, Friedrich AW, Soulanoudjingar S, Ngoa UA, von Eiff C, et al Population structure of *Staphylococcus aureus* from remote African Babongo Pygmies. PLoS Negl Trop Dis. 2011;5(5):e1150 Epub 2011/05/17. 10.1371/journal.pntd.0001150 21572985 PMC3091839

[38] Monecke S, Coombs G, Shore AC, Coleman DC, Akpaka P, Borg M, et al A field guide to pandemic, epidemic and sporadic clones of methicillin-resistant *Staphylococcus aureus*. PLoS ONE. 2011;6(4):e17936 10.1371/journal.pone.0017936 21494333 PMC3071808

[39] Monecke S, Jatzwauk L, Muller E, Nitschke H, Pfohl K, Slickers P, et al Diversity of SCC*mec* elements in *Staphylococcus aureus* as observed in South-Eastern Germany. PLoS ONE. 2016;11(9):e0162654 Epub 2016/09/21. 10.1371/journal.pone.0162654 . 27648947 PMC5029946

[40] Monecke S, Slickers P, Ehricht R. Assignment of *Staphylococcus aureus* isolates to clonal complexes based on microarray analysis and pattern recognition. FEMS Immunol Med Microbiol. 2008;53:237–51. 10.1111/j.1574-695X.2008.00426.x 18507678

[41] Wildeman P, Tevell S, Eriksson C, Lagos AC, Söderquist B, Stenmark B. Genomic characterization and outcome of prosthetic joint infections caused by *Staphylococcus aureus*. Sci Rep. 2020;10(1):5938 Epub 2020/04/05. 10.1038/s41598-020-62751-z 32246045 PMC7125104

[42] Monecke S, Slickers P, Gawlik D, Müller E, Reissig A, Ruppelt-Lorz A, et al Molecular typing of ST239-MRSA-III from diverse geographic locations and the evolution of the SCC*mec* III element during its intercontinental spread. Frontiers in Microbiology. 2018;9(1436). 10.3389/fmicb.2018.01436 30087657 PMC6066798

[43] Herrmann M, Abdullah S, Alabi A, Alonso P, Friedrich AW, Fuhr G, et al Staphylococcal disease in Africa: another neglected 'tropical' disease. Future Microbiol. 2013;8(1):17–26. Epub 2012/12/21. 10.2217/fmb.12.126 . 23252490

[44] Strauß L, Ruffing U, Abdulla S, Alabi A, Akulenko R, Garrine M, et al Detecting *Staphylococcus aureus* Virulence and Resistance Genes: a Comparison of Whole-Genome Sequencing and DNA Microarray Technology. Journal of Clinical Microbiology. 2016;54(4):1008–16. 10.1128/JCM.03022-15 26818676 PMC4809937

[45] Dunne WM, Pouseele H, Monecke S, Ehricht R, van Belkum A. Epidemiology of transmissible diseases: Array hybridization and next generation sequencing as universal nucleic acid-mediated typing tools. Infection Genetics and Evolution. 2018;63:332–45. 10.1016/j.meegid.2017.09.019 WOS:000442167600043. 28943408

[46] Feil EJ, Cooper JE, Grundmann H, Robinson DA, Enright MC, Berendt T, et al How clonal is *Staphylococcus aureus*? J Bacteriol. 2003;185(11):3307–16. 10.1128/jb.185.11.3307-3316.2003 . 12754228 PMC155367

