# Supplementary material for: Molecular investigations on a chimeric strain of Staphylococcus aureus sequence type 80
Source: PLoS One. 2020 Oct 14;15(10):e0232071. doi: 10.1371/journal.pone.0232071 (PMC7556507; doi:10.1371/journal.pone.0232071)
Supplement: S1 File — (PDF) [file pone.0232071.s001.pdf]

Detailed MINION protocol

Material for sequencing:

- AMPure Beads RT (Beckman Coulter GmbH, A63882)
- Blunt-End Ligase (New England Biolabs GmbH, M0367L)
- NEB Next FFPE DNA Repair mix (New England Biolabs GmbH, M6630)
- NEB Next Ultra II End repair/dA tailling Module (New England Biolabs GmbH, E7546)
- Barcodes RT (Oxford Nanopore Technologies, EXP-NBD103)
- Sequencing kit (Oxford Nanopore Technologies, SQK-LSK 109)

DNA isolation

- DNA isolation with NucleoBond AXG 20 (MACHEREY-NAGEL, REF 740544)
- DNA concentration measurement using Qubit 3.0 with DNA broad range kit (ThermoFisher, Q32850)

DNA Cleanup and size selection

- add 0.5 v/v AMPure Beads directly to DNA sample
- incubate at room temperature for 5 min on Hula mixer
- bind beads using magnetic rack
- wash twice with 200µl 80% Ethanol (prepared fresh)
- eluate in 50µl molecular grade water (nuclease free)
- use 1 µl for DNA measurement on Qubit 3.0

DNA outcome

- minimum DNA·concentration 1µg within 48µl

DNA repair and end-prep

- prepare NEBNext FFPE DNA Repair Mix and NEBNext End repair / dA-tailing Module reagents
- mix the following in a 0.2 ml thin-walled PCR tube:
    - *48.0 µl DNA*
    - *3.5 µl NEBNext FFPE DNA Repair Buffer*
    - *2.0 µl NEBNext FFPE DNA Repair Mix*
    - *3.5 µl Ultra II End-prep reaction buffer*
    - *3.0 µl Ultra II End-prep enzyme mix*
- incubate at 20° C for 30 minutes and 65° C for 30 minutes
- 30 seconds on ice

Clean up

- Add 60.0 µl AMPureBeads per sample
- incubate at room temperature for 10 min on Hula mixer
- bind beads using magnetic rack
- wash twice with 200 µl 80% Ethanol (prepared fresh)
- eluate in 26.0 µl molecular grade water (nuclease free)
- use 22.5 µl DNA for barcoding

Barcoding

- mix the following in a 0.2 ml thin-walled PCR tube
  - *22.5 µl DNA*
  - *2.5 µl Barcode*
  - *25.0 µl Bluntligase*
- Mix gently by flicking the tube, and spin down
- Incubate at room temperature for 20 min
- Standard cleanup procedure using only 50.0 µl AMPure XP Beads (see CleanUp section)
- Eluate DNA in 26.0 µl
- use 1 µl for DNA measurement on Qubit 3.0
- pool all barcoding samples at an equal DNA concentration to reach in total 700 ng in a final volume of 60 µl

Adapter ligation and clean-up

- mix the following in a 0.2 ml thin-walled PCR tube
  - *60.0 µl pooled DNA*
  - *25.0 µl NEBNext Quick Ligation Reaction Buffer (5X)*
  - *10.0 µl NEB next T4 Ligase*
  - *5.0 µl Barcode Adapter Mix (BAM 1D)*
- mix gently by flicking the tube, and spin down
- Incubate the reaction for 10 minutes at room temperature
- Cleanup procedure using 50.0 µl AMPure XP beads and 200 µl Long Fragmentation Buffer for resuspending
- Final eluate DNA in 15.0 µl Elution Buffer

Priming Solution for FlowCell

- Mix 30.0 µl FLT to one tube FLB
- Vortex for 30seconds

Library

- Mix the following in a new 1.0 ml low bind tube:
  - *37.5 µl Sequencing Buffer (SQB)*
  - *25.5 µl Loading Beads (LB), mixed immediately before use*
  - *12.0 µl DNA library*
- Mix the prepared library gently by pipetting up and down just prior to loading
- Add 75 µl of sample to the flow cell via the SpotON sample port in a dropwise fashion

Setting sequencing run

- Without real time basecalling
- 100,000 reads per FAST5
- Saving on external USB-C 2 TB SSD (Sandisk)
- Running time 24 h
