# Supplementary material for: Molecular investigations on a chimeric strain of Staphylococcus aureus sequence type 80
Source: PLoS One. 2020 Oct 14;15(10):e0232071. doi: 10.1371/journal.pone.0232071 (PMC7556507; doi:10.1371/journal.pone.0232071)
Supplement: S2 File — (PDF) [file pone.0232071.s002.pdf]

STRAIN / ISOLATE	SPECIES MARKER							STAPHYLOXANTHIN BIOSYNTHESIS OPERON					REGULATORY GENES										
	rrnD1 (S. aureus)	gapA	katA	CoA	nuc1	spa	sbi	crtM-nonST93	crtM-ST93	crtN	crtO	crtP	sarA	saeS	vraS	agrI (total)	agrII (total)	agrIII (total)	agrIV (total)	agrV-ST1850	agrV-ST2198	agrV-other	hld
	Domain 1 of 23S-rRNA	glyceraldehyde 3-phosphate dehydrogenase, locus 1	katalase A	coagulase	thermostable extracellular nuclease	staphylococcal protein A	IgG-binding protein	dehydrosqualene synthase		dehydrosqualene desaturase	staphylo-xanthin acyltransferase	diaponeurosporene oxidase	staphylococcal accessory regulator A	histidine protein kinase, sae locus	sensor protein	accessory gene regulator allele I	accessory gene regulator allele II	accessory gene regulator allele III	accessory gene regulator allele IV	accessory gene regulator alleles from S. argenteus gro			haemolysin delta
**CC1-MRSA-IV (PVL+), USA400**																							
MW2	POS	POS	POS	POS	POS	POS	POS	POS	NEG	POS	POS	POS	POS	POS	POS	NEG	NEG	POS	NEG	NEG	NEG		POS
Strain MW2 GenBank BA000033.2: predicted hybr. pattern	POS	POS	POS	POS	POS	POS	POS	POS	NEG	POS	POS	POS	POS	POS	POS	NEG	NEG	POS	NEG	NEG	NEG	NEG	POS
**ST567-MSSA (PVL+)**																							
Örebro_02T-671	POS	POS	POS	POS	POS	POS	POS	POS	NEG	POS	POS	POS	POS	POS	POS	NEG	NEG	POS	NEG	NEG	NEG	NEG	POS
Örebro_02T-671 : predicted hybridisation pattern	POS	POS	POS	POS	POS	POS	POS	POS	NEG	POS	POS	POS	POS	POS	POS	NEG	NEG	POS	NEG	NEG	NEG	NEG	POS
**CC80 [ORF CM14+]-MSSA (PVL+)**																							
ANR570100	POS	POS	POS	POS	POS	POS	POS	POS	NEG	POS	POS	POS	POS	POS	POS	NEG	NEG	POS	NEG	NEG	NEG	NEG	POS
ANR570100 : predicted hybridisation pattern	POS	POS	POS	POS	POS	POS	POS	POS	NEG	POS	POS	POS	POS	POS	POS	NEG	NEG	POS	NEG	NEG	NEG	NEG	POS
SAMEA3671725: predicted hybridisation pattern	POS	POS	POS	POS	POS	POS	POS	POS	NEG	POS	POS	POS	POS	POS	POS	NEG	NEG	POS	NEG	NEG	NEG	NEG	POS
SAMEA48342418: predicted hybridisation pattern	POS	POS	POS	POS	POS	POS	POS	POS	NEG	POS	POS	POS	POS	POS	POS	NEG	NEG	POS	NEG	NEG	NEG	NEG	POS
**CC80-MRSA-IVc (PVL+)**																							
V16073 (isolate from DOI: 10.1111/j.1469-0691.2006.01420.x)	POS	POS	POS	POS	POS	POS	POS	POS	NEG	POS	POS	POS	POS	POS	POS	NEG	NEG	POS	NEG	NEG	NEG	NEG	POS
11819-97: predicted hybridisation pattern	POS	POS	POS	POS	POS	POS	POS	POS	NEG	POS	POS	POS	POS	POS	POS	NEG	NEG	POS	NEG	NEG	NEG	NEG	POS

STRAIN / ISOLATE	METHICILLIN RESISTANCE AND SCCmec TYPING																													
	ugpQ	mecA	Delta mecR1	mecR1	mecI	fudoh-PSM	cstB-SCC1 (Q2G1R6)	xylR/mecR2	mecC	mecR-SCCmec XI	mecR-S04009	mecI (SCCmec XI)	mecI (S04009)	blaZ (SCCmec XI)	mecA-vitulinus	mecR2-vitul (-aureus)	mecR2-lent	mecA-sciuri	mecR2-sciur	pls-SCC (COL)	mvaS-SCC	Q5HJW6	Q7A207	cstB-SCC2 (Q2G1R6)	Q9S0M4	kdpA-SCC	kdpB-SCC	kdpC-SCC	kdpD-SCC	kdpE-SCC
	Glycerophosphoryl diester phosphodiesterase. Accompanies mecA in nearly all SCCmec sequences.	Modified penicillin binding protein (PBP2a)	Truncated methicillin resistance operon repressor 1. Truncated mecR1 is present in SCCmec I, IV, V, VI, VII; complete absence of mecR1 from	Methicillin resistance operon repressor 1. Un-truncated sequence in SCCmec II, SCCmec III, SCCmec VIII	Methicillin-resistance regulatory protein. Present in SCCmec II (although absent from Irish SCCmec II variants C and E), SCCmec III, SCCmec VIII	Phenol soluble modulin from SCCmec	CsoR-like sulfur transferase-regulated genes B/metallo-beta-lactamase superfamily protein. Pseudogene containing two stop codons. Subtyping SCCmec II. Also	Methicillin resistance operon repressor 2. Located next to mec operon downstream of mecI (not present if mecI is truncated). Present in SCCmec II, III, VIII	Alternate gene encoding a modified penicillin binding protein. SCCmec XI					Beta-lactamase from SCCmec XI						Plasmin-sensitive surface protein, prevents bacterial adhesion in vitro, located in SCC, close to mec operon. Subtyping SCCmec I	Truncated 3-hydroxy-3-methylglutaryl CoA synthase. Subtyping SCCmec I, II, IV, V	Putative protein next to dru	Putative protein	CsoR-like sulfur transferase-regulated genes B/metallo-beta-lactamase superfamily protein. Present in SCCmec IVa (truncated) and SCCmec X, variably present in SCCmec I	Putative protein. Subtyping SCCmec I, SCCmec/ACME composites and SCCmec from WA40	Potassium-translocating ATPase A, chain 2	Potassium-transporting ATPase B, chain 1	Potassium-translocating ATPase C, chain 2	Sensor kinase protein	KDP operon transcriptional regulatory protein
**CC1-MRSA-IV (PVL+), USA400**																														
MW2	POS	POS	POS	NEG	NEG	NEG	NEG	NEG		NEG	NEG	NEG	NEG	NEG	NEG	NEG	NEG	NEG	NEG	NEG	POS	AMB	POS	POS	NEG	NEG	NEG	NEG	NEG	NEG
Strain MW2 GenBank BA000033.2: predicted hybr. pattern	POS	POS	POS	NEG	NEG	NEG	NEG	NEG	NEG	NEG	NEG	NEG	NEG	NEG	AMB	NEG	NEG	NEG	NEG	NEG	POS	POS	AMB	POS	NEG	NEG	NEG	NEG	NEG	NEG
**ST567-MSSA (PVL+)**																														
Örebro_02T-671	NEG	NEG	NEG	NEG	NEG	NEG	NEG	NEG	neg	NEG	NEG	NEG	NEG	neg	NEG	NEG	NEG	NEG	NEG	NEG	NEG	NEG	NEG	NEG	NEG	NEG	NEG	NEG	NEG	NEG
Örebro_02T-671 : predicted hybridisation pattern	NEG	NEG	NEG	NEG	NEG	NEG	NEG	NEG	NEG	NEG	NEG	NEG	NEG	NEG	NEG	NEG	NEG	NEG	NEG	NEG	NEG	NEG	POS	NEG	NEG	NEG	NEG	NEG	NEG	NEG
**CC80 [ORF CM14+]-MSSA (PVL+)**																														
ANR570100	NEG	NEG	NEG	NEG	NEG	NEG	NEG	NEG	neg	NEG	NEG	NEG	NEG	NEG	NEG	NEG	NEG	NEG	NEG	NEG	NEG	NEG	AMB	NEG	NEG	NEG	NEG	NEG	NEG	NEG
ANR570100 : predicted hybridisation pattern	NEG	NEG	NEG	NEG	NEG	NEG	NEG	NEG	NEG	NEG	NEG	NEG	NEG	NEG	NEG	NEG	NEG	NEG	NEG	NEG	NEG	NEG	POS	NEG	NEG	NEG	NEG	NEG	NEG	NEG
SAMEA3671725: predicted hybridisation pattern	NEG	NEG	NEG	NEG	NEG	NEG	NEG	NEG	NEG	NEG	NEG	NEG	NEG	NEG	NEG	NEG	NEG	NEG	NEG	NEG	NEG	NEG	POS	NEG	NEG	NEG	NEG	NEG	NEG	NEG
SAMEA48342418: predicted hybridisation pattern	NEG	NEG	NEG	NEG	NEG	NEG	NEG	NEG	NEG	NEG	NEG	NEG	NEG	NEG	NEG	NEG	NEG	NEG	NEG	NEG	NEG	NEG	NEG	NEG	NEG	NEG	NEG	NEG	NEG	NEG
**CC80-MRSA-IVc (PVL+)**																														
V16073 (isolate from DOI: 10.1111/j.1469-0691.2006.01420.x)	POS	POS	POS	NEG	NEG	NEG	NEG	NEG	neg	NEG	NEG	NEG	NEG	NEG	NEG	NEG	NEG	NEG	NEG	NEG	POS	AMB	POS	NEG	NEG	NEG	NEG	NEG	NEG	NEG
11819-97: predicted hybridisation pattern	POS	POS	POS	NEG	NEG	NEG	NEG	NEG	NEG	NEG	NEG	NEG	NEG	NEG	AMB	NEG	NEG	NEG	NEG	NEG	POS	POS	AMB	NEG	NEG	NEG	NEG	NEG	NEG	NEG

STRAIN / ISOLATE	METHICILLIN RESISTANCE AND SCCmec TYPING													
	Q93IB7	D1GU38	D1GU38 (TW20)	Q933A2	D1GU55	F9JXC0 (full)	F9JXC0 (trunc)	B2Y834	B6VQU0	Q3YK51	ydhK (FPR3757)	hp1473_ydhK	hp1472_ydhK	C1PH94
	LytTR domain DNA-binding regulator. Subtyping SCCmec III (present in, e.g., TW20 GenBank FN433596.1, but absent in, e.g., Bmb9393 GenBank CP005288.1)	Putative protein. Subtyping SCCmec III, identification of SCCmec VT, SCCmec ZH47, SCCmec VII because of an association with (additional/second) ccrC copies	Putative protein	Putative ADP-ribosyltransferase. Subtyping SCCmec III and SCCmec IX	Putative membrane protein. Subtyping SCCmec III, additional marker for SCCmec VII	KDP operon transcriptional regulatory protein		Abortive phage resistance protein. Subtyping SCCmec IV, i.e., identification of SCCmec IV A, G, c and SCCmec MRSAZH47	Putative protein. Subtyping SCCmec IV, i.e., identification of SCCmec IVh/j	Putative protein. Subtyping SCCmec IV, i.e., identification of SCCmec IV g	Putative lipoprotein. Present in some composite elements comprising SCCmec and heavy metal resistance genes including the one in FPR3757, GenBank: CP000255.1			Putative protein
**CC1-MRSA-IV (PVL+), USA400**														
MW2	NEG	NEG	NEG	NEG	NEG	NEG	NEG	NEG	NEG	NEG	NEG	NEG	NEG	NEG
Strain MW2 GenBank BA000033.2: predicted hybr. pattern	NEG	NEG	NEG	NEG	NEG	NEG	NEG	NEG	NEG	NEG	NEG	NEG	NEG	NEG
**ST567-MSSA (PVL+)**														
Örebro_02T-671	NEG	NEG	NEG	NEG	NEG	NEG	NEG	NEG	NEG	NEG	NEG	NEG	NEG	NEG
Örebro_02T-671 : predicted hybridisation pattern	NEG	NEG	NEG	NEG	NEG	NEG	NEG	NEG	NEG	NEG	NEG	NEG	NEG	NEG
**CC80 [ORF CM14+]-MSSA (PVL+)**														
ANR570100	NEG	NEG	NEG	NEG	NEG	NEG	NEG	NEG	NEG	NEG	NEG	NEG	NEG	NEG
ANR570100 : predicted hybridisation pattern	NEG	NEG	NEG	NEG	NEG	NEG	NEG	NEG	NEG	NEG	NEG	NEG	NEG	NEG
SAMEA3671725: predicted hybridisation pattern	NEG	NEG	NEG	NEG	NEG	NEG	NEG	NEG	NEG	NEG	NEG	NEG	NEG	NEG
SAMEA48342418: predicted hybridisation pattern	NEG	NEG	NEG	NEG	NEG	NEG	NEG	NEG	NEG	NEG	NEG	NEG	NEG	NEG
**CC80-MRSA-IVc (PVL+)**														
V16073 (isolate from DOI: 10.1111/j.1469-0691.2006.01420.x)	NEG	NEG	NEG	NEG	NEG	NEG	NEG	POS	NEG	NEG	NEG	NEG	NEG	NEG
11819-97: predicted hybridisation pattern	NEG	NEG	NEG	NEG	NEG	NEG	NEG	POS	NEG	NEG	NEG	NEG	NEG	NEG

STRAIN / ISOLATE	METHICILLIN RESISTANCE AND SCCmec TYPING													
	DUF1958	Q4LAG7 (SCC consensus)	Q4LAG7 (SCCmecV, SO385)	Q4LAG7 (SCCfus 45394F/MSSA476)	Q4LAG7 (plasmid SK933)	Q4LAG7 (plasmid pCDC25)	C5QAP8 (SCCmec XI)	Q8CU82	D3JD07	cas1 (MSHR1132)	cas1 (M06-171)	fusC (Q6GD50)	tirS	yeeA
	Putative protein. Subtyping SCCmec VT. Present, e.g., in PM1, GenBank BAFA but absent, e.g., in Strain 3957, GenBank AOFU	Putative protein located within SCCmec type V/SCCfus elements	Putative protein located within SCCmec type V elements	Putative protein located within SCCfus elements			Putative protein. Identification of SCCmec XI	Putative protein. Present in some SCCmec/fus composite elements such as, e.g., CMFT120, GenBank HF569094.1 and CMFT2, GenBank HF569101.1	Putative protein. Present in some composite SCC elements	CRISPR-associated endonuclease 1. Present in MSHR1132	CRISPR-associated endonuclease 1. Present in M06/0171, GenBank HE980450.1	SCC-associated fusidic acid resistance gene. Present alone in "SCCfus" elements or together with mecA in composite elements. Most common in CC1 and CC5 strains	Staphylococcal TIR-protein binding protein. Subtyping SCCfus because it is frequently, but not always, accompanying fusC	Putative DNA methyltransferase. Subtyping SCCmecIV/fus composite elements
**CC1-MRSA-IV (PVL+), USA400**														
MW2	NEG	NEG	NEG	NEG	NEG	NEG	NEG	NEG	NEG	NEG	NEG	NEG	NEG	NEG
Strain MW2 GenBank BA000033.2: predicted hybr. pattern	NEG	NEG	NEG	NEG	NEG	NEG	NEG	NEG	NEG	NEG	NEG	NEG	NEG	NEG
**ST567-MSSA (PVL+)**														
Örebro_02T-671	NEG	NEG	NEG	NEG	NEG	NEG	NEG	NEG	NEG	NEG	NEG	NEG	NEG	NEG
Örebro_02T-671 : predicted hybridisation pattern	NEG	NEG	NEG	NEG	NEG	NEG	NEG	NEG	NEG	NEG	NEG	NEG	NEG	NEG
**CC80 [ORF CM14+]-MSSA (PVL+)**														
ANR570100	NEG	NEG	NEG	NEG	NEG	NEG	NEG	NEG	NEG	NEG	NEG	NEG	NEG	NEG
ANR570100 : predicted hybridisation pattern	NEG	NEG	NEG	NEG	NEG	NEG	NEG	NEG	NEG	NEG	NEG	NEG	NEG	NEG
SAMEA3671725: predicted hybridisation pattern	NEG	NEG	NEG	NEG	NEG	NEG	NEG	NEG	NEG	NEG	NEG	NEG	NEG	NEG
SAMEA48342418: predicted hybridisation pattern	NEG	NEG	NEG	NEG	NEG	NEG	NEG	NEG	NEG	NEG	NEG	NEG	NEG	NEG
**CC80-MRSA-IVc (PVL+)**														
V16073 (isolate from DOI: 10.1111/j.1469-0691.2006.01420.x)	NEG	NEG	NEG	NEG	NEG	NEG	NEG	NEG	NEG	NEG	NEG	NEG	NEG	NEG
11819-97: predicted hybridisation pattern	NEG	NEG	NEG	NEG	NEG	NEG	NEG	NEG	NEG	NEG	NEG	NEG	NEG	NEG

STRAIN / ISOLATE	METHICILLIN RESISTANCE AND SCCmec TYPING																									
	ACME total	arcA-SCC	arcB-SCC	arcC-SCC	arcD-SCC	opp3B	opp3B (FPR3757)	opp3B (C427)	opp3C (FPR3757)	opp3C (C427)	adhC	speG (FPR3757)	ccrA-1	ccrB-1	ccrA-2	ccrB-2	ccrA-3	ccrB-3	ccrAA (MRSAZH47)_probe 1	ccrC (85-2082)	ccrA-4	ccrB-4	Q9XB68-dcs	SCCmec Terminus 1 to 6	SCCmec Terminus 7	SCCmec Terminus 9 to 14
	ACME total	Arginine deiminase	Ornithine carbamoyltransferase	Carbamate kinase	Arginine/ornithine antiporter	Oligopeptide permease, channel-forming protein			Oligopeptide permease, channel-forming protein		Alcohol dehydrogenase, zinc-containing	Spermidine N-acetyltransferase. Usually associated with ACME or composite SCCmec/ACME elements	Cassette chromosome recombinase A, type 1	Cassette chromosome recombinase B, type 1	Cassette chromosome recombinase A, type 2	Cassette chromosome recombinase B, type 2	Cassette chromosome recombinase A, type 3	Cassette chromosome recombinase B, type 3	Cassette chromosome recombinase homologue	Cassette chromosome recombinase C	Cassette chromosome recombinase A, type 4	Cassette chromosome recombinase B, type 4	Located at the terminus of SCCmec directly next to orfX	SCC integration site alternate to dcs		
**CC1-MRSA-IV (PVL+), USA400**																										
MW2	NEG	NEG	NEG	NEG	NEG	NEG	NEG	NEG	NEG	NEG	NEG	NEG	NEG	NEG	POS	POS	NEG	NEG	NEG	NEG	NEG	NEG	POS	NEG	NEG	NEG
Strain MW2 GenBank BA000033.2: predicted hybr. pattern	NEG	NEG	NEG	NEG	NEG	NEG	NEG	NEG	NEG	NEG	NEG	NEG	NEG	NEG	POS	POS	NEG	NEG	NEG	NEG	NEG	NEG	POS	NEG	NEG	NEG
**ST567-MSSA (PVL+)**																										
Örebro_02T-671	NEG	NEG	NEG	NEG	NEG	POS	AMB	POS	NEG	POS	NEG	NEG	POS	NEG	NEG	NEG	NEG	NEG	NEG	NEG	NEG	NEG	NEG	NEG	POS	NEG
Örebro_02T-671 : predicted hybridisation pattern	NEG	NEG	NEG	NEG	NEG	POS	AMB	POS	NEG	POS	NEG	NEG	POS	AMB	NEG	NEG	NEG	NEG	NEG	NEG	NEG	NEG	NEG	NEG	POS	NEG
**CC80 [ORF CM14+]-MSSA (PVL+)**																										
ANR570100	NEG	NEG	NEG	NEG	NEG	POS	AMB	POS	NEG	POS	NEG	NEG	POS	NEG	NEG	NEG	NEG	NEG	NEG	NEG	NEG	NEG	NEG	NEG	POS	NEG
ANR570100 : predicted hybridisation pattern	NEG	NEG	NEG	NEG	NEG	POS	AMB	POS	NEG	POS	NEG	NEG	POS	AMB	NEG	NEG	NEG	NEG	NEG	NEG	NEG	NEG	NEG	NEG	POS	NEG
SAMEA3671725: predicted hybridisation pattern	NEG	NEG	NEG	NEG	NEG	POS	AMB	POS	NEG	POS	NEG	NEG	POS	AMB	NEG	NEG	NEG	NEG	NEG	NEG	NEG	NEG	NEG	NEG	POS	NEG
SAMEA48342418: predicted hybridisation pattern	NEG	NEG	NEG	NEG	NEG	NEG	NEG	NEG	NEG	NEG	NEG	NEG	NEG	NEG	NEG	NEG	NEG	NEG	NEG	NEG	NEG	NEG	NEG	NEG	POS	NEG
**CC80-MRSA-IVc (PVL+)**																										
V16073 (isolate from DOI: 10.1111/j.1469-0691.2006.01420.x)	NEG	NEG	NEG	neg	NEG	NEG	NEG	NEG	NEG	NEG	NEG	NEG	NEG	NEG	POS	POS	NEG	NEG	NEG	NEG	NEG	NEG	POS	NEG	NEG	NEG
11819-97: predicted hybridisation pattern	NEG	NEG	NEG	NEG	NEG	NEG	NEG	NEG	NEG	NEG	NEG	NEG	NEG	NEG	POS	POS	NEG	NEG	NEG	NEG	NEG	NEG	POS	NEG	NEG	NEG

STRAIN / ISOLATE	HEAVY METAL RESISTANCES, SCCmec AND OTHERWISE																										
	merA	merB	mco-plasmid (MRSA252)	copA2_pla	copA2-SCC	mco-SCC	arsA	arsD	arsD2	arsB (SCC)			arsB (chromosomal)			arsB (chromosomal or plasmidic - crossreactive)		arsB (chromosomal-argenteus)		arsB (plasmidic)							
	mercury resistance operon		Multi copper oxidase	Copper exporting ATPase	Copper exporting ATPase	Multi copper oxidase	arsenical pump-driving ATPase	Putative dehydrogenase	trans-acting repressor of arsenic resistance operon	arsenical pump membrane protein																	
**CC1-MRSA-IV (PVL+), USA400**																											
MW2	NEG	NEG	NEG	NEG	NEG	NEG	NEG	NEG	NEG	NEG	NEG	NEG	POS	POS	POS	POS	POS	NEG	NEG	NEG	NEG	NEG	NEG	NEG	NEG	NEG	NEG
Strain MW2 GenBank BA000033.2: predicted hybr. pattern	NEG	NEG	NEG	NEG	NEG	NEG	NEG	NEG	NEG	NEG	NEG	NEG	POS	POS	POS	NEG	NEG	NEG	NEG	NEG	NEG	NEG	NEG	NEG	NEG	NEG	NEG
**ST567-MSSA (PVL+)**																											
Örebro_02T-671	NEG	NEG	NEG	NEG	NEG	NEG	NEG	NEG	NEG	NEG	NEG	NEG	POS	POS	POS	POS	POS	NEG	NEG	NEG	NEG	NEG	NEG	NEG	NEG	NEG	NEG
Örebro_02T-671 : predicted hybridisation pattern	NEG	NEG	NEG	NEG	NEG	NEG	NEG	NEG	NEG	NEG	NEG	NEG	POS	POS	POS	NEG	NEG	NEG	NEG	NEG	NEG	NEG	NEG	NEG	NEG	NEG	NEG
**CC80 [ORF CM14+]-MSSA (PVL+)**																											
ANR570100	NEG	NEG	NEG	NEG	NEG	NEG	NEG	NEG	NEG	NEG	NEG	NEG	POS	POS	POS	POS	POS	NEG	NEG	NEG	NEG	NEG	NEG	NEG	NEG	NEG	NEG
ANR570100 : predicted hybridisation pattern	NEG	NEG	NEG	NEG	NEG	NEG	NEG	NEG	NEG	NEG	NEG	NEG	POS	POS	POS	NEG	NEG	NEG	NEG	NEG	NEG	NEG	NEG	NEG	NEG	NEG	NEG
SAMEA3671725: predicted hybridisation pattern	NEG	NEG	NEG	NEG	NEG	NEG	NEG	NEG	NEG	NEG	NEG	NEG	POS	POS	POS	NEG	NEG	NEG	NEG	NEG	NEG	NEG	NEG	NEG	NEG	NEG	NEG
SAMEA48342418: predicted hybridisation pattern	NEG	NEG	NEG	NEG	NEG	NEG	NEG	NEG	NEG	NEG	NEG	NEG	POS	POS	POS	NEG	NEG	NEG	NEG	NEG	NEG	NEG	NEG	NEG	NEG	NEG	NEG
**CC80-MRSA-IVc (PVL+)**																											
V16073 (isolate from DOI: 10.1111/j.1469-0691.2006.01420.x)	NEG	NEG	NEG	NEG	NEG	NEG	NEG	NEG	NEG	NEG	NEG	NEG	POS	POS	POS	POS	POS	NEG	NEG	NEG	NEG	NEG	NEG	NEG	NEG	NEG	NEG
11819-97: predicted hybridisation pattern	NEG	NEG	NEG	NEG	NEG	NEG	NEG	NEG	NEG	NEG	NEG	NEG	POS	POS	POS	NEG	NEG	NEG	NEG	NEG	NEG	NEG	NEG	NEG	NEG	NEG	NEG

STRAIN / ISOLATE	HEAVY METAL RESISTANCES, SCCmec AND OTHERWISE																				RESISTANCE : PENICILLINASE		
	arsC (chromosomal)		arsC (SCC or plasmidic)				czrB	czrC	cadA (cons)	cadA (pTW20)	cadA (TN554)	cadA (Sepi)	cadC (TN554)	cadC (pI258)	cadD (total)	cadD (R35, SCC)	cadD (Smas)	cadX (plasmid probe I)	cadX (plasmid probe II)	cadX (JCSC6943)	blaZ	blaI	blaR
	arsenate reductase						zink and cobalt transporter protein	cadmium and zinc resistance gene C, heavy metal translocating P-type ATPase. Frequently associated with SCCmec elements from livestock MRSA	Cadmium transport protein D						cadmium efflux adenosine triphosphatase			Putative regulator of cadmium efflux			beta-lactamase	beta lactamase repressor (inhibitor)	beta-lactamase regulatory protein
**CC1-MRSA-IV (PVL+), USA400**																							
MW2	NEG	NEG	NEG	NEG	NEG	NEG	POS	NEG	NEG	NEG	NEG	NEG	NEG	NEG	POS	NEG	NEG	POS	POS	NEG	POS	POS	POS
Strain MW2 GenBank BA000033.2: predicted hybr. pattern	NEG	NEG	NEG	NEG	NEG	NEG	POS	NEG	NEG	NEG	NEG	NEG	NEG	NEG	POS	NEG	NEG	POS	POS	NEG	POS	POS	POS
**ST567-MSSA (PVL+)**																							
Örebro_02T-671	NEG	NEG	NEG	NEG	NEG	NEG	POS	NEG	NEG	NEG	NEG	NEG	NEG	NEG	NEG	NEG	NEG	NEG	NEG	NEG	NEG	NEG	NEG
Örebro_02T-671 : predicted hybridisation pattern	NEG	NEG	NEG	NEG	NEG	NEG	POS	NEG	NEG	NEG	NEG	NEG	NEG	NEG	NEG	NEG	NEG	NEG	NEG	NEG	NEG	NEG	NEG
**CC80 [ORF CM14+]-MSSA (PVL+)**																							
ANR570100	NEG	NEG	NEG	NEG	NEG	NEG	POS	NEG	NEG	NEG	NEG	NEG	NEG	NEG	POS	NEG	NEG	POS	POS	NEG	POS	POS	POS
ANR570100 : predicted hybridisation pattern	NEG	NEG	NEG	NEG	NEG	NEG	POS	NEG	NEG	NEG	NEG	NEG	NEG	NEG	POS	NEG	NEG	POS	POS	NEG	POS	POS	POS
SAMEA3671725: predicted hybridisation pattern	NEG	NEG	NEG	NEG	NEG	NEG	POS	NEG	NEG	NEG	NEG	NEG	NEG	NEG	POS	NEG	NEG	POS	POS	NEG	POS	POS	POS
SAMEA48342418: predicted hybridisation pattern	NEG	NEG	NEG	NEG	NEG	NEG	POS	NEG	NEG	NEG	NEG	NEG	NEG	NEG	POS	NEG	NEG	POS	POS	NEG	POS	POS	POS
**CC80-MRSA-IVc (PVL+)**																							
V16073 (isolate from DOI: 10.1111/j.1469-0691.2006.01420.x)	NEG	NEG	NEG	NEG	NEG	NEG	POS	NEG	POS	POS	NEG	NEG	NEG	POS	NEG	NEG	NEG	POS	NEG	NEG	POS	POS	POS
11819-97: predicted hybridisation pattern	NEG	NEG	NEG	NEG	NEG	NEG	POS	NEG	POS	POS	NEG	NEG	NEG	POS	NEG	NEG	NEG	NEG	NEG	NEG	POS	POS	POS

STRAIN / ISOLATE	RESISTANCE : MLS-ANTIBIOTICS																						
	ermA	ermA-43	ermB	ermC	ermC-GM	ermF	ermT	linA/lnuA	lnuB	lsa-B	lsa-E	msrA	mefA	mph(C)	vat(A)	vat(B)	vga(A)	vga(A) (BM 3327)	vgaB	vgaC	vgaD	vgaE	vgb
	rRNA adenine N-6-methyl-transferase, erythromycin/clindamycin resistance							Lincosamid-Nucleotidyltransferase	lincosamide nucleotidyltransferase	lincosamide ABC transporter		energy-dependent efflux of erythro-mycin	macrolide efflux protein A	probable lysylphos-phatidyl-glycerol synthetase	virginia-mycin A acetyltransferase	acetyl-transferase inactivating streptogramin A	ATP binding protein, streptogramin-A-resistance		streptogramin A /	streptogramin A ABC transporter		streptogramin A /	virginia-mycin B hydrolase
**CC1-MRSA-IV (PVL+), USA400**																							
MW2	NEG	NEG	NEG	NEG	NEG	NEG	NEG	NEG	NEG	NEG	NEG	NEG	NEG	NEG	NEG	NEG	NEG	NEG	NEG	NEG	NEG	NEG	NEG
Strain MW2 GenBank BA000033.2: predicted hybr. pattern	NEG	NEG	NEG	NEG	NEG	NEG	NEG	NEG	NEG	NEG	NEG	NEG	NEG	NEG	NEG	NEG	NEG	NEG	NEG	NEG	NEG	NEG	NEG
**ST567-MSSA (PVL+)**																							
Örebro_02T-671	NEG	NEG	NEG	NEG	NEG	NEG	NEG	NEG	NEG	NEG	NEG	NEG	NEG	NEG	NEG	NEG	NEG	NEG	NEG	NEG	NEG	NEG	NEG
Örebro_02T-671 : predicted hybridisation pattern	NEG	NEG	NEG	NEG	NEG	NEG	NEG	NEG	NEG	NEG	NEG	NEG	NEG	NEG	NEG	NEG	NEG	NEG	NEG	NEG	NEG	NEG	NEG
**CC80 [ORF CM14+]-MSSA (PVL+)**																							
ANR570100	NEG	NEG	NEG	NEG	NEG	NEG	NEG	NEG	NEG	NEG	NEG	NEG	NEG	NEG	NEG	NEG	NEG	NEG	NEG	NEG	NEG	NEG	NEG
ANR570100 : predicted hybridisation pattern	NEG	NEG	NEG	NEG	NEG	NEG	NEG	NEG	NEG	NEG	NEG	NEG	NEG	NEG	NEG	NEG	NEG	NEG	NEG	NEG	NEG	NEG	NEG
SAMEA3671725: predicted hybridisation pattern	NEG	NEG	NEG	NEG	NEG	NEG	NEG	NEG	NEG	NEG	NEG	NEG	NEG	NEG	NEG	NEG	NEG	NEG	NEG	NEG	NEG	NEG	NEG
SAMEA48342418: predicted hybridisation pattern	NEG	NEG	NEG	NEG	NEG	NEG	NEG	NEG	NEG	NEG	NEG	NEG	NEG	NEG	NEG	NEG	NEG	NEG	NEG	NEG	NEG	NEG	NEG
**CC80-MRSA-IVc (PVL+)**																							
V16073 (isolate from DOI: 10.1111/j.1469-0691.2006.01420.x)	NEG	NEG	NEG	NEG	NEG	NEG	NEG	NEG	NEG	NEG	NEG	NEG	NEG	NEG	NEG	NEG	NEG	NEG	NEG	NEG	NEG	NEG	NEG
11819-97: predicted hybridisation pattern	NEG	NEG	NEG	NEG	NEG	NEG	NEG	NEG	NEG	NEG	NEG	AMB	NEG	POS	NEG	NEG	NEG	NEG	NEG	NEG	NEG	NEG	NEG

STRAIN / ISOLATE	RESISTANCE : AMINOGLYSOSIDES									RESISTANCE : MISCELLANEOUS GENES																			
	aacA-aphD	aadD	ant9-C2944	ant9	ble	aad6	aadA-var1	aadE	aphA3	sat	dfrA	dfrG	dfrG-Tn559	dfrG-K	dfrG-D	far1	mupA	mupB	tetK	tetL	tetM (com-bined)	tetM-O	tetM-S	cat	cfr	fexA	apmA	fosB	fosB (plasmid)
	bifunctional enzyme Aac/Aph, gentamicin resistance	amino-glycoside adenyl-transferase,tobramycin resistance	adenyltransferase AAd9		bleomycin resistance protein	aminoglycoside 6-adenylyltransferase	aminoglycoside-3'-adenylyltransferase	streptomycin aminoglycoside 6-adenyltransferase	3'5'-aminoglycoside phospho-transferase, neo-/kanamycin resistance	strepto-thricine-acetyl-transferase	dihydro-folate reductase type 1	dihydrofolate reductase				fusidic acid resistence	mupirocin resistence protein	plasmidic isoleuc	tetracycline resistance	Tetracycline resis	tetracycline resistance			chlor-amphenicol acetyltransferase	23S rRNA methyltransferase	chloramphenicol/florfenicol exporter	aminocyclitol acetyltransferase, confers apramycin resistance	metallothiol transferase	
**CC1-MRSA-IV (PVL+), USA400**																													
MW2	NEG	NEG	NEG	NEG	NEG	NEG	NEG	NEG	NEG	NEG	NEG	NEG	NEG	NEG	NEG	NEG	NEG	NEG	NEG	NEG	NEG	NEG	NEG	NEG	NEG	NEG	NEG	NEG	NEG
Strain MW2 GenBank BA000033.2: predicted hybr. pattern	NEG	NEG	NEG	NEG	NEG	NEG	NEG	NEG	NEG	NEG	NEG	NEG	NEG	NEG	NEG	NEG	NEG	NEG	NEG	NEG	NEG	NEG	NEG	NEG	NEG	NEG	NEG	NEG	NEG
**ST567-MSSA (PVL+)**																													
Örebro_02T-671	NEG	NEG	NEG	NEG	NEG	NEG	NEG	NEG	NEG	NEG	NEG	NEG	NEG	NEG	NEG	NEG	NEG	NEG	NEG	NEG	NEG	NEG	NEG	NEG	NEG	NEG	NEG	NEG	NEG
Örebro_02T-671 : predicted hybridisation pattern	NEG	NEG	NEG	NEG	NEG	NEG	NEG	NEG	NEG	NEG	NEG	NEG	NEG	NEG	NEG	NEG	NEG	NEG	NEG	NEG	NEG	NEG	NEG	NEG	NEG	NEG	NEG	NEG	NEG
**CC80 [ORF CM14+]-MSSA (PVL+)**																													
ANR570100	NEG	NEG	NEG	NEG	NEG	NEG	NEG	NEG	NEG	NEG	NEG	NEG	NEG	NEG	NEG	NEG	NEG	NEG	NEG	NEG	NEG	NEG	NEG	NEG	NEG	NEG	NEG	NEG	NEG
ANR570100 : predicted hybridisation pattern	NEG	NEG	NEG	NEG	NEG	NEG	NEG	NEG	NEG	NEG	NEG	NEG	NEG	NEG	NEG	NEG	NEG	NEG	NEG	NEG	NEG	NEG	NEG	NEG	NEG	NEG	NEG	NEG	NEG
SAMEA3671725: predicted hybridisation pattern	NEG	NEG	NEG	NEG	NEG	NEG	NEG	NEG	NEG	NEG	NEG	NEG	NEG	NEG	NEG	NEG	NEG	NEG	NEG	NEG	NEG	NEG	NEG	NEG	NEG	NEG	NEG	NEG	NEG
SAMEA48342418: predicted hybridisation pattern	NEG	NEG	NEG	NEG	NEG	NEG	NEG	NEG	NEG	NEG	NEG	NEG	NEG	NEG	NEG	NEG	NEG	NEG	NEG	NEG	NEG	NEG	NEG	NEG	NEG	NEG	NEG	NEG	NEG
**CC80-MRSA-IVc (PVL+)**																													
V16073 (isolate from DOI: 10.1111/j.1469-0691.2006.01420.x)	NEG	NEG	NEG	NEG	NEG	NEG	NEG	POS	POS	POS	NEG	NEG	NEG	NEG	NEG	POS	NEG	NEG	POS	NEG	NEG	NEG	NEG	NEG	NEG	NEG	NEG	NEG	NEG
11819-97: predicted hybridisation pattern	NEG	NEG	NEG	NEG	NEG	NEG	NEG	POS	POS	POS	NEG	NEG	NEG	NEG	NEG	POS	NEG	NEG	NEG	NEG	NEG	NEG	NEG	NEG	NEG	NEG	NEG	NEG	NEG

STRAIN / ISOLATE	RESISTANCE : MISCELLANEOUS GENES									VIRULENCE : TOX.SCHOCK.TOXIN			VIRULENCE : ENTEROTOXINS		
	qacA	qacC	smr (AL1)	smr (KACC16562)	smr (OJ82)	smr (VCU012)	vanA	vanB	vanZ	tst1 (consens.)	tst1 ("human" allele)	tst1 ("bovine" allele)	entA	entA (320E)	entA (N315) / entP
	quaternary ammonium compound resistance protein A	quaternary ammonium compound resistance protein C					vancomycin resistance gene	vancomycin resistance gene from enterococci and Clostridium	teicoplanin resistance gene from enterococci	toxic shock syndrome toxin 1			Enterotoxin A	Enterotoxin A, allele from strain 320E	Enterotoxin A, allele from strain N315 = Entero-toxin P
**CC1-MRSA-IV (PVL+), USA400**															
MW2	NEG	NEG	NEG	NEG	NEG	NEG	NEG	NEG	NEG	NEG	NEG	NEG	POS	NEG	NEG
Strain MW2 GenBank BA000033.2: predicted hybr. pattern	NEG	NEG	NEG	NEG	NEG	NEG	NEG	NEG	NEG	NEG	NEG	NEG	POS	NEG	NEG
**ST567-MSSA (PVL+)**															
Örebro_02T-671	NEG	NEG	NEG	NEG	NEG	NEG	NEG	NEG	NEG	NEG	NEG	NEG	NEG	NEG	NEG
Örebro_02T-671 : predicted hybridisation pattern	NEG	NEG	NEG	NEG	NEG	NEG	NEG	NEG	NEG	NEG	NEG	NEG	NEG	NEG	NEG
**CC80 [ORF CM14+]-MSSA (PVL+)**															
ANR570100	NEG	NEG	NEG	NEG	NEG	NEG	NEG	NEG	NEG	NEG	NEG	NEG	NEG	NEG	NEG
ANR570100 : predicted hybridisation pattern	NEG	NEG	NEG	NEG	NEG	NEG	NEG	NEG	NEG	NEG	NEG	NEG	NEG	NEG	NEG
SAMEA3671725: predicted hybridisation pattern	NEG	NEG	NEG	NEG	NEG	NEG	NEG	NEG	NEG	NEG	NEG	NEG	NEG	NEG	NEG
SAMEA48342418: predicted hybridisation pattern	NEG	NEG	NEG	NEG	NEG	NEG	NEG	NEG	NEG	NEG	NEG	NEG	NEG	NEG	NEG
**CC80-MRSA-IVc (PVL+)**															
V16073 (isolate from DOI: 10.1111/j.1469-0691.2006.01420.x)	NEG	NEG	NEG	NEG	NEG	NEG	NEG	NEG	NEG	NEG	NEG	NEG	NEG	NEG	NEG
11819-97: predicted hybridisation pattern	NEG	NEG	NEG	NEG	NEG	NEG	NEG	NEG	NEG	NEG	NEG	NEG	NEG	NEG	NEG

STRAIN / ISOLATE	entB	entC	entD	entD2	entE	entH	entJ	entK	entL	entN2	entQ	entR	entS	entT	entU2	entW
	Enterotoxin B	Enterotoxin C	Enterotoxin D	Enterotoxin similar to EntD	Enterotoxin E	Enterotoxin H	Enterotoxin J	Enterotoxin K	Enterotoxin L	Putative Enterotoxin N2	Enterotoxin Q	Enterotoxin R	Enterotoxin S	Enterotoxin T	Enterotoxin U2	Putative Enterotoxin
**CC1-MRSA-IV (PVL+), USA400**																
MW2	NEG	POS	NEG	NEG	NEG	POS	NEG	POS	POS	NEG	POS	NEG	NEG	NEG	NEG	NEG
Strain MW2 GenBank BA000033.2: predicted hybr. pattern	NEG	POS	NEG	NEG	NEG	POS	NEG	POS	POS	NEG	POS	NEG	NEG	NEG	NEG	NEG
**ST567-MSSA (PVL+)**																
Örebro_02T-671	NEG	NEG	NEG	NEG	NEG	NEG	NEG	NEG	NEG	NEG	NEG	NEG	NEG	NEG	NEG	NEG
Örebro_02T-671 : predicted hybridisation pattern	NEG	NEG	NEG	NEG	NEG	NEG	NEG	NEG	NEG	NEG	NEG	NEG	NEG	NEG	NEG	NEG
**CC80 [ORF CM14+]-MSSA (PVL+)**																
ANR570100	NEG	NEG	NEG	NEG	NEG	NEG	NEG	NEG	NEG	NEG	NEG	NEG	NEG	NEG	NEG	NEG
ANR570100 : predicted hybridisation pattern	NEG	NEG	NEG	NEG	NEG	NEG	NEG	NEG	NEG	NEG	NEG	NEG	NEG	NEG	NEG	NEG
SAMEA3671725: predicted hybridisation pattern	NEG	NEG	NEG	NEG	NEG	NEG	NEG	NEG	NEG	NEG	NEG	NEG	NEG	NEG	NEG	NEG
SAMEA48342418: predicted hybridisation pattern	NEG	NEG	NEG	NEG	NEG	NEG	NEG	NEG	NEG	NEG	NEG	NEG	NEG	NEG	NEG	NEG
**CC80-MRSA-IVc (PVL+)**																
V16073 (isolate from DOI: 10.1111/j.1469-0691.2006.01420.x)	NEG	NEG	NEG	NEG	NEG	NEG	NEG	NEG	NEG	NEG	NEG	NEG	NEG	NEG	NEG	NEG
11819-97: predicted hybridisation pattern	NEG	NEG	NEG	NEG	NEG	NEG	NEG	NEG	NEG	NEG	NEG	NEG	NEG	NEG	NEG	NEG

STRAIN / ISOLATE	VIRULENCE : ENTEROTOXINS											VIRULENCE : LEUKOCIDINS															
	egc (total)	seg	sei	selm	seM/I/V	seln (consens.)	seln (other than RF122)	seln-argenteus	selo	selu	ORF CM14_probe2	lukF	lukS	lukS (ST22+ST45)	hlgA	lukF (int)	lukS (int)	lukF-PV	lukS-PV	lukF-PV (P83)	lukM	lukD	lukE	lukX	lukY	lukY (ST30+ST45)	lukY (ST1850)
	egc cluster	Enterotoxin G	Enterotoxin I	Enterotoxin M		Enterotoxin N			Enterotoxin O	Enterotoxin U and/or Y	Enterotoxin-like protein ORF CM14	Haemolysin gamma / leukocidin, component B	Haemolysin gamma / leukocidin, component C		Haemolysin gam	intermedius group leukocidin		Panton Valentine leukocidin F component	Panton Valentine leukocidin S component	F component from ruminant leukocidin	S component from ruminant leukocidin	leukocidin D component	leukocidin E component	leukocidin/ haemolysin toxin family protein	leukocidin/haemolysin toxin family protein		
**CC1-MRSA-IV (PVL+), USA400**																											
MW2	NEG	NEG	NEG	NEG	NEG	NEG	NEG	NEG	NEG	NEG	NEG	POS	POS	NEG	POS	NEG	NEG	POS	POS	NEG	NEG	POS	POS	POS	POS	NEG	NEG
Strain MW2 GenBank BA000033.2: predicted hybr. pattern	NEG	NEG	NEG	NEG	NEG	NEG	NEG	NEG	NEG	NEG	NEG	POS	POS	AMB	POS	NEG	NEG	POS	POS	NEG	NEG	POS	POS	POS	POS	NEG	NEG
**ST567-MSSA (PVL+)**																											
Örebro_02T-671	NEG	NEG	NEG	NEG	NEG	NEG	NEG	NEG	NEG	NEG	POS	POS	POS	NEG	POS	NEG	NEG	POS	POS	NEG	NEG	POS	POS	AMB	POS	NEG	NEG
Örebro_02T-671 : predicted hybridisation pattern	NEG	NEG	NEG	NEG	NEG	NEG	NEG	NEG	NEG	NEG	POS	POS	POS	AMB	POS	NEG	NEG	POS	POS	NEG	NEG	POS	POS	POS	POS	NEG	NEG
**CC80 [ORF CM14+]-MSSA (PVL+)**																											
ANR570100	NEG	NEG	NEG	NEG	NEG	NEG	NEG	NEG	NEG	NEG	POS	POS	POS	AMB	POS	NEG	NEG	POS	POS	NEG	NEG	POS	POS	POS	POS	NEG	NEG
ANR570100 : predicted hybridisation pattern	NEG	NEG	NEG	NEG	NEG	NEG	NEG	NEG	NEG	NEG	POS	POS	POS	AMB	POS	NEG	NEG	POS	POS	NEG	NEG	POS	POS	POS	POS	NEG	NEG
SAMEA3671725: predicted hybridisation pattern	NEG	NEG	NEG	NEG	NEG	NEG	NEG	NEG	NEG	NEG	POS	POS	POS	AMB	POS	NEG	NEG	POS	POS	NEG	NEG	POS	POS	POS	POS	NEG	NEG
SAMEA48342418: predicted hybridisation pattern	NEG	NEG	NEG	NEG	NEG	NEG	NEG	NEG	NEG	NEG	POS	POS	POS	AMB	POS	NEG	NEG	POS	POS	NEG	NEG	POS	POS	POS	POS	NEG	NEG
**CC80-MRSA-IVc (PVL+)**																											
V16073 (isolate from DOI: 10.1111/j.1469-0691.2006.01420.x)	NEG	NEG	NEG	NEG	NEG	NEG	NEG	NEG	NEG	NEG	NEG	POS	POS	NEG	POS	NEG	NEG	POS	POS	NEG	NEG	POS	POS	POS	POS	NEG	NEG
11819-97: predicted hybridisation pattern	NEG	NEG	NEG	NEG	NEG	NEG	NEG	NEG	NEG	NEG	NEG	POS	POS	AMB	POS	NEG	NEG	POS	POS	NEG	NEG	POS	POS	POS	POS	NEG	NEG

STRAIN / ISOLATE	VIRULENCE : HAEMOLYSINS								VIRULENCE : HLB-CONV PHAGES			VIRULENCE : OTHER FACTORS									VIRULENCE : PROTEASES									
	corB (=hl)	hla	hlIII (cons)	hlIII (other than RF122)	hlb-probe 1	hlb-probe 2	hlb-probe 3	un-truncated hlb	sak	chp	scn	etA	etB	etD	etD2	edinA	edinB	edinC	esxA	esxB	aur (cons)	aur (other than MRSA252)	aur (MRSA252)	splA	splB	splE	sspA	sspB	sspP (cons)	sspP (other than ST93)
	Putative membrane protein	Haemolysin alpha	Putative membrane protein		haemolysin beta				staphylo-kinase	chemotaxis-inhibiting protein (CHIPS)	Staphyl. Comple-ment inhibitor	exfoliative toxin serotype A	exfoliative toxin serotype B	exfoliative toxin D	exfoliative toxin D2	epidermal cell differen-tiation inhibitor	epidermal cell differen-tiation inhibitor B	epidermal cell differen-tiation inhibitor C	virulence factor esxA	virulence factor esxB	aureolysin			serin-protease A	serin-protease B	serin-protease E	glutamylendopeptidase	Staphopain B, protease	Staphopain A (Staphylopain A), protease	
**CC1-MRSA-IV (PVL+), USA400**																														
MW2	POS	POS	POS	POS	AMB	POS	AMB	NEG	POS	NEG	POS	NEG	NEG	NEG	NEG	NEG	NEG	NEG	POS	POS	POS	POS	NEG	POS	POS	NEG	POS	POS	POS	POS
Strain MW2 GenBank BA000033.2: predicted hybr. pattern	POS	POS	POS	POS	POS	POS	POS	POS	POS	NEG	POS	NEG	NEG	NEG	NEG	NEG	NEG	NEG	POS	POS	POS	POS	NEG	POS	POS	NEG	POS	POS	POS	POS
**ST567-MSSA (PVL+)**																														
Örebro_02T-671	POS	POS	POS	POS	POS	POS	NEG	NEG	POS	NEG	POS	NEG	NEG	NEG	NEG	NEG	NEG	NEG	POS	POS	NEG	NEG	POS	POS	POS	POS	POS	POS	POS	POS
Örebro_02T-671 : predicted hybridisation pattern	POS	POS	POS	POS	POS	POS	POS	POS	POS	NEG	POS	NEG	NEG	NEG	NEG	NEG	NEG	NEG	POS	POS	POS	NEG	POS	POS	POS	POS	POS	POS	POS	POS
**CC80 [ORF CM14+]-MSSA (PVL+)**																														
ANR570100	POS	NEG	POS	POS	POS	POS	AMB	NEG	POS	NEG	POS	NEG	NEG	NEG	NEG	NEG	NEG	NEG	POS	POS	POS	POS	NEG	POS	POS	NEG	POS	POS	POS	POS
ANR570100 : predicted hybridisation pattern	POS	NEG	POS	POS	POS	POS	AMB	POS	POS	NEG	POS	NEG	NEG	NEG	NEG	NEG	NEG	NEG	POS	POS	POS	POS	NEG	POS	POS	NEG	POS	POS	POS	POS
SAMEA3671725: predicted hybridisation pattern	POS	POS	POS	POS	POS	POS	AMB	POS	POS	NEG	POS	NEG	NEG	NEG	NEG	NEG	NEG	NEG	POS	POS	POS	POS	NEG	POS	POS	NEG	POS	POS	POS	POS
SAMEA48342418: predicted hybridisation pattern	POS	NEG	POS	POS	POS	POS	AMB	POS	POS	NEG	POS	NEG	NEG	NEG	NEG	NEG	NEG	NEG	POS	POS	POS	POS	NEG	POS	POS	NEG	POS	POS	POS	POS
**CC80-MRSA-IVc (PVL+)**																														
V16073 (isolate from DOI: 10.1111/j.1469-0691.2006.01420.x)	POS	POS	POS	POS	AMB	POS	NEG	NEG	POS	NEG	POS	NEG	NEG	POS	NEG	NEG	POS	NEG	POS	POS	POS	POS	NEG	POS	POS	NEG	POS	POS	POS	POS
11819-97: predicted hybridisation pattern	POS	POS	POS	POS	POS	POS	AMB	POS	POS	NEG	POS	NEG	NEG	POS	NEG	NEG	POS	NEG	POS	POS	POS	POS	NEG	POS	POS	NEG	POS	POS	POS	POS

STRAIN / ISOLATE	VIRULENCE : STAPHYLOCOCCAL SUPERANTIGEN/ENTEROTOXIN-LIKE GENES (SET/SSL)																													
	setC / selX	ssl01/set6 (COL)	ssl01/set6 (Mu50+ N315)	ssl01/set6 (MW2+ MSSA476)	ssl01/set6 (MRSA252)	ssl01/set6 (RF122)	ssl01/set6 (other alleles)	ssl02/set7	ssl02/set7 (MRSA252)	ssl03/set8_ probe 1	ssl03/set8_ probe 2	ssl03/set8 (MRSA252, SAR0424)	ssl04/set9	ssl04/set9 (MRSA252, SAR0425)	ssl05/set3_ probe 1	ssl05/set3 (RF122, probe-611)	ssl05/set3_ probe 2 (612)	ssl05/set3 (MRSA252)	ssl06/set21	ssl06 (NCTC8325 + MW2)	ssl07/set1	ssl07/set1 (MRSA252)	ssl07/set1 (AF188836)	ssl08/set12 _probe 1	ssl08/set12 _probe 2	ssl09/set5_ probe 1	ssl09/set5_ probe 2	ssl09/set5 (MRSA252)	ssl10/set4	ssl10 (RF122)	ssl10/set4 (MRSA252)
	Staphyl. exotoxin-like protein	Staphylococcal superantigen-like protein 1						Staphylococcal superantigen-like protein 2		Staphylococcal superantigen-like protein 3			Staphylococcal superantigen-like protein 4		Staphylococcal superantigen-like protein 5				Staphylococcal superantigen-like protein 6		Staphylococcal superantigen-like protein 7			Staphylococcal superantigen-like protein 8		Staphylococcal superantigen-like protein 9			Staphylococcal superantigen-like protein 10		
**CC1-MRSA-IV (PVL+), USA400**																															
MW2	POS	NEG	NEG	POS	NEG		NEG	POS	NEG	POS	POS	NEG	POS	NEG	POS	NEG	POS	NEG	POS	POS	POS	NEG	NEG	POS	POS	POS	POS	NEG	POS	NEG	NEG
Strain MW2 GenBank BA000033.2: predicted hybr. pattern	POS	NEG	NEG	POS	NEG	NEG	NEG	POS	AMB	POS	POS	NEG	POS	NEG	POS	NEG	POS	NEG	POS	POS	POS	NEG	NEG	POS	POS	POS	POS	NEG	POS	NEG	NEG
**ST567-MSSA (PVL+)**																															
Örebro_02T-671	POS	NEG	NEG	POS	NEG		NEG	POS	NEG	POS	POS	NEG	POS	NEG	POS	NEG	POS	NEG	POS	POS	POS	NEG	NEG	POS	POS	POS	POS	NEG	POS	NEG	NEG
Örebro_02T-671 : predicted hybridisation pattern	POS	NEG	NEG	POS	NEG	NEG	NEG	POS	AMB	POS	POS	NEG	POS	NEG	POS	NEG	POS	NEG	POS	POS	POS	NEG	NEG	POS	POS	POS	POS	NEG	POS	NEG	NEG
**CC80 [ORF CM14+]-MSSA (PVL+)**																															
ANR570100	POS	NEG	NEG	NEG	NEG	NEG	POS	POS	NEG	POS	POS	NEG	POS	NEG	POS	POS	AMB	NEG	NEG	NEG	POS	AMB	NEG	POS	POS	POS	POS	NEG	POS	NEG	NEG
ANR570100 : predicted hybridisation pattern	POS	NEG	NEG	NEG	NEG	NEG	POS	POS	AMB	POS	POS	NEG	POS	NEG	POS	POS	NEG	NEG	NEG	NEG	POS	NEG	NEG	POS	POS	POS	POS	NEG	POS	NEG	NEG
SAMEA3671725: predicted hybridisation pattern	POS	NEG	NEG	NEG	NEG	NEG	POS	POS	AMB	POS	POS	NEG	POS	NEG	POS	POS	NEG	NEG	NEG	NEG	POS	NEG	NEG	POS	POS	POS	POS	NEG	POS	NEG	NEG
SAMEA48342418: predicted hybridisation pattern	POS	NEG	NEG	NEG	NEG	NEG	POS	POS	AMB	POS	POS	NEG	POS	NEG	POS	POS	NEG	NEG	NEG	NEG	POS	NEG	NEG	POS	POS	POS	POS	NEG	POS	NEG	NEG
**CC80-MRSA-IVc (PVL+)**																															
V16073 (isolate from DOI: 10.1111/j.1469-0691.2006.01420.x)	POS	NEG	NEG	NEG	NEG		POS	POS	NEG	POS	POS	NEG	POS	NEG	POS	POS	POS	NEG	NEG	NEG	POS	NEG	NEG	POS	POS	POS	POS	NEG	POS	NEG	NEG
11819-97: predicted hybridisation pattern	POS	NEG	NEG	NEG	NEG	NEG	POS	POS	AMB	POS	POS	NEG	POS	NEG	POS	POS	NEG	NEG	NEG	NEG	POS	NEG	NEG	POS	POS	POS	POS	NEG	POS	NEG	NEG

STRAIN / ISOLATE	VIRULENCE : STAPHYLOCOCCAL SUPERANTIGEN/ENTEROTOXIN-LIKE GENES (SET/SSL)									CAPSULE AND BIOFILM-ASSOCIATED GENES							ADHAESION FACTORS / GENES ENCODING MICROBIAL SURFACE COMPONENTS RECOGNIZING ADHESIVE MATRIX MOLECULES (MSCRAMM GENES)						
	ssl11/set2 (COL)	ssl11+ set2(Mu50 + N315)	ssl11+ set2(MW2+ MSSA476)	ssl11/set2 (MRSA252)	setB3	setB3 (MRSA252)	setB2	setB2 (MRSA252)	setB1	cap 1	cap 5	cap 8	icaA	icaC	icaD	bap	bbp	bbp (cons)	bbp (COL+ MW2)	bbp (MRSA252)	bbp (Mu50)	bbp (RF122)	bbp (ST45)
	Staphylococcal superantigene-like protein 11				Staphylococcal exotoxin-like protein, second locus					Capsule type 1	Capsule type 5	Capsule type 8	intercellular adhesion protein A	intercellular adhesion protein C	biofilm PIA synthesis protein D	Surface protein involved in biofilm formation	Bone sialoprotein-binding protein						
**CC1-MRSA-IV (PVL+), USA400**																							
MW2	NEG	NEG	POS	NEG	POS	NEG	POS	NEG	POS	NEG	NEG	POS	POS	POS	POS	NEG	POS	POS	POS	NEG	NEG	NEG	NEG
Strain MW2 GenBank BA000033.2: predicted hybr. pattern	NEG	NEG	POS	NEG	POS	NEG	POS	NEG	POS	NEG	NEG	POS	POS	POS	POS	NEG	POS	POS	POS	NEG	NEG	NEG	NEG
**ST567-MSSA (PVL+)**																							
Örebro_02T-671	NEG	NEG	POS	NEG	POS	NEG	POS	NEG	POS	NEG	NEG	POS	POS	POS	POS	NEG	POS	POS	POS	NEG	NEG	NEG	NEG
Örebro_02T-671 : predicted hybridisation pattern	NEG	NEG	POS	NEG	POS	NEG	POS	NEG	POS	NEG	NEG	POS	POS	POS	POS	NEG	POS	POS	POS	NEG	NEG	NEG	NEG
**CC80 [ORF CM14+]-MSSA (PVL+)**																							
ANR570100	NEG	NEG	NEG	NEG	POS	NEG	POS	NEG	POS	NEG	NEG	POS	POS	POS	POS	NEG	POS	POS	NEG	NEG	POS	NEG	NEG
ANR570100 : predicted hybridisation pattern	NEG	NEG	NEG	NEG	POS	NEG	POS	NEG	POS	NEG	NEG	POS	POS	POS	POS	NEG	POS	POS	NEG	NEG	POS	NEG	NEG
SAMEA3671725: predicted hybridisation pattern	NEG	NEG	NEG	NEG	POS	NEG	POS	NEG	POS	NEG	NEG	POS	POS	POS	POS	NEG	POS	POS	NEG	NEG	POS	NEG	NEG
SAMEA48342418: predicted hybridisation pattern	NEG	NEG	NEG	NEG	POS	NEG	POS	NEG	POS	NEG	NEG	POS	POS	POS	POS	NEG	POS	POS	NEG	NEG	POS	NEG	NEG
**CC80-MRSA-IVc (PVL+)**																							
V16073 (isolate from DOI: 10.1111/j.1469-0691.2006.01420.x)	NEG	NEG	NEG	NEG	POS	NEG	POS	NEG	POS	NEG	NEG	POS	POS	POS	POS	NEG	POS	POS	NEG	NEG	POS	NEG	NEG
11819-97: predicted hybridisation pattern	NEG	NEG	NEG	NEG	POS	NEG	POS	NEG	POS	NEG	NEG	POS	POS	POS	POS	NEG	POS	POS	NEG	NEG	POS	NEG	NEG

STRAIN / ISOLATE	ADHAESION FACTORS / GENES ENCODING MICROBIAL SURFACE COMPONENTS RECOGNIZING ADHESIVE MATRIX MOLECULES (MSCRAMM GENES)																									
	clfA	clfA (cons)	clfA (COL+ RF122)	clfA (MRSA252)	clfA (Mu50+ MW2)	clfB	clfB (cons)	clfB (COL+ Mu50)	clfB (MW2)	clfB (RF122)	cna	ebh (cons)	ebpS	ebpS_probe 612	ebpS_probe 614	ebpS (01-1111)	ebpS (COL)	eno	efb / fib	efb / fib (MRSA252)	fnbA	fnbA (cons)	fnbA (COL)	fnbA (MRSA252)	fnbA (Mu50+ MW2)	fnbA (RF122)
	Clumping factor A					Clumping factor B					Collagen-binding adhesin	Cell wall associated fibronectin-binding protein	cell surface elastin binding protein					enolase	fibrinogen binding protein (19 kDa)		fibronectin-binding protein A					
**CC1-MRSA-IV (PVL+), USA400**																										
MW2	POS	POS	NEG	NEG	POS	POS	POS	NEG	POS	POS	POS	POS	POS	POS	POS	NEG	NEG	POS	POS	NEG	POS	POS	NEG	NEG	POS	NEG
Strain MW2 GenBank BA000033.2: predicted hybr. pattern	POS	POS	NEG	NEG	POS	POS	POS	NEG	POS	AMB	POS	POS	POS	POS	POS	NEG	NEG	POS	POS	NEG	POS	POS	NEG	NEG	POS	NEG
**ST567-MSSA (PVL+)**																										
Örebro_02T-671	POS	POS	NEG	NEG	POS	POS	POS	NEG	POS	AMB	NEG	POS	POS	POS	POS	NEG	NEG	POS	POS	NEG	POS	POS	NEG	NEG	POS	NEG
Örebro_02T-671 : predicted hybridisation pattern	POS	POS	NEG	NEG	POS	POS	POS	NEG	POS	AMB	NEG	POS	POS	POS	POS	NEG	NEG	POS	POS	NEG	POS	POS	NEG	NEG	POS	NEG
**CC80 [ORF CM14+]-MSSA (PVL+)**																										
ANR570100	POS	POS	NEG	NEG	POS	POS	POS	POS	NEG	NEG	NEG	POS	POS	POS	POS	NEG	NEG	POS	POS	NEG	POS	POS	NEG	NEG	NEG	NEG
ANR570100 : predicted hybridisation pattern	POS	POS	NEG	NEG	POS	POS	POS	POS	NEG	NEG	NEG	POS	POS	POS	POS	NEG	NEG	POS	POS	NEG	POS	POS	NEG	NEG	NEG	NEG
SAMEA3671725: predicted hybridisation pattern	POS	POS	NEG	NEG	POS	POS	POS	POS	NEG	NEG	NEG	POS	POS	POS	POS	NEG	NEG	POS	POS	NEG	POS	POS	NEG	NEG	NEG	NEG
SAMEA48342418: predicted hybridisation pattern	POS	POS	NEG	NEG	POS	POS	POS	POS	NEG	NEG	NEG	POS	POS	POS	POS	NEG	NEG	POS	POS	NEG	POS	POS	NEG	NEG	NEG	NEG
**CC80-MRSA-IVc (PVL+)**																										
V16073 (isolate from DOI: 10.1111/j.1469-0691.2006.01420.x)	POS	POS	NEG	NEG	POS	POS	POS	POS	NEG	NEG	NEG	POS	AMB	POS	POS	NEG	NEG	POS	POS	NEG	POS	POS	NEG	NEG	NEG	NEG
11819-97: predicted hybridisation pattern	POS	POS	NEG	NEG	POS	POS	POS	POS	NEG	NEG	NEG	POS	POS	POS	POS	NEG	NEG	POS	POS	NEG	POS	POS	NEG	NEG	NEG	NEG

STRAIN / ISOLATE	ADHAESION FACTORS / GENES ENCODING MICROBIAL SURFACE COMPONENTS RECOGNIZING ADHESIVE MATRIX MOLECULES (MSCRAMM GENES)																											
	fnbB	fnbB (COL)	fnbB (COL+ Mu50+ MW2)	fnbB (Mu50)	fnbB (MW2)	fnbB (ST15)	fnbB (ST45-2)	map	map (COL)	map (MRSA252)	map (Mu50+ MW2)	sasG	sasG (COL+ Mu50)	sasG (MW2)	sasG (other than252+ 122	sasX / sesI	sdrC	sdrC (cons)	sdrC (B1)	sdrC (COL)	sdrC (Mu50)	sdrC (MW2+ MRSA252+ RF122)	sdrC (other than 252+ RF122)	sdrD	sdrD (cons)	sdrD (COL+ MW2)	sdrD (Mu50)	sdrD (other)
	Fibronectin-binding protein B							Major histocompatibility complex class II analog protein (=Extracellular adherence protein, eap)				Staphylococcus aureus surface protein G					Ser-Asp rich fibrinogen-/bone sialoprotein-binding protein C							Ser-Asp rich fibrinogen-/bone sialoprotein-binding protein D				
**CC1-MRSA-IV (PVL+), USA400**																												
MW2	POS	NEG	POS	NEG	POS	NEG	NEG	NEG	AMB	NEG	AMB	POS	NEG	POS	POS	NEG	POS	POS	NEG	NEG	NEG	POS	POS	POS	POS	POS	NEG	NEG
Strain MW2 GenBank BA000033.2: predicted hybr. pattern	POS	NEG	AMB	NEG	AMB	NEG	NEG	POS	AMB	NEG	POS	POS	NEG	POS	POS	NEG	POS	POS	NEG	NEG	NEG	POS	POS	POS	POS	POS	NEG	NEG
**ST567-MSSA (PVL+)**																												
Örebro_02T-671	POS	NEG	AMB	NEG	POS	NEG	NEG	POS	NEG	NEG	POS	POS	NEG	POS	POS	NEG	POS	POS	NEG	NEG	NEG	POS	POS	POS	POS	POS	NEG	NEG
Örebro_02T-671 : predicted hybridisation pattern	POS	NEG	AMB	NEG	AMB	NEG	NEG	POS	AMB	NEG	POS	POS	NEG	POS	POS	NEG	POS	POS	NEG	NEG	NEG	POS	POS	POS	POS	POS	NEG	NEG
**CC80 [ORF CM14+]-MSSA (PVL+)**																												
ANR570100	POS	NEG	AMB	NEG	POS	NEG	NEG	POS	NEG	NEG	POS	POS	NEG	POS	POS	NEG	POS	POS	NEG	POS	NEG	NEG	POS	POS	POS	NEG	NEG	POS
ANR570100 : predicted hybridisation pattern	POS	NEG	AMB	NEG	POS	NEG	NEG	POS	AMB	NEG	POS	POS	NEG	POS	POS	NEG	POS	POS	NEG	POS	NEG	NEG	POS	POS	POS	NEG	NEG	POS
SAMEA3671725: predicted hybridisation pattern	POS	NEG	AMB	NEG	POS	NEG	NEG	POS	AMB	NEG	POS	POS	NEG	POS	POS	NEG	POS	POS	NEG	POS	NEG	NEG	POS	POS	POS	NEG	NEG	POS
SAMEA48342418: predicted hybridisation pattern	POS	NEG	AMB	NEG	POS	NEG	NEG	POS	AMB	NEG	POS	POS	NEG	POS	POS	NEG	POS	POS	NEG	POS	NEG	NEG	POS	POS	POS	NEG	NEG	POS
**CC80-MRSA-IVc (PVL+)**																												
V16073 (isolate from DOI: 10.1111/j.1469-0691.2006.01420.x)	POS	NEG	POS	NEG	POS	NEG	NEG	POS	AMB	NEG	POS	POS	NEG	POS	POS	NEG	POS	POS	NEG	POS	NEG	NEG	POS	POS	POS	NEG	NEG	POS
11819-97: predicted hybridisation pattern	POS	NEG	AMB	NEG	POS	NEG	NEG	POS	AMB	NEG	POS	POS	NEG	POS	POS	NEG	POS	POS	NEG	POS	NEG	NEG	POS	POS	POS	NEG	NEG	POS

STRAIN / ISOLATE	ADHAESION FACTORS / MSCRAMM GENES						IMMUNOD.AG.B		DEFENSIN RESIST.		TRANSFERRIN BINDING PROT			PUTATIVE TRANSPORTER					TYPE I RESTRICTION-MODIFICATION SYSTEM, SINGLE SEQUENCE SPECIFICITY PROTEIN												
	vwb	vwb (cons)	vwb (COL+ MW2)	vwb (MRSA252)	vwb (Mu50)	vwb (RF122)	isaB	isaB (MRSA252)	mprF (COL+ MW2)	mprF (Mu50+ 252)	isdA (cons)	isdA (MRSA252)	isdA (other than MRSA252)	lmrP (other than RF122)	lmrP (other than RF122)	lmrP (RF122)	lmrP (RF122)	lmrP-pWBG762	hsdS1-RF122	hsdS2-ST5+ ST8	hsdS2-MW2+ 476	hsdS2-RF122	hsdS2-MRSA252	hsdS3-Allother thanRF122 + 252	hsdS3-ST8+ ST1+ RF122	hsdS3-Mu50+ N315	hsdS3-CC51+ 252	hsdS3-MRSA252	hsdSx-CC25	hsdSx-CC15	hsdSx-etd
	van Willebrand factor binding protein						immunodominant antigen B		defensin resistance protein		transferrin-binding protein			hypothetical protein, similar to integral membrane protein LmrP					type I site-specific deoxyribo-nuclease subunit, 1st locus	type I site-specific deoxyribonuclease subunit, 2nd locus				type I site-specific deoxyribonuclease subunit, 3rd locus					type I site-specific deoxyribonuclease subunit, unknown locus		
**CC1-MRSA-IV (PVL+), USA400**																															
MW2	POS	POS	POS	NEG	NEG	NEG	POS	NEG	POS	NEG	POS	NEG	POS	POS	POS	NEG	NEG	NEG	NEG	NEG	POS	NEG	NEG	POS	POS	NEG	NEG	NEG	POS	NEG	NEG
Strain MW2 GenBank BA000033.2: predicted hybr. pattern	POS	POS	POS	NEG	NEG	NEG	POS	NEG	POS	NEG	POS	NEG	POS	POS	POS	NEG	NEG	NEG	NEG	NEG	POS	NEG	NEG	POS	POS	NEG	NEG	NEG	POS	NEG	NEG
**ST567-MSSA (PVL+)**																															
Örebro_02T-671	POS	POS	POS	NEG	NEG	NEG	NEG	POS	POS	NEG	POS	NEG	POS	POS	POS	NEG	NEG	NEG	NEG	NEG	AMB	NEG	NEG	POS	POS	NEG	NEG	NEG	POS	NEG	NEG
Örebro_02T-671 : predicted hybridisation pattern	POS	POS	POS	NEG	NEG	NEG	NEG	POS	POS	NEG	POS	NEG	POS	POS	POS	NEG	NEG	NEG	NEG	NEG	POS	NEG	NEG	POS	POS	NEG	NEG	NEG	POS	NEG	NEG
**CC80 [ORF CM14+]-MSSA (PVL+)**																															
ANR570100	POS	POS	NEG	POS	NEG	NEG	POS	AMB	AMB	POS	POS	NEG	POS	POS	POS	NEG	NEG	NEG	NEG	NEG	NEG	NEG	NEG	NEG	NEG	NEG	NEG	NEG	NEG	NEG	NEG
ANR570100 : predicted hybridisation pattern	POS	POS	NEG	POS	NEG	NEG	POS	NEG	NEG	POS	POS	NEG	POS	POS	POS	NEG	NEG	NEG	NEG	NEG	NEG	NEG	NEG	NEG	NEG	NEG	NEG	NEG	NEG	NEG	NEG
SAMEA3671725: predicted hybridisation pattern	POS	POS	NEG	POS	NEG	NEG	POS	NEG	NEG	POS	POS	NEG	POS	POS	POS	NEG	NEG	NEG	NEG	NEG	NEG	NEG	NEG	NEG	NEG	NEG	NEG	NEG	NEG	NEG	NEG
SAMEA48342418: predicted hybridisation pattern	POS	POS	NEG	POS	NEG	NEG	POS	NEG	NEG	POS	POS	NEG	POS	POS	POS	NEG	NEG	NEG	NEG	NEG	NEG	NEG	NEG	NEG	NEG	NEG	NEG	NEG	NEG	NEG	NEG
**CC80-MRSA-IVc (PVL+)**																															
V16073 (isolate from DOI: 10.1111/j.1469-0691.2006.01420.x)	POS	POS	NEG	POS	NEG	NEG	POS	NEG	NEG	POS	POS	NEG	POS	POS	POS	NEG	NEG	NEG	NEG	NEG	NEG	NEG	NEG	NEG	NEG	NEG	NEG	NEG	NEG	NEG	POS
11819-97: predicted hybridisation pattern	POS	POS	NEG	POS	NEG	NEG	POS	NEG	NEG	POS	POS	NEG	POS	POS	POS	NEG	NEG	NEG	NEG	NEG	NEG	NEG	NEG	NEG	NEG	NEG	NEG	NEG	NEG	NEG	POS

STRAIN / ISOLATE	MISCELLANEOUS GENES																							HYALURONATE LYASE							
	ear2 = Q2FXC0	Q2YUB3	Q7A4X2	Q931R4 (CC5, CC15, CC30, CC97, CC188, ST1850)	Q9RL82 (consens.)	Q9RL82 (CC8)	Q9RL82-CC10/361	Q2G1R6-genomic island / cstB	sau3AI	sauUSI	sauRF122	sauSO385	sau96I	G7ZRU6	ycjY = C5Q1F1 ("Argenteus/ST1850-like", CC12, CC361, CC398)	sagD	G7ZTC1	G7ZTC1-argenteus	sdrM / tetEfflux	sdrM (argen-teus)	Q2YUB3 (RF122)	Q2YUB3 (Swar)	Q2YUB3 (Sepi)	hysA1 (MRSA252)	hysA1 (MRSA252+RF122) and/or hysA2 (cons)	hysA1 (MRSA252+RF122) and/or hysA2 (COL+USA300)	hysA2 (All other than MRSA252)	hysA2 (COL+USA300+NCTC8325)	hysA2 (All other than COL+USA300+NCTC8325)	hysA2 (All other than COL+USA300+NCTC8325)	hysA2 (MRSA252)
	Putative protein	Multidrug resistance transporter	Putative protein	major facilitator superfamily transporter	Putative protein				type II restriction-modification system endonuclease					acetyltransferase, GNAT family, "Argenteus/ST185 0-like"		Putative bacteriocin biosynthesis associated protein	TetR family regulator		Multidrug resistance transporter		Multidrug resistance transporter			Hyaluronate lyase, variable first / second locus			Hyaluronate lyase, second locus				
**CC1-MRSA-IV (PVL+), USA400**																															
MW2	POS	NEG	NEG	NEG	NEG	NEG	NEG	POS	NEG	POS	NEG	NEG	NEG	NEG	NEG	NEG	NEG	NEG	POS	NEG	NEG	NEG	NEG	NEG	POS	NEG	POS	NEG	POS	POS	NEG
Strain MW2 GenBank BA000033.2: predicted hybr. pattern	POS	NEG	NEG	NEG	NEG	NEG	NEG	POS	NEG	POS	NEG	NEG	NEG	NEG	NEG	NEG	NEG	NEG	POS	NEG	NEG	NEG	NEG	NEG	POS	NEG	POS	NEG	POS	AMB	NEG
**ST567-MSSA (PVL+)**																															
Örebro_02T-671	POS	NEG	NEG	NEG	NEG	NEG	NEG	NEG	NEG	POS	NEG	NEG	NEG	NEG	NEG	NEG	NEG	NEG	POS	NEG	NEG	NEG	NEG	NEG	POS	NEG	POS	NEG	POS	POS	NEG
Örebro_02T-671 : predicted hybridisation pattern	POS	NEG	NEG	NEG	NEG	NEG	NEG	NEG	NEG	POS	NEG	NEG	NEG	NEG	NEG	NEG	NEG	NEG	POS	NEG	NEG	NEG	NEG	NEG	POS	NEG	POS	NEG	POS	AMB	NEG
**CC80 [ORF CM14+]-MSSA (PVL+)**																															
ANR570100	NEG	NEG	NEG	NEG	POS	POS	AMB	NEG	NEG	POS	NEG	NEG	NEG	NEG	NEG	NEG	NEG	NEG	POS	AMB	NEG	NEG	NEG	NEG	POS	NEG	POS	NEG	POS	POS	NEG
ANR570100 : predicted hybridisation pattern	NEG	NEG	NEG	NEG	POS	POS	NEG	NEG	NEG	POS	NEG	NEG	NEG	NEG	NEG	NEG	NEG	NEG	POS	NEG	NEG	NEG	NEG	NEG	POS	NEG	POS	NEG	POS	AMB	NEG
SAMEA3671725: predicted hybridisation pattern	NEG	NEG	NEG	NEG	POS	POS	NEG	NEG	NEG	POS	NEG	NEG	NEG	NEG	NEG	NEG	NEG	NEG	POS	NEG	NEG	NEG	NEG	NEG	POS	NEG	POS	NEG	POS	AMB	NEG
SAMEA48342418: predicted hybridisation pattern	NEG	NEG	NEG	NEG	POS	POS	NEG	NEG	NEG	POS	NEG	NEG	NEG	NEG	NEG	NEG	NEG	NEG	POS	NEG	NEG	NEG	NEG	NEG	POS	NEG	POS	NEG	POS	AMB	NEG
**CC80-MRSA-IVc (PVL+)**																															
V16073 (isolate from DOI: 10.1111/j.1469-0691.2006.01420.x)	NEG	NEG	NEG	NEG	POS	POS	NEG	NEG	NEG	POS	NEG	NEG	NEG	NEG	NEG	NEG	NEG	NEG	POS	NEG	NEG	NEG	NEG	NEG	POS	NEG	AMB	NEG	POS	POS	NEG
11819-97: predicted hybridisation pattern	NEG	NEG	NEG	NEG	POS	POS	NEG	NEG	NEG	POS	NEG	NEG	NEG	NEG	NEG	NEG	NEG	NEG	POS	NEG	NEG	NEG	NEG	NEG	POS	NEG	POS	NEG	POS	AMB	NEG
