# Supplementary material for: Molecular investigations on a chimeric strain of Staphylococcus aureus sequence type 80
Source: PLoS One. 2020 Oct 14;15(10):e0232071. doi: 10.1371/journal.pone.0232071 (PMC7556507; doi:10.1371/journal.pone.0232071)
Supplement: S3 File — (PDF) [file pone.0232071.s003.pdf]

Gene: dnaA (chromosomal replication initiator protein)

Position: 517 to 1878, length: 1362 nt, orientation: FORWARD

Perfect match to: (CIG1213-AHVF01000003-[490323:491684], allele observed in CC5+CC8+CC97)

Sequence:

ATGTCGGAAAAAGAAATTTGGGAAAAAGTGCTTGAAATTGCTCAAGAAAAATTATCAGCTGTAAGTTACTCAACTTTCCTAAAAGATACTG
AGCTTTACACGATTAAAGATGGTGAAGCTATCGTATTATCGAGTATTCCTTTTAATGCAAATTGGTTAAATCAACAATATGCTGAAATTATCC
AAGCAATCTTATTTGATGTTGTAGGCTATGAAGTTAAACCTCACTTTATTACTACTGAAGAATTAGCAAATTATAGTAATAATGAAACTGCTA
CTCCAAAAGAAACAACAAAACCTTCTACTGAAACAACTGAGGATAATCATGTGCTTGGTAGAGAGCAATTCAATGCCCATAACACATTTGAC
ACTTTTGTAATCGGACCTGGTAACCGCTTTCCACATGCAGCAAGTTTAGCTGTAGCCGAAGCACCAGCCAAAGCGTACAATCCATTATTTAT
CTATGGAGGTGTTGGTTTAGGAAAAACCCATTTAATGCATGCCATTGGTCATCATGTTTTAGATAATAATCCAGATGCCAAAGTGATTTACA
CATCAAGTGAAAAATTCACAAATGAATTTATTAAATCAATTCGTGATAACGAAGGTGAAGCTTTCAGAGAAAGATATCGTAATATCGACGT
CTTATTAATCGATGATATTCAGTTCATACAAAATAAAGTACAAACACAAGAAGAATTTTTCTATACTTTTAATGAATTGCATCAGAATAACAA
GCAAATAGTTATTTCGAGTGATCGACCACCAAAGGAAATTGCACAATTAGAAGATCGATTACGTTCACGCTTTGAATGGGGGCTAATTGTT
GATATTACGCCACCAGATTATGAAACTCGAATGGCAATTTTGCAGAAGAAAATTGAAGAAGAAAAATTAGATATTCCACCAGAAGCTTTAA
ATTATATAGCAAATCAAATTCAATCTAATATTCGTGAATTAGAAGGTGCATTAACACGTTTACTTGCATATTCACAATTATTAGGAAAACCAA
TTACAACTGAATTAACTGCTGAAGCTTTAAAAGATATCATTCAAGCACCAAAATCTAAAAAGATTATCATCCAAGATATTCAAAAAATTGTA
GGCCAGTACTATAATGTTAGAATTGAAGATTTCAGTGCAAAAAAACGTACAAAGTCAATTGCATATCCGCGTCAAATAGCTATGTACTTGTC
TAGAGAGCTTACAGATTTCTCATTACCTAAAATTGGTGAAGAATTTGGTGGGCGTGATCATACGACCGTCATTCATGCTCATGAAAAAATAT
CTAAAGATTTAAAAGAAGATCCTATTTTTAAACAAGAAGTAGAGAATCTTGAAAAAGAAATAAGAAATGTATAA

Gene: dnaN (DNA polymerase III subunit beta)

Position: 2156 to 3289, length: 1134 nt, orientation: FORWARD

Perfect match to: (N315-BA000018-[2156:3289], allele observed in CC5+CC8)

Sequence:

ATGATGGAATTCACTATTAAAAGAGATTATTTTATTACACAATTAAATGACACATTAAAAGCTATTTCACCAAGAACAACATTACCTATATTA
ACTGGTATCAAAATCGATGCGAAAGAACATGAAGTTATACTAACTGGTTCAGACTCTGAAATTTCAATAGAAATCACTATTCCTAAAACTGT
AGATGGCGAAGATATTGTCAATATTTCAGAAACAGGCTCAGTAGTACTTCCTGGACGATTCTTTGTTGATATTATAAAAAAATTACCTGGTA
AAGATGTTAAATTATCTACAAATGAACAATTCCAGACATTAATTACATCAGGTCATTCTGAATTTAATTTGAGTGGCTTAGATCCAGATCAAT
ATCCTTTATTACCTCAAGTTTCTAGAGATGACGCAATTCAATTGTCGGTAAAAGTACTTAAAAACGTGATTGCACAAACGAATTTTGCAGTGT
CCACCTCAGAAACACGCCCAGTACTAACTGGTGTGAACTGGCTTATACAAGAAAATGAATTAATATGCACAGCGACTGACTCACACCGCTT
GGCTGTAAGAAAGTTGCAGTTAGAAGATGTTTCTGAAAACAAAAATGTCATCATTCCAGGTAAGGCTTTAGCTGAATTAAATAAAATTATG
TCTGACAATGAAGAAGACATTGATATCTTCTTTGCTTCAAACCAAGTTTTATTTAAAGTTGGAAATGTGAACTTTATTTCTCGATTATTAGAA
GGACATTATCCTGATACAACACGTTTATTCCCTGAAAACTATGAAATTAAATTAAGTATAGACAATGGGGAGTTTTATCATGCGATTGATCG
TGCCTCTTTATTAGCGCGTGAAGGTGGTAATAACGTTATTAAATTAAGTACAGGTGATGACGTTGTTGAATTGTCTTCTACATCACCAGAAA
TTGGTACTGTAAAAGAAGAAGTTGATGCAAACGATGTTGAAGGTGGTAGCCTGAAAATTTCATTCAACTCTAAATATATGATGGATGCTTT
AAAAGCAATCGATAATGATGAGGTTGAAGTTGAATTCTTCGGTACAATGAAACCATTTATTCTAAAACCAAAAGGTGACGACTCGGTAACG
CAATTAATTTTACCAATCAGAACTTACTAA

Gene: yaaA (RNA-binding S4 domain protein)

Position: 3670 to 3915, length: 246 nt, orientation: FORWARD

Perfect match to: (RF122-AJ938182-[3670:3915], highly conserved allele)

Sequence:

GTGATTATTTTGGTTCAAGAAGTTGTAGTAGAAGGAGACATTAATTTAGGTCAATTTCTAAAAACAGAAGGGATTATTGAATCTGGTGGTC
AAGCAAAATGGTTCTTGCAAGACGTTGAAGTATTAATTAATGGAGTGCGTGAAACACGTCGCGGTAAAAAGTTAGAACATCAAGATCGTAT
AGATATCCCAGAATTACCTGAAGATGCTGGTTCTTTCTTAATCATTCATCAAGGTGAACAATGA

Gene: recF (DNA replication and repair protein F)

Position: 3912 to 5024, length: 1113 nt, orientation: FORWARD

Perfect match to: (TCH70-ACHH02000005-[484707:485819:r], allele observed in CC1+CC5+CC8+CC30+CC97)

Sequence:

ATGAAGTTAAATACACTCCAATTAGAAAATTATCGTAACTATGATGAGGTTACGTTGAAATGTCATCCTGACGTGAATATCCTCATTGGAGA
AAATGCACAAGGAAAGACAAATTTACTTGAATCAATTTATACCTTAGCTTTAGCAAAAAGTCATAGAACGAGTAATGATAAGGAACTCATA
CGTTTTAATGCTGATTATGCTAAAATAGAAGGTGAGCTTAGTTATAGACACGGCACGATGCCATTAACAATGTTTATAACTAAAAAAGGTAA
ACAAGTCAAAGTGAATCACTTAGAGCAAAGTCGTCTAACTCAATATATTGGACACCTTAATGTGGTTCTATTTGCGCCAGAAGATTTGAATA
TTGTAAAAGGCTCTCCTCAAATAAGACGACGCTTTATAGATATGGAGTTGGGCCAAATTTCTGCTGTTTACTTAAATGATTTAGCTCAATACC
AACGTATTTTAAAGCAAAAGAATAATTACTTAAAGCAGTTACAATTAGGCCAAAAAAAGGACTTAACAATGTTGGAAGTATTAAATCAGCA
GTTTGCTGAATATGCAATGAAAGTAACTGATAAACGTGCACATTTTATTCAAGAGCTAGAGTCGTTAGCTAAACCGATTCATGCTGGTATCA
CAAATGATAAAGAAGCGTTGTCGCTGAATTATTTACCTAGTCTTAAATTTGATTATGCTCAAAATGAAGCGGCACGACTTGAAGAAATTATG
TCTATTCTTAGCGATAATATGCAAAGAGAAAAAGAACGAGGCATTAGCTTATTCGGACCACATCGAGATGATATAAGTTTTGATGTGAATG
GCATGGATGCTCAAACATATGGTTCTCAAGGACAGCAACGTACAACGGCTTTGTCCATTAAATTAGCTGAAATTGAGTTAATGAATATCGA
AGTTGGGGAATATCCCGTCTTATTATTAGATGATGTACTCAGTGAATTAGATGATTCGCGTCAAACGCATTTATTAAGTACGATTCAGCATA
AAGTACAAACATTTGTCACTACGACATCTGTAGATGGTATTGATCATGAAATCATGAATAACGCTAAATTGTATCGTATTAATCAAGGTGAA
ATTATAAAGTAA

Gene: gyrB (DNA topoisomerase II, subunit B)

Position: 5034 to 6968, length: 1935 nt, orientation: FORWARD

Perfect match to: (N315-BA000018-[5034:6968], allele observed in CC5+CC15+CC25+CC188-ST582+CC630)

Sequence:

ATGGTGACTGCATTGTCAGATGTAAACAACACGGATAATTATGGTGCTGGGCAAATACAAGTATTAGAAGGTTTAGAAGCAGTACGTAAA
AGACCAGGTATGTATATAGGATCGACTTCAGAGAGAGGTTTGCACCATTTAGTGTGGGAAATTGTCGATAATAGTATCGATGAAGCATTAG
CTGGTTATGCAAATAAAATTGAAGTTGTTATTGAAAAAGATAACTGGATTAAAGTAACGGATAACGGACGTGGTATCCCAGTTGATATTCA
AGAAAAAATGGGACGTCCAGCTGTCGAAGTTATTTTAACTGTTTTACATGCTGGTGGTAAATTCGGCGGTGGCGGATACAAAGTATCTGGT
GGTTTACATGGTGTTGGTTCATCAGTTGTAAACGCATTGTCACAAGACTTAGAAGTATATGTACACAGAAATGAGACTATATATCATCAAGC
ATATAAAAAAGGTGTACCTCAATTTGACTTAAAAGAAGTTGGCACAACTGATAAGACAGGTACTGTCATTCGTTTTAAAGCAGATGGAGAA
ATCTTCACAGAGACAACTGTATACAACTATGAAACATTACAGCAGCGTATTAGAGAGCTTGCTTTCTTAAACAAAGGAATTCAAATCACATT
AAGAGATGAACGTGATGAAGAAAACGTTAGAGAAGACTCCTATCACTATGAGGGCGGTATTAAATCGTACGTTGAGTTATTGAACGAAAA
TAAAGAACCTATTCATGATGAGCCAATTTATATTCATCAATCTAAAGATGATATTGAAGTAGAAATTGCGATTCAATATAACTCAGGATATG
CCACAAATCTTTTAACTTACGCAAATAACATTCATACGTACGAAGGTGGTACGCATGAAGACGGATTCAAACGTGCATTAACGCGTGTCTTA
AATAGTTATGGTTTAAGTAGCAAGATTATGAAAGAAGAAAAAGATAGACTTTCTGGTGAAGATACACGTGAAGGTATGACAGCAATTATAT
CTATCAAACATGGTGATCCTCAATTCGAAGGTCAAACGAAGACAAAATTAGGTAATTCTGAAGTGCGTCAAGTTGTAGATAAATTATTCTCA
GAGCACTTTGAACGATTTTTATATGAAAATCCACAAGTCGCACGTACAGTGGTTGAAAAAGGTATTATGGCGGCACGTGCACGTGTTGCTG
CGAAAAAAGCGCGTGAAGTAACACGTCGTAAATCAGCGTTAGATGTAGCAAGTCTTCCAGGTAAATTAGCCGATTGCTCTAGTAAAAGTCC
TGAAGAATGTGAGATTTTCTTAGTCGAAGGGGACTCTGCCGGAGGGTCTACAAAATCTGGTCGTGACTCTAGAACGCAGGCGATTTTACCA
TTACGAGGTAAGATATTAAATGTTGAAAAAGCACGATTAGATAGAATTTTGAATAACAATGAAATTCGTCAAATGATCACAGCATTTGGTA
CAGGAATCGGTGGCGACTTTGATCTAGCGAAAGCAAGATATCACAAAATCGTCATTATGACTGATGCCGATGTGGATGGAGCGCATATTA
GAACATTGTTATTAACATTCTTCTATCGATTTATGAGACCGTTAATTGAAGCAGGCTATGTGTATATTGCACAGCCACCGTTGTATAAACTGA
CACAAGGTAAACAAAAGTATTATGTATACAATGATAGGGAACTTGATAAACTTAAATCTGAATTGAATCCAACACCAAAATGGTCTATTGCG
CGATACAAAGGTCTTGGAGAAATGAATGCAGATCAATTATGGGAAACAACAATGAACCCTGAGCACCGCGCTCTTTTACAAGTAAAACTTG
AAGATGCGATTGAAGCGGACCAAACATTTGAAATGTTAATGGGTGACGTTGTAGAAAACCGTAGACAATTTATAGAAGATAATGCAGTTTA
TGCAAACTTAGACTTCTAA

Gene: gyrA (DNA topoisomerase II, subunit A)

Position: 7005 to 9668, length: 2664 nt, orientation: FORWARD

Perfect match to: (Strain_21283-AHJZ01000002-[15746:18409], allele observed in CC8+CC1+CC5+CC97)

Sequence:

ATGGCTGAATTACCTCAATCAAGAATAAATGAACGAAATATTACCAGTGAAATGCGTGAATCATTTTTAGATTATGCGATGAGTGTTATCGT
TGCTCGTGCATTGCCAGATGTTCGTGACGGTTTAAAACCAGTACATCGTCGTATACTATATGGATTAAATGAACAAGGTATGACACCGGATA

AATCATATAAAAAATCAGCACGTATCGTTGGTGACGTAATGGGTAAATATCACCCTCATGGTGACTCATCTATTTATGAAGCAATGGTACGT
ATGGCTCAAGATTTCAGTTATCGTTATCCGCTTGTTGATGGCCAAGGTAACTTTGGTTCAATGGATGGAGATGGCGCAGCAGCAATGCGTT
ATACTGAAGCGCGTATGACTAAAATCACACTTGAACTGTTACGTGATATTAATAAAGATACAATAGATTTTATCGATAACTATGATGGTAAT
GAAAGAGAGCCGTCAGTCTTACCTGCTCGATTCCCTAACTTGTTAGCCAATGGTGCATCAGGTATCGCGGTAGGTATGGCAACGAATATTC
CACCACATAACTTAACAGAATTAATCAATGGTGTACTTAGCTTAAGTAAGAACCCTGATATTTCAATTGCTGAGTTAATGGAGGATATTGAA
GGTCCTGATTTCCCTACTGCTGGACTTATTTTAGGTAAGAGTGGTATTAGACGTGCATATGAAACAGGTCGTGGTTCAATTCAAATGCGTTC
TCGTGCAGTTATTGAAGAACGTGGAGGCGGACGTCAACGTATTGTTGTCACTGAAATTCCTTTCCAAGTGAATAAGGCTCGTATGATTGAA
AAAATTGCAGAGCTCGTTCGTGACAAGAAAATTGACGGTATCACTGATTTACGTGATGAAACAAGTTTACGTACTGGTGTGCGTGTCGTTA
TTGATGTGCGTAAGGATGCAAATGCTAGTGTCATTTTAAATAACTTATACAAACAAACACCTCTTCAAACATCATTTGGTGTGAATATGATTG
CACTTGTAAATGGTAGACCGAAGCTTATTAATTTAAAAGAAGCGTTGGTACATTATTTAGAGCATCAAAAGACAGTTGTTAGAAGACGTAC
GCAATACAACTTACGTAAAGCTAAAGATCGTGCCCACATTTTAGAAGGATTACGTATCGCACTTGACCATATCGATGAAATTATTTCAACGA
TTCGTGAGTCAGATACAGATAAAGTTGCCATGGAAAGCTTGCAACAACGCTTCAAACTTTCTGAAAAACAAGCTCAAGCTATTTTAGACATG
CGTTTAAGACGTCTAACAGGTTTAGAGAGAGACAAAATTGAAGCTGAATATAATGAGTTATTAAATTATATTAGTGAATTAGAAGCCATCTT
AGCTGATGAAGAAGTGTTATTACAGTTAGTTAGAGATGAATTGACTGAAATTAGAGATCGTTTTGGTGATGATCGTCGTACTGAAATCCAA
TTAGGTGGATTTGAAGATTTAGAAGATGAAGATCTCATTCCAGAAGAACAAATAGTAATTACTTTGAGCCATAATAACTACATTAAACGTTT
GCCGGTATCTACATATCGTGCTCAAAACCGTGGTGGTCGTGGTGTTCAAGGTATGAATACATTGGAAGAAGATTTTGTCAGTCAATTGGTA
ACTTTAAGTACACATGACCATGTATTGTTCTTTACTAACAAAGGTCGTGTATACAAACTTAAAGGTTATGAAGTGCCTGAGTTATCAAGACA
GTCTAAAGGTATTCCTGTAGTGAATGCTATTGAACTTGAAAATGATGAAGTCATTAGTACAATGATTGCTGTTAAAGACCTTGAAAGTGAA
GACAACTTCTTAGTGTTTGCAACTAAACGTGGTGTCGTTAAACGTTCAGCATTAAGTAACTTCTCAAGAATAAATAGAAATGGTAAGATTGC
GATTTCGTTCAGAGAAGATGATGAGTTAATTGCAGTTCGTTTAACAAGTGGTCAAGAAGATATCTTGATTGGTACATCACATGCATCATTAA
TTCGATTCCCTGAATCAACATTACGTCCTTTAGGCCGTACAGCAACGGGTGTGAAAGGTATTACACTTCGTGAAGGTGACGAAGTTGTAGG
GCTTGATGTAGCTCATGCAAACAGTGTTGATGAAGTATTAGTAGTTACTGAAAATGGTTATGGTAAACGTACGCCAGTTAATGACTATCGTT
TATCAAATCGTGGTGGTAAAGGTATTAAAACAGCTACGATTACTGAGCGTAATGGTAATGTTGTATGTATCACTACAGTAACTGGTGAAGA
AGATTTAATGATTGTTACTAATGCAGGTGTCATTATTCGACTAGATGTTGCAGATATTTCTCAAAATGGTCGTGCAGCACAAGGTGTTCGCT
TAATTCGCTTAGGTGATGATCAATTTGTTTCAACGGTTGCTAAAGTAAAAGAAGATGCAGAAGATGAAACGAATGAAGATGAGCAATCTAC
TTCAACTGTATCTGAAGATGGTACTGAACAACAACGTGAAGCGGTTGTAAATGATGAAACACCAGGAAATGCAATTCATACTGAAGTGATT
GATTCAGAAGAAAATGATGAAGATGGACGTATTGAAGTAAGACAAGATTTCATGGATCGTGTTGAAGAAGATATACAACAATCATCAGAT
GAAGAATAA

Gene: nnrD (ADP-dependent (S)-NAD (P)H-hydrate dehydratase)

Position: 9755 to 10585, length: 831 nt, orientation: REVERSE

Perfect match to: (11819-97-CP003194-[9755:10585:r], allele observed in CC80+CC6+CC1156)

Sequence:

CTAACTTTCTAATTGTTTCATTGCGTAAGGTATTTCATTGATAAGTCTTGATGGTGGCACCACATACATATCTTTTGCAAGGTTTTCGCCAATA
AAACTATGTGTATATGTGGCACTCATAACCGCTTCTTTTAAGTTATCAAATTGACCGACAAAGCTTGTAATCATACCAGCAAGTGTATCGCCC
ATACCACCAGTCGCCATTGCTGGGCTACCGATTGTCAATTTAAAGTCTTCATCTTTAAAGAAAATTTCAGTACCATGTTTTTTAAGTACAACA
GTTGCACCTAAACGATCAACTGCTTCACGATTACGCTCATATGTCTGTTCCTCAATAGGAATACCACTTAATCGTTCCCATTCTTTGAGGTGT
GGTGTAAAGATCACACGACATGTAGGTAATTGCGGTTTCAGTTTACTAAAGATTGTAATCGCATCGCCGTCTACGATTAAATTTTGATGCGG
TTGTATATTTTGTAGTAGGAATGTAATGGCATTATTTCCTTTGAAATCAACGCCAAGACCTGGACCAATTAGTATACTGTCAGTCATTTCAAT
CATTTTCGTCAACATTTTCGTATCATTAATATCAATAACCATCGCTTCTGGGCAACGAGAATGTAATGCTGAATGATTTGTTGGATGTGTAGC
TACAGTGATTAAACCACTACCGCTAAATACACATGCACGAGCCGCTAACATAATGGCACCACCTAAGTTAGCAGATCCACCAATTAATAAAA
TTTTGCCATAATCACCTTTATGTGAATCTTCTTTGCGCTTAGGAATGTTAATAGAATTTAACGTTTCCATAGTGATATAACCTCCCAT

Gene: hutH (histidine ammonia lyase)

Position: 10893 to 12407, length: 1515 nt, orientation: FORWARD

Perfect match to: (11819-97-CP003194-[10893:12407], allele observed in CC80+CC1156)

Sequence:

ATGACTTTATATTTAGATGGTGAAACACTAACAATTGAGGATATTAAATCATTTTTACAACAACAATCAAACATTGAAATTATTGATGATGCG
TTAGAACGTGTCAAAAAAAGTAGAGCGGTAGTTGAACGTATTATTGAAAATGAGGAAACGGTTTACGGTATCACTACAGGTTTTGGGTTAT
TTAGTGATGTACGTATAGACCCGACGCAATATAATGAATTACAAGTGAATCTGATACGCTCACATGCCTGTGGGCTAGGTGAGCCATTTTCA
AAAGAAGTAGCATTAGTCATGATGATTTTACGATTGAATACATTATTAAAAGGTCATTCAGGTGCCACTTTAGAATTAGTGAGACAATTACA
ATTTTTTATAAATGAACGTATTATACCGATAATCCCACAGCAAGGCTCTCTCGGTGCATCAGGAGATTTAGCGCCATTATCACACTTAGCATT

AGCATTAATTGGTGAAGGGAAAGTATTGTACAGAGGGGAAGAAAAGGATAGTGACGATGTATTAAGAGAATTAAATAGACAACCTTTGAA
CCTTCAGGCTAAAGAAGGTTTAGCATTGATTAATGGTACGCAAGCTATGACAGCTCAAGGTGTCATTAGTTATATAGAAGCAGAAGATTTA
GGTTACCAATCTGAATGGATTGCTGCATTAACGCATCAGTCTCTTAATGGCATTATAGATGCATATCGACATGATGTGCACGCAGTTCGTAA
TTTTCAAGAACAGATTAATGTGGCAGCGCGTATGCGTGATTGGTTAGAAGGATCAACATTAACGACGCGACAAGCAGAAATACGTGTACA
AGATGCATATACGTTGCGTTGTATACCACAAATCCATGGCGCGAGTTTTCAAGTATTCAATTATGTTAAACAGCAATTAGAATTTGAAATGA
ATGCGGCTAATGATAATCCACTTATATTTGAGGAAGCAAATGAAACGTTTGTTATTTCAGGTGGTAACTTCCATGGACAACCTATTGCTTTT
GCATTAGATCATCTTAAATTAGGTGTAAGTGAATTAGCAAACGTATCGGAACGTCGTCTAGAGCGACTAGTAAATCCTCAATTAAATGGTG
ATTTACCAGCATTTCTTAGTCCAGAGCCAGGATTGCAAAGTGGCGCGATGATTATGCAATATGCTGCTGCAAGTCTCGTTTCTGAAAATAAA
ACTTTAGCGCATCCAGCGAGTGTTGATTCTATCACTTCATCTGCGAACCAAGAAGATCACGTATCTATGGGAACTACAGCTGCTAGACATGG
TTATCAAATTATTGAAAATGCAAGACGTGTGTTGGCAATCGAATGTGTTATTGCATTACAAGCAGCAGAGTTGAAAGGTGTCGAAGGATTA
TCACCAAAAACACGTCGCAAGTATGATGAGTTTCGAAGTATCGTGCCATCCATTACACATGATCGTCAATTTCATAAAGATATTGAAGCGGT
TGCACAGTATTTAAAGCAATCAATTTATCACACGACTGCATGTCACTAA

Gene: tbox01 (T-box leader element)

Position: 12485 to 12695, length: 211 nt

Sequence:

CATTGTTCGTAGGACAAGTAATATATAGTGTTCGATATCAGAGAGCTTGTGGTTAGTGTGAACAAGAATCAACATATATATGAATCTACCTA
CTTATTTAAAAGAACAATCGGTGATAACCGTTATTTTAGTGAAGTGCAATTTAGGTTTAGTGTATCTTTATAACTTAAATTGTTAAATAGGGT
GGCAACGCGTAGACCACGTCCCTTGT

Gene: serS (seryl-tRNA synthase)

Position: 12785 to 14071, length: 1287 nt, orientation: FORWARD

Perfect match to: (CIGC93-AHVD01000002-[64773:66059], allele observed in CC15+CC8+CC72)

Sequence:

ATGTTAGACATTAGATTATTCAGAAATGAGCCTGACACAGTTAAGAGCAAAATTGAATTACGTGGAGATGATCCAAAAGTTGTAGATGAAA
TTTTAGAATTGGATGAGCAACGACGTAAATTAATTAGTGCAACAGAAGAAATGAAAGCACGTCGTAATAAAGTAAGCGAAGAAATCGCAT
TAAAAAAACGTAATAAAGAAAATGCTGATGATGTGATTGCTGAAATGCGCACATTAGGTGACGATATTAAAGAAAAAGATAGTCAATTAA
ATGAAATTGATAATAAAATGACAGGTATCCTTTGTCGTATTCCAAATTTAATAAGTGATGATGTACCTCAAGGTGAATCTGATGAAGATAAC
GTTGAAGTTAAAAAGTGGGGTACACCACGTGAGTTTTCATTTGAACCAAAAGCACATTGGGATATTGTAGAAGAATTGAAAATGGCTGATT
TTGATCGTGCAGCAAAAGTTTCAGGCGCGCGTTTTGTATATTTAACAAATGAAGGTGCGCAATTAGAGCGTGCTTTAATGAACTATATGATT
ACAAAACATACAACACAACATGGTTATACAGAAATGATGGTACCACAGCTTGTGAACGCAGATACAATGTATGGTACAGGTCAATTACCTA
AATTTGAAGAAGATTTATTTAAAGTAGAAAAAGAAGGATTATATACAATTCCAACTGCTGAAGTACCATTAACGAATTTCTACCGTAATGAA
ATTATTCAACCAGGTGTACTTCCTGAAAAATTCACTGGTCAATCTGCATGTTTCCGTAGTGAAGCAGGATCAGCAGGTAGAGATACAAGAG
GATTAATTCGTTTACATCAATTCGATAAAGTGGAAATGGTACGTTTTGAACAACCTGAAGATTCATGGAATGCTTTAGAAGAAATGACAACA
AACGCAGAAGCAATTCTAGAAGAGTTAGGTTTACCATATCGTCGTGTTATTTTATGTACAGGTGATATTGGATTTAGTGCAAGCAAAACATA
TGATTTAGAAGTTTGGTTACCAAGCTACAATGATTATAAAGAAATTAGTTCATGCTCAAACTGTACGGATTTCCAAGCGCGTCGTGCTAACA
TCCGCTTCAAGCGTGACAAAGCAGCTAAACCAGAATTAGCACATACATTAAATGGTAGTGGTTTAGCAGTTGGACGTACATTTGCTGCTATT
GTTGAAAATTACCAAAATGAAGATGGAACAGTAACAATTCCAGAAGCATTAGTACCATTTATGGGTGGTAAAACACAAATTTCAAAACCAG
TTAAATAA

Gene: azlC (branched-chain amino acid ABC transporter, transmembrane permease subunit)

Position: 14721 to 15416, length: 696 nt, orientation: FORWARD

Perfect match to: (FPR3757-CP000255-[14749:15444], allele observed in CC8)

Sequence:

ATGACAACACATTTAAGTTTTAGACAAGGCGTGCAAGAGTGTATCCCAACATTATTGGGTTATGCCGGTGTTGGTATTTCATTTGGTATTGT
GGCTTCGTCTCAAAACTTTAGTATTTTAGAAATTGTCTTGTTATGTCTTGTTATATATGCCGGTGCTGCGCAATTTATTATGTGCGCGTTGTTT
ATAGCAGGTACACCTATATCAGCGATTGTACTAACTGTATTTATCGTAAATTCAAGAATGTTCCTTTTAAGTATGTCGCTTGCACCAAACTTC
AAGACATATGGGTTTTGGAACCGTGTTGGATTAGGTTCATTAGTAACTGACGAAACGTTTGGCGTCGCCATTACACCTTATTTAAAAGGAG

AAGCTATCAATGATCGTTGGATGCATGGTCTTAACATCACAGCATATTTATTTTGGGCAATTTCATGTGTAGCTGGGGCTTTATTTGGCGAA
TATATCTCAAATCCGCAAACGCTAGGGTTAGATTTTGCTATCACGGCTATGTTTATCTTTTTGGCCATTGCGCAATTTGAATCAATTACTAAAT
CGCGATTAAGAATTTACATAGTACTCATTATTGCCGTCATAGTAATGATGTTATCGCTAAGTATGTTTATGCCTTCATATCTAGCAATATTAAT
TGCAGCCACAATTTCAGCAGCGTTAGGAGTGATGATGGAACGATGA

Gene: azlD (branched chain amino acid ABC transporter, transmembrane permease subunit)

Position: 15413 to 15742, length: 330 nt, orientation: FORWARD

Perfect match to: (MW2-BA000033-[15416:15745], highly conserved allele)

Sequence:

ATGATAACTCATATGAACATGTTAATACTTATTTTATTGTGTGGTATCGTAACGCTATTAATTCGAATTATACCTTTTATCATGATTTCAAAAG
TGCAATTGCCTGATGTCGTGGTTCGATGGCTATCATTTATCCCAATCACACTATTTACGGCACTTGTCATAGACAGCATTATTCAACAGACGC
CTCATGGTGAGGGGTATACATTAAACATCCCTTACATTATCGCGCTCATTCCGACGGTTATTTTATCTATAATCACGCGTAGTTTAACTATTA
CAATTATTAGTGGGATTGTTATCATGGCAGCATTACGATTTTTCTTTTAA

Gene: metX (homoserine O-acetyltransferase)

Position: 16103 to 17071, length: 969 nt, orientation: FORWARD

Perfect match to: (MW2-BA000033-[16108:17076], highly conserved allele)

Sequence:

ATGACAAATTACACAGTAGATACTTTAAATTTAGGGAAATTTATTACAGAATCTGGGGAAGTCATAGATAACTTGCGTTTGAGATATGAGC
ATGTCGGTTATCATGGACAACCATTAGTTGTAGTTTGTCATGCATTAACTGGCAATCATTTAACATATGGAACAGATGATTATCCGGGTTGG
TGGCGAGAAATTATTGATGGGGGATATATACCCATTCACGATTATCAATTTTTAACATTTGATGTTATTGGTAGTCCTTTCGGTTCAAGCTCA
CCTTTAAACGACACTCATTTTCCTAAAAAATTAACATTAAGAGATATTGTCAGAGCGAATGAACGAGGTATACAAGCCCTTGGTTATGACAA
GATTAATATTTTAATAGGGGGAAGTCTTGGAGGAATGCAAGCAATGGAACTACTTTACAATCAACAGTTTGAAGTAGATAAAGCCATTATC
CTTGCTGCAACAAGCCGAACATCATCTTATAGTAGAGCTTTTAATGAAATTGCAAGGCAAGCCATTCATCTTGGTGGTAAGGAAGGTCTAA
GTATTGCACGCCAATTAGGTTTTTTGACATATCGATCATCAAAAAGTTATGATGAACGTTTCACGCCGGATGAAGTAGTCGCATACCAACAA
CATCAAGGTAATAAATTTAAAGAACATTTTGATTTGAATTGTTATCTGACACTGCTAGATGTATTGGATAGTCACAACATTGACCGAGGCCG
CACAGACGTAACGCATGTTTTTAAAAATTTAGAAACAAAAGTGTTAACGATGGGGTTCATAGATGATTTGCTATATCCGGACGATCAAGTTC
GTGCATTAGGTGAACGTTTTAAATATCATCGTCATTTCTTCGTGCCTGATAATGTTGGGCATGATGGATTCCTACTAAACTTTAGTACCTGGG
CACCTAACTTATATCATTTCTTAAATTTAAAGCATTTTAAGCGTAAGTAG

Gene: yybS (putative transmembrane helix protein)

Position: 17381 to 18304, length: 924 nt, orientation: FORWARD

Perfect match to: (CA347-CP006044-[17407:18330], highly conserved allele)

Sequence:

GTGTTTTCAAAAATACAACCTAAAGCAACAATAATTGCAACGATTACGTTGGTATTTGTCGCTTTAGCTTTATATCTAGTGCCTGGTTTAGGA
CTAATATTTGCATTATTTGCAACCATACCAGGTATCGTTTTATGGAATAAATCAATACAATCTTTCGGGATTAGTGCACTTATTACAGTAATTA
TAACAACTGTTTTAGGTAATACTTTCGTTTTAAGTGCCATCATATTAGTCTTAATTGCAAGTTTAATTATTGGTCAATTGCTCAAAGAAAGAA
CGTCTAAAGAAAGAATATTATACGTAACAACAGTAGCGATGAGCTTAATTTCATTAATCGCTTTTATGTTACTACAAACATTCGGAAGAATT
CCACCATCAGCGAGCATAGTAAAACCTTTCAAGCAAACATTACATGAAGCGATTACGATGAGCGGTGCCGATGCGAATATGACCCAAATAT
TAGAAGAAGGGTTTAGACAAGCGACCGTTCAATTACCAGGTTTCATCATTATCATTACATTTTTAATCGTCTTAATTAACTTAATCGTTACATT
TCCGATTTTACGAAAATTTAAAATCGCTACACCTGTATTTAAGCCACTTTTCGCGTGGCAAATGAGCGGTATTTTATTATGGATATACATTAT
TGTTATCATATGTTTATTATTTACAGGTCAACCGAGTGTGTTCCAGAGCATTCTTTTAAACTTCCAACTTGTGTTATCATTAGTAATGTATATT
CAAGGTTTAAGTGTTATTCATTTCTTTGGTAAAGCGAAAGGTTTGCCGAATGCAGTAACGATTTTACTATTGATTATCGGTACAATACTGAC
ACCTACGACACATATTGTAGGACTACTTGGTGTTATCGATTTAAGTTTGAATTTGAAGCGAATCATGAAAAATAATTCTAAAAAGTGA

Gene: gdpP (cyclic di-AMP phosphodiesterase)

Position: 18319 to 20286, length: 1968 nt, orientation: FORWARD

Perfect match to: (JKD6159-CP002114-[17792:19759], allele observed in CC93+CC7+CC72+CC692+CC772)

Sequence:

ATGAATCGGCAGTCCACTAAGAAAGCTTTACTAATACCATTTGTCATCATGATCATCACAGCAATTGTTTTAATGGGTGTATGGTTTATCTTT
AATAGTCTTATAGCATTAATTGCATCTATCGTTCTTGTCGTGATGATTATTGTTAGCATCATTTTATTCAGACAAGCTTTAATGAAAATGGATA
GTTATGTAGATGGTTTGAGTGCTCAAATTTCAACAACAAATAATAAAGCAATCAAACATTTACCAATTGGTATCATTGTTTTAGATGAAAAT
GATCACATCGAATGGGTTAACCAATTTATGACAGATCATATGGAAGCAAATGTCATTTCTGAATCTGTAAATGAAGTATTTCCAAACATTTT
AAAGCAATTAGATAGAGTGAAATCCGTTGAAATAGAATATAATCAGTATCATTTCCAAGTACGTTATTCTGAGAATGATCACTGCCTCTATT
TCTTTGATATAACTGAACAAGTACAAACAAATGAACTATATGAAAATTCTAAACCAATCATTGCGACATTATTTTTAGATAACTACGATGAGA
TTACTCAAAATATGAATGATACGCAGCGTTCGGAAATCAACTCAATGGTAACGCGTGTCATTAGTCGATGGGCAACTGAGTATAATATATTC
TTTAAAAGGTATAGTTCCGATCAATTCGTAGCCTATTTAAATCAAAAAATATTAGCTGACTTAGAAGAATCTAAATTTGATATCTTGAGTCAA
TTACGTGAAAAAAGTGTTGGTTATCGTGCCCAATTAACATTAAGTATCGGTGTTGGTGAAGGCACTGAAAATTTAATCGACTTAGGTGAATT
ATCACAATCAGGCCTAGACTTAGCATTAGGACGCGGTGGCGACCAAGTTGCAATTAAAAGTATTAATGGTAATGTGCGCTTCTATGGCGGT
AAGACTGACCCGATGGAGAAACGTACTCGTGTAAGAGCACGTGTGATCTCACATGCGTTAAAAGATATCCTTGCAGAGGGTGACAAAGTC
ATTATCATGGGACATAAACGTCCTGACTTAGATGCAATTGGTGCAGCAATCGGTGTGTCTAGATTTGCAATGATGAATAATTTAGAAGCATA
CATCGTATTAAATGAGACTGACATTGATCCAACATTACGACGCGTGATGAACGAAATAGATAAAAAGCCAGAGTTAAGAGAGCGATTTATT
ACATCAGATGATGCTTGGGATATGATGACATCTAAGACAACCGTAGTGATTGTTGATACGCATAAACCGGAACTGGTTTTAGATGAAAATG
TCTTAAATAAAGCAAACCGTAAAGTTGTTATCGATCATCATAGACGTGGTGAAAGCTTCATCTCTAATCCATTGTTGATATATATGGAACCAT
ACGCAAGTTCGACAGCTGAATTGGTAACAGAGTTACTGGAATATCAACCAACAGAACAACGTTTAACACGTCTTGAATCAACAGTGATGTA
TGCAGGTATTATTGTAGATACAAGAAACTTTACATTACGAACAGGATCAAGAACATTCGATGCAGCGAGTTATTTACGTGCACATGGTGCA
GATACGATTTTAACGCAACATTTCTTAAAAGATGATGTGGATACTTACATTAATCGATCTGAATTAATTCGAACTGTAAAAGTTGAAGATAA
TGGCATAGCCATTGCGCATGGTTCAGACGATAAAATTTATCATCCAGTAACAGTTGCACAAGCAGCAGATGAACTGTTAAGTTTAGAAGGT
ATTGAAGCATCATATGTTGTTGCGAGACGTGAAGATAATCTGATTGGTATATCTGCGCGTTCACTCGGTTCAGTAAATGTCCAGTTAACAAT
GGAAGCACTTGGTGGCGGTGGACATTTAACCAATGCGGCAACACAACTTAAAGGTGTGACAGTCGAAGAGGCGATAGCACAATTACAACA
AGCAATTACAGAACAATTAAGTAGGAGTGAAGATGCATGA

Gene: rplI (50S ribosomal protein L9)

Position: 20283 to 20729, length: 447 nt, orientation: FORWARD

Perfect match to: (N315-BA000018-[20292:20738], highly conserved allele)

Sequence:

ATGAAAGTAATTTTTACACAAGATGTTAAAGGTAAAGGTAAAAAAGGTGAAGTTAAAGAAGTACCAGTAGGTTATGCAAATAACTTCTTAT
TGAAAAAGAATTATGCTGTAGAAGCAACACCAGGTAACCTTAAACAATTAGAGTTACAGAAAAAACGTGCAAAACAAGAACGCCAACAAG
AAATTGAAGATGCTAAAGCATTAAAAGAAACGTTATCAAACATTGAAGTTGAAGTATCAGCAAAAACTGGTGAAGGTGGTAAATTGTTTG
GGTCAGTAAGTACAAAACAAATTGCCGAAGCACTAAAAGCACAACATGATATTAAAATTGATAAACGTAAAATGGATTTACCAAATGGAAT
TCATTCCCTAGGATATACGAATGTACCTGTTAAATTAGATAAAGAAGTTGAAGGTACAATTCGCGTACACACAGTTGAACAATAA

Gene: dnaC (replicative DNA helicase)

Position: 20761 to 22161, length: 1401 nt, orientation: FORWARD

Perfect match to: (11819-97-CP003194-[20761:22161], allele observed in CC80)

Sequence:

ATGGATAGAATGTATGAGCAAAATCAAATGCCGCATAACAATGAAGCTGAACAGTCTGTCTTAGGTTCAATTATTATAGATCCAGAATTGA
TTAATACTACTCAGGAAGTTTTGCTTCCTGAGTCGTTTTATAGGGGCGCCCATCAACATATTTTCCGTGCAATGATGCACTTAAATGAAGATA
ATAAAGAAATTGATGTTGTAACATTGATGGATCAATTATCGACGGAAGGTACGTTGAATGAAGCGGGTGGCCCGCAATATCTTGCAGAGTT
ATCTACAAATGTACCAACGACGCGAAATGTTCAGTATTATACTGATATCGTTTCTAAGCATGCATTAAAACGTAGATTGATTCAAACTGCAG
ATAGTATTGCCAATGATGGATATAATGATGAACTTGAACTAGATGCGATTTTAAGTGATGCAGAACGTCGAATTTTAGAGCTATCATCTTCT
CGTGAAAGCGATGGCTTTAAAGACATTCGAGACGTCTTAGGACAAGTGTATGAAACAGCTGAAGAGCTTGATCAAAATAGTGGTCAAACA
CCAGGTATACCTACAGGATATCGAGATTTAGACCAAATGACAGCAGGGTTCAACCGAAATGATTTAATTATCCTTGCAGCGCGTCCATCTGT
AGGTAAGACTGCGTTCGCACTTAATATTGCACAAAAAGTTGCAACGCATGAAGATATGTATACAGTTGGTATTTTCTCGCTAGAGATGGGT
GCTGATCAGTTAGCCACACGTATGATTTGTAGTTCTGGAAATGTTGACTCAAACCGCTTAAGAACGGGTACTATGACTGAGGAAGATTGGA
GTCGTTTTACTATAGCGGTAGGTAAATTATCACGTACGAAGATTTTTATTGATGATACACCGGGTATTCGAATTAATGATTTACGTTCTAAAT

GTCGTCGATTAAAGCAAGAACATGGCTTAGACATGATTGTGATTGACTACTTACAGTTGATTCAAGGTAGTGGTTCACGTGCGTCCGATAA
CAGACAACAGGAAGTTTCTGAAATCTCTCGTACATTAAAAGCATTAGCCCGTGAATTAGAATGTCCAGTTATCGCATTAAGTCAGTTATCTC
GTGGTGTTGAACAACGACAAGATAAACGTCCAATGATGAGTGATATTCGTGAATCTGGTTCGATTGAGCAAGATGCTGATATCGTTGCGTT
CTTATACCGTGATGATTACTATAACCGTGGCGGCGATGAAGATGATGACGATGATGGTGGTTTTGAGCCACAAACGAATGATGAAAACGG
TGAAATAGAAATCATCATTGCTAAGCAACGTAACGGTCCAACAGGCACAGTTAAGTTACACTTTATGAAACAATATAATAAATTTACAGATA
TCGATTATGCACATGCTGATATGATGTAA

Gene: txbi_dnaC_var1 (bidirectional rho-independent terminator of dnaC variant 1)

Position: 22291 to 22329, length: 39 nt

Perfect match to: (Strain_21310-AFNP01000044-[36102:36140:r], allele observed in CC22)

Sequence:

AAAGGGTTTTTAGGACGTTGAAGACGATTTGTTAAAATG

Gene: purA (adenylosuccinate synthase)

Position: 22440 to 23723, length: 1284 nt, orientation: FORWARD

Perfect match to: (08-02119-CP015645-[2168200:2169483:r], highly conserved allele)

Sequence:

ATGTCATCAATCGTAGTAGTTGGGACACAATGGGGAGACGAAGGAAAAGGAAAAATAACGGATTTCTTGGCAGAACAGTCAGATGTTATC
GCGCGTTTTTCAGGTGGTAATAATGCAGGCCATACCATTCAATTTGGCGGAGAAACATATAAATTACATTTAGTACCATCTGGTATCTTTTAC
AAAGACAAATTAGCGGTAATCGGTAACGGTGTCGTTGTTGATCCAGTTGCACTATTGAAAGAATTAGACGGATTAAATGAACGTGGCATTC
CTACAAGTAATTTACGTATATCTAATCGTGCGCAAGTGATTTTACCATATCACTTAGCACAAGATGAATATGAAGAACGTTTACGCGGTGAC
AATAAGATTGGTACAACTAAAAAAGGTATCGGTCCAGCATATGTAGACAAAGTTCAACGTATCGGTATTCGTATGGCAGATTTACTTGAAA
AAGAAACATTCGAAAGATTATTAAAATCAAACATTGAATATAAACAAGCATATTTCAAAGGTATGTTTAACGAAACATGTCCATCATTTGAT
GATATCTTTGAAGAATACTATGCTGCAGGTCAACGTTTAAAAGAATTTGTAACAGACACATCAAAAATCTTAGACGATGCATTTGTAGCAGA
TGAAAAGGTACTTTTCGAAGGTGCGCAAGGTGTAATGTTAGATATCGACCATGGTACATATCCATTCGTTACATCAAGTAATCCAATTGCAG
GTAACGTTACTGTTGGTACAGGTGTAGGTCCTACATTCGTTTCAAAGGTAATTGGTGTATGTAAAGCTTATACATCACGTGTTGGTGATGGT
CCATTCCCTACTGAATTATTCGATGAAGATGGACATCATATTAGAGAGGTTGGTCGTGAATACGGTACAACAACAGGACGTCCACGTCGTG
TAGGTTGGTTTGATTCAGTTGTATTACGTCACTCTCGTCGTGTAAGTGGTATTACAGATTTATCTATTAACTCAATCGATGTTTTAACAGGCC
TAGACACAGTGAAAATCTGTACAGCTTATGAATTAGACGGTAAAGAAATTACTGAGTACCCAGCAAACTTAGATCAATTAAAACGTTGTAA
ACCAATCTTTGAAGAGTTACCAGGTTGGACAGAAGACGTAACAAATGTGCGTACTTTAGAAGAATTACCTGAAAATGCACGTAAATATTTA
GAGCGTATTTCAGAATTATGTAATGTACAAATTTCTATCTTCTCAGTTGGTCCAGATAGAGAACAAACAAACCTATTAAAAGAATTGTGGTA
G

Gene: walR (two component sensor/regulator of autolysis, transcriptional regulator)

Position: 24927 to 25628, length: 702 nt, orientation: FORWARD

Perfect match to: (11819-97-CP003194-[24927:25628], highly conserved allele)

Sequence:

ATGGCTAGAAAAGTTGTTGTAGTTGATGATGAAAAACCGATTGCTGATATTTTAGAATTTAACTTAAAAAAAGAAGGATACGATGTTTACT
GTGCATACGATGGTAATGATGCAGTCGACTTAATTTATGAAGAAGAACCAGACATCGTATTATTAGATATCATGTTACCTGGTCGTGATGGT
ATGGAAGTATGTCGTGAAGTGCGCAAAAAATACGAAATGCCAATTATAATGCTTACTGCTAAAGATTCAGAAATTGATAAAGTGCTTGGTT
TAGAACTAGGTGCAGATGACTATGTAACGAAACCGTTTAGTACGCGTGAATTAATCGCACGTGTGAAAGCGAACTTACGTCGTCATTACTC
ACAACCAGCACAAGACACTGGAAATGTAACGAATGAAATCACAATTAAAGATATTGTGATTTATCCAGACGCATATTCTATTAAAAAACGT
GGCGAAGATATTGAATTAACACATCGTGAATTTGAATTGTTCCATTATTTATCAAAACATATGGGACAAGTAATGACACGTGAACATTTATT
ACAAACAGTATGGGGCTATGATTACTTTGGCGATGTACGTACGGTCGATGTAACGATTCGTCGTTTACGTGAAAAGATTGAAGATGATCCG
TCACATCCTGAATATATTGTGACGCGTAGAGGCGTTGGATATTTCCTCCAACAACATGAGTAG

Gene: walK (two component sensor/regulator of autolysis, sensor histidine kinase)

Position: 25641 to 27467, length: 1827 nt, orientation: FORWARD

Perfect match to: (TCH130-ACHD01000027-[18070:19896:r], allele observed in CC72+CC5)

Sequence:

ATGAAGTGGCTAAAACAACTACAATCCCTTCATACTAAACTTGTAATTGTTTATGTATTACTGATTATCATTGGTATGCAAATTATCGGGCTG
TATTTTACAAATAATCTTGAAAAAGAGCTGCTTGATAATTTTAAGAAGAATATTACGCAGTACGCTAAACAATTAGAAATTAGTATTGAAAA
AGTATATGACGAAAAGGGCTCCGTAAATGCACAAAAAGATATTCAAAATTTATTAAGTGAGTATGCCAACCGTCAAGAAATTGGAGAAATT
CGTTTTATAGATAAAGACCAAATTATTATTGCGACGACGAAGCAGTCTAACCGTAGTCTAATCAATCAAAAAGCGAATGATAGTTCTGTCCA
AAAAGCACTATCACTAGGACAATCAAACGATCATTTAATTTTAAAAGATTATGGCGGTGGTAAGGACCGTGTCTGGGTATATAATATCCCC
GTTAAAGTCGATAAAAAGGTAATTGGTAATATTTATATCGAATCAAAAATTAATGACGTTTATAACCAATTAAATAATATAAATCAAATATTC
ATTGTTGGTACAGCTATTTCATTATTAATCACAGTCATCCTAGGATTCTTTATAGCGCGAACGATTACCAAACCAATCACCGATATGCGTAAC
CAGACGGTTGAAATGTCCAGAGGTAACTATACGCAACGTGTGAAGATTTATGGTAATGATGAAATTGGCGAATTAGCTTTAGCATTTAATA
ACTTGTCTAAACGTGTACAAGAAGCGCAGGCTAATACTGAAAGTGAGAAACGTAGACTGGACTCAGTTATCACCCATATGAGTGATGGTAT
TATTGCAACAGACCGCCGTGGACGTATTCGTATCGTCAATGATATGGCACTCAAGATGCTTGGTATGGCGAAAGAAGACATCATCGGATAT
TACATGTTAAGTGTATTAAGTCTTGAAGATGAATTTAAACTGGAAGAAATTCAAGAGAATAATGATAGTTTCTTATTAGATTTAAATGAAGA
AGAAGGTCTAATCGCACGTGTTAACTTTAGTACGATTGTGCAGGAAACAGGATTTGTAACTGGTTATATCGCTGTGTTACATGACGTTACTG
AACAACAACAAGTTGAACGTGAGCGTCGTGAATTTGTTGCCAATGTATCACATGAGTTACGTACACCTTTAACTTCTATGAATAGTTACATT
GAAGCACTTGAAGAAGGTGCATGGAAAGATGAGGAACTTGCGCCACAATTTTTATCTGTTACCCGTGAAGAAACAGAACGAATGATTCGA
CTGGTCAATGACTTGCTACAGTTATCTAAAATGGATAATGAGTCTGATCAAATCAACAAAGAAATTATCGACTTTAACATGTTCATTAATAA
AATTATTAATCGACATGAAATGTCTGCGAAAGATACAACATTTATTCGAGATATTCCGAAAAAGACGATTTTCACAGAATTTGATCCTGATA
AAATGACGCAAGTATTCGATAATGTCATTACAAATGCGATGAAATATTCTAGAGGCGATAAACGTGTCGAGTTCCACGTGAAACAAAATCC
ACTTTATAATCGAATGACGATTCGTATTAAAGATAATGGCATCGGTATTCCTATCAATAAAGTCGATAAGATATTCGACCGATTCTATCGTGT
AGATAAGGCACGTACGCGTAAAATGGGTGGTACTGGATTAGGACTAGCCATTTCGAAAGAGATTGTGGAAGCGCACAATGGTCGTATTTG
GGCAAACAGTGTAGAAGGTCAAGGTACATCTATCTTTATCACACTTCCATGTGAAGTCATTGAAGACGGTGATTGGGATGAATAA

Gene: walH (extracellular negative effector of the regulator of autolysis)

Position: 27460 to 28794, length: 1335 nt, orientation: FORWARD

Perfect match to: (JKD6159-CP002114-[26919:28253], allele observed in CC93+CC5)

Sequence:

ATGAATAATAAGGAACATATTAAATCTGTCATTTTAGCGCTACTCGTCTTGATGAGTGTCGTATTGACATATATGGTATGGAACTTTTCTCCT
GATATTGCAAATGTCGACAATACAGATAGTAAGAAGAGTGAAACGAAACCTTTAACGACACCTATGACAGCCAAAATGGATACAACTATTA
CGCCATTTCAGATTATTCATTCGAAAAATGATCATCCAGAAGGTACGATTGCGACGGTATCTAATGTGAATAAACTGACGAAACCTTTGAAA
AATAAAGAAGTGAAGTCCGTGGAACATGTTCGTCGGGATCATAACTTGATGATTCCTGATTTGAGCAGTGATTTTACATTATTCGATTTTAC
GTATGATTTACCGTTATCAACATATCTTGGCCAAGTACTGAACATGAATGCGAAAGTACCAAATCATTTCAATTTCAATCGTTTGGTCATAGA
TCATGATGCTGATGATAATATCGTGCTTTATGCTATAAGCAAAGATCGCCACGATTACGTAAAATTAACAACTACAACGAAAAATGATCATT
TTTTAGATGCATTAGCAGCAGTGAAAAAAGATATGCAACCATACACAGATATCATCACAAACAAAGATACAATTGATCGTACGACGCATGT
TTTTGCACCAAGTAAACCTGAAAAGTTAAAAACATATCGCATGGTATTTAACACGATTAGTGTTGAGAAAATGAATGCTATACTATTTGACG
ATTCAACCATCGTTCGTAGTTCAAAGAGTGGTGTTACTACTTACAACAATAATACAGGTGTCGCAAACTATAACGATAAAAATGAAAAATAT
CATTATAAAAACTTATCCGAAGATGAAGCAAGTTCCAGCAAAATGGAAGAAACGATTCCAGGAACCTTTGATTTTATTAATGGTCATGGTG
GTTTCTTAAACGAAGACTTCAGATTGTTTAGTACGAATAATCAGTCAGGCGAGCTAACATATCAACGTTTCCTTAATGGTTATCCAACGTTTA
ATAAAGAAGGCTCTAATCAAATTCAAGTCACTTGGGGTGAAAAAGGCGTCTTTGACTATCGTCGTTCGTTATTACGCACCGACGTTGTTTTA
AACAGTGAAGATAATAAAACGTTGCCGAAATTAGAGTCTGTACGTTCAAGCTTAGCGAACAATAGTGATATTAATTTTGAAAAAGTAACAA
ACATCGCTATCGGTTACGAAATGCAGGATAACTCAGATCATAATCACATTGAAGTGCAGATTAACAGTGAACTCGTACCGCGTTGGTATGT
AGAATATGATGGCGAATGGTATGTTTATAACGATGGGAGGCTTGAATAA

Gene: walI (extracellular negative effector of the regulator of autolysis)

Position: 28795 to 29583, length: 789 nt, orientation: FORWARD

Perfect match to: (11819-97-CP003194-[28795:29583], allele observed in CC80)

Sequence:

ATGAACTGGAAACTGACGAAGACACTTTTCATTTTCGTGTTTATTCTTGTCAACATCGTGTTAGTATCGATTTATGTTAATAAAGTCAATCGC
TCACACATTAATGAGGTTGAGAGTAACAATGAAGTTAATTTTCAGCAAGAAGAAATTAAAGTACCGACTAGTATATTGAATAAATCAGTTA
AAGGTATAAAATTAGAGCAAATTACGGGGCGTTCGAAAGACTTTAGTTCTAAAGCTAAGGGCGATTCGGATTTGACCACATCAGATGGTG
GAAAATTATTAAATGCGAACATTAGTCAATCGGTAAAGGTCAGTGACAATAACTTAAAAGATTTGAAGGATTATGTTAACAAACGTGTGTT
CAAAGGTTCAGAATATCAATTAAGTGAGATTAGTTCTGGTTCTGTAAAATACGAACAAACGTATGATAATTTCCCGATTTTGAATAATAGTA
AAGCGATGTTGAACTTTAATATAGAAGATAACAAAGCGGCTAGTTATAAACAATCAATGATGGATGACATTAAGCCCACAGATGGTGCAGA
TAAGAAGCATCAAGTTATTGGAGTGAGAAAAGCAATCGAGGCATTATATTATAATCGTTACTTGAAAAAAGGTGATGAAGTCATTAATGCT
AGACTCGGTTACTACTCAGTCGTGAACGAAACGAATGTTCAATTGTTACAACCAAACTGGGAAATTAAAGTGAAGCATGACGGTAAGGAC
AAAACGAATACTTACTATGTCGAAGCGACAAATAATAACCCTAAAATTATTAATCATTAA

Gene: walJ (exonuclease associated with regulator of cell wall autolysis)

Position: 29971 to 30771, length: 801 nt, orientation: FORWARD

Sequence:

ATGAGCCGCTTGATACGCATGAGTGTATTAGCAAGTGGTAGTACAGGTAACGCCACTTTTGTAGAAAATGAAAAAGGTAGTCTATTAGTTG
ATGTTGGTTTGACTGGCAAGAAAATGGAAGAATTGTTTAGTCAAATTGACCGTAATATTCAAGATTTAAATGGTATTTTAGTAACCCATGAA
CATATTGATCATATTAAAGGATTAGGTGTTTTGGCGCGTAAATATCAATTGCCAATTTATGCGAATGAAAAGACTTGGCAGGCAATTGAAA
AGAAAGATAGTCGCATCCCTATGGATCAGAAATTCATTTTTAATCCTTATGAAACGAAATCTATTGCAGGTTTCGATGTTGAATCGTTTAACG
TGTCACATGATGCGATAGATCCGCAATTTTATATTTTCCATAATAACTATAAAAAGTTTACGATTTTAACGGATACGGGTTACGTGTCTGATC
GTATGAAAGGTATGATACGTGGCAGTGATGCGTTTATTTTTGAGAGTAATCATGACGTCGATATGTTGAGAATGTGTCGTTATCCATGGAA
GACGAAACAACGTATTTTAGGCGATATGGGTCATGTATCTAATGAGGATGCGGGTCATGCGATGACAGACGTGATTACAGGTAACACGAA
ACGTATTTACTTATCACATTTATCACAAGATAATAACATGAAAGATTTGGCGCGTATGAGTGTTGGCCAAGTATTGAACGAACACGATATTG
ATACGGAAAAAGAAGTATTGCTATGTGATACGGATAAAGCTATTCCAACGCCAATATATACAATATAA

Gene: sasH (cell-wall attached adenosine synthase A)

Position: 30998 to 33316, length: 2319 nt, orientation: FORWARD

Sequence:

ATGAAAGCTTTATTACTTAAAACAAGTGTATGGCTCGTTTTGCTTTTTAGTGTGATGGGATTATGGCATGTCTCGAACGCGGCTGAGCAGCA
TACACCAATGAAAGCACATGCAGTAACAACGATAGACAAAGCAACAACAGATAAGCAACAAGTAACGCCAACAAAGGAAGCGGCTCATCA
TTATGGTGAAGAAGCGGCAACCAACGTATCAGCATCAGCACAGGGAACAGCTGATGAAATAAACAATAAAGTAACATCCAACGCACCATC
TAACAAACCATCTACAGCAGTTTCAACAACAGTAAACGAAACGCGCGATGTAGATACACAACAAGCCTCAACACAAAAACCAACTCGCACA
GCAACATTCAAATTATCAAATGCTAAAACAGCATCACTTTCACCACGAATGTTTGCTGCCAATGTACCACAAACAACAACACATAAAATATTA
CATACAAATGATATCCATGGCCGACTAGCCGAAGAAAAAGGGCGTGTCATCGGTATGGCTAAATTAAAAACAGTAAAAGAACAAGAAAAG
CCTGATTTAATGTTAGACGCAGGAGACGCATTCCAAGGTTTACCACTTTCAAATCAATCTAAAGGTGAAGAAATGGCTAAAGCGATGAATG
CAGTAGGTTATGATGCTATGGCAGTCGGTAACCACGAATTTGACTTTGGATACGACCAGTTGAAAAAGTTAGAGGGTATGTTAGACTTCCC
GATGTTAAGTACTAACGTTTATAAAGATGGAAAACGCGCATTTAAACCATCAACGATTGTAACGAAAAATGGTATTCGTTATGGAATTATTG
GCGTAACGACACCAGAAACAAAGACGAAAACAAGACCTGAAGGCATTAAAGGTGTTGAATTTAGAGATCCATTACAAAGTGTGACAGCAG
AAATGATGCGTATTTATAAAGACGTAGATACATTTGTTGTTATATCACATTTAGGGATTGATCCTTCAACACAAAAAACATGGCGTGGTGAT
TACTTAGTGAAACAATTAAGTCAAAATCCACAATTGAAGAAACGTATTACAGTCATTGATGGTCATTCACATACCGTACTTCAAAATGGTCA
AATTTATAACAATGATGCATTAGCACAAACAGGTACAGCACTTGCGAATATCGGTAAGGTTACATTTAATTACCGCAATGGAGCGGTATCG
AATATTAAGCCGTCATTGATTAATGTTAAAGACGTTGAAAATGTAACACCGAACAAAGCATTAGCTGAACAAATTAATCAAGCTGATCAAAC
ATTTAGAGCACAAACAGCAGAGGTTATTATTCTAAATAATACGATTGATTTCAAAGGAGAAAGAGATGACGTTAGAACGCGTGAAACAAAT
TTAGGAAACGCGATTGCAGATGCTATGGAAGCATATGGCGTTAAGAATTTCTCTAAAAAGACTGACTTTGCCGTGACAAATGGTGGAGGTA
TTCGCGCCTCTATCGCAAAAGGTAAGGTGACACGCTATGATTTAATTTCAGTATTACCATTTGGAAATACGATTGCGCAAATTGATGTAAAA
GGTTCAGACGTCTGGACGGCTTTTGAACATAGTTTAGGCGCTCCAACAACACAAAAAGATGGTAAGACAGTATTAACAGCGAATGGCGGT
TTACTACATATCTCTGATTCAATCCGTGTTTACTATGATATGAATAAACCGTCTGGCAAACGAATTAACGCTATTCAAATTTTAAATAAAGAG
ACAGGTAAGTTTGAAAATATTGATTTAAAACGTGTATATCACGTAACGATGAATGACTTCACAGCATCAGGTGGCGACGGATATAGTATGT
TCGGTGGCCCTAGAGAAGAAGGTATTTCATTAGATCAAGTACTAGCAAGTTATTTAAAAACAGCTAACTTAGCTAAGTATGATACGACAGA
ACCACAACGTATGTTATTAGGTAAACCAGCAGTAAGTGAACAACCAGCTAAAGGACAACAAGGTAGCAAAGGTAGTGAGTCTGGTAAAGA
TACACAACCAATTGGTAAAGACAAAGTGATGAATCCAGCGAAACAACCAGCGCCAAGTAAAGTTGTGTTGTTGCCAGCGCATAGAGGAAC
TGTTAGTAGTGGTAGAGAAGGTTCTGATCGCGCATTGGAAGGAACTGCTGTATCAAGTAAGAGTGGGAAACAATTGGCTAGCATGTCAGC
GCCTAAAGGTAGCGCACATGAGAAACAGTTACCAAAAACTGGAACTGATCAAAGTTCAAGCCCAGCAGCGATGTTTGTATTAGTAGCAGG
TATAGGTTTAATCGCTACTGTACGACGTAGAAAAGCTAGCTAA

Gene: orfX (23S rRNA methyltransferase)

Position: 33683 to 34162, length: 480 nt, orientation: FORWARD

Sequence:

ATGAAAATCACCATTTTAGCTGTAGGGAAACTAAAAGAGAAATATTGGAAGCTAGCCATAGCAGAATATGAAAAACGTTTAGGCCCATACA
CCAAGATAGACATCATAGAAGTTCCAGACGAAAAAGCACCAGAAAATATGAGCGACAAAGAAATTGAGCAAGTAAAAGAAAAAGAAGGC
CAACGAATACTAGCCAAAATCAAACCACAATCCATAGTCATTACATTAGAAATACAAGGAAAGATGCTATCTTCCGAAGGATTGGCCCAAG
AGTTGAACCAACGCATGACCCAAGGGCAAAGCGACTTTGTATTCGTCATTGGCGGATCAAACGGCCTGCACAAGGACGTCTTACAACGCA
GTAACTACGCGCTATCATTCAGTAAAATGACATTCCCACATCAAATGATGCGGGTTGTGTTAATTGAGCAAGTGTATAGAGCGTTTAAGATT
ATGCGTGGAGAAGCATATCATAAGTGA

Gene: sRNA6 (antisense RNA associated with orfX)

Position: 33863 to 34146, length: 284 nt

Sequence:

CAACGAATACTAGCCAAAATCAAACCACAATCCATAGTCATTACATTAGAAATACAAGGAAAGATGCTATCTTCCGAAGGATTGGCCCAAG
AGTTGAACCAACGCATGACCCAAGGGCAAAGCGACTTTGTATTCGTCATTGGCGGATCAAACGGCCTGCACAAGGACGTCTTACAACGCA
GTAACTACGCGCTATCATTCAGTAAAATGACATTCCCACATCAAATGATGCGGGTTGTGTTAATTGAGCAAGTGTATAGAGCGTTTAAGATT
ATGCGTGGAGA

Gene: DR_SCC (direct repeat of SCC)

Position: 34144 to 34162, length: 19 nt

Perfect match to: (JS395-CP012756-[1418304:1418322])

Sequence:

AGAAGCATATCATAAGTGA

Gene: dam5 (type II restriction-modification system, endonuclease and methyltransferase)

Position: 34457 to 37204, length: 2748 nt, orientation: FORWARD

Sequence:

ATGGGAAAAAAAAAGAACTTAAGTATTACAGAGATAGAGAATTCTGTAAATACTTTGATTAATCATTTGAAAGAGAAAGATTTTATAACTG
AATTTTTAAGTTTCTATGATATACCAAAAACTTCAATTACACGAGCAAAAGTAAAATTTGATAAAGGTGAACCATTTATAATTAAAAATAAA
GTATACTATACAGAAATTCAAGGTGAAGTTATTACAGCAATAGATGCTATTGAGCATGAAATATTAAATCAGAAATCCAAACCGAGATATCT
TATTGCAAATAATTATTCTGAAATTGCAGCTTTAGATATTAAAACTCGAGACACACTTAACATTCCATTAAGTGAGTTACCATCAAAAGCTGA
TTTCTTTTTAGCGTGGAATGGTATCGAAAAGTCAGATTATCAATCTGAACATCCAGCCGATAGAAAAGCAGCTGAACGGTTTGCAAAACTTT
ACGATGTGCTTGAAAAGGATAATCCTAATGTTAAAGAACACTCATTTAACACATTTTTAATTCGTATATTATTTTTATTGTTTGCAGAAGATA
CGGGAATTATGAAGAAAGGTATATTTACTAATACACTTAAAATAAGAACAAAAGAAGATGGCAGTAATTTTAATGAAGTTATTGAAGAGTT
ATTTGAAATTTTAAATACTAATGAACTAAATCGAGATAAAAAATCAGATTGGTTAAAAATATTTCCTTATGTTAATGGTAAACTTTTTAGTGA
GCCTCACGTATCCTTAATTTTTACAAAGAAATCTAGAAAGTTACTGATTGAAGCTGGCGAGCTTCTAAATTGGAACGAAATTAATCCTGATA
TTTTGGGTTCAATGATTCAAACAGTATCCTCTTCTAAAAAAAGGCAAGTATCAGGAATGCACTACACTAGTGTTCCTAATATTATGAAAGTA
ATAAAGCCTCTTTTTTTAGATGAACTTTATAAAATATTTAATGATTTATCTGCTCAATATGAAGAAAATAAAATTAAAAATATTACAGAGAAA
ACAAAAAAAACCTATAATAAGAAAATAATTAACTCTTTATCGGATTTGTTAGAAAGAATTTCAAGGATTAATTTTTTAGATCCTGCATGTGGT
TCAGGAAATTTTTTAATTATAGCATATAAAGAAATCAGACGTCTTGAAATAAAAATACTTGTTTTATTAGATGAAATACAGCAATCAGACAC
TATGCCAATGACAGCGATACATTTAGAAAATTATAATGGTATTGAAGTTGATGACTTTGCGCATGAAGTAGCGAAATTATCTCTCTGGATTG
CTGAACATCAAATGAATGAAGAAATGGAAAAAGCTTTACCTGGTTATATTTCGGCTCTACTACCCTTAAAAGATTCTGGAAATATAGTTTTA
GGAAATGCGTTGAGGATTGATTGGAATAATATTATACCTCAAAATAAAAATGAGGAAATTTATATATTTGGTAACCCACCCTATATTGGAGC
AGCTAATAAAAACGAAAACCAAAAAAACGATTTAGCTTTTGTGTTTCATGATACAGATATTTCATTTGGAAAATTGGATTATATCACGGGAT
GGTTTTATAAATCTGTTAAATTTATGGAAAAAAGAAAATCTGTCTTTGCATTTGTTTCAACAAATTCAATTGTCCAAGGAGAACAAGTTTCTT

TAATTTGGCCTGAATTATTTAAAACTGCACAAATAAGTTTTGCTTATCCATCTTTTAAATGGTCTAATAATGCAAAATCAAATGCTGGTGTTA
CAGTAGTGATAATTGGTTTCGAATATAAAGATTATTTAGGACCAAAAAGTATATATTCTTCAAATGGAACAGTAAAAAAGGTGGATAATATT
ACACCTTATTTAGTTGAAGGGGTAAATATAATTGTTGAAAAAGAAAATAAATCAATAAATGGCTTTACAGAGATGGTTAAAGGAAGTTCTC
CAACTGATGATGGTGGATTAATATTTTCAGAGTATGAATATCAACAAGCTATAGAACTTTATCCAAATTTGAAAGATATTTTAAAAAAATAT
CAAGGATCAAGAGAATATATTAATGATATAAGCAGATATGTTCTTTGGATGTCTGATGATGACGCTAAATTATTTAAAAATAATCCTATAAT
TTCAAAAAGACTTGAACACGTACGTCAATTTAGATTAAATAAAAAGGGAAACACCTTGAAAAAAGCTGAAACACCTTGGGAATTCGTTTCT
AATGGTAAAAGAAAAGCAGCTTTGAAAAAAAAATAAAAATATGAAACAAATCTTAATACCACGAGTTTCTTCAGAAAATAGATATTACGTTC
CAATGGGATATGTTAATAAAGATACTATAATATCTGATTCATCTATGGCAATTTATGATGCTCCATTATGGCTTTTAGGATTACTTCAATCAC
GAATGCATATGGTTTGGCTTAGAGCAATTGGTGGAAAATTAAAAACTGATTATAGATACTCCTCAGGGCTAGTTTATAATACTTTTCCAATA
CGAAGATTATCTCCTCAACGATTAAAGGAAATTGAACGTGTGATAACGGATATTTTAGATTTAAGGGAGTATGAAGGTGGTAGTTTAGCTT
ACCTTTATAATAGTAAAACTATGCCAATTAGTTTAAAAGAAAAACATCAAGAACTTGATGGTATAGTGGAACGCGCATACCGACAAAAACC
TTTTAATAGTGATGAAGAACGTTTGAGTACCTTACTAACATTATATAAAGAAAAGAAGGTAATAGTAGATGACAACTAA

Gene: helicase5 (DNA helicase, associated with dam)

Position: 37194 to 39159, length: 1966 nt, orientation: FORWARD

Sequence:

ATGACAACTAATTTATTCGAAGTTAATTTTGAAGAAAAAAATGAAAAAAATACTAATAATCTTGGTATGAGAGAAATGCAAGAAAAAGTTT
ATAAAAAACGCTTTGCACGTTATTTATTAGTTAAAGCACCTCCAGCCTCTGGTAAATCGCGTGCTTTAATGTTTGTGGGTTTAGATAAATTAA
ATACTCAAGGTCTAAAAAAAATAGTTATTGCAGTTCCAGAAAAATCAATCGGAAAATCGTTTCGTAATACTAAACTAACAAATTATGGTTTT
TATTGGGATTGGAATGTAAAACCTTGTAATAATCTAACTATTGGAGTAGGTGAAACTTCAAAAGTTAAAAGATTTGTTGAATTTATGAAATC
TAATGATAAAGACGACAATATTCTCATTGCAACACATGCCACTTTGCGTTATGCATTCGAAGAATTAGATGATTCAGCTTTTGATAATTCATT
ACTTGCAATTGATGAGTTTCATCATGTCAGTCGAGATGACAGTTCAGTGCTTGGTAATGCTTTGCGCAGTATTATGAGTAATTCAACTGCAC
ACATTTTAGCCATGACCGGATCCTATTTTCGAGGTGATTCTGTGCAAATTTTAGAGCCGAAAGATGAAGATAAGTTTGAAAAAGTAACTTAC
ACATATTATGAGCAATTAAATGGCTATCAATATTTAAAATCTTTTGCTATGGGTTATAGTTTTTATCGAGGACAATATACAGAAGCTTTAGAT
GAAGTTATAGATGTGTCGAAGAAATCGATTATTCACATACCTAATATTAATTCTAGTGAATCAACTAAAGATAAATATGACGAAGTTGATCG
AATTATGGATTTAATTTCTGATGGTGGAAATATTTATCAAAATAGTGATGGTATATATGAAGTTAATCGGCCTGATGGAAAAGAATTATTAG
TTGCCGATTTAGTCAATGAAGATTCAAGAGAAAAGGTTACATCTTATTTAGCTAATATAACTGATGATATAAAGGATTTAGATAAATTAGAT
ATTATCATTGCACTTGGTATGGCTAAAGAGGGTTTTGATTGGCCCTTTGCAGAATACGCTTTGACGATTGGTTATCGTAATTCTTTGACAGA
AATTATTCAAATTATCGGACGAGTAACTCGAGATAGTGCTAATAAAAATCATGCTCAATTCACAAATTTAATTGCACAGCCTGATGCTCAAG
ATGATGAAGTATTATATGCAGTTAATAGTCTTATGAAAGCAATAACTGCGTCATTACTTATGGAGCAAGTTTTAGCACCTGTTTATAATTTTA
AACCTAAGGATAAAAAAATAGACAATCCTGATGATATATCTATAAAAGGATTGAAACCTTCTAATACAACAGAACGAACTAAAAAAATAAT
TAAAGATGATATGGCTGACTTGAAGTCAAGTATATTACAAAGTGAGCATATTCAAAATGCTATTGTATCTGGTTCTGATGCTCAAATGATTA
ACAAGACTTTAGTCCCGCGTGTGATTATTGAACGTTATCCTGATTTAACAGAGGAAGAATTGGAAACAGTTCGTCAACATACAGTTGCTAAC
GTTAATTTTTCAGCAAGTTCTAAAGTGATTTCAAATGATGGTAGTCGTGAAATTATAAAAATGGCTGATAGATTTATTAATATAGATGAATT
AAATATAGATTTAATTGATTCTATAAATCCATTTCAGCGCGCGTATGAAATGATAAGTCGTGATATTAATGCTCCAACGTTACAATTTATTCA
AGATTATATGTCATCGAAAAAATATGAATTTACCAATGAACAACTAGTGAATGCTTATCAACGTGCAAAACAATTTCGAATTGAAAATGGGC
GTAATCCAGAAAGAAATAGTAAAGATGAGGGAGAAAGATATTTAGCTTTTGCACTTTTAAGATTAGCTGAAATGAAACGTGAACGAGAGG
CTGATAAAAAATAATAATGAGTTACTTAGATGA

Gene: YeeC-like (putative protein from SCC/ACME elements)

Position: 39162 to 40602, length: 1441 nt, orientation: TRUNCATED

Sequence:

TTTTTTCAGATGAACTTTTTGAAGATTTAACAAAACAAGAAAAAGAGAAAAAAATTGAAAAGTTAGATCCAGAAATCGGTAAATTTCAAGA
AATAATAAATTTTGTAAAAAATAAAAATAGAGAACCTGAAAAGACAAATCGATGGTCAGAAGAACGTGCTTTGTGGGCAAGACTTCAAGG
ATTTCGTAATAAAAAAGAACGTTCAGATAAAGTAAAACATATTGATGAATTAAATCTATTAAATAAGCAAGGAGATTTTTTAGATGAATTGA
ACTTGACTGTTAATAGAGAATCAGTAAGTAATATTGATGAAGTACTAAATGATAACCAATTACTTGATAACTTTTCGGATTTACTTGATACTT
CAAGATATAAAAAGACTGTTAATGCATTGAACAAAAAAAGTTCACGTAAACGAGCTGATAATTTTAATGAATATAAAGAATTATTTAAAAA
AGTACATGAAGAAATAGCAAGTGGTAAAAGAAAAATTTTACCATTTAAGAAATATGATATTGAAGAAGGTAGATTTTATATACAAAATGGT
GTAATGCTGTATATTGTATCAATCTCTGATGAAAAATTTATCGGAGACAATGGTAAAGAGAATGCCCGCATGCATGTTGTATATGAAAATG
GAACAGAGAACAAACAGCTATTACTGCAATCGTTAGCGTCTTCATTATACTCTAATGAACGTCATGGTAGAATGGTAACAGAAATTATTGAT
GAAAATTCATTATCTGAAAGTTTTGGCGCAGAATTTACGACAGGATACATTTACGTTTTAAAATCGTTAAACACGAATCCAGAAATTAGTCA
ATTAACTCATTTATATAAAATTGGCTTTACTAAAAACAGTATAGAGTCTAGAATTGTTAATGCAGAAAATGAAGTTACTTATTTAAATGCTCC
AGTTCGTATAGTATTGTCTGTAGAGGTAAAAAATCTTAATGCACAGTTGTTAGAGCGTACGTTACATCATACATTTCACGATAAACAAGTTA

TATTTCAAAATAGTAAATATAAAAAAGCAACAGAATGGTATATTTTACCACTTGAAGAAATAGAGAGCAGAATTAATGAAATATTATCTCAT
ATTCAATTTTAGTTTAGAAGGTTTTTGATTGAATAAAAAATAAACTAATAAGAGCATCCCTCACCGCAAAGTGAAGGATGCTCTTTTTTTATT
AGAATATAGTTTTCATTTTGATAGATAATGGTTAATAATTATCTGTAAATCATTATTAAGTACCGTTGTTATAGAGTCATCATTCATAAAAGCT
TCATAGATTTTATCAAGTATTTCCTCATCTTCAATCGCTGTAAAATGATGTACTAAACCTTTTACTATTGATACATAATTTTGAGATGCTAAGC
AACTAGCAACCGTTCGCCAGTCTGATTGTGTAGTTTCATGGTTCATAGATAATCCTCCTT

Gene: A9UFT0 (LPXTG protein homologue)

Position: 40367 to 40588, length: 222 nt, orientation: REVERSE

Sequence:

TCATTTTGATAGATAATGGTTAATAATTATCTGTAAATCATTATTAAGTACCGTTGTTATAGAGTCATCATTCATAAAAGCTTCATAGATTTTA
TCAAGTATTTCCTCATCTTCAATCGCTGTAAAATGATGTACTAAACCTTTTACTATTGATACATAATTTTGAGATGCTAAGCAACTAGCAACC
GTTCGCCAGTCTGATTGTGTAGTTTCATGGTTCAT

Gene: Q9KX75 (putative protein)

Position: 40603 to 41105, length: 503 nt, orientation: REVERSE

Sequence:

TTATTTCAATGTCCATTTTTGACGTGCTTTAGGATTGAGTGGATGCATGATTTCATTTGTTGCGGGGTTCATGAGTTGCTTCACTTTCTTTTTA
TGTGGTTTCAACATTTCTATTACTTCATTGACACGTGCTTCAACAACGGGTTTTTAGCGTAAGCGCCCCAAGCTAATATAACTTCATCCGATT
CTTTCACAGCCTTCATAATTTGAATATTTGTATGATTATCAAAAGCATTTTTTGAATGTTTAATATTAATAGGTGTTGTAATATTAGAATATAG
ATTCACAAAATTGATTGAACCAAATGCATCCATTTCTGAAACTTTGTTCATAATGAGCTGAGTTGTTAGGTCGATGTTGAGAATGCCATCATA
ATGTGGATACATTGTAATGATTGTAATTGTTTGTTTTTCACTATCCCATTTTTTCTTAAGTAGATAGCGATGTTCTTGATCATCACTGAATATA
GCTTCGGTTTCTAATGTACTTTTGATTGTTTTCAT

Gene: Q7A207 (putative protein)

Position: 41121 to 41432, length: 312 nt, orientation: REVERSE

Sequence:

TTAGTATTCTTCTGGTAAAAGCATCACATAATAAAGAAAGTCTACATCATCTTCACGAATGACGTAGACTTTTTTAGGTAATGCATTTTGTTT
TTTTACATAGTTTGTATAGTGATATTCCAATTTGTATGCGGGTTGTTCTTGTTCATGTGTGATTGACAGTATATTCTCATCTTCTTGTAGTCTA
AAAATGTGTAGGTAATCTGTATGAGGTTTATTATCTCTTTCTTTCACCATATTCCAAAGTAAGATTTGAAGGTCTAGAGATAGGTGTTCACTA
ATGCCTCTTGTGATGTATCGATTGATTTTCAT

Gene: Q7A206 (putative protein)

Position: 41434 to 41520, length: 87 nt, orientation: TRNC-RVRS (no start codon)

Perfect match to: (G265-LCL_10074-[66452:66538:r])

Sequence:

CTATTTTCCTCCATTTTGCTTTTCTTTCATGATGTCAATCACTTCGTTAATGACTGTAACAGATATTTGTGCCACTTTGATCCAATT

Gene: ccrB-1 (cassette chromosome recombinase B, type 1)

Position: 42336 to 43964, length: 1629 nt, orientation: REVERSE

Sequence:

TTAGATTTGAAAATAGAGTGTTTGAGGCAAAAACAAAGACGAAGTGCTGAGGAGCACTTCGTCTAGATTATTATTATTGAAAAGTTGTTTA
ATAATTTCATTATTAAGTTTTAGTGTAACGTAGAATTGCTTTTTATGATTCTCATCTTTACGAATGTCAATACGGTCAATAACCGTTAGGTACA

AAGCTTTGAGCTGTGATTTATCCATGGATTCTATATTTTGAAATATTCGTTGTAATAGGGCAGCGATTTGTTTCGTATCATAAGATGGTTTCT
CTTGATTTTGTTGCTGCTTGAGTTGATTAATTTGGTTTGTAATGTCATTGAGTTGTGTTTCATATTGATGAATGGTTGGTTTGAGTGCAGATG
TTAAGTCTGGATTGTCTTCGAGGGTTTGAATTAGATTTTTTAGTTTAGTGTTTATTTCATCAAATTGTTGTTGCTTATATGCGATATCATGGTT
AAGTGCAGCCATATCAATTTGACTATCTTGATTAACACGTTCGACAACTTGTTTGAGAACTTTATCGCTTTTGACAATTTCAAGTATTTGATCC
ATGACGTATTTCTCAATCACATCAGCTCTAACACTATTGGCTGAACATACCTTTGATCCTTTGTTGCGAAAGTTACTACAAGAATAATAGCGA
ATACGTTTCTTAGTTCCATCTTTGAGTGTATTAGTAGTATTACTCGCGGCCATAGGTGCTCCGCATTGGGGACAATGAACGATACCGGTTAA
TAGATTAGTTCCTTTACCGTGAACTTGGGGTTTTTGACTGACTTGCTTTTTACGTGCTTGCACTTTATCCCATAATGCTTGGCTAATAATAGGG
GCGTGCTTACCCTCAGCTATGATCGGTTTATCATTCAGTCCTTTACGACGTTTTTCATTCCAATCTTTGTACTTCGCAAATTGAATTTTACCCAT
ATAGAAGGGATTGGATAGAATATAAGTAATAGAACTTATACTGAAAGGCTTCCCTTTTTTAGTGACATAGCCTTTATGATTGAGTGTATTGG
CAATTTTACGATAGCCATGCCCTTTGGCATAAGATTCGAAAATATATTTTACAATATTAGCTTCATGTTGATTAATCATCAATTCGTGTTTACT
GTCGGGTATTTTGTCATAGCCTAGCGGTAAATTTCCTTGATAATAGCCTTCTTGGGCACGTCTGGTTTGGCCCATGAATACGTTCTCGACAAT
GTTATTACGTTCAAATTCTGAGAAACTCGCAAGTATTTGTAACATGAGTTTTCCAGAAGAAGTATTGACTTCCATACGTTCAGACAAACTGA
AAAATTCGACATTTTGCTTATGCAAATCTTCGACAATTTTGAGTAAGTCTGATGTATTACGTGCCAGTCGGTTTGTTTTGTACACCATGACAC
AATCGATATGACCTTCATTCGCATCCTTCAACATACGTTGGAGCTCAGGACGGTTCATAGATTTTCCGGAAATACCACGATCTGCGTACACA
TCTACCACTTTGAAGTTATTAAATTGACAGTATTCTTCAATCTGATTGATCTGGCCTGTTATCGAGTAGCCTTCAGTACTTTGGATTTCCGTTG
ATACACGAACATAGATAGCGACACGTTTTTGTTTGAGTTGTTGCAT

Gene: ccrA-1 (cassette chromosome recombinase A, type 1)

Position: 43985 to 45334, length: 1350 nt, orientation: REVERSE

Sequence:

TTAAGCAATCGATGATTGCGCCGTTTGATTGACGATGTTTAAGGGCTCATTTTTGAAATAGATACCCGTAAGTGTTTTATTTTTGGTAATATG
AATTTCATCAATGTAAGGGTACAACATGTTTAACGTGAAACGTTGTTGTATGATATTTTGAAAAACCTTTCGAATTTGATGTGCATTGATTGA
TGTTATAGGGTTTGACTGTTGTCGAAATGATTGCGATTGTTCTCTGAACGTATCTGCATCAATTTTGCCTTGGGCTAATTTTTCTATCAGCTG
CTCGTGATTGAGTGTAGTTTTAGTTTCTATATCTCTTTGTCTTTTGAGTCGTTGTTGAATAGTATGATTTATTTTTGAATAGAGCTGTTGATTTT
GAAAGAAGTCCTGACAAGTCGCTAAAACACTTGTTTCTAATTCTTGTGCGTTGATTCCTTTAAATTCACAAACAAAGCGAGCGTTATTCATAT
TTTTCGGACAGACATAGTAACGCAATGAATGATTTGGTTTTCGGACGGTCATGTTTGTGAGTGTCGAATGGCAATAGGGACATTTGATTTTT
TGTTTGAGTTGATTTTCTGACGGCTTACGTTTGACTTGTTTCTGAGTTCGGGTAACTTGAGCTTCTTCGTATATCGTTGTACTGACAATAGCT
GGGAACATGTTTTCATATTGTCCGTATTGATTGATAACACGGCCACAGTAATTAGGGTTAAGGATAATATTACGCACTTGATAGGGCTTGCG
ATTAATGAATTTATCATCAGCTTCTAAGTATTGCGCAATTTTTTTATAACCATAACCTTGAAGGTAATAATTGAACACAGCTTTTACTGTTGGT
GATTTTACTGTGTCTATTGTGAAAGTACCATTATGATAGTGATACCCAAAGGGTGCATGTGTTGTAATTAGTTTACCTTGTCTCGCTTTTTCTT
TGATTCCATTTTTGACTTGTTCGCCTATATTATCAGATTCTAGTTCGGCCAAGCTGATGAAAATATTGAGTTTGAGCCGATCGAATGCTTTAT
CCATGTCGAAGTAGCCATCATGAACGCTTAAGATATGGATATGGTATTTTTGACACAATTTCATGAGTTTTAATGCATTTTTAAGATTGCGAT
GAAGTCGGTTTAATCTGTAACAACATAATACGTTACATTGTCCTTGTTGAATCAGTTCAGTAATTTGTTGATAACCATTCCGCTTATCAGTAC
GTCCTGATTGCTTATCGCTATAAAAGGTAATGTATGTAATATTATGTTTTTTGGCTAATGCCTCTATGGTTTGTTTTTGTGCTGCTAGGGATT
GTTGCTTTGTAGTGCTCTGTCGTAAGTAACCTATTGCTTGTTTCAT

Gene: ORF-NoKK12 (undescribed ORF from SCC/ACME elements)

Position: 45337 to 45867, length: 531 nt, orientation: TRUNCATED

Sequence:

TATTTCCTCCTTCCACATTGATAATATATATTTATGAACGAATTTATGCATATGCCCAACGCCATCAGGCGTTGGGCATTAACTTTAGTCATCA
GCGTGATTGATTTCTTCAATCACTAAATCAGCGAGTAATGTGATTAATTCGTCCATATTCTTCCTCCTAATTGACTTTGATAGTAGTAAAAGT
GTTATACCAACTTTGGAAATATTGTAATGAACTCAATTTTTCGCTTTGAGCAGATTTGCTCCAACTCAGGATAATCTTCATAAATGCCCCAAA
AGACGATACGTTTAGGTTTGACGTGCTTCAGTTTAATATCATTTATTAATTGGTTATCGTCGTTATAATCTTTTGTGATTTTATAACCATGTTC
TTTACAATATGCTTTTACAGTATCAATAGGTTCGTCTAAGTAGACTTCTCTTTCATCTGTAATATTATGTGCTAATGCAACTACATTCATAGTT
AATACCTCCGGTTGATTTAATGAGCTACAACGCATAGAGCGTTGTAGCGGTATAATATTCAAA

Gene: cch (cassette chromosome helicase)

Position: 45869 to 47656, length: 1788 nt, orientation: REVERSE

Sequence:

TTATAGTTCTAAATCGTCACTTGCTTCTTTTTCTTTCGCTTGTTTAATAAAATTATTAAGCAAATCTGGATTTGTATTATCATCAAATTTCGGTG
GTGTGAAAGTTTCTGTATTGGAACTTAAGCCAAAAATAGATGCAAGTTCTTTATCCAATTTAAGATGATAGAATACTATCGTTTTTGATTTGC
CCGTTGCATCTTTGACACTTCGTTTTGTTGTCTTACGATCTCGGTCCGATTCCATATAACCTTTTTCTATCAAAGCTTCAACTACATTGTTGGTG
TCCTGAAATTGATTATTTTTAAGCATCTCTTTGAAAACTTTGGCAATTATCTTAACTTGTATATGGTCATCTTTAAGTTCTATGAGACCGAACC
CCTCCAGCATATTTGATAATCGACCATCATCTGAAAATTTATTTCTGTACTGTGCTACAAATTGGACAATTAGTTCGATTGCTTTATCGGACA
GAGACCTTTCGCTCACCGAATCTGAGTGGTAATTCACTAGGTATTCTCTAACTGCATTCAAATCGATAGGGGTTGCGATAACACGTTCTAAT
ATTCGTGCTGATGTCGTAATGGTGGCGTAGCGCTTGAACATACGTATGCCTACATTGTTTGTTTCATTGTTAAGTTGTGTTTTGAACCAATCG
TGTTCTCTATGAAACCAATTGATTACTTCAGATTCACGATTTAGAAGATATTCAGCTACTAACGGCATTATGTGCCCATAGTTTTTTGATGTTC
CTTTTTTAATGGCGTCTGCATTGTCAGCACTTGTTGTAAAGACATCAGAAATTTCTATACATCTTGCTCTGAGACCATCATTCGCACTAGCATC
CCTGAAAATCGTGTGTTCTGCTGTACTAATAACACTAGATCCAAAGTGGTGAGGATTTTTTACATTTCCTTCTCGATTAGCACGTAGACGACC
TTGCCCCTCTGCTAGGGAATAAAGTAAACCATTTGTATCTTTAAAGGTAGCTGCTGATAATTCATCTAAGGCAATAGGAACACCAAAGTTGT
TACTTAAGTATCCTTCAATTGCATTACGGGTGCCGTTCCAACCTCTGAATAATGTTTGATCACCTTTAGATGGATTACCTGCTATTGAAACTG
CTAATGCCGCTGCAGTCGATTTACCCGTACTAGAGTTCCCCATAAATGAAAATATCGTGCCAAAATATTCGACTTCATGCTTTGTTTTTAAGA
ACGCAGTCACTAATGAAGAAACACCAAATATCACAGCAAGCTCCAGTAACAAGTTTCCTTTTACTTCATCAAGATACATTTGCCACCAGCTTT
CAAATGTACCTTTAGGTTGTAAGTCATACTTTGTTTCACAAATGATTTCATCAGCCTGAGACTGCTCGATGTCTTTTGAAAGATATGGTTCAT
CTAATGAAATGACAGTACCTTCATCAGTACTTAAGACACCTACACCTGTATACAATGTAGAAATGGGCAGTGATTGACGCATTAATTGTAGT
GCATTACTTAGCGATTTAATGTACGTTTCATTGATGCTGAAGCTAAACATAACCAGTCTAGGTAACTTTTGTGTCGTTAGAATATCTGACGTT
TCAATACGTTTAATATTTTTGCCATCCGTGATAGTCAATTTCTCAACCCCTGTAGTAGGGTCGAGAAATTTGTTTTCTATGATGATTGGACTA
GATAAACGAATTATCTTATCTTCGTCGTTTTTGTTTTTTGCTCGAATAAGTTCATACCATGCAGTTGAATCTAACCAGTATGGATATTGCTCGA
AGATGTTGTTAGCCAT

Gene: orf7795 (putative protein)

Position: 48080 to 49639, length: 1560 nt, orientation: REVERSE

Sequence:

TTATTTTTTAAAATATTTTTGTCTCAAGTTTAGATTATCAATTACATTCATAGAATTAATTAGAGATAGATGAGTTTCACTAGAAATTTTAAAG
TCATAAATGAATAAGTTTAGGTCGTACTTATTTGATTTACTATGAGTTAGGTCATTTTTAGCATGGAGAGGTGTTATTGTATTACTCCATTTTT
TTGTCGTTTTTATAATTTGATTACTCAAATTAAGAACTTCTATAGGCGCATTTGTATGAAAGAAATTCATGTTCTCGCAAATATGAGCGTAAA
CACCTGATAAAGTACTAAGCAAAGTTTCCATAAATGGTAGCTGATCCATATAAAATAATTTATGTATAAACAAATTTGATGCATTTATAAGTT
CATTATCGATGATATGAAAAGATGAAAAATCACCTTCATCATCTTCATTTCCTAATTTGTCATCTGTGAAGACAATGTGTAAGTGATTTTCAG
TTAATTCATCTATGTCGTTATGTAGTTGTTGAAAATCAGCGTTGAATTTTAATTTTTGTTCCTTAAAATCTTGGAGTTCTGAATAATTAACTTTT
TGCTTTTTTAATATATTTAATTCTTGCTTGTTAAATAGTAAAGCAAAATAGCTATTGTTGCCAGAGGCATTTTTGTGTTTAGCATGTGTATTAC
TATCTATGTACAAATATCTAAAATAAAAAACAGACAAAATTGTTAGTACATCACTAAAAGGGAACCTTCCACGAATGTTGTCTTTTGATAATT
TACCAATTAATGAATTTAGATTTGCAGTTATCTGTTTTTCTTCATCTGTGTTGATATCAGGGAACCTATTTAATAGTGTCTGTATTATAGATGA
AATAAGTTGTTCTTTTGTGAAATATCTATGGAATATCTCATTATTTTTGTCATTTAAATCTATATTGATTTGTGTACTTATATCAGCAATCTTAT
TTAAGTTTTCCCATTCTTCATGTGAATATGATGAGGAGTAGTTATTTGAATAATTATAGTTTTTACGTTCGAGACCATTTTTAATTGTAAAAGC
AGTGTTTAGCATATTATCCAGTGATGTGATATTCGGATTATTTTCATCGTAAAATCTAAAAGGTATATCATTTTTATAACCAAAAGTAGCATT
TACAGTAGCTCTTTTAAAAGTAGTGTCGTCAAGAGCTATAGATCCATCATTATGTAATTTTAATAGTACATGTTCCTTTAGTTTGCCTTTAGAT
GTGAACAAATAAGTGAAAATTGCATTTTTATATTCTTCAAATTGATCATCTAAGTTTGAAGCATCCATGAAACTTGTTAAGACTTCATAAAAT
CCTTCGTTGAATCCACGCATAAAAGTATCTACACCATATTCAATACCTAACAAATCTATTAAATTTGATAAAATATTTTTTATATAAAGTTTAT
TATTTTGAAATTGATAACGTGTGTAATTGCCTTTATTATCGAATTCGACTTCAATTGATGATGAACCTTCAATACCTTCTAACGATATGTTTAT
TTGTTTGATTATATCCTGCATGAATTCAGAGTTATACTTTGAAATAAGTTTTAGTGTCAT

Gene: IR_IS431 (inverted repeat of IS431)

Position: 49906 to 49921, length: 16 nt

Perfect match to: (Plasmid_pHKK701-L38972.1-[631:646])

Sequence:

GGTTCTGTTGCAAAGT

Gene: tnpIS431-06 (transposase for IS431)

Position: 49966 to 50640, length: 675 nt, orientation: REVERSE

Perfect match to: (Strain_21342-AHKU01000078-[56395:57069:r])

Sequence:

TTAACTTGTTAGCATGATGCTAATTTCATGGCATGGCGAAAATCCGTAGATCTGAAGAGACCTGCGGGTCTTTTTATATAGACCGTAAATAC
ATTCAACACCTTTTAAAGTATTCTTTGCCGTATTGATACTTTGATATCTTGTCTTTCTTACTTTAATATGACGATGATCTTGCTCAATGAGGTTA
TTCAGATATTTCGATGTACAATGACAGTCGGGATTCAGTTTAAATGCTTTAATTACTTTAGCCATTGCTACCTTCGTTGAAGGTGCCTGATCT
GTAATCACCTTTTGAGGTTTACCAAATTGTTTAATGAGACGTTTGATAAACGCATATGCTGCATGATTATCTCGTTGCTTACGCAACCAAATA
TCTAATGTATGTCCCTCTGCATCAATGGCACGATATAAATAGCTCCATTTTCCTTTTATTTTGATGTATGTCTCATCAATACGCCACTTATAAT
AGGCTTTTTTATGCTTTTTCTTCCAAATCTGATACAAAATTGGGGCATATTCTTGAACCCAACGGTAGAGCGTTGAATGATGAACGTTTACAC
CACGTTCCCTTAATATTTCAGATATATCACGATAACTCAATGCATATCTTAGATAGTAGCCAACGGCTACAGTGATAACACCTTTACTGAATT
GTTTATATCTGAAATAGTTCAT

Gene: DLJ55-14705 (Unidentified ORF from SCC/ACME elements)

Position: 50925 to 57955, length: 7031 nt, orientation: TRNC-FRWD (no stop codon)

Sequence:

ATGAGAGACAAGAAGTTGACAATGGTCAACAACAAAAGTTAAATAAAGCTGATAATGATAGACAAGAAGTTGACAATGGTCAACAACAAA
AGTTAAATGAAGCTGATAATGATAGACAAGAAGTTGACAATGGTCAACAACAAAAGTTAAATAAAGCTGATAATGAGAGACAAAAAGTTG
ACAATAGTCAACAACAAAAGTTAAATAAAGCTGATAATGATAGACAAGAAGTTGACAATGGTCAACAACAAAAGTTAAATAAAGCTGATA
ATGAGAGACAAAAAGTTGACAATAGTCAACAACAAAAGTTAAATAAAGCTGATAATGAGAGACAAGAAGTTGACAATAGTCAACAACAAA
AGTTAAATAAAGCTGATAATGATAGACAAGAAGTTGACAATAGTCAACAACAAAAGTTAAATAAAGCTGATAATGAGAGACAAGAAGTTG
ACAATAGTCAACAAGAGTTGTTAAAAAATAAATGAATCAACAAATAGAGAATCAACGGTGAAGAGACTTTTAGAAGATGTTTATTCTAAAG
AGAATGCTCGAGGTATTCTTAATAAAATAAAACCAGAAGATTATAATTTAAGTTCTAAAGAATTAATAAATAAAATAATTAAAGCCGGAATA
GATTATTCTGAAGAAAATAATAGTAAGTACAAAGTCTTTGCTTCTGTTCCTCAAGCTAGTATTACTTTGAAAAGTGAGTATAATATTGGTAAT
TCTTTAGTGGTTAGAAGCGATAAAGTTATAAAACCAGAAGATTATGTTAATTATCCAGAAGGGTATCACGTTCAAAGTTATGTTTCCAAAGG
AAAAGAATATCCTAGTTTAGATGATATAGTTAAAAAGAGTCACAGGTAGTTATAAAATTACGTATCGATTATTAGATAGTAATGATGAACCT
GTTCACGGAGAAGATGGTCAAGCGATTGTAAGATCATTAAATATTTATTTCTATAATCCAGAACCAGCTTTAGATATAATTAAAGAAATCGA
AGAAGATATAGATGAAAATAATTATCAAAATGGCGAGGAGATAAAAGAAGATTTAGAAAAATAAAAATGAACTAGAAAACCAACTAAAAT
ACCAGATGTACCTGCTTTGATTGATGAAATAAATAATAAGAAAACCAGCTTGAAAATTTAGATACGACGGCACCAGACGCACCAAAAGTTA
AGGATACAGAGTCAGGCAGCAAGAAAATCACTGGTGAAGGATCAGAACCAGGCAATGACATTACCGTAACGTTCCCTAGCGGAAAAACAA
GTCAAGGTAAAGTTGGTCAAGATGGCCAATGGGAAGTCGACGTACCAGCAGGTGAAGAGTTAAAACCTGGAGACAAAGTTACAGCAAAA
GAAACAGATCCATCAGGTAATGAATCACCAAGTGGTACAGGTAACGTCATAGATACGACGGCACCAGACGCACCAAAAGTTAAGGATACA
GAGTCAGGCAGCAAGAAAATCACTGGTGAAGGATCAGAACCAGGCAATGACATTACCGTAACGTTCCCTAGCGGAAAAACAAGTCAAGGT
AAAGTTGGTCAAGATGGCCAATGGGAAGTCGACGTACCAGCAGGTGAAGAGTTAAAACCTGGAGACAAAGTTACAGCAAAAGAAACAGA
TCCATCAGGTAATGAATCACCAAGTGGTACAGGTAACGTCATAGATACGACGGCACCAGACGCACCAAAAGTTAAGGATACAGAGTCAGG
CAGCAAGAAAATCACTGGTGAAGGATCAGAACCAGGCAATGACATTACCGTAACGTTCCCTAGCGGAAAACAAGTCAAGGTAAAGTTGGT
CAAGATGGCCAATGGGAAGTCGACGTACCAGCAGGTGAAGAGTTAAAACCTGGAGACAAAGTTACAGCAAAAGAAACAGATCCATCAGG
TAATGAATCACCAAGTGGTACAGGTAACGTCATAGATACGACGGCACCAGACGCACCAAAAGTTAAGGATACAGAGTCAGGCAGCAAGAA
AATCACTGGTGAAGGATCAGAACCAGGCAATGACATTACCGTAACGTTCCCTAGCGGAAAAACAAGTCAAGGTAAAGTTGGTCAAGATGG
CCAATGGGAAGTCGACGTACCAGCAGGTGAAGAGTTAAAACCTGGAGACAAAGTTACAGCAAAAGAAACAGATCCATCAGGTAATGAATC
ACCAAGTGGTACAGGTAACGTCATAGATACGACGGCACCAGACGCACCAAAAGTTAAGGATACAGAGTCAGGCAGCAAGAAAATCACTG
GTGAAGGATCAGAACCAGGCAATGACATTACCGTAACGTTCCCTAGCGGAAAAACAAGTCAAGGTAAAGTTGGTCAAGATGGCCAATGGG
AAGTCGACGTACCAGCAGGTGAAGAGTTAAAACCTGGAGACAAAGTTACAGCAAAAGAAACAGATCCATCAGGTAATGAATCACCAAGTG
GTACAGGTAACGTCATAGATACGACGGCACCAGACGCACCAAAAGTTAAGGATACAGAGTCAGGCAGCAAGAAAATCACTGGTGAAGGA
TCAGAACCAGGCAATGACATTACCGTAACGTTCCCTAGCGGAAAAACAAGTCAAGGTAAAGTTGGTCAAGATGGCCAATGGGAAGTCGAC
GTACCAGCAGGTGAAGAGTTAAAACCTGGAGACAAAGTTACAGCAAAAGAAACAGATCCATCAGGTAATGAATCACCAAGTGGTACAGGT
AACGTCATAGATACGACGGCACCAGACGCACCAAAAGTTAAGGATACAGAGTCAGGCAGCAAGAAAATCACTGGTGAAGGATCAGAACC
AGGCAATGACATTACCGTAACGTTCCCTAGCGGAAAAACAAGTCAAGGTAAAGTTGGTCAAGATGGCCAATGGGAAGTCGACGTACCAGC
AGGTGAAGAGTTAAAACCTGGAGACAAAGTTACAGCAAAAGAAACAGATCCATCAGGTAATGAATCACCAAGTGGTACAGGTAACGTCAT
AGATACGACGGCACCAGACGCACCAAAAGTTAAGGATACAGAGTCAGGCAGCAAGAAAATCACTGGTGAAGGATCAGAACCAGGCAATG
ACATTACCGTAACGTTCCCTAGCGGAAAAACAAGTCAAGGTAAAGTTGGTCAAGATGGCCAATGGGAAGTCGACGTACCAGCAGGTGAAG
AGTTAAAACCTGGAGACAAAGTTACAGCAAAGAAACAGATCCATCAGGTAATGAATCACCAAGTGGTACAGGTAACGTCATAGATACGAC
GGCACCAGACGCACCAAAAGTTAAGGATACAGAGTCAGGCAGCAAGAAAATCACTGGTGAAGGATCAGAACCAGGCAATGACATTACCG
TAACGTTCCCTAGCGGAAAAACAAGTCAAGGTAAAGTTGGTCAAGATGGCCAATGGGAAGTCGACGTACCAGCAGGTGAAGAGTTAAAAC
CTGGAGACAAAGTTACAGCAAAAGAAACAGATCCATCAGGTAATGAATCACCAAGTGGTACAGGTAACGTCATAGATACGACGGCACCAG
ACGCACCAAAAGTTAAGGATACAGAGTCAGGCAGCAAGAAAATCACTGGTGAAGGATCAGAACCAGGCAATGACATTACCGTAACGTTCC
CTAGCGGAAAAACAAGTCAAGGTAAAGTTGGTCAAGATGGCCAATGGGAAGTCGACGTACCAGCAGGTGAAGAGTTAAAACCTGGAGAC

AAAGTTACAGCAAAAGAAACAGATCCATCAGGTAATGAATCACCAAGTGGTACAGGTAACGTCATAGATACGACGGCACCAGACGCACCA
AAAGTTAAGGATACAGAGTCAGGCAGCAAGAAAATCACTGGTGAAGGATCAGAACCAGGCAATGACATTACCGTAACGTTCCCTAGCGGA
AAAACAAGTCAAGGTAAAGTTGGTCAAGATGGCCAATGGGAAGTCGACGTACCAGCAGGTGAAGAGTTAAAACCTGGAGACAAAGTTAC
AGCAAAAGAAACAGATCCATCAGGTAATGAATCACCAAGTGGTACAGGTAACGTCATAGATACGACGGCACCAGACGCACCAAAAGTTAA
GGATACAGAGTCAGGCAGCAAGAAAATCACTGGTGAAGGATCAGAACCAGGCAATGACATTACCGTAACGTTCCCTAGCGGAAAAACAA
GTCAAGGTAAAGTTGGTCAAGATGGCCAATGGGAAGTCGACGTACCAGCAGGTGAAGAGTTAAAACCTGGAGACAAAGTTACAGCAAAA
GAAACAGATCCATCAGGTAATGAATCACCAAGTGGTACAGGTAACGTCATAGATACGACGGCACCAGACGCACCAAAAGTTAAGGATACA
GAGTCAGGCAGCAAGAAAATCACTGGTGAAGGATCAGAACCAGGCAATGACATTACCGTAACGTTCCCTAGCGGAAAACAAGTCAAGGTA
AAGTTGGTCAAGATGGCCAATGGGAAGTCGACGTACCAGCAGGTGAAGAGTTAAAACCTGGAGACAAAGTTACAGCAAAGAAACAGATC
CATCAGGTAATGAATCACCAAGTGGTACAGGTAACGTCATAGATACGACGGCACCAGACGCACCAAAAGTTAAGGATACAGAGTCAGGCA
GCAAGAAAATCACTGGTGAAGGATCAGAACCAGGCAATGACATTACCGTAACGTTCCCTAGCGGAAAAACAAGTCAAGGTAAAGTTGGTC
AAGATGGCCAATGGGAAGTCGACGTACCAGCAGGTGAAGAGTTAAAACCTGGAGACAAAGTTACAGCAAAAGAAACAGATCCATCAGGT
AATGAATCACCAAGTGGTACAGGTAACGTCATAGATACGACGGCACCAGACGCACCAAAAGTTAAGGATACAGAGTCAGGCAGCAAGAA
AATCACTGGTGAAGGATCAGAACCAGGCAATGACATTACCGTAACGTTCCCTAGCGGAAAAACAAGTCAAGGTAAAGTTGGTCAAGATGG
CCAATGGGAAGTCGACGTACCAGCAGGTGAAGAGTTAAAACCTGGAGACAAAGTTACAGCAAAAGAAACAGATCCATCAGGTAATGAATC
ACCAAGTGGTACAGGTAACGTCATAGATACGACGGCACCAGACGCACCAAAAGTTAAGGATACAGAGTCAGGCAGCAAGAAAATCACTG
GTGAAGGATCAGAACCAGGCAATGACATTACCGTAACGTTCCCTAGCGGAAAAACAAGTCAAGGTAAAGTTGGTCAAGATGGCCAATGGG
AAGTCGACGTACCAGCAGGTGAAGAGTTAAAACCTGGAGACAAAGTTACAGCAAAAGAAACAGATCCATCAGGTAATGAATCACCAAGTG
GTACAGGTAACGTCATAGATACGACGGCACCAGACGCACCAAAAGTTAAGGATACAGAGTCAGGCAGCAAGAAAATCACTGGTGAAGGA
TCAGAACCAGGCAATGACATTACCGTAACGTTCCCTAGCGGAAAAACAAGTCAAGGTAAAGTTGGTCAAGATGGCCAATGGGAAGTCGAC
GTACCAGCAGGTGAAGAGTTAAAACCTGGAGACAAAGTTACAGCAAAAGAAACAGATCCATCAGGTAATGAATCACCAAGTGGTACAGGT
AACGTCATAGATACGACGGCACCAGACGCACCAAAAGTTAAGGATACAGAGTCAGGCAGCAAGAAAATCACTGGTGAAGGATCAGAACC
AGGCAATGACATTACCGTAACGTTCCCTAGCGGAAAACAAGTCAAGGTAAAGTTGGTCAAGATGGCCAATGGGAAGTCGACGTACCAGCA
GGTGAAGAGTTAAAACCTGGAGACAAAGTTACAGCAAAAGAAACAGATCCATCAGGTAATGAATCACCAAGTGGTACAGGTAACGTCATA
GATACGACGGCACCAGACGCACCAAAAGTTAAGGATACAGAGTCAGGCAGCAAGAAAATCACTGGTGAAGGATCAGAACCAGGCAATGA
CATTACCGTAACGTTCCCTAGCGGAAAAACAAGTCAAGGTAAAGTTGGTCAAGATGGCCAATGGGAAGTCGACGTACCAGCAGGTGAAGA
GTTAAAACCTGGAGACAAAGTTACAGCAAAAGAAACAGATCCATCAGGTAATGAATCACCAAGTGGTACAGGTAACGTCATAGATACGAC
GGCACCAGACGCACCAAAAGTTAAGGATACAGAGTCAGGCAGCAAGAAAATCACTGGTGAAGGATCAGAACCAGGCAATGACATTACCG
TAACGTTCCCTAGCGGAAAACAAGTCAAGGTAAAGTTGGTCAAGATGGCCAATGGGAAGTCGACGTACCAGCAGGTGAAGAGTTAAAACC
TGGAGACAAAGTTACAGCAAAAGAAACAGATCCATCAGGTAATGAATCACCAAGTGGTACAGGTAACGTCATAGATACGACGGCACCAGA
CGCACCAAAAGTTAAGGATACAGAGTCAGGCAGCAAGAAAATCACTGGTGAAGGATCAGAACCAGGCAATGACATTACCGTAACGTTCCC
TAGCGGAAAAACAAGTCAAGGTAAAGTTGGTCAAGATGGCCAATGGGAAGTCGACGTACCAGCAGGTGAAGAGTTAAAACCTGGAGACA
AAGTTACAGCAAAGAAACAGATCCATCAGGTAATGAATCACCAAGTGGTACAGGTAACGTCATAGATACGACGGCACCAGACGCACCAAA
AGTTAAGGATACAGAGTCAGGCAGCAAGAAAATCACTGGTGAAGGATCAGAACCAGGCAATGACATTACCGTAACGTTCCCTAGCGGAAA
ACAAGTCAAGGTAAAGTTGGTCAAGATGGCCAATGGGAAGTCGACGTACCAGCAGGTGAAGAGTTAAAACCTGGAGACAAAGTTACAGC
AAAAGAAACAGATCCATCAGGTAATGAATCACCAAGTGGTACAGGTAACGTCATAGATACGACGGCACCAGACGCACCAAAAGTTAAGGA
TACAGAGTCAGGCAGCAAGAAAATCACTGGTGAAGGATCAGAACCAGGCAATGACATTACCGTAACGTTCCCTAGCGGAAAAACAAGTCA
AGGTAAAGTTGGTCAAGATG

Gene: IR_IS431 (inverted repeat of IS431)

Position: 58285 to 58300, length: 16 nt

Perfect match to: (Plasmid_pHKK701-L38972.1-[631:646])

Sequence:

GGTTCTGTTGCAAAGT

Gene: F8WKF9 (putative membrane protein)

Position: 58503 to 58855, length: 353 nt, orientation: FORWARD

Sequence:

ATGTGATAGCGAATAGACATCAATATACGATGTATTTGAATGGTGAAGAAGTAGGCACACTTGAGATGAAACAGTTCTTCAAAAGAGGGG
GAAAGCAACAAATTCCTTATATGTTTAATTACAAATTTGAAGTGTTTGATGTAAGCAATCCGTTTTTTAGTAATGAAACGAAAATCACATTTT
AGAATGACGTATTATTAGCCGCAAAGCGTAGTTTTTTAGATATTTTAAAAAGCAAACGAACTAAAAAATGAGGCGAAAAACATACTATACA
CATTTACAGCACTAGAGTAGAGAAAGAAATATTAATAACTATTTACTTACAATGCATAATCAATAAGCAAACACAATAA

Gene: EHQ67276 (putative protein)

Position: 59113 to 59538, length: 426 nt, orientation: FORWARD

Perfect match to: (C427_ST42-ACSQ01000050-[1158:1583])

Sequence:

TTGATATATTGGAATTTATATAAAATTTTTAAATATAAAATAAGTCATATATTACTCGCTTGTATCTTCACTAATGTCGGATTAACTAAAATTA
TTATGTATTCAACGCCAGTATTAATGTTTATCTATCCGTTAGCGATTACACTGATTTTATTAACGCTTGTCAGTCCATTAATTAATCATTCGAC
AATTGTACATAAATTTACAACGTTCTTTACAATGTTTGCAGCGTTCTTTGATGGTTTAAACGCATGCCCAGAATTCTTTGCAAAGACATCATTT
GCACAAGCGTTAATAATTTTCGCTGAACGTTATTTACCGTTCTTCACAATTGGAATGGGTTGGATTGTTCCGGCAATCATAGGATTTGTAGT
AGGACTTATCGTTTATTTAATTCGTTCTCGTAGACAAACTCAAACACAATAA

Gene: opp3A-A8YYZ6 (putative S-adenosyl-L-methionine-dependent methyltransferase)

Position: 59947 to 60585, length: 639 nt, orientation: FORWARD

Perfect match to: (C427_ST42-ACSQ01000050-[1992:2630])

Sequence:

ATGGCGTGTGACCATCAAGAAGCTTGGGAGAGAACTATAGAAATGCTTGATACATCGGATATCAAAGGGAAGACAATCTTAGATGTGGGA
TGTAATCAAGGCGGATTTTTAAGAAAATTATATGACACAACGCCATTTAAAGAAGGTGTCGGCATAGATTTAGCACGTTTATCTTTAGAAAA
GGCAGAAACTTTAAAAGGTGAACGACCACTTACATATTATTTAACGGATAAACCACAAGAGACAAATAGAACATTTGATACTGCTGTAAGT
ACATCTGTTTTGTACTTAATCGAAGACATTCCACAACATGCACAAGATTTAAAAGAAGTGTTGAAACCGGGAGGCGTTTACTATGCTTCATT
CGCCGATTTAACCAATAATCCTAGTCGTCAATTTATGGATGATACGATTAATCAATACGGCGCAACACCTTCTCAGAACCATTCTTTAAAACA
TATCGTTGATAGCTTTGTTGATGCAGGGTTTGAAGTTGCAGTAATGAAAGAACCTGTGCCTGTCGTGATTGATTTAACACATTATAGTGATT
TTTATCTATCACCAAATGATTATCTACAAACACTTTATGAGGAATCATTTTTAATTAAAGCAAGCGTGAAAGAAGGTATCGGGAAATGA

Gene: opp3B (oligopeptide permease, channel-forming protein)

Position: 62138 to 63094, length: 957 nt, orientation: FORWARD

Sequence:

ATGCTTAAACGAACAATAAAATTAATACTTTACTTAATCGTGAGTTCGTTTATTATATTCGTTTTAGTCGAGAAGACATCAGGTAATCCAGCG
ATACTATACTTACAACGTCATGGTTATACGTCGATTACCCAGGAAAATATTGAAGCAGCACAACATAAACTTGGTTTGGGCCAGCATTTTTT
ATTAAGATATATCGATTGGGTTGGACATGCACTTACTGGTAACTTGGGATACAGTTTTAGTACAAATGAGCCAGTAACAACTATGATTATGG
AGGCCGTCATTCCAACATTAATATTAATCGTTGTTTCAAGTTGTATCATGTTGCCATTGGGTTATATGGTTGGTTATTTTATTGGGACGCGCC
CACATACTCGATACGCGAATGGTATTCGTGGATTCGCTCAAGTGATGACTTCTATGCCGGAATATTGGTTAGCCATTTTGTTTATATATTATT
TAGGAGTACGTTGGCAGTTGCTACCTTTTGTAGGTAGCGATTCCTGGCAACATTTCGTGTTACCCATCTTCACCATTGTTGTTATAGAAGGAT
GTCATATCTTATTGATGACATCGCACCTTATTGCGCCAACGTTAGATAACGATGCGTATCAACTTGCTCAGTTAAGACATTACTCATTAAAGG
CACGTATCATTGTCCAGATTAAAGAGATATTTGCACCACTCATGACCATTTCAATTAACAGTGTGATTCATTTAATTGGAAAAGTAGTCATAC
TTGAAGTAATCTTCAGCATGTCTGGTATAGGGAAATTATTAATTAATGCTATCAATCAACGAGATTATCCACTTATTCAAGGCATTGTGGTCT
TTATCATTGTCTTAATTATGTTCATTAATTATTTAGGCGACATTATTATTTTAAACAATGAACCTAGACTTCGACGACGACATACCAAACGCCA
AGCCCATGAGAAAAGAGGTGTGTCGTGA

Gene: opp3C (oligopeptide permease, channel-forming protein)

Position: 63094 to 63861, length: 768 nt, orientation: FORWARD

Sequence:

ATGAAAAAATATCAAACTTACATCGCCATAAGTGCAATATTAAGTGTGATGTTTGTGCTAATTGTATATTGCTTTATGCAAGACACACAAAA
CTTGGACCCACTTCAATCACCTAATAGCCAACATTGGTTAGGCACGGATCAATTAGGCAGAGATTTCTTAGTCAGACTGATTGTAGGTAGCC
TAGTCACATTAAGCTTAACTAGCGTGGTAATTCTGTTAAGTGTTTGTATTGGACTGGTCTTTGGGTTAATTGCAGGTATAGAAAGAAAATGG
TTAGACCAAATCATCATGTTTATTGCTGATATGCTATTGGCAATTCCTTCGTTTATTATTGCATTAGTCATCTTAAGCTTAGTAAGTAATTCAA
TGCTAGGTTTGATACTTGCTTTAACGATTGGATGGATAGGCCGTTATTTACGTTATTTCAGAAATTTAACACGAGATATTCAAAAACGTCCCT

TTGTCAGTTATGCAAGACTAAGTGGTAACTCAACGTTTAAAACGACAGTGACGCACATGATTCCGCATTTACTAAGTAATATATTTGCTTTG
GTCACGGCTGACTTTGGCAAAATGATGCTAAGTATATCTGGACTTGCTTTTTTAGGCCTAGGTATTAAACCACCGACGCCCGAGTTAGGAAC
CATTCTCTTTGACGGGAAAAGTTATTTCAATGGAGCACCTTGGCTCTTCTTCTTCCCTGGTGTATTGTTAGGAGGTTTCGCCTTATTATGTCAA
ATGATCAACAAAAAAATAACGCAATGA

Gene: opp3D-A8YZ00 (nickel/peptide ABC superfamily ATP binding cassette transporter, ABC protein)

Position: 63828 to 64595, length: 768 nt, orientation: FORWARD

Sequence:

ATGTCAAATGATCAACAAAAAAATAACGCAATGAATACTGTAGTTAAAGTCAATCAATTATCCATCTTAGATCATAATCAGTCATTGTTAAAC
GATGTGAATTTGACAATAACTAAAGGTGCATTTCATTGCATTATAGGTGAGAGTGGTAGTGGGAAGTCATTATTGACGAGAACTATTCTTG
GAATGAAGCCTTCACAACTAAGCTATCAAGGAGATATCAAGATTGATTTAAATAAGGTGGATGCCGTTTTTCAAGATGTTCAAAGTAATAT
GTTTCAAAACGTGAACTTGGCTAAACATTTCCAATATATTTATGAAGCTAACCATTCACAACTATCTAAACAAGATATCAAGGAAGACGTCC
TAGATAAAATGCAATTACTCGGTTTAAATCAAGGTGAACAATTACTTAAACGATATCCCTTTGAACTTAGTGGAGGTATGGCACAACGTATA
GCCTTTATAATGTCATTAATTAGACGCCCGGACTACTTATTTTTAGATGAACCGACGAGTGCGCTAGATCAAGAGAATGTTAAAAAGTTTAT
GCACTATTTGATTAAGGCACAGGAACATTATCAAATGACAATTGTCTTTATTACACATGATATTAACTTAGTTAAGGATTATGCCACTCATAT
TAGTATTATGCAGCAAGGTCAATTGATTGAGAGTGGTGAGGCATCATCCATTTTAGCTAACCCCATACATAGCTATACGAAAAATTTAATTG
CTATCGCACATCGGAGACAGGCCTATGCTTAA

Gene: opp3E-A8YZ01 (nickel/peptide ABC superfamily ATP binding cassette transporter, ABC protein)

Position: 64588 to 65222, length: 635 nt, orientation: TRNC-FRWD (no stop codon)

Sequence:

ATGCTTAAAATTGAAAGATTAACTAAATACATTGATGGGAAACTTATCTTCAAAGATATATCATGTGCAATTAATGACCAACATTTACTTATA
AGTGGTGAAAGTGGCTGCGGTAAGTCTACGTTAGCCAAGATTATCGCCGGATTAGATATGAATTATCAAGGTAACTTATACTTTAATGGCC
AATTACGTAAGTCATATACGGCAAAAGAATGGATGAAACACATCCAATATGTGCCCCAGTATCAACGTGACACATTAAATAAGCGTAAAAC
CGTATTATCTACGTTATTAGAACCACTTAAGAATTACAAATTTGATAAGCAGAGTTACACATCAAGAATAGAAGCGGTACTTAGACAATGCA
AGTTACCACAAAGCATACTTAATCAAAGCATTTCGACGCTAAGTGGTGGACAATTTCAACGTGTATGGATAGCAAAGGCACTAATTCTAGA
GCCGGAGATATTGATATTAGATGAAGCAACGACAAACTTAGATGTGATTAACGAGGAAGAAATACTTCAAATGTTAATTACGTTAAAGCAA
ACGCAATTAATCATTATTTCACATGATGCGTACGTCTTGAGTCGCTTTAAAGGCGTTCAGTTACAACTCAAACAATTGAATAATTA

Gene: tnp_A8YYY6 (transposase)

Position: 65309 to 65975, length: 667 nt, orientation: TRNC-RVRS (no start codon)

Sequence:

CTAGGCTATTAATTCTCTGTATTTTACAGGGGATAAGTAGCCTAGTTTTTGTTGAATTCGATTATTATTATAGTTTTTAATATTCTTTTCGACA
ATATCTATTACAAATATGATTAGAGCGATTAAGCTCATTATTGATGTAAAAAGTTTCAGACTTTAGGGAGGAATGGAAACTTTCTATCGGGG
CGTTATCGGCAGGTGTTCCCTTTCGGGACATACTTCTGATAATGCCTTTTTCTTCGCACAATTGATAATAAGCATAAGATGTATAAACGTTGC
CTTGATCACTATGTAATATACAGCCCTCAGGTATATCGATTTGATTTAATGTATCATTAACTAAACTTTGGTCTTGTTTATCATCTATTTTATAC
GCCACAATTTCTCCGTTATAAATATCCATTATCGAAGATAAATACAACATAGAATGACCAAATGGTAAATAAGTAATATCGGTTGTTAATAC
TTCCATTGGACAACTCGCTTTAAATTGTCTTTGTAATAAATTGTCCGTTTTATAATACGTTTTACCTATTCTTGTCGTCTTTTTAGGTCTAACTC
GGCAGTTCAAATGATGCTTCTGCATCATTCTCTGTACTTTCTTATGATTAATTGGTGATGTATAACATTGATTAATCAATGCTGTAATCTTACG
ATAACCGTAGGT

Gene: DR_SCC (direct repeat of SCC)

Position: 67365 to 67383, length: 19 nt

Perfect match to: (JS395-CP012756-[1418304:1418322])

Sequence:

AGAAGCATATCATAAGTGA

Gene: Q6GKL1 (HTH transcriptional regulator)

Position: 81852 to 82427, length: 576 nt, orientation: FORWARD

Sequence:

ATGGAAAAAAATCAACGAAAAAAACGCAGTGATGCCGAATATAACCAACAGATTATCTTAACTACAATGGAAGATTTACTAGAACAGGGT
GAAGATATAAGTGCTAAGAAAATGTCAGATATTGCTAAAATATCAGGTGTTGGAGTTGGTACATTATATCGTCATTTTGAAAGTAAAACATT
GTTATGTCAGGCTATTATGGATAAAAAAGTCGATCAAATGTTTATAGAAATAGAAGATGTTTTAGCGGAAAATACACAGTGGCCCGTGCGA
GATAAAATCAATATTATATTAACAAAGTATCTAGATTTAAAAGAAGCAAATTTCACAACATTAAATTTTATAGAAAAATCCAGTTCACACTCT
AGTTCTGTAATTAATATTCCATTCTTTGAGCGACTAAAAAACTTATTAATTCAACAATTCGAAAATGTTAATTCCATAGCAGACTTGGATTTTA
AACTTAATCTAATGCTCAATGCATTTTCTTCAGATTTCTATTATTTTGTAAAACATAACCAAAAATTAACAAAACAACAATTTTTAGACAAATT
ACTAGATTTATTTCTAAAATAA

Gene: ORF CM14 (enterotoxin-like protein)

Position: 83580 to 84359, length: 780 nt, orientation: FORWARD

Sequence:

ATGAGAAAAAGTACTTATACTAATCACTTTGTTATTTGGTTACAGTTGCTATTTGTTGTTGGAAGCTAAAGCAGAGACACAAAATGATCCAAA
TATAAGTGAATTAAACAAAGCTAGTCAATATACGGGTTCATGGCATAATATATGGTATTTATATAATAGCGATCCAGTCAATGCTAAAAAAA
TTAAGCTGAGTGACAAATTTTTGAGTCATGATTTCATTGTTCCAATAAATAGCCCTGGTCATTACGACTATGTTAAAACTGAGTTAAAAGACA
GTACGATGGCGAGTTCATTTGATGGGAAAGAAGTCGATATTTTTGGCGTCAATTATTTTCATCAATGCTATTTTTCAAATGAAAATATACAG
TGTGATAGTAATCAAGGAGCTGGAAGTAAAAAAACTTGTATGTATGGTGGTATAACATTAAATGAGAACAATACCAATAATAGAATTCAGC
CAATTGTTGTAAAAGTTTACGAGAATGACAGCGTTACGCTTTCTTTTGATATCAATATTGATAAAGAGACTGTAACTATTCAAGAGTTAGATT
ATAAAGTGAGAAATAAATTAATTTCTAAAATCAATTTATACCATTTAGGTGGCACTTCATATGAAACGGGATACATTAAATTTATAGAAAAT
GCTAATCGTTATTATTGGTATGATATGATGCCAGACCCTAGCTTTACTCAGTCTAAATATTTGATGATATATCGAGGTAATGAAACAGTTGA
ATCAGCCAAAACGGAAATAGAAGTGCACTTAACTAAAAAGTAG

Gene: dusC (tRNA-dihydrouridine synthase C)

Position: 84426 to 85412, length: 987 nt, orientation: REVERSE

Sequence:

CTATAATTCTATTTTAACGTCTTCGTCCATTTGGGCTTCAAATTCATCTAGTAGTGCTCGTGCTTCTGCAATTGATTGTGTGTTCATCAATTGA
TGGCGAAGTTCGCTAGCGCCTCTTATGCCACGCACATAGATTTTAAAGAATCTACGCAAGCTCTTGAATTGTCGTATTTCATCTTTTTCATATT
TGTTAAACAATGATAAATGCAATCTCAACAGATCTAATAGTTCTTTGCTTGTATGTTCGCGTGGTTCTTTTTCAAAAGCGAATGGATTATGGA
AAATGCCTCTACCAATCATGACGCCATCAATGCCATATTTTTCTGCAAGCTCAAGTCCTGTTTTTCTATCGGGAATATCACCGTTAATTGTTAA
CAATGTGTTTGGTGCAATTTCGTCACGTAAATTTTTAATAGCTTCGATTAATTCCCAATGCGCATCTACTTTACTCATTTCTTTACGTGTACGA
AGATGAATAGATAAATTTGCAATGTCTTGTTCGAAGACATGCTTCAACCAATCTTTCCATTCATCGATTTCATAGTAGCCAAGGCGTGTTTTA
ACACTTACCGGAAGTCCACCTGCTTTAGTTGCTTGAATAATTTCGGCAGCGACGTCAGGTCTTAAGATTAAGCCGGAACCCTTACCTTTTTTA
GCAACATTTGCTACAGGACATCCCATATTTAAGTCAATGCCTTTAAAGCCCATTTTAGCTAGTTGAATACTCGTTTCACGGAACTGTTCTGGC
TTATCTCCCCATATGTGAGCGACCATCGGCTGTTCATCTTCGCTAAAAGTTAAGCGTCCGCGTACACTATGTATGCCTTCAGGGTGACAAAA
GCTCTCGGTATTTGTAAATTCAGTGAAAAACACATCCGGTCTAGCTGCTTCACTTACAACGTGTCGAAAGACGATATCTGTAACGTCTTCCAT
TGGCGCCAAAATAAAAAATGGACGTGGTAATTCACTCCAAAAATTTTCCTTCAT

Gene: A6TXM6 (putative protein)

Position: 85721 to 85924, length: 204 nt, orientation: FORWARD

Sequence:

ATGGAAGATTTAAAACACTCTTTAAAAGATTTAGGTTGGTATGACTTATTTTTTACAGTACCTATGTTTCTACTATTCGTGTATCTGCCGAATT
ATAATTTTATAACTATATTTATTAACATTGTTATCATTATTTTCTTTTCCATAGGTTTGATTTTAACTACTCATATAATTATAGATAACATTAAG
AACAACTCTAAATGA

Gene: A6QD71 (putative protein)

Position: 85974 to 86270, length: 297 nt, orientation: TRNC-FRWD (no stop codon)

Sequence:

ATGGCGACTGAGAAAGATGTAAATGATTTATTTTTAAATCATGTGAATTCAAATGCTGTTAAAACTAGAAAGATGATGGGAGAATATATTG
TTTATTATGATGGTGTGGTTATAGGTGGTTTGTATGATAATAGATTATTGGTCAAGGCGACTAAAAGTGCACATCATCAATTTCAAGATAAT
ACATTAGTATCGCCATATCCTGGTGCCAAGGAAATGGTTTTAATTCCACACTTTGCCGAAGTAACAAATCTAAATGATTTATTTGAGCTCATA
AAAAATGATTTGAAAAACACA

Gene: Q6GKK6 (putative protein)

Position: 86470 to 87080, length: 611 nt, orientation: FORWARD

Sequence:

GTGAACGATATGTTAATTAGTCTTGTAATCCCAGTTTTTGCTTTGTTAGTTATTGGTGGTATTATTTGGATGATTATAGAAGGTATAGTACAT
ATTTCAAAAAAGAATAAAGCAATTGATAACTTTTTTAACAAAGTTAATAAAGTAAGTGAGACATATAAATTCGCTACTACTTTTTTATTTCTA
ATCTTGGCTACGGCTGGTATTTCTCAATTTTATCTATATTATATAGTATCAGAGTTTCTTTTTTGCTGGTTATATTTACCTTTGGTATTGCAGGT
ATCATTTTTTTAATGCCATATGGCTTATGTTTCCTACCACTTTATAAACAAAAAAAGAAAAAACAGACATTTAAAAAATACATGGCTTACACT
ACGATTGGTTTGTCAATTTATCTAGGCTTATCTCTAGTTTTGGTTCACACTACGAAAATTTATATGGACGAAGGTGGCGTAAGATACTATTAC
GGTAGTTTTGTAATGAAACAAGCGGGCGGTTATGCTTATTTAGCTTTAGCAGTACTTTCAACGTTGTTAATTGTTGCGAAAAAAGCTACAAA
TAAAAATAAAAAAAATCGAAACCGTCGACAATACAAATATAACGGAAAGATAA

Gene: Q7A890 (putative NTPase)

Position: 87348 to 90500, length: 3153 nt, orientation: FORWARD

Sequence:

ATGAGTCAATTGCTAAATGATACGTTATCGGCTTGGTTGTTAATTGAATCTTTAAGTCCAGGAGAAGTAAATTTTACAGCGGAAGATATACT
CTCAGCTGAAAATTTTAAAAATGGTGCAAAGCAAGCGCAACTTCAAAGTTTTGATGAATATTTTGAAATATGGAATGATGAACGCTTTATTA
TATCAGAAGAAAAATCAGAGATTGGCGAAATCATTTTTAAATTTTACAGACACTGTTTCCGCTATAATGAAATTAATTTGAAAATTCAGGAT
ATTTTTGATGATTATTCAGATATTCATAATCCAAATGGGACACACTGTTATGGTTACACATTTAATATAGATAAACACGGTCAAGTGATAGTT
GATTCTATACATATTCCGATGATTATGAGTGCATTGAAAGAAATTGAAAAGAATAAAAATGCTAATATAGAAGAAAAATTTAATGATTCTGT
TGAAAAATTTGTTCAAAAAGTAAAAGAAATTTTAGTGGATGAACCAATTAATGAATTTAAATTGAAGAAGATGGACATAGCTTATGATGAG
TATTTTTCTGTATTAAATTCAAAGAAAGATGGATTATTTGCACATTATGTAGCAATAGAATATGTGAAAGATAGTGATTTACCACAGCCAGA
ATTTAATAGCTTCTTCATAAGTGATATTGAGAAAGCAAGAAAATCTCCCAATCAAACTTTAATTGATTACATTGAGGGTGTAGAAGAAAGTA
AGCGCATAGAAGTGGATGAAAATAAAGAAATGTTCGACAAATTTTTACATCCTTCACGTTTGCCTGATGGACGCTGGCCATCACAGACTGA
GTTTAGATTGTCATTAATGCAACAACTTGCTGTAAACCAAATTACGAGTGGTAATGAAAGGATAAGTTCAGTTAATGGGCCACCAGGGACA
GGTAAGACTACTTTATTAAAAGATATATTTGCGCATCTAGTAGTTGAAAGAGGTAAAGAGTTAGCCAAACTAAATAATCCTAAAGATACATT
TGTCAAAACAAAAACTCATGAAACAGATGATAAATATGTCTATTTACTTAAGGAATCTATTGCTAAATATAAGATGGTAGTCGCATCTAGTA
ATAATGGAGCTGTTGAAAATATATCTAAAGATTTACCGAAAATTGAAGAAATTATAAGAAATCCCGAAAAATGTAAATTCCCTAAATATGAA
CAGAATTATGCAAATTTAGCACATGAATTAAAAGATTTTGCTGAAATAGCTGAAGATTTGATTGGTGAAAGTGCCTGGGGCTTATTTTCTGG
AGTATTTGGTAAAAGTACTAATATTAACCAAGTATTGAGTCATATGTTAAAACAAGATGCGAATGATATTGGCTTTGCTAAATTACTACAAA
ATGAGAATAATCGTATGAGTTATAACGAGTTAATGAGTGAATGGCAATCACATCAACGTGCATTTTTAGAAGAGTTGAGGCATGTTGAAAT
GTTAAAAGAAGAATCTATTAGAGCCTATGATGTTTATAAAAATTGTGAGTCTTACTCTAAGGTTGAACATGAACTAAATAGCAAAAAAATGA
ATGTTAAGGAAAAGTTGAATCATTTAGAAATTCAAATATCTTGTGACAATAAAGAAATTGAAGATTTGGATGATCGAATTAATTATAATACA
AAGCAACTCGAAACTTTAAATGAGTTAATTAAATCCATCAGAGATAGTAACAAAGGGTTTGTTAACAAGCTGAAGGCGATATTTAATTCAG
AAGAAGATGAAAGATATAAAAAACATAATGCAGAGAAGCAACAATTATTAGGACAACAGATAGAGTTAGAGAAATGTAAAAAAATTAAAA
ATGAAGACCTTGTTAGCAAACTAAAAGAAAAAGAGAAATTAATTAAACAATTAACTAAAGTACAGTTGCAATTAGACGAGTTAAATTCACA
GTTACAAGAGTTAGAAGCATATCGTATTGAGTCAAAAATTACAATTCCAGAAAAAGATTTTTGGAGTGACAACAATTATGATGAGCGCCAA
GTTACTAATCTGTGGACGAGTGACGAACTTCAATACAGACGTGCCATGCTCTTTTTAAGAGCAATGATATTGCATAAATTATTATTGATTGCT
AATAATACAACTATTTATTATGCGATTAATGATTTTAAAGATAGAAGGAAATTAATTGATGCAAATCCAGATAAAGTACACAACGCATGGAA
TGTGATGCATTTAATATTTCCAGTAGTTAGTACGACGTTTGCAAGCTTTAAATCTATGTATGGGGGCATACCAAAAGATTTCATAGACTACTT
ATTTATTGATGAAGCAGGACAAGCAATACCTCAAGCAGCTGTGGGAGCATTATATCGTTCAAAAAAAGTTGTAGCTGTAGGTGATCCGATT
CAAATAGAACCGGTTGTGACTTTAGAAAGTCATTTAATTGATAACATTCGTAAAAATTATCATGTTCCGGAATATCTAGTTTCTAAAGAAGCT

TCTGTGCAGTCTGTTGCAGACAACGCCAATCAATATGGTTTTTGGAAATCTGATGCTACTGATAGTAATCAAAAAACCTGGATAGGCATACC
TTTATGGGTGCACAGACGATGTTTAAAACCTATGTTCACGATAGCTAACCAAATCGCTTATAATAATAAAATGGTGTTGCCAAGTAATATTA
CAAAAGTAGGTAAAACAGGTTGGTATGACGTTAAAGGAAACGCAGTTCAAAAACAATTTGTGAAAGAGCATGGTGAAAAAGTAGTGGGA
TTATTAGCTGATGATTGGATTGAAGCAATTAAGGAAGGTAAAAATGAACCGAGCTCATTTGTAATATCGCCTTTTTCAGCAGTACAGCAACA
GATTAAACGTATGTTAAAGCAACAACTACCGACTAGAATTGATATTGAACGTACAAAAATTAATCAATGGGTCGATAAATCCATTGGTACTG
TTCATACTTTTCAAGGTAAAGAGGCTCAGAAGGTGTATTTTGTAATAGGTACTGATAATACCCAAGATGGTGCTGTGAACTGGTCATGCGA
AAAACCAAACTTGTTAAACGTTGCAGTGACAAGAGCTAAGAAAGAGTTTTATGTAATTGGCGACATTCAAAGAATACAGATGAAACCATTT
TATGAGACGATTTTTAAAGAAAGAAATGTAAAATAA

Gene: Q2YUT2 (putative protein)

Position: 90557 to 91039, length: 483 nt, orientation: FORWARD

Sequence:

ATGGAGAAATTTAACAACTGGATATTAAATGCAATAAGTGGATCTCAAACAGACAAGAATGAAACAACTGAAGAATTAAAAGGGGCAAAA
TTTATCATTTTATATGCATATTCAATGCTCGTTTTGCTTGCGTTAGTAATTTCTAACATATTCATTCACATTTTGGAGCCTAAACTATCAATCAC
CACTCAAATCATCATCGTTTTGATTTTAATTGAAGCACTAATTGGACTGCGTTTCTTGAAAGCGTACGATGTTAAGCGTGGCAAAGATAAAG
AAAATAAGAAAAATAGTAAGGATTTCGTTAAACTAAAATCAATTTTAGTAGCAATTTTATTTACATCATTGGCGCTGACAGCAGGTACTGTA
GCTGATATATACGGTTTCACTGACTTAGGAAATACTAGAAGTGATTTAATCGTTTGGAGCATAGGTGGTATTATATTTGGCCTCGTATTTTAC
ACAATGGAAGATAAAAGATAA

Gene: plc (phosphatidylinositol-specific phospholipase C)

Position: 91244 to 92230, length: 987 nt, orientation: FORWARD

Sequence:

ATGAAAAAGTGTATTAAGACTTTGTTTTTAAGTATCATTTTAGTAGTGATGAGTGGTTGGTATCATTCAGCACATGCGTCAGATTCGTTGAG
TAAAAGTCCAGAAAATTGGATGAGTAAACTTGATGATGGAAAACATTTAACTGAGATTAATATACCGGGTTCACATGATAGTGGCTCATTC
ACTTTAAAGGATCCAGTAAAATCAGTTTGGGCAAAGACTCAAGATAAAGATTACCTTACCCAAATGAAGTCGGGAGTCAGGTTTTTTGATA
TTAGAGGTAGAGCAAGTGCTGATAATATGATTTCAGTTCATCACGGCATGGTTTATTTGCATCATGAATTAGGAAAATTTCTCGATGATGCT
AAATATTACTTGAGTGCTTATCCAAACGAAACAATTGTGATGTCTATGAAAAAGGACTACGATAGCGATTCTAAAGTTACGAAGACATTTGA
AGAAATTTTTAGAGAATATTATTATAATAACCCGCAATATCAGAATCTTTTTTACACAGGAAGTAATGCGAATCCTACTTTAAAAGAAACGA
AAGGTAAAATTGTCCTATTCAATAGAATGGGGGGTACGTACATAAAAAGTGGTTATGGTGCTGACACGTCAGGTATTCAATGGGCAGACA
ATGCGACATTTGAAACGAAAATTAATAATGGTAGCTTAAATTTAAAAGTACAAGATGAGTATAAAGATTACTATGATAAAAAAGTTGAAGC
TGTTAAAAATTTATTGGCTAAAGCTAAAACGGATAGTAACAAAGACAATGTATATTTGAATTTCTTGAGTGTAGCGTCTGGAGGCAGCGCA
TTTAATAGTACTTATAACTATGCATCACATATAAATCCTGAAATTGCAAAAACGATTAAAGCAAATGGGAAAGCTAGAACGGGTTGGCTGA
TTGTTGACTATGCAGGATATACGTGGCCTGGATATGATGATATCGTAAGTGAAATTATAGATAGTAATAAATAA

Gene: lpl-locus0080-SAOUHSC_00052 (tandem lipoprotein-like protein, first locus)

Position: 92451 to 93221, length: 771 nt, orientation: FORWARD

Perfect match to: (COL-CP000046-[89394:90164], highly conserved allele)

Sequence:

ATGATGAAACGATTAAACAAATTAGTGTTAGGCATTATTTTTCTGTTTTTAGTCATTAGTATCACTGCTGGTTGTGGCATAGGTAAAGAAGC
GGAAGTTAAGAAAAGCTTTGAAAAAACATTGAGTATGTACCCTATTAAAAATCTAGAGGATTTATACGATAAGGAAGGCTATCGTGATGAT
CAGTTTGATAAAAATGATAAAGGTACATGGATTATAAATTCTGAAATGGTTATTCAACCTAATAATGAAGATATGGTAGCTAAAGGCATGG
TTCTATATATGAATAGAAATACCAAAACAACAAATGGTTACTACTATGTCGATGTGACTAAGGACGAGGATGAAGGAAAACCGCACGACAA
TGAAAAAAGATATCCGGTTAAAATGGTCGATAATAAAATCATTCCAACAAAAGAAATTAAAGATGAAAAAATAAAAAAAGAAATCGAAAA
CTTTAAGTTCTTTGTTCAATATGGCGACTTTAAAAATTTGAAAAATTATAAAGACGGAGATATTTCATATAATCCAGAGGTGCCGAGTTATTC
GGCTAAATATCAATTAACTAATGATGATTATAATGTAAAACAATTACGCAAAAGATATGATATACCGACGAGTAAAGCTCCAAAGTTATTGT
TAAAAGGTTCAGGGAATTTAAAAGGCTCATCAGTTGGATATAAAGATATTGAATTTACGTTTGTAGAGAAAAAAGAAGAAAATATATACTT
TAGTGATAGCTTAGATTATAAAAAAAGCGGAGATGTATAA

Gene: lpl-locus0080-SAOUHSC_00053 (tandem lipoprotein-like protein, first locus)

Position: 93273 to 94043, length: 771 nt, orientation: FORWARD

Perfect match to: (NN50-BAEA01000031-[49388:50158:r], allele observed in CC4803+CC8)

Sequence:

ATGATAAAACGTGTAAATAAATTAGTGCTTGGTATTAGTCTTCTGTTTTTAGTCATTAGTATCACTGCTGGTTGTGGCATGGGTAAAGAAGC
GGAAATAAAGAAAAGTTTTGAAAAAACATTGAGTATGTATCCGATTAAAAATCTAGAGGATTTATACGATAAAGAAGGATATCGTGATGAT
CAATTTGATAAAAATGATAAGGGAACATGGATTGTAAATTCTCAAATGGCAATTCAAAATAAAGGAGAAGCTCTAAAAATAAAAGGCATGC
TTTTGAAGATAGATAGAAATACAAGAAGTGCAAAAGGATTTTACTATACTAATGAAATAAAGACGGAGAAATACGAAGTAGCTCAGGATA
ATCAAAAAAAAATATCCAGTTAAAATGATTAATAATAAATTCATTTCTACTGAGGAAGTTAAAGAAGAAAACATAAAAAAAGAAATCGAAAA
CTTTAAGTTTTTTGCGCAATATAGCAATTTTAAAGATTTAATGAATTATAAAGATGGAGATATATCATATAATCCAGAGGTGCCGAGTTATTC
AGCTCAATATCAATTAACTAATGATGATTATAATGTAAAACAATTACGTAAAAGATATGACATACCAACAAATAAAGCGCCGAAGCTGTTGT
TGAAAGGTACAGGGAATTTAAAAGGTTCATCAGTTGGATATAAAAAAATTGAATTTACTTTTTTAGAGAATAAAAATGAAAATATTTACTTT
ACTGATAGTCTACATCTTAAACCGAGCGAGGATAAATAA

Gene: lpl-locus0080-MW0073 (tandem lipoprotein-like protein, first locus)

Position: 94394 to 95086, length: 693 nt, orientation: FORWARD

Perfect match to: (MW2-BA000033-[84355:85047], allele observed in CC1)

Sequence:

ATGGGTAAAGAAGCGGAAATTAAAAAAAGTTTTGAAAAAACGTTGAGTATGTATCCGATTAAAAATCTAGAAGATTTATACGATAAAGAA
GGATATCGTGATGATCAATTTGATAAAAAAGATAAAGGCACATGGATTATTAATTCTGAAATGGCGACTCAAAATAAGGGAGAAGCTTTGA
AAATTAAAGGTATGGTCTTATACATGAATAGAAATACAAAAACAACAAAAGGATATTATTATGTAAATGCAATAAAGAATGATAAAGACGG
AAGACCCCAAGAGAATGAAAAAAGGTACCCAGTTAAAATGATTGATAATAAAGTTATTCCAACAAAAGAAATTAAAGATGAAAACATAAA
AACAGAAATCAAAAACTTTAAGTTCTTTGTTCAATATGGCAACTTTAAAGATTTGAAAAATTATAAAGACGGAGATATTTCATATAATCCAG
AAGCCCCGATTTATTCAGCGAAATATCAACTAACTAATGATGATTATAATGTAAAACAATTACGTGAAAGATATGATATACCGACGAATAAA
GCGCCAAAGTTATTGTTAAAAGGGTCAGGGAACTTAAAAGGTTCATCAGTTGGATATAAAAATATAGAATTTACTTTTGTAGAGGAAAAAG
GTAAAAATATATACTTTAGTGATAGCTTAGATTATAAAAAAAGTGGAGAAGTATAA

Gene: lipC3-MW0074 (putative lipase class 3)

Position: 95089 to 96465, length: 1377 nt, orientation: FORWARD

Perfect match to: (MW2-BA000033-[85050:86426], allele observed in CC1+CC1290)

Sequence:

ATGGCTAAAGCAGAATATGAAATTGATCCTGGAAAAATAACAAGTAATTCGGAAGAAACTAGCGCAATATCTAATATTAGTTATGAAATAG
AAAATGCAAATGATAATAATTTAGATAATGAAAAAATTAGAGGGCAAATTACTAAATTAAAAAAGGGTGGGAATTTTCCTAAAAACTTAGA
CTACATAGATAGTTATACAGACCCCAGTACGGGGACCACGGCAACGGCATTTTTAAATAAAGATACAGGTAAAGTTACTGTTGGAATGGCT
GGTACAAACTTTCACGGCGATCAACTTAAAAGAGTAGCACTTAGTTCGATGTCTCCGTTATTATTCCCACCCTCTAAGCAAGATATGAAAGA
TGTACGAGGCACTATGAAGGATGGTGTAGCGGATTTAGCCATTGGAGTAGGAATGGTCAATTATAAAGGAAAACATTTTGCAAATACACA
GCAGTTTATTGAGAATTTACAAAAAAAGTACGAAATTGATACCGTTACTGGTCATTCTTTAGGAGGGAGAGATGCTATCTTTCTTGGGCTTC
GTTATAACATCAAAAATGTTGTGGCATACAATCCTGCTCCGTTAGAAGTAAAAAGCATTCGTAATAAATTTGGTGGTCAATTGTTTAGAAAT
ACGACATTTCCAGATGAAAAATATTTAAAAGAGTTAATGGATAATTATGATGGAGATATTACTAAGGTTATAACTCAAAAGGATGGATTAG
ACTATTTAGTGAAGCGTACAGATCATTTAACTTGTGGTGATGTGTTACGTATAAACAATGGTCAGGGCCATGCGATGGAGAATTTTTTAGG
AGAAAAAGAACAACGTGAAATAATCGAAGAACTAATGTTAGTAAAAGGATATCGCGATGCAAATGACAAAGCATTTAAGGCTTTGAAAAA
AAATACTGAGAAAAAACTCGGTAAAATAGATGAAATCAAATCGAAGTTACTGCAAACGAACGGTGGCGCCCTCTCGTCTTCTCAACAAAAA
TTGCTTGAAACTTTAGTGGCATTTTCGGTAGCTGAAGGACTAAGTAAAATGGTTGACCAAGAACTTCAGCAGTTGAAAAATATGTTCAATCT
TATGGATGAGAAATTTGAAGCAAATTGGAAAGACGCACAAGAAGCTAGTGATATTGTAGGTAAACATCTTTCATACCCAGAAAAGGTAAG
TGCATTAGATAACGGTGGTGTCAATGAAAGTAAACTTGCTACCGAACCACATAATGAAATAAAAGACAAACTAAATAAAATTACGGACTTA
TCTAAGAAATATAATTCGTATTTACAGCAAATCGAAAAAAGTATTAATGAAATAGTTGCTAAGGATCAACAATTGGCAGGGCAAATAGGTG
ATTTAATATGA

Gene: Q8NYT6 (HTH-type transcriptional regulator)

Position: 97358 to 99595, length: 2238 nt, orientation: FORWARD

Sequence:

ATGCAACGTGATTATTTAATTCGAGTAGAAACTGAAAGCATGCCAGATTTCAAAAGGCTCAATGGTTTAATGATTGGTTTTGTTATTAAAGG
TGAGGCACATATTTATGATGAAAATAACATGACGCAATGCAACAGTGGCGACATTTTCATCATTAACCACCGTGACTTGTATCGATTTCAAC
TTCAACAAGATGGCATCATATGTTATATCCAATTCCAAATGAAATATTTGGCAGACAAGTTTGATGATGCGCATTGTCTATATTTTCACTTAA
CAGATGCGACCACAACCAAGAATATACATCAACTGAGAAATATAATGGCAAGACTGGTTTCAACACACATTCGACATAATGAGTTGTCTAA
ATTGACTGAGCAACAACTTGTGATTCAGTTGCTTATGCATATGATTCATTATGTCCCGCGTACATATCATTCGAACCAAAGTATCTTAAATGA
TGATAAAGTGAATCAAGTATGCGACTATATCGAGTTACATTTTCATGAAGATTTAAGCCTTTCAGAATTAAGCGAATACGTTGGGTGGTCAG
AGAGCCATCTGTCTAAAAAGTTTACAGAATCGCTAGGTGTAGGATTCCAACATTTCTTAAATACGACGCGAATTGAGCATGCGAAACTCGA
TTTAACATACACAGATGAAACGATTACTGATATTGCATTGCAAAATGGCTTTTCAAGTGCAGCGAGCTTTGCGAGAACATTTAAACACTTTA
CGCATCAAACGCCTAAACAATATCGAGGTGATCGTCCAGCAATCACTGAAAATAAACAATCGGCACAACATAATTATCACGACCGTGAATT
GATATTACTTTTAAATGACTACATTGAAGAAATGAATCATTTCATTGAAGATATTGAAAAGATGAACTATAAAGAGATTGCCTTTAAACCAA
CTAATCAACAACTAAATCAATTTAATCATATTATTCAAGTGGGCTATTTGAGGAATTTGCTCAATACACAGTATCAATCACAGTTGCTTACAT
GTCATCATGATTTTCAAGTCAATGAAGTATTAGCATATGATGTGATGCCATATATTATGAAAAAGCTCAATGCGCCATTCACGTATGATGCA
GAGATTTCGAATATATTTTATGATATCGATTTGTGTTTAGACTTTTTATTAGATCATAATTTTAGTCTAACCATGCATTTGAATCAGTATGACT
CACGAGATTATATCGATGCATTCAAAGTATTTATCCATCACGTTGCCCTGCATGTCAGTCATAGAAAAGATTTGAAGTTCAACTTGTATGTGA
CGACATTGCACAATGCTTTGATTGAAATGATTGATTATTTTAAAGCGTTATTCCCTAATGGTGGCTTGTACATTCACTTAGATCAAGCTACGG
AAAGACATCTACCATTGTTGAAACGACTTGAGCCACACATCGACCATTTTGTATTTGATGCCAATTCAAATGATGCTGTTGATTTTAATAAAA
TGAATGATGATGAATTTAAAACCGCGAGTCAAATGATTATTAATAAAACGAATTACCTTATCGACTTAATACATCGTCATAACCTAAAGCGT
CCACTCATTTTACTCAATTGGAATACATTGACGGGTGATACATTTATAACCAACGGCGAATATTTTAGAGGTGGTATCATCATTGAGCAGTT
ATTAAAGTTAAGCTCTAAAGTAGAGGGTATCGGGTATTGGTTGAATTATGATTTGCACGTTAGTCATTGTAAAAATGAACGGGATTATATG
AATTCTATTGAACTGTTTCATCAATATAATGGAAAACGTCCGGTCTATTTCACGGCATTGCTATTTAATAAATTAACAAGCAATATTTTGTATT
CTGATGATACATGTATTGTCACGGGAACTGATTCAAATTTTCAAATATTGTTATATGATGCAAAGCATTTTAATCCGTACTTAGCGTTGGACA
ATCAAATGAATATGCGTGCAACGGAAATGATCCATTTGAACATTAATGCCTTGGAAGAAGGTATGTATAAGATTAAACATTTTACCTTAGAT
AAAGAAAATGGTGCGTTATTTAATCTTTGGCGCAAACATCATACGATACATGGCATGGACAAGGACTCTATAGATTACGTTAATCGAATGA
GTTTTCCGAAATTAGAAGTATATGATATAGATATCACGGACACACTGGCATTAAACATTAAAATGATTACGAATGGGATTCACTTAATTGAA
GTAAAACGTTACCCAAGTTCATAA

Gene: Teg15as (antisense RNA)

Position: 99386 to 99629, length: 244 nt

Sequence:

GATAAAGAAAATGGTGCGTTATTTAATCTTTGGCGCAAACATCATACGATACATGGCATGGACAAGGACTCTATAGATTACGTTAATCGAA
TGAGTTTTCCGAAATTAGAAGTATATGATATAGATATCACGGACACACTGGCATTAAACATTAAAATGATTACGAATGGGATTCACTTAATT
GAAGTAAAACGTTACCCAAGTTCATAAAATGATCACAAATCACAAATTTTGATATACATAA

Gene: Q8NYT5 (Mn (2+) binding amidohydrolase)

Position: 99746 to 100924, length: 1179 nt, orientation: FORWARD

Perfect match to: (DAR4145-CP010526-[95066:96244], highly conserved allele)

Sequence:

ATGAATCAACAATTAATTGAAACTTTAAAATCTAAAGAAGGCAAAATGATTGAGATCAGACGTTATTTACATCAGCATCCAGAATTATCTTT
TCATGAAGATGAAACGGCGAAATACATCGCTGAATTTTACAAAGGTAAAGATGTGGAAGTAGAAACGAATATCGGACCACGTGGAATTAA
AGTAACGATTGATTCAGGGAAACCTGGTAAAACATTAGCAATCCGTGCAGACTTTGATGCATTACCTATTACTGAAGATACAGGATTATCTT
TTGCATCACAAAATAAAGGTGTTATGCACGCATGTGGTCACGATGCACATACAGCATACATGCTTGTATTAGCAGAGACGCTTGCTGAAAT
GAAAGATAGTTTTACAGGAAAAGTCGTTGTGATACATCAACCAGCTGAAGAAGTACCACCAGGTGGTGCTAAAGCAATGATTGAAAATGG
TGTATTAGACGGTGTTGATCATGTATTAGGTGTACACGTCATGAGCACAATGAAAACAGGTAATGTGTATTACAGACCTGGTTATGTTCAAA
CAGGACGCGCATTTTTCAAATTGAAAGTTCAAGGTAAAGGTGGTCATGGTTCATCACCCCATATGGCCAATGATGCCATTGTTGCAGGTAG
CTACTTCGTCACAGCGTTACAAACAGTTGTATCTAGACGACTAAGTCCATTTGAAACCGGTGTTGTCACAATCGGTTCATTTGACGGTAAAG
GTCAATTCAATGTCATTAAAGATGTTGTTGAAATTGAAGGTGATGTACGTGGATTAACAGATGCTACAAAAGCAACAATTGAAACAGAAAT
TAAACGTTTATCAAAAGGATTAGAAGCATTGTATGGTGTAACTTGCACATTAGAATATAACGATGATTATCCTGCATTATATAATGATCCAG

AGTTTACTGAGTACGTGGCTAAGACGTTAAAAGAAGCAAACCTTGATTTTGGTGTCGAAATATGTGAACCACAACCACCTTCAGAAGACTTT
GCATACTATGCTAAAGAACGTCCAAGTGCCTTTATTTATACAGGTGCAGCTGTGGAAGATGGTGAAATTTACCCACATCATCATCCTAAATT
TAACATTTCAGAAAAATCATTACTTATTTCGGCAGAAGCTGTAGGGACAGTTGTTTTAGATTACCTTAAAGGAGATAACTAA

Gene: norC (multidrug efflux pump)

Position: 100926 to 102314, length: 1389 nt, orientation: FORWARD

Perfect match to: (MW2-BA000033-[90887:92275], allele observed in CC1+CC772+CC1290)

Sequence:

ATGAATGAAACGTATCGCGGGGGCAACAAGTTAATCTTAGGTATTGTATTAGGTGTTATTACATTTTGGTTGTTTGCACAATCACTTGTAAA
TGTTGTACCAAATTTACAACAAAGTTTTGGTGCAGACATGGGAACAATTAGTATTGCGGTAAGTCTAACCGCACTATTTTCAGGCATGTTTG
TTGTTGGAGCAGGCGGCCTAGCAGATAAAATTGGGCGCGTGAAAATGACGAATATCGGTTTATTGTTAAGTATTATTGGTTCAGCATTAAT
TATTATTACGAATTTACCGGCATTATTAATTTTAGGTCGTATTATACAAGGCGTATCAGCAGCGTGTATTATGCCTTCCACATTGGCCATTAT
GAAAACTTATTATGAGGGTGCTGAACGTCAGCGCGCCTTAAGCTACTGGTCTATCGGTTCTTGGGGTGGAAGTGGTATCTGTTCACTCTTCG
GTGGGGCAGTTGCGACAACTATGGGTTGGAGATGGATTTTCATCTTCTCAATTATCGTTGCCGTACTTTCAATGTTACTCATCAAAGGGACG
CCTGAAACGAAATCAGAAGTTACCAATACACATAAATTTGACGTTGCAGGGCTAATTGTTCTAGTAGTTATGTTGCTAAGTTTAAACGTTGT
CATTACTAAAGGTGCAGCACTTGGTTACACATCATTATGGTTCTTTGGTTTAATTGCAATCGTAATTGTAGCATTCTTTATTTTCTTAAAAGTT
GAGAAAAAAGTAGATAATCCGCTTATTGATTTTAAATTATTTGAAAATAAACCATATACAGGTGCAACGATTTCGAACTTCTTATTAAACGG
TGTTGCAGGTACATTAATTGTAGCGAATACATTCGTGCAACAAGGTTTAGGTTATACAGCATTGCAGGCAGGATACTTATCAATTACTTATT
TAATCATGGTGTTATTGATGATTCGAGTTGGTGAAAAATTATTACAAAAAATGGGTTCTAAGCGACCAATGTTATTAGGTACATTCATTGTG
GTCATTGGTATTGCACTTATTTCATTAGTATTCTTACCAGGCATATTTTATGTTATTAGTTGTGTCGTAGGATATTTATGTTTCGGACTAGGCT
TAGGTATTTATGCAACACCTTCTACAGATACAGCTATTTCGAATGCACCGTTAGATAAAGTTGGCGTTGCTTCAGGTATTTATAAAATGGCTT
CATCACTTGGTGGTGCATTCGGTGTCGCAATTAGTGGTGCAGTATATGCCGGTGCAGTTGCTGCAACTAGCATTCATACAGGTGCGATGAT
TGCACTTTGGGTTAACGTATTAATGGGAATCATGGCATTTATCGCAATTTTATTCGCGATTCCTAATGATGATAAACGTGTCAAAGATGCGA
AATAA

Gene: nptA (putative sodium:phosphate symporter)

Position: 102801 to 104462, length: 1662 nt, orientation: REVERSE

Sequence:

TTAAATTTCAGTTGTTGCAATTTCTTCATCTGTAGGTACATCATCGTTAAGGCCAACAAGTGCTTCAGAAACATTTCGTGAATGATAACCGAT
ACGTTCAAGAACACCAATCATATCGATATATAGTAATCCGCCTTTTGTTGTACATTCACCACGATTAAGGCGTTTAATATGACCTTTGCGTAG
TTTATGTTCAATATTAAATGATTCTCTACTACGTTCTACAATTTCATCTTTTTTCGTTTTGTCATAAACATCTAACATGTCGATGGCTTTATCAA
ATGACTCAGCAACATGGTTGAATAATTTATCCATACCGCGTTGTGCATCTTCTGTAATGCGAATATCTTCATCATGTTGGCGTTTTAATTGAG
CGACATACTCTTCTGTTAGCTCTGCTACTTTTAAAATAGAGCGATTGACATCAAACATAACTGCTAAACGCTCAACGTCAGCCTTCGTTATGG
CTTTTGTAGAAATTCTAACTAAATAATTTCGAATGCTATCATTGATTGTTTCAACAGCTTGGTGCTTTTGTTCAAGCTTTTTGATCAATTTTTTA
TCGTCTTTTGTAATTTCGCGAATGTCTTCAAACATTGATAAGACAATCTGACCAACATTTTGTAATTCTTTTTGAGTTTCTTGTAATGCAACAC
CAGGTGCGTGATAAACAAGATCTTTGTTTAAGTGCTGAGGTTTATAGTCATCAGCAATATCTTTACCTGGGACAAGCTTTGTAACAATCCAT
GCTAAACCTGCTACAAATGGTAATTGAATCAAAGTATTTGTTATGTTGAAGATACCATGTGATACTGCAATCGTCATCGCTGGTTTTAAGTG
CCATACATCTTGTAACAAACTAATCAAATGAATCACAACTGGCAAGAAAATTGTGAAGATAATTACCCCGATTAAGTTAAAGATGACGTGTA
CAAGCGCCGCACGTTTTGCAGCGATTGAGCCGGCTAAACTAGCTAAGATAGCTGTAATCGTTGTACCAATATTATCGCCTAGTAACACAGG
GATTGCTGCGTTTAAGCTAATTAAATCTTGTTGATAAAATTCTTGTAAAATACCAATCGTCGCACTTGAACTTTGAACTAGTGCTGTTAACCC
TGCGCCGACAATAACAGCAAGTATTGGATTTGATGACATATCAAGCATTAATTGCTTAAATCCATCTAATGATGCTAAAGGTTTAACGGCAT
CACCCATAAATTCTAGACCGAAGAATAGAGAACCGAAACCGAATAGTATGCGGCCAATGTTATTAATTTTAGAGCGTTTAAAGAAAAAGAT
TAAAAATGCACCTAATGCTAAAATTGGCATTGCATATTCGCCTAAATCTATACCGATAATAAATGCAGTTACCGTTGTTCCGATATTAGCACC
CATTATCACTCCAATGGCTTGTTTCAATGTCATAAATCCAGCTGTTACCAGTCCGATTGTGATAACTGTCGTACCTGAACTACTTTGTATTAAA
ATAGTTACAACGATACCTGCAATAACACCTAATACTGGATTTGATGTAAATTTGTTTAAAATATCTCGTAGCCTGTCTCCTGCTGATGCTTGA
AGCCCGTCTCCCATGATTTTTAAGCCGTAAAGGAAAATACCTAAACCACCTAAAAAGGAGAAAATGACTTCTGTAACCGACAT

Gene: Q2YUS5 (67 kDa myosin-crossreactive streptococcal antigen homologue)

Position: 104786 to 106561, length: 1776 nt, orientation: REVERSE

Sequence:

TTATAACAATTTGTGTTCTTTTAATAATGACTCAATGTACGTACCTTTTATCTTTTTAAGGAATCCTGCTAATGCGAGTTTTTGCATTTTCGAAT
CTTTAGTAATCTCGCGCAAATCTTGATGGTCATTCAGTTCGTATATGGCATCCATTAAGACGCGAAGATCAAATGTACTATTGATGACCTCTG
GAATACCACGATCTATATTTAGTAATTGATAAACAGCTTCCATGGCAGTACGAACCGAATATTCTGTTGTAAATACAGTATCTCGCTCTGTTT
CTGCAAAGTTACCAATAAATGCTAAATTTTGTGATTGATGCGGGACGACTAAAGGTCTGTCGCCGATAGCACGCGTCATGAAATAAGAGGT
AATATATGGCATATAAACAGGAATCGTATTAGATGCATGTTTTGCTAAGTCTTCAATTTTGTCAGTTGGTACACCTAAGTGATACAGCCATTC
TTGACATATTTCATTACCACTACATTCTGTGATTGGCTTTTTAATATAATCGCCGTTTACATCTGAATATAAGGCATAAATCCATGTAGATATT
TCATTTTTAGGTTGGTCTTTAAACTGTTGCTGACGATTGATTGTAAAACTGATTTGCCATGCAGAATCATTGATTGTAATAATTCCGCCAGTA
ACCGTTTTGCCTGCAAGAGGGTCACATTTACAAATGCTTTCTATTGTATCGATAATATCTTTATTGTTTGTTGTCGATGTTGCAGAAACAAAC
CAACTCTTTTGAGGAATATTTTGGCAAAACTTATCAGGATTACCAAATTCAGGACTTTGTCGCGCTAAATTTTTCCATAGTGTCCAACTACCA
CCTAATTCGTCAGTTGGTGGTGCTGGTGTATCATTATCACCATACGTAGAGCTTTCCGTAATACTACCGTTTGTCACAAAGACAAGATCATCT
ACAGTCAGTTTAATTGACTCTGCTTTGCCATGTCGGTGAATTAATATTTCTCGGGCAATTTTTTGACTTGTCGTAACATCTACTTTAATATCTT
CTACTTTTACACCGTATTCAAATTGAACCCCATGCGATTTTAAATATTCAACCATAGGTAATACTAAAGATTCATATTGATTATATTTAGTGAA
TTTTAAAGCTGAAAAGTCTGCGAGACCACCAATATGATGAACGAATCGCATTAGATAGCGACGCATTTCCATAGCAGAATGCCACGGTTCA
AATGCAAACATCGTTTTCCAGTAAATCCAAAAGTTTGAATTAAAGAAGTCATCGGAAAATACATCTGTTATTTTGACATCATCTAAATCTTCT
TCATTCGTTAAGCATAAATCTAAAATTTCTTTAATCGCTTTTTTGGTCAAAGTGAAGTCTCCATCGGTAACTAAACGTTGACCCTGTTTTTCAA
TAACTCGACAGCGAGAATAGTTAGGGTCTTCTTTGTTTAGCCAATAGAACTCATCTAATACAGACGCGTTATCGATTTCTAATGAAGGGATA
GATCTGAATAAGTCCCACAAACATTCAAAGTGGTTCTCCATTTCACGACCACCTCGGACAACATAGCCTTTTAAAGGCATATTTTCACCATCA
AGACTACCACCTGCTTTAGGTAACTCTTCTAAAATATGAATCTTCGAACCTTCCATTTGACCATCCCTTATTAAAAAACAAGCTGCAGCAAGT
GAAGCTAGACCAGATCCGATTAAGTAAGCGGATTTGTTTTCTACATTTTCAGGTTTTTTAGGGCGCGCAAATGCTTCATAATTTCCATAACTG
TAATACAT

Gene: DUF1648 (putative protein)

Position: 106912 to 107385, length: 474 nt, orientation: FORWARD

Perfect match to: (MW2-BA000033-[96873:97346], allele observed in CC1+CC80+CC445)

Sequence:

ATGTCTAAAATCAGGTCTTTTACAATATTAAGTCTACTTATTTACTTAGCTATGATGTGCTATACAGTAGTGACCTATTCAAAATTACCAACCA
AAGTACCTATTCATTATAATTTAGCAGGGGATGCTGATAACTTTGCTGATAAATGGGTGCTGCTTTTGATTAATAGCGCATTTATAGTGATTT
GGCTTATATTTTTCATTGCAGGTAGATACTATGAACGATTTGCCAAATGGTCACATTATAATCATACACCACGTGAAATTCGAGCGATTAAAT
TATTTTTAAGTACGTTAAATTTAGAGATTATGAGCTATATGTCTATCTTCACAGTATTAGAAATTTGGCAAATACAACACCATCATCAATTCA
ATTTACTATGGTTTAATATGATATTTATCATTATCATTGGTTTGACGCTTGTCATATTTTGTTTACTTCCTACAATTCATAAAATGAGAGATTCT
CAATAA

Gene: lctP-locus1 (L-lactate permease)

Position: 107647 to 109239, length: 1593 nt, orientation: FORWARD

Perfect match to: (MW2-BA000033-[97608:99200], allele observed in CC1)

Sequence:

ATGACACTACTTACTGTAAATCCATTCGATAATGTTGGATTATCAGCCTTAGTTGCAGCAGTACCTATTATTTTATTTTTATTATGCTTAACCG
TTTTTAAAATGAAAGGCATTTATGCAGCATTGACAACTTTGGTTGTTACATTGATTGTGGCTTTATTTGTATTTGAATTACCAGTGCGTGTAT
CAGCAGGTGCGATTACAGAAGGCGTTGTTGCCGGTATTTTCCCAATAGGATATATCGTTTTAATGGCAGTTTGGTTATATAAAGTTTCTATT
AAAACAGGACAATTTTCTATTATTCAAGATAGTATTGCAAGTATTTCAGAGGACCAAAGAATCCAACTATTATTAATTGGATTTTGTTTCAAC
GCATTTTTAGAAGGTGCAGCAGGATTTGGTGTGCCAATTGCGATTTGTGCAGTATTATTAATTCAACTTGGATTTGAACCATTAAAAGCAGC
GATGTTATGCTTAATTGCTAATGGTGCGGCGGGTGCCTTTGGTGCAATTGGTTTACCAGTTAGTATTATTGATACGTTTAACTTAAGTGGAG
GCGTTACAACATTAGATGTTGCGAGATATTCAGCATTAACACTTCCAATTTTAAACTTTATTATCCCATTTGTTTTAGTATTCGTTATAGATGG
CATGAAAGGTATTAAAGAAATTTTACCTGTCATTTTAACAGTGAGTGGTACATATACTGGATTACAATTATTATTAACAATATTCCATGGTCC
AGAACTAGCAGACATTATCCCATCACTAGCAACAATGGTGGTGTTAGCATTTGTTTGTCGTAAATTTAAACCGAAAAACATTTTCAGGTTGA
AAGAATCAGAACATAAAATTCAAAAACGAACGCCTAAAGAAATTGTCTTTGCTTGGAGTCCGTTCGTCATTTTAACTGCCTTTGTTTTAGTAT
GGAGTGCACCATTCTTCAAAAAATTATTCCAACCTGGAGGTGCACTTGAAAGTTTAGTAATAAAATTGCCAATTCCAAATACTGTGAGTGAT
TTATCGCCTAAAGGAATTGCGTTGCGTCTCGATTTAATTGGTGCAACTGGGACAGCGATTTTATTAACAGTAATTATTACAATTTTAATTACG
AAGCTAAAATGGAAAAGTGCAGGTGCTTTATTGGTCGAAGCAATTAAAGAATTATGGTTACCGATCCTTACAATTTCAGCTATCCTAGCTAT
TGCTAAAGTTATGACATACGGTGGTTTGACTGTAGCAATTGGACAAGGTATTGCTAAAGCTGGAGCAATTTTCCCATTATTCTCTCCAGTAT
TAGGTTGGATTGGTGTGTTTATGACTGGTTCAGTTGTAAATAACAATACTTTATTCGCACCTATTCAAGCGACAGTAGCACAACAAATTTCA
ACAAGCGGTTCATTACTTGTGGCAGCTAACACTGCAGGTGGTGTAGCAGCGAAACTTATTTCACCACAATCAATTGCCATTGCGACTGCAGC

TGTTAAAAAAGTTGGTGAAGAATCTGCATTATTAAAAATGACGCTAAAATACAGTATTATATTTGTTGCTTTTATTTGTGTTTGGACGTTTAT
ACTAACGTTAATATTCTAA

Gene: spa (immunoglobin G binding protein A)

Position: 109568 to 110800, length: 1233 nt, orientation: REVERSE

Sequence:

TTATAGTTCGCGACGACGTCCAGCTAATAACGCTGCACCTAACGCTAATGATAATCCACCAAATACAGTTGTACCAATGAATGGATTTTCTTC
ACCAGTTTCTGGTAATGCTTGAGCTTTGTTAGCATCTGCATGGTTTGCTGGTTGCTTCTTATCAACAACAAGTTCTTGACCAGGTTTGATCAT
GTTTTTATCAGCTAATTTGTTATCTGCAGCAATTTTGTCAGCAGTAGTGCCGTTTGCTTTTGCAATGTCATTTACTGTATCACCAGGTTTAACG
ACATGTACTCCGTTGCCGTCTTCTTTACCAGGTTTGTTGTTGTCTTCTTTGCCAGGCTTGTTGCCATCTTCTTTACCAGGTTTTTTGTTGTCTTC
TTTGCCAGGTTTGTTGCCGTCTTCTTTACCAGGTTTGTTGTTGTCTTCCTCTTTTGGTGCTTGAGCATCGTTTAGCTTTTTAGCTTCTGCTAAAA
TTTCTTTGCTCACTGAAGGATCGTCTTTAAGGCTTTGGATGAAGCCGTTACGTTGTTCTTCAGTTAAGTTAGGTAAATGTAAAATTTCATAGA
AAGCATTTTGTTGTTCTTTGTTGAATTTGTTGTCAGCTTTTGGTGCTTGTGCATCATTTAGCTTTTTAGCTTCTGCTAAAAGGTTAGCGCTTTG
GCTTGGGTCATCTTTTAAGCTTTGGATGAAACCATTGCGTTGTTCTTCGTTTAAGTTAGGTAAATGTAAGATTTCATAGAAAGCATTTTGTTG
TTCTTTGTTGAAATTGTTGTCAGCTTTCGGTGCTTGAGATTCGTTTAATTTTTTAGCTTCACCTAAAACGTTAGTGCTTTGGCTTGGATCGTCT
TTAAGACTTTGAATGAAACCATTGCGTTGCGCTTCGTTTAAGTTAGGCATGTTCAAGATTTCATAGAAGGCGCTTTGTTGATCTTTGTTGAAG
TTATTTTGTTGCGCATCAGCTTTTGGAGCTTGAGAGTCATTAAGTTTTTGAGCTTCACCTAAAACGTTAGCACTTTGGCTTGGATCATCTTTAA
GGCTTTGGATAAAACCATTACGTTGATCAGCGTTTAAGTTAGGCATATTTAACACTTGATAAAAAGCATTTTGTTGAGCTTCATCGTGTTGC
GCAGCATTTGCAGCAGGTGTTACGCCACCAGATATAAGTAATGTACCTAAAGTTACAGATGCAATACCTACACCTAGTTTACGAATTGAATA
AATGTTTTTCTTTTTCAA

Gene: sarS (HTH-type transcriptional regulator)

Position: 111221 to 111973, length: 753 nt, orientation: REVERSE

Perfect match to: (RF122-AJ938182-[67420:68172:r], highly conserved allele)

Sequence:

TTATTCAAAAACAAGATGTAAATGATCTTTATCTGCTAATAATTGATTCACTTGAGCTAATAATTGTTCAGCATGGTCTTGCTGCGCGTCATC
CATATGAATTAAAATTTTTCTTTCATCTTCAGTTGAGCGTTCTTTTATTAGATAGCCTTGCTTTTTTAAATTATTGAGAGCTCTAACAGTTTGAG
GGTATTTATGGTGGATTGTTTCAATTAAATCTTTAAGAAGAACGATGTTTTTATTTTGAGAAGTGATAATAGCTAGAATTGTGAATTCTACAA
AACTTAATGTTAGATGTTTTTTGATAATATTCTTGAAATACATTGTATACATCATCAAGTTTAGAAATTCTTTACTATCTTTTGGTATCATCTGT
GATTCACTTTGATCTGCAAGGTTAAATTGTTTAATGATTTGATCAAACAATGTAACACGTTCTGCAATTTTCTCTCGTTGTTCTTCAGATATTG
AAATGTAAGTATTACGCTCATCAATTTTACTTCGAACTTTACTAATATATGAATGTTTCACAAGTACTTTTATATGCTGTACTAAATCCGATTG
TTTATAACATAAATCTGAAACAATCTTCTTAAATGGAAGTGTGTTTTCTTGCTGATGAAATAAATAAGTCAGTAATATAAATTCTTTTATAGTC
ATATCGACTTCAGGCTTGACTTTTTTCTTAAAACGAAACATATATGCTTCAATGATTATAAAATCTCTAATTTTGTCATGGTTATTATATTTCAT

Gene: sirC (siderophore 'staphylobactin' ABC transporter, transmembrane permease subunit)

Position: 112342 to 113340, length: 999 nt, orientation: REVERSE

Perfect match to: (MW2-BA000033-[102549:103547:r], allele observed in CC1+CC6+CC20)

Sequence:

TTATAACTTTTTCACGGTTAATAATAAGTATATGAAGAATGGGGCACCAAAAGCAGCAATAAATACACCTGCTGGCACTTCTTTAGGCAAGA
ATAAGGTACGCCCAATTAAGTCTGCAATAACAATTGATATGGCACCAATCATTGCTGACATTAGTAACTTTTTAGCATAACTTCCGCGAACG
ATTGTTTTCGCGATATGTGGTGCGATTAAACCGACAAACCCAATGTTACCTACTAAACTGATTGCCATAGATACGAGTATAGTAGAAGTGAT
TAATTGGATTAGTTTCATACGTTGTACATGTAAGCCTAAGCCAATCGCTACAGGGTCATCAAGTATAGATATTTTCATTTTTGGTATAACAAG
AAATAACAACGGCACAACAGCTAAAATAACCATACCCAAGATGATTGTATCTTTAAACGTAGCACCGTAAAGACTTCCGACTAGCCATGTAT
AAGCTTTGGCAGCAGATAATTGCTTCGTTGTAATGAGTAATCCTTGGACAAGCGCAATAAACAACGTTTGCATCGAAATACCGATGATTATG
AGTGTTGTCGGGCGTATTTGTCCTTTCGTTTGAAACACTAATAGTATCATCATTGCAATTGCGCCACCTAATACAGCGAATAGTGGAAGTAA
ATGTATCGTTAAATGGCTGAAAAATGCAATAAAGACAACGGCACTTAAGCTAGCACCACCTGTGATACCGATAATATCAGGTGAGGCAATT
GGATTTTTTAATACATTTTGCAACATTAAACCACTCATTCCTAGTGCGGCACCTGCTAAAATCGCAAGTGTAATGCGAGGTAAGCGTAATAC

TTCTAAAGTGAATTGATCCATACTGTCATTTGGATTTATAAAGTACATCAGTACGCGTTGTAATGGTATAAAGCTTGAACCACTCATCATACT
TACCACTGAAACGATGGCTAAAAAGATTAACGCGAAGATGAGATGGTAATTGTCTTTTTTATTAATCTTTTCGGTCAT

Gene: sirB (siderophore 'staphylobactin' ABC transporter, transmembrane permease subunit)

Position: 113337 to 114332, length: 996 nt, orientation: REVERSE

Sequence:

TCATAAGCGTTGACGTCCTTTCTTCATAATATAGATTAAGACAATAGCGCCAATGACAGCGGTAACGACACCGATAGGCAACTCTAGTGGCT
TAATTATTATACGAGCAACAATGTCTGAAATGATCATTAGGATTGCTCCAGCTAATGCAGTAAAAGGAATTAAATACTTATAGTTTGGTGGT
AATAATCGTTTGCTAATATTCGGTACGATAAGACCCACAAAGACGATTGCTCCAGCTACGGCTACCGAAATACCGGCTAACATACTGATGA
GCATAATAATCATCCATTTGATTAATTTTATGTTTTGACCGAGGCCGATTGCAATGTCGTCACTTGTCATCAAGATGTTGATGTGTGCAGCCA
TGCTAAATGCAATTAAAATAAGTATCAATACAAGCGAAATAATCCATGGGATATCCCAAATATTACGTAATGAAACGGAGCCACTTAACCA
AAATAATAGGCCTTGTAAGTCTGTTTCGTTCATAATAAGTATGCCTTGAGTAAAGGCTGTAAATAGCATCGCAATCGCAGCACCTGCCAAAA
TGACACGGTGAGGTGAGAATAGTGTTTGTCTAAACATACCTAGCGCAACAACTAATACAGTAACAACAATAGCCCCCAAAAATGCAATAAC
TACAATCATTTTAAAAGATTGAATTTGGATAAATGTAATACTAAAAATGACAAAAAATACTGCGCCTGCATTGACACCGAAAAGCCCTGGTG
AGGCTATTGGGTTTCGTGTAAGTGCTTGCATCAACAAACCTGAGACAGCAAGGGCAGCACCAGTCAATAACGCAATGATTGTTCTCGACGC
CCGTGCACCAGTGACAACATCATGTAAATCGTTTTCACTATCAAAGTTGAATAACGCCTGTATCACCGTACCTGGTGACACAAGCGTATTTC
CAATCATTAAACTTAAGATAGCTACTATTGCAAGACATAAACCAGCAATAACGATTTGGTATTTTGGTTTAAGTAGCAT

Gene: sirA (siderophore 'staphylobactin' ABC transporter, substrate-binding protein)

Position: 114348 to 115340, length: 993 nt, orientation: REVERSE

Perfect match to: (RF122-AJ938182-[70544:71536:r], highly conserved allele)

Sequence:

TTATTTTGATTGTTTTTCAATATTTAACTTTTCATATAAATCGTCAATAAGTTTTAATGAAGATTTATATCCGCCAGCTAAGTTCCAAGTGATTT
CATCTAAATCATCAGATACTTGGTTGTTTTTAACTGCGTCTAAATTTTTCCACTCTTTACTTGAAGTCCATTCGCTTTCAGTCTTTTTAACTAAT
GCAGCATCTTTCGCATTTGGATCTGATTTTACTACAAAAATATGATCAGCGTTCATTAATGGAATGCTTTCTTTAGATGTAAGTTGGATAATA
TCTTTACCATTATCAACTTGTTTTTGTAAGTCTTTATTACGTTTGAATCCTAAATCATTTAAGATTTCACCAGCATATCCACCAGCATAAATTCT
TGTATGATCAGCACGGAAGTTAACAACTGAAGCTTTCAATGGCCATGCATCTTTATACTTTGCTTTTGCATCTTTTTGGAATGCAGCTACTTT
ATCATCGTACTTTTTAAGTAAATCTTCAGCTTCTTTTTCTTTCCCTAAAGCTTTCCCCATTAACTTAGTTGTATCTTTGAATTTGAAAACTGTATC
AGTAGAAACTGTTGGTGCGATTTTAGATAATTGATCGTAAACTTTTTCATTTCTAACTTTTGACGCGACAATTAAGTCCGGTTTTAATTTAGA
GATTTCCTCTAAGTTAGGTGCAGGTTCTTGACCTACAATCTTAGTATCTTTTAAATCATTTTTTATGTATTCGAATTTCGGTTTTTGTGTCCATG
ATTCTACAGCACCTACAGGTTTAACACCTAAAGATACAGCGACGTCAGTGGCACCTTGATATAGCGTAACAACACGCTTTGGTTTCCCTTTA
ATTTCAGTTGTACCCATTGCATGTTTAATTGAAGTTGTTTCCTTATCTTTGTTATCAGATGATTGTTTATTTGAATTCCCACTACATCCTGCTAA
AACAAGTAGGAAAGCAAGCGTAACAACAAGCATTTTAATTACTTTATTCAT

Gene: sbnA (siderophore biosynthesis protein A)

Position: 115571 to 116551, length: 981 nt, orientation: FORWARD

Perfect match to: (11819-97-CP003194-[121982:122962], allele observed in CC80)

Sequence:

TTGATTGAAAAAAGTCAAGCATGTCACGATTCATTGATAGATTCTGTAGGGCAAACACCTATGGTTCAACTTCATCAACTATTTCCGAAACA
TGAAGTGTTTGCAAAGTTAGAGTATATGAATCCTGGAGGCAGCATGAAAGATCGACCTGCCAAGTACATCATTGAACATGGTATTAAACAT
GGTTTAATCACTGAGAATACACATTTAATTGAAAGTACTTCTGGTAATTTAGGCATTGCGTTGGCAATGATAGCTAAAATCAAGGGATTAAA
ACTCACGTGTGTTGTTGATCCTAAAATATCACCAACAAATTTGAAAATTATTAAAAGTTATGGTGCCAATGTAGAAATGGTTGAAGAACCTG
ATGCACATGGGGGTTATTTAATGACTCGTATTGCAAAGGTGCAAGAACTGTTAGCCACTATTGACGATGCATATTGGATTAATCAATATGCG
AATGAGTTAAATTGGCAATCCCATTATCATGGTGCAGGCACAGAGATTGTTGAAACAATTAAGCAACCTATAGATTATTTTGTCGCGCCAGT
CAGCACGACAGGTAGCATTATGGGTATGAGTAGAAAAATAAAAGAAGTGCATCCAAACGCACAAATTGTTGCTGTTGATGCGAAAGGGTC
AGTCATTTTTGGTGACAAACCTATTAATAGAGAATTACCTGGTATCGGTGCTAGTCGTGTACCCGAAATATTGAATAGATCAGAAATTAATC
AAGTGATCCATGTAGATGATTATCAATCTGCTTTGGGCTGTCGAAAACTGATTGATTATGAAGGCATATTTGCCGGAGGTTCAACAGGTTC

GATTATTGCAGCGATTGAGCAGTTGATAACGTCAATTGAAGAAGGTGCAACAATTGTCACGATTTTACCAGATCGAGGCGATCGTTACTTA
GATTTAGTTTATTCAGATACATGGTTAGAAAAAATGAAATCAAGACAAGGAGTTAAATCAGAATGA

Gene: sbnB (siderophore biosynthesis protein B)

Position: 116548 to 117558, length: 1011 nt, orientation: FORWARD

Sequence:

ATGAATAGAGAGATGTTGTATTTAAATAGATCAGATATTGAACAAGCGGGAGGTAATCATTCACAAGTTTATGTGGACGCATTAACAGAAG
CATTAACAGCCCATGCGCACAATGATTTTGTACAACCGCTTAAGCCGTATTTAAGACAGGATCCTGAAAATGGACACATCGCAGATCGAAT
AATTGCAATGCCAAGTCATATCGGTGGTGAACACGCAATTTCAGGTATTAAGTGGATAGGTAGTAAGCACGATAATCCATCGAAACGTAAT
ATGGAGCGTGCAAGTGGTGTCATTATTTTGAATGATCCAGAAACGAATTATCCAATTGCAGTTATGGAAGCAAGTTTAATTAGTAGTATGC
GTACTGCAGCAGTTTCAGTGATTGCAGCTAAGCATTTGGCTAAAAAAGGATTTAAAGACTTAACAATCATTGGATGCGGGCTAATCGGAGA
CAAGCAATTACAAAGTATGTTAGAGCAATTCGATCATATTGAACGCGTGTTTGTTTACGATCAATTCTCTGAAGCATGTGCACGCTTTGTTG
ATAGATGGCAACAACAGCGTCCGGAAATTAATTTTATTGCGACAGAAAATGCTAAAGAAGCAGTATCAAATGGTGAAGTAGTCATTACATG
TACCGTAACGGATCAACCATACATTGAATATGATTGGTTACAAAAGGGTGCATTTATTAGCAACATTTCTATCATGGATGTGCATAAAGAAG
TCTTTATTAAAGCTGACAAAGTCGTAGTAGACGACTGGTCACAATGTAATCGAGAAAAGAAAACTATTAACCAATTGGTGTTAGAAGGTAA
ATTCAGCAAAGAAGCACTTCATGCTGAACTAGGGCAACTTGTGACAGGTGACATACCAGGACGTGAAGACGATGATGAAATCATATTACTT
AATCCGATGGGTATGGCTATCGAAGATATTTCAAGTGCTTACTTTATTTATCAACAGGCACAACAACAAAATATTGGGACAACATTGAACCT
ATATTAA

Gene: sbnC (siderophore biosynthesis protein C)

Position: 117579 to 119333, length: 1755 nt, orientation: FORWARD

Sequence:

TTGCAGAATCATACAGCAGTCAATACAGCACAAACGATAATATTAAGAGATTTAGTTGATGCATTATTATTTGAAGATATAGCCGGTATTGT
ATCGAATAGTGAGATTACTAAAGAAAATGGACAAACCATTTTGATATACGAACGTGAAACACAACAAATAAAGATACCTGTTTATTTTAGTG
CTTTAAATATGTTTCGTTATGAAAGTTCACAACCAATTACGATAGAGGGAAGGGCGTCTAAGCAACCTTTAACGGCAGCTGAATTTTGGCAA
ACAATTGCTAATATGAATTGTGATTTAAGTCAAGAATGGGAAGTGGCTCGAGTTGAAGAAGGACTGACTACTGCTGCCACACAGCTTGCTA
AACAATTATCAGAATTAGATTTAGCGTCGCATCCTTTTGTGATGTCCGAGCAGTTTGCAAGTTTAAAAGATCGTCCATTCCATCCATTAGCTA
AAGAAAAAAGAGGATTAAGAGAAGCGGATTATCAAGTGTATCAAGCTGAATTAAATCAATCATTTCCTTTAATGGTTGCAGCAGTTAAAAA
GACACATATGATTCATGGCGATACTGCAAATATCGATGAATTAGAAAATTTGACAGCACCTATAAAAGAACAAGCGACAGACATGTTAAAT
GATCAAGGGTTATCAATAGATGACTATGTACTATTTCCGGTACATCCTTGGCAATATCAGCATATTTTGCCGAACGTCTTTGCGAAAGAGAT
TAGTGAAAAGTTGGTTGTACTATTACCGTTAAAATTTGGAGATTATCTGTCGTCTTCAAGTATGCGTTCATTAATTGATATTGGCGCACCGTA
TAACCATGTCAAAGTACCATTTGCAATGCAGTCATTAGGGGCATTAAGGCTAACGCCTACGCGTTACATGAAAAACGGAGAACAAGCAGAA
CAATTATTACGTCAGCTTATAGAAAAAGATGAAGCACTAGCTAAGTATGTCATGGTTTGTGATGAAACAGCTTGGTGGTCATATATGGGTC
AAGATAATGATATTTTCAAAGATCAATTAGGTCATCTAACTGTTCAGCTAAGAAAATATCCCGAAGTGCTAGCCAAAAATGATACGCAACAG
CTAGTGTCAATGGCAGCACTCGCGGCAAATGATCGCACTTTATATCAAATGATTTGTGGAAAAGATAATATTTCTAAAAATGATGTCATGAC
GTTATTTGAAGATATCGCGCAAGTCTTTTTAAAGGTAACACTATCATTTATGCAATACGGCGCATTACCAGAGTTGCATGGTCAAAATATATT
GTTGTCATTTGAAGATGGACGTGTACAAAAATGCGTGTTACGTGATCATGATACTGTCAGAATTTATAAACCATGGCTAACAGCACATCAGC
TTTCATTGCCGAAGTATGTCGTCAGAGAAGATACACCTAATACGCTAATTAATGAAGATTTGGAAACATTCTTTGCTTATTTTCAAACATTAG
CTGTATCGGTAAATCTATATGCCATTATTGATGCAATTCAAGATTTATTTGGTGTAAGTGAGCATGAACTTATGTCGTTGTTAAAACAAATTT
TAAAGAATGAAGTGGCAACTATTTCCTGGGTTACAACTGATCAGCTAGCTGTCAGACACATTTTATTTGATAAACAGACGTGGCCATTCAAA
CAAATTTTATTACCATTGCTATATCAACGTGATAGTGGTGGAGGTAGTATGCCTTCAGGTTTAACTACCGTACCAAATCCAATGGTGACATA
TGATTAA

Gene: sbnD (siderophore biosynthesis protein D)

Position: 119326 to 120582, length: 1257 nt, orientation: FORWARD

Perfect match to: (ATCC51811-ADVP01000042-[20093:21349:r], allele observed in CC1)

Sequence:

ATGATTAATCAGTCTATATGGCGCAGTAACTTTCGCATTTTATGGCTCAGTCAGTTTATAGCGATTGCTGGACTGACAGTACTTGTGCCATTA
TTGCCAATTTATATGGCATCACTACAAAATCTATCAGTTGTAGAAATACAGTTGTGGAGTGGTATAGCGATTGCTGCTCCAGCTGTAACGAC

GATGATAGCTTCGCCGATATGGGGGAAGCTAGGTGATAAGATTAGCCGGAAATGGATGGTGTTAAGAGCGTTACTTGGTTTGGCGGTATG
CTTATTTTTAATGGCATTGTGTACGACACCATTACAGTTTGTACTTGTGAGGTTATTGCAGGGACTATTTGGTGGTGTTGTTGATGCATCAAG
TGCGTTTGCGAGTGCAGAGGCGCCAGCTGAAGATCGTGGAAAGGTATTAGGAAGACTGCAAAGTTCAGTCAGCGCAGGGTCTCTTGTGG
GGCCATTAATTGGCGGTGTTACAGCTTCGATATTAGGTTTTAGTGCGTTACTGATGAGTATTGCCGTTATTACTTTTATTGTCTGTATTTTCG
GTGCATTAAAATTGATTGAAACGACACATATGCCAAAATCACAAACACCAAATATTAATAAAGGTATTCGCCGTTCATTTCAATGTCTATTAT
GCACACAACAAACATGTCGATTTATTATCGTTGGCGTTTTAGCAAACTTTGCTATGTATGGCATGCTAACTGCATTATCACCACTTGCTTCAT
CAGTGAATCATACAGCGATAGATGACCGTAGTGTGATTGGATTTTTACAGTCCGCATTTTGGACGGCTTCGATATTAAGCGCGCCTTTATGG
GGACGCTTTAATGATAAATCATATGTTAAATCAGTATATATATTTGCCACGATTGCATGTGGTTGTAGTGCGATACTGCAAGGTTTAGCGAC
GAATATAGAGTTTTTAATGGCTGCAAGAATACTTCAAGGATTAACATATAGTGCATTGATTCAAAGTGTCATGTTTGTTGTCGTGAATGCGT
GTCATCAACAACTTAAAGGCACATTTGTTGGAACGACGAACAGTATGTTAGTTGTTGGTCAAATTATTGGCAGTCTTAGTGGCGCTGCCATT
ACAAGTTATACTACACCAGCTACTACGTTTATCGTTATGGGCGTAGTATTTGCAGTAAGTAGTTTATTTTTAATTTGTTCAACCATCACTAATC
AAATCAACGATCACACATTAATGAAATTATGGGAGTTGAAACAAAAAAGTGCAAAATAA

Gene: sbnE (siderophore biosynthesis protein E)

Position: 120572 to 122308, length: 1737 nt, orientation: FORWARD

Sequence:

GTGCAAAATAAAGAATTAATACAACATGCAGCGTATGCGGCTATCGAACGCATTTTAAATGAATATTTTAGAGAAGAAAATTTATATCAAG
TACCACCTCAAAATCATCAATGGTCTATACAATTATCAGAGCTCGAAACTTTAACGGGTGAATTTCGCTATTGGTCTGCGATGGGGCATCAT
ATGTATCATCCAGAGGTATGGCTTATCGATGGAAAAAGTAAAAAAATAACAACTTATAAAGAAGCAATTGCGCGTATTTTGCAACATATGG
CTCAAAGTGCAGATAATCAAACGGCAGTGCAACAACATATGGCGCAAATTATGTCTGACATCGATAATAGCATTCATCGCACGGCGCGTTA
TTTGCAAAGTAACACAATAGACTACGTAGAGGATCGTTATATCGTTTCAGAACAATCTTTATACTTAGGTCATCCATTTCATCCGACTCCTAA
GAGTGCAAGTGGGTTTTCAGAAGCAGATTTAGAGAAATATGCACCCGAATGTCATACATCATTCCAATTGCATTATTTAGCTGTGCATCAAG
ATGTTCTGCTCACGCGCTATGTAGAAGGTAAAGAAGATCAGGTTGAGAAAGTGTTGTATCAATTAGCAGATATAAACATATCAGAGATACC
CAAAGATTTTATTTTATTACCAATACATCCTTATCAAATCAATGTGTTGCGACAGCATCCACAGTATATGCAATATAGTGAACAAGGTTTAAT
AAAAGACCTTGGCGTTTCCGGTGATTTAGTGTACCCGACGTCTTCGGTTAGAACTGTATTTTCAAAAGCATTAAACATTTATTTAAAATTACC
GATACACGTTAAAATTACTAATTTTATACGTACGAATGATCTTGAACAAATTGAACGAACAATTGATGCCGCGCAAGTTATCGCATCAGTCA
AAGATGAGGTTGAAACACCCCATTTTAAATTGATGTTTGAAGAAGGATATCGTGCATTGTTACCGAATCCATTAGGGCAAACAGTTGAACC
TGAAATGGATTTATTAACAAATAGTGCCATGATTGTTCGTGAAGGGATACCGAATTACCATGCTGATAAAGATATTCATGTATTGGCGTCAT
TATTTGAAACGATGCCTGATTCACCGATCTCTAAGTTATCACAAGTGATTGAGCAAAGTGGTTTAGCACCAGAAGCATGGCTTGAATATTAT
TTGGATCGTACATTATTGCCGATATTAAAGCTGTTTAGTAACACAGGCATTAGTCTAGAAGCACATGTACAAAATACATTAATTGAATTAAA
AGATGGCATACCCGATGTATGCTTTGTCAGAGATCTTGAAGGCATTTGTCTATCTAGAACGATTGCTACTGAAAAACAGCTTGTGCCAAATG
TTGTGGCAGCATCAAGCCCTGTTGTATATGCACATGATGAAGCATGGCATCGTCTTAAATATTATGTTGTAGTAAATCACTTAGGACAATTA
GTATCAACTATTGGTAAAGCGACTAGAAATGAAGTTGTGTTATGGCAACTTGTAGCGCATCGTCTTATGACTTGGAAAAAAGAATACGCGA
ATAACGCAGTATTTGTTGACTGTGTAGAAGATTTATATCAAACGCCGACCATTGCGGCTAAAGCGAATTTGATGAGTAAATTGAATGATTGT
GGTGCAAACCCTATTTATACACATATACCAAATCCAATTTGTCATAACAAGGAGGTATCGTATTGTGAATCAAACAATTCTTAA

Gene: sbnF (siderophore biosynthesis protein F)

Position: 122328 to 124067, length: 1740 nt, orientation: FORWARD

Perfect match to: (11819-97-CP003194-[128739:130478], allele observed in CC80+CC361+CC779)

Sequence:

ATGCATCAACTGGTATCATCACTTATTTATGAGAATATTGTTGTGTATAAAGCGTCATATCAAGACGGTGTCGGTCATTTTACAATAGAAGG
ACATGATTCAGAGTATCGTTTTACTGCTGAAAAGACACATAGCTTTGATCGTATACGTATCACATCACCAATTGAGCGTGTCGTAGGAGATG
AGGCAGATACAACAACAGACTATACACAATTATTGAGAGAGGTTGTATTTACATTTCCTAAAAATGATGAAAAGCTAGAACAATTTATTGTT
GAGTTATTACAGACAGAATTAAAAGATACACAAAGTATGCAGTATCGAGAATCAAACCCACCAGCAACACCTGAGACATTTAACGACTATG
AATTTTATGCGATGGAAGGGCATCAGTATCATCCAAGTTACAAATCACGTTTAGGATTTACGTTGAGTGATAATTTGAAATTTGGTCCTGAT
TTTGTACCAAACGTTAAACTGCAGTGGTTAGCTATCGACAAAGATAAAGTAGAAACGACGGTATCAAGAAATGTTGTAGTTAACGAAATGT
TACGTCAACAAGTTGGCGATAAGACTTATGAACATTTTGTACAGCAAATTGAAGCGTCTGGCAAACATGTAAATGATGTTGAGATGATACC
TGTACACCCATGGCAGTTTGAACATGTCATCCAAGTTGATTTGGCTGAAGAAAGGCTTAATGGCACAGTACTATGGTTAGGGGAAAGTGAT
GAGCTATATCACCCTCAACAATCGATTCGTACGATGTCGCCAATAGACACGACAAAATATTATTTAAAGGTACCAATAAGTATAACGAACAC
TTCAACGAAACGAGTGTTGGCGCCTCATACAATTGAAAATGCAGCGCAAATTACGGATTGGTTAAAGCAGATACAGCAACAAGATACGTAT
TTAAAAGATGAATTAAAGACAGCTTTTCTAGGGGAAGTCTTAGGACAGTCTTATTTAAATACACAACTTTCGCCTTATAAACAAACTCAAGT
TTATGGTGCGTTAGGTGTTATATGGCGTGAAAATATATATCATATGTTAATCGATGAAGAGGATGCGATACCATTTAATGCACTTTATGCAA

GTGATAAGGATGGTGTACCATTCATTGAAAAGTGGATTAAACAATATGGTTCTGAAGCTTGGACAAAGCAATTTTTAGCTGTAGCGATTCG
TCCAATGATTCATATGCTTTATTATCACGGTATTGCCTTTGAATCGCATGCACAAAATATGATGCTCATTCATGAAAATGGTTGGCCTACACG
TATTGCCTTAAAAGATTTCCACGATGGTGTTCGTTTTAAGCGTGAGCATTTAAGTGAAGCAGCTTCACACCTGACATTAAAGCCAATGCCAG
AAGCACATAAAAAAGTGAATAGTAATTCATTTATTGAAACAGATGACGAACGTTTAGTACGCGACTTTTTACATGATGCATTTTTCTTTATTA
ATATCGCCGAAATCATCTTATTTATTGAAAAGCAATATGATATCGATGAGCAGCGACAATGGCAATGGGTTAAAGACATTATCGAGGCGTA
TCAAGAAGCATTTCCAGAGTTGAATAACTATCAACATTTCGATTTGTTTGAACCTACGATTCAAGTTGAAAAGTTAACGACACGTCGATTATT
AAGTGACTCAGAGTTAAGAATTCATCATGTTACAAATCCATTAGGTGTAGGAGGTATCAATGATGCAACAACTATCTCTGAAACATAG

Gene: sbnG (siderophore biosynthesis protein G)

Position: 124042 to 124818, length: 777 nt, orientation: FORWARD

Perfect match to: (M1060-AQGA01000003-[700661:701437], allele observed in CC5)

Sequence:

ATGCAACAACTATCTCTGAAACATAGATTAAACAATGGTGATTCAGTTTATGGCATTTTTAATTCTATACCGGACCCATTGATGATCGAGGTT
ATCGCAGCAAGCGGGTATGACTTTGTTGTGATTGATACAGAACACGTGGCGATTAATGATGAGACACTAGCGCATTTAATTCGTGCAGCTG
AAGCAGCGCATATTATACCAATTGTACGTGTCACTGCAGTGATAGATAGAGATATCATTAAAGTGTTAGATATGGGTGCGAGAGGTATTAT
TGTGCCACATGTTAAAGATCGTGAGACAGTTGAGCATATTGTGAAATTAAGTCGTTATTACCCGCAAGGATTAAGAAGTTTGAATGGTGGT
CGCATGGCAAGATTTGGACGTACACCATTACTTGATGCAATGGAGATGGCTAATGAGCATATTATGGTGATTGCCATGATAGAAGATGTTG
AAGGGGTTATGGCCATTGACGATATAGCACAAGTCGAAGGTTTAGACATGATAGTCGAAGGTGCCGCAGATTTATCCCAGTCACTTGGCAT
ACCATGGCAAACGCGTGATGATCAAGTAACATCACATGTTCAACATATTTTTGAAGTTGTGAATGCACATGGTAAACATTTTTGTGCATTAC
CACGTGAAGATGAAGATATTGCAAAATGGCAGGCACAAGGTGTACAAACATTTATTTTAGGTGATGATCGCGGAAAAATATATCGCCATTT
AAGTGCATCTCTAGCGACGTCTAAACAGAAAGGGGATGAAGGCTAA

Gene: sbnH (siderophore biosynthesis protein H)

Position: 124818 to 126020, length: 1203 nt, orientation: FORWARD

Perfect match to: (Strain_21310-AFNP01000053-[13535:14737:r], allele observed in CC22+CC5+CC22+CC80+CC97)

Sequence:

ATGCGTATAGTTCAACCTGTTATTGAACAATTAAAAGCACAATCTCATCCAGTTTGTCATTATATCTATGATTTAGTCGGACTGGAACATCAT
TTGCAACATATTACATCGTCATTGCCGAGTAATTGTCAAATGTACTATGCAATGAAAGCAAATAGTGAACGAACAATCCTAGATACAATTAG
TCAGTATGTTGAAGGATTCGAAGTTGCATCTCAAGGTGAAATAGCAAAAGGTCTTGCTTTTAAACCAGCAAATCATATTATTTTTGGTGGCC
CTGGTAAGACAGACGAGGAACTAAGATATGCAGTAAGTGAAGGTGTTCAGCGTATTCATGTTGAAAGTATGCATGAATTACAACGGCTAA
ATGCCATCTTAGAAGATGAAGATAAGACACAACACATTTTATTGCGTGTTAATTTAGCAGGACCATTTCCCAATGCAACGTTGCATATGGCA
GGACGCCCAACACAATTTGGTATTTCTGAAGACGAAGTTGATGATGTCATTGAAGCTGCGCTAGCAATGCCAAATATTCATCTAGATGGCTT
TCATTTTCATTCTATTTCTAACAATTTAGACTCGAATTTACATGTCGATGTAGTGAAACTTTATTTTAAAAAAGCAAAATCATGGTCTGAAAAA
CATCGATTTCCACTCAAACATATCAATCTTGGTGGTGGTATAGGGGTTAACTATGCAGATTTAACTAGCCAATTTGAGTGGGATAATTTTGT
AGAAAATTTTAAAACACTTATCGTTGAGCAAGAAATGGAAGATGTGACATTGAACTTTGAATGTGGGCGCTTTATTGTGGCACATATTGGTT
ACTATGTGACAGAAGTGCTAGATATTAAGAAAGTGCATGGTGCTTGGTATGCCATTTTAAGAGGAGGTACGCAACAATTTAGACTGCCGGT
ATCTTGGCAGCATAACCATCCTTTTGAAATTTATCGCTATAAGGACAATCCATATTCATTTGAAAAAGTTTCAATTTCGAGACAGGACACAAC
GTTAGTCGGTCAATTATGTACACCGAAAGATGTCTTTGCTAGAGAAGTACAGATAGACGCAATCAGTACAGGCGACGTTATTGTTTTCAAAT
ATGCAGGTGCATACGGATGGTCTATTTCACATCACGATTTCTTAAGCCATCCACATCCTGAATTTATTTATTTAACGCAAACAAAGGAGGAT
GAATAA

Gene: sbnI (siderophore biosynthesis protein I)

Position: 126024 to 126788, length: 765 nt, orientation: FORWARD

Perfect match to: (Newbould_305-AKYW01000002-[342023:342787], highly conserved allele)

Sequence:

TTGAATCATATTCATGAACATTTAAAATTGGTACCAGTAGATAAGATTGATCTTCACGAAACATTCGAACCTTTAAGATTGGAAAAAACGAA
AAGTAGTATTGAAGCAGATGATTTTATACGTCATCCTATTTTAGTGACAGCGATGCAACATGGTAGATATATGGTTATAGATGGTGTGCATC
GGTATACAAGTTTGAAAGCGTTAGGATGTAAGAAAGTTCCAGTGCAAGAAATCCATGAAACACAATATTCAATTAGTACATGGCAACATAA

AGTTCCATTTGGTGTGTGGTGGGAAACGTTACAACAAGAACATCGCTTGCCATGGACTACTGAGACAAGACAAGAAGCGCCATTTATTACA
ATGTGTCATGGTGATACAGAACAATATTTGTATACAAAAGATTTAGGCGAAGCACATTTTCAAGTATGGGAAAAGGTTGTCGCAAGTTATA
GTGGTTGTTGTTCTGTAGAGAGAATTGCACAAGGTACATATCCTTGTCTTTCTCAACAAGATGTACTCATGAAGTATCAGCCATTGAGTTAT
AAGGAAATTGAAGCGGTTGTTCATAAAGGGGAAACTGTGCCAGCAGGTGTGACACGCTTTAATATTTCAGGACGATGTCTTAATCTTCAAG
TACCACTGGCATTACTTAAACAAGATGATGATGTTGAACAACTGCGCAATTGGAAGCAGTTTTTAGCAGATAAGTTTGCCAATATGAGATG
CTATACTGAAAAAGTATACTTGGTGGAGCAATAG

Gene: Q5HJP3 (putative protein)

Position: 126984 to 127609, length: 626 nt, orientation: FORWARD

Perfect match to: (11819-97-CP003194-[133395:134020], allele observed in CC80+CC5+CC8+CC97)

Sequence:

ATGGAAAAAATGTAGAAAAATCATTCATAAAGATAGGTTTATATTTTCAAATAGCTTATATAGTACTCATGGCTATAACTTTATGTGGGTTTG
TAATTTGCTATGGACTAATTTTCGGCCTTTTCTATTTATTATCAGGTAGCAGAGCTGATTATTTAATAGTAACAATAGTTATATCGGCAATAAT
TTCTATATTTGTAATTATACTTTCAATCGTACCTGTCATCGTATTGGCATCTGACTTATTTAAAGAAAGGATTTCAAAAGGTGTCATATTAATT
GTATTGGCTATTATTGCTTTAGTATTATGCAACTTTGTATCTGCAATACTCTGGTTTGTTTCAGCCATATCTATTTTAGGTAGAAAAAAATTAG
TAGCTGCAGCAGATACTACCACTATTCAAAAAAGTAAAGGGAACGCAAATCAAGCATCACATAAAGACACGTGTAAAAAGGAACTTGATA
GTCAAGACATGATGGAACATCCTGAGGTTAAAAATCCCACGACTAAAAACCTTGAAGGATTTAACGAAGAAATACATAAAGATGAAGCTAC
AACTAAAGTTGTCAGTGATAACACGGAACCGCCGATTGAATCAAAAGACCATGTCTCGAAAAAAGATTGA

Gene: butA (acetoin (diacetyl) reductase)

Position: 127821 to 128597, length: 777 nt, orientation: FORWARD

Perfect match to: (11819-97-CP003194-[134232:135008], allele observed in CC80+CC50)

Sequence:

ATGACAAACAACAAAGTAGCATTAGTAACTGGCGGAGCACAAGGGATTGGTTTTAAAATTGCAGAACGTTTAGTGGAAGATGGTTTCAAA
GTAGCAGTTGTTGATTTCAATGAAGAAGGGGCAAAAGCAGCTGCACTTAAATTATCAAGTGATGGTACAAAAGCTATTGCTATCAAAGCAG
ATGTATCAAACCGTGATGATGTATTTAATGCAGTAAGACAAACTGCAGCTCAATTTGGCGATTTCCATGTCATGGTAAACAATGCCGGCCTT
GGACCAACAACACCAATCGATACAATTACTGAAGAACAGTTTAAAACAGTATATGGCGTGAACGTTGCAGGTGTGCTATGGGGTATTCAAG
CCGCACATGAACAATTTAAAAAATTCAATCATGGTGGTAAAATTATCAATGCAACATCTCAAGCAGGCGTTGAGGGTAACCCAGGCTTGTC
TTTATATTGCAGTACAAAATTCGCAGTGCGAGGTTTAACACAAGTAGCCGCACAAGATTTAGCGTCTGAAGGTATTACTGTGAATGCATTCG
CACCTGGTATCGTTCAAACACCAATGATGGAAAGTATCGCAGTGGCAACAGCCGAAGAAGCAGGTAAACCTGAAGCATGGGGCTGGGAA
CAATTTACAAGTCAGATTGCTTTGGGCAGAGTTTCTCAACCAGAAGATGTTTCAAATGTAGTGAGCTTCTTAGCCGGTAAAGACTCTGATTA
CATTACTGGACAAACAATTATTGTAGATGGTGGTATGAGATTCCGTTAA

Gene: galE (UDP-glucose 4-epimerase)

Position: 128941 to 129912, length: 972 nt, orientation: FORWARD

Perfect match to: (11819-97-CP003194-[135352:136323], allele observed in CC80)

Sequence:

TTGGAAAGAGTTTTGATAACTGGTGGGGCAGGATTTATTGGGTCGCATTTAGTAGATGATTTACAACAAGATTATGATGTTTATGTTCTAGA
TAACTATAGAACAGGTAAACGAGAAAATATTAAAAGTTTGGCTGACGATCATGTGTTTGAATTAGATATTCGTGAATATGATGCAGTTGAA
CAAATCATGAAGACATATCAATTTGATTATGTTATTCATTTAGCAGCATTAGTTAGTGTTGCTGAGTCGGTTGAGAAACCTATCTTATCTCAA
GAAATAAACGTCGTAGCAACATTAAGATTGTTAGAAATCATTAAAAAATATAATAGTCATATAAAACGTTTTATCTTTGCTTCGTCAGCAGCT
GTTTATGGTGATCTTCCTGATTTGCCTAAAAGTGATCAATCATTAATCTTACCATTATCACCATATGCAATAGATAAATATTACGGCGAACGG
ACGACATTAAATTATTGTTCGTTATATAACATACCAACAGCGGTTGTTAAATTTTTTAATGTATTTGGGCCAAGACAGGATCCTAAGTCACAA
TATTCAGGTGTGATTTCAAAGATGTTCGATTCATTTGAGCATAACAAGCCATTTACATTTTTTGGTGACGGACTGCAAACTAGAGATTTTGTA
TATGTATATGATGTTGTTCAATCTGTACGCTTAATTATGGAACACAAAGATGCAATTGGACACGGTTATAACATTGGTACAGGCACTTTTACT
AATTTATTAGAGGTTTATCGTATTATTGGTGAATTATATGGAAAATCAGTCGAGCATGAATTTAAAGAAGCGCGAAAAGGAGATATTAAGC
ATTCTTATGCAGATATTTCTAACTTAAAGGCATTAGGATTTGTTCCTAAATATACAGTAGAAACAGGTTTAAAGGATTACTTTAATTTTGAGG
TAGATAATATTGAAGAAGTTACAGCTAAAGAAGTGGAAATGTCGTGA

Gene: wcaJ-tuaA (capsular polysaccharide biosynthesis glycosyltransferase)

Position: 129875 to 130567, length: 693 nt, orientation: FORWARD

Perfect match to: (11819-97-CP003194-[136286:136978], highly conserved allele)

Sequence:

TTGAAGAAGTTACAGCTAAAGAAGTGGAAATGTCGTGAAAATGACATTGAAGCTGTCCATAATAATAAGGGTTATGCCTATCAAAGAAAAT
TAGACAAACTAGAAGAAGTGAGAAAAAGCTATTACCCAATTAAACGTGCGATTGACTTAATTTTAAGCATTGTTTTATTATTTTTAACTTTAC
CGATTATGGTTATATTCGCCATTGCTATCGTCATAGACTCACCAGGAAACCCTATTTATAGTCAGGTTAGAGTTGGGAAGATGGGTAAATTA
ATTAAAATATACAAATTACGTTCGATGTGTAAAAACGCAGAGAAAAATGGTGCGCAATGGGCTGATAAAGATGATGATCGTATAACAAAT
GTCGGGAAGTTTATTCGTAAAACACGCATTGATGAATTACCACAACTAATTAATGTTGTTAAAGGGGAAATGAGTTTTATTGGACCACGCCC
GGAACGTCCGGAATTTGTAGAATTATTTAGTTCAGAAGTGATAGGTTTCGAGCAAAGATGTCTTGTTACACCAGGGTTAACAGGACTTGCG
CAAATTCAAGGTGGATATGACTTAACACCGCAACAAAAACTGAAATATGACATGAAATATATACATAAAGGTAGTTTAATGATGGAACTAT
ATATATCAATTAGAACATTGATGGTTGTTATTACAGGGGAAGGCTCAAGGTAG

Gene: epsF (capsular polysaccharide biosynthesis glycosyltransferase)

Position: 130777 to 131943, length: 1167 nt, orientation: FORWARD

Perfect match to: (KPL1845-AZJA01000015-[35775:36941], allele observed in CC96+CC5)

Sequence:

TTGAAAATTATATATTGTATTACTAAAGCAGACAATGGTGGTGCACAAACACATCTCATTCAACTCGCCAACCATTTTTGCGTACACCATGAT
GTTTATGTCATTGTAGGCAATCATGGACCAATGATTGAACAACTAGATGCAAGAGTTAATGTAATTATTCTCGAACATTTAGTAGGTCCAAT
TGACTTTAAACAAGATATTTTAGCTGTCAAAGTGTTAGCACAGTTATTCTCGAAAATTAAGCCTGATGTTATCCATTTACATTCTTCTAAAGCT
GGAACGGTCGGACGAATTGCGAAGTTCATTTCGAAATCGAAAGACACACGTGTAGTTTTTACTGCGCATGGATGGGCTTTTACAGAGGGT
GTTAAACCAGCTAAAAAAATTTCTATATCTAGTTATCGAAAAATTAATGTCACGTATTACAGATAGCATTATTTGTGTTTCAGATTTCGATAAA
CAGTTAGCGTTAAAATATCGATTTAATCGATTGAAATTAACCACAATACATAATGGTATTGCAGATGTTCCCGCTGTTAAGCAAACGCTAAA
AAGTCAATCACATAACAATATTGGCGAAGTAGTTGGAATGTTGCCTAATAAACAAGATTTACAGATTAATGCCCCGACAAAGCATCAATTTG
TTATGATTGCAAGATTTGCTTATCCAAAATTGCCACAAAATCTAATCGCGGCAATAGAGATATTGAAATTACATAACAGTAATCATGCGCAT
TTTACATTTATAGGCGATGGACCTACATTAAATGATTGTCAGCAACAAGTTGTACAAGCTGGGTTAGAAAATGATGTCACATTTTTGGGCAA
TGTCATTAATGCGAGTCATTTATTATCACAATACGATACGTTTATTTTAATAAGTAAGCATGAAGGTTTGCCAATTAGCATTATAGAAGCTAT
GGCTACAGGTTTGCCTGTTATAGCCAGTCATGTTGGCGGTATTTCAGAATTAGTAGCTGATAATGGTATATGTATGATGAACAACCAACCCG
AAACTATTGCTAAAGTCCTGGAAAAATATTTAATAGACAGTGATTACATCAAAATGAGTAATCAATCTAGAAAACGTTATTTAGAATGTTTT
ACTGAGGAGAAAATGATTAAAGAAGTGGAAGACGTTTATAATGGAAAATCAACACAATAG

Gene: wzy (capsular polysaccharide polymerase)

Position: 131924 to 133162, length: 1239 nt, orientation: FORWARD

Perfect match to: (394_SAUR-JVIV01000033-[7343:8581:r], allele observed in CC6+CC5+CC8)

Sequence:

ATGGAAAATCAACACAATAGTAAATTACTAACATTGTTACTTATCGGTTTAGCGGTTTTTATTCAGCAATCTTCGGTTATTGCCGGTGTGAAT
GTTTCTATAGCTGACTTTATCACATTACTAATATTAGTTTATTTACTGTTTTTCGCTAACCATTTATTAAAGGCAAATCATTTTTTACAGTTTTTC
ATTATTTTGTATACATATCGTATGATTATTACGCTTTGTTTGCTATTTTTTGATGATTTGATATTTATTACGGTTAAGGAAGTTCTTGCATCTAC
AGTTAAATATGCATTTGTAGTCATTTATTTCTATTTAGGGATGATCATCTTTAAGTTAGGTAATAGCAAAAAAGTGATCGTTACCTCTTATATT
ATAAGCAGTGTGACTATAGGTCTATTTTGTATTATAGCTGGTTTGAACAAGTCCCCTTTACTAATGAAATTGTTATATTTTGATGAAATACGT
TCAAAAGGATTAATGAATGACCCTAACTATTTCGCGATGACACAGATTATTACATTGGTACTTGCTTACAAGTATATTCATAATTACATATTC
AAGGTCCTTGCATGTGGTATTTTGCTATGGTCTTTAACTACAACGGGGTCTAAGACTGCGTTTATCATATTAATCGTCTTAGCCATTTATTTCT
TTATTAAAAAGTTATTTAGTAGAAATGCGGTAAGTGTTGTGAGTATGTTAGTGATTATGCTGATATTACTTTGTTTTACCTTTTATAATATCAA
CTACTATTTATTCCAATTAAGCGACCTTGATGCCTTACCGTCATTAGATCGAATGGCGTCTATTTTTGAAGAGGGCTTTGCATCATTAAATGA
TAGTGGGTCTGAGCGAAGTGTTGTATGGATAAATGCCATTTCAGTAATTAAATATACACTAGGTTTTGGTGTCGGATTAGTGGATTATGTAC
ATATTGGCTCGCAAATTAATGGTATTTTACTTGTTGCCCATAATACATATTTGCAGATCTTTGCGGAATGGGGCATTTTATTCGGTGCATTAT
TTATCATATTTATGCTTTATTTACTGTTTGAATTATTTAGATTTAACATTTCTGGGAAAAATGTAACAGCAATTGTTGTAATGTTGACGATGCT

GATTTACTTTTTAACAGTATCATTTAATAACTCAAGATATGTCGCTTTTATTTTAGGAATTATCGTCTTTATTGTTCAATATGAAAAGATGGAA
AGGGATCGTAATGAAGAGTGA

Gene: wzx (capsular polysaccharide extrusion protein)

Position: 133152 to 134582, length: 1431 nt, orientation: FORWARD

Perfect match to: (TCH959-AASB02000032-[4863:6293], allele observed in CC7+CC15+CC97+CC692)

Sequence:

ATGAAGAGTGATTCACTAAAAGAAAATATTATTTATCAAGGGCTATACCAATTGATTAGAACGATGACACCACTGATTACAATACCCATTAT
TTCACGTGCATTTGGTCCCAGTGGTGTGGGTATTGTTTCATTTTCTTTCAATATCGTGCAATACTTTTTGATGATTGCAAGTGTTGGCGTTCA
GTTATATTTTAATAGAGTTATCGCGAAGTCCGTTAACGACAAACGGCAATTGTCTCAGCAGTTTTGGGATATCTTTGTCAGTAAATTATTTTT
AGCGTTAACAGTTTTTGCGATGTATATGGTCGTAATTACTATATTTATAGATGATTACTATCTAATTTTCCTACTACAAGGAATCTATATTATA
GGTGCAGCACTCGATATTTCATGGTTTTATGCTGGAACTGAAAAGTTTAAAATTCCTAGCCTCAGTAATATTGTTGCGTCTGGTATTGTATTA
AGTGTAGTTGTTATCTTTGTCAAAGATCAATCAGATTTATCATTGTATGTATTTACTATTGCTATTGTGACGGTATTAAACCAATTACCTTTGT
TTATCTACTTAAAACGATACATTAGCTTTGTTTCGGTTAATTGGATACACGTCTGGCAATTGTTTCGTTCGTCATTAGCATACTTATTACCAAA
TGGACAGCTCAACTTATATACTAGTATTTCTTGCGTTGTTCTTGGTTTAGTAGGCACATACCAACAAGTTGGTATCTTTTCTAACGCATTTAAT
ATTTTAACGGTCGCAATCATAATGATTAATACATTTGATCTTGTAATGATTCCGCGTATTACCAAAATGTCTACCCAGCAATCACATAGTTTA
ACTAAAACGTTAGCTAATAATATGAATATTCAATTGATATTAACAATACCTATGGTCTTTGGTTTAATTGCAATTATGCCATCATTTTATTTAT
GGTTCTTTGGTGAGGAATTCGCATCAACTGTCCCATTGATGACCATTTTAGCGATACTTGTATTAATCATTCCTTTAAATATGTTGATAAGCA
GGCAATATTTATTAATAGTGAATAAAATAAGATTATATAATGCGTCAATTACTATTGGTGCGGTAATGAATTTAGTATTATGTCTTGTTTTGA
TATATTTTTATGGAATTTACGGTGCGGCTATTGCACGTTTAATTACAGAGTTTATCTTACTTATTTGGCGATTTGTTGATATTACTAAAATCAA
TGTGAAGTTGAATATTGTAAGTACGATTCAATGTGTCATTGCTGCCGTTATGATGTTTATTGTGCTTGGTGTGGTCAATCATTATTTGCCCCC
TACAATGTACGCTACGCTGCTATTAATTGCGATTGGTATAGTAGTTTATCTTTTATTAATGATGACTATGAAAAATCAATACGTATGGCAAAT
ATTGAGGCATCTTCGACATAAAACAATTTAA

Gene: sodA-L1 (superoxide dismutase, locus 1)

Position: 134850 to 135449, length: 600 nt, orientation: FORWARD

Perfect match to: (CN1-CP003979-[115432:116031], highly conserved allele)

Sequence:

ATGGCATTTAAATTACCAAATTTACCATATGCATATGATGCATTGGAACCATATATAGATCAAAGAACAATGGAGTTTCATCACGACAAACA
TCACAATACGTACGTGACGAAATTAAACGCAACAGTTGAAGGAACAGAGTTAGAGCATCAATCATTAGCGGATATGATTGCTAACTTAGAC
AAGGTACCGGAAGCGATGAGGATGTCAGTCCGTAATAATGGCGGTGGTCATTTTAACCATTCATTATTCTGGGAAATACTATCACCTAATTC
TGAAGAAAAAGGTGGTGTAATAGATGACATCAAAGCGCAGTGGGGCACTTTAGATGAATTTAAAAATGAATTTGCAAATAAAGCAACAAC
ATTATTTGGATCAGGTTGGACTTGGTTAGTTGTTAATGATGGCAAATTAGAAATTGTGACAACGCCAAACCAAGATAATCCATTAACAGAA
GGCAAAACACCAATCTTACTATTTGATGTTTGGGAGCATGCCTACTATCTGAAATATCAAAATAAACGTCCAGACTATATGACTGCATTTTG
GAATATTGTTAACTGGAAAAAAGTTGATGAATTATACCAAGCAGCAAAATAA

Gene: sasD (Staphylococcus aureus surface protein D)

Position: 135818 to 136543, length: 726 nt, orientation: FORWARD

Perfect match to: (Strain_21333-AHKA01000042-[112710:113435:r], allele observed in CC80)

Sequence:

ATGAAAAAATTAGCAACAGTAGGTTCTTTAATTGTAACAAGCACTTTAGTATTCTCAAGTATGCCTTTTCAAAATGCGCATGCCGACACAACT
TCAATGAATGTGTCGAATAAACAAAGCCAAAATGTACAAAATCATCGTCCTTATGGCGGAGTAGTACCACAAGGAATGACGCAAGCACAAT
ATACTGAATTAGAGAAAGCTTTACCCCAATTAAGCGCTGGCAGTAATATGAAAGACTATAATATGAAATTGTATGATGCGACGCAAAATAT
TGCTGATAAATACAATGTGATAATTACAACTAATGTAGGGGTATTTAAACCACATGCTGTTAGAGATATGAATGGCCATGCGTTACCTTTAA
CAAAAGATGGCAATTTTTATCAAACGAATGTAGATGCAAATGGTATTAATCATGGTGGTAGTGAAATGGTGCAAAATAAAACAGGTCATAT
GAGTCAACAAGGCCATATGAATCAGAACACACACATGAACCAACAGCCACACATGCAACAAGGTCATATGCAATCATCAAACCATCAAATG
ATGAGTCCAAAAGCAAATATGCAATCATCAAATCATCAAATGAACCAAAGTAACAAAAAAGTTTTACCAGCTGCTGGTGAAAGTATGACAT
CAAGTATACTTACTGCAAGTATTGCCGCACTACTATTAGTATCTGGTTTATTCTTAGCATTTAGACGACGTTCAACAAATTAATAA

Gene: Q5HJN3 (transcriptional regulator, GntR family)

Position: 136734 to 137489, length: 756 nt, orientation: REVERSE

Perfect match to: (11819-97-CP003194-[143145:143900:r], allele observed in CC80)

Sequence:

TTAATAATCCGATTGTCTTATACGTGTCAGTGTTAATTCAGATATTTCCTGTGGAATATACCACTTATTAATCATAATTGGATAAGGTGTTTGT
GCGTACAATGTTTCAATAATCAGCCAACAATGTGTATCACCATCAAACACGTGACTATGATTTTTGAAGTGGGGTGCTTTGGTAATAGACAT
TTTTAAATCTGATTGATATGCATTGTTATAAATCGTTTGCTCAACGAACGTCTTCATGTCGTCTTCGTTTTGTGTATTCACTTTAAATGTGTCA
ATGACATTTAACGGTATAAAGGTAAAACAAAATGCATCAGCTTGCTTAGAATGATTGTCCTTTTTTTGATAATAGCGTTCCATTGCAATGAC
GGCAGAAGGATGGTTTGCAAACAAATGATTTGTATATTCACTTTCTAAATCAACACGATAATTAATTGATGACATAGATACGCGAGCTAGCA
ATATTTGATCAAGTGGATGCTTAAATTGATCCATACTTGAAGCGTGTTGGGCATTTGTTTGTGGAATAACAAAGTGTCCTTTCCCTCTTGTAC
TCTCTACGATGCCATCTTCGGCTAACAATTTTATAGCTTGGCGCAAAGTCATACGACTGACATCAAAGCGCGCACAAAGTTCCTTTTCAGTA
GGTAATGCATGGCCACTCGGATATTTTCCTATTTGAATTTCTTTATATAACGTATTATAAATCGTTAAAAATTTTGGTTGTGTTTGCGTCACGT
AGACAACCTCCAT

Gene: deoD-L1 (purine nucleoside phosphorylase locus 1)

Position: 137725 to 138432, length: 708 nt, orientation: FORWARD

Perfect match to: (N315-BA000018-[150926:151633], highly conserved allele)

Sequence:

ATGAAATCAACACCACACATTAAACCAATGAATGACGTCGAAATTGCAGAAACGGTTCTATTGCCAGGAGATCCGTTAAGAGCTAAGTTCA
TTGCAGAAACTTATTTGGATGATGTGGAACAGTTCAATACAGTGCGAAACATGTTTGGTTTTACCGGAACATATAAAGGTAAAAAAGTTTCT
GTCATGGGTTCAGGTATGGGTATGCCATCTATTGGCATTTACTCTTATGAATTAATTCATACATTTGGTTGTAAAAAATTAATTCGCGTTGGC
TCTTGTGGCGCGATGCAAGAAAACATTGATTTATATGATGTGATTATTGCACAAGGTGCCTCTACTGATTCAAATTACGTTCAACAATATCA
ATTACCAGGTCATTTTGCGCCAATTGCTTCTTATCAATTATTAGAAAAAGCAGTTGAAACAGCACGTGACAAAGGTGTACGTCATCATGTAG
GTAATGTGTTATCAAGTGATATTTTCTATAACGCGGATACAACAGCGAGTGAACGTTGGATGCGTATGGGTATTTTAGGTGTAGAAATGGA
ATCAGCTGCATTATACATGAATGCAATTTACGCTGGTGTCGAAGCATTAGGTGTGTTCACAGTGAGCGATCATTTAATTCATGAAACGTCAA
CAACACCTGAGGAAAGGGAACGTGCATTTACAGATATGATTGAAATTGCACTGTCATTGGTGTAG

Gene: tet38 (major facilitator superfamily permease)

Position: 138439 to 139791, length: 1353 nt, orientation: FORWARD

Perfect match to: (COL-CP000046-[136988:138340], highly conserved allele)

Sequence:

ATGAATGTTGAATATTCTAAAATAAAGAAAGCAGTACCTATTTTATTATTCTTATTTGTATTCAGTTTGGTTATAGACAACTCATTTAAATTGA
TTTCTGTAGCCATTGCTGATGACTTAAACATATCTGTAACGACAGTAAGTTGGCAAGCGACATTAGCCGGTTTAGTAATTGGTATTGGCGCT
GTAGTATACGCTTCATTATCTGATGCCATTAGTATACGCACACTATTTATTTATGGCGTGATATTAATCATTATCGGATCAATTATTGGTTACA
TTTTCCAACATCAATTCCCATTACTTTTAGTTGGACGTATTATTCAAACTGCCGGTTTAGCTGCTGCAGAGACATTATATGTGATATATGTTGC
AAAGTATCTTTCTAAAGAGGACCAGAAGACTTACCTTGGCTTAAGTACGAGCAGTTATTCCTTGTCATTAGTTATCGGTACATTATCAGGTG
GATTTATTTCTACGTATTTACACTGGACAAATATGTTTTTAATTGCATTAATCGTAGTATTTACGTTGCCATTCCTATTTAAATTATTACCAAAA
GAAAATAATACGAATAAAGCTCATTTAGATTTTGTTGGCTTAATTCTAGTGGCAACTATTGCTACAACAGTCATGCTGTTTATTACGAACTTT
AATTGGTTATATATGATTGGTGCCTTAATTGCGATTATCGTTTTTGCGCTATATATTAAAAATGCGCAACGTCCATTAGTAAATAAATCATTTT
TCCAAAATAAACGTTATGCTTCATTTTTATTTATAGTATTTGTAATGTATGCTATCCAATTGGGTTATATTTTTACGTTCCCATTCATAATGGA
GCAAATTTATCATCTGCAACTAGACACAACATCACTGTTATTAGTACCGGGTTATATAGTAGCAGTCATTGTTGGTGCACTAAGTGGTAAAA
TCGGCGAATATCTGAATTCAAAACAAGCGATTATCACAGCAATTATTTTAATAGCACTGAGCTTGATTTTACCTGCATTTGCAGTAGGTAATC
ACATTTCAATCTTCGTCATTTCTATGATATTCTTTGCAGGTAGCTTTGCTTTAATGTATGCACCTTTACTTAACGAAGCCATTAAAACAATAGA
TCTTAATATGACAGGTGTGGCTATTGGTTTTTATAATTTAATTATTAATGTGGCGGTATCTGTAGGTATTGCGATTGCTGCGGCTCTAATCGA
TTTTAAAGCATTAAATTTCCCAGGCAATGATGCATTAAGTTCACATTTCGGTATTATTTTAATTATTTTAGGTTTAATGAGTATTGTCGGATTA
GTTTTATTCGTCAGCTTAAATCGTTGGACACAATCTGAAAAATAA

Gene: deoC-L1 (deoxyribose-phosphate aldolase locus 1)

Position: 139872 to 140534, length: 663 nt, orientation: FORWARD

Perfect match to: (MW2-BA000033-[130537:131199], allele observed in CC1+CC8+CC25+CC97+CC4803)

Sequence:

ATGAAATTTGAGAAATATATAGATCACACTTTATTGAAGCCTGAGTCAACACGTACGCAAATCGATCAAATCATCGATGAAGCGAAAGCAT
ACAATTTTAAATCTGTATGTGTGAATCCAACACATGTTAAATATGCAGCAGAGCGACTAGCTGATTCAGAGGTGCTCGTTTGTACGGTAATA
GGATTCCCATTAGGTGCGTCGACAACTGCAACGAAAGCATTTGAAACAGAAGATGCAATTCAAAATGGTGCAGATGAAATTGACATGGTC
ATCAACATCGGCGCATTAAAAGATGGACGTTTTGATGATGTACAACAAGACATTGAAGCAGTGGTTAAAGCTGCGAAAGGTCACACAGTA
AAAGTGATTATTGAGACGGTATTGTTGGACCATGACGAAATTGTAAAAGCGAGTGAATTAACAAAAGCGGCTGGTGCGGACTTCGTTAAA
ACTTCAACAGGTTTTGCAGGTGGCGGTGCGACTGCAGAAGACGTTAAATTAATGAAAGATACAGTAGGTGCTGATGTAGAAGTAAAAGCA
TCAGGTGGCGTACGTAATTTAGAAGATTTCAATAAAATGGTTGAAGCAGGTGCGACACGTATTGGTGCGAGCGCAGGTGTTCAAATTATGC
AAGGTTTAGAAGCAGATTCAGATTACTAA

Gene: deoB (phosphopentomutase)

Position: 140562 to 141740, length: 1179 nt, orientation: FORWARD

Perfect match to: (N315-BA000018-[153763:154941], highly conserved allele)

Sequence:

ATGACAAGACCATTTAATCGTGTACATTTAATCGTAATGGATTCAGTAGGTATTGGTGAAGCGCCAGACGCAGCTGATTTTAAAGATGAAG
GTTCACATACTTTAAGACATACCTTAGAAGGTTTCGATCAAACTTTACCAAACCTTGAAAAGTTAGGTCTAGGGAACATCGATAAATTACCA
GTAGTAAATGCAGTTGAACAACCAGAAGCATACTATACTAAATTGAGTGAAGCTTCAGTTGGTAAAGATACAATGACTGGTCACTGGGAAA
TTATGGGATTAAATATTATGCAACCTTTTAAAGTATACCCTAATGGATTCCCTGAAGAGTTAATTCAACAAATTGAAGAAATGACAGGTCGT
AAAGTTGTTGCTAACAAACCGGCATCGGGTACGCAAATTATCGATGAGTGGGGCGAGCACCAAATGAAAACTGGTGACTTAATTGTTTATA
CAAGTGCAGACCCAGTATTGCAAATTGCTGCACATGAAGACATTATCCCATTAGAAGAGTTATATGATATTTGTGAAAAGGTTCGTGAGTT
GACAAAAGACCCTAAATATTTAATTGGTCGTATTATCGCACGTCCATATGTTGGTGAACCAGGAAACTTTACACGTACATCTAATCGACATG
ACTATGCGTTAAAACCTTTTGGTAAAACTGTCTTAGATCATTTGAAAGACGGTGGTTATGATGTTATTGCCATCGGTAAAATTAATGACATTT
ATGATGGTGAAGGTGTAACAGAAGCGGTTCGTACGAAGAGTAACATGGACGGTATGGATCAATTGATGAAAATTGTTAAGAAAGATTTCA
CAGGTATTAGCTTCTTAAACTTAGTAGACTTTGATGCATTATACGGTCATCGTCGTGATAAACCAGGTTATGCACAAGCAATTAAAGATTTC
GATGATCGCTTGCCAGAACTGTTTAGCAACTTAAAAGAAGACGATTTAGTAATTATTACAGCAGACCATGGTAATGACCCGACAGCGCCAG
GTACGGACCATACGAGAGAATATATCCCAGTAATTATGTACAGTCCGAAATTTAAAGGTGGTCATGCACTAGAAAGTGATACTACATTCAG
TTCTATCGGTGCAACTATAGCAGATAATTTCAACGTAACATTACCAGAGTTCGGTAAAAGTTATTTAAAGGAATTGAAATAG

Gene: phnE2 (phosphonate ABC transporter, transmembrane permease)

Position: 141871 to 142686, length: 816 nt, orientation: REVERSE

Perfect match to: (502A-CP007454-[1013764:1014579], allele observed in CC5)

Sequence:

TTAAACAATACGTTTTCGGATTGAACCGGAAATTAAATCGACAATTGCGACCATTAGTACTAAACCGATTAATATAATACCTACACGGTCCC
AAGAACGTGTTTGAATGGCAAATATGAGTGGTGTCCCGATACCACCAGCCCCAATTAGCCCCAGTATAGAAGCTGAACGTAAGTTTAGTTC
AAAGCGATAAAGTATGAGTGATAGAAAGGCAGGCATAATTTGTGGTATGACTGCAAATACGAGTGTTTTAATTTTATTCGCACCACTGGCC
TTTAATGATTCTACAGCACTGAAATCTAGACCTTCAATATCTTCAGCTAAAAGTTTCCCAAGCATACCTACGGAATGGATACCTAAAGCTAAT
ACACCTGAAAATGAACCTGGGCCAACAGCTTTGATAAATATAAGTGCCATTACAATTTCTGGGAAGACACGTATAACACTTAAAATAAATTT
GCTAACACCTGAAACCGGGCGTAACTTTACCATATTATTTGCACCTAAAAATGCTAATGGAATACAGATAATTGCGGCGATGAAAGTACCTA
CAACGGCTATCGCAAAGGTTTCAAGTAAACCACGTAATAAGTCTTCGCCATCTGGTATATAGATATAGCTGATGTCAGGATGGAATAATCC
GCTGAATATGGATTTTAAGATTTCTAATGATTTACTTTTAAGTTCTAAACTTGGTACACCTGCAAATGCCCAGATGATAATAGCTAAGACGAC
AATTGCAATAAGCCATCTTTTAATCAATTTTCGTTTGTGTGCTTTTGTGTGAACATTATATTTTGCTATTTCCTGTGTCAT

Gene: phnE1 (phosphonate ABC transporter, transmembrane permease)

Position: 142683 to 143483, length: 801 nt, orientation: REVERSE

Perfect match to: (ED133-CP001996-[105204:106004:r], highly conserved allele)

Sequence:

TCATGCGAGATGTGCCCTCACTTTCGTACTGATGTAATCAATGACGACGACGATAACTAAAGTAAATAAAATAATCGTTGCTGTTTTTGGAT
ATTGAAATAAACCAAGTGTTTGATCATAAAACAATCCAATACCGCCAGCGCCGACTAATCCAAGCACAGCTGAAGCTCGTATATTTACTTCA
AATGCATATAATACGTATGACATAAATGACGATATGGCTTGTGGTACAACACCGAAAACAATCCATTTTATTTTATTAGCGCCAACAGCCGT
CATTGCTTCCATTGGACCTGGATCTATCGTTTCCAATGATTCATATAATAATTTTCCAATAATACAGATAGTTAAAATAAACAATGCTAATATC
CCTGGAATTTGACCGATTCCAAATACAGCCACAAAGATTGCTGCTAATAACAAATCTGGAATAGTACGAACTATATTTAAAATAAAGCGCGA
GGGTATTGAAATCCACTTTTGATGAACGATATTGCTAGCACATAATAACGCAATTGGTATTGAAACGATGCTACCTAATACTGTACTTACGA
TAGCCATTCGAATGGTATCTAACATTGGCGTTGTAATTTGTTGTAAATACTCGAAATCAGGTGGAATCATTTGTTTGAATAGATCACCTATTT
GAGGTATTCCTATCATTAAATCTCCAAAATTAAACCCCGTATAAATGAAGCTCCAAATGATAAGCACAATGATTAACATGAAGGTAAAACTC
GTTTTTAAAGAAACCTTTTTCTTTAAAAGGGAGTCATACTTTGTAGGTATTTCTAAAGGCAT

Gene: phnC (phosphonate ABC transporter, ATP-binding protein)

Position: 143485 to 144258, length: 774 nt, orientation: REVERSE

Sequence:

TTAGTTCACTCCTAGCTTTTCATCTTCTTTAATTGTACGTCCATATATTTCACTAAATACGTCATCTGTTGCTTCAGATGCAGGACCATCATAG
ACAACTTCACCATCACGTAAACCAATGATGCGTGTACCATATTCTTTTGCCAAGTCAACAAAATGTAAATTAATTAAAATTGTGATGCCTAAT
TCTTGGTTGATTTTTCTTAAATCATCCATAACCTGTTTCGTTGTTAATGGGTCTAATGAAGCAACTGGTTCATCTGCAAGAATAATTTCGGATT
CTTGGCATAGCGCACGTGCAATAGATATACGTTGTTGTTGGCCACCTGATAATTCATCAGAGCGTTGATTGTATTTATCTAAGATATTGACG
CGTTCTAGTGCATCCATTGCTTTAATTTTGTCTTCTTTTGGGAATAAACCTAATACCATTTTCCAAGTAGGGTGATAACCTACACGTCCACTTA
GTACATTTCGTAATACACTTGACCGTTTAACTAAATTAAAATGTTGGAAAATCATACCTATATTTCGGCGCATTTCTAATAATGCTTTACCATG
GGCTTTAGTGATGGATTTACCTTGGATGAAAATTTCACCTGACGTGATATCATGCAAACGATTTACAGATCTTAATAACGTGGATTTCCCAG
CACCAGATAGTCCGACAATAACTGCAAATTCACCTTTTTCAATATTTAAGTTAATATTTTTCAAGCCTACATGACCGTTAGGATAGACTTTACT
GACGTTTTTAAATTCGATTTGACTCAT

Gene: phnB (phosphonate ABC transporter, substrate-binding protein)

Position: 144453 to 145409, length: 957 nt, orientation: REVERSE

Perfect match to: (11819-97-CP003194-[150864:151820:r], allele observed in CC80+CC88+CC692)

Sequence:

TTATTTCATATCTTTAACTAATTTTTCGTACTCTCTTACAATGTCGAAATTTGAATCTTTCGTTTCTGTGTATCCTTCATGTGAATAAACTTCGCT
AATAATTTTGTGACCTTCTTTTGATTTAGCAATGTCTATAAAAGCTTTTTTCAATTTTTCTTGGAAATCTTTATCCATATCTGGTCTTACAGAAA
TTGTGTCATTCGGAATAGCTTGTGTTAATTTTAAAATTCGTGTGTCTTTAAATACATTTGGTTGGTCTTTTTTCACAGTATTACGTGCATCGTT
AAATACAGCCGCAGCATCTACATCTCCATTTAATAATGAGATAACTGCTTGGTCATGACCTTTAACATTCACAATTTTCATATCTTTAGTTGCA
TTAATACCTGCTTCGTTTTTTAACATCGCAAGTGGGAATGTATATCCAGCAGTTGATGTTACATCTTGTAAGGCAATTTTCTTACCTTTTAAAT
CTTTCAAGCTTTTAATTTTTGAGTCTTTTTTAACAAGAATTTCTGATTTATAGCTATCTACAAGTTCTTTACTTGCTGAACCATCTTCTTTTACAC
CGAAACGTTGTGCTTGTAATAATAAATCAGCTGCTTTTTGATCATGTGCTAATGTGTATGCCGTTGGTGGTAAGAAACCAACATCAACTTTTT
TAGACTTCATAGCTTCAACAATTGTATTGTAGTTAGTTGATACAGACACTTTAACTGGAATCCCTAATTCTTTAGATAGTAATTTTTCTAATGG
TTTTGCTTTAGCTTCTAATGTTCCAGCATTTTGCGAAGGTACAAATTGAACGGTTAATTCTTTAGGTTTGTATCCTCCTGATTTAGAATCCGAA
TCATTACTAGCGTTCTTTTGATTATCTAAAGAACTTGAATTTCCACATGCTGCTGCAAAAACAATGACTGCTAACATTAATACAAATAAACAC
TTAAAATTTTTCAT

Gene: Q5HJM4 (putative exported protein)

Position: 145638 to 147182, length: 1545 nt, orientation: FORWARD

Perfect match to: (Strain_16125-HE579067-[134680:136224], allele observed in CC5+CC1+CC8+CC97)

Sequence:

ATGAAAAAAATATATAAGTCATTAACTGTCTCTGCAATTGTTGCAACGGTATCATTAAGTGCTTTACCGCAATCTTTAGCTATAACGCATGAA
TCGCAACCTACAAAGCAACAGCGAACGGTATTATTCGATCGTTCTCATGGTCAAACAGCTGGTGCTGCAGATTGGGTTAGTGATGGTGCAT

TTTCAGATTATGCGGATTCAATACAAAAACAAGGTTATGACGTTAAAGCTATTGATGGTCATTCGAACATAACAGAAGCAAGTTTGAAAAG
TTCCAAAATATTTGTAATTCCTGAGGCTAACATTCCTTTCAAAGAATCAGAACAGGCAGCAATTGTTAACTATGTGAAACAAGGTGGCAATG
TTGTCTTTATTTCAGATCATTACAATGCTGACCGAAATTTAAATCGTATTGATTCATCGGAGGCAATGAATGGTTATCGACGTGGAGCATAT
GAAGATATGTCGAAAGGTATGAATGCAGAAGAAAAAAGTTCTACTGCAATGCAAGGTGTGAAAAGTTCAGATTGGTTATCTACAAACTTTG
GCGTACGTTTTCGATATAATGCACTAGGTGATTTAAATACGAGCAATATTGTTTCTTCAAAAGAAAGTTTCGGTATTACTGAAGGTGTGAAA
TCTGTCTCTATGCATGCCGGATCGACATTAGCAATTACTAATCCAGAGAAAGCAAAAGGTATTGTGTATACACCAGAACAATTGCCAGCGA
AAAGTAAATGGTCACATGCTGTAGATCAAGGTATTTATAATGGGGGCGGTAAAGCAGAAGGCCCCTATGTAGCAATTTCTAAAGTTGGAA
AAGGTAAAGCAGCATTTATCGGTGATTCATCACTTGTGGAAGATAGTTCGCCCAAATATGTAAGAGAAGATAATGGAGAAAAGAAGAAAA
CATATGATGGTTTTAAAGAACAAGACAACGGTAAGCTATTAAATAATATAACGGCTTGGATGTCTAAAGATAATGATGGGAAATCACTTAA
GGCGAGTGGCCTAACATTAGATACAAAGACTAAGTTGCTTGATTTTGAACGACCAGAGCGTTCAACTGAGCCTGAAAAAGAGCCATGGTC
ACAACCGCCGAGTGGTTATAAATGGTATGATCCAACAACATTTAAAGCAGGTAGTTATGGCAGCGAAAAAGGCGCAGATCCTCAGCCAAA
CACACCAGATGATCATACGCCACCAAATCAGAACGAAAAAGTAACATTTGATATCCCGCAAAATGTTTCTGTAAATGAGCCATTTGAAATGA
CAATACATTTAAAAGGATTTGAAGCAAATCAAACACTTGAAAATCTTAGAGTTGGTATTTACAAAGAAGGCGGACGTCAAATCGGACAATT
TTCAAGTAAAGATAACGATTATAACCCACCAGGTTACAGTACTTTGCCAACAGTTAAAGCAGATGAAAACGGAAATGTCACAATTAAGGTC
AATGCTAAAGTACTTGAAAGTATGGAAGGTTCAAAGATTCGTTTAAAACTCGGTGACAAAACCTTGATTACAACAGACTTCAAATAA

Gene: cpdB (putative 5' nucleotidase)

Position: 147233 to 148768, length: 1536 nt, orientation: FORWARD

Sequence:

ATGTCAAACATAGCATTTTATGTCGTGAGTGACGTACATGGTTATATTTTCCCAACAGATTTTACGAGTAGAAATCAATATCAACCTATGGG
ATTGTTACTAGCGAATCATGTTATAGAACAAGACAGAAGGCAGTATGACCAAAGTTTTAAAATAGATAATGGTGATTTTTTGCAAGGGTCA
CCATTTTGTAATTACTTAATCGCGCATAGCGGCAGTAGCCAGCCTTTAGTTGATTTTTATAATCGAATGGCATTCGACTTTGGTACGCTTGGT
AATCATGAATTTAATTATGGATTGCCATACTTAAAAGACACTTTACGCAGACTCAATTATCCAGTTTTGTGCGCTAATATATATGAAAATGAT
AGTACATTGACTGATAACGGTGTGAAGTATTTTCAGGTTGGAGATCAAACGGTTGGTGTGATAGGTTTAACGACACAATTTATTCCCCATTG
GGAACAACCAGAGCATATTCAGTCACTTACGTTTCATAGTGCTTTTGAAATACTTCAACAATACTTACCTGAAATGAAGCGACATGCAGATA
TCATTGTGGTTTGTTACCATGGTGGATTTGAAAAGGATTTAGAAAGTGGTACGCCGACCGAAGTATTAACGGGTGAAAATGAAGGATATG
CCATGTTAGAAGCGTTTTCTAAAGATATAGATATCTTTATTACGGGTCACCAACATCGACAAATTGCTGAAAGGTTTAAGCAAACGGCTGTG
ATTCAACCTGGTACGAGAGGTACAACTGTAGGCAGAGTAGTCTTGAGTACTGATGAATATGAAAATTTATCCGTTGAATCATGTGAATTACT
TCCTGTTATAGATGATTCCACATTTACTATTGATGAAGATGACCAACATTTACGAAAGCAGTTAGAGGACTGGTTAGATTACGAAATTACTA
CATTGCCATATGATATGACGATTAATCATGCATTTGAGGCAAGTGTGGCACCGCATCCTTTTACAAATTTTATGAATTACGCTTTATTAGAAA
AAAGTGGAGCAGATGTTGCCTGTACAGCTTTGTTTGATTCTGCTAGTGGTTTCAAGCAAGTCGTGACGATGCGAGATGTTATTAACAATTAC
CCATTTCCAAATACATTTAAAGTTTTAGCTGTAAGTGGTGCCAAACTTAAAGAAGCCATTGAACGATCAGCAGAATATTTTGACGTGAAAAA
TGATGAAGTAAGTGTGAGCGCAGACTTCCTTGAACCCAAACCACAACACTTTAATTATGATATATATGGTGGCGTAAGTTATACCATTCATG
TTGGAAGACCAAAGGGACAACGTGTGAGCAATATGATGATACAAGGTCACGCAGTTGATTTAAAACAGACATATACAATTTGTGTAAATAA
TTATCGTGCAGTTGGCGGTGGTCAGTATGATATGTATATCGACGCGCCAGTTGTAAAAGATATTCAAGTTGAAGGCGCACAACTACTTATT
GATTTTTTATCAAATAATAAATTGATGCGCATCCCGCAAGTTGTTGATTTTAAAGTTGAAAAGTGA

Gene: Q1Y4B9 (putative DNA-binding protein)

Position: 148835 to 149049, length: 215 nt, orientation: TRNC-FRWD (no start codon)

Sequence:

CTTTAGACGACGTGATTGAAATGTCACGTCCTGTCAAAATGAATAGTTTATTAAAGTTGTATTTTGATAGAAATTTACTGACACCACAAAAAT
TATTGAATTATTTAAAAGTTGATGAAACATTTTTGAATCATCTAGCAGGTATTAATTTAAAACTCTTTAAGGATTATGTTAATGAAAATAGAG
AATATAATATAACGAATCTATATAAATAA

Gene: repD-chr (pseudogene similar to plasmid replication initiation protein)

Position: 149308 to 150200, length: 893 nt, orientation: TRUNCATED

Sequence:

TTTGTAAAACTGGATACTCTAATATCCGGTAATAGCCGGTTAAATCGACATAGGATGTCACTAGCTATTCCAAAATTGTCTTTTGACGCTATA
ACGATTGTTGGGAATCTTAGCATGTCTAACGCAGAGAGACTTTCACATTTTATGAGTACTAATCCTGAAATTCGGCTTTGGGATATTTTACA
AACAAACTTTAAAGCTAAAGCTCTTAAAGAAAAAGTTTATATTGAATATGACAAAATAAAAGCAACTCTTTGGAATAGACGTAGTATGCGC

GTTGAATTTAATCCTAATAAGCTTTCGCATGATGAAGTGCTTTGGTTAAAACAAAATATCATCAGTTATTTGGACGATGTTAGTTTTACGAGA
TTAGATTTGGCTTTTGATTTTGAATTTGATTTAAATGACTATTATGCATTGTCAGATAAGTCGGCAAAGAAAACTATATTTTATGGACGTAAT
GTAAAACCAGAAACAAAATATTTTGGTGTGCGTAATAGTGATAGGTTTATTCGGATTTATAATAAAAACAAGAACGTAAAGATAATGCAGA
TGTTGAAATTGATTCAACATTTCTATGGCGTGTGGAAATTGAATTAAAACGAGATATGGTTGATTGTTGGAAAGATTGTTTTGATGATTTGC
ATATTTTAAAACCGAATTTAAAAATGATTGAAAATATACAAGAACGAGCAATGCTTCATTTATTAACTCACGAAGAAGAGGAATGGGGAAA
TTTAGAAAGACGTACTAAAAATAAATATAGAGATAAGTTGAAAAATATAGCGTCTATTGATTTGACAGATTTAATGAAAATATCTTTAAGAG
GAAATGAAAACCAATTGCAAAAACAAATCGACTTTTGGTTGAATTAATTATTATAACTAAAGAAC

Gene: Q2G1L1 (putative protein)

Position: 150371 to 150658, length: 288 nt, orientation: FORWARD

Perfect match to: (MW2-BA000033-[141055:141342], allele observed in CC1+CC8+CC25+CC72+CC80)

Sequence:

ATGTTTATAATAATATTGAAAGATATTTCTGTTTTCCTTTTGAATAATAAACTTAAAAGAGAATATAAAATAATGCTTATTAAAATTAGGGGT
GGAAATATGAAAAGAGATTTTTTTGAAAAGTGGTCGTTTATTTTAAATTTAATTTTATTTTGTGCTTTTTTGTTTTTAGTGGTTCTGATGTTTT
ATAAGGATGTCGATTTGATTTATATTTTTTGTACATTATTAATATCATTATTACCTTTGCTAAATATTATAAAAAGATATAAGAAAAAGACGG
ATAATTAA

Gene: tnpIS200 (transposase of IS200)

Position: 150987 to 151471, length: 485 nt, orientation: FORWARD

Perfect match to: (11819-97-CP003194-[157398:157882])

Sequence:

ATGTCATCTGACACAAACAGTTTAGCACATACAAAATGGAAGTGTAAGTAACATATTGTCTTTGCACCTAAATACAGAAGACAAGTGATATA
TGGAAAAATAAAAAAAGATATAGGGATTATATTACGTCAATTATATGAAAGAAAAGGTGTAGAGATAATTGAAGCAGAGGTATGTAAAGA
TCATATCCATATGTTAGTAAGTATACCACCCAAACTTGGGGTATCATCATTTGTTGGCTATTTAAAAGGAAAAGTAATTTAATGATATTTGAT
AGACATGCTAACTTAAAGTATAGATATGGAAATAGAAAGTTTTGGTGTAAAGGATTTTATGTGGATACAGTAGGTGGAAATAAAAAAGTG
ATTGAAAATTATATTCGTAATCAATTACAAGAGGATATCGTTGCAGACGAAATCTCAATGGAAGAATATTTAGATCCTTTCACTGGAGAGAA
AAATAAAAAAAGAAAGAAAAAAGAGTAA

Gene: adhE (alcohol-acetaldehyde dehydrogenase)

Position: 151921 to 154530, length: 2610 nt, orientation: FORWARD

Perfect match to: (CIG1770-AHVN01000004-[730686:733295], allele observed in CC8+CC5+CC97)

Sequence:

ATGTTAACTATACCTGAAAAAGAAAATCGTGGATCGAAAGAACAAGAAGTGGCAATTATGATTGATGCTCTAGCTGACAAAGGGAAAAAA
GCATTAGAAGCATTATCTAAAAAGTCACAAGAAGAAATTGATCATATTGTTCATCAAATGAGCTTAGCAGCTGTTGATCAACATATGGTGCT
AGCAAAATTAGCACATGAAGAAACTGGAAGAGGTATATACGAAGATAAAGCGATTAAAAATTTATACGCTTCTGAATATATATGGAATTCA
ATAAAAGACAATAAGACAGTAGGGATTATTGGTGAAGATAAAGAAAAAGGATTAACGTATGTAGCGGAACCAATTGGTGTTATTTGTGGT
GTTACGCCAACAACAAATCCTACGTCGACAACTATTTTTAAAGCGATGATTGCAATTAAGACAGGAAATCCAATCATTTTTGCATTCCATCCA
AGTGCACAAGAATCGTCGAAGCGTGCAGCAGAAGTTGTATTAGAAGCGGCAATGAAGGCAGGTGCACCTAAAGATATTATTCAGTGGATT
GAAGTGCCTTCTATCGAAGCAACAAAACAATTAATGAATCACAAAGGTATTGCATTAGTTCTAGCAACAGGTGGTTCGGGCATGGTTAAGT
CTGCATATTCAACTGGCAAACCGGCATTAGGTGTGGGACCAGGTAACGTGCCGTCTTACATTGAAAAAACAGCACACATTAAACGTGCAGT
AAATGATATCATTGGTTCAAAAACATTTGATAATGGTATGATTTGTGCTTCTGAACAAGTTGTAGTCATTGATAAAGAAATTTATAAAGACG
TTACTAATGAATTTAAAGCACATCAAGCATATTTTGTTAAAAAAGATGAATTACAACGCTTAGAAAATGCAATTATGAATGAACAAAAAACA
GGTATTAAGCCTGATATTGTCGGTAAATCTGCAGTTGAAATAGCTGAATTAGCAGGTATACCTGTCCCCGAAAATACAAAACTTATCATAGC
CGAAATTAGCGGTGTAGGTTCAGACTATCCGTTATCTCGTGAAAAATTATCTCCAGTATTAGCCTTAGTAAAAGCCCAATCTACAAAACAAG
CATTTCAAATTTGTGAAGACACACTACATTTTGGTGGATTAGGACACACAGCCGTTATCCATACAGAAGATGAAACATTACAAAAAGATTTT
GGACTAAGAATGAAAGCTTGTCGTGTACTTGTAAATACACCATCAGCGGTTGGAGGTATTGGTGATATGTATAACGAATTGATTCCGTCTTT
AACATTAGGTTGTGGTTCCTACGGTAGAAACTCAATTTCACATAATGTTAGTGCGACAGATTTATTAAACATTAAAACGATTGCTAAACGAC
GTAATAATACTCAAATTTTCAAGGTGCCTGCTCAAATTTATTTTGAAGAAAATGCAATCATGAGTCTAACAACAATGGACAAGATTGAAAAA

GTGATGATTGTCTGTGACCCTGGTATGGTAGAATTCGGTTATACAAAAACAGTTGAGAATGTATTAAGACAAAGAACGGAACAGCCTCAAA
TTAAAATATTTAGCGAAGTCGAACCGAACCCATCAACTAATACAGTATATAAAGGTCTGGAAATGATGGTTGATTTCCAACCGGATACAATC
ATTGCACTTGGTGGTGGTTCAGCGATGGATGCTGCAAAAGCAATGTGGATGTTCTTTGAACACCCTGAGACATCATTCTTCGGTGCTAAAC
AAAAGTTCCTAGACATCGGTAAACGTACTTATAAAATAGGCATGCCTGAAAATGCGACGTTCATTTGTATCCCTACGACATCAGGTACAGGT
TCAGAAGTAACACCATTTGCAGTTATCACAGATAGTGAAACAAATGTAAAATATCCGTTGGCTGATTTTGCTTTAACACCTGACGTTGCAAT
TATTGACCCTCAATTTGTGATGAGTGTGCCAAAAAGCGTTACAGCAGATACAGGAATGGATGTACTAACGCATGCAATGGAATCATATGTA
TCTGTAATGGCTTCAGACTATACAAGAGGTTTGAGTCTACAAGCGATTAAATTGACGTTCGAATATTTAAAATCATCTGTTGAAAAGGGTGA
TAAAGTTTCAAGAGAGAAAATGCATAACGCATCAACTTTGGCTGGTATGGCATTTGCAAATGCATTCTTAGGTATTGCACACTCAATTGCAC
ATAAAATTGGTGGCGAATATGGTATTCCGCATGGTAGAGCGAATGCGATATTACTACCGCATATTATCCGTTATAATGCCAAAGACCCGCA
AAAACATGCATTATTCCCTAAATATGAGTTCTTCAGAGCAGATACAGATTATGCAGATATTGCCAAATTCTTAGGATTAAAAGGTAATACGA
CAGAAGCACTCGTAGAATCATTAGCTAAAGCTGTCTACGAATTAGGTCAATCAGTCGGAATTGAAATGAATTTGAAATCACAAGGTGTGTC
TGAAGAAGAATTAAATGAGTCAATTGATAGAATGGCAGAGCTCGCATTTGAAGATCAATGTACAACTGCTAATCCTAAAGAAGCACTAATC
AGTGAAATCAAAGATATCATTCAAACATCATATGATTATAAGCAATAA

Gene: capA-L1 (capsular polysaccharide biosynthesis protein A, locus 1)

Position: 154875 to 155543, length: 669 nt, orientation: FORWARD

Perfect match to: (MW2-BA000033-[145619:146286], highly conserved allele)

Sequence:

ATGGAAAGTACATTAGAATTAACAAAAATTAAAGAAGTATTACAAAAAAACTTGAAGATTTTAATTATTTTACCGCTATTATTTTTAATTATT
AGCGCTATTGTTACATTTTTCGTCTTATCACCTAAATATCAAGCTAATACTCAAATCTTAGTGAATCAAACTAAGGGTGACAATCCTCAGTTT
ATGGCACAAGAGGTTCAAAGTAATATTCAACTTGTAAATACGTATAAAGAAATTGTTAAAAGTCCTAGAATTTTAGATGAGGTGTCAAAGG
ACTTAAATGATAAGTATTCACCATCTAAATTGTCGAGTATGTTGACAATTACAAACCAAGAAAATACGCAACTTATCAACATCCAAGTTAAA
AGTGGTCATAAACAAGATTCGGAAAAAATTGCGAATAGCTTCGCTAAAGTTACAAGTAAACAAATTCCGAAGATTATGAGTGTGGATAACG
TATCAATTTTATCTAAAGCAGACGGTACAGCAGTTAAAGTCGCACCAAAAACTGTAGTGAATCTAATCGGTGCATTCTTTTTAGGATTAGTT
GTCGCGCTTATATATATCTTCTTCAAAGTAATTTTCGATAAGCGAATTAAAGATGAAGAAGATGTAGAGAAAGAATTAGGATTGCCTGTATT
GGGTTCAATTCAAAAATTTAATTAA

Gene: capB-L1 (capsular polysaccharide biosynthesis protein B, locus 1)

Position: 155559 to 156245, length: 687 nt, orientation: FORWARD

Perfect match to: (Mu50-BA000017-[168054:168740], allele observed in CC5+CC8+CC97)

Sequence:

ATGTCAAAAAAGGAAAATACGACAACAACACTATTTGTATATGAAAAACCAAAATCAACAATTAGTGAAAAGTTTCGAGGTATACGTTCAA
ACATCATGTTTTCAAAAGCAAATGGTGAAGTAAAGCGCTTATTGGTTACTTCTGAAAAGCCTGGTGCAGGTAAAAGTACAGTTGTATCGAA
TGTAGCGATTACTTATGCACAAGCAGGCTATAAGACATTAGTTATTGATGGCGATATGCGTAAGCCAACACAAAACTATATTTTTAATGAGC
AAAATAATAATGGACTATCAAGCTTAATCATTGGTCGAACGACTATGTCAGAAGCAATTACGTCGACAGAAATTGAAAATTTAGATTTGCTA
ACAGCTGGCCCTGTACCTCCAAATCCATCTGAGTTAATTGGGTCTGAAAGGTTCAAAGAATTAGTTGATCTGTTTAATAAACGTTACGACAT
TATTATTGTCGATACACCGCCAGTTAATACTGTGACTGATGCACAACTATATGCGCGTGCTATTAAAGATAGTCTGTTAGTAATTGATAGTG
AAAAAAATGATAAAAATGAAGTTAAAAAAGCAAAAGCACTTATGGAAAAAGCAGGCAGTAACATTCTAGGTGTCATTTTGAACAAGACAA
AGGTCGATAAATCTTCTAGTTATTACCACTATTATGGAGATGAATAA

Gene: capC-L1 (capsular polysaccharide biosynthesis protein C, locus 1)

Position: 156248 to 157012, length: 765 nt, orientation: FORWARD

Perfect match to: (11819-97-CP003194-[162659:163423], allele observed in CC80+CC1+CC97+CC772+CC779)

Sequence:

ATGATTGATATTCATAACCATATATTGCCTAATATCGATGACGGTCCGACAAATGAAACAGAGATGCTGGATCTTTTAAAACAAGCGACAAC
ACAAGGTGTTACAGAAATCATTGTAACATCACATCACTTACATCCTCGATATACCACACCTATAGAAAAAGTGAAATCATGTTTAAACCATAT
TGAAAGCTTAGAGGAAGTACAAGCACTAAATCTAAAGTTTTATTATGGTCAGGAAATAAGAATTACCGATCAAATTCTTAATGATATTGATC
GAAAAGTTATTACCGGTATTAATGATTCACGCTATTTACTAATAGAATTTCCATCAAATGAAGTTCCACACTATACTGATCAATTATTTTTCGA

ATTACAGAGTAAAGGCTTTGTACCGATTATTGCACATCCAGAGCGGAATAAAGCAATAAGTCAAAACCTTGACATACTATACGATTTAATTA
ACAAAGGTGCTTTAAGTCAAGTGACAACGGCGTCATTAGCGGGTATTTCCGGTAAAAAAATTAGAAAATTAGCAATTCAAATGATTGAAAA
CAATCTGACACATTTCATCGGTTCAGATGCGCATAACACAGAAATCAGACCGTTCTTAATGAAAGACTTATTTAATGATAAGAAATTACGTG
ATTATTATGAAGATATGAACGGATTTATTAGTAATGCGAAGTTAGTTGTTGATGATAAAAAAATTCCTAAACGAATGCCACAACAAGATTAT
AAACAGAAAAGATGGTTTGGGTTATAA

Gene: capD (capsular polysaccharide biosynthesis protein D)

Position: 157032 to 158855, length: 1824 nt, orientation: FORWARD

Perfect match to: (KLT6-APFH01000001-[124539:126361], allele observed in CC12+CC8)

Sequence:

ATGGCACATTTATCTGTGAAATTGCGGCTTTTAATACTAGCATTAATCGATTCACTGATAGTGACATTTTCAGTATTCGTAAGTTATTACATTT
TAGAACCGTATTTCAAAACATATTCTGTCAAATTATTAATATTGGCAGCTATATCACTATTCATATCGCATCATATTTCAGCATTTATTTTTAAT
ATGTATCATCGAGCGTGGGAATATGCCAGTGTGAGTGAATTGATTTTAATTGTTAAAGCTGTGACGACATCTATCGTTATTACGATGGTGGT
CGTGACAATTGTTACAGGCAATAGACCGTTTTTTAGATTGTATTTAATTACTTGGATGATGCACTTGATTTTAATAGGTGGCTCAAGGTTATT
TTGGCGTATTTATCGGAAATACCTTGGAGGTAAGTCATTTAATAAGAAGCCAACTTTAGTTGTTGGTGCTGGTCAAGCAGGTTCAATGCTGA
TTAGACAAATGTTGAAAAGTGACGAAATGAAACTTGAACCGGTATTAGCAGTCGATGATGACGAACATAAACGCAATATCACAATTACTGA
GGGTGTAAAAGTCCAAGGTAAAATTGCAGATATTCCAGAACTAGTGAGGAAATATAAGATTAAAAAAATCATCATTGCAATTCCAACTATT
GGTCAAGAGCGTTTGAAAGAAATTAATAATATTTGCCATATGGATGGCGTTGAGTTATTGAAAATGCCAAATATAGAAGACGTCATGTCTG
GTGAGTTAGAAGTGAACCAACTTAAAAAAGTTGAAGTAGAAGATTTACTAGGCAGAGATCCTGTTGAATTAGATATGGATATGATATCAAA
TGAATTGACGAATAAAACTATTTTAGTTACGGGTGCAGGTGGTTCAATAGGATCAGAAATTTGTAGACAAGTTTGTAATTTCTATCCAGAAC
GTATTATTCTACTTGGCCATGGTGAAAACAGTATTTATTTAATCAATCGTGAATTGCGAAATCGCTTCGGAAAAAAATGTTGATATCGTTCCTA
TTATAGCGGATGTGCAAAATAGAGCGCGTATGTTTGAAATTATGGAAATGTATAAACCATACGCAGTTTATCATGCAGCAGCACACAAGCA
CGTGCCGTTAATGGAAGACAACCCTGAAGAAGCAGTACGTAATAATATTTTAGGTACGAAAAATACTGCTGAAGCTGCTAAAAATGCAGA
GGTAAAGAAATTCGTTATGATTTCTACGGATAAAGCCGTTAATCCGCCTAATGTCATGGGCGCTTCAAAGCGAATTGCAGAAATGATTATTC
AAAGTTTAAATGATGAAACGCATCGAACAAATTTTGTTGCAGTGAGATTTGGTAATGTACTTGGATCGAGAGGATCTGTGATTCCACTTTTC
AAAAGTCAAATTGAAGAAGGTGGGCCAGTTACTGTGACACATCCTGAAATGACACGTTACTTTATGACAATTCCTGAAGCTTCTAGACTAGT
TTTGCAGGCAGGGGCATTAGCAGAAGGTGGCGAAGTATTTGTGCTAGATATGGGAGAACCAGTGAAAATTGTTGATTTGGCACGTAATTT
AATTAAGCTAAGTGGTAAAAAAGAGGACGACATACGCATTACTTATACAGGGATTAGACCCGGCGAAAAAATGTTTGAAGAGCTTATGAA
TAAAGATGAAGTTCATCCTGAACAAGTATTTGAAAAAATTTATCGTGGCAAAGTACAACATATGAAATGTAATGAAGTTGAAGCCATTATTC
AAGACATCGTCAATGACTTTAGTAAAGAAAAAATTATTAACTATGCCAATGGCAAAAAGGGAGATAATTATGTTCGATGA

Gene: capE (capsular polysaccharide biosynthesis protein E)

Position: 158845 to 159873, length: 1029 nt, orientation: FORWARD

Perfect match to: (MRSA252-BX571856-[172628:173656], allele observed in CC30+CC772)

Sequence:

ATGTTCGATGACAAAATTTTATTAATTACTGGGGGCACAGGATCATTCGGTAATGCTGTTATGAAACGGTTTTTAGATTCTAATATTAAAGA
AATTCGTATTTTTTCACGCGATGAGAAAAAACAAGATGACATTCGAAAAAAATATAATAATTCAAAATTAAAGTTCTACATTGGTGATGTGC
GTGATAGTCAAAGTGTAGAAACAGCAATGCGAGATGTTGATTACGTATTCCATGCAGCAGCTTTAAAACAAGTGCCGTCATGTGAATTCTTT
CCAGTTGAGGCAGTGAAGACAAATATTATTGGTACAGAAAATGTCTTACAAAGTGCTATTCATCAAAATGTTAAAAAAGTCATATGTTTATC
TACAGATAAGGCAGCGTATCCTATTAATGCTATGGGTATTTCAAAAGCAATGATGGAAAAAGTATTCGTAGCCAAATCAAGAAATATTCGT
AGTGAACAAACGCTTATTTGTGGTACAAGATACGGTAATGTGATGGCTTCAAGAGGATCAGTAATACCTTTGTTTATCGACAAAATCAAAG
CTGGAGAACCTTTAACGATTACAGATCCTGATATGACAAGATTTTTAATGAGCTTAGAAGATGCGGTAGAACTAGTTGTTCATGCATTTAAG
CATGCAGAGACAGGAGATATTATGGTTCAAAAAGCACCAAGCTCAACGGTAGGGGATCTTGCGACCGCATTATTAGAATTGTTTGAAGCTG
ATAATGCAATTGAAATCATTGGTACGCGACATGGAGAAAAAAAAGCAGAAACATTGTTGACGAGAGAAGAATACGCACAATGTGAAGATA
TGGGTGATTATTTTAGAGTGCCGGCAGACTCCAGAGATTTAAATTATAGTAATTATGTTGAAACCGGTAACGAAAAGATTACGCAATCTTAT
GAATATAACTCCGATAATACACATATTTTAACGGTGGAAGAGATAAAAGAAAAACTTTTAACACTAGAATATGTTAGAAACGAATTGAATG
ATTATAAAGCTTCAATGAGATAG

Gene: capF (capsular polysaccharide biosynthesis protein F)

Position: 159886 to 160995, length: 1110 nt, orientation: FORWARD

Perfect match to: (11819-97-CP003194-[166297:167406], allele observed in CC80)

Sequence:

TTGAATATTGTAATTACAGGAGCAAAAGGTTTTGTAGGAAAAAACTTGAAAGCAGATTTAACATCAACGACAGATCATCATATTTTCGAAGT
ACATCGACAAACTAAAGAGGAAGAATTAGAGGCGGCGTTGTTGAAAGCAGACTTTGTCGTGCATTTAGCGGGAGTTAATCGACCTGAACA
CGACAAAGAATTCAGCTTAGGAAACGTGAGTTATTTAGATCATGTACTTGATATATTAACTAGAAATACGAAAAAGCCGGCGATATTATTAT
CGTCTTCAATACAAGCAACACAAGATAATCCTTATGGTGAGAGTAAGTTGCAAGGGGAACAGCTATTAAGAGAGTATGCCGAAGAGTATG
GCAATACGGTTTATATTTATCGATGGCCAAATTTATTCGGTAAGTGGTGTAAGCCAAATTATAACTCAGTGATTGCAACATTTTGTTACAAAA
TTGCACGTAACGAAGAGATTCAAGTTAATGATCGGAATGTTGAACTGACGCTAAACTACGTGGATGATATCGTCGCTGAAATAAAACGTGC
TATTGAAGGAACTCCAACGATTGAAAATGGTGTACCTACAGTACCAAATGTATTTAAAGTGACATTGGGAGAAATTGTAGATTTATTATACA
AGTTCAAACAGTCACGTCTCGATCGAACATTGCCGAAATTAGATAACTTATTTGAAAAAGATTTGTATAGTACGTATTTAAGCTATCTACCTA
GTACAGACTTTAGTTATCCCTTACTTATGAATGTGGATGATAGGGGTTCTTTTACAGAATTTATAAAAACACCGGATCGTGGTCAAGTTTCT
GTAAATATTTCTAAGCCAGGTATTACTAAAGGTAATCACTGGCATCATACTAAAAACGAAAAATTTCTAGTCGTATCAGGTAAAGGGGTAAT
TCGTTTTAGACATGTTAATGATGATGAAATCATTGAATATTATGTTTCTGGCGACAAATTAGAAGTTGTAGACATACCAGTAGGATACACAC
ATAATATTGAAAATTTAGGCGACACAGATATGGTAACTATTATGTGGGTGAATGAAATGTTTGATCCAAATCAGCCAGATACGTATTTCTTG
GAGGTATAG

Gene: capG (capsular polysaccharide biosynthesis protein G)

Position: 160999 to 162123, length: 1125 nt, orientation: FORWARD

Perfect match to: (11819-97-CP003194-[167410:168534], allele observed in CC80+CC5+CC7+CC22+CC80)

Sequence:

ATGGAAAAACTGAAATTAATGACAATAGTTGGTACAAGGCCTGAAATCATTCGTTTATCATCAACGATTAAAGCATGTGATCAATATTTTAA
TCAGATATTAGTACACACTGGTCAAAATTATGATTATACATTGAATCAAATTTTCTTTGATGATTTGGAATTAAGACAACCGGACCACTACTT
AGAGGCAGTTGGAAGTAACCTTGGAGAAACGATGGGGAATATTATTGCGAAGACATATGACGTTTTATTACGCGAACAACCAGATGCACT
TTTAATTCTTGGTGATACAAATAGTTGTTTAGCAGCAGTATCTGCTAAACGATTAAAGATTCCTGTTTTCCACATGGAAGCGGGTAATAGAT
GCTTTGATCAGAATGTACCTGAAGAAATCAATCGTAAAATTGTTGACCATGTCAGTGATGTGAATCTACCTTATACGGAACATAGCAGACGT
TATTTATTAGATGAAGGCTTCAATAAAGCGAATATCTTTGTGACAGGATCACCGATGACAGAAGTGATAGAAGCGCATCGAGATAAAATTA
ATCACAGTGACGTTTTAAATAAACTAGGATTAGAACCGCAACAATACATTTTAGTATCTGCGCATAGAGAAGAGAATATCGATAATGAAAA
GAATTTTAAATCATTAATGAATGCGATAAATGATATTGCCAAAAAGTATAAAATGCCTGTGATTTATTCAACGCATCCAAGAAGTTGGAAGA
AAATTGAAGAAAGTAAATTTGAATTTGATCCATTAGTTAAACAGTTAAAGCCATTTGGTTTCTTTGATTATAATGCATTGCAAAAAGATGCAT
TTGTTGTGCTATCAGATAGTGGAACATTGTCAGAAGAGTCGTCTATTTTGAAGTTCCCTGGTGTCCTTATTCGAACTTCCACAGAAAGACCG
GAAGTACTAGATAAAGGTACGGTTATTGTAGGTGGTATTACCTATAACAATCTAATCCAATCCGTTGAACTAGCAAGAGAGATGCAAAACA
ATAACGAACCGATGATTGATGCTATTGATTATAAAGACACTAACGTTTCGACAAAGGTAGTTAAAATTATTCAAAGCTATAAAGATATTATC
AATCGAAATACTTGGAGGAAATGA

Gene: capH8 (capsular polysaccharide synthesis enzyme Cap8H)

Position: 162126 to 163205, length: 1080 nt, orientation: FORWARD

Sequence:

ATGAGGATAGCGATTCTTGGCGCTACTAACATTAAGCATATGTCATTATTATCACATTATTTAAACCACATTGATTTGAATATCAATGAGGTG
GACATTATATACACTGACAAATATGATATCGAAGAACATATCCAAGGCATCAATAATTACTATAAATATAAAGTAGATATTAAAGAAGATTG
GACATTTATCAAAAAAGCTATTGCTTACTATCGATTTAGGCCATACGCTATGAAAATTCTTAAAGAAAATCATTATGATTTTGTCATAGTATG
GGGAAGTTATACAGGACACTTATTTAGAAGTTTTTTAGAAAAACACTATAAAAATAAATTCATTTTAAATATAAGGGACTACTTTTTTGAAA
ATAATAAACTTATTAAGTATAGAATGAAAAAAATCGTTGATGCTAGCAGGCTGACAACATTATCTTCAGAAGGTTTTCTTAAATTTTTACCTA
AATCTGAAAAAATATAGAATTATTTATAGTTATAACATGAGTATTATTAGAGAAAGTAATGTAACCGATGGATTTAAAAAAAGATGGCCAATT
AATATAGGTTTTATTGGTAATGTTAGATTTAATGAGATAAATCAAAAATTGATAAAGGAACTGGCAAATGATTCAAGGTTCCATATGCAATA
TTTTGGAACAGGGTCGGAAAAAATTAGAAGTCTTTGCTCGAGAAAATTTTATTAATAATATTACATTTTCTGGCGGCTTTGACTTGAAAGAAA
CACCGAAATATTTAAATGAAATTGATATACTTAATAATTTGTTTGGTAATCAAAATATTGCTTTAGATACTGCTTTATCCATAAGAATGTATTA
TGCGTTGTTTTTAAACAAACCTATTATTACAACAGACGATACATTCACCGCTACAGAAGCTAATAAATTCGGACTGGGTTTTAGTATTAATCC
AGAAAATTTAAAAGGTATTGGTGATGAATTGATGGATTGGTATAACAATTTGGATGTAATGGACATTAATCATAAAAGAGAAGCTTATAGA
AACGATGTAATTGAAAATAATAAACAGTTCTATCAAGAGATAGGCAGGATATTTAATGAATAA

Gene: capI8 (capsular polysaccharide synthesis enzyme Cap8I)

Position: 163198 to 164592, length: 1395 nt, orientation: FORWARD

Perfect match to: (11819-97-CP003194-[169609:171003], allele observed in CC80+CC88)

Sequence:

ATGAATAAGATATATAATGTCACTTCATATGTTATTGCCATTTTAATGTTTCCTTGCCTTATGTTAGGTGATAAACCTTTATTATTTTTAGCACC
TATAAGTTATGGAGTAGGAAAGCTCTTTATAAGCTTCTCGAATAATCCGAATTTTAAATTTTCGAAAATTGTATACGATGTTTTAGGTTTTCT
TAGATTAGTATTTATACCTGCTATGATAGTGTTTTTCCAGGATTCAACTATAGATAATTTGCCATTAGGACAAGCTTATTTTAATCAAGCGGT
TATTTATATGAGTGTGGAGTTTATCATAGGCTCGCTATTTATATTGATACTATCTAAATTATTCAAACATGAAGAGGTATCAAGAAATAGCTT
TACACTTTCTGGATCATCAATTTATTACATTGTGTTTGGTCTTGTTATTTGTGGGATTTTTGTAGCTTTTCCCGAAGTGCGCAAAAACATATCA
TTTTTAATTATTAAAACAGATGCAATGGGAAGAGGAACCGAAGCAACAAGTGGTTTAAATGTTCTTTTTGTAATGCTATTTCAACTTGCCTTA
GCGTTATTATTCTTAATAATCGCATATGCTTCATATAAAAAGTATAAAGAGAATCCTAAAATTATTTATGTTGTATTACCGCTAGCTATAGGA
ATTTTAAATATTAGTTTAATTGTTGGTGAAAGAAGAAGTTATCAACTTTATACAATGGTTGCTGTTTTAACAGTGGTGTCAATCTTGTTTTCTA
AGCATAAAAGACGTATCAATATCATTATTATATCTGTAGGTATTTTCGTATTAGCATTGATGACACTATATAAAGAATTGTATGTGTTTAATT
ATAGTTCGTATAGCGAGGCGTTAAATAGTACAAGTGTAAGTAATCTTAAAATAGTTGACACGTTGCAGTCATATTTTTATGGACCTAGCAAT
ATTGCAGCTTCTATAGACTATTTGAATTACTATAATGGTTCGTTCAAACAATATTTATTCGATAATACCAGAGCTGTTTTTGGGTTTAATTTCT
TTTTAGATAAAAAGCAGTTGATTACTAGCCAACTTTTTAACCAATTGATATATGGTAGTAAACAATTGACTGGTCATCTAATTTCGAGTGCTG
GATATGGAATTATTTATTTTGGACCCTTATTCTTCTATTTGAATTTAATTGCAAATATCTTTTTTGCATTTCTGAGTGAGTATATTATTCGGAAG
AGTCATTCGTTAGAAGTGATATTTATTGGTACATATATTTATATGAGACTTATTACGAGTATTTTTAGTCACCCGACACCATTAATCACATTG
ATTTCTATGATTTTAGTTGTATATGTTATAGCTATTATCCCAGGCATTATAATAAAAAAATTTACTAAAAAAGTAGGGATAGAATGA

Gene: capJ8 (capsular polysaccharide synthesis enzyme Cap8J)

Position: 164589 to 165146, length: 558 nt, orientation: FORWARD

Perfect match to: (11819-97-CP003194-[171000:171557], allele observed in CC80+CC101)

Sequence:

ATGATTGTAAAAACATTTATGAAATCGAAAATATTTAGATTAATGAATACACCACTATTATTATTTTATAAGAAAGAATATTTAACTGGATAT
TATTTTGAAAATAAAGTGGCTGGATGGTTATGGGCGTGGAAAGCTGTTCCGTTCAAGTTGTTAGGAATAAATATGAGTTTGCCATTTCCTGC
AGATATAACTGTTAGAATGCATAACCCTAATAACATTGTTTTTGATAAAAATGATATTCATATTTTTCAATCGCCCGGGACGTATTTTAATAA
TTTTTCAGCAGTTATATATATATAGGTAGAGGTGTTTATATAGCGCCTAACGTAGGTATTATTACAGCTAATCATAATATTAAAAATTTGAAGTC
ACATGCACCAGGTGAAGATGTCAAAATAGGGAATTATAGTTGGATTGGAATGAACTCAGTTATATTACCAGGAGTAGAATTGGGGGAACA
TACAATTGTAGGGGCTGGGTCGGTTGTAACAAAAAGTTTTCCAGAAGGTAACGTTGTTATAGGTGGCAATCCAGCGAAAATCATCAAGAA
AATCTGA

Gene: capK8 (capsular polysaccharide synthesis enzyme Cap8K)

Position: 165155 to 166393, length: 1239 nt, orientation: FORWARD

Perfect match to: (11819-97-CP003194-[171566:172804], allele observed in CC80+CC1+CC96)

Sequence:

ATGAGATTAAATAAATTTATTGGCGATTCGTTTTTAATGATTTTAAGCAGTGGCATCGCTCAAGTCATATTAATCATCACTACCCCAATTATTA
CAAGACTATATTCACCTACAGAATTTGGTGAGTTTACAATTTTTTCAAATATCGCAATGATTTTAATACCAATAATAAATGCAAGATACGATT
TGTTGATTGTGAATACCAAAAATGACCGTAGTGCTAATATACTTTCACAAATCAGTTTTTTGATATCATTGCTTATTTTATTAATACTGATACC
AATATTTGCGATTAGTGCATGTTTATACCCAAACTTTATATTAGATTTTATTTTCATTATTATTATGTTGTTTTTGGTAAGTTTAACAAACATTT
TTACAAATTATCTAAATAAGGAAAGAAAGTATAAAGTGTTAAGTTTGATTAATGTGTTTAGAGCTGGATCAATGGCTTTACTTCAAATCATTT
TCGGACTTTTAGCATTAGGAAGTTTAGGATTAATTATTGGTTTTTCATTATCCTATATCGCAGGCATTACACTAGGATATAAAACGTTTAAAA
AGCACTTTAATATTGTGAGAGATAAAGAAGAAACTAAAGCATTATTTTTAGAAAATAAAAATCAGTTAGTTTATTCAACACCATCAATATTAT
TAAATAGTTTGTCTTTCTCGGTTGTTGTGTTCTTTATAGGTATTTTGTATACCAATACAGAAGTGGGTATTTATGGTATGGCCATAAGAGTAC
TAGGCATACCAGTGACAATTATTTCATTAGGGTTATCAAAAATATTTATGCAACAAGCCAATGACTATTATATTGAACATGGTAACTTCCGA
AATTTATTACTTAAATTTAGTTCCATACTGGTTATAGTTTCTATAATTCTTTATGTGCCACTTTATTTGTTCAGTGAAGAATTAGTCAATATATT
ATTAGGACACAGCTGGGTTGACGCAATTACAGTTATAAAAATTGTTATCCCATTATTTGTTATAAGGCTGATTGTATCAACGGTATCACTTTC
TGTGATTGTATTACAAAAACAACAGTTAGAATTAATACTACAAGCGTTATTTTTAATAGGTACTACTGCAACATTTGTTATATCAAAAATGCT

TAATTTAACTTTTTTAAACTTTGTATCTATTAATACAATTGTTTTAATCGTATCGTACATGATATTTTTCATAGCACTCTATTATTTTGCTAAAAA
TAAACAGTTCAAAAATTCTTAG

Gene: capL (capsular polysaccharide synthesis protein L)

Position: 166427 to 167632, length: 1206 nt, orientation: FORWARD

Perfect match to: (11819-97-CP003194-[172838:174043], allele observed in CC80)

Sequence:

ATGAGTGAAAAAAAGATTTTGATTTTATGTCAGTATTTTTATCCGGAATATGTATCTTCTGCGACGTTACCAACTCAATTGGCGGAAGATTTA
ATTGCGAATCACATTAATGTCGATGTCATGTGTGGATGGCCATATGAATATAGTAATCATAAACAGGTTGCTAAAACCGAGATGCATCGTG
GAATTCGCATTCGACGTCTCAAGTATTCGAGGTTTAATAACAAAAGTAAGGTTGGAAGGATCATCAATTTCTTTAGTTTATTTTCAAAATTCG
TGATTAATATACCTAAAATGTTGAAATATGATCAGATTCTTGTTTACTCTAATCCACCAATCTTGCCATTAATACCAGACATTTTACACAGACT
GCTTAAGAAAAAATATTCTTTTGTGGTGTATGATATAGCACCTGATAATGCGATTAAGACAGGTGCAACTCGTCCAGGTAGCATGATTGATA
AACTGATGCGTTACATTAATAGACATGTCTACAAGAATGCTGAAAATGTCATTGTCCTTGGTACGGAAATGAAAAACTACTTACTAAATCAT
CAAATTTCTAAAAATGCTGACAATATTCATGTGATTCCTAACTGGTATGACATGCGTCAGTTACAAGACAATCGTATCTATAATGACACATTT
AAAGCTTGCCGTGAGCAATACGACAAAATTTTATTGTATAGCGGTAATATGGGGCAGTTACAGGATATGGAGACACTTATCTCATTTTTAAA
ATTAAATAAGGATCAGCCTCAAACGTTAACAATACTTTGTGGTCATGGTAAGAAATTTGCAGATGTCAAAACGGCAATAGAAGACCATCGT
ATTGAAAATGTTAAAATGTTTGAGTTTTTAACAGGTACAGACTATGCTGACGTATTAAAAATTGCGGATGTATGTATTGCATCGCTGATTAA
AGAAGGCGTCGGTTTAGGCGTGCCGAGCAAGAATTATGGCTATCTTGCAGCTAAGAAAGCGTTGGTACTCATCATGGATAAGCAATCTGAT
ATCGTTCAACATGTTGAACAATATGATGCGGGTATCCAAATTGATAATGGCGATGCACATGCCATTTATAACTTCATCAACACTCACTCGAG
TAAGGAATTGCACGAGATGGGTGAGCGCGCACATCAACTGTTTAAAGATAAATATACGAGAGAAATTAATACTATGAAGTATTACAATCTG
TTGAAGTGA

Gene: capM (capsular polysaccharide synthesis protein M)

Position: 167643 to 168200, length: 558 nt, orientation: FORWARD

Perfect match to: (CIGC93-AHVD01000003-[83009:83566], highly conserved allele)

Sequence:

ATGAAGCGATTATTCGATGTAGTGAGTTCAATATATGGTTTAGTAGTTTTAAGTCCGATTCTGTTAATTACAGCATTACTAATTAAAATGGAA
TCACCTGGACCAGCCATTTTCAAACAAAAAAGACCGACGATTAATAATGAATTGTTTAATATTTATAAGTTTAGATCAATGAAAATAGACAC
ACCTAATGTTGCAACTGATTTAATGGATTCAACATCGTATATAACAAAGACAGGGAAGGTCATTCGTAAGACCTCTATTGATGAATTGCCAC
AATTATTGAATGTTTTAAAAGGAGAAATGTCAATTGTAGGTCCTAGACCAGCGCTTTATAATCAATACGAATTAATCGAAAAACGTACAAAA
GCGAACGTGCATACGATTAGACCAGGTGTGACAGGACTAGCTCAAGTGATGGGGAGAGATGATATCACTGATGATCAAAAAGTAGCGTAT
GATCATTATTACTTAACACATCAATCTATGATGCTTGATATGTATATCATATATAAAACAATTAAAAATATCGTTACTTCAGAAGGTGTGCAT
CACTAA

Gene: capN (capsular polysaccharide synthesis protein N)

Position: 168200 to 169087, length: 888 nt, orientation: FORWARD

Perfect match to: (11819-97-CP003194-[174611:175498], allele observed in CC80+CC15)

Sequence:

ATGAGAAAAAATATTTTAATTACAGGCGTACATGGATATATCGGTAATGCTTTAAAAGATAAGCTTATTGAACAAGGACATCAAGTAGATC
AAATTAATGTTAGGAATCAATTATGGAAGTCGACCTCGTTCAAAGATTATGATGTTTTAATTCATACAGCAGCTTTGGTTCACAACAATTCAC
CTCAAGCAAGGCTATCTGATTATATGCAAGTGAATATGTTGCTGACGAAACAATTGGCACAAAAGGCTAAAGCTGAAGACGTTAAACAATT
TATTTTTATGAGTACTATGGCAGTTTATGGAAAAGAAGGTCATGTTGGTAAATCAGATCAAATTGATACACAAACACCAATGAATCCTACGA
CCAACTATGGTATTTCCAAAAAGTTCGCTGAACAAGCATTACAAGAGTTGATTAGTGATTCGTTTAAAGTAGCAATTGTGAGACCACCAATG
ATTTATGGTGCACATTGCCCAGGAAATTTCCAACGGTTAATGCAATTGTCAAAGCGACTGCCAATCATTCCCAATATTAACAATCAGCGCAG
TGCATTATATATTAAACATCTGACAGCATTTATTGATCAATTAATATCATTAGAAGTGACAGGCGTGTATCATCCTCAAGATAGTTTTTACTTT
GATACATCGTCAGTAATGTATGAAATACGTCGCCAATCACATCGTAAAACGGTATTGATCAACATGCCTTCAATGCTAAATAAGTATTTTAA
TAAGTTGTCGGTCTTTAGAAAATTATTCGGCAATTTAATATACAGCAATACGTTATATGAAAATAATAATGCACTTGAAGTTATTCCTGGAAA
AATGTCACTTGTTATTGCGGACATCATGGATGAAACGACAACCAAAGATAAGGCATAA

Gene: capO (capsular polysaccharide synthesis protein O)

Position: 169141 to 170403, length: 1263 nt, orientation: FORWARD

Sequence:

ATGAAGTTAACAGTAGTTGGCTTAGGTTATATTGGTTTACCAACATCAATTATGTTTGCAAAGCATGGCGTCGATGTGCTTGGTGTTGATAT
TAATCAGCAAACGATTGATAAGTTACAAAGTGGTCAAATTAGTATTGAAGAACCTGGATTACAAGAGATTTATGAAGAGGTTCTGTCATCG
GGAAAATTGAAGGTATCTACAACGCCAGAAGCATCTGATGTTTTTATCATTGCCGTTCCGACGCCGAATAATGATGATCAGTACCGGTCATG
TGACATTTCGCTAGTTATGCGTGCATTAGATAGTATTTTACTATTTTTAAAAAAAGGGAATACTATTATTGTAGAGTCGACAATTGCGCCTAA
AACGATGGATGATTTTGTAAAACCAGTCATTGAAAATTTAGGGTTTACAATAGGTGAAGATATTTATTTAGTGCATTGTCCAGAACGTGTAC
TGCCAGGAAAAATTTTAGAAGAATTAGTTCATAACAATCGCATCATTGGCGGTGTGACTAAAGCTTGTATTGAAGCGGGTAAACGTGTCTA
TCGCACATTTGTTCAGGGAGAAATGATTGAAACAGATGCACGTACTGCTGAAATGAGTAAGCTAATGGAAAACACATATAGAGACGTGAA
CATTGCTTTAGCTAATGAATTAACAAAAATTTGCAACAACTTAAATATTAATGTATTAGATGTGATTGAAATGGCAAACAAACATCCGCGTG
TTAATATCCATCAACCTGGTCCAGGTGTAGGCGGTCATTGTTTAGCTGTTGATCCGTACTTTATTATTGCTAAAGACCCTGAAAATGCAAAGT
TAATTCAAACTGGACGTGAAATTAATAATTCAATGCCGGCCTATGTTGTTGATACAACGAAGCAAATCATCAAAGCGTTGAGCGGGAATAA
AGTCACAGTCTTTGGTTTAACTTATAAAGGTGATGTTGATGATATAAGAGAATCACCAGCATTTGATATTTATGAACTATTAAATCAAGAAC
CAGACATAGAAGTATGTGCTTATGATCCACATGTTGAATTAGATTTTGTGGAACATGATATGTCACATGCTGTCAAAGACGCATCGCTAGTA
TTGATTTTAAGTGATCACTCAGAATTTAAAAATTTATCGGACAGTCATTTTGATAAAATGAAGCATAAAGTGATTTTTGATACAAAAAATGTT
GTGAAATCATCATTTGAAGATGTATCGTATTATAATTATGGCAATATATTTAATTTTATCGACAAATAA

Gene: capP (capsular polysaccharide synthesis protein P)

Position: 170450 to 171625, length: 1176 nt, orientation: FORWARD

Perfect match to: (11819-97-CP003194-[176861:178036], allele observed in CC80+CC72+CC88)

Sequence:

ATGTGTTTGAACTTCAGAGAGGATAATGTTATGAAAAAAATTATGGTTATTTTCGGTACGAGACCCGAAGCAATAAAAATGGCACCATTAG
TAAAAGAAATTGATCATAATGGGAACTTTGAAGCGAACATTGTGATTACAGCACAACATAGAGATATGTTAGATAGTGTGTTAAGTATATT
TGATATTCAAGCTGATCATGATTTAAATATTATGCAAGATCAACAAACATTAGCAGGCCTTACGGCGAATGCGCTTGCTAAACTTGATAGCA
TCATTAATGAGGAACAGCCGGATATGATTTTAGTACATGGTGATACTACAACGACTTTTGTAGGAAGTTTGGCAGCATTTTATCATCAAATT
CCGGTCGGACATGTAGAAGCTGGACTTCGAACACATCAGAAATACTCACCATTTCCTGAAGAGTTAAATCGAGTCATGGTAAGTAATATTG
CTGAATTGAATTTTGCGCCAACAGTAATTGCAGCTAAAAATTTACTTTTTGAAAACAAAGACAAAGAGCGTATCTTTATTACTGGAAATACA
GTTATTGACGCATTGTCAACAACAGTTCAAAATGATTTTGTTTCAACGATTATTAATAAACATAAAGGCAAGAAAGTTATTTTACTAACAGC
GCATCGTCGTGAAAATATTGGGGAACCGATGCATCAGATTTTTAAAGCAGTAAGAGATTTGGCAGATGAATATAAAGATGTTGTCTTCATT
TATCCAATGCATCGTAATCCAAAGGTAAGAGCGATTGCCGAAAAATATTTATCTGGGAGAAATCGGATTGAATTAATTGAGCCATTAGATG
CGATTGAGTTCCATAATTTTACAAATCAATCGTACCTCGTGCTGACAGATTCTGGTGGTATTCAAGAGGAGGCTCCTACATTTGGAAAACCT
GTGTTGGTATTAAGGAATCATACAGAGCGTCCCGAAGGCGTTGAGGCGGGAACATCGAGAGTAATTGGCACAGATTATGACAATATTGTT
CGAAATGTGAAACAATTGATTGAGGATGATGAAGCGTATCAACGTATGAGTCAAGCGAATAATCCATATGGTGATGGACAAGCATCACGA
CGTATTTGTGAAGCAATAGAATATTATTTTGGATTGCGCACAGACAAGCCGGATGAATTCGTACCTTTACGTCACAAATAA

Gene: isdI (heme oxygenase)

Position: 171690 to 172016, length: 327 nt, orientation: REVERSE

Perfect match to: (COL-CP000046-[170038:170364:r], highly conserved allele)

Sequence:

TTATTTTTGATAGTGGTAGCCAATATCATATTTGAATACTTTATTTGATAATATTGGACTTTGCTGTCCATCGTCATCACTTTTTAAACGTACAT
TTTTATGAGCTTCTTTAAATACATCGGAATTCAACCAATTATTAAAGCTATCTTCAGATTCCCAAATAGTTAAGATTTTAACTTCGTCTGTATC
CTCGGTATTTAATGTTTTAGTGACAAACATTTGTTGGAAGCCTTCAATAGTTTCAATACCTTGTCTATTGTAAAAACGTTCAATCGTTTCTTCC
GCACTGCCTTTTTGTAATTGTAATCTATTTTCTGCCATAAACAT

Gene: ybaN (putative membrane spanning protein)

Position: 172023 to 172406, length: 384 nt, orientation: REVERSE

Perfect match to: (GR1-AJLX01000027-[30877:31260:r], allele observed in CC361+CC80)

Sequence:

TCACTCCTCTATTTTATTATTTGATTTGGGTAATGTTTTTACAAATGTAAAGAGTACAGCGGTTTGTATGATAACCATTATGATTAATCCTACA
CGGACTGCAAGAACATCCACCATATAAATTGAAAAACCTATTACAATGTATAAGCTAATTAAAATTTTAATTTTCTGTTGTAGCGTGTAGCCT
CGATGTAAATAAAAGTTTTCTACATATTCTTTATAAATTTTTTGATTAATAAGCCAATTGTAAAAGCGATCTGAACTTCGAGCAAAACAAAAA
ACTGCTACGAGTAAAAAAGGGGTCGTTGGCAGTAAAGGTAATACGGCACCTGCAATACCAAGCGCTGTAAATATTAAGCCAATAACGATT
AAAATAAGTCGCAT

Gene: aldA1 (aldehyde dehydrogenase, locus 1)

Position: 172833 to 174320, length: 1488 nt, orientation: FORWARD

Perfect match to: (11819-97-CP003194-[179244:180731], allele observed in CC80+CC361+CC4803)

Sequence:

ATGGCAGTAAACGTTCGAGATTATATTGCAGAGAATTATGGTTTATTTATCAATGGGGAATTTGTTAAAGGTAGCAGTGACGAAACAATCG
AAGTGACTAATCCAGCAACTGGAGAAACACTATCACATATTACAAGAGCAAAAGATAAAGATGTCGATCATGCAGTCAAAGTGGCGCAAG
AGGCATTTGAATCATGGTCATTAACTTCTAAATCAGAACGTGCACAAATGTTGCGTGATATTGGTGATAAATTAATGGCACAAAAAGATAA
AATTGCAATGATTGAAACATTAAATAATGGTAAACCGATTCGTGAGACAACAGCAATTGATATTCCATTTGCTGCAAGACATTTCCATTATTT
CGCAAGTGTTATTGAAACAGAAGAAGGTACAGTGAATGATATCGATAAAGACACAATGAGTATCGTACGACATGAGCCGATTGGCGTCGT
AGGTGCTGTTGTTGCTTGGAACTTCCCAATGCTATTAGCTGCATGGAAGATTGCGCCAGCCATTGCTGCAGGTAATACAATTGTGGTTCAAC
CTTCGTCTTCAACACCATTAAGTTTATTGGAAGTTGCTAAAATTTTCCAAGAGGTATTACCTAAAGGTGTTGTCAATATACTAACGGGTAAAG
GTTCAGAATCAGGTAATGCAATTTTCAATCATGATGGTGTAGATAAATTATCATTTACGGGCTCAACTGATGTAGGTTATCAAGTTGCCGAA
GCTGCAGCAAAACATCTAGTACCCGCTACATTAGAGCTTGGTGGTAAAAGCGCCAATATCATATTAGATGATGCTAATTTAGACATTGCAGT
TGAAGGTATTCAGTTAGGTATTTTATTCAACCAAGGTGAAGTATGTAGTGCAGGTTCTCGATTATTAGTTCATGAAAAAATTTATAATCAATT
GGTGCCACGTTTACAAGAGGCATTTTCAAATATTGAAGTTGGAGACCCACAAGATGAAGCTACACAAATGGGTAGTCAAACTGGTAAGGA
TCAATTAGATAAAATTCAATCATATATTGATGCAGCAAAAGAATCAGATGCACAAATTTTAGCAGGCGGTCATCGCTTAACTGAAAATGGAT
TAGATAAAGGGTTCTTCTTTGAGCCGACATTAATTGCTGTGCCAGACAATCATCACAAATTAGCACAAGAAGAAATATTTGGACCAGTGTTA
ACAGTTATTAAAGTGAAGGACGATCAAGAAGCAATTGATATAGCTAATGATTCTGAGTATGGTTTAGCAGGCGGTGTATTTTCTCAAAATA
TCACACGTGCATTAAATATTGCTAAAGCTGTACGTACAGGACGTATTTGGATTAACACTTACAACCAAGTACCAGAAGGCGCACCATTTGGT
GGTTATAAAAAATCAGGTATCGGTCGAGAAACTTATAAAGGTGCGTTAAGTAACTATCAACAAGTTAAAAATATTTATATTGATACAAGCA
ATGCTTTAAAAGGTTTGTACTAG

Gene: czcD (Co/Zn/Cd efflux potassium/proton antiporter)

Position: 174967 to 175926, length: 960 nt, orientation: FORWARD

Perfect match to: (DSM_20231_T-AMYL01000013-[386330:387289], allele observed in CC8)

Sequence:

ATGAATGGGAAAAAGGCGAATACGATAAACAGATACAAATATTTTCATCATGTCAATCATCAAAAAATTCAACAAAGTTCTAAAAAGACGC
TGTGGGCATCACTAATCATCACATTGTTATTTACAGTGATTGAATTTGTCGGAGGTTTAGTATCTAATTCATTGGCATTACTGTCAGATTCAT
TTCATATGCTTAGTGATGTATTAGCACTTGGTTTATCTATGTTGGCCATTTATTTTGCAAGTAAAAAGCCGACTGCACGATACACATTTGGAT
ATTTAAGATTTGAGATATTAGCTGCATTTTTAAATGGTTTAGCATTAATTGTAATTTCAATCTGGATTTTATATGAAGCTATTGTACGTATTAT
TTATCCGCAACCAATTGAAAGTGGCATTATGTTTATGATTGCTAGTATTGGTTTACTCGTTAATATTATTTTGACAATTATCCTTGTAAGGTCT
TTAAAACAAGAAGACAATATCAATATTCAAAGTGCATTATGGCATTTCATGGGAGACTTATTGAACTCTATTGGTGTCATCGTTGCAGTTGT
ATTGATTTACTTTACAGGATGGCGCATCATCGACCCAATCATTAGTATTGTAATTTCACTCATCATTTTACGTGGTGGTTATAAAATTACGCG
TAATGCGTGGTTAATTTTAATGGAAAGTGTGCCTCAACATTTGGATACTGATCAAATTATGGCAGATATTAAAAACATAGATGGCATATTAG
ATGTACATGAATTTCATTTGTGGAGTATTACAACAGAGCATTATTCATTAAGTGCCCATGTTGTGTTAGATAAAAAATATGAGGGTGATGAT
TATCAAGCGATTGATCAAGTATCATCATTGTTGAAAGAAAAATATGGCATTGCACATTCAACGTTGCAAATTGAAAACTTGCAATTGAATCC
ATTAGACGAGCCATACTTCGACAAATTAACATAA

Gene: tx_universal2 (rho-independent terminator)

Position: 175998 to 176030, length: 33 nt

Perfect match to: (Strain_21194-AGTU01000149-[993:1025], allele observed in CC45I)

Sequence:

CTGAACGAAAATGCGCTTGTAACAAGCTTTTTT

Gene: tx_universal2 (rho-independent terminator)

Position: 176000 to 176038, length: 39 nt

Perfect match to: (Strain_21331-AGTV01000040-[104403:104441], allele observed in CC398)

Sequence:

GAACGAAAATGCGCTTGTAACAAGCTTTTTTCAATTCTA

Gene: STAR (Staphylococcus aureus repeat element)

Position: 176043 to 176262, length: 220 nt

Sequence:

GGGGCCCCAACACAGAGAATTTCGAAAAGAAATTCTACAGGTAATGCAAGTTGGGGTGTGGGCCCCAACAAAGAGAAATTGGATTCCCAA
TTTCTACAGACAATGCAAGTTGGCGGGGCCCCAACATAGAAGCTGGCGGAAAGTCAGCTTACAATAATGTGCAAGTTGGGGTGGGACAAC
GAAATAAATTTTGCGAAAATATCATTTCTGTCCCACTCCC

Gene: SIRU01 (staphylococcal interspersed repeat unit 1)

Position: 176045 to 176211, length: 167 nt

Sequence:

GGCCCCAACACAGAGAATTTCGAAAAGAAATTCTACAGGTAATGCAAGTTGGGGTGTGGGCCCCAACAAAGAGAAATTGGATTCCCAATT
TCTACAGACAATGCAAGTTGGCGGGGCCCCAACATAGAAGCTGGCGGAAAGTCAGCTTACAATAATGTGCAAGTTGG

Gene: Q5HJK0-srpF (alpha-helical coiled-coil protein)

Position: 176658 to 177170, length: 513 nt, orientation: FORWARD

Perfect match to: (N315-BA000018-[188955:189467], highly conserved allele)

Sequence:

ATGACATTATTTTTATTAGAAGCTAACAATCTTGATTTTGCATCAACGAAAGAAGAACTAGAAGCAAAGGCAGCATCACTATCTACGAAGAC
AATTCCAACATTAATTGAAGTACAAGCTACTGAAAATTTAACTCATGGTTATTTTATTGTGGAAGCAAATGACGAAGCAGAAGCTAAACAAT
TTTTAACAGAAGCAGATATTAGTATTCAATTAGTCAAAGAAGTACGCTTAGTTGGTAAAGATTTAGATGAAGTTAAAAATGGTGATGCACA
TGTTGATTACCTTGTAACTTGGAACATTCCGGAAGGCATTACGATGGATCAATATTTAGCACGTAAAAAGAAAAATTCTGTTCATTATGAAG
AAGTGCCAGAAGTTGAATTTAAACGCACATATGTATGTGAAGATATGTCTAAATGTATTTGTTTATACAACGCACCTGATGAAGAAGCGGT
ACGTCGCGCGCGCAAAGCAGTTGATACACCGATTGATGGCATCGAAAAACTTTAA

Gene: tauB (taurine ABC transporter, ATP-binding protein)

Position: 177512 to 178252, length: 741 nt, orientation: FORWARD

Sequence:

ATGATTAAAATACAACAATTACAACATCACTTTGGATCACATAAAGTAATTCATAACTTTAATTTGGACATTAGCAAAGGAGAAATAGTCAC
TTTCATAGGAAAAAGTGGTTGCGGAAAGTCTACTTTACTCAATATCATCGGTGGATTTATTCATCCATCATCTGGACGTGTGTCATTATTGATAA

CGAAATTAAACAACAGCCGTCTCCAGATTGTTTAATGCTATTTCAACATCATAATTTGCTGCCATGGAAAACGATTAATGACAACATTAGGA
TTGGATTTCGACAGAAAATTAGTGATGAAGAGATTAACGCACAGCTTAAATTAGTTGATTTAGAAGGCAGGGGAAAGCATTTTCCCGAGCA
ACTGTCCGGGGGTATGAAACAACGTGTGGCACTATGTCGAGCGCATGTGCATAAGCCTAACGTTATATTGATGGATGAGCCATTAGGTGCA
TTAGATGCATTTACACGTTATAAACTTCAGGACCAACTAGTGCAACTAAAACATAAAACGCAATCAACTATTATTTTAGTGACGCATGACATT
GATGAAGCTATTTATCTTTCCGACCGCATTGTTCTGTTAGGTGAAGGGTGCAATATTATTTCTCAATATGAAATTACAGCATCACATCCACGC
AGTCGTAATGATAGCCACCTACTTAAGATTCGTAATGAAATTATGGAAACATTTGCATTGAATCATCATCAAGTTGAACCTGAATATTATTTA
TAA

Gene: tauA (taurine ABC transporter, substrate-binding protein)

Position: 178266 to 179240, length: 975 nt, orientation: FORWARD

Perfect match to: (N315-BA000018-[190563:191534], allele observed in CC5+CC1+CC72+CC80)

Sequence:

ATGAAAAGGTTAAGCATAATCGTCATCATTGGAATCTTTATAATTACAGGATGTGATTGGCAAAGGACGTCTAAAGAACCGTCTAAAAATG
CCCAAAATCAGCAAGTGATTAAAATTGGATATTTGCCGATTACACATTCAGCTAATTTGATGATGACTAAAAAATTATTATCACAATACAATC
ATCCGAAATATAAACTAGAATTAGTTAAATTCAATAATTGGCCAGATTTAATGGACGCATTAAACAGTGGTCGTATTGATGGTGCATCAACT
TTAATAGAGCTAGCGATGAAATCAAAACAGAAGGGCTCAAATATAAAGGCTGTGGCATTGGGCCATCATGAAGGCAATGTCATTATGGGA
CAAAAAGGTATGCACTTAAATGAATTTAATAATAATGGCGATGATTACCATTTTGGTATACCACATCGTTATTCAACACATTATCTTTTACTT
GAGGAATTACGTAAACAATTAAAGATTAAACCGGGGCATTTTAGCTATCATGAAATGTCGCCAGCAGAAATGCCAGCCGCATTGAGTGAAC
ACAGAATTACAGGGTATTCTGTAGCCGAACCATTCGGTGCACTGGGTGAAAAGTTAGGCAAAGGTAAGACTTTGAAACATGGTGATGACG
TTATACCTGATGCGTATTGCTGTGTGCTAGTACTGAGAGGGGAATTGCTTGATCAACACAAGGATGTAGCGCAAGCATTTGTACAAGATTA
TAAAAAGTCTGGCTTTAAAATGAATGATCGCAAGCAAAGTGTAGACATTATGACGCATCATTTTAAACAAAGTCGTGACGTTTTAACACAGT
CAGCGGCATGGACATCCTATGGTGATTTAACAATTAAGCCATCCGGCTATCAAGAAATTACGACATTGGTAAAACAACATCATTTGTTTAAT
CCACCTGCATATGATGACTTTGTTGAACCGTCATTGTATAAGGAGGCATCGCGTTCATGA

Gene: tauC (taurine ABC transporter, transmembrane permease)

Position: 179237 to 179998, length: 762 nt, orientation: FORWARD

Perfect match to: (MW2-BA000033-[169923:170684], allele observed in CC1+CC5)

Sequence:

ATGACACGTCCCACAAATAACAAATTTATATTACCTATTATCACATTTATTATTTTCTTAGGCATTTGGGAAATGGTCATTATTATTGGGCATT
ACCAACCTGTATTGTTACCTGGTCCTGCTCTTGTAGGAAAAAGTATATGGACTTTCATTGTTACTGGAGAAATTTTCCAACATTTAGCAATTA
GTTTATGGAGATTTGTAGCGGGCTTTGTTGTCGCATTGTTGGTTGCTATTCCATTGGGCTTCTTGCTTGGAAGGAATCGTTGGCTATACAAC
GCTATCGAACCGCTATTTCAATTGATTAGACCGATATCTCCGATAGCATGGGCACCATTTGTTGTTCTATGGTTTGGTATTGGTAGTTTGCCA
GCGATTGCGATTATTTTTATCGCCGCTTTTTTCCCAATTGTGTTCAATACTATTAAAGGCGTTAGAGACATTGAACCTCAATATTTAAAAATA
GCGGCAAATTTAAATTTAACTGGGTGGTCATTGTATCGCAATATATTATTTCCCGGGGCATTTAAACAAATCATGGCTGGGATACATATGGC
GGTAGGAACAAGTTGGATATTTTTAGTTTCTGGTGAAATGATTGGTGCACAATCGGGATTAGGTTTTTTAATCGTTGATGCACGAAATATGT
TGAACTTAGAAGATGTTTTAGCAGCAATATTCTTTATCGGATTATTTGGTTTTATTATTGATCGATTCATTAGTTATATTGAGCAGTTTATACT
TAGAAGATTTGGTGAATAA

Gene: Q5HJJ8 (putative acyl-CoA dehydrogenase)

Position: 180011 to 181042, length: 1032 nt, orientation: FORWARD

Sequence:

ATGACTTTAGAAACGCTTATCAAAGAACAATTAGATCCTCATTTAGTAGAAGTTGATGAAGGGACGTATTATCCGAGAACATTTATTCAACA
ATTATTTGTAGATGGTTATTTCGGTGAGGCGGCATTGAGAAAAAATGCTGAAGTAATCGAAGCTGTATCGCAGTCTTGTTTGACAACAGGA
TTTTGTTTATGGTGCCAATTAGCTTTTTCAACGTATTTAGAAAATGCCACGCAGCCACATTTAAATAATGACTTACAACAGCAATTGTTATCT
GGAGAAATATTAGGTGCTACCGGATTGTCTAATCCGATGAAGTCATTTAATGATTTAGAAAAGTTGAACCTTGAACACACTTATGTTGATGG
ACAATTGGTTGTCAGTGGACGTATGCCAGCTGTAAGTAATATTCAAGAAGACCATTATTTTGGTGCGATTTCGAAACATGAATCATCAGATG
AATTTGTCATGTTCATTCTACGTGCCAATCAAGATGGTATCACTCTCGTTGAAAAAACAAATTTTTTAGGGGTAAATGGCTCGGCTACGTATC
AAATTACTTTGAATCAAGTCGTAGTGCCACAATCACAAATTATCACGCATGATGCGAAGCAGTTTGCGGCAACTATTCGCCCACAATTTATT

GCTTATCAAATTCCAATAGGATTAGGCTCAATTAAAAGTTCTTTAGAGTTAATTGATGCATTTTCAAATGCGCAAAACGGAATAAATCAATA
TTTAGAGTATGATGTTGAAGCTTTTAAAAAACGTTATCGTCAACTTAGAGAAGAATATTATGCAATATTAGATGACGGTAACTTAACTTCAC
ATTTAAATGAATTAATATCATTGAAGAAGGACATCGGCTATTTATTGCTAGATGTAAATCAAGCTTCTGTTGTCAATGGTGGTTCAAGAGCG
TACACACCATATTCGCCACAAGTTCGCAAGTTAAAAGAAGGAGTCTTCTTTGCAGCATTGACACCAACATTAAGACATTTAGGTAAACTTGA
AGCAGAGTTGAAGGGGTAA

Gene: yrhF (putative regulator protein)

Position: 181259 to 181618, length: 360 nt, orientation: FORWARD

Perfect match to: (MW2-BA000033-[171945:172304], highly conserved allele)

Sequence:

GTGAATACTATAGATACGCATACTAAAGAACAACAATTCTCGAATCTAGTAAGATCTTATCGTAAAGAATACGTGGGTAAAGGACCCAATA
GTATTCGAGTGTCGTTTAAAGATAATTGGGCGATTGCACATATGACAGGTGTTTTGAGTAAAGTTGAGAGTTTTTACCTAAACGACAAACG
CAATGAATCGATGCTCCATTATACACGCACAGAGAAGATTAAACAGATGTATAAAGAAATAGATGTAAATGAGATGGAAAGTCTTGTAGG
CGCTAAGTTTGTAAAATTATTTACAGATATTGATTTGAATGATGATGAAGTCATTTCAATATTTGTTTTCGATAAGTCAATAGAATAA

Gene: fdh (NAD-dependent formate dehydrogenase)

Position: 181792 to 182817, length: 1026 nt, orientation: FORWARD

Perfect match to: (MW2-BA000033-[172478:173503], highly conserved allele)

Sequence:

ATGAAAATCGTAGCATTATTTCCAGAAGCAGTAGAAGGTCAAGAAAATCAATTACTTAATACTAAAAAAGCATTAGGATTAAAAACATTTTT
AGAGGAAAGAGGACATGAGTTCATTATATTAGCAGATAATGGTGAAGACTTAGATAAACATTTACCAGATATGGATGTGATTATTAGTGCG
CCATTTTATCCTGCATATATGACTCGTGAACGTATTGAAAAAGCACCGAACTTGAAATTAGCAATTACAGCAGGTGTAGGATCTGACCATGT
AGATTTAGCGGCAGCAAGTGAACACAATATTGGTGTCGTTGAAGTTACAGGAAGTAATACAGTTAGTGTGGCAGAACATGCGGTTATGGA
TTTATTAATACTTCTTAGAAACTATGAAGAAGGTCATCGTCAATCAGTAGAAGGTGAATGGAACTTGTCTCAAGTAGGTAATCATGCGCATG
AATTACAACACAAAACAATTGGTATTTTTGGATTTGGTCGAATTGGACAACTTGTTGCTGAAAGATTAGCGCCATTTAATGTAACATTACAA
CACTATGATCCAATCAATCAACAAGACCATAAATTGTCTAAATTTGTAAGCTTTGATGAACTTGTTTCAACAAGTGATGCGATTACAATTCAT
GCACCATTAACACCAGAAACTGATAACTTATTTGATAAAGATGTTTTAAGTCGTATGAAAAAACACAGTTATTTAGTGAATACTGCACGTGG
TAAAATTGTAAATCGCGATGCGTTAGTTGAAGCGTTAGCATCCGAGCATTTACAAGGATATGCTGGTGATGTTTGGTATCCACAACCTGCAC
CTGCTGATCATCCATGGAGAACAATGCCTAGAAATGCTATGACGGTTCACTATTCAGGTATGACTTTAGAAGCACAAAAACGTATTGAAGA
TGGAGTTAAAGATATTTTAGAGCGTTTCTTCAATCATGAACCTTTCCAAGATAAAGATATTATTGTTGCAAGTGGTCGTATTGCTAGTAAAA
GTTATACAGCTAAATAG

Gene: lmrP (integral membrane transporter)

Position: 183203 to 184453, length: 1251 nt, orientation: FORWARD

Sequence:

ATGAAACGCTTAAGTACGACTTTGAAAGTACGATTGATTAGCAATTTTTTACAGCTAATTATTACGACAGCATTTATACCGTTTATAGCACTA
TATTTAACAGATATGTTAAGTCAATCAATTGTCGGTATATATCTTGTTGGTTTAGTGGTTCTAAAATTTCCATTGTCCATTATATCTGGTTACC
TTATTGAGATTTTTCCGAAAAAGTTGCTAGTACTTATTTATCAAGCGACGATGGTGATAATGCTTGTGTTCATGGGCGTATTTGGGTCACATC
AATTGTGGCAAATTATTGGTTTTTGTGTTGCATATGCCATATTTACAATCGTTTGGGGATTACAATTTCCAGTTATGGACACATTAATTATGG
ATGCAATTACCGAAGACGTGGAACATTATATTTACAAGATTAGCTATTGGATGACGAACTTATCGGTAGCTATTGGGGCATTGTTAGGTGG
CTTGATGTATGGCTACAGTATGTTACTACTTTTCTTAATAGCAGCTTGTATATTTTTAATTGTACTCTTTATTTTATATATTTGGTTACCTCAAG
ACCGAAATCAAGTAAAGCAAAGTGATGACAAGAGGCATGCAAGTCGTTATCAAAAATTACAAATAATGAATATATTTCGCAGTTATAAATT
AGTTTTGAAAGACCGTAATTATATGTTATTGATTTCGGGGTTCAGTATCATCATGATGGGTGAATTTTCAATCTCCTCATATATTGCTATTAG
ACTAAAGGATCAGTTTGAAACAATAAGTATAGGTTCATATGATATTACAGGTGCTAAGATGTTAGCAATCTTGCTAATGATTAATACGGTCG
TCGTCATTTTACTCACGTATTCAATCTCGAAAGTTGTATTGAAAATAGATTTTAAAAAAGCTTTAATCACTGGTTTGCTGATTTATATTGTTGG
CTATAGTGGTCTAACCTATCTTAATCAGTTTGGCTTATTAGTTGTTTTTATGATAATTGCGACTGTAGGTGAAATTATTTATTCGCCTATAGTT
TCAGAACAACGCTTTAAAATTATTCCTAAAGCTAAAAGAGGAACATATAGTGCAGTTAATGCATTAGGTATTCATTTTTCAGAAACACTAGC

TAGGTTAGGGATTGTGTTGGGTGTTTTCTTAACGTCATTACAAATGGGACTGTATATGTTTATCGTTTTAACAATTGGTGCTAGCATGCTTGT
TGCTGGTGTATTTGGGGGGCAAAAACAAGTGAATACAAATTGA

Gene: ausA (nonribosomal peptide synthase)

Position: 184900 to 192075, length: 7176 nt, orientation: FORWARD

Perfect match to: (08-02119-CP015645-[2047574:2054749:r], allele observed in ST582+CC15+CC188)

Sequence:

ATGATTATGGGTAATTTGAGATTTCAACAGGAATATTTTCGTATATACAAAAATAATACAGAATCAACGACACACCGTAATGCGTATTGGGT
TAAACTCGCTAAAAATGTTGAAGCTACTAAAATGATGTATGCATTATCGACAATTGTGCAACAACATGCATCTATAAGACATTTTTTTGATGT
TACTACCGATGACAATTTAACAATGATACTTCATGAATTTCTGCCTTTTATTGAGATAAAACAAGTTCCATCTTCTTCCGCAAACTATGATTTA
GAAGCTTTTTTTAAGCAAGAATTAAGTACTTACCATTTTAATGATTCACCTTTATTCAAAGTTAAATTGTTTCAGTTCGCTGATGCTGCATATA
TACTATTAGATTTTCATGTGTCCATTTTCGATGATAGTCAAATTGATATTTTTCTTGATGATTTATGCAATGCATATCGTGGCAATACTGTTAT
TAACAATACTCGACAGCATGCACATATAAATAGAAATGATGATAAAGACAATCAAGATGCATCGCATATAGCATTAGACTCAAACTATTTTC
GGTTAGAGAATAACTCTGACATCCATATTGATAGTTATTTTCCAATTAAGCATCCATTTGAACAAGCTTTATATCAAACGTATTTGATTGATG
ATATGACATCAATAGATATGGCATCGTTGGCTGTTAGTGTGTATTTAGCTAATCATATAATGAGTCAACAACATGATGTCACATTAGGTATA
CATGTACCATCACATTTACCAAATGATTTACACGGAAATATTGTGCCGTTAACGTTAACAATCGATGCAAAAGATGTATGTCAACGTTTTACA
ACAGATTTTAATAAATGTGTGTTGCAAAATATGTCGCAATTACAGTGCGCGAAGTCTTCGCTTTCACTAGAGACTATTTTTCATTGTTATCAT
CATATGATGTCTTGTTGTAATGATGTTATTGAGGATGTACATCAAATACATGATGCACATACATCTTTAGCGGATATTGAAATTTTTCCACAT
CAACACGGGTTCAAAATTATATATAACAGTGCAGCATATGATTTGCTCTCAATCGAGACGCTGAGTGACTTAGTTCGAAATATTTATTTGCA
AATTACTGAAGAAAATGGAAATAAACGAACAACTGTAGATGAACTTAATTTGATGACAGAACGTGATATTCAATTATATGACGATATCAATT
TAAGTTTGCCTGAGATAGATGATGCGCAAACAGTTGTTACCTTATTTGAGCAACAAGTTGAAGCAACGCCGAATCATGTCGCTGTGCAATTT
GACGGAGTGTTTATAACATATCAAACATTGAATGCACGCGCGAATGATTTAGCACACCGTTTGAGAAACCAGTATGGTGTTGAACCTAATG
ATCGTGTCGCTGTCATAGCTGAAAAAAGTATTGAGATGATAATAGCGATGATAGGTGTGTTGAAAGCTGGTGGGGCTTACGTGCCAATTG
ATCTGAACTATCCAAGTGATCGTCAGGAGTACATTTTAAAAGATGTAACGCCTAAAGTTGTAATAACGTACCAAGCTTTATATGAAAATGGT
AAACAAAATATTAATCACATTGATTTGAATAAGATAGCGTGGAAAAATATTGATAATCTTTCTAAATGTAACACGTTAGAAGATCATGCTTA
TGTTATTTACACGTCGGGGACAACTGGTAACCCTAAAGGGACACTAATTCCGCACCGAGGTATTGTTCGCTTGGTCCATCAAAATCATTATG
TACCATTAAATGAAGAGACGACGATTTTGTTATCAGGAACTATAGCCTTTGATGCTGCAACATTTGAAATATATGGTGCATTGCTCAATGGT
GGAAAGCTGATTGTTGCTAAAAAAGAACAATTATTAAATCCAATAGCGGTAGAACAATTAATCAATGAAAATGACGTTAATACTATGTGGT
TAACCTCCTCATTATTTAATCAGATTGCTAGTGAACGAATAGAAGTATTGGTACCGTTAAAGTATTTATTAATTGGTGGAGAAGTATTGAAT
GCTAAGTGGGTGGATTTGCTTAATCAAAAACCGAAGCATCCTCAAATTATTAATGGTTATGGACCAACTGAAAATACAACATTTACAACGAC
GTATAATATACCTAACAAAGTTCCAAATCGTATTCCTATTGGTAAACCGATTCTGGGTACTCATGTTTATATCATGCAAGGCGAGCGTCGGT
GTGGCGTTGGTATTCCTGGAGAATTATGTACAAGTGGCTTTGGGTTAGCTGCAGGTTATTTAAATCAGCCAGAATTGACAGCAGATAAATT
TATCAAAGATTCAAATATAAATCAGCTGATGTATAGAAGTGGTGATATCGTTCGTTTGTTACCCGATGGCAACATAGATTATTTATATCGAA
AGGACAAACAAGTTAAGATTCGAGGGTTTAGGATTGAGTTGTCAGAGGTTGAGCATGCGCTCGAGCGTATACAAGGTATTAATAAAGCAG
TTGTTATTGTTCAAAATCATGATCAAGATCAGTATATCGTTGCTTATTATGAAGCGATGCATACATTATCACATAATAAGATTAAATCACAAT
TACGTATGACCTTACCGGAGTACATGATACCAGTTAATTTCATGCATATTGAGCAAATTCCTATTACTATTAATGGGAAATTAGATAAGAAG
GCATTGCCTATCATGGACTATGTCGATACGGATGCCTATGTAGCACCGAGTACAGATACCGAACACTTGCTATGCCAAATTTTTGCAGATAT
TTTACATGTGAATCAAGTAGGTATTCATGATAATTTCTTTGAATTAGGTGGCCATTCATTAAAAGCAACGTTAGTGGTGAATCGGATAGAGG
CATCTACTGGGAAACGATTACAAATTGGTGATTTATTACAAAAGCCAACTGTATTTGAACTAGCACAAGCGATTGCTAAGGTTCAAGAACA
AAACTATGAAGTGATTCCAGAAACTATAGTTAAAGATGATTATGTGCTGAGCTCTGCACAAAAGCGTATGTATTTATTATGGAAATCAAACC
ATAAAGATACGGTGTATAACGTACCTTTTTTATGGCGGTTATCATCAGAACTTAATGTAGCTCAATTGCGACAAGCAGTGCAGCGTTTGATA
GCGCGACATGAGATTTTACGAACACAATATATTGTTGTAGATGATGAGGTTCGACAACGTATTGTGGCAGATGTTGCAGTTGACTTTGAAG
AAGTTAACACGCATTTTACGGATGAACAAGAAATCATGCGCCAATTTGTAGCACCTTTTAATTTGGAAAAGCCAAGTCAAATTAGAGTGAG
ATACATTAGAAGTCCCTTACATGCATACCTCTTTATAGATACGCATCATATCATTAATGACGGTATGAGTAATATACAATTAATGAATGATCT
TAACGCACTTTATCAACATAAATTATTGTTACCACTTAAATTGCAATATAAAGACTATAGTGAGTGGATGTCGCATCGTGATATGACGAAAC
ATAGACAATATTGGTTATCTCAATTCAAAGATGAAGTACCTATTTTAAGCTTACCGACAGACTATGTTAGACCAAATATTAAAACGACAAAT
GGAGCAATGATGTCATTTACAATGAATCAACAAATGAGACAGCTACTTCAAAAGTATGTAGAAAAGCATCAAATTACTGATTTTATGTTCTT
TATGAGTGTGGTCATGACGTTGTTAAGTAGATATGCTCGAAAAGATGATGTTGTTGTCGGTAGTGTGATGAGTGCGCGTATGCATAAAGGC
ACGGAGCAAATGCTAGGCATGTTTGCTAATACGTTGGTATATAGAGGGCAACCGTCACCTGATAAAATGTGGACACAGTTTTTACAAGAGG
TTAAGGAAATGAGTTTGGAGGCATACGAGCATCAAGAATACCCATTCGAATGTTTAGTAAATGACTTAGATCAATCACATGATGCCTCACG
GAATCCATTATTTGATGTCATGTTAGTACTACAAAACAATGAAACGAATCATGCTCATTTTGGGCATAGTAAATTAACACACATTCAACCCAA
ATCAGTGACGGCGAAATTTGATTTATCTTTCATCATTGAAGAAGATCGCGATGACTATACAATCAATATCGAGTATAATACCGATTTATATCA
CTCAGAAACAGTTCGTCACATGGGTAATCAATGTATGATTATGATTGATTATATTTTGAAGCATCAAGATACACTACAAATTTGTGATATACC
AAACGGCACGGAGGAACTTCTAAATTGGGTCAATACGCATGTTAACGATCGAATGCTTAATGTCCCGGGAAATAAATCTATCATAAGTTAC
TTTAATGAAGTTGTCTCACGACAAGGTAATCATGTTGCGCTAGTCATGAATGATTTGACAATGACGTATGAAACATTACGCAACTATGTGGA
TGCCATTGCGCACATGCTCCTATCAAATGGTGTGGGCAATGGTCAACGGGTTGCCTTGTTTACAGAACGTAGTTTTGAAATGATTGCGGCG

ATGTTGGCGACAGTTAAAGTAGGTGCATCTTATATACCTATCGATATTGATTTTCCGAATAAACGACAAGGTGCAATTTTGGAGGATGCTAA
AGTAACTGCAGTCATGTCTTACGGCGTTGAAATTGAAACGACATTACCAGTCATTCAATTGGAAAATGCTAAAGGCTTTGTTGAATCAAAG
GAAAATGAACAATATGATGATTTACATGGCAATCAACTTGAAAACACAGCGATGTTAGATAATGAGATGTATGCTATTTACACATCTGGTAC
GACCGGGATGCCTAAAGGGGTTGCCATACGACAACGAAATTTGTTGAATTTAGTGCATGCATGGTCAACTGAATTGCAATTAGGCGACAAT
GAAGTATTTTTGCAACATGCAAATATTGTTTTTGATGCATCAGTTATGGAGATTTATTGTTGTTTGTTAAATGGTCATACGCTTGTGATTCCA
GATAGAGAGGAACGTGTTAATCCAGAACAGTTACAACAACTCATTAATAAGCATCGTGTGACGGTTGCGTCGATTCCGTTACAGATGTGTA
GTGTTATGGAAGACTTTTATATTGAAAAGTTGATTACAGGCGGGGCAACTAGTACGGCATCCTTTGTTAAATATATTGAGAAGCATTGTGG
CACGTATTTCAATGCCTATGGACCATCTGAGTCAACAGTCATCACATCGTATTGGTCACATCAATGTGGTGATTTGATACCTGAGACGATTC
CAATTGGCAAACCCTTATCTAACATCCAAGTGTATATTATGTCAGATGGTTTGTTATGCGGTATTGGTATGCCAGGCGAGTTGTGTATTGCA
GGTGATAGTTTAGCGATAGGATATATTAACCGTCCAGAATTAATGGCTGATAAATGGCAAAATAATCCATTTGGTAAAGGAAAGTTGTATC
ATAGTGGTGATTTAGCACGTTATACATCTGATGGTCAAATTGAATTTTTAGGAAGAATAGATAAACAAGTGAAAGTTAACGGGTACCGTAT
TGAACTTGATGAAATTGAAAATGCAATATTAGCTATTCGTGGTATATCTGATTGTGTTGTTACAGTAAGTCACTTTGATACGCATGATATATT
GAATGCTTATTATGTCGGAGAGCAACAAATGGAGCAGGATTTGAAGCAATATTTAAATGATCATCTGCCTAAGTATATGATTCCTAAGACTA
TAACGCATATCGATTATATGCCATTAACCACGAATGACAAGGTGGATACTACGCGTTTGCCAAATCCATCAACTATACAACAGTCTAATAAA
GTGTATAGCGAACCATCTAATGAAATTGAGCAGACATTTGTTGATGTATTCGGAGAGGTATTGAAACAAAATGATGTCGGTGTTGACGATG
ATTTCTTTGAACTTGGTGGTAACTCATTAGAGGCGATGTTAGTTGTCTCGCATTTAAAACGATTTGGCCATCATATTTCAATGCAGACATTAT
ACCAATATAAAACCGTGCGACAGATTGTTAATTATATGCACCAAAATCAACAATCATTAGTTGCATTACCGGATAATCTTTCGGAATTACAA
AAGATTGTTATGTCTCGTTATAATTTGGGTGTTTTAGAGGATAGTCTAAGTCATCGACCTATAGGAAATACACTATTGACTGGCGCGACAGG
GTTTTTAGGTGCTTATCTGATTGAAGCACTACAAGGATACAGTCATCGCATTTATTGTTTCATACGTGCTGATAATGAGGAAATAGCAAGGT
ATAAGTTGATGACGAATTTAAATGATTATTTTTCAGAAGAGACGGTTGAAATGATGTTATCAAACATTGAAGTCATTGTTGGTGATTTCGAG
TGTATGGATGATGTTGTTTTACCAGAAAACATGGATACGATTATTCATGCAGGTGCTCGTACAGATCACTTTGGTGATGATGATGAATTTGA
AAAAGTAAATGTTCAAGGTACTGTTGATGTCATACGTTTGGCACAACAACATCATGCAAGGTTAATATATGTGTCTACGATAAGTGTGGGA
ACTTATTTTGATATAGACACAGAAGATGTGACATTTTCAGAAGCGGATGTCTATAAAGGGCAACTACTAACATCACCATATACACGGAGCA
AATTTTATAGTGAATTAAAAGTATTAGAAGCTGTAAATAATGGCTTAGATGGTCGGATTGTACGTGTTGGTAATTTGACGAGTCCTTACAAT
GGAAGATGGCATATGAGAAATATAAAGACTAACCGTTTTTCAATGGTAATGAATGATTTGTTACAACTGGATTGTATCGGGGTTAGCATGG
CTGAAATGCCTGTAGATTTTTCTTTTGTGGATACGACTGCAAGACAAATTGTCGCATTAGCACAGGTCAACACACCACAAATCATTTACCAT
GTGCTATCACCTAATAAAATGCCGGTGAAATCTTTGTTAGAATGCGTTAAGCGCAAAGAAATTGAACTCGTCAGCGATGAATCATTTAATGA
AATTTTACAGAAACAAGACATGTACGAAACGATTGGATTAACTAGTGTTGACCGTGAACAACAACTAGCAATGATAGATACAACATTAACA
TTAAAAATAATGAATCACATCAGTGAAAAATGGCCAACGATAACTAACAATTGGCTGTATCATTGGGCACAATATATCAAAACAATATTCAA
TAAGTAA

Gene: ausB (holo-[acyl-carrier-protein] synthase)

Position: 192088 to 192732, length: 645 nt, orientation: FORWARD

Perfect match to: (08-02119-CP015645-[2046917:2047561:r], allele observed in ST582+CC25+CC361)

Sequence:

ATGACAGTATTTGTAATGCAATTACAGAGTAACTTGAAAAGTATTGAAGAATTAATATCACAAAGTCGTTGGTCATATAAAAAACCGCGTAC
AGTCAACTATAGATACAATCAAGATAAACTCATGCACAGATTGGGAGATATTTTAGTGCAATATGGAATTCAACATGACACAGGTTTATTAC
CACATGAATGGCATTATCACATTTCGCCACGAGGTAAGGCAGATATTGTTCAACACAATCGTGATGGACAGCCCATCTATGTGAGCTTATCA
TATAGTTATCCTTATATCGTGTGTGTTGTCGATAAAGAACCAGTTGGTATTGATATCGAAAAGATATCACAACGTTTAGACTGGCGTACGTT
AGTGACGTGTTTCTCTACAAACGAAGCACATCAAATATGTAGTTTAAATGATTTTTATCAAATATGGACACAAAAAGAAAGTTTTACAAAAT
TGATTGGTGAAGGTTTAATCAAAGGATTGGACATTTATGATATGACACAATCACACTTTTATCAATCACGTGAAGTGAAGTTCAAACAATTT
ATTTTTGATCAGTTTATGGTACAGGTATGTTTCTTAGGACAGGCACCCTGGGGGTATAAAAAAGTGTCTGTATTTCAGTTATTGAGTAGTTA
A

Gene: Q5HJJ2 (putative protein)

Position: 193060 to 193554, length: 495 nt, orientation: REVERSE

Perfect match to: (MW2-BA000033-[183746:184240:r], highly conserved allele)

Sequence:

TTAATTTAATTTATAGCGATAGACAAAATGTTGTCGCACTAATTCAATTGTCATCATCCAGACGATATGGCCAAAGAATTCTGACAGATGCTC
TTGGAATGGTTGATCCCACACAGCAGGTACAGTATGCATGATTGGCATAATGATAAGGTGGAATAATACCCAAATAGCAATACCAAAAACA
GCACCTTGTCCCATTGCTAAGTAAGCGTATTTTTTAACTAATATGCAGTAAATAATTGCAATGACGATAGAAAAACTAAAGTGGACAATAAA
GCTTACCCAAGGCAATTCCATATTTGAAAATGTATATGTTTGATGCGTAAACTCACTACTAAATCCTAATTGTTGCAATAACTCTTGAGGTGG

GTTCGTTGCATTACGTTCTGGTGTGCGAGGTGGAAACATGACCTCCCAACCTAATTTTACAATTCCAGATAACAAGCCACCGATAATTCCAG
CATAAATGAATATACCCATTTGTCGTTTCGTCAA

Gene: argB (acetylglutamate kinase)

Position: 193803 to 194573, length: 771 nt, orientation: REVERSE

Sequence:

TCATAGTTATGAATTAAGGATTGTTGTGCCAACAAAATCATTATTGTAAATAGATTCAATGATATTTGGCTTGTTTCCTGATGCAATGATAAC
TTTAGGACAGCCATTTTCAATCGCATTTTTGGCATCTAGCACTTTGGGAATCATACCTCCATAAATATCACCATGTTCAATATATTGATGAATA
TCGACTAATGGCAATTGAGGTATAACAACATCATTGATGAGTACACCTGCAATATTACTTAATACATAAATAGGCGCTTTTAATGATGATGC
AATAAAATAGGCAAGCGTGTCAGCATTAATATTGTAAAATTCTCCATCATGGTTATTGAAACCAATCGAATTGATGATAGGTACAAATTTAG
TACATAAATACTGTAAAGCATCCTTATTTAAAGCGGTCGGAACACCGACATATCCATATTGTTGATCAAAAGATGTAATTTCAAACAGCTGT
GCATCCAAACCACATAAGCCTATTGCAGAACATTGGTGCTGGTTAAATTGAGCTACTAATGCAGTGTTAACGTCTGCAATGAGCGTGTGTTT
AGTAATGGTCATGGTTGCTTTATCAGTCACTCTTAGGCCATTAACAAAGTGTGGCTCGATTTGCTGGTTTGATAATGCTTCATTAATAAATGG
GCCACCGCCATGAACGATAATGGGGTAGATGTTGTTTGATCGTAAATGCTTAATGTTGTTAATAATTGATGGATGCATGTCACTAAGTGTAC
TGCCACCAATTTTAATGACAATAAATTTCAT

Gene: argJ (arginine biosynthesis bifunctional protein)

Position: 194589 to 195830, length: 1242 nt, orientation: REVERSE

Perfect match to: (MW2-BA000033-[185275:186516:r], highly conserved allele)

Sequence:

TTATGTTCGATATGATGCGTTGATACGCACATAATCATAGGATAAATCACAACCGTATGCAGTCGCTGCAGCGTTACCTAAACCAAGCTGAA
CGTCAATTGTGACATTTTCATGAGTTAATGTATTCGACATAGCTTGCTCATCAAATAGTACAGCCATACCTTTATCAACGACAGGTATTTGGT
TCAGTTGAACATATGTGCAGTTAGGATCAATTTCACATCCGCTGTAGCCAATAGCTGTAATGATTCGACCAAAATTGGCATCTTCGCCAAAA
ATAGCTGATTTTACTAGATTTGAACTTACGATAGTTTTACCGATTTTTCTTGCATCTGATATTGATTTAGCGCCTGACACATTGACGCTGATTA
ACTTTGTTGCGCCTTCGCCATCTCTGGCTATAGCTTTAGCTAAAAATGTACAGACAAAATTGAATGCATCAACAAATGTTTCCCATTGTGGAT
GGTCTTGACTAAGTATTTGGTGTTCAACTTGGTGATTTGCCATGACTAATACCATGTCATTTGTACTTGTATCGCCATCAACAGTAATCATAT
TAAATGTATGGTCAGTCGAAGATTTTAATAATTGATGAAGTGTATTCGATTCAATCGATGCATCGGTTGTTATAAAAGCAAGCATGGTAGCC
ATATTTGGGTGAATCATACCTGAACCTTTGGTGCTACCACCAATTGTAACGGTTTTACCATCGATTTTTAGTGATACAGCGATATGTTTTGTA
CAGGTATCAGTTGTTAAAATTGCCTCGTTAAACGCACCTGGCGTTGCAAAATTAGCATCCTTAATATGTTCGGTCCCAGTCTTAATTTTATCC
ATAGGCAAATATTCACCAATGACCCCAGTTGAAGCAACAGCAACATGCTCAGATGGTATTTGAAGTTGTTGAGCAACCCATGTTTGTGTTTG
TCGTGCATCATCTATGCCTTGTTGACCGGTACAAGAATTTGCATTAGCTGAATTAACAACAAGTGCTTGTAATTTTCCTTTAGACTTTTGTAA
AGTGTCTTCAGTGACAATAAGTGGTGCAGCTTTAAACTGATTTAAAGTATATACGGCAGCTGCACTTGCCAAAGACGATGAGTAAATCCAC
CCAAAGTCTTTTTTGTTAGCGCGTAAACCGATGTGCATACCACCAGCCGTGAAGCCTTGAGGTGTACTGATATCGCCATGTTTAATAATTGA
AAAGTTATATTGTTGTGATGTCGTTTCTTGATGTTTCAT

Gene: argC (N-acetyl-gamma-glutamyl-phosphate reductase)

Position: 195842 to 196876, length: 1035 nt, orientation: REVERSE

Perfect match to: (MW2-BA000033-[186528:187562:r], highly conserved allele)

Sequence:

TTATGGATAAACTGGTGATTGATTTAGGCCAGTCGTCACTTCAAAATCATATAATATATTTAAATTTTGAATGGCTTGCCCACTTGCGCCTTT
GACAAGGTTATCAATCACTGATACTAAAATTGCTGTTTGCGTTGTTTCATCTACATAGATGCCGATATCGCAGTAGTTACTACCGAGTACTTC
TTTTGTGGTTGGAAAAGTCCCAATATCTCTAATTCTGACAAATGGCTGATTAGCATAATAAGAGGTCATTAATTTATGTAATGATTCAGTCGT
ATATTCAGATGATAATTTGACATATATTGTTGATAAAATACCTCGTGTCATTGGTACGAGATGTGGTGTAAATATGACTGATACATCTTGACC
CGCAATGATAGATAAATATTGCTCGATTTCCGGTTTGTGTTTATGGTTTCCGATTGCATAAGCGCTTAGATTTTCATTCATTTCTGAAAAATG
AACACGTTGTGATAATGAACGACCAGCACCTGACACGCCGGTCTTAGCATCAATAATAATAGATGACAAATCTACTATTTTTTCGCTAATAA
GTGGATGTAATGCTAATAATGTTGCTGTAGGGAAACAGCCAGGATTAGAAATGAGCTTCGTTCCATTGTTATCAAACGATTGCCATTCTGAA
ATGCTGTAAATAGCATGATTCAAATCATCTTGTGCTGCAGCAGTTTCTTTGTAATATGCTTCATATATTTCACGATTCTTAATTCTAAATGCGC
CAGATAAATCGATAACATGAATACCTTTTTCTACTAAGGGAGGGATACATGTTTTACTTACGGGTGCTGGTGTCGCAAAGAAAATTACATCA

CAGTCATTATTGTCCACTGTAAGTGCTTCGAAATGTTGCATAATATGTTGTAAATGTGGAAATGTTAATTTCAACGGTTCATCTACTTTTGAA
TGTGAGTAGATGTGTGCAATCGTTACATGAGGATGTGTTTGTAACAATCGAATTAATTCAATTGCGCCATAACCGCTACCGCCAACGATACC
TACTTTAATCATCAT

Gene: rocD1 (ornithine aminotransferase 1)

Position: 196912 to 198096, length: 1185 nt, orientation: REVERSE

Perfect match to: (COL-CP000046-[194970:196154:r], highly conserved allele)

Sequence:

TTAATTTTTAAAAACGTCTTGAAAAGCTGCAACAATTTGATGGATTTCCTCTTTATCAATGACTAGAGGTGGAGACAATCGAATGATAGTAC
GATGCGTGTCTTTGCATAAGATTCCACGTTGAATCAGTTGATCCACAAAAGGTGCAGCATCTGTGTTAAGCTCTATGCCTATAAATAAACCA
CGACCTCTAATTTCTTTAATACTAGGATGTTTAAGTTGTAGCAACGCTTTTAATAAAAATGAACCTAAGCGTTCTGATCGTTCAACCAGTTGT
TCATCTTTAAGTACATCAAGCGCTGCCGTCGATATTGCAATGGCTAAAGGGTTACCACCAAATGTTGAACCATGTGTACCTGGTGTTAGAAC
ACGCATGACATCATTATTTGCAAGTACAGCAGATACAGGGTATAAGCCGCCACCCAATGCCTTACCTAAAATATAAATGTCTGGAACGACTT
GCTCCCATTCCATAGCAAACCATTTCCCAGTTCTACCAAGACCAACTTGAATTTCATCTGCAATCAATAATATTTGATGTTTATCACATAGTTG
ACGCACAGCTTGAATATATCCTTTCGGTGGTATATTAACGCCACCTTCACCTTGAATTGGTTCCAAAATAATTGCTGCTGTATTCGGTGAAAT
AGCTTGTGTTAATTGTTCAATGTCTCCAAAATCTACTGTTGTAGTGCCTTGAAGTAGGGGGTGAAATCCTGCTTTATATGCGTCGTGGTTAG
ATAGTGATAATGAGCCAAGTGTACGACCGTGAAAATTGTTATTCATAGCGATGATTTCAACTTGTCCGTCAGTAATGCCTTTAACTTCAGAG
CCCCATTTTCTAGCAATTTTAATGGCTGCTTCAACAGCTTCAGTACCAGAGTTAAGGGGGAGTACTTTGTCTTTCTTAGCAAGATGACAAATT
TTTTCTTCCCATTTCCCGAGATTGTCACTATAAAGGACACGTGAAATGATAGACAACTTTGAAGCTTGTTCTGTCATCGCTTTAACAATTGTT
GGATGACAATGGCCTTGGTTTGCAACTGAAAAACCCGAAATGCAATCTATATATTGTTTGCCATCAGTATCCCAAACTTTGACACCTTTACCT
TTAGAAATGACAAGCTTAAGTGGTGCATAATTATTAGAGCTATAATAATCAGTTAATTCAATGATTGAATTCAT

Gene: brnQ1 (branched-chain amino acid transport system II carrier protein)

Position: 198349 to 199704, length: 1356 nt, orientation: REVERSE

Sequence:

TTATGCTTGTGGAATTTTACGATGCTTAATTTTATAAATAATGAAGCCGATAATGAAACCAATCAAACTGAGAACAACCCAGCCCATACCAA
TGTCTGATAATGGTAAATATTTTTGGCTGAAATTAATCAAAGTTTGTGAGAATGATGTGCTTGAAATGAACTCTGGACTAGCTTTTAATCCAT
CTACTAATGCAGCAATCATTGTAAAGAAAATGGTACATTGATAAATAAGTTTTGAATGATGGAATTTGCTACTAAATAATGTTAGTACAATC
AAGGCAATTGCTAATGGATATAAGAACATTAACACTGGGACTGAGTACATAATAATCTTAGTTAAACCAACATTCGCGAATAAGAACGAAA
TAAAGCTTACAACTGTTGCAATCGCTAGGTAATTCATTTTAGGGAAAAGGTGTTCGAATGTTTCTGAAAATGCCGTAATCAAACCGATGGCT
GTTTTTAAACAAGCAACCATAACGATAAGTGACAACAGGACGATACCGTAGTTACCTAAGTAGTATTGAGTAATTTGCGCTAAGGCAATAC
CACCATTTTCACTAAGTTTGAAATGACCAATACTTAATGTACCCATGATTGCTAGTAGGGTATAAATGATCCCCATCATAATGATACTGATAG
TACCAGACTTAATTGTTTCTTTAGCGATATCAGTTGGATTTTCGATACCTAACTTTTTAATCGTTGCAACAATGATAATACCAAATGCCAATGA
CGCTAGCGCATCTAAGGTATTGTATCCATCTAAAAAGCCGTTAAATAAGGCATGTGATTGATATTGTTTACTAATAGGTGCATCAGATATGC
CACCTAATGGATGGATAAAAGCAAATAATAAAATAATTGCTAATAATACTAAGAATACCGGATTTAAAAATTTACCGATATATTCTAAAATT
CTTGATGGCTTTCTCGCAAAAAACCATGCAATCACAAAGAAGACGAAGATAAAAATAAATAAATATAAAGTGATTTGCTTTGGTGATAAAA
ATGGCGAAAATGCAATTTCAAATGATGTCGTTGCCAGTCTAGGTAAGGCGAAAAATGGTCCGATAACTAGATATAAGGCAATCGTGAAAA
TGTAAGCATATGTTTTATTAACACGCGATGCAATTTCAAATAAACCAGATGTCTTTGAAATGCCAATAGCAATGATACCTAGAAATGGTAAG
CCAATTGCTGTAATTAAAAATCCTAAGTTAGCGATAAAAACGTTAGAACCAGCAGCTTGACCCAAGTGTATTGGGAAGATAAGATTGCCGG
CACCAAAGAATAAACCAAATAACATAGAACCTATAAACATGTTTTCTTTAAATGTTAGTTTCTTCTTCAT

Gene: ywoC2 (isochorismatase)

Position: 199980 to 200477, length: 498 nt, orientation: REVERSE

Perfect match to: (11819-97-CP003194-[206390:206887:r], allele observed in CC80+CC5+CC22+CC80+CC772)

Sequence:

TTAGTTCAATACATTTAGAAAGTCGTTTGTAGTTATTGTTTGTCCCATCAATGGGAATACATTATCTATTGGAAATTGATGTAGCGTTTCGTTT
TGTGCACTCATCATATCTGTAACAAAAAACTGATTGTAGTTTAATTGATAGGCATCTCGCGCTGTCGTATCTACGCCAATATGCGTTGCGAC
ACCACCAAGAACAATCGTATCAATTCCTCGACGTCGCAATTGTAAGTCCAAATCTGTTCCTACAAATGCACTAAAATGTCGTTTGTCTATGAC

AAAATCGTCATCTCTCTTGTCTAATAAATGATGGAAACGACTGTAGTCGTCGCCTTCTTTTGGTGGTAATGAGATCATTGCATTTGGTTGCAA
TACATCTTTACCATCATAGAAATTCACGCGAACAAAAGCGATAAAGCCATTGTTTTTCTAAAAACATCTATTAATTTATTAGCGTTTTGAAC
GACATTTTCAGCTGTATGTGGCGCATAATCCAT

Gene: ipdC (indole-3-pyruvate decarboxylase)

Position: 200604 to 202244, length: 1641 nt, orientation: REVERSE

Perfect match to: (GR1-AJLX01000027-[59399:61039:r], highly conserved allele)

Sequence:

TTATTTATTTTGTTGTGAAAAAGCTTTAGCGATATCGATGAGTTTCTTCGGTGCGTCTTCGACAGACATTTTGACTTCGACAAAATGCATCAC
ATCGGGATGACCATTAATTGCATTAAACGTGTCTTGTAAATCTTTTGATGATTCAACGTCATGAATTTCAACATTTTTACCACCAAATACAGC
TGGTAAAGCTTTATAATCCCACATGTGAATTTCATTATAAGGTTCATACATGCCGTGAATAAGTCGTTCTACCGTATAGCCGTCATTATTAAT
CACAAATAATACCGGTTTAATATGCTGTCTAATCATAGTTGAAATAGCTTGAACAGTTAGTTGCAATGAGCCATCACCAATTAATAATAAGT
TACGACGATCTTTGTCTGCTAATTGTGAACCTAATGTTGCAGGTAATGTATAGCCGATAGAACCCCATAACGGTTGCCCTATAAAAGTATTG
TTTTTGTATAATGCTAAATCATAAGCACCAAAGAATGATGTACCTTGATCAGCAATAATGACATCATTTGGTTTTAAGAAATTTTGCATCATT
TTAAAATAAGTTTGTTGTGTTAATGGTTCTGTGCCAACTGTATAATCGGGTGATGTTGGACGATGATACGCAGGGAACGTTGCGTTATTCGT
ATGTGAAATATTGGATAACTGTTTTAACAATGATGGTAGAGATATTTCATCATTTGTAACATCGTCAATTTTGATATTGTGATGATTTAACAT
AACGACATCATCGATATTGAATTGGTATGAAAAACCTGCTGTTGCTGAATCTGTTAATTTGGCTCCAATATTTAAAATTAAATCGCTGTTGTC
CACATAATCTCGTATTTTATCTTCGGCAATTTTCCCATCGTAAATACCCATATAATATGGATTTTCCTCATTAAAAGCACCTTTTCCTAATGAAA
GTTGTGCTACTGGTATCTGTGTTTGATTTACAAAATCTTCTAATTCTTGATGAAGGTGAAAACTGTTAATTTCATGTCCAGTAATGATGATAG
GCTGCTTCGATTGATGCAATTTAGATGTTAATAACTCTATATATGTTGATGCATCCGTATCTTTGGCTGCCGTTACTTCAAATGGTGTCGGTA
TCTCAATTTCAGAGATTGCGACATCGATTGGTAAATGTAAATGAACTGGGCGTCTTTCGGCGATTGCTGTATTAATTAAACGTGGTATTTCG
GTTGTTGCATTTTCAGGTGTGATATAACCTTGTGCAACGGTTATATGTGCAAACATTTTTCGGTAGTCGTCAAATGTACCTTCACCAAGTGAG
TGATGTACATATTTACCGGCATGTTCAACAGCACGTGTCGGCGCACCTGTAATCGCAATGACAGGTATGCGTTCAGCATATGAACCTGCGAT
ACCGTTGACGGCACTTAATTCGCCAACACCAAATGTAGTAACTAATGCAGCGAGTCCATTAAGACGGGCATAACCGTCCGCTGCGTAACTT
GCGTTTAATTCATTTGTATTTCCTACCCAATCTACATTGGGATTGCTGATAATATCGTCTAGAAAAGCGAGATTAAAATCACCAGGAACACCA
AAAATTTTATCGACGCCTGCTCGATGAATAGCGTCAATTAAGTAAGCTCCAATGCGTTGTTTCAT

Gene: glcA (glucose-specific PTS transporter protein II, ABC component)

Position: 202517 to 204562, length: 2046 nt, orientation: REVERSE

Perfect match to: (M0239-AIWE01000019-[115836:117881:r], allele observed in CC188+CC8+CC12+CC80)

Sequence:

TTATTTAGCTTCAAATAATTGATCGCCAAATGAAATGTTGCCATGTTCACCTTGTTTAAAATCAAGGTTTGTAATGTTTCCTTGTGTCACGATA
ATAGGCGTAATATCACTCTTTGCATGATTGCGGATGTAGTCTAAATCAAAGTTGATTAATAAATCACCTTGTTTAACTTCTTGACCTTCCTCA
ACATGTAAAGTAAAGCCTTCTCCGTTTAATTTAACAGTGTCTAAACCGATGTGGATTAATAGTTCTAAACCACTATCTGATACAAGACCAATT
GCATGTTTTGTTGGGAAAATCATTTGTACTTTACCGTTGAATGGTGCACGAACTTCACCTTGTGAAGGTTTAATAGCGATACCGTCACCCATC
ATTTTTTCGCTGAACACTTGATCAGGCACTTCTGATAATGGTGTTACTTCACCAGTTAATGGTGCATGCACGATATGGCTCAATTCGCTAGTT
GCAGATTTATCTTCTGCAACAACAACAGTTTCGTCTTTATCGTCTTCCATAGTAGTAGGGTTTTCTACTACTTGACCATTCATAATCTGTTGCA
TTTCATGTTTGATTTGGTCAGATTTTGGACCAAAAATCGCTTGCATATTATTGCCGACTTCTAATACACCGGATGCACCTAAATCTTTCAAACC
AGGAACATCAACTTTAGATTTGTCGTTAACTTCAACACGTAGACGTGTGATACAAGCGTCTAAATGTTTAATGTTTGCTTTACCACCCATAGC
TTCTAATACTGCATATGGTAATTCAGTTGCTGAAGCAGTAGCCGCTTGTGATTGTTTATCTTCACGACCTGGTGTTTTGTATTTTAATTTTACA
ATTAAGAATCGGAATACGAAGTAGTAAATAACTGCGTATACAAGACCTACAGGAATAACTAACCACCATTGTGTCTTATTAGGTAGTATACC
GAGTAAGAAGTAGTCGATGAAACCACCTGAGAATGTATAACCTAGATGAAGATCTAATAAGTACAATGTTAAGAATGATAAACCATCAAGT
ACTGCGTGAATAAAGAATAATAATGGTGCTACAAATAAGAATGAGAATTCTAATGGTTCTGTAATACCAGTTAAGAATGATGTTAAAGCAG
CAGAACCCATTAAACCTGCTACTACTTTCTTATTTTCAGGTTTAGCTGTGTGATAAATTGCTAAAGCTGCTGCAGGTAAACCGAACATCATAA
CAGGGAATTCACCTTGCATGAATTTACCAGCTGTCAAATGTGCGCCTTCACGAATTTGTTCGATAAAGATACGTTGGTCACCGTGAATAATT
TCACCAGCTGCATTTTTCCATGAACCAAACTCGAACCAGAACGGTGCGTGGAAAATGTGATGTAGACCGAATGGAATTAATAAACGCTTGA
TGAAACCAAATAAGAATACGGCAACACCAGTATTTGAATCTAATAATCCTGTACTGAATGCATTTAATCCTGATTGAATCGTTGGCCAAATT
AATGCCATTGGGAATGCTAAAATAAATGATGTTGTAGCCATCATAATAGGTACGAAACGCTTACCAGCGAAGAAACCTAAATAAGATGGTA
AGTTAATGTTATAGAACTTGTTATAACACCAAGCTGCCAGGGCCCCGATTATAATACCGCCGAACACACCTGTTTGTAATGTTGGGATACCT
AAAATGCTAGCGTAACCACTCGCTGGATCACCAATATTCTTAGGTGTAACTTGTAAAAAGTCGCCCATTGTTTTGTTCATGATTATGTAACCG
ACGAATGCTGCGATAGCTGCTACGCCATCACCGCCAGCTAATCCGATTGCGACACCTAATGCGAAAATCATAGGCAAGTTATCAAAAATGA
TACCACCAGCACCTGTCATTAATTTAGCGACAGTTTGTACGCCACCATTTTGTATAAACGGCAAGTAGTGTTGTAATGATTCACCTTGCATAG

CTGTACCGATAGCTAATAACAGACCAGCTGCTGGTAAAATCGCAACAGGTAACATTAGCGCTTTACCAATACGTTGCAATTGACCGAAAAG
TTTCTTCCTCAC

Gene: rsaK (ncRNA of Staphylococcus aureus K)

Position: 204622 to 204833, length: 212 nt

Sequence:

AATAGACTGTGTTGGTAACCATCACAGCTTAATTTAACTCATGCCTAATCTTACTTAGTAACACGTTGGTGTATGTAATTAATGTAAAAGAAG
CAATTGACTAAGTAGTATATAAAACTGATACGTTAGTTTATCTAGCTTACCATCACATCTTATTGAATCTTATTTGTTTGCGACTCCTATTTTA
GCATGGTGAAGTATGCGTTTTCAAT

Gene: DUF871 (putative protein)

Position: 205148 to 206203, length: 1056 nt, orientation: FORWARD

Perfect match to: (Strain_21269-AFTU01000052-[42683:43738:r], allele observed in CC121+CC80)

Sequence:

TTGACAGGCTTTTCAGTGTATTTAGGACAACCTTTAGATGAAGCGTATATTAAGCGAATGATTAAACAAGGTTACCAAATGATTTTTACATCT
GTACAAATACCAGAAGAAGATGACGAGACAAAATATCATTATTTCACAAAACTACTCAATTTATTAAAACATGAACAAGTGACTTACCTCAT
AGATGCTAATCCATCTATTTTAACACCATCTTTTTATGAGCATCTTCGACAATATGATGCACAATTTATGATTCGTATCGATCATAGTACATCA
ATTGAGGCAATCGAAGCGATAATGGCACAGGGTTTAAAGTGCTGTTTGAATGCAAGTATTATTTCCCGGGAATTGTTAACAAGCTTACATC
AACAATTGAATGATTTTACATTACTTTCATTTTGTCATAACTATTATCCAAGACCAGATACGGGATTATCTGTTGACTTGGTCAATAAGAAAA
ATGAACTCATTTATCAATTTAATCCAAAGGCACAAATATATGGTTTTATTGTAGGGAGTGGTTTGCGAGGTCCTTTGCATAAAGGCTTGCCA
ACAATTGAAGCAACGAGACATAGTCATCCTGTCGTTGCAGCTAAATTATTACAAGAAACTGGTGTATCTGAAGTGTTAGTTGGAGACTCATT
GATTGAAATGAGGCAGGCAAAACAACTTATAGATTTTTGCAAGCATAGGCATTTCACGTTATGTATTGAAGAAGTGTTTGATACGACAGTG
ACTTACCTTTTCGATATGTGTCATAAAGTACGCCCGGATAATCCGGAAAATGTCATTCGTTCGGAAACGTCAAGACAAATATGTCCACATTC
GATTCAACCACAGTTTACGACGCAACGACGCATTGGTTCAGTAACCGTTGATAATTTGAATAACGGACGTTATCAAGGCGAAATGCAAATT
GTGAGACAAACGCTTAGTGCACATGACAATGTGAATGTTGTTGCACAAATTATTAAAGAAGACTTACCACTGTTAAGTTGTATCGAGCCGA
ATGATACATTTGATTTTCAAAAAACTAGGGAGTGTAAGAAGTGA

Gene: murQ (N-acetylmuramic acid 6-phosphate etherase)

Position: 206200 to 207099, length: 900 nt, orientation: FORWARD

Perfect match to: (Strain_21334-AGTW01000044-[77128:78027:r], allele observed in CC9+CC22+CC80)

Sequence:

GTGATGGAAAATAGTACGACCGAAGCGCGTAATGAAGCGACGATGCATCTTGATGAAATGACTGTGGAAGAGGCTTTAATTACGATGAAT
AAAGAAGATCAGCAAGTCCCGTTAGCAGTTCGAAAGGCAATACCACAATTGACAAAAGTAATTAAAAAAACAATTGCACAGTATAAAAAG
GGTGGACGATTGATCTATATCGGTGCAGGTACAAGTGGAAGATTGGGTGTCTTAGATGCAGCGGAGTGTGTACCTACATTCAATACTGACC
CTCATGAAATTATAGGTATTATTGCTGGTGGACAACATGCTATGACGATGGCTGTAGAAGGTGCGGAAGATCACAAAAAATTAGCGGAAG
AAGATTTGAAAAATATAGATTTAACATCAAAAGATGTCGTTATAGGAATTGCCGCGAGTGGCAAAACGCCATATGTTATAGGCGGTTTAAC
ATTTGCTAACACAATCGGTGCTACAACAGTATCTATTTCATGCAATGAACATGCAGTTATAAGTGAAATTGCGCAGTATCCAGTAGAAGTTA
AAGTTGGTCCAGAAGTATTAACTGGTTCAACGCGTTTAAAGTCTGGTACAGCACAAAAAATTAATTTTAAATATGATTTCAACCATCACAATG
GTTGGTGTCGGAAAAGTTTATGATAACCTCATGATTGATGTTAAAGCAACCAATCAAAAACTGATCGACCGTTCAGTGCGTATTATTCAAGA
AATATGTGCTATCACATATGATGAAGCAATGGTGTTATATCAGGTGTCTGAGCATGATGTGAAAGTTGCGACAGTTATGGGTATGTGTGGC
ATTTCTAAGGAAGAAGCAACAAGACGGTTATTAAACAATGGTGACATTGTTAAACGAGCAATCAGAGATAGACAACCTTAG

Gene: Q5HJI0 (phosphotransferase system EIIC component)

Position: 207111 to 208565, length: 1455 nt, orientation: FORWARD

Perfect match to: (CN1-CP003979-[187412:188866], allele observed in CC72+CC7+CC80+CC188)

Sequence:

ATGACCAAAGAACAACAACTTGCAGAACGAATTATTGCTGCAGTAGGTGGTATGGATAATATAGATAGTGTCATGAACTGTATGACACGTG
TGCGTATTAAAGTATTAGATGAAAATAAAGTAGATGACCAAGAACTAAGGCATATTGATGGTGTCATGGGTGTTATACACGATGAACGCAT
TCAAGTTGTGGTTGGACCTGGTACAGTCAATAAAGTGGCTAATCATATGGCGGAATTAAGTGGTGTTAAACTAGGTGACCAAATACCACAC
AATCACAATGATAGTGAAAAAATGGACTATAAATCATATGCAGCTGATAAAGCAAAGGCGAATAAAGAAGCACATAAAGCAAAACAAAAG
AATGGTAAGTTGAATAAAGTATTGAAATCAATTGCCAATATCTTTATACCGTTGATTCCTGCATTTATTGGAGCTGGATTAATTGGTGGTATT
GCAGCAGTACTGAGTAACTTAATGGTGGCAGGCTATATTTCAGGTGCTTGGATTACGCAACTTATAACAGTATTTAATGTCATTAAAGACGG
TATGTTAGCATACTTAGCTATTTTCACTGGTATTAATGCGGCTAAAGAATTTGGTGCGACACCAGGACTTGGTGGCGTGATTGGTGGTACAA
CGTTATTAACGGGTATTGCTGGTAAAAATATTTTAATGAATGTCTTCACTGGAGAACCATTGCAACCTGGACAAGGTGGGATTATTGGCGTT
ATTTTTGCCGTTTGGATTTTAAGTATTGTCGAAAAGAGATTGCATAAAATTGTGCCAAATGCGATTGATATTATTGTAACGCCGACTATTGCA
TTGTTGATTGTAGGACTATTAACTATCTTTATCTTTATGCCATTAGCAGGTTTTGTTTCAGACAGTTTAGTTTCAGTAGTTAACGGAATTATTA
GTATTGGTGGCGTATTTAGTGGATTTATCATTGGAGCAAGCTTCCTACCATTAGTTATGTTAGGGCTTCATCATATTTTTACGCCAATTCATA
TAGAAATGATTAACCAATCTGGTGCTACTTACTTATTGCCAATTGCAGCGATGGCTGGTGCTGGACAAGTAGGTGCCGCATTAGCACTTTG
GGTAAGATGTAAACGCAACACAACATTACGTAATACTTTAAAAGGTGCATTGCCAGTTGGTTTCCTAGGTATCGGAGAACCATTAATCTATG
GTGTGACTTTGCCGTTAGGTCGACCTTTCTTAACTGCTTGTATTGGTGGTGGTATTGGTGGCGCTGTAATAGGTGGAATTGGACATATTGGT
GCCAAAGCAATAGGCCCAAGTGGTGTGTCACTATTACCATTAATCTCAGATAATATGTATTTAGGTTATATTGCAGGATTACTTGCTGCGTA
TGCTGGTGGATTCGTTTGTACATATTTATTTGGAACGACAAAGGCGATGCGACAGACAGATTTGTTGGGTGATTAA

Gene: rpiRB (putative phosphosugar-binding transcriptional regulator)

Position: 208565 to 209443, length: 879 nt, orientation: FORWARD

Perfect match to: (COL-CP000046-[206624:207502], highly conserved allele)

Sequence:

ATGATGACAAATATTTTATATCGCATTGATAAGCAGTTGAGTGATTTTACGAAGACAGAAAAGATAATCGCTGATTACATTTTAAAGAATCC
ACATAAAATCATTGATATGACTGTGAATGATTTGGCAGATGTTACGAATGTTAGTACAGCATCAATTGTTAGATTTAGTCGGAAAATGACAC
ATCAAGGTTTTCAAGAGCTAAAGATTGCGATATCTCGATACTTACCCGAAGATATTGCAACCAATCCACATTTAGAATTGATTGAAAATGAA
TCTGTAGAAACTTTGAAAAATAAAATGATTGCTAGAGCAACGAATACGATGCGATTTGTAGCTACTAATATTATGGATGCGCAAATTGATG
CAATTTGTGATGTGTTGAAAAATGCCAGGACAATATTTTTATTTGGATTTGGCGCATCGAGTTTGACTATTGGTGATCTTTTTCAAAAGTTAT
CTCGTATTGGCTTAAATGTCAGGTTATTACATGAAACGCATTTACTTGTGTCAACATTTGCGACGCATGATGATAGAGATTGCATGATTTTTG
TGACGAATCAAGGTAGTCATAGTGAATTGCAGTCAATTGCACAGGTGGCCACACATTACAGTATTCCCATCATAACTATATCTAGTACAGCT
AATAATCCAGTGGCTCAAATTGCAGACTATGCATTGATTTATGGCAGAACTGATGAAAATGAAATGCGTATGGCGGCTACAACGTCACTAT
TTGCACAGTTATTCACGGTAGATATATTGTACTATCGATTTGTAGCATTAAATTATCATGCGATTCTAGATTGTATAACCCAATCGAAAATGG
CACTTGATAATTACAGGAAGCATCTTGCGACGATAGATTTTAAACATTAG

Gene: A5IP63 (putative protein)

Position: 209637 to 209918, length: 282 nt, orientation: REVERSE

Perfect match to: (N315-BA000018-[221933:222214:r], highly conserved allele)

Sequence:

TTAAATGCTGAAGAATAATTTTAATGCTAAATGAACTAATACGAAGGTGCTAAAAGCAATTATTATCAAATTAGTGATATGAATGAAATGTT
TAAATCGTTTGCGTTGATTGTATTCGGGACTTTTATAGAGTAGGTAGTAGTACATAAAGAGTATGGCTGCGAACATGCCGAATATTGAAGT
GAAGTTACCTACAGTGACTGCCATGTAATGATTTAAAGTTAAATAGTAACTTAGACTAATAATAAAAAATAAAATGATTTGATAGGTAATTA
ACACCAT

Gene: hsdR (type I restriction-modification system endonuclease)

Position: 210131 to 212920, length: 2790 nt, orientation: FORWARD

Perfect match to: (11819-97-CP003194-[216541:219330], allele observed in CC80)

Sequence:

ATGGCATACCAAAGTGAATACGCATTAGAAAATGAAATGATGAATCAACTTGAACAATTGGGTTACGAAAGAGTAACAATACGTGATAATA
AGCAATTGCTTGATAATTTTAGAACGATTTTAAATGAGCGTCATGCGGACAAATTAGAAGGCAATCCCTTAACAGATAAAGAATTTCAACGT
CTGTTAACGATGATTGATGGGAAAAGTATCTTCGAGAGTGCCCGTATTTTACGTGATAAATTACCACTTAGACGTGATGATGAGTCTGAGG
TTTATTTGTCGTTTTTAGATACGAAAAGTTGGTGTAAAAATAAGTTTCAAGTGACGAATCAAGTATCTGTCGAGGATACATATAAAGCACGT
TATGATGTAACGATATTAATCAACGGACTACCCCTTGTCCAAGTTGAATTGAAACGTCGAGGTATTGATATTAATGAGGCGTTTAACCAAGT
AAAACGTTACCGCAAACAAAATTACACAGGCTTATTCCGCTACATACAAATGTTTATCATTAGTAATGGTGTTGAAACGCGATACTTTTCTAA
TAATGATAGCGAACTATTGAAGAGTCACATGTTTTATTGGAGTGATAAACAGAATAACCGAATCAATACATTACAATCGTTTGCTGAGTCGT
TTATGAGACCTTGTCAATTAGCTAAGATGATATCGCGCTATATGATTATTAATGAAACAGATAGAATACTGATGGCAATGCGTCCGTATCAA
GTGTATGCGGTAGAAGCACTTATTCAACAAGCGACTGAGACAGGGAATAATGGATATGTATGGCATACAACTGGAAGTGGTAAGACATTG
ACTTCTTTTAAAGCGAGTCAGATTTTATCACAGCAAGATGACATTAAGAAAGTTATCTTTTTGGTTGACCGTAAAGACTTGGATAGTCAAAC
AGAAGAGGAATTTAATAAATTTGCTAAGGGTGCTGTAGACAAAACTTTTAATACCTCGCAACTGGTACGCCAACTAAATGATAAAAGTTTG
CCACTTATTGTAACGACGATACAAAAAATGGCTAAAGCGATTCAAGGTAATGCCCATTTATTAGAACAGTATAAAACGAATAAAGTTGTATT
TATTATTGATGAGTGTCATCGCAGTCAATTTGGTGACATGCATCGTCTAGTTAAACAACATTTCAAAAATGCCCAATACTTTGGATTCACTGG
TACGCCACGTTTTCCAGAAAATAGTAGTCAAGATGGTAGAACAACTGCAGATATTTTCGGTAGATGCTTACATACGTATTTAATTAGAGATG
CCATTCATGATGGTAATGTACTTGGTTTCTCAGTTGACTATATTAATACTTTTAAAAATAAAGCTTTAAAAGCAGAAGATAACAGCATGGTTG
AAGCAATTGATACGGAAGAAGTATGGTTAGCGGATAAACGTGTGGAATTAGTAACACGACATATCATCAATAATCATGATAAATATACACG
TAATCGTCAATATTCAAGTATATTTACAGTCCAAAGTATTCACGCGCTTATTAAATATTATGAGACATTTAAGCGACTTAACAAAAAGTTGGA
ACAACCGTTAACGATAGCTGGTATATTTACGTTTAAACCTAATGAAGATGATCGTGATGGTGAAGTGCCATATCATTCACGTGAAAAATTAG
AGATAATGATTAGTGATTATAATAAAAAGTTCGAGACGAATTTTTCAACAGACACAACTAATGAGTATTTTAATCATATTTCAAAAAACGTT
AAAAAGGGCGTTAAAGATAGTAAAATTGATATCTTAATCGTTGTTAATATGTTCTTAACTGGTTTTGATAGTAAAGTACTGAACACTTTATAT
GTTGATAAGAATTTAATGTATCATGATTTAATTCAAGCGTATTCACGTACAAATAGGGTTGAAAAAGAATCAAAGCCATTTGGTAAAATTGT
AAACTATCGTGACTTGAAAAAAGAGACAGACGATGCACTGAGAGTATTCTCACAAACAAATGATACGGATACAATTTTAATGCGCAGTTAT
GAAGAGTATAAAAAAGAATTTATGGACGCTTATCGTGAGCTTAAAATGATTGTGCCGACACCACACATGGTTGATGACATTCAAGATGAAG
AAGAGCTAAAGCGCTTTGTTGAAGCTTATCGTTTATTAGCTAAAATAATATTACGTTTAAAAGCATTTGACGAGTTTGAGTTTACAATTGATG
AAATTGGAATGGATGAACAAGAGAATGAAGACTATAAAAGTAAATATTTAGCTGTGTACGATCAAGTAAAAAGAGCGACGGCTGAGAAA
AATAAAGTATCCATTTTAAATGATATTGATTTCGAAATAGAAATGATGCGTAATGATACGATTAATGTGAATTATATTATGAATATATTGAG
ACAAATTGATCTTGAAGACAAAGCGGAACAACGTCGTAACCAAGAACAAATTAGACGCATTTTAGATCATGCAGATGATCCGACATTGAGG
TTAAAACGAGATCTAATTAGAGAATTCATCGACAATGTTGTACCTTCTTTAAATAAGGATGATGATATCGATCAAGAATATGTTAATTTCGA
AAGTATTAAAAAAGAAGCGGAGTTCAAAGGATTTGCTGGAGAGAGATCTATCGATGAACAAGCCCTAAAAACAATTTCAAATGACTACCA
GTATAGTGGTGTTGTAAACCCACATCACCTTAAAAAAATGATTGGTGATTTGCCATTGAAAGAAAAGCGTAAAGCAAGAAAAGCCATTGAA
TCTTTCGTGGCAGAAACAACTGAAAAATACGGTGTGTAA

Gene: Q5HJH7 (putative protein)

Position: 213125 to 213967, length: 843 nt, orientation: FORWARD

Perfect match to: (11819-97-CP003194-[219535:220377], highly conserved allele)

Sequence:

ATGGATAATAGAAATATGATTAATCGTGTTTTTAGTCAAAAGATATTACATCAAATTGCAATCAAAAATAAAAGTGATGTTGTTGATGAGGC
ATATGATTTTTATATACAGGGACCTAAAAATATCAATGTAATACAGAAGATGAAATCTTTATATAATTATCTTAAAAAGTCTTATCGTAACGA
ATATTTTTACAAAAACACAATACTTAATAAACTCCTTCTAGGACGACATTCTATTAATACAACTACTGCACTTTCTGAGATGCCCATAGGGAA
AAGTATTGCTGATTTTATATTGTTAAACGGCAAAGGCGTTGTCTATGAGATTAAAACAGAATTAGATAAGCTGGATAGATTAGATAATCAA
ATTAATGATTATTATGAAGTGTTTAATTATGTAGTAGTTATTACAAATGACAAACATCTGAATAAAGTTATGGCTAGATACAAAGATACAAC
AGTTGGAATTTTAGTGTTAACAACTAGAAATACACTGAGTGAAGTTCAAAAACCTAAAGAAAACAATAGTCTCTTAAACACAAAAGCGATG
TATAACTTTTTACGAAAAGAAGAAAGAAAAAGAGTTATTGCACAAAATCATATGGATGTGCCAACTTATAATGATTTCACAGAGTATGATGT
GTTATTTGACGTGTTTAAAGAAATACCAATGACGAAACTGCATAACAATATGATTTCTGAGTTGAAAAAAAGAGGCAACATGAAAGAATAC
AAAGATGAATTTTTAGCAGCGCCGGCTGAAATTAAGTTCTTGTTATATTTCGCAAAAATGACAAAGAAAGATAAAAATAAACTATATCATTT
TCTTAAGGAGGATTAA

Gene: Q9RL82 (putative protein)

Position: 213969 to 214892, length: 924 nt, orientation: FORWARD

Perfect match to: (11819-97-CP003194-[220379:221302], highly conserved allele)

Sequence:

ATGTATTATCCTTATTTGCGTGGGAAACAAAATGAACTATTTGCAATTAGAGAATTGTTAGAGAAAGGTTTGATTGGTGATTGTATTCAACC
TATAATTGAACCGATTAAGTATACAACCACGTTTAAAAATGTTTTGAAATATTGTGGTGAAAAAGAATTTCGTATAAATTTAGTAGTAAATTC
GAAGTTAACTGAAGAAGAGATTAGTAACGAAACTGTTGAAGACTTAAGCGAAATAATAACAAAAAACAAAAGTGTTATTCAAAAAGCTTA
CTTGGGTCCTTCTGATGAAGGCGATGATAGGTTGAAACAGCAATTTTCAAGTAATAGTTTAGCTATTTTAACAAGTGTAGATGACTGGGAA
ATGTTTGGAGATAAAAATAAACTTGAAATGGTTTTTGTACCAGATGATAGACACATTAAACGTAAATTGCGTAATATTCCAAACAAAGGCAT
CATTATGGATCCTTTTAATAAACTAAGTCGTAATGTTGATTATTTAGATAATGATGACGAGTTTTATAGCGACGATCACCTTTATTATAAGGA
AGATGGATACGTAGCATTTTCAGACTATTCTGTTATAGGTGGAGAATATGTAGACGGTGGCTTTTCGCCATTAGCTATTGCGATACATATTG
TCTATTTTGATGAGGCTAATGAGTTAAGAGTTAAACATTTTGTCTCTGATTCTAATAACGATAGATTAAATCCAGCTAAAAAGTTTTTTGAGG
CTGTAGATAAATTAGTAACTTGGTCTAAAAACTTAGATAATAAAAATAGATCTTATGCAATCGGACAATTTGAAGAATTAAATGAAAATAAT
AAGTATCCAGGATTAGGTTTAATTAAAAGGTTATCTATCATGCATCATCTAGAAATTATGAATAGATACTTGGAGTCTCAAAATGAAAATAT
GTGA

Gene: A6QDI2 (RES domain protein)

Position: 214879 to 215991, length: 1113 nt, orientation: FORWARD

Perfect match to: (11819-97-CP003194-[221289:222401], highly conserved allele)

Sequence:

ATGAAAATATGTGAAAAAATGTTTTAATAATACTGAAATCGTAGAAATCATTGTAAATGATAATAGCAAATTTGACAATTGTGATATTGATAA
CGATCATCTTGGTGTTAAAATATTTGATACGACTCGACACATAGATAAATTAGAGCAGATTAGAGATTATTTAAGACCAGCGTTAGAATTAT
ATGATATTAGTATAAATTTACCAGATACTTTTAGCCAAAAAGAAGGTAAAAAAATTGAAACAGCATTAAAAGATGATTGGAGTATATTTAAT
GTTGAAGAAGCTCAAATAAGTTGTATTTTAAATGAACTTTTTAAAGATGATGAAAATATAGATAGACGGGTGTTGGAAGGTTTAGTAAGCG
CTAAAATCATTAAAGATAAAAAATATACAAATGAAAATCTAATTGTAGCAAATAATGACTGGGATGGATTTTGTGAGAGCTTGAAGTATAA
AAATAGATTTCATAATAATATGATCAATTTAGAGAATTTAGCGTTCTTTCTCGATATCACTACTAATTACTATAGTATTGAGGAATTTAGAGA
AAGATTTGAACCATTATATAGATCTAGAATAGTTAAGTTCATTACAGAGAAAAGTGAACTAATGTCACCACCAAAGGGATTTGCAACGGCA
GGAAGATTGAATTCTAAATGGATAAGCGTATTATACCTTAGTACAAAAGAACAGGTCAGTATAGAAGAAGTTAAACCTAAGCATAATGACA
TTATTTATATAGGTAAGGTTAAGTTACAAAAAAACGTTACAAAAGAGAAATTGAAAATTGCGAATTTAACTAATTTAACTAGTAATGCAATC
AAAGCTGGCGATGATGGATTTAGAAAGTATTTTGTAAATCACCAAACATTAAAAAAGATTCATAAAGGAATAACAAATCCAAGCGATGAAC
AAGGAATAGATTATTTGCCATTTCAATATTTAGCTGATTATATTCGAAGCCTTAAATATGACGGGATAATGTATGAGAGCATATTGCAAGAT
GGTACTTTTAACTTTGTGTTTTTTGATCAAAATTTATTTGAATGTGTTAATTATGAAAGAAAAAGAGTAAGTGATGTTAAATATACATTGACT
AAGCTAAGTTGA

Gene: oppF2 (oligopeptide ABC transporter 2, ATP-binding protein)

Position: 216255 to 217847, length: 1593 nt, orientation: REVERSE

Sequence:

TTATTTAGCAATAACTGCTACTTCTGAAATAAGTTGCTTTGCATAGTCTGACTGCGGATGTTTAATAATATCTTCTGTGTTATTCAGTTCAACG
ATTTCGCCATTTTTCATAACTGCAACGCGATCACATATTTCATTGATAACACCCATGTCATGTGTGATGAATAAATAAGTGATGCCGAAGTCT
AACTGTAATTGTTTTAATAACTCGATGATATCTTTTTGAATTGAAACGTCTAAAGCGGACACTGCCTCGTCGCAAACAATCACTTTAGGTTCT
ACAGCAAGTGCTCTCGCGATACTTACACGCTGACGTTGCCCACCAGATAATTCGTGTGGATAGCGATATAAGAAACTTTGATCTAGGCCAA
CCTTTTCTAACAACGATACGACAGTTTTAATAATGTCATCATTATCTTTGACTTTCCCATGAATGATTAGTGGTCGTTTAATCACATCAATGAC
TTTAAATCTTGGATTAATAGATGCGAATGAATCTTGAAAAATCATTTGTATCTCTTGTCGTAAAGATTTCAATTCATCATCTTTAAATAAACTT
AATGGTAATTCGTTATACCAAATAAAGCCTTCTGACACTTCCTTTAGACCGACGACCGTCTTAGCTAATGTCGATTTCCCTGACCCTGACTCA
CCAACAATGCCTAATGTTTCGCCTTTTCTAATAGCCAAGTTAATATCATTAACTGCTCGGTATAGGCTGCCACTTGGTGACTTGTAATCCACG
CTCACACGATCGAATTTTAATAAAATATCATTGTTTAACAATCTTGGCGGACGCGTTTGATGAATATCAGGAATTGCATCTATTAAGCGTTTC
GCATAGGTATGTTGTGGCGATTTAAAAATACTTTCAACCGTGCCACTTTCAACGACACTTCCATCTTTCATTACAATCACATCGTCGCAAAAT
TGATACACAGCACCTAAATCGTGAGTGATAAAAATAATAGACGTTTCTGTGTACTCATAAAGGGACTTCATTAACTGCAGTAATTGATTTTG
TGTACTGGCATCTAATGCCGTTGTTGGTTCATCTGCGATTAAAATTTGTGGCTTTAAAATCAATGCCATTGCTATCATGACACGTTGACGCAT
ACCACCAGAAAGTTCATGTGGATAAGCATCAAATTGTCGAGTTGCATGTTTTATACCTACTTTTTCTAAAATGTCTATTGTCATCGACTTTGCT
TCAGATTTAGATACACGTTTATGTTGAAATATTACTTCTGTAATTTGTTTGCCAATCGTTAATCTTGGATTCAACGAAGAGAGTGGATCTTGA
AAAATCATTGAAATATCCTTACCTCGAATTTGTTGTAACGCTGAAGTTGATAAATTATTTAACGATTGCCCATTAAAAATAATTTCTCCTGTTA
ATGTGTGATCTGGATAATCTGGTAGTAGCCCTAAAATAGATTTAGCGGTAATACTTTTTCCTGATCCTGATTCACCAACAATACCTAGGATAT
GTTTTTTTCGTAATTCGAAAGAGACGTTTTTTACCGCTTGAACTGTAGTTTCATCATAATTGAATTGTACATTCAGACTGTTGACTTCTAATAA
ATTTGACAT

Gene: oppB2 (oligopeptide ABC transporter 2, transmembrane permease)

Position: 217975 to 219282, length: 1308 nt, orientation: FORWARD

Perfect match to: (11819-97-CP003194-[224385:225692], allele observed in CC80+CC96)

Sequence:

TTGTATCACAAGGCATTTTACAAGTTTGTATTATCGGTATTAAGCTTGCCAATATTTTTATATGCAGTGATAAAGTTTTTCTTTTCTGCTAAGA
GAAAAAATTTTTACGCTAATAATTCTGAAATTTCAGAAATTGAACAGGCGTTACATCAAAAATATAAATATTTATCGCAGCAAAAGTCATCC
ACACAAATACATAAAGAAGCATTAAAAATATTCAAGGCACAAAGTTCTAATACGAGTTCAAAGAATATTGAACAAGCACATTTTTCAACATA
CTTTGAAAATGTATTATTTCATAAGTTCATCATGATCAAAGTGATATTGGCCTTGCCGATGTTCATCTTATTGACTTTTTATTTACAGCCATTA
GTTAGATATATTTTTGAACGAATTGTCATGGCTGTGATTGTCATCATTGGTGTTATTGTCAGTGTGTTTACCATTCTGTATTTTTCACCGCTTG
ATGCGGCTTATAGCATACTGGGACAAAATGCAACAAAGGCACAGATACATCAATTCAATGTATTACATCATCTTAACGAACCTTATTTTATTC
AATTGTGGGATACCATCAAGGGTGTTTTTACCTTTGACTTAGGTACGACTTACAAAGGGAATGAGGTTGTGACTAAAGCAGTTGGCGAAAG
AATTCCAATTACAATAATTGTCGCAGTATTAGCGCTAATTGTGGCATTAATTATTGCAATACCAATTGGTATTATCAGTGCGATGAAGCGAA
ATAGTTGGCTTGATATCACGTTAATGATAATTGCATTAATTGGTTTATCTATTCCAAGTTTCTGGCAAGGGCTATTATTCATTTTAGCGTTCTC
ATTGAAATTGGATATTTTGCCACCATCTTATATGCCAGAACATCCAATATCGTTGATTTTACCTGTACTTGTCATTGGAACAAGTATTGCTGCT
TCTATCACGCGTATGACAAGGTCTTCTGTACTTGAAGTAATGCGCAGCGATTATGTTTTAACTGCTTATGCAAAAGGATTATCGACGACACA
AGTTGTTATTAAACATATTTTGAAAAATGCCATTATTCCAATTGTAACGTTAGTTGGTCTTCTAGTGGCAGAGTTACTAGGCGGTTCAGCAGT
GACGGAACAAGTATTTAACATTAATGGTATCGGGCGTTATATCGTCCAAAAACAACTAATACCTGATATCCCAGCAGTCATGGGTGGGGTC
GTATATATATCAATTGTAATATCTTTAGCAAACTTAATTATTGATATATTTTATGCTTTAATCGATCCAAAATTACGTAGTGAAATTAACGAAA
GGAAGTGA

Gene: oppC2 (oligopeptide ABC transporter 2, transmembrane permease)

Position: 219288 to 220451, length: 1164 nt, orientation: FORWARD

Sequence:

ATGGTAAAACTTACAACAAAGATAGCTTCCTTAAAACTATTCGCAAGTTATGCTATAGCAACTTATATTTTAGTTATATTAACGAGTGCATTA
AATCTTTTTAAAGGTTATGTGGCCGATACGTTCTATATTGCTGAAACATTGCTAATCGTTTTAACCATCATTTTAATTATCATTTTAACAACGG
AACAAACATGGAAGCATCATGACTTATGGCGACGTATCGTCGAAGTGTTGTTATTGTTGATGACATTAACAGGCAACGTATTTACATTATTA
ATGTTTGTAAGTATTAGACGTTACCAACGTACATCGCAAATACATAGTTATAACGGGTGGGAATCGTTTATACGAAAAACTACTAGACATCG
TATTGCGATTATCGGGTTACTTATTTTAGTCTACATGCTGACATTATCAATTGTGTCACAATTTACATTTGATACGACATTGGCTACTAAAAAT
CAGTTCAATGCACTGTTACATGGACCGAGTCTAGCCTATCCGTTTGGTACTGATGATTTCGGTAGAGACTTATTTACACGCGTAGTTGTCGG
AACGAAACTGACATTTTCAATTTCAATTATTTCAGTAGTTATTGCAGTTATTTTTGGTGTGTTACTAGGCACTATCGCAGGTTATTTTAATCAT
ATTGATAATTTAATAATGCGAATTTTAGATGTAGTGTTTGCAATTCCATCATTATTGTTAGCGGTGGCAATTATTGCATCATTTGGAGCAAGT
ATTCCAAATTTAATTATTGCTTTAAGTATCGGTAATATACCATCATTTGCACGGACAATGCGTGCCAGTGTTTTAGAAATTAAACGCATGGAA
TATGTAGATGCAGCACGTATCACTGGTGAAAACACTTGGAATATCATATGGCGTTATATTTTACCGAATGCGATTGCGCCTATGATTGTACG
TTTTTCATTAAATATAGGTGTGGTTGTATTAACAACAAGTAGTTTAAGTTTCCTAGGACTTGGTGTTGCACCTGATGTAGCTGAATGGGGCA
ACATTTTACGTACCGGTAGTAACTACTTGGAAACGCACAGTAATTTAGCTATTGTACCTGGTGTTTGTATTATGTTCGTCGTTTTAGCATTTA
ATTTTATAGGTGATGCAGTGCGTGATGCACTAGATCCAAGAATTCATTAA

Gene: oppA2 (oligopeptide ABC transporter 2, substrate-binding protein)

Position: 220468 to 222243, length: 1776 nt, orientation: FORWARD

Sequence:

GTGAAGAAAATCATTAGTATCGCAATTATAGTTTTAGCGTTGGTATTAAGTGGTTGTGGTGTCCCTACGAAATCAGAAGTGGCTCAAAAGT
CATCGAAAGTTGAAGTGAAAGGCGAGCGACCAACAATACATTTCCTAGGACAAGCAAGTTATGAAAATGATATGAATACCGTTAAAGATC
AATTGGAAAATGCAGGATTTAACGTGAAGATGAATATCCAACCAGATTATGGTAGCTATCGTACACAACGTCAAGCCGGCAATTATGATAT
CCAAATTGATGACTGGATGACAGTGTTTGGTGACCCGAACTATGCTATGACGGCATTATTTAGTTCTACAGGATCAAATAGTTTATTGAAAG
ATAAACATGTAGACCAGTTGTTAAATAAAGCTTCTACTCAAAATGAAGCAGATGTTAAACAAACATATAAGCAAATTGAAGATGAAGTTGT
ATTTGATAAAGGGTATATGGCGCCTTTATATGGATCAAAAAAGAATTTAGTATATGACAATAAAGTGTTAGATAAAAATAGTGTTGGATTG
CCAAATTCACGTGCATTAATATGGCAACAATTTGATTACAACAATAGTAGAGAACGAGATACGCGGCCACTTGTGATGACACAACAAGATG
GTGAAATTCCTACATTGGATCCAATACGTTCAATTGCGCCGTCAGTATATTCAATTAATATGAATATGTACACAAGGTTATTATTATTAGATG
AAAATGATCATTTAACAACGAAAGGTTCGTTAAGTCATGATTATGCTGTGAATAAGGACAATAAAGCATTTTATTTCTTGTTAAGAGATGAT
GATTATTTTGCGAAAGTGGTCAATGGACAAGCACGTAATACTGGAGAGCGTGTATCGGCTGAAGATGTTAAGTTTTCTTTAGATAGAGCAC

GTGATAAAAAGTCTGTGCCTAACAATAATACTTACAATATGCACAAACATATAAATGACATCAAGATATTAAAAGATGAGGACATCGATCA
GTTGCGTAAAGAGAAAGACAAGGACGATAAATCAATCTATGATAAGTTGATTAAAGCTTATAACGTCAAATCGTTAACGACAGATGGTCAA
AAAGTAAATAATAAAGACGGTATTTATCAAATTGTTAAAATTACGACAGATCAATCGATGCCTCGAGAGGTAAATTACTTAACACACTCTTC
GGCAGGCATTTTATCTAAAAAATTTGTTAATCAAGTAAATCAAGAATATCCAAAAGGATATGGGGATAGCAGTACAATTCCTGCAAATTCA
GATTGGAAAAATGCGCTGTATGCAAGTGGCGCATACATTATGACACAGAAAAATGCATATCAAGCAACGTTTCAACGTAATCCAGGATTCA
ACGAAACAGAAAAAGGTAGTTATGGACCAGCTAAAATTAAAAATATTACATTGAAGTTTAATGGTGACCCGAATAATGCATTGTCAGAACT
TAGAAATCATTCAATTGATATGTTGGCAGATGTGAATCAAAAACATTTTGATTTAATTAAGTCGGATAAAAATTTAAGCATTATTCGCAAAA
ATGGACGCAAGTCAGTCTTTTTAATGCTAAATATTAAAAAAGGTATATTTATGACGCATCCAAACTTGAGACAAGCAGTAGTTAATGCGATA
GATCAGGATCAATTTATTAAGTTTTATCGTGGCGATAAATTTAAAATTGCATCACCGATTACACCACTTGTCGATACTGGTAACGAGCAACG
TCAAGATTTAGAAAAAGTAGAAAAAGCCATCAATCAATAA

Gene: ggt (gamma-glutamyltranspeptidase)

Position: 222281 to 224287, length: 2007 nt, orientation: FORWARD

Perfect match to: (11819-97-CP003194-[228691:230697], allele observed in CC80+CC72+CC188)

Sequence:

ATGGTCATTAACTTAAATGACAAACAGACAAAAACATCTAAAGAAGGGTTAATTTCCGTATCACATCCTCTTGCGGCTAAAATTGGTAAGGA
TGTATTAGATCAAGGTGGCAACGCCATGGATGCAGTGATTGCAATTCAACTGGCATTGAATGTGGTAGAACCATTTGCATCAGGTATTGGT
GGTGGCGGGTATTTGCTATATTATGAGCAAAGTACTGGCAGTATAACTGCGTTTGATGCGCGTGAGACAGCCCCTGCACATGTAGATAAAC
AATTTTATCTAGATGATTCAGGCGAATATAAATCATTTTTTGATATGACTACACATGGTAAAACTGTCGCTGTGCCAGCAATTCCAAAGCTGT
TTGATTATATTCACAAGCGTTATGCTAAGTTGTCATTGGAAGATTTAATTAATCCTGCAATTGAACTAGCTATTGAAGGTCATGCAGCCAATT
GGGCTACTGAAAAATATTCGCGCCAGCAACACGCACGATTGACAAAGTATTATGAAACGGCACAAGTATTTACGCATGAAAATCAATATTG
GCGTGAAGGTGATTGGATTGTACAACCCGAATTAGGTAAGACATTTCAAATATTAAGAGAACAAGGGTTTAATGCATTTTATAAAGGTGAC
ATTGCGAAACAATTAGTCAATGTTGTCAAAGCATGTGGTGGGACAATCACTTTAGAGGATCTAGCCAAATATGACATTCAGATTAAAGCGC
CAATCAGTGCAACATTTAAAGACTATGACATTTATTCAATGGGACCATCTAGTTCTGGCGGTATCACGGTAATTCAAATATTGAAGTTATTA
GAACATGTCGATTTACCATCTATGGGTCCAAGATCTGTCGATTACTTGCATCATTTGATACAAGCGATGCATTTAGCATATAGTGATCGCGC
GCAATACTTGGCGGATGATAATTTTCATGAGGTGCCTGTACAGTCATTAATTGATGACGATTATTTAAAAGCACGCAGTACGCTCATTGATA
GCAATAAAGCAAATATTGATATAGAGCATGGTGTTGTGTCTGATTGCATTAGTCATACAGATGTTGAAGAAAATCATACCGAAACAACTCA
TTTTTGTGTGATTGATAAGGAAGGTAATATTGCTTCATTTACGACATCAATCGGTATGATTTACGGATCGGGTATTACGATTCCAGGGTACG
GCGTGTTATTGAATACCACAATGGATGGTTTTGATGTAGTAGATGGTGGTATTAACGAAATTGCACCATATAAACGACCACTAAGTAACAT
GGCTCCAACGATTGTGATGCATCACGGGAAGCCGATATTAACAGTAGGTGCACCTGGTGCCATAAGTATCATTGCTAGTGTTGCGCAAACA
TTAATCAATGTATTAGTGTTTGGCATGGATATTCAGCAGGCTATAGATGAACCTAGAATTTATAGTAGCCATCCTAATCGCATTGAATGGGA
GCCTCAATTTTCACAATCTACAATATTAGCATTGATTGCACGTGGACATGCAATGGAACATAAACCAGATGCCTATATTGGAGATGTACATG
GGTTACAGGTTGACTTGAATACACGTGACGCGTCGGGAGGTGCTGATGATACGAGGGAAGGCACAGTGATAGGTGGCGATGTATTATCA
ATTAGAAAACAACCATTACCTAGTCCGAAAATATACGATAATGACACCCATCGAGTATATTTCAATGATATGCAGTTGCCATTATATGCTGA
ACAAGTGCGGTGGATGCATGACAAATATTGGGTTGATGAAAGTGTTATTAGAATCATTTTCCCTGAAGTTAGTGTGCATATTGAAGATTTA
AGAAGTTATGAAATCGCCGGAAAAAATTATATAGATATTGCCTGGTTGGCACGGAAGAAAGGTTATCAAGTTACCTTAAAGGATGACAGTT
TATACTTAACTGATGAAACATATCATTCAGTGAAAGCAAACACAAATGCATACTATAGATATGATCGAGATAGTATCACAAGATAA

Gene: DUF576_Saur (putative tandem lipoprotein)

Position: 224585 to 225358, length: 774 nt, orientation: REVERSE

Perfect match to: (MW2-BA000033-[219721:220494:r], highly conserved allele)

Sequence:

TTAATGTTCACTTAATTTATTATAAGTAAGGCTTTCTTTGTATATAATTTGGTCTTTTCCAGGACTTGAAAAATAAAAATAAAGTTTCTGATCT
TGATGATTATTGCCTTTTAAATCACTCATTCCCTTTAATTCCACCTTTGTTGATGCATTTCTGGGAATATTATATCTACGTTTTAATTGTTTAAC
GTTTGGATTATCATTCGACAACTTACCAGATAAATTATAATAATCAGTGGTAGGATCATGTGTGACTTTCAAATCATTGCTATTTAAAATGCT
TGTTAAATCACCACTTTGAATCAAAAATTGATTGTTTTCGATTTTTTGTTTCAGCGCGGGATCTTTTACGTCTTTTGTGAAAACGATTTTATTA
TTAACTACTTTTACTGGATAACTTTTGTATGTCGAGTCAGTAGCATTTTTTCTATCGTTTGTAGTTGTGTCATATTCACCAGTTATTTTATGTGT
GTTCTTATCTACCTTTAACAACATACGGTCTTCTTTTAAAAGCTCATCTGATCCAACAACTGAATAAGAGGATTCTATATACCATGTGTCTTGA
TCATTATTTTCATAATGGGGATTATCGTGACCATCAATTTCATAAAGCGTTTCTAAGTTTTTAATAGGATACGTACTTAGTACTTTTTTAAGAC
CATCTTTCAAATGAATTTGTTCCCACTTCATTGCCAAAAACATACCGCCACTGACTACAATTGAAATAATAATAATTGCTGCTAAGTTTAACC
AGAAAATTTTATGTGCTTTCAT

Gene: acpD (FMN-dependent NADH-azoreductase)

Position: 225547 to 226173, length: 627 nt, orientation: REVERSE

Perfect match to: (Strain_21334-AGTW01000044-[53274:53900], highly conserved allele)

Sequence:

TTAAAATGTAGTTGCTAATTTTTCAGCATTATTAATACTAGTTGCTTTAATTTCTTCAGTCTTATGAGGTTCAGCATTGTGTCCTTCAATAATG
ATTGTTTCATATGATGGCACACCTAAGAATGTCATAATTGTTCTTAAATAACGGTCACCCATTTCAAAATCAGCAGCAGGTCCTTCAGTATAA
TATCCACCACGTGATTGAATGTGTAATACTTTTTTGTCAGTTAACAAACCTTGAGGTCCTTCAGCAGAATATTTAAAAGTTTTACCTGCAATT
GAAATAGCATCAATATATGCTTTAACTACAGGTGGGAAAGAAAGGTTCCACATAGGCGTTACAAATACATATTTATCTGCACTTAAAAATTC
TTCTAAAATGTCACTCAATCTTGAAACTTTCATTTGTTCATCATCAGTTAACGTTTCGCCATTACTCATTTTTCCCCAACCAGTTAATACATCTT
TGTCAATAACTGGAATATAAGTTTCAAATAAATCAATATGTTTCACTTCATCATCAGGATGTTGTTGTTGATATGTTTCGATAAATGCTTTACC
AGCCGCCATAGAATTTGATACCAGTTCATTAAAAGGGTGTGCTGTAATATATAATACTTTTGCCAT

Gene: Q5HJG7 (M23/M37 family peptidase)

Position: 226382 to 226960, length: 579 nt, orientation: FORWARD

Perfect match to: (MW2-BA000033-[221519:222097], allele observed in CC1+CC7+CC8+CC22+CC80)

Sequence:

ATGACAAAGCGACCAAAACGTATTTTGGCAACAATTATCATTTTTCTTTCACTATTATTTACGATTATTTATATAGATGACATTCAAAAATGGT
TTAACCAATATACCGATAAATTGACACAAAATCATAAAGGACAAGGACACTCAAAATGGGAAGACTTTTTTAGAGGGAGTCGGATTACTGA
GACTTTTGGTAAATATCAACATTCACCATTTGATGGTAAGCATTATGGCATTGATTTTGCATTGCCAAAAGGTACACCAATTAAAGCGCCGA
CGAATGGTAAAGTAACACGTATCTTTAATAATGAATTGGGCGGCAAGGTATTACAGATTGCCGAAGACAATGGAGAATATCACCAGTGGT
ATCTACACTTAGACAAATATAATGTCAAAGTAGGTGATCGAGTCAAAGCAGGTGATATTATTGCATATTCAGGCAATACAGGTAAACAAAC
GACAGGCGCACATTTACATTTTCAAAGAATGAAGGGTGGCGTAGGTAATGCATATGCAGAAGATCCAAAACCGTTTATCGATCAGTTACCT
GATGGGGAACGTAGCCTATATGATTTGTAG

Gene: malK (maltose ABC transporter, ATP-binding protein)

Position: 227344 to 228441, length: 1098 nt, orientation: FORWARD

Perfect match to: (GR1-AJLX01000027-[86137:87234], allele observed in CC361+CC5+CC8+CC34+CC96)

Sequence:

ATGGCAGAACTAAAGTTAGAGCATATTAAAAAGACGTATGATAACAACAATACTGTAGTGAAAGATTTTAATCTACATATTACTGACAAAG
AATTCATTGTATTTGTTGGACCATCGGGATGTGGTAAATCAACAACATTACGAATGGTTGCTGGACTAGAGTCTATCACATCTGGAGATTTT
TATATTGATGGGGAACGCATGAACGATGTTGAACCAAAGAATAGAGATATTGCGATGGTATTTCAAAACTATGCATTATATCCACATATGA
CTGTTTTTGAAAATATGGCATTTGGGCTAAAGCTACGTAAAGTAAATAAAAAAGAGATTGAACAAAAAGTCAATGAAGCAGCTGAAATATT
AGGATTAACTGAGTATCTTGGTCGTAAACCAAAAGCGTTATCTGGTGGACAGCGTCAACGTGTTGCTTTGGGCAGAGCTATTGTTAGGGAT
GCGAAAGTCTTTTTAATGGATGAACCATTATCGAATCTTGATGCGAAGCTTCGAGTACAAATGCGCACAGAAATATTGAAATTACATAAGC
GACTTAATACTACGACAATTTATGTTACACATGATCAAACTGAAGCATTGACGATGGCTAGTCGAATTGTTGTTTTGAAAGATGGCGACATT
ATGCAAGTCGGCACACCTAGAGAAATATATGATGCCCCTAATTGCATATTTGTGGCGCAATTTATCGGCTCACCAGCAATGAATATGTTGAA
TGCTACAGTTGAAATGGACGGATTGAAGGTAGGAGCACACCATTTTAAATTACATAATAAAAAATTTGAAAAGTTAAAAGCTGCTGGCTAC
TTAGACAAGGAAATTATTTTAGGTATTCGAGCTGAAGACATTCATGAAGAACCAATATTTATTCAAACTTCTCCAGAGACACAATTTGAATC
TGAAGTAGTTGTATCCGAACTGTTAGGTTCAGAAATCATGGTACATAGTACATTCCAAGGAATGGAATTGATTTCTAAATTAGATTCAAGAA
CCCAAGTGATGACGAACGACAAGATTACACTAGCATTTGATATGAATAAGTGTCACTTTTTTGATGAAAAAACAGGAAATCGTATCGTCTAA

Gene: malE (maltose ABC transporter, substrate-binding protein)

Position: 228454 to 229725, length: 1272 nt, orientation: FORWARD

Perfect match to: (11819-97-CP003194-[234864:236135], allele observed in CC80+CC15+CC25+CC80)

Sequence:

ATGTCTAAAATTTTAAAATGTATCACGTTAGCCGTGGTAATGTTATTAATCGTAACTGCATGTGGCCCTAATCGTTCGAAAGAAGATATTGA
TAAAGCATTGAATAAAGATAATTCTAAAGACAAGCCTAACCAACTTACGATGTGGGTGGATGGCGACAAGCAAATGGCGTTTTATAAAAAA
ATTACGAATCAATATACTAAAAAAACCGGCATCAAAGTAAAGCTTGTAAATATTGGTCAAAATGATCAACTAGAAAATATTTCGCTAGACGC
TCCTGCAGGAAAAGGTCCAGATATCTTTTTCTTAGCACATGATAATACTGGAAGTGCCTATCTACAAGGCTTAGCTGCTGAAATCAAATTAT
CAAAAGATGAGTTGAAAGGTTTCAATAAGCAAGCACTTAAAGCGATGAATTATGACAATAAGCAACTAGCATTGCCAGCTATCGTAGAAAC
AACCGCACTTTTCTATAATAAAAAATTAGTGAAAAATGCACCGCAAACGTTAGAAGAAGTTGAAGCTAATGCTGCCAAGCTAACTGATAGT
AAAAAGAAACAATACGGTATGTTATTTGATGCTAAAAATTTCTATTTTAATTATCCGTTTTTATTCGGCAATGATGATTATATTTTCAAGAAA
AATGGCAGTGAATATGATATTCATCAGCTAGGACTAAATTCAAAACATGTCGTCAAGAATGCTGAACGATTACAAAAATGGTACGACAAAG
GGTATCTTCCTAAGGCAGCAACACATGATGTCATGATTGGTCTTTTTAAAGAAGGAAAAGTAGGACAATTTGTCACTGGACCGTGGAACAT
TAATGAATATCAAGAAACGTTTGGTAAAGATTTAGGAGTAACAACATTACCTACAGATGGTGGCAAACCTATGAAACCATTTCTCGGTGTA
CGTGGTTGGTATTTATCTGAATATAGTAAACATAAGTATTGGGCTAAAGATTTAATGCTGTATATCACTAGTAAAGATACATTACAAAAATA
TACAGATGAAATGAGCGAAATTACTGGACGTGTTGACGTGAAATCATCTAATCCAAATTTAAAAGTGTTTGAAAAGCAAGCACGTCATGCT
GAACCGATGCCTAATATTCCTGAAATGCGACAAGTTTGGGAGCCAATGGGCAATGCAAGCATATTTATCTCAAATGGTAAGAATCCTAAAC
AAGCGTTAGATGAGGCGACGAATGATATAACGCAAAATATTAAGATTCTTCATCCGTCACAAAATGATAAGAAAGGAGATTAG

Gene: malC (maltose ABC transporter, transmembrane permease)

Position: 229728 to 230996, length: 1269 nt, orientation: FORWARD

Perfect match to: (SA40-CP003604-[210564:211832], allele observed in CC59+CC15+CC59+CC80)

Sequence:

ATGACGAAACGTAACCCTAAATTAGCGGCATTATTATCTGTTATACCTGGTTTGGGACAGTTTTATAATAAAAGACCCATTAAAGGGACGAT
ATTTTTTATCTTTTTCATCAGTTTTATTTCTGTATTTTATAGCTTTTTAAATATTGGTTTTTGGGGATTGTTCACATTAGGGACAGTACCTAAGT
TAGACGATTCTCGTGTCTTACTTGCACAAGGTATTATTTCTATCTTACTCGTTGCTTTTGCAATCATGCTATATGTCATTAATATTTTAGATGCA
TATCGTAATGCTGAACGATTTAATCGCAATGAGGAAATAAAGGATCCGAAGGCACGTATGGTTGCAACATGGGACAAGACGTTCCCATACT
TATTAATCTCACCAGGTACATTCTTATTGATATTTGTAGTTGTATTTCCATTAATATTTATGTTTGGAGTAGCATTTACAAATTACAATTTATAC
AACGCGCCTCCGAGACACACATTAGAATGGGTTGGTTTAGATAACTTTAAAACGTTATTCACAATTGGCGTTTGGCGTAAAACATTTTTCAG
TGTTATTACTTGGACATTAGTATGGACGCTTGTTGCAACGACACTTCAAATTGCATTAGGGCTGTTTTTGGCAATTATTGTAAATCACCCTGT
CGTCAAAGGTAAGAAATTTATCCGTACTGTGTTAATCCTACCTTGGGCTGTACCATCATTTGTGACAATTTTAATATTTGTAGCGTTATTTAAT
GATGAATTTGGTGCGATAAATAATGATATTTTGCAACCTTTATTAGGTGTAGCACCAGCATGGTTAAGTGATCCGTTTTGGGCAAAAGTGG
CATTAATCGGCATTCAAGTATGGCTTGGATTCCCATTTGTCTTTGCACTGTTCACTGGAGTACTGCAAAGTATTTCATCAGATTGGTACGAAG
CAGCAGATATGGATGGTGCGTCTAGTTGGCAAAAGTTTAGAAACATCACATTCCCGCATGTCATTTACGCCACAGCGCCATTGTTAATTATG
CAATATGCAGGTAATTTCAATAATTTTAATCTTATTTATCTATTTAATAAAGGCGGTCCACCAGTGTCAGGACAGAATGCTGGTAGTACAGA
TATCTTGATATCTTGGGTGTATAATCTGACATTTGAGTTTAACAACTTCAACATGGGTGCAGTTGTGTCATTAATTATTGGATTTATTGTTGCT
ATTGTCGCATTTATTCAATTCAGACGTACAAGTACGTTTAAAGATGAGGGAGGTTTATAA

Gene: malD (maltose ABC transporter, transmembrane permease)

Position: 230998 to 231837, length: 840 nt, orientation: FORWARD

Perfect match to: (MRSA252-BX571856-[241712:242551], highly conserved allele)

Sequence:

ATGACAAAGAAGAAAAACATATTAAAAGCAATCGGTATTTACAGTTTTATAGCGATGATGTTTGTCATCATTTTATATCCACTACTGTGGAC
ATTTGGCATTTCCCTTAATCCAGGTACGAACTTGTATGGTGCCAAAATGATACCAGACAATGCAACATTTAAAAATTATGCATTCTTACTATT
CGATGACAGTAGTCAATACCTGACTTGGTATAAAAATACGCTTATCGTAGCATCTGCAAATGCACTGTTTAGTGTGATATTTGTCACGTTAA
CAGCATATGCTTTTTCTAGATATCGCTTTGTTGGTCGTAAATACGGGCTGATTACATTTTTGATTTTACAAATGTTCCCTGTATTAATGGCAAT
GGTCGCAATCTATATTTTGCTAAATACAATTGGATTATTAGATTCTTTATTTGGACTAACACTGGTATATATTGGTGGATCAATACCGATGAA
TGCCTTTTTAGTGAAAGGTTACTTCGATACGATTCCAAAAGAACTTGATGAATCTGCCAAAATTGATGGTGCAGGGCATATGCGTATTTTCT
TACAAATTATGCTTCCATTAGCTAAGCCGATTTTAGCAGTTGTTGCTTTGTTCAATTTTATGGGGCCATTTATGGACTTTATATTACCTAAAAT
ACTATTAAGAAGTCCTGAAAAATTCACATTAGCAGTTGGATTGTTCAACTTTATTAATGATAAGTATGCAAATAATTTCACAGTGTTTGCAGC
AGGGGCAATTATGATTGCAGTACCTATAGCAATCGTATTCTTGTTCTTGCAACGCTATTTAGTATCAGGTTTAACAACAGGTGCGACAAAAG
GTTAG

Gene: yrbE (putative NAD-binding oxidoreductase)

Position: 231911 to 232987, length: 1077 nt, orientation: FORWARD

Perfect match to: (NN50-BAEA01000012-[170307:171383:r], allele observed in CC4803+CC22)

Sequence:

ATGACGATTAAAGTTGGAATCATTGGGTGTGGTGGTATTGCGAATGGCAAGCACATGCCAAGTTTACAAAAAGTTGAAAATGTTGAAATG
ATCGCATTTTGTGACGTAGACATTTCGAAAGCAGCGAGTGCGGCAGAAGCATACGGAACTGACAATGCAAAGGTTTATGATGATTACAAA
GCATTGTTAAAAGATGACACGATTGATGTTATCCATGTTTGTACGCCAAATGACTCGCATTGTGAAATTACTGTAGCAGGGTTGCATGCCGG
TAAACATGTGATGTGTGAAAAACCAATGGCTAAAACGACAGCAGAAGCTCAAAAAATGATAGATACAGCTAAATCAACAGGTAAAAAATT
AACAATAGGTTATCAAAATCGCTTCAGACCAGATAGTCAATTTTTATATAAATCAGCGCAACGTGGCGACTTAGGAGACATTTACTTCGGAA
AGGCACATGCCATTCGTCGTCGAGCAGTACCAACATGGGGTGTCTTTCTAGACGAAGAAGCTCAAGGTGGAGGACCATTAATCGATATCG
GTACACACGCTTTAGATTTAACGTTATGGATGATGGATAATTATGAGCCAGAATCAGTGATGGGTTCAACATTCCATAAATTAAATAAACAG
CATCATGCGGCAAACGCTTGGGGTTCATGGAATCCAGATGAATTTACAGTTGAAGATTCTGCGTTTGGATTTATTAAAATGAAGAATGGAG
CGACGATCATTTTAGAATCCGCTTGGGCGATTAATTCTTTAGAAGTGGATGAGGCAAAATGTTCATTATCAGGAACTAAAGCAGGTGCTGA
TATGAAAGATGGTCTACGTATTCATGGTGAAGACATGGGTACACTTTATACCAAACACGTTGAATTGGAAAACAAAGGCGTCGACTTTTAT
GAAGGTAATGAAGTGGATGAAGCTGAAGAAGAAGCAAAAGCTTGGATTGATGCAGTTGTAAATGATACTGAACCAGTTGTGAAACCGGA
ACAAGCAATGGTAGTTACAAAAATTCTTGAAGCGATTTATCAGTCTGCAAAATCAGGCAAAGCAATTTACTTTGAATAA

Gene: mviM (NADH-dependent dehydrogenase)

Position: 233012 to 234052, length: 1041 nt, orientation: FORWARD

Perfect match to: (TCH130-ACHD01000256-[56736:57776], allele observed in CC72+CC80)

Sequence:

ATGACAAAATTAAAAGTTGGTGTGATAGGTGTTGGTGGCATTGCACAAGACCGTCATATTCCAGCATTGCTGAAACTCAAAGACACAGTCT
CATTAGTTGCAGTACAAGATATTAATACAGTTCAGATGATTGATGTTGCGAAGCGCTTTAATATACCTCAGGCAGTTGAGACACCTAGCGA
GCTGTTTAAACTTGTTGATGCGGTGGTCATTTGTACACCCAATAAATTCCATGCTGATCTTTCTATAGAAGCATTGAACCATGGTGTCCATGT
CTTATGCGAAAAGCCAATGGCAATGACGACGGAAGAGTGTGATCGCATGATTGAAGCGGCTAATAAAAATCACAAATTATTAACTATCGCT
TATCATTATCGTCACACAGATGTCGCTATGACTGCTAAAAAAGCAATTGAAGCAGGTGTGGTTGGTAAACCTTTAGTAGCACGTGTACAAG
CGATGCGTAGGCGTAAAGTACCTGGGTGGGGTGTTTTTACCAATAAAGCGTTGCAAGGTGGCGGTAGTTTAATCGATTATGGTTGCCACTT
GTTAGACTTATCTTTGTGGCTATTAGGTAAAGATATGGTGCCGCATGAAGTGCTAGGAAAAACATATAATCAATTGAGTAAACAACCGAAT
CAAATTAATGATTGGGGAACATTTGATCATACTAAATTTGATGTCGATGATCATGTTACTAGTTATATGACATTTGCCAATCGAGCAAGCAT
GCAGTTTGAATGTTCGTGGTCTGCAAATATAAAGGAAGATAAGGTGCACGTTAGTTTATCAGGCGAAGATGGCGGTATCAATTTATTTCCA
TTTGAAATATATGAGCCCCGCTTTGGAACTATTTTTGAAAGCAAAGCTAATGTTGAGCATAACGAAGACATTGCTGGTGAGAGACAGGCGC
GTAACTTTGTCAATGCGTGTTTAGGGATAGAAGAGATTGTGGTGAAACCGGAAGAAGCACGCAATGTAAATGCCCTTATAGAAGCGATTT
ATCGTAGCGATCTTGATAACAAGAGCATACAACTTTAA

Gene: iolE (sugar phosphate isomerase/epimerase)

Position: 234107 to 235075, length: 969 nt, orientation: FORWARD

Perfect match to: (11819-97-CP003194-[240517:241485], allele observed in CC80+CC72+CC80)

Sequence:

ATGAAAATAGGTGTATTTTCAGTATTATTTTACGATAAAAATTTTGAAGATATGTTAGATTATGTCGCAGAATCTGGATTGGATATGATTGA
AGTTGGAACAGGTGGTAACCCAGGAGATAAATTTTGTAAGTTAGATGAGTTGTTAGAAAATGAAGACAAGCGCCAAGCATTTATGAAGTC
AATCACAGACAGAGGCTTACAAATAAGTGGTTTCAGTTGTCATAACAATCCAATTTCTCCAGATCCGACAGAAGCGAAAGAAGCCGATGAA
ACGTTACGTAAAACAATCCGTTTAGCAAATCTATTAGACGTGCCAGTTGTTAATACATTTTCTGGCATTGCAGGATCAGATGATACCGCTAA
AAAGCCTAATTGGCCTGTTACACCTTGGCCAACAGCCTACTCTGAAATTTATGATTATCAGTGGAATGAAAAGTTGATACCATATTGGCAAG
ATTTAGCTGAGTTTGCAAAAGAGCAAGATGTAAAAATTGCCATAGAGTTGCATGCAGGATTTTTAGTTCATACACCATATACGATGTTGAAG
TTACGTGAAGCTACAAATGAATATATCGGTGCTAACTTAGATCCTAGTCATTTATGGTGGCAAGGTATTGACCCAATTGCTGCGATTCGCAT
ATTAGGCCAAGCAAATGCAATTCATCACTTCCATGCTAAAGATACGTATATTAATCAAGAAAATGTAAATATGTATGGTCTAACTGATATGC
AACCATATGGTAACGTTGCAACAAGAGCATGGACATTCCGTACAGTTGGTTATGGACATAGTCCATATGTATGGGCAGATATCATAAGTCA
ACTTATTATTAATGGATATGATTATGTATTAAGTATTGAACATGAAGATCCTATTATGTCAGTAGAAGAAGGTTTCCAAAAAGCTTGTCAAA
CTTTGAAATCTGTTAATATTTACGACAAGCCAGCAGACATGTGGTGGGCATAA

Gene: Q7A1X1 (isoprenylcysteine carboxyl methyltransferase)

Position: 235436 to 235930, length: 495 nt, orientation: REVERSE

Perfect match to: (11819-97-CP003194-[241846:242340:r], allele observed in CC80)

Sequence:

TTAAATATTCATTAATTTCTCTTCTTGTTTAATACGTACATATAAGAAATACGCATACGGTACTAATAAAATAGTTGTATATGTTGCGTGTGTT
AATAATAATACACCGATTAATTCAGGAATGATGTTTAAGAAGTAATTTGGGTGTTTTGTAATTTTATATAATCCAGATTTAATAATAGGATG
GTTAGGTAAAATGAATAATTTTAATGTCCAAATACCACCTAAAGTTTTAATAACCATAAATAACATGATATAAGCAAAGATTAATATAACTA
AGCCAATACCATTTGCAAAGCTAAATGTATCTTTATTAATAAATGCCTCTACACCAGCCAATACATAAATTAAAACGTGTGTTATTGCTAAAA
ACTTCGAATTTTTAACGCCATATTCAACTGCACCGTCTGCTTTTAATTGTTTTGAGTGATTAATAGATATCTTTAAGCTGACAAGTCTGATACA
GAAAAAGATAAGTAATATAGATAAAATCAT

Gene: uhpT (hexose phosphate transporter, major facilitator superfamily)

Position: 236163 to 237542, length: 1380 nt, orientation: FORWARD

Perfect match to: (11819-97-CP003194-[242573:243952], allele observed in CC80)

Sequence:

ATGAACTTTTTTGATATCCATAAGATTCCGAACAAAGGCATTCCATTATCGGTACAACGTAAATTATGGCTTAGAAACTTTATGCAAGCTTTC
TTTGTAGTGTTCTTTGTTTATATGGCTATGTATTTAATTCGAAACAACTTTAAGGCGGCACAACCGTTTTTAAAAGAGGAAATTGGATTATCT
ACATTAGAACTTGGTTATATCGGATTAGCATTTAGTATCACGTACGGTTTAGGAAAAACATTACTTGGATATTTTGTCGATGGACGTAACAC
AAAACGTATTATCTCATTCTTACTTATCTTATCTGCGATTACAGTTTTAATTATGGGATTTGTGTTAAGTTACTTTGGTTCTGTAATGGGATTA
TTAATTGTACTTTGGGGACTTAACGGGGTGTTCCAATCAGTTGGTGGACCTGCAAGTTATTCAACGATTTCAAGATGGGCGCCAAGAACGA
AACGTGGCCGATACTTAGGATTTTGGAATACATCACATAATATCGGTGGTGCCATAGCAGGTGGTGTTGCACTTTGGGGTGCTAATGTATT
CTTCCATGGAAATGTTATAGGGATGTTCATTTTCCCATCGGTGATTGCATTACTTATTGGTATCGCAACATTATTTATCGGAAAAGATGATCC
GGAAGAATTAGGATGGAATCGTGCTGAAGAAATTTGGGAAGAGCCGGTTGATAAAGAAAATATTGATTCTCAAGGTATGACGAAATGGG
AGATCTTTAAAAAATATATCCTGGGAAATCCTGTTATATGGATTCTATGTGTTTCAAACGTCTTTGTATACATTGTACGAATCGGTATTGATA
ACTGGGCACCGTTATATGTGTCAGAGCATTTACACTTTAGTAAAGGCGATGCAGTTAATACGATATTCTACTTTGAAATTGGTGCTTTAGTT
GCAAGTTTATTATGGGGCTACGTATCAGACTTATTAAAAGGTCGTCGTGCAATTGTAGCTATTGGCTGTATGTTTATGATTACATTTGTTGTC
TTATTCTACACAAATGCTACAAGTGTCATGATGGTTAACATTTCATTGTTTGCATTAGGTGCGTTAATCTTTGGTCCGCAATTATTAATTGGT
GTATCATTGACTGGTTTTGTTCCTAAAAATGCCATCAGTGTAGCAAACGGAATGACAGGTTCATTCGCGTATCTATTCGGTGACTCAATGGC
GAAAGTTGGTTTGGCGGCTATTGCTGATCCAACACGTAACGGTTTAAACATCTTTGGATATACATTAAGTGGATGGACAGATGTTTTCATCG
TCTTCTATGTTGCATTATTCCTAGGCATGATTCTATTAGGAATCGTTGCTTTCTATGAAGAAAAGAAAATTAGAAGTTTAAAAAATTTAA

Gene: rsaG (ncRNA of Staphylococcus aureus G)

Position: 237597 to 237790, length: 194 nt

Sequence:

CCCCGACGGCATGTGCGTGAAGAGATGAAAGATACTGCTTCTACCCTTGCAAATATATCATCTCTATGTCTCGGGGCAGATCATAATTCCCT
GTTATGAAGTATCCTTATTTGCCCGACTTAGGGTGACTCAATGAATTTACTCCTTACAATAAAGACATATAGCGGTGTCAATATTGTAGGGA
GTATTGTTTT

Gene: yesN (two component sensor/regulator, transcriptional regulator)

Position: 237901 to 238659, length: 759 nt, orientation: REVERSE

Perfect match to: (GR1-AJLX01000027-[96694:97452:r], allele observed in CC361+CC80)

Sequence:

CTATTTTGCTTGCTTACAATAATCACTTGGCGACATTTGTAAATATTTTTTAAAATGATAGCTAAACATTTTATACTCTGAAAAGCCTACTTTG
TCTGCAATTTCATAGTGTTTGTAATGTCGATCTAACAATTGCAGAGATTGTAAAATACGATAGCGATTTAAATAATCGACAATTGTAATACCA

ACATGATCTTTAAATGTTCGCATCGCATACGATTCACTAACATCGATATGTTGAATTAAATCTGAAACAGTCACTTTCGTTTGATAAGATTGC
TTAATTTGATCAACAATCTGGTTTACATAATAATCATCGTATTCTACTTTTAATAGTGGTTGGAAGGCATCATGACAAGATGCTAAGCTACGG
CCGTTCTGTGATTGTTGCTCTAATAAGGTACGGACAAGTCTTCCTAAAATAACTTCTAATTGTGCATGGTCTACTGGTTTTAATAAATAATCA
AGAACATGATGTTGAATGCCGGCTTTCATATATTCAAAGTCATCGTAACTCGATAATATGATGACATTACAATCTAGATGCGCAATATCATT
GAGTAAATCGACGCCATTTTTACGTGGCATACGAATATCAGTAATTACTAATTCTGGCTGATGTTGTTGAATTAGTGATAATGCTTCAACACC
ATCTTTAGCAGTGTATATTGTATTGAAATGATAGTCTCCCCAAGGAATGATTTGCTTTAATCCTTCTCGAATAATTCGTTCATCATCACAAATA
ACTACCTTAAACAT

Gene: yesM (two component sensor/regulator, sensor histidine kinase)

Position: 238652 to 240208, length: 1557 nt, orientation: REVERSE

Perfect match to: (Mu3-AP009324-[255716:257272:r], allele observed in CC5+CC80+CC772)

Sequence:

TTAAACATCTACATTCCCCCTTGAAAGTGGTATTTTATAACAAATTAACGTACCTTGATTACGCTTTGAAAAAATATGAAGTCGTGCATGTGA
ACCATATTGAATCATTGCTTTATTGTGTAAATGATTTAATCCCAAATGCTTAGTATCAAATACATCATTATTAAGAGATTGGCGTACATATTG
CAGGCGAGATGACGACATCCCGATACCATTGTCGCAAACTAAAACATGTAAATTCTGACGTGCCAATGTCAGGCGTATAGTAATGTCCAAT
GACTCAGTATCTCTACCATGTTTAATAGCATTTTCTATGAGTGGCTGAAGCATCATTTTACCAATTGTCTGGTGACGCGCTTCTTCAGAACTTT
CGATATGGAGCTTAATCATGTCATCAAAACGGATGTTTTGTATCGCAACATACTGTTCAATGTAGTTCAACTCTTCGTTTAATTCCACTGTAT
GTGAGTTTGTACGTAATGAGTAACGTAACATTTGCGATAATTGTTGGACCACAGTTTGTGCTAATTTAGGAGATAACGTAATTAAATATTGT
ATTGTTTGCATCGTATTGAATAGGAAATGAGGTTGGAATTGGCGCTCTATTTCCTTTAACTGAATATCACGCAAGCGACGTTCTGTATGCTC
GATAGAATGGATTAGTTGCTCATTTGATTCAAATAAATCGTAAATATAATTATTAATTTCTTCTAGTTCACTGTTGTTTTTTAAAGGCGTATAT
GTACCTAGATGACGATTTTTGGCATAGTAAATTTTTTGAATAATCGTTTCGATATCTTTTGTTTGTCGTTTAGCCATATTATCTGCGCTAATGA
AACCAAATATTACTAGTAAAACAAGAACTACGGCCATAACAATTAACAACGTGATACCATCTTCAATGTTTTCATGTATATCTTTATAAATAA
TGAGACGATGGTCAGCATGGTTTAATTTTACAGATTCATTCATAAATCCGAATTGTTGCGGTCTATACTTTTCACCTATAGTAAAACGGTCAT
CGTTGGCGTATAAAATATTGTCATATTGATCAACGATAAGTGCGAATTGTCGGCTATCTTTCTTAATTTCACTTAAACGTGGGGTGTTAGCCA
TATAAATTTTAAGCATATATGTACTATTATTGAATTTAAGCTGATGCGTTGAAAATAAATACATATTTTTAGTGTTTAAATGTTCATAATTATT
GGTTATAAACTGATTTGGTCCAGATAATTCATAATAAAGTGTTGCGGGCTGTTGGTGTATTAATTTTAATAATTCACGTTTTGTAGCGGTAAC
ATCATGATGATTTGTTAAATCGAGCTCTTGAAACGAATTATTATGCTGTGTAATAAATGTCTGAATCTGCTTTTCAGTGTGATGTAAAGATGA
CTGACTTTCATCAACATGTTGATGAATCGTACGATGCTCAATCCAAATATAGATGGCATAGAAGCTTACTAGTCCAATAATAATGACTAAAA
ATACTGGAAAAATAGTAGACGCAAATAACGATCGTCTCAATTGATGTCTATAAGGTTTGTATGCCGTCAT

Gene: hptA (phosphate sensing protein)

Position: 240205 to 241173, length: 969 nt, orientation: REVERSE

Perfect match to: (MW2-BA000033-[235341:236309:r], allele observed in CC1+CC80+CC772)

Sequence:

TCATTGAATCATCTCCAAAAATTTATGATGTGGAATGTCCGGTAATTTAGATTTCGGTATTAAAGGTATGTTCTTAAGATTTTCGATAGACTG
ATCGCTTTGTTCACTAACATCCTTTCGAATTGACTTGGCATCGAACTCTGCAACTAATCGTTGTTGTACTGAGCGGCTTGTTAAATATTGCAC
TAACTTTTTACGCTTAGGATGAGGGTGTGCATTTTTAACTAAAGCAATACCATCAACATTTAACATTGTTCCTTCAATTGGATAAACGATTGA
TACAGGATAACCTTTGTTTTTCCATGTGCGTGCATCTTGTTCGTAGCTTAGACCTGCGTAATATTTACCTTTTGCAACATCTTCAATGACTTTA
GACGTCTTTGACAGTTGCATCGCATGGTTTTGGAATTGATGCACATCACTTACTCGATGATGCATGCTATAAATAGCGCGCATATGTTGATA
GCCTGTCGTTGTTGTATTTGGATTTGAGTACGCAATTTTACCTTTAAGTATAGGTTGTAATAAATCTTGATAACCTCGAATCTTAATATCTCCT
TGTAAATCTGAATTCACTACTATAACTGTTGGCATTAATAGAAAACTAGTAACATATTTATTGTTCGAGCGATAATCCTCTAATTGCTGTGTT
ACAGATGTATCTTGATAGGGAACAAAATCTTCTGGATGATCAATTGTTTCTGATAACACACCACCCATAAAGACGTCACCACGCTCCGAAAA
ATCTTCGTTATGCAAGTTTGAAAGCAGTACTTGAGTAGATCCATGTTTGATTTCAATTTTGACATGCTCTTGTTTTTCAAATTCATTTAAAATT
GGACGAATCAAGTTTGATTGATACGGAGAATAAACTGTTAATACATTTTTATCTGATTCAGAGTGACGCGTATTAGCGCATGCTGATAAAAA
AATGAGAAATAATAGCAAGATATAAATTTTTGATTTCAT

Gene: pflB (formate acetyltransferase)

Position: 241761 to 244010, length: 2250 nt, orientation: FORWARD

Perfect match to: (11819-97-CP003194-[248171:250420], allele observed in CC80+CC239+CC4803)

Sequence:

ATGTTAGAAACAAATAAAAATCATGCAACAGCTTGGCAAGGATTTAAAAATGGAAGATGGAACAGACACGTAGATGTAAGAGAGTTTATC
CAATTAAACTACACTCTTTATGAAGGTAATGATTCATTTTTAGCAGGACCAACAGAAGCAACTTCTAAACTTTGGGAACAAGTAATGCAGTT
ATCGAAAGAAGAACGTGAACGTGGCGGCATGTGGGATATGGACACGAAAGTAGCTTCAACAATCACATCTCATGATGCTGGTTATTTAGA
CAAAGATTTAGAAACAATTGTAGGTGTACAAACTGAAAAGCCATTCAAACGTTCAATGCAACCATTCGGTGGTATTCGTATGGCGAAAGCA
GCTTGTGAAGCTTACGGTTACGAATTAGACGAAGAAACTGAAAAAATCTTTACAGATTATCGTAAAACACATAACCAAGGTGTATTCGATG
CATATTCTAGAGAAATGTTGAACTGCCGTAAAGCAGGTGTAATCACTGGTTTACCTGATGCATACGGACGTGGACGTATTATCGGTGACTA
TCGTCGTGTAGCTTTATATGGTGTAGATTTCTTAATGGAAGAAAAAATGCACGACTTCAACACGATGTCTACAGAAATGTCAGAAGATGTA
ATTCGTTTACGTGAAGAATTATCAGAACAATATCGTGCATTAAAAGAATTAAAAGAACTTGGACAAAAATATGGTTTCGATTTAAGCCGTCC
AGCAGAAAACTTCAAAGAAGCAGTTCAATGGTTATACTTAGCATACCTTGCTGCAATTAAAGAACAAAACGGTGCAGCAATGAGTTTAGGT
CGTACATCAACATTCTTAGATATCTATGCTGAACGTGACCTTAAAGCAGGCGTTATTACTGAAAGCGAAGTTCAAGAAATTATTGACCACTT
CATCATGAAATTACGTATTGTTAAATTTGCTCGTACACCTGATTACAATGAATTATTCTCTGGAGACCCAACTTGGGTAACTGAATCTATCGG
TGGTGTAGGTATTGACGGACGTCCACTTGTTACGAAAAACTCATTCCGTTTCTTACACTCATTAGATAACTTAGGTCCAGCACCAGAACCAA
ACTTAACAGTATTATGGTCAGTACGTTTACCTGACAACTTCAAAACATACTGTGCAAAAATGAGTATTAAAACAAGTTCTATTCAATATGAA
AATGATGACATTATGCGTGAAAGCTATGGCGATGACTATGGTATCGCATGTTGTGTATCAGCGATGACAATTGGTAAACAAATGCAATTCT
TCGGTGCACGTGCGAACTTAGCTAAAACATTACTTTACGCTATCAATGGTGGTAAAGATGAAAAATCTGGTGCACAAGTTGGTCCAAACTTC
GAAGGTATTAACAGCGAAGTATTAGAATATGACGAAGTATTCAAGAAATTTGATCAAATGATGGATTGGCTAGCAGGTGTTTACATTAACT
CATTAAATGTTATTCACTACATGCACGATAAATACAGCTATGAACGTATTGAAATGGCATTACATGATACAGAAATTGTACGTACAATGGCA
ACAGGTATCGCTGGTTTATCAGTAGCAGCTGACTCATTATCTGCAATTAAATATGCACAAGTTAAACCAATTCGTAACGAAGAAGGTCTTGT
AGTAGACTTTGAAATCGAAGGCGACTTCCCTAAATACGGTAACAATGACGACCGTGTAGATGATATCGCAGTTGATTTAGTAGAACGCTTC
ATGACTAAATTACGTAGTCATAAAACATATCGTGATTCAGAACATACAATGAGTGTATTAACAATTACTTCAAACGTTGTATACGGTAAGAA
AACTGGTAACACACCAGACGGACGTAAAGCTGGCGAACCATTTGCACCAGGTGCAAACCCAATGCATGGCCGTGACCAAAAAGGTGCATT
ATCTTCATTAAGTTCTGTAGCTAAGATCCCTTACGATTGCTGTAAAGATGGTATTTCAAATACATTCAGTATCGTACCAAAATCATTAGGTAA
AGAACCAGAAGATCAAAACCGTAACTTAACTAGTATGTTAGATGGTTACGCAATGCAATGTGGTCACCACTTAAATATTAACGTATTTAACC
GTGAAACATTAATAGATGCAATGGAACATCCAGAAGAATATCCACAGTTAACAATCCGTGTATCTGGTTACGCTGTTAACTTCATTAAATTA
ACACGTGAACAACAATTAGATGTAATTTCTCGTACATTCCATGAAAGTATGTAA

Gene: pflA (pyruvate formate-lyase-activating enzyme)

Position: 244033 to 244788, length: 756 nt, orientation: FORWARD

Perfect match to: (11819-97-CP003194-[250443:251198], allele observed in CC80+CC772)

Sequence:

ATGCTTAAGGGACACTTACATTCTGTCGAAAGTTTAGGTACTGTCGATGGACCGGGATTAAGATATATATTATTTACACAAGGATGCTTACT
TAGATGCTTGTATTGCCATAATCCAGATACTTGGAAAATTAGTGAGCCATCAAGAGAAGTCACAGTTGATGAAATGGTGAATGAAATATTA
CCATACAAACCATACTTTGATGCATCGGGTGGCGGTGTAACAGTCAGTGGTGGCGAACCATTGTTACAAATGCCATTCTTAGAAAAATTATT
TGCAGAATTAAAAGAAAATGGTGTGCACACTTGCTTAGACACATCGGCTGGATGTGCTAATGATACAAAAGCATTTCAAAGGCATTTTGAA
GAATTACAAAAACATACAGACTTGATATTATTAGATATAAAACATATTGATAATGACAAACATATTAGATTGACAGGAAAGCCTAATACACA
CATCCTTAACTTCGCGCGCAAACTGTCAGATATGAAACAACCTGTATGGATTCGACATGTCCTTGTGCCTGGTTATTCTGATGATAAAGACG
ATTTAATTAAACTAGGGGAATTTATTAATTCTCTTGATAACGTCGAAAAGTTTGAAATTCTGCCATATCATCAGTTAGGTGTTCATAAGTGGA
AAACATTGGGCATTGCATATGAATTAGAAGATGTCGAAGCGCCCGATGATGAAGCTGTTAAAGCAGCCTACCGTTATGTTAACTTCAAAGG
GAAAATTCCCGTTGAATTATAA

Gene: Q2YV51 (putative protein)

Position: 244778 to 245072, length: 295 nt, orientation: FORWARD

Sequence:

TTGAATTATAAATACAATTCAGACCGAAAAGAAAGCATATGCAACTTCAAGAGTGAAGGGGCATATGCTTCTTTTTCAATTGAGTATTGAGT
ATTAGCAAGACGTAGTAGGTATATGAGACAACTTCTACAATGGTTGAAGGAAGACGTTTTTGTAAGTAGCTATGCTGATAAAGAATGTGAT
GTCTTGTTAAAGGTGAGGTTCCAATATCATCGAATGGGTTATTATTTGCTACTTGCATATGAATATGAGTCTTTTCAAATTTTTATTGACCCTG
AGTAATGAAAAATATTAA

Gene: glpQ1 (glycerophosphoryldiester phosphodiesterase)

Position: 245110 to 246873, length: 1764 nt, orientation: FORWARD

Perfect match to: (11819-97-CP003194-[251520:253283], allele observed in CC80+CC361+CC772)

Sequence:

ATGAAGAGAATTAGTAAAGATATATGGGCAGTATTTAAATTACTGTATCAAAATAAAGGGCGTTTTAGCATTAATGCCTTACTATTGCAGTT
AATCATGATTTTTATTAGTAGTACATACTTAATTTTACTATTTAATATGATGTTAAAAGTAGCTGGGCAAAGCCAACTTACGATTAACAATTG
GACGGAAATCATAAGTCATCCTGCCAGTGTGATACTTCTTATTATATTCATATTAAGTGTTGCTTTTCTGATTTATGTAGAGTTTTCATTGTTA
GTTTATATGGTTTATGCCGGCTTTGATCGACAGATTATTACATTTAAATCCATTTTTAAAAATGCCTTTGTAAATGTGCGTAAACTCATAGGT
GTACCAGTTATTTTCTTTGTCATTTATTTAATGTTAATGATACCCATTGCCAACCTAGGACTAAGTTCAGTATTAACAAAAAATATTTACATAC
CTAAATTTTTAACGGAAGAACTTATGAAAACGACGAAAGGTATAATCATTTACGGTACCTTTATGATTGCTGTATTTATATTAAACTTTAAAT
TAATATTTACGTTACCGTTAACGATTTTAAACCGCCAGTCGTTATTTAAAAAATATGAGACTAAGTTGGCAAATTACGAAGCGAAATAAGTTT
CGACTTGTTATAGAAATAGTTATATTGGAACTCATCATTGGTGCGATTTTAACATTAATTATTTCAGGAGCAACATATCTTGCTATTTGTGTA
GATGAAGAAGGAGATAAGTTTTTAGTCTCATCAATTTTATTTGTTGTATTGAAAAGCGCATTGTTCTTCTATTATTTATTTACGAAATTATCAT
TAATCAGTGTGTTAGTACTGCACTTAAAACAAGAGAATGTATTAGACCAACCGGGCTTAGAATTTAAATACCCAAAACCGAAACGGAAGTC
TAGGTTCTTTATAATTTCAATGGTGCTTGCAGTGACATGTTTTATCGGTTATAACATGTACTTACTTTACAATAATACTATCAATACAAATATC
TCCATTATTGGCCATCGTGGTTTCGAAGATAAAGGCGTTGAAAATTCTATTCCGTCATTGAAAGCTGCTGCAAAAGCGAATGTCGAATACGT
TGAGTTAGATACAATTATGACGAAAGATAAACAATTTGTTGTTAGTCATGATAACAATTTGAAACGTTTAACAGGTGTTAATAAAAATATTT
CTGAATCTAATTTCAAAGATGTCGTCGGTTTGAAAATGCGTCAAAATGGACATGAAGCAAAACTTGTATCCTTAGACGAATTTATTGAAACG
GCTAAACAATCAAATGTGAAGCTACTAGTAGAGTTAAAGCCACATGGTAAAGAACCAGCAGATTATACACAACGTGTTATTGATATTTTGA
AAAAGCATGGTGTTGAACATCAATATCGTGTGATGTCTTTGGATTATGATGTGATGACTAAGTTGAAAAAAGAAGCGCCATATCTCAAGTG
TGGTTATATCATTCCGTTGCAGTTTGGTCATTTTAAAGAAACATCATTAGATTTCTTTGTCATCGAAGATTTTTCTTATTCGCCAAGACTTGTT
AATCAAGCGCACTTGGAAAATAAAGAAGTCTATACTTGGACCATTAACGGCGAAGAAGATTTAACGAAATACTTACAAACCAATGTTGATG
GTATTATCACAGATGACCCAGCATTAGCTGATCAGATTAAAGAAGAAAAGAAAGACGAAACATACTTCGATCGTTCTATAAGAATTTTGTTT
GAATAA

Gene: Q2YV49 (putative protein)

Position: 247037 to 247381, length: 345 nt, orientation: REVERSE

Perfect match to: (11819-97-CP003194-[253447:253791:r], allele observed in CC80+CC9+CC72+CC80)

Sequence:

TTAATTGTTTTGTGAATGCAAAGGGTTAGAAATTGAATTGTAAATACTTTCTAATCTTTGTTTCGCTTTAGTCATTTGATCCAAATTTTTAGTG
CGTATAGCGGATTTTGCAATATAGTGCGCAGCTAAAATATCGCGTTTTTGAAACGCATCTAAATTTAGGTACGATAATTTATTTAAGTCAGT
GTTTGCTATTAATTCATGTAATTGATCTACAAGCGCTTGATGTTGATACGTATGTGATGTAGTTTCAGGTTTGCTTGCTAATTTAATACCAGT
CGTATCAAGGAGCGCCGCTTTAATACCAGCAACTAAATATGTTTTGATTTTCATTTGTGTTGTCAT

Gene: coa (staphylococcal coagulase)

Position: 247571 to 249568, length: 1998 nt, orientation: FORWARD

Sequence:

ATGAAAAAGCAAATAATTTCGCTAGGCGCATTAGCAGTTGCATCTAGCTTATTTACATGGGATAACAAAGCAGATGCGATAGTAACAAAGG
ATTATAATGGGAAATCACAAGTTAAAAAAGAGAGTAAAAATGGGACATTAATAGATAGCAGATATTATTGGGAAAAAATAGAAGCTCTAG
AAAAGCAGTTTTCAAGTGCATTGGCATTAACAGATGAGTATCAATATGGTGGTAATGAATATAAAGAGGCAAAAGATAAATTAATGGAAA
GGATATTAGGAGAAGATCAATATTTATTAAAGAAAAAAATAGATGAATACGATTATTATAAAAAAATGGTATAAAGCAACTTACCCAAACGA
TAATTCAAAAATGTATTCATTTCATAAGTATAATGTTTACTATTTAACAATGAATGAATATAATGAAATCTCAAATTCATTAAAAGACGCTGT
GGAAAAATTTAATAATGAAGTAAGAGATATTCAATCTAAAAATGAAGACTTAAAGCCTTATGACGAAAATACTGAAAAACAAGAAACTGAT
AAAATATATGAATTTGTTAGTGAAATAGATACAGTTTTTGCAGCGTATTATAGTCATGAAAAATTTGGTATACATGCTAAAGAATTACGAGC
AAAATTAGACATAATACTAGGTGACGTGCATAACCCGAATAGAATCACTAATGAACGTATAAAAAAAGAAATGATGGAAGACTTAAATTCA
ATAGTCGATGATTTCTTTATGGAAACAAATCAGAATAGACCTACAACAATAAAGAAATATGACCCCAATATTCATGATTATACAAAGAAAAA
AGAGAATAAAGAAAATTTTGATAAGTTAGTTAAAGAAACAAGAGAAGCGGTTGCAAATGCAGATGCATCTTGGAAAAAGAAAACTGTCAA
AAAATACGGAGAAACTGAAACAAAATCGCCAGTAGTAAAAGAAGAGAAGAAAGTTGAAGAACCTCAAGCACCTCAAGCACCTAAAGTTGA
TAACCAACAAGAGGTTAAAACTACGGCTGGTAAAGCTGAAGAAACAACACAACCAGTTGCACAACCTCTAGTTAAGATTCCAGAAGGTACA
ATTCAAGGTGTAACTGTAGAAGGGCCAAAATACCCAACGATGGAACAGCACACAATCTATGGTGAAATTGTAAAAGGTCCGGAATATCCA
ACGATGGAAAATAAAACGTTACAAGGTGAAATCGTTCAAGGTCCAGATTTTCTAACAATGGAACAAAACAGACCATCTTTAAGTGACAATT

ATACACAACCATCTGTGACTTTACCGTCAATTACAGGTGAAAGTACACCAACGAACCCTATTTTAGAAGGTCTTGAAGGTAGCTCATCTAAA
CTTGAAATAAAACCACAAGGTACTGAATCAACGTTGAAAGGTACTCAAGGAGAATCAAGTGATATTGAAGTTAAACCTCAAGCAACTGAAA
CAACAGAAGCTTCTCAATATGGTCCGAGACCGCAATTTAACAAAACACCAAAGTATGTGAAATATAGAGATGCTGGTACAGGTATCCGTGA
ATACAACGATGGAACATTTGGATATGAAGCGAGACCAAGATTCAACAAGCCAAGTGAAACAAATGCATACAACGTAACGACAAATCAAGA
TGGCACAGTAACATATGGCGCTCGCCCAACACAAAACAAGCCAAGCAAAACAAATGCATACAACGTAACAACACATGCAAATGGTCAAGT
ATCATATGGCGCTCGCCCGACACAAAAAAAGCCAAGCAAAACAAATGCATACAACGTAACAACACATGCAAATGGTCAAGTATCATATGGC
GCTCGCCCGACACAAAAAAAGCCAAGCGAAACAAATGCATACAACGTAACAACACATGCAAATGGTCAAGTATCATATGGCGCTCGCCCG
ACACAAAAAAAGCCAAGCGAAACAAACGCATATAACGTAACAACACATGCAGATGGTACTGCGACATATGGGCCTAGAGTAACAAAATAA

Gene: fadA (3-ketoacyl-CoA transferase)

Position: 250171 to 251355, length: 1185 nt, orientation: REVERSE

Sequence:

CTAACGCACATACTCAAATATAGCAGCTGCACCCATGCCGACACCAATACACATCGTAACCATGCCGTAACGGCTATCGGGACGTCTACCCA
TTTCATTAAGTAAACGCGCGGTTAACATTGCCCCTATAGCACCTAATGGATGACCTAAAGCAATAGCGCCACCATTCACATTCGTACGTGAT
ATATCTAGACCTACTTCTTTAATAGATGCAATCGTTTGAGAAGCAAATGCTTCGTTCAATTCGATCAAATCAATGTCTTCAACAGATAGATTG
CTGAGTGACAATACTTCAGGAATCGCATATGCAGGCCCAATACCCATAATTTTCGGGTCAACGCCTACTGCCTTATAACCAACGAATCGTGC
AATAGGTGTCACGCCTAGTTCTTTCACTTTATCTCCAGACATTAAAACTACAAATCCTGCACCATCAGAAAGTGGGGCAGATGTTCCCGCAG
TCACAGTGCCGTCAGCTTTAAATACTGTACGTAATTTGGCTAATGCCTCCATCGTGGTGTCAGGGCGTATAAATTCATCTTGGTCAAAGATA
TTTGTGTGTACTTTTGGTCCTGCGTTTGTATATTCAACTGAGTTTACCCGTATTGGAATAATTTCATCTTTGAACCGACCATCACGTTGTGCGT
CATAGGCACGTTGATGACTTCTGACAGCATAAGCATCTTGATCTTCGCGTGATACGTCAAATTGGGATGCTACATTTTCAGCAGTTAAACCC
ATAGGATATGACGCACCTATATCATCATATTGTAAGGTTGGATTGTTTGTGGGCTCGTTGCCACCCATTGGTACGGCACTCATCAATTCAAC
GCCACCAGCTACAAGTATATCTCCTTGACCAGCCATAATTTGATTGGCTGCAATCGCGATGGTTTGTAATCCTGATGAGCAGTAGCGATTCA
CTGTTTGACCCGGTACCGTGTCAGATAATCCCGCACGCAATGCAATCGTTCGTGCAATGTTTTGTCCTTGTAATCCTTCTGGAAAAGCCGTAC
CAACAATGACATCTTCAATCATATTCTTATTGAATTTTCCGTCAATACGTTTCAATACGCCTTGTAATACTTTGGCTGCGACATCATCAGGTCT
TTCGTGGAATAATGCGCCTTGCTTTGCTTTCGCTGCGGCTGAACGCCCATAAGCTACAATGTATGCTTCTTGCAT

Gene: fadB (NAD binding 3-hydroxyacyl-CoA dehydrogenase)

Position: 251385 to 253646, length: 2262 nt, orientation: REVERSE

Perfect match to: (11819-97-CP003194-[257804:260065:r], allele observed in CC80+CC8+CC80)

Sequence:

TTAATTACGTAATGGCTTACCAGTTTTTAACATATGTGCAATTCTTTCATATGATTTTTTAGATTTTAGTAAGTCAATAAAGCCAATTTTCTCCA
ACGATTGAATGTAACGTTGATTTATAAATGTATTTCTTGGTAAATCACCACCCGCTAAAATTGTGGCGATATTTAAGGCGATATGATAATCAT
GGTCGCTAATAAAATGCCCCCGTCTTTGCGCATCTAATTGTCCTTGGATCAATGCTTTGAAGTCTTCACCTAAAGCGATATATTGATGTCTAG
GATTCGGAATATAGTTTGTTTCTGCTTCATATTTCGCACGTTTGAGCGCAACTTCGACACGTTGTGCTGTATTGAAAATAATCGTATCTGTAT
CACGTAAATAACCATAACGACGTGCCTCAAAGGCATTTGTAGAGACTTTCGCAAATGCGATATTCGTCAGTACTTTTGTCATGGAAGCTTGT
TTGTCATCAAACTTATGCGATGTGCGTAATATGCGATCAGCCATTTCTGCAAGGCCACCGCCACTCGGTAATAAGCCAACGCCTGCTTCAAC
AAGACCTATATATGTTTCACTTGCAGCGACAACAATAGGTGAGTAAAGTACAAGCTCACAGCCACCGCCTAAGGCACGACCTTGAACAGCT
GTGACTACTGGTTTCAAACTATACTTCAAACGATTAAAGCTATAATGTAATTTATCAATTGATTGTGCAACGACATCATCTACAAGACCGTCT
TCATGCGCCTTTTTCATTAAGAAAAGGTTAGCACCCACACTGAAATTGTTACCATCTGCATAAATGACCATACTTGTGTAATGTTCATTTTCCA
GTAAATCAATGGCATCAACTAACGCATCGTTGAATTCATCGGTAATGACATTATTTTTACTTTGTAATTTCAGTAACAGTTGATCATCATGAG
TTACGGAAAGTTTGGCATCACCTTTATCCCAAAGTTCATCTTTTACGAAGTGAGAAATAGGTGTTGCATATTCAATGGTCTCATCTTGTTTAT
AAAAGCCACCATCTAAATCACTAATCCATTGTGGTAAGTCTCCAAGTTCGTCTTCCATACGTGTTTTAACACGTTCGTATCCCATTGCATCCCA
TAATTGGAATGGACCAAGTTTCCAGTTGAACCCCCAGACAAGCGCACGGTCTATGTCTCGGAAATCATCGGTAGCTTTAGGTACATTGATA
GCAGAGTAATAGAAATTATTACGTAATGTCTCCCATAAAAATAGTCCCGCTTCGTCTTGCGCATTGAATATGGTATCAAGGTTATGCACTAA
GTCTTTATTAAATTCATTTAAAATTGGTAATTGTGGTTGCGATACAGGTACATAATCTTGTTTTTCAACATCGTAAACAAGTCGAGCTTTAGT
TTCTTTATCCTTTTTGTAAAATCCTTGTTTCGTTTTACGTCCGAGTGCGCCATTGTCAAACAACGTATTTACAATTTTGACATCATGAAAATAA
GGTGTTTCTTCAGGTACTTGTTGCATGCCTTTAATTACAGACACTGCAATATCTAAACCGACTAGGTCAGATAGCGCATATGTACCTGTTTTA
GGACGACCAATCGCTTGCCCAGTTAAAGCATCCACATCTACAATGCTTATCTTGTGTTGCTCGGCGCGATACATAATATCATTCATTGTTTGC
GTGCCGACTCTATTTGCGACAAAGCCAGGCACATCATTGACGACAATGACACCTTTACCTAACACATTTTGCGCGAAATTTTTTACATCTAAT
ATAATAGATTCCTTCGTGTGTGACGTAGGTATTAACTCCACTAATTTCATAATACGTGGTGGGTTAAAGAAATGTAGACCAAAGAATCGTTC
TTGATCCTTCTCGTTAAATGCTTGAGCAATCGCATTAATTGGAATACCTGATGTATTTGTAGCGAATAAAGCATCTTCTTTAGCATGTTGTAG
AACTTGTTGCCAAACAGCATGCTTAATTTCAATATCTTCTTTGACTGCTTCGATATATAAATCAGCATCATCATTTACCAAGTCATCATCAAAA

TTACCATATGTTAAATGACTCGCTAGATTTAAGTCGAATAGTAGCGGCCGTTTCTTATCTGTAATTTTATCGTAAGATTTTTTCGCAATGAGA
TTTGGATCGTTTTTGTCCACTACAATATCTAATAGTTTTACTTTAAGTCCAGCATTCACAAAAAGTGCTGCCAGTTGAGCGCCCATTGTGCCT
GCGCCAAGAACGGTTACTTTATTAATTGTCAT

Gene: fadD (putative acyl-CoA dehydrogenase)

Position: 253833 to 255044, length: 1212 nt, orientation: REVERSE

Perfect match to: (11819-97-CP003194-[260252:261463:r], allele observed in CC80)

Sequence:

TTATACGAAAGCAGAATCTCCAGTCAAAGCGCGTCCAATTACTAAGGCATTAATTTCATGTGTACCTTCGTACGTGTAAATCGCTTCTGCATC
AGAGAAGAAACGTGCAATATCATAATCGTCAGCTAGTATGCCATTACCACCTGTAATACCACGGCCCATAGCTACTGTCTCACGCAAACGTA
AGGCATTCATCATCTTCGCCGTTGAAGTTGCAACCTCGTCATATTCACCATGTGCTTGCATATTAGCTAATTGAGCACATGTTGCCATTGCTT
GAGCTAAATTACCTTGCATCATTGCTAGCTTTTCTTGTATTAACTGATATTTACTAATTGGTTTGCCGAATTGCTTACGCTCAGTGACATAATC
TAATGTGGCACGTAAAGCGCCAGCCATACCACCTGTAGCCATATAAGCAACGCCTGCTCTCGTTGAATAAAGAATTTTGGCAATATCTTTAA
AGCTTGTTATGTTTTGTAAGCGATCCGCTTCATCTACTTTGACATTAGTTAATTTAATTAGGGCGTTAGGAACAATGCGAAGTGCGATTTTAT
TATCAATGACTTCAATATCGACGCCATCTTGTTCTGGTCTGACTACAAAGCAATGGGGTTTGCCAGTTTCTTTATTTACTGCGAATACTGGAA
TGACATCAGATACATGTGCACCACCAATCCATTTCTTTTCACCATTGATAACCCAAGTATCGCCTTGGCGTTCAGCGACTGTTTCAAGACCTC
CCGCAACGTCCGAACCGTGTTCTGGTTCAGTTAAAGCAAAGCATGTACGCAGTTCATGTGACTGTAATTTAGGTACATATTTCGCAATTTGT
TCTTTGCTACCTCCGAAATAGAAAGTGTTATGCCCTAAACCTTGGTGAACACCGAGTAGGGTAGCTAAGGAAATATCAAATCGCGCGAGTA
GGTAAGACATGAAAAACTGAAATAGTTGACTAGGCATTTTGGCGTTTGGACGATCCTTGTAAAGTAATGGATTGTTAAAATAATTTAATTCT
CCAAGATCTTTAAAATAGTCCTCGGGTACAGTAGCGTCTATCCAATGTTGATTAATATTTTCACGGTACTTACTTTCTAGCAATGAATCTACTT
GTTGTAAAAAATTCGACTTCACCGTCTGTTAAACCTTTAGCAATACTAAGTACATCTTCAGGAAATAATGTTTTTAAGACCGTTTCTTTTTCAAA
TGTCAT

Gene: fadE (putative long-chain-fatty-acid--CoA ligase)

Position: 255156 to 256661, length: 1506 nt, orientation: REVERSE

Perfect match to: (Strain_21333-AHKA01000033-[69379:70884], allele observed in CC15+CC80)

Sequence:

TTAAAGTGTTTGTGAGACTTTGTCATTCATCATTTGTCGAATCGCAAGTTTATCTGGTTTCTGCGTACTGTTTAACGGCATATGTGTCACTGG
TACATACATTCTTGGGACTTTATAACCTGCTAAACGACTTCGCATATGTTGATCTAAAATTTCAGCGTAATGAGGTTCATCTTCGCGAAGTAT
AATGGCTGCAGCAATTGATTCACCATATTTTGGATGATCATAGCCAACGACCACACACCGGTCTACTAGTGGATGCTCAGCTAAAGCATTTT
CGACTTCGGATGGTAAGACATTTTCGCCACCAGTTATGATTAATTCTTTTTTGCGGTCAATAATAAATATATCGCCATCGTCGTCCATCTTCG
CTAAGTCACCAGTTAATAAATAACGACCATGAAATGCTTTGGCAGTCTCTGCTGGTTTATTCCAATATCCTGGCGTGACATTTTTAGCCTTAA
TTGCAAGTTCGCCAATCTCACCAGTAGGTACTTCCTCACCGTTATCATCAAGGATACGTGCATCAACGAACATGACTGCTTTACCAATACTCA
TTGGCTTACGTTTTGAATTTTCCGGTGTATTAACAAGTACAAGAGGTGCTTCAGTTAAACCATAGCCGTTAATAATGTTTATGCCATATTGTT
TAAAAGCTGCTTGGATACTTGGTAATGGTTGTGAACCACCTTGGATGATATAATCCATAGCTTTAAAATTTTTAGGATTAAAATTACTAGCAC
GTAGCGTACTATAATACATTGTCGGAATCATGATAATAAATGTAGGGTGATATTGTGCAATCATGTCATTCAATTCTTCGCCGTTAAAGTAA
CGTTGAAGAATAAGTGTGCCACCTGACATTAATACTGGTAATACAGTATCGTTAAACCCTAAAACATGGAACATTGGTGTTGATACAATCGT
AATATAGTTTGAATTGAACTTATACGTCAGCTCTAAGTTTGCGCCGTTATGAACAAATGATTCATATGAGAACATCACACCTTTAGGTGATCC
GGTTGTACCACTTGTATAAATTAATGCTGCAAGATCTTGTGGTTCAACAGGTGTTGCTTGAAAAGGTTGGTGATAATCTGGATTTACGATTT
CATCATATTGCGCTACATCAATATCCATATGCAATAAGTTTTGGTCAATATCGGTGAGTGAACTTAAATGTTTTTCAGCATAGAAGAGCAGTT
TTAATTGTGCATCTTCCACAATGGCTGCAATTTCTTTTGGGTTAAGCCGCCAATTCAATGGTAAAAAAACCGCACCTGTTTTAAAACAAGCAA
ATAATAAATCTAATATTGCAATATCATTTGGCGCAAAAAATACCGATAACATCGCCTTTTTTAACACCTTGAGATGTTAAATAATGTGCCATAT
TATCAGCACGTGCATTGAGTTGTTGGTATGTCCAAGATGTTTGTTTTGCGTGATCAATAACGGCAGGCTTGTCATCATCGAAGTCTGAACGC
GTTTTTATCCAATCGAAATTCAT

Gene: fadX (putative acetyl-CoA/acetoacetyl-CoA transferase)

Position: 256687 to 258249, length: 1563 nt, orientation: REVERSE

Perfect match to: (11819-97-CP003194-[263106:264668:r], allele observed in CC80+CC22+CC72+CC80)

Sequence:

TCATACTTTATGAATTGATTGTTTAAGTTGTCCCCATTTTTCTTGTAAATGCTGGTATCAATTAATTTTAAATGATCAGCAATAATTGGTTTA
AAAGCCATTTGATTCAAAATATCTTTATGCAAATCAAGACCTGGTGCAATTTCAATTAGTTTCAAGCCTTGATTGGTGAGTTCGAATACTGCA
CGATCAGTAACAAAATAGACTTCTTGCTCGAGTGATTGTGAATATTGTGCATTAAAGTCGATATGGCTCACATCTGATACAAATTTCTGGTTT
TGTCCTTCAGTTTCAATGTTTAATCGTTGATTATGGCATGAGACATGACTGCCAGCTACAAAAGTACCTGAAAAGATAATTTTATTTACAGAT
TGCGTAATGTCTATAAAGCCACCACATCCATTTAGTCGGTCATTGAAGTAAGACACGTTGACATTGCCGTATTGATCAACCTCAGCAAAGCT
AAGATAGGCAACTGATACACCATTGTTATAAATAAAATCCCATGCTCGATCATGAGGCATGCGCACATCTGCATTGTAATTCATACCAAAAT
GTTCACGACTCCCAACGAATCCACCGAAAATGCCAACATCTAGAATCGGTTGCACATCATGTTCAACACATTCTTCGTGCAATAAATTAGAG
AGTTCATTATTGATGCCATAACCGATGCTAATTGTATCGCCATAAGTTAAAAACTGAGCAGCACGTCGGAGAATCAATTTGCGACTATTAAA
AGGTAATGCGGGTTCAGGTATTCCATCAATTCGTTCTTCTCCAGACAAGGCTGGTAAATAATGACTCTGAATTACTTGGCGGTGATTCTTTTC
ATCTTCTGTGACGTATACATAATCGACAAGATTTCCTGGGATAACAACTTCATTCGGTTTTAGTTGATAGTCGTCAACTAAAGCTTTAACTTG
TACAATAACTTTCCCATGATTGGCTTTCGTGTTTAATGCGACATGATAACACTCGCTCAAGTACGCTTCTTGAGTTAAATAAATGTTACCTTG
TTGATCTGCGTATGTTCCTCTCAGTAGTGCCACATCAACGCTAGGGAATGTGTAATGTAAGTATGTTTCATCGTTGATGGTTACTAATGAAA
CTAAATCATCCGTTGTTCGTGTATTTACTTTACCGCCACCGTATCTAGGATCAACAGCTGTGTTTAATCCGATTTTAGTAATAACTCCAGGTA
ATAATTGATTACTCTGACGATAATGAGTTGCAATGATACCTTGTGGTAAAAAATAAGCTTCAATGTCATTATTTTTCATTGCTTGTGCCGTTTT
GGAAGAAGCCGTTAAAATACTCATAATGACACGTTTAATCATGCGACGTTCTATAAAATCATCTAAATCCGGTGCGGCACCTAAACTATGGA
TATCATTCGCTAATATAAACGTTAAATCATTGGGCGTATGATATGTGTCATGTTGCGCTAACACAGCACGTAGAACTTCGGCGGGTAAGTTG
GCTACAGCTAATGCTGGTAAACCAATCACATCACCATCTTTAATGATATGTTGTAAGTCGTGCCATGTGATTTGTTTCAA

Gene: prsW-prsS (protease responsible for activating sigma-S)

Position: 258705 to 259847, length: 1143 nt, orientation: FORWARD

Perfect match to: (11819-97-CP003194-[265124:266266], allele observed in CC80)

Sequence:

ATGTCATCAGCAAGTACACAATCAACTAAGACGTCCGACATACATAATGAATCTATCGATAAACAAATGGAAGCCAAAGCGCATGAAACAG
CGCAAAATACAGATTTAAAAAACGAAGCAAGAAGTTTATTTGATAATGCAACCAAATCAATCGGTAGACTAGCGGGCAATGATGAAAGCTT
AAATCTTAATTTAAAAGATATGTTTTCTGAAGTATTTAAGCCGCATACTAAAAACGAAGCAGATGAAATATTTATAGCGGGTACTGCTAAAA
CTACGCCAGCAATTTGTGACATATCAGAAGAATGGGGGAAGCCATGGCTCTTTTCTCGAGTATTCATCGCTTTCACAGTAACATTTATTGGA
TTATGGGTCATGGCAGCAATTTTTAATAACACTAACGCGATTCCGGGGCTCATTTTTATAGGGGCTTTAACAGTACCATTATCGGGTTTGTTC
TTCTTTTATGAATCAAATGCGTTTAAAAATATTAGCATTTTTGAAGTTATTATCATGTTCTTTATTGGCGGCGTATTTTCATTACTAAGTACGA
TGGTATTATATAGATTTGTCGTTTTTAGTGATCAATTCGAAAGGTTTGGTTCTTTAACATTTTTCGATGCATTTTTAGTAGGATTAGTTGAAGA
AACTGGAAAAGCACTCATTATTGTTTATTTCGTCAATAAATTGAAAACTAATAAGATTTTGAATGGATTATTAATCGGTGCTGCTATTGGTGC
AGGGTTCGCAGTTTTTGAATCAGCAGGTTATATTTTGAATTTCGCTTTAGGAGAAAATGTCCCATTATTAGATATTGTCTTCACACGTGCGTG
GACTGCGATTGGTGGTCATTTAGTTTGGTCAGCGATTGTTGGTGCTGCAATAGTTATTGCGAAAGAACAGCATGGCTTTGAATTCAAAGAT
ATTTTTGATAAACGCTTTTTAATATTCTTTTTATCAGCCGTTGGTTTACATGGCATTTGGGATACATCATTAACGATTCTTGGCAGTGATACGT
TGAAAATATTTATTTTAATCGTTATTGTGTGGATACTTGTATTCATTTTAATGGGGGCAGGTTTAAAACAAGTGAATTTACTGCAGAAAGAA
TTTAAAGAACAACAGAAAAAAGTAGACGAATAA

Gene: nikA (nickel ABC transporter, substrate-binding protein)

Position: 260161 to 261636, length: 1476 nt, orientation: REVERSE

Sequence:

TTATCGTTCAATCGTTGTTCGATAATCGATTAAATAGATACCTTCAGGTGTTACTTTATAATTTTTAACCTTAGAGTTAGCAGCGACTATTTGA
TCGTTGTAAGCAATATAACTGTTTGGTACATCTCGACTTGATAATTTAATAATATCATTAGAAATATTGTGACGTTCCTTAACATCTACAGTAT
GATTCAATTGATTAATTAAATCATCGACGTTGCTATTATTGTAGTCTCCTTTATTAATAGCACCATCTTTTTTATATGCTTGATTAAAGAAATA
ACCTGTATCTCCACGAGGAATTGTTCCGAAACTATACATCGTTGCATCCCATGCAGAACGGTCTTTTAAGTAACCTTCTATGTCATCAACACT
TTTAATGTCGATTTCAATATTTGCTTTTTTAGCATCTGATTGTAATACTTGCGCAATTTTCGATAGCTCTGGACGACCGTCATACGTAATTAAC
TTAATTTTTAAAGGGTGTTCTTTTGTATAACCATCTTTAGCTAATAACATTTTTGCTTGTTCGATATTTTGTTTGGTTAACTTAGGTTCTTTAAT
ATATGGAATTTTTATCATTAAATGGACTCGTTGCAGGTTTCGCATAACCTTGATAAATATGATCTGCAATACCTTGTCTATCAATGATATGATC
TAATGCTTCACGAACGGATTTAGTCATTTTTTTTATTAGTATGATTATACATAAGTAAAGAAGTTCTAAATCCAGATTCTTTTGACACTTTTAAA
TTTTGATTATTTTCTATGTCTTGAACTTTATTAACTGGGACATCAGTTATTAAATCATCTTTTTGAGATTCTAAATTTCTGACGCGATTATTGCC
GTCTTCTTGGTACGTCACAGTAATATGATCAAGTTTCGGTTTACCTTGCCAATAGTCCTTAAAATTCGACAATGATATTTTTCGAGATTGCTTA
TAATCTTTTATTTGGTAAGGGCCTGTACCAACAGGAATTTGATTAACATCTGATTTAGCATCTGTATCATAAATTGCCATAAAAGGATTAGCT
AATTCAGATACAAGTTCAGGGTAAGCGGAGTTGGTTTTAATTGTCAGTTTTTGACCTTTAGCGGTAATTGATGATATTGGTAATGAATATTT

GACCAAGTCGCTTTTTTTCATGCTATTTTCAAGGCTAGATTTCACTTTTTCTGCAGTCAATTTTTGACCGTTTTGAAATTTAATATTATCTTTTA
ATTCTATATCTAACGTTGTATCATTTGGTTGATGATACGATTTCACTAATGCTTTTTCTATTTTTCCTTGATCATTTGTTTTAAATAATGATTCT
GCAGCACCAATCTTAACTGGTACATCTGTTTCATAAGGTGCAATAGACTTTGTTTTTAACGGTAACGAAATATTTAAGTCTTTGCCAGATGAA
TGCATTGAGCCACATCCTGATAACACTAATACTGCTGAAAATATAGTTGCTAGTCTTTTAAACTTCAT

Gene: DUF488 (putative protein)

Position: 261834 to 262190, length: 357 nt, orientation: REVERSE

Perfect match to: (N315-BA000018-[278901:279257:r], highly conserved allele)

Sequence:

CTAAGTATTGAGCAACTGCTGTAGTACTACAGCTTGGTTATGTTTAGTATCTTTTGCTGCATATAACAATAGAACATGATTATGCTGATTTAC
AATATCCTTTAATTTTTCAAAAGCATCTTTTTGCGCATCCTGATCACGTAATTCTTTTTCATATTTTTCTTTAAAAGCTCCAAAAAGTTTAGGAT
CATGTTGGAACCATTGTCGCAACTCAGTAGAAGGGGCAATGTCTTTTAACCAATAATCTAGGTTAGCAGTTCTTTTCGAAATACCTCTCGGC
CAGACTCTATCGACTAGGATACGAATAGCGTCGGTATTATCTTTATTGTCATAAATCCGTCCAATATCTACGGTCAT

Gene: Q5HJD9 (putative protein)

Position: 262347 to 262520, length: 174 nt, orientation: REVERSE

Perfect match to: (RF122-AJ938182-[223774:223947:r], highly conserved allele)

Sequence:

TCATTTATTTTTACGTTTAGTTAAATACTTCAAACCAACTGCAAAGACCGTGTACCCGGCTATGGTTTGTATCAATGTTTTAATTAAATTATTA
TTTTTAACAATAATATTTGCAGTAACAATACTTACGAAATATAATGCAAATACTTTTACGTAACGTTGATTAAGTTTCAT

Gene: hmp (radical nitric oxide-detoxifying flavohemoglobin)

Position: 262546 to 263691, length: 1146 nt, orientation: REVERSE

Perfect match to: (COL-CP000046-[260532:261677:r], highly conserved allele)

Sequence:

TTATACTGCAACGCTTAGTCTTGGAATAAATGTTTCGTAGTGTACGCGATCCATATCGTAATTTAAAGATTTAAGTGCTTCGATCATAGATTG
TAAGAATTTTGTACCACCACAGATATAAATTTCAGGTTTATTTGCTAAAAATACTTGTAATTCTTCAGCACCAATATAGCCTTGTTTATCTTTT
AAGTGTGTATATAATTTAGCGTTGTCATGATGGCTTGCGATACTGTTGAAGTTGTCTTTGAAAGGTAAATGTTGTTCATTTTCAGCAACTTGA
ACCATCTGTGTATCTAAACCTTTGGCAGAGGCAGCTTCATACATAGCTACTAAAGGTGTAACACCAATACCTGAACCTAAGAAAAGTTGTGG
TTCAGTCGTATTCTCTAATACGAATCCACCTACAGGCGCAGCTAAATTAATCATATCGCCTTCTTTAATCTCATCGTGTAAAATTGTTGAAACT
TCGCCTTCATGTTCTGTTGTGACATCACGTTTAACGCCAAAAGTTAAATGGTTTTTTTCACCTGATACGATAGAATAGTGACGTTTAGCTCTA
TATGGAAGTTTATCACTAGAAACATCAACTGTGATGTATTGGCCTGGTGTAAATTCACTAAAGTCATATTCTTCAGTTTCAACTGTAAATGAT
TTAATGTCTTCAGATTCTTGTTTAATATTGGTAATTTTGAATGGTTTAAAACCAATCCACATCATTTGATCATAAATTTCTTTTTCAATTTGGAT
GAACACATCCGCAATAACGCCATATGCTTTTGCCCAAGCTTGAATGACAGGGTCATTTTCTTCTAATCCTGTCACGTCTTGAATGGCTTTTAA
TAAATTTTTCCCCACAATTGGATAATGTTCAGCATAAACTTGTAGTGCGCAGTGTTTATATGCGACTGGCATAATGACTGGTTTAATAACACT
TAAGTTATCGATATTAACCGCTGCGGCCATTACAGCTTGTGCTAATGCTGAAGATTGCATGCCTCGTTTTTGGTTCGTTTGATTAAACATGTT
TAAAAGTTCAGGATGCGCTTTAAACATTTTTGGATAAAAGATTGACGTAATTTCTGTCCCTTTCTCTTTAAGTAAAGGCACCGTTTGTTTGAT
AATGTCTTTCTCTTGTTCTGTAAGCAT

Gene: lctE (L-lactate dehydrogenase 1)

Position: 264264 to 265217, length: 954 nt, orientation: FORWARD

Perfect match to: (930918-3-ABFA01000001-[112317:113270], allele observed in CC8+CC1+CC8+CC97)

Sequence:

ATGAACAAATTTAAAGGGAACAAAGTTGTATTAATAGGTAATGGTGCAGTAGGTTCAAGCTACGCATTTTCATTAGTGAACCAAAGCATTG
TTGATGAATTAGTCATCATTGATTTAGACACTGAAAAAGTTCGAGGAGATGTTATGGATTTAAAACATGCCACACCATATTCTCCAACAACA
GTTCGTGTGAAAGCTGGCGAATACAGTGATTGTCATGATGCGGATCTAGTTGTCATCTGTGCTGGTGCTGCACAAAAACCTGGAGAAACAC
GTTTAGATTTAGTATCTAAAAACTTGAAAATATTCAAATCAATTGTTGGTGAAGTAATGGCATCAAAATTTGATGGTATTTTCTTGGTAGCTA
CAAATCCTGTTGATATTTTAGCGTATGCAACATGGAAATTCTCTGGTTTACCTAAAGAACGCGTTATAGGTTCTGGTACAATTTTAGACTCTG
CACGCTTTAGATTATTGTTAAGCGAAGCGTTCGATGTTGCGCCACGTAGCGTCGATGCTCAAATTATTGGTGAACATGGTGACACTGAATTA
CCAGTATGGTCACACGCTAATATTGCGGGTCAACCTTTGAAGACATTACTTGAACAACGTCCTGAGGGCAAAGCGCAAATTGAACAAATTT
TTGTTCAAACACGTGATGCAGCATATGACATTATTCAAGCTAAAGGTGCCACTTATTATGGTGTTGCAATGGGATTAGCTAGAATTACTGAA
GCGATTTTCAGAAATGAAGATGCCGTATTGACTGTATCAGCATTATTAGAAGGCGAATATGAGGAAGAAGATGTTTATATTGGTGTTCCAG
CAGTCATCAATAGAAACGGTATTCGCAACGTCGTAGAAATCCCATTAAACGACGAGGAACAAAGCAAGTTCTCACATTCAGCTAAAACATT
AAAAGATATTATGGCTGAAGCAGAAGAACTTAAATAA

Gene: ptsIIBC (putative PTS transport system, IIBC component)

Position: 265538 to 267067, length: 1530 nt, orientation: REVERSE

Perfect match to: (11819-97-CP003194-[271957:273486:r], allele observed in CC80)

Sequence:

TTAATCCCCGAGCAATTCTTCAATTTCATTTTTGATAACTGTAACGTGAGGCCCATAAATTACTTGCACACCAGTGCCTTGCTGGATTACACC
TTTGGCACCAGTACTTTCGAGTAATACTTTATCGACTTTGTCATTTTGATGAAGTGTGACGCGTAGTCTCGTTGCACAACAGTCAACGATTTC
AATGTTATCTTTGCCTCCCAAACCAGCAACAATTGTTTGTGCTCTTTCAGTAGCCTCAACTTGTTGTGCTGCAGCTTTATCTTCTCGACCAGGT
GTTTTGAAATTAAATTTCGTAATTAAGAATCTGAAAACGATGTAATACAAACAGAACCACACAATTCCAATAGGTATGACGTATAGGTAGTT
TGTTTTACTATTACCTTGTAGCACACCAAAAAGTAAGAAATCGATAAAGCCTCCACTGAAGGTTTGACCAATTGTAATGTTGAAAATGTCTG
CCATCATAAATGCTAATCCATCAAAGAAGGCATGGATTACATAAAGAATAGGTGCGACAAACAAGAAACTAAACTCTAAAGGTTCGGTAAT
ACCTGTTAAAAATGAAGTGAGTGCAGCGGATAACATTAAACCGCCGACAACTTTTTTATGTTCAGGTTTAGCTGTGTGATAAATTGCAAGTG
CGGCACCACATAAGCCGAACATCATCGTAATAAAACGGCCTGACATAAAGCGTGACACACCTGAATAATACTTCGTCACATCTGGATCACC
AAGTTGAGCAAAGAAGATGTTCTGCGTACCTTGAACTAAGTGCCCTTTGACTTCTAAAGTACCACCAAGTGCCGTCTGCCAAAACGGTAAG
TAAAAAATATGGTGTAAACCGAGTGGACCTAACAATCTTAAGATGAAGCCATAAACAAAAGTACCGATGGCACCTGTTTTCGTTACAAATC
CACCAACATGGTAAATGCCGGCTTGTATGCTTGGCCAAATGAAAAACATCAATACACCTAAAAAGATTGCGGCAAATGCTGTGACAATAGG
GACAAATCTAGAGCCACCAAAGAAACCTAAATACGGTGGTAATACCACTTTGTGATATTTGTTGTGAAGTATTGCAGTCATAATACCTGTGA
TAATCCCGCCAAAAACACCGGTTTCAACCGTTTGTATACCGAGCACCATGCCTTGTCCATTTTGTGCAAGCTGATCTTTTGCCAATGTGCCCG
TGATAGTTAATAAGCCATTCATAGTTGCGTTCATAATTAAGAAACCGAGCAGTGCAGCTAAACCTGCAGTACCTTTATCGCTTCTAGATAAT
CCGATTGCGACACCAATGGCAAAGATGACCGGTAAATTTTGGAAAACAATACTACCTGCAGCTGACATTAATGTAAAAATATTTTGTAATAA
GGTAATATCTAAAATAGGGTATGCTTTAACGGTGTTTGGATTACTTAATGCACCACCGATACCCAACAATAGACCTGCAGCTGGTAAGATTG
CGATAGGTAACATAAAGGACTTGCCGAACTGCTGTGCTTTTTCAAATAAAGATTTCAT

Gene: rihA (inosine-uridine preferring nucleoside hydrolase)

Position: 267429 to 268364, length: 936 nt, orientation: FORWARD

Perfect match to: (TCH959-AASB02000034-[89218:90153], allele observed in CC7+CC25)

Sequence:

ATGAAAAGAAAGATTATTATGGATTGTGATCCAGGACACGATGATGCAATAGCATTAATTTTAGCGGGGGCAATTGACAGTCCACTAGAG
ATATTAGCTGTAACCACAGTCGCAGGTAATCAATCAGTTGACAAGAATACGACAAACGCCTTGAACGTATTGGATATTATGGGACGCCAAG
ATATAGCAGTAGCGAAAGGTGCGGATAGGCCGTTAATTAAACCAGCTGCCTTTGCTTCTGAAATACATGGGGAATCTGGATTAGATGGTCC
GAAACTACCGTCGACACCATCACGTCAAGCAGTTGCAATGCCAGCATCAGATGTGATTATAAACAAAGTGATGACGAGTGATACACCTGTA
ACAATTGTAGCGACAGGTCCTCTTACGAATGTAGCAACGGCATTGATTCGTGAGCCAAGAATCGCTGAGCATATTGAATCTATTACTTTGAT
GGGTGGTGGTACATTTGGAAATTGGACGCCTACAGCAGAATTCAATATTTGGGTAGATGCTGAAGCAGCGAAGCGTGTTTTTGAAAGTGG
GATTACTATAAATGTGTTTGGTTTAGATGTAACACATCAAGTTTTAGCCGACGATCACGTGATTGAACGCTTTGAAAGTATCAATAATTCTGT
TGCACAATTCGTCGTAGAATTATTGCAATTCTTTAAGAAGACATACAAGACTCACTTTAATATGGACGGTGGTCCAATACATGATGCTTGTA
CAATTTTGTATTTGTTACAACCAGAATTGTTTACAATGGTACCCGTTAATATCGACATTGAACATCAAAGTCCACTAACTTATGGTACTATGG
CTGTCGATTTAAATCATGTTACAGGTAAGCCTGCCAATGCTTATTTTGCTACAGCAGTTGATGTTGAAGAAGTGTGGAACTTGATAGACCAT
AAGTTACGTACATACGAATAA

Gene: bglG (transcriptional antiterminator, PTS regulator)

Position: 268701 to 270797, length: 2097 nt, orientation: FORWARD

Perfect match to: (11819-97-CP003194-[275120:277216], allele observed in CC80)

Sequence:

ATGAACGGGGATAATCAGCAAATACTCAGAGAAATTGTATTGAATCCTACTATTCATGGTAAAGAACTTGAATCGATATTTGGTTTGTCTCG
TAGACAACTAGGATATCGCATTCAAAAAATCAATTTGTGGCTTGAACAAGAGGGTTATCCAAAACTTGAAAGAACAAGCCAAGGAAATTTT
ATTGTAAGTTCTTAAATCATGACGTTATTCAAACGAGATGTATCAGAGCAGCAAATGTTAAACGGCAACAATGTCATTTTTAGCATAGAAAC
ACGTCGTTATTATTTAATGCTCATGCTTTTTAGTAAGGAAAACGCAATGTCTCTAAACCATTTTTCAATTGATTTACAAGTCAGTAAAAATACT
GTCATTCACGATATAAATCATGTGAAAGAGCAATTGGAAAATCATGGTTTGTCAATTAAGTATTCTCGAAAACATGGTTATGAAATTGTTGG
TGATGAATTTGAAGTTCGCCGTTTCTTCATTAAGTTGATTGATCAAAGGTTGAATCATGATATTACTAAAAGTGAAGTTTTAAAGGCGCTCA
ACTTAACATTCGAAGATATCGCATATCAAAAAGACAAAATCAAACAGGTAGAACAATTTTTGAAGAGTCGCTTTATAGACAAATCACTTAGT
TCATTGCCTTATGTCCTTTGTGTGATTCGTAGACGAATTCAAAGTGGTCATGTGATGAATCCATTAAATATTAATTATCAGTATTTGAGGGAT
ACGAAAGAATATCAAGCAACGGAGATTATGACGCAACTTGAGCCGGATTTGCCAGAAGCGGAAAAGTTATATTTGACATTACACTTACTTT
CAACAAGTGTGCAATGGACTGATTTGCAGGAATCAGATAACATATCGAATTTAACGATGGCTATCGCTCAAATGATTCACCATTTTGAACAA
ATCACTTTTATTAACATTGAAGATAAGGAGAAATTATCACAGCAACTCTTGTTACATTTAACGCCTGCTTTTTATAGGATTAAATATAACTTA
ACGGATCGTGATGAATTAATAAATCCTTTACAAGGAAATTATCAATCCTTATTTCATATGGTGAAACAATCATGTCAATCGTTAACTGAATAT
TTCGGAAAATCGTTGCCTGATAATGAAATAGCATATTTAACCATGTTGTTCGGAGGTAGTTTGAGACGTCAAGATGAAAACTTCGATGGCA
AGATAAAAGCTATTATCGTGTGTACACAAGGCACGTCAGTATCACAAATGATGTTATACGAGTTGCGAAACTTATTTCCAGAAATTATTTTC
TTAGATGCGATTTCACTTAGAACATTTGAAAATTACACATTAGATTATGACATCGTCTTTTCACCAATGTTTGTCCTAACACATAAAAAATTAT
TTATCACAAAAGTAGCTTTATCTGAAAATGAGCAACGAAAGTTACGTAAAGAAGTGATGAAGTACATTAATAAGGAATCGGCTGACATTGA
TAAGGAAATAAACAAGTTAATGGCATTAATTGAACGCACTACGACAGTTAATGACATTACAGAACTACGTGATGGTTTAGAAGATTTTATT
GCGAATTATAATTCAATTTCAACCATTAATGGATCGATTGTCACACAAAATAAGACATTAGATTTAGCTGACTTGATACCGGCAAGGCACGT
GAAAAGAATGCATCATGTTGAAAATATTGTTGAAGCTATTGCTAAAGCAAGTGATGTGTTAGTTGCTAATCATTTTATTGATATTAAATATAT
TCATGAGATGCAACAGGTATTTGATGATTCGTATATGGTTATCATGCAAAATATTGCTATTCCACATGCATACTCTGAAAAGCATGTACATA
AAACAGCGATGAGTATGTTGATATTACAAGAACCAATATACATGTCAGATGGCACAGCAATCCATATTATTGTACCTATTGCTGCTGTTGAT
AAAGTGACACACTTAAGAGCGTTACTACAATTGAGAGATGTGGCGCAAGACAATGACGCAATTAAGCGCATCATACAAAGTCGCAAAAAT
TCTGATGTAAATGAGATTTTAAAAAATTATTCAAATAAAGAAGCGAGGGAAAATGGATGGGACAGCAATTAG

Gene: Q5HJD2 (phosphotransferase system sugar-specific component IIA)

Position: 270782 to 271249, length: 468 nt, orientation: FORWARD

Perfect match to: (11819-97-CP003194-[277201:277668], allele observed in CC80+CC9+CC80)

Sequence:

ATGGGACAGCAATTAGTGCATAAAGAAAATATAATGCTCAATTTGTCGGCAACTGATAAAGAATCCGTATTGTCACAAATGTCAGAAGTGT
TATTTCAAAATGGGTTCGTGAAGTCAACGTTTAAAGATGCAGTCATCGACAGAGAAAAAGAATTTGCTACTGGTTTACCAACGCATCTATGT
TCGGTCGCTATACCGCATACAGATGTCGAACATATTAACCATAGAACGATAGGTGTGGCTGTTCTAGAAAAAGAAGTGCCGTTTATTGAAA
TGGGAACACTTGATCAACAGACAGAAGTGAAAATCGTTTTTATGTTAGCAATGGATAAAGTAGATGATCAACTTAAGTTATTACAACAGTT
GATGCAAATTTTTCAAAGTGAAGAAAAATTGGAGCAGATTCTGCGAACGAAAGATGAAACAATTTTAGCAACACTAATCAATGATTATTTG
GAATATAACTAA

Gene: Q5HJD1 (phosphotransferase system sugar-specific component IIB)

Position: 271272 to 271550, length: 279 nt, orientation: FORWARD

Perfect match to: (11819-97-CP003194-[277691:277969], highly conserved allele)

Sequence:

ATGAAACAAGTATTAGTAGCGTGTGGTGCAGGTATTGCAACGTCAACAGTAGTAAATAATGCAATTGAGGAAATGGCAAAGGAACACAAT
ATTAAAGTAGATATTAAACAAATCAAAATTACAGAAGTTGGACCTTATGAAGACACTGCAGATTTATTAGTTACAACTGCAATGACAAAAAA
AGAATATAAATTCCCAGTTATCAACGCACGTAATTTCTTAACTGGTATTGGTATTGAAGAAACAAAACAACAAATCTTAACAGAGTTACAAA
AATAA

Gene: gatC1-Q2G2C8 (PTS system transmembrane permease)

Position: 271777 to 273036, length: 1260 nt, orientation: FORWARD

Perfect match to: (11819-97-CP003194-[278196:279455], allele observed in CC80)

Sequence:

ATGAGTTACTTCACTGATTTTGTAAGGGGATTTTTAGATTTAGGTGCAACTGTTATTTTACCGGTTGTCATATTCTTGCTTGGCCTATTCTTTA
GGCAGAAAATTGGAGCGGCATTTAGATCTGGTTTAACAATAGGTGTGGCTTTTGTAGGGATTTTCTTAGTCATCGATTTATTAGTTAAAAAT
TTAGGGCCAGCAGCACAAGCGATGGTTAAAAATTTAGGCGTCAGTCTGAATGTGATTGATGTAGGTTGGCCAGCAACATCATCTATCGCTT
GGGCATCATCTGTCGCAGCATTTATTATTCCACTCGGAATCATAGTTAACGTTGTATTGCTAGTAACTAAAGTGACAAAGACGATGAATGTA
GATATTTGGAATTTTTGGCATTATACGTTTACAGCAGCAATGGTTTATGCCGTATCAGGCAGTATTTGGCAAGCGTTATTAGCAGCAGTTAT
TTTCCAAGTTATCTGTTTGAAAGTAGCAGATTGGACAGCACCGATGATGAGTGAGTTCTTTGATTTACCAGGTGTATCGATTGCGACAGGA
AGCACAATTTCTTATGCACCAGGTATTTACTTAGTTAAATTGTTACAAAAAGTACCCGGTCTGAATAAGTTAGATGCTGATCCTGAAACAATT
CAAAAACGTTTTGGCGCATTTGGAGAGTCTATCTTTGTCGGCTTAATTTTAGGTTTAGGTATTGGTGTGTTAGCAGGTTACAAACCTGGAGA
CATCATTAATTTAGGAATGTCAATGGCTGCAGTAATGGTATTAATGCCTAGAATGGTAAAAATCTTAATGGAAGGTTTAATGCCAGTTTCAG
AGTCTGCAAGAACATGGCTAAATAAACGTTTTGGCGAACGTGAAATTTATATTGGATTGGATGCGGCTGTAGCATTAGGTCATCCAGCGGT
TATTTCGACAGCATTAATTTTAGTACCTATCACTGTTTTATTAGCTGTTATTTTACCAGGAAACCAAGTACTACCTTTTGGTGACTTAGCAACG
ATACCATTTGTTGTCGCGTTTATTGTTGGTGCAGCAAGAGGAAACATTATTCATTCTGTCATTGTGGGTACGATTATGATTGCAATTTCACTA
TATATTGCAACAGACGTAGCACCTATTTTCACAGATATGGCGAAAGGTACGAATGTACAAATGCCAAAAGGTTCATCTGAAATTTCAAGTAT
TGATCAAGGTGGTAATATCGTTAACTATCTTATCTTTAAACTATTTAGTCTATTCAATTAA

Gene: gutB (L-iditol 2-dehydrogenase)

Position: 273054 to 274109, length: 1056 nt, orientation: FORWARD

Perfect match to: (11819-97-CP003194-[279473:280528], allele observed in CC80)

Sequence:

GTGAAAGCTTTAGTAAAAACAAGAGAAGGACATGGCAACTTAGAACTTCTTGATAAAGAAGTTGCAACACCGCTAGATGATAAAGTAAAG
ATTAAAGTTCATTATGCAGGAATTTGTGGCACAGATATTCATACTTATGAAGGTCATTATAAAGTTAATTTTCCAGTGACATTAGGTCATGAA
TTTTCTGGTGAAATCGTTGAAGTTGGAGCAGACGTTAAAGATTTTAAAGTTGGTGACCGTGTCACTTCTGAAACGACATTCTATGTTTGTAA
TGAGTGTGAATACTGTGAATCAAAAGACTATAATTTATGCAACCATCGAAAAGGTATTGGAACACAAGTTGATGGCGCATTTACTAATTAT
GTCATTGCACGTGAAGAAAGTTTGCATCATATTCCAGATGAAGTATCGTATCAGTCTGCAGCTATGACAGAACCATTAGCATGTGCACATCA
TGGCGTTTCTAAGATTCAAGTGAACTCTGGCGATGTAGCAGTTGTAATGGGACCTGGGCCAATCGGATTACTTGTAGCACAAGTGTTAAAA
AGTAAAGGCGCAACTGTTGTGGTAACTGGATTGGACAATGACAAAGTCAGATTAGATAAAGCAGAAGCATTGCACATGGATTATGTAGTC
AATTTACAACAAACAGACTTAAAAACGTATATCAATGGAATTACAGACGGTTACGGTGCAGATGTTGTTGTTGAATGTTCAGGTGCAGTTCC
AGCAGCACGACAAGGTTTGGATATTTTACGCAAAAAAGGTTACTACAGTCAAATAGGTATTTTTAAGGATGCTGAAATTCCATTTGATATGG
AAAAAGTGATTCAAAAAGAAATAACAGTTGTTGGTAGTAGAAGTCAAAAGCCAGCAGATTGGGAACCTTCATTGCAACTTATGGCGGATG
GTTTAGTAAATGCTGAAGCTTTGGTGACAAAAATATATGATATTTCGAAATGGGACGAGGCGTATCAACATTTAAAATCTGGCGAAGGTAT
TAAAGCATTACTTAAGCCGCTCGATTTAGATGAAAATGAAGGAGAGAATTAA

Gene: Q5HJC7 (putative protein)

Position: 274111 to 274257, length: 147 nt, orientation: FORWARD

Perfect match to: (MW2-BA000033-[269164:269310], highly conserved allele)

Sequence:

ATGGTAGAATCAATGCTAACTTTTATGCTTGGGCCATTAAGACAAATCACTGATTTTTATATGGAACATTTACTCGTAAGTAATTCCATTGTC
ATTGCAGGTTATTTTGCGACAGGTATTTTTAAAAAGAAAAAAGTTGTGAATTAA

Gene: Q5HJC6 (putative sugar-phosphate dehydrogenase)

Position: 274281 to 275324, length: 1044 nt, orientation: FORWARD

Perfect match to: (LGA251-FR821779-[264439:265482], allele observed in CC425+CC80)

Sequence:

GTGAAAGCATTGAAATTATATGGCGTGGAAGATTTACGGTATGAGGATAATGAAAAGCCAGTCATTGAAAGTGTGAATGACGTTATTGTTA
AAGTACGAGCGACTGGCATATGTGGTTCAGACACGTCACGATACAAAAAAATGGGGCCATACATTAAAGGTATGCCATTTGGTCATGAATT
TTCAGGTGTTGTAGATGCCATTGGAAGTGATGTTACGCATGTTAATGTAGGCGACAAAGTGACAGGTTGCCCAGCAATACCTTGTTATCAA
TGCGAGTATTGTTTAAAAGATGAATATGCACGATGTGAAAAGTTGTTCGTCATTGGCTCATATGAACCTGGATCGTTCGCGGAATATGTCA
AATTGCCAGCGCAAAATGTTTTAAAGGTACCAGACAATGTTGATTACATTGAAGCAGCAATGGTTGAGCCATCAGCCGTTGTTGCGCATGG
GTTTTATAAATCGAATATACAACCTGGTATGACTGTTGCAGTAATGGGGTGTGGCAGTATAGGATTGTTAGCTATTCAATGGGCACGAATA
TTTGGTGCTGCGCATATCATCGCTATAGATATAGATGCGCATAAACTAGATATTGCAACATCATTGGGCGCACATCAAACAATTAATTCAAA
AGAAGAAAATCTTGAGAAATTCATCGAAAATCATTACGCCAATCAAATCGATTTAGCTATAGAATCATCAGGTGCTAAAGTTACGATTGGTC
AAATATTGACGCTACCGAAAAAAGGTGGCGAGGTGGTATTACTCGGAATACCATATGATGATATTGAGATTGATCGCGTTCATTTTGAAAA
AATTCTGCGTAACGAGTTGACAATATGTGGCTCTTGGAACTGTTTGTCCAGTAATTTTCCGGGCAAAGAGTGGACGGCAACCTTACATTATA
TGAAGACGAAAGATATTAATGTAAAGCCTATTATTTCTCATTTTTTACCGTTAGAAAAAGGCCCGGAGACATTTGATAAATTAGTTAACAAG
AAAGAACGATTTGATAAAGTCATGTTTACGATTTATTAG

Gene: tarI1 (2-C-methyl-D-erythritol 4-phosphate cytidylyltransferase 1)

Position: 275852 to 276568, length: 717 nt, orientation: FORWARD

Perfect match to: (11819-97-CP003194-[282271:282987], allele observed in CC80+CC96)

Sequence:

ATGATTTATGCAGGTATTTTAGCAGGAGGTATTGGTTCGAGAATGGGGAACGTGCCATTACCAAAACAATTTTTAGATATTGATAATAAACC
GATTTTAATTCATACAATTGAGAAGTTCATTTTAGTGAGTGAATTTAATGAGATTATTATCGCAACACCAGCACAGTGGATTTCCCATACACA
GGATATTTTAAAAAAATATAACATTACAGATCAACGTGTCAAAGTAGTTGCAGGTGGTACGGATCGAAACGAAACAATTATGAACATTATC
GACCATATTCGCAATGTAAATGGAATTAATAATGATGATGTGATTGTAACTCATGATGCCGTAAGACCATTTTTAACTCAACGTATTATTAAA
GAGAACATTGAAGTAGCGGCAAAATATGGTGCAGTAGATACAGTCATTGAAGCAATTGATACGATTGTAATGTCTAAAGATAAACAGAAC
ATACACAGTATCCCTGTAAGGAATGAAATGTATCAAGGCCAAACACCACAATCATTTAATATTAAATTATTACAAGATAGTTATCGCGCCTT
AAGTAGTGAACAAAAAGAAATCTTATCAGATGCATGTAAAATCATTGTCGAATCTGGACATGCAGTTAAATTGGTACGTGGAGAACTATAC
AACATTAAAGTGACAACACCGTATGATTTAAAAGTAGCAAATGCCATTATTCAAGGTGATATTGCCGATGATTAA

Gene: tarJ1 (CDP-ribitol synthase)

Position: 276561 to 277586, length: 1026 nt, orientation: FORWARD

Perfect match to: (Mu3-AP009324-[293586:294611], allele observed in CC5)

Sequence:

ATGATTAATCAAGTATATCAACTCGTTGCACCGAGACAGTTCGACGTCACATATAATAATGTTGATATTTATGGTAATCATGTCATCGTAAG
ACCTTTATACTTGTCTATTTGTGCAGCTGATCAAAGGTATTACACAGGTCGAAGAGATGAAAATGTACTGCGCAAAAAATTGCCAATGTCAT
TAGTTCATGAAGCTGTTGGTGAAGTTGTATTCGATAGTAAAGGAGTATTTGAAAAAGGTACGAAAGTAGTAATGGTGCCGAATACACCTAC
AGAGCAACATCATATTATCGCGGAGAATTACTTAGCCTCTAGTTATTTTAGATCTAGTGGTTATGATGGTTTTATGCAAGACTACGTTGTGAT
GGCACATGATCGTATCGTTCCGCTGCCTAATGACATTGATTTGAGTACGATTTCATACACAGAGTTAGTGTCAGTAAGTTATCATGCTATAC
AACGATTTGAACGTAAATCTATACCTTTGAAAAACAGCTTTGGTATTTGGGGTGATGGTAACTTAGGTTATATTACTGCTATTTTGCTACGTA
AGTTGTACCCAGAAGCTAAAATTTATGTATTTGGTAAGACAGACTATAAATTAAGTCATTTTTCATTTGTAGATGACATCTTTACAGTAAATC
AAATACCAGATGATCTTAAAATTGATCATGCATTTGAATGTGTTGGAGGTAAAGGAAGTCAAGTTGCACTTCAACAAATAGTTGAACATATT
TCACCAGAAGGCAGTATTGCTTTGTTAGGCGTAAGTGAATTACCCGTGGAAGTGAATACACGATTAGTACTTGAAAAAGGATTAACGTTGA
TTGGTAGTAGTCGAAGCGGCTCTAAAGATTTTGAGCAAGTTGTTGATTTATATCGTAAGTACCCAGACATTGTTGAAAAGTTAGCATTATTA
AAAGGACATGAAATTAATGTATGTACGATGCAAGATATCGTCCAAGCGTTTGAAATGGATTTATCGACATCTTGGGGAAAAACAGTATTGA
AATGGACGATTTAA

Gene: tarL1 (glycosyl/glycerophosphate transferase)

Position: 277608 to 279302, length: 1695 nt, orientation: FORWARD

Perfect match to: (NN50-BAEA01000012-[124147:125841:r], allele observed in CC4803+CC49)

Sequence:

ATGACAAAAACGAAACAAGCAATACATATTGATAACATATACTGGGAACGTGTTCAGTTATATATTGAAGGACATAGTGAAGGTGTCGATT
TAACATCAGGACAATTTGTTCTGAGGAATTTAACCGAAACAAAAACATTAGAAGCAAATGAAATGAAAATAGACGGTAATACATTTATATG
TAGATTCAACGTCGCAATATTAGACGATGGGTATTATTTACCAATGGATAAATATTTATTTGTTTATCATGACCAGTTAGAGTATATTGGACA
ACTTAATCCAAATATTATTAATCAAGCTTATGCGGCATTAAATGAAGAGCAAATTGAAGAATACAATGAGCTGACTACACAAAATGGAAAA
GCAAACTATGTGTTAGCCAACGAAGCTAAAGTTTTCCGCAAAGGTGGCGTATCACAACATACGGTCTATACCATTACTCCGGAAATAGCAA
GTGACGTTAACGAATTTGTATTTGATATTGAAATCACCTTACCTCAAGAGAAATCAGGGGTCATTGCGACAAGTGCACACTGGCTTCATAAA
CAAGGTCATAAAGCTTCATTTGAAAGTAGAAGTTTCTTATTTAAAGCTATTTTTAATATTACAAAGTTACTACATATTAAAAGAAGCAAAACA
ATATTATTCACATCAGATTCGCGTCCGAATTTATCAGGGAATTTCAAGTATGTATATGATGAGTTACTACGCCAAAAAGTAGATTTTGATTAT
GATATTAAAACGGTATTTAAGGCGAATATTACGGATAGACGTAAATGGAGAGACAAGTTTAGATTGCCATATTTACTTGGTAAGGCAGATT
ATATTTTTGTTGATGATTTCCATCCATTAATTTATACGGTTCGCTTTAGACCATCACAAGAAATTATTCAAGTGTGGCATGCCGTTGGTGCTTT
TAAAACAGTTGGCTTTAGTCGTACAGGTAAAAAAGGTGGTCCGTTTATCGATTCATTAAACCATCGTAGTTACACGAAAGCATATGTTTCAT
CAGAAACCGATATTCCATTTTATGCTGAAGCATTTGGAATTAGAGAAGAAAATGTTGTACCAACAGGTGTACCACGTACTGATGTACTATTT
GATGAAGCTTATGCAACACAAATTAAACAAGAGATGGAAGATGAATTGCCAATTATAAAAGGTAAGAAAGTTATTCTATTCGCACCGACAT
TTAGAGGTAATGGTCACGGTACGGCACATTATCCATTTTTTAAAATTGATTTTGAACGTTTAGCAAGATACTGCGAGAAGCATAATGCAGTT
GTGTTATTCAAAATGCATCCGTTCGTAAAAAATAGACTTAATATTTCACGTGAACATAGACAATACTTTATCGATGTGTCAGATCATCGTGAA
GTTAACGATATTCTCTTTGTTACAGACTTGTTGATTAGTGATTATTCATCTTTAATATATGAATATGCAGTATTTAAAAAGCCAATGATTTTCT
ATGCATTTGACTTAGAAGATTATATTACGACGCGTGATTTCTATGAACCATATGAATCATTTGTTCCAGGTAAAATTGTACAATCCTTTGATG
CATTAATGGATGCTTTGGACAATGAAGATTATGAGGTTGAAAAAGTTGTGCCATTTTTAGATAAACATTTTAAATATCAAGATGGTCGCTCA
AGTGAACGTTTAGTCAAAGATTTGTTTAGACGCTAA

Gene: tx_universal2 (rho-independent terminator)

Position: 279909 to 279947, length: 39 nt

Perfect match to: (Strain_21331-AGTV01000040-[104403:104441], allele observed in CC398)

Sequence:

TAGAATTGAAAAAAGCTTGTTACAAGCGCATTTTCGTTC

Gene: tarF (CDP-glycerol:poly (Glycerophosphate) glycerophosphotransferase)

Position: 280270 to 281439, length: 1170 nt, orientation: FORWARD

Perfect match to: (11819-97-CP003194-[286689:287858], allele observed in CC80)

Sequence:

ATGATTAAAACTACAATTAAAAAATTGATAGAACACAGTATATATATGACATTTAAATTACTATCAAAATTGCCAAACAAGAATCTAATTTAT
TTTGAAAGCTTTCATGGTAAACAATACAGCGACAACCCCAAAGCATTATATGAATACTTAACTGAACATAGCGATGCCCAATTAATATGGGG
TGTGAAAAAAGGATATGAACACATATTCCAACAGCACAATGTACCATATGTTACAAAGTTTTCAATGAAATGGTTTTTAGCGATGCCAAGA
GCGAAAGCGTGGATGATTAACACACGTACACCAGATTGGTTATATAAATCACCGCGAACGACGTACTTACAAACATGGCATGGCACGCCAT
TGAAAAAGATTGGTTTGGATATTAGTAACGTTAAAATGCTAGGAACAAATACTCAAAATTACCAAGATGGTTTTAAAAAAGAAAGCCAACG
TTGGGATTATCTAGTGTCACCTAATCCATATTCGACATCGATATTTCAACATGCATTTCATGTTAGTCGAGATAAGATTTTGGAAACAGGTTA
TCCAAGAAATGATAAATTATCACATAAACGCAATGATACTGAATATATTAACGGTATTAAGACAAGATTAAATATTCCATTAGATAAAAAAG
TGATTATGTACGCGCCAACTTGGCGTGACGATGAAGCGATTCGAGAAGGTTCATATCAATTTAATGTTAACTTTGATATAGAAGCTTTGCGT
CAAGCGCTGGATGATGATTATGTTATTTTATTACGCATGCATTATTTAGTTGTGACACGTATTGATGAACATGATGATTTTGTGAAAGACGTT
TCAGATTATGAAGACATTTCGGATTTATACTTAATCAGCGATGCGTTAGTTACCGACTACTCATCTGTCATGTTCGACTTCGGTGTATTAAAG
CGTCCGCAAATTTTCTATGCGTATGACTTAGATAAATATGGCGATGAGCTTAGAGGTTTTTACATGGATTATAAAAAAGAGTTGCCAGGACC
AATTGTTGAAAATCAAACAGCACTCATTGATGCATTAAAACAAATCGATGAGACTGCAAATGAGTATACTGAAGCACGAACGGTATTTTAT
CAAAAATTCTGTTCATTAGAAGATGGACACGCGTCACAACGAATTTGCCAAACGATTTTTAAGTGA

Gene: tarI2 (2-C-methyl-D-erythritol 4-phosphate cytidylyltransferase 2)

Position: 281715 to 282431, length: 717 nt, orientation: FORWARD

Perfect match to: (ST1464-ANIT01000018-[5331:6047], allele observed in CC1464+CC8+CC97)

Sequence:

ATGAAATACGCTGGTATTCTAGCTGGAGGTATAGGCTCAAGAATGGGTAACGTACCTTTACCTAAACAATTTTTAGATTTAGACAACAAACC
GATTTTAATCCATACATTAGAAAAATTTATTTTAATTAATGATTTTGAAAAAATTATTATCGCGACGCCACAACAATGGATGACGCATACGAA
AGATACACTTAGAAAATTCAAAATTTCTGATGAAAGAATTGAAGTCATTCAAGGTGGTAGCGATCGTAACGATACAATTATGAATATCGTTA
AACATATTGAATCAACAAATGGTATTAACGATGACGATGTTATTGTGACACATGATGCAGTTAGACCATTTTTAACACATCGTATTATTAAA
GAAAATATTCAAGCTGCTTTAGAGTACGGTGCAGTAGACACAGTGATTGATGCTATAGATACGATTGTTACATCTAAAGATAATCAAACGA
TTGATGCAATTCCAGTGCGTAATGAAATGTACCAAGGTCAAACACCTCAATCGTTTAATATTAATTTATTAAAAGAAAGCTATGCACAGTTG
AGTGATGAGCAAAAGAGTATTTTATCTGATGCTTGTAAGATTATTGTAGAAACAAACAAACCGGTTCGACTTGTAAAAGGTGAGTTATATA
ACATTAAAGTAACAACACCTTACGATTTAAAAGTAGCGAATGCTATTATTCGAGGTGGTATTGCCGATGATTAA

Gene: tarJ2 (CDP-ribitol synthase)

Position: 282424 to 283449, length: 1026 nt, orientation: FORWARD

Sequence:

ATGATTAATCAAGTATATCAATTAGTTGCACCTAGACAATTTGAAGTTACGTATAACAACGTAGATATTTACAGTGACTATGTCATTGTACGT
CCTTTATATATGTCAATTTGTGCTGCCGATCAAAGATATTATACTGGTAGCCGTGATGAGAATGTCTTATCTCAGAAATTGCCAATGTCTTTA
ATTCATGAAGGTGTTGGTGAGGTCGTATTTGACAGTAAAGGTGTGTTTAATAAAGGTACAAAAGTAGTTATGGTACCGAATACGCCGACAG
AAAAAGACGATGTCATTGCTGAAAACTATTTAAAATCGAGCTACTTCAGATCAAGTGGACATGATGGGTTTATGCAAGATTTTGTGTTGCTA
AATCATGATAGAGCTGTACCACTACCTGATGATATTGATTTAAGTATTATTTCATATACAGAGCTTGTAACAGTAAGTTTGCATGCTATTCGT
CGTTTTGAAAAGAAATCTATTTCAAATAAAAATACATTTGGTATTTGGGGTGATGGTAACTTAGGTTACATTACAGCCATTTTATTACGTAAA
TTATATCCAGAGTCTAAAATATATGTTTTTGGTAAAACAGATTATAAATTGAGTCACTTCTCATTTGTTGATGATGTCTTCTTTATTAATAAAA
TACCTGAAGGCTTAACATTTGATCATGCATTTGAGTGTGTGGGTGGTCGCGGTAGTCAATCAGCCATAAATCAAATGATCGATTGCATTTCA
CCAGAAGGAAGCATTGCACTGTTAGGTGTAAGTGAGTTCCCAGTAGAAGTTAATACACGTCTAGTATTGGAAAAAGGACTAACGTTGATTG
GTAGTAGTCGAAGTGGTTCAAAAGATTTCCAAGATGTTGTAGACTTATACATTCAATACCCAGATATTGTAGATAAATTAGCGCTGTTAAAA
GGTCAAGAATTTGAAATTGCAACAATTAATGATCTTACAGAAGCTTTTGAAGCAGACCTGTCTACATCTTGGGGTAAAACAGTATTAAAATG
GATTATGTAA

Gene: tarL2 (glycosyl/glycerophosphate transferase)

Position: 283471 to 285159, length: 1689 nt, orientation: FORWARD

Perfect match to: (PSP1996-ANHU01000033-[41265:42953:r], allele observed in CC8)

Sequence:

TTGGTTAAAAGTAAGATATATATAGATAAAATCTATTGGGAACGTGTTCAGTTATTCGTTGAAGGACATAGTGAAAACCTAGATTTAGAAG
ATAGTAATTTTGTATTAAGAAATTTAACTGAGACACGTACAATGAAGGCGAATGATGTCAAAATAGATGGGAATCAATTCGTTTGTCGTTTC
AATGTAGCTATCTTAGATAATGGTTATTACTTACCTGAAGATAAGTACTTATTAGTGAATGAGCAAGAACTTGATTATATTGCACAGTTAAA
CCCAGATGTGATTAATGATGCATATCAAAATCTAAAGCCAGAACAAGAAGAAGAATACAACGAATTAGAAACACAAAATGGTAAAATCAA
TTTCTTATTGCAGACTTACCTAAAAGAGTTTAGAAAAGGTGGCATTTCGAAGAAAACGGTTTATACTGTTACACCTGAAATTTCTAGCGATG
TTAATGAATTTGTCCTTGATGTTGTTGTAACGACTCCGGAAGTTAAAAGTATTTATATCGTTCGTAAATATAAAGAATTACGTAAGTATTTTC
GCAAACAATCATTTAATACAAGACAATTTATTTTTAAAGCGATATTTAATACGACGAAATTTTTCCACTTGAAAAAAGGAAATACGGTGTTG
TTCACATCAGACTCTAGACCAACGATGTCTGGAAACTTTGAATACATCTATAACGAAATGTTACGTCAAAATTTAGATAAAAAGTATGATAT
TCACACTGTTTTTAAAGCGAATATTACAGATAGACGTGGCATCATCGACAAGTTTAGATTGCCATATTTACTTGGGAAGGCAGACTACATTT
TTGTTGATGACTTTCACCCATTGATTTATACAGTGCGTTTTAGACGTTCTCAAGAAGTTATTCAAGTATGGCATGCCGTTGGTGCCTTTAAAA
CAGTTGGCTTTAGTCGTACTGGTAAAAAGGGTGGACCATTTATTGATTCATTAAATCATCGTAGCTATACAAAAGCTTATGTATCATCTGAA
ACCGATATTCCATTCTACGCTGAAGCATTTGGTATTAAAGAGAAAAATGTAGTGCCTACAGGTGTTCCACGTACTGATGTACTATTTGATGA
AGATTATGCGACACAGATCAAACAAGAGATGGAAGATGAATTACCAATTATTAAAGGTAAGAAAGTCATTCTTTTCGCACCAACATTTAGA
GGTAGTGGTCATGGTACAGCACATTACCCATTTTTCAAAATTGATTTCGAACGTTTAGCAAGATATTGCGAAAAAAATAACGCGGTTGTATT
ATTTAAAATGCATCCATTTGTGAAAAATAGACTTAATATTGCAGACAAACATAAACAATATTTTGTTGACGTTTCTGACTTTAGAGAAGTTAA
TGATATACTGTTCATAACAGATTTATTAATTAGTGACTATTCATCTTTAATATATGAATATGCAGTATTTAAAAAGCCAATGATTTTCTATGCA
TTTGATTTAGAAGATTATATTACGACGCGTGATTTTTATGAACCATATGAATCATTTGTTCCAGGTAAAATTGTGCAATCATTTGACGCATTA
ATGGACGCCTTGGACAATGAAGATTATGAAGGAGAAAAAGTCATTCCATTCTTAGATAAACATTTTAAATATCAAGATGGCCGATCAAGTG
AGCGTTTAGTCAGAAATTTATTTGGTAGCTAA

Gene: tarS (beta-GlcNAc appending glycosyltransferase)

Position: 285192 to 286917, length: 1726 nt, orientation: FORWARD

Sequence:

ATGATGAAATTTTCAGTAATAGTTCCAACATACAATTCAGAAAAGTATATAACAGAATTACTTAATAGCCTTGCGAAACAAGATTTTCCGAA
AACTGAATTTGAAGTGGTTGTAGTTGATGACTGTTCAACAGATCAAACGTTACAAATAGTTGAAAAGTATCGTAATAAATTGAACTTGAAA
GTAAGTCAACTCGAAACAAATTCTGGTGGTCCAGGTAAACCTAGAAATGTGGCTTTAAAACAAGCAGAAGGTGAATTTGTATTATTTGTGG
ACTCCGATGACTATATAAACAAAGAGACTTTAAAGGATGCAGCAGCATTTATTGATGAACATCACTCAGATGTCTTGTTGATTAAAATGAAA
GGTGTTAATGTTCGTGGTGTACCACAATCTATGTTTAAAGAAACAGCACCTGAAGTTACTTTGTTAAATTCAAGAATTATCTATACTTTAAGC
CCAACTAAAATCTATAGAACAGCATTACTAAAAGATAATGACATTTATTTTCCAGAAGAATTAAAGAGTGCAGAAGATCAATTATTTACAAT
GAAAGCATATTTGAATGCAAATCGAATCAGTGTGTTAAGTGATAAAGCGTATTATTATGCTACAAAGCGTGAAGGTGAACATATGAGTAGT
GCGTATGTTTCACCTGAAGACTTTTACGAAGTCATGAGATTGATTGCTGTAGAAATATTAAATGCAGATTTAGAAGAAGCCCATAAAGATCA
AATCTTAGCAGAATTTTTAAATCGTCATTTTAGTTTTTCTCGTACGAATGGCTTCTCACTTAAAGTTAAACTAGAAGATCAACCGCAATGGAT
TAATGCTCTAGGAGACTTTATACAAGCAGTTCCAGAACGTGTAGATGCATTGGTGATGAGTAAATTACGACCATTGTTGCACTACGCGAGA
GCGAAAGATATAGACAACTATAGAACTGTAGAAGAAAGTTACCGTCAAGGTCAATACTACCGTTTTGATATTGTAGATGGTAAATTAAACA
TTCAATTCAATGAAGGCGAACCATACTTTGAAGGCATTGATATCGCTAAGCCAAAAGTGAAAATGACAGCATTTAAATTTGATAATCATAAA
ATTGTTACAGAGCTAACGTTAAATGAATTTATGATTGGCGAAGGACATTATGATGTCAGACTTAAATTACATTCACGAAACAAGAAGCACA
CAATGTATGTACCTTTAAGTGTCAATGCGAATAAACAATATCGTTTTAACATTATGTTAGAAGATATTAAAGCGTATTTACCTAAAGAAAAA
ATTTGGGATGTTTTCTTAGAAGTCCAAATAGGTACGGAAGTATTTGAAGTGCGTGTTGGTAATCAACGTAATAAATATGCATATACTGCAGA
AACAAGTGCATTAATTCATTTGAATAATGATTTTTATAGATTAACACCGTATTTCACAAAAGACTTTAATAACATTTCGTTATACTTTACAGCT
ATTACATTAACGGATTCAATCTCAATGAAGTTAAAAGGTAAGAACAAAATCATTTTAACTGGTCTGGATCGTGGTTATGTATTTGAAGAAGG
TATGGCTAGTGTCGTGCTAAAAGACGACATGATTATGGGGATGTTAAGCCAAACGTCAGAAAACGAAGTGGAAATCTTACTTAGTAAAGA
TATTAAGAAGCGAGACTTCAAAAATATTGTTAAGTTAAACACTGTACATATGACTTACTCGCTAAAATAAATAA

Gene: scdA (iron-sulfur cluster repair protein)

Position: 287057 to 287731, length: 675 nt, orientation: FORWARD

Perfect match to: (TCH959-AASB02000035-[7243:7917], allele observed in CC7+CC25+CC80+CC88+CC2970)

Sequence:

ATGATAAATAAAAATGACATAGTAGCAGATATAGTAACAGATTATCCGAAAGCAGCAGATATTTTTAGAAGCGTAGGTATAGACTTTTGTT
GCGGTGGACAAGTAAGTATAGAAGCAGCATCCTTAGAAAAGAAAAATGTAGATTTGAACGAATTATTACAGCGTCTCAATGACGTTGAAC
AAACGAATACACCAGGTTCGCTTAACCCTAAATTTTTAAATGTTTCGTCACTTATTCAATATATTCAAGCAGCATATCATGAACCTCTTAGAG
AAGAATTTAAAAATTTAACACCTTATGTGACGAAATTATCGAAAGTACATGGACCTAACCATCCATATTTAGTCGAGTTAAAAGAAACATAT
GATACATTTAAAAATGGCATGTTAGAGCATATGCAAAAAGAAGATGATGTTGATTTTCCAAAACTAATTAAATATGAACAAGGTGAAGTAG
TAAACGATATTAATACAGTGATTGATGATTTAGTATCTGATCACATTGCAACGGGACAATTGTTAGTGAAAATGAGCGATTTAACATCTAGC
TATGAACCACCGATAGAGGCATGTGGTACGTGGCGACTCGTTTATCAGAGATTAAAAGCACTTGAAGTGTTAACACATGAGCATGTTCATT
TAGAGAATCATGTTTTATTTAAAAAAGTATCATAA

Gene: lytS (two component sensor/regulator, sensor histidine kinase)

Position: 287976 to 289730, length: 1755 nt, orientation: FORWARD

Perfect match to: (ED133-CP001996-[248297:250051], allele observed in CC133+CC8+CC97)

Sequence:

GTGCTATCGCTAACAATGTTATTACTTGAGCGTGTAGGTTTAATTATTATTTTGGCCTATGTGTTGATGAATATTCCATATTTTAAAAACTTAA
TGAATCGTCGACGTACATGGAAAGCACGTTGGCAATTATGTATTATTTTCAGTTTGTTTGCCTTAATGTCTAATTTAACTGGTATCGTCATCG
ATCATCAACATAGTTTGTCAGGAAGTGTGTACTTCCGTTTAGATGATGATGTATCTTTAGCTAACACACGTGTATTAACGATAGGTGTCGCA
GGATTAGTTGGTGGCCCTTTTGTAGGTCTATTTGTTGGCGTTATTTCAGGTATTTTCAGAGTGTATATGGGTGGGGCGGATGCACAAGTTTA
TCTTATCTCATCTATATTTATCGGTATAATTGCTGGTTATTTTGGCTTACAAGCTCAAAGACGCAAGCGTTACCCGAGTATTGCGAAAAGTGC
CATGATTGGAATTGTTATGGAAATGATTCAAATGTTGAGCATTTTAACATTTTCCCACGACAAAGCATATGCGGTTGACCTCATATCATTAAT
TGCACTACCAATGATTATTGTTAATAGCGTCGGTACGGCGATTTTTATGTCTATTATCATTTCAACATTAAAGCAAGAAGAGCAAATGAAGG
CGGTTCAAACACACGACGTACTGCAATTGATGAACCAGACATTGCCGTATTTTAAAGAAGGATTGAATAGAGAATCGGCACAGCAAATTGC
GATGATTATTAAAAATTTAATGAAAGTATCTGCCGTAGCAATTACAAGCAAAAATGAAATCTTATCGCATGTAGGTGCAGGTAGTGATCATC
ACATACCAACAAATGAAATATTAACAAGTCTGTCTAAAGATGTATTGAAATCAGGGAAGTTGAAAGAAGTTCATACTAAAGAAGAGATTGG
TTGTAGTCATCCGAATTGCCCGCTTAGAGCAGCTATCGTGATACCACTTGAGATGCATGGTTCTATCGTCGGTACATTGAAGATGTATTTTA

CAAACCCTAATGATTTAACTTTTGTGGAACGTCAACTTGCAGAAGGATTGGCAAATATTTTTAGTAGCCAAATTGAACTTGGTGAAGCCGAA
ACGCAAAGTAAGTTATTGAAAGATGCTGAGATTAAGTCATTACAGGCACAAGTGAGTCCACATTTTTTCTTCAATTCAATTAACACGATCTC
AGCTTTAGTTAGAATAAATAGCGAAAAGGCACGAGAGTTACTATTAGAATTGAGTTATTTTTTCAGAGCGAATTTACAAGGCTCTAAGCAA
CATACGATTACTTTAGATAAAGAGTTAAGTCAAGTGCGTGCATACTTATCACTCGAACAAGCACGTTATCCAGGAAGATTTAATATCAATAT
TAATGTTGAAGACAAATATCGCGATGTGCTTGTACCACCATTTTTAATTCAAATTTTAGTTGAAAATGCCATCAAACATGCGTTTACGAATCG
AAAGCAAGGTAACGATATTGACGTGTCAGTGATTAAAGAAACTGCAACACATGTACGTATTATTGTACAAGATAATGGTCAGGGTATTTCT
AAAGATAAAATGCATTTGTTGGGAGAAACATCTGTAGAATCAGAGTCTGGAACTGGTAGTGCTTTAGAAAATTTAAACTTACGCCTAAAAG
GATTATTTGGAAAATCCGCAGCATTACAATTTGAATCGACATCGAGCGGTACCACTTTTTGGTGTGTACTTCCTTATGAAAGACAAGAGGAG
GAATAA

Gene: lytR (two component sensor/regulator, transcriptional regulator)

Position: 289733 to 290473, length: 741 nt, orientation: FORWARD

Perfect match to: (CIGC345D-AHVO01000006-[143308:144048:r], allele observed in CC8+CC1+CC8+CC97)

Sequence:

ATGAAAGCATTAATCATAGATGATGAGCCATTAGCACGTAATGAATTAACATATTTATTAAATGAAATTGGTGGTTTTGAAGAAATTAATGA
GGCAGAAAATGTAAAAGAAACATTGGAAGCACTACTGATCAATCAATATGACATTATATTTTTAGATGTCAATTTAATGGATGAAAATGGG
ATCGAATTAGGAGCTAAGATTCAAAAGATGAAAGAGCCACCTGCGATTATTTTTGCAACTGCACATGACCAATACGCAGTACAGGCATTTG
AATTAAATGCGACAGACTATATTTTGAAACCGTTTGGTCAAAAACGTATTGAACAAGCAGTCAATAAAGTGCGTGCGATTAAAGCCAAAGA
TGATAATAACGCAAGTGCAATTGCGAATGATATGTCGGCGAATTTTGATCAAAGCTTACCTGTTGAAATTGACGATAAAATTCACATGTTAA
AGCAACAAAATATTATTGGGATTGGCACACATAATGGTATTACAACCATACATACAACGAATCATAAATACGAAACAACAGAGCCATTGAA
TCGTTATGAAAAACGATTGAATCCCACTTATTTTATACGTATTCATCGTTCATATATTATTAACACGAAACACATTAAAGAAGTGCAACAATG
GTTTAACTACACTTATATGGTAATATTGACAAATGGTGTCAAGATGCAAGTTGGACGTTCATTTATGAAAGATTTTAAAGCGTCGATAGGAT
TACTTTAA

Gene: lrgA (antiholin-like protein)

Position: 290586 to 291029, length: 444 nt, orientation: FORWARD

Perfect match to: (RF122-AJ938182-[251476:251919], highly conserved allele)

Sequence:

ATGGTCGTGAAACAACAAAAAGACGCATCAAAACCAGCACACTTTTTCACCAAGTCATTGTAATTGCTTTAGTACTCTTTGTATCGAAAATA
ATTGAATCATTTATGCCAATTCCTATGCCTGCATCAGTAATCGGTTTAGTATTATTATTTGTATTATTATGTACTGGTGCTGTTAAGTTAGGCG
AAGTCGAAAAAGTAGGAACGACACTAACAAATAACATTGGCTTACTCTTCGTACCAGCCGGTATCTCAGTTGTTAACTCTTTAGGTGTCATT
AGCCAAGCACCATTTTTAATCATTGGACTAATAATCGTCTCAACAATACTATTACTTATTTGTACTGGCTATGTCACACAAATTATTATGAAA
GTTACTTCGAGATCTAAAGGTGACAAAGTCACAAAAAAGATCAAAATAGAGGAGGCACAAGCTCATGATTAA

Gene: lrgB (antiholin-like protein)

Position: 291022 to 291723, length: 702 nt, orientation: FORWARD

Perfect match to: (11819-97-CP003194-[297441:298142], allele observed in CC80)

Sequence:

ATGATTAACCACTTAGCACTAAACACACCTTACTTCGGAATACTGTTATCCGTTATACCATTTTTCTTAGCGACCATATTATTTGAAAAAACAA
ATCGTTTCTTCTTATTCGCACCGCTATTTGTCAGTATGGTATTTGGTGTGGCCTTCCTCTATTTAACAGGCATTCCGTATAAGACTTACAAAAT
AGGTGGAGACATTATTTACTTCTTCTTAGAACCGGCAACAATCTGTTTTGCGATTCCGTTATATAAAAAGCGTGAAGTGCTTGTTAAACATT
GGCATCGTATCATCGGAGGTATTGGTATCGGTACAGTTGTAGCGTTATTAATTATTTTAACTTTTGCGAAGTTAGCACAATTTGCCAATGAT
GTTATTTTTATCAATGTTACCTCAAGCAGCAACTACAGCGATTGCGTTACCAGTATCAGCTGGTATCGGTGGTATAAAAGAATTAACATCATT
AGCAGTTATTTTAAATGGTGTCATTATTTATGCACTAGGTAATAAATTCTTGAAGCTTTTCCGAATTACTAACCCTATTGCCCGAGGATTAGC
ACTTGGAACAAGTGGTCACACATTAGGTGTAGCACCAGCCAAAGAATTAGGACCTGTAGAAGAATCAATGGCAAGTATAGCTTTAGTGTTA
GTTGGTGTAGTTGTTGTAGCAGTTGTGCCTGTCTTTGTAGCAATATTCTTCTAA

Gene: Q5HJB2 (putative transcriptional regulator, GntR family)

Position: 291831 to 292535, length: 705 nt, orientation: REVERSE

Perfect match to: (FPR3757-CP000255-[309956:310660:r], allele observed in CC8+CC80)

Sequence:

TTACTTTTTACTAGGAATATAAAACTGTGCATGACGATAATGAAATACGATGTCAGACGAATCAAAGGGTTTGCCAGTCATTGTATAAAAAG
TCTGGTGGTAACGTAAACATGGTTCACCTGTAGACAATTGTAGTAATGAAGCTTCACTTGAAGTGAGTTTATCTACATTAAAGAAAATATCT
GAAAAACCAATACGAAGTTTCATGTTTGATTCTAAATAATCGAAGATAGAGCCCTTAGCAATATCATCATTTAAATATTTCACGATTTCTTTA
TGATAATAAGAATATTCGATACATAAAACATCATCGTCCACGAATCTTAATCGCTCTAAATAGTAGACGGTATCATCTGCATTTAATTGGAG
CTCATCTTGTACAGATTTAGGTGGCGTTGCAATCTCCTTAAAAACAAGTACCTTACTTGTCATTCGGTGTTCACCTAAACTTTTAGAGAAACC
ATTAGTCTTAAAGACGTTGATACGATTGGCATCAGCAATATTTCTCACATAAATACCACTGCCTTGTGCTTGATAGATCAAACCATCTTGTTC
CAATAAGCCTAATGCTTTAATGATAGTACTCTTACTTACTTGATAACGTTCTTTTAATTGCGTCACGCTTGGCAATTTATCACCGGGTTTGAAA
TTAGATTGATGTATAAACGCATTAAGTTGCTTAGCAATATGTTCATACTTTAACAA

Gene: Q1YAD5 (PTS system, IIA component)

Position: 292684 to 293475, length: 792 nt, orientation: FORWARD

Perfect match to: (JH1-CP000736-[303063:303854], allele observed in CC5+CC80)

Sequence:

ATGAGCAATAAATATAAAGAACAAGCCCAAGACATTCTTACAGCTGTAGGTGGTGTCGAAAACATTGTTGATGCAACGTATGATACGAAGT
GCATTACAATTCATATGCAACATACAATTCCTTCTACAGCAAATGAAGTGAAACAAATAGTTGATGTGACATCTGTAGCAGAAAATGATGCG
CAGTTAGTCATAAAATTAAATGGAAATGTCGATGAAGTGTATCAGCAATTACAGCGATTAATTAAGAATGCTAATGTTGAAGATGGTACGA
ATACTGACAATATTAATAGCCAAGATACAAGTTATACACCTCAAGTAAAAGTAACAACACCAATTTTAGTGAAAGCACCAATCGCTGGTCGT
CGTATTTTACTTAAAGAAGTAAGAGATTCAATTTTTAGAGAGAAAATGGTAGGTGAAGGCTTGGCAATCAAAGCTCATGAAGAATCCAAAG
TAATCGCACCGGTCAATGGTTTAATATCTATGATTGTACCAACTAAGCATGCAGTTGGTATTCAATCAGAAGACGGTGTGGACATAGTCATT
CATATTGGCGTGAATACAGTTGACTTGGAAGGTAAAGGGTTTAAGTGCTTTGTAAAGCAAAATGATCGTGTTGAAGCAGGGCAAACGTTG
TTACAATTCGACCAGCAATATATACAACAACAAGGCTACAATGCTGACGTTATTGTCGTTATTAGCAACTCTGCCGATTTAGGAAAAGTAGA
ACTGACAATGAATGAAATCATTACGACTGAAGATGTTATTTTTAAAATATTTAAAAACTAG

Gene: bglA (putative 6-phospho-beta-glucosidase)

Position: 293491 to 294926, length: 1436 nt, orientation: FORWARD

Sequence:

ATGACCAAATTACCGCAAAATTTTATGTGGGGTGGCGCTCTTGCCGCAAATCAATTTGAAGGTGGATATGATAAAGGTGGCAAAGGATTAA
GTGTAATTGATGTTATGACGAGTGGTGCACATGGCAAAGCACGTCAGATTACAGAATCTATAGATCCCAATCACTATTATCCAAATCATGAA
GGTATTGATTTTTATCATCGTTATAAGGAAGATATTGCCTTGTTTAAAGAAATGGGATTGAAATGTTTACGTACGTCGATTGCGTGGACACG
TATCTTTCCGAATGGGGATGAAGATGTGCCAAATGAAGAAGGACTCGCCTTTTATGATCGTATCTTTGATGAATTAATTGCACAAGGTATTG
AACCTGTTGTGACGTTATCACATTTTGAGATGCCACTTCATTTAGCGAAACATTATGGTGGATTTAGAAATAGAGAAGTTGTCGATTATTTT
GTGCATTTTGCGCGTGTTGTATTTGAAAGATATAAAGATAAAGTGACATATTGGATGACGTTTAATGAAATTAATAATCAGATGGACACATC
AAATCCTATCTTTTTATGGACGAATTCTGGAGTAGCATTGACAGAAAATGATAATCCCGAAGAAGTCTTGTATCAAGTAGCACATCATGAAC
TTTTAGCCAGTGCTTTAGCGGTTCGTCTTGGTAAAGAGATCAATCCGAAGTTTAAGATTGGAACAATGATTTCACATGTACCCATTTATCCAT
ATTCGTGTCATCCGAAAGATATGATGGAAGCACAAATTGCGAATCGCTTACGTTTCTTTTTCCCGGATGTTCAAGTAAGAGGTTATTATCCA
AGCTATGCTAAAAAATGTTGGCACGAAAAGGATATGATGTTGGATGGCAAGAAGGGGACGACAGTATTTTACAGCAGGGCACGGTTGATT
ATATTGGCTTTAGTTATTACATGTCTACGGCTGTAAAACATGATGTTGATACTACAGTTGAAAACAACATCGTCAACGGTGGTTTGAATCATT
CTGTGGAGAATCCGCATATCGCAACGAGTGATTGGGGTTGGGCGATTGATCCAGATGGCTTAAGATATACATTGAATGTGTTATATGATCG
TTATCAGTTGCCACTTTTTATTGTGGAAAATGGTTTTGGTGCAGTTGATGAAGTGGTAGATGGACATATTCATGATGATTATCGCATTGAAT
ATTTAAAAGCACATATTACAGCAGCGATGGAAGCAGTTGATCAAGATGGTGTAGATTTAATCGGTTATACGCCATGGGGAATTATTGATAT
TGTTTCATTTACAACCGGTGAAATGAAGAAACGCTATGGTTTAATATATGTTGATCGAGATAATGATGGTCATGGCACGATGGAACGCTTG
AAAAAAGATTCGTTCTATTGGTATCAACAAGTGATAGCATCAAATGGAGATAAATTATAA

Gene: Q5HJA9 (putative methyltransferase)

Position: 295388 to 296149, length: 762 nt, orientation: REVERSE

Sequence:

TTAGTCCTCTTTTTTAGCGACAAAAGTAATATAATTCATATCTTTACGCAATTTAGTCATCGTTTTAAACATTTTACAAAACATTGATCGATTTT
CTTTTTTTAAAGCATTGTTGATAATCTTTATAGTTCCAACAATACCTTCGTCATAAATTAAACCTTTTGGTGTCATTAAACTCATTGGACCAGT
ATGATAATGCACATGATTAAAACCAGCTTGATTATATAAATCTAACCAGCCAAGTTTCGTCTGCGGTGAGACATTGACATTAATAGCTGCAG
ATAATGATTTAACTACATGTGTGGCATGTGATTCATTAACAATGACAATATCATGTGTTAACAAGATACCCCCAGGCTTTAAGACTCGGTAG
TACTCGCGTAATGCTTTTTCCTTTATGGCGATGGGTAACATTGTTAACATTGCTTCATTTAAAACGATATCGAATTGATTGTCATCAAAGGGC
AATTTAACAGCATTCGCTTGTTGAACTTGAATATATGATTCAAGACCTGCTGCTGAAATATTTTCCTGTGCTTTTTCTAATGCTTTCTTATTTAT
ATCAACGCCTTGAATGTGACAGCCATATGTATGAGCTAGATAAATAGATGTTGTGCACATATTACATGCCACTTCTAACACTTGTTTATCTTG
TGAAAATGCCCCTTGTTGTATTAACCAATCTGTTGCTTCTTTACCACCGGGGCGTAGACGAGTTTTTCCTAATTTAGCTAAAAATGTATGACC
AGCTTCTTTAGACAT

Gene: rbsK (ribokinase)

Position: 296400 to 297314, length: 915 nt, orientation: REVERSE

Perfect match to: (M1216-AIYW01000016-[119977:120891:r], highly conserved allele)

Sequence:

TTAAACTTGATTTACTTCTTCTAGTAGAGGAATAGATGCTTGCGCGCCGTGTTTTTGTACAGTGAGTGAGCTCGCTTTATTACCAAAATCAAT
AGCATCTGCTAAGTTATCTTGCGACTTGTTTAAGCGACTGACAAATGCACCAATAAATGTGTCGCCTGCAGCAGTTGTATCAATCGCATTTA
CTTTATAAGCTTCGATGTGTTGGCTTTGATTTTTAGTAGCAAAATATGTACCTTGCTTACCTAGCGTAATCAAAACAGTCTTAATGCCTATAG
ATAAAAAGTAATTGGCATTGTCTTTCATAGATTGTTCATTAGTTACTTTAATCCCAGATAACAATTCGGCTTCTGTTTCGTTTGGCACAATAAT
ATCGATTAATGATAATAATTCATTAGGTAATGCTTTCGCTGGTGCAGGATTTAATACTGTCGTCACACCATGTGCCTTGGCAATTTCAAATGC
AGATATAATAGCCGGGATGGGTACTTCTAATTGTGCAACGACAAAGTCTGCATTGATTATAGCGTCTTTTGCGTTAATAACATCTTCAGGTG
TCATCGTCATATTCGCACCACCATAAACATAGATGGTGTTTTGTCCTTCTGCATTCACAGTGATAAAGGCTTGGCCCGTTTTTGCTTCAGCTG
TTTTGATAATATATGATGTATCAATATGAGCTGCTTTAAAATCTTCTAAGATGAAATCAGCAACGCCATCAGTGCCAATTTTAGTAATAAATG
TTGTGTCTGCTTGCATGCGTGCAGTGGCAATAGCCTGGTTGGCACCTTTACCTCCGCCGAATGCTTTTTGTGCTTCTTCAACATGTAATGTTT
CGCCTGGTTGTGCATATCTTTCAACTGTTAAAAATTGATCGACATTCGTTGAACCTAAAATAACAACTTTGTTGGTCAT

Gene: rbsD (D-ribose pyranase)

Position: 297342 to 297746, length: 405 nt, orientation: REVERSE

Perfect match to: (Strain_21333-AHKA01000033-[28293:28697], allele observed in CC80+CC1)

Sequence:

TTAAAAAGTAACATTCGATTCTAATGCAATATTAGAGTAGGGCGTTGTTTCACCAGTACGAATATTACCTTTATTTAATGTGTGAGCTAAGTT
ACTTTTCATTTCTTCGTGAGGAATGAAAATGATTTCTATTTCCGATGAAATCAATTGTTTAATTTGTTGCAATTGTGTAGGGTTATGTTCTTTT
ATTTCTTCTGCTAAGTATATTTTTTGGATTTCCATTTCTTCTAACACTGTAGCTAAGACATCAATAAAGCGTGGTAAGTTTTTAGTTACAGCTA
GGTCGATACGACGATGATCATTTGGAATTGGCATGCCAGCGTCATTAATCGTTAATAAATCAAAATGACCAATTGTCGCGATTGCTTTTGAA
ATATGTTCATTTAAAACAGCTGATTTTTTCAT

Gene: rbsU (ribose uptake protein)

Position: 297761 to 298642, length: 882 nt, orientation: REVERSE

Perfect match to: (N315-BA000018-[314313:315194:r], highly conserved allele)

Sequence:

TTATTTTATAAATACTGTAACAGAAGCGGCTACTAAAATGAGTACTAAGCCGATGATTGTAATAACCATTTCTTTTGACGTTTTATGTTGTTTT
AAGAAATAAATACCAGTTAATGTAGCAAGCACAACGGATGTTTGAGAAAGAATAAATCCAGTTGCTAAACCATTCATATTAGGTTGTGCTG
AAATAAGATATGTTAAAGCACCAAATGCAAAGAAGAAACCTGAAATAATTTGTAACCACGTAATTTTATTACGGAATGGATTCTCTGCTTTC
ATATTCATAAAGCCATAAATGACTGCAACAATTACCATACCCATTGCTTGAGGTAAAAAGGCAGTTAGGCCATCAATAGAAGTTGCTTGCG
GTGCAGCTGAATATAACCAGTATCCAAATTCACCAATTAACAGAAGTACCACTGCACGACGTAAATTTTTGGCGTTACTTGCTTCTTTGCGTT

CACTCCAAACTGTCATACGCGCTCCAATTAGAATAACGACTAAAGCTGTAAATCCAATGATTTTATGACCAATGCCTGGCCAATTTCCTAATG
CAAAGACACCCCATAAAGATGCGCCTAATAATTGGAATGCTGTTGTGACTGGCATGGCACGAGATGAGCCGACTAATTCGAACGCTTTAAA
TGTAATGATTTGTCCGAATCCCCATCCTGCACCTGATAATAAGGCGAATAGCAAATTGGTTCCAGTAGGGAAGCCACTTGATGTGACTACG
GCTAATATAATAGCGAAGATTAACGTACCTACAGTAGCACCGATAATTTGATGTACAGGTTTACCACCAAACTTTGAAGCGACTGTTGGGA
AGAAGCCCCAGCCAATTAAGGGGCCTAACCCGATAAGTAATGCAACAATGCTCAT

Gene: rbsR (ribose operon transcriptional repressor)

Position: 298874 to 299872, length: 999 nt, orientation: REVERSE

Perfect match to: (N315-BA000018-[315426:316424:r], allele observed in CC5+CC7+CC80)

Sequence:

TTAGTTTGAAAGATGATAGCCAGTTGTTGCACGAATTTTTAAAGTCGTTGGTAATTCAATCATATCAATGGATTTATCTAAGTGCTGTAATCG
TTGAAGTAATAAGGTTAAAGATGTTTTGCCAATATCAGTTATAGGTTGTGCCACAGTAGTTAAAGGTGGCGAGACGTACGCTGCATAATCA
ATGTCGTCATAACCTATTAATGAGATATCTTTCGGAATACTGATGCCATGTTCAATTAGTCCTCGTAAAATGCCAATAGCGAGTTCATCGTTA
ATAGCGAAGATTGCAGTGGCAGATTGAACCATGATGTCATCAACAATGGTTAGCCCACCGCGCTTAGATAATTCAGTATGGACGATTTGTG
GTTCTGGCAATTGATTCGCGCGCAAAGTATCGACAAATCCAGCGACACGAGTCGACATATTCGCCATCATGTCATATGGTGCAACAATTATC
ATATTGTTGTGACCGAGTTCTATTAAATGTTGTGCTGCAAGTTGTCCACCTTGATATTCATTTGTCCGAACAAAATCTGTATAGCCTTGATGG
TCATTTTGATCCAGTACGACATAAGGTACATGATGTTTCTTTAGATAGTTATTTAGGGCGTCCGGGGATGATATGTATTGTGCGATAATTAA
TCCGTCAATACCTCGATCAATTAAATGTTTAATATTGTCATACAAATCAGTTGCTGTAGATGTTAAAAAGCATAAATCAACATCAGATGGTTT
ATGGTCATGAATACTTTGCATCAGTGCTGAGAAAAACGGATTTGTTAAGCTAGGTAAAATGACGCCAATAGTTTGAATTTTACTGCCGCGCA
ATTGTTTTGCATGTTTATTAGGGGCATAGCCTAAACGTTCTGAAACAGCATGTACGTTTTTTATCGTTGTTGCGGAAAAACGACTATCATTAT
GATTTAAAATATGTGACACAGTTGTAACTGATACACCAGCTTCTCTAGCAACATCTTTAATTGACACTTTTTTCAT

Gene: Q5HJA4 (putative protein)

Position: 300023 to 300163, length: 141 nt, orientation: FORWARD

Perfect match to: (S1800-CAWB010000034-[1269:1409], allele observed in CC80)

Sequence:

TTGAGGCGAGAGAATATATTATTAAGGTTAAATAACTTTTAATGATTATGATAACAAGAATTTTAAAATGTTTATTGTGATGAAAAATACAA
AAGTCATTATTTTAAAGAATAAAATATTTGTTTTTGAAAAAAGTATTAA

Gene: Q5HJA3 (putative protein)

Position: 300254 to 300646, length: 393 nt, orientation: FORWARD

Perfect match to: (11819-97-CP003194-[306673:307065], allele observed in CC80+CC50+CC1153)

Sequence:

ATGCAATTCAAATTAAAAGAAGAAGAGATTATTAGTTTTTTAGAATTGAAATATCCAGAAAAAGAGTTCGAATATGGTCGTTTGTTAGTTGG
ACAACATAAACGTGATGATTTAGATGTTTATTACTTTGGTGATACGTTTTTAATGTGCACGATTATTTCATTCAAAACATTTGAAATTAAAGA
AACAGTAGAATTATCATATGATGCTGTTAATCGTATTGTGTTAAAAGATGGATGGTTATTTAGAAAAATGAGAATAGAAACAATGCAAAAA
GTGTTAAAATACGGTACTTCTAAATTAATGTTAACTGATTTTCAAAAGGACAATTATAATAAATATATTCAAGGTCAGAAACAACGCGTGAT
ATTTGAAAATGGCCATTTTGTCTAA

Gene: yusP (major facilitator superfamily transporter)

Position: 301319 to 302695, length: 1377 nt, orientation: REVERSE

Perfect match to: (NN50-BAEA01000012-[101322:102698], allele observed in CC4803+CC8+CC188)

Sequence:

TTAAGATTTAACCGTTTTTGGAATGATCATGTATGCAGACAATGAGCCGAGTATCATTAATACAATGCTGACGATAAATGTTACAGTTGCCG
CTACACTTGGTGCATAGTTTAGTTGTAATATACTGAAAACTGTAGTACTTAGTGCTATACCAAAGGCGCCACCTAATGTACCACTCATTTTAT
ATAATCCTGTAGCTAAACCAACTTTTTCATTTGGCATACTGAAAATTGCAATTGTAAGGCCGGGTGTTGCGACTAAACCATTTCCAATTGCGC
AAATGACAAACCCAATAACAACAGCAATGATATATTGTGATGGTTGAAGTGATGTCATGCTAATAATAGTGATGCCGATGACAGGGAACAA
CGGACCAATGATGAGCATCAATTTGCCACCGAAACGTAATGTTGCTTTTTCACCTAAACGAATCATCGCAACTGCCACAATGGCATATGGCA
ATGTAACAAGTCCAGATTGCGCAGCTGATAAACCAAGGTGTGTTTGAGCATATATGAAAAAGACCACTGTTACGCCTAGACCGCTATTTAA
AACAAAGTTATTTAAAAATGCACCAATGAACGGACGGTTGCGTAATACTGAGAAATCAATAAAAGGTACTTCATGTCGACGTTCGATGATG
ATGAATATCAACGTAGTGATGATAAAAATGCTCAGACAAATGATTGAAAATGTACTAAACCAACCTTGTTCGAATCCTTGTGTTAACAATAA
TGTAAAGCTACCAATCATAACAGCGAAAATCGACATACCTTTGTAATCGAATGGATGACGGTGGCTATGTTGACTTACTTTTTCAGGTGTGC
CTTTTAGAAGCAATATGGCAATGAAAGCAATGACTATACTAATGATGAAATTCGTTTGCCATCCGAAATTTGAGGCAATTAAACCGCCGATA
ACACCAGCTAGGCCGATGCCACCAACAGTACTAATCATTAGATAACTAATCGCTCGTCTTAAATGTTCTCCTTTAAATTGATTATTTAACACG
CCAACTGTTGAAGGTAACAAGATAGCTGCTGATAGACCTTGTAAAATTCTACCGATGATGAGCAGTGCAGTGATGTCCGATATAATTAATA
GAAGAGATGCAAACATACTGATTATGAGACCCATGTATGTCATTCTCAGTTGTCCTATTTTATCAGCAATATCACCTGCAGCCACCATGAAG
ATACCTGTGGCGAAGGAAGTTAAACTAATAGATAAATTTAACACGGCAGGAGAGGTTTGATACGTTTGACCAACGAGAGGTCCTATATTAA
TAAATGATTGTGCAAACAACCAATATGTTAATGCAGACAACATAATCGCAATAATAATATTACTGCGTGGTGAAGATTGTGTGTTATTCAC

Gene: yxeI (choloylglycine hydrolase family protein)

Position: 302929 to 303921, length: 993 nt, orientation: FORWARD

Perfect match to: (Strain_21266-AFTT01000027-[140521:141513:r], allele observed in CC12+CC80+CC772)

Sequence:

ATGTGCACAGGATTCACAATACAAACTTTAAATAATCAAGTACTTCTTGGACGCACGATGGATTATGATTATCCATTAGATGGTTCGCCAGC
AGTGACGCCTAGAAATTATCGTTGGAAATCTCGCACTGGCACGACAGGCCAAACGCAATATGGCTTTATTGGCACAGGAACAGATATGGA
AGGTTTTATTTATGGTGATGGTGTTAATGAACATGGCGTTGCCATTTCAACACAATATTTCCGAGGTTATAGTTCATATGGATCAACACACA
AAGCGGACGCGATGAATATTACGCAAAATGAAATTGTGACATGGATTTTGGGATATACAACAAGCATTGAAGATATGAAACAACAAGCAT
CCCAAATACATGTTGTAGCTGTATATTTAAATGACATCGGTGAAGTTCCGCCATTGCATTATCATGTTTCCGATGCAACTGGACATACAGTC
GAAGTTTCATTTAAAGAGGGTGAAGTGGTTATAAAAGATAATCCTATTGGTGTCTTAACAAATCATCCAGACTTAAATTGGCATTATAGTAA
TTTAAGACAATATATCAATATTTCTCCTTATCCAGCAACAGCAAATTTATTGGAAGGTGTAACGATTGAACCTTTAGGCAATGAAGCAGGTA
CATTTGGATTGCCAGGTGGATTTACTTCAACTGAGCGCTTTGTGAGAATGGCATTTATGAAAGCAAACATTGCTCAAAACAATGATAAAGA
AATGGATTTAATGAATGCATTTTATTTATTAGATGCGGTAAATATACCGATTGGAATTGTACGTCCGCATGATGCTGACAATCACTATACGA
TGTATCAGACCGTAATAAATTTAACTACAAGAACGTTATATATTAAGTATTATGGCAGCAATGAATTAGTAGCATTAAAGCTCACAGATGAT
TTAATTAATAGAAAAGATATGACGATTTTTAAGCCTGAGAAGCATATCATTATTAGAAAGTTGAATGACAATCAATAG

Gene: lytM (lysostaphin)

Position: 304247 to 305197, length: 951 nt, orientation: FORWARD

Perfect match to: (NN50-BAEA01000012-[98820:99770:r], allele observed in CC4803+CC1+CC8+CC80)

Sequence:

ATGAAAAAATTAACAGCAGCAGCGATTGCAACGATGGGCTTCGCTACATTTACAATGGCGCATCAAGCAGATGCAGCAGAAACGACAAAC
ACCCAACAAGCACATACACAAATGTCAACACAATCACAAGACGTATCTTATGGTACTTATTATACAATTGATTCTAATGGGGATTATCATCAC
ACACCTGATGGTAACTGGAATCAAGCAATGTTTGATAATAAAGAATATAGCTATACATTCGTAGATGCTCAAGGACATACGCATTATTTTTA
TAACTGTTATCCAAAAAATGCAAATGCCAATGGAAGCGGCCAAACATATGTGAATCCAGCAACAGCAGGAGATAACAATGACTACACAGC
GAGTCAAAGCCAACAGCATATTAATCAATATGGCTATCAATCAAATGTAGGTCCAGACGCGAGCTATTATTCACATAGTAACAACAACCAA
GCGTATAACAGCCATGATGGTAATGGAAAGGTCAATTCCCCTAATGGCACGTCTAATCAAAATGGCGGATCAGCAAGTAAAGCGACAGCT
AGTGGTCATGCGAAAGACGCAAGCTGGTTAACAAGTCGTAAACAACTACAACCATATGGACAATATCACGGTGGTGGTGCGCATTACGGT
GTCGACTATGCAATGCCTGAAAATTCACCAGTTTACTCATTAACTGATGGTACAGTAGTACAAGCAGGTTGGAGTAACTATGGTGGTGGTA
ATCAAGTAACGATTAAAGAAGCGAACAGTAATAACTACCAATGGTATATGCATAATAATCGTTTAACTGTTTCAGCTGGTGATAAAGTCAA
AGCTGGTGACCAAATTGCATATTCAGGTAGTACGGGTAATTCAACAGCACCTCACGTACACTTCCAACGTATGTCTGGTGGCATCGGTAATC
AATATGCAGTAGACCCAACGTCATACTTGCAAAGTAGATAA

Gene: Q5HJ98 (putative ABC transporter, ATP-binding protein)

Position: 305249 to 305908, length: 660 nt, orientation: REVERSE

Sequence:

TTAATAATAGGTATAAGCCCATTGCTTGTCCTCTAAGACATGTTGATAAAAAGGATCTTGGCCAATTAACTTGATATCATCTGCAAGTGCTTC
AACTTCATCTAAATGATGGGTAGTTAATATAATTAAACATTTAGATTTCATGATGTTAAGTAGTTGGTGGATGTCATGTCTAGATTTTAAATC
AATACCAACTGTCGGTTCATCTAAAATGAGAATTCGAGGTTGACCTAGTAAACCTACTAATATATTAATTTTACGTTTATTCCCACCGGACAA
TGTAGATACTTTGGCAGACGTATCATCAAAATTTAATTGCTGTAAATATTCATTGATAGTTGTATCGTTAATTGGATTTTTACAAAGTGATTT
AAAAAATTTAATGTTTTCAGCCACTGTCATGTGTTCAAATAACGCAATGTCTTGTGGCACATAACCGATGTGATTTTGTATTTGTCTTTGATTC
CATTTTTCGTCGAAATAGTTGATAGTTCCATCATTAGCTTTTTCAATACCAGCAATCATACGAAGTAATGTTGATTTTCCAGCGCCATTATCGC
CAAGTAATACGGTTAAACGATTACTATCAAAGGACATGGTTAAATGATTGAAAATCTGTTTGTTACGGTAACGCTTTGAAAGGTTATTAATT
TCTATCAT

Gene: Q5HJ97 (putative ABC transporter, transmembrane permease)

Position: 305922 to 306842, length: 921 nt, orientation: REVERSE

Perfect match to: (Strain_21333-AHKA01000033-[19198:20118], allele observed in CC80)

Sequence:

TTACATGAAAATAATCAAGTATACGATACCCATAGCAAGTGCATATATAAATGTCATGAATAATCGATGACTTATTGTTTGAATATGGAATA
AGATAAAGACGATACCTATCTCATAAATCAATATAAGTAACAGTGATTTTAAGTAAAATATTAAGCTGAGTGGTTGAGACAAATATAGACTA
ACTGCCAATAGTACCAACAATAACAAGATCGTATGTGTCATTACATAAGTACTATATAGTTTGAAACGGCTTAAATGATATTGTGATAATCG
TTGCAATGCTGCTTGTTGGTTTAAACGATAATGAAGTACTACTTGAACAGCGCTAACAAATAAAATCACCGCAAAGATTAAGCTAATTGAAA
TAGAGTGTTGTGCTTGTTTAGTAAGCGACACAAATTTGATTTTAGATTCAGGTGTATGTTTATGATAGGACTTGTTGATAGCATCGATGGAT
TGATGCTGTTTCATATCCTCAAAGTGTTCATAAATAATGTTAGGAATTTGCTGTTCATATAATGAACTACTAACAATTTCTACAGCAATACCA
CCTATAAAGTCATCTCTACCATATAACTGTATCGTTTCTTTTAAACGGTTCTCTTTTAATTTTTGAGAGAAACCTTTAGGAATTTGCATACTTA
AAATAGCTTCCTTTTTAGTAACATCATCTTCAATATAGCTTTCATCTTCATCGACTTTTTTAATAGTTACATAGTCAGATTGTTTAATTTTATTG
ACGAATGATTTTGATGCAGTGGTTTGGTCTAAATCTTGAATGGTAATCGGTATTTTGAAGTTGTCATGTGCTACACGGTAACCGATACCAAT
AAGTACGAGTGCGATGACAATGGTTGTTACGAGCAAGATGTATTGTAACCATTGCTTGAACACAACAAGTTGTATATAAGGCTTCAT

Gene: Q5HJ96 (putative ABC transporter, ATP-binding protein)

Position: 306839 to 308005, length: 1167 nt, orientation: REVERSE

Perfect match to: (TCH70-ACHH02000005-[187071:188237], allele observed in CC1+CC80)

Sequence:

TCATTGACGATACCTCCAAACCAATACAGCTAAATTAATTATCAAAAGTGCGATGAAGCTAAGATAGAAACTAGGGTGTAGTTCTAAAATG
TAGTTGTTTAAAATAATTTCTAACAATTGATTTGTTACAACTGCGAACGGTTGAATATTGAAAACGCCATTTGCTATATGTTGTAAAAAAATC
GTAGGTATTGTTAAACCAGATAACACCAGGATGACAATAGCTAATATGACTTTACTAATACTATTCAACAAGCCTGTTGTTAAAAGTTCGAT
GAGTAATAACCACAGTATTAAAAAGGTAACATAATAGCTTAAATGAATGGCTAACGTTGGCCAATTATATAATTCAAAGGTATTCGGAATA
CTGAACACAATCCAAACTACACCAACGATACTCCATAACATAGTATAAAACCATGTAATCAACGCACGAATGATTAATAAACGCTCTTTAGA
AAAATGAAACATTTTCAATCGCGCTTTCAATACAGTATCTTGATTCATTTTCAAAACTGTAAATAAAGATAGTGCAAAGATGAAAACCGTTG
CTAAAAATCCTGTAATTGCATAATAACTGCCCGTATCGTATAAATGAATCGGTTCTAAGTTAAATGCACCTGAACGGTTTAATCCTGTAATCA
GCAAATCAGTCATAACATTGATACTGTCAGAATGTGATGCTTTCGGTGCTAAGTCTTGAAAAGCTAAGATGCCACCCATTGATCGCATAAGA
CGTTGGTAAACAGAATCTGTTAGCTGAGATAGCACGACACTTTTCATGGATTGTTGATCATATGTATATACTGAAATTGGTAGTTCGCCTTG
TTTATAAAATGCCTTGGTCATACCTTTATCAAAAACAAAATAGCCTTGAAGTTTATGTTTTTTAACAAAGTATGTGCTTGCTTATCATCATAT
GCCTTAATGCTCACGTTTTTTCCTAGGTTACTCCCTTTACCAATAGAGTTTAAGATTAATTTCGTTTCACTTGATTGATCTTTATCTACGACACC
TATATTAAAATGATTGTCATCTTCTGTTACATGTTGGATCGTCGTTAATGTAATAACGAGTGCCGCTAATATAAATAGTAAATAGATAATCAA
ATACCACTTTTTCAATAAAAAAGAGTGGTAGATGCGAAACAAATGTATTGTTTTCAT

Gene: Q5HJ95 (putative membrane protein)

Position: 308073 to 309596, length: 1524 nt, orientation: REVERSE

Perfect match to: (Strain_21269-AFTU01000005-[15070:16594], allele observed in CC121+CC80)

Sequence:

TTATTTAGATGCCTTTTTTAAAATTGATTCAAACATTTTGCCGCCATTTTTTTCGATTTCTTTTTCAAGTTTTTCACGGTCTTTTGAAGATAGAC
TATTGAAATCTTTCGCACCACTATCATCAAAATCAATATCTGCTTTCAATTTTGTGCTAGATTTTAAAATGAAATTAATTGGTTCTTCAGCATA
TTTGATGCCGATATTTAACGTAGATTTCTGAGTGTTATTTTTTACGTCAGAATCTATATTATTTTCAAAAGTGAATTCATTTTCGTCGCTATATT
TATCTAATGCGACAGTAATTTTACCTTTATCTTGACGTTTTGTGCCATCTACTTTTTCTTGGTTATCTAATTTGATTTTTGATTCATCATATTCTG
TCTTTTTACCAAATTCGTATTTATCACTGTATTTATTGTCTTTTTCCTTAGAAGATACGCCTTTAATTGTATATTTCGCTTCAGCATACGTGTATT
TATCTTGATCGAAATCAAGTGCGTAATCTAGTTTTAACTTATCGTCTTCTAAAGTATTAGTACCTTTGATTTTAGTTTTATTATTTTCTTTGTCT
GTAATAGTAATTTCTCGTTTTACAATCGTATGTTTTTCGGTATAAATTTTAGATTGAATTTTAGCAAATTCATCCTTTTTAGTTTCTTTGACATC
GTCAATTGCTTTTTTGATGTCTTTTTCAAAGTCTTTTGTAGCACCTTGTTCTTCCATTAATTTTTTAAGGTCTTTATCCTTTTTAGCTTCTTCTAAT
ACAGCTAATGTAATTTTTTTAGTGTCAGCTCTGCTAAGTGTTAACGTGACAGGTCTAACTTTGTACTTTTCACCATTAACCTTAATTTCTTCTTT
TTTACCTTTATCAAAATTATCGTCATCTAATTTGTCGACAATAAGTTCGGAATATTTTTCGGCAATTTTGCTGTAGTCACTTTGTTGTGCTTGA
GCATTACTAAAAAGAGTATTTAAATTTAGTTGTTGGTTTGTAATACCATTTTCTTTTGCTGTTTCTTCATCTTCACCTGTAAGTTTTGAATAAGT
TGATAATAAATCAGAATTATTAACACTATATTTCCCTTTAAATAATGGTGATTCGAAATAATGCTTATCTTTATCTGCAGCTAACTGGAATTTC
CCTAATTCAGAGTCTGCTATTGTTGGTTCAAGATTAATCATTGATTTCTCTTTTTTAGGATCATGTCCATATGACATTTTAATTTTCGAAGCATT
AACAACAGATTTAGGAATACCAAGCCCCTTAACAATTTCATCTGATGCATCTGCGCTTAATTCTAATGAAGATAAAAATGAATTATCTTTCAT
CTTTTCTTGGAACTTCACTTCGTTTTCAAAACGGTCATTAAAATAATCTTTATACATTTTTGCAGTTTGTTGTTCGCTTTTTAAGTATGTATTTTT
CGGTGTGTTTGCAAAAAATGCATAAACTCCCCATGCGATTCCACCTATTAATAATAAGACAATAATAATAGGAATTATAATTTTTAACTTTTT
AGACAT

Gene: ssaA (staphylococcal secretory antigen A)

Position: 309926 to 310828, length: 903 nt, orientation: REVERSE

Perfect match to: (N315-BA000018-[325932:326834:r], allele observed in CC5+CC8+CC9+CC80)

Sequence:

TTAATGGATGTAATTATATGATGAAACTTCTGAAGCAGAGATGGTTCTTGATGAAACGATATATTCACCAATCCAGTTCATTTCTGAAATTA
GAATACTTCCATCAATATTAACTTTTTCAACGTAGGCTACATGACCAAATGGACCATTTACTGTTTGTAAAATTGATCCTCGTGTTGGGTGTC
TATCTACTTTGAAGCCATTGCTTGAAGCTTGGCCTGCCCAGTTTTTAGCATCTCCCCAAAATGTACTAATCGTGTGTCCATCTTTGGCACGTTT
ATCAAAGACATACCATGTACATTGTCCAGCAGTATATAAGTTGTTCTTACTTGTGATAAGAGGCTGATCAATGATTTTACCGTTACCTAATGC
TAAAGGTTTACCGTCAGCAGTCTTTGCTTTGTCATTAAATTCGGCGATTTGTAATTCGTCATACAATTCGTCTAAATCGATGCCTGTAATAAG
CCCTTTGTTATCTTTTGAAAAAGCGTTATTTAATTCATCGTCATTGTCTTCGACATTCGGTATTGCTGGTGTCAAAGGATTGCTTGGTGACGTT
TGAGGCGGTGTGTGTGAATCAATTGCGTCATTAATGTGCGTATACTGACCACTTAATGAAGAATGGTACTGATTGTTGTTAAAATCACGTTG
ATTTGCGTGTTGATGATTGTCGTTTGTACGTGACTGGTTTTGATGATTGTTGTTTGGCGTGTTGTTTTTGTCATATGTATAAGTATACGCGCC
GGTGTCTTTATTCACTTTGAACTGTGCGTTTGGGTGTGCTCTCTTTGCTTCTTCTAATGTTTTGCTATCATTCGTATATGCTTGAGCCGAGTTA
GGCGACATACTAAATAAAGTAAGAGTTGTCATCGTCAGTAAAATTGTTTTCTTCATAATAACCAT

Gene: esxA (virulence factor esxA)

Position: 311068 to 311361, length: 294 nt, orientation: FORWARD

Perfect match to: (N315-BA000018-[327073:327366], highly conserved allele)

Sequence:

ATGGCAATGATTAAGATGAGTCCAGAGGAAATCAGAGCAAAATCGCAATCTTACGGGCAAGGTTCAGACCAAATCCGTCAAATTTTATCTG
ATTTAACACGTGCACAAGGTGAAATTGCAGCGAACTGGGAAGGTCAAGCTTTCAGCCGTTTCGAAGAGCAATTCCAACAACTTAGTCCTAA
AGTAGAAAAATTTGCACAATTATTAGAAGAAATTAAACAACAATTGAATAGCACTGCTGATGCCGTTCAAGAACAAGACCAACAACTTTCTA
ATAATTTCGGTTTGCAATAA

Gene: esaA (protein esaA)

Position: 311444 to 314473, length: 3030 nt, orientation: FORWARD

Perfect match to: (11819-97-CP003194-[317863:320892], allele observed in CC80+CC5)

Sequence:

ATGAAAAAGAAAAATTGGATTTATGCATTAATTGTCACTTTAATTATTATAATTGCCATAGTTAGTATGATATTTTTTGTTCAAACAAAATAT
GGAGATCAATCAGAAAAAGGATCCCAAAGTGTAAGTAATAAAAATAATAAAATACATATCGCAATTGTTAACGAGGATCAACCAACGACAT

ATAATGGTAAAAAAGTTGAGCTGGGTCAAGCATTTATTAAAAGGTTAGCAAATGAGAAAAACTATAAATTTGAAACAGTAACAAGAAACG
TTGCTGAGTCTGGTTTGAAAAATGGTGGATACCAAGTCATGATTGTTATCCCAGAAAACTTTTCAAAATTGGCAATGCAATTAGACGCTAAA
ACACCATCGAAAATATCGCTACAGTATAAAACAGCTGTAGGACAAAAAGAAGAAGTAGCTAAAAACACAGAAAAAGTTGTAAGTAATGTA
CTTAACGACTTTAACAAAAACTTAGTCGAAATTTATTTAACAAGCATCATTGATAATTTACATAATGCACAAAAAAATGTTGGCGCTATTATG
ACGCGTGAACATGGTGTGAATAGTAAATTCTCGAATTACTTATTAAATCCAATTAACGACTTCCCGGAATTATTTACAGATACGCTTGTAAAT
TCAATTTCTGCAAACAAAGACATTACAAAATGGTTCCAAACATACAATAAATCATTATTGAGTGCGAATTCAGATACGTTCAGGGTGAACAC
AGATTATAATGTTTCGACTTTAATTGAAAAACAAAATTCATTATTTGACGAGCACAATACAGCGATGGATAAAATGTTACAAGATTATAAAT
CGCAAAAAGATAGTGTGGAACTTGATAACTATATCAATGCATTAAAACAGATGGACAGCCAAATTGATCAACAATCAAGTATGCAAGATAC
AGGTAAAGAAGAATATAAACAAACTGTTAAAGAAAACTTAGATAAATTAAGAGAAATCATTCAATCACAAGAGTCACCATTTTCAAAAGGT
ATGATTGAAGACTACCGTAAGCAATTAACAGAATCACTGCAAGATGAGCTTGCAAATAACAAAGACTTACAAGATGCGCTAAATAGCATTA
AAATGAACAATGCTCAATTCGCTGAAAACTTAGAAAAACAACTTCATGATGATATTGTCAAAGAACCTGATACAGATACAACATTTATCTAT
AACATGTCTAAACAAGACTTTATAGCTGCAGGTTTAAATGAGGATGAAGCTAATAAATACGAAGCAATTGTCAAAGAAGCAAAACGTTATA
AAAACGAATATAATTTGAAAAAACCGTTAGCAGAACACATTAATTTAACAGATTACGATAACCAAGTTGCGCAAGACACAAGTAGTTTGAT
TAATGATGGTGTCAAAGTGCAACGTACTGAAACGATTAAAAGTAATGATATTAATCAATTAACTGTTGCAACAGATCCTCATTTTAACTTTG
AAGGCGACATTAAAATTAATGGTAAAAAATATGACATTAAGGATCAAAGTGTTCAACTCGATACATCTAACAAGGAATATAAAGTTGAAGT
CAATGGCGTTGCTAAATTGAAAAAGGATGCTGAGAAAGATTTCTTAAAAGATAAAACAATGCATTTACAATTGTTATTTGGACAAGCAAAT
CGTCAAGATGAACCAAATGATAAGAAAGCAACGAGTGTTGTGGATGTAACATTGAATCATAACCTTGATGGTCGCTTATCGAAAGATGCAT
TAAGCCAGCAATTGAGTGCATTATCTAGGTTTGATGCACATTATAAAATGTACACAGATACAAAAGGCAGAGAAGATAAACCATTCGATAA
CAAACGTTTAATTGATATGATGGTTGACCAAGTTATCAATGACATGGAAAGTTTCAAAGACGATAAAGTAGCTGTGTTACATCAAATTGATT
CAATGGAAGAAAACTCAGACAAACTGATTGATGACATTTTAAATAACAAAAAGAATACAACAAAAAATAAAGAAGATATTTCTAAGCTGAT
TGATCAGTTAGAAAACGTTAAAAAGACTTTTGCTGAAGAACCACAAGAACCAAAAATTGACAAAGGCAAAAATGATGAATTCAACACAATG
TCTTCTAACTTAGATAAAGAAATTAGCAGAATTTCTGAAAAGAGCACGCAATTGCTATCTGATACACAAGAATCAAAAACAATTGCAGATTC
AGTTAGTGGACAATTAAATCAATTAGATAATAATGTGAATAAACTACATGCGACAGGTCGAGCATTAGGCGTAAGAGCGAATGATTTGAAC
CGTCAAATGGCTAAAAACGATAAAGATAATGAGTTATTCGCTAAAGAGTTTAAAAAAGTATTACAAAATTCTAAAGATGGCGACAGACAAA
ACCAAGCATTAAAAGCATTTATGAGTAATCCGGTTCAAAAGAAAAACTTAGAAAATGTTTTAGCTAATAATGGTAATACAGAGGTGATTTC
ACCGACATTATTCGTATTATTGATGTATTTACTATCAATGATTACAGCATATATTTTCTATAGTTATGAACGTGCCAAAGGTCAAATGAATTTC
ATTAAAGATGATTATAGTAGTAAAAACCATCTTTGGAATAATGTCATTACGTCAGGTGTTATTGGTACAACTGGTTTGGTAGAGGGATTAAT
TGTCGGTTTAATTGCAATGAATAAGTTCCATGTATTAGCTGGCTATAGAGCGAAATTCATCTTAATGGTGATTTTAACTATGATGGTCTTTGT
ACTTATTAATACGTATTTACTAAGACAGGTAAAATCTATCGGTATGTTCTTAATGATTGCTGCATTGGGTCTATACTTTGTAGCTATGAATAA
TTTGAAAGCGGCTGGACAAGGTGTGACTAATAAAATTTCACCATTGTCTTATATCGATAACATGTTCTTCAATTATTTAAATGCAGAGCATCC
TATAGGCTTGGCACTAGTAATATTAACAGTACTTGTGATTATTGGTTTTGTACTGAACATGTTTATAAAACACTTTAAGAAAGAGAGATTAAT
CTAA

Gene: essA (protein essA)

Position: 314473 to 314931, length: 459 nt, orientation: FORWARD

Perfect match to: (N315-BA000018-[330478:330936], highly conserved allele)

Sequence:

ATGTTGATGAATAGCGTGATTGCTTTAACTTTTTTAACAGCATCTAGCAATAATGGCGGACTTAATATTGATGTGCAACAAGAAGAGGAAA
AGCGAATCAATAATGATTTAAATCAATATGATACAACGCTATTTAATAAAGATAGTAAAGAAGTCAACGACGCGATTGCTAAGCAGAAAAA
AGAACGACAACAACAAATAAAAAATGATATGTTTCAAAATCAAGCGAGTCACTCGACTCGCTTGAATGAAACTAAAAAAGTGTTATTTTCCA
AATCTAACTTAGAAAAGACTTCGGAGAGTGATAAAAGCCCCTATATTCAAAACAAGCAGGAGAAAAAAATATTCCCGTACATTTTGATGTCT
GTAGGGGCTTTTTTGACTTTAGGATTTGTCATTTTTTCAATTCATAAAGGGAGACGAACGAAAAATGAATCAGCACGTAAAAGTAACATTTG
A

Gene: esaB (protein esaB)

Position: 314903 to 315145, length: 243 nt, orientation: FORWARD

Perfect match to: (N315-BA000018-[330908:331150], highly conserved allele)

Sequence:

ATGAATCAGCACGTAAAAGTAACATTTGATTTTACTAATTATAATTACGGCACATATGACTTAGCAGTACCAGCATATTTACCGATAAAAAA
TTTAATAGCTTTAGTATTGGATAGTTTGGACATTTCAATATTTGATGTCAATACACAAATTAAAGTGATGACGAAAGGTCAATTACTTGTTGA
AAATGATCGACTCATTGATTATCAAATCGCTGATGGAGATATTTTGAAGTTACTATAG

Gene: essB (protein essB)

Position: 315158 to 316492, length: 1335 nt, orientation: FORWARD

Sequence:

ATGGTTAAAAATCATAACCCTAAAAATGAAATGCAAGATATGTTAACGCCTTTAGATGCTGAAGAAGCAGCTAAAACAAAATTACGCTTAG
ATATGAGAGAGATTCCTAAGTCTTCAATTAAACCAGAACATTTTCATTTAATGTACTTATTAGAACAACATTCTCCATATTTTATAGATGCTGA
ATTAACTGAACTACGTGACAGTTTCCAAATACATTATGACATTAATGACAATCATACACCTTTTGATAATATTAAATCATTTACTAAAAATGA
AAAATTACGATACTTACTCAATATCAAAAATTTAGAAGAAGTAAATCGTACACGTTACACATTTGTTTTGGCACCAGATGAGTTATTTTTCAC
AAGAGATGGATTACCAATTGCTAAAACAAGAGGTTTACAAAATGTTGTTGATCCATTACCGGTGTCAGAAGCTGAATTTTTAACAAGATATA
AAGCACTGGTTATCTGTGCATTCAATGAGAAACAATCATTTGATGCTTTAGTTGAAGGAAACTTAGAACTACATAAAGGAACGCCATTTGAA
ACTAAAGTTATTGAAGCGGCAACGTTAGATTTACTAACGGCATTTTTAGATGAACAGTATCAGAAACAAGAACAAGATTATAGTCAAAATT
ATGCATATGTACGCAAAGTAGGGCATACCGTTTTCAAATGGGTTGCTATCGGTATGACAACGTTAAGTGTTTTATTAATTGCATTCTTAGCCT
TTTTATATTTTTCAGTAATGAAGCATAATGAGCGCATTGAAAAAGGATACCAAGCATTTGTAAAGGATGATTATACGCAAGTACTAAATACG
TATGATGATTTAGATGGTAAAAAATTAGATAAAGAGGCACTTTACATTTATGCCAAAAGTTATATCCAAACAAATAAACAAGGTTTAGAAAA
AGATAAGAAAGAAAATTTACTTAATAACGTGACACCAAATTCAAACAAAGACTACTTATTATATTGGATGGAATTAGGACAAGGACATCTT
GATGAAGCGATTAATATTGCCACTTATTTAGATGATAACGATATTACAAAGTTAGCGTTGATTAATAAATTAAATGAGAATAAAAATAACGG
AGATTTATCGAATGATAAACGTTCTGAAGAAACGAAAAAGTATAACGATAAATTGCAAGATATTTTAGACAAAGAAAAACAAGTTAAAGAT
GAAAAAGCGAAATCTGAAGAAGAGAAAGCAAAAGCAAAAGATGAGAAATTAAAGCAACAAGAAGAGAACGAAAAGAAACAAAAAGAAC
AAGCACAAAAAGATAAAGAAAAACGTCAAGAGGCTGAAAGAAAAAAATAG

Gene: essC (protein essC)

Position: 316514 to 320953, length: 4440 nt, orientation: FORWARD

Perfect match to: (NN50-BAEA01000012-[81706:86145:r], allele observed in CC4803+CC8)

Sequence:

ATGCATAAATTGATTATAAAATATAACAAACAATTGAAGATGCTCAATTTGCGAGATGGTAAGACATATACTATTAGCGAAGACGAGCGTG
CAGATATTACGTTGAAATCGTTAGGCGAAGTCATTCATTTAGAACAAAATAATCAAGGTACTTGGCAAGCGAATCATACTTCTATTAATAAG
GTGCTTGTTAGAAAAGGTGACCTTGATGACATTACATTACAGCTTTATACAGAAGCTGATTATGCATCATTTGCTTATCCTTCAATTCAAGAT
ACGATGACAATTGGGCCAAATGCGTATGATGATATGGTTATTCAAAGCTTGATGAATGCCATCATTATTAAAGATTTTCAATCAATACAAGA
ATCACAATACGTACGCATTGTGCACGATAAAAATACAGATGTGTATATTAACTATGAACTACAAGAGCAACTAACGAACAAAGCTTACATTG
GTGATCATATTTATGTTGAAGGGATATGGCTCGAAGTACAAGCTGATGGTTTAAATGTATTGAGTCAGAATACAGTGGCATCGTCATTAATT
CGCTTAACACAAGAGATGCCACATGCACAGGCAGATGATTACAATACGTACCATCGTTCGCCAAGGATTATTCACCGTGAACCGACCGATG
ATATTAAGATTGAAAGACCACCACAGCCAATACAGAAGAACAATACAGTGATATGGCGTTCCATTATACCGCCATTAGTAATGATTGCTTTA
ACTGTTGTCATCTTTTTAGTGAGACCAATTGGTATTTATATTTTAATGATGATTGGTATGAGTACAGTAACGATAGTATTTGGTATTACAACG
TATTTCTCTGAAAAGAAAAAGTATAACAAAGATGTTGAAAAACGAGAGAAAGATTACAAAGCTTATTTGGATAATAAATCTAAAGAAATTA
ATAAAGCGATTAAAGCACAACGTTTTAGTTTGAATTACCATTATCCAACGGTTGCTGAAATTAAAGATATCGTTGAAACGAAAGCACCAAG
AATATATGAAAAAACATCGCATCATCACGATTTCTTACATTATAAGTTAGGTATTGCGAATGTAGAAAAGTCATTCAAATTAGATTACCAAG
AAGAAGAATTTAACCAACGTCGTGATGAACTATTCGACGATGCTAAAGAATTGTATGAATTTTACACAGATGTAGAACAAGCACCATTAAT
CAATGATTTAAATCATGGACCAATTGCATATATTGGTGCACGACATCTCATTTTAGAAGAATTGGAGAAAATGCTAATCCAATTGTCAACAT
TCCATAGTTATCATGATTTAGAGTTTCTATTTGTGACACGTGAAGATGAAGTTGAAACATTGAAATGGGCACGTTGGTTGCCACATATGACA
TTGAGAGGGCAAAACATTAGAGGATTTGTTTACAATCAACGAACACGTGACCAAATTTTAACGTCAATCTATAGCATGATCAAAGAACGTA
TCCAAGCTGTGCGTGAACGCAGCAGAAGTAATGAGCAAATTATTTTCACACCGCAATTAGTGTTTGTCATTACAGATATGTCATTAATTATT
GATCACGTCATTTTAGAATATGTAAACCAAGATTTATCAGAATATGGTATTTCATTAATCTTTGTTGAAGATGTGATTGAAAGTTTGCCAGAG
CATGTAGATACCATTATTGATATCAAGTCTCGTACTGAAGGCGAACTGATTACAAAAGAAAAAGAATTAGTTCAATTGAAATTTACACCGGA
AAATATTGATAACGTCGATAAAGAATATATCGCGCGACGTTTGGCGAATTTGATACACGTCGAACATTTGAAAAATGCAATTCCTGATAGTA
TTACATTTTTAGAGATGTATAACGTGAAAGAAGTAGATCAGCTTGATGTAGTTAATCGATGGAGACAAAACGAAACATACAAAACGATGGC
AGTACCTTTAGGCGTAAGAGGTAAAGATGATATTTTATCATTGAACTTACATGAAAAAGCACACGGGCCACATGGTTTAGTTGCTGGTACC
ACTGGTTCAGGGAAATCTGAGATTATCCAATCATACATTTTATCTTTAGCTATTAATTTTCACCCTCATGAAGTTGCATTCCTATTGATTGACT
ATAAAGGTGGGGGTATGGCGAACTTATTTAAAGATTTAGTCCATCTAGTTGGTACGATTACAAACTTAGATGGCGATGAAGCGATGCGTGC
CTTAACATCAATCAAAGCCGAATTGAGAAAACGTCAACGTTTATTCGGAGAGCATGATGTTAACCATATTAATCAATACCATAAGTTATTTA
AAGAAGGTGTTGCGACAGAACCAATGCCACATTTATTCATTATTTCCGATGAGTTTGCCGAATTAAAATCAGAACAACCTGATTTTATGAAA
GAACTTGTATCAACGGCACGTATTGGACGTTCGTTAGGTATTCATTTAATACTTGCGACACAAAAACCATCGGGTGTTGTTGATGACCAAAT
TTGGTCTAACTCTAAATTTAAGTTGGCATTAAAAGTACAAGATAGACAAGACAGTAATGAAATTTTAAAAACACCAGATGCAGCAGACATT
ACATTACCAGGTCGTGCGTATTTACAAGTTGGTAATAATGAAATTTATGAATTGTTCCAATCTGCATGGAGTGGAGCAACATATGACATCGA

AGGCGATAAATTAGAAGTTGAAGATAAGACGATTTACATGATTAATGACTATGGTCAACTGCAAGCGATCAACAAAGACTTGAGTGGACTT
GAAGATGAAGAAACGAAAGAAAACCAAACTGAGTTAGAAGCGGTTATCGATCATATCGAATCTATTACAACACGATTAGAAATTGAAGAA
GTTAAGCGTCCATGGTTACCACCATTACCAGAAAATGTATATCAAGAAGATTTAGTAGAAACAGATTTTAGAAAATTATGGTCAGATGATG
CAAAAGAAGTGGAATTAACATTAGGACTTAAAGACGTACCAGAAGAACAATATCAAGGGCCGATGGTATTGCAATTGAAAAAAGCTGGTC
ACATCGCGTTAATCGGAAGTCCAGGATATGGTAGAACAACGTTCTTACACAACATTATTTTCGATGTTGCAAGACACCATCGTCCTGATCAA
GCACACATGTACTTGTTCGATTTCGGAACTAATGGTTTGATGCCAGTCACAGACATACCACATGTCGCTGATTACTTTACAGTAGATCAAGA
AGACAAGATTGCGAAGGCGATACGTATATTTAATGATGAAATTGATCGTCGTAAGAAGATTTTAAGTCAGTATCGTGTCACTAGTATTTCTG
AATATCGAAAATTAACTGGTGAAACAATTCCGCATGTCTTTATTCTTATTGATAACTTTGACGCAGTAAAAGATTCACCTTTCCAAGAAGTTT
TTGAAAATATGATGATTAAAATGACACGTGAAGGGCTAGCATTAGACATGCAAGTAACCTTAACTGCCTCAAGAGCTAACGCTATGAAAAC
ACCAATGTACATTAATATGAAAACGCGTATCGCCATGTTTTTATATGATAAATCAGAGGTGTCGAACGTAGTAGGACAGCAAAAATTTGCG
GTTAAAGATGTAGTGGGTCGAGCATTGTTAAGTAGCGATGACAACGTATCATTCCATATTGGCCAACCATTTAAACATGATGAGACCAAAT
CATATAATGATCAAATTAATGATGAAGTATCGGCGATGACAGAATTTTATAAAGGTGAAACACCAAGTGATATTCCTATGATGCCAGATGA
AATTAAATATGAAGATTACAGAGAATCATTAAGCTTACCGGATATAGTTGCAAATGGTGCTTTACCAATTGGATTAGATTATGAAGGTGTTA
CACTACAAAAAATTAAATTAACTGAACCAGCAATGATTTCATCAGAAAATCCGAGAGAAATTGCGCATATTGCTGAAATTATGATGAAAGA
AATTGACATATTAAATGAAAAATATGCGATTTGTATCGCAGACTCAAGTGGAGAGTTTAAAGCTTATAGGCATCAAGTGGCTAACTTTGCC
GAAGAAAGAGAAGACATTAAAGCGATTCATCAACTAATGATTGAAGACTTAAAACAAAGAGAAGTGGACGGCCCATTTGAAAAAGATTCA
CTTTATATTATCAATGATTTTAAAACATTTATTGATTGCACGTATATTCCGGAAGATGATGTTAAAAAACTTATTACAAAAGGACCAGAACTT
GGCTTGAACATTTTATTTGTCGGCATTCATAAAGAATTAATAGATGCTTATGATAAACAGATTGATGTTGCACGTAAAATGATTAACCAATTT
AGTATAGGTATTCGTATTTCAGACCAACAATTCTTTAAATTTAGATTTATTCAACGAGAACCTGTTATTAAAGAAAATGAAGCATATATGGTC
GCAAACCAAGCTTATCAAAAGATTAGATGGTTTAAATAG

Gene: esxC (protein esaC)

Position: 320983 to 321375, length: 393 nt, orientation: FORWARD

Perfect match to: (COL-CP000046-[319760:320152], highly conserved allele)

Sequence:

ATGAATTTTAATGATATTGAAACAATGGTTAAGTCGAAATTTAAAGATATTAAAAAGCATGCTGAAGAGATTGCGCATGAAATTGAAGTTC
GTTCTGGATATTTAAGAAAAGCTGAACAATATAAGCGATTAGAATTTAATTTGAGTTTTGCACTAGATGATATTGAAAGCACAGCAAAGGA
CGTACAAACTGCAAAATCTAGTGCTAATAAGGACAGTGTAACTGTTAAGGGAAAGGCGCCCAATACGTTATATATTGAAAAAAGAAATTTG
ATGAAACAAAAGCTTGAAATGTTGGGTGAAGATATCGATAAAAATAAAGAATCCCTCCAAAAAGCTAAGGAAATTGCTGGCGAAAAGGCA
AGTGAATATTTTAATAAAGCAATGAATTAA

Gene: esxB (virulence factor esxB)

Position: 321391 to 321705, length: 315 nt, orientation: FORWARD

Perfect match to: (11819-97-CP003194-[327810:328124], allele observed in CC80+CC1290)

Sequence:

ATGGGTGGATATAAAGGTATTAAAGCAGATGGTGGCAAGGTTGATCAAGCGAAACAATTAGCGGCAAAAACAGCTAAAGATATTGAAGT
ATGTCAAAAGCAAACGCAACAGCTCGCTGAGTATATCGAAGGTAGTGATTGGGAAGGACAGTTCGCCAATAAGGTGAAAGATGTGTTACT
CATTATGGCAAAGTTTCAAGAAGAATTAGTACAACCGATGGCTGACCATCAAAAAGCAATTGATAACTTAAGTCAAAATCTAGCGAAATAC
GATACATTATCAATTAAGCAAGGGCTTGATAGGGTGAACCCATGA

Gene: esaE (putative protein)

Position: 321702 to 322379, length: 678 nt, orientation: FORWARD

Perfect match to: (11819-97-CP003194-[328121:328798], allele observed in CC80)

Sequence:

ATGATGAAAGATGTTAAGCGAATAGATTATTTTTCTTACGAAGAATTAACAATTTTAGGTGGTAGTAAATTGCCTCTCGTAAATTTTGAATTG
TTTGATCCATCAAATTTTGAAGAAGCTAAAGCTGCTTTAATTGAAAAGGAATTAGTAACAGAGAATGACAAGTTAACTGATGCAGGTTTTAA
AGTGGCGACATTAGTAAGAGAGTATATTAGCGCCATTGTAAATATTCGAATTAATGATATGTATTTTGCACCATTTAGCTATGAAATAGATG
AATATATTTTGTTAAGCCGGTTTAAAAATAATGGATTTCAAATACGAATTATAAGTAAAGACATTGCATGGTGGTCGATTGTACAATCATAT

CCTTTATTAATGAGACAAGAAAAGTCCAATGATTGGGACTTTAAACAAATTGACGATGAAACATTGGAGAACTTAAATAATGAAAGTATCG
ATACGATTGAGCGTGTGTTAGAAATTGAAATCTACAATCATCAAGGTGACCCTCAACAAAGTTTATATAACATTTATGAACAAAATGATTTG
CTACTCATTCGATACCCATTAAAAGATAAAGTACTGAATGTTCATATTGGTGTCATTAATACATTTATACGAGAATTATTTGGATTCAATACT
GATGAAAATCATATTAATAAGACAGAGGAGTAA

Gene: esxD (putative protein)

Position: 322379 to 322696, length: 318 nt, orientation: FORWARD

Perfect match to: (11819-97-CP003194-[328798:329115], allele observed in CC80+CC121)

Sequence:

ATGACGTTGAGTGGGAAAATTAGTGTTAAAGCTGAAACGATTGCACATGTTGTAAAAGAATTAGAAAGCATTAGTCAAAAGTATGATGAA
ATAGCTCAAAACTTTGGAAAAATAGCGCAATTAAATTATTACAGTAGTGAAAAAGCTGCACATTCTATGGAAAATGGCTATAGTAGTGCTG
CAACAGTCATTAGTGGTCTCAAAGGTCCACTGAGCACACTCGGTGGTGGCGTCATGAATTCAGCACAAAAGTTCTTTGAAGCAGATGAACA
TTGGGGTACGGAATTTGCCAAGCTTTACTATAATATTGAGGGATAG

Gene: essD (putative protein)

Position: 322706 to 324550, length: 1845 nt, orientation: FORWARD

Perfect match to: (11819-97-CP003194-[329125:330969], allele observed in CC80-ST291)

Sequence:

ATGACAAAAGATATTGAATATCTAACAGCTGATTATGACAATGAAAAGTCATCTATCCAAAGTGTAATAGATGCAATAGAGGGGCAAGACT
TCTTAGATGTAGATACAACAATGGATGATGCGGTAAGCGATGTCAGTTCTTTAGACGAAGATGGCGCAATATCATTAACAAGTAGTGTAGT
AGGTCCACAAGGATCTAAATTAATGGGTTATTATCAAAATGAGCTATATGATTATGCATCTCAATTAGATTCGAAAATGAAAGAAATTATTG
ACACGCCATTTATAGAAGATATAGATAAAGCATTCAAAGGTATAACGAATGTTAAATTGGAAAATATATTAATAAAAAATGGCGGTGGTCA
TGGTAGAGATACCTATGGGGCTTCTGGGAAAATTGCAAAGGGAGATGCCAAGAAAAGTGACAGCGATGTTTATAGCATCGATGAAATATT
AAAATCGGATCAAGAATTTGTAAAAGTAATTGATCAGCATTACAAAGAAATGAAAAAAGAAGATAAGAAATTATCTAAGAGTGATTTTGAA
AAAATGATGACTCAGGGCGCTTCTTGTGATTACATGACAGTAGCTGAAGCGGAAGAGCTAGAGGAGCAAAAGAAAAAAGAAGAAGCTAT
AGAGATTGCAGCACTAGCTGGTATGGTAGTTTTATCTTGTATTAATCCTGTTGCTGGAGCAGTAGCTATTGGTGCTTATTCCGCCTATTCAGC
AGCAAATGCAGCCACAGGAAAAAATATTGTAACTGGAAGAAAGCTATCTAAAGAAGAACGAATCATGGAAGGACTTTCGCTTATTCCATTG
CCAGGTATGGGCTTCCTCAAAGGTGCTGGGAAAAGTTTAATGAAATTAGGCTTCAAAGGCGGAGAAAAAATTTGCAGTTAAAACAGGATTG
CAAAAGACAATGCAACAAGCAGTTAGTCGTATTTCACCTAAAATGGGAATGATGAAAAACAGTGTGTTGAATCAATCTCGTAACTTTGCTC
AAAATACTCATGTTGGACAAATGCTGAGTAACATGCGTGGTCAAGCAACTCATACTGTTCAACAAAGTAGAAATTGGATTGGACAACAAGC
ACAAAACGTCAAGCGAATAGTGAATAATGGACTTGATAAAGAAATAGCACATCCATTTAAACAACAACTTGCACCAGCGGGAATGGGTGG
TATAAAATTTGCTGAAACAACTACTTTGAGAAACATGGGTCAAAAAATGAAACGTGCTGTTACACCACAAAATCACGTGACACATGGTCCG
AAAGATAGTATGGTGAGAAGTGAAGGTAAACATAGTGTAAGTAGCCATGAAATAAATTCATCAAAATATGTTGAATCACCAAACTACACCA
AGGTTGAATTCGGAGAACACTATGCAAGACTTAGACCTAAGAAACTAAAAGCAAATATAGAATATACAACACCAACTGGTCACATATATCG
GACCGATCATAAAGGGCGCATAAAAGAAGTTTATGTAGACAATCTTTCTCTAAAAGATGGCGGTCGTAATAACCATGCACAAAGAACTGTG
GGGGGAGAGGATAGATTACCAGACGATGATGGAGGTCACTTAATCGCTAGAATGTTTGGTGGGTCAAAAGACATAGATAACCTTGTAGCA
CAAAGTAAATATATCAACCGTTCATTTAAGGAAAATGGTGATTGGTACAAGTTGGAAAAACTATGGGAAAAGGCAATCAAGTCTGGAAAA
CCAATAGAAAATATTAAAATAGAAGTTAAATATAAAGGTAATAGTCAAAGGCCAACTATATTTAAGGTTGGATATGAGATTAATAATGAAC
GAAAAGTTAAAACAATAGAAAATATATAG

Gene: DUF600-locus5 (putative protein)

Position: 324561 to 325052, length: 492 nt, orientation: FORWARD

Perfect match to: (CN1-CP003979-[306859:307350], allele observed in CC72)

Sequence:

ATGACATTTGAAGAGAAGCTTAGCAAAATATACAATGAAATTGCGAATGAGATTAGCAGTATGATACCGGTAGAGTGGGAAAAAGTATAT
ACAATGGCTTATATAGATGATGGAGGAGGTGAAGTATTCTTTAATTATACTAAACCAGGTAGCGATGAATTGAATTATTACACGAATATATC
TAAAGATTATAATGTTTCAGAGGAAATATTTGATGATTTATGGATGAATCTGTATTACTTGTTTAAGAATTTAAGGAATTTATTTAAAGAAGA
AGGTCTTGAGCCATGGACATCATGTGAATTTGATTTTACAAGAGAGGGTAAATTGAAAGTATCATTTGATTATATAGATTGGATAAATACA

GAGTTTGATCAATTGGGCCGTGAAAATTATTATATGTACAAAAAATTTGGGGTTATACCAGAAATGGAATATGAAATGGAGGAGGTTAAAC
AAATCGAGCAATATATTAAAGAGCAAGAAGAATAG

Gene: Q1Y4R3 (putative protein)

Position: 326088 to 326699, length: 612 nt, orientation: FORWARD

Perfect match to: (Newbould_305-AKYW01000002-[547002:547613], allele observed in CC97+CC72+CC80+CC97)

Sequence:

ATGGAGTTCTTATTATTAATTGTCGTAGCCGGACTGTATTATATTATATATTTAACTGCTGTGATGTATTCTGAAAAAATAGTAGTATTGCCT
ATAATCATCTATGCCATTGTGTTTGTAATAATTGGTATCACTTATATCTTTATAGGCGACAGCTATGATCAATTAACAAATTTCAATGTGATTT
TGTATATGGGGAGTTTGTTTTATGCATGGATGGCTTTTAGAAATCTTTGGAACAGACCATTATTATTAAAATATAAGAATATTACAGATAGT
TCAAGTGGAATAGTTAATAAATCTGAATACAATTCAGTTGAAAGCTTACGTATAAATATTGAAATAGCTAAGTATAAAGGGATTATTTCTTT
GATAGTAGCTATAGTACTAACGGTATTAATGACATTAAAGTCAACACCTCAAATTACTGCGGAAACACGTGACTTAAGTATCTCATTTTTCAT
ACTCAGCTTATTTATCATTGTTATATTTGCTGTTTGGGATTTAATTATTAGAGTTAGAAAAGGAGCGTTTGCTTTTGTTGTAATAAGGCCAAT
ATTATTCAGTTGTTGGTTATTTATTCTGAATATGATTTTATCGAGATTATTATAA

Gene: Q9L3N6 (putative protein)

Position: 326834 to 327052, length: 219 nt, orientation: FORWARD

Perfect match to: (11819-97-CP003194-[333253:333471], allele observed in CC80+CC97)

Sequence:

ATGGAAAACCAAAATCAAGGCAATGGCTTAAAAATTGCAACATGGGTATTTATTGTATTAACAATAGTTACACCGTTATTTGGTATTGGAAG
TATTGTTTGTAGTATTAATTACAAAAAATACGATGCTGAAAAAGGTTCGAAGTTATTGCAAATTGCAATTATCGTAACAATAATTGCTTTTGT
TTTAAATTTATTAGCATATTTAGGTTTAAGATAA

Gene: DUF600-other (putative protein)

Position: 327142 to 327642, length: 501 nt, orientation: FORWARD

Perfect match to: (11819-97-CP003194-[333561:334061], allele observed in CC80)

Sequence:

ATGACTTTCGAAGAAAAACTAAGTCAAAAGTACAACGAGATTGCGAATGAGATCAATGGAATGATACCGGTAGAGTGGGAAAAGGTATAT
GTAATTGCATATGTGGACGATGGAGGTGGGCAAGTTATATTTTATTATACCAAGCCCGGAAATGATGAGCTGTATTATTATTCGAGTATCGT
TGAAGATTATAATGTTTTAGAAGAAGTCTTTGATGATTTATGGATGGAACTTTATAGATCATTTAAAAAATTAAGAAATATATTTAAAGAAG
AAAGTCTTGAACCATGGACATCATGTGAATTTGACTTTACAAGCGACGGTAAATTAAACGTATCTTTTGATTATATAGATTGGATGAATTCA
GAATTTGGACAAATAGCAAAAGAAAATTATTATATGTACAAAAAATTTGGAATTGTACCTGAAACGGAATATGAAATTAATAAAGTTAAAG
AAATCGAACAATATATTATAGAGCAAGAAGAAGCTGAACAATAG

Gene: Q1Y4R2 (putative protein)

Position: 328357 to 329040, length: 684 nt, orientation: FORWARD

Perfect match to: (11819-97-CP003194-[334776:335459], allele observed in CC80+CC97)

Sequence:

ATGACTACTAAGGAAAATATAGACATTCTTCGAAAGCCAGGTGGACAAGCCTTAAGTTTAGCATCATTATTTATGATACTTTTTTCATGTCTA
ACTTTCTTTTTTGGTTTAGATTATGAAAGGTTTCCAAATTATTTAAAGATAACGACAATTATAGAATTAATAATTATTGTAATTAGTTTACTTC
AATGGATTAGATTTATAGATTTCGAAAAGGAAAGCGCACAGAAATATAAAAAAATATATGCCCGATTTTTAGTTGTTATAAATGTGCTAACT
ACTATCACCGCAGTATTTGCAACATGTAATCTTTACTATTTTGTTGCTGTGCAAAATCATTATGATTTATTTAATTATTGGTTAATGGGTACTA
TTTCAATCATAATTAGCTATTTGTTATTAGTAATTGGCGGAATGTTCACGTTATTAAAATTACCTAAAGTAACAAAACGTTGGGGTGGTAAA
ACTAAAACACATTTCGGTTTATTATTAACCGCGTTGAGCGCATTTATATATATTGAAAGAATTATCGAATATATATTGGTTCCCAATGTCGTA

GAATCCAAGTTTGTCATAATGGTAAGTATCATTATTATTGCTTGTACACAATTTGTAGCCTTTCAATTTATTATGCAATACAGTAGATTCTATA
TTTTTGAATTAAACACTGAAGATGATGACTAA

Gene: DUF600 (putative protein)

Position: 329617 to 330105, length: 489 nt, orientation: FORWARD

Sequence:

ATGACTTTCGAAGAAAAACTAAATGAAATGTACAACGAGATTGCGAATAAGATCAGTGGGATGATACCAGTAGAATGGGAGCAAGTATAT
ACGATAGCCTATGTAAATGATAGAGGTGGAGAGGTCATTTTTAATTATACTAAACCAGGTAGCGATGAATTGAATTATTACACGGATATAT
CTAGAGATTATAATGTTTCGGAAGAAATATTTGATGATTTATGGATGAATCTGTATTACTTGTTTAAGAATTTAAGGAATTTATTTAAAACAG
AAGGACATGAACCATGGACATCATGCGAATTTGACTTTACAAGAGATGGTAAATTAAACGTATCTTTTGATTATATTGATTGGATTAAATTA
GGCTTAGGTCCGTTAGCTAGAGAAAATTACTATATGTATAAAAAATTTGGGGTTATACCAGAAATGGAAGAAATTAAAGAAATCGTGCAAT
ATATTAAAGAGCAAGATGAAGCTGAAATATAG

Gene: DUF600 (putative protein)

Position: 330116 to 330612, length: 497 nt, orientation: FORWARD

Sequence:

ATGACTTTCGAAGAAAAACTAAGTCAAATGTACAACAAGATTGCGAATGAGATTAGTGGGATGATACCAGTAGAGTGGGAAAAGGTATAT
ACAATTGCCTACCTAAATGATGAAGGTGGAGAGGTCGTTTTTAATTATACTAAACCAGGTAGCGATGAATTGAATTATTACACATATATCCC
TAGAGAGTATAATGTCTCTGAAAAGTATTTTATGATTTGTGGACGGATTTATATAGATTGTTTAAGAAGTTAAGAGATTTATTTAAAGAAGA
TTTAGAACCATGGACATCATGTGAATTTGATTTTACAAGAGATGGTAAATTAAACGTATCTTTTGATTATATTGATTGGATGAATTCAGAATT
TGGACCAATGGGAAGAGAAGATTATTACATGTATAAAAAGTTTGGAATTTGGCCTGAAAAAGAATATGCAATAAATAGGGTTAAAAAAT
AGAAGATTATGTTAAAGAGCAAGAAGAAGCTGAACTATAG

Gene: DUF600 (putative protein)

Position: 330623 to 331111, length: 489 nt, orientation: FORWARD

Perfect match to: (11819-97-CP003194-[336547:337035], allele observed in CC80)

Sequence:

ATGACTTTCGAAGAAAAACTAAGTGAAATATACAACAAGATTGCGAATGAGATTAGTGGGATGATACCAGTAGAGTGGGAAAAAGTATAT
ACAATTGCTTATGTAGATGATGAAGGTGGAGAGGTCGTTTTTAATTATACTAAACCAGGTAGCGAGGACTTGAATTATTACACATATATCCC
TAGAGAGTATAATGTCTCTGAAAAAGTATTTTATGATTTGTGGACGGATTTATATAGATTGTTTAAGAAGTTAAGAAACGCATTTAAAGAAG
AAGATTTAGAACCATGGACATCATGTGAATTTGACTTTACAAGAGAGGGAAATTTGAAAGTATCATTTGATTATATAGATTGGATTAAATTA
GGTTTTGGCCCATCTGGAAAGGAAAACTACTATATGTATAAAAAGTTTGGTGTTTTACCAGAAATGGAATATGAAATGGAAGAAATTCGAG
AAGTAGAGAAGTATGTTAAAGACCAAGAGTAG

Gene: Q1Y4S0 (putative protein)

Position: 331736 to 332110, length: 375 nt, orientation: FORWARD

Perfect match to: (11819-97-CP003194-[337660:338034], allele observed in CC80)

Sequence:

ATGAAAAGAATATTGGTAGTATTTTTAATGTTAGCAATTGTATTAGCAGGTTGTTCTAATAAAGGTGAAAAGTATCAAAAAGATATTGATAA
AGTGTACAAAGAACAGAATCAAATGAATAAAATTGCCTCGAAAGTACAAAACACTATTAAAACAGACATTAAACAAGAAGACAGTAATACA
CATGTTTATAAAGATGGTAAAGTCATTATTATTGGTATTCAATTGTATAAAGATCGTGAAAAAATGTATTATTTCGCATATGAAATAAAAGA
TGGTAAGGCAGAGATCAATAGAGAAATAGACCCAATTAAGTATATGAAAGACCATAAAGCAGATTATGAAGATGAAAATGTAGAAGTGG
AAAAAGATTAA

Gene: Q2G173 (putative membrane protein)

Position: 332266 to 332664, length: 399 nt, orientation: FORWARD

Perfect match to: (11819-97-CP003194-[338190:338588], allele observed in CC80)

Sequence:

ATGGAAAAATCGATCAAAATAATGACAGTAATAGGAATTGTTGTTCAGGGTTTAGCAACGGTATTTAGTTTACTATTGATGGTTTTAGCAGC
ATCAGGTGTAATGACTACAGATGTGTCAACAACAGTTAATGGTGAGGTTGACCCAGTTGATGCAGAAACAGCAGCAGCAATTTTCACTGTA
TTATTCTTATCCCTATTCATATTTGGAATCATTTCAATTATTTTAGGTGCAATCGGTATGTTTAAAGCATCTAAAAACAAAAAAATGAGTGGT
ATATTGTTGATTATTGGAGCTGTAATAAGTGGTAACATAATTACATTTGCTTTATGGTTAATCAGTGGTATTAAACTACTTACTAATAACAAG
CCTAAAGATGAAATAAGCGACTTATCATAA

Gene: nirC-focA-L1 (formate/nitrite transporter, locus 1)

Position: 332880 to 333704, length: 825 nt, orientation: REVERSE

Perfect match to: (11819-97-CP003194-[338804:339628:r], allele observed in CC80)

Sequence:

TTAATTATTTTTATAAAATTGATGATGATCATTCAAGTAAGCATAGAATAAACCTATAATGAGTCCGCCTCCAATATAGTTACCGATAAAAGC
CGCAGCGATATTCGAAATAGCTGGTATGAAGTGCAATGTATCAACTTGATAAATTAAACCACCCATAAATAAGCAACTGTTGTAAACGACA
TGTTCATAACCCATAAAGGCAAATATGGTAACACCGAACATCATGACAAACATTTTTGCGAGTACATCGTCAATTTGCATGGCAATAACTAA
TGAAATATTGATAAAGAAATTGGCGAATATCGCTTTCATTAATATACTTACAAAACCAGTAGACAACGTTTTGTGCTCAATAACTGCTGATA
ACTGATTTAACATATCTGGCGTCATTACATTTGAAAAACGCATGAAACTAAATAAAATAGCAGCACCTAAAATATTTCCTGCAAAGCATAAT
AAAAATATTTTCAATACTCTAGTTGGTTTAATTACTTTATAATACAGGCCTACAGTAAAGTACATGAAGTTACTGGTTAGTAGTTCGGAGTTT
GTAAATAAAATGAGTACTAACGCAAAGCTGAATGTAATGGCACTGGCCATATTCACAATGCCTGGCGGTAAATCTGGTTCGTGTGTTGCTTT
AACTGATAATACGAAGACCGTAATAATCCCGATAATAAATCCTGCCATCATCGCGCGTAATAAATAACGTTTTAAATAAACGCTTTGTAATA
TATCTTTCGTTCTTATCGTTTCGACTACGTTATTTACCCAGTCGTCCCCATAAAATATTTTATCCCATTTAATATGTTTCTCCTTCAC

Gene: brnQ2 (branched-chain amino acid transport system II carrier protein)

Position: 333941 to 335248, length: 1308 nt, orientation: REVERSE

Perfect match to: (11819-97-CP003194-[339865:341172:r], allele observed in CC80)

Sequence:

TTAAGTTGTCGCTTGTTTCGGACGACGTATAACTACATCGATGATGAAACCGATAATAGCAAAGAGCATGAATGGTACAAGCCAAGCTAAA
TCGATATCTGCTAAAGGTAACATCATAAACCATTTCAAAATAACACCGTGTAATAAGTTGAAACTATTTAGTATTTGTAAAATTGAAATAATC
AATGTAATAACAGTTGCGAGTCGATAGGCCCAACTGAATCTGAATTTGCTAAACATGTTAGCAAATGATATGAGTACAAGTGCAATCGACA
CGGGATATATTAAAGTCAATAATGGGACAGCAATTTTTAAAATCATTTCTAAACCAAGTGTTGTAAATAAGAACCCTATGATAGAGAAAATA
AGTGCGAATATTTTATAAGAAAACTTAGGTACGTGTTTCTTAGTAAATGTGGCGCAAGCATTGACGAGTCCTATACATGTTGTTAAACATGC
AAGTATCACTGTCATACCAAATACTATGTTGCCAAATGAACCAAATACTCGTAATGAGTTATACGTCAATATATCTGTACCATCTTTAAAGTT
TCCTGGAGCCGTTGATGCCCCAACGTATGCAAGTGCAAAGTAAATCATTCCAAGTAATATGGCTGCAATAAGACCTGAAAAGCAGACATAT
TTTAAAATTTTCATGCGATCTGTGAGGCCTTTAAACTTATAGCCATTGACAATGACTACGGAAAAAGCTAACGCAGCAACAAGATCCATTGT
AAAATAGCCTTCCAAACTTCCTGAAATGAAAGGATGTGTTATATATTTATCCTTAGGTGCACTTAGTGCAGATTCAGGGTTGAAAATGACAG
CAATACTTAATAGAGCGACCATTAATAGTAATAATGGTGTTAATAATTTACCTAAATTATCAACGATTTTCGATGGATTTAAACTAATCCAGT
AAACGATGGCAAAAAAGACTGCTGCGAATATAATTAAAGTCCATTGGTTGTGCACAGGTAAAATGTGTCTTGTACCAATTTCGTACGCGAC
ATTTGCAGCACGTGGAATACCGTAAAATGCTCCGATAGACATGTAAATCACGACAGCAAAAATAAACCCGAACCATGGATGTATACGATTG
CCTACACTTTCAACACCTTCATCATAAAATGCAACAACAATAACAGTAATAAAGGGGAGTAATATGCCTGTAAGGGCAAAGCCTAACATACC
AATCCACATATTTTGACCTGCTGTATGGCCAAGCATGGGCGGGAATATTAAATTTCCGGCTCCAAAAAATAGTGAAAATAACATGAGGCCC
GAAATAATAACTTGTCTTTTCAA

Gene: Q99WS0-sapS (secreted acid phosphatase)

Position: 335832 to 336722, length: 891 nt, orientation: FORWARD

Perfect match to: (11819-97-CP003194-[341756:342646], allele observed in CC80+CC7+CC12+CC80)

Sequence:

ATGAATAAAATTTCAAAGTATATTGCAATAGCATCATTATCGGTAGCGGTTACAGTTTCGGCACCACAAACGACAAATTCTACAGCGTTTGC
CAAAAGTTCTGCTGAAGTTCAACAAACACAACAAGCTTCTATACCAGCATCACAAAAGGCGAATCTTGGTAATCAAAATATTATGGCAGTG
GCTTGGTATCAAAATTCAGCTGAAGCAAAAGCATTATATTTACAAGGTTATAACAGTGCAAAGACACAGTTAGATAAAGAGATTAAAAAGA
ATAAAGGTAAACATAAGTTAGCTATTGCTTTGGATTTAGATGAAACAGTTTTAGATAATTCTCCATATCAAGGCTATGCATCAATACATAATA
AACCTTTCCCAGAAGGTTGGCATGAATGGGTACAAGCTGCTAAAGCTAAACCTGTCTATGGCGCAAAAGAATTCTTGAAATATGCTGACAA
AAAAGGTGTCGATATCTACTATATTTCTGATAGAGATAAAGAAAAAGATTTAAAGGCAACACAAAAGAACTTAAAACAACAAGGTATCCCT
CAAGCTAAGAAGAGTCATATTTTACTAAAAGGTAAAGATGATAAGAGTAAAGAATCACGCAGACAAATGGTTCAAAAGGATCATAAACTT
GTCATGCTATTTGGAGATAATTTATTAGACTTTACAGATCCAAAAGAAGCTACAGCTGAATCTCGTGAAGCATTAATTGAAAAACATAAAGA
CGATTTCGGTAAGAAATATATCATTTTCCCTAACCCAATGTATGGTAGTTGGGAAGCTACGATTTACAACAATAACTATAAAGCAAGTGACA
AAGCAAAAGATAAATTACGTAAAAATGCTATTAAGCAATTCGATCCTAAAACAGGCGAAGTTAAATAA

Gene: Q2G168 (ABC transporter, transmembrane permease)

Position: 336971 to 338020, length: 1050 nt, orientation: FORWARD

Perfect match to: (NN50-BAEA01000012-[65672:66721:r], allele observed in CC4803+CC8+CC97)

Sequence:

ATGTTTTTAGCTTGGAATGAAATACGGCGCAACAAATTGAAGTTTGGACTAATTATTGGTGTGTTAACGATGATTAGTTACTTGCTATTTTTA
TTATCTGGATTGGCGAATGGTCTTATCAATATGAATAAAGAAGGCATTGATAAGTGGCAAGCAGATGCCATTGTTCTAAATAAAGATGCCA
ATCAAACTGTGCAACAATCTGTTTTTAACAAGAAAGATATTGAAAATAAATACAAGAAGCAAGCTACTTTGAAGCAAACAGGGGAAATTGT
GTCTAATGGCCATCAAAAAGACAATGTTTTAGTGTTCGGTGTTGAAAAGTCATCATTTTTAGTTCCGAGTTTAATAGAAGGGCATAAAGCGA
CTAAAGATAATGAAGTGTTAGCTGATGAAACACTTAAAAATAAAGGATTTAAAATTGGCGACACATTATCACTATCTCAATCAGATGAAAA
ATTGCATATCGTAGGTTTTACAGAAAGTGCAAAATATAATGCGTCACCAGTCATTTTCACGAATGACGCTACCATTGCCAAAATCAATCCTA
GATTGACTGGAGATAAAATTAATGCAGTTGTTGTACGTGATACAAATTGGAAAGACAAAAAATTAAACCAAGAGCTTGAAGCGGTAAGTA
TTAATGACTTTATTGAAAATTTACCAGGTTATAAACCACAGAACTTAACATTAAACTTTATGATTTCATTCTTATTTGTCATTTCAGCTACAGT
TATAGGCATTTTCTTATATGTCATGACATTACAAAAAACAAGTTTATTTGGCATATTAAAGGCTCAAGGATTTACGAATGGCTATTTAGCGAA
CGTAGTTATTTCGCAGACGCTCATATTAGCATTATTTGGTACGGCATTTGGCTTACTGTTAACAGGCGTTACAGGTGCATTTTTACCTGATGC
AGTACCTGTCAAATTCGATGTACTAACATTGCTCGTATTTGCAATTGTGTTAATGATTGTCTCTGTATTAGGAAGTTTATTCTCCATTTTAACA
ATTAGAAAAATAGATCCGTTAAAGGCGATCGGGTAG

Gene: A5IPH6 (ABC transporter, ATP binding protein)

Position: 338033 to 338710, length: 678 nt, orientation: FORWARD

Perfect match to: (11819-97-CP003194-[343957:344634], highly conserved allele)

Sequence:

ATGTTGAAATTTGAAAATGTAACAAAGTCATTTAAAGATGGGAATCGTAACATTGAAGCGGTTAAAGATACAAATTTTGAGATAAATAAAG
GTGATATTATAGCATTGGTTGGACCTTCTGGCTCTGGTAAAAGTACATTTCTAACTATGGCAGGTGCTTTACAAACACCGACATCTGGGCAC
ATTTTAATCAATAACCAAGATATTACGACAATGAAGCAAAAAGCATTGGCAAAAGTTAGAATGTCTGAAATAGGTTTTATTTTACAAGCTAC
AAACCTTGTACCATTTTTAACGGTAAAGCAACAATTTACATTATTGAAAAAGAAAAATAAGAATGTTATGTCTAATGAAGACTATCAGCAAC
TTATGTCACAATTAGGTCTAACTTCATTGCTTAATAAGTTACCTTCAGAAATTTCAGGTGGTCAGAAACAACGTGTGGCGATAGCCAAAGCG
TTATATACGAATCCGTCGATTATTTTAGCGGATGAACCTACCGCGGCGTTAGATACTGAAAATGCGATTGAAGTCATTAAAATTCTACGTGA
TCAAGCCAAACAAAGAAAGAAAGCATGTATTATTGTTACACATGATGAACGACTTAAAGCATATTGTGATCGTTCATATCATATGAAAGAT
GGCGTCCTTAATCTTGAAAATGAAACAGTAGAATAG

Gene: pfoR (putative perfringolysin O regulator protein)

Position: 338918 to 339949, length: 1032 nt, orientation: FORWARD

Perfect match to: (M1216-AIYW01000019-[7979:9010], allele observed in CC445+CC80)

Sequence:

ATGGATTTATTGATAGGTACTTTATTTTTATTTTTGGTCTTAGTGATTTTTACATTATTTACATATAAAGCACCTAATGGTATGCGTGCCATGG
GAGCATTAGCTAATGCAGCAATCGCAACATTTTTAGTGGAAGCATTTAATAAATATGTTGGTGGCGAAGTATTCGGTATTAAATTTTTAGAA
GAGCTAGGAGACGCTGCGGGAGGTCTAGGTGGTGTCGCTGCCGCTGGATTAACAGCATTAGCTATCGGTGTGTCACCAGTATATGCATTA
GTTATAGCAGCCGCGTGCGGTGGTATGGATTTATTACCAGGTTTCTTTGCGGGTTATATGATTGGATATGTGATGAAATATACAGAGAAAT
ATGTGCCGGATGGTGTCGACTTAATTGGATCGATTGTCATCTTAGCGCCATTAGCTCGTCTTATTGCAGTATTATTAACGCCAGTAGTGAAT
AGTACATTGATTCGAATTGGTGATATTATCCAAAGTAGTACGAATACGAATCCAATTATCATGGGTATCATTTTAGGTGGTATTATTACGGT
TGTCGGCACAGCGCCATTGAGTTCAATGGCATTGACAGCATTATTAGGTTTAACGGGTGTACCTATGGCTATTGGTGCCATGGCAGCATTTA
GTTCGGCATTTATGAATGGGACGCTATTCCATCGCTTAAAATTAGGTGATCGTAAGTCTACGATTGCAGTAAGTATTGAACCTTTATCACAA
GCAGATATTGTATCAGCCAATCCAATTCCAATCTATATTACAAATTTCTTTGGTGGTGCGATTGCTGGTTTAATTATTGCTATGTCAGGTTTA
ATTAACGATGCGACAGGTACAGCTACACCGATTGCAGGATTTTTAGTAATGTTTGGATTTAATCATCCAATGACTATTGTAATTTATGGTGT
AGTAATGGCGATTGTAGGTGCGCTTGCAGGTTATCTTGGTTCAATCGTATTTAAAAAATATCCAATTGTTACTAAGCAAGACATGATTAATC
GAGGTGCAGTTGACGCATAG

Gene: Q5HJ57 (PfkB family carbohydrate kinase)

Position: 340292 to 341410, length: 1119 nt, orientation: FORWARD

Perfect match to: (11819-97-CP003194-[346216:347334], allele observed in CC80)

Sequence:

ATGAGCGATTCTGAGAAAGAAATTTTAAAAAGAATTAAAGATAATCCGTTTATTTCACAACGTGAACTTGCTGAGGCAATTGGATTATCTAG
ACCCAGCGTAGCAAACATTATTTCAGGATTAATACAAAAGGAATATGTTATGGGAAAGGCATATGTTTTAAATGAAGATTATCCTATTGTTT
GTATTGGCGCAGCGAATGTAGATTGTAAGTTTTATGTGCATAAAGATTTAGTTGCAGAAACATCAAATCCTGTAACGTCAACACGCTCTATT
GGTGGCGTAGCAAGAAATATTGCTGAGAACTTAGGTAGGCTTGGCGAAACGGTCGCTTTTTTATCTGCTAGTGGACAAGATAGTGAATGG
GAAATGATTAAACGATTGTCCACACCATTTATGAATTTGGATCATGTTCAACAATTTGAAAATGCGAGTACAGGTTCATATACAGCTTTAATT
AGTAAAGAAGGCGACATGACATATGGCTTAGCAGATATGGAAGTGTTTGACTACATTACGCCTGAATTTTTAATTAAGCGTTCACACTTATT
GAAAAAGGCTAAGTGCATTATTGTCGATTTGAATTTAGGCAAAGAGGCATTAAACTTCTTATGTGCCTATACCACGAAACATCAAATCAAAT
TAGTTATCACCACGGTTTCTTCCCCAAAAATGAAAAATATGCCTGATTCATTACATGCTATTGATTGGATTATCACGAATAAAGATGAAACA
GAAACATACTTAAATTTAAAAATAGAATCTACTGATGATTTAAAAATAGCTGCTAAACGCTGGAATGATTTAGGTGTTAAAAATGTTATTGT
GACAAATGGCGTGAAAGAACTCATTTATCGAAGTGGTGAGGAAGAAATCATCAAGTCAGTTATGCCATCAAATAGTGTGAAAGATGTTAC
AGGTGCAGGCGATTCATTCTGTGCTGCAGTAGTATATAGCTGGTTAAATGGGATGTCTACTGAAGATATATTAATTGCTGGTATGGTTAAC
GCAAAGAAAACGATAGAAACGAAATATACAGTTAGGCAAAACCTAGATCAACAGCAACTTTATCACGATATGGAGGATTATAAAAATGGC
AAATTTACAAAAGTATATTGA

Gene: psuG (pseudouridine-5'-phosphate glycosidase)

Position: 341385 to 342308, length: 924 nt, orientation: FORWARD

Perfect match to: (11819-97-CP003194-[347309:348232], highly conserved allele)

Sequence:

ATGGCAAATTTACAAAAGTATATTGAGTATTCTCGAGAAGTTCAGCAAGCACGGGAGAACAATCAACCGATTGTAGCATTAGAATCAACAA
TTATTTCGCATGGTATGCCGTACCCACAAAATGTTGAAATGGCAACAACAGTAGAGCAAATTATCAGGAATAATGGTGCCATTCCAGCAAC
CATAGCCATTATAGATGGCAAAATTAAAATTGGTTTAGAAAGCGAAGATTTAGAAATACTGGCAACTAGTAAAGACGTTGCTAAAGTATCT
AGAAGGGATTTAGCAGAAGTTGTTGCGATGAAGTGTGTTGGTGCTACTACTGTAGCGACGACGATGATATGTGCTGCAATGGCTGGTATT
CAATTTTTTGTTACAGGAGGTATTGGGGGCGTCCATAAAGGTGCAGAACATACGATGGACATTTCAGCAGACTTAGAAGAACTGTCTAAAA
CAAATGTCACTGTTATCTGTGCAGGTGCCAAATCAATTTTAGACTTACCTAAGACGATGGAGTATTTAGAAACAAAAGGCGTTCCAGTTATT
GGATATCAAACGAATGAATTGCCAGCATTCTTCACTCGCGAAAGCGGTGTTAAGTTAACAAGTTCGGTTGAAACGCCAGAACGACTTGCTG
ACATTCATTTAACAAAACAGCAGTTAAATCTTGAAGGTGGCATTGTTGTTGCTAATCCAATTCCATATGAGCATGCCTTATCAAAAGCATATA
TTGAGGCAATCATAAATGAAGCTGTTGTTGAAGCGGAAAATCAAGGTATTAAAGGTAAGGACGCCACACCGTTCTTGTTAGGGAAAATTGT
AGAAAAAACGAATGGTAAAAGTTTAGCAGCAAATATAAAACTTGTTGAAAACAATGCGGCGTTGGGTGCTAAAATTGCTGTCGCTGTTAAT
AAATTATTGTAG

Gene: nupC2 (nucleoside permease C, locus 2)

Position: 342319 to 343539, length: 1221 nt, orientation: FORWARD

Perfect match to: (11819-97-CP003194-[348243:349463], allele observed in CC80+CC5+CC15+CC80+CC772)

Sequence:

ATGAATATTTTATTCGCTATCACAGGGATAGCATTTGCACTATTTGTTGCGTTTTTATTCAGTTTTGATCGTAAAAACATAGACTTCAAAAAG
ACGTTAATAATGATATTTATTCAAGTGTTGATTGTGTTATTTATGATGAACACAACGATTGGTTTAACAATCTTAACTGCATTAGGCTCATTTT
TTGAAGGGTTAATAAATGTTAGTAAAGCAGGTATAAACTTTGTTTTTGGAGATATACAAAATAAAAATGGCTTTACGTTCTTTTTAAATGTGT
TACTGCCATTAGTGTTCATTTCTGTATTAATAGGCATTTTTAATTATATTAAGGTATTACCATTTATTATCAAATATGTAGGTATCGCTATTAAT
AAAATAACGAGAATGGGGCGCTTAGAAAGTTACTTTGCTATTTCAACAGCAATGTTTGGACAACCAGAAGTATATTTAACAATAAAAGATA
TTATTCCAAGATTATCTAGAGCGAAATTATATACAATTGCGACGTCTGGTATGAGTGCTGTTAGTATGGCAATGTTGGGTTCATATATGCAG
ATGATTGAGCCCAAGTTCGTAGTTACAGCTGTAATGTTAAATATTTTTAGTGCGCTTATCATCGCCAGTGTAATCAATCCCTATAAATCTGAT
GATAGTGATGTTGAAATTGATAACTTAACTAAATCAACGGAAACGAAATCAGTGAATGGAAAAACAGGAAAACCTAAGAAAGTTGCCTTTT
TCCAAATGATTGGTGATAGTGCGATGGATGGGTTTAAAATCGCTGTTGTAGTAGCCGTGATGTTGTTAGCGTTTATTTCATTAATGGAAGCA
ATCAATATCATTTTTGGTAGTGTTGGTTTGAATTTTAAACAGTTAATTGGTTATTTGTTTGCACCTATCGCATTCTTAATGGGGATTCCTTGGA
GTGAAGCTGTTCCAGCTGGCTCTTTAATGGCTACAAAATTAATTACAAATGAGTTTGTAGCAATGCTAGATTTTAAAAAATGTTTTGGGTGAC
GTATCAGCTAGAACACAAGGTATTATCTCAGTTTACTTAGTGAGTTTTGCTAATTTCGGTACTGTTGGTATCATCGTTGGTTCAATTAAAGGA
ATTAGTGATAAGCAAGGAGAAAAAGTTGCATCCTTTGCAATGAGGTTGCTACTTGGTTCAACTCTAGCTTCAATCATTTCAGGATCAATTAT
TGGCTTAGTATTGTAA

Gene: nanT (N-acetylneuraminic acid transporter)

Position: 343644 to 345176, length: 1533 nt, orientation: REVERSE

Perfect match to: (11819-97-CP003194-[349568:351100:r], allele observed in CC80+CC12+CC25+CC80+CC188)

Sequence:

CTACTTTTTCGTAGCCGTTTTTGAAATGTATGTTGATGGTTTATCTTTTTCAAAAATTGTTAATCCCGTTATATCTTTTTTATGTTTTGAAGGGA
CAATGAAGCTAAGTATATAAGCAAAGACAAAAGCAACTGTAAATGAAATGGTAGATACATAGAAAGGTGAGTTACCTTTGCCAACATCATT
ATAGACGTAAGCAAAGATGATACCCAATATTAATCCACAAATAACACCGAATGTATTCGTACGTTTAGTGAAAATACCAACTGCAAATACAC
CAGCCAATGGAACGCCGAATAATCCAGTCACAAACAAGAATAAATCCCATAAGTCATTTGAATTAGAAGCAATTAAGTATAGTGACATTCC
AAAACCGAAAATACCTGCAATGATGATGACCAAACGAGCAAAGTTAACTTCGTGTCGCTCGCTACCTTTTCCGAAGAAGCGTTGCTTAATGT
CGATTGAAATACAAGCAGATATAGAATTTAAACTAGATGAAATGGTAGACTGTGCAGCGGCGAAAATGGCTGCAATAAGTAATCCTGCTA
CAAATGGTGGCATCTCAGTCAAAATGAAATATGGCACTACAGATGATGTATTGAAGCCTTTTGGTAAAACAGCTTCATGTGTATAAAATGA
ATACAGCATTGTACCCATACCATAAAATAAGGGTGCTGAAATTAAAGCTAGGATACCATTTGTCCATAACGATTTATTTGTTTCTTTTAAACT
ATCAGAAGCTTGATAACGCTGCACGACGTCTTGACTCGCTGTGTATTGATACAAGTTGTTGAAAATATTTCCTAGGAAAATAATTGGAATGG
CAGCTGCCGCAGTGTTTAGCTTCCAATTATCTGCACTAATTAATTTTTTGTGCTCAATCGCATCTGCAAAGACAGTGCCAAAACCGCCTTTAA
TGTTCATAACACCTAGAATAATAATAACTAAAGCGCCGCCTAATAAAATGACGCCTTGAATGAAATCACTCCAAACCACACCTTCGAATCCA
CCTAAAAATGTATATAAAATACATAGTAAACCAACGAGTGATGCAACGATATAAGGGTTCATGTCTGATACAGATGTGATTGCTAATGTTG
GTAAGTAGATAACGATTGCAACACGCCCTAAATGGTAAACGACAAATAATAATGAGCCAATGACACGTATGCTAGGGCCAAATCTAGCTTC
TAAATATTCATATGCAGATGTTACCTTTAACTTTTTAAAGAAAGGGACATAGAAATAAATAAGTAATGGAATAATTGCTACGATAGCGATGT
TACCTGCAATATATGACCAATCTGTTAAAAATGCTTTCTCTGGTGTCGACATAAACGTAATTGCACTTAATGTAGTAGCATAAATTGAAAAG
CCAACTACCCAAGATGGCAAGCGACCACTTGCAGTAAAGAAACTATTCGTACTTTGACTCGCGCGCTTGGTAAAATAAACGCCAATGAGTA
ACATAGCTAGTAGATAAATGATAACGGCAACCCAGTTTAGTGTGCCAAATCCAACTTCTTTCAT

Gene: nanA (N-acetylneuraminate pyruvate-lyase)

Position: 345216 to 346097, length: 882 nt, orientation: REVERSE

Perfect match to: (TCH70-ACHH02000005-[150618:151499], allele observed in CC1+CC80)

Sequence:

CTATAAATCGTATTTTGCAATGAGTTGATCTAATGTTTGTCGATGTGCTTCGTTAAAAGGTTTGAAAGGTCTTTTCGGTAATCCTGCATCAAT
GCCACGGTGACGTAGTATTTCTTTCAATGTTGGATAAATCCCCGTTGATAACACTGTTTCGATGATGTCGTTCGAATCATGTTGTAGTTGGTA
AGCTTCTTGAATTTGACCTTGTCGTGCTAAGTCGAAGATTTTTCTAGCACGGCGACCATTAACGTTATATGTAGAACCAATTGCACCATCTAC
GCCAGAAATCGTAGCTTGAACTAACATTTCATCAAAGCCAGATAAGATTAATTTATCTGGGAATGCTTTTCTAATACGTTCTAGTAAGAAGA
AGTTCGGTGCTGTATATTTAACACCAACAATTTTTTCGTGATTAAATAGCTCGCTGAATTGTTCAATAGAAATATTCACACCTGTTAAATCTG
GTATTGCATAAATAATCATATTGTTCTGTGTTGCTTCGATAATATCGAAATAGTAATCTCTAATTTCTTCAAAAGTAAATGGATAGTAGAATG
GTGTTACGGCAGAAAGTGCATCATAACCGAGTTCTGTGGCATATTTTCCGAGTTCAATGGCTTCATTTAAATCTAACGAACCTACTTGAGCA

ATCAATTTCACTTTATCCCCAACTGCCTCTTTGGCAACTTTGAAAACTTGCTTCTTCTGCTCTGTATTTAATAAAAAGTTTTCGCCTGAGCTACC
ATTTACATAAAGACCGTCTAATTCTTCAGTTTCAATGGCATTTTGAGCAATTTGTTTGAGTCCTTGTTCATTTACTTGACCATTTTCATCAAAA
GGAACGAGTAACGCTGCATATAAACCTTTTAAATCTTTGTTCAT

Gene: nanK (N-acetylmannosamine kinase)

Position: 346255 to 347115, length: 861 nt, orientation: FORWARD

Perfect match to: (11819-97-CP003194-[352179:353039], allele observed in CC80+CC97)

Sequence:

GTGTATTACATCGCAATCGATATTGGAGGCACTCAAATTAAATCGGCAGTTATTGATAAGCAATTGAATATGTTTGACTATCAACAAATACC
AACGCCGGACAACAAAAGTGAGCTTATTACTGACAAAGTATATGAGATTGTAACAGGATATATGAAGCAATATCAGTTGATCCAACCTGTC
ATAGGTATTTCATCAGCAGGCGTTGTTGATGAACAAAAAGGCGAAATTGTATACGCAGGGCCAACCATTCCGAATTATAAAGGTACTAATT
TTAAGCGATTATTAAAATCATTGTCTCCTTATGTCAAAGTAAAAAATGATGTAAACGCTGCATTACTAGGCGAATTGAAATTACATCAATATC
AAGCAGAACGGATCTTTTGTATGACGCTTGGTACAGGCATTGGGGGTGCGTACAAGAATAATCAAGGTCATATTGATAATGGTGAGCTTCA
TAAGGCAAATGAAGTTGGGTATTTATTGTATCGTCCAACTGAAAATACAACGTTTGAGCAACGTGCTGCAACGAGTGCATTGAAAAAGCGC
ATGATTGACGGAGGATTTACGAGAAGCACACATGTGCCAGTATTGTTTGAAGCAGCTGAAGAAGGTGATGATATTGCAAAACAAATATTG
AATGAGTGGGCAGAAGATGTAGCAGAAGGGATTGCCCAAATACAAGTCATGTATGATCCAGGGCTTATATTAATTGGTGGCGGTATATCT
GAACAAGGAGACAATCTCATTAAATATATCGAGCCGAAAGTTGCACACTATTTACCAAAAGACTATGTTTATGCACCGATACAAACGACTAA
GAGTAAAAATGATGCAGCATTATATGGCTGTTTGCAATGA

Gene: nanR (repressor of nan gene cluster)

Position: 347393 to 348193, length: 801 nt, orientation: REVERSE

Perfect match to: (11819-97-CP003194-[353317:354117:r], allele observed in CC80)

Sequence:

TTATTTGTTGTCTAGGATAATAGATTTAGTATGTTGATAAGTTTGACTCAGATTCGTATTTTCTAATAAATGATAACTCACGATATCGATTAA
AAAGAGTGTCGCAATTTGTGTGTTGATAAATTGATGGTCGGTATTACGCGATTGATCCGTTGTTAAAAGTACTAAATCTGCACAATCTGTAA
GTTTACTACCTTCAAAATTTGTGATGGCAACGACATATGCACCATGAGATTTGGCGACTTCCGCTGCAGAAATTAATTCCGAAGTATTACCA
CTATTTGACATAGCAATAAACATATCTGAATGAGATAGTAGGGATGCCGATATTTTCATTAAATGTGAATCGGTAGTAACATTACCTTTTAG
CCCCATACGAATCATACGATAATAAAATTCAGTCGCTGATAAACCAGAGCTACCTAGTCCAGCAAAGAGTATATGTCGACTTGATTGAAGTT
TGTCGATAAAGGTTTGGATAATGTCGTTATCAATAAATTCACCAGTTTGTTGAATGATTTGTTGATGATATTTATGAATTCTTTGAATGATTG
GGCTATTTTCAATAACTGTCTCTGTCATTTCTTGTTGAATATTAAATTTTAAATCTTGGAAATTCTCATAATCCAGCTTATGACTAAAGCGTGT
CATTGTTGCTGGTGATGTACCAATCGCATGGGCTAAGGAGTTAATCGTTGAAAAGGCATCGCTATAACCATTTTGTCTTATATAATTGACGA
TGCGTTTATCAGTTTTTGTAAATAAATGTTGATAACGTTGAACACGATTCTCAAATTTCAT

Gene: nanE (N-acetylmannosamine-6-phosphate 2-epimerase)

Position: 348333 to 349001, length: 669 nt, orientation: FORWARD

Perfect match to: (11819-97-CP003194-[354257:354925], allele observed in CC80)

Sequence:

ATGTTACCACATGGATTAATAGTATCTTGTCAGGCACTACCAGATGAACCATTGCATTCATCTTTTATTATGTCGAAAATGGCATTAGCTGCG
TATGAAGGTGGTGCTGTTGGTATTCGCGCAAATACTAAGGAAGACATTTTAGCAATTAAAGAAACGGTAGATTTACCAGTTATTGGCATTG
TGAAACGTGACTATGATCACTCAGATGTTTTTATTACTGCAACGTCAAAAGAAGTTGATGAACTGATAGAAAGCCAATGTGAAGTCATTGC
ATTGGATGCAACGTTACAGCAACGTCCAAAAGAAACGTTAGACGAATTAGTATCATATATTAGAACACATGCACCGAACGTTGAAATTATG
GCTGATATCGCGACCGTTGAAGAAGCTAAAAATGCCGCACGACTTGGCTTTGATTATATTGGCACGACGTTACATGGCTATACTAGTTATAC
GCAAGGACAATTACTTTATCAGAATGACTTCCAATTTCTAAAAGATGTACTACAAAGTGTTGATGCAAAAGTTATTGCGGAAGGTAATGTCA
TTACACCGGATATGTATAAACGCGTGATGGACTTAGGCGTTCATTGTTCAGTCGTTGGTGGTGCGATAACACGACCAAAAGAAATTACGAA
ACGTTTTGTTCAAGTTATGGAAGATTAA

Gene: Q5HJ49 (nucleoside recognition membrane gate protein)

Position: 349132 to 350445, length: 1314 nt, orientation: REVERSE

Perfect match to: (11819-97-CP003194-[355056:356369:r], allele observed in CC80+CC8+CC22+CC4803)

Sequence:

TTATCCAAAAATAAGTAAAGCGACGGGGATGGTGATTAATAGCGAGAATGCCACGCGTAAAAACCAAATGATGATGAGTTTCCAGACAGG
TATTTTAATTTCAGTTGCTAGTATACATGGCACTAATGCTGAGAAAAAGATAATGGCTGATACGCTTACTACACCGACGACAAATTTAGTAC
TCATTGCAGCTTTAGTTACTAACAAAGATGGTAGAAACATCTCTACAATAGAAATCGCTGACGCTTTTGCAAGTAAAGCCTGATCAGCAATT
GGGAAAATATAAATAAATGGATAGAATATATAGCCAAGCCAATCAATGAATGGTGTATAGTTCGCTACAATCAGTCCTAAAAAACCAATCG
ATAATATAGAAGGTAAAATACCAACAGTCATTTCTAAACCGTCTTTCAAATTGTCCCAAACGTTCTTCACGAGGGATGGTGTTAATGCATTTT
GTTTCATCGCCTCTGCATATGCAGTTTTCAGTCTGCTTCCTTCAATAGCAACTTCTTGTTCTCCTTCTTGTCCGTTATAATATTCTGTTGATTCA
TTGGTGATTGGCGGTAGCCATGCAGTAATTGCAGTCACGACAAATGTGATGACTAAAGTTATCCAAAAGTATAAATTCCAATGCGGCATTA
ATCCTAAAGTTTTAGCAACGATAATCATAAAAGTTGCTGAAACTGTTGAAAAGCCAGTCGCAATAATCGTGGCTTCTCGTTTGTTGTACATC
CCTTGCTTATAGACACGATTAGTAATCAATAATCCTAAGGAATAACTGCCGACAAACGAAGCTACTGCATCGACAGCGGATTTTCCTGGTGT
TTTAAAAATAGGTCTCATAATAGGCTCCATATAAACACCGACAAATTCTAATAAGCCATAGCCTACTAATAAAGAAAGCGCAATTGCACCTA
CTGGAATTAAGATACTTAATGGCATCATTAATTTTTCAAACAAGAACGGACCATAGTTAGCTTTAAATAGTATTGATGGACCGATTTTAAAT
ACATACATTATACCAATCATTGCACCTGCAACTTTAAATAATGTAATGACCAAGTTTGTGATTGAAGTCATAAAAGTACGTCTCACTATTGGT
AACGCTGTACCAATTAAAATCATAATCAGTGCAACATAGGGCATAAGTGGACCTATGATTGAGCGAATGGCTAGATGAACATGATCGACG
AAAATAGTGTTGTTACCATTAATCGTAAAAGGAATAAAGAAACATAGTATGCCCACTAAACTATAGACAAAAAAACGCCATGCACTTGGTT
GTTGTGCATTAGAATGATATTGATTCAT

Gene: lip2 (lipase 2)

Position: 350863 to 352938, length: 2076 nt, orientation: FORWARD

Sequence:

ATGTTAAGAGGACAAGAAGAAAGAAAGTATAGTATTAGAAAGTATTCAATAGGCGTGGTGTCAGTGTTAGCGGCTACAATGTTTGTTGTG
TCATCACATGAAGCACAAGCCTCGGAAAAAACACCAACGTCCAATGCAGCGGCACAAAAAGAAACACTAAATCAACCGGGAGAACAAGG
GAATGCGATAACGTCACATCAAATGCAGTCAGGAAAGCAATTAGACGATATGCATAAAGAGAATGGTAAAAGTGGAACAGTGACAGAAG
GTAAAGACACGCTTCAATCATCGAAGCATCAATCAACACAAAATAGTAAAACAATCAGAACGCAAAATGATAATCAAGTAAAGCAAGATTC
TGAACGACAAGGTTCTAAACATTCACACCAAAATAATGCGACTAATAATACTGAACGTCAAAATGATCAGGTTCAAAATACCCATCATGCTG
AACGTAATGGATCACAATCGACAACGTCACAATCGAATGATGTTGATAAATCACAACCATCCATTCCGGCACAAAAGGTATTACCCAATCAT
GATAAAGCAGCACCAACTTCAACTACACCCCCGTCTAATGATAAAACTGCACCTAAATCAACAAAAGCACAAGATGCAACCACGGAAAAAC
ATCCAAATCAACAAGATACACATCAACCCGCGCATCAAATCATAGATGCAAAGCAAGATGATACTGTTCGCCAAAGTGAACAGAAACCACA
AGTTGGCGATTTAAGTAAACATATCGATGGTCAAAATTCCCCAGAGAAACCGACAGATAAAAATACTGATAATAAACAACTAATCAAAGAT
GCGCTTCAAGCGCCTAAAACACGTTCGACTACAAATGCAGCAGCAGATGCTAAAAAGGTTCGACCACTTAAAGCGAATCAAGTACAACCAC
TTAACAAATATCCAGTTGTTTTTGTACATGGATTTTTAGGATTAGTAGGCGATAATGCACCTGCTTTATATCCAAATTATTGGGGTGGAAATA
AATTTAAAGTTATCGAAGAATTGAGAAAGCAAGGATATAATGTACATCAAGCAAGTGTAAGTGCATTTGGTAGTAACTATGATCGCGCTGT
AGAACTTTATTATTACATTAAAGGTGGTCGCGTAGATTATGGCGCAGCACATGCAGCTAAATACGGACATGAGCGCTATGGTAAGACTTAT
AAAGGAATCATGCCTAATTGGGAACCTGGTAAAAAGGTACATCTTGTAGGGCATAGTATGGGTGGTCAAACAATTCGTTTAATGGAAGAG
TTTTTAAGAAATGGTAACAAAGAAGAAATTGCCTATCATAAAGCGCATGGTGGAGAAATATCACCATTATTCACTGGTGGTCATAACAATAT
GGTTGCATCAATCACAACATTAGCAACACCACATAATGGTTCACAAGCAGCTGATAAGTTTGGAAATACAGAAGCTGTTAGAAAAATCATG
TTCGCTTTAAATCGATTTATGGGTAACAAGTATTCGAATATCGATTTAGGATTAACGCAATGGGGCTTTAAACAATTACCAAATGAGAGTTA
CATTGACTATATAAAACGCGTTAGTAAAAGCAAAATTTGGACATCAGACGACAATGCTGCCTATGATTTAACGTTAGATGGCTCTGCAAAAT
TGAACAACATGACAAGTATGAATCCTAATATTACGTATACGACTTATACAGGTGTATCATCTCATACTGGTCCATTAGGTTATGAAAATCCTG
ATTTAGGTACATTTTTCTTAATGGATACAACGAGTAGAATTATTGGTCACGATGCAAGAGAAGAATGGCGTAAAAATGATGGTGTCGTACC
AGTAATTTCGTCATTACATCCGTCCAATCAACCATTTGTTAATGTTACGAATGATGAACCTGCCACACGCAGAGGTATCTGGCAAGTTAAAC
CAATCATACAAGGATGGGATCATGTCGATTTTATTGGTGTGGATTTCCTAGATTTCAAGCGTAAAGGTGCAGAACTTGCCAACTTCTATACA
GGTATTATAAATGACTTGTTGCGTGTTGAAGCAACTGAAAGTAAAGGAACACAATTGAAAGCAAGTTAA

Gene: A5IPQ2 (esterase/lipase-like protein)

Position: 353180 to 354007, length: 828 nt, orientation: REVERSE

Perfect match to: (COL-CP000046-[398650:399477:r], allele observed in CC8+CC7+CC12+CC72+CC80)

Sequence:

TTACACCATTGTTATAGCGTTTAAGAAATCAACAACTTTACGATAAATAGTGATTGCTTCGTCATTAGGTCTACGATCAAAATCATGCTCGTT
TTTATTCACGCGTTCAAATGTTGAATGTGGAACATGATTCATGATATGTTCGCTTTCCTCAACGGGAACATCATAATCGCTATTACAATGCGC
AATGAAAACAGGTGGAAGTGTTTTAAGTTCATCTGGTGCAATATTATATTTTGAATCAGTATAATCAGCAATGTTAATCATATTTATCCATTT
ACCTGTGCCACGTGCATAAACGTAGATTAAAAAACGTTGTGCGATTTGATCTTGAACAACCGGTGTTGGTGAAGTGAGTTGTGCAATCATT
GTTTCGTTTACGCTTTGAGCTATTTTTGCGTAATAACTATTAGTTGTTTTAAAAGGTTCAGTGTTGATGCGACTATAACCATAAAAATCAATA
ACACCATCAATATCTCTGTCTCGTGCAATTAATAGACTTAAATATGCACCTGATGATCTGCCAAAGGTAAAAATAGGGCAATTAGAATATTG
TGATTGAATCGCATCGAATGATGCGTAGACATCCTCAATAATGCAATCGAGACTTACTTCTGGTAATAAACGATAACTTAGTTGAATTAAAT
CGTAATGTTCCGTAAGGATATCGATATACTGTGGGGATAAATCGTTAGCTTTACCGAACATTAATCCACCACCGTGGATGTAGACAATAGC
GCCTTTTGTTGGTTGATTTTTTGCTTTAATAATTGTGTAAGGTAATGCAAATGCATCTTTAGTAATTACTTTATCTTTAATTTCAGTCAC

Gene: Q6GJZ4 (putative NADH:flavin oxidoreductase)

Position: 354080 to 355279, length: 1200 nt, orientation: REVERSE

Perfect match to: (11819-97-CP003194-[360004:361203:r], allele observed in CC80+CC12+CC72+CC80)

Sequence:

TTAAGACGATGAGTCATGATAATTCTGTTCCAATTGACGTAAAGCGTCACGGGTATGCTTCTTTAGACCTTCCCCATAATCCATCATTTTAAC
AATATCTTTAAAAGCAGCATGTGGAATGGCTAAATCTTCTAAATCTGCCATAGAAAATTCAAGATTGATATCATGTGGTCGCTGTTCAGCAA
GTTTATGCACAAAGTCAGGTTCTGTGACAAAAGGCGAAGACATGCCGACCATATCTGCATGTTGTAAAGCATCTAAAGCAGACTCTGGAGA
ATTAATCCCGCCACTTGCAATTAAAGGGATACGACCTGCTAAATGTTCATAGACAATTTGGTTAACTGGTCGACCGAAATGATCACCTGGTG
TACGAGACGTATTTTGATAAATATGTCGACCCCAGCTAGCGATTGCTAAGTATTGGATGTTTGAAACGTCCATGACCCAATCGATTAATTGG
TTGAACTCGTCAATGGTATATCCTAAATCACTGCCTCTGGTTTCTTCTGGCGTTGCTCGAAATCCTAAAATAAAATTGTCAGGTGCTTCTTTAT
CAATCACTTCTTGTACCGCACGCATAACTTCTAAACATAATCTTGCACGATTTTTTAATGAGTCGGCACCGTAATGGTCTGTACGTTTATTCG
AAAAAGTTGAGAAAAATGTTTGAATCAGCAAACGTTGTGCAATCGAAATTTCCACACCATCAAAACCTGCTTTAATCGCGCGTAATGTAGCA
TCGCGATACTGCTGAATGATGCTATTGATTTTCTCATGAGACATGGCGATAACATCGTGTTCAATCGGTGAATGCAATGTCATAGGGCTTGG
TCCATACACCTTTCCAAAATTTAAAATGGCTTGATTTGAAAAACGACCAGCATGCGCTAGCTGGATAATAGCGAGGCTACCATGTTGTTTCA
TCGTAGATGCCATGTTAGTTAATCCAGGGATACAAGCATCATGATCAATGTTGAACCCATATTCAAACAATTGACCATAAGGTTCAATGTAA
GCAGCGCCGGTGACTTGCATACCAGCTGAATTAGAGCGACGTGCAGCATAAGCCAAGTCTGCTTTTGTAATATAGCCTTCTTTTGTTGATGC
GTTTACGGTCATTGGTGATAATACAAAGCGATTCGAAATTTTGATGCCATTAGGTAAGTGGATTGATTGTAAAAGTGGTTTGTATCGGTACA
T

Gene: limB1 (putative monooxygenase locus 1)

Position: 355581 to 356582, length: 1002 nt, orientation: FORWARD

Perfect match to: (11819-97-CP003194-[361505:362506], allele observed in CC80+CC12+CC72+CC80+CC779)

Sequence:

TTGGTTAAATTAAGCGTATTAGACTATGCCTTAATAGATGAAGGTAAGGATGCACAAAAGGCATTGCAAGATTCAGTGACACTTGCAAAAT
TAGCAGATCGACTTGGCTTTAAGCGAATTTGGTTTACGGAACATCATAATGTACCAGCGTTTGCATGTAGTAGTCCAGAACTTTTGATGATG
CATACATTGGCGCAGACAAATCACATACGAGTTGGCTCTGGTGGTGTGATGTTGCCGCACTATCGACCTTATAAAATTGCTGAGCATTTTAG
AATGATGGCAGCGTTATATCCAAATCGTATTGATTTAGGTATTGGCAATAATCCGGGTACTACTATGGTAAAGCAAGCTTTAGATGGAATA
AATCCTACATATGATAGTTACGATGAATCGATTTCGTTATTACGTGATTATCTTACAATAAAGGATAAACCAAGTGCGCATACGTTAGGTGT
CCAACCACACATTGATCATTTTCCAGAAATGTGGTTATTAAGTAGTAGCGCAACATCTGCCAAAATAGCTGCCGAACTAGGTATAGGGCTTT
CTGTTGGCACATTTTTGCTACCAGATATAAATGCGATACATGCAGCGAAGGATAACATTGATATTTACAAAAAACATTTCCAAGCATCAACG
ATTAAAATGGACGCAAAGGTGATGGCATCTGTATTTGTCATTGTAGCTGATAATGAAGCGGAAGTAGCAGCATTGCAACATGCCTTAGATG
TTTGGTTATTAGGTAAATTACAATTTGCAGAATTTGAACATTTTCCATCGGTAGACACAGCGCAAAAGTATAAGCTTAATGATCGAGACAAA
GAGATGATTCAAGCACATCAAGCACGCATCATTGCAGGTACACAAGAACAGGTTAGAGCACAATTAGATGATTTCATTGCTACGTTTGAAG
TTGATGAGGTGTTAGTAGCACCGCTTATTCCAGGCATTGAACAGCGTTGTAAAACATTAAAATTACTCGCGGAAATCTATTTGTAG

Gene: gcvH1 (glycine cleavage system H protein, locus 1)

Position: 356616 to 356948, length: 333 nt, orientation: FORWARD

Perfect match to: (11819-97-CP003194-[362540:362872], allele observed in CC80+CC12+CC72+CC80)

Sequence:

ATGAAAAAGTTAGCTAATTATTTATGGGTAGAAAAAGTAGGAGATTTGTATGTGTTTAGTATGACACCTGAATTGCAAGATGATATTGGGA
CAGTAGGTTATGTTGAATTCGTAAGTCCAGATGAAGTTAAAGTGGATGATGAAATTGTGAGTATCGAAGCATCGAAAACGGTCATTGATGT
GCAAACGCCATTGTCAGGAACGATTATTGAGCGAAATACAAAAGCGGAAGAAGAACCGACAATTTTAAACTCTGAAAAACCAGAAGAAAA
TTGGTTGTTCAAATTGGATGATGTCGATAAAGAAGCATTCCTAGCATTACCGGAGGCTTAA

Gene: UPF0189 (macro domain protein)

Position: 356949 to 357749, length: 801 nt, orientation: FORWARD

Perfect match to: (Newbould_305-AKYW01000002-[578371:579171], allele observed in CC97+CC22+CC80+CC96+CC97)

Sequence:

ATGGAAACGTTAAAATCAAATAAAGCGAGACTTGAATATTTAATCAATGATATGCGTCGAGAGAGAAATGACAATGACGTATTGGTAATGC
CATCTTCATTTGAAGATTTGTGGGAATTATATCGAGGCTTAGCAAATGTCAGACCGGCATTACCTGTAAGTGATGAATATTTAGCTGTACAA
GATGCTATGTTAAGTGATTTGAATCGTCAACATGTTACGGATTTGAAGGATTTGAAGCCGATAAAAGGTGACAATATCTTTGTTTGGCAAG
GTGATATCACGACGTTAAAAATCGATGCTATTGTTAATGCTGCAAATAGTCGTTTTCTAGGATGTATGCAAGCTAATCATGACTGCATTGAT
AATATTATTCATACAAAAGCGGGTGTTCAAGTTCGACTTGATTGTGCAGAGATCATTCGACAACAAGGGCGCAATGAAGGTGTAGGTAAA
GCCAAAATAACACGTGGATATAATTTGCCAGCAAAGTATATAATTCATACGGTTGGTCCGCAAATACGTCGATTGCCTGTTTCAAAGATGAA
TCAGGACTTGTTAGCTAAATGTTATCTTAGCTGTCTTAAATTGGCTGATCAACATAGTTTAAATCATGTCGCTTTTTGCTGTATATCTACAGGT
GTATTTGCTTTTCCTCAAGATGAAGCAGCAGAAATTGCTGTTCGAACAGTAGAAAGCTATCTCAAAGAAACAAATTCAACATTGAAAGTCGT
GTTCAATGTATTTACAGATAAGGATTTACAACTGTATAAGGAGGCATTGAACCGTGATGCAGAGTAG

Gene: sir2 (NAD-dependent protein deacetylases, SIR2 family)

Position: 357739 to 358683, length: 945 nt, orientation: FORWARD

Perfect match to: (Strain_21343-AHKV01000004-[129689:130633:r], allele observed in CC88+CC12+CC80+CC188)

Sequence:

ATGCAGAGTAGTAAATGGAATGCAATGTCTCTTTTAATGGATGAAAAGACAAAGCAGGCTGAAGTATTGCGTACTGCGATTGATGAAGCA
GATGCGATAGTGATTGGAATTGGTGCAGGCATGTCTGCATCTGACGGATTTACATATGTTGGAGAGCGTTTTACGGAAAATTTCCCAGATT
TTATTGAAAAATATCGCTTCTTTGATATGTTGCAAGCGAGTTTACATCCTTATGGCAGTTGGCAAGAATATTGGGCATTTGAGAGTCGTTTTA
TTACATTAAACTATTTAGATCAACCTGTAGGTCAGTCTTACCTCGCTTTAAAATCCTTGGTGGAAGGTAAACAGTACCACATTATAACTACGA
ATGCAGATAATGCTTTCGATGTAGCTGATTATGATATGACGCATGTATTTCATATACAAGGGGAGTATATACTGCAACAGTGTAGTCAGCAT
TGTCATGCTCAAACGTATCGCAATGATGATTTAATTCGTAAAATGGTTGTTGCGCAACAAGATATGCTTATACCTTGGGAGATGATTCCAAG
ATGTCCAAAATGTGATGCCCCAATGGAAGTGAATAAACGTAAAGCGGAAGTTGGGATGGTTGAAGATGCTGAATTTCATGCGCAACTACA
TCGTTATAATGCTTTTCTAGAGCAACATCAAGATGATAAAGTGTTGTATTTGGAAATTGGAATTGGTTATACTACACCACAATTTGTGAAGC
ATCCTTTTCAGCGTATGACACGTAAAAATGAAAATGCACTTTATATGACGATGAATAAAAAGGCATATCGCATTCCGAATTCAATTCAAGAA
CGTACCATACATTTGACTGATGATATCTCAACATTGATTACAACAGCACTCCGGAACGATAGCACAACGAAAAATAACAACATTGGAGAGA
CAGAAGATGTACTTAATAGAACCGATTAG

Gene: lplA1 (lipoate-protein ligase A, locus 1)

Position: 358661 to 359683, length: 1023 nt, orientation: FORWARD

Perfect match to: (Strain_21266-AFTT01000027-[85733:86755:r], allele observed in CC12+CC80)

Sequence:

ATGTACTTAATAGAACCGATTAGGAATGGAGAATATATTACTGATGGTGCGATTGCACTCGCTATGCAAGTTTATGTTAACCAGCATATCTT
TTTAGATGAAGATATTTTATTCCCTTATTATTGTGATCCAAAAGTGGAAATTGGACGTTTTCAAAATACTGCTATAGAAGTGAATCAAGATTA
TATAGATAAACACAGTATTCAAGTAGTTCGCCGAGATACTGGTGGTGGCGCTGTGTATGTTGATAAAGGTGCCGTTAATATGTGTTGTATTT
TAGAACAAGACACTTCAATTTATGGTGATTTTCAACGATTTTATCAACCAGCTATAAAGGCATTGCATACATTAGGTGCAACAGATGTGATA
CAAAGCGGTAGAAATGATTTAACATTGAACGGCAAAAAAGTGTCAGGCGCCGCAATGACATTAATGAACAATCGTATTTATGGCGGTTATT

CGCTATTACTTGATGTTAATTATGAAGCAATGGATAAAGTGTTAAAGCCTAATCGCAAAAAGATTGCATCGAAAGGGATTAAATCTGTGCG
TGCACGTGTTGGTCATCTTAGAGAAGCACTGGATGAAAAGTATCGTGATATAACAATTGAAGAATTTAAAAATTTAATGGTGACGCAGATT
TTGGGAATCGATGACATTAAAGAGGCAAAACGATACGAATTATCTGATGCGGATTGGGAAGCGATTGATGAATTAGCTGATAAAAAGTAT
AAAAATTGGGATTGGAATTATGGTAAGTCACCTAAATATGAATATAATCGAAGTGAAAGATTATCATCAGGTACTGTAGATATTACCATTTC
TGTTGAACAAAATCGTATCGCAGATTGTCGTATTTATGGGGATTTCTTTGGACAAGGTGATATAAAAGATGTGGAAGAAGCATTACAAGGA
ACAAAAATGACAAGAGAAGATTTAATGCATCAGTTAAAGCAATTAGACATCGTTTATTATTTTGGTAATGTTACGGTAGAATCATTGGTTGA
GATGATTTTAAGTTAA

Gene: Q5HIW6-v1 (putative NAD-dependend oxidoreductase)

Position: 359999 to 361024, length: 1026 nt, orientation: FORWARD

Perfect match to: (11819-97-CP003194-[365923:366948], allele observed in CC80+CC12+CC15+CC80)

Sequence:

ATGAATAATAAAGTATTAGTAACCGGTGGTACAGGGTTTGTTGGCATGCGAATTATTTCACGATTATTAGAACAAGGTTATGAGGTACAAA
CGACGATACGTGATTTAAGTAAAGCTGATAAAGTAATTAAAACAATGCAAGACAATGGCATTTCCACAGAGCGATTAATGTTTGTCGAAGC
GGATTTATCACAAGATGAACATTGGGATGAAGCAATGAAAGATTGTAAATATGTCTTGAGTGTAGCATCTCCGGTGTTTTTCGGTAAAACA
GACGATGCAGAAGTGATGGCGAAGCCTGCCATTGAAGGCATACAACGTATTTTAAGAGCTGCAGAACATGCTGGCGTGAAGCGTGTGGTG
ATGACTGCAAACTTTGGTGCAGTTGGTTTTAGCAATAAAGATAAAAAATTCAATCACAAATGAAAGTCATTGGACAAATGAAGATGAACCAG
GCTTATCAGTATATGAAAAATCAAAATTGTTAGCTGAAAAGGCAGCGTGGGATTTTGTTGAGAATGAAAATACAACAGTAGAATTTGCCAC
AATCAATCCAGTTGCAATTTTTGGGCCATCATTAGATGCACACGTTTCAGGAAGCTTTCATTTATTAGAAAATTTATTGAATGGTTCAATGAA
ACGTGTACCGCAAATTCCATTGAATGTTGTTGATGTGAGAGACGTAGCTGAACTACACATTTTGGCAATGACAAATGAACAGGCTAATGGC
AAGCGATTTATTGCTACAGCTGATGGACAAATTAATTTGTTGGAAATTGCAAAATTAATTAAAGAAAAGAGACCTGAAATAGCTCAAAAAG
TTTCTACTAAAAAAATTACCAGACTTTGTTTTGAGTCTAGGTGCTAAATTTAATCATCAAGCTAAAGAAGGTAAACTTTTATTAGATATGAATC
GAAATGTAAGTAACGAACGTGCAAAAATACTTCTTGGTTGGGAACCGATTGCGACACAAAAAGAAGCAATTTTAGCAGCTGTCGATAGTAT
GGCTAAGTATCATTTAATATAA

Gene: ulaA (putative sugar-specific permease, SgaT/UlaA)

Position: 361117 to 362463, length: 1347 nt, orientation: REVERSE

Perfect match to: (Strain_21334-AGTW01000029-[30170:31516:r], allele observed in CC9+CC12+CC80)

Sequence:

CTATTGCTGTGGCTTTTGACGGCCTTTTAATAAAATTGCTGTCGCACCTACGATAATAATAAATAGAATCGCACCAAATAATCCCATATATTT
TACTGCGTTACCGAACACGATACCGACAGCTAAAAAGTCTGTATCTGAGAATGTTGTTGCAGCACCACCTAATTCGCCTAAAAATGGCAAG
AATAATAATGGTAAAAACGTGATTAGGACACCATTTAGAGCGGCGCCAGCAACAGCACCTTTAATACCGCCTCTTGCATTACCGAATACAG
CAGCCGTTGCACCTAAGAAGAAGTGTGCAACTACGCCAGGTAAAATGACGACGCCACCAAATAAGAATAAGATAAACATACCGATGACAC
CTGTAATAAAGCTGACAAAGAATCCAATTAATACTGCATTTTGTGCATAAGGGAACACAATAGGGCAGTCTAATGCAGGTTTAGAATTTGG
TACAAGCTTTTCAGAAATCCCTTTAAATGCTGGGACGATTTCAGCTAAGATTAAGCGAACGCCCGTTAAAATAATAAATACACCAGCAGCAA
ATGTCACACCTTGAATTAATGAAAAAACAATAAAGTTTTGACCATTACTAATAGATTCGTGTACATAACTAACGCCTGCAAATAAGCATGCG
ATGAAGTAAAGTAATGCCATCGTAATCGAGATACTAATTGTACTTTCTCGTAAGAAACTTAAGCCTTTTGGAAATTTAATCTCTTCCGTTGAT
TTAGACTTACCTTTGAATAATTGACCTACAGCACCTGCGGCAAAGTAACTGATTGAGCCAAAATGACCTAAAGCTACTTGATCATTCCCTGT
AATTTTTCGCATCGTAGGTTGGAGTAATGCAGGTAAGACTGCCATGATTAATCCTAATACGAGTGCGCCGATAACAATCGTTAGCCAGCCTT
TAATATGACTGACTGTTAAAATGATTGCTAAAAACGCAGCCATGTAAAATGTATGATGACCTGTTAAAAAGATATATTTTAAATTAGTGAAG
CGGGCAATTAAAATATTAACAATCATGCCACAGACCATGATGAGTGCAGCTGTTGTACCAAAATCTTTTAAGGCTAGTGAGACGATAGCTT
CGTTGTTAGGTACGATACCTTGCACACCGAATGCGTGTTGGAATATTTTGCCGAATGGCTCAAGAGATCGAACGACGACATCAGCACCTGC
ACTTAAAATTAAGAAGCCTAATATCGTTTTAATGGTTCCTGAAGTGATCGTTGCGGCAGGTTTTTTCTGAACGATTAAACCTATAAAGGCAA
TCAGTGCAACAAGAATGGCTGGTTGACTTAAAATATCGACTATAAAATTAAGGATTGCTTGCAT

Gene: Q5HIW4 (putative phosphotransferase system protein)

Position: 362478 to 362762, length: 285 nt, orientation: REVERSE

Perfect match to: (11819-97-CP003194-[368402:368686:r], highly conserved allele)

Sequence:

TTAAATCATGTTAAGTTGTTGTAATTTTTCTGAGAGCTTTTGTTGTAATTCAGCTTTGTCTAAAATATTATCAAGAACTAAGACATCCCCTAGA
CGTTCGGCATTTTCAGCTAAATCTCTACCACAAATAAACAAGTCAGCCATCTCTGGACTTGCTGTCATAATGTCACTATGTTCAACTTCGATA
TCAGATGGTGCATTAAGTTGCCTAAGTGCTTCTTGTGCGTTCATTTCTACCATAAAACTACTTCCTAAACCGTGGCCACATACTACTAAAATT
TTCAT

Gene: Q5HIW3 (putative phosphotransferase system protein)

Position: 362764 to 363207, length: 444 nt, orientation: REVERSE

Perfect match to: (Strain_21333-AHKA01000028-[81597:82040], allele observed in CC80+CC22+CC80)

Sequence:

TTAATCATGCTCCTTTAAAATGTTTTTAATGTCTTGTGCGTTTGTTGCAGTTAATAGTTGCTGGACTGTTTGGTTATCGCCCAGTACGGTTGCT
AAATTTTGTAATACAGATAAGTGTGAATGATTGTCGATGGCACTCAATACAAAAATGAGTGATGCGTAGTGATTTTCATCACAGAATGCCAC
ATGTTGGTTCAACTTTAATAGACTTAAACCAACTTGATGTACGTCATTGTTCGGTCTTGCATGTGCAATTGCAATTTCAGGTGCGATAACGAT
ATAAGGTCCAAGTTCATTAACGCTATCAATCATTGCTTGAACATAGCCTTGTTCAATAATTTGTTCTTGTAGTAATGGCTGAGAAGCTATAGT
TATAGCTTCAGTCCAATCATTTACTTGTTCTTTTACAATGATGCGTGTTGTTGACAAAATGTCTAATGACAC

Gene: A8YZF7 (transcriptional antiterminator, BglG family)

Position: 363212 to 365167, length: 1956 nt, orientation: REVERSE

Sequence:

TTAAGCCTCCTTTGTCATAGTTAAAGCAATGTGTTGTTTAATTTTAAAAATATTCCCATCTAAGAAATCTTGTCGATATAAGTCGCTGCTTAAG
CATTCGCTTAACTGTCCCAATGCCTTTAAATGTGCATTGGGGTGGTCCGTTGCTAATGTAATTACAAGGTGAACGGGATCGTTAGCTTTACT
ACCAAAGATAATCCCTTCAGTGAAATATGTTAGTGCGAAACCTACACCATTCTGTACATAATCAGTACCAGCGTGAATAAGTGCAATATGTG
GACTAATGACCATATATGACCCGAATTGTTCAAATTGTTTTAAAATTGCAGCTGTATAATTTGAATAGACAATGCCATCATTGATTAAAGGTT
GCACAGCCACTGCAATTGCGGATTCAATTGATAATGGTTGTTTATTTATAATGATGCGATGTTCAGGCAATAAATCTGCGAGTGACTTGCCA
TCAGTTGCCATTTTCATGACTCGTTGTTCTCTTGAGTCATTGATAATTTGATTCAATTTTTGACGAGATTGTTGATTGATAAATGGATCGACAT
GAATAACTGGTACAGCTGATATTTCACAAGGTACTGTTGAAATGACATAATCAATGTTATCTTGCAATAATCGACTTTCTTCCAATTGATAAA
TGGAATAGGCATCCCAAATGTGAAACTCAGGATACAGGTGATTTAGTTTTGATTTTAAAAGTTGTGACGTGCCTATACCAGAACCACATAGT
AAGACAACCTTAATCATTGATTGTTTATGTGTTGCAACACGCTCTATACTTGATGCGAAGTGAATTGTAATGTATGTTAATTCATCTTCGTTG
AAGCGAATAGCAGCATCTTGTTCAATTGGACTAATATGCTTGCTAACGGCTTCAATGATTTGAGGATAGCGACGCATAACTTCTTGCCTCAA
AGGATTAGGTTGTAGCATATCGTATTTAATACGATGTATAGCTGGTTTGATATGTGTGATCAGACTGGTATGTAACTTGTTATCTTTTGACAT
ATCAATGCCTAATTCTTGGCTAACACAAGTAATCAATTCATGTATATTTTGCGATAAATCATGGTATTCAAAGGTAATTGAAGATGCTGTATG
TTCAGTCATTTTAGAGCCTAGTAAATGTAACGTGATAAAGATAATTTCAGACTCTGGAAATGTGACATTACAACTGCGTTCTAAGTTTTCTAT
CATTTTTGAAGCAATAGCATACTGACTAGTATGTCGCCATTTATCAATTTCATTGATAGGTATATCGAACGAAAAATTTTCATTTAGACGCTG
AATGGCAATGAGTATATGATAGATTAAGCCATCGATAGCCGACTGAACTAAATGATAATTTTCACTATTTAATGTCTTAATAATGGCACGGC
GAACCAATGCGATTGATTCTGAATTAAAGATATCCGCCTCTATAAAAGGTGCAGCTTGTTTCATATATTGATGTATAAAGTGTGCATACGCT
TTACGATAGTGATCTTCCTCACCAATAATATTGAATCCTTTATTGTGGACATAATTTAACTTTAAATGGTATTGATCTAGTTGGGCCTGTATCA
TTTTAATATCATCTGCAATTGTCCGACGCGAAACATTAACATCTTGCGCAAGTTGCTTTGTTGAAACAGGATCGGTTGTTTCGAATAACTTTA
AAGCGATATGTGTGAGTCGTTCATCTTTTGAAAAATGAATTTGATTTGTTAGATTGTGCTCTAATTCATTTAATAGTGTCGCGTGAGCTGTTG
TTACTTTGATGCCTGCAGCTTTATTACGGCTGACTTGGTAATGATAAGTTTCAGCATATTGCTCAATATATGCTATATCATATTGAATGGTAC
GAGGTGATACACCAAGTTGATTAGCAATGGTATTGATTGGAATAAACGTTTGCTCATGAATTAAAAGATACAAAATTTCGATTTGTCTATAA
CTTAACAA

Gene: mepR (MATE regulatory protein)

Position: 365379 to 365798, length: 420 nt, orientation: FORWARD

Perfect match to: (11819-97-CP003194-[371303:371722], allele observed in CC80)

Sequence:

ATGGAATTCACTTATTCGTATTTATTTAGAATGATTAGTCATGAGATGAAACAAAAGGCTGATCAAAAGTTAGAGCAATTTGATATTACAAA
TGAGCAAGGTCATACGTTAGGTTATCTTTATGCACATCAACAAGATGGACTGACACAAAATGATATTGCTAAAGCATTACAACGAACAGGT

CCAACTGTCAGTAATTTATTAAGAAACCTTGAACGTAAAAAGCTGATCTATCGCTATGTCGATGCACAAGATACGAGAAGAAAGAATATAG
GACTGACTACCTCTGGGATTAAACTTGTAGAAGCATTCACTTCGATATTTGATGAAATGGAGCAAACACTCGTATCGCAGTTATCTGAAGAA
GAAAATGAACAAATGAAAGCAAACTTAACTAAAATGTTATCTAGTTTACAATAA

Gene: mepA (multidrug and toxin extrusion protein)

Position: 365905 to 367260, length: 1356 nt, orientation: FORWARD

Perfect match to: (11819-97-CP003194-[371829:373184], allele observed in CC80)

Sequence:

ATGAAAGACGAACAATTATATTATTTTGAGAAATCGCCAGTATTTAAAGCGATGATGCATTTCTCATTGCCAATGATGATAGGGACTTTATT
AAGCGTTATTTATGGCATATTAAATATTTACTTTATAGGATTTTTAGAAGATAGCCACATGATTTCTGCTATCTCACTAACACTGCCAGTATTT
GCTATCTTAATGGGGTTGGGTAATTTATTTGGCGTTGGTGCAGGAACTTATATTTCACGCTTATTAGGTGCGAAAGACTATAGTAAGAGTAA
ATTTGTAAGTAGTTTCTCTATTTATGGTGGTATTGCACTAGGACTTATCGTGATTTTAGTTGCTTTACCATTCAGTGATCAAATCGCAGCAATT
TTAGGAGCGAGAGGTGAAACGTTAGCTTTAACAAGTAATTATTTGAAAGTAATGTTTTTAAGTGCACCTTTTGTAATTTTGTTCTTCATATTA
GAACAATTTGCACGTGCAATTGGGGCACCAATGGTTTCTATGATTGGTATGTTAGCTAGTGTAGGCTTAAATATTATTTTAGATCCAATTTTA
ATTTTTGGTTTTGATTTAAACGTTGTTGGTGCAGCTTTGGGTACTGCAATCAGTAATGTTGCTGCTGCTCTGTTCTTTATCGTTTATTTTATGA
AAAATAGTGACGTTGTGTCAGTTAATATTAAACTTGCGAAACCTAATAAAGAAATGCTTTCTGAAATCTTTAAAATCGGTATTCCTGCATTTT
TAATGAGTATCTTAATGGGATTCACAGGATTAGTTTTAAATTTATTTTTAGCACATTATGGAAACTTCGCGATTGCAAGTTATGGTATCTCAT
TTAGACTTGTGCAATTTCCAGAACTTATTATCATGGGATTATGTGAAGGTGTTGTACCATTAATTGCATATAACTTTATGGCAAATAAAGGCC
GTATGAAAGACGTTATCAAAGCAGTTATCATGTCTATCGGCGTTATCTTTGTTGTATGTATGAGTGCTGTATTTACAATTGGACATCATATGG
TCGGACTATTTACTACTGATCAAGCCATTGTTGAGATGGCGACATTTATTTTGAAAGTAACAATGGCATCATTATTATTAAATGGTATAGGTT
TCTTGTTTACTGGTATGCTTCAAGCGACTGGGCAAGGTCGTGGTGCTACAATTATGGCCATTTTACAAGGTGCAATTATCATTCCAGTATTAT
TTATTATGAATGCTTTGTTTGGACTAACAGGTGTCATTTGGTCATTATTAATTGCTGAGTCACTTTGTGCTTTAGCAGCAATGTTAATCGTCTA
TTTATTACGTGATCGTTTGACAGTTGATACATCTGAATTAATAGAAGGTTAA

Gene: mepB (putative protein)

Position: 367364 to 367804, length: 441 nt, orientation: FORWARD

Perfect match to: (COL-CP000046-[412833:413273], allele observed in CC8+CC80+CC239+CC4803)

Sequence:

ATGTGTAAATCTAAAATACTGTTGAAAAATATTTTTAGTGAAGAATCAGAAGTTAAAGATTTAACTGAAGAAAAATATAATCAAGATTACGA
AGCATTAACATTTAGCTTTAAAGAGGAAACATATCAAAGTAGGTTAGCTAAGAAAACACCGACTAAATCGGGATATTTCGTGACATGTTGG
ACAAAAGACGAAGATAATTATAATCGGCCATACAAAATTGAAGAGTTTGCTGATTACCTGATTGTTGCTGTTATCGATGATGAATTAAATGG
CTACTTTCTATTTCCTAGGGAATTATTGGTAGAAAAAGGTATCTTAGCTTCATCTAAGTATCAAGGGAAAATGGCTTTTAGAGTTTATCCTAA
GTGGTGTAATCAATTGAATAAAACAGCAGGGCAAACACAAAAGTGGCAATGTAAATATTTTTTTGAATACTAA

Gene: glpT (glycerol-3-phosphate transporter)

Position: 367887 to 369245, length: 1359 nt, orientation: REVERSE

Perfect match to: (Strain_21259-AFTS01000010-[126649:128007], allele observed in CC72+CC80)

Sequence:

TTAATGATGAACGGTTTCTTGTCCTACTTTATTCCAAGTGAGGATAAAGCTCAACATTGCAAACACACTGATTGCTGTTAATAAAATAAAACC
GACATCCCATCCGAATTTATCAACTACAGCACCTAAGACGATATTGGCCATTACAGCACCAAACAGATAACCAAATAATCCTGTTAATCCAG
CTGCTGTGCCAGCTGCTTTTTTAGGTACATAATCTAATGCTTGTAAACCAATTAACATAACTGGTCCATATATTAAGAAACCAATGGCAATTA
ATGAGACATTGTCTAACCAAGCATTGCCTGGAGGATTTAACCAATAAATTAATACAAATACTGTGACACCTAACATAAAGAAGAAACCTGC
AGGTCCACGACGACCTTTGAATAATTTATCAGAAATGTAACCACATAATAATGTACCAGGAATTCCAGCCCATTCGTATAAGAAGTATGCCC
AACCTGATGCTTTTAAGTCGAAATGTTTTTCTTCACTTAAGTAGACTGGCGCCCAATCAAGTACACCATAACGCACGAAATAAACAAATATA
TTTGCAAAGGCAATTGCCCATACCCATTTATTGTTTAGTACATATTTAAATAAAATTTCTTTTGTAGTTAATTCTGTTTCTAATGTTTTCTTATC
GCTTGTAGCAAAGTCATTTTTATAAATTTCGATTGGAGGTAAACCTTGAGATTGAGGTGTGTCTCTAATCAATACGTATGAAATTGCTGCAA
TGATAAGTGCTAAGAGTGCAGGGTAAATGAATACACCTTCGAAACCTTTTAAATAACCAAAGTTGATAAATGCTGTTGTTGTAATACCCCAA
GCAGCAATAGGTGCCATAATACCTCCACCAACATTATGCGCAACGTTCCAAAGGGCAGTCTTACTTCCGCGTTCACTTACACTAAACCAGTG

AACGAGAACACGGCCTGAAGGTGGCCAGCCCATACCTTGAAACCATCCATTTAAGAATAATAGGACAAACATAATACCGATACCTGATGTA
AAGAACGGTACAAATCCCATTAACAAATTGACGATAGCAGTGAGTGCTAATCCAAGAACTAAGAATATCCGAGCATTGCTCCGATCACTTA
CAGTACCCATAAAGAACTTACTAAATCCATATGCGATGGAAACAGCAGAAAGTGCAAAACCTAGTTCCGCTTTTGTAAAACCTTGCTCTTGC
AATGCCGGCATCGCTAACGAAAAGTTTTTACGTAATAAATAGTACCCAGCGTAACCGATGAAAATACCAAGAAATACTTGGAGACGTAATC
GTTTATAGGTATCATCTATCTGATTTTCTGGCAAAGGCTTAATATGCTTTGCAGGTTTAAGAAAATTCAT

Gene: mhqA (putative hydroquinone-specific extradiol dioxygenase)

Position: 369557 to 370417, length: 861 nt, orientation: TRNC-FRWD (no stop codon)

Perfect match to: (11819-97-CP003194-[375481:376341], allele observed in CC80+CC72+CC80)

Sequence:

ATGAATATAGTAGGGCATCATCACATATCCATGTATACAAAAGATGCAAAACGTAATAAGGATTTTTACACAAATGTCCTTGGATTACGATT
AGTTGAAAAGTCGGTTAATCAAGACAATCCTTCAATGTATCATTTGTTTTATGGGGACGAAGTAGGTACAGCCGGAACAATTTTAAGCTTTT
TTGAAATTCCCAATGCGGGTCATAAGCAGCCAGGCACTGAAACGATTTATCGATTTTCTTTATTAGTACCAAATCAAGCGGCACTTCATTATT
TTGAAAAACGCCTTGATAATAATGGTATTACGTCTGAACGTTTGTACTATCTTGGACAAGAAGGTGTTGTCTTTAAAGATGAAGACGACTTA
GAAATCATATTGCTTGTTAATGATAGTTTTGAAGTACCACATCAATGGCAACATAACGTTTATAGTGAAATACCTCAAGCATATCAAATTTTA
GGAATAGGGCCAGTCGAATTAAGAGTTAGAAATGCAGCGCGTACGGTAGAATTTTTGGAAAATGTCTTAGGTTATCGCAAAAGAGATAAT
AAATCATTCGATGTGCTGACATTAGCACCACAAGGTTTATATTCGGATTTTGTAGTTATTGAGCAACAGGGACAACGTGAAAGACCTGGAC
GAGGTTATATCCATCATATTGCAGTTAATACACCACTAATGAGTGATTTAGATGCAATTTACAAGAAATTACAACAACAACCACAAAGTAAT
TCAGGTATAATTGATCGCTATTTCTTTAAATCATTATACTATCGCCATAATTCAATTATGTATGAATTTGCGACTGAAGCGCCTGGATTTACTA
TTGATACACCTGTTGAACAATTAGGAAGTCAA

Gene: ssuD (flavin utilizing monoxygenases, luciferase family)

Position: 370497 to 371558, length: 1062 nt, orientation: FORWARD

Perfect match to: (Newman-AP009351-[381636:382697], allele observed in CC8+CC80+CC239+CC772)

Sequence:

ATGGCCAAATTAGAAATGAATAAAAATACGCCTCTTGAGTTTGGTTTGTATTCCTTAGGTGATCATTTATTGAATCCATTGAAAGGTGAAAA
AGTTAGTTATGAGCAACGTATTAATGAAATTATTGAAGCAAGTAAATTAGCAGATGAAGCAGGTATTGATGTTTTTGCAGTTGGTGAAAGT
CATCAGGAGCATTTTACAACACAGGCACATACGGTTGTGTTAGGTGCAATTGCCCAAGCGACAAAGCATATTAAAGTTTCAAGTTCTTCAAC
GATTATTAGTGCAACAGATCCTGTAAGAGCATTTGAAGACTTCGCAACATTAGATTTGATTTCTCATGGTAGAGCCGAAATTGTGGCTGGTA
GAGCATCAAGAACAGGTATTTTTGACTTGTTTGGCTATGATTTAAAAGACTATGATGAATTGTTTGAAGAAAAATTAGGTTTACTTTTAGAG
TTAAATAAAACTGAGCGTATTACTTGGTCTGGAAAATATCGTCCAGAACTTAGAAATATGAAAATATTCCCAAGACCAATCGATAATACATT
GCCAATATGGCGTGCTGTTGGTGGTCCACCTGCAAGTGCTATCAAAGCGGGAAAACAAGGTGTGCCAATGATGATTACAACCCTTGGTGG
CCCAGCAATGAACTTTAAAGGTTCTATAGATGCTTATCGTCAAGCAGCAACTGAAGCAGGTTTCGATGTTTCGTCTAAGTCTTTACCAGTAA
GTACAGCGAGTCTGTTTTATACAGCTGAAACAACTCAGGATGCTATGAGAGAATTTTTATCCACATTTGAATACAGGGATGTCATTTATTCGT
GGCGTTGGTTATCCAAAACAGCAATTTGCTAATTCGTCAGATTATCGAGAAGCGCTAATGGTTGGAAGTCCGCAACAAATTATTGAAAAGA
TATTGTATCAACACGAGTTGTATGGTCATCAACGTTTTATGGCACAGCTTGATTTTGGCGGTGTGCCATTTGAAAATGTTATGAAGAATATT
GAGTTAATTGGCAACGAAATTATACCGGCGATTAAAAAGCATTTATCAAAATAG

Gene: ssuE (flavin mononucleotide reductase)

Position: 371572 to 372138, length: 567 nt, orientation: FORWARD

Perfect match to: (11819-97-CP003194-[377496:378062], highly conserved allele)

Sequence:

ATGAATATTGTATTATTGTCAGGTTCCACAGTAGGTTCTAAAACGAGAATTGCTATGGATGATTTAAAAAATGAACTAGAAGTCATCAATGA
GGGACATCAAATAGAGTTGATGGATTTACGAGAACTTGAATTAGAATTTAGCGTTGGAAAGAATTATCTAGATACTACAGGAGATGTATAT
AAATTAACGACGTCGTTAATGCAGGCTGATGTGATTTTTATTGGTTTTCCAATTTTTCAAGCTTCCATCCCTGGTGCTTTGAAAAATGTATTTG
ATTTGCTTCCGGTCAATGCGTTTCGTGACAAGGTAATAGGACTTGTAGCGACAGCAGGTTCTAGTAAACATTATTTAATTCCTGAAATGCAT
TTAAAACCAATATTGAGTTACATGAAAGCACATACGATGCAAACGTATGTATTTATTGAAGAGAAAGATTTTTCAAATCAACAAATTGTCAA

TGATGATGTTGTATTTCGTTTAAAAGCGTTGGCACAATCCACAATGCGAACTGCCAAAGTACAACAACAAGTGTTTGAAGAAGAAAACAAC
CAATACGACTTTTAA

Gene: yeiH (UPF0324 membrane protein)

Position: 372198 to 373193, length: 996 nt, orientation: REVERSE

Sequence:

TTAACTATATAGCCAATGAACGACGATAAAGGCAAGTGATGACAAGCATATTGAGGTAATAATGATTGTCATAAGCGGTTTAAGTGCGCGA
TTTTTAAGATCTTTAAATGCCACATTTAACCCTAAAGCAACCATGGCCATTAATAAGCAAATTGTTGATACAGTATTTAAAATATTTAGCAAT
GCTGACGGAATAGTTACATATGTATTCACTAATGCCATAATGACAAATCCAATTAAAAAGTATGGAATGCTTATTCGACCTTTGCTAGATGA
TTCTGATGAACGGAAACGCATAATTAAAATAAGTACGATGGTTAATGGAATCAGTAAGAATACTCTGCCAAGTTTGCCCAGCAGTGCAATT
TTAAGTGCATCACTACCACCAAAGCCGCCAGCTAAGACAACATGCGCAATTTCATGAAGACTAACGCCGGACCAAGCGCCATAGACAGTTG
TCGTCATTGAAAAGATAGCATAGATAGCTGTATATATAAGTGAAAATATCGTACCAATCAATGCGATGATACCGATACTAATAGCTGTATCC
TTTTCACGTGATTTGAATATTGGAGCTACTGCAGCAATAGCGGCAGCACCACAAACGCCTGTACCTACACCTAGTAATAATGCGATGTTTTT
GTCACCATGTAACAGTTTGTTGACAAAGAGCATCATTACAATACTAAAAATAACGACACCTACATCGATGGCTAATAGTCTACTACCTTGAC
CGATAATATCGAATATATTGAGTTTAAGTCCATATAGGATGATTGCAAATCTTAATAAATATTTAGATGAAAACGTAATACCTGAGCTATATT
GCTCAGGATATCCTCTAAAGTGACGATATAGAATAGCGATTAATATCGCGATAGTTAATGCGCCAACCTTATCTAAGATTGGCAATTTAGCT
GCTAAAAAGCTAAATAATGCGACTATAAATGTTAATGATAGTCCAATCATAAAATGCTTGTTTTTCAATGATGCCAT

Gene: rimL (ribosomal protein N-acetyltransferase)

Position: 373667 to 374212, length: 546 nt, orientation: FORWARD

Perfect match to: (CIGC93-AHVD01000006-[48902:49447], allele observed in CC15+CC80)

Sequence:

ATGTTTGGAATGAAAGTGAATGAACAAATAACATTAAAAATTTTAGAAGCTCATGACACAGAAGCGCTTTTCAATTTAGTCAATCGTTCAAG
AAATTCACTTAGGGAATGGTTACCTTGGGTAGATGCAACTGAGCAACCATCAGATACGCGTGCATTTATTAAAAGAGGACTTTTGCAATTTG
CTGATGGTAATGGATTTCAGTGTGGCATTTGGTATGAAGGAACGTTAGTTGGTGTCATCGGTTTACATGAAATTAATCACATTCACAGAAA
AACTTCATTAGGATATTATTTAGATAAACAATTTGAGTGTCATGGGATTATGACACAAGCAGTTGAGGCATTGATAAAGTATTGTTTCGAAG
AGCTTGACTTAAACCGAATTGAGATTAGTGCCGCAGTTAATAATGAAAAAAGCCGAGCTATTCCTGAAAGGCTGGGATTTACTAGAGAAG
GTATGTTACGTGACAATGAATTACTAAATGGTATTTATTCATCGAGTTACATCTATAGTTTATTAAAATCAGAATACGACCAAAAATGA

Gene: fepA (lipoprotein)

Position: 374480 to 375334, length: 855 nt, orientation: FORWARD

Perfect match to: (NN50-BAEA01000012-[28425:29279:r], allele observed in CC4803+CC8+CC80)

Sequence:

ATGAAAAAGTTAACAACGCTATTATTAGCATCAACGTTATTAATTGCTGCATGTGGGAACGACGATAGTAAGAAGGATGATTCAAAGACAT
CGAAAAAAGATGATGGTGTTAAAGCAGAATTAAAACAAGCAACAAAAGCATATGATAAATATACTGATGAACAGTTAAATGAATTTTTAAA
AGGTACAGAAAAATTTGTTAAAGCGATTGAAAATAATGATATGGCCCAAGCAAAAGCGTTATATCCAAAAGTTCGTATGTATTATGAACGC
TCTGAACCAGTTGCAGAAGCATTTGGAGATTTAGATCCTAAAATTGATGCACGTCTTGCAGATATGAAAGAAGAGAAAAAGGAAAAAGAA
TGGTCAGGATATCATAAGATTGAAAAAGCATTATACGAAGATAAGAAAATTGATGATGTGACTAAAAAAGATGCACAACAATTATTGAAA
GATGCAAAAGAATTGCATGCCAAAGCTGATACATTAGATATCACACCAAAATTAATGTTACAAGGTTCTGTTGACCTATTAAATGAAGTTGC
AACTTCTAAAATCACAGGTGAAGAAGAAATTTATTCACATACAGATTTATATGATTTTAAAGCGAACGTTGAAGGCGCACAAAAAATTTATG
ACTTATTTAAACCTATTTTAGAGAAAAAAGATAAAAAATTAAGTGATGATATTCAAATGAACTTCGATAAAGTGAATCAATTATTGGATAAA
TATAAAGATAACAACGGCGGTTATGAGTCATTTGAAAAAGTATCTAAGAAAGACCGTAAAGCATTTGCGGATGCTGTTAATGCATTAGGAG
AGCCACTAAGTAAAATGGCTGTGATTACTGAATGA

Gene: fepB (iron-dependent Dyp-type peroxidase)

Position: 375331 to 376560, length: 1230 nt, orientation: FORWARD

Perfect match to: (Strain_21340-AGTX01000031-[71892:73121], allele observed in CC188+CC80)

Sequence:

ATGACAAATTATGAAAAAGTTAACGATAGTACGCAATTTTCAAGACGTACATTTTTGAAAATGTTAGGTATTGGCGGTGCCGGTGTTGCAAT
TGGCGCAAGTGGTGTTGGTAGCATGTGGTCTTTCAAATCAATGTTCAATACACCAGAAGATCCGGAAAAAGATGCGTATGAATTTTATGGT
AAAGTGCAACCAGGCATTACCACACCCACGCAAAAAACATGCAATTTCGTTGCGTTAGATTTGAAGTCAAAAGATAGAGATGCAATTAAGG
CAATGTTTAAAAAGTGGACGGTTATGGCTGATCGTATGATGGATGGTGATACAGTTGGCAAGACGAGTAACAATCCTTTAATGCCACCAGT
AGATACCGGTGAATCGATAGGATTAGGTGCAAGCAAGTTAACGATTACCTTTGGGATTAGTAAGTCTTTGATGAAGAAAATTGGGTTATCT
AGTAAAATTCCCGATGCCTTTAAAGATTTACCGCATTTTCCGAATGATCAGTTAATAGACGATTACAGCGATGGTGATATTATGATTCAAGC
ATGCTCAAATGATTCGCAAGTATCCTTTCATGCGGTTCATAATTTAGTTCGTCCATTTCGAGATATTGTTAAGGTACGTTGGGCGCAATCTGG
TTTTATCTCTGCTAAAGGTAAGGAAACACCTAGAAATTTAATGGCATTTAAAGATGGAACAATTAATCCTAGAAAGAGTAATCAACTTAAAG
ATTATGTGTTTATTGATGACGGATGGGCGAAACATGGAACTTATTGTGTTGTCAGACGTATTCAAATACACATTGAAACGTGGGATCGTACT
GCGCTGGAAGAACAAGAGGCTACATTTGGTCGGAAACGACATAGTGGTGCGCCGTTAACAGGTGGGAAAGAGTTTGATGAAATTGACTTA
AAAGCGAAAGATAGTCATGGCGAGTATATTATTGATAAAGATGCCCATACGAGACTAGCGAAAGAAGCAAATACGTCAATTTTACGTAGA
GCCTTTAATTATGTTGATGGCACGGATGACCGCACAGGTAACTTCGAAACAGGCTTGTTGTTCATTGCTTTTCAAAAAGCGACAAAACAATT
TATCGATATACAAAATAATTTAGGTAGTAATGATAAATTAAATGAATATATTACACATAGAGGTTCTGCTTCATTTTTAGTATTACCAGGTGT
TAGTAAGGGAGGATACCTTGGTGAAACATTATTTGACTAA

Gene: fepC (high-affinity iron transporter)

Position: 376541 to 378253, length: 1713 nt, orientation: FORWARD

Perfect match to: (11819-97-CP003194-[382465:384177], allele observed in CC80+CC15+CC72+CC80)

Sequence:

GTGAAACATTATTTGACTAAATTTGTAGCAATGCTAATAACTGCTGCTATGGTGTGTAGCTTTGGGTTACTGAAAAGTCAGGCAGCAGAAC
AACAAAGTATTAGTGATGTATATAGTGTGATAACGGATGCGAAATCTGCACTTTCTAATAATTCGATATCGAATGACAATAAGCAGAAAGC
AATTGAGCAAGTGGTAAGTGCAGTTAAGAAATTATCGCTTGAAGATAATAGTGAAGGTAATGCTGTCAAATCAGATGTGAGAAAGCTTGA
AGATGCAAAAGCGAATGATGATCAAAAAGATACACTTTCGCAATTAACGAAGTCATTAATTGCTTATGAAGAGAAATTGGCTAGTAAAGAT
GCGGGTTCTAAAATTAAACTATTACAACAGCAAGTCGATGCCAAAGATGCTGCGATGACAAAAGCGATTAAAGATAAAAATAAAGCGGAA
TTAGAATCTTTGAACAATAGTTTGAATCAGATTTGGACAAGTAATGAAACAGTGATTCGCAATTATGACGCAAATCAATATGGACAAATTGA
AGTCGCATTATTACAACTTAGAATTGCAATTCATAAGTCACCATTAGATACGGCAAAAGTGTCACATGCTTGGACAACTTTTAAATCAAATAT
TGATCATGTCGATAAAAAAAGTGATACGTCTGCAAATGATCAATACCGTGTATCACAATTAAATGATGAGTTAGAGAAGGCGATTAAAGCT
ATCGACGACAATCAATTGTCGGATGCTGATGCTGCGCTTACACATTTTATAGAAACTTGGCCGTATGTTGAAGGTCAAATTCAAACTAAAGA
CGGTGCTTTGTATACGAAAATTGAAGATAAAATACCATATTATCAAAGTGTATTAGACGAACATAATAAAGCACATGTGAAAGATGGCTTA
GTAGATATAAACAATCAAATTAAAGAGGTTGTTGGCCATAGTTATAGCTTTGTCGATGTGATGATTATCTTTTTACGTGAAGGGCTAGAAGT
GTTGTTAATTGTAATGACATTGACTACCATGACGCGTAATGTAAAAGATAAGAAAGGGACTGCAAGTGTGATTGGTGGTGCAATTGCCGG
ACTTGTACTGAGTATTATCATAGCAATTACGTTTGTAGAAACTTTAGGGAATAGTGGCATTCTTCGTGAAAGTATGGAAGCGGGATTAGGT
ATCGTTGCGGTCATATTAATGTTTATCGTTGGTGTTTGGATGCACAAACGTTCAAATGCAAAACGTTGGAATGACATGATTAAAAATATGTA
TGCTAATGCGATTAGTAATGGTAATTTGGTATTGTTAGCGACGATTGGTTTAATATCTGTGTTGCGTGAAGGTGTCGAGGTTATCATTTTCT
ATATGGGGATGATAGGTGAGCTAGCGACCAAAGATTTTATTATTGGTATTGCTTTAGCTATCGTTATTTTAATCATCTTTGCGTTATTATTTA
GATTTATCGTCAGATTGATACCTATATTCTATATATTTAGAGTGTTGTCGATTTTTATTTTTATTATGGGATTCAAAATGCTTGGCGTAAGTAT
TCAAAAGTTACAATTATTAGGTGCTATGCCGAGACATGTCATTGAAGGATTCCCAACGATTAACTGGTTAGGATTCTATCCAACTTATGAAC
CATTGATAGCACAAGCTGCCTATATTATGGTAGTTGCTATCTTAATCTTTAAATTTAAAAAAATAA

Gene: tatC (twin-arginine translocation protein C)

Position: 378345 to 379043, length: 699 nt, orientation: TRNC-RVRS (no start codon)

Perfect match to: (11819-97-CP003194-[384269:384967:r], allele observed in CC80)

Sequence:

TTAATGTGTTGGCGGTTTGCCTCGGCATGTGAACTTAACAATGAACATGCTGAACTCAAAGAGCAATATGAGTGGCAATGTAAGTAATATA
TTTAATGTTAAATCGGGTGGTGCAATGATACTTGCTAATACAAAGCAAGCGAAATAAATATATTTTCGATAATGCTTCAATGATGTGGTATC
TATAAGACCGAATTTTGCAAGTCCTATGAATAATATTGGCAATTGGAATAAAATGCCAAAAGTGAATAACCAACGTATAAGTTCAACTAAAT
ACGCTTTAAAGCCAATCACTGGCGAAATGTTCAAAGTGGTTGATAATTTTAACGCGAATTGAATGATCATTGGAAAGCCAACATAAAATGC
AAAAGCGACACCAAAAATAAATAAAAGTACGCTAAATAAGCTATATTTATAAATAAATTGGCGCTCATTATTATGCAAACCAGGCGCAACA

AACGCCCACAATTGATAAAACATAACCGGTGAAATGAGACAAAACGAGATGAAAAATATAATCATCACGTATATTTGGATCATTTCTGTGA
ATGAAAATGCATGTAAGGACACATGTGCTCGAGTAATATATGATATGACTAATGTCATCCACCAAAATGATGAAACATAAACGATGATGAC
TGTGACGACGAACGACAATAGTATTTTAACTAACCGTTGGCGTAGTTCGCTAAAATGAAC

Gene: tatA (twin-arginine translocation protein A)

Position: 379063 to 379278, length: 216 nt, orientation: REVERSE

Perfect match to: (N315-BA000018-[393696:393911:r], allele observed in CC5)

Sequence:

CTATTGCTCTCGCTGTTGTTTCGATTCCTTACTGGGTGTGTCGTGAGACTCTTTGTCTAAATCTTCTGTTGCAGATTTAAATTCTTTTAAAGTA
GAACCGATAGCACGACCAAATTGTGGTAATTTTTTCGGACCAAAAATAATTAAAGCGATAATGCTAATGACGACAAGACTTGTTGGGCCTG
TGATGCCTAAAATAAAAGTGTTAGTTATCAT

Gene: DUF1398 (putative protein)

Position: 379387 to 379776, length: 390 nt, orientation: REVERSE

Perfect match to: (11819-97-CP003194-[385311:385700:r], allele observed in CC80+CC15+CC80+CC361)

Sequence:

TTATTGAGGGATTAATTCTGAAATAACAGCTTGGTCTTGCAAGTCGATATAAGTACAAGTGCCCGCTTGAATATCGATATGCCATTTATAAA
TGCCAGCTTCAGCCATTTCATCACAAAATGTTTCAAAATCTGTTTGCCCTTGTTGATGTCTTGTTAAGACGTCTTGAACTATTGTTTTGTTTGA
TTTTTGAGCAACAGGATGATTACTTTTTACAGATGACGTAACGATATCATCTTCTGATTGATGTACGTATGTTGCTGTACCATCTTGAATATT
AACAATATTGTACGTCATACCCATATCCTTAAATGCTTTGAATAGTTTTGGAAAATCAACACCAGTAAATTGTTGATGTGCTTGTTGAATTGC
AGATAATGTAAATGCCAT

Gene: Q5HIU5 (putative transcriptional regulator)

Position: 380017 to 380220, length: 204 nt, orientation: FORWARD

Perfect match to: (11819-97-CP003194-[385941:386144], allele observed in CC80+CC15+CC80+CC88+CC96)

Sequence:

GTGCGTAATCGATTGAAAGAATTACGAGCACGAGATGGCTTAAACCAAACGCAACTTGCTAAACAAGCGGGCGTTTCAAGACAAACCATAT
CGCTAATTGAGCGAAACAATTTTATGCCATCAGTATTAACGGCAATAAAAATTGCTCGAATTTTCAATGAAACGGTGGAAACTGTTTTTATT
ATTGAGGAGGATGAGGTATGA

Gene: DUF3169 (ABC transporter, transmembrane permease)

Position: 380217 to 380930, length: 714 nt, orientation: FORWARD

Perfect match to: (TCH70-ACHH02000005-[115957:116670:r], allele observed in CC1+CC80)

Sequence:

ATGAAAATACTAAGATATATCGGATATCTTTTACTAGGTGGACTTGTAGGGGGTATCATTGGTGGAATTTTAGGTAATTTTGATGGATTGG
GTATTGAGAACTTGACGTTTGCGACATATACCAATGTCGTTGTAATATCGATTGTTGCGACGATTATTATCATATTGGTAGAAGCCATTGTTT
TGATGAATCAAAGACGTGCATTGAAGTATAAGCGACTTGTAGATGAAGAGGTAGATATCGATGCAACAGATCAATATGAATTGCTTGCGA
ATCGTTATGTTTTAAATGGAAGTATATTAAGCGTTATACAGACAATTATTGCCTTTGTAGTGTTACTAATTTTTGTGGTAGGGCAAGCTGCAG
CAAATGCAATGCTATTCTTTTTAATACCATTTTTTGCTAGTGCTATTTTCAATACACAATTTACACTGTTTAATAGAAAATTTGATGACAGAAT
GCCAAAAAATTGCAGATAAGAATTACACTGAAAAGCGATTGGAAATATTGGATGAGGGTGAACGCCATATAGAATTAATTGCATTATTTAAA
ACATATGCGATCAACTTATCCATATTGATACTAGCCATTATTTTTATAGGGTCTTATTCAATTGCTACAGGAATTAATCAAAGCTTTAGTTTGC
TACTTATCATTGCTATTTTCATATATAACGCCTTTAGTTATTTATTGAAGAGAAGACGTTTTTATTAA

Gene: Q5HIU3 (ABC transporter, ATPase)

Position: 380955 to 381797, length: 843 nt, orientation: FORWARD

Perfect match to: (11819-97-CP003194-[386879:387721], allele observed in CC80)

Sequence:

ATGACAACATTGTTAAACGTAGATAGTGTGAACAAACAATACAAAGATTCGGATTTTAAATTGCAAGATGCATCTTTAACGATTTCTACTAA
TGAGACAGTTGGATTAATTGGGAAAAATGGCTCAGGTAAATCGACATTAATTAATATTCTAGTAGGCAATCGACATAAAGATAACGGTAGT
ATTGCATTTTTTGGAGAAGAACATGCTGCGGATGATGTCGAATATAAAGAACACATAGGTGTTGTGTTTGATGATCTGAGAGTACCTAATA
AATTGACTATTAAAGATATTGATAAAGTATTTCAATCTATTTATACGACTTGGAATAGTCAAAAATTCTTTGATTTAATCAAATATTTCGAGTT
ACCACTACAAACTAAAATTAAAACTTTTTCAAGAGGGATGCGAATGAAGATAGCTTTAACAATTGCGCTTTCTCATGATGTGAAGTTATTAA
TCTTAGATGAAGCAACTGCAGGTATGGATGTTTCTGGACGAGAAGAAGTAATGGAACTATTAGAAGATTTTGTCGCTCAAGGTGGAGGCA
TCTTAATATCATCGCATATTTCTGAAGATATAGAACAATTAGCGGATAAATTAGTGTTTATGAAAGATGGACGAATGATTTTAACTGAACAG
AAAGATATACTTTTAGCACAATATGGAATTGTTACGACAGGAGATAAAGATGTTGAAATTCCTAAGCATTTAATCATTGCTTCTAGATTGTC
AAAGGGGAAATATCAAATTTTAGTTAAAGATTATGCAGAAATTGAAAATGCAGAGCCTTTAAAACACATTGATGACGCTACGAAAATCATA
ATGCGAGGTGAAGTATAA

Gene: Q5HIU2 (ABC transporter, transmembrane permease)

Position: 381797 to 382426, length: 630 nt, orientation: FORWARD

Perfect match to: (TCH959-AASB02000040-[52275:52904], allele observed in CC7+CC80)

Sequence:

ATGAAAGGTATGTTCCTAAGTAGTTTTTATGCAACGAGAAAGCAAACATATATTTATTTTATAGTCGCTATCATAGCTGCGGGATACTTTGC
AGTATTTAATCCGTTGATGAGTTCTGCAATGGCTGGGGTTATGTTAATCACACCCATTACTGATAATATTAAACATGAAAAAGACTCAAGAT
GGATGTATTATGTATCTACGTTACCGGTTAAACGTAGTGATTATATTAAGTCATACTTTGCCTTTTATTTAATCTTATTCGGTGCAAGTTTAAT
GATTGGATTAGTTGTGACTACAATCGTGACCCAAAGTGTGATGATTGGTATTATGTCAGGTTTAATGAGTTTTGGTATCATAGGGGCATACT
CTATCATTTTCCCATTGACATTTAAATTTGGCGCTGAAAACTCTAATGTCATTATGATATTTGCATCTATACTACTACTTATTTCTTTCGTGGTC
TTTTTCTTTATATATGGTATGGTTAGTGGTGCATCTGCATTAGAATTTGAAAAAATTAGCACTGAAGGATGGCTAGTTGTCATAGCATATGC
GGTCATTGGTATAGTTATAACGAGCGTTTCTTATATATTGTCTATTAAAATTTTTAACAAACAAGAACTATAA

Gene: ltrA-L1 (low temperature requirement A, locus 1)

Position: 382807 to 383940, length: 1134 nt, orientation: REVERSE

Perfect match to: (11819-97-CP003194-[388731:389864:r], allele observed in CC80)

Sequence:

TTATTCATTTTTATTACGTAGACGTTGATTTTTAAAATAGATGAGCATGATGCAGAAAGTCGCAACGGATACCGAAATAATCATTACTTGGTC
ATGCCCTTTAAAGATAAGGCTGATGATATATGAAACTACAAGTATGCCTAGTGAAGAAGAAATATATTTTGCGTTTGTCAGTTCATTATGGA
AATAAGGCGTGATTAACCATAATCCAGTATAGAATATTAAAACACTGATATACATCATATTAATTTCAAACAAGTCATTTAGTTTATTGTTAT
TACTAAAAACAATTGCGGCATTAATCACACCTAAAGCGATATTGATTAATAGATGCGTATACGATAAACGGAAACCGATAGATGACAATTT
ATGATTAATGTAATTTTCAGTAATGATCCAATAAACACCGAAAAGACTAATTAAAATCATAAATTGGAATATATAAATGTAACTAAAATGAT
CAATGCTAAATGATGACGAAGCTAAACCAACCAGTACCTCGCCAAAGATAATAATTGTTAGTAACGAAAAACGTTCTACTAAATGCATCATA
TTAACAGGTGATAATACAAGATATTTCTGAAATGGAATAAGTCCTGTCGCTGCAATGAATACGCCTAAAAATCCAGGGATGTAATGGATAC
TTTGTGGTAGTACTAATGATAGAAATGATAAAAATGAAATCACAAAGGCTACGCTCGCAAAAGCTTGACATGTACGCTTATCGCCATAATCT
AACCCTGTACGTATATGTAATAAATACTGTAATCCGATACTTAAATACATAATTGCCACGCATAAGAAGAATGGGAAGAATGTCTTTTCAAA
GTCCGGATATAGGCTGTTAGATAGGAAGACCATGATGAACATATTAAACATCATAAACGAGACGTCTTTGAATGTAACTTGACCAAATCGA
TTTGTAAAAAAATGTTTGATGAGACCACATTAACCATAAGAACAAACTCATGACGATGTATTTGAAAAATAAATCAGCTGAAATGGAACCATT
TTGTGTTGTTAAAATCACATGTGCAATTTTTTGAATGGCATAGACGAAAATCAAATCAAAGAACAACTCATGGAATCCTGCACGCTTTTCAG
CTAAATGTTTTGGTGTTAATGCATTAACCAT

Gene: thlA-yqil (acetyl-CoA acetyltransferase)

Position: 384479 to 385660, length: 1182 nt, orientation: FORWARD

Perfect match to: (KLT6-APFH01000002-[53720:54901], allele observed in CC12+CC8+CC80+CC772)

Sequence:

ATGACGAGAGTCGTATTAGCAGCAGCATACAGGACACCTATTGGCGTTTTTGGAGGTGCGTTTAAAGACGTGCCAGCCTATGATTTAGGTG
CGACTTTAATAGAACATATTATTAAAGAGACGGGTTTGAATCCAAGTGAGATTGATGAAGTTATCATCGGTAACGTACTACAAGCAGGACA
AGGACAAAATCCAGCACGAATTGCTGCTATGAAAGGTGGCTTGCCAGAAACAGTACCTGCATTTACAGTGAATAAAGTATGTGGTTCTGGG
TTAAAGTCGATTCAATTAGCATATCAATCTATTGTGACTGGTGAAAATGACATCGTGCTAGCTGGCGGTATGGAGAATATGTCTCAGTCACC
AATGCTTGTCAACAACAGTCGCTTCGGTTTTAAAATGGGACATCAATCAATGGTTGATAGCATGGTATATGATGGTTTAACAGATGTATTTA
ATCAATATCATATGGGTATTACTGCTGAAAATTTAGTAGAGCAATATGGTATTTCAAGAGAAGAACAAGATACATTTGCTGTAAACTCACAA
CATAAAGCAGTACGTGCACAGCAAAATGGTGAATTTGATAGTGAAATAGTTCCAGTATCGATTCCTCAACGTAAAGGTGAACCAATAGTTG
TCACTAAGGATGAAGGTGTACGTGAAAATGTATCAGTCGAAAAACTAAGTCGCTTAAGACCAGCTTTCAAAAAAGACGGTACAGTTACAGC
AGGTAATGCATCAGGAATTAATGATGGTGCTGCGATGATGTTAGTCATGTCAGAAGACAAAGCTAAAGAATTAAATATCGAACCATTGGCA
GTGCTTGATGGCTTTGGAACTCATGGTGTAGATCCTTCTATTATGGGTATTGCACCAGTTGGCGCTGTTGAAAAGGCTTTGAAACGTAGTAA
AAAAGAATTAAGCGATATTGATGTATTTGAATTAAATGAAGCATTTGCAGCACAATCATTAGCTGTTGATCGTGAATTAAAATTACCTCCTG
AGAAGGTGAATGTTAAAGGTGGCGCTATTGCATTAGGACACCCTATTGGTGCATCTGGTGCTAGAGTATTAGTGACATTATTGCATCAACT
GAATGATGAAGTTGAAACTGGTTTAACATCATTGTGTATTGGTGGCGGTCAAGCTATCGCTGCAGTTGTATCAAAATATAAATAA

Gene: mdh (cyclase family protein)

Position: 385743 to 386495, length: 753 nt, orientation: REVERSE

Perfect match to: (11819-97-CP003194-[391667:392419:r], highly conserved allele)

Sequence:

TTAGTCATTTGATGGTTTAATTGCAAATGCTCTAACAGGGAACCCAGGTGCATCTTTTGGTTTAGGGCTGATAGCGTAAATGATGGCGCCAC
GAGTTGGTAATTGATCTAAATTAGTTAATAACTCGACTTGGTATTTATCCTGACCAAGAATATAACGTTCGCCAACTAAATCGCCATTTTTTA
CAACGTCCACAGATGCATCGGTATCGAATGTTTCATGACCAACAGCTTCAACACGGCGTTCTTCAATTAAGTACTTCAAAGCATCTAATCCCC
AACCCGGTGCATGTTGTTGTCCGTTCGCATCTTTGTTTTCAAACTTTTCAATATTAGGCCAACGTTTTGACCAATCGGTACGAAGTGCAACAA
AAGTGCCAGGTTCAATAGTACCATGCTCTTTTTCCCATGCTTCTATATGCGCACGTGTTACGATGAAATCATTGTTGTTCGCTACTTCTGTTGA
AAAGTCTAATACAATTAACGGCAATACCAATTCTTTTAAATCAATGTCTTCTAAATAACGTTTATTCTCGACAAAGTGAATTGGTGCATCAAT
GTGAGTACCATATTGCGTTACAATATTCCAACGTTGCACATAGAAACCATGATCTTTAACCGTGAATAAAGTTGAAACTTCGCCTTTTTCAAA
CTCACTAAAACGTGGTATTTCCGGATCAAATGTATGCGTTAAATCAACCCAAGTTGCTTGTTTTAAAGTATTTAATTGTTGCCATAAAGGATA
TTGTGTCAT

Gene: metE (5-methyltetrahydropteroyltriglutamate--homocysteine methyltransferase)

Position: 386538 to 388766, length: 2229 nt, orientation: REVERSE

Perfect match to: (11819-97-CP003194-[392462:394690:r], allele observed in CC80)

Sequence:

TTATTCTTGGCGTTTAGCTTTAACAGCATTCACAAGCACAGTCAATGCATCTTTAACTTCTTCTTCTTTTCGCGTTTTCAAACCACAGTCAGGG
TTTACCCAGAATAATGAACGGTCGATTTGTTGTAGTGAACGATTGATTGCTGTAGTAATTTCTTCTTTTGTTGGAATACGTGGACTATGAATA
TCATACACACCTAGACCAATACCTAAATCATAATTAATATCTTCAAAGTCTTTAATTAAATCACCATGGCTACGAGATGTTTCAATTGAAATA
ACATCAGCATCTAAGTCATGAATAGCATGAATGATTTGACCGAATTGAGAATAACACATATGTGTATGGATTTGAGTTTCATCACGAACTGA
AGACGTTGCAAGTTTAAATGATAAAACAGCATCTTTAAGATATTGTTCGTGATATTCAGAGCGTAATGGTAAGCCTTCACGTAATGCAGGTT
CGTCAACTTGGATAACTTTGATTCCTGCAGCTTCAAGTGCTAATACTTCTTCGTTGATTGCTAAAGCAATTTGATCTTGAACGACTTTACGTG
GTAAATCAACACGTTCAAATGACCAGTTTAGAATTGTTACAGGTCCAGTTAACATACCTTTAACTGGTTTATCTGTTAAGCTTTGTGCATAAA
CTGTTTCATCAACAGTTAAAGGCGCTGTCCATTTTACATCACCATAAATGATTGGTGGTTTTACGGCACGTGAACCATATGATTGCACCCAAC
CGAATTTAGTTACTAAGAAACCTTGTAATTTTTCTCCGAAGAATTCAACCATGTCATTACGTTCAAATTCACCGTGAACTAATACATCTAAGC
CAATTTCTTCTTGAATTTTAATCCATCGAGCAATTTCATTTTTTAAGAATGTTTCATATGCTTCGTCTGTAATGCGTTTGTTCTTCCAATCTGCA
CGGTATTTTCGAACTTCTCGGCTTTGTGGGAATGATCCAATAGTTGTTGTTGGTAAATCCGGTAAGTTCAAACGTTTTTGTTGTTGTTCAATA
CGTTGCGCGAATGGTGATTGTCTTGAAGTGCGCACGCTTTCGAAATCATAATCTAAGTTTTTAAATGATTGATTTTGGAAACGCTCATAACG
TGCTTTTAATTTATCATATTTAACACTATCGTTTTGATTAAATAGGCGACGCAATGCATCTAATTCGTCTAATTTTTCAGTTGCAAAGCTTAAG
CCTTCGCCAACACTTGTATCTAATGTTTCATCATCTAAAGATACTGGAACATGTAATAATGAAGATGATGGTTGAATGACAAGTTCATTAGT
GTGTGCTAACAATTTATCGATTAAGACTTTTTTAGCTTCAATGTCACTTGCCCATACATTACGACCATCAATAATTCCAGCGTATAATGTTTTT
GATTTATCAAAATCTCCAGCTTCAATTTGTTTAAGGTTATAGCCATTATCATGGACAAAGTCTAAACCTATACCACCAACAGGTAAAGAACTT

AAGAATTTAAGATGTGCACGTTCAAAGTATGTTTGAATGACTAATTTTTTAGCAACACCAGCTTTTTCGAAATAGTCATAAGCTTCACGTGTA
ATATTTTCATAGCTTTCACTGTCGTCTGTAACTAAGATTGGCTCATCAACTTGAATGTACTCAGCACCTGCATCAATTAATGATTCAAACACTT
CTTTATAAAGTGGTAATAACGTTTTAACTTTTTCTTCAAAAGTTTGGTGACCGCCTTTTGATAATTTAACAAAAGTAATCGGACCAACAATGA
CAGGGTGAGCGTTAACGTTTAAAGATTGGGCATATTTAAAGCGATCTAATAATACGTTGCGACTCACTTTAGGCTCAACATTGTCCCATTCA
GGTACGATGTAATGATAGTTAGTGTTAAACCATTTTATAAGTGCACTTGCAACATGGTCTTTATTACCGCGAGCAATATCAAATAATAAATC
ATCATCAATAGTTCTTCCTTGGAAACGTTCAGGGATGATGTTGAATAATAATGACGTATCTAAAATATGGTCATATAAAGAGAAATCACCAA
CTGGGATGCTATCTAAGTGATAGTACTTTTGTAATAATAAATTTTCTTTATGTAGATCAGTTAATGTTTGATCTAATTCTTCTTTAGAAATCTT
TTTTGCCCAATAACTTTCGATGGCTTTTTTCCATTCTCTTTTTCTACCTAATCTTGGGAATCCTAAGTTTGATGTTTTAATTGTTGTCAT

Gene: metF (methylenetetrahydrofolate reductase)

Position: 388763 to 390604, length: 1842 nt, orientation: REVERSE

Perfect match to: (MW2-BA000033-[383143:384984:r], allele observed in CC1+CC6+CC80+CC96+CC361)

Sequence:

TCATAATATTGCCTCCTTGTGAGCAGTAATAGATTTTGAGTATGCTGCAAGTTCTAATGAATCTTCGACATTTTGAAACGGTGTGATAATGTA
TAAACCATTAAAATATTCATGAACAGTATCGATTAAATCCTTTGAAAGCTTAAGACTTAGTTCTCGTGTTTTGGCTTTATCATCTTTAACTGCT
TCAAATTGTTGTAAAATTTCATCTGACATCTTGATTCCTGGCACTTCATTATGCAAAAAGAGTGCGTTTTTGTAACTTGCGATAGGCATAATG
CCTATGAAAAATGGTTTGTTCAAGTGCTTAGTGGCATGGTAAATTTCAATGATTTTCTCTTTGCTGTACACGGGTTGTGTTATAAAATAAGAC
ATTCCGCTTTCTATCTTTTTCTCTAATCTTTTGACGGCACCATCTAATTTTCGAACATTAGGATTAAAGGCGCCAGCGATGTTGAAGTGTGTAC
GTTTCTTCAGCGCATCACCGTCAGTGTTAATACCTTGATTAAATCTTAGAGCGAGTTCAGTTAATCCTTTAGAATTAACATCATAGACATTGG
TTGCACCTGGTAAGTGACCAACTTTTGAAGGATCACCAGTTATGGCTAATATTTCGTTAACGCCAATGAGCGATAATCCAAGTAAATGGGAC
TGCAAGCCGATTAAGTTTCGGTCTCGACAAGTAATATGTACGAGTGGTTCAATATTGTAATATTGTTTAATTAAGCTTGCAGCAGCAATGTT
GCTAATTCTGACAGTTGCCAATGAATTATCTGCGAGTGTTACCGCATCTACATTAGCTTTGTCAAGTTTAGCGATATTTTCAAAAAATCTATC
CGTGTCTAAATGTTTCGGTGTATCCAATTCGATAATAACGGTTGGACGTTCTTGAACCTTAGATGTTAATGATTGTCTAACTTTATTTTGAGA
TGGATTGAAAAGTGCTTTCTTTGGTATCGGAATCACTTTTTTGTCATTAACAGGTTTAAGTGTCTGAATAGATTCTTTAATAAATTTGATGTG
CTCTGGCGTTGTACCACAGCAACCACCAATTAAACGAACACCTTCGCGAATTAGATTTTGAGCAACTTGACCGAAATATTGTGCATTGTCAC
TATACTTAAATTCACTATTTTCAATATCTAATAAGCTGGCATTTGGATAACAAGATAAGAATGCGTGCTCTGGTAATTCAATATGTGTGAAAG
ACTCTTGCATATGGTGCGGGCCATGATGACAATTGAGTCCCACGATGTTTGCACCACATTGAACGAGTTGTTTTAATCCTTCATTGATTGCCT
GACCATTAACTAAGTAATTTGTGTTTGAAGCGGTTAATTGAGCAATGATTGGAATGTCGTATTTCTTTCTCGTTCGTGAAATGACATTTGTTA
GCTCTTCTAGGTCGTAATACGTTTCGAAAAGTAGCGCGTCAACGCCTTCTTCAATTAAGGTGTCTATTTGAATTTCAGTATGATAAAGAATA
GTTTGTAAGCTGATATCCTCTTGTTTGATACCTCTAAACCCACCAACTGTGCCTAATATATACGTATCTTTATTTGCTGCTTTTTTTGCGATGC
GTACGGCGGCTTGATGTATAGCTTTAACTTTATCTTCAAGACCGAATCGTTTTAACTTTTCAAAATTTGCACCATAAGTATTGGTTTGAATGA
CATCAGCACCAGCTTCGATATATGAACGATGGATACGTTCAACTTTATCTGGATGGCTAAGATTATATGCTTCTGGACAGGTGTCTAATCCT
TCAGAGTATAAAATGGTTCCAATAGCGCCATCAGCTACTAAAACATTATCTTTCAATTGTGTGAGGAATTGACTCAT

Gene: metC (cystathionine beta-lyase)

Position: 390573 to 391733, length: 1161 nt, orientation: REVERSE

Perfect match to: (IS-105-AHLR01000110-[943:2103:r], allele observed in CC22+CC8+CC398)

Sequence:

TTATCTTTCAATTGTGTGAGGAATTGACTCATTGAATGCCTCCTTTAATGCGTATTTGATGTCTGCAATGAGTTCATCAGGATCTTCGAGACC
AACACTTAATCGGAATAGACCGAAAGTGATACCACGTTCTTGTCTCACTTCTTCAGGTAGTGCAGCGTGAGACATTGTTGCTGGATGTGAAA
GGATCGTTTCAACACCGCCCAGACTCACTGAAACGAGTGGTAATGTCAGTGCATCGACAAATTGTTGTGCTTTAGACTCATCAGCTAAACGA
AAGCCAATAACGGCACCGCCATTTTTAGCTTGTTCTAAATGAGCAGTAGTGAGTCCCGGATAATAAACTTCTGAAATTTCATCTTGCTTTATT
AAAAATGACACGATTTTTTGAGCGTTTTCGACAGATTGTTTAAATCTGATTGGAAAAGTTTTTAAATGTTTAGCAAGTGTCCAGCTATCCTGA
GCAGATAACATATTGCCTGTACCATTTTGTATTAAATAAAGAGCGTCACTAATTGCCTCATTATTAGTTATGACAGCACCAGCAATTAAATCG
CTATGTCCACTTAAAAATTTTGTAGCACTATGAATGACAATATCAGCGCCAAGTAATAAAGGTGATTGACCTAACGGTGTCATAAATGTATT
GTCCACAGCTACCAGTAGTTCATGCTTTTCGGCTATTTTAGAAACAGCTTTGATATCAGTAATTTTAAAACAGGGATTCGATGGTGTTTCGAT
ATAAATTAATTTTGTGTTTGATTGAATGGCACCCTCGATTTGTTCGAGCTTGGTAGTATCTACGGTTGTAAATTCAATATTAAATCGATTCAA
AATTTGCTCAGTGAGGCGAAAAGTACCGCCATATACATCATCGGGTAAGATGACATGATCACCAGATTTGAAAGTCAAAAGTACTGCTGAA
ATAGCAGCAATACCTGATGCAAAAGCAAAAGCGAATTTTCCCTGTTCTAATCGTGCTAACTTCTCTTCTAAAAGTTCACGGTTAGGGTTGCC
ACTTCGTGCATAATCATATTTAACATCGCCACCAAGACTTGTTTGATGGAATGTTGAAGAATCATAGAGTGGTGGGTTAGCTGAATGATATT
CCACACCTCTACGCCAATCGAATATCACTTCTGTCTCTTTTGAAAGTGTCAT

Gene: metI (cystathionine gamma-synthase/cysteine synthase)

Position: 391730 to 392833, length: 1104 nt, orientation: REVERSE

Sequence:

TCATACAATCTCTCCAATCTGAGCTTTATCTAATGCTTGGATGATATCGCGTTCGATGTCTTCATAATTTTCAACACCTAGTGATAAGCGGATT
AAATACTCATCAATGCCACGTTTATCTTTTTCAGCATCTGGCATATCAACATGTGTTTGGGTGTAAGGGAAGGTCACTAATGTTTCAGTACCT
CCTAAACTTTCTGCAAAAATGCAAATGTCTAAATTTTCTAATAATTCAGCGACGCTATAGGCCTTGTTAAGTCTTAAACTAAGCATGCCAGTT
TGCCCGCTATATAGTACTTCGTCAATTGCTTGAAGTGACTGACATTTTTTAGCAAGTTTTCTAGCGTTTGATTGCGCACGCTCAATGCGTAAA
TGCAAAGTTTTAAGTCCACGTAACAACAAATAACTATCTATTGGTGAAAGTGTTGCGCCAGTCATGTTGTGAAAATCAAAAAACTGTTGCGC
TAGTGATTCATCTTTGACAGTTACGACACCTGCTAGTACATCGTTATGTCCGCCAATATATTTCGTGGCTGAATGTAAGACTATATCAGCACC
TTCTGCTAGTGGTGTTGAAAGATAAGGTGTTAAAAAAGTATTGTCGATAATTGACAATAAGCCTTTAGCTTTACAAAGTTGATAGTATGGCT
TTACATCAATAGCAATCATTTGTGGGTTAGATATTGGTTCAATGAATAATGCAACTGTTTTATCAGTGATTTCTTTTTCAACTTGTTCATAATC
TGTAAAATCAACGTACTTAAATTTGATATTGTATTGTTGCTCGTAAAATTCAAATAATCTAAATGTGCCACCATATAAATCGAATGAAACTAA
AATTTCATCATGAGGTTTAAATAGATTACATATTAATTGAATGGCTGACATTCCACTTGATGTAGCGAATGATGCAATACCATGCTCAAGTTT
GGCAAAACAGGTTTCAAATGTTGAGCGTGTAGGATTTTTAGTACGTGTATAATCAAATCCTGTCGATTGTCCTAGTTTTGGATGTTTGTAGG
CAGTAGATAAATGGATTGGATTCGCTATAGCACCGGTTGAATCATCAGTTAATGTGATTTGGGCTAACTGTGTATCCTTCAT

Gene: tbox02 (T-box leader element)

Position: 392897 to 393266, length: 370 nt

Sequence:

AAAAAGGACGAAAGCTTATGTTTCGCGGTACCACCTTTATTTGTTATTCCATCGCTGAAATAACCTTATTCAGTACGCATTAAAAGTAAATAT
GCTTACTGAACAATTATCACAATTTAAGTCGTTAAGTAAGGATATAGTAATGTGCTATCCCATACTTATTAACAAAAAATCGTGCGTAAAGA
ATCCAGTACGCCATTTAACATCAATGTTAATACTGTATCGCTATAACGGGCGAACCCGTAGACACCTCATATTGGCATCAACACTCCAAGGC
CATTTTCAAACACGCTTTCAAAATCTTCTCTCAGCTACTAAAGACTCTCTGTATAAGCAGGGTGTGTTTTACTTTCCTCTTTATTGTGTTTAC

Gene: spo0J (chromosome partitioning protein)

Position: 393495 to 394340, length: 846 nt, orientation: FORWARD

Perfect match to: (BU_N22_t6-LFNS01000002-[8497:9342:r], allele observed in CC3019+CC10+CC45I+CC80+CC88)

Sequence:

GTGAGTGAATTGTCAAAAAGTGAAGATCAACGTATTACTAAAACAAAAGATGAACAAATTAAGCAAATAGATATATCGGATATCAAACCGA
ATCCGTATCAGCCCCGAAAAACTTTCGATGAAAATCATTTAAATGATTTGGCAGATTCAATTAAGCAATATGGTATTTTGCAACCAATTGTGC
TTAGAAAAACAGTTCAAGGTTATTACATTGTAGTTGGTGAAAGAAGGTTTAGAGCTTCGAAAATTGCTGGTCTAAAATACGTATCAGCGAT
TATCAAAGATTTAACAGATGAAGATATGATGGAACTGGCGGTCATCGAAAATTTACAACGAGAAGACTTAAATGCGATTGAAGAAGCTGA
AAGTTATCAACGTTTGATAACAGATTTGAAAATTACACAACAAGAAGTAGCGAAACGATTGAGTAAGTCGCGCCCGTATATAGCGAATATG
TTGAGGTTATTACATTTGCCGAAAAAGATTGCTGACATGGTAAAAGATGGGCGACTGACAAGTGCACATGGACGAACGTTATTGGCAATTA
AAGATGAACAACAAATGCTTAGGTTAGCGAAACGGGTTGTTAAAGAAAAGTGGAGTGTCAGATATTTAGAAAACCATGTTAATGAATTAA
AAAATGTTTCGTCAAAGTCGGAAACAGACAAAGTAGATATAACTAAGCCTAAATTTATAAAGCAGCAAGAACGACAGTTGCGAGAACAGT
ATGGTACCAAAGTAGATATATCAATAAAAAAATCGGTTGGTAAAATCTCATTTGAGTTTGATTCACAAGAAGATTTTCTAAGAATAATTGAA
CAATTAAATCGTAGGTATGGTAAATAG

Gene: ykuT (putative small-conductance mechanosensitive channel)

Position: 394497 to 395378, length: 882 nt, orientation: FORWARD

Perfect match to: (11819-97-CP003194-[400421:401302], allele observed in CC80+CC88)

Sequence:

ATGAATCAAGTCATGAATATTATTTCATCTCTATTTGAGCCATTAACAAAAATAGAAACATATGAAAACATTGCAACTAAAATCGCTATGATT
GTTATTTATATTATCGTAGCCCTCATAGTTATTAAAATACTGAATAAAATGATTGAACAGGGATTTAAGATTCAAAATAAGAGTAAAAAGAG

TAACAAAAAGCGATCTAAAACTTTAATATCTCTTGTTCAAAATGTAGTGAAGTATATCGTTTGGTTTATAGTTATTACGACGATTTTAAGTAA
ATTTGGCATTAGTGTTGAAGGTGTTATTGCAAGTGCTGGTGTCGTAGGTTTAGCAGTAGGTTTTGGTGCTCAAACCATTGTTAAAGACGTTA
TTACTGGATTTTTCATTATATTTGAAAGTCAATTTGATGTAGGTGATTATGTTAAAATAAACAATGGTGGTACGACTGTGGCAGAGGGAACG
GTTAAATCAATAGGACTTCGTTCAACACGAATCAATACAATTTCAGGAGAATTAACAATTTTACCAAATAGTAGTATGGGTGAAATAACGAA
CTACTCAATTACAAATGGTACAGCTATCGTTAAAATTCCAGTGTCTGTCGAAGAAAACATTGATAATGTTGATAAAAAACTAAACAAACTAT
TTACTTCTTTACGTAGTAAATATTACTTATTTGTTAGTGATCCGGTTGTTATTGGTATTGATGCTATTGAAGATACAAGAGTAATATTGAGAA
TATCTGCAGAAACAATTCCAGGTGAAGGATTTGCTGGAGCTCGAATTATTCGCAAAGAAGTACAAAAAATGTTTTTACAAGAAGGTATTAA
AACACCTCAACCAATTATGACTGCTTATAATCATAGTGAAAACGGTGTTTAG

Gene: yyzM (putative DNA binding protein)

Position: 395408 to 395611, length: 204 nt, orientation: FORWARD

Perfect match to: (MW2-BA000033-[389790:389993], highly conserved allele)

Sequence:

ATGGCGTCAAAATATGGAATAAATGATATAGTAGAAATGAAAAAACAACATGCGTGTGGAACAAACCGTTTTAAGATTATTAGAATGGGT
GCAGACATAAGAATTAAATGTGAAAATTGTCAAAGAAGTATTATGATTCCACGTCAAACGTTTGATAAAAAACTTAAAAAAATCATCGAATC
TCATGATGATACACAAAGATAG

Gene: yyaF (ribosome-binding ATPase)

Position: 395623 to 396720, length: 1098 nt, orientation: FORWARD

Perfect match to: (11819-97-CP003194-[401547:402644], highly conserved allele)

Sequence:

ATGGCTTTAACAGCAGGTATCGTTGGATTGCCAAACGTTGGTAAATCAACATTATTTAATGCAATAACAAAAGCAGGTGCTTTAGCAGCGA
ACTATCCATTCGCTACGATTGATCCTAATGTAGGGATAGTAGAAGTGCCAGATGCTAGATTACTTAAATTAGAAGAAATGGTTCAACCTAAA
AAGACATTGCCGACTACATTTGAATTTACAGATATCGCTGGTATTGTGAAAGGTGCTTCAAAGGGAGAAGGGTTAGGTAATAAATTCTTAT
CACATATTAGAGAAGTAGATGCGATTTGTCAGGTCGTTCGTGCATTTGATGATGATAACGTAACTCATGTTGCTGGTCGAGTAGACCCTATT
GATGATATTGAAGTTATTAATATGGAATTAGTACTAGCGGACTTAGAATCTGTTGAGAAACGTTTGCCTAGAATTGAAAAATTAGCACGTCA
AAAAGATAAGACTGCTGAAATGGAAGTACGTATTTTAACAACTATTAAAGAAGCTTTAGAAAATGGTAAACCCGCTCGTAGTATTGACTTT
AATGAAGAAGATCAAAAATGGGTGAATCAAGCGCAATTACTGACTTCTAAAAAAATGCTTTATATCGCTAATGTTGGTGAAGATGAAATTG
GTGATGATGATAATGATAAAGTAAAAGCGATTCGTGAATATGCAGCGCAAGAAGACTCTGAAGTGATTGTTATTAGTGCAAAAATTGAAG
AAGAAATTGCTACATTAGATGATGAAGATAAAGAAATGTTCTTAGAAGATTTAGGTATCGAAGAACCAGGATTAGATCGATTAATTAGAAC
AACTTATGAATTATTAGGATTATCAACATATTTTACTGCTGGTGTGCAAGAAGTACGTGCTTGGACATTTAAACAAGGTATGACTGCACCTC
AATGTGCTGGTATCATTCATACTGATTTTGAACGTGGATTTATCCGTGCCGAAGTAACAAGTTATGACGACTATGTACAATATGGTGGCGAA
AGTGGCGCTAAAGAAGCGGGCAGACAACGATTAGAAGGTAAAGAATATATTATGCAAGATGGCGATATCGTTCATTTCAGATTTAATGTAT
AA

Gene: Q5HIT0 (putative bacteriophagal protein)

Position: 396806 to 396997, length: 192 nt, orientation: REVERSE

Perfect match to: (RF122-AJ938182-[365662:365853:r], highly conserved allele)

Sequence:

TTAAAAGAATGGAATAATTTTACTCGCGTTAATAATATCTTGAGTGCTGAAAAATTGTTTGCCTTCGCCAGTATAAGCAGGCTCTAAAACAA
GATTAGCCTTTGCACAATAAAGCCATTCAGGATGAATGCCACTATTAAGTATCTCTTGGAATTCTTGAAAATCTTTAGACCAATCAATATTTA
AATTCAT

Gene: rpsF (30S ribosomal protein S6)

Position: 397259 to 397537, length: 279 nt, orientation: FORWARD

Perfect match to: (MW2-BA000033-[391641:391919], highly conserved allele)

Sequence:

ATGTACATCGTACGCCCAAACATTGAGGAAGATGCTAAAAAAGCGTTAGTTGAACGTTTCAACGGCATCTTAGCTACTGAAGGTGCAGAAG
TTTTAGAAGCAAAAGACTGGGGTAAACGTCGCCTAGCTTATGAAATCAATGATTTCAAAGATGGCTTCTACAACATCGTACGTGTTAAATCT
GATAACAACAAAGCTACTGACGAATTCCAACGTCTAGCTAAAATCAGTGACGATATCATTCGTTACATGGTTATTCGTGAAGACGAAGACA
AGTAA

Gene: ssb (single-stranded DNA-binding protein, chromosomal)

Position: 397558 to 398061, length: 504 nt, orientation: FORWARD

Perfect match to: (RF122-AJ938182-[366414:366917], highly conserved allele)

Sequence:

ATGCTAAATAGAGTTGTATTAGTAGGTCGTTTAACGAAAGATCCGGAATACAGAACCACTCCCTCAGGTGTGAGTGTAGCGACATTCACTC
TTGCAGTAAATCGTACGTTCACGAATGCTCAAGGGGAGCGCGAAGCAGATTTTATTAACTGTGTTGTTTTTAGAAGACAAGCAGATAATGT
AAATAACTATTTATCTAAAGGTAGTTTAGCTGGTGTAGATGGTCGCTTACAATCCCGTAATTATGAAAATCAAGAAGGTCGTCGTGTGTTTG
TTACTGAAGTTGTGTGTGATAGCGTTCAATTCCTTGAACCTAAAAATGCGCAACAAAATGGTGGCCAACGTCAACAAAATGAATTCCAAGA
TTACGGTCAAGGATTCGGTGGTCAACAATCAGGACAAAACAATTCGTACAATAATTCATCAAACACGAAACAATCTGATAATCCATTTGCAA
ATGCAAACGGACCGATTGATATAAGTGATGATGACTTACCATTCTAA

Gene: rpsR (30S ribosomal protein S18)

Position: 398113 to 398355, length: 243 nt, orientation: FORWARD

Perfect match to: (RF122-AJ938182-[366969:367211], highly conserved allele)

Sequence:

ATGGCAGGTGGACCAAGAAGAGGCGGACGTCGTCGTAAAAAAGTATGCTATTTCACAGCAAATGGTATTACACATATCGACTACAAAGAC
ACTGAATTATTAAAACGTTTTATCTCAGAACGCGGTAAAATTTTACCACGTCGTGTAACTGGTACTTCAGCTAAATATCAACGTATGTTGACT
ACAGCTATCAAACGTTCTCGTCATATGGCATTATTACCATATGTTAAAGAAGAACAATAA

Gene: Q6GJS9 (anti bacteriophage infection-like protein)

Position: 398592 to 399599, length: 1008 nt, orientation: REVERSE

Perfect match to: (Strain_21266-AFTT01000027-[45979:46986], allele observed in CC12)

Sequence:

TTAATATGTTTTTAGATAAAAATCAAGGTTATTGATAAAGTTCTTTAAACTTCTTAATTTAGGTCTTGCTCGAACACCTATAATTTTATCATAA
GTCGACTCGCTTATCTTTGATCGTAAAAAATCAAGGTTTTGTTCGAATTGCTTTATAATTCTTTGGAGTTCTGTTTTAGAATAAGAGAGACCA
GAACAAATTAAAATTAATAAAATGAGATAATCAATTATTCTGTCGAAATCTAAATTGTGTATATCCATTTTTACTGCTAAATATTCTTTTAATC
TATTACTTGTCTCTCCAGTTTTAAATCTACAGTCATAAAGTACACCATTGTGAGCTATAGCATTTCTTAAGCCTTTCATACAATCAATGATGTC
ACCTAGTATATCTTTACTTTGATCAAATGCTGGGGAGTATAATTTTAGTTGCTTTTGAGCTTCTATATTCAAATTTTGGGACATGCAACGAGT
GAAAAATACCAGATTTCCCAAAGTGAATAATTCAAATACGGCATATAGTGGCACGGTTCTATCTTGATGTATGTAATGATAAATGTAAGGTT
TCGAATTACAATGTTCATGGATCATGTCGTAAATATCTCTTCTTAGTCTAGTAGTATTTTTTAAATGTTTTTTATATTTTGAGCTACCGAGACT
ATAACTTTTATAATATGTTAATGATTTTTGAAATAAACTATCTAAATCAAAGCCAGAGTTTACACAAACTATTTCTAGCAATCTATTTTTAATA
GCCGTTTCACAGAACATAACGTGTTTATAGAAAAGGCTTTTAATTTCTGTATCAAAACTGTATAAAGCTTGTAATTCGCTAAATTTTTCAAAA
TTAAGTTCTTCCTCTTTGTTTAAAAAGAAATTATACCCTTTGAATCCATGAAAATAGCCGATGTTTCGTAACTGGATTTTTTCCGTGCTACCAT
TTATTTGGATACCTTTATTGTCTCTTATATGTCTCATTAAACCATCAGTAGTTTTAGGTGTTTTTCTGTTCAT

Gene: int5-setC (truncated integrase, associated with setC)

Position: 399605 to 400314, length: 710 nt, orientation: TRNC-RVRS (no start codon)

Sequence:

TTAACTCCCCAATCATTCAAATTTATTCATCATATCTTTCGCCATCTGATTAGTAACATGTGTGTATATCTCTAGATTTTTTTATAATCTGAAT
GACCTACATGCTCTTGCATTGCTTTTAAGTTAATTCCTAATTGAGCAAGTGTAGATATATGCGAATGATGTAATGTATGCGTCGTTACAGGTT
TCTTAATAGAACTAATATCAGCGCCCCCTTTAATAATGTGGCTAATTTTGTTCGAGTCGATAGGGCTACCAGCCGTATTTGTGAATATGTACT
CTCTATCAATAAACTTATCATTCCAAGCATAAGTGTTCTTAATAAGTCGATGCTTTGGGTAGTGAGCCCTATGGCCTTATAGCTATTACTTCTT
TCAGTTGTCTCCTTTACTCCGAATGCTCCCGTCTTTTTTCAGTTATCCAATTAATTGTACCATCGATATCTAGCGTTTTATCTTCATAGTTTATA
TTTTCTCTCTTTATTGCAAGTAGCTCACCGATACGCATGTCATTAGCAATTTGAAACTGTACCATAGCTTTTACCATTTTATAATTACGTTTTGT
CGTTGGATATTTTTATACTTAATTACATAGTCGAAACAATCCAGTAACTCCTTATCTTCATTATCTTCATTATCTTCTAAAGTGTTATTACGTTT
AGCTAGTAACGCATCACTATAGGGATATCTATTTTATCTATTACACATAT

Gene: setC (staphylococcal enterotoxin-like toxin X)

Position: 400520 to 401131, length: 612 nt, orientation: FORWARD

Perfect match to: (11819-97-CP003194-[406444:407055], allele observed in CC80)

Sequence:

ATGTTCAAAAAAAAATGACTCGAAAAATTCAATCGTATTAAAATCTATTCTATCGCTAGGTATCATCTATGGGGGAACATTTGGAATATATCC
AAAAGCAGACGCGTCAACACAAAATTCCTCAAGTGTACAAGATAAACAATTACAAAAAGTTGAAGAAGTACCAAATAATTCAGAAAAAGCT
TTGGTTAAAAAACTTTACGATAGATACAGCAAGGATACAATAAATGGAAAATCTAATAAATCTAGGAATTGGGTTTATTCAGAGAGACCTT
TAAATGAAAACCAAGTTCGTATACATTTAGAAGGAACATACAGAGTTGCTGATAGAGTGTATACACCTAAGAGGAACATTACTCTTAATAA
AGAAGTTGTCACTTTAAAAGAATTGGATCATATCATAAGATTTGCTCATATTTCTTATGGCTTATATATGGGAGAACATTTGCCTAAAGGTAA
CATTGTCATAAATACAAAGAATGGTGGTAAATATACATTAGAGTCGCATAAAGAGCTACAAAAAGATAGGGAAAATGTAAAAATTAATACT
GATGATATAAAAAATGTAACTTTCGAACTTGTGAAAAGTGTTAATGACATTGAACAAGTTTGA

Gene: Q2G106 (putative exported protein)

Position: 401500 to 401868, length: 369 nt, orientation: FORWARD

Perfect match to: (N315-BA000018-[416142:416510], allele observed in CC5)

Sequence:

TTGAAAACGATTTTAAAAACAATAACATATCTTGCACTTACTATCATTGGCGCTTATGCTGCTTTATTCATTTTAAAAACAATAGACTCTCATG
GTATAACAGATCAATTTAACCCATTAGTAAAGGAAGATGATTCTTATGTTAAAACGACAGAGGTGTCTACTAGAATGGATGATCAACTCCG
AAGTTATACTCAAAGTGCTTTTAATAAAGAAGGGAAAGAGACGCAATTAATGTATACTGCTACATTTGATGTTAAACCGCATAGATACTTGA
AAATTACACATAAAGGTCATCATGTAGAAACTTTTGAAGAAGTTGAAAAGGAAGAAGTACCTAAAAAAGCATTAGACAAACTGAGTCGAT
AA

Gene: Q1Y9M1 (putative protein)

Position: 402049 to 402621, length: 573 nt, orientation: FORWARD

Perfect match to: (Strain_21178-AGRN01000068-[53467:54039:r], allele observed in CC239)

Sequence:

ATGAAATTAAAATCATTAGCAGTGTTATCAATGTCAGCGGTGGTGCTTACTGCATGTGGCAATGATACTCCAAAAGATGAAACAAAATCAA
CAGAGTCAAATACTAATCAAGACACTAATACAACAAAAGATGTTATTGCTTTAAAAGATGTTAAAACAAGCCCAGAAGATGCTGTGAAAAA
AGCTGAAGAAACTTACAAAGGCCAAAAGTTGAAAGGAATTTCATTTGAAAATTCTAATGGTGAATGGGCTTATAAAGTGACGCAACAAAA
ATCTGGTGAAGAGTCAGAAGTACTTGTTGCTGATAAAAATAAAAAAGTGATTAATAAAAAGACTGAAAAAGAAGATACAGTGAATGAAAA
TGATAACTTTAAATATAGCGATGCTATAGATTACAAAAAAGCCATTAAAGAAGGACAAAAAGAATTTGATGGTGATATTAAAGAATGGTCA
CTTGAAAAAGATGATGGCAAACTTGTTTACAATATCGATTTGAAAAAAGGTAATAAAAAACAAGAAGTTACTGTTGATGCTAAGAACGGTA
AAGTATTAAAGAGTGAGCAAGATCACTAA

Gene: Q5HIS2 (putative protein)

Position: 402759 to 403022, length: 264 nt, orientation: REVERSE

Perfect match to: (COL-CP000046-[448197:448460:r], highly conserved allele)

Sequence:

CTATTTATCCATGCGAATATCGACTTCTTCTAAATGTTTCTGATATTCTTTAACCTTACTTTCTAAAAACATTTCATATGGTGCATCAAAGAAG
TCAGCTAAATGCATGGCATCTTTAAATTTAGGTTCATGGTGATGATTCTCCCATTCCCAAATTTGATGTGCTTCATATTTAGTACCATATTTCT
CATTTAATTGCTGTGCTAATTCGTCAATTTCTAAATTATGTTTAGTTCGTAAGTTATATAAAATATGCATATTCAT

Gene: Q5HIS1 (putative protein)

Position: 403322 to 403573, length: 252 nt, orientation: TRNC-FRWD (no stop codon)

Perfect match to: (11819-97-CP003194-[409246:409497], allele observed in CC80+CC25+CC80+CC97)

Sequence:

ATGTTTGGATTTATTGGAATGTTAATTGTCGGTGGCTTAATTGGATGGGCTGCTGGTGCTATTATGGGTAAAGATATCCCAGGTGGTATTTT
AGGTAATATTATCGCGGGTATTATTGGATCATGGGTAGGTGGCAAACTATTCGGACAATGGGGTCCTGAATTAGGAAGTATTTACATCTTA
CCAGCTTTAATTGGTTCAATTATCTTTATTGCAATTGTAACGTTAATTTTAAGAGCTGTTCGTAAAAAA

Gene: Q7A7J4 (putative protein)

Position: 404001 to 404582, length: 582 nt, orientation: FORWARD

Perfect match to: (11819-97-CP003194-[409925:410506], allele observed in CC80)

Sequence:

ATGACGATTTATTTAGTTAGACATGGTGAATCACAATCGAATTATGATAATAAACATTTTAGATCTTATTTTTGTGGACAATTAGATGTGCCG
TTAACGGATACTGGCACAAAAAGTGCAGACGATTTATGTGATTATTTTAAAGAGAAACAGATTAAACATGTATATGTTTCAGACTTATTAAG
AACACAGCAAACGTTTGAACATATTTTTCCATATGACATTGCATCAACGACTACGCCTCTATTAAGAGAACGTTCACTTGGCGTATTTGAGG
GTGAATATAAAGATGAAATCAGTGCGAATCCGAAATATGAAAAATATTTCAATGATCCAAACTTTAAAGACTTTCGTCATAGTTTTTCACAA
AAAGCGCCTGAAGGAGAAAGTTATGAAGATGTATATCAACGCGTAGAACATTTTATGAATCATGTTGTCAATGAAGATACACAAAAAGATG
ATATCGTCATTGTTGCACATCAAGTTGTCATTCGTTGTTTAATGGTTTATTTTAATAACGTTTCACGGGAAGAAGCTGTGGATTTAAAAGTTG
AAAATTGCAAACCATATATCATTGAATAG

Gene: Q5HIR9 (putative membrane protein)

Position: 404648 to 405031, length: 384 nt, orientation: REVERSE

Perfect match to: (11819-97-CP003194-[410572:410955:r], allele observed in CC80)

Sequence:

TTAATGGGAATCATTCTTATCAACAGACTTACCACCAATAGATAATGTGATAATTGTGCTTGCGAACAAAATAATTGAAGTTATAAATGACG
CTAATGTAAAAGGTGTAAATTGTGTGAACGATAATATCAATGATAAAAAGAACCAAGAGATAGCCCCAAGAAATTGTAAAATAGCAGAAG
AAATTCTAAAACTCGTATAATCATATATAGCTAAAACAATTGAAGGTAATGCGACTAATACCATTATAATGATGTTGAGGGTGAATAAATAT
GGCTGTTCAAAAGTTACTGTGTTTGGACCTGTTGGATGCATGGCTGCTAAAAATAAGCAAACAATCGTTGATAAAATTGATAAGATAATAA
AAGTAATTCGAACTTTCAT

Gene: A5IPV8 (putative lipoprotein)

Position: 405286 to 405912, length: 627 nt, orientation: REVERSE

Perfect match to: (11819-97-CP003194-[411210:411836:r], allele observed in CC80+CC8+CC239+CC4803)

Sequence:

TTATTTATCGATAACATCACTCTTGATACCTTTAGATTTTAAGAAATCTTTAATTTTATCTTGTTGCTTTTTATTAACATCACCGGCATATTTTGT
TGGCACGTCGACTACATTGATTTTATTTTGCGGTTGATAGCTAAGCTTTTCAATATCTTCATCAACATTGGCGATTGTACTATTTAAAGCTTTG
AAGTAATTCATCATTAATTCAACGGGTTTCTTATATTCTTTAGGAATATTGTTTTCAGTGACAAATTTCTTGAAATGCAAATCGTTTTTAACAG
CTAAGTTAGATAAGTGGCTAAGTGTTTCTGCTTGTTTTTCAGTCACTTTTGTTTGACTGTCAATTTGTTTATCTAGTTTATGTTGCATAATATAT
TTGTTATCAAGTATATCGCTATTTACAGACAAATACTTTTCTATAGCTTGCTTCATCTCTGCATCACTAATATCACTATTTTTCTTATCTGAGTT
AAAGATATCTTTTGTTTCTAATTTTTTAGCGCTTTTAGGTGCATGGATGCCAGTACTTGTATGATGATCTTCGTTATCAGATTGATCGGACGC
GCAACCTGTAAGAATTAATGTCGATGCTAAAAATGTACTTAGTAGTAATCTCTTTTTCAT

Gene: Q6GJR9 (putative protein)

Position: 405985 to 406221, length: 237 nt, orientation: TRNC-RVRS (no start codon)

Sequence:

TTAGTTTGGAATTTCACGATGACCTTGCAATGACCATAGACGTAAATGATTACGTGCATGAGTTACTTTTTCTATCAATAATGCGTCGTTTTG
AACATTGTTAAGGATAGCATTATCTATAAATAACTGCATAATTGGTTGTATCAATTTAGATGTAGGTATCGTACGTAAAAGCATAATGATTTC
GTTCACATACTTTTCCTTCTCAATATCATTTTTCATATTGATTTGTTTGCG

Gene: ahpF (alkyl hydroperoxide reductase subunit F)

Position: 406356 to 407879, length: 1524 nt, orientation: REVERSE

Perfect match to: (TW20-FN433596-[473223:474746:r], allele observed in CC239+CC8+CC4803)

Sequence:

TTAGTTTCTGATAATATAGTCAAAGGCATTTAATGCTGCATTTGCACCAGCGCCCATTGAAATAATAATTTGTTTGTTCTTCTGATCTGTTACA
TCGCCAGCAGCAAATATTCCAGGTACGTTAGTATTATTGTTACGATCAATAACAATTTCACCACGTTCGTTTAATTCAACAGCATCGTTTAAC
CATGATGTGTTTGGAAGTAAACCAATTTGAACAAAGATACCATCTAAGTTAAGTAGATGTTCTTCGCCGGTGCTCATGTCTTCGTAACGTAT
ACCTGTAACATGGTCTTCTCCGACAACTTCAGTAGTTTTGGCATTTGTTTTGATATCAACATTTGATAAAGAACGTAAACGATCTTGTAACAC
GTTGTCTGCTTTTAATTCGCTAGCGAATTCGAATAATGTAACATGATTAACGATACCAGCAAGGTCAATTGCTGCTTCAACCCCAGAGTTACC
GCCACCGATAACTGCTACGTCTTTATTTTCGAATAGAGGTCCGTCACAGTGAGGGCAGAATGCAACACCTTTATTAATCAATTGCTCTTCACC
TGGAATGTTTAGCTTACGCCAACCTGCACCAGTAGCAATAATGACTGTTTTACTTTCTAAGACAGCACCGTTTTCTAACGTAACTTTAATTGC
TTCGTCAGTCTTTTCGATATCTGTAGCACGTATACCTGTCATTGCATCAATGTCATATTGATCAATGTGCGCTGCTAAGTTAGAAGAAAATTC
AGAACCAGTTGTTTCTTTAACAGTAATGAAGTTCTCAATACCAGCAGTATCATTAACTTGGCCACCGATACGATCAGCAACTATACCAGTAC
GTAAACCTTTACGTGCTGTGTAAATCGCTGCACTACCACTAGCAGGACCACCACCAACGATTAAGACATCATAAGGTTCTTTATTTTCAAACT
CAGATGCATCTGCCGTACTGCCTAGTTTCGAAAGAATATCTTGGATTGTCATACGACCATTGCCAAATTCTTCGCCATTTAAAAAGACAGCA
GGGACTGCCATGATGTTTTCAGATTCTTCACGGAACACTGCACCATCAATCATAGAATGCGTGATGTTAGGGTTGATCACACTCATTAAGTT
AAGTGCTTGAACGACATCAGGACATTTTTGACACGTTAAACTAATGAATGTTTCAAAATGGAATGAACCTTCTAATTTTTTAATTTGGTCAAT
GATTGACTGTTTTTCTTTAGGTGCACGACCACTAACCTGTAAAATTGCTAAAACAAGTGAGTTAAACTCGTGACCTAATGGAATACCTGCAA
ATGTTACACCTGTTTCTTCGCCAGGACGATTGACTGAGAAACTTGGTGTACGTTTTAAAGATTTTTCAGAAAGAGATAGTCTAGGTGACATA
TCAGTAATTTCTGTCAACAAATCTTTAAGTTCTTTGGATTTATCATCTGAACCAAGGCTGGCAACGAATTCAACGTTGCCCTCCATTAGTTCTA
ATAGTTGTTTAAGTTGTTGTTTTAAATCAGCATTAAGCAT

Gene: ahpC (alkyl hydroperoxide reductase subunit C)

Position: 407895 to 408464, length: 570 nt, orientation: REVERSE

Perfect match to: (N315-BA000018-[422549:423118:r], highly conserved allele)

Sequence:

TTAGATTTTACCTACTAAATCTAAACCAGGTTGCAATGTTTTAGCGCCTTCTTCCCATTTAGCTGGGCATACTTCGCCAGGGTTTTTACGAAC
ATATTGAGCTGCTTTGATTTTGTGAGCTAATGTACTAGCGTCACGGCCAATTCCGTCAGCGTTAATTTCAGATGCTTGTACAACACCGTCTGG
GTCGATAATGAATGTACCACGTTGAGCTAAACCAGTAGCTTCATCTAATACATCAAAATTACGAGTGATTGTTTGTGATGGGTCACCAATCA
TAGTGTAAGTGATTTTGCTAATTGCATCTGAATGGTCATGCCATGCTTTGTGTACGAAGTGAGTATCAGTTGATACTGAGAATACATTTACG
CCTAATTTTTGTAATTCTTCATATTGGTTTTGTAAGTCTTCTAATTCAGTTGGACAAACGAATGAGAAGTCAGCAGGATAGAAGCATACTACG
CTCCAAGAACCTTTTAAATCTTCTTGTGTAACTTCTTTAAATTGATCTTTTTTTGGATCGAAAGCTTGCGCTGTAAATGGTAAGATTTCTTTGT
TAATTAATGACAT

Gene: nfrA (NADPH-dependent oxidoreductase)

Position: 408956 to 409711, length: 756 nt, orientation: FORWARD

Perfect match to: (MW2-BA000033-[404682:405437], highly conserved allele)

Sequence:

GTGTCAGAACATGTATATAATCTTGTGAAAAAGCATCATTCTGTTAGAAAATTTAAGAATAAACCTTTAAGTGAAGACGTTGTTAAGAAATT
GGTAGAAGCTGGACAAAGCGCTTCGACGTCAAGTTTCCTGCAAGCATACTCAATTATTGGTATCGACGATGAGAAGATTAAAGAAAATTTA
CGAGAAGTTTCTGGACAACCTTATGTTGTAGAAAATGGCTATTTATTCGTCTTTGTTATTGATTATTATCGTCATCATTTAGTTGATCAACATG
CTGAAACTGATATGGAAAATGCATATGGTTCAACGGAAGGTTTGCTAGTAGGTGCAATCGATGCAGCATTAGTTGCCGAAAATATTGCGGT
AACTGCTGAAGATATGGGGTATGGCATTGTCTTTTTAGGATCATTAAGAAATGATGTTGAACGCGTTCGAGAAATTTTAGACTTACCTGACT
ATGTCTTCCCGGTATTTGGTATGGCAGTAGGGGAACCCGCAGATGACGAAAATGGTGCAGCCAAGCCACGCTTACCATTTGACCATGTCTT
CCATCATAATAAGTATCATGCTGATAAGGAAACACAGTATGCACAAATGGCAGATTACGACCAGACAATCAGCGAGTACTATGATCAACGT
ACAAACGGGAATCGCAAAGAAACATGGTCGCAGCAAATTGAGATGTTCCTAGGAAACAAAGCAAGATTAGATATGTTAGAACAATTGCAA
AAATCAGGCTTAATACAGCGATAG

Gene: tcyP (L-cysteine uptake protein)

Position: 409792 to 411180, length: 1389 nt, orientation: REVERSE

Perfect match to: (11819-97-CP003194-[415716:417104:r], allele observed in CC80)

Sequence:

TTAGTGTGAAGTTAATGCAGCATTATCATTTGAATCGAAAGTATCTTTATCCCAATGTTTAGTTAACTTGGCGGTACCTGTACCAGCTAGCAT
TGAATCGTTCACGTTTAATGCTGTTCTACCCATGTCAATCAATGGTTCAACGGAGATGAGCACGCCGGCTAAAGCGACTGGCAAGTTTAAC
GTTGACAACACCAATATGGATGCAAATGTAGCCCCGCCACCGACGCCAGCAACGCCGAATGAACTAATAATCACGACAGCGATTAACGTTA
CAATAAATTGTAAATCAATTTCTACATTAGCGACGGGTGCGACCATAATTGCAAGCATGGCAGGGTAAATGCCTGCACAACCATTTTGTCCA
ATCGACAATCCAAATGTCGCAGCGAAATTGGCAATACCTTCTGGCACGCCTAGACGTCTTGTTTGTGTTTGTACATTCAATGGTAAGGCACC
CGCGCTTGAGCGTGATGTGAATGCAAAGATTAATACTTCCAAAGTCTTTTTAACATAGCGAATTGGGCTAATACCTAACAGGCTTAAAATAA
TTAAGTGAATGATATACATCGTAATTAATGCAGCGTACGATGCGATTAAGAATTTTCCTAAAGTCCAAATGGCGCCAAAGTCACTTGTCGAT
AATGTGTTGGCCATAATTGCTAATACACCGTATGGCGTTAAACGTAAGACGAACGTCACAATCGCCATTACTAGTGAATAGATAGCGTCAA
TCGCACGCTTAAGCAATTCACCATGATCAGGTTGTTTGCGTGCTACGCGTAAATAAGCAAATCCTATAAACGAAGCAAATATCACGACAGC
AATCGTGGAAGTTGCACGTTGTCCAGTGAAATCTAAGAATGGATTTTTAGGCAATAATTCCAAAATTTGTTGTGGTAACGTATGTGCTGTTA
AATCTTTCGCTTGTTTAGCAATTTCGCTTCCACGTGCTTGTTCAGCGTTACCAAGGTTAATTGTTGATGCATCTAAACCAAACACCAAGGCAT
ACACAACACCAACAATCGCAGCAATGGTGACAGTGCCAATTAAAAAGATAAAAATGAGACTACCAATTTTAGCAAACTTTTCTCCGATTTGA
ATTTTAGTGAATGCAGCTACAATAGAAATGAAAATTAAAGGCATCACAATCATTTGCAACAATGCAACGTAACCTTGTCCGACAATGTTGAA
CCAGTCACTTGTTGATGTAATAACATTCGAATGTGTGCCATAAATAAGATGCAATAACACACCGAATACTATACCAATCCCTAAAGCTGTAA
ACACACGTTTCGCAAAAGATATATGTTTGCGAGCCATCATGTGCAATATTACGATGAAAATCACCAATACAATAATATTAATCAGTGTAAGA
AAAGCATTCAT

Gene: Q5HIR2 (putative protein)

Position: 412157 to 413101, length: 945 nt, orientation: REVERSE

Perfect match to: (11819-97-CP003194-[418081:419025:r], allele observed in CC80+CC72+CC188)

Sequence:

TTACAGGTTGAATAAACGTGCGAAGAAACCTTTTTTCTCTTCTTGAGGTTTAGGTTCTGCCATTTCAGACTGTGAAGCTGTGCTGTCATTTTC
TGCTTTCGCTTCCAATGAATCGTTAGAAGTAGCTTGTTTAGAAGCTTCTGCATGGGCTTCTGCTACTTGTTGTTCAGCATCATGTTGCGCTTC
TGCATCTACTGTAGCTTCTTTATTTGAAGGCTCAGCTGCATTGTCATTTTTAGACTGCTCAACGCTGGCTTTTTCAATTGCTTCAGTTGCAGAA
ATTTGATCCTCTTTATCTACTTTAGTAGCAGTGCCATCGCCTGCATTTTCAGCATTAGTATCCGTTGCAGCATTTGCTGTTGACTCTTTCGAAT
CTTTACTGTCAGCTTTATCTTCAGTAACAGCATTATTTTCAACTTTATTTGTTGATTCAGCCAATACTTTAGCTTGAGATGCTTGTGCTTCTTGG
ATAGCCTCTTGTGATGTTTGGATGGCTTTTAAGGATTCGCTATTTGAATCGATTTTAGATGTAAGTTGATTTTGAAGTTCTTTTAATTCTTGTT
GTTGCTGATGTACTTGATTCATCATTTGACCAAGTAGGTGACGTTCTTCGCGCATTTTCTGGATTTCTAGGCGTAAATCTTCTACTAGTTGAG
CGACATTTTGATTAGTAGGTAAGTTTTTGTCATCGTTTTTGACAATGACTTGCAGGAAGTCTTTTTCTTTTTCTAATTCATCAAACGCTAGATC

ATAACTATTTGTTTGTTTTACTTTGTCGGCAATGTCTTTGAAAAGCTCGATATCTTCTTCTTTGAAATCAGTTGCTTCACGACCACGATATTCA
GTCTTGCTTAGTTGGTAACCACGTTCTTCTAAATGTTGAACAATTTTACGGACTTGCTTCTCACTTAGTTCTACGCGTTGTGCAAATTCTTTAG
TTAACAT

Gene: Q5HIR1 (putative protein)

Position: 413219 to 413881, length: 663 nt, orientation: REVERSE

Perfect match to: (NN50-BAEA01000003-[77330:77992], allele observed in CC4803+CC8+CC188)

Sequence:

TTAAAATGGTTTACGTAAATCCATTTCTTCTAATAAAGCATAAATAATTTCACGTGCGATACCATTGGCAAATACATACTTCAAAATATAATTT
GATTTAACACTTGTACCGTAGATATCCTTTGTCAGTTTAATATTTTTCTTTAAAAATAAGTCCACTTTTTTAGTGTGACCATCATTAGTTGGAT
GAATGTAATCAATTTCAGTTTGTCTTTCAAATTGATGTTCGTATAAAAACTGTCTACGGTGTTCAATAATGAGTCCATTATCTTGTGTTAAATC
TGCAGGTTCTTTAGGGACTTCCATCATTAAAAAGTTAATACTTCTAGAATGAACAAGGTTTGATAATAAGTTTAGTTCATGTTCAATCTGATG
TAATGTTTGCGTCATTAACTCATCATGGTTGGGTAGGTCTTTTGCGAATAAATAAATTAAAAATGCTGAATTGGTTGTGGCTTCATAATGTG
CAGTTGCTAGACTGACAACAACATCGTTTTCTAAGCCTACGATAAAGACATAATCGTTTTCGGTTTTGTTGTTTTCTAATGAGCGTTTGATGA
TACGTTTATCTTCAGTTAAAGATAAATTTAACTTAGTATCGTAAAGTTTAAGTGCTTCGTTGTAATGTGGATCTTTGACAGATTGAATGGTTT
TAAATTCCAT

Gene: yflT (heat induced stress protein)

Position: 414024 to 414431, length: 408 nt, orientation: REVERSE

Perfect match to: (N315-BA000018-[430210:430617:r], highly conserved allele)

Sequence:

CTAAGCTTTGGGACCTTTAGATGCTTCAGCAAAATGTGTAATATCAATCTCTTCATAAGCTGAATTATTTTCATGCACTTCTTGATGTGATGA
TTTGTCACGAACCGCTACAACTAACATTTTATCGTCTAAAATAAGTTGTTTATATTTTTCTAATTCATCAGGCGCTAAGTTGTAGCGTGATAA
AACTGCATGTTCACCATCTTCTCCTGTTAACAGTTTAGTCATTCTATCACTAAATGTTCCACTTGTTGAGATAAGGGAGATTTCAGAGTCGTG
TAAGTCATTTAGGTGTAATTTACTTTTACTAATAATTGTTAGCTCTGATTCTAAATAACCTTCAGATTTCTTTTGATTGATTACGTTGTATAATT
CGCCAGTGTCATTTACTACAGTAATATCTGCCAT

Gene: xpt (xanthine phosphoribosyltransferase)

Position: 414944 to 415522, length: 579 nt, orientation: FORWARD

Perfect match to: (M0239-AIWE01000023-[85261:85839], allele observed in CC188+CC8)

Sequence:

GTGGAGTTACTAGGACAAAAAGTAAAGGAAGACGGCGTTGTCATTGATGAGAAGATTTTAAAAGTCGATGGATTTTTAAATCATCAAATTG
ATGCAAAGTTAATGAATGAAGTTGGTCGCACTTTTTACGAGCAATTTAAAGATAAAGGGATTACTAAAATCTTAACCATTGAAGCTTCCGGT
ATCGCACCTGCAATCATGGCTGCACTGCATTTTGATGTGCCATGTTTATTTGCGAAAAAAGCAAAACCTAGCACTTTGACGGATGGTTATTA
TGAAACATCTATTCATTCATTTACTAAAGATAAAACAAGTACGGTCATTGTTTCAAAAGAGTTTTTATCAGAAGAAGATACTGTACTTATTAT
CGATGACTTTTTAGCAAATGGTGATGCTTCATTAGGATTATACGATATCGCACAGCAAGCGAATGCTAAGACAGCTGGTATTGGTATTGTTG
TTGAAAAGAGTTTCCAAAATGGGCATCAACGTTTAGAAGAAGCAGGTTTAACAGTTTCGTCTCTCTGCAAGGTTGCTTCACTAGAAGGAAA
TAAAGTGACATTGGTGGGAGAAGAATAA

Gene: pbuX (xanthine permease)

Position: 415522 to 416790, length: 1269 nt, orientation: FORWARD

Perfect match to: (MW2-BA000033-[411457:412725], highly conserved allele)

Sequence:

ATGAAAAAATTTAATCCTAAGTGTTCAACATCTTTTAGCTATGTACGCAGGTGCCATCTTAGTTCCAATCATTGTTGGTACAAGTTTGAAGTTT
ACACCTGAACAAATCGCTTACTTAGTTACAGTAGATATATTTATGTGTGGGGTTGCCACATTTTTACAAGCCAATAAAGTAACAGGAACAGG
ATTACCAATCGTTCTTGGATGTACATTCACGGCTGTTGCGCCCATGATTTTAATTGGTCAAACGAAAGGAATAGATGTACTTTATGGTTCGCT
ATTTTTATCAGGGATATTAGTTATTATCATCGCGCCTTTCTTTTCACATCTTGTAAAATTCTTCCCACCAGTAGTAACGGGTAGTGTTGTTACT
ATCATTGGTATCAATTTAATGCCAGTAGCAATGAATTACTTAGCTGGAGGTCAAGGTGCAAAGGACTATGGAGATGTTAAGAACATTTTGT
TAGGTTTAATGACATTAATCATTATTCTTGTTTTACAAAGATTCACAACTGGATTTATTAAGAGTATTGCCATATTAATTGGACTCGTTTTAGG
AACGATAGGTGCTGGCTTACTTGGGATGGTCGATATTAATCAAGTCAATCATGCCGGTTGGTTAGGCATCCCAGTGCCGTTTAGATTCTCTG
GATTTAGCTTTGATGTGACATCGACGTTAGTGTTCTTTATTGTAGCTATCGTTAGTTTAATTGAGTCGACAGGTGTCTATCATGCGTTAAGTG
AAATTACCGGTAAGAAGTTAGAAAGAAAAGATTTTCGTAAAGGTTATACTGCGGAAGGTCTAGCGATAGTGTTAGGTTCTATATTCAATTC
ATTTCCGTATACAGCCTATTCGCAAAATGTAGGACTTGTTTCTTTATCCGGCGCTAAGAAAAATAATGTTATATACGGCATGGTCGTGTTATT
ACTTATATGTGGTTGTATACCTAAGCTTGGTGCATTAGCAAATATCATACCGCTACCTGTGTTAGGTGGTGCGATGATAGCTATGTTTGGCA
TGGTAATGGCATATGGTGTTAGTATATTAGGACATATCGATTTTAAAAATCAAAACAATTTATTAATTATCGCTGTATCAGTAGGATTAGGT
ACTGGTATAAGCGCTGTACCACAAGCATTTAAAGGTTTAGGTGAACAATTTGCATGGTTGACTCAAAACGGAATTGTTTTAGGCGCAATCTC
TGCAATTATTCTTAATTTCTTTTTAATGGAATAAAGTATAAACAAACGGAAGAAAATGTGAAATAA

Gene: guaB (inosine-5'-monophosphate dehydrogenase)

Position: 416828 to 418294, length: 1467 nt, orientation: FORWARD

Perfect match to: (O46-AEUR01000018-[18406:19872:r], allele observed in CC130+CC8+CC97)

Sequence:

ATGTGGGAAAGTAAATTTGCAAAAGAATCATTAACGTTTGATGATGTGTTATTAATTCCAGCACAATCTGATATTTTACCGAAAGACGTTGA
TTTAAGCGTACAATTATCAGACAAAGTTAAATTAAATATTCCAGTTATTTCTGCTGGTATGGATACTGTAACTGAATCTAAAATGGCGATTGC
TATGGCTCGTCAAGGTGGTTTAGGTGTTATTCATAAAAATATGGGCGTTGAAGAACAAGCGGACGAAGTTCAAAAAGTAAAACGCTCAGA
AAATGGTGTCATTTCAAACCCATTTTTCTTAACGCCAGAAGAAAGCGTTTATGAAGCAGAAGCATTAATGGGTAAATACCGTATTTCAGGTG
TACCAATTGTTGATAATAAAGAAGATCGCAACTTAGTAGGTATTTTAACAAACCGTGACTTACGTTTTATTGAAGACTTCTCGATTAAAATTG
TAGATGTAATGACGCAAGAAAATTTAATTACAGCTCCAGTGAATACAACACTTGAAGAAGCAGAAAAAATTCTCCAAAAACATAAGATTGA
AAAGTTACCATTAGTTAAAGACGGACGTCTAGAAGGTCTTATTACTATTAAAGATATTGAAAAAGTAATCGAATTCCCTAATGCAGCAAAA
GATGAACATGGTCGTCTACTTGTAGCCGCAGCAATCGGTATTTCAAAAGACACTGATATTCGTGCTCAAAAATTAGTCGAAGCAGGTGTGG
ATGTCTTAGTTATCGATACAGCACATGGTCACTCTAAAGGCGTTATCGATCAAGTGAAACATATTAAGAAGACTTACCCAGAAATCACATTA
GTAGCTGGTAACGTAGCAACTGCAGAAGCAACAAAAGATTTATTTGAAGCGGGTGCAGATATTGTTAAAGTTGGTATTGGCCCAGGTTCAA
TTTGTACGACGCGTGTTGTAGCAGGTGTTGGTGTACCACAAATTACAGCAATTTATGATTGTGCAACTGAAGCACGCAAACATGGTAAAGC
TATCATTGCTGATGGCGGTATTAAATTCTCAGGAGATATCATTAAAGCATTAGCTGCTGGTGGACATGCGGTTATGTTAGGTAGCTTATTAG
CAGGTACTGAAGAAAGTCCAGGCGCAACAGAAATTTTCCAAGGTAGACAATATAAAGTATACCGTGGTATGGGCTCTTTAGGTGCGATGG
AAAAAGGTTCAAACGACCGTTACTTCCAAGAGGACAAAGCGCCTAAGAAATTTGTTCCTGAAGGTATCGAAGGACGTACGGCATATAAAG
GTGCTTTACAAGATACAATTTACCAATTAATGGGCGGAGTGCGTGCTGGTATGGGTTATACTGGTTCACACGATTTAAGAGAATTACGCGA
AGAAGCACAATTCACACGTATGGGTCCTGCTGGTTTAGCAGAAAGCCACCCACATAATATTCAAATTACGAAAGAATCACCGAACTACTCAT
TCTAA

Gene: guaA (GMP synthase [glutamine-hydrolyzing])

Position: 418319 to 419860, length: 1542 nt, orientation: FORWARD

Perfect match to: (NN50-BAEA01000003-[71351:72892:r], allele observed in CC4803+CC1+CC8+CC97)

Sequence:

ATGGAAATGGCAAAAGAACAAGAGTTAATCCTTGTCTTAGACTTTGGTAGCCAATACAACCAATTAATTACACGCCGAATTCGTGAAATGG
GCGTTTATAGTGAATTACACGATCATGAAATTTCAATTGAAGAAATTAAGAAAATGAATCCAAAAGGTATTATCTTATCAGGTGGTCCAAAT
TCAGTTTATGAAGAAGGTTCATTTACAATTGATCCGGAAATATATAATTTAGGAATTCCAGTACTTGGTATTTGTTACGGCATGCAATTAACT
ACTAAATTATTAGGTGGTAAAGTTGAACGTGCCAATGAACGTGAATACGGTAAAGCAATCATTAATGCGAAGTCAGATGAGTTATTCGCTG
GCTTACCAGCAGAACAAACTGTTTGGATGAGTCATTCTGATAAAGTTATTGAAATTCCAGAAGGCTTTGAAGTTATCGCTGATAGCCCAAGT
ACAGACTATGCAGCAATCGAAGATAAGAAACGTCGCATTTATGGTGTTCAATTCCATCCAGAAGTACGTCATACAGAATATGGTAATGATTT
ATTAAATAATTTTGTCCGTCGTGTTTGTGATTGTAAAGGTCAATGGACAATGGAAAACTTTATCGAAATCGAAATTGAAAAGATTCGTCAAC
GCGTAGGAGACCGTCGTGTATTATGTGCGATGAGTGGCGGCGTAGATTCATCTGTTGTAGCTGTACTATTGCATAAAGCAATTGGGGATCA
ACTAACATGTATCTTTGTAGACCATGGCTTACTTCGTAAAGGTGAAGGCGACATGGTTATGGAACAATTCGGTGAAGGGTTCAACATGAAT
ATTATTCGTGTTAATGCGAAAGATCGTTTTATGAATAAATTAAAAGGTGTTTCAGATCCTGAACAAAAACGTAAAATCATTGGTAATGAATT
TGTATATGTATTTGATGATGAAGCATCAAAACTTAAAGGTGTAGACTTCCTTGCGCAAGGAACACTATATACAGACGTCATCGAATCAGGTA

CTAAGACAGCACAAACAATCAAATCACACCACAATGTTGGTGGATTACCAGAAGACATGGAATTCGAATTAATCGAACCAATCAATACATT
GTTTAAAGATGAAGTACGTAAATTAGGTATTGAATTAGGTATTCCAGAACATTTAGTGTGGAGACAACCATTCCCAGGACCTGGTCTTGGT
ATTCGTGTACTTGGAGAAATTACTGAAGATAAACTAGAAATCGTTAGAGAATCAGACGCGATTTTACGCCAAGTGATTAGAGAAGAAGGTC
TTGAAAGAGAAATTTGGCAATACTTCACAGTGTTACCAAACATTCAATCAGTAGGTGTTATGGGAGACTACCGTACGTATGATCACACAGT
AGGTATCCGTGCAGTAACATCTATCGACGGTATGACAAGTGATTTTGCACGCATCGATTGGGAAGTCCTACAAAAGATTTCTAGTCGTATCG
TAAACGAAGTAGATCACGTCAACCGCGTAGTCTATGACATTACATCAAAACCACCAAGCACAATCGAGTGGGAATAA

Gene: Q8NY68 (putative protein)

Position: 420430 to 421536, length: 1107 nt, orientation: FORWARD

Perfect match to: (SA40-CP003604-[401195:402301], allele observed in CC59+CC25)

Sequence:

ATGGATAAATCACTTGAAATTAACTTAAAAGAATTAGAAAGCATAATGTATAAAGAAAAAGTAATCGACGTTAAAGAGTATGATAATTTAA
AAGTAACTTTTGAGACACTAAACGATGCTCCTAAAAGTGAAATACCCACTCCCTTTACAATAGAACAAATGTTATATAAATTAGAAACTAAA
AATTTATATTTTCCTGAAATTAAGTCAAAAGAAAAATGTGTGGAGCTTTTACATAGTGTTGGCTATTATAATTTGAAGCATTTTATGTATAAT
GAATTTGGTAAAGATGAGCCGAAAAATTTTGATGAAGTATATATACTTTATAAATTTGATAGATATTTATTGAAACAATTATTTAGTTTAGTA
AATATGTTGGAAACTCATATAAGAAATATTATTTTAGAAGTATATATGTTAGAAATTGAGTATAATAACAAGCCTTCGACACTGTTCTATTTA
GATAAAGATTTATATTTTGAGAAAGTTAATGATGAGTATAAATATTCTGCCAAAAAGCTAAAAGAGTTCAATAGGTTACAAAATGTTTTTTG
GAGAGCTATAGAAAAAAAGAAAAGTAACGATAACGTTAAACATAATATTAAAAAATACAATATTATACCAGCTTGGGTTTTATTCCAAAATT
TTAGTTTTGGAGATTTGTCTACTTTTTATAGAACTACATTACCAACTTATAGAAATAAAGTAAGTAAGCGAATTGAAAATTTAATAGAAAAA
AATACTGGTATAAGCATAAAACTTCCAGAAAAGTTATTGTGTGCATGGTTGAATAGTATCAGGTTTTTGAGAAATAGAATAGCTCACACAG
ATATAATTTATGGAATTAATTTTACAAACACCTGTGCAAAACATCATAGTGATGAGGAAATGTATGTAAATATAGAAAAGTATAAATATCAG
CAAAGGTTAGTTACATTTTTATTAGCTATGAAGAAAATATTTATGAGTATGCCAGAAAATAATATTATTGAATGGAATGAGACTCTAACTAA
GATAGAGAATAAATGTTCAGAACATAACTTTATTAAACTTTCTAGGTTAGGAGTTATTGAAAATAACCTTTCTTACTTTAAAATAACTAAATA
A

Gene: Q6GC78 (putative protein)

Position: 422162 to 422554, length: 393 nt, orientation: REVERSE

Perfect match to: (RF122-AJ938182-[404861:405253:r], allele observed in CC705+CC49+CC80)

Sequence:

TTAAAAATCTATACTATCTATATTGAAATTTTTATTTATGGTTATGCCATAACTACTTTGTATTTCCGGTACTAAACTTTGCAATATTGGATAAT
AGTTATTAGCGAAACCAATAATAATGGAATTAATCACATTGATACTAAGTTCTTTATCTTCAGGTAATGCATTTTCTATTATTTTGATTAAGTC
TATATATTTTATTTCATCAGAATTTTTATGTGCTAAATACTTCATAAAATCAGTTTGCACTTTTTCGAAAACGCTATATCTTAAGTCCATTTGTA
CAAATATTAAGCTGACAGTTTTAGTTTCGAATTGACTTTGACCCAAAACTGAAGCAAGTTCTTCTATTGATTGATATAGCAATCTGATATCGC
CGTTATCTTCAAGCAT

Gene: Q8NY63 (putative protein)

Position: 423981 to 424313, length: 333 nt, orientation: FORWARD

Perfect match to: (TCH70-ACHH02000005-[71068:71400:r], allele observed in CC1)

Sequence:

GTGGCAACACTAAAAGATGTAAAGACTGGTAAAAAGGAAATTTTGCCATCAACTGCAAAAGATAAAAATGGTAAAAATGTTACGTTAATTT
ATTTTGAAAAAGACGGTAAATTAGGTTTTTATGTGCAGAAAACACAAAAAGAACGTGGAGTAGGGAAATGCGTTTCTGGTATAGCGGGTG
GCGCAGTGACAGGAGGCACTACTTTAGGTCTTGCAGGTGCAGGAGTAGGAACAGTTACTATTCCAGTAATTGGGACAGTTAGTGGAGGCG
TAGTTGGAGCTGTTGGTGGTGCTGTCGGCGGGGGTCTAACCGGTGGAGCCACATTCTGCTAA

Gene: Q2YVN4 (putative protein)

Position: 425008 to 425190, length: 183 nt, orientation: TRUNCATED

Sequence:

ATCTATAATTAAATACGCACGTAAGTAATTTTGATCGTTCATTGTTATTGTTTTATGTTTTAGGAAATGTGCTATTGAAAGGTTTAAAGATGA
TTGGAATTTCTTGGTTTCTTGACTTATATAGTTATGAAAGAGGGCGTTGTCCTTAATGGCATTTCTGAGATCAGAAATGCTATTAATAAA

Gene: Q6GC74 (putative protein)

Position: 425419 to 427245, length: 1827 nt, orientation: FORWARD

Sequence:

ATGGGATATAGAATAGAAACAAGAACGTTTGAAGAGTTAGAAGGAAAATTAAAGTTACCAACTTTTCAAAGAGCACTAGTTTGGTCTAATA
ATCAAAAGCAAGAATTTTTCTCAACGCTGAAAGATGGTTTTCCTTTTGGTTCTATTCTGTTATACGAATATGAAAATGATAATAAATACAGTC
TTATTGATGGCCTGCAAAGGTATTCTACAATGGTAGATTTCATGGAGAATCCAACAAACTATATTGAATTTGAAGATTTTACATTGAAAATT
GCTGAATTGTATAAAGGGGCATCAGAGAGTACAAAAGCAGAAGTAGTTAATTATGCCGAAGATATTCTGCTGAAAATTATTGAAAAATATA
TTGATTCTACAAAAGAAAATGTAAAAGTAAATGAACTAGCTAAAAAAATGGTGGATAAATTTCCAGTATTAGGTGAAACAGATATTAGAGA
TGAAATCACTCAAATACAGTCAGATGTTATTACTTATTTTAATAATCAACTAGATATTAGTAATGTCAAGATTCCGTGTATCTTCTTTGAAGG
AGAAGAGACTGAATTGGCAGAAGTTTTTCAAAGGTTAAACAGTGGAGGTTAAAAGTTATCGAAGTATCAAGTTTTTGCTGCACATTGGGAC
AGATATGAAGTTGAATTGGGAGATTTAAAGTATAGTGATGAGATACTTAAAAATGTAATAGAAAGGTACGAAAAATTAAATAATGATAGA
GGTATCTTAATTGAAAATTTTGACCCTAAAGAAATGGAGACGTCAAGAGTAATTAATTTATCTGAATTATGTTATGGACTTGGTAAAGTGAT
TTCTGATGAATTACAAGCATTTTTTAAAGAGTCAACAGAAGATATATGCAATGAATTAGGATTTCAAACAATGCTAATTGTATTTGGCATTCC
TACAAATAAGATGAATGAGTTACCATATAAATATCAATATATGAAGAATAAAAATAATATAGAAATCTTGTTTGAAAAAATTGTGAAGATAT
ATAAAATAATAAATAACAAGTTTAAAGAAGCTTTCTCTATGGTAGTATCGTCAAGAGAAGATAAATATGAAACTAAATCTTTTACTAGATTA
CAAATAATGTCATTCTTTTCATCTTTATGGATCTCATCGTATAGTATTGAATTAGATGAAAATAAAAACACATTTTCTATTAAACAAACTAAAT
CTTTGAAAAAAAATCAACAAGTGATTTTGTCTAATATCATAATTTATGCAATTTATGATGTGATAAGAAAGTACTGGTCAGGAACAGGTGAT
CGAAAATTAATGGATATATACATATCTCAAAACAATAGATATTTAAAGCCGTTAGCAAAAGAAGTGTTTAGAAATGAGCTACTTAGATGGA
ATGAAGAGAATATAAATAAGACAAGTATTAATATTCAAACGGATGAGAAGATGATCATTACTTTTATTGCTAATAAATTTAGAAGCTTCTAT
TCATCTTTGAATGATAATTTAGATTATGAACATATTTTTTCAAGAAAATTATACAAGCTGCATAAAGGTAATGCAATAATCCCTGCTGGATCT
TTAGGTAACATCATGTTACTTGACTCTGGTATTAATAGAAGAAAAAAAGAAAAATTTCTTTATTCAGCAATAGATATTGATAAGGCATCTATT
ACGGGAACTATAGAAAATCAGTATGTTAACAACAGTTTTTATCCAGATAAAAGAACAATAGATACTATTGAAATAGATATAATTGATGAAA
AATATGATAGTTTAATAAAAATGATCAAAAATCGTGGTGATAAATTAATAGAAGAAGTTGTTAGAGAATTATATAAATAA

Gene: Q6GC73 (putative protein)

Position: 427783 to 428205, length: 423 nt, orientation: REVERSE

Perfect match to: (MW2-BA000033-[423659:424081:r], allele observed in CC1+CC12+CC25+CC188)

Sequence:

TTAGTTAATATATTTAATTGCTAACTTATTATTAGGGTCTCTTTCAAGAATTTTATTTGCGACCAATTGAATATTCTCATGTGAAATCATATTA
AAGTCTGGTTCTCCAAGGATTTCTAAAAATTCGTATAATAAATTAGCATTATTAAATTGCGAAATCTCTAACGATTGCCAATAATGATAGTAT
GCTAAATCATAGGCATTATCTATGTAATTGAAATGAAAGTGATAGATGCTTGCGACCATTTCATGATATTCAATTGTATTTATTTGATTCGCA
AGATTTGATAAATATGATACTAATTTTTCATCTGAAATTTCGTTCGACATAAAGTATGTTAGAATATTCTCCATATCTTTATTCTCTAACATATT
TTTTAATTCAGTTGGTAATAAATTTAATAGAATGTTAACGCTTTTCAT

Gene: Q2YVN4 (putative protein)

Position: 428787 to 429078, length: 292 nt, orientation: TRNC-RVRS (no stop codon)

Sequence:

TTTTCGTTTTTCCACTAACGTCTTTTTCAAGCTTTATATTTACAATTTTTAAGCAGAAGTATCCCATTATTTCAAGGTCTGATTTATCTATAATTA
AATACGTACGTATGGAATTTTGATCGTTCATTTTTATTGCTTTATGTTTTAGGAAATGTGCTATTGAAGGGTTTAAAGATGTTTGGAATTTCTT
GGTTTCTTGACTTATATAGTTATGAAAGAGGGCGTTGTCCTTTATTAAATTTCTGAGATCAGAAATGCTATAAATAAAGTGATTAAGATTTTT
TGTTTTCAT

Gene: Q6GC70 (putative protein)

Position: 429574 to 429981, length: 408 nt, orientation: FORWARD

Perfect match to: (Strain_21266-AFTT01000027-[14229:14636:r], allele observed in CC12)

Sequence:

ATGGAGCTATCTATTTTTTACAATGGGCAATTTTTTGTAGCATTGGTAGAATATAAAATGGGAAATAAATCAAAATTTATCCAATACACATTT
GGGAATGAACCTGATGATATAGAGGTATTGGATTTTATTCATCATCAATTAATGAAAATGATTGATGATATGCAAACTATTGTTTATACGAA
AAATATTTCTAGAAAAGTAAACCCGAAAAGGCTACAAAGACAAATTGCTAAGGAGCAAAAGAAACCTAAATACTCTACCCAAGCACAAATA
GCTATTAAGAAAGAATTAGAATTAAAGAAAAAGCAAAAGCGGAAGAACTATAAAGAAAAACGTGATGCATTTCAAAAAAGAAAAAGAGA
AATTAAAAAGGTTAAAGCAAAAGAGAAGCATAAGGGGCATTAG

Gene: DUF523 (putative protein)

Position: 430028 to 430511, length: 484 nt, orientation: FORWARD

Perfect match to: (11819-97-CP003194-[435951:436434], allele observed in CC80)

Sequence:

ATGATTTTAATTAGTTCGTGTTTGATAGGCGAAAATGTAAGATACGATGGTGGCAATCAATTGGATGTTAGATTGAAAAAATTAATAGACA
GTGGAAAAGCTATTCACGCATGTCCAGAATTGCTTGGTGGATTGTTAATTCCTAGAGAACCTGCAGAAATTATTGGTGGAGATGGTTTTGA
TGTGTGGAATAACGCTGCAAAGGTTGTTACTATTTCTGACAAAGACGTAACTGACGATTATAAACATGGAGCAATAGTTACATTAAAAATTT
TGAAAAAATATCAATGTGATACAGTTATTTTAAAAGCAAATAGCCCATCATGTGGATCACAAGAGATATATGATGGGAATTTTACAGGAAA
TAAGAAAAAGGGTGTAGGTGTGGCAACTGCTTTACTCATTAATGAAGGTATAAAAGTTTATGATGAAAATACGTTTTTTGACCAAAACCAT
GATTGAAACGATAGTACATGAAAAGTAA

Gene: Q6GC68 (putative protein)

Position: 430711 to 431400, length: 690 nt, orientation: FORWARD

Perfect match to: (11819-97-CP003194-[436634:437323], allele observed in CC80+CC1+CC12+CC188)

Sequence:

ATGGAAAAAGTAGCCATTTTAGTAGATGGTGGATACTATAGAAAGATAAGCGCAAAAGTCTACGGAAAAGTTACAGCGAAAGAAAGAGCT
GACGAGTTATATAGTTATTGCAATAGACACTTAAAAGAGACACATTTCAAAGAAGAAATATATAATAAATTATACAGAATATTTTACTATGA
TTGTCCTCCTATTGATAAGATAGTTTATCACCCCTTATTAAAAAAGAATGTAAATTTTTCCAATACTGATACGAAAAAGTGGACAGAAGACTT
TTTCAAAGAAATGAGCAAAAAAAGAAAGGTAGCATTGAGGTTAGGAGAGTTGAGTGAGTATTCAGTTGAATACAACTTAAAATACTCTATT
ACAAAAAAATTATTAAATGGAAGTATCGATTTAAATGATTTGAAAGAAAAAGATTTTTCCCTATCATTACAACAAAAAGGTGTTGATATGAA
AATAGGTTTAGACATAGCTTCATTATCATTTAAACATCAAGTAGATAAAATTATTTTAATAGCTGGTGATAGCGATTTTGTACCTGCTGCTAA
ACTAGCAAGAACAGAAGGAATTGATTTTGTATTAGATTCTTTAGGGGCAGATATTAGAAACAACTTGTCATTACACATTGATGGTAGGCGT
ACTTGTGATGAAAAATTTAAATTTTATTTAAATCAACTATTTAAATAA

Gene: Q5HIQ1 (putative membrane protein)

Position: 431657 to 432016, length: 360 nt, orientation: REVERSE

Perfect match to: (11819-97-CP003194-[437580:437939:r], allele observed in CC80+CC12)

Sequence:

TTACCAAAGCATTGATATGAGTGCCAATACTGGCAATGTGCCTTGTTTGAAAAAGATACTAATATTGCTTGAAAGGCCACCATAAATAGCAA
CGCCAATGATATACACTAAAATAGCTGCGCATATTTCTTTTGGATTACTGCTGATAAACAAACCGTATATTAGCAAAATTCCGATTAAACCGT
TATATACGCCTTGGTTCTTCAAAAGTAGGTTAATATTTTTGTCTTTCAATTTATCGACGCTTATATTAAATGTCTCGCTAGTCTTTTTGGAAGTT
GTAGCAATCGTTTCAAGGTACATAATATAGAAAAACTCTAATGCCACAAATATGATTAAAATTATCGATATGATATTCAC

Gene: Q2G0Y0 (putative protein)

Position: 432035 to 432880, length: 846 nt, orientation: REVERSE

Perfect match to: (11819-97-CP003194-[437958:438803:r], allele observed in CC80+CC12)

Sequence:

TTAAATATTTTCTTGTAAAAATGATTGTAGTGTCTGTGGTTGATCATTGACTAATTGTTGGAAATCATTGGATTCTTGGTCTAATAGTCCTCTT
GCTCCTGCGTTGTACATTGATGCCAATAATGCACCAAAGCCTTTAGGTTCATCGTACATTTCTGCAAATGTCTCTAATGAAACGGGCTCATAT
TTAATTTCTGTGCCTGATGCCTCAGATAAAATAGCAGCAAGTTCTTTCATATCATAACTGTAGCCTGATAATAAATAGCGTTTGCCCCAAGTA
TCTGGATTTTTAATAATAGCAATGACACCTCTAGCAATATCATTTCTAGTAATATAATTAATACGACCATCACCAGCTGGATAAATCAGTTTA
TGCATATTCATCAATTCTGGTAAATATGGTTTAAGTGGATCCATGTACATTGCCATTCTTACATACGTATAGTCAATGCCACTTGTTGCCAAT
AAACGTGCTGCATAACCAAAATAAGGACTCATATGGAATGGATTATTATGCTGATCTGCGTAATAACCTATGAAAATGATATGAGCAACGC
CACTTTGCTTTGCCGCATATACTAAATTTTCCACTTCAGGAATACGTTTGAATGATGGATGAATAATACTTGGAATAAACACAACGGTATCCA
TTTCTTTAAATGCTTCTACCATGCTTTCTTGATTAAAATAATCTAATTGTCGAACAGAAACTTTTCCACGCCAATCTTCTGGAACTTTCTCAACA
TTTCTAACACCAATGTGAAAATGATCTATGTGATTTGCAATGGCTTGATTTGTAATATGTGTGCCTAAATGACCTGTAGCACCTGTTAACATA
ATATTCAT

Gene: ssl01 (staphylococcal superantigene like protein locus 1)

Position: 433352 to 434032, length: 681 nt, orientation: FORWARD

Perfect match to: (11819-97-CP003194-[439275:439955], allele observed in CC80+CC12)

Sequence:

ATGAAATTTAAAGCGATAGCAAAAGCAAGTTTAGCATTGGGAATGTTAGCAACAGGTGTAATAACGTCGAATATACAATCAGTACAAGCG
AAAACAGAAGTTAAACAACAAAGTGAATCAGAGTTGAAACACTATTATAATAAACCATTTTTCGAGCGTAAAAATGTGACTGGATATAAAT
ATACTGAAAATGGTAAAGATTATATGGAAGTCGCAACAGATCATCAGTATTATCAAATATCGTTACTAGGTCCGGATAAAGATAAATTTAA
AGAAGGAAATAATCCAGGTCTAGATATATTTGTCGTTAGGGAAGGTGACAGTAGGCAAGCTGCGAATTACTCAATTGGTGGCGTAACAAA
AACAAACAGTCAACCTTTTATTGACTATATACACACACCAATCCTTGAAATCAAGAAAGGTAAAGAAGAACCACAAAGTAGTCTATACCAAA
TTTATAAAGAAGACATCTCATTAAAAGAACTTGATTATAGATTAAGAGAACGTGCGATTAAACAACACGGCTTGTATTCAAATGGTCTTAAA
CAAGGTCAAATTACAATTACAATGAATGATGGCACAACACATACAATCGATTTAAGTCAAAAACTTGAAAAAGAACGTATGGGTGAGTCAA
TCGACGGCACTAAGATTAATAAAATTCTAGTAGAAATGAAATAA

Gene: ssl02 (staphylococcal superantigene like protein locus 2)

Position: 434318 to 435013, length: 696 nt, orientation: FORWARD

Perfect match to: (11819-97-CP003194-[440241:440936], allele observed in CC80)

Sequence:

ATGAAAATGAAAAATATTGCAAAAATAAGTTTGTTATTAGGAATATTAGCAACAGGTGTAAATACTACAACGGAAAAACCAGTTTATGCCG
AAAAGAAACCTATTGTAATAAGTGAAAATAGCAAAAAATTAAAAGCTTATTATAATCAACCTAGTATTGAATATAAAAATGTGACAGGTTAT
ATCAGTTTCATTCAACCAAGTATTAAATTTATGAATATCATAGATGGTAATTCTGTTAATAATCTTGCTTTAATTGGCAAAGATAAGCAACAT
TATCATACGGGTGTACATCGTAATCTTAATATATTTTACGTTAATGAGGATAAGAGATTTGAAGGTGCAAAGTACTCTATTGGCGGTATCAC
TAGTGCAAACGATAAAGCTGTCGACCTAATAGCAGAAGCAAGAGTTATTAAAGCAGATCATATTGGTGAATATGATTATGACTTTTTCCCAT
TTAAAATAGATAAAGAAGCAATGTCATTGAAAGAGATTGATTTTAAATTAAGAAAATACCTTATTGATAATTATGGTCTTTACGGTGAAATG
AGTACAGGGAAAATTACCGTCAAAAAGAAATACTACGGAAAGTATACATTTGAATTGGATAAAAAGTTACAAGAAGACCGGATGTCCGAT
GTTATCAATGTCACAGATATTGATAGAATTGAAATCAAAGTTAGAAAAGCATAA

Gene: ssl03 (staphylococcal superantigene like protein locus 3)

Position: 435304 to 436374, length: 1071 nt, orientation: FORWARD

Sequence:

ATGAAAATGAGAACAATTGCTAAAACCAGTTTAGCACTAGGGCTTTTAACAACAGGCGCAATTACAGTAACGACGCAATCAGTCAAAGCAG
AAAAAGTACAATCAACTATAGTTGACAAAGTACCAACGCTTAAAGCAGAGCGATCAGCAATGATAAACATAACAACAGGTGCAAATACAG
TGACAACACAAGCAGCTAACACAGGACAAGAACGCACGCCTAAACTCGAAAAGGCACCAAATACTAATGAGGAAAAAACTTCAACTTCCA
AAATAGAAAAAGTATCACAACCTAAACAAGAAGCGCAGAAATTGCTTAATATATCAGCAACGCCAGCGCCTAAACAAGAACAATCACAAAC
GACAACCGAATCCACAACGCCGAAAACTAAAGTGACAACACCTCCATCAATAAACACGCCACAACCAATGCAATCTACTAAATCAGACACA

CCACAATCTCCAACCATAAAACAAGCACAAACAGATATAACTCCTAAATATGAAGATTTAAGAGCGTATTATACGAAACCAAGTTTTGAATT
TGAAAAGCAGTTTGGATTTATGCTCAAACCATGGACGACGGTTAGGTTTATGAATGTTATTCCAAATAGGTTCATCTATAAAATAGCTTTAG
TTGGAAAAGATGAGAAAAAATATAAAGATGGACCTTACGATAATATCGATGTATTTATCGTTTTAGAAGACAATAAATATCAATTGAAAAA
ATATTCTGTCGGTGGCATCACGAAGACTAATAGTAAAAAAGTTAATCACAAAGCAGAATTAAGCATTACTAAAAAAGATAATCAAGGTATG
ATTTCACGTGATGTTTCAGAATACATGATTACTAAGGAAGAGATTTCCTTGAAAGAGCTTGATTTTAAATTGAGAAAGCAACTCATTGAAAA
ACATAATCTTTACGGTAACATGGGTTCAGGAACAATCGTTATTAAAATGAAAAACGGTGGGAAGTATACGTTTGAATTACACAAAAAACTG
CAAGAGCATCGTATGGCAGATGTCATAGAAGGTATAAACATTGATAAAATTGAAGTGAATATAAAATAA

Gene: ssl04 (staphylococcal superantigene-like protein locus 4)

Position: 436739 to 437578, length: 840 nt, orientation: FORWARD

Perfect match to: (11819-97-CP003194-[442662:443501], allele observed in CC80)

Sequence:

ATGAAAATAACAACGATTGCTAAAACAAGTTTAGCACTAGGCCTTTTAACAACAGGTGTAATCACAACGACAACGCAAGCAGCAAATGCGA
CAACACCACCTTCAACTAAAGTGGAAGCACCGCAACAAACACCGAACGCGACAACACCATCTTCAACTAAAGTAGAAGCACCGCAATCAAA
ACCAAACGCGACAACACCATCTTCAACTAAAGTGGAAACGCCACAATCGCCAACCACAAAACAAGTACCAACAGAAATAAATCCTAAATTT
AAAGATTTAAGAGCGTATTATACGAAACCAAGTTTAGAATTTAAAAATGAGATTGGTATTATTTTAAAAAAATGGACGACAATAAGATTTAT
GAATGTTGTCCCAGATTATTTCATATATAAAATTGCTTTAGTTGGTAAAGATGATAAAAAATATGGTGAAGGAGTACATAGGAATGTCGAT
GTATTTGTCGTTTTAGAAGAAAATAATTACAATCTGGAAAAATATTCTGTCGGTGGTATCACAAAGAGTAATAGTAAAAAAGTTGATCACAA
AGCAGGAGTAAGAATTACTAAGGAAGATAATAAAGGTACAATCTCTCATGATGTTTCAGAATTCAAGATTACTAAAGAACAGATTTCCTTG
AAAGAACTTGATTTTAAATTGAGAAAACAACTTATTGAAAAAAATAATCTTTACGGTAACGTTGGTTCAGGTAAAATTGTTATTAAAATGAA
AGACGGTGGAAAGTACACGTTTGAATTGCACAAAAAATTACAAGAAAATCGCATGGCAGATGTCATAGATGGCACTAATATTGATAACATT
GAAGTGAATATAAAATAA

Gene: ssl05 (staphylococcal superantigene like protein locus 5)

Position: 437942 to 438646, length: 705 nt, orientation: FORWARD

Perfect match to: (11819-97-CP003194-[443865:444569], allele observed in CC80)

Sequence:

ATGAAAATGACAGCAATTGCGAAAGCAAGTTTAGCATTAGGTATTTTAGCAACAGGAACAATAACATCAACGCATCAAACTGTAAATGCGA
GTGAACATGAAGCAAAATATGAAAATGTGACAAAAGATATTTTTGACTTAAGAGATTACTATAGTGGCGCAAGTAAGGAACTTAAAAATGT
TACTGGTTATCGTTATAGCAAAGGTGGCAAGCATTACCTTATCTTTGATAAAAATAGAAAATTCACAAGAGTACAGATATTTGGTAAAGATA
TTGAAAGATTTAAAGCACGCAAAAATCCGGGATTAGACATATTTGTTGTTAAAGAAGCGGAAAACCGTAATGGCACAGTGTTTTCATATGG
TGGTGTCACTAAGAAAAATCAAGACGCTTATTATGATTATATAAACGCACCAAGATTTCAAATCAAGAGAGATGAAGGTGACGGTATTGCT
ACGTACGGTAGAGTACACTACATTTATAAAGAAGAGATTTCACTTAAAGAACTCGACTTTAAATTGAGACAGTATTTAATTCAAAATTTTGA
TCTGTATAAAAAGTTTCCTAAAGATAGTAAGATAAAAGTGATAATGAAAGATGGCGGCTATTATACGTTTGAACTTAATAAAAAATTACAAA
CAAATCGCATGAGTGACGTCATTGACGGTAGAAATATTGAAAAAATAGAAGCCAACATTAGATAA

Gene: ssl07 (staphylococcal superantigene like protein locus 7)

Position: 439078 to 439773, length: 696 nt, orientation: FORWARD

Perfect match to: (11819-97-CP003194-[445001:445696], allele observed in CC80)

Sequence:

ATGAAATTAAAAACGTTAGCTAAAGCAACATTGGCATTAGGCTTATTAACTACTGGTGTGATTACATCAGAAGGCCAAGCAGTCCACGCAA
AAGAAAAGCAAGAGAGAGTACAACATTTATATGATATTAAAGACTTACATCGATACTACTCATCAGAAAGTTTTGAATTCAGTAATATTAGT
GGTAAGGTTGAAAACTATAACGGTTCTAACGTTGTACGCTTTAACCAAGAAAAACAAAATCACCAATTATTCTTATTAGGAGAAGATAAAG
CTAAATATAAACAAGGACTTCAAGGTCAAGATGTCTTTGTGGTAAAAGAATTAATTGATCCAAACGGTAGATTATCTACTGTTGGTGGTGTA
ACGAAGAAAAATAACCAATCTTCTGAAACTAATATACATTTATTAGTTAATAAATTGGACGGAGGAAATTTAGACGCTACAAATGACTCATT
TTTAATTAATAAAGAAGAAGTTTCACTGAAAGAACTTGATTTCAAAATTAGAAAGCAATTAGTTGAAAAATACGGTTTATATCAAGGTACGT
CTAAATACGGTAAAATCACTATCATCTTGAACGGCGGGAAAAAGCAAGAAATTGATTTAGGTGATAAATTGCAATTCGAGCGCATGGGCG
ATGTGTTGAATAGTAAGGATATTAATAAGATTGAAGTGACTTTGAAACAAATTTAA

Gene: ssl08 (staphylococcal superantigene like protein locus 8)

Position: 440112 to 440810, length: 699 nt, orientation: FORWARD

Perfect match to: (GR1-AJLX01000043-[65615:66313:r], allele observed in CC361+CC80)

Sequence:

ATGAAATTTACAGCGATAGCTAAAGCGATATTTGTATTAGGAATATTAACAACAAGTGCAATGATAACAGAAAATCAATCGGTTAATGCAA
AAGGAAAGTATGAAAAAATGAACCGTTTATATGATACAAACAAGTTACATCAATACTATTCAGGACCTAGTTATGAGTTAACAAATGTTAGT
GGCCAAAGTCAAGGTTATTATGACTCTAACGTTTTGCTTTTTAACCAACAAAATCAAAAGTTCCAAGTGTTTTTATTGAGAAAAGATGAAAA
TAAATACAAAGAAAAAACACATGGTTTAGATGTCTTTGCGGTACCGGAATTAGTAGATTTAGATGGAAGAATATTTAGTGTTAGTGGTGTA
ACAAAGAAAAATGTAAAATCAATATTTGAGTCTCTAAGAACGCCGAACTTACTAGTTAAAAAAATAGACGATAAAGACGGTTTTTCGTATG
ATGAATTTTTCTTTATTCAAAAGGAAGAAGTATCATTGAAGGAACTTGATTTCAAAATAAGAAAACTGTTAATTAAAAAATACAAATTGTAT
GAAGGGGCAGCTGATAAAGGTAGAATTGTTATTAATATGAAAGATGAAAATAAGTATGAAATTGATTTAAGTGATAAATTAGGTTTCGAG
CGTATGGCAGATGTCATTAATAGTGAACAAATTAAAAACATCGAAGTGAATTTGAAATAA

Gene: ssl09 (staphylococcal superantigene like protein locus 9)

Position: 441191 to 441889, length: 699 nt, orientation: FORWARD

Perfect match to: (MW2-BA000033-[438308:439006], allele observed in CC1+CC80+CC188)

Sequence:

ATGAAATTTACAGCATTAGCAAAAGCAACATTAGCATTAGGAATATTAACTACAGGTGTGTTTACAACAGAAAGTAAAGCTGTTCACGCGA
AAGTAGAACTTGATGAGACACAACGCAAATATTATATCAATATGCTACATCAATACTATTCTGAAGAAAGTTTTGAACCAACAAATATTAGT
GTTAAAAGCGAAGATTACTATGGCTCTAACGTTTTAAACTTTAAACAACGAAATAAAGCTTTTAAAGTATTTTTACTTGGTGACGATAAAAA
TAAATATAAAGAAAAAACACATGGCCTTGATGTCTTTGCAGTACCTGAATTAATAGATATAAAAGGTGGCATATATAGCGTTGGCGGTATA
ACAAAGAAAAATGTGAGATCAGTGTTTGGATTTGTAAGTAATCCAAGTCTACAAGTTAAAAAAATCGATCCTAAACATGGCTTTTCGATAAA
TGAGTTGTTCTTTATTCAAAAGGAAGAAGTATCGTTGAAGGAACTGGATTTTAAAATAAGAAAAATGTTAGTCGAAAAATATAGATTGTAT
AAAGGCGCGTCAGATAAAGGTAGAATCGTTATTAATATGAAAGACGAAAAGAAATATGTAATTGATTTAAGTGAAAAATTAAGTTTTGATC
GTATGTTTGATGTAATGGATAGTAAGCAAATTAAAAATATTGAAGTGAATTTGAATTAA

Gene: ssl10 (staphylococcal superantigene like protein locus 10)

Position: 442247 to 442929, length: 683 nt, orientation: FORWARD

Sequence:

ATGAAATTTACAGCATTAGCAAAAGCAACATTAGCTTTAGGAATTTTAACAACAGGAACTTTAACAACAGAAGTTCATTCAGGTCATGCAAA
ACAAAATCAAAAGTCAGTAAATAAACATGACAAGGAGGCACTATACCGATACTACACTGGAAAGACTATGGAAATGAAAAATATTAGTGC
TTTGAAACATGGTAAAAACAACTTACGTTTTAAGTTTAGAGGTATTAAGATTCAAGTTTTACTGCCTGGAAATGATAAAAGTAAATTTCAAC
AGCGTAGTTATGAGGGGTTAGATGTTTTCTTTGTTCAAGAAAAAAGAGATAAGCACGATATATTTTATACTGTTGGTGGTGTAATACAGAAT
AATAAAACATCTGGAGTTGTCAGTGCCCCAATATTAAATATTTCAAAAGAAAAGGGTGAAGATGCTTTTGTGAAAGGTTACCCTTATTACAT
TAAAAAAGAAAAATAACATTAAAAGAGCTGGATTATAAGTTGAGAAAGCATCTAATTGAAAAATACGGACTTTATAAAACAATCTCAAAAG
ATGGTAGGGTCAAAATTAGCTTGAAAGATGGCAGTTTTTATAACCTTGATTTAAGGTCTAAATTAAAATTTAAATATATGGGGGAAGTCATA
GAAAGCAAACAAATTAAAGATATTGAAGTTAACTTAAAGTAA

Gene: hsdM (type I restriction-modification system DNA methylase)

Position: 443183 to 444739, length: 1557 nt, orientation: FORWARD

Perfect match to: (11819-97-CP003194-[449105:450661], allele observed in CC80)

Sequence:

ATGTCTATTACTGAAAAACAACGTCAGCAACAAGCTGAATTACATAAAAAATTATGGTCGATTGCGAATGATTTAAGAGGGAATATGGATG
CGAGTGAATTCCGCAATTACATTTTAGGCTTGATTTTCTATCGCTTCTTATCCGAAAAAGCGGAACAAGAATACGCAGATGCCTTGTCAGGT

GAAGACATCACGTATCAAGAAGCATGGGCAGACGAAGAATACCGTGAAGACTTAAAAGCAGAATTAATTGATCAAGTCGGTTACTTCATT
GAGCCACAAGATTTATTCAGTGCGATGATTCGTGAAATTGAAACGCAAGATTTCGATATCGAACATTTGGCGACGGCGATTCGTAAAGTTG
AAACATCAACGCTAGGTGAAGAAAGTGAAAATGACTTTATCGGTCTGTTCAGCGATATGGATTTGAGTTCAACGCGACTAGGTAACAATGT
CAAAGAACGTACTGCACTAATTTCCAAAGTCATGGTTAATCTTGACGATTTACCATTTGTTCACAGTGATATGGAAATTGATATGTTAGGTG
ATGCATACGAATTTCTTATCGGGCGCTTTGCGGCGACAGCGGGTAAAAAAGCAGGTGAGTTCTATACACCACAACAAGTATCTAAGATACT
GGCGAAGATTGTCACAGACGGTAAAGATAAATTACGTCACGTGTATGACCCAACATGTGGTTCAGGTTCATTACTGTTACGTGTTGGTAAA
GAGACACAAGTGTATCGTTATTTCGGACAAGAACGTAACAATACTACATACAACTTGGCGCGTATGAACATGTTGTTACATGATGTGCGCT
ATGAGAACTTCGATATCCGCAATGATGACACGTTGGAAAATCCAGCCTTTTTAGGCAATACATTTGATGCGGTTATTGCGAACCCACCATAT
AGTGCGAAATGGACAGCTGATTCAAAGTTTGAAAATGACGAACGCTTTAGTGGTTACGGCAAGCTTGCGCCAAAATCCAAAGCAGACTTTG
CCTTTATTCAACACATGGTACATTACCTAGACGATGAAGGTACCATGGCAGTTGTACTACCGCATGGTGTATTATTCCGAGGTGCTGCCGAA
GGTGTCATTCGTCGTTATTTAATAGAAGAAAAGAACTACTTAGAAGCCGTGATTGGTTTGCCAGCGAATATTTTCTATGGGACAAGTATTCC
AACATGTATCTTAGTATTTAAAAAATGTCGCCAACAAGACGACAATGTATTATTTATCGATGCATCCAATGATTTTGAAAAAGGAAAAAATC
AAAACCATTTAAGCGATGCCCAAGTCGAACGTATTATAGACACATATAAGCGTAAAGAAACGATTGATAAATATAGCTACAGCGCGACATT
ACAAGAGATCGCCGATAACGATTACAACTTAAACATACCGCGATATGTTGATACATTCGAAGAAGAAGCACCGATTGATTTAGATCAAGTC
CAACAAGATTTGAAAAATATCGACAAAGAAATCGCAGAAATCGAACAAGAAATCAATGCATACCTGAAAGAGCTTGGGGTGTTGAAAGAT
GAGTAA

Gene: hsdS (type I restriction-modification system site-specificity determinate)

Position: 444732 to 444798, length: 67 nt, orientation: TRNC-FRWD (no stop codon)

Perfect match to: (ST1464-ANIT01000097-[1:67:r], allele observed in CC1464)

Sequence:

ATGAGTAATACACAAAAGAAAAATGTGCCAGAGTTGAGATTCCCAGGGTTTGAAGGCGAATGGGAAG

Gene: ssl11 (staphylococcal superantigene like protein locus 11)

Position: 446330 to 447028, length: 699 nt, orientation: FORWARD

Perfect match to: (11819-97-CP003194-[452252:452950], allele observed in CC80)

Sequence:

ATGAAATTAAAAAATATTGCTAAAGCAAGTTTAGCACTAGGGATTTTAACAACAGGGATGATTACAACTACTGCTCAGCCAGTAAAAGCAG
ACGAAGCTAGTAGCAGATTATCAGTTACTTCAAAAGATACACAAAATTTAAAGACGTATTATACTGGAGCAAGTTTTGATCTTAGAGGATTA
AGCGGATATAAAGAAGGAGATAAAGTAATATTTCCTCAAAATGGTCAACAAATTGATGTTACATTAACAGGTAAAGAGGGAGACGTGGTT
CAATCTAATGACGACGTTACAAATGTTGATGTGTTTGCTGTTCCTGAAGGTACAGGTCGTTCTGCCATAATGACTACAATTGGTGGGATTAC
TACACCAAACACTCAAAGTTATAAAGACACTACAAATAATGTGAACTTAAGAGTATCAAAGAGTACAAGTCCCAATACCATATCTGTGTCTG
TAAAAAATTATGATATTTTTAAAGAAAAAAATTTCATTAAAAGAACTTGATTTCAAATTAAGAAAGCATTTAATTGATAATCATGATCTTTATA
AAACAGAACCTAAAGACAGTAAAATTAGAATTAATATGAAAGATGGCGGCTACTATACGTTTGAATTAAATAAAAAGTTACAAACTCATCG
TATGGGTGACGTAATTGATGGTACTAAAATAAAAGAAATTAATGTTGAATTAAACTAA

Gene: slaP (exported SSL-associated protein)

Position: 447048 to 448610, length: 1563 nt, orientation: FORWARD

Perfect match to: (11819-97-CP003194-[452970:454532], allele observed in CC80+CC772)

Sequence:

ATGAGGGAAAATTTTAAGTTACGTAAAATAAAAGTCGGTTTAGTATCAGTTGCAATTACAATGTTATATATCATAACAAACGGACAAGCAG
AAGCATCTGAAAATCAAAATATAGAATCTAAAAATTCTTCTAGTAATATAGCATCACAGCCAAAACAAATTAATGAGAATTATACAGGACAT
AAAGAAAATGAAAAAGGTGAAGATCAAAATAAACCTGCTAACTTTGTAAAATTAGGAAGTGTTAATCCTGGAGATACATCTGTCAAAGGTA
CAACATTACCACATCAAATTATACTTTTAAATATTGATAAACAGAGTGTAGAACCAGTGGAAGATAATAATGGTGGATTTGTAATGTCGGAT
GAAAAAGGAGAATTTGAATATAAACTTAAAGATCGTAAAATAGTTCATAACCAAGAAATAGAAGTTTCATCATCTTCCTTAGATGGATTAGA
AGAGGATGACGAAGAGGAAGAAATTGAAAAAAAATCTTCGGATAACAAAGGTGAAAGTATAGAATCACCTATAGAGAAAAATGAAGAAG
TTGAAGAGTCAAGTAATGATGCTACAGCTACATACACTACACAAAGATACGAAGGTGCGTATAAGATTCCTGATAAGCAATTAGAAAAGAA
AGGCGATCATCACCAGATTTTAGTGGAACCTATTACTGAATGGACAGGCATAATTAAAGGACATACTTCAGTGAAAGGAAAAGTTGCATTA

TCAATTAATAATAAATTTATTAATTTTGAAGAAAGTGGTAAAAGCAAAAAAACTTTAACTGAAGAAGAAGGTAAATCAAGAATTGAAGGTA
TCTGGAAGCACATTGATGATAAAGGATATTTTGATTTTGATTTCAAAAAGAAAAGATTTGATAATTTAAACTTAAAGAAAGATGATATTGTT
TCTTTGACATTTTTACCAGAAGATGAAGATGAGGCGTTAAAACCAATTATTTTCAAAACAAAAGTAACAAGCTTTGATAACATTGCTTCTGC
ATATACAGAATATAATCCTGAAAAGGTAGAAAAAGTAAAAACTCTAAATAATGGTTTAGAGGACTTGAATGTCAAAGATATTTATGGTTTT
GTATACGAGAGTGAGCGTGGTATTGGTATTCCGCAAGCAAATAATAACGCCACTAAAGTAATTGAAGGAAAAACGAAATTTGCCAATGCT
GTCGTAAAAGTTTACTCAAGTTTAGGGGATGGGCAAGAATTCCCTGATTTACAAGTAAATGAAAAAGGAGAATTCAGCTTCGACTCTTTTG
ATGCAGGTTATAGGTTATACAATGGTGAGAATTTAAACTTTACTGTTGTTGATCCTGTTACTGGTAAACTATTAAGCAAATTGGTAACTAAA
GAAATTGATATTTATGAATCACCTGAACAAAAAGCAGACCGTGAATTTGATGAAAGACTTGAAAATACACCTGCTTACTACAAGTTATATGG
TGATAAAATAGTTGGTTTCGATACTAATGATTTCCCAATTACTTGGTTCTATCCATTAGGTGAAAAGAAAGTTGAACGTAAGGCACCAAAAT
TAGAAAAATAA

Gene: Q2G0X2 (putative protein)

Position: 448717 to 449025, length: 309 nt, orientation: REVERSE

Perfect match to: (11819-97-CP003194-[454639:454947:r], allele observed in CC80+CC361+CC445)

Sequence:

TTATTTTACGTGTTCATATTTTGAAACATCAAAGCCGTCTTGCTTAGCTTTGTTGATGATGTCTTTGATTGAATGTAATCCTTTATCGGCGAAG
TATGATCTTAAGTTGTCTTTTGTAGCTTGGTCAGCATTCTTATCTAGTAACACATCGATATAACTTAACTCGTGTTCTAAGAAGTTTGCGTCAT
CATGTAGTACGAGTCCATTTTGAGAATACACTTTCGCATCTGCTTGATTACCATATCCAACAACGCCAGTTGCTAATACACCTACCATTGCCG
TAGCTACTAAAACCTTTTTAAATTTCAT

Gene: lpl-locus0450-SAB0396 (tandem lipoprotein-like protein, second locus)

Position: 456164 to 456952, length: 789 nt, orientation: FORWARD

Sequence:

TTGTACATGAGTGTATTACTTTTAATCATTTTTATTGGGGGATGTGGAAATATGAAAGATGAACAGAAAAAAGAGGAACAAACGAATAAAA
CAGATTCAAAAGAAGAGCAAATCAAAAAGAGTTTTGCGAAAACGTTAGATATGTATCCAATTAAGAATCTCGAGGATCTATATGACAAAGA
AGGATATCGAGATGGTGAGTTTAAAAAAGGCGATAAAGGTACGTGGACGATATCAACAGATTTTGCTAAAAGCAACAAACCGGGTGAAAT
GGATAGTGAAGGTATGGTACTGCATTTGAATAGAAATACGAGGACGGCAACTGGGTATTATACTATAAGAACAACTTATGATGAAGTGGG
TAAGATGACACGCGAAAAAAATTATCGTGTTGAACTTAAAAATAATAAGATAGTACTTTTAGATAAAGTAGAAGATGAAAACCTTAAACAT
AAAATAGAGAATTTTAAATTTTTCGGGCAATATGCCGATTTTAAAGACTTGAAAAATTATAAAAATGGAAGAATAACCATCAATGAAAATGT
TCCATATTATGAGGCAGAGTACAAAATTAATAATAGTGATGGAAATGTAAAAAAACTTAGAGAAAATTACCCAATTACAGCCAAAAAGTCT
CCGATATTAAAACTGCATATAGACGGAGATATTAAAGGTAGCTCAGTTGGATATAAACAGATAGAATACAAGTTTTCTAAGGATAAAGAGG
ATGAGACTTTTATGAGTGATTTTCTAAATTTCGGACCAACTAAAGGTGGAAATAATGACTAA

Gene: lipC3-SAA6159_00398 (putative lipase class 3)

Position: 456945 to 458267, length: 1323 nt, orientation: FORWARD

Perfect match to: (11819-97-CP003194-[462867:464189], allele observed in CC15+CC80)

Sequence:

ATGACTAAACAATTTGAAGTGGATTATGGCAAACTAAATAGTGAAACAGAAGCAACGAGTGCTGTTTCAAAATTAGCTTATGAGGTTGAAA
ATGCAAATAATAATAAAATATCAAATCGAGAATTGAAAACAAAATTTGAAGTATTAAAAAAACGGGGAAAATTCCCTTCTAATCTTGAGTAT
ATAGATAGTCACACAGATAAAAAACACGGTGTGACAGCATCAGCTTTCCTAAACAAAGATACTGGGAAAGTGATTATTGGCATGACTGGTA
CAAATTTCAAAAAGGAAGCTCTTGCGAAGCAACTTTTGCTACCAGATTTTGCAACAACAAAACAGGATAGAATTGACTCAGCTGAGACTAA
AAAGGATTTAATTTCCGATGCCCAAATTGGAACATTCCCTCCTTCTGATAACAGTAAACATTTTGAAGAAACACAAGAGTTCATCAAAAAAA
TAAAAAATAAGTATGATATTGATTTTATAACTGGGCATTCATTAGGTGGTAGAGAAGCGGTAATATTAGGAATGAGTAATGGTATTCCGAA
TATTGTTGTTTATAATCCAGCTCCTATAGCAGCTAGGTATTTAAATCATAACTCCCGATTATTTAAACTATATAAAGGTTATAAAGGTAACATT
ACTAGAATTGTATCTGATAAGGATTGGTTAACTAATATTATAAAGGGATATACGAATTATACTTATTTCGGTAATGAAATTGTGGTTCATAA
CGGCAAGTCACATGATATGGCTGGATTTTTAACTGAACAAGAACAAAAAGCTATAAAAAAAGAACTTAAAAAAGTACAAGGTTATGTAGA
AGAAAATAATAAGTCGTTCGTAAAAAATTCTAATAATGCTATGTCTAAATTAGCTAGTATAGAATTACTTAGAGCCAATATGATGACTACAA
ATGGTGGTTGGTTATCTTCTTCGCAACAGAAAGTTTTAGAAAGTTTAACAGCTTTAACAATTGCGCAGTCATTCAATCAACTGATAGAGGAC

GAAATTAATCAAATCAAAAAAATGTATAATGAAAAGAAAAAGAAATTTGAAAAAAATTGGGAAGACGCGCAAAAAGCTGGAAACGCTGTA
GGTAAAGATATAACCGTGAATGAAGTGCTAGAAGCTTTAGATGAAGGTCATGTGAATGAAAGTAGTATGGTAGGGGAACCTAAACAAATG
ATATCTGCAAAAGAAAAACAACTTTCAACAATAGGTTCATCTATATCAAATTATATTACAAGAGTTAGATCTAGTATTAATGAAATCGTTGAT
AAAGATCAAGCACTTGCATCGCAAATAGGTGGATTACTATGA

Gene: lipC3 (putative lipase class 3)

Position: 456945 to 458267, length: 1323 nt, orientation: FORWARD

Sequence:

ATGACTAAACAATTTGAAGTGGATTATGGCAAACTAAATAGTGAAACAGAAGCAACGAGTGCTGTTTCAAAATTAGCTTATGAGGTTGAAA
ATGCAAATAATAATAAAATATCAAATCGAGAATTGAAAACAAAATTTGAAGTATTAAAAAAACGGGGAAAATTCCCTTCTAATCTTGAGTAT
ATAGATAGTCACACAGATAAAAAACACGGTGTGACAGCATCAGCTTTCCTAAACAAAGATACTGGGAAAGTGATTATTGGCATGACTGGTA
CAAATTTCAAAAAGGAAGCTCTTGCGAAGCAACTTTTGCTACCAGATTTTGCAACAACAAAACAGGATAGAATTGACTCAGCTGAGACTAA
AAAGGATTTAATTTCCGATGCCCAAATTGGAACATTCCCTCCTTCTGATAACAGTAAACATTTTGAAGAAACACAAGAGTTCATCAAAAAAA
TAAAAAATAAGTATGATATTGATTTTATAACTGGGCATTCATTAGGTGGTAGAGAAGCGGTAATATTAGGAATGAGTAATGGTATTCCGAA
TATTGTTGTTTATAATCCAGCTCCTATAGCAGCTAGGTATTTAAATCATAACTCCCGATTATTTAAACTATATAAAGGTTATAAAGGTAACATT
ACTAGAATTGTATCTGATAAGGATTGGTTAACTAATATTATAAAGGGATATACGAATTATACTTATTTCGGTAATGAAATTGTGGTTCATAA
CGGCAAGTCACATGATATGGCTGGATTTTTAACTGAACAAGAACAAAAAGCTATAAAAAAAGAACTTAAAAAAGTACAAGGTTATGTAGA
AGAAAATAATAAGTCGTTCGTAAAAAATTCTAATAATGCTATGTCTAAATTAGCTAGTATAGAATTACTTAGAGCCAATATGATGACTACAA
ATGGTGGTTGGTTATCTTCTTCGCAACAGAAAGTTTTAGAAAGTTTAACAGCTTTAACAATTGCGCAGTCATTCAATCAACTGATAGAGGAC
GAAATTAATCAAATCAAAAAAATGTATAATGAAAAGAAAAAGAAATTTGAAAAAAATTGGGAAGACGCGCAAAAAGCTGGAAACGCTGTA
GGTAAAGATATAACCGTGAATGAAGTGCTAGAAGCTTTAGATGAAGGTCATGTGAATGAAAGTAGTATGGTAGGGGAACCTAAACAAATG
ATATCTGCAAAAGAAAAACAACTTTCAACAATAGGTTCATCTATATCAAATTATATTACAAGAGTTAGATCTAGTATTAATGAAATCGTTGAT
AAAGATCAAGCACTTGCATCGCAAATAGGTGGATTACTATGA

Gene: Q5HIM7 (putative protein)

Position: 459237 to 459431, length: 195 nt, orientation: REVERSE

Sequence:

TTATAAACCATAAAGCGCTGCAGTTAAGTATTGAATGGCATAGCCAATTGAAATGAAAATAGCAATCGTTATAATGGCACTACCAATTGTAT
ATAAAGTATGTTTCGTAGATAAACCTTCCGTCTTAGAACCAAAAATACTTAGTAGAATAAATGCAATGCCAATGATATATAAAACTAAAATG
ACAAATAACAT

Gene: cobW1 (cobalamin biosynthesis protein)

Position: 459629 to 460831, length: 1203 nt, orientation: FORWARD

Perfect match to: (112808A-AHZK01000055-[33861:35063], allele observed in CC8+CC398)

Sequence:

ATGGCTAAAATTCCAGTTACGGTATTAAGTGGTTATTTAGGCTCGGGGAAGACAACGTTGTTAAATCATATTTTACAAAATCGAGAAGGTC
GACGTATCGCGGTAATTGTAAATGATATGAGTGAAGTAAATATCGATAAAGATCTTGTCGCAGATGGTGGGGGACTATCGCGTACAGATG
AAAAATTAGTCGAACTTTCTAATGGTTGTATCTGTTGTACACTTAGAGACGATTTATTAAAAGAAGTTGAGCGTTTAGTGAAAAAAGGTGGC
ATCGATCAAATTGTTATTGAGTCAACAGGGATTTCAGAGCCAGTACCTGTTGCACAAACTTTCTCATATATTGATGATGAACTTGGCATTGAT
CTTACAGCGATTTGCCTTTTAGATACAATGGTTACAGTTGTGGATGCTAACCGCTTCGTACATGACATCAACTCAGAAGATTTATTGATGGA
TCGTGATCAAAGCGTTGATGAAACAGATGAACGTTCGATTGCTGATTTATTAATTGACCAAGTTGAATTTTGTGATGTATTGATTATTAATA
AAATTGATTTAATTAGTGAAGAAGAACTTGCAAAGTTAGAAAAAGTGTTAAGCGCATTGCAACCTACTGCTAAAATTATTAAGACAACAAA
TTCTGAAGTAGATTTAAAAGAAGTCTTAAATACGCAGCGTTTTGATTTTGAAAAAGCGAGCGAGTCAGCAGGATGGATCAAAGAACTTGAG
TCTGGTGGTCATGCATCGCATACGCCTGAAACAGAAGAATATGGCATATCATCATTTGTATATAAACGTCGTCTACCTTTCCATGCTAAAAG
GTTCAATGATTGGTTAGAAAGCATGCCAAATAATGTCGTTCGATCAAAAGGTATCGTATGGTTAGCACAATACAATCATGTAGCATGTTTAT
TATCTCAAGCAGGGTCATCTTGCAATATTCATCCAGTTACATATTGGGTGGCTAGTATGTCTGAAGCGCAACAAACACAAATATTAGCAGAA
CGTCAAGATGTCGCAGCTGAATGGGATCCAGAATATGGCGATCGTCATACACAATTTGTCATTATTGGTACAGAATTAGATGAAGAAAAAT
TAACAAAAGAACTCGACGCATGCTTAGTCAATGCGCAAGAAATTGATGCAGATTGGCAACAATTTGAAGATCCATATCAATGGCAAATTAG
ACCAGCACGATAA

Gene: psmA4 (phenol-soluble modulin alpha 4)

Position: 461489 to 461551, length: 63 nt, orientation: REVERSE

Perfect match to: (N315-BA000018-[469998:470060:r], highly conserved allele)

Sequence:

TTATTTTGCGAAAATGTCGATAATTGCTTTGATGATTTTAATGATAGTACCTACAATAGCCAT

Gene: psmA3 (phenol-soluble modulin alpha 3)

Position: 461615 to 461683, length: 69 nt, orientation: REVERSE

Perfect match to: (RF122-AJ938182-[437079:437147:r], highly conserved allele)

Sequence:

TTAGTTGTTACCTAAAAATTTACCAAGTAAATCTTTAAAGAATTTGAATAATTTTGCTACGAATTCCAT

Gene: psmA2 (phenol-soluble modulin alpha 2)

Position: 461735 to 461800, length: 66 nt, orientation: REVERSE

Perfect match to: (RF122-AJ938182-[437199:437264:r], highly conserved allele)

Sequence:

TTACTTACCAGTGAATTTCTCAATTAATCCTTTAATGAATTTAATGATTCCTGCAATGATACCCAT

Gene: psmA1 (phenol-soluble modulin alpha 1)

Position: 461832 to 461897, length: 66 nt, orientation: REVERSE

Perfect match to: (RF122-AJ938182-[437296:437361:r], highly conserved allele)

Sequence:

TTATTTACCAGTGAATTGTTCGATTAAGCTTTTGATAACTTTAATGATGCCAGCGATGATACCCAT

Gene: nuoL-mpsA (membrane potential-generating system subunit A)

Position: 462418 to 463902, length: 1485 nt, orientation: FORWARD

Perfect match to: (79_S10-CP010944-[393657:395141], allele observed in CC772+CC1+CC772)

Sequence:

GTGTTAAGTTTTCAATTGCTATTTTCACTGTTTGTTATTGCGCTTATCATTGCATTGATAAGTGGCTTGTTGTTTTTAGCACCAGTTATGCCAA
TGAGATATATTAAATTACATTTATACATACTAGTCATGCCAGTATTATTTGCAGTCATTGGCTTTTTCGGTATTCATGGTCAACATGTCTTAGG
TCCATTTAAAATAGATCGTTTATCTTGGTTATTAGCTGGCTTTGTAATGGCGCTTGGTTTTATTATTCAAAAGTTTTCAATGCGATATTTACTA
GGTGATCATCATTATAGACATTACTTTCCATTGTTCACTGCGATTACGTCGTTTGCATCTTTAGCATGGATGTCTGAAGACTTAAGACTGATG
GCACTCTGCTGGGGTATGACATTATTATGTTTAACACTGCTGATGAACGTTAATCGTTTTTGGAAAGTGCCACGTGAGTCTGCGAAATTATC
AAGCATGACATTTTTATGTGGTTGGCTTGCATTCGTTGGAGCAATTGTAACTATTTATATTGCGACTGGCGAGTGGCGCGTGCCTCAACATA
TGTCTAATTCGACATGGTCATTGTTGACGAATGTACTACTTGTATTAGCTGTCATGATACCAGCAGCACAATTTCCATTTCATCGATGGTTGA
TTGAATCTGTAACAGCACCAACGCCAGTATCGGCAATTATGCATGCAGGAATTGTGAATGCAGGTGGTGTTATTCTAACTCGTTTTGCGCCG
ATATTTGATAATGGATTTGCGTTATCATTATTACTTATCCTTTCTAGTATTTCTGTATTGTTAGGATCGGGTATTAGCTTGGTTCAAGTGGATT
ACAAAAGACAGTTAGTCGGCTCTACGATGAGTCAAATGGGCTTTATGTTAGTTCAGTGTGCGCTAGGTGTCTATTCAGCAGCGATTATTCAT
TTAATATTGCACGGTATTTTTAAAGCAACATTATTTTTACAATCAGGTTCTATCGTGAAGCGATTCAATATTCCAAAACAAGCATCTGCTAAA

GACGCTTATGGCTGGATTGTCATGGGACGTGTATTAGCTATTATCGTGGCATTTCTGTTTTGGATGAGTAGTGACAGAAGTGCATATGAAG
TGTTAAGTGCACTCATTCTAGCATGGTCATTACTTGTATCTTGGAATCAAATGGTAGCCTTTAGTAAAGGGCGTATGGCACGTCTGGTTGGC
ATGATTTTGATTTCAATTGTGACATTTATCTACATCATCACACATAATTATTTTTACGATGTATTACAAAATATAACAACATATGCGACAACAC
CGCCTACAATTAGTGTCATCATTAGTGTTGTCATATTAATATTTGGTAGTTTATTAAGTATCTGGGTAGCGCGTCATCGATACTCTAAGACTT
TTGCGGTATTGTACGTGTGGTTAGTTAATCTAGGTGAAGCACGCTCGAAAGCGATAGAAAGTCATCCGAATTATTTGAAGAAGTATTTATA
G

Gene: ybcC-mpsB (membrane potential-generating system subunit B)

Position: 463915 to 466620, length: 2706 nt, orientation: FORWARD

Perfect match to: (11819-97-CP003194-[469837:472542], allele observed in CC80+CC7+CC72+CC80)

Sequence:

ATGACAACACAGTTAAATATCAATTCAGTCATTGAAAATGCGAAACGTGTTATTACACCATTATCACCGATTTCGATTTTTGCAGCACGTAAC
CCCTGGGAAGGATTAGAAGCGGATACGTTTGAAGATGTCGCAAAATGGTTACGTGACGTTCGCGATGTGGATATTTTCCCAAATAAAGCAT
TAATAGAAAGTGCTGTGGCACGTGGTGAATTAGATGAAAGTGTCTTTAATCAACTTGTTACTGATATGTTACTTGAACATCACTACAATATC
CCGCAACACTACATCAATCTTTATATTGATAACATTAAAACATTAAAAGACGTACCTGCATCATATATGAATCATTCAAATGTTGATGTTGTT
GCTGATCTACTATTAGAAAAAATCAAAACGTGATATGGCTGAATCATATCATCACTATGATGTACGTCCGATGAGTGATGCAATAATAGATGA
ACAAGGTGAGCCACTTAGCGAACAAGTGAATCGTCAAATGATTAAATGGACGAAACTTTATATCGATCAATTTCTATCGAGTTGGACAATG
CCGAAGCGTGAGCAAAGTTTTTACCATGCATGGTTGCATTTAGCGCAACATGACCATAGTTTTACTAAAGCACAGCGCCAAGTGATTAAAG
GCTTACCCAATGATCCTGAAATGACGATAGAGTCAGTATTAACTTATTTTTCAATAGATCAGGAAGACTACCAAGCTTATGTTGAAGGACAT
CTTTTGGCGTTACCGGGTTGGGCAGGTATGTTGTATTACCGTTCACAACAGCATCACTTTGAACAACATTTGTTAACGGATTATTTGGCAATT
CGGTTAGTTGTCGAACAATTGCTAGTTGGTGATGAGTTTAAGTCAGTCACTAAAGACTGTGAAAGTAGATCGGAAAATTGGTTTAAGCAAA
CTGTTGCATCATGGTGTTACTACAGTGATATGCCTAGCGATGTATTACTACAACATGACGTCAATGAAATTCAAACGTTTATTCATTTTGCAG
CAACTATGAATAAAAATGTATTTAAAAATTTATGGCTAATTGCCTGGGAAATGACATACGAATCTCAGTTAAAACAAAAAATTAAAGCAGGT
CATGAAAGTGTGGCGGGCGCATTAGATGCAAATCAAGTAAATGTTTCGGAAAATGACAACGCTAATCAGTTACATTCAGTATCGTTAAATG
ATACACAAGCAGTTGATGAAAATAATAGCGAGCTAAATCAGGTGGGCACATCAACGAAAGTGCAAATTGCATTTTGTATAGATGTTCGTTC
AGAACCATTTCGTAGACATATCGAAGCAGCAGGTCCCTTTGAAACGATTGGTATTGCAGGTTTCTTTGGATTACCTATTCAAAAAGATGCCG
TAGACGAACAATTCAAACATGATTCATTACCTGTCATGGTACCGCCGGCATATCGCATTAAAGAATTTGCAGACCGCTACGATATGAATGTT
TATCGACAACAGCAACAGACAATGTCATCGATGTTTTACACATTTAAATTGATGAAAAATAATGTTATGCCTAGTCTGTTATTGCCTGAATTA
AGTGGGCCATTTTTAAGTTTGAGTACCATTGTCAATTCGATTATGCCTAGAAAAAGTCGCGCGTCTTTACAAAAAATAAAACAAAAATGGTT
GAAAAAGCCTGAAACAAAGTTAACGATTGATCGTGAGTTTGACCGAACATCAGACTTACCTGTTGGATTTACTGAGCAAGAGCAAATTGAT
TTCGCGTTACAAGCGTTGAAATTGATGGATTTAACCGAAGCATTTGCGCCGTTCGTTGTGTTAGCAGGTCATGCTAGTCATTCTCACAATAA
TCCACATCATGCATCACTTGAATGTGGGGCTTGTGGTGGCGCATCAAGCGGTTTTAATGCTAAGTTATTAGCGATGATATGTAATCGTCCAA
ATGTCAGACAAGGATTAAAACAATCAGGTGTGTATATTCCAGAGACAACTGTTTTTGCGGCAGCAGAACATCATACGTCTACTGATACGTT
GGCATGGGTATATGTGCCAGACACATTATCAGCTTTAGCTCTAGATGCATATGAATCATTGAATGACGCGATGCCGATGATTTCTGAACAA
GCGAATCGCGAACGTTTGGACAAACTGCCAACGATTGGTCGTGTGAATCATCCAGTGGAAGAAGCACAGCGGTTTGCAAGTGATTGGAGT
GAGGTACGTCCAGAATGGGGCTTGGCTAAAAATGCATCATTTATAATTGGACGACGCCAATTAACAAAAGGCATTGATTTAGAAGGGCGG
ACATTTTTACACAATTATGATTGGCGTAAAGATAAAGATGGCACATTATTAAATACCATCATTTCTGGTCCGGCACTTGTGGCACAATGGAT
TAATTTACAATATTATGCGTCGACAGTTGCGCCGCATTTTTACGGAAGTGGGAATAAAGCGACACAAACCGTCACGTCAGGTGTTGGTGTC
ATGCAAGGTAATGCGAGTGATCTGATGTATGGCTTATCATGGCAATCTGTTATGGCTGCTGATCGAACGATGTATCATTCGCCAATTCGTTT
GCTTGTCGTTATTCAGGCACCCGACTATGTTGTAGCTAGACTACTCGCGAATAATGAGCATTTCGCTAGGAAGGTGTCTAATCATTGGCTGC
GTTTAATGAGCGTTAATGAGGAAGGGCGTTTTAAAAGTTGGATTTAA

Gene: ybcI-mpsC (membrane potential-generating system subunit C)

Position: 466782 to 467144, length: 363 nt, orientation: FORWARD

Perfect match to: (11819-97-CP003194-[472704:473066], allele observed in CC80+CC445)

Sequence:

ATGAAAAGAACGAAAGGTGAAATCGAAGCTGAAATCAGTAAAGCCATTACGCAATGGGAAAAAGATTTCCTTGGCAGAGGTTCCTTGTCA
GTTAAATCAGACATTTTAAGAGATATGGTGATTATTAGTTTACAAGGTATCTTAACGCCAGCAGAATATCGTGTATGTAGTACGAATGAAG
GATTATTAAATATTAAACGAACACGTTCTGAATTAGTTGAATCCGGTGAGCAAGATTTGAATGATATCATTTTTAAAATTACAGGTATCAAA
GTGATGAGCTTCCATAGTGATTTAAGTACAGTTACAGGTGAACGTATTATCGTATTCAAACTTGAGGATAATTTGGAAAAGCATATTTAA

Gene: Q5HIM2 (putative protein)

Position: 467373 to 467720, length: 348 nt, orientation: REVERSE

Perfect match to: (MW2-BA000033-[461173:461520:r], highly conserved allele)

Sequence:

TTACATAAAGAGTATATGTGTGACGTAGGCATATAATCGATAAAGTATTCCTAAAAAATTAGGTATAATGACCATTGTTGCAATAAGTTTTT
CGGCCTTGAAACCGATAACGCATAGTATAATAGGAATAAAATATAATACAAATATCCAGTAAATCAAATTTGAAAACGATGGATGAATAGG
GGTAAAGAATCTAACTAATGTAATGATTAACGCTATATAGACAAAAATAACTCTATTGATACGCCTTACCCCCTCTGTATAATAAATATAGAT
CGTACTAAATTGAAAATAGCAAAATATTGTTATTTCAATTATACAATGTTTTATTTGCAATATACATAACAA

Gene: Q5HIM1 (lipid phosphate phosphohydrolase 2 family protein)

Position: 467827 to 468501, length: 675 nt, orientation: FORWARD

Perfect match to: (Strain_21178-AGRN01000090-[29064:29738:r], allele observed in CC239+CC80)

Sequence:

ATGATAGATAAAAAATTAACATCACCGAAAATGACAGTGCCTTTATTTTTAATCGCGCTGATTGTATTTATAGGTATGTTTTACAGTGTAGTG
ACAAATCAAGAATGGCTTAAAAATATAGATATGGGATCATTAACATGGTTTACAGATTATTTCGGTGAGCCACAACGTCAGTATGTTAACAA
TTTGTTTAATTACTATATGACGTTTAGTGCGGAAATTGGAGATGTCAAAGGTGTCGTGTTGATTTCCATTATCGTCACAATCATACTGTTTAT
TAAACAGAGGCATTTAGCGGTTTGGTTTGTGACATATTTGGTTTCAGGTGTCATCATGAACAAATTAATTAAAGATACTGTATTACGTCCAA
GACCATATAATCATTTAGCCGTTGATACAGGCTTTTCATTTCCAAGTGGACATTCCAACGCCAGCACATTATTATATTTCGCCTTAATGATCAT
AATTATTTCACTTGCTGCTAAGACAATAACAAAAGTGTTGAGTGCGCTAGTTATGGGAATATTATGGCTTAGCATATTATTTTGTCGCCTTTA
TTTTCATGCGCATTACTTTTCAGATGTCATTGGCGGCACGTCACTAGCAATCATTTGGGTAGCGTTATTCTTAATGGTATACCCATACTTTATT
AATCATCGACGACAACGCGTTTAG

Gene: Q5HIM0 (putative esterase)

Position: 468538 to 469272, length: 735 nt, orientation: FORWARD

Perfect match to: (11819-97-CP003194-[474460:475194], allele observed in CC80)

Sequence:

ATGAGAATTAAAACACCGAGTCCATCTTATTTAAAAGGTACAAATGGACATGCGATATTATTATTACATTCATTTACAGGTACAAATCGGGA
TGTGAAGCATCTTGCAGCTGAGTTAAATGCACAAGGATTCAGTTGTTATGCACCGAATTATCCAGGTCATGGTTTATTGTTGAAAGATTTCA
TGACATATAATGTAGATGATTGGTGGGAAGAAGTTGAGAAAGCTTACCAATTTTTAGTCAATGAAGGTTATGAATCTATCAGTGCAACTGG
TGTGTCTTTAGGTGGATTAATGACATTAAAATTGGCGCAACACTATCCTTTGAAACGTATCGCTGTCATGTCAGCACCAAAGGAAAAGAGT
GACGATGGTTTAATAGAACATTTAGTTTATTATAGTCAACGCATGTCGGATATTTTAAATTTAGATCAGCAAGCATCGAGTGCGCAATTAGC
AGCAATTGATGATTATGAAGGTGAAATTACGAAGTTTCAACATTTTATTGATGATATCATGACAAATTTAAATGTTATTAAAATGCCAGCTA
ATATATTATTTGGTGGTAAAGATGCGCCATCCTATGAAACAAGTGCACATTTTATTTATGAACATTTAGGATCAGTAGACAAAGAATTAAAT
GGTCTGAAGGATTCGCATCATTTAATGACGCATGGAGAAGGCAGAGATATTTTAGAAGAAAATGTTATTCGCTTTTTCAATGCTTTAACATA
A

Gene: Q5HIL8 (transporter)

Position: 469777 to 471114, length: 1338 nt, orientation: FORWARD

Sequence:

ATGAAAAGACAACAATCACAATGGAAGTCATCAACTGGATTTATTTTAGCTAGTGCGGGTTCTGCAATCGGTCTTGGTGCCATGTGGAAAT
TCCCATATATGGCAGGGATTTATGGCGGCGGTGCCTTTCTAGCTATGTTCTTAATATTCACCATTTTTGTTGGGTTGCCATTACTCATTATGG
AATTCACTGTTGGGAAAATGGGACGGACATATACAACACAAATATATAGTAAATTAACTGGTAAAAAATGGCTCAATATCATTGGCTGGAA
CGGTAATTTGGCAGTGTTTATTTTATTTGGCTTCTATAGTGTTATCGGTGGTTGGATTGTCATTTACATCGGACAAGTTTTATGGCAATTAGT
TATATTTCAACGCATCAATCATCTCCAAGAAATGAATTTTGAAGCGGTAATATCAAATCCTTGGTTAACCGTTCTAGGGCAAGGTATATTCAT
ATTCGCTACGATGATTATTGTCATGTTAGGTGTTGAAAAAGGATTAGAAAAGGCATCAAAAGTTATGATGCCATTGCTGTTTGTCTTTTTAA
TCATCATTGTGATTAAGTCTTTAACATTAGATGGTGCCTTAGAAGGTGTGAAATTTATTTTACAACCAAGAGTATCAGAGATTACTGCTGGC

GGTATCTTGTTTGCGCTAGGCCAATCATTCTTTACGTTATCATTAGGAACTACAGGTATGATTACTTATGCGAGTTATGCCTCTAAAGACATG
ACGATTAAGTCATCAGCTATTTCTATCGTTGTTATGAATATCTTTGTATCTGTATTGGCAGGTCTAGCTATATTTCCGGCGTTACATAGTTTTG
GCTATGAACCACAAGAAGGGCCTGGATTATTATTTAAAGTACTACCAATGGTCTTTAGTCAAATGCATCTAGGCACATTATTCTATTTGGGA
TTCTTAGTGCTGTTCTTATTTGCGGCTTTAACGTCATCTATTTCTTTATTAGAATTAAATGTTTCTAACTTCACGAAGAATGACAATACAAAAC
GTAAAAAAGTCGCAGTGATTGGTAGTATTTTAGTATTTATCATTAGTATTCCAGCAACCTTATCTTTTGGTATCTTAAAAGATGTAAGATTCG
GTGCGGGAACGATTTTTGATAATATGGATTTCATCGTTTCGAATGTATTGATGCCATTAGGCGCATTAGGTACTACGCTTGTCGTAGGACAA
TTATTAGATAAAAAATTATTACAACAATATTTTGGTAAAGATCGATTTAGATTATTCAGTGGTTGGTATTACTTAATTAAGTATGCGATGCCT
GCCGTTATTATTTTAGTCTTTATCGTGCAATTATTTAGTTAA

Gene: mccA (O-acetylserine-thiol-lyase)

Position: 471331 to 472236, length: 906 nt, orientation: FORWARD

Perfect match to: (11819-97-CP003194-[477253:478158], allele observed in CC80+CC5+CC22)

Sequence:

ATGATTACTTATGATTTAATTGGCAATACACCATTAGTACTGTTAGAACATTATAGTGATGATAAAGTTAAAATTTATGCCAAGCTTGAACAA
TGGAATCCTGGAGGCAGTGTTAAAGACAGACTCGGGAAATATTTAGTAGAGAAGGCAATTCAAGAAGGGCGTGTGCGTGCAGGTCAAAC
TATTGTTGAAGCGACTGCTGGTAATACAGGCATAGGGTTAGCTATTGCAGCGAATAGACATCATTTGAAATGTAAGATCTTTGCGCCGTAT
GGTTTTTCAGAAGAAAAGATTAATATTATGATAGCGCTTGGTGCAGATGTTTCAAGGACGAGTCAGTCTGAAGGTATGCACGGGGCACAAT
TAGCTGCACGTTCCTATGCTGAAAAATATGGTGCCGTTTATATGAATCAATTTGAATCCGAACATAATCCGGATACATATTTTCATACATTGG
GACCCGAATTGACTTCAGCATTACAGCAAATTGATTATTTTGTGGCTGGTATTGGCTCTGGCGGTACATTTACAGGTACCGCACGTTATTTA
AAGCAACATCACGTGCAATGTTTTGCCGTTGAGCCAGAAGGGTCCGTGTTAAATGGAGGGCCAGCTCATGCACATGACACTGAAGGTATC
GGTTCTGAGAAATGGCCGATATTTTTAGAGAGACGTCTTGTAGATGGGATATTTACGATTAAAGATCAAGATGCCTTTCGAAATGTCAAAA
GTTTGGCTATAAATGAAGGGTTGTTAGTAGGCAGTTCTTCAGGTGCAGCATTACAAGGTGCATTGAATTTAAAAGCGCAACTAACTGAAGG
TACGATTGTTGTCGTATTTCCAGATGGTAGCGATCGATATATGTCTAAGCAAATATTTAATTATGAGGAGAATGATTATGAATAA

Gene: mccB (cystathionine gamma-synthase)

Position: 472229 to 473371, length: 1143 nt, orientation: FORWARD

Perfect match to: (11819-97-CP003194-[478151:479293], allele observed in CC80)

Sequence:

ATGAATAAGAAAACTAAATTAATTCACGGTGGACACACAACGGACGACTATACAGGTGCAGTTACTACACCAATTTATCAAACAAGTACAT
ATTTACAAGATGATATTGGTGATTTACGTCAAGGATATGAATATTCTCGTACTGCGAATCCAACAAGAAGTTCTGTAGAAAGCGTTATTGCG
GCATTAGAAAATGGCCAACATGGCTTTGCATTTAGTTCAGGTGTTGCAGCAATCAGTGCAGTTGTTATGCTGTTGGACAAAGGTGATCATA
TTATTTTAAATTCAGATGTATACGGCGGTACTTATCGCGCATTGACAAAAGTATTTACACGATTTGGCATTGAAGTGGATTTTGTAGATACA
ACTCATACAGATTCAATTGTACAAGCGATACGCCCAACAACAAAGATGTTGTTTATTGAAACACCTTCTAATCCATTATTACGTGTTACTGAC
ATTAAAAAAGTCTGCTGAAATTGCGAAAGAACACGGTTTGATTTCAGTCGTTGATAACACATTTATGACACCTTATTATCAGAATCCATTAGAT
TTAGGCATCGATATTGTCTTGCATTCTGCAACGAAATATTTAGGTGGACATAGTGATGTCGTTGCTGGTTTAGTTGCAACATCGGATGACAA
GCTTGCAGAACGTTTAGCATTTATTTCAAATTCAACAGGTGGCATTTTAGGACCTCAAGATAGCTATTTACTTGTGAGGGGTATTAAAACAT
TAGGTTTACGTATGGAACAAATTAATCGCAGTGTTATTGAAATTATTAAAATGTTACAAGCACATCCAGCTGTGCAACAAGTGTTCCATCCA
AGTATTGAAAGTCATTTAAATCATGATGTCCATATGGCTCAAGCGGATGGCCATACAGGTGTGATTGCATTTGAAGTGAAAAATACAGAAA
GTGCCAAACAATTGATTAAAGCAACATCGTATTACACATTAGCTGAAAGTTTAGGTGCAGTGGAAAGTTTAATTTCAGTACCTGCATTGATG
ACACATGCATCCATTCCAGCAGATATTCGAGCTAAAGAAGGTATTACAGACGGACTTGTAAGAATTTCTGTAGGTATTGAAGATACTGAAG
ATTTAGTCGATGATTTAAAACAAGCACTAGATACGTTATAA

Gene: metN2 (methionine ABC transporter locus 2, ATP-binding protein)

Position: 473667 to 474692, length: 1026 nt, orientation: FORWARD

Perfect match to: (11819-97-CP003194-[479589:480614], allele observed in CC80+CC50+CC130+CC707)

Sequence:

ATGATTGAGTTTCGACAAGTTAGTAAATCATTTCATAAGAAAAAGCAAACAATAGATGCTTTGAAGGACGTATCATTTACGGTCAATCGCAA
TGATATTTTTGGTGTGATTGGATATAGTGGTGCAGGAAAAAGTACGTTGGTAAGACTCGTGAATCATCTTGAAGCTGCCTCGAATGGACAA

GTGATTGTAGATGGACATGATATTACGAATTATAGCGATAAAATGATGAGGGATATTAAGAAAGATATCGGTATGATATTTCAGCATTTCA
ATTTATTAAATTCAGCTACGGTATTTAAAAATGTAGCAATGCCACTCATTTTAAGTAAGAAAAGCAAAACAGAAATTAAGCAACGAGTAACA
GAAATGCTTGAATTTGTAGGATTGAGTGATAAAAAAGACCAATTTCCTGATGAATTATCTGGTGGGCAGAAGCAAAGGGTGGCTATTGCA
AGAGCGCTTGTTACTAATCCGAAAATACTCCTATGCGATGAAGCAACAAGCGCATTGGATCCAGCAACGACTGCTTCGATATTGACGTTATT
AAAGAATGTCAATCAAACCTTTGGCATTACAATTATGATGATTACACATGAAATGCGCGTTATTAAAGACATTTGTAATCGTGTTGCTGTAA
TGGAAAAGGGGCAAGTGGTTGAAACAGGAACTGTTAAAGAGGTGTTTAGTCATCCTAAAACGACGATTGCTCAAAATTTTGTGTCTACAGT
TATACAGACTGAGCCAAGTACATCATTGATTCGTCGATTGAATGACGAACAAGTTGGCGATTTTAAAGATTATAAAATCTTCGTCGAGGAA
ACTCAGGTGACACAACCGATTATAAATGACTTGATTCAAATTTGTGGCAGAGAGGTTAAAATTTTATTTTCATCTATGTCAGAAATACAAGG
TAACACCGTATGTTATATGTGGCTTCGATTTAATATAGATCAACAATTTGATGACACGGCAATAAATCAATATTTCAAAGAGAAAAATATTC
AATTTGAGGAGGTGCATTAA

Gene: metP2 (methionine ABC transporter locus 2, transmembrane permease)

Position: 474696 to 475355, length: 660 nt, orientation: FORWARD

Perfect match to: (N315-BA000018-[483071:483730], allele observed in CC5+CC80)

Sequence:

ATGTTTGGTTCTGATTTAGACAGTGCACAGTTATTACAAGCATTGTACGAAACGTTATATATGGTATCTATTGCTTTATTTTTAGGAGCAGTG
ATTGGTATTCCATTAGGTGTCTTATTGGTAATTACTCGAAAACAAGGCATATGGCCCAATATAGTGATACATCAAGTTTTAAATCCTTTAATC
AATATTTTAAGGTCACTACCATTTATTATTTTGTTAATTGCGATTGTGCCATTCACAAAATTAGTAGTAGGTACTTCAATTGGTACGACTGCT
GCCATCGTGCCTTTAACAGTATATGTGGCACCTTACATTGCAAGACTTGTTGAAAACTCATTATTGGAAGTAGACGAGGGGATTATTGAAGC
GGCGAAAGCGATGGGCGCTTCACCACTACAAATCATTAGATATTTTTTAATTCCTGAAGCGTTAGGTTCATTAGTATTAGCAATTACCACTG
CGATTATTGGACTTATTGGAAGTACGGCGATGGCAGGAGCTGTTGGCGGTGGTGGTATAGGAGACTTAGCTTTAGTGTATGGTTATCAAA
GATTTGATACGACGGTCATTATTATTACCGTTATTGTATTAGTCATTATTGTCCAAGTGATTCAAACGCTAGGGAATGTCCTAGCTAGATTCA
TACGTAGACATTAA

Gene: metQ2 (methionine ABC transporter locus 2, substrate-binding protein)

Position: 475392 to 476234, length: 843 nt, orientation: FORWARD

Perfect match to: (ATCC51811-ADVP01000040-[21444:22286:r], allele observed in CC1+CC80)

Sequence:

ATGAAAAGATTGATTGGGTTAGTTATCGTAGCACTTGTATTATTAGCAGCGTGTGGTAGTAACAATGATAAAAAAGTAACAATTGGTGTCG
CATCAAATGACACTAAGGCTTGGGAGAAGGTTAAAGAATTAGCTAAAAAAGATGATATTGATGTGGAGATTAAGCACTTTTCCGATTACAA
TTTACCGAATAAAGCATTAAACGATGGTGATATTGATATGAATGCATTCCAACATTTTGCATTTTTAGATCAGTATAAGAAGGCACATAAAG
GAACAAAGATTTCAGCATTAAGTACAACAGTTTTAGCACCGTTGGGCATTTACTCAGATAAAATTAAAGATATCAAAAAGGTTAAAGATGG
TGCTAAAGTTGTCATTCCAAATGATGTATCAAACCAAGCACGTGCTCTTAAACTATTAGAAGCAGCTGGTTTAATAAAACTGAAAAAAGATT
TCGGTTTGGCAGGCACGGTGAAAGATATAACGTCAAATCCAAAACATTTAAAAATTACTGCAGTAGATGCACAGCAAACTGCGCGTGCTTT
ATCTGATGTCGATATTGCAGTTATTAATAACGGTGTAGCAACTAAAGCGGGGAAAGACCCTAAAAATGATCCGATATTTTTAGAAAAATCA
AATTCAGATGCTGTAAAGCCATATATTAATATTGTTGCAGTTAATGACAAAGACTTGGATAACAAAACATATGCTAAAATCGTAGAATTGTA
TCATTCAAAAGAAGCTCAAAAAGCGTTGCAGGAAGATGTCAAAGATGGAGAGAAACCTGTTAATTTATCTAAAGATGAGATTAAGGCAAT
AGAAACGTCATTAGCAAAATAA

Gene: aaa (N-acetylmuramoyl-L-alanine amidase)

Position: 476566 to 477570, length: 1005 nt, orientation: FORWARD

Perfect match to: (IS-88-AHLO01000041-[20067:21071:r], allele observed in CC8+CC5+CC97)

Sequence:

GTGCAAAAAAAAAGTAATTGCAGCTATTATTGGGACAAGCGCGATTAGCGCTGTTGCGGCAACTCAAGCAAATGCGGCTACAACTCACACA
GTAAAACCGGGTGAATCAGTGTGGGCAATTTCAAATAAGTATGGGATTTCGATTGCTAAATTAAAGTCATTAAACAATTTAACATCTAATCT
AATTTTCCCAAACCAAGTACTAAAAGTATCTGGCTCAAGTAATTCTACGAGTAATAGTAGCCGTCCATCAACGAACTCAGGTGGCGGATCAT
ACTACACAGTACAAGCAGGCGACTCATTATCATTAATCGCATCAAAATATGGTACAACTTACCAAAACATTATGCGACTTAATGGTTTAAAT
AATTTCTTTATTTATCCAGGTCAAAAATTAAAAGTATCAGGTACTGCTAGCTCAAGTAACGCTGCGAGCAATAGTAGCCGTCCATCAACGAA

CTCAGGCGGTGGATCATACTACACAGTACAAGCAGGTGACTCATTGTCATTAATCGCATCAAAATATGGTACAACTTATCAAAAAATTATGA
GCTTAAATGGCTTAAATAATTTCTTTATTTATCCGGGTCAAAAATTGAAAGTAACTGGTAATGCATCTACGAACTCAGGATCTGCAACAACG
ACAAATAGAGGTTACAATACACCAGTATTCAGTCACCAAAACTTATATACATGGGGTCAATGTACATATCATGTATTTAATCGTCGTGCTGA
AATTGGTAAAGGTATTAGTACTTATTGGTGGAATGCTAATAACTGGGATAACGCAGCGGCAGCAGATGGTTACACTATCGACAATAGACCT
ACTGTAGGTTCTATCGCTCAAACAGATGTAGGTTACTATGGTCATGTTATGTTTGTAGAACGTGTAAATAACGATGGTAGTATTTTAGTTTC
AGAAATGAACTATTCAGCTGCACCAGGTATTTTAACTTACAGAACGGTACCAGCTTACCAAGTAAATAATTATAGATATATTCACTAA

Gene: Q5HIL1 (putative protein)

Position: 477755 to 478024, length: 270 nt, orientation: REVERSE

Perfect match to: (N315-BA000018-[486130:486399:r], highly conserved allele)

Sequence:

TTAGTATTCATGCTTTACTTTGCGAATGCTTGTAAATAATCTAGCACCGTTTGTTATTAAAGTAACAACTGCCACTGCTTTTTTGAACTTACGT
GGTGACTTAATTGAAATGTAAAAGTCTAACACGGTTCCTACAGTGCTTAAACCTAATGAAAGATATACTAATTTTTTATTTTTAGCATGATAT
TTATAGCCATTGTAGCCGTCGACTATGAAACCTGCGACATTTAGTAAACTTGATAAACGTTGTGATTTGGAACGTTTTGCCAT

Gene: nudG (nucleoside diphosphate phosphohydrolase G)

Position: 478173 to 478568, length: 396 nt, orientation: FORWARD

Perfect match to: (MW2-BA000033-[471925:472320], highly conserved allele)

Sequence:

ATGATTAAATGTGTCTGTTTAGTTGAAGAAACAGCTGATAAAATATTACTTGTTCAAGTAAGGAATCGCGAAAAGTATTATTTCCCAGGTGG
TAAAATAGAAGAAGGGGAATCACGAGTACAAGCGCTGTTAAGAGAAGTAAAAGAAGAATTAAATTTAACATTAACAATGGATGAAATTGA
ATATATCGGGACAATTGTAGGTCCTGCATATCCACAACAGGATATGTTAACTGAGTTAAATGGATTTCGCGCATTAACCAAAATCGATTGGG
AAAACGTAACTATCAATAATGAAATTACGGATATACGCTGGATTGATAAAGATAATGATGCGTTGATTGCGCCTGCTGTCAAAGTTTGGATT
GAAACGTATGGTGGTAAACATGACAAATAA

Gene: bltD (acetyltransferase, GNAT family)

Position: 478558 to 479043, length: 486 nt, orientation: FORWARD

Perfect match to: (N315-BA000018-[486933:487418], highly conserved allele)

Sequence:

ATGACAAATAATGACACCATCATGTTACGACATTATGTCCCACAAGATTATTCGATGTTAGAAGCTTTTCAATTAAGTGAAAGTGATTTGAA
GTTTGTTAAAACGCCAGAGGAAAATATTACAGCTGCAATGTCTGATAATGAAAGGTATCCCATCGTTGTAATGGATGGCAGGCAATGTGTG
GCCTTTTTTACATTACATCGTGGAAAAGGGGTCGCACCATTTAGCGATAACCAAGATGCAGTATTTTTCAGGTCATTTAGTGTTGATCAACG
TTATCGTAATAGAGGAATAGGTAAAGTGGTAATGGAAAAATTGGCGTCATTTATCACTTCAACATTTCAGGATATTAATGAGATTGTGTTAA
CGGTTAATACTGACAATCCACATGCCATGGCACTTTATCGCCAACAAGGATATCAATATATGGGAGATAGTATGTTCATCGGAAGACCTGTT
CATATTATGGCGTTAACTATAAAATAA

Gene: yibF (putative membrane protein)

Position: 479212 to 479994, length: 783 nt, orientation: REVERSE

Perfect match to: (MRSA252-BX571856-[495564:496346:r], highly conserved allele)

Sequence:

TTAATTGATAGACTCATCATTTTTGCGCTGTCGAGATGGTCTTTTTATTAAAAATGCCGTAATCCAAGCCGTAATCGGAATACTGATTGCAAC
GGCAATACCGCCTAAAATAATAGAAATAAATTCTTGGGCAAATATTTTCGAGTTTATAATATGACCAAATGAATATTTAAGTTTGAAAAACC
AAATAAATAAAGCAAGTTGGCCACCAAAAAAGGCAAGGTAAATCGTGTTCGCAGATGTCGCTAAAATTTCTCTACCAACACGCATGCCAGA
TTGGAATAATTCGTATTGCGTAAGTGTTGGATTTACTTGATGCAATTCATAAATGGGTGAACTAATGGTAATCGTTAAATCTATCACAGCTG

CAATAACAGCAAGAATAATAGTGAACACCATAAATTGAACCATATCAATGCCAATATTCATTGAATACACATATGTTTCATCTTGTTGTTCGG
TTGAAAAGCCTTGTAGATGACCGAAGTAGACAGATAAATAAATGAGTGTAATCAACAATATTGTTGTAACGATAGTGCTGATAAATGCAGC
TTGTGTTTTAACATTGTAACTATTGAGTACGAATAAATTACAAGCGCCAATAATAATGCAGAAAAAGAATGTGACGACATAAATCGGTACG
CCAAAAATAATCAATACAATACTAATAATTAAAATAGCGAAATTTAAAAATAGGGTTAAATAAGAGATGAATCCCTTTTTACCTCCGAAAAT
TATCATCAGAAAGAGGAGCAATAACGCCAATATAAATACAGCATTCAT

Gene: yibE (putative membrane protein)

Position: 479991 to 481103, length: 1113 nt, orientation: REVERSE

Perfect match to: (11819-97-CP003194-[485913:487025:r], allele observed in CC80+CC188)

Sequence:

TCATTGTTTCGCCCTCCTTAATGTTTCAAATACTTCCATAAACAATATTGTGATAGGAATTGTAAGTACGATACCTATACCGCCTGTTAGTGC
GCGCGCAATTTCTAACGACCAATTCATCGAAATAGTATAAGTCACAGTATTTGCATTTTTTAAAAAGATTAAAAACATAGGTAGTGCACCGG
ATAAATATGAGAATAATAAGATGTTAGTCATTGTTCCCATAATATCTTGGCCGATGTTTCGCCCAGCAAGCGCCCATCTCCTCATTGAAATGT
GTGGCGTACGCTGTAAAATTTCATGCATACCACTAGCAATTGTAATTGCAACATCCATAATAGCGCCAAGTGAACCTATTAACACTGAGGCT
AGGAAGATATCTTTCGGTGGTAATGATAAAAAGTTCATCGTTTCATATTTAATGCCTTTACCATCTGTCATATATATGATTAATTCTGTTAAAC
CTATACTCAAAAAAGTTCCGATAATTGTACTGGCTATGGTAATGAGTGTACGCATATGCCAGCCTGTAACGAGCAATAAAGTGAGTATTGTT
GAACAGATCATGGCAATGGTCATGAGTAAGAATAAATTAATATTGCTATGTTGAATATGAATGTAAATTGCGATTAATATGGCAATAGAAT
TCAAGATTAACGATAAAATCGATTGCAGTCCGACTTTGCGACCAACCAATAATACAGTTAATAAGAACAAACCAGTGATGATAACCGTTAA
GGTATCACGCTTCTTTTCTATAATATAAGCATCACTCGGCTTGTTAGAAATATGTAATAATACTTTTTCGTGTGTGCGAAATGCCTCAGAATC
TGCTTGCGATTTGACGTACTGATGATTAATCGTCGTCGTTTCTCCAGCAAATTGACCATTTAATATTTTGACTTTTAATTGATTTTTATATTTAA
TATCACGATTATTTTGTGCATCTTTTGTAGGTGTCGAAGAAACATGTTTGACATCTATAATTTGACCAATTGGTTTGTTGTAAAAGTTCTCATT
ATTGAATGTAAATAAAATAGCACCAATGAATGCGATGCAGAACAAACCTAAAATTATATTAAATGGCTTTGTAAATAAATTTCTATATTTCA
A

Gene: gltC (transcription activator of glutamate synthase operon)

Position: 481224 to 482108, length: 885 nt, orientation: REVERSE

Perfect match to: (11819-97-CP003194-[487146:488030:r], allele observed in CC80+CC239+CC4803)

Sequence:

TTAATGGAATGTAGACGTTTTAGTCATTAATTGCTGAATAAGTGTTAATAAGATACCAATATCACTCTTTGTATAAGGCTCCTTTGTAATAGC
ACATATCGTTCTTTTTAATTCAGTATGATCTAATTTTATATCTATCCATGATTTAGATTCTGGTAAATGTATATTTTGTGATGAAATGATATAA
CCTTCTTTTTGACGAAGGAGATACTGCGCAAGTGGTTGGCTACTGATTGTGTATACATCTGATTTAGTAATCTTGCGCAATTGTTTTTTTACA
GTTTCGGCAAATGGTGCCAAGCAATAAATATGACTATGCTCAAACTGAATTAATGGTGGGTGTGTCGCCATCGTAATTGGATCGTCTGAAG
GCGCATATAAATGATAGTGCTCTTCGAATAAAGGTAGCATATGTAATTGTTTGTGTTTACGTATTTCTGGTGTAAGTTCCGTGAAACCAATG
TCTATATTCCCATTTAATACGCTATTTATAATTGTGTCATGTTCTAATAAGCTCGGTATGACATGTGTATCATTTTGTAAATGAAACGTTTGGA
TAAGTGGTAGTAACATGTGGGATGCGTCACTCTCATCATAGCCAATGTAGATACTTTTATTTTTAGTTAATCCATGGCTTTGAAATTGTTCAA
TCGTGCTATCTAAATGTTCAATAATACGCAGAGCTTCATTAAATAATAATTTCCCTTCAGATGTGAGCGTAATATTGCGTCCTTGCTTTTTAAA
TAAAGACACATTAAGTTCTTGTTCTAATAATGTAATTTGACGGCTTATCGCTGATTGAGCAATGTTTAGTTCAAGTGCTGTTTCGGAGATATG
TTCTCTTTTAGCGACCTCGATAAAATATCTTAATTGTTTAATTTCCAT

Gene: gltB (NADH-glutamate synthase large subunit)

Position: 482290 to 486789, length: 4500 nt, orientation: FORWARD

Sequence:

ATGCACAATGAGAAATTAATTAAAGGCTTATATGACTATCGTGAGGAACATGATGCGTGTGGTATTGGTTTTTATGCGAATATGGATAATA
AAAGGTCTCACGACATCATTGATAAATCTCTTGAAATGTTGCGACGCTTAGATCACAGGGGCGGGGTCGGCGCAGATGGCATCACTGGTG
ATGGCGCAGGTATTATGACTGAAATACCTTTTGCATTTTTCAAACAACATGTAACGGACTTTGATATCCCAGGTGAAGGTGAATATGCCGTG
GGGTTATTTTTTTTCCAAAGAACGCGTTTTAGGTTCTGAACATGAAGCAGTTTTTAAAAAATATTTTGAAGGCGAAGGGTTATCAATTCTTGG
TTATCGTAATGTACCAGTTAATAAAGATGCCATTGCTAAACATGTAGCAGATACGATGCCAGTCATTCAACAAGTGTTTATTGATATTAGGG
ACATTGAAGATGTTGAAAAGCGTTTATTTTTAGCGAGAAAACAATTAGAGTTCTATTCGACTCAGTGCGATTTAGAATTGTATTTTACGAGC

TTATCACGTAAAACAATTGTATATAAAGGTTGGTTACGATCAGACCAAATTAAAAAACTCTATACAGATTTATCGGATGATTTATATCAATCA
AAGCTAGGGTTAGTGCATTCGAGATTTAGTACGAATACATTCCCGAGTTGGAAAAGGGCGCATCCTAACCGTATGTTAATGCATAATGGTG
AGATTAACACGATAAAAGGTAATGTGAACTGGATGCGAGCACGCCAACATAAATTAATCGAAACATTATTTGGCGAGGATCAACATAAAGT
GTTTCAAATTGTCGATGAGGATGGTAGTGACTCTGCTATTGTAGATAATGCGCTAGAGTTCTTATCGTTAGCCATGGAGCCTGAAAAGGCA
GCGATGTTACTCATACCTGAACCTTGGTTATACAATGAAGCGAATGATGCAAATGTACGTGCGTTTTATGAATTTTATAGTTATTTAATGGA
ACCGTGGGATGGTCCTACAATGATTTCGTTCTGTAACGGTGACAAACTTGGCGCGCTTACAGATAGAAATGGATTACGTCCAGGTCGTTAT
ACGATTACTAAAGATAACTTTATTGTCTTTTCATCTGAAGTGGGTGTTGTGGACGTACCTGAAAGTAATGTTGCTTTTAAAGGTCAATTGAAT
CCTGGAAAGTTATTGCTTGTTGATTTTAAACAGAATAAAGTCATTGAAAATAATGATTTAAAAGGTGCGATTGCTGGAGAATTACCATATAA
AGCGTGGATTGATAACCATAAAGTTGACTTTGATTTTGAAAATATACAATATCAAGATTCGCAATGGAAAGATGAGACGTTATTTAAATTAC
AACGTCAATTTGCATACACGAAAGAAGAGATTCATAAGTATATTCAGGAACTTGTAGAAGGTAAGAAGGATCCTATCGGTGCAATGGGAT
ATGATGCGCCAATTGCAGTGTTGAACGAGCGACCAGAATCACTATTTAATTACTTTAAACAGCTGTTTGCACAAGTTACGAATCCACCAATT
GATGCGTATCGTGAAAAAATCGTAACGAGTGAACTTTCTTATTTAGGTGGCGAAGGTAACTTACTAGCACCTGACGAAACGGTTTTAGATC
GTATTCAATTGAAAAGGCCGGTATTGAATGAATCACACTTAGCAGCGATTGATCAGGAACATTTTAAATTAACTTATTTATCAACGGTATAT
GAAGGGGATTTGGAAGATGCGTTAGAAGCATTAGGCCGAGAAGCAGTGAATGCTGTAAAGCAAGGCGCTCAAATTCTAGTGTTAGATGAT
AGTGGATTAGTTGATAGCAATGGCTTTGCAATGCCGATGTTACTCGCAATAAGTCATGTGCATCAATTACTTATTAAAGCAGATTTACGTAT
GTCTACAAGTTTAGTCGCTAAATCTGGTGAGACACGAGAAGTGCATCATGTTGCTTGTTTACTCGCATATGGCGCGAATGCAATTGTGCCAT
ACCTAGCGCAACGTACAGTTGAACAACTGACATTGACAGAAGGGTTACAAGGCACCGTTGTCGATAATGTTAAGACATATACGGATGTATT
GTCAGAAGGTGTCATTAAAGTAATGGCTAAGATGGGAATTTCGACAGTGCAAAGTTATCAAGGTGCACAAATATTTGAAGCGATTGGCTTG
TCTCATGATGTGATTGATCGTTATTTTACTGGAACACAGTCTAAGTTATCTGGTATTTCGATTGATCAAATTGATGCTGAAAATAAAGCACGT
CAACAAAGTGATGATAATTATCTTGCATCAGGTAGTACATTCCAATGGAGACAACAAGGTCAACATCATGCTTTTAATCCGGAATCTATTTT
CTTATTGCAGCACGCATGTAAAGAAAATGACTATGCGCAATTTAAAGCATACTCTGAAGCGGTGAACAAAAATAGAACAGATCACATTAGA
CATTTACTTGAATTTAAAGCATGTACACCGATTGACATCGACCAAGTTGAACCGGTAAGTGACATTGTCAAACGCTTTAATACAGGGGCGAT
GAGTTATGGATCGATTTCAGCGGAAGCACATGAAACGTTAGCACAAGCCATGAACCAATTAGGTGGAAAGAGTAATAGTGGTGAAGGTG
GCGAAGATGCAAAACGTTATGAAGTACAAGTTGATGGAAGCAACAAAGTAAGTGCGATTAAACAAGTTGCTTCTGGGCGTTTTGGTGTAA
CTAGTGATTATTTACAACATGCCAAAGAAATTCAAATTAAAGTTGCGCAAGGTGCAAAGCCTGGTGAAGGTGGTCAATTACCTGGTACTAA
GGTATATCCGTGGATTGCGAAGACAAGAGGGTCAACGCCAGGTATCGGTCTGATTTCACCACCGCCACATCATGATATTTATTCAATAGAA
GATTTAGCGCAACTGATACATGATTTGAAAAATGCGAATAAAGATGCAGATATCGCGGTAAAATTAGTTTCGAAAACAGGTGTTGGTACCA
TTGCATCTGGGGTGGCAAAAGCATTTGCAGATAAAATTGTCATCAGTGGTTACGATGGTGGTACAGGGGCTTCACCTAAAACGAGTATTCA
GCATGCCGGTGTTCCTTGGGAGATTGGTTTAGCAGAAACACATCAAACATTAAAACTAAATGACTTAAGAAGTCGTGTTAAGTTAGAAACA
GACGGTAAGTTATTAACTGGTAAAGATGTAGCGTACGCATGTGCGCTTGGAGCGGAAGAATTTGGATTTGCAACTGCACCATTAGTGGTGT
TGGGCTGTATTATGATGCGTGTATGCCATAAAGATACATGTCCAGTAGGAGTTGCAACTCAAAACAAAGATTTACGTGCTTTATATAGAGG
TAAAGCACATCATGTTGTTAATTTTATGCATTTTATTGCACAAGAATTAAGAGAAATTTTAGCATCTTTAGGTTTGAAACGTGTAGAAGACTT
AGTTGGAAGAACTGATTTATTACAACGATCATCAACATTAAAAGCGAATAGCAAAGCGGCTAGTATTGATGTTGAAAAACTGTTATGTCCTT
TCGATGGGCCAAACACAAAAGAAATTCAACAAAATCATAATCTTGAGCATGGATTTGATTTAACAAATTTATATGAAGTAACGAAGCCATAT
ATTGCTGAAGGACGTCGCTATACAGGTAGCTTTACAGTAAATAATGAACAACGTGATGTAGGAGTTATTACAGGTAGTGAGATTTCGAAAC
AATATGGAGAAGCAGGACTTCCTGAAAATACAATTAATGTTTATACGAATGGTCATGCTGGTCAAAGTCTTGCAGCATACGCACCGAAAGG
CTTAATGATTCATCATACTGGAGATGCGAATGACTATGTTGGTAAAGGATTATCTGGTGGTACGCTCATTGTCAAAGCACCTTTTGAAGAAC
GACAAAATGAAATTATTGCTGGTAACGTCTCATTCTATGGTGCGACAGGTGGTAAGGCATTTATTAACGGTAGTGCAGGAGAAAGATTCTG
TATTAGAAATAGTGGTGTAGATGTTGTCGTTGAAGGTATCGGTGACCATGGATTAGAGTACATGACTGGTGGACATGTCATTAATTTAGGG
GATGTAGGTAAGAACTTCGGTCAAGGTATGAGTGGTGGTATTGCTTACGTTATCCCGTCTGATGTAGAAGCTTTTGTTGAAAATAATCAACT
AGATACGCTTTCGTTTACAAAGATTAAACACCAAGAAGAAAAAGCGTTCATTAAGCAAATGCTGGAAGAACATGTGTCACACACGAATAGT
ACGAGAGCGATTCATGTGTTAAAACATTTTGATCGCATTGAAGATGTCGTCGTTAAAGTTATTCCAAAAGATTATCAATTAATGATGCAAAA
AATTCATTTGCACAAATCATTACATGACAATGAAGATGAAGCGATGTTAGCTGCATTTTACGATGACAGTAAAACAATCGATGCTAAACATA
AACCAGCCGTTGTGTATTAA

Gene: gltD (NADH-glutamate synthase small subunit)

Position: 486807 to 488270, length: 1464 nt, orientation: FORWARD

Perfect match to: (CIGC93-AHVD01000006-[153640:155103], allele observed in CC15+CC80)

Sequence:

ATGGGTGAATTTAAAGGATTTATGAAGTATGACAAACAGTACTTAGGTGAATTATCACTGGTAGACCGTTTGAAGCATCATAAAGCATATC
AACAACGATTTACTAAAGAAGATGCCTCTATCCAAGGTGCACGATGTATGGATTGTGGAACGCCGTTTTGTCAAACCGGACAACAGTATGG
TAGGGAAACAATAGGTTGTCCAATTGGAAACTACATTCCTGAATGGAACGACTTAGTGTATCATCAAGATTTTAAAACTGCTTATGAACGCT
TAAGCGAAACAAATAACTTTCCTGACTTTACAGGGCGTGTATGTCCTGCACCATGCGAAAGTGCTTGTGTGATGAAGATTAATAGAGAATC
GATTGCGATTAAAGGTATTGAACGCACAATTATTGATGAAGCTTTTGAAAATGGTTGGGTAGCACCGAAAGTTCCGAGTCGTCGTAGAGAT
GAAAAAGTGGCAATCGTTGGAAGCGGTCCAGCAGGATTAACTGCTGCTGAAGAACTTAATCTACTAGGATATCAAGTAACTATTTATGAAC
GTGCTAGAGAATCAGGCGGTTTATTAATGTATGGTATTCCGAATATGAAACTTGATAAAGATGTGGTTCGACGTCGTATTAAGTTAATGGA

AGAAGCGGGCATTACTTTCATTAATGGTGTTGAAGTAGGCGTTGATATTGATAAAGCAACGTTAGAATCTGAGTATGATGCCATTATATTAT
GTACTGGTGCACAAAAAGGTAGAGATTTACCTTTAGAAGGACGCATGGGTGATGGTATACATTTCGCTATGGATTATTTAACTGAACAAAC
GCAGCTGTTAAATGGAGAAATTGATGATATAACAATAACTGCAAAAGATAAGAATGTCATTATCATTGGTGCTGGTGATACAGGGGCAGA
CTGTGTAGCGACAGCATTAAGAGAAAATTGTAAATCGATTGTTCAATTTAATAAATATACGAAATTGCCAGAAGCAATTACATTTACAGAAA
ATGCATCATGGCCTTTAGCAATGCCGGTGTTTAAAATGGGCTATGCGCACCAAGAGTACGAAGCTAAGTTTGGTAAGGAACCACGTGCATA
TGGTGTTCAAACAATGCGTTACGATGTTGACGATAAAGGACACATACGTGGTTTGTATACTCAAATTTTAGAGCAAGGCGAAAATGGTATG
GTCATGAAAGAAGGACCTGAAAGATTTTGGCCTGCTGACCTTGTATTATTATCAATCGGCTTCGAAGGTACAGAACCAACAGTACCGAATG
CTTTTAACATTAAAACGGATAGAAATCGAATCGTGGCGGATGATACAAACTATCAAACTAATAATGAAAAGGTATTTGCTGCTGGAGATGC
TAGACGTGGTCAAAGTTTAGTTGTATGGGCAATTAAAGAAGGTAGAGGCGTAGCGAAAGCAGTAGATCAGTATTTAGCGAGTAAAGTTTG
TGTATAA

Gene: treP (PTS system, trehalose-specific IIBC component)

Position: 489101 to 490528, length: 1428 nt, orientation: FORWARD

Perfect match to: (COL-CP000046-[520470:521897], highly conserved allele)

Sequence:

ATGGCTGTAAAAAGAGAAGATGTAAAAGCCATCGTAACCGCTATTGGGGGAAAAGAAAATCTTGAAGCTGCAACGCATTGTGTAACACGA
TTACGTTTAGTGCTGAAGGATGAAAGTAAAGTTGATAAAGACGCATTAAGTAATAACGCGTTGGTCAAGGGGCAGTTTAAAGCAGACCAT
CAATATCAAATTGTCATTGGTCCAGGAACAGTCGATGAAGTGTATAAGCAGTTTATTGATGAAACAGGTGCTCAAGAAGCTTCGAAAGATG
AAGCGAAACAAGCAGCTGCACAAAAAGGGAATCCAGTACAACGTTTGATCAAATTGTTGGGGGATATTTTTATACCAATATTACCTGCGAT
TGTGACAGCTGGTTTGTTAATGGGAATCAATAATTTACTTACAATGAAAGGTTTATTTGGTCCAAAAGCACTTATTGAGATGTATCCACAAA
TTGCTGATATTTCAAACATCATTAATGTGATTGCGAGTACGGCATTTATTTTCTTACCAGCATTAATTGGTTGGAGTAGTATGCGTGTATTTG
GTGGTAGTCCGATTCTAGGCATAGTCTTAGGTTTGATTTTAATGCATCCGCAATTAGTATCTCAGTATGATTTGGCAAAAGGGAATATTCCG
ACGTGGAACTTATTTGGCTTAGAGATTAAGCAGTTGAATTACCAAGGTCAAGTGTTGCCAGTTTTAATTGCAGCTTACGTTCTAGCTAAAAT
TGAAAAAGGATTAAATAAAGTCGTTCACGATTCGATAAAAATGTTGGTCGTTGGACCCGTAGCGCTTTTAGTTACTGGATTTTTAGCATTTA
TTATCATTGGACCAGTTGCGTTATTGATTGGTACAGGTATTACATCTGGTGTTACATTTATATTCCAACATGCAGGATGGCTTGGCGGAGCA
ATATATGGATTGTTATATGCACCACTTGTAATTACAGGACTACACCATATGTTTTTAGCAGTAGATTTCCAATTGATGGGTAGCAGCTTAGG
CGGTACGTATTTATGGCCAATTGTTGCGATTTCCAATATTTGTCAGGGCTCTGCAGCATTTGGAGCATGGTTTGTCTATAAACGTCGTAAAA
TGGTTAAAGAAGAAGGCTTGGCATTAACATCTTGTATTTCTGGTATGTTAGGTGTTACTGAACCAGCCATGTTCGGTGTGAACTTACCTCTG
AAATATCCATTTATCGCTGCGATATCAACGTCTTGTGTATTGGGGGCAATCGTTGGTATGAATAACGTACTTGGAAAAGTTGGTGTTGGTG
GCGTGCCAGCATTCATTTCAATTCAAAAAGAATTTTGGCCAGTATATCTTATTGTGACAGCTATTGCTATTGTTGTACCATGTATACTAACAA
TTGTGATGTCTCATTTTAGTAAACAAAAAGCGAAAGAAATTGTTGAAGATTAA

Gene: treC (alpha,alpha-phosphotrehalase)

Position: 490592 to 492232, length: 1641 nt, orientation: FORWARD

Perfect match to: (11819-97-CP003194-[496514:498154], allele observed in CC80+CC96)

Sequence:

GTGTCGAAAGAAATAGATTGGAGAAAATCCGTTGTATATCAAATTTATCCTAAGTCGTTTAATGATACGACGGGGAATGGTATAGGAGATA
TCAACGGAATTATAGAAAAAATTGGATTATATCAAGTTATTGGGTGTTGATTATATTTGGTTAACACCAGTGTATGAATCACCTATGAATGAT
AATGGCTATGATATCAGCAATTATTTAGAAATCAATGAAGACTTTGGAACGATGGATGATTTTGAAAAGTTAATCAAAGTTGCTCATCAAAA
AGACTTGAAAGTAATGTTAGATATTGTCATTAATCATACGTCGACGGAGCATGAATGGTTTAAAGAAGCCCGTAAATCTAAAGATAACCCTT
ATAGAGATTATTACTTTTTCAGATCACCTGAAGACGGGCCGCCAACAAATTGGCATTCTAAATTCGGTGGTAATGCATGGAAGTATGATTCT
GAGACAGATGAATATTATTTACATTTATTTGATGTCAGTCAAGCTGATTTAAATTGGGATAATCCGGAAGTACGTCAATCGTTATATCGCAT
AGTCAATCATTGGATAGACTTCGGCGTTGATGGTTTTCGATTTGATGTCATTAACTTAATTTCTAAAGGTGAATTTAAGGACTCTGACAAAAT
AGGTAAAGAATTTTATACGGATGGTCCTAGAGTGCATGAGTTTCTGCATGAATTAAATCGTCAAACGTTTGGTAACACTGACATGATGACT
GTAGGAGAAATGTCTTCGACGACGATTGAAAATTGTATTAAGTATACACAACCAGAACGCCAAGAATTGAATAGTGTTTTTAATTTTCATCA
TCTAAAGGTTGATTATGTTGATGGTGAAAAGTGGACAAATGCGAAGCTTGATTTCCATAAGTTAAAGAAAATTCTGATGCAATGGCAACGA
GGTATTTATGACGGTGGCGGATGGAACGCGATTTTCTGGTGTAATCATGATCAGCCACGGGTAGTGTCTAGATTTGGTGATGATACGTCGG
AAGAGATGAGGATACAAAGTGCTAAAATGTTAGCTATCGCACTGCATATGTTGCAAGGGACGCCATATATTTACCAAGGTGAAGAAATTG
GTATGACGGACCCACATTTTACATCAATAGCACAATATCGCGATGTTGAATCGATTAATGCCTACCATCAGTTGTTAAGTGAAGGGCATGCT
GAAGCGGATGTGTTAACGATTTTAGGACAGAAGTCACGAGACAATTCGAGAACGCCTATGCAATGGAGTGATGATGTTAATGCTGGATTT
ACAGCTGGTAAGCCTTGGATTGATATTTCGGAAAATTATCATCAGGTCAACGTTAGACAAGCACTTCAGAATAAAGAGTCTATTTTCTATAC
GTATCAAAAAATTAATACAATTAAGACATACGCATGATATTATTACGTATGGAGACATTGTGCCACGTTTTATGGATCATGATCATTTATTTGT

TTATGAACGTCATTATAAGAATCAACAATGGCTAGTAATTGCGAATTTCTCAGCATCGGCTGTTGATTTGCCAGAAGGATTGGCTAGAGAA
GGTCGTGTTGTGATTCAAACAGGCACAGTGGAAAATAATACGATAAGCGGGTTTGGTGCAATTGTAATCGAAACAAACGCGTAA

Gene: treR (trehalose operon repressor)

Position: 492257 to 492985, length: 729 nt, orientation: FORWARD

Perfect match to: (MW2-BA000033-[486009:486737], allele observed in CC1+CC80+CC96)

Sequence:

ATGGCGAAACAAAAAAAGTTTATGAAGATTTATGAGGCGTTGAAAGAAGATATATTAAACGGGCAGATTCAATATGGTGAACAAATTCCG
TCTGAACATGATTTGGTGCAATTGTACCAGTCATCTCGAGAGACCGTGCGTAAGGCATTAGATTTGTTGGCATTAGACGGCATGATTCAAA
AGATTCATGGTAAAGGGTCACTTGTCATTTATCAGGAGGTTACAGAGTTTCCATTTTCTGAACTGGTTAGTTTTAAAGAAATGCAAGAAGAA
ATGGGCGTCGCATATTTAACTGAAGTTGTTGTGAATGAGGTTGTTGAAGCGCATGAAGTTCCAGAAGTTCAACATGCTTTAAACATCAATTC
TAGTGAATCACTCATTCATATTGTTAGAACTCGTCGGCTTAACCAACATGTGAAGATTGTTGATGAAGATTATTTTCTAAAGTCGATTGTTTC
AGACATAGGTAATGATGTTGCGAGTGATTCTATTTATGATTATTTGGAAAAGGTATTAAATCTTAATATTAGTTATTCAAGTAAGTCTATTAC
TTTTGAACCGTTTGATGAACAAGCATATCAATTGTTTGGTGATGTATCGGTGGCTTATTCAGCAACAGTTCGAAGTATTGTGTATTTAGAAA
ATACAATGCCGTTTCAATATAATATTTCAAAACATCTTGCAAATGAATTTAAATTTAACGACTTCTCAAGACGTCGTATAAAGTAA

Gene: ffs (signal recognition particle RNA subunit)

Position: 493047 to 493316, length: 270 nt

Sequence:

CCGTGCTAGGTGGGGAGGTAGCGGTTCCCTGTACTCGAAATCCGCTTTATGCGAGGCTTAATTCCTTTGTTGAGGCCGTATTTTTGCGAAGT
CTGCCCAAAGCACGTAGTGTTTGAAGATTTCGGTCCTATGCAATATGAACCCATGAACCATGTCAGGTCCTGACGGAAGCAGCATTAAGTG
GATCATCATATGTGCCGTAGGGTAGCCGAGATTTAGCTAACGACTTTGGTTACGTTCGTGAATTACGTTCGATGCTTAGGTGCACGG

Gene: Q5HIK0 (acetyltransferase, GNAT family)

Position: 493628 to 494152, length: 525 nt, orientation: FORWARD

Perfect match to: (MW2-BA000033-[487380:487904], allele observed in CC1+CC80)

Sequence:

ATGCAAATATATTTAAGTACTTTAACAGAGTTAGATTATGATAAATCTTTAAATAGTATTGAAGAAAGTTTTGATGATAATCCTGAAACGAG
TTGGCAAGCACTTGCGAAAGTAAAACATTTAAGAAAATCTCCTTGCTATAATTTTGAATTAGAAGTAATAGCGAAAAATGAAAATAACGAT
GTCGTTGGACACGTTTTATTAATTGAAGTAGAAATTAATAGTGATGATAAGACGTATTATGGTTTGGCGATTGCCTCTTTATCAGTTCATCCT
GAATTACGTGGACAAAAATTAGGTCGTGGCTTGGTTCAAGCAGTAGAAGAGCGTGCCAAAGCACAAGAGTATAGTACGGTTGTTGTAGAC
CATTGTTTTGACTACTTTGAAAAGTTGGGTTATCAAAATGCTGCTGAGCATGACATTAAATTAGAATCTGGTGATGCACCGTTACTTGTAAA
ATATTTATGGGATAATTTGACGGATGCACCACACGGAATCGTAAAATTTCCAGAACATTTTTATTAA

Gene: dnaX (DNA polymerase III gamma and tau subunits)

Position: 494221 to 495918, length: 1698 nt, orientation: FORWARD

Perfect match to: (11819-97-CP003194-[500143:501840], allele observed in CC80+CC1+CC7+CC97+CC101)

Sequence:

TTGAATTATCAAGCCTTATATCGTATGTACAGACCCCAAAGTTTCGAGGATGTCGTCGGACAAGAACATGTCACGAAGACATTGCGCAATG
CGATTTCTAAAGAAAAACAGTCGCATGCTTATATTTTTAGTGGTCCGAGAGGCACGGGGAAAACGAGTATTGCCAAAGTGTTTGCTAAAGC
AATCAACTGTTTAAATAGCACTGATGGAGAACCTTGTAATGAATGTCATATTTGTAAAGGCATTACGCAGGGGACTAATTCAGATGTGATA
GAAATTGATGCTGCTAGTAATAATGGCGTTGATGAAATAAGAAATATTAGAGACAAAGTTAAATATGCACCAAGTGAATCGAAATATAAAG
TTTATATTATAGATGAGGTGCACATGCTAACAACAGGTGCTTTTAATGCCCTTTTAAAGACGTTAGAAGAACCTCCAGCACACGCTATTTTTA
TATTGGCAACGACAGAACCACATAAAATCCCTCCAACAATCATTTCTAGGGCACAACGTTTTGATTTTAAAGCAATTAGCCTAAATCAAATT

GTTGAACGTTTAAAATTTGTAGCAGATGCACAACAAATTGAATGTGAAGATGAAGCCTTGGCATTTATCGCTAAAGCGTCTGAAGGGGGTA
TGCGTGATGCATTAAGTATTATGGATCAGGCTATTGCATTTGGTGATGGTACGTTAACATTGCAAGATGCGTTGAATGTCACAGGTAGCGT
ACATGATGAAGCGTTGGATCACTTGTTTGATGATATTGTACAAGGTGACGTACAAGCATCTTTTAAAAAATACCATCAGTTTATAACAGAGG
GTAAAGAAGTGAATCGCCTAATAAATGATATGATTTATTTTGTCAGAGATACGATTATGAATAAAACATCTGAGAAAGATACTGAGTATCG
AGCACTGATGAACTTAGAATTAGATATGTTATATCAAATGATTGATCTTATTAATGATACATTAGTGTCGATTCGTTTTAGTGTGAATCAAAA
CGTTCATTTTGAAGTGTTGTTAGTAAAATTAGCTGAGCAGATTAAGGGTCAACCACAAGTGATTGCGAATGTAGCTGAACCAGCACAAATT
GCTTCATCGCCAAACACAGATGTATTGTTGCAACGTATGGAACAGTTAGAGCAAGAACTAAAAACACTAAAAGCACAAGGAGTGAGTGTC
GCTCCTGTTCAAAAATCTTCGAAAAAGCCTGCGAGAGGCATACAAAAATCTAAAAATGCATTTTCAATGCAACAAATTGCAAAAGTGCTAG
ATAAAGCGAATAAGGCAGATATCAAATTGTTGAAAGATCATTGGCAAGAAGTGATTGATCATGCCAAAAACAATGATAAAAAATCACTCGT
TAGTTTATTGCAAAATTCGGAACCTGTGGCGGCAAGTGAAGATCACGTACTTGTGAAATTTGAGGAAGAGATCCATTGTGAAATCGTCAAT
AAAGACGACGAGAAACGTAGTAGTATAGAAAGTGTTGTATGTAATATCGTTAATAAAAACGTTAAAGTTGTTGGTGTACCATCAGATCAAT
GGCAAAGAGTTCGAACGGAGTATTTACAAAATCGTAAAAACGAAGGCGATGATATGCCAAAGCAACAAGCACAACAAACAGATATTGCTC
AAAAAGCAAAAGATCTTTTCGGTGAAGAAACTGTACATGTGATAGATGAAGAGTGA

Gene: yaaK (DNA binding protein)

Position: 496008 to 496325, length: 318 nt, orientation: FORWARD

Perfect match to: (RF122-AJ938182-[471380:471697], highly conserved allele)

Sequence:

ATGCGCGGTGGCGGAAACATGCAACAAATGATGAAACAAATGCAAAAAATGCAAAAGAAAATGGCTCAAGAACAAGAAAAACTTAAAGA
AGAGCGTATTGTAGGAACAGCTGGCGGTGGCATGGTTGCAGTTACTGTAACTGGTCATAAAGAAGTTGTCGACGTTGAAATCAAAGAAGA
AGCTGTAGACCCAGATGATATTGAAATGCTACAAGACTTAGTGTTAGCAGCTACTAATGAAGCGATGAATAAAGCTGATGAGCTTACTCAA
GAACGTTTAGGTAAACATACTCAAGGCTTAAACATCCCTGGAATGTGA

Gene: recR (recombination protein R)

Position: 496332 to 496928, length: 597 nt, orientation: FORWARD

Perfect match to: (MW2-BA000033-[490084:490680], highly conserved allele)

Sequence:

ATGCATTATCCAGAACCTATATCAAAACTTATTGATAGCTTTATGAAATTGCCAGGCATTGGTCCAAAGACAGCCCAACGTCTGGCTTTTCAT
ACCTTAGATATGAAAGAAGACGATGTTGTTCAGTTTGCCAAAGCATTAGTAGATGTTAAGAGAGAATTAACATATTGTAGCGTATGTGGTC
ACATTACTGAAAATGATCCATGTTATATTTGTGAAGATAAGCAAAGAGATCGTTCAGTTATTTGTGTTGTGGAAGATGACAAAGATGTCATA
GCTATGGAAAAAATGAGAGAATACAAAGGTTTATATCACGTTTTACATGGGTCTATTTCGCCTATGGATGGCATTGGACCAGAAGATATTA
ATATTCCTTCATTGATTGAACGCTTGAAAAACGATGAAGTTAGCGAATTAATCTTAGCTATGAACCCGAACTTAGAGGGGGAATCTACAGCC
ATGTATATTTCTAGATTAGTTAAGCCTATAGGTATCAAAGTGACGAGATTAGCACAAGGGTTATCGGTAGGTGGCGATTTAGAGTATGCTG
ACGAAGTAACATTATCTAAAGCAATCGCAGGTAGAACAGAAATGTAA

Gene: rrs (16S ribosomal RNA)

Position: 497782 to 499336, length: 1555 nt

Sequence:

TTTTATGGAGAGTTTGATCCTGGCTCAGGATGAACGCTGGCGGCGTGCCTAATACATGCAAGTCGAGCGAACGGACGAGAAGCTTGCTTCT
CTGATGTTAGCGGCGGACGGGTGAGTAACACGTGGATAACCTACCTATAAGACTGGGATAACTTCGGGAAACCGGAGCTAATACCGGATA
ATATTTTGAACCGCATGGTTCAAAAGTGAAAGACGGTCTTGCTGTCACTTATAGATGGATCCGCGCTGCATTAGCTAGTTGGTAAGGTAAC
GGCTTACCAAGGCAACGATGCATAGCCGACCTGAGAGGGTGATCGGCCACACTGGAACTGAGACACGGTCCAGACTCCTACGGGAGGCA
GCAGTAGGGAATCTTCCGCAATGGGCGAAAGCCTGACGGAGCAACGCCGCGTGAGTGATGAAGGTCTTCGGATCGTAAAACTCTGTTATT
AGGGAAGAACATATGTGTAAGTAACTGTGCACATCTTGACGGTACCTAATCAGAAAGCCACGGCTAACTACGTGCCAGCAGCCGCGGTAA
TACGTAGGTGGCAAGCGTTATCCGGAATTATTGGGCGTAAAGCGCGCGTAGGCGGTTTTTTAAGTCTGATGTGAAAGCCCACGGCTCAACC
GTGGAGGGTCATTGGAAACTGGAAAACTTGAGTGCAGAAGAGGAAAGTGGAATTCCATGTGTAGCGGTGAAATGCGCAGAGATATGGAG
GAACACCAGTGGCGAAGGCGACTTTCTGGTCTGTAACTGACGCTGATGTGCGAAAGCGTGGGGATCAAACAGGATTAGATACCCTGGTAG
TCCACGCCGTAAACGATGAGTGCTAAGTGTTAGGGGGTTTCCGCCCCTTAGTGCTGCAGCTAACGCATTAAGCACTCCGCCTGGGGAGTAC

GACCGCAAGGTTGAAACTCAAAGGAATTGACGGGGACCCGGACAAGCGGTGGAGCATGTGGTTTAATTCGAAGCAACGCGAAGAACCTT
ACCAAATCTTGACATCCTTTGACAACTCTAGAGATAGAGCCTTCCCCTTCGGGGGACAAAGTGACAGGTGGTGCATGGTTGTCGTCAGCTC
GTGTCGTGAGATGTTGGGTTAAGTCCCGCAACGAGCGCAACCCTTAAGCTTAGTTGCCATCATTAAGTTGGGCACTCTAAGTTGACTGCCG
GTGACAAACCGGAGGAAGGTGGGGATGACGTCAAATCATCATGCCCCTTATGATTTGGGCTACACACGTGCTACAATGGACAATACAAAG
GGCAGCGAAACCGCGAGGTCAAGCAAATCCCATAAAGTTGTTCTCAGTTCGGATTGTAGTCTGCAACTCGACTACATGAAGCTGGAATCGC
TAGTAATCGTAGATCAGCATGCTACGGTGAATACGTTCCCGGGTCTTGTACACACCGCCCGTCACACCACGAGAGTTTGTAACACCCGAAG
CCGGTGGAGTAACCTTTTAGGAGCTAGCCGTCGAAGGTGGGACAAATGATTGGGGTGAAGTCGTAACAAGGTAGCCGTATCGGAAGGTG
CGGCTGGATCACCTCCTTTCT

Gene: rrl (23S ribosomal RNA)

Position: 499637 to 502320, length: 2684 nt

Sequence:

GATTAAGTTATTAAGGGCGCACGGTGGATGCCTTGGCACTAGAAGCCGATGAAGGACGTTACTAACGACGATATGCTTTGGGAGCTGTAA
GTAAGCTTTGATCCAGAGATTTCCGAATGGGGAAACCCAGCATGAGTTATGTCATGTTATCGATATGTGAATACATAGCATATCAGAAGGC
ACACCCGGAGAACTGAAACATCTTAGTACCCGGAGAAGAGAAAGAAAATTCGATTCCCTTAGTAGCGGCGAGCGAAACGGAAGAGCCCA
AACCAACAAGCTTGCTTGTTGGGGTTGTAGGACACTCTATACGGAGTTACAAAGGACGACATTAGACGAATCATCTGGAAAGATGAATCAA
AGAAGGTAATAATCCTGTAGTCGAAAATGTTGTCTCTCTTGAGTGGATCCTGAGTACGACGGAGCACGTGAAATTCCGTCGGAATCTGGGA
GGACCATCTCCTAAGGCTAAATACTCTCTAGTGACCGATAGTGAACCAGTACCGTGAGGGAAAGGTGAAAAGCACCCCGAAGGGAGTGAA
ATAGAACCTGAAACCGTGTGCTTACAAGTAGTCAGAGCCCGTTAATGGGTGATGGCGTGCCTTTGTAGAATGAACCGGCGAGTTACGATTT
GATGCAAGGTTAAGCAGTAAATGTGGAGCCGTAGCGAAAGCGAGTCTGAATAGGGCGTTTAGTATTTGGTCGTAGACCCGAAACCAGGTG
ATCTACCCTTGGTCAGGTTGAAGTTCAGGTAACACTGAATGGAGGACCGAACCGACTTACGTTGAAAAGTGAGCGGATGAACTGAGGGTA
GCGGAGAAATTCCAATCGAACCTGGAGATAGCTGGTTCTCTCCGAAATAGCTTTAGGGCTAGCCTCAAGTGATGATTATTGGAGGTAGAGC
ACTGTTTGGACGAGGGGCCCTCTCGGGTTACCGAATTCAGACAAACTCCGAATGCCAATTAATTTAACTTGGGAGTCAGAACATGGGTGAT
AAGGTCCGTGTTCGAAAGGGAAACAGCCCAGACCACCAGCTAAGGTCCCAAAATATATGTTAAGTGGAAAGGATGTGGCGTTGCCCAGAC
AACTAGGATGTTGGCTTAGAAGCAGCCATCATTTAAAGAGTGCGTAATAGCTCACTAGTCGAGTGACACTGCGCCGAAAATGTACCGGGG
CTAAACATATTACCGAAGCTGTGGATTGTCCTTTGGACAATGGTAGGAGAGCGTTCTAAGGGCGTTGAAGCATGATCGTAAGGACATGTG
GAGCGCTTAGAAGTGAGAATGCCGGTGTGAGTAGCGAAAGACGGGTGAGAATCCCGTCCACCGATTGACTAAGGTTTCCAGAGGAAGGC
TCGTCCGCTCTGGGTTAGTCGGGTCCTAAGCTGAGGCCGACAGGCGTAGGCGATGGATAACAGGTTGATATTCCTGTACCACCTATAATCG
TTTTAATCGATGGGGGACGCAGTAGGATAGGCGAAGCGTGCGATTGGATTGCACGTCTAAGCAGTAAGGCTGAGTATTAGGCAAATCCGG
TACTCGTTAAGGCTGAGCTGTGATGGGAGAAGACATTGAGTCTTCGAGTCGTTGATTTCACACTGCCGAGAAAAGCCTCTAGATAGAAAAT
AGGTGCCCGTACCGCAAACCGACACAGGTAGTCAAGATGAGAATTCTAAGGTGAGCGAGCGAACTCTCGTTAAGGAACTCGGCAAAATGA
CCCCGTAACTTCGGGAGAAGGGTGCTCTTTAGGGTTAACGCCCAGAAGAGCCGCAGTGAATAGGCCCAAGCGACTGTTTATCAAAACACA
GGTCTCTGCTAAACCGTAAGGTGATGTATAGGGGCTGACGCCTGCCCGGTGCTGGAAGGTTAAGAGGTGGTTAGCTTCTGCGAAGCTACG
AATCGAAGCCCCAGTAAACGGCGGCCGTAACTATAACGGTCCTAAGGTAGCGAAATTCCTTGTCGGGTAAGTTCCGACCCGCACGAAAGG
CGTAACGATTTGGGCACTGTCTCAACGAGAGACTCGGTGAAATCATAGTACCTGTGAAGATGCAGGTTACCCGCGACAGGACGGAAAGAC
CCCGTGGAGCTTTACTGTAGCCTGATATTGAAATTCGGCACAGCTTGTACAGGATAGGTAGGAGCCTTTGAAACGTGAGCGCTAGCTTACG
TGGAGGCGCTGGTGGGATACTACCCTAGCTGTGTTGGCTTTCTAACCCGCACCACTTATCGTGGTGGGAGACAGTGTCAGGCGGGCAGTTT
GACTGGGGCGGTCGCCTCCTAAAAGGTAACGGAGGCGCTCAAAGGTTCCCTCAGAATGGTTGGAAATCATTCATAGAGTGTAAAGGCATA
AGGGAGCTTGACTGCGAGACCTACAAGTCGAGCAGGGTCGAAAGACGGACTTAGTGATCCGGTGGTTCCGCATGGAAGGGCCATCGCTC
AACGGATAAAAGCTACCCCGGGATAACAGGCTTATCTCCCCAAGAGTTCACATCGACGGGGAGGTTTGGCACCTCGATGTCGGCTCATCGC
ATCCTGGGGCTGTAGTCGGTCCCAAGGGTTGGGCTGTTCGCCCATTAAAGCGGTACGCGAGCTGGGTTCAGAACGTCGTGAGACAGTTCG
GTCCCTATCCGTCGTGGGCGTAGGAAATTTGAGAGGAGCTGTCCTTAGTACGAGAGGACCGGGATG

Gene: IGR_rrl_rrf (intergenic spacer between rrl and rrf)

Position: 502321 to 502613, length: 293 nt

Sequence:

GACATACCTCTGGTGTACCAGTTGTCGTGCCAACGGCATAGCTGGGTAGCTATGTGTGGACGGGATAAGTGCTGAAAGCATCTAAGCATG
AAGCCCCCTCAAGATGAGATTTCCCAACTTCGGTTATAAGATCCCTCAAAGATGATGAGGTTAATAGGTTCGAGGTGGAAGCATGGTGACA
TGTGGAGCTGACGAATACTAATCGATCGAAGACTTAATCAAAATAAATGTTTTGCGAAGCAAAATCACTTTACTTACTATCTAGTTTTGAAT
GTATAATTTACATTCATATG

Gene: yaaO (putative Orn/Lys/Arg decarboxylase)

Position: 503632 to 504969, length: 1338 nt, orientation: FORWARD

Perfect match to: (11819-97-CP003194-[509575:510912], allele observed in CC80+CC8+CC188)

Sequence:

ATGAAGCAACCTATTTTAAATAAATTAGAAAGTTTAAATCAAGAAGAAGCGATTTCTTTGCATGTTCCGGGTCATAAAAATATGACTATCGG
TCATTTATCTCAATTATCAATGACAATGGATAAAACTGAAATACCTGGATTAGATGATTTACATCATCCTGAAGAAGTCATTTTGGAAAGTAT
GAAGCAGGTGGAGAAACATTCAGATTATGATGCTTATTTCTTAGTGAATGGCACCACTTCAGGAATATTATCTGTCATCCAGTCTTTTTCACA
GAAAAAAGGCGATATCTTAATGGCAAGAAATGTACATAAATCTGTATTACATGCGCTCGATATTAGCCAACAAGAAGGGCATTTTATTGAA
ACGCATCAAAGTCCGTTAACGAATCATTATAATAAAGTTAATTTAAGCCGTTTGAATAATGACGGTCACAAACTTGCTGTGTTGACTTATCCT
AACTATTACGGTGAAACATTTAATGTAGAAGAGGTTATCAAATCTTTGCACCAATTAAATATTCCTGTACTCATTGACGAAGCACACGGCGC
GCACTTTGGATTGCAAGGATTTCCAGATTCTACATTAAATTATCAAGCTGACTATGTTGTTCAATCTTTTCATAAAACGTTACCAGCTTTAAC
GATGGGCTCGGTACTTTATATTCATAAAAATGCACCTTATAGAGAAACTATTATAGAATATCTAAGCTACTTCCAAACATCTAGTCCTTCGTA
TTTGATTATGGCTAGTTTAGAGTCAGCTGCCCAGTTCTATAAAACATATGATAGTACCGTGTTTTTTGATAAGAGAGCACAATTAATCGAAT
GTTTGGAGAATAAAGGTTTTGAAATGCTTCAAGTTGATGATCCGTTGAAGTTGTTGATAAAATATGATGGCTTTACAGGACATGATATTCAA
AGTTGGTTTATGAATGCACATATCTATTTAGAGTTAGCAGACGACTATCAAGCTTTAGCAATATTGCCGTTATGGCATCATGATGATACGTA
TTTATTTGATTCGCTTTTACGTAAAATTGAAGATATGATTTTACCGAAAAAATCAGTTTCTAAAGTTAAACAAACACAACTTTTAACAACTGA
AGGTAACTATAAACCAAAACGCTTTGAATATGTTACTTGGTGTGATTTGAAAAAGGCAAAAGGTAAAGTTCTGGCGCGACATATTGTCCCG
TATCCGCCAGGGATTCCTATTATTTTCAAAGGAGAAACAATAACTGAAAATATGATAGAATTGGTAAATGAATATCTGGAAACTGGAATGA
TAGTTGAAGGAATTAAAAATAATAAAATTTTAGTTGAGGATGAATAA

Gene: tmk (thymidylate kinase)

Position: 504971 to 505588, length: 618 nt, orientation: FORWARD

Perfect match to: (MW2-BA000033-[498743:499360], allele observed in CC1+CC80+CC101)

Sequence:

ATGTCAGCTTTTATAACTTTTGAGGGCCCAGAAGGCTCTGGAAAAACAACTGTAATTAATAAAGTTTACCATAGATTAGTAAAAGATTATGA
TGTCATTATGACAAGAGAACCGGGCGGCGTTCCTACTGGTGAAGAAATACGTAAAATTGTATTAGAAGGCAATGATATGGACATTAGAACT
GAAGCAATGTTATTTGCTGCATCTAGAAGAGAACATCTTGTATTAAAGGTCATACCAGCTTTAAAAGAAGGTAAGGTTGTGTTGTGTGATC
GCTATATCGATAGTTCATTAGCTTATCAAGGTTATGCTAGAGGGATTGGCGTTGAAGAAGTAAGAGCATTAAACGAATTTGCAATAAATGG
ATTATATCCAGACTTGACGATTTATTTGAATGTTAGTGCTGAAGTAGGCCGCGAACGTATTATTAAAAATTCAAGAGATCAAAATAGATTAG
ATCAAGAAGATTTAAAGTTTCACGAAAAAGTAATTGAAGGTTACCAAGAAATCATTCATAATGAATCACAACGGTTCAAAAGCGTTAATGC
AGATCAACCTCTTGAAAATGTTGTTGAAGACACGTATCAAACTATCATCAAATATTTAGAAAAGATATGA

Gene: darA (cyclic-di-AMP receptor)

Position: 505616 to 505945, length: 330 nt, orientation: FORWARD

Perfect match to: (RF122-AJ938182-[480866:481195], highly conserved allele)

Sequence:

ATGAAAATGATTATAGCGATCGTACAAGATCAAGATAGTCAGGAACTTGCAGATCAACTTGTTAAAAATAACTTTAGAGCAACAAAATTGG
CAACAACAGGTGGGTTTTTAAGAGCAGGTAATACAACATTCTTATGTGGTGTCAATGATGACCGTGTAGATGAAATATTGTCTGTGATTAAT
CAAACGTGTGGTAATAGAGAACAGTTGGTTTCACCTATTACACCTATGGGAGGCAGTGCGGATTCGTACATTCCATATCCAGTTGAAGTTG
AAGTTGGCGGTGCTACTGTATTTGTTATGCCAGTTGATGCATTCCATCAATTTTAA

Gene: holB (DNA polymerase III, delta' subunit)

Position: 506159 to 507085, length: 927 nt, orientation: FORWARD

Perfect match to: (MW2-BA000033-[499931:500857], allele observed in CC1+CC8+CC30+CC80)

Sequence:

ATGGATGAACAGCAACAATTGACGAATGCATATCATTCAAATAAATTATCGCATGCCTATTTATTTGAAGGTGATGATGCACAAACGATGA
AACAAGTTGCGATTAATTTTGCAAAGCTTATTTTATGTCAAACAGATAGTCAATGTGAAACAAAGGTTAGTACATATAATCATCCAGACTTT

ATGTATATATCAACAACTGAGAATGCAATTAAGAAAGAACAAGTTGAACAACTTGTGCGTCATATGAATCAACTTCCTATAGAAAGCACAA
ATAAAGTGTACATCATTGAAGACTTTGAAAAGTTAACTGTTCAAGGGGAAAACAGTATCTTGAAATTTCTTGAAGAACCACCGGACAATAC
GATTGCTATTTTATTGTCTACAAAACCTGAGCAAATTTTAGACACAATCCATTCAAGGTGTCAGCATGTATATTTCAAGCCTATTGATAAAGA
AAAGTTTATAAATAGATTAGTTGAACAAGACATGTCTAAGCCAGTAGCTGAAATGATTAGTACTTATACTACGCAAATAGATAATGCAATG
GCTTTAAATGAAGAATTTGATTTATTAGCATTAAGGAAATCAGTTATACGTTGGTGTGAATTGTTGCTTACTAATAAGCCAATGGCACTTATA
GGTATTATTGATTTATTGAAACAGGCTAAAAATAAAAAACTGCAATCTTTAACTATTGCAGCTGTGAATGGTTTCTTCGAAGATATCATACAT
ACAAAGGTAAATGTAGAGGATAAACAAATATATAGTGATTTAAAAAATGATATTGATCAATATGCGCAAAAGTTGTCGTTTAATCAATTAAT
TTTGATGTTTGATCAACTGACGGAAGCACATAAGAAATTGAATCAAAATGTAAATCCAACGCTTGTATTTGAACAAATCGTAATTAAGGGTG
TGAGTTAG

Gene: yaaT (protein related to DNA replication)

Position: 507086 to 507889, length: 804 nt, orientation: FORWARD

Perfect match to: (JKD6159-CP002114-[498949:499752], allele observed in CC93+CC80+CC398)

Sequence:

ATGCCAAATGTAATAGGTGTTCAGTTTCAAAAAGCGGGAAAATTAGAATATTATACACCTAATGATATACAAGTAGATATAGATGACTGGG
TAGTTGTCGAATCTAAAAGAGGCATAGAGATAGGTATTGTTAAAAATCCATTAATGGATATTGCTGAAGAGGATGTTGTGTTACCTCTTAAA
AATATTATTCGCATTGCTGATGACAAAGATATTGATAAATTTAATTGTAATGAACGAGATGCTGAAAATGCATTAATACTATGTAAAGACAT
TGTAAGAGAACAAGGTTTGGACATGCGTTTAGTCAATTGCGAATATACATTAGATAAATCGAAAGTTATTTTTAATTTTACGGCGGATGATC
GTATTGATTTTAGAAAATTAGTAAAAATATTAGCGCAACATTTAAAAACACGTATCGAGTTGAGACAAATTGGTGTAAGGGATGAAGCCAA
ATTGCTTGGCGGTATCGGACCTTGTGGTAGGTCGTTATGTTGTTCTACATTTTTAGGGGATTTTGAACCAGTATCGATTAAGATGGCTAAGG
ATCAAAATTTATCATTAAATCCAACTAAAATTTCCGGTGCATGTGGTCGTTTGATGTGTTGTTTAAAATATGAAAATGACTACTATGAGGAA
GTACGTGCACAATTACCTGATATCGGTGAAGCAATTGAAACGCCTGATGGTAACGGGAAAGTAGTTGCTTTAAATATATTAGACATTTCTAT
GCAGGTGAAGCTTGAGGGACATGAACAGCCACTTGAATATAAATTAGAAGAAATAGAAACTATGCATTAA

Gene: yabA (initiation-control protein)

Position: 507906 to 508253, length: 348 nt, orientation: FORWARD

Perfect match to: (MRSA252-BX571856-[525697:526044], highly conserved allele)

Sequence:

TTGGATCGCAATGAAATATTTGAAAAAATAATGCGTTTAGAAATGAATGTCAATCAACTTTCAAAGGAAACTTCAGAATTAAAGGCACTTGC
AGTTGAATTAGTAGAAGAAAATGTAGCGCTTCAACTTGAAAATGATAATTTGAAAAAGGTGTTGGGCAATGATGAACCAACTACTATTGAT
ACTGCGAATTCAAAACCAGCAAAAGCTGTGAAAAAGCCATTACCAAGTAAAGATAATTTGGCTATATTGTATGGAGAAGGATTTCATATTT
GTAAAGGCGAATTATTTGGAAAACATCGACATGGTGAAGATTGTCTGTTCTGTTTAGAAGTTTTAAGTGATTAA

Gene: yabB (putative O-methyltransferase)

Position: 508527 to 509252, length: 726 nt, orientation: FORWARD

Perfect match to: (VCU006-AGTZ01000012-[32355:33080:r], allele observed in CC15+CC80)

Sequence:

ATGTTAAAAGAGAATGAACGATTTGATCAACTAATCAAAGAAGATTTTAGTATTATTCAAAATGATGATGTTTTTTCGTTTTCAACGGATGCT
TTGTTGTTAGGGCATTTTACAAAACCTAGAACAAAAGATATAGTGTTGGACTTATGTTCAGGCAATGGGGTGATACCCTTGTTATTGTTTGC
GAAACATCCACGACATATAGAAGGTGTTGAGATTCAAAAAACACTTGTCGATATGGCGCGACGCACATTTCAATTCAATGATGTTGATGAA
TATTTAACAATGCATCACATGGATTTGAAAAACGTTACTAAAGTATTTAAACCTTCACAATATACTTTAGTAACGTGTAATCCGCCTTATTTTA
AAGAGAATCAGCAACACCAACATCAAAAAGAAGCACATAAGATAGCGAGACATGAGATTATGTGTACACTTGAAGATTGCATGATTGCAG
CCCGTCATTTATTAAAAGAAGGTGGCAGGCTAAACATGGTACATCGTGCAGAGAGACTAATGGATGTCTTGTTTGAAATGAGAAAAGTGA
ATATTGAACCTAAGAAAGTCGTTTTTATATATAGTAAAGTAGGGAAATCAGCACAAACGATAGTAGTAGAAGGTCGAAAAGGTGGAAATC
AAGGTTTAGAAATCATGCCCCCATTTTATATTTATAATGAAGATGGTAATTATAGCGAAGAAATGAAGGAAGTATATTATGGATAG

Gene: yazA (putative UvrC-type endonuclease)

Position: 509245 to 509493, length: 249 nt, orientation: FORWARD

Perfect match to: (RF122-AJ938182-[484495:484743], highly conserved allele)

Sequence:

ATGGATAGTCATTTTGTATATATTGTAAAATGTAGTGATGGAAGTTTATATACAGGATACGCTAAAGACGTTAATGCACGTGTTGAAAAACA
TAACCGAGGTCAAGGAGCCAAATATACGAAAGTAAGACGTCCGGTGCATTTAGTTTATCAAGAAATGTATGAGACAAAGTCTGAAGCATT
GAAGCGTGAATATGAAATTAAAACTTATACCAGACAAAAGAAATTGCGATTAATTAAGGAGCGATAG

Gene: rsmI (ribosomal RNA small subunit methyltransferase I)

Position: 509495 to 510334, length: 840 nt, orientation: FORWARD

Perfect match to: (11819-97-CP003194-[515438:516277], allele observed in CC80+CC130)

Sequence:

ATGGCTGTATTATATTTAGTGGGCACACCAATTGGTAATTTAGCAGATATTACTTATAGAGCAGTTGATGTATTGAAACGTGTTGATATGAT
TGCTTGTGAAGACACTAGAGTAACTAGTAAGCTGTGTAATCATTATGATATTCCAACTCCATTAAAGTCATATCACGAACATAACAAGGATA
AGCAGACTGCTTTTATCATTGAACAGTTAGAATTAGGTCTTGACGTTGCGCTCGTATCTGATGCTGGATTGCCCTTAATTAGTGATCCTGGAT
ACGAATTAGTAGTGGCAGCCAGAGAAGCTAATATTAAAGTAGAGACTGTGCCTGGACCTAATGCTGGGCTGACGGCTTTGATGGCTAGTG
GATTACCTTCATATGTATATACATTTTTAGGATTTTTGCCACGAAAAGAGAAAGAAAAAAGTGCTGTATTAGAGCAACGTATGCATGAAAAT
AGCACATTAATTATATACGAATCACCGCATCGTGTGACAGATACATTAAAAACAATTGCAAAGATAGATGCAACACGACAAGTATCACTAG
GGCGTGAATTGACTAAGAAGTTCGAACAAATTGTAACTGATGATGTAACACAATTACAAGCATTGATTCAGCAAGGCGATGTACCATTGAA
AGGCGAATTCGTTATCTTGATTGAGGGTGCTAAAGCGAACAATGAGATATCGTGGTTTGATGATTTATCTATCAATGAGCATGTTGATCATT
ATATTCAAACTTCACAGATGAAACCAAAACAAGCTATTAAAAAAGTTGCTGAAGAACGACAACTTAAAACGAATGAAGTATATAATATTTAT
CATCAAATAAATTAA

Gene: metS (methionyl-tRNA synthetase)

Position: 510619 to 512592, length: 1974 nt, orientation: FORWARD

Sequence:

ATGGCTAAAGAAACATTTTATATAACAACCCCAATATACTATCCTAGTGGGAATTTACATATAGGACATGCATATTCTACAGTGGCTGGAGA
TGTTATTGCAAGATATAAGAGAATGCAAGGATATGATGTTCGTTATTTGACTGGAACGGATGAACACGGTCAAAAAATTCAAGAAAAAGCT
CAAAAAGCTGGTAAGACAGAAATTGAATATTTGGATGAGATGATTGCTGGAATTAAACAATTGTGGGCTAAGCTTGAAATTTCAAATGATG
ATTTTATCAGAACAACTGAAGAACGTCATAAACATGTCGTTGAGCAAGTGTTTGAACGTTTATTAAAGCAAGGTGATATCTATTTAGGTGAA
TATGAAGGTTGGTATTCTGTTCCGGATGAAACATACTATACAGAGTCGCAATTAGTAGACCCACAATACGAAAACGGTAAAATTATTGGTG
GCAAAAGTCCAGATTCTGGACACGAAGTTGAACTAGTTAAAGAAGAAAGTTATTTCTTTAATATTAGTAAATATACAGACCGTCTATTAGAA
TTCTATGACCAAAATCCAGATTTTATACAACCACCATCAAGAAAAAATGAAATGATTAACAACTTCATTAAACCAGGACTTGCTGATTTGGCT
GTTTCTCGTACATCATTTAACTGGGGTGTCCATGTTCCGTCTAATCCAAAACATGTTGTTTATGTTTGGATTGATGCGTTAGTTAACTATATTT
CAGCATTAGGCTATTTATCAGATGATGAGTCACTATTTAACAAATACTGGCCAGCAGATATTCATTTAATGGCTAAGGAAATTGTGCGATTC
CACTCAATTATTTGGCCTATTTTATTGATGGCATTAGACTTACCGTTACCTAAAAAAGTCTTCGCACATGGTTGGATTTTGATGAAAGATGGA
AAAATGAGTAAATCTAAAGGTAATGTCGTAGACCCTAATATTTTAATTGATCGCTATGGTTTAGATGCTACACGTTATTATCTAATGCGTGA
ATTACCATTTGGTTCAGATGGCGTATTTACACCTGAAGCATTTGTTGAGCGTACAAATTTCGATCTAGCAAATGACTTAGGTAACTTAGTAA
ACCGTACGATTTCTATGGTTAATAAGTACTTTGATGGCGAATTACCAGCGTATCAAGGTCCACTTCATGAATTAGATGAAGAAATGGAAGCT
ATGGCTTTAGAAACAGTGAAAAGCTACACTGAAAGCATGGAAAGTTTGCAATTTTCTGTGGCATTATCTACGGTATGGAAGTTTATTAGTA
GAACTAATAAGTATATTGACGAAACAACGCCTTGGGTATTAGCTAAGGACGATAGCCAAAAAGATATGTTAGGCAATGTAATGGCTCACTT
AGTTGAAAATATTCGTTATGCAGCTGTATTATTACGTCCATTCTTAACACATGCGCCGAAAGAGATTTTTGAACAATTGAACATAAACAATCC
TCAATTTATGGAATTTAGTAGTTTAGAGCAATATGGTGTGCTTACTGAGTCAATTATGGTTACTGGGCAACCTAAACCTATTTTCCCAAGATT
GGATAGCGAAGCGGAAATTGCATATATCAAAGAATCAATGCAACCGCCTGCTACTGAAGAGGAAAAAGAAGAGATTCCTAGCAAACCTCA
AATTGATATTAAAGACTTTGATAAAGTTGAAATTAAGGCAGCAACGATTATTGATGCTGAACATGTTAAGAAGTCAGATAAGCTTTTAAAA
ATTCAAGTAGACTTAGATTCTGAACAAAGACAAATTGTATCAGGAATTGCCAAATTCTATACACCAGATGATATTATTGGTAAAAAAGTAGC
AGTTGTTACTAACCTGAAACCAGCTAAATTAATGGGACAAAAATCTGAAGGTATGATATTATCTGCTGAAAAAGATGGTGTATTAACCTTAG
TAAGTTTACCAAGTGCAATTCCAAATGGTGCAGTGATTAAATAA

Gene: tatP (deoxyribonuclease)

Position: 512623 to 513396, length: 774 nt, orientation: FORWARD

Perfect match to: (TW20-FN433596-[597867:598640], highly conserved allele)

Sequence:

ATGTTAATCGATACACATGTCCATTTAAATGATGAGCAATACGATGATGATTTGAGTGAAGTGATTACACGTGCTAGAGAAGCAGGTGTTG
ATCGTATGTTTGTAGTTGGTTTTAACAAATCGACAATTGAACGCGCGATGAAATTAATCGATGAGTATGATTTTTTATATGGCATTATCGGTT
GGCATCCAGTTGACGCAATTGATTTTACAGAAGAACACTTGGAATGGATTGAATCTTTAGCTCAGCATCCAAAAGTGATTGGTATTGGTGA
AATGGGATTAGATTATCACTGGGATAAATCTCCTGCAGATGTTCAAAAGGAAGTTTTTAGAAAGCAAATTGCTTTAGCTAAGCGTTTGAAGT
TACCAATTATCATTCATAACCGTGAAGCAACTCAAGACTGTATCGATATCTTATTGGAGGAGCATGCTGAAGAGGTAGGCGGGATTATGCA
TAGCTTTAGTGGTTCTCCAGAAATTGCAGATATTGTAACTAATAAGCTGAATTTTTATATTTCATTAGGAGGACCTGTGACATTTAAAAATGC
TAAACAGCCTAAAGAAGTTGCTAAGCATGTGTCAATGGAGCGTTTGCTAGTTGAAACCGATGCACCGTATCTTTCGCCACATCCGTATAGA
GGGAAGCGAAATGAACCGGCGAGAGTAACTTTAGTAGCTGAACAAATTGCTGAATTAAAAGGCTTATCTTATGAAGAAGTGTGCGAACAA
ACAACTAAAAATGCAGAGAAATTGTTTAATTTAAATTCATAA

Gene: rnmV (ribonuclease M5)

Position: 513565 to 514101, length: 537 nt, orientation: FORWARD

Perfect match to: (11819-97-CP003194-[519508:520044], allele observed in CC15+CC80)

Sequence:

ATGAAAATCAATGAGTTTATTGTTGTTGAAGGTCGAGATGATACTGAACGTGTTAAACGAGCTGTAGAATGTGATACGATTGAAACAAATG
GCAGTGCAATCAATGAGCAAACTTTGGAAGTAATTAGAAATGCACAACAAAGTCGTGGTGTCATTGTACTGACAGATCCAGATTTTCCTGG
AGATAAAATTAGAAGTACAATTACGGAGCATGTAAAAGGTGTTAAACACGCATATATTGATAGAGACAAAGCTAAAAATAAAAGAGGAAA
AATCGGTGTTGAACATGCTGAATTAGAAGATATAAAGGAAGCGTTAATGCATGTGAGTTCACCATTTGAAGAAGCATATGAGTCAATTGAT
AAAACCGTGTTAATTGAGTTAGGACTAATTGTCGGTAAAGATGCTAGACGTCGTCGTGAAATTTTAAGTAGGAAATTACGAATTGGTCACT
CAAACGGAAAGCAATTATTGAAGAAGTTAAATGCTTTTGGTTATACGGAAACAGATGTAAGGCAAGCTTTAAAAGATGAATAA

Gene: ksgA (ribosomal RNA small subunit methyltransferase A)

Position: 514115 to 515005, length: 891 nt, orientation: FORWARD

Perfect match to: (VCU006-AGTZ01000012-[26602:27492:r], allele observed in CC15+CC80)

Sequence:

TTGGATAATAAAGATATTGCAACGCCATCTCGAACACGAGCATTATTAGATAAATATGGATTTAATTTTAAGAAAAGTTTAGGTCAGAACTT
TTTAATAGACGTTAATATTATAAATAATATTATCGATGCAAGTAATATTGATGAACGTACTGGTGTTATTGAAATTGGACCTGGTATGGGGT
CATTAACTGAACAGTTGGCGAAACACGCTAAAAAAGTATTGGCATTTGAAATAGACCAACGCTTAATACCTGTACTTAAGGACACATTATCA
CCATATGATAATGTGACAGTGATTAACGAAGATATTTTAAAAGCAAATATTAAAGCAGCAGTTGAAAACCATTTACAAGACTGCGAAAAAA
TAATGGTTGTCGCGAATTTACCGTATTACATTACAACGCCGATACTTTTAAATTTGATGCAACAAGATATACCAATTGATGGCTACGTGGTG
ATGATGCAAAAAGAAGTGGGCGAACGCTTAAATGCTGAAGTAGGTTCAAAAGCATATGGTTCGTTATCAATTGTCGTACAATACTATACAG
AGACTAGTAAAGTATTAACGGTACCTAAATCTGTATTTATGCCACCACCTAATGTTGATTCAATAGTTGTAAAACTGATGCAGAGAACTGAA
CCGTTAGTAACAGTAGATAACGAGGAAGCATTCTTTAAGTTAGCAAAAGCAGCATTTGCACAAAGAAGAAAGACAATTAACAATAACTATC
AAAATTATTTTAAAGATGGTAAACAACACAAAGAAGTGATTTTACAATGGTTGGAACAAGCAGGTATTGATCCAAGACGTCGCGGTGAAAC
GCTATCTATTCAAGATTTTGCTAAATTGTATGAAGAAAAGAAAAAATTCCCTCAATTAGAAAATTAA

Gene: veg (conserved hypothetical protein)

Position: 515105 to 515368, length: 264 nt, orientation: FORWARD

Perfect match to: (RF122-AJ938182-[490353:490616], highly conserved allele)

Sequence:

ATGCCAAAATCAATTTTGGACATCAAAAATTCTATTGATTGTCATGTAGGAAATCGTATTGTACTGAAAGCCAATGGAGGCCGTAAGAAAA
CAATAAAACGTTCTGGAATTTTAAAAGAAACATATCCGTCAGTTTTCATTGTTGAGTTAGATCAAGACAAACACAACTTTGAGAGAGTATCT
TATACATACACTGATGTGTTAACTGAAAATGTTCAAGTTTCATTTGAAGAGGATAATCATCACGAATCAATTGCACACTAA

Gene: ispE (4-diphosphocytidyl-2-C-methyl-D-erythritol kinase)

Position: 515678 to 516526, length: 849 nt, orientation: FORWARD

Perfect match to: (IS-105-AHLR01000113-[622:1470], allele observed in CC22+CC8)

Sequence:

ATGATATATGAAACGGCACCAGCCAAAATTAATTTTACGCTCGATACACTTTTTAAAAGAAATGATGGCTATCATGAGATTGAAATGATAAT
GACAACAGTTGATTTAAATGATCGTTTAACTTTTCATAAAAGAAAAGATCGAAAGATAGTTGTTGAGATTGAACATAATTATGTGCCTTCTA
ATCATAAAAATCTCGCATATCGTGCAGCGCAACTATTTATTGAGCAATATCAACTAAAGCAAGGTGTAACAATTTCTATCGATAAAGAAATA
CCTGTTTCTGCTGGCTTAGCTGGAGGTTCGGCTGATGCAGCAGCAACTTTAAGAGGATTGAATCGACTTTTTGATATAGGGGCGAGTTTGG
AAGAATTGGCTCTACTAGGCAGTAAAATTGGGACAGATATTCCGTTTTGTATTTATAATAAAACTGCACTATGTACTGGAAGAGGAGAGAA
AATCGAGTTTTTAAATAAACCACCTTCAGCTTGGGTGATTCTTGCTAAACCAAACTTAGGCATATCATCACCAGATATATTTAAGTTGATCAA
TTTAGATAAGCGTTACGACGTACATACGAAAATGTGTTATGAGGCCTTAGAAAATCGAGATTATCAACAATTATGTCAAAGTTTGTCTAATC
GATTAGAGCCAATTTCTGTTTCAAAACACCCACAAATCGATAAATTAAAAAATAATATGTTGAAAAGTGGTGCAGATGGTGCGTTAATGAG
TGGAAGCGGACCGACTGTGTATGGGCTAGCACGAAAAGAAAGCCAAGCAAAAAATATTTATAATGCAGTTAACGGTTGTTGTAATGAAGT
GTACTTAGTTAGACTATTAGGATAG

Gene: purR (purine operon repressor)

Position: 516540 to 517364, length: 825 nt, orientation: FORWARD

Perfect match to: (RF122-AJ938182-[491788:492612], highly conserved allele)

Sequence:

ATGAGATATAAACGAAGCGAGAGAATTGTTTTTATGACGCAATATTTGATGAACCATCCGAATAAATTGATTCCATTAACTTTTTTTGTGAA
AAAATTTAAACAGGCGAAGTCTTCAATAAGTGAAGATGTCCAAATTATAAAAAATACATTCCAAAAAGAAAAGTTAGGTACAGTAATTACT
ACTGCTGGCGCAAGTGGTGGTGTTACGTATAAACCAATGATGAGTAAAGAAGAGGCGACTGAAGTTGTTAATGAGGTCATTACTCTATTAG
AAGAGAAAGAACGTTTGTTACCTGGCGGATATTTATTTTTATCAGATTTGGTAGGTAATCCATCGCTACTAAACAAAGTTGGTAAGTTAATT
GCCAGTATTTACATGGAAGAAAAATTAGATGCTGTTGTTACCATTGCGACAAAAGGTATTTCATTGGCAAATGCGGTTGCTAATATTTTAAA
TTTACCAGTAGTAGTGATTAGAAAAGACAACAAGGTGACTGAAGGTTCTACAGTTTCAATTAATTACGTTTCAGGATCTTCAAGAAAAATAG
AAACAATGGTACTTTCGAAGAGAACTTTAGCAGAAAATTCAAATGTTTTAGTTGTCGATGATTTTATGAGGGCTGGTGGCTCTATTAATGGT
GTTATGAATTTAATGAATGAGTTTAAAGCCCATGTAAAAGGGGTATCAGTACTTGTAGAATCAAAAGAAGTTAAACAAAGATTGATTGAAG
ATTATACTTCCTTAGTGAAATTATCTGATGTAGATGAATATAATCAAGAGTTTAACGTAGAACCTGGCAACAGTTTATCTAAGTTTTCATAA

Gene: yabJ (translation initiation inhibitor)

Position: 517381 to 517761, length: 381 nt, orientation: FORWARD

Perfect match to: (11819-97-CP003194-[523324:523704], allele observed in CC80)

Sequence:

ATGAAAATCATTAACACAACAAGATTACCGGAAGCACTTGGACCATATTCGCATGCAACAGTTGTGAATGGTATGGTTTATACTTCTGGTCA
GATTCCATTGAATGTTGATGGAGAAATCGTAAGCGCTGATGTTCAAGCACAGACAAAGCAAGTTTTAGAAAATTTAAAGGTTGTTTTGGAA
GAAGCAGGATCTGATTTGAATTCTGTTGCGAAAGCGACCATTTTCATTAAAGATATGAATGATTTCCAAAAAATAAATGAAGTGTATGGTCA
ATATTTTAATGAACACAAGCCAGCGCGTAGTTGTGTAGAGGTTGCGCGTTTGCCAAAAGATGTGAAAGTAGAAATTGAATTAGTAAGTAAA
ATTAAGGAATTATAA

Gene: spoVG (septation protein spoVG)

Position: 517833 to 518135, length: 303 nt, orientation: FORWARD

Perfect match to: (RF122-AJ938182-[493081:493383], highly conserved allele)

Sequence:

ATGAAAGTGACAGATGTAAGACTTAGAAAAATACAAACAGATGGACGAATGAAAGCACTCGTTTCCATTACATTAGATGAAGCTTTCGTAA
TTCATGATTTACGTGTAATTGAAGGAAACTCTGGCTTGTTCGTTGCAATGCCAAGTAAACGTACACCAGATGGTGAATTCCGCGACATCGCG
CATCCTATTAATTCAGATATGAGACAAGAAATTCAAGATGCAGTGATGAAAGTATATGATGAAACAGATGAAGTAGTACCAGATAAAAACG
CTACATCAGAAGATTCAGAAGAAGCTTAA

Gene: glmU (UDP-N-acetylglucosamine pyrophosphorylase)

Position: 518478 to 519830, length: 1353 nt, orientation: FORWARD

Perfect match to: (TCH959-AASB02000183-[428:1780], allele observed in CC7+CC8+CC97+CC707)

Sequence:

ATGCGAAGACACGCGATAATTTTGGCAGCAGGTAAAGGCACAAGAATGAAATCTAAAAAGTATAAAGTGCTACACGAGGTTGCTGGGAAA
CCTATGGTCGAACATGTATTGGAAAGTGTGAAAGGCTCTGGTGTCGATCAAGTTGTAACCATCGTAGGACATGGTGCTGAAAGTGTAAAA
GGACATTTAGGCGAGCGTTCTTTATACAGTTTTCAAGAGGAACAACTCGGTACTGCGCATGCAGTGCAAATGGCGAAATCACACTTAGAAG
ACAAGGAAGGTACGACGATCGTTGTATGTGGTGACACACCGCTCATCACAAAGGAAACATTAGAAACATTGATTGCGCATCATGAGGATG
CTAATGCTCAAGCAACTGTATTATCTGCATCGATTCAACAACCATATGGATACGGAAGAATCGTTCGAAATGCGTCAGGTCGTTTAGAACGC
ATAGTTGAAGAGAAAGATGCAACGCAAGCTGAAAAGGATATTAATGAAATTAGTTCAGGTATTTTAGCGTTTAATAATAAAACGTTGTTTG
AAAAATTAACACAAGTGAAAAATGATAATGCGCAAGGTGAATATTACCTCCCTGATGTATTGTCGTTAATTTTAAATGATGGCGGCATCGTA
GAAGTCTATCGTACCAATGATGTTGAAGAAATCATGGGTGTAAATGATCGTGTAATGCTTAGTCAGGCTGAGAAGGCGATGCAACGTCGT
ACGAATCATTATCACATGCTAAATGGTGTGACAATCATCGATCCTGACAGCACTTATATTGGTCCAGACGTTACAATTGGTAGTGATACAGT
CATTGAACCAGGCGTACGAATTAATGGTCGTACAGAAATTGGCGAAGATGTTGTTATTGGTCAGTACTCTGAAATTAACAATAGTACGATT
GAAAATGGTGCATGTATTCAACAGTCTGTTGTTAATGATGCTAGCGTAGGAGCGAATACTAAGGTCGGACCGTTTGCGCAATTGAGACCAG
GCGCGCAATTAGGTGCAGATGTTAAGGTTGGAAATTTTGTAGAAATTAAAAAAGCAGATCTTAAAGATGGTGCCAAGGTTTCACATTTAAG
TTATATTGGCGATGCTGTAATTGGTGAACGTACTAATATTGGTTGCGGAACGATTACAGTTAACTATGATGGTGAAAATAAATTTAAAACTA
TCGTCGGCAAAGATTCATTTGTAGGTTGCAATGTTAATTTAGTAGCACCTGTAACAATTGGTGATGATGTATTGGTGGCAGCTGGTTCCACA
ATCACAGATGACGTACCAAATGACAGTTTAGCTGTGGCAAGAGCAAGACAAACAACAAAAGAAGGATATAGGAAATAA

Gene: prs (ribose-phosphate pyrophosphokinase)

Position: 519977 to 520942, length: 966 nt, orientation: FORWARD

Perfect match to: (MW2-BA000033-[513748:514713], allele observed in CC1+CC15+CC80+CC96+CC772)

Sequence:

ATGTTAAATAATGAATATAAGAATTCGTCATTAAAGATTTTTTCATTGAAAGGAAACGAAGCATTAGCGCAAGAAGTTGCTGACCAAGTAG
GAATTGAACTAGGTAAATGTTCAGTTAAACGTTTTAGTGATGGAGAAATTCAAATTAATATCGAAGAGAGTATTCGTGGTTGTGACGTATTT
ATTATTCAACCAACATCATATCCTGTGAATCTACATTTAATGGAATTATTAATTATGATTGACGCTTGTAAACGTGCTTCTGCAGCAACAATC
AATATTGTAGTGCCATATTATGGATATGCAAGACAAGATAGAAAAGCCCGTAGCCGTGAGCCAATCACAGCTAAATTAGTTGCAAACTTAA
TCGAAACAGCTGGCGCAACTCGTATGATTGCGTTAGACTTACATGCACCACAAATTCAAGGATTCTTTGATATTCCAATTGACCACTTAATG
GGTGTGCCAATTCTTGCTAAACATTTCAAAGATGATCCGAATATTAACCCAGAAGAATGTGTCGTTGTTTCACCAGACCATGGCGGTGTTAC
ACGTGCACGTAAATTAGCTGACATTTTAAAAACTCCAATTGCAATTATAGATAAACGTCGTCCTAGACCAAATGTTGCTGAAGTGATGAACA
TTGTTGGTGAGATTGAAGGACGTACGGCAATTATTATTGACGATATTATTGATACAGCAGGTACAATCACTTTAGCTGCACAAGCATTAAAA
GATAAAGGTGCTAAAGAAGTATATGCTTGTTGTACACACCCTGTTTTATCAGGACCGGCTAAAGAACGTATCGAAAATTCTGCTATAAAAG
AATTAATCGTAACAAACTCAATTCATTTAGATGAAGACCGCAAACCATCTAACACTAAAGAATTATCTGTTGCTGGTTTAATCGCACAAGCT
ATCATTCGTGTATACGAAAGAGAATCAGTTAGCGTATTATTTGACTAA

Gene: rplY (50S ribosomal protein L25)

Position: 521092 to 521745, length: 654 nt, orientation: FORWARD

Perfect match to: (N315-BA000018-[529681:530334], highly conserved allele)

Sequence:

ATGGCTTCATTAAAGTCAATCATCCGTCAAGGTAAACAAACACGTTCAGATCTTAAACAATTAAGAAAATCTGGTAAAGTACCAGCAGTAGT
ATACGGTTACGGTACTAAAAACGTGTCAGTTAAAGTTGATGAAGTAGAATTCATCAAAGTTATCCGTGAAGTAGGTCGTAACGGTGTTATC
GAATTAGGCGTTGGTTCTAAAACTATCAAAGTTATGGTTGCAGACTACCAATTCGATCCACTTAAAAACCAAATTACTCACATTGACTTCTTA
GCAATCAATATGAGTGAAGAACGTACTGTTGAAGTACCAGTTCAATTAGTTGGTGAAGCAGTAGGCGCTAAAGAAGGCGGCGTAGTTGAA
CAACCATTATTCAACTTAGAAGTAACTGCTACTCCAGACAATATTCCAGAAGCAATCGAAGTAGACATTACTGAATTAAACATTAACGACAG
CTTAACTGTTGCTGATGTTAAAGTAACTGGCGACTTCAAAATCGAAAACGATTCAGCTGAATCAGTAGTAACAGTAGTTGCTCCAACTGAAG
AACCAACTGAAGAAGAAATCGAAGCTATGGAAGGCGAACAACAAACTGAAGAACCAGAAGTTGTTGGCGAAAGCAAAGAAGACGAAGA
AAAAACTGAAGAGTAA

Gene: pth (peptidyl-tRNA hydrolase)

Position: 522056 to 522628, length: 573 nt, orientation: FORWARD

Perfect match to: (11819-97-CP003194-[527999:528571], allele observed in CC80+CC1+CC96)

Sequence:

ATGAAATGTATTGTAGGTCTAGGTAATATAGGTAAACGTTTTGAACTTACAAGACATAATATCGGCTTTGAAGTCGTTGATTATATTTTAGA
GAAAAATAATTTTTCATTAGATAAACAAAAGTTTAAAGGTGCATATACAATTGAACGAATGAACGGCGATAAAGTGTTATTTATCGAACCAA
TGACAATGATGAATTTGTCAGGAGAAGCAGTTGCACCGATTATGGATTATTACAATGTTAATCCAGAAGATTTAATTGTCTTATATGATGAT
TTAGATTTAGAACAAGGACAAGTTCGCTTAAGACAAAAAGGAAGTGCGGGCGGTCACAATGGTATGAAATCAATTATTAAAATGCTTGGT
ACAGACCAATTTAAACGTATTCGTATTGGTGTGGGAAGACCAACGAATGGTATGACGGTACCTGATTATGTTTTACAACGCTTTTCAAATGA
TGAAATGGTAACGATGGAAAAAGTTATCGAACACGCAGCACGCGCAATTGAAAAGTTTGTTGAAACATCACGATTTGACCATATTATGAAT
GAATTTAATGGTGAAGTGAAATAA

Gene: mfd (transcription-repair-coupling factor)

Position: 522628 to 526134, length: 3507 nt, orientation: FORWARD

Perfect match to: (PSP1996-ANHU01000063-[15764:19270:r], allele observed in CC8+CC22)

Sequence:

ATGACAATATTGACAACGCTTATAAAAGAAGATAATCATTTTCAAGACCTTAATCAGGTATTTGGACAAGCAAACACACTAGTAACTGGTCT
TTCCCCGTCAGCTAAAGTGACGATGATTGCTGAAAAATATGCACAAAGTAATCAACAGTTATTATTAATTACCAATAATTTATACCAAGCAG
ATAAATTAGAAACAGATTTACTTCAATTTATAGATGCTGAAGAATTGTATAAGTATCCTGTGCAAGATATTATGACCGAAGAGTTTTCAACA
CAAAGCCCTCAACTGATGAGTGAACGTATTAGAACTTTAACTGCGTTAGCTCAAGGTAAGAAAGGGTTATTTATCGTTCCTTTAAATGGTTT
GAAAAAGTGGTTAACTCCTGTTGAAATGTGGCAAAATCACCAAATGACATTGCGTGTTGGTGAGGATATCGATGTGGACCAATTTCTTAAC
AAATTAGTTAATATGGGGTACAAACGGGAATCCGTGGTATCGCATATTGGTGAATTCTCATTGCGAGGAGGTATTATCGATATCTTTCCGCT
AATTGGGGAACCAATCAGAATTGAGCTATTTGATACCGAAATTGATTCTATTCGGGATTTTGATGTTGAAACGCAGCGTTCCAAAGATAATG
TTGAAGAAGTCGATATCACAACTGCAAGTGATTATATCATTACTGAAGAAGTGATCAGCCATCTTAAAGAAGAGTTAAAAACTGCATATGA
AAATACAAGACCCAAAATAGATAAATCAGTGCGCAATGATTTGAAAGAAACGTATGAAAGCTTTAAATTATTCGAAAGTACATACTTTGATC
ATCAAATACTACGTCGCTTAGTAGCGTTTATGTATGAAACACCTTCGACAATTATTGAGTATTTCCAAAAAGATGCAATCATTGCAGTTGATG
AATTTAATCGTATTAAAGAAACTGAAGAAAGTTTAACAGTAGAGTCTGATTCGTTTATTAGCAATATTATTGAAAGTGGTAATGGATTTATA
GGACAAAGTTTTATAAAATATGATGATTTTGAAACATTGATTGAAGGCTATCCTGTCACTTATTTTTCATTATTCGCTACAACAATGCCGATA
AAACTAAATCATATTATTAAATTTTCATGTAAACCTGTCCAACAATTTTATGGGCAATATGACATTATGCGTTCTGAATTTCAACGATATGTTA
ATCAAAACTATCATATCGTGGTTTTGGTCGAAACCGAAACTAAAGTTGAACGTATGCAAGCGATGTTAAGTGAAATGCATATTCCATCAATA
ACAAAATTGCATCGCTCAATGTCATCGGGACAAGCAGTGATTATTGAGGGCAGTTTATCTGAAGGATTTGAACTACCTGATATGGGATTAG
TTGTCATTACTGAGCGTGAGCTTTTTAAATCAAAACAGAAAAAGCAACGAAAACGTACGAAAGCTATCTCAAATGCTGAAAAAATTAAGTC
TTACCAAGATTTAAATGTGGGAGATTATATTGTTCATGTGCATCATGGTGTTGGTAGATATTTAGGTGTTGAGACGCTCGAAGTGGGGCAA
ACGCATCGTGATTATATTAAATTGCAATATAAAGGTACGGATCAACTATTTGTTCCAGTAGATCAAATGGATCAAGTTCAAAAATATGTAGC
TTCGGAAGATAAGACGCCAAAATTAAATAAACTCGGTGGCAGTGAATGGAAAAAAACAAAAGCTAAAGTTCAACAAAGTGTTGAAGATAT
TGCTGAAGAGTTGATTGATTTATATAAAGAAAGAGAAATGGCAGAAGGTTATCAATATGGGGAAGACACAGCTGAGCAAACAACATTTGA
ATTAGATTTTCCATATGAACTTACGCCTGACCAAGCTAAATCTATCGATGAAATTAAAGATGACATGCAAAAATCGCGTCCAATGGATCGCT
TGCTATGTGGTGATGTTGGTTATGGTAAAACTGAAGTTGCAGTGAGAGCAGCATTCAAAGCTGTAATGGAAGGAAAGCAGGTTGCATTTTT
AGTTCCTACAACTATTTTAGCTCAGCAACATTATGAGACGTTAATTGAGCGTATGCAAGATTTTCCTGTTGAAATTCAATTAATGAGTCGTTT
TAGAACGCCTAAAGAGATAAAACAAACTAAGGAAGGACTTAAAACTGGATTTGTTGACATAGTTGTTGGTACACACAAATTACTTAGTAAA
GATATACAGTATAAAGATTTAGGGCTGTTGATTGTAGATGAAGAACAACGATTTGGTGTACGCCATAAAGAGCGTATTAAAACATTAAAAC
ATAATGTAGATGTACTAACATTGACTGCAACCCCAATACCTAGAACATTGCATATGAGTATGCTAGGTGTGCGCGATTTGTCAGTGATTGAA
ACGCCGCCAGAAAATCGTTTCCCAGTTCAAACATATGTATTAGAACAGAACATGAGTTTTATCAAAGAAGCTTTAGAAAGAGAACTATCCC

GTGATGGCCAAGTGTTTTATCTTTATAATAAAGTGCAATCCATTTATGAAAAACGAGAACAACTCCAGATGTTAATGCCAGATGCTAACATT
GCAGTTGCTCATGGACAAATGACAGAGCGCGATTTAGAAGAAACGATGTTAAGTTTTATCAATAATGAATATGATATTTTAGTAACGACGA
CGATTATTGAAACAGGTGTCGATGTCCCAAATGCAAATACTTTGATCATTGAAGATGCAGATCGCTTTGGATTGAGTCAGTTGTATCAATTA
AGAGGTCGTGTTGGTCGTTCAAGTCGTATTGGTTATGCATACTTCTTACATCCAGCAAATAAGGTACTAACTGAGACTGCAGAAGATCGATT
ACAAGCGATTAAAGAATTTACGGAGTTAGGCTCAGGATTTAAGATTGCGATGCGTGATTTGAACATTCGTGGTGCTGGTAATTTGTTAGGT
AAACAACAGCACGGCTTTATTGATACAGTTGGATTTGATTTGTACAGTCAAATGTTAGAAGAAGCTGTAAATGAAAAACGTGGTATTAAGG
AACCAGAATCTGAGGTGCCAGAAGTCGAAGTTGATTTAAACTTGGATGCATATTTGCCAACAGAATATATTGCAAATGAACAAGCTAAAAT
TGAAATTTATAAAAAGCTACGAAAAACTGAAACATTTGATCAAATTATCGACATTAAAGATGAATTAATTGATCGTTTCAATGATTATCCTGT
TGAAGTAGCACGTTTGCTTGATATAGTGGAAATAAAAGTACACGCATTACATTCAGGTATCACGTTGATTAAAGATAAAGGGAAAATAATT
GATATTCATTTATCTGTAAAAGCCACTGAAAATATTGATGGCGAAGTGCTGTTCAAAGCAACACAACCTTTAGGTAGAACAATGAAGGTTG
GTGTTCAAAATAATGCAATGACAATTACTTTAACGAAACAAAATCAATGGCTTGATAGTTTGAAGTTTTTAGTTAAGTGCATTGAAGAAAGT
ATGAGAATCAGTGATGAAGCATAA

Gene: yabM (putative teichoic acid exporter)

Position: 526124 to 527650, length: 1527 nt, orientation: FORWARD

Perfect match to: (11819-97-CP003194-[532067:533593], allele observed in CC80)

Sequence:

ATGAAGCATAAAGATGCATTTAATGGCGTTGTCGTGTTAACTGCTGCATTAATTGTCATTAAAATTCTGAGTGCTGTATATCGAATTCCATAT
CAAAATATATTAGGCGATACAGGTTTGTATGCATATCAACAAGTGTATCCAATTGTAGCATTAGGAATGATATTATCGATGAATGCCATTCC
TAGTGCAATTACACAAAATATAGGAAAGTATCATAGTGACGAAGCATATGCAAAAGCAGTCGCTTATATACAATTAGTTGGTATATTAATAT
TTATTGCTATTTTTGTGTTTGCAAACAATATTGCACATATGATGGGTGATGGCCATTTAACACCAATGATTCAAGCTGCAAGTTTAAGCTTTA
TATTTATAGGTATGCTTGGCGTGTTAAGAGGTTATTATCAATCTGCAAATAATATGACAGTTCCGGCTATTTCCCAGGTTATAGAACAAGTT
ATACGAGTAGGTATTATCATTGTTACTATTGTTATTTTTGTAGACAGAGGTTGGACGATATATGAAGCGGGAACAATTGCTATTTTAGCATC
AACGATAGGTTTTTTAGGTTCTTCAATTTATTTAGTAGCGCACCGACCTTTTAAGTTTAAAATGGTAAATAACACTGCAAAGATTGTTTGGAA
ACAGTTCGCACTTTCGGTTTTGATTTTCGCTATCAGTCAATTAATCGTAATTTTATGGCAAGTGATTGATAGTGTTACTATTATTAAGTCACTT
CAAGCGATACGCGTGCCATTCGATGTTGCCATAACTGAAAAAGGAGTCTATGACCGTGGTGCATCATTTATTCAGATGGGATTGATTGTAA
CTACAACATTTAGTTTTGCGCTCATTCCTCTGTTAAGTGACGCAATCAAAATGAATAATCAGGTACTTATGAATCGTTATGCAAATGCGTCAT
TAAAGATTACGATTTTAATAAGTACAGCAGCGGGAATAGGATTAATTAATTTATTGCCTTTAATGAACGGTGTGTTTTTTAAGACGAATGAT
TTAACCTTAACGTTAAGTGTTTATATGATTACGGTCATTTGTGTATCGTTAATTATGATGGATATGGCTTTATTACAAGCGCAACATGCTGTG
AGACCTATTTTTGTTGGTATGACGGCAGGATTGGTTATTAAATTTATACTTAATATCATTTTGATTCGTTTAAGTGGCATTATTGGTGCGAGC
ATTAGTACTGTTGTATCATTAATTATATTCGGTACGATTATCCATATTGCTGTCACGAGAAAATACCACTTACATGCGATGAGACGATTTTTT
ATCAATGTTGTTTTAGGTATGGTATTTATGTCGATTGTTGTTCAATGCGTGTTAAACATAGTGACAACACACGGTAGATTCACTGGACTCATT
GAATTATTATGTGCAGCAGTATTAGGTATCATTGCATTGTTTTTCTATATTTTTAGATTTAATGTTTTGACATATAAAGAGTTAACTTATTTAC
CATTTGGTTCAAAGTTGTATCAAATTAAGAAAGGAAGACGTTGA

Gene: mazG (tetrapyrrole methylase)

Position: 527650 to 528843, length: 1194 nt, orientation: FORWARD

Perfect match to: (Strain_21266-AFTT01000012-[13041:14234:r], allele observed in CC12+CC80)

Sequence:

ATGGCACATACCATTACGATTGTTGGCTTAGGAAACTATGGCATTGATGATTTGCCGCTAGGGATATATAAATTTTTAAAGACACAAGATAA
AGTTTATGCAAGAACGTTAGATCATCCAGTTATAGAATCATTGCAAGATGAATTAACATTTCAGAGTTTTGACCATGTTTATGAAGCACATG
ACCAATTTGAAGATGTCTATAATGATATTGTGGCGCAATTGGTTGAAGCTGCTAATGAAAAAGATATTGTCTATGCGGTTCCGGGTCATCCT
AGAGTTGCTGAGACAACTACAGTGAAATTACTGGCTTTAGCAAAGGACAATACTGATATAGATGTGAAAGTTTTAGGTGGTAAAAGCTTTA
TTGATGATGTGTTTGAAGCAGTTAATGTAGATCCAAATGATGGCTTCACACTGTTAGATGCGACATCATTACAAGAAGTAACACTTAATGTT
AGAACGCATACATTGATTACGCAAGTTTATAGTGCAATGGTTGCTGCTAATTTGAAAATCACTTTAATGGAACGATATCCTGATGATTACCC
TGTTCAAATTGTCACTGGTGCACGAAGCGATGGTGCGGATAACGTTGTGACATGCCCATTATATGAATTGGATCATGATGAAAATGCATTC
AATAATTTGACGAGTGTATTCGTACCAAAAATCATAACATCGACATATTTGTATCATGACTTTGATTTTGCAACGGAAGTGATTGATACTTTA
GTTGATGAAGATAAAGGTTGTCCATGGGATAAAGTGCAAACGCATGAAACGCTTAAGCGTTATTTACTTGAAGAAACATTTGAATTGTTCG
AAGCTATTGACAATGAAGATGATTGGCATATGATTGAAGAGCTAGGAGATATTTTATTACAAGTGTTATTGCATACTAGTATTGGTAAAAA
AGAAGGGTATATCGACATTAAAGAAGTGATTACAAGTCTTAATGCTAAAATGATTCGTAGACACCCACACATATTTGGTGATGCCAATGCT
GAAACTATCGATGACTTAAAAGAAATTTGGTCTAAGGCGAAAGATGCTGAAGGTAAACAGCCAAGAGTTAAATTTGAAAAAGTATTTGCA

GAGCATTTTTTAAATTTATATGAGAAGACGAAGGATAAGTCATTTGATGAGGCCGCGTTAAAGCAGTGGCTAGAAAAAGGGGAGAGTAAT
ACATGA

Gene: hslR (ribosome-associated heat shock protein Hsp15)

Position: 528840 to 529103, length: 264 nt, orientation: FORWARD

Perfect match to: (11819-97-CP003194-[534783:535046], allele observed in CC80+CC12+CC72+CC80+CC97)

Sequence:

ATGAGATTAGATAAATATTTAAAAGTATCACGGTTAATAAAGCGACGTACGCTAGCAAAAGAAGTAAGTGATCAAGGTAGAATTACAATAA
ATGGTAATGTTGCTAAAGCTGGATCGGATGTTAAAGTTGAAGATGTGCTGACGATTCGCTTTGGTCAAAAATTAGTAACAGTTAAAGTAAC
TGCATTAAATGAACATGCATCTAAAGATAACGCAAAGGGCATGTATGAAATCATTGAAGAGCGTCGACTTGAAGAAGCGTAA

Gene: divIC (cell-division protein)

Position: 529121 to 529513, length: 393 nt, orientation: FORWARD

Perfect match to: (11819-97-CP003194-[535064:535456], highly conserved allele)

Sequence:

ATGAAAAAATAAAGTAGAACATATAGAAAATCAGTACACGTCGCAAGAGAACAAGAAAAAACAACGTCAAAAAATGAAAATGCGTGTTGTT
CGTAGGCGTATTACAGTATTTGCGGGCGTATTACTTGCGATAATTGTTGTTTTATCAATCTTGCTTGTTGTCCAAAAACATCGCAATGATATC
GATGCACAGGAGCGAAAAGCGAAAGAAGCACAGTTTCAAAAGCAACAAAATGAAGAAATTGCGTTAAAAGAAAAGTTGAATAATCTGAA
TGACAAAGATTATATTGAAAAAATTGCGCGTGATGATTATTACTTAAGCAACAAAGGTGAAGTGATTTTTAGGTTGCCAGAAGACAAAGAT
TCGTCTAGCTCAAAATCTTCGAAAAAAATAA

Gene: yabR (putative RNA degradation protein)

Position: 529618 to 530019, length: 402 nt, orientation: FORWARD

Perfect match to: (MW2-BA000033-[523389:523790], highly conserved allele)

Sequence:

ATGTCAATCGAAGTTGGAAATAAGCTTAAAGGTAAAGTCACTGGTATTAAAAAGTTTGGTGCATTCGTAGAATTACCTGAAGGAAAAAGTG
GTTTAGTTCACATTAGTGAAGTCGCAGATAATTATGTTGAAAACGTAGAAGAGCACCTTTCTGTTGGTGATGAAGTAGACGTAAAAGTATT
ATCTATTGCTGATGATGGAAAAATTAGTCTTTCAATTAAGAAAGCTAAAGACCGTCCACGTAGACAACATACGAGTAAACCAAGTCATCAA
AAACCAGTGCAAAAAGCCGAAGATTTTGAAAAGAAATTAAGCAATTTCTTAAAAGATAGTGAAGATAAATTAACTTCAATCAAACGTCAAA
CAGAATCTAGACGCGGTGGCAAAGGTTCAAGACGTTAA

Gene: tilS (tRNA (Ile)-lysidine synthase)

Position: 530199 to 531494, length: 1296 nt, orientation: FORWARD

Perfect match to: (KLT6-APFH01000003-[82827:84122], allele observed in CC12+CC72+CC80)

Sequence:

ATGCAGTTAAATAGTAATGGTTGGCATGTTGATGACCATATTGTTGTCGCTGTTTCTACAGGTATTGATAGTATGTGTTTATTGTATCAACTA
CTAAATGATTATAAAGATAATTATAGAAAACTAACATGCTTACATGTCAATCATGGCGTTAGGTCAGCTTCAATCGAGGAAGCCAGATTTTT
AGAAGCATACTGCGAACGTCATCACATCGATTTACATATCAAAAAGTTAGATTTGTCGCATAGTCTCGACCGAAATAACAGCATTCAGAATG
AAGCTCGAATTAAACGTTACGAATGGTTTGATGAAATGATGAATGTATTAGAAGCGGATGTATTGCTAACGGCGCATCATTTGGACGATCA
ATTAGAAACTATTATGTATCGTATTTTTAATGGGAAATCGACGCGTAATAAACTAGGATTTGATGAGTTATCGAAGCGAAAAGGTTATCAG
ATTTATCGACCACTTTTAGCTGTCTCTAAAAAAGAAATAAAACAATTCCAAGAGAGATATCATATTCCATATTTTGAAGATGAATCTAATAAA
GATAACAAATATGTTAGAAATGATATTCGTAATAGAATTATTCCAGCTATTGATGAAAATAATCAACTTAAAGTATCGCATTTATTAAAATTA
AAACAATGGCATGATGAACAATATGATATTTTGCAATATTCAGCTAAACAATTTATTCAAGAATTTGTGAAGTTTGATGAAAAGTCAAAATA
TTTAGAGGTTTCTAGACAAGCTTTTAATAACTTACCAAACTCATTAAAGATGGTTGTGTTGGATTGCCTATTATCAAAGTATTATGAGTTGTT

TAATATTAGTGCTAAAACATACGAAGAGTGGTTTAAACAATTTAGTAGTAAGAAAGCACAATTCAGTATTAATCTCACGGATAAATGGATA
ATTCAAATCGCATATGGTAAATTAATAATAATGGCTAAAAATAATGGCGATACATATTTTAGAGTTCAAACTATTGAAAAGCCAGGTAATTA
TATTTTTAACAAATATCGATTAGAGATACATTCTAATTTACCAAAATGTTTATTTCCGCTTACAGTGAGAACACGACAAAGTGGCGATACATT
TAAACTGAATGGGCGCGATGGTTATAAGAAAGTGAATCGCCTGTTTATAGATTGTAAAGTGCAACAGTGGGTTCGGGATCAAATGCCAATC
GTATTGGATAAACAACAGCGCATTATTGCGGTAGGAGATTTATATCAACAACAAACAATAAAACAATGGATTATAATTAGTAAAAATGGAG
ATGAATAG

Gene: hpt (hypoxanthine-guanine phosphoribosyltransferase)

Position: 531499 to 532038, length: 540 nt, orientation: FORWARD

Perfect match to: (RF122-AJ938182-[506747:507286], highly conserved allele)

Sequence:

ATGCATAATGATTTGAAAGAAGTATTGTTAACTGAAGAAGATATTCAAAATATCTGTAAGGAATTGGGAGCACAATTAACAAAGGATTATC
AAGGTAAACCATTAGTATGCGTGGGTATCTTAAAAGGCTCAGCAATGTTTATGTCAGATTTAATTAAACGAATTGATACCCATTTATCAATT
GATTTCATGGATGTTTCTAGTTATCACGGAGGCACTGAGTCAACTGGTGAAGTTCAAATCATTAAAGATTTAGGTTCTTCTATTGAAAATAA
AGACGTATTAATTATTGAAGATATCTTAGAGACTGGTACTACACTTAAGTCAATTACTGAATTATTACAATCTAGAAAAGTTAATTCATTAGA
AATAGTTACTTTATTAGATAAACCAAACCGTCGTAAAGCGGACATTGAAGCTAAGTATGTAGGTAAAAAAATACCAGATGAATTTGTTGTT
GGTTACGGTTTAGATTATCGTGAATTATACCGAAACTTACCATATATCGGTACGTTAAAACCTGAAGTGTATTCAAATTAA

Gene: ftsH (cell-division protein H)

Position: 532295 to 534389, length: 2095 nt, orientation: FORWARD

Sequence:

ATGCAGAAAGCTTTTCGCAATGTGCTAGTTATCGTAATAATAGGCGTTATTATTTTTGGTCTATTTTCATATTTAAACGGTAATGGAAATATG
CCGAAACAGCTTACATATAATCAATTTACTGAGAAGTTGGAAAAAGGTGACCTTAAAACTTTAGAAATCCAACCACAACAAAATGTCTATAT
GGTAAGTGGTAAAACGAAAAATGATGAAGACTATTCATCAACTATTTTATATAACAACGAAAAAGAATTACAAAAAATTACTGATGCTGCT
AAAAAGCAAAACGGTGTAAAATTAACGATTAAAGAAGAAGAAAAACAAAGTGTCTTTGTGAGTATACTTTCAACATTAATTCCAGTTGTAG
TCATAGCGTTATTATTTATTTTCTTCCTAAGCCAAGCACAAGGTGGCGGTAGTGGCGGTCGTATGATGAACTTTGGTAAATCTAAAGCGAAA
ATGTACGATAATAACAAACGTCGTGTTCGTTTCTCTGATGTAGCAGGGGCAGATGAAGAAAAACAAGAATTAATTGAAATTGTTGATTTCTT
GAAAGATAATAAAAAATTCAAAGAAATGGGATCTAGGATTCCTAAAGGTGTCTTACTTGTTGGACCTCCAGGTACTGGTAAAACATTACTT
GCTAGAGCGGTTGCAGGTGAAGCTGGCGCACCATTCTTCTCTATTAGTGGTTCAGACTTTGTAGAGATGTTTGTTGGTGTTGGTGCGAGCC
GTGTTCGTGACTTATTCGATAATGCTAAGAAAAACGCGCCTTGTATTATCTTTATCGATGAGATTGATGCTGTTGGTCGTCAACGTGGTGCA
GGTGTTGGTGGCGGTCATGATGAACGTGAACAAACCCTAAACCAATTATTAGTTGAAATGGATGGATTCGGTGAAAATGAAGGTATCATTA
TGATAGCTGCTACAAACCGTCCTGATATCCTTGACCCAGCCTTATTACGTCCAGGTCGTTTTGATAGACAAATTCAAGTTGGTCGTCCAGAT
GTGAAAGGCCGTGAAGCAATTCTTCATGTTCATGCTAAAAACAAACCACTTGATGAAACGGTTGATTTAAAAGCAATTTCACAACGTACACC
TGGTTTCTCAGGTGCTGATTTAGAGAACTTATTAAATGAAGCATCTTTAATTGCTGTACGTGAAGGTAAAAAGAAAATTGACATGAGAGAT
ATCGAAGAGGCAACGGATAGAGTTATAGCCGGACCTGCTAAGAAATCTCGAGTTATTTCTAAGAAAGAACGTAATATTGTTGCTCATCACG
AAGCTGGTCATACAATTATCGGTATGGTACTTGATGAGGCAGAAGTAGTGCATAAAGTTACTATTGTTCCACGTGGACAAGCAGGTGGTTA
TGCAATGATGCTACCTAAACAAGATCGTTTCTTAATGACTGAACAAGAGTTATTAGATAAAATCTGTGGTTTACTTGGTGGACGTGTATCAG
AAGATATTAACTTTAACGAAGTATCAACAGGTGCTTCAAATGACTTCGAACGTGCAACACAAATCGCACGCTCAATGGTTACGCAATATGGT
ATGAGTAAAAAATTAGGACCATTACAGTTCGGTCATAGCAATGGTCAAGTATTCTTAGGTAAAGATATGCAAGGTGAGCCTAATTATTCAA
GCCAAATCGCATATGAAATTGATAAAGAAGTTCAACGAATCGTTAAAGAACAATACGAACGTTGTAAACAAATTTTATTAGAGCACAAAGA
ACAATTAATTTTAATTGCTGAAACATTATTAACAGAAGAAACATTAGTTGCTGAACAAATTCAATCATTATTCTACGAAGGTAAATTACCTGA
AATTGATTATGATGCAGCTAAAGTTGTTAAAGATGAAGATTCTGAATTTAATGATGGTAAATTCGGTAAATCTTATGAAGAGATTCGTAAAG
AGCAATTAGAAGATGGACAACGTGACGAAAGTGAAGATCGTAAAGAAGAAAAAGATATTGCTGAGGATAAAAAAAGAAGCTGATAAATC
TGATGAAAAAGATGAACCAGCACATCGACAAGCCCCAAATATCGAAAAACCTTACGATCCAAATCACCCAGACAATAAATAA

Gene: hslO (33 kDa chaperonin)

Position: 534617 to 535498, length: 882 nt, orientation: FORWARD

Perfect match to: (11819-97-CP003194-[540559:541440], highly conserved allele)

Sequence:

ATGACACACGATTATATTGTTAAAGCATTAGCATTTGATGGGGAGATTAGGGCTTATGCTGCTTTGACAACTGAAACTGTTCAAGAAGCAC
AAACGAGACATTATACATGGCCGACAGCATCTGCTGCAATGGGAAGAACAATGACAGCAACAGCTATGATGGGCGCAATGTTGAAAGGTG
ATCAAAAATTAACTGTCACTGTAGATGGCCAAGGACCTATTGGACGAATTATTGCCGATGCAAATGCTAAAGGCGAGGTGCGTGCTTATGT
AGACCATCCACAAACTCATTTTCCATTAAATGAGCAAGGTAAACTTGATGTAAGACGAGCAGTAGGGACAAATGGATCTATTATGGTTGTT
AAAGACGTTGGAATGAAAGACTATTTCTCTGGAGCAAGTCCAATTGTTTCAGGAGAACTTGGTGAAGATTTTACTTATTATTATGCTACAAG
TGAACAAACACCTTCATCGGTAGGTCTTGGTGTATTGGTAAATCCTGATAATACGATTAAAGCAGCAGGAGGATTTATCATTCAAGTTATGC
CAGGTGCCAAAGATGAAACAATTTCAAAATTAGAAAAAGCAATTAGTGAAATGACACCAGTTTCTAAATTAATTGAACAAGGATTAACGCC
AGAAGGATTACTAAACGAAATCTTAGGTGAAGACCATGTGCAAATTTTAGAGAAAATGCCTGTTCAATTTGAATGTAATTGTAGTCATGAG
AAATTTTTAAATGCTATTAAAGGATTGGGCGAGGCTGAGATTCAAAATATGATTAAAGAAGATCATGGTGCTGAAGCAGTATGTCATTTCT
GTGGAAATAAATATAAATATACTGAAGAAGAATTAAACGTGTTGCTAGAAAGTTTAGCGTAA

Gene: cysK (cysteine synthase)

Position: 535677 to 536609, length: 933 nt, orientation: FORWARD

Perfect match to: (RF122-AJ938182-[511167:512099], highly conserved allele)

Sequence:

ATGGCACAAAAACCAGTAGATAATATTACTCAAATTATTGGCGGTACACCGGTAGTCAAATTGAGAAATGTAGTAGATGACAATGCAGCAG
ATGTTTATGTAAAATTGGAATATCAAAATCCAGGTGGTTCTGTAAAGGATAGAATTGCTTTAGCAATGATTGAAAAAGCAGAGCGAGAAG
GCAAAATTAAACCTGGCGATACAATTGTAGAACCAACAAGTGGTAATACAGGTATCGGTTTAGCATTTGTATGTGCTGCTAAAGGATATAA
AGCAGTATTTACTATGCCCGAAACAATGAGCCAAGAGCGTCGTAATTTATTAAAAGCATACGGTGCGGAATTAGTTTTAACGCCTGGATCA
GAAGCGATGAAAGGTGCAATTAAAAAAGCTAAAGAATTGAAAGAAGAACATGGTTACTTCGAGCCACAACAATTTGAAAACCCTGCGAAC
CCTGAAGTTCATGAGTTAACTACAGGTCCTGAGTTATTACAACAATTTGAAGGGAAAACTATCGATGCGTTCCTAGCTGGTGTTGGTACTGG
TGGTACGTTATCTGGTGTAGGTAAAGTTCTGAAAAAAGAATATCCTAACATCGAAATTGTTGCTATAGAGCCTGAGGCTTCTCCAGTATTGA
GCGGTGGTGAGCCAGGTCCACATAAATTACAAGGTTTAGGTGCTGGATTTATTCCAGGCACTTTGAATACAGAAATCTATGACAGTATTATT
AAAGTAGGAAATGATACAGCGATGGAAATGTCTCGTCGAGTTGCTAAAGAGGAAGGTATTTTAGCAGGTATTTCATCAGGTGCTGCGATTT
ATGCTGCCATTCAAAAAGCAAAAGAATTAGGAAAAGGTAAAACAGTAGTAACAGTATTGCCGAGTAATGGTGAACGCTACTTATCAACACC
TTTATATTCATTCGATGACTAA

Gene: folP (dihydropteroate synthase)

Position: 536825 to 537628, length: 804 nt, orientation: FORWARD

Perfect match to: (MW2-BA000033-[530596:531399], highly conserved allele)

Sequence:

ATGACTAAAACAAAAATTATGGGCATATTAAACGTCACACCTGATTCATTCTCAGATGGTGGAAAATTTAATAATGTTGAATCAGCTATAAA
TAGAGTGAAAGCCATGATAGATGAAGGTGCTGACATTATAGATGTTGGAGGTGTTTCAACGAGACCAGGTCATGAAATGGTTTCATTAGA
AGAAGAGATGAACAGAGTATTACCTGTTGTTGAAGCTATTGTCGGTTTTGATGTAAAAATTTCAGTCGATACATTTCGAAGTGAGGTTGCT
GAAGCATGTTTAAAATTAGGCGTTGATATCATTAATGATCAATGGGCTGGGCTGTATGATCATCATATGTTCCAAATTGTAGCTAAATATGA
CGCGGAAATTATTTTAATGCATAATGGAAATGGTAATCGTGATGAACCGGTTGTCGAAGAAATGTTAACATCTTTGTTAGCACAAGCACATC
AAGCTAAAATAGCTGGTATACCTTCAAATAAAATTTGGCTAGATCCAGGTATAGGTTTCGCTAAAACTAGAAATGAAGAAGCCGAAGTTAT
GGCAAGACTGGATGAACTTGTTGCAACAGAATATCCAGTTTTATTAGCGACAAGCCGGAAACGTTTCACTAAAGAGATGATGGGTTATGAT
ACAACACCGGTTGAAAGAGATGAAGTAACTGCAGCTACGACTGCATATGGTATTATGAAAGGCGTTAGAGCAGTACGCGTTCATAATGTC
GAGTTGAATGCTAAATTAGCTAAAGGTATAGATTTTTTAAAGGAGAATGAAAATGCAAGACACAATCTTTCTTAA

Gene: folB (dihydroneopterin aldolase)

Position: 537606 to 537971, length: 366 nt, orientation: FORWARD

Perfect match to: (MW2-BA000033-[531377:531742], highly conserved allele)

Sequence:

ATGCAAGACACAATCTTTCTTAAAGGTATGCGCTTTTATGGATATCATGGTGCTTTATCAGCTGAAAATGAAATAGGGCAAATTTTCAAAGT
GGATGTAACTTTGAAAGTAGACTTAGCTGAAGCTGGGCGTACTGATAATGTTATTGATACAGTTCATTATGGTGAAGTGTTCGAAGAGGTT
AAATCAATTATGGAAGGTAAGGCCGTTAATTTACTTGAGCATCTAGCTGAACGTATTGCAAATCGTATAAATTCACAATATAATCGTGTAAT

GGAAACGAAAGTGAGAATCACTAAAGAAAACCCACCGATTCCGGGTCATTATGATGGAGTAGGTATCGAAATAGTGAGGGAGAATAAATGA

Gene: folK (2-amino-4-hydroxy-6-hydroxymethyldihydropteridine pyrophosphokinase)

Position: 537968 to 538444, length: 477 nt, orientation: FORWARD

Perfect match to: (COL-CP000046-[569392:569868], highly conserved allele)

Sequence:

ATGATTCAAGCATACTTAGGATTAGGTAGTAATATTGGTGATAGAGAAAGCCAGTTAAACGATGCTATAAAGATTTTGAATGAATATGATG
GTATTAACGTATCTAATATTTCTCCGATTTATGAAACAGCACCAGTTGGGTATACTGAGCAACCTAACTTTTTAAATTTGTGTGTTGAAATTC
AAACAACACTCACAGTATTACAACTGTTGGAATGTTGTTTGAAGACAGAAGAATGTTTACACCGTATTAGAAAGGAACGATGGGGTCCTAG
AACTTTAGATGTGGATATTTTGTTGTATGGAGAAGAAATGATAGATTTACCAAAACTGTCGGTGCCACATCCGAGAATGAATGAACGTGCA
TTTGTTTTAATCCCATTAAATGATATAGCAGCAAATGTCGTAGAACCACGTTCGAAATTGAAAGTGAAAGATTTAGTTTTTGTCGATGACAG
TGTAAAGAGATATAAATAA

Gene: lysS_leader (lysyl-tRNA synthetase leader peptide)

Position: 538838 to 538930, length: 93 nt, orientation: FORWARD

Perfect match to: (N315-BA000018-[547426:547518], highly conserved allele)

Sequence:

ATGGTTAATGATAAAGTATTAGAAACATCGAAAGAGATGTATGTTGAGCAAAAATGTCTGATATTTTATAAAACTTTAAAGGAAAATGTTT
GA

Gene: lysS (lysyl-tRNA synthase)

Position: 538983 to 540470, length: 1488 nt, orientation: FORWARD

Perfect match to: (CIGC93-AHVD01000007-[38689:40176], allele observed in CC15+CC8+CC30)

Sequence:

ATGTCAGAAGAAATGAATGACCAAATGTTGGTTCGACGTCAAAAATTACAAGAATTATATGATCTTGGTATAGACCCGTTTGGTTCTAAATT
TGACCGTTCAGGTTTATCTAGTGATTTGAAAGAAGAGTGGGACCAGTATTCTAAAGAAGAATTGGTAGAAAAAGAAGCGGATAGTCATGT
CGCTATAGCTGGACGATTAATGACTAAGCGTGGTAAAGGTAAAGCAGGATTTGCACACGTTCAGGACTTAGCTGGACAAATTCAAATTTAC
GTTCGTAAAGATCAAGTTGGCGATGACGAATTTGATTTATGGAAAAATGCTGATTTAGGCGATATCGTTGGTGTTGAAGGTGTAATGTTCA
AAACAAATACTGGCGAATTATCGGTTAAAGCGAAGAAATTCACGCTACTAACTAAATCATTGCGACCATTACCGGATAAATTCCACGGTTTA
CAGGATATTGAACAGAGATATCGTCAAAGATATTTAGATTTAATTACGAACGAAGATAGCACTCGTACATTTATTAATCGTAGTAAAATCAT
TCAAGAAATGCGTAATTATTTAAATAATAAAGGTTTCTTGGAAGTAGAAACACCTATGATGCACCAAATTGCTGGTGGAGCAGCTGCTAGA
CCATTTGTAACACATCATAATGCATTAGATGCAACGTTATACATGCGTATTGCTATTGAGTTGCATTTAAAACGTTTAATTGTCGGTGGACTT
GAAAAAGTATATGAAATTGGTAGAGTATTCCGTAATGAAGGTGTATCAACTAGACATAACCCTGAATTCACAATGATTGAATTATATGAAG
CATATGCAGATTATCATGACATTATGGATTTAACAGAATCTATGGTGAGACATATTGCCAATGAAGTATTAGGTTCTGCAAAAGTACAATAC
AATGGGGAAACGATTGATTTAGAATCTGCTTGGACTCGTTTGCATATTGTTGATGCTGTAAAAGAAGCTACTGGTGTGGATTTTTATGAAGT
TAAAAGTGATGAAGAAGCTAAAGCTTTAGCTAAAGAACATGGTATTGAAATTAAAGATACAATGAAATATGGTCATATTTTAAATGAATTCT
TTGAGCAAAAAGTTGAAGAAACACTTATTCAGCCAACGTTTATCTATGGTCATCCGACTGAAATTTCACCTTTAGCGAAGAAAAATCCTGAA
GATCCTAGATTTACTGATCGTTTCGAATTGTTCATTGTAGGTAGAGAGCATGCAAATGCATTTACTGAATTAAATGATCCTATTGATCAAAAA
GGTCGTTTTGAAGCGCAACTTGTTGAAAAAGCGCAAGGTAATGATGAAGCGCATGAAATGGATGAAGATTACATTGAAGCGTTAGAATAT
GGTATGCCTCCGACAGGTGGTCTTGGTATCGGTATTGACAGATTGGTTATGTTATTAACTGATTCTCCATCAATCAGAGACGTGTTATTATTC
CCTTATATGAGACAAAAATAA

Gene: rrs (16S ribosomal RNA)

Position: 541906 to 543446, length: 1541 nt

Sequence:

TTTTATGGAGAGTTTGATCCTGGCTCAGGATGAACGCTGGCGGCGTGCCTAATACATGCAAGTCGAGCGAACGGACGAGAAGCTTGCTTCT
CTGATGTTAGCGGCGGACGGGTGAGTAACACGTGGATAACCTACCTATAAGACTGGGATAACTTCGGGAAACCGGAGCTAATACCGGATA
ATATTTTGAACCGCATGGTTCAAAAGTGAAAGACGGTCTTGCTGTCACTTATAGATGGATCCGCGCTGCATTAGCTAGTTGGTAAGGTAAC
GGCTTACCAAGGCAACGATGCATAGCCGACCTGAGAGGGTGATCGGCCACACTGGAACTGAGACACGGTCCAGACTCCTACGGGAGCAG
CAGGGAATCTTCCGCAATGGGCGAAAGCCTGACGGAGCAACGCCGCGTGAGTGATGAAGGTCTTCGGATCGTAAAACTCTGTTATTAGGG
AAGAACATATGTGTAAGTAACTGTGCACATCTTGACGGTACCTAATCAGAAAGCCACGGCTAACTACGTGCCAGCAGCCGCGGTAATACGT
AGGTGGCAAGCGTTATCCGGAATTATTGGGCGTAAAGCGCGCGTAGGCGGTTTTAAGTCTGATGTGAAAGCCCACGGCTCAACCGTGGAG
GGTCATTGGAAACTGGAAAACTTGAGTGCAGAAGAGGAAAGTGGAATTCCATGTGTAGCGGTGAAATGCGCAGAGATATGGAGGAACAC
CAGTGGCGAAGGCGACTTTCTGGTCTGTAACTGACGCTGATGTGCGAAAGCGTGGGGATCAAACAGGATTAGATACCCTGGTAGTCCACG
CCGTAAACGATGAGTGCTAAGTGTTAGGGGTTTCCGCCCCTTAGTGCTGCAGCTAACGCATTAAGCACTCCGCCTGGGGAGTACGACCGCA
AGGTTGAAACTCAAAGGAATTGACGGGGACCCGCACAAGCGGTGGAGCATGTGGTTTAATTCGAAGCAACGCGAAGAACCTTACCAAATC
TTGACATCCTTTGACAACTCTAGAGATAGAGCCTTCGGGGACAAAGTGACAGGTGGTGCATGGTTGTCGTCAGCTCGTGTCGTGAGATGTT
GGGTTAAGTCCCGCAACGAGCGCAACCCTTAAGCTTAGTTGCCATCATTAAGTTGGGCACTCTAAGTTGACTGCCGGTGACAAACCGGAGG
AAGGTGGGGATGACGTCAAATCATCATGCCCCTTATGATTTGGGCTACACACGTGCTACAATGGACAATACAAAGGGCAGCGAAACCGCG
AGGTCAAGCAAATCCCATAAAGTTGTTCTCAGTTCGGATTGTAGTCTGCAACTCGACTACATGAAGCTGGAATCGCTAGTAATCGTAGATCA
GCATGCTACGGTGAATACGTTCCCGGGTCTTGTACACACCGCCCGTCACACCACGAGAGTTTGTAACACCCGAAGCCGGTGGAGTAACCTT
TTAGGAGCTAGCCGTCGAAGGTGGGACAAATGATTGGGGTGAAGTCGTAACAAGGTAGCCGTATCGGAAGGTGCGGCTGGATCACCTCC
TTTCT

Gene: rrl (23S ribosomal RNA)

Position: 543782 to 546465, length: 2684 nt

Sequence:

GATTAAGTTATTAAGGGCGCACGGTGGATGCCTTGGCACTAGAAGCCGATGAAGGACGTTACTAACGACGATATGCTTTGGGAGCTGTAA
GTAAGCTTTGATCCAGAGATTTCCGAATGGGGAAACCCAGCATGAGTTATGTCATGTTATCGATATGTGAATACATAGCATATCAGAAGGC
ACACCCGGAGAACTGAAACATCTTAGTACCCGGAGGAAGAAAAGAAAATTCGATTCCCTTAGTAGCGGCGAGCGAAACGGAAGAGCCCA
AACCAACAAGCTTGCTTGTTGGGGTTGTAGGACACTCTATACGGAGTTACAAAGGACGACATTAGACGAATCATCTGGAAAGATGAATCAA
AGAAGGTAATAATCCTGTAGTCGAAAATGTTGTCTCTCTTGAGTGGATCCTGAGTACGACGGAGCACGTGAAATTCCGTCGGAATCTGGGA
GGACCATCTCCTAAGGCTAAATACTCTCTAGTGACCGATAGTGAACCAGTACCGTGAGGGAAAGGTGAAAAGCACCCCGGAAGGGTGAAA
TAGAACCTGAAACCGTGTGCTTACAAGTAGTCAGAGCCCGTTAATGGGTGATGGCGTGCCTTTGTAGAATGAACCGGCGAGTTACGATTTG
ATGCAAGGTTAAGCAGTAAATGTGGAGCCGTAGCGAAAGCGAGTCTGAATAGGGCGTTTAGTATTTGGTCGTAGACCCGAAACCAGGTGA
TCTACCCTTGGTCAGGTTGAAGTTCAGGTAACACTGAATGGAGGACCGAACCGACTTACGTTGAAAAGTGAGCGGATGAACTGAGGGTAG
CGGAGAAATTCCAATCGAACCTGGAGATAGCTGGTTCTCTCCGAAATAGCTTTAGGGCTAGCCTCAAGTGATGATTATTGGAGGTAGAGCA
CTGTTTGGACGAGGGCCCTCTCGGGTTACCGAATTCAGACAAACTCCGAATGCCAATTAATTTAACTTGGGAGTCAGAACATGGGTGATAA
GGTCCGTGTTCGAAAGGGAAACAGCCCAGACCACCAGCTAAGGTCCCAAAATATATGTTAAGTGGAAAAGGATGTGGCGTTGCCCAGACA
ACTAGGATGTTGGCTTAGAAGCAGCCATCATTTAAAGAGTGCGTAATAGCTCACTAGTCGAGTGACACTGCGCCGAAAATGTACCGGGGCT
AAACATATTACCGAAGCTGTGGATTGTCCTTTGGACAATGGTAGGAGAGCGTTCTAAGGGCGTTGAAGCATGATCGTAAGGACATGTGGA
GCGCTTAGAAGTGAGAATGCCGGTGTGAGTAGCGAAAGACGGGTGAGAATCCCGTCCACCGATTGACTAAGGTTTCCAGAGGAAGGCTC
GTCCGCTCTGGGTTAGTCGGGTCCTAAGCTGAGGCCGACAGGCGTAGGCGATGGATAACAGGTTGATATTCCTGTACCACCTATAATCGTT
TTAATCGATGGGGGACGCAGTAGGATAGGCGAAGCGTGCGATTGGATTGCACGTCTAAGCAGTAAGGCTGAGTATTAGGCAAATCCGGT
ACTCGTTAAGGCTGAGCTGTGATGGGAGAAGACATTGTGTCTTCGAGTCGTTGATTTCACACTGCCGAGAAAAGCCTCTAGATAGAAAATA
GGTGCCCGTACCGCAAACCGACACAGGTAGTCAAGATGAGAATTCTAAGGTGAGCGAGCGAACTCTCGTTAAGGAACTCGGCAAAATGAC
CCCGTAACTTCGGGAGAAGGGTGCTCTTTAGGGTTAACGCCCAGAAGAGCCGCAGTGAATAGGCCCAAGCGACTGTTTATCAAAACACAG
GTCTCTGCTAAACCGTAAGGTGATGTATAGGGGCTGACGCCTGCCCGGTGCTGGAAGGTTAAGAGGAGTGGTTAGCTTCTGCGAAGCTAC
GAATCGAAGCCCCAGTAAACGGCGGCCGTAACTATAACGGTCCTAAGGTAGCGAAATTCCTTGTCGGGTAAGTTCCGACCCGCACGAAAG
GCGTAACGATTTGGGCACTGTCTCAACGAGAGACTCGGTGAAATCATAGTACCTGTGAAGATGCAGGTTACCCGCGACAGGACGGAAAGA
CCCCGTGGAGCTTTACTGTAGCCTGATATTGAAATTCGGCACAGCTTGTACAGGATAGGTAGGAGCCTTTGAAACGTGAGCGCTAGCTTAC
GTGGAGGCGCTGGTGGGATACTACCCTAGCTGTGTTGGCTTTCTAACCCGCACCACTTATCGTGGTGGGAGACAGTGTCAGGCGGGCAGT
TTGACTGGGGCGGTCGCCTCCTAAAAGGTAACGGAGGCGCTCAAAGGTTCCCTCAGAATGGTTGGAAATCATTCATAGAGTGTAAAGGCA
TAAGGGAGCTTGACTGCGAGACCTACAAGTCGAGCAGGGTCGAAAGACGGACTTAGTGATCCGGTGGTTCCGCATGGAAGGGCCATCGC
TCAACGGATAAAAGCTACCCCGGGATAACAGGCTTATCTCCCCAAGAGTTCACATCGACGGGAGGTTTGGCACCTCGATGTCGGCTCATCG
CATCCTGGGGCTGTAGTCGGTCCCAAGGGTTGGGCTGTTCGCCCATTAAAGCGGTACGCGAGCTGGGTTCAGAACGTCGTGAGACAGTTC
GGTCCCTATCCGTCGTGGGCGTAGGAAATTTGAGAGGAGCTGTCCTTAGTACGAGAGGACCGGGATG

Gene: IGR_rrl_rrf (intergenic spacer between rrl and rrf)

Position: 546466 to 546758, length: 293 nt

Sequence:

GACATACCTCTGGTGTACCAGTTGTCGTGCCAACGGCATAGCTGGGTAGCTATGTGTGGACGGGATAAGTGCTGAAAGCATCTAAGCATG
AAGCCCCCTCAAGATGAGATTTCCCAACTTCGGTTATAAGATCCCTCAAAGATGATGAGGTTAATAGGTTCGAGGTGGAAGCATGGTGACA
TGTGGAGCTGACGAATACTAATCGATCGAAGACTTAATCAAAATAAATGTTTTGCGAAGCAAAATCACTTTACTTACTATCTAGTTTTGAAT
GTATAAATTACATTCATATG

Gene: rrs (16S ribosomal RNA)

Position: 547079 to 548622, length: 1544 nt

Sequence:

TTTTATGGAGAGTTTGATCCTGGCTCAGGATGAACGCTGGCGGCGTGCCTAATACATGCAAGTCGAGCGAACGGACGAGAAGCTTGCTTCT
CTGATGTTAGCGGCGGACGGGTGAGTAACACGTGGATAACCTACCTATAAGACTGGGATAACTTCGGGAAACCGGAGCTAATACCGGATA
ATATTTTGAACCGCATGGTTCAAAAGTGAAAGACGGTCTTGCTGTCACTTATAGATGGATCCGCGCTGCATTAGCTAGTTGGTAAGGTAAC
GGCTTACCAAGGCAACGATGCATAGCCGACCTGAGAGGGTGATCGGCCACACTGGAACTGAGACACGGTCCAGACTCCTACGGAGGCAG
CAGTAGGGAATCTTCCGCAATGGGCGAAAGCCTGACGGAGCAACGCCGCGTGAGTGATGAAGGTCTTCGGATCGTAAAACTCTGTTATTA
GGGAAGAACATATGTGTAAGTAACTGTGCACATCTTGACGGTACCTAATCAGAAAGCCACGGCTAACTACGTGCCAGCAGCCGCGGTAAT
ACGTAGGTGGCAAGCGTTATCCGGAATTATTGGGCGTAAAGCGCGCGTAGGCGGTTTTAAGTCTGATGTGAAAGCCCACGGCTCAACCGT
GGAGGGTCATTGGAAACTGGAAAACTTGAGTGCAGAAGAGGAAAGTGGAATTCCATGTGTAGCGGTGAAATGCGCAGAGATATGGAGG
AACACCAGTGGCGAAGGCGACTTTCTGGTCTGTAACTGACGCTGATGTGCGAAAGCGTGGGGATCAAACAGGATTAGATACCCTGGTAGT
CCACGCCGTAAACGATGAGTGCTAAGTGTTAGGGGGTTTCCGCCCCTTAGTGCTGCAGCTAACGCATTAAGCACTCCGCCTGGGAGTACGAC
CGCAAGGTTGAAACTCAAAGGAATTGACGGGGACCCGCACAAGCGGTGGAGCATGTGGTTTAATTCGAAGCAACGCGAAGAACCTTACCA
AATCTTGACATCCTTTGACAACTCTAGAGATAGAGCTTTCCTTCGGGGACAAAGTGACAGGTGGTGCATGGTTGTCGTCAGCTCGTGTCGT
GAGATGTTGGGTTAAGTCCCGCAACGAGCGCAACCCTTAAGCTTAGTTGCCATCATTAAGTTGGGCACTCTAAGTTGACTGCCGGTGACAA
ACCGGAGGAAGGTGGGGATGACGTCAAATCATCATGCCCCTTATGATTTGGGCTACACGTGCTACAATGGACAATACAAAGGGCAGCGAA
ACCGCGAGGTCAAGCAAATCCCATAAAGTTGTTCTCAGTTCGGATTGTAGTCTGCAACTCGACTACATGAAGCTGGAATCGCTAGTAATCGT
AGATCAGCATGCTACGGTGAATACGTTCCCGGGTCTTGTACACACCGCCCGTCACACCACGAGAGTTTGTAACACCCGAAGCCGGTGGAGT
AACCTTTAGGAGCTAGCCGTCGAAGGTGGGACAAATGATTGGGGTGAAGTCGTAACAAGGTAGCCGTATCGGAAGGTGCGGCTGGATCA
CCTCCTTTCT

Gene: rrl (23S ribosomal RNA)

Position: 548940 to 551623, length: 2684 nt

Sequence:

GATTAAGTTATTAAGGGCGCACGGTGGATGCCTTGGCACTAGAAGCCGATGAAGGACGTTACTAACGACGATATGCTTTGGGAGCTGTAA
GTAAGCTTTGATCCAGAGATTTCCGAATGGGGAAACCCAGCATGAGTTATGTCATGTTATCGATATGTGAATACATAGCATATCAGAAGGC
ACACCCGGAGAACTGAAACATCTTAGTACCCGGAGGAAGAGAAAGAAAATTCGATTCCCTTAGTAGCGGCGAGCGAAACGGAGAGCCCA
AACCAACAAGCTTGCTTGTTGGGGTTGTAGGACACTCTATACGGAGTTACAAAGGACGACATTAGACGAATCATCTGGAAAGATGAATCAA
AGAAGGTAATAATCCTGTAGTCGAAAATGTTGTCTCTCTTGAGTGGATCCTGAGTACGACGGAGCACGTGAAATTCCGTCGGAATCTGGGA
GGACCATCTCCTAAGGCTAAATACTCTCTAGTGACCGATAGTGAACCAGTACCGTGAGGGAAAGGTGAAAAGCACCCCGGAAGGGTGAAA
TAGAACCTGAAACCGTGTGCTTACAAGTAGTCAGAGCCCGTTAATGGGTGATGGCGTGCCTTTGTAGAATGAACCGGCGAGTTACGATTTG
ATGCAAGGTTAAGCAGTAAATGTGGAGCCGTAGCGAAAGCGAGTCTGAATAGGGCGTTTAGTATTTGGTCGTAGACCCGAAACCAGGTGA
TCTACCCTTGGTCAGGTTGAAGTTCAGGTAACACTGAATGGAGGACCGAACCGACTTACGTTGAAAAGTGAGCGGATGAACTGAGGGTAG
CGGAGAAATTCCAATCGAACCTGGAGATAGCTGGTTCTCTCCGAAATAGCTTTAGGGCTAGCCTCAAGTGATGATTATTGGAGGTAGAGCA
CTGTTTGGACGAGGGGCCCTCTCGGGTTACCGAATTCAGACAAACTCCGAATGCCAATTAATTTAACTTGGGAGTCAGAACATGGGTGATA
AGGTCCGTGTTCGAAAGGAAACAGCCCAGACCACCAGCTAAGGTCCCAAAATATATGTTAAGTGGAAAAGGATGTGGCGTTGCCCAGACA
ACTAGGATGTTGGCTTAGAAGCAGCCATCATTTAAAGAGTGCGTAATAGCTCACTAGTCGAGTGACACTGCGCCGAAAATGTACCGGGGCT
AAACATATTACCGAAGCTGTGGATTGTCCTTTGGACAATGGTAGGAGAGCGTTCTAAGGGCGTTGAAGCATGATCGTAAGGACATGTGGA
GCGCTTAGAAGTGAGAATGCCGGTGTGAGTAGCGAAAGACGGGTGAGAATCCCGTCCACCGATTGACTAAGGTTTCCAGAGGAAGGCTC
GTCCGCTCTGGGTTAGTCGGGTCCTAAGCTGAGGCCGACAGGCGTAGGCGATGGATAACAGGTTGATATTCCTGTACCACCTATAATCGTT
TTAATCGATGGGGACGCAGTAGGATAGGCGAAGCGTGCGATTGGATTGCACGTCTAAGCAGTAAGGCTGAGTATTAGGCAAATCCGGTAC
TCGTTAAGGCTGAGCTGTGATGGGAGAAGACATTGTGTCTTCGAGTCGTTGATTTCACACTGCCGAGAAAAGCCTCTAGATAGAAAATAGG

TGCCCGTACCGCAAACCGACACAGGTAGTCAAGATGAGAATTCTAAGGTGAGCGAGCGAACTCTCGTTAAGGAACTCGGCAAAATGACCC
CGTAACTTCGGGAGAAGGGTGCTCTTTAGGGTTAACGCCCAGAAGAGCCGCAGTGAATAGGCCCAAGCGACTGTTTATCAAAACACAGGT
CTCTGCTAAACCGTAAGGTGATGTATAGGGGCTGACGCCTGCCCGGTGCTGGAAGGTTAAGAGGAGTGGTTAGCTTCTGCGAAGCTACGA
ATCGAAGCCCCAGTAAACGGCGGCCGTAACTATAACGGTCCTAAGGTAGCGAAATTCCTTGTCGGGTAAGTTCCGACCCGCACGAAAGGC
GTAACGATTTGGGCACTGTCTCAACGAGAGACTCGGTGAAATCATAGTACCTGTGAAGATGCAGGTTACCCGCGACAGGACGGAAAGACC
CCGTGGAGCTTTACTGTAGCCTGATATTGAAATTCGGCACAGCTTGTACAGGATAGGTAGGAGCCTTTGAAACGTGAGCGCTAGCTTACGT
GGAGGCGCTGGTGGGATACTACCCTAGCTGTGTTGGCTTTCTAACCCGCACCACTTATCGTGGTGGGAGACAGTGTCAGGCGGGCAGTTT
GACTGGGGCGGTCGCCTCCTAAAAGGTAACGGAGGCGCTCAAAGGTTCCCTCAGAATGGTTGGAAATCATTCATAGAGTGTAAAGGCATA
AGGAGCTTGACTGCGAGACCTACAAGTCGAGCAGGGTCGAAAGACGGACTTAGTGATCCGGTGGTTCCGCATGGAAGGGCCATCGCTCA
ACGGATAAAAGCTACCCCGGGGATAACAGGCTTATCTCCCCAAGAGTTCACATCGACGGGGAGGTTTGGCACCTCGATGTCGGCTCATCGC
ATCCTGGGGCTGTAGTCGGTCCCAAGGGTTGGGCTGTTCGCCCATTAAAGCGGTACGCGAGCTGGGTTCAGAACGTCGTGAGACAGTTCG
GTCCCTATCCGTCGTGGGCGTAGGAAATTTGAGAGGAGCTGTCCTTAGTACGAGAGGACCGGGATG

Gene: IGR_rrl_rrf (intergenic spacer between rrl and rrf)

Position: 551624 to 551916, length: 293 nt

Sequence:

GACATACCTCTGGTGTACCAGTTGTCGTGCCAACGGCATAGCTGGGTAGCTATGTGTGGACGGGATAAGTGCTGAAAGCATCTAAGCATG
AAGCCCCCTCAAGATGAGATTTCCCAACTTCGGTTATAAGATCCCTCAAAGATGATGAGGTTAATAGGTTCGAGGTGGAAGCATGGTGACA
TGTGGAGCTGACGAATACTAATCGATCGAAGACTTAATCAAAATAAATGTTTTGCGAAGCAAAATCACTTTACTTACTATCTAGTTTTGAAT
GTATAAATTACATTCATATG

Gene: pdxR (transcriptional regulator of pyridoxine metabolism)

Position: 553389 to 554771, length: 1383 nt, orientation: REVERSE

Perfect match to: (Newbould_305-AKYW01000003-[749:2131:r], allele observed in CC97+CC1+CC5+CC97)

Sequence:

TTACTTTATAATTAATGATTTTATTAGAGCGTCTACATGCGGTTTTAAAGCATCATCGTCTATACCGCCAAAGCCTAATATAAATTTAGGGGT
TTTCTTATAGTCTTGATCATCATCAAAATTATAAACTTGTAATTTTAACTTTACTTTGTTTGCTCTATCAAGACACTCTTGTAATGTTAATCCAT
TTTTTACTGTAATTGTAAAATGCATACCCGTTTCAGCACCTTGAATATCAAGCTGCTCTTTGTAAGGTTTCAATCTTTTTAAAATATAGGTTAG
TTTTCTACGATAAATTCGTCTCATTTTATTTAAATGCCTTTCAAAACCACCGGAAGATATAAACGTTGCAATAAGGTTTTGCATATGAACAGG
TACAGTGTTGCCTTCAATGTGATTTTGAGAATGATATTTTTTCATTATAGAATAGGGTAACACCATATATGCAACTCGACAGCTAGGAAAAA
TAGACTTTGAAAATGTACTGATATAAATCACTTTTTCTCCTCTTGAATATAGACCTTGAATTGCTGGAATGGGTTTGCCGAAATATCTAAACT
CGGAATCATAATCATCTTCTATAATAAATCGTTCTTCTTTTTCTTGAGCCCATTGTATTAATTGAGTTCGCTTTTTTAAGTCCATCACATATCCA
GTTGGAAATTGATGGGAAGGCGTTATATATACTATATTTTTTTGTGATTTAATAACTTCATCTACGTTTATTCCATTATCTTCAACTTCAATTT
GTTCATATTCAACTTGTTTTTTATCTAAAATATTTTTGATTGGTGGATAACTAGGTTTTTCGATAATAAATGTTGAAGTATAAAGTAAATCGAC
TAATTGATTTACTAATTGTTCGGTAGATGAGCCAATTATAATTTGATTAGGATCACAAATTACACCACGATTAGTAAATAAATAAAATGCCA
GTTGAAATCTTAAATGTAATTCTCCTTGAAAATGTCCTCTACGTAATTGATTTAAATGATTTGTATCATAAAGATCTTTGGAATACTTTCTGAA
AAGTTCTATCGGGAAATGTTTCGTATCTATTTCATCCAAATTAAAAGCATAATCATAAGCTTCATCACTCGCTTTTGGTTTATATGAATCATCA
TCAAAAAGAGAGGGGATAGGTTGATTGTTTAAAATTGTTAAAGATTCAATTTCGGACACAAAATATCCAGAGCGAGGTCTTGAATAAATGT
AACCTTCGTCTAATAGAAGTTGATATGCATGCTCTACGGTTGTTTGGCTGATAGATAAATGTTTGCTTAATTGTCTTTTAGAATAAAATTTAT
CGCCTTCTTTAAATTGACCTTCAATTATTTGTTTTTTTAATTTTTCATAAAGTTGATGGTATAAAGTGTTTTTCAA

Gene: pdxS (pyridoxal synthase subunit)

Position: 554875 to 555762, length: 888 nt, orientation: FORWARD

Perfect match to: (Newbould_305-AKYW01000003-[2235:3122], allele observed in CC97+CC772)

Sequence:

ATGAGTAAAATTATTGGATCAGACAGAGTCAAAAGAGGTATGGCTGAAATGCAAAAAGGCGGCGTTATTATGGATGTCGTTAATGCTGAG
CAAGCAAGAATTGCAGAAGAAGCTGGCGCGGTAGCAGTTATGGCATTAGAACGAGTACCTTCTGATATTAGAGCTGCTGGTGGCGTTGCA
CGTATGGCAAACCCTAAAATTGTAGAAGAAGTAATGAATGCTGTTTCTATTCCAGTCATGGCTAAAGCACGTATTGGTCATATCACTGAAGC

AAGAGTATTAGAGGCGATGGGTGTTGACTATATTGATGAATCAGAAGTGTTAACACCAGCAGATGAGGAATATCACTTAAGAAAAGACCA
ATTTACAGTACCATTTGTATGTGGATGTCGTAATTTAGGTGAAGCTGCGCGTAGAATTGGTGAAGGTGCTGCTATGTTACGTACTAAAGGT
GAACCAGGTACAGGTAATATTGTTGAAGCTGTAAGACATATGAGACAAGTTAATTCAGAAGTTAGTCGTTTGACTGTAATGAATGATGATG
AGATTATGACTTTTGCGAAAGATATCGGTGCGCCTTATGAAATTTTAAAACAAATTAAAGACAATGGTCGTTTACCGGTAGTTAACTTTGCA
GCTGGTGGCGTTGCGACTCCTCAAGATGCTGCTTTAATGATGGAATTAGGTGCCGACGGTGTATTCGTTGGATCAGGTATTTTTAAATCAG
AAGATCCAGAAAAAATTTGCTAAAGCAATTGTTCAAGCAACAACACATTACCAAGACTATGAACTAATTGGAAGATTAGCAAGTGAACTTGG
CACTGCTATGAAAGGTTTAGATATCAATCAATTATCATTAGAAGAACGTATGCAAGAGCGTGGTTGGTAA

Gene: pdxT (pyridoxal synthase subunit)

Position: 555766 to 556326, length: 561 nt, orientation: FORWARD

Perfect match to: (C427_ST42-ACSQ01000009-[2712:3272], allele observed in CC34+CC5+CC8+CC30+CC772)

Sequence:

ATGAAAATAGGTGTATTAGCATTACAAGGTGCAGTACGTGAACATATTAGACATATTGAATTAAGTGGTCATGAAGGTATTGCAGTTAAAA
AAGTTGAACAATTAGAAGAAATCGAGGGCTTAATATTACCTGGTGGCGAGTCTACAACGTTACGTCGATTAATGAATTTATATGGATTTAA
AGAGGCTTTACAAAATTCAACTTTACCTATGTTTGGTACATGCGCAGGATTAATAGTTCTAGCGCAAGATATAGTTGGTGAAGAAGGATAC
CTTAACAAGTTGAATATTACTGTACAACGAAACTCATTCGGTAGACAAGTTGACAGCTTTGAAACAGAATTAGATATTAAAGGTATCGCTAC
AGATATTGAAGGTGTCTTTATAAGAGCGCCACATATTGAAAAAGTAGGCCAAGGCGTAGATATCCTATGTAAGGTTAATGAGAAAATTGTA
GCCGTCCAGCAAGGTAAATATTTAGGCGTATCATTCCATCCTGAATTAACAGATGACTATAGAGTAACTGATTACTTTATTAATCATATTGTA
AAAAAAGCATAG

Gene: nupC1 (nucleoside permease C, locus 1)

Position: 556534 to 557748, length: 1215 nt, orientation: REVERSE

Perfect match to: (N315-BA000018-[559257:560471:r], highly conserved allele)

Sequence:

TTAAATAAAGAATCCAGCGATTGCAGCTGAAATGAAAGATACTAGTGTTGCACCGAATAATAATTTCAAACCAAAGCGGGCAACTGTATCT
CCTTTTTTGTCATTAAGTGATTTAATCGCACCTGAAATAATACCGATAGAGCTAAAGTTAGCAAATGATACTAAGAATACAGATGTAACACC
TTTTGCGTGTTCAGATAAATCACTAAGTTTACCAAGTGCTTGCATTGCTACAAATTCGTTAGATAATAGTTTTGTCGCCATAACTGAACCGGC
TTGAACTGCATCTTGCCATGGCACACCGACTAAGAATGCAAATGGTGCAAAGACAAAACCAATTAATGTTTGGAAATCCCAAGAAATAGCG
CCACCTGAAACTGTACTAAAGATATTGCTTACAATTCCATTTAATAGAGCGATAATGGCAATGTATCCGATTAACATTGCGCCTACAATGAC
AGCTACTTTAAATCCATCTAAAATATATTCTCCTAGCATTTCGAAGAATGATTGTTGTCTTTCTTCAGTTTCTTCAACTAATAATTTGTCATCTT
CTTCATTAACTTTATAAGGGTTAATAATTGAAGCGATGATGAAACCACCAAATAAGTTTAAGACAACAGCCGTTACAACATATTTAGGTTCA
ATTAAGGTAAAGTATGCACCGATAATTGAAGCAGAAACAGTCGACATTGCTGAAGCTGTTAATGTGTATAAACGTTGCTTAGGTATGTATG
GTAATTGTTTTTTAATTGAAATAAATACTTCAGATTGTCCCAAAATTGCTGCAGCAACTGCATTGTATGATTCTAAACGTCCCATACCATTAAT
TTTAGAAATTAAGAATCCTAAAACATTAATGATTAAAGGTAAAATCTTTGTGTATTGAAGGATACCGATAATCGCTGAAATAAATACGATAG
GTAATAATACACTGAAGAAGAATGGTGGTTGCTTAGGATCGATATATTGAATACCACCGAATACAAAGTTAACACCATCTGCTGCTTTTAAT
AATAAGTAGTTAAAACCGTTTGAAATACCACCAATAACCTTGATTCCCATTGTAGTTTTAAGCAAGATAAATGCAAAGATAAGCTGAATTGC
AAGTAAAATTCCTACATATTTCCAGCGAATATTTTTCCTGTCTGAGCTAAATAGAAACGCAAGTGCTAAAAAGAAGATAATTCCGATAATCC
CAATTAGAATATGCAT

Gene: ctsR (transcriptional regulator)

Position: 557906 to 558367, length: 462 nt, orientation: FORWARD

Perfect match to: (COL-CP000046-[587996:588457], highly conserved allele)

Sequence:

ATGCACAATATGTCTGACATCATAGAACAATACATCAAACGTTTATTTGAAGAGTCGAATGAAGATGTCGTTGAAATTCAGAGAGCGAATA
TCGCACAGCGTTTTGATTGCGTACCATCACAATTAAATTATGTAATCAAAACACGATTCACTAATGAACATGGTTATGAAATCGAAAGTAAA
CGTGGTGGTGGTGGTTACATCCGAATCACTAAAATTGAAAATAAAGATGCAACAGGTTATATTAATCATTTGCTTCAGCTGATTGGACCTTC
TATTTCTCAACAACAAGCTTATTATATTATTGATGGGCTTTTAGATAAAATGTTAATAAATGAACGTGAAGCTAAAATGATTCAAGCAGTTAT

TGATAGAGAAACGCTATCAATGGATATGGTTTCTAGAGATATTATTAGAGCAAATATTTTAAAACGTTTGTTACCAGTTATAAATTATTACTA
A

Gene: mcsA (modulator of CtsR-dependent repression)

Position: 558386 to 558952, length: 567 nt, orientation: FORWARD

Perfect match to: (N315-BA000018-[561109:561675], allele observed in CC5+CC7+CC80+CC97)

Sequence:

GTGCTTTGTGAAAATTGTCAACTTAATGAAGCGGAATTAAAAGTTAAAGTTACAAGTAAAAATAAAACAGAAGAAAAAATGGTGTGTCAAA
CTTGTGCTGAGGGGCACCATCCGTGGAATCAAGCTAATGAACAGCCTGAATATCAAGAACATCAAGATAATTTCGAAGAAGCATTTGTTGT
TAAGCAAATTTTACAACATTTAGCTACGAAACATGGCATTAATTTTCAAGAAGTAGCGTTTAAAGAAGAAAAACGTTGCCCATCATGTCATA
TGACTTTGAAAGATATTGCACATGTTGGTAAATTTGGGTGTGCTAATTGTTATGCAACATTTAAAGATGACATCATTGATATCGTCCGCAGA
GTTCAAGGTGGACAATTTGAGCACGTTGGAAAGACACCACATTCTTCACATAAAAAGATAGCTTTAAAGCGAAAAATCGAAGAAAAGAAT
GAATATTTGAAAAAACTTATTGAAATCCAAGATTTTGAGGAAGCAGCCATTGTTAGAGATGAAATTAAAGCACTAAAAGCTGAGAGTGAG
GTGCAACATGATGACGCATAA

Gene: mcsB (protein arginine kinase)

Position: 558942 to 559949, length: 1008 nt, orientation: FORWARD

Perfect match to: (11819-97-CP003194-[559737:560744], highly conserved allele)

Sequence:

ATGACGCATAATATTCATGATAATATCAGCCAATGGATGAAAAGTAATGAAGAAACACCAATTGTTATGTCTTCTAGAATTCGGTTAGCGCG
TAATTTAGAAAATCATGTGCATCCACTAATGTATGCTACTGAAAATGATGGATTTAGAGTTATAAATGAGGTACAAGATGCCTTGCCAAATT
TTGAATTAATGCGTCTTGATCAAATGGATCAACAAAGTAAAATGAAAATGGTTGCAAAGCATTTGATTAGTCCTGAACTAATAAAACAACCA
GCAGCCGCAGTATTAGTGAATGATGATGAATCTTTAAGTGTCATGATAAATGAAGAGGACCATATTCGTATTCAAGCTATGGGAACTGACA
CGACATTACAGGCTTTATATAATCAAGCTTCATCAATTGATGATGAATTAGATCGAAGCCTTGATATAAGTTATGATGAACAACTTGGTTATT
TAACTACATGTCCTACCAATATAGGTACTGGTATGAGAGCAAGCGTGATGCTACATTTACCAGGTCTATCTATTATGAAAAGAATGACACGG
ATTGCTCAAACCATTAATCGTTTTGGATATACAATCAGAGGTATTTACGGTGAAGGTTCGCAAGTTTATGGACATACTTATCAAGTATCCAA
CCAACTTACACTTGGTAAATCTGAGTTAGAAATCATAGAAACATTAACAGAAGTTGTTAATCAAATCATTCATGAAGAAAAACAAATACGAC
AAAAGTTAGACACTTATAATCAATTAGAAACACAAGACCGTGTTTTTCGCTCGCTAGGTATTTTACAAAACTGTAGAATGATAACTATGGAA
GAGGCTTCTTATAGATTAAGCGAAGTTAAACTTGGTATAGATTTAAATTACATTGAATTACAAAACTTTAAATTTAATGAATTGATGGTAGCT
ATACAGTCACCATTTTTATTAGATGAAGAAGATGACAAATCTGTAAAAGAAAAACGAGCAGATATACTAAGAGAACATATAAAGTAG

Gene: clpC (chaperone-like protein C)

Position: 559963 to 562419, length: 2457 nt, orientation: FORWARD

Perfect match to: (Strain_21333-AHKA01000020-[114391:116847:r], allele observed in CC80+CC1)

Sequence:

ATGTTATTTGGTAGATTAACTGAGCGTGCACAGCGCGTATTAGCACATGCACAAGAAGAAGCAATTCGTTTAAATCATTCAAATATAGGAA
CAGAACACCTATTATTGGGATTAATGAAAGAACCTGAAGGAATTGCTGCAAAAGTATTAGAAAGTTTTAATATCACTGAAGATAAAGTAAT
TGAAGAAGTTGAAAAATTAATCGGACATGGTCAAGATCATGTTGGTACATTGCATTATACACCTAGAGCTAAAAAAGTTATTGAATTATCG
ATGGATGAAGCTAGAAAATTACATCACAATTTTGTTGGAACGGAACATATTTTATTAGGCTTGATTCGTGAAAATGAAGGTGTTGCAGCAA
GAGTTTTTGCAAATCTAGATTTAAATATTACTAAAGCACGTGCACAAGTTGTGAAAGCTTTAGGAAACCCTGAAATGAGTAATAAAAATGC
ACAAGCTAGTAAGTCAAATAATACTCCAACTTTAGATAGTTTAGCTCGTGACTTAACAGTCATTGCCAAAGACGGTACATTAGATCCTGTTA
TAGGACGTGATAAAGAAATTACACGTGTAATTGAAGTATTAAGTAGACGTACGAAAAACAATCCTGTACTTATTGGAGAGCCAGGTGTTGG
TAAAACTGCTATTGCTGAAGGTTTAGCGCAAGCCATAGTGAATAATGAGGTACCAGAGACATTAAAAGATAAGCGTGTTATGTCTTTAGAT
ATGGGAACAGTAGTTGCAGGTACTAAATATCGTGGTGAATTTGAAGAGCGTCTGAAAAAGGTTATGGAAGAAATCCAACAAGCAGGTAAT
GTCATCCTATTTATTGATGAGTTGCATACTTTAGTTGGTGCTGGTGGTGCTGAAGGTGCTATCGATGCTTCGAATATTTTGAAACCGGCATT
AGCACGTGGTGAATTACAATGTATTGGTGCTACTACATTAGATGAATATCGCAAAAATATTGAAAAAGACGCGGCTTTAGAACGTCGTTTC
CAACCTGTACAAGTTGATGAACCTTCAGTAGTAGATACAGTTGCTATTTTAAAAGGATTAAGAGATCGTTACGAAGCACACCATCGTATTAA
TATTTCAGACGAAGCTATTGAAGCAGCTGTTAAATTAAGTAACAGATACGTTTCAGATCGTTTCTTACCAGATAAAGCAATTGATTTAATTG

ATGAAGCAAGTTCTAAAGTAAGACTTAAGAGTCATACGACACCTAATAATTTAAAAGAAATTGAACAAGAAATTGAAAAAGTTAAAAATGA
AAAAGATGCTGCAGTACATGCTCAAGAGTTTGAAAATGCTGCTAACCTGCGTGATAAACAAACAAAACTTGAAAAGCAATATGAAGAAGCT
AAAAATGAATGGAAGAATGCACAAAATGGCATGTCAACTTCATTGTCAGAAGAAGATATTGCTGAAGTTATTGCAGGATGGACAGGTATC
CCATTAACTAAAATCAATGAAACAGAATCTGAAAAACTTCTAAGTCTAGAAGATACATTACATGAGAGAGTTATTGGGCAAAAAGATGCTG
TTAATTCAATCAGTAAAGCGGTTAGACGTGCCCGTGCAGGGTTAAAAGATCCTAAACGACCAATTGGTAGCTTTATCTTCCTTGGACCAACT
GGTGTTGGTAAAACTGAATTAGCTAGAGCTTTAGCTGAATCAATGTTTGGCGATGATGATGCGATGATCCGTGTAGACATGAGTGAATTTA
TGGAAAAACACGCAGTGAGCCGATTAGTTGGTGCTCCTCCAGGATATGTTGGTCATGATGATGGTGGACAATTAACTGAAAAAGTTAGAC
GTAAACCATATTCTGTAATTTTATTTGATGAAATTGAAAAAGCTCATCCAGATGTATTTAATATTCTATTACAAGTTTTAGATGATGGACATTT
GACAGATACAAAAGGACGTACAGTTGATTTCAGAAATACAATTATCATAATGACATCAAACGTTGGGGCACAAGAATTACAAGATCAACGA
TTTGCTGGATTCGGTGGTTCAAGTGATGGACAAGATTATGAAACAATTCGAAAAACGATGTTAAAAGAATTAAAAAATTCATTCCGTCCAG
AATTTTTAAACCGTGTAGATGATATCATTGTATTCCATAAACTAACAAAAGAAGAATTAAAAGAAATTGTAACAATGATGGTTAATAAATTA
ACAAATCGATTATCTGAACAAAACATAAATATTATTGTTACTGATAAAGCGAAAGACAAAATCGCAGAAGAAGGATATGATCCAGAATATG
GTGCAAGACCATTAATTAGAGCGATACAAAAAACTATCGAAGATAATTTAAGTGAATTAATATTAGATGGTAATCAAATTGAAGGTAAGAA
AGTTACAGTAGATCATGATGGTAAAGAGTTTAAATATGACATTGCTGAACAAACTTCAGAAACTAAAACACCATCGCAAGCATAA

Gene: radA (DNA repair protein A)

Position: 562903 to 564267, length: 1365 nt, orientation: FORWARD

Sequence:

TTGGCCAAGAAAAAAGTGATTTTTGAATGTATGGCTTGTGGTTATCAATCTCCTAAATGGATGGGGAAATGCCCTAATTGTGGCGCTTGGA
ATCAAATGGAGGAAATTGTTGAAAAAGCAGCCAATCCTAAACATGGAGTTAAAACCAAGGAATTAGCAGGTAAAGTACAAAAATTAAATA
GTATTAAACATGAAACAACGCCGAGAGTGTTAACAGATTCAGCAGAATTCAACCGTGTATTAGGTGGAGGTATTGTGAGCGGATCGTTAGT
ACTTATTGGTGGGGATCCAGGTATTGGTAAGTCAACGTTACTTTTACAAATTTGTGCATCGTTATCTCAAAAGAAAAAAGTACTATATATTAC
TGGAGAAGAATCGCTTAGTCAGACTAAATTACGTGCAGAGCGATTAGATGAAGATTCAAGTGAATTGCAAGTATTAGCTGAAACAGATCTT
GAAGTTATTTATCAAACAGTAAAAGAAGAACAACCTGATTTATTAGTAGTGGATTCGATTCAAACAATATATCATCCTGAAATCAGCTCTGC
GCCAGGTTCTGTTTCACAAGTTCGTGAAAGTACACAAAGTTTAATGAATATTGCTAAACAAATGAACATTGCAACTTTTATAGTGGGTCATG
TAACGAAAGAAGGTCAAATTGCTGGCCCAAGATTGCTAGAACACATGGTTGATACTGTGCTTTATTTTGAAGGCGATGAACACCACGCATA
TCGAATTTTGCGAGCTGTTAAAAACCGTTTTGGTTCAACGAATGAAATGGGAATCTTCGAAATGAAGCAAAGTGGATTAAAAGGTGTAAAT
AATCCATCTGAAATGTTTTTAGAAGAACGTTCAACAAATGTTCCAGGTTCAACAATTGTTGCAACCATGGAGGGAATCAGACCACTTTTAAT
AGAAGTTCAAGCGCTGGTAACTCCAACGACTTTTAACAATCCGAGACGAATGGCAACAGGGATTGATCATAATCGATTAAGTTTGTTGATG
GCTGTTTTGGAAAAGAAAGAAAATTATCTATTACAACAACAAGATGCTTATATCAAAGTAGCTGGCGGTGTAAAGTTAACGGAGCCAGCAG
TTGATTTAAGTGTAATTGTAGCAACTGCATCTAGCTTTAAAGATAAAGCTGTCGACGGATTAGATTGCTATATTGGAGAAGTTGGTTTAACG
GGTGAGGTACGTCGTGTATCTCGGATAGAACAACGCGTGCAAGAGGCTGCAAAACTAGGTTTCAAACGTGTAATTATTCCTAAAAATAATA
TAGGCGGATGGACATATCCTGAAGGTATACAAGTAATAGGTGTAACTACTGTACATGAAGCATTGTCATTTGCTCTTCATTCATAA

Gene: pilT (PIN/TRAM domain protein)

Position: 564292 to 565365, length: 1074 nt, orientation: FORWARD

Perfect match to: (MW2-BA000033-[557578:558651], highly conserved allele)

Sequence:

GTGAATATCGTTAAACTAATGGTTATTATTATTTACTTAATTATTGGGAGCGCATTAGGAATAATTATTATTCCTGAAATTGCAAATGATCTT
GGATTACAAAACTCCAGCTTTTTAAAAAATCACTATGTAGATGGCATTATCGGTAGTATTTTTATGTTCTTAATTTTTGGTGTATTTATTAGAC
GAGTTACTAACGCTATAAAAGGTTTAGAACATTTTATTATGCGTAGAAGTGCTGTTGAAATACTATTCGCAACAATAGGTTTAATAATCGGA
TTACTTATTTCTGTTATGGTGTCGTTTATATTAGAATCAATTGGTAACTCTATTTTTAATCATTTCATTCCTGTCATAATTACGATATTACTATG
TTATTTCGGTTTCCAATTTGGCCTTAAAAAACGAGATGAAATGTTAATGTTTTTACCTGAGAATATAGCGCGTTCCATGTCACAACATACTAA
AAGTGCTACGCCAAAAATTATCGACACAAGCGCAATTATTGATGGTCGTATTTTAGAAGTCATTCGTTGCGGTTTTATCGATGGCAATATTT
TAATTCCACAAGGTGTTATTAATGAATTACAAATTGTTGCAGATTCAAATGACAGTGTTAAACGTGAAAAGGGTAAAAGAGGCTTAGATAT
TTTAAATGAATTGTATGATTTAGACTATCCTACAAAGGTTATACATCCAACTAAAACACATAGTGATATTGATACGATGTTATTAAAACTAGC
AAAACAATATCATGCAAGTATTATAACGACAGATTTCAACCTAAATAAAGTTTGTCATGTACATGGTATTAAAGCATTAAATGTTAATGATTT
ATCAGAAGCAATCAAACCTAATGTACATCAAGGTGATCAACTGCATATTTTACTGACAAAAATGGGTAAGGAGCCTGGTCAGGCAGTAGG
ATATCTAGATGATGGTACGATGGTGGTTGTTGATAATGCTAAAAATCTTATTGGCAGTCATGTCAATTTAGAAGTAGTCAGCTTATTGCAAA
CATCTTCAGGAAGAATTGTTTTTGCTAAAAAAAATCGAAGATACAGTATCATTATAA

Gene: gltX (glutamyl-tRNA synthetase)

Position: 565922 to 567376, length: 1455 nt, orientation: FORWARD

Perfect match to: (MW2-BA000033-[559208:560662], allele observed in CC1+CC30+CC80+CC188)

Sequence:

ATGAGCGATCGTATAAGAGTAAGATATGCACCAAGTCCAACTGGTTATCTTCATATTGGTAATGCAAGAACAGCATTATTCAATTACTTGTA
TGCTAAACATTACAACGGAGATTTTGTGATTCGAATTGAAGATACTGATAAAAAACGTAATTTAGAAGATGGAGAAACATCACAATTTGAT
AATCTTAAATGGTTAGGATTAGATTGGGATGAGTCTGTAGATAAAGACAATGGCTACGGACCATATCGTCAATCTGAACGTCAACATATCT
ACCAACCATTAATAGATCAGTTACTAGCAGAAGATAAAGCATATAAATGCTATATGACAGAAGAAGAATTAGAAGCTGAACGTGAAGCAC
AAATCGCTCGTGGTGAAATGCCTCGCTATGGTGGACAACATGCGCATTTGACTGAAGAACAACGTCAACAATTTGAAGCAGAAGGACGCC
AACCATCAATTCGTTTCCGAGTACCTCAAAACCAAACGTATTCATTTGATGATATGGTAAAAGGAAATATTTCATTTGATTCAAATGGTATTG
GTGACTGGGTTATCGTAAAAAAAGATGGCATTCCAACGTACAATTTTGCAGTAGCTATAGATGATCATTACATGCAAATTTCAGATGTAATT
CGTGGTGATGATCATATTTCAAACACGCCTAAACAAATTATGATTTATGAAGCATTTGGCTGGGAGCCACCTCGTTTTGGTCATATGTCATT
AATTGTTAATGAAGAACGTAAAAAGTTAAGTAAACGTGATGGGCAAATTTTACAATTTATTGAGCAATATCGTGACTTAGGTTATTTACCTG
AAGCGTTATTTAATTTTATTGCGTTATTAGGTTGGTCTCCTGAAGGTGAAGAAGAAATCTTTTCTAAAGAAGAATTTATCAAAATCTTTGATG
AAAAGCGTTTGTCAAAATCACCAGCATTTTTCGATAAGCAAAAATTAGCATGGGTTAATAACCAATATATGAAACAAAAAGATACTGAAAC
AGTATTCCAATTAGCATTACCTCATTTAATTAAAGCAAATTTGATTCCTGAGGTGCCGTCAGAAGAGGATTTATCTTGGGGACGCAAATTAA
TTGCGCTTTATCAAAAAGAAATGAGTTATGCCGGTGAAATTGTACCTTTATCAGAAATGTTCTTTAAAGAAATGCCAGCTCTTGGTGAAGAA
GAACAACAAGTGATTAATGGAGAGCAAGTACCAGAGTTAATGACGCACTTATTCAGTAAATTAGAAGCACTTGAACCATTTGAAGCGGCTG
AAATTAAAAAGACAATTAAAGAAGTTCAAAAAGAAACAGGAATAAAAGGCAAGCAATTATTTATGCCTATTCGTGTTGCTGTAACAGGCCA
AATGCATGGTCCTGAATTACCAAATACAATTGAAGTACTTGGTAAAGAAAAAGTGCTAAACCGTTTAAAACAATATAAGTAA

Gene: tbox03 (T-box leader element)

Position: 567481 to 567706, length: 226 nt

Sequence:

ATAAAACGAAGGATTAGTAATTAAATTTATACGATGCAGAGAGTGTACGGTTGCTGTGAGTACAACGTAGAAATTAATGAATGCACCTTCG
TAAAATGAATTAAATATATAATGAGAGTGATGAGCATTAAGTTGACTTAGTTTCCTTGATAATTTGGAAGCGCCCGCAATATTATTAATGTT
ATTCGCTAAATTCAGAGTGGAACCGTGCGGAAGCGCCTCTAAC

Gene: cysE (serine acetyltransferase)

Position: 567805 to 568446, length: 642 nt, orientation: FORWARD

Perfect match to: (CN1-CP003979-[544489:545130], highly conserved allele)

Sequence:

TTGTTAAAAAGAATGAGAGACGATATAAAAATGGTATTTGAGCAGGATCCAGCGGCACGTTCAACATTAGAAGTCATTACAACGTATGCAG
GTTTACATGCAGTTTGGAGTCATTTGATTGCACATAAGTTATACAACCAAAAAAAATATGTTGCAGCACGCGCGATATCTCAAATTTCAAGA
TTTTTCACAGGTATAGAAATCCATCCAGGTGCTAAAATTGGAAAGCGTCTATTTATAGATCATGGTATGGGCGTTGTAATAGGAGAAACAT
GTACAATTGGTGATAATGTGACAATCTATCAAGGCGTGACACTTGGTGGGACAGGGAAAGAAAGAGGGAAAAGACACCCAGATATAGGA
GACAATGTTTTAATAGCAGCGGGTGCGAAAGTTTTAGGAAATATTAAAATAAATTCAAATGTAAATATTGGTGCAAATTCAGTTGTTTTACA
ATCAGTTCCAAGCTATTCAACGGTTGTTGGTATACCAGGACATATTGTTAAGCAAGATGGTGTTCGAGTTGGAAAAACATTTGATCATCGCC
ATCTACCTGATCCAATTTATGAACAAATTAAGCATTTAGAACGACAACTTGAAAAGACTAGGAATGGAGAGATTCAAGATGATTACATTATA
TAA

Gene: cysS (cysteinyl-tRNA synthase)

Position: 568430 to 569830, length: 1401 nt, orientation: FORWARD

Perfect match to: (11819-97-CP003194-[569225:570625], allele observed in CC80)

Sequence:

ATGATTACATTATATAATACGCTTACACGTCAAAAAGAAGTGTTCAAGCCTATAGAACCAGGAAAAATAAAAATGTATGTATGTGGTCCTAC
TGTATATAACTACATTCATATTGGTAACGCAAGACCAGCAATTAATTATGACGTAGTGAGACGTTACTTTGAATACCAAGGATATAATGTAG
AATATGTATCAAATTTTACAGACGTAGATGATAAATTAATTAAACGTTCTCAAGAATTAAATCAGTCTGTTCCCGAAATTGCAGAAAAATAT
ATCGCAGCTTTTCATGAAGATGTTGGTGCGTTAAATGTTAGAAAAGCGACTTCAAATCCAAGGGTAATGGACCATATGGATGACATTATTC
AATTTATTAAAGATTTGGTGGATCAAGGTTATGCATATGAAAGTGGTGGCGATGTTTACTTTAGAACACGTAAATTTGAAGGTTATGGTAA
ATTAAGTCATCAATCCATAGATGACTTAAAAGTGGGTGCTCGTATAGATGCAGGAGAGCATAAAGAAGATGCACTTGATTTTACATTGTGG
AAAAAAGCGAAGCCTGGCGAGATTAGTTGGGATAGCCCATTTGGTGAAGGTAGACCAGGATGGCATATAGAATGTTCTGTAATGGCATTT
CATGAGCTAGGACCTACAATTGATATACATGCGGGTGGTTCAGATTTACAATTTCCACATCATGAAAATGAAATAGCACAATCAGAAGCAC
ATAATCATGCGCCATTTGCTAATTATTGGATGCATAATGGTTTCATTAATATTGATAATGAAAAAATGAGTAAATCACTAGGCAACTTTATTT
TAGTTCACGATATTATTAAAGAAGTTGATCCAGATGTACTAAGATTCTTTATGATTAGCGTACATTATAGAAGCCCAATTAACTATAATCTAG
AATTGGTAGAATCAGCACGTAGTGGACTAGAGCGTATTCGCAATAGTTATCAATTAATTGAAGAGCGCGCACAAATTGCTACTAATATTGA
AAATCAACAGACATATATTGATCAAATTGATGCGATTTTAAATCGTTTTGAAACAGTTATGAATGATGATTTTAATACAGCTAATGCAATTAC
AGCTTGGTATGATTTAGCAAAACTTGCGAATAAATATGTACTAGAGAACACAACATCAACAGAAGTAATTGATAAATTTAAAGCAGTTTATC
AAATTTTCAGCGATGTTTTAGGTGTACCGTTAAAATCTAAAAATGCAGATGAATTATTGGATGAAGATGTTGAAAAATTAATCGAAGAGCG
TAATGAAGCAAGGAAAAACAAAGATTTTGCACGAGCAGATGAAATTCGAGACATGCTGAAATCACAAAACATTATATTAGAAGACACACCT
CAAGGGGTTAGATTTAAACGTGGATAA

Gene: mrnC (minimal RNase III)

Position: 569823 to 570227, length: 405 nt, orientation: FORWARD

Perfect match to: (N315-BA000018-[572547:572951], highly conserved allele)

Sequence:

GTGGATAATCAACAAGATAATCACATTAAATTATTGAATCCATTGACCTTAGCATATATGGGAGACGCAGTCTTAGATCAATATGTACGTAC
CTATATCGTTTTAAAGCTTAAAAGTAAGCCTAATAAACTACATCAAATGTCTAAAAAATATGTATCTGCCAAAAGTCAGGCGCAAACGTTAG
AATATTTAATGGAGCAAGAATGGTTTACAGACGAAGAAATGGATATTTTGAAGCGAGGGCGTAACGCGAAAAGTCATACTAAAGCTAAAA
ACACTGATGTTCAAACATATCGTAAAAGTTCAGCGATAGAAGCAGTGATAGGTTTTCTTTATTTAGAAAAAAGAGAAGAACGATTAGAGGC
ATTATTAAATAAAATAATAACAATAGTAAACGAAAGGTAG

Gene: yacO (tRNA/rRNA methyltransferase)

Position: 570235 to 570981, length: 747 nt, orientation: FORWARD

Perfect match to: (11819-97-CP003194-[571030:571776], allele observed in CC80+CC5+CC97)

Sequence:

GTGGAAGATACGGTTATTGTTGGTAGGCATGCTGTTAGAGAAGCGATTATTACTGGGCATCCGATAAATAAGATATTGATTCAAGAAGGTA
TTAAAAAGCAACAAATTAATGAAATTTTAAAAAATGCAAAAGATCAAAAAAATTATTGTTCAAACTGTACCAAAATCTAAATTAGATTTTTTAG
CAAATGCACCACATCAGGGTGTTGCAGCGCTTATTGCACCATATGAATATGCTGACTTCGATCAATTTTTAAAACAGCAAAAAGAAAAAGA
AGGTTTATCGATAGTACTTATATTAGACGGCTTAGAAGACCCGCATAACTTGGGATCAATTTTAAGAACAGCCGATGCAACGGGAGTTGAT
GGTGTTATTATTCCTAAACGTCGTTCAGTTACACTAACGCAAACAGTTGCAAAAGCCTCAACAGGTGCAATTGAACATGTACCAGTTATTCG
AGTGACAAATTTAGCTAAAACTATCGATGAACTAAAAGATAATGGCTTTTGGGTAGCTGGCACTGAAGCTAATAATGCAACAGATTATAGA
AATCTAGAAGCGGACATGTCATTGGCTATTGTAATTGGTAGCGAAGGACAGGGTATGAGTCGCCTAGTAAGTGATAAATGCGATTTTTATA
TTAAGATTCCAATGGTTGGACATGTAAACAGTTTGAATGCTTCGGTTGCAGCAAGTTTAATGATGTACGAAGTATTTCGAAAAAGACATGAT
GTTGGAGAAATATAA

Gene: yacP (putative protein)

Position: 570981 to 571505, length: 525 nt, orientation: FORWARD

Perfect match to: (N315-BA000018-[573705:574229], highly conserved allele)

Sequence:

ATGAAAGAACGTTACTTAATCATTGATGGATACAATATGATAGGACAATCACCAACGCTAAGCGCCATTGCAAAAGAGAATTTAGAAGAAG
CTAGAATGCAATTAATAGATGCAATTGCAAATTATAATGCAGTTATTTCAGATGAAATTATTTGTGTTTTCGATGCTTATGACCAATCGGGTG
TTGAAAGAGAATACATGTATCATGGCGTTAAAACGATTTTTACCAAGGAAAAAGAAACAGCTGATAGTTTCATAGAACGTTATGTTTATGA

ACTTTATGACAAGCATACTAAGCATATTACAGTTGTAACAAGTGATATGAGTGAGCAACATGCTATCTTTGGATCAGGTGCATATAGAATAT
CATCTCGCGAAATGTGGAGAGATTTAAAAGAAAATGAAATTGATGTGAGTAAATCATTAGATGATATAAGTGAAAACAAGCCAAGAACTC
GAATTCCGTTATCTTCTGAAATCCTTGCAGAATTTGAAAAAATACGAAGAGGACATCATAAGAAATGA

Gene: sigH (alternative sigma subunit of RNA polymerase)

Position: 571586 to 572155, length: 570 nt, orientation: FORWARD

Perfect match to: (11819-97-CP003194-[572381:572950], allele observed in CC80+CC772)

Sequence:

TTGAAATACGATTTGACAACTCAAGACAGTACAATCAAACGTAACAATGCAATAAATGATAAAGACTTCGAAAAGTTAGTAATGGATCTAA
AACCATTAATTATTCGACGCATCAAAACATTTGGATTTAATCATTATGATTTAGAAGACTTATATCAAGAAATACTTATACGGATGTATAGGT
CGGTCCAAACATTTGATTTTAGTGGAGAGCAGCCTTTCACAAATTATGTTCAATGTTTAATTACGTCTGTAAAGTATGATTATTTGAGAAAAT
ATTTAGCTACAAATAAAAGAATGGATAATTTGATTAATGAATATAGAGTTACGTATCCATGTGCAATAAAGCGTTATGATGTTGAAAACAAT
TATTTGAATCAATTAGCAATTAAAGAGTTGATTCGTCAGTTTAAGTATTTGAGTGCATTTGAAAAAGATGTCATGTATTTAATGTGTGAACA
ATATAAGCCGAGAGAAATTGCTCAACTGATGCATGTAAAAGAGAAAGTGATTTATAATGCCATACAACGATGTAAAAATAAAATAAAACGT
TATTTCAAAACGATTTGA

Gene: rpmG3 (50S ribosomal protein L33, locus 3)

Position: 572270 to 572413, length: 144 nt, orientation: FORWARD

Perfect match to: (N315-BA000018-[574994:575137], highly conserved allele)

Sequence:

GTGAGAAAAATACCTTTAAATTGTGAAGCTTGTGGCAATAGAAATTATAATGTTCCTAAGCAAGAAGGCTCGGCAACAAGATTAACCTTAA
AGAAATATTGTCCAAAATGTAACGCGCACACAATTCATAAAGAATCGAAATAA

Gene: secE (preprotein translocase subunit E)

Position: 572469 to 572651, length: 183 nt, orientation: FORWARD

Perfect match to: (RF122-AJ938182-[543743:543925], highly conserved allele)

Sequence:

ATGGCTAAAAAAGAAAGTTTCTTTAAAGGCGTTAAGTCTGAAATGGAAAAAACAAGTTGGCCGACGAAAGAAGAGCTATTTAAATATACT
GTAATTGTAGTTTCTACTGTTATATTCTTCTTAGTCTTTTTCTATGCCTTAGATTTAGGAATTACAGCATTGAAAAATTTATTATTTGGTTAG

Gene: nusG (transcription antitermination protein)

Position: 572664 to 573212, length: 549 nt, orientation: FORWARD

Perfect match to: (MRSA252-BX571856-[586599:587147], highly conserved allele)

Sequence:

ATGTCTGAAGAAGTTGGCGCAAAGCGTTGGTATGCAGTGCATACATATTCTGGATATGAAAATAAAGTTAAAAAGAATTTAGAAAAAAGA
GTAGAATCTATGAATATGACTGAACAAATCTTTAGAGTAGTCATACCGGAAGAAGAAGAAACTCAAGTAAAAGATGGCAAAGCTAAAACG
ACTGTTAAAAAAACATTCCCTGGATATGTTTTAGTGGAATTAATCATGACAGATGAATCATGGTATGTGGTAAGAAATACACCAGGTGTTAC
TGGTTTTGTAGGTTCTGCAGGTGCAGGGTCTAAGCCAAATCCATTGTTACCAGAAGAAGTTCGCTTCATCTTAAAACAAATGGGTCTTAAAG
AAAAGACTATCGATGTTGAACTCGAAGTTGGCGAGCAAGTTCGTATTAAATCAGGTCCATTTGCGAATCAAGTTGGTGAAGTTCAAGAAAT
TGAAACAGATAAGTTTAAGCTAACAGTATTAGTAGATATGTTTGGCCGAGAAACACCAGTAGAAGTTGAATTCGATCAAATAGAAAAGCTT
TAA

Gene: rplK (50S ribosomal protein L11)

Position: 573393 to 573815, length: 423 nt, orientation: FORWARD

Perfect match to: (N315-BA000018-[576117:576539], highly conserved allele)

Sequence:

GTGGCTAAAAAAGTAGATAAAGTTGTTAAATTACAAATTCCTGCAGGTAAAGCGAATCCAGCACCACCAGTTGGTCCAGCATTAGGTCAAG
CAGGTGTGAACATCATGGGATTCTGTAAAGAGTTCAATGCACGTACTCAAGATCAAGCAGGTTTAATTATTCCGGTAGAAATCAGTGTTTAT
GAAGATCGTTCATTTACATTTATTACAAAAACTCCACCGGCTCCAGTATTACTTAAAAAAGCAGCTGGTATTGAAAAAGGTTCAGGCGAACC
AAACAAAACTAAAGTTGCTACAGTAACTAAAGATCAAGTACGCGAAATTGCTAACAGCAAAATGCAAGACTTAAACGCTGCTGACGAAGA
AGCAGCTATGCGTATTATCGAAGGTACTGCACGTAGTATGGGTATCGTTGTAGAATAA

Gene: rplA (50S ribosomal protein L1)

Position: 574023 to 574715, length: 693 nt, orientation: FORWARD

Perfect match to: (11819-97-CP003194-[574818:575510], allele observed in CC80+CC20+CC80+CC772)

Sequence:

ATGGCTAAAAAAGGTAAAAAGTATCAAGAAGCAGCTAGTAAAGTTGACCGTACTCAGCACTACAGTGTTGAAGAAGCAATTAAATTAGCT
AAAGAAACAAGTATTGCTAACTTTGACGCTTCTGTTGAAGTTGCATTCCGTTTAGGAATTGATACACGTAAAAATGACCAACAAATCCGTGG
TGCAGTTGTATTACCAAACGGAACTGGTAAATCACAAAGTGTATTAGTATTCGCTAAAGGTGACAAAATTGCTGAAGCTGAAGCAGCAGGT
GCTGACTATGTAGGTGAAGCAGAATACGTTCAAAAAATCCAACAAGGTTGGTTCGACTTCGATGTAGTAGTTGCTACACCAGACATGATGG
GTGAAGTTGGTAAATTAGGTCGTGTATTAGGACCAAAAGGTTTAATGCCAAACCCTAAAACTGGAACTGTAACAATGGATGTTAAAAAAGC
TGTTGAAGAAATCAAAGCTGGTAAAGTAGAATACCGTGCTGAAAAAGCTGGTATCGTACATGCATCAATTGGTAAAGTTTCATTTACTGAT
GAACAATTAATTGAAAACTTCAATACTTTACAAGATGTATTAGCTAAAGCTAAACCATCATCTGCTAAAGGTACATACTTCAAATCTGTTGCT
GTAACTACAACAATGGGTCCTGGAGTTAAAATTGATACTGCAAGTTTCAAATAA

Gene: rplJ (50S ribosomal protein L10)

Position: 574987 to 575487, length: 501 nt, orientation: FORWARD

Perfect match to: (ED133-CP001996-[589915:590415], highly conserved allele)

Sequence:

ATGTCTGCTATCATTGAAGCTAAAAAACAACTAGTTGATGAAATTGCTGAGGTACTATCAAATTCAGTTTCAACAGTAATCGTTGACTACCG
TGGATTAACAGTAGCTGAAGTTACTGACTTACGTTCACAATTACGTGAAGCTGGTGTTGAGTATAAAGTATACAAAAACACTATGGTACGTC
GTGCAGCTGAAAAAGCTGGTATCGAAGGCTTAGATGAATTCTTAACAGGTCCTACTGCTATTGCAACTTCAAGTGAAGATGCTGTAGCTGC
AGCGAAAGTAATTTCTGGATTTGCTAAAGATCATGAAGCATTAGAAATTAAATCAGGCGTTATGGAAGGCAATGTTATTACAGCAGAAGAA
GTTAAAACTGTTGGTTCATTACCTTCACACGATGGTCTTGTATCTATGCTTTTATCAGTATTACAAGCTCCTGTACGCAACTTCGCTTATGCGG
TTAAAGCTATTGGAGAACAAAAAGAAGAAAGCGCTGAATAA

Gene: rplL (50S ribosomal protein L7/L12)

Position: 575530 to 575898, length: 369 nt, orientation: FORWARD

Perfect match to: (N315-BA000018-[578254:578622], highly conserved allele)

Sequence:

ATGGCTAATCATGAACAAATCATTGAAGCGATTAAAGAAATGTCAGTATTAGAATTAAACGACTTAGTAAAAGCAATTGAAGAAGAATTTG
GTGTAACTGCAGCTGCTCCAGTAGCAGTAGCAGGTGCAGCTGGTGGCGCTGACGCTGCAGCAGAAAAAACTGAATTTGACGTTGAGTTAA
CTTCAGCTGGTTCATCTAAAATCAAAGTTGTTAAAGCTGTTAAAGAAGCAACTGGTTTAGGATTAAAAGATGCTAAAGAATTAGTAGACGG
AGCTCCTAAAGTAATCAAAGAAGCTTTACCTAAAGAAGAAGCTGAAAAACTTAAAGAACAATTAGAAGAAGTTGGAGCTACTGTAGAATTA
AAATAA

Gene: ybxB (16S rRNA methyltransferase C)

Position: 576073 to 576681, length: 609 nt, orientation: FORWARD

Perfect match to: (MW2-BA000033-[569359:569967], highly conserved allele)

Sequence:

ATGAGTCATTATTACGATGAAGATCCAAGTGTAATTAGCAATGAACAACGTATTCAATATCAATTAAACCATCATAAAATTGATTTAATAACT
GATAATGGAGTGTTTTCGAAAGATAAAGTAGATTATGGTTCAGATGTTCTTGTTCAAACTTTTTTAAAAGCGCATCCACCTGGTCCAAGTAA
GCGAATTGCCGATGTTGGTTGTGGTTACGGACCAATTGGTTTGATGATTGCTAAAGTATCACCACATCATTCAATTACAATGCTAGATGTTA
ATCACAGAGCGCTAGCCTTAGTTGAAAAAAACAAAAAAATAAATGGTATTGATAATGCGATCGTAAAGGAAAGTGATGCTTTGTCTGCTGT
GGAAGACAAAAGTTTTGATTTTATTTTAACCAATCCACCAATAAGAGCAGGGAAAGAAACCGTGCATCGTATATTCGAGCAAGCATTACAT
AGATTAGACTCGAACGGTGAACTATTCGTTGTAATTCAGAAGAAGCAAGGTATGCCATCTGCAAAGAAAAGAATGAATGAACTTTTTGGAA
ATGTAGAAGTGGTAAATAAAGATAAAGGATATTACATTCTGAGAAGTATAAAAGCTTGA

Gene: rpoB (DNA-directed RNA polymerase beta subunit)

Position: 576896 to 580447, length: 3552 nt, orientation: FORWARD

Perfect match to: (RF122-AJ938182-[548167:551718], highly conserved allele)

Sequence:

TTGGCAGGTCAAGTTGTCCAATATGGAAGACATCGTAAACGTAGAAACTACGCGAGAATTTCAGAAGTATTAGAATTACCAAACTTAATAG
AAATTCAAACTAAATCTTACGAGTGGTTCCTAAGAGAAGGTTTAATCGAAATGTTTAGAGACATTTCTCCAATTGAAGATTTTACTGGTAATT
TGTCATTAGAGTTTGTGGATTACCGTTTAGGAGAACCAAAATATGATTTAGAAGAATCTAAAAACCGTGACGCTACTTATGCTGCACCTCTT
CGTGTAAAAGTGCGTCTAATCATTAAAGAAACAGGAGAAGTTAAAGAACAAGAAGTCTTTATGGGTGATTTCCCATTAATGACTGATACAG
GTACGTTCGTTATCAATGGTGCAGAACGTGTAATCGTATCTCAATTAGTTCGTTCACCATCCGTTTATTTCAATGAAAAAATCGACAAAAATG
GTCGTGAAAACTATGATGCAACAATTATTCCAAACCGTGGTGCATGGTTAGAATATGAAACAGATGCTAAAGATGTTGTATACGTGCGTAT
TGATAGAACACGTAAACTACCATTAACAGTATTGTTACGTGCATTAGGTTTCTCAAGCGACCAAGAAATTGTTGACCTTTTAGGTGACAATG
AATATTTACGTAATACTTTAGAGAAAGACGGCACTGAAAACACTGAACAAGCGTTATTAGAAATCTATGAACGTTTACGTCCAGGTGAACC
ACCAACTGTTGAAAATGCTAAAAGTCTATTGTATTCACGTTTCTTTGATCCAAAACGCTATGACTTAGCAAGCGTGGGTCGTTATAAAACAA
ACAAAAAATTACATTTAAAACATCGTTTATTTAATCAAAAATTAGCTGAGCCAATTGTAAATACTGAAACTGGTGAAATTGTAGTTGAAGAA
GGTACAGTGCTTGATCGTCGTAAAATCGACGAAATCATGGATGTACTTGAATCAAATGCAAACAGCGAAGTGTTTGAATTGCATGGTAGCG
TTATAGACGAGCCAGTAGAAATTCAATCAATTAAAGTATATGTTCCTAACGATGATGAAGGTCGTACGACAACTGTAATTGGTAATGCTTTC
CCTGACTCAGAAGTTAAATGCATTACACCAGCAGATATCATTGCTTCAATGAGTTACTTCTTTAACTTATTAAGCGGTATTGGATATACAGAT
GATATTGACCATTTAGGTAACCGTCGTTTACGTTCTGTAGGTGAATTACTACAAAACCAATTCCGTATCGGTTTATCAAGAATGGAAAGAGT
TGTACGTGAAAGAATGTCAATTCAAGATACTGAGTCTATCACACCTCAACAATTAATTAATATTCGACCTGTTATTGCATCTATTAAAGAATT
CTTTGGTAGCTCTCAATTATCACAATTCATGGACCAAGCAAACCCATTAGCTGAGTTAACGCATAAACGTCGTCTATCAGCATTAGGACCTG
GTGGTTTAACACGTGAACGTGCTCAAATGGAAGTACGTGACGTTCACTACTCTCACTATGGCCGTATGTGTCCAATTGAAACACCTGAGGG
ACCAAACATTGGATTGATTAACTCATTATCAAGTTATGCACGTGTAAATGAATTCGGCTTTATTGAAACACCATATCGTAAAGTTGATTTAGA
TACACATGCTATCACTGATCAAATTGACTATTTAACAGCTGACGAAGAAGATAGCTATGTTGTAGCACAAGCAAACTCTAAATTAGATGAAA
ATGGTCGTTTCATGGATGATGAAGTTGTATGTCGTTTCCGTGGTAACAACACAGTTATGGCTAAAGAAAAAATGGATTATATGGATGTATC
GCCGAAGCAAGTTGTTTCAGCAGCGACAGCATGTATTCCATTCTTAGAAAATGATGACTCAAACCGTGCATTGATGGGTGCGAACATGCAA
CGTCAAGCAGTGCCTTTGATGAATCCAGAAGCACCATTTGTTGGTACAGGTATGGAACACGTTGCAGCACGTGATTCTGGTGCGGCTATTA
CAGCTAAGCACAGAGGTCGTGTTGAACATGTTGAATCTAATGAAATTCTTGTACGTCGTCTAGTTGAAGAGAACGGCGTTGAGCATGAAGG
TGAATTAGATCGCTATCCATTAGCTAAATTTAAACGTTCAAACTCAGGTACATGTTATAACCAACGTCCAATCGTTGCAGTTGGAGATGTTGT
TGAGTATAACGAGATTTTAGCAGACGGACCATCTATGGAATTAGGAGAAATGGCATTAGGTAGAAACGTAGTAGTTGGTTTCATGACTTGG
GACGGTTACAACTATGAGGATGCCGTTATCATGAGTGAAAGACTTGTGAAAGATGACGTGTATACTTCTATTCATATTGAAGAGTATGAAT
CAGAAGCACGTGATACTAAGTTAGGACCTGAAGAAATCACAAGAGATATTCCTAATGTTTCTGAAAGTGCACTTAAGAACTTAGACGATCG
TGGTATCGTTTATATTGGTGCAGAAGTAAAAGATGGAGATATTTTAGTTGGTAAAGTAACGCCTAAAGGTGTAACTGAGTTAACTGCCGAA
GAAAGATTGTTACATGCAATCTTTGGTGAAAAAGCACGTGAAGTTAGAGATACTTCATTACGTGTACCTCACGGCGCTGGCGGTATCGTTC
TTGATGTAAAAGTATTCAATCGTGAAGAAGGCGACGATACATTATCACCTGGTGTAAACCAATTAGTACGTGTATACATCGTTCAAAAACGT
AAAATTCATGTTGGTGATAAGATGTGTGGTCGACATGGTAACAAAGGTGTCATTTCTAAGATTGTTCCTGAAGAAGATATGCCTTACTTACC
AGACGGACGTCCGATCGATATCATGTTAAACCCTCTTGGTGTACCATCTCGTATGAACATCGGACAAGTATTAGAGCTACACTTAGGTATGG
CTGCTAAAAATCTTGGTATTCACGTTGCATCACCTGTATTTGACGGTGCAAACGATGACGATGTATGGTCAACAATTGAAGAAGCTGGTATG
GCTCGTGATGGTAAAACTGTACTTTATGATGGACGTACAGGTGAACCATTCGATAACCGTATTTCAGTAGGTGTAATGTACATGTTGAAACT
TGCGCACATGGTTGATGATAAATTACATGCGCGTTCAACAGGACCATATTCACTTGTTACACAACAACCACTTGGCGGTAAAGCGCAATTCG
GTGGACAACGTTTCGGTGAGATGGAGGTATGGGCACTTGAAGCATATGGTGCTGCATACACATTACAAGAAATCTTAACTTACAAATCCGA
TGATACAGTAGGACGTGTGAAAACATACGAGGCTATTGTTAAAGGTGAAAACATCTCTAGACCAAGTGTTCCAGAATCATTCCGAGTATTG

ATGAAAGAATTACAAAGTTTAGGTTTAGATGTAAAAGTTATGGATGAGCAAGATAATGAAATCGAAATGACAGACGTTGATGACGATGAT
GTTGTAGAACGCAAAGTAGATTTACAACAAAATGATGCTCCTGAAACACAAAAAGAAGTTACTGATTAA

Gene: rpoC (DNA-directed RNA polymerase beta' subunit)

Position: 580584 to 584207, length: 3624 nt, orientation: FORWARD

Perfect match to: (11819-97-CP003194-[581379:585002], allele observed in CC80+CC1+CC97)

Sequence:

TTGATTGATGTAAATAATTTCCATTATATGAAAATAGGATTGGCTTCACCTGAAAAAATCCGTTCTTGGTCTTTTGGTGAAGTTAAAAAACCT
GAAACAATCAACTACCGTACATTAAAACCTGAAAAAGATGGTCTATTCTGTGAAAGGATTTTCGGACCTACAAAAGACTGGGAATGTAGTT
GTGGTAAATACAAACGTGTTCGCTACAAAGGCATGGTCTGTGACAGATGTGGAGTTGAAGTAACTAAATCTAAAGTACGTCGTGAAAGAA
TGGGTCACATTGAACTTGCTGCTCCAGTTTCTCACATTTGGTATTTCAAAGGTATACCAAGCCGTATGGGATTATTACTTGACATGTCACCAA
GAGCATTAGAAGAAGTTATTTACTTTGCTTCTTATGTTGTTGTAGATCCAGGTCCAACTGGTTTAGAAAAGAAAACTTTATTATCTGAAGCTG
AATTCAGAGATTATTATGATAAATACCCAGGTCAATTCGTTGCAAAAATGGGTGCAGAAGGTATTAAAGATTTACTTGAAGAGATTGATCTT
GACGAAGAACTTAAATTGTTACGCGATGAGTTGGAATCAGCTACTGGTCAAAGACTTACTCGTGCAATTAAACGTTTAGAAGTTGTTGAAT
CATTCCGTAATTCAGGTAACAAACCTTCATGGATGATTTTAGATGTACTTCCAATCATCCCACCAGAAATTCGTCCAATGGTTCAATTAGATG
GTGGACGATTTGCAACAAGTGACTTAAACGATTTATACCGTCGTGTAATTAATCGAAATAATCGTTTGAAACGTTTATTAGATTTAGGTGCA
CCTGGTATCATCGTTCAAAACGAAAAACGTATGTTACAAGAAGCCGTTGACGCTTTAATTGATAATGGTCGTCGTGGTCGTCCAGTTACTGG
CCCAGGTAACCGTCCATTAAAATCTTTATCTCATATGTTAAAAGGTAAACAAGGTCGTTTCCGTCAAAACTTACTTGGTAAACGTGTTGACTA
TTCAGGACGTTCAGTTATTGCAGTAGGTCCAAGCTTGAAAATGTACCAATGTGGTTTACCAAAAGAAATGGCACTTGAACTATTTAAACCAT
TCGTAATGAAAGAATTAGTTCAACGTGAAATTGCAACTAACATTAAAAATGCGAAGAGTAAAATCGAACGCATGGATGATGAAGTTTGGG
ACGTATTGGAAGAAGTAATTAGAGAACATCCTGTATTACTTAACCGTGCACCAACACTTCATAGACTTGGTATTCAAGCATTTGAACCAACT
TTAGTTGAAGGTCGTGCGATTCGTCTACATCCACTTGTAACAACAGCTTATAACGCTGACTTTGACGGTGACCAAATGGCGGTTCACGTTCC
TTTATCAAAAGAGGCACAAGCTGAAGCAAGAATGTTGATGTTAGCAGCACAAAACATCTTGAACCCTAAAGATGGTAAACCTGTAGTTACA
CCATCACAAGATATGGTACTTGGTAACTATTACCTTACTTTAGAAAGAAAAGATGCAGTAAATACAGGCGCAATCTTTAATAATACAAATGA
AGTATTAAAAGCATATGCAAATGGCTTTGTACATTTACACACTAGAATTGGTGTACATGCAAGTTCGTTCAATAATCCAACATTTACTGAAG
AACAAAACAAAAAGATTCTTGCTACGTCAGTAGGTAAAATTATATTCAATGAAATCATTCCAGATTCATTTGCTTATATTAATGAACCTACGC
AAGAAAACTTAGAAAGAAAGACACCAAACAGATATTTCATCGATCCTACAACTTTAGGTGAAGGTGGATTAAAAGAATACTTTGAAAATGA
AGAATTAATTGAACCTTTCAACAAAAAATTCTTAGGTAATATTATTGCAGAAGTATTCAACAGATTTAGCATCACTGATACATCAATGATGTT
AGACCGTATGAAAGACTTAGGATTCAAATTCTCATCTAAAGCTGGTATTACAGTAGGTGTTGCTGATATCGTAGTATTACCTGATAAGCAAC
AAATACTTGATGAGCATGAAAAATTAGTCGACAGAATTACAAAACAATTCAACCGTGGTTTAATCACTGAAGAAGAAAGATATAATGCAGT
TGTTGAAATTTGGACAGATGCAAAAGATCAAATTCAAGGTGAATTGATGCAATCACTTGATAAAACTAACCCAATCTTCATGATGAGTGATT
CAGGTGCCCGTGGTAACGCATCTAACTTTACACAGTTAGCAGGTATGCGTGGATTGATGGCCGCACCATCTGGTAAGATTATCGAATTACC
AATCACATCTTCATTCCGTGAAGGTTTAACAGTACTTGAATACTTCATCTCAACTCACGGTGCACGTAAAGGTCTTGCCGATACAGCACTTAA
AACAGCTGACTCAGGATATCTTACTCGTCGTCTTGTTGACGTGGCACAAGATGTTATTGTTCGTGAAGAAGACTGTGGTACTGATAGAGGT
TTATTAGTTTCTGATATTAAAGAAGGTACAGAAATGATTGAACCATTTATCGAACGTATTGAAGGTCGTTATTCTAAAGAAACAATTCGTCA
TCCTGAAACTGATGAAATAATCATTCGTCCTGATGAATTAATTACACCTGAAATTGCTAAGAAAATTACAGATGCTGGTATTGAACAAATGT
ATATTCGCTCAGCATTTACTTGTAACGCACGACATGGTGTTTGTGAAAAATGTTACGGTAAAAACCTTGCTACTGGTGAAAAAGTTGAAGTT
GGTGAAGCAGTTGGTACAATTGCAGCCCAATCTATCGGTGAACCAGGTACACAGCTTACAATGCGTACATTCCATACAGGTGGGGTAGCA
GGTAGCGATATCACACAAGGTCTTCCTCGTATTCAAGAGATTTTCGAAGCACGTAACCCTAAAGGTCAAGCGGTAATTACGGAAATCGAAG
GTGTCGTAGAAGATATTAAATTAGCAAAAGATAGACAACAAGAAATTGTTGTTAAAGGTGTTAATGAAACAAGATCATACCTTGCTTCAGG
TACTTCAAGAATTATTGTAGAAATCGGTCAACCAGTTCAACGTGGTGAAGTATTAACTGAAGGTTCTATTGAACCTAAGAATTACTTATCTG
TTGCTGGATTAAACGCGACTGAAAGCTACTTATTAAAAGAAGTACAAAAAGTTTACCGTATGCAAGGTGTAGAAATCGACGATAAACACGT
TGAGGTTATGGTTCGACAAATGTTACGTAAAGTTAGAATTATCGAAGCAGGTGATACGAAGTTATTACCAGGTTCATTAGTTGATATTCATA
ACTTTACAGATGCAAATAGAGAAGCATTTAAACACCGTAAGCGTCCTGCAACAGCTAAACCAGTATTACTTGGTATTACTAAAGCATCACTT
GAAACAGAAAGTTTCTTATCTGCAGCATCATTCCAAGAAACAACAAGAGTTCTTACAGATGCAGCAATTAAAGGTAAGCGTGATGACTTAT
TAGGTCTTAAAGAAAACGTAATTATTGGTAAGTTAATTCCAGCTGGTACTGGTATGAGACGTTATAGCGACGTAAAATACGAAAAAACAGC
TAAACCAGTTGCAGAAGTTGAATCTCAAACTGAAGTAACGGAATAA

Gene: ybxF (RNA binding protein)

Position: 584344 to 584598, length: 255 nt, orientation: FORWARD

Perfect match to: (RF122-AJ938182-[555615:555869], highly conserved allele)

Sequence:

TTGTCTAAGGAAAAAGTTGCACGCTTTAACAAACAACATTTTGTAGTTGGTCTTAAAGAAACGCTTAAAGCGTTAAAGAAAGATCAAGTTAC
ATCTTTGATTATTGCTGAAGACGTTGAAGTATATTTAATGACTCGCGTGTTAAGCCAAATCAATCAGAAAAATATACCTGTATCTTTTTTCAA
AAGCAAACATGCTTTGGGTAAACATGTAGGTATTAACGTCAATGCGACAATAGTAGCATTGATTAAATGA

Gene: rpsL (30S ribosomal protein S12)

Position: 584696 to 585109, length: 414 nt, orientation: FORWARD

Perfect match to: (MW2-BA000033-[577982:578395], highly conserved allele)

Sequence:

ATGCCAACTATTAACCAATTAGTACGTAAACCAAGACAAAGCAAAATCAAAAAATCAGATTCTCCAGCTTTAAATAAAGGTTTCAACAGTAA
AAAGAAAAAATTTACTGACTTAAACTCACCACAAAAACGTGGTGTATGTACTCGTGTAGGTACAATGACACCTAAAAAACCTAACTCAGCGT
TACGTAAATATGCACGTGTGCGTTTATCAAACAACATCGAAATTAACGCATACATCCCTGGTATCGGCCATAACTTACAAGAACACAGTGTT
GTACTTGTACGTGGTGGACGTGTAAAAGACTTACCAGGTGTGCGTTACCATATTGTACGTGGAGCACTTGATACTTCAGGTGTTGACGGAC
GTAGACAAGGTCGTTCATTATACGGAACTAAGAAACCTAAAAACTAA

Gene: rpsG (30S ribosomal protein S7)

Position: 585175 to 585645, length: 471 nt, orientation: FORWARD

Perfect match to: (N315-BA000018-[587899:588369], highly conserved allele)

Sequence:

ATGCCTCGTAAAGGATCAGTACCTAAAAGAGACGTATTACCAGATCCAATTCATAACTCTAAGTTAGTAACTAAATTAATTAACAAAATTAT
GTTAGATGGTAAACGTGGAACAGCACAAAGAATTCTTTATTCAGCATTCGACCTAGTTGAACAACGCAGTGGTCGTGATGCATTAGAAGTA
TTCGAAGAAGCAATCAACAACATTATGCCAGTATTAGAAGTTAAAGCTCGTCGTGTAGGTGGTTCTAACTATCAAGTACCAGTAGAAGTTC
GTCCAGAGCGTCGTACTACTTTAGGTTTACGTTGGTTAGTTAACTATGCACGTCTTCGTGGTGAAAAAACGATGGAAGATCGTTTAGCTAAC
GAAATTTTAGATGCAGCAAATAATACAGGTGGTGCCGTTAAGAAACGTGAGGACACTCACAAAATGGCTGAAGCAAACAAAGCATTTGCT
CACTACCGTTGGTAA

Gene: efg (translation elongation factor G)

Position: 585768 to 587849, length: 2082 nt, orientation: FORWARD

Perfect match to: (Strain_21282-AMPF01000064-[25142:27223:r], allele observed in CC8+CC22)

Sequence:

ATGGCTAGAGAATTTTCATTAGAAAAAACTCGTAATATCGGTATCATGGCTCACATTGATGCTGGTAAAACGACTACGACTGAACGTATTCT
TTATTACACTGGCCGTATCCACAAAATTGGTGAAACACACGAAGGTGCTTCACAAATGGACTGGATGGAGCAAGAACAAGACCGTGGTATT
ACTATCACATCTGCTGCAACAACAGCAGCTTGGGAAGGTCACCGTGTAAACATTATCGATACACCTGGACACGTAGACTTCACTGTAGAAG
TTGAACGTTCATTACGTGTACTTGACGGAGCAGTTACAGTACTTGATGCACAATCAGGTGTTGAACCTCAAACTGAAACAGTTTGGCGTCAG
GCTACAACTTATGGTGTTCCACGTATAGTATTTGTAAACAAAATGGACAAATTAGGTGCTAACTTCGAATACTCTGTAAGTACATTACATGA
TCGTTTACAAGCTAACGCTGCTCCAATCCAATTACCAATTGGTGCGGAAGACGAATTCGAAGCAATCATTGACTTAGTTGAAATGAAATGTT
TCAAATATACAAATGATTTAGGTACTGAAATTGAAGAAATTGAAATTCCTGAAGACCACTTAGATAGAGCTGAAGAAGCTCGTGCTAGCTT
AATCGAAGCAGTTGCAGAAACTAGCGACGAATTAATGGAAAAATATCTTGGTGACGAAGAAATTTCAGTTTCTGAATTAAAAGAAGCTATC
CGCCAAGCTACTACTAACGTAGAATTCTACCCAGTACTTTGTGGTACAGCTTTCAAAAACAAAGGTGTTCAATTAATGCTTGACGCTGTAATT
GATTACTTACCTTCACCACTAGACGTTAAACCAATTATTGGTCACCGTGCTAGCAACCCTGAAGAAGAAGTAATCGCGAAAGCAGACGATTC
AGCTGAATTCGCTGCATTAGCGTTCAAAGTTATGACTGACCCTTATGTTGGTAAATTGACATTCTTCCGTGTGTATTCAGGTACAATGACATC
TGGTTCATACGTTAAGAACTCTACTAAAGGTAAACGTGAACGTGTAGGTCGTTTATTACAAATGCACGCTAACTCACGTCAAGAAATCGATA
CTGTATACTCTGGAGATATCGCTGCTGCGGTAGGTCTTAAAGATACAGGTACTGGTGATACTTTATGTGGTGAGAAAAATGACATTATCTTG
GAATCAATGGAATTCCCAGAGCCAGTTATTCACTTATCAGTAGAGCCAAAATCTAAAGCTGACCAAGATAAAATGACTCAAGCTTTAGTTAA
ATTACAAGAAGAAGACCCAACATTCCATGCACACACTGACGAAGAAACTGGACAAGTTATCATCGGTGGTATGGGTGAGCTTCACTTAGAC
ATCTTAGTAGACCGTATGAAGAAAGAATTCAACGTTGAATGTAACGTAGGTGCTCCAATGGTTTCATATCGTGAAACATTCAAATCATCTGC
ACAAGTTCAAGGTAAATTCTCTCGTCAATCTGGTGGTCGTGGTCAATACGGTGATGTTCACATTGAATTCACACCAAACGAAACAGGCGCA
GGTTTCGAATTCGAAAACGCTATCGTTGGTGGTGTAGTTCCTCGTGAATACATTCCATCAGTAGAAGCTGGTCTTAAAGATGCTATGGAAA
ATGGTGTCTTAGCAGGTTATCCTTTAATTGATGTTAAAGCTAAATTATATGATGGTTCATACCATGATGTCGATTCATCTGAAATGGCCTTCA

AAATTGCTGCATCATTAGCACTTAAAGAAGCTGCTAAAAAATGTGATCCTGTAATCTTAGAACCAATGATGAAAGTAACTATTGAAATGCCT
GAAGAGTACATGGGTGATATCATGGGTGACGTAACATCTCGTCGTGGACGTGTTGATGGTATGGAACCTCGTGGTAATGCACAAGTTGTT
AATGCTTATGTACCACTTTCAGAAATGTTCGGTTATGCAACATCATTACGTTCAAACACTCAAGGTCGCGGTACTTACACTATGTACTTCGAT
CACTATGCTGAAGTTCCAAAATCAATCGCTGAAGATATTATCAAGAAAAATAAAGGTGAATAA

Gene: tuf (translation elongation factor Tu)

Position: 588066 to 589250, length: 1185 nt, orientation: FORWARD

Perfect match to: (MW2-BA000033-[581352:582536], highly conserved allele)

Sequence:

ATGGCAAAAGAAAAATTCGATCGTTCTAAAGAACATGCCAATATCGGTACTATCGGTCACGTTGACCATGGTAAAACAACATTAACAGCAG
CAATCGCTACTGTATTAGCAAAAAATGGTGACTCAGTTGCACAATCATATGACATGATTGACAACGCTCCAGAAGAAAAAGAACGTGGTAT
CACAATCAATACTTCTCACATTGAGTACCAAACTGACAAACGTCACTACGCTCACGTTGACTGCCCAGGACACGCTGACTACGTTAAAAACA
TGATCACTGGTGCTGCTCAAATGGACGGCGGTATCTTAGTAGTATCTGCTGCTGACGGTCCAATGCCACAAACTCGTGAACACATTCTTTTA
TCACGTAACGTTGGTGTACCAGCATTAGTAGTATTCTTAAACAAAGTTGACATGGTTGACGATGAAGAATTATTAGAATTAGTAGAAATGG
AAGTTCGTGACTTATTAAGCGAATATGACTTCCCAGGTGACGATGTACCTGTAATCGCTGGTTCAGCATTAAAAGCTTTAGAAGGCGATGCT
CAATACGAAGAAAAAATCTTAGAATTAATGGAAGCTGTAGATACTTACATTCCAACTCCAGAACGTGATTCTGACAAACCATTCATGATGCC
AGTTGAGGACGTATTCTCAATCACTGGTCGTGGTACTGTTGCTACAGGCCGTGTTGAACGTGGTCAAATCAAAGTTGGTGAAGAAGTTGAA
ATCATCGGTTTACATGACACATCTAAAACAACTGTTACAGGTGTTGAAATGTTCCGTAAATTATTAGACTACGCTGAAGCTGGTGACAACAT
TGGTGCATTATTACGTGGTGTTGCTCGTGAAGACGTACAACGTGGTCAAGTATTAGCTGCTCCTGGTTCAATTACACCACATACTGAATTCA
AAGCAGAAGTATACGTATTATCAAAAGACGAAGGTGGACGTCACACTCCATTCTTCTCAAACTATCGTCCACAATTCTATTTCCGTACTACTG
ACGTAACTGGTGTTGTTCACTTACCAGAAGGTACTGAAATGGTAATGCCTGGTGATAACGTTGAAATGACAGTAGAATTAATCGCTCCAAT
CGCGATTGAAGACGGTACTCGTTTCTCAATCCGTGAAGGTGGACGTACTGTAGGATCAGGCGTTGTTACTGAAATCATTAAATAA

Gene: yhaA (M20 family peptidase)

Position: 589532 to 590707, length: 1176 nt, orientation: REVERSE

Perfect match to: (11819-97-CP003194-[590327:591502:r], allele observed in CC80+CC239+CC4803)

Sequence:

TTAATTTGTTTTAAATACTTGCTCTAATTCAATGATTTTTAAAAATACAGCTACAGCGTATTTTAATGATTTTTCATCAATATCAAATTTGGGAT
TATGGTGTGGCGCTGTAATACCTTTACTTTCATTACCACAACCAGTCAGAAAGAATGCACCTGGTCGTACTTTCAAATAATGTGAAAAATCTT
CTCCAATCATCATTAAATCTGATTCATTAAAGCGTACATGTAAGTCATTTGTTGCTTCTTTAATAACTTGATATGCTTTCTCGTTATTATGGAC
AGGCAAATACCCTTTAATATAATTCAAATCATAGTTAATATCATTTGCTATTGCTAAACCTTGTAAAAGCTTATCCATTTTGTCCATTACATGA
TTCTGTATATCTGAATCGAAAGTTCTAACTGTACCTTTACAAAATGCTTGATCAGGAATAACGCTATCTGTGGTGCCTGCTTGAATCATTCCA
AATGAAAGTACAGCTTGTTTAACTGGATCGATCGTACGTGAAATTATTTTTTGTGCACTTAAAATGAACTCTGCCATGATTACTATTGGGTCA
ATGGTTTCATGAGGTTTGGCACCATGACCGCCACGACCTTTAATTGTGACGCTAAATTCATCTGGAGAGGCCATGATTGCCCCCGCACGTGA
ATGAATAGTTCCAGTAGGATAACCACTCCATAAATGTGTACCGTAAATTCTATCTACATTTTCCAGACATCCAGCATCTATCATTTCTTGAGA
ACCACCTGGCATGATTTCTTCACCGTACTGGAATATTAATACAACATTACCTTCTAATAAATGTTTATGTTCATCTAAAATCTCTGCTACAGTA
AGTAAAATTGCTGTATGACCATCATGCCCACACGCATGCATACATCCTGGATTTTTAGACTTATAAGGCACATCGTTTAATTCCTCGACAGGT
AACGCATCAAAGTCAGCTCTTAATGCAATGGTAGGTCCTGTGCCCAAGCCTTTAAATGTGGCTTTGATACCATTGCGGCCGATAGGAGTTTC
AATATCACAAGATAACTGGCTTAATTGGTTAACAATATAATCATGTGTTTGAAATTCTTCAAAAGATAACTCAGGATATTGGTGTAAATAAC
GTCTGAGTTGAATTGTTTTATTTTCTTTATTATTTGCTAGTTGGAACCAATCTAACAC

Gene: kbl (2-amino-3-ketobutyrate coenzyme A ligase)

Position: 590878 to 592065, length: 1188 nt, orientation: FORWARD

Perfect match to: (MRSA252-BX571856-[604812:605999], highly conserved allele)

Sequence:

GTGGTTCAATCATTACATGAGTTTTTAGAGGAAAATATAAATTATCTAAAAGAAAATGGTTTGTATAATGAAATAGATACAATTGAAGGTGC
AAACGGACCAGAAATCAAAATCAATGGGAAATCATACATTAACTTATCTTCAAATAATTATTTAGGACTAGCAACAAATGAAGATTTGAAAT
CAGCTGCAAAAGCAGCTATTGATACACATGGTGTAGGTGCAGGCGCTGTTCGTACAATCAATGGTACATTAGATTTACACGACGAATTAGA

AGAAACACTAGCAAAATTTAAAGGAACAGAAGCTGCAATAGCTTATCAGTCAGGATTTAATTGTAATATGGCTGCTATTTCAGCTGTCATGA
ATAAAAATGATGCTATTTTATCAGATGAGCTTAATCATGCATCAATTATTGATGGATGTCGCTTATCTAAAGCTAAAATTATTCGAGTTAACC
ATTCAGACATGGATGATTTACGTGCGAAAGCAAAAGAAGCAGTTGAATCAGGTCAATACAATAAAGTGATGTATATCACTGATGGCGTTTT
TAGTATGGATGGTGATGTGGCTAAATTACCTGAAATTGTAGAAATTGCAGAAGAATTTGGTTTATTAACTTATGTTGACGACGCTCATGGTT
CAGGTGTTATGGGTAAAGGCGCTGGTACGGTTAAACATTTTGGTTTACAAGATAAAATCGATTTCCAAATAGGTACGCTTTCTAAAGCAATT
GGTGTCGTTGGCGGCTATGTAGCAGGTACAAAAGAGTTAATAGATTGGTTAAAAGCACAATCACGACCATTCTTATTCTCTACATCATTAGC
ACCTGGGGATACCAAAGCAATAACTGAAGCAGTTAAAAAGTTAATGGATTCAACTGAATTACATGATAAATTATGGGACAATGCACAATAT
TTAAAAAATGGATTGTCAAAATTAGGATATGATACAGGTGAGTCAGAAACTCCAATTACACCAGTAATTATTGGTGATGAAAAAACAACTC
AAGAATTTAGTAAGCGTTTAAAAGACGAAGGTGTCTATGTGAAATCTATCGTTTTCCCAACAGTACCAAGAGGTACAGGACGTGTAAGAAA
TATGCCTACAGCTGCACATACAAAAGACATGTTAGATGAAGCAATTGCGGCTTATGAAAAAGTAGGAAAAGAAATGAAGTTGATTTAA

Gene: hchA (chaperone Hsp31 and glyoxalase 3)

Position: 592331 to 593209, length: 879 nt, orientation: FORWARD

Perfect match to: (N315-BA000018-[595059:595937], highly conserved allele)

Sequence:

ATGTCACAAGATGTAAATGAATTAAGTAAGCAACCAACGCCAGATAAAGCAGAAGATAACGCATTTTTCCCATCACCATATTCCCTTAGTCA
ATATACAGCACCTAAAACAGATTTTGATGGTGTTGAACACAAAGGTGCCTATAAAGATGGTAAATGGAAAGTATTGATGATTGCTGCTGAA
GAGAGATATGTATTATTGGAAAATGGAAAAATGTTCTCTACGGGTAATCATCCTGTTGAAATGTTATTACCTTTACATCATTTAATGGAAGC
AGGTTTTGACGTTGATGTTGCGACATTATCTGGTTATCCAGTTAAATTAGAATTATGGGCTATGCCAACTGAAGACGAGGCAGTTATAAGTA
CTTATAATAAATTGAAAGAAAAATTAAAACAGCCAAAAAAATTAGCAGATGTGATTAAAAATGAATTAGGACCTGATTCAGACTATTTATCT
GTCTTTATCCCAGGCGGACATGCTGCAGTTGTTGGTATTTCTGAAAGTGAGGACGTTCAACAAACATTAGATTGGGCATTAGACAATGACC
GCTTTATAGTTACATTATGTCATGGACCAGCAGCACTACTTTCAGCAGGGCTTAACAGAGAAAAATCTCCATTAGAAGGATACTCTGTTTGT
GTCTTCCCTGACTCATTAGATGAAGGTGCAAATATTGAAATAGGTTATTTACCTGGACGCTTGAAATGGTTAGTTGCTGATTTATTAACTAA
ACAAGGATTAAAAGTAGTTAACGACGATATGACAGGAAGAACGTTAAAAGATCGTAAATTATTAACAGGTGACAGTCCTTTAGCTTCAAAT
GAGTTAGGAAAATTAGCAGTTAATGAAATGTTAAATGCAATACAAAATAAATAA

Gene: araB (ribulokinase)

Position: 593367 to 595004, length: 1638 nt, orientation: FORWARD

Perfect match to: (11819-97-CP003194-[594162:595799], allele observed in CC80+CC12+CC72+CC80+CC772)

Sequence:

ATGTCTTATAGCATTGGAATTGATTATGGAACAGCTTCAGGCCGTGTGTTTTTAATTAATACAACTAACGGTCAAGTAGTATCAAAATTTGT
GAAACCATATACACATGGTGTCATTGAGAGTGAATTAAATGGTTTGAAAATACCACATACATATGCACTTCAAAATAGTAATGATTATTTAG
AAATTATGGAAGAAGGAATATCATATATAGTACGTGAATCAAAAATAGATCCAGTCAATATAGTAGGTATTGGTATAGACTTTACTTCATCT
ACTATTATTTTTACTGACGAAAACCTTAACCCGGTACATAATTTAAAACAATTTAAAAACAATCCACATGCGTATGTGAAACTTTGGAAACAT
CATGGTGCATATAAAGAAGCAGAGAAATTATATCAAACTGCTATTGAAAATAATAATAAGTGGTTAGGCCATTATGGATATAATGTTAGTA
GTGAATGGATGATTCCCAAAATAATGGAAGTCATGAATCGAGCACCAGAAATTATGGAAAAAACGGCTTATATTATGGAAGCGGGCGATT
GGATTGTAAATAAATTAACTAATAAAAATGTACGCTCGAATTGTGGATTAGGTTTCAAAGCATTTTGGGAAGAAGAAACAGGGTTTCATTA
TGATTTATTTGATAAAATAGACCCCAAATTATCAAAAGTAATTCAAGATAAAGTATCTGCACCGGTTGTTAATATTGGTGAAGTAGTAGGGA
AACTGGACGATAAAATGGCACAGAAATTAGGATTATCAAAAGAGACTATGGTAAGTCCTTTTATTATTGATGCCCATGCTAGTTTATTAGGT
ATTGGGTCTGAAAAAGATAAAGAAATGACTATGGTGATGGGAACAAGCACATGCCATCTTATGTTAAATGAAAAGCAACATCAAGTGCCA
GGTATATCAGGTTCTGTAAAAGGAGCAATTATTCCAGAATTATTTGCTTATGAAGCGGGGCAATCAGCAGTAGGTGATTTGTTTGAGTATG
TCGCTAAGCAAGCACCAAAGTCATATGTAGATGAAGCAGCAAATAGAAATATGACTGTATTTGAATTAATGAATGAAAAGATAAAACATCA
AATGCCAGGTGAAAGTGGGCTCATTGCTCTTGATTGGCATAATGGAAATCGAAGTGTATTAAGTGATAGCAATTTAACAGGTTGTATCTTT
GGATTAACTTTACAAACTAAGCATGAGGATATTTATAGAGCATATTTAGAAGCTACAGCATTTGGTACTAAGATGATTATGCAACAGTATCA
AGATTGGCATATGGAAGTAGAAAAGGTATTTGCATGTGGCGGTATACCTAAAAAGAATGCTGTTATGATGGATATCTATGCGAATGTACTG
AATAAAAAACTAATTGTTATGGATAGTGAGTATGCACCAGCAATAGGCGCAGCAATATTAGGTGCAGTCAGTGGTGGCGCACATAATTCAA
TTAATGATGCAGTTGATGCTATGAAAGAGCCAATTTTATACGAAATTAATCCAGAAGCGGAAAAAGTACAAAGGTATGAAACATTATTTAA
AGCTTATAAGGCTTTACATGATATCCATGGTTATAAAAAAGCTAATATAATGAAAGATATCCAGAGTTTAAGAGTTGAGGGATAA

Gene: Q5HIC2 (uncharacterized epimerase/dehydratase)

Position: 595218 to 596183, length: 966 nt, orientation: FORWARD

Perfect match to: (CN1-CP003979-[571907:572872], allele observed in CC72+CC25+CC72+CC80)

Sequence:

ATGAAAAAAATTATGATTACTGGTGCATTAGGACAAATTGGTACAGAATTAGTTGTTAAGTGCAGAGAAATTTATGGGACAGATAATGTTC
TTGCTACAGATATTAGGGAACCTGAAGCAGACTCACCTGTACAAAATGGACCATTTGAAATCTTAGACGTAACAGATCGTGACCGTATGTTT
GAGTTAGTTAGGGACTTTGAAGCGGATAGTCTAATGCATATGGCAGCATTATTATCAGCAACTGCAGAGAAAAATCCAATTCTAGCTTGGG
ATTTAAATATGGGTGGATTAATGAATGCATTAGAAGCTGCAAGAACTTATAATTTGCACTTTTTCACACCAAGTTCAATTGGTGCATTTGGA
GACTCAACTCCTAAAGTTAATACGCCACAAGTAACAATTCAGCAACCTACGACAATGTATGGTGTAAATAAAGTAGCTGGAGAATTATTGT
GTCAATACTATTTCAAACGTTTTGGTGTAGATACAAGAAGTGTTAGATTCCCAGGTTTAATCTCGCATGTTAAAGAGCCAGGTGGCGGTACT
ACAGACTATGCTGTTGAAATATACTTCAAAGCAGTAAGAGAAGGTCATTATACAAGCTTCATAGATAAAGGCACGTATATGGATATGATGT
ATATGGATGATGCAATTGAAGCAATTATTAAACTTATGGAAGCAGACGACGCTAAATTAGAAACTAGAAACGGTTATAATTTGAGCGCAAT
GAGTTTTGATCCAGAGATGGTAAAAGAAGCAATTCAAGAATACTATCCAAATTTTACATTAGATTATGATGTTGATCCTATTAGACAAGGTA
TCGCTAATAGTTGGCCGGATTCTATTGATACAAGCTGTTCACGTGGCGAATGGGGATTTGATCCTAAATATGATTTAGCGAGCATGACTAA
ATTAATGTTAGAAGCTATTGAACAAAAAGATACTGTTAAAAATAATAACTAA

Gene: ilvE (branched-chain-amino-acid aminotransferase)

Position: 596519 to 597595, length: 1077 nt, orientation: FORWARD

Sequence:

ATGTCACAAGCAGTTAAAGTTGAACGACGAGAAACATTAAAACAAAAACCAAATACATCTCAACTAGGTTTTGGTAAATATTTTACTGATTA
TATGTTGAGTTATGATTATGATGCAGATAAAGGATGGCATGATTTGAAGATAGTACCTTATGGTCCTATTGAAATTTCACCTGCTGCACAAG
GTGTTCATTATGGTCAATCGGTATTCGAAGGATTAAAAGCATATAAAAGAGATGGGGAAGTTGCACTTTTCCGTCCAGAAGAAAATTTTAA
GCGTCTTAATAACTCGTTAGCACGATTAGAAATGCCTCAAGTAGACGAAGCAGAATTGTTAGAGGGGCTAAAACAATTAGTTGATTTAGAA
AGAGATTGGATTCCTGAAGGGGAAGGTCAATCATTATATATTCGTCCATTTGTTTTTGCAACAGAAGGGGCACTTGGCGTTGGTGCATCAC
ATCAGTATAAATTATTAATTATTTTATCTCCTTCAGGTGCATATTATGGTGGTGAAACTTTAAAACCAACTAAAATCTATGTAGAAGATGAAT
ATGTGCGTGCTGTTCGTGGCGGTGTAGGGTTTGCGAAAGTTGCAGGTAACTATGCGGCAAGTTTATTAGCACAAACAAATGCAAATAAATT
AGGTTATGACCAAGTATTATGGCTTGATGGTGTTGAACAGAAATATATCGAAGAAGTTGGTAGCATGAACATTTTCTTCGTTGAAAATGGA
AAAGTAATTACACCAGAGTTGAATGGCAGTATTTTACCTGGTATTACACGTAAATCTATTATCGAATTAGCTAAAAACTTAGGATATGAAGT
CGAAGAGCGCCGCGTTTCAATCGATGAATTATTCGAATCATATGATAAAGGTGAGTTAACAGAAGTATTTGGTAGTGGTACTGCAGCAGTT
ATTTCACCTGTGGGTACATTGAGATACGAAGATCGTGAAATCGTTATTAATAATAATGAGACTGGTGAAATTACTCAAAAATTATACGACGT
CTATACTGGTATTCAAAATGGTACTTTAGAAGATAAAAATGGTTGGAGAGTCGTTGTACCAAAATATTAA

Gene: ppaX1 (P-Ser-HPr phosphatase)

Position: 597845 to 598528, length: 684 nt, orientation: FORWARD

Perfect match to: (COL-CP000046-[627938:628621], highly conserved allele)

Sequence:

ATGGAATGGATATTATTTGATAAAGATGGTACGTTAATTGAATTTGATAGAAGTTGGGAAAAAAATAGGGGTACGATTTGTACAATCATTGC
TTGAGACTTTCCCAGTACATAATAAAGAAGCTGCTTTAAGACAACTCGGTGTCATTAAAGAATCTATTGATCCAAAATCAGTGATGGGTTCA
GGATCTTTACAACAAATTATCCAGGCATTTAATGATGTGACGGGACAAGATACAACCGACTGGTCCAAGTCAACAAGTCAAAAGCTGGTAG
ATGAACGTATTCCTGAAATTAATTGGGTAGAAGGTGTTAAAGAAGCACTTATCGATTTGAAAGCAAAAGGCTATCAACTTGGTATTGTTAC
GAGTGATACTAAAAAAGGTGTAGAACAATTTTTAGCACATACCAATGCTATCTCGTTGTTCGATTTGATCATTTCTACCGAAGCGGATGCCT
ATGAGAAGCCAAATCCTAAAGTATTATCGCCTTTATTTGAGCAATATAATGTAGATCCTCAGAAAGTAGCTATAGTAGGAGACACTGCTAAT
GATATGAAGACAGCAAGTAATGCAAATTTAGGTATGGCAATAGGTGTATTAACAGGTATTGCAACAAAAGAAGAATTACATGAAGCTGAT
ATTATTTTAAATAGTGCGGCAGATATTTTAGAAGCTTTAAATTAA

Gene: dck (deoxyadenosine/deoxycytidine kinase)

Position: 598644 to 599306, length: 663 nt, orientation: REVERSE

Perfect match to: (N315-BA000018-[601373:602035:r], allele observed in CC5+CC72+CC80)

Sequence:

TTATCGTGTATCTACTTGTCGATATGTTTGAATAATTCGAGCAATTTTGTCTATCATAGGATTTAAAGATTCGGGGTCCTTATGGATATCATA
TTCATTAATATTGATACGTACAACTGGACATGCATTAAAGCTATTAATCCAATCGTCATAGCGTTTAAATAGCTTTTTCCAGTATTCAGGGTC
TGTATTAATTTCCATTTCGCGACCACGTTCAATAATACGATCAATGACCTCATCATAGTTACATTCTAAATAAATCATTACATCAGGTTTAGGA
AAATAAGGTGTCATGACCATGGCATTAAATAAGTCTGAATATGTTTTGAAATCTTCTTTACTCATTGTGCCTTCTTCTTCATGCATTTTTGCAA
AAATATCAACATCTTCATAAATTGATCGATCTTGGACAAAGCCACCACCATATTCAAACATACGCTTTTGTTCTTTAAAACGTTCAGCTAAGA
AGTAAATTTGCAAATGGAAACTCCATCGTTCAAAATCGCTATAAAATTTATCTAAATATGGATTATGTTCGACATTTTCAAAAGACGTTTTAA
AGTTTAATTTATCTGCAAGTGCTTGCGTTAGTGTTGATTTTCCAACACCAACTGTACCTGCAATGGTTATAATGGCATTTTGTGGAATACCGT
AATTATTCAT

Gene: dgk (deoxyguanosine kinase)

Position: 599299 to 599916, length: 618 nt, orientation: REVERSE

Perfect match to: (MRSA252-BX571856-[613243:613860:r], highly conserved allele)

Sequence:

TTATTCATTGGTAATATCTCCTATCATAGGTAATATAATATGTAATATATCTTCGTAATCTTGTTCATTTTTAAGAAAATCAATAGAAGTTGTA
TCGATTAAAACTACATTTGAACCATTACTTTGTAAGGACTCATAATACTCACGATAATCTTTTTTTAACTTTAACAGATATTCATCTTCTATTTG
ATGCTCAAAACTACGGTTACGTTTAGCAATTCTAGATTTTAACACATCAAGGTCTGCATCTAAAAAGATAATCATATTCGGCATAATCATATC
TTCAGTTAAAATATCATAAATTTTACTGAATTTCTGAAATTCAACAGGACTCAAAGTATTTTTAGCAAATATCTTATTTTTATGTATATGATAA
TCACTAACTACACCTTGATTTAGTTGTGTTACATCTTGAAATTGCTTATATCTATTGCATAAAAAGAACATTTCAGTTTGAAAACTCCATTTAG
AGATATCTTCATAAAAGTCTGATAAAAATGGATTTTCTGTGATGATTTCTTTTTCTTCATAAAAATCTAAAGTTTGACTTAATTTGTGTGCAAG
TGAAGATTTACCTACGCCAATAGGACCTTCAATTGCTATAAAAGGTTTGTTCAT

Gene: tadA (nucleoside deaminase)

Position: 599983 to 600453, length: 471 nt, orientation: FORWARD

Perfect match to: (11819-97-CP003194-[600778:601248], allele observed in CC80+CC22)

Sequence:

ATGACAAATGATATATATTTTATGACATTAGCGATTGAAGAAGCTAAAAAAGCAGCTCAACTAGGCGAAGTACCTATAGGTGCTATCATCA
CTAAAGATGATGAAGTTATCGCTAGAGCACATAATTTAAGAGAAACACTACAACAACCAACGGCGCATGCTGAACATATTGCAATTGAACG
TGCAGCCAAAGTGTTAGGTAGTTGGCGTTTAGAAGGTTGCACATTATATGTAACCTTAGAACCATGTGTCATGTGCGCAGGAACAATTGTA
ATGAGTCGCATTCCAAGAGTCGTCTATGGCGCAGATGATCTTAAAGGTGGTTGTAGTGGCAGTTTAATGAATTTATTGCAACAATCTAATTT
TAATCATCGTGCAATTGTTGATAAAGGTCTACTTAAAGAAGCATGTAGCACATTATTAACAACATTTTTTAAAAACTTAAGAGCCAATAAGA
AATCCACCAATTAG

Gene: A5IQB3 (putative hydrolase, haloacid dehalogenase-like family)

Position: 600600 to 601469, length: 870 nt, orientation: FORWARD

Perfect match to: (11819-97-CP003194-[601395:602264], allele observed in CC80+CC5+CC80)

Sequence:

ATGATAAAACTAATAGCCACTGATATGGATGGCACGCTACTTAATGCAGCACATGAAATTTCTCAACCTAATATTGATGCGATTAAATACGC
TCAAGAACAAGGGATAACAGTTGTTATCGCGACAGGTCGAGCATTTTATGAAGCACAAGCACCAGTTGCTGACACAGATTTAACAGTACCA
TATATTTGTTTGAATGGTGCTGAAGTACGTGATGAAACTTTCAATGTAATGAGCACTTCACACCTTAATAAATCGTTAGTACACAAAATTACA
AATGTTTTAAAAGATGCAGGTATTTATTATCAAGTATACACGAGTCGTGCGATTTATACTGAAGATCCACAAAGAGATTTAGACATTTACAT
AGATATTGCTGAGCGTGCAGGTCAACATGCAAACGTTGAGCGTATTAAAAATGGTATTCAAAGACGCATAGATAATGGTACGTTGAAAGTT
GTTGATAATTATGATGCTATTGAAAACATACCTGGTGAATTAATTATGAAAATATTAGCATTTGATGGAAATTTAGAAAAAAATTGACAAAGC
TAGTAAAATTTTAGCTGAATCTCCGAATTTAGCTATATCATCATCTTCGAGAGGAAATATAGAAATAACGCATTCAGATGCACAAAAAGGTA
TTGCGCTAGAAACAATTGCCGAAAGATTAGGGATTGAAATGAAAGATGTCATGGCAATAGGTGACAATTTAAATGACTTATCAATGTTAGA
GAAAGTTGGCTATCCAGTTGCGATGGAAAATGGTGCAGAAGAAGTTAAAAAAATAGCGAAATATGTCACAGATACGAATGAAAATAGTGG
TGTTGGAAAAGCTATTATGAAATTATTACGTGAACAACAAGTTTAA

Gene: azo1 (FMN-dependent NADPH-azoreductase)

Position: 601490 to 602056, length: 567 nt, orientation: FORWARD

Perfect match to: (RF122-AJ938182-[572763:573329], highly conserved allele)

Sequence:

ATGAAAGGATTAATTATTATTGGCAGTGCACAAGTGAATTCACATACAAGTGCACTAGCAAGATACTTAACTGAGCATTTTAAAACACATGA
TATTGAAGCGGAAATATTCGATTTAGCAGAAAAACCGTTAAATCAATTAGATTTTTCAGGAACAACACCGTCTATTGATGAAATCAAACAAA
ATATGAAAGATTTAAAAGAGAAAGCAATGGCGGCGGACTTTTTAATATTAGGAACGCCAAACTATCATGGTTCATATTCTGGAATATTGAA
AAATGCATTAGATCATCTAAATATGGATTATTTTAAAATGAAACCTGTAGGCTTAATAGGAAATAGTGGTGGTATTGTTAGTTCAGAGCCAT
TGTCACATTTAAGAGTAATCGTCAGAAGTTTACTAGGCATTGCTGTACCAACTCAAATAGCAACACATGATTCTGATTTTGCTAAAAATGAA
GATGGTTCATATTACTTAAATGATAGTGAATTCCAATTACGAGCAAGATTATTTGTCGATCAAATTGTATCTTTTGTGAATAATAGTCCATAT
GAACATTTAAAATAA

Gene: sdrC (serine aspartate repeat protein C)

Position: 602487 to 605384, length: 2898 nt, orientation: FORWARD

Sequence:

ATGAATAATAAAAAGACAGCAACAAATAGAAAAGGCATGATACCAAATCGATTAAACAAATTTTCGATAAGAAAGTATTCTGTAGGTACTG
CTTCAATTTTAGTAGGGACAACATTGATTTTTGGGTTAAGTGGTCATGAAGCTAAAGCGGCAGAACATACGAATGGAGAATTAAATCAATC
AAAAAATGAAACGACAGGCACAAGTGAGAATAAAACAACTGAAAAAGTTGATAGTCGTCAACTAAAAGACAATACGCAAACTGCAACTGC
AGATCAGCCTAAAGTGACAATGAGTGATAGTGCAACAGTTAAAGAAACTAGTAGTAACATGCAATCACCACAAAACGCTACAGCTAATCAA
TCTACTACACAAACTAGCAATGTAACAACAAATGATAAATCAGCAACTACATATAGTAACGATACTAGTAAAAGCAATTTAACGCAGGCAA
AAGACGTTTCAACTAAGCCTAAAACAACAACTATTAAACCAAGAACATTAAACCGTATGGCAGTGAATACTGTTGCAGCACCACAACAAGG
TACAAATGTTAATGATAAAGTACATTTTAAGAATATTGATATTGCGATTGATAAAGGACATGTTAATAAAGATACTGGTAAAACTGAATTTT
GGGCAACTTCAAGTGATGTTTTAAAATTAAAAGCAAATTATACAATCGATGATTCTGTTAAAGAGGGCGATACATTTACTTTTAAATATGGT
CAATATTTCCGTCCAGGATCAGTAAGATTACCATCGAAAACACAAAATTTATATAATGCTCAAGGTAACATTATTGCTAAAGGTATTTATGAT
AGTGCAACAAATTCAACAACATATACATTTACAAACTATGTTGATCAATATACAAATATTAGTGGTAGTATTGAACAAGTTTCATTTGCCAAA
CGTGAAAATGCAACAACTGATAAAACTGCTTATAAAATGGAAGTTACATTAGGAAACGACTCTTATAGTAAAGAAGTTATTGTCGATTATG
GTAATAAAAAAGCACAACCGCTTATTTCAAGTACAAATTATATCAACAATGAAGATTTGTCACGAAATATGACTGCATATGTAAATCAACCT
AAAAATACATATACTAAACAAACGTTTGTTACTAATTTAACTGGATATAAATTTAATCCAAATGCAAAAAACTTCAAAATATACGAAGTGACT
AACCAAAATCAGTTTGTGGATAGTTTCACACCAGATACTTCAAAACTTACAGATGTTACTGATCAATTCGATATTAATTATAGTAATGATAAT
AAAACAGCTACAGTCGATTTAATGAAAGGCCAAAAAAGTAGTAATAAACAATACATCATTCAACAAGTTGCTTATCCAGATAATAGTTCAAC
AGATAATGGGAAAATTGATTATACTTTAGACACTGACAAAACTAAATATAGTTGGTCAAATAGTTATTCAAATGTGAATGGCTCATCAACTG
CAAATGGCGATCAAAAAAAATACAATCTTGGTGATTATGTTTGGGAAGATACAAATAAAGATGGTAAACAAGATGCTAACGAAAAGGGTA
TTAAAGGCGTGTATGTCATTCTTAAAGATAGCAATGGTAAAGAATTAGATCGTACGACAACAGATGAAAATGGTAAATATCAGTTCACAGG
TTTAAGCAATGGAACATATAGTGTAGAATTTTCAACACCAGCCGGTTATACACCAACAACTGCAAATGCAGGTACAGATGACGCAATAGAT
TCTGATGGACTAACTACAACAGGTGTCATTAAAGATGCTGACAACATGACATTAGACAGTGGATTCTATAAAACACCAAAATACAGTTTAG
GTGATTATGTTTGGTATGACAGTAATAAAGATGGTAAACAAGATTCGACTGAAAAAGGAATTAAAGGTGTTAAAGTTACATTGCAAAATGA
AAAAGGCGAAGTAATTGGTACAACTGAAACAGATTCAAATGGTAAATATCGTTTTGATAATTTAGATAGTGGTAAATACAAAGTTATCTTTG
AAAAGCCTGCTGGCTTAACTCAAACAGGTACAAATACAACTGAAGATGATAAAGATGCTGATGGTGGCGAAGTTGACGTAACAATTACGG
ATCATGATGATTTCACACTAGATAATGGCTACTACGAAGAAGAAACATCAGATAGCGACTCAGATTCAGACAGCGATTCAGACTCAGATAG
CGACTCAGATTCTGACAGCGATTCAGACTCAGATAGCGACTCAGATTCAGACAGCGACTCAGACTCAGATAGCGACTCAGACTCAGACAGC
GACTCAGACTCAGATAGCGACTCAGACTCAGACAGCGACTCAGATTCAGATAGCGATTCAGACTCAGACAGCGATTCAGACTCAGATAGC
GACTCAGACTCAGATAGCGATTCAGACTCAGACAGCGATTCAGACTCAGACAGCGACTCAGACTCAGATAGCGATTCAGACTCAGACAGT
GATTCATATTCAGACAGCGATTCAGAATCAGACAGTGACTCAGATTCAGATAGCGATTCGGACTCAGATAGCGACTCAGATTCAGACAGCG
ACTCAGATTCAGATAGCGATTCGGACTCAGACAACGACTCAGATTCAGATAGCGATTCAGATTCGGACAGTGACTCAGACTCAGATAGCGA
TTCAGACTCAGATAGCGACTCAGACTCAGATAGCGATTCAGACTCAGATAGCGATTCAGACTCAGATGCAGGTAAACATACTCCGACTAAA
CCAATGAGTACGGTTAAAGATCAGCATAAAACAGCTAAAGCATTACCAGAAACAGGTAGTGAAAATAATAATTCAAATAATGGCACATTAT
TCGGTGGATTATTCGCGGCATTAGGATCATTATTGTTATTCGGTCGTCGTAAAAAACAAAATAAATAA

Gene: sdrD (serine aspartate repeat protein D)

Position: 605751 to 609968, length: 4218 nt, orientation: FORWARD

Perfect match to: (11819-97-CP003194-[606486:610703], allele observed in CC80)

Sequence:

ATGCTAAACAGAGAAAATAAAACGGCAATAACAAGAAAAGGCATGGTATCCAATCGATTAAATAAATTTTCGATTAGAAAGTACACAGTG
GGAACAGCATCAATTTTAGTAGGTACAACATTAATTTTTGGTCTGGGGAACCAAGAAGCAAAGGCTGCAGAAAGTACTAATAAAGAATTG
AACGAAGCGACAACTTCAGCAAGTGATAATCAATCGAGTGATAAAGTTGATATGCAGCAACTAAATCAAGAAGACAATACTAAAAATGAT
AATCAAAAAGAAATGGTATCATCTCAAGGTAATGAAACGACTTCAAATGGGAATAAATTAATAGAAAAAGAAAGTGTACAATCTACCACTG
GAAATAAAGTTGAAGTTTCAACTGCCAAATCAGATGAGCAAGCTTCACCAAAATCTACGAATGAAGATTTAAACACTAAACAAACTATAAG
TAATCAAGAAGCGTTACAACCTGATTTGCAAGAGAATAAATCAGTGGTAAATGTTCAACCAACTAATGAGGAAAACAAAAAGGTAGATGC
CAAAACTGAATCAACTACATTAAATGTTAAAAGTGATGCTATCAAGAGTAATGATGAAACTCTTGTTGATAACAATAGTAATTCAAATAATG
AAAATAATGCAGATATCATTTTGCCAAAAAGTACAGCACCTAAACGTTTGAATACAAGAATGCGTATAGCAGCAGTACAGCCATCATCAAC
AGAGGCTAAAAATGTTAATGATTTAATCACATCAAATACAACATTAACTGTCGTTGATGCAGATAAAAACAATAAAATCGTACCAGCACAA
GATTATCTAGCATTAAAATCACAAATTAAAGTTGATGACAAAGTTAAATCAGGTGATTATTTCACAATAAAATACTCAGATACAGTACAAGT
ATATGGATTGAATCCGGAAGATATTAAAAATATTGGAGATATTAAAGATCCAAATAATGGTGAAACAATTGCGACTGCAAAACATGATACT
GCAAATAATTTAATTACATATACATTTACAGATTATGTAGATAGATTTAATTCTGTACAAATGGGAATTAATTATTCAATTTATATGGATGCT
GATACAATTCCTGTTAGTAAAAACGATGTTGAGTTTAATGTTACGATAGGTAATACTACAACAAAAACAACTGCTAACATTCAATATCCAGA
TTATGTTTCTAGAGATAATAATTCTATAGGTTCAGCATTTACTGAAACTGTTTCACATGCAGGTAATGCAGAAGACCCGGGTTACTATATACA
GACTGTTTATGTAAATCCATCGGAAAAAACTTTAACAAATGCTAAATTAAAAGTTGAAGCTTATCATAAGGATTATCCTGACAATGTAGGTC
AAATAAATAAAAATGTAACTAAAATTAAAATTTATCAAGCACCAAAAGACTATGTTTTAAACAAAGGTTATGATGTTAATACAAATCAACTT
ATAGATGTGACTGAACAATTTAAAGATAAGATTACATACGGAACTAATGACAGTGTAAATGTTGATTTTGGTAGCATTAACAATTCTTATGT
AGTTATGGTGGATACAAAATTTGAATTTACAACTAGTGAAAGTCCAACACTTGTTCAAATGGCAACTTTGACTTCTGATGGAAACAGATCCG
TTTCTACTGGCAATGCTTTAGGATTTACTAATAACCAAAGTGGCGGAGCTGGTCAAGAAGTATATAAAATTGGTAACTACGTATGGGAAGA
TACTAATAAAAACGGTGTTCAAGAATTAGGAGAAAAAGGCGTTGGCAATGTAACTGTAACTGTATTTGATAATAATACAAATACAAAAGTA
GGAGAAGCAGTTACTAAAGAAGATGGGTCATACTTGATTCCAAACTTACCTAATGGAGATTACCGTGTAGAATTTTCAAACTTACCAAAAG
GTTATGAAGTAACCCCTTCAAAACAAGGTAATAACGAAGAATTAGATTCAAACGGCTTATCTTCAGTTATTACAGTTAATGGCAAAGATAAC
TTATCTGCAGACTTAGGTATTTACAAACCTAAATACAACTTAGGTGACTATGTCTGGGAAGATACAAATAAAAATGGTATCCAAGACCAAG
ATGAAAAAGGTATATCTGGCGTAACGGTAACATTAAAAGATGAAAACGGTAACGTGTTAAAAACAGTAACAACAGACGCTGATGGCAAAT
ATAAATTTACTGATTTAGATAATGGTAATTATAAAGTTGAATTTACTACACCAGAAGGCTATACACCGACTACAGTAACTTCTGGAAATGAT
ACTGAAAAAGACTCTAATGGTTTAACAACAACAGGTGTTATTAATGGTGCTGATAACATGACATTAGATAGTGGATTCTACAAAACACCAA
AATATAATTTAGGTAATTATGTATGGGAAGATACAAATAAAGATGGTAAGCAGGATTCAACTGAAAAAGGTATTTCAGGCGTAACAGTTAC
ATTGAAAAATGAAAACGGTGAAGTTTTACAAACAACTAAAACAGATAAAGATGGTAAATATCAATTTACTGGATTAGAAAATGGAACTTAT
AAAGTTGAATTCGAAACACCATCAGGTTACACACCAACACAAGTAGGTTCAGGAACTGATGAAGGTATAGATTCAAATGGTACATCAACAA
CAGGTGTCATTAAAGATAAAGATAACGATACTATTGACTCTGGTTTCTACAAACCGACTTACAACTTAGGTGACTATGTATGGGAAGATACA
AATAAAAACGGTGTTCAAGATAAAGATGAAAAGGGTATTTCAGGTGTAACAGTTACGTTAAAAGATGAAAACGACAAAGTTTTAAAAACA
GTTACAACAGATGAAAATGGTAAATATCAATTCACTGATTTAAACAATGGAACTTATAAAGTTGAATTCGAGACACCATCAGGTTATACACC
AACTTCAGTAACTTCTGGAAATGATACTGAAAAAGATTCTAATGGTTTAACAACAACAGGTGTCATTAAAGATGCAGATAACATGACATTA
GACAGTGGTTTCTATAAAACACCAAAATATAGTTTAGGTGATTATGTTTGGTACGACAGTAATAAAGACGGCAAACAAGATTCAACTGAAA
AAGGTATCAAAGATGTTAAAGTTACTTTATTAAATGAAAAAGGCGAAGTAATTGGAACAACTAAAACAGATGAAAATGGTAAATATCGTTT
CGATAATTTAGATAGCGGTAAATACAAAGTTATTTTTGAAAAGCCTGCTGGCTTAACACAAACAGGTACAAATACAACTGAAGATGATAAA
GATGCAGATGGTGGCGAAGTTGACGTAACAATTACGGATCATGATGATTTCACACTTGATAACGGATACTTCGAAGAAGATACATCAGATA
GCGACTCAGATTCAGATAGCGATTCAGATTCAGATAGCGACTCAGACTCAGACAGCGACTCAGATTCAGACAGCGACTCAGACTCGGATA
GCGATTCAGATTCAGACAGTGATTCAGATTCAGACAGCGATTCGGATTCAGACAGCGACTCAGACTCGGATAGCGACTCAGACTCAGATAG
CGACTCAGATTCGGATAGCGACTCAGACTCAGACAGCGACTCAGATTCAGACAGCGACTCAGACACAGATAGCGACTCAGACTCAGACAG
CGACTCAGACAGTGATTCAGACTCAGATAGCGATTCAGATTCAGACAGCGACTCAGACTCAGATAGTGACTCAGATTCAGATAGTGACTCA
GACTCAGACAGTGATTCAGACTCAGACAGTGATTCAGACTCAGATAGCGACTCAGACTCAGACAGCGACTCAGACTCAGATAGTGACTCAG
ATTCAGATAGCGATTCGGACTCAGACAGTGATTCAGATTCGGACAGCGACTCAGACTCAGACAGTGATTCAGATTCAGATAGCGACTCAGA
CTCAGATAGCGACTCAGATTCAGACAGCGATTCAGACTCAGATAGTGACTCAGATTCGGACAGCGATTCAGACTCAGATAGCGACTCAGAT
TCAGACAGTGATTCAGACTCAGATGCAGGTAAGCACACACCTGTTAAACCAATGAGTACTACTAAAGACCATCACAATAAAGCAAAAGCAT
TACCAGAAACAGGTAATGAAAATAGCGGCTCAAATAACGCAACGTTATTTGGCGGATTATTCGCAGCATTAGGATCATTATTGTTATTCGGT
CGTCGTAAAAAACAAAATAAATAA

Gene: bbp (bone sialoprotein-binding protein)

Position: 610363 to 613818, length: 3456 nt, orientation: FORWARD

Sequence:

ATGATTAACAGGGATAATAAAAAGGCAATAACAAAAAAGGGTATGATTTCAAATCGCTTAAACAAATTTTCGATTAGAAAGTATACTGTAG
GAACTGCATCGATTTTAGTAGGTACGACATTGATTTTTGGTCTAGGGAACCAAGAAGCTAAAGCTGCTGAAAACACTAGTACAGAAAATGC

AAAACAAGATGATGCAACGACTAGTGATAATAAAGAAGTAGTGTCGGAAACTGAAAATAATTCGACAACAGAAAATAATTCAACAAATCC
AATTAAGAAAGAAACAAATACTGATTCACAACCAGAAGCTAAAAAAGAATCAACTTCATCAAGTACTCAAAAACAGCAAAATAACGTTACA
GCTACAACTGAAACTAAGCCTCAAAACATTGAAAAAGAAAATGTTAAACCTTCAACTGATAAAACTGCGACAGAAGATACATCTGTTATTTT
AGAAGAGAAGAAAGCACCAAATAATACAAATAACGATGTAACTACAAAACCATCTACAAGTGAAATTCAAACAAAACCAACTACACCTCAA
GAATCTACAAATATTGAAAATTCACAACCGCAACCAACGCCTTCAAAAGTAGACAATCAAGTTACAGATGCAACTAATCCAAAACAAACGG
TAAATGTATCAAAAGAAGATCTTAAAAATGATCCTGAAAAATTAAAAGAATTAGTTAGAAGTGAAAATAACACTAATCATTCAACTAAACCA
GTTGCTACAGCACCAACAAGTGTTGCACCAAAACGATTAAATGCGAAAATGCGTTTTGCAGTTGCACAACCAGCAGCAGTTGCGTCAAATA
ATGTAAATGATTTAATTACAGTGACGAAACAGACGATCAAAGTTGGCGATGGTAAAGATAATGTGGCAGCAGCGCATGACGGTAAAGATA
TTGAATATGATACAGAGTTTACAATTGACAATAAAGTCAAAAAAGGCGATACAATGACGATTAATTATGATAAGAATGTAATTCCTTCGGAT
TTAACAGATAAAAATGATCCTATCGATATTACTGATCCATCAGGAGAGGTTATTGCCAAAGGAACATTTGATAAAGCGACTAAGCAAATCA
CATATACATTTACAGATTATGTAGATAAATATGAAGATATAAAAGCACGTTTAACTTTATACTCATATATTGATAAGCAAGCAGTACCTAATG
AAACTAGTTTGAATTTAACGTTTGCAACAGCAGGTAAAGAAACTAGCCAAAACGTTTCTGTTGATTATCAAGACCCAATGGTTCATGGTGAT
TCAAACATTCAATCTATCTTTACAAAGTTAGATGAAAACAAACAAACTATTGAACAACAAATTTATGTTAATCCTTTGAAAAAAACAGCAACT
AACACTAAAGTTGATATAGCTGGTAGTCAAGTAGATGATTATGGAAATATTAAACTAGGAAATGGTAGTACCATTATTGACCAAAATACAG
AAATAAAAGTTTATAAAGTTAACCCTAATCAACAATTGCCTCAAAGTAATAGAATCTATGATTTTAGTCAATACGAAGATGTAACAAGTCAA
TTTGATAATAAAAAATCATTTAGTAATAATGTAGCAACATTGGATTTTGGTGATATTAATTCAGCCTATATTATCAAAGTTGTTAGTAAATAT
ACACCTACATCAGATGGCGAACTAGATATTGCTCAAGGTACTAGTATGAGAACAACTGATAAATATGGTTATTATAATTATGCAGGATATTC
AAACTTCATCGTAACTTCTAATGACTCTGGCGGTGGTGACGGTACTGTTAAACCTGAAGAAAAGTTATACAAAATTGGTGACTATGTATGG
GAAGACGTTGATAAAGACGGTGTCCAAGGTACAGATTCGAAAGAAAAGCCAATGGCAAACGTTTTAGTTACATTAACTTACCCAGACGGT
ACTACAAAATCAGTAAGAACAGATGCTAACGGTCATTATGAATTCGGTGGTTTGAAAGACGGAGAAACTTATACAGTTAAATTCGAAACGC
CAGCTGGATATCTTCCAACAAAAGTAAATGGAACAACTGATGGTGAAAAAGACTCAAATGGTAGTTCTGTAACTGTTAAAATTAATGGTAA
AGATGATATGTCTTTAGACACTGGTTTTTATAAAGAACCTAAATATAATCTTGGTGACTATGTATGGGAAGATACAAATAAAGATGGTATCC
AAGATGCTAATGAACCTGGTATCAAAGATGTTAAGGTTACATTAAAAGATAGTACTGGAAAAGTTATTGGTACAACTACTACTGATGCCTC
GGGTAAATATAAATTTACAGATTTAGATAATGGTAACTATACAGTAGAATTTGAAACACCAGCAGGTTACACGCCAACGGTTAAAAATACT
ACAGCTGATGATAAAGATTCTAATGGTTTAACAACAACAGGTGTCATTAAAGATGCAGATAATATGACATTAGACAGTGGTTTCTATAAAA
CACCAAAATACAGTTTAGGTGATTATGTTTGGTACGACAGTAATAAAGACGGCAAACAAGACTCAACTGAAAAAGGTATTAAAGATGTGAC
AGTTACATTGCAAAACGAAAAAGGCGAAGTAATTGGAACAACTAAAACAGATGAAAATGGTAAATATCGTTTCGATAATTTAGATAGCGGT
AAATACAAAGTTATTTTTGAAAAGCCTGCTGGCTTAACACAAACAGTTACAAATACAACTGAAGATGATAAAGATGCAGATGGTGGCGAAG
TTGACGTAACAATTACGGATCATGATGATTTCACACTTGATAACGGATACTACGAAGAAGAAACATCAGATAGCGACTCCGATTCGGACAG
CGATTCAGACTCAGATAGCGACTCAGATTCAGACAGTGATTCAGACTCAGATAGTGACTCAGACTCAGACAGCGACTCAGATTCAGATAGC
GACTCAGACTCAGACAGCGACTCAGATTCAGATAGTGACTCAGACTCAGATAGCGACTCAGATTCAGACAGCGATTCAGATTCAGATAGTG
ACTCAGACTCAGATAGCGATTCAGATTCAGACAGCGACTCAGACTCAGACAGTGATTCAGATTCAGATAGCGATTCAGACAGCGACTCAGA
CTCGGATAGCGACTCAGACTCAGACAGCGATTCAGACTCGGATAGCGATTCAGATTCGGACAGCGACTCAGACTCAGACAGTGATTCAGAT
TCAGATAGCGACTCAGACTCAGATAGCGACTCAGACTCAGACAGTGATTCAGACTCAGATAGCGATTCGGACTCGGATGCAGGAAAACAT
ACACCTGTTAAACCAATGAGTACTACTAAAGACCATCACAATAAAGCAAAAGCATTACCAGAAACAGGTAGTGAAAATAACGGCTCAAATA
ACGCAACGTTATTTGGTGGATTATTTGCAGCATTAGGTTCATTATTGTTATTCGGTCGTCGCAAAAAACAAAACAAATAA

Gene: sdgA (serine-aspartate repeat glucosyltransferase A)

Position: 613937 to 615409, length: 1473 nt, orientation: FORWARD

Perfect match to: (MW2-BA000033-[606990:608462], highly conserved allele)

Sequence:

ATGAATTACATTTTAGGAACAATTTTAGAAAGTAAAATTACAGGTGTAGAAAAAGCGCAAATAAATAGATTGAAGTTGTTCAAACAACACG
GCATATCTTCAAAATGTGTATATGTTAAATGGAATCCTTATTCATACACATATGCGAAGCAACATCAGATTGAAAATGATGTATTTACAATGT
ATGACTATTTTCAAAAAGCAATCAATTATAAAAAGACAAAGCAAGTTAACTGGATACAGTATTGGGAAAAGTCATGTAGGTACACATTGAA
ATTTGTGGAAAATTCAAATGATGTCAGAATATATGATGAAGAGCAATTTATAATGTATGCTCATTTTTTAGATAAACAGTATCATCATTTAAA
CTATGTGAATTATTTTGATCATAAAAGAAGAAAAGTAAAACGCGAATTGTATGATGGAAGAGGCTTTTTAAGTTGTTCTCGAATTTTAGGTG
AAGGACAACGGATTGTACTCGAAAATTACTATACACCTAATGGGGAAATCGTCATCCAAAAATATTTCGACGATATAAAAGGGAAAAACAC
GCTCACAAAGGTTATCTTAAATGAAGATCAGCATCAACAATTTTTTGATACAGAAGATGAATTAGTTCAATATTTTCTCCATCAATTATGTAA
AAATAATGATCAAATCATATTAGATCGTCCTCATGAATTAGGAAATGTTATAGCGGGATTAAATCAAAGTATTCCAGTTGTTGTTGTGCTCC
ATAGTACACATTTATTCGGTACCGGTAATGGTATAAAAAGTTTTTATAAAACAGTATTTAATAATTTAACACGTTATAAAGCGATTGTTGTAT
CAACAGAAAAGCAATGCCAAGATATTTCACAATATATTGAAAATAAAATACCAGTTATCAATATTCCGGTTGGCTACGTGGCAAATTTAAAG
TATCAATTTGACATCAATCAAAAGGAGAAAAATCATATCATATCAATTGCTCGCCTCGTTGAAAATAAACAAATTAAACATCAAATTGAAGT
GATCAAGCAATTAGTAACAAAACATCCCAATATTCAATTGAATATTTATGGACATGGAAATGGTTTGTCAGAATATCGACAACTTGTAGAAG
ATTATCATTTATCGGAACATGTTAAATTTCATGGTTTTAAGACGCATATTAATGAAGAGATTGCTAAAGCAGAACTGATGTTATCGACAAGT
AAAATGGAAGGTTTTGGCTTAGCAATTTTAGAGTCGCTTTCAGTAGGTACACCAGTGATCAGTTATGATGTAGATTATGGTCCATCAGAACT

GATTCAAGATGGATTTAATGGCTATTTAGTACCTCAAGGTGACATCAATCAAATGGTTGAAAAGGTCGACCAATTACTAAATAATACTCAAA
AATTGCAACAGTTTTCAATTAATAGCATAGAATCTGCACAACAGTACAATGCAACTACTATCAGTACAAAGTGGCAAAATATTTTAAACTAA

Gene: sdgB (serine-aspartate repeat glucosyltransferase B)

Position: 615534 to 617024, length: 1491 nt, orientation: REVERSE

Sequence:

TTATATAAGACGAACAAATTGGTCCCATTGTTTAATTAACGACGCTTTACTATATTGTTGCGCTTTTGCCAAACTACCTTTTGACAGTCGTTGT
TGTACTTCAGGATGATCAATCACATATTTTACTTTATCAAATAGGGCATCTTCATCATTTTTAGTAATTAAATAACCATTGAAATCAGGCGTA
ATTAATTCGTTAGGTCCATATTTAATATCATAACTAATAACTGGAACACCATGTGCTAAAGATTCAAGTAGCGCTAAAGAGAAACCTTCCAT
GTTACTTGTTATTAAACTCAAATAGGCATCGCTATATTCTTGGTCTAGATTGCTTAAAAAGCCGCGTAAGTAAACATGATTTTCCAATCTATA
TTTTTGTATCAATTCATTTAATTTTTTACTTTCAGAACCAAAACCATACATATGAAGCTCTATTTTTGGGACATACGATACTAAGCGTTTAATT
AATTCAATTTGTTGATGTAATTGTTTTTCAGGTGAATAACGAGCAACGGAAATTAATTTAACACTGCGCTGATCTAATGTTTGGACTGGTGT
ATCAATTGTTTCACTATAGCCGACAGGAATATTAACAACTGGAATAGTATGGTTAATACGTTTTTCAACATCTAATTTTTGCTGCTCAGTAGA
AACGATAATTGCACGATATCGAGATAAATTTTCAAACATCGCTTTATATACATTTTTAAATGGCGATGAATCTAATGCATCAATATTTTTAAT
GTGTGTACTGTGAAGCACAGCTACTACTGGGATTGACTCAGGCGTTAAGTTGAAAATAGGTGCTGTGTACACATTACGGTCACTGAAAAAT
AAATCCCCATGTTGATATAGTTGTTTAATGAAAAATGCGCCTAATTCCGTTTCATTATTAAAGAAATATTGTTTGTTAGCATAGTAAACAATA
ATTTTTTGTACTTCTGGTTTGCCATCTTTGTAAGAAAAATACTTTTCTAATTTTGTGTCACCTTCTGGATTATAGAAAAATTCACATAATGTTTG
TTGTTTATCAACAAGAATCCTACTACAACTTAAAAAGCCACGCACATCATAAAAATCACGTTTTACTTTTCGTCTTTGACTATCAAAATGATTC
ACATAATCTAATATACGATATTTAGGATCTTGAAAATGGGCATACATTAAGAAACGCTCTTGATCATATATTCTAAAGTCATGACTATTTTCA
ACATGTTTTAAAGTATAATGACATTCATCAGTCCAATACGACAACCAGTCAAATGGTTCATTGCGTTCTAAATATGTTGCTTCTTGGAAGAAA
TCATACATATTAATATAGTCAGAACTAGTAATATAATTTTGGGCATTTCTATATAAATATCTATTCCATGACAGAAATACACATTGCGCTGGT
CTTCCCATTTCTTTAAATAAATTTAAACGATTAATAATTGCTTTTTCTATCCCAGTTAAATTAACACCTAAACTATTACCTACAAAATAATTCAT

Gene: folE2 (GTP cyclohydrolase)

Position: 617306 to 618184, length: 879 nt, orientation: REVERSE

Perfect match to: (Strain_21334-AGTW01000032-[8167:9045:r], allele observed in CC9+CC8+CC88+CC239+CC4803)

Sequence:

TTATTTGCGATACTTTAATTTAGCGAAAGCATCATGTTGATGGATAGACTCTTCATTACGACATTCGATATCGAAACCGTCTAACCAATCAAA
TCCAACTAAGTCCGCGGCAATTAAACGAATTAAGTCTTCGACAAAACGTGGATTTTCATATGCACGCTCTGTCACACGTTTTTCATCAGGAC
GTTTTAAAATAGGGTATAGAATTGAACTTGCATTAGCTTCCATTGCATCTAAAATTTTATTTTTATAGTCATCAACTATGTCTTGATCTTTATT
AATATATGTTTTAACAGTGACAACACCACGTTGGTTGTGCGCTGAATACTCACTTATTTCTTTTGAACAAGGGCATAGCGTTGTGACAGTTG
CTTCAATAGTAAGTTCTTTACGTGTAACTTTATCACCGTCAATTGCTAATCCATAAGTGACATCGGCATTACCAACTGCTTTAATATTTGTGGT
TGGACTATAGCGATCAAAGAACCATTTCCCAGAAACATCAACGCCTGCCGCATTTTGTTTCATATTCGTTTGTAAAGTGCGTAACACCTGATA
AAGTGTATTAAATTCAAGTTCAATACCATTATCATAGTGCTTTTCAACACTTTCAATTATACGGCTCATATTAATACCTTTTTCGTCTTTTGTTA
AACTTGTTGAAAAACTAAATGTGCCAGCTGTTTGATACTGGTCAACAAGTACAGGGTACACTAAGTTTTTAATACCAACTTCTTCTATTTCAA
ATAAAAAATCTTTATGTGTACTTTGTAAATCTGTCATTTCGTTCTTAGTAGTAGGTTTCGTGCCTTCAATAGGATCTACGGAACCAAAGTGTT
TCCAACGACCTTCTCGTGTCGATAAATCAAATTCAGTCAT

Gene: bshB2 (bacillithiol biosynthesis deacetylase)

Position: 618197 to 618862, length: 666 nt, orientation: REVERSE

Perfect match to: (N315-BA000018-[620800:621465:r], highly conserved allele)

Sequence:

TTAAGATTTAAAGTGATATGTCCAATATGGTTCGACTGTTAAAAAGCTGTGTTGTTTACCATCGATTTCAGGACTTGCTAATTGTTTTAAAAA
TGGACCTGTTTGAGAAGCATGTGCTTCAAATGCCTTAATTTTAAGTTCTTTAAAATCTGTAATATCATTTTGAATATCAGGTTCTCCAAGAGC
TTCGGTTGCATCATTACTGAACGCAACTAAAGTTAAACGAGGGCGTTCTTCTTTAGGCATGCGTTCAACCGTTCGAATTACAGCGTCTGCTG
TTGCTTCGTGATCAGGATGTACTGCATATCCAGGATAAAATGAAATAATCAATGATGGATTTGTATCATCGATTAAAGATTTAATCATACCA
TCTATATGTTCGTAGGGTTCAAATTCGACAGTTTTGTCACGTAAACCCATTTTTCTTAAATCAGTAATACCAATAACTTTACAAGCTTCTTCTA
GTTCACGCTCACGAATACTTGGTAATGATTCGCGTGTTGCAAATGGGGGATTACCTAAATTTCTGCCCATTTGTCCTAGGGTTAAACATGCA

TATGTTACAGGTATGCCTTTTTGGATATAACTTGCTAATGTGCCTGCAGATGAGAAGGTTTCATCATCAGGATGTGGAAATATTACTAATAC
ATGTCTTTCGTCAGTCAT

Gene: yojF (protein of unknown function, DUF1806)

Position: 618876 to 619238, length: 363 nt, orientation: REVERSE

Perfect match to: (N315-BA000018-[621479:621841:r], highly conserved allele)

Sequence:

CTATAAATTAAATGGTCGCTCACTAATTTGAAGTGCTGCAGCGAGTTGACCTTCGTAATTAAAACCTGCAATTAAAAATTCATCATGTTCATT
GACCTCAAAATGCGTTAGACCTTGTACATAAACCCAACCACCATTTGATAGTTTAAGACCAATGCGATAAGGTTCTTTATTACCACCTTTTAG
TTGTGCATGCGTATATGTTACTTGTATGTTTCTTAAAAATGTACCAGCATTAAAAACACGTTGATCGAAATGGTTCGCATAGGCCCCATTTGT
CGTTTCAACATGCAGATACACAGGTTTATGTTCAAAAGAAGCAAGTAAATCTATAACTTCTTGTTCTTTAATTGGTTCCAACAC

Gene: nagB (glucosamine-6-phosphate deaminase)

Position: 619514 to 620272, length: 759 nt, orientation: FORWARD

Perfect match to: (11819-97-CP003194-[620249:621007], allele observed in CC80+CC1+CC361)

Sequence:

ATGAAAGTATTAAACTTAGGATCGAAAAAACAAGCATCATTCTATGTTGCATGTGAGTTATATAAAGAGATGGCATTTAATCAGCACTGTAA
ACTTGGTTTAGCAACTGGTGGTACAATGACAGATTTGTATGAGCAACTTGTTAAGTTATTAAATAAAAATCAGTTAAACGTAGACAATGTAT
CCACGTTTAATTTAGACGAATATGTAGGTTTAACCGCATCACATCCGCAAAGTTATCACTATTATATGGATGACATGCTTTTCAAACAATATC
CTTATTTTAATAGAAAGAACATTCATATTCCAAATGGAGATGCCGATGATATGAATGCGGAAGCGTCAAAATATAATGACGTTTTAGAACA
ACAAAGTCAACGTGATATTCAAATTTTAGGTATTGGAGAAAATGGTCATATTGGATTTAATGAACCTGGTACGCCGTTTGATAGTGTTACTC
ATATCGTTGATTTGACTGAAAGTACTATTAAGGCTAATAGTCGATATTTTGAAAACGAAGATGATGTTCCAAAGCAAGCCATTTCGATGGGA
CTTGCTAATATTCTTCAAGCCAAACGTATCATTTTACTCGCATTTGGTGAAAAGAAACGTGCTGCTATTACACATTTATTAAATCAGGAAATT
TCTGTTGATGTTCCAGCCACATTACTTCACAAACACCCGAATGTTGAGATATATTTAGACGACGAAGCTTGCCCGAAAAATGTTGCGAAAAT
TCATGTCGATGAAATGGATTGA

Gene: hxlA (3-hexulose-6-phosphate synthase)

Position: 620349 to 620981, length: 633 nt, orientation: FORWARD

Perfect match to: (MW2-BA000033-[613401:614033], highly conserved allele)

Sequence:

GTGGAATTACAATTAGCAATTGATTTATTAAACAAAGAAGACGCGGCTGAGTTAGCAAATAAAGTAAAAGATTATGTAGATATCGTAGAAA
TCGGTACGCCAATCATTTACAACGAAGGTTTACCAGCAGTTAAACATATGGCAGACAACATTAGTAATGTAAAAGTATTAGCAGACATGAA
AATTATGGATGCAGCTGATTATGAAGTTAGCCAAGCAATTAAATTTGGCGCGGATGTAATTACAATACTAGGTGTTGCAGAAGATGCATCA
ATTAAAGCAGCTATTGAAGAAGCTCATAAAAATAATAAACAATTACTAGTTGATATGATTGCTGTTCAAGATTTAGAAAAACGTGCAAAAG
AACTAGATGAAATGGGTGCGGATTATATTGCAGTACACACTGGTTATGATTTACAAGCAGAAGGGCAATCACCATTAGAAAGTTTAAGAAC
CGTTAAATCTGTTATTAAAAATTCTAAAGTTGCAGTAGCAGGTGGAATTAAACCAGATACAATTAAAGAAATTGTCGCTGAAAGTCCTGATC
TTGTTATTGTTGGTGGCGGAATCGCAAATGCAGATGATCCAGTAGAAGCTGCAAAACAATGTCGCGCTGCAATCGAAGGTAAGTAA

Gene: sis (sugar isomerase)

Position: 620983 to 621531, length: 549 nt, orientation: FORWARD

Perfect match to: (MW2-BA000033-[614035:614583], allele observed in CC1+CC80+CC361)

Sequence:

ATGGCTGAATTTAGTGACTATCAATTAATTCTAGATGAATTAAAGATGACTTTGTCACATGTTGAAGCGGATGAGTTTTCAACGTTTGCATC
CAAAATACTACATGCTGAACATATATTTGTAGCTGGTAAAGGACGTTCAGGATTCGTGGCGAATAGTTTTGCAATGCGCTTAAATCAGCTCG

GCAAACAGGCACATGTTGTTGGAGAATCAACGACACCTGCGATTAAGTCGAATGATGTATTTGTAATTATCTCTGGTTCAGGTTCCACGGA
ACATTTAAGATTATTAGCAGACAAAGCAAAATCAGTAGGTGCTGACATCGTATTAATTACTACAAATAAAGATTCTGCAATAGGCAATCTAG
CTGGGACGAACATCGTTTTGCCTGCAGGTACAAAATATGATGAACAAGGCTCGGCACAACCATTAGGAAGTTTGTTTGAACAAGCATCTCA
ATTATTTTTAGATAGTGTTGTAATGGGATTGATGACTGAAATGAATGTTACGGAACAAACGATGCAACAAAATCATGCTAATTTAGAATAA

Gene: Q2FJ68 (putative haloacid dehalogenase-like hydrolase)

Position: 621644 to 622291, length: 648 nt, orientation: FORWARD

Perfect match to: (COL-CP000046-[651653:652300], allele observed in CC8+CC5+CC7+CC8+CC239)

Sequence:

TTGAAGTTTGACAATTATATTTTTGATTTTGATGGTACGTTGGCAGACACGAAAAAATGTGGTGAAGTAGCAACACAAAGTGCATTTAAAG
CATGTGGCTTAACGGAACCATCATCTAAAGAAATAACGCATTATATGGGAATACCTATTGAAGAATCATTTTTAAAATTAGCAGACCGACCA
TTAGATGAAGCAGCATTAGCAAAGTTAATCGATACATTTAGACATACATATCAATCTATTGAAAAGGACTATATTTATGAATTTGCGGGTAT
AACTGAAGCCATTACAAGTTTGTATAACCAAGGGAAAAAACTTTTCGTGGTGTCTAGTAAGAAGAGTGATGTATTAGAAAGAAATTTATCG
GCTATTGGATTAAATCACTTGATTACCGAAGCTGTTGGATCCGATCAAGTAAGTGCATATAAACCAAATCCTGAAGGCATACACACAATTGT
GCAACGCTACAATTTAAATAGCCAACAAACGGTTTATATTGGTGATTCAACATTTGATGTTGAGATGGCGCAACATGCTGGTGTACCATCTG
CAGCAGTAACATGGGGAGCACATGATGCAAGGTCATTACTTCATTCAAATCCAGATTTTATAATCAATGATCCATCAGAAATTAATACAGTA
TTATAA

Gene: proP (putative proline/betaine symporter)

Position: 622793 to 624193, length: 1401 nt, orientation: FORWARD

Perfect match to: (11819-97-CP003194-[623528:624928], allele observed in CC80+CC8+CC9+CC80+CC188)

Sequence:

ATGGATTTTAATAAAGAGAATATTAACATGGTGGATGCAAAGAAAGCTAAAAAAACCGTTGTTGCAACCGGTATCGGTAATGCAATGGAA
TGGTTCGATTTTGGTGTCTATGCATATACAACTGCGTACATTGGAGCGAACTTCTTCTCTCCAGTAGAGAATGCAGACATTCGACAAATGTT
GACTTTCGCAGCATTAGCCATTGCGTTTTTATTAAGACCAATTGGTGGTGTCGTATTTGGTATTATTGGTGACAAATATGGACGTAAAGTTG
TATTAACATCTACAATTATTTTAATGGCATTTTCAACATTAACCATTGGATTATTGCCAAGCTATGATCAAATTGGACTTTGGGCACCAATACT
ATTATTGCTTGCAAGAGTACTGCAAGGATTTTCAACAGGTGGAGAGTATGCGGGGGCAATGACATATGTTGCCGAATCATCTCCAGATAAG
CGTCGTAACTCATTAGGTAGTGGACTAGAAATTGGGACATTATCAGGTTACATAGCTGCTTCAATTATGATTGCTGTATTAACATTCTTTTTA
ACAGATGAACAAATGGCATCATTTGGTTGGAGAATCCCATTCTTACTCGGTTTATTCCTAGGATTATTTGGCTTATATTTACGTCGTAAGCTG
GAAGAATCACCAGTTTTCGAAAATGATGTTGCAACACAACCAGAAAGAGATAACATTAACTTTTTACAAATCATCAGATTTTATTACAAAGA
TATATTTGTATGTTTTGTAGCTGTTGTATTCTTCAATGTTACGAACTATATGGTAACTGCATATTTACCAACCTATTTAGAACAAGTTATTAAA
TTAGATGCAACGACAACAAGTGTATTAATTACTTGTGTCATGGCAATAATGATTCCATTAGCATTAATGTTTGGTAAGTTAGCGGATAAAAT
AGGTGAAAAGAAAGTATTTCTAATTGGTACTGGTGGGCTAACATTATTCAGTATCATCGCATTTATGTTATTACATTCACAATCATTTGTTGT
AATAGTAATCGGTATATTTATATTAGGATTTTTCTTATCAACTTACGAAGCGACAATGCCAGGGTCGTTACCAACGATGTTTTACAGTCATAT
AAGATATCGAACTTTATCAGTAACATTTAATATCTCTGTTTCGATATTTGGTGGTACGACGCCATTAGTTGCAACATGGTTAGTTACGAAAAC
TGGAGATCCATTAGCACCTGCGTATTATTTAACAGCAATCAGTGTTATTGGCTTTTTAGTTATTACATTCTTACATTTAAGTACAGCAGGAAA
ATCTCTAAAAGGTTCGTATCCAAATGTAGATAACGAGCAAGATAGAGCTTATTATGCAGAACATCCAAAAGAAGCATTATGGTGGGTTAAA
GAACGTAAGAATTAG

Gene: rsaOI (structural RNA)

Position: 624194 to 624527, length: 334 nt

Sequence:

AGATTTTAATAAAAAGTATAAATCAATCGTATATAAGCACTTTAAAGCTAGTAGGTTCTGCTAACTTTAAAGTGCTTTTTAAATTGAGAACTG
TAATTAGCCGTAATAAAGTTTTTGTATATACATAAACCCCCACTGCAATGATTATCGCAATGGGGGAAAGAGGGGACTTAAAGCATATGTTT
AGCTTTGAATACTTAAAATTCTCTTGCTATTGAAATGTTAGGATGTAAATATGTCTTAGAGTATTTTGTCCAACGCAATTAATATTGAGACTC
TAACCTTCAATATTATTATAGAGAACACAAACTTAAATAGATTGGGTGACTTATTT

Gene: vraA (long chain fatty acid CoA ligase)

Position: 624737 to 626113, length: 1377 nt, orientation: FORWARD

Perfect match to: (COL-CP000046-[654746:656122], highly conserved allele)

Sequence:

ATGAACGTAATTTTAGAACAGTTGAAAACACATACTCAAAATAAACCTAATGACATAGCATTACATATCGATGATGAAACAATTACATATAG
TCAACTAAATGCCCGCATCACTAGCGCAGTTGAATCTTTGCAGAAATATTCACTTAACCCTGTCGTTGCTATTAATATGAAATCACCGGTGCA
AAGTATTATTTGTTATTTAGCTTTGCATCGTTTACATAAAGTGCCTATGATGATGGAAGGTAAATGGCAAAGTACTATACATCGTCAATTGAT
TGAAAAATATGGTATTAAAGATGTAATTGGAGATACAGGTCTCATGCAGAATATAGACTCACCGATGTTTATTGATTCAACGCAATTACAGC
ACTACCCCAATTTATTACATATTGGTTTTACTTCAGGGACAACTGGACTGCCAAAAGCATATTATCGTGATGAAGATTCATGGTTGGCTTCTT
TTGAAGTTAATGAAATGTTGATGTTAAAAAATGAAAATGCAATAGCAGCCCCTGGACCACTATCGCACTCGTTAACATTATATGCGTTATTG
TTTGCTTTAAGTTCCGGTCGTACTTTTATAGGACAGACCACTTTTCATCCTGAAAAGTTACTTAATCAATGTCATAAAATATCATCATACAAAG
TTGCTATGTTTCTTGTTCCAACGATGATTAAATCATTATTGTTAGTTTACAACAATGAACATACAATCCAATCATTTTTTAGCAGTGGAGATAA
GCTGCATTCTTCTATTTTTAAAAAGATAAAAAATCAAGCAAATGACATAAATTTGATTGAATTTTTTGGTACATCGGAAACCAGTTTTATCAG
CTATAACTTGAATCAGCAAGCACCAGTTGAATCAGTAGGTGTGCTATTTCCAAATGTGGAATTGAAAACAACGAATCACGATCACAATGGT
ATAGGAACTATTTGTATAAAAAGTAATATGATGTTTAGTGGCTATGTAAGTGAACAATGTATAAATAATGATGAATGGTTTGTTACTAATGA
TAATGGCTATGTAAAAGAGCAGTATTTATATTTAACGGGACGTCAACAGGATATGTTAATTATTGGTGGTCAAAATATATATCCAGCACATG
TTGAACGCCTTTTAACGCAATCTTCGAGCATTGATGAAGCAATTATCATCGGTATTCCAAATGAGCGTTTTGGTCAAATAGGCGTATTGCTTT
ATTCTGGTGATGTGACACTTACACATAAAAATGTAAAACAATTTTTAAAAAAGAAAGTGAAACGCTATGAAATTCCATCGATGATTCATCAT
GTAGAAAAGATGTATTACACTGCAAGTGGTAAAATTGCTAGAGAAAAAATGATGTCGATGTATTTGAGAGGTGAATTATAA

Gene: vraB (acetyl-CoA c-acetyltransferase)

Position: 626115 to 627254, length: 1140 nt, orientation: FORWARD

Perfect match to: (N315-BA000018-[628718:629857], highly conserved allele)

Sequence:

ATGAATCAAGCAGTCATAGTTGCAGCTAAACGAACTGCATTTGGGAAATATGGTGGCACTTTAAAACATTTAGAGCCAGAACAATTGCTTA
AACCTTTATTCCAACATTTTAAAGAGAAGTATCCAGAGGTAATATCTAAAATAGATGATGTAGTTTTAGGTAATGTTGTTGGGAATGGTGGC
AATATTGCAAGAAAAGCATTGCTTGAAGCGGGGCTTAAAGATTCAATACCTGGCGTCACAATCGATCGGCAATGTGGGTCTGGACTTGAAA
GTGTTCAATATGCATGTCGCATGATCCAAGCCGGAGCTGGCAAGGTATATATTGCAGGTGGTGTTGAAAGTACAAGTCGAGCACCTTGGA
AAATCAAACGACCGCATTCTGTGTACGAAACAGCATTACCTGAGTTTTATGAGCGTGCATCATTTGCACCTGAAATGAGCGACCCATCAATG
ATTCAAGGTGCTGAAAATGTGGCCAAGATGTATGATGTTTCAAGAGAATTACAAGATGAATTTGCTTATCGAAGTCATCAATTGACAGCGG
AAAATGTAAAGAATGGAAATATTTCTCAGGAAATATTACCTATAACCGTTAAAGGAGAAATATTCAACACTGATGAAAGTCTAAAATCACAT
ATTCCGAAAGATAACTTTGGCCGATTTAAGCCCGTGATCAAAGGTGGGACCGTTACCGCTGCGAATAGTTGTATGAAAAATGATGGTGCAG
TTTTATTGCTTATTATGGAAAAAGATATGGCATACGAATTAGGTTTCGAGCATGGTTTATTATTTAAAGATGGTGTTACGGTAGGTGTTGAT
TCTAATTTTCCTGGCATTGGTCCAGTACCAGCCATTTCCAACTTACTAAAAAGAAATCAATTAACGATAGAAAATATTGAAGTCATTGAAATT
AACGAAGCGTTCAGTGCACAGGTAGTTGCCTGCCAACAAGCTTTAAATATTTCAAATACGCAATTAAATATATGGGGTGGTGCATTAGCAT
CAGGTCATCCATACGGTGCAAGCGGTGCCCAATTAGTGACTCGATTATTTTATATGTTTGACAAAGAGACTATGATTGCATCTATGGGGATA
GGGGGAGGTCTAGGAAATGCAGCATTATTTACTCGATTCTAA

Gene: vraC (putative protein)

Position: 627229 to 627594, length: 366 nt, orientation: FORWARD

Perfect match to: (RF122-AJ938182-[593815:594180], highly conserved allele)

Sequence:

ATGCAGCATTATTTACTCGATTCTAACCAGCGATTAAATGTGTCATTTTCTAAGGATAGTGTGGCTGCATATTATCAGTGTTTTAACCAACCT
TATAGAAAAGAAGTACCACCATTAATGTGTGCGTCATTATGGCCAAAATTTGATTTATTTAAAAAATATGCAAATAGCGAACTGATTTTAAC
AAAATCAGCAATTAATCAAACTCAAAAGATAGAAGTAGACACAATATATGTAGGGCATTTAGAAGATATTGAATGCCGACAGACTCGCAAT
ATCACACGTTATACAATGGCTTTAACATTAACTAAAAATGATCAACATGTCATAACGGTTACACAAACTTTTATTAAGGCGATGAAGTAG

Gene: Q5HI98 (putative protein)

Position: 627597 to 627866, length: 270 nt, orientation: FORWARD

Perfect match to: (N315-BA000018-[630200:630469], highly conserved allele)

Sequence:

ATGAAGTTTAATGAGATATGGATAAATGAATATTTGGCGCTCGTAAATGATGATAATCCAATACATAATGAGATTGTGCCAGGACAATTAG
TGAGTCAAATGATGCTGATGGCTATGTCATTAGAGACAAACCAGTGTCAAATTAACTACGTTAAACCTATTTTAATAAATGAAAATATCGAA
TTCATTGAACAACACGAACACGAAATTATAGCAATTAATGACGATGGAGAGATTAAAATAAAAATTTCTTTGAGCACAAAAAAATAA

Gene: ssr128 (small stable RNA 128)

Position: 627861 to 628253, length: 393 nt

Sequence:

AAATAACCGATATTAGCTGCATGAACGCATATTAATTAGGAGATGAAAGGACAGCTAATATCAGTTATGTATTGTTATTATTATTGGGAACA
GAGATGAATATAGGTTACGTTTCTTTCTTTGCACGGGGATGCATTAATCTAAAATAATAATAACAACTATATCAATGTTTAATAAATTCTGGA
TTATTGGAACGATTAGTCAATTTAACTAACTTTCATATGATCTATATCGTCTTGTAATAAAGAGAGCAATTTGAATATTTCAGTATCACTAAAT
GAATCGTCACATTTAATTGAAACATGCTGAAACGTTTTGGTTATAATTTCATAAACTGGTGCGCCTTCATGGTGATACTGTCGATAAATAATC
ATAACCTATATTACCTCCTTT

Gene: thiD1 (phosphomethylpyrimidine kinase)

Position: 628741 to 629571, length: 831 nt, orientation: REVERSE

Perfect match to: (MRSA252-BX571856-[637913:638743:r], highly conserved allele)

Sequence:

TTAAACCTCTGTTACTTCAACATCGATATGTTCAATACGGTTGTATGCACCGTGATCCACAGGACCAACAAAATCATTCATTTTCCAACCGTTT
TTAATAGCAGAAGCGACGAAAGCTTTCGCGCTAATCACAGCTTCTTTCGGTGACTTACCGTTAGCTAAATATGCAGTTGTTGCCGCAGCAAA
TGTACAACCAGCACCATGGTTATAACTTTGTTGGAACATGTCTGTTGTTAGTTGATAAAATGTTTGACCATCATAGTATAAGTCATACGATTT
ATCTTGATCTAAAGCTTTGCCACCTTTAATGATGACATGCTGTGCGCCTTTATCAAAGATAATTGTTGCAGCCTTTTTCATATCTTCAATTGAA
TTTAATTTACCTAATCCTGATAATTGACCCGCTTCAAATAAGTTTGGTGTCACTACCGTTGCTTTAGGTAGTAAATATTTAATCATCGCCTCAG
TATTTCCAGGATTAAGCACTTCATCTTCGCCTTTACAAACCATGACAGGATCTACTACAAAATATTGTGCATTAGATGCCTCATATACTTCTCC
AGCACGTTTGATTATCTCCTCAGTACCTAACATACCTGTTTTAATAGCATCAGGTCCGATTGATAAAGCCGTTTCAAGTTGCTTTTCAAATAC
GTCCATAGGTAATGGTGTGACATCGTGTGACCATGTATCTTTATCCATAGTAACGATGGCAGTTAAAGCGACCATGCCATACGTATCTAATT
CTTGGAACGTTTTCAAATCTGCTTGCATACCTGCGCCAGCACTTGTGTCAGAACCGGCAATTGTTAAAACTTTCTTTAAAGCCAT

Gene: ung (uracil-DNA glycosylase)

Position: 629755 to 630411, length: 657 nt, orientation: FORWARD

Perfect match to: (COL-CP000046-[659764:660420], highly conserved allele)

Sequence:

ATGGAATGGTCGCAAATTTTTCATGACATAACAACGAAACATGACTTTAAAGCTATGCATGATTTTTTAGAAAAAGAATATTCGACTGCAAT
CGTATACCCTGATAGGGAAAATATATATCAAGCGTTTGATTTAACACCGTTTGAAAATATCAAAGTTGTTATATTAGGACAAGACCCGTATC
ATGGTCCAAACCAAGCACATGGATTAGCATTTTCAGTGCAACCTAACGCAAAATTCCCTCCATCTTTACGTAATATGTATAAAGAATTAGCA
GATGATATTGGATGCGTTAGACAAACACCGCATTTACAAGATTGGGCAAGAGAAGGCGTCTTGTTATTGAATACAGTTTTAACCGTAAGAC
AGGGTGAAGCAAATTCTCATCGTGATATTGGTTGGGAAACATTTACTGATGAAATTATTAAAGCAGTGTCTGATTATAAAGAACATGTTGTC
TTTATTTTGTGGGGGAAACCTGCACAGCAAAAAATAAAGCTTATCGATACATCTAAACATTGTATTATAAAATCAGTGCATCCTAGTCCACT
GTCTGCATATAGAGGATTCTTTGGATCAAAACCGTATTCCAAAGCGAATGCCTATTTAGAGTCAGTAGGAAAATCACCAATTAATTGGTGTG
AAAGTGAGGCGTAG

Gene: ywdI (putative protein)

Position: 630412 to 630792, length: 381 nt, orientation: FORWARD

Perfect match to: (MW2-BA000033-[623464:623844], highly conserved allele)

Sequence:

ATGTTGAATAGAGAAACTTTAATAGCACGAATTGAGCAAGAATTAGTACAAGCAGAGCAGGCACAGCATGACCATGACTTTGAAAAACAT
ATGTATGCCATACATATATTAACATCTTTATATGCTTCAACATCAAATACACCACATATTGGTGAACAACAAATGAATCGTCGTATTGCTAAC
CATAATCAAATGCCACAATCACAAATAACGCAGCCAACTCATCAAGTGACAGCTGCTGAAATTGAAGCGATGGGTGGTAAAGTAAATACGC
ATTCAGCACATCATCATAATAAGTCATATTCACAACCTTCAAACCAACAACAAAGATTAGCGACAGATGATGACATTGGCAATGGTGAATCC
ATATTTGATTTTTAA

Gene: ywdK (putative small membrane protein)

Position: 630925 to 631293, length: 369 nt, orientation: FORWARD

Perfect match to: (RF122-AJ938182-[597536:597904], highly conserved allele)

Sequence:

ATGAAATTATTTATTATTTTAGGTGCATTAAACGCGATGATGGCTGTCGGAACAGGTGCATTTGGTGCGCATGGTTTACAAGGAAAAATAA
GTGATCACTATTTATCAGTATGGGAAAAAGCAACGACGTATCAAATGTACCATGGCTTAGCATTATTAATTATAGGTGTAATTAGTGGTACA
ACTTCAATCAATGTTAACTGGGCTGGCTGGTTAATATTTGCTGGTATTATTTTCTTTAGTGGATCATTATATATTTTAGTATTAACTCAAATTA
AAGTTTTAGGTGCGATTACGCCAATTGGTGGCGTATTGTTCATCATTGGATGGATAATGTTAATCATTGCGACATTCAAATTTGCTGGTTAA

Gene: yfnA2 (cationic amino acid/polyamine transporter, locus 2)

Position: 631414 to 632898, length: 1485 nt, orientation: FORWARD

Perfect match to: (11819-97-CP003194-[632149:633633], allele observed in CC80+CC239+CC4803)

Sequence:

ATGGAAAAAAAGAATAAGCAAATAGATAGAGGCGATTTAAAACAAAACCTATCTGAAAAGTTTGTATGGGCGATTGCATATGGTTCATGTA
TCGGATGGGGCGCATTCATCTTACCAGGAGACTGGATTAAGCAGTCAGGTCCGATTGCAGCATCAATTGGTATAGTTATTGGTGCATTATT
AATGATATTAATTGCGGTTAGTTATGGCGCATTAGTAGAGAGATTTCCAGTATCAGGGGGCGCGTTTGCCTTTAGTTTCTTAAGTTTCGGCA
GATATGTGAGTTTCTTCTCATCATGGTTTTTAACTTTTGGTTATGTCTGTGTCGTTGCTTTAAATGCGACCGCATTCAGTTTACTAGTTAAATT
CTTATTGCCAGATGTCTTAAATAATGGGAAACTATACACCATTGCGGGCTGGGACGTTTATATTACGGAAATCATTATTGCGACCGTATTAC
TACTTGTATTCATGCTAGTAACGATTCGTGGCGCAAGTGTATCTGGATCATTACAATATTATTTCTGTGTGGCGATGGTAATCGTCGTATTAT
TGATGTTCTTTGGTTCATTCTTTGGTAATAATTTTGCACTTGAAAATTTACAACCGTTAGCTGAACCTAGCAAAGGATGGTTAGTGTCTATTG
TGGTTGTTGTATCCGTGGCACCATGGGCATATGTTGGATTTGATAATATTCCACAAACAGCAGAAGAGTTTAACTTTGCACCAAACAAGACA
TTTAAGCTTATCGTGTACAGTTTATTAGCAGCATCATTAACTTATGTTGTCATGATTTTATACACTGGTTGGTTATCAACAAGTCATCAAAGTT
TAAATGGGCAGTTGTGGTTAACAGGTGCTGTTACACAAACAGCATTTGGTTATATTGGATTAGGTGTATTAGCAATTGCAATTATGATGGG
TATATTTACTGGTTTAAATGGATTCTTGATGAGTTCAAGTCGCTTGTTATTTTCTATGGGACGTTCAGGTATTATGCCAACAATGTTTAGTAA
ATTACATAGTAAATACAAAACACCATATGTCGCAATCATATTCCTAGTAGGTGTGTCGTTAATTGCACCTTGGCTAGGAAGAACTGCATTGA
CTTGGATTGTAGATATGTCATCTACAGGTGTATCCATTGCCTACTTTATTACATGTTTGTCTGCAGCGAAATTATTCAGTTATAACAAACAAA
GTAATACGTATGCACCGGTTTACAAAACGTTTGCTATTATCGGCTCATTTGTATCATTCATTTTCTTAGCGTTGTTATTAGTGCCAGGTTCTCC
TGCAGCACTGACTGCACCGTCTTATATTGCATTACTTGGATGGTTAATCATCGGTTTAATATTCTTTGTGATTCGATATCCTAAATTGAAAAA
TATGGATAATGATGAATTAAGTCGCTTGATTTTAAATAGAAGTGAAAATGAAGTTGATGATATGATTGAAGAACCTGAAAAAGAAAAAACT
AAATAA

Gene: DUF3815 (protein of unknown function)

Position: 633039 to 633491, length: 453 nt, orientation: REVERSE

Perfect match to: (11819-97-CP003194-[633774:634226:r], allele observed in CC80+CC12+CC88+CC772)

Sequence:

TTATAATCGTTTAAAAGATTTCACGAACCCAGAAACAATTAATTTGGAAATTTGGTCGGCGAATAATAAACCTAATGCGATGGCTCCTGCAA
TAAGTGTTACTTCTAACATTGTATTTATTGCTGTGCTGAAATTTAATAAGACTAAATTTTTTGTAGCATCGTATGCTAATCCACCTGGTACTAA

TGGAATGATACCCGTTACCATAAAAATGATGGCAGGTTCTTTTTGTTTACGAGCCATATAATGACTTAACAAGCCTAATGCTAAACTACCAA
AGAAACTAGAGTATATAGTGTGCACATTAAAGCCGTTGAAGAATAAGGTGTAAACCATCCATCCACATGTACCAACGAAACCACATGATAG
ATATAATTTTCTAGGTGCATCAAAAATGACGCAGAAGAACATTGAAGCTAAAAAGCTAAAGATAAAGTTTAAGATCCAAAACAT

Gene: DUF1212_L1 (protein of unknown function DUF1212, locus 1)

Position: 633504 to 634268, length: 765 nt, orientation: REVERSE

Perfect match to: (COL-CP000046-[663514:664278:r], allele observed in CC8+CC22+CC80+CC772+CC4803)

Sequence:

CTATACTAAAATTAATACGCTACCAACGCCAGCACCGATGCCAAACGCAGTAACCAATGCTTCTAATGATTTCGTTGTGAACATCAACATGT
GTCCACCAAATAAATCTTGTATTGCGTTTGTTATTAATACACCAGGAACAATAGGCATGACTGCCGCAATGATAATAGTTGCCAAGTCACCT
GTTGGAATAAGTGTATGTCCAATAACGGCGATAATCCCAATAACTAATGAACCAATGAATTCTGGGATAAACTGTGCGTGTAACTTACGAT
CTAAAATCTCAGTGACTAGGTATCCTAGACTACCTGCTAATATCGCAGTTAAAACATCAATCAATCTACCACCTTGTAAATATAAGAAACTCA
TTGCAATCATTGCTGCAGCAAAACCTTTAAAGGGAAGACTGCTGTCACGCTTAGCAACATATATTTTTTCAAGTTGCGTTTTTGCTTCGGCTA
AAGAAATTTCATTATTTGTAATTTGACGCGAAATTTTATTAGCTTGCGAAATTTTTATTAAGTTTGTATCTCGAGAGGTAATTCTAAATATTCT
AGGAAACGATTCCGAATGTAACGTAAACTGGATGACAGTGTTTGTAACAAAGCTGTTACTTTCACTGTAACCAAGTTTTTTTGCAATACGTG
TCATGGTATCTTCTACACGCGTACCTTCTGCACCAGATTCTAATAGTATGCGAGCAGCAAGCATGACAACGTCTTTGATAAGTACCTCTTGTT
TGTATTCTTCTGAATTTATGTCCAT

Gene: hemQ (Fe-coproporphyrin oxidase)

Position: 634817 to 635569, length: 753 nt, orientation: REVERSE

Perfect match to: (11819-97-CP003194-[635552:636304:r], allele observed in CC80)

Sequence:

TTAAGAAATCGCAAAGAATTGATCGAATTCGTTTGTGTTAATAATATGTCCTACAAAGAAACTACCGAATTCACCGTATCGTGCTGTTGTTTC
ATCAAAGCGCATTTCGTATACAATTTTTTTGAATTGTAATACGTCATCTGAGAACAATGTTACGCCCCATTCGAAATCATCAAACCCTACAGA
ACCAGTAATAAATTGTTTGATTTTGCCAGCATATTTTCTACCAATCATACCATGGTCATACATTAATTTTTGGCGTTCTTCCATAGTTAACATG
TACCAGTTATAAGTTTCATTACGACGTTTGTTCATTGGATAGAAACAAATATAATCAGAATGTGGTAATTCTGGGTATAATCTTGCTTTGATA
TGAGGGTTCTCATAAGGATCTTCATCAGATTTACCAGCTAAATAATTGCTCAATTCAATGACTGATACATATGAATATGTAGGGATTAAGAA
GTCAGCAATGCGCAATTTGTTAAATTCATTTTCAATATGATTTAAAGACTTCATTTCAGGACGTAAGTACCATAATAACAAATCTGCTTTTTG
ACCAGTTATATTATAAATAGCTTGATCACCAGATTTTGATGATCTTACAGTTGCTGTATTTTCTAAAAATGATTGAAATTCAGTGACAAGTGC
ATCGCGTTCGTCCTTTGGAACTATACGTAATGATGCCCAATCAACTGCATAAAATAAATGTAGACTATACCAACCATCTAATGTTTCGGCTGC
TTGACTCAT

Gene: pta (phosphotransacetylase)

Position: 635737 to 636723, length: 987 nt, orientation: FORWARD

Perfect match to: (11819-97-CP003194-[636472:637458], allele observed in CC80)

Sequence:

ATGGCTGATTTATTAAATGTATTAAAAGACAAACTTTCTGGTAAAAACGTTAAAATCGTATTACCTGAAGGAGAGGACGAACGTGTTCTAAC
AGCTGCAACACAATTACAAGCAACAGATTATGTTACACCAATCGTGTTAGGTGATGAGACTAAGGTTCAATCTTTAGCGCAAAAACTTAATC
TTGATATTTCTAATATTGAATTAATTAATCCTGCGACAAGTGAATTGAAAGCTGAATTAGTCCAATCATTTGTTGAACGACGTAAAGGTAAA
GCGACTGAAGAACAAGCGCAAGAATTATTAAACAATGTGAACTACTTCGGTACAATGCTTGTTTATGCTGGTAAAGCAGATGGTTTAGTTA
GTGGTGCAGCACATTCAACAGGCGACACTGTGCGTCCAGCTTTACAAATCATCAAAACGAAACCAGGTGTATCAAGAACATCAGGTATCTT
CTTTATGATTAAAGGTGATGAACAATACATCTTTGGTGATTGTGCAATCAATCCAGAACTTGATTCACAAGGACTTGCAGAAATTGCAGTAG
AAAGTGCAAAATCAGCATTAAGCTTTGGCATGGATCCAAAAGTTGCGATGTTAAGCTTTTCAACAAAAGGGTCTGCTAAATCAGACGACGT
GACAAAAGTTCAAGAAGCTGTCAAATTAGCACAACAAAAAGCTGAAGAAGAAAAATTAGAAGCAATCATTGATGGCGAATTCCAATTTGA
TGCTGCGATTGTACCAGGTGTTGCTGAGAAAAAAGCGCCAGGTGCTAAATTACAAGGTGATGCAAATGTCTTTGTATTCCCAAGTTTAGAA
GCTGGTAATATTGGTTACAAAATTGCACAACGTTTAGGTGGATATGATGCAGTTGGTCCAGTATTACAAGGTTTAAATTCTCCAGTAAATGA
CTTATCACGTGGCTGCTCAATTGAAGATGTATACAATCTTTCAATTATTACAGCAGCGCAAGCCTTACAATAA

Gene: lipL (octanoyl-[GcvH]:protein N-octanoyltransferase)

Position: 636726 to 637562, length: 837 nt, orientation: FORWARD

Perfect match to: (Strain_21340-AGTX01000008-[256737:257573:r], allele observed in CC188+CC80)

Sequence:

ATGGATTTAGCGAGTAAATATTTTAATGGCGTCAACTGGCGATATATCGATCATTCTTCTGGATTAGAACCTATGCAATCTTTCGCATTCGAT
GATACATTTTGCGAAAGTGTGGGCAAAGATATATCAGATAATGTTGTGCGTACTTGGATTCATCAACATACTGTTATTCTTGGTATTCATGA
TTCAAGATTGCCGTTTTTAAAAGATGGCATTGATTATTTAACGAATGAGATTGGTTATAATGCCATTGTTAGAAATTCTGGTGGCTTAGGTG
TCGTTCTAGATCAAGGTGTATTAAATATATCGCTGATGTTCAAAGGACAAACAGAAACAACGATTGATGAAGCGTTTACTGTGATGTACCTC
TTAATTAGCAAAATGTTCGAAGATGAGAATGTTGATATTGATACGATGGAAATTGAACATTCTTATTGCCCAGGAAAATTTGACTTAAGTAT
CGATGGTAAGAAATTTGCAGGCATATCGCAACGAAGAGTTAGAGGCGGTATTGCTGTACAAATTTATCTTTGTGTTGAAGGCTCTGGTTCA
GAACGTGCATTGATGATGCAAACATTTTATGAACATGCTTTAAAAGGTGAAGTGACTAAATTTAAATATCCTGAAATTGAACCATCTTGTAT
GGCCTCATTAGAGACATTGCTTAACAAAACGATTACTGTTCAAGATGTAATGTTTTTACTATTATATGCAATCAAAGATCTTGGCGGTGTATT
AAATATGACGCCAATTACTCAAGAAGAATGGCAAAGATACGATACGTATTTTGATAAAATGATTGAAAGAAACAAGAAAATGATAGATCA
AATGCAATAG

Gene: mvaK1 (mevalonate kinase 1)

Position: 638149 to 639069, length: 921 nt, orientation: FORWARD

Perfect match to: (79_S10-CP010944-[544889:545809], allele observed in CC772+CC8)

Sequence:

ATGACAAGAAAAGGATATGGGGAATCGACAGGTAAGATTATTTTAATAGGAGAACATGCTGTTACATTTGGAGAGCCTGCTATTGCAGTA
CCGTTTAACGCAGGTAAAATCAAAGTTTTAATAGAAGCCTTAGAGAGCGGGAACTATTCGTCTATTAAAAGCGATGTTTACGATGGTATGTT
ATATGATGCGCCTGACCATCTTAAGTCTTTGGTGAACCGTTTTGTAGAATTAAATAATATTACAGAGCCGCTAGCAGTAACGATCCAAACGA
ATTTACCACCATCACGTGGATTAGGATCGAGTGCAGCTGTCGCGGTTGCTTTTGTTCGTGCAAGTTATGATTTTTTAGGGAAATCATTAACG
AAAGAAGAACTCATTGAAAAGGCAAATTGGGCAGAGCAAATTGCACATGGTAAACCAAGTGGTATTGATACGCAAACGATTGTATCAGGC
AAACCAGTTTGGTTCCAAAAAGGTCATGCTGAAACATTGAAAACGTTAAGTTTAGACGGCTATATGGTTGTTATAGATACTGGTGTGAAAG
GTTCAACAAGACAAGCGGTAGAAGATGTTCATAAACTTTGTGAGGATTCTCAGTACATGTCACATGTAAAACATATCGGTAAGTTAGTTTTA
CGTGCGAGTGATGTGATTGAACATCATAACTTTGAAGCCTTAGCGGATATTTTTAATGAATGTCATGCGGATTTAAAGGCGTTGACAGTTA
GTCATGATAAAATAGAACAATTAATGAAAATTGGTAAAGAAAATGGTGCGATTGCTGGAAAACTTACTGGTGCTGGTCGTGGCGGAAGTA
TGTTATTGCTTGCCAAAGATTTACCAACAGCGAAAAATATTGTAAAAGCTGTAGAAAAAGCTGGTGCAGCACATACTTGGATTGAGAATTT
AGGAGGTTAA

Gene: mvaD (mevalonate diphosphate decarboxylase)

Position: 639074 to 640057, length: 984 nt, orientation: FORWARD

Perfect match to: (SA40-CP003604-[615944:616927], highly conserved allele)

Sequence:

TTGATTAAAAGTGGCAAAGCACGTGCACATACGAATATTGCACTTATAAAATATTGGGGTAAAAAAGATGAAGCACTAATCATTCCAATGA
ATAATAGCATATCTGTTACATTAGAAAAATTTTACACTGAAACGAAAGTCACTTTTAACGACCAGTTAACACAGGATCAATTTTGGTTGAAT
GGTGAAAAGGTTAGTGGCAAAGAATTAGAGAAAATTTCAAAATATATGGATATTGTCAGAAATAGAGCTGGCATCGATTGGTATGCAGAA
ATTGAAAGCGACAATTTTGTACCAACAGCAGCAGGGTTGGCTTCATCAGCAAGCGCATATGCAGCTTTAGCAGCAGCTTGTAATCAAGCAC
TAGACTTGCAGCTGTCAGATAAGGATTTATCGAGATTGGCGCGAATTGGTTCGGGTTCTGCATCGCGTAGTATTTATGGTGGATTTGCAGA
ATGGGAAAAAGGGTATAATGATGAGACGTCATATGCCGTTCCACTTGAATCGAATCATTTTGAAGATGACCTTGCCATGATATTTGTTGTGA
TTAATCAACATTCTAAAAAGGTACCTAGTCGATATGGTATGTCGTTGACACGAAACACATCAAGGTTTTATCAATATTGGTTAGATCATATTG
ATGAAGATTTAACTGAAGCAAAAGCAGCGATTCAAGACAAAGATTTTAAACGCCTTGGTGAAGTAATTGAAGAAAATGGTTTGCGTATGCA
TGCCACGAATCTAGGATCAACACCGCCGTTCACATATCTTGTGCAAGAAAGTTATGATGTCATGGCGCTCGTTCACGAATGCCGAGAAGCG
GGATATCCGTGTTATTTTACGATGGATGCGGGTCCTAATGTGAAAATACTTGTAGAAAAGAAAAACAAGCAACAGATTATAGATAAATTAT
TAACACAGTTTGATAATAACCAAATTATTGATAGTGACATTATTGCCACAGGAATTGAAATAATTGAGTAA

Gene: mvaK2 (phosphomevalonate kinase 2)

Position: 640070 to 641146, length: 1077 nt, orientation: FORWARD

Perfect match to: (Strain_21269-AFTU01000041-[5532:6608:r], allele observed in CC121+CC8+CC88)

Sequence:

ATGATTCAGGTCAAAGCACCCGGAAAACTTTATATTGCTGGAGAATATGCTGTAACAGAACCAGGATATAAATCTGTACTTATTGCGTTAGA
TCGTTTTGTAACTGCTACTATTGAAGAAGCAGACCAATATAAAGGTACCATTCATTCAAAAGCATTACATCATAACCCAGTTACATTTAGTAG
AGATGAAGATAGTATTGTCATTTCAGATCCACATGCAGCAAAACAATTAAATTATGTGGTCACAGCTATTGAAATATTTGAACAATACGCGA
AAAGTTGCGATATAGCGATGAAGCATTTTCATCTGACTATTGATAGTAATTTAGATGATTCAAATGGTCATAAATATGGATTAGGTTCAAGT
GCAGCAGTACTTGTGTCAGTTATAAAAGTATTAAATGAATTTTATGATATGAAGTTATCTAATTTATACATTTATAAACTAGCAGTGATTGCA
AATATGAAGTTACAAAGTTTAAGTTCATGCGGAGATATTGCTGTGAGTGTATATAGTGGATGGCTAGCGTATAGTACTTTTGATCATGAAT
GGGTTAAGCATCAAATTGAAGATACTACGGTTGAAGAAGTTTTAATCAAAAACTGGCCTGGATTGCACATCGAACCATTACAAGCACCTGA
AAATATGGAAGTACTTATCGGTTGGACTGGCTCACCGGCGTCATCACCACACTTTGTTAGCGAAGTGAAACGTTTGAAATCAGATCCTTCAT
TTTACGGTGACTTCTTAGAAGATTCACATCGTTGTGTTGAAAAACTTATTCATGCTTTTAAAACAAATAATATTAAAGGTGTGCAAAAGATG
GTGCGTCAGAATCGTACAATTATTCAACGTATGGATAAAGAAGCTACAGTTGATATAGAAACTGAAAAGCTAAAATATTTGTGTGATATTG
CTGAAAAGTATCACGGCGCATCTAAAACATCAGGCGCTGGTGGTGGAGACTGTGGTATTACAATTATCAATAAAGATGTAGATAAAGAAA
AAATTTATGATGAATGGACAAAACATGGTATTAAACCATTAAAATTTAATATTTATCATGGGCAATAA

Gene: DUF1450 (protein of unknown function)

Position: 641323 to 641664, length: 342 nt, orientation: FORWARD

Perfect match to: (N315-BA000018-[643926:644267], highly conserved allele)

Sequence:

ATGAAAAATACATTCCTTATTTGTGATGAATGTCAGGCAGTCAATATAAGAACGTTACAAAAGAAGTTGGAAAAATTAGATCCCGATGCTG
AAATCGTGATAGGTTGTCAATCTTATTGTGGACCTGGACGCCGAAAAACATTCACTTTTGTTAATAACCGCCCACTGGCTGCGCTTACTGAA
GAAGAATTAATCGAAAAAGTTTCTCAACAATTAAAGAAACCACGTGATCCTGAAGAAGAAGAGCGTTTAAGAAAACGACATGAAGAACGT
AAACGTCGTAAAGAAGAACAAGATAGAAAGCTTAAAGAAAAATTAGAAAAGCGAAAAGCACAACAATAA

Gene: ykgC (FAD-dependent pyridine nucleotide-disulphide oxidoreductase)

Position: 641716 to 643038, length: 1323 nt, orientation: REVERSE

Sequence:

CTAGAAATTAAATAAATCATTAAATGATTCGGCCATCGTAGGATGCGTATAAATATTATCTCGTAATACGGTATATGGAATGTTTTGATCAA
TCGCAAGTTTAATTATATTAATTAATTCTTCAGATTGCTTACCATATAATGTAGCACCTAAAATCATATTATTTTCATTATTAATGACTACTTTA
AATAAACCTCTTGGATCATTGTTAATTTTATGACGAGGTATAGCACTTACTAAAAGTTGATGTTCAGTGTAATCATAATGTTGAGCGGCAGC
TTCTTTACTAGTTAATCCAACACGTGATAATGGTGGATCTATAAATACTGTATAAGGCACGCTGCCTCTATTGTCAGTCGTACGTGACTGATT
ACCATATAACGCTGATTTGATAATTCGATAATCATCTAAAGATATATACGTAAATTGAAGTCCGCCTTTAACATCACCTGCAGCATAAATATG
CGGCACAGTTGTTTGAAGATGAGCATTGACTTTAATTTCGCCTCTGTCGCCTAATTCGATATCAGTATTTTCTAAAGCTAAATCCGTATTCGG
TTTGCGCCCGATAGCCAAAAGTACTGCATCAGCCTCAAAGTTACCAACGTTGGTATGGACTGTTGTATGATGATTGTCAGATGACAATTCAG
TCGTTTCAACATTTGTATGCAATGCAATGCCTTTATTTTCTAAGTCAGTAATACCATGTGCAACGACATCTTGATCTTCGCGTGGCATAAATG
ATTCGCCACGTTCTAATACTGTTACCTTACTACCTAAATTCGCAAACATTGAAGCAAATTCTAAGGCGATATAACCGCCACCTACAATAACGA
GGTGCTTAGGTTGATAGCTAATGTTTAATAAACCTGTCGAATCGAAGACGTGTTTAGCTTGATCAAGGCCTTTAATGTTAGGAATGACAGA
GGTAGCACCGGTATTAATAATGATATGAGGTGCAGTAATACTATCGACGATATCGTCATGTTGATCTAATAAATTCACTTCAGTATTAGATT
TAAACTGCGCTTTAAAATCCAGTACATCAATGTTGTTATCGTCTGCTAATAAGTGGTAATTTTTATTGTTTAGCGCATTGACAACATCGTTTTT
ACGGTTATAACTTGCTTCAAAAGATTTGCCTTCTAATCCATCATGTACAAGTGTCTTCGAAGGTATACATCCTATATTTATACAAGTGCCTCCA
TACATTTTCGAAGATTGTTCGATAACTGCGACGTGTTGACCTGTTGATGCAGCGTATTTCGCTAAAGTTTTACCAGCTTTCCCAAATCCTATT
ACAATTAAATCATATGTTTTCAT

Gene: Q5HI81 (transcriptional regulator)

Position: 643025 to 643465, length: 441 nt, orientation: REVERSE

Perfect match to: (N315-BA000018-[645628:646068:r], allele observed in CC5+CC8+CC239)

Sequence:

TCATATGTTTTCATGACATAAATCCTCCTTTTGAATGTCTTCAATGACATCTTTGATTGTTTTTCCATTATAAAAATTAATAATGATATTCTGTT
CGTCTTGCTGATAATGTGACATGGTAGTTGCAATATTACGAGCAATTTGACAGTGACTGCCTTCGTCGCCAGTAAATAGACGTGTGTGGTGT
TCTTTCTCTAAGACAAAATGTTTATATAATGTTGCTAGAGAGACATCAGCACTTTGATCATTTGCTAAATAACCGCCATCTTTACCTCGTATTG
TGTCAATCATTTTTAAATCGACAAGTTGAGTCGTCACGCGTCGTAATTGAACAGGATTTAAACAAGTTAATTCTGCTAATGAACTACTATTGA
ATTTTTCTGAATGATGCTTAGTTAAAAAAGCTAATACATGCACGGCAATGTTAAATTCTAAATTCAA

Gene: Q5HI79 (putative protein)

Position: 644090 to 645496, length: 1407 nt, orientation: FORWARD

Sequence:

ATGAGTATTGACATGTATTTAGACAGATCTCGAAACCAAGCTTCAAGTGTGGGGAATTTGAGTCAAACAATGAATTCAAATTATGATGCGTT
GGAAAAAGCAATTACTCAATTTATTAATGATGATGCGCTTAAAGGGAAAGCGTATACGTCAGCTAAGCAATTTTTTAGTACGGTGTTAATTC
CATTATCAACAAGTATGAAAACATTGAGTGATTTAACGAAGCAAGCTTGCGATAATTTTGTGTCACGTTATACGAGTGAGGTTGATAGCATA
TCTTTAAAAGAATCAGAGCTTGAAGAAGATATCAGATCATTAAGTCAACAAATTACGCGATATGAAAATTTGAATAACAATTTGAAAAAGC
ATGCTTCCGATAATCAGCAAGCCATTTCATCGAACCAACAAATAATACGAACATTAGGTCAACAAAAACATGAATTAGAAGAGAAGCTACG
CAAATTGCGTGAGTTTAATCAAAAATCACCAGAAATATTTAAAGAAGTTGAAGAATTTCAAAAAAATTGTCCAACAAGGACTTACCCAAGCG
CAGAATTTTTGGAACTTTTCAACAAATCAATTTAATATACCTTCAGGTAAAGAACTTGATTGGGCTAAAGCAAGTCATGAAAAATATTTGAA
AGTTGCTATGGGGAAAATTGAACATAAAGCAGAGAAAGAAACTTTAAATAAAGCAGACTTTGCTGTTATAAAGGCATATGCCAAAGAGCA
TCCAGAAGACGATATCCCGAAAAGTATAATGAAATATATAAATGACAATAAAGACAGTATTAAAAGAGATATAGGATTAGATATCACTTCA
ACACTTTTAGAGCAAGGCGGTATAAATGCAAGTAAATTCGGTGTATTTATCAATACAGCAGGTGGAGTGAAAGGCCCAGCCGGTCCAAATT
CATTTGTGGAAGTCAAACGTACATCAGGTAATGTGTTTATAGAAAATGGTAGTAAATTTGCTAAAGGTGGAAAATATCTAGGTAAAGGTGT
TGCAGGTGTAGGATTTGGTATAGGTATGTATGATGACCTTGCAAATGATGATAAAACATTTGGGGAGGCGTTGTCGCATAATGCTTTAACT
ACAGGAATAGGTACTGGTGCTGGAATTGCTACTTCAGCAACAATAGTCGCACTTGCTAGTAACCCAGTTGGTTGGGCTGCTTTAGGAGGTT
TGGCAGTAGGTATTGGAGCTACCCACCTTGCTGATTGGGCTTACCAAGCAAATATTTTGGGGTTAAAAGATAAAACTGATTGGGTTGGACA
TAAGATAGATGAAGGTATTGATAATTTCAAAAAGTCAACGGTAAATACAGTAGATAGCATTGGAAATGTCGTTGGTGAAGCTACAAAAAG
TATTAGCAATCATATAAACCCTATGAAATGGGAGTGGTAA

Gene: DUF443-var9 (putative protein)

Position: 646195 to 646824, length: 630 nt, orientation: FORWARD

Perfect match to: (TCH959-AASB02000218-[37841:38470], allele observed in CC7)

Sequence:

GTGCTTTGTGAATCTCGAGTCATTAATAAAAACCCAAAATACAGGATCATTAAATATAATGAGGAGTATTTCATGGTTGATTTAGTAAGTAC
TTGGATTGCTTATTTTTTGCCCATGATTAATTGGTTTATTCCCAAAAAGTACGTGAAAATTAGTAGAGAAGAATTTGAAAGTTTAAATATTGT
CAAACCCGCTAAAAATAATACTTTCTGGCCTGTTGCAGGATTTGCAGTGTTATTAACAACCTTAACAAGAAAATATATTTATTTGCTTAATAT
CCATTTAGAAAAAGAATTAGTTATATTAACATGCTGCATGATACTTCTAGGTGTTTTCGCATTGTTTATATATATAAATACAAAATTGAAGTT
ACATATTTTTGATAAAAATAAAAGTAATAACGAAAAGATCATATTAATACCTACATTTAAAAATATTTGTTTATCTTTATTTGCTTATATATTA
TTTGGTGGATTGTCAACAATGGCTCTGAGTATGTTAGTAACTTCATCCCCTCAAAATATAATAGAATTTCTTGCTTTAATTGGCATGACTGCA
TGCTTCTTTCTACTGAATATGTCATCGGTTTTAGATAAAAATATTCATGTTATTTTAAAAACAAATAAGTAG

Gene: DUF443-var12 (putative protein)

Position: 647020 to 647650, length: 631 nt, orientation: FORWARD

Perfect match to: (TCH959-AASB02000218-[38666:39296], allele observed in CC7)

Sequence:

ATGCTATGCGAATCTAAAATCATCAATAAAAATCCAAAATATAGAATTATTAAATATAATGATGAGTACTTAATGATCGATATAATAAGCAC
TTGGATTAGTTTGTTTTTTCCTTTTATTAATTGGTTCATCACCAAAAGATACGTCAAAATTAGTAGAGAAGAGTTTGAAAATTTAAATATTGTT
AAACCTGCTAAAAAGAATGTTTTTTGGCCAGTTGCAGGTATCTCTACTTTATTCGCAGTTACATTAAGAAAGTATACGCATTTACTTGACACT

CAACTTGATAAAAAAATTAGTTATTGCCATATGTTGTATCACATTTATAGGGATTTTAATATTTTATGTACGCCTAATTAAAAAATCATCTTTAA
ATATTTATAATACTAAAAATAAAAGGTCGAAAATCATCTTAATACCAACACTAAAAAATTTTTGTTTCACATTGTTCGGATATGTTTTTTTAAT
TTTTTGGACAGTGGTTTTCTCATATGCTCTATTATCAATGAGTTATCAAAACATAATAGTATATTTTGCTTGGATTACAGCAATAATGGGTTTT
TTTCTAGTGAATATAGCTTTAATTATAGATAAAAACATTCATGTCATACTTAAAAATTAAATGGTGGTAA

Gene: DUF443-var6 (putative protein)

Position: 647780 to 648412, length: 633 nt, orientation: FORWARD

Perfect match to: (Mu3-AP009324-[675224:675856], allele observed in CC5+CC72)

Sequence:

TTGGTTTGCGAGGTAATAGCTATATACAAAAATCCCAAATATAGAATTATTAAATATAATGATGAATATTTGATGGTGAATATAATAAATAA
TTGGTTAGTTTTATTCATTCCATTGCTTAATTGGTTGACACCAAAAAGATACATCAAGATCAGTCAAGAAGAATTAGAAAGTTTAAATACTTT
CAAACCTGCTAAAAACAATGCATTTTGGCCAGCATTAGGTAGTTCAGTTTTATTTAGTGTGACTTTTAGAAAGTATATGCCTTTATTTAATGT
TAGATTGGAAAAAACAATAGTAATAGCCATATTTTTTGTTGTATTTCTTGGTATTTTATTTTTCTACCTAAATTTAAATCGTAGGCTAGCATTA
AGTGTTTTTACTATGAATAAAGAAAAAAGTCAGAAAATGATATTGCTACCTAATTTAAAACATTTTTTCTTTATAATATTTGCATATTTATATT
TCGGTGGTTTTTCATTTTTCACTTTATATGCCCTGATAACTATAGATGTTCAAAATATAATTATATTTATCTGTTGGGGTGCAGTAACTATGTT
TTTCTTTTTTATAAACATTGTTCCCATTGGCATTAAAAAAGCTCATGTAATACTTCTTAAAAAAGCTGAATAA

Gene: Q5HI67 (oxidoreductase)

Position: 648860 to 649798, length: 939 nt, orientation: FORWARD

Sequence:

ATGAAGCAATTTGTAAACTTAGGTAAATCTGATGTTGAAGTGTTTCCAATCGCACTTGGGACGAACGCAGTAGGTGGGCATAATTTATATCC
GAACTTAGATGAAGAACAAGGAAAAGATGTTGTTCGTCAAGCCATTAATCATGGTATTAATTTATTAGATACGGCATATATTTATGGGCCA
GAACGATCAGAAGAATTGGTTGGAGAAGTTGTTAAAGAATATCCGCGAGAGCAAATTAAAATTGCTACAAAAGGGTCTCGTGAATTTGAT
GAAAATCAAGAAGTACATCAGAACAATCAACCTGAATATTTAAAACAACAAGTTGAGAATAGTTTGAAACGTCTACAAACTGATTATATCG
ATTTATATTATATTCATTTTCCGGATGACAACACTCCGAAAGATCAAGCAGTTGCAGCATTACAAGAGCTTAAGGAACAAGGGAAGATTAA
AGCCATTGGTGTATCAAATTTCACATTAGATCAACTTAAAGAAGCAAATAAAGATGGTTACGTTGATGTTGTACAGTTAGAATATAATTTAT
TGCACCGCGAAAATGAGGCAGTATTGCAATATTGTGTTGATCACCAAATCACATTTATTCCTTATTTCCCATTAGCATCCGGTATTTTAGCTG
GAAAATATGATGAGAACACTAAATTTAGTGACCATCGTACTACACGTCGTGATTTTATACCTGGTGTTTTTGAAGAGAATGTGCGTCGCGTA
AAAGCTTTGGAAAGCCTAGCTGCAGCACATCAAATTTCAATTGCGAACATTGTATTAGCATTTTATTTAACGAGACCAGCTATCGATGTGAT
TATTCCTGGTGCAAAACGTGCAGAACAAGTCGTTGAAAATATTAAAGCTGCAGATATCGTTTTATCAGATGATGAGATTCAATATATCGATG
AACTGTTTCCGATTGAAGACTAA

Gene: Q5HI66 (NADPH-dependent FMN reductase)

Position: 650007 to 650543, length: 537 nt, orientation: FORWARD

Perfect match to: (RF122-AJ938182-[617538:618074], highly conserved allele)

Sequence:

ATGATTACTGTTTTGTTTGGTGGGAGTAGACCAAACGGTAATACTGCACAATTAACAAAATTCGCTTTGCAAGATTTAGAGTATCAATGGAT
TGACGTGACACAACATCAGTTTAAACCGATACGTGACGTGAGACATACAGCAGAGACTATTACTTCATATGACGATGACTATTTGCCGATTC
TAGATAAAATATTGGCTAGTGATACAATTATTTTTGCATCACCAGTGTATTGGTATAGCATTTCAGCACCATTGAAAGCATTTATCGAACATT
GGTCAGAAACATTACAAGATAAACGATATCCTAATTTTAAGGCACAAATGGCCGAAAAGGATTTTAGAGTTATTTTAGTTGGTGGAGATTG
TCCAAAAAATAAAAGCGAAGCCAGCAATTACGCAAATGAAATATAGTTTAGACTTTTTAGGTGCCACTTTAAATGGTTATATTATTGGAACTG
CTGAAAAGCCTGGTGACATCATGAAAGACAACTATGCCTTAGCACGTGCAACTGAGTGGAATAGTATATTGCAATAA

Gene: Q8NXU4 (GNAT family acetyltransferase)

Position: 650711 to 651187, length: 477 nt, orientation: FORWARD

Perfect match to: (RF122-AJ938182-[618243:618719], highly conserved allele)

Sequence:

ATGTTCAAGGTAAGACAAGCAACTGAAAAAGATGTTGTTCAAATTAGAGATGTCGCAACTAAAGCTTGGTTTAATACATACTTAAATATATA
CGCTGCGACAACAGTTAATCACTTGTTAGAAGCTTCATATAATGAACATCATTTAAAGAAAAGACTTCAAGAACAATTATTCTTAGTCGTTG
AAGAAGGTAATGACATCGTTGGCTTTGCTAACTTTATTTACGGTGAAGAATTATATTTATCAGCTCATTATGTTAAACCAGAATCGCAACATA
CAGGTTATGGTACAGCATTGTTAAATGAAGGATTATCACGTTTTGAAGATAAATTTGAAGGTGTTTACTTAGAAGTAGATAATAAAAATGA
AGAAGCAGTAGCTTACTATAAAGAGCAAGGTTTTACAATCTTACGCTCTTATCAGCCAGAAATGTATGGCGAAAAGTTAGACTTAGCACTTA
TGTACAAAGCATTTTAA

Gene: Q5HI65 (phosphohydrolase)

Position: 651227 to 652522, length: 1296 nt, orientation: FORWARD

Perfect match to: (11819-97-CP003194-[695213:696508], allele observed in CC80)

Sequence:

ATGACTAACGCATTTGTAGATTTAAAATTAGTAGAAGAAAAAGTTTTTAAAGACCCGATACATCGATATATTCATGTTGAAGATCAATTGAT
ATGGGATTTAATTAAAACTAAGGAATTCCAAAGGTTACGTCGAATTAGACAACTAGGAACACTGTACCTATCTTTTCACACAGCAGAACATA
GTCGCTTTGGACATTCTTTAGGTGTGTATGAAATAGTTAGACGATTAATTGATGAGTCATTTATTGGTCATGATGCATGGGACAATAAAGAT
AGACCGTTGGCATTATGTGCTGCATTATTACATGATTTAGGACATGGTCCATTTTCACATAGTTTTGAAAAAATATTTAATACAGACCATGAA
GCATACACACAAGCGATTATTACTGGAGATACTGAGGTGAATGCTGTATTACGTAAAGTGGCGCCTGAGTTTCCAAGAGAAGTTGCGGAA
GTAATTAATAAAACGCATCATAATAAATTGGTCATTTCGATGATTTCGTCACAAATCGATGCGGATAGAATGGATTATTTACAACGTGATGC
GTATTTCACAGGTGTATCATATGGTGCTTTTGATATGGAACGTATTTTAAGATTAATGCGACCTTCTAAAGATGAAGTACTAATTAAAGAAA
GTGGTATGCATGCAGTTGAAAACTTTATTATGAGTCGTTATCAAATGTATTGGCAAATTTATTTCCACCCAGTTAGTCGTGGTGGAGAAGTG
CTGCTTAATAATTGTTTGAAACGCGCAAAACAGCTTTATAATGAAGGCTATGAATTTAAGTTGCATCCACATGATTTTATTCCATTTTTTGAA
GAGACAGTTACGATTGAACAATATGTTGAACTCGATGAAGCGGTAGTTACGTATTATTTGGAAAAATGGACAAAAGAAGATGATGCTATTT
TAAGTGATTTAGCAAGTCGATTTATTAATAGAGACTTATTTAAATATATTCCATTTGATGGCTCAATTATTACAATATCAGAACTGCAAGAAC
TGTTTGAAGCAGGTGGTATTAATCCAGATTATTATTTTGTGAGTGAAGCATTTTCGGATTTGCCATATGACTATGATCGACCTGGGTCAAAT
CGCAAACCGATTCATTTATTAAGACAAGATGGTACGATTAGAGAAATAAGCAATCAATCTTTAGTCATTCATAGTATTACAGGTATTAATCG
CCAAGACTATAAATTATATTATCCTAGAGAAATGGTTGCAAAGATTAAAGATAAGACAATTAGAGAAGCTATTGAAAATTTGATTAATGAG
CTTAATTAA

Gene: ywhD (putative protein)

Position: 652550 to 653071, length: 522 nt, orientation: FORWARD

Perfect match to: (11819-97-CP003194-[696536:697057], allele observed in CC80+CC22+CC72+CC80)

Sequence:

ATGGAGGTTATATCATTGTCTGAGAAAAAAGGCTTTAATTTTAATATCATAAAAAATGACCCTCTAGATGGTCATAAAGGTACAAATATTGG
TTCAATTAGCTTAGACAATATTGCACCAGTTTTTATCGATGTTGCTAACAAAGAAGCATTTATTGATATTGGAGGCATGCATGCTCGCGCCA
AAGTTGAAAAAGGCGTGAAATGGATTACTGATAAAGCTGCTGTTGAAGGCGATGAAGCTAAAGAATATTGGTTGTGTTGGGTAACAACAG
AACGTAATGAACAAGGACCATATTATGCTGGTTTAACAGCGTGCTATTTATTAGTGAATAAAGCAATTCGTCGTGGTTATAAAAGTATGCCT
GAACATGTTAATATGATGGATAAATCAATGAAACATCATATTATCATTGATCAAATTGGTGACGAGAATAAAGCTATTTTAAAAGACTTTTT
AATGAACCATGATGAAGGTATGTGGAAGCTTTCTTCTGATGCTTTACATCAAGCATTTAATTAA

Gene: adhA (alcohol dehydrogenase I)

Position: 653570 to 654580, length: 1011 nt, orientation: FORWARD

Perfect match to: (11819-97-CP003194-[697556:698566], allele observed in CC80+CC5+CC50+CC59+CC80)

Sequence:

ATGAGAGCAGCAGTTGTAACGAAAGATCACAAAGTAAGTATTGAGGACAAAAAGTTAAGAGCTTTAAAACCTGGTGAAGCATTGGTACAA
ACGGAATATTGTGGTGTTTGTCATACCGATTTACATGTTAAGAATGCTGATTTTGGTGATGTTACAGGCGTTACTTTAGGTCATGAAGGTAT
TGGTAAAGTCATCGAAGTTGCAGAAGATGTAGAATCATTAAAAATTGGAGACCGTGTGTCTATCGCTTGGATGTTCGAAAGCTGTGGAAG
ATGTGAATATTGTACAACAGGTCGTGAAACACTTTGCCGTAGTGTGAAAAATGCTGGTTATACAGTAGATGGTGCAATGGCTGAACAAGTT

ATTGTTACTGCAGACTATGCTGTGAAAGTACCTGAAAAATTAGATCCAGCAGCAGCGTCTTCTATTACATGCGCAGGTGTGACAACTTATAA
AGCTGTAAAAGTAAGTAATGTAAAACCTGGTCAATGGTTAGGTGTTTTTGGTATAGGTGGTTTAGGTAACCTAGCTTTACAATATGCTAAAA
ACGTTATGGGGGCTAAAATTGTTGCCTTCGACATTAATGATGATAAATTAGCATTCGCGAAAGAATTAGGTGCAGATGCTATTATTAATTCT
AAAGATGTTGATCCAGTTGCAGAAGTTATGAAATTAACTGATAACAAAGGATTAGATGCAACAGTGGTAACTTCAGTTGCTAAGACGCCAT
TTAACCAAGCGGTTGATGTTGTAAAAGCTGGTGCAAGAGTTGTTGCCGTTGGTTTACCTGTTGATAAAATGAACTTAGATATCCCAAGATTA
GTGCTTGACGGTATTGAAGTAGTAGGTTCACTTGTTGGTACAAGACAAGACTTACGTGAAGCGTTTGAATTTGCTGCTGAAAATAAAGTAA
CACCTAAAGTTCAATTAAGAAAATTAGAAGAAATCAATGATATTTTTGAAGAAATGGAAAATGGTACTATAACTGGTAGAATGGTTATTAA
ATTTTAA

Gene: ywiB (putative protein)

Position: 654844 to 655272, length: 429 nt, orientation: FORWARD

Perfect match to: (N315-BA000018-[656831:657259], highly conserved allele)

Sequence:

GTGAGTAGTAAATTGGATAAAAAAGTAAGTATTCAAACAAAGCAAGTGTTGAAACAGCACAACGAAAAAGAAAAATTTGAATTTACTACT
GAAGGAACTTGGCAACAAAGGCAATCTAACTTTATTCGGTATGTAGAACAAATTGAGGATGCAACAGTTAATGTTACAATAAAAGTGGATG
ATGATAGCGTTAAGTTGATTCGTAAAGGCGACATTAATATGAATTTGCATTTTGTTGAAGGACAAACGACAACAACTTTTTACGATATATCG
GCTGGACGAATTCCACTAGAAGTTAAAACATTACGCATTTTACATTTCGTAAGTGGAGACGGTGGCAAGCTAAAGATTCATTATGAATTATA
TCAAGATAATGAAAAAATGGGTTCTTATCAATATGAAATTAACTATAAGGAGATAGGCGAATGA

Gene: argS (arginyl-tRNA synthase)

Position: 655269 to 656930, length: 1662 nt, orientation: FORWARD

Perfect match to: (HIF003_B2N-C-AIVK01000002-[50841:52502], allele observed in CC8)

Sequence:

ATGAATATTATTGATCAAGTGAAACAAACATTAGTAGAAGAAATTGCAGCAAGTATTAACAAAGCAGGATTAGCAGATGAGATTCCTGATA
TTAAAATTGAAGTTCCTAAAGATACAAAAAATGGAGATTATGCTACTAATATTGCGATGGTACTGACTAAGATTGCAAAGCGTAATCCTCGT
GAAATTGCTCAAGCGATTGTTGATAACTTAGATACTGAAAAAGCACATGTAAAACAAATTGACATTGCTGGTCCAGGATTCATTAATTTTTA
CTTAGATAATCAGTATTTAACAGCAATTATTCCTGAAGCAATTGAAAAAGGTGATCAATTTGGACATGTAAATGAATCAAAAGGTCAAAAT
GTATTGCTTGAGTATGTTTCAGCTAACCCTACAGGAGATTTACATATTGGTCATGCTAGAAATGCAGCAGTTGGTGATGCTTTAGCTAATAT
TTTAACTGCAGCTGGCTATAATGTAACACGTGAATATTATATTAATGATGCTGGTAATCAAATTACTAACTTAGCGCGTTCGATTGAAACAC
GTTTCTTTGAAGCTTTAGGTGACAATAGTTATTCAATGCCAGAAGATGGCTATAATGGAAAAGATATTATTGAAATAGGTAAAGATTTAGCA
GAGAAACACCCTGAAATTAAAGATTATTCTGAAGAAGCACGTTTGAAAGAATTTAGAAAATTAGGCGTAGAATACGAAATGGCTAAATTGA
AAAATGATTTAGCAGAGTTCAATACGCATTTTGATAATTGGTTTAGTGAAACATCTTTATATGAAAAAGGAGAAATTCTTGAAGTTTTAGCA
AAAATGAAAGAATTAGGTTATACGTATGAAGCTGATGGCGCTACATGGTTACGTACAACTGATTTTAAAGATGACAAAGACAGAGTATTAA
TTAAAAATGACGGTACATATACGTATTTCTTACCAGATATTGCGTACCACTTCGATAAAGTTAAACGTGGTAATGACATTTTAATCGATTTAT
TTGGTGCTGATCATCATGGTTATATTAATCGTTTGAAAGCATCTCTTGAAACGTTTGGTGTAGATAGTAATCGTTTAGAAATTCAAATCATGC
AAATGGTTCGTTTAATGGAAAATGGTAAAGAAGTGAAGATGAGTAAACGTACTGGTAATGCGATTACATTAAGAGAAATTATGGACGAAG
TTGGCGTTGACGCTGCACGTTATTTCTTAACTATGCGTAGTCCTGATAGTCACTTTGATTTTGATATGGAATTAGCGAAAGAGCAATCTCAA
GACAATCCAGTTTACTATGCTCAATATGCACATGCGCGTATTTGTTCAATTTTAAAACAAGCGAAAGAGCAAGGAATTGAGGTAGCTGCTG
CGAATGATTTTACAACGATTACAAATGAAAAAGCGATTGAATTGTTGAAAAAAGTAGCTGATTTCGAACCTACAATTGAAAGTGCTGCTGA
GCATAGATCAGCACATAGAATTACTAACTATATTCAAGACTTGGCTGCTCATTTCCATAAATTCTATAATGCTGAAAAAGTGTTAACAGATG
ATATTGAAAAAACAAAAGCACATGTTGCTATGATTGAAGCGGTCAGAATTACATTGAAAAATGCATTGGCAATGGTCGGTGTAAGCGCACC
TGAATCAATGTAA

Gene: tx_universal2 (rho-independent terminator)

Position: 657140 to 657179, length: 40 nt

Perfect match to: (LGA251-FR821779-[1071185:1071224:r], allele observed in CC425)

Sequence:

TGAACGAAAATTCGCTTGTAACAAGCTTTTTTCAATTCTA

Gene: nth2 (putative endonuclease III, locus 2)

Position: 657264 to 657899, length: 636 nt, orientation: FORWARD

Perfect match to: (COL-CP000046-[692691:693326], highly conserved allele)

Sequence:

ATGTTAGGAACTGATGAATTATATAAAGTTTTATATGAACATCTCGGACCACAATTTTGGTGGCCTGCTGATAATGACATTGAAATGATGTT
AGGTGCAATTTTAGTTCAAAATACTAGATGGCGAAATGCAGAAATTGCATTGAATCAGATTAAAGAACATACGCATTTTAATCCAAATCATA
TATTAGAACTACCTATTGAAACGTTACAATCATTGATACATTCAAGTGGCTTTTATAAAAGTAAATCACTGACGATTAAAACATTATTAACAT
GGTTAGCACGACATCATTTCAATTATCAAGAGATTAATGAGCGATATAAAGGTGGATTAAGAAAAGAATTATTATCTTTGAAAGGTATTGG
AAGTGAAACAGCAGATGTCTTACTTGTTTATATATTCGGACGTATTGAATTTATTCCAGATAGCTATACAAGAAAAATATATGATAAATTAG
GATATGAAAACACTAAAAATTATGATCAATTAAAAAAAGTAGTCACATTACCAAATCATTTTACAAATCAAGATGCTAATGAATTTCATGCTC
TGTTAGATGTATTTGGTAAACATTACTTTAGAGACAAAGATATAAAGAATTATGATTTTTTAGAACCTTACTTTAAAAAGTAA

Gene: A5IQG3 (similar to iron-binding transport protein)

Position: 658216 to 659103, length: 888 nt, orientation: FORWARD

Perfect match to: (COL-CP000046-[693705:694592], highly conserved allele)

Sequence:

GTGAAGAAATCGTTAATTGCTTTTATTTTGATTTTTATGCTTGTCCTGAGTGGCTGTGGTATGAAAGATAATGATAAACAAGGTAGCAATGA
TAATGGCTCGTCTAAATCGCCGTACCATAGAATTGTTTCGTTAATGCCTAGTAATACTGAAATTTTATATGAATTAGGATTAGGTAAATACAT
AGTTGGTGTTTCAACGGTTGATGATTATCCAAAAGATGTGAAAAAGGGTAAGAAACAATTTGATGCTTTGAATCTAAATAAAGAGGAACTT
TTAAAGGCAAAGCCAGATCTAATTCTTGCGCATGAGTCGCAAAAGGCAACTGCTAATAAAGTATTGTCATCATTAGAGAAACAAGGCATCA
AAGTAGTGTATGTTAAAGATGCACAATCAATTGATGAAACTTACAACACATTTAAGCAAATTGGGAAATTAACGCATCATGATAAGCAGGC
TGAACAACTTGTTGAGGAAACTAAAGATAATATCGATAAAGTCATAGATTCAATTCCTGCTCATCATAAAAAATCAAAAGTATTTATTGAGG
TTTCATCAAAGCCTGAAATATATACAGCAGGGAAGCATACATTTTTTAATGATATGTTAGAAAAATTAGAAGCCCAAAATGTGTATAGTGAC
ATTAATGGTTGGAACCCTGTAACGAAGGAAAGTATTATTAAAAAGAACCCAGATATATTAATTTCGACGGAAGCTAAGACAAGATCAGATT
ATATGGATATCATCAAAAAAAGAGGTGGATTCAATAAAATTAATGCTGTCAAGAATACACGTATTGAAGTTGTAAATGGTGATGAAGTATC
AAGACCAGGTCCACGTATTGATGAAGGATTAAAAGAATTAAGAGATGCAATTTATAGAAAATAA

Gene: yvrB (putative vitamin B12 permease)

Position: 659251 to 660201, length: 951 nt, orientation: FORWARD

Perfect match to: (11819-97-CP003194-[703237:704187], highly conserved allele)

Sequence:

ATGACATTTAATAAAGTATTATTGAGCTGGATAGTCATATTGATTATAACAACTAGCATATATCTATTTTGGCAGTTGGGCGATATCAATGAT
GTATTTAACCAGTCTATTTTAATCAATGTTAGATTACCGAGATTATTAGAAGCATTGTTGACAGGTATGATATTAACTGTTGCAGGCCTTATA
TTTCAAACAGTTTTAAATAATGCATTGGCAGATAGCTTTACATTAGGATTGGCAAGCGGCGCTACATTTGGTTCAGGATTAGCATTATTTTTA
GGTTTAACAACGTTATGGATTCCTGTATTTTCAATAACATTTAGTTTGATAACATTAATAACTGTATTAGTCATTACGTCGGTATTGAGCCAA
GGCTATCCAGTTAGAATCTTAATATTAAGTGGTTTAATGATTGGTGCGTTATTCAATTCACTTCTATATTTTTTGATTTTATTAAAACCTCGCA
AATTAAATACAATTGCCAATTATCTGTTTGGTGGTTTTGGTGATGCAGAATACTCAAATGTATCTATAATAGCAATCACATTTATCATTGCAT
TGTTTGGTATATTTATCATTCTTAATCAACTAAAGTTATTGCAATTAGGAGAACTAAAAAGTCAGTCACTAGGCTTAAATGTTCAATTGATTA
CATATATCGCGTTATGTATAGCTTCTATGATAACGGCGATAAATGTCGCATATGTTGGCATCATTGGATTCATTGGTATGGTGATACCGCAA
CTCATTAGAAAATGGCAGTGGAAACAATCATTAGGAAGACAATTGGCTTTAAATATTGTAACTGGAGGACAAATAATGGTTATGGCAGATT
TTATTGGTAGCCATATATTGTCACCAGTACAAATACCGGCAAGTATTATCATTGCATTAATTGGTATACCAGTGTTATTTTACATGCTAATAT
CTCAGTCGAAACGGTTACACTAG

Gene: ppaX2 (haloacid dehalogenase-like hydrolase)

Position: 660256 to 660975, length: 720 nt, orientation: FORWARD

Perfect match to: (N315-BA000018-[662115:662834], highly conserved allele)

Sequence:

ATGGATTTGAATCAAATTAAAGCAGTTGTATTTGATTTAGAAGGTACGTTGTTGGACAGAGTTAAATCTCGAGAGAAATTTATCGAAGAGC
AATATGAACGATTTCATGACTACTTAATTCATGTTCAACTGGCAGATTTTAAAAAAGCATTTATTGAGCTAGATGACGATGAAGATAATGAT
AAACCTGATTTATATAAAGAAATCATTAAACGTTTCCATGTAGATAGGTTAACTTGGAAAGACTTATTTAATGATTTTGAAATGCATTTTTAT
CGTTATGTATTTCCTTATTACGATACTTTGTATACACTAGAAAAGCTATCGCAAAAAGGCTTTCAAATTGGTGTTATCGCAAATGGTAAATCT
AAGATTAAACAATTTCGATTACATTCACTTGGTTTGATGCATGTTATTAATTATTTATCAACATCAGAAACAGTTGGTTTTCGTAAACCACATC
CTAAAATTTTTGAAGATATGATTGATCAACTAGGGGTATTACCTGAGCAAATTATGTATGTTGGCGATGATGCGTTAAATGATGTAGCTCCA
GCACGAGCTATGGGCATGGTTAGTGTATGGTATAAACAAGAAGATGCTGAAATTGAACCACTCGAAGAAGAAGTTGATTTTACAATTACAA
CAGTGGAAGAATTATTAACCATTTTACCAATAAAAAATGATAATAAAGGAGAAAATTATGGATCTATTTACTAG

Gene: ydjP (alpha/beta fold hydrolase)

Position: 660959 to 661759, length: 801 nt, orientation: FORWARD

Perfect match to: (COL-CP000046-[696448:697248], highly conserved allele)

Sequence:

ATGGATCTATTTACTAGAAAAGATGGAACATCGATACATTACAGTACATTAGGTGAAGGCTATCCTATCGTATTGATTCATACTGTACTTGA
TAATTATTCTGTGTTTAATAAATTAGCAGCAGAATTAGCAAAATCATTTCAAGTTGTGTTAATTGATTTACGTGGACATGGCTATTCTGATAA
ACCTCGTCACATTGAAATAAAAGATTTTTCTGATGACATTGTTGAATTACTTAAATATTTATATATTGAAGAAGTTGCATTTGTATGCCATGA
AATGGGTGGAATCATTGGTGCGGATATTTCAGTACGTTATCCTGAATTTACATCATCACTTACGTTGGTAAATCCAACATCTATTGAAGGTG
AATTACCGGAAGAACGTTTATTTAGAAAATATGCCCATATTATTCGAAACTGGGATCCTGAAAAACAAGATAAATTTTTAAATAAGCGTAAG
TATTATCGTCCGAGAAAAATGAATCGATTCCTCAAACATGTCGTAGATACAAATGAAATATCAACTAAAGAAGAAATTCAAGCAGTTAAAG
AGGTATTCAAAAACGCTGATATTTCTCAAACTTATAGAAATGTCGTAGTACCGACAAAAATTATTGCAGGAGAATTCGGTGAAAGAACAAC
AAGATTAGAAGCTAAAGAAGTAGCTGATTTAATCCAAAATGCGGACTTTGAAGTATATCAAGAATCAAGTGCATTCCCATTTGTTGAAGAG
CAAGAAAGATTCGTCGAAGATACAGCTGCATTTATCAACAAACATCACGATGAAAAGCATGTTTAA

Gene: Q5HI54 (putative protein)

Position: 661895 to 662401, length: 507 nt, orientation: FORWARD

Perfect match to: (COL-CP000046-[697384:697890], highly conserved allele)

Sequence:

ATGAAAAAGCTACTAACGGCAAGTATAATTGCATGTTCTGTTGTAATGGGAGTAGGCTTAGTGAACACTAGTGCCGAAGCAGCAAGTGGC
AACTCTATTGATACTGTTAAACAATTAATTAAGGGTGATCAGTCATTAGAAAATGTGAAAATTGGCGAATCTATTAAAGATGTTTTAACTAA
GTACAAAAATCCGATGTATTCTTACAATGAAGATGGAACTGAACATTATTACGAATTCCATACTAAAAAAGGTATGTTATTAGTAACTACTG
ATGGTAAGAAAAACAATGGTAAAGTAACTCATATTTCAATGATGTATAATGATGCTAATGGTCCAACATATCAAGCTGTTAAAAATTATGTT
GGCAAAGCAGTAACACATACGGAATATAGCAAAGTTGCTGGTAATTTTGGATATATTGAAAAAGGCAAAACGACTTATCAATTTGCCTCAG
CACCAAAAGATAAAAACATAAAATTATATCGTATTGATTTGGAAAAATAA

Gene: Q5HI53 (putative protein)

Position: 662563 to 663279, length: 717 nt, orientation: FORWARD

Perfect match to: (Strain_21334-AGTW01000032-[57949:58665], allele observed in CC9+CC15+CC80)

Sequence:

ATGCGTATTAACGATAAAATTTTACTAGAAAATATAGAAGATTACTTTAATCATAAAGGTTTATCACCGCATTTGATTGATGATATTAAAGA
GAAAGTAATTACTGATATAAAAAATTCTGAAAAGAAAGATCAAGATTATATTGAATATAAAAGAAAATCTCCAGCACAAATCATATTAATG
ATTCAAAGGAATTTGTTTGCTTTACAAATGAATCCAGTTATTTTCTTTATTATAAACTTCATTCTCATATCCTATTTATATGATAAACAGTATGT
TCAGTTTCAAGCTATTACTGGAATGAGTCTATTTTACTGTTTAGTGATTTTTCCAATGACTATTGTTGTATACCTAAGGGTGTCTCAAAAGAA
TTACTTGCGTAGTAATAAAATAGAAATGATTATGGGTACAATTATCGCTATCATATCCTTGTTATTAATTATATTACAAGCATTTAATATTACT
TGGGGCGTTATACCAATTACAAATTTTGGACATCAATTTTTCTTTTTCATTGGTATTATTTTAGTAATTGCCGGCATATTTTATAAGCGACTTG

AGTTTTCGGGAATCGGGTTATTATTTTGTCAAAAAACCGTCGATGCAATGATTCATAATCCACAATCAGCCCAGATTTTTTCATTAATTATAT
GGATATTATTAGTAGTTCTAGTTATATATTTCACAATTAGATTATCTTCACGTACAAGATTATAA

Gene: Q5HI52 (alpha/beta hydrolase fold protein)

Position: 663448 to 664236, length: 789 nt, orientation: FORWARD

Perfect match to: (11819-97-CP003194-[707434:708222], allele observed in CC80+CC239)

Sequence:

ATGAATAAAGTCACAATTAATCCTCAAATCCAATTAACTTATCAAATTGAAGGTAAAGGGGATCCTATAATATTACTTCATGGATTGGATGG
TAATTTAGCTGGATTTGAAGATTTGCAACATCAACTAGCATCATCATATAAAGTACTTACTTACGATTTAAGAGGTCATGGCAAGTCTTCTAA
AAGTGAATCATACGATTTAAACGATCACGTTGAGGATTTAAAAATTCTAATGGAGAAGTTAAATATTCATGAGGCACATATTCTAGGACAT
GATTTAGGTGGGGTAGTTGCTAAGTTATTTACAGATAAATATGCTTATCGTGTAAAATCATTAACTACCATTGCATCGAAGAAAGATGACTT
AATACACAGCTTTACTCAATTGTTAATTCAATATCAAGATGATATAGCGGGTTTTAATAAGTCTGAAGCGTATATTCTTTTATTTTCTAAATTG
TTTAGAAATCAAGAGAAGACGATGAAATGGTATCAAAAACAAAGAATATATAGCATTAAGTCTGAGGATGATAGTGCGGTGGCAATTCGT
TCATTAATTTTGCATAAAGATGAACCTATGTATTTAAAAAAACGTACATGTGTGCCTACTTTGTTAATTAATGGGGAACATGATCCTTTGATT
AAAGATAAAAATCATTTTAAATTGGAAGCGCATTTTTTAAATGTTACGAAAAAAATTTTCGAACATTCAGGACATGCACCGCATATTGAAGA
ACCAGAAGCATTTATGAATTATTATTTAAATTTTTTGAAAAGCGTATCATAA

Gene: sarA (staphylococcal accessory regulator A)

Position: 664485 to 664859, length: 375 nt, orientation: REVERSE

Perfect match to: (RF122-AJ938182-[633329:633703:r], highly conserved allele)

Sequence:

TTATAGTTCAATTTCGTTGTTTGCTTCAGTGATTCGTTTATTTACTCGACTCAATAATGATTCGATTTTTTTACGTTGTTGTGCATTAACAAGAA
TTAATACAGTTCTTTCATCATGCTCATTACGTTTTTTATCGAAGTAATCTTCTTGAGATAAAATTTTAACTGCTTTAACAACTTGTGGTTGTTTG
TAGTTTAAATGATTAATAATATCTTTAAGATAGTATTCTTTCTCTTTGTTTTCGCTGATGTATGTCAATACAGCGAATTCTTCAAAGCTAATTG
AAAATTCCTTTTTAATTAAACTTTTTAATTTGTCAGCATAAGTGACCATTGATAACAACTCAAAGCAATCATTGATTTTTGTAATTGCCAT

Gene: teg49 (small RNA upstream of sarA)

Position: 665023 to 665218, length: 196 nt

Sequence:

AAAAATGAGAAGTAAACAAAAAAGTGTTTATAAAACAAATTATTCATTAGACACAGTGATTGTATTTCTGGGTTAGCATTTGGTTTAGTCAA
AAATATCAGCATTTTTTATAATTTACCTTAATTTAATCGCCAATACAAGGTAATTTATTTAATGAAACGATTTAGCGCAATTAAATGTTTCGAT
ATTCAATGTA

Gene: Q2YSV8 (putative protein)

Position: 665807 to 666736, length: 930 nt, orientation: REVERSE

Perfect match to: (11819-97-CP003194-[709793:710722:r], allele observed in CC80+CC239+CC4803)

Sequence:

TTAAAATAATCTTAAAAGTATAATGCCTGTCAAAATACATAATAGACCAATAGTTTTTCTGGATGTCATTGCTATTTTAGGTGAACCAAATAA
TCCAAAGTGATCTATCAATATGCCCATTAGAATCTGGCCAAACATCCCAATAAGTGTTGTTAATGCTGCACCCATATGAGGCATTAAGATAA
TGTTAGCTGTTACAAAAGCCATGCCAAGTATACCGCCAGTAAAATAGATAGGCTTTAATTTACCGAATTTTAAATGACTTGTTTTCAGTTTTA
AAGAACGATTAAAAATAGCGGTTAAAATCAATAGCGCTATTGACCCAATTGTAAATGATACTAATGATGCAAAGGCTGGTGAATGAGTATG
ACTTGCTAAAGCACTATTAATTGTCGTTTGAATAGGTGGAAAGAAACCAAAAATAAATCCTAAGAGAAGCCAAAACAGTAAATACTTTTGA
TCAGTTAGTAATAAATTATTCTTGTTAAATTGATTCATTATGACGATGCCGATAATGAGTAACAATACTCCAATTGCTTTAATTAAATTAAAAT
CATGAATTGTAGCGCCAAATAAGCCAAATGTATCAATAATGACACCCATAATAATTTGACCCGCAACTGTTGCAATTACAGTTAATGTTGCA

CCTAATTTTGGCAATAACAATAAATTGCCAGTTAAAAAGCTAACCCCAAGCAAACCACCGACTACCCATGTGTAGTTAAATGATTGATTATT
GTAAAAGTGAATAGTAAATACTTCTGGATTGATAATGATATTTAAAATAATTAAACAAATTGTTCCAACTGAAAATGAAATGAATGAAGTAT
AGAAAGGCGATTTAGTATACAGTGATAGTCTTGAATTGACAGATGTTTGGATAGGAATAAGCATTCCAACTAGGACACCAATGATATAGAA
AAGAAGCAT

Gene: A6QET1 (putative protein)

Position: 666931 to 667155, length: 225 nt, orientation: REVERSE

Perfect match to: (RF122-AJ938182-[635775:635999:r], highly conserved allele)

Sequence:

CTATTTCTTATCTTCAAATAAAACAGTTGTTGTTTTATCAATAATTTGAGCAGAATGTACTTTGGTGATAGTACCACTATTTGACTTTATAATA
TTTAAATCTACTAATGCATTCATACTATCACGTGCAACTTGCTCGGTCACTACGTTGTTTAATTGTGGTAATTGCAGTTTTACAGGCTTGTCTA
GTGAAGATTTAAAACTTAATTCAAGTGTTTTAGTCAT

Gene: Q7A1N3 (putative protein)

Position: 667172 to 667375, length: 204 nt, orientation: REVERSE

Perfect match to: (COL-CP000046-[702663:702866:r], allele observed in CC8+CC7+CC72+CC239)

Sequence:

TTAAATTGATAAAGATTTGATGAGTTCGATATTGCTATATGTTTCTCCAGTAAGTCGCTCAATAAGTTTGCTGAATGTTTTAATTTGGTCGTTT
GATGCATCAGGGTTAATGTTAGCGAATCGACGCTTAAATTCTGTTTGTTTGCCGTTAGCGTCTACTTTAGTAAATGATAATACAATAGTGAT
GTGGTTTATTTTACTCAT

Gene: A5IQH4 (putative phage integrase family protein ??)

Position: 667534 to 668094, length: 561 nt, orientation: FORWARD

Perfect match to: (N315-BA000018-[669396:669956], highly conserved allele)

Sequence:

ATGAATAAAGTAGAAGCGATTAAATTTAATGATGATATTGTTAAAATGTATGAAGCGCTCAAGATAAAATCTGAACGTGACTATTTATTCTT
TAAGTTAGCTATACATAGTGGATTGAAAGTATCAGAATTATTAACAATTACAGTCTCTCAAGTTAAGAGACTAATTGAAAAGTGTACGTTAT
CAGAAATGTGTAAAGCACATTTTCATTCGTTGATTAAAATTAGGTTACCAGAAACATTATCGAAAGAACTACTTCAATATATAGAGGACAGG
AGTCTTTCGAATGAAGACGTTCTTTTTCAATCACTACGAACAAATCAAGTATTATCTAGACAGCAAGCATATCGAATAATTCACCAAGCATCA
ATTGAAGCTGGTATAGATAATGTAGGACTAACGACATTGCGTAAGACATTTGCATATCATGCTTATCAAAAAGGTATACCTATACCAGTCAT
TCAAAAGTATTTAGGGCATCAATCTGCTATTGAAACACTAAATTTTATCGGTTTAGAAAATGAGTGTGAACATAGTATTTATATTTCATTACA
ATTATAG

Gene: mrpA (monovalent cation/proton antiporter protein A)

Position: 668113 to 670515, length: 2403 nt, orientation: FORWARD

Perfect match to: (930918-3-ABFA01000056-[10960:13362], allele observed in CC8)

Sequence:

ATGAGTTTGGTTTATTTATTAATTGCTATACTTGTGATTATGGCGATGATACTTCTAATGTCTAAACGTAGAGCATTGGCTAAATATGCCGGG
TACATAGCGTTGGTTGCACCTGTAATTTCATCTATCTATTTTTTGATTCAAATACCATCAGTAGCTAAACTGCAATATCTTTCTACCTCTATTCC
ATGGATTAAGACATTAGATATTAATTTAGATTTACGTTTAGATGGTTTAAGTTTAATGTTTTCTCTTATTATTTCACTTATTGGAGTTGCAGTA
TTCTTCTATGCAACTCAATATTTATCCTCTCGAAAAGACAATTTACCAAGGTTTTATTTTTATTTAACGTTATTTATGTTCAGTATGATTGGTAT
TGTATTATCAGACAATACGATATTGATGTACATTTTTTGGGAATTAACGAGTGTATCATCATTTTTATTGATTTCATATTGGTATAACAACGG
AGATAGTCAATTTGGTGCGATGCAATCATTTATGATTACAGTATTTGGTGGTTTGGCGTTATTAGTTGGTTTTATCATGTTGTATATTATGAC
AGGAACGAATAACATCACAGAGATATTAGGACAAGCAGATCATATTAAGAATCATGGATTGTTTATCCCTATGATTTTTATGTTTTTATTAG

GTGCATTTACAAAATCAGCACAATTTCCATTTCATATTTGGCTACCTAGAGCAATGGCTGCACCTACACCTGTAAGTGCTTATTTACATTCAG
CCACGATGGTAAAAGCTGGTATCTTTTTATTACTTCGATTTACACCATTATTAGGTCTTAGCAATATGTACGTATATATCGTTACGTTTGTTGG
TTTAATAACAATGTTATTTGGTTCAATTACAGCTTTAAAACAATGGGATTTAAAAGGTATCCTAGCGTACTCTACAATCAGTCAACTTGGGAT
GATTATGGCTATGGTGGGTATAGGTGGCGGATATGCTCAACACCAACAAGACGCAATAGCATCTATTTATGTATTTGTATTATTTGGTGCGC
TATTTCATCTAATGAATCATGCCATCTTTAAATGTGCGCTTTTCATGGGAGTAGGTATTTTAGATCATGAAGCAGGTTCAAGGGATATACGA
ATTTTAAGTGGAATGCGTCAACTATTTCCTAAAATGAATCTAGTCATGACGATAGCGGCTCTATCTATGGCTGGAGTACCATTTTTAAATGG
ATTTTTAAGTAAAGAAATGTTTTTAGATGCATTAACACAAACTGGACAATTATCCCAATTTAGTTTGATTTCAATGATAGCTATCGTGTTTGTT
GGTGTTATTGCGAGTGTTTTTACATTCACATATGCACTATACATGGTAAAAGAAGTATTTTGGACAAAATATGATTCTAAGGTTTTTACTAAA
AAAAATATCCACGAACCATGGTTGTTTAGTTTACCATCTCTTATATTAATGGTGCTAGTACCTGTAATCTTTTTTGTACCAAATATATTTGGGA
AGGGGATTATCGTTCTAGCATTAAGAGCTGTATCAGGTGGTAATCATCAAATTGATCAATTGGCACCACATGTTTCGCAATGGCATGGATTT
AACATACCGCTTCTTTTAACCATCATCATTATTTTATTGGGTAGTGTACTAGCAATCAAAGTAGATTGGAAAAAAGTGTTCACAGGTAAAATT
AGACAGATTTCAGTTTCAAAAAGCTATGAGATGGTATATCGACATTTTGAAAAGTTTGCTACGAAGCGATTTAAACGTGTTATGCAAGATCG
TTTAAACCAATACATTATTATGACCTTAGGCATATTTATGATTATCATTGGATATGGTTATATTCGAATTGGACTTCCTAAAGTACATCAGTTA
CATGTTTCTGAATTTGGGGCATTAGAAATTATATTAGCAATCGTAACTGTCACAATTGGTATTTCTTTAATTTTTATACGTCAACGACTGACA
ATGGTCATTTTAAATGGAGTCATCGGATTTGTTGTGACCTTATTCTTTATAGCAATGAAAGCCCCTGATTTAGCATTGACTCAGCTAGTAGTT
GAAACAATAACGACGATACTATTTATTGTCAGTTTTTCAAGATTACCAAACGTGCCAAGATCTAACGCTAACAAAAAAAGAGAAATAATTAA
AATTTCTGTATCACTCTTGATGGCACTTATTGTTGTATCATTAATTTTTATTACACAACAAACAGATGGTTTATCATCAATATCAGACTTTTATT
TAAAAGCTGACAAACTAACAGGTGGTAAAAATATTGTAAATGCGATACTTGGTGACTTTAGAGCATTAGATACATTATTTGAAGGATTAGT
GTTAATTATTACTGGGCTAGGTATTTACACATTATTAAATTATCAAGATCGGAGGGGACAAGATGAAAGAGAATGA

Gene: mrpB (monovalent cation/proton antiporter protein B)

Position: 670502 to 670927, length: 426 nt, orientation: FORWARD

Perfect match to: (MW2-BA000033-[668971:669396], highly conserved allele)

Sequence:

ATGAAAGAGAATGATGTCGTGTTAAGAACGGTCACGAAACTTGTTGTATTTATTTTATTGACTTTCGGATTCTATGTCTTCTTCGCAGGTCAT
AATAATCCTGGTGGTGGGTTTATTGGTGGTTTAATATTTAGTTCAGCGTTTATTTTAATGTTTCTGGCTTTTAATGTTGAAGAGGTTTTAGAA
AGTTTACCGATTGATTTTAGAATTTTAATGATTATTGGAGCATTGGTATCATCTATTACTGCGATAATACCTATGTTTTTTGGAAAACCATTTT
TGTCTCAATATGAAACAACTTGGATACTTCCAATTTTAGGACAAATTCATGTAAGTACAATAACACTTTTTGAATTAGGTATTTTATTCTCAGT
TGTTGGTGTTATTGTCACAGTGATGTTGTCGCTTAGCGGAGGTCGATCATGA

Gene: mrpC (monovalent cation/proton antiporter protein C)

Position: 670924 to 671268, length: 345 nt, orientation: FORWARD

Perfect match to: (MW2-BA000033-[669393:669737], highly conserved allele)

Sequence:

ATGAATTTAATATTATTACTAGTTATAGGATTTTTAGTGTTTATAGGAACATATATGATTTTATCAATCAATTTAATTCGTATTGTAATCGGAA
TTTCAATATATACTCATGCTGGTAATCTCATTATTATGAGTATGGGAACGTATGGTTCTAGTAGATCAGAACCACTAATAACTGGTGGAAAC
CAATTGTTTGTTGATCCCTTGTTACAAGCTATTGTACTAACTGCAATAGTTATAGGGTTTGGGATGACTGCGTTTTTACTTGTACTTGTTTATA
GAACTTATAAAGTAACAAAAGAAGATGAAATTGAAGGCCTAAGGGGGGAAGATGATGCTAAGTAA

Gene: mrpD (monovalent cation/proton antiporter protein D)

Position: 671258 to 672754, length: 1497 nt, orientation: FORWARD

Perfect match to: (COL-CP000046-[706749:708245], highly conserved allele)

Sequence:

ATGCTAAGTAACTTATTGATTTTACCAATGTTATTACCATTCCTTTGTGCCTTAATCCTTGTATTTTTAAAAAATAATGATCGTATTTCTAAATA
TTTATACTTAGGTACAATGACTATCACCACAATTATTTCATTAATGCTATTAATTTATGTTCAGCGTCACCGTCCAATTACGCTAGACTTTGGA
GGATGGTCAGCGCCCTTTGGTATACAGTTTTTAGGAGATTCTTTAAGTTTAATTATGGTTACAACCGCTTCGTTTGTGATTACTTTAATTATG
GCATACGGATTTGGGCGTGGCGAACATAAAGCAAATCGTTATCACTTGCCATCGTTCATATTATTTTTAAGTGTTGGCGTGATAGGCTCTTT
TCTAACATCAGATTTATTTAATTTATACGTCATGTTTGAAATTATGTTACTAGCGTCATTTGTACTCATTACACTTGGACAATCTGTAGAACAA

TTACGTGCTGCAATTATTTATGTTGTCTTGAATATTATTGGTTCATGGCTATTCTTATTAGGTATAGGTTTACTTTATAAAACAGTAGGTACAT
TAAACTTTTCACATATTGCAATGCGTTTGAATGACATGGGAGATAATCGCACTGTTACAATGATTTCATTAATCTTCTTAGTCGCATTTAGTG
CGAAAGCAGCGCTGGTCCTTTTTATGTGGCTACCCAAAGCCTACGCTGTGTTAAATACTGAGCTTGCAGCATTATTTGCAGCGTTAATGACC
AAAGTAGGGGCCTATGCATTAATTCGATTCTTCACTTTACTATTTGATCAACATAATGATCTCATACATCCATTGCTAGCAACTATGGCTGCT
ATAACTATGGTCATCGGCGCTATAGGTGTCATTGCTTATAAAGATATTAAAAAGATTGCAGCTTACCAAGTCATAATCTCAATAGGATTTAT
CATTTTAGGTTTAGGAACAAACACGTTTGCAGGTATTAATGGTGCAATATTTTATTTGGTAAATGACATTGTTGTAAAAACATTGCTATTTTT
TATTATTGGTAGTTTAGTTTACATTACAGGCTATCGACAATATCAATATTTGAATGGCTTAGCTAAAAAAGAACCTTTATTTGGAGTTGCGTT
TATTATAATGATTTTTGCTATTGGCGGCGTGCCTCCATTTAGTGGCTTTCCGGGGAAAGTACTTATTTTCCAAGGTGCATTGCAAAATGGCA
ATTATATTGGACTAGCGTTAATGATTATTACTAGTCTAATTGCAATGTACAGTTTATTTAGGATACTTTTTTATATGTATTTTGGAGATAAAGA
TGGGGAGGAAGTTAATTTTAAGAAAATCCCGCTATATCGAAAAAGAATTTTAAGTATTTTAGTAGTTGTGGTTATCGCAATCGGAATTGCTG
CACCTGTTGTGTTAAATGTTACAAGTGATGCAACTGAGTTGAACACGAGTGATCAATTATATCAAAAACTTGTAAATCCGCATTTGAAAGGA
GAGGACTAA

Gene: mrpE (monovalent cation/proton antiporter protein E)

Position: 672755 to 673237, length: 483 nt, orientation: FORWARD

Perfect match to: (N315-BA000018-[674617:675099], highly conserved allele)

Sequence:

ATGAATCAAATAGTTTTAAATATTATCATTGCATTCTTATGGGTATTATTTCAAGATGAAGATCATTTTAAATTCTCGACTTTCTTTTCTGGAT
ATCTAATTGGTTTAATTGTCATTTATATATTACACAGGTTTTTCAGCGATGATTTTTATGTTAGAAAAATATGGGTAGCTATTAAATTTTTAGG
TGTTTATTTATATCAATTAATAACATCTAGCATTAGCACGATTAATTATATTCTTTTTAAAACAAAAGATATGAACCCTGGATTACTTTCATAT
GAAACAAGACTAACAAGTGATTGGTCAATAACATTTTTAACAATTTTAATTATTATAACTCCAGGGTCTACAGTAATACGAATTTCTCAAGAC
TCTAAAAAGTTTTTTATTCATAGTATCGACGTGTCAGAAAAAGAAAAAGATAGTTTGTTAAGAAGTATTAAGCATTATGAAGACTTAATATT
GGAGGTGTCGCGATGA

Gene: mrpF (monovalent cation/proton antiporter protein F)

Position: 673234 to 673536, length: 303 nt, orientation: FORWARD

Perfect match to: (RF122-AJ938182-[642078:642380], highly conserved allele)

Sequence:

ATGATACAAACAATAACACATATTATGATTATTAGTTCACTCATTATTTTTGGAATTGCATTAATCATCTGTTTATTTAGATTAATCAAGGGAC
CTACAACAGCAGATCGTGTCGTTACATTTGATACAACAAGTGCTGTCGTAATGTCAATTGTGGGTGTGTTAAGTGTACTTATGGGCACCGTT
TCTTTCTTAGATTCAATCATGCTCATTGCCATTATATCTTTTGTAAGTTCTGTTTCAATATCACGCTTTATTGGTGGGGGGCATGTGTTTAATG
GAAATAACAAAAGAAATCTTTAG

Gene: mrpG (monovalent cation/proton antiporter protein G)

Position: 673511 to 673948, length: 438 nt, orientation: FORWARD

Perfect match to: (COL-CP000046-[709002:709439], highly conserved allele)

Sequence:

ATGGAAATAACAAAAGAAATCTTTAGTCTTATTGCTGCTGTGATGTTGTTGTTAGGTAGTTTTATTGCTCTTATTAGTGCAATAGGTATCGTG
AAATTCCAAGATGTTTTCTTAAGAAGTCACGCTGCGACAAAAAGTTCAACTTTATCCGTGTTATTAACTTTAATCGGTGTATTAATTTATTTTA
TTGTGAATACAGGATTTTTCAGTGTGCGTTTATTACTGTCACTTGTTTTTATTAATTTAACTTCACCAGTCGGCATGCACTTAGTCGCTCGCGC
TGCTTATCGCAACGGCGCTTATATGTATCGAAAAAATGATGCTCACACACATGCATCAATATTATTAAGTTCAAATGAACAAAACTCTACAG
AAGCATTACAATTACGTGCTGAAAAACGAGAAGAGCATCGTAAGAAATGGTATCAAAACGATTGA

Gene: nhaK1 (Na+/H+ antiporter locus 1)

Position: 674288 to 676330, length: 2043 nt, orientation: FORWARD

Perfect match to: (N315-BA000018-[676150:678192], allele observed in CC5+CC80+CC239)

Sequence:

TTGGAAATATTTGAAACAATTCTTATATTTATAGCTGTTGTGATACTAAGTTCGTTTGTCCATACTTTCATACCTAAAGTACCCCTAGCATTTA
TACAAATTTTCTTGGGCATGTTACTATTTATTACCCCAATCCCTGTTCAATTTAATTTTGATTCTGAATTGTTTATGGTAACAATGATTGCGCCT
TTGTTATTTGTAGAAGGTGTTAATGTTTCTAGAGTCCATTTAAGGAAATATATTAAGCCAGTGATGATGATGGCATTAGGATTAGTCATTAC
TACTGTGATAGGTGTAGGTTTATTTATTCATTGGATTTGGCCAGATTTACCTATTGGAGCAGCATTTGCAATTGCTGCCATTCTTTGTCCTACT
GATGCAGTAGCAGTGCAAGCAATCACTAAAGGAAAGGTCTTGCCAAAAGGAGCAATGACAATTCTTGAAGGTGAGTCATTATTGAATGAT
GCTGCTGGTATTATTTCATTTAAAATAGCTGTTGGAGTATTAGTTACAGGTGCTTTTTCACTTGTTGATGCTGTTCAGTTGTTTTTAATTGCAT
CAATTGGTGGCGCAGTGGTTGGTTTACTTATAGGTATGGCATTAGTAAGGTTCCGATTAACATTGATGCGTCGAGGATATGAAAACATTAA
TATGTTTACAATTATTCAATTGTTAACACCATTTGTTACGTATTTAATTGCTGAATTGTTTCACGCATCAGGAATCATTGCAGCAGTAGTTGCA
GGACTTGTACATGGTTTCGAACGTGACAGAATTATGCAAGTACGTACACAACTGCAAATGAGTTACAATCATACATGGAATATACTAGGTT
ATGTTTTAAATGGCTTTGTTTTTTCAATATTAGGATTTTTAGTACCTGAAGTTATTATTAAAATTATCAAAACAGAACCGCACAATTTAATCTT
TTTAATAGGCATCACTATTGTTGTTGCTTTAGCTGTCTATCTATTTAGATTTGTTTGGGTTTATGTCTTATATCCTTATTTTTATTTAGCCATCA
GTCCATTCCAAAAAATGATGACTAAAAATGATGATGATAATCCAACGACTGAGAAACCACCAAAGCGAAGTTTATACGCTTTAATTATGAC
GTTATGTGGTGTGCATGGAACAATTTCTTTAGCAATCGCATTAACGTTACCGTATTTTTTAGCAGGGCATCATGCTTTTACGTATAGAAACGA
CTTATTATTTATTGCATCTGGTATGGTTATTATTAGTTTGGTAGTTGCGCAAGTATTATTGCCATTATTAACGAAACCTGCACCTAAAACAGT
AATTGGCAATATGTCGTTTAAAGTTGCTAGAATTTATATATTAGAACAAGTTATTGATTATCTAAATCAAAAATCTACTTTCGAAACAAGTTT
TAAATATGGTAACGTGATTAAAGAATATCATGATAAATTAGCATTTTTAAAAACTGTAGAGAAAGATGATGAAAACTCTAAAGAATTAGAA
CGTCTACAAAAAATTGCTTTTAATGTAGAAACAAAAACATTAGAGTCTTTAGTAGATGAAGGACAAATAACGAATAGTGTACTTGAAAACT
ATATGCGTTATGCTGAAAGAACACAGGTATATAGACAAGCATCATTAATAAGAAGAATGATTGTATTATTACGAGGTGCTTTATTAAAACG
AAGAGTACAAACGAGAGTGAACTCCGCATCTTCACTTAGTGTTACGGATAACTTAATGGAATTAAATAAAATTAATAAATTAGTCCATTATA
ATGTGGTTAGTCGTTTGTCTAAGGAAACAACAAAAGATAATACACTTGAAGTTGGAATGGTTTGTGACGGTTATTTAATGCGAATTGAAAA
CTTAACACCATCAAATTTCTTCAACTCAGCAAGTGAAGATACGATTACTAAAATTAAATTAAATGCATTGAGAGAACAACGTCGCATTTTAC
GTGAATTGATAGATACAGACGAAGTATCTGAAGGTACTGCTTTGAAATTAAGAGAGGCAATCAATTACGATGAAATGGTTATTGTAGATAG
TATGACGTAG

Gene: txbi_nhaK1 (bifunctional rho-independent terminator of nhaK1)

Position: 676334 to 676364, length: 31 nt

Perfect match to: (N315-BA000018-[678196:678226:r], allele observed in CC5+CC8)

Sequence:

CTAATTATGCTAAAAGGGATTGATGAAAAAC

Gene: rsaC (ncRNA of Staphylococcus aureus C)

Position: 676529 to 677122, length: 594 nt

Sequence:

AATAGCCACACTCATATGACATCGGATGAGTGTGGCTTAAGGATCTATGGGGGGAGGAAACCATAGATGTTTACTTTGATAGGCCAGATTA
AATATCAAAGTATGCGATTATTTATAGCTTGATGCAAAAGTGGTATGCCTATTAAAAGTTACTGCACATAGCTTTTAATATTCCGTTCAAAGG
AAAGGGGCATACAATTGAACAATCTGTAATAGTACTTTTAACCAGCTATGCTAAAAGTCTAGTAGGGAGAACAGTTGTCCAATCACATAAG
AACCTCTAACTTCGTTAGTACGATTAAGAAAAGCTTTTTAGTTAGTATGTAATACAATTTATTGACGCGCGTGAATCTCTTTTATAAGAGTGT
GTAGGGAATGGCGTTGTATAAATTGTATTAGAAGAACTTCTAACGCATCTCTGTGGTTAAAAGAGATGAAGGGAACGACAGTTTAATTAAA
ACTGCATAAGAACTTCTAGCTTTTCTCTTTCGTTCAAAGAGAAGCAGCTGTTCGCAGTTTAATCAAAACCACATAAAGCTTTTAACTTTACTCT
TTGATTTAAAGAGTGACAAATGTTTACAGTTTAATTAAAAC

Gene: mntC (manganese ABC transporter, substrate-binding protein)

Position: 677490 to 678419, length: 930 nt, orientation: REVERSE

Perfect match to: (11819-97-CP003194-[721476:722405:r], allele observed in CC80+CC97)

Sequence:

TTATTTCATGCTTCCGTGTACAGTTTCAATATTTGATTTCATCATTTTGTAGTAAGAGTCACCTTTAGTGCCTTCTTTACCGATTGAATCTGTGT
ACACTTCACCAAAGATATCTTTCTTCGTTTCTTCAGATAAACTTTCCATTGCTTTCTTATCAACACTTGTTTCTACTAATAAGTGTTTTAATTTGT
GCTTTTTAACAAACTCAATAGCTTGTCTCATTTGTTCAGGTGTACCTTGTTTTTCAGTGTTAATTTCCCAAATATAACCTGGTGTAATACCGTA
TTGTTTTGAGAAGTACTTGAAGGCACCTTCACTTGTAATCATGGCACGTTGTTCTTTTGGAATGTCATTAAATTTGTCTTTACTGTCATTATTT
AATTTTTCCAATTGAGCAATGTATTTGTTACCTTGCTTTTCATAATCTGCTTTATGTTTTTTGTCGTTATCGATAAATGTTTGTTGAATTGTTTTT
ACGTATTTAATACCGTTATCTAAACTTAACCATGCGTGTGGATCTTGTTTATCTTTGTTGCCTTCTTCACCGTTTAAATAGATAGGTTTAACAT
CTTTTGATACTGCGATAACTTTTTTATCTTTTAATGATTTACCAGCCTGTTCTAAGGCTTTTTCAAACCAACCGTTACCAGTCTCTAAATTTAAT
CCGTTGTATAAAATAACGTCAGCGTCAGTTAACTTTTTAATATCTTTAGGTTTAACTTCATATTCATGAGGATCTTGACCAACAGGTACAATA
CTATGAATATCGACGTTGTCCCCACCAACATTTTTAGCCATATCATATAAAATTGAATTCGTCGTTACTACTTTTAATTTGCCATTTGACTTATC
ACTGCTTTGTTTACCACCAGTACCACATGCAGCAACTAGAAGTAATAAGGCTAATAATAAAGGTACTAATTTTTTCAT

Gene: mntB (manganese ABC transporter, transmembrane permease)

Position: 678416 to 679252, length: 837 nt, orientation: REVERSE

Perfect match to: (N315-BA000018-[681665:682501:r], highly conserved allele)

Sequence:

TCATGTTAAACTTCCTCGTTTCTTTCTATTCGTAAATTTTGTGAAAAATAATGTGATGATATAAATTACAAACGTACAAAGTACGATTGTCGC
ACCACTAGGAATGTTGTAAATATAGCTGTAATAAAGTCCGACAATTGAACTTATGACACTTATTAAACTTGCTATAATCATCATTGAGTATAG
TTTTTTACTAATTAAAAATGCTGTAGATGCAGGTGTAATTAATAATGCAACTACAAGAATAATACCTACCGTTTGAATACTTGCTACTGTTAC
TAATGAGAGTAACAACATCACAAAGTAATGTAATAACGTCGTATTTAGACCACTCATTCTACTAAACGTTGGATCGAATGTAGAAATCATTA
ATGGACGATAGAAAATAATGATTAGAATAAGGACGATTGAACCAATCACAATAGTTGTTAAAAATGCACTATTTGTGATTGCCAGTAAATT
ACCAAACAGAATATGGTACAAATCTGTCGTAGTGTTTATTAAGCTAATAATAATAATCCCCGAAGCTAAGAAAGCGGTAAAACTAATTCCAA
TAGCGGCGTCAGGTTTCGTTTTACTACTAGATGTGATATAACCGATAAAAATACTTGCGATCATACCAGTTATAAGTGCGCCTACAAACATT
GGAATACCAAATAAGAATGATAGGGCAACACCAGGTAATACTGCGTGACTCATTGCATCTCCCATTAATGAAAGACCACGTAATACAATTA
AACTACCAACTGTACCACAAACTATCCCTACAATAATTGAAGTTATCAATGCTCGATTCAAGAATTGATATGTAAATAAATGTTCGACAAACT
CTAACAT

Gene: mntA (manganese ABC transporter, ATP-binding protein)

Position: 679246 to 679989, length: 744 nt, orientation: REVERSE

Perfect match to: (FPR3757-CP000255-[693267:694010:r], allele observed in CC8+CC1+CC8+CC97)

Sequence:

CTAACATGTTATATTGCTCCTTTGACTAGGGTCACTACAGTCAGTGCTACTCATAAATGTTTCGTTTAAGCGAGTGACACTCATAGCCTCTTC
ACTATCACCAAAGTATCGTAATGTTTGATTTAATAGAATAATGCGATCAAAGTATTGCTTTACTTTTGATAGATCATGGTGGATGATAAGAA
TAAGTTTTCCTTGTTGTTTTAAGTTCTCGATTTTTGTCATGATTAATTTTTCGCTACTAAAATCAATTCCGACAAACGGCTCATCTAGAAAATA
AACTTCACTTTCGGACATCAATGCTCTTGCTACTAGCACACGTTGTAATTGTCCACCACTTAATTCTGAAATTTGTCGATGACGTAAAGATTC
TAATTCTAAATCGCTTAATAACTGTTTGAGTTTATCCCTTGCTGATTTATTAGGTCGTCTAAACCATCCAATTTCTTTGTAGCAACCTGATAAA
ATCACTTGTTCCACACTTATAGGAAAATCTAAATCAATATGTGCTTTTTGTGGAATATATGTAATATGTTGCAGTTGTTGTTGTATAGGTTTG
TTATATAACAATTTAGTACCGGTAGCATTAAATTCACCAATTAAAGACTTGATAAGGGAAGATTTACCAGCACCATTCGGGCCCATGATACC
AATTATTTCGCCGCGTACTGGTATCGATAAGGAAATGTTTTTAAGTACATGCTTATTACCTAAAAACAGATTTAAATCTTTTGTTTCTAACAA

Gene: mntR (manganese-dependent transcriptional repressor)

Position: 680111 to 680755, length: 645 nt, orientation: FORWARD

Perfect match to: (M0239-AIWE01000001-[129719:130363], highly conserved allele)

Sequence:

ATGTTAACTGAAGAAAAAGAGGACTATTTAAAGGCAATCCTTACGAATAATGGCGATAAAAACTTTGTGACAAATAAAATCTTATCTCAATT
TTTAAATATTAAGCCTCCATCTGTAAGTGAAATGGTAGGACGTCTTGAAAAAGCAGGCTATGTTGAAACAAAACCATACAAAGGTGTTAGA
TTAACAGAGGATGGTTTAACGCATACGCTTGATATCATTAAGAGACATCGACTATTAGAATTATTTTTAATAGAAATATTGAAATATAATTG
GGAAGAAGTACATCAAGAAGCAGAAATTTTAGAACATCGAATTTCAGATTTATTTGTTGAAAGGCTGGATAGCTTGTTAAATTTCCCAGAA
ACTTGCCCGCACGGCGGTGTGATTCCTAGAAATAATGAATATAAAGAGAAATATATAACAACGATATTGAATTATGAACCTGGCGATATCG

TTACAATCAAACGTGTGAGAGATAAGACCGATTTGCTAATATATTTGTCTAGTAAAGATATTTCTATTGGTAATGAAGTGGAAATTGTATCG
AAAGATGAAATGAATAAAGTAATTATCATTAAACGTAATGATAATGTAATTATTGTCAGTTACGAAAATGCAATGAACATGTTTGCTGAAAA
ATAA

Gene: Q5HI33 (putative membrane protein)

Position: 680835 to 681587, length: 753 nt, orientation: REVERSE

Perfect match to: (COL-CP000046-[716326:717078:r], allele observed in CC8+CC239+CC772)

Sequence:

TTAATTTATCAAGTGAGTATATTTGAGTAAAATATTCACTGCATAAAGATTGAAGATAATCCAGATTGTACTATAAATGAAGATAGGTACAT
GACTGAGTTCTTTAAGTGCACTACCATCCCACTGTGGACTCGGACGCTGGAAAGTCAATTTAGCAATCGTCCAACTAGATTGTAGAACTTCG
CCTAATAATACACCTAAAATATATTGATAACTCATTGTGACAAGTAGTTGAATTTCTACTATATTTTCATCTTTTAATATAAAATACAACATGA
TAGAAATTAAAGTTATAACAACAATGGGTGAGCCTTTTCTAGATGTTAAAATTAAAAAATAAATAAATATCAATAAATAGGTAAATATAAAG
AAACTAGGTATCTGATAATGGCTCGACGCTAAACCTATCAATAACATAATAGGAGGCATAAAATAACCACCAATCGTTGTAAGCCATTGGC
CTGCTAGATGTCTAGATTGTGTAATTGCGAATCCTTGTTGTAATGTCTGTTGTCGCTCTCGTGGACTTGTTACAATGACTAAATCTTTTGCAC
GGCCACCAGCGAGTTTATTAAACAGTACATGACCAAATTCATGTGTTAAAACAGGGATATAGTTTAAAATGACATCTAAATAGTTCAAAACA
GGCTTATGTCTATATTGATGAATAGCAATATAACAAGCTGCAACAATAACGATAATGTATATATTAAGTTGAATTGTCGTATTAAAAAAGTT
TGATAAATAATTCAT

Gene: tarA (N-acetylmannosamine transferase)

Position: 681823 to 682587, length: 765 nt, orientation: FORWARD

Perfect match to: (PFESA1303-FKUO01000003-[169347:170111:r], allele observed in CC1156+CC8+CC97)

Sequence:

ATGACTGTTGAAGAAAGATCCAATACAGCCAAAGTTGACATTTTAGGGGTCGATTTTGATAATACAACAATGTTGCAAATGGTTGAAAATA
TTAAAACCTTTTTTGCAAATCAATCAACGAATAATCTTTTTATAGTAACAGCCAACCCTGAAATAGTGAATTACGCGACGACACATCAAGCGT
ATTTAGAGTTAATAAATCAAGCGAGCTATATTGTTGCTGATGGGACAGGAGTAGTCAAAGCTTCGCATCGTTTAAAGCAACCTCTAGCGCA
TCGTATACCTGGTATTGAGTTGATGGATGAATGTTTGAAAATTGCTCATGTAAATCATCAAAAAGTATTTTTGCTAGGGGCAACTAATGAAG
TTGTAGAAGCGGCACAATATGCATTGCAACAAAGATATCCAAACATATCGTTTGCACATCATCACGGTTATATTGATTTAGAAGATGAGACA
GTAGTGAAACGAATTAAACTGTTTAAACCTGATTACATATTTGTAGGTATGGGATTCCCTAAACAAGAAGAATGGATTATGACACATGAAA
ACCAATTTGAATCTACAGTGATGATGGGCGTAGGTGGTTCTCTTGAAGTATTTGCTGGGGCTAAAAAGAGAGCGCCTTATATCTTTAGAAA
ATTAAACATTGAATGGATATATAGAGCATTAATAGATTGGAAACGTATTGGTAGATTGAAGAGTATTCCAATATTTATGTATAAAATAGCCA
AAGCAAAAAGAAAAATGAAAAAAGCGAAATAG

Gene: tarH (teichoic acid ABC transporter, ATP-binding protein)

Position: 682648 to 683442, length: 795 nt, orientation: REVERSE

Sequence:

TTATTTAATAACGAAGCGGGCCTCATCGAGTTTGTTTCTAAATTCTTTTTGTTCGGCTTTGGATTTCTTTTTAAAATCGTTAAGGAAAGCGTCA
TATTTAGGTAATACATCATCAAGTTCACCGTAATCTTTTAACTTTCCGCCTTCAATCCAAGCAATCTTAGTACAAAATTGTCTCACTTGGCCTA
AGTTATGACTAACGAAAAAGATGGTTTTGTTTTGCTCTTTAAACTCGTAAATTTTATCTAAACATTTTTGTGCAAAAGTTTGGTCACCTACAG
ATAAAGCTTCGTCAATGACTAAGATATCTGGATTAACTGTGATATTAATTGAAAAACCAAGTTTTGCACGCATACCACTTGAATACTTTTTAA
CTGGTTGATAAATAAACTCACCAAGTTCACTAAATTCAATAATCTTAGGTGTCATCGCTTTAATTTCTTTTCGCTTAAAGCCCATACATAACAT
TTTAAATTCGATATTTTCAATCCCTGTAAGTTGTCCACTCAAGCCAGCACTAATTGCGATAACGCTGACTTCACCATTACGATCCACTTTGCCA
ACAGTAGGCGACAAAGAACCGCCAATGATATTGCTCAACGTTGATTTGCCGGAACCATTGATGCCAACAAGCCCTATGACGTCGCCTTCAT
ATGCTTTTAAACTAATGTCATCTAAAGCGAAAAATGTTTTGTTTTTATGTTTGGGAATGAGCGCATCTTTCATACGTTCTTTATTTGTACGATA
AATACGATATTCTTTTGTTACATTTTTAATGTTTACCGAAACGTTCAT

Gene: tarG (teichoic acid ABC transporter, transmembrane permease)

Position: 683768 to 684601, length: 834 nt, orientation: FORWARD

Perfect match to: (11819-97-CP003194-[727754:728587], allele observed in CC80+CC6+CC72+CC80)

Sequence:

TTGAAAGTGTGGTTTAATGGAATGTCAGCAATAGGAACAGTTTTTAAAGAACATGTAAAGAACTTTTATTTAATTCAAAGACTGGCTCAGTT
TCAAGTTAAAATTATCAATCATAGTAACTATTTAGGTGTTGCTTGGGAATTAATTAATCCTGTTATGCAAATTATGGTTTACTGGATGGTTTT
TGGATTAGGAATAAGAAGTAATGCACCAATTCATGGTGTACCTTTTGTTTATTGGTTATTGGTTGGTATCAGTATGTGGTTCTTCATCAACCA
AGGTATTTTAGAAGGTACTAAAGCAATTACACAAAAGTTTAATCAAGTATCGAAAATGAACTTCCCGTTATCGATAATACCGACATATATTG
TGACAAGTAGATTTTATGGACATTTAGGCTTACTTTTACTTGTGATAATTGCATGTATGTTTACTGGTATTTATCCATCAATACATATCATTCA
ATTATTGATATATGTACCGTTTTGTTTTTTCTTAACTGCCTCGGTGACGTTATTAACATCAACACTCGGTGTGTTAGTTAGAGATACACAAAT
GTTAATGCAAGCAATATTAAGAATATTATTTTACTTTTCACCAATTTTGTGGCTACCAAAGAACCATGGTATCAGTGGTTTAATTCATGAAAT
GATGAAATATAATCCAGTTTACTTTATTGCTGAATCATACCGTGCAGCAATTTTATATCACGAATGGTATTTCATGGATCATTGGAAATTAAT
GTTATACAATTTCGGTATTGTTGCCATTTTCTTTGCAATTGGTGCGTACCTACACATGAAATATAGAGATCAATTTGCAGACTTCTTGTAA

Gene: tarB (teichoic acid biosynthesis protein B)

Position: 684700 to 685803, length: 1104 nt, orientation: FORWARD

Perfect match to: (11819-97-CP003194-[728686:729789], allele observed in CC80)

Sequence:

ATGAACGTTTTAATAAAGAAATTTTATCATTCGGTAGTTCGAATACTTTCTAAAATGATTACGCCTCAAGTGATTGATAAACCGCATATCGTA
TTTATGATGACTTTTCCAGAAGATATTAAGCCTATCATCAAAGCATTAAATAATTCGTCGTATCAGAAAATTGTTTTAACAACACCAAAACAA
GCGCCTTATTTATCTGATCTTAGCGACGATGTTGATGTGATAGAAATGACTAATCGAACATTGGTAAAACAAATTAAGGCTTTGAAAAGCGC
GCAGATGATTATTATCGATAATTATTACCTATTACTAGGTGGATATAATAAGACTTCTAATCAACACATTGTTCAAACGTGGCATGCAAGTG
GTGCATTAAAAAACTTTGGATTAACAGATCATCAAGTCGATGTGTCTGACAAGGCAATGGTTCAGCAGTACCGTAAAGTTTATCAAGCGAC
GGATTTTTACTTAGTGGGTTGTGAACAAATGTCACAATGTTTTAAACAGTCTTTAGGTGCAACAGAAGAGCAAATGCTGTATTTTGGGCTTC
CGAGAATTAATAAATATTACACAGCTGATAGAGCAACGGTTAAGGCAGAGTTAAAGGATAAATATGGAATTACAAATAAGTTGGTATTATA
TGTACCAACATATAGAGAAGATAAAGCAGATAATAGGGCTATTGATAAAGCTTATTTTGAAAAATGTTTACCAGGATATACACTGATTAATA
AATTACATCCATCAATTGAAGATTCAGACATTGATGACGTATCTTCAATCGACACGTCTACATTAATGCTAATGTCAGATATAATTATTAGCG
ACTATAGTTCGCTGCCAATAGAAGCTAGCTTGTTAGATATTCCAACTATATTTTATGTGTATGATGAAGGAACATATGATAAGGTGAGAGGC
CTGAATCAATTTTACAAAGCAATACCGGATAGCTACAAAGTGTATACTGAAGAAGATTTAATAATGACGATACAAGAAAAAGAACATCTAT
TAAGTCCGTTATTTAAAGATTGGCATAAGTATAATACTGATAAAAGTTTACATCAGCTCACAGAATATATAGATAAGATGGTGACAAAATGA

Gene: tarX (teichoic acid biosynthesis protein X)

Position: 685800 to 686861, length: 1062 nt, orientation: FORWARD

Perfect match to: (11819-97-CP003194-[729786:730847], allele observed in CC80)

Sequence:

ATGAGGTTTACGATAATCATACCTACATGTAATAATGAGGCAACAATTCGACAATTGTTAATATCTATTGAGAGTAAAGAACACTATAGAAT
CCTTTGTATTGATGGTGGTTCTACTGATCAAACAATTCCTATGATTGAACGGTTACAAAGAGAACTCAAGCATATTTCATTAATACAATTACA
AAATGCTTCGATAGCTACGTGTATTAATAAAGGTTTGATGGATATCAAAACGACAGATCCACATGATAGTGACGCATTTATGGTCATAAAAC
CAACATCAATCGTATTGCCAGGTAAATTAGATAGGTTAACTGCTGCTTTCAAAAATAATGATAATATTGATATGGTAATAGGGCAGCGCGCT
TTCAATTACCATGGTGAATGGAAATTGAAAAGTGCTGATGAGTTTATTAAAGACAATCGAATCGTTACATTAACGGAACAACCATATTTGTT
ATCAATGATGTCTTTTGACGGAAAGTTATTCAGTGCTAAATTTGCTGAATTACAGTGTGACGAAACTTTAGCTAACACTTACAATCACGAAA
TACTTGTCAAGGCGATGCAAAAAGCTACGGATATACATTTAGTTTCACAGATGATTGTCGGAGATAACGATATAGATACACATGCTACAAG
TAACGATGAAGATTTTAATAGATATATCACAGAAATTATGAAAATAAGACAACGAGTCATGGAAATGTTACTATTACCTGAACAAAGGCTA
TTATATAGTGCTATGGTTGATCGTATTTTATTCAATAATTCATTAAAATATTATATGAACGAACACCCAGCAGTAACGCACACGACAATTCAA
CTCGTAAAAGACTATATTATGTCTATGCAGCATTCTGATTATGTATCGCAAAACATGTTTGACATTATAAATACAGTTGAATTTATTGGTGAG
AATTGGGATAGAGAAATATACGAATTGTGGCGACAAACATTAATTCAAGTGGGCATTAATAGGCCGACTTATAAAAAATTCTTGATACAAC
TTAAAGGGAGAAAGTTTGCACATCGAACAAAATCAATGTTAAAACGATAA

Gene: tarD (glycerol-3-phosphate cytidylyltransferase)

Position: 686924 to 687322, length: 399 nt, orientation: FORWARD

Perfect match to: (N315-BA000018-[690173:690571], highly conserved allele)

Sequence:

ATGAAACGTGTAATAACATATGGCACATATGACTTACTTCACTATGGTCATATCGAATTGCTTCGTCGTGCAAGAGAGATGGGCGATTATTT
AATAGTAGCATTATCAACAGATGAATTTAATCAAATTAAACATAAAAAATCTTATTATGATTATGAACAACGAAAAATGATGCTTGAATCAA
TACGCTATGTCGATTTAGTCATTCCAGAAAAGGGCTGGGGACAAAAAGAAGACGATGTCGAAAAATTTGATGTAGATGTTTTTGTTATGGG
ACATGACTGGGAAGGTGAATTCGACTTCTTAAAGGATAAATGTGAAGTCATTTATTTAAAACGTACAGAAGGCATTTCGACGACTAAAATC
AAACAAGAATTATATGGTAAAGATGCTAAATAA

Gene: pbpD (penicillin binding protein D)

Position: 687439 to 688734, length: 1296 nt, orientation: REVERSE

Perfect match to: (NN50-BAEA01000016-[71979:73274], allele observed in CC4803+CC5+CC8+CC239)

Sequence:

TTATTTTCTTTTTCTAAATAAACGATTGATTATCATATGAACAATAAGTGCTAATCCAGCGACTAGGCATGCACCACCAATGATAGTGAATAA
TGGATGTTCTGCCCACATACTTTTAGCAACAGTATTTGCCTTTTGAATAATTGGCTGATGAACTTCTACAGTTGGAGGTCCATAATCTTTATT
AATAAATTCTCTTGGATAGTCCGCGTGTACTTTACCATCTTCGACTACAAGTTTATAATCTTTTTTACTAAAATCACTTGGTAAAACATCGTAA
AGATCGTTTTCAACATAATATTTCTTACCATTTATCCTTTGCTCACCTTTAGACAATATTTTTACATATTTATACTGATCAAATGAGCGTTCCAT
TAATGCATTCCCCATCATATTACGTTGCTTCTCGCCACCAAGGTTTTTATAGTCTCCCGCACCCATGATAACTTGATTAATTCTAAATTTACCTC
GTTTGGTAGTAATCGTATGGTTGTAATTTGCTGTATCACTTGATCCAGTTTTTAAACCATCTGTACCAGGCAAACTCATTTTTGCACCTTCCAA
TGAAAAGTTGAATGTGTAATACGTAACTGCATGCGTTGTTGGTGCTAACTGCTTTGTAAAGTCTAATATTTTAGGTGTCTCTTTAATCACGTG
TAAATCTAAAATGGCATAGTCTCTAGCAGTCGTTACAGTACGTTCTTGGTCTTTATACTTTGTTGGTGCAAATGAACGTAATCTTGAATTTTC
AGCACCCGTTGGATTGACGAAATGTGTATTTTTCATTCCGATAGCTTTAGCTTTGTTATTCATTAAATCAACGAAATCGCTGGTGTTTTTTGAT
ACCTTCTTAGCTAAAATTAATGCCGCGGCATTACTAGAATTAGATACTGTAATTTGTAATAGGTCTGCGATTGTCCATACTTGTCCAGGATAT
AGTTTCGTATTACTCAACTCAGGTAGTGTAGACATAATATATTCTTTGTTCGTCATTGTGACTGTGTCATCAAGTGAAAGCTGCCCCTTATTT
ACAGCTTCCAATGTTAAGTACATTGTCATTAATTTAGTCATAGACGCTGGATTCCACTTAGTATCGATATTGTATTGATACAGTAATTGTCCA
GTTTGACTTACATTAACAGCACTCGTCGGTTCGTATGCAGCCGACAAACCTGCATAACCATATTGATTTGCTGCTTGTACAGGGGTTACGTC
ACTGTTAGTAGCTTGTGCATATGGTGTCATAATACTTAATGTTAAACATAAAATGATGATAATAGATATTAAATTTTTCAT

Gene: msbA1 (lipid A ABC transporter, fused ATPase and transmembrane permease)

Position: 689155 to 690882, length: 1728 nt, orientation: FORWARD

Perfect match to: (ED133-CP001996-[703482:705209], highly conserved allele)

Sequence:

ATGAAACGAGAAAATCCATTGTTTTTCTTATTTAAAAAACTATCATGGCCAGTGGGTCTTATCGTTGCAGCTATCACTATTTCATCACTAGGG
AGCTTAAGTGGACTATTAGTGCCACTGTTTACTGGACGAATTGTAGATAAATTTTCCGTGAGCCATATCAATTGGAATCTAATCGCATTATTT
GGTGGTATCTTTGTCATCAATGCTTTATTAAGCGGATTAGGTTTATATTTATTAAGTAAAATTGGTGAAAAGATTATTTATGCGATACGCTCA
GTTTTATGGGAGCATATCATACAATTAAAAATGCCATTCTTTGACAAAAATGAAAGTGGTCAATTAATGAGTCGATTAACTGACGATACGAA
AGTGATAAATGAATTTATTTCACAAAAGCTACCTAACTTATTACCATCAATCGTTACATTAGTTGGGTCACTAATCATGTTATTTATTTTAGAT
TGGAAAATGACATTATTAACATTTATAACGATACCGATATTCGTTTTAATTATGATTCCTCTAGGTCGTATTATGCAAAAGATATCGACAAGT
ACACAATCTGAAATTGCAAACTTCAGTGGTTTGTTAGGGCGTGTCCTAACTGAAATGCGTCTTGTTAAAATATCAAATACAGAGCGTCTTGA
ATTAGATAATGCACATAAAAATTTGAATGAAATATATAAATTAGGTTTAAAACAGGCTAAAATTGCGGCAGTTGTACAACCAATTTCAGGTA
TAGTTATGTTGCTAACAATTGCAATTATTTTAGGTTTTGGTGCATTAGAAATTGCGACTGGTGCAATCACTGCAGGTACATTAATTGCAATG
ATATTTTATGTTATTCAGTTATCTATGCCTTTAATCAATCTTTCCACGTTAGTTACAGATTATAAAAAGGCAGTCGGTGCAAGTAGTAGAATA
TACGAAATCATGCAAGAACCTATTGAACCGACAGAAGCTCTTGAAGATTCTGAAAATGTATTAATTGATGACGGTGTATTGTCATTTGAACA
TGTAGACTTTAAATATGATGTGAAGAAAATATTAGATGATGTGTCGTTCCAAATCCCACAAGGTCAAGTGAGTGCTTTTGTAGGCCCTTCTG
GGTCTGGTAAAAGTACGATATTTAATCTGATAGAACGTATGTATGAAATTGAGTCAGGTGATATTAAATATGGCCTTGAAAGTGTCTATGAT
ATCCCGTTATCTAAGTGGCGACGCAAAATTGGATATGTTATGCAATCAAATTCGATGATGAGTGGTACAATTAGAGACAATATTTTATACGG
AATTAATCGTCATGTTTCAGATGAAGAACTTATTAATTATGCTAAATTAGCGAACTGTCATGATTTTATCATGCAATTTGATGAAGGATATGA
CACGCTTGTAGGTGAACGAGGATTGAAACTGTCTGGCGGACAACGTCAACGTATTGATATTGCTAGAAGTTTTGTTAAAAATCCTGATATTT
TGTTACTTGATGAAGCAACAGCTAATCTCGATAGTGAAAGTGAATTGAAAATTCAAGAAGCTTTAGAAACATTGATGGAAGGTAGAACAAC

GATTGTCATTGCGCATCGTTTGTCTACAATTAAAAAAGCCGGTCAAATTATATTCTTAGACAAAGGACAGGTAACAGGTAAAGGTACGCATT
CAGAACTGATGGCATCACATGCGAAGTATAAAAACTTTGTAGTGTCTCAAAAATTAACAGATTAA

Gene: nupG (purine nucleoside transporter)

Position: 691241 to 692470, length: 1230 nt, orientation: FORWARD

Perfect match to: (N315-BA000018-[694488:695717], highly conserved allele)

Sequence:

ATGTTTTTATTAATCAACATTATTGGTCTAATTGTATTTCTTGGTATTGCGGTATTATTTTCAAGAGATCGCAAAAATATCCAATGGCAATCAA
TTGGGATCTTAGTTGTTTTAAACCTGTTTTTAGCATGGTTCTTTATTTATTTTGATTGGGGTCAAAAAGCAGTAAGAGGAGCAGCCAATGGT
ATCGCTTGGGTAGTTCAGTCAGCGCATGCTGGTACAGGTTTTGCATTTGCAAGTTTGACAAATGTTAAAATGATGGATATGGCTGTTGCAG
CCTTATTCCCAATATTATTAATAGTGCCATTATTTGATATCTTAATGTACTTTAATATTTTACCGAAAATTATTGGAGGTATTGGTTGGTTACT
AGCTAAAGTAACAAGACAACCTAAATTCGAGTCATTCTTTGGGATAGAAATGATGTTCTTAGGAAATACTGAAGCATTAGCCGTATCAAGT
GAGCAACTAAAACGTATGAATGAAATGCGTGTATTAACAATCGCAATGATGTCAATGAGCTCTGTATCGGGAGCTATTGTAGGTGCGTATG
TACAAATGGTACCAGGAGAACTGGTACTAACGGCAATTCCACTAAATATCGTTAACGCGATTATTGTGTCATGCTTGTTGAATCCAGTAAGT
GTTGAAGAGAAAGAAGATATTATTTACAGTCTTAAAAACAATGAAGTTGAACGTCAACCATTCTTCTCATTCCTTGGAGATTCTGTATTAGC
AGCAGGTAAATTAGTATTAATCATCATCGCATTTGTTATTAGTTTTGTAGCGTTAGCTGATCTATTTGATCGTTTTATCAATTTGATTACAGGA
TTGATAGCAGGATGGATAGGCATAAAAGGTAGTTTCGGTTTAAACCAAATTTTAGGTGTGTTTATGTATCCATTTGCGCTATTACTCGGTTT
ACCTTATGATGAAGCGTGGTTGGTAGCACAACAAATGGCTAAGAAAATTGTTACAAATGAATTTGTTGTTATGGGTGAAATTTCTAAAGAT
ATTGCATCTTATACACCACACCATCGTGCGGTTATTACAACATTCTTAATTTCATTTGCAAACTTCTCAACGATTGGTATGATTATCGGTACAT
TGAAAGGCATTGTTGATAAAAAGACATCAGACTTTGTATCTAAATATGTACCTATGATGCTATTATCAGGTATCCTAGTTTCATTATTAACAG
CAGCTTTCGTTGGTTTATTTGCATGGTAA

Gene: rsaD (ncRNA of Staphylococcus aureus D)

Position: 692620 to 692796, length: 177 nt

Sequence:

AAAAACCAAGTGCACATGGTAATACACTTGGCTTTTATGGGAAATGAATATTATTGTACATATGACAGTAAGGACTAGGTACAGTCATAGT
ACTTCGAGCAAAATTTGTTTTGTTATTATAAACAACACAAAGGAGATAACTTCTCTATTGAAGAAGTTAAAAACATTATAGCAGAC

Gene: yxkD (putative membrane protein)

Position: 692995 to 693828, length: 834 nt, orientation: FORWARD

Perfect match to: (COL-CP000046-[728486:729319], highly conserved allele)

Sequence:

GTGAATAAAACGGTTAAAGATTTAATACTAGTTGTCTTAGGTTCATTTATCTTTGCTGCAGGTGTAAATGCATTTATTATTTCTGGTAACTTA
GGTGAAGGCGGGGTTACAGGTTTAGCAATTATTTTATATTATGCGTTTCATATTTCACCAGCCATCACTAACTTCTTGGTCAACGCAGTATTG
ATTGCCATAGGTTATAAATTTTTGAGTAAGAGAAGTATGTACTTAACTATTCTTGTAACAATTCTTATTTCAATATTTTTGAGTTTAACAGAAT
CATGGCAAGTAGAAACTGGAAACAGCATTGTGAATGCCATTTTTGGTGGTGTAAGCGTTGGACTAGGAATCGGAGTAATTATCCTTGCAGG
CGGTACAACAGCAGGTACAACAATTTTGGCGAGAATTGCAACGAAATACCTCGATGTAAGCACGCCATATGCTTTGCTTTTCTTCGATATGA
TCGTTGTTGCAATTTCACTTACAGTTATTCCACTTGATAAAGTATTAGTAACAGTAATATCACTTTATATAGGAACAAAAGTGATGGAATATG
TCATAGAAGGTTTAAACACTAAAAAAGCTATGACGATTATTTCAACTAATCCCGACAAACTTGCCAAAGCAATAGACGAGCAAATTGGAAG
AGGTTTAACCATTTTAAACGGACATGGCTATTATACGCGTGAAGAAAAAGATGTCTTATACGTTGTTATTTCTAAAACACAAGTTTCAAAAG
CAAAGCGATTAATTAAACAAATCGATAAAGATGCATTCCTCGTAATTCATGATGTAAGAGATGTCTATGGTAATGGCTTTCTTGCAGATGAA
TAA

Gene: fhuC (iron-III-hydroxamate ABC transporter, ATPase)

Position: 694110 to 694907, length: 798 nt, orientation: FORWARD

Perfect match to: (N315-BA000018-[697357:698154], highly conserved allele)

Sequence:

ATGAATCGTTTGCATGGACAACAAGTTAAAATTGGTTACGGGGATAACACGATTATAAATAAATTAGATGTTGAAATACCAGATGGCAAAG
TGACGTCAATCATTGGTCCTAACGGCTGCGGGAAATCTACTTTGCTAAAGGCATTGTCACGTTTATTGGCAGTTAAAGAAGGCGAAGTATTT
TTAGATGGTGAAAATATTCATACACAATCTACGAAAGAGATTGCAAAAAAAATAGCCATTTTACCTCAATCACCTGAAGTAGCAGATGGCTT
AACTGTTGGGGAATTAGTTTCATATGGTCGTTTTCCACATCAAAAAGGATTTGGTAGATTAACTGCTGAGGATAAGAAAGAAATTGATTGG
GCAATGGAAGTTACAGGAACTGATACATTCCGACACCGTTCAATCAATGATTTAAGTGGTGGTCAAAGACAACGTGTTTGGATTGCAATGG
CATTAGCACAAAGAACTGATATTATCTTTTTAGACGAACCAACAACATATTTAGATATCTGTCATCAATTAGAAATACTAGAATTAGTTCAGA
AGCTAAATCAGGAACAAGGTTGTACAATTGTCATGGTTCTTCATGATATCAACCAAGCGATTCGTTTCTCAGATCATCTTATTGCGATGAAA
GAAGGGGATATCATCGCTACAGGTTCAACAGAAGACGTATTAACACAGGAAATTTTAGAAAAAGTTTTCAATATTGATGTTGTTTTAAGTA
AAGATCCTAAAACTGGAAAACCTTTATTGGTAACTTATGACTTATGTCGCAGAGCTTATTCTTAA

Gene: fhuB (iron-III-hydroxamate ABC transporter, transmembrane permease subunit)

Position: 694943 to 695947, length: 1005 nt, orientation: FORWARD

Perfect match to: (MW2-BA000033-[693545:694549], highly conserved allele)

Sequence:

ATGACAAATAGAGAGAATTCAACGCCATTGAAGTTTTTAGCCTATATTATAGGTTTAAGTATGATACTACTAATCACACTATTTATTTCTACA
TTAATAGGTGACGCCAAAATTCAAGCCTCTACAATTATAGAGGCTATTTTTAATTATAATCCTAGCAATCAACAGCAAAACATCATCAATGA
GATTAGGATTCCCAGAAATATAGCAGCAGTAATTGTGGGTATGGCGCTTGCAGTTTCTGGTGCGATTATACAAGGTGTTACCCGTAATAGT
CTTGCTGATCCGGCGCTCATAGGTTTAAATTCAGGTGCTTCATTTGCTTTAGCATTAACATATGCAGTTTTACCAAACACTTCATTTTTAATAT
TGATGTTTGCTGGATTTTTAGGTGCTATTCTAGGAGGTGCTATTGTATTAATGATAGGCCGATCTAGACGTGATGGATTTAATCCGATGCGT
ATTATTTTAGCGGGTGCAGCAGTAAGTGCTATGTTAACAGCGCTAAGTCAAGGTATTGCATTAGCTTTTAGACTAAATCAAACAGTAACATT
TTGGACTGCTGGAGGCGTTTCAGGCACAACATGGTCACACCTTAAGTGGGCAATTCCATTAATTGGTATTGCGTTATTCATTATATTAACAA
TTAGTAAACAACTTACCATTTTAAATCTTGGTGAATCATTAGCTAAAGGTTTAGGTCAAAATGTAACAATGATCAGAGGCATATGTTTAATTA
TTGCTATGATTCTAGCAGGTATTGCAGTTGCTATCGCTGGACAAGTTGCATTTGTAGGTTTGATGGTACCTCATATAGCAAGATTTTTAATTG
GAACTGATTATGCTAAAATTCTACCATTAACAGCCTTGTTAGGTGGGATACTCGTGCTTGTTGCCGATGTGATAGCACGATATTTAGGAGAA
GCGCCTGTTGGTGCAATCATTTCATTTATCGGTGTTCCTTACTTTTTATATTTAGTTAAAAAAGGAGGACGCTCAATATGA

Gene: fhuG (iron-III-hydroxamate ABC transporter, transmembrane permease subunit)

Position: 695944 to 696960, length: 1017 nt, orientation: FORWARD

Perfect match to: (11819-97-CP003194-[739930:740946], allele observed in CC80)

Sequence:

ATGATTAGTTCAAATAATAAACGCAGACAATGGATTGCACTGGCTGTTTTTAGCATTCTACTATTTCTAGGTTGTACTTGGAGTATTACCTCA
GGTGAATACAACATACCTGTTGAAAGATTTTTCAAAACTTTAATTGGACAAGGTGATGCCATTGATGAGTTAATCTTATTAGATTTCAGATTA
CCTCGGATGACGATTACTATTTTGGCTGGCGCAGCGCTTAGTATTAGTGGTGCAATAGTGCAAAGTGTCACGAAAAATCCAATAGCTGAAC
CCGGTATATTAGGTATTAACGCAGGTGGCGGATTTGCAATCGCATTATTTATTGCAATTGGTAAAATTAATGCTGACAACTTTGTTTATGTAC
TGCCGTTAATAAGTATACTAGGTGGTATCGCCACTGCATTGATTATTTTTATTTTCAGTTTTAATAAAAATGAAGGTGTTACACCTGCGAGTA
TGGTATTAATAGGTGTAGGTTTACAAACAGCATTATATGGTGGCTCAATTACAATTATGTCAAAATTTGATGATAAGCAATCTGATTTCATC
GCTGCTTGGTTTGCAGGTAATATTTGGGGTGACGAATGGCCATTTGTCATTGCATTTTTACCGTGGGTGTTGATTATTATTCCTTACTTACTA
TTTAAATCGAATACACTAAATATTATTCATACGGGTGATAATATTGCACGAGGTCTAGGTGTAAGGTTAAGCAGAGAACGTTTAATATTATT
CTTTATCGCAGTGATGTTATCATCTGCTGCTGTAGCAGTAGCAGGTTCGATTTCGTTTATCGGATTAATGGGTCCGCATATTGCCAAACGTAT
CGTTGGACCACGTCACCAGTTGTTTTTACCAATTGCCATTTTAGTAGGGGCATGTTTACTTGTTATAGCTGATACAATTGGCAAAATTGTATT
ACAACCAGGTGGGGTTCCAGCAGGTATTGTCGTAGCAATTATTGGTGCACCGTATTTCTTATATTTAATGTACAAAACGAAAAATGTATAG

Gene: dakK (putative dihydroxyacetone kinase, K subunit)

Position: 697227 to 698195, length: 969 nt, orientation: FORWARD

Sequence:

ATGATGAAAAAGTTAATCAATAAAAAAGAAACATTTTTAACTGATATGCTTGAAGGATTGTTAATTGCGCACCCAGAGTTAGATCTGATTGC
TAATACAGTTATTGTAAAAAAAAGCTAAGAAAGAACATGGTGTAGCAATAGTCTCTGGAGGTGGAAGCGGTCATGAACCTGCGCATGCCGA

TTTTGTTGCAGAAGGTATGCTAGATGCAGCGGTTTGTGGCGAAGTATTTACATCACCTACACCAGATAAAATATTAGAAGCTATTAAAGCA
GTAGATACTGGTGATGGTGTATTACTAGTTGTAAAAAACTATGCAGGTGACGTGATGAATTTCGAAATGGCACAAGAGCTTGCAGAAATG
GAAGGTATAAATGTTCAAACTGTTATTGTTCGTGACGACATTGCTGTGACAAACGAAGCACAACGTCGTGGTGTTGCAGGAACAGTGTTTG
TTCATAAGCTTGCTGGTTATCTTGCTGAAAAAGGTTATTCATTAACAGAGATAAAATCGCGTGTAGAAGCGTTGTTACCTGAAATTAAAAGT
ATTGGTATGGCAATTGAGCCACCGCTTGTTCCAACTACTGGAAAATATGGCTTTGATATTGAAGACGACAAAATGGAAATCGGTATTGGTA
TACATGGTGAAAAAGGTATTCATAGGGAAGAAGTAAAGGATATTGATCATATTGTTGGAACATTGTTAGACGAATTGTATAAAGAAGTTAC
TGCCAATGATGTCATATTAATGGTAAATGGTATGGGTGGTACGCCGTTATCTGAATTAAATATCGTAACTAAATATATTCAACAAAATTTAG
CTGCAAGAACGGTTAATGTTGCTAAATGGTTTGTTGGTGATTATATGACATCTTTAGACATGCAAGGTTTTTCTATAACTATCGTGCCTAATA
AACCAGAATATTTGGAAGCATTTTTAGCACCAACAACAAGTCAATACTTTAAATAA

Gene: dakL (putative dihydroxyacetone kinase, L subunit)

Position: 698237 to 698821, length: 585 nt, orientation: FORWARD

Perfect match to: (11819-97-CP003194-[742223:742807], allele observed in CC15+CC80)

Sequence:

ATGAAAGTGAATGATATGAAAGCACGTTTATTAAATTTAGAAGAAACGTTTAAAAAACATGAATCTGAATTAACTGAATTAGATCGAGCAA
TTGGTGATGGTGACCACGGGGTTAACATGGTTCGTGGGTTTAGTAGTCTTAAAGACAAACTTGATGATAGCTCAATGCAATCATTGTTCAA
ATCAACTGGTATGGCATTGATGTCAAATGTTGGGGGTGCATCTGGACCACTGTATGGCTTTAGCTTTGTTAAAATGTCTGCAGTCACGAAA
GATGATATGGATAATCAAGATTTCATTACACTAATTCAGGCATTTGCCGAAGCGGTTGAATCACGTGGTAAAGTTACTTTAAATGAAAAGAC
AATGTATGATGTAATAGCGCGAGCAGCAGAGAAGCTTAAAAATGGTGAAACTTTAACATTCAATGATTTACAGCAATTAGCAGATAATACA
AAAGATATGGTAGCAACGAAAGGTAGAGCTGCATATTTTGGAGAAGAATCAAAAGGTTATATTGATCCAGGTGCTCAAAGTATGGTTTATA
TTTTAAACGCTTTGATTGGAGATGAAGATAATGCCTAA

Gene: dakP (putative dihydroxyacetone kinase, phosphotransferase subunit)

Position: 698814 to 699176, length: 363 nt, orientation: FORWARD

Perfect match to: (Strain_21193-AFEG01000023-[142066:142428:r], highly conserved allele)

Sequence:

ATGCCTAAAATTATACTTGTTAGCCACAGTAAAGAAATTGCAAGTGGTACAAAATCTTTGTTAAAGCAAATGGCAGGTGACGTTGATATTAT
ACCAATCGGGGGATTACCAGATGGTTCAATTGGAACTTCATTTGATATCATCCAAGAAGTTTTGACTAAATTAGAGGATGATGCATTGTGTT
TTTACGATATTGGATCTTCAGAAATGAATGTAGATATGGCAATTGAAATGTATGATGGTAATCATCGTGTGTTAAAAGTTGATGCACCAATT
GTTGAAGGCAGTTTTATCGCAGCAGTAAAGCTATCAATCGGCGGTTCAATTGATGATGCATTAGCAGAAATGAAACAATCATTTTAG

Gene: Q5HI15 (putative protein)

Position: 699292 to 699789, length: 498 nt, orientation: FORWARD

Perfect match to: (RF122-AJ938182-[667831:668328], highly conserved allele)

Sequence:

ATGCAACACCTTATAAAAAAACATGTATTGAATGGCGAGTTTGATTTAGTACGACAATTGATGTCCGAAACAGATTTTATGGAATTTGAAGA
AGCATATATTTCAAGTGCGCATGAAGTAGAAAGTATGATGTTTTATACATGTATTTTAGATATGATTAAGTACGAAGAATCATCTGAAATGC
ATGACTTAGCATTTTTATTGCTTGTGTATCCACTAAGTGAATATGAAGGTGCTTTGGATTCTGCTTATTATCATGCAGACGCTTCCATAAAAC
TTACTGACGGCAAAGAAGTTAAAAGTTTGTTACAAATGTTATTATTGCATGCGATACCAACACCTGTTATTTCAGATAAGAAGGCTTTTGAT
ATCGCCAAGCAAATTTTAAAATTAGATCCTAATAATAATGTTGCTCGTAACGTCTTAAAAGACACTGCCAAACGTATGGACAACGTTGTTGT
TGATATAAATGAATTACACCAACGTAATGCACGTTAA

Gene: Q5HI14 (putative membrane protein)

Position: 700246 to 701313, length: 1068 nt, orientation: FORWARD

Perfect match to: (MW2-BA000033-[698815:699882], highly conserved allele)

Sequence:

ATGATGATGAATAAAGAAGCAACAAAAATTGGATTTGCCTACGTCGGCATTGTAGTGGGCGCAGGATTTTCAACTGGACAAGAAGTTATG
CAATTTTTCACTAAATATGGCTTGTGGGCTTATTTAGGTGTTATTATATCTGGTTTTATTTTAGCTTTTATTGGGCGCCAAGTAGCAAAAATTG
GTACTGCCTTTGAAGCGACAAATCATGAATCAACATTACAATACGTATTCGGTGAAAAGTTTAGTAAAGTCTTTGATTATATTTTAATCTTCT
TCTTATTTGGTATAGCTGTAACCATGATAGCTGGTGCAGGCGCAACATTTGAAGAAAGTTATAACATACCTACATGGCTAGGTGCTTTAATT
ATGACATTAGCGATTTATATTACGTTGCTATTAGACTTTAATAAAATAGTACGTGCACTAGGTATCGTTACACCATTTTTAATTGTTTTAGTTG
TATTAATCGCTGGCGTTTATTTATTTAAAGGTCATGTTTCATTAGCAGAAGTTAACCAAGTAGTGCCTGAAGCAAGTATTTGGAAGGGAATC
TGGTTTGGTACAATATATGGTGGATTAGCTTTTTCTGTAGGTTTTAGTACCATCGTAGCAATCGGTGGGGATACTGAAAAGCGTACAGTGTC
AGGTGCAGGCGCGATGTATGGTGGTATTATCTATACTGTATTACTAGCATTGATCAACTTTGCATTGCAAAGTGAATATCCAACTATTAAAA
ATGCCTCAATTCCTACATTGACGTTAGCAAATAATATCCATCCTTTAATAGCAACAGTGTTATCTGTTATTATGCTGGCGGTTATGTATAATA
CTATTCTAGGACTAATGTATTCATTTGCAGCACGTTTTACAGAACCATACAGTAAAAATTATCATATCTTTATTATTATAATGATGGTAGCAG
GTTATTTATTAAGTTTCGTAGGATTTGCTGAATTAATTAATAAGTTATATACAATTATGGGATATGTAGGCTTATTTATTGTAGTAGCTGTAA
TTATTAAATATTTCAAACGTAAAAATGCGGATAAAAAACATATTGCTTAA

Gene: lip4 (putative lipase)

Position: 701464 to 702507, length: 1044 nt, orientation: FORWARD

Perfect match to: (11819-97-CP003194-[745450:746493], allele observed in CC80+CC5+CC15+CC80+CC97)

Sequence:

ATGAATAAAGATAATAAATGGACGATGATAACTGCGCTTTTTATAACTGTAATCAGTGTATTGTTAGCATTTCATCTGAAACAACATTATGA
CCAAATTACAAATGAGAACCATGCTAATAAAGACAAAATTAATATTAAAAATAAAAATGTGCGCATTTATCAAAACCTTACATACAATAGAG
TTTTCCCTAACAGTAAATTAGATATTATTACACCTGTTGATATGTCTTCTAATGCCAAACTGCCAGTTATTTTTTGGATGCACGGTGGTGGTTA
TATTGCGGGTGATAAGCAGTATAAAAACCCATTATTAGCGAAAATTGCTGAACAAGGGTACATTGTTGTGAATGTAAATTATGCATTGGCG
CCACAATATAAATATCCCACACCATTAATTCAAATGAATCAAGCAACTCAATTCATTAAAGAAAATAAAATGAATTTACCTATTGATTTTAAT
CAAGTAATTATTGGCGGTGATTCTGCAGGTGCTCAATTAGCTAGCCAATTTACGGCAATACAGACGAATGATCGCTTAAGAGAAGCCATGA
AATTTGATCAGTCATTCAAACCATCGCAAATTAAAGGTGCTATACTATTTGGTGGTTTTTATAATATGCAAACAGTTAGAGAAACTGAGTTTC
CAAGAATACAGTTATTTATGAAAAGTTATACTGGCGAAGAAGATTGGGAAAAGAGTTTTAAAAACATTTCACAAATGTCGACAGTAAAACA
ATCGACAAAAAAATTATCCACCAACATTTTTATCTGTTGGAGATAGCGATCCATTCGAAAGTCAAAATATAGAATTCAGTAAGAAATTACAAG
AATTGAATGTACCAGTAGATACTTTGTTTTATGATGGTACGCATCATTTACATCATCAGTATCAATTTCACCTTAATAAACCAGAATCGATAG
ATAATATCAAAAAAGTGTTACTTTTCTTAAGTCGTAATACATCTTCTAGTGGTATTCAAACTGAAGAGAAACCACAAATAGAAAATCCGAGT
AATGAATTACCGTTAAATCCTTTAAACTAA

Gene: Q5HI12 (putative protein)

Position: 702836 to 703264, length: 429 nt, orientation: FORWARD

Perfect match to: (11819-97-CP003194-[746822:747250], highly conserved allele)

Sequence:

ATGACAAGGTCAGCATTAAAACCATTTAAAAATAAACGCGTTATGGTTACTGGACGTATACAACGTGTTTTGTTTAAAAATTATTTAGATAG
ACATAGCACATTTAAGCCGAATGTAAGGATATTATTAAAAGATGTATTTGTTTCAGGTGTATCAATAGATCATTTATGGTTATATGAGACAA
ATAAATACTATGCATTGGCAATGGAACTTATTCATCAACGAGTAAAATTTAGTGCGAATGTTGTACCGTATTACAAAATAAATAGAAATAAT
AATTTATTCGTACAAGATTATGGAATTAAGCGTAAAGGTAGGTTAATTACTGAAGAAGCTTACAATCAAAACAATCAGTATCAGGATAAGA
TATATGAAAAATTACCGGATATAGATTTTAGACTCGAAGATTTTTATAGTAAGGAAAACTAA

Gene: Q5HI11 (N-acetyltransferase)

Position: 703473 to 703979, length: 507 nt, orientation: REVERSE

Perfect match to: (ED133-CP001996-[717766:718272:r], highly conserved allele)

Sequence:

TTAATTAAGAATTTTAGCCATCATATAGTCATCGTAATATTTACCATCGATAAATAACTTATCTTTTAAAACGCCTTCGATTTGAAAATCGGCA
CTTTTAAAAAGCTCGAGGGCAGGTTGGTTATTGAGTGGTACATTTGCTTCAATTCGGTGTATTTGATTGTTTAAACACCAAGCCATAATGGC

ATCAAGAAGTGCTTGGCCAATTCCACGATGTTGATATAATTTCTTTACACCTAAATCAATTTTAGCAACATGTTTAATGCGTTGAAATGGTGT
CGTATTAACAAAGGCAAAGCCAACGAGTTGTTCATCACTTTCAGCAACAAAGATGACTTTATGTGGAGAAGTGATATATTCTTCTAATTGTT
TACTAGCCGATGTGACGCTAGGATCATATTCTCCTGGTGTGTAGAACATATACGGAGATTCGTCGTATATTTTCGCTAACATTGAAATGAAA
TTTTCTACATCTTTGATACTAACTCTACGTATAATATGGGCCAT

Gene: graX (cofactor of GraRS)

Position: 704092 to 705015, length: 924 nt, orientation: FORWARD

Perfect match to: (N315-BA000018-[707306:708229], highly conserved allele)

Sequence:

ATGAAACCTAAAGTTTTATTAGCAGGTGGAACAGGATATATTGGTAAGTATTTAAGTGAAGTGATTGAAAATGATGCTGAACTTTTTACTAT
ATCAAAATATCCAGACAATAAAAAAACAGATGATGTTGAAATGACTTGGATTCAGTGTGATATATTTCATTACGAACAGGTTGTTGCAGCA
ATGAATCAAATAGATATTGCTGTATTCTTTATCGACCCAACAAAGAATTCTGCCAAAATAACACAATCATCAGCAAGAGATTTAACATTAATC
GCAGCAGATAATTTTGGTCGAGCAGCGGCTATCAATCAAGTAAAAAAAGTAATCTACATACCTGGGAGTCGTTATGATAATGAAACAATTG
AACGCCTAGGTGCATATGGCACACCTGTAGAAACAACAAATTTAGTTTTTAAACGTTCTTTAGTTAATGTAGAATTACAAGTTTCAAAGTAT
GATGATGTTAGATCAACGATGAAGGTAGTTTTACCAAAGGGATGGACATTAAAGAACGTTGTAAACCATTTTATTGCATGGATGGGTTACA
CTAAAGGAACTTTTGTGAAAACAGAAAAATCACATGATCAATTTAAGATATATATTAAGAATAAGGTGCGACCGCTCGCAGTATTTAAAATA
GAAGAAACAGCTGACGGAATAATAACTTTAATTTTATTGAGTGGAAGTTTAGTGAAAAAATACACAGTTAATCAAGGGAAGTTAGAATTTA
GATTAATCAAAGAGTCGGCAGTCGTTTATATACATCTATACGATTATATCCCTCGATTATTTTGGCCGATTTATTACTTTATACAAGCACCAAT
GCAAAAAATGATGATTCATGGCTTTGAAGTTGACTGCCGGATTAAAGATTTTCAAAGTCGATTAAAATCAGGAGAAAATATGAAATATACT
AAATGA

Gene: graR (two component sensor/regulator, transciptional regulator)

Position: 705031 to 705705, length: 675 nt, orientation: FORWARD

Perfect match to: (NN50-BAEA01000016-[55042:55716:r], allele observed in CC4803+CC239)

Sequence:

ATGCAAATACTACTAGTAGAAGATGACAATACTTTGTTTCAAGAATTGAAAAAAGAATTAGAACAATGGGATTTTAATGTTGCTGGTATTGA
AGATTTCGGCAAAGTAATGGATACATTTGAAAGTTTTAATCCTGAAATTGTTATATTGGATGTTCAATTACCTAAATATGATGGGTTTTATTG
GTGCAGAAAAATGAGAGAAGTTTCCAACGTACCAATATTATTTTTATCATCTCGTGATAATCCAATGGATCAAGTGATGAGTATGGAACTTG
GCGCAGATGATTATATGCAAAAACCGTTCTATACCAATGTATTAATTGCTAAATTACAAGCGATTTATCGTCGTGTCTATGAGTTTACAGCTG
AAGAAAAACGTACATTGACTTGGCAAGATGCTGTCGTTGATCTATCAAAAGATAGTATACAAAAAGGTGATCAGACGATTTTCCTGTCCAA
AACAGAAATGATTATATTAGAAATTCTTATTACCAAAAAAAATCAAATCGTTTCGAGAGATACAATTATCACTGCATTATGGGATGATGAAG
CATTTGTTAGTGATAATACGTTAACAGTAAATGTGAATCGTTTACGAAAAAAATTATCTGAAATAAGTATGGATAGTGCAATCGAAACAAAA
GTAGGAAAAGGATATATGGCTCATGAATAA

Gene: graS (two component sensor/regulator, sensor histidine kinase)

Position: 705698 to 706738, length: 1041 nt, orientation: FORWARD

Perfect match to: (11819-97-CP003194-[749684:750724], highly conserved allele)

Sequence:

ATGAATAATTTGAAATGGGTAGCTTATTTTTTGAAATCTCGCATGAACTGGATATTTTGGATATTGTTTTTAAACTTCCTTATGTTAGGCATTA
GTCTAATCGATTATGATTTTCCAATAGACAGTTTATTTTATATTGTTTCTTTGAATTTAAGTTTAACAATGATTTTTCTTATATTGACATATTTTA
AAGAAGTAAAATTATATAAGCATTTTGACAAAGATAAAGAAATAGAAGAAATTAAACATAAAGATTTAGCGGAAACGCCATTTCAACGTCA
TACAGTTGATTATTTATATCGTCAAATCTCAGCGCACAAAGAAAAGGTTGTTGAGCAACAGTTACAATTGAACATGCATGAACAAACCATTA
CAGAATTTGTGCACGACATAAAAACACCTGTGACAGCCATGAAATTATTAATTGATCAAGAAAAAAATCAAGAAAGAAAACAGGCATTACT
ATATGAATGGTCTCGTATAAACTCGATGCTGGATACACAGCTGTATATTACTAGATTAGAATCTCAACGCAAAGATATGTATTTTGATTACG
TGTCACTTAAACGCATGGTCATTGACGAAATACAATTAACAAGACATATTAGTCAGGTTAAAGGTATTGGTTTTGATGTTGACTTTAAAGTG
GATGATTATGTTTATACAGATATAAAATGGTGCCGTATGATTATTAGACAGATTTTGTCAAACGCATTGAAATATAGTGAGAATTTTAATATT
GAAATTGGGACAGAATTAAATGATCAACATGTTTCGTTATATATTAAAGACTATGGCAGAGGTATTAGTAAAAAAGATATGCCGCGAATAT
TTGAACGAGGATTTACGTCAACGGCTAACAGAAATGAAACGACGTCTTCAGGTATGGGTCTATATTTAGTAAATAGTGTAAAGGATCAATT

AGGTATTCACCTGCAAGTCACGTCGACTGTTGGTAAGGGGACAACTGTCAGATTGATTTTCCCATTACAAAATGAAATTGTTGAACGCATGT
CGGAAGTGACAAATTTGTCATTTTAA

Gene: vraF (ABC transporter, ATP-binding protein)

Position: 706882 to 707643, length: 762 nt, orientation: FORWARD

Perfect match to: (COL-CP000046-[742339:743100], allele observed in CC8+CC15+CC101+CC121+CC772)

Sequence:

GTGGCAATTTTAGAAGTAAAACAATTAACAAAAATATATGGAACTAAAAAAATGGCACAAGAAGTGTTGCGAGATATCAATATGTCTATTG
AAGAAGGCGAGTTTATTGCTATTATGGGTCCCTCTGGATCTGGGAAAACGACATTATTAAATGTTTTAAGTTCAATTGATTATATTTCACAA
GGTTCTATTACATTAAAAGGAAAAAAAATTAGAAAAGCTTTCAAACAAGGAATTATCTGATATACGCAAGCATGATATTGGTTTTATTTTTCA
AGAGTATAATTTACTGCATACATTGACTGTTAAAGAAAACATAATGTTACCACTAACGGTTCAGAAGTTAGATAAAGAACATATGTTAAATC
GTTATGAAAAAGTAGCAGAAGCATTAAATATATTGGATATTAGTGATAAATACCCTTCTGAATTGTCTGGTGGACAAAGACAACGAACATC
TGCTGCAAGAGCGTTTATTACATTACCTTCTATTATATTTGCTGACGAACCAACAGGTGCACTGGATTCTAAAAGTACTCAAGATTTATTAAA
ACGATTAACAAGAATGAATGAAGCATTTAAGTCTACAATTATTATGGTAACGCATGATCCTGTTGCAGCAAGTTATGCCAATAGAGTAGTG
ATGCTAAAAGATGGTCAAATTTTCACTGAATTATACCAAGGGGATGACGATAAACATACCTTTTTCAAAGAAATAATACGTGTACAAAGTGT
TTTAGGTGGCGTTAATTATGACCTTTAA

Gene: vraG (ABC transporter, transmembrane permease)

Position: 707633 to 709522, length: 1890 nt, orientation: FORWARD

Sequence:

ATGACCTTTAACGAGATAATATTTAAAAATTTCCGTCAAAATTTATCACATTATGCCATCTATCTTTTTTCATTAATTACGAGTGTAGTATTGT
ATTTTAGCTTTGTAGCATTAAAATACGCGCATAAACTAAACATGACAGAGTCATATCCAATTATAAAGGAAGGCTCACAAGTCGGAAGCTA
CTTTCTATTTTTCATCATAATTGCATTTTTGTTATATGCCAATGTGTTATTTATTAAACGACGAAGTTATGAGCTTGCATTATATCAAACATTAG
GTTTATCTAAATTCAACATTATTTATATACTAATGCTCGAACAATTACTAATATTTATAATTACGGCAATATTAGGTATTATTATTGGTATTTTT
GGTTCGAAACTGTTATTAATGATTGTCTTTACATTATTAGGAATTAAAGAAAAGGTTCCAATTATTTTTAGTTTGAGGGCGGTATTTGAAACA
TTAATGTTAATCGGTGTCGCTTATTTTTTAACATCTGCTCAAAATTTTATATTAGTGTTCAAACAATCTATTTCACAGATGTCAAAGAATAACC
AGGTTAAAGAAACAAATCATAATAAAATTACATTTGAAGAGGTTGTTTTAGGCATCTTAGGTATAGTATTGATTACCACAGGATACTATCTA
TCTTTGAACATTGTTCAATATTATGATTCTATCGGTACACTTATGTTTATTTTATTGTCAACTGTGATTGGGGCATACTTATTTTTTAAAAGCTC
TGTTTCTCTAGTTTTTAAAATGGTAAAGAAGTTTAGAAAAGGTGTTATAAGTGTAAATGATGTCATGTTCTCATCATCTATTATGTATCGTAT
TAAGAAAAATGCTTTTTCACTTACGGTCATGGCAATCATTTCAGCGATTACTGTTTCAGTTCTTTGCTTTGCTGCTATAAGTAGAGCGTCCTTA
TCAAGTGAAATAAAATATACTGCACCACACGACGTTACAATTAAAGACCAACAAAAAGCTAATCAGTTAGCAAGTGAATTAAACAATCAAA
AAATTCCTCATTTTTATAATTATAAAGAAGTAATTCATACGAAATTGTATAAAGATAATTTATTTGATGTAAAAGCGAAAGAACCATACAATG
TAACAATTACTAGTGATAAATACATCCCTAATACTGATTTGAAACGTGGGCAAGCTGATTTGTTTGTAGCGGAAGGTTCTATCAAAGATTTA
GTGAAACATAAGAAGCATGGTAAGGCAATTATAGGAACGAAAAAACATCATGTTAATATTAAGTTACGTAAAGATATTAATAAAATCTATT
TTATGACAGATGTTGATTTAGGTGGACCAACGTTTGTCTTAAATGACAAAGACTATCAAGAAATAAGAAAGTATACAAAGGCAAAGCATAT
CGTCTCTCAATTTGGATTCGATTTGAAACATAAAAAAGATGCTTTAGCATTAGAAAAAGCGAAAAATAAAGTTGATAAATCTATTGAAACAA
GAAGTGAAGCGATAAGCTCAATATCAAGTTTAACCGGAATATTATTATTTGTAACATCATTTTTAGGTATTACATTCTTGATTGCTGTATGTT
GCATTATATACATAAAGCAAATAGATGAAACCGAAGATGAGTTAGAGAATTATAGTATTTTGAGAAAGCTTGGATTTACACAAAAAGATAT
GGCAAGGGGACTAAAGTTTAAAATTATGTTTAATTTTGGGTTACCTTTAGTTATTGCACTATCACATGCATATTTTACATCATTAGCATATAT
GAAATTAATGGGTACAACGAATCAAATACCGGTTTTCATAGTAATGGGATTATACATTTGTATGTATGCTGTTTTTGCAGTGACGGCTTATA
ATCATTCCAAGCGAACAATTAGACATTCCATATAA

Gene: ykaA-pitR (regulator of inorganic phosphate transporter)

Position: 710246 to 710863, length: 618 nt, orientation: FORWARD

Perfect match to: (11819-97-CP003194-[754233:754850], allele observed in CC80)

Sequence:

ATGTTTAGTAAGAAAAAAGATAAGTTTATGGTTCAATTAGAAGAGATGGTTTTCAATCTGGATCGTGCTGCTATTGAATTCGGTAAAATGG
ATTTCAATACACATTTAGATTTAAAAGCATACTCAGACAACATTAAAACTTATGAGTCACATGGTGACGAATTGGTACATCAAGTAATTACT

GATTTAAATCAAACATTTATCACACCAATTGAACGTGAAGATATTTTATCATTATGTGATGCAATTGATGATGTTTTAGATGCAATTGAAGAA
ACGGCAGCTATGTTTGAAATGTATTCAATCGAATACACAGATGAATATATGGCTGAGTTTGTTGATAACATTCAAAAAGCAGTTGCAGAAA
TGAAACTTGCTGTCGGCTTATTAGTCGATAAAAAATTATCACATATGCGTATTCATTCAATTAATATTAAAGAATTTGAAACAAACTGTGATG
GTATTTTAAGACAGTCAATTAAACATATTTTCAATAGCGAAACAGATCCAATCACATTAATTAAAATAAAAGATATTTATGAAAGCATGGAA
GAAATTGCTGATAAATGTCAAATCGTAGCAAATAATTTTGAAACTATTATTATGAAAAATAGCTAA

Gene: pitA (inorganic phosphate transporter)

Position: 710879 to 711886, length: 1008 nt, orientation: FORWARD

Perfect match to: (Strain_132-ACOT01000023-[33549:34556], allele observed in CC8+CC97)

Sequence:

ATGTCATATATAATCATCGTCACTATAGCTGTAGTTATTTTCTCGCTGATATTTGACTTTATCAATGGATTCCATGATACAGCCAATGCAGTA
GCTACTGCTGTATCTACTAGAGCGTTAACGCCTAAAACGGCAATTTTAATGGCAGCAGTGATGAACTTTATAGGTGCTTTAACATTTACGGG
CGTTGCAGGCACCATTACTAAAGACATTGTCGATCCATTTAAATTGGAAAATGGATTAGTTGTTGTGTTAGCTGCAATACTTGCGGCTATTA
TTTGGAATTTAGCTACTTGGTTTTACGGAATTCCAAGTTCGTCTTCACATGCACTTATAGGTTCAATTGCGGGTGCAGCAATCGCATCTGAAG
GCTCATTTGGAGTGTTACATTACCAAGGTTTCACAAAAAATTATTATTGTATTAATCGTTTCACCGATAATCGCATTTTGTGTTGGTTTCTTGAT
GTATTCAATTTTTAAAGTTATCTTTAAAAATGCAAATTTAACAAGAGCGAATCGTAACTTTAGATTTTTCCAAATTTTCACAGCAGCGTTACA
ATCATTCTCTCACGGTACGAATGATGCGCAAAAGTCAATGGGTATTATTACGTTGGCATTAATTGTTGCTAATGTACAGAATGATGGCAGCG
TTGAACCACAGTTATGGGTAAAATTTGCCTGTGCGACAGCAATGGGGCTTGGTACTGCAATTGGTGGCTGGAAAATTATCAAAACTGTAGG
TGGTAATATTATGAAAATACGTCCAGCAAATGGTGCTGCGGCCGATTTATCATCTGCATTAACAATTTTTGTTGCATCATCGCTACATTTCCC
ATTATCAACAACTCACGTTGTGTCATCATCAATCTTAGGTGTTGGTGCTTCTAACCGAGCTAAAGGTGTAAAATGGAGTACTGCGCAACGAA
TGATTATTACATGGGTGATTACATTACCTATTTCAGCATTGTTAGCAGGTTTACTATTCTATATACTTAACTTATTTTTCTAA

Gene: ssaA5 (staphylococcal secretory antigen A5)

Position: 712474 to 713271, length: 798 nt, orientation: REVERSE

Perfect match to: (11819-97-CP003194-[756461:757258:r], allele observed in CC80)

Sequence:

TTAATGGATGAATGCATAGCTAGAAACTTCTGAAGCTGGAATTGTACGGTAGTTCATATTGTATGGACCATATGTGTAATTCATTTCAGAAA
TCAAGATACTACCATCACCATTGACACGTTCAACATAAGCAACATGACCATATGGACCAGGTGTGCTTTGCATAATTGAACCAACTGATGGT
GTGTTGTTTACTTGGTAACCATCATTAGCTGCGTTACCAGCCCAATACTTAGCGTCTGACCAATATGTGCTAATTGGACTACCAGCTTGAGCA
CGACGGTCAAATACGTACCATGTACATTGACCAGCAGTGTATAAATTTTGGTGATTAAAAGATGATGCATTGCCATTGCTACCTGTTGTAGC
TGTTGGTGTTGTACCACCTGATCCACCATTAGGAATTTGTAATGTTTGGTTAGGCATAATTAAATAACCACGTAAGTTATTGGCTGCCATTAA
TTGATCAACTGAAACACCATATCTGCTAGCAATGATATTTAATGATTCACCAGCTTGTACAGTATGAGATGATGCTGAACCTGCTTGTGGAG
AAGTGTTTGACGTATTTTGTGCATCACTTCCACCTACTGAGATAACTTGACCAGGGAATACCAAGTTGTTATCTAATTGGTTATTTTGTTTAA
TACTCTCTACTGAAGTGTTGTATTTTTGAGCAATACTCCATAATGATTCACCAGATTGTACTGTATGTTGTGTAGAAGCTTGTGCATCATGAT
GCGTTAAAAATGCAGCTGCACCAGATGTTGCTGTTATTGCAAATGCTAATTTTTTCAA

Gene: yetJ (pH-dependend calcium efflux protein)

Position: 713632 to 714276, length: 645 nt, orientation: FORWARD

Perfect match to: (11819-97-CP003194-[757619:758263], allele observed in CC80)

Sequence:

TTGTCGCAAAATACAAATCATTCATATTATCATCAAAATCAGCATGCTCAATCAATAAGTAAAGTGTGGCTTTATTTTATGTATTATTGGATT
ATATTTGGCATAGGATGCTATCTAGGTCAGTTTTTACCATTAAGTTGGCGACAACCCTTGTCATTTGGATTACTGATTATTATTTTAGCAACA
CTTGTTTTTGAAAGAGCGAGACGGTTCGGTTTAATTATTTCACATATTTACGCTGTAGTGATCGGCTTATTGTCATACGCAACGTTTACCACG
TATTTACAAAATTTAGGACCAGATATTTTCTATAAAAATATCGCATTAGCAATTTTTGCATTTATAGCATTTGGTATTATTGGTTATTTCTTCGT
TGGAGATGCATCGAGTATAGGCAAATATTTATTCGTTACATTAATAACATTAATTATTGCGAGTCTAATTGGTATTTTTCTTCAAAATCCTATT
TTTTACACTATTATTACCGTCGTTAGTCTGTTGTTATTTCTACTTTATACTTTGTATGATTTTAATCGTTTAAAAAGAGGTGACTATTCACCAAG
AGAAATGGGATTTAATCTATTTATTAATTTGTTGAATATTATTAAGGATATACTTTATCTTGCTAATATGTTCAGAAGATAA

Gene: rbf (regulator of biofilm formation)

Position: 714973 to 717123, length: 2151 nt, orientation: FORWARD

Perfect match to: (NN50-BAEA01000016-[43623:45773:r], allele observed in CC4803+CC30)

Sequence:

ATGGCAAAATCATGCTTGCATATACTTACTAATAATGAATATGCGACAACGCGTTGCCAAGATGGCATAGTCTTATTTTGGCCAATTGACGG
GGAAATCGAACTACAAAAATTTCGTAAAAGTAAAATAATTGAAGATGATATATATATTATTAATCATCTGGATGTATTTAGTATTAAGAATA
ATAAAAAAACGATCATGTTGTATTTGAGTAGCGATTGGTTTGCGGAATTAGGCTTTACTTTCTTTAATTACCACTATACAGCAAAGTTGATTA
AATCATCCTATAATTTGAAATGTCTACTATTAAAATTGACCTATCGATACCTTGATAATCAGCCTCTTAATGACGCTGATATTAGAAAATTAC
AGGATATTATTAAAATCATTGCAAAAGAAGCAAGTATGGATAAAAAGATTGCACAAAATCAATATCGATATGCTTATTATGGTGATTTGCGT
GATGAGCTCGAATATATTTATCAAAATGTAAATCAACGATTGACATTAAAAAGTGTCGCTGATAAATTATTTGTCTCAAAGTCAAATTTGTCA
TCACAATTCCACTTACTTATGGGCATGGGTTTTAAAAAAATATATTGATACTTTGAAAATTGGTAAATCGATTGAAATTCTACTTACTACTGAT
AGTACTATTAGCAACATAAGTGAACATTTAGGTTTTAGTAGTAGCTCCACTTACTCTAAAATGTTTAAAAGTTATATGGATATAACACCGAAT
GAATATCGTAATTTATCAAAATATAATAAATGTTTAATGCTAAAGCCAGAACCACTAGTAGGCAAAATGGTGCAAGAAGTAAAAGAAATCA
TATTGAATTATATTGAACATTATAAAAACCACCTAACTGATGTTATACATATTGATGAAGACAAATTTGAAACACCTAAATTGTTTCAAACGG
TTATTCAAATAAATACTTATACAGAAATGAAATTAGTTTTCTTAGAAGGAATCTTTAAAACCTTATTGAATAAGAACAGTCAAGTTGTCTTTT
TCATCATGCCATCGATTCTAAAAAGTAAAAATACCATGTCCGAAGAAGAAAAATTCACAATCATTAAAACAATAATTGAAAGTGATCTAAAG
ATAGCATTTAATATAAATGATATTGAAACAACTTATTTTGTTGAAGAAGCTTTTATGAGTGTTTTCAGACAAATATCTCCAAACGAATTAAGT
AATCATAATAATTACGAAGTGCATTTTGTTTTTGATTTATCATTGATGGAAATTAGAACAATTTATCGAATGATATTAAAATTACATAACATC
ATGTTGAATGTGAAATTAGGATTGAACATTACCTGTTTATTTGAAAAACCTTCAGTTTTTAAATCACTAGTATCACAAATAAAGCGACTTAAA
TTCGATTCGTTAATAATAGATAATGCAAATTTAAGTAGCCCTTATTTGATGGGGGAAAGTGATGAGTTACTATTGAAAAATATTTTGCATTTT
AAAAATTTAAAACAAGTAATTAATGAATTGGATATTGAACAAGAAAAGCTTATTTTTCTAAATGTTGAAAATCATAAACTGCTTAATAATAA
AGAACGAGATTTAAGTAATAGTGCTCCATTAATTTATAAGACATTAAGTGCGCTGTATCACAACTTTGATGGCTTTGGATTAAACATTTTTGA
TAATCATCATACATTTAATGCGATGCATCTATATGATAAAAATGGATTTAAAACAACACTAGGTCTTATATTGGAAAAATTTATCGAATATGT
CTCGAAACCAAAATACGAAAACAGTTATTATTCTATTTTTGATATAGAGAATTATTATTGTCTTGTTATTTATGATTGGCGAGTGATAGAGAG
CGAGACAATTATGAGTAATTTTGAGGATAGTCAAGTTTATATAAATTTTAAAAACAATGTTTTAAACGATAAATATCTAATTGTAATAGAAA
CATTGGACGAAAATAGTGGCAACATTAATCATTTGATTTCTAAAGAATTAAGAGATAAATATGAATGGAACCCTAGTTTACTATCTAAAATT
GACAACTACCTTAAACCAGCAATAGAGATTAAAGAGCATAATTTTAGTGATAATTCTTTGAATATTAACGTTACTTTTAATGCGTTATACATA
ATTAAAATAGGAAAAAAAATAA

Gene: sarX (HTH-type transcriptional regulator)

Position: 717203 to 717628, length: 426 nt, orientation: FORWARD

Perfect match to: (N315-BA000018-[720416:720841], highly conserved allele)

Sequence:

ATGGCAAAAAAATTTAAGATAATAATGACAGAAGCATTGTCTTTATATATTTGGGGGTGCAACATTTTGAATACTGAGAAATTAGAAACATT
GCTTGGCTTCTATAAACAATATAAAGCATTATCTGAATATATTGATAAAAAATATAAGTTGTCGCTAAATGATTTAGCAGTCTTAGATTTAAC
GATGAAGCATTGCAAAGATGAAAAAGTACTTATGCAATCATTTTTAAAAACTGCAATGGATGAGCTAGATTTAAGTAGGACAAAATTATTA
GTTTCTATAAGAAGACTAATTGAAAAAGAAAGACTTAGTAAAGTTAGATCATCTAAAGATGAGCGTAAAATTTATATTTATTTAAATAATGA
TGATATATCTAAATTTAATGCTTTATTTGAAGATGTAGAACAATTTTTAAATATTTAA

Gene: yeeI (putative transcriptional regulator)

Position: 717818 to 718534, length: 717 nt, orientation: FORWARD

Perfect match to: (N315-BA000018-[721031:721747], allele observed in CC5+CC1+CC49+CC80+CC239)

Sequence:

ATGGGACGTAAATGGAATAACATTAAAGAAAAAAAGGCCCAAAAAGATAAAAACACAAGTAGAATATATGCGAAATTTGGTAAGGAGATT
TATGTTGCAGCAAAATCTGGTGAACCCAATCCAGAATCTAACCAAGCTTTAAGGTTGGTGCTTGAACGCGCTAAGACATATTCAGTGCCGA
ATCATATTATTGAAAAAGCAATAGATAAAGCTAAGGGTGCTGGAGACGAAAACTTTGATCACCTAAGATATGAAGGATTTGGCCCAAGCG
GATCAATGCTAATTGTTGATGCGTTAACAAATAATGTAAATCGTACTGCCTCTGATGTGCGAGCTGCTTTTGGTAAAAAATGGCGGTAATATG

GGTGTATCTGGATCAGTTGCTTATATGTTTGATCATGTGGCAACATTTGGTATTGAAGGAAAGTCTGTTGACGAAATACTTGAAACATTAAT
GGAACAAGATGTAGATGTAAATGATGTGATTGACGATAATGGATTGACAATAGTCTATGCTGAACCAGATCAATTTGCAGTCGTTCAAGAT
GCGCTTCGTGCAGCAGGTGTTGAAGAATTTAAAGTTGCTGAATTTGAAATGTTACCTCAAACAGATATTGAACTTTCTGAAGCGGACCAAG
TAACATTTGAAAAATTAATCGATGCATTAGAAGATTTAGAAGATGTACAAAACGTATTCCATAATGTGGATTTGAAATAA

Gene: DUF985 (protein of unknown function DUF985)

Position: 718534 to 719007, length: 474 nt, orientation: FORWARD

Perfect match to: (MW2-BA000033-[717104:717577], allele observed in CC1+CC80+CC239)

Sequence:

ATGAAATCAGCAGAACAATGGATTGATGAATTGCAACTTGAATCACATCCTGAAGGTGGTTTCTATAGAGAGACAATTCGAGAAGTATTGA
AAGATGGACGCAGAGCGCCGTTTAGTAGTATTTATTTTTTACTTACAGATGACAATATTTCGCATTTTCATCGAATTGATGCTGATGAAGTAT
GGTACTATCATGCTGGCGATTCTCTAACAATTCATATGATAAATCCGGATGGGGAATATACGACTGCAACATTGGGTACTGATATCCAAAAT
GGAGATGTATTGCAATATGTAGTGCCTAAAGGAACAATTTTTGCTTCTTCAATCGAATTTTCAAATACTTATAGTTTAGTAGGTTGTATGTGT
CAACCGGCATTTGAGTTTAAGCAGTTTGAATTGTTTAAGCAATCTGAATTAATTACACAATATCCGCATCTTAAATCAGTGATTGAAAAATAT
GCTTTAAAATAA

Gene: A5IQM5 (putative protein)

Position: 719360 to 720004, length: 645 nt, orientation: FORWARD

Perfect match to: (N315-BA000018-[722573:723217], allele observed in CC5+CC1+CC239+CC4803)

Sequence:

GTGAAAGTAAAGTATATAGATAAACGTCACTGGCGTCGCCTAATTGATAGGGAATACACAGAGGTAAAAGTTAATAATAATAGGTTTAAG
GGTATTATAGGCTTAGTCACGATGAAAAAGGTTCGTGATCCTTTAGAGGTGACGGTAGTTGGACAAAATATCATTGTCGCAGATGACAATT
ATAAATGGTTGCAAATACTACCTGAAAAGAAACGTTATAGTATAACTGTAATGTTTGATAATAAAGGCAATCCATTAGAATATTATTTTGAT
ATAAATATCAAAAATATAACGCAAAAAGGTAATGCGCGTACAGTAGATTTATGTTTAGATGTTTTAGCGTTACCAAGTGGTGAATATGAGTT
GGTAGATGAAGATGACTTAATGTTTGCATTAGAAAGTGAGCAAATTACAAAAAAGCAATTTCATGAAGCATATATGATTGCACATCAAATT
ATGGCAGAGTTAGAAAATGATTTTAAAGGATTCCAAAAGAAAATCATGTACTGCTTTAATAAAATTAATGCAAAGGCTCAAAAAAATCATC
AAAAGCCACAAAATAAAACTAATATTGAAAAAAGCAAACAAATAAAGCCTAAGCAATATAATCAAACTAAAAATCACCAACAACAAAAGAA
AAACTAA

Gene: ccpE (catabolite control protein E)

Position: 720120 to 720986, length: 867 nt, orientation: FORWARD

Sequence:

ATGAAGATTGAAGACTATCGTTTACTAATAACATTAGACGAAACGAAAACGTTACGTAAAGCGGCTGAAATTTTATATATATCTCAACCTGC
TGTTACACAAAGACTAAAAGCTATTGAAAATGCTTTTGGAGTAGATATTTTTATCAGAACAAAAAAACAATTGATTACAACAACTGAAGGAA
CAATGATTATTGAGCATGCCCGTGACATGTTGAAAAGAGAGCGATTATTTTTTGACAAAATGCAGGCACATATTGGTGAAGTGAATGGAAC
AATATCAATCGGGTGTTCTTCTTTGATTGGACAAACCTTACTTCCTGAAGTTTTGAGCCTATATAATGCCCAATTTCCTAATGTTGAAATACA
AGTGCAAGTTGGTTCAACTGAACAAATTAAAGCAAATCATAGAGATTATCATGTTATGATAACTCGTGGAAATAAAGTAATGAATTTAGCT
AACACACATTTATTTAATGATGATCATTATTTTATTTTTCCAAAAAATAGACGAGATGATGTTACAAAGTTACCATTTATAGAGTTTCAAGCT
GATCCGATTTATATAAATCAAATAAAACAATGGTATAACGATAATTTAGAACAAGATTACCATGCAACTATTACAGTGGATCAAGTAGCAAC
TTGCAAAGAAATGTTGATTAGTGGTGTAGGTGTTACAATTTTGCCGGAAATTATGATGAAAAATATCAGCAAAGAACAATTTGAGTTTGAA
AAAGTAGAAATTGATAATGAACCGCTGATTCGTTCGACATTTATGAGTTATGATCCGAGCATGTTGCAATTGCCACAAGTTGATTCTTTTGT
AAATCTCATGGCGAGCTTTGTTGAACAACCAAAGGCATAG

Gene: A8Z196 (putative sugar:cation symporter)

Position: 721324 to 722544, length: 1221 nt, orientation: FORWARD

Perfect match to: (T0131-CP002643-[742303:743523], allele observed in CC239)

Sequence:

ATGGATATGTTTGCAGCGTTATTACAAATAAAGAATTATAAACTCTTTGTTGCTAATATGTTTCTACTAGGTATGGGTATTGCGGTTACGGTC
CCATATCTTGTTCTTTTTGCAACTAAAGATTTAGGTATGACAACAAATCAGTATGGATTACTTCTAGCATCTGCAGCGATTAGCCAGTTTACA
GTAAATTCAATTATTGCTAGATTTTCGGATACGCATCACTTTAATAGAAAAATTATTATTATTCTCGCATTATTAATGGGTGCGCTTGGTTTTT
CAATATACTTTTTTGTAGATACAATCTGGTTATTCATATTACTATATGCGATTTTTCAAGGATTATTTGCACCAGCAATGCCCCAACTTTACGC
ATCTGCTAGAGAATCTATCAATGTTTCAAGCTCTAAAGATAGAGCTCAATTTGCCAACACAGTATTACGTTCAATGTTCTCATTGGGCTTTTT
ATTTGGTCCATTTATTGGTGCCCAATTAATCGGATTAAAAGGCTATGCTGGATTGTTTGGTGGAACAATAAGTATCATTTTATTTACTTTAGT
ACTTCAAGTGTTTTTCTATAAGGATTTAAACATTAAACACCCTATTAGTACGCAACAACATGTTGAAAAAATTGCTCCTAATATGTTTAAAGA
CAAAACACTTTTATTACCATTTATTGCATTTATTTTATTACACATTGGACAATGGATGTATACGATGAATATGCCTTTATTTGTTACTGATTATT
TAAAAGAAAATGAGCAACATGTCGGTTATTTAGCTAGTTTATGTGCTGGTTTAGAAGTGCCATTTATGATCATTCTTGGCGTTTTATCATCTA
GATTACAGACTCGAACATTGTTGATTTATGGAGCGATTTTTGGTGGTTTATTCTACTTCAGCATTGGGGTATTTAAAAACTTCTATATGATGT
TAGCAGGACAGGTGTTTTTAGCTATTTTCTTAGCGGTTCTTTTAGGAATTGGTATTAGTTATTTCCAAGATATCTTACCAGATTTTCCAGGAT
ACGCCTCAACACTATTTTCTAATGCAATGGTTATTGGACAGTTAGGCGGTAACCTATTAGGTGGTGCTATGAGTCACTGGGTAGGTTTGGA
AAATGTATTTTTTGTATCAGCAGCATCAATCATGTTAGGTATGATACTTATATTCTTTACTAAAAATCAAAAAATTACAAAAGAGGATGTGAT
ATCAACATGA

Gene: Q2YSN2 (putative membrane protein)

Position: 722541 to 723029, length: 489 nt, orientation: FORWARD

Perfect match to: (MW2-BA000033-[721182:721670], highly conserved allele)

Sequence:

ATGACAATTATTTTATGGCTACTTATCATCGCTGCCTTCATGTTAGCATTTGTTGGGTTGATTAAGCCGATTATTCCTTCTGTTTTAGTATTAT
GGGTTGGCTTTTTAATCTATCAATTTGGCTTTCATAATCAGCATTTATCATGGGTGTTTTATGTATCTATGGCATTGCTAACAATATTAATTTT
ATGTGCCGACTTTTTAGCTAATAAATATTTTGTGAATCGCTTCGGTGGTTCTAAGTTTGGAGAGTATGCAGCTTTAATTGGTGTGGTTATTGG
ATGTTTTGTTTTACCGCCATTTGGAATTATTATTATACCTTTTATTTTGGTATTCATAGTTGAATTAATACAAGGCTATTCATTTGAAAGAGCA
GTTAAAGTAAGTATAGGTTCAATCGTAGCATTTTTAACAAGTAGTATAGCTCAAGCAATCATTATGTTTATAATGATTGTATGGTTCTTTATA
GATGCTTTATTGATTAATTAA

Gene: Q2YSQ6 (putative uncharacterized protein)

Position: 723128 to 723817, length: 690 nt, orientation: REVERSE

Sequence:

TTATAAAAATGTATGAGAATGTTTTTTCGAAATATTTCTTTCAATGCGTAATCCAATAGGCATAACTATAAATGAAAATATAATAAATACCCA
ATTGCTTACCTGAATATATGGTCCAAAGGCAAGTAATGATTGTGGAATAAAGAATACATAGAAAAATCCTACAATTAGTAGAATGATTGCT
AAATAGGTGAATATCCATATCCAATTTGAATTGTTAAAACACTGCAGTTGTATTGTTTTAAAACTAAACCATATAAATAAAAAGACAATAAA
GAATCCAACGACTACTGAAAACGGGAATGAAACAAAATATAAATTACTTCCATTTTTTTCCATGAAAAATCCTAAAAATCCTTTGAGAAAAC
TAACAATCCCAATTAATAGAACGATGTGTTGATAGATATATTTAAAAATATTTTTAAATGTTTCATTAGGCATCGCTTTTAGTTCTTTTATTGC
ATGTGCTTTTGGGTCGTGATTGAAAAAATCTAAGGCTAATAAACCATGTTGTTCTGCGCTTAATAATTGTTTAAGTATACGGTTAATAATTAA
CTCTGTATCATGAGGATTGACGCGAAAGTCAGAGCGCATATAAGTCATATAATTCTCGAAGATTTCTCTATCAGTATTGCTTAATCTTAATGA
TTTAACATTATTTTCTTTTGTTAATTGCGCAGTACTTTTCAT

Gene: A5IQN0 (putative acetyltransferase)

Position: 723944 to 724387, length: 444 nt, orientation: FORWARD

Sequence:

ATGAGGACACTTAATAAAGATGAACATAATTATATCAAGCAAATAGCTAATATACATGAGACATTATTGTCGCAAGTAGAATCCAACTATAA
ATGTACTAAACTGAGTATTGCTCTTAGGTACGAGATGATATGTTCAAGATTAGAACATACAAATGATAAAATTTATATATATGAAAATGAAG
GTCAATTAATAGCGTTTATTTGGGGACATTTTAGTAATGAAAAAAGTATGGTTAACATTGAACTGCTATATGTTGAACCACAATTTCGCAAA
CTGGGAATAGCTACGCAACTGAAGATTGCGCTTGAAAAATGGGCAAAAACTATGAATGCAAAGCGAATAAGCAGTACAATTCATAAAAAT
AATTTGCCAATGATATCTTTGAATAAAGATTTAGGTTATCAAGTGAGTCATGTGAAAATGTATAAAGATATTGATTAG

Gene: A8YZU8 (putative lipoprotein)

Position: 724454 to 724849, length: 396 nt, orientation: FORWARD

Sequence:

ATGAAGAAATTAATCATCAGTATTATGGCGGTCATGCTATTTTTAACAGGTTGTGGTAAAAGTCAAGAGAAAGCCACTCTGGAAAAGGATA
TCGATAATTTACAAAAAGAAAATAAAGAATTAAAAGATAAAAAAGAAAAGCTTCAACAAGAAAAAGAAAAATTAGCAGATAAGCAAAAAG
ACCTTGAAAAAGAAGTGAAAGATTTAAAACCTTCAAAAGAAGATAACAAGGATGATAAAAAAGACAAAGACAAAAATGAAGATAAAGAC
AAAGATAAAGAGGCATCACAAGATAAGCAATCAAAAGATCAAACTAAGTCATCGGATAAAGATAATCACAAAAAGCCTACATCAACAGAT
AAAGATCAAAAAGCTAATGACAAACACCAATCATAA

Gene: Q5HHZ0 (putative protein)

Position: 724987 to 725286, length: 300 nt, orientation: FORWARD

Perfect match to: (N315-BA000018-[727994:728293], highly conserved allele)

Sequence:

ATGCATGAACAAGATTTTAGAATTTTAGAGGGTCAAGATATTACTTTGCCAGAATTAGGTAGAGAATTAGAAAATATTACAGGACATACGA
TTGCTGATTCTACTGGCGAAATTAAGCGTGTAATTGCACATTTACCAAACTTTGAGTCCGATACAGATACTTTTGTTGCTACATATCGTTTAA
ACCATCAACAAGATTTTATAGATGCAACTTTTACTGCGCTGAAATCAGATAGAGCACGTTTAAAAGAAGTGCCAGTTCATGTTGAACTTATA
AGTTATATTTCTAAATCAAAATAA

Gene: ykkB (putative acetyltransferase, GNAT family)

Position: 725366 to 725908, length: 543 nt, orientation: FORWARD

Perfect match to: (11819-97-CP003194-[769285:769827], allele observed in CC80+CC12+CC25+CC80)

Sequence:

ATGATTTATTGTGAAACGGAGCGTTTAATATTAAGAGACTGGCATGAAGATGATCTGTTACCTTTTCAAAAAATGAATGCGAATTATGACGT
ACGTAAATACTTTCCAAGTTTATTGAGTTATCGTCGTTCAGAATTAGATATGAGAACTATGGATGCGGTTATTAAAGATTATGGCATTGGAT
TATTTGCTGTAGAAGATAAAGAGACACATCAATGGATAGGCTTTATAGGTTTGAATTATATTCCAGAAACAAGCGATTATCCATTTAAAGAA
TTACCGCTTTATGAAATAGGTTGGCGCTTGTTGCCAGAATTTTGGGGAAAAGGATTAGCAACTGAAGGCGCAAAGGCAACGTTGAAGTTA
GCAGAAGAACATCAAATATACGATGTCTATAGTTTTACAGCAGAAGCAAATAAAGCTTCACAACGTGTAATGGAAAAAATTGGCATGACAG
TGTATGATCATTTCGAATTACCCAATCTAAGTAAGTATCATTTATTAAAAAGGCAAGTGCGCTATTACATTAATCTTCGAAAGTGA

Gene: yvdD (decarboxylase family protein)

Position: 726004 to 726570, length: 567 nt, orientation: REVERSE

Perfect match to: (MW2-BA000033-[724645:725211:r], highly conserved allele)

Sequence:

TTAATCGTATGAACGAGTACCCAGAGGTTTGAAATTTAATATTGATTCAATTAATGATTCCTTAGTGTCGCATAACGGTGCAAGAGCACGAT
ACTTAGGATCAATAAAACCTTCCTCAATCATATGGTCAATCATTGTTTGTAGTGGATTGAAAAAGCCATTAATATTATAAATGGCAATAGGC
TTTTCATGGATACCTATTTGAGCCCAACTATACATTTCGAAAAATTCTTCTAGTGAACCTGCGCCACCAGGAGCCATGACAAATGCATCTGCA
AGTTCTGCCATTTTATTTTTACGTTCATGCATAGAATCAACTAAAATTAATTCAGTTAAACGTTGGCTTGTGATTTCATGTTCATCTAACATTTT
AGGCATGACGCCAATAGCTTTGCCGCCATGATCTAATACACCATCTTGAATGGCACCCATAATGCCAATTGACCCTGCACCAAATACTAATT
CATAACCTTGTTCAGCAAAATATTTACCTAAATCGTATGCTTTTTGTACATATGAAGGGTCATGACCTTTGCTTGCACCACAATAAACTGCGA
TTCGTTTCAT

Gene: yqxD (conserved hypothetical protein)

Position: 726572 to 727030, length: 459 nt, orientation: REVERSE

Perfect match to: (N315-BA000018-[729579:730037:r], highly conserved allele)

Sequence:

TTAATCCAGCTCCTTAATTCGATGAATGACTTTTAATAGTGATTGTTCAAACACTTTTTGATCTTGCTTTGTAAAAGGTGGGGGACCTTTGTG
GCGACCACCTTGTTTTCTAATTTGTGCATTCATATATCGTTTATCTAATAGTTGTTGAATATTTTTGGAATTGTATATCTTCCCATTATGATGCA
TGACAATTAAGACTTTGTCGACTAATAAACTTGCGAGTCCATAATCTTGAGTGACTACGATATCATCCTTCGTTGATAATTGAACAATTTTGT
AATCAACTGCATCTGGTCCATCATCAACATATAATGTTGATACATGTGGAGGATATAATTGGTTCGAAAAATGGCTGAAGCTCCGAATAATT
GTCACAAAAATGCCTGTCTCAGTTGTTAAATCTATAATAGAATCAACAACAGGACAAGCATCTCCATCAATAATAATATGTGTCAC

Gene: Q5HHY6 (putative protein)

Position: 727033 to 727716, length: 684 nt, orientation: REVERSE

Perfect match to: (11819-97-CP003194-[770952:771635:r], allele observed in CC80+CC4803)

Sequence:

TTATGCCTCTGTATTGTTTTCTTTATTTTGTTGAGAGGCGCTTTTGGCAACATAATCTTTATATTTTTTAAATGACTTGATGCGTGCTTTATCAG
CTTCTTGTTGGCGTTTTTGTTCTTCTTTGTGTCGTTTTTCAATATTTTTTTGTAACTTTTTATTCATTTTAGCGATTTCTTTGCGATTTTTTTCAGC
TAGTTTATCGCCTTTTTTCTCAGTTTTCTCATCTAATTTATTAGGTGTTAAGCCTGCTTTTTCTTCGTATTTTTGTGATTTTTTCATATCTTTAATA
CGTTGTATTTCATTCTTTTCGCGGGCTTTTTGCTCTTCTTTATGACGCTTTTCGATATTTTTTTGAAGTATTTTATTCATTTTATTAGCGTCTTTA
CGATTTTGTTTAGCTAATTTTTCGCCTTTTTTCTCAATATAGGCAGGATCATGTTCTCTAGCAAACTTTTTAAGTTCACGTTTATTTTCAAAATC
TTGTTTTTTATCGCCGACATATTCTTTAACATCACTCGCTGTGTTACTGATTGCTGCAGATGTTTTTGAAGCAACTTTACTTGTAGCATCTGTA
ACTTTTTGTACGTCCGGATGTTGTTTGATACGTTTACGTTCAACAATTAACGGTACCAATACAATTGGTAATACATTAATCATAAATTTGATG
ACTTTTTTCTTATCCAT

Gene: uppP (undecaprenyl-diphosphatase)

Position: 727888 to 728763, length: 876 nt, orientation: REVERSE

Perfect match to: (11819-97-CP003194-[771807:772682:r], allele observed in CC80+CC188+CC4803)

Sequence:

TTAAATACCTTTACCAATGCCAAATCCGAAGTAAAGTATAGCAATAAAGATTACTAATACAATTCTGTAAATGGCAAATGGAATTAGTTTGA
TTTTGTTAATTAGATGCAAGAATGTTTTGATTGCAATTAGTCCAACAGTAAATGCAGCTAAAAAGCCTAAAATATAAAAAGGTATATCAGCA
ATCTGAATATCTTGATAATGTTTTAATAAAGATAAACCACTAGCTGCTAACATAATTGGAACAGCCATAATAAATGTAAAGTCCGATGCTGC
TTTATGATTTAATTTCATTAATACCCCAGTTGAAATTGTTGAGCCTGAACGGCTGAAACCAGGCCACATAGCTACTGCTTGAGAGATACCAA
TTACAAATGCTTGGAAATAACTGATTTGATCTACTGTTTGTGGGTTTTTAACTTTAGCTGAGTATTTATCAGCAATAATCATATAGATAGCAC
CTACGAATAAGCCAATCATAACAGTTGGCACACTAAATAAATGTTCTTCGATGAAATCATCAAATAGTAAGCCTAAAATACCTGCTGGCACC
ATACCCACTAATACATGTAATAAATTTAAACGTCTTGGCTTTGAACGTCTTTGTTGATCGTTATCTCCTTCAACATGTTTGTGTTTACCAATAT
GTAAAATCTCTAAGAAGCGTTCGCGGAACACCCATGCTGCTGCAAAGACGGATCCTAATTGGATGACGATTTTAAATGTAAATGCTGACTG
AGAACCTAAAAATTCAGATGATTTTAACCACATATCATCAACTAGGATCATATGTCCAGTAGAGGAAACAGGTGCAAATTCTGTTAATCCTT
CGACGACCCCTAAGATAATACCTTTTATTAATTCAATGATAAACAT

Gene: cydD (cysteine ABC transporter, ATPase subunit 2)

Position: 728982 to 730613, length: 1632 nt, orientation: FORWARD

Perfect match to: (11819-97-CP003194-[772901:774532], allele observed in CC80)

Sequence:

GTGAAAAAAATTAACAACGATACTGTTTCAATATAAAATTTTTCCGGTACTCATGTTCTTGGTCAGTACTGGTCTCGGCATACTCGTTATAACG
CAAAATATTTTAATAGCAGATTTTTTAGCTAAAATTATAAGACATCAATTTCAAGGTTTATGGATTGTATTATTTATTTTATTAGGTGTTTTAT
TTTTAAGAGCAACTGTGCAATTTCTAAATCAATGGTTAGGTGATACATTAGCATTTAAAGTTAAGCATATGCTTAGACAGCGGGTTATTTAT
AAAAATAATGGTCATCCAATCGGTGAACAAATGACTATACTCACAGAAAACATTGATGGTCTAGCACCTTTTTATAAGAGTTATTTGCCTCA
AGTGTTCAAATCAATGATGGTTCCGCTCATCATAATCATTGCAATGTTTTTCATCCATTTCAATACCGCATTAATTATGTTAATAACTGCACCA
TTTATTCCTTTGTTTTATATTATTTTCGGTTTGAAAACCCGAGATGAGTCAAAAGATCAAATGACTTATTTGAATCAATTTAGTCAACGGTTTT

TAAATATTGCTAAAGGTTTAGTGACGTTAAAGCTATTTAATCGTACAGAGCAAACAGAGAAGCATATTTACGACGATAGTACTCAGTTTAGA
ACTTTAACAATGCGCATTTTACGCAGTGCTTTTTTATCGGGTTTAATGCTCGAATTTATAAGTATGTTAGGTATTGGATTGGTTGCATTGGAA
GCAACGCTAAGCTTAGTAGTATTTCATAATATTGATTTTAAAACTGCGGCAATTGCGATTATTTTAGCGCCTGAATTTTATAATGCAATTAAG
GACTTAGGGCAAGCGTTCCATACTGGAAAACAAAGTGAAGGTGCCAGTGACGTTGTGTTTGAGTTTTTAGAACAACCGAACAATAATAATG
AATTTCTATTAAAGTATGAGGAAAATCAAAAGCCATTTATTCAGTTAACAGACATATCATTTCGATATGATAATTCTGATAGATTGGTATTAA
ATGATTTAAATTTGGAAATATATAATGGTGATCAAATTGCACTTGTAGGTCCAAGCGGGGCAGGTAAATCCACTTTGACACATCTAATTGCA
GGTGTTTATCAGCCAACAATAGGTACTATAAGTACAAACCAGCGTGATTTAAATATAGGAATACTTAGTCAACAGCCATATATTTTCAGTGC
TTCTATAAAAGAGAATATTACGATGTTTAAAGATATAGAAAATAATACTATTGAAGAAGTGCTAGACGAAGTAGGTTTATTAGACAAAGTG
CAATCTTTCACAAAAGGCATTAACACAATAATAGGTGAAGGAGGCGAAATGTTATCTGGTGGACAGATGAGACGCATAGAACTTTGCCGTC
TTTTAGTTATGAAGCCAGATCTCGTTATATTTGATGAGCCTGCAACTGGTTTAGATATTCAAACAGAACACATGATTCAGAACGTTCTGTTTC
AACATTTTAAAGATACAACGATGATTGTCATTGCACATAGAGATAATACAATTCGCCATTTACAACGACGTTTGTATATAGAAAATGGAAGA
CTGATTGCTGATGATCGCAATATTTCAGTAAATATAACAGAAAATGGTGATGACTTATGA

Gene: cydC (cysteine ABC transporter, ATPase subunit 1)

Position: 730610 to 732283, length: 1674 nt, orientation: FORWARD

Sequence:

ATGAAAACACGACTAAAATTTCAAGTAGATAAGGATTTATTGTTAGCTATAGTTGTTGGTGTTTGTGGAAGTTTAGTTGCGCTCGCCATGTT
TTTCTTAAGTGGTTATATGGTGACACAAAGTGCACTTGGTGCGCCACTATACGCTCTGATGATTTTAGTCGTTACAGTAAAATTGTTTGGGTT
TTTAAGAGCTATTACTCGATACGTAGAGCGCCTTATTTCTCATAAAGCTACATTTACAATGCTACGTGATATTCGGGTACAGTTTTTCGGTAA
ATTAGTAAATGTCATTCCTAATGTTTACCGTAAACTGAGTTCTAGTGATTTAATTTCACGTATGATTAGTCGTGTTGAGGCATTACAAAATAT
ATATTTACGTGTTTATTATCCACCAGTCGTCATCGGTTTGACAGCGCTAGTTACAGTAATAGTATTGGCGTTCATTTCAATCGGCCATGCGCT
ATTGATTATGGTTAGCATGTTGTTTACTTTACTCATTGTTCCTTGGTTAAGCTCAAAAAAAGCACGTACTTTAAAGAAACATGCAGCTAATGA
ACAGGCCCGATTTTTAAATCATTTTTATGATTATAAAGCTGGTATGGATGAACTACGTCGATTTAATCAAATTAATCATTATCGAGATAATTT
GATGGCTAAATTAAATCATTTTGATAAATTACAACTTAAAGAGCAACGCTTTTTAACGATTTATGATTTTATATTAAATATTATTGCTATGCTT
TCGATTTTTGGTAGTTTAGTTCTAAGATTAATTCAAATTAATGCAGGCCAACTAAATATTATTTATATGACGAGTATAGTTTTAATGGTCTTA
ACTTTATTTGAACAAGCTGTACCAATGACAAATGTCGCGTATTATAAAGCGGATACTGACCAAGCATTGCATGATATTAATGAAGTGATATC
TGTACCTTCTACTAATGGAAAAAACCGTCTTAATGATAAGTATGATGCAACGAACATTTATGAAGTTAAGGACGCTAGTTTTAAGTATTGGA
ACCAGCAAACGTATGTGTTGTCGGATATTAATTTTAATGTTAATAGAGGCGAAAAGATTGCGATTGTGGGGCCTTCTGGTTCAGGAAAAAG
TACATTACTACAAATTATGGCTGGGTTATATCAATTAGATAGTGGCTCTATTCGTTTCGAAAATATGGATATGTTTGAAATAGATGACAAAG
ATAAGTTTGAATCGTTAAATGTCTTGCTACAATCTCAACAATTATTTGATGGTACAATACGTCAAAATTTATTTACCGATGAAAAAGATGAAG
CGGTGCAAGCAATATTTAAGCAATTAGATTTAGAACATTTGGCACTAGAACGTCAAATTGACTTAGATGGTCATACATTATCTGGCGGAGA
AATTCAGCGTTTAGCGATTGCAAGGATGTTATTAAAAGATACTGCATCAACATGGATTTTAGATGAACCAACAACTGCATTAGATAAACAAA
ATAGTTTAAAAGTTATGGATTTAATTGAAGCACATGCAGAAACATTAATTGTTGCTACACACGATTTAACTTTATTGTCACGTTTTGAGACCA
TCATTGTGATGATAAATGGTAAAATAGTTGAAAAGGGAAACTATCAACAATTACTCGCTAATCAAGGTGCTTTATGGAATATGATTCAATAT
AATGCATAA

Gene: mgrA (regulator of autolytic activity)

Position: 732410 to 732853, length: 444 nt, orientation: REVERSE

Perfect match to: (RF122-AJ938182-[700739:701182:r], highly conserved allele)

Sequence:

TTATTTTTCCTTTGTTTCATCAAATGCATGAATGACTTTACCTAATAAGCGATTAAGTTCTTTAACTTCATCTTGAGATAAAGAAGAAGCTGAA
GCGACTTTGTCAGATGCATTACTTAATTCTGGTCTAATAGTTTCACTTTTGTCAGTCAAGTGAATAAATACTTCACGTTGATCGACTTCGGAA
CGTTCACGCTTAATTAAGTCTACTTGTTCCATTCGTTTTAATAATGGTGATACTGTACCAGTATCGAGTGCTAATTCAGTTACGACTTTCTTGA
CGTTTACAGGAGATTCATCCCATAAAATTGTTAAGACAAGAAATTGTGGGTATGTTAGATTGTACTTCTTAAAAACTTTGTTAGAGTAGTAG
CGATTAACTTGTCTTTGAGCATTGTACAAACTAAAGCATAGCTGTTCTTTTAAATTATGTTGATCAGACAT

Gene: cobW2 (cobalamin (vitamin B12) biosynthesis protein)

Position: 733080 to 734006, length: 927 nt, orientation: FORWARD

Perfect match to: (11819-97-CP003194-[776999:777925], allele observed in CC80+CC22+CC4803)

Sequence:

ATGAAAAATAATAAAGATGAAAAAATAAGAATATCCATAATTAACGGATTTTTGGGTAGTGGTAAAACCACGTTACTGACACATTATATTA
GTGAATTATTAAAAAATGATGAGAAAATTAAAATCATCATGAATGAATTCGGTACTTTTGATATTGATAGCAATAGTATTTCAAATGAAATT
GAAGTCCATTCATTGATTAATGGTTGTGTTTGTTGCGATCTTAAACAAGAACTTGTCTATGAACTAAAAGCCATTGCTTTAAAAGGGGACGT
TAATCATGTCATCATAGAAGCGACAGGCATTGCGCATCCTTTGGAATTACTAGTTGCATGTCAAGATCCGCAAATCGTTAATTTCTTTGAAA
AGCCGATTATTTATGGTGTATTAGATGCGACTCGATTTTTAGAACGTTATCAATATACCGAAAATACAGTTTCGCTGATGGAAGATCAGTTG
AAACTAAGTGACATGATTATTATTAATAAAATTGATCTTATAACTGATGACAGTCTTGAGAAAATTGATAAGCAATTAGGTATGATTTGTGC
AAGTATTCCAACTTATAAAACAACCTATGGAAAAGTTTCGTTGGAAGAATTGGACTTAACTGTTAAAGACAGAGAGATATCGTCTCATCATC
ACCATCATCATGGGATTAAAAGTATGACTTACACGTTTACAGGTCCGATTGATCGTCAATTGTTTTATCAATTTATAATGAAATTACCGGAAT
CTGTTCTACGTTTGAAAGGTTATGTGTCATTTAGAGATCAACCAAATGCAATTTATGAATTTCAATATGCATATGGTTTACCAGACTATGGAA
TAATTGACATGCAATTACCATTAACGATTGTTATTATTGGTGAAACTTTAGATACAAATCACATACGTAATCAATTGGATATGCTACAATTTA
CGTAA

Gene: ycsN (putative oxidoreductase)

Position: 734109 to 735017, length: 909 nt, orientation: FORWARD

Perfect match to: (11819-97-CP003194-[778028:778936], highly conserved allele)

Sequence:

ATGGAACAAATAATGATTAATCACTATGTTCATTTTTCTAGGCTTGTACAAGGTTTTTGGCGTGCAAATGAATGGAAGATGACTGCGAAAGA
GTTAAATTATTTTATAAATGAATTAGTTGAACGTGGAATTACAACGATGGATCATGCTGATATTTATGGGGATTATCAATGTGAATCACTGT
TTGGTAATGCTTTGGATTTATCACCCGAATTAAGAAATAAAATTCAAATTGTTACGAAATGTGGTATCATTTTGCCTTCTAAGCAATTTGATT
TTACAAATGGACATCGTTATGATTTGAGTAGTAAGCACATCGTGAAATCTGTTGAACAGTCATTAATCAATTTGAATGTAGATTATTTAGAT
AGTCTACTCATTCATCGTCCTTCACCATTGATGGATCCAGAACAAGTTGCTGATGCATTAACTAAACTTGTTAAACAAGGTAAGTTGAAGTC
ATTCGGGGTGTCGAATTTTAATCATTCACAATACCAATTGTTAAATCAATATATTATGAAAGAAAGACTACATATTAGCATCAATCAATTAGA
ATTATCGCCATATCACGTTGATAGTTTACAAGATGGAACAATGGATTCAATGTATCAAAACCATGTTCAAATTATGGCTTGGAGTCCTTTTGC
AGGCGGTAAAATTTTCGACAAGGAAGATATTAAAGCGCAACGTATTATGAAAGTTGTTCAATCAATAGCTGACAAATATGGTGTGAGTGAC
ACAGCTGTGATGATAGCATGGTTAGTAAAAATACCGCATCGTATCATGCCGATACTTGGAACAAGTCAGTTAAAGCGTATTGATCAAGCAA
TCGAAGGGCTACAACTTAATTTAGATGATCAGTCGTGGTTTGACATTTACACCGCTATTATCGGACAAGATATTCCGTAA

Gene: Q5HHX9 (putative protein)

Position: 735052 to 735339, length: 288 nt, orientation: FORWARD

Perfect match to: (N315-BA000018-[738059:738346], highly conserved allele)

Sequence:

ATGACAAACGAAGATAAACGTTTCGAACAATTAAGATTTGAACGCAAATTTATAGTTATTCCGTATTTAATTTATGCAGTCATTGTATTACTA
TTAAATATTTTCTATTCTGATTTGAAAATAACAATGACATTATTCGGACTTTTCTTTGCGTATAATGTAGTCATTTTGTTCATAGCATTTATTAA
ACATTATAAACGCACATTGTTACTAAGTCTTATATTAACAGTGCTTAGTGGCGCGGCATTCTTTGGAATTATTTATGTTTATGGCATTAATCA
TTTTTAA

Gene: yflS (putative malate transporter)

Position: 735622 to 737175, length: 1554 nt, orientation: FORWARD

Perfect match to: (11819-97-CP003194-[779541:781094], allele observed in CC80)

Sequence:

ATGTCAGAAGAAAAACATGTAGTTGAACATGAACAACAAAAGAAAGAAAAGACAAAAAAGCAATACAAGACATTTTGGATTGTCATGAGT
TTTATAATACTTATAGTTGTACTATTACTCCCGGCACCTTCAAGTCTGCCGATAATGGCTAAGGCAGTACTAGCTATTTTAGCTTTTGCAGTTA
TTATGTGGGTAACGGAAGCTGTATCATATCCGGTGTCAGCAACTTTAATTATTGGCTTAATGATATTACTTTTAGGATTTAGCCCGGTTCAAA
ATTTAGGGGGAGAAGCTAGGTAATCCGAAAAGTGGCAGTGCTGTTTTAGCTGGAAGTGACCTTCTAGGAACTAATCATGCATTATCATTAGC
GTTTAGTGGATTTGCAACTTCAGCTGTAGCTCTCGTTGCAGCCGCATTATTTTTGGCTGCTGCTATGCAAGAAACGAATTTGCATAAAAGAC
TAGCTCTTTTAGTGTTATCAATTGTTGGTAATAAAACTAGAAATATAGTTATTGGAGCAATTATCGTTTCAATTGTACTTGCATTTTTCGTTCC

TTCTGCAACAGCTAGAGCAGGGGCAGTTGTACCAATCTTGCTGGGTATGATTGCGGCATTTAAAGTTTCCAAAGATAGCAAGTTAGCGTCT
TTATTAATAATTACTTCGGTACAAGCTGTGTCAATTTGGAATATTGGTATCAAAACGGCGGCAGCACAAAATATCGTAGCGATTAATTTTAT
AAACCATCAATTAGGATTTGATGTTTCATGGGGCGAGTGGTTCTTATATGCAGCGCCTTGGTCCATAGTTATGTCTATAGCTTTATATTTCAT
CATGATTAAAGTGATGCCTCCCGAAATTAATACAATAGAGGGTGGCAAAGATTTAATAAAGAAGAATTGCATAAACTTGGCCCCGTTAGC
GCACGTGAATGGCGTTTAATTGTTATATCGATGTTATTATTACTGTTTTGGTCAACTGAAAAAGTATTACATCCGATTGACTCTGCATCCATT
ACTATTATTGCTTTAGGTGTTATGTTAATGCCGAAAATTGGTGTCATGACATGGAAACATGTTGAAAATAAAATACCATGGGGAACAATTAT
CGTGTTTGGTGTAGGTATTTCACTAGGTAACGTTCTTTTGAAAACAGGTGCAGCTCAATGGTTAAGTGATCAAACTTTTGGTGTTTTAGGTTT
AAAACATTTACCTATTATCGCGACAATTGCACTTATCACGCTTTTTAATATATTGATTCATTTGGGCTTTGCGAGTGCAACAAGTTTATCATCA
GCGTTAATACCTGTTTTTATTTCGCTAACCTCTACGTTACACTTAGGAGACCAGTCTATAGGATTTGTTTTAATTCAACAATTTGTTATTAGTT
TTGGTTTCTTATTACCTGTTAGTGCACCTCAAAATATGTTGGCTTATGGCACTGGTACTTTTACGGTTAAAGATTTCTTGAAGGCAGGTATAC
CATTGACAATTGTAGGGTATATTCTAGTGATAGTTTTTAGCATGACTTATTGGAAATGGTTAGGTTTGCTTTAA

Gene: phrB (deoxyribodipyrimidine photo-lyase)

Position: 737261 to 738634, length: 1374 nt, orientation: FORWARD

Perfect match to: (Strain_21333-AHKA01000037-[167703:169076:r], allele observed in CC80+CC239)

Sequence:

ATGGCAATTGCTGTGTTATTAAATCGAATGTTTCGAATGGAACACAATCCATTATTTGAATATATTTATCAACAAAAAGAAGACATTGATGC
ATGTTATTTTTATCATTCCGGAAGAGGACATGTCTTCAGCTTCTGATTTGAAAGCACAGTTTTATCGCGGTACTTTGCAGCGCTTTTACCAATC
GTTGCACGCAGAAAAGCTTACACCTTATGTTATGTCTTATGACGATATCATTTCATTTTGTAAAGAAAACAATATCTCTGAAGTAGTGACTGC
GGGTGATATTATGAGTTATCATTTTGAAGAGTATGATATTTTACATCAACGTTCTTTATTCAATGAAGCACGCATTGCCGTTACTTTGATACG
TGGGAATCATTACTTTAAAGCGAGTAAAACAATGAATCAACAAGGGGAGCCATACAAAGTTTTTACTAGTTTCTATAAAAAAATGGCGACCT
TACTTGAGGCATAGAGACGTATATCACTATGATTTAAAATCATTCGAAGACTTCGTCATTGCATCACCTGATGATTTAGTGTTTGATGACATA
GCATTTGGATCCTCACAAATAATTGAACAGAATAAATGGCAACATTTTTTAGATCAAGATATACAGAATTACGAAAGCGGAAGAGACTATT
TACCTGAAGTATTAACAAGTCAGCTAAGTGTTGCTTTAGCATATGGATTATTAGATATTATTGAAATTTTTAATGATTTATTGGCGCGTTATG
ATGAAGATGAGGCAAACTATGAAGCATTTATACGTGAACTCATTTTTAGAGAATTTTATTATGTGTTAATGACACAGTATCCTGAAACCTCA
TACCAAGCTTTCAAACCTAAATATCGACAGATAAAATGGTCGCAAAATGAAGCGAATTTTAATGCATGGTGCGAAGGGCAAACAGGATTTC
CAATCATTGATGCAGCAATAATGGAATTGACACAAACTGGTTTTATGCATAATCGAATGAGAATGGTTGTGTCGCAATTTTTAACCAAAGAT
TTATTTATAGATTGGACATGGGGAGAAAAATTCTTTAGAAAGCACCTTATTGACTATGATGCAGCATCAAATATTCATGGATGGCAATGGTC
TGCTTCTACAGGTACGGATGCAGTGCCGTATTTTAGAATGTTTAATCCAATAAGACAGAGTGAACGCTTTGATGCTAAAGCTTTGTATATCA
AAACATATCTTCCGATTTTTAATCAAATTGATGCAAAATATTTGCATGATACGCAACGCAATGAGTCCAACCTTTTTGAACAGGGGATTGAA
TTAGGTCGTCATTATCCAAAACAAATAGTGGATCATCAAGAAAAGAGATCTCAAGTTTTAGCGACATTTAAAGCATTAGACTAA

Gene: Q2G0A5 (putative membrane protein)

Position: 738789 to 739073, length: 285 nt, orientation: REVERSE

Sequence:

TTATCTTAGTATGTCCGTAAATAAAGTGAGGTATAGTACGACATACTCTAAAAACGTAGTGAGATAAATATATTTCAATCTAACTTTTATGTT
TTGAGGCACTTGCCATTTAGGATATTGTCGTTCGTAATACGACACTTGTTGTATAAATACACCTAGTCCAAATGGCAGCATCATGAGTAAGA
TACTTCTTAAATAACTTAAACCAATATCATGCCATATGTGTCCAATAATCAATTGAAAGCCAATGATAGATACTATTAAAACGATTATATTTA
TTGTCAC

Gene: Q5HHX6 (putative protein)

Position: 739070 to 739687, length: 618 nt, orientation: REVERSE

Perfect match to: (Newbould_305-AKYW01000003-[184443:185060:r], allele observed in CC97+CC80+CC97+CC772)

Sequence:

TCACTTGTTCAAACGCACTCCTTTTCCAAATAATAGAATTGCTACTTGCATGACAACCATAAAACATACAAACATAGCAGTTTTAAGCGTTAG
ACTTTCTAGAATGTGATTTAAAACATGTAAGGGCTCATTAAAGAAATAAACGGAATGTAAGCGTAAGAAACGACCAATATAAATTCCGAAT
CCATTTAAAAACATTAGCACGACAACAATTAATCTATTAAGCCAACGGTGAGAAGTCAATGTTAGTATTTCAAAATAGATTAAAATCATCAC
ATAAACCGCTAAGAAGACACCAAGCAGTAAATAGGTAAAGTATTTCCACTCACTTAAATTTAGTCCTGCGTAAAAGTTGAATTGAAATTGGT

TCAAATGGATTAAATCAGTTACCATATAAAATGTATTTGGTAAAAGTAACACAAATATAAAACTATATATGATAAATAGTGGCCATTCATAC
TTTTTATTCGGTTTGAATAATCGTAATAATAGACTAAGCTCAAAAGGTATATATGCTAAAAACAAATTTAAAGTCATAAATTGAAAAATTTTA
GTCTCAAAAAGTGAGACGATAAATAAAATTAAAAAGTAAATTCTAGCGATGTATCGAGATTGCAT

Gene: Q5HHX5 (putative protein)

Position: 739873 to 740295, length: 423 nt, orientation: REVERSE

Perfect match to: (11819-97-CP003194-[783792:784214:r], allele observed in CC80)

Sequence:

CTATTGAGGATCAAGTAAATTTAAACCGACTGCATCTTTGAATGAACGTTGAATGTCCAAATCAGTTACAGTAAGAATGATTTCAGCAGTTG
CAGAAACAATTGCTTTAGCGAGATGTCCGCTAAAAGTTTCATATGATTGAGTACCGAAAGTAGCATGCAAATGTGCAAAATGACCATTGTC
TAGATGAGAAATATTACCTAATAAGCTCGTCAATTCCAGTGGCTCAGTAATATGTTTTTCTTCGTATTGTTTCGTTGTTAAATTGAAAAATTTT
AATACAACGTCATCACATGCACCAATGCCGCTGACAGATGTAAATGTTAAGTCTTGGTCATCTGCAAAGGTTGTTATACATTCAACGATATC
TTCTCCTTTTTCCAACACTAGTAGTATAGTATGATTACTTTTTTGCAATTTCAT

Gene: norA (multidrug efflux pump)

Position: 740504 to 741670, length: 1167 nt, orientation: FORWARD

Perfect match to: (COL-CP000046-[775963:777129], highly conserved allele)

Sequence:

ATGAATAAACAGATTTTTGTCTTATATTTTAATATTTTCTTGATTTTTTTAGGTATCGGTTTAGTAATACCAGTCTTGCCTGTTTATTTAAAAGA
TTTGGGATTAACTGGTAGTGATTTAGGATTACTAGTTGCTGCTTTTGCGTTATCTCAAATGATTATATCGCCGTTTGGTGGTACGCTAGCTGA
CAAATTAGGGAAGAAATTAATTATATGTATAGGATTAATTTTGTTTTCAGTGTCAGAATTTATGTTTGCAGTTGGCCACAATTTTTCGGTATT
GATGTTATCGAGAGTGATTGGTGGTATGAGTGCTGGTATGGTAATGCCTGGTGTGACAGGTTTAATAGCTGACATTTCACCAAGCCATCAA
AAAGCAAAAAACTTTGGCTACATGTCAGCGATTATCAATTCTGGATTCATTTTAGGACCAGGGATTGGTGGATTTATGGCAGAAGTTTCACA
TCGTATGCCATTTTACTTTGCAGGAGCATTAGGTATTCTAGCATTTATAATGTCAATTGTATTGATTCACGATCCGAAAAAGTCTACGACAAG
TGGTTTCCAAAAGTTAGAGCCACAATTGCTAACGAAAATTAACTGGAAAGTGTTTATTACACCAGTTATTTTAACACTTGTATTATCGTTTGG
TTTATCTGCATTTGAAACATTGTATTCACTATACACAGCTGACAAGGTAAATTATTCACCTAAAGATATTTCGATTGCTATTACGGGTGGCGG
TATATTTGGGGCACTTTTCCAAATCTATTTCTTCGATAAATTTATGAAGTATTTCTCAGAGTTAACATTTATAGCTTGGTCATTATTATATTCA
GTTGTTGTCTTAATATTATTAGTTTTTGCTAATGGCTATTGGTCAATAATGTTAATCAGTTTTGTTGTCTTCATAGGTTTTGATATGATACGAC
CAGCCATTACAAATTATTTTTCTAATATTGCTGGAGAAAGGCAAGGCTTTGCAGGCGGATTGAACTCGACATTCACTAGTATGGGTAATTTC
ATAGGTCCTTTAATCGCAGGTGCGTTATTTGATGTACACATTGAAGCACCAATTTATATGGCTATAGGTGTTTCATTAGCAGGTGTTGTTATT
GTTTTAATTGAAAAGCAACATAGAGCAAAATTGAAAGAACAAAATATGTAG

Gene: yedL (putative N-acetyltransferase)

Position: 741963 to 742415, length: 453 nt, orientation: FORWARD

Perfect match to: (COL-CP000046-[777422:777874], highly conserved allele)

Sequence:

ATGACAGTAAAAAATTTATTTTTAGGCTTTGTTGCTGTAATATTAACCGTTTGTTTAATTGGTTTATTAATATTAGCAACAAATGAAGATGCG
CTTGCTAAGGTACATAAAACAATTAATACGCTTAACGCGATAAATGTATCAACTGAAGATACTTATAAAAAGAAAATGGATATTCTCAATAT
TCATACTGCTAAAGCATCTGAAGTGAATGAAAATGTGAAAAAGCAAAATCATTTTAAACATCGTGTGAATGCAAATAAATCAAATTCTTTTA
ACGAACAAGAGTGCCAAGTTATTGCTGATCGTTATGCAGATAAGCATATCAATGATAATTATGGTTTAGAAAGAATTTCTAAGACAAATCAT
GGATATAATTATGTGTATTCCAATGATAATTCAACTAGTAAGCAACATGTAAGTATTTCAAATCAAGGCATAATAACGAAATAA

Gene: ybaK (transcriptional regulator)

Position: 742589 to 743071, length: 483 nt, orientation: FORWARD

Perfect match to: (11819-97-CP003194-[786508:786990], allele observed in CC80+CC8+CC188)

Sequence:

ATGGCTAAAAATAAGAAAACGAACGCGATGCGTATGCTTGATCGTGCAAAAATTAAATACGAAGTTCATAGCTTTGAGGTACCAGAAGAA
CATTTATCTGGTCAAGAAGTCGCAGAACTCATACAAGCAAATGTTAAAACAGTATTTAAAACGCTTGTTCTAGAAAATACAAAACATGAACA
TTTTGTATTTGTTATCCCAGTAAGTGAAACTTTAGATATGAAAAAGGCAGCTGCTTTGGTTGGAGAGAAGAAATTGCAGCTTATGCCTTTAG
ATAATTTGAAAAATGTAACGGGGTACATTCGTGGTGGGTGTTCGCCTGTTGGTATGAAAACATTGTTTCCAACAGTCGTTGACAAATCGTGT
GAAAATTATAGTCATATCAGTGTGAGTGGTGGGCTTCGAACAATGCAAATCACAATAGCTGTTGAGGATTTGATTACAATAACTAAAGGCA
AAATTGGAGCAGTTATCCATGAATGA

Gene: fruR (transcriptional regulator of fructose operon)

Position: 743324 to 744085, length: 762 nt, orientation: FORWARD

Perfect match to: (11819-97-CP003194-[787243:788004], allele observed in CC80+CC772)

Sequence:

ATGATAATTACAGAAAAAAGACACGAGTTAATTTTAGAAGAACTTTCGCACAAAGATTTTTTGACTTTACAAGAATTAATAGATCGAACTGG
GTGCAGTGCTTCAACAATACGAAGAGATTTATCTAAACTACAACAATTAGGGAAATTGCAACGTGTGCATGGTGGTGCAATGTTAAAAGAA
AATCGTATGGTTGAGGCGAATTTAACTGAAAAATTAGCAACGAATCTTGATGAAAAGAAAATGATTGCTAAAATAGCAGCTAATCAAATCA
ACGATAATGAATGCTTATTTATCGATGCTGGTTCATCTACATTGGAGCTAATTAAATATATTCAAGCGAAAGATATCATTGTGGTAACCAAT
GGTTTAACACATGTAGAAGCTTTACTTAAAAAAGGTATTAAAACAATTATGCTAGGTGGTCAAGTTAAAGAAAATACACTTGCTACGATTG
GTTCTAGTGCCATGGAGATATTAAGACGATATTGTTTCGATAAAGCTTTTATCGGGATGAATGGATTAGATATTGAACTTGGATTAACTACT
CCCGATGAGCAAGAGGCATTAGTTAAACAAACAGCAATGTCATTAGCCAATCAATCATTTGTACTTATTGATCATTCTAAGTTTAATAAAGT
ATATTTTGCTCGTGTACCTTTGCTAGAAAGTACGACAATCATCACATCTGAAAAAGCATTAAATCAAGAATCGTTAAAAGAATACCAACAAA
AGTATCACTTTATAGGAGGGACTTTATGA

Gene: fruB (fructose 1-phosphate kinase)

Position: 744082 to 745002, length: 921 nt, orientation: FORWARD

Perfect match to: (11819-97-CP003194-[788001:788921], allele observed in CC80+CC772)

Sequence:

ATGATTTATACAGTGACTTTCAATCCTTCAATTGACTATGTCATTTTTACGAATGATTTTAAAATTGATGGTTTGAACAGAGCAACAGCAACA
TATAAATTCGCTGGGGGGAAAGGTATTAATGTCTCGCGCGTCTTAAAGACATTGGATGTTGAGTCAACTGCCTTGGGATTTGCAGGTGGAT
TTCCTGGAGAATTCATTATAGATACATTAAATAACAGTGCAATTCAATCGAATTTTATTGAAGTTGATGAAGATACACGTATTAATGTGAAA
TTAAAAACAGGACAAGAAACAGAAATCAATGCACCGGGTCCTCATATAACGTCAACACAATTTGAACAACTGTTACAACAAATTAAAAATA
CAACAAGCAAAGATATAGTTATTGTTGCTGGAAGTGTACCAAGTAGTATTCCAAGCGATGCGTATGCGCAAATTGCACAAATTACAGCACA
GACAGGTGCTAAATTAGTAGTCGACGCTGAAAAAGAATTGGCTGAAAGCGTTTTACCATATCACCCACTATTTATTAAACCTAATAAAGATG
AATTAGAAGTGATGTTTAATACAACAGTGAACTCAGACGCAGATGTTATTAAATATGGTCGTTTGTTAGTTGATAAAGGTGCGCAATCTGTT
ATTGTCTCGCTTGGCGGTGATGGTGCTATTTATATTGATAAAGAAATCAGTATTAAAGCAGTTAATCCACAAGGGAAAGTGGTTAATACAG
TTGGCTCTGGTGATAGTACAGTTGCAGGCATGGTGGCTGGAATTGCTTCAGGTTTAACGATTGAAAAAGCATTCCAACAAGCAGTCGCATG
CGGTACTGCCACGGCATTTGATGAGGACTTAGCAACACGGGACGCTATAGAAAAAATAAAATCACAAGTTACGATTAGCGTACTTGATGG
GGAGTGA

Gene: fruA (fructose specific permease)

Position: 745008 to 746966, length: 1959 nt, orientation: FORWARD

Perfect match to: (11819-97-CP003194-[788927:790885], allele observed in CC80)

Sequence:

ATGAGAGTAACAGAGTTATTAACAAAAGATACGATAGCAATGGATTTAATGGCAAATGACAAAAATGGTGTTATTGATGAGTTAGTAAATC
AATTAGACAAAGCAGGTAAATTAAGTGATGTCGCGTCATTTAAGGAAGCGATTCACAATAGAGAATCACAAAGTACAACTGGTATCGGCG
AAGGTATTGCCATTCCACATGCCAAAGTGGCCGCAGTTAAGTCACCAGCTATTGCATTTGGTAAATCTAAAGCAGGTGTAGATTATCAAAGT
TTGGATATGCAACCAGCACACTTATTCTTTATGATAGCAGCGCCAGAAGGTGGCGCCCAAACACATTTAGATGCTTTAGCTAAGTTGTCTGG
TATTTTAATGGATGAAAATGTACGTGAGAAATTATTACATGCTTCATCACCTGAAGAAGTACTAGCGATTATAGATGAGGCTGATGATGAA

GTGACAAAAGAAGAAGAGGCAGAAGCTGAAGCACAACAAGTTGCAACTGCAGAACAATCATCTAAACAATCTAACGAGCCATATGTGTTA
GCAGTAACTGCTTGTCCAACAGGTATTGCACATACGTACATGGCACGTGACGCATTGAAAAAACAAGCGGATAAGATGGGTATTAAAATTA
AAGTAGAAACGAATGGTTCAAGCGGCATTAAAAACCATTTAACTGAACAAGATATTGAAAATGCAACAGGTATCATTGTTGCTGCTGATGT
TCATGTTGAGACGGATCGCTTCGATGGTAAAAATGTCGTAGAAGTACCAGTAGCAGATGGTATTAAACGCCCAGAAGAATTAATTAATAAA
GCATTAGATACAAGTCGTAAACCTTTTGTTGCCCGTGATGGTCAAAGAAAAGGTAACTCAAATGACAGTCAAGAAAAAATTAAGCCCAGGTA
AAGCATTCTATAAACACTTAATGAACGGTGTTTCTAACATGTTGCCACTTGTAATATCTGGTGGTATTTTAATGGCAATTGTATTTTTATTTGG
AGCAAATTCATTTAATCCAAAAAGCTCAGAGTACAATGCGTTTGCAGAGCAGCTTTGGAACATAGGTAGTAAAAGTGCATTCGCGTTAATC
ATTCCAATTTTATCTGGATTCATTGCACGTAGTATTGCGGATAAACCTGGTTTCGCTTCAGGTCTTGTAGGTGGTATGTTAGCAATTTCAGGT
GGTTCAGGATTTATTGGTGGTATTATTGCAGGTTTCTTAGCAGGTTACTTAACACAAGGTGTTAAAGCGATGACACGTAAGTTACCACAAGC
ATTAGAGGGATTAAAACCAACATTAATTTACCCACTATTGACAGTAACAGCTACAGGCTTATTGATGATTTATGCCTTTAATCCACCAGCATC
TTGGTTAAATCATTTGTTATTAGATGGATTAAACAATTTATCAGGTTCTAATATTGTATTATTAGGTTTAGTTATTGGCGCTATGATGGCGAT
TGATATGGGCGGTCCATTCAACAAAGCGGCATATGTTTTTGCAACAGGTGCGTTGATTGAAGGTAATGCAGCACCAATTACAGCTGCAATG
ATTGGTGGTATGATTCCACCGTTAGCAATTGCGACAGCGATGTTAATTTTTAGACGTAAATTTACAAAAGAACAACGTGGTTCAATTATCCC
TAACTATGTGATGGGTATGTCATTTATTACAGAAGGTGCGATTCCATTTGCAGCTGCCGATCCATTACGTGTTATTCCTTCAATGATGATTGG
TTCAGGTATAGGTGGCGCAATTGCTTTAGGCTTAGGTTCACGAATTACTGCGCCACATGGTGGTATTATTGTAATTGTTGGTACTGATGGTG
CACACTTACTTCAAACTCTTATTGCACTTCTAGTTGGCACATTAGTTTCAGCATTAATTTACGGTTTAATCAAACCAAAGTTAACTGAAACAG
AAATCGAAGCTTCAAAATCAATGGACGAGTAG

Gene: nagA (N-acetylglucosamine-6-phosphate deacetylase)

Position: 747274 to 748455, length: 1182 nt, orientation: FORWARD

Perfect match to: (COL-CP000046-[782732:783913], allele observed in CC8+CC80+CC239)

Sequence:

GTGTCAGAATTAATTATATATAACGGCAAAGTTTATACTGAAGATGGCAAAATCGATAATGGTTACATTCATGTGAAAGATGGACAGATTG
TTGCAATTGGAGAAGTGGATGATAAAGCAGCAATTGATAATGATACGACAAATAAAATTCAAGTGATTGATGCTAAAGGTCATCATGTATT
ACCAGGTTTTATTGATATACATATTCATGGTGGTTATGGTCAAGATGCAATGGATGGGTCATACGATGGCTTAAAATATCTATCCGAAAATT
TGTTGTCTGAAGGGACGACATCATACTTGGCCACTACAATGACGCAATCGACTGATAAAATAGATAATGCACTTACAAATATTGCTAAATAT
GAAGCGGAGCAAGATGTTCACAATGCAGCGGAAATTGTAGGTATACATTTAGAAGGACCATTTATATCTGAAAATAAAGTTGGTGCTCAAC
ATCCGCAATACGTTGTACGCCCATTTATCGATAAAATTAAACATTTTCAAGAGACTGCTAACGGATTAATAAAGATTATGACGTTTGCACCT
GAAGTTGAAGGTGCAAAAGAAGCGCTTGAAACGTATAAAGATGACATTATTTTTTCAATTGGTCATACAGTAGCAACATACGAAGAAGCA
GTTGAAGCTGTTGAGCGAGGAGCTAAACATGTCACGCATTTATATAATGCAGCGACGCCATTCCAACATAGAGAACCAGGTGTTTTTGGAG
CAGCATGGTTGAATGATGCTCTACATACCGAAATGATTGTTGATGGCACTCATTCTCATCCGGCATCGGTTGCAATTGCTTACCGTATGAAA
GGTAATGAACGTTTTTATTTAATTACCGATGCAATGCGTGCAAAAGGTATGCCTGAAGGAGAATATGATTTGGGTGGACAAAAAGTAACTG
TTCAATCGCAACAAGCACGTCTTGCAAATGGTGCGCTTGCTGGTAGTATTTTAAAAATGAATCATGGGTTACGTAACTTAATATCATTTACA
GGTGATACATTAGATCATTTATGGCGAGTAACAAGTTTAAATCAAGCCATTGCATTAGGTATCGATGATAGAAAAGGTAGTATTAAAGTAA
ATAAGGATGCAGATCTTGTTATTCTAGATGATGATATGAATGTAAAATCTACAATAAAACAAGGCAAGGTTCACACATTTAGCTAA

Gene: corC-mpfA (magnesium protection factor A)

Position: 748676 to 750025, length: 1350 nt, orientation: FORWARD

Perfect match to: (MW2-BA000033-[747316:748665], highly conserved allele)

Sequence:

TTGGAAACTTCGACCATAATTAGTTTGATTATATTTATTCTATTAATTGCATTAACCACTGTATTTGTTGGTTCAGAATTTGCATTAGTAAAAA
TTAGAGCAACAAGAATTGAACAGCTAGCAGATGAAGGAAATAAACCTGCTAAAATAGTAAAAAAGATGATTGCTAATCTAGATTATTATCT
TTCTGCTTGTCAGTTAGGTATAACAGTAACATCTTTAGGGTTAGGTTGGCTTGGTGAACCAACGTTTGAAAAGCTATTACACCCAATATTTG
AAGCAATCAATTTACCAACTGCATTAACGACGACGATTTCGTTTGCAGTGTCATTTATAATCGTTACGTATTTGCATGTAGTACTTGGTGAAT
TAGCGCCTAAATCTATAGCTATTCAACATACTGAAAAGCTTGCTTTAGTATATGCAAGACCATTGTTCTATTTCGGTAACATTATGAAACCAT
TGATTTGGCTGATGAATGGTTCTGCACGTGTTATTATTAGAATGTTTGGTGTAAATCCTGATGCCCAAACTGATGCAATGTCAGAAGAAGAA
ATCAAAATTATTATTAACAATAGTTATAATGGTGGAGAAATCAACCAAACTGAATTGGCATATATGCAAAATATCTTTTCATTCGATGAAAG
ACATGCAAAAGATATAATGGTACCTAGAACTCAAATGATTACACTAAATGAACCTTTTAATGTAGACGAATTACTAGAAACAATAAAAGAA
CATCAATTTACGCGTTATCCAATTACTGATGATGGTGATAAAGACCACATTAAAGGATTTATTAACGTCAAAGAATTTTTAACTGAATACGCT
TCTGGAAAAACGATTAAAATAGCAAACTATATACATGAGTTGCCAATGATTTCAGAGACAACACGTATCAGTGATGCATTAATTAGAATGC
AACGTGAACATGTACATATGAGTCTTATTATAGATGAATATGGTGGAACGGCAGGTATTTTAACGATGGAAGATATTTTAGAAGAAATCGT
TGGAGAAATTCGTGATGAATTTGATGATGATGAAGTGAATGATATCGTTAAAATTGATAATAAGACATTCCAAGTAAATGGCAGAGTACTA

TTGGATGATTTAACTGAAGAGTTCGGTATAGAATTTGATGACTCTGAGGATATTGATACGATAGGTGGATGGTTACAATCTCGTAATACCA
ATTTACAAAAAGATGATTACGTGGATACAACTTATGATCGCTGGGTTGTTTCAGAAATCGATAACCACCAAATTATTTGGGTGATATTAAAC
TATGAATTTAATGAAGCGAGACCTACTATCGGACAGTCTGATGAAGATGAAAAATCAGAATAG

Gene: yvgN1 (glyoxal/methylglyoxal reductase, locus 1)

Position: 750247 to 751086, length: 840 nt, orientation: FORWARD

Perfect match to: (MW2-BA000033-[748887:749726], highly conserved allele)

Sequence:

ATGTTGAATGAGATACAAATATTAAATAATGGATACCCGATGCCTTCAGTTGGATTAGGTGTTTATAAAATCTCTGACGAAGATATGACTAA
AGTTGTAAATGCTGCAATTGACGCAGGCTATAGAGCGTTTGATACAGCATACTTTTATGATAATGAGGCTTCACTAGGACGAGCATTAAAG
GATAATGGCGTCGATAGAGAAGATTTGTTTATAACAACGAAGTTATGGAATGACTATCAAGGTTATGAGAAAACATTCGAATATTTCAACA
AATCGATTGAAAATTTACAAACTGATTATCTTGATTTATTTCTAATACATTGGCCTTGTGAAGCAGATGGTCTATTTTTAGAAACATATAAAG
CTATGGAAGAACTTTACGAGCAAGGTAAGGTAAAAGCAATAGGTGTATGTAATTTTAATGTTCATCATCTAGAAAAATTAATGGCTCAATC
AAGTATCAAACCAATGGTGAATCAAATTGAGGTACATCCATATTTTAACCAACAAGAATTACAAGAATTTTGTGATCGTCACGATATTAAAG
TGACTGCATGGATGCCTTTGATGAGAAATAGAGGACTACTAGACAACCCTGTCATTGTTAAAATTGCTGAAAAATATCATAAAACACCAGC
ACAAGTTGTATTACGTTGGCATTTAGCACACAATAGAATTATTATTCCAAAATCTCAGACACCTAAACGCATTCAAGAAAATATAGATATTTT
AGATTTTAATTTAGAATTAACAGAAGTAGCTGAAATTGATGCTTTAAATAGAAATGCAAGACAAGGTAAAAATCCAGATGATGTGAAAATT
GGGGATTTAAAATAA

Gene: csbB (putative glycosyl transferase)

Position: 751218 to 752201, length: 984 nt, orientation: FORWARD

Perfect match to: (Bmb9393-CP005288-[803317:804300], allele observed in CC239+CC80)

Sequence:

ATGAAAATTAGAGTCGTCATTCCTTGTTTTAATGAAGGGGAAGTCATTACACAAACACATCAACAATTAACTGAAATACTTTCACAAGATAG
TAGTGTGAAAGGCTATGATTATAATATGCTTTTCATAGATGATGGTAGTACGGATACCACTATAGATGAAATGCAACATCTTGCCACAATAG
ATAGGCATGTCAGCTTTATTTCTTTTAGTAGAAATTTTGGAAAAGAAGCAGCTATGATTGCAGGTTACCAGCATAGTACTGAATTTGATGCA
GTCATCATGATAGATTGTGATTTGCAACATCCACCTGAATATATTCCGAAAATGGTTGAAGGTTTTATGGAAGGCTATGATCAAGTGATTGC
AAAGCGTGATAGAAGTGGTGAAAATTTTAGTCGCAAAACATTAAGCCATTTGTACTATAAGTTAGTTAATTGCTTTGTAGAAGAAGTACAAT
TTGATGATGGTGTTGGTGATTTTAGACTTTTAAGCCAAAGAGCTGTTAAATCCATTGCATCACTTGAAGAATATAATCGATTTTCAAAAGGG
TTATTTGAATGGATAGGCTATAATACTAAAGTGTTTACGTATCAAAATGTTGAGAGACAAAAAGGGGAATCTAAGTGGTCCTTTAAAAAGT
TATTTAATTATGGTATTGATGGATTGATTTCCTTTAATAGTAAACCTTTGAGAATGATGATTTATCTTGGCTTGTTTATCTTTTCAATAAGCGT
GCTATATATTATCTATTTATTCATCAATATTATGATATCTGGTGTTAATATTCCAGGATATTTTTCAACGATTGCAGCTATTTTATTATTAGGCG
GCATACAGTTAATTTCAATTGGTGTTGTAGGTGAATATATTGGCAGGATATATTATGAAGTTAAGGCACGTCCTAAATATATTATTCAAGCT
ACAAATCTTTCAAGTATTGAAAATGATGAGAAGGATACCCATAAAGTTTATTCTAAATAA

Gene: saeS (histidine protein kinase, sae locus)

Position: 752273 to 753328, length: 1056 nt, orientation: REVERSE

Perfect match to: (Strain_21343-AHKV01000061-[61570:62625], highly conserved allele)

Sequence:

TTATGACGTAATGTCTAATTTGTGTAATGTTACAGTCATCGTAGTTCCTACATCTATATCACTGCTTACACTGATTTTTGCGTTATTTTGTTGC
GCGAGTTCATTAGCTATATATAAGCCTAATCCAGAACCACCCGTTTTTGTATTACGAGAGTTTTCTACTCTGAATGTACGTTCGAATATACGT
TCTTGTAGTTCTGGTATAATGCCAATACCTTCATCGCTAATAGCAATGTCGATAGTATCTTGATCTTTGTTTTCACTAATATTAATATCAATGC
GACTACCAACATTTGAAAATTTTAGCGCATTATCAAGTAAGTTTGTTAAAATACGCTCAAGTGGCGTTCGATATTGATAAAATGCATCAATTT
CGCTACAGAAATTCACTTCTAATGTGCGGTTTTCATGTTTGATACGTTGCTCATATGGTTGCAATATTGATACAAGTAATTGGTCTAGTTGTA
TTAATTCTGGGGGATATGTTTTACCTGTATTTAAAGTGATAATATGAGTCATATCATCAAATAATGTTGATAATCTGTTTGCTTGTTTAATTAA
TATGTCGTATGACTCTTTAATCTCATGATCCTTAGTGATTATACCATCACGTAGTCCTTCAGAATATGAAATAATGCTTGCTAAAGGTGTTTTT
AAATCATGGGCTAAGTTTTGAATCAGTTCTGTTTTTTCTTGTTGTTCGGATTTAATTTGATTCATTTGTTGCGTAATTTCAGAAGCCATTTTATT
AAAAGATTGATTTAATTCATAAATTTCTTTTGGTGAATTAAACGTTTTATCATTGCTTGCGTAATTTCCGTTAGCAAATTGCTTAGTTTTTATAT

TAAACTGCTTAATTTTTTGTATAAGTGGATTAATAAAAATACTACATATTAATAAGGTTAAACAGCTTGTAATTATTGTCGTTAAGGTCAAAG
TTAGTGTCATATGGCCGTTAAACCACATTAAAATATATGCAATTGCTAAAATAGTTGAAGTTAATAGTATACTCGATACGACGCCAATAATG
ATTTGACTTCTAATTGATAACACCAT

Gene: saeR (response regulator, sae locus)

Position: 753328 to 754014, length: 687 nt, orientation: REVERSE

Perfect match to: (RF122-AJ938182-[721582:722268:r], highly conserved allele)

Sequence:

TTATCGGCTCCTTTCAAATTTATATCCTAATCCCCATACAGTTGTGATGGTATATGTTGTAAAGCTCTCTTTTTCTAATTTTTCTCTAATACGGT
GTATATGGACATTCACGGTATTAGCATCTTCGTAATAGTCATATCCCCAAACTTTTTCAAGTAATTCTGATTTAGAAATAACTTCATTTTCTCT
AGAAGCTAAATACCACAATAACTCAAATTCCTTAATACGCATAGGGACTTCGTGACCATTTACAGTCACAACTTTACTTAAGTTAATAAGTGT
TAATTCATCAAACGACAGTTGTTCAACTGGTTGATGATGGTATTTCTTCATTCTTGTAAGTAAATTATTAATACGTAAAACGAGTTCCCTTGG
ACTAAATGGTTTTTTGACATAGTCATCTGCACCTAAAGTTAAGGCGTAAATGGTATCATGTTCTTGTGTTTTGGCAGTTAAATAGATAAAGG
GGATATCTAATTTTTGCCTTTTCATTTCTTTGACAATGTCGTAACCATTAACTTCTGGCATCATGATATCAAGTACCATGATATCAATATCATTT
GATAGTAAAGAAATTGCTTCTTTACCGCTAGTTGTCGTTGTTACTTTGTAACCTTCATATTCAAAATAGGTTTGACAAATGTCTACAATGTCTT
GTTCATCATCCACGATCAGTAAGTGGGTCAT

Gene: saeQ (putative protein)

Position: 753989 to 754462, length: 474 nt, orientation: REVERSE

Perfect match to: (MW2-BA000033-[752629:753102:r], highly conserved allele)

Sequence:

TCATCCACGATCAGTAAGTGGGTCATCTATTTTTTCACCTCTGTTCTTACGACCTCTAAAGTAATTAATGATTTCTTTAAGTGAAATCTGTTTT
AACAATGAGTGACTCATTAGTAAAAGGATAAAGAAAGTTAATTGAAGAGGATACGTAAATATCATATCTGCTAAGATATAATTTATCATAA
CAAAGGCTCCAAAGAAACTAGCAGCATATGCAAAAACTCCAAAAATTAAACCTAATCCAATTGCAATCTCTCCGAGTGGGACAACAATATC
AAATAATGACGTCGTATGTGCAACTATATTTGCGAAAAACCACTTATACCACTCTGGTGAATCAGTATTGTTAGCGATGACTGGTACTAAAC
CTTTCAGCGTAAATCCGCCCGTTAATTTTTCGTAGCCTTGCATTAACATAACAATACCTGAACCCACACGAATGATAAATGTAACGAGTAGC
AATAATTTATTCAT

Gene: saeP (putative lipoprotein)

Position: 754804 to 755244, length: 441 nt, orientation: REVERSE

Perfect match to: (MW2-BA000033-[753445:753885:r], allele observed in CC1+CC5+CC96+CC772)

Sequence:

TTATTTTAATTTAGCGCCGCCGAAGATGACGTGAGCTTTTTTACAATAGATTGTTACTTCATCATATTTGCTTAAATCTATATTTTTAAGATCA
AATGTTTGTTTTTCTTTATCGTAGTCAACCATTGCGATTTCTTTACCGTTTTTAATGTCGCCATTTTTTGTTAGGTAGACGTATAAATCTGGACC
TTTTGATGATTTGTAGTTAGTAAGCATTAATTTACCATTTTTAATCTCAGCTTTACCTTCAACAGTTTCACCGTTTTTAGAACTGAATGTACCTG
TTAGGTGTTTTGTTTTATCAGTTTTAACATTGCTATCTTCTGACTTTGTTTTTTGTTCAGTTTTGTTACCTTGATCTTGTGAATTAGAATTACCAC
AAGCGCCTAAAGTTAATACAGAGGCAACAGCACCAACTGCTAAAAAATATTTTGTATTCAT

Gene: Q5HHW1 (putative membrane protein)

Position: 755524 to 756108, length: 585 nt, orientation: REVERSE

Perfect match to: (N315-BA000018-[758532:759116:r], highly conserved allele)

Sequence:

TTAAAATTTATAAACCTTTTGCCACAATAAAAACAACAAGAAAATAAGATAGACGATTAAATACAAGTACCAATATGGTGTTAAAACCATCA
TAACGATAAATGAAATGTTAATAATGATAAGTCCTTTAATCATTAGTTTCTTTATTTTGTTATTATTTTCAGCAGTTTCATCTAAGCTATTAAAT

GTTGAAATAAACAACATAATACCACCAATGATGAAAATTGCAGGGGCTAAAAATGGAATAGACATATATATTGATTGTGTACTTGAAAAGT
CGGTAACACGTTGCGTTAAACTGACAACAATCGTTAATATGATAGCAATAAATTTTTGGTCTGTAATGTATACAGGTTTTAATTTTTTATCAA
TGTAAAACTGTAGTATCGTCCAAATGATAAACATCGTTATAGTACACAGAGTAATAAAGTTATATTTATCAATTATGAATGTATTTATAAAG
GCGTTTATCGTAATAGATAACAACATTGCACTTAATGATAAATGTTGCATTTGATGCCTATATAAATTGAATCCAGTTATAAGTACGACGATA
GCAATATTAATTAATATATATATAAAATCAT

Gene: queE (7-cyano-7-deazaguanosine biosynthesis protein E)

Position: 756207 to 756920, length: 714 nt, orientation: REVERSE

Perfect match to: (KPL1845-AZJA01000002-[215402:216115:r], allele observed in CC96+CC5)

Sequence:

TTATACACCTTTTTTATTACTCCAAAGTAATGTATGAAGTTGTGGTAACACATAAACGTGATTCATATCATTACTTTGCATAACTAAATCCACC
AACTGCTCGTAGCGTTCTAACAACTTTTCGGTATGATTATCTACGCTGTCTGATAAATATGGGTTACCAACTTGTAAATAGAAGGGAATATCT
GGATAACGGTGGTGTATCATTTTGGCAAAATCATAATCTTTATCGTCGAATACAACTACTTTTAAGTTTAATGAGGAAGGTACGCATTGTGT
AATCACTTCATCTAACTTTTTTAAATCAGGTGTCATAGTTGAACTTGGTGGTTTTGGACTAATCGTTAAATCATCAATTTGTGTCATCCAAAGT
TGGAATTTACTGCCTTGTGTCTCCAGTGCGCTGAAAATACCTTTATCTTGAAATAAGTCAACTAACTCTTGGATACCTTTAATTAATGCTGGG
TTACCACCAGAAATTGTAACGTGATTAAATAAATCGCCACCAATTCGTTTTAATTCATCATAAATTTCTTCAGCGGTCATGAGTTTTATATCGC
CTTTAGCACTACCATCCCAAGTAAATGCAGAATCACACCAGCTACAGCGATAATCACATCCAGCTGTTCTCACAAACATCGTTTTTCTACCGA
TTACTCGACCTTCACCCTGAATGGTTGGACCGAATATTTCGAGTACAGGAATTTTAGCCAT

Gene: queD (queuosine biosynthesis protein D)

Position: 756924 to 757343, length: 420 nt, orientation: REVERSE

Perfect match to: (CN1-CP003979-[734133:734552:r], allele observed in CC72+CC80)

Sequence:

TTACACCTGTTCCTTTGGTCTAAATACAACATAACTTGTTGGTGTTTCTCTTACAAATACTTGAATACATTTTGGTTGGTGTTCGAGCGATGCC
AAATTTTCTTTAACAATTTGATAAATTGTTTCCGCTACGATTTCAGTTGAAGGGATTTTATTTTTAAAAGCAGGTAAGTTATTTAACAGTTGAT
GGTCAAATTTACCGTGTATCATCTTTTTCAAATGGCTAAAGTTCACTAAGAAGCCAGTGTTATCTAGTTTATCACCGACAATTGTTAAATTAA
CAAAGTAAGTATGACCATGGACATTTTGACAAATACCTGCTTCTTCACAAGGAATGTGATGTGCAGCTGAAAAATTAAAATCTTTATTTAAT
TCGAATTGATATGGATGCGTTGTACTAGGATAGATTTGTTGTAACAT

Gene: queC (7-cyano-7-deazaguanine synthase)

Position: 757345 to 758013, length: 669 nt, orientation: REVERSE

Perfect match to: (11819-97-CP003194-[801264:801932:r], highly conserved allele)

Sequence:

TTAAAGCGCTCCTTTACTTTCAAGATATTGATTTAGTCCACGTTGACGTAAATGACAAGCTGGACATTCACCACAGCCATCCCCAATGATACC
GTTATAGCATGTTAATGTTTTTGTACGAATATAATCTAAAACTTCGAGTTCATCACTTAATTTCCACGTTTCTGCTTTGTTTAACCACATTAAA
GGAGTATGAATGACAAAATCTTTGTCCATAGCTAGGCTTAATGTTACGTTCATTGATTTTATAAAACTATCACGACAGTCTGGGTAGCCTGA
AAAGTCTGTTTCACATACGCCTGTAATAATATGCTTAGCCCCAATTTGATAAGCTAGAGCGCCTGCAAACGACAAGAAAAGTAAATTTCTAG
CTGGAACAAATGTATTAGGTATACCATCTTCATTATTAGTAATTTCCATATCATGTTGTGTTAATGCGTTTGGAGTAAGTTGTGATAATAATG
ACATATCTAAAACGTGATGTTTCATTCCTTGATCTTGTGCAATTTGTTTTGCGACTTCAATTTCAGTATCATGTCTTTGGCCATAATTAAACGT
TACGAGTTCAACTTCTTTGAAATGTTTTTTTGCATAAAAGAGACATGTTGTACTGTCTTGACCACCACTAAAGACAACGATGGCTTTTTCATT
ATTTAATACACTTTCCAT

Gene: pabA (putative para-aminobenzoate synthase, glutamine amidotransferase, component II)

Position: 758364 to 758957, length: 594 nt, orientation: FORWARD

Perfect match to: (MW2-BA000033-[757005:757598], allele observed in CC1+CC80+CC96+CC188)

Sequence:

ATGATTCTAGTCATAGATAATAATGATTCATTTACATATAATTTAATAGACTATATTAAGACTCAAACGAAACTAACAGTTCAAGTTGTTGGT
ATTGATAATCTGCTGATAGAAGACGTCATTAATATGAAGCCAAAAGCAATTGTTATTTCACCTGGGCCGGGTAATCCGGATGATTATCCTAT
ATTGAATGAAGTGTTAGAACAATTTTATCAGCGTGTACCTATACTAGGTGTATGCTTAGGATTTCAATGTATCGTGTCTTATTTTGGTGGAAA
TATCATTCACGGCTATCATCCTGTACACGGACATACTACACAGTTACGCCATACCAATGAAGGTATTTTTCAAGGACTGCCTCAAAATTTCAA
TGTAATGCGTTATCATTCATTAATTGCTGACGGAGCGACTTTTCCAAATTGCTTAAAGATTACAGCCAAAAACGATGAAGCGATTATTATGG
CATTTGAGCATATTACATTTCCGGTTTTTGGTGTGCAATATCATCCTGAATCTATTTTGAGTGAATACGGTTATCGACAAGTTGAATTATTTTT
ATCGAAGGTAGGTGATTACTGTGAGAATAGAATATAA

Gene: pabB (anthranilate/para-aminobenzoate synthase component I)

Position: 758941 to 760092, length: 1152 nt, orientation: FORWARD

Perfect match to: (11819-97-CP003194-[802860:804011], allele observed in CC80+CC1+CC5+CC361+CC772)

Sequence:

GTGAGAATAGAATATAATTATCGCTACTATTTAACTGAAAATGAATATAAGCAATACCATATTCAATTAAAGGGATTTATAAAGAAGTATGT
TGCTACTAAGTTGGCTGATGTGGGAGAAGTGATACACTTTGCACAAGCGCAGCAACGACAAGGTAGATATGTCTCGTTATATTTAAGTTAC
GAAGCGGCAAAGTATTTTAATCATGTTATGTGTACACATTCATTAGCTAAAGATGATATTTATGCAGTAGCTTATAGTTTTGAAAAAGCGGA
AAGCATAAATTCAACATATGAACATCAAACTTCTTATGTATCAAAGCATCATTTTTCATTTGTTGAATCTTCTGAGGTTATGATGACTAATATT
AAACGTGTCCAACAAGCAATTGTTGAAGGCGAAACGTATCAAGTGAACTATACGGCGCGCTTAACGGATAACATTTATTATCCTATTAGTAC
TTTATATGAACGATTAACTCAATTTAGTAATGGTAATTATACTGCGTTATTACAAACAGATGAAATCCAAGTAGCGTCTATCTCACCAGAATT
ATTTTTTCAAAAAGGACAGTTTAACAATGTCGATAACGTTATCATAAGCAAACCGATGAAAGGGACAATGCCTAGAGGTAAAACGGAAGCT
GAAGATCAACAGTATTATAAAACATTGCAAACTTCTTCGAAAGATCGTGCAGAAAATGTCATGATTGTTGATTTACTAAGAAACGATATAG
GGAGAATATCACAGAGTGGCTCAATTAAGGTGTATAAACTATTTTTTATTGAGGCATATAAAACTGTATTTCAAATGACTTCGATGGTAAGT
GGTACTTTAAAAACTAATACAGACCTAACTCAAATTTTAACATCGTTATTTCCTTGTGGTTCGATTACAGGTGCACCGAAACTGAATACAATG
AAATATATTAAACAATTAGAAAGTTCACCTCGTGGTATATACTGCGGAGCAATTGGACTATTACTTCCAACTGAAGATGATAAAATGATTTT
TAATATTCCGATTCGTACTATTGAGTATAAATATGGACAAGCGATTTATGGAGTCGGAGCAGGTATTACAATTGATTCTAAGCCAAAAGATG
AAGTGAATGAATTTTACGCAAAAACCAAGATTTTGGAGATGTTATAA

Gene: pabC (4-amino-4-deoxychorismate lyase)

Position: 760092 to 760700, length: 609 nt, orientation: FORWARD

Perfect match to: (CIGC93-AHVD01000008-[214865:215473], allele observed in CC15+CC22+CC80+CC97)

Sequence:

ATGCAATTATTTGAAACAATGAAAATTGATAATGGACATATCCCTAGACTTACTTATCATACTAATCGCATAAAATGTTCTTCTGAGCGATTA
AACTTTAAATTTGATGAACATGCATGGCGAAATGAATTAAACGATGTAACAACAAAGTATCACAGTGGCCAATATAGACTTAAAATCGTATT
AAATGCTGAAAGCGAATTTGAAACGATAGTGTCACCTTTACCTGAGAAAAGTAGTTTTACAGCAAAATTTCAAGTGTTGCCCAAAGTAGTTA
ATCCAACTTTTATAAATAATAAAACGACAGAACGAAAGCATTTAGCTCACAATCATGAAACAGATTTAATATTGCTAACTTCAGAGGACGGC
AAGGTCCTTGAATTTGATATTGGCAACATTGTCATTGAAGAGGATGGAAAATGGTACACACCAAGTTATAAAGATGATTTCTTAAAAGGAT
GCATGCGTGATTATTTAATAGATAGTGACAAACTTGTTGAAAAAGACTTTAATAAAAACGAATTGATTTATAAATATCATAACAATGAGATA
CGTTTATTTTTGATAAATAGTTTACGAGAGGTTGCCGATGTCCACCTTTGCCTTTAA

Gene: Q6GIS6 (putative membrane protein)

Position: 760766 to 760972, length: 207 nt, orientation: FORWARD

Perfect match to: (MW2-BA000033-[759407:759613], highly conserved allele)

Sequence:

ATGATTATTGTTTATATTGTGCTGTTGTTAATTCTTGTATACGTAAATTATCGATTAGTGAATCGATTGCTATCTGAAAATAGAATATATGTTG
TTCGTTTGATAGCAACAATTACTACTGTTATAAGCTTTATCCTTGTATACGCATTAATTCACGAACTCATGCCTTTTGTTGTGCGGGCAATGG
ATTTAATGTATCACCAGTAA

Gene: ahs1 (allophanate hydrolase subunit 1)

Position: 761060 to 761770, length: 711 nt, orientation: FORWARD

Perfect match to: (MW2-BA000033-[759701:760411], highly conserved allele)

Sequence:

ATGAAAATATATAGTCAAGGTGACCAAGCCATTGTAGTCGCAATTGAAAAAGAAGTATCTAAAAGTTTAACCGAAGATTTATTAACACTTC
GCTCATATTTAATTGAACAAAATTATCCATTTATTATAGAAATTGTGCCATCAGAATCAGACATGATGATTGTCTATGACGCAAGGGATATG
ATTAAACACCATAATATACAATCACCTTTTTTATACATGAAAGCACTAATAGAATCGATTCATTTAAACATAAAACATGATTTTAACCAGCAA
GATTTGATTGAAATACCAATTGTGTATGGTTCGAAATATGGTCCGGATTTAGAATCACTTTTAAAACATTACAAAATCAAGCTAGAAACTTTT
ATTGAATTACATTCTAAGGCGCAATATTTTGTTTCGATGATGGGATATTCACCTGGGTTTCCTTATTTAACTGGATTAAATAAGAAATTGTAT
ATTAATCACACGAGTAAACAGAAAAAATTCATTCCAGCTGGTTCTGTAGTACTTGAAGGGAAAAAATGCGGTATTGTAACTACGGATACAA
TTAATGATTGGTTAGTTATTGGTTATACACCATTATCACTTTTTAATCCGAAAGAATCAGATTTCGCACGCTTAAAGTTAGGCGATAATATTA
AATTTAGACCTATCAATGAAAATGAATTAGAAGTAGGAGCGTTTAAAGATGTCAATCATAATTGA

Gene: ahs2 (allophanate hydrolase subunit 2)

Position: 761754 to 762758, length: 1005 nt, orientation: FORWARD

Perfect match to: (Strain_21340-AGTX01000008-[125880:126884:r], allele observed in CC188+CC80)

Sequence:

ATGTCAATCATAATTGAAAAAAGTGGCTTATTCAGTAGCTTTCAGGACTTTGGCAGAAGGGGATATGAACATGATGGTGTAATTCCATGTG
GTGCACTTGATACTTTAGCACATGAAATTGCTAATCGATTAGTTGCTAATGACAAGAATGAAGCAACTTTGGAAATGACTAATAAAATGGC
AACGATTCGTTTTACAGAACCTACGCTGATTGCATTAGCAGGGGGTAATGTCAAAGCTTACACTGAGCATATGACTATATCTCCATATAAAT
TGTATTTGTTAGATAAAGGCGATGTTTTAAAGTTTAGAGAAACAAGTTATACATCGCGAGTGTATTTAGCTGTGGGAGGTGGATTTGAATT
AGATGCATGGTTAGGATCTAATTCAACCGACTTTAATGTAAAAATTGGTGGTTTTAAAGGTAGAACATTACAAGATGGCGATGAAATAAAG
CTTAAGAGAGATTATACAGCTCGTCATCATAAGTTATTTGAAAACCTTGCTCACACGAAACAAACAGATTGGGGTATTGATGGATACGCCTT
GTCATTTAATTATATGTCTGATGTATTTCATGTCGTTAAAAATAAAGGTACGGAAGATTTTAAAGAAGATGCCATTCAAAGATTTGTGAAAC
ATGATTATAAAGTAACGAGCAAAGCAAATCGCATGGGGATGATGCTTGAAGGTGAAAAAATCAAAGCTTTTTATGAAGATATGCCACCGT
ATCAGACTGTCAAAAAAGGAACGATACAAATTAAGCGTGATGGCACACCTATTATCCTATTAAATGATCATTATACGCTAGGTAGCTACCCG
CAAATCGGTACAATCGCAAGTTATCATTTAACGAAATTAGCACAAAAACCGCAAGGATCACGTTTGAAATTTCAATTTATAGATATTTTAAC
GGCTGAAAAGAACCTTGTTAAGTATAGTAACTGGTTAAACCAATTATTCCATGGAATAGAATATAGAATGCAATTAGAAATGATGAAATAA

Gene: ltaS (glycerol phosphate lipoteichoic acid synthase)

Position: 763232 to 765172, length: 1941 nt, orientation: FORWARD

Perfect match to: (CIGC128-AHVY01000003-[218793:220733], allele observed in CC1+CC8+CC97)

Sequence:

ATGAGTTCACAAAAAAAGAAAATTAGTCTTTTTGCGTTCTTCTTATTAACCGTAATAACGATTACCTTGAAGACGTATTTTTCTTATTATGTTG
ATTTTTCTTTAGGTGTTAAAGGTTTAGTACAAAACTTAATATTATTGATGAATCCTTATAGTTTAGTAGCACTGGTTTTAAGTGTGTTCCTATT
CTTTAAAGGCAAAAAAGCATTTTGGTTCATGTTCATAGGCGGCTTCTTATTGACGTTCCTATTATATGCCAATGTTGTGTACTTTAGATTCTTC
TCGGATTTTTTAACGTTTAGTACTTTAAACCAAGTAGGTAACGTAGAATCTATGGGTGGTGCGGTTAGTGCATCATTCAAATGGTATGACTT
TGTTTATTTCATTGATACGTTAGTTTACTTATTCATTTTAATATTTAAAACAAAATGGTTAGACACAAAAGCATTTAGTAAGAAATTTGTTCCT
GTCGTAATGGCGGCTTCAGTAGCATTATTCTTCTTAAACTTAGCTTTTGCTGAAACTGACAGACCAGAATTATTAACACGTACATTTGACCAT
AAATATTTAGTGAAATATTTAGGACCGTATAACTTTACAGTATACGATGGTGTTAAAACTATCGAAAATAATCAACAAAAAGCGTTAGCATC
TGAAGATGACTTAACAAAAGTATTAAATTATACGAAACAACGTCAAACAGAGCCTAACCCAGAATATTATGGGGTGGCAAAGAAGAAAAA
TATTATTAAGATTCATTTAGAAAGTTTCCAAACCTTCTTAATTAATAAAAAGGTTAATGGTAAAGAAGTAACACCGTTTTTAAACAAATTATC
AAGTGGGAAAGAGCAATTCACATACTTCCCTAACTTTTTCCATCAAACAGGTCAAGGTAAAACATCTGACTCTGAATTTACAATGGATAACA
GTTTATACGGTTTACCGCAAGGTTCTGCCTTTTCATTAAAAGGAGATAATACGTATCAGTCACTACCAGCAATTTTAGATCAAAAGCAAGGC
TACAAATCTGATGTCATGCACGGTGACTATAAAACATTCTGGAACAGAGACCAAGTATATAAACACTTTGGTATCGATAAATTCTATGATGC
AACATACTATGACATGTCAGATAAAAACGTTGTAAACTTAGGCTTGAAAGACAAAATTTTCTTTAAAGATTCTGCTAATTATCAAGCTAAGA
TGAAATCACCATTCTATTCTCATTTAATTACATTGACTAACCACTATCCATTCACATTAGATGAAAAGGATGCGACTATTGAGAAGTCAAACA

CAGGTGATGCAACAGTTGATGGTTATATTCAAACAGCGCGTTATTTAGACGAAGCATTAGAAGAATATATTAATGACTTGAAGAAAAAAGG
ATTATATGACAATTCAGTGATTATGATTTATGGTGACCACTATGGTATCTCTGAAAACCATAACAATGCCATGGAAAAACTATTAGGTGAAA
AAATCACACCAGCTAAATTTACAGATTTAAACAGAACTGGTTTCTGGATTAAAATCCCTGGTAAATCTGGTGGTATCAATAATGAATATGCT
GGTCAAGTCGATGTAATGCCAACAATTTTACATTTGGCTGGTATAGATACGAAGAATTATTTAATGTTCGGTACTGATTTATTCTCTAAAGGT
CATAATCAAGTAGTTCCATTCAGAAATGGTGACTTTATAACAAAAGATTATAAATATGTTAATGGTAAGATTTATTCTAATAAAAATAATGA
ACTCATAACTACTCAACCAGCTGATTTCGAAAAGAATAAAAAGCAAGTTGAAAAGGATCTCGAAATGAGTGACAACGTGCTTAATGGTGAT
TTGTTTAGATTCTACAAAAATCCAGACTTCAAAAAGGTAAATCCTTCGAAGTATAAATATGAAACAGGACCTAAAGCAAACTCTAAAAAATA
A

Gene: uup (putative ABC protein involved in excision of transposons)

Position: 765448 to 767325, length: 1878 nt, orientation: FORWARD

Perfect match to: (11819-97-CP003194-[809367:811244], allele observed in CC80+CC12)

Sequence:

ATGGAAGCATATAAAATTGAACATTTAAATAAATCTTATGCCGATAAGATTATATTCGATAATCTAGATTTATCAATTTCAGAAGGTGAAAA
AATAGGTTTAGTAGGCATAAATGGTACAGGGAAAAGTACGTTGTTAAAAGTAATTGGTGGTATTGATGATGATTTTACAGCCAATGTTATG
CATCCAAATCAATATCGAATTCGATATTCGTCTCAGAAACAGGACCTTAATGAAGATATGACAGTTTTTGATGCAGTATTAAGTTCTGATAC
AACAACTTTACGCATCATCAAGCAATATGAGCAGACAGTACAAGCTTATGCGGATGACCAAAGTGATAAATTGTTCAAGCGAATGATGGAT
GCGCAAGATGCTATGGATCAACATGATGCTTGGGACTATAACGCTGAAATTAAAACAATCCTCTCAAAACTAGGTATACATGATACTACTAA
ATACATTAAAGAATTATCCGGCGGACAACAAAAACGTGTTGTACTTGCTAAAACATTAATAGAACAACCAGATTTATTGTTATTAGATGAAC
CTACGAACCATTTAGACTTCGAATCAATCAGCTGGTTGATCAATTATGTGAAGCAATATCCTCATACAGTTTTATTCGTAACCCATGATCGAT
ATTTTTTAAATGAAGTTTCCACTAGAATTATTGAACTAAACAGAGGTAAGTTAGCGTCATATCCTGGCAACTATGAATCTTATATTGAAATGC
GCGCTGAAAGAGAAGTAACACTTCAAAAGCAACAACAAAAGCAACGAGCTTTATATAAGGAAGAACTTGCTTGGATGAGGGCTGGAGCTA
AGGCTCGTACTACAAAGCAACAAGCTAGAATTAATCGATTTAATGACCTAGAAAATGAAGTTAACCAGCAATATAAAGACGATAAAGGTGA
ATTGAATCTTGCTTATTCAAGATTAGGTAAGCAAGTGTTCGAATTAGAAGACTTATCAAAGGCTATTAATGATAAAGTATTATTTGAACATCT
GACGGAAATTATTCAAAAAGGTGAGCGTATTGGTGTTGTTGGGCCAAATGGAGCTGGTAAAACAACACTCTTAAATATTTTGAGTGGAGA
AGACCAACAATTCGAAGGTAAATTGAAGACTGGGCAGACGGTTAAAGTAGCTTATTTTAAGCAAACTGATGAGACCCTGGATAGAGATAT
TCGTATGATTGATTATTTAAGAGAAGAAAGTGAGATCGCAAAAGAAAAAGATGGAACCTCGGTATCTATTACACAACTTCTTGAACGATTTT
TATTTCCAAGTGCAACTCATGGTAAAAAAGTTTATAAATTATCTGGTGGAGAGCAAAAGCGCTTGTATTTATTACGTCTACTCGTACACCAG
CCAAATGTTCTGTTGTTAGATGAACCGACAAATGATTTAGATACTGAGACTTTAACAATACTTGAAGATTATATTCATACTTTCGGTGGTACA
GTGATTACCGTAAGCCATGATCGCTACTTCTTAAATAAAGTTGCACAGTCATATTGGTTTATTCATGATGGTCAGATGGAAAAGATTATCGG
AACTTTTGAAGATTATGAAAGTTATAAAAAATCATTAGATAAAAATAAATCCACATTGAAGCAACAATCTAAATCTTCTACAACTGTACGTA
AGAAAAATGGTTTATCATATAAAGAAAAATTAGAATATGAACAATTGATGAAACGCATAGAACAAGCGGAAGTAAGAATGGAAGAAATTG
ATGTGCTCATGATTGAGGCAAGTGCAGATTATGGGAAAATTAAAGAACTAAACGAAGAAAAAGAACAACTTGAAATTCAATATGATTTAG
ACATCACAAGATGGAGTGAGTTAGAAGAAATTAAAGAACAACAATAA

Gene: recQ1 (ATP-dependent DNA helicase)

Position: 767337 to 769118, length: 1782 nt, orientation: FORWARD

Perfect match to: (21333-AHKA01000037-[137219:139000:r], allele observed in CC80)

Sequence:

ATGATGCAACAAACATTATCGCATTACTTTGGGTATGAAACGTTTCGACCAGGACAAGAAGAAATTATTAGCAAAGTATTAGACCATCGTA
ATGTGCTTGGTGTCTTACCAACTGGTGGAGGTAAGTCTATATGCTATCAAGTACCAGGTTTATTGTTAGGTGGTACAACAATTGTAATAAGT
CCACTAATATCATTAATGAAAGATCAAGTGGATCAATTAAAAGCGATGGGAATTCAAGCTGCTTTTTTAAATAGTAGTTTGACTCAAAAAGA
GCAACAACGTATTGAAAAAGCATTATCAAATGGAGAAATTCAATTTTTGTATGTTGCACCAGAACGATTTGAAAACCGATATTTTTTAAATA
TGCTTCAGCGTATAAAGATTCACTTAGTCGCGTTTGATGAAGCGCATTGTATTTCTAAATGGGGTCATGATTTCAGACCGAGTTATCAAAAC
GTCATTTCTAAAGTATTTACGTTACCTCAAGATTTTACAATAATAGCGTTGACAGCAACTGCCACAGTTGAAGTACAGCAAGATATTAGAGA
AAAGTTAAATATCGCTCAAACTGATCAAATTAAAACGAGTACTAAGCGTAGAAACTTAATTTTTAAAGTAAATCCTACTTATCAACGTCAAA
AATTTATATTGGATTATATTAAAACACACGATGAAGATGCAGGTATTATTTATTGTTCTACACGTAAGCAAGTTGAAGAGCTTCAAGAAGCT
TTAGAAAGCCAGAAAATTGAAAGTGTTATATATCATGCAGGTTTGAGCAATAAAGAAAGAGAAGAAGCGCAGAATGATTTCTTATTTGATC
GTGTTAAAGTAGTTGTTGCTACAAATGCTTTTGGTATGGGTATTGATAAATCCAATGTACGCTTTGTTATTCATTATAATATGCCTGGAGATT
TAGAATCTTATTATCAAGAAGCGGGTCGTGCAGGTCGTGACGGGTTAAAAAGTGAATGTATTTTGTTATTTAGCGAACGCGATATCAATTT
ACACGAGTATTTTATAACAGTCTCTCAAGCTGATGATGACTATAAAGATAAAATGGGCGAAAAGTTAACTAAAATGATTCAATATACAAAA
ACAAAAAAATGTTTAGAAGCAACAATTGTCCATTATTTTGAACCGAATGAAAAATTAGAAGAATGTGAACAATGTAGTAATTGTGTTCAACA

AGATAAATCATATAATATGACACAAGATGCTAAGATGATTATTAGTTGCATCGCTCGTATGAAACAACAAGAGAGTTATAGTGTTATCATTC
AAGTGCTAAGAGGAGAGTCAACAGATTATATTAAGTACAAAGGTTATGACCAAATTTCAACCCATGGTTTAATGAAAGGTTACACAACATC
AGAATTAAGTCACTTAATAGATGAATTAAGATTCAAAGGGTTCTTAAATGAAAATGATGAAATATTAATGTGTGATACTTCAATTAAAAAAT
TACTCAGTAATGAAGTAGAAGTATTCACAACACCATTTAAGCAAAAAGCGACTGAAAAAGTATTTATAAATACGGTTGAAGGGGTTGACCG
AGTATTATTCAGTCAGTTGGTAGAAGTTCGTAAAAAGTTAAGTGACAAATTAACGATAGCACCTGTAAGTATATTTTCTGATTACACGTTGG
AGGAATTTGCTAAACGTAAGCCTGCTTCGAAACAAGATATGATTAATATTGATGGCGTAGGTAGTTACAAATTAAAACATTATTGTCCAGCA
TTTTTAGAAACGATTCAAAATTATAAAGCCAAAGTATAG

Gene: opuBA (osmoprotectant ABC transporter, ATP-binding protein)

Position: 769341 to 770318, length: 978 nt, orientation: FORWARD

Perfect match to: (Strain_21266-AFTT01000025-[75107:76084:r], allele observed in CC12+CC8)

Sequence:

GTGATTAAGTTTAAAAATGTAACTAAGCGTTATGGCAAACATGTTGCTGTCGATAACATTAGTTTCAATATTAATGAGGGTGAATTTTTTGT
GCTAATTGGACCTTCAGGTTGTGGAAAAACTACGACATTAAAAATGATTAATCGACTCATTCACTTAAGTGAAGGTTATATTTATTTTAAAG
ATAAACCAATAAGTGATTATCCAGTATACGAAATGCGTTGGGATATTGGATACGTATTGCAGCAGATTGCATTATTCCCACATATGACAATC
AAAGAAAATATTGCACAAGTGCCACAAATGAAAAAGTGGAAAGAAAAAGATATAGATAAAAGAGTAGATGAATTACTTGATATGGTTGGA
TTAGAACCTGAAAAATATAAAAACAGAAAACCTGATGAATTGTCAGGGGGGCAACGACAACGTGTAGGAGTTATACGTGCGTTAGCAGCT
GATCCACCAGTTATTTTAATGGATGAACCGTTTAGTGCATTAGACCCAATCAGCCGAGAAAAACTTCAAGATGATTTAATTGAATTACAAAC
TAAAATTAAGAAGACAATCATATTTGTTACACATGATATTCAAGAGGCGATGAAACTTGGTGATAAGATTTGTCTTTTGAATGAAGGCCATA
TTGAACAAATTGACACACCAGAAGGATTTAAAAATAATCCTCAAAGTGAATTTGTTAAACAATTTATGGGTAGTCATTTAGAAGATGATGCG
CCATGTGTTGAAGAGAACGCAATTATCCGTGACTTGGATATTATGAAACCAATCGATGAGGTTACATCTATGAGCGCTTATCCAATTGTTTA
TGACAATCAACCAATTGAAGTATTGTATCAACTTTTATCAGAGAGCGAGCGTGTCATTGTCATGCAAGAAGATAGCGTAGGTCAATATGTTA
TTGATAGGAAAGATATCTTCAAATATTTGTCCCAGAAAAAGGAGGTAGCTCAACATGACTAA

Gene: opuBB (osmoprotectant ABC transporter, transmembrane permease)

Position: 770311 to 771825, length: 1515 nt, orientation: FORWARD

Perfect match to: (11819-97-CP003194-[814230:815744], highly conserved allele)

Sequence:

ATGACTAACTTTTTCGACATATTGAGTGAACGTAAGGGGCAACTCTTTTCGACAATGATAGAACATATTCAAATATCATTTATCGCATTATTG
ATTGCAACTGCTATTGCGGTACCATTAGGTATTTTATTAACGAAGACTAAAACGATATCTGAAATCGTAATGAATATTGCGGCAATTCTTCA
AACCATACCATCGTTGGCATTATTAGGTTTAATGATTCCTTTATTTGGTATCGGTCGTGTGCCAGCAATTATTGCACTTGTAGTGTATGCGTT
GTTACCAATTTTAAGGAATACGTATACTGGAATTAAAGAAGTTGATCCATCACTCATTGAAGCGGCTAAAGGTATAGGTATGAAACCATTTA
GACGTTTAACTAAAGTCGAACTTCCGATAGCAATGCCTGTTATAATGGCTGGTGTAAGAACGGCTATGGTATTAATTATAGGTACAGCAAC
ACTAGCAGCATTAATTGGTGCAGGCGGACTAGGAGATTTAATTTTATTAGGTATAGACCGTAACAATGCATCGTTGATATTATTAGGTGCAA
TTCCAGCAGCCTTATTGGCAATTATATTTGATTTAATTTTAAGATTTATGGCTAAATTATCTTATAAAAAGTTATTGATGACGTTAGGTGTTAT
AGTGATGATTATTATACTGGCTATCGCTATTCCTATGTTTGCACAAAAAGGTGATAAAATTACGTTAGCTGGAAAGCTTGGCTCCGAGCCAT
CGATTATTACAAATATGTATAAAATTTTAATAGAAGAAGAGACCAAAAATACTGTAGAAGTGAAAGATGGTATGGGCAAAACAGCATTTTT
ATTTAATGCTTTAAAATCTGACGATATAGATGGGTATTTAGAATTTACTGGAACAGTTTTAGGTGAATTAACAAAAGAACCATTGAAGTCAA
AAGAAGAGAAAAAAGTTTATGAACAAGCTAAGCAAAGTCTTGAAAAGAAATATCAAATGACTATGTTAAAACCAATGAAGTATAACAATA
CGTATGCTTTAGCTGTAAAACGTGATTTTGCTAAACAACATAATATACGTACAATTGGTGATTTAAATAAGGTTAAAGATCAACTTAAACCA
GGATTTACATTGGAATTTAATGATCGTCCAGATGGTTACAAAGCTGTTCAAAAGGCTTATAATTTAAATTTAGATAACATACGTACAATGGA
ACCTAAGTTGAGATATCAAGCGATCAATAAAGGTAATATTAATTTAATAGATGCATATTCAACTGACGCTGAATTAAAACAATATGATATGG
TTGTGTTAAAAGATGATAAGCACGTATTTCCACCATATCAAGGAGCACCATTATTTAAAGAAAGCTTTTTAAAGAAACATCCAGAAATTAAG
AAACCGTTAAACAAACTAGAAAACAAAATATCTGATGAAGATATGCAAATGATGAACTATAAAGTAACAGTTAAAAATGAAGACCCATATA
CAGTTGCGAAAGATTATTTAAAAGCAAAAGGGTTAATCAAATAA

Gene: hisC2 (histidinol-phosphate aminotransferase locus 2)

Position: 772065 to 773123, length: 1059 nt, orientation: FORWARD

Perfect match to: (NN50-BAEA01000007-[70771:71829:r], allele observed in CC4803+CC1+CC8+CC97)

Sequence:

ATGAAAGAACAACTTAATCAACTATCAGCATATCAGCCTGGTTTATCTCCAAGGGCATTGAAAGAAAAGTATGGCATTGAAGGAGATTTAT
ATAAACTTGCATCAAATGAAAATTTGTATGGACCATCGCCTAAAGTTAAAGAAGCGATATCAGCACACTTAGATGAGTTATATTATTATCCT
GAAACAGGATCACCGACATTAAAAGCGGCGATTAGTAAACATTTAAATGTAGATCAATCACGCATTTTATTTGGTGCGGGATTAGATGAAG
TTATATTAATGATTTCTAGAGCTGTATTAACGCCAGGGGATACTATTGTTACAAGTGAAGCGACATTCGGTCAATATTATCACAATGCGATT
GTTGAATCAGCTAATGTGATACAAGTACCTTTAAAAGATGGTGGCTTCGATTTAGAAGGTATTTTAAAAGAAGTTAATGAAGATACGTCATT
GGTATGGTTATGTAATCCAAATAATCCTACAGGTACATATTTTAATCATGAGAGCGTAGATTCGTTTTTATCTCAAGTACCTCCACATGTACC
AGTAATTATAGATGAAGCTTATTTTGAATTTGTGACAGCAGAGGACTACCCGGATACACTTGCTTTGCAACAAAAATATGACAATGCTTTCT
TATTACGTACATTTTCAAAGGCGTATGGATTAGCGGGTTTACGTGTAGGATATGTGGTAGCAAGTGAACATGCGATTGAAAAATGGAACAT
CATTAGACCACCATTTAATGTGACACGTATATCTGAATACGCAGCAGTTGCAGCACTTGAAGATCAACAATATTTAAAAGAGGTAACACATA
AAAATAGTGTTGAACGCGAAAGATTTTATCAATTACCTCAAAGTGAGTATTTCTTGCCAAGTCAAACGAATTTTATATTTGTAAAAACAAAG
CGGGTAAATGAACTTTATGAAGCACTTTTAAATGTAGGGTGTATTACGCGACCATTTCCAACTGGTGTTAGAATTACAATTGGTTTTAAAGA
ACAAAATGATAAAATGTTAGAAGTTTTATCAAACTTTAAATACGAATAG

Gene: tx_universal2 (rho-independent terminator)

Position: 773239 to 773277, length: 39 nt

Perfect match to: (Strain_21331-AGTV01000040-[104403:104441], allele observed in CC398)

Sequence:

TAGAATTGAAAAAAAGCTTGTTACAAGCGCATTTTCGTTC

Gene: yorS (5' (3')-deoxyribonucleotidase)

Position: 773462 to 774004, length: 543 nt, orientation: FORWARD

Perfect match to: (N315-BA000018-[776469:777011], allele observed in CC5+CC8+CC80+CC88+CC96)

Sequence:

ATGACCCGTAAATCAATCGCGATTGATATGGATGAAGTATTGGCAGATACATTAGGAGAAATCATTGATGCTGTCAATTTTAGAGCGGATT
TAGGTATTAAAATGGAAGCTTTGAATGGTCAAAAACTTAAACATGTTATTCCTGAACATGATGGATTAATTACAGAAGTATTGAGAGAACC
AGGCTTCTTCAGACATCTTAAAGTGATGCCACATGCACAAGAAGTTGTGAAAAAATTAACTGAACATTATGATGTATATATTGCTACAGCAG
CAATGGATGTACCAACATCATTTAGTGATAAATATGAATGGTTACTAGAGTTCTTTCCATTTTTAGATCCTCAGCATTTTGTTTTTTGTGGTAG
AAAAAACATCGTTAAAGCTGATTATTTAATAGATGACAATCCTAGACAGCTTGAAATTTTTACTGGTACACCGATTATGTTTACAGCAGTGC
ATAATATTAATGATGATCGATTTGAACGCGTAAATAGCTGGAAAGATGTAGAACAGTATTTTTTAGATAATATTGAGAAATAA

Gene: dgkA1-bmrU (putative lipid kinase)

Position: 774571 to 775488, length: 918 nt, orientation: REVERSE

Perfect match to: (JS395-CP012756-[2165152:2166069:r], highly conserved allele)

Sequence:

TTACAAATCATTGACAGTAAGTAACTGAATGGCATTTGGTATAACCTCAATATCAATAGGTGTTTCTAATGAAATTTCGCCATCAATATCAAC
TTTCATTGCTGGATCTGTTGTAAGTGAAATCTTTTTACCAGGTATATGCTCAATACCTTGAGTGATTTCATTCCAATTCATGCTATCACGCTTT
TTAAAAATATCATTTAAAATACTGAAACTTTGTTCATTAAAAATGAAAGTGTTCAGTTCACCATCTTGAGGAGACAAATCAGTCAATGGTAT
ACGACTACCACCAATGAATGGGCCATTTGCTGTTAGTATCATGGTCGTTTCGCCAGAATATGTCTTATCATCTATTGATAATTGATAATTAAA
TTGTGTTGGATTTAGCAGTGTTTTGACAGTTGATCCAATATAACTCAATTTACCAAATATATCTTTTGAACCATCTTGTACGTTTTCAGCGTTT
TGAACAATTAGACCTAACCCAACAAAGTTGAGTGCATATTGATTATTTATTTTAATTACATCGTATGTACCAACTTGTGCAGAAATCATTTGT
TCACTAGCTTGTTTATGATTAGGTGCTATATTTAGCGTTTTTGTAAAATCATTAAAAGTACCGCCTGGTAAAATGCCAATAGGGAGTTGAAG
GTCATGTGACATAACACCATTTATAAGTTCGTTAACCGTGCCATCACCGCCAAGAATAAATAATATATCTACATCTTTTGCATAGTTTTTAGTT
TTGATTTCTTGGCAATATTTAATAATGTCACCTTCGTTTTCACTCAATTGAATAGAAAGATGCTTACAAATTGAACTTAATGCTGTTGTAACTT
CCCCAATACCTTGATTAATATTTTTTAATCCACTGTGTTCATGGTAAAAGAGGACACCATGTGTATATTTATTTTCCAT

Gene: dtpT (proton-dependent di- and tripeptide transporter)

Position: 775758 to 777263, length: 1506 nt, orientation: REVERSE

Perfect match to: (Strain_21193-AFEG01000023-[64293:65798], highly conserved allele)

Sequence:

TTAACGTATACCTTTCATCGCTTTGATGATTAAAGGTGAGAATGCTAATACAATTGTTGTAACAATAATTGCAACAACACCTAGGAAAATAA
AGTAATTTGTTTGACCTAGTGGTTCTATTAACTTAACTAAAGTACCATTGATTGCTTGTGCAGAAGCGTTAGTTAAGTACCAAATACTCATCA
TTTGGGCATTAAATGCTTTAGGTGCTAACTTAACAGCAGCACTATTACCCGTTGGTGATAAGCATAGCTCACCGATAACACAAATAATGTAC
GATAAAATAACCCAGTTAACTGAGAAGTTTGATGAACCTGATGCATAACCTACAATACCAATTAGTATGTATGACGCACCTGCTAAGAACGT
ACCAATTGCAAATTTTACTGGCAGGCTAGGTTGTTTAGTTCCAAGCTTTTGCCATAAAAGTGAAATAATTGGAGCTAGTAATAAAATAAATA
ATGGGTTAATTGATTGGAAGATCGCTTCACCAAAGTTTGTTTTCCAACCAAATAAGTTTAATTTCATATCTGAATGTTCAATTCCATATATGTT
TAATACATTAGACCCTTGTTCTTGAATAGCCCAGAACACCATTCCAAGAATAAATAATGGAATAAATGCTTTAACACGAGAACGTTCAGTAT
CAGTGACATCTTTACTTCTAATAATTAAAGTGAAGTAAATGATTGGTAATGCAATACCTAATACTAAAACAGTATTACTAACTAAGTTAAATG
ATAATGAATTAGTTAATGCACCAATAACGATAATTAATACAATTGCTAAAACAACACTTCCGATAATAAGACCATACTTTTTCTTTTCAGCTG
GTGTCAATGGGTTAGTAGGTTTCATACCAACGCTACCTAAGTTTTTGCGGTTGAAAAGTACATACCATACTAAACCTAATGCCATACCAACT
GCTGCAATCAAGAATCCGCCGTGGAAGTTTTTAACATTAACAAAGTGTTGCAAAATAATAGGTGATAATAATGCACCCATATTAACTGACAT
ATAGAAAATAACAAAACCTGCATCCATACGTCTATCATTTTCAGGATATAAACGGCCAACGATATTTGAAATGTTTGGCTTCATTAAACCTG
AACCAATAATGATGAAGAACATTGATGTGAATAAGCCGATTAATGCAAATGGTAAGCTTAAACAAATATGTCCGATAATAATAAAGACTGC
ACCTAATAAAGTAGCGCCTCTAGTACCTGTAATTCTGTCAGCAATCCATCCGCCTGGTATTGATGTCATATAGATTAATGAACCATAAACTGA
CATAATTGACATAGCTGTTGTTTTATCAATTCCAAGGCCATTATCTGTTACGGCAAAGTACATGTAGAAAATGAGTAGGGCACGCATGCCAT
AATAACTAAACCTTTCCCAGAACTCTACAAAGAAGAGTACGCCTAGTCCTCGAGGATGCCCGAAAAATCCTGTTTGAGGTATGTCTTGAATT
TGATTTCCATGGGAGTTTTGTTGTGTCAT

Gene: queF (NADPH-dependent 7-cyano-7-deazaguanine reductase)

Position: 777604 to 778104, length: 501 nt, orientation: REVERSE

Perfect match to: (N315-BA000018-[780923:781423:r], highly conserved allele)

Sequence:

TTAACGATTATCTATTTTTTCGGGATATAAATCATGATTCATCAAACGATGCTCAGCCATTTTTTCATATTTAGAATTTGGACGTCCATAGTTT
GTATAAGGATCAATAGAAATTCCACCACGTGGTGTGAACTTGCCCCAGACTTCAATATAATGTGGGTCCATAAGCTCTATCAAATCATTCAT
AATAATATTCATACAATCTTCGTGAAAATCACCGTGATTTCTGAAACTAAATAAGTATAATTTCAAAGATTTTGATTCAACCATTTTAACATTT
GGAATATATGAAATATAGATAGTTGCAAAATCTGGTTGCCCAGTAATTGGACATAATGATGTAAATTCTGGACAGTTGAATTTTACGAAATA
GTCACGACCTTGATGCTTATTATCAAACGATTCTAATACATCAGGACGATAGTCAAAATTGTAAGTATTGTCTTGATTTCCTAATAAAGTTAT
ATCTTGTAATTCATCTTGTTGACGGCCATGTGCCAT

Gene: yxxF (putative transporter)

Position: 778124 to 778990, length: 867 nt, orientation: REVERSE

Perfect match to: (MW2-BA000033-[776834:777700:r], highly conserved allele)

Sequence:

TTATTTTTTTATTATTTTGGCGTCTCGGCGTGCTTTTTCAAACATGTAATAACTTGCACCGATAATAACGACGTAACCTAATGTTGCATAGAAA
TCTGGAGATTCTCCGAATAGAATAAATCCAAGTATTGCTGTGAAAATTATAGATGCATACGTGAAAATAGAAATATCTTTTGCTGCTGCAAA
ACTATATGCTAATGTAACACCAATTTGACCCACAGCGGCAGCTAAGCCAGCCCCTAATAGATAAAGTATTTGCATCTGACTCATTGGTTCAT
AGGTATATGCAGTGAAAGGTATTAAAACGATGACAGAAAATAAGGAGAAGTAAAATACTATAGTATATGGTGCTTCTCTTGTACTAAGTGC
TCGAACACATGTATATGCTGATGCTGCAAAAATACCTGAGAATAAGCCAGCTAATGATGGAATCATAGATGATGAAAATTCAGGTTTCACT
ATTAAAAGCATACCTAAAATAGCAATTATCATTGCTGTAATTTGATACTTCCTTACCTTTTCATGTAAGAAAACAATGCTTAATAAAATCGTCC
AGAAAGGATTGAGTTTCATTAATGAATCGGCATCACTAAGTACCATATGATCAATGGCATAAATATTTAACAATACACCAATAAGTCCAAGT
GTTGATCGTGTTATTAATAAGGGTTGACTTGAAAGTCTGCCAAACATTGGCTGATGGTATTTATATATAAAAAATAATGGAATAAACATTGC
TACTAAGTTTCGTGCTAATGATTTTTGAAAAACAGGAAGGTCACCTGCAAGTCTGAAAAACACTGACATAAAACTGAAACCAATAGCCGAA
ATTAAAATGGCAATGATACCTTTTACTTTAGGATTCAA

Gene: rnaIV (regulatory RNA IV)

Position: 779401 to 779630, length: 230 nt

Sequence:

TAATATAGTGTTGGTGAAACGAAAAAGACACAATATCTTGTGTTTTGTATGCAAATGCTTTATTTATGAAGAAATTACATTTAAAAGTAATTT
AACACAGAAATTTAATAGTTATTATCAATTAATAGTCATATTTTTAGAAAATGTACTGAGCAAATGGAAGATATCCAATGATGTAAACACTA
CATATAGTGATTTTTATACATTCAACCCATATAAGCTACTATTTT

Gene: nrdI (stimulator of ribonucleotide reduction)

Position: 779791 to 780189, length: 399 nt, orientation: FORWARD

Perfect match to: (RF122-AJ938182-[747913:748311], highly conserved allele)

Sequence:

ATGAAAATAATATATTTTTCATTTACTGGAAATGTCCGTCGTTTTATTAAGAGAACAGAACTTGAAAATACGCTTGAGATTACAGCAGAAAA
TTGTATGGAACCAGTTCATGAACCGTTTATTATCGTTACTGGCACTATTGGATTTGGAGAAGTACCAGAACCCGTTCAATCTTTTTTAGAAGT
TAATCATCAATACATCAGAGGTGTGGCAGCTAGCGGTAATCGAAATTGGGGACTAAATTTCGCAAAAGCGGGTCGCACGATATCAGAAGA
GTATAATGTCCCTTTATTAATGAAGTTTGAGTTACATGGAAAAAACAAAGACGTTATTGAATTTAAGAACAAGGTGGGTAATTTTAATGAAA
ACCATGGAAGAGAAAAAGTACAATCATATTGA

Gene: nrdE (ribonucleotide-diphosphate reductase alpha chain)

Position: 780152 to 782257, length: 2106 nt, orientation: FORWARD

Perfect match to: (11819-97-CP003194-[824071:826176], highly conserved allele)

Sequence:

ATGAAAACCATGGAAGAGAAAAAGTACAATCATATTGAATTAAATAATGAGGTCACTAAACGAAGAGAAGATGGATTCTTTAGTTTAGAA
AAAGACCAAGAAGCTTTAGTAGCTTATTTAGAAGAAGTAAAAGACAAAACAATCTTCTTCGATACTGAAATCGAGCGTTTACGTTATTTAGT
AGACAACGATTTTTATTTCAATGTGTTTGATATTTATAGTGAAGCGGATCTAATTGAAATCACTGATTATGCAAAATCAATCCCGTTTAATTT
TGCAAGTTATATGTCAGCTAGTAAATTTTTCAAAGATTACGCTTTGAAAACAAATGATAAAAGTCAATACTTAGAAGACTATAATCAACACG
TTGCCATTGTTGCTTTATACCTAGCAAATGGTAATAAAGCACAAGCTAAACAATTTATTTCTGCTATGGTTGAACAAAGATATCAACCAGCG
ACACCAACATTTTTAAACGCAGGCCGTGCGCGTCGTGGTGAGCTAGTGTCATGTTTCTTATTAGAAGTGGATGACAGCTTAAATTCAATTAA
CTTTATTGATTCAACTGCAAAACAATTAAGTAAAATTGGGGGCGGCGTTGCAATTAACTTATCTAAATTGCGTGCACGTGGTGAAGCAATTA
AAGGAATTAAAGGCGTAGCGAAAGGCGTTTTACCTATTGCTAAGTCACTTGAAGGTGGCTTTAGCTATGCAGATCAACTTGGTCAACGCCC
TGGTGCTGGTGCTGTGTACTTAAATATCTTCCATTATGATGTAGAAGAATTTTTAGATACTAAAAAAGTAAATGCGGATGAAGATTTACGTT
TATCTACAATATCAACTGGTTTAATTGTTCCATCTAAATTCTTCGATTTAGCTAAAGAAGGTAAGGACTTTTATATGTTTGCACCTCATACAGT
TAAAGAAGAATATGGTGTGACATTAGACGATATCGATTTAGAAAAATATTATGATGACATGGTTGCAAACCCAAATGTTGAGAAAAAGAAA
AAGAATGCGCGTGAAATGTTGAATTTAATTGCGCAAACACAATTACAATCAGGTTATCCATATTTAATGTTTAAAGATAATGCTAACAGAGT
GCATCCGAATTCAAACATTGGACAAATTAAAATGAGTAACTTATGTACGGAAATTTTCCAACTACAAGAAACTTCAATTATTAATGACTATG
GTATTGAAGACGAAATTAAACGTGATATTTCTTGTAACTTGGGCTCATTAAATATTGTTAATGTAATGGAAAGCGGAAAATTCAGAGATTCA
GTTCACTCTGGTATGGACGCATTAACTGTTGTGAGTGATGTAGCAAATATTCAAAATGCACCAGGAGTTAGAAAAGCTAACAGTGAATTAC
ATTCAGTTGGTCTTGGTGTGATGAATTTACACGGTTACCTAGCAAAAAATAAAATTGGTTATGAGTCAGAAGAAGCAAAAGATTTTGCAAA
TATCTTCTTTATGATGATGAATTTCTACTCAATCGAACGTTCAATGGAAATCGCTAAAGAGCGTGGTATCAAATATCAAGACTTTGAAAAGT
CTGATTATGCTAATGGCAAATATTTCGAGTTCTATACAACTCAAGAATTTGAACCTCAATTCGAAAAAGTACGTGAATTATTCGATGGTATG
GCTATTCCTACTTCTGAGGATTGGAAGAAACTACAACAAGATGTTGAACAATATGGTTTATATCATGCATATAGATTAGCAATTGCTCCAAC
ACAAAGTATTTCTTATGTTCAAAATGCAACAAGTTCTGTAATGCCAATCGTTGACCAAATTGAACGTCGTACTTATGGTAATGCGGAAACAT
TTTACCCTATGCCATTCTTATCACCACAAACAATGTGGTACTACAAATCAGCATTCAATACTGATCAGATGAAATTAATCGATTTAATTGCGA
CAATTCAAACGCATATTGACCAAGGTATCTCAACGATCCTTTATGTTAATTCTGAAATTTCTACACGTGAGTTAGCAAGATTATATGTATATG
CGCACTATAAAGGATTAAAATCACTTTACTATACTAGAAATAAATTATTAAGTGTAGAAGAATGTACAAGTTGTTCTATCTAA

Gene: nrdF (ribonucleotide-diphosphate reductase beta chain)

Position: 782375 to 783346, length: 972 nt, orientation: FORWARD

Perfect match to: (N315-BA000018-[785694:786665], highly conserved allele)

Sequence:

ATGATAGCTGTTAATTGGAACACACAAGAAGATATGACGAATATGTTTTGGAGACAAAATATATCTCAAATGTGGGTTGAAACAGAATTTA
AAGTATCAAAAGACATTGCAAGTTGGAAGACTTTATCTGAAGCTGAACAAGACACATTTAAAAAAGCATTAGCTGGTTTAACAGGCTTAGA
TACACATCAAGCAGATGATGGCATGCCTTTAGTTATGCTACATACGACTGACTTAAGGAAAAAAGCAGTTTATTCATTTATGGCGATGATGG
AGCAAATACACGCGAAAAGCTATTCACATATTTTCACAACACTATTACCATCTAGTGAAACAAACTACCTATTAGATGAATGGGTTTTAGAG
GAACCCCATTTAAAATATAAATCTGATAAAATTGTTGCTAATTATCACAAACTTTGGGGTAAAGAAGCTTCGATATACGACCAATATATGGC
CAGAGTTACGAGTGTATTTTTAGAAACATTCTTATTCTTCTCAGGTTTCTATTATCCACTATATCTTGCTGGTCAAGGGAAAATGACGACATC
AGGTGAAATCATTCGTAAAATTCTTTTAGATGAATCTATTCATGGTGTATTTACCGGTTTAGATGCACAGCATTTACGAAATGAACTATCTGA
AAGTGAGAAACAAAAAGCAGATCAAGAAATGTATAAATTGCTAAATGACTTGTATTTAAATGAAGAGTCATACACAAAAATGTTATACGAT
GATCTTGGAATCACTGAAGATGTGCTAAACTATGTTAAATATAATGGAAACAAAGCACTTTCAAACTTAGGCTTTGAACCTTATTTTGAGGA
ACGTGAATTTAACCCAATCATTGAGAATGCCTTAGATACAACAACTAAAAACCATGACTTCTTCTCAGTAAAAGGTGATGGTTATGTATTAG
CATTAAACGTAGAAGCATTACAAGATGATGACTTTGTATTTGACAACAAATAA

Gene: sstA (iron compound ABC transporter, transmembrane permease subunit)

Position: 783721 to 784692, length: 972 nt, orientation: FORWARD

Sequence:

ATGAAGTATTTATTAAAGGGAAATATTTTGCTTCTATTACTAATATTGTTGACAATTATTTCGTTGTTCATAGGTGTGAGTGAACTATCAATT
AAAGATTTACTACATTTAACTGAGTCACAGCGGAATATTTTATTCTCAAGCCGAATACCAAGGACGATGAGTATTTTAATTGCTGGAAGTTC
GTTGGCTTTAGCAGGCTTGATAATGCAACAAATGATGCAAAATAAGTTTGTTAGTCCGACTACAGCTGGAACGATGGAATGGGCTAAACTA
GGTATTTTAATTGCTTTATTGTTCTTTCCAACCGGTCATATTTTATTAAAACTAGTATTTGCTGTTATTTGCAGTATTTGCGGTACGTTTTTATT
TGTTAAAATCATTGATTTTATAAAAGTGAAAGATGTCATTTTTGTACCGCTTCTAGGAATTATGATGGGTGGGATTGTTGCAAGTTTCACAA
CCTTCATCTCATTGCGCACGAATGCTGTTCAAAGCATTGGTAACTGGCTTAACGGGAACTTTGCCATTATCACAAGTGGACGCTATGAAATT
TTATATTTAAGTATTCCTCTTTTAGCATTGACATATCTTTTTGTTAATCATTTCACGATTGTAGGAATGGGTAAAGACTTTACTAATAATTTAG
GTTTGAGTTACGAAAAATTAATTAACATCGCATTGTTTATTACTGCAACTATTACAGCATTGGTAGTGGTGACTGTTGGAACATTACCGTTCT
TAGGACTAGTAATACCAAATATTATTTCAATTTATCGAGGTGATCATTTGAAAAATGCTATCCCTCATACGATGATGTTAGGTGCCATCTTTG
TATTATTTTCTGATATAGTTGGCAGAATTGTTGTTTATCCATATGAAATAAATATTGGGTTAACAATAGGTGTATTTGGAACAATCATTTTCCT
TATCTTGCTTATGAAAGGTAGGAAAAATTATGCGCAACAATAA

Gene: sstB (iron compound ABC transporter, transmembrane permease subunit)

Position: 784679 to 785635, length: 957 nt, orientation: FORWARD

Perfect match to: (N315-BA000018-[787998:788954], highly conserved allele)

Sequence:

ATGCGCAACAATAATAAAAAAATAATGCTTTTAATTGCAGTAACGTTATTAATTAGTATGCTGTACTTATTTGTAGGTATTGATTTTGAAATA
TTTGAATATCAATTTTCAAGTCGTTTAAGAAAGTTCATATTAATTATTTTAGTAGGTGCTGCCATTGCAACTTCAGTGGTGATTTTTCAAGCG
ATTACAAATAACCGTCTATTGACACCATCAATAATGGGGTTAGATGCAGTTTATTTATTTATCAAAGTATTGCCAGTCTTTTTATTTGGAATTC
AATCGGTATGGGTTACTAATGTATATTTGAACTTTATATTAACACTTATAACGATGGTGTTATTCGCACTAATCCTATTCCAAGGTATCTTTAA
AATTGGACATTTTTCAATTTATTTTATCTTACTTATTGGTGTCCTTTTAGGAACATTTTTTAGAAGCATAACAGGTTTTATTCAACTGATTATGG
ATCCTGAGTCATTTTTAGCAATACAAAGTAGTATGTTTGCTAATTTTAATGCTTCTAATTCGAATTTAGTTACTTTCTCAGCAGTGCTATTAGT
AATCTTATTAGTCATTACAATTTTACTATTGCCTTATTTAGATGTATTGCTTTTAGGTCGTGCTGAAGCAATTAATCTTGGGATATCGTATGAA
AAATTAACGCGAATTCTACTTGTAATAGTCTCAGTTTTAGTTTCTGTGTCAACTGCATTAGTAGGACCAATTACATTTTTAGGTTTATTAACTG
TAAATCTAGCGCATGAACTAATGAAGACGTATGAACATAAGTATATTTTAATTGCGACAATTTGCTTGAGTTGGATTAGTTTATTTAGTGCG
CAATGGGTAGTTGAAAATGTGTTTGAAGCTACGACAGAAATGAGTATACTTATTGATTTGATTGGTGGAAGTTATTTCATTTATCTATTAGT
TAGAAGGAGAAATGCGCAATGA

Gene: sstC (iron compound ABC transporter, ATP-binding protein)

Position: 785632 to 786393, length: 762 nt, orientation: FORWARD

Perfect match to: (N315-BA000018-[788951:789712], highly conserved allele)

Sequence:

ATGATTCAAGTTGAAAATTTAACTAAAACTATAAATAATCAAATGATATTGGAAGATATTAGCATAGATATCGAAAAAGGTAAATTGACTTC
TTTAATTGGACCTAATGGTGCGGGTAAGAGTACTTTACTTTCAGCGATATGTAGGTTAATTCGTTTTGATAACGGTGAAGTGAAAATAGATG
GACAGCTCATGTCTGATTATAAAAATAATGACTTGTCGAAAAAAATATCTATATTAAAACAAACAAACCATACTGAAATGAATATTACGGTA
GAGCAGTTGGTAAACTTTGGACGATTCCCTTATTCTAAAGGTCGTTTGACGAAAGAGGATCATGATATTGTCAATGATGCGCTAGATTTGTT
GCAACTACAAGATATCAGAAATCGTAATATTAAGTCATTATCTGGTGGACAACGTCAGCGTGCATACATTGCAATGACAATAGCACAAGAT
ACTGAATATATTTTGCTAGATGAACCATTAAATAATTTAGATATGAAGCATGCTGTTCAAATTATGCAAACGTTAAAAATGTTAGCGCATAA
AATGAATAAAGCGATTGTCATTGTGTTACATGATATTAACTTTGCGTCCTGTTATTCAGATCAGATTGTAGCATTGAAAAACGGACAACTAG
TTAAGTCAGATTTGAAAGATAATGTCATTCAAAGTAGTGTTTTAAGTGATTTATATGACATGAATATTCAAATTGAACATATAAGAAATCAA
AGGATTTGTTTATATTTTAAGGATTGA

Gene: sstD (iron compound ABC transporter, substrate-binding protein)

Position: 786512 to 787540, length: 1029 nt, orientation: FORWARD

Perfect match to: (TCH130-ACHD01000007-[32447:33475:r], allele observed in CC72+CC80)

Sequence:

ATGAAGAAAACAGTCTTATATTTAGTATTAGCAGTAATGTTTTTATTAGCGGCATGCGGTAACAATTCTGATAAAGAACAATCAAAATCAGA
AACTAAAGGTTCTAAAGATACAGTGAAAATTGAAAATAACTATAAAATGCGTGGCGAGAAAAAAGATGGTAGTGACGCTAAAAAAGTTAA
AGAAACTGTTGAAGTACCAAAAAATCCTAAAAATGCAGTTGTGTTAGACTATGGCGCATTAGATGTAATGAAAGAAATGGGCTTATCAGAT
AAAGTAAAAGCATTACCTAAAGGGGAAGGCGGTAAGTCATTACCGAATTTCTTAGAATCATTTAAAGATGATAAATATACAAACGTTGGTA
ATTTAAAAGAAGTGAATTTTGATAAAATTGCTGCGACGAAACCCGAAGTAATCTTTATCTCTGGACGTACAGCTAATCAAAAGAATTTAGAT
GAATTCAAAAAAGCTGCACCTAAAGCGAAAATTGTTTATGTTGGTGCAGATGAAAAGAACTTAATTGGTTCAATGAAACAAAACACTGAAA
ATATCGGTAAAATTTACGATAAAGAAGATAAAGCTAAAGAATTAAATAAAGATTTAGATAACAAAATTGCTTCAATGAAAGATAAAACGAA
AAACTTCAATAAAACTGTTATGTATTTACTAGTTAACGAAGGTGAATTATCAACATTTGGACCTAAAGGTCGTTTTGGTGGATTAGTTTACG
ATACATTAGGATTCAATGCAGTTGATAAAAAAGTAAGTAATAGCAATCATGGACAAAATGTTTCTAACGAATATGTTAATAAAGAAAATCC
AGATGTTATTTTAGCGATGGATAGAGGTCAAGCGATAAGTGGTAAATCAACTGCGAAACAAGCATTAAATAATCCTGTATTAAAAAATGTT
AAAGCAATTAAAGAAGACAAAGTATATAATTTAGATCCTAAATTATGGTACTTTGCTGCTGGATCAACTACAACTACAATTAAACAAATTGA
GGAACTTGATAAAGTTGTAAAATAA

Gene: Q5HHT3 (putative protein)

Position: 787857 to 788171, length: 315 nt, orientation: REVERSE

Perfect match to: (N315-BA000018-[791176:791490:r], highly conserved allele)

Sequence:

TTAAATTTCAAAATAAATATGATAGTGATATTTACAGCGATTGTTAAACCGAGATTGGCAATTTGGACAACGCTCTACCATCATATATTCATT
GATTGTTAATTCGTGTTTGCATACACCGCATAAGATTGCTTTTTCGTTAAATGAAGGCTCAGACCAACGCTTAATGGCGTGCTTTTCAAACTC
ATTATGGCACTTATAGCATGGATAGTATTTATTACAACATTTAAATTTAATAGCAATAATATCTTCTTCGGTAAAATAATGGCGACAGCGTGT
TTCAGTATCGATTAATGAACCATAAACTTTAGGCAT

Gene: murB (UDP-N-acetylenolpyruvylglucosamine reductase)

Position: 788189 to 789112, length: 924 nt, orientation: REVERSE

Perfect match to: (ED133-CP001996-[802255:803178:r], highly conserved allele)

Sequence:

TTACGATTCCTTTGGATGTTCACCAATAATGCGAACTTCACGATTTAATTCAATGCCAAATTTTTCTTTGACGGTCTTTTGTACATAATGAATA
AGGTTTTCATAATCTGTAGCAGTTCCATTGTCTACATTTACCATAAAACCAGCGTGTTTAGTTGAAACTTCAACGCCGCCAATACGGTGACCT
TGCAAATTAGAATCTTGTATCAATTTACCTGCAAAATGACCAGGCGGTCTTTGGAATACACTACCACATGAAGGATACTCTAAAGGTTGTTT
AGATTCTCTACGTTCTGTTAAATCATCCATTTTAGCTTGTATTTCAGTCATTTTACCAGGAGCTAAAGTAAATGCAGCTTCTAATACAACTAAG
TGTTCTTTTTGAATAATGCTATTACGATAATCTAACTCTAATTCTTTTGTTGTAAGTTTAATTAACGAGCCTTGTTCGTTTACGCAAAGCGCAT
AGTCTATACAATCTTTAACTTCGCCACCATAAGCGCCAGCATTCATATACACTGCACCACCGATTGAACCTGGAATACCACATGCAAATTCAA

GGCCAGTAAGTGCGTAATCACGAGCAACACGTGAGACATCAATAATTGCAGCGCCGCTACCGGCTATTATCGCATCATCAGATACTTCGAT
ATGATCTAGTGATAATAAACTAATTACAATGCCACGAATACCACCTTCACGGATAATAATATTTGAGCCATTTCCTAAATATGTAACAGGAA
TCTCATTTTGATAGGCATATTTAACAACTGCTTGTACTTCTTCATTTTTAGTAGGGGTAATATAAAAGTCGGCATTACCACCTGTTTTAGTATA
AGTGTATCGTTTTAAAGGTTCATCAACTTTAATTTTTTCATTTGGGATAAGTTGTTGTAAAGCTTGATAGATGTCTTTATTTATCAC

Gene: grpB (putative nucleotidyltransferase)

Position: 789239 to 789757, length: 519 nt, orientation: REVERSE

Perfect match to: (MW2-BA000033-[788282:788800:r], allele observed in CC1+CC80)

Sequence:

TTATAAGTATTTTTTACATAGTTTTTCAAAGTATTGTTGCTTTTGCATCTCATATTGTCTAATTGTTAAGCTATGTTGCAATATTTGGTGCTTTT
TTGTATGGAATTGCAAAGCAATATCATCATTAGTTGATAAGAGGTAATCAAGTGCAAGATAAGATTCAAATGTTTGGGTATTCATTTGAATG
ATATGTAGACGCACCTGTTGTTTTAGTTCATGAAAATTGTTAAACTTCGCCATCATAACTTTCTTATTATATTTATGATGCAAGCGATAAAACC
CTACATAATTTAAGCGTTTTTCATCTAAGGATGTAATATCATGCAAATTTTCTACACCTACTAAAATATCTAAAATTGGCTCTGTTGAATATTT
AAAATGATGCGTACCGCCAATATGTTTTGTATATTTTACTGGGCTGTCTAAGAGGTTGAATAATAATGATTCAATTTCAGTGTATTGTGATTG
AAAACAATTAGTTAAATCACTATTAATGAATGGTTGAACATTTGAATACAT

Gene: Q5HHT0 (putative lipoprotein)

Position: 789877 to 790755, length: 879 nt, orientation: FORWARD

Perfect match to: (MRSA252-BX571856-[831583:832461], highly conserved allele)

Sequence:

ATGAAAAAAATTGTTATTATCGCTGTTTTAGCGATTTTATTTGTAGTAATAAGTGCTTGTGGTAATAAAGAAAAAGAGGCACAACATCAATT
TACTAAGCAATTTAAAGATGTTGAGCAAAAACAAAAAGAATTACAACATGTCATGGATAATATACATTTGAAAGAAATTGATCATCTAAGTA
AAACTGATACAACTGATAAAAATAGTAAAGAATTTAAGGCACTACAAGAAGATGTTAAAAACCATCTCATACCTAAATTTGAAGCATATTAT
AAGTCAGCAAAAAAATTTGCCTGATGATACAATGAAAGTTAAGAAATTAAAAAAAGAATATATGACGCTTGCAAATGAGAAGAAGGATGCG
ATATATCAATTAAAAAAATTCATAGGTTTATGTAATCAATCTATCAAGTATAACGAAGACATTTTAGATTATACGAAACAATTTGAAAAAAAT
AGATACAAAGTTGAATCAGAAATTAAATTAGCTGATAATAAAAGTGAAGCAACTAATCTTACGACAAAATTAGAACATAATAATAAAGCGT
TAAGAGATACTGCGAAGAAGAACCTAGATGATAGTAAAGAAAATGAAGTAAAAGGCGCGATTAAAAATCACATTATGCCAATGATTGAAA
AGCAAATTACCGATATTAACCAAACTAATATTAGTGATAAGCATGTTAATAATGCAAGGAAAAACGCAATAGAAATGTATTACAGTCTGCA
GAACTATTATAATACACGTATTGAAACAATAAAGGTTAGTGAGAAGTTATCAAAAGTCGATGTAGATAAGTTGCCGAAAAAGGGTATAGAT
ATAACTCACGGCGATAAAGCCTTTGAAAAAAAGCTTGAAAAATTAGAAGAAAAATAA

Gene: ytxJ (thioredoxin-like protein)

Position: 790909 to 791229, length: 321 nt, orientation: FORWARD

Perfect match to: (RF122-AJ938182-[759206:759526], highly conserved allele)

Sequence:

GTGGCTATAAAGCTAAGTTCAATTGACCAATTTGAACAGGTTATTGAGGAAAATAAATATGTTTTTGTATTAAAACATAGTGAAACTTGTCC
AATATCGGCAAATGCGTACGATCAATTTAATAAATTTTTATATGAACGCGATATGGACGGTTATTATTTGATTGTCCAACAAGAACGCGATT
TGTCAGATTATATTGCTAAAAAAACGAACGTTAAACATGAATCACCTCAAGCATTTTATTTTGTAAATGGTGAAATGGTTTGGAATCGAGAC
CACGGTGATATCAATGTGTCGTCATTAGCACAAGCAGAAGAATAA

Gene: glxK2 (glycerate kinase, locus 2)

Position: 791654 to 792778, length: 1125 nt, orientation: FORWARD

Perfect match to: (11819-97-CP003194-[835573:836697], allele observed in CC80+CC15+CC30+CC72)

Sequence:

ATGAAAGTATTAGTAGCCATGGATGAGTTTCATGGAATTATTTCGAGTTATCAAGCTAATAGATATGTTGAAGAGGCAGTTGCAAGCCAAA
TTGAAACTGCAGATGTAGTTCAAGTACCATTGTTTAATGGAAGACATGAATTATTAGATTCTGTATTTTTATGGCAATCTGGGCAAAAGTAT
CGTATACCAGTACATGATGCAGATATGAATGAAGTTGAAGGTGTTTACGGACAAACTGATACAGGGATGACCGTTATCGAGGGGAATTTA
TTTTTAAAAGGTAAAAAACCAATTGTTGAACGAACAAGTTATGGTTTAGGAGAAATGATTAAACATGCATTAGATAATGACGCAAAACATG
TTGTAATTTCACTAGGTGGGATTGATAGTTTTGATGCTGGTGCAGGTATGTTACAAGCATTAGGTGCTCAATTCTATGATGACGAAGGGCG
TGTCGTAGATATGAGACAAGGTGCTGGTGTAATTAAATATATTCGTCGTATGGATATGTCGAACTTACACCCTAAAATGGAAACAGCAAGA
ATTCAAGTAATGTCGGATTTTTCAAGTCGATTATATGGTAAGCAAAGTGAAATCATGCAAACTTATGATGCGCATCAGTTGAATCATAATCA
AGCAGCAGAAATCGATAATTTAATTTGGTATTTTAGTGAGTTATTTAAAAGTGAATTGAAAATTGCAATTGGTCCAGTTGAACGTGGTGGTG
CTGGTGGTGGAATTGCAGCAGTCTTGAATGGACTGTATAAAGCTGAAATATTAACCAGTCATGCATTAGTAGACCAACTAACACATTTAGA
AAATTTAGTTGAACAAGCGGATTTAATTATTTTTGGAGAAGGATTAAATGAAAATGATCAGTTGCTAGAAACGACAACATTGCGTATTGCA
GAACTTTGTCATAAACATCAAAAGGTTGCCATTGCAATTTGTGCAACTGCTGAAAAGTTTGATTTATTTGAATCACAAGGGGTTACAGCAAT
GTTTAATACATTTATCGATATGCCAGAAACTTATACTGACTTTAAAATGGGATTACAAATTAGGCATTATACGGTTCAGTCTTTAAAACTGTT
GAAAACACATTTTAATGTTGAGGTTTAG

Gene: pepT (peptidase T)

Position: 792960 to 794186, length: 1227 nt, orientation: REVERSE

Perfect match to: (CN1-CP003979-[770193:771419:r], highly conserved allele)

Sequence:

TTAGTGATTTTCAGCGATATCTTCTACAATTCCAATGATTACTTGTACTGCTTTTTCCATAACATCAATGGATGCATATTCATATGGGCCGTGG
AAGTTACCGCAACCTGTAAAGATGTTTGGAGTTGGTAACCCCATAAATGACAATTGTGAACCATCTGTACCACCGCGAATAGGTTCAGTGTT
TGCTGGAATATCTAATTTGGCAAAGACACGTTTAGGTATATCAATAATATGAGGCAATGGTAATATTTTTTCTGCCATATTGAAATATTGATC
CGATATATCAACTTTAACTGGATAATTTTCAAAATGGGCATTGATATCGTCACGTATTTCTAAAATACGTTTCTTACGCAATTCGAATTGTTTT
TTATCATGATCACGAATAATGTATTGCAAAGTTGCTTTTTCAACAGTTCCTTCAAAGTTCATTAAGTGATAAAAGCCTTCGTATCCTTCTGTTC
GCTCCGGAACTTCACTATCAGGTAGCAAACTATCGAATTGTTCACCTAAACGTATTGCGTTTACCATTGCATTTTTAGCTGAACCAGGATGAA
CATTTACACCGTGGCATGTAATAACCGCTTCAGCAGCGTTAAAGCTTTCATATTGTAATTCTCCATATTGACTACCATCCATAGTATAAGCAA
AATCAGCATTGAAGCGGTCAACATCAAATTTATGTGGACCACGACCGATTTCTTCGTCTGGTGTAAATCCAATGCGAATGGTACCATGTTTA
ATTTCTGGATGTTCTTGTAAATAACAAATAGCTTCCATAATTTCCACAATACCCGCTTTATCGTCTGCACCTAGTAACGATGTACCATCAGTTA
CCATTAATGTATGACCAACTAAACTGTTAAGTTCTGGAAATACTTTAGGATCTAAGACACGTTTAGTATTGCCTAGTTTGTATGGCTTACCAT
CATAGTTTTCAATAATTTGCGGTTTAACATTTGAAGCATTGAAATCAGGTGATGTATCAACATGCGCCAAAAATCCAACTGTTGGGACGTCG
ACATCGATGTTACTTTCTAATGTAGCAAATAAGTAGCCATTTTCATCTAAATCAGTTGGCAATCCTAATTGTTGTAATTCTTTTTCTAATAAAT
GTAACAAATCCCATTGCTTTTCAGTTGAAGGTGTTGTTGTAGATTTTGGATCAGATTGCGTATCAATTGTCGTATATCTTGTTAATCTATCTAT
CAATTGGTTCTTCAT

Gene: Q5HHS6 (putative membrane protein)

Position: 794200 to 794694, length: 495 nt, orientation: REVERSE

Perfect match to: (11819-97-CP003194-[838119:838613:r], highly conserved allele)

Sequence:

TTAAACTCTATTATTCATGTTGTAAGATTTTTTATATGTCTTACCTTTGATTTTACCATACAGTTGTTTGATACGTGTGTATAGGTAATATAGA
ATTTCAGAAACTAATATACCGAAAGCAATCGCACCTGAAATCAGTGTAACTTCTAAAAATGTATTTACAGCACTTGTATAATCATTTGATACT
AAAAAACGAGTCGCTTGATAAGCTGCACCACCAGGTACTAATGGTATAATGCCTGGCACTATGAATATAATTACCGGTCGTTTATATCTGCG
ACTCATAGTATGACTCATTAAGCCTAAAATTAAGCTTCCCAAAAATGAAGCGCCAACTTTTCCAAACTCTAAATCTACCGTTAATTGGTAAAT
CGTCCATGCAATGGCACCCACAAATCCACATGCTACTAAGAGGCGTTTGGGTGCATTGAAAATGATAGAGAAAAGTACTGTTGATATAAAG
CTGATTGTAAAATGAAATAAATAAAATAGCAT

Gene: DUF1212_L2 (protein of unknown function DUF1212, locus 2)

Position: 794712 to 795473, length: 762 nt, orientation: REVERSE

Perfect match to: (11819-97-CP003194-[838631:839392:r], highly conserved allele)

Sequence:

TTAAATGATTAATAAAACGATTGCGACACCAGCACCGATTGCGAATGCTGTTAATGCAGCTTCAACACCACGAGACATACCTGCAAGTAATT
CACCCGCTAATAAATCTCGAATGGCATTGGTAATTAATATACCAGGGACAAGTGGCATGACACTGGCTATAGTAATGATATCTTGATTGGTT
GCAATGCCTAATTTAGTAAATGTGGCTGCAATGGATATGACCACAGCGGCTGCAACAAACTCTGAGAAAAATTTAATTTGTATATAGCGTT
GCACAAAGCTGAATGTTAAAAATGCGGATCCGCCAGCAATGACTGCAATCCAACAATCTGATGCGACACCACCAAACATAAATAGGAAGA
AGCCACATGCAATGGCAGCTGCAAAGAAATTCGTTAAAAAAGAATATTGTAATGATGCATGCTGTAAATGAATAAATTCAGATTTAGCTTC
ATCAATTGTGAGTTCTTTATTTGATATTTTACGTGAAAGACTATTCGTTAAAGCAATTTTCTCTAAATCTGTTGTACGCTCTTGTACACGAATT
AATCTTGTACTTGTTCGATCATTTAATGAAAAAATAATTGCAGTTGAACTGACAAAACTATATGTATTATGAAGACCATAACTATGTGCGATA
CGGTTCATTGTATCTTCAACTCGATATGTTTCAGCACCTGATTCAAGTAAAATTCTACCTGCAATTAATACAACATCAATCACTTTGTTTTCAT
CTATAATTGTGATTGAATCTGGCAT

Gene: gdpS (membrane bound diguanylate cyclase GGDEF domain-containing protein)

Position: 795669 to 796739, length: 1071 nt, orientation: REVERSE

Perfect match to: (NN50-BAEA01000007-[46859:47929], allele observed in CC4803+CC8+CC80+CC188)

Sequence:

TTATAAATTGATAATAGGGTTAAACATTACTTTGTTTCGCCCTTGATTTTTGGCTACATGCACCATATCGTCTGCATCTTTAAACACTTTACGC
TGTGATTTTGGATCGTCATCTGTTAAATAACCAACACCGATAGACACTGACAATTTAATAACTTCTTTGTTTGGTAAATGGAATGATGATTTT
TCAACACCCGAACGAATATTTTCAGCTAATTTAACACTTTGATCAAGTGAATAATTGTGAATGACAACTGAGAACTCTTCGCCACCATTTCTA
AAAATTTTAAATTGATTCGGCACATAGTTTTTAAGTAATTGAGACATTTGTTTTAATACAGCATCACCTGATTTGTGTGAGTAGGTATCATTG
ACATCTTTAAATCCATCGATATCGATTAATAATAAAGCGATACTTTGATGTTCTTTTTCAGCTTTTCGTGAAATTTCATTTAAATGTCTATCAA
ATTCTTTTACATTACCTAAGCCTGTTAAGTAATCATATTTATCTTCGTTTTCATAACGATTTACGAGTGAGAAGAAATGCCAAATATCGACAA
ATGTTATCGCTGAAGCTAAAGTGATAATTAATGAAATTGGTATTAAAATGATAACTTCCGATAGTGTGTAAATAGGACTCACTAACGCGACA
CCAAATAAAATGATTATTGTAACAACATTAAGTATTAATAATGATAGCACATCATTTTGTTTTAAAAATGGTCCAATAGCACTTGTTACTGCA
GCAATAACAATCAACGTAACACCGTACATAATCGAGTTGTTAAATACTACAATTTCAACAATTGCTACAATTACTGTGGCAGATAATGTATA
GACCATATTTGTAAATCTACCTAAAAACAATAAAGGAACGAATGTTAAGTGAATTAAATAATCTTCACGATAAGGGATAGGGTAGACAGAT
AATAATAATGATACGATTGTCATTAAAACAGTGACATAAGCCTTAGAAAAAACCATACGTTTGTTTTCTGAATACTGTAAGCGATGGAATAA
ATAGATTCCAGCGACTATAACAGATATATTGTATATAAATGCTTCGAACAT

Gene: tarO (N-acetylglucosamine-1-phosphate transferase)

Position: 797057 to 798112, length: 1056 nt, orientation: FORWARD

Perfect match to: (CA347-CP006044-[810751:811806], highly conserved allele)

Sequence:

ATGGTTACATTATTACTAGTTGCAGTAACAATGATTGTCAGTTTGACGATAACACCAATTGTTATTGCAATATCGAAAAGATTAAATTTAGTT
GATAAACCAAATTTTAGAAAAGTACACACTAAACCTATTTCAGTTATGGGTGGTACAGTGATTCTCTTTTCATTTTTAATAGGTATTTGGATT
GGTCATCCTATTGAAACAGAAATCAAACCACTTATTATTGGTGCGATTATTATGTACGTACTTGGGCTTGTAGATGATATCTACGATTTGAAA
CCGTATATAAAATTGGCTGGTCAAATTGCCGCTGCCTTAGTAGTTGCTTTTTATGGTGTGACTATTGATTTTATTTCGTTGCCAATGGGTACA
ACGATTCATTTTGGATTTCTTAGTATTCCAATTACTGTGATTTGGATTGTTGCTATTACAAATGCAATTAACTTAATTGATGGACTCGATGGTT
TGGCGTCGGGTGTTTCTGCAATCGGACTCATTACAATAGGATTCATTGCAATTTTACAAGCTAATATTTTCATAACGATGATTTGTTGTGTTT
TATTAGGCTCTTTAATTGGGTTTTTATTTTACAATTTCCATCCTGCCAAAATATTTTTAGGTGATAGTGGGGCTTTAATGATTGGATTTATCAT
CGGATTCCTTTCTTTACTCGGATTCAAAAATATTACAATTATTGCATTGTTCTTCCCAATTGTTATCTTAGCAGTTCCATTCATTGATACTTTGT
TCGCAATGATTCGACGTGTGAAAAAAGGGCAGCATATAATGCAAGCTGATAAATCGCATTTGCATCATAAACTATTAGCTTTAGGCTACAC
ACATAGACAAACAGTATTATTAATCTATTCAATCTCTATTTTATTTAGTCTTTCGAGCATTATTTTGTATGTATCGCCACCATTAGGTGTTGTAT
TAATGTTTGTATTAATCATATTTAGTATTGAATTAATTGTTGAATTTACAGGATTAATAGATAACAACTACCGACCAATATTAAATTTAATTAG
TCGGAAATCGTCACACAAAGAGGAATAG

Gene: Q5HHS2 (putative protein)

Position: 798284 to 798925, length: 642 nt, orientation: REVERSE

Perfect match to: (11819-97-CP003194-[842203:842844:r], allele observed in CC80)

Sequence:

TTATTCAGTTGGTATATCGAAAGGTAACTGCTTTGGAGTTTCTTCAGTCAAATCGAAATTTCCTGCAGTCATTTGATTTAAAAAGTTAATAAA
CGCTTCATAGTCACTTTTAACGACATCGATATAGTAGCTTACCTTATCAGTGTAAGTTTGGTTTCTTAACATAAAATGAGTTGAAGCTAATTC
ATATTCAAATTTACCAGTTTGATCATAATTCAGTGTTACTATACATGGTACTGCTTCTCGTAGTTCGACACGTCCGACATCATAAATGACGTC
TCTAACAGCACCGCTATAGGCGCGAATTAAACCGCCACCACCTAATTTAATACCACCAAAATATCTTGTTACTACGACACACGCATTATGAA
CATCGAGCTTTTTTAATATGTCTAACATTGGGACACCGGCAGTTCCTGTCGGTTCACCATCATCATTCGCTTTTTGAATATTCATTTCAGGTCC
AATAGTATATGCAGAACAATTATGAGTGGCATCTTTATGTTCTTTTTTTATTGCAGCAATAAATGCTTTAGCTTCATCTTCATTTTGAACAGGC
TTGATGTGAGCAATGAATCTTGATTTACTAATCACATTTTCAATAATGTGTTCTTTTTTAACAGTAATGATATTTTGTGTCAT

Gene: degV1 (DegV domain-containing fatty-acid-binding protein)

Position: 799069 to 799935, length: 867 nt, orientation: FORWARD

Perfect match to: (11819-97-CP003194-[842988:843854], allele observed in CC80)

Sequence:

ATGAAAATTGCTGTGATGACCGATTCTACAAGTTATCTTTCGCAGGACTTAATCGATAAATATAATATTCAAATAGCGCCATTAAGTGTGAC
TTTTGATGATGGGAAGAACTTTACAGAAAGTAATGAAATAGCAATTGAAGAATTTTATAATAAAATGGCATCGTCTCAAACGATTCCAACA
ACAAGCCAACCAGCAATTGGCGAATGGATTACTAAATATGAAATGCTAAGAGATCAAGGTTACACAGATATCATTGTCATTTGCTTATCAA
GTGGAATTAGTGGAAGTTATCAATCTAGTTATCAAGCAGGGGAAATGGTTGAAGGTGTTAATGTACATGCATTTGATAGTAAGCTTGCAGC
AATGATTGAAGGATGCTATGTATTACGTGCTATTGAAATGGTTGAAGAAGGATACGAACCACAGCAAATTATTGATGATTTAACTAATATG
CGTGAACACACAGGTGCATATTTAATTGTTGATGACTTAAAGAATTTACAAAAAAGTGGTCGAATTACTGGTGCTCAAGCATGGGTTGGAA
CATTATTGAAAATGAAGCCAGTTCTTAAGTTTGAAGATGGCAAGATTATACCAGAAGAAAAAGTTCGTACTAAAAAGCGTGCCATTCAAAC
ATTAGAAAAGAAAGTATTAGATATTGTAAAAGACTTTGAAGAAGTAACTTTATTTGTCATAAATGGAGATCATTTCGAAGATGGTCAAGCG
TTATACAAAAAGTTACAAGATGATTGTCCTTCAGCTTATCAAGTAGCATACTCTGAGTTTGGTCCAGTTGTTGCAGCACATTTAGGTTCTGGT
GGATTAGGTTTAGGCTATGTTGGCAGAAAAATAAGATTAACATAA

Gene: comFA (type II DNA/RNA helicase)

Position: 800299 to 801381, length: 1083 nt, orientation: FORWARD

Perfect match to: (Strain_21305-AFNO01000011-[18885:19967:r], allele observed in CC25+CC80)

Sequence:

ATGGATAATGTAACAAGATATAAAATAACAGAGAGTTCGCAAAGTTCATCACAAGCATATTATCATCTCTCATTTGCATTGTCGGAACAGCA
GTCTTATGCCTCAGAACATATTGTTCGAGCCATTAGAAAGAGACAAACGATTTTGTTATATGCCGTAACAGGTGCAGGTAAGACAGAAATG
ATGTTTCAAGGCATTCAATATGCAAGAATACAGGGAGATAATATAGCTATTGTGTCACCACGTGTAGATGTTGTTGTAGAAATTAGTAAAC
GTATTAAAGACGCATTTCTTAATGAAGATATAGACATACTACACCAGCAATCAAGACAACAATTTGAAGGGCATTTTGTTGTATGCACAGTG
CATCAACTTTACCGATTCAAACAGCACTTTGATACTATTTTTATTGATGAAGTCGATGCCTTTCCTATATCAATGGATAAAAATCTACAACAA
GCATTGAAGTCATCTTCTAAAGTTGAACATGCAACAATTTATATGACAGCAACACCACCGAAACAACTTCTGTCAGAGATTCCCCACGAAAA
TATAATTAAATTGCCAGCTCGCTTTCATAAAAAATCACTTCCAGTTCCTAAATATTGCTATTTCAAACTTAATAATAATAAGATTCAGAAAATG
TTATACCGAATTTTACAAGATCAAATTAATAATCAACGTTATACACTGGTGTTTTTTAACAATATAGAAACAATGATTAAAACATTTTCGGTTT
ATAAGCAGAAAATTACTAAATTAACATACGTCCATAGCGAGGATGTTTTTCGCTTTGAAAAAGTTGAACAATTAAGGAATGGACATTTCGAT
GTCATTTTTACTACGACAATATTAGAACGTGGATTTACAATGGCAAATTTGGATGTTGTTGTTATCGATGCACATCAATATACTCAAGAGGCT
TTAATACAAATTGCTGGACGTGTTGGACGAAAATTAGAATGTCCTACTGGAAAAGTATTGTTTTTCCATGAAGGAGTAAGTATGAATATGAT
TCAAGCTAAAAAAGAGATTCAAAAGATGAACAAATTAGCATTAAAAAGAGGTTGGATTGATGAATAA

Gene: comFC (type II DNA/RNA helicase)

Position: 801371 to 802048, length: 678 nt, orientation: FORWARD

Perfect match to: (CA347-CP006044-[815066:815743], allele observed in CC45I+CC25+CC80+CC2249)

Sequence:

TTGATGAATAATTGTTTGAGTTGTGGTGCTAAGTTATATGAAAATATAACCATTTATAATTTGTTCAAGAAACCTAATAGATTATGTGACAGA
TGCAAAGAGAATTGGGACAATATTAAACTTGATATTAAAGCAAGGCGATGTTCAAGGTGCTTAAAACACTTAAATCAAAATGAAGCGTATT
GTTTAGACTGCAAGTTTCTATCGGCACACTTTAATTTAATGGAACAATTATATTGTCAATTTCAATATGACGGTTTAATGAAAGAGATGATAC
ATCAGTATAAATTTTTGAAAGACTATTATTTATGTGAATTATTGGCACATTTGATTGAAATACCACAAACATCTTATGACTATATTGTGCCAAT

TCCTTCTTCGCCGGCACATGATTTATCTAGAACATTTAACCCGGTAGAAGCAGTACTAAAAGCTAAAGGGATTCGCTTTGATAAGATTTTAA
AGATGTCAAATAGACCAAAACAGTCTCATTTAACTAAGAAAGAGCGTCTGGCAGATGAAAATCCATTTATTATTGATACGGAATTAGATTTA
AATGGCAAGGAAATATTACTCGTTGACGATATTTATACAACTGGATTAACAATTCATCGTGCAGGGTGTAAATTATATGCTAAAAATATCAG
AAAATTCAAAGTGTTTGCGTTTGCACGATAG

Gene: yfiA (sigma-54 modulation protein)

Position: 802109 to 802681, length: 573 nt, orientation: FORWARD

Perfect match to: (N315-BA000018-[805425:805997], highly conserved allele)

Sequence:

ATGATTAGATTTGAAATTCATGGAGATAACCTCACTATCACAGATGCTATTCGCAACTATATTGAGGAAAAAATTGGTAAGTTGGAACGTTA
TTTTAATGACGTACCAAATGCAGTGGCGCATGTTAAAGTTAAAACTTATTCAAATTCAGCTACTAAAATTGAAGTAACAATTCCATTGAAAA
ATGTTACGTTAAGAGCTGAAGAGCGAAACGATGATTTATACGCAGGTATTGATTTAATTAATAATAAACTTGAAAGACAAGTTCGAAAATA
TAAAACACGTATTAATCGTAAGAGCCGTGATCGAGGAGATCAAGAAGTGTTTGTTGCCGAATTACAAGAAATGCAAGAAACACAAGTTGA
TAATGACGCTTACGATGATAACGAGATAGAAATTATTCGTTCAAAAGAATTCAGCTTAAAACCAATGGATTCAGAAGAAGCGGTATTACAA
ATGAATCTATTAGGTCATGACTTCTTTGTATTCACAGACAGAGAAACTGATGGAACAAGTATCGTTTACCGCCGTAAAGACGGTAAATATG
GCTTGATTCAAACTAGTGAACAATAA

Gene: secA1 (preprotein translocase subunit A)

Position: 803095 to 805626, length: 2532 nt, orientation: FORWARD

Perfect match to: (NN50-BAEA01000007-[37974:40505:r], allele observed in CC4803+CC5+CC8+CC22)

Sequence:

ATGGGATTTTTATCAAAAATTCTTGATGGCAATAATAAAGAAATTAAACAGTTAGGTAAACTTGCTGATAAAGTAATCGCTTTAGAAGAAAA
AACGGCAATTTTAACTGATGAAGAAATTCGTAATAAAACGAAACAATTCCAAACAGAATTAGCTGACATTGATAATGTCAAAAAGCAAAAT
GATTATTTAGATAAAATTTTACCAGAAGCATATGCACTTGTTAGAGAAGGCTCTAAACGTGTATTCAATATGACACCATATAAAGTTCAAAT
TATGGGTGGTATTGCAATTCATAAAGGTGATATCGCTGAGATGAGAACAGGTGAAGGTAAAACATTAACAGCGACAATGCCAACATACTT
AAATGCATTAGCTGGTAGAGGTGTTCACGTTATTACAGTCAATGAATACTTATCAAGTGTTCAAAGTGAAGAAATGGCTGAGTTATATAACT
TCTTAGGTTTGACTGTCGGATTAAACTTAAACAGTAAGACGACAGAAGAAAAACGTGAAGCATACGCACAAGACATTACTTACAGTACTAA
TAATGAGCTAGGTTTTGATTACTTACGAGATAACATGGTGAATTATTCTGAAGATAGAGTAATGCGTCCATTACATTTTGCAATCATTGATG
AGGTTGACTCAATTTTAATCGACGAGGCACGTACGCCATTAATTATTTCTGGTGAAGCTGAAAAGTCAACGTCACTTTATACACAAGCAAAT
GTTTTTGCGAAAATGTTAAAACAGGACGAAGATTATAAATACGATGAAAAAACGAAAGCTGTACATTTAACAGAACAAGGTGCGGATAAA
GCAGAACGTATGTTCAAAGTTGAAAACTTATATGATGTACAAAATGTTGATGTTATTAGTCATATCAACACAGCTTTACGTGCGCACGTTAC
ATTACAACGTGACGTAGACTATATGGTTGTTGATGGCGAAGTATTAATTGTCGATCAATTTACAGGACGTACAATGCCAGGCCGTCGTTTCT
CGGAAGGTTTACACCAAGCTATTGAAGCGAAGGAAGGCGTTCAAATTCAAAATGAATCTAAAACTATGGCGTCTATTACATTCCAAAACTA
TTTCAGAATGTACAATAAACTTGCGGGTATGACAGGTACAGCTAAAACTGAAGAAGAAGAATTTAGAAATATTTATAACATGACAGTAACT
CAAATTCCGACAAATAAACCTGTGCAACGTAACGATAAGTCTGATTTAATTTACATTAGCCAAAAAGGTAAATTTGATGCAGTAGTAGAAG
ATGTTGTTGAAAAACACAAGGCAGGGCAACCAGTGCTATTAGGTACTGTTGCAGTTGAGACTTCTGAATATATTTCAAATTTACTTAAAAAA
CGTGGTATCCGTCATGATGTGTTAAATGCGAAAAATCATGAACGTGAAGCTGAAATTGTTGCAGGCGCTGGACAAAAAGGTGCCGTTACTA
TTGCCACTAACATGGCTGGTCGTGGTACAGATATCAAATTAGGTGAAGGCGTAGAGGAATTAGGCGGTTTAGCAGTAATAGGTACAGAAC
GACATGAATCTCGTCGTATTGATGACCAGTTACGTGGTCGTTCTGGACGTCAAGGTGATAAAGGGGATAGTCGCTTCTATTTATCATTACAA
GATGAATTAATGATTCGTTTTGGTTCTGAACGTTTACAGAAAATGATGAGCCGACTAGGTTTAGATGACTCTACACCAATTGAATCAAAAAT
GGTATCAAGAGCTGTAGAATCAGCACAAAAACGTGTAGAAGGTAATAACTTCGACGCGCGTAAACGTATCTTAGAATACGATGAAGTATT
ACGTAAACAACGTGAAATTATCTATAACGAAAGAAATAGTATTATTGATGAAGAAGACAGCTCTCAAGTTGTAGATGCAATGCTACGTTCA
ACGTTACAACGTAGTATCAATTACTATATTAATACAGCAGATGACGAGCCTGAATATCAACCATTCATCGACTACATTAATGACATCTTCTTA
CAAGAAGGTGACATTACAGAGGATGATATCAAAGGTAAAGATGCTGAAGATATTTTCGAAGTCGTTTGGGCTAAGATTGAAGCAGCATAT
CAAAGTCAAAAAGATATCTTAGAAGAACAAATGAATGAGTTTGAGCGTATGATTTTACTTCGTTCTATTGATAGCCATTGGACTGATCATAT
CGACACAATGGATCAATTACGTCAAGGTATTCACTTACGTTCTTATGCACAGCAAAATCCATTACGTGACTATCAAAATGAAGGTCATGAAT
TATTTGATATCATGATGCAAAATATTGAAGAAGATACTTGTAAATTCATTTTAAAATCTGTAGTACAAGTTGAAGATAATATTGAACGTGAA
AAAACAACAGAGTTTGGTGAAGCGAAGCACGTTTCAGCTGAAGATGGTAAAGAAAAAGTGAAACCGAAACCAATCGTTAAAGGCGATCA
AGTTGGTCGTAACGATGATTGTCCATGTGGTAGTGGTAAAAAATTCAAAAATTGCCATGGAAAATAA

Gene: prfB (peptide chain release factor 2, programmed frameshift)

Position: 805941 to 807051, length: 1111 nt, orientation: FORWARD

Perfect match to: (MW2-BA000033-[804984:806094], allele observed in CC1+CC22+CC80+CC239+CC4803)

Sequence:

ATGGAATTATCAGAAATCAAACGAAATATAGATAAGTATAATCAAGATTTAACACAAATTAGGGGGTCTCTTTGACTTAGAGAACAAAGAA
ACTAATATTCAAGAATATGAAGAAATGATGGCAGAACCTAATTTTTGGGATAACCAAACGAAAGCGCAAGATATTATAGATAAAAATAATG
CGTTAAAAGCAATAGTTAATGGTTATAAAACACTACAAGCAGAAGTAGATGACATGGATGCTACTTGGGATTTATTACAAGAAGAATTTGA
TGAAGAAATGAAAGAAGACTTAGAGCAAGAGGTCATTAATTTTAAGGCTAAAGTGGATGAATACGAATTGCAATTATTATTAGATGGGCCT
CACGATGCCAATAACGCAATTCTAGAGTTACATCCTGGTGCAGGTGGCACGGAGTCTCAAGATTGGGCTAATATGCTATTTAGAATGTATC
AACGTTATTGTGAGAAGAAAGGCTTTAAAGTTGAAACTGTTGATTATCTACCTGGGGATGAAGCAGGGATTAAAAGTGTAACATTGCTCAT
CAAAGGGCATAATGCTTATGGTTATTTAAAAGCTGAAAAAGGTGTACACCGACTAGTACGAATTTCTCCATTTGATTCATCAGGACGTCGTC
ATACATCATTTGCATCATGCGACGTTATTCCAGATTTTAATAATGATGAAATAGAGATTGAAATCAATCCGGATGATATTACAGTTGATACAT
TCAGAGCTTCTGGTGCAGGTGGTCAGCATATTAACAAAACTGAATCGGCAATACGAATTACCCACCACCCCTCAGGTATAGTTGTTAATAAC
CAAAATGAACGTTCTCAAATTAAAAACCGTGAAGCAGCTATGAAAATGTTAAAGTCTAAATTATATCAATTAAAATTGGAAGAGCAGGCAC
GTGAAATGGCTGAAATTCGTGGCGAACAAAAAGAAATCGGCTGGGGAAGCCAAATTAGATCATATGTTTTCCATCCATACTCAATGGTGAA
AGATCATCGTACGAACGAAGAAACAGGTAAGGTTGATGCAGTGATGGATGGAGACATTGGACCATTTATCGAATCATATTTAAGACAGAC
AATGTCGCACGATTAA

Gene: lysM (LysM domain protein)

Position: 807460 to 808299, length: 840 nt, orientation: FORWARD

Perfect match to: (MW2-BA000033-[806503:807342], highly conserved allele)

Sequence:

ATGAAAAAAACTCTTACAGTGACGGTTTCGTCAGTGTTAGCTTTTTTAGCTTTAAATAATGCAGCACATGCACAACAACATGGCACACAAGT
AAAAACACCTGTTCAACATAATTATGTATCAAATGTTCAAGCACAAACGCAATCACCGACAACTTATACAGTAGTTGCTGGCGATTCATTAT
ATAAGATTGCATTAGAGCATCACTTAACGTTGAATCAATTATATTCATACAATCCTGGTGTAACACCTTTAATTTTTCCTGGTGACGTGATTTC
ACTTGTGCCTCAAAATAAAGTGAAACAAACTAAAGCGGTTAAAACACCAGTAAGAAAAGCAAGCCAAGCTAAAAAGGTAGTAAAACAACC
TGTACAACAAGCGTCTAAAAAAGTAGTAGTTAAGCAAGCACCTAAGCAAGCAGTAGCTAAGACAGTTAATGTAGCATACAAACCTGCTCAA
GTACAAAAATCAGTACCAACTGTACCTGTTGCACATAACTACAATAAATCAGTTGCTAACAGAGGGAACTTATATGCTTATGGAAACTGCAC
ATATTATGCTTTCGATCGTCGTGCACAATTAGGTAGAAGTATAGGAAGTTTATGGGGCAATGCAAATAACTGGAATTACGCAGCAAAAGTT
GCAGGATTTAAAGTAGATAAAACACCAGAAGTTGGCGCTATTTTCCAAACAGCTGCTGGCCCATATGGACATGTTGGTGTTGTTGAATCTG
TAAACCCTAATGGAACAATTACTGTTTCTGAAATGAACTATGCTGGATTTAATGTTAAATCTTCAAGAACAATTTTAAATCCAGGAAAATATA
ATTACATCCACTAA

Gene: yfbR (nucleotidase)

Position: 808473 to 809123, length: 651 nt, orientation: FORWARD

Perfect match to: (M0239-AIWE01000001-[258412:259062], highly conserved allele)

Sequence:

ATGGGTGTACATCAATATTTTAAAAGATTATCAGATATGGAAAGACTTATAAGATTACCTGGAAAATTTAAATATTTTGAACACAATGTTGC
AGCACACTCCTTTAAAGTAACTAAAATTGCTCAATATCTAGCAACAGTTGAAGAATATCATGGACGAAAGATTAATTGGAAAAGCTTATATG
AAAAAGCATTAAATCATGATTTCGCCGAAGTGTTTACTGGTGATATAAAAACACCTGTTAAATATGCGAGTAGTGAATTAAAAAAATTATTT
TCGCAAGTTGAAGAAGAAATGGTAGAGACCTTTATTGAAGAAGAAATTCCACTACAATATAGAGATGTTTATAAGCAACGACTGCAAGAA
GGTAAAGATGATTCATTAGAAGGCCAAATACTTTCAGTTGCTGATAAAATTGATTTGCTTTATGAAACATTTGGAGAAATACAAAAACGTAA
TCCCGAAGAATTATTTTTCGAAATTTATGAAATGAGTCTAGAAACAATTATTCAATTTGACCATTTAGCATCTGTACAAGATTTTATTAATAAT
ATCATTCCAGAAATGTTGACTGAAAACTTTATACCTAGAACAGAATTAAGAGAAACAACCATGAACATTTTAAATAAAAGAAAAGAGGAAA
ATGAATGA

Gene: DUF2198 (putative membrane protein)

Position: 809120 to 809356, length: 237 nt, orientation: FORWARD

Perfect match to: (N315-BA000018-[812436:812672], highly conserved allele)

Sequence:

ATGATATGGTATTTTAGCGCAGCATTCTTTCCATGTGTCTTGGTAGTATTATTTAGTGTAATAACAAGAAGTAAATGGGTCGGTACTATTCTG
ACATTAATTTTAATTGGTGCCTCAATCTATAAAGAGTATTTCCATAACGAGTGGATTATTTTTATTGATGTAGTGTCATTATTAGCTGGTTATT
TAATTATAGATCAACTCGAATTTCATAAACATCAAGATGAAGATCGCTAA

Gene: uvrB (ultraviolet response system subunit B)

Position: 809619 to 811610, length: 1992 nt, orientation: FORWARD

Perfect match to: (11819-97-CP003194-[853538:855529], allele observed in CC80)

Sequence:

GTGACAATGGTTGAACATTATCCTTTTAAAATACATTCTGATTTTGAGCCTCAAGGTGATCAACCGCAAGCAATTGAAGAAATCGTGGAAG
GTATTAAAGCGGGGAAAAGACATCAAACTTTATTAGGTGCTACTGGCACAGGGAAAACATTTACGATGAGTAATGTTATTAAAGAAGTTG
GGAAACCAACGTTAATTATCGCGCATAACAAAACATTAGCAGGACAATTATATAGTGAGTTTAAAGAATTTTTTCCTGAAAACAGGGTGGA
ATACTTTGTAAGTTACTATGATTATTATCAACCAGAGGCATACGTACCGTCTACTGACACTTTTATTGAAAAAGATGCCTCAATCAATGATGA
AATTGATCAACTACGACATTCTGCTACAAGTGCATTATTTGAACGCGATGATGTAATTATTATTGCTAGTGTAAGTTGTATATATGGTTTAGG
TAATCCTGAAGAATATAAAGATTTAGTAGTAAGTGTTCGAGTTGGTATGGAAATGGATAGAAGTGAATTACTTAGAAAACTTGTAGATGTG
CAATATACACGAAATGACATCGATTTCCAACGAGGAACGTTTCGAGTGCGTGGTGATGTAGTTGAAATATTCCCAGCCTCTAAAGAAGAAC
TTTGTATAAGGGTTGAGTTTTTCGGCGATGAGATTGACCGTATCCGAGAAGTTAACTACCTAACAGGTGAAGTGTTGAAAGAAAGAGAACA
TTTTGCGATATTCCCAGCTTCTCACTTCGTAACACGTGAAGAAAAGTTGAAAGTTGCGATTGAACGTATTGAAAAAGAATTGGAAGAACGA
TTGAAAGAATTACGAGATGAGAATAAATTACTAGAAGCGCAAAGGTTAGAACAGCGTACCAACTATGATTTAGAAATGATGCGAGAGATG
GGATTCTGTTCAGGAATTGAAAACTATTCCGTACATTTAACTTTGCGACCACTGGGTTCGACACCATATACTTTATTGGATTACTTTGGCGAT
GATTGGTTAGTAATGATTGATGAATCACATGTGACATTACCGCAAGTTCGAGGCATGTATAACGGAGACAGAGCGCGTAAACAAGTTTTGG
TGGATCATGGGTTTAGATTACCGAGTGCATTAGATAACCGTCCACTTAAATTTGAAGAATTTGAAGAAAAGACAAAACAACTTGTGTATGT
ATCTGCAACGCCTGGACCATACGAAATTGAACATACGGATAAGATGGTTGAACAAATTATTCGTCCTACTGGTTTACTGGATCCTAAGATTG
AGGTTAGACCTACTGAAAATCAAATTGACGATTTATTAAGTGAAATTCAAATAAGGGTTGAGCGTAATGAACGCGTACTTGTTACAACGCT
CACTAAAAAGATGAGTGAAGATTTAACCACATACATGAAAGAAGCGGGTATTAAAGTTAATTATCTGCATTCAGAAATCAAGACATTAGAA
CGAATCGAAATAATTAGAGACTTACGAATGGGTACATATGATGTTATCGTAGGTATTAATTTATTAAGAGAGGGTATTGATATACCAGAAG
TTTCTCTAGTTGTCATATTAGATGCAGATAAAGAAGGATTTTTACGTTCTAACCGCTCATTAATTCAAACAATAGGTAGAGCTGCGCGTAAC
GATAAAGGTGAAGTCATTATGTATGCCGATAAAATGACTGATTCGATGAAGTATGCAATTGATGAGACACAACGTCGTCGAGAAATACAG
ATGAAACATAATGAAAAACATGGTATTACACCTAAAACAATTAATAAAAAAATACATGATTTAATTAGTGCTACTGTTGAAAATGACGAAAA
TAATGACAAAGCACAAACTGTGATACCTAAGAAGATGACGAAAAAAGAACGTCAAAAGACAATCGACAATATAGAAAAAGAAATGAAACA
AGCAGCGAAAGATTTAGATTTCGAGAAAGCTACAGAATTAAGAGATATGTTATTTGAATTAAAAGCAGAAGGGTGA

Gene: uvrA (ultraviolet response system subunit A)

Position: 811618 to 814464, length: 2847 nt, orientation: FORWARD

Perfect match to: (M0239-AIWE01000001-[261557:264403], allele observed in CC188+CC8)

Sequence:

ATGAAAGAACCATCCATAGTAGTAAAAGGTGCTCGTGCGCATAACTTGAAAGATATTGATATCGAACTACCTAAAAATAAATTAATTGTTAT
GACAGGTTTATCTGGGTCAGGTAAATCGTCATTAGCATTCGATACTATATATGCTGAAGGACAACGACGTTATGTTGAATCATTAAGTGCCT
ATGCGCGTCAATTTTTAGGCCAAATGGACAAACCAGATGTTGATACAATTGAAGGATTATCGCCAGCAATTTCAATAGATCAAAAAACAAC
AAGTAAAAATCCAAGATCAACTGTAGCAACAGTAACAGAAATATATGATTATATACGTTTGTTATATGCACGTGTTGGTAAACCTTACTGTC
CAAATCACAATATAGAAATTGAATCGCAAACAGTACAACAAATGGTTGACCGCATTATGGAATTAGAGGCACGTACAAAGATTCAATTATT
AGCACCTGTCATCGCTCATCGTAAAGGTAGTCATGAAAAGCTAATCGAAGATATTGGTAAAAAAGGTTATGTACGTTTAAGAATCGATGGC
GAAATTGTTGATGTAAATGATGTACCTACTTTAGATAAGAACAAGAATCATACAATAGAAGTTGTTGTAGACCGATTAGTTGTTAAAGATG
GAATTGAAACACGACTAGCTGACTCTATAGAAACTGCCTTAGAGCTTTCAGAAGGACAATTAACAGTCGATGTCATTGACGGGGAAGACCT
TAAGTTTTCAGAAAGCCATGCTTGTCCTATATGTGGATTTTCAATCGGAGAGTTAGAACCAAGAATGTTTAGCTTTAACAGTCCTTTTGGTGC
TTGTCCGACATGTGATGGCTTAGGCCAAAAGTTAACAGTCGATGTAGACTTGGTTGTTCCCGACAAAGATAAGACGCTAAACGAAGGTGCA
ATAGAACCTTGGATACCGACGAGTTCTGATTTTTATCCAACATTGTTAAAACGTGTTTGTGAAGTTTATAAAATCAATATGGATAAACCTTTT
AAAAAGTTAACAGAACGTCAACGTGATATTTTATTGTATGGTTCTGGTGACAAAGAAATTGAATTTACATTTACACAACGTCAAGGTGGTAC

TAGAAAACGAACAATGGTTTTCGAGGGTGTAGTTCCTAATATAAGTAGACGATTCCATGAATCTCCTTCAGAATATACACGTGAAATGATG
AGTAAATATATGACTGAACTACCTTGCGAAACTTGTCATGGAAAGCGATTGAGTCGTGAAGCGTTATCTGTTTATGTAGGTGGTTTAAATAT
TGGTGAAGTAGTCGAATATTCAATCAGTCAAGCGCTGAACTATTATAAAAACATTGATTTGTCAGAACAAGATCAAGCGATTGCAAATCAA
ATATTGAAAGAAATTATTTCCCGACTTACTTTTTTAAATAATGTGGGACTTGAATATTTAACGTTAAACAGAGCTTCAGGTACACTTTCAGGT
GGTGAAGCACAACGTATTCGATTAGCAACGCAAATTGGATCGCGTTTGACTGGTGTCTTATATGTATTAGATGAGCCATCAATTGGACTGC
ATCAAAGAGATAATGATCGATTAATTAATACACTTAAAGAAATGAGAGATTTAGGAAATACTTTAATTGTAGTTGAACACGATGATGATAC
AATGCGTGCGGCTGATTACTTAGTGGATATAGGTCCTGGTGCTGGTGAACATGGAGGACAGATTGTGTCTAGTGGTACTCCTCAAAAGGTA
ATGAAAGATAAAAAATCATTAACAGGACAATACTTGAGTGGTAAGAAACGTATTGAAGTACCTGAATATCGCAGACCGGCTTCAGATCGTA
AAATTTCTATACGTGGAGCTAGAAGCAACAATCTTAAAGGGGTTGATGTGGACATACCACTATCAATCATGACGGTTGTTACAGGTGTATC
AGGTTCTGGTAAAAGCTCATTAGTAAATGAAGTATTATACAAATCATTAGCTCAAAAAATTAATAAATCTAAAGTAAAGCCAGGATTGTACG
ATAAGATTGAAGGTATTGATCAACTTGATAAAATTATTGATATTGATCAATCACCGATAGGTAGAACGCCACGCTCTAATCCAGCAACATAT
ACTGGTGTGTTTGATGATATACGTGATGTGTTTGCGCAAACAAATGAAGCTAAAATTCGAGGATATCAAAAAGGGCGTTTTAGTTTTAATG
TAAAAGGTGGACGCTGTGAAGCTTGTAAAGGTGACGGTATTATTAAAATTGAAATGCATTTTTTACCTGATGTTTATGTTCCTTGTGAAGTG
TGTGATGGTAAACGATATAATCGTGAGACACTAGAGGTTACTTACAAAGGTAAAAATATTGCTGACATTTTAGAAATGACTGTTGAAGAAG
CAACACAATTTTTTGAAAATATTCCTAAGATTAAGCGCAAGTTACAAACACTAGTTGATGTTGGTCTTGGATACGTCACATTAGGTCAACAA
GCTACAACGTTATCAGGTGGTGAGGCTCAACGTGTGAAACTTGCATCTGAACTTCATAAACGTTCAACTGGTAAATCTATTTATATCCTAGA
TGAACCGACAACAGGGTTACATGTTGACGATATTAGTAGATTATTAAAAGTATTAAACCGATTAGTTGAAAATGGTGATACTGTTGTAATTA
TTGAACATAACCTAGATGTTATCAAAACAGCAGACTATATTATCGACTTAGGTCCTGAAGGTGGTAGTGGCGGTGGTACTATTGTTGCGAC
TGGCACACCCGAAGATATTGCTCAGACAAAGTCATCATATACAGGAAAGTATTTAAAAGAAGTACTTGAACGAGATAAACAAAATACTGAA
GATAAATAA

Gene: STAR (Staphylococcus aureus repeat element)

Position: 814607 to 814828, length: 222 nt

Sequence:

GGGAGTGGGACAGAAATGATAAAGAATCACTAATGATTTATTATGTAGTGGTTCTTTGTCATTAGCCACAGCTATTGTGTACTTAAAAATAG
GAATGCATGAGTGCAACTCATGCATAAGAAATACTAATTTCTAAAGAAAAAGTATTTCTTTATGTTGGGGCCCACCCCAACTTGCATTGTTT
GTAGAATTTCTTTTCGAAATTCTCTGTGTTGGGGCCCC

Gene: hpr (bifunctional kinase and phosphorylase)

Position: 815073 to 816005, length: 933 nt, orientation: FORWARD

Perfect match to: (Newbould_305-AKYW01000003-[260862:261794], allele observed in CC97+CC8+CC59)

Sequence:

ATGTTAACGACAGAAAAACTAGTTGAAACATTAAAGTTAGATTTAATCGCTGGTGAAGAAGGACTATCGAAGCCAATTAAAAATGCTGATA
TATCAAGACCGGGCTTAGAGATGGCAGGTTATTTTTCACATTATGCGTCAGATAGAATACAACTATTAGGAACAACGGAACTATCGTTTTAC
AATTTATTACCAGATAAGGATCGCGCAGGTCGTATGCGTAAACTATGCAGACCAGAAACGCCTGCAATTATTGTGACACGTGGATTGCAGC
CACCAGAAGAATTAGTTGAAGCTGCAAAAGAATTAAATACCCCACTTATAGTTGCTAAAGATGCGACTACAAGTTTAATGAGTCGCTTAAC
AACGTTTTTAGAGCATGCACTTGCAAAGACGACATCTTTACATGGTGTTCTAGTAGATGTTTACGGTGTTGGTGTACTAATTACCGGTGATT
CAGGAATAGGTAAAAGTGAGACTGCGTTGGAATTAGTTAAACGTGGGCATAGATTAGTAGCAGATGATAATGTAGAAATACGTCAAATTA
ATAAAGATGAACTAATAGGGAAACCACCAAAGTTAATAGAACATCTATTAGAAATACGTGGACTAGGTATTATCAATGTTATGACTTTATTT
GGCGCGGGTTCAATATTAACTGAAAAACGAATTAGATTAAATATTAATTTGGAAAACTGGAACAAGCAAAAGTTATATGACCGCGTAGGTC
TTAATGAAGAGACGCTAAGTATTTTAGATACTGAAATCACTAAAAAAACAATCCCTGTAAGACCTGGTAGAAATGTTGCGGTAATTATTGA
GGTCGCTGCAATGAACTATCGATTAAATATCATGGGCATTAACACGGCCGAAGAATTTAGTGAAAGATTAAATGAAGAAATTATCAAGAAC
AGTCATAAGAGTGAGGAGTAG

Gene: lgt (prolipoprotein diacylglyceryl transferase)

Position: 816011 to 816850, length: 840 nt, orientation: FORWARD

Perfect match to: (JKD6009-ABSA01000057-[1281:2120], allele observed in CC239+CC8+CC239)

Sequence:

ATGGGTATTGTATTTAACTATATAGATCCTGTGGCATTTAACTTAGGACCACTGAGTGTACGATGGTATGGAATTATCATTGCTGTCGGAAT
ATTACTTGGTTACTTTGTTGCACAACGTGCACTAGTTAAAGCAGGATTACATAAAGATACTTTAGTAGATATTATTTTTATAGTGCACTATTT
GGATTTATCGCGGCACGAATCTATTTTGTGATTTTCCAATGGCCATATTACGCGGAAAATCCAAGTGAAATTATTAAAATATGGCATGGTGG
AATAGCAATACATGGTGGTTTAATAGGTGGCTTTATTGCTGGTGTTATTGTATGTAAAGTGAAAAATTTAAACCCATTTCAAATTGGTGATA
TCGTTGCGCCAAGTATAATTTTAGCGCAAGGAATTGGACGCTGGGGTAACTTTATGAATCACGAGGCACATGGTGGACCTGTGTCACGCGC
TTTTTTAGAACAATTACATTTGCCAAATTTTATAATAGAAAATATGTATATTAACGGCCAATATTATCATCCAACATTCTTATATGAATCCATT
TGGGATGTCGCTGGATTTATTATCTTAGTTAATATTCGTAAACATTTAAAATTAGGAGAAACATTCTTTTTATATTTAACTTGGTATTCAATTG
GTCGATTCTTTATAGAAGGATTACGTACAGATAGCTTAATGCTCACAAGTAATATTAGAGTTGCACAATTAGTATCAATTCTTTTAATTTTAA
TAAGTATAAGTTTAATTGTATATAGAAGGATTAAGTATAATCCACCGTTGTATAGCAAAGTTGGGGCGCTTCCATGGCCAACAAAAAAAGT
GAAGTAG

Gene: yvoF (putative acetyltransferase)

Position: 816858 to 817343, length: 486 nt, orientation: FORWARD

Perfect match to: (COL-CP000046-[852777:853262], highly conserved allele)

Sequence:

TTGAGGAAATTTTTATCAAAAACACATCATCATACAAACCCTTTATGGCGTGTATACCGTCTTGTTAAATTTTCGAAAGTTTTTAAGAATGTA
ATTATCATTGAATTTTCGAAATTTATTCCAAGTATGGTACTGAAAAGACATATATATAAACAACTTTTAAATATTAATATCGGTAATCAATCG
TCGATAGCTTATAAAGTAATGTTAGATATTTTTTACCCAGAACTGATTACGATTGGTAGTAACAGTGTTATTGGTTACAATGTAACAATTTTG
ACGCATGAAGCATTAGTTGATGAATTTCGTTATGGACCAGTGACGATAGGATCTAACACTTTGATTGGTGCAAATGCTACCATTTTACCCGG
TATAACGATTGGTGACAATGTAAAAGTTGCAGCTGGTACGGTTGTTTCAAAAGATATACCGGATAATGGATTTGCATATGGCAACCCTATG
TATATAAAAATGATTAGGAGGTGA

Gene: yvcD (tetratricopeptide repeat protein)

Position: 817351 to 818790, length: 1440 nt, orientation: FORWARD

Perfect match to: (11819-97-CP003194-[861270:862709], allele observed in CC80+CC12+CC4803)

Sequence:

ATGGCGCAAAAGAATAATAATGTAATTCCAATGACTTTTGATGATGCATTTTATCGTAAAATGGCTAAACAGAAGTTTAAACAAAGAGAAT
ATAAACGAGCTGCTGAATACTTTGAAAAAGTGTTAGAATTGTCACCTGATGATCTGGAAATTCAAATTGATTATGCACAATGTCTAGTGCAA
CTTGGTATTGCTAAAAAAGCAGAACATTTATTTTATGACAATATTATTTATAATAGGCATCTAGAAGATAGCTTTTATGAATTGAGTCAGCTC
AACATTGAAGTTAACGAACCAAACAAGGCATTCTTGTTTGGTATTAATTATGTTATTGTTAGCGACGACCAAGATTATAGAGATGAATTAGA
TCAAATGTTTGATGTGAAATATCAAAGTGAAGAACAAATTGAACTTGAAGCTCAATTGTTTGTAGTTCAAATACTATTCCAATATCTTTTTTC
TCAAGGTCGATTAAAAGATGCAAAGAATTATGTCTTACATCAACCACAAGAAGTTCAAGATCATCGTGTCGTACGTAATTTATTGGCAATGT
GTTATTTATATCTCGGCGAATATGATACGGCTAAAGCATTGTACGAAGCACTATTACAAGAGGATAGTACAGATATATATGCATTATGCCAT
TATACTTTGCTACTTTATAACACTAAGGAAAATGAACAATACCAAAAATATTTAAAAATATTAAATAAAGTTGTACCTATGAATGACGATGA
AAGTTTTAAATTAGGTATTGTTTTAAGTTATTTAAAGCAGTATCGTGCATCACAACAATTGTTGTACCCTTTATATAAAAAAGGAAAATTTTT
ATCAATTCAAATGTACAATGCTTTAGCATATAATTATTATTATTTAGGTGAAGAAGACGAAAGTCATTACTACTGGGATAAATTGAAGCAAA
TTTCTAAAGTGGAAATTGGACATGCGCCTTGGGTAATTGAAAATAGCAAAGAAGTTTTTGACCAACATATTTTGCCATTACTTCAAAGTGAT
GACAGTCATTATCGTTTATATGGTATTTTTTTATTGGATCAATTAAATGGTAAAGAAATTGTGATGACGGAAAGTATTTGGCAGGTTCTGGA
AAATCTAAATAATTATGAGAAATTGTATTTAACGTATTTAGTTCAAGGTTTAACGCTCAATAAATTAGACTTCATTCATCGCGGCTTGTTAAC
GCTTTACCAAAATGAATTATTTGTAAGTGAAAATGATTTAATGGTTGCATGGATTAATCAAGGTGAACTCATAATTGCTGAAAAAGTAGATT
TAACTGATGTTGAGCCATATATCGGTGCGTTTATTTATTTGTATTTTAAAAATCAACCTCGAAACGTTACAAAGAAGCAAATTACAACATGGT
TAGGCATAACACAATATAAACTGAACAAAATGATTGAATTTCTCTTGAGCATATAG

Gene: trxB (thioredoxin reductase)

Position: 818857 to 819792, length: 936 nt, orientation: FORWARD

Perfect match to: (RF122-AJ938182-[788917:789852], highly conserved allele)

Sequence:

ATGACTGAAATAGATTTTGATATAGCAATTATCGGTGCAGGTCCAGCTGGTATGACTGCTGCAGTATACGCATCACGTGCTAATTTAAAAAC
AGTTATGATTGAAAGAGGTATTCCAGGCGGTCAAATGGCTAATACAGAAGAAGTAGAGAACTTCCCTGGTTTCGAAATGATTACAGGTCCA
GATTTATCTACAAAAATGTTTGAACACGCTAAAAAGTTTGGTGCAGTTTATCAATATGGAGATATTAAATCTGTAGAAGATAAAGGCGAAT
ATAAAGTGATTAACTTTGGTAATAAAGAATTAACAGCGAAAGCGGTCATTATTGCTACAGGTGCAGAATACAAGAAAATTGGTGTTCCGGG
TGAACAAGAACTTGGTGGACGCGGTGTAAGTTATTGTGCAGTATGTGATGGTGCATTCTTTAAAAATAAACGCCTATTCGTTATCGGTGGT
GGTGACTCAGCAGTAGAAGAGGGAACATTCTTAACTAAATTTGCTGACAAAGTAACAATCGTTCACCGTCGTGATGAGTTACGTGCACAGC
GTATTTTACAAGATAGAGCATTCAAAAATGATAAAATCGACTTTATTTGGAGTCATACTTTGAAATCAATTAATGAAAAAGACGGCAAAGTG
GGTTCTGTGACATTAACGTCTACAAAAGATGGTTCAGAAGAAACACACGAGGCTGATGGTGTATTCATCTATATTGGTATGAAACCATTAA
CAGCGCCATTTAAAGACTTAGGTATTACGAATGATGTTGGTTATATTGTGACAAAAGATGATATGACAACATCAGTACCAGGTATTTTTGCA
GCAGGAGATGTTCGTGACAAAGGTTTACGCCAAATTGTCACTGCTACTGGCGATGGTAGTATTGCAGCGCAAAGTGCAGCGGAATATATT
GAACATTTAAACGATCAAGCTTAA

Gene: repeat_nySAgamma (repeat element)

Position: 820083 to 820263, length: 181 nt

Sequence:

TTGGGGCCCCGCCAACTTGCATTGTCTGTAGAAATTCTTTTCGAAATTCTCTGTGTTGGGGCCCACACCCCAACTTGCATTGTCTGTAGAAAT
TGGAAATCCAATTTCTCTGTGTTGGGGCCCACACCCCAACTCGCATTGCCTGTAGAATTTCTTTTCGAAATTCTCTGTGTTGGGGCCC

Gene: SIRU01 (staphylococcal interspersed repeat unit 1)

Position: 820094 to 820262, length: 169 nt

Sequence:

CCAACTTGCATTGTCTGTAGAAATTCTTTTCGAAATTCTCTGTGTTGGGGCCCACACCCCAACTTGCATTGTCTGTAGAAATTGGAAATCCAA
TTTCTCTGTGTTGGGGCCCACACCCCAACTCGCATTGCCTGTAGAATTTCTTTTCGAAATTCTCTGTGTTGGGGCC

Gene: tx_universal2 (rho-independent terminator)

Position: 820269 to 820307, length: 39 nt

Perfect match to: (Strain_71193-CP003045-[770837:770875:r], allele observed in CC398)

Sequence:

TAGAGTTGAAAAAAGCTTGTTGCAAGCGCATTTTCATTC

Gene: yvcJ (P-loop NTPase family protein)

Position: 820500 to 821411, length: 912 nt, orientation: FORWARD

Perfect match to: (MW2-BA000033-[819598:820509], highly conserved allele)

Sequence:

ATGGATAATAATGAAAAAGAAAAAAGTAAAAGTGAACTATTAGTTGTAACAGGTTTATCTGGCGCAGGTAAATCTTTGGTTATTCAATGTTT
AGAAGACATGGGATATTTTTGTGTAGATAATCTACCACCAGTGTTATTACCTAAATTTGTAGAGTTGATGGAACAAGGAAATCCATCCTTAA
GAAAAGTGGCAATTGCAATTGATTTAAGAGGTAAGGAACTATTTAATTCATTAGTTGCAGTAGTGGATAAAGTTAAAAGTGAAAGTGACGT
CATCATTGATGTTATGTTTTTAGAAGCAAGTACTGAAAAATTAATTTCAAGATATAAGGAAACGCGTCGTGCACATCCTTTGATGGAACAAG
GTAAAAGATCCTTAATTAATGCAATTAATGATGAGCGAGAGCATTTGTCTCAAATTAGAAGTATAGCTAATTTTGTTATAGATACTACAAAG
TTATCACCTAAAGAATTAAAAGAACGCATTCGTCGATACTATGAAGATGAAGAGTTTGAAACTTTTACAATTAATGTCACAAGTTTCGGTTTT
AAACATGGGATTCAGATGGATGCAGATTTAGTATTTGATGTACGATTTTTACCAAATCCATATTATGTAGTAGATTTAAGACCTTTAACAGG
ATTAGATAAAGACGTTTATAATTATGTTATGAAATGGAAAGAGACGGAGATTTTCTTTGAAAAATTAACTGATTTGTTAGATTTTATGATAC
CCGGGTATAAAAAAGAAGGGAAATCTCAATTAGTAATTGCCATCGGTTGTACGGGTGGACAACATCGATCTGTAGCATTAGCAGAACGAC
TAGGTAATTATCTAAATGAAGTATTTGAATATAATGTTTATGTGCATCATAGGGACGCACATATTGAAAGTGGCGAGAAAAAATGA

Gene: mgfK (putative gluconeogenesis factor)

Position: 821408 to 822403, length: 996 nt, orientation: FORWARD

Perfect match to: (11819-97-CP003194-[865327:866322], highly conserved allele)

Sequence:

ATGAGACAAATAAAAGTTGTACTTATCGGTGGTGGCACTGGCTTATCAGTTATGGCTAGGGGATTAAGAGAATTCCCAATTGATATTACGG
CGATTGTAACAGTTGCTGATAATGGTGGGAGTACAGGGAAAATCAGAGATGAAATGGATATACCAGCACCAGGAGACATCAGAAATGTG
ATTGCAGCTTTAAGTGATTCTGAGTCAGTTTTAAGCCAACTTTTTCAGTATCGCTTTGAAGAAAATCAAATTAGCGGTCACTCATTAGGTAAT
TTATTAATCGCAGGTATGACTAATATTACGAATGATTTCGGACATGCCATTAAAGCATTAAGTAAAATTTTAAATATTAAAGGTAGAGTCAT
TCCATCTACAAATACAAGTGTGCAATTAAATGCTGTTATGGAAGATGGAGAAATTGTTTTTGGAGAAACAAATATTCCTAAAAAACATAAAA
AAATTGATCGTGTGTTTTTAGAACCTAACGATGTGCAACCAATGGAAGAAGCAATCGATGCTTTAAGGGAAGCAGATTTAATCGTTCTTGG
ACCAGGGTCATTATATACGAGCGTTATTTCTAACTTATGTGTCAATGGTATTTCAGATGCGTTAATTCATTCTGATGCGCCTAAGCTATATGT
TTCTAATGTGATGACGCAACCTGGGGAAACAGATGGTTATAGCGTGAAAGATCATATCGATGCGATTCATAGACAAGCTGGACAACCGTTT
ATTGATTATGTCATTTGTAGTACACAAACTTTCAATGCTCAAGTTTTGAAAAAATATGAAGAAAAACATTCTAAACCAGTTGAAGTTAATAA
GGCTGAACTTGAAAAAGAAAGCATAAATGTAAAAACATCTTCAAATTTAGTTGAAATTTCTGAAAATCATTTAGTAAGACATAATACTAAAG
TGTTATCGACAATGATTTATGACATAGCTTTAGAATTAATTAGTACTATTCCTTTCGTACCAAGTGATAAACGTAAATAA

Gene: whiA (transcription regulator)

Position: 822514 to 823458, length: 945 nt, orientation: FORWARD

Perfect match to: (CIG290-AIES01000015-[48469:49413], highly conserved allele)

Sequence:

ATGAGCTTTGCATCAGAAATGAAAAATGAATTAACTAGAATAGACGTCGATGAAATGAATGCAAAAGCAGAGCTCAGTGCACTGATTCGA
ATGAATGGTGCACTTAGTCTTTCAAATCAACAATTTGTTATAAATGTTCAAACGGAAAATGCAACAACGGCAAGACGTATTTATTCGTTGAT
TAAACGTGTCTTTAATGTGGAAGTTGAAATATTAGTCCGTAAAAAAATGAAACTTAAAAAAAATAATATTTATATTTGTCGTACAAAGATGA
AAGCGAAAGAAATTCTTGATGAATTAGGAATTTTAAAAGACGGCATTTTTACGCATGAAATTGATCATTCAATGATTCAAGATGACGAAAT
GAGACGCAGTTACTTGAGAGGAGCTTTTCTGGCAGGTGGCTCAGTGAATAACCCTGAAACATCTTCGTACCATTTGGAAATTTTTTCTCAAA
ATGAGAGTCATGCAGAAGGCTTAACGAAACTAATGAATAGTTATGAGTTGAATGCCAAACATTTAGAGCGAAAAAAAGGAAGTATTACGT
ATTTAAAAGAAGCGGAAAAGATTTCGGATTTTCTTAGTTTGATAGGTGGCTATCAAGCGTTATTAAAATTTGAAGACGTACGTATTGTAAGA
GATATGCGTAATTCTGTTAACCGACTCGTTAATTGTGAAACGGCCAATCTAAATAAAACAGTTAGTGCTGCGATGAAACAAGTTGAGAGCA
TTAAATTGATTGATAAAGAAATTGGTATTGAAAATTTACCAGACAGGTTGAGAGAGATTGCTAGAATTCGAGTAGAACATCAAGAAATTTC
GTTGAAAGAGCTTGGAGAAATGGTATCAACTGGTCCAATTTCAAAATCAGGTGTAAATCACCGATTAAGAAAACTTAATGATTTAGCCGAT
AAGATTAGAAATGGTGAACAAATAGAATTATAA

Gene: clpP (ATP-dependent chaperone protease, proteolytic subunit)

Position: 824022 to 824609, length: 588 nt, orientation: FORWARD

Perfect match to: (KLT6-APFH01000004-[266418:267005], allele observed in CC12+CC8+CC188+CC772+CC4803)

Sequence:

ATGAATTTAATTCCTACAGTTATTGAAACAACAAACCGCGGTGAACGTGCATATGATATATACTCACGTTTATTAAAAGACCGTATTATTATG
TTAGGTTCACAAATTGATGACAACGTAGCAAATTCAATCGTATCACAGTTATTATTCTTACAAGCGCAAGACTCAGAGAAAGATATTTATTT
ATACATTAATTCACCAGGTGGAAGTGTAACAGCTGGTTTTGCGATTTATGATACAATTCAACACATTAAACCTGATGTTCAAACAATTTGTAT
TGGTATGGCTGCATCAATGGGATCATTCTTATTAGCAGCTGGTGCAAAAGGTAAACGTTTCGCGTTACCAAATGCAGAAGTAATGATTCAC
CAACCATTAGGTGGTGCTCAAGGACAAGCAACTGAAATCGAAATTGCTGCAAATCACATTTTAAAAACACGTGAAAAATTAAACCGCATTTT
ATCAGAGCGTACTGGTCAAAGTATTGAAAAAATACAGAAAGACACAGATCGTGATAACTTCTTAACTGCAGAAGAAGCTAAAGAATATGG
CTTAATTGATGAAGTGATGGTACCTGAAACAAAATAA

Gene: yfcH (cell-division inhibitor)

Position: 824852 to 825754, length: 903 nt, orientation: REVERSE

Perfect match to: (T0131-CP002643-[846527:847429:r], allele observed in CC239+CC80)

Sequence:

TTATTCTTTGATTAAATCTTCAAGTGCCATTTTTAAATTACTATATTTAAATTGGAATCCCAATGCTTGAATTTTATTAGGTAATACTTTTTGAG
TATCCAATACTACTGTTGACATTTGACCAAGTATGAGACGCATTGCAAGACTTGGTGCCCAAGTTTCATGGGGCTTATGCATAGCTCTTGCT
AAAGTGTAGCCAAATAAATTTTGACGCTCAGGTATAGGTGCAGTTAAATTAAACGGACCACTAGCTGACTCGTTATTTATTAAAAATAAAAT
AGCTTGAATTAAATCATTGATATGAATCCATGAATACCATTGTTGACCAGAACCTAATTTACCACCAATGTAATATTTGTATGGTAGTTTCAT
TGTTTGTAACGCACCGCCTTCATTCGATAAAATCATACCGAAACGACCGATGACAACTCGCGTACCTAATTGTTCAAATTGTTGTGCGAAAC
GTTCCCATTGATACACAATATCTGATAAGAAATCAAATGGTAAAGTTTTATAAACTTCTGTGTAACTCATAAATAAATCAGGAGGATAGTAA
CCAGTGGCACTAGCATTAAATAAAACTTTAGGTGCTTTATTGCGTGATTTAAACAATTCATATAAAGCTTGCGTAGATTGAATTCTACTTAGC
ATTAGCGTTTGTTTATATTCCGGTGTCCATCGTTTATTCAATGTAGCACCTGCTAAGTTGATGACCACATCGATATTTTGAGGAACTTTGTGTT
CCCACCCAGATTTAGCCCAGTTGACATATGAAATTTTCTTATCATTTGAAATTTGGTCGTGTCGCGTTAATATCGTGATATGTGAATCTGATT
TTTTAATTTCATTAACTAATTGAGATCCAACCATACCAGTCCCACCAGTAATTAAGTATTGTTTCAT

Gene: Q5HHP8 (putative exported protein)

Position: 826457 to 827086, length: 630 nt, orientation: FORWARD

Perfect match to: (RF122-AJ938182-[798354:798983], highly conserved allele)

Sequence:

ATGTCGAATCAAAATTACGACTACAATAAAAATGAAGATGGAAGTAAGAAGAAAATGAGTACAACAGCGAAAGTAGTTAGCATTGCGACG
GTATTGCTATTACTCGGAGGATTAGTATTTGCAATTTTTGCATATGTAGATCATTCGAATAAAGCTAAAGAACGTATGTTGAACGAACAAAA
GCAGGAACAAAAAGAAAAGCGTCAAAAAGAAAATGCAGAAAAAGAGAGAAAGAAAAAGCAACAAGAGGAAAAAGAGCAGAATGAGCT
AGATTCACAAGCAAACCAATATCAGCAATTGCCACAGCAGAATCAATATCAATATGTGCCACCTCAGCAACAAGCACCTACAAAGCAACGT
CCTGCTAAAGAAGAGAATGATGATAAAGCATCAAAGGATGAGTCGAAAGATAAGGATGACAATGCATCTCAAGATAAATCAGATGATAAT
CAGAAGAAAACTGATGATAATAAACAACCAGCTCAGCCTAAACCACAGCCGCAACAACCAACACCAAAGCCAAATAATAATCAACAAAATA
ATCAATCAAATCAGCAAGCAAAACCACAAGCACCACAACAAAATAGCCAATCAACAACAAATAAACAAAATAATGCTAATGATAAGTAG

Gene: gapR (regulator of glyceraldehyde 3-phosphate dehydrogenase)

Position: 827788 to 828801, length: 1014 nt, orientation: FORWARD

Perfect match to: (M1015-ACST01000009-[106584:107597], allele observed in CC30+CC8+CC30+CC188)

Sequence:

GTGAAAGACTTATTGCAAGCACAGCAAAAGCTTATACCGGATCTCATAGATAAAATGTATAAACGTTTTTCTATTCTTACTACTATCTCAAAA
AATCAGCCTGTCGGACGTCGAAGTTTAAGCGAACATATGGATATGACTGAACGTGTACTGCGTTCTGAAACAGATATGCTTAAGAAACAAG
ATTTGATAAAAGTTAAGCCTACCGGAATGGAAATTACAGCTGAAGGTGAGCAACTGATTTCGCAATTGAAAGGTTACTTTGATATCTATGC
AGATGACAATCGTCTGTCAGAAGGTATTAAGAATAAATTTCAAATTAAGGAAGTTCATGTTGTTCCTGGTGATGCTGATAATAGTCAATCTG
TTAAAACAGAATTAGGTAGACAAGCAGGTCAATTACTTGAAGGCATATTACAAGAAGACGCGATAGTTGCTGTAACTGGCGGATCCACGA
TGGCATGTGTTAGTGAAGCAATTCATTTATTACCATATAATGTATTCTTCGTACCAGCCAGAGGTGGACTAGGCGAAAATGTTGTCTTTCAG
GCAAACACAATTGCAGCCAGTATGGCACAACAAGCTGGCGGTTATTATACGACGATGTATGTACCTGATAATGTCAGTGAAACAACATATA
ACACATTGTTGTTAGAGCCATCAGTCATAAACACTTTAGACAAAATTAAACAAGCAAACGTTATATTACACGGCATTGGTGATGCGCTGAAG
ATGGCGCATCGACGTCAATCACCTGAAAAGGTCATTGAACAACTTCAACATCATCAAGCTGTCGGAGAGGCATTTGGTTATTATTTTGATAC
ACAAGGTCAAATTGTCCATAAGGTTAAAACAATTGGACTTCAATTAGAAGACCTTGAATCAAAAGACTTTATTTTTGCAGTTGCAGGAGGCA
AATCGAAAGGTGAAGCAATTAAAGCATACTTGACGATTGCACCCAAGAATACAGTGTTAATCACTGATGAAGCCGCAGCAAAGATAATACT
TGAATAA

Gene: gapA (glyceraldehyde 3-phosphate dehydrogenase, locus A)

Position: 828854 to 829864, length: 1011 nt, orientation: FORWARD

Perfect match to: (TW20-FN433596-[913640:914650], highly conserved allele)

Sequence:

ATGGCAGTAAAAGTAGCAATTAATGGTTTTGGTAGAATTGGTCGTTTAGCATTCAGAAGAATTCAAGAAGTAGAAGGTCTTGAAGTTGTAG
CAGTAAACGACTTAACAGATGACGACATGTTAGCGCATTTATTAAAATATGACACTATGCAAGGTCGTTTCACAGGTGAAGTAGAGGTAGT
TGATGGTGGTTTCCGCGTAAATGGTAAAGAAGTTAAATCATTCAGTGAACCAGATGCAAGCAAATTACCTTGGAAAGACTTAAATATCGAT
GTAGTATTAGAATGTACTGGTTTCTACACTGATAAAGATAAAGCACAAGCTCATATTGAAGCAGGCGCTAAAAAAGTATTAATCTCAGCAC
CAGCTACTGGTGACTTAAAAACAATCGTATTCAACACTAACCACCAAGAGTTAGACGGTTCTGAAACAGTTGTTTCAGGTGCTTCATGTACT
ACAAACTCATTAGCACCAGTTGCTAAAGTTTTAAACGATGACTTTGGTTTAGTTGAAGGTTTAATGACTACAATTCACGCTTACACAGGTGA
TCAAAATACACAAGACGCACCTCACAGAAAAGGTGACAAACGTCGTGCTCGTGCAGCAGCAGAAAACATCATCCCTAACTCAACAGGTGCT
GCTAAAGCTATCGGTAAAGTTATTCCTGAAATCGATGGTAAATTAGATGGTGGTGCACAACGTGTTCCTGTAGCTACAGGTTCATTAACTGA
ATTAACAGTAGTATTAGAAAAACAAGACGTAACAGTTGAACAAGTTAACGAAGCTATGAAAAATGCTTCAAACGAATCATTCGGTTACACT
GAAGACGAAATCGTTTCTTCAGACGTTGTAGGTATGACTTACGGTTCATTATTCGACGCTACACAAACTCGTGTAATGTCAGTTGGCGACCG
TCAATTAGTTAAAGTTGCAGCTTGGTATGATAACGAAATGTCATATACTGCACAATTAGTTCGTACATTAGCATACTTAGCTGAACTTTCTAA
ATAA

Gene: pgk (phosphoglycerate kinase)

Position: 830003 to 831193, length: 1191 nt, orientation: FORWARD

Perfect match to: (11819-97-CP003194-[873922:875112], allele observed in CC80)

Sequence:

ATGGCTAAAAAAATTGTTTCTGATTTAGATCTTAAAGGTAAAACAGTCTTAGTACGTGCTGATTTTAACGTACCTTTAAAAGACGGTGAAAT
TACTAATGACAACCGTATCGTTCAAGCTTTACCTACAATTCAATACATCATCGAACAAGGTGGTAAAATCGTACTATTTTCACATTTAGGTAA
AGTAAAAGAAGAAAGTGATAAAGCAAAATTAACTTTACGTCCAGTTGCTGAAGACTTATCTAAGAAATTAGATAAAGATGTTGTTTTCGTA
CCAGAAACACGCGGTGAAAAACTTGAAGCAGCTATTAAAGACCTTAAAGAAGGCGACGTATTATTAGTTGAAAATACACGTTATGAAGATT
TAGACGGTAAAAAAGAATCTAAAAATGATCCAGAATTAGGTAAATACTGGGCATCTTTAGGTGATGTGTTTGTAAATGATGCTTTTGGTACT
GCGCATCGTGAGCATGCATCTAATGTTGGTATTTCTACACATTTAGAAACTGCAGCTGGATTCTTAATGGATAAAGAAATTAAGTTTATCGG
TGGCGTAGTTAACGATCCACATAAACCAGTTGTTGCTATTTTAGGTGGAGCAAAAGTATCTGACAAAATTAATGTCATCAAAAACTTAGTTA
ACATAGCTGATAAAATTATCATCGGCGGAGGTATGGCTTATACTTTCTTAAAAGCGCAAGGTAAAGAAATTGGTATTTCATTATTAGAAGAA
GATAAAATCGACTTCGCAAAAGATTTATTAGAAAAACATGGTGATAAAATTGTATTACCAGTAGACACTAAAGTTGCTAAAGAATTTTCTAA
TGATGCCAAAATTACTGTAGTACCATCTGATTCAATTCCAGCAGACCAAGAAGGTATGGATATTGGACCAAACACTGTAAAATTATTTGCAG
ATGAATTAGAAGGTGCGCACACTGTTGTATGGAATGGACCTATGGGTGTATTCGAGTTCAGTAACTTTGCACAAGGTACAATTGGTGTATG
TAAAGCAATTGCAAACCTTAAAGATGCAATTACGATTATCGGTGGCGGTGATTCAGCTGCAGCAGCAATCTCTTTAGGTTTTGAAAATGACT
TCACTCATATTTCAACTGGTGGCGGAGCGTCATTAGAGTACCTAGAAGGTAAAGAATTGCCTGGTATCAAAGCAATCAATAATAAATAA

Gene: tpi (triosephosphate isomerase)

Position: 831315 to 832076, length: 762 nt, orientation: FORWARD

Perfect match to: (JKD6159-CP002114-[821637:822398], allele observed in CC93+CC692+CC772+CC1290)

Sequence:

ATGAGAACACCAATTATAGCTGGTAACTGGAAAATGAACAAAACAGTACAAGAAGCAAAAGACTTCGTCAATGCATTGCCAACATTACCAG
ATTCAAAAGAAGTAGAATCAGTAATTTGTGCACCAGCAATTCAATTAGATGCATTAACTACTGCAGTTAAAGAAGGAAAAGCACAAGGTTT
AGAAATCGGTGCTCAAAATACGTATTTCGAAGATAATGGTGCGTTCACAGGTGAAACGTCTCCAGTTGCATTAGCAGATTTAGGCGTTAAA
TACGTTGTTATCGGTCATTCTGAACGTCGTGAATTATTCCACGAATCAGATGAAGAAATTAACAAAAAAGCGCACGCTATTTTCAAACATGG
AATGACTCCAATTATATGTGTTGGTGAAACAGACGAAGAGCGTGAAAGTGGTAAAGCTAACGATGTTGTAGGTGAGCAAGTTAAGAAAGC
TGTTGCAGGTTTATCTGAAGATCAACTAAAATCAGTTGTAATTGCTTATGAACCAATCTGGGCAATCGGAACTGGTAAATCATCAACATCTG
AAGATGCGAATGAAATGTGTGCATTTGTACGTCAAACTATTGCTGACTTATCAAGCAAAGAAGTATCAGAAGCAACTCGTATTCAATATGG
TGGTAGTGTTAAACCTAACAACATTAAAGAATACATGGCACAAACTGATATTGATGGGGCATTAGTAGGTGGCGCATCACTTAAAGTTGAA
GATTTCGTACAATTGTTAGAAGGTGCAAAATAA

Gene: gpmI (2,3-bisphosphoglycerate-independent phosphoglycerate mutase)

Position: 832079 to 833596, length: 1518 nt, orientation: FORWARD

Perfect match to: (IS-24-AHLM01000008-[13387:14904:r], allele observed in CC8+CC97)

Sequence:

ATGGCTAAGAAACCAACTGCGTTAATTATTTTAGATGGTTTTGCGAACCGCGAAAGCGAACATGGTAATGCGGTAAAATTAGCAAACAAGC
CTAATTTTGATCGTTATTACAACAAATATCCAACGACTCAAATCGAAGCGAGTGGCTTAGATGTTGGACTACCTGAAGGACAAATGGGTAA
CTCAGAAGTTGGTCATATGAATATCGGTGCAGGACGTATCGTTTATCAAAGTTTAACTCGAATCAATAAATCAATTGAAGACGGTGATTTCT
TTGAAAATGATGTTTTAAATAATGCAATTGCACACGTGAATTCACATGATTCAGCGTTACACATCTTTGGTTTATTGTCTGACGGTGGTGTGC
ACAGTCATTACAAACATTTATTTGCTTTGTTAGAACTTGCTAAAAAACAAGGTGTTGAAAAAGTTTACGTACACGCATTTTTAGATGGTCGTG
ACGTAGATCAAAAATCCGCTTTGAAATACATCGAAGAGACTGAAGCTAAATTCAATGAATTAGGCATTGGTCAATTTGCATCTGTGTCTGGT
CGTTATTATGCAATGGACCGTGACAAACGTTGGGAACGTGAAGAAAAAGCTTACAATGCTATTCGTAATTTTGATGCCCCAACTTATGCAAC
TGCCAAAGAAGGTGTGGAAGCAAGTTATAATGAAGGCTTAACTGACGAATTCGTAGTACCATTCATCGTTGAGAATCAAAATGACGGTGTT
AATGATGGAGATGCAGTGATCTTCTATAATTTCCGACCTGATAGAGCAGCACAATTATCGGAAATTTTTGCGAACAGAGCATTCGAAGGCT
TTAAAGTTGAACAAGTTAAAGACTTATTCTATGCAACATTCACTAAGTATAACGACAATATCGATGCGGCTATCGTTTTCGAAAAAGTTGAT
TTAAATAATACAATTGGTGAAATTGCACAAAATAACAATTTAACACAATTACGTATTGCAGAAACTGAAAAATATCCTCACGTTACTTACTTT
ATGAGTGGTGGACGTAATGAGGAATTTAAAGGTGAACGCCGTCGTTTAATTGATTCACCTAAAGTTGCAACGTATGACTTGAAACCAGAAA
TGAGTGCTTATGAAGTTAAAGATGCATTATTAGAAGAGTTAAATAAAGGTGACTTGGACTTAATTATTTTAAACTTTGCTAACCCTGATATG
GTTGGACATAGTGGTATGCTTGAGCCGACAATCAAAGCAATCGAAGCGGTTGATGAATGTTTAGGAGAAGTGGTTGATAAGATTTTAGAC
ATGGACGGTTATGCAATTATTACTGCTGACCATGGTAACTCTGATCAAGTATTGACGGATGATGATCAACCAATGACTACGCATACAACGA
ACCCAGTACCAGTGATTGTAACAAAAGAAGGCGTTACACTTAGAGAAACTGGTCGCTTAGGTGACTTAGCACCTACATTATTAGATTTATTA
AATGTAGAACAACCTGAAGATATGACAGGTGAATCTTTAATTAAACACTAA

Gene: eno (enolase)

Position: 833726 to 835030, length: 1305 nt, orientation: FORWARD

Perfect match to: (Strain_21343-AHKV01000010-[13632:14936], highly conserved allele)

Sequence:

ATGCCAATTATTACAGATGTTTACGCTCGCGAAGTCTTAGACTCTCGTGGTAACCCAACTGTTGAAGTAGAAGTATTAACTGAAAGTGGCGC
ATTTGGTCGTGCATTAGTACCATCAGGTGCTTCAACTGGTGAACACGAAGCTGTTGAATTACGTGATGGAGACAAATCACGTTATTTAGGT
AAAGGTGTTACTAAAGCAGTTGAAAACGTTAATGAAATCATCGCACCAGAAATTATTGAAGGTGAATTTTCAGTATTAGATCAAGTATCTAT
TGATAAAATGATGATCGCATTAGACGGTACTCCAAACAAAGGTAAATTAGGTGCAAATGCTATTTTAGGTGTATCTATCGCAGTAGCACGT
GCAGCAGCTGACTTATTAGGTCAACCACTTTACAAATATTTAGGTGGATTTAATGGTAAGCAGTTACCAGTACCAATGATGAACATCGTTAA
TGGTGGTTCTCACTCAGATGCTCCAATTGCATTCCAAGAATTCATGATTTTACCTGTAGGTGCTACAACGTTCAAAGAATCATTACGTTGGG
GTACTGAAATTTTCCACAACTTAAAATCAATTTTAAGCAAACGTGGTTTAGAAACTGCAGTAGGTGACGAAGGTGGTTTCGCTCCTAAATTT
GAAGGTACTGAAGATGCTGTTGAAACAATTATCCAAGCAATCGAAGCAGCTGGTTACAAACCAGGTGAAGAAGTATTCTTAGGATTTGACT
GTGCATCATCAGAATTCTATGAAAATGGTGTATATGACTACAGTAAGTTCGAAGGCGAACACGGTGCAAAACGTACAGCTGCAGAACAAG
TTGACTACTTAGAACAATTAGTAGACAAATATCCTATCATTACAATTGAAGACGGTATGGACGAAAACGACTGGGATGGTTGGAAACAACT
TACAGAACGTATCGGTGACCGTGTACAATTAGTAGGTGACGATTTATTCGTAACAAACACTGAAATTTTAGCAAAAGGTATTGAAAACGGA
ATTGGTAACTCAATCTTAATTAAAGTTAACCAAATCGGTACATTAACTGAAACATTTGATGCAATCGAAATGGCTCAAAAAGCTGGTTACAC
AGCAGTAGTTTCTCACCGTTCAGGTGAAACAGAAGATACAACAATTGCTGATATCGCTGTTGCTACAAACGCTGGTCAAATTAAAACTGGTT
CATTATCACGTACTGACCGTATTGCTAAATACAATCAATTATTACGTATCGAAGATGAATTATTTGAAACTGCTAAATATGACGGTATCAAAT
CATTCTATAACTTAGATAAATAA

Gene: Q2YSE7 (putative membrane protein)

Position: 835381 to 835839, length: 459 nt, orientation: FORWARD

Perfect match to: (N315-BA000018-[839200:839658], highly conserved allele)

Sequence:

ATGGCTGATAGAACGAATAAAGAAATTAAAACAGGACGCTTTATTGCAACTGCATCAATCGTATTCTCAATATTATTGATTATTCATTACTTT
GTTTCGTTGGATAATGCGACTGCCAAAGCATTACTTAATTTAACGAATCAAAACACTTCAGATAAAGCGATTGATTACATTTTAAACAGCTTT
AGATTCACTGGTATTATGTATATTTTGGCTTATCTAGCAGGCTTCATCACTTTTTGGAATCGACATACTTATGTGTGGTGGTTTATGTTTGCA
GTTTATGTATCAAATAGTTTGTTTACGTTGATTAATTTATCAATCACAATTCAAGCAATAAAAGCTGCACACGGTGCGTACTTAACATTGCCA
ATTTTAATCGTTATTATAGGTTCGGTTGCATTAGCGATTTATATGCTTGTTGTTTCTATCAAACGTAAAAGTACATTTAATCGCTAG

Gene: secG (preprotein translocase subunit G)

Position: 835906 to 836139, length: 234 nt, orientation: FORWARD

Perfect match to: (N315-BA000018-[839725:839958], highly conserved allele)

Sequence:

ATGCATACATTTTTAATCGTATTATTAATCATTGATTGTATTGCATTAATAACTGTTGTACTACTCCAAGAAGGTAAAAGCAGTGGACTTTCA
GGTGCCATCAGTGGTGGTGCTGAGCAGTTATTCGGTAAACAAAAACAACGTGGCGTCGATTTATTCTTAAATAGATTAACAATTATTTTATC
AATATTATTTTTTGTACTTATGATTTGCATAAGTTATCTTGGTATGTAA

Gene: est (putative carboxylesterase)

Position: 836255 to 836995, length: 741 nt, orientation: FORWARD

Perfect match to: (TCH959-AASB02000221-[15964:16704], highly conserved allele)

Sequence:

ATGCAGATAAAATTACCAAAACCTTTCTTTTTTGAAGAAGGCAAACGTGCCGTGTTATTATTACATGGATTTACAGGCAATTCGTCTGATGTA
CGTCAATTAGGTCGATTTTTACAAAAAAAGGGATATACTTCGTATGCACCACAATATGAAGGTCACGCGGCACCACCAGAGGAAATACTGA
AATCTAGTCCTTTCGTTTGGTTTAAAGATGCGTTAGATGGTTATGATTATCTTGTTGAACAAGGTTATGATGAAATTGTTGTTGCTGGTCTAT
CATTAGGTGGGGATTTTGCTTTAAAATTAAGCTTAAATAGAGATGTAAAGGGTATTGTAACGATGTGTGCACCAATGGGTGGCAAAACTGA
AGGTACCATTTACGAAGGCTTTTTAGAGTATGCACGTAACTTTAAAAAGTATGAAGGTAAAGATCAAGAGACTATTGATAATGAAATGGAT
CATTTTAAACCAACTGAAACTTTAAAAGAACTAAGTGAATCATTAGATACGATTAAAGAGCAAGTTGATGAAGTGTTGGATCCTATTTTAGT
GATTCAAGCAGAAAACGACAATATGATTGATCCACAATCCGCAAATTATATATATGACCATGTAGATTCTGATGACAAAAATATCAAGTGGT
ACAGTGAATCTGGACATGTTATTACGATTGATAAAGAGAAAGAACAAGTATTTGAAGATATTTATCAATTTTTAGAGTCATTAGACTGGTCA
GAATAA

Gene: rnr (ribonuclease R)

Position: 837029 to 839401, length: 2373 nt, orientation: FORWARD

Perfect match to: (11819-97-CP003194-[880948:883320], allele observed in CC80+CC97)

Sequence:

ATGAATTTAAAGCAATCTATAGAAGAGATTATTAATCAACCTGAATATGAACCTATGTCAGTGTCAGATTTTCAAGATGCATTAGGTTTAAG
CAGTGCCGACTCGTTTAGAGATTTAATTAAGGTGCTTGTGGAGTTAGAACAATCAGGATTAATCGAACGTACAAAAACAGACAGATACCAA
AAAAAGCATAGTTCTAGAGGTCAATCAAAATTGATAAAAGGAACGTTAAGTCAAAATAAAAAAGGCTTTGCATTCTTAAGACCTGAAGATG
AGGATATGGAAGATATATTTATTCCCCCGACGAAAATTAATCGTGCCTTGGATGGAGATACTGTTATTGTAGAAACACATCAATCTAAAGGT
GAACATAAAGGTAAAATTGAAGGGGAAGTTAAATCGATTGAGAAGCATTCAGTAACTCAAGTTGTTGGTACGTATAGTGAAGCTAGACAT
TTCGGATTTGTTATTCCAGATGATAAACGTATTATGCAAGATATTTTCATTCCTAAAGGACAAAGTTTAGGTGCAGTAGATGGGCATAAGGT
ACTTGTTCAAATTACTAAATATGCTGATGGTTCAGATAATCCAGAAGGACATATTTCTGCTATTTTAGGACATAAAAATGATCCTGGCGTAG
ATATTTTATCTATTATCTATCAACATGGCATAGAAATTGAATTTCCTGATGAAGTGTTACAAGAAGCTGAAGCAGTACCTGATCATATTGAAA
ATACTGAAATTAAAGGCCGTCATGATTTACGTGATGAATTGACAATCACAATTGATGGTGCTGATGCTAAAGACTTAGATGACGCAATTAG
TGTTAAAAAGTTAGCGAACGGTAATACGCAGTTGACTGTAAGTATTGCTGATGTCAGTTATTATGTAACTGAAGATTCAGCTTTAGATAAAG
AAGCTTATGATAGAGCGACAAGTGTATATCTTGTTGACCGTGTGATTCCGATGATTCCACATCGATTAAGCAATGGTATTTGTTCATTAAAT
CCTCACGTTGATCGTTTAACGTTAAGCTGTCGCATGGAAATTGATGCTAGTGGTCGCGTTGTTAAACATGAAATTTTTGATAGTGTTATACAT
TCTGATTATCGAATGACGTATGATGCGGTAAATCAGATTATTACTGAAAAGGATCCTAACATTCGCGAACAATATAAAGAAATTACGCCTAT
GTTAGATTTAGCACAAGATTTATCTAATCGTTTGATTCAAATGAGAAAACGACGTGGTGAAATCGATTTTGATATTAGTGAAGCAAAAGTAT
TAGTTAACGAGGACGGTATACCAACAGATGTTCAATTAAGACAACGTGGCGAGGGTGAACGTCTAATTGAATCATTTATGTTAATTGCAAA
TGAAACAGTTGCTGAACATTTTAGTAAGTTAGATGTACCTTTTATTTACCGAGTGCATGAGCAACCTAAATCAGATCGCTTAAGACAATTCTT
TGATTTTATTACAAACTTTGGCATCATGATTAAGGGTACTGGCGAAGATATTCATCCAACAACACTTCAAAAGGTTCAAGAAGAAGTAGAA
GGTCGACCTGAACAAATGGTCATTTCAACAATGATGTTACGTTCAATGCAACAAGCGCATTATGATGATGTGAACTTGGGACATTTTGGCTT
ATCAGCTGAATATTATACGCATTTTACATCACCAATTAGACGTTATCCTGATTTAACAGTTCATCGTTTAATCCGTAAGTATTTAATTGAGAAA
TCAATGGATAACAAAGAAGTGAAGCGTTGGGAAGACAAATTGCCTGAGTTAGCTGAACATACTTCTAAACGTGAACGTCGTGCTATTGAG
GCAGAACGTGATACTGATGAATTGAAAAAAGCAGAATATATGATTCAACATATTGGTGATGAATTTGAAGGTATTGTCAGCTCAGTAGCTA
ACTTCGGTATGTTCATTGAATTGCCAAATACGATAGAAGGTATGGTTCATATTGCGAATATGACTGATGATTATTACCGTTTTGAAGAGCGT
CAAATGGCATTAATTGGTGAGCGTCAAGCTAAAGTATTTAGAATTGGTGACACAGTTAAGGTTAAAGTGACGCATGTTGATGTAGATGAAC

GATTAATTGATTTTCAAATTGTTGGAATGCCTTTACCTAAAAATGACCGCTCACAGCGACCAGCAAGAGGTAAAACGATTCAAGCTAAAAC
GCGTGGCAAATCTTTAGATAAATCGAAATCTGATGATAAGGGTCGTAAGAAAAAAGGTAAGCAACGTAAAGGTAAAAACCAACGTAATAA
TGATAAATCAGGTAATAGTAAGCATAAGCCATTTTATAAAGATAAAAGTGTGAAAAAGAAAGCACGTCGTAAGAAAAAATAA

Gene: ssrP (tmRNA binding protein)

Position: 839423 to 839887, length: 465 nt, orientation: FORWARD

Perfect match to: (11819-97-CP003194-[883342:883806], allele observed in CC80+CC72+CC80+CC97+CC188)

Sequence:

ATGGCTAAGAAGAAATCACCAGGTACATTAGCGGAAAATCGTAAAGCAAGACATGATTATAATATTGAAGACACGATTGAAGCGGGAATC
GTATTACAAGGTACAGAAATAAAATCGATTCGCCGAGGTAGTGCTAACCTTAAAGATAGTTATGCGCAAGTTAAAAACGGTGAAATGTATT
TGAATAATATGCATATAGCACCATACGAAGAAGGGAATCGTTTTAATCACGATCCTCTTCGTTCTCGAAAATTATTATTGCACAAGCGTGAA
ATCATTAAATTGGGTGATCAAACACGTGAGATTGGTTATTCGATTGTGCCGTTAAAGCTTTATTTGAAGCATGGACATTGTAAAGTATTACT
TGGTGTTGCACGAGGTAAGAAAAAATATGATAAACGTCAAGCTTTGAAAGAAAAAGCAGTCAAACGAGATGTTGCGCGCGATATGAAAGC
CCGTTATTAA

Gene: ssrA (tmRNA, regulatory RNA)

Position: 839978 to 840339, length: 362 nt

Sequence:

GGGGACGTTCATGGATTCGACAGGGGTCCCCCGAGCTCATTAAGCGTGTCGGAGGGTTGTCTTCGTCATCAACACACACACAGTTTATAATAA
CTGGCAAATCAAACAATAATTTCGCAGTAGCTGCCTAATCGCACTCTGCATCGCCTAACAGCATTTCCTATGTGCTGTTAACGCGATTCAACC
TTAATAGGATATGCTAAACACTGCCGTTTGAAGTCTGTTTAGAAGAAACTTAATCAAGCTAGCATCATGTTGGTTGTTTATCACTTTTCATGA
TGCGAAACCTTTCGATAAACTACACACGTAGAAAGATGTGTATCAGGACCTCTGGACGCGGGTTCAAATCCCGCCGTCTCCATAT

Gene: A8Z1B0 (putative membrane protein)

Position: 840661 to 840942, length: 282 nt, orientation: FORWARD

Perfect match to: (N315-BA000018-[844480:844761], highly conserved allele)

Sequence:

ATGGCTACTGAAAAAGAACTTTTCATTTTTGTTACGTCACTAAGTGGGTGTAGTTATAAAGAGATGAGCCGAGTTTTGATATTTTCATTAGA
ATCAATATGCCTATTAACACAATCAGCAATAGTTGACGAGACGGAAATAAAAGAAGTCGTAGTTAAGAAATGCATTTCACAACATACCATT
GTAGCCATTTTTATTGTTTTGGATGATAAACTCTTTTTGGAATTTTTAGTTTTTATAATTTGCAACTACACTACTTCTTTTACTAATATTAATGTC
TAA

Gene: repeat_Sepi (repeat element)

Position: 840841 to 841204, length: 364 nt

Sequence:

ATTGTAGCCATTTTTATTGTTTTGGATGATAAACTCTTTTTGGAATTTTTAGTTTTTATAATTTGCAACTACACTACTTCTTTTACTAATATTAAT
GTCTAAGTAATCGATAAAAAAATTTTCCATTGAATAAATGAGAAGTTAAAAACTTTACTTAACCTTTCTCATTGCATTTTCCTATTCACGATTTT
AAGAACCCAACATACTACAAACGAATTTTAAAAGGCGAGAGTAAAGCTTACTTGTTTATTATACATATTTAAAATCCAAGAGTCAGAACAGA
CTACTCCTCTTTATAACTATAAAAAATAGCTATGAAAAAATCTATCGTCATAGATTCCTTCATAGCTAATCTTAGTATGTTT

Gene: Q2G021 (putative protein)

Position: 841209 to 841532, length: 324 nt, orientation: REVERSE

Perfect match to: (N315-BA000018-[845028:845351:r], highly conserved allele)

Sequence:

TTATTTTAGGATGCTATTTATCAACTCAACATATAACTCACTATTTTTATAACCTTCTAATATATCATTAACTTGTCTAATAGGTATTTCTGGTA
CTTCTCTAATGTTTTCCAATTTTGTTTTAAATTGTTTTTTTGTTATTTGCTCTTTATTTGTAGCCAATTGGAACAAGTAAGAATCTAGCATATTA
ATTTCTTTATATGAATACATATATCTTAATAACACTAAATCTCTAGTTTTTAAGTTAGGCGCTAGTTCTTCTTGTAATTGTTCTATTGATTGTTT
CATTAATAACAATCTCATTTCTAATTCTTCATTATTCAT

Gene: Q1YB79 (putative lipoprotein)

Position: 841886 to 842614, length: 729 nt, orientation: REVERSE

Perfect match to: (Tager_104-CP012409-[745638:746366], allele observed in CC49+CC8+CC49)

Sequence:

TTATACTTCATCTAAACCACTGTGGTCGTCATCTTTTTGCTTTTCTTTTTCTTTCTCTCGTTCTTGTTCTTTTTTGTACTCTTCTTCAAATTCTTTTT
CTTTCTTTTCTACTTCTTCTCTTGTTTCCGCTCTATGAGAAAAATCTTCGGTTTTGAGTTTACTAAATTTGAATGATTTAGAATCAACTGTTTTA
TCTTCTGAGTATTTATGGACATTTAAATTAATATTTCCATCACCTCTTAACTCATAGATAAACATGGCTTGTGCAGTTTTGCCTTTTTTAATTTG
ATCTTGGTTATGTTCTGTCCAATCTTTATATTTTTTATCACTTAAAAGATAACCATCTCTTAATTTATTTACTGTATTTTTATCATCTTGAGTGAT
ATTAATATAGTCATGAGAAATAGAAGATGGATTTAAATCTTTATCGTCTTTTTTAGCAGTAATTTCCATTTTAAAAGCGATATATTTCTTTTTC
TCATCTTTTTCATTGATGATAAACGGTTCTTTTATTTTAGCTTCAAATTTGTCACTAACAATAGTATCGCCTTTAATTTTTATATCCATATTTTTT
TTGCTTTTAAATTCTTTAAGTTCTTCATTTAATTCTTCATTGTCATTTTCTTTCTTTTTGTGACTAGTGCTCTCTTTTTTTGCACTATCTTGATGAT
GTCCACAAGCACCTAAGATAAGTGTACTTGCTAATAATATCCCCATTACTTTTTTCAT

Gene: Q5HHN0 (putative membrane protein)

Position: 843212 to 843734, length: 523 nt, orientation: FORWARD

Sequence:

ATGATTAATATTATTTCAGCTATAGGATCTATTGGAACATTTATTATGGCTTTATTTTATTTTGTATCAGTTTCAGTTCAACTTTATCAAATGAA
AATTAGCTTTCTGCCAGCTTTAGGTTTTAACCAAATTTTATTAGAAAGGGAGGAGGATCAACTTAATATAATGAATTCGGCAACAGAAGAGC
ATCATCATAAAGATTATATTAAACTATATAATTTAGGTGGCGGTGCTGCTAAAAAAAATTGCAATAGAGGTTTTATTGGGTAATGATAAAGTC
ATTCAGAAAAAAATACGTGAATATTTTACCTAGTAAAGAAGGGTACATGTTACCAATTAATAAAAATGTGTACGAAGAATTAGAAAAGAACG
ATTGAGAACAATGGTTATGAAGCTGATTTGAATGTACGTATGACTTATTATCATAATGTAAGTCGCAAACAACAGGAAGTTATATTAAAAG
GTCAAATCGACCGTTTTAATACTTATAATAATAAAGAAATTTATGATTTGCAGTTTATCTAA

Gene: Q1XZI1 (putative acetyltransferase, GNAT family)

Position: 843873 to 844403, length: 531 nt, orientation: FORWARD

Perfect match to: (11819-97-CP003194-[887791:888321], highly conserved allele)

Sequence:

ATGCAAATTAGACAAATACATCAACATGACTTTGCTCAAGTTGACCAGTTAATTAGAACGGCATTTGAAAATAGTGAACATGGTTATGGTAA
TGAATCAGAGCTAGTAGACCAAATTCGTCTAAGTGATACGTATGACAATAACTTAGAAATAGTAGCTGTTCTTCAAAACGAAGTTGTAGGG
CACGGTTTACTAAGTGAAGTTTATCTTGATAACGGAGCACAACGGGAAATTGGATTAGTGTTAGCACCTGTATCTGTTGATATTCATCATCA
AAATAAAGGTATTGGGAAGCGATTGATTCAAGCATTAGAACGAGAAGCAATATTAAAAGGATATAATTTTATCAGTGTATTAGGATGGCCG
ACGTATTATGCCAATCTAGGATATCAACGCGCAAGTATGTACGACATTTATCCACCATATGATGGTATACCAGACGAAGCGTTTTTAATTAA
AGAATTAAAAGTGAACAGTTTAGCGGGAAAAACAGGTACCATAAATTACACATCTGCTTTTGAAAAAATATGA

Gene: clfA (clumping factor A)

Position: 844666 to 847578, length: 2913 nt, orientation: FORWARD

Sequence:

ATGAATATGAAGAAAAAAGAAAAACACGCAATTCGGAAAAAATCGATTGGCGTGGCTTCAGTGCTTGTAGGTACGTTAATCGGTTTTGGA
CTACTCAGCAGTAAAGAAGCAGATGCAAGTGAAAATAGTGTTACGCAATCTGATAGCGCAAGTAACGAAAGCAAAAGTAATGATTCAAGT
AGCGTTAGTGCTGCACCTAAAACAAACGACACAAACGTGAGTGATACTAAAACATCGTCAAACACTAATAATGGCGAAACGAGTGTGGCG
CAAAATCCAGCACAACAGGAAACGACACAATCAGCATTAACAAATGCAACTACGGAAGAAACTCCGTTAACTGGTGAAGCTACTACGGCA
ACGAATCAAGCTAATACACCGGCAACAACTCAATCAAGCAATACAAATGCGGAGGAATTAGTGAATCAAACAAGTAATGAAACGACTTCTA
ATGATACTAATACAGTATCATCTGTAAATTCACCTCAAAATTCTACAAATGCGGAAAATGTTTCAACAACGCAAGATACTTCAACTGAAGCA
ACACCTTCAAACAATGAATCAGCTCCACAGAGTACAGATGCAAGTAATAAAGATGTAGTTAATCAAGCGGTTAATACAAGTGCGCCTAGAA
TGAGAGCATTTAGTTTAGCGGCAGTAGCTGCAGATGCACCGGCAGCTGGCACAGATATTACGAATCAGTTGACAGATGTGAAAGTTACTAT
TGACTCTGGTACGACTGTGTATCCGCACCAAGCAGGTTATGTCAAACTGAATTATGGTTTTTCAGTGCCTAATTCTGCTGTTAAAGGTGACA
CATTCAAAATAACTGTACCTAAAGAATTAAACTTAAATGGTGTAACTTCAACTGCTAAAGTGCCTCCAATTATGGCCGGAGATCAAGTATTG
GCAAATGGTGTAATCGATAGTGATGGTAATGTTATTTATACATTTACAGACTATGTTGATAATAAAAACGATGTTAAAGCAACACTAACTGT
TCCTGCATACATTGATCCAGAAAATGTAACGAAAACAGGTAATGTAACATTGACAACTGGCATAGGAACCAATACTGCTAGTAAGACAGTT
TTAATCGACTATGAGAAATATGGACAATTCCATAATTTATCAATTAAAGGTACAATTGATCAAATCGACAAAACAAACAATACGTATCGTCA
AACGATTTATGTCAATCCAAGTGGAGACAATGTTGTATTACCAGTGTTAACTGGTAATCTAATTCCTAAGAGTAATAGTAATGCTTTAATAG
ATGCCAACAATACTAATATTAAAGTTTATAAAGTGGATAATGCTAATGATTTATCTGAAAGTTATTATGTGAATCCTAGCGATTTTGAAGAT
GTAACTAATCAAGTTAGAATTTCATTTCCAAATGCTAATCAATACAAAGTAGAATTTCCTACGGACGATGACCAAATTACAACACCGTATATT
GTAGTTGTTAATGGCCATATTGATCCTGCTAGTACAGGTGATTTAGCACTACGTTCGACATTTTATGGTTATGATTCTAATTTTATATGGAGA
TCTATGTCATGGGACAACGAAGTAGCATTTAATAACGGATCAGGTTCTGGTGACGGTATCGATAAACCAGTTGTTCCTGAACAACCTGATG
AGCCTGGTGAAATTGAACCAATTCCAGAGGATTCAGATTCTGACCCAGGTTCAGATTCTGGCAGCGATTCTAATTCAGATAGCGGTTCAGA
TTCGGGTAGTGACTCTACATCAGATAGTGGTTCAGATTCAGCAAGCGATTCAGATTCAGCAAGTGATTCAGATTCAGCGAGTGATTCAGAT
TCAGCGAGTGATTCAGATTCAGCAAGTGATTCAGACTCAGCAAGTGATTCAGATTCAGCAAGTGATTCAGACTCAGCAAGTGATTCAGATT
CAGCGAGCGATTCAGATTCAGCGAGTGATTCAGATTCAGCGAGTGATTCAGATTCAGACAGCGATTCAGACTCAGACAGTGACTCAGATTC
CGATAGTGACTCAGACTCAGATAGCGACTCAGATTCCGATAGTGACTCAGACTCAGACAGCGATTCAGATTCAGATAGTGATTCAGATTCA
GATAGCGATTCGGATTCAGATAGTGACTCAGATTCAGATAGCGACTCAGATTCAGATAGCGATTCAGATTCCGACAGTGACTCAGATTCCG
ACAGCGATTCAGATTCCGACAGTGACTCAGACTCAGACAGTGATTCGGATTCAGATAGCGATTCAGACTCAGACAGTGACTCGGATTCAGA
TAGCGATTCCGACTCAGACAGTGACTCAGATTCCGACAGCGATTCAGATTCCGACAGTGACTCAGACTCAGACAGTGATTCGGATTCAGAT
AGCGATTCAGACTCAGACAGTGACTCGGATTCAGATAGCGATTCCGACTCAGACAGTGACTCGGATTCAGATAGCGATTCTGACTCAGATA
GCGATTCAGACTCAGACAGTGACTCAGACTCAGACAGCGATTCAGATTCCGATAGCGATTCAGATTCCGACAGTGACTCAGACAGCGATTC
AGACTCAGACAGTGACTCGGATTCAGATAGCGATTCTGACTCAGATAGTGACTCCGACTTAGACAGCGACTCAGATTCAGATAGCGATTCA
GATTCCGACAGTGACTCAGATTCGGATAGCGATTCAGAATCAGATAGTGACTCCGACTCAGGTTCTAACAATAATGTAGTGCCGCCTAATTC
ACCTAAAAATGGTACTAATGCTTCTAATAAAAATGAGGCTAAAGATAGTAAAGAGCCATTACCAGATACAGGTTCTGAAGATGAAGCGAAT
ACGTCACTAATTTGGGGATTATTAGCATCATTAGGTTCATTACTACTTTTCAGAAGAAAAAAAGAAAATAAAGATAAGAAATAA

Gene: nuc1 (thermostable extracellular nuclease locus 1)

Position: 849042 to 849728, length: 687 nt, orientation: FORWARD

Perfect match to: (11819-97-CP003194-[895780:896466], allele observed in CC80+CC188)

Sequence:

ATGACAGAATACTTATTAAGTGCTGGCATATGTATGGCAATCGTTTCAATATTACTTATAGGGATGGCTATCAGTAATGTTTCGAAAGGGCA
ATACGCAAAGAGGTTTTTCTATTTCACTACTAGTTGCTTAGTGTTAACTTTAGTTGTAGTTTCAAGTCTAAGTAGCTCAGCAAATGCATCACA
AACAGATAATGGCGTAAATAGAAGTGGTTCTGAAGACCCAACAGTATATAGTGCAACTTCAACTAAAAAATTACATAAAGAACCTGCGACA
TTAATTAAAGCGATTGATGGTGATACGGTTAAATTAATGTACAAAGGTCAACCAATGACATTCAGACTATTATTGGTTGATACACCTGAAAC
AAAGCATCCTAAAAAAGGTGTAGAGAAATATGGTCCTGAAGCAAGTGCATTTACGAAAAAAATGGTAGAAAATGCAAAGAAAATTGAAGT
CGAGTTTGACAAAGGTCAAAGAACTGATAAATATGGACGTGGCTTAGCGTATATTTATGCTGATGGAAAAATGGTAAACGAAGCTTTAGTT
CGTCAAGGCTTGGCTAAAGTTGCTTATGTTTATAAACCTAACAATACACATGAACAACTTTTAAGAAAAAGTGAAGCACAAGCAAAAAAAG
AGAAATTAAATATTTGGAGCGAAGACAACGCTGATTCAGGTCAATAA

Gene: cspC-L1 (RNA chaperone locus1)

Position: 850085 to 850285, length: 201 nt, orientation: FORWARD

Perfect match to: (RF122-AJ938182-[825949:826149], highly conserved allele)

Sequence:

ATGAATAACGGTACAGTTAAATGGTTTAATGCAGAAAAAGGTTTTGGTTTCATCGAAAGAGAAGATGGTAGCGACGTATTCGTACACTTCT
CAGCAATCGCTGAAGATGGATACAAATCATTAGAAGAAGGCCAAAAAGTTGAATTCGACATCGTTGAAGGCGACCGTGGCGAGCAAGCTG
CAAACGTAGTTAAAATGTAA

Gene: Q1Y1Z8 (putative protein)

Position: 850780 to 850998, length: 219 nt, orientation: REVERSE

Perfect match to: (11819-97-CP003194-[897518:897736:r], allele observed in CC80+CC25+CC80+CC97)

Sequence:

TTAATTTACAGCGTCAAATATACTTATTTCTAATGCTTTGGGGTCTACTGAAACAAGTAGAGAATGATCGATGTTACTAACATTGCCATTCTC
CAAATCTATTTCTGTGAGTATTTGGAAGCTACCATTAGGCAATGGTTTAACAATAGACAATTGCTTTTCCGCTTGTTGTATTAAAAAAGGTTT
TGTAGATTGATTATTAATATGCCATTCACTCAT

Gene: Q2G007 (putative protein)

Position: 851060 to 851344, length: 285 nt, orientation: REVERSE

Perfect match to: (11819-97-CP003194-[897798:898082:r], allele observed in CC80+CC97+CC188)

Sequence:

TCAACCAAAATTTTGTTCAAATTCAATAAATGTCTTGTTTAAAATAGAAATATTGTAAATGTTATCGTCCAAAATTTCACCAGTTAAGTATTTG
TTTTGAATTAAAATTTGGCAGTTAGTTAAGAAGTCTTGATAATCACGATCGCAAAAATAGTTTTCACGTGCATCTTTAGCATCGCCAAAAAA
GTTAGCGACTGTTTCTGTTTCTCCTTTATTCGAACGTTCAATATATAATTTGTAAAATTTAGCTATTGTATACTTTTGTTCTTTAGTTAGTTCAT
TCAA

Gene: Q1YB69 (putative exported protein)

Position: 851432 to 852001, length: 570 nt, orientation: REVERSE

Perfect match to: (GR1-AJLX01000011-[7035:7604:r], allele observed in CC361+CC80+CC772)

Sequence:

TTAATAAGCTTTAACAAGCCTTAGTTTGTATGGATCTATAAAATTATCTTTAATTGCATAGGGTGAAATAATATGTAGTCCATAACTTTTAACT
GATTTTTCACTTACACCAAATTTATAAGCTTGATAGATAATTTTAGTACAATACGTAAATTTTTTGCTGTTCAAATTTAATGTAACTAGATAAC
GATGATTTGTATTTTCATAGTTTTTCTTAACCCAGTCAGCCGCTTTTTTACCTGCACCAGGATAGCTGCAACGATAAACTTTCATCCAATCATT
TTTGCCACTTGCATAATTATATTTAAAAGATTCGAAGGATTGTGTAGTTGGTTTGTCGCCAGGCCCCTCAATTTGTAAAATCGTTTTATCATC
AATCGCGATACTACAATGACCAAAAAATCCCCACATGACAGGGCCTTTTGTAACAATAATATCACCAGGTTGTAATTGGAATTTGTCATCTT
GAATTTCTGAATACTTATTATCTGCAATTGTTTTTGGTGAGTTTATAGGGGATACGACAACGAATAATATAAGTAAAATTATCGTTCGTTTAA
TATAGTTCAC

Gene: Q6GIJ6 (putative protein)

Position: 852189 to 852377, length: 189 nt, orientation: REVERSE

Perfect match to: (N315-BA000018-[858892:859080:r], highly conserved allele)

Sequence:

TTATAGCACACCAAATATAAATGTGTATATTAATATAGCGAATCCAAAAATATAGAGAATGACAGTGAAACTTAAATAGGACTCTTTTTTAG
ATTCTTTGCCAGTTTTTTTCATTAATACGAGTATAAACGTAGCGGCAACTAAGAAAATTAATGCTAGCCAAAACAGAATTGCAAAATGTAAA
GACAT

Gene: Q6GIJ5 (putative membrane protein)

Position: 852406 to 852666, length: 261 nt, orientation: REVERSE

Perfect match to: (11819-97-CP003194-[899144:899404:r], allele observed in CC80+CC9+CC80+CC97+CC188)

Sequence:

TTAACCTATAAAACTATAAAAAATAGGATAAATGTAATATAGAACGAGTACGATATTTATAAAAAATAATATTTCACTTAACCAGTTTTTAGT
TATCATTGCAATGGTAAAGGATACGATGAGTATCACACCACAAATGATAATACCAGGCAGGAGCCAACATAAATCATCTAAATCTTTATTAT
ATGTGATTAAAATATTAAAGATAACAAAAGTGGTAGTAATAACTATATTGATAGCATTTAACAAAATGTTATTCAT

Gene: DUF1250 (PF06855 family protein)

Position: 853007 to 853210, length: 204 nt, orientation: FORWARD

Perfect match to: (RF122-AJ938182-[828871:829074], highly conserved allele)

Sequence:

ATGACGTTTTACAATTTCATCATGGGTTTTCAAAATGATAACACACCATTTGGTATATTGGCCGAACACGTTAGTGAAGATAAAGCATTCCCT
CGATTAGAAGAAAGACACCAAGTAATTAGAGCATATGTGATGTCTAATTACACAGATCATCAATTAATTGAAACTACAAATAGAGCTATTA
GCTTATATATGGCAAATTAA

Gene: Q6GIJ3 (putative protein)

Position: 853207 to 853443, length: 237 nt, orientation: REVERSE

Perfect match to: (MW2-BA000033-[868324:868560:r], highly conserved allele)

Sequence:

TTAATTTGAGTAGTACCAATTATGATGTATTAGTGCATCCCAAATATCTTTTGTTTTAAAGTTTATTTCATCATTTCTTATCGAAAATGGTGTA
ATAATGTCTTTATCTAACCAAGTGTTGATAAGTTCATTTGGTACACCATCTAACAACATTTCACTTTTACTAATTATAAAACATTCCCAGTCAA
GTGAAACATTTTGTGGATTCACATAATTACATTGATTATGATTATCCAT

Gene: gpmA1 (putative phosphoglycerate mutase)

Position: 853665 to 854249, length: 585 nt, orientation: FORWARD

Perfect match to: (11819-97-CP003194-[900403:900987], allele observed in CC80+CC15+CC80+CC4803)

Sequence:

TTGTTGGCTAAAACGTTATATTTAATGCGCCACGGACAAACTTTGTTTAATTTTAAGGGACTAATTCAGGGATTTGGAGATTCGCCGCTAAC
AGAACTTGGAATTGCTCAAGCTCAAAAGGCACGTAGTTATTATGAAACTAAGGGGATAAACTTCGATTTATATGCATCATCAACGCAAGAA
CGCGCAAGTGACACACTTGAAAATGTTGCACCTAACCAATCGTATCAACGTTTTAAGGGACTGAAGGAATGGCATTTTGGATTATTTGAAG
GTGAGTCAGTCTATCTATTTGATAATCTATACAAGCCTGAGGACTTATTCGGAGATCGAATTGTTCCTTTCAAAGGAGAGGCAAGGCAACA
AGTTGAGGAACGCATTGTGAAAACTTTACATGACATTATGTCTCAAACAAAGAATAATGCATTAGTCGTGAGTCATGGAACAATAATGGGA
GTATTTTTAAGATATTGCCTTAAACTAGATGAAGCATTAAAGCATAATATCGGTAATTGTAATATCCTGAAATTTGAATATGACAATGGAAC
ATTTAAATTTGTTGAGTTAATTGATCCAAATTTATAA

Gene: yisU1 (putative aminoacid related metabolite efflux transporter)

Position: 854322 to 854939, length: 618 nt, orientation: REVERSE

Perfect match to: (11819-97-CP003194-[901060:901677:r], allele observed in CC80)

Sequence:

TTAGAATATTAATTGAATAAGTTTTTGTAATATCATTAGAGCAACAATAATAATTATTATGCTTGATATTTTATTGATTATTGTTAATAATTTTC
CGGTTTTATCAATTGATCCAACCATTTTTCCTAAAATTGCGAGTAAAAAGAACCATAACCATGAAACGCTAATACAAGCGATTGTAAACGCA
ATTTTATTGCTGCCACTATATAATGCAGCACTACTACCAATTACACCAATTGTATCTAAAATAGCATGTGGATTGAGTAATGAAACTGATAAA

GCAAAACTTACTTGTTTCATTGGAGACATAATTTGAGCTTCTCCATCTGTTGAGGGTTTATCATGCCAAATGGTCCAAGCCATATACATCAAG
AAAATTAAACCAACTATATAAATAATTGCTTGAAGTACAGGTAAAGACATAATAATGATAGATACTCCTACCACTGCAATAATAATAAGTAA
GCTGTCAGACAACCCGGCTGTAATTATTGCAGGCAATACATATCTATATTTTGGTTGATTAGCTCCTTGGTTAAAAATAAATACATTTTGTGC
ACCTAAAGGTAAAATTAGACCAATCGCTAAGATAAATCCATGAATAATTGCGGTTACCAT

Gene: Q5HHL4 (putative acetyltransferase)

Position: 855095 to 855589, length: 495 nt, orientation: REVERSE

Perfect match to: (N315-BA000018-[861797:862291:r], highly conserved allele)

Sequence:

TTACTTATTAATCAAATCATAAAAAAGCCAATGTTCATCGAAATAGGTATTTCCGATTTTAATTGCATTTTTCTCAAAACCAAGAATGTCGAA
TCCTAGTGCACTATAGAAAACTTTAGCACCTATGTTGTTTGACACAATTGATGTAAGTAAAGATTCGTAATTGTTCTGCCGTGCATAATTAAT
AATGAAATTAATGAGCTCGCTATTAATCGATTTATCGTTATTAGTAACAAAGTTGTATTTAATTAATGATTTATGTTCTTTTCCAACGTATCGA
ATTTGTTCTAAGGCAGCTGTTGCGATAAGTGTTTCGTTTTCATAACAGCCAAATACAATACATTTCGGTGAACCTTGATTTAAAATGTCATGT
ATTAAGGTGTCAGTCATTGTTTGACTATAATGTGAATCTTGAGTGAATTCTTCATTGACTGTCGAAACAAGTCTCTTATACTCATTAAAATCA
TTATTCGTTAATGTTTTAATTTCAATCAT

Gene: ohrB (peroxiredoxin)

Position: 855988 to 856410, length: 423 nt, orientation: REVERSE

Perfect match to: (11819-97-CP003194-[902726:903148:r], allele observed in CC80)

Sequence:

CTAATCTACAACATTTACATTTAAATCGACATTAATATTTCCTTGAGTCGCTTTTGAATATGGACAAAATTCATGAGCCATTTGTAAATATTTT
TCAGCTTCTTCTTGAGATATAACATTTTTAATTGTTGCATCAATTGAAACACTTAATTTAGGACTTTCTGAGTCTGAATCATCTTCTAGTCTCA
CTGTTAGTGTTACTTCTGGATGAGCATCACGCACTTTGTTTTGCTTTAAAATTAGGTCGAAGGCACCGTTGAAGCAAGATGCATAACCTGCT
GCAAATAATTGTTCGGGGTTAGTAGCTTTACCATCTGCTTGAGCAGGCGGAACGATATCAATATCTAACGTTCGATCATCAGTATAAACATG
TCCTTTACGTCCGCCAACATTCGTCGCTTTAGTTTCATAATGTATTGCCAT

Gene: aroD (3-dehydroquinate dehydratase type I)

Position: 856558 to 857274, length: 717 nt, orientation: FORWARD

Perfect match to: (11819-97-CP003194-[903296:904012], allele observed in CC80)

Sequence:

ATGACACATGTGGAAGTAGTAGCGACTATCGCGCCACAATTATCTATCGAAGAAACTTTAATTCAAAAAATTAATCATCGTATTGATGCAAT
AGACGTATTAGAATTACGAATTGATCAAATTGAAAATGTCACAGTTAATCAAGTGGCAGAAATGATTACAAAGCTGAAGGTTATGCAAGAT
TCATTCAAATTATTAGTTACGTATCGTACAAAGTTACAAGGTGGCTATGGGCAATTTACAAATGACTCGTATCTTAATTTAATATCAGACTTA
GCAAATATCAATGGTATAGATATGATTGATATAGAATGGCAAGCAGATATTGACATTGAAAAACATCAACGAATCATTACACATTTGCAAC
AGTATAATAAAGAGGTGGTTATATCACATCATAATTTCGAAAGTACGCCTCCGTTAGATGAATTGCAATTTATATTTTTTAAAATGCAAAAAT
TCAACCCAGAATACGTTAAATTAGCAGTAATGCCACATAATAAAAATGATGTGTTAAATTTATTGCAGGCAATGTCTACATTTTCAGATACTA
TGGACTGCAAAGTTGTTGGTATTTCAATGTCTAAACTTGGACTAATAAGTAGAACGGCTCAAGGCGTTTTTGGTGGTGCGTTGACTTATGGT
TGTATCGGAGTACCACAAGCTCCAGGACAGATTGATGTTACTGATTTAAAAGCACAAGTGACTTTATACTAA

Gene: ntrA (iron-regulated nitroreductase)

Position: 857357 to 857896, length: 540 nt, orientation: FORWARD

Perfect match to: (Strain_132-ACOT01000026-[12907:13446], highly conserved allele)

Sequence:

ATGGAATTACAACAAGCAATAGCTAATAGAAGAAGTGTGAAAAAATTTAAAAGAGATATGCACATAGATGACGCATTGCTATATCAAGCA
ATTGAGAAAGCTGCTGATGCTCCAAATCACGGAATGAGGGAACCATGGAGAGTTGTGCATGTTCCGAAAGACAGATTAGGAGATATGAGT
AAGGATATTTCTAAATTTGCATTTCCTAATGAATTAGATAAGCAACAATGTCATTATGATGCAGTTACGAAACTAGGTGGCATGTTATTGCTT
ATTTTAAAAACAGATCCAAGACAACGTCAAAATGATGAAAACTACTTTGCATTTGGTGCATATGCACAAAATCTTATGTTGTTACTTTATGAA
GCGGGAATAGGTACATGTTGGAAATCGCCATTATATATCTATGATCCTAAAGTAAGAAAAACACTTGGTATAAAGAAAGATGAAGTTCTTG
CTGGATTCTTATATTTAACGGATTTAGAAGAAGATATGCCTAAAGCACCACGTAAAAATAGAAACTTAATTACATTATATTAA

Gene: trxA2 (thioredoxin)

Position: 858046 to 858366, length: 321 nt, orientation: REVERSE

Perfect match to: (11819-97-CP003194-[904784:905104:r], allele observed in CC15+CC80)

Sequence:

TTATTTAAAAGTTTCTGCTAAAAATGATTCAACTTGATCAGGTGACTTAGCATTTGCTGAATGAAGGTGTGCAATTTTGTCGCCGTTTTTAAA
TACTAGCAAGCTAGGGATACCCATAACTTCATTTTCAACAACTACATCCTCTAATTCATCACGATTAACAGTATACCATTGGTAATCATTATAT
TGTTCTACGATTGGGTCAATCCATAAATCCATAGCACGACAGTCTGGGCACCATCCTGCCTCAAATTTAACAATTACAGGTGTATCGCTATTA
ATTACAGATTTAAATGATTCATTACTTTTGATTGATTGCAT

Gene: yusI (putative oxidoreductase)

Position: 858510 to 858866, length: 357 nt, orientation: FORWARD

Perfect match to: (COL-CP000046-[897178:897534], highly conserved allele)

Sequence:

ATGATTAAATTTTACCAATATAAGAATTGTACAACTTGTAAAAAGGCAGCAAAGTTTTTAGATGAATATGGCGTAAGTTATGAACCAATTGA
TATCGTTCAACATACACCTACAATAAATGAATTTAAAACAATAATTGCAAATACAGGCGTAGAAATTAATAAATTGTTTAATACACACGGCG
CGAAATATCGTGAGCTTGATTTGAAAAATAAATTACAAACTTTATCAGATGATGAAAAGTTAGAGTTGTTATCATCTGATGGTATGTTAGTA
AAGCGTCCTCTAGCAGTAATGGGCGATAAGATAACATTAGGATTTAAAGAAGATCAATATAAAGAGACTTGGTTAGCGTAA

Gene: gcvH2 (glycine cleavage system protein H, locus 2)

Position: 859024 to 859404, length: 381 nt, orientation: FORWARD

Perfect match to: (N315-BA000018-[865727:866107], highly conserved allele)

Sequence:

TTGGCAGTACCAAATGAATTGAAATATTCAAAAGAGCATGAATGGGTTAAAGTTGAAGGTAATGTAGCAACAATTGGAATCACAGAATAC
GCACAAAGCGAGTTAGGTGATATTGTTTTCGTTGAATTACCAGAAACAGATGATGAAATTAATGAAGGGGATACGTTTGGTAGCGTAGAAT
CAGTTAAAACTGTATCAGAATTATATGCACCAATCTCTGGTAAAGTAGTTGAAGTCAACGAAGAACTAGAAGATAGTCCCGAATTTGTAAA
TGAATCTCCATACGAAAAAGCATGGATGGTAAAAGTAGAAATTAGTGATGAAAGTCAGATTGAAGCTTTATTAACAGCTGAAAAATATTCA
GAAATGATTGGTGAATAA

Gene: ywqG (putative protein)

Position: 859583 to 860461, length: 879 nt, orientation: FORWARD

Perfect match to: (11819-97-CP003194-[906321:907199], allele observed in CC80)

Sequence:

ATGCTAAACATTCAAGACGTTAGTCATCTTTCTAAAAAGGAGCAAAAAGCATATAACCGTTTCGTAGAATCTGTAGAAAACGGTAATTTACC
AGTACTACCATGTATTGAAATGGATCTAAAAGAGATGAAAGAAGAAACATTAAACCAGAGTAAGATTGGTGGAATGCCATTTTTAAAATCT
TTTAAAGATATACCATTAGATGAAAATAATGTACCAATGGTATTGTTAGCACAGATTAATTTGGATAATCTTCCAGAACAACAAGAATTATTT
CCTGTAAAAGAAGGGATATTGCAGTTTTGGATTAGTTCAGAAGATCAAATGTATGGTATGTCTGAAAATTTAAAGGGAAACAATATAAACT
CAAGGCTTGTTTATATAAAAGAGCCAATTACAGATTTATCTCTCGAAAATATTCAAGCGCATTTAAAGTCATTAGATGCTGATAATGAGGAT

ATCCCGTTCAGTGGAGCATTTTCTATAGAATTTAGATTGTCGAAACAAACTATTACATGTACTGATTATAAGTACGATGAGGACGTGCTTGC
ATTGTGGAATAAAGTCAATCCATCCTTCGCGCTAAAATCAATGTTTGGTGGTTATGATGAATTGATGGAACCTGTGTGTAACACATTTACTG
CTAAGGAACCATTTAATCAACTTGGTGGTTATCCATATTTTGACCAAATAGATCCAAGAACGAACGATCAAGAACTGAAAATGTATGATAG
AGTCTTACTGCAAATTGATTCTACAAGAGATGGTAATTCTTCGATTATATGGGGTGATTTAGGTATTGCCAATATCTTAGTAAAATCTACTGA
CCTTGAGGCTATGAAGTTTGATGATTACATGTATTCATGGGATTGCAGCTAA

Gene: yusF (small primase-like protein (toprim domain))

Position: 860876 to 861262, length: 387 nt, orientation: FORWARD

Perfect match to: (JKD6159-CP002114-[854094:854480], allele observed in CC93+CC45I+CC80+CC130+CC395)

Sequence:

ATGGCTATTGTAAATAAAGTGATAATTGTTGAAGGAAAATCTGATAAAAAAAGGGTGCAACAGGTTATTGCAGAACCAGTCAATATTATTT
GTACTCATGGAACAATGAGTATAGATAAGCTTGATGATATGATAGAATCACTATATGATAAACAAGTTTTTGTATTAGCCGATTCTGATGAC
GAAGGAGATCGAATTAGAAATTGGTTTAAACGTTATTTGAGTGAAAGTGAACATATATTTATTGATAAAACTTACTGTCAAGTTGCGAATTG
CCCCAAACAATATTTGGCGCATGTACTTTCAAAACATGGCTTTACTTGTAAGAAAGAAACACCTCTTTTACCGAATATAAATAATGAAAGGT
TAGTTTTAGTAAATGAATAA

Gene: yusE (putative thioredoxin)

Position: 861255 to 861551, length: 297 nt, orientation: FORWARD

Perfect match to: (N315-BA000018-[875052:875348], highly conserved allele)

Sequence:

ATGAATAATTCATTAGACATCAAAGATGTAACTACATTTTATGAGGAAGACAAACATTTAATCTTTGGTTATACACCAACGTGTGGTACTTGT
AAGGTTTCAGAAAGAATGTTAGACATTGCTAATGAAATATTGCAGTTACCATTATTGAAAATAGATTTAAACTTTTATCCTCAGTTTTGTAAA
GATATGCAAATCATGTCTACGCCGATTTTATTGTTGATGAATAAAGATAAAGAAGTAAAACGAATTTATGCATTTAAATCGGTGACTGATTT
GTTAGAAAATTTAAAATAG

Gene: metN1 (methionine ABC transporter locus 1, ATP-binding protein)

Position: 861801 to 862826, length: 1026 nt, orientation: FORWARD

Perfect match to: (11819-97-CP003194-[908539:909564], allele observed in CC80+CC361+CC1290+CC2970)

Sequence:

GTGATTGAATTAAAAGAAGTTGTTAAAGAATATCGGACTAAAAATAAAGAAGTCCTTGCTGTAGATCACGTTAATTTATCGATTCGAGCAG
GATCGATTTATGGCGTCATTGGTTTTTCTGGAGCAGGAAAAAGTACTTTGATTCGAATGTTTAATCATTTAGAAGCGCCTACATCAGGCGAA
GTTATTATAGATGGGGACCATATAGGTCAATTGTCCAAAAATGGATTAAGAGCAAAAAGACAAAAAGTAAGTATGATCTTCCAACATTTTA
ATTTGTTATGGTCAAGGACTGTGTTAAAAAATATTATGTTTCCGCTTGAAATTGCAGGTGTCCCTAGAAGGAGAGCTAAGCAAAAAGCATT
AGAACTTGTCGAACTCGTCGGTTTAAAAGGTAGAGAAAAGGCTTATCCATCAGAGTTATCAGGTGGACAAAAGCAACGTGTTGGGATTGC
ACGAGCGTTAGCTAATGATCCAACGGTCTTGCTTTGTGATGAGGCAACAAGTGCACTTGATCCGCAAACAACAGATGAAATTTTAGATCTA
CTACTAAAAATTAGAGAACAACAAAATTTAACAATTGTACTAATTACGCATGAAATGCATGTCATTCGTCGTATTTGTGATGAAGTTGCAGT
TATGGAAAGTGGTAAAGTGATAGAACAAGGACCGGTGACACAGGTTTTTGAAAATCCGCAACACACTGTGACAAAACGATTTGTGAAAGA
CGATTTAAATGATGATTTCGAAACATCTTTAACAGAATTAGAGCCATTAGAAAAAGATGCTTATATCGTTAGATTAGTTTTCGCTGGTTCAAC
AACAACCGAGCCTATTGTATCGAGTCTATCAACTGCCTATGATATTAAAATTAATATTTTAGAAGCAAATATTAAAAATACAAAAAATGGAA
CAGTCGGCTTTTTAGTTCTGCATATTCCATATATTTCAAGTGTAGATTTCGGAAAATTCGAAAAAGAGTTAATTGAGCGACAAGTTAAAATG
GAGGTGTTAAGACATGGGTAA

Gene: metP1 (methionine ABC transporter locus 1, transmembrane permease)

Position: 862819 to 863514, length: 696 nt, orientation: FORWARD

Perfect match to: (JKD6159-CP002114-[856037:856732], allele observed in CC93+CC5+CC80+CC361+CC772)

Sequence:

ATGGGTAAATCATTTAGTGAAATTATAAATGAAATGATTACAATGCCTAATATTCAGTGGCCAGAAGTTTGGACTGCAATAGTCGAAACACT
ATACATGACAGTCGTCTCAACTATATTTGCATTTATACTCGGTCTTATTTTAGGTGTGTTATTATTCTTGTCTGCTAAAGGTAAGTCTATCGGT
GCAAGGTTATTTTATTCTATCGTTTCTTTCATTGTTAACTTATTTAGAGCGATACCATTTATTATTTTAATTTTATTATTAATTCCATTTACAAGT
TTGATACTTGGAACGATAAGTGGTCCGACAGGTGCGTTACCAGCCTTGATTATTGGCGCAGCGCCGTTTTATGCAAGGCTCGTAGAAATTG
CTTTTAAAGAAATTGATAAAGGTGTCATCGAAGCGGCTTGGTCAATGGGTGCTAATACTTGGACAGTAATTCGTAAAGTCCTTTTACCTGAA
GCCATGCCAGCGCTAGTGTCTGGCATTACAGTTACAGCAATCGCTTTAGTTGGTTCAACAGCAGTTGCAGGTGTAATTGGTGCCGGTGGTT
TAGGAAATTTAGCATACTTAACAGGTTTCACTCGAAATCAAAATGATGTCATTTTAGTATCAACAGTTTTTATTTTAATTATTGTATTTATAAT
CCAATTCATTGGGGATTGGCTTACAAATAAACTTGATAAACGATAA

Gene: metQ1 (methionine ABC transporter locus 1, substrate-binding protein)

Position: 863532 to 864353, length: 822 nt, orientation: FORWARD

Perfect match to: (MRSA252-BX571856-[910328:911149], allele observed in CC30+CC59+CC80)

Sequence:

ATGAAAAAATTATTTGGTCTTATTTTAGTATTAACATTTGCAGTTGTATTAGCAGCTTGCGGTAATGGAAGCAAAAGTGGCAGTGACGACAA
GAAAATAACAGTAGGTGCTTCACCAGCACCACATGCTGAAATTTTAGAAAAAGCAAAACCATTATTAGAGAAAAAAGGTTATGAACTAGAT
ATTAAAACAATTAACGATTACACTACGCCTAATAAATTACTAGACAAAGGTGAAATTGACGCAAACTATTTCCAACATACACCATATTTAAAC
ACAGAGAAAAAGGATAAAGGTTACAAAATCGTAAGTGCCGGTGATGTTCACTTAGAACCTATGGCTGTATACTCTAAAAAGTATAAAAGTT
TAAAAGAATTACCAAAAGGTGCAACAGTTTATGTGTCTAATAATCCAGCTGAACAAGGACGCTTCTTAAAATTCTTCGTTGATGCAGGTTTA
ATTAAGATCAAAAAAGGCGTAAAAATTGAAGATGCTAAGTTTAGTGATATTACAGAGAATAAAAAAGATATTAAGTTTAATAATAAACAAT
CAGCAGAATTCTTACCTAAAATTTATCAAAATGAAGACGCTGATGCTGTTATCATTAATTCGAACTTTGCAATCGAACAAAAACTAAATCCTA
AAAAAGATTCTATTGCTGTAGAAAGTGCGAAAGATAATCCTTATGCAAACTTAATTGCTGTTAAAGAAGGACATCAAGATGATAAGAAAAT
CAAAGCATTAATTGAAGTATTACAATCTAAAGATATTCAAGACTTCATTAATGAAAAATACAATGGTGCAGTTATTCCTGCTAAATAA

Gene: csbD-L1 (stress response protein, locus 1)

Position: 864707 to 864901, length: 195 nt, orientation: FORWARD

Perfect match to: (N315-BA000018-[878504:878698], highly conserved allele)

Sequence:

ATGGCAGACGAAAGTAAATTTGAACAAGCAAAAGGTAATGTTAAAGAAACAGTAGGTAATGTTACTGATAATAAAAATTTAGAAAACGAA
GGTAAAGAAGATAAAGCTTCTGGTAAAGCGAAAGAATTCGTTGAAAATGCAAAAGAAAAAGCAACTGATTTTATTGATAAAGTAAAAGGT
AACAAAGGCGAGTAA

Gene: DUF368 (putative integral membrane protein)

Position: 865135 to 865986, length: 852 nt, orientation: REVERSE

Perfect match to: (NN50-BAEA01000008-[342040:342891], highly conserved allele)

Sequence:

TTATTCATTTTCAGCGGTAATTCGACCTAAAGTCAAACTTACAATAAAACCGATGATAAATACTACTAATGAAACGAACCACATCACGATGTT
AGTTGGTAAACCTGGAAATACTGCAAAGAGGGAGCCAACAACAAAACCAATGATTAATGCAAAAGTCATTAGTTTATGATGTGTTAGGAA
ATACTGGATAATTTTGCTTGAAATAATGAATCCAGCAAGCACGCCAAATCCGACTGCAAGTAATATAGGAAGACCGGTAAAGTTAAGTTTA
ACAACTTCAGATATTGCTAGCATGACCGTACCATAGACGCCAAATACTAATAACATAAATGATCCTGAAATACCTGGGAGTAACATAGCGCT
AGATGCACACATACCTGCAATAAAGTATTTAATAATAAGACCAGTTGATAGAGTAAGTGTTTCTCCAGCATGTTTATCGCCATTATTCATTAA
TGTAATAACAATTAAGATAGCGATACCAGCTATAACCATCATGTAATGTTTAGTTGTAAATGACGTTTTATAGTTAGAAATTTTCAATAAATA
TGGAACGATACCAATGATTAATCCACCAAAGAAAAACATAGTTGGAATATGGTGTTGGCTTAATAAATAATTAAAAAGATTACTTAGTGAT
CCCATTGCCAGTAACATTCCAATTATAATGGGGATTAAAAATGTAAAACTTGGCCAAAAACGTCGTGAGAATATGCCGCTAATTGAAGCGA
TAAATTGATTGTAAATACCTAACAATAATGCGATAGTCCCACCGCTAACACCAGGTACCAAGTCACTCGTTCCCATAGCAAAACCTTTTAGA
ATATTAATCCATTTAAATTGTTGCAT

Gene: sufC (iron-sulfur cluster assembly ATPase)

Position: 866314 to 867075, length: 762 nt, orientation: FORWARD

Perfect match to: (11819-97-CP003194-[913052:913813], allele observed in CC80+CC72+CC80+CC361)

Sequence:

ATGGCATCAACATTAGAAATCAAAGACCTACATGTGTCTATTGAGGATAAAGAAATCTTAAAAGGTGTTAACTTGACAATTAACACTGATG
AAATACATGCGATTATGGGACCAAACGGGACAGGTAAATCAACTTTATCATCTGCAATTATGGGACATCCAAGCTATGAAGTAACTAAAGG
AGAAGTACTTTTAGACGGTGTAAATATTTTAGAATTAGAAGTTGATGAAAGAGCAAAAGCAGGATTATTCTTGGCAATGCAATATCCATCA
GAAATTACAGGTGTTACAAATGCTGATTTCATGCGTTCAGCAATCAATGCGAAACGTGAAGAAGGACAAGAAATCAACTTAATGCAATTTA
TTAAGAAATTAGATAAAAACATGGATTTTCTAGACATAGATAAAGACATGGCACAACGTTATTTAAATGAAGGTTTCTCAGGTGGAGAGAA
GAAACGTAACGAAATCTTACAATTAATGATGTTAGAACCTAAGTTTGCAATCTTAGATGAAATCGATTCAGGATTAGACATCGATGCATTAA
AAGTTGTATCTAAAGGTATTAACCAAATGCGTGGGGAAAACTTTGGTGCATTAATGATTACACACTATCAACGATTATTAAATTACATTACT
CCTGATAAAGTACATGTAATGTATGCTGGTAAAGTCGTTAAATCTGGTGGTCCAGAATTAGCAAAACGTCTTGAAGAAGAAGGATATGAAT
GGGTTAAAGAAGAGTTCGGTTCAGCTGAATAA

Gene: sufD (iron-sulfur cluster assembly protein D)

Position: 867173 to 868480, length: 1308 nt, orientation: FORWARD

Perfect match to: (CN1-CP003979-[846511:847818], highly conserved allele)

Sequence:

ATGACAACTGATATTTTGAACATTTCTGAAGAACAACTTGTTGATTATTCTAAAGCCCACAATGAACCTTCTTGGATGACAGAATTACGTAAA
AAAGCTTTGAAATTAACAGAAACTTTAGAAATGCCAAAACCTGATAAAACAAAATTAAGAAAATGGGATTTTGATTCTTTTAAACAACACGA
TGTAAAAGGTGATGTTTATCAATCTTTATCACAATTACCTGAGTCAGTAAGAGAAATTATTGACGTAGATCATTCTAAAAACTTAGTAATTCA
ACATAATAATACGATTGCGTACACACAAGTTGATGATAATGCATCGAAAGATGGCGTTATCGTTGAAGGTTTAGCAGACGCTCTTATGAAC
CATAGTGATTTAGTACAAAAGTACTTTATGAAAGATGCAGTAACAGTAGATGAACATCGTATCACAGCGCTACACACGGCATTAGTTAATG
GTGGCGTATTTGTTTATGTTCCTAAAAATGTAGTTGTAGAACATCCAGTACAATACGTTGTGTTGCACGACGACGAAAATGCAAGCTTTTAT
AACCATGTTATCATCGTTACTGAAGAAAGCGCCGAAGTCACATATGTTGAAAATTACTTATCAAATGCATCTGGTGAAGGAAATCAATTAAA
TATTATTTCTGAAGTGATTGCTGGTGCAAATTCAAATATCACATATGGCTCAGTGGACTATATGGATAAAGGCTTTACAGGTCATATCATTC
GACGTGGTATTACTGAAGCGGATGCCTCAATTAATTGGGCACTAGGTTTAATGAATGAGGGTAGCCAAATTATTGATAATACAACAAATTT
ATTTGGTGATCGTTCAACAAGTTCACTTAAATCAGTAGTTGTAGGTACAGGCGAACAAAAAATCAATCTAACATCTAAAATCGTACAATATG
GTAAAGAAACAGATGGTTATATCCTTAAACATGGTGTTATGAAAGAACATGCATCATCTGTATTTAATGGTATCGGCTACATTAAGCATGGT
GGAACTAAATCAATTGCTAATCAGGAATCACGTGTATTAATGTTATCTGAACATGCTCGTGGTGACGCGAATCCTATTTTTATTAATTGATGA
AGATGATGTACAAGCTGGTCATGCTGCATCAGTAGGTCGTGTTGATCCAGATCAACTTTACTATTTAATGAGTCGTGGTATTTCTCAAAGAG
AAGCGGAACGTCTTGTTATACATGGTTTCTTAGATCCAGTAGTACGTGAATTACCTATCGAAGACGTTAAACGTCAATTGAGAGAAGTAATT
GAACGCAAAGTTTCTAAATAA

Gene: sufS (iron-sulfur cluster assembly cysteine desulfurase)

Position: 868595 to 869836, length: 1242 nt, orientation: FORWARD

Perfect match to: (11819-97-CP003194-[915333:916574], allele observed in CC80+CC22+CC59+CC72)

Sequence:

GTGGCCGAACACTCATTTGACGTTAATGAAGTAATCAAGGATTTTCCGATATTAGATCAAAAAGTCAATGGCAAACGTTTAGCATATCTTGA
TTCAACAGCGACAAGTCAAACGCCTGTGCAAGTGTTAAATGTTTTAGAGGATTACTACAAGCGTTATAATTCAAACGTTCATCGTGGTGTTC
ATACATTAGGATCATTGGCAACTGATGGTTATGAAAATGCCCGTGAAACCGTTCGTCGTTTTATTAATGCGAAGTATTTTGAAGAAATCATT
TTCACACGCGGAACAACTGCATCGATTAACCTTGTAGCACATAGCTATGGTGATGCAAATGTTGAAGAGGGCGATGAAATTGTTGTCACTG
AAATGGAACATCATGCCAATATTGTTCCTTGGCAACAGTTAGCAAAGCGTAAAAATGCGACATTGAAATTTATACCAATGACAGCTGACGG
TGAATTAAACATCGAAGATATTAAGCAAACGATTAATGATAAAACAAAGATCGTTGCTATTGCACATATTTCTAATGTACTCGGTACAATTA
ATGATGTTAAAACCATTGCAGAAATAGCTCATCAACATGGTGCAATTATCAGTGTTGATGGGGCGCAAGCAGCACCACATATGAAACTTGA
TATGCAAGAAATGAATGCTGATTTTTATAGTTTTAGTGGTCATAAAATGCTTGGACCAACAGGTATTGGCGTATTATTTGGTAAACGTGAGT
TACTACAAAAAATGGAACCGATTGAGTTCGGTGGCGACATGATTGATTTTGTAAGTAAGTATGATGCAACATGGGCTGATTTACCTACTAA

ATTTGAGGCGGGCACTCCATTAATTGCACAAGCAATTGGGCTTGCAGAAGCTATTCGCTATTTAGAACGCATAGGATTTGATGCAATTCATA
AATATGAACAAGAATTAACGATATATGCTTATGAGCAAATGTCTGCAATTGAAGGAATTGAAATTTATGGCCCGCCAAAGGATCGTCGTGC
AGGTGTAATAACGTTTAATTTACAAGATGTACATCCACACGATGTTGCTACAGCCGTAGATACAGAAGGTGTAGCGGTTAGAGCTGGGCAT
CATTGTGCGCAACCGTTAATGAAATGGTTAAATGTGTCTTCAACAGCTAGAGCGAGTTTTTATATATACAACACGAAAGAAGACGTTGATCA
GTTAATAAATGCCTTGAAACAAACGAAGGAGTTTTTCTCTTATGAATTTTAA

Gene: sufN (iron-sulfur cluster assembly protein N)

Position: 869826 to 870290, length: 465 nt, orientation: FORWARD

Perfect match to: (11819-97-CP003194-[916564:917028], allele observed in CC80)

Sequence:

ATGAATTTTAATAATCTAGATCAATTATATAGATCTGTCATTATGGATCATTATAAAAATCCTAGAAATAAAGGTGTATTAGATAACGGGAC
TATGACAGTAGATATGAATAACCCGACATGCGGTGACCGTATACGACTAACATTTGATATAGAAGACGGCATTATAAAAGATGCTAAGTTT
GAAGGTGAAGGTTGTTCGATTTCAATGGCAAGTGCATCGATGATGACACAAGCTGTTAAAGGTCATTCACTTGGAGAAGCAATGCAAATG
AGCCAAGAATTTACGAAAATGATGCTTGGTGAAGACTATGTGATTACAGAAGAAATGGGAGATATTGAAGCATTGCAAGGTGTATCTCAA
TTCCCAGCTCGTATTAAATGTGCCACATTAGCTTGGAAAGCATTGGAAAAAGGTACTGTTGCTAAAGAAGGTAAAGCAGAAGGTACGACTG
AAGAAGAATAG

Gene: sufB (iron-sulfur cluster assembly protein B)

Position: 870441 to 871838, length: 1398 nt, orientation: FORWARD

Perfect match to: (Strain_16035-HE579065-[862888:864285], allele observed in CC5)

Sequence:

ATGGCTAAAAAAGCACCTGATGTTGGGGATTATAAATATGGATTCCACGACGATGATGTATCCATTTTCAGATCAGAACGTGGTTTAACTG
AGAATATCGTTAGAGAAATTTCTAACATGAAAAATGAGCCGGAATGGATGTTAGATTTCCGTCTTAAATCATTAAAATTGTTTTATAAAATG
CCAATGCCTCAATGGGGTGGCGACTTATCAGAATTGAATTTCGATGACATTACTTACTATGTAAAGCCTTCAGAACAAGCTGAACGTTCATG
GGATGAAGTGCCAGAAGAAATTAAAAGAACTTTCGATAAATTAGGAATTCCTGAAGCTGAACAAAAATATTTAGCTGGTGTTTCTGCTCAA
TATGAATCTGAAGTTGTTTACCATAATATGGAAAAAGAACTTGAAGAAAAAGGTATTATCTTTAAAGATACAGATAGTGCTTTACAAGAAA
ATGAAGAATTATTCAAAAAATACTTTGCTTCTGTAGTACCTGCAGCAGATAACAAATTTGCGGCGTTAAACTCAGCAGTATGGTCAGGTGGT
TCGTTCATTTATGTACCTAAAAATATCAAACTAGATACGCCACTACAAGCTTATTTCCGTATTAACTCTGAGAACATGGGTCAATTTGAACGT
ACATTAATCATTGCTGATGAAGGTGCTTCTGTACATTACGTAGAAGGTTGTACTGCACCAGTTTATACAACTAGTTCTTTACACTCTGCTGTT
GTGGAAATCATTGTGCATAAAGATGCGCACGTTCGTTATACTACGATTCAAAACTGGGCGAACAATGTATACAATTTAGTTACAAAACGTAC
TTTTGTTTATGAAAACGGAAATATGGAATGGGTAGATGGTAACTTAGGTTCTAAGTTAACGATGAAATATCCAAACTGTGTTCTTTTAGGTG
AAGGTGCAAAAGGTAGTACATTATCTATTGCATTTGCTGGTAAAGGACAAGTTCAAGATGCCGGTGCTAAAATGATTCATAAAGCGCCAAA
TACATCTTCAACGATTGTTTCTAAATCTATTTCTAAAAATGGTGGTAAAGTTATTTATCGCGGTATTGTTCATTTTGGACGTAAAGCAAAAGG
TGCTCGTTCAAATATTGAATGTGATACATTAATCTTAGATAACGAATCAACATCAGATACAATTCCATATAACGAAGTATTCAACGATCAAAT
ATCATTAGAACATGAAGCCAAGGTTTCAAAAGTTTCTGAAGAACAATTATTCTATCTAATGAGTCGTGGTATTTCTGAAGAAGAAGCGACA
GAAATGATTGTTATGGGATTCATCGAACCATTTACAAAAGAACTTCCAATGGAATACGCGGTCGAAATGAACCGTTTAATCAAGTTCGAAA
TGGAAGGTAGTATTGGATAG

Gene: Q2YWM5 (putative protein)

Position: 872168 to 872482, length: 315 nt, orientation: REVERSE

Perfect match to: (MW2-BA000033-[887608:887922:r], highly conserved allele)

Sequence:

TTAAAATAAGAAAACTATTAATAATATTAAGTTCACTACAGATGTTGCTAATGGACCATAAGTTTTAAAGACATCTTCACTTTTATAACCAAC
AATCGCATCTAAAAATTGAACTAAGATCATTGCAATGGATATAGTTATCAAAAATATAGCACTATGAATGACTAAAGAAAAAATAGCTAATA
AAAATAAAGGTAAGCTTCGACTAAGTGCATAATATGCATTTATATTATGGCTAGATGCACATGCTTGAATTGAATAACCTAAACTTACACTG
GCACTGATTATTGTAAATATTGCTAAAACAAAATACAT

Gene: corB (CBS domain comprising Mg2+ and Co2+ transporter)

Position: 872847 to 873887, length: 1041 nt, orientation: FORWARD

Perfect match to: (NN50-BAEA01000008-[334139:335179:r], allele observed in CC4803+CC188)

Sequence:

GTGATCATTGCCATAATTATATTGATATTTATTTCGTTTTTCTTTTCAGGAAGCGAGACGGCATTAACGGCTGCCAATAAAACAAAATTTAAA
ACTGAAGCTGACAAAGGTGATAAAAAAGCAAAAGGCATTGTAAAGTTACTTGAAAAACCAAGTGAGTTTATTACAACGATTCTAATTGGGA
ATAATGTCGCGAATATTTTATTACCAACACTTGTTACAATTATGGCTTTACGTTGGGGGATTAGCGTTGGTATTGCATCAGCTGTTTTAACAG
TTGTTATCATATTGATTTCCGAAGTGATTCCCAAGTCTGTCGCTGCAACATTTCCAGATAAAATAACAAGGCTTGTATATCCAATTATTAATAT
TTGTGTCATTGTGTTCCGTCCTATCACATTACTTTTAAATAAGTTGACGGACAGTATTAATCGAAGTTTATCTAAGGGCCAACCTCAAGAACA
TCAATTTTCAAAAGAAGAATTTAAAACAATGTTAGCAATTGCTGGACATGAAGGTGCTTTAAATGAAATTGAGACGAGTAGGTTGGAAGGT
GTCATTAATTTTGAAAATTTAAAAGTAAAAGATGTAGATACAACACCTAGAATTAATGTGACGGCATTTGCTTCAAATGCGACATACGAAGA
AGTTTATGAAACGGTTATGAATAAGCCATACACTAGATATCCAGTGTACGAGGGAGATATTGATAACATTATTGGAGTGTTTCATTCTAAAT
ATCTGTTGGCTTGGAGTAATAAAAAAGAAAATCAAATTACAAACTATTCAGCTAAGCCATTATTTGTGAATGAACACAATAAAGCTGAATG
GGTATTACGTAAGATGACTATTTCTAGAAAACATTTAGCAATTGTGTTGGACGAATTTGGTGGTACCGAAGCGATAGTGTCACATGAAGAC
TTAATTGAAGAATTATTAGGTATGGAAATTGAAGATGAGATGGATAAAAAGGAAAAAGAAAAACTTTCTCAACAGCAAATTCAATTTCAAC
AACGGAAAAATCGCAACGTATCTATATAA

Gene: npd (putative 2-nitropropane dioxygenase)

Position: 873901 to 874968, length: 1068 nt, orientation: FORWARD

Perfect match to: (11819-97-CP003194-[920644:921711], allele observed in CC80+CC15+CC80+CC361+CC772)

Sequence:

ATGTGGAATAAGAATCGACTTACTCAAATGTTAAGTATTGAATATCCAATTATACAAGCAGGTATGGCAGGAAGTACGACACCGAAATTAG
TTGCATCAGTAAGTAACAGTGGTGGATTAGGCACAATAGGCGCAGGTTACTTTAATACGCAGCAATTAGAAGATGAAATAGATTATGTACG
CCAATTAACGTCAAATTCTTTTGGTGTAAATGTCTTTGTACCAAGTCAACAATCATATACCAGTAGTCAAATTGAAAATATGAATGCATGGTT
AAAACCTTATCGACGCGCATTACATTTAGAAGAGCCGGTTGTAAAAATTACCGAAGAACAACAATTTAAGTGTCATATTGATACGATAATTA
AAAAGCAAGTGCCTGTATGTTGTTTTACTTTTGGAATTCCAAGCGAACAGATTATAAGCAGGTTGAAAGCAGCGAATGTCAAACTTATAGG
TACAGCAACAAGTGTTGATGAAGCTATTGCGAATGAAAAAGCGGGTATGGATGCTATCGTTGCTCAAGGTAGTGAAGCAGGTGGACATCG
TGGTTCATTTTTAAAACCTAAAAATCAATTACCTATGGTTGGAACAATATCTTTAGTGCCACAAATTGTAGATGTCGTTTCAATTCCGGTCAT
TGCCGCTGGTGGAATTATGGACGGTAGAGGAGTTTTGGCAAGTATTGTCTTAGGTGCAGAAGGGGTACAAATGGGCACTGCATTTTTAAC
ATCACAAGATAGTAATGCATCAGAACTACTACGAGACGCAATTATAAATAGTAAAGAAACAGATACAGTCATTACAAAAGCGTTTAGTGGA
AAGCTTGCACGCGGTATCAACAATAGGTTTATCGAAGAAATGTCCCAATACGAAGGCGATATCCCAGATTATCCAATACAAAATGAGCTAA
CAAGTAGCATAAGAAAAGCCGCAGCAAACATCGGCGACAAAGAGTTAACACATATGTGGAGTGGACAAAGCCCGCGACTAGCAACAACG
CATCCCGCCAACACCATCATGTCCAATATAATCAATCAAATTAATCAAATCATGCAATATAAATAA

Gene: yunF (putative protein)

Position: 875255 to 876103, length: 849 nt, orientation: FORWARD

Perfect match to: (11819-97-CP003194-[921998:922846], allele observed in CC80+CC8+CC22+CC772+CC4803)

Sequence:

ATGATAAACATCGGATTAACAGGTTGGGGTGATCACTATTCATTATATGAAGATTTAGAACGCCAAACCGATAAACTTAAAACATATGCTG
GACATTTTCCGGTTGTCGAATTAGATGCGACATACTATGCGATACAACCGGAAAGAAATATATTGAAATGGATAAAAGAAACGCCTGATAC
ATTTGAATTTGTGGTCAAAATTCATCAAGCACTCACATTGCATGCAGACTACAAAACATTTGCAGATACAAGGCAAGAACTATTTGATCAAT
TTAAGAATATGTTAGAGCCCTTACATACACAGAAAAAAATTAGCAATGGTATTGGTTCAATTTCCGCCATGGTTTGACTGCAATGCACAAAAT
ATCAAATATATTTTGTATGTAAGACAGCAATTACAAGCATTTCCAATATGTGTAGAATTTAGGCATCAATCATGGTTTAGTGATGCATTTAAA
GAACAAACATTGGCATTTTTAACAGAACATCAAATCATTCATGCAGTAGTTGATGAACCACAAGTGAAAGATGGCAGTGTACCTTTAGTCA
ATCGAATCACAAATGAAATTGCGTTTGTACGTTATCATGGACGTAATCATTACGGTTGGACTAAGAAAGATATGTCAGATCAAGAATGGCG
AGATGTACGCTATTTATATGATTATAATGAGCAAGAATTAATAGACTTGGCACAAAAGGCACAAATATTAGCACAAAAAGCTAAGAAAGTT
TACGTCATATTTAACAATAATTCTGGTGGTCATGCAGCAAATAATGCCAAAACATATCAGCGATTATTGAATATAGAATATGAAGGGTTAGC
ACCACAACAATTAAAATTATTTTAA

Gene: yunE (putative membrane protein)

Position: 876116 to 876943, length: 828 nt, orientation: FORWARD

Perfect match to: (11819-97-CP003194-[922859:923686], allele observed in CC80+CC7+CC80)

Sequence:

ATGTTATTAACAATTACATTATTAGTTTTAATCGGAGGTTTGTCAGCGATTATAGGTTCAATCGTAGGCATTGGAGGCGGTATTATTATCGTT
CCAACAATGGTTTACCTTGGTGTTGAACATGGATTACTACATAATATTACAACACAAGTAGCGATAGGGACGTCTTCAGTCATTCTAATTGT
GACAGGACTTTCTTCATCACTTGGATATTTAAAAACAAAACAAGTTGATATTAAAAATGGTTCCATCTTTTTATTTGGACTATTACCAGGTTC
ATTGCTTGGGTCCTTCATTAGTAGATATTTAACATTTGAGTCATTTAATTTATATTTTGGTATCTTTTTAATTTTCGTAGCCATTTTTATTAATGG
TAAGAAATAAGATTAAACCGTTTAAAATTTTTGATAAACCCAAGTATGAAAAGACTTATGTAGATGCTAAAGGTAAAACATATCATTATAGT
GTGCCACCATTGTTTGCTTTTATTACAACGTTTTTAATTGGTATATTGACAGGTTTATTTGGTATTGGAGGTGGCGCACTAATGACGCCACTA
ATGCTTATTGTATTTAGATTTCCACCTCATGTAGCTGTTGGAACAAGTATGATGATGATTTTCTTTTCAAGTATCATGAGTTCTATAGGGCAC
ATTGCTCAAGGTCACGTAGCTTGGGGGTATGCAATCATTTTAATTATTTCTAGTTATTTTGGTGCGAAAATCGGTGTCAAAGTGAATCAATC
AATTAAGTCAGATACGGTAGTAACATTATTGAGAACAGTAATGTTGTTAATGGGTATATATTTAATTATTCGTGCGTTGATTTAA

Gene: yunD (putative 5'-nucleotidase)

Position: 876970 to 878289, length: 1320 nt, orientation: FORWARD

Perfect match to: (Strain_103564-AHZI01000051-[46968:48287:r], allele observed in CC80)

Sequence:

TTGAGGCTTACAATTTATCATACGAACGATATTCATAGTCATTTACATGAATACGAACGCATTAAAGCATATATGGCAGAACATCGGCCACG
ACTTAACCATCCTTCTTTATATGTTGATCTAGGTGATCATGTAGATTTATCCGCACCTATAACTGAAGCAACTTTAGGTAAAAAGAATGTGGC
ATTACTAAATGAAGCAAAATGTGATGTTGCAACAATCGGTAATAATGAAGGGATGACCATTTCACACGAAGCTTTAAATCACCTTTACGAC
GAAGCAAAATTTATAGTGACATGTAGCAATGTTATAGATGAATCAGGTCATTTACCAAATAATATCGTTTCTTCTTATATTAATGACATAGAC
GGTGTGAAAATACTATTCGTTGCAGCGACAGCACCTTTTACCCCATTTTATCGTGCACTAAATTGGATTGTTACCGATCCACTTGAATCTATA
AAAGAAGAAATTGAACTTCAACGAGGTAAATTTGATGTATTAATCGTGCTAAGTCATTGTGGCATTTTCTTCGATGAAACATTATGCCAAGA
ATTGCCTGAAATTGATGTCATTTTTGGTAGTCATACGCATCATTATTTTGAACATGGTGAAATCAATAATGGTGTACTGATGGCGGCAGCTG
GAAAGTATGGTAATTATCTTGGAGAGGTTAATTTAACTTTTGAGGCACATAAAGTAGTACATAAAACTGCAAAGATTATTCCTTTAGAAACA
TTACCTGAAGTTGAAACTTCATTTGAAGAAGAAGGAAAAACGTTAATGTCCAATTCAGTAATTCAACATCCAGTAGTGCTTAAGCGTAGTAT
GAATCACATAACTGAAGCTGCATACTTATTAGCTCAAAGTGTTTGTGAGTATACACATGCACAATGTGCCATCATCAATGCTGGCTTACTCG
TTAAAGATATTGTAAAAGATGAAGTGACAGAATATGACATTCATCAAATGTTACCGCATCCGATTAATATGGTAAGGGTTAGACTTTCTGGT
GTGAAATTAAAAGAGATTATAGCCAAAAGTAATAAACAAGAATATATGTATGAACATGCACAAGGTTTGGGTTTCAGAGGGAATATATTTG
GAGGATATATTCTTTATAATTTAGGGTACATTCATTCTACAGGGCGTTACTATCTGAATGGAGAAGAAATCGAAGACGACAAAGAATATGT
ACTAGGTACGATAGATATGTATACGTTCGGTCGTTATTTCCCAACATTGAAAGAATTACCAAAAGAGTATTTAATGCCAGAGTTTTTAAGAG
ATATATTTAAAGAAAAATTATTGGAATATTAA

Gene: lipA (iron-sulfur cluster comprising lipoyl synthase)

Position: 878373 to 879290, length: 918 nt, orientation: FORWARD

Perfect match to: (11819-97-CP003194-[925116:926033], allele observed in CC80+CC8+CC30+CC239+CC4803)

Sequence:

ATGGCGACAAAAAACGAGGAAATATTACGTAAACCGGATTGGTTGAAAATAAAATTAAATACCAACGAAAACTATACAGGACTTAAGAAG
ATGATGAGGGAAAAAAATCTTAATACTGTATGTGAAGAAGCTAAATGTCCTAATATACATGAATGTTGGGGTGCACGTCGTACAGCGACAT
TTATGATTTTAGGTGCCGTATGTACAAGAGCTTGTCGTTTTTGTGCGGTTAAGACAGGTTTACCTAATGAACTTGATTTAAATGAGCCTGAA
CGTGTAGCTGAATCAGTTGAATTAATGAACTTGAAACACGTTGTTATCACTGCTGTTGCGCGTGATGATTTAAGAGATGCTGGTTCAAATGT
TTATGCTGAGACAGTACGTAAAGTTAGAGAAAGAAATCCATTTACAACGATTGAAATTTTACCATCAGATATGGGCGGGGACTATGATGCG
TTAGAAACATTAATGGCTTCAAGACCTGACATTTTAAACCATAATATTGAAACTGTTCGTCGCTTAACACCGAGAGTTCGTGCGCGTGCGAC
TTACGACAGAACATTAGAGTTTTTACGTCGTTCAAAAGAATTACAACCGGATATCCCAACTAAATCAAGTATTATGGTTGGATTAGGTGAAA
CTATAGAAGAAATTTATGAAACGATGGATGATTTACGTGCGAATGATGTAGATATTTTAACGATTGGTCAATATTTACAACCTTCACGTAAA
CATTTAAAGGTTCAAAAATATTACACGCCTTTAGAGTTTGGTAAATTAAGAAAAGTGGCAATGGATAAAGGGTTTAAACATTGCCAAGCTG

GACCTTTAGTACGTAGTTCTTATCATGCGGATGAGCAAGTAAATGAAGCTGCTAAAGAAAAGCAACGCCAAGGTGAGGCACAGTTAAATA
GTTAA

Gene: DUF1027 (protein of unknown function)

Position: 879411 to 879803, length: 393 nt, orientation: FORWARD

Perfect match to: (MRSA252-BX571856-[926687:927079], highly conserved allele)

Sequence:

GTGAAGAATTTGATAAAAGTAGATCAACATTACTTTGAATTAATAGAAAATTATCGCGAATGTTTTAATGAAGAACAATTTATTGCTAGGTA
TTCAGATATTTTAGATAAATATGATTACATTGTTGGTGACTATGGTTACGATCAATTACGATTAAAAGGTTTTTACAAAGATTCTAATAAAAA
AGCAGAGATGAGTAAACGTTTTTCAAATATTCAAGATTACATATTTGAATATTGTAACTTTGGTTGTCCTTACTTTGTATTAAGACATTTGTCT
AAACAAGAGGTTAAAAAGTTAATCGAAGAAGTTCATCCGTCTGATGTGATAGATGACGACAATAAACTTCAAGATGTGAAGATTAAGCCAA
CCATTCAAGATACTGAACATTAA

Gene: DUF3055 (protein of unknown function)

Position: 879882 to 880139, length: 258 nt, orientation: REVERSE

Perfect match to: (ED133-CP001996-[898380:898637:r], allele observed in CC133+CC7+CC72+CC80)

Sequence:

TTAATGAATGACTTCATTTAAATACTCAGTAATTTCATCGCCTTCTTCAGCATTTACACCTAAAATATGAGCGATATAGCCTTCTTCTTTTAAA
TCATCAGTACCGATAATACCGAATTTATTTGTTTGCATATTAAGTACGAGTGTCTTACCATAATGTCTATTTGTATGGACTAACATTAAATCAT
ATCGACTATGCTCGCCAACAAAACCAACAAACTGAACTTGACTCTCTTCGTTGTCATCATATAAATACAT

Gene: yutE (conserved hypothetical protein)

Position: 880246 to 880680, length: 435 nt, orientation: FORWARD

Perfect match to: (Newman-AP009351-[888280:888714], allele observed in CC8+CC15+CC80+CC772)

Sequence:

ATGTATTTTGTAGACAAAGATAAACTAACTCAGAAATTAGCCTATTTACAAGCATTAACTGATGATTATCATGAGAGCAAGCACAATCATTA
TGCATTTGAACGCATTGCTCAAATGTTGATAGAATCATCGGTAGATATAGGGAATATGATTATCGATGCATTTATTTTAAGGGATCCTGGTA
ATTATAAAGATGTGATTGATATATTAGAACTAGAAAATGTTATTACTAAAGAAACACAGCAGGCGATTAATAAAACTGTCGATATTCGTAAA
CAATTTACATATGATTACACAGCCTTAGATATTAAGGTTATCATGCCAATGTTTGATGACGCATTACCTTATTACAAACAATTTATTACAGAA
GTAACGACATTTTTACATCAAGAAAATGTACCAGTAACAGCTTTTGGTAAAGGAGAAAATCAATAA

Gene: yutF (putative p-nitrophenyl phosphatase)

Position: 880680 to 881459, length: 780 nt, orientation: FORWARD

Perfect match to: (11819-97-CP003194-[927423:928202], allele observed in CC80)

Sequence:

ATGAAACAGTATAAAGCGTATTTAATCGATTTAGATGGCACAATGTATATGGGAACAGATGAGATTGATGGAGCAAAACAATTCATCGATT
ATTTAAATGTAAAAGGCATTCCTCATTTATACGTAACAAATAATTCAACAAAAACACCTGAGCAAGTAACTGAAAAATTACGTGAAATGCAC
ATTGATGCGAAACCAGAAGAGGTTGTAACGTCAGCGTTAGCCACTGCTGATTATATTTCAGAACAATCACCAGGAGCATCAGTATATATGT
TAGGTGGGAGTGGTTTAAATACTGCGTTAACCGAAGCGGGACTTGTCATTAAAAATGACGAGCATGTTGATTATGTAGTTATTGGACTTGA
CGAACAAGTTACATATGAAAAGCTTGCGATTGCAACGTTAGGTGTAAGAAATGGTGCAACATTTATTTCTACAAATCCTGATGTATCAATTC
CTAAAGAGCGTGGTTTATTACCTGGTAATGGTGCTATTACAAGTGTTGTAAGTGTATCGACAGGTGTATCGCCACAATTTATTGGTAAACCA
GAACCGATTATTATGGTTAAAGCATTAGAAATTTTAGGATTAGATAAATCCGAAGTTGCTATGGTAGGCGATTTGTACGATACCGATATTAT
GTCTGGTATTAACGTAGGTATGGATACGATTCATGTACAAACAGGTGTATCTACGTTAGAAGATGTGCAAAATAAAAATGTGCCACCAACG
TATTCTTTTAAAGATTTAAATGAAGCAATAGCTGAATTAGAAAAATAG

Gene: gyaR (2-ketogluconate reductase)

Position: 881492 to 882451, length: 960 nt, orientation: FORWARD

Perfect match to: (N315-BA000018-[896909:897868], highly conserved allele)

Sequence:

TTGGTAAAAATAGTTGTTTCGAGGAAAATTCCAGATAAATTTTATCAACAATTAAGTAAACTTGGTGACGTTGTTATGTGGCAAAAATCATT
AGTGCCTATGCCTAAAGATCAATTTGTGACAGCACTTCGTGACGCAGATGCTTGTTTTATTACATTAAGTGAACAGATCGATGCAGAAATTT
TAGCACAATCACCAAATTTAAAAGTAATTGCGAATATGGCTGTAGGATATGACAACATCGATGTTGAAAGTGCAACAGCGAATAACGTGGT
TGTCACGAATACACCAAATGTACTTACTGAAACAACTGCAGAATTAGGATTTACATTAATGCTTGCTATAGCACGCCGTATTGTAGAAGCTG
AAAAATATGTAGAAGCAGATGCATGGCAAAGCTGGGGTCCTTATTTATTGTCAGGTAAAGATGTCTTCAATTCAACTATTGGAATATATGGT
ATGGGAGATATTGGTAAAGCTTTTGCAAGAAGGTTGCAAGGGTTTAATACTAATATTCTTTATCATAATCGATCAAGACATAAAGATGCAG
AGGCGGACTTTAATGCAACATATGTTTCTTTTGAAACGTTGTTAGCAGAAAGTGATTTTATCATCTGTACAGCGCCACTTACAAAAGAAACA
CATCATAAATTTAATGCTGAAGCATTTGAACAAATGAAAAATGATGCAATTTTTATTAATATCGGTAGAGGGCAAATTGTAGATGAAACAG
CATTAATCGATGCACTAGACAATAAAGAAATTTTAGCATGTGGTTTAGATGTATTAGCAAATGAACCGATTGATCATACACATCCATTAATG
GGACGTGATAATGTTCTGATTACACCACACATTGGTAGCGCATCAGTAACAACACGGGACAATATGATTCAATTATGTATTAATAATATAGA
AGCGGTTATGACAAATCAGGTACCACATACTCCAGTAAATTGA

Gene: dltX (putative protein located in teichoic acid biosynthesis gene cluster)

Position: 882863 to 883015, length: 153 nt, orientation: FORWARD

Perfect match to: (RF122-AJ938182-[864187:864339], highly conserved allele)

Sequence:

ATGAAATCTAAAAGTAAACAGCCACCTAATAAATATGTTGAAGCATTCAAACCATATTTATTAACACTATTGTATTTGGCAATATTTATTACT
TTATATTTAATTTATGGCAGTGGCGACACACACAATAACTTCATTTATAATGAGTTCTAA

Gene: dltA (D-alanine-D-alanyl carrier protein ligase)

Position: 883031 to 884488, length: 1458 nt, orientation: FORWARD

Perfect match to: (Strain_21193-AFEG01000001-[41687:43144:r], allele observed in CC25+CC80)

Sequence:

ATGACAGATATTATTAACAAGCTGCAAGCGTTTGCGGATGCAAATCCACAAAGCATTGCTGTTAGACACACAACTGATGAATTAACTTATCA
ACAGTTAATGGATGAGTCTAGTAAATTAGCACATCGATTACAAGGTAGTAGGAAACCGATGATTTTATTCGGTCACATGTCACCATATATGA
TTGTTGGGATGATTGGTGCCATTAAAGCAGGATGTGGATATGTACCTGTAGACACTTCAATTCCTGAAGACCGTATTAAAATGATTATTAAC
AAGGTTCAACCAGAGTTTGTATTTAATACGACTGATGAATCATTTGAAAGTTTAGAAGGCGAAGTATTTACAATAGAAGATATTAAAACATC
TCAAGACCCAGTAATTTTTGATAGTCAGATTAAAGATAACGACACAGTATACACAATCTTTACATCTGGTTCTACTGGGGAACCTAAAGGTG
TTCAAATTGAATATGCAAGTTTAGTTCAATTTACTGAGTGGATGTTAGAACTTAATAAATCAGGAAATGAACAACAATGGCTTAACCAAGCG
CCATTTTCATTTGATTTATCTGTAATGGCTATTTATCCATGTTTAGCATCAGGCGGTACATTAAATCTTGTAGATAAAAACATGATTAATAAAC
CTAAATTATTAAATGAAATGCTAACAGCAACACCGATTAACATTTGGGTATCAACACCATCATTTATGGAAATGTGTTTATTATTACCAACGC
TTAATGAAGAACAATATGGTAGTCTTAACGAATTCTTCTTCTGTGGTGAAATTCTACCTCACAGAGCAGCAAAAGCGTTAGTAAGCCGTTTC
CCAAGTGCGACGATTTACAACACATATGGTCCAACTGAAGCTACGGTAGCAGTTACAAGTATTCAAATTACACAAGAAATCTTAGATCAATA
TCCGACATTACCTGTTGGCGTTGAAAGACCAGGCGCAAGATTATCTACTACAGATGAAGGTGAACTTGTTATCGAAGGTCAAAGTGTAAGT
TTAGGATACTTAAAAAATGACCAAAAAACAGCTGAAGTATTTAATTTCGATGACGGTATTCGTACTTATCACACTGGTGATAAAGCGAAGTT
TGAAAATGGTCAATGGTTCATTCAAGGTCGTATTGATTTCCAAATCAAATTGAATGGCTACAGAATGGAATTAGAAGAAATTGAAACACAA
TTACGCCAGTCTGAGTTCGTAAAAGAAGCGATTGTTGTACCTGTATATAAAAAATGATAAAGTTATTCATTTAATTGGTGCAATTGTGCCAAC
GACTGAAGTTACGGACAATGCAGAAATGACTAAAAAATATTAAAAATGACTTGAAATCACGCTTACCAGAGTATATGATTCCTAGAAAGTTT
GAATGGATGGAACAATTGCCATTGACTTCAAATGGTAAAATTGACAGAAAGAAAATTGCAGAGGTAATTAACGGATGA

Gene: dltB (putative membrane transporter)

Position: 884485 to 885699, length: 1215 nt, orientation: FORWARD

Perfect match to: (MRSA252-BX571856-[931760:932974], highly conserved allele)

Sequence:

ATGATTCCATATGGTGATTTTACATTCTTCTTAATTGCTTTAATTGCATTATTACCAGTCATTATACTTGGATTTTTAGGTAAGCGAAGTTACA
TTTATAATGGCGTAGTTACAGCATTTATGATTGTGTTAATCTTTTCTTCTGATAAACATAATCTGTTTGACCAAAAGTATTTAAGTGTTCAATT
AATTAGTTTTATTATTTACGTCGTATGGCAAGTTTTATTGATAATGTTTTATTATCATTCAAAACCAAAAAATAATTCATTTTCAAAATTTGTA
ACTGTAATGGTTTTATCAATATTGCCATTAGCACTTGTGAAAGTGTTACAAAGTACATGGTTAGGTGGACATCAGATTCACTTCCATGAAAG
TAAATTAATTGAATTTGTTGGTTTCTTAGGAATTTCTTATGTTACATTCAAAAGTGTGCAGTTAATTATGGAAATTCGTGATGGTTCTATCAA
AGAAATTAAAGTATGGAAATTAATTCAATTTATTTCATTCTTCCCAACGATTTCATCTGGACCAATCGATCGTTACAAACGTTTCGTTAAAGA
CGATAAAAAAGTACCAACAGGCAATGAATATCGTGAATTAGTATTAAAAGCAATTCACATGATTATGCTTGGTTTCTTGTATAAATATATTG
TTGCTTACTTTATTAACACATATGCAATCATGCCGTTACAATTAGACTTACATGGCTTTGTCAATTTGTGGTTATATATGTACGCATACAGCTT
ATATTTATTCTTTGACTTTGCAGGTTATAGTTTATTTGCGATAGCATTTAGTTATTTATTCGGTATTAAAACACCACCAAACTTCGATAAACCT
TTCAAAGCGAAAAATATTAAAGATTTCTGGAATAGATGGCATATGACATTATCATTCTGGTTCAGAGATTGTATTTACATGAGATCTTTATTC
TACATGTCTCGTAAAAAATTATTGAAGAGTCAATTTGCAATGTCTAACGTGGCATTCTTAATCAACTTCTTCATAATGGGAATTTGGCATGGT
ATCGAAGTGTATTACATTGTTTATGGTTTATACCATGCAGCATTGTTTATAGGTTATGGCTATTATGAACGTTGGCGTAAGAAACATCCGCC
ACGTTGGCAAAATGGTTTCACAACAGCACTTAGCATTGTGATTACATTCCACTTTGTAACATTTGGCTTTTTAATCTTCTCAGGTAAACTTATA
TAA

Gene: dltC (D-alanine--poly (phosphoribitol) ligase subunit 2)

Position: 885717 to 885953, length: 237 nt, orientation: FORWARD

Perfect match to: (RF122-AJ938182-[867041:867277], highly conserved allele)

Sequence:

ATGGAATTTAGAGAACAAGTATTAAATTTATTAGCAGAAGTAGCAGAAAATGATATTGTAAAAGAAAATCCAGACGTAGAAATTTTTGAAG
AAGGTATTATTGATTCTTTCCAAACAGTTGGATTATTATTAGAGATTCAAAATAAACTTGATATCGAAGTATCTATTATGGACTTTGATAGAG
ATGAGTGGGCAACACCAAATAAAATCGTTGAAGCATTAGAAGAGTTACGATGA

Gene: dltD (putative exoprotein)

Position: 885950 to 887125, length: 1176 nt, orientation: FORWARD

Perfect match to: (VRS3a-AHBM01000021-[6384:7559], allele observed in CC5+CC8+CC59+CC97+CC1290)

Sequence:

ATGAAATTAAAACCTTTTTTACCCATTTTAATTAGTGGAGCGGTATTCATTGTCTTTCTATTATTACCTGCTAGTTGGTTTACAGGATTAGTAA
ATGAAAAGACTGTAGAAGATAATAGAACTTCATTGACAGATCAAGTACTAAAAGGCACACTCATTCAAGATAAGTTATACGAATCAAACAA
GTATTATCCTATATACGGCTCTAGTGAATTAGGTAAAGATGACCCATTTAATCCTGCAATTGCATTAAATAAGCATAACGCCAACAAAAAAG
CATTCTTATTAGGTGCTGGTGGTTCTACAGACTTAATTAACGCAGTTGAACTTGCATCACAGTATGATAAATTAAAAGGTAAGAAATTAACA
TTTATTATTTCACCACAATGGTTTACAAACCATGGTTTAACGAATCAAAACTTTGATGCTCGTATGTCTCAAACTCAAATTAATCAAATGTTCC
AGCAGAAAAACATGTCTACTGAATTAAAACGTCGTTATGCACAACGTTTATTACAGTTTCCACATGTACACAATAAAGAATACTTGAAATCT
TATGCTAAAAACCCTAAAGAAACTAAAGATAGTTATATTTCTGGTTTTAAAGAGAATCAATTGATTAAAATAGAAGCGATTAAATCATTGTT
TGCAATGGATAAATCTCCATTAGAACATGTTAAACCTGCTACAAAACCAGATGCTTCTTGGGATGAGATGAAACAAAAAGCAGTTGAAATT
GGTAAAGCTGATACTACATCGAATAAATTTGGTATTAGAGATCAATACTGGAAATTAATTCAAGAAAGTAAGCGTAAAGTTAGACGTGACT
ACGAATTCAATGTTAATTCTCCAGAATTCCAAGATTTAGAATTACTTGTAAAAACAATGCGTGCTGCTGGTGCAGATGTTCAATATGTAAGT
ATTCCATCAAACGGTGTATGGTATGACCACATTGGTATCGATAAAGAACGTCGTCAAGCAGTTTATAAAAAAATCCATTCTACTGTTGTAGA
TAATGGTGGTAAAATTTACGATATGACTGATAAAGATTATGAAAAATATGTTATCAGTGATGCCGTACACATCGGTTGGAAAGGTTGGGTT
TATATGGATGAGCAAATTGCGAAACATATGAAAGGTGAACCACAACCTGAAGTAGATAAACCTAAAAATTAA

Gene: nfuA (Fe-S cluster carrier protein)

Position: 887388 to 887609, length: 222 nt, orientation: REVERSE

Perfect match to: (MW2-BA000033-[902717:902938:r], highly conserved allele)

Sequence:

TTAGAATACTTGTTCTACTTCTATTACACCAGGCACTTCTTCGTGTAATGCACGCTCAATACCAGCTTTAAGAGTGATTGTAGAACTTGGGCA
TGTACCACATGCACCATGTAATTGTAATTTAACAATACCGTCTTCCACGTCAATCAATGAGCAGTCGCCACCATCACGTAATAAAAATGGAC
GAAGACGTTCAATAACTTCTGCTACTTGATCAAACAT

Gene: yuzD (putative sulfur oxido-reduction management enzyme)

Position: 887731 to 888054, length: 324 nt, orientation: FORWARD

Perfect match to: (11819-97-CP003194-[934474:934797], allele observed in CC80)

Sequence:

ATGGAGCACGTGAGTGTGGTAGTATATGGGGCAGATGTTATTTGTGCAAGTTGCGTTAATGCGCCAACATCGAAAGATATTTATGACTGGC
TACAGCCGCTATTAAAAAGAAAATACCCAAATATATCATTTAAATATACGTATATAGATATTACAAAAGATAATGACAACTTAACAGATCAT
GATTTACAATTTATTGAAAGAATAGAACAAGATGAACTATTTTATCCATTAATTACAATGAACGATGAATATGTAGCAGATGGTTATATACA
AACAAAGCAAATCACTCGATTTATAGACCAAAAGCTTGTGAATGAATAA

Gene: yutJ-ndhF (type-II NADH:quinone oxidoreductase)

Position: 888113 to 889177, length: 1065 nt, orientation: REVERSE

Perfect match to: (Strain_21333-AHKA01000037-[14352:15416], allele observed in CC80+CC72)

Sequence:

TTAACCATTATGATATTTATATAACCAAAGTACGCCGGATTTTAAAATAGAAGCGAGTCGTCCTGTTACAGTGCGATCCATGATATATGCAA
AACCTTGTTTATCTCCTAATGACCCAACGATACCTTGTACCTTTAGTTCCGGCATTTTGTCAGGTAATGGTTCATTTAGCCATTGCTTTTTAAG
CACATCGGCAATTTGATCACCTTGAACTTCGGCTAACTGAGCACTTGGCGCATGTGGTAAATCAGCACAATCACCAACTACATAGACGTTAC
GATATGTTGGTACTTGATGATACTGGTTAACTATCACGCGTCCATTACTATTTATATCAATCGGCAAGTTACGAACAACTTCAACAGGTTGAA
TTCCTGCTGTCCATACAACTAAATCAATATCTTTAGGTTCATCACAGTTATATATTTTACCAGGTTCAACTTTATTAATATTTGAATTTGGAAC
AACAGTAACATTATTTTTGGCGAACCATTTCGCAACATACTTACTTAATTTTTCTGGAAAATTTCTTAAAATTCGCGGCCCACGGTCATAAAG
ATATATTTCCAAGTCTGATCTACTTTCTCTTAATTCGCTGGCAAGTTCTATGCCGCTTAATCCAGCACCAACGATACCAACTTTAGCACCTTCT
GGTAGTTCACTAATACTATGGAAAGTATCCCGAGCCTTTGAGAGTGTTTGAATACTATGTGTATATTCTTCGGCTCCTGGAACGTTATGATA
TTTATCTTCACATCCTAAACCAATGATTAGCTCATCATAATCAATTTTAGAATTACCGACTGAGACAATTTGAGCATCTAAATCTATGTCGTTA
ATTTCACCATAAACTGTATTCACTTGTGGATGATTAGGGAATTTCATGCGAACATCTTTATCTGATTTCGTGCCCGCAGCTAAAGCATAAAAT
TCTGGTTTCAATCCATGAAATGGCATACGATCAACTAATGTGACTGTATAATCTTGTGGTAAAGAAGTAGTTAAAATGCGTGACATGATACG
CATATTACCATATCCGCCGCCTAACAAAACTAAGTTTTTCAT

Gene: yuzB (conserved hypothetical protein, UPF0349 family)

Position: 889493 to 889729, length: 237 nt, orientation: FORWARD

Perfect match to: (MW2-BA000033-[904823:905059], highly conserved allele)

Sequence:

ATGAATCCGATCGTAGAATTTTGTCTCTCTAACATGGCAAAAGGTGGAGATTATGTTTTTAATCAACTGGAAAATGACCCAGATGTCGATGT
GTTAGAGTATGGTTGCCTGACACATTGTGGTATATGTTCAGCCGGGTTGTATGCTTTAGTAAATGGTGATATTGTTGAAGGTGATTCGCCG
GAAGAATTATTACAAAATATATATGCACATATAAAAGAAACTTGGATTTTTTAA

Gene: sufA (chaperone involved in Fe-S cluster assembly)

Position: 889742 to 890101, length: 360 nt, orientation: FORWARD

Perfect match to: (11819-97-CP003194-[936485:936844], allele observed in CC80+CC25+CC80+CC96)

Sequence:

ATGCCAACAGTTATATTAACAGAAGCAGCTGCTTACGAAGTAAAAGATATGCTTAAAGCAAATGAAATGCCAGATGGCTATTTAAAAATTA
AAGTGAATGGTGGCGGGTGCACTGGTTTAACATACGGTATGGGTGCAGAAGAAGCGCCTGGTGAAAATGATGAAATCTTAGAATACTTTG
GATTAAAAGTTTTAGTAGACAAAAAAGATGCACCCGTATTAAATGGTACGACTATTGATTTTAAGCAATCATTAATGGGTGGCGGTTTCCAA
ATCGACAATCCTAATGCAATTGCTTCATGTGGCTGTGGTAGTTCATTTAGAACTGCAAAAGTTGCAGGTAATCCTGAAAATTGCTAA

Gene: yumB-ndhC (type-II NADH:quinone oxidoreductase)

Position: 890554 to 891762, length: 1209 nt, orientation: FORWARD

Perfect match to: (11819-97-CP003194-[937297:938505], allele observed in CC80)

Sequence:

ATGGCTCAAGATCGTAAAAAAGTACTTGTACTTGGTGCTGGTTATGCAGGTTTACAGACTGTAACTAAATTGCAAAAAGCGATATCAACAG
AAGAAGCAGAAATTACGCTTATTAATAAAAATGAATATCACTATGAAGCAACATGGTTACATGAAGCATCAGCAGGTACACTAAACTATGA
AGATGTATTATATCCTGTGGAAAGTGTCTTGAAGAAAGACAAAGTGAACTTTGTTCAAGCAGAAGTAACAAAAATTGACCGTGATGCTAAA
AAGGTAGAAACAAATCAAGGTATTTATGACTTTGATATTTTAGTAGTAGCATTAGGTTTCGTTAGTGAAACATTCGGCATCGAAGGTATGAA
AGATCATGCTTTCCAAATTGAAAATGTTATCACAGCACGTGAATTATCACGTCATATCGAAGACAAATTTGCTAACTATGCAGCATCAAAAG
AAAAAGATGATAACGATTTATCTATCTTAGTTGGTGGTGCTGGATTCACTGGTGTTGAATTCTTAGGTGAATTAACAGACAGAATTCCTGAA
TTATGTAGCAAATATGGTGTGGATCAAAATAAAGTTAAAATCACTTGTGTTGAAGCAGCACCTAAAATGTTACCAATGTTCTCAGAAGAATT
AGTTAACCACGCAGTTAGCTACTTAGAAGACCGCGGTGTTGAATTTAAAATTGCTACACCAATCGTTGCTTGTAACGAAAAAGGTTTTGTAG
TTGAAGTAGATGGTGAAAAACAACAATTAAATGCAGGTACTTCAGTATGGGCAGCTGGTGTACGTGGTAGTAAATTAATGGAAGAATCAT
TTGAAGGCGTTAAACGTGGACGTATCGTTACAAAGCAAGATTTAACAATCAATGGTTACGACAACATTTTTGTTATTGGTGACTGTTCAGCG
TTTATCCCAGCTGGAGAAGAACGTCCATTACCAACTACAGCACAAATTGCAATGCAACAAGGTGAAAGTGTTGCTAAAAACATTAAACGCA
TCTTAAACGGTGAATCAACTGAAGAATTCGAATACGTTGATCGTGGAACTGTTTGTTCTTTAGGTTCACATGACGGTGTAGGTATGGTATTT
GGTAAACCTATCGCTGGTAAAAAAGCAGCATTCATGAAAAAAGTGATTGATACACGTGCGGTATTCAAAATCGGTGGTATCGGTTTAGCAT
TCAAAAAAGGTAAATTCTAG

Gene: pepZ (cytosolic leucine aminopeptidase)

Position: 891893 to 893368, length: 1476 nt, orientation: FORWARD

Perfect match to: (11819-97-CP003194-[938636:940111], allele observed in CC80+CC5+CC7+CC22)

Sequence:

ATGAATTTTAAATTAAATAACACACTAAGCAACGAAATAAATACATTGATTATTGGTATACCAGAACATTTAAATCAGTTAGAGCGCATTAG
TTTTAATCATATCGATATTACAGAATCACTTGAAAGACTAAAACATCAACATATTATTGGTAGTAAAGTTGGGAAGATTTATACAACTGCATT
TGATGTACAAGATCAAACATATCGTTTAATTACAGTTGGTTTAGGAAACTTAAAAGCACGTAGTTATCAAGATATGTTGAAAATATGGGGA
CATCTTTTCCAATACATAAAGTCAGAACATATTGAAGATACGTATTTACTTATGGATTCATTTATTTCAAAATATGATCAGTTATCAGATGTAT
TAATGGCATGCGGTATTCAAAGTGAGCGTGCAACATATGAATTCGATCATTATAAATCAAGTAAGAAGGCACCGTTTAAGACGAATTTAAA
CCTTATTAGTGAATCATTAATTGAATTAGATTTCATTCATGAGGGTATCAGTATTGGCCAATCCATTAATTTGGCAAGAGACTTTAGTAATAT
GCCACCGAATGTATTAACACCACAAACATTTGCAGAAGATATTGTTAATCATTTTAAAAATACAAAGGTCAAAGTAGATGTTAAAGATTATG
ACACTTTAGTTTCTGAAGGATTCGGACTTTTACAAGCAGTAGGTAAAGGTAGTAAGCATAAACCGAGATTAGTAACCATCACATATAATGG
CAAAGACAAAGATGTAGCACCAATTGCCTTAGTTGGTAAAGGTATAACGTATGATTCTGGTGGTTATAGTATTAAAACGAAGAATGGCATG
GCTACAATGAAGTTTGACATGTGTGGCGCTGCGAATGTCGTTGGTATCATTGAAGCGGCTAGTCGTTTACAACTGCCTGTAAATATTGTCG
GAGTGCTTGCTTGTGCTGAAAATATGATAAATGAAGCATCAATGAAGCCAGATGATGTATTTACAGCATTAAGTGGTGAAACTGTAGAAGT
AATGAATACAGACGCTGAAGGTAGATTAGTCCTTGCAGATGCTGTGTATTATGCAAATCAATATCAGCCTAGTGTGATTATGGACTTTGCTA
CATTAACGGGTGCAGCCATCGTTGCACTAGGCGATGATAAAGCTGCTGCATTTGAATCGAATAGTAAAGTGATATTAAACGATATATTACA
AATAAGTTCTAAAGTCGATGAAATGGTATTTGAATTACCGATTACTGCAACCGAACGTGCAAGTATTAATCACAGTGATATCGCTGATTTAG
TTAACCATACGAATGGACAAGGTAAAGCGCTATTTGCGGCAAGTTTTGTAACACATTTTAGTGGTCAAACACCTCACATTCATTTCGATATT
GCAGGTCCAGCAACGACTAATAAAGCTTCATATAATGGTCCAAAAGGGCCAACAGGATTTATGATTCCGACGATAGTACAATGGTTAAAAC
AACAATAA

Gene: nhaC3 (Na+/H+ antiporter)

Position: 893779 to 895095, length: 1317 nt, orientation: FORWARD

Perfect match to: (N315-BA000018-[909198:910514], allele observed in CC5+CC80)

Sequence:

ATGATAAATGCAGTAGTAATAGCAGTAATATTAATGATTGTGCTATGTTTATGTCGATTAAACGTAGTTATAAGCTTATTTATCAGTGCGCTA
GTTGGTGGCTTAATTTCAGGCATGAGCATTGAAAAAGTTATAAATGTATTTGGGAAAAATATAGTCGATGGTGCTGAGGTAGCATTAAGCT
ATGCATTATTAGGTGGATTTGCAGCATTAATTTCATACAGTGGTATCACAGACTATTTAGTAGGAAAAATTATAAATGCAATTCACGCTGAA
AATAGTCGATGGTCAAGAGTTAAAGTAAAAGTGACAATAATCATTGCATTATTAGCTATGAGTATCATGAGTCAAAACTTAATTCCTGTACA
TATTGCATTCATTCCAATTGTCATCCCACCATTGTTAAGTCTGTTTAATGACTTAAAAATAGATAGACGTTTAATCGGTTTGATTATCGGTTTT
GGTTTATGTTTCCCGTATGTGTTATTACCATATGGATTCGGTCAAATTTTCCAGCAAATTATTCAAAGTGGCTTTGCAAAGGCGAATCACCCA
ATTGAGTTTAATATGATTTGGAAAGCAATGCTTATTCCTTCAATGGGGTATATTGTTGGCTTACTTATCGGTTTATATGTATATCGTAAACCA
CGTGAATATGAAACACGTAAAATTTCAGATAGTGACAATGTTACAGAGTTAAAACCATATATCTTAATAGTAACAATTGTGGCAATACTAGC
TACATTTTTAGTACAAACATTTACAGATTCAATGATTTTTGGTGCACTGGCAGGGGTACTCGTATTCTTTATTTCACGTGCATATAATTGGTAT
GAATTAGATGCTAAGTTTGTTGAAGGTATTAAAATTATGGCTTATATTGGTGTAGTTATTTTAACAGCAAATGGGTTTGCTGGTGTAATGAA
TGCTACTGGTGATATAGATGAATTAGTTAAAACTTTAACAAGTATTACTGGTGATAATAAATTATTTAGCATTATCATGATGTATGTGATAG
GTTTAATTGTCACTTTAGGTATTGGATCATCATTTGCAACAATTCCTATTATCGCATCATTATTCATTCCTTTTGGAGCGTCAATTGGACTAGA
TACAATGGCATTAATCGCATTGATTGGAACAGCGAGTGCATTAGGTGACTCAGGTTCACCTGCAAGTGATTCAACATTAGGACCAACTGCG
GGATTAAATGTTGATGGCCAACATGATCATATACGCGATACATGTGTACCAAACTTCTTGTTTTATAATATTCCTTTAATGATTTTCGGTACT
ATTGCTGCTATGGTACTATAA

Gene: yuxO (putative esterase)

Position: 895114 to 895488, length: 375 nt, orientation: FORWARD

Perfect match to: (N315-BA000018-[910533:910907], allele observed in CC5+CC80)

Sequence:

ATGACTCATTTATTAGAGACATTTGAGATGTCAATAGATCACCAGGAAGATGGTTTAGTTGTTATTTCTATGCCTGTCACTGATAAAGTAAA
ACAACCATTTGGATATTTACATGGTGGGGCTTCGATTGCTTTAGGTGAAACAGCATGTTCATTAGGATCTGCTAATTTAATTGATACAACCA
AATTTATTCCATTAGGTTTAGAGATGAATGCTAACCATATTCATTCTGCTAAAGATGGTCGTGTTACTGCGACAGCTGAAATTATTCATCGAG
GTAAGTCGACACATGTATGGGATATAAAAATTAAGAATGACAAAGAACAATTAATTACAGTTATGCGTGGTACAGTTGCTATTAAACCTTTA
AAATAA

Gene: Q5HHE0 (putative pyridine nucleotide-disulfide oxidoreductase family protein)

Position: 895544 to 896698, length: 1155 nt, orientation: REVERSE

Perfect match to: (GR1-AJLX01000010-[53180:54334:r], allele observed in CC361+CC80)

Sequence:

TTATGCGCTATATTGTTGTAGTTTTAGAAATGCTTGTTCAATGCGTTCGGCAGCTTTACGGCCACCCATAACATTTCTACCAAATGGTCCTAA
TTCTAAGTCTGCAAAGCATCCTGCGACAAATAGATTTGGTATCCATTCTAATTTTTCGGAAATAACAGGGTAATTACATTCGTTGATAGGTGC
ATCATAATTTTGTATTAATTGCTTAATAAGTGGTTGTGACATAAAATCTTGTTCAAAACCAGTTGCAACCATAATCTGTTGATATGGAACAGA
ATTATTTTCAGTGTTAATTAAACCATCACTAATTTGAGTGATAGGTGTTTTATGCACATTTATACGACCATTTTTAATATGTTTTTTAAGGCGT
AAGTACAGTTCGTGAGGCATTGATCCTTTATGACGTTCACGTTGTACAATGGCATTTCTTTCAGGCATGCTTTTAGTACTTAAAAATGAAGAC
ATATTTTTCGGACCTAACCAACCAGGATCAGCATCAAAGTCATGTATTTCAATATCTTTATTTAGCCATAAATGAATCTTTTTATCGTTATCAT
GATTTAACAATTTAAGTGCAAGATGTGCAGCAGTAATGCCACTACCAACGATATGATCGGTCTTATCATATACTACTTGATCATGTTCTTTCT
CGAAAATATGATTTACATTCTGTTTGTCTTTTAAAATGTCAGGCATAAACGGAATATTTGTACTGCCTATTGCAATAACGACGCAATCTGTAG
TGATAATTTGTCCATCTTCTAACTTGATATGCCATTTGTCTTCTTGTTTATCTAAAGTTTGAACTAAACCTTGAACCAAGCAATCCTCTAATTG
ATATTGTTTAGAAGCATGTGCAATATGATCCATAAACATTGTCAATTCAGGTCGTTGATAAGGACCATAAAAAGCATTTGTATATTGGTGCT
GTTTAGCGAATTGTTTTAGATGGAACGGTTGTGGATGTACGTGATGTACAATCGGTGATCTTAAATAAGTCATTTCTATTCGATTTGTATAT
GAGTTAAACCTTTGGCAAAAAGTTTCGTATGGGTCAATGATTGTTAATCGGTCTGTTGTTAATCCGCTTGATAATAGTTTTTGTGCGATTGCA
GTTCCCTGTATGCCACCGCCGATAATTGTCCAATGCAT

Gene: mnhG (Na+/H+ antiporter, subunit G)

Position: 896946 to 897302, length: 357 nt, orientation: REVERSE

Perfect match to: (COL-CP000046-[951804:952160:r], highly conserved allele)

Sequence:

CTACAATTTTGTGTCTTTTAAGTCTTCCGAAATTTCATCGACTTTAGTCTTTTTAGTATAAGGCGTTTTAATATTATATGCTGCTTTCATAATCA
TATGACTTGAAAGAGGACCTGTAATTAATACAAAGATAATCGCAACGATTAATTGCATATTTACAAAACCTTGAGTAGCAATAAAATATAAA
AACGTACCAAATAGTAATGACATTGCACCTAATGTTGATGCTTTTCCGGCAGCATGTGCACGTGAATATACATCTTCAAGTCTCAATAATCCT
ATAGCTGCTAGGGCGCTAATTAAAGCACCGATGATAACAAAGATAAGTGCAAGACTAATCAGTATGATTTTGATCAT

Gene: mnhF (Na+/H+ antiporter, subunit F)

Position: 897280 to 897573, length: 294 nt, orientation: REVERSE

Perfect match to: (MW2-BA000033-[912612:912905:r], highly conserved allele)

Sequence:

CTAATCAGTATGATTTTGATCATGTTCAATCACCTTACCTTTGTCCATAAATTTAGAGAATACTGCAGTACCTAAAAAAGCTAATATACCAAT
CATCATAATAACGACAATCATGTATTTAATATTTAATAAAATACTGAATAATGCTATAACTGCCATTAATTGAAGACCAATCGCATCTAATGC
GACAACACGATCGGCAAGTGATGGGCCTAGCACAACGCGAATGAGCATAGCTAACATAGAAATGACAACTATGATTAATGCAATAACGAT
AATAACATTATGATTCAT

Gene: mnhE (Na+/H+ antiporter, subunit E)

Position: 897573 to 898052, length: 480 nt, orientation: REVERSE

Perfect match to: (11819-97-CP003194-[944316:944795:r], highly conserved allele)

Sequence:

TTATATTTCGCCCACCTCTCTTACAATTTTCTCTAATGATGTTTTAATACTTTCTACTTCTTGCTCTTTAGTTGAAAAATCTATGGCATGAATAT
AAATTTTTGTACGATCGTCACTTACACCAAGCACTACAGTACCAGGTGTTAATGTAATTAAATTAGACAGCAAGACAATTTGCCAATCTTTTT
TTAAATCTGTGTGATAAACAAAGAATCCTGGTTCATTTTTAATCGAAGGTTTAATAATAATTTTCAAAACATCAAAATTAGCTTTAATCAGTT
CGATTAAGAAAATAATAACTAATTTAATAATACGATATAGCGTGATGACATAAAATCTACCTGGTAACACTCTGTGTAAGAGGTAAACAAG
AACTAGGCCAAAGATGAAACCTAACACAAAGTTATTTGTTGTGTAACTATTTGTTACAAACAACCAAAACACTGCGATAATAAAGTTTAATA
CTAATTGTACAGCCAT

Gene: mnhD (Na+/H+ antiporter, subunit D)

Position: 898054 to 899550, length: 1497 nt, orientation: REVERSE

Perfect match to: (11819-97-CP003194-[944797:946293:r], allele observed in CC80+CC5+CC22+CC80+CC4803)

Sequence:

TTATTTACCTCCTAATACAGCTTTAACGTAGGTTGATGGATTGTAGAATGTTTCTGCACCAGCTTTTACCATTGGATATAAGTAATCTGCTGA
CAATCCATATAAAACAGTTATCACAACTGCAACGATTGCAATCGTAGTTAAATATTTGACGTCGACTTTGTTATTAAGATCATATCCTTTTGG
TTGACCGAAAAAGCCTTGTAGGAATATGCGAATGACAGAATATAATACGACTAAACTTGATAATAAGACGATGACACCACTTAAATAAAAT
CCTCTTTCAAATGTTGATTGGACAATGAAAAATTTTCCATAAAAGCCACTGAGTGGGGGAATGCCAGCTAAACTTAATGCTGCGATAAAGA
ATGACCAACCAAGTACAGGATATCGTTTAATTAAGCCACCAAATTGTCTTAAATCAGCAGTGCCTGTAATTTTAATCATAATTCCGATAAGCA
AGAATAATGCAAGTTTTACTAACATGTCGTGCAATGTATAGTAAATAGCCCCAATCATACCTGACTCTGTCATCATTGCAACGCCGACTAAG
ATCACACCTACAGCAATCATGACATTGTATAGGATGATTTTTTTAATGTTGGCATATGCAACAGCACCGACACAACCAAAGATGATCGTTAA
TAGTGCTAAGAATAAAATGACATAATGTGAAAAGCTTACATTATCACTAAAGAATAGGCTCAATGTTCTAGCGATTGCATAAACACCAACTT
TTGTTAACAAAGCACCAAAGAATGCAATGATTGGAATTGGTGGTGCATAGTATGCACTAGGTAACCAAACAAACATTGGGAATACGCCAG
CTTTTGTAGCAAAAACAAAGATAAATAGTATGAAAACGATATTGACTAAGCCACTGTCATGCGCTGAAAGGTTAGCTAATTTATTGCTTATA
TCTGCTAGATTCAATGTTCCTACTACTGAATATAAAATCGCTACACCCATTACGAAGAAGGATGACGATACAACGTTAACAAGAACATATTT
GATTGTTTCTTGTAGTTGAATTTTTGTAGAACCAATTACTAATAAGAAATAAGATGACATTAAAAATACTTCGAAAAATACGAATAGGTTGA
AAATGTCACCAGTTGTGAATGCACCAATGATACCTATTAACATAAATAGTACTGAAAAATAATAATAATATCTTTCACGTTCAATACCAATTG
TTTGGTATGAATATAAAATCACAATAGCTGTAATAATAATACTAGTAATTATTAGTAGGGCACTGAATATGTCTAATACAAAGACAATACTG
TATGGTGCTTTCCATGAACCTAGCTCTACGCGTATTGGTCCATGTTTAACAACATTTGCTAAATTGATAATTGCCGCGACCAAGGTTAATAAT
GTACCGCCTAGTGCGACATAACGCTTTATAATAGGACGCTTTCCAATAAAGACAAGTAATATGGCTGTAATTACTGGAATAACTAGCGTTAA
CACAAGCATATTACTTTCAATCAT

Gene: mnhC (Na+/H+ antiporter, subunit C)

Position: 899543 to 899884, length: 342 nt, orientation: REVERSE

Perfect match to: (11819-97-CP003194-[946286:946627:r], allele observed in CC80+CC7+CC12+CC80)

Sequence:

TCAATCATCTTCTGGAACTCCTTTCATACACTCAACGTTATCTGTGCCTAATTCTTTATATGTTCTAAATGCTAATACTAAGAAAAAGGCTGTT
GTCGCAAAGGCGATAACGATTGCTGTTAAAATAAGTGCTTGCGGGATAGGATCAACATAGCTTTTTACGTTCGCTTCATAAATTGGAACAG
TACCATGTTTAAGTCCGCCCATAGTTATTAAAAATAAGTTTGCTGCATGTGTTAATAGTGTAGTTCCCATAACAATTCGTATCAGACTTTTAG
ACAAAACGAGATAGACACTTATTGCTGTGAGAATACCACTAACAAAAATCATAATAATTTCCAC

Gene: mnhB (Na+/H+ antiporter, subunit B)

Position: 899884 to 900312, length: 429 nt, orientation: REVERSE

Perfect match to: (MW2-BA000033-[915216:915644:r], highly conserved allele)

Sequence:

CTATTCGTTCTCTCCAATCGAAATAATAATTGTCATGACAGTACCAACTACTGCACATAAAACACCGAAATCAAAGAATACTGCTGTTGTCAT
ATGAACAGGTTCTAATATAAATAACGGTATATCAAATGTGACATGCGTAAAGAAATTTTTGCCTAAAAACCAACTTGCGATAGGCGTCGCA
ATACAAAAAACTAATCCGATACCTATCAAGATTTTAAAATCTAATGGGAAAATTTTACGCATTGTTTCTATATCAAATGCAATCGTAATGATA
ACAAGTGAACTTGCGAATAATAATCCGCCGACGAAACCGCCACCAGGTGTATAATGTCCTGCTAAGAAAAGTGAAAAACCAAAGACCATTA
CCATGAAAAAGATAATAACTGCAGCAAATTGCAAAATTAGATCATTTTGTTGTCTATTCAT

Gene: mnhA (Na+/H+ antiporter, subunit A)

Position: 900305 to 902710, length: 2406 nt, orientation: REVERSE

Perfect match to: (MW2-BA000033-[915637:918042:r], highly conserved allele)

Sequence:

CTATTCATGATTTTTCACCTCGTTACCTTGCGTTTGACGCTTTTTACGTAATTTAATCATTGTATATACAGCTAATCCTGCGATACCAAGCACA
GATGACTCGAATAAAGTATCCATACCACGGAAATCAACAAGTATGACGTTTACCATGTTTTTACCGTGAGCTAAATCATAAACGTGCTCTTG
ATAAAACTTAGATATCGATTCAAAATGTCTATTTCCGTATGCAATTAAACCGATAATAATGACGGACAAACCAACACCACCAGCAATTAAAG
CATTAGTAAGCTGGAATGAGCGCTTTTCATTATAACGATTTAAATTTGGTAAGTGGTAGAAGCATAATAAGAACAATGCTGTTGAAATAGA
TTCAACGACAAACTGTGTCAATGCTAAGTCGGGTGCTTTAAAGAATATAAACAATACAGACACAGCATATCCAACTGCACTTAACATAATGA
TGCTAAATAATCTTGATTTAGCGAAAAGAATTAAAAAGGCAGCACTTAATAATAAAATTACGATACAAACTTCGAAAATTCTAATCGGACTA
ACGTCTTTAAAATTAATGTTGAAAGGTACTGAGAATATAGTGACAAATGTTAATAAAATTAATGCACCAAAAATGATAACTAAATTATTACG
TGAATAATCGGTAACATAGCTATTCGTCATCTTTTCAGAGTAGTTTGGAATAACATTTGCACTTCTGTTGTACCAATAATTGAATGTTAGTTT
ACCAGGTTGTCGTTGCAACAATTTCACCCAATAACTAAATGTCACAATTAGTAAGATACCTAAAATATAAATCACTAATGTTGATAAAAAGG
CAGGCGTTAATCCATGGAACATATGGAATTCAACATCATCAATTACCGTATGATTAATCGAAGATGTAGCTGGTTCAATAATCGAATTAGTT
AAAATGCCAGGGAATAAACCAAATACAATTACTAATGTAGCTAAAATAGCTGGTGATAAAAGCATTAATATTGATACTTCGTGTGCTTTTTT
AGGTAATTGTTCAGGTTTATATTGTCCGAAAAATATATGCATTATAAATTTAATTGAATATACAAATGTGAAGACACTGCCCACTATACCAAT
GATTGGGAATAGGTAGCCTAATGTATCAACACTGAATAAATTTGCTTGGCTTGCTGTAAATGTTGTTTCTAAAAATGATTCTTTTGATAAGA
AACCATTGAACGGTGGTACACCAGCCATACTTAATGCTGTAATAACAGTGATTGTAAATGAAATAGGCATAATTGTTAGTAAGCCACCTAAT
TTCTTAACATCACGTGTACCAGTAGAATGATCCACTGCACCTGTAATCATAAATAGGGCACCTTTAAATGTTGCATGGTTGATTAAATGGAA
TATTGCAGCCGTAAATGCAGCAGCATATATTTTGCTATCATCGCCTTGATAGTGATAACTAATGGCACCGATTCCAAGCATCGCCATAATCAT
ACCTAATTGGGATACTGTTGAAAATGCCAGTATACCTTTCAAGTCTTGTTGTTTTGTTGCGTTTAGCGAAGCCCAGAATAATGTAATTAAACC
AACGAGTGTGACAGTCCATACCCAACCTTGCGATGCTGCGAAGATTGGTGTCATTCGAGCGATTAAATATAACCCTGCTTTAACCATTGTTG
CTGAATGAAGATAAGCACTGACTGGTGTAGGTGCTTCCATTGCATCTGGTAGCCAAATATAAAATGGAAACTGAGCAGATTTTGTAAAAGC
ACCAATCATGATTAAAATCATCGCAAAAATGAAGAATGGGCTATTTTGAATTTCAGAAGCATGTTGAATCATGTACTGAATGCTAAATGATT
GTGTTGGTATAGCGAGTAAGATGATACCACCTAATAATGATAGACCACCAAATACTGTGATTATGAGCGATTTTTGAGCACCATATATAGAT
GCTTGTCGTTCGCGCCAGAATGAAATAAGTAAAAAACTAGAAAATGACGTTAGCTCCCAGAATAAATATAGAATAATAACATTATCTGAAA
GTACGACACCTAACATTGCACCCATAAATAGTAATAAATAACAATAAAAATTCCCTAGTTGTTCTGACTTACTTAAGTAGCCGATTGAATATA
ATACTACTAAACTGCCGATTCCTGAAATAAGCAAACTAAAGAGTAAACCTAAGCCATCAAGATATAAATCAAAGTTCATACCAAAATGAGG

CATCCAATTTAAGGTTTTCATTACAGTATTACCTGACATCGTCGTTTTAATTAATGTAAGCATATAAATAAATATGACGATAGGGACAGGTAA
TACGAACCATCCTAAATGTATACGTTTAAAAAATCTATACAGGATAGGAATAATGAGTGCGAATATTAACGGTAATATCACCGCAATATGTA
ACAAACTCAC

Gene: kapB (kinase associated protein B)

Position: 902841 to 903224, length: 384 nt, orientation: REVERSE

Perfect match to: (COL-CP000046-[957699:958082:r], allele observed in CC8+CC80+CC239+CC445+CC4803)

Sequence:

TTATTTAAAATTTTCTTTGTATTGAAGTGAATAATCTTCTTTTAAGCGTGCTAAACTAGCTAAAGACATTTCAGCATGTTTTGTTTGCTGAGCT
TTAAGTTTAGTTTCTAAATCTGTAATTGCTTGTTGAAGTGAATCTTCATAGCGCAATACATCAACATTGAAGTCGCGTAATTGTGAACGTTTC
GTATAGCGTTTTTCAAAATGGCTTAATGCTTTGCGGTCATGGAAAAATACACCTTCAGTTTCAGTAGGGTTATGTAAATCACCTTGTTTCGG
GTGTTTGATAACTTGTTCAACTTTAACAAGGACATCGTCTCCATTTTCTTCAACAATCGTGACACCATAGCTACCTGTTTTGTGTGAAAATCG
ATATAGCTTCAT

Gene: prsA1 (extracellular chaperone)

Position: 903288 to 903881, length: 594 nt, orientation: FORWARD

Perfect match to: (JKD6159-CP002114-[896672:897265], highly conserved allele)

Sequence:

ATGGCTAACTATCCACAGTTAAACAAAGAAGTACAACAAGGTGAAATTAAAGTGGTTATGCACACAAATAAAGGTGACATGACATTCAAAT
TATTTCCAAATATTGCACCAAAAACAGTTGAAAATTTTGTGACACATGCAAAAAATGGTTATTATGATGGAATAACATTCCACCGTGTCATTA
ATGACTTCATGATTCAAGGTGGCGATCCAACAGCTACTGGTATGGGTGGCGAAAGTATTTATGGCGGTGCTTTTGAAGATGAATTTTCATT
AAATGCATTTAACTTATATGGCGCATTATCAATGGCTAACTCAGGACCTAATACTAATGGTTCACAATTTTTCATTGTTCAAATGAAAGAAGT
ACCTCAAAATATGTTAAGTCAACTTGCAGATGGTGGTTGGCCACAACCAATCGTTGATGCATATGGCGAGAAGGGTGGTACACCATGGTTA
GATCAAAAACATACAGTATTCGGTCAAATCATTGATGGTGAAACTACATTAGAAGATATTGCAAATACAAAAGTGGGACCACAAGATAAAC
CACTTCATGATGTTGTAATTGAATCTATTGATGTTGAAGAATAA

Gene: yugI (putative RNA degradation protein)

Position: 904297 to 904674, length: 378 nt, orientation: FORWARD

Perfect match to: (RF122-AJ938182-[887150:887527], highly conserved allele)

Sequence:

TTGAATAACTACAAAATTGGCCAACATATCAAGGTGCGTGTAACTGGTATTCAACCATACGGTGCGTTTGTTGAGACCCCTAATCATACTGA
AGGACTGATTCATATATCAGAAATTATGGATGACTACGTTCATAATTTGAAGAAATTTCTATCAGAAGGCCAAATTGTTAAAGCTAAAATTT
TGTCTATAGATGATGAAGGAAAGCTTAATCTATCATTAAAGGATAATGATTACTTCAAAAATTATGAGCGTAAGAAGGAAAAACAATCAGT
ATTAGATGAAATCAGAGAAACAGAAAAATATGGGTTTCAAACACTTAAAGAACGCTTACCAATCTGGATAAAACAGTCAAAGCGAGCAATT
CGAAACGACTAA

Gene: namA (NADH:flavin oxidoreductase)

Position: 905036 to 906163, length: 1128 nt, orientation: FORWARD

Perfect match to: (11819-97-CP003194-[951779:952906], allele observed in CC80+CC188)

Sequence:

ATGAAAAGTAAATACGAACCATTGTTTGATAAAGTAGAATTACCAAATGGAGTAGAGTTGAGAAATCGATTTGTGTTAGCCCCTTTAACAC
ATATTTCTTCAAATGATGATGGTACTATTTCAGATGTAGAACTTCCTTATATTGAAAAGCGTTCACAAGATGTTGGTATTACAATTAATGCTG
CGAGTAATGTGAGTGATGTCGGAAAAGCATTTCCAGGACAGCCGTCAATCGCGCATGACAGTGATATTGAAGGACTAAAACGATTAGCTA
CAGCAATGAAGAAAAACGGTGCCAAAGCACTCGTACAAATACATCATGGTGGTGCACAAGCATTGCCTGAATTAACACCTGATGGAGACG

TTGTAGCACCAAGTCCAATTTCTTTAAAAAGTTTCGGTCAGAAACAAGAACATAGTGCTAGAGAAATGACGAATGAAGAGATTGAACAAGC
AATCAAGGATTTTGGTGAAGCAACACGACGTGCAATTGAAGCAGGTTTTGATGGTGTTGAAATACATGGCGCGAATCATTACTTAATTCAT
CAATTTGTATCACCATACTATAATAGAAGAAATGATGTATGGGCAAATCAATATAAATTCCCGGTCGCTGTGATTGAAGAAGTGCTTAAAGC
GAAAGAAGCGTATGGCAATAAAGACTTTATAGTTGGATACAGATTATCTCCAGAGGAAGCGGAGTCTCCAGGAATCACAATGGAAATTAC
AGAGGAACTCGTTAATAAAATTAGCCATATGCCAATCGACTATATTCATGTTTCAATGATGGATACGCATGCAACGACACGTGAAGGTAAA
TACGCTGGACAAGAAAGACTACCTTTAATTCACAAATGGATAAATGGTCGTATGCCACTTATCGGTATTGGTTCAATTTTCACAGCTGACGA
AGCTTTAGATGCAGTTGAAAATGTTGGTGTTGACTTAGTAGCCATTGGTAGAGAGCTACTACTGGATTATCAATTTGTTGAAAAAATTAAAG
ATGGACGGGAAGATGAAATTATTAATTACTTTGATCCAGAGAGAGAAGATAATCATCACTTAACTCCTAATTTATGGCATCAATTTAATGAA
GGATTCTATCCATTACCACGTAAAGATAAATAA

Gene: rocD2 (ornithine aminotransferase 2)

Position: 906471 to 907661, length: 1191 nt, orientation: FORWARD

Perfect match to: (MW2-BA000033-[921802:922992], highly conserved allele)

Sequence:

ATGACTAAATCTGAAAAAATTATTGAGTTAACAAATCATTACGGAGCACATAATTATTTACCATTGCCAATTGTCATTTCAGAAGCTGAAGG
GGTATGGGTTAAAGATCCTGAAGGCAATAAATATATGGATATGTTATCTGCATATTCCGCTGTTAACCAAGGTCATAGACATCCGAAAATTA
TTCAAGCATTAAAAGATCAAGCTGATAAAGTGACTCTAGTTTCACGTGCTTTTCATAGTGATAACTTAGGTGAATGGTACGAAAAAATTTGT
AAACTGGCAGGTAAAGATAAAGCTTTACCAATGAATACAGGTGCTGAAGCAGTAGAAACAGCTTTGAAAGCAGCACGACGCTGGGCATAC
GATGTTAAAGGAATTGAGCCAAATAAAGCAGAAATCATTGCATTTAATGGTAACTTCCATGGTCGAACAATGGCGCCAGTTTCATTATCTTC
AGAAGCAGAATACCAACGTGGTTATGGTCCGTTATTAGATGGATTTAGAAAAGTTGATTTTGGAGATGTAGATGCATTGAAAGCTGCAATT
AATGAAAATACTGCAGCAGTTTTAGTAGAACCAATTCAAGGTGAAGCGGGTATAAATATACCGCCAGAAGGATATTTGAAAGCAATTAGA
GAATTATGTGATGAACATAATGTCTTATTTATTGCTGACGAAATCCAAGCAGGATTAGGTCGTTCGGGTAAATTATTTGCTACGGATTGGGA
TAATGTAAAACCTGATGTCTATATTTTAGGTAAAGCACTAGGTGGTGGTGTCTTCCCAATTTCTGTTGTATTAGCAGATAAAGAAGTATTAG
ATGTCTTTACACCTGGCTCACATGGTTCAACATTTGGTGGTAATCCACTTGCTTGTGCTGCATCAATTGCTGCATTAGATGTTATCGTTGATG
AGGATTTACCAGGGCGCTCTTTAGAATTAGGAGATTATTTTAAAGAACAATTAAAGCAAATTGATCATCCATCAATTAAAGAAGTCCGTGG
ACGTGGTTTGTTTATAGGTGTGGAACTTAATGAAAGTGCTAGACCATATTGTGAAGCTTTGAAAGAAGAAGGCTTATTATGTAAAGAAACG
CATGATACTGTCATTCGTTTTGCACCACCATTAATTATTACTAAAGAAGAATTGGACCTTGCACTTGAAAAAATAAGACATGTATTTCAATAA

Gene: gluD (NAD-specific glutamate dehydrogenase)

Position: 907770 to 909014, length: 1245 nt, orientation: FORWARD

Perfect match to: (Strain_21305-AFNO01000003-[17080:18324:r], allele observed in CC25)

Sequence:

ATGACTGAGAACAATAATTTAGTAACTTCTACTCAAGGAATTATTAAAGAAGCATTGCATAAATTGGGATTTGACGAAGGAATGTACGATTT
AATTAAAGAACCTTTAAGAATGTTACAAGTGCGTATCCCTGTACGAATGGATGATGGCACAGTTAAAACATTCACAGGTTACCGTGCGCAA
CATAATGATGCTGTTGGACCAACAAAAGGGGGCGTGCGTTTCCACCCAGATGTTGATGAAGAAGAAGTAAAAGCATTATCAATGTGGATG
ACTTTGAAATGTGGCATTGTAAACTTACCATACGGTGGTGGTAAGGGTGGTATCGTTTGTGATCCACGTCAAATGAGCATTCATGAAGTTG
AACGTTTATCACGCGGATATGTAAGAGCAATTTCACAATTCGTAGGTCCGAACAAAGATATTCCAGCACCAGATGTATTTACAAACTCACAA
ATTATGGCTTGGATGATGGATGAATATAGTGCATTAGATAAATTTAATTCACCAGGTTTCATCACAGGTAAACCAATTGTATTGGGTGGTTC
TCATGGACGCGACAGATCAACTGCACTAGGTGTAGTTATTGCAATTGAACAAGCTGCAAAACGTCGTAATATGCAAATTGAAGGTGCCAAG
GTTGTTATTCAAGGTTTCGGTAATGCCGGAAGTTTCTTAGCTAAATTCTTATATGATTTAGGTGCAAAAATTGTAGGTATCTCTGATGCTTAC
GGTGCATTACACGATCCAAATGGCTTAGATATAGATTATTTATTAGACCGTCGTGATAGTTTTGGTACGGTAACAAATTTATTTGAAGAAAC
AATCTCAAATAAAGAATTGTTTGAATTAGATTGTGACATTTTAGTACCAGCGGCTATTTCAAACCAAATTACAGAAGACAATGCACATGATA
TTAAAGCTAGTATCGTTGTTGAAGCTGCTAATGGACCTACAACACCAGAAGCAACACGTATTTTAACTGAACGTGGTATATTATTAGTTCCA
GACGTATTAGCAAGTGCTGGTGGTGTAACGGTTTCTTACTTCGAATGGGTACAAAATAATCAAGGTTATTATTGGTCTGAAGAAGAAGTAA
ATGAAAAACTACGTGAAAAATTAGAAGCGGCATTTGACACGATTTACGAATTGTCTCAAAATCGAAAAATAGATATGAGACTTGCAGCATA
TATCATAGGTATTAAACGTACAGCAGAAGCAGCTAGATATCGTGGTTGGGCATAA

Gene: SIRU01 (staphylococcal interspersed repeat unit 1)

Position: 909142 to 909308, length: 167 nt

Sequence:

GGCCCCAACACAGAGGCTGGTGGAAAGTCAGCTTACAATAGTGTGCAAGTTGGCGGGGCCCCAACACAGAGGCTGGCGGAAAGTCAGCT
TACAATAGTGTGCAAGTTGGGGTGGAGCCCCGACACAGAGAAATTAGCTCCTCAATTTCTACAGACAATGCAAGTTGG

Gene: glpQ2 (glycerophosphodiester phosphodiesterase)

Position: 909465 to 910394, length: 930 nt, orientation: REVERSE

Perfect match to: (11819-97-CP003194-[956208:957137:r], allele observed in CC80+CC88+CC361)

Sequence:

CTACTTAATTACTTCTTTATATTTATCAGCGAAATTTGTAAAGACACCATCAACGCCATATTTATTTAATCGTAACATATCAGCTTGTTCATTCA
CTGTATAAGGATGTACTATAAATCCTAAGTCTTTTAAATGATGGGTATTTTGTTCAGTTAAATCTGTATAATCTGGACCTAATCCAATCGCAT
AAGAGCGTATCTCTTTTAAGCGTTGGTCGTTAAATTGTTGTAGTTCACCTTTATCAACTAATTTTACTAATGGCACATTCTTATTTTGACGATG
AATTTTCTTTAAACTTTCGTCAGAAAATGATTGAATCATTACATGTCCATTTTTAAATTTATTGTTATTTAAAAGGTGATGCTTTTTCAATGAA
GCTAATAATTGTTCTTCCATTCCTGGGTATACATCAGGTGACTTTGTTTCAATATAATAGTTTGCATTCGGACCATAACGTTCTAAAATTTCAT
CTAAAGTGGGTACTTTAGCATTTTTATAACTTGCTCTTGCGTATTTTGGATATTTTTTATTAAACCAACTTCCTGCATCTAACTGTTTTAATTCA
TCAAGGGTATAATCCTCAACTTTACCGTGTCCATTTGTTGTACGGTTAACAGTTTCATCATGCATAGCAACTAAATGGCCATCTTTGGTACGT
TGTAAATCAATTTCGATATACGATGCTTTTAACTCATTATGACTCTTATCATATGCTTGAAACGTATGCTCGGGTGCATAGCCACTTGCGCCA
CGATGAGCGATAGTAGTGAATCGCTCATTCGTTAAATTTGTATGCCATTGAATAGCCTGAGGTTTATTTGCAATTTGATTTGTTTGTTCAGCG
CCAGCAGTAGGTACTGATAAAAATCCCATAGTAAAAACAGCAGAAGCAGCCATAAATTTAGTGAAGCTTTTCGAAGAGTTAGTCAT

Gene: argH (argininosuccinate lyase)

Position: 910636 to 912015, length: 1380 nt, orientation: REVERSE

Perfect match to: (Strain_21343-AHKV01000006-[15927:17306], allele observed in CC88+CC8+CC22+CC80)

Sequence:

TTATTGTGATAGTAATTGTTTAGCAACATCAAGTTGTTGTTTGACCGATGATTGACCTGTTGAACCGTAACTTTGACGTCGTTTTAAACAATT
TTCAGGCTGCAAATAATCGTAAATATCGGCATCAATACTAGAATGATGTTGTTGATATGTTGCTAAAGGAACATCTAATAAATAATGACCTT
GTTGTATACATTCTAAGACGATTTTTCCTACAATTTCATGTGCAGTTCTAAATGGAATATTTTTAGTTACTAAATAATCTGCTAGTTCCGTTGC
ATTTGAAAAATCTTCTTTAACAGTTTGATTGAGTCGTTCTTTATTAACTGTCATCGTTTGAATCATACCTTCGAAAATACGTAAAGAACCTTTA
ATTGTATGGACAGCATCGAATAAACCTTCTTTATCTTCCTGCATATCTTTGTTATATGCTAGAGGTAATCCTTTTAAAGTCATAAGCATGCTCA
TTAAATGACCAGTCGTTCGACCAACTTTACCTCTAATTAATTCTGCCATATCAGGATTTTTCTTTTGTGGCATAATAGATGAGCCAGTTGAAA
ATGCATCTGATAATGTAATGAATTTAGCTTCGTCTGTGGACCAGAAAATAATTTCCTCTGCAAAGCGTGATAAGTGAACCATCGTTAAAGAA
ATATTATGCAATGTTTCAATAATATAGTCTCTGTCACTAACAGCATCTAGGCTATTCTCATAGAGACTGCCAAAGTTCAACAATGCTGTTGTC
TCGTGTCTATCGATAGGGTATGTGGTACCACTTAAGGCTGCTGCACCTAAAGGATTAATATCGATTCGTTTTAAACTATCTTCAAATCGTTGT
TGGTCTCGTTGTAACATCCAAAAATAAGTCATAATATGATGTGCAAATGAAATTGGCTGTGCACGCTGTAAATGAGTATAACCAGGCATAA
TTGTATCAACATTATTGGAAGCGATGTCTACAATTACACTTTGTAACGACTTAATTAATGCGATGATATCTTGCACTTGTTTCTTAGTGTACA
AGTGCATGTCTGTTGCAACTTGATCGTTTCTACTGCGTCCAGTATGCAACTTACCACCAGCATCACCAATACGTTTAATTAATTCATGTTCAAT
ATTTAAATGAATATCTTCTAATGATGCACTAAATTGAATTTGATCTTGATGATAATCATGTTGAATAGATTTTAGTCCTTGTATAATTTGTTCG
CTGTCTTGTTGACTAATAATGCCTTGATTCGCAAGCATAGTTGCATGTGCAATGCTGCCTTCGATATCTTGATCTATGAGCGTTTGATCAAAA
GTAATGGATGCGTTAAAGTCGTCAACCCACTCTTCAGGTTGTACTTCAAATCTACCGCCCCAAGCTTTATTGCTCAT

Gene: argG (argininosuccinate synthase)

Position: 912005 to 913210, length: 1206 nt, orientation: REVERSE

Perfect match to: (11819-97-CP003194-[958748:959953:r], allele observed in CC80+CC239)

Sequence:

TTATTGCTCATTGCTATAGCCTCCATGCAACATCGCATTTACTTGAGTAGGTAAACCATAGATATCGATAAAGCCAACAGCAGCGTCTTGATT
AAATGCATCTTCTTTTGTATAAGTTGCTAATTTTTCATCATATAATGTGTAAGGTGATTTTCTACCATTTACGATGGCATTACCTTTGAATAAT
TTAATTCTGACATCACCACTTACGTATTGCTGAGTACTATCAATAAATAATTTTAAGCTATCAGTTAAAGGTGAGAACCAAAGTCCATTGTAT
AGTTGTTCAGCAAATTGCTTCTCGATGATTGGTTTAAAGTGTGCGACATCTTTCGTTAACGTAATCGTTTCTAATGCTTTATGCGCTTTTAAAA

TAACTTCTGCAGCAGGTGCCTCATAAATTTCTCTTGATTTGATACCTACAAGTCTATTTTCTACATGGTCAATTCTTCCGATACCATGCTTACC
AGCTAATGCATTCAACGTTAAAATTAAATCGTCTAATTCATATGTTTTGCCATCAATTTGAACTGGGATGCCTTTATCAAACGTTAAAATGAT
TTCATCAGCATCATCTGGTGTTTCTTCTAAAGCATTTGTTAGATCGAACGCATCCTCTGGTGGCGCAGCATAAGGATCTTCTAAAATACCACA
TTCATTCGCTCTGCCCCATAGATTTTGATCGATAGAATAAGGTGAATCATGGTTGATTGATACAGGGATATTATGTTTAATTGCATAATCGAT
TTCTTCTTCACGACTCCATGCCCACTCACGTACAGGTGCGAATGCTTTCAATGATGGGTTTAATGCTTTAATGGCAACTTCGAAACGTACTTG
GTCATTCCCTTTACCAGTACAACCATGTGCAATACCTACTGAATTTGTTTTCTCAGCAATCTCTACTAATTTTTTAGCGATTAATGGTCTTGAT
AAAGCTGAAACTAATGGATATGCATTTTCATACATTAAATTTCCTTTGATTGCATAACTTACATACTCATCACTAAATTCTTTTGTTGCATCAA
TAATATGACATTCAACTGCTCCCATATCTAAAGCTTTTTTATAAACGATGTCTAAATCTTTACCTTCACCAACATCTAGGCAACAAGCTACAAC
GTCGTATCCTTTGTCGATAAGCCATTGAACGGCCACACTTGTATCTAGTCCTCCTGAATATGCTAAAACAATTTTCTCTTTCAT

Gene: pgi (glucose-6-phosphate isomerase)

Position: 913561 to 914892, length: 1332 nt, orientation: FORWARD

Perfect match to: (11819-97-CP003194-[960304:961635], highly conserved allele)

Sequence:

ATGACTCATATTCAATTAGATTTTAGTAAAACGTTAGAATTTTTCGGTGAACACGAATTAAAACAACAACAAGAAATTGTTAAATCAATTCAC
AAAACAATTCATGAAGGTACTGGTGCAGGTAGTGACTTCTTAGGCTGGGTTGATTTACCAGTTGATTACGACAAAGAAGAATTTTCAAGAA
TTGTTGAAGCATCAAAACGCATTAAAGAAAATTCTGATGTTTTAGTAGTCATCGGTATTGGTGGTTCTTACTTAGGTGCACGTGCAGCAATC
GAAATGTTAACGTCATCATTTAGAAACAGCAATGAATACCCTGAAATTGTATTTGTTGGTAATCACTTATCATCAACATATACGAAAGAGTT
AGTTGATTATTTAGCAGACAAAGATTTCTCTGTAAACGTTATTTCTAAATCTGGTACAACTACAGAACCAGCAGTTGCATTTAGATTGTTCAA
ACAATTAGTTGAAGAAAGATACGGTAAAGAAGAAGCACAAAAACGTATATTTGCAACAACGGATAAAGAAAAAGGTGCTTTAAAACAGTT
GGCTACAAACGAAGGTTATGAAACGTTTATCGTACCTGATGATGTAGGTGGAAGATATTCTGTTTTAACAGCAGTAGGATTATTACCAATTG
CAACAGCTGGAATTAACATCGAAGCTATGATGATTGGTGCTGCAAAAGCACGTGAAGAATTATCTTCAGATAAATTAGAAGAAAACATTGC
ATACCAATATGCGACAATTCGAAACATTTTATATGCAAAAGGTTATACAACAGAAATGTTGATTAACTATGAACCATCTATGCAATACTTTAA
TGAATGGTGGAAACAATTATTTGGTGAATCAGAAGGTAAAGACTTCAAAGGTATCTATCCTTCAAGTGCCAACTACACAACTGATTTACATT
CTTTAGGTCAATATGTACAAGAAGGCCGTCGCTTCTTATTCGAAACAGTTGTAAAAGTAAATCATCCTAAATATGATATTACTATTGAAAAA
GATAGTGATGATCTAGACGGATTAAATTATTTAGCTGGTAAAACAATCGACGAAGTTAACACAAAAGCATTCGAAGGTACATTATTAGCGC
ATACTGATGGTGGTGTTCCTAACATGGTAGTGAACATTCCACAATTAGATGAAGAAACTTTCGGTTACGTCGTATACTTCTTCGAACTTGCTT
GTGCAATGAGTGGATACCAATTAGGCGTAAATCCATTTAACCAACCTGGTGTAGAAGCATATAAACAAAACATGTTCGCATTATTAGGTAA
ACCTGGTTTTGAAGACTTGAAAAAAGAATTAGAAGAACGTTTATAA

Gene: yhjE (putative membrane protein)

Position: 915217 to 915792, length: 576 nt, orientation: FORWARD

Perfect match to: (Strain_21178-AGRN01000032-[54770:55345], allele observed in CC239+CC80)

Sequence:

TTGTCGTTTCATCAAGTAGAAGAATGGTTTGAGATATTTCGACAGTTTGGTTATTTACCTGGATTTATATTGTTATATATTAGAGCTATAATTC
CAGTATTTCCTTTAGCACTCTATATTTTAATTAACATTCAAGCTTATGGACCTATTTTAGGTATATTGATTAGTTGGCTTGGATTAATTTCTGG
AACATTTACAGTCTATTTGATCTGTAAACGATTGGTGAACACTGAGAGGATGCAGCGAATTAAACAACGTACTGCTGTTCAACGCTTGATTA
GTTTTATTGATCGCCAAGGATTAATCCCATTGTTTATTTTACTTTGTTTTCCTTTTACGCCAAATACATTAATAAATTTTGTAGCGAGTCTATCT
CATATTAGACCTAAATATTATTTCATTGTTTTGGCATCATCAAAGTTAGTTTCAACAATTATTTTAGGTTATTTAGGTAAGGAAATTACTACAA
TTTTAACGCATCCTTTAAGAGGGATATTAATGTTAGTTGTGTTGGTTGTATTTTGGATTGTTGGAAAAAAGTTAGAACAGCATTTTATGGGG
TCGAAAAAGGAGTGA

Gene: spsA (signal peptidase I subunit A)

Position: 915797 to 916321, length: 525 nt, orientation: FORWARD

Perfect match to: (TCH959-AASB02000223-[98050:98574], allele observed in CC7+CC72+CC80+CC361+CC772)

Sequence:

GTGAAAAAAGTTGTAAAATATTTGATTTCATTGATACTTGCTATTATCATTGTACTGTTCGTACAAACTTTTGTAATAGTTGGTCATGTCATTC
CGAATAATGATATGTCACCAAGCCTTAACAAAGGGGATCGTGTTATTGTAAATAAAATTAAAGTTACATTTAATCAATTGAATAATGGTGAT

ATCATTACATATAGGCGTGGTAACGAGATATATACTAGTCGAATTATTGCCAAACCTGGTCAATCAATGGCGTTTCGTCAGGGACAATTATA
CCGTGATGACCGACCGGTTGACGCATCTTATGCCAAGAACAGAAAAATTAAAGATTTTAGTTTGCGCAATTTTAAAGAATTAGATGGAGAT
ATTATACCGCCTAACAATTTTGTTGTGCTAAATGATCATGATAACAATCAACATGATTCTAGACAATTTGGTTTAATTGATAAAAAGGATATT
ATTGGTAATATAAGTTTGAGATATTATCCTTTTTCAAAATGGACGATTCAGTTCAAATCTTAA

Gene: spsB (signal peptidase I subunit B)

Position: 916337 to 916912, length: 576 nt, orientation: FORWARD

Perfect match to: (TCH959-AASB02000223-[98590:99165], allele observed in CC7+CC72+CC80+CC361)

Sequence:

TTGAAAAAAGAATTATTGGAATGGATTATTTCAATTGCTATTGCGCTAGTTATATTTGCTATAATAGGTAAATTTATTGTTACACCATATACG
ATTAAAGGTGAATCAATGGATCCAACTTTGAAAGATGGCGAGCGAGTAGCTGTAAACATTATTGGATATAAAACAGGTGGTTTGGAAAAA
GGTAATGTAGTTGTCTTCCATGCAAACAAAAATGATGACTATGTTAAACGTGTCATCGGTGTTCCTGGCGATAAAGTAGAATATAAAAATG
ATACATTATATGTCAATGGTAAAAAACAAGATGAACCATATTTAAACTACAATTTAAAACATAAACAAGGTGATTACATTACTGGGACTTTC
CAAGTTAAAGATTTACCGAATGCGAATCCTAAATCAAATGTCATTCCAAAAGGTAAATATTTAGTGCTTGGAGATAATCGTGAAGTAAGTA
AAGATAGCCGTGCGTTTGGCCTCATTGATGAAGACCAAATTGTTGGTAAAGTTTCATTTAGATTCTGGCCATTTAGTGAATTTAAACATAAT
TTCAATCCTGAAAACACTAAAAATTAA

Gene: rexB (ATP-dependent nuclease subunit B)

Position: 917072 to 920548, length: 3477 nt, orientation: FORWARD

Perfect match to: (TCH959-AASB02000223-[99325:102801], allele observed in CC7+CC80)

Sequence:

ATGACATTACATGCTTATTTAGGTAGAGCGGGAACAGGTAAGTCTACGAAAATGTTAACTGAAATAAAACAAAAAATGAAACAGGATCCAC
TCGGCGATCCAATCATTTTAATTGCGCCAACTCAAAGTACATTTCAATTAGAACAAGCCTTTGTCAATGATCCGGAATTAAATGGTAGTTTAA
GAACAGAAGTGTTGCATTTTGAACGATTAAGTCATCGTATTTTCCAAGAAGTTGGTAGTTATAGCGAACAAAAGTTATCTAAAGCTGCAAC
GGAAATGATGATTTATAACATTGTTCAAGAACAACAAAAGTATTTAAAACTTTATCAATCACAAGCAAAATATTATGGGTTTAGTGAAAAAT
TAACAGAACAAATTCAAGATTTTAAAAAATATGCAGTAACGCCTGAACATTTAGAACACTTTATTGCTGATAAAAATATGCAAACTCGAACT
AAAAATAAGTTAGAGGATATTGCTTTAATATACCGTGAGTTCGAACAACGCATTCAAAACGAGTTTATTACGGGTGAGGATTCATTACAATA
TTTTATTGATTGTATGCCGAAATCAGAGTGGCTAAAACGTGCTGATATATATATTGATGGTTTTCACAACTTTTCAACGATTGAGTATTTAAT
AATCAAAGGATTAATTAAATATGCGAAGAGTGTCACAATTATATTGACGACAGATGGTAACCACGATCAATTTAGTTTATTTAGAAAACCAT
CGGAAGTGTTACGACATATTGAAGAAATAGCAAATGAACTCAATATTTCTATTGAACGTCAATATTTCAAGCATTTATATCGCTTCAATAATC
AAGATTTAAAGCATCTTGAACAAGAATTTGATGCACTTCAAATCAATCGAGTGGCATGTCAAGGTCATATCAATATTTTAGAATCTGCGACT
ATGAGAGAGGAAATAAATGAAATTGCGCGACGTATCATCGTTGATATTCGTGATAAGCAATTACGATATCAAGATATTGCAATTTTATATCG
TGACGAGTCTTATGCTTATTTATTTGATTCCATATTACCGCTTTATAATATTCCTTATAACATTGATACAAAGCGTTCGATGACACATCATCCG
GTCATGGAAATGATTCGTTCATTGATTGAAGTTATTCAATCTAATTGGCAAGTGAATCCAATGCTACGCTTATTGAAGACTGATGTGTTAAC
GGCATCATATCTAAAAAGTGCATACTTAGTTGATTTACTTGAAAATTTTGTACTTGAACGTGGTATATACGGTAAACGTTGGTTAGATGATG
AGCTATTTAATGTCGAACATTTTAGCAAAATGGGGCGTAAAGCGCATAAACTGACCGAAGATGAACGTAACACATTTGAACAAGTCGTTAA
GTTAAAGAAAGATGTCATTGATAAAATTTTACATTTTGAAAAGCAAATGTCACAAGCGGAAACTGTAAAAGATTTTGCAACTGCTTTTTATG
AAAGTATGGAATATTTCGAACTGCCAAATCAATTGATGACAGAGCGAGATGAACTTGATTTAAATGGTAATCATGAAAAGGCGGAGGAAA
TTGATCAAATATGGAATGGCTTAATTCAAATCCTTGATGACTTAGTTCTAGTATTTGGAGATGAACCAATGTCGATGGAACGTTTCTTAGAA
GTATTTGATATTGGTTTAGAACAATTAGAATTTGTTATGATTCCGCAAACATTGGACCAAGTAAGTATTGGTACGATGGATTTGGCTAAAGT
CGATAATAAGCAACATGTTTACTTAGTAGGTATGAATGATGGTACGATGCCACAACCAGTAACTGCGTCAAGCTTGATTACAGATGAAGAA
AAGAAATACTTTGAACAGCAGGCTAATGTCGAGTTAAGTCCAACATCAGATATTTTACAGATGGATGAAGCATTTGTTTGTTATGTTGCTAT
GACTAGAGCTAAGGGAGATGTTACATTTTCTTACAGTCTAATGGGATCAAGTGGTGATGATAAGGAGATCAGCCCATTTTTAAATCAAATTC
AATCATTGTTCAACCAATTGGAAATTACTAACATTCCTCAATACCATGAAGTTAACCCATTGTCACTAATGCAACATGCTAAGCAAACCAAAA
TTACATTATTTGAAGCATTGCGTGCTTGGTTAGATGATGAAATTGTGGCTGATAGTTGGTTAGATGCTTATCAAGTAATTAGAGATAGCGAT
CATTTAAATCAAGGTTTAGATTATTTAATGTCAGCATTAACGTTTGACAATGAAACTGTAAAATTAGGTGAAACGTTGTCTAAAGATTTATAT
GGTAAGGAAATCAATGCCAGTGTATCTCGTTTTGAAGGTTATCAACAATGCCCATTTAAACACTATGCGTCACATGGTCTGAAACTAAATGA
GCGAACGAAGTATGAACTTCAAAACTTTGATTTAGGTGATATTTTCCATTCTGTTTTAAAATATATATCTGAACGTATTAATGGCGATTTTAA
ACAATTAGACCTGAAAAAAATAAGACAATTAACGAATGAAGCATTGGAAGAAATTTTACCTAAAGTTCAGTTTAATTTATTAAATTCTTCAG
CTTACTATCGTTATTTATCAAGACGCATTGGCGCTATTATAGAAACAACACTAAGCGCATTAAAATATCAAGGCACGTATTCAAAGTTTATGC
CAAAACATTTTGAGACAAGTTTTAGAAGGAAACCAAGAACAAATGACGAATTAATTGCACAAACATTAACGACAACTCAAGGTATTCCAAT
TAATATTAGAGGGCAAATTGATCGTATCGATACGTATACAAAGAATGATACAAGTTTTGTTAATATCATTGACTATAAATCCTCTGAAGGTA

GTGCGACACTTGATTTAACGAAAGTATATTATGGTATGCAAATGCAAATGATGACATACATGGATATCGTTTTACAAAATAAACAACGCCTT
GGATTAACAGATATTGTGAAACCAGGTGGATTATTATACTTCCATGTACATGAACCTAGAATTAAATTTAAATCATGGTCTGATATTGATGA
AGATAAACTAGAACAAGATTTAATTAAAAAGTTTAAGCTGAGTGGTTTAGTGAATGCAGACCAAACTGTTATTGATGCATTGGATATTCGTT
TAGAACCTAAATTCACTTCAGATATTGTACCAGTTGGTTTGAATAAAGATGGCTCTTTGAGTAAACGAGGCAGCCAAGTGGCAGATGAGGC
AACGATTTATAAATTCATCCAACATAACAAAGAGAATTTTATAGAAACAGCTTCAAATATTATGGATGGACATACTGAAGTTGCACCATTAA
AGTACAAACAAAAATTGCCATGTGCTTTTTGTAGTTATCAATCGGTATGTCATGTAGATGGCATGATTGATAGTAAGCGATATCGAACTGTA
GATGAAACAATAAATCCAATTGAAGCAATTCAAAATATTAACATTAATGATGAATTTGGGGGTGAGCAATAG

Gene: rexA (ATP-dependent nuclease subunit A)

Position: 920549 to 924202, length: 3654 nt, orientation: FORWARD

Sequence:

ATGACAATTCCAGAGAAACCACAAGGCGTGATTTGGACTGACGCGCAATGGCAAAGTATTTACGCAACTGGACAAGATGTACTTGTTGCA
GCCGCGGCAGGTTCAGGTAAAACAGCTGTACTAGTTGAGCGTATTATCCAAAAGATTTTACGTGATGGCATTGATGTCGATCGACTTTTAG
TCGTAACGTTTACAAACTTAAGCGCACGTGAAATGAAGCATCGTGTAGACCAACGTATTCAAGAGGCATCGATTGCTGATCCTGCAAATGC
ACACTTGAAAAACCAACGCATCAAAATTCATCAAGCACAAATATCTACACTTCATAGTTTTTGCTTGAAATTAATTCAACAGCATTATGATGT
ATTAAATATTGACCCGAACTTTAGAACAAGCAGTGAAGCTGAAAATATTTTATTATTAGAACAAACGATAGATGAGGTCATAGAACAACATT
ACGATATCCTTGATCCTGCTTTTATTGAATTAACAGAGCAATTGTCTTCAGATAGAAGTGATGATCAGTTTCGAATGATTATTAAACAATTGT
ATTTCTTTAGCGTTGCAAATCCAAATCCTATAAATTGGTTGGATCAATTGGTGACACCATACGAAGAAGAAGCACAACAAGCGCAACTTATT
CAACTACTAACAGACTTATCTAAAGTATTTATCACAGCTGCCTATGATGCTTTAAATAAGGCGTATGATTTGTTTAGTATGATGGATAGCGTC
GATAAACATTTAGCTGTTATAGAAGATGAACGACGTTTAATGGGGCGTGTTTTAGAAGGTGGCTTTATTGATATACCTTATTTAACTGGTCA
CGAATTTGGCGCGCGTTTGCCTAATGTAACAGCGAAAATTAAAGAAGCAAATGAAATGATGGTCGATGCCTTAGAAGATGCTAAACTTCAA
TATAAAAAATATAAATCATTAATTGATAAAGTGAAGAGTGATTACTTTTCAAGAGAAGCTGATGATTTGAAAGCTGATATGCAACAATTGG
CGCCACGAGTAAAGTACCTTGCGCGTATTGTGAAAGATGTTATGTCAGAATTCAATCGAAAAAAGCGTAGCAAAAATATTTTGGATTTTTCT
GATTATGAACATTTTGCATTACAAATTTTAACTAATGAGGATGGTTCGCCTTCAGAAATTGCCGAATCATACCGTCAACACTTCCAAGAAATA
TTGGTCGATGAGTATCAAGATACGAACCGAGTTCAAGAGAAAATACTATCTTGCATCAAAACGGGTGATGAACATAATGGTAATTTATTTA
TGGTTGGAGATGTTAAGCAATCCATTTATAAATTTAGACAAGCTGATCCAAGTTTATTTATTGAAAAGTATCAACGCTTTACTATAGATGGA
GATGGCACTGGACGTCGAATTGATTTGTCGCAAAACTTCCGTTCTCGAAAAGAAGTACTGTCAACGACTAACTATATATTCAAACATATGAT
GGATGAACAAGTCGGTGAAGTAAAATATGATGAAGCGGCACAGTTGTATTATGGTGCACCATATGATGAATCGGACCATCCAGTAAACTT
AAAAGTGCTTGTTGAAGCGGATCAAGAACATAGTGATTTAACTGGTAGTGAACAAGAAGCGCATTTTATAGTAGAACAAGTTAAAGATATC
TTAGAACATCAAAAAGTTTATGACATGAAAACAGGAAGCTATAGAAGTGCGACATACAAAGATATCGTTATTCTAGAACGCAGCTTTGGAC
AAGCTCGCAATTTACAACAAGCATTTAAAAATGAAGATATTCCATTCCATGTGAATAGTCGTGAAGGTTACTTTGAACAAACAGAAGTCCGC
TTAGTATTATCATTTTTAAGAGCGATAGATAATCCATTACAAGATATTTATTTAGTTGGGTTAATGCGCTCCGTTATATATCAGTTCAAAGAA
GACGAATTAGCTCAAATTAGAATATTGAGTCCAAATGATGACTACTTCTATCAATCGATTGTAAATTACATTAATGACGAAGCAGCAGATGC
TATTTTAGTTGATAAATTAAAAATGTTTTTATCAGATATTCAAAGTTACCAACAATATAGTAAAGATCATCCGGTGTATCAGTTAATTGATAA
ATTTTATAATGATCATTATGTTATTCAATACTTTAGTGGACTTATTGGTGGACGTGGACGACGTGCAAACCTTTATGGTTTATTTAATAAAGC
TATCGAGTTTGAGAATTCAAGTTTTAGAGGTTTATATCAATTTATTCGTTTTATCGATGAATTGATTGAAAGAGGCAAAGATTTTGGTGAGG
AAAATGTAGTTGGTCCAAACGATAATGTTGTTAGAATGATGACAATTCATAGTAGTAAAGGTCTAGAGTTTCCATTTGTCATTTATTCTGGA
TTGTCAAAAGATTTTAATAAACGTGATTTGAAACAACCAGTTATTTTAAATCAGCAATTTGGTCTCGGAATGGATTATTTTGATGTGGATAAA
GAAATGGCATTTCCATCTTTAGCTTCGGTTGCATATAAAGCTGTTGCCGAAAAAGAACTTGTGTCAGAAGAAATGCGATTAGTCTATGTAGC
ATTAACAAGAGCGAAAGAACAACTTTATTTAATTGGTAGAGTGAAAAATGATAAATCGTTACTAGAACTAGAGCAATTGTCTATTTCTGGTG
AGCACATTGCTGTCAATGAACGATTAACTTCACCAAATCCGTTCCATCTTATTTATAGTATTTTATCTAAACATCAATCTGCGTCAATTCCAGA
TGATTTAAAATTTGAAAAAGATATAGCACAAGTTGAAGATAGTAGTCGTCCGAATGTAAATATTTCAATTATATACTTTGAAGATGTGTCTA
CAGAAACCATTTTAGATAATAATGAATATCGTTCGGTTAATCAATTAGAAACTATGCAAAATGGTAATGAGGATGTTAAAGCACAAATTAAA
CACCAACTTGATTATCAATATCCATATGTAAATGATACTAAAAAGCCATCAAAACAATCTGTTTCTGAATTGAAAAGGCAATATGAAACAGA
AGAAAGTGGCACAAGTTACGAACGAGTAAGACAATATCGTATCGGTTTTTCAACGTATGAACGACCTAAATTTCTAAGTGAACAAGGTAAA
CGAAAAGCGAATGAAATTGGTACGTTAATGCATACAGTGATGCAACATTTACCATTCAAAAAAGAACTCATATCTGAAGTTGAGTTACATC
AGTATATCGATGGATTAATCGATAAACATATTATCGAAGCAGATGCGAAAAAAGATATCCGTATGGATGAATTAATGACATTTATCAATAG
TGAGTTATATTCGATTATTGCTGAAGCAGAGCAAGTATATCGTGAATTACCGTTTGTAGTTAACCAAGCATTAGTTGACCAATTGCCACAAG
GAGATGAAGACGTCTCAATTATTCAAGGTATGATTGACTTAATCTTTGTTAAAGATGGTGTGCATTATTTTGTAGACTATAAAACCGATGCA
TTTAATCGTCGTCGTGGGATGACAGATGAAGAAATTGGTACACAATTAAAAAATAAATATAAGATACAGATGAAATATTATCAAAATACGC
TTCAAACGATTCTTAATAAAGAAGTTAAAGGTTATTTATACTTCTTCAAATTTGGTACATTGCAACTGTAG

Gene: Q5HHB6 (putative fumarylacetoacetase)

Position: 924368 to 925270, length: 903 nt, orientation: FORWARD

Perfect match to: (RF122-AJ938182-[907654:908556], allele observed in CC705+CC22+CC80+CC88)

Sequence:

ATGAAATTCTTATCATTCAAGTATAATGACAAAACTTCATATGGCGTTAAAGTAAAACGCGAAGATGCTGTATGGGATTTAACACAAGTATT
TGCTGACTTTGCAGAAGGAGATTTCCATCCTAAAACATTGTTAGCTGGTTTACAACAAAATCATACTTTAGATTTTCAAGAACAAGTACGTA
AAGCAGTTGTAGCAGCAGAAGATAGCGGCAAAGCTGAAGACTATAAAATTTCATTTAATGACATTGAATTCTTACCACCAGTAACACCTCC
GAATAATGTGATTGCTTTTGGTAGAAATTACAAAGATCATGCGAACGAATTAAATCATGAAGTAGAAAAATTATATGTATTTACAAAAGCA
GCGTCATCTTTAACAGGAGATAATGCAACAATTCCAAATCATAAAGATATTACTGATCAATTAGATTATGAAGGTGAATTAGGTATTGTTAT
TGGTAAGTCTGGTGAAAAGATTCCAAAAGCATTAGCTTTAGATTATGTTTACGGCTATACAATTATTAACGATATCACTGATCGCAAAGCAC
AAAGTGAACAAGATCAAGCATTTTTATCAAAAAGTTTAACTGGCGGTTGCCCAATGGGTCCTTATATCGTTACTAAAGACGAACTACCATTA
CCTGAAAATGTAAATATTGTTACAAAAGTTAACAATGAAATTAGACAAGATGGTAACACTGGCGAAATGATTCTTAAAATTGATGAATTAAT
AGAAGAAATTTCAAAATATGTTGCACTACATCCGGGAGATATTATTGCAACTGGTACACCAGCAGGCGTTGGTGCAGGTATGCAACCACCT
AAATTTTTACAACCAGGTGATGAAGTTAAAGTGACTATTGATAATATTGGAACACTGACAACTTATATCGCTAAATAA

Gene: UPF0344 (unknown protein fold UPF0344)

Position: 926087 to 926476, length: 390 nt, orientation: FORWARD

Perfect match to: (11819-97-CP003194-[972834:973223], allele observed in CC80+CC9+CC80+CC88+CC97)

Sequence:

ATGTTACATTTACATATATTAAGTTGGGTATTAGCGATTATTTTATTTATCGCTACATACTTAAACATTTCAAAAAATCAAGGTGGTACGCCAT
ATTTCAAACCGTTGCACATGATTTTACGCTTATTTATGCTGTTGACGTTAATTTCAGGATTTTGGATATTAATTCAGTCATTTATGAATGGCGG
GGCAAATCATATGTTGCTTACATTGAAAATGCTGTGTGGTGTTGCAGTAGTTGGATTGATGGAAGTGTCGATTGCTAAAAGAAAGAGACAT
GAACAAAGTCACACAATGTTTTGGATAACAATTGCATTAATTATCATCACAATGGTATTAGGTGTCATTCTACCGTTAGGGCCTATATCAAA
ATTATTCGGTATTGGCTAA

Gene: cdr (coenzyme A disulfide reductase)

Position: 926647 to 927963, length: 1317 nt, orientation: REVERSE

Perfect match to: (CN1-CP003979-[908705:910021:r], allele observed in CC72+CC8+CC772)

Sequence:

TTATTTAGCTTTGTAACCAATCATATTGATTAAATCTTTAGGGTGGCTATATGGTGGTGCATAAGCCACTTCAAACTCAGTTAACTCATCTAC
AGTTAGCTGGTTCATCATTGCCATCGATAGTACATCAATACGTTTATCTGCACCTTCTTTTCCTACTGCAGCTGCTCTTAAAATCTGACGGTTT
GAAGTGTCATAATATACCCTTAAGTGTAAAGGGGAATTTCCTGGGTAATAATTCGCGTGTGCACCTTGAGTGACTTCCACCATTTTATAGTC
AAATTGCTTTAGTTCATTTGGTTTAACGCCGACACTCGCAAATGTATAATCAAAGAACTTCACAATATTGTTGCCTAAGAAGCCTTTGAATTC
AATAGTGTCATTTCCAGCAATTTGTTCGGCAACAATACTTGCTGCACGGTGAGCGCCCCAAGCTAAAGGAACACTAGCCGGTAGATCGACA
TGTCGATAATGTGATGTTGCAATATCGCCTATTGCATAAATGTTTGGAACATTTGTTTCAAATTTATCGTTTACCGGTATGAAACCTTTTCGAT
CAAGTTTGATATTTGAACTTTCGATAAATTTTGAATTGGGGTGAGTACCGACACCTTCAATAATCATATCGTAATGTTCAACTTTTCCTGATTT
AAATGTAATTTCATTTCCATTGATAGCATCAATTTCCTCATTTAAACGGTATGGAATCTCCAGCTTATCTAATTCATCAAGTATAGGTTGATTC
ATGTCAGCATCCATTAATTTATTTATCTTATCAGATCGATGAATTAAAGTAGGGTGTAAACCACGTTCGTAAAGATTTTCAAGAACTTCTAAT
GAAACATACCCTGCACCTACAACTAATACTTTATCAACTTGATTTGCTTTGATGAATTGATCGATAGCATCAGTGTCTTCTAAATTTCTAAGTG
TAAATGTAATATCACTTTCAAAGCCAAGGCTATTTGCACTTGCACCAGGGCTTAAAATGAGTTTATCGTAAGATTCTTCAAATTGTTCGTTGG
TCTTTCTATTTAATACAGTTACAGTTTGTCTTTCATCATTGATTGCAATAACTTCATGATAAGTTTTTACTGTAATTTGCTTTCTATCATAAAATT
TTTCAGGTGTATACGCTAAAGCATATTTTCTATCTTCAACAACTTCGCCAATGACATAAGGCAATGCACAATTAGCAAAGCTCATATCACGAT
CTTTTTCAAAAATAATAATGTCACTTTCTTTATCTAAACGTCGAATTTGGCTGGCACATGTTGCACCGCCAGCGACTGCTCCGACTACGACTA
TTTTGGGCAT

Gene: yitU (putative phosphatase)

Position: 928015 to 928839, length: 825 nt, orientation: REVERSE

Perfect match to: (11819-97-CP003194-[974762:975586:r], allele observed in CC80+CC239+CC4803)

Sequence:

TTAACAGTAATATCTAATATTTAAATTAAAGAAATCATTTAAATATCGACCAATGCCATCTTCATTATTGTTGAATGTAATATTGTTCGCTACA
TCTTTAAGTTCTTGCAAACCATTTTCCATAGCAACACCATGGCGGGCGTACTCAATCATTTCAATATCATTATCTTCATCACCGAATGCAATAA
TATTATTTCGGTCAATATTTAAAAATTGTCTAACTTGCTCAATGCCTCTTGCTTTATTAATACCAAGTTTTACAATTTCAATGACAGGGAATGG
TGCGCCCCAGCGTCGATGCTCAATATGATCGGCATAAAAATGAGTAAGCATATTTTTGATTTCAGGTATTTTACTTTCTTCGGCTTCAATTAA
AATTGAGGTAGGGGATTCTTTCAAGTGGACAAGTAAATTACCAGTTTGAATTCTTGGATTACCCATTGAAAAACCTTCAAATAATCTTGGAT
CATGATTGTTAATGAAAACATAATCTTTCACTTCTGCTATAATATTCGATACTTGATATTGTTGTAATCCTTGAATAATGTTTTGTGCGATGCC
TAAATCTAAAATTTCATGGCAAGTTTTGAAGTTTTTATCTTTAGGGTGATGTACGTAAGCGCCATTAAAATTAACAATTGGTGTCGTTAAATT
TAATTCATGATAATACATTTGACTTGCACGATAAGGTCTGCCAGTCGCAATCATAATTTGGTGTCCACGTTGTTGTAATTCATTTAATACTTG
TTTAGTATATGATGAAATTTCTTTGTTATCGTTTAATAATGTTCCGTCTAAGTCTAGACATATTAAATGTGGTTGCAT

Gene: paaD-sufT (factor essential for maturation of holo-LipA)

Position: 928953 to 929261, length: 309 nt, orientation: FORWARD

Perfect match to: (MW2-BA000033-[943736:944044], allele observed in CC1+CC15+CC80)

Sequence:

ATGGAAGAGGCATTGAAAGATAGTATCTTAGGTGCATTAGAAATGGTAATTGACCCTGAATTAGGAATTGATATCGTTAATTTAGGTTTAG
TATACAAAGTGAATGTTGATGATGAAGGCGTATGTACAGTTGATATGACTTTAACATCAATGGGATGTCCAATGGGACCTCAAATTATTGAT
CAAGTTAAAACAGTATTAGCAGAGATTCCTGAAATACAGGATACTGAAGTGAATATCGTATGGAGTCCACCTTGGACAAAAGATATGATGT
CACGTTACGCTAAGATTGCACTTGGTGTGAGCTAA

Gene: Q5HHB1 (putative O-acetyltransferase)

Position: 929809 to 931623, length: 1815 nt, orientation: FORWARD

Perfect match to: (11819-97-CP003194-[976556:978370], highly conserved allele)

Sequence:

ATGAACAAAACAAAGGGTTTTACAAAGTATAAGAAAATGAGATATATGCCAGGGCTCGATGGTTTGAGGGCAATCGCTGTTCTAGGAATT
ATTATTTACCACTTAAACAAGCAATGGTTGACAGGTGGCTTTTTAGGTGTGGATACATTTTTTGTGATCTCTGGTTATTTAATTACAAGCTTA
TTACTCAAAGAGTATGATGACACAGGTATCATTAAATTGAAAAGCTTTTGGATACGTCGTTTAAAACGTTTATTACCAGCAGTCATAGTTTTA
TTAATGGTTGTAGGGACAGCAACCTTATTATTAAAATCAGATAATATCATTAGGGTTAAACATGATATTATTGCTGCGATATTTTATGTATCA
AACTGGTGGTATATAGCAAAAGATGTTAATTATTTTGAGCAATTTTCATTTATGCCATTAAAGCATTTATGGTCTTTAGCAATTGAAGAACAG
TTTTACATATTTTTCCCAGTTATTTTGGTTACATTATTGTTAACAATTAAAAAGCGATACAAAATAGGATTTATTTTTTGGGGAGTATCAATAA
TTTCTTTAGGGTTAATGATGTTTATCTACAGTATTAATGGGGATCATTCACGAGTGTATTTTGGTACAGATACTAGATTACAGACATTGTTAC
TGGGTGTTATTTTAGCTTTTTTATGGCCACCGTTTAAATTGAAAAATGATCCACCTAAAGTTGTAAAATATGTTATTGATAGCATAGGTAGTT
TATCATTTATAGTACTTATATTATTATTTTTCATTATTAATGATGAGACGAATTGGATATATGATGGTGGTTTCTATTTAATATCCATATTAAC
GTTATTTATTATTGCCAGTGTCGTTCATCCATCTACATGGATAGCGAAGATATTTTCAAATCCAGTGTTAGTATTTATCGGGAAAAGGTCTTA
TAGTTTATATTTATGGCATTTTGCAGTAATTAGTTTCGTACATAGTTACTATGTAGATGGACAGATACCTGTATATGTGTACTTTATAGATAT
AAGTTTAACAATTATATTTGCAGAGCTATCATATCGCTTTATAGAAACTCCATTTAGAAAAGAAGGTATTAAAGCTTTAAATTGGCGACCTTC
TTATATACCACAATTTATAAGAATGGCAATTGTAGTAACCTTGTTAATTCCATTTATGTTGATTTTAGTAGGTGCATTCAATAAATATGGTAA
AGACATTATTGGAGAAAAAGCGAATAGCTTTGATACCACTATTGAAGATAATTATTTAATGCGGATAGCACCAATTGATAACATTCACATTG
ATGGCTTAGTAAGTGAGAAGAAAAAGGAATCTTCCGACGTATATAATAATATTAAACCTCTTTTAATCGGTGATTCAGTAATGGTTGATATC
GGTGAGTCATTTAAGTCATCAGTTCCTAAGTCTAGAATTGATGGAAAAGTAGGGCGTCAATTGTATCAAACCTTACCTTTAGTTAAAGCGAA
TTATTCACAATATAAAAAATCATCTGATCAAGTCGTATTAGAATTAGGTACAAATGGCGACTTTACTGTCAAACAGCTCGACGATTTACTTAA
TCAATTTGGAAAAGCCAAGATTTATTTAGTTAATACACGTGTTCCAAGAATTTATGAGGCAAATGTAAATCGATTATTAGCTGACGCGGCGA
AACGAAAGTCCAATGTCACATTAATTGATTGGAATAAGCGATCACAAGGACATAGTGAATATTTTGCACCAGACGGTGTACATTTAGAGTA
CAAAGGAGTCCTAGCTTTAAAAGATGAAATATTAAAAGCACTTAAAAAGAAATAA

Gene: clpB (chaperone-like protein B)

Position: 931826 to 934435, length: 2610 nt, orientation: FORWARD

Perfect match to: (11819-97-CP003194-[978573:981182], allele observed in CC80+CC239+CC4803)

Sequence:

ATGGATATAAATAAAATGACATATGCTGTTCAAAGTGCTTTACAACAAGCAGTTGAACTGAGTCAGCAACATAAATTACAAAATATAGAAA
TTGAGGCAATTTTAAGCGCTGCCTTAAATGAAAGTGAAAGCTTATATAAAAGTATTTTAGAACGAGCAAATATTGAGGTAGATCAATTAAA
CAAAGCTTATGAAGACAAACTAAACACGTATGCATCTGTAGAAGGTGACAATATACAATATGGTCAATATATTAGCCAACAAGCAAACCAA
TTGATAACTAAGGCTGAATCATACATGAAAGAATATGAAGATGAATATATTTCAATGGAGCATATTTTACGTTCGGCAATGGACATTGATCA
AACAACAAAACATTATATAAATAATAAAGTAGAAGTTATCAAAGAAATTATTAAAAAAGTAAGAGGGGGAAATCACGTGACATCACAAAAT
CCAGAAGTTAATTACGAAGCATTAGCTAAATATGGCCGCGACTTAGTAGAAGAAGTTAGACAAGGTAAAATGGATCCTGTTATAGGAAGA
GATGAAGAAATTCGAAATACGATTCGTATTTTAAGTCGTAAAACTAAAAACAACCCTGTGCTCATTGGTGAACCAGGTGTTGGTAAAACTG
CAATTGTTGAAGGATTAGCGCAACGTATAGTTAAGAAAGATGTGCCAGAATCATTATTAGATAAAACTGTTTTTGAGTTAGATTTAAGCGCA
TTAGTAGCGGGCGCTAAATATCGTGGTGAATTTGAAGAGAGATTAAAAGCAGTCCTAAAAGAAGTTAAAGAGTCTGATGGTAGAATTATA
TTATTTATTGATGAAATCCATATGCTTGTAGGTGCTGGTAAAACAGATGGTGCCATGGATGCAGGCAACATGCTAAAACCAATGTTAGCAC
GAGGAGAGTTACATTGTATTGGTGCAACAACTTTAAATGAATATCGAGAATATATTGAAAAAGATTCGGCATTAGAGCGTCGTTTCCAAAA
AGTAGCAGTTAGTGAGCCTGATGTTGAAGATACAATTTCAATTTTACGTGGTTTAAAAGAACGATATGAAGTGTATCATGGTGTGCGTATTC
AAGATAGAGCCTTAGTTGCTGCCGCTGAATTGTCTGATCGTTACATCACTGATCGTTTTTTACCAGATAAAGCGATTGATTTAGTTGACCAA
GCATGTGCAACAATTCGTACGGAAATGGGATCAAATCCAACTGAATTGGATCAAGTTAATAGACGTGTCATGCAATTAGAAATTGAAGATA
GCGCACTTAAAAATGAATCTGACAATGCGAGCAAACAGAGATTACAAGAACTACAAGAAGAGCTTGCCAATGAAAAAGAGAAACAAGCA
GCACTTCAATCTCGTGTAGAATCAGAAAAAGAAAAAATAGCAAATTTACAAGAAAAACGTGCGCAACTAGATGAAAGTAGACAAGCGTTG
GAAGATGCACAAACAAATAACAATTTAGAAAAAGCTGCTGAACTACAATATGGAACAATTCCTCAATTGGAAAAAGAACTTAGAGAATTAG
AGGATAATTTCCAAGATGAGCAAGGTGAAGATACAGATCGAATGATTCGTGAAGTTGTAACAGACGAAGAAATTGGCGATATTGTCAGCC
AATGGACAGGCATACCAGTTTCAAAATTAGTTGAAACAGAACGTGAAAAATTACTTCACTTAAGTGACATCTTGCATAAACGTGTTGTAGGT
CAAGATAAAGCAGTTGACCTGGTTTCAGATGCAGTAGTTAGAGCAAGAGCAGGTATTAAAGATCCAAACAGACCTATTGGTAGTTTCTTAT
TCCTAGGTCCAACTGGAGTAGGTAAAACTGAATTAGCTAAATCATTAGCTGCATCATTATTTGATTCTGAAAAACATATGATTCGTATTGATA
TGAGTGAATATATGGAAAAACATGCAGTATCAAGATTGATAGGGGCACCTCCAGGATATATTGGACATGATGAAGGGGGTCAATTAACTG
AAGCGGTTCGTCGTAATCCATACTCAGTTATTTTATTAGATGAGGTTGAAAAAGCGCATACTGACGTCTTTAATGTATTATTGCAAATTTTAG
ATGAAGGCCGTTTAACTGATTCTAAAGGACGTAGCGTTGATTTTAAAAATACTATTATTATTATGACAAGTAATATTGGATCTCAAGTTTTAT
TAGAAAACGTAAAAGAGACTGGTGAAATTACAGAATCAACAGAAAAAGCTGTTATGACAAGTTTAAATGCATATTTCAAACCAGAAATTTT
GAATCGTATGGATGATATCGTATTATTTAAACCATTATCTATTGATGACATGAGTATGATTGTAGATAAAATCTTAACGCAATTAAATATAAG
ATTATTAGAACAACGAATCTCAATTGAAGTTTCTGATGATGCTAAAGCTTGGCTAGGTCAAGAAGCTTATGAACCTCAATACGGTGCAAGA
CCATTAAAACGTTTTGTACAACGCCAAATTGAAACACCATTAGCACGTATGATGATTAAAGAGGGATTCCCAGAAGGTACAACGATTAAAG
TTAATTTAAATTCAGACAATAACTTAACGTTTAATGTTGAAAAAATTCATGAATAA

Gene: Q5HHA9 (transcriptional regulator, LysR family)

Position: 934494 to 935363, length: 870 nt, orientation: REVERSE

Perfect match to: (MW2-BA000033-[949277:950146:r], highly conserved allele)

Sequence:

TTATAATTGTTCATTGGCAATATACTTTTTAAATGATTTAATAAACACCAATATTTCTGGGCTTTCTTTTTTGCTGTATATATATGTAAATGAAA
TAGGTGCTTGTAAAATTTTCGTGTTAATAACCGAAATATTGTAATCACTATTTGTTGTTATATAAAGAGGCAGGAATGATATACCTTGATTCA
TTTCGATTAATTTAATTGAAGTATGCACATCATTGATAGATAGAAATTGTGCTTTTTCATAAATATTTAAAATATTATTTTTAAGTGATGACCA
ATATTCTGGATGGTTATCACTTATTATTTTGTATTTTTCAAATAAAGATGCCTCAGTTAGAAGATGATTATTCTCTTTATTGGGAGCAATCAAT
ACAATTTTACCTTCGCATACTTTTTCAGAATGAACTTCTCTTAGTTTAGGTTGATTTCTGCTAATCCCGATGTCATACGTATGATTATTAATATC
TTTTTCAATATTTTCATTTTTGACATGAAGAGAAACATCGATAAAAGGATGCTCGTTAAAGAAAGATTTTAAAAATTTGGGCATAATGAATG
TCGCGATATATGAAGACACGACAACATTTAATTTCGATTGAAACATCGTTTTTTTAAGTTGGATATGTTTGATGCCACTTTCATATTGTTCAAT
AAAACTTTGCGCAATTGGAAGAAATGTATGACCATCTTCAGTCAAGATAATTTGATTTTTATAAGTTTCAAATAGTTTCACATTGAGATGCTG
TTCTAAATTTTTAATTTGCTTATGTATAGAAGGTATAGTGAGATTAATTTCTTCACTAGCTAATCGATAGTTTAACGTCTTCGCTAAAATGACA
AATGTATAGTACCAATCTAAATTCAT

Gene: leuA2 (2-isopropylmalate synthase, locus 2)

Position: 935473 to 936618, length: 1146 nt, orientation: FORWARD

Perfect match to: (Strain_21189-AFEE01000041-[2761:3906:r], allele observed in CC8+CC97)

Sequence:

ATGATTCGAATTCAAGATAATACAATAAGGGATGGTATGCAACAAAGTAATGTTCGAAAAAGTCTAATTATAAAAAAAGAAGTATTGAAAC
AAATTAACAAGTTAAATATAAATTCTGTTGAAGTAGGCATGTGTACAACTATCGAGGATGAATTTAATATTCATCAATTCAGAGACATTTTA
AGTCCTGAAAAAGAATTAGTAGTATTGACTAGGCTTAATGAAAAAGAAATAAAAAAAATAGTCAAATTGAAAATTCATAATTTAGTGGTAA

AAATACTATTGCCAATATCTGACTTGCATATAAAAGAAAAGCTTAATTTTTCAAATAAATATTATATTCAGAAAATCAAAGACTGCTTGGATA
TATTAAAGAAAGATAAAAAAGGAGTAGATATTTGTTTTGAAGATGCAACAAGGACTTCTAGAGAAAAATTGAAAGAATACATGGAAATTAT
TTCAAAATATCAAGTTAGAACAGTTACATTTGCGGACACTGTAGGATGTTCGACACCATTAGAATACGGAGATATTTTTAATTACTTTGTAA
AAAAATATTCTAACATAATTTTTTCTGCTCATTGTCATAACGATCTAGGGTTGGCTACTGCAAATACATTAGCTGCAATTTTAAATGGTGCAA
AGCAAATAGAAACTACATTTTTGGGAATTGGTGAGAGAGCGGGTAATGCTTCTATTGAGGAAATAATTACTATTTTGACAAAAAAACAAAT
AGAAAGTACGGAATTCACTTTACCCGACGTATATAAAACTAGTATTAATATTTCTAAAATTCTCGATTTTCAAATATCAGAAAACAAACCTAT
AATTGGTGAAAATATATTTAAACATGAATCAGGAATTCATCAAGATGGTTCTAAAAAAAATATAAATATGTATCAATATTTAGTTCCTAGTG
ATTTAGGATTGAAAAATTCACAAGTTGTTCAGTTTCCAATAAGTAATATTTCTAGTAAGAAAATCTTGCACAATAAATTTAAATCAATAGTTA
ACACTGAAGAAATTGATGAAAGTATTTCTTTCTATAAACTTGTGAACAAGTTTTCACCTGAAGTAGCACCTGAAGATACAGTGGATTTACTT
CAGATAATAAAAAGGAGGAGTAAAGATGGAAATTTTAAATGA

Gene: Q5HHA7 (Sua5/YciO/YrdC/YwlC family protein)

Position: 936602 to 937243, length: 642 nt, orientation: FORWARD

Perfect match to: (MW2-BA000033-[951385:952026], highly conserved allele)

Sequence:

ATGGAAATTTTAAATGATTCTGTAAAAAGTTTTGAAAAACTTTATTTACAATTACAAGATGGTTTTCCAGTGATTGTTCCTACTGATACTAATT
ATAATTTATGTAGTTTACCTAACAACGATCTTTGTATCGATAAAATATTTGAATATAAAAAGCGATCGAAAGATAAGCCATTATCATTATTTA
TTGATAAGCCAGAGGATTGGAAATTGTATGGAGATAATCAAAATACGGAAATAGTTGATAAACTAGTTGAAATATTTTGGCCTGGACCATT
AAATATTATTTTAAAAAATAAAACAAGCTATAATTATATGCTCAATAATTCAGATAGTATAGCTATAGGATGTGTACAAAATAAAACGATGA
GAAGATTTATTTCATATATTAATTCACCTATAGCAATTACTTCAGCAAATATATCTGGAACTGCCGATGATATTTTAATAACTGAAAATGAAG
CAATTAAGCACATGGGCGAAAATGTAAGATATATGCTAAGAAGTCAAAATAAAACTAACTATAAAACATCTAGTACGATTATTAAAGTGAC
AGATAATAAAATTGAATTATTAAGAGAAGGAGATATAAAGTTTGAAGAAATAAAAGAAAGACTAGGTACAGGTATTATTTATGAATAA

Gene: Q5HHA6 (putative protein)

Position: 937236 to 938354, length: 1119 nt, orientation: FORWARD

Sequence:

ATGAATAAGTTAATACTTGGGATTTATTTATACCGAATTTTTTCACGAGCATACTTTTATTTACCGTTTTTATTAATTTACTTTTTGATTCAAGG
TTATTCCATAATACAATTAGAAATATTAATGGCGTCTTATGGCATTGCAGCATTTTTATTCTCTCTATACAAAGAGAAGTGTTTTAAAATTTGT
AACTTAAAAGATTCTAATAAATTAGTTGTTAGTGAAATATTCAAAATCATCGGTTTATTGTTGTTATTATATCAAAATCAATATTTAATTTTAG
TAGTGGCACAAATATTATTAGGGTTAAGTTACTCAATGATGGCGGGTGTTGATACCGCAATAATTAAAAGAAATATAACAAATGAGAAATA
CGTACAAAATAAGTCAAATAGCTATATGTTCCTATCATTATTAATTTCAGGGATTATAGGTAGTTATCTTTATGGAATAAATATTAAATGGCC
TATAATAATGACTAGTATATTTTCAACTCTAACAATTATAATTATTCGATGCACATTAGTTGAAAATAGGGAATTAAATTTAATAGGAGAAAC
AAAGGGAAAGATAAAGAAATTTCTACCAGAAGAGAAGTTTTGGATATTGCATTATTCTTTTTTAAGAGCGTTAATATTAGGATTTTTTATAG
GATTTATTCCAATTAATATATATAATGATTTAAAACTGAATAATTTACAATTTATTTCAGTATTAACTTGTTACACAGTTATGGGTTTTGTATCT
TCACGTTATTTAACTAAATACTTGAATTATAAGTTTGTGTCAGAAATTTGTTTAGTAATATTTTTAATAATATATACATATCAAAGTTTCATAG
CAGTTACTATTTCTATGATATTTTTAGGTATTTCTTCAGGGTTAACTCGTCCACAAACTATAAATAAACTTTCTAGCAGTAGTAACTTAAGAGT
GATGCTTAATTATGCAGAAACGTTATATTTTATTTTTAATATCGCATTTTTACTTATGGGTGGTTACTTATATACAATAGGAACTATTCAATAC
TTAATATTATTTATTTCGTTATTAATTTTTATATATTTAATAATAATATTTTATTTTACAAGGAGAGAGCAACATGAAAATAAAAACTGA

Gene: Q5HHA5 (putative protein)

Position: 938338 to 938853, length: 516 nt, orientation: FORWARD

Perfect match to: (Strain_21266-AFTT01000017-[616823:617338:r], allele observed in CC12+CC80)

Sequence:

ATGAAAATAAAAACTGAATTTAAAGGGAACAATATACCATATGAATACGCAGCAGGTGCAGATGTGAGTGATTCTATTAACGGGAATCCAA
TTAAGTCATTTCCATTTGAAGTAATTGAATTACCGGAAGGGACTAAATATCTTGCTTGGTCTTTAATTGACTATGATGCAATTCCTGTGTGTG
GCTTTGCATGGATTCATTGGAGTGTTGCTAATGTAAGTGTTAGTGGCAATTCAATTTCTATAAAAGCAGATTTATCAAGAACAAAGGGTGAC
TATGTACAAGGTAAAAATAGCTTTACTAGTGGGTTGTTGGCTGAAGATTTTTCAGAAATAGAAAATCACTATGTAGGACCTACACCACCTGA

TCAAGATCATCAATATGAATTAACAGTTTATGCGTTAGATCATTCTTTAAATTTGAAGAATGGGTTCTACTTGAATGAATTTTTAAAAGAAGT
AAATCAACATAAAATTGATCAAACAAGTATTAACCTTATAGGAAGAAAAATTTAA

Gene: eapH-2 (extracellular adherence protein homolog, locus 2)

Position: 939164 to 939598, length: 435 nt, orientation: FORWARD

Perfect match to: (11819-97-CP003194-[985911:986345], highly conserved allele)

Sequence:

ATGAAATTAAAATCATTTATAACTGTAACTTTGGCACTGGGCATGATCGCAACGACTGGCGCTACTGTGGCAGGTAATGAGGTATCTGCAG
CAGAAAAGGACAAACTACCGGCAACTCAAAAAGCTAAAGAAATGCAAAATGTTCCATATACAATTGCAGTAGATGGCATTATGGCTTTCAA
TCAATCTTACTTAAATTTACCAAAAGATAGCCAATTATCATATTTAGATTTAGGAAATAAAGTTAAAGCTTTATTATATGATGAACGCGGTGT
AACACCTGAGAAGATTCGAAATGCAAAATCTGCCGTTTACACGATTACTTGGAAAGATGGTAGTAAAAAAGAAGTGGATCTTAAGAAAGA
TAGCTACACAGCAAACTTGTTTGATTCAAATTCAATTAAACAAATTGATATTAATGTAAAAACTAAATAA

Gene: A6U088 (putative protein)

Position: 939855 to 940040, length: 186 nt, orientation: REVERSE

Perfect match to: (N315-BA000018-[954670:954855:r], highly conserved allele)

Sequence:

TTATTGTGATGAATCTTTCGGCGGTTTAATTACTGCAGCAAAAATTGCTGTGAAAATCGTGAACAATACTGCCATGATAATTGGATTCACTA
CATTTAAGCTGTCTCCACCTACTAGGCTATTAAGTACAAAGTTAACCATTTGCATTAATAATAATGCCCAAAAGAATGTTACGAGGTGTTTCA
T

Gene: fabH (beta-ketoacyl-ACP synthase III)

Position: 940335 to 941276, length: 942 nt, orientation: FORWARD

Perfect match to: (11819-97-CP003194-[987082:988023], allele observed in CC80+CC692)

Sequence:

ATGAACGTGGGTATTAAAGGTTTTGGTGCATATGCACCAGAAAAGATTATTGACAATGCCTATTTTGAGCAATTTTTAGATACATCTGATGA
ATGGATTTCTAAGATGACTGGAATTAAAGAAAGACATTGGGCAGATGACGATCAAGATACTTCAGATTTAGCATATGAAGCAAGTATAAAA
GCAATCGCTGACGCTGGTATTCAGCCCGAAGATATAGATATGATAATTGTTGCCACAGCAACTGGAGATATGCCATTTCCAACTGTCGCAA
ATATGTTGCAAGAACGTTTAGGGACGGGCAAAGTTGCCTCTATGGATCAACTTGCAGCATGTTCTGGATTTATGTATTCAATGATTACAGCT
AAACAATATGTTCAATCTGGAGATTATCATAATATTTTAGTTGTCGGTGCAGATAAATTATCTAAAATAACAGATTTAACTGACCGTTCTACT
GCAGTTCTATTTGGAGATGGTGCAGGTGCGGTTATCATCGGTGAAGTTTCAGAAGGCAGAGGTATTATAAGTTATGAAATGGGTTCTGATG
GCACTGGTGGTAAACATTTATATTTAGATAAAGATACTGGTAAACTGAAAATGAATGGTCGAGAAGTATTTAAATTTGCTGTTAGAATTATG
GGTGATGCATCAACACGTGTAGTTGAAAAAGCGAATTTAACATCAGATGATATAGATTTATTTATTCCTCATCAAGCTAATATTAGAATTAT
GGAATCAGCTAGAGAACGCTTAGGTATTTCAAAAGACAAAATGAGTGTTTCTGTAAATAAATATGGAAATACTTCAGCTGCGTCAATACCTT
TAAGTATCGATCAAGAATTAAAAAATGGTAAACTCAAAGATGATGATACAATTGTTCTTGTCGGATTCGGTGGCGGCCTAACTTGGGGCGC
AATGACAATAAAATGGGGAAAATAG

Gene: fabF (3-oxoacyl-[acyl-carrier-protein] synthase 2)

Position: 941288 to 942532, length: 1245 nt, orientation: FORWARD

Perfect match to: (CIG1770-AHVN01000007-[420550:421794], allele observed in CC8+CC97)

Sequence:

ATGAGTCAAAATAAAAGAGTAGTTATTACAGGTATGGGAGCCCTTTCTCCAATCGGTAATGATGTCAAAACAACATGGGAGAATGCTCTAA
AAGGCGTAAATGGTATCGATAAAATTACACGTATCGATACTGAACCTTATAGCGTTCACTTAGCAGGAGAACTTAAAAACTTTAATATTGAA
GATCATATCGACAAAAAAGAAGCGCGTCGTATGGATAGATTTACTCAATATGCAATTGTAGCAGCTAGAGAGGCTGTTAAAGATGCGCAAT

TAGATATCAATGATAATACTGCAGATCGAATCGGTGTATGGATTGGTTCTGGTATCGGTGGTATGGAAACATTTGAAATTGCACATAAACA
ATTAATGGATAAAGGCCCAAGACGTGTGAGTCCATTTTTCGTACCAATGTTAATTCCTGATATGGCAACTGGGCAAGTATCAATTGACTTAG
GTGCAAAAGGACCAAATGGTGCAACAGTTACAGCATGTGCAACAGGTACAAATTCAATCGGAGAAGCATTTAAAATTGTGCAACGCGGTG
ATGCAGATGCAATGATTACTGGTGGTACAGAAGCACCAATTACTCATATGGCAATTGCTGGTTTCAGTGCAAGTCGAGCGCTTTCTACAAAT
GATGACATTGAAACAGCATGTCGTCCATTCCAAGAAGGTAGAGACGGTTTTGTTATGGGTGAAGGTGCTGGTATTTTAGTAATTGAATCTT
TAGAATCAGCACAAGCTCGAGGTGCCAATATTTATGCTGAGATAGTTGGCTATGGTACTACAGGTGATGCTTATCATATTACAGCGCCAGCT
CCAGAAGGTGAAGGTGGTTCTAGAGCAATGCAAGCAGCTATGGATGATGCTGGTATTGAACCTAAAGATGTACAATACTTAAATGCCCAT
GGTACAAGTACTCCTGTTGGTGACTTAAATGAAGTTAAAGCTATTAAAAATACATTTGGTGAAGCAGCTAAACACTTAAAAGTTAGCTCAAC
AAAATCAATGACTGGTCACTTACTTGGTGCAACAGGTGGAATTGAAGCAATCTTCTCAGCGCTTTCAATTAAAGACTCTAAAGTCGCCCCGA
CAATTCATGCGGTAACACCAGATCTAGAATGTGATTTGGATATTGTTCCAAATGAAGCGCAAGACCTTGATATTACTTATGCAATGAGTAAT
AGCTTAGGATTCGGTGGACATAACGCAGTATTAGTATTCAAGAAATTTGAAGCATAA

Gene: Q2YWV0 (putative protein)

Position: 942587 to 942958, length: 372 nt, orientation: REVERSE

Perfect match to: (MW2-BA000033-[957370:957741:r], highly conserved allele)

Sequence:

TTATACTAAGATGAGCGACAGCACAATCGTCATAATAAAATATAAAATATTTATTAATAATAAGGGGATTATCCATGTAGAAACAAAGTAAT
GCTCTTTTTTTACCTCTTGTGGGTTGAAAAATGGATCATCAGAGATAGACTTCTTCTTTTTCGAAGATGACATTTGATACTTTAATCTTCTAAA
ACCATAACTTGTCGCATCAAAAATGCCTTCTTGTACAAGTAAAATCAAAAATATGCTAATAAAAATAATTAATGAAACATAAAACAATATATT
TAAATATGTAATGATAGTATGGCTATTAAAAAGCCATATAATAAACGTTAATATTGGCGTTATTAGTGCCATTCCAAGCCATTTTTTCAACAT

Gene: oppB (oligopeptide ABC transporter, transmembrane permease subunit)

Position: 943201 to 944127, length: 927 nt, orientation: FORWARD

Perfect match to: (COL-CP000046-[997622:998548], allele observed in CC8+CC239+CC425)

Sequence:

ATGGGGAAATATATTTTCAAACGATTTATTTATATGCTTATTTCTTTATTTATTATTATTACAATTACATTTTTCTTAATGAAATTAATGCCAGG
TTCGCCATTTAACGATGCTAAATTAAATGCTGAACAAAAAGAAATTTTAAATGAAAAATATGGATTAAATGATCCTGTAGCTACGCAGTATT
TACATTATTTAAAAAATGTTGTTACAGGCGATTTTGGTAATTCATTCCAGTATCATAATCAACCTGTGTGGGATTTGATTAAACCGAGACTAC
TACCTTCTTTTGAAATGGGTCTTACAGCAATGTTCATCGGTGTGATACTGGGACTTATTTTAGGTGTTGCAGCAGCTACTAAACAAAATTCTT
GGGTTGACTATACAACTACAGTTATTTCAGTTATTGCTGTATCTGTACCATCTTTTGTACTTGCTGTACTTTTACAATATGTATTTGCAGTTAA
ATTAAGATGGTTCCCAGTAGCTGGATGGGAAGGTTTTTCGACCGCGGTATTACCGTCACTTGCATTATCTGCAGCTGTTTTAGCAACTGTCG
CCAGATACATAAGAGCAGAGATGATAGAGGTATTAAGTTCAGACTATATTTTATTAGCGAGAGCTAAAGGTAATTCGACAATGCGTGTACT
TTTTGGACATGCACTTAGAAATGCTTTAATTCCAATTATTACAATTATCGTTCCCATGTTAGCAAGTATTTTAACAGGCACTTTAACAATTGAA
AATATTTTTGGGGTTCCTGGATTAGGGGATCAATTCGTACGTTCAATTACAACAAATGATTTCTCAGTAATCATGGCAATCACACTATTATTT
AGCACACTGTTTATCGTTTCTATTTTTATTGTAGATATTTTGTACGGTGTGATAGATCCACGAATTCGTGTTCAAGGAGGTAAAAAATAA

Gene: oppC (oligopeptide ABC transporter, transmembrane permease subunit)

Position: 944127 to 945197, length: 1071 nt, orientation: FORWARD

Perfect match to: (Strain_21193-AFEG01000025-[98733:99803:r], allele observed in CC25+CC80)

Sequence:

ATGGCTGAAAATAAAAACAATTTGTCGATTAACGACGATCATTCTAATGCAGCTATGACGCATACCTCTGACGCTATCGCATCATCTGATTTT
ATTATTAGAGATTTAGATTTGAATCAGGAACCTGAAATGCAACGAGAAAGCAAAAACTTTTGGCAAGATGCTTGGGCTCAGTTAAAACGAA
ATAAGTTAGCTGTTGTCGGTATGATAGGTTTAATTATCATTGTAATATTTGCTTTTATCGGTCCAGTTATAAATAAACATGATTATGCTGAAC
AAAATGTAGAACATAGAAATCTTCCGGCAAAAATACCTGTATTAGACAAAGTTCCATTTTTACCTTTTGATGGTAAAGATGCAGATGGCAAG
GATGCTTATAAAGCAGCAAATGCTAAAGAAAATTATTGGTTTGGTACTGATCAGTTGGGTCGAGATTTATGGACAAGAACATGGAAAGGT
GCTCAAATTTCATTGTTTATCGGTGTTGTTGCAGCGATGTTAGATATTTTTATTGGCGTTGTATATGGTGCGATTTCTGGATTCTTCGGTGGA
CGTGTCGATACGATTATGCAACGTATACTTGAAGTCATAGCATCTATTCCGAATTTAATTGTCGTAATTTTATTTGTATTAATTTTTGAACCAT
CCATTTGGACAATTATATTGGCTATGTCTATCACAGGCTGGTTAGGCATGAGCAGAGTTGTACGTGGAGAATTTTTAAAATTAAAAAATCAA

GAGTTTGTCATGGCTTCGAAAACATTGGGGGCTTCAAAATTCAAATTGATATTTAAGCATATTTTACCTAATACATTAGGTGCTATCGTGGTT
ACATCAATGTTTACAGTACCTAGTGCTATTTTCTTCGAAGCATTTTTAAGTTTCATTGGTATAGGTGTACCCGCACCTCAAACATCGTTAGGG
TCATTAGTAAATGATGGGCGAGCAATGTTATTAATTTATCCACATGAATTATTTATACCAGCAATGATTTTAAGTTTATTAATTCTATTCTTTT
ACTTATTTAGTGATGGATTACGTGATGCATTTGATCCGAAAATGCGTAAATAA

Gene: oppD (oligopeptide ABC transporter, ATPase subunit)

Position: 945213 to 946295, length: 1083 nt, orientation: FORWARD

Perfect match to: (11819-97-CP003194-[991960:993042], allele observed in CC80+CC1+CC72)

Sequence:

ATGACTGAAAGAATATTAGAAGTAAATGATTTGCATGTTTCCTTTGATATTACAGCAGGGGAAGTGCAGGCAGTGAGAGGTGTAGATTTTT
ATTTAAACAAGGGGGAAACATTGGCAATTGTTGGTGAATCAGGTTCAGGTAAATCTGTAACAACAAAAGCAATTACAAAATTATTCCAAGG
GGACACAGGAAGAATTAAAAAGGGAGAAATTTTATTTTTAGGGGAAGATTTAGCAAAAAAACCTGAAAATGAGTTAATTAAATTACGTGG
CAAAGATATTTCAATGATCTTTCAAGATCCAATGACATCTTTAAACCCAACTATGCAAATTGGTAAACAAGTCATGGAACCATTAATTAAGCA
CAAAAATTATAGTAAAGCACAAGCTAAAAAGCGCGCATTGGAAATACTAAATCTTGTAGGTTTACCAAATGCAGAAAAAAGATTTAAAGCA
TATCCACATCAATTTTCAGGTGGACAAAGGCAAAGAATTGTTATTGCAACCGCATTAGCTTGTGAACCTAAAGTGCTCATTGCTGATGAACC
AACGACTGCATTAGACGTAACGATGCAGGCACAAATTTTAGATTTAATGAAAGAACTACAACAAAAAATCGATACAGCAATTATTTTTATAA
CGCATGATTTAGGGGTTGTTGCGAATATTGCTGATAGAGTGGCAGTTATGTATGGTGGTCAAATGGTTGAAACAGGAGATGTTAACGAAA
TATTTTATGATCCAAAGCATCCATATACATGGGGATTATTATCGTCAATGCCTGATTTATCAACAACAAATGACACACCATTACTAGCGATTC
CTGGAGCGCCACCTGATTTATTACACCCACCTAAAGGTGATGCATTTGCGAGACGTAGTCAATATGCATTAGATATTGATTTTAAAGTAGAA
CCACCGTGGTTTAAAGTTTCACCGACACATTTTGTGAAATCTTGGTTATTAGACGCACGTGCACCAAAAGTTGAACTACCCGAGCTGGTAAA
ACAACGTATGAAACCGATGCCTAATAATTATGAAAAACCACTCAAGGTAGAAAGGGTGTCGTTCAATGAAAAATGA

Gene: oppF (oligopeptide ABC transporter, ATPase subunit)

Position: 946285 to 947226, length: 942 nt, orientation: FORWARD

Perfect match to: (11819-97-CP003194-[993032:993973], allele observed in CC80)

Sequence:

ATGAAAAATGATGAAGTGCTATTATCTATTAAAAATTTAAAGCAATATTTTAACGCAGGAAAGAAAAACGAAGTGAGAGCGATTGAAAATA
TTTCGTTTGATATATACAAAGGGGAAACATTAGGCTTAGTAGGAGAATCGGGGTGTGGTAAATCTACAACTGGTAAATCAATTATTAAACT
TAATGATATTACAAGTGGAGAAATTTTGTATGAGGGTATTGATATACAAAAGATTCGTAAACGTAAAGATTTGCTTAAATTTAATAAAAAGA
TACAGATGATTTTTCAAGACCCATATGCGTCTTTAAATCCTAGGTTAAAAGTAATGGATATAGTAGCTGAAGGTATTGATATCCATCATTTAG
CAACTGATAAACGTGACCGTAAAAAACGTGTCTATGATTTACTTGAAACTGTTGGATTAAGTAAAGAACATGCCAATCGTTATCCTCATGAA
TTTTCAGGCGGACAACGCCAACGTATTGGAATTGCCCGTGCATTAGCCGTTGAACCAGAATTTATTATAGCAGATGAACCTATTTCAGCATT
GGATGTTTCGATTCAAGCTCAAGTAGTCAATTTGTTATTAAAATTACAGCGTGAAAAAGGTATTACATTCCTATTTATTGCACATGATTTATC
AATGGTGAAGTATATCTCTGATCGAATTGCAGTCATGCATTTTGGGAAAATTGTAGAAATCGGACCAGCAGACGATATTTATCAATATCCAT
TACATGATTATACTAAGTCTTTATTGTCGGCAATTCCTCAACCGGATCCTGAATCGGAACGTAGTCGCAAACGATTTAGTTATATTGATGATG
AAGCAAATAATCATTTAAGACAATTACATGAAATAAGACCACAGCATTTTGTCTTTAGTACTGAAGAAGAAGCGGAACGACTACGAAAAAA
TTTGTTGGTAACACAAGATTAA

Gene: oppA (oligopeptide ABC transporter, substrate-binding protein)

Position: 947245 to 948900, length: 1656 nt, orientation: FORWARD

Perfect match to: (Strain_21333-AHKA01000006-[28686:30341:r], allele observed in CC80)

Sequence:

ATGACAAGAAAGTTAAAAACGCTGATTTTAATATTGGTTGCCACAATTGCATTAAGTGGTTGTGCTAATGATGATGGTATTTATTCAGATAA
AGGTCAAGTATTCAGAAAAAATTTTGTCAGCAGACTTAACATCCCTTGATACATCATTAATAACGGATGAAATATCTTCTGAAGTGACTGCGC
AAACATTCGAAGGTTTATACACATTAGGAAAAGGTGACAAACCGGTGTTAGGTGTTGCGAAAGCTTTTCCTGAAAAGAGTAAAGATGGGA
AAACTTTAAAGGTTAAATTAAGAAGCGATGCTAAATGGAGCAATGGTGACAAAGTGACTGCACAAGACTTTGTTTATGCTTGGAGAAAAAC
AGTTGACCCTAAAACTGGTTCTGAATTTGCATACATTATGGGGGACATTAAAAATGCGAGTGATATTAGTACTGGTAAGAAACCTGTAGAG
CAATTAGGTATCAAAGCATTAGATGATGAAACATTACAAATTGAATTAGAAAAGCCGGTTCCATATATTAATCAATTATTAGCACTTAATAC

ATTTGCACCTCAAAATGAAAAAGTTGCCAAAAAATATGGTAAAAATTATGGCACAGCAGCTGATAGAGCGGTATACAATGGTCCATTTAAA
GTTGATGATTGGAAACAAGAAGATAAAACGTTACTATCTAAAAATCAGTATTATTGGGATAAAAAGAATGTAAAGTTAGATAAAGTGAATT
ATAAAGTTATTAAAGACTTACAAGCCGGTGCTTCATTGTATGATACTGAATCAGTAGATGACGCAATTATTACTGCGGATCAAGTGAATAAA
TATAAAGATAATAAAGGATTAAACTTTGTATTAACGACTGGGACATTTTTTTTGAAAATGAATGAAAAACAATATCCTGATTTTAAAAATAA
AGAATTAAGACTTGCAATCGCGCAAGCAATAGATAAAAAAGGCTACGTTGATTCAGTGAAAAACAATGGCTCAATTCCTTCCGATACATTA
ACAGCCAAAGGCATTGCGAAAGCGCCTAATGGCAAAGATTATGCGAGTACCATGAATTCGCCTTTAAAATATAATCCTAAAGAAGCAAGAG
CACACTGGGACAAAGCTAAAAAAGAGTTAGGTAAAAATGAATTGACATTTTCAATGAACACAGAAGATACACCAGATGCAAAAATATCTGC
TGAATATATCAAATCGCAAGTTGAGAAAAAATTTACCAGGAGTTACTTTGAAAATTAAGCAATTGCCGTTTAAACAAAAAGTATCACTAGAAC
TGAGTAACAATTTTGAAGCATCACTTAGTGGTTGGTCTGCAGATTACCCTGATCCTATGGCTTACTTAGAAACAATGACCACAGGCAGCGCA
CAAAATAATACAGACTGGGGTAATAAAGAATACGATCAATTACTTAAAGTAGCGAGAACCAAATTGGCACTTCAACCGAACGAACGATATG
AAAATTTGAAAAAAGCAGAAGAAATGTTCCTAGGAGATGCACCGGTAGCACCAATTTATCAAAAAGGTGTTGCACATTTAACAAATCCTCA
AGTAAAAGGATTAATTTACCATAAATTTGGTCCAAATAACTCACTTAAACATGTCTATATTGATAAATCGATAGATAAAGAAACAGGTAAGA
AGAAAAAAATAA

Gene: appA (oligopeptide ABC transporter, substrate-binding protein)

Position: 949112 to 950827, length: 1716 nt, orientation: FORWARD

Perfect match to: (11819-97-CP003194-[995858:997573], allele observed in CC80)

Sequence:

ATGGGGAAGCTAATTAAATATATTTCAATACTTCTTATTGTCGTTTTAGTGTTGAGTGCTTGCGGAAAAAGCAGTAATAAAGATGAAGGAGT
AAAAGATGCTACTAAAACGGAAACCTCAAAACATAAAGGTGGTACTTTAAATGTAGCATTAACAGCACCGCCAAGTGGTGTTTATTCTTCGT
TATTAAATAGTACACATGCAGATTCTGTAGTTGAAGGATATTTTAACGAAAGCTTATTAGCAACTGATAAAAAAATACGTCCTAAGGCATAT
ATTGCTTCATGGAAGGACATCGAGCCGGCTAAGAAAATAGAATTTAAAATTAAAAAAGGTATTAAATGGCATGATGGTAATGAATTGAAA
ATTGATGATTGGATTTATTCAATTGAAGTCTTAGCTAACAAGGACTACGAAGGTGCTTATTATCCAAGTGTAGAAAATATCCAAGGTGCGAA
AGATTATCATGAAGGAAAAACTGATCATATTAGCGGATTGAAGAAAATAGATGACTACACTATGCAGGTTACATTTGATAAAAAACAAGAA
AATTACTTAACAGGATTTATTACTGGACCTTTATTAAGTAAAAAATATTTATCAGATGTACCAATTAAAGATTTAGCGAAATCAGATAAAATC
CGAAAATATCCTATTGGTATTGGACCGTATAAAGTTAAGAAAATCGTTCCAGGTGAGGCTGTTCAACTCATTAAATTTGATGATTATTGGCA
AGGTAAGCCTGCACTAGACAAAATCAATTTAAAAGTTATTGATCAAGCTCAAATTATTAAGGCAATGGAAAAAGGCGATATTGATGTTGCG
AATGATGCTACCGGTGCAATGGCAAAAGATGCTAAGTCATCTAATGCTGGTCTCAAGGTATTATCTGCGCCAAGCTTAGACTACGGTTTAAT
AGGATTCGTATCTCATGATTACGATAAAAAAGCTAATAAAACTGGTAAAGTGAGACCAAAATATGAAGACAAAGAATTACGTAAAGCAAT
GCTTTATGCAATTGATAGAGAAAAATGGATTAAAGCGTTTTTCAATGGTTACGCTAGTGAAATCAATAGTTTTGTACCATCTATGCATTGGA
TAGCAGCCAATCCTAAGGACCTAAATGATTACAAATATGATCCTGAAAAAGCTAAAAAAATCTTAGATAAGTTAGGTTATAAAGATAGAGA
TGGTGACGGATTTAGAGAAGATCCTAAAGGTAATAAATTTGAGATTAACTTTAAACATTATTCAGGTTCAAATCCTACTTTTGAACCAAGAA
CTGCTGCGATAAAAGATTTCTGGGAAAAAGTTGGCTTGAAAACAAATGTGAAGTTAGTAGAATTCGGTAAATATAATGAAGACTTAGCAAA
TGCATCTAAAGATATGGAAGTGTACTTCAGATCATGGGCAGGAGGTACAGATCCAGATCCATCAGATTTATACCACACTGATAGACCTCAA
AATGAAATGAGAACAGTTTTACCAAAATCAGATCAATATTTAGATGATGCATTAGACTTCGAAAAAGTAGGCATTGATGAAAAGAAACGTA
AAGATATTTATGTTAAATGGCAAAAATATATGAATGATGAGTTACCTGGATTACCAATGTTCCAAGGTAAATCGATAACTATTGTTAACGAT
AAAGTACGAAACTTAGACATTGAAATTGGAACTGATCAAAGTTTATATAATTTAACTAAAGAAGCTTAG

Gene: appD (oligopeptide ABC transporter, ATPase subunit)

Position: 950878 to 951864, length: 987 nt, orientation: FORWARD

Perfect match to: (O11-CP024649-[989450:990436], allele observed in CC130+CC8+CC30+CC239)

Sequence:

ATGAATAATGTATTGTTAGAGGTTAAAGATTTAGAAACATCATTAAAAATAAATAATGAATGGTTAGCAACTGTTGAAAATATTTCTTTTGA
ATTATCTAAAGGAGAAGTTTTGGGTATAGTAGGGGAATCTGGTTGCGGTAAGTCCATATTAAGTAAGTCAATTATTAAATTATTACCAGAA
AAGATATCTAAACTAAGTAATGGAGAAGTTATATTTGATGGTAAACGAATCGATACGCTCAATGAGAAGCAATTGCTAGATATTCGAGGAA
ATGATATTGCTATGATTTTTCAAGAACCTATGACTGCTTTAAATCCTGTATTTACCATAAAAAATCAACTTGTGGAATCTATAAAATCACATA
AAAAAATTTCTAAAAAAGAAGCAAATAACTTAGCAAAAGATTTACTAAAAAAAGTTGGAATTGCTAGACAAGATGAAATATTAAATAGCTA
TCCTCATCAATTATCTGGTGGTATGAGACAAAGAGTAATGATTGCAATGGCCATTTCATGTTCTCCTAAATTATTAATTGCTGATGAACCTAC
AACAGCATTGGATGTCACGATTCAAGCGCAAATATTAGACTTATTAAAAGAATTGCAAAAGGAAACGCAAATGGCAATTATGATGATTACA
CATGATTTGAGTGTAGTTGCTGAGTTTTGCGATAAAGTCTTAGTTATGTATGCAGGTCAAATTGTAGAATTTGGAGGCATAAAAGAAATACT
ACACAATCCGAAACATCCTTATACCCAAAAATTATTATCAACAATTCCAAAACTTAAAGAAGAGCAGAAACGACTTGAAACGATAGAAGGA

ATTGTGCCATCAATCCAAGCATTTCACGTTAATAAGTGCAGATTTGCAAATAGATGTAACAAAAAAATGGATATTTGTAATAATCAATCTCCT
AAAATGCATGTTTGTGAAGACGTCATTGTACGTTGTCATTTGTACAAAAATGAATATAAGGAGATATAA

Gene: appF (oligopeptide ABC transporter, ATPase subunit)

Position: 951867 to 952847, length: 981 nt, orientation: FORWARD

Perfect match to: (11819-97-CP003194-[998613:999593], allele observed in CC80)

Sequence:

ATGGAAAATATTTTAGAAGTCAACCAAATAAAAAAATACTACAAAATTAAAACTGGATTATTACAAAAAACTCAGTACGTTAAAGCTGTTGA
TGACGTATCGTTTTCAATAAAAAAAGGACAAACTTTTGGATTAGTAGGAGAATCGGGTTGTGGTAAGTCAACGTTAGGTAAAGTGATTATC
AGGCTTGAAGATGCAACATCAGGCTCAATAATTGTTAATGGTGAAGATATAACAAGATTACAAGGTAAAAAACTCAGAAAATCACGACAAC
AATATCAGATGATATTTCAAGATCCGTATGCATCATTGAATCCGATGCAAATGGTTGGAGATATCATTTCAGAACCTATTTTAAATTATAAAA
AATTGCCAAAAGAAGAAATAAAAAAAGAAGTACTATATTTATTAAAATGTGTTGGCCTAAGTGAAGATGCATATTATAAATATGCACATGA
ATTTTCAGGTGGACAGAGACAAAGAGTGGGAATTGCAAGAGCATTGGCTTTGCGTCCGAGTTTAATTGTTGCTGATGAGCCTGTAAGTGCA
TTAGATGTATCTGTTCAATCTCAAGTACTGAATTTATTAAAAGATTTACAAGAACAATTTAACTTAAGCTATTTATTTATCGCACATGATTTAA
GTGTAGTAAAACATATAAGTGATGTCATTGGAGTTATGTATTTAGGTCATATAGTTGAAATCGCATCTGATAAAGAAATTTATGAAAATCCC
AAACATCCATATACAAAAGCGTTGATTTCATCAATACCACAAATTGATAAACATAATAACAATAGAATTATATTAAAAGGAGAATTACCTTC
GCCAAGTAATCCGCCGCAAGGTTGTCCTTTTCATACAAGATGTCCGATTGCAAAAGATAAATGTAAAGAAAATATACCACAATTAAAGGAC
ATTGGTGATGAACATCAAGTTGCTTGTTTTTATGTAAATAAAGTAGGTGATTTAAATGGTTAA

Gene: appB (oligopeptide ABC transporter, transmembrane permease subunit)

Position: 952840 to 953802, length: 963 nt, orientation: FORWARD

Perfect match to: (MW2-BA000033-[967623:968585], highly conserved allele)

Sequence:

ATGGTTAAATTAATATTAAAGAGATTAGGTTTAATGATTCCGTTACTAATTTTAATTTCTATTGTTGTATTTTCATTAGCTATCATTCAACCAG
GAGATCCATTTTCAGATTTACAAAACGGAAAAATAAAACAAGAAGCGATAAATGCACAAAGGGAAAAGTTAGGCCTCAACGACTCTATATC
ACATCAATACATTAGATGGGTCAATCATGTTATACATGGTGATTTAGGGGAGTCAATCAAATATAAAAGGCCGGTAATTGATGTTATTGAG
GAAAGAATTCCAAATACAATATTACTCGGTGCTATGTCATTAATTATTACTTATATTATCTCATTTGCTTTAGGAATAACGTCAGGTAGATATT
CTTACAGTTTGACTGATTATACTGTGCAAATATTTAATTATTTGATGTTAGCTATTCCATCTTTTATTGCGGGAGTATTTGCAATTTTTATTTTT
TCTTTTGAATTACAATGGTTTCCGTTTCAAGGTTCTGTTGATATTAACCTTAAAGAAGGTACTTTTGAATATTATATGAGTAAAATTTATCATA
CATTTTTGCCTGCATTAACTTTAGGATTATTATCTACTGCTGGTTATATTCAATATTTACGTAATGATATTATTGAAAATTCTAAAAAAGATTA
TGTATTGACGGCAAGGTCAAAAGGATTATCTATGAATAAAATTTATAATAAACATATATTGAGAAATTCTTTAATACCTATTATTACATTTTT
AGGTGCTGATATTGTAAGTATTTTAGGTGGAGCTGTGATTACTGAGACTATCTTTTCATATAACGGTATCGGTAAATTATTTTTAGAATCGGT
AATAGGTCAAGACTATCCATTAATGATGGCATTAACGTTGTTTTTCTCATTTTTAGGTTTACTGGGTAATTTGATTTCTGATATTACTTATGGA
TTTATAGATCCAAGAATTAGAAGTAACTAG

Gene: appC (oligopeptide ABC transporter, transmembrane permease subunit)

Position: 953814 to 954695, length: 882 nt, orientation: FORWARD

Perfect match to: (11819-97-CP003194-[1000560:1001441], allele observed in CC80)

Sequence:

ATGCAAAATAAGTCAAAATCGCCTTTTAAAATTGCATTGTCTAGATTTATTCATAATAAAATTGCAATGTTATCGATTATTTTTTTATTAATCA
TAACTATTATATCAATTATAGCGCCATTAATAGCTCCTCTTCCAGTGAACCAACAAGATTTATTAAATATAAAAGGTGAAATGACAGCACAA
AACATTCTTGGTACAGACTCTGGTGGTAGGGATAACTTTAGTCGTTTGTTATATGCAGGTCGTATTTCATTATCCATTGGAATTACATCTACA
ATAGGAATGCTTTTGATTGGAATTACAGTTGGAGTGATTTCTGGTTATTTTGGAGGTATTGTTGATACATTATTAATGAGAATAACCGAATT
TGTTATGTTATTTCCATTTTTAATATTTGCAATTGTATTAAATGCTGCACTTGGAGATAAAATTAAAAATCCTTATGGATCTGCCATAATTCTT
GTTCTAGTTATTATCGTATTAAGTTGGGGAGGTATTGCAAGACTTGTTCGTGGTAAAGTACTTCAAGAAAAAGAAAATGAATACTTTTTGGC
AGCAAAATCAATTGGTACACCCACATATAAAATTATTTTGAAACATCTTTTGCCGAATATATTAAGTGTAGTTATCGTACAAGCAACATTGTT
ATTTGCCGGTATGATTGTAGTGGAATCAGGATTGAGCTTTTTAGGATTCGGAATTAGTAAAGCAATACCATCTTGGGGTAATATGTTGAGT

GATGCTCAAGAAGGGGATGTTATAAGTGGTAAACCGTGGATATGGATGCCACCTGCTATAATGATTACATTAACTATATTAAGTATAAACTT
TGTAGGGGAAGGGCTTAAAGATGCTTTTAATCCTAGAGGTAGACGTTAA

Gene: trpS (tryptophanyl-tRNA synthase)

Position: 954737 to 955726, length: 990 nt, orientation: REVERSE

Perfect match to: (MW2-BA000033-[969520:970509:r], highly conserved allele)

Sequence:

TTATCTCTTACGTCCTAAACCCATCGCTTTTTCCATTTTTTTGACAGTTTTAAATGAAACTTTGTGTGCTTTATCTCTACCTTGATCTAAAATATC
ATCAAGTTTATCTGAGTTATAGAAACTTTCGTATTTTTCTTGGAATTCTACTAAAAATGCTTTAACTATTTCAGCAAGGTCACCTTTAAATTTA
CCATAACCTTCGCCCTCATATTTTGCCTCAATATCTTTAATTGGCATGTCTGTTAATCCAGCGTATATTGAAATTAAATTTGTTATACCTGGCTT
GTTGTCACGATCAAATTTAATAATACCATCTGAATCAGTTACTGCGCTTTTAATTTTTTTAGCTGCAACATTCGGCTCGTCTAATAATGAAATG
AAGTTTTTAGCATTATCATCACTCTTACTCATTTTTCTTGTTGGGTCTTGTAAACTCATGACACGTCCACCAACTTTAGGCATACGAATTTCAG
GTTTCACAAGCACATCATTATAGCGACTATTAAATCTATCTACAAGGTTACGAGTCAATTCGATATGCTGCTTTTGGTCATCTCCAACTGGAA
CGATATTAGTATTGTAAAGAACAATATCAGCTGCCATTAAAGGTGGATATGTTAATAGACCAGCAGGTATACCTTCAACTGCTTTCTGAGCT
TTATCTTTGTATTGCGTCATACGCTCTAATTCTCCAACAGAAGCAATCGTAGTTAACATCCATCCTGCTTGTACGTGTGCAGGGACTTCAGAT
TGTATGAACAATGTTGCTTTGTCTGGATCTATACCAGAAGCTAAATAAATCGCTGCTAATTGTCTGGTCTGTTTACGTAATTTTAAACGATCT
TGTGGCATTGTAATTGCATGTTGATCTACGATACAGAAATAACAATCATAGTCATTTTGCACATCAACAAATTGTTTTAGTGCGCCAATATAA
TTTCCAATAGTAGGAATTCCACTAGGTTGGATGCCTGAAAATAATGTCTCCAT

Gene: spxA (thiol/oxidative stress global regulator)

Position: 956021 to 956416, length: 396 nt, orientation: FORWARD

Perfect match to: (N315-BA000018-[970835:971230], highly conserved allele)

Sequence:

ATGGTAACATTATTTACTTCACCAAGTTGCACATCTTGCCGTAAAGCGAAAGCATGGTTACAAGAACATGACATTCCGTATACGGAGCGTAA
TATTTTTTCTGAACATTTAACAATTGATGAAATTAAGCAAATATTAAAAATGACTGAAGACGGTACTGATGAAATCATTTCTACACGTTCTAA
AACATACCAAAAATTAAATGTTGATATTGATTCACTACCATTACAAGACTTATATTCAATCATTCAAGATAATCCTGGCTTATTACGTCGTCCA
ATTATTTTAGATAATAAACGACTACAAGTTGGTTATAATGAGGACGAGATTCGACGTTTCTTACCTAGAAAAGTTCGTACGTTCCAATTACA
AGAAGCACAACGTATGGTTGACTAA

Gene: trfA (adapter protein)

Position: 956787 to 957506, length: 720 nt, orientation: FORWARD

Perfect match to: (11819-97-CP003194-[1003533:1004252], highly conserved allele)

Sequence:

ATGAGAATAGAACGAGTAGATGATACAACTGTAAAATTGTTTATAACATATAGCGATATCGAGGCCCGTGGATTTAGTCGTGAAGATTTAT
GGACAAATCGCAAACGTGGCGAAGAATTCTTTTGGTCAATGATGGATGAAATTAACGAAGAAGAAGATTTTGTTGTAGAAGGTCCATTATG
GATTCAAGTACATGCCTTTGAAAAAGGTGTCGAAGTCACAATTTCTAAATCTAAAAATGAAGATATGATGAATATGTCTGATGATGATGCA
ACTGATCAATTTGATGAACAAGTTCAAGAATTGTTAGCTCAAACATTAGAAGGTGAAGATCAATTAGAAGAATTATTCGAGCAACGAACAA
AAGAAAAAGAAGCTCAAGGTTCTAAACGTCAAAAGTCTTCAGCACGTAAAAATACAAGAACAATCATTGTGAAATTTAACGATTTAGAAGA
TGTTATTAATTATGCATATCATAGCAATCCAATAACTACAGAGTTTGAAGATTTGTTATATATGGTTGATGGTACTTATTATTATGCTGTATAT
TTTGATAGTCATGTTGATCAAGAAGTCATTAATGATAGTTACAGTCAATTGCTTGAATTTGCTTATCCAACAGACAGAACAGAAGTTTATTTA
AATGACTATGCTAAAATAATTATGAGTCATAACGTAACAGCTCAAGTTCGACGTTATTTTCCAGAGACAACTGAATAA

Gene: trfB (putative protein)

Position: 957627 to 958613, length: 987 nt, orientation: FORWARD

Perfect match to: (CN1-CP003979-[939731:940717], highly conserved allele)

Sequence:

ATGTTAGTAGCTTTAAATGAAGAAAAGGAACGTGTGTTAGCAACTACTGCATTGAGAAAGACACAATATTTTTGTCCGGTGTGTGGCAAGC
AAGTTATTTTAAAGCGTGGGCTCAAAGTAATTAGTCATTTTGCACATAAACATTTAGCGGAACAAAAATGTTTTAATAATGAAACGATTAAA
CATTATAAAAGTAAATTGATTTTAGCACAGATGATACAGCAACAAGGATGTAAAGTAGAGATAGAGCCATTTTTAAAAGAAATAAAACAAA
TTCCGGATATTTTGATTAATAATAAATATGTTATTGAGCTACAGTATTCGCCAATTCCTTATAAACAGATTCTTCAACGAACGGAAGGTTTAA
AGAAAATGGGATATAAAGTAAGTTGGTTATTAAATGATGTTGATTATTGTCATAATAAAGTGAAGTTCAATCATTTTCAAAGTATGTTTATT
AATCCAATCACTCGAAAACTTCATACGTTCAATTTAGAGAAAAAACAAATAATGATGTTTCAACAAATACAATATTTAGGCGGGCACAAATA
TGTCGCTGAAAAAAGGAATGCCAAAATTAGTGAGTTGTTTAATGAGGCGCCTTGTGATTATCATGCTGTTTATAAATTATCAAAGTTCGCAA
TTAATCAATATATCAAATATTGTCGCTGGCAAAATTCTGTTTTAGAACCCACTTTAAGTGCAATATATCAATTACAGTTAACTGATCAAGAAG
TAGTGCACAATTATGGTTATATTTTTCCAGAGCAAATTTATATTGAAAATCATCCAATTGAGTGGCAATTACAAGTTGATTTATGGTTAAAGA
ATGGAAAAAGCAAATTAGTAAGTGACAATCTTAATTATTTTAAACTGAAAAAGTTTATTGTTGGTCTAGAAAGTAAAACAGCAATTATAGAA
AAACTTATTAACAATTATTTAAATATTTGTTCAGATAGAGGTAATGACGTGCAAATTTTGTTCTAA

Gene: pepF1 (oligoendopeptidase F)

Position: 958661 to 960469, length: 1809 nt, orientation: FORWARD

Perfect match to: (ATCC51811-ST1-ADVP01000010-[199829:201637:r], allele observed in CC1+CC80)

Sequence:

ATGAGTCAACAATTATCTAGAGAAGAACAGGAACGTAAATATCCTGAATATACATGGGACTTAACAACAATTTTCAAAGATGATGAAGCTT
TTGAGGCTGCATTTAAAGAAGTTGAAAATGAGTTAGGCAAAGAAGAACAATTTAAAGGACACATTGGTGATAGTGCTGAGACATTATACA
ATGCATTAGAATTAGAAGATACATTAGGTACTAAATTAGAAAAAGTATATGTATACGCGCACCTAAAACAAGACCAAGATACAACGAACGA
CAAGTATACTGGTATGGAGTCAAGAGCACATCAATTAATTATTAAATTTAGCTCGGCATGGAGTTTCTTAGTGCCAGAGATTTTACAAATTG
ATGAAGATAAAATTCAATCATTTGTAAATTCATATGATAAATTACAAAAATTCGCATTTGATTTGAAGTTGATTAATGAAAAACGTCCTCATA
TTTTAGATGCTGAAACTGAAAAGTTATTAACAGAAGCGCAGGACGCGTTATCAACGCCATCAAATGTATACGGTATGTTTAGCAACGCTGA
TTTAGTATTTGAAGATGCGATAGATAAAGATGGAAATGCACACCCGTTAACACAAGGTACATTTATTAAGTATTTAGAAACAGATGATCGC
AAACTAAGAGAAAGTGCTTTTAGAAATGTATATAAAGCATATGGTGCTCATAATAATACGCTTGGCGCTACGCTAGCAGGTGAAGTGAAGA
AAAATGTATTTAATGCTCGTACACACAATTACAAAACTGCAAGAGAAAAAGCATTGAGTAATAATCATATTCCAGAAAATGTATATGACAAT
CTAGTAAAAACTGTACATAAATATTTACCATTGCTACATAGATATACTGAATTGCGCAAAGAATTGCTAGGTTTAGATGACTTGAAAATGTA
TGATTTATATACACCATTAATTAAAGATATTAAGTTTGAAATGCCTTATGAAGAAGCTAAAGAGTGGATGTTAAAAGCTTTAGAACCAATGG
GTGAAGAATATTTAAATGTAGTTAAAGAAGGCTTAAACAATCGTTGGGTCGATGTCTATGAGAATAAAGGTAAACGTTCAGGTGGCTATTC
ATCAGGTGCACATTTAACTAATCCATTTATTCTACTTAACTGGTCTAATACTATTTCAGACTTATACACATTAGTTCATGAATTTGGGCATTCA
GCACATAGTTACTTCAGTAGAAAATTCCAACCGTCAAATTCTAGTGACTACACTATTTTTGTCGCTGAAGTTGCATCAACTTGTAACGAAGCA
CTTTTAAGTGATTATATGGATAAACATCTTGATGATGAAAAACGCTTATTATTATTAAACCAAGAATTAGAACGTTTCAGAGCTACATTATTC
CGACAAACAATGTTCGCAGAATTTGAGCATAAAATTCATGCAATTGAAGAAGCAGGTGAACCATTAACGCCAACTAGAATGAATGAAGAAT
ATGCCAAATTAAATAAATTATACTTCGGTGATTCTGTAGAAACTGATGAAGATATTAGTAAGGAATGGTCACGTATTCCACACTTCTATATG
AATTATTATGTATATCAATACGCAACTGGTTACAGTGCAGCTCAAAGCTTAAGTCATCAAATTTTAACAGAAGGTAAGCCAGCAGTAGATAG
ATATATTAATGAATTCTTGAAAAAAGGTAGCTCAAATTATCCAATTGAGATATTAAAAAATGCTGGTGTAGATATGACAACACCTGAACCAA
TTGAACAAGCTTGTGAAGTTTTTGAACAAAAATTGAACGCTTTTGAAAAATTAATGAAAGCTTAG

Gene: yjbH (SpxA binding thioredoxin domain protein)

Position: 960929 to 961735, length: 807 nt, orientation: REVERSE

Perfect match to: (RF122-AJ938182-[942535:943341:r], highly conserved allele)

Sequence:

TTATTTTGATTTGATTTTAGGCATTTTAGATTTCCAGAAATCGCCATCTGGATATTTAAGTTTTTCAATTTTTTGTTGAATGGCTAACTTCTTTA
ACTCTTTGTTTAAAAGTTTTTCTGGCCATTCATAAATAGTAAGTAATTCTTCCATCGTTACAAGTTGTTGTTGCTGTATATAAGTTTCTAATTTA
GGAGGAAGATTCTTTTCGATAGGTTTACCCATCAATTCATTAATTATATAAGTATAGATGTGATATGGGTATAATCCTTCGACTTTTAAACCT
TCTTCATGAACATCTTCACTAAAGAAAACGAGAGAAGGGGCTTGTTCGATTTCCATTTCTCTTGCAATATGCAAATCAATTTTCAAGCTTTCG
GTTAGTTTACTTTTTTGTAAGTCGTCTTTAAATACTTCTAAATCAATACCTGCATTTTGAATACAGTCACAAATCATTGATTCTGTAATAATATC
TCTTTTAGGTATGATTTCATTTTGCATTAAATGTATAAATCGTTCGGCACGTACACGACCTTGTAACTCAGCTGCTTTATAAGCTAGGGCGAT
GTTATCAAAGTTGGATGTACTTTGAGCTTGGCATTTCGTTAATACTTTTAACGAAGGATTTAATATATGTCTGATACGTATATATTGATTATA
TTCAATTCTTAATTTGGATAAGATTGCTGATAATTTGAAGCAATCGGAGCTAAATGGATCGAAAAATGAATAAATTTCGATTTTACTTACAG
GTGATAGATTAATATCTTCACGACTCTTATTTTCCATTATTCGTAATTCTCCAGCCAT

Gene: yjbI (putative thiol management oxidoreductase component)

Position: 961758 to 962123, length: 366 nt, orientation: REVERSE

Perfect match to: (N315-BA000018-[976572:976937:r], highly conserved allele)

Sequence:

TTAGGAATTCACCATATGATTAGCAGTTAATCTTAAGCGCTCAAATAAATAATCTCCAACACCTTGTGGAAACGCAGCGCGATTAATTGCTG
TCTGCATATTTTCTAACCATGCATCTCTTTCAAATTCAGTGATTGTAAAATCCATATGTCTTTTTCTTAGCATAGGATGTCCGTGTTCTTCGGTA
TAAATGTTTGGACCGCCCAAAAACTGTGTTAAAAATTGTTTTTGTTTACGACTTGTTTCTGCAAAATCTCCTGGAAACAGGTGATTAAGTCGT
TCATCTTTTTCTACAAGGGTGTAAAAATAATCAATCATATCGTATAACGCTTCTTTACCAATGATGTCATATGGTGTTGTTGTCAT

Gene: yjbK (putative RNA/thiamine triphosphatase)

Position: 962227 to 962820, length: 594 nt, orientation: REVERSE

Perfect match to: (MRSA252-BX571856-[1015274:1015867:r], highly conserved allele)

Sequence:

TTAATTTATATTGTTTGAAAGTGTTTCTTTTTTCTTGAAAAAACGTTGAACTTTATTTAAAGGTTGATGATGTTCGAGGTTTAGTTCGTTTAAT
AAAGATTGGAACTTTTGTAAACCTTGATTATAGTCTTTAACTTCGAACTCTAACTCATAATCCGCAGTATCGAAATACTCACTTTTATCTAAAA
CGAGTAAATCACCTTTATATTTAGTTTCTTGGCGATATGTCGTTAATGCACCAAGTATTGATAAAGTTGTATCTTTTACACCAAACTGTTCAAC
TATAATTTGACGAATGTCATCTGGAAGATTGTCGTTTGAAATAATCAAGTTCATCTCTGGTTTAATGTCGACGATATAGTTGTATTCTAATAG
ACCAACCTTTGCTGGTGTCTTTAAAGTCATTTCATATTGATTGTCTTTAACTCTTATGCGTAGTGCAGAGCGATGTTCCTTTAATTTGAAATCG
GGTGTATCAATATAGTAATTGACTTGCTTAAAAAGCACACTGTCTTTAAAATATTTCTCTTGCAATTTATTATAGATTGATGCAGTTATCATTT
GTTTAAATTCTATTTCATGATTTGTTGCCAT

Gene: yjbL (putative phosphatase)

Position: 963006 to 963353, length: 348 nt, orientation: FORWARD

Perfect match to: (MW2-BA000033-[977789:978136], highly conserved allele)

Sequence:

ATGCGTTTATATATTAATGAAATTAAAATTAAAGATGACATACTTTATTGTTATACAGAAGATTCTATTAAAGGATTATCTGAAGTAGGACA
AATGCTCGTTGATAGTGATAATTATGCCTTTGCGTATACATTAGATGATGGTAAAGCGTATGCTTATCTCATTTTCGTACAAGAAACATGGA
CGATGTTGCATGAAAACATGACTAAAAAAATTATTATCAATGATGAACTAGAATTGACTGAATTCCACCAAGAACTTACTTATATTTTAGAC
AACATAAAAGGGAATAATAATTATGGTAAGGAATTTGTTGCAACCGTTGAAGAAACATTCGACATTGAATAA

Gene: relQ (ppGpp synthase III)

Position: 963370 to 964005, length: 636 nt, orientation: FORWARD

Perfect match to: (MW2-BA000033-[978153:978788], highly conserved allele)

Sequence:

ATGAATCAATGGGATCAGTTCTTAACACCTTATAAGCAAGCGGTTGATGAGTTGAAAGTGAAACTTAAAGGCATGCGCAAACAATATGAAG
TTGGTGAACAAGCGTCGCCAATAGAATTTGTTACTGGTCGTGTTAAACCAATCGCTAGTATTATAGATAAGGCAAACAAACGACAAATACC
ATTTGATAGGTTAAGAGAAGAAATGTACGATATCGCTGGTTTAAGAATGATGTGCCAATTTGTTGAAGATATTGATGTTGTCGTCAATATTT
TAAGACAAAGAAAAGATTTTAAAGTAATTGAAGAACGAGATTATATTCGTAACACTAAAGAAAGTGGTTACCGCTCGTATCATGTCATTATT
GAATATCCAATTGAAACATTACAAGGCCAAAAATTTATATTGGCTGAGATTCAGATTCGTACATTAGCAATGAATTTCTGGGCAACGATTGA
ACATACTTTACGATATAAATATGATGGTGCTTATCCGGATGAAATTCAACATCGTTTGGAAAGAGCGGCAGAAGCAGCGTATTTACTTGAT
GAAGAGATGTCTGAAATTAAAGATGAAATTCAGGAAGCTCAAAAATATTACACGCAAAAACGTTCTAAAAAACATGAAAATGATTAA

Gene: ppnK (putative inorganic polyphosphate/ATP-NAD kinase)

Position: 964022 to 964831, length: 810 nt, orientation: FORWARD

Perfect match to: (COL-CP000046-[1018443:1019252], highly conserved allele)

Sequence:

ATGCGTTATACAATTTTAACTAAAGGTGACTCCAAGTCTAATGCCTTAAAGCATAAAATGATGAACTATATGAAAGATTTTCGCATGATTGA
GGATAGTGAAAATCCTGAAATTGTTATTTCAGTTGGTGGTGACGGTACATTACTACAAGCATTCCATCAGTATAGCCACATGTTATCAAAAG
TGGCATTTGTTGGAGTTCATACAGGTCATTTAGGATTTTATGCGGATTGGTTACCTCATGAAGTTGAAAAATTAATCATCGAAATTAATAATT
CAGAGTTTCAGGTCATTGAATATCCATTGCTTGAAATTATTATGAGATACAACGACAACGGCTATGAAACAAGGTATTTAGCATTAAATGAA
GCAACGATGAAAACTGAAAATGGCTCAACACTTGTTGTGGATGTTAACTTAAGAGGGAAACACTTTGAGCGATTTAGAGGCGATGGATTAT
GTGTATCAACACCTTCGGGTTCAACGGCTTATAACAAAGCGCTAGGTGGCGCACTGATACATCCTTCACTTGAAGCAATGCAAATTACAGA
AATTGCCTCGATAAATAATCGTGTGTTTAGAACGGTAGGATCACCACTTGTATTACCAAAGCATCATACATGTTTAATATCACCAGTTAATCA
TGATACCATTAGAATGACGATAGATCATGTTAGTATCAAACATAAAAATGTTAATTCAATACAATACCGTGTAGCAAATGAAAAAGTGAGG
TTTGCACGTTTTAGACCATTCCCATTCTGGAAACGTGTGCACGATTCTTTCATATCAAGTGATGAAGAACGATGA

Gene: rluE (ribosomal large subunit pseudouridine synthase)

Position: 964828 to 965682, length: 855 nt, orientation: FORWARD

Perfect match to: (11819-97-CP003194-[1011574:1012428], allele observed in CC80+CC239)

Sequence:

ATGAAATTTAAGTATCATATATCACAACAAGAAACTGTTAAAACTTTTTTAGCACGACATGATTTTTCTAAGAAGACAGTGAGCGCCATTAA
AAATAATGGCGCTTTAATTGTTAATGATGAACCAGTCACAGTGCGTAAGCAATTAATGACAAATGATATATTAGAAATTCATTTACCGCGAG
AAATACCGAGTGTTAATTTAATACCTTATGCTCGTAAGCTAGAAGTATTGTATGAAGATGCTTTTATCATCATAGTTACTAAACCAAACAATC
AAAATTGTACGCCTTCGAGAGAACATCCTCATGAAAGTTTAATCGAACAAGTACTATATCATTGTCAGGAACATGGTGAAAATATTAACCCA
CATATTGTTACGCGTCTAGATCGTAATACAACTGGTATTGTGATATTCGCTAAATATGGACATATCCATCATTTATTTTCTAAAGTAAACTTG
AAAAAAATATATACTTGCCTTGTATATGGTAAAACCCATACATCTGGTATTATTGAAGCTAATATTAGACGGTCAAAGGATAGTATTATAAC
TAGAGAAGTTGCCTTGGATGGTAAATACGCTAAAACATCTTATGAAGTAATAAATCAGAATGATAAATACAGTTTATGCAAAGTTCATTTGC
ATACGGGACGTACACATCAAATTCGTGTACATTTTCAACATATTGGGCATCCAATTGTGGGAGATTCTTTGTATGATGGTTTTCATGACAAA
ATTCATGGTCAAGTACTGCAATGTACGCAAATATATTTTGTTCATCCAATCAATAAGAACAATATTTATATTACAATTGATTATAAGCAATTA
CTTAAATTATTCAATCAACTCTAA

Gene: mgtE (magnesium transporter E)

Position: 965703 to 967088, length: 1386 nt, orientation: FORWARD

Perfect match to: (11819-97-CP003194-[1012449:1013834], allele observed in CC80+CC8+CC80+CC239+CC4803)

Sequence:

ATGTCAATGAACACAGATGAAAAAGAGCGTGTTCAAGAGGAATTATACGACCAAACTTTATTAGATCAATATTTAGAAAATGATGATATTG
ATCAATTTAGAGATGAATTTCTAGCATTACACACATATGAACAAAGTGAGTATTTTGAAGATACTACCGATGAAAATAGACAAAAGATTTTT
CAATATTTATCACCTGAAGAAGTTGCAAATTTCTTTGATCAATTAGATATTGATGACGATGAATATGAGTTGCTATTTGATAAGATGAATGC
GACATACGCAAGTCACATATTAGAAGAAATGTCATACGACAATGCAGTAGATATTTTAAATGAGTTGACTAAACCAAAAGTTGCTAGTCTTT
TAACATTGATGAATAAAGATGACGCGAATGAAATCAAAGCATTACTTCACTATGATGAGGATACGGCCGGCGGTATTATGACGACGGAGT
ATTTATCACTTAAAGCGCATACGCCTGTTAAAGAAGCATTATTATTGGTCAAAGCGCAAGCACCAGACGCAGAAACAATATATGTTATATTT
GTCGTTGATGATGATGGTAAATTAGTAGGTGTTTTATCGCTAAGAGATTTAATTGTAGCTGAAAATGATGCTTATATTGAAGATATTATGAA
TGAACGTGTCATTAGTGTGAATGTAGCAGACGACCAAGAAGATGTTGCTCAAGTTATGAGAGACTATGATTTCATGGCTGTACCTGTTATA
GATTACCAAGAACATTTGCTTGGTATCATCACGATTGATGATATTTTAGACGTTATGGATGAAGAGGCTAGTGAAGACTACTCTCGTTTAGC
CGGGGTATCAGATATCGATTCGACTAATGATTCAATCATTAAAACAGCATTAAAACGTTTACCATGGTTGATTATTTTAACATTTTTAGGAAT
GATTACTGCGACAATTTTAGGGAGATTCGAAAAAACATTAGAAAATGTAGCGCTACTCGCAGCGTTTATTCCTATTATTAGTGGTATGTCAG
GAAATTCAGGTACACAATCTTTAGCCGTTTCAGTTCGTAACATTACGACAGGGGAAATTAATGAGCAAAGTAAATTTAGAATTGCATTAAG
AGAAGCAGGAAGTGGTGTATTATCGGGTGTTGTATGTTCAACAATATTATTTACAATTATTGTTGCAATATATCATCAGCCACTTTTAGCATT
AATCGTTGCAGGAAGTTTAACTTGTGCGATGACGGTGGGGACGTTTGTAGGTTCGATGATTCCATTATTGATGAATAAATTAAATATCGATC
CAGCAGTGGCTAGTGGACCATTTATTACAACAATTAATGATATTATTAGTATGTTGATTTATTTTGGTTTAGCTACATCATTTATGGCTTACTT
AATTTAA

Gene: cpaA (monovalent cation:H+ antiporter)

Position: 967098 to 968942, length: 1845 nt, orientation: FORWARD

Perfect match to: (RF122-AJ938182-[948704:950548], highly conserved allele)

Sequence:

ATGGAGTTTTTATCTTTAGTTATTGTTGTTTTAGCAGCGTTTTTAACTCCAATAATTGTCAATCGATTAAATATTAATTTCTTGCCAGTTGTTGT
TGCAGAAATTTTGATGGGGATTGTGATTGGAAATTCATTTCTAAATATAGTAGAAAGGGATTCAATTCTAAATATTTTATCAACGTTAGGCT
TTATCTTTTTAATGTTTTTAAGTGGTTTAGAAATTGATTTTAAAGCTTTTAAAAAAGATAAACGCGCACGTCAAGGACAAAATGATGATGAAT
CCTCAATTCCAGGGCATCTTAATCTAGCGTTAACTGTATTTGCATTTATTATGATTATTTCGATTCTTTTAGCGTATGTATTTAAATGGCTTGG
ATTAGTGGATGATGTGTTATTAATGGTCATTATCATTTCAACTATTTCCTTAGGCGTAGTTGTTCCAACTTTAAAAGAAATGAATATTATGAG
AACAACTATAGGGCAATTTATCCTATTAGTAGCAGTACTTGCGGACTTAGTAACTATGATTTTATTAACGGTCTATGGCGCAATCAATGGTC
AAGGCGGCAGTACAATATGGTTAATAGGTATATTAGTTGTTTTCACAGCAATTTCATATATTTTAGGTGTTCAATTTAAAAGAATGTCATTTT
TACAAAAATTGATGGATGGTACGACGCAAATCGGTATTCGTGCGGTATTTGCATTAATAATATTATTAGTAGCCCTAGCAGAGGGAGTTGG
CGCAGAAAATATATTAGGTGCATTCTTAGCAGGTGTCGTTGTTTCATTATTAAATCCAGATGAAGAAATGGTTGAAAAGTTAGACTCATTTG
GTTATGGGTTCTTTATTCCTATTTTCTTTATAATGGTTGGTGTAGATTTAAACATACCTTCATTAATTAAAGAACCGAAATTACTAATTATCAT
ACCGATTTTAATCGTTGCATTTATCATTTCAAAATTAATTCCAGTCATGTTTATTCGACGTTGGTTTGATATGAAAACAACGATTGCATCAGC
ATTTTTATTAACATCAACATTATCGCTCGTGATAGCTGCAGCCAAAATTTCAGAAAGATTAAATGCTATTTCAGCTGAAACGTCAGGTATATT
AATTTTAAGCGCAGTCATTACATGTGTATTCGTTCCGATTATTTTCAAAAAACTGTTTCCAGTTCCAGATGAGTTTAACCGTAAAATTGAAGT
TAGTTTAATTGGTAAAAATCAATTAACGATTCCTATAGCGCAAAATTTAACATCTCAGTTATATGACGTGACATTATATTATCGCAAAGACTT
GAGTGATCGTCGTCAATTGTCAGATGATATCACGATGATAGAAATTGCTGATTATGAACAAGATGTTTTAGAACGACTAGGTCTGTTTGACC
GAGACATAGTTGTTTGTGCTACGAATGACGATGATATTAACCGAAAAGTTGCTAAATTAGCCAAAGCACATCAAGTTGAGCGTGTCATTTG
CAGACTTGAAAGCACAACGGACGATACAGAGTTAGTTGATTCAGGTATTGAAATTTTCAGTAGCTACTTAAGTAATAAAATCTTATTAAAAG
GTTTAATTGAAACACCTAACATGTTGAATTTATTAAGTAATGTTGAAACGTCACTATATGAAATTCAAATGTTAAATTATAAATATGAAAATA
TTCAATTACGTAATTTCCCATTCGGAGGAGACATCATCTTCGTGCGTATTATCCGTAATAATGAGTCGATTGTTCCGCATGGAGATACACAAT
TGCGATATGGAGATCGCTTAATTGTTACCGGTGCTAAAGAATACGTTGATGAATTGAAGCAAGAGTTAGAATTTTATTTTTAA

Gene: fabI (enoyl- (acyl-carrier-protein) reductase)

Position: 969220 to 969990, length: 771 nt, orientation: FORWARD

Perfect match to: (NN50-BAEA01000008-[238657:239427:r], allele observed in CC4803+CC5+CC8+CC88+CC239)

Sequence:

ATGTTAAATCTTGAAAACAAAACATATGTCATCATGGGAATCGCTAATAAGCGTAGTATTGCTTTTGGTGTCGCTAAAGTTTTAGATCAATT
AGGTGCTAAATTAGTATTTACTTACCGTAAAGAACGTAGCCGTAAAGAGCTTGAAAAATTATTAGAACAATTAAATCAACCAGAAGCGCAC
TTATATCAAATTGATGTTCAAAGCGATGAAGAGGTTATTAATGGTTTTGAGCAAATTGGTAAAGATGTTGGCAATATTGATGGTGTATATCA
TTCAATCGCATTTGCTAATATGGAAGACTTACGCGGACGCTTTTCTGAAACTTCACGTGAAGGTTTCTTGTTAGCTCAAGACATTAGTTCTTA
CTCACTAACTATCGTTGCTCATGAAGCTAAAAAATTAATGCCAGAAGGTGGTAGCATTGTTGCAACAACATATTTAGGTGGCGAATTCGCA
GTTCAAAACTATAATGTGATGGGTGTTGCTAAAGCGAGCTTAGAAGCAAATGTTAAATATTTAGCATTAGACTTAGGTCCAGATAATATTCG
CGTTAATGCAATTTCAGCTGGTCCAATCCGTACATTAAGTGCAAAAGGTGTGGGTGGTTTCAATACAATTCTTAAAGAAATCGAAGAGCGT
GCACCTTTAAAACGTAATGTTGATCAAGTAGAAGTAGGTAAAACTGCGGCTTACTTATTAAGTGATTTATCAAGTGGCGTTACAGGTGAAA
ATATTCATGTAGATAGCGGATTCCACGCAATTAAATAA

Gene: UPF0118 (putative membrane protein)

Position: 970185 to 971270, length: 1086 nt, orientation: REVERSE

Perfect match to: (11819-97-CP003194-[1016931:1018016:r], allele observed in CC80)

Sequence:

TTAGTCCTTAACATTACTGTTTGCTTTATCAATAATGCGTTGGCGGTATTTGAAAATATTACTAACAACCGTTTTAAGTACAGCATATAATGG
CACTGCAATCAGAACTAATGTAAAGCCACCTAAATCTCCTGCTGCTAAAATAACAACGATAATTGTTAAAGGATGGATACTTAAAGATTTAC
CCATTACATTTGGTGTAATGATATTACCTTCAAGTTGATGTGCAATTAATGTAATGATACAAACCCATATAAATGTAGTAGGACTATCTATAA
TACCGAGTATTGCTGCAGGTGCAAATGATAACCATGAACCTAAGAAAGGAATTAAGTTTGCGACACCAGCAAATAGTACTAATAAAGGAAT

ATATGGTAAGTCAATAATTGAATAACCGATATATAAGAATATACCTAAAATAACACTGACAGTTACTTGACCTTGAATGTAAGATTTTAATG
TAAAGTTTAAATCAGTTAATAAATCTACGAAAAATACTTTACGTTCACCTTTGAAAAATTTAGCAACAGCTGGGATAAATTTTTCATGGTCTT
TTAACATATAAATTAAGAAGAATGGAACCATAATCAATAAGAAGATGGTTGAAATTAATGATGTAATGTACTGTAATGAATTAGATAAAAT
ATTAGTAACGCCATCACCCATTGATTTAACCATATTTGTAATTCTACTTGTTACATCTTCAGGTAATTTATCCATTTGAGCTAGTGCGAATTTA
ATAATTTGCTCTGCTTCTTTTTGTAGGGCAGGTGTCTGACTAATTAAATTGTTGATATTTGAAATGATGATTGGTGCAACAAACGCAACAATT
AAGCCGATAATAGCAAACAAAGCTAACATGATTGTTGTTATACTAGCCCATCTTGGAAAACCGACTTTTTCAAGTAAGTTTTGAAAAGGTAG
ACAGATATAAAATAAAAAACCACTAATTAAAAATGGAAGAAATACAGAACCGATGATTGTAGCTATTGGAGTAAATACTTCGTGCACTTCC
ATAAATAGTTTGATGAGAATGAACAGCATAATAAGAGCGATGCCAGTTCGGAACCAAACCTTGTTTAACAT

Gene: yrbD (alanine or glycine carrier protein)

Position: 971612 to 973180, length: 1569 nt, orientation: FORWARD

Perfect match to: (Bmb9393-CP005288-[1045867:1047435], allele observed in CC239+CC8)

Sequence:

ATGATTGAAAAATTAGTAACCTTTTTAAATGAGGTTGTTTGGAGTAAGCCATTAGTTTATGGTTTGCTAATTACTGGTGTGCTATTTACATTG
CGTATGCGATTTTTTCAAGTTAGACATTTTAAAGAAATGATTCGATTAATGTTTCAAGGAGAGAAGTCTCCTAATGGTATTTCAAGTTTTCAA
GCGATAGCCATGTCTTTAGCAGGCAGGGTTGGTACAGGTAATATTGTCGGTGTATCTACTGCAATATTTATAGGAGGACCTGGTGCAGTAT
TTTGGATGTGGATTACTGCGTTTTTAGGTGCAAGTAGTGCTTTTATTGAATCTACACTTGGTCAAATATTCAAGAGAGTTGAAAATAATGAA
TACCGTGGTGGACCAGCGTATTATATTGAATATGGTATTGGTGGTAAATTTGGTAAAATTTACGGAATTATCTTTGCTATTGTTACGATTATC
TCAGTAGGTCTATTGCTTCCTGGTGTGCAATCTAACGCTATAGCAAGTTCTATGCATAATGCGATTCATGTTCCACAATGGTTAATGGGTGCT
ATTGTTGTAGTTATTTTGGGATTAATTATTTTTGGTGGTGTACGTAGTATTGCCAATGTTGCAACAGCCGTTGTACCATTTATGGCAATTATT
TACATACTGATGGCTGTCATTATCATTTGTATCAATATACAAGAAGTGCCAGCGTTATTTGCATTAATTTTCAAATCAGCATTTGGATTACAA
TCTGCTTTTGGTGGTATCGTTGGCGCAATGATAGAGATTGGTGTTAAACGTGGATTATACTCAAATGAGGCTGGTCAAGGTACAGGTCCAC
ACGCAGCAGCGGCAGCAGAAGTATCACATCCAAGTAAACAAGGTCTAGTACAAGCATTTTCAGTTTATATTGATACATTATTTGTATGTACT
GCAACTGCTCTGATTATACTTATTTCTGGTACATATAATGTGACTGATGGTACGGTTAATGCGAATGGCACACCGCATTTAATTAAAGATGG
CGGTATTTATGTTGAAAATGCAACAGGTAAAGATTATTCAGGTACTGCGATGTATGCACAAGCCGGCATTGATAAAGCGTTCCATGGCAGT
GGTTATCAATTTGATCCTACTTTCTCTGGCGTAGGTTCATACTTTATTGCATTTGCTTTATTCTTCTTTGCATTTACTACTATTTTGTCGTACTAC
TACATTACAGAAACAAATGTTGCTTATTTAACGCGTAATCAAAATAATCAAGTTTCATCGATATTTATTAATATTGCTCGTGTGATTATTTTGT
TCGCTACATTTTACGGTGCAGTTAAAACAGCTGATGTAGCATGGGCATTCGGTGATTTAGGTGTAGGTCTAATGGCTTGGTTAAATATCATT
GCGATTTGGATTTTACATAAGCCTGCCGTAAATGCTTTAAAAGATTATGAAATTCAAAAGAAACGTTTAGGCAACGGTTATAATGCAGTTTA
TCAACCTGATCCGAATAAATTACCTAATGCTGTCTTTTGGTTGAAGACGTATCCAGAACGTTTAAAACAAGCACGTACCAAAAAGTAA

Gene: yjcH (putative esterase)

Position: 973324 to 974082, length: 759 nt, orientation: FORWARD

Perfect match to: (11819-97-CP003194-[1020070:1020828], allele observed in CC80)

Sequence:

ATGTTGGAATTTAAAGCTGGAAAGATTAATAAACTTGTTTTATATAGTGATATTTTAAATAGAGATGTCACACTAAGTATTTATTTACCAGAA
TCTTATAATCAACTTGTTAAATATAATGTCATTCTTTGCTTTGACGGATTAGATTTTTTGCGTTTCGGGAGAATACAACGTACATATGAATCGT
TAATCAAAGAAGCGCGTATTGATGATGCGATCATTGTCGGATTCCATTATGAAGACGTTGATAAGCGTAGGGAGGAATTTCATCCACAAGG
AAGTCGTTCTCATTTAACTATTCAATCAGTCGGTAAAGAAATATTGCCATTTATTGACTCGACGTTTTCTACACTGAAAGTAGGTAATGCAAG
GTTATTAGTAGGGGATAGTTTAGCGGGTAGTATTGCCTTATTAACGGCGTTGACCTATCCAACGATTTTTAGTCGTGTAGCAATGTTAAGTC
CACATTCAGATGACAAAGTATTAGATAAGCTAAATCAATGTGCAAATAAAGAACAATTGACAATTTGGCATGTCATTGGTCTAGATGAAAA
AGATTTTACTTTACCAACAAATGGTAAGCGTGCCGATTTCTTAACACCGAATAGAGAATTAGCTGAACAAATTAAGAAATATAATATAACTT
ATTATTACGATGAATTTGATGGTGGTCACCAATGGAAAGATTGGAAACCATTGCTGTCAGATATATTATTGTATTTTTTAAGTAAAAACACA
GATGATCAACTTTATGAATAA

Gene: UPF0477 (putative protein)

Position: 974276 to 974785, length: 510 nt, orientation: FORWARD

Perfect match to: (COL-CP000046-[1028695:1029204], highly conserved allele)

Sequence:

ATGATTTTAGGATTAGCATTAATTCCATCAAAGTCATTTCAAGAAGCGGTGGATTCTTACCGTAAAAGATATGATAAACAGTATTCACGAAT
TAAACCACATGTGACAATTAAAGCGCCATTTGAAATTAAAGATGGTGATTTAGATTCTGTCATTGAACAGGTTAGAGCTCGTATTAATGGTA
TACCAGCAGTAGAAGTTCATGCTACAAAAGCTTCTAGCTTCAAACCAACGAACAATGTGATTTACTTTAAAGTTGCGAAGACGGACGACTTA
GAAGAATTGTTTAATCGCTTTAATGGAGAAGATTTCTATGGAGAAGCTGAACATGTTTTTGTGCCACACTTTACAATAGCACAAGGACTATC
TAGCCAAGAATTCGAAGATATTTTTGGTCAAGTAGCATTAGCTGGGGTAGACCATAAAGAAATTATCGATGAATTAACTTTGTTACGTTTTG
ACGATGACGAAGATAAATGGAAAGTTATTGAAACGTTTAAATTAGCTTAA

Gene: ltaA (glycolipid permease)

Position: 974898 to 976088, length: 1191 nt, orientation: REVERSE

Perfect match to: (11819-97-CP003194-[1021644:1022834:r], allele observed in CC80)

Sequence:

TTACTTAGCTTTTTCTCTATTTACTATAAAGTAGCTTCCATAAAATACAGCTAAGACTAAAAAGATTAATGCCGAGAAATAAAATGTATTGTT
TAAATTGTTGGTAAATTGTGTAATTAATCCGCCAAATAATGGCCCTATCATTGAGCCGAATCCTTGGATACTATTAAAAACACCCCAAGTTTC
TTCTTGTTCATCTGATTTGATAAATCGTGCCATAAAGGTATTCCATGCTGGTAATAAGATGCCATACATTAGACCGATAGCTAAAGCGATAA
TCCACAAGATGTGAATATTAACAATCATAGATAGAGTAAAAATTAATATCATGTATAAAATAAATCCGCTTAGAATAACACCATACATAAAG
TTTCTGCTGCGGTTATCTATTAGTTTCGATAAAAATAGCATCGAAACTGCACAGCCGATACCACCAATAATGATTGCAACAGTATATTCAATT
GTGCTTACGTTAATAACCTTAGTAGCATATGTTGGTAATATAGGAACTAGGGCAGCAATTGCGGCACCTTGTAAAAGAATACCAGGGAACA
ACAATAAATGGCGCTTTGTCACATCAACAATTTGTCTCAATTGAGCTTTAACTGGACGAGTATTATAATTTGTTAACTTTACATCGACAAAAT
AATATAATATCCATGCAATTAAAACGACTAAAGACATCATGAAGGCAAAGCGTGTTGGGTGCACTTTGATAAGTAGATTCATAAAAACCAT
ACCTACCAATAGGCCTAACAACCATGAAAAATAAACATAGCCCATTTGTTTGCCACGTTTATCTTCTTCAACACTGGATAACATAATGACCCA
AATAGGACTAACTGCAATACCGAGCATCATAGCACTAAATATGATTACAAAAGGTGATGCTGGAAACCAAATAACTAAAAATAAACTTGTA
AATGCTAAAATAAATCCAGTCGTTAAAACGATTTTTGTGCCGAATTTTTTCAGTAAAAATCCTATAACAAAGTTTGTAGATGCATCAGCAATA
AAATGTATTGAAAATGCTAGAGACGTTATTGCTACAGCAATGGTTGTAACTGTTGGCAAGAAATTAATATAGCTTAGGATATACATGCCTCT
CGCAAATTCCATTAAAAATAAGATAATAAGCATTAAAATGAAATTTTTATGATTAGCGTAATTATTTAACGAAGAATCTTGCAT

Gene: ugtP (processive diacylglycerol glucosyltransferase)

Position: 976066 to 977241, length: 1176 nt, orientation: REVERSE

Perfect match to: (COL-CP000046-[1030485:1031660:r], highly conserved allele)

Sequence:

TTATTTAACGAAGAATCTTGCATATAAAGGAACCTTTCCATAAATCTCTTGTGGTTGTGATGAATGACCGATTAAATCAAGTAAGTCTCGAC
ATATTGTCTGTGTAGCATACTTAATTTTATCTTGTTCCATTGTACTAATCATGTTAGTTAATTGCTCATTACCGTTAGTTAAACTTGCTACAATT
TTTATTGCTTCTTCTGGAGTATCAGCGATTTTACCAAAACCTTTTTCTTCAAAGTAAAGGGCATTTTCAAGCTCTTGACCAGGTGCAGGATTT
AGGAAAATCATTGGAATACAACGGGCGAAACCTTCAGTTATTGTGATACCACCAGGTTTCGTAATCATAAGTTGACTTGATGCCATCCATTC
ATTCATGTGTTTGGTATAACCTAGAATCAATACATTCTCGTTAGATTTAAACTTAGCTGTTAAAGAACGCTTTAGCTCTTTGCTCTTACCACAA
ATCATAACTACTTGTGCATTTGCACTTTTCGCTAATATATCAGTAATCATCGTGTCAAAACCTTTAGATACACCAAATGCACCAGCTGACATT
AAAATAGTTTGCTTATCTGGATCTAAGTTGTTGTCTATTAACCACTGCTTTTGATTAATAGGCGTTTCAAATTTGTTATCAATAGGAATACCTG
TCACTTTAACTGTTGAAGGATCAATACCTACGTCTATGAAGTCTTGTTTCGTTTCTTTTGTTGCCACATAATATCTTGTTGAATACGGCGTAAT
CCAGTTTTTATGTAAGCGATAGTCTGTCATCACTGTAGCAACTGGAATATTAATGTTAAATTGCTCAGTTAGTACCGACATAACTGGTGTAG
GAAACGTTAATAATATTAAATCTGGCTTTTCTTTTATCAATAAATTAATTAACTTATTAAGTCCATAGTATTTGTAAAAACATTTGTCTAGTTT
ATCTGGGCGGCTGTAATAAAACCCTTTGTACATATTTCTAAAATATTTAAAGCTATTGATATACCATTTTTTACAAATAGAAGTCAAAATTGG
ATGAGCTTCCATAAATAAATCGTGCTCAATGACGCTTAAATGGTCTAGATTCATATCATTAAGTTGATTAACGATACTCTGTGTAACTTGCAT
ATGACCGTTACCGAATGAGCCAGTAATAATCAATATCTTTTTATTTTGAGTAACCAT

Gene: murE (UDP-N-acetylmuramoylalanyl-D-glutamate--2,6-diaminopimelate ligase)

Position: 977674 to 979158, length: 1485 nt, orientation: FORWARD

Perfect match to: (N315-BA000018-[992491:993975], highly conserved allele)

Sequence:

TTGGATGCAAGTACGTTGTTTAAGAAAGTAAAAGTAAAGCGTGTATTGGGTTCTTTAGAACAACAAATAGATGATATCACTACTGATTCAC
GTACAGCGAGAGAAGGTAGCATTTTTGTCGCTTCAGTTGGATATACTGTAGACAGTCATAAGTTCTGTCAAAATGTAGCTGATCAAGGGTG
TAAGTTGGTAGTGGTCAATAAAGAACAATCATTACCAGCTAACGTAACACAAGTGGTTGTGCCGGACACATTAAGAGTAGCTAGTATTCTA
GCACACACATTATATGATTATCCGAGTCATCAGTTAGTGACATTTGGTGTAACGGGTACAAATGGTAAAACTTCTATTGCGACGATGATTCA
TTTAATTCAAAGAAAGTTACAAAAAAATAGTGCATATTTAGGAACTAATGGTTTCCAAATTAATGAAACAAAGACAAAAGGTGCAAATACG
ACACCAGAAACAGTTTCTTTAACTAAGAAAATTAAAGAAGCAGTTGATGCAGGCGCTGAATCTATGACATTAGAAGTATCAAGCCATGGCT
TAGTATTAGGACGACTGCGAGGCGTTGAATTTGACGTTGCAATATTTTCAAATTTAACACAAGACCATTTAGATTTTCATGGCACAATGGAA
GCATACGGACACGCGAAGTCTTTATTGTTTAGTCAATTAGGTGAAGATTTGTCGAAAGAAAAGTATGTCGTGTTAAACAATGACGATTCATT
TTCTGAGTATTTAAGAACAGTGACGCCTTATGAAGTATTTAGTTATGGAATTGATGAGGAAGCCCAATTTATGGCTAAAAATATTCAAGAAT
CTTTACAAGGTGTCAGCTTTGATTTTGTAACGCCTTTTGGAACTTACCCAGTAAAATCGCCTTATGTTGGTAAGTTTAATATTTCTAATATTAT
GGCGGCAATGATTGCGGTGTGGAGTAAAGGTACATCTTTAGAAACGATTATTAAAGCTGTTGAAAATTTAGAACCTGTTGAAGGGCGATTA
GAAGTTTTAGATCCTTCGTTACCTATTGATTTAATTATCGATTATGCACATACAGCTGATGGTATGAACAAATTAATCGATGCAGTACAGCCT
TTTGTAAAGCAAAAGTTGATATTTTTAGTTGGTATGGCAGGCGAACGTGATTTAACTAAAACGCCTGAAATGGGGCGAGTTGCCTGTCGTG
CAGATTATGTCATTTTCACACCGGATAATCCGGCAAATGATGACCCGAAAATGTTAACGGCAGAATTAGCCAAAGGTGCAACACATCAAAA
CTATATTGAATTTGATGATCGTGCAGAAGGGATAAAACATGCAATTGACATAGCTGAGCCTGGGGATACTGTCGTTTTAGCATCAAAAGGA
AGAGAACCATATCAAATCATGCCAGGGCATATTAAGGTGCCACATCGAGATGATTTAATTGGCCTTGAAGCAGCTTACAAAAAGTTCGGTG
GTGGCCCTGTTGATCAATAA

Gene: yueH (putative protein)

Position: 979148 to 979399, length: 252 nt, orientation: FORWARD

Perfect match to: (N315-BA000018-[993965:994216], highly conserved allele)

Sequence:

TTGATCAATAAAAGATTTATTGATGAAGGTAAAACTATTGATGTTTATTTATTCGAAGCATTAAATAACCAGATAATCATTGCTATACCAGAT
TGGTTTTGGTCATATCAGATGGCAATGACATTAGATGAAGAAACTTGTTTTGAAGCAATACTCATGCAATTGTTTGTTTTTAAAGAAGAGGA
AGAGGCAGAATCGATTGCATCACAACTAACAGATTGGATAGAAACATATAAAAAGGAGAAAGACTAA

Gene: prfC (peptide chain release factor 3)

Position: 979399 to 980961, length: 1563 nt, orientation: FORWARD

Perfect match to: (N315-BA000018-[994216:995778], highly conserved allele)

Sequence:

ATGAACTTAAAGCAAGAAGTTGAGTCTAGAAAGACTTTTGCGATTATTTCACATCCCGATGCAGGGAAAACAACGTTAACTGAAAAACTAT
TGTACTTCAGTGGTGCTATTCGTGAAGCGGGTACAGTTAAAGGGAAGAAGACTGGTAAATTTGCGACAAGTGACTGGATGAAAGTTGAAC
AAGAGCGTGGTATTTCTGTAACTAGTTCAGTAATGCAATTTGATTACGATGATTATAAAATCAATATCTTAGATACACCAGGACATGAAGAC
TTTTCAGAAGATACGTATAGAACATTAATGGCAGTTGACAGTGCTGTCATGGTCATAGACTGTGCAAAAGGTATTGAACCACAAACATTGA
AGTTATTTAAAGTTTGTAAAATGCGTGGTATTCCAATCTTTACATTCATTAATAAATTAGACCGAGTAGGTAAAGAACCATTTGAATTATTAG
ATGAAATCGAAGAGACATTAAATATTGAAACATACCCTATGAATTGGCCAATTGGTATGGGACAAAGTTTCTTTGGCATCATTGATAGAAA
GTCTAAAACAATTGAACCATTTAGAGATGAAGAAAATATATTACATTTGAATGATGATTTTGAGTTGGAAGAAGATCATGCAATTACAAAT
GATAGTGCTTTTGAACAAGCGATTGAAGAATTAATGTTGGTTGAAGAAGCGGGTGAAGCCTTTGATAATGACGCGCTGTTGAGTGGAGAC
TTAACACCTGTATTTTTCGGTTCAGCTTTAGCTAACTTTGGTGTACAAAATTTCTTAAATGCATATGTTGATTTTGCGCCAATGCCAAATGCGA
GACAAACAAAAGAAGACGTTGAAGTAAGCCCGTTTGATGATTCATTTTCAGGATTTATCTTTAAAATTCAAGCCAACATGGACCCTAAACAC
CGTGATAGAATTGCCTTTATGCGTGTCGTTAGTGGTGCATTTGAACGTGGTATGGATGTTACTTTGCAACGTACTAATAAAAAGCAAAAGAT
CACACGTTCAACGTCATTTATGGCAGACGATAAAGAAACTGTGAATCATGCTGTAGCAGGCGATATCATTGGACTATATGATACTGGTAATT
ATCAAATTGGAGATACTTTAGTTGGTGGAAAACAAACCTACAGTTTCCAAGATTTACCACAATTTACGCCAGAAATTTTTATGAAAGTTTCTG
CTAAAAACGTCATGAAACAGAAGCATTTCCATAAAGGTATTGAACAATTAGTACAAGAAGGTGCGATTCAATACTATAAAACATTACACAC
AAACCAAATTATTTTAGGTGCTGTTGGTCAGTTACAATTTGAAGTTTTCGAACATAGAATGAAAAACGAATATAATGTTGATGTTGTTATGG
AGCCAGTAGGCCGTAAAATTGCACGTTGGATTGAAAATGAAGACCAAATTACAGATAAGATGAACACATCAAGATCGATTTTAGTGAAAG
ATAGATATGACGATTTAGTATTCTTATTTGAAAATGAATTTGCAACAAGATGGTTTGAAGAGAAATTCCCTGAAATTAAATTGTATAGTTTAC
TTTAA

Gene: terC (putative integral membrane protein, TerC family)

Position: 981263 to 982066, length: 804 nt, orientation: FORWARD

Perfect match to: (N315-BA000018-[996080:996883], highly conserved allele)

Sequence:

ATGTTAATGGATCCAAGTTTGATCTTACCTTATTTATGGGTACTTGTCGTTTTAGTATTTTTAGAAGGCTTATTAGCAGCAGATAACGCGATT
GTTATGGCTGTAATGGTTAAGCACTTACCACCCGAACAACGTAAAAAAGCTTTGTTTTACGGTTTGTTAGGTGCATTTGTATTTAGATTTTTA
GCATTATTCTTAATTAGTATTATCGCGAACTTTTGGTTTATTCAAGCTGCAGGAGCGGTTTACTTAATTTATATGTCAATCAAAAATCTGTGG
CAGTTCTTTAAACACCCAGAAATTGAAAGTCCTGAAGCTGGAGATGATCATCATTATGATGAATCTGGTGAAGAGATTAAAGCAAGTAACA
AATCATTCTGGGGAACTGTGTTGAAAATAGAATTTGCAGATATCGCATTTGCCATTGATTCTATGCTTGCTGCTTTAGCTATTGCTGTAACAC
TTCCTAAAGTTGGTATTCACTTTGGTGGTATGGACTTAGGTCAGTTCGTAGTCATGTTCCTAGGTGGAATGATTGGTGTTATTCTAATGCGTT
ATGCAGCAACATGGTTTGTAGAGCTATTAAACAAATATCCAGGACTTGAAGGTGCAGCCTTCGCGATCGTTGGTTGGGTAGGTGTTAAATT
AGTTGTCATGGTATTAGCGCACCCAGACATCGCTGTATTGCCTGAGCACTTCCCACATGGCGTATTATGGCAATCTATTTTCTGGACAGTACT
AATTGGATTAGTAATTATCGGTTGGTTAGGTTCAGTTGTTAAAAATAAAAAATCGCATAAATAA

Gene: htrA (serine protease)

Position: 982300 to 984609, length: 2310 nt, orientation: FORWARD

Perfect match to: (CIGC345D-AHVO01000006-[870790:873099], allele observed in CC8+CC15)

Sequence:

GTGGATATTGGTAAAAAACATGTAATTCCTAAAAGTCAGTACCGACGTAAGCGTCGTGAATTCTTCCACAACGAAGACAGAGAAGAAAATT
TAAATCAACATCAAGATAAACAAAATATAGATAATACAACATCAAAAAAAGCAGATAAGCAAATACATAAAGATTCAATTGATAAGCACGA
ACGTTTTAAAAATAGTTTATCATCGCATTTAGAACAGAGAAACCGTGATGTTAATGAGAATAAAGCTGAAGAAAGTAAAAGTAATCAGGAT
AGTAAGTCAGCATATAACAGAGATCATTATTTAACAGACGATGTATCTAAAAAACAAAATTCATTAGATTCAGTGGACCAAGATACAGAGA
AATCAAAATATTATGAGCAAAATTCTGAAGCGACTTTATCAACTAAATCAACCGATAAAGTAGAATCAACTGAAATGAGAAAGCTAAGTTC
AGATAAAAACAAAGTTGGTCATGAAGAGCAACATGTACTTTCTAAACCTTCAGAACATGATAAAGAGACTAGAATTGATTCTGAGTCTTCA
AGAACTGATTCAGACAGCTCGATGCAGACAGAGAAAATAAAAAAAGACAGTTCAGATGGAAATAAAAGTAGTAATCTGAAATCTGAAGTA
ATATCAGACAAATCAAATACAGTACCAAAATTGTCGGAATCTGATGATGAAGTAAATAATCAGAAGCCATTAACTTTACCGGAAGAACAGA
AATTGAAAAGACAGCAAAGTCAAAATGAGCAAACAAAAACCTATACATATGGTGATAGCGAACAAAATGACAAGTCTAATCATGAAAATG
ATTTAAGTCATCATATACCATCGATAAGTGATGATAAAGATAACGTCATGAGAGAAAATCATATTGTTGACGATAATCCTGATAATGATATC
AATACACCATCATTATCAAAAACAGATGACGATCGAAAACTTGATGAAAAAATTCATGTTGAAGATAAACATAAACAAAATGCAGACTCGT
CTGAAACGGTGGGATATCAAAGTCAGTCAACTGCATCTCATCGTAGCACTGAAAAAAGAAATATTTCTATTAATGACCATGATAAATTAAAC
GGTCAAAAAACAAATACAAAGACATCGGCAAATAATAATCAAAAAAAGGCTACATCAAAATTGAACAAAGGGCGCGCTACGAATAATAAT
TATAGTGACATTTTGAAAAAGTTTTGGATGATGTATTGGCCTAAATTAGTTATTCTAATGGGTATTATTATTCTAATTGTTATTTTGAATGCCA
TTTTTAATAATGTGAACAAAAATGATCGCATGAATGATAATAATGATGCAGATGCTCAAAAATATACGACAACGATGAAAAATGCCAATAA
CACAGTTAAATCGGTCGTTACAGTTGAAAATGAAACATCAAAAGATTCATCATTACCTAAAGATAAAGCATCTCAAGACGAAGTGGGATCA
GGTGTTGTATATAAAAAATCTGGAGATACGTTATATATTGTTACGAATGCACACGTTGTCGGTGATAAAGAAAATCAAAAAATAACTTTCTC
GAATAATAAAAGTGTTGTTGGGAAAGTGCTTGGTAAAGATAAATGGTCAGATTTAGCTGTTGTTAAAGCAACTTCTTCAGACAGTTCAGTG
AAAGAGATAGCTATTGGAGATTCAAATAATTTAGTGTTAGGAGAGCCAATATTAGTCGTAGGTAATCCACTTGGTGTAGACTTTAAAGGCA
CTGTGACAGAAGGTATTATTTCAGGTCTGAACAGAAATGTTCCTATTGATTTCGATAAAGATAATAAATATGATATGTTGATGAAAGCTTTC
CAAATTGATGCATCAGTAAATCCAGGTAACTCGGGTGGTGCTGTCGTCAATAGAGAAGGAAAATTAATAGGTGTAGTTGCAGCTAAAATTA
GTATGCCAAACGTTGAAAATATGTCATTTGCAATACCTGTTAATGAAGTACAAAAGATTGTAAAAGATTTAGAAACAAAAGGTAAAATTGA
CTATCCCGATGTAGGTGTTAAAATGAAGAATATTGCCAGTCTAAATAGTTTTGAAAGACAAGCAGTTAAATTGCCAGGAAAAGTTAAGAAC
GGTGTTGTTGTAGATCAAGTTGACAACAATGGTTTAGCAGATCAATCTGGTCTGAAAAAAGGTGATGTAATTACTGAATTAGATGGCAAAC
TTTTAGAAGATGATTTACGCTTTAGGCAGATTATATTTAGTCATAAAGATGACTTGAAATCAATTACAGCGAAGATTTATAGAGATGGTAAA
GAGAAAAAAATTAATATTAAACTAAAATAA

Gene: ktrD (potassium uptake protein D)

Position: 984626 to 985984, length: 1359 nt, orientation: FORWARD

Perfect match to: (Strain_21333-AHKA01000047-[175249:176607:r], allele observed in CC80)

Sequence:

GTGTCAATTTTTTAGCCAGTTTTTAAAAAGATCAAGCCCTCAACAAGGTATTGTATTGTACTATATCGTCGCAATTGTCATTGCATTTTTATTAT
TAAACTTACCGTATGTTCATAAACCAGGTGTAGAAGTAAATCCAATTGACACATTATTTGTTGCCGTATCCGGAATTAGTGTTACTGGATTGT

CTCCGATAAGTATTGTCGATACCTATTCTACATTTGGACAATTAATTATCCTCGTGATATTAAATATTGGTGGAATTGGCGTCATGGCAATTG
GTACGATGTTATGGGTGGTACTAGGTAAACATATTGGAATTAGAGAACGTCAGTTAATTATGTTAGATAATAACAAAAACACTATGAGTGG
TACCGTCAAATTGATTATTGATATTGTAAAATCAATATTTGTAATCGAACTCGTAGGAGCCATGTTATTAGCATTTTACTTTTATCGAGATAA
TCCAGATTTAAAATATGCAATCATGCAAGGTGTTTTTGTTTCTATTTCTGCCACTACCAATGGTGGATTAGATATTACAGGTAAGTCATTAAT
TCCTTATGCACATGATTATTTTGTACAAGCGATAGTTATATTTTTAATAATTTTAGGATCAATCGGCTTCCCAGTATTATTAGAAGTTAAAGCT
TATATTCAAAATAGGGTTACTAATTTTAGATTTTCATTATTTACTAAAATTACGACATCAACATATTTATTCCTATTTATTGTTGGGGTATTAG
CCATTCTATTATTTGAACATAACCATGCGTTCAAAGGTTTAAGTTGGCATCAATCGTTATTCTATTCGCTGTTTCAATCAGCGACTACAAGAA
GTGCGGGTCTTCAAACAATTGATGTGACAACACTAAGTGACCCCACTAATATTATCATGGGTATTTTAATGTTTATAGGATCTTCGCCAAGTT
CGGTTGGTGGCGGTATTCGTACAACAACTTTCGCTATTTTAATTTTGTTTTTAATTAACTTTAGTAATAATGCCGATAAAACATCCATTAAAGT
TTACAATAGAGAAGTACACATTATGGATATTCAACGTTCATTTGCAGTATTTACAATGGCGACAATTTTAACATTTTTAGGAATGCTAATTAT
ATCAGCTACTGAAAATGGTAAGCTTACATTTTTACAAGTATTTTTTGAAGTCATGTCTGCATTTGGAACTTGTGGACTATCGCTTGGTGTCAC
AAGTGATATTAGTGATATTTCTAAGGTCGTACTAATGATACTCATGTTTATAGGACGTGTTGGCTTAATATCATTTATCATTATGATAGCAGG
ACGTCGAGAACCAGATAAATTCCATTATCCAAAAGAACGTATTCAAATAGGATAA

Gene: Q1XY52 (putative 5'-nucleotidase)

Position: 986120 to 987634, length: 1515 nt, orientation: FORWARD

Perfect match to: (11819-97-CP003194-[1032866:1034380], allele observed in CC80+CC239)

Sequence:

ATGATGGAAAAAAATGAAAACATTAATGTAGAGATTTTAACTACGTCAGATATGCATAGTCATTTCTTAAATGGTGATTATGGTTCAAATAT
TTATAGAGCTGGTACTTATGTTAACCAAGTAAGAGCACAAAATCATCGCGTCATTTTATTAGATAGTGGCGGAAGTTTAGCTGGCTCGTTAG
CGGCCTATTATTATGCTATTGTTGCACCTTATAAACGACATCCAATGATAAAGTTAATGAACAGAATGCATTATGATGCTAGCGGTGTGAGT
CCAAGTGAATTCAAGTTTGGTTTATCATTTTTAACTCGTTCAATTGCTTTGGCACGTTTTCCATGGTTATCAGCAAATATTGAATACAATGTTA
CTAAGGAGCCTTATTTTTCAACTCCATATTGTATTAAACATTTTGGTGACTTAAAAATTGCTATCGTAGGCGTCACAGCAGATGGTTTAATGG
AAAATGAGTATTCTGAAATGGAGCAAGATGTATCTATTGAAAAGACATTAGTGGCATCAAAACGTTGGATTAGATATATCCATGAAGTTGA
AGAGCCAGATTTTTTGATTGTAATTTATCATGGTGGATTGAATAAAATTAGTAATAGTACGAAAAATAAAAAGGCAAGTTCGAATGAAGCT
GAAAAATTAATGGAAGAACTCGGTGTTATAGATTTAATGATTACAGCTCATCAGCATCAAACAATAGTAGGTCAAGATCATGAAACGTATT
ATGTTCAGGCTGGTCAAGATGCCAAAGAGCTTGTACATCTTTCGATTAATTTTAAAAAGAGAACAACAACTTATGATGTTGAAAGCATTGAT
TCTAAAGTGATTGACTTAAATGAGTATGAAGAGGATCAAGAATTATTAGATTTAACATTCTATGATAGAAAAGCAGTGGCTTATTGGTCACA
GGAAATCATAAGTGATAAAGGTTTGATGTTATCAGTAAATGGGTTACAAGATTTAGTCTGTCAAACACATCCATTTTCGCAATTATTACATG
ATGCAATTCACCTTGCATTTGATAATGATATAACATGTGTCCACGTGCCTATGAACGGAGAGAAGGGGTTGAGTGGACAGATTCGAAATGA
AGATTTGTATCATGCATACCCATATCCAGATAAGCCAATGGATATGACAATTAGTGGTCAAAATATCAAAGATATATTGGAGTATAGTTATT
CACATTTAGATTTTGTTAACGAGCAATTAAGCTTAACAATTATTGATGAAACGTTATGTACAATGTGGCAAGGATTCAATTATGAGATTGAT
ATGAATCAAGAACCTGGGCAACGAGTAATGTTAGATCAAATTGATTTGACTAAGAGTTATAGAGTTACAATGACTGACTATTGTTATCGTAA
CTACAAGAATTATTTGAAAAATGCTATTATACATGAATCATACGATGAAACAATGAGTACATTAATTGCAGAGAAGTTAAGAGATCCGAATT
ATCGTATTTCATGTAGTGATAATTTTGTAGTTAAAAACAGGTAA

Gene: comK (putative competence transcription factor)

Position: 988121 to 988690, length: 570 nt, orientation: REVERSE

Perfect match to: (11819-97-CP003194-[1034867:1035436:r], highly conserved allele)

Sequence:

TTATTTATCTTCAAATAAAGAATAGCGTGTCAATGCTTCAAACACATTCAATGTGGCTTTATTATAATCTATTGGTTGGTCGGGATTTTTTGAT
ATCATGCGAGATTGTTTATCTACCATGTAATAGTAAAAAATGGAATTGTTATATTGATGCCATAAACTGTGGTATGAAACATGAAGAATGAT
AGATTCATTATTAATAAATGTCACTTTACATTTACGATTTTTTAATTCTTTAATACTTTCGATATAATGCATATTTAACCAAATATTTTCATTTTG
TCTGTCAGAATGTGTTGGGAAAAAATAAGTTGGAAATAATGGTGTTAGTAAAATAGGTGGTTTACTAGAAATGCCAGTAATGCGGTTTGTC
TCTGCTTTCTTGCCAAGATAAGTATTACCATAAAATTTGCAAGAGCGTTCAATGATTTTCTGGACTTTAAAAGGATTTTGAATACGCGTTTTG
TCAAACCGAATTATTTCACTACCGTTTCTTTGATCGTCATCATCAAATGCTGGTCGAATAACCATGTCTCCTTTGCGTATCACATAAATATTTT
GAGAATACAT

Gene: Q6GI58 (putative protein)

Position: 988891 to 989118, length: 228 nt, orientation: FORWARD

Perfect match to: (N315-BA000018-[1003708:1003935], highly conserved allele)

Sequence:

GTGGTTTCTATGAAATACAATACTAATGTTAAACATACAACTTTAGAAGCGTTTGTCACAACTGTCAATGATTTGGGTATTGAATTAATTATC
AATGAAGCACTTCGAGAGGTAAGAAAACGACAGCTCATAGAACTTATAGATGACGCACTCGTCAATAAAGATGAAGCAGCATTTAATCAAT
ATACGGCAGAATACAAAAATTTGGAGGCATTTCTCGGTGAATAA

Gene: lplA2 (lipoate-protein ligase A, locus 2)

Position: 989199 to 990185, length: 987 nt, orientation: REVERSE

Perfect match to: (11819-97-CP003194-[1035945:1036931:r], allele observed in CC80+CC59+CC80+CC772+CC2249)

Sequence:

TTATGACATTAATCTAATTAATTCATGTCTATCTATGTCACCGAAATAATGATATAAATCATATTCTGATAGTGCTTCTTCTATGTGCTCAAAG
TCGTGTAGACAGCCAACTAATGCATTTTCAAGATCAGTGACATCTCCGACACCAAAGAAATCACCGAAAATTTTTGCATGTTCGATTTTACCT
CGTTTAACATCAAACTTAATTTGTACAAATCCTTTTTCAAATTTTTCCTCGCGTTCAAAGTTATATTTAGGGTTTCTACCATAATTCCATTCCCA
AGTTCTATATTTGTCGTTACTTAACTTTTCAATATTTTCCCAATCTTCATCCGTTAATTTATATTCTTCTACTTCAGTTTCTCCAAAGATAGTTTT
CAAGATGATTTTTTTAAATTCTTCAATTTCTAAAGGGTCATTTAAAAATTCTTGAATGTTTGCTACTCGTTTACGAACAGATTTAATACCTTTT
GATTTAATCTTAGCTGGATTAACTTTTAGTGCATTCTGAACTTCGTCTAAATCACTATTTAACATCAACGTACCATGACTAAACATTCTATTTT
TAACTTTAACCATAGCATTCCCTGAGATTTTCGCTTGTCCAACTTGAATATCGTTACGACCAGTTAATTCAGCATTTACGCCTAAAGATTGTAA
TGCTTGAACAATTGGTTCAGTGAATTTTTGGAAATTGTGGAAACTGTTACCATCATCATCTGTTATAAAACTAAAGTTTAAATTGCCAGTATC
ATGATAAACAGCGCCACCACCAGAAATTCTTCTAACTACATCGATGTTATGAGCATCTATATATGTCTGATTTACTTCCTCTATCGTATTTTGA
TTCTTTCCAACAATGATAGATGGTCTATTTATGTAAAATAAAAAGTAACTTTCTTCTGCTGGTAAATTTTTTAAAACATATTCTTCCATTGCTA
AGTTTAAAGTTGGATCTGTAATATTATTATTACTAATGAATTTCAT

Gene: Q6GI56 (putative protein)

Position: 990384 to 990560, length: 177 nt, orientation: FORWARD

Perfect match to: (RF122-AJ938182-[972000:972176], highly conserved allele)

Sequence:

ATGACTGTTGCAGAAGTGGGTAACATTGTTGAGTTTATGGATGGATTAAGAGGTCGTGTTGAAAAAATCAACGATAACTCTGTTATTGTTG
ACTTAACAATTATGGAAAATTTTAATGACCTTGATTTACCGGAAAAAACTGTTATCAATCATAAACGATATAAGATTGTTGAATAA

Gene: Q5HH56 (putative protein)

Position: 990575 to 991177, length: 603 nt, orientation: FORWARD

Perfect match to: (11819-97-CP003194-[1037321:1037923], allele observed in CC80)

Sequence:

ATGAATAAAATCTCGAAGGCTTTAACTTGGTTTATTATAAGTTTCATTATATTTCATCTCATATTATTTATTATGTGGGGCGAACACCAAGAA
TACTGGTATTTATATACAGGTATAATGCTAATTGCTGGTATCAGTTATGTATTTTATCAAAGAGATATTGAATCTAAGCGATTGCTTACATCA
ATTGGTGTTGGTATTATTACGGCAATTATTTTAATTATGCTTCAACTTTTATTCTCACTTATAACTTCTAATTTAAGTTATAGTTCATTAATTAA
AGAATTAGCAAGAACAGGTGTCAATTGGAAGTGGCAAATGTTAGTAACTTTACTTTTTGTCATTCCATGTCACGAGTTATATATGAGAACTG
TTTTACAAAAAGAATTAACACATTTTTCAATACCGAAATGGATAGCTATATTAATAACTGCAATTTGTTCTAGTTCATTATTTATTTATTTAGA
TAATTGGTGGATAGTAACATTTATATTTGTTGCACAAGTGATATTATCATTAAGTTATGAGTATACTCGACGCATAGCAACAACATCCGTTGC
GCAAATTGTTGCTATTATATTGTTATTAATTTTTAATGCTTAA

Gene: rli28-L1 (Listeria sRNA rli28 homolog)

Position: 991339 to 991521, length: 183 nt

Sequence:

ATAAAAATGGTATAATATTTTTGAGCAAGTTGAATTGATGGTGGCTATCTGAGTAAAGGGGGGTGGTGCCTATGGCATTACTTAACACTTTT
GAAAGGAAAAGCCTATTGTGATATCTATTGCAAACGCATTACATTTAATGTTAAGTTTCGGTATGTTTATCGTCACTTTCATTGGTATAGT

Gene: txpA-var1 (toxin involved in plasmid maintenance)

Position: 991448 to 991555, length: 108 nt, orientation: FORWARD

Perfect match to: (N315-BA000018-[1006264:1006371], highly conserved allele)

Sequence:

GTGATATCTATTGCAAACGCATTACATTTAATGTTAAGTTTCGGTATGTTTATCGTCACTTTCATTGGTATAGTAGTAGCAATAATAAATTTA
AGCAATAAAAAATAA

Gene: Q7A194 (putative exported bacteriocin)

Position: 992182 to 992477, length: 296 nt, orientation: TRNC-FRWD (no start codon)

Perfect match to: (11819-97-CP003194-[1038928:1039223], highly conserved allele)

Sequence:

AGGTGTTTTTATAGAAGAATAAAATTTTTGGCACTATATTATATTTAACTTTAGCACTTGGATTATCAACAGCAGCTTATGCATCTACAGAAT
ACGCAGAAGGAGGCACTTGGAGTCATGGTGTCGGCAGTAAGTATGTTTGGTCGTATTATTATCATGGTCATAAAGGACATGGTGCAACAG
CTATTGGAAAATATAGATCATTTAGTGGTTATACAAGAGCTGGTGTAAAAGCAAAAGCATCAGCTACTAAACATAATTGGTGGGTCAATAG
AGCGTATTATAACATTTATTAA

Gene: yujE (putative bacteriocin-associated integral membrane protein)

Position: 992521 to 994485, length: 1965 nt, orientation: FORWARD

Perfect match to: (11819-97-CP003194-[1039267:1041231], highly conserved allele)

Sequence:

ATGAAATGGTTCAAATTAATACTTGATGTTACAATCTTCATTCTGATTGCTATATTGTTATTTGTTTATACATATAAAGAAAACGAAGAAATA
TTGCCTGATACTAAATACCCTATAGCGGTAACTGACTGGAATAAAAAATATAGTAAAAATGAGATTTATAAACGTATAGATCAATTCGCTAA
AAATGAGAACGTAGCAATCTATAAATCAACTTCAAATTATACAAACAAAAACGTAGATAAAGATATATATGTATTTAATAAATCAAAAGCAG
CAACTATCACTCCTTTTAATGCTAAATATAACATTCATTATTTAAGTGACGACGAAGTATTAAAAAAAGATATCAAAGGGAGTTATTTTGTAA
AAGACAAAAATTTTGACGTGTCTAAATTCATAAATTTTTTAAAAGAATATGGTGTTACTGCTGAATCATACAAAATAGATCATATGATGATT
GCCGTTGGTGTCATTAAACAAATGAATATAGTAGTTCCATTATCCTCACTTTTAATCGTTTATTTTATTTATTATATATTTCGAAAAGAACATTA
ATTTCAAAGCGTATGCGATTAAGTATTTAAATGGTTTTACATTAAGAAAAATAATTTTCGAAAATTTTTCAAAGAAATGCACGTATTGGGTA
ACGTTAATCATAACTCAAATTCTTTTAACTACAAGTGTACTTTGGATATTAAATTACACGGGTAATTTAGATTTATTCATATTAAGAATAGTTC
TGCTTTCATGTCTTTTCATTTTAACGATTAGTGTTATTAATTTATGGACTTTCTTAATGTTACTAAATTTAAATATTGCAAATATGATTAAAGGT
AAGCAGCACTTTAAAACAATTCGTTTTATTAATACAGTTTGTAAAAGTATTCTCTTAGTACTAATAGCTAATGTAATGATAGAAAATACTAGT
GTTATTAAAGATTTGAATAAAATAAAAGAAACTGAGAAATATTGGAATGTATTAGATGATTATTACACGATTGAATTTGCACCTTATCACGA
AACAAAACAAAGTTTGATTGATAATATGGTGCGATCAGAACAATTAGTAAAGGCTAGTGAAGCAGAAAATAATGCGATTTTATTCAAACCA
AAGGGTGACTCCGTTGACAATGACAACTTTTCGCCTGATGAGGGGAATGTAATATTAGTAAATAATCAGTTCTGGTCGATTTATTACAAGCA
GTTTCAACCTGATATTCCGATAAAAAATCAAAAAAATAATGTCGAAGTAATTATTCCACAAAAGTTTCATGCAATGCGTAATGAAATCAATC
AAGCATATCATTCATGGTTTGAATTTGTACAAAATAAAAATAATAAAGAGAATAAGTTATCTATACAGTTTATCAACAAAAATGATTGTCGA
ATTTTTTCATTTGATGCACGAGATAGTCGCCATTTGTCATTTATAGAGGCGCCAATCATTGTGAATGTTCAGGCATCAGATTTATCGAATGAT
TTTTATTATGCCATGATCAGTCAAGGCGGGTATTTATTCAAAAATTATGACGCGCTAGTAAAAAATATTGAAAAGTATCATCTTGATGGGGA
AATCAGTGGAATAACCAATTATAAAGATAGCGTGATGGAAATGTATCATGAAAACAATTTGAAATTAACAGTACTCAACTTTTCACAAATCA
TTATCGCAATCATTTTAATAATTATTATTTTATTTGATGTGAAATATTATTTTGAACAGCATCGAAAATTACTCGTAATCAAAAAGCTATATGG
TTATTCAACATTAAGAGCCAATTACCAATACTTATTAATAAATAATATAGTTGTTATTTTTATTGGAATATTGACGAATGTAATTTTACATTCT
CACTATATAATGATGTTATTTGCAACGATTCTTGTCGTTCAAATATTATTGCAGATTTGCAGCCTATACTATCATGGCCGACGTTTTAATGAA
GTTATCAAGGAGTTTTAA

Gene: yxeA (putative protein)

Position: 994488 to 994808, length: 321 nt, orientation: FORWARD

Perfect match to: (COL-CP000046-[1048908:1049228], highly conserved allele)

Sequence:

ATGAAATTTATCATTGCAATATTATTAGGATTAATATTATCCATTACTATTGCTTTTACAATCATACATCATCCTATACTTGATCGTTTTAATCC
TTTCTTAAAAACGGAGTATAGTTATGCCAAAGTGCCAAAAGGTACGCAACAATATGTTAATATTACGGCTTATAGTGAAAGAGGGGAAAAG
CTTGATTATAAATTAACATTTAATGGATTTTCACCTAGTAGAACGTATGTCGAAATAAAGCATAAAGGGCAATATGTCATATCGATCACATA
TGTTGAAAAAGAGGATACACCAAAAGAGGTAAGACAAGAATGA

Gene: yujD (ABC transporter, ATP-binding protein)

Position: 994805 to 995446, length: 642 nt, orientation: FORWARD

Perfect match to: (COL-CP000046-[1049225:1049866], allele observed in CC8+CC72+CC80+CC239)

Sequence:

ATGATAGAATTAAAAGATTTAACCATACAAAAAGGTAATACACATATTTTGAAGAAACTTAATTTGAAATTTCAATGTGGAAAATCATATGC
ACTTATTGGTAAAAGTGGATGCGGTAAATCTACATTATTAAATACTATTGCTGGACTTGAAAAAACAGGAAAACAATATGTCTATTTTAATG
GTCAATTAGAACAATTTAAATCTAATTTTTACAGAGATAAATTAGGATATTTATTTCAAAATTATGGATTAATCGATAATTTGACAGTAAATG
AAAATTTAGATATTGGATTAGCATATAAAAAAATAAGTAAGAAAGAAAAAGAACAAATAAAGATACGTTATATAGAACAGTTTGGTCTGTC
AAACAGTTTAAAAAGAAAAGTTCATACGCTAAGTGGAGGTGAACAACAACGTGTCGCTTTAATTAGAATGATGTTAAAAGATCCGATTGTT
ATGTTAGCTGATGAACCAACGGGTGCGTTAGATCCTAAAACAGGACAGATGATTATTCAATCATTATTTGGTTTGGTCGATGAAAATAAAG
TGCTGATTTTAGCAACACATGATATGGCTATTGCAAATCAATGTGACGAAATAATAGATTTAGAACAGTATAGAAAAGTAGCATCTATGTG
A

Gene: Q6GI49 (putative membrane protein)

Position: 995534 to 995824, length: 291 nt, orientation: TRNC-RVRS (no start codon)

Perfect match to: (11819-97-CP003194-[1042280:1042570:r], allele observed in CC80)

Sequence:

TTATGAAAGTAACAACGGCAAACTGAAAAAAAGCAATATAATAATATTTAATACGATTAGTAATAAATGTAATTGATTTTTCGTTTTAATATA
GCAAAAAACAATGACTAAAGATAACAACAATATCATGATTGCACCTGGGAAGAACCATAATGCTGAAGCTGTGTACCAAATCATTGGTAAC
GCCATAATAATAAAAGATATAAGACAAGAAAGGATACCAATAATATTATAAGTTCTAGTCAAAATTACACCTCCAATTTTCACTTTAAAATG
ACATGCGTAAAAAAA

Gene: Q6GI47 (putative protein)

Position: 996103 to 996390, length: 288 nt, orientation: FORWARD

Perfect match to: (COL-CP000046-[1050523:1050810], highly conserved allele)

Sequence:

ATGGAAAAGGGGATTTTCAATTACGATAATGCGAATGTATTAAAATTAGATACTAATCAGTTAAATGAAAACATAAAAGTCATTGACGATA
TATTTAAAAATTATGAGCAGATAGAGCCAACTATAGAAGTCGAAAATGGAAATACAAAATTAAAATTAAATGGATATTTTATTGCGTCAATT
ATAAGCCCATTAAATTTAAATAAACTTAATAATTTATATGTTGAAGAAGAATTTTATCATACATATAACGAATTAATTGTTAAATATACTGAG
GTAAAGGAATAA

Gene: tarM (alpha-GlcNAc appending glycosyltransferase)

Position: 996396 to 997877, length: 1482 nt, orientation: FORWARD

Perfect match to: (IS-111-AHLS01000124-[10865:12346], allele observed in CC8)

Sequence:

ATGAAAAAAATATTTATGATGGTACATGAGTTAGATGTGAATAAAGGTGGTATGACCTCTTCGATGTTCAATAGAAGTAAAGAGTTTTATG
ATGCGGACATACCTGCTGATATTGTTACTTTCGATTACAAAGGAAACTATGATGAAATTATTAAAGCTTTGAAAAAACAAGGTAAAATGGAT
CGAAGAACGAAAATGTATAATGTATTTGAGTATTTTAAACAAATTTCAAATAATAAACATTTTAAGTCTAATAAATTGTTATATAAACATATT
TCAGAAAGACTAAAAAATACGATTGAAATTGAAGAGAGTAAAGGTATTTCAAGATATTTTGATATAACGACTGGTACATATATTGCCTACAT
TAGAAAAAGTAAATCTGAAAAAGTGATTGATTTCTTTAAAGATAATAAACGAATTGAACGGTTTAGTTTTATAGATAATAAAGTGCATATGA
AGGAAACATTTAATGTAGATAATAAAGTTTGTTATCAAGTATTTTATGATGAAAAGGGATACCCATATATTTCAAGGAATATTAATGCTAAT
AATGGTGCTGTAGGTAAAACTTATGTGTTAGTTAATAAAAAAGAATTTAAAAACAATTTAGCACTGTGTGTTTACTATTTAGAAAAACTAAT
AAAAGATTCTAAAGATAGTATTATGATTTGTGATGGACCAGGGAGTTTTCCAAAAATGTTTAATACAAATCATAAAAATGCTCAGAAATATG
GCGTTATTCATGTTAATCATCATGAAAATTTCGATGATACGGGTGCATTTAAAAAAAGTGAGAAATATATTATTGAGAATGCGAATAAAATT
AACGGTGTAATTGTATTAACAGAGGCACAAAGATTAGATATTCTTAATCAATTTGATGTAGAAAATATTTTCACTATTAGCAATTTTGTTAAG
ATACATAATGCTCCAAAACATTTTCAAACTGAAAAAATCGTAGGTCATATTTCTAGAATGGTACCAACGAAGCGAATTGATTTGCTTATTGA
AGTGGCTGAGTTAGTCGTAAAAAAAGATAATGCTGTTAAATTTCATATATATGGAGAAGGATCTGTCAAAGATAAAATAGCTAAAATGATT
GAAGATAAAAATTTAGAAAGAAATGTTTTTCTTAAAGGATATACAACAACTCCACAAAAAATGCTTGGAAGATTTTAAATTAGTCGTTTCTAC
ATCTCAATATGAAGGTCAAGGGTTAAGTATGATAGAAGCAATGATTTCTAAAAGGCCTGTTGTTGCCTTTGATATCAAATACGGGCCAAGT
GATTTTATAGAAGATAATAAAAATGGTTATTTAATAGAAAACCATAATATTAACGACATGGCTGATAAAATATTAAAGCTTGTTAATAATGA
TGTTTTAGCAGCGGAGTTTGGTTCGAAAGCGAGAGAAAACATTATAGAAAAATATTCAACGGAATCAATATTAGAAAAATGGTTAAATCTT
TTCAATAGCTAA

Gene: Q6GI46 (putative membrane protein)

Position: 997966 to 998322, length: 357 nt, orientation: REVERSE

Perfect match to: (N315-BA000018-[1011007:1011363:r], highly conserved allele)

Sequence:

TTAATAAAATTGTAGCCATTTCTTCCAGCTGTAACTACTAATACAAGCTAAGTTCATTACGATATGTATGATTTCAAATTCATAACCACTTACA
TATCCTCTTGTAAGTTTAAGTGTGAAGATTGAGCCTAACATAACACAAATATAAAAAACATTAACATAAGGCGTTAAAAAGCCGATAATAAG
TGCTAAACCTCCAACAACTTCACCAATTGACATCAATACTGCGATGGTAGGTGGTAAACCTAAAGAGCCTAAGAAGTGAATTGTTCCATCTA
AATCAACAAATTTCAAAGTACCATGCACTAAGAATGATGTTGCTAACATCCATCTAATTAAAAGCATACCTAATTTCAT

Gene: Q5HH47 (ABC transporter, substrate-binding protein)

Position: 998810 to 999769, length: 960 nt, orientation: FORWARD

Perfect match to: (11819-97-CP003194-[1045556:1046515], allele observed in CC80)

Sequence:

GTGAATAGGAATATCGTTAAACTAGTTGTGTTTATGCTAATTTTAGTTGTAGCAGTAGCGGGTTGTGGTCAAAAAGATACTGAAGAGAAAA
CTGAAATGACGACAATAAAAGATGAATTAGGAACTGAAAAAATTAAGAAAAATCCTAAACGTGTTGTTGTATTAGAATATAGTTTTGCTGA
TTATTTAGCGGCATTAGATATGAAACCTGTTGGTATTGCAGATGATGGCAGCACTAAAAATATAACAAAGTCAGTAAGAGATAAGATTGGG
GCATATGAATCGGTTGGATCTAGACCGCAACCGAATATGGAAGTGATAAGTAAATTAAAACCGGATTTGATCATTGCAGATGTTAGCAGAC
ATAAGAAAATCAAATCAGAATTGAGCAAAATTGCTCCGACAATCATGTTAGTTAGCGGTACGGGAGATTATAATGCAAATATTGAAGCATT
TAAAACAGTCGCTAAAGCAGTAGGCAAAGAGAAAGAAGGCGAGAAGCGTCTGGAAAAGCATGATAAAATATTAGCGGAGATTAGAAAGA
AAATTGAACAGAGTACGTTAAAATCTGCATTTGCATTCGGTATCTCAAGAGCAGGTATGTTTATTAATAATGAAGATACATTTATGGGACAA
TTCTTAATTAAAATGGGTATTCAACCTGAAGTCACAAAAGACAAAACTACGCATATTGGTAAACGCAAGGGTGGTCCTTATATATATTTAAA
TAATGAAGAACTTGCCAATATCAATCCAAAAGTTATGATTTTAGCCACTGACGGAAAAACGGACAAAAATAGAACGAAATTCATTGATCCT
GCAGTTTGGAAATCATTAAAAGCTGTGAAAGATAACAAAGTTTATGACGTTGACCGAAATAAGTGGTTGAAATCAAGGGGGATTATCGCA
AGTGAAAGTATGGCAGAAGATTTAGAAAAAATTGCAGAAAAAGCAAAATAA

Gene: sbrA (sigma-B regulated small RNA A)

Position: 999764 to 999960, length: 197 nt

Sequence:

AAATAAAAATACAGCGCTACTCGTAAATCATATAAGAGTGGCGCTGTATTTTTAATTATGTTTATTATCTGTCGGATGTGATGATTTACCTGA
AAGTTTATTTCGAATAAATTTAATTACATAACCGACAAGGATTGTTTTAACAATTCTTTTAATGAATTGGCGCATCGTTACATACCTCATTTCT
CTATATCTTA

Gene: shpA (small basic peptide)

Position: 999815 to 999931, length: 117 nt, orientation: REVERSE

Perfect match to: (RF122-AJ938182-[982280:982396:r], highly conserved allele)

Sequence:

TTAATTATGTTTATTATCTGTCGGATGTGATGATTTACCTGAAAGTTTATTTCGAATAAATTTAATTACATAACCGACAAGGATTGTTTTAACA
ATTCTTTTAATGAATTGGCGCAT

Gene: Q7A191 (short two-transmembrane helix protein)

Position: 1000010 to 1000225, length: 216 nt, orientation: REVERSE

Perfect match to: (N315-BA000018-[1013051:1013266:r], highly conserved allele)

Sequence:

TTAATTTTTAGGGAATATAATATAACCATCTTTATCTGCTTTTTTAGTAAAAATGACAAAAATTGCATGTATTATTGAGATGATGGTAGGGAT
ACCTGTCCAGAAAAATAATAAGTGAAAAAGACCTTGTCCAAATTTATCAGCATAAAATTTATGAATACCTAAACCTCCAAGAAATAATGCAA
CAATAACATAAATGGCTTTATTGACTTTCAT

Gene: Q6GAH2 (GNAT family acetyltransferase)

Position: 1000390 to 1000941, length: 552 nt, orientation: FORWARD

Sequence:

GTGATACGTCAAGCACGTCCAGAGGACCGATTTGATATTGCGAAGTTAGTTTATATGGTTTGGGATGATATGGAATTAGAATTGGTAAAGC
ATCTACCTAAAGACATGGTATTAGATGCAATTGAAAAAAGCTGTGTTGATGCAACATATCGAACTTTTTATCAGCATATTTTAGTTTATGAAG
TAGAAAATAAAGTAGCAGGTTGTATTATTAGCTATAGTGGTGAAAATGAATTGAAATACGAAAAAGCATGGGAACTACTTGACTTGCCAGA
AGAAATAAAACAATATGGCACGCCATTACCTGTAAAAGAAGCTAAAGACGATGAGTATTATATAGAAACAATTGCGACATTTGCAGCATAT
AGAGGTAGAGGCATCGCGACAAAGTTATTAACGTCATTACTTGAATCAAATACACATGTTAAATGGAGTTTGAATTGTGATATTAATAATG
AAGCAGCATTAAAGTTATATAAAAAAGTAGGCTTTATATCTGATGGACAGATTGAATTATACAAGCACATGTATCATCATTTAATTGTTAAA
TAA

Gene: menA (1,4-dihydroxy-2-naphthoate octaprenyltransferase)

Position: 1000993 to 1001931, length: 939 nt, orientation: REVERSE

Perfect match to: (N315-BA000018-[1014034:1014972:r], highly conserved allele)

Sequence:

TTAAATGCCTGCAAATAATGCACTAATATAAATACCTAATGCATATAATAAACCGAAAAATGTATTTGTTTTACCAGCAGCAGCCATTGCTG
GCATCATTGTAGGCGGTGTATCATTCTTCTTGAAACGTCTGATAACTTTAACAGGCATTGGGAATGATAACAACGCAAGTAAGTAAAATAAT
GAGCCACCAGGTTTAATAATGATCGTAAGTACAATAAAGGCATAAGCGATAAAGTACATGATTGCCATAAATGTTAAAGAAGCATTTTTAC
CTAATAGAATGGGTAAAGTTTTGCGACCACTTGCTTTATCTTTGACACGGTCGCGAATATTGTTAGCCATATTAATTAAACCGATAGTGATTA
CTATAGGTACACTTAACCAAATTACATAACTTTGAATATTGCCAGTTTGAATAAAGAATGCAATAACGATAATAAACATACCCATAAATACG
CCTGAGAATAATTCACCGAAAGGCGTCCATGAAATAGGGAAAGGGCCACCTGTATATAGGTAACCAACAGCCATACATACTAATCCAACTG
GTAATAACCAAAATGAAGAGTTAGCAGCTAAAAACAAACCTAATATTGCTGCTAAGATGTAAAATGCAATGGCTAATCGTAGCACAAGCTC
TGGGCTCATACCGTTGCGAACAATGGCACCACCAATGCCTACAGATTCATGATCATCAAGGCCTTTTTTATAATCATAGTATTCATTAAACAT
ATTAGTTGCTGCTTGAATAAGTAAGCATGCTAGTAACATGGCAATGAATAGGCTGATTTTAATATGATCTTCGCTACCAAGAAAATATATTT

TAGATGCTGCTGTACCAACTAAAACGGGTACTACGGAAGCAGTTAATGTATGAGGACGCATTAAATGCCAATATTTCTTAACTGTAGAATAT
TGCTGATATTGATTACTCAT

Gene: menF (putative menaquinone-specific isochorismate synthase)

Position: 1002113 to 1003474, length: 1362 nt, orientation: FORWARD

Perfect match to: (ED98-CP001781-[1026345:1027706], allele observed in CC5)

Sequence:

ATGGCTACGGGCGTATTAGAGGACGATATTGTCAAAGAGATATATGGCAGCTCAAAGGAATGGGTTTCAGTTGAAGTGAAATTATCACAG
TCACTAGACCCGAGCACATTATTTCATCTCACTGACAATGAGGCAGGAGATCGCTTTTATATGCGTTTGAATGATAATCGAACGTCATATTTT
GGCTACAAAGCAATTCAATTATTCAAAAATAATTCTAAAAATAAACAATCTATTTTTAAAGACTGGGAAAAATTAAAACATAACATCACATTT
ATACATCCGCAATCTGAGAAACATCATCTTCGAGTTGTTGGAGGGTTCCAATTTTCAAGTCATAAATCAGATGATGAATGGCGAGAGTTTG
GACTAAATCATTTTGTATTACCTGAAGTTTTAATTTCAACTGATAAAAATGGGACATTTTTAACTTATACAGTTAAAAGGGAAAGTTTTACTG
TTGAGGCATTGAACGATTTAATGGATTTGTTCAACAATATATCGGACATAGATGTGGACGAGCAAATTGGGGAAATTACTAGAAATGAAGA
TATTTATAAAGATGACTGGCGTCAACTTGTAGTAGAAGCTATAGAATCTATTAATAATGAAGAAAAAAATTGTACTAGCACGTAGACGGTTA
ATAAAGTTCGATAAAGATATCAGTATTCCATATATTCTAAAGCAAGCATATTCTAAAGAAAAAAACAGTTATATATTCTTGTTAGAATCACAA
GATTCTATATTCTTTTCACAAACACCTGAACAATTAATAAAGGTCAATAATAAAATACTATCGACTAAAGCTGTAGCAGGTACAATTAAACGT
TCACAAGATGAGGACGAAGATACAAAAAATGTTGAAGCATTTTTAAAAGATAATAAAAACTTAATCGAACATCGATTTGTTGTTGACAGTA
TTTTACATGATATTAAACCTTATATCACTGAATTACATTATGATAAGACGCCTAAAATTCTAAAAAATGATCATTTATATCACTTGTACACTGA
AATAAAGGCGCCACTGAAGGATGATTCGTATATTAGTTTAATTGATAATTTACATCCAACACCTGCTTTAGGTGGCTATCCAAAAGAATTTG
CGATGGATTTTATTGAACAGAAAGAATTTGGTACACGAGGATTATATGGTGCGCCGGTTGGCTATATAGATATATATGATGATTGTGAATT
TATTGTTGCAATTCGTTCGATGCTTATTAAGAAAGCACAAGCAACTTTATTTGCTGGGTGTGGCATTGTTAAAGATTCTGATCCAGATAGTG
AATTGGCAGAAACGAACCTTAAGTTCACACCTATGATGAATGCATTAGGAGTCGATATGAATGGGAAATCATAA

Gene: menD (2-succinyl-6-hydroxy-2, 4-cyclohexadiene-1-carboxylic acid synthase/2- oxoglutarate decarboxylase)

Position: 1003461 to 1005134, length: 1674 nt, orientation: FORWARD

Sequence:

ATGGGAAATCATAAAGCAGCTTTAACGAAGCAAGTTTTTACATTTGCATCTGAGTTATATGCGTACGGCGTAAGGGAAGTAGTTATCAGTC
CGGGATCACGCTCAACGCCACTTGCACTTGCATTTGAAGCACATCCAAATATTAAAACATGGATACACCCCGATGAGCGAAGTGCAGCGTT
TTTTGCAGTTGGGTTAATTAAAGGTAGTGAAAGACCTGTCGCTATATTATGTACGTCAGGTACAGCAGCAGCGAATTATACGCCTGCAATT
GCTGAAAGCCAAATTAGTAGAATTCCATTAATCGTTTTAACAAGTGACCGTCCGCATGAATTAAGAAGTGTAGGCGCACCACAAGCGATTA
ATCAAGTAAATATGTTTAATAATTATGTAAGTTATGAGTTCGATATGCCTATTGCGGATGATAGTAAAGAGACCATTGATGCAATTTACTAT
CAAATGCAAATTGCTAGTCAATATTTATATGGACCACATAAAGGGCCAATTCATTTTAACTTGCCATTTAGAGATCCGTTAACACCTGATTTG
AATGCAACAGAATTGTTAACTTCTGAGATGAAAATTTTACCGCACTATCAAAAAAGTATAGATGCATCGGCATTAAGACACATTTTAAATAA
GAAAAAAGGTTTAATTATTGTAGGGGATATGCAGCACCAAGAAGTTGATCAAATACTAACGTATTCAACGATATATGATTTGCCTATTTTAG
CTGATCCTTTAAGTCATTTAAGAAAATTTGATCATCCGAATGTTATCTGTACATATGATTTGCTGTTTAGAAGCGGCTTAGACTTAAATGTGG
ATTTCGTAATTCGTGTTGGGAAACCAGTGATTTCTAAAAAGTTGAATCAATGGTTAAAGAAAACTGATGCATTTCAAATATTAGTGCAAAAC
AATGATAAGATTGATGTCTTTCCAATAGCACCAGATATTTCATATGAGATTTCTGCGAATGATTTCTTTAGGTCATTAATGGAAGACACGAC
AGTTAATCGCGTAAGTTGGTTAGAAAAATGGCAATGCTTAGAGAAAAAAGGGCGTAAAGAAATTAAATGTTATTTGGAACAAGCTACAGA
TGAGAGTGCATTCGTTGGTGAATTGATTAAGAAAACATCTGAAAAAGATGCGTTATTTATTAGTAATAGTATGCCTATCAGAGATGTAGAT
AACTTGTTATTGAATAAAAATATAGATGTCTATGCGAATCGTGGTGCGAATGGTATTGATGGTATCGTTTCAACTGCACTGGGTATGGCTGT
GCATAAACGAATAACATTATTGATAGGTGATTTGTCATTTTATCATGATATGAATGGACTATTAATGTCAAAATTAAATAATATTCAGATGAA
TATTGTATTATTGAACAACGATGGTGGCGGTATTTTTTCATATTTACCACAAAAAGAAAGTGCAACCGACTATTTTGAACGGTTGTTTGGCA
CACCGACGGGATTGGATTTCGAGTATACAGCTAAGTTATATCAATTCGATTTTAAACGTTTTAACAGTGTTTCAGAATTTAAAAATGCCACAT
TATTATCTGAAACTTCGACGATTTATGAATTGATAACGAATCGCGAAGATAACTTTAAACAGCATCAAATTTTATATCAGAAATTGAGTGAA
ATGATTCATGACACATTATAA

Gene: menH (2-succinyl-6-hydroxy-2, 4-cyclohexadiene-1-carboxylate synthase)

Position: 1005121 to 1005924, length: 804 nt, orientation: FORWARD

Perfect match to: (11819-97-CP003194-[1051867:1052670], allele observed in CC80+CC25+CC72+CC80)

Sequence:

ATGACACATTATAAATACTATGAAGCAAACGTTGAGACCAATCAAGTTTTAGTATTACTGCATGGTTTTCTTAGCGACAGTCGTACTTATTAT
AATCACATCGATAAATATACTGATATCTGTCATGTCATCACTATAGACTTACCAGGACATGGCGAAGATCAGTCTTCAATGGATGAAACGTG
GAATTTTGATTATATTACGACGTTGTTAGACCGAATTTTAGATAAATATAAAGATAAATCAATAACATTGTTTGGATATTCAATGGGTGGGC
GTGTTGCATTATATTATGCAATTAATGGTCACATCCCTATATCTAATTTGATATTAGAAAGTACGTCACCAGGTATTAAAGAAGAAGCAAAT
CAATTGGAACGCCGTCTTGTTGATGATGCACGTGCTAAAGTATTAGACATAGCAGGTATTGAATTATTTGTTAATGATTGGGAAAAGTTGCC
ATTATTTCAATCGCAACTAGAATTACCAGTTGAAATACAACATCAAATAAGACAACAACGATTGTCTCAATCGCCACATAAAATGGCCAAAG
CATTAAGAGATTATGGTACAGGTCAAATGCCAAACTTATGGCCGCGCCTGAAAGAAATTAAAGTACCAACATTAATATTAGCTGGAGAATA
TGATGAAAAATTTGTACAGATTGCGAAAAAAATGGCAAATTTAATTCCTAATAGTAAATGTAAATTAATTTCTGCTACAGGTCATACAATTC
ATGTGGAAGATAGTGATGAATTTGATACAATGATATTAGGATTTTTAAAGGAGGAGCAAAATGACTAA

Gene: menB (1,4-dihydroxy-2-naphthoyl-CoA synthase)

Position: 1005917 to 1006738, length: 822 nt, orientation: FORWARD

Perfect match to: (ED133-CP001996-[1022683:1023504], highly conserved allele)

Sequence:

ATGACTAACAGACAATGGGAAACACTTAGAGAATATGATGAAATCAAATATGAATTTTACGAAGGGATTGCTAAGGTAACAATAAATCGCC
CTGAAGTACGCAATGCGTTTACACCTAAAACAGTTGCTGAAATGATTGACGCATTTTCACGTGCACGTGATGATCAAAACGTTTCAGTTATC
GTATTAACTGGTGAAGGTGATTTAGCATTCTGTTCTGGTGGTGACCAGAAGAAACGTGGACATGGTGGTTATGTAGGTGAAGACCAAATC
CCTCGCTTAAATGTATTAGATTTACAGCGTTTAATTCGTATTATTCCAAAACCGGTTATCGCGATGGTAAAAGGTTATGCTGTAGGTGGCGG
TAATGTACTAAATGTTGTTTGTGACTTAACGATTGCTGCTGATAATGCTATTTTTGGACAAACTGGTCCTAAAGTAGGTTCATTTGATGCGG
GTTATGGTTCAGGATATTTAGCACGTATCGTTGGACATAAGAAAGCACGTGAAATTTGGTACTTATGTCGTCAATACAATGCACAAGAAGC
TTTAGATATGGGTCTAGTAAATACAGTGGTACCTTTAGAGAAAGTTGAAGATGAAACTGTGCAATGGTGTAAAGAGATTATGAAACACTCA
CCAACAGCGTTACGATTCCTTAAAGCAGCTATGAATGCTGACACAGATGGTTTAGCTGGTTTACAACAAATGGCTGGGGATGCAACATTGC
TTTATTACACAACTGATGAAGCGAAAGAAGGCCGTGATGCGTTTAAAGAAAAACGTGATCCTGACTTCGATCAATTCCCTAAATTCCCATAA

Gene: sspC (staphostatin B)

Position: 1006977 to 1007306, length: 330 nt, orientation: REVERSE

Perfect match to: (MW2-BA000033-[1021760:1022089:r], highly conserved allele)

Sequence:

TTATACTAAGCGCTCATAAACGATTGGTCGCGAAGTGCCAATACCTTGTTTGGATGACGTTTGCATGAGGATATGCTGTGTATCATCATAAT
CAAGAATATAAATGATTTCTTCATTATCAATAGATGAAAATTTAATGCGTTGATGTGCCGTATCTATAAAAAGAATATGATATTGATTGTGAC
TTGTATCATCGCCATGACGTATACAAATTGATTTTTGTAATTGATGATTACTCCAATTACCAATGAATAAATTGATATTGGTTTGTTCTAGATG
TGTGAGTTTGGTTGTGTCGTAAACTAAATTTATAAATTGTAGTTGATACAT

Gene: sspB (staphopain B)

Position: 1007344 to 1008525, length: 1182 nt, orientation: REVERSE

Perfect match to: (11819-97-CP003194-[1054090:1055271:r], allele observed in CC80)

Sequence:

TTAGTAACCTATCATTGAACCATACCAGTTATAATCACGATTGAATGATAAATGTAATAGGCTTGAATCTGCATCTTGGATTGATAATTCTGT
ATCCCAAGGATTCCAGTAAATAAGTTTTTCTTGGTCATTAATTTTAGCATTACCAACAACTGCTAGCGCATGTCCTAAATGTGGGTCATTAGG
GTTTTGAGATACACTTTGTGCAAGGATCATAATTCCTACATTATCTTTTGTAAGTTGATCAACTTGTTCATATGATGGTACGCCTTCTTGATAA
TGAATATCTCTGCCTTGTGATTTACCGTATTCAATCATTTGATTAGGGAATGTTGCACAATTAGGAAGGTCTTGCTCACTTACTTCAGGGTAT
AATGTACGCATAATATCATGTGCATTATAAGTGTCTGTATTTTTAGTTGCATTTAATAATGCTGCCATACTGAATCCTGCACACCATGAGTTA
TCGAATTGTTGTTCTCTAATTTTGAAGTTTTTTAATGTATTTTCATATTGAACTTGATCTTCTTGTATCGCTTCGTTTTCTTCAACTTTAGTAGGT
GTTACTGTATTTTTTAACTCTTGTTTCAATTTTGCTGAAACTGTTTTAGCACTTTCTTTTTCTTTTACATTACCAGGTAGAGGCGTAGCTTTTAC
TAATCTAACTTTGCCATCTTCTTCAAAATAAAACCCTTTTTCATCAGTAAGAACAGTGATATTTGAATTTTTATCTTTAATTTGGTCTAAATCTT
TAGCGATGAAGTTTGAAATTTTAACACTGTAATTCATGTCTTCTTTTGATTTGTTTAAATCATCTTTATTTTTAGGGCTAAGTGTTAAAGTATA

AACAATTTTACCGTCTTTTATAACTGGATAATAGTAACTATTATCTTCTTCACCATTAAATTTATAAATTTTAAAAGCTTCGCCAAGTTCATATT
TACCAGTTTTTGCATTACTTTGTTTATCTAATGCTTTAGCATAACCAGCAAATTGTTGTTGTGCTAGATCTTTTACTTTTTGAGGTACTTTGTCA
CTCTTAACATTAATTTCTAGCTGTTTAGAGTGTGTATCGGCTTTGGCTTTATTGTTATTAGCAAATGCGCCTAGTGATAAAATAAGCATTGAA
ACCATTATGATGCTTATAATATTGAATACTCTAGATTTACATGAACTATTCAT

Gene: sspA (glutamyl endopeptidase)

Position: 1008607 to 1009617, length: 1011 nt, orientation: REVERSE

Sequence:

TTATGCAGCGTCAGGGTTGTCTGAATTATTGTTATCGCTATTGTCTGGATTATCAGGGTTGTTAGGGTTGTCAGGGTTATTTGGTTCATCTG
GGTTGTTAGGGTTATCAGGATTGTTAGGGTTATCAGGATTATCTGGGTTATTAGGTTGGTCATCGTTGGCAAAATGGATATCTTCAATATTT
TGTTTTAAGAAGTTGCGTACATTTTCATTAATAAATACCGCACCATTAAATTCATTTGGTACACCGCCCCAATGAATTCCGATCACTTCATTTT
TTTCATTAAATACAGGTGAACCTGAGTTACCACCAGTTGTACTTAAATCATATTGCATAGCTTCGCCTTTGAGGTAAGTGATTTTTCCTTTACT
TTCCCACATTGTTGCTACAGGTTTATCACCAGGATATCCTGTTACAGTAATATTTTGGTTAACTTGTGTTTCAGCATTATTACTCATTGTTGCT
GGTTTAACTACTTCACCAATATGTTTGTTTTGTTCATTAGGGGAGAATTTAACTATTGCTAAATCACCTTCGCCTGAATATTTAGTGATTTGTT
CAGCAGTGAAACCACCATTTGGATAATTGTCTTGGTTAATTGCAGAAGGGAATGCTTTTAAAGCATGAGGATCACCGTGCGTAGCATCTAC
GACGTGTTTATTTGTTAAAAGAGTATCTTTACCTACAACTACACCGGAAGCAATAAATGTACCAGTAGGTGCTTCAACTTGAATATAAGTTA
CGGGTGCATAATGACCATTCATTGTATCTGTGATTTGGTGACGATCATTATTTGGTAATATAACATTTGCGTGTTCACGTTGTTCTAATGGTT
TAAGGTTACCGCCTTTTTGAATCTTAGGTGTTTGCTGTTTGCTTGACTGCGTTTGTTGTGGATGATTGTCCATAGCCTTTGATGATAACGCGT
TTGCTGCTGGAGAACTCACAAGTGTCGCTGTTGTCAAAGTTGCAACGAATAAAGAACTAACTTTTAAAAATTTACCTTTCAT

Gene: aspC (putative aspartate transaminase)

Position: 1010145 to 1011299, length: 1155 nt, orientation: REVERSE

Perfect match to: (N315-BA000018-[1023203:1024357:r], allele observed in CC5+CC1+CC80+CC239)

Sequence:

TTATTTAGTATTTAAATATTTAGCAAGTCTCTTCATACCTTCTTTTAAAACATCCATTTCATAAGCATAGGAAATCCTTACAAATCCTTTACCGA
ATTCTGTAAAGGATGAGCCTGGAACGATTGCTAAATGTGTTGATTCAAGTAAATCGACACAAAATTCGAAATCGTCATCGGTGATATGTTTA
ATACTTGGGAAAATATAAAACGCGCCTTCAGGTTGAGCGGTAATCTCAAAACCTAATTTAGTTAATTCAGATACTAAATAATTTCTTCGTTCT
ACATAAGCTTCGTTCATATATTTAGGAGCTTCTAATCCTTCGTTAAGTGCTGTTATACATGCTATTTGAGCTGGAACATTGGCACAAATACAA
TTATAGGCGTGCATAAATGTTAATTTATCAATCAAATATTGAGGTCCTAATAGAAAACCAATTCTTATTCCGGTTGCTGAGTGTGATTTACTT
AAACCACCAATTAATATTAATTGATCACGAATGTCTTCAAATTCAGCGAAGGATACATGTTTACCACTAAATGTATTTTCAGCATAAATCTCA
TCGCTAATAATAAATATCGGATATTTTTTTAATACATTTACGATATTTAAAACTTCATTTCTTTTTAAAACTACGCCAGTTGGATTAGTCGGAT
AATTTAACAAGACAGCTTTTGTCTTTGGAGAAATATGACTTTCTAATGCATCAGGTGTAATTTTAAATTGTGTTGCTGTTGTATCAATATAAA
TTGGTTTACCACCTAGTACTTCGATGAGTGGTATGTAGCCTGCATAAATTGGTCCCGGTATTATAATTTCATCTCCAGGCTCTATGATAGAAC
GTAACGTTGTGTCTATTGCTTCACTTGCTCCATTTGTCACAATAATTTCTTCAGGATCATAGGAAAAATGATAACGATTTTTGAAGTATTGAC
TAATTGCTTCGCGAGTTTCTAATAACCCTTTATTGTGAGAGTATGATGTCTTGTCATTGTTAATAGCATCAATATATGCCTTTTTCACAACATC
AGGCATCGGGAAGTCTGGTTGGCCAATAGTTAAATTAACACAATCATCCAAATTATTCATACGATTTGAAAATTGGCGAATACTTGGTGCTC
TTAAATATTTAGAATTAGAATTTAAAGAAAGTTTCAT

Gene: ykrP (putative membrane bound acyltransferase)

Position: 1011495 to 1012505, length: 1011 nt, orientation: REVERSE

Perfect match to: (M0239-AIWE01000002-[166017:167027:r], highly conserved allele)

Sequence:

TTATGAATCTTTAAACTGTGATGGTCTTTGTAAATTAATTATTGGATTTGTCCATTTACAAACAAAGTTTGTAGATAATACATATACGATGATT
ACAGATATACTTATTAAATAAAGATAAGTCATTAATGATATAGGATTATCGAATGGGTACCATTCAAATCCTCTAACAATGCCAATAATTAA
ACCATGTAATAAATATACGTATAGCGTACGACTACCAATATAAGTATATAATTTTTTCTTTGTTGACATTAAATTTAGAAACGCAGTCATTGC
GATTAATATAATTCCATATAATATAAGTCGTTTAAAAGGACTGAATATACTCTGTCCTTCATTTTCAAGTGAAGTATATGGTGAACTTCCCAA
TAACCAATCTGCATTGATAGGATGAATCACGTAAACGATAAAAAACAAAATAAAGGTAATGATAGATACTGGTATTAGTTTTTTATTTTTAA
AAATAGCCGTATGTTTTTTGGTGAAAATGTAACCTAGATAAAATATTGGGAAAAATACGATTGTCCTTGAAATGCTTAAGTAGCTATCGATG

TTATCTGAAAAACCTGCTCCAATAGATATAATAATTGAAACTGATAGCACTTTATATGGATTAAATCTTCTAACTATTACTAAAATGACATGA
AAGAAAAATAGCGTGATCAAAAACCATAACGCAAATACTGGGTTAAAAGGATCAAGTTGTAATTCGTCACTTTTACCTGTTAAGAAATAAT
AAATTGAAAAGAATGCAAAAAATATCATATAAGGTACTATCAAACGTTTTGAAATTTTTTCTAAATAGTATGGTTTATCAATATTTTTCGCGA
AATAACCAGATATAAACAAAAATGTTGGCATATGGAAACTATAAATAACTAGGTATAATGCTGATAAATATTTATCGCCACTAGTGTAGGG
TTGTAACATATGTCCAAATACGACTAATAATATTAAAATTGCTCTTGCGTTGTCAAAAAAATAATCTCTCTCTTTTAATGAAGTCAT

Gene: Q5HH32 (helix-turn-helix transcriptional regulator)

Position: 1012658 to 1013077, length: 420 nt, orientation: FORWARD

Sequence:

ATGTATAAACAACTTGAAAAACTTATTACACTGACTAACAATGACTTAAACTTAGTGAATAGAAGATTTGGACAACGCACGGATATCACATC
TGAACAGCTAGAACTTCTCCGTATTTTATTTAATTACGATCGCTTATCACAGTATGATTTGACGATGAAGATTAGCAGGGAACAATCTATAGT
TTCAAGGTGGATTAAGAAATTAGTTTTGAAAGGGTACATCACAAGTCAACAATCTAGCGAAGATTTAAGATGTAAAGAATTAATTTTGACT
GATCAAGCACGTACATTAATTTCACAAATAAATAATGAACGTTGCGAATTGATTGAAGCAAGATGTCAATGTTTATCGGAAGTCGAATTAG
ACAATTTAAATCAATTACTTGATAAGTTAAATCAACGACGCATATCGTTGTAA

Gene: atl (autolysin)

Position: 1013284 to 1017054, length: 3771 nt, orientation: REVERSE

Sequence:

TTATTTATATTGTGGGATGTCGAAGTATTTGCCGACTTCGCCAATTTTATCATAGTAGCCTTTGATGATTTTAGCATTGATGTTAGCCCAATCT
ACATCTGTAGCATATTGGTGTGTTCCTGGATGTGCAGGATTCCATCTCATTTTGTAAAGTGTATTTTGACCAGCTTTTACATATGAGTTGCCG
ATGAATTTAGCACCACCAACGATTGCTTTTGATACTGTGTCCCAACCAGCTTGTTTAGCATATTTAATACCTTCACGTAAAGGATCGTTATCA
TATGCAGCAATACCAAATACGTTATGGTATTTCGTGTTTGAGTTAGTTACAACTTTGTTGTTCACTACATCTGCACCTTTCGCTAATTGAGAA
GTACCGTTACCTGTTTCTAATAGGGCATGTGAGATAAGATAAACTTCATTAATGCCATACATTTGAGCAGCTTTGTTAAATGCAGCACCTTG
GTTTTCTAATACACCTTTACCTTTTAAGAATTGATTAATTTTATCAATAGAAATATTTTGTGGTTGGTCTAAGCGTAAGAATTGATATTTTAAT
GCTGGATCTTGAGCTAAACGCTTCGTATCCATTGCATGCTTAACATCATTAAAGTTAGCATCTGTCCACTTACCTGGTACACGTTGTACTTGT
GGTTTATATTGTAAACCAGCTTGTATTTGAGCAACTTGGTTTAATGTCATACCTGTTTGATTATACTTAATTAATTCTTTAGCTAAATCAGTTG
ATTTAATCCATGCTAATTTACCGTTAGATAATTTACCATAGTACCAAGTTTGTCCATTAATGACTTGTTCTTTAACAACTGCGAATGGTTGTTC
ATTAAATGCTTTTAATGAGTATTTAGCTGTATCAGAATTTGGTGTTACATAGTAATAACCATTACCATTTTTAATTACATAAGTGTAGTTATAA
TCTTTGGCAGCTGATGTAGTTGGTTTCACAGCAGTTGGTGCAGTTAAATCTTTTGCATTTACCCAACCAGTGCGGTTATTAATAGTACCGTAT
AAATAAACATCTTTGCCTACAGATACTTGTTTCGTTGCATTAAATGTACCTTGAGCAATGTTATTGCCTGTTAAAATGACTTGGTTTTTAGTAC
CCCAAGGAACCATTGATAAGCCGTTATTTGATTTATTAACAGTATATTTTTGAGTCGTTTTAACTTCTTTGCCTAAGTTTTGAACATTTAAGTC
TTTTACATTGAACCAACCTAATGGGATGTTATGGCTTGTATTGTTTAATAATACATACGTTTCATTACCATGAGCACGCTCTTTTGTTACATAG
AACGTACGGTCTGCATATTTCGCACCGTTTTTCGCTGTTTTTTCATAAACAGAAGCACGAATACCAGTGTTGTTTGGTTTAACTTGAGCAATC
TTGCTAACTGTTTGAGTCGTTTGTGGTTTAGTAACAGTATAAGCTTTTACAGCTGTTTTTGGTTGTGCTACTGCTTTTTTAGGTGCAGCAGGT
ACAGCTAAATATGCTTTACTAACCCAACCAGATTTACCATTTACAGTTCCAAATAAATAGATAGATTTATCAATTTGTTGTTGCTTAGTCGCTT
TAAAAGTTTGGTTACCTGTACCAGAAACCGCACCAGCTTCTTGTTTATAAGTGCCCCAAGGTACTGAATATAATTTAGTGCCTGGTTTTACTG
TATATGTTTGCATTACATTTACAGGTGATTTTGCATTGTTATAAATAACGTCACCTTGTTTAACCCAACCAATTAAAGTTGGACTATTGTAATC
TTTAACTAAGTAGAATTTGTTTCCACCTAGACTTGCTTCTTTTGTTACAGCAAATGTTTTTTGAACTTCTTTCGTTGGCTTACCAGTTTTGTCAT
AAACTGTAGTGAATAAGCCATTGTTTTTAGCATTAATTTGAGCAACACCGTTTAATGATGAAACTGTTAATTTATTATTTGTTGTAGGTGTTG
ATGGCTTAGGTGTTGGTGTAGGCGTAGGTTTAGCAGTATCAACTAAATATGCTTTACTTACCCAACCAGATTTACCATTCACAGAGCCATAT
AAATAAATTGATTTATCAATTTGTTGTTGCTTTGAAGCCTTAAATGTTTGGTTTCCAGAGCCAGACACGCTACCAGCAACTTGTTTAGATGTA
CCCCAAGGTACTGTATAAAGTTTCGTACCAGGTTTGATTGAATATGATTGATTTACATTTACAGGTGATTTAGCTGTGTTGTAAACCCACATCG
CCTTCTTTAACCCAACCAAATTTATTACCAGAATTGTAATCTTGAACAAGATAGAATTTTTGATTACCTAATGTAGCTGTTTTAGATACAGCAA
ATGTTTTTTGAACTTCATTAGTTGCTTTACCAGTTTTGTCGTAAACAGTAGTATATAAACCACTATTTGTTGGTTTGATTTGTGCGACACCATT
GTTTGCTGCAACTGTTAATTTACCAGTTGATGGTTTCGACGGTGTTGTTGGTTTTGATGGTGTAGTAGGGGGTAGTTGAAGATTGCGTACCCC
ATGGCGCCACTTTACCCATTTTTATTAAATATTTTTCATTAATTAAGTCATATAATTGATCATAACTATAATTATGACTTCTTAAATATCCATGT
GGATCGGCATGGTCCGTACCACCTAAATATTTACTTACAGCGTAGTGAGTCCATACTGTACCATTTCCATCATACTCAGCACTGTCTGGTTTT
AAACCATAATATTGTAATTGTGTAGCTGCATAGTCAGCATAGTTATTCATTGAACGTGCAAATGAAGCATAGTCGTGTGTGTGTACGATTTC
AACATTGATGAATCTAGGGTTACCGACTGCACCGACACCCCAAGATAAGTAATCCGTTGGTGCTGTTTCGATTATACGATCCCCATCAACAA
ATGCATGTACGAATGCGTTTTGATAGTTATTTTTCATATAACTAATTTCACCATTTATCGTCGAACGATCATTAGCTGTATCATGAACTACGAT
ACCTTCAGGACGGCCAACACCGTTACGGTATGCGTATTTAGGGAAGTAAGATGTGTAATCTTCTTCGATTTTAGGTGCTTTTAAATTATTTTT
ACGAATATAATCATTAATTGATGAATTTACCTGAGGTTTATATTTAGGTAAAGACGTCTTTGGTGCTGCAGCAGCTGATCTTGGTTGTGCTG
AAGCGCTAAAAGTAGTTACTTTAGGTGTCGCTTCAGTTTTAGCTTTAGGTGCTGATGTAGTTGCAGCTTTAGGTGCTGCGGCTTTATATTGC

GTTTCAAGAGCTGCAGGTTTAGCAGCTGATTTAATTAATTCTGGATTAATTTGATTTTCTGAATTATCATCTTCATCATCAACTAAACTATAAC
CAGCATTTGTAACATTAGTGTTAGTTTTAGGTGCTGTAGTGCTTGTTGACTTTGTAACAGGCTGCGTATTATTTGTAGTCGCTGATTGATTAG
CACGAGTGTCACCATTTACTTGTGCAGTATCAACTTTTTGACTTACTTGAGCATTGCCTGTTTTGTTATTTGCTGTTTTTGGTTGGACAATAGC
AGGGTCTTGATATACTTGAGTGCCAGAAATGTTTTGCGTTGGATTTTTTACCTCAGCTTTTGCTTGTTCAGTAGTTGATTTAACTTTATTACTA
TCTAAAACGTTTTTATTAGTAGTTTGATCTTGTGTCGTCTCAGCTGCTTGAACTTGATGTGCAGTGACTGCTGAACCTACAAGCGTTAATGCA
ACCATTGATGGTAGTTTGTAATTGAATTTTTTCGCCAT

Gene: UPF0039 (putative N-acteyltransferase)

Position: 1017282 to 1017716, length: 435 nt, orientation: REVERSE

Perfect match to: (N315-BA000018-[1030316:1030750:r], highly conserved allele)

Sequence:

TTAATTAAGCGAGGTTAACTTTTTTGTCATTTCAATATGCTCGATGCCTTCCTCAAGAAATATATTACCTCTCATTTTAAAGTTTAAACTTTCAT
AAAATGGGATAGCATGACATTGGGCATTCATAGTAGCTACGTAAAAACCTTCATCTTTAGCTAATGATTCTACAGCTTGCATAAGCATTCTA
CCCATTCCTTGTCCACGATGTGATTTCATCACAGCTACTCGTTCTATTTTGACAGTTGTTTCATTAATAGGGCGTATTCGAGCAGTGGCAACT
GGCTGTCCATTATCATATCCAATGAGGTGAATAGATTCAGATTCATATTCATCAATTTCACTTTCCTCAGGGACGCCTTGTTCTTCTACAAAC
ACTTTCTTTCTTATATAGAAGCAATCTTCTAACATCTTTTGATTGTTTACTTTTGAAAACAT

Gene: Q5HH29 (putative protein)

Position: 1017871 to 1018341, length: 471 nt, orientation: REVERSE

Perfect match to: (11819-97-CP003194-[1064617:1065087:r], highly conserved allele)

Sequence:

TTATAGTGCTTCTTTTCTAATAATTGCTGTATATTGCCAAAAGATTCGCATTTGAGCAATATTCCAATTATTCATTCCCATTGTAAATCCTGAA
GGCTTAAATATATTCACATCAGTTACTTCTAAAGCTGCTGCAACTAAATATTGTAATGGAATGCTAAGTGATTTCATTGTTTCGGTAATGCCA
TTTTCATCATATACCCATGAAAATGGTAACTCTGATTCGAAAACATCAACACTATTGAAAATTTCAGGTAGGGCTAAAATATAACATGCACC
ATCTACAATAGGATTGTCGATACTATTTTTGTAATAAATTAGGAGTGCTTCATAGTTTTCTCGATGCTCATGATTGACGTAAAAAAATTCACT
TCTAAAATGAGCCATATCTTGGCTATGAATGATTTGTTGTTCTAACATATTAAACATGCCATTTATATTTGCAATCTTTTCGTATGTTTTTCTAC
TCAT

Gene: tagV-lcpB (phosphotransferase)

Position: 1018389 to 1019615, length: 1227 nt, orientation: REVERSE

Perfect match to: (CIG547-AHVQ01000006-[491258:492475:r], allele observed in CC8+CC5+CC97)

Sequence:

TTAATTTACAACACCATTTTGGTTATTTGAAGCTTGTGGCGTTTGTTGAATATTACCTTGATCTTGGTTAGTGTTTTGACCCGTAGTAGGTTGT
TGCTGATTATTGACACCATTGTTGTTATTACTATTATTACTGTCGTCAGTATTATTCGAAGATTTATCTTTATCTGTCGTATCATTTTGTTCTTTT
CTCAATAAACTACTATCTAAAGGCGTTAACGGTATTAATGAACCATAATGATTAATGACACGTTGATCTAAGAAATCATTTTTATCATTAATA
GGTGATAATTCTAAGTCTTTACGAAGTAAGTTTGCATATTTTTGAATGCTTTCAACACTTGGATGATAATAGTAAATACCATTTAACATATCA
TCTTTACCTTTTAATTGCGCAGTTTTAATTTCAACATCATTTGTTAAGTACATTTTTGCTAAAGCTTTAATTTCAGAGTTAGTTAAATTATGCTT
TGCATTTTTACCTACAATTTGAATCACGTTATCAAGTTTATCAATAGAATCAACTTCCTGTGCTTTTTGGAATAAAATCTTAATTAATTCCATTT
GACGTTGTCCACGTTTTAAGTCTGAATCATGATGTCTAGTTCTAGCAACTGCTAAAGCCTCATCACCATTTAATTTTTGGTACCCTTTTTTAAT
TTTAATCTTACCAGTATCATCTGTGTTAGGTTCATTTAAGTCGTATGGCACATCATAGTATATGCCACCAAGCTCATTTACAGCCTCGACAAA
TGCTTTCATATTGACTCTCACATAATAATCAACAGGTACATTCATGGTAGCTTCTACCGAATCCATTGCGGCAATTGGACCACCATATGCATG
TGCGTGGGTAATCTTATCGTAATAGCCAACTTTAGGAATGTAGCTGATAGTATCACGTGGAATACTAAGCATTCTAATTTGATGTTTTGATT
GATTAAAAGTAGTTAAAATCATAGCGTCTGATCTAGAGTGTTCAGCATCCTGTCCTTTTTTTCTTCTTCCATCGTTATCATCGATACCTAAGAA
AAGAATAGAGATAGGTTGTTCTTCGGGATTGACTTTATTATCTCTTAAGTTGGATTGACGATTAGCATTTTTGCTGTCTTGAGAAGATTCGA
ATGCATCTTGGGACGTTTTAAAAAGTAACGTAGCGAAGACTATTGGAACAACAATGAGAACCAATGCTAGAAGGATCAAAAAGTATTTTAA
AAATTTATTCAT

Gene: fmtA (autolysis and meticillin resistant-related protein)

Position: 1020066 to 1021259, length: 1194 nt, orientation: FORWARD

Perfect match to: (N315-BA000018-[1033091:1034284], highly conserved allele)

Sequence:

ATGAAATTTAATAAAGTAAAACTAGTTATACATGCGTGTGTACTATTATTTATCATTATTTCTATAGCGTTAATTTTTCATCGATTACAGACGA
AGACACATTCTATAGACCCAATACATAAGGAAACAAAATTATCAGACAATGAAAAATATTTAGTGGATCGTAATAAGGAAAAGGTTGCGCC
GTCTAAACTAAAAGAGGTATATAATAGCAAGGATCCTAAATATAAGAAAATTGACAAGTATTTACAAAGTTCATTATTTAACGGTTCAGTAG
CTATATATGAAAATGGCAAATTGAAAATGAGTAAAGGTTATGGATATCAAGATTTTGAAAAAGGTATTAAAAACACACCGAATACGATGTT
TTTAATAGGTTCAGCTCAAAAATTTTCAACAGGGTTACTGTTAAAACAGTTAGAAGAAGAACATAAAATAAATATCAATGATCCAGTAAGTA
AATACCTTCCATGGTTTAAAACATCTAAGCCTATCCCATTGAAAGATTTAATGTTGCATCAAAGTGGATTATATAAATATAAATCCTCAAAAG
ATTATAAAAATTTAGATCAAGCAGTTAAAGCGATTCAAAAACGTGGTATTGATCCTAAGAAATACAAAAAGCATATGTATAACGATGGGAA
TTATTTAGTACTTGCGAAAGTAATTGAAGAAGTTACAGGTAAATCTTATGCTGAGAATTATTATACAAAAATAGGAGATCCTTTAAAACTTC
AGCACACAGCATTTTATGATGAACAACCTTTTAAAAAATATCTAGCAAAAGGTTATGCTTATAATAGTACAGGACTTTCATTCTTAAGACCTA
ATATTTTGGACCAATACTATGGTGCAGGTAATTTATATATGACACCAACAGATATGGGTAAATTAATTACTCAAATACAACAATATAAATTAT
TCAGTCCTAAAATAACCAATCCATTATTACATGAGTTTGGTACGAAAAAGTATCCAGATGAATATCGATATGGTTTCTATGCTAAGCCAACA
TTGAATAGACTTAACGGGGGATTCTTTGGACAAGTCTTTACTGTTTACTATAATGATAAGTATGTAGTTGTACTTGCATTAAATGTAAAAGG
AAACAATGAAGTTCGAATCAAACATATTTATAATGATATTTTAAAACAAAATAAACCTTACAATACGAAGGGTGTTATTGTTCAATAA

Gene: qoxD (cytochrome aa3 quinol oxidase subunit 4)

Position: 1021796 to 1022086, length: 291 nt, orientation: REVERSE

Perfect match to: (MW2-BA000033-[1036543:1036833:r], allele observed in CC1+CC12+CC15+CC80+CC772)

Sequence:

TTATAAGTGTGAAGAGTGACCGCCTTGCATAACCCAATATGTTCCGACAACGAAACAAAGTGTAATTACAAGAGCAAAGATAACTTTGAAT
GTTTGTAAACGTCCATCTTTACCTTCAGTTAAATGCATGAACATTAATAATTGAAGTCCTGCTTGGACGAATGCAAAGCCAAAGATAATTGT
CAACTTCGCGTGGAATGTTAATGACGTGTATAGTGTTACGTATACTGCTAAAAGCGTTAATACGATAGATGCGATAAATCCTACAGTATGTT
TCATTATTGTACTCAT

Gene: qoxC (cytochrome aa3 quinol oxidase subunit 3)

Position: 1022083 to 1022688, length: 606 nt, orientation: REVERSE

Perfect match to: (RF122-AJ938182-[1004510:1005115:r], highly conserved allele)

Sequence:

TCATCCGCTATACACCATCCCTATCATATATACGGCAGTAAAGATGAAAACCCAAACAACATCTAAGAAGTGCCAGTATAAACTTACTATAA
ATAATTTTGGCGCATTATATTTGTCTAATCCGCGTCGTTGGATTTGGATTAATAAACAAATGGCCCAAACGATACCTAGCGATACGTGACAA
CCATGCGTTCCTAATAGGATAAAGAAACTAGACCAGTAAGAACCAATTGTTGGGTTAACGCCTTCTGATGCATAGTGTGCGAATTCATAAAT
TTCGAATCCAACAAAGACTAAACCTAAAAGTAACGTAATGATCATCCAAAACATCATTAACTTTTGTTTTTCTTGGCGCATGTAGTAAATAGC
AATACCACATGTGTAAGAACTGAATAATAATGCAAACGTCATTATTAAAACAAGAGGCAATTCAAATAACTCAGTAGTCATTTTACCTGCAT
AATCGCCACCATGTTGCAAAGTTAATAGTGTCGCAAATAGGGTACCGAATAACGCAAATTCGGCTGTAATGAAAATCCAAAAGCCAAGCTT
ATTTAATTCGCCTTCATGTGTGCGTGAATCAATAGTGTTTGTATCATGACTCAT

Gene: qoxB (cytochrome aa3 quinol oxidase subunit 1)

Position: 1022678 to 1024666, length: 1989 nt, orientation: REVERSE

Perfect match to: (MW2-BA000033-[1037425:1039413:r], highly conserved allele)

Sequence:

TCATGACTCATGACTTACAGCCTCCCTTTCTTTAATACGCGCTTCTCTTAATCTAGCTTCAGTTTCAGCAACTTCAGCAGCAGGGATATGATAT
CCGTGATCGATTTGGAAACTGCGATAAATCATAGTACCAAAAATACCGAATAAACAAATTAATGCTGGAATTACAGTTTCGAAAATTAAGA

AGAAACCGCCGATAGTCATAAAGATACCAATCCAGAATCCAACAGGAGTATTGTTTGGCATATGAATGTCTTTGTAATTATGGTTGTCTAAG
TAATGACGACCATGTTCTTTCATATCAACAAATGTGTCGTAGTCATTCCAATCTGGTGTAATGGCAAAGTTGTATTTAGGTGGAATTGCTGA
TGCAGTAGTCCACTCTAGAGTACGACCAAGGCCATCCCAGTTATCTCCAGTTGCTTCACGTGGAGATTTGAAGTGACTGTATACGATACTAA
CAACAAGGAATAAGAATCCGATTGCCATTAATAATGCACCGATAGTTGAAATTAAGTTTAATAAGAACCAACCATCTGATGGCATATAAGT
GTATAAACGACGTGGCATACCATCTAATCCAAGAATGAATTGTGGTAAGAAACAAACGTTAAATCCGATCATGAAGAACCAGAAGCACCAT
TTGTTTAATGTTTCGTTTAATTTGTAACCCATCATCTTTGGATACCAGAAGATTAAACCAGCTAAGCAGGCAAATACAACACCAGTAACCAAT
GTATAGTGGAAGTGAGCTACTAAGAAGTACGTATTGTGATATTGATAGTCAGCTGATGCCATTGCTAACATTACACCCGTAACACCACCTAA
AAGGAAGTTAGGGATAAATGCTAATGAGAATAGCATTGGTGACTCAAATGTAATACGTCCTTTATATAATGTTAATAACCAGTTAAACAATT
TCACACCAGTTGGAATACCAATCAGCATTGTTGAAATTGAGAAGAATGAGTTGATTAACGCACCATTACCCATTGTGAAGAAATGGTGAAC
CCAAACTAAGAAACTAAGGAACGCGATACCGGCAGTTGCCCATACCATACTTTGATGTCCGAATAAACGCTTACGAGCGAATGTCGGGATA
ATTTCTGAGTAAATACCAAATGCTGGAAGGATAACGATATAAACTTCAGGGTGCCCCCATACCCAGAAGAAGTTAGCCCAAAGCATTGGCA
TACCGCCATGTGCAACTGTGAAGAATGCTGTGTCAAATATTCTATCAGTTGTCATTAATGCTAACGCTACTGTTAAAGGAGGGAAAGCAAG
AATAACAATTAATGTAGTAATAAATGTTGTTACTGTAAACATTGGCATTTGCATAAACTTCATAGTTGGTGTTTTACATCTTAAAATTGTTAC
AAAGAAGTTGATACCTGTAGCTAAGGTACCAAGCCCTGAAATTTGTATAGCTATTAAGTAATAGTTAACACCCGGACCAGGACTGAATTCA
CCTGCTAGTGGCGCATAGTTTGTCCAACCAGCTGCTGGTGAACCACCAATAATAAATGACAGGTTGAATAAAATCATACCTGCAAAGAATA
GCCAGAAACTTACGTTGTTTAATACTGGGAATGCAACATCACGTGCTCCAATTTGTAATGGAACAACGATATTCCATAAACCAAAGATAAAT
GGCATTGCCATGAAGATAATCATGATTACACCATGTGTACTAAAAATTTCGTTATAGTGGTTAGATTCTAAAAATTTGTTATCAGGTACTGTT
AATTGCGCACGAATAAGTAACGCATCAATACCACCACGGACGAACATTAATACGGCACAGATTAAATACATAATACCGATTTTCTTATGGTC
TACAGATGTGAACCATTCTTTGTAAAGATATTTCCATAATTTAAAGTAAGTAATTACTGCGATTAAACCAATAACTAAGAATGGGGCACCAA
TTTGTGCCATTGTAATCATCCAGTTACCTTTAACTAGTAATTGATCCCATGGAAAATTCAT

Gene: qoxA (cytochrome aa3 quinol oxidase subunit 2)

Position: 1024666 to 1025766, length: 1101 nt, orientation: REVERSE

Perfect match to: (11819-97-CP003194-[1071412:1072512:r], highly conserved allele)

Sequence:

TTAATGTCCACCTCCATGATCATCATTGTCTTGATCTTGCGCATCTTTTGAAATTTTCTTCATTTCTTTCGCATTTTTCGATTCATCTTTCTTGAA
CTCATTGTTATATGGTTCGTCATTTCCAAGAATCATCAACTTCATACCATGTCGTTTATAGTTCGCATTTGTAATTTGAGCTTTACGAGCAGGT
ATTAATGGTTTGTCTGATACATCTTTAAACATATTTTCTTCACTAGTGAAGTTTGGATCTTTCAATTCGAAATTGAAACGTTTATATGCATAGA
AGATGTATTCTGGATCGGCTGCTGGATCAACAAACGCCATATGTGTACCATTAAATTCTAAAGCTTTATTAGGTGTGCTTGGTAATAATTGT
TTATCAAATGTATCTTGATCTAACGTTTTCTTACCTTTAACTTCTTTCACCCATTTGTCGTAGTCTTTTTGACTAACGGCATTTACTTTAAATGTT
TGACGTGAGAATCCTTCACCATTGAAGTTAGAGTTACGACCTCTGAACGTACCAGTTTGAGATGCTTCTAACGTCCAATTCATTGTCATGCC
AGTCATGGCATATTTTTGACCACCTAATTGTGGAATCCAGAAACTTGTCATTGTATCCATAGCTTGAAGCTTAAATACAACAGGACGATCTTT
AGGGATTGTTAATGTATTAACAGTCTCTATATGTTCATCTGGATAAGCAAAGAACCATTTGTATCCTGCACTTACTGCATATACAACCATTGG
ATCTTTCTCACTCTTCGGTGGTTTTTCGTAATCGTATAAAGTTTTAACTGTAGGAATAGCTAAAGCAGCAACGATTATGATAGGTATTACAAA
CCATATTGTTTCAATGATGGCATTATGGTGCATCTTACCAGATTCGGCATTCTTATTATAACTATACTTGTAAATAAAAATGGCGAACATGCC
AAGTACAACGAAACAAATAACAAGCATGAAGACGATTGAATAAAGAATCAAGAACTTCTGACTACTTGCTACTGGCCCTTTTGCGTTGAAA
ATTTCTATATTTGAACAACCACTAAGTAAAATTAGTGTGCCAAATAATAGAAGCAAAGACTTAAATTTTGACAC

Gene: iraE (chitinase B)

Position: 1026338 to 1026655, length: 318 nt, orientation: REVERSE

Sequence:

CTATTTAACCTCAATATACCTAGTGGAAATTTTAATTGCGCCATCTGAATCAGTGACATGATATTTAATTTGATATTTACCAGATTTAGATGTA
TCAATTTGGCCATCGACTTTAATTTTATCGGTTAAATCTCCATCTTCTTTATCAAATGCACTTATGCCGTTTAGAAGGTTATAATCTTGACCTTT
CTTAATAACGATATCATTAGCGCCTTTAATTTGTGGTGTAGTATTCGTCGTTGCATCTGCATTTAAATTTGGTGTTACTAGTGTAGCAGAAAC
ACCGAGGGCTGATAATGACTGTAATAGTTTATTCAT

Gene: SIRU01 (staphylococcal interspersed repeat unit 1)

Position: 1027061 to 1027229, length: 169 nt

Sequence:

CCAACTTGCACATTATTGTAAGCTGACTTTTCGTCAGCTTCTTTGTTGGGGCCCATACCCCAACTTGCATTGCCTGTAGAATTTCTTTTCGAAA
TTCTCTGTGTTGGGGCCCCTCCCCAACTTGCACATTATTGTAAGCTGACTTTTCGTCAGCTTCTTTGTTGGGGCC

Gene: tx_universal2 (rho-independent terminator)

Position: 1027593 to 1027632, length: 40 nt

Perfect match to: (LGA251-FR821779-[1071185:1071224:r], allele observed in CC425)

Sequence:

TAGAATTGAAAAAAGCTTGTTACAAGCGAATTTTCGTTCA

Gene: folD (bifunctional methylenetetrahydrofolate dehydrogenase and methenyltetrahydrofolate)

Position: 1027693 to 1028553, length: 861 nt, orientation: REVERSE

Perfect match to: (11819-97-CP003194-[1074439:1075299:r], allele observed in CC80+CC5+CC80+CC239+CC772)

Sequence:

TTACGAATCAATACCTCGACGCATTTTTTCTGCAAGCAAAGTATTATTTAATACCATAGTAATTGTTAATGGACCAACGCCACCAGGAACTG
GTGTAATAGCTCCAGCAATTTCTTTAACCGCATCATAATCAACGTCACCTTTTAATTTGCCATTTTCATCTGGCGTATTGCCAACATCGATAAT
TACTGCTCCTTCTTTGACCACATCTTTTGTTACTAAACCAGGCTTACCAACTGCACTGACAATGACATCAGCATCCTTTAAATATGATGCCATA
TCTTTTGAACGAGAATGTAAGATTGTTACTGATGCATTTTTTTGAAGTAGTAACTTAGAAACTGGTTGTCCGACAATATGACTTCGTCCAATT
ACAACTGCATTTTTACCTTCTAAATCAATATCAGCATGTTTTAATATTTCCATGATGCCGAGCGGTGTGCAAGGTACAAAAGTTTGTTCATCG
ATATATAATTTCCCTATATTTATTGGATGAAATCCGTCCACATCTTTTTCAGGATTGATTGCTTCTAATATTTTCTGTTCGCTAACTTGTTTTGG
TAATGGTACTTGTACCAAAATACCACTTACAGAATCATCATTATTTAGTCTATTTAGTTCGTTTAATACTTCTTCTTCAGTAGCTGTTTCTTCCA
AATGTACGATTTCTGAAATCATACCAATTTTTTCAGCTGCTTTCTTTTTTGATCTAACATAACTTTGACTAGCGCCATCATTACCAACTAATATA
ACGGATAATTTAGGTGTAAAACCCTTTTCTTTTAGCGCTTCAACTTGATCTTGTAACCCCTGTCTGTAGTCTTTGGCAATTTGTTTACCATCTA
AAATTTTAGCAACCAT

Gene: purE (phosphoribosylaminoimidazole carboxylase catalytic subunit)

Position: 1028754 to 1029236, length: 483 nt, orientation: FORWARD

Perfect match to: (COL-CP000046-[1082922:1083404], allele observed in CC8+CC7+CC72+CC239)

Sequence:

GTGAAAGTAGCAGTCATTATGGGCAGTTCTTCCGATTGGAAAATTATGCAAGAGAGTTGTAACATGTTGGATTATTTTGAAATTCCGTACG
AAAAACAAGTAGTATCCGCACATCGTACGCCAAAAATGATGGTTCAATTTGCTTCTGAAGCGAGAGAAAGAGGTATAAACATTATCATTGC
AGGCGCTGGCGGTGCGGCACATTTACCAGGTATGGTTGCATCATTAACGACGCTACCAGTTATTGGAGTGCCGATTGAAACAAAAAGTTTA
AAGGGTATAGATTCTTTATTATCAATTGTTCAAATGCCAGGAGGTATTCCGGTTGCAACGACTGCAATTGGTGCAGCAGGTGCTAAAAACG
CAGGTATACTTGCAGCAAGAATGTTAAGTATTCAAAATCCTTCTTTAGTTGAAAAACTAAATCAGTATGAATCTTCGTTAATTCAAAAAGTG
GAGGACATGCAAAATGAACTTCAATAA

Gene: purK (phosphoribosylaminoimidazole carboxylase ATPase subunit)

Position: 1029223 to 1030347, length: 1125 nt, orientation: FORWARD

Sequence:

ATGAACTTCAATAAATTAAAGTTTGGTGCGACTATTGGCATTATTGGTGGTGGTCAGCTTGGAAAGATGATGGCACAATCAGCTCAAAAAA
TGGGTTATAAAGTGGTTGTATTGGATCCTGCTGAAGATTGTCCATGTAGATACGTTGCACACGAATTTATACAAGCCAAGTATGACGATGA
AAAGGCACTCAATCAATTAGGACAAAAAATGTGATGTGATTACTTATGAATTTGAAAACATTTCAGCCCAACAATTAAAACTATTATGTGAAA
AGTACAATATTCCGCAAGGTTACCAAGCTATACAGTTATTACAAGACCGCTTAACTGAAAAAGAAACATTAAAAAGTGCTGGTACCAAAGT
TGTCCCGTTCATTTCAGTAAAAGAATCTACAGATATTGACAAAGCAATTGAAACATTAGGATATCCTTTTATTGTAAAAACTAGATTTGGTG
GCTACGATGGCAAAGGTCAAGTTTTAATTAACAACGAAAAAGACTTACAAGAAGGTTTTAAATTAATTGAAACTAGTGAATGCGTAGCTGA

AAAATATTTGAATATCAAGAAAGAAGTATCTCTTACTGTTACAAGAGGAAACAACAATCAAATCACTTTTTTCCCATTACAAGAAAATGAGC
ATAGAAATCAAATACTTTTCAAAACAATTGTTCCAGCGAGAATAGATAAAACAGCTGAGGCGAAAGAGCAAGTTAATAAAATTATCCAATC
GATTCATTTCATTGGAACATTTACAGTGGAATTTTTTATAGATAGTAACAACCAATTGTATGTGAACGAGATTGCACCAAGGCCTCACAATTC
CGGACATTATTCAATTGAAGCATGTGATTATTCACAATTTGATACTCATATTTTAGCAGTTACCGGACAATCATTACCAAATTCAATTGAATT
ATTAAAGCCTGCAGTCATGATGAACTTACTAGGTAAAGACTTAGATTTATTGGAAAATGAATTTAATGAACATCCAGAGTGGCACTTACATA
TTTATGGTAAGTCTGAGCGTAAAGATAGCAGAAAAATGGGGCATATGACTGTACTAACGAATGATGTAAACCAAACTGAACAAGATATGT
ACGCTAAATTTGAGGGGAGTAATTAA

Gene: purC (phosphoribosylaminoimidazole-succinocarboxamide synthase)

Position: 1030351 to 1031055, length: 705 nt, orientation: FORWARD

Perfect match to: (TCH1516-CP000730-[1074991:1075695], allele observed in CC8)

Sequence:

ATGACATTATTATATGAAGGAAAAGCGAAGCGCATTTTCTCAACAAATCAAGAAAATGAATTAAGAGTTGAATATAAAGATGAAGTTACTG
CTGGAAACGGGGCTAAGAAAGACACAATGGCAGGTAAGGGGCGATTAAATAATCAAATTACTTCTATTATATTTAAATATTTACAAGAAAA
TGGAATAGAAAGTCACTTTATTAAACAATTATCTGAAACAGAACAATTAGTTAAGCCTGTGAAAATAATTCCATTAGAAGTAGTTGTTCGTA
ATATTGCTAGTGGATCTATTACAAAGCGTTTAGGTTTTGAAAATGGTGAAGTTTTTAGAGAACCACTTGTGGAATTTTTCTATAAAAATGAT
GCGTTAAATGATCCGTTGATAACGGATGACCATGTTAAATTGCTCAATATAGCATCAGATGAAGATATTGAAATACTAAAATCCAAAGCATT
AAAGATTAATAATGTGTTGAAACAATTAATGGATGCTATGAATTTAAAATTAGTAGATTTTAAAATCGAATTTGGAAAGACTGAGACTGGTC
AAATTTTGTTAGCGGATGAAATATCTCCAGATACATGTCGAATTTGGGATAAAGCTACCAATGCAAACTTTGATAAAGATGTATATAGAAAT
AACACTGGATCACTGATTGAAACATATCAAATATTTTTAAACAAATTGGAGGATTTAAAATAA

Gene: purS (phosphoribosylformylglycinamidine synthase)

Position: 1031055 to 1031318, length: 264 nt, orientation: FORWARD

Perfect match to: (N315-BA000018-[1043905:1044168], highly conserved allele)

Sequence:

ATGAAAACAATTGAACTACATATCACATTACAACCACAAGTATTAGATACGCAAGGACAAACGCTTACTCGAGCTGTACATGACTTAGGTTA
TGCACAAGTGAATGATATTCGTGTAGGAAAAGTATTATATATGACAGTGGATGAGGTTAGTGATGAAAAGGTACACAACATTATTACAACT
CTAAGTGAAAAATTGTTTGCAAATACAGTGATTGAAGAATATAGCTATAAAGTGTTAGATGATGAAAAGGAGAATGCATAA

Gene: purQ (phosphoribosylformylglycinamidine synthase 1)

Position: 1031320 to 1031991, length: 672 nt, orientation: FORWARD

Sequence:

ATGAAATTTGCGGTTCTTGTTTTTCCAGGTTCGAATTGTGATAGAGACATGTTTAATGCTGCTATTAAAAGTGGTGTTGAAGCGGAATATGT
AGATTATAGAGAAACATCACTAAGTGGATTTGATGGCGTACTTATTCCTGGTGGATTTTCATTCGGGGATTACTTAAGATCTGGGGCAATG
GCTAGTGTAGCGCCGATTATTTCGGAAGTTAAACGTCTTGCAGCAGAAGGTAAGCCAGTATTAGGTGTTTGTAATGGGTTTCAAATTTTAAC
TGAAATAGGCTTATTACCTGGTGCATTATTGCATAACGATTCACATTTATTTATTAGTAGAAATGAAGAGTTAGAAATAGTGAATAATCAAA
CGGCATTTACAAATCTTTATGAACAAGGTGAAAAAGTTATATATCCTGTAGCTCACGGTGAAGGTCATTATTATTGTACTGATGAAATATAT
CAACAATTAAAAGCTAACAATCAAATTATTCTGAAATATGTGAATAATCCGAACGGTTCATATGATGATATTGCAGGAATTGTTAACGAAAA
AGGCAATGTATGTGGCATGATGCCACATCCTGAAAGAGCTTTAGAAACGTTGTTAGGTACTGATAGTGGTGTGAAATTATTTGAAGCGATG
GTAAAAAGTTGGAGGGAACAACATGTCTAA

Gene: purL (phosphoribosylformylglycinamidine synthase 2)

Position: 1031984 to 1034173, length: 2190 nt, orientation: FORWARD

Perfect match to: (MW2-BA000033-[1046476:1048665], highly conserved allele)

Sequence:

ATGTCTAAATTTATCGAACCAAGCGTTGAAGAAATTAAACTTGAAAAAGTATATCAAGATATGGGATTAAGTGATCAAGAATATGAAAAAG
TTTGCGATATTTTAGGCAGACAACCTAACTTTACAGAAACAGGTATCTTTTCTGTTATGTGGAGTGAACATTGCTCTTATAAACATTCTAAAC
CGTTTTTAAAGCAATTTCCTACGTCAGGTGACCATGTGCTTATGGGGCCTGGTGAAGGTGCAGGGGTAGTCGATATAGGTGATAATCAAGC
CGTAGTATTTAAAGTAGAGTCTCACAATCATCCATCAGCAATTGAACCATATCAAGGGGCTGCTACAGGCGTTGGTGGAATCATTCGTGAC
ATTGTCTCTATTGGGGCTAGACCTATTAATTTGTTAAACAGTCTTAGATTTGGAGAATTAGATAATAAACAAAACCAAAGATTACTTAAAGG
TGTTGTAAAGGGTATCGGAGGTTATGGTAACTGCATTGGTATTCCAACAACTGCTGGTGAAATCGAATTTGATGAACGTTATGATGGCAAT
CCACTTGTTAATGCAATGTGTGTTGGTGTTATCAATCACGACATGATTCAAAAAGGCACAGCAAAAGGTGTAGGTAATTCGGTCATTTATGT
TGGTTTGAAAACTGGTCGAGATGGTATTCATGGTGCTACTTTTGCATCTGAAGAATTGACGGAAGAAAGCGAAAGTAAACGACCTTCTGTA
CAAATCGGTGATCCATTTGTAGGTAAAAAATTAATGGAAGCAACACTTGAAGCAATTACATTTGATGAATTAGTTGGTATTCAAGATATGG
GTGCTGCTGGTTTAACATCTTCATCGTCTGAAATGGCGGCAAAAGGTGGTAGTGGGTTACATTTGAGATTAGAACAAGTGCCAACACGTGA
GCCAGGTATTTCTCCTTATGAAATGATGCTTTCAGAAACTCAAGAACGTATGTTACTAGTTGTTGAAAAAGGTACTGAACAAAAATTCTTAG
ATTTATTTGATAAGCACGAATTGGATAGTGCTGTTATAGGTGAAGTTACAGATACAAATCGTTTTGTTTTAACATATGATGACGAAGTTTAT
GCTGACATTCCAGTTGAACCACTAGCTGATGAAGCACCTGTATATATTTTAGAAGGAGAAGAAAAAGATTATAATACTTCTAAAAATGATTA
TACACACATCGATGTTAAAGATACTTTCTTTAAATTACTTAAGCATCCGACTATAGCATCTAAACACTATTTATATGATCAATACGACCAACA
AGTTGGTGCCAATACGATAATTAAGCCAGGACTTCAAGCATCGGTAGTACGTGTGGAAGGCACAAATAAGGCAATTGCTTCAACAATTGAT
GGTGAAGCGCGTTATGTATATAACAATCCATATGAAGGTGGAAAGATGGTAGTAGCTGAAGCTTATCGAAATTTAATTGCCGTGGGTGCA
ACACCATTAGCAATGACAGATTGTTTAAATTATGGTTCTCCTGAAAAGAAAGAAATCTATCAACAGTTGATAGATTCAACGAAAGGTATGG
CAGAAGCATGCGACATTCTTAAGACACCAGTAGTTTCTGGTAATGTATCTTTATATAACGAAACGAAAGGTACTTCTATTTTCCCAACACCA
GTTGTTGGAATGGTAGGTTTGATTGAAAATGTAAATTATTTAAATGATTTTGAACCTCAAGTTGGAGATAAATTATATTTAATCGGTGATAC
TAAGGACGACTTTGGTGGTAGTCAACTTGAAAAGTTAATTTATGGCAAAGTTAATCATGAATTTGAGTCATTAGATTTGAGTTCAGAAGTTG
AAAAAGGTGAATCAATCAAGACCGCTATTCGTGAAGGACTATTATCACATGTTCAAACAGTTGGTAAAGGTGGCTTACTGATTACCTTAGCT
AAACTAAGTGCGCATTACGGTTTAGGATTAAAATCTTCAATAGATATAACAAATGCACAATTGTTTAGTGAGACGCAAGGCCGATATGTTGT
TTCTGTTAAATCAGGTAAAACTTTAAATATTGATAATGCAATAGAAATTGGACTTTTAACAGATAGTGATAATTTCAAGGTAACAACACCAT
ATACAGAGATTAGTGAAAATGTTTCAGATATTAAACAAATATGGGAAGGGGCAATTGCTCAATGTTTAACTACTCAGGATTAA

Gene: purF (phosphoribosylpyrophosphate amidotransferase)

Position: 1034152 to 1035636, length: 1485 nt, orientation: FORWARD

Sequence:

ATGTTTAACTACTCAGGATTAAACGAAGAATGTGGCGTGTTTGGTATTTGGAATCATCCTGAAGCAGCGCAACTAACATATATGGGACTTCA
TAGTTTGCAACATCGTGGTCAAGAAGGTGCAGGTATAGTTGTTTCTGATCAAAATGAATTAAAAGGCGAGCGAGGATTAGGCTTACTAACT
GAAGCAATTAAAGATGATCAAATGGAACGATTAAAAGGATATCAACATGCAATTGGTCACGTCCGTTATGCTACTTCAGGTAATAAAGGTA
TTGAAAATATTCAACCGTTTCTGTATCACTTTTATGATATGAGTGTAGGTATTTGTCATAATGGTAACCTCATTAATGCTAAATCATTGCGTCA
GAATTTAGAAAAACAAGGTGCTATCTTCCATTCGTCTTCTGATACTGAAGTCATTATGCATTTGATACGTCGAAGTAAAGCTCCTACTTTTGA
GGAAGCGTTGAAAGAAAGTTTGCGAAAAGTTAAAGGCGGTTTTACATTTGCGATTTTAACTAAAGATGCGTTATATGGCGCAGTAGATCCA
AATGCTATCAGACCACTTGTTGTAGGTAAAATGAAAGATGGGACATACATCCTTGCAAGTGAAACATGTGCAATAGATGTGTTAGGTGCAG
AATTTGTTCAAGATATTCATGCAGGTGAATATGTCGTGATTAACGATAAAGGTATTACAGTTAAATCTTATACACATCATACGACAACTGCA
ATTTCTGCGATGGAATATATTTATTTTGCTAGACCAGACTCAACAATAGCTGGTAAAAATGTCCATGCAGTACGTAAAGCTTCTGGTAAAAA
ATTAGCCCAAGAAAGCCCTGTAAATGCTGATATGGTCATCGGTGTACCCAATTCATCGCTATCAGCTGCGAGTGGTTATGCTGAAGAAATA
GGTTTGCCATATGAAATGGGACTAGTTAAAAATCAATATGTTGCAAGAACATTTATTCAACCAACTCAAGAATTACGTGAGCAAGGTGTGA
GAGTGAAGTTATCTGCGGTAAAAGATATAGTAGATGGGAAAAATATCATTCTTGTTGATGATTCCATTGTTCGCGGTACGACAATTCGACG
CATTGTGAAAATGTTAAAAGATTCTGGTGCAAATAAAGTACATGTGCGTATAGCATCACCGGAATTTATGTTCCCAAGTTTTTATGGAATCG
ATGTTTCAACTACGGCAGAATTAATTTCTGCAAGCAAATCACCTGAAGAAATTAAAGATTATATTGGCGCTGATTCATTAGCATATCTATCTG
TAGATGGGTTAATTGAATCAATTGGTTTAGATTATGACGCGCCATATAGTGGCTTATGTGTAGAAAGTTTCACTGGAGATTATCCTGCAGG
GTTATATGATTATGAAGCAAATTATAAAGCGCATTTAAGTCATCGACAAAAGCAATATATTTCTAAAAACAAACACTTTTTTGATAGCGAGG
GAAATTTAAATGTCTAA

Gene: purM (phosphoribosylformylglycinamidine cyclo-ligase)

Position: 1035629 to 1036657, length: 1029 nt, orientation: FORWARD

Sequence:

ATGTCTAAAGCATATGAACAATCTGGTGTAAATATTCATGCTGGTTATGAAGCTGTAGAAAGAATGTCTAGTCATGTTAAACGTACGATGC
GTAAAGAAATTATCGGTGGTTTAGGTGGATTCGGTGCTACATTTGATTTATCACAATTAAATATGACAGCGCCAGTTTTAGTTTCTGGAACA
GACGGAGTAGGTACGAAATTAAAACTAGCTATCGACTATGGGAAACATGATTCGATAGGTATCGATGCAGTTGCAATGTGTGTTAATGATA
TTTTAACGACAGGTGCAGAACCATTGTATTTTTTAGATTACATCGCTACAAATAAAGTTGTTCCTGAAGTTATTGAACAAATTGTTAAAGGTA

TTAGTGATGCATGCGTTGAAACGAATACTGCACTTATCGGTGGAGAGACTGCTGAAATGGGTGAAATGTATCACGAAGGTGAATATGATG
TAGCCGGATTTGCTGTTGGAGCAGTTGAAAAGGATGACTATGTAGATGGTTCAGAAGTGAAAGAGGGACAAGTTGTTATAGGACTTGCGT
CAAGTGGCATTCATTCAAATGGATACAGTTTAGTGCGCAAATTAATTAATGAATCAGGCATTGATTTGGCATCAAACTTCGATAATCGTCCA
TTTATAGATGTCTTTTTAGAACCAACTAAATTATATGTCAAACCTGTACTTGCTTTGAAAAAAGAAGTTTCTATTAAGGCAATGAATCATATT
ACTGGTGGAGGTTTTTATGAAAATATTCCACGTGCATTGCCAGCCGGATATGCTGCTAGAATTGATACTACATCATTTCCAACGCCAAAAAT
ATTTGATTGGTTACAACAACAAGGCAATATAGACACAAATGAAATGTATAACATTTTTAACATGGGTATTGGCTATACGGTTATCGTTGATG
AAAAAGATGCATCACGCGCTTTGAAGATTTTAGCAGAACAAAATGTGGAAGCTTATCAAATTGGTCATATTGTGAAAAATGAGTCAACTGC
AATTGAATTGTTGGGGGTATAA

Gene: purN (phosphoribosylglycinamide formyltransferase)

Position: 1036660 to 1037226, length: 567 nt, orientation: FORWARD

Sequence:

ATGGTTAAAATTGCGATTTTTGCATCAGGTTCAGGAAGTAACTTTGAAAATATAGTTGAGCATATTGAATCAGGAAAACTTGAAAATATTGA
AGTTACGGCGCTATATACGGATCATCAAAATGCGTTTTGTATAGATAGAGCAAAAAAGCACGGTATTCCTGTTTATATTAATGAACCAAAAC
AATTTGATTCAAAAGCAGCGTATGAACAACATTTAGTATCACTATTAAATGAAGATAAGGTAGAGTGGATTATTTTAGCTGGCTACATGCGT
CTAATTGGTCCAGATTTATTAGCTTCATTTGAAGGTAAAATATTGAATATACATCCATCTCTATTGCCGAAATATAAGGGGATTGACGCAATA
GGCCAAGCATATCATAGTGGCGATACTATTACTGGTTCGACAGTACATTATGTTGATTGTGGTATGGATACGGGAGAAATTATTGAACAGA
GACAATGTGATATTAGACCGGACGATTCAAAAGAACAATTAGAAGAGAAAGTAAAAAAATTGGAATATGAGTTATATCCAAGTGTTATTGC
TAAAATTGTAAAATAA

Gene: purH (bifunctional phosphoribosylaminoimidazolecarboxamide formyltransferase/IMP cyclohydrolase)

Position: 1037241 to 1038719, length: 1479 nt, orientation: FORWARD

Sequence:

ATGAAGAAAGCTATTTTGAGCGTATCAAATAAAACAGGTATTGTAGAGTTTGCTAAAGCGTTAACGCAATTAAATTATGAATTATATTCAAC
TGGTGGTACTAAACGTATATTAGATGAAGCAAATGTACCAGTTCGTTCTGTTTCAGACTTAACACATTTTCCAGAAATAATGGATGGCCGTG
TTAAAACATTACATCCGGCTGTTCATGGTGGCATTTTAGCTGATCGAAATAAACCGCAGCATTTAAAGGAATTATCAGAACAACATATAGAT
TTAATTGATATGGTAGTAGTTAATTTATATCCATTCCAACAAACTGTTGCAAACCCTGATGTAACGATGGACGAAGCAATTGAAAATATTGA
TATTGGTGGTCCAACAATGTTACGTGCTGCAGCTAAAAACTATAAACATGTAACAACAATTGTACATCCTGCAGATTATCAAGAAGTATTGA
CGCGATTAAGAAACGGTTCGTTAGATGAGTCATATAGACAGTCATTAATGATCAAAGTTTTTGAGCATACTGCAGAATATGATGAAGCGAT
TGTACGTTTCTTTAAAGGGGATAAAGAAACTTTAAGATATGGAGAAAACCCACAACAATCAGCGTATTTTGTGAGAACTTCGAATGCTAAG
CACACGATTGCAGGCGCTAAACAATTACATGGGAAACAATTAAGCTATAACAATATTAAAGATGCAGATGCTACACTAGCTTTAGTTAAAA
AGTTTGATACACCTGCTGCAGTTGCGGTTAAACACATGAATCCATGTGGTGTTGGTATCGGTGACACGATAGAACAAGCATTTCAACATGC
GTATGAAGCGGATAGTCAATCAATATTTGGTGGAATTGTTGCATTAAACCGAGCTGTAACACCTGAGTTAGCAGAGCAATTGCATAGTATC
TTTTTGGAAGTCATTATTGCACCAAAATTTACAGATGAAGCATTAGATATTTTAAAACAAAAGAAGAATGTAAGATTATTAGAAATTGATAT
GACTATAGACAGTAACGAAGAAGAGTTTGTTTCAGTATCTGGCGGATATTTAGTTCAAGATAAAGACAATTATGTCGTGCCAAAAGAAGAA
ATGAAAGTTGTTACAGAAGTAGCACCTACTGATGAACAATGGGAAGCAATGTTATTAGGATGGAAAGTTGTACCATCAGTAAAAAGTAAT
GCAATTATTTTAAGTAATAATAAACAAACTGTAGGTATAGGTGCTGGACAAATGAATCGTGTCGGTGCTGCTAAAATTGCGTTAGAGAGAG
CTATTGAAATCAATGATCATGTAGCGTTAGTATCTGATGGATTTTTCCCTATGGGAGATACAGTTGAACTTGCAGCACAACATGGTATAAAG
GCAATTATCCAACCGGGTGGTTCGATTAAAGATCAAGATTCAATTGATATGGCTAATAAACATGGTATTGCAATGGTAGTCACAGGCACTC
GACATTTTAAACACTAA

Gene: purD (phosphoribosylamine-glycine ligase)

Position: 1038741 to 1039988, length: 1248 nt, orientation: FORWARD

Perfect match to: (TW20-ST239-FN433596-[1140260:1141507], allele observed in CC239+CC7+CC8+CC239)

Sequence:

ATGAATGTATTAGTAATTGGTGCTGGTGGACGAGAACATGCACTTGCATATAAACTTAATCAATCGAATCTAGTTAAACAAGTGTTTGTCAT
TCCAGGTAATGAGGCAATGACACCTATAGCTGAAGTACACACTGAAATTTCAGAATCTGATCATCAAGCGATACTAGATTTTGCTAAACGG
CAAAATGTTGATTGGGTAGTTATAGGTCCAGAACAGCCGCTAATTGATGGATTAGCAGACATTTTACGAGCGAATGGTTTCAAAGTGTTTG
GTCCAAATAAGCAAGCAGCTCAAATCGAAGGCTCAAAATTATTTGCTAAAAAGATAATGGAAAAATATAATATTCCAACTGCTGATTATAAA

GAAGTTGAGCGAAAAAAGGATGCTTTAACATATATTGAAAACTGTGAATTGCCCGTTGTTGTCAAGAAAGATGGGTTAGCTGCTGGGAAA
GGCGTTATTATTGCAGATACTATTGAAGCAGCCAGAAGTGCTATTGAGATTATGTATGGTGATGAAGAAGAAGGTACTGTTGTATTTGAAA
CGTTTTTAGAAGGTGAAGAGTTCTCGCTAATGACATTTGTTAATGGTGATTTAGCAGTACCTTTCGACTGTATTGCACAAGATCATAAACGC
GCATTTGATCATGATGAAGGACCAAATACTGGTGGTATGGGGGCTTATTGTCCAGTACCACATATTAGTGACGATGTTTTAAAACTTACAAA
TGAAACAATTGCACAACCCATTGCAAAGGCAATGCTTAATGAAGGTTATCAATTCTTCGGTGTATTATACATTGGTGCTATTTTAACTAAAG
ATGGTCCAAAAGTAATAGAATTTAATGCCCGTTTTGGTGATCCTGAAGCTCAAGTATTATTAAGTCGCATGGAAAGTGATTTAATGCAGCAT
ATTATTGATTTAGATGAAGGAAAACGTACTGAATTCAAATGGAAAAATGAATCTATTGTAGGGGTCATGTTGGCATCAAAAGGATATCCTG
ATGCATATGAAAAAGGGCATAAAGTAAGTGGCTTTGATTTAAATGAAAACTATTTTGTTAGTGGATTAAAGAAGCAAGGTGATACCTTTGT
TACTTCAGGTGGTAGAGTTATACTTGCCATCGGAAAAGGTGACAATGTACAAGATGCACAGCGAGACGCATACAAAAAAGTATCACAAAT
ACAAAGTGACCATTTATTCTATCGTCATGACATTGCGAATAAAGCACTACAACTTAAATAA

Gene: ykoC (hydroxy-methyl-pyrimidine ABC transporter, transmembrane permease)

Position: 1040253 to 1041059, length: 807 nt, orientation: REVERSE

Perfect match to: (11819-97-CP003194-[1086999:1087805:r], allele observed in CC80)

Sequence:

TTAACCTACACGACCAAGACGAACATCATCTATGCCCGTGATGGGTAAGGTGATTGAACAATAATATGCCATAGTAATAATGGCAATTAAA
ACTATAATAAAGATTATATCTTTATATGAGAAAGGTACGTTGTAATAGTAAGTACGAGGACCATCTCTAAATCCTTTCGACTCCATCGCAACT
GATAATTGATGTGCCTTTCTAATATTTTGGCTTAATAGAGGTATAATTAAATGCTTAAATCGCTTTAACCCTCTATAATTTGCCGCGTCTATCA
TCTGATAGCGCATTTTTAAAGATCTGCGAAGCTGTATTAAAGAACTAATCATTAAAGGTATCATACGAATGGCAGCCATGAATGCATAAGC
AACTTTTGATTTAACCTTTAAATGTTGCATTAAACTATAAAATATCATGACAACCTGAGATGTAAGTGCGATTAAGATACCGAAAAATGAAA
TAGCAATGGTTCTTAATGATACATGTAAACCACGAACTAAACTTTCTGTTGTAATATGGATAAATCCGAATTTCAAAATTGTATGGCTACCAT
TCCCGTATAAAATCATGAACAGGGAAGAGAGTAATGCAAAGCCAATACTTATAGTTATAAAAATTGCTGTAATTTTAAACTGAGTACCATTA
AACATCAATAAGAAAACTAACATTAAGATATTGATATAAAGCATAAAATCGAAATTATGCACAAATATAATAAAGAAAAATAGTATAATTCC
AAGAAATAGTTTCGTTATAATGTTGACATCATCAACAAATGATTGCCGAACTTTCCATTGCTCATACAT

Gene: ykoD (hydroxy-methyl-pyrimidine ABC transporter, ATPase)

Position: 1041052 to 1042452, length: 1401 nt, orientation: REVERSE

Perfect match to: (COL-CP000046-[1095220:1096620:r], highly conserved allele)

Sequence:

TCATACATTCGTATCACCATCACAATCTAGTAACGCACCATCTGAAATTTTAAGTCTTCTTGATGGATAACGTTCAATTATTTCATCGTCATGT
GTAACCATGACAATACTTTGTCCCAAATTAATTCGCTTTTGGAAAAGTTTGATCAACTGGAATGTATTATGGCTATCAAGTCCAAATGTCGGT
TCATCTAAAAAGATAATATCAGCTTTAGAACTTAGTGCGGTAGCTACGCTAAGGCGTCGTTTTTGACCAATAGACAACTCATAAGGATGTTG
ATCTTTTACATTTTGTAAATCTAAAAGTTTTAAAAGTTGTATCGTTTCATCATCACTTTGATCTTTAGAAAGGTGATTAAAATGAATGTTAATT
TCATCATAAACCGAATTTGTTATAAATTGTAATTCTGGGTTTTGATAAACTAGGTACATGTGTTTTGCTGCATGTTTAATTTTTGTTAAACGCT
GATTTTCAAAATAAACATCACCTTGATATTTAATCAATTGCATAATTGATTCAAGCAAGGTTGTTTTACCACTACCATTTGCCCCTGTAATTGT
AATCCACTCACCTAGACCAATTTCTAAATCTGAGAATGAGAGCAATGTTGATTTACCGCGAATAATACGTCCATTTTTAAATTGTAATAAGTG
TGAGTTTGTTGTTGGAAAGTCAACACGACTTGGTGCGAATTCCCATGCACGTGGATGCCACACACCATATTCACTGAGTAAATGAACATACT
TCTGTAATATGATTTCAGGACATTCATCGGCAATGATATTTCCGTTATAATCCATCAAAATGACGCGGTCGACATGATTCCAGATGTGTTTAA
CTTTATGTTCAACGATTACAACCGTTTGATCTTCCCAAAGTTCAATTAGTTTAGTCCATAAATCTTCTGTTGCTTGAACATCTAACATTGCTGT
CGGTTCATCTAAAAACAATGTTTTTGATTGTTGAAGAATGGTTTCAACAATTGCCAATTTCTGTTTCATCCCGCCACTTAAATCTTTGATATAC
GTTTCAGGGGTAACATTTAAATTGACCATATTTAAAGCATTGATAATTAACGCATCCATGTCTTCACGTGGTAATTGTCTATTTTCTAAAACG
AATGCAAGTTCTTCGTATACTTTTGGCATACAAAACTGGCTATCAGGGTCTTGGAAAATAACGCCACTTAATGGGTCAACGATTAGTTCATC
ATATTTCATAGGTAATTCAATTAAATTAGGAACAATACCACTTAATACATTCAGAAGTGTACTTTTACCGCAACCAGAAGGACCGAGTAAAA
GTACTTTTTCTTTGTCTTGAATAGTGATATTTAAATGATCGAAAATTTTACGTTGACCACTTGGATATTTTAATCGTAAATCACTTACTTTTAAC
AC

Gene: ykoE (hydroxy-methyl-pyrimidine ABC transporter, substrate-binding protein)

Position: 1042467 to 1043042, length: 576 nt, orientation: REVERSE

Perfect match to: (IS-111-AHLS01000092-[5179:5754], allele observed in CC8+CC239)

Sequence:

TTATAAGTTGTCGTAATCTTCTTTAGCAGCTGGTCTAAATAATTTTGTTACGCCTGTCTTATCTAAAGCTTTTACTAAAAGGTAAGATAGGAC
GCCGGCGACTACTGCACCACTAATTAATCTAAATACGATGAATAATGTTAAGTTCCAACCTGCAACTTCATTTAAATAACCATAGAAATAATC
TATCGGGAAAGCCGCGATTGCTGTACAAAAACCTGCTAACATAGCTACTATAACTGAACGTGATTGATATTTAAAAATTGCAAAGACAAGT
TCACACGCTAAACCTTGTATAAAAGCGTAAACGATTGTCGGAATATCGAAACGACCCATAATGATAGTTTCGCCGGCACCTGCAGCAAATTC
AGCCAGTAAAGCAATACCTGGTTTTGGAATAATTAGATAGCAGACAATCGCTGCCATGAACCAAACCCCGTTTGTTAATTGTTCGAGGTGA
AGGCCTGTAGCTTGCACACCATTGTAAACAAACCACCATAAATTGTAAATAACTGCGAATACTACTGAAATAAGTACGGTTACTAGTATTTC
AGATAGCTTTAAACCTTTTGACAT

Gene: graF (putative protein)

Position: 1043752 to 1043886, length: 135 nt, orientation: REVERSE

Perfect match to: (N315-BA000018-[1056731:1056865:r], highly conserved allele)

Sequence:

TTAATTTAGTGTATCTTGGATATCTTGTTTTGTTTGATTAATATCTTCTGTTTTTTCTTCTTTTTTATCTTTTAATTTTTCTTCAACTTCTTTAGCTT
TTTCTGCTGCTTTTTTATTTTGATTTTCATTAGACAT

Gene: Q5HH05 (polytopic membrane protein)

Position: 1044038 to 1045333, length: 1296 nt, orientation: FORWARD

Perfect match to: (11819-97-CP003194-[1090721:1092016], highly conserved allele)

Sequence:

ATGTCTTTTCTTAGGAAACACGCCGAAATTATTTTTAGCTATTTAATCGGTATCGTTTCACTCTTCACTGGTCTCATTATTTTAATTAACTTGCC
ATTAATTAAACAATTAAATGGTGGTAAAAAAGTTGATACACATGTTCATAATGTGTGGGAATTTCTGAATGCATTTTTCAGTGAAATTATTA
AAGTAATGAGTCGATTTATAGGTAATTTCCCTATAGTTAGTGCAATTGTGATAATTATATTCGGTATTTTAGTTATGTTGATTGGTCATACAT
TACTTAGAACTATTAAGTATGACTATGATATTTCTATCTTTTTCTTAGTTATCGGTATCATGTACTTTATTATTACTCTTATATTAATGACTCAA
GTTTATGGATTCTTTGCAGTGATTTTCATTATTCCATTTACAATTCATATAGGATATATCGTCTATAAAGATGAATTGAATCAGGAAAATGTA
AAAAATCATTTCATGTGGATAATTGTGAGTTATGGTATAAGTTACTTAATTACACAAATTGCATTGTATGGCAGAATTGATGCTAATGAAAT
AGAGTCAATTGATATCTTAAGTGTCAATGCTTTCTTTATAATTATGTGGTTACTTGGTCAAATGGCTATTTGGAATTTCTTGTTCTTGCGCCGA
GCTTTACCTTTAACAAAGCAAGAATTAGGTGAAGAGGAGCCAGAATTATCAAGAACAAGTAAAGGGAATGTCACGAATCAAACTAAAATT
CACTTGAAACAACTCCAAGATAAGACTACAGAATATGCACGTAAGACAAGAAGAAGTGTCGATTTAGATAAAATTAGAGCTAAAAGAGAT
AAATTCAAAAAGAAAGTTAATGATATTATCGATATTCAAGAAGACGATATTCCTGATTGGATGAGAAAACCGAAATGGGTTAAACCAATGT
ATGTCGAACTATTTTGTGGTGTCGTCATCTTTTTATTCACATTTTTAGAATTTAATAATCGTAATGCATTATTTGTATCTGGTGATTGGAAATT
ATCACAGACACAATATGTTATTGAATGGGTTACATTATTAATTCTGTTATTCATTATTATCGCATATATCGCTACAACGTTAACTTTCCACTTG
AAAGGTAAGTTTTATTATTTACAACTATTTATGGGGAGCATTTTATTCTTTAAATTGTTAACGGAATTTATAAATATAATGATTCATGGACTAT
TACTTTCAGTGTTCATTACGCCAACATTACTATTAATGTTATTGGCAATCATCATTTCTTATTCGTTACAATTACGAGAGCGACCATAA

Gene: ywbD (S-adenosylmethionine-dependent methyltransferase)

Position: 1045758 to 1046930, length: 1173 nt, orientation: FORWARD

Perfect match to: (11819-97-CP003194-[1092441:1093613], allele observed in CC80)

Sequence:

ATGAAAATAGCAACTCTGAACAAAGGCAAAGAAACAAAATATTTTAATGGATATCCTTTAATTGAAGAAGAGGATATCTATTCACAAGATC
ATTTAAAAGAAGGAGATATTTTTCAAATTGTGACTGATAAATCACAATATGTTGCAACGGCTTATGTTGGTCGCCAACATAAAGGATTAGGT
TGGGTTCTAACATACGATAAAGCTCAAGAAATCAACACAGCTCTCTTTGTGAAATTGTTTAATACTGCATTAGCAGAACGTGATTATTATTTT
AATATAGATGGAACAAATGCTTTTAGATTATTTAATGCTGAAGGTGATGGTGTTGGGGGATTAACAATCGACAATTACGATGGTCATTTGTT
GATTCAATGGTACTCAAAAGGTATTTATAAATTTAAATATGCCATTCTTGAAGCGGTTAGAAAAGTATTTGATTATAAATCTATTTACGAAAA
AGTAAGATTTAAAGACAGCGAATATAGTGGTGGTTTTGTTGAAGGAGATGCACCTGAGTTTCCAATTGTTATCGAAGAGAACTTCACATTTT
ATAATGTAGACCTTGAAGATGGTTTGATGACAGGTATCTTTTTAGATCAAAAAGAAGTGCGCAAGAAATTAAGGGATCAATATGCCAAAGA
ACGCCATGTTTTAAACTTATTTAGTTATACAGGTGCTTTTTCTGTAATAGCAGCAAGTGAGGCATCTTCAACAACAAGTGTAGATTTGGCTAA

TCGTTCTCGTAGTTTAACTGAAGAAAATTTTGGATTAAATGCTATTGATCCTAAATCCCAATATATTTATGTCATGGACACTTTTGATTTCTAT
AAATATGCTGCACGACATGGACATAGTTATGACACGATCGTGATTGATCCACCTAGCTTTGCGCGTAACAAAAAACGTACATTTTCAGTGCA
AAAAGATTATGACAAATTAATTAATGGCGCCTTAAATATCTTATCATCTGAAGGAACATTATTGTTATGTACAAACGCAAGTGTATATCCATT
AAAGCAATTTAAAAATACTATTAAAAAGACGCTTGAAGAGAGTGGCGTTGATTATGAATTAACTGAAGTTATGGGATTACCAAAAGATTTT
AAAACGCATCCACATTATAAGCCATCTAAATATTTAAAAGCTGTTTTTGTAAATATTAGACATTAA

Gene: Q5HH03 (putative protein)

Position: 1046984 to 1047526, length: 543 nt, orientation: FORWARD

Perfect match to: (MW2-BA000033-[1061287:1061829], allele observed in CC1+CC72+CC80+CC188+CC772)

Sequence:

ATGGGATTCAAAAACAATTTAACATCAAATTTAACAAATAAAATTGGTAATTCAGTCTTTAAAATAGAAAATGTTGACGGAAAAGGTGCAAT
GCCAACGACGATTCAAGAATTGAGAGAAAGACGACAACGTGCTGAAGCAATTGTAAAGAGAAAATCTTTAATGTCATCAACAATGAGCGT
TGTTCCAATTCCGGGTTTAGATTTTGGTGTTGATTTAAAATTAATGAAAGATATTATCGAAGATGTAAATAAAATATACGGATTAGATCATA
AGCAAGTTAATAGCCTTGGGGATGATGTGAAAGAAAGAATTATGTCTGCAGCAGCAATTCAAGGTAGTCAATTTATTGGTAAAAGAATTTC
AAGTGCATTTTTAAAAATAGTTATTAGAGATGTAGCTAAACGTACTGCTGCAAAACAAACAAAATGGTTTCCTGTTGTAGGACAAGCTGTGT
CTGCATCTATTAGTTACTATTTTATGAATAAAATTGGAAAAGATCACATTCAAAAATGCGAAAATGTTATTAAAAATGTCATGTAG

Gene: ptsH (phosphocarrier protein HPr)

Position: 1047680 to 1047946, length: 267 nt, orientation: FORWARD

Perfect match to: (N315-BA000018-[1060659:1060925], highly conserved allele)

Sequence:

ATGGAACAAAATTCATATGTAATCATCGACGAGACTGGTATTCACGCTAGACCAGCAACAATGTTAGTACAAACAGCTTCAAAATTCGATTC
TGATATTCAATTAGAATATAACGGTAAGAAAGTAAACTTAAAATCAATCATGGGTGTTATGAGCCTTGGTGTTGGTAAAGATGCTGAAATT
ACAATTTATGCTGACGGTAGTGATGAATCTGACGCCATTCAAGCAATCAGTGACGTCTTATCAAAAGAAGGATTGACTAAATAA

Gene: ptsI (phosphoenolpyruvate-protein phosphotransferase)

Position: 1047949 to 1049667, length: 1719 nt, orientation: FORWARD

Perfect match to: (RF122-AJ938182-[1029931:1031649], allele observed in CC705+CC1+CC8+CC239)

Sequence:

ATGTCTAAATTAATTAAAGGTATTGCCGCATCTGATGGTGTCGCAATTGCTAAAGCTTATTTATTAGTTGAGCCAGACTTAACATTCGACAAA
AATGAAAAAGTCACTGATGTTGAAGGAGAAGTTGCAAAGTTCAATAGCGCTATCGAAGCTTCTAAAGTTGAGTTAACTAAAATTAGAAATA
ATGCAGAGGTTCAACTAGGTGCTGATAAAGCTGCTATCTTTGATGCACATTTATTAGTTTTAGATGACCCTGAATTAATTCAACCAATCCAAG
ATAAGATTAAAAATGAAAACGCTAATGCTGCTACAGCATTAACGGATGTAACAACACAATTTGTTACAATTTTTGAATCTATGGATAACGAA
TACATGAAAGAACGTGCGGCTGATATTCGCGACGTTTCTAAACGTGTGTTATCACATATTTTAGGTGTAGAATTACCGAATCCGAGTATGAT
TGATGAAAGCGTTGTTATTGTAGGGAATGACTTAACGCCATCTGATACTGCTCAATTAAATAAAGAATTCGTACAAGGTTTTGCTACAAACA
TTGGCGGAAGAACAAGTCACTCTGCAATTATGAGTCGTTCTTTAGAAATTCCAGCAATTGTTGGTACAAAATCAATTACTCAAGAAGTTAAA
CAAGGCGACATGATTATCGTAGATGGATTAAATGGTGATGTAATCGTTAATCCAACTGAAGATGAGTTAATCGCTTATCAAGATAAACGTG
AGCGTTATTTTGCTGACAAGAAAGAATTACAAAAACTACGTGATGCTGATACTGTTACAGTTGATGGTGTTCACGCAGAGCTTGCTGCAAAT
ATTGGTACACCTAATGATTTGCCAGGTGTTATTGAAAATGGTGCACAAGGTATCGGCTTATATAGAACTGAGTTTTTATATATGGGTCGTGA
CCAAATGCCTACAGAAGAAGAACAATTTGAAGCTTATAAAGAAGTATTAGAAGCAATGGGCGGTAAACGTGTTGTTGTACGTACTTTAGAT
ATAGGTGGAGATAAAGAATTATCATACTTAAACTTGCCTGAAGAAATGAATCCATTCTTAGGTTACCGTGCGATTCGTTTATGCCTTGCGCA
ACAAGATATTTTCAGACCACAGCTACGTGCATTATTACGTGCATCAGTTTATGGTAAGTTAAATATCATGTTCCCAATGGTTGCAACAATTAA
CGAATTTAGAGAAGCTAAAGCTATATTATTAGAAGAAAAAGAAAACCTTAAAAATGAAGGTCATGACATTTCGGATGATATAGAATTAGGA
ATCATGGTAGAGATACCTGCAACAGCAGCATTAGCTGATGTCTTTGCTAAAGAAGTAGATTTCTTCAGTATCGGTACAAATGATTTAATTCA
ATACACATTAGCTGCTGACCGTATGTCAGAGCGTGTATCATATCTATACCAACCATATAACCCTTCAATCTTACGTTTAGTTAAACAAGTTAT
TGAAGCGTCACATAAAGAAGGTAAATGGACAGGTATGTGTGGTGAAATGGCTGGAGATGAAACAGCTATTCCATTATTGCTTGGTTTAGG
TTTAGATGAGTTCTCTATGAGTGCAACGTCTATTCTGAAAGCAAGAAGACAAATTAATGGTTTAAGTAAAAATGAAATGACTGAACTTGCTA
ACCGTGCAGTCGACTGTGCAACGCAAGAAGAAGTTATTGAATTAGTTAACAACTACGTAAAATAA

Gene: nrdH (glutaredoxin)

Position: 1049904 to 1050137, length: 234 nt, orientation: REVERSE

Perfect match to: (COL-CP000046-[1104009:1104242:r], allele observed in CC8+CC72+CC239)

Sequence:

TTAATCTTGGATATTTAATACTTTGTTGATTTCATCAAGATCAACATGGTACATTGGATTGCCATTCAACAAAATAAACGGAGTTGAAAAAGC
ATCAAAATCTATCATTTCGTTTCGATATTGTTGATTGTTGATATTTCTCTCTTCAAAATCAATGTGATGCTCATTTAGATAATTTTTTACAAATG
TACAAGGTGGACAATCATTCTGCGTATAAACGATTATTTCTGACAT

Gene: cydA (cytochrome bd quinol oxidase subunit 1)

Position: 1050335 to 1051696, length: 1362 nt, orientation: FORWARD

Perfect match to: (N315-BA000018-[1063314:1064675], highly conserved allele)

Sequence:

ATGGATACAGTTGAAATCAGTCGGTTTTTGACAGCTATGACTTTAGCAGTTCATATCATTTTTGCAACGATTGGTGTTGGTATGCCTTTAATG
TTCGCAATTGCAGAATTTTTAGGTATTCGCAAAAATGATCTTCAATATATAGCTATGGCCAAAAGATGGGCTAAAGCTTATACAATTACTGT
AGCAGTGGGAGTTGTTACAGGTACAATTATAGGACTTCAATTATCATTGATTTGGCCTACATTTATGGAAATGGGTGGACACGTTATTGCAC
TTCCATTATTTATGGAAACATTTGCGTTCTTCTTTGAAGCTATTTTCTTAAGTATATATTTATATACTTGGGATCGTTTTAAAAATAAATGGAC
ACATTTCTTAATTAGTATACCAGTAATTATTGGTGGCTCTTTCTCAGCATTCTTCATTACTTCAGTGAATTCATTTATGAATACGCCTGCAGGT
TTTGAGTTGAAGAATGGAAAGATGGTCAATGTTCAACCTATAGAAGCGATGTTTAACCCATCGTTTATAGTTCGATCATTTCACGTAATTACT
ACAGCCGGTATGACGATGGCATTTGTTATTGCATCAATAGCAGCTTTTAAATTATTGCGTAATCGTCAACCTAAAGATACTGTCTACCATAA
GAAAGCTTTGAAAATGTCTATGATAGTTGGATTCTTTTCAACATTACTTTCTATGTTGGCAGGGGATTTATCTGCAAAATTTTTGCATAAATT
CCAACCTGAAAAATTAGCAGCTTATGAATGGCATTTCGATACATCTTCCCATGCCAAATTATTATTATTTGGTGTGTTAGATGAAAAGACTCA
GCAAGTTAAAGGTGCGATTGAATTACCTGGACTATTGAGTTTCTTAGCAGATAATAGTGTCAAAACTAAAGTACAAGGGTTAAATGATTTTC
CAAAAAGTTTACATCCGCCTATGATCGTCCATTATTTCTTTGATTTAATGGTAACGATGGGAATTTTATGTTTTGTCATTTCAGGTGTTTATGT
CTTAACTTTAATGTTTAAAAAGCTTAGAAAGTTTTCTACTCATAAATGGATGCTTTATGGAATATTATTAACAGGCCCAGCTTCAATGCTAGC
TATAGAATTTGGATGGTTCTTAACAGAGATGGGTAGACAGCCTTGGATTGTTCGTGGTTATATGCGCGTGGCAGAAGCAGCAACACAAGC
AGGCGGAATAACCTTCGTTACAATTTTATTTGGCATATTGTACATCATTTTAATGTATACATGTGCATACGTATTAATTCGTATGTTTAAAAAT
AAACCGGCGTATGAAGATGTAAATCGTTTAGCCAAGAAGCAAGGAGGAGAAATAGAAAAATGA

Gene: cydB (cytochrome bd quinol oxidase subunit 2)

Position: 1051693 to 1052712, length: 1020 nt, orientation: FORWARD

Perfect match to: (COL-CP000046-[1105798:1106817], allele observed in CC8+CC1+CC7+CC72+CC239)

Sequence:

ATGATTTATGCATTTATAGGTATAACAGTGTTATGGTTGTTTTTATTTTGCTATATCATTATTGCTTCTATAGATTTTGGGGCAGGTTTCTTCG
CATTGCATTCAAAGTTAACTGGTGATGAAAAGAAAATTAATCACTTAATTTCACGTTATTTAAACCCAGTTTGGGAAGTTACGAATGTATTCT
TTGTTTTCTTCTTCGTAGGATTCGTAGGTTTCTTTCCAGAGTCAATCAAATATTTAGGCACGGTATTGTTAATACCAGGTTCAATAGCACTGA
TTATGATATCGTTGAGAAACAGTTTTTATGCATTTGAAAATTATGGTCAAGATACAAAATTAGCATGGATGATCATGTATGGGGTAAGTGG
ATTATTAATTCCAGCTTCATTATCTACTGCTTTAACTATTACAGAAGGTGGCTATATTAATGTTCGAAACAATGTTATCGATCTAGATTGGGT
GCAGTTATTATTAAGCCCATTTGCTTGGTCTGTAGTATTCTTGGCAATTATTTCAGTTTTATATATTTCATCAGGATTTTTGACATATTATGCTA
AAAAAGCAAATGACGAACCAGCATATAATTTAACAAGACAATGGCACATATTTTTAGGGCCGCCGATGATTATCATTTGTTTATTCGTATTT
CTATCATTACGCATTCAAAATTCTGAACATTTTTATTCAGCTGTTTTTGACTATTGGTGGATGTTTGGTATAAGTTTCTTATTCTTTGCATTAGC
TTCATTGTTAACATTCTTTAAGAAAAAACATGGTTTGGCTTTTGTATTTGTTATTTTACAAATGATGTTCGCGTTCTTTGGCTATGGAATTAGT
AAATTGCCATACCTTTTATATCCGTTTGTAAAAATTACAGATGCATATGTTAATCCAGAAATGGGCTGGACATTAGTGATTGTCTTTATTTTA
GGTTTACTTTTATTACTTCCATCGTTAATATTATTATTAAGATTATTTGTTTTCGACAAAGAATATGTTGAAGGAAAGAAATCATAA

Gene: ktrA (potassium uptake protein A)

Position: 1052845 to 1053507, length: 663 nt, orientation: FORWARD

Perfect match to: (BAA-39-ST239-AEEK01000068-[241812:242474:r], allele observed in CC239+CC80)

Sequence:

ATGGGTAAAGAATATGTAGTCATCGGTCTAGGTCGTTTTGGAGGTAGTATCGTTCGTGAGTTGAATGCATTAGACATGGATGTAATGGCCA
TCGACCATGATGAAAATAGAGTGAATGAATATAGTGATATCGCTACTCATGCGGTTGTTGCAGACACTACAGATGAAGCAGTTATGAAAAG
TTTAGGTATCCGTAACTTTGATCATGTCATTGTGGCAATTGGTGAAAATATTCAATCAAGTACGTTGACGACTTTAATTTTAAAAGAGTTAG
GTGTAAAAAAAGTAACTGCTAAAGCACAAAATGATTATCATGCAAAGATTTTAAATAAAATTGGAGCAGATACGGTTGTGCACCCTGAGCG
TGATATGGGTAGACGTATTGCGCATAATGTTGCGAGTGCAAGTGTACTTGATTATCTTGAGTTGGCAGACGAGCATTCTATTGTAGAATTG
AAAGCAACTGAAAAGATGGCGGGGCAGTCTATCATTGATTTAGATATAAGAGCACAATATGGAATTAACATTATTGCAATTAAACGAGGCA
AAGAGTTCATCATATCACCAAATCCAAATATTAATTTAGAAATAGGTGATATTTTAATCATGATTGGACATGATAATGATTTAAATCGCTTTG
AAAAAAATATTGCGACGAGATAA

Gene: tx_universal2 (rho-independent terminator)

Position: 1053619 to 1053657, length: 39 nt

Perfect match to: (Strain_21331-AGTV01000040-[104403:104441], allele observed in CC398)

Sequence:

TAGAATTGAAAAAAGCTTGTTACAAGCGCATTTTCGTTC

Gene: rnjA (ribonuclease J1)

Position: 1053797 to 1055494, length: 1698 nt, orientation: REVERSE

Perfect match to: (11819-97-CP003194-[1100480:1102177:r], allele observed in CC80+CC8+CC30+CC239+CC4803)

Sequence:

TTATTTATTGTTTGATTCTTTTTGTTCGTTTACCTTCATAATGACTGGTAAAATCATTGGTTTTCTAGCTGTTTTTTCAAATAAATAAGGTTGTA
ATGTTTCAATGATAGAAGATTTAATCTGATGCCATTGAATATCTTTATTTTGATTTAACTTACTAATAACATCAGTTTTGATTTTGCGTTGTGC
ATCATAAATTAATTGACCTGATTCCCTCATATATACAAATCCTCGAGAAATAATGTCTGGACCAGAAAGTAATTTATTTGTATTAAAATCAAT
ACTAACAACAACGATAACTAAACCTTCTTCAGATAATAGCTTACGGTCTCTTATTACAACATTACCGATATCACCGATACCACTACCATCAAC
AAGTACATTACCAGATGGAATGCGACCAGCTTTACGTGCTGAATCGTGTGTTAAAGCTAAGACATCTCCAATATCAAAGATGAAGACATTA
TCTTCTTCAACGCCGCATTCAACACCAGTCTCACCATGTGCTTTTAACATACGGTATTCACCATGAATAGGTAAGAAATATTTCGGCTTGATT
AATCGAAGCATTAATTGTTGATCACCTTGAGAACCATGCCCTGAAGTATGGATGTTAGAAATCTTGCTATGGATAACATCTGCACCAGCTTT
ATACAAGGAATTAATAGTTCTGTTAATACTTTTTGTATTACCTGGGATAGGTGATGAACTAAATACAACGGTATCTTCAGGTATAATTTTAAT
TTGCTTATGAGTACCATTAGCAATTCTAGATAATGCTGCCATTGGTTCACCTTGTGAACCAGTACATAGTATCAATAACTCATGCTTCGGTAC
GGTATTAATTTTATTAGGTTCAATAAATGTTTCAGGTGGTGCTTTAATATAACCAAGTTCCATACCTATTTTAATATTGTTTTCCATCGAACGA
CCGAACGTAACAATTTTACGGTTATTTTTGATAGCAGCTTCAACTGCTTGTTGAACTCGGTAAATATTAGAAGCGAAGGTAGCAAATATAAT
ACGACCTTTACAATTACGGAAGATCTTATCTACGTTTTGACCAACTTCACGTTCGCTTAAAGTAAAATCAGGCACAAGTGAATTTGTTGAGTC
TGAAAGTAAACATAGAACGCCTTCTTCGCCTAATTGAGCCATTTTAGCAATGTTTGCTGGTTTGCCTACAGGTGTAAAATCAAATTTAAAGTC
ACCGGTATGAACTACTTTTCCTTCAGGTGTATCTACGATGACGCCATAAGTTTCAGGAATACTATGCGTAGTTAAGTAGAAAGAAATCGTAA
AGTGCTTAGATTTAATCACACTGTCCTCATTGATTTCATTTAGTTTAGCAGTACGTAATAAATGATGTTCTTCAAGTTTATTACGGATTAAACC
TAATGCTAAAGGACCACCATAAATAGGTATATTAAGTTGTTTTAATAGGAAGGGCACACCGCCTATATGGTCTTCGTGACCATGTGTTATAA
ATAGGCCAACAATTTTTATCTTGGTTTTGAACTAGATATGTGTAGTCAGGTATAACATAATCAATCCCTAATAAGTTATCATCAGGGAATTTGA
TACCTGCATCGATAATGACAATTTCGTCTTTATACTCAACTGCATAAGTATTTTTACCGATTTCACCTAGACCTCCAAGTGCATATACACCTAC
TTCATTTGGATGTAATTGTTTCAT

Gene: rnpZA (DNA-directed RNA polymerase subunit omega 1)

Position: 1055494 to 1055712, length: 219 nt, orientation: REVERSE

Perfect match to: (N315-BA000018-[1068429:1068647:r], highly conserved allele)

Sequence:

TTATTTAGCAATCTCCACATTAAAGTGTTCTGAGTTTTCTTTTTCGTAATCTAAATGTGCGCCCTCTAATTTAGTGATAAATTCGATATTAAAA
TTACGATCTTTCAAGTAACGACGTACTTGTTCTTCTGTTTGAGCTTCAACATAAAGTGATTGTGTATTTTCACGCACAATTACCTCGTCTCTGT
TATGTTGATAAAAAACTTTAAATACTGCCAT

Gene: def1-defB (N-formylcysteine deformylase)

Position: 1056194 to 1056745, length: 552 nt, orientation: REVERSE

Perfect match to: (MW2-BA000033-[1070556:1071107:r], highly conserved allele)

Sequence:

TTAAACTTCTACTGCATCTGTATGTGGTTGTAATGGGTGGTCTTTGTCAATGTGATCATAGAACATTACACCATTTAAATGATCAATTTCATG
TTGGAAAACAATTGCTGGATATCCTTTTAGTCGTAATTGTATATCATTACCTTCGATGTCTTTGGCTTTAATTGTAATTCTATTATGACGGTGA
ACTAGACCAGCAACATTATCATCGACACTAAGGCAACCTTCACCAGTTGGTAAATAAGCTTCTTGAACGCTATGACTTACAATTTTTGGGTTC
ACAAGCATATAGTCATAAGATTTGCCACTGCCATCATCTGGTATTAAAACAGCAATCATACGTTTAGAAATATTAATTTGAGGTGCAGCCAA
ACCAACGCCTGAACGTAAACCATATCGTTTCGCGATTTCCTCATCTTGACTATTTACTAAAAACTCTCTCATGGCGATTAATGTTTCTTTTTCTT
CTTTAGTTAATGGTAATTCTAACTCAGCTGCTTTTTGACGCAAAGTTGGATGACCATCTCTAATGATGTCTTTCATTGTTAACAT

Gene: ykyA (putative cell-wall binding lipoprotein)

Position: 1057110 to 1057736, length: 627 nt, orientation: FORWARD

Perfect match to: (MW2-BA000033-[1071472:1072098], allele observed in CC1+CC80)

Sequence:

ATGAAATTTGGAAAAACAATCGCAGTAGTATTAGCATCTAGTGTCTTGCTTGCAGGATGTACTACGGATAAAAAAGAAATTAAGGCATATT
TAAAGCAAGTGGATAAAATTAAAGACGATGAAGAACCAATTAAAACTGTTGGTAAGAAAATTGCTGAATTAGATGAGAAAAAGAAAAAAT
TAACTGAAGATGTCAATAGTAAAGATACAGCAGTTCGCGGTAAAGCAGTAAAGGATTTAATTAAAAATGCCGATGATCGTCTAAAGGAATT
TGAAAAAGAAGAAGACGCAATTAAGAAGTCTGAACAAGACTTTAAGAAAGCAAAAAGTCACGTTGATAACATTGATAATGATGTTAAACG
TAAAGAAGTAAAACAATTAGATGATGTATTAAAAGAAAAATATAAGTTACACAGTGATTACGCGAAAGCATATAAAAAGGCTGTAAACTCA
GAGAAAACATTATTTAAATATTTAAATCAAAATGACGCGACACAACAAGGTGTTAACGAAAAATCAAAAGCAATAGAACAGAACTATAAAA
AGTTAAAAGAAGTATCAGATAAGTATACAAAAGTACTAAATAAGGTTGGTAAAGAAAAGCAAGACGTTGATCAATTTAAATAA

Gene: pdhA (pyruvate dehydrogenase E1 component, alpha subunit)

Position: 1057907 to 1059019, length: 1113 nt, orientation: FORWARD

Sequence:

ATGGCTCCTAAGTTACAAGCCCAATTCGATGCAGTAAAAGTTTTAAATGATACTCAATCGAAATTTGAAATGGTTCAAATTTTGGATGAGAA
TGGTAACGTCGTAAATGAAGACTTAGTACCTGATCTTACGGATGAACAATTAGTGGAATTAATGGAAAGAATGGTATGGACTCGTATCCTT
GATCAACGTTCTATCTCATTAAACAGACAAGGACGTTTAGGTTTCTATGCACCAACTGCTGGTCAAGAAGCATCACAATTAGCGTCACAATA
CGCTTTAGAAAAAGAAGATTACATTTTACCGGGATACAGAGATGTTCCTCAAATTATTTGGCATGGTTTACCATTAACTGAAGCTTTCTTATT
CTCAAGAGGTCACTTCAAAGGAAATCAATTCCCTGAAGGCGTTAATGCATTAAGCCCACAAATTATTATTGGTGCACAATACATTCAAGCTG
CTGGTGTTGCATTTGCACTTAAAAAACGTGGTAAAAATGCAGTTGCAATCACTTACACTGGTGACGGTGGTTCTTCACAAGGTGATTTCTAC
GAAGGTATTAACTTTGCAGCAGCTTATAAAGCACCTGCAATTTTCGTTATTCAAAACAATAACTATGCAATTTCAACACCAAGAAGCAAGCA
AACTGCTGCTGAAACATTAGCTCAAAAAGCAATTGCTGTAGGTATTCCTGGTATCCAAGTTGATGGTATGGATGCGTTAGCTGTATATCAAG
CAACTAAAGAAGCACGTGACCGCGCAGTTGCAGGTGAAGGTCCAACATTAATTGAAACTATGACATATCGTTATGGTCCTCATACAATGGC
TGGTGACGATCCAACTCGTTATAGAACTTCAGACGAAGATGCTGAATGGGAGAAAAAAGACCCATTAGTACGTTTCCGTAAATTCCTTGAA
AACAAAGGTTTATGGAATGAAGACAAAGAAAATGAAGTTATTGAACGTGCAAAAGCTGATATTAAAGCAGCAATTAAAGAGGCTGATAAC
ACTGAAAAACAAACTGTTACTTCTCTAATGGAAATTATGTATGAAGATATGCCTCAAAACTTAGCAGAACAATATGAAATTTACAAAGAGAA
GGAGTCGAAGTAA

Gene: pdhB (pyruvate dehydrogenase)

Position: 1059023 to 1060000, length: 978 nt, orientation: FORWARD

Perfect match to: (N315-BA000018-[1071958:1072935], highly conserved allele)

Sequence:

ATGGCACAAATGACAATGGTTCAAGCGATTAATGATGCGCTTAAAACTGAACTTAAAAATGACCAAGATGTTTTAATTTTTGGTGAAGACG
TTGGTGTTAACGGCGGTGTTTTCCGTGTTACTGAAGGACTACAAAAAGAATTTGGTGAAGATAGAGTATTCGATACACCTTTAGCTGAATC
AGGTATTGGTGGTTTAGCGATGGGTCTTGCAGTTGAAGGATTCCGTCCGGTTATGGAAGTACAATTCTTAGGTTTCGTATTCGAAGTATTTG
ATGCGATTGCTGGACAAATTGCACGTACTCGTTTCCGTTCAGGCGGTACTAAAACTGCACCTGTAACAATTCGTAGCCCATTTGGTGGTGGC
GTACACACACCAGAATTACACGCAGATAACTTAGAAGGTATTTTAGCTCAATCTCCAGGTCTAAAGGTTGTTATTCCTTCAGGCCCATACGA
TGCGAAAGGTTTATTAATTTCTTCTATTAGAAGTAATGACCCAGTCGTATACTTAGAGCATATGAAATTGTATCGTTCATTCCGTGAAGAAGT
ACCTGAAGAAGAATATACAATTGACATTGGTAAGGCTAATGTGAAAAAAGAAGGTAATGACATTTCAATCATCACATACGGTGCAATGGTT
CAAGAATCAATGAAAGCTGCAGAAGAACTTGAAAAAGATGGTTATTCTGTTGAAGTAATTGACTTACGTACTGTTCAACCAATCGATGTTG
ACACAATTGTAGCTTCAGTTGAAAAAACTGGTCGTGCAGTTGTAGTTCAAGAAGCACAACGTCAAGCTGGTGTTGGTGCAGCAGTTGTAGC
TGAATTAAGTGAACGTGCAATCCTTTCATTAGAAGCACCTATTGGAAGAGTTGCAGCAGCAGATACAATTTATCCATTCACTCAAGCTGAAA
ATGTTTGGTTACCAAACAAAAATGACATCATCGAAAAAGCAAAAGAAACTTTAGAATTTTAA

Gene: pdhC (dihydrolipoyllysine-residue acetyltransferase component of pyruvate dehydrogenase complex)

Position: 1060091 to 1061383, length: 1293 nt, orientation: FORWARD

Perfect match to: (N315-BA000018-[1073026:1074318], allele observed in CC5+CC1+CC8)

Sequence:

GTGGCATTTGAATTTAGATTACCCGATATCGGGGAAGGTATCCACGAAGGTGAAATTGTAAAATGGTTTGTTAAAGCTGGAGATACTATTG
AAGAAGACGATGTTTTAGCTGAGGTACAAAACGATAAATCAGTAGTAGAAATCCCATCACCAGTATCTGGTACTGTAGAAGAAGTTATGGT
AGAAGAAGGTACAGTAGCTGTAGTTGGTGACGTTATTGTTAAAATCGATGCACCTGATGCAGAAGATATGCAATTTAAAGGTCATGATGAT
GATTCATCATCTAAAGAAGAACCTGCGAAAGAGGAAGCGCCAGCAGAGCAAGCACCTGTAGCTACTCAAACTGAAGAAGTAGATGAAAAC
AGAACTGTTAAAGCAATGCCTTCAGTACGTAAATACGCACGTGAAAAAGGTGTTAACATTAAAGCAGTTTCTGGATCTGGTAAAAATGGTC
GTATTACAAAAGAAGATGTAGATGCATACTTAAATGGTGGTGCACCAACAGCTTCAAATGAATCAGCTGCTTCAGCTACAAGTGAAGAAGT
TGCTGAAACTCCTGCAGCACCTGCAGCAGTAACATTAGAAGGCGACTTCCCAGAAACAACTGAAAAAATCCCTGCTATGCGTAGAGCAATT
GCGAAAGCAATGGTTAACTCTAAGCATACTGCACCTCATGTAACATTAATGGATGAAATTGATGTTCAAGCATTATGGGATCACCGTAAGA
AATTTAAAGAAATCGCAGCTGAACAAGGTACTAAGTTAACATTCTTACCTTATGTTGTTAAAGCACTTGTTTCTGCATTGAAAAAATACCCAG
CACTTAACACTTCATTCAATGAAGAAGCTGGTGAAATCGTTCATAAACATTACTGGAATATCGGTATTGCAGCAGACACTGATAGAGGATTA
TTAGTACCTGTTGTTAAACATGCTGATCGTAAGTCTATTTTCCAAATTTCAGATGAAATTAATGAATTAGCTGTTAAAGCACGTGATGGTAAA
TTAACAGCCGATGAAATGAAAGGTGCTACATGCACAATCAGTAATATCGGTTCAGCTGGTGGACAATGGTTCACTCCAGTTATCAATCACCC
AGAAGTAGCAATCTTAGGAATTGGCCGTATTGCTCAAAAACCTATCGTTAAAGATGGAGAAATTGTTGCAGCACCAGTATTAGCATTATCAT
TAAGCTTTGACCACAGACAAATTGATGGTGCAACTGGCCAAAATGCAATGAATCACATTAAACGTTTATTAAATAATCCAGAATTATTATTA
ATGGAGGGGTAA

Gene: pdhD (dihydrolipoamide dehydrogenase)

Position: 1061387 to 1062793, length: 1407 nt, orientation: FORWARD

Perfect match to: (11819-97-CP003194-[1108070:1109476], allele observed in CC80)

Sequence:

ATGGTAGTTGGAGATTTCCCAATTGAAACAGATACTATAGTAATCGGAGCAGGTCCTGGTGGATACGTTGCAGCAATTCGTGCAGCTCAAT
TAGGACAAAAAGTAACAATTGTTGAGAAAGGTAATCTTGGTGGTGTTTGCTTAAACGTAGGATGTATTCCTTCAAAAGCATTACTACATGCT
TCTCACCGTTTTGTTGAAGCACAACATTCTGAAAACTTAGGTGTTATTGCTGAAAGTGTTTCTTTAAACTTCCAAAAAGTTCAAGAATTCAAA
TCATCAGTTGTTAATAAATTAACTGGTGGTGTTGAAGGCTTACTTAAAGGTAACAAAGTTAACATCGTTAAAGGTGAAGCATATTTCGTAGA
TAACAATAGCTTACGTGTTATGGACGAAAAGAGCGCACAAACATACAACTTTAAAAATGCAATCATTGCAACAGGTTCAAGACCAATTGAA
ATTCCTAATTTCAAATTCGGTAAACGTGTTATCGACTCAACAGGTGCTTTAAACTTACAAGAAGTACCAGGTAAATTAGTTGTAGTTGGTGG
AGGATACATTGGATCAGAATTAGGTACAGCATTTGCTAACTTTGGTTCAGAAGTAACCATCCTTGAAGGTGCTAAAGATATCTTAGGTGGCT
TCGAAAAACAAATGACACAACCTGTTAAAAAAGGTATGAAAGAAAAAGGTGTTGAAATCGTTACTGAAGCTATGGCTAAATCAGCTGAAG
AAACAGATAACGGAGTTAAAGTTACTTATGAAGCTAAAGGCGAAGAGAAAACAATCGAAGCTGATTATGTATTAGTAACTGTAGGTCGTC
GTCCAAACACAGACGAATTAGGCCTAGAAGAATTAGGTGTTAAATTCGCTGACCGTGGATTATTAGAAGTTGATAAACAAAGCCGTACGTC
TATCAGCAATATCTATGCAATTGGTGATATCGTTCCAGGTTTACCACTTGCTCACAAAGCTAGCTATGAAGCTAAAGTTGCTGCTGAAGCAA
TTGATGGTCAAGCTGCTGAAGTTGATTACATTGGTATGCCAGCAGTATGCTTTACTGAACCAGAATTAGCTACAGTTGGTTATTCAGAAGCG

CAAGCTAAAGAAGAAGGTTTAGCAATTAAAGCTTCTAAATTCCCATATGCAGCAAATGGTCGTGCATTATCATTAGATGATACTAACGGATT
TGTTAAACTTATTACACTTAAAGAAGATGATACTTTAATCGGTGCTCAAGTAGTTGGTACTGGTGCATCAGATATTATCTCTGAATTAGGTTT
AGCAATTGAAGCTGGTATGAATGCTGAAGATATCGCATTAACAATCCATGCACATCCAACATTAGGTGAGATGACTATGGAAGCAGCAGA
AAAAGCTATCGGATACCCAATCCATACAATGTAA

Gene: UPF0223 (putative protein)

Position: 1062961 to 1063236, length: 276 nt, orientation: FORWARD

Perfect match to: (N315-BA000018-[1075896:1076171], highly conserved allele)

Sequence:

ATGGAATATGAGTATCCAATTGATTTAGACTGGAGTAATGAAGAGATGATTTCAGTGATAAATTTCTTTAATCATGTAGAGAAGTATTATGA
ATCCGGCGTGACGGCAGGCGACTTTATGGGTGCATATAAAAGATTTAAAGAAATTGTGCCTGCTAAAGCAGAGGAAAAACAAATTTTTAAT
ACTTTCGAAAAAAGTAGTGGCTATAATAGTTACAAAGCAGTTCAAGATGTAAAAACTCACTCTGAAGAACAAAGAGTAACAGCTAAAAAAT
AA

Gene: puuR (transcriptional regulator)

Position: 1063380 to 1063919, length: 540 nt, orientation: FORWARD

Perfect match to: (MW2-BA000033-[1077742:1078281], highly conserved allele)

Sequence:

ATGAACATAGGTAATAAAATTAAAAATCTTAGAAGAATTAAAAATTTAACGCAAGAAGAACTTGCTGAACGTACAGACTTATCGAAAGGCT
ACATTTCACAAATAGAAAGTGAACATGCCTCACCAAGTATGGAAACTTTCTTAAATATTATAGAGGTGTTAGGAACGACGCCAAGTGAATTT
TTTAAAGACAGTGAAAATGAAAAAGTATTATACAAGAAGGAAGAACAAGTTATTTATGATGAGTATGATGAAGGTTATATATTAAATTGGT
TAGTTTCAAAGTCAAATGAATATGATATGGAGCCATTAATATTAACTTTAAAGCCTGGAGCATCATATAAAAATTTTAATCCATCAGAGTCT
GATACGTTTATTTATTGTATGTCAGGTCAGATAACACTTAATTTAGGCAAAGAGATATATCAAGCACAAGAAGAAGACGTTTTGTATTTTAA
AGCACGAGATAATCATCGTTTGTCAAACGAATCAAACAATGAAACACGAATACTTATTGTAGCGACAGCTTCATATTTATAG

Gene: potA (spermidine/putrescine ABC transporter, ATP-binding protein)

Position: 1063932 to 1065026, length: 1095 nt, orientation: FORWARD

Perfect match to: (MW2-BA000033-[1078294:1079388], highly conserved allele)

Sequence:

TTGGAACCGTTATTATCATTAAAATCAGTTAGTAAAAGCTATGATGATCTTAATATCTTAGATGACATAGATATTGATATTGAATCAGGATAC
TTTTATACATTATTAGGTCCTTCAGGTTGTGGTAAAACAACAATTTTAAAATTAATTGCAGGGTTTGAATATCCTGACAGTGGTGAAGTGATT
TATCAAAACAAACCAATTGGTAATTTACCACCAAATAAACGTAAAGTGAATACAGTCTTTCAAGATTATGCATTATTTCCACACTTAAACGTC
TATGATAATATCGCTTTTGGTTTGAAATTAAAAAAATTATCAAAAACCGAAATTGATCAAAAAGTAACTGAGGCATTAAAATTAGTAAAACT
TTCAGGTTATGAAAAAAGAAATATTAATGAAATGAGTGGCGGACAAAAGCAACGTGTTGCAATTGCACGTGCTATCGTAAATGAACCAGA
AATATTATTGTTAGATGAATCTTTATCCGCATTAGATTTGAAATTGCGTACTGAAATGCAATATGAATTACGAGAATTGCAATCTAGATTAG
GTATTACATTTATATTTGTAACACATGATCAAGAAGAAGCGTTAGCATTAAGTGACTTTCTTTTTGTATTAAAAGATGGGAAAATTCAACAAT
TTGGCACACCAACAGATATATATGACGAACCAGTGAATCGATTTGTAGCTGATTTTATTGGAGAATCTAATATTGTTGAAGGGCGCATGGTT
AGAGATTATGTCGTGAATATTTATGGGCAAGATTTCGAATGTGTCGATATGGGTATTCCTGAAAATAAAAAAGTAGAAGTCGTTATTCGAC
CAGAAGATATATCATTAATCAAAGCTGAAGAAGGATTATTTAAAGCAACTGTTGATTCTATGTTATTTAGAGGGGTCCACTATGAAATATGT
TGTATAGACAATAAAGGTTATGAATGGGTAATACAAACGACTAAAAAAGCTGAAGTAGGCAGTGAAGTTGGTCTTTATTTTGATCCTGAAG
CCATTCATATTATGGTTCCTGGAGAAACAGAAGAAGAATTTGATAAACGTATTGAAAGCTATGAGGAAGTAGACAATGCGTAA

Gene: potB (spermidine/putrescine ABC transporter, transmembrane permease)

Position: 1065019 to 1065816, length: 798 nt, orientation: FORWARD

Sequence:

ATGCGTAATACTAATAAATTTCTCTTAATCCCGTATTTACTATGGATGGTTATATTTATTATTGTACCAGTTGTATTACTCATTTATTTTTCATTT
TTAGATATCAATGGACGTTTTAGTTTCACGAATTATCAACAAATTTTTACTACAAAATATTTGAAAATGTTTGCATATTCAATTTTATATGCCG
CTTCAATAACAATTATTACCTTGACTATCAGTTATCCAGCTGCCTACTATATTACTCGTTCGAAATTTCAAAATATCTTATTAATGATAATGATT
ATTCCAACATGGATAAATTTATTGTTAAAGACATATGCTTTTATAGGTTTATTAAGTCATGATGGCGTGATTAATCAATTTTTCCACTTATTTA
ATTTACCATCATTCAATTTGTTATTTACAACTGGTGCGTTTTTAGTAGTGGCAAGTTACATTTATATACCATTTATGATTTTACCTATATTTAAT
AGCATGAAAGCAATTCCTAATAATTTATTGCAGGCCTCAAGTGATTTAGGTGCCAGTCCTTTCTATACTTTTAGAAAAGTAATCATGCCGTTA
ACAAAAGAAGGTGTTATGACTGGGATTCAAGTAACCTTTATTCCATCACTTTCATTATTTATGATTACAAGATTAATTGCAGGTAATAAAGTC
ATAAATATAGGTACGGCAATAGAGGAACAATTTTTAACAATTCAAAATTATGGTATGGGATCAACTATAGCTATATTCTTAATTGTATTTATG
GCATTCATTTTAATCATTACAAAATCATCTAATGGGAGAGGGTGA

Gene: potC (spermidine/putrescine ABC transporter, transmembrane permease)

Position: 1065822 to 1066631, length: 810 nt, orientation: FORWARD

Perfect match to: (N315-BA000018-[1078757:1079566], allele observed in CC5+CC8+CC22+CC239)

Sequence:

ATGAAATGGTATGGAAAGCTGTATATCGGGATACTTTTAGCGATTTTATACATCCCAATATTCTTTTTAATGTTCTATTCATTCAATTCGGCTG
GTAATATGATTCACTTTGAACATTTTACATTAGAGCATTATCAATCATTATTTCAAAATGATCATTTAATGTCGGTCATTTTTAATACGATAGC
TGTAGCACTTTTAGCAGCCTCAATTTCTACAGTTATTGGTACATTTGGTGCCATTGCTATTTATTATTTAAGAAATAAAAAGTTTAAAGTAACT
TTACTAACATTGAATAATGTCTTGATGGTATCATCCGACGTTGTCATAGGTGCATCATTCTTAATTATGTTTACAACGATTGGCCATTTTACTG
GTCTTGGTTTAGGATTTTGGACAGTTCTAATATCTCATATTGCATTTTGCATACCTATAGTTGTGATTATCGTCTTACCACAACTGTATGAAAT
GAATAATAATATGTTAAATGCTGCAAGAGATTTAGGAGCGACTGAACCACAATTATTAAGCAACATTATTATTCCTAATATTTTACCTTCTAT
TATAGGAGGATTCTTTATGGCTTTAACTTATTCACTAGACGATTTCACAGTAAGTTTCTTCGTTACTGGTAATGGCTTTAGTGTGTTATCAGTT
GAAGTTTATGCTATGGCGAGAAAAGGAATTAGTATGGAAATTAATGCGATTTCAACATTATTGTTTGCTGTTATTGTATTAGGAATACTAGG
ATATTATTTGATTCAATACGTGATAAATAAGAAAAAACTAATCAAGCGAGGTGTAAAATAA

Gene: potD (spermidine/putrescine ABC transporter, substrate-binding protein)

Position: 1066631 to 1067704, length: 1074 nt, orientation: FORWARD

Perfect match to: (Strain_2016-MLQA01000001-[200000:201073], allele observed in CC580+CC772)

Sequence:

ATGAAACGTTTTTTACAACTCATTATAGGTGCATTAGTTGTGGGTATGCTTTGTCTTACTTTAAGTCATTGGTTTAAATCTAAAGAACAAGTG
CATACAAATCAAAAAATTTACGTATACAATTGGGGCGAATATATTGATCCAGAGTTAATTAAGAAATTTGAAAAAGAAACTGGCATTCAAG
TCGTTTATGAAACTTTCGATTCAAATGAAGCGATGGAAGCCAAAATTCGCAATGGCGGTACACATTATGATGTTGCTTTTCCTAGTGAATAT
ACAGTTCAAAAATTGAAAAGAGATCATATATTGTTACCAATAGATCATAATAAGGTACCTAATATTAAAAATTTAGATTCAGATTATATGAA
TATGTCATTTGATAAAGGCAATAAATATTCTTTACCTTATTTCTTTGGAACTGTAGGTATTTTATATAATAAAGAAAAGTATCCAAATGAATC
ATTTGATAGTTGGAAGTCATTGTATAATCCTAAATTTAAAAACCAAATTTTACTAGTTGACGGTGCTAGAGAGATTATAGGCATGAGTTTGA
ATAAACTTGGGTATAATCTTAATGACCGTAATTCGCACCATTTAAAAGAAGCAGAGCGAGATTTAACCAAACTAGCACCACAAGTAAGAGG
TGTCGTAGGTGATGAAATTACCATGATGCTTCAACAAAATGAAGGTAACATAGCGGTTGTTTGGAGTGGTGTTGCAGCACCTCTAGTGCAA
GAAGGGGATAAATATAATTATGTTATTCCTAAAGAAGGATCGAATTTATGGTTCGACAATATGGTAATTCCAAAAACGGCACAAAATAAAG
AGGGTGCATATAAATTTATGAATTTTTTATTAGATGCTAAAAATAACAAGCAAAATACAGAATTCGTAGGCTATGCAACGCCAAACAAGGC
TGCTCGACAATTGTTGCCTAAAGAGATTAAAGACGACCATCGTTTTTATCCGACTAAGAAAGAGCAAGAACGCCTTGAAGTTTATAAAGATT
TAGGACCTGAAGTTTTAAGTGAATACAATGAAAACTTTTTGAATTTCAAAATGTCATTAAAATAA

Gene: Q5HGY1 (polytopic membrane protein)

Position: 1067778 to 1068797, length: 1020 nt, orientation: FORWARD

Perfect match to: (11819-97-CP003194-[1114461:1115480], allele observed in CC80)

Sequence:

ATGACTGGAGAACAATTTACTCAAATTAAACGTCCAGTAAGTAGATTAACTGAAAAAGTTCTAGGTTGGTTATGTTGGGTGATGTTATTAGT
GCTTACTGTAATTACGATGTTTATAGCACTTGTTTCATTCAGTAATAACACATCGATTGCTAATCTTGAAAATACATTAAACAATAATGCATTT
ATCCAGCAATTATTAGCTGGAAATGGCTATAATACAACACAATTTGTAATATGGTTACAAAATGGTATATGGGCTATTATCGTTTACTTTATT

GTTTGTTTGTTGATTTCATTTTTAGCTCTCATATCTATGAATATAAGAATCTTGTCAGGTTTCTTATTCTTAATATCAGCAATTGTAACGATTCC
TTTAGTTTTACTTATCGTTACTTTAATCATTCCGATATTATTCTTTATTATTGCGATGATGCTATTTATAAGAAAAGATAAAGTTGAAATGGTT
GCGCCACAATATTATGAAGAGTATAACGGACCGATTTATGATTATCGTGAACCTGTGTATGAGCGCCCCCAACCGAAAGATGATTATTATG
ATGTGCCTAAATATGAAAAAGAATTGGATAAATCAAATACTGTATATGATCAAGAACAGGAAAGAGATAAATATGATCAATTTCCTAAACG
TGCAGTTGAAAGTGAATATAATTATGATGAGCGCACTGAGGAAGAACCATCAGTATTATCCAGACAGGCTAAATACAAACAAAAAAGTACT
GAAGAACTAGGTATTGAAGATGATGGTTATTATGCAGAACCTGAAGTTGATCCAAAAGAATTGAAAGCACAACAAAAGCGAGAAAAAGCT
GAAATTAAAGCTAAGAAAAAAGAAAAACGTAAAGCTTATAACCAACGAATGAAAGAACGTAGGAAAAATCAACCTAGCGCAGTTAGTCAA
CGTCGAATGAATTTTGAAGAGCGACGTCAAATTTACAACAATGATATTTCTGAAGAACGCAATTCAAGTGAAGTTAAGGACAAAAAAGAGC
AAGAATAA

Gene: DUF4064 (protein of unknown function)

Position: 1069080 to 1069499, length: 420 nt, orientation: FORWARD

Sequence:

ATGAATAGAAAACCTGAATTAATCATGGCTTGGATTGCAAATAGTATTAGTATTATTTATTTATTAGTTATGGGGCTATCCTATTTTTCTTTAA
AAAGTGGTAATGCATCACAACGTGAAGAATTAGCGAAGCAATTATCTCAGAACGGTGGCAATGTTTCTTTAGATATGCTTCAGACAACAAT
GGGTGCATTAGCAATTATTTTATTAATTTCAACACTTTATGGTATATTTGCGACAATTTGTATTAAAGGGCGTAGAAAATTATCGATTATACT
TTTTGTTATCGCGATAATTGTAAGTTTGATGGCTCTTAATTTAATTGCAATTGTCTTATGGGTTATCGTGATGATTATGTTGATTTCTAAAAAA
GAATCAAAAGAAACAACACATAAGGACGATGAGTATATTTATCATTAA

Gene: mntH (divalent metal cation transporter)

Position: 1069581 to 1070933, length: 1353 nt, orientation: REVERSE

Perfect match to: (11819-97-CP003194-[1116264:1117616:r], allele observed in CC80+CC97)

Sequence:

TTAACTTTGTAGTTCTTGGAATGTTTGAACGATTAAATAGACATTTAAAATACTTAGAATAATAATAAGTGTCCATGAAATGATATTAACCCA
TGTTTTATTATAGAAAGGTCCCATCAAGTCTTTATTACTAGTAGCTAATTGTAATGGTATTAAACAGAATGGCAGAGCAATACTTAAAAATA
CTTGAGAGAATACAAGTAGTTGCTCAATTTTAGCTGCATTACCTTTAAAAATGATTAAGCATACGATAACAGGAATGACAGCAAGGGAACG
TGTAATTAAACGTCTTAACCAATTTGGTATGTGTAATCTTAAAAATCCTTCCATTACAATTTGTCCTGCTAAAGTACCAGTAATCGTTGAATTT
TGACCTGACGCTAATAATGCAACTGCAAATAATGTACTCATGATTGCACCCATTGTTGCACCTAGTACAGGTTCAGTTTTTAAGGCGTGATA
TAAATCATAGAAACCACCTAAATCGTCAGCATTTGAGTTGAAAAATAGTGATGCTCCTAACACTAATAATAAGCAATTGACTACAAATGCGA
TTGATAACTGAATGTTCGAATCTATCGTAGCAAATTTAATCGCTTGCGCCTTTTCTTCATTGTTATGTCTTGAGTATGTTCTAGATTGTACAAT
TGATGAATGTAAGTACAAATTATGAGGCATAATTGTAGCGCCAATAATACCTAATGCAATATAGAGAATGCCGTTATTTGTAATGATTTCAC
TATGTGGTATAAATCCATTTAACACAGCATTCAACTGTGGTGATGAAATATAGACTTCAAATATAAAGATGAATAACACTGTGAAAATTAAT
GTACCAACAATAGCTTCAATTTTTCTAAAACCATATTTCATTATAAATAGTAGTAAAAATACATCAAGTACAGTTATTAGTGCACCGACGATT
AAAGGTATGTTAAATAGGAGATTAAGAGCAATAGCACTACCAATAACCTCAGCAATATCTGTAGCGATAATTGCTAGTTCTGCAATGATCC
AAAAGATTATAGCAATAGGTCTTGATAAATAATGTCGTGTCATTTGAGCTAAGTCCATACCTGTTGCTATTCCTAATCTCACTGTCATGCTTT
GAAGTAACATTGCTGATAAACTTGAAATAAGAATTACGAATAGCAAAGTATAGCCATATTGCGCGCCACCTTGCATTGATGTTATCCAGTTT
CCGGGATCCATGTAACCAACAGCGACTAATAACCCAGGCCCAAGAAATGATAAAAATTTCTGTTTATTTGAACTGCGATGATCGAATTTAAT
TGTATTGTTTATTTCGTCTAAACTTAATTGTTCATTTGTTGAATGTCGTTTATTATTCAT

Gene: yktB (conserved hypothetical protein)

Position: 1071117 to 1071731, length: 615 nt, orientation: REVERSE

Perfect match to: (11819-97-CP003194-[1117800:1118414:r], allele observed in CC80+CC97)

Sequence:

TTATGCAGAATAAAATGGTAAGAACTGATCGAAGGTTTCTTCTAAAAATGCAATAAATGCTTTGTCACTTTTTAATCTTTTATCTTGTGGTGT
GATTGCACGCGCTATAAAGAATTCACCTTTTTTCACATTGATGGCTCTTTGTATCGCTTCTATTAAATCATCATCCGTTAAATCTTTAATAAAT
GGTTTATCAGGTTTCATATGATCTAAGCAAACACGATAATCATCAGGTAATTGTTGAATAGCTTTAAATTTTCTTTCAAAAACTTTTGCACGT
GTTGCTTTATCTTTTGCTTCATGCATGATTCCAAACATAACAAACAGTTGATCTTCAAACATACCAATTTGGAAATGAGGTAACATTTTATAG
CCTCTTTTGTTTGTTGCAAAAGCAACCCATGTATCTTTAGGAGGATTCACACTTCTTCTAGCATGTTTTGCTACGTGAGGATAAAATGTTTCA

CCAGTTTGACTTGTAAAGAAGTCACTAAAATATTCTCCTAATTCACGGAGTTGTGGTCGTATGTATTCGTTTAAAGCTTCCATTCGTGCGTCT
AAGCCTTCTACGTTGAACGCTTTGAAATCTTTAGGTTTAAATGTATATTTTGTCAT

Gene: suhB1 (inositol monophosphatase)

Position: 1071888 to 1072715, length: 828 nt, orientation: FORWARD

Perfect match to: (11819-97-CP003194-[1118571:1119398], allele observed in CC80+CC5+CC7+CC72+CC188)

Sequence:

ATGGCACTTTATGGATTTGCCCAAGGACTTATTCAAGAAGCAGGAATTAGAATTAAACAATTGATGGAGCAAAATTTAACAATTGAAACAA
AGTCAAATCCGAATGACCTTGTTACAAATGTAGATAAAGCAACAGAAGATTTCATTTTTGATACAATTTTAGAAACATATCCCAATCATCAA
GTATTAGGTGAAGAAGGGCATGGTCATGACATCGATACTTCCAAAGGTACGGTATGGGTTGTTGACCCAATAGACGGTACATTGAATTTTG
TTCATCAACAAGAAAATTTCGCAATTTCAATTGGTATTTATATCGATGGTAAACCTTATGCAGGTTTTGTATATGATGTTATGGCTGACGTCT
TATATCATGCTAAAGTAGGGGAAGGTGCATATCGTGGTAGCCAACCCTTGAAACCATTGAATGATTCTAATCTAAGACAAAGCATTATTGG
GATCAATCCGAACTGGTTAACTAAACCAATTTTAGGAGAAATCTTTAAAGAAATTGTTAATGATTCTAGAAGTGCAAGGGCATATGGTAGT
GCAGCGCTTGAAATCGTTTCAGTTGCTACAGGTAATTTAGAAGCATATATGACGCCAAGACTTCAACCATGGGATTTTGCTGGCGGATTGG
TTATTTTATATGAAGTAAATGGACAAGCTTCCAATTTACTAGGAGAACCATTAACAATTAGTGGTCCAAATTCAATCTTAGTTGGAAATCGT
GGTCTCCATCAAGAAATTAGCAACGATTATTTAGAGCCCCACCATGATGCGTTAATACAATTACATGAACAACGATTTAAAAGAAAATCAAA
ATAA

Gene: ylaF (putative membrane protein)

Position: 1072869 to 1073060, length: 192 nt, orientation: REVERSE

Perfect match to: (N315-BA000018-[1085801:1085992:r], highly conserved allele)

Sequence:

TTATAGCCAATCGTTTTCTCGATATTTTTCTTTGTAGTAAATCCAATTCCAAAGGTTGCAACGAGTAATATAAATGTTAAAATCATCATTGGT
ACATTTGATGCACCAACAGCAAAACTAAATAATACTAAGAAAACAACAGCTAATATAGAAAATACCCAAAAGATATTTTTAGATTTTTTTTGT
TTCAT

Gene: typA (GTP-binding protein)

Position: 1073162 to 1075009, length: 1848 nt, orientation: FORWARD

Perfect match to: (NN50-BAEA01000008-[134010:135857:r], allele observed in CC4803+CC45I+CC239)

Sequence:

ATGACTAATAAAAGAGAAGATGTCCGCAATATAGCAATTATTGCTCACGTTGACCATGGTAAAACAACTTTAGTAGATGAGTTGTTAAAAC
AATCTGGTATATTCAGAGAAAATGAACATGTCGATGAACGTGCAATGGACTCTAACGATATCGAAAGAGAGCGTGGAATTACGATTCTAGC
CAAAAATACGGCTGTTGATTATAAAGGTACACGTATTAATATTTTGGATACACCAGGACATGCAGACTTTGGTGGAGAAGTAGAACGTATT
ATGAAAATGGTTGATGGGGTTGTCTTAGTAGTAGATGCGTATGAAGGTACAATGCCTCAAACACGTTTTGTACTTAAAAAAGCGCTAGAAC
AAAACCTGAAACCTGTTGTTGTTGTTAATAAAATTGATAAACCATCAGCACGTCCAGAGGGTGTTGTAGATGAAGTTTTAGATTTATTTATT
GAATTAGAAGCAAACGATGAACAATTAGAATTCCCTGTTGTTTATGCTTCAGCAGTAAATGGTACAGCTAGCTTAGATCCTGAAAAGCAAG
ATGATAATTTACAATCATTATATGAAACAATTATTGATTATGTACCAGCTCCAATTGATAACAGTGATGAGCCATTACAATTCCAAGTAGCAT
TGTTGGACTACAATGATTATGTTGGACGTATTGGTATTGGTCGTGTATTCAGAGGTAAAATGCGTGTCGGAGATAATGTATCACTAATTAAA
TTAGACGGTACAGTGAAAAACTTCCGTGTAACTAAAATCTTTGGTTACTTTGGATTAAAACGTTTAGAAATTGAAGAAGCACAAGCTGGAG
ATTTAATTGCTGTTTCAGGTATGGAAGACATTAATGTTGGTGAAACTGTAACACCACATGACCATCAAGAAGCATTGCCAGTTCTACGTATT
GATGAGCCTACTCTTGAAATGACATTTAAAGTTAACAATTCTCCATTTGCTGGCCGTGAAGGTGACTTTGTAACAGCGCGTCAAATTCAAGA
ACGTTTAAATCAACAATTAGAAACAGATGTATCTTTGAAAGTTTCTAACACAGATTCTCCAGATACATGGGTAGTTGCTGGTCGCGGTGAAT
TGCATTTATCAATCCTTATTGAAAATATGCGTCGTGAAGGTTATGAATTACAAGTTTCAAAACCACAAGTAATTATTAAAGAAATAGATGGT
GTAATGTGTGAACCATTTGAACGTGTGCAATGTGAAGTGCCACAAGAAAATGCAGGTGCTGTTATTGAATCATTAGGTGCACGTAAAGGTG
AAATGGTTGATATGACTACAACTGATAATGGACTTACACGTTTAATCTTTAATGTACCGGCTCGTGGTATGATTGGTTATACGACTGAATTT
ATGTCAATGACAAGAGGTTACGGTATTATTAACCATACATTTGAAGAATTTAGACCACGTATTAAAGCACAAATTGGCGGTCGTCGTAATG
GTGCATTAATTTCAATGGATCAAGGTTCTGCAAGTACTTATGCCATTTTGGGACTTGAAGATAGAGGTGTAAACTTCATGGAACCTGGTACT
GAAGTTTATGAAGGTATGATTGTTGGTGAACATAATCGTGAAAATGATTTAACTGTTAACATCACTAAAACAAAACATCAAACTAACGTACG

TTCTGCAACGAAAGACCAAACACAAACAATGAATAGACCGCGTATTCTAACATTGGAAGAAGCGTTACAATTCATTAATGATGATGAACTT
GTTGAGGTTACACCAGAAAGCATACGTTTAAGAAAGAAAATTTTAAACAAAAATGTTCGTGAAAAAGAAGCAAAGCGTATCAAACAAATG
ATGCAAGAAAACGAATAA

Gene: ylaL (protein of unknown function)

Position: 1075318 to 1075800, length: 483 nt, orientation: REVERSE

Perfect match to: (KLT6-APFH01000004-[532050:532532:r], highly conserved allele)

Sequence:

TTAAATCACCTCAATATGAGTTACATGGTTGATTCGATATGAATAACCATCTTCTTGATTGTAAATAAAACTATCTACACCATTATCGCTGTAA
AGTCGTTTACCGTCTTTTGCAAATTGGAAAAATAAATAGGGTAATAGATCTATCGGTATATCTAAATGATTATGCTCATTTGATAATCGAATA
GTAGTTGCAGAGTCATGTGGTTCGGAATGTTTGAAAAAAGGTGTCATATTAATGACAAATGAACCTTCTAACATGGCACGTTTTTTATATTT
TATTTCTGAATTTAAAGTAGGCGGATTAGTTTGTCCTTCTAGGATAGCACGATTCCATTCATGATTATCTTCAAAGTCGATTGGTTTTGAACC
ATCAAATACACCTTTTTCTAAATCTTCGATGCTAACTTTTCTATCATCGAAAATCCAAGTCGTACTATCTAATGTTATAGGAAACTTTACGGCT
CCTTTAATTTGTATCAT

Gene: ylaN (putative conserved protein)

Position: 1075941 to 1076216, length: 276 nt, orientation: FORWARD

Perfect match to: (N315-BA000018-[1088873:1089148], highly conserved allele)

Sequence:

ATGGCGAAACAAGCAACAATGAAAAATGCAGCTTTGAAACAATTGACTAAAGATGCTGATGAAATCTTGCATCTGATTAAAGTTCAACTAG
ATAATTTAACATTACCTTCATGCCCATTATATGAAGAAGTACTAGATACACAAATGTTTGGACTTCAAAAAGAAGTTGATTTTGCTGTTAAAT
TAGGTTTAGTTGACCGCGAAGATGGCAAACAAATTATGTTACGTCTTGAGAAAGAACTTTCAAAATTACATGAAGCTTTTACACTTGTTTAA

Gene: ftsW2 (cell division protein W2)

Position: 1076530 to 1077756, length: 1227 nt, orientation: FORWARD

Perfect match to: (11819-97-CP003194-[1123213:1124439], allele observed in CC80+CC5+CC8+CC239)

Sequence:

ATGAAGAATTTTAGAAGTATTTTACGGTATATTGGTAAAACCTCAAAGTTTATTGATTATCCGTTATTAGTTACATATATTGTATTGAGTTTA
ATTGGATTAGTGATGGTATATAGTGCAAGTATGGTTCCAGCAACTAAAGGCACATTGACTGGTGGTATCGATGTTCCAGGAACGTATTTTT
ACAACCGACAATTAGCATATGTCATAATGAGTTTTATAATTGTATTTTTTATTGCATTTTTAATGAATGTTAAATTACTGAGTAATATTAAAGT
GCAAAAAGGTATGATTATAACTATCGTCTCACTATTATTACTGACGTTAGTAATAGGTAAAGATATTAATGGTTCTAAAAGTTGGATAAACT
TAGGATTTATGAACTTACAGGCATCTGAGTTATTAAAAAATTGCAATCATATTATATATCCCATTTATGATCAGTAAAAAAATGCCTAGAGTAT
TAAGTAAACCAAAATTAATTTTAAGTCCTATTGTATTAGCATTAGGTTGTACGTTTTTAGTTTTCCTACAAAAAGACGTAGGGCAAACATTAC
TAATATTAATTATTTTAGTTGCGATCATTTTTTTATTCAGGAATTGGGGTAAACAAAGTCCTAAGATTTGGTATACCAGCAGTGCTAGGATTCT
TAGTAGTATTTGTCATTGCATTAATGGCTGGTTGGTTACCAAGTTATTTAACTGCCAGATTTAGTACGCTAACAGATCCATTCCAATTCGAAT
CAGGAACTGGATACCATATTTCCAATTCATTGCTTGCGATAGGTAACGGTGGCGTATTTGGAAAAGGATTAGGAAATAGTGCAATGAAATT
GGGCTATTTACCAGAACCACATACAGATTTTATTTTTGCAATTATTTGCGAAGAATTAGGTTTAATCGGAGGATTGCTAGTTATTACTTTAGA
GTTCTTTATTGTATATCGTGCCTTCCAGTTTGCAAATAAAACATCATCATATTTTTATAAACTTGTGTGTGTTGGGATTGCCACATACTTTGGA
AGTCAAACGTTTGTAAACATTGGCGGTATTTCGGCAACAATTCCATTAACTGGTGTGCCATTGCCATTTATCAGCTTTGGTGGATCATCAAT
GATTAGTTTAAGTATTGCTATGGGATTACTTCTGATTGTAGGTAAACAAATCAAAGTAGACCAGCAACGAAAGAAACAACAACAAAAAGTT
GATATAAGAAGACAATTTAATTAA

Gene: pycA (pyruvate carboxylase)

Position: 1078310 to 1081762, length: 3453 nt, orientation: FORWARD

Perfect match to: (COL-CP000046-[1132473:1135925], highly conserved allele)

Sequence:

TTGAAACAAATAAAAAAGTTACTTGTTGCTAACCGTGGAGAAATTGCAATTCGTATATTCAGAGCGGCGGCAGAATTAGACATCAGCACAG
TTGCAATTTATTCGAATGAAGACAAAAGTTCATTACATAGATATAAAGCAGATGAATCCTATTTAGTTGGAAGTGATTTAGGTCCTGCTGAA
AGTTATTTAAATATTGAGCGTATCATTGATGTAGCAAAACAAGCGAATGTGGATGCGATTCATCCTGGCTATGGATTTTTAAGTGAAAATGA
ACAATTTGCGCGTCGTTGTGCAGAAGAAGGAATTAAATTTATTGGTCCTCATTTAGAACATTTAGATATGTTTGGAGATAAAGTTAAAGCTC
GTACAACGGCTATCAAGGCAGATTTACCAGTTATTCCTGGTACAGACGGTCCAATTAAATCATATGAATTAGCAAAAGAATTTGCAGAAGA
AGCTGGTTTCCCGCTAATGATTAAAGCCACAAGTGGTGGCGGCGGTAAAGGTATGAGAATCGTTCGTGAAGAAAGTGAATTAGAAGATGC
TTTCCATAGAGCAAAATCAGAAGCTGAAAAATCATTTGGTAATAGTGAAGTTTACATAGAAAGATACATTGATAATCCAAAGCATATTGAA
GTACAAGTTATAGGTGACGAACATGGAAATATCGTACACTTATTTGAACGTGATTGTTCAGTACAACGTCGTCATCAAAAAGTTGTAGAAG
TTGCACCATCAGTTGGATTATCACCAACATTACGTCAACGTATTTGTGATGCTGCAATTCAATTGATGGAAAATATTAAATATGTCAATGCAG
GTACTGTTGAATTTCTAGTATCTGGTGACGAATTCTTCTTTATAGAAGTTAACCCTCGTGTACAAGTTGAGCATACAATTACAGAGATGGTA
ACAGGAATTGATATTGTTAAGACACAAATTTTAGTTGCAGCAGGTGCCGATTTATTTGGTGAAGAAATTAATATGCCGCAACAAAAAGATA
TTACAACATTGGGCTATGCCATCCAATGTCGTATTACAACAGAAGATCCGTTAAATGATTTCATGCCGGATACTGGAACAATCATTGCTTATC
GTTCAAGCGGTGGCTTTGGTGTACGTCTCGATGCTGGAGATGGTTTCCAAGGTGCTGAGATATCACCTTATTATGATTCATTACTCGTAAAA
TTATCTACACACGCGATATCATTTAAACAAGCAGAAGAAAAAATGGTACGTTCATTACGAGAAATGCGTATTCGTGGTGTTAAAACTAATAT
TCCATTCTTAATTAATGTAATGAAGAATAAAAAGTTCACAAGTGGTGATTACACAACTAAATTTATTGAAGAAACACCAGAACTTTTCGACA
TTCAGCCGTCTCTAGATAGAGGTACTAAAACATTAGAATATATAGGTAATGTAACAATTAATGGTTTCCCAAATGTTGAGAAACGTCCGAAA
CCAGACTATGAATTAGCATCAATTCCAACTGTATCTTCAAGTAAAATCGCTTCATTTAGTGGTACGAAACAATTGCTTGATGAAGTAGGTCC
AAAAGGTGTAGCTGAATGGGTTAAAAAGCAGGATGATGTCTTACTAACAGATACAACCTTTAGAGATGCACACCAATCATTATTAGCTACA
CGAGTTAGAACTAAGGATATGATTAATATCGCATCCAAAACAGCGGACGTATTTAAAGATGGTTTCTCACTTGAAATGTGGGGCGGTGCTA
CATTTGATGTGGCATATAATTTCTTGAAGGAAAACCCATGGGAACGACTTGAACGTCTACGTAAAGCTATTCCAAATGTATTATTCCAAATG
TTGTTACGTGCTTCAAACGCAGTTGGTTATAAAAACTATCCTGATAATGTTATTCATAAATTCGTACAAGAAAGTGCTAAAGCAGGCATAGA
TGTCTTTAGAATTTTCGATTCTTTAAACTGGGTAGATCAAATGAAAGTTGCCAATGAAGCAGTACAAGAAGCGGGCAAAATCTCTGAAGGT
ACTATTTGTTATACAGGTGACATTTTAAATCCTGAGCGATCAAACATTTATACTTTAGAGTATTATGTCAAACTAGCTAAAGAGTTAGAACGT
GAAGGTTTCCATATTTTAGCGATTAAAGATATGGCAGGCTTATTAAAACCTAAAGCCGCTTACGAATTGATTGGTGAGTTAAAATCAGCTGT
AGATTTACCAATTCATCTTCACACTCATGATACAAGTGGTAATGGTTTATTAACATACAAACAAGCAATAGATGCTGGTGTCGATATCATTG
ATACTGCTGTTGCTTCAATGAGTGGTTTAACAAGTCAGCCAAGCGCCAATTCGTTATATTATGCATTAAATGGCTTCCCACGCCACCTTAGAA
CTGATATTGAAGGTATGGAGTCACTTAGTCATTATTGGTCAACTGTACGTACTTATTATTCAGACTTTGAAAGTGATATCAAATCACCGAATA
CTGAAATTTATCAACATGAAATGCCTGGTGGACAGTATTCGAATTTAAGTCAACAAGCTAAAAGTTTAGGTTTAGGCGAAAGATTTGATGA
AGTCAAAGATATGTATCGCAGAGTGAATTTCTTATTTGGTGATATCGTAAAAGTAACTCCATCGTCTAAAGTAGTTGGTGATATGGCACTTT
ATATGGTACAAAATGATCTTGATGAACAATCCGTGATTACAGATGGCTATAAATTAGATTTCCCAGAATCAGTAGTGTCGTTCTTCAAAGGC
GAAATAGGACAACCTGTAAATGGTTTTAATAAAGATTTACAAGCGGTTATTTTAAAAGGCCAAGAAGCACTAACAGCTCGTCCAGGTGAAT
ATCTAGAGCCAGTTGATTTTGAAAAAGTCCGTGAGTTGCTTGAAGAAGAGCAACAAGGTCCTGTTACGGAGCAAGATATTATTAGTTATGT
ATTATATCCAAAGGTATATGAACAATATATTCAAACTAGAAATCAATACGGAAACTTATCGTTACTTGATACGCCTACATTCTTCTTTGGAAT
GCGTAATGGTGAAACAGTAGAAATCGAAATCGATAAGGGTAAACGATTAATTATTAAACTAGAAACGATTAGTGAACCAGATGAAAATGG
TAATAGAACGATTTACTATGCGATGAATGGTCAAGCGAGACGTATTTACATTAAAGATGAGAATGTGCATACAAATGCGAACGTTAAGCCA
AAAGCAGATAAGAGTAATCCAAGTCATATCGGTGCGCAAATGCCAGGTTCAGTAACTGAAGTCAAGGTTAGTGTAGGTGAATCTGTGAAA
GCTAACCAGCCGTTGCTAATTACTGAAGCTATGAAAATGGAAACAACAATTCAAGCACCATTTGACGGTGTGATTAAACAAGTAACTGTAA
ATAATGGTGACACAATAGCGACAGGCGATTTATTAATCGAAATTGAAAAAGCAACTGACTAA

Gene: ctaA (heme A synthase)

Position: 1081902 to 1082813, length: 912 nt, orientation: REVERSE

Perfect match to: (N315-BA000018-[1094834:1095745:r], highly conserved allele)

Sequence:

TTATTGCTTGTCACTTCTTACTGATCGTAACATTAGCATGATAAAGTATGTTGTCATACCAAATAAATAAGTGATAAATAATGCATGGAAAA
GTGCGATTATCAGGTTAACATTTGTCATAATAGATAATGCACCTGTGATAACTTGTAATATAACAAGTATAAATGCAGCTGTATAACCATAA
TGAACAGTACGGTTATTTGGATAATTTTTAACAGCGTGAATATAAGTAATCATAATAATCGTAAACACTATAAAAGCCATGATACGATGCGT
GAGTTGAACCCAATCTTGTTCTGAATGTGGTACAAGATCGTGGAATGGCAATGGCCAACCACCATATGCTAAACTTGCATCCGCATGTCTCA
CTAGTGCACCAGTATAAACACCACAATAAATGATGATTGCCATTAACCATGTTAAACGTCTTAATGGCTTTTTGATATATAATTCGTCAGCTT
CATATTTTTGATCTATAGAGAAAATAATCAATGTTATTAAAAATACAGATGAGAAACTGATTAATGATATACCAAAGTGCAATGCTAAAACG
TAATCGTTTTGTTGCCAAATAACAGCAGCAGCTCCGATTAATGCTTGCAATAATAAGAATCCAACACTAATGATTGATAAAGGTTTAATTTCT
TTAATATAGCCTATATGTTTCCATGCAGTGATAACTAACCATAAGACCATTAATAAAGACAAAGCTGAAACGGCTCTATGACTTAACTCAAT
AATCGTATCAATAGGAAAGAATTCTGGAATCAACGCACCATGACATAGTGGCCAAGAAGAACCACAACCATCAGCTGATCCGGTTTTGGTA
ACTAAGGCTCCACCAAGTTGTACAAATGTCATCATTAACGTTGCTACGACACCTAACCATTTTAAATTCTTTTTGCCAAACAA

Gene: ctaB (protoheme IX farnesyltransferase)

Position: 1083253 to 1084164, length: 912 nt, orientation: FORWARD

Perfect match to: (11819-97-CP003194-[1129936:1130847], allele observed in CC80)

Sequence:

ATGAGCAAAGAGCATACTTTGTCACAAAATATTAGCAGAGTTAACTTCAAAGAATTGCAACAGATAATTAAAATGGGACTTGTTCAAGGCA
ACTTAATTCCGGCTTTTGCGGGCGCATGGCTAGCAGTTGTAATGACAAATCATTCCTTCTTATCATCAATACCTCAAATTTTATTAATGCTATT
TGGATCTACTTTAATTATGGGTGGCGCATGTGCGTTAAATAATTATTACGACCAAGATATTGATCGTATTATGCCTAGTAAACAAAATAGAC
CAACTGTAAATAATAGAATTACAGATCAAAATTTATTACTATTAAGTTTTGGTATGATGTTAGTTGGAGAAATTTGTTTGTTTTTATTGAATA
TACCATCAGGCGTACTTGGTCTTATGGGGATTGTAGGTTATGTGTCTTATTACTCAATATGGTCTAAAAGACATACAACATGGAACACAGTC
ATTGGGAGTTTTCCTGGAGCAGTACCACCACTAATTGGATGGGTTGCAATTGAAGGACAAATTAGTTTAACAGCGATTGCGCTGTTTTTAGT
TGTATTTTGTTGGCAACCAATTCATTTTTATGCCTTAGCTATTAAACGTAAAGATGAATATGCACTTGCAAATATTCCAATGTTACCATCAGTT
AAGGGCTTTAAACGTACACGTGTCAGTATGTTTATCTGGTTGATTATTTTATTGCCAGTACCTTTATTACTAATAAATTTAGGTGTCGTATTCG
TAGTGTTAGCTACATTATTAAATTTAGGATGGATTGCATTAGGTTTAACAACATTTAAGAAAAATTCAGATCAAACAAAATGGGCAACACAA
ATGTTTATATATTCACTAAATTATTTAGTGATCTTTTTCGTGTTAGCTGTGATTGTTTCATTACTTACTTTGATCTAG

Gene: yozB-ctaM (factor required for functioning of QoxABCD)

Position: 1084189 to 1084650, length: 462 nt, orientation: FORWARD

Perfect match to: (RF122-AJ938182-[1065986:1066447], highly conserved allele)

Sequence:

ATGGGCGTTCCAATTTTACCAACGATTAGTACGACATGTATTGTCATTAGTGCAATTTTAATTGCCATTGGTTGGAGACTTATTTGGAAAAG
GGAAATAAATAAACACAAAAATGTTATGTTAGCTGCAGCTGTTTTTGCTTTAACTTTTTTCTTAATCTATGCAAGTAGAACGATTTTTATCGG
TAATACAGCATTTGGCGGACCAGCATCAATTAAGAAATATTATACGATTTTCTTATTTTTCCACATTAATTTAGCAACAATTGGTGGTATTCTA
GGCCTAGTTCAAATTATTACTGCATTTAAAGATAAATATAATGTACACCGCAAATTCGGGCCATTCGCTTCAGTTATATGGTTCTGTACTGCA
ATTACAGGTGTAGCAGTTTACTTATTATTATATGTATTATATCCAGGTGGAGAAACGACATCACTGATTAAAGCAACATTTGGTCATTAA

Gene: ylbC2 (putative protein)

Position: 1085115 to 1086014, length: 900 nt, orientation: FORWARD

Perfect match to: (MW2-BA000033-[1099477:1100376], highly conserved allele)

Sequence:

GTGAATAAATCAAATAACCATGCTGAAAATCCAAAGCCTAAAGAAGGTGTTGGTACATGGGTAGGTAAAGATATTAAAGTGCTTACTTCTA
AATTTGGACAAGCAGATCGTGTCTACCCTTTTAGAGATGGTTACAAAAATTATGTGTTTAAAGACAAAAACAGTTATTACATTGTTTCAACTA
AACGTGAAGAAATCGTTTCAGTGTATGCTACAGGTGAGAAAGTCAATGTTAGTCCGTTAAAAATAGGACAACATTCTGCAGAAATTTTTAA
TCATACAAGTATTAATCCAGAACCGTCCTTTAAAGTTGATGGTAAAAAATATGAATTTGAACTTTCAGATGAAGATTTAAAAACACAAACAC
TGATTAAATATGGCGACATATATGCACAAGTGTATTCTGATCAACAATCGAAAAAGGTGCTAAGTGTACGATTTTTAACAAAAGAAATGTTA
GCAGATATTGAACCTTATCAATTAAATTCTAATTCTACGTCAGAAGAGCATAATAAGCGTCCAGTTGAGCAAAATCCAAATCAATTAATTTCT
CTTTATGAAGTAACGAATGAAATGAGAAAATTAAAAGGATTAAAACCATTGAAAATCAATAGCGATTTAGCACATATTGCATCTAATAACTT
ATATGAAGCGACCTCTAATGGTTCTGATAGTGTTGAATTTACAGAGGACGCATTAAGAGGGCAATTAGATAAAAATCACGTTACTTATAAA
ACAACTGCTCAAAATGTTGGTTATGCGTTTAATGATGTACCAACATTAATCCATAGTTGGATGAATTCAGATATACATCGATCTCGTCTATTA
AATTCAAAATACGATGAGATGGGTGGAGATGTAATGAGAGATTATTATTCACTAATTTTCTTAGAAAAATAA

Gene: ylbF (putative regulator protein)

Position: 1086030 to 1086464, length: 435 nt, orientation: FORWARD

Perfect match to: (COL-CP000046-[1140193:1140627], highly conserved allele)

Sequence:

ATGATTAATGAAGCTTCACTAGCGATATTAGATGATATTGATGAACTAGCTGATATGATAGTTGCATCAGATATTTATGCATCATTTGAGCA
AGCTAAACAGGCGCTCGAAAACAATGACGAAGCACATTTACTTTATCAATCATTTTTAAAATCAAAAGAGAAATATGATGAAGTAATGCGA
TTCGGGAAATATCATCCTGATTATAAGAAAGTTATGTTAGAGACAAGACAACGCAAAAGAGCATATGAGATGCTTGACGTTGTGATGCATT
ACAAAGCTAGGGAAATGGCACTTCAACATTTAATAGATGAAGTTGTTACGAAAATTGCGTACGCTGTTTCAGAACATGTCAAAATAGAAAC
AGGTAATCCATTCTTTCAAACATCACATAGTGGTTGTGCGACGGGCGGATCCTGTAATTGTTCATTATAA

Gene: yhdW (putative glycerophosphodiester phosphodiesterase)

Position: 1086506 to 1087453, length: 948 nt, orientation: REVERSE

Sequence:

CTAGCATCTGACTCGATGTTTTTATTTATTCGGGATTGTTTGTTTGAATTGTTGTGCTAAATCTGGTCGATCTGTCACAATCGTGTGTGCACCT
TTTTGGTATAAATCATTCATCAGATCTATACTATTTACGCCATAATAGCCTGGAATGATATTCATATCATTTAACCATTTGATAAAACGAGAT
GAAGTCAAATCAATGCCTTTAAAATGAGTAGGCATTTGGAACGTTTGTGCTAATGGTTGGTAGTACCTACCACCTAATAAATGATATTTTAA
AAATGCTTCTGTAACTTCCTGTTGGCTAGCACCAATTGCGACGGATCCTTGTGCAATTTTATTAAAACGAACGATTTGTTCTTTATAAAAACT
TGTCACAAGAACGCGGTCAAATGCTTGATTTTCTGCAATTGTATCAAACATAATTTGTGGTGCGATTGAGCTTTCATAGGATTCAGGAGCAT
CTTTTAAGTCTACGTTTATATACATATCAGGATATTGCTTCAGCAACTCATCGAAGGTTAGTATAGCTGTGTGTGCATGACCACGATATGGT
GTTAGTCCATTGATATCTTTGAAGTGATAAGCTGCGTCTAATTTCTTTAATTCTGCTAATGTATGGGCACTAACTTTTCCAGAGCCGTTCGTC
GTTCTATCAACAGTTGCGTCATGAAAAACGATAAGCTGTTGATCTTTTGTGAGTCTCACATCTGTTTCAAAGCCATCAACGCCTAATTGTTTA
GCATAGTCAAATGCAAGTTGCGTTTGCTCTGGTCTTAAAGCCATACCACCGCGATGCGCAAATATATATGGTGCATTGCCTTTGAAAAAAGC
AGGGATGGTTTGCTTTTTAGTAATCACTTTATTTTTATTGATCATTAATAGACTACTTAAAAATCCAGCACCGACTAGTACCGCATTTAAAAT
GTTTCTGTTTACTTTTTTCAT

Gene: ylbG (putative conserved protein)

Position: 1087692 to 1087946, length: 255 nt, orientation: FORWARD

Perfect match to: (N315-BA000018-[1100624:1100878], highly conserved allele)

Sequence:

ATGAATTTAATCCCAAGAACTAGTATTGTAGTTTATTTAAAACATATGAAACATGAACGACAAATCCGAAAATATGGACATATCGTTCATTC
AAATAGAGATCGTAAATTTGTAATTATGTATGTGAATGAGCAGGATGTTGATCAAATTGTACATAAACTAATGCAACTTAAATACGTTAGAC
ACATTGATGGCTCACCATATAAATACTTAAAGAAAACTTACGAAAAAGAGAAACACGAAATATATAATTAA

Gene: Q5HGW1 (putative protein)

Position: 1087949 to 1088338, length: 390 nt, orientation: REVERSE

Perfect match to: (MW2-BA000033-[1102311:1102700:r], highly conserved allele)

Sequence:

TTATTGAAGTTCTGGTATCCATTTTTGGAGCCTTAACACACTAATTAAATAATTATAATATAACGATAAATCATGAAATGCTTGAGGGGGCT
GTACATCGATACCTAAAATTGGTATGTCGAATTTTTCAGCACAATCATTAATCATTTGAAACTTTTTATTTGAATCTGGATATGGATATTTAAT
AGCGTTAATCATGATATAAAATGCTTGGAAATGATTGCCAGTTGTAGGGTTCATAATGATTGCTACAGATGACTTTTTATTTTCAGCTTGTG
GTGAATGAAAATAGATAATTTTATCAATACGTTGATGGTTATATTCAATCATCGTCATGAATTCGAACAAATCTGTTAGACCTTCACCAAGTG
TAATAAATGTTTGTTTCAT

Gene: rsmD (RNA methyltransferase)

Position: 1088408 to 1088950, length: 543 nt, orientation: FORWARD

Perfect match to: (N315-BA000018-[1101340:1101882], highly conserved allele)

Sequence:

ATGCGCGTCATTGCAGGTAAACATAAAAGTAAAGCTTTAGAAAGTATGGAAGGCCGTAATACGAGACCAACTATGGATAAAGTTAAAGAA
GGTATCTTTAATAGTTTATATGATGTGTCAGGTATAGGTTTAGATTTATTTGCAGGAAGCGGGGCGCTTGGAATAGAAGCACTCTCTCGAG
GTATGGATAAGGTAATCTTTGTTGATCAAAATTTTAAAGCTGTAAAAGTTATTAAATCAAATCTTGCGAATTTGGATTTAGAGGCACAATCT
GAAGTTTATAAAAATAATGCAGATAGAGCTTTAAAAGCATTGTCAAAACGTGATATTCAATTTGATGTCATTTTCTTAGATCCACCTTATAAT
AAAGGTCTCATTGATAAAGCTTTAAAACTAATTTCAGAGTTTAATTTATTGAAAGAAAATGGTATCATCGTTTGTGAATTTAGCAATCATGAA
GAAATAGACTATCAACCGTTTAATATGATTAAACGTTACCATTATGGGTTGACAGACACATTGTTATTAGAAAAGGGAGAATAG

Gene: coaD (phosphopantetheine adenylyltransferase)

Position: 1088952 to 1089434, length: 483 nt, orientation: FORWARD

Sequence:

ATGGAACATACAATAGCGGTCATTCCGGGTAGTTTTGACCCCATTACTTATGGTCATTTAGACATTATTGAGAGAAGTACAGATAGATTTGA
TGAAATTCATGTCTGTGTTCTTAAAAATAGTAAAAAAGAAGGTACGTTTAGTTTAGAAGAGCGTATGGATTTAATTGAACAATCTGTTAAAC
ATTTACCTAATGTCAAGGTTCATCAATTTAGTGGTTTACTAGTCGATTATTGTGAACAAGTAGGAGCTAAAACAATCATACGTGGTTTAAGA
GCAGTCAGTGATTTTGAATATGAATTACGCTTAACTTCAATGAATAAAAAGTTGAACAATGAAATTGAAACGTTATATATGATGTCTAGTAC
TAATTATTCATTTATAAGTTCAAGTATTGTTAAAGAAGTTGCAGCTTATCGAGCAGATATTTCTGAATTCGTTCCACCGTATGTTGAAAAGGC
ATTGAAGAAGAAATTTAAGTAA

Gene: ylbM (HIGH nucleotidyl transferase)

Position: 1089496 to 1090635, length: 1140 nt, orientation: REVERSE

Perfect match to: (11819-97-CP003194-[1136179:1137318:r], highly conserved allele)

Sequence:

TTAACGATATTGCTGTATGACAGGCGTGTTGAAATCTGTTTGTTGTTGCCCGCTTATTGCATTGTATATGTGTGTTGCTTTGATTTCATTTGTG
AAGTAATGTGCATTGCTTTTGTTAATATTGGTTATATATTGTCTTTCTGGGAACGCTGTTTTTAAATGCTTTAAATATTGTCTGCCACGGTCGT
TCATCGCTAATACTTTAACTGCGTGAATGTTTCTCGTAACATCTGTAGGTTTAATGTTTAATAATACATTCATTAACAGTCTTTGGATATGCGT
ATATGTATAACGCTTTGTTTTTAGTAATTTTACAAAATGATGAAAATCAGTTGCTTCATAAATGTTAGATTTCAAACGATTTTCAAAACCTTCA
GTAACAGTATAAATATTTTTTAATGAATCTGTAGTCATAGCTATGATTTGATATTTCAAATATGGAAATATTTGATTTAATGTTATATGAGGT
GTTACGTACAAGTGTTGAATATCTTTAGGTACCACATGATGCCAATGATCATCTTGACTAATGATTGATGTTCTAATAGATGTACCACTTGCA
AACTGATGGTGTTGAATTAATGAATCATGATGTTGAGCATTTTCTCGTTTGATAGAAATTGCATTGATGTTTTTAGCATTTTTAGCAATTGCT
TTCAGATAACTAATACCAAGTATGTTGTTAGGACTTGCTAGTGCTTCATGATGCTCTAATAATTCGCTAATGATACGAGGGTAGCTTTTACCT
TCTTTTACTTTTTGTGAAAAGGATTCAGATTGTTCAATTTCATTAATGCTGTGTGCTAATTGCTTTAATGTTTTGATATCATTATTTTCACTACC
AAATGCAATTGTATCGACACTCATATAATCTGCGACTTTAACTGCTAGTTCGGCAAAATGATCGCCAGATGATAAACTGGCAGTTGCTGGTA
GTTCGATAACTAAATCAGCTGTTGATAATGCCATTTTTGCACGAGTAAACTTATTATAGATTGCTGGTTCGCCACGCATGACAAAGTTACCAC
TCATTATTGCAATAGTAACGTCAGCATTTGTAAGTTTTTTAGATTGATTAATATGATATTGATGCCCATTATGAAAGGGATTATATTCTGTGA
TTAAGCCAACGCTTTTCAT

Gene: Q4L5E0-ylbN (putative DNA binding protein)

Position: 1090762 to 1091319, length: 558 nt, orientation: FORWARD

Perfect match to: (11819-97-CP003194-[1137445:1138002], allele observed in CC80+CC72+CC80+CC188+CC2885)

Sequence:

ATGAAATGGTCAATTACGCAATTAAGGAAATATCAAGGTAAGCCATTTGAATTTGATCAAACGGTGAGTTTTGACAATTTAAAAGAATCATT
AGATTTAATTGATTTATCTCCAATTACAATCCAAGGTCAGTTAACCATTAAGTCAACAGAAGTCGTTGCGAATATTCACATTACTGGAACGTA
TACAATGCCTTGTGCACGTACTCTTGTACCAGTTAAAGTCCCACTAGATGTAACTACTACAGAAGTATTTGATTTAGAAGGGTACAATCAGT
ATAACGATGATCAAGATGATGTAGATGAACACTATCACATTATTAAAGATGGTATGGTTAATCTTCAGGATATTGTCGAGGATATAGTTATT
ATTGAGAAACCAATGAGAGCTTATTCAGAGCAAAGTGACCAAATGTTGACAGTAGGTAATGGTTGGGAAGTAATCGATGAGGATCAATTA
GATGAGCTTGCTAAACAGCAAGAACAAGATGATTCAGAATCACGACAAGTTGATCCAAGGCTTCAAAAATTACAACAATTATATGATAAAG
AGCAATAA

Gene: rpmF (50S ribosomal protein L32)

Position: 1091399 to 1091572, length: 174 nt, orientation: FORWARD

Perfect match to: (RF122-AJ938182-[1073196:1073369], highly conserved allele)

Sequence:

ATGGCAGTACCAAAAAGAAGAACTTCTAAAACTAGAAAAAACAAACGTCGTACGCATTTCAAAATTTCAGTACCAGGTATGACTGAATGCC
CAAACTGTGGCGAATACAAATTATCACACCGTGTATGTAAAAACTGTGGTTCTTACAATGGCGAAGAAGTAGCAGCTAAATAA

Gene: isdB (extracellular hemoglobin-binding protein)

Position: 1091726 to 1093942, length: 2217 nt, orientation: REVERSE

Sequence:

TTAGTTTTTACGTTTTCTAGGTAATACGAATGCAACGATGCTACTTAAAGCTAATAATGCCATTAATGGTAATGTCATATCTTTATTTGATTCT
TCACCAGTTTGTGGTAATGATTTTGCTTTATTTTCTTGTGTGTTTTTATTGTTTTGGCTTTGAGTGTGTCCATCATTTGTGTTTTTAATGTTTGC
TTTTTGTAATGGAGCACTATCTTTTGCTTCTCTAGAACCTGCTGAAGTTTGAACAACATCTTTTGTTGTTTTTGATGAAGCAGTTGTTGGTTTT
ACAACATTTTGAGTCGTAGATACTACCTTAGTTGGAGTTGTACTACTTGATTCTACTTCACCTTTAGCTGGTTTTGTAGCAGGCGTTTTGTCTT
TACCTGACTCACTAGATGCGTCATTTTCTTTTTCAACACTTGGTAATTGTTTATTGTCATCTTTTTGGCTGTCTTGTTTTTGTGATTCTTTTTCAA
CAGGTGATGGTGTTGGTTTGCTAGGCGTAGCTGAAGTTTGAACAACATCTTTTGTTGTTTTTGATGAAGCAGTTGTTGGTTTTACAACATTTT
GAGTCGTAGATACTACCTTAGTTGGAGTTGTACTACTTGATTCTACTTCACCTTTAGCTGGTTTTGTAGCAGGCGTTTTGTCTTTACCTGACTC
ACTAGATGCGTCATTTTCTTTTTCAACACTTGGTAATTGTTTATTGTCATCTTTTTGGCTGTCTTGTTTTTGTGATTCTTTTTCAACAGGTGATG
GTGTTGGTTTGCTAGGCGTAGCTGGAGTAGCTTCCTTCTTAGCTGAGTTATCTTGTTGTTCTTTTTTGTTAGATTTATCGGTATTGGCTTTTGT
AAATGCTTCTTTATCAACGATTCTGACATGGTATTGTCCATCATAATCAATCGTTTTTACGTGAACTTTAACGATAGCATCATATAGAGTTTTA
CCTTCAACATATGGGAAAATAATTGTTCTAGTATTATTTTTAGCATCTTTGCTTATAGTTCTAACACGTTGACCTTCAACCATGAAATCTTTCC
AGTAATCGTCATTAGTAGTTTCCATGACCATATATTTTTTGCCGTTAAGCATACCTGTTTTAATAGGGTGTTTAACAAAAGTATCCATCATAG
ATTCGTTATTCTCAACACTTTCATAAACAACATATTTTGTATCTTGTAAATCAGTCATTTTTTCATTTGTTGGTTGTACATTTTGGAATTCAGTA
ATAGCTGATTTCACTTGCTCATCTAAAGCTTTCTTTGTATCCTCTAATTTCTTCTTGTACTCAGCCTTTAATTTTTCAGGAAGTTTATCTTGAATT
TTATTTAATTCATAAACTTGTCTTTCTAGTGTTTTCGCTTTTTTATATGGCGCTAATAATTTTTCAGCTTTATAATCTTCTTCAGTTTTGAATTTA
TCTGCACTGTTATAAATTGGTTGTGCGAATTCCATTAATGTGTAATCGTATTTTTCTTCTTTGTTATTGAAGTGTGTTGAACTAACAATTTTAA
CAGCTTTTGTTCCATTTGATACAGAGAAGCGAATGTAAGCATAATCTTTAACAGTATCGTATGATACTAATTTAATTGGCAACTTTTTGTCAC
CTTCATAAACTTCAAATTTTCTCCAAAATTGACCTGATTGTAATCCTAATTCAATTTCTGGTTTTGAATCAGTGAAAATAACTCTAGCAGGTTT
AACAGAACTTGCATAATGATAAAACTGTTGAGTTCCATCTTTCTTTTTCATTTCAAAATCAATTGGACGAGAGTTTGGTGCGCTATGATCTTT
GTCTTTTATTGCAGGGTTTTTAATCGCTTCTCTAAGTTCCTGATTCAAAATAGGATATGTATTGTTAGTGGCTTTTGCTGCTGGTTTAACTTCT
TTTGTTTCCTTAGGGGCTTTAACTTCTTTAACTTCTTTAACTTCTTTAGCTTCTTTTGTTTCAGAAGTAGGGGCCTCAACTTCTTTATTAGATAC
TGAGACAGCATTAGCTACTGGTTTAGTTTCTGGAGCTTTTTCAGATGTTGTTGTTGGACTTGCAACTGCTTCAGTTTTTGGTTGTGCTTCTGT
ATTTGTACCACCTGTTTCTTCAGCTGCTTGTGCTTCGCCATTTGACATTAATAATAAAAGTGTACTAATCGCTACAGATGCAACGCCTAGTGA
TGACTTTCTAATTGAATAAAATGATTTAAATTCTTTTTGCTGTTTGTTCAT

Gene: isdA (extracellular transferrin-binding protein)

Position: 1094146 to 1095198, length: 1053 nt, orientation: REVERSE

Perfect match to: (11819-97-CP003194-[1140555:1141607:r], highly conserved allele)

Sequence:

TTATTTAGATTCTTTTCTTTTGAAAAGTAATAAAGATAATGAACCTAAAAGGGCAAGTGTTGCGAAGGCAACTGTGCTAATAAAGTTATCAA
CTGAAGTTAAACCAGTTTTTGGTAATTCTTTAGCTTTAGATGCTTGTTTAGGCGTTTCGTTATGTTTTGTAACTTTGTTAGTTTGTTGTGATTT
ATTATCACTTACAGCTTGATTGTTGCTTTCAGATTTCGCTGTTGCAACATCTTTAACAGGTGTTTGAACTTTATTTTGTTCTTGAGCAGTTTGT
GCTGTTTTAACTGTATGAGCAGTTTGTGTTTTAGTTTGATCTTTTGTTGTGTCAGTACTTACAACTTTAGTAGAGTGATTGTCTTCAACTTTGC
TTGTTGTAGTTACAGTAGGTTTAACTTTTTCAACTTTAGGTTGAACTGGTTTAGTTTGCTCAGTAGGTGTTTTAGGTTGAGCTGGTTTTGGTT
GAACCGGTTTAACATTGTTTGGTTTTGCTGCGTCAGCTAATGTAGGAATTGCTTTTTCAAATTCCAAATGCGTAGTATATCTATGATTGTAAT
TAATTTGTGGCACGACAATATGTACTTTAGTAGTTAAGCTCTTATATCCAGGTTCAACTGCAACATTGATTGTTCTAGTATCCGCTTTTTTATT
ATCGTTAACAACAGTTGTTGCTAATTCTTGATTGTTTGCATTGTAAAATTTGTATTCTTTCCAGAATGATGCATTGTTTAACACGGTTTGGAAA
TAATATTTATTATTTTGTTTAATTACTTTACCAGGGTGTTGCATATAGTCATCCATGTGTGACTTCTCTGAAGAGCCATCTTTTTGCACTTGGA
AATTAATTGGTTGTGATGTTGCTTGAGAAACTTGTGTGCTTTGATTATTAGTTGCGTTCGTAGCTTCTGTTGCCGCATTGACTTGTTGGCTGT

CTGCGCCTATGTATACAAGGGAACCTAAAATGATAGATGCTGTACCCATTGTAATCTTTTTCATAGCTGATGAACGTTGTTCTGATTGATACT
TACTGTTTAAATAATGTTTTGTCAT

Gene: isdC (extracellular heme-binding protein)

Position: 1095407 to 1096090, length: 684 nt, orientation: FORWARD

Perfect match to: (A9299-ACKH01000021-[6297:6980], allele observed in CC5+CC8+CC188)

Sequence:

TTGAAAAAATATTTTAAAAGTTTTTAATACAACGATTTTAGCGTTAATTATCATCATCGCGACATTCAGTAATTCTGCAAATGCCGCAGATAGC
GGTACTTTGAATTATGAGGTTTACAAATACAATACCAATGACACGTCAATTGCTAATGACTATTTTAATAAACCGGCAAAGTACATTAAGAA
AAATGGTAAATTGTATGTTCAAATAACTGTCAACCACAGTCATTGGATTACTGGAATGAGTATCGAAGGACATAAAGAAAATATTATTAGT
AAAAACACTGCCAAAGATGAACGCACTTCTGAATTTGAAGTAAGTAAGTTGAACGGTAAAATAGATGGAAAAATTGACGTTTATATCGATG
AAAAAGTAAATGGTAAGCCATTCAAATATGACCATCATTACAACATTACATATAAATTTAATGGACCAACTGATGTAGCAGGTGCTAATGCA
CCAGGTAAAGATGATAAAAATTCTGCTTCAGGTAGTGACAAAGGATCTGATGGAACGACTACTGGTCAAAGTGAATCTAACAGTTCGAATA
AAGACAAAGTAGAAAATCCACAAACAAATGCTGGTACACCTGCATATATATATGCAATACCAGTTGCATCCGTAGCATTATTAATCGCAATC
ACATTGTTTGTTAGAAAAAAATCTAAAGGCAATGTGGAATAA

Gene: isdD (heme ABC transporter, membrane component)

Position: 1096090 to 1097166, length: 1077 nt, orientation: FORWARD

Perfect match to: (11819-97-CP003194-[1142499:1143575], allele observed in CC80+CC9+CC80+CC97+CC101)

Sequence:

ATGAGAAATGTTAAACAAATTGCTACAAAATCTATTATAGCTATTATTAGCATAGGTATACTTACTTATACAACAATGATTGGTAGCGTGTTG
GCTGATGAGATAAAAATATCCATCAGCCAAATTTAATCAACCTGAAGCAAAAGATAAAACAGAATTAACTACATCAATTTTTGATGAAAAGAT
AAAAGAGAATAAAGCGTTAGAGTTACTAATTTTTAATCAAGAAAATAAAAATGTAACTGAGGAACAACAACTAGTTGACGAAAAGGCGCA
ATTGATTTCAGATATGACTGGTAAAATTTACTTGCAAGTAAAGCTAAAAGGTCAAATTGATAAAGAACAACTTGTTTTTCAAAATGACAAAA
ATGAAGAATTTCCATTTGTTATAAAAGATGAAAAGAATGACACAATAGTAAGAATTTTAATTGAACAGCATATGGATAAAATCGATATGCAT
GTTAAAACATTGGCTGAAAAGAAAGATCTAGATAACAAAGAAATGGTGTATTCTATTCATTTTAAAGAGAAAAAAGTTCAACATGATGATG
CAAAAGAAGTGCCTTCAAAACATCAAAATCAAGAAAATAATCAAGATCAGCTTAAAAAAGATATTGATGACAAAAAAGATAGTCAAAAATC
AGACATTAAGGAAAGACGTACTAGCCTTTTAACTGAAAAAGGATTAAATGATATTCCTGTACAAAAAGATAAAGTGCAACAAGACAGTAAT
AAAAAGATTGAAAATGAGCGACCTAAAGCATCAGGTACATTAAAAGTTGAAAATAGCCCTCCAACAGTAAAAAAGGTTGAAAATAATCAC
AAAGAGCAACCGAAACATAAAGATGAAAAATCAAAAAAGGAAAAGAAAAAAGTAGTTGAAAAAGAAAAAGCGTTACCAGCTTTTAATAG
AGATGATGATAGCAAGAATAATAGTCAATTATCTAGTGATATTAAAGAACTTGATGAACCAAATCATAAAAAGCAATATATTTTATTTGCAG
CTGGCATTGTGTTAGCAACTATTTTACTTATTTCGGCACATTTATACAGCAGAAAGAGAGGTAACCAAGTTTGA

Gene: isdE (heme ABC transporter, substrate-binding protein)

Position: 1097163 to 1098041, length: 879 nt, orientation: FORWARD

Perfect match to: (M0239-AIWE01000003-[53300:54178], allele observed in CC188+CC8)

Sequence:

TTGAGAATCATAAAGTATTTAACCATTTTAGTGATAAGCGTCGTTATCTTAACCAGCTGTCAATCTTCCAGTTCTCAAGAATCAACTAAATCC
GGCGAATTCAGAATCGTACCAACAACTGTTGCATTGACAATGACATTGGACAAATTGGATTTACCAATTGTCGGCAAACCCACGTCATATAA
GACATTGCCTAATCGTTATAAAGATGTACCGGAAATTGGTCAACCAATGGAGCCGAATGTTGAAGCTGTTAAAAAGTTAAAACCAACACAT
GTTTTGAGTGTGTCAACGATTAAAGATGAAATGCAACCATTTTACAAACAATTAAATATGAAAGGCTACTTTTATGATTTTGATAGTTTAAAA
GGGATGCAAAAGTCGATTACACAATTAGGTGATCAATTTAATCGTAAAGCACAAGCAAAAGAATTAAATGACCATTTAAATTCTGTAAAGC
AAAAAATTGAAAATAAAGCAGCTAAACAAAAGAAACATCCCAAAGTATTAATATTAATGGGTGTACCGGGTAGCTATTTAGTAGCAACTGA
TAAATCATATATTGGTGATTTAGTTAAAATAGCAGGTGGAGAAAATGTTATTAAAGTGAAAGATCGTCAATATATTTCGTCTAATACTGAAA
ATTTGTTGAATATCAATCCAGATATTATTTTAAGATTACCACACGGAATGCCTGAAGAAGTTAAGAAAATGTTTCAAAAAGAATTTAAACAG
AATGATATTTGGAAACATTTTAAAGCTGTGAAAAATAATCATGTTTATGACTTAGAGGAAGTGCCATTCGGTATTACAGCAAATGTTGATGC
TGATAAGGCAATGACTCAATTATATGATTTATTTTATAAGGATAAAAAATAG

Gene: isdF (heme ABC transporter, transmembrane permease)

Position: 1098054 to 1099019, length: 966 nt, orientation: FORWARD

Perfect match to: (11819-97-CP003194-[1144463:1145428], allele observed in CC80+CC97)

Sequence:

ATGATAAAAAAATAAAAAGAAACTACTATTTTTATGTTTGTTAGTCATTTTAATCGCAACTGCTTATATTTCGTTTGTAACCGGTACAATTAAAT
TGTCATTTAATGACCTAATTACAAAATTTACAACTGGTAGCAATGAAGCAGTGGATTCAATCATTGATTTGCGATTGCCACGTATATTAATTG
CATTAATGGTTGGCGCAATGTTAGCAGTTTCTGGAGCATTATTACAAGCAGCACTACAAAATCCTTTGGCAGAGGCGAATATCATTGGCGTT
TCTTCAGGTGCACTTATAATGAGAGCACTTTGTATGTTGTTTATTCCACAATTGTACTTTTACTTACCATTATTAAGTTTTATTGGAGGTTTAA
TACCATTTTTAATAATTATATTGTTGCATTCTAAATTTAGATTCAATGCTGTAAGTATGATATTAGTAGGTGTTGCGTTATTCGTATTATTAAA
TGGTGTTTTAGAAATTTTAACTCAAAACCCTTTAATGAAAATTCCTCAAGGCTTAACAATGAAAATATGGAGCGACGTATACATATTAGCAG
TATCAGCATTATTGGGATTAATATTAACATTACTATTGTCCCCTAAATTGAATTTACTAAATTTAGACGACATACAAGCGCGAAGTATCGGTT
TTAATATTGATCGTTACAGATGGTTAACAGGTTTATTAGCAGTATTTTTAGCAAGTGCAACTGTTGCGATTGTTGGACAACTAGCCTTTTTAG
GTATTATTGTGCCACATGTGGTTAGAAAGCTAGTTGGGGGCAATTACAGAGTACTTATTCCGTTTTCTACAGTTATTGGTGCATGGCTATTG
TTAGTGGCTGATTTATTAGGACGAGTGATACAGCCTCCTTTAGAAATTCCAGCCAATGCTATTTTAATGATTGTCGGTGGTCCAATGCTAATT
TACTTAATTTGTCAAAGTCAACGAAATCGAATCTAA

Gene: srtB (sortase B, NPQTN specific)

Position: 1099081 to 1099815, length: 735 nt, orientation: FORWARD

Perfect match to: (GR1-AJLX01000008-[172906:173640], allele observed in CC361+CC80)

Sequence:

ATGAGAATGAAGCGATTTTTAACTATTGTACAAATTTTATTGGTTGTAATTATTATCATTTTTGGTTACAAAATTGTTCAAACATATATTGAAG
ACAAGCAAGAACGCGCAAATTATGAGAAATTACAACAAAAATTTCAAATGCTGATGAGCAAACATCAAGCACATGTGAGACCACAATTTGA
ATCACTTGAAAAAATAAATAAAGACATTGTTGGATGGATAAAATTATCAGAAACATCATTAAATTATCCAGTACTACAAGGTAAGACAAATC
ACGATTATTTAAATTTGGATTTTGAGCGAGAACATCGACGTAAAGGTAGTATTTTTATGGATTTTAGAAATGAATTGAAGAATTTAAATCAT
AATACTATTTTATACGGGCACCATGTCGGTGATAATACGATGTTTGATGTGTTAGAAGATTATTTAAAGCAATCGTTTTATGAAAAACACAA
GATAATTGAATTTGACAATAAATATGGTAAATATCAATTGCAAGTATTTAGTGCATATAAAACTACTACTAAAGATAATTACATACGTACAG
ATTTTGAAAATGATCAAGATTATCAACAATTTTTATATGAGACAAAACGTAAATCTGTAATTAATTCAGATGTTAATGTAACGGTAAAAGAT
AAAATAATGACTTTATCAACCTGCGAAGATGCATATAGTGAAACAACGAAAAGAATTGTTGTTGTCGCAAAAAATAATTAAGGTAAGTTAA

Gene: isdG (heme oxygenase)

Position: 1099834 to 1100157, length: 324 nt, orientation: FORWARD

Perfect match to: (11819-97-CP003194-[1146243:1146566], allele observed in CC80+CC96+CC97)

Sequence:

ATGAAATTTATGGCAGAAAATAGGCTGACGTTAACAAAAGGAACAGCAAAAGATATTATAGAACGATTTTATACGAGACATGGGATTGAA
ACATTAGAAGGCTTTGATGGCATGTTTGTTACACAAACTTTAGAACAAGAAGATTTTGATGAAGTGAAAATTTTAACAGTTTGGAAATCAAA
GCAAGCTTTTACGGATTGGTTAAAATCTGATGTCTTTAAAGCAGCGCATAAACATGTTAGAAGTAAAAATGAAGATGAAAGTAGCCCGATT
ATCAATAACAAAGTAATTACATATGATATAGGCTATAGTTACATGAAATAA

Gene: spoU (putative tRNA/rRNA methyltransferase)

Position: 1100541 to 1101281, length: 741 nt, orientation: FORWARD

Sequence:

ATGGAACAAATTACTTCTGCACAAAATAATAGAATTAAACAAGCGAACAAGCTAAAAAAGAAACGTGAGAGGGATAAAACTGGATTAGCT
TTAATTGAAGGTGTGCATTTAATTGAAGAAGCTTATCAAAGTGGAATTGTAATTACACAATTATTTGCAATTGAACCGGTAAGATTAGATCA
GCAAATTATCGCATACGCGCAAGAAGTTTTTGAAATAAACATGAAAGTTGCTGAATCTTTATCAGGTACAGTGACACCACAAGGGTTTTTCG

CAATCATTGAGAAGCGGCATTATGATATTTCTAAAGCACAACAAGTATTGCTCATCGATCGTGTTCAAGATCCTGGAAATTTAGGCACATTA
ATTAGAACTGCGGATGCAGCTGGAATGGATGCTGTAATAATGGAGAAGGGTACGACAGATCCTTATCAAGATAAAGTGTTGCGAGCGAGT
CAAGGTAGTGTTTTCCATTTGCCAGTTATGACACAAGATCTCGATACGTTTATTACTCAATTTAATGGTCCTGTTTATGGTACAGCACTTGAA
AACGCAGTGGCATACAAAGAAGTTACTTCAAGTGATTCTTTTGCATTACTATTAGGTAATGAGGGAGAAGGTGTTAATCCTGAATTATTAGC
ACATACTACACAAAATTTAATCATACCTATTTATGGTAAAGCTGAAAGTTTAAATGTAGCGATTGCAGGTAGTATTTTACTTTATCATTTGAA
AGGTTGA

Gene: tbox04 (T-box leader element)

Position: 1101362 to 1101573, length: 212 nt

Sequence:

TAAACCAATAAAAAGGCATGGACATTTATATAAATAATTGTTTTAGGGAGAATAATCGTGACTGCAAGTTATTCCAATTATTTAAAGTCTTTT
CACCTTTTTGGTTACTTAAAGAGATTTAAGTCGGAAAGACAATCCGTTATCAATATTAAACAAGTGTATGCTTAGGCATAAATTTGGGTGGT
ACCACGGAAATGACTTTCGTCCCTTAT

Gene: pheS (phenylalanyl-tRNA synthase alpha chain)

Position: 1101662 to 1102720, length: 1059 nt, orientation: FORWARD

Perfect match to: (ED133-CP001996-[1158568:1159626], highly conserved allele)

Sequence:

ATGTCTGAACAACAAACAATGTCAGAGTTAAAACAACAAGCGCTTGTAGATATTAATGAAGCAAATGATGAACGTGCACTGCAAGAAGTTA
AAGTGAAATACTTAGGTAAAAAAGGGTCAGTTAGCGGACTAATGAAATTGATGAAGGATTTGCCGAATGAAGAGAAACCTGCGTTTGGTC
AAAAAGTGAATGAATTGCGTCAAACAATTCAAAATGAATTAGATGAAAGACAACAGATGTTAGTTAAAGAAAAATTAAATAAGCAATTGG
CTGAAGAAACAATTGATGTATCATTACCAGGTCGTCATATTGAAATCGGTTCAAAGCATCCATTAACACGTACAATAGAAGAAATTGAAGA
CTTATTCTTAGGTTTAGGTTATGAAATTGTGAATGGATATGAAGTTGAACAAGATCATTATAACTTCGAAATGCTGAATTTACCTAAATCACA
CCCTGCACGTGATATGCAAGATAGTTTCTATATTACGGATGAAATTTTATTACGTACGCATACATCACCAGTGCAGGCACGTACGATGGAAT
CACGTCATGGTCAAGGTCCAGTTAAAATTATTTGCCCTGGTAAAGTGTATCGTCGTGACTCTGATGATGCGACACATAGTCATCAATTTACA
CAAATCGAAGGATTAGTTGTTGATAAAAACGTTAAAATGAGTGATTTGAAAGGCACTTTAGAATTGTTAGCTAAGAAATTATTTGGTGCTG
ATCGTGAAATTCGTTTACGTCCAAGTTACTTCCCATTCACTGAACCTTCTGTAGAAGTTGATGTGTCATGTTTTAAATGTAAAGGAAAAGGTT
GTAATGTGTGTAAACACACAGGATGGATTGAAATTTTAGGTGCTGGAATGGTACATCCTAATGTATTAGAAATGGCTGGTTTTGATTCTTCA
GAGTACTCTGGATTTGCATTTGGTATGGGACCAGACCGTATTGCAATGTTGAAATATGGTATAGAAGATATTCGTCATTTCTATACTAATGA
TGTGAGATTTTTAGATCAATTTAAAGCGGTAGAAGATAGAGGTGACATGTAA

Gene: pheT (phenylalanyl-tRNA synthase beta chain)

Position: 1102720 to 1105122, length: 2403 nt, orientation: FORWARD

Sequence:

ATGTTGATATCAAATGAATGGTTGAAAGAATATGTAACAATCGATGATTCTGTAAGTAATTTGGCAGAACGTATTACGCGCACAGGTATTG
AAGTGGATGATTTAATTGACTACACAAAAGATATCAAAAATTTAGTTGTCGGCTTCGTTAAGTCAAAAGAGAAACATCCTGATGCTGATAA
ATTAAATGTTTGCCAAGTTGATATCGGAGAAGACGAACCTGTACAAATCGTTTGTGGTGCACCGAACGTTGATGCAGGACAATATGTCATT
GTTGCTAAAGTAGGTGGCAGATTGCCTGGTGGTATTAAAATTAAGCGTGCCAAATTACGCGGTGAACGTTCAGAAGGTATGATTTGTTCGT
TACAAGAAATTGGTATTTCAAGTAATTATGTACCGAAAAGTTTTGAATCAGGCATTTATGTTTATAGTGAATCCCAAGTTCCAGGAACAGAT
GCCTTACAAGCTTTATATTTAGATGATCAAGTAATGGAATTTGATTTAACGCCGAATCGTGCAGATGCTTTAAGTATGATAGGTACTGCTTA
TGAAGTTGCAGCATTATATAATACAAAAATGACTAAGCCAGAGACAACATCAAATGAGCTTGAGTTATCTGCAAATGATGAATTGACTGTG
ACAATTGAAAATGAAGATAAAGTACCATATTATAGTGCACGTGTTGTTCACGACGTGACAATTGAACCCTCGCCAATTTGGATGCAAGCAC
GCTTAATAAAAGCGGGTATACGTCCTATTAATAATGTTGTTGACATTTCAAATTATGTGTTATTAGAATACGGTCAACCATTGCACATGTTTG
ATCAAGATGCGATTGGTTCACAACAAATTGTTGTTCGTCAAGCTAATGAAGGTGAAAAAATGACAACATTAGATGATACAGAACGTGAATT
ATTAACGAGCGATATTGTCATTACTAATGGACAAACTCCAATTGCATTAGCTGGTGTTATGGGTGGCGATTTTTCAGAAGTTAAAGAACAAA
CATCAAATATAGTGATTGAAGGTGCTATTTTTGATCCAGTTTCAATTCGTCATACATCAAGACGTTTAAATTTACGCAGTGAATCATCTAGTC
GTTTTGAAAAAGGAATAGCTACTGAATTTGTAGATGAAGCAGTCGACCGTGCATGTTATTTATTACAAACTTATGCAAACGGAAAAGTGCT
AAAAGATAGAGTGTCTTCAGGAGAGCTTGGTGCATTTATTACACCAATCGACATCACTGCTGATAAAATTAATCGCACTATTGGATTTGATT
TGTCACAAAATGATATTGTTACTATTTTTAATCAACTAGGGTTTGATACAGAAATAAATGATGATGATATTACAGTGCAAGTACCATCACGTC

GTAAAGATATTACAATTAAAGAAGATTTAATTGAAGAAGTTGCACGTATATATGGTTACGACGATATTCCATCAACGTTACCTGTCTTCGAA
AAAGTTACTAGTGGTCAGCTAACTGATCGCCAATATAAAACTAGAATGGTTAAAGAAGTGTTAGAAGGTGCTGGATTAGATCAAGCTATTA
CGTATTCGTTAGTTTCTAAAGAGGATGCTACTGCATTTTCGATGCAACAGCGTCAAACAATTGATTTATTGATGCCAATGAGTGAAGCGCAT
GCGTCATTACGTCAAAGTTTATTACCACATTTAATCGAAGCGGCATCATATAATGTGGCACGCAAAAATAAAGATGTAAAATTATTTGAAAT
CGGCAATGTCTTCTTTGCTAATGGAGAAGGTGAACTACCAGATCAAGTTGAATATTTAAGTGGTATTTTAACTGGAGATTATGTAGTCAATC
AATGGCAAGGTAAGAAAGAAACGGTTGATTTCTATTTAGCAAAAGGTGTCGTGGATCGAGTATCTGAAAAGTTAAACCTTGAATTTAGTTA
TCGTCGTGCTGATATTGATCGATTACATCCAGGTCGTACTGCTGAAATCTTATTAGAGAATAAAGTTGTTGGTTTTATTGGTGAATTACATCC
AACATTAGCAGCTGATAATGATTTAAAACGTACGTATGTTTTTGAGTTGAATTTTGATGCATTAATGGCTGTGTCGGTAGGTTACATTAATTA
CCAGCCAATTCCGAGATTCCCAGGCATGTCTCGTGACATTGCATTAGAAGTAGATCAAAATATTCCAGCAGCTGATTTATTATCAACGATTC
ATGCACACGGTGGCAATATATTAAAAGATACACTTGTCTTTGATGTATATCAGGGCGAACATTTAGAAAAAGGTAAAAAATCAATTGCAAT
ACGTTTAAATTATTTAGACACAGAAGAAACATTGACAGATGAGCGCGTTTCAAAAGTACAAGCGGAAATTGAAGTAGCATTAATTGAACAA
GGTGCTGTTATTAGATAA

Gene: rnhC (ribonuclease HIII)

Position: 1105357 to 1106295, length: 939 nt, orientation: REVERSE

Perfect match to: (Strain_21333-AHKA01000047-[55211:56149], allele observed in CC80+CC772)

Sequence:

TCAAAGAGGCTTTAAAATTTTTTGGGCTTTTTCACGATTTTTAAAATGCTTTTTTGAAATGGTATCTAAACGTGAAAGACCGTATTTTTTTATA
ATTTTGGCGGCGATTACATCGACTTTAGCACCGGCACCTTTAGGAATCGTCATATTAATATTTTTTGATATTTGATCCATATATGTAACAAAT
GCGTATCGAGAAATTATGCTTGCCACTGCAATGGCTAATGACTTCGATTCTCCTTTTGTTTCAAATTTTGTTTTCTTTGGAAGTGGTATATCG
GATAATGCGTAATGGTTATACACTTCGCGTTTTGCGAACTGATCAATGACGATATAGTCTAATTGAGACGAATCAATTTTTTCAAGTACATTT
TTGATGGCTTCATTATGAAGAACAGCTTTCATTTTTACTTGAGTCCAGCCTTTTGCTTGCTGAATATTATATATTTTCATTGTGTAGTGTTAATA
GTGAATGTGGGATGAAAGTAACCAATTGCTCAGCAAGTTCTACAATTTTGGTATCGGTTAATTTTTTTGAGTCATCTACACCCAAAGTTTTTA
AAATAGGGACATGCTCTTTGGTAACGAAAGCAGCACACACAGTCAACGGACCAAAGTAATCGCCACTTCCAGCTTCATCACTACCAATACA
GTTAAATTGATCATACATTAAAGTTTGTTCAAGAAAAGAATTAGCCATATTTTTCTTTTTAGTTTTATTAGTATTCAATTGAGAATGCTGCGGT
AGAAGTTCTTCAGACACAGCTTCTGCATGATTACCCTGAAACATGACTTTACCTGATTGGTAAATATTTACAGTTGTATTTTGATACTTTGCA
CGTGCTTTCATACCTTGAGGTAAATTCTCAGTATCAAAAGAAATGCGTGACATTAATGTCGTTATGTCTTTATCCGACAATTTAAAAACGATA
TTCGCCAT

Gene: zapA (regulator of Z-ring formation)

Position: 1106670 to 1106936, length: 267 nt, orientation: FORWARD

Perfect match to: (N315-BA000018-[1119513:1119779], highly conserved allele)

Sequence:

ATGGCACAGTTTAAAAACAAGGTAAATGTATCAATTAATGATCAGCTTTTTACAATTGTTGGGGAAGATAACCCAGAGCACATACGATATG
TAGCACATTTAGTTGATGATAAAATAAAAGAATTAGGGTATAAAGCAGCAGGTTTAGATACTTCAAGAAAAGCAATACTAACTGCTGTGAA
TATTATGCATGAAAAAGTACTACTAGAAGAAGAAAATCGACGTTTGAAACAACAAATTCACAAATTGCAGCAGCGTGAGCAATAA

Gene: yshB (uncharacterized transmembrane protein)

Position: 1106937 to 1107458, length: 522 nt, orientation: FORWARD

Perfect match to: (N315-BA000018-[1119780:1120301], highly conserved allele)

Sequence:

ATGGTCATTGATTTTATCATAATCATTTTCTTTGTGTATTTTGTCATCGTTGGATTCAGACGAGGTTTTTGGTTATCTATGATACATTTGAGTG
CAACGATTGTATCATTGTGGATTGCCAGTCAATTTTACAAATCTATTGTAGAAAGATTAATTGTATTTATTCCATATCCTAAAACAACAGCAT
TTAATACAACTTTTGCGTTTCATTTTAATCATCTACAAAATCGATTTGAAGCGATTGTAGCTTTTTTAATGATTACATTGTTTTGTAAGTTCATT
TTATATCTAATTATCGTAACTTTTGATAAAATAATAGCGTATCAAAACATTCATATTTTCAGTCGTGCAATGGGAATGATAGTTGGTGTGTTT
ATGACGATAATTGTCTTACACTTTACGTTATATCTATTGGCATTATATCCTAACGAAGCATTACAACATCAGCTTAAAATATCTATTGTGAGTC
ATTCATTGATTTTTCACATCCCATATTTATCGGCTTTCACCATTAATTTATAA

Gene: polX (DNA-directed DNA polymerase IV beta subunit)

Position: 1107531 to 1109243, length: 1713 nt, orientation: FORWARD

Perfect match to: (CIG2018-AHVV01000008-[58260:59972], allele observed in CC8+CC5+CC97)

Sequence:

ATGACAAAAAAAGATGTTATCAAACTATTAGAACAAATTGCTACTTATATGGAATTAAAAGGGGAAAATACTTTTAAAATATCAGCGTATCG
AAAAGCAGCTCAAAGTCTTGAATTAGATGAACGACCATTAGATGAAATATCTGATGTAACGGAGTTAAAAGGCATTGGTAAAGGTGTTGC
AGAAGTAATCAATGATTACCGTGAGACCGGTGAATCTCAGTATTTACAGCAATTACAGGAAGAAGTTCCGGAAGGTCTTATTCCACTTTTGA
AAATTCAAGGACTTGGAAGTAAGAAAATTGCTAAGCTATATAAAGAGTTGAATATTGTTGATAAAGCGTCACTTCAAGTTGCTTGTGAAAA
TGGAAAAGTTAGTGAATTAAGCGGATTTGCTAAGAAAACGGAACAAAACATATTAGAAGCTGTGAAACAACTTAGTGCTAAGAAAGATAG
ATATCCAATTGATCAAATGAGAAGACTTAATCAAGAAATCATTGATTATATAGATACATTAAATTATATCGATCAATATTCATCTGCAGGAA
GCTTCCGTCGTTTTAAAGAAATGAGCAAAGATTTAGATTTCATAATAAGTACCGATAACCCAAAAGCAGTGCAGCAGCAATTATTAAATATT
CCCAATAAAGTAAAAGAAGTTGCAGTGGGGAACACAAAAGTTTCATTAGAATTAGCGTATGATGATGAAACGATTGGTGTCGATTTTCGAT
TAATTGAACCAAGTGCTTTTTATCATACATTGCAGCATTTTACTGGGTCAAAAGAACATAATATAAGAATTCGACAACTTGCTAAAGCACGT
GATGAAAAAGTAAGTGAATATGGAATTGAACAAGCTGATGGTACATTAATTCAATATGATAGTGAAGCCAAGATATATGAACATTTTAATG
TGAATTTTATACCACCTGCTATGCGAGAAGATGGTAGCGAATTTGATAAAGATCTAAGTAATATCATTACATTAGATGATATTAATGGTGAT
ATTCATATGCATACAACGTATAGTGATGGTGCGTTTTCTATTCGAGACATGGTAGAAGCAAATATCGCAAAAGGTTATAAATTCATGGTAAT
TACTGATCATTCACAAAGTTTACGTGTTGCTAATGGCTTACAAGTGGAAAGACTTTTAAGACAAAATGAAGAAATTAAGGTTTTAGATAAAG
AATATAGTGAAATTGATATTTATTCAGGTACAGAAATGGATATATTACCTGATGGCTCGCTGGATTATGATGATGAAATTTTAGCACAACTT
GATTATGTAATTGGAGCTATTCATCAAAGCTTTAACCAATCAGAAGAACAAATTATGGAACGATTAGCTAATGCATGTCGCAATCCATACGT
GCGACATATAGCGCATCCAACAGGGCGTATTATAGGTAGAAGAGATGGTTATAAACCGAATATTGAACAATTAATGGCATTAGCTGAAGA
AACGAATACAGTATTAGAAATTAATGCCAATCCACATCGACTGGATCTGAACGCTGATATCGTTCGTAAATATCCAAATGTGAAATTAACTA
TTAACACTGATGCGCATCATACAAATCATTTAGATTTTATGAATTATGGCGTAGCAACTGCGCAAAAAGGATTTGTAACAAAAGATAGAGT
GATTAACGCATTATCGCGTGAAGCTTTTAAAGACTTTATTGAAAATAATATAAAACTTAAGAAATAG

Gene: mutS2 (DNA mismatch repair ATPase)

Position: 1109253 to 1111601, length: 2349 nt, orientation: FORWARD

Sequence:

ATGAGACAAAAAACATTAGACGTCTTAGAATTCGAAAAAATAAAATCACTCGTTGCCAATGAAACTATTAGTGACTTAGGCTTGGAAAAGG
TCAATCAAATGATGCCAGCTACTAATTTTGAAACGGTTGTTTTTCAAATGGAAGAAACGGATGAGATTGCTCAAATCTATAATAAGCATCGT
TTACCAAGCTTGAGTGGCTTATCTAAAGTATCAGCATTCATTCATCGCGCTGATATTGGCGGCGTTTTAAATGTATCAGAGCTTAACTTGATA
AAAAGATTAATTCAAGTACAAAATCAATTCAAGACATTTTATAATCAATTGGTTGAAGAAGATGAAGGTGTTAAATACCCAATATTAGATGA
CAAGATGAATCAATTACCTGTGTTAACTGATCTTTTTCAACAAATAAATGAAACATGCGATACGTATGATTTATATGATAATGCGAGTTATG
AATTGCAAGGGATTAGAAGTAAAATTTCTAGCACGAATCAACGTATTAGACAAAATTTGGACCGTATTGTTAAAAGCCAAGCAAATCAGAA
AAAATTATCAGATGCTATTGTAACAGTTAGGAATGAAAGAAACGTTATACCTGTCAAAGCTGAATATCGACAAGATTTTAATGGGATTGTA
CATGATCAATCTGCTTCAGGACAAACATTGTATATTGAGCCATCATCAGTTGTTGAAATGAATAATCAAATTAGTCGATTACGTCATGACGA
AGCAATTGAAAAAGAACGCATTTTAACGCAACTAACTGGTTATGTGGCTGCGGACAAAGATGCACTACTTGTGGCAGAACAAGTCATGGG
TCAGTTAGATTTTTTAATCGCAAAAGCGAGATATAGTAGAAGTATTAAAGGAACAAAGCCGATATTTAAAGAGGACCGTACTGTATATTTA
CCTAAAGCATACCATCCATTATTAAATCGTGAGACTGTTGTAGCTAATACCATCGAATTTATGGAAGATATTGAAACGGTAATTATTACAGG
ACCGAATACAGGTGGTAAAACTGTAACATTAAAAACATTAGGTTTAATTATTGTTATGGCTCAATCAGGATTGTTGATTCCCACACTTGATG
GTAGTCAGTTAAGTGTATTTAAAAATGTATATTGCGATATCGGAGATGAACAATCAATAGAACAATCATTATCAACTTTTTCATCTCATATGA
CGAATATAGTTGAAATTTTAAAGCATGCAGACAAACATAGTTTAGTTTTATTTGATGAATTAGGTGCAGGTACAGATCCGAGTGAAGGTGC
TGCATTAGCAATGAGCATTTTAGATCATGTTAGAAAAATTGGTTCTCTAGTAATGGCAACGACGCACTATCCCGAACTTAAAGCATATAGTT
ATAATCGAGAAGGCGTTATGAATGCGAGTGTAGAATTTGATGTAGATACTTTGAGTCCAACGTATAAGTTATTAATGGGTGTGCCGGGTCG
TTCAAATGCTTTTGACATTTCTAAAAAGTTAGGTCTTAGTTTGAATATTATTAATAAGGCTAAGACGATGATTGGTACTGATGAAAAAGAAA
TAAATGAAATGATTGAATCATTAGAGCGTAATTACAAACGTGTAGAGACACAGAGGTTAGAACTGGACCGTCTTGTAAAAGAAGCGGAGC
AAGTGCATGATGATTTATCTAAGCAGTATCAACAATTCCAAAATTATGAAAAGTCTCTAATAGAGGAAGCGAAAGAAAAAGCAAATCAGAA
GATTAAGGCTGCAACAAAAGAAGCTGACGATATTATTAAAGACTTAAGACAATTGCGTGAACAAAAAGGTGCAGATGTTAAAGAACATGA
ATTGATTGATAAGAAGAAACGATTAGATGATCATTATGAAGCGAAATCTATAAAGCAAAATGTACAAAAGCAAAAATACGATAAAATTGTT
GCTGGTGATGAAGTAAAAGTATTATCTTACGGTCAAAAGGGTGAAGTTTTAGAAATTGTCAATGATGAAGAAGCAATTGTTCAAATGGGA
ATTATTAAAATGAAGTTACCTATTGAAGATTTAGAGAAAAAACAAAAAGAAAAAGTTAAGCCAACGAAAATGGTTACACGTCAAAATCGTC
AAACAATTAAAACTGAACTTGACTTACGAGGCTATCGTTATGAGGATGCTTTAATTGAACTAGATCAATATTTAGATCAAGCCGTTTTAAGT

AATTACGAACAAGTTTATATCATTCATGGTAAAGGTACAGGTGCACTTCAAAAAGGTGTACAACAACATTTGAAAAAGCATAAAAGTGTTA
GTGACTTTAGAGGTGGTATGCCAAGCGAAGGTGGATTTGGCGTTACCGTTGCAACACTAAAATAA

Gene: trxA1 (thioredoxin)

Position: 1111774 to 1112088, length: 315 nt, orientation: FORWARD

Perfect match to: (11819-97-CP003194-[1158183:1158497], allele observed in CC80+CC239+CC4803)

Sequence:

ATGGCAATCGTAAAAGTAACAGATGCAGATTTTGATTCAAAAGTAGAATCTGGTGTACAACTAGTAGATTTTTGGGCAACATGGTGTGGTC
CATGTAAAATGATCGCTCCGGTATTAGAAGAATTAGCAGCTGACTATGAAGGTAAAGCTGACATTTTAAAATTAGATGTTGATGAAAATCC
ATCAACTGCAGCTAAATATGAAGTGATGAGTATTCCAACATTAATCGTCTTTAAAGACGGTCAACCAGTTGATAAAGTTGTTGGCTTCCAAC
CAAAAGAAAACTTAGCTGAAGTTTTAGATAAACATTTATAA

Gene: uvrC (ultraviolet response system subunit C)

Position: 1112412 to 1114193, length: 1782 nt, orientation: FORWARD

Perfect match to: (N315-BA000018-[1125255:1127036], highly conserved allele)

Sequence:

TTGGAAGACTATAAGCAACGAATTAAAAATAAATTAAATGTCGTACCTATGGAACCAGGCTGCTATTTAATGAAAGATCGTAATGATCAAG
TGATATATGTTGGCAAAGCTAAAAAGCTAAGAAATCGATTGAGATCATATTTTACGGGTGCTCATGATGCTAAAACAACGAGACTGGTTGG
TGAAATACGTCGTTTTGAGTTTATTGTCACGTCAAGTGAAACAGAGTCACTTTTACTGGAATTGAATCTGATAAAACAATATCAACCAAGAT
ATAATATATTATTAAAGGATGATAAAAGTTATCCATTTATTAAAATTACGAAGGAGAAATATCCTAGACTACTAGTGACGAGAACTGTAAAA
CAAGGTACTGGCAAATATTTCGGACCGTATCCGAATGCATATTCTGCTCAAGAAACTAAAAAGTTATTAGACAGAATATATCCATATCGCAA
ATGTGATAAGATGCCAGATAAATTATGTCTTTATTACCATATTGGACAATGTTTAGGACCATGTGTATATGACGTTGATTTGAGTAAATACG
CACAAATGACGAAGGAAATTACTGATTTTCTGAATGGGGAAGACAAAACAATTTTAAAAAGTTTAGAAGAGCGAATGTTAACTGCAAGTG
AATCACTTGATTTTGAACGGGCTAAAGAATATAGAGATTTAATTCAACATATTCAAAATCTGACAAACAAACAAAAAATTATGTCATCAGAT
AAAACGATTCGTGATGTCTTTGGTTATTGTGTTGATAAAGGATGGATGTGTATCCAAGTTTTCTTTATACGACAAGGTAATATGATAAAGCG
AGATACAACGATGATTCCATTACAGCAAACAGAAGAAGAAGAATTTTATACATTTATTGGACAATTTTATAGCTTAAACCAACATATTTTACC
TAAGGAAGTTCATGTACCACGTAATTTGGATAAAGAAATGATTCAATCTGTTGTGGACACTAAAATCGTTCAACCCGCGCGAGGTCCCAAA
AAAGATATGGTTGACCTAGCTGCACATAACGCTAAAGTATCCTTAAATAATAAATTTGAATTAATATCACGTGATGAGTCAAGAACGATTAA
AGCTATTGAAGAACTTGGAACACAAATGGGAATTCAAACACCAATTAGAATTGAAGCATTCGATAATTCTAATATTCAAGGTGTGGATCCA
GTGTCAGCAATGGTTACATTTGTCGACGGTAAACCAGATAAGAAAAATTATAGAAAGTATAAAATCAAAACGGTTAAAGGTCCAGATGATT
ACAAATCAATGAGAGAAGTAGTAAGACGACGATATTCTCGCGTTTTAAACGAAGGATTACCATTACCTGATTTAATAATAGTCGATGGTGG
TAAAGGACATATGAACGGGGTTATTGATGTGCTACAAAACGAATTAGGTCTTGATATCCCTGTTGCAGGTTTGCAGAAAAATGATAAACAC
CAAACATCTGAATTATTATATGGCGCTAGTGCAGAAATTGTACCACTGAAGAAAAATAGCCAGGCATTTTATTTGTTGCATCGTATCCAAGA
TGAGGTTCACAGATTCGCAATCACATTTCATAGACAAACACGTCAAAAGACAGGCTTGAAATCAATACTTGATGATATAGATGGTATCGGT
AACAAACGTAAAACATTATTATTGCGTTCATTCGGTTCAATCAAGAAAATGAAGGAAGCTACACTTGAAGATTTTAAAAATATAGGTATTCC
TGAAAACGTCGCAAAGAACCTACATGAACAATTGCATAAATAA

Gene: sdhC (succinate dehydrogenase cytochrome b-558 subunit)

Position: 1114517 to 1115131, length: 615 nt, orientation: FORWARD

Perfect match to: (MW2-BA000033-[1128594:1129208], highly conserved allele)

Sequence:

TTGGCTCAATCAAAAAATGAATTTTATCTAAGACGTATTCACTCGTTATTAGGTATTATCCCAATAGGTGCATTTTTGGTCGTTCATTTATTAG
TGAATCACCAAGCAACACAAGGTGCTGAAGCGTTTAATAAGGCATCTAACTTTATGGAATCATTACCATTTCTAATTATTGTAGAATTTTTAT
TTATATACATTCCGTTGTTATATCACGGTTTGTTTGGTATACACATTGCATTTACAGCAAAAGAAAATGTTGGACATTACTCGATTTTTAGAA
ACTGGATGTTCTTCTTCCAAAGAGTGAGTGGTATCTTAACATTTATCTTTATTGGTATCCATTTATGGCAAACACGTTTACAAAAAGCATTTT
ACGGCAAAGAAGTGAATTACGATTTAATGCACGAAACATTGCAACATCCTGGATGGGCAATATTTTATATTATTTGTATTATTGCTGTTGTG
TTCCACTTTGCAAATGGCTTATGGTCATTCTTAGTTACTTGGGGTGGACTTCAATCTCCAAAATCACAACGAGTATTTACATGGGTTTCATTA
ATCGTATTTTTAGTTATTTCGTATATTGGTGTTACTGCAATTATTGCCTTTATGTAA

Gene: sdhA (succinate dehydrogenase flavoprotein subunit)

Position: 1115183 to 1116949, length: 1767 nt, orientation: FORWARD

Perfect match to: (11819-97-CP003194-[1161592:1163358], allele observed in CC80+CC772)

Sequence:

ATGGCAGAGAAACATCTTATTGTTGTCGGAGGTGGCCTAGCGGGCTTAATGTCAACAATTAAAGCGGCAGAAAAAGGTGCACATGTAGAT
TTGTTCTCAGTTGTACCAGTAAAGCGTTCGCACTCTGTTTGTGCCCAAGGTGGCATTAATGGTGCGGTCAATACTAAAGGGGAAGGCGATT
CTCCTTGGATTCACTTTGATGATACAGTGTATGGTGGCGACTTCCTTGCAAACCAACCACCTGTTAAAGCGATGACAGAGGCAGCACCTAAA
ATTATTCATTTATTAGACCGTATGGGCGTAATGTTCAATAGAACAAATGAAGGTCTATTAGATTTTAGACGTTTCGGTGGTACATTACACCAC
AGAACAGCATATGCAGGGGCAACAACTGGACAACAATTATTATATGCATTGGATGAACAGGTTCGTGCATATGAAGTAGATGGATTAGTT
ACGAAGTATGAAGGATGGGAATTCCTTGGCATAGTTAAAGGTGACGATGATAGTGCAAGAGGTATCGTTGCACAAAATATGACAACTGCT
GAGATTGAAACATTTGGTTCAGATGCAGTTATTATGGCAACGGGTGGCCCTGGTATTATTTTCGGTAAAACAACAAACTCAATGATTAATAC
AGGATCAGCGGCTTCCATTGTTTACCAACAAGGCGCTATTTATGCTAATGGTGAGTTCATTCAAATTCATCCTACTGCAATCCCTGGTGATG
ATAAACTGCGACTAATGAGTGAATCAGCACGTGGTGAAGGTGGACGAATTTGGACATATAAAGATGGTAAGCCTTGGTACTTCTTAGAAG
AGAAATATCCTGATTATGGTAACTTAGTACCTCGTGATATCGCAACGCGTGAAATTTTCGATGTATGTATTAACCAAAAATTAGGTATAAAT
GGCGAAAACATGGTATATCTTGATTTGTCACATAAAGATCCACATGAGTTAGATGTAAAACTAGGTGGTATCATTGAGATTTATGAAAAATT
CACTGGTGATGACCCACGCAAAGTACCAATGAAGATTTTCCCAGCTGTTCACTATTCAATGGGTGGTCTATATGTAGATTATGATCAAATGA
CAAATATTAAAGGGTTATTTGCAGCTGGAGAATGTGACTTCTCTCAACATGGTGGTAACCGCTTAGGTGCCAATTCATTGTTATCAGCGATT
TATGGTGGTACAGTAGCAGGTCCAAACGCGATTGATTATATTTCAAATATTGATCGATCATATACTGATATGGACGAAAGTATTTTTGAAAA
GCGTAAAGCTGAAGAGCAAGAACGTTTTGATAAATTATTAGCTATGCGCGGTACAGAAAATGCCTATAAATTACACCGTGAACTTGGTGAA
ATTATGACAGCAAATGTAACTGTTGTTCGTGAAAATGAAAAACTGTTAGAAACAGATAAAAAGATTGTTGAATTGATGAAACGTTATGAAG
ATATTGATATGGAAGATACTCAAACTTGGAGTAACCAAGCGGTATTCTTTACTCGTCAATTATGGAACATGTTAGTACTTGCACGTGTTATTA
CGATTGGTGCATATAACCGTAACGAATCACGCGGTGCCCATTATAAACCAGAATTCCCAGAGCGTAATGATGAAGAGTGGTTAAAAACGAC
AATGGCCTCATTCCAAGGCGCATTTGAAAAACCACAGTTTACTTATGATGACGTCGATGTGAGTTTAATACCACCTCGTAAACGTGATTACA
CAAGTAAGTCTAAAGGGGGTAAAAAATAA

Gene: sdhB (succinate dehydrogenase iron-sulphur protein subunit)

Position: 1116949 to 1117764, length: 816 nt, orientation: FORWARD

Perfect match to: (NN50-BAEA01000008-[91604:92419:r], allele observed in CC4803+CC5+CC8+CC239)

Sequence:

ATGACTGAACAATCAGTGAAAAACACTCCACAACATGAAACACAATCTAAACCGAAACAAAAAACAGTAAAATTAATTATTAAACGACAAG
ATACAAGTGATTCTAAGCCTTATGAAGAAACATTTGAAATTCCATATCGTGAAAATTTAAACGTCATTGCTTGTTTAATGGAAATTAGACGT
AACCCAGTTAATATTAAAGGTGAAAAAACAACACCTGTTGTCTGGGATATGAACTGCTTAGAAGAAGTATGTGGAGCATGTTCTATGGTTA
TCAATGGTCGTGCAAGACAATCTTGTTCTGCGATTGTTGATCAATTAGAACAACCTATTCGTTTAGAGCCAATGAATACTTTCCCAGTTATCC
GTGACTTACAAGTTGATCGTTCTAGAATGTTCGATAACTTAAAACGTATGAAAGCATGGATCCCAATTGATGGAACGTATGATTTAGGTCCG
GGACCACGTATGCCAGAGAAAAAACGTCAAACAGCTTATGAATTATCTAAATGTATGACATGTGGTGTATGTTTAGAGGTTTGTCCTAATGT
TACTGAAAATAATAAATTCGTTGGTGCGCAAGCAATCTCGCAAGTTCGTTTGTTTAATTTGCACCCAACAGGATCTATGACTAAAGATGAAC
GTTTAAATGCATTAATGGGTACTGGTGGCTTACAGCAGTGTGGAAATTCACAAAACTGTGTTAATGCTTGCCCTAAAGGTATTCCATTAACA
ACATCCATTGCAGCAATGAACAGAGAAACAACATTCCACATGTTTAAATCATTCTTTGGTTCAGACCATGAAGTAGAATAA

Gene: murI (glutamate racemase)

Position: 1118005 to 1118805, length: 801 nt, orientation: FORWARD

Perfect match to: (Strain_21310-AFNP01000036-[104477:105277], allele observed in CC22+CC80)

Sequence:

ATGAATAAACCAATAGGTGTAATAGACTCTGGTGTCGGAGGTTTGACAGTAGCTAAAGAAATTATGCGTCAGTTGCCAAATGAGACGATTT
ATTACTTAGGTGATATTGGGCGATGCCCATATGGGCCAAGACCAGGAGAACAAGTAAAACAATATACAGTTGAAATCGCTCGTAAATTAAT
GGAATTTGATATAAAAATGCTCGTGATTGCTTGTAATACAGCAACTGCTGTAGCTTTAGAATATTTACAAAAGACCTTATCAATCCCAGTGA
TTGGCGTAATTGAACCAGGTGCTAGAACAGCAATAATGACTACTAGAAATCAAAATGTATTAGTACTAGGAACGGAAGGCACAATTAAATC

TGAAGCATATCGTACGCATATTAAACGTATCAATCCACATGTAGAGGTACATGGCGTTGCCTGTCCAGGTTTTGTGCCACTTGTAGAACAAA
TGAGATATAGTGATCCAACAATTACAAGCATTGTCATTCATCAAACACTGAAACGTTGGCGTAATAGTGAGTCTGATACTGTCATTTTAGGA
TGTACCCACTATCCATTGCTCTATAAACCTATCTATGATTATTTTGGTGGTAAAAAGACAGTGATTTCGTCTGGATTAGAAACGGCTCGTGAA
GTTAGTGCATTGCTAACATTTAGTAATGAACATGCAAGTTATACTGAAAATCCAGATCATCGATTTTTTGCAACAGGTGATCCTATTCACATT
ACTAACATTATCAAAGAGTGGTTAAATTTATCTGTCAATGTGGAACGTATATCAGTGAATGACTAG

Gene: ntpA (nucleoside-triphosphatase)

Position: 1118817 to 1119404, length: 588 nt, orientation: FORWARD

Perfect match to: (11819-97-CP003194-[1165226:1165813], highly conserved allele)

Sequence:

ATGAAAGAGATTGTTATTGCATCGAATAATCAAGGGAAAATAAATGACTTTAAAGTAATATTTCCAGATTACCACGTAATAGGTATTTCAGA
ACTAATACCAGATTTTGATGTGGAAGAAACAGGATCAACATTTGAAGAAAATGCTATATTAAAATCAGAAGCTGCTGCAAAAGCATTGAAT
AAAACGGTCATAGCTGATGACAGTGGACTAGAAGTTTTTGCATTAAATGGTGAGCCAGGTATATACTCTGCACGTTATGCTGGTGAAAATA
AAAGCGATGAAGCAAATATTGAAAAAATTATTAAATAAGCTTGGTAATACAACTGATCGTCGTGCGCAATTTGTTTGTGTCATAAGTATGAGT
GGCCCTGATATGGAAACAAAAGTATTTAAAGGTACTGTTTCAGGTGAAATTGCAGATGGAAAATATGGCGAAAATGGTTTCGGATATGATC
CGATATTTTATGTACCGAAATTAGATAAAACCATGGCTCAACTTTCAAAAGAACAAAAAGGGCAAATTAGTCATAGACGAAATGCGATTAA
TTTACTTCAAGCTTTTCTTGAAGGTGATAAAAATGTCTAA

Gene: ysnB (metallophosphatase)

Position: 1119397 to 1119900, length: 504 nt, orientation: FORWARD

Perfect match to: (11819-97-CP003194-[1165806:1166309], allele observed in CC80)

Sequence:

ATGTCTAAATGGATCATTGTGAGTGATAACCATACAGAATCAGGCGTTTTATATCAAATTTATGAAATGCACCCAGATGCAGATGTATATTT
ACATTTAGGAGATTCAGAATTCGCGTATGATGATACGGAACTTAGCTTATTTAATAGAGTAAAAGGCAATTGTGATTTTTACCCAGAATTTG
AAAATGAAGCGGTCGCAAAATATAATGACGTGAAAGCATTTTATACTCATGGACATTTATATCAAGTCAATCGAACAAGAGATTTATTAGCT
GAAAAAGGACTTGAATTAGGTTGTATGTTTGCATTTTATGGACATACACATGTGGCAAAATATGAGTATATTAATGGTGTTCATGTTATTAA
CCCTGGAAGTATATCTCAATCTAGAAGTTCAATGGAAGAAACATATGCTGAAGTTATTATTGATGATCAAACTTTACATGGCACCATCAATT
TCAAAAATCGACATCACGAAACAATCAGTCATACTACTTTTTAA

Gene: Q6GA61 (putative protein)

Position: 1120028 to 1120195, length: 168 nt, orientation: FORWARD

Perfect match to: (11819-97-CP003194-[1166437:1166604], allele observed in CC80+CC72+CC80+CC772)

Sequence:

ATGAAAAGAATTGTAATTTTAGAGAATGTGATTGTTGATGACCCTATATGGTTGAATCATGTTGATTATTTGGTTAAAAGTGTATTATTAAA
AAGTATAAAATTAAGATTCCAAGAAGTCAGGAAATTTGAAATTTTAGTTAAAATCATGATTCGTATACATATTTAA

Gene: ecb (extracellular complement-binding protein)

Position: 1120389 to 1120706, length: 318 nt, orientation: FORWARD

Perfect match to: (11819-97-CP003194-[1166798:1167115], allele observed in CC80+CC7+CC15+CC80+CC96)

Sequence:

ATGAAAAAAGAATTTTATTGGGAAATCAATTTTAAGCATAGCTGCTATTAGTTTAACGGTATCAACGTTTGCCGGTGAATCTCATGCACAAAC
TAAGGTTGAAAAAATATAACGAGTATCAAACAAACTTTAAAAAACAAGTAAATAAAAAAGTTATGGACGCACAAAAAGCTGTAAACTTGTTC
AAACGTACAAGAACTGTTGCAACACACCGTAAAGCACAAAGAGCTGTTAATTTAATTCATTTCCAACACAGCTATGAAAAGAAAAAATTAC
AAAGACAAATCGATCTAGTTTTAAAATATAATACTTTAAAATAA

Gene: flr (formyl peptide receptor inhibitory protein)

Position: 1121075 to 1121477, length: 403 nt, orientation: TRNC-RVRS (no stop codon)

Sequence:

ATTAATCCCAATAAATCGAGTCAATTTCACCGTTTTTAACAACTTTAATATTTTTTCTGTTCTCTTCTAAAGGACGAGTTAAGTCAAAAGTATA
GTAATCTCTAGGACCACCATCTTTAATTCTGACAACTGCTTTCTTCACATCACCTTGGCTTAATTTTTTAACAATAAGGTACAGATCATTAACA
GTTTCAGCTTTGTAAGGAGTTAGATTTTTATCAGACTCTTTCATTAACTTATCAATACGTTCATCGTCCTTTTTAGCTTGATCTGCTAAATTTTT
TGCGATTTCTAAACCTTTCCATTCATAACTAAAGAAAGCTTTAGCATCATTAGTTTGAGTTGAAAGACCTGCAGCGATTACTGTAGATGCGAT
AATAGTTTTTGTGATATTTTTTTTCAT

Gene: Q2YXB9 (putative membrane protein)

Position: 1122148 to 1122654, length: 507 nt, orientation: FORWARD

Perfect match to: (MW2-BA000033-[1136217:1136723], allele observed in CC1+CC72+CC80+CC188)

Sequence:

ATGATAATTAAAATTTTAACAATTCTATTACTACTTTGTATATTGAGCTATTTGGTTACAAATAGAAAGAAGCCTTTTCTGTTCTTAAAAACAC
TCTTTATGGGTGTGGTATTTATCTTTATAGGATATATTTCACTGGCAATATCTGCCGTAATTATTTATGGCATTATTCAATTTATCACAATTGA
TTTTGGTAGTTTTTTCTTAATGGGTATTATATTAATCTTGATTTCAAGTATATTCCAATTATTTATAGTTAGATTACTTTTTAGAAAAAAGAATG
TCGATTTGACAGAGGTTGTCGTTTTAGAACATTTAATTCAATGGTTCTTAGTTTACTTTGCGATCTATCAAGCAGTAAATGAAAAAATGGACA
TTAATGATATTAATATCGACAATTTTCAATCTGTCTTTTTTGACGTGTCTAATTTGAATTTAGTAATTCTACCAACGTTAATCATTAGCTGGGT
CACAATATTTAACTATAGAATGAGAAGTTACAAATAA

Gene: efb (extracellular fibrinogen-binding protein)

Position: 1122913 to 1123410, length: 498 nt, orientation: FORWARD

Perfect match to: (11819-97-CP003194-[1169322:1169819], allele observed in CC80)

Sequence:

ATGAAAAATAAATTGATAGCAAAACCTTTATTAACATTAGCGGCAATAGGTATTACTACAACTACAATTGCGTCAACAGCAGATGCGAGCG
AAGGATACGGTCCAAGAGAAAAGAAACCAGTGAGTATTAATCACAATATCGTAGAGTACAATGATGGTACTTTTAAATATCAATCTAGACC
AAAATTTAACTCAACACCTAAATATATTAAATTCAAACATGACTATAATATTTTAGAATTTAACGATGGTACATTCGAATATGGTGCGCGTCC
ACAATTTAATAAACCAGCAGCGAAAACTGATGCAAATATTAAAAAAGAACAAAAATTGATTCAAGCTCAAAATCTTGTGAGAGAATTTGAA
AAAACACATACTGTCAGTGCACACAGAAAAGCACAAAAGGCAGTCAACTTAGTTTCGTTTGAATACAAAGTTAAGAAAATGGTCTTACAAG
AGCGAATTGATAATGTATTAAAACAAGGATTAGTTAAATAA

Gene: scc (similar to complement inhibitor)

Position: 1123564 to 1123914, length: 351 nt, orientation: FORWARD

Perfect match to: (N315-BA000018-[1136409:1136759], highly conserved allele)

Sequence:

ATGAAATTTAAAAAATATATATTAACAGGAACATTAGCATTACTTTTATCATCAACTGGGATAGCAACTATAGAAGGGAATAAAGCAGATG
CAAGTAGTCTGGACAAATATTTAACTGAAAGTCAGTTTCATGATAAACGCATAGCAGAAGAATTAAGAACTTTACTTAACAAATCGAATGTA
TATGCATTAGCTGCAGGAAGCTTAAATCCATATTATAAACGTACGATTATGATGAATGAATATAGAGCTAAAGCGGCACTTAAGAAAAATG
ATTTCGTATCAATGGCTGATGCTAAAGTTGCATTAGAAAAAATATACAAAGAAATTGATGAAATTATAAATAGATAA

Gene: repeat_nySAgamma (repeat element)

Position: 1124286 to 1124468, length: 183 nt

Sequence:

GGCGGGGCCCCAACATAGAGAAATTGGATCACCAATTTCAACAGACAATGCAAGTTGGGGTGGGGCCCAACACAGAAGCTGGCGAAAAG
TCAGCATACAAAAATGTGCAAGTTGGGGATGGGCCCCAACACAGAGAAATTCAAAAAGAAATTCTACAGACAATGCAAGTTGGCGGGGCC
CCAA

Gene: setB3 (staphylococcal enterotoxin-like toxin B locus 3)

Position: 1124689 to 1125405, length: 717 nt, orientation: REVERSE

Perfect match to: (11819-97-CP003194-[1175123:1175839:r], allele observed in CC80+CC15+CC80+CC772)

Sequence:

CTAATCAAAGAGGACGTCAACAGTTATTTTATTAGGATTTTTAACATAAACATTTGCTAGATCTGAATGTAATCTTTTGCTTAAATCAATAGT
GTAGTTATTACCGCCACCGGTGATCTTAAGCTTACCTTTATTACGATTTTCGTTATATAATATTTTATTTTTTATTAACGCTTCTCGTGCACGGA
AATCGATTTCTTTCAATGTTAATACTGGTTTATTGCCTTGGTATATTTTATGCGCACCAATAATCGTTTGTAGTTTATCTTTGTATTGCACAAA
AAGATTATAAGTTTTATCAGAAGGTTTTGCGGCTGGTGTAACGCCACCTGTAAATGTCTCTCTATAAGACCACCATAACTGATCAGTATCTTT
GTCTTTTAGTCCAAACACATCTACGTAACGATCTTTTAACTGATTAATATTTCCCCAACTTTCAGCGCCCCATAAAGATATATGTGCTGACCAT
GAATATCTCTTAAGTTCAACATAAATGTTTCCATTATCATATTGATATAGCCATTTATTTGAAAATGAAAAATGAGGCTGTGTGTAATATTTA
ATTAATTCATTGATGTTAGTTTCATCTTGACCAATACTATAAGCTTTAGCTTCAGAGTAAAAACTAAATACTGGTTTTTGATTTTGAGGTAATA
CAGTACCTAATAGTAATAATGTTGTCGTTAAAATTATATTTTTCGTGATGTTCTTACTCAT

Gene: setB2 (staphylococcal enterotoxin-like toxin B locus 2)

Position: 1125513 to 1126238, length: 726 nt, orientation: REVERSE

Sequence:

TTAGTTTGATTTTTCGAGGATAACTTCAATTTTTGCATTTTGAGGTTTTTTAACATATCTATTTGCATCAGTTGATGGCAACCTTTTACTTAAAT
CTATTGTGTAGTTATTGTCTGCACCTGTTATTTTAATTTGTCCTTTATTATAAGAATTATTATATAATTTTTTACTTTTAATTAATGTTTGACGAA
TACGAAAATCTAATTCTTTTAAAGTTAAAACAGGCTTATTGCCTTCATAAACTGGAAATCCGCCAGTAAACGTTTCTGCTTTATCTTTATATGT
TACATTCAGTTTATAGTGTTTATCGTTAGATGTTGCTGCAGGAGTAACACCACCAGTAAACGTTTCTTGAGATAATGCAAAAGAGTCAATGG
TTTCTTGGTCTTTTATGCCAAAAATATCAACGCTTTTATTTCTTAATTGGTTGATATTGCCCCAACTTTCAGGTCCATAAACTTGAATATGACT
ATACCAAGAAAACTGTAACAACGTTGCATGAATCGTACCGTTATCTTTTTGCCATAACGTACTGTTTGAGAAGGTTAAATATTTTTGCGAGTA
ATATTTAGTTAACTCATTTACGTTAGTTTCGTTTTGATTTATATAATAAGCTTTCGCTTCAGATGAAGAATTGATAGGTGTATTAGGAAATTGT
GTAGATGCTGTACCTAATAGTAACAATGTTGTTGATAAAATAATTTTTTTCGTGATGTTATTGTTCAT

Gene: setB1 (staphylococcal enterotoxin-like toxin B locus 1)

Position: 1126333 to 1127058, length: 726 nt, orientation: REVERSE

Perfect match to: (11819-97-CP003194-[1176767:1177492:r], allele observed in CC80)

Sequence:

TTAGTTAGATTTTTCGAGTATGACTTCAATTTGTGCATTTTTAGGATTTTTAACATAACGGTTTGTGTCAGTTAATTTTAACTTTTTACTTAAAT
CAATCGTGTAATTATTTCCATCAGCAGTTATCTTAATTTGACCTTTATTAAATTCTCCGTTATATAACTTTTTATTCTTTATTAATGTTTGACGA
ATACGGAAGTCTAATTCTTTTAGAGTTAATACTGGTTTATTTCCTTTGTAAAATTCATGTCCACCGATGATAGTTTGTTGTTTATCACTATATTT
TAAAAATAGTCTATAAGGTTTATCAGATGAAGTAGCTGCTGGCGTAACACCACCAGTAAATGTTTCATCATAAGTCCAGTAACCTTCAACTG
TGTCCTCATCTTTAGTTCCAAATATATCAACGTATTTATTTCTTAACTGATTAATGTTTCCCCAACTCTCGGATCCAAACACTTGAATATGACTA
TACCAAACCCACGTTTGCAATGTTGCATGAATGCTACCATTGGGCTTTTGCCATAACCATTTATTTGATAGTGATAAATGAGGCTGTGTATAA
TACTTTATTAACTCATTGATATTAGTCTCTTTTTCACTGATATTATAGGCTTTTGCTTCAGATGAAAAACTGATTGGTGTTTTAGGAAGTTGTG
TAGATGTGGTTCCTAAAAGTAACAATGCTGTTGATAAAACTAATTTATTCATGATGTTCTTTTTCAT

Gene: arcB-L1 (ornithine carbamoyltransferase locus 1)

Position: 1127495 to 1128496, length: 1002 nt, orientation: FORWARD

Perfect match to: (11819-97-CP003194-[1177929:1178930], allele observed in CC80)

Sequence:

ATGAAAAATTTACGAAACAGAAGTTTTTTAACTTTATTAGACTTTTCACGACAAGAGGTAGAATTCTTATTAACACTCTCCGAGGATTTAAAA
CGTGCTAAATATATTGGCACTGAAAAGCCTATGTTAAAAAATAAAAATATTGCACTGTTATTTGAAAAAGATTCTACAAGAACACGATGTGC
ATTTGAAGTTGCAGCGCATGATCAAGGTGCAAATGTAACTTATTTAGGCCCAACTGGATCACAAATGGGTAAAAAAGAAACAACTAAAGAT
ACTGCACGTGTGCTTGGTGGAATGTATGATGGCATTGAATACCGTGGTTTTTCACAAAGAACAGTAGAAACTTTAGCTGAATATTCAGGCG
TACCGGTGTGGAATGGTTTAACTGATGAAGATCATCCTACTCAAGTTCTTGCTGATTTCTTAACAGCAAAAGAAGTCTTAAAAAAAGATTAT
GCAGATATTAACTTTACATATGTTGGAGATGGTCGTAATAACGTTGCAAATGCATTAATGCAAGGTGCTGCCATTATGGGTATGAACTTCCA
TTTAGTTTGTCCAAAAGAATTAAATCCAACAGATGAATTATTAAATCGCTGTAAAAATATTGCCGCTGAAAATGGTGGCAACATATTAATCA
CAGATGATATTGACCAAGGTGTAAAAGGTTCGGATGTAATTTACACTGATGTTTGGGTATCAATGGGTGAACCTGATGAAGTATGGAAAG
AACGACTTGAATTATTGAAACCATATCAAGTAAATAAAGAAATGATGGATAAAACTGGTAATCCAAATGTTATTTTTGAGCATTGCTTACCA
TCTTTCCATAATGCTGATACGAAAATTGGTCAACAAATTTTTGAAAAATATGGAATTCGAGAAATGGAAGTTACAGATGAAGTATTCGAAA
GTAAAGCTTCAGTTGTATTCCAAGAAGCTGAGAACAGAATGCATACAATCAAAGCAGTGATGGTTGCTACATTGGGTGAATTTTAA

Gene: arcC-L1 (carbamate kinase, locus 1)

Position: 1128519 to 1129451, length: 933 nt, orientation: FORWARD

Perfect match to: (KLT6-APFH01000004-[588874:589806], allele observed in CC12+CC8+CC97)

Sequence:

ATGGCGAAAATAGTAGTAGCATTAGGTGGTAATGCTTTAGGAAAATCACCTCAAGAACAACTCGAGCTTGTTAAAAATACTGCGAAATCAT
TAGTAGGATTAATAACAAAAGGACATGAGATTGTTATTAGTCATGGTAATGGACCACAGGTTGGAAGCATTAATTTGGGACTTAACTATGC
TGCAGAACATAACCAAGGTCCGGCATTTCCATTTGCTGAATGTGGCGCAATGAGTCAAGCTTACATCGGCTATCAATTACAAGAAAGCTTAC
AAAATGAATTGCATTCTATTGGAATAGATAAACAAGTGGTAACACTAGTGACACAAGTTGAAGTTGATGAAAATGATCCGGCATTTAACAA
TCCTTCAAAACCAATTGGGTTATTTTACAACAAAGAAGAAGCTAAACAAATTCAAAAAGAAAAAGGATTTATATTTGTTGAAGATGCTGGA
AGAGGATATAGACGTGTTGTTCCTTCACCACAACCCATCTCTATTATTGAATTAGAGAGTATTAAAACACTTATTAAAAATGATACACTCGTT
ATTGCTGCTGGCGGTGGAGGTATACCAGTAATTAGAGAGCAACATGATGGTTTTAAAGGTATTGATGCAGTTATAGACAAAGATAAAACA
AGTGCATTGTTGGGTGCTAATATTCAATGCGATCAATTGATTATTTTAACAGCAATTGATTATGTATATATTAATTTTAACACTGAAAACCAA
CAGCCTTTGAAAACAACAAATGTTGATGAATTAAAACGATATATAGACGAAAATCAATTTGCAAAAGGAAGTATGTTACCAAAAATTGAAG
CAGCCATATCATTTATTGAAAACAATCCAAAAGGAAGTGTGCTTATAACATCATTAAATGAATTAGATGCTGCCTTAGAGGGTAAAGTAGG
TACTGTGATTAAAAAGTAA

Gene: arcD1 (C4 transporter, TRAP family)

Position: 1129623 to 1131179, length: 1557 nt, orientation: FORWARD

Sequence:

GTGGAAAATACAATTAATGAAAGTGAAAAGAAAAAACGATTTAAATTAAAAATGCCAGGTGCATTTATGATTTTATTCATTTTAACGGTTGT
TGCAGTTATAGCAACATGGGTTATTCCTGCTGGTGCATATTCTAAACTTTCTTACGAACCTTCATCCCAAGAACTAAAGATAGTTAACCCTCA
TAACCAAGTGAAAAAGGTTCCGGGTACGCAACAGGAACTAGACAAAATGGGGGTTAAAATTAAGATTGAACAATTTAAATCAGGTGCAAT
TAATAAGCCAGTATCAATTCCGAATACTTATGAAAGATTAAAGCAACATCCAGCTGGACCAGAACAAATAACAAGTAGCATGGTTGAAGGT
ACGATAGAAGCGGTCGATATCATGGTATTCATTCTTGTACTAGGGGGACTTATTGGCGTAGTTCAAGCCAGTGGTTCTTTTGAATCGGGATT
GTTAGCTTTAACGAAGAAAACAAAAGGGCATGAATTTATGCTAATTGTGTTTGTATCAATACTAATGATTATCGGCGGGACGTTATGTGGTA
TTGAAGAAGAAGCTGTAGCATTCTATCCGATTTTAGTCCCTATATTTATAGCGTTAGGATACGATTCTATCGTTTCAGTTGGTGCCATATTCC
TTGCCAGCTCTGTCGGTAGTACATTTTCAACTATTAACCCGTTCTCGGTTGTAATTGCCTCTAATGCCGCTGGTACAACTTTTACGGATGGCT
TGTATTGGAGAATAGGTGCTTGTATTGTCGGTGCGATTTTTGTTATTAGTTATTTATATTGGTACTGTAAAAAAATTTAAAACGATCCTAAAG
CGTCATATTCTTATGAAGACAAAGATGCTTTTGAACAGCAATGGTCTGTATTAAAAGATGACGATAGTGCCCATTTTACTTTGCGTAAGAAG
ATAATCCTTACATTATTTGTACTACCATTTCCAATTATGGTATGGGGAGTTATGACGCAAGGTTGGTGGTTCCCAGTTATGGCTTCAGCATTT
TTAATATTTACAATTATAATAATGTTTATTGCTGGGACAGGTAAATCTGGATTGGGAGAAAAAGGAACTGTAGATGCATTTGTCAATGGCG
CATCAAGTTTAGTAGGTGTATCTTTAATTATTGGCTTAGCTCGAGGTATTAATTTAGTGTTGAATGAAGGTATGATTTCAGATACAATCTTAC
ACTTTTCATCATCTTTAGTTCAACATATGAGTGGACCATTATTTATCATCGTATTACTATTTATTTTCTTCTGTTTAGGTTTTATCGTGCCATCAT
CTTCTGGATTAGCGGTATTATCAATGCCTATCTTTGCACCACTAGCTGATACAGTAGGTATACCAAGATTCGTCATCGTTACGACATATCAAT
TTGGTCAATATGCGATGTTATTCTTAGCGCCGACTGGACTTGTTATGGCCACACTACAAATGTTAAACATGCGATATTCACATTGGTTCCGAT
TTGTATGGCCGGTAGTTGCTTTTGTATTGATTTTCGGTGGCGGAGTACTAATTACGCAAGTACTAATTTATTCATAA

Gene: A5IS57 (putative membrane protein)

Position: 1131486 to 1131713, length: 228 nt, orientation: FORWARD

Perfect match to: (N315-BA000018-[1149809:1150036], highly conserved allele)

Sequence:

ATGACACATTTGACAAAGGTTTTAGATACACTAACTGGAATATGCGTAGTATTATTATTTAGTAAATATTTTGTGGCGTATGCAAATATGGT
GTTTGATTGGAATTTAAGATGGTATTTGCTAGAAAACATACCACATTTGCCAATTATATTATTTATTCTGATGTTTATTTTCGGAGTACCTTCT
GAAATGATAAAAGATAGGCAAAGGAAAAATAACGGTGTTTAA

Gene: per (putative permease)

Position: 1132018 to 1132965, length: 948 nt, orientation: REVERSE

Perfect match to: (NN50-BAEA01000008-[72540:73487], allele observed in CC4803+CC239)

Sequence:

TTACTGAGACGCATTACGAAGAAATTTATCTTGATTTTCCTTTTTTAAAAAGAAGTGAAGATATCCTATAAAGACTCTAAGTACTATTACAGT
GGCTAATAACACTTCGATAAACAAAAGACCTTTCCAAATGTCTGGAAACATAAGTACAGGCAAACTGTTCTTTAAAGCAGTTGCTGAGATTA
CTAAAGGGAATGTGAAAGCTGAAAATACGGGCGAAAAAGGTTCTTTTAGTAATTTAGGCAATTGTATAATGATATAAAAATAAAATATTTG
AGCTAATATTAAAAATATAATTACGATAAAAGCATTCGCCTTAGGAAAGGCTATAACATATGCTGCAGCGACTAAAGAAAATGGTGCACAA
ATTGTCGATGTGTTCGGTTTGATTGACGTCTGCAAAGGAAATGCTTTTAATCGCTTGAAAACTATAGGTAAGACAATACAAGTAGCTACAAA
GCCATATATTACTGTTAATTGACCTATGAAAAAATATCCGCTAACGGGTGCCGTCAATCCTGCGATAGCAATACCAATAAAAAGTACAGTCC
ACGAAGGATAAACATTTTCAAGTGAAAAATCTTTTAAATATTTTATTGAAAAAATAATCATATGCGTCATAATTCCCACAAGGCATAAAATCC
AAATAGGCGTTATTAAGCTATTGATAAAAGTTATGTTACTAAAAAATGTATTTAAATAAGTAGTACCTAAAAAGCCAGACATGAAAAATGTT
GTGAACACTGATGAAACTAGAGGACTGTTTAATTGTTCTTTCACATTATTAAAATATTTGATCATAGTACATAAAAGGTGAATCCAAATCAA
GAAAGCAAAGATTCCGCAAACAGCGTTTAAAGTAAGAGATAAGTCTTTTAATAGATTACCCAGGCCTAATAATCCTAAGACTAGTCCTGAC
GTTACTAGAGGTGCTTTTTGAAGTCTCAT

Gene: A6QG73 (putative DNA-binding protein)

Position: 1133214 to 1133402, length: 189 nt, orientation: FORWARD

Perfect match to: (MW2-BA000033-[1151305:1151493], highly conserved allele)

Sequence:

ATGAGAAATCAAATTCAAAAACTATTAGACAGTGATTTGAGCAGTTTACATATATCGAAACAAACAGGAGTTCCACAAAGCACAATACACA
GAATGAGAAAAAATGAAAGATCATTAGACAATATGTCATTGAAAAACGCTGAACTACTTTATAAATTTGCCAATAGTATATTTAGCAATGAA
AATTAA

Gene: psmB1 (phenol soluble modulin beta 1)

Position: 1134308 to 1134442, length: 135 nt, orientation: FORWARD

Perfect match to: (N315-BA000018-[1152631:1152765], highly conserved allele)

Sequence:

ATGGAAGGTTTATTTAACGCAATTAAAGATACCGTAACTGCAGCAATTAATAATGATGGCGCAAAATTAGGCACAAGCATTGTGAGCATCG
TTGAAAATGGCGTAGGTTTATTAGGTAAATTATTCGGATTCTAA

Gene: psmB2 (phenol soluble modulin beta 2)

Position: 1134499 to 1134633, length: 135 nt, orientation: FORWARD

Perfect match to: (N315-BA000018-[1152822:1152956], highly conserved allele)

Sequence:

ATGACTGGACTAGCAGAAGCAATCGCAAATACTGTGCAAGCTGCACAACAACATGATAGTGTGAAATTAGGCACAAGTATCGTAGACATC
GTTGCTAACGGTGTGGGTTTACTAGGTAAATTATTTGGATTCTAA

Gene: Q1Y2B3 (putative haloacid dehalogenase-like hydrolase)

Position: 1134757 to 1135452, length: 696 nt, orientation: FORWARD

Perfect match to: (11819-97-CP003194-[1185191:1185886], allele observed in CC80)

Sequence:

ATGGGGAAATTGGGATATAAAAATATTTTGATAGACTTTGATGATACAATTGTTGATTTTTATGATGCAGAAGAATGGGCGTTTTACTATAT
GGCGAATGTTTTTAATCATAAAGCAACAAAGGATGATTTTTTAACATTTAAAAAAATCAATCACCAACATTGGGAAGCTTTTCAACAAAATA
AATTAACGAAGTCTGAAGTATTATCAGAACGATTTGTGAATTACTTCAAACATCATCAAATGGAAGTTGATGGGCATCGTGCAGATGTGTTA
TTTAGAAATGGATTAGCAGAAGCTAAAGTTAAATACTTTGATCAAACATTAGAAACAATTGTCGAATTATCGAAAAGACATGATTTATATAT
TGTTACTAATGGTGTAACCGAAACGCAAAAGAGAAGGTTAAATCAGACGCCGTTACATAAATATATTAAAAAGATATTTATATCTGAGGAA
ACAGGATATCAAAAACCTAATCCGGAATTTTTTAATTATGTTTTTAATGATATTGGTGAGGATGAAAGACAGCACTCGATTATAGTTGGAGA
TTCTTTAACATCTGACATTCTAGGTGGAATCAATGCGGGTATAGCTACTTGCTGGTTTAATTTTAGAGGGTTTGATCATAATCCAGGAATTAT
ACCTGATTATGAAATTAATTCATGGAAACAACTAAATGATATTGTACGTTAA

Gene: Q931T2 (N-acetyltransferase, GNAT family)

Position: 1135561 to 1136001, length: 441 nt, orientation: REVERSE

Perfect match to: (HE681097-[1122874:1123314:r], allele observed in CC22+CC5+CC22)

Sequence:

TTATGGTTTCCACAATAAGACATCATGTCCCTCTATATTTTTAGCTTCCACATCTGTAAATCCATGATGTAAAAAGAAATCCTTAGAATCATTT
CTGCCAATGGCTTTAATTGGCATGTTGAAACTCTTTGCAAAATCAATCAATTCTGAAGCGTACCCTCTGTTTTGATATTTTGGTAATACTTCTA
ACTTCCATAATAATATATAATCTTCAAAATCTGGGAAGTAGATTTCTTCGACATCACCTTTTTTTAGTAATGCCATTCTAGCTCCTAATTGATC
TCCGACAAATATGCCATAAAATGGTGAATCTGAACTTGCATCAATCATTTGACCGTTTAACTCATTGACCATGTATAAGTCTTTGTTGCCAAA
CGCTCTAAAGTTTTCGAATAATTCGTCAGTTTTGTAATTAATTTCAAGACGTTTGATTTCACTCAT

Gene: bshC (bacillithiol cysteine-adding enzyme)

Position: 1136196 to 1137809, length: 1614 nt, orientation: FORWARD

Sequence:

ATGGACTGTAAAGTAGTTAGTTTAAATGAAAAAGATCAGTTTATACCAAAAATAAAGAGCAGTGACCCTGTAATAACAGGATTATTTCAAT
ATGATGCAGCTCAACAAACTAGTTTTGAAAAAAGGATGTCTAAAGAAAATAATGGAAGAGAAGCGGCATTAGCGAATGTTATTCGTGAAT
ATATGAGTGATTTAAAGCTTTCAAGTGAACAAGAATTAAACATACAACATTTAGCTAATGGTTCAAAAGTTGTGATTGGTGGACAACAAGC
AGGGCTTTTCGGGGGACCATTGTATACATTCCATAAAATATTTTCAATCATTACTTTATCTAAGGAATTAACGGATACACATAAGCAACAAG
TAGTACCAGTTTTTTGGATTGCAGGAGAAGATCATGATTTCGATGAAGTGAATCATACATTTGTTTATAACGAAAATCATGGGTCGCTGCAT
AAGGTTAAATATCATACAATGGAGATGCCAGAGACGACTGTCTCTAGATATTATCCTGATAAGGCTGAATTGAAACAAACTTTAAAAACGA
TGTTCATTCATATGAAAGAAACTGTTCATACACAAGGTCTACTGGAGATTTGTGACAGAATTATTGACCAATATGACTCGTGGACTGATATG
TTTAAAGCACTACTGCATGAAACATTTAAAGCATATGGCGTTCTATTTATAGATGCGCAGTTTGAGCCGTTAAGAAAAATGGAAGCGCCTAT
GTTTAAAAAGATTTTGAAAAAACATCAGTTGCTTGATGATGCTTTTAGAGCAACACAACAACGTACTCAAAATCAAGGCTTGAATGCGATG
ATACAAACAGATACAAATGTTCATTTATTCTTACATGATGAAAATATGCGTCAATTAGTTTCGTATGATGGTAAACATTTTAAATTAAATAAA
ACAGATAAGACATATATAAAGGAAGAAATTATAAATATTGCGGAAAATCAACCTGAATTATTTTCTAATAATGTAGTGACAAGACCATTAAT
GGAAGAATGGTTATTTAACACGGTGGCATTTGTTGGAGGACCGAGTGAAATTAAATACTGGGCTGAACTAAAAGATGTATTTGAACTATTT
GATGTTGAAATGCCTATCGTGATGCCAAGGCTTAGAATTACTTATTTAAATGACCGTATTGAAAAATTACTTTCGAAATACAATATTCCATTA
GAAAAAGTGTTAGTCGATGGTGTTGAAGGAGAAAGAAGTAAGTTTATTAGAGAACAAGCATCACATCAATTTATTGAAAAGGTAGAAGGT
ATGATTGAACAACAGCGTCGTCTAAACCAAGACTTATTAGATGAAGTGGCGGGGAATCAAAATAATATTAACCTTGTGAATAAAAATAATG
AAATTCATATACAACAGTATGATTATTTGTTAAAACGTTATCTTTTAAACATTGAAAGAGAAAACGACATCAGTATGAAGCAATTTAGAGAA

ATTCAAGAAACACTCCATCCAATGGGAGGATTACAAGAAAGAATATGGAATCCACTTCAAATTTTGAATGATTTTGGGACAGATGTGTTCA
AGCCCTCCACCTATCCACCACTTTCTTACACTTTTGATCATATTATTATAAAACCTTAA

Gene: mraZ (putative transcriptional regulator)

Position: 1137953 to 1138384, length: 432 nt, orientation: FORWARD

Perfect match to: (RF122-AJ938182-[1123275:1123706], highly conserved allele)

Sequence:

ATGTTCATGGGAGAATACGATCATCAATTAGATACAAAAGGACGTATGATTATACCGTCCAAGTTTCGTTATGACTTAAATGAGCGTTTTAT
TATCACAAGAGGCCTTGATAAATGTTTATTCGGTTACACTCTAGACGAATGGCAACAGATTGAAGAGAAAATGAAAACCTTACCTATGACA
AAAAAAGACGCACGTAAGTTTATGCGTATGTTCTTCTCTGGTGCTGTTGAAGTAGAACTTGATAAGCAAGGGCGTATTAACATCCCTCAAAA
CTTGAGGAAATACGCTAATTTAACTAAAGAATGTACAGTAATCGGTGTTTCAAATCGTATTGAGATTTGGGATAGAGAAACTTGGAATGAT
TTCTATGAAGAATCTGAAGAAAGTTTCGAAGATATTGCTGAAGATTTAATAGATTTTGATTTTTAA

Gene: rsmH-mraW (16S rRNA methyltransferase H)

Position: 1138421 to 1139335, length: 915 nt, orientation: FORWARD

Perfect match to: (D139-ST145-ACSR01000039-[18831:19745], allele observed in CC10+CC8)

Sequence:

ATGTTAAACGAAACCATTGATTATTTAAATGTAAAAGAAAATGGTGTGTACATTGACTGTACGCTAGGTGGAGCGGGACATGCCCTTTATTT
ACTAAATCAATTAAATGACGACGGAAGATTAATAGCAATCGATCAAGACCAAACTGCAATTGATAATGCTAAAGAGGTATTAAAGGATCAT
TTGCATAAGGTGACTTTTGTTCATAGCAACTTCCGTGAATTAACTCAAATATTAAAAGACTTAAACATTGAAAAAGTAGATGGAATTTATTAC
GACTTGGGTGTTTCAAGCCCACAACTCGACATTCCAGAACGAGGATTCAGTTATCACCATGACGCAACATTAGACATGCGTATGGACCAAA
CACAAGAACTAACAGCATATGAAATTGTTAACAATTGGTCATATGAAGCGTTAGTGAAGATTTTTTATCGCTATGGCGAGGAGAAATTTTCA
AAACAGATAGCTCGAAGAATCGAAGCACATCGCGAACAACAACCAATAACAACAACATTAGAATTAGTTGACATTATAAAAGAAGGTATTC
CTGCGAAAGCAAGAAGAAAAGGCGGACATCCTGCAAAACGAGTATTTCAAGCACTACGAATTGCAGTAAACGATGAATTGTCAGCTTTTG
AAGATTCAATAGAACAAGCGATTGAATTAGTGAAAGTAGACGGCAGGATTTCGGTAATCACTTTCCATTCTTTAGAAGATCGTTTATGTAAA
CAGGTGTTCCAAGAATATGAAAAAGGTCCAGAGGTACCAAGAGGATTACCAGTTATACCAGAAGCATATACACCTAAGTTAAAACGTGTTA
ATCGTAAACCGATTACCGCTACAGAAGAAGATTTAGATGACAATAACAGAGCACGAAGCGCGAAATTACGTGTAGCTGAAATACTTAAATA
A

Gene: ftsL (initiator of cell division)

Position: 1139349 to 1139750, length: 402 nt, orientation: FORWARD

Perfect match to: (N315-BA000018-[1157672:1158073], highly conserved allele)

Sequence:

ATGGCTGTAGAAAAAGTGTACCAACCATATGACGAACAAGTTTATAATAGTATACCGAAGCAACAACCACAAACTAAGCCCGAAAAGAAG
ACTGTTTCGAGAAAAGTGGTTGTACAATTAACTAAATTTGAAAAAGTTTTATACATAACTTTGATTACTGTAATTGCTATGTTAAGTATTTAT
ATGCTATCTTTAAAAATGGATGCGTATGATACGCGAGGAAAGATTGCAGATTTAGATTATAAAATAGATAAACAATCAAGTGAAAACAGTG
CTTTACAATCTGAAATCAAAAAGAATTCTTCTTATGAACGCATATACGAAAAGGCTAAGAAACAGGGGATGAGCCTTGAGAACGATAATGT
AAAGGTAGTGCGTAGTAATGGCGAAGCAAAAAATTAA

Gene: pbpA (penicillin binding protein A)

Position: 1139731 to 1141965, length: 2235 nt, orientation: FORWARD

Perfect match to: (T0131-CP002643-[1204300:1206534], allele observed in CC239+CC8)

Sequence:

ATGGCGAAGCAAAAAATTAAAATTAAAAAAAATAAAATAGGGGCAGTCCTACTTGTTGGTTTATTCGGACTGCTCTTTTTTATATTGGTTTT
AAGAATTTCATATATCATGATTACTGGACATTCTAATGGTCAAGATTTAGTCATGAAGGCAAATGAAAAGTATTTAGTTAAGAATGCACAAC
AACCAGAACGAGGAAAGATATATGATCGTAATGGTAAAGTGCTAGCAGAAGATGTAGAAAGATATAAACTTGTTGCAGTAATAGATAAAA
AGGCGAGTGCCAATTCTAAAAAGCCTAGGCATGTAGTTGATAAAAAAGAGACTGCAAAGAAATTATCTACAGTCATTGATATGAAGCCAG
AGGAAATTGAAAAGAGACTTAGTCAAAAGAAAGCTTTCCAAATTGAATTTGGACGCAAAGGAACAAATTTAACGTATCAGGATAAATTGA
AAATAGAGAAAATGAATTTGCCTGGTATTTCTTTATTGCCTGAAACAGAACGCTTTTATCCAAATGGCAATTTTGCATCACACTTAATTGGTA
GAGCTCAGAAAAATCCGGATACTGGTGAACTTAAAGGTGCGCTTGGAGTTGAAAAGATTTTTGATAGTTATTTAAGTGGATCTAAAGGATC
ATTGAGATATATTCATGATATTTGGGGATATATTGCACCAAATACTAAAAAAGAGAAGCAGCCTAAACGTGGTGATGATGTCCATTTAACA
ATCGATTCAAATATTCAAGTATTTGTTGAAGAAGCTTTAGATGGCATGGTTGAAAGATACCAGCCGAAAGATTTATTTGCGGTTGTCATGGA
TGCTAAAACTGGAGAAATTTTAGCATACAGTCAGCGACCAACATTTAATCCTGAAACTGGTAAAGACTTTGGTAAAAAGTGGGCAAATGAC
CTTTATCAAAACACATACGAGCCTGGATCAACATTTAAATCATATGGGTTAGCAGCTGCTATTCAAGAAGGTGTTTTTGATCCTGATAAGAA
ATATAAATCTGGACATAGAGATATTATGGGTTCACGTATTTCAGACTGGAATAGAGTCGGTTGGGGTGAAATCCCAATGTCACTCGGATTT
ACTTATTCATCTAATACATTGATGATGCATTTACAAGATTTAGTTGGTGCAGACAAAATGAAATCTTGGTATGAACGATTTGGATTTGGAAA
ATCAACTAAAGGTATGTTTGATGGAGAAGCACCTGGTCAAATTGGATGGAGTAATGAGTTACAACAAAAAACGTCATCATTTGGTCAATCG
ACAACTGTAACACCTGTTCAAATGTTACAGGCGCAATCAGCGTTCTTTAATGATGGTAATATGTTAAAACCATGGTTTGTGAATAGCGTTGA
AAATCCTGTTAGTAAAAGACAATTTTATAAAGGGCAAAAACAAATCGCAGGCAAGCCAATAACAAAAGATACTGCTGAAAAAGTTGAAAA
GCAATTGGATTTAGTTGTGAATAGTAAGAAGAGTCACGCTGCAAACTATCGTATTGATGGTTATGAGGTCGAAGGTAAGACTGGTACAGC
ACAAGTCGCTGCACCTAATGGTGGTGGATACGTTAAAGGTCCAAACCCATATTTTGTAAGTTTTATGGGTGACGCGCCGAAAAAAAATCCT
AAAGTTATTGTATACGCTGGTATGAGCTTGGCACAAAAAAATGACCAAGAAGCTTATGAATTAGGTGTTAGTAAAGCGTTTAAACCAATAA
TGGAAAATACTTTGAAATATTTAAATGTAGGTAAATCAAAAGATGACACATCTAATGCAGAGTATAGTAAAGTGCCAGATGTTGAAGGTCA
AGACAAACAAAAAGCTATTGATAATATGAGTGCAAAATCATTAGAACCAGTTACTATCGGTTCTGGCACACAAATAAAAGCACAATCTATA
AAAGCAGGGAATAAAGTCTTACCTCATAGTAAAGTACTGTTATTAACAGACGGAGACATAACTATGCCTGACATGTCAGGATGGACGAAA
GAAGATGTCATTGCTTTTGAAAACCTAACAAATATTAAAGTAAATTTAAAAGGTAGCGGTTTTGTGTCCCACCAATCAATTAGTAAGGGACA
AAAACTTACTGAAAAAGATAAAATAGACGTAGAATTTTCATCAGAGAATGTAGACAGCAATTCGACGAATAATTCTGATTCAAATTCAGAT
GATAAGAAGAAATCTGACAGTAAAACTGACAAGGATAAGTCGGACTAA

Gene: mraY (phospho-N-acetylmuramoyl-pentapeptide-transferase)

Position: 1142257 to 1143222, length: 966 nt, orientation: FORWARD

Sequence:

ATGATTTTTGTATATGCGTTATTAGCGCTAGTGATTACATTTGTTTTGGTACCTGTTTTAATACCTACATTAAAAAGGATGAAATTTGGTCAA
AGTATTCGAGAAGAAGGTCCACAAAGCCATATGAAGAAGACTGGTACACCAACGATGGGTGGACTAACATTTCTATTAAGTATTGTGATAA
CGTCTTTGGTGGCTATTATATTTGTAGATCAAGCTAATCCAATCATACTGTTATTATTTGTGACGATTGGTTTTGGGTTAATTGGTTTTATAGA
TGATTATATTATTGTTGTTAAAAAGAATAACCAAGGTTTAACAAGTAAACAGAAGTTTTTGGCGCAAATTGGTATTGCGATTATATTCTTTGT
TTTAAGTAATGTGTTTCATTTGATGAATTTTTCTACGAGCATACATATTCCATTTACGAATGTAGCAATCCCACTATCATTTGCATATGTTATTT
TCATTGTTTTTTGGCAAGTAGGTTTTTCTAATGCGGTAAATTTAACAGATGGTTTAGATGGATTAGCAACTGGACTGTCAATTATCGGATTTA
CAATGTATGCCATCATGAGCTTTGTGTTAGGAGAAACGGCAATTGGTATTTTCTGTATCATTATGTTGTTTGCACTTTTAGGATTTTTACCAT
ATAACATTAACCCTGCTAAAGTGTTTATGGGAGATACAGGTAGCTTAGCTTTAGGTGGTATATTTGCTACCATTTCAATCATGCTTAATCAG
GAATTATCATTAATTTTTATAGGTTTAGTATTCGTAATTGAAACATTATCTGTTATGTTACAAGTCGCTAGCTTTAAATTGACTGGAAAGCGT
ATATTTAAAATGAGTCCGATTCATCATCATTTTGAATTGATAGGATGGAGCGAATGGAAAGTAGTTACAGTATTTTGGGCTGTTGGTCTGAT
TTCAGGTTTAATCGGTTTATGGATTGGAGTGCATTAA

Gene: murD (UDP-N-acetylmuramoylalanine-D-glutamate ligase)

Position: 1143224 to 1144573, length: 1350 nt, orientation: FORWARD

Sequence:

ATGCTTAATTATACAGGGTTAGAAAATAAAAATGTATTAGTTGTCGGTTTGGCAAAAAGTGGTTATGAAGCAGCTAAATTATTAAGTAAATT
AGGTGCGAATGTAACTGTCAATGATGGAAAAGACTTATCACAAGATGCTCATGCAAAAGATTTAGAATCTATGGGCATTTCTGTTGTAAGT
GGAAGTCATCCATTAACGTTGCTTGATAATAATCCAATAATTGTTAAAAATCCTGGAATACCTTATACAGTATCTATTATTGATGAAGCAGTG
AAACGAGGTTTGAAAATTTTAACAGAAGTTGAGTTAAGTTATCTAATCTCTGAAGCACCAATCATAGCTGTAACGGGTACAAATGGTAAAA
CGACAGTTACTTCTCTAATTGGAGATATGTTTAAAAAAAGTCGCTTAACTGGAAGATTATCCGGCAATATTGGTTATGTTGCATCTAAAGTA
GCACAAGAAGTAAAGCCTACAGATTATTTAGTTACAGAGTTGTCGTCATTCCAGTTACTTGGAATCGAAAAGTATAAACCACACATTGCTAT
AATTACTAACATTTATTCGGCGCATCTAGATTACCATGAAAATTTAGAAAACTATCAAAATGCTAAAAAGCAAATATATAAAAATCAAACGG
AAGAAGATTATTTGATTTGTAATTATCATCAAAGACAAGTGATAGAGTCGGAAGAATTAAAAGCTAAGACATTGTATTTCTCAACTCAACAA
GAAGTTGATGGTATTTATATTAAAGATGGTTTTATCGTTTATAAAGGTGTTCGTATTATTAACACTGAAGATTTAGTATTGCCTGGTGAACAT

AATTTAGAAAATATATTAGCAGCTGTGCTTGCTTGTATTTTAGCTGGTGTACCTATTAAAGCAATTATTGATAGTTTAACTACATTTTCAGGA
ATAGAGCATAGATTGCAATATGTTGGTACTAATAGAACTAATAAATATTATAATGATTCCAAAGCAACAAACACGCTAGCAACACAGTTTGC
CTTAAATTCATTTAATCAACCAATCATTTGGTTATGTGGTGGTTTGGATCGAGGGAATGAATTTGACGAACTCATTCCTTATATGGAAAATGT
TCGCGCGATGGTTGTATTCGGACAAACGAAAGCTAAGTTTGCTAAACTAGGTAATAGTCAAGGGAAATCGGTCATTGAAGCGAACAATGT
CGAAGACGCTGTTGATAAAGTACAAGATATTATAGAACCAAATGATGTTGTATTATTGTCACCTGCTTGTGCGAGTTGGGATCAATATAGTA
CTTTTGAAGAGCGTGGAGAGAAATTTATTGAAAGATTCCGTGCCCATTTACCATCTTATTAA

Gene: ftsQ (cell division protein Q)

Position: 1144589 to 1145908, length: 1320 nt, orientation: FORWARD

Perfect match to: (11819-97-CP003194-[1195023:1196342], allele observed in CC80+CC8+CC80+CC239)

Sequence:

ATGGATGATAAAACGAAGAACGATCAACAAGAATCAAATGAAGATAAAGATGAATTAGAATTATTTACGAGGAATACATCTAAGAAAAGA
CGGCAAAGAAAAAGATCAAAGGCTACACATTTTTCTAATCAAAATAAAGATGATACATCTCAACAAGCTGATTTTGATGAAGAAATTTACTT
GATAAATAAAGACTTCAAAAAAGAAGAAAGCAATGATAAAAATAATGATTCTGCTTCTAGTCATGCGAATGATAATAATATCGATGATTCT
ACAGACTCTAATATTGAAAATGAGGATTATAGATATAATCAAGAAATTGACGACCAAAATGAATCGAATGTAATTTCAGTCGACAACGAAC
AACCTCAATCAGCTCCTAAAGAACAAAATAGCGACTCGATTGATGAGGAAACAGTAACGAAAAAAGAACGAAAAAGTAAAGTAACACAAT
TAAAGCCATTAACACTTGAAGAAAAGCGGAAGTTAAGACGTAAGCGACAAAAGCGAATCCAATACAGTGTTATTACAATATTGGTATTGTT
GATTGCTGTTATATTAATTTACATGTTTTCACCACTTAGTAAAATTGCGCATGTAAATATAAATGGAAATAATCACGTTAGTACTTCAAAGAT
AAACAAAGTTTTAGGTGTTAAAAATGATTCAAGGATGTATACGTTTAGTAAAAAAAATGCTATTAATGATCTCGAAGAGGATCCATTAATCA
AAAGTGTTGAGATACACAAGCAATTACCAAACACATTAAACGTAGATATCACAGAAAATGAAATTATTGCTTTAGTGAAATATAAAGGTAA
ATATTTACCTTTATTAGAAAATGGTAAATTGCTTAAAGGTTCAAATGATGTCAAAATTAATGATGCACCTGTCATGGATGGTTTCAAAGGTA
CAAAAGAAGATGATATGATTAAGGCGTTATCTGAAATGACACCTGAAGTTAGACGATATATTGCCGAAGTGACATACGCCCCAAGTAAAAA
CAAACAAAGCAGAATTGAATTGTTTACGACAGATGGACTTCAAGTAATCGGTGATATTTCGACGATATCTAAGAAAATGAAATATTATCCG
CAGATGTCACAATCATTATCAAGGGATAGTTCGGGTAAACTAAAAACACGAGGCTATATTGATTTATCAGTCGGTGCTTCATTTATCCCATA
CCGTGGAAACACGTCTAGTCAATCAGAAAGCGATAAAAATGTGACTAAATCATCTCAAGAGGAAAATCAAGCAAAAGAAGAATTACAAAG
CGTTTTAAACAAAATTAACAAACAATCAAGTAAGAATAATTAA

Gene: ftsA (cell division protein A)

Position: 1146014 to 1147420, length: 1407 nt, orientation: FORWARD

Perfect match to: (CIGC93-AHVD01000014-[22423:23835], allele observed in CC15+CC5+CC8+CC72+CC772)

Sequence:

ATGGAAGAACATTACTACGTAAGTATTGATATTGGATCATCAAGCGTAAAAACAATAGTAGGCGAGAAATTTCACAATGGTATAAATGTGA
TAGGTACAGGACAAACCTACACGAGCGGTATAAAAAATGGTTTAATTGATGATTTTGATATTGCGCGACAAGCAATCAAAGACACAATTAA
AAAGGCATCAATCGCTTCGGGTGTTGATATTAAAGAAGTTTTCCTGAAATTACCTATCATTGGAACGGAAGTTTATGATGAATCAAATGAAA
TCGACTTTTATGAGGATACAGAAATCAACGGTTCACATATCGAAAAAGTATTAGAAGGTATTAGAGAAAAAAATGATGTGCAAGAAACAG
AAGTAATTAATGTGTTCCCGATTCGTTTTATAGTCGATAAAGAAAATGAGGTTTCAGACCCTAAAGAATTAATTGCCAGACATTCATTAAAG
GTTGAAGCAGGCGTAATTGCTATTCAAAAATCGATTTTAATTAATATGATTAAATGCGTAGAAGCATGTGGTGTTGATGTATTAGATGTTTA
CTCTGATGCATATAACTATGGTTCAATCCTAACAGCTACTGAAAAAGAGTTAGGTGCATGTGTCATTGATATTGGTGAAGACGTTACGCAAG
TTGCTTTTTATGAACGCGGTGAATTAGTAGATGCTGATTCTATCGAAATGGCAGGGCGTGATATTACAGACGATATTGCACAAGGATTAAA
CACTTCTTATGAAACTGCTGAAAAAGTTAAACACCAATATGGTCATGCATTCTATGATTCTGCTTCAGATCAAGATATCTTCACTGTTGAACA
GGTTGATAGTGATGAAACAGTACAGTATACTCAAAAAGATTTGAGTGACTTTATTGAAGCGCGTGTAGAAGAAATATTCTTCGAAGTATTT
GATGTTTTACAAGATTTAGGATTAACAAAAGTAAATGGTGGGTTTATTGTAACTGGTGGATCTGCAAACTTACTTGGCGTAAAAGAATTATT
ATCAGATATGGTAAGTGAAAAAGTTAGAATTCACACGCCATCACAAATGGGAATTAGAAAACCTGAATTTTCTTCAGCAATTTCTACAATTT
CTAGTAGTATCGCTTTTGATGAGTTATTAGATTATGTTACAATTAATTATCATGATAATGAAGAAACTGAAGAAGATGTTATTGATGTGAAA
GACAAAGATAACGAATCTAAATTAGGCGGATTTGATTGGTTTAAACGTAAAACAAACAAAAAAGATACTCATGAAAATGAAGTAGAGTCA
ACAGATGAAGAAATTTATCAATCAGAAGATAATCATCAGGAACATAAACAGAATCATGAACATGTTCAAGACAAAGATAAAGAAGAAAGT
AAATTCAAAAAACTAATGAAATCTCTATTTGAATGA

Gene: ftsZ (cell division protein Z)

Position: 1147453 to 1148625, length: 1173 nt, orientation: FORWARD

Perfect match to: (Strain_21333-AHKA01000047-[8855:10027:r], allele observed in CC80+CC5)

Sequence:

ATGTTAGAATTTGAACAAGGATTTAATCATTTAGCGACTTTAAAGGTCATTGGTGTAGGTGGTGGCGGTAACAACGCCGTAAACCGAATGA
TTGACCACGGAATGAATAATGTTGAATTTATCGCTATCAACACAGACGGTCAAGCTTTAAACTTATCTAAAGCTGAATCTAAAATCCAAATC
GGTGAAAAATTAACACGTGGTTTAGGAGCAGGAGCTAACCCTGAAATCGGTAAAAAAGCTGCAGAGGAATCTCGTGAACAAATTGAAGAT
GCAATCCAAGGTGCAGACATGGTATTTGTTACTTCTGGTATGGGTGGCGGAACTGGTACTGGTGCAGCACCAGTCGTTGCTAAAATTGCAA
AAGAAATGGGCGCATTAACTGTTGGTGTTGTAACTCGTCCATTTAGTTTTGAAGGACGTAAACGTCAAACTCAAGCTGCTGCTGGAGTAGA
AGCTATGAAAGCTGCAGTAGATACATTAATCGTTATACCAAATGACCGTTTATTAGATATCGTTGACAAATCTACGCCAATGATGGAAGCAT
TTAAAGAAGCTGACAACGTGTTACGCCAAGGTGTACAAGGTATCTCAGACTTAATCGCTGTTTCTGGTGAAGTAAACTTAGACTTTGCAGAC
GTTAAGACAATTATGTCTAACCAAGGTTCTGCATTAATGGGTATTGGTGTTTCTTCTGGTGAAAATAGAGCGGTAGAAGCTGCTAAAAAAG
CAATCTCTTCTCCATTACTTGAAACATCTATCGTTGGTGCACAAGGTGTGCTTATGAATATTACTGGTGGCGAGTCATTGTCATTATTTGAAG
CACAAGAGGCTGCTGATATTGTCCAAGATGCTGCAGATGAAGACGTTAATATGATTTTCGGTACAGTTATTAATCCTGAATTACAAGATGA
GATTGTTGTAACAGTTATTGCAACTGGTTTTGATGACAAACCAACATCGCATGGTCGTAAATCTGGTAGCACTGGATTCGGAACAAGCGTA
AATACTTCTAGCAATGCAACTTCTAAAGATGAATCATTCACTTCAAATTCATCAAATGCACAAGCAACTGATAGTGTAAGTGAAAGAACGCA
TACAACTAAAGAAGATGATATTCCAAGCTTCATTAGAAATAGAGAAGAAAGACGTTCAAGAAGAACAAGACGTTAA

Gene: ylmD (laccase domain protein)

Position: 1148883 to 1149674, length: 792 nt, orientation: FORWARD

Perfect match to: (JKD6159-CP002114-[1147103:1147894], highly conserved allele)

Sequence:

GTGAATGATAATTTTAAAAAGCAACCGCATCATTTAATATATGAAGAATTATTACAACAAGGTATTACTCTAGGTATTACAACTAGAGGCGA
TGGTTTAAGTGACTATCCTAAAAATGCTTTTAATATGGCGAGATATATTGATGATCGCCCATATAATATTACTCAACATCAATTGCAATTAGC
TGAAGAAATTGCGTTTGATAGAAAAAATTGGGTGTTTCCCATCCAAACACATGAAAATAAAGTCGCTTGTATTACAAAGGATGATATAGGC
ACAAATATAGACACTTTAACTGATGCGCTTCATGGTATTGATGCGATGTACACATATGATAGTAATGTCTTATTAACGATGTGTTATGCAGA
CTGTGTACCAGTATATTTTTATAGTACAAAACATCATTTTATTGCATTGGCGCATGCAGGTTGGCGTGGTACCTATACTGAAATTGTAAAAG
AAGTGCTCAAACATGTGAACTTTGATTTGAAAGACTTACATGTCGTTATTGGACCATCTACATCATCAAGTTATGAAATTAATGATGATATTA
AAAGTAAATTTGAAACATTGCCAATTGATAGTGCAAACTATATTGAAACTAGAGGACGAGATCGTCATGGTATTGATTTGAAAAAAGCCAA
TGCTGCATTATTAAATTATTATGGTGTTCCTAAAGAAAATATTTATACGACAGCGTATGCTACATCTGAACATTTAGAATTATTTTTCTCTTAT
CGATTAGAAAAAGGTCAAACAGGACGCATGTTAGCATTCATTGGTCAACAGTAA

Gene: ylmE (putative protein similar to Bacillus subtilis YlmE)

Position: 1149692 to 1150366, length: 675 nt, orientation: FORWARD

Perfect match to: (16K-BABZ01000044-[6793:7467:r], allele observed in CC239+CC8)

Sequence:

TTGCGTGTGAAAGATAATTTACAACAAATCTCAACACAAATTAATGACAAAAGTGAAAAAAATAATTTTTCAACAAAACCAAACGTGATTGC
AGTTACAAAATATGTTACAATAGAGCGAGCTAAAGAAGCGTATGAGGCTGGAATAAGACATTTTGGTGAGAATAGATTGGAAGGCTTTTTT
CAAAAGAAAGAAGCATTACCATCAGATGCGGTGATCCATTTTATAGGATCATTACAATCTCGAAAAGTTAAGGGCGTTATAAACGACGTAG
ATTATTTCCATGCTTTAGATCGATTGAGCTTAGCCAAAGAAATTAACAAACGTGCAGAACATAAAATTAAATGTTTCTTGCAAGTGAACGTT
TCGGGAGAAGCTTCTAAACATGGTATTGCTTTAGAAGATGTTGATCAGTTTATAGATGATCTTAAAAAATATGACAAAATCGAAATTGTAG
GTTTAATGACGATGGCACCATTGACAGATGATGAAGCATATATTAGATCGTTATTTAAACAGTTACGTTTGAAAAAAGAAGAAATACAACG
ACTCAATTTAGAATATGCGCCTTGTGATGAATTATCAATGGGAATGAGTAATGACTATCTTATTGCAGTTGAAGAAGGTGCGACGTTTGTTA
GAATTGGGACTAAACTTGTAGGAGAAGAGGAGTGA

Gene: sepF (cell division protein)

Position: 1150363 to 1150926, length: 564 nt, orientation: FORWARD

Perfect match to: (11819-97-CP003194-[1200797:1201360], allele observed in CC80)

Sequence:

GTGAGCCACTTGGCTTTAAAAGATTTATTTAGTGGATTTTTTGTAATAGATGATGAAGAGGAAGTAGAAGTACCTGACAAACAACAACAGG
TAAATGAAGCGCCAGCAAAAGAGCAGTCACAACAAACAACAAAACAAAACGCAATCAAATCAGTCCCTCAGAAATCTGCATCAAGATATAC
AACAACGTCAGAAGAAAGGAATAACCGTATGTCTAATTATTCAAAAAATAATTCACGTAATGTTGTAACTATGAACAATGCTACACCAAACA
ATGCATCACAAGAAAGTTCAAAAATGTGTTTATTCGAACCACGTGTTTTTTCAGATACACAAGATATTGCTGATGAGCTTAAAAACCGCCGT
GCGACACTTGTCAATTTACAACGTATTGATAAAGTATCAGCGAAAAGAATTATTGATTTTTTAAGCGGTACTGTTTATGCAATCGGTGGAGA
TATCCAACGTGTAGGTACTGATATTTTCTTATGTACGCCTGATAATGTGGAAGTAGCTGGAAGCATTACAGACCATATTGAAAATATGGAAC
ATTCATTCGACTAA

Gene: ylmG (putative protein)

Position: 1150938 to 1151228, length: 291 nt, orientation: FORWARD

Perfect match to: (11819-97-CP003194-[1201372:1201662], allele observed in CC80+CC188)

Sequence:

ATGGATATAAATGTGCTAGCTACAATATTTAAATTTATCCTTTTTGTTGTTGAAATTTATTATTTCGGCATGATTATATATTTCTTTACATCTTG
GGTACCAAGTATTAGAGAAACTAAGATAGGTTATTTTTTAGCGAAAATATATGAACCTTTCTTACAACCATTTAGAAAAGTAATTCCACCTAT
TGGAATTATCGACATATCATCAATCGCTGCAATTTTCGTTTTAGTATTATTCCAAAAAGGGTTACTCCAAATCTTTAATTGGATTTTAATTCAA
TTACAATAA

Gene: ylmH (putative protein similar to Bacillus subtilis YlmH)

Position: 1151311 to 1152117, length: 807 nt, orientation: FORWARD

Perfect match to: (MW2-BA000033-[1169416:1170222], highly conserved allele)

Sequence:

ATGGTATTTTATAATTTTTTTAAGGTAGTGATTAACATAGATATTTATCAACACTTTAGACAGGAAGAATACGAATTAATTGATCAGCTAACG
GATAAATGTGATCAAGCGGAACAGCATTATGCACCAGTATTAACGCATTTTTTAGATCCAAGAGGGCAATATATATTGGAAGTGATTTGTG
GCAGTTATGAAGATTTAAACGTATCTTTTTATGGTGGACCTAATGCTGAAAGAAAAAGAGCAATCATTTCGCCGAACTATTATGAACCTAAA
GAAAGCGACTTTGAATTAACTTTAATGGAAATAGATTATCCTGAAAAATTCGTCACTTTAAAACATCAACATATTTTAGGGACATTAATGTCT
TTAGGTATCGAACGCGAACAAGTTGGAGATATAATTGTGAATGAACGAATTCAATTTGTTTTGACAAGTAGATTGGAATCATTTATTATGTT
AGAATTACAACGTATTAAAGGCGCATCAGTTAAACTTTATACTATTCCAGTAACAGATATGATACAATCTAATGAGAATTGGAAAAATGAAA
GTGCAACAGTTAGTTCTTTAAGGTTAGATGTTGTTATTAAAGAAATGATACGTAAATCACGTACGATTGCGAAACAACTAATCGAAAAAAA
ACGTGTTAAAGTGAATCACACTATTGTTGATTCAGCAGATTTTCAATTACAAGCAAATGATTTAATATCCATCCAAGGTTTTGGTAGAGCAC
ACATTACTGACTTAGGTGGTAAAACTAAAAAAGATAAAACGCACATTACCTATAGAACATTATTCAAATAG

Gene: divIVA (cell-division initiation protein)

Position: 1152141 to 1152758, length: 618 nt, orientation: FORWARD

Perfect match to: (JKD6008-CP002120-[1212643:1213260], allele observed in CC239)

Sequence:

ATGCCTTTTACACCAAATGAAATTAAGAATAAAGAGTTTTCACGTGTAAAGAATGGTTTAGAACCTACTGAAGTTGCTAATTTTTTGGAGCA
ACTAAGCACTGAAATTGAACGTCTTAAAGAAGATAAAAAACAACTTGAAAAAGTAATCGAAGAGAGAGATACTAATATTAAGTCTTATCAA
GACGTGCATCAATCTGTAAGTGATGCTTTGATACAAGCTCAAAAAGCTGGTGAAGAAACTAAGCAAGCTGCAGAAAAACAAGCTGAAGCG
ATTATAGCTAAGGCAGAAGCGCAAGCTAATCAAATGGTTGGTGACGCGGTAGAAAAAGCACGCCGTTTAGCATTCCAGACTGAAGATATG
AAACGTCAATCAAAAGTATTTAGATCGCGTTTCCGTATGTTAGTTGAAGCGCAATTAGACTTATTAAAAAACGAAGATTGGGATTACTTGTT
GAATTATGATTTAGACGCTGAACAAGTGACGCTTGAAAATATTCATCATTTGCATGAAAATGATTTAAAGCCAGATGAAGTTGCAGCAAAT
GCACAAAATAATGCATCAAATACATCAGACAATAATCAACAATCCAATGATTCAGAAACAACTAAGAAGTAG

Gene: tbox11 (T-box leader element)

Position: 1152764 to 1152936, length: 173 nt

Sequence:

AAATAAAGACAGACGCGTAATATACATTTAACTTTTCACAGCGAATTAGGTAATGGTGAGAGCCTAGTAAAAGCATGTATGTTATATCACT
GGCTTTTTAATATTTAAATAATGTAATGAGAGAACTCTAAGTTGAGTTAATAAGGGTGGTACCGCGAGCAATCGTCCCTTTT

Gene: ileS (isoleucyl-tRNA synthetase)

Position: 1152979 to 1155732, length: 2754 nt, orientation: FORWARD

Perfect match to: (11819-97-CP003194-[1203413:1206166], allele observed in CC80)

Sequence:

ATGGATTACAAAGAAACGTTATTAATGCCTAAAACAGATTTCCCGATGCGAGGTGGTTTACCAAACAAGGAGCCGCAAATTCAAGAAAAAT
GGGATGCAGAAGATCAATACCATAAAGCGTTAGAAAAAAATAAAGGTAACGAAACATTCATTTTACATGATGGCCCACCATACGCGAATG
GTAACTTACATATGGGACATGCCTTGAACAAAATTTTAAAAGACTTTATTGTACGTTATAAAACTATGCAAGGGTTCTATGCACCATACGTA
CCAGGTTGGGATACACATGGTTTACCAATTGAACAAGCATTAACGAAAAAAGGTGTTGACCGAAAGAAAATGTCAACAGCTGAATTCCGT
GAGAAATGTAAAGAATTTGCTTTAGAACAAATTGAATTACAGAAAAAAGATTTTAGACGTTTAGGTGTTCGTGGTGACTTTAATGATCCATA
TATTACATTAAAACCTGAATACGAAGCTGCACAAATTCGTATTTTTGGAGAAATGGCAGATAAAGGTTTAATTTATAAAGGTAAAAAGCCA
GTTTATTGGTCTCCTTCAAGTGAGTCTTCATTAGCAGAAGCAGAAATTGAATATCACGATAAACGTTCAGCATCAATTTACGTTGCATTTGAC
GTTAAAGATGACAAAGGTGTCGTAGATGCAGATGCTAAATTTATTATCTGGACAACAACGCCATGGACAATTCCATCAAATGTTGCGATTA
CCGTTCATCCTGAATTAAAATATGGTCAATACAATGTAGGTGGCGAAAAATATATTATTGCAGAAGCCTTATCTGACGCTGTAGCAGAAGC
ACTGGATTGGGATAAAGCATCAATCAAATTAGAAAAAGAATACACAGGTAAAGAATTAGAGTATGTTGTAGCACAACATCCATTCTTAGAC
AGAGAATCGTTAGTGATTAATGGCGATCATGTTACTACAGATGCTGGTACAGGTTGTGTACATACAGCACCAGGTCACGGGGAAGATGAC
TATATTGTTGGTCAAAAATATGAATTGCCAGTAATTAGTCCAATCGATGATAAAGGTGTATTTACTGAAGAAGGCGGCCAATTTGAAGGGA
TGTTCTATGATAAAGCTAATAAAGCCGTTACTGATTTATTAACAGAAAAAGGTGCACTATTAAAATTAGACTTTATTACACATAGCTATCCAC
ACGACTGGAGAACAAAAAAACCTGTAATCTTCCGTGCTACACCACAATGGTTTGCCTCAATCAGTAAAGTAAGACAAGATATTTTAGATGC
AATCGAAAATACAAACTTCAAAGTAAATTGGGGTAAAACACGTATTTACAATATGGTTCGTGACCGTGGCGAATGGGTTATTTCTCGTCAAC
GTGTGTGGGGTGTACCGTTACCAGTATTTTATGCTGAAAATGGCGAAATTATCATGACGAAAGAAACAGTGAATCATGTTGCTGATTTATTT
GCAGAACACGGTTCAAATATTTGGTTTGAAAGAGAAGCGAAAGACTTACTACCAGAAGGATTTACACATCCAGGCAGCCCTAACGGTACAT
TTACTAAAGAAACAGACATTATGGACGTTTGGTTTGATTCTGGTTCATCACACCGTGGCGTGTTGGAAACAAGACCGGAATTAAGTTTCCCA
GCGGATATGTATTTAGAAGGTAGTGACCAATATCGTGGTTGGTTCAACTCTTCTATCACAACTTCAGTTGCTACAAGAGGAGTATCACCTTA
TAAATTCTTACTTTCTCATGGTTTTGTTATGGACGGTGAAGGTAAGAAAATGAGTAAATCTTTAGGTAATGTGATTGTACCTGACCAAGTGG
TTAAACAAAAAGGTGCTGATATTGCGAGACTTTGGGTAAGTAGTACGGACTATTTAGCTGATGTTAGAATTTCTGATGAAATTTTAAAACAA
ACATCTGATGTTTATCGTAAAATCAGAAATACATTAAGATTTATGTTAGGTAACATTAACGATTTCAATCCTGACACAGATAGCATTCCTGAA
TCAGAGTTATTAGAAGTGGATCGTTACTTGCTAAATCGTTTACGTGAATTTACTGCAAGTACGATTAACAACTATGAAAACTTTGACTACTTA
AATATTTATCAAGAAGTTCAAAACTTTATCAATGTTGAGTTAAGTAATTTCTATTTGGATTACGGTAAAGATATTTTATATATTGAACAACGT
GATTCTCATATCCGTCGTAGTATGCAAACAGTGTTATATCAAATTTTAGTTGATATGACGAAGTTGTTAGCACCAATCTTAGTGCATACAGCT
GAAGAAGTTTGGTCTCATACACCACATGTTAAAGAAGAAAGTGTTCACTTAGCAGACATGCCTAAAGTTGTAGAAGTAGATCAAGCTTTAT
TGGATAAATGGCGTACATTTATGAATTTACGTGATGATGTGAACCGTGCATTAGAAACTGCTCGTAATGAAAAAGTTATTGGTAAATCATTA
GAAGCTAAAGTTACGATTGCTAGTAACGATAAATTTAATGCATCTGAATTCTTAACTTCATTTGATGCATTACATCAATTATTTATCGTGTCAC
AAGTTAAAGTTGTAGATAAGTTAGACGATCAGGCAACAGCTTATGAACATGGTGATATTGTCATCGAACATGCAGATGGTGAAAAATGTG
AAAGATGTTGGAACTATTCAGAGGATCTTGGTGCTGTTGATGAATTGACGCATCTATGTCCACGATGCCAACAAGTTGTAAAATCACTTGTA
TAA

Gene: PF00903 (glyoxalase family protein)

Position: 1156022 to 1156819, length: 798 nt, orientation: FORWARD

Perfect match to: (Bmb9393-CP005288-[1572120:1572917:r], allele observed in CC239+CC80)

Sequence:

ATGTATCATAACAGTAACGCAAACTTTGTCAATGGTATCACTTTAAATGTGAGAGATAAGAATGAATTAAAGCCATTTTATGAGGACATATT
AGGATTAAATATTATAAATGAGACATTAACATCGATACAATATGAAGTAGGTCAAAATAATCATGTCATTACACTTGTTGAATTACAAAATG
GACGTGAACCTTTAATGTCCGAAGCGGGACTGTTTCATATCGCAATTAAACTACCTCAAATTAGTGATTTAGCTAATTTACTAATTCATTTAA
GCGAATATGATATTCCAGTTAACGGAGGTATACAGCCTGCTTCGTTATCATTATTTTTTGAAGACCCAGAAGGAAACGGTTTTAAATTTTAT
GTTGATAAAGACGAAGCGCAATGGACGAGGCAAAATGATTTAGTAAAAATTGATATTAGACCATTAAATGTACCGAGATTAGTGAGTCAT
GCAACAAAATTGTTATGGTTAGGTATTCCAGATGACGCTATTATAGGTGCATTGCATATTAAGACAATTCATTTATCAGAGGTAAAAGAGTA
CTACCTCGATTATTTTGGATTAGAGCAATCGGCATATATGGATGATTATTCAATATTTTTAGCATCGAATGGCTATTATCAACATTTGGCCAT

GAATGATTGGGTATCAGCAACGAAACGTGTAGAAAATTTTGATACGTATGGATTAGCAATTGTTGACTTTCATTATCCTGAAACAACACATT
TAAATTTACAAGGTCCGGATGGTATCTATTATCGCTTTAATCATATCGAAGTTGAAGATTAG

Gene: lspA (lipoprotein signal peptidase)

Position: 1157716 to 1158207, length: 492 nt, orientation: FORWARD

Perfect match to: (MRSA252-BX571856-[1217944:1218435], highly conserved allele)

Sequence:

ATGCACAAAAAATATTTTATTGGCACTTCCATTTTAATAGCAGTATTTGTCGTTATATTTGACCAAGTTACTAAATATATTATAGCTACTACAA
TGAAAATTGGAGATTCATTTGAAGTGATACCGCACTTTTTAAACATAACATCACATCGAAATAATGGTGCTGCATGGGGAATATTGAGTGG
AAAAATGACATTTTTCTTTATTATTACCATTATTATATTAATAGCCTTAGTATATTTCTTTATTAAAGATGCTCAATATAATTTGTTTATGCAAG
TTGCTATTAGTTTACTTTTTGCAGGTGCACTTGGAAACTTTATTGATAGAATTTTAACAGGAGAAGTTGTTGACTTTATTGATACAAATATTTT
TGGTTATGATTTTCCAATATTTAATATCGCAGATTCAAGTTTAACAATTGGTGTAATATTAATTATTATTGCCTTATTAAAGGATACTTCCAAT
AAAAAGGAGAAGGAGGTTAAGTAA

Gene: ylyB (ribosomal large subunit pseudouridine synthase)

Position: 1158207 to 1159124, length: 918 nt, orientation: FORWARD

Perfect match to: (Strain_21266-AFTT01000017-[394984:395901:r], allele observed in CC12+CC80)

Sequence:

ATGGAGACTTATGAATTTAACATTACAGATAAAAAACAAACAGGTATGCGTGTAGATAAGTTGCTGCCTGAATTAAATAGTGATTGGTCTC
GTAACCAGATACAAGATTGGATTAAAGCAGGTTTAGTCGTTGCAAACGATAAAGTTGTTAAATCTAATTATAAAGTGAAACTTAATGATCAT
ATAGTTGTCACTGAAAAAGAAGTGGTTGAAGCTGATATTCTACCTGAAAATTTAAATTTAGATATTTATTATGAAGATGACGATGTTGCAGT
TGTATATAAACCGAAAGGCATGGTAGTTCATCCATCACCAGGGCATTATACCAATACATTAGTTAATGGTTTAATGTATCAAATTAAAAATTT
GTCAGGTATTAATGGAGAAATTCGTCCAGGTATTGTTCACCGTATAGATATGGATACTTCTGGTTTATTAATGGTTGCTAAAAATGATATTG
CTCATCGTGGGCTTGTAGAACAATTAATGGATAAATCTGTTAAAAGAAAATATATCGCTTTAGTTCACGGGAATATTCCTCATGATTACGGT
ACAATCGATGCGCCAATTGGTAGAAACAAAAATGATCGTCAATCTATGGCTGTTGTTGATGATGGTAAGGAAGCAGTGACACATTTTAACG
TACTAGAACATTTTAAAGATTATACGCTTGTTGAATGTCAACTTGAAACAGGACGTACGCATCAAATCCGTGTGCACATGAAATATATTGGC
TTCCCATTAGTTGGTGATCCAAAGTATGGACCGAAAAAGACATTGGATATTGGTGGTCAAGCTCTACATGCTGGACTTATTGGATTCGAACA
TCCAGTAACAGGTGAATATATTGAAAGACATGCTGAATTACCACAAGACTTTGAAGATTTATTAGATACAATTCGAAAAAGAGATGCATAA

Gene: pyrR (pyrimidine operon regulator and uracil phosphoribosyltransferase)

Position: 1159524 to 1160051, length: 528 nt, orientation: FORWARD

Perfect match to: (SA40-CP003604-[1167670:1168197], allele observed in CC59+CC22+CC59+CC80+CC361)

Sequence:

ATGTCTGAACGTATCATAATGGATGATGCCGCAATACAACGTACAGTGACGAGAATCGCTCATGAAATTTTGGAGTATAACAAAGGTACTG
ATAATTTAATTCTTTTAGGTATCAAAACAAGAGGTGAATATTTAGCGAATCGTATACAAGATAAAATTCATCAAATTGAGCAACAACGTATA
CCTACTGGAACAATTGATATTACATACTTTAGAGATGATATAGAGCACATGTCATCACTTACGACAAAAGACGCAATAGACATCGACACAG
ATATTACAGATAAAGTAGTCATCATTATTGACGATGTGCTGTATACTGGTCGAACGGTTCGTGCTTCACTTGATGCTATTTTGCTAAATGCTA
GACCTATTAAAATTGGTTTAGCTGCTTTGGTTGATCGAGGACATCGTGAGTTACCAATTCGAGCAGATTTTGTTGGTAAAAATATACCTACT
TCTAAAGAGGAAACGGTAAGTGTCTATTTAGAAGAAATGGATCAAAGAAATGCAGTTATAATTAAATAA

Gene: pyrP (uracil:cation symporter)

Position: 1160269 to 1161576, length: 1308 nt, orientation: FORWARD

Perfect match to: (MW2-BA000033-[1178330:1179637], highly conserved allele)

Sequence:

ATGCAAAATGATGAAATGTTTGAACGAACAGTAAAACCTGTACTAGATGTAAATGAAAAACCACAACCAGCGCAATGGGCATTTTTAAGCT
TACAACATTTATTTGCGATGTTTGGCGCAACAGTACTAGTACCATTCTTAACAGGATTACCAATATCCGCAGCGTTACTAGCTTCGGGAATC
GGTACATTACTTTATATCTTAATAACGAAGGCGCAAATACCAGCATACTTGGGATCTAGCTTTGCATTTATCACGCCAATTATCACGGGATTA
AGTACGCATAGCTTAGGAGACATGCTTGTAGCATTATTCATGAGTGGTGTTATGTACGTCATCATCGGGATTCTAATCAAATTAAGTGGGAC
AGCATGGTTAATGAAATTATTACCACCAGTTGTTGTTGGACCAGTCATCATGGTAATTGGTTTAAGCTTAGCGCCTACTGCAGTCAATATGG
CAATGTATGAAAATCCTGGAGATATGAAAGGTTACAATATCAGTTTCTTAATTGTTGCAATGATAACCTTGCTTGTAACAATAGTCGTTCAA
GGATTTTTTAAAGGATTCCTATCTTTAATTCCAGTACTTGTAGGTATTATCGTAGGTTATGTGGTAGCGATTTTTATGGGGATTGTGAAATTT
GATGCAATTATGTCAGCAAAATGGATAGATTTCCCTCATATTTATCTGCCATTTAAAGATTATGTACCTTCATTTCACTTAGGACTTGTACTG
GTAATGATTCCGATTGTGTTTGTAACAGTAAGTGAACATATTGGGCACCAAATGGTATTGAATAAAATCGTAGGTAGAAACTTCTTTGAAAA
GCCAGGACTTGATAAATCAATCATTGGTGATGGTGTTTCTACAATGTTTGCCAGTATTATTGGTGGACCACCAAGTACAACATACGGTGAAA
ATATCGGTGTATTAGCGATTACCAGAATATACAGTATTTACGTCATTGGTGGTGCAGCAGTTATAGCAATTGTTTTAGCATTCATTGGTAAG
TTCACTGCATTAATTTCTTCTATACCTACACCAGTTATGGGAGGAGTATCTATATTACTTTTCGGTATTATTGCAGCAAGTGGCTTAAGAATG
CTAGTTGAAAGCAAAGTAGATTTTGCGAACAATCGAAATTTAGTTATAGCTTCAGTAATTTTAGTTGTAGGTATCGGTAATTTAGTATTTAAC
TTAAAAGAAATTGGTATCAACCTTCAAATTGAGGGGATGGCATTAGCTGCACTTTCAGGAATTATTTTGAACTTAATCTTACCTAAAGAGAA
AAAACAAAACAATTAA

Gene: pyrB (aspartate carbamoyltransferase)

Position: 1161604 to 1162485, length: 882 nt, orientation: FORWARD

Perfect match to: (Bmb9393-CP005288-[1566527:1567408:r], allele observed in CC239+CC8)

Sequence:

ATGAATCATTTATTATCAATGGAACATTTATCTACAGATCAAATATACAAACTTATCCAAAAGGCAAGTCAATTTAAATCTGGTGAACGTCAA
CTACCAAACTTTGAAGGGAAATATGTCGCAAATTTATTCTTTGAAAATTCTACTCGAACAAAATGTAGTTTTGAAATGGCAGAACTTAAGCT
AGGGTTAAAAACGATTAGCTTTGAAACATCAACATCATCTGTTTCAAAAGGTGAATCTTTATATGACACATGTAAAACTTTAGAAAGTATTG
GCTGTGATTTATTAGTCATTAGACATCCGTTTAATAACTACTATGAAAAATTAGCGAATATTAACATCCCAATTGCGAATGCTGGTGATGGT
AGTGGACAACATCCAACACAAAGTTTACTTGATTTAATGACGATATATGAAGAATATGGATATTTTGAAGGCTTGAATGTATTGATTTGTGG
AGACATTAAAAATTCACGTGTCGCACGTAGTAATTACCATAGTTTAAAAGCATTAGGTGCAAACGTAATGTTTAATAGCCCAAATGCTTGGA
TTGATGATTCTTTAGAAGCACCTTATGTAAATATAGATGATGTTATAGAAACAGTAGATATAGTTATGTTATTAAGAATTCAACATGAAAGA
CATGGGCTTGCAGAAGAAACTAGATTTGCAGCAGATGATTATCATCAAAAGCATGGCTTAAATAAAGTGCGCTATAACAAATTACAAGAAC
ATGCTATTGTTATGCATCCGGCACCTGTGAATAGAGGAGTAGAAATACAAAGCGATTTAGTAGAAGCTTCAAAATCAAGAATTTTTAAGCA
AATGGAAAATGGCGTTTACTTAAGAATGGCAGTCATTGATGAATTATTAAAATAG

Gene: pyrC (dihydroorotase)

Position: 1162503 to 1163777, length: 1275 nt, orientation: FORWARD

Perfect match to: (COL-CP000046-[1220347:1221621], allele observed in CC8+CC9+CC80+CC239)

Sequence:

ATGAAATTAATTAAAAACGGTAAAGTATTACAAAATGGCGAATTACAACAAGCAGATATTTTAATTGATGGTAAGGTAATTAAACAAATTG
CACCTGCAATTGAACCAAGCAATGGTGTTGACATCATAGATGCGAAAGGTCACTTTGTGTCACCTGGATTTGTCGATGTTCATGTTCATTTA
CGTGAACCTGGTGGTGAATATAAAGAGACAATTGAAACTGGTACTAAAGCTGCTGCTAGAGGCGGATTTACAACTGTATGTCCAATGCCTA
ACACAAGACCGGTACCAGATTCTGTAGAACATTTTGAAGCTTTACAAAAATTAATCGATGACAATGCTCAAGTACGTGTATTACCTTATGCT
TCAATTACAACACGTCAATTAGGTAAAGAATTGGTTGATTTCCCAGCACTAGTAAAAGAAGGTGCCTTTGCGTTTACAGATGACGGTGTAG
GAGTACAAACTGCAAGCATGATGTATGAAGGTATGATTGAAGCTGCAAAAGTAAACAAAGCCATCGTAGCACACTGTGAAGATAATTCATT
AATCTATGGTGGTGCAATGCATGAAGGGAAACGCAGTAAAGAGTTAGGTATACCAGGTATTCCAAACATTTGTGAATCTGTTCAAATCGCA
AGAGATGTACTATTAGCTGAAGCAGCAGGTTGTCATTATCATGTATGTCATGTTTCTACTAAAGAAAGTGTTAGAGTCATTCGTGATGCTAA
ACGCGCAGGCATTCATGTTACAGCTGAAGTTACACCACATCATTTATTGTTAACAGAAGATGATATTCCTGGTAATAATGCCATTTATAAAAT
GAATCCACCATTGAGAAGTACTGAAGATAGAGAGGCTTTGTTAGAAGGGTTACTAGACGGTACAATTGACTGTATCGCAACAGACCATGC
ACCACATGCACGTGATGAAAAAGCGCAACCAATGGAAAAAGCACCATTCGGAATTGTTGGTAGTGAAACAGCATTCCCATTATTATATACG
CATTTTGTAAAAAATGGTGATTGGACATTACAACAATTAGTAGATTACTTAACAATTAAACCATGTGAGACATTTAATTTAGAATACGGCAC
ATTAAAAGAAAATGGTTATGCAGATTTAACAATCATTGATTTAGATAGTGAACAAGAAATTAAAGGAGAAGATTTCTTATCAAAAGCAGAT
AATACACCATTTATCGGCTATAAAGTTTATGGAAATCCGATCTTAACAATGGTTGAAGGCGAAGTTAAATTTGAGGGGGATAAATAA

Gene: pyrAA (carbamoyl-phosphate synthase short chain)

Position: 1163779 to 1164879, length: 1101 nt, orientation: FORWARD

Perfect match to: (11819-97-CP003194-[1214157:1215257], allele observed in CC80+CC9+CC15+CC25+CC80)

Sequence:

ATGCAAAGCAAACGTTATCTAGTGTTAGAAGACGGTTCTTTTTACGAAGGCTACCGTTTAGGATCTGATAACTTAACTGTAGGAGAAATTGT
ATTTAATACAGCGATGACAGGTTATCAAGAAACTATTTCAGATCCATCATATACAGGTCAGATCATTACTTTTACGTATCCATTAATCGGTAA
TTATGGTATCAATAGAGACGATTTTGAATCATTAGTACCTACATTAAACGGTATTGTTGTGAAAGAAGCGAGTGCGCATCCAAGTAATTTTA
GACAGCAAAAGACACTTCATGACGTTTTAGAATTGCATCAAATTCCAGGGATTGCAGGTGTTGATACAAGAAGTATTACGCGTAAAATTCG
ACAACACGGTGTGTTAAAAGCTGGTTTTACTGATCGAAAAGAAGATATTGATCAACTTGTCAAACATTTACAACAAGTAGAATTACCTAAAA
ACGAAGTAGAAATCGTTTCGACAAAAACACCGTATGTTTCGACAGGTAAGGATCTAAGTGTTGTACTTGTAGACTTTGGTAAGAAGCAAAA
TATTGTTCGAGAATTAAACGTCAGAGGTTGTAACGTCACAGTTGTACCATATACAACTACTGCCGAAGAAATTTTAGCAATGGCTCCAGATG
GCGTTATGCTATCAAACGGACCAGGTAATCCTGAAGTTGTAGAATGTGCGATTCCAATGATTCAAGGAATTTTAGGGAAAATTCCGTTCTTT
GGTATCTGTCTAGGACATCAACTTTTTGCATTATCTCAAGGAGCAAGCTCATTTAAAATGAAGTTTGGTCATCGTGGTGCGAACCATCCAGT
TAAAAATTTAGAGACTGGAAAAGTTGATATTACGAGTCAAAACCATGGATATGCAATAGATATAGATTCGTTAAAAAGTACTGATTTAGAA
GTTACTCATCTTGCATTAAATGATGGTACTGTAGAAGGTTTAAAACATAAAACATTACCAGCATTTTCTGTTCAATACCATCCTGAAGCAAAT
CCAGGACCGTCAGATTCAAACTATCTATTTGATGATTTTGTAGCAATGATGACTAATTTTAAGGAAAAGGAGCGTCATATCAATGCCTAA

Gene: pyrAB (carbamoyl-phosphate synthase large chain)

Position: 1164872 to 1168045, length: 3174 nt, orientation: FORWARD

Perfect match to: (Strain_21333-AHKA01000002-[208245:211418:r], allele observed in CC80+CC22)

Sequence:

ATGCCTAAACGTAATGATATCAAAACAATTTTAGTAATAGGGTCTGGGCCAATTATCATAGGTCAAGCAGCTGAATTTGATTATGCTGGAAC
ACAAGCATGTCTAGCTTTAAAAGAAGAGGGATACCGAGTTATTCTTGTAAATTCAAATCCAGCGACAATCATGACTGATAAGGAAATTGCG
GATAAAGTATATATCGAACCGTTAACTCATGATTTTATAGCGCGAATTATACGTAAAGAGCAACCTGACGCTTTACTCCCAACTTTAGGTGG
TCAAACAGGTTTAAACATGGCGATTCAACTACACGAAAGTGGTGTGCTTCAAGATAATAACGTCCAATTATTAGGAACTGAGCTAACATCA
ATTCAACAAGCAGAAGACCGTGAAATGTTTAGAACATTAATGAATGATTTAAACGTTCCTGTACCAGAGAGTGACATTGTAAATACAGTAG
AGCAAGCCTTTAAATTCAAAGAGCAAGTGGGATACCCGCTAATTGTTAGACCGGCATTTACGATGGGGGGTACCGGAGGCGGTATTTGTC
ATAATGATGAAGAATTACATGAAATCGTCTCAAATGGTCTTCATTATAGTCCAGCAACGCAATGTTTATTAGAAAAATCTATCGCAGGTTTT
AAAGAAATCGAATACGAAGTAATGCGTGATAAAAACGATAATGCCATCGTTGTATGTAACATGGAAAATATTGATCCAGTTGGTATTCATA
CAGGCGATTCAATTGTTGTGGCTCCTAGCCAAACATTATCAGATGTTGAGTATCAAATGTTACGTGATGTTTCATTAAAAGTTATTCGAGCTT
TAGGTATCGAAGGTGGTTGTAATGTTCAATTAGCATTAGATCCCCATTCATTCGATTATTATATTATAGAAGTAAATCCGCGTGTATCACGTT
CATCAGCGTTAGCTTCAAAAGCAACAGGATATCCTATTGCAAAATTAGCTGCTAAAATCGCGGTTGGTCTAACATTAGATGAAATGTTAAAT
CCAATTACAGGAACATCTTATGCAGCGTTTGAACCAACTTTAGACTATGTGATTTCAAAAATACCAAGATTTCCTTTTGATAAATTTGAAAAA
GGAGAACGAGAGCTTGGCACACAAATGAAAGCAACAGGTGAAGTTATGGCCATTGGTCGAACTTACGAAGAATCATTGTTAAAAGCAATT
CGATCACTTGAGTATGGTGTGCATCACTTAGGATTACCAAATGGTGAAAGCTTCGATCTTGATTATATTAAAGAACGTATTTCACACCAAGA
TGATGAACGATTATTTTTCATCGGCGAAGCAATTAGAAGAGGCACAACATTAGAAGAAATTCATAATATGACTCAGATTGATTACTTCTTCT
TACACAAGTTCCAAAACATTATTGATATTGAGCATCAATTAAAAGAGCATCAAGGTGATTTAGAATATCTTAAATATGCAAAAGATTATGGA
TTTAGTGATAAAACAATAGCGCATCGCTTTAATATGACGGAAGAAGAAGTATATCAATTGCGTATGGAAAATGATATTAAACCTGTTTACAA
GATGGTTGATACTTGCGCAGCTGAATTTGAATCTTCAACACCATATTATTATGGTACATACGAAACTGAAAATGAATCCATAGTTACTGACA
AAGAAAAAAATCTTAGTATTAGGCTCTGGACCAATTCGAATCGGCCAAGGTGTAGAATTTGACTATGCGACAGTTCACGCCGTTTGGGCAAT
TCAAAAAGCAGGGTACCAAGCGATAATTGTGAATAACAATCCAGAAACAGTTTCAACAGACTTCTCAATTTCTGACAAATTATACTTTGAAC
CTTTAACTGAAGAAGATGTGATGAATATCATTAATTTAGAAAAACCTAAAGGTGTCGTTGTACAATTTGGAGGACAAACAGCGATTAATTTA
GCAGACAAATTGGCTAAACATGGTGTTAAAATACTTGGTACTTCACTAGAAAATCTAAATCGTGCTGAAGATAGAAAAGAATTTGAAGCAC
TATTAAGAAAAATTAACGTGCCACAGCCACAAGGGAAAACAGCTACATCACCTGAGGAAGCATTAGCGAATGCTGCAGAAATCGGATATC
CGGTTGTAGTAAGACCTTCTTATGTATTAGGTGGTCGCGCAATGGAAATTGTAGACAATGACAAAGAGTTAGAAAACTATATGACCCAGGC
TGTAAAAGCGAGTCCGGAACATCCGGTACTAGTCGATAGATATTTAACTGGTAAAGAAATTGAAGTTGATGCGATTTGTGATGGAGAAAC
GGTCATTATTCCAGGAATCATGGAACATATTGAACGTGCTGGTGTACATAGTGGTGACTCAATCGCTGTATATCCGCCACAAACTTTGACAG
AAGACGAGTTAGCAACACTTGAGGACTATACTATAAAATTAGCTAAAGGTTTAAACATCATTGGCTTAATCAACATTCAATTCGTTATAGCT
CACGATGGTGTATATGTTTTAGAAGTAAACCCACGTTCTAGTAGAACGGTACCATTCTTAAGTAAAATTACTGATATTCCAATGGCACAATT
AGCTATGCGAGCAATCATTGGGGAAAAACTAACAGATATGGGTTATCAAGAAGGGGTTCAACCATATGCTGAGGGTGTCTTTGTGAAAGC
ACCGGTATTTAGTTTTAATAAATTGAAAAATGTTGATATTACTTTAGGACCTGAAATGAAGTCAACAGGTGAAGTGATGGGGAAAGATACT
ACATTAGAAAAGGCGTTATTCAAAGGGTTAACAGGTAGTGGCGTTGAAGTTAAAGATCACGGTACAGTATTAATGACCGTCAGTGACAAA
GATAAAGAGGAAGTTGTTAAATTGGCACAACGCTTAAATGAAGTTGGCTATAAAATTTTAGCAACGTCTGGAACAGCTAATAAATTAGCTG

AGTATGACATACCTGCAGAAGTAGTAGGCAAAATTGGTGGCGAAAATGATTTATTAACACGTATTCAAAATGGTGATGTTCAAATCGTTAT
AAATACAATGACTAAAGGTAAAGAAGTAGAAAGGGATGGCTTCCAAATTAGACGTACTACAGTTGAAAATGGTATTCCATGTTTGACATCT
TTAGATACAGCTAATGCCTTAACGAATGTAATTGAAAGTATGACATTTACAATGCGTCAAATGTAA

Gene: pyrF (orotidine 5'-phosphate decarboxylase)

Position: 1168155 to 1168847, length: 693 nt, orientation: FORWARD

Perfect match to: (MW2-BA000033-[1186216:1186908], highly conserved allele)

Sequence:

ATGAAAGATTTACCAATTATTGCATTAGATTTTGAATCAAAAGAAAAAGTAAATCAATTTTTAGATTTATTTGATGAATCATTATTCGTAAAA
GTAGGTATGGAACTTTTTTATCAAGAAGGTCCTCAATTAATTAATGAGATAAAAGAAAGAGGCCATGATGTATTTTTAGATTTAAAATTGCA
TGATATTCCTAATACAGTAGGTAAGGCGATGGAAGGACTAGCTAAATTGAATGTTGATCTGGTAAATGTTCATGCTGCTGGTGGCGTAAAA
ATGATGTCTGAGGCCATTAAAGGATTAAGAAAACATAATCAACATACAAAAATTATTGCAGTAACACAGCTTACGTCAACAACAGAAGACA
TGTTACGACACGAACAAAATATACAAACATCGATTGAAGAGGCCGTTTTAAATTATGCCAAGTTAGCAAATGCAGCTGGTTTAGATGGCGT
TGTTTGTTCACCTCTTGAAAGTCGTATGTTGACTGAAAAGTTAGGTACATCATTTTTAAAAGTAACACCAGGTATTAGACCTAAAGGTGCAT
CTCAAGATGACCAACACCGTATTACGACACCGGAAGAAGCAAGACAGCTTGGTTCGACGCATATTGTAGTCGGTAGACCGATTACACAAA
GTGACAATCCAGTCGAAAGTTATCATAAAATTAAAGAAAGTTGGTTAGTATAA

Gene: pyrE (orotate phosphoribosyltransferase)

Position: 1168847 to 1169458, length: 612 nt, orientation: FORWARD

Perfect match to: (COL-CP000046-[1226691:1227302], highly conserved allele)

Sequence:

ATGGCTAAAGAAATTGCAAAATCATTATTAGATATTGAAGCTGTAACATTATCACCAAATGATTTATATACATGGAGTTCAGGTATTAAATC
ACCGATTTACTGTGATAACCGTGTTACGTTAGGTTATCCTTTAGTTCGAGGCGCAATCCGCGATGGTTTAATTAACTTAATTAAAGAACACTT
TCCTGAAGTAGAAGTTATTTCTGGTACTGCAACAGCTGGTATTCCACATGCAGCTTTTATTGCTGAAAAATTAAAATTACCAATGAATTATGT
TCGTTCATCAAATAAGAGTCATGGTAAGCAAAATCAAATCGAAGGTGCTAAAAGTGAAGGTAAAAAAGTAGTTGTGATAGAAGATTTAATT
TCGACAGGGGGATCTTCAGTCACAGCAGTTGAAGCCTTAAAACAAGCAGGTGCAGAAGTATTAGGTGTTGTAGCTATCTTTACTTACGGTT
TGAAAAAAGCAGATGATACATTTAGCAATATTCAACTACCTTTTTACACTTTAAGTGATTACAATGAATTAATTGAAGTAGCTGAAAATGAA
GGTAAAATTTCTAGTGAAGATATCCAAACATTAGTTGAATGGAGAGACAACTTAGCATAA

Gene: Q5HGM6 (putative protein)

Position: 1169488 to 1169700, length: 213 nt, orientation: FORWARD

Sequence:

ATGAATGACAAAACATCTAATGATTTATATGGGAAGATAAAACATTGTAACGAATTTATCAATCATTCAAATGATTCCAATCTATCTAGTAGT
CACGATGTCGACGAAAGTTCAACGAAGAAAAAACATATAAAAAATAAAACAACTATAGATCATAATGATGATTTATTTAAACATGTAAAGG
ATATATTACGTAAACAAGGACAAATTTAA

Gene: Q5HGM5 (putative protein)

Position: 1170137 to 1170538, length: 402 nt, orientation: FORWARD

Perfect match to: (TCH959-AASB02000232-[12979:13380], allele observed in CC7+CC8+CC80+CC239+CC361)

Sequence:

ATGGATATTCCAAAAATCACGACATTTTTAATGTTTAATAACCAAGCTGAAGAAGCTGTTAAACTATACACAAGCTTATTTGAAGATAGTGA
GATTATAACAATGGCTAAGTATGGTGAAAATGGACCTGGTGATCCCGGGACTGTACAACACTCAATATTTACATTAAATGGACAAGTATTC
ATGGCGATTGATGCTAATAGTGGCACAGAATTACCAATGAATCCTGCGATTTCATTATTTGTTACAGTAAAAGATACTATTGAAATGGAACG

GCTATTTAATGGATTAAAAGATGAAGGTGCCATTTTAATGCCAAAAACGAATATGCCACCATACAGAGAGTTTGCTTGGGTTCAAGATAAG
TTTGGAGTAAGTTTTCAATTAGCTTTACCTGAGTAA

Gene: fbpA (putative fibronectin/fibrinogen-binding protein)

Position: 1170801 to 1172498, length: 1698 nt, orientation: REVERSE

Perfect match to: (KPL1845-AZJA01000004-[329586:331283:r], allele observed in CC96+CC1+CC80)

Sequence:

TTATGATTGTTTCATTTTTTGAATAAGTTCATAATCAGGTGTAGCATACAAAGTTTTTTGATTGTCATATGTTACAAACCCAGGCTTTGCACCT
GATGGTTTGTGCACATTTTTAATTAATGTGTAATCAACAGGTATTTGTCCAGAATTACCAGCTTTTGAAAAGTATCCTGCTAACATAGCCGCT
TCCTTGATTGTCGTATCACTTGGTGCATCATTAAATATAACGACATGTGAACCAGGAATATCTTTTGTGTGTAACCATGTGTGAGTTTTTTTA
GCTTTTTTATTTGTTAAATAATCATTTTGCTTGTTATTCTTGCCAACATATATATCGTCGCCATCAGTTGATACATAATGTTGTAATTGAATCTG
CGCTTTCTTTTTCTTAGTTTGATTTTTACGCTGTTTCATAAAGCCTTGTTCTGCTAATTCATCTCTAATTTCATCAATGTCATGGACAGAAATAT
GATGTAATTGTTGTTCGATTGTTGAAAAAATAATCTATATTGTCTTTCGTCAATTGAATTTGATGTTGTAATTCACGTTCTCTCGTTTTCATACG
ATTATATTGTTTATAATAATATTGAGCATTTGCTGATGGGGATTTTGTAGGATTTAAAGGAATGACAACTTCTTCATTCGTATAATAATTCAA
TGCCGTCACTTCTTTATCGCCTTGCTTAATTCGATATATATTAGCAGTGATCAATTCACCATATAACTGTTCAGTATCTTTATTTTTAGACTGCT
CATATTCTTCAATCAACTTCGCTAATTTATTTTGATATTTGTGCAACTGCTGTTGAACAAATCGAACTAAATCATTCGCACGTTGTTTAACGCG
TTCACGTTCGCCACGCGCATCATAAAAACGATCAAGTAAATCATTTAATGAATCGTATGTAACTGTATCATCATTAAATTGATTTAACTTTAT
AAAATAGAAATCCTCTTTACCTGTTTCATGATTTTTATGAAAAATAGGAGTAGGTGGTAACTTGGTTTCTGCCATTACTTCGTCAAATGCTTC
TGGTAATGTTGATGAAGTCATAAATTGACGACGACTAACGATTTCATTCGTAATTAAAGGGCTAAATCCTTCAAACTGATTCAATAATTGTTT
AGCAATATTACCTGCGTTAAAATCGATATATTTCAACACCTCTGCACCTGTAATATCATACGGATTTATTTTGTGCTGAGTAGGTGGTGCTTC
ATAATTAAATCCTGGCATTACTGTACGATAGTGATTCGTATTTGGTGTTAAGTGTTTAAATCCTTCAATTATTTTGCGATTTTCATCTACTAAA
ATTAAGTTACTATGTTTACCCATAATCTCAAGGATGACAGTGCGGTAAATAGTATCGCCAATTTCATCTTTACTCTTTATTTCGATTTCAATGC
GACGATCATTACCAATTTGCTTAATCGATTCGATAATACCACCTTCTAAGTGTTTTCTAAAAACACGCGCAAACATGGGTGGATTAAATGGA
TTATCATATTTTTTAGTAGTTAATTGTAATCTTGAAAAGTTTGGATGGATTGACAATAACAATTGATGGTTTTGTCTATTTTGACGTACAACCA
TTAGTATCGTGTCATTATCAGGTTGATTGATTTTGTGAACGCGTCCTGTTGTTAAAAATTGTAGAGACTCAACCATTTTCTTTGTAAATAAGC
CATCATAAGCCAT

Gene: gmk (guanylate kinase)

Position: 1172773 to 1173396, length: 624 nt, orientation: FORWARD

Perfect match to: (Strain_3989-ALWH01000033-[48448:49071], allele observed in CC772+CC97+CC772)

Sequence:

ATGGATAATGAAAAAGGATTGTTAATCGTTTTATCAGGACCATCTGGAGTAGGTAAAGGTACTGTTAGAAAACGAATATTTGAAGATCCAA
GTACATCATATAAGTATTCTATTTCAATGACAACACGTCAAATGCGTGAAGGTGAAGTTGATGGCGTAGATTACTTTTTTAAAACTAGGGAT
GCGTTTGAAGCTTTAATCAAAGATGACCAATTTATAGAATATGCTGAATATGTAGGCAACTATTATGGTACACCAGTTCAATATGTTAAAGA
TACAATGGACGAAGGTCATGATGTATTTTTAGAAATTGAAGTAGAAGGTGCAAAGCAAGTTAGAAAGAAATTTCCAGATGCGCTATTTATT
TTCTTAGCACCTCCAAGTTTAGAACACTTGAGAGAGCGATTAGTAGGTAGAGGAACAGAATCCAATGAGAAAATACAAAGTCGTATTAACG
AAGCGCGTAAAGAAGTTGAAATGATGAATTTATACGATTACGTTGTAGTTAATGATGAAGTAGAACTTGCGAAGAATAGAATTCAATGTAT
TGTAGAAGCTGAGCACTTAAAAAGAGAGCGCGTAGAAGCTAAGTATAGAAAAATGATTTTGGAGGCTAAAAAATAA

Gene: rpoZ (DNA-directed RNA polymerase subunit omega)

Position: 1173396 to 1173614, length: 219 nt, orientation: FORWARD

Perfect match to: (11819-97-CP003194-[1223774:1223992], highly conserved allele)

Sequence:

ATGTTAAATCCACCATTAAACCAATTAACGTCACAAATTAAATCAAAGTATTTAATTGCAACAACTGCAGCGAAAAGAGCGCGTGAAATTGA
TGAACAACCTGAAACTGAATTATTAAGTGAATATCATTCATTTAAACCAGTTGGTAGAGCGTTAGAAGAAATTGCTGACGGTAAAATTCGCC
CTGTTATTTCAAGTGATTATTATGGTAAAGAATAG

Gene: coaBC (bifunctional phosphopantothenoylcysteine decarboxylase/phosphopantothenate--cysteine ligase)

Position: 1173830 to 1175029, length: 1200 nt, orientation: FORWARD

Sequence:

ATGAAGAAAATATTATTAGCCGTTACAGGTGGCATTGCGGCATATAAAGCGATTGATTTGACAAGTAAGTTAACACAATCTGGGTATGAAG
TTCGCGTTATGTTAACGAATCACGCACAAAAATTTGTGACACCATTAGCATTTCAAGCAATAAGTCGAAATGCTGTTTATACAGATACTTTTA
TAGAAGAAAATCCTTCAGAAATACAGCATATTGCATTAGGTGATTGGGCAGATGCAATCATTGTTGCACCTGCAACGGCAAATACAATTGC
AAAATTGAGTGTAGGTATTGCTGATGATTTGGTGACATCAACGTTGCTAGCAACAGAGACACCGAAATTTATTGCGCCTGCTATGAATGTG
CATATGTATGAAAATAAACGTACGCAGCAAAATATTAATATTTTAAAAGAAGATGGGTATCATTTTATCGAACCAGGAAGCGGATTTCTAG
CATGTGGTTATGTTGCTAAAGGACGTATGGAAGAACCGCTTCAAATCGTTTCTGTTATTGATGCTCATTTTCAAAATAGTAATCGTTTAGCTA
ATAGTTCATTTCAAGATAAACGCGCATTGGTTACAGCAGGACCAACTATTGAAGTTATCGATCCAGTCAGATTTGTATCCAATCGTTCTTCTG
GAAAAATGGGTTATGCAATAGCTGAAGCATTGCGAAATCGAGGAGCTATCGTGACGTTAGTTGCTGGTCCTACAACACTAGAGGATCCAA
AAGATATTGAAGTTATTCATGTTCAAAGTGCTGAAGAAATGTTTGAACAAGTGACAAGCCGATTTGACGAACAAGATATTGTTGTAAAAGC
AGCAGCCGTATCTGACTATACACCGGTTGATGTATTAGAACATAAGATGAAAAAGCAGGATGGTGATTTATCAGTATCTTTTAAACGTACTA
AAGACATTCTTAAATATTTAGGTGAACATAAAACATCACAGTATTTAATAGGCTTTGCAGCAGAGACTGAAGATATTGAAAATTATGCACAA
CAAAAATTACGCAAGAAAAATGCAGATGTGATTATTTCAAATAATGTTGGGGATATGTCTATCGGATTTAGTTCTGATGATAATGAATTGAC
AATGCATTTTAAAAATAATGAAAAGGTAAATATCAAGAAAGGAAAAAAAGTAGTATTAGCTGCACAAATTTTAGATGAACTAGAAACTAGG
TGGCAATAA

Gene: priA (primosomal protein N')

Position: 1175029 to 1177438, length: 2410 nt, orientation: FORWARD

Sequence:

ATGATAGCGAAAGTCATAGTCGATGTCGCGTCGAAGAGCGTTGACTATAAATTTGATTATATAATTCCTGAACAACTCGAATCTGTCATCCA
ACCTGGTGTGCGTGTGATTGTACCTTTTGGACCAAGAACGATTCAAGGTTATGTAATGGAAGTAACAGCAGAACCTGATGCACAACTTGAC
GTTTCGAAGTTAAAAAAAATCATAGAAGTGAAAGATATACAACCTGAATTAACATCAGAATTAATAGCTTTAAGTGAGTGGATGGGTTCAA
CTCATGTCATTAAACGTATTTCTATGCTAGAAGTGATGCTTCCTGAGTGCTATTAAAGCGAAGTATAAAAAAGCATTTAAGATGAAAGATGA
CAAAGAGGTACCTTCAGCTTTATTACAAAAATTTGATAAGCATGGTTACTATTATTATAAAGATGCGCAAAAAAATAATGATATTCAATTGC
TTATGAAGTTGTTAAAAGATGATATCGTTGAAGAAAAAACGATTCTCACACAAAATATAACTAAAAAAACCAAGCGTGCTGTTCGTGTCATT
GAAGGGTATCATCCTGATGAAGTATTAGCTAAGTTGGAGAAAGTTATTAAACAATACGATTTGTATGCTTACTTGTCTGAAGAACAACATAA
AACAATATTTTTAACTGATATTGAGGATATGGGCTTTTCAAAATCCAGTTTAGATGGACTTATCAAAAAAGGTTATGTTGAAAAATATGACG
CGGTTGTTGAAAGAGACCCATTTAAAGATCGTGTTTTCGAACAAGAATCAAAACAGCAATTAACAGAAGACCAATATAAAGCATATGAAGC
GATTAAAGCTAAAATTGTAAGCCAAGAGCAAGAAACATTTTTACTTCATGGTGTGACGGGATCAGGTAAAACAGAAGTATATTTACAAACG
ATAGAAGATGTTTTAAGCCAAGGAAAACAGGCGATGATGTTAGTTCCTGAAATCGCTCTAACACCGCAAATGGTTTTACGCTTCAAACGTC
GATTTGGTGATGACGTTGCTGTATTACATTCTGGCTTATCTAATGGGGAACGTTATGATGAGTGGCAAAAAATTAGGGATGGTCGTGCGAG
AGTAAGTGTTGGTGCAAGGTCAAGTGTGTTCGCACCTTTCAAAAATTTAGGGTTAATCATCATTGATGAAGAACATGAATCTACATATAAGC
AAGAAGATTATCCGAGATATCATGCTAGAGAAATTGCCCAATGGCGAAGTGAATATCATCACTGCCCAGTCATTTTAGGAAGTGCAACACC
ATGTCTTGAAAGTTATGCACGAGCTGAAAAAGGCGTTTATCATTTGCTATCATTACCAAACAGAGTGAACCAACAAGCTTTACCTGAAATTG
ATATAGTAGATATGCGTGAAGAATTGAGTGAAGGTAATCGGTCAATGTTTTCAAAAGATTTACGTGAAGCCATACAATTAAGATTAGATCG
ACAGGAACAAGTTGTTTTATTTTTAAATCGACGTGGTTATGCATCGTTTATGTTATGTCGGGATTGTGGATATGTACCGCAATGTCCAAACT
GTGATATTTCATTAACGTATCATAAAACGACAGACTTATTAAAATGTCACTATTGTGGTTACCAAGAGACGCCACCGAATCAATGTCCAAAT
TGTGAGAGTGAACACATTCGACAAGTAGGTACTGGTACTCAGAAAGTTGAAGAACTATTGCAACAAGAATTTGAAGATGCGCGCATAATT
AGGATGGATGTAGATACAACCTCAAAGAAAGGTGCACATGAAAAGTTATTGACTGAATTCGAAAAAGGTAACGGTGATATTTTACTAGGT
ACTCAGATGATTGCGAAAGGATTAGATTATCCAAATATTACTTTAGTTGGTGTGCTGAATGCAGATACAATGTTAAATTTACCTGATTTTCG
GGCGAGCGAACGTACTTATCAACTATTAACGCAAGTGGCTGGTAGAGCTGGTCGTCATGAAAAGGCAGGTCAAGTCATCATTCAAACGTAT
AATCCGGATCATTATTCAATATTGGATGTTCAAAAAAATGATTATTTAACATTTTATCGTCAGGAAATGGAATATCGTAAATTAGGAAAGTA
TCCACCGTATTATTATTTGATTAATTTCACAATCTCACATAAAGAAATGAAGAAGGTTATGGAAGCATCGCAGCATGTTCATAAAATTTTATT
ACAGCATTTAACAGAAAAAGCGCTTGTACTAGGTCCATCTCCGGCAGCACTTGCGAGAATCAACAATGAATTTAGATTCCAAATTTTAGTGA
AATATAAAAGTGAACCTGGATTATTACAAGCCATTCAGTTTTTAGATGACTATTACCATGAAAAATTTATAAAAGAAAAAATTAGCATTGAAG
ATTGATATTGATCCACAGATGATGATGTAA

Gene: Q5HGL9 (putative lipoprotein)

Position: 1177944 to 1178897, length: 954 nt, orientation: FORWARD

Perfect match to: (N315-BA000018-[1196202:1197155], highly conserved allele)

Sequence:

ATGAAAAAGACACTGGGATGTTTACTTTTAATTATGCTTTTAGTCGTAGCAGGTTGTTCTTTTGGTGGGAATCATAAATTATCATCAAAGAA
ATCAGAAGAATCAAAACAAGAAACTGTAAAAAAAGAATCGGAAGAAGAGAAAGATCCAGATTTAGAGAAATATGAAGAAATAGAGAAGA
AAATGAAAGGAATTAAAGATGCGCCATCTCTTGATAAGTTGGATCCATTAATGACAGAAAAGTCGTTTACGAATAGTAAAGGGATTCAAGG
ATGGAAAGATTACAAAGAATTAATGGGTAAAGTGGAACTTGCAGATTATAGATTTACTAAAGATTCAAAAGGATCTTCAATAAAAGATGTT
GATGCATTCTTTAAAGGTAAGAAAGGTATAAAAAGGAAAGTGATTGAAACACACGATGATGTAAAACAAGTTGATTATTGGTATGTAGATC
CAGATGGAAAGAAAATTGGCAATTCAAACACACCTGTTTTTACGCAGAAATTATGACAAAATATAAAGATGGAAAGTTAGTTTATGCATC
AGTCGAACCAGGATCTTACGTAATACATAAAGATGATGCAATTAAATATGACGATTATTCTAAGTTAAAAAAATTAAGTCAGCTAACTAAAC
TTGATCATCCAAAACCAGTTCCATATAGCGTAGCTCAAATCAAATCTTTCGGAGTACCTTTAACAAGCGTTTCATTTATGACACATGGATCAA
AGGATACTAAAGATGAAGTGTTGCCGGCATTGGCCTATTTCACTTTTTCACCAAAAAATTATGAAGACAAGTCTAATCCAGATCCAAAAGTT
TTAAATTTAGTACATATGGATTTCTTAAATGCATCTAGTGATTTTGGTAACGCACATTTTGTTGTTTTAAGTAAATATATTAAAGAGTATGAA
TCAAACTATGAAACAGCGTCAGATGATTCTTTAAAATAG

Gene: Q5HGL8 (short two-transmembrane helix protein)

Position: 1179060 to 1179338, length: 279 nt, orientation: REVERSE

Perfect match to: (MW2-BA000033-[1197117:1197395:r], highly conserved allele)

Sequence:

TTATAAAGTGACATTACCTTGTTCATCAGCAGGTTTGAAAACAGTAATCACTGCACTAATAATTGCTAAAATGTGTGGGATACCTGTCCAAC
AGAATATTAAGTGTAGAATACCTTGCATATTCTTGCCGGCATAAAATTTATGAATACCAAAACTACCTAAGAACAATGCTAATAAAATATAA
ATAACTTTGTTTACTTGCATTTCTTTCCCTCCAGTTGAATTGCTTATAATGACATTAGCTTCTCTTTTTATTATACCCACTTTTAGTTCAAACAT

Gene: def2-defA (formylmethionine deformylase)

Position: 1179562 to 1180050, length: 489 nt, orientation: FORWARD

Perfect match to: (MW2-BA000033-[1197619:1198107], allele observed in CC1+CC22+CC80+CC188+CC772)

Sequence:

ATGGCGATTAAAAAGTTAGTACCAGCATCGCATCCTATTTTAACGAAAAAAGCGCAAGCAGTTATAAAATTTGATGATTCGTTAAAAAGATT
ATTACAAGATTTAGAAGATACAATGTATGCACAAGAAGCAGCTGGCTTATGTGCACCTCAAATTAATCAGTCATTGCAAGTGGCAATCATTG
ATATGGAAATGGAAGGATTATTACAACTTGTTAATCCGAAAATTATTAGTCAATCAAATGAAACGATAACAGACTTAGAAGGTTCAATTACA
TTGCCAGATGTTTACGGCGAAGTGACAAGAAGTAAAATGATAGTTGTCGAAAGTTATGACGTCAATGGGAACAAAGTTGAACTAACTGCA
CATGAAGATGTAGCAAGAATGATTTTGCATATTATAGATCAAATGAACGGTATCCCTTTTACAGAACGTGCGGACCGTATTTTAACAGATAA
AGAAGTGGAGGCATATTTTATAAATGACTAA

Gene: fmt (methionyl-tRNA formyltransferase)

Position: 1180043 to 1180978, length: 936 nt, orientation: FORWARD

Perfect match to: (Strain_21343-AHKV01000037-[153460:154395:r], allele observed in CC88+CC1+CC72)

Sequence:

ATGACTAAAATAATATTTATGGGTACACCAGACTTTTCAACAACTGTTTTAGAAATGCTTATTGCAGAACATGATGTCATTGCAGTCGTAAC
GCAACCAGATCGACCTGTTGGACGTAAACGTGTTATGACACCACCACCAGTTAAAAAAGTTGCAATGAAATATGATTTACCTGTATATCAGC
CTGAAAAATTAAGTGGATCAGAAGAATTAGAACAATTGCTTCAATTAGATGTAGATTTAATTGTAACTGCTGCTTTTGGACAATTATTACCT
GAATCATTGTTGGCATTACCAAAACTTGGGGCAATTAATGTACATGCATCATTGTTACCGAAGTATAGAGGTGGTGCACCAATTCATCAGGC
AATTATCGATGGTGAACAAGAAACCGGCATAACAATTATGTATATGGTTAAAAAATTAGATGCAGGTAATATTATTTCGCAACAAGCAATT
AAAATAGAAGAAAATGATAATGTCGGTACGATGCATGATAAATTAAGTGTATTAGGGGCAGATTTATTAAAAGAAACTTTACCATCTATTA
TAGAGGGCACAAATGAAAGCGTACCTCAAGATGATACGCAAGCAACATTTGCTTCCAATATTCGACGCGAAGATGAGCGAATTAGCTGGA
ATAAACCAGGAAGACAAGTGTTTAATCAAATTCGTGGATTATCCCCATGGCCAGTTGCTTATACAACTATGGATGACACTAACTTGAAAATA
TACGATGCTGAACTCGTTGAGACTAATAAGATAAACGAGCCTGGAACCATTATAGAAACGACTAAAAAAGCCATTATTGTTGCTACAAATG
ATAATGAAGCTGTTGCAATTAAAGATATGCAATTAGCTGGGAAAAAGAGAATGTTAGCTGCCAATTATTTAAGTGGTGCGCAAAACACACT
AGTAGGGAAGAAACTTATATGA

Gene: sun-rsmB (ribosomal RNA small subunit methyltransferase B)

Position: 1180975 to 1182282, length: 1308 nt, orientation: FORWARD

Perfect match to: (11819-97-CP003194-[1231352:1232659], allele observed in CC80+CC12+CC96+CC97)

Sequence:

ATGATAGAAAACGTGAGAAGTCTTGCTTTTGACACGATTCAAGATATATTAAATGAAGGTGCGTATAGTAACTTGCGTATCAATGAAGTGT
TGTCAGAAAATGAATTAAATGCAATGGATAAGGCTTTATTTACAGAAATTGTCTACGGAACCGTTAAAAGAAAATATACGTTAGATTTTTAT
TTAAAGCCTTTTGTGAAAACAAAAATTAAGGCATGGGTTAGGCAATTATTATGGATGAGTATTTATCAATATGTTTATTTAGATAAAGTTCC
AAATCACGCCATCATTAATGAAGCAGTTGAAATAGCAAAAGAACGTGGTGGTTATCATAATGGTAATGTCGTAAATGGTATTTTACGTACA
ATGATGCGTAGTGACTTACCTGATTTTAATGAAATTGCAGATCCTAAAAAAAGAATGGCAATCGAGTATAGTATGCCGAAGTGGATTATAG
ATCATTGGGCAACACATTATGGTCTCGAAAAAACTGAAACAATTTTACAGTCATTTTTAGAAACGACATCAACAACTGTGCGTGCCAACCTG
ACGCGAGCATCATTAGATGATATTATTGAAAAGTTGCAAGACGAAGGTTATGACGTTGAAAAAGATCATGACTTACCTTATTGTCTCCATAT
AGGAGGACAACCAATTATTCATTCTCGTTCATTTAAAGATGGATTCGTTTCAATTCAAGATAAAAGCTCAATGTTTGTTGCACACATTATGAA
TGTAGACCGACATGATCACGTATTAGATGCATGTAGTGCACCTGGCGGTAAAGCTTGTCACATTGCTGAAGTTTTAATGCCAGAAGGGCAA
GTTGACGCTTCAGATATACATGATCACAAAATAGACTTAATTAATTTTAATATAAAAAAATTACGATTAACAAATATTAAAGCCTTTCAACAT
GATGCGACAAAACCTTATGATAAAACATACGATAAGATACTTGTTGATGCACCATGTAGCGGATTAGGTGTAATGAGACATAAGCCGGAG
ATTAAGTATACTCAAAGCAAACAACATATTGAGTCACTAGTTGATTTACAGCTTGAAATATTGGAAAATGTAAAAAACAATGTAAAAATAG
GTGGCGAAATCATCTATTCCACATGTACAATAGAGCAACTAGAAAATGAAAACGTGATTTATACGTTTTTGAAAAATAATAAAAACTTCGAA
TTTGAACCGTTTCAACATCCGATAACTGGAGAGTTGGTCAAAACGTTACAAATCATGCCGCAAGACTTTAATTCAGATGGATTCTTTATCACT
AAGATAAAAAGAAAGGACAATTAG

Gene: rlmN (ribosomal RNA large subunit methyltransferase)

Position: 1182285 to 1183379, length: 1095 nt, orientation: FORWARD

Perfect match to: (COL-CP000046-[1240128:1241222], allele observed in CC8+CC97)

Sequence:

ATGATAACTGCTGAAAAGAAAAAGAAGAATAAATTTCTTCCAAATTTTGACAAGCAATCAATATATTCATTGCGATTTGACGAAATGCAAAA
CTGGCTCGTTGAACAAGGGCAACAAAAATTTCGAGCGAAACAGATTTTTGAATGGTTATATCAAAAAAGAGTAGATTCGATTGATGAAATG
ACGAACTTATCGAAAGACTTACGACAGCTTTTAAAAGATAACTTTACTGTTACAACTTTAACAACTGTAGTAAAACAAGAAAGTAAAGACGG
TACAATTAAATTCTTATTTGAATTACAAGATGGCTATACAATTGAAACTGTTTTAATGAGACATGATTATGGAAATTCAGTATGTGTAACGAC
ACAAGTAGGTTGTCGTATCGGATGTACGTTTTGTGCTTCTACACTTGGCGGCTTAAAAAGAAACCTTGAAGCTGGCGAAATTGTTTCACAAG
TTTTAACAGTTCAAAAAGCCCTTGATGCTACAGAAGAGCGCGTATCTCAAATTGTCATAATGGGTATCGGTGAACCATTTGAAAATTATGAT
GAAATGATGGACTTTTTAAGAATCGTCAATGATGATAATAGTTTAAATATTGGTGCACGTCACATTACAGTATCAACATCAGGTATCATTCCT
AGAATATATGACTTTGCGGATGAAGATATTCAAATTAATTTTGCTGTAAGCTTACACGCCGCAAAAGATGAAGTGCGATCACGCTTGATGCC
AATTAACCGTGCATATAATGTTGAGAAGTTAATCGAAGCAATTCAATATTATCAAGAAAAAACAAATCGTCGTGTTACTTTTGAATATGGTC
TGTTTGGTGGTGTGAATGACCAACTAGAACATGCAAGAGAATTAGCACATTTAATAAAAGGCTTAAACTGCCATGTTAACTTAATTCCTGTC
AACCATGTTCCAGAAAGAAATTATGTGAAAACGGCTAAAAATGATATCTTTAAATTTGAAAAAGAATTAAAGAGACTAGGAATTAATGCCA
CAATACGTCGTGAACAAGGTTCGGATATTGACGCAGCTTGTGGTCAATTAAGAGCAAAGGAACGACAAGTAGAAACGAGGTAA

Gene: stp1-prpC (serine/threonine protein phosphatase)

Position: 1183386 to 1184129, length: 744 nt, orientation: FORWARD

Perfect match to: (CN1-CP003979-[1171119:1171862], allele observed in CC72+CC8+CC20+CC97+CC239)

Sequence:

ATGCTAGAGGCACAATTTTTTACTGATACTGGACAACATAGAGATAAGAATGAAGATGCGGGTGGTATTTTTTATAATCAAACTAATCAACA
ACTTTTAGTTCTGTGTGATGGTATGGGTGGCCATAAAGCAGGAGAAGTTGCAAGTAAATTTGTTACAGATGAGTTGAAATCCCGTTTTGAA
GCGGAAAATCTTATAGAACAACATCAAGCTGAAAATTGGTTGCGTAATAATATAAAAGATATAAATTTTCAGTTATATCACTATGCACAAGA
AAATGCAGAATATAAAGGTATGGGTACAACATGTGTTTGTGCACTTGTTTTTGAAAAATCAGTTGTGATAGCAAATGTCGGTGATTCTAGA
GCCTATGTTATTAATAGTCGACAAATTGATCAAATTACTAGTGATCACTCATTTGTTAATCATCTTGTTTTAACGGGTCAAATTACGCCGGAA
GAAGCATTTACACATCCACAACGTAATATTATTACGAAGGTGATGGGCACAGATAAACGTGTGAGTCCAGATTTGTTTATTAAGCGATTAA

ATTTTTATGATTATTTATTATTAAATTCAGATGGATTAACTGATTATGTTAAAGACAATGAAATTAAGCGTTTGTTAGTAAAAGAAGGTACAA
TAGAAGATCATGGTGATCAATTAATGCAATTGGCATTAGATAACCATTCGAAAGATAACGTTACTTTCATACTCGCGGCTATTGAAGGTGAT
AAAGTATGA

Gene: pknB-prkC (muropeptide sensing serine/threonine protein kinase)

Position: 1184126 to 1186120, length: 1995 nt, orientation: FORWARD

Perfect match to: (IS-111-AHLS01000064-[22854:24848:r], allele observed in CC8+CC1+CC8+CC97)

Sequence:

ATGATAGGTAAAATAATAAATGAACGATATAAAATTGTAGATAAGCTTGGCGGCGGTGGCATGAGTACCGTTTATCTTGCTGAAGATACGA
TACTTAACATTAAAGTTGCAATTAAGGCGATTTTTATACCACCTAGAGAAAAAGAAGAAACATTAAAACGTTTTGAACGAGAAGTACATAAC
TCATCACAGCTATCACATCAAAATATAGTAAGTATGATCGATGTTGATGAAGAAGATGACTGTTACTACTTAGTAATGGAATATATTGAAGG
TCCGACTTTGTCTGAGTATATTGAAAGTCATGGGCCATTAAGTGTTGACACGGCGATTAATTTTACGAATCAAATATTGGATGGTATTAAAC
ATGCGCATGATATGCGTATTGTACATAGAGATATTAAGCCACAAAATATATTAATTGACAGCAATAAAACGTTGAAAATATTTGATTTTGGA
ATTGCTAAAGCTTTAAGTGAGACGTCTTTAACTCAGACTAATCATGTGTTAGGTACTGTGCAGTACTTTTCGCCAGAACAAGCAAAAGGTGA
GGCAACGGATGAATGTACAGATATTTATTCTATAGGTATTGTGTTATATGAAATGCTTGTTGGTGAACCACCCTTTAATGGAGAAACTGCAG
TTAGCATTGCGATTAAACATATTCAGGATTCTGTGCCAAATGTGACAACAGATGTACGTAAGGATATTCCGCAATCTTTAAGTAATGTCATTT
TACGCGCTACAGAAAAAGACAAAGCGAATCGTTACAAAACAATTCAAGAAATGAAAGATGATTTGAGTAGTGTTTTACATGAAAATCGAGC
GAATGAAGATGTCTATGAACTCGATAAAATGAAAACGATAGCGGTACCTTTGAAAAAAGAAGATCTAGCAAAGCATATTAGTGAACATAA
GTCGAATCAACCTAAACGTGAAACGACGCAAGTACCTATTGTAAATGGGCCTGCTCATCATCAGCAATTCCAAAAGCCAGAAGGTACGGTT
TACGAACCAAAACCTAAAAAGAAATCAACACGAAAGATTGTGCTCTTATCACTAATCTTTTCGTTGTTAATGATTGCACTTGTTTCTTTTGTG
GCAATGGCAATATTTGGTAATAAATACGAAGAGACACCTGATGTAATCGGGAAATCTGTAAAAGAAGCAGAGCAAATATTCAATAAAAAC
AACCTGAAATTGGGTAAAATTTCTAGAAGTTATAGTGATAAATATCCTGAAAATGAAATTATTAAGACAACTCCTAATACAGGTGAACGTGT
TGAACGTGGTGACAGTGTTGATGTTGTTATATCAAAAGGCCCTGAAAAGGTTAAAATGCCAAATGTCATTGGTTTACCTAAGGAGGAAGCC
TTGCAGAAATTAAAATCGTTAGGTCTTAAAGATGTTACGATTGAAAAAGTATATAATAATCAAGCGCCAAAAGGATACATTGCAAATCAAA
GTGTAACCGCAAATACTGAAATCGCTATTCATGATTCTAATATTAAACTATATGAATCTTTAGGCATTAAGCAAGTTTATGTAGAAGACTTTG
AGCATAAATCCTTTAGCAAAGCTAAAAAAGCCTTAGAAGAAAAAGGGTTTAAAGTTGAAAGTAAGGAAGAGTATAGTGACGATATTGATG
AGGGTGATGTGATTTCTCAATCTCCTAAAGGAAAATCAGTAGATGAGGGGTCAACGATTTCATTTGTTGTTTCTAAAGGTAAAAAAAGTGA
CTCATCAGATGTCAAAACGACAACTGAATCGGTAGATGTACCATACACTGGTAAAAATGATAAGTCACAAAAAGTTAAAGTTTATATTAAA
GATAAAGATAATGACGGTTCAACTGAAAAAGGTAGTTTCGACATTACTAGTGATCAACGTATAGACATTCCTTTAAGAATTGAAAAAGGAA
AAACAGCAAGTTATATTGTTAAAGTTGACGGTAAAACTGTAGCTGAAAAAGAAGTCAGCTATGATGATGTATAA

Gene: cpgA-engC (ribosome small subunit-dependent GTPase A)

Position: 1186348 to 1187223, length: 876 nt, orientation: FORWARD

Perfect match to: (N315-BA000018-[1204606:1205481], highly conserved allele)

Sequence:

TTGAAGACAGGTCGAATAGTGAAATCAATTAGTGGGGTATATCAAGTAGACGTTAATGGCGAACGTTTCAATACAAAACCACGAGGATTAT
TTAGAAAGAAAAAATTTTCACCGGTAGTTGGTGATATAGTGGAATTTGAAGTACAAAACATTAACGAAGGCTATATTCATCAAGTGTTTGA
GCGGAAAAATGAGTTGAAAAGACCACCTGTAAGTAATATAGATACACTAGTAATTGTAATGAGTGCTGTCGAGCCAAATTTTTCAACGCAA
TTATTAGATCGATTTTTAGTTATTGCACATTCGTATCAGTTAAATGCGAGAGTTTTGGTGACTAAAAAAGATAAAACACCAATTGAAAAGCA
GTTCGAAATTAATGAGTTGTTGAAAATATATGAAAATATTGGCTATGAGACTGAATTTATTGGAAATGATGATGATCGAAAAAAAATTGTA
GAAGCTTGGCCAGCTGGACTTATAGTACTTAGTGGTCAATCAGGTGTCGGTAAGTCCACTTTCTTAAATCATTATCGTCCAGAACTTAATCTT
GAGACAAATGATATATCAAAATCATTAAATCGAGGAAAGCATACTACAAGACATGTCGAACTATTCGAACGTCAAAACGGTTATATTGCAG
ACACACCTGGATTCAGTGCTTTAGATTTTGATCATATAGATAAAGATGAAATAAAAGATTATTTTCTTGAATTAAATCGATATGGTGAAACA
TGTAAGTTTAGGAATTGTAATCATATCAAAGAACCTAATTGTAATGTTAAGCATCAATTAGAGATAGGGAATATTGCGCAATTTAGATACGA
CCATTATTTACAACTATTTAATGAAATTTCAAATAGAAAGGTTAGATATTAA

Gene: rpe (ribulose-5-phosphate 3-epimerase)

Position: 1187224 to 1187868, length: 645 nt, orientation: FORWARD

Perfect match to: (MW2-BA000033-[1205281:1205925], highly conserved allele)

Sequence:

ATGACAAAACTATATCCATCATTATTATCTGTTGATTTTTTGGATTTACAACATGAATTAAAACGACTTGAAGAAGCAGGTGTCGACGGAGT
TCATTTTGATGTTATGGATGGTCAATTTGTGCCTAATATATCTATTGGTTTACCAATATTAGATGCAGTAAGAAAAGGCACAACATTACCTAT
AGACGTACATTTGATGATTGAAAATCCAGAAAAGTATATTGCATCATTTGCAGAACATGGTGCCGATATGATTTCAATTCATGTCGAATCAA
CGCCTCATATTCATCGTGCTATTCAAATGATTAAACATTTAGATAAAAAAGCTGGTGTAGTAATTAATCCTGGTACACCAATATCACAAATTG
AACCTATTTTAGACATTGTTGATTATGTACTAGTGATGACAGTTAACCCAGGGTTTGGTGGTCAATCATTTATTGATCAATGCGTAGAAAAA
ATAGCGGGTCTTAATGCTATTAAAATGGAACGTCAATTAAACTTTGATATTGAAGTTGATGGAGGCGTAAATACCGATACAGCGAAAGTTT
GTGTTGAAAATGGTGCTACAATGCTAGTAACAGGTTCATTTTTCTTTAAACAAGAGGATTATAAAAAAGTCACACAAGCATTGAAAGGTTG
A

Gene: thiN (thiamine pyrophosphokinase)

Position: 1187875 to 1188516, length: 642 nt, orientation: FORWARD

Perfect match to: (MW2-BA000033-[1205932:1206573], highly conserved allele)

Sequence:

ATGCATATAAATTTATTATGTTCTGATCGACACTTGCCGCAAGATATTTGGGCCAAAAGTAATGAAGGTAAATGGGGCGGCGTTGATAGAG
GTGCTTTGATTTTATTGAAGCATCAAATTATCCCTTTTTTCTCAGTGGGAGACTTTGATTCAGTCAGTAAAGAAGAACGCCAACTTCTAACAG
AACAGTTACAAATCAAACCAGTTCAAGCTGAAAAAGCTGATACGGATTTAGCTTTAGCGGTTGATAAAGCTGTTGCACTTGGATTTGATAGT
ATTACAATTTATGGTGCAACAGGCGGACGATTAGATCACTTTTTTGGGGCAATTCAGTTATTATTGAAAAAAGCATATTATAAACATGATGT
TCATATAGAAGTTATCGATCAACAAAATAAAATTGAATTATTGCCTAAAGGTCAACATACAGTTGAAAAAGATAAGAGTTATCCGTACATTT
CATTTATACCGATGACTGATGATGTAGAACTTTCTCTAGCAGGTTTTAAATATAATTTAGCTAGACAAATGCTTAATATAGGTTCTACTTTAA
CTATTTCAAATGAAATTGAGTCTTTGCAAGCGAAAGTAACTGTACATGATGGGTTGATTTTGCAAATTAGAAGTACAGATTTAAATTAA

Gene: rpmB (50S ribosomal protein L28)

Position: 1188897 to 1189085, length: 189 nt, orientation: REVERSE

Perfect match to: (RF122-AJ938182-[1174752:1174940:r], highly conserved allele)

Sequence:

TTAAACTCTAGTTACTTTACCAGATTTTAAAGCACGTGCAGAAACCCAAACTTTTTTAGGTTTACCGTCAACTAGGATTCTAACTTTTTGAAG
GTTAGCGTTCCATCTACGTTTAGTAGAGTTTAAAGCGTGTGAACGTCTGTTACCAGTCGAAGCTTTACGACCTGTTACGAAACATTGTTTACC
CAT

Gene: yloU (similar to alkaline-shock protein)

Position: 1189528 to 1189902, length: 375 nt, orientation: FORWARD

Perfect match to: (11819-97-CP003194-[1239905:1240279], allele observed in CC80)

Sequence:

ATGACATTAGAGATTTCAAATGATTACGGTAAAATTGATATTTCAAACGAAGTGATTGCTTCGGTTGTAGGTGGAAAGGCCGTTGAATGTT
ATGGTATTGTAGGTATGGCATCTAGACAACAAGTTAGAGATGGTATTGCGGAAATACTAGGACATGAAAATTATGCTAAAGGCATTAAAGT
AACTGAAAATAATGGCGTAGTGGATATAGATATGTACATTATTGTAAGTTACGGTGTGAAAATATCTGAAGTTGCCAATAATGTACAATCA
ACAGTGAAATATACTTTGGAAAAATCACTTAATGTATCAGTAAATTCAATCAATATATATGTACAAGGTGTACGTGTGAATAATACAGGCAA
GAAAGCTTAG

Gene: yloV (putative glycerone kinase)

Position: 1189917 to 1191563, length: 1647 nt, orientation: FORWARD

Perfect match to: (H19-ST10-ACSS01000036-[61540:63186], allele observed in CC10+CC8+CC97)

Sequence:

ATGATTAGCAAAATTAATGGTAAATTATTTGCCGATATGATTATACAAGGGGCACAAAATTTATCTAACAATGCAGATTTGGTAGATTCTTT
GAATGTGTATCCAGTGCCAGATGGTGATACAGGAACAAATATGAATCTTACTATGACTTCAGGTCGCGAAGAAGTAGAGAATAATTTGTCG
AAAAATATCGGCGAATTAGGTAAAACATTCTCGAAAGGTTTACTAATGGGTGCAAGAGGTAACTCTGGTGTCATCTTGTCACAATTATTCAG
AGGATTTTGTAAAAATATTGAAAGTGAATCTGAAATTAATTCAAAATTGTTAGCTGAAAGTTTTCAAGCTGGTGTTGAAACGGCATATAAAG
CTGTTATGAAACCAGTTGAAGGTACAATACTTACAGTTGCAAAAGATGCTGCGCAAGCTGCAATAGAAAAAGCAAATAATACTGAAGATTG
TATAGAATTAATGGAGTACATTATTGTAAAAGCCAATGAATCACTTGAAAACACACCAAACTTATTAGCTGTACTTAAAGAAGTTGGTGTTG
TTGATAGTGGCGGTAAAGGTTTGTTATGCGTTTACGAAGGATTCTTAAAAGCGCTTAAAGGTGAAAAAGTTGAAGCTAAAGTTGCAAAGAT
AGATAAAGATGAATTTGTACATGATGAACATGATTTCCATGGTGTAATTAATACTGAAGATATTATTTATGGCTATTGTACTGAAATGATGG
TTCGTTTTGGAAAGAATAAAAAAGCCTTTGATGAACAAGAATTCAGGCAAGATATGAGTCAATTTGGTGATTCTTTATTAGTCATTAATGAT
GAAGAAATTGTGAAAGTTCACGTGCATACCGAATACCCAGGTAAAGTGTTTAATTATGGTCAACAATATGGTGAATTAATTAAACTTAAGG
TTGAAAATATGAGAGAACAGCATCGTGAAGTGATTCGAAAAGAACAGCACACAGCTAAACCGAAAATGGAAACGGTTGAAACAGCGATTA
TTACTATTTCTATGGGTGAAGGTATTTCAGAGATATTTAAATCAATGGGTGCCACACATATCATTAGTGGTGGACAAACGATGAATCCTTCT
ACAGAAGATATCGTTAAAGTCATTGAACAATCAAAATGTAAACGTGCAATTATTTTACCGAATAATAAAAATATTTTAATGGCAAGTGAACA
AGCAGCGAGTATTGTTGATGCAGAAGCTGTTGTTATTCCAACGAAATCTATTCCTCAAGGTATAAGCGCACTATTCCAATATGATGTGGACG
CAACACTTGAAGAAAATAAAGCGCAAATGGCTGATTCAGTAAATAACGTTAAATCTGGTTCATTAACGTACGCTGTTCGTGATACGAAAATT
GATGGCGTTGAGATTAAAAAAGACGCGTTTATGGGCTTGATTGAAGATAAGATTGTAAGCAGCCAAAGTGATCAATTAACAACGGTTACT
GAGTTGTTAAATGAGATGTTAGCAGAAGATAGTGAAATATTGACTGTGATTATTGGTCAAGATGCAGAGCAAGCAGTTACAGATAACATG
ATAAACTGGATTGAAGAGCAATATCCAGATGTAGAAGTGGAAGTTCATGAAGGTGGACAACCAATTTATCAATATTTCTTTTCAGTAGAAT
AA

Gene: recG (ATP-dependent DNA helicase)

Position: 1191753 to 1193813, length: 2061 nt, orientation: FORWARD

Perfect match to: (NN50-BAEA01000008-[11749:13809:r], allele observed in CC4803+CC239)

Sequence:

TTGGCTAAAGTAAACTTAATAGAAAGTCCATATTCTCTTTTACAATTAAAAGGTATAGGTCCTAAGAAAATAGAAGTATTGCAACAACTAAA
TATTCATACAGTGGAAGATCTTGTTCTTTATTTGCCAACTAGATATGAAGATAATACAGTGATTGATTTGAATCAAGCAGAAGATCAATCTA
ACGTTACGATAGAAGGACAAGTATATACAGCTCCAGTAGTTGCATTTTTTGGAAGAAATAAATCAAAATTAACCGTTCATTTAATGGTAAAT
AATATTGCTGTCAAATGTATTTTTTTCAATCAACCGTATTTAAAAAAGAAAATCGAATTAAATCAAACTATAACTGTTAAAGGTAAGTGGAAT
AGGGTTAAACAGGAAATTACTGGTAATAGGGTTTTCTTTAATTCACAAGGGACACAAACTCAAGAAAACGCAGATGTTCAATTAGAACCAG
TCTATCGTATTAAGGAAGGTATTAAACAAAAGCAAATACGAGACCAAATTAGACAAGCGTTAAATGATGTGACAATTCATGAATGGTTAAC
TGATGAACTAAGAGAAAAATATAAATTAGAGACCTTGGACTTTACTTTGAACACATTACATCATCCTAAAAGTAAAGAGGATTTATTACGTG
CTCGTAGAACCTATGCATTTACTGAACTGTTTTTATTCGAATTACGTATGCAATGGCTAAATAGATTAGAAAAGTCATCTGACGAAGCAATT
GAAATTGATTATGACATAGACCAAGTTAAATCATTTATTGATCGTTTACCTTTTGAACTAACTGAAGCACAGAAATCCAGTGTTAATGAAATT
TTTAGAGATTTAAAAGCACCAATACGTATGCATCGATTACTTCAAGGTGATGTAGGTTCAGGAAAAACAGTAGTTGCTGCAATTTGTATGTA
TGCATTAAAAACGGCTGGTTATCAATCAGCATTGATGGTACCAACTGAAATTTTAGCAGAGCAACATGCTGAAAGTTTAATGGCTTTATTTG
GAGATTCTATGAACGTTGCATTGTTAACTGGGTCAGTAAAAGGTAAGAAACGAAAGATACTTTTAGAACAACTTGAAAATGGTACGATTGA
TTGTTTAATTGGAACCCATGCTTTGATTCAAGATGATGTGATTTTCCATAATGTTGGTTTAGTAATTACAGATGAACAACATCGATTTGGTGT
GAATCAACGCCAGCTTTTAAGAGAAAAAGGTGCAATGACGAATGTGTTATTTATGACAGCAACGCCGATACCAAGAACACTAGCAATATCA
GTTTTTGGTGAGATGGATGTGTCTTCAATTAAACAATTACCAAAAGGTCGTAAACCTATCATTACTACTTGGGCAAAGCATGAGCAATACGA
TAAAGTTTTGATGCAAATGACCTCAGAGTTGAAAAAAGGTCGTCAAGCATATGTCATTTGCCCGCTTATAGAAAGTTCTGAGCATCTCGAAG
ATGTTCAAAATGTTGTCGCATTGTACGAGTCTTTACAACAGTATTATGGTGTTTCCCGTGTAGGGTTATTGCATGGTAAGTTGTCTGCCGAT
GAAAAAGATGAGGTCATGCAAAAGTTTAGCAATCATGAGATAGATGTTTTAGTTTCTACTACTGTTGTTGAAGTAGGTGTTAATGTACCGA
ATGCAACTTTTATGATGATTTATGATGCGGATCGCTTTGGATTATCAACTTTACATCAGTTACGCGGACGTGTGGGTAGAAGTGACCAGCAA
AGTTACTGTGTTTTAATTGCATCCCCTAAAACAGAAACAGGAATTGAAAGAATGACAATTATGACACAAACAACGGATGGATTTGAATTGA
GTGAACGAGACTTAGAAATGCGTGGTCCTGGCGATTTCTTTGGTGTTAAACAAAGTGGATTGCCAGATTTCTTAGTTGCCAATTTAGTTGAA
GATTATCGTATGTTAGAAGTTGCTCGTGATGAAGCAGCTGAACTTATTCAATCTGGCGTATTCTTTGAAAATACGTATCAACATTTACGTCAT
TTTGTTGAAGAAAATTTATTACATCGTAGTTTTGACTAA

Gene: fapR (fatty acid and phospholipid biosynthesis transcriptional regulator)

Position: 1194031 to 1194588, length: 558 nt, orientation: FORWARD

Perfect match to: (RF122-AJ938182-[1179874:1180431], highly conserved allele)

Sequence:

TTGAAACTAAAGAAAGATAAACGTAGAGAAGCAATCAGACAACAAATTGATAGCAATCCCTTCATCACAGACCATGAACTAAGCGACTTAT
TTCAAGTGAGTATACAAACAATTCGTTTAGATCGCACTTATTTAAACATACCAGAATTAAGGAAGCGTATTAAATTAGTTGCTGAAAAGAAT
TATGACCAAATAAGTTCTATTGAAGAACAAGAATTTATTGGTGATTTGATTCAAGTCAATCCAAATGTTAAAGCGCAATCAATTTTAGATATT
ACATCGGATTCTGTTTTTCATAAAACTGGAATTGCGCGTGGTCATGTGCTGTTTGCTCAGGCAAATTCGTTATGTGTTGCGCTAATTAAGCA
ACCAACAGTTTTAACTCATGAGAGTAGCATTCAATTTATTGAAAAAGTAAAATTAAATGATACGGTAAGAGCAGAAGCACGAGTTGTAAAT
CAAACTGCAAAACATTATTACGTCGAAGTAAAGTCATATGTTAAACATACATTAGTTTTCAAAGGAAATTTTAAAATGTTTTATGATAAGCG
AGGATAA

Gene: plsX (glycerol-3-phosphate acyltransferase)

Position: 1194593 to 1195579, length: 987 nt, orientation: FORWARD

Perfect match to: (COL-CP000046-[1252436:1253422], highly conserved allele)

Sequence:

ATGGTTAAATTAGCAATTGATATGATGGGTGGCGACAATGCGCCTGATATCGTATTAGAAGCCGTACAAAAGGCTGTTGAAGACTTTAAAG
ATCTAGAAATTATACTTTTCGGTGACGAAAAAAAGTATAATCTGAACCATGAACGAATCGAATTTAGACATTGTTCTGAAAAGATTGAAATG
GAAGATGAGCCTGTTAGAGCGATTAAACGTAAAAAAGATAGCTCAATGGTAAAAATGGCTGAAGCTGTGAAATCTGGTGAAGCAGATGG
ATGTGTGTCAGCAGGTAATACTGGTGCTTTAATGTCAGCTGGTTTATTCATTGTTGGACGTATTAAAGGTGTAGCTAGACCGGCTTTAGTAG
TAACATTGCCAACGATTGATGGAAAAGGTTTTGTCTTTTTAGACGTTGGTGCAAATGCTGATGCTAAACCTGAACACTTATTACAGTATGCG
CAACTAGGGGATATTTATGCTCAAAAAATTAGAGGTATTGATAATCCGAAAATCTCATTATTAAATATAGGAACCGAGCCAGCTAAAGGTA
ATAGTTTAACGAAAAAATCATATGAGTTATTAAATCATGATCATTCATTGAATTTTGTTGGGAATATTGAAGCGAAGACATTAATGGATGGC
GATACAGATGTTGTAGTTACCGATGGCTATACTGGGAACATGGTCCTTAAAAATTTAGAAGGTACTGCAAAATCAATCGGTAAAATGTTAA
AAGATACGATTATGAGTAGTACTAAAAATAAATTAGCAGGTGCAATATTGAAGAAAGATTTAGCTGAATTCGCTAAAAAGATGGATTACTC
AGAATACGGTGGTTCCGTATTATTAGGATTGGAAGGTACTGTAGTTAAAGCACACGGTAGTTCAAATGCTAAAGCTTTTTATTCTGCAATTA
GACAAGCGAAAATCGCAGGAGAACAAAATATTGTACAAACAATGAAAGAGACTGTAGGTGAATCAAATGAGTAA

Gene: fabD (malonyl CoA-acyl carrier protein transacylase)

Position: 1195572 to 1196498, length: 927 nt, orientation: FORWARD

Perfect match to: (CF_Marseille-CABA01000048-[38219:39145], allele observed in CC5)

Sequence:

ATGAGTAAAACAGCAATTATTTTTCCGGGACAAGGTGCCCAAAAAGTTGGTATGGCACAAGATTTGTTTAATAACAATGATCAAGCAACTG
AAATTTTAACTTCAGCAGCAAAGACGTTAGACTTTGATATTTTAGAGACAATGTTTACTGATGAAGAAGGTAAATTGGGTGAAACTGAAAA
CACGCAACCAGCTTTATTGACGCATAGTTCGGCATTATTAGCAGCGCTAAAAAATTTGAATCCTGATTTTACTATGGGGCATAGTTTAGGTG
AATATTCAAGTTTAGTTGCAGCTGACGTATTATCATTTGAAGATGCAGTTAAAATTGTTAGAAAACGTGGTCAATTAATGGCGCAAGCATTT
CCTACTGGTGTAGGAAGCATGGCTGCAGTATTGGGCTTAGATTTTGATAAAGTCGATGAAATTTGTAAGTCATTATCATCTGATGACAAAAT
AATTGAACCAGCAAACATTAATTGCCCAGGTCAAATTGTTGTTTCAGGTCACAAAGCTTTAATTGATGAGCTAGTAGAAAAAGGTAAATCAT
TAGGTGCAAAACGTGTCATGCCTTTAGCAGTATCTGGTCCATTCCATTCATCGCTAATGAAAGTGATTGAAGAAGATTTTTCAAGTTATATTA
ATCAATTTGAATGGCGTGATGCTAAGTTTCCTGTAGTTCAAAATGTAAATGCGCAAGGTGAAACTGACAAAGAAGTAATTAAATCTAATAT
GGTCAAGCAATTATATTCACCAGTACAATTCATTAACTCAACAGAATGGCTAATAGACCAAGGTGTTGATCATTTTATTGAAATTGGTCCTG
GAAAAGTTTTATCTGGCTTAATTAAAAAAATAAATAGAGATGTTAAGTTAACATCAATTCAAACTTTAGAAGATGTGAAAGGATGGAATGA
AAATGACTAA

Gene: fabG1 (beta-ketoacyl-[acyl-carrier-protein] reductase)

Position: 1196491 to 1197225, length: 735 nt, orientation: FORWARD

Perfect match to: (RF122-AJ938182-[1182334:1183068], highly conserved allele)

Sequence:

ATGACTAAGAGTGCTTTAGTAACAGGTGCATCAAGAGGAATTGGACGTAGTATTGCGTTACAATTAGCAGAAGAAGGATATAATGTAGCA
GTAAACTATGCAGGCAGCAAAGAGAAAGCTGAAGCAGTAGTCGAAGAAATCAAAGCTAAAGGTGTTGACAGTTTTGCGATTCAAGCAAAT
GTTGCCGATGCTGATGAAGTTAAAGCAATGATTAAAGAAGTAGTTAGCCAATTTGGTTCTTTAGATGTTTTAGTAAATAATGCAGGTATTAC
TCGCGATAATTTATTAATGCGTATGAAAGAACAAGAGTGGGATGATGTTATTGACACAAACTTAAAAGGTGTATTTAACTGTATCCAAAAA

GCAACACCACAAATGTTAAGACAACGTAGTGGTGCTATCATCAATTTATCAAGTGTTGTTGGAGCAGTAGGTAATCCGGGACAAGCAAACT
ATGTTGCAACAAAAGCAGGTGTTATTGGTTTAACTAAATCTGCGGCGCGTGAATTAGCATCTCGTGGTATCACTGTAAATGCAGTTGCACCT
GGTTTTATTGTTTCTGATATGACAGATGCTTTAAGTGATGAGCTTAAAGAACAAATGTTGACTCAAATTCCGTTAGCACGTTTTGGTCAAGA
CACAGATATTGCTAATACAGTAGCGTTCTTAGCATCAGACAAAGCAAAATATATTACAGGTCAAACAATCCATGTAAATGGTGGAATGTAC
ATGTAA

Gene: SIRU13 (staphylococcal interspersed repeat unit 13)

Position: 1197412 to 1197440, length: 29 nt

Perfect match to: (MW2-BA000033-[1215597:1215625], allele observed in CC1)

Sequence:

TAAGAAACACTAATCAATAAATTGATAAG

Gene: SIRU13 (staphylococcal interspersed repeat unit 13)

Position: 1197540 to 1197568, length: 29 nt

Perfect match to: (MW2-BA000033-[1215597:1215625], allele observed in CC1)

Sequence:

TAAGAAACACTAATCAATAAATTGATAAG

Gene: acpP (acyl carrier protein)

Position: 1197659 to 1197892, length: 234 nt, orientation: FORWARD

Perfect match to: (RF122-AJ938182-[1183276:1183509], highly conserved allele)

Sequence:

GTGGAAAATTTCGATAAAGTAAAAGATATCATCGTTGACCGTTTAGGTGTAGACGCTGATAAAGTAACTGAAGATGCATCTTTCAAAGATG
ATTTAGGCGCTGACTCACTTGATATCGCTGAATTAGTAATGGAATTAGAAGACGAGTTTGGTACTGAAATTCCTGATGAAGAAGCTGAAAA
AATCAACACTGTTGGTGATGCTGTTAAATTTATTAACAGTCTTGAAAAATAA

Gene: rnc (ribonuclease 3)

Position: 1198008 to 1198739, length: 732 nt, orientation: FORWARD

Perfect match to: (MW2-BA000033-[1216065:1216796], highly conserved allele)

Sequence:

ATGTCTAAACAAAAGAAAAGTGAGATAGTTAATCGTTTTAGAAAGCGCTTTGATACTAAAATGACAGAGTTAGGCTTTACTTATCAAAATAT
TGATTTATACCAACAAGCATTTTCGCATTCGAGTTTTATTAATGATTTTAATATGAATCGTTTAGACCATAATGAGCGTTTAGAGTTTTTGGG
TGATGCGGTATTAGAATTGACGGTTTCACGATATTTATTTGATAAACATCCCAACTTGCCAGAAGGGAATTTAACAAAAATGCGTGCCACTA
TTGTATGTGAGCCCTCACTTGTAATATTTGCGAATAAAATTGGATTGAACGAAATGATTTTACTTGGTAAAGGTGAAGAGAAAACAGGGGG
ACGTACAAGACCATCATTAATATCAGATGCATTCGAAGCATTTATTGGGGCATTGTATTTGGATCAAGGACTAGATATAGTTTGGAAATTTG
CTGAGAAAGTCATTTTCCCACATGTAGAACAAAATGAGTTATTAGGCGTGGTAGATTTTAAAACACAATTCCAAGAATATGTGCACCAGCA
AAATAAAGGTGATGTAACCTATAATTTAATAAAAGAAGAGGGACCGGCACATCATCGTCTATTCACTTCAGAAGTTATTCTGCAAGGGGAA
GCAATAGCTGAAGGTAAAGGGAAAACGAAAAAAGAATCAGAACAACGTGCTGCTGAAAGTGCCTATAAGCAATTAAAACAAATTAAATAG

Gene: smc (chromosome segregation protein SMC)

Position: 1198886 to 1202452, length: 3567 nt, orientation: FORWARD

Perfect match to: (CIG1770-AHVN01000007-[682591:686157], allele observed in CC8+CC5+CC97)

Sequence:

ATGGTTTATTTAAAATCAATAGATGCCATTGGATTTAAGTCTTTTGCAGATCAAACCAATGTTCAATTCGATAAAGGTGTAACTGCAATTGTT
GGTCCAAATGGAAGCGGTAAAAGTAATATTACAGATGCTATTAAATGGGTGTTGGGTGAACAATCGGCTAAATCATTACGTGGCTCAAAA
ATGGAAGATATTATCTTCTCAGGTGCAGAACATCGCAAAGCTCAAAATTATGCTGAAGTACAGTTAAGATTAGATAATCATTCTAAAAAGCT
CAGTGTTGATGAAAACGAAGTTATTGTAACAAGAAGATTGTATCGAAGTGGTGAAAGTGAGTACTACATAAATAATGACCGTGCAAGATT
AAAAGATATTGCCGATTTATTTTTAGATTCTGGATTGGGAAAAGAAGCGTATAGCATTATCTCGCAAGGTAGAGTTGATGAAATACTAAAT
GCTAAACCAATTGATAGACGTCAAATTATTGAAGAATCGGCTGGTGTACTTAAATATAAAAAACGTAAGGCTGAATCATTAAATAAACTTG
ACCAAACAGAAGATAATTTAACGAGAGTAGAAGACATTTTATATGATTTGGAAGGTCGCGTAGAACCTCTAAAAGAGGAGGCAGCTATAG
CTAAAGAATATAAGACACTTTCACATCAAATGAAACATAGTGACATTGTAGTTACAGTGCACGATATTGATCAATATACAAATGACAATAGA
CAATTAGATCAACGTTTAAATGATTTACAAGGCCAACAAGCAAATAAAGAAGCTGACAAGCAACGTTTAAGCCAACAAATTCAACAATATA
AAGGTAAACGTCATCAACTTGATAATGATGTTGAATCGCTTAATTATCAATTAGTAAAAGCTACGGAAGCCTTTGAAAAATATACGGGACA
ATTAAATGTTTTAGAAGAACGTAAGAAAAATCAATCTGAAACAAATGCACGATATGAAGAAGAACAAGAAAATTTAATGGAGCTTTTAGAA
AATATATCAAATGAGATTTCTGAAGCTCAAGATACTTATAAGTCTCTGAAAAGTAAACAAAAAGAACTCAATGCTGTCATTCGTGAACTTGA
AGAACAACTATATGTTTCAGACGAAGCACATGATGAAAAATTGGAAGAAATTAAAAACGAATACTATACATTAATGTCAGAGCAATCAGAT
GTTAACAATGATATTCGTTTTTTAAAGCATACTATAGAAGAGAATGAGGCTAAAAAATCAAGACTAGATTCTCGATTAGTTGAAGTTTTGA
GCAATTGAAAGATATTCAGGGTCAAATAAAAACGACAAAAAAAGAATATCAACAGACCAACAAAGAACTTTCTGCTGTAGATAAAGAAATT
AAAAATATAGAAAAAGACCTCACTGATACAAAAAAAGCACAAAATGAATACGAAGAGAAATTGTATCAAGCATATCGATATACCGAAAAA
ATGAAAACACGTATTGATAGTTTGGCAACGCAAGAGGAAGAATATACTTATTTTTTCAATGGCGTCAAACATATTTTGAAAGCTAAAAATAA
AGAATTAAAGGGTATTCATGGTGCAGTTGCGGAAATTATTGATGTGCCATCTAAATTAACTCAGGCAATTGAAACAGCATTAGGTGCTTCAT
TACAACATGTCATTGTAGATTCAGAAAAAGATGGACGCCAGGCTATTCAATTTTTAAAAGAACGTAATTTAGGTCGTGCGACGTTTTTACCA
TTAAATGTTATACAGAGTAGAGTGGTAGCGACTGATATTAAATCTATTGCTAAAGAGGCAAACGGATTTATTAGTATTGCTTCGGAAGCAG
TTAAAGTAGCACCAGAATATCAAAATATTATCGGGAATTTATTAGGTAATACGATTATCGTTGATCATTTAAAGCATGCAAATGAATTGGCA
CGTGCGATTAAATATCGAACTCGTATTGTTACTTTGGAAGGTGATATTGTAAATCCTGGTGGTTCTATGACTGGTGGTGGCGCTCGTAAGTC
AAAAAGTATTCTGTCTCAAAAAGACGAGTTGACAACAATGAGACACCAATTAGAAGATTACTTGCGTCAAACAGAATCATTTGAACAACAA
TTTAAAGAGTTGAAGATAAAAAGTGATCAATTAAGTGAACTGTATTTTGAAAAAAGTCAAAAGCATAATACACTTAAAGAGCAAGTGCATC
ATTTTGAAATGGAGCTCGATAGATTAACTACACAAGAAACACAAATAAAAAATGATCATGAAGAATTCGAATTTGAAAAAAATGATGGTTA
TACGAGTGACAAAAGTCGACAAACTTTGAGTGAAAAAGAAACTCATTTAGAAAGTATTAAAGCATCTTTAAAACGACTAGAAGATGAAATT
GAACGCTACACAAAACTTTCTAAAGAAGGTAAGGAAAGCGTAACTAAAACACAACAAACGTTACATCAGAAACAATCTGATCTTGCTGTGG
TTAAAGAGCGTATTAAAACACAACAACAGACAATAGATCGATTAAATAATCAAAGTCAACAAACCAAACATCAATTAAATGATGTTAAAGA
AAAAATAGCATTCTTTAATTCGGATGAAGTGATGGGCGAGCAAGCTTTTCAAAATATTAAAGATCAAATTAATGGTCAACAAGAAACGAGA
ACACGCTTATCTGATGAATTAGATAAATTGAAACAACAACGTATTGAGTTGAATGAACAAATCGATGCGCAAGAAGCTACACTACAAGTTT
GTCACCAAGATATTTTAGCTATCGAAAATCACTACCAAGATATTAAAGCTGAACAATCAAAGCTAGATGTATTAATTCATCATGCGATAGAT
CATTTAAATGATGAATATCAATTGACTGTTGAACGTGCGAAATCTGAATATACGAGTGATGAATCGATTGACGCATTACGTAAAAAAGTTA
AGTTAATGAAGATGTCGATTGATGAACTAGGTCCTGTAAACTTAAATGCAATTGAACAATTTGAAGAGTTAAATGAACGTTATACATTTTTA
AGTGAACAACGTACAGATCTTCGTAAAGCTAAAGAAACATTAGAGCAAATTATAAGTGAAATGGATCAAGAGGTTACTGAAAGATTTAAA
GAAACTTTCCATGCTATTCAAGGACATTTTACAGCTGTGTTCAAACAATTGTTTGGTGGAGGCGATGCAGAATTGCAATTAACTGAAGCCGA
TTATTTAACAGCTGGTATTGATATTGTGGTACAACCACCGGGTAAAAAGTTGCAACATTTATCGTTACTGAGTGGTGGTGAGCGTGCATTAA
CTGCTATTGCTTTACTATTTGCAATTTTAAAAGTAAGATCTGCACCTTTTGTTATATTAGATGAGGTTGAAGCTGCACTAGATGAAGCAAATG
TTATTAGATACGCAAAATATTTAAATGAGTTATCAGACGAAACACAATTCATTGTTATTACACACCGTAAAGGAACAATGGAATTTGCAGAT
AGGTTATACGGTGTAACAATGCAAGAATCAGGTGTTACTAAACTTGTGAGTGTGAATTTAAATACAATAGATGATGTGTTGAAGGAGGAG
CAATAA

Gene: ftsY (signal recognition particle receptor)

Position: 1202452 to 1203702, length: 1251 nt, orientation: FORWARD

Perfect match to: (11819-97-CP003194-[1252829:1254079], highly conserved allele)

Sequence:

ATGAGCTTTTTTAAACGCTTAAAAGATAAGTTTGCAACAAATAAAGAAAATGAAGAAGTTAAATCCTTAACAGAAGAACAAGGTCAAGACA
AATTAGAAGATACACATTCTGAAGGTTCAACGCAGGACGTAAATGATTTAGCAGAAAATGCTGAAGTGAAAAAGAAGCCACGCAAGTTGA
GTGAAGCGGATTTTGATGACGATGGCTTAATATCAATTGAAGATTTTGAAGAAATTGAAGCTCAAAAAATGGGGGCTAAATTTAAAGCAG
GACTCGAAAAATCTCGTCAAAATTTCCAAGAACAATTAAATAATTTGATAGCGAGATATCGTAAAGTAGATGAAGACTTTTTTGAAGCTTTA
GAAGAAATGTTAATCACTGCAGACGTCGGTTTTAATACAGTGATGACGTTAACTGAAGAATTACGTATGGAAGCACAACGACGTAATATTC
AAGATACTGAAGATTTGCGTGAAGTCATTGTTGAAAAAATCGTAGAGATTTACCATCAAGAAGATGATAATTCAGAAGCTATGAACTTAGA
AGATGGTCGTTTGAATGTCATTTTAATGGTCGGTGTGAATGGTGTTGGTAAAACAACAACAATTGGAAAATTAGCTTACCGATATAAAATG

GAAGGTAAAAAAGTAATGTTAGCTGCGGGCGATACTTTTAGAGCGGGTGCTATTGATCAATTGAAAGTTTGGGGCGAACGTGTTGGTGTA
GACGTAATTAGCCAAAGTGAAGGTTCTGATCCAGCTGCTGTTATGTATGATGCGATTAATGCCGCTAAAAACAAAGGTGTTGATATTTTAAT
CTGTGATACCGCTGGACGTTTACAAAATAAAACAAATCTAATGCAAGAATTAGAAAAAGTTAAGCGTGTAATTAATCGAGCAGTGCCAGAT
GCGCCTCATGAAGCATTACTATGTTTAGATGCTACAACTGGTCAGAATGCGTTGTCACAAGCTAGAAACTTTAAAGAAGTAACAAATGTTAC
AGGTATTGTATTAACGAAATTAGATGGTACAGCCAAAGGTGGTATCGTATTAGCCATTCGTAATGAATTGCACATTCCAGTTAAATATGTAG
GTTTAGGTGAGCAATTAGATGACTTACAACCATTTAACCCTGAAAGTTATGTCTACGGCTTATTCGCTGATATGATTGAACAAAATGAAGAA
ATAACAACAGTTGAAAATGATCAAATTGTAACAGAAGAAAAGGACGATAATCATGGGTCAAAATGA

Gene: ylxM (modulator of signal recognition partical GTPase activity)

Position: 1203689 to 1204021, length: 333 nt, orientation: FORWARD

Perfect match to: (11819-97-CP003194-[1254066:1254398], highly conserved allele)

Sequence:

ATGGGTCAAAATGATTTAGTTAAAACGTTACGAATGAATTATTTGTTTGATTTTTATCAATCCTTATTGACGAATAAACAACGTAATTATTTG
GAATTATTTTATCTCGAAGATTATTCTTTAAGTGAAATCGCAGATACTTTTAATGTGAGTAGACAAGCAGTTTATGATAATATAAGAAGAAC
TGGCGATTTAGTTGAAGATTATGAAAAGAAATTGGAATTATACCAGAAATTTGAGCAACGCCGAGAAATATATGATGAAATGAAACAACAT
TTAAGTAATCCAGAACAAATACAACGTTATATTCAACAATTAGAAGACTTAGAATAG

Gene: ffh (signal recognition particle protein subunit)

Position: 1204047 to 1205414, length: 1368 nt, orientation: FORWARD

Perfect match to: (Strain_21193-AFEG01000003-[161805:163172:r], highly conserved allele)

Sequence:

ATGGCATTTGAAGGCTTATCAGAACGCCTGCAAGCGACGATGCAAAAAATGCGTGGTAAGGGTAAACTTACTGAAGCTGATATAAAGATA
ATGATGCGTGAAGTAAGATTAGCGTTACTTGAGGCTGACGTAAACTTTAAAGTGGTAAAAGAATTTATTAAAACAGTATCAGAACGCGCAT
TAGGTTCCGATGTAATGCAATCATTAACACCAGGGCAACAAGTTATTAAAATAGTTCAAGATGAATTAACGCAGTTGATGGGTGGAGAAAA
TACGTCGATTAATATGTCAAATAAACCACCTACTGTTGTTATGATGGTTGGTTTACAAGGTGCTGGTAAAACAACAACTGCAGGTAAATTAG
CATTATTGATGCGTAAAAAATACAACAAAAAACCTATGTTAGTTGCAGCAGATATTTATCGTCCAGCAGCGATAAATCAATTACAAACAGTA
GGGAAACAAATTGATATTCCTGTATACAGTGAAGGAGATCAAGTAAAGCCACAACAAATTGTAACTAATGCATTAAAACATGCTAAAGAAG
AACATTTAGACTTTGTAATCATTGATACAGCAGGTCGATTACACATCGATGAAGCATTGATGAACGAATTAAAAGAAGTAAAAGACATTGC
TAAACCAAACGAAATTATGTTAGTTGTCGATTCAATGACGGGTCAAGATGCTGTCAATGTTGCAGAATCTTTTGACGATCAACTTGATGTCA
CAGGTGTTACCTTAACTAAATTAGATGGTGATACACGTGGTGGTGCAGCTTTATCTATTCGTTCGGTGACACAAAAACCAATTAAATTTGTT
GGTATGAGTGAAAAGTTAGATGGTTTAGAGCTATTCCATCCTGAACGTATGGCATCACGTATTTTAGGTATGGGTGATGTGTTAAGTTTAAT
TGAAAAAGCGCAACAAGATGTGGATCAAGAAAAAGCAAAAGATTTAGAGAAAAAGATGCGTGAGTCATCGTTTACTTTAGATGATTTTTTA
GAACAACTTGATCAGGTGAAAAATCTAGGACCACTGGATGATATTATGAAAATGATTCCAGGTATGAATAAAATGAAAGGGCTAGATAAG
CTTAATATGAGTGAAAAGCAAATTGATCATATTAAAGCGATTATCCAGTCAATGACGCCGGCTGAAAGAAACAATCCAGACACATTGAATG
TATCACGTAAAAAGCGTATTGCTAAAGGGTCTGGTCGTTCATTACAAGAAGTCAATCGTTTGATGAAACAATTTAACGATATGAAGAAAAT
GATGAAACAATTCACTGGTGGCGGTAAAGGTAAAAAAGGTAAACGCAATCAAATGCAAAATATGTTAAAAGGTATGAATTTACCGTTTTAA

Gene: rpsP (30S ribosomal protein S16)

Position: 1205849 to 1206124, length: 276 nt, orientation: FORWARD

Sequence:

ATGGCAGTTAAAATTCGTTTAACACGTTTAGGTTCAAAAAGAAATCCATTGTATCGTATCGTAGTAGCAGATGCTCGTTCTCCACGTGACGG
ACGTATCATCGAACAAATCGGTACTTATAACCCAACGAGCGCTAATGCTCCAGAAATTAAAGTTGACGAAGCGTTAGCTTTAAAATGGTTA
AATGATGGTGCGAAACCAACTGATACAGTTCACAATATCTTATCAAAAGAAGGTATTATGAAAAAATTTGACGAACAAAAGAAAGCTAAGT
AA

Gene: rimM (16S rRNA processing protein)

Position: 1206312 to 1206815, length: 504 nt, orientation: FORWARD

Perfect match to: (11819-97-CP003194-[1256689:1257192], allele observed in CC80+CC72+CC80)

Sequence:

ATGAGAGTTGAAGTTGGTCAAATTGTTAACACACATGGTATTAAAGGTGAAATTAAAGTAAAATCCAATTCAGACTTTACAGACGTTCGTTT
TCAACCCGGTCAAGTGCTGACAGTTGTGCATAACAATAACGACCTTGAATATACTGTTAAGTCACATAGAGTGCATAAAGGGCTTCATATGC
TCACATTTGAAGGTATTAATAATATTAATGATATTGAGCATCTAAAAGGGAGTTCTATTTATCAAGAACGTGATCATGAAGATATCGTACTT
GAGGAAAATGAATTTTATTATTCAGATATTATAGGATGTACAGTTTTTGATGATCAAGAAACACCAATAGGTCGTGTAATTAATATATTTGA
AACAGGTGCGAATGATGTGTGGGTGATTAAAGGATCTAAAGAATATTTGATTCCTTATATTGCTGATGTTGTAAAAGAAGTGGATGTTGAA
AATAAAAAAATTATCATCACGCCAATGGAAGGATTGTTGGATTAA

Gene: trmD (tRNA (guanine-N (1)-)-methyltransferase)

Position: 1206815 to 1207552, length: 738 nt, orientation: FORWARD

Perfect match to: (KPL1845-AZJA01000004-[365535:366272], allele observed in CC96+CC1+CC5+CC8+CC239)

Sequence:

ATGAAAATTGATTATTTAACTTTATTTCCTGAAATGTTTGATGGTGTTTTAAATCATTCAATTATGAAACGTGCCCAAGAAAACAATAAATTA
CAAATCAATACGGTTAATTTTAGAGATTATGCAATTAACAAGCACAACCAAGTAGATGATTATCCGTATGGTGGCGGACAAGGTATGGTGT
TAAAGCCTGAACCTGTTTTTAATGCGATGGAAGACTTAGATGTCACAGAACAAACACGCGTTATTTTAATGTGTCCACAAGGCGAGCCATTT
TCACATCAGAAAGCTGTTGAATTAAGCAAGGCTGACCACATCGTTTTCATATGTGGACATTATGAAGGTTACGATGAACGTATCCGAACAC
ATCTTGTCACAGATGAAATATCAATGGGTGACTATGTTTTAACAGGTGGAGAATTGCCAGCGATGACCATGACTGATGCCATTGTTAGACT
GATTCCAGGTGTTTTAGGTAATGAACAGTCACATCAAGACGATTCATTTTCAGATGGATTATTAGAGTTTCCGCAATATACACGTCCGCGTG
AATTTAAGGGTCTAACAGTTCCAGATGTTTTATTGTCTGGAAATCATGCCAATATTGATGCATGGAGACATGAGCAAAAGTTGATCCGCACA
TATAATAAAAGACCTGACTTAATTGAAAAATATCCATTAACTAATGCAGATAAGCAAATATTAGAAAGATATAAAATAGGATTGAAAAAAG
GTTAG

Gene: rplS (50S ribosomal protein L19)

Position: 1207655 to 1208005, length: 351 nt, orientation: FORWARD

Perfect match to: (MRSA252-BX571856-[1267753:1268103], highly conserved allele)

Sequence:

ATGACAAATCACAAATTAATCGAAGCAGTAACTAAATCACAATTACGTACAGACTTACCAAGTTTCCGTCCTGGTGATACTTTACGTGTACA
CGTACGTATCATTGAGGGTACTCGTGAGCGTATCCAAGTATTCGAAGGCGTTGTAATTAAACGTCGTGGCGGTGGCGTTTCTGAAACGTTT
ACAGTTCGTAAAATTTCATCAGGTGTTGGCGTGGAACGTACATTCCCATTACACACACCAAAAATTGAAAAAATCGAAGTTAAACGTCGTG
GTAAAGTACGTCGTGCTAAATTATATTACTTACGTAGTTTACGTGGTAAAGCTGCTAGAATCCAAGAAATTCGTTAA

Gene: yfhO (putative membrane protein)

Position: 1208459 to 1211065, length: 2607 nt, orientation: REVERSE

Perfect match to: (06BA18369-ARXY01000021-[15183:17789:r], allele observed in CC5)

Sequence:

TTATTTTTGTCTTGCCCATCTCGTGAAAATAATACTACATATAATGCCGAATATAGTAATTGTTATAAGTAAATAATAGTATGGTGGGGTATA
GCTCAATTGAATCTTTGTTATATTTTTAGGTGCTTTAATGCCGGTCATAATACCATTTACTTGTTCAACTTTAAGACTTTGATCACCTGATGTC
GCTTTCATACCTTGATTATATGCTGTTGGCAAAACAATATACCCAGATGAATTTTTATTTTTAGTAATAGTATAACCTTGCTTTGTCTTACTAA
CTTTGACAGCCTCTAATGAATTTGAAGCGTCTTTAAGCGTGGTATAATCTTCGCCGTATATCCCTTTTAAATTTACACGATACTTACCTTTAGG
CAATGATAATCTAATCCTATCTGAAGCTTTAATGCGTATTGTTACGGGTGTTACAAAGCGTCGATATTTATAAGTGAGTTTATTTCTTTCTTGT
GTATATTCATTCACTTTAACATCATGAGCTTTATCCGGCGAAAGTAATTCTAAATCCATTTCAAAATACAAATCTTTAAATTGATTAGAAACTG
ATTTTGGCAACTGTACAGTTAGACCACCATTATTTTGTTTAACTTGTAATAAATGTTTTGTAGGAGATTGCCAGGCTGCACTATTTAATTTAAT
TGTTGAATCTGATAGTAAATTTTTATTGGCTTTAAAATGTGTATTAACATCTTTAGTATTGTTAGAAACAATCCCTTGCAACATTGCTTGTTCT
TTATCTAATGGAGATTTTAATTCTTTATTGGAAAAGACCTTATTTGTAATATGTGCACTTGGATAATGGATGGTATTTTTAGAATGAATCCAA

CGAACTTTATTGTCTTTGTGTTCAGAGTTAATTTTAAATCCATATGGTAAGTTGTCATCATGATTTACTCTAATTCGATCATTCACATTCCAAA
GCGATAGTAAATTTTGACGATTGCCAAGTAATCTATAAGTGCTGTTTTTATCGATTGGCATATTAATTTGGAGTGTCTTGTCATAATATTTTA
AAATGTCTCCATTAAAAATACTAGAATATAATGAAATGCCATTATAATGATATATAAATGGTGAATTTAATGCATAGTCTGACATATAATCAA
TGCGATTAAATGGGCCTGTTGCATTTTGATTTATCTTTTTTATTAGCTGGTTTACATAGTTACTATGATAATCATGTTGTTTCAACGTTGATAT
TGATTGTTGATAAGGTTTGATTGCCATATTTTTGTTGTTATCTAAAATGGCGATTTGTTGAATCATAACGATTAATACTAATATTGCAACGGT
TAATTTTTTATAACGCCATAAACTAAATTTTAAAATAACGGCAAGCACCATTAGCAGGATAATACCTACTATAAGTGCAAGTGGGTGTGTCG
GTGATGGTAATACATAAAGTAATGCGATGATGCTTACTGGTATTGTTCTGATTAAATAATATTTCATATTTAATGTTGATAAATGTTGAATAA
ACAATCCGCAAAGAGCACTTGATGATAGTGCTAAGATATACACCCAACGCCTTTCTGGAAATGAAAAACCATTAAAAGCACTGTCGAAATA
CTGTGATAATGAACCTATAAATAATATCCATGTTACTATTGCGAAAAGTCTATAAAAGTAAAAACGATACAGTTTGAATGACAATAATGCAA
CGATAGTAAGAATTGAAATCGTAATATAAAATCCATCGCTAAAGAAAAAATAATGATAATCAAGTGGTGTCAAAAACGGTATATCAACATT
GGGATTTTGCTTTCTGTCATTTTCCAAAAACGCAGAAATGCCAGTGAATAAACCAAATACACTTGATAACACACTCAAAACTGTAGCAGATA
TGACGCAAATTAATTTTTGTGTTCTAGAGACAATGTCATATTTGTAAGTGAAAATGAGTCGATATAAATAGTAGCAACCTATAATAATAGCT
TGATAATAACTGAAATAAAAATTGCTAAATAGTGTTAAGGCTATCGCAACAATGAAAATACCGATTTTGCGTTGTTGAAAATATCTTTCCAA
ACCAAGAATCGATAATGGTAATAAATATAATAAATTTCCATAAAATGACCAAGTAAAATTAAAGTATATAACGACAGTTGACATGCCGTATA
AAATCGTAGCGATCATATTTGTTGAGCGTTTAAAGTGTAATATTTTAAATAAGTAGAAGGTCACGACAAATGTTATGATAGCTCGTATCATA
GCCATAATAAGTTGGTTTGTCGGCCAAAAATGTATTGTTGTCGGATTAAATATACCAACCGTTTCTCCTATTTTAATGAATAGAAAATTTAGC
CACATTAAAGGTGACAGCGAATAATAATATGATAGTCCTTTCATATAATCGCCACCTAATCCAAACGATGCATCATATAAACTAGAAAAACT
ACTTAGATGTTCATACAAATACATTTGAAATGGCATCATTTGACGGAATCCATCTCCAGCCCCACTAAAAACAGTACCATTCACAATATAATC
ATAGATATGAGTAGAAAATAAAATAAGCGTTAATATTACACTAATGAAAGTTATAACAAAGAATTGTTTGACGTTTGAATTTAGCCACTTTT
TTAACAC

Gene: rbgA (putative GTP-binding protein)

Position: 1211466 to 1212350, length: 885 nt, orientation: FORWARD

Sequence:

ATGGTTATTCAATGGTATCCAGGACATATGGCGAAAGCCAAAAGAGAAGTAAGTGAACAATTAAAAAAAGTAGATGTAGTGTTTGAACTA
GTAGATGCAAGAATTCCATATAGTTCAAGAAACCCTATGATAGATGAAGTTATTAACCAAAAACCACGTGTTGTTATATTAAATAAAAAAGA
TATGTCTAATTTAAATGAGATGTCAAAATGGGAACAATTTTTTATTGATAAAGGATACTATCCTGTATCAGTGGATGCTAAGCACGGTAAAA
ATTTAAAGAAAGTGGAAGCTGCAGCAATTAAGGCGACTGCTGAAAAATTTGTACGCGAAAAAGCGAAAGGACTTAAACCTAGAGCGATAA
GAGCGATGATTGTTGGAATTCCAAATGTTGGTAAATCCACATTAATAAATAAACTGGCAAAGCGTAGTATTGCACAGACTGGTAATAAACC
AGGTGTGACCAAACAACAACAATGGATTAAAGTTGGTAATGCATTACAACTATTAGACACACCAGGGATACTTTGGCCTAAATTTGAAGAT
GAAGAAGTCGGTAAGAAGTTGAGTTTAACTGGTGCGATAAAAGATAGCATTGTGCACTTAGATGAAGTAGCTATATATGGATTGAACTTTT
TAATTCAACATGACATTGAACGCTTAAAAGCACATTATAAAATTGACGTTCCAGAATATGCAGAAATTTTAGCGTGGTTTGATGCGATAGGG
AAAAAACGTGGCTTAATTCGACGTGGTAATGAAATTGATTACGAAGCAGTCATTGAACTGATTATTTATGATATTCGAAATGCTAAAATAG
GAAATTATTGTTTTGATATTTTTAAAGATATGACTGAGGAATTAGCAAATGACGCTAACAATTAA

Gene: rnhB (ribonuclease HII)

Position: 1212334 to 1213101, length: 768 nt, orientation: FORWARD

Perfect match to: (11819-97-CP003194-[1262712:1263479], allele observed in CC80)

Sequence:

ATGACGCTAACAATTAAAGAAGTTACGCAGTTGATTAATGCGGTTAATACAATAGAAGAATTAGAAAATCATGAATGCTTTTTAGATGAGC
GAAAAGGTGTTCAAACGGCAATTACTAGGCGCAGAAAAGCGTTAGAAAAAGAACAAGCTTTAAAAGAAAAGTATGTTGAAATGACTTACT
TTGAAAATGAAATATTAAAAGAGCATCCTAATGCTATTATTTGTGGTATTGATGAAGTTGGAAGAGGACCTTTAGCAGGTCCAGTCGTTGC
ATGCGCAACAATTTTAAATTCAAATCACAATTATTTGGGCCTTGATGACTCGAAAAAAGTACCTGTTACGAAACGTCTAGAATTAAATGAAG
CACTAAAAAATGAAGTTACTGCTTTTGCATATGGTATCGCGACAGCTGAAGAAATAGATGAATTTAATATTTATAAAGCTACTCAAATCGCC
ATGCAGCGAGCTATTGATGGATTATCAGTACAACCAACGCATTTATTGATAGACGCGATGACGCTTGATAATGCACTGCCTCAAGTATCTTT
AATCAAGGGTGATGCAAGAAGTGTGTCCATTGCAGCTGCAAGTATCATGGCGAAGGTTTTTCGTGATGATTATATGACACAGTTATCTAAA
GATTATCCTGAATACGGTTTTGAAAAAAACGCGGGTTATGGTACCAAACAACATTTACTAGCAATCGATGATATTGGCATTATGAAAGAGC
ATAGAAAAAGCTTTGAACCTATAAAATCGTTACTGTAA

Gene: sucC (succinyl-CoA synthase subunit beta)

Position: 1213210 to 1214377, length: 1168 nt, orientation: FORWARD

Sequence:

ATGAATATCCACGAGTATCAAGGTAAAGAAATATTTCGTTCAATGGGCGTTGCAGTTCCAGAAGGACGAGTAGCATTTACTGCTGAAGAAG
CGGTGGAGAAAGCAAAAGAATTAAATTCTGATGTTTATGTTGTAAAAGCACAAATTCATGCTGGAGGTAGAGGTAAAGCAGGCGGAGTAA
AAATTGCTAAATCTTTATCTGAGGTAGAAACATATGCAAAAGAATTATTAGGGAAAACTTTGGTGACACATCAAACTGGTCCAGAAGGTAA
AGAAATTAAGCGTTTATATATCGAAGAAGGTTGTGCTATTCAAAAAGAATATTACGTTGGATTCGTTATTGATCGTGCGACTGACCAAGTAA
CATTGATGGCGTCTGAAGAAGGGGGCACTGAGATTGAAGAAGTTGCTTCGAAGACTCCTGAAAAGATCTTCAAAGAAACTATCGATCCAG
TAATCGGACTTTCACCATTCCAAGCAAGACGAATTGCGTTTAATATTAATATTCCTAAAGAATCTGTTAACAAAGCTGCTAAATTCTTATTAG
CACTTTATAATGTATTCATTGAAAAAGATTGTTCAATCGTAGAAATCAACCCATTAGTTACAACAGCTGATGGTGATGTATTGGCATTAGAT
GCTAAAATTAATTTTGATGATAATGCATTATTCAGACATAAAGATGTTGTAGAATTACGTGATTTAGAAGAAGAAGATCCGAAAGAGATTG
AAGCGTCTAAACATGATTTATCATACATTGCATTAGATGGTGGCATCGGATGTATGGTTAATGGTGCAGGTTTAGCCATGGCAACAATGGA
TACGATTAATCATTTCGGTGGAAACCCAGCCAATTTCTTAGATGCAGGCAGGAAGCGCTACTAGAGAAAAAGTAACTGAAGCATTTAAAAT
CATTTTAGGTGATGAAAATGTTAAAGGTATTTTTGTAAATATTTTCGGTGGCATTATGAAATGTGATGTTATCGCAGAAGGTATCGTTGAAG
CTGTAAAAGAAGTAGATTTAACTTTACCACTAGTTGTACGCTTAGAAGGTACAAATGTTGAGTTAGGTAAAAAAATCTTAAAAGACTCAGG
ATTAGCAATTGAACCAGCAGCAACAATGGCTGAAGGTGCACAAAAAATTGTTAAACTAGTCAAAGAAGCATAA

Gene: sucD (succinyl-CoA synthase subunit alpha)

Position: 1214399 to 1215307, length: 909 nt, orientation: FORWARD

Perfect match to: (Strain_21333-AHKA01000002-[160983:161891:r], allele observed in CC80+CC1+CC97)

Sequence:

ATGAGTGTATTTATAGATAAGAATACTAAAGTAATGGTACAAGGTATTACAGGGTCTACTGCCCTTTTCCATACAAAACAAATGCTTGATTA
TGGTACGAAAATAGTAGCAGGTGTGACGCCTGGTAAAGGTGGTCAAGTTGTTGAAGGCGTTCCTGTTTTCAACACTGTTGAAGAAGCTAAA
AATGAAACCGGGGCAACGGTTTCAGTCATTTACGTTCCAGCACCATTTGCTGCAGACTCAATTTTAGAAGCAGCTGATGCAGACTTAGATAT
GGTTATTTGTATCACTGAACATATTCCTGTATTAGACATGGTTAAAGTTAAACGCTACTTACAAGGTAGAAAAACACGTTTAGTTGGTCCAA
ACTGTCCAGGTGTGATTACAGCAGATGAATGTAAAATTGGTATTATGCCTGGCTATATTCACAAAAAAGGTCATGTTGGTGTAGTATCACGT
TCAGGTACACTAACATATGAAGCAGTGCACCAATTGACTGAAGAAGGTATTGGTCAAACTACAGCTGTTGGTATTGGTGGAGACCCAGTCA
ACGGAACAAACTTTATTGATGTTTTAAAAGCATTCAATGAAGATGACGAAACGAAAGCAGTTGTTATGATTGGTGAAATCGGTGGTACAGC
TGAAGAAGAAGCAGCTGAATGGATTAAAGCGAATATGACAAAACCAGTTGTAGGCTTTATCGGTGGACAAACAGCACCTCCTGGAAAACG
TATGGGACATGCTGGTGCAATCATTTCAGGTGGTAAAGGTACTGCTGAAGAGAAAATTAAAACATTAAATAGTTGTGGTGTGAAAACAGC
GGCAACACCTTCAGAAATTGGTTCAACATTAATTGAAGCTGCTAAAGAAGCAGGTATTTATGAATCATTATTAACTGTTAATAAATAA

Gene: lytN (cell-wall hydrolase)

Position: 1215501 to 1216652, length: 1152 nt, orientation: FORWARD

Sequence:

ATGTTTATATATTATTGTAAGGAGTGTTTCATCATGAATAAACAACAAAGTAAAGTACGCTATTCAATTAGAAAAGTTAGTATTGGAATTTT
GTCAATTTCAATAGGTATGTTTTTGGCATTGGGTATGTCGAACAAAGCATATGCAGATGAAATTGATAAATCTAAAGATTTTACAAGAGGGT
ATGAGCAAAATGTATTCGCGAAATCAGAGTTAAATGCTAATAAAAATACGACAAAAGACAATATAAAAAATGAAGGTGCTGTTAAAACATC
GGACACAAGTTTAAAGTTAGACAACAAATCAGCAATTTCAAACGGAAATGAAATTAATCAAGATATAAAGATTTCAAATACTCCGAAAAAC
TCAAGCCAAGGTAACAATCTAGTTGTTAATAACAATGAACCTACTAAAGAAATTAAAATTGCAAACTTGGAAGCTCAAAATTCTAATCAGGA
GAAAATGAATAAAGTTACTAATAATTACTTTGGTTACTACAGTTTTAGAGAAGCTCCAAAAACACAAATCTATACTGTAAAAAAAGGAGACA
CACTTAGTGCTATAGCATTAAAATACAAAACTACAGTTTCAAATATTCAAAATACAAATAATATAGCAAATCCTAATTTAATATTTATTGGTC
AAAAATTAAAAGTGCCAATGACACCATTAGTAGAACCAAAACCAAAAACAGTGTCTTCAAATAATAAAAGTAATAGTAATAGCAGTACATT
AAATTATTTGAAAACATTAGAGAATAGAGGATGGGATTTCGACGGTAGTTATGGATGGCAATGTTTCGATTTAGTTAATGTATATTGGAAT
CATCTTTATGGTCATGGATTAAAAGGATATGGAGCTAAAGATATACCATATGCAAATAATTTTAATAGTGAAGCTAAAATTTATCACAACAC
ACCAACTTTCAAAGCTGAACCTGGGGACTTAGTGGTTTTTAGCGGAAGATTTGGTGGAGGATATGGTCATACAGCTATTGTCTTAAATGGT
GATTATGATGGAAAATTAATGAAGTTCCAAAGTTTAGATCAAAACTGGAATAATGGTGGATGGCGTAAAGCAGAGGTTGCACATAAAGTT
GTTCATAATTATGAAAATGATATGATTTTTATTAGACCATTTAAAAAAGCATAA

Gene: fmhC (endopeptidase resistance gene)

Position: 1216680 to 1217924, length: 1245 nt, orientation: FORWARD

Perfect match to: (N315-BA000018-[1234728:1235972], highly conserved allele)

Sequence:

ATGAAATTTTCAACTTTAAGTGAAGAAGAATTTACCAACTACACCAAAAAGCACTTCAAACATTATACACAGTCTATAGAATTATATAATTAT
AGAAATAAAATAAATCATGAAGCACATATTGTGGGAGTGAAGAATGATAAAAATGAAGTTATAGCTGCATGTTTATTAACAGAGGCACGA
ATTTTTAAATTCTACAAATATTTCTACTCTCATAGAGGTCCTTTACTTGATTATTTCGATGCTAAATTAGTTTGTTACTTTTTTAAAGAATTATC
TAAATTCATTTATAAAAATAGAGGAGTATTTATTCTTGTTGATCCATATTTAATAGAGAATTTAAGAGATGCAAATGGTAGGATAATAAAGA
ATTATAATAATTCAGTGATAGTAAAGATGCTAGGGAAAATTGGGTATCTCCATCAAGGTTATACAACAGGATATTCAAATAAAAGTCAAATT
AGGTGGATTTCTGTATTGGATTTAAAAGATAAAGATGAGAATCAACTTTTAAAAGAAATGGAATACCAAACTAGAAGAAATATAAAAAAGA
CTATTGAGATTGGTGTTAAGGTTGAAGATTTATCTATTGAAGAAACAAATCGATTTTATAAATTGTTTCAAATGGCTGAAGAAAAACATGGT
TTTCATTTCATGAATGAAGATTATTTTAAACGAATGCAAGAAATATATAAAGATAAGGCAATGTTAAAGATAGCTTGTATAAATCTTAATGA
ATATCAAGATAAATTAAAAATACAATTATTGAAAATCGAAAATGAAATGATGACTGTGAACAGAGCATTAAATGAAAATCCAAATTCTAAA
AAAAATAAATCAAAATTAAATCAGTTAAATATGCAATTATCTAGTATTAATAATAGAATTAGTAAAACCGAAGAACTAATATTTGAAGATGG
ACCTGTTTTGGATTTAGCTGCTGCTTTATTTATATGTACTGATGATGAAGTTTATTATCTATCAAGTGGATCAAATCCGAAATATAATCAGTA
TATGGGTGCATATCATCTACAATGGCATATGATAAAATATGCAAAATCACATAATATTAATAGGTATAATTTTTATGGAATAACAGGCGTCT
TTAGTAATGAGGCGGATGATTTTGGTGTTCAACAATTTAAAAAGGGTTTTAATGCACATGTTGAAGAATTAATTGGTGATTTCATCAAACCA
GTAAGACCAATTCTATATAAATTTGCAAAACTTATTTATAAGGTTTAA

Gene: dprA (DNA processing protein)

Position: 1218097 to 1218969, length: 873 nt, orientation: FORWARD

Perfect match to: (MW2-BA000033-[1236282:1237154], highly conserved allele)

Sequence:

TTGATTAAACTATTTTTGCTTAAGTTATACTGGGCACACTTTTCGACTAAACAAATTCATCAATTTTTAATGGCATATCCTAATGTAATTAAAG
AGGAGGGAAGAAAAAAAGATAGTTATTTATGTGAATGGGTGAATAGGGAAGAAAATGTTCATTTATTACGTAAATACTATGCTTTTATAAA
ACTTGATCATAACGATATTATTAAAGAACTGCAGAAATTAAAAGTAAGTTACATTACATATATGGATTCTGAATACCCAGTGCTATTAAAAG
AAATATATCAATTTCCATTACTTCTTTTCTATAAAGGGAACATCAAATTAATAAATAATATGCATCATTTGGCAGTAGTAGGTGCAAGAGATT
CTACAAGTTATACCCAACAGTCTTTAGAATTTTTATTATCAAATGATAAAAGCAAATATTTAACAATTGTTTCCGGCCTTGCTCAAGGAGCTG
ATGCAATGGCACATCAAATAGCTTTAAAATACAATCTCCCTACAATTGCAGTTTTAGCCTTTGGCCATCAAACACATTATCCCAAAAGTACAT
TAGCATTAAGAAATAAAATAGAAGAAAAAGGTTTAGTTATATCCGAATATCCACCACATACACCAATTGCTAAATATAGATTTCCTGAGCGC
AATAGAATTATCAGCGGTTTGTCAAAAGGGGTTTTAATTACTGAGGCTAAGGAACAAAGTGGCAGTCACATCACGATAGATTTTGCATTAG
AGCAAAATAGAAATGTTTATGTTTTACCTGGATCTATGTTTAATCCTATGACAAAAGGTAATTTATTACGTATCCAAGAAGGTGCTAAGGTA
GTATTAAACGCTAATGATATATTTGAAGACTACTATATTTAA

Gene: topA (DNA topoisomerase I (type IA))

Position: 1219149 to 1221218, length: 2070 nt, orientation: FORWARD

Perfect match to: (MW2-BA000033-[1237334:1239403], highly conserved allele)

Sequence:

TTGGCAGATAATTTAGTCATTGTTGAATCGCCTGCAAAAGCAAAAACCATTGAAAAGTATTTAGGTAAGAAATATAAAGTTATAGCTTCAAT
GGGACACGTCAGAGACTTACCAAGAAGTCAAATGGGTGTCGACACTGAAGATAATTACGAACCAAAATATATAACAATACGCGGAAAAGG
TCCTGTTGTAAAAGAATTGAAAAAACATGCAAAAAAAGCGAAAAACGTCTTTCTCGCAAGTGACCCCGACCGTGAAGGTGAAGCAATTGCT
TGGCATTTATCAAAAATTTTAGAGCTTGAAGATTCTAAAGAAAATCGCGTTGTTTTCAACGAAATAACTAAAGACGCTGTTAAAGAAAGTTT
TAAAAATCCTAGAGAAATTGAAATGAACTTAGTCGATGCACAACAAGCGCGTCGAATATTAGATAGATTGGTTGGCTATAACATCTCGCCA
GTTCTATGGAAAAAAGTAAAAAAAGGTTTGTCAGCGGGTCGAGTTCAATCTGTTGCACTTCGTTTAGTCATTGACCGTGAAAATGAAATTC
GAAACTTTAAACCAGAAGAATATTGGACTATTGAAGGAGAATTTAGATACAAAAAATCAAAATTCAATGCTAAATTCCTTCATTATAAAAAT
AAACCTTTTAAATTAAAAACGAAAAAAGATGTTGAGAAAATTACAGCTGCACTAGATGGAGATCAATTCGAAATTACAAACGTGACTAAAA
AAGAAAAAACGCGTAATCCAGCAAACCCATTTACAACTTCTACATTACAACAAGAGGCGGCACGTAAATTAAACTTTAAAGCAAGAAAAAC
AATGATGGTCGCACAACAATTATATGAAGGTATAGATTTGAAAAAACAAGGTACGATTGGTTTAATAACATATATGAGAACCGATTCTACA
CGTATTTCAGATACTGCCAAAGCTGAAGCAAAACAGTATATAACTGATAAATACGGTGAATCTTACACTTCTAAACGTAAAGCATCAGGGA
AACAAGGTGACCAAGATGCCCATGAGGCTATTAGACCTTCAAGTACTATGCGTACGCCAGATGATATGAAGTCATTTTTGACGAAAGACCA
ATACCGATTATACAAATTAATTTGGGAACGATTTGTTGCTAGTCAAATGGCTCCAGCAATACTTGATACAGTCTCATTAGACATAACACAAG

GTGACATTAAATTTAGAGCGAATGGTCAAACAATCAAGTTTAAAGGATTTATGACACTTTATGTAGAAACTAAAGATGATAGTGATAGCGA
AAAGGAAAATAAACTGCCTAAATTAGAGCAAGGTGATAAAGTCACAGCAACTCAAATTGAACCAGCTCAACACTATACACAACCACCTCCA
AGATATACTGAGGCGAGATTAGTAAAAACACTAGAAGAATTGAAAATTGGGCGACCATCAACTTATGCACCGACAATAGATACGATTCAAA
AGCGTAACTATGTCAAATTAGAAAGTAAGCGTTTTGTTCCTACTGAGTTGGGAGAAATAGTTCATGAACAAGTGAAAGAATACTTCCCAGA
GATTATTGATGTGGAATTCACAGTGAATATGGAAACGTTACTTGATAAGATTGCAGAAGGCGACATTACATGGAGAAAAGTAATCGACGG
TTTCTTTAGTAGCTTTAAACAAGATGTTGAACGTGCTGAAGAAGAGATGGAAAAGATTGAAATCAAAGATGAGCCAGCCGGTGAAGACTG
TGAAGTTTGTGGTTCTCCTATGGTTATAAAAATGGGACGCTATGGTAAGTTCATGGCTTGCTCAAACTTCCCGGATTGTCGTAATACAAAAG
CGATAGTTAAGTCTATTGGTGTTAAATGTCCAAAATGTAATGATGGTGACGTCGTAGAAAGAAAATCTAAAAAGAATCGTGTCTTTTATGG
ATGTTCGAAATATCCTGAATGCGACTTTATCTCTTGGGATAAGCCGATTGGAAGAGATTGTCCAAAATGTAACCAATATCTTGTTGAAAATA
AAAAAGGCAAGACAACACAAGTAATATGTTCAAATTGCGATTATAAAGAGGCAGCGCAGAAATAA

Gene: gid-trmFO (methylenetetrahydrofolate--tRNA- (uracil-5-)-methyltransferase)

Position: 1221374 to 1222681, length: 1308 nt, orientation: FORWARD

Perfect match to: (MW2-BA000033-[1239559:1240866], highly conserved allele)

Sequence:

ATGACTCAAACTGTAAATGTAATAGGTGCTGGTCTTGCCGGTTCAGAAGCGGCATATCAATTAGCTGAAAGAGGAATTAAAGTTAATCTAA
TAGAGATGAGACCTGTTAAACAAACACCAGCGCACCATACTGATAAATTTGCGGAACTTGTATGTTCCAATTCATTACGAGGAAATGCTTTA
ACTAATGGTGTGGGTGTTTTAAAAGAAGAAATGAGAAGATTGAATTCTATAATTATTGAAGCGGCTGATAAGGCACGAGTTCCAGCTGGT
GGTGCATTAGCAGTTGATAGACACGATTTTTCAGGTTATATTACTGAAACACTTAAAAATCATGAAAATATCACAGTTATTAATGAAGAAAT
TAATGCCATTCCAGATGGATACACAATTATCGCAACAGGACCACTTACTACAGAAACCCTTGCGCAAGAAATAGTGGACATTACTGGTAAA
GATCAACTTTATTTCTATGATGCGGCTGCTCCAATTATTGAAAAAGAATCTATTGATATGGATAAAGTTTACTTAAAGTCCCGTTATGATAAA
GGTGAAGCTGCATATTTAAACTGTCCTATGACTGAGGATGAATTTAATCGCTTTTATGATGCAGTATTAGAAGCTGAAGTTGCGCCTGTAAA
TTCATTTGAAAAAGAAAAATATTTCGAGGGTTGTATGCCTTTTGAAGTAATGGCAGAACGCGGACGCAAGACATTACTATTTGGACCAATG
AAACCAGTAGGATTAGAAGATCCAAAGACTGGGAAACGTCCTTATGCAGTGGTTCAATTAAGACAAGATGATGCTGCTGGCACACTCTACA
ATATTGTCGGCTTCCAAACGCATTTAAAATGGGGAGCTCAAAAAGAAGTTATTAAATTAATTCCAGGTTTAGAAAATGTTGATATTGTTAGA
TATGGTGTGATGCATAGAAATACCTTCATTAACTCACCGGACGTATTAAACGAGAAATATGAATTGATTTCACAACCAAACATACAGTTTGC
GGGACAAATGACTGGTGTTGAAGGTTATGTAGAAAGCGCAGCTAGCGGCTTAGTTGCAGGTATCAATCTTGCGCATAAAATATTAGGCAA
AGGTGAGGTAGTATTTCCTAGAGAAACGATGATTGGAAGTATGGCTTACTATATTTCTCATGCTAAAAATAATAAGAATTTCCAACCTATGA
ATGCTAACTTCGGGTTATTACCATCTTTAGAAACTAGAATTAAAGATAAAAAAGAACGCTATGAAGCACAAGCTAATAGAGCTTTGGATTAC
TTAGAAAATTTCAAAAAAACTTTATAA

Gene: xerC (site-specific recombinase C)

Position: 1223098 to 1223994, length: 897 nt, orientation: FORWARD

Perfect match to: (N315-BA000018-[1241147:1242043], allele observed in CC5+CC1+CC15)

Sequence:

TTGAATCATATTCAAGAAGCGTTTTTAAATACATTGAAAGTTGAACGGAATTTTTCGGAACATACATTGAAATCATATCAAGATGACTTAATT
CAGTTTAATCAATTTTTAGAACAAGAACATTTACAGTTGAAAACTTTTGAATACAGAGATGCTAGAAATTATTTGAGCTATTTATATTCAAAT
CATTTGAAAAGAACATCTGTTTCTCGTAAAATCTCAACGTTAAGAACTTTCTATGAATATTGGATGACGCTTGATGAGAACATTATTAATCCA
TTTGTTCAATTAGTACATCCGAAAAAAGAAAAATATCTTCCGCAATTCTTTTACGAAGAAGAAATGGAAGCGTTATTTAAAACTGTAGAAGA
GGACACTTCAAAAAGTTTACGGGATCGAGTTATTCTTGAATTGTTATATGCTACAGGTATCCGTGTTTCGGAATTAGTTAATATTAAAAAAC
AAGATATAGATTTTTACGCGAATGGTGTTACCGTATTAGGAAAAGGGAGCAAAGAGCGCTTTGTACCGTTTGGTGCTTATTGTAGACAAAG
CATCGAAAATTATTTAGAACATTTCAAACCAATTCAGTCATGCAATCATGATTTTCTTATTGTAAATATGAAGGGTGAAGCAATCACTGAAC
GCGGTGTACGATATGTTTTAAATGATATTGTTAAACGAACAGCAGGCGTAAGTGAGATTCATCCCCACAAGCTCAGACATACATTTGCAAC
GCATTTATTGAATCAAGGTGCAGACCTAAGAACAGTACAATCGTTATTAGGTCATGTTAATTTGTCAACAACTGGTAAATATACACACGTAT
CTAACCAACAATTAAGAAAAGTGTATCTAAATGCACATCCTCGAGCGAAAAAGGAGAATGAAACATGA

Gene: hslV (heat shock protein, ATP-dependent protease)

Position: 1223991 to 1224536, length: 546 nt, orientation: FORWARD

Perfect match to: (11819-97-CP003194-[1274368:1274913], allele observed in CC80+CC5+CC188)

Sequence:

ATGAGTAATACAACATTACATGCAACAACAATTTATGCTGTAAGACATAATGGGAAAGCAGCTATGGCTGGAGATGGACAAGTAACGCTT
GGTCAACAAGTCATCATGAAACAAACGGCAAGAAAAGTGCGACGTTTATATGAAGGTAAAGTGTTAGCTGGTTTCGCAGGTAGTGTAGCA
GATGCGTTTACGTTATTTGAAAAATTTGAAACAAAATTACAACAGTTTAGTGGTAACTTAGAAAGAGCTGCTGTTGAATTGGCACAAGAAT
GGCGAGGCGATAAACAATTACGTCAATTAGAAGCTATGCTAATTGTAATGGATAAAGATGCTATTTTAGTTGTCAGTGGAACTGGCGAAGT
TATTGCCCCAGATGATGACCTTATCGCTATTGGATCAGGAGGCAACTACGCATTAAGCGCAGGACGTGCATTGAAACGCCATGCATCGCAT
TTGTCTGCTGAAGAAATGGCATATGAGAGCTTGAAAGTAGCGGCTGATATTTGTGTCTTTACCAACGATAATATTGTTGTTGAAACACTATA
A

Gene: hslU (heat shock protein, ATPase subunit)

Position: 1224602 to 1226005, length: 1404 nt, orientation: FORWARD

Perfect match to: (Strain_21340-AGTX01000048-[153458:154861:r], allele observed in CC188+CC80)

Sequence:

ATGGATACAGCTGGAATAAGATTAACTCCAAAAGAAATCGTATCTAAATTAAATGAATATATCGTTGGACAAAATGATGCTAAACGTAAAG
TGGCAATTGCCCTACGTAATCGATACAGAAGAAGTTTATTAGATGAGGAATCAAAGCAAGAAATTTCACCTAAAAATATTTTGATGATTGG
ACCAACCGGCGTTGGTAAAACTGAAATTGCAAGAAGAATGGCCAAAGTTGTTGGCGCGCCATTTATAAAAGTAGAAGCTACTAAATTTACT
GAGGTAGGTTATGTAGGACGAGATGTTGAAAGTATGGTTAGAGATCTTGTTGATGTTTCAGTAAGATTAGTCAAGGCGCAGAAAAAATCA
TTGGTACAAGATGAAGCAACAGCTAAGGCCAATGAAAAACTTGTTAAGTTATTAGTTCCAAGTATGAAAAAGAAAGCGTCTCAAACGAATA
ATCCTTTAGAGTCACTTTTCGGAGGTGCAATTCCAAATTTCGGACAAAATAACGAAGATGAAGAAGAACCACCTACTGAGGAAATTAAAAC
AAAACGTTCTGAAATTAAGAGACAGCTAGAAGAAGGCAAACTTGAAAAAGAAAAGGTAAGAATTAAAGTCGAACAAGATCCTGGTGCTTT
AGGTATGCTAGGTACAAATCAAAATCAGCAAATGCAAGAGATGATGAATCAATTAATGCCTAAAAAGAAAGTTGAGCGAGAAGTTGCTGT
TGAGACGGCAAGGAAAATCTTAGCTGATAGTTATGCGGATGAACTAATTGATCAAGAAAGCGCTAACCAAGAAGCGCTTGAATTAGCAGA
ACAAATGGGTATCATCTTTATAGATGAAATCGACAAAGTTGCGACGAATAATCATAATAGTGGTCAAGATGTCTCAAGACAAGGTGTTCAA
AGAGATATTTTACCTATACTTGAAGGTAGCGTTATTCAAACCAAATATGGTACTGTGAATACTGAACATATGCTGTTTATAGGTGCTGGAGC
TTTCCATGTATCTAAGCCGAGTGACTTGATACCAGAATTGCAAGGTCGTTTTCCGATTAGAGTTGAACTTGATAGTTTATCGGTAGAAGATT
TTGTAAGAATTTTGACAGAACCAAAATTGTCATTAATTAAACAATATGAAGCATTGCTTCAAACAGAAGAAGTTACTGTAAACTTTACCGAT
GAAGCAATTACTCGCTTAGCTGAGATTGCTTATCAAGTAAATCAAGATACAGACAACATTGGTGCACGTCGACTTCATACAATTTTAGAAAA
GATGCTAGAAGATTTATCATTCGAAGCACCAAGTATGCCGAATGCAGTTGTAGATATTACCCCACAATATGTTGATGATAAATTAAAATCAA
TTTCAACAAATAAAGATTTAAGTGCATTTATTCTATAA

Gene: codY (GTP-sensing transcriptional pleiotropic repressor)

Position: 1226030 to 1226803, length: 774 nt, orientation: FORWARD

Perfect match to: (RF122-AJ938182-[1209102:1209875], highly conserved allele)

Sequence:

ATGAGCTTATTATCTAAAACGAGAGAGTTAAACACGTTACTTCAAAAACACAAAGGTATTGCGGTTGATTTTAAAGATGTAGCACAAACGA
TTAGTAGCGTAACTGTAACAAATGTATTTATTGTATCGCGTCGAGGTAAAATTTTAGGGTCGAGTCTAAATGAATTATTAAAAAGTCAAAGA
ATTATTCAAATGTTGGAAGAAAGACATATTCCAAGTGAATATACAGAACGATTAATGGAAGTTAAACAAACAGAATCAAATATTGATATCG
ACAATGTATTAACAGTATTCCCACCTGAAAACAGAGAATTATTCATAGATAGTCGTACAACTATCTTCCCAATTTTAGGTGGAGGAGAAAGA
TTAGGTACATTAGTACTTGGTCGAGTACATGATGATTTTAATGAAAATGATTTGGTACTAGGTGAATATGCTGCTACAGTTATTGGTATGGA
AATCTTACGTGAGAAGCATAGTGAAGTAGAAAAAGAAGCGCGCGATAAAGCTGCTATTACAATGGCAATTAATTCATTATCTTATTCTGAA
AAAGAAGCGATTGAACATATCTTTGAAGAACTTGGCGGTACGGAAGGCCTATTAATCGCATCAAAAGTTGCAGATAGAGTTGGTATTACTA
GATCTGTAATTGTAAATGCACTACGTAAATTAGAAAGTGCTGGTGTAATTGAATCACGTTCTTTAGGAATGAAAGGTACTTTCATTAAAGTT
AAAAAAGAAAAATTCTTAGATGAATTAGAAAAAAGTAAATAA

Gene: rpsB (30S ribosomal protein S2)

Position: 1227145 to 1227912, length: 768 nt, orientation: FORWARD

Perfect match to: (N315-BA000018-[1245199:1245966], highly conserved allele)

Sequence:

ATGGCAGTAATTTCAATGAAACAATTACTAGAAGCGGGTGTTCACTTCGGTCACCAAACACGTCGTTGGAACCCAAAAATGAAAAAATATA
TCTTCACTGAGAGAAATGGTATTTATATCATCGACTTACAAAAAACAGTGAAAAAAGTAGACGAGGCATACAACTTCTTGAAACAAGTTTCA
GAAGATGGTGGACAAGTCTTATTCGTAGGAACTAAAAAACAAGCACAAGAATCAGTTAAATCTGAAGCAGAACGTGCTGGTCAATTCTACA
TTAACCAAAGATGGTTAGGTGGATTATTAACTAACTATAAAACGATCTCAAAACGAATCAAACGTATTTCTGAAATTGAAAAAATGGAAGA
AGATGGTTTATTCGAAGTATTACCTAAAAAAGAAGTAGTAGAACTTAAAAAAGAATACGACCGTTTAATCAAATTCTTAGGCGGAATTCGT
GATATGAAATCAATGCCTCAAGCATTATTCGTAGTTGACCCACGTAAAGAGCGTAATGCAATTGCTGAAGCTCGTAAATTAAATATTCCTAT
CGTAGGTATCGTTGACACTAACTGTGATCCTGACGAAATTGACTACGTTATCCCAGCAAACGACGATGCTATCCGTGCGGTTAAATTATTAA
CTGCTAAAATGGCAGATGCAATCTTAGAAGGTCAACAAGGCGTTTCTAATGAAGAAGTAGCTGCAGAACAAAACATCGATTTAGATGAAA
AAGAAAAATCAGAAGAAACAGAAGCAACTGAAGAATAA

Gene: tsf (translation elongation factor Ts)

Position: 1228094 to 1228975, length: 882 nt, orientation: FORWARD

Perfect match to: (COL-CP000046-[1285544:1286425], highly conserved allele)

Sequence:

ATGGCAACTATTTCAGCAAAACTTGTTAAAGAATTACGTGAAAAAACTGGCGCGGGTATGATGGATTGTAAAAAAGCGCTAACTGAAACTG
ATGGTGACATCGATAAAGCGATTGACTACCTACGTGAAAAAGGTATTGCTAAAGCAGCTAAAAAAGCAGACCGTATTGCGGCTGAAGGTT
TAGTACATGTAGAAACTAAAGGTAACGACGCAGTTATCGTTGAAATCAACTCTGAAACAGACTTTGTTGCTCGTAACGAAGGTTTCCAAGA
GTTAGTTAAAGAAATCGCTAATCAAGTATTAGATACAAAAGCTGAAACTGTTGAAGCTTTAATGGAAACAACTTTACCAAATGGTAAATCA
GTTGATGAAAGAATTAAAGAAGCAATTTCAACAATCGGTGAAAAATTAAGTGTTCGTCGTTTTGCTATCAGAACTAAAACTGATAACGATG
CTTTCGGCGCTTACTTACACATGGGTGGACGCATTGGTGTATTAACAGTTGTTGAAGGTTCAACTGACGAAGAAGCAGCAAGAGACGTTGC
TATGCATATCGCTGCAATCAACCCTAAATATGTTTCTTCTGAACAAGTTAGCGAAGAAGAAATCAACCACGAAAGAGAAGTTTTAAAACAAC
AAGCATTAAATGAAGGTAAACCAGAAAACATCGTTGAAAAAATGGTGGAAGGACGTTTACGTAAATACTTACAAGAAATTTGTGCTGTAG
ATCAAGACTTCGTTAAAAACCCTGATGTAACAGTTGAAGCTTTCTTAAAAACAAAAGGTGGAAAACTTGTTGACTTCGTACGCTATGAAGTA
GGCGAAGGTATGGAAAAACGCGAAGAAAACTTTGCGGATGAAGTTAAAGGACAAATGAAATAA

Gene: pyrH (uridylate kinase)

Position: 1229112 to 1229834, length: 723 nt, orientation: FORWARD

Perfect match to: (TW20-FN433596-[1335110:1335832], highly conserved allele)

Sequence:

ATGGCTCAAATTTCTAAATATAAACGTGTAGTTTTGAAACTAAGTGGTGAAGCGTTAGCTGGAGAAAAAGGATTTGGCATAAATCCAGTAA
TTATTAAAAGTGTTGCTGAGCAAGTGGCTGAAGTTGCTAAAATGGACTGTGAAATCGCAGTAATCGTTGGTGGCGGAAACATTTGGAGAG
GTAAAACAGGTAGTGACTTAGGTATGGACCGTGGAACTGCTGATTACATGGGTATGCTTGCAACTGTAATGAATGCCTTAGCATTACAAGA
TAGTTTAGAACAATTGGATTGTGATACACGAGTATTAACATCTATTGAAATGAAGCAAGTGGCTGAACCTTATATTCGTCGTCGTGCAATTA
GACACTTAGAAAAGAAACGCGTAGTTATTTTTGCTGCAGGTATTGGAAACCCATACTTCTCTACAGATACTACAGCGGCATTACGTGCTGCA
GAAGTTGAAGCAGATGTTATTTTAATGGGCAAAAATAATGTAGATGGTGTATATTCTGCAGATCCTAAAGTAAACAAAGATGCGGTAAAAT
ATGAACATTTAACGCATATTCAAATGCTTCAAGAAGGTTTACAAGTAATGGATTCAACAGCATCCTCATTCTGTATGGATAATAACATTCCGT
TAACTGTTTTCTCTATTATGGAAGAAGGGAATATTAAACGTGCTGTTATGGGTGAAAAGATAGGTACGTTAATTACAAAATAA

Gene: frr (ribosome recycling factor)

Position: 1229853 to 1230407, length: 555 nt, orientation: FORWARD

Perfect match to: (N315-BA000018-[1247907:1248461], highly conserved allele)

Sequence:

ATGAGTGACATTATTAATGAAACTAAATCAAGAATGCAAAAATCAATCGAAAGCTTATCACGTGAATTAGCTAATATCAGTGCAGGAAGAG
CTAATTCAAATTTATTAAACGGCGTAACAGTTGATTACTATGGTGCACCAACACCTGTACAACAATTAGCAAGCATCAATGTTCCAGAAGCA
CGTTTACTTGTTATTTCTCCATACGACAAAACTTCTGTAGCTGACATCGAAAAAGCGATAATAGCGGCTAACTTAGGTGTCAACCCCAACAAG
TGATGGTGAAGTGATACGTATTGCAGTACCTGCCTTAACAGAAGAGCGTAGAAAAGAGCGCGTTAAAGATGTTAAGAAAATTGGTGAAGA
AGCTAAAGTATCTGTTCGAAATATTCGTCGTGATATGAATGATCAGTTGAAAAAAGATGAAAAAAATGGCGACATTACTGAAGATGAGTTG

AGAAGTGGTACTGAAGATGTTCAGAAAGCAACAGACAATTCAATAAAAGAAATTGATCAAATGATTGCTGATAAAGAAAAAGATATTATG
TCAGTATAA

Gene: uppS (undecaprenyl pyrophosphate synthase)

Position: 1230780 to 1231550, length: 771 nt, orientation: FORWARD

Perfect match to: (MW2-BA000033-[1248965:1249735], highly conserved allele)

Sequence:

ATGTTTAAAAAGCTAATAAATAAAAAGAACACTATAAATAATTATAATGAAGAATTAGACTCGTCTAATATACCTGAACATATCGCTATTATT
ATGGATGGTAATGGGCGATGGGCTAAGAAGCGAAAAATGCCTAGAATTAAAGGTCATTACGAAGGTATGCAAACAATAAAAAAAATTACT
AGGGTAGCTAGTGATATTGGTGTTAAGTACTTAACTTTATACGCCTTTTCCACTGAAAATTGGTCAAGACCTGAAAGTGAAGTAAATTATAT
TATGAATTTGCCTGTCAATTTCTTAAAGACATTCTTACCGGAACTAATTGAAAAAAATGTCAAAGTTGAAACAATTGGATTTACTGATAAGTT
GCCAAAATCAACGATAGAAGCAATTAATAATGCTAAAGAAAAGACAGCTAATAATACCGGCTTAAAATTAATATTTGCAATTAATTATGGT
GGCAGAGCAGAACTTGTTCATAGTATTAAAAATATGTTTGACGAGCTTCATCAACAAGGTTTAAATAGTGATATCATAGATGAAACATATAT
AAACAATCATTTAATGACAAAAGACTATCCTGATCCAGAGTTGTTAATTCGTACTTCAGGAGAACAAAGAATAAGTAATTTCTTGATTTGGC
AAGTTTCGTATAGTGAATTTATCTTTAATCAAAAATTATGGCCTGACTTTGACGAAGATGAATTAATTAAATGTATAAAAATTTATCAGTCAC
GTCAAAGACGCTTTGGCGGATTGAGTGAGGAGTAG

Gene: cdsA (phosphatidate cytidylyltransferase)

Position: 1231557 to 1232339, length: 783 nt, orientation: FORWARD

Perfect match to: (MW2-BA000033-[1249742:1250524], highly conserved allele)

Sequence:

ATGAAAGTTAGAACGCTGACAGCTATTATTGCCTTAATCGTATTCTTGCCTATCTTGTTAAAAGGCGGCCTTGTGTTAATGATATTTGCTAAT
ATATTAGCATTGATTGCATTAAAAGAATTGTTGAATATGAATATGATTAAATTTGTTTCAGTTCCTGGTTTAATTAGTGCAGTTGGTCTTATC
ATCATTATGTTGCCACAACATGCAGGGCCATGGGTACAAGTAATTCAATTAAAAAGTTTAATTGCAATGAGCTTTATTGTATTAAGTTATACT
GTCTTATCTAAAAACAGATTTAGTTTTATGGATGCTGCATTTTGCTTAATGTCTGTGGCTTATGTAGGCATTGGTTTTATGTTCTTTTATGAAA
CGAGATCAGAAGGATTACATTACATATTATATGCCTTTTTAATTGTTTGGCTTACAGATACAGGGGCTTACTTGTTTGGTAAAATGATGGGT
AAACATAAGCTTTGGCCAGTAATAAGTCCGAATAAAACAATCGAAGGATTCATAGGTGGCTTGTTCTGTAGTTTGATAGTACCACTTGCAAT
GTTATATTTTGTAGATTTCAATATGAATGTATGGATATTACTTGGAGTGACATTGATTTTAAGTTTATTTGGTCAATTAGGTGATTTAGTGGA
ATCAGGATTTAAGCGTCATTTCGGCGTTAAAGACTCAGGTCGAATACTACCTGGACACGGTGGTATTTTAGACCGATTTGACAGCTTTATGT
TTGTGTTACCATTATTAAATATTTTATTAATACAATCTTAA

Gene: rseP (membrane-associated zinc metalloprotease)

Position: 1232551 to 1233837, length: 1287 nt, orientation: FORWARD

Perfect match to: (11819-97-CP003194-[1282928:1284214], allele observed in CC80+CC1+CC15+CC80)

Sequence:

GTGAGCTATTTAGTTACAATAATTGCATTTATTATTGTTTTTGGTGTACTAGTAACTGTTCATGAATATGGCCATATGTTTTTTGCGAAAAGA
GCAGGCATTATGTGTCCAGAATTTGCGATCGGTATGGGACCGAAAATTTTTAGTTTTAGAAAAAACGAAACACTTTACACTATTAGGTTATT
GCCTGTTGGTGGATATGTTCGTATGGCAGGAGATGGCTTAGAAGAGCCACCAGTCGAGCCCGGTATGAACGTTAAAATTAAACTGAATGA
AGAAAATGAAATAACACATATCATATTAGATGATCATCATAAGTTTCAACAAATTGAAGCAATCGAAGTTAAAAAATGTGATTTTAAAGATG
ACTTATTCATAGAAGGTATCACTGCTTATGATAACGAAAGACATCATTTTAAAATTGCTAGAAAGTCTTTCTTTGTTGAAAATGGTAGCTTAG
TTCAAATTGCTCCGAGAGACAGACAATTTGCACATAAAAAGCCATGGCCGAAATTTTTAACATTATTTGCGGGACCGTTATTTAACTTTATAT
TAGCTTTAGTCCTATTTATTGGTCTTGCATATTATCAAGGCACGCCTACGTCTACTGTAGAACAAGTCGCAGATAAGTATCCAGCTCAACAAG
CAGGATTACAAAAAGGTGATAAGATCGTCCAAATTGGCAAATATAAAATATCTGAATTTGATGATGTTGATAAGGCGTTAGATAAAGTTAA
AGATAATAAGACGACTGTTAAATTTGAACGTGATGGTAAAACAAAGTCAGTTGAATTAACACCTAAAAAGACTGAAAAAAAACTGACTAAA
GTAAGTTCAGAGACGAAGTATGTTCTCGGATTCCAACCAGCGAGTGAACATACACTTTTTAAACCAATTGTATTCGGATTTAAAAGCTTTTT
AATCGGTAGTACTTATATTTTTACAGCTGTAGTCGGTATGTTGGCTAGTATATTTACGGGCGGATTCTCATTTGATATGTTAAATGGTCCGGT
TGGTATTTATCATAACGTCGACTCAGTTGTTAAAGCAGGTATCATTAGCTTAATTGGTTACACTGCGTTGTTAAGTGTAAACTTAGGTATTAT

GAATTTAATTCCTATTCCTGCACTAGACGGTGGTCGTATTTTATTTGTTATATATGAAGCGATTTTCAGAAAACCAGTTAATAAAAAAGCGGA
AACAACGATTATTGCTATTGGTGCCATTTTCATGGTCGTTATTATGATATTAGTAACGTGGAATGATATTCGACGATATTTCTTATAA

Gene: proS (prolyl-tRNA synthase)

Position: 1233857 to 1235560, length: 1704 nt, orientation: FORWARD

Perfect match to: (Strain_6850-CP006706-[1229600:1231303], allele observed in CC50+CC1+CC59+CC97)

Sequence:

ATGAAGCAATCCAAAGTTTTTATACCAACGATGCGTGATGTGCCATCAGAAGCAGAAGCACAAAGTCATCGTTTATTATTGAAATCGGGTTT
GATAAAACAAAGTACAAGTGGGATTTATAGTTATTTACCGCTAGCAACACGTGTGTTAAATAATATTACTGCAATTGTGCGACAAGAAATG
GAACGTATCGATTCTGTTGAAATTTTAATGCCAGCGTTACAACAAGCTGAATTATGGGAAGAATCAGGACGTTGGGGTGCATATGGCCCAG
AATTAATGCGTTTACAAGATAGACATGGAAGACGATTTGCATTAGGTCCAACACATGAAGAATTAGTTACATCAATAGTAAGAAATGAATT
GAAATCATACAAACAATTACCGATGACATTATTCCAAATTCAATCTAAATTCCGTGATGAAAAGAGACCACGTTTTGGTTTACTTCGTGGGC
GTGAATTTATTATGAAAGATGCGTATTCATTCCATGCTGACGAGGCATCATTAGATCAAACGTATCAAGATATGTATCAAGCGTATAGCCGT
ATTTTTGAGAGAGTGGGCATTAACGCAAGACCAGTAGTTGCAGATTCAGGTGCTATAGGCGGTAGCCATACACATGAATTTATGGCATTAA
GTGCTATCGGTGAGGATACAATCGTTTACAGTAAAGAAAGTGACTATGCTGCTAATATCGAAAAAGCAGAAGTCGTTTACGAACCAAATCA
TAAGCATACTACTGTGCAACCTTTAGAAAAAATTGAAACACCAAATGTTAAGACTGCGCAAGAATTGGCAGACTTCTTAGGTAGACCAGTA
GATGAAATCGTTAAAACGATGATTTTCAAAGTTGATGGCGAATATATTATGGTTTTAGTGCGTGGCCATCATGAAATTAATGACATTAAATT
AAAATCTTATTTCGGCACAGATAATATTGAATTAGCAACACAAGACGAAATTGTTAATTTAGTTGGTGCAAATCCGGGTTCACTAGGTCCTG
TTATTGATAAAGAAATCAAAATTTATGCAGATAATTTTGTGCAAGATTTAAATAATTTAGTTGTCGGTGCTAACGAAGATGGCTATCACTTA
ATTAATGTAAATGTAGGTAGAGACTTCAACGTTGATGAATATGGCGATTTCCGTTTTATTTTAGAAGGCGAAAAGTTAAGTGATGGTTCAG
GCGTTGCACATTTTGCTGAAGGTATTGAAGTTGGTCAAGTATTCAAATTGGGTACTAAGTATTCAGAATCAATGAATGCTACATTCTTAGAT
AACCAAGGAAAAGCTCAACCTTTAATTATGGGTTGTTACGGAATTGGAATTTCTAGAACGCTAAGTGCGATTGTTGAACAAAATCACGATG
ATAATGGAATTGTTTGGCCTAAATCAGTTACTCCGTTTGATTTACATTTAATTTCTATTAATCCTAAGAAAGATGATCAACGAGAACTAGCAG
ATGCACTATATGCTGAATTTAATACTAAATTTGATGTGTTGTACGATGATCGTCAGGAACGTGCAGGTGTCAAATTTAATGATGCCGATTTA
ATTGGTTTACCACTGCGAATTGTTGTTGGTAAACGTGCATCGGAAGGTATTGTAGAAGTTAAAGAACGTTTAACAGGTGATAGCGAAGAAG
TTCACATTGATGACTTAATGACTGTCATTACAAATAAATATGATAACTTAAAATAA

Gene: polC (DNA-directed DNA polymerase III alpha chain)

Position: 1235824 to 1240134, length: 4311 nt, orientation: FORWARD

Perfect match to: (11819-97-CP003194-[1286201:1290511], allele observed in CC15+CC80)

Sequence:

ATGACAGAGCAACAAAAATTTAAAGTGCTTGCTGATCAAATTAAAATTTCAAATCAATTAGATGCTAAAATTTTAAATTCAGGTGAACTGAC
ACGTATAGATGTTTCTAACAAAAACAGAACATGGGAATTTCATATTACATTACCACAATTCTTAGCTCATGAAGATTATTTATTATTTATAAA
TGCAATAGAGCAAGAGTTTAAAGATATCGCCAACGTTACATGTCGTTTTACGGTAACAAATGGCACGAATCAAGATGAACATGCAATTAAA
TACTTTGGGCACTGTATTGACCAAACAGCTTTATCTCCAAAAGTTAAAGGTCAATTGAAACAGAAAAAGCTTATTATGTCTGGAAAAGTATT
AAAAGTAATGGTATCAAATGACATTGAACGTAATCATTTTGATAAGGCATGCAATGGAAGTCTTATCAAAGCGTTTAGAAATTGTGGTTTTG
ATATCGATAAAATCATCTTCGAAACAAATGATAATGATCAAGAACAAAACTTAGCTTCTTTAGAAGCACATATTCAAGAAGAAGACGAACA
AAGTGCACGATTGGCAACAGAGAAACTTGAAAAAATGAAAGCTGAAAAAGCGAAACAACAAGATAACAACGAAAGTGCTGTCGATAAGT
GTCAAATTGGTAAGCCGATTCAAATTGAAAATATTAAACCAATTGAATCTATTATTGAGGAAGAGTTTAAAGTTGCAATAGAGGGTGTCATT
TTTGATATAAACTTAAAAGAACTTAAAAGTGGTCGTCATATCGTAGAAATTAAAGTGACAGACTATACTGACTCATTAGTTTTAAAAATGTTT
ACTCGTAAAAACAAAGATGATTTAGAACATTTTAAAGCGCTAAGTGTTGGTAAATGGGTTAGGGCTCAAGGTCGTATTGAAGAAGATACAT
TTATTAGAGATTTAGTTATGATGATGTCTGATATTGAAGAGATTAAAAAAGCGACGAAAAAAGATAAGGCTGAAGAAAAGCGTGTAGAAT
TCCACTTGCATACTGCAATGAGCCAAATGGATGGTATACCCAATATTGGTGCGTATGTTAAACAGGCAGCAGACTGGGGACATCCAGCCAT
TGCAGTTACAGACCATAATGTTGTGCAAGCATTTCCAGATGCTCACGCAGCAGCGGAAAAACATGGCATTAAAATGATATACGGTATGGAA
GGTATGTTAGTTGATGATGGTGTTCCGATTGCATACAAACCACAAGATGTCGTATTAAAAGATGCTACTTATGTTGTGTTCGACGTTGAGAC
AACTGGTTTATCAAATCAGTATGATAAAATCATCGAGCTTGCAGCTGTGAAAGTTCATAACGGTGAAATCATCGATAAGTTTGAAAGGTTTA
GTAATCCGCATGAACGATTATCGGAAACGATTATCAATTTGACGCATATTACTGATGATATGTTAGTAGATGCCCCTGAGATTGAAGAAGTA
CTTACAGAGTTTAAAGAATGGGTTGGCGATGCGATATTCGTAGCGCATAATGCTTCGTTTGATATGGGCTTTATCGATACGGGATATGAAC
GTCTTGGATTTGGACCATCAACGAATGGTGTTATCGATACTTTAGAATTATCTCGTACGATTAATACTGAATATGGTAAACATGGTTTGAATT
TCTTAGCTAAAAAATATGGCGTAGAATTAACGCAACATCACCGTGCCATTTATGATACAGAAGCAACAGCTTACATTTTCATAAAAAATGGTT
CAACAAATGAAAGAATTAGGCGTATTAAATCATAACGAAATCAACAAAAAACTCAGTAATGAAGATGCATATAAACGTGCAAGACCTAGTC
ATGTCACATTAATTGTACAAAACCAACAAGGTCTTAAAAATCTATTTAAAATTGTAAGTGCATCATTGGTGAAGTATTTCTACCGTACACCTC

GAATTCCACGTTCATTGTTAGATGAATATCGTGAGGGATTATTGGTAGGTACAGCGTGTGATGAAGGTGAATTATTTACGGCAGTTATGCA
GAAGGACCAGAGCCAAGTTGAAAAAATTGCCAAATATTATGATTTTATTGAAATTCAACCACCGGCACTTTATCAAGATTTAATTGATAGAG
AGCTTATTAGAGATACTGAAACATTACATGAAATTTATCAACGTTTAATACATGCAGGTGACACAGCGGGTATACCTGTTATTGCGACAGGA
AATGCACACTATTTGTTTGAACATGATGGTATCGCACGTAAAATTTTAATAGCATCACAACCCGGCAATCCACTTAATCGCTCAACTTTACCG
GAAGCACATTTTAGAACTACAGATGAAATGTTAAACGAGTTTCATTTTTTAGGTGAAGAAAAAGCGCATGAAATTGTTGTGAAAAATACAA
ACGAATTAGCAGATCGAATTGAACGTGTTGTTCCTATTAAAGATGAATTATACACACCGCGTATGGAAGGTGCTAACGAAGAAATTAGAGA
ACTAAGTTATGCAAATGCGCGTAAACTGTATGGTGAAGACCTGCCTCAAATCGTAATTGATCGATTAGAAAAAGAATTAAAAAGTATTATC
GGTAATGGATTTGCGGTAATTTACTTAATTTCGCAACGTTTAGTTAAAAAATCATTAGATGATGGATACTTAGTTGGTTCCCGTGGTTCAGT
AGGTTCTAGTTTTGTAGCGACAATGACTGAGATTACTGAAGTAAACCCGTTACCGCCACACTATATTTGTCCGAACTGTAAAACGAGTGAAT
TTTTCAATGATGGTTCAGTAGGATCAGGATTTGATTTACCTGATAAGACGTGTGAAACTTGTGGAGCGCCACTTATTAAAGAAGGACAAGA
TATTCCGTTTGAAACATTTTTAGGATTTAAGGGAGATAAAGTTCCTGATATCGACTTAAACTTTAGTGGTGAATATCAACCGAATGCCCATA
ACTACACAAAAGTATTATTTGGTGAGGATAAAGTATTCCGTGCAGGTACAATTGGTACTGTTGCTGAAAAGACTGCTTTTGGTTATGTTAAA
GGTTATTTGAATGATCAAGGCATCCACAAAAGAGGTGCTGAAATAGATCGACTCGTTAAAGGATGTACAGGTGTTAAACGTACAACTGGA
CAGCATCCAGGGGGTATTATTGTAGTACCTGATTACATGGATATTTATGATTTTACGCCGATACAATATCCTGCCGATGATCAAAATTCAGC
ATGGATGACGACACATTTTGATTTCCATTCTATTCATGATAATGTATTAAAACTTGATATACTTGGACACGATGATCCAACAATGATTCGTAT
GCTTCAAGATTTATCGGGCATTGATCCAAAAACGATACCTGTAGATGACAAAGAAGTCATGCAAATATTTAGTACACCTGAAAGTTTAGGT
GTTACTGAAGATGAAATTTTATGTAAAACAGGTACGTTTGGGGTTCCAGAATTCGGTACAGGGTTCGTGCGTCAAATGTTAGAAGATACAA
AGCCAACAACATTTTCTGAATTAGTTCAAATCTCAGGATTATCTCATGGTACAGATGTGTGGTTAGGCAATGCTCAAGAATTAATTAAAACC
GGTATATGTGATTTATCAAGTGTAATTGGTTGTCGTGATGATATCATGGTTTATTTAATGTATGCTGGTTTAGAACCATCAATGGCTTTTAAA
ATAATGGAGTCAGTACGTAAAGGTAAAGGTTTAACTGAAGAAATGATTGAAACGATGAAAGAAAATGAAGTGCCGGATTGGTATTTAGAT
TCATGTCTTAAAATTAAGTACATGTTCCCTAAAGCCCATGCAGCAGCATACGTTTTAATGGCAGTACGTATCGCATATTTCAAAGTACATCAT
CCACTTTATTACTATGCATCTTACTTTACAATTCGTGCGTCAGACTTTGATTTAATCACGATGATTAAAGATAAAACAAGCATTCGAAATACT
GTAAAAGACATGTATTCTCGCTATATGGATCTAGGTAAAAAAGAAAAAGACGTATTAACAGTCTTGGAAATTATGAATGAAATGGCGCATC
GAGGTTATCGCATGCAACCGATTAGTTTAGAAAAGAGTCAGGCGTTCGAATTTATCATTGAAGGCGATACACTTATTCCGCCGTTCATATCA
GTGCCTGGGCTTGGCGAAAACGTTGCGAAACGAATTGTTGAAGCTCGTGACGATGGCCCATTTTTATCAAAAGAAGATTTAAACAAAAAAG
CTGGATTATCTCAGAAAATTATTGAGTATTTAGATGAGTTAGGCTCATTACCGAATTTACCAGATAAAGCTCAACTTTCGATATTTGATATGT
AA

Gene: rimP (ribosome maturation factor)

Position: 1240424 to 1240891, length: 468 nt, orientation: FORWARD

Perfect match to: (MW2-BA000033-[1258609:1259076], highly conserved allele)

Sequence:

ATGAGTAAAATTACAGAACAAGTAGAAGTGATTGTTAAACCAATTATGGAAGACTTGAATTTTGAACTTGTAGACGTTGAATATGTCAAAG
AGGGTAGAGATCATTTTCTTAGAATCTCTATTGATAAAGAAGGTGGCGTAGATTTAAATGATTGTACGCTAGCTTCTGAAAAAATAAGTGA
AGCTATGGATGCAAATGATCCTATTCCTGAAATGTATTATTTAGACGTAGCGTCACCTGGTGCAGAACGTCCAATTAAAAAAGAACAAGATT
TCCAAAATGCAATAACTAAACCTGTATTTGTTTCTTTATATGTACCAATTGAAGGTGAAAAGGAATGGTTAGGCATTTTACAAGAAGTCAAT
AATGAAACAATTGTAGTACAAGTTAAAATCAAAGCAAGAACGAAAGATATAGAGATACCGAGAGACAAAATAGCAAAAGCACGTCACGCA
GTTATGATTTAA

Gene: nusA (transcription antitermination protein)

Position: 1240912 to 1242087, length: 1176 nt, orientation: FORWARD

Perfect match to: (MRSA252-BX571856-[1300799:1301974], allele observed in CC30+CC8+CC97+CC1156)

Sequence:

GTGTCAAGTAATGAATTATTATTAGCTACTGAGTATTTAGAAAAAGAAAAGAAGATTCCTAGAGCAGTATTAATTGATGCTATTGAAGCAG
CTTTAATTACTGCATACAAAAAGAATTATGATAGTGCAAGAAATGTCCGTGTGGAATTAAATATGGATCAAGGTACTTTCAAAGTTATCGCT
CGTAAAGATGTTGTTGAAGAAGTATTTGACGACAGAGATGAAGTGGATTTAAGTACAGCGCTTGTTAAAAACCCTGCATATGAAATTGGTG
ATATATACGAAGAAGATGTAACACCTAAAGATTTTGGTCGTGTAGGTGCTCAAGCAGCGAAACAAGCAGTAATGCAACGTCTTCGTGATGC
TGAACGTGAAATTTTATTTGAAGAATTTATAGACAAAGAAGAAGACATACTTACTGGAATTATTGACCGTGTTGACCATCGTTATGTATATG
TGAACTTAGGTCGTATCGAAGCTGTTTTATCTGAAGCAGAAAGAAGTCCTAACGAAAAATATATTCCTAACGAACGTATCAAAGTATATGTT
AACAAAGTGGAACAAACGACAAAAGGTCCTCAAATCTATGTTTCTCGTAGCCATCCAGGTTTATTAAAACGTTTATTTGAACAAGAAGTTCC
AGAAATTTACGATGGTACTGTAATTGTTAAATCAGTAGCACGTGAAGCTGGCGATCGCTCTAAAATTAGTGTCTTCTCTGAAAACAATGATA
TAGATGCTGTTGGTGCATGTGTTGGTGCCAAAGGCGCACGTGTTGAAGCTGTTGTTGAAGAGCTAGGTGGCGAAAAAATCGACATCGTTC

AATGGAATGAAGATCCAAAAGTATTTGTAAAAAATGCTTTAAGCCCTTCTCAAGTTTTAGAAGTTATTGTTGATGAAACAAATCAATCTACA
ATAGTTGTTGTTCCTGATTATCAATTGTCATTAGCGATTGGTAAAAGAGGACAAAACGCACGTCTAGCTGCTAAATTAACCGGCTGGAAAAT
TGATATTAAATCAGAAACAGATGCGCGTGAAGCGGGTATCTATCCAGTAGTTGAAGCTGAAAAAGTAACTGAAGAAGATGTTGCTTTAGA
AGATGCTGACACAACAGAATCAACCGAAGAGGTAAATGATGTTTCAGTTGAAACAAATGTAGAGAAAGAATCTGAATAA

Gene: ylxR (putative protein)

Position: 1242108 to 1242392, length: 285 nt, orientation: FORWARD

Perfect match to: (COL-CP000046-[1299583:1299867], highly conserved allele)

Sequence:

ATGAAAAAGAAAAAAAATTCCGATGCGAAAATGTATTCTTTCAAATGAAATGCATCCCAAAAAAGATATGATTCGTGTTGTTGTTAATAAAGA
AGGCGAAATCTTTGCGGATGTTACTGGAAAGAAACAAGGCCGTGGCGCATATGTTTCTAAAGATGTTGCTATGGTTGAAAAAGCACAACA
AAAAGAAATTTTAGAAAAATATTTTAAAGCATCTAAAGAGCAATTGGATCCTGTTTACAAAGAAATTATTAGATTAATTTATAGAGAAGAGA
TCCCAAAATGA

Gene: ylxQ (RNA binding protein)

Position: 1242389 to 1242706, length: 318 nt, orientation: FORWARD

Perfect match to: (N315-BA000018-[1260443:1260760], highly conserved allele)

Sequence:

ATGAGTATAGATCAAATATTAAACTTTTTAGGATTAGCAATGAGAGCTGGTAAAGTAAAAACAGGTGAATCAGTCATTGTTAATGAGATTA
AAAAAGGAAATTTGAAGCTCGTTATTGTTGCAAATGATGCGTCTGATAATACAGCTAAATTAATTACAGATAAATGTAAGAGTTACAAAGTT
CCATTCAGAAAGTTTGGAAATCGAAATGAATTGGGAATAGCACTTGGAAAAGGTGAGCGTGTTAATGTAGGGATTACTGACCCAGGCTTT
GCTAAAAAGTTGCTATCAATGATAGATGAATATCATAAGGAGTGA

Gene: infB (translation initiation factor 2)

Position: 1242711 to 1244828, length: 2118 nt, orientation: FORWARD

Sequence:

ATGAGTAAACAAAGAATTTACGAATATGCGAAAGAATTAAATCTAAAGAGTAAAGAGATTATAGATGAGTTAAAAAGCATGAATATTGAG
GTTTCAAATCATATGCAAGCTTTGGAAGATGACCAAATTAAAGCAATAGATAAAAAGTTCAAAAAAGAACAAAAGAACGACAATAAACAA
AGCACTCAAAATAATCACCAAAAATCAAACAATCAAAACCAAAATAAAGGGCAACAAAAAGATAACAAAAAGAATCAACAACAAAATAAT
AAAGGCAACAAAGGCAATAAAAAGAATAATAGAAATAATAAGAAAAATAACAAGAATAATAAACCACAAAATCAACCAGCTGCTCCAAAA
GAAATACCATCAAAAGTGACATATCAAGAAGGTATTACAGTAGGCGAATTTGCGGATAAATTAAATGTTGAATCATCAGAAATTATCAAAA
AATTATTCTTACTTGGTATTGTTGCTAATATCAATCAATCATTAAATCAAGAAACAATCGAATTAATTGCCGATGATTATGGCGTTGAGGTTG
AAGAAGAAGTTGTGATTAATGAAGAAGATTTATCAATCTACTTCGAAGACGAAAAAGATGATCCAGAAGCAATTGAGAGACCAGCAGTTG
TAACAATTATGGGACATGTTGACCATGGTAAAACTACTTTATTAGATTCAATTCGTCATACAAAAGTTACAGCAGGTGAAGCAGGCGGAAT
CACTCAACATATTGGTGCATATCAAATTGAAAACGATGGCAAAAAAATCACTTTCTTAGATACACCGGGACATGCTGCATTTACAACGATGC
GTGCGCGTGGTGCACAAGTAACAGATATTACAATTTTAGTAGTTGCAGCTGACGATGGTGTTATGCCACAAACAATTGAAGCAATTAACCA
TGCTAAAGAAGCAGAAGTACCAATTATTGTTGCAGTAAATAAAATTGATAAACCAACTTCAAATCCTGATCGAGTTATGCAAGAATTAACTG
AATATGGTTTAATTCCTGAAGATTGGGGCGGCGAAACAATTTTCGTTCCACTTTCTGCATTAAGTGGTGATGGTATCGACGATTTATTAGAA
ATGATAGGATTAGTTGCAGAAGTTCAAGAACTTAAAGCAAATCCTAAAAACCGTGCTGTTGGTACAGTTATCGAAGCTGAATTAGATAAAT
CACGTGGTCCTTCTGCATCATTATTAGTACAAAACGGTACATTAAATGTTGGTGATGCGATTGTAGTTGGTAATACTTACGGCCGTATCCGT
GCAATGGTTAATGACTTAGGTCAAAGAATCAAAACGGCTGGTCCATCAACGCCTGTTGAAATTACAGGTATTAATGATGTGCCACAAGCTG
GGGATCGCTTTGTTGTATTTAGTGATGAAAAACAAGCTCGTCGTATTGGTGAATCAAGACACGAAGCTAGCATTGTACAACAACGTCAAGA
AAGTAAAAATGTTTCATTAGATAACCTGTTTGAACAAATGAAACAAGGTGAAATGAAAGATTTAAACGTTATTATTAAAGGTGATGTTCAA
GGTTCTGTTGAAGCTTTAGCTGCATCATTAATGAAAATTGATGTTGAAGGCGTAAATGTTCGTATCATTCATACAGCGGTTGGTGCAATTAA
TGAGTCAGACGTGACACTTGCTAATGCCTCAAATGGTATTATCATTGGTTTCAATGTTCGTCCAGACAGTGGTGCAAAACGTGCTGCAGAA
GCTGAAAATGTTGATATGCGTTTACACAGAGTTATTTATAATGTTATCGAAGAAATTGAATCAGCGATGAAAGGTTTACTTGATCCAGAATT
TGAAGAACAAGTTATCGGACAAGCTGAAGTTCGTCAAACATTCAAAGTTTCTAAAGTTGGTACTATTGCTGGATGTTATGTTACTGAAGGTA
AAATTACGCGAAATGCTGGTGTACGTATTATCCGTGATGGTATTGTTCAATATGAAGGCGAATTAGATACACTTAAACGTTTCAAAGATGAT

GCTAAGGAAGTTGCAAAAGGTTATGAATGTGGTATTACAATTGAAAACTACAATGACCTTAAAGAAGGCGATGTTATCGAAGCATTTGAAA
TGGTTGAAATTAAGCGTTAA

Gene: rbfA (ribosome-binding factor A)

Position: 1245214 to 1245564, length: 351 nt, orientation: FORWARD

Perfect match to: (N315-BA000018-[1263268:1263618], highly conserved allele)

Sequence:

ATGAGCAGTATGAGAGCAGAGCGTGTTGGTGAACAAATGAAGAAGGAATTAATGGATATCATCAACAATAAAGTCAAAGATCCTCGAGTT
GGTTTTATTACAATTACAGATGTTGTTTTAACAAATGATTTATCGCAGGCTAAAGTATTTTTAACTGTATTAGGTAACGATAAAGAAGTAGA
AAATACATTTAAAGCACTTGATAAAGCAAAAGGCTTCATTAAGTCTGAATTAGGTTCTAGAATGCGATTACGTATTATGCCGGAATTAATGT
ATGAATATGATCAATCAATCGAATATGGTAATAAAATTGAACGAATGATTCAAGATTTACACAAACAAGATAGATAA

Gene: truB (tRNA pseudouridine synthase B)

Position: 1245733 to 1246650, length: 918 nt, orientation: FORWARD

Perfect match to: (MW2-BA000033-[1263919:1264836], highly conserved allele)

Sequence:

ATGTATAATGGGATATTACCAGTATATAAAGAGCGCGGTTTAACAAGTCATGACGTTGTATTCAAATTGCGTAAAATATTAAAAACTAAAAA
AATAGGTCACACGGGTACGCTTGATCCCGAAGTTGCAGGCGTGTTACCGGTATGTATAGGTAATGCAACGAGAGTTAGTGATTATGTTATG
GATATGGGCAAAGCTTATGAAGCAACTGTATCGATAGGAAGAAGTACAACGACTGAAGATCAAACGGGTGATACATTGGAAACAAAAGG
TGTACACTCAGCAGATTTTAATAAGGACGATATTGACCGATTGTTAGAAAATTTTAAAGGTGTTATTGAACAAATTCCGCCAATGTATTCATC
AGTCAAGGTGAATGGTAAAAAATTATATGAATATGCGCGTAATAATGAAACAGTTGAAAGACCAAAGCGTAAAGTTAATATTAAAGACATT
GGGCGTATATCTGAATTAGATTTTAAAGAAAATGAGTGTCATTTTAAAATACGCGTCATCTGTGGTAAAGGTACATATATTAGAACGCTAGC
AACTGATATTGGTGTGAAATTAGGCTTTCCGGCACATATGTCGAAATTAACACGAATCGAGTCTGGTGGATTTGTGTTGAAAGATAGCCTT
ACATTAGAACAAATAAAAGAACTTCATGAGCAGGATTCATTGCAAAATAAATTGTTTCCTTTAGAATATGGATTAAAGGGTTTGCCAAGCAT
TAAAATTAAAGATTCGCACATAAAAAAACGTATTTTAAATGGGCAGAAATTTAATAAAAATGAATTTGATAACAAAATTAAAGACCAAATTG
TATTTATTGATGATGATTCAGAAAAAGTATTAGCAATTTATATGGTACACCCTACAAAAGAATCAGAAATTAAACCTAAAAAAGTCTTTAATT
AA

Gene: ribF (riboflavin biosynthesis protein)

Position: 1246665 to 1247636, length: 972 nt, orientation: FORWARD

Perfect match to: (11819-97-CP003194-[1297042:1298013], allele observed in CC80+CC97+CC1156)

Sequence:

ATGAAAGTCATAGAAGTGACACATCCTATACAATCTAAACAGTATATTACAGAGGATGTTGCAATGGCATTCGGATTTTTCGATGGCATGCA
TAAAGGTCATGACAAAGTCTTTGATATATTAAATGAAATAGCTGAGGCACGCAGTTTAAAAAAAGCGGTGATGACATTTGATCCGCATCCA
TCTGTCGTGTTGAATCCTAAAAGAAAACGAACAACGTATTTAACGCCACTTTCAGATAAAATCGAAAAAATTAGCCAACATGATATTGATTA
TTGTATAGTGGTTAATTTTTCATCTAGGTTTGCTAATGTGAGCGTTGAAGATTTTGTTGAAAATTATATAATTAAAAATAATGTAAAAGAAGT
CATTGCTGGTTTTGATTTTACTTTTGGTAAATTTGGAAAAGGTAATATGACTGTTCTTCAAGAATATGATGCGTTTAATACGACAATTGTGAG
TAAGCAAGAAATTGAAAATGAAAAAATTTCTACAACTTCTATTCGTCAAGATTTAATCAATGGTGAGTTGCAAAAAGCGAATGATGCTTTAG
GCTATATATATTCTATTAAAGGCACTGTAGTGCAAGGTGAAAAAAGAGGAAGAACTATTGGCTTCCCAACAGCTAACATTCAACCTAGTGA
TGATTATTTGTTACCTCGTAAAGGTGTTTATGCTGTTAGTATTGAAATCGGCACTGAAAATAAATTATATCGAGGGGTAGCTAACATAGGTG
TAAAGCCAACATTTCATGATCCTAACAAAGCAGAAGTTGTCATCGAAGTGAATATCTTTGACTTTGAGGATAATATTTATGGTGAACGAGTG
ACCGTGAATTGGCATCATTTCTTACGTCCTGAGATTAAATTTGATGGTATCGACCCATTAGTTAAACAAATGAACGATGATAAATCGCGTGC
TAAATATTTATTAGCAGTTGATTTTGGTGATGAAGTAGCTTATAATATTTAG

Gene: rpsO (30S ribosomal protein S15)

Position: 1247751 to 1248020, length: 270 nt, orientation: FORWARD

Perfect match to: (RF122-AJ938182-[1230825:1231094], highly conserved allele)

Sequence:

ATGGCAATTTCACAAGAACGTAAAAACGAAATCATTAAAGAATACCGTGTACACGAAACTGATACTGGTTCACCAGAAGTACAAATCGCTG
TACTTACTGCAGAAATCAACGCAGTAAACGAACACTTACGTACACACAAAAAAGACCACCATTCACGTCGTGGATTATTAAAAATGGTAGG
TCGTCGTAGACATTTATTAAACTACTTACGTAGTAAAGATATTCAACGTTACCGTGAATTAATTAAATCACTTGGTATCCGTCGTTAA

Gene: pnpA (polyribonucleotide nucleotidyltransferase)

Position: 1248390 to 1250486, length: 2097 nt, orientation: FORWARD

Perfect match to: (MW2-BA000033-[1266574:1268670], highly conserved allele)

Sequence:

ATGTCTCAAGAAAAGAAAGTTTTTAAAACTGAATGGGCAGGAAGATCTTTAACGATTGAAACAGGGCAATTAGCTAAACAAGCAAATGGC
GCTGTATTGGTTCGTTATGGAGATACAGTCGTGTTATCGACGGCAACTGCATCAAAAGAACCTCGTGATGGAGATTTCTTCCCATTAACAGT
GAACTATGAAGAAAAAATGTACGCTGCGGGTAAAATTCCTGGTGGATTTAAAAAGAGAGAAGGACGTCCTGGTGACGATGCAACATTAAC
TGCGCGATTAATTGATAGACCAATTAGACCTTTATTCCCTAAAGGATATAAGCATGATGTTCAAATTATGAACATGGTATTAAGTGCAGATC
CTGATTGTTCACCACAAATGGCTGCAATGATTGGTTCATCTATGGCGCTTAGTGTGTCGGATATTCCATTCCAAGGGCCAATCGCCGGTGTA
AATGTGGGTTATATTGACGGTAAATATATCATTAACCCAACAGTAGAAGAAAAAGAAGTTTCTCGTTTAGACCTTGAAGTAGCTGGTCATA
AAGATGCAGTAAACATGGTAGAGGCAGGCGCTAGTGAGATTACTGAACAAGAAATGTTAGAGGCGATTTTCTTTGGTCATGAAGAGATTC
AACGTTTAGTTGATTTCCAACAACAAATCGTCGACCACATTCAACCTGTTAAACAAGAATTTATTCCAGCAGAGCGTGATGAAGCGCTAGTT
GAACGTGTAAAATCTTTAACCGAAGAAAAAGGACTTAAAGAAACAGTTTTAACATTTGATAAACAACAACGAGATGAAAATCTTGATAACT
TAAAAGAAGAAATCGTCAATGAATTTATCGATGAAGAAGATCCAGAAAATGAATTACTTATTAAAGAAGTTTATGCAATTTTAAATGAATTA
GTGAAAGAAGAAGTTCGACGTTTAATTGCAGATGAAAAAATTAGACCAGACGGCCGTAAACCTGATGAAATCCGTCCATTAGATTCTGAAG
TTGGTATTTTACCTAGAACGCATGGTTCAGGTCTATTTACACGTGGTCAGACTCAAGCACTTTCAGTTTTAACATTAGGTGCTTTAGGCGATT
ATCAATTAATTGATGGTTTAGGACCTGAAGAAGAAAAAAGATTCATGCATCATTACAACTTCCCGAATTTTTCAGTAGGTGAAACTGGTCCA
GTACGTGCGCCAGGTCGTCGTGAAATTGGACATGGTGCGTTAGGTGAAAGAGCATTAAAATATATTATTCCTGATACTGCTGATTTCCCATA
TACAATTCGTATTGTAAGTGAGGTACTTGAATCAAACGGTTCATCATCTCAAGCGTCAATTTGTGGATCAACATTAGCATTAATGGATGCGG
GCGTACCGATTAAAGCACCAGTTGCTGGTATTGCTATGGGTCTTGTTACACGTGAAGATAGCTATACGATTTTAACTGATATCCAAGGTATG
GAAGATGCATTAGGTGATATGGACTTTAAAGTCGCTGGTACTAAAGAAGGTATTACAGCAATTCAAATGGATATTAAAATTGACGGTTTAA
CGCGTGAAATTATCGAAGAGGCTCTAGAACAAGCGAGACGTGGTCGTTTAGAAATAATGAATCATATGTTACAAACAATTGATCAACCACG
TACTGAATTAAGTGCTTACGCGCCAAAAGTTGTAACTATGACAATTAAACCAGATAAGATTAGAGATGTTATCGGACCTGGTGGTAAAAAA
ATTAACGAAATTATTGATGAAACAGGCGTTAAATTAGATATTGAACAAGATGGTACTATCTTTATTGGGGCCGTTGATCAAGCTATGATAAA
TCGTGCTCGTGAAATCATTGAGGAAATTACACGTGAAGCGGAAGTAGGTCAAACTTATCAAGCCACTGTTAAACGTATTGAAAAATACGGT
GCGTTTGTAGGCCTATTCCCAGGTAAAGATGCGTTGCTTCACATTTCACAAATTTCAAAAAATAGAATTGAAAAAGTGGAAGATGTATTAAA
AATCGGTGACACAATTGAAGTTAAGATTACTGAAATTGATAAACAAGGTCGAGTAAATGCTTCACATAGAGCATTAGAAGAATAA

Gene: rnjB (ribonuclease J2)

Position: 1250723 to 1252396, length: 1674 nt, orientation: FORWARD

Sequence:

TTGAGTTTAATAAAGAAAAAGAATAAAGATATTCGCATTATACCATTAGGCGGTGTTGGCGAAATTGCTAAAAATATGTATATCGTTGAAG
TAGACGATGAAATGTTTATGTTAGATGCTGGACTTATGTTTCCAGAAGACGAAATGCTAGGTATTGATATTGTTATACCAGACATTTCATAC
GTACTTGAAAATAAAGATAAATTGAAGGGTATATTCCTTACACACGGACATGAGCACGCGATTGGTGCAGTGAGTTATGTTTTAGAACAAT
TAGATGCACCAGTATATGGATCTAAATTGACAATAGCGTTAATTAAAGAAAATATGAAAGCCCGTAATATTGATAAAAAAGTTCGCTACTAC
ACAGTTAATAATGATTCAATTATGAGATTCAAAAACGTGAATATTAGTTTCTTTAATACGACACACAGTATTCCTGATAGTCTAGGTGTTTGT
ATTCATACTTCATATGGTGCCATTGTGTATACAGGTGAATTTAAGTTTGACCAAAGTTTACATGGACATTATGCACCAGATATTAAACGTATG
GCAGAGATTGGTGAAGAAGGCGTATTTGTCTTAATCAGTGATTCTACTGAGGCAGAGAAACCTGGATATAATACTCCGGAAAATGTGATTG
AACATCATATGTATGACGCTTTTGCAAAAGTGCGAGGTCGCTTGATTGTTTCATGTTATGCTTCGAACTTTATACGTATTCAGCAAGTTTTAA
ATATTGCTAGCAAGCTAAATCGTAAAGTGTCATTTTTAGGAAGATCACTTGAAAGTTCATTTAATATTGCTCGTAAAATGGGGTATTTCGAC
ATTCCTAAAGATTTGCTAATTCCTATAACAGAAGTTGATAATTATCCTAAAAATGAAGTGATAATTATAGCTACTGGTATGCAAGGAGAACC
TGTAGAAGCCTTAAGTCAAATGGCGCAACATAAGCATAAAATTATGAATATCGAAGAAGGCGATTCTGTATTTTTAGCAATTACGGCTTCTG
CAAATATGGAAGTTATCATTGCGAATACATTAAATGAGCTTGTACGTGCTGGCGCACATATTATTCCAAATAACAAGAAGATTCATGCTTCA
AGTCATGGTTGCATGGAAGAATTAAAAATGATGATTAATATTATGAAACCTGAATACTTTATTCCTGTACAAGGTGAATTTAAAATGCAGAT
AGCACATGCGAAGCTAGCAGCTGAAGCAGGTGTTGCACCAGAAAAGATTTTCCTTGTGGAAAAAGGAGATGTCATTAATTACAACGGTAA

AGATATGATATTAAATGAAAAGGTAAATTCAGGAAATATTTTAATAGATGGTATTGGTATTGGGGATGTAGGAAATATCGTGTTGAGAGAC
CGTCATCTTTTAGCAGAAGATGGTATCTTTATTGCTGTAGTAACGTTAGATCCTAAAAATAGACGTATAGCTGCGGGACCTGAAATTCAATC
TCGTGGGTTTGTATATGTACGTGAAAGTGAAGACTTATTACGTGAAGCAGAAGAGAAAGTACGTGAAATAGTAGAGGCTGGTTTACAAGA
AAAACGCATAGAATGGTCTGAAATTAAGCAAAATATGCGTGATCAAATTAGTAAACTATTATTCGAAAGTACAAAACGTCGTCCTATGATTA
TTCCAGTAATTTCTGAAATTTAA

Gene: ftsK (cell division protein K)

Position: 1252778 to 1255021, length: 2244 nt, orientation: FORWARD

Perfect match to: (Strain_21333-AHKA01000002-[121270:123513:r], allele observed in CC80+CC22+CC80)

Sequence:

ATGGTGTTGGGTGTTTTCCAATTAGGAATAATAGGTCGTCTAATTGACAGCTTCTTTAATTATTTATTTGGGTACAGTAGATATTTAACATAT
ATTTTAGTACTCTTAGCAACTGGTTTTATTACATACTCTAAACGTATTCCTAAAACTAGACGAACGGCTGGTTCGATTGTATTGCAAATTGCA
TTGCTATTTGTATCACAGTTAGTTTTTCATTTTAATAGTGGTATCAAAGCTGAAAGAGAACCTGTACTTTCTTATGTGTATCAGTCATACCAAC
ACAGTCATTTCCCAAATTTTGGTGGCGGTGTATTAGGCTTTTATTTATTAGAGTTAAGCGTACCTTTAATTTCATTATTTGGTGTATGTATTAT
TACTATTTTATTATTATGCTCAAGTGTTATTTTTATTAACAAACCATCAACATCGTGAAGTTGCAAAAGTTGCACTGGAAAATATAAAAGCTTG
GTTTGGTTCATTTAATGAAAAAATGTCGGAAAGAAACCAAGAAAAACAATTGAAGCGTGAAGAAAAAGCAAGACTTAAAGAAGAACAAAA
GGCACGTCAAAATGAACAGCCACAAATAAAAGATGTGAGTGATTTTACGGAAGTGCCTCAAGAAAGAGATATTCCAATTTATGGGCATACT
GAAAATGAAAGTAAAAGCCAGAGTCAACCAAGTCGAAAAAAACGAGTGTTTGATGCAGAGAATAGTTCGAATAACATCGTAAATCATCAT
CAAGCAGATCAGCAAGAACAATTAACAGAACAAACTCATAACAGTGTTGAAAGTGAAAACACTATTGAAGAAGCTGGTGAAGTTACGAAT
GTATCGTATGTTGTTCCACCGTTAACTTTACTTAATCAACCTGCAAAACAAAAAGCAACATCTAAAGCTGAAGTGCAACGTAAAGGACAAGT
ACTAGAGAATACATTAAAAGATTTTGGGGTAAATGCAAAAGTGACACAAATTAAAATTGGTCCTGCAGTAACTCAATATGAAATTCAACCA
GCTCAAGGGGTTAAAGTGAGTAAAATTGTAAACTTGCATAATGATATTGCATTAGCTTTAGCAGCAAAAGATGTTAGAATCGAAGCGCCAA
TACCTGGTCGCTCTGCAGTAGGTATTGAAGTGCCAAATGAGAAAATTTCATTAGTTTCACTAAAAGAAGTTTTAGATGAAAAATTCCCGTCT
AATAATAAACTAGAAGTTGGATTAGGAAGAGATATATCAGGTGATCCAATTACTGTTCCACTAAATGAAATGCCACACTTATTGGTGGCAG
GATCGACGGGTAGTGGTAAATCTGTTTGTATAAATGGTATTATTACAAGTATTTTATTAAATGCTAAGCCGCATGAAGTTAAACTTATGTTA
ATCGATCCGAAAATGGTTGAACTAAATGTTTATAACGGAATTCCACATTTATTAATTCCGGTTGTTACAAATCCTCATAAAGCTGCTCAAGCT
TTAGAAAAAATTGTAGCTGAGATGGAAAGACGTTATGATTTATTCCAACATTCATCAACTAGAAATATTAAAGGTTATAACGAATTAATCCG
TAAGCAAAATCAAGAATTAGATGAGAAGCAACCAGAATTACCTTATATCGTTGTTATTGTAGATGAGCTTGCAGATTTAATGATGGTAGCT
GGTAAAGAAGTTGAAAATGCGATTCAACGTATCACACAAATGGCACGTGCAGCAGGTATACATTTGATTGTAGCAACACAAAGACCTTCTG
TGGATGTAATTACAGGTATCATTAAAAATAACATTCCATCTAGAATTGCTTTTGCTGTGAGTTCTCAAACAGATTCAAGAACTATTATTGGTA
CTGGCGGCGCAGAAAAGTTACTTGGTAAAGGTGACATGTTATACGTTGGAAATGGTGACTCATCACAAACACGTATTCAAGGGGCGTTTTT
AAGTGACCAAGAGGTGCAAGATGTTGTAAATTATGTAGTAGAACAACAACAGGCAAATTATGTAAAAGAAATGGAACCAGATGCACCAGT
GGATAAATCGGAAATGAAAAGTGAAGATGCTTTATATGATGAAGCGTATTTGTTTGTTGTTGAACAACAAAAGGCAAGTACATCATTGTTA
CAACGCCAATTTAGAATTGGTTATAATAGAGCATCTAGGTTGATGGATGATTTAGAACGCAATCAGGTAATCGGTCCACAAAAAGGAAGCA
AGCCTAGACAAGTTTTAATAGATCTTAATAATGACGAGGTGTAA

Gene: ymfC (putative transcriptional regulator, GntR family)

Position: 1255026 to 1255739, length: 714 nt, orientation: FORWARD

Perfect match to: (COL-CP000046-[1312500:1313213], highly conserved allele)

Sequence:

ATGTCAGAAATGAATGCGGTATATAACGTTAAACAATACATTTTAAATTTGATTAAGCAAAATAAATTGGAATATGGTGACCAACTTCCAAG
TAATTTATCAATTGCCAGAGAATTAAATGTAAAAACCGACGATGTTTATGAAGCAATTCAGGCATTGATTACTGAACAAGTCATTAAAGATA
ATTTTGAAGAGGGCACAAGTGTTAAGTCACTGCCCCCTTTCTTTTATCCATTGAATGAACTTATAAGTATTGGGCAAATGATTAAAAATGCA
GGATTTGAATGCGGAACTGAATACTTAAATTTTGATGAGCAACCAGCAACTATGTTAGATGCAAATTTGTTGAGCGTTGAAGAAGGATATC
CAGTAACCATTATAGAACGATTACGAACTGCCGATGGAGAACCGGTCGTCTATTGTTTAGATAAAATTGCTAAAAAAGAATTAACATGTAC
AGAGTATCAAATGAGCAATGGATCGATTCTAAGTGCGATAAAAGAACAAAGTAATCATAATATTTGTTATGCTGATACAGAAATTGAAGCG
GTAAATTATGAACCTCGAATATCCGAAGTACTGAATGCTTCGCCACACGAAGGTTTGATTTTATTAAAAATTACGCACTATAATGAATCAGA
TGAACCTATTTTGTATTCATTAAATTATATGAAAAATAGCTTAGTTCAATTTAAAATCACTAGAAAAATATAG

Gene: ymfF (putative metalloprotease)

Position: 1255770 to 1257035, length: 1266 nt, orientation: FORWARD

Perfect match to: (Strain_21178-AGRN01000105-[35540:36805:r], allele observed in CC239+CC80)

Sequence:

TTGAGTAGACAATCTCAACCAAATATACATATCAAAGTTTCACCAACAACTAAATTTAAAACAACTACTATAGTTTTTAAATTTATGGCACCT
TTAGAATATGACACAATAACAGCTAGATCATTATTAAGCAAATTATTAGTTCGAGCAACTAAGAAATGGCCAACCGATAAGTCGTTTAATAA
TCACTTAGCCGATTTATATGGTGCGTATGTGAATAGTACAATTTCAAAATTCAAAGATCAGCATGTCATTACATTTTCATTAGAAATTGTTAA
TGAACGTTATTTAAGAAACGGTGAATCATTATTTAATCAAGGATTAGATTTATTACAAGAAATCATTTGGAATCCATTAATTGAAAATAAAG
CATTCAATGATAATTTTGTTAACCAAGAGAAAACATTATTAGCAAAAAAAATAGAAGCAATGGTAGATAATAAAGCACAATATTCGTTTTTA
AAATTACTCGACCATATGTTTGAAAATGAAGCATATAAATACTTATCTACAGGACAACTAGAACAAATCCCACATATTACTGCTGAAACACT
ATATCATACATATCAATCAATGATTAATAATGATCAATGTTCTGTTTATGTTGTCGGCAATGTAGAACCTGAAAGTGTTGAGAAACAAATAC
GTGAAAAATTTGCACTTAAACCATTCGATAAACATCAATTCCAACATTCTACTCATCATTTACACGATGAAGAAGTTGATTATATTGTTGAAT
ATGATGACGTGGATCAAGCTAAATTAAATATGGGATACCGTTTTCCAACACAATATGGACAAAGTGGATATGCTGCCTTTGTTGTATTTAAC
ATGATGTTTGGAGGAGATCCTTCATCTGTTTTATTTAATGAAGTGCGAGAAAAGCAAAGTTTAGCGTACTCTATACATTCACAAATTGATGG
CAAAAATGGCTATTTATTTGTTTTGAGTGGGGTTTCAAGTGATAAGTACGAAACTGCAAAAGACACTATTATAAGTGAATTTGAAAAAAATAA
AAGCAGGAGATTTCACTGAAGAAAAATTAGAGTTAGCTAAAAAAGTAATCATTTCTCATCGATATGAATCTGAAGATCGTCCGAAAAGTAT
TATAGAGATTATGCATAACCAAATATTATTAGAGCAACCACAAAGCAAAGAAACATTTATAAATGATATACAGAAGGTAAGTCGCGAAGAT
ATTGTTTCTGTTGCTGAAAAAGCATTTTTAGATACAATCTATGTGTTGACAAAAGGAGGGGATAAATAA

Gene: ymfH (processing protease)

Position: 1257035 to 1258321, length: 1287 nt, orientation: FORWARD

Perfect match to: (IS-125-AHVC01000117-[37112:38398:r], allele observed in CC239+CC8)

Sequence:

ATGAAAGAGCGTTATTATGAATTAATAGACGAAAGAGTATTCGAACAAGAATTAGAAAATGGTTTACGATTATTTATTATCCCCAAACCAG
GTTTTCAAAAGACATTTGTCACTTACACTACACAATTTGGTTCATTAGATAATCAATTCAAACCCCTTGGACAAGACCAATTTGTTACTGTACC
TGATGGAGTTGCTCACTTTTTAGAACATAAATTATTTGAAAAAGAAGAAGAAGACTTATTTACTGCGTTTGCTGAAGATAATGCACAAGCAA
ATGCGTTTACAAGCTTTGATCGTACAAGCTACTTGTTCAGTGCAACTGATAATATTGAAAACAACATTAAACGTTTACTTACAATGGTTGAAA
CGCCTTATTTTACAAAAGAAACTGTTGATAAAGAAAAAGGTATTATTGCAGAAGAAATAAAAATGTATCAAGAACAACCTGGATATAAATT
AATGTTTAATACATTGCGTGCAATGTATCAACAACATCCAATACGTGTTGATATTGCCGGTAGTGTAGAAAGTATATACGATATTACAAAAG
ATGATTTGTATCTATGTTATGAAACGTTTTATCATCCATCAAATATGGTTTTATTTGTTGTTGGTGATGTGAATCCAGAAGAAATATGTCGAA
TAGTAAAACAACACGAGGATGCTCGTAATAAAGTTAACCAACCCAAAATCGAACGAGGACTTGTTGATGAACCGGAGGATGTTAAAGAAG
CATTTGTTACTGAATCTATGAAAATTCAATCACCAAGACTAATGCTTGGTTTTAAAAATAAACCATTACAAGAAGCGCCTCAAAAATATGTAC
AACGTGATTTAGAAATGTCATTATTCTTTGAGTTAATTTTTGGGGAAGAAACAGATTTTTATCAGAATTTATTAAACGAAGGACTTATCGATG
ATACATTTGGTTATCAATTTGTACTAGAGCCGACGTATAGTTTTTCAATCGTGACAAGCGCTACTGAAGAACCAGATAAATTAAAAAAATTA
TTATTAGATGAGTTGCGTGATAAAAAAGGCAATTTCCAAGATGCAGAAGCATTTGAACTTTTGAAAAAGCAATTTATAGGTGAGTTCATATC
AAGTTTAAACTCACCTGAATATATTGCTAATCAATATACTAAATTGTATTTTGAAGGTGTTAGTGTGTTTGACATGTTAGACATTGTTGAAAA
CATCACGTTAGATAGTATTAATGAAACGTCATCGTTATATTTAAATTTAGATCAGCAAGTCGATAGTCGTTTGGAGATTAAAAAGTAA

Gene: fabG2 (3-oxoacyl-[acyl-carrier-protein] reductase)

Position: 1258321 to 1259025, length: 705 nt, orientation: FORWARD

Perfect match to: (MW2-BA000033-[1276505:1277209], allele observed in CC1+CC5+CC8+CC80+CC239)

Sequence:

ATGAAAGCATTAGTATTAGGTGGTTCTGGTTCAATTGGTTCTGAGATAGTCAAACAATTATTAACTGATGGATTTGAAGTTTATGTGCAATA
TTATCGTACTGATATAAATGAATTAACTAGCAAATTTAATGATGATAAAGTTCGTTTTATACAAGCGGATTTATCTCAAACAATTGATATTGA
CAAAACATTTGGTGACATTAAATCATTAGACTGTTTAATATATGCAAGTGGTCAGTCTTTATATGGTGTTTTACAAGATATGAAAGACCATG
ATATTGATGCATGTTATCAGTTAAATGTCTTGCAATTAATTCGATTATGTAGATATTTCGTTGATGTTTTACGTCAAAGTGACAATGGAAGAA
TTATTGTAATTTCATCAATTTGGGGTGAGACAGGAGCTAGTATGGAAACTATTTATTCGACGATGAAAAGTGCACAATTAGGTTTCGTTAAG
GCGCTTAGTCAAGAGCTTGCACTAACATCAGTGACAGTAAATGCTATCGCACCTGGATTTGTAGCCGGTAATATGGCAAGTGAGTGGCAAG
AAGATGAACTTCAAGCAATGATAACTGAATTACCACAACAGCGATTGATTTTACCGAGTGAGGTTGCTCATACATGCGCCTATTTATATCAC
CCAAATGCTAGAAGTGTTACTGGAACTATACAGAAAGTTAATGGTGCTTGGTATATTTAA

Gene: ymfK (ACT domain regulatory protein)

Position: 1259130 to 1259957, length: 828 nt, orientation: FORWARD

Perfect match to: (MW2-BA000033-[1277314:1278141], highly conserved allele)

Sequence:

ATGACAGTTGCAGAGAAAAAAGAATGGTACCTAGAATACGAAATTGAAATTAATAGACCGGGTCTTTTAGGTGATGTATCTAGTTTATTAG
GTATGTTAGGTATAAGTATTGTTACAATTAATGGTGTTGATCAAGGTAAACGAGGCCTTTTAATTAAAACAGACAATCTTGAAAAAGTTGAA
CGTTTTGAGCAAATAGCTCGTGGTATAAATGAAATTGAAATAACAAAGCTTAAAAAACCAGAATTAAGAGACCGTCTTGCAGTAAGACATG
GTAGATATATTGAGCAAGATGCAAAAGATAAGAAAACCTTTCGATTTGAGCGTGAAGATTTAGGCTTGTTAGTAGACTTTTTAGCTGAATT
GTTCAAAGAAGAAGGTCATAAGTTGATTGGCATTAGAGGTATGCCACGAGTTGGTAAAACTGAATCAATTGTTGCGGGAAGTGTTTGTGC
ACATAAGAGATGGTTATTTATTAGTTCTACTTTAATAAAACAAACTGTACGTAGCTCTCTAATTAAAGGGGAATATGATGCCAATCATGTAT
ACATTATTGATGGTGCAGTTACTGCCAGAGAATCTAATCCAAAACATCAAGAGCTTGTTAACGAAGTTATGACGTTACCATCAATCAAAGTC
GTTGAACATCCAGATTTATTTGTTGAAACAAGTACTTGTACAATGGAAGATTTTGACTATATTATCGAATTGAGAGAAAATGAAAATCAAGA
AATACATTACGAAGAAATGAAGAAACAGACAGTCCAAAGTAAGAATAATTTAGATTTTGGAGATCCGTTTGGTGGTGGTTTTGGTTTCTTC
GAGTAA

Gene: ymfM (transcriptional regulator)

Position: 1259976 to 1260368, length: 393 nt, orientation: FORWARD

Perfect match to: (N315-BA000018-[1278026:1278418], highly conserved allele)

Sequence:

TTGAAAACGGTCGGTGAAGCGCTAAAAGGTAGACGTGAAAGGTTAGGAATGACTTTAACAGAATTAGAGCAACGTACTGGAATTAAACGT
GAAATGCTAGTGCATATTGAAAATAATGAATTCGATCAACTACCGAATAAAAATTACAGCGAAGGATTTATTAGAAAATATGCAAGCGTAG
TAAATATTGAACCTAACCAATTAATTCAAGCTCATCAAGATGAAATTCCATCGAACCAAGCCGAATGGGACGAAGTAATTACAGTTTTCAAT
AATAATAAAGACTTAGATTATAAGAGTAAATCAAAAGAGCCAATACAATTATTAGTAATCATGGGTATTACAGTTTTAATAACTTTATTGTTA
TGGATCATGTTAGTTTTAATATTTTAA

Gene: pgsA (CDP-diacylglycerol--glycerol-3-phosphate 3-phosphatidyltransferase)

Position: 1260402 to 1260980, length: 579 nt, orientation: FORWARD

Perfect match to: (N315-BA000018-[1278452:1279030], highly conserved allele)

Sequence:

ATGAATATTCCGAACCAGATTACGGTTTTTAGAGTAGTGTTAATACCAGTTTTTATATTGTTTGCGTTAGTTGATTTTGGATTTGGCAATGTG
TCATTTCTAGGAGGATATGAAATAAGAATTGAGTTATTAATCAGTGGTTTTATTTTTATATTGGCTTCCCTTAGCGATTTTGTTGATGGTTATT
TAGCTAGAAAATGGAATTTAGTTACAAATATGGGGAAATTTTTGGATCCATTAGCGGATAAATTATTAGTTGCAAGTGCTTTAATTGTACTT
GTGCAACTAGGACTAACAAATTCTGTAGTAGCAATCATTATTATTGCCAGAGAATTTGCCGTAACTGGTTTACGTTTACTACAAATTGAACA
AGGATTCGTAAGTGCAGCTGGTCAATTAGGTAAAATTAAAACAGCAGTTACTATGGTAGCAATTACTTGGTTGTTATTAGGTGATCCATTG
GCAACATTGATTGGTTTGTCATTAGGACAAATTTTATTATACATTGGCGTTATTTTTACTATCTTATCTGGTATTGAATACTTTTATAAAGGTA
GAGATGTTTTTAAACAAAAATAA

Gene: cinA (competence-damage inducible protein)

Position: 1261207 to 1262358, length: 1152 nt, orientation: FORWARD

Perfect match to: (08-02119-CP015645-[893272:894423:r], highly conserved allele)

Sequence:

ATGTCAATTGCCATTATTGCTGTAGGCTCAGAACTATTGCTAGGTCAAATCGCTAATACCAACGGACAATTTCTATCTAAAGTATTTAATGAA
ATTGGACAAAATGTATTAGAACATAAAGTTATTGGAGATAATAAAAAACGTTTAGAATCAAGTGTACGTCATGCGCTAGAAAAATATGATA

CTGTTATTTTAACAGGTGGCTTAGGTCCTACGAAAGATGACTTAACGAAGCATACAGTGGCCCAGATTGTTGGTAAAGATTTAGTTATTGAT
GAGCCTTCTTTAAAATATATTGAAAGCTATTTTGAGGAACAAGGACAAGAAATGACACCTAATAATAAACAACAGGCTTTAGTAATTGAAG
GTTCAACTGTATTAACAAATCATCATGGCATGGCTCCAGGAATGATGGTGAATTTTGAAAACAAACAAATTATTTTATTACCAGGTCCACCG
AAAGAAATGCAACCAATGGTGAAAAATGAATTGTTGTCACATTTTATAAACCATAATCGAATTATACATTCTGAACTATTAAGATTTGCGGG
AATAGGTGAATCTAAAGTAGAAACAATATTAATAGATCTTATCGATAAACAGACTAATCCTACGATTGCGCCTTTGGCGGGAAGTCATGAA
GTATATATTAGATTGACTGCAAATGCCGACTCAAAAGAACAAGCACAATCATTGATTCAACCTGTTAAACAAGAAATTCTTGATCGTATTGG
AGAATATTATTATGGTTCAGATGACACATTAATTGAGCAAGCTGTAATAAAGAAAATTCATGAACCTTTTGTAATATATGATGGTATTACTA
ATGGTGCTTTATATCATCGATTGAAAGAAGTGGATTTAAACGATGTTCTAAAGGGTATGATTAATCACAATGAAAACTTTGTTGATATTAAT
AAACCTATTGAGCAGCAATTAAAAGATGCAGTGCAATTTGTTAATAAATTGTTTAATGTGTCATCAGCAATTATTCTATTAGAGTATGATGG
TGTAGTCCATATAGGCTATGATAATAACTTTGAATTTAAAACTGAGCAATTTAAAATGTCTAAATCTAGAAATTTATTAAAGAACAGAAGTC
AAAATTATGCGCTCATAAGATTATTAAATTGGCTTAGAACAACAAATTAA

Gene: recA (recombinase A)

Position: 1262523 to 1263566, length: 1044 nt, orientation: FORWARD

Perfect match to: (RF122-AJ938182-[1245844:1246887], highly conserved allele)

Sequence:

TTGGATAACGATCGTCAAAAAGCTTTAGATACAGTAATTAAAAATATGGAGAAATCTTTCGGTAAAGGTGCCGTAATGAAGTTGGGTGACA
ATATAGGTCGCCGAGTTTCAACTACATCAACTGGTTCAGTTACATTAGATAATGCGCTAGGTGTAGGTGGCTATCCTAAAGGACGAATTATT
GAAATTTATGGTCCTGAAAGTTCTGGTAAGACAACAGTAGCGCTTCACGCTATTGCTGAAGTACAAAGTAATGGCGGGGTGGCAGCATTTA
TCGATGCTGAACATGCTTTAGATCCAGAATATGCTCAAGCATTAGGCGTAGATATCGATAATTTATATTTATCGCAACCGGATCATGGTGAA
CAAGGTCTTGAAATCGCCGAAGCATTTGTTAGAAGTGGTGCAGTTGATATTGTAGTTGTAGACTCAGTTGCTGCTTTAACACCTAAAGCTGA
AATTGAAGGAGAAATGGGAGACACTCACGTTGGTTTACAAGCTCGTTTAATGTCACAAGCGTTACGTAAACTTTCAGGTGCTATTTCTAAAT
CAAATACAACTGCTATTTTCATCAACCAAATTCGTGAAAAAGTTGGTGTTATGTTCGGTAATCCAGAGACTACACCAGGTGGACGTGCATTA
AAATTCTATAGTTCAGTAAGACTAGAAGTACGTCGTGCAGAACAGCTTAAACAAGGACAAGAAATTGTAGGTAATAGAACTAAAATTAAAG
TCGTTAAAAATAAAGTGGCACCACCATTTAGAGTAGCTGAAGTTGATATTATGTATGGACAAGGTATTTCTAAAGAGGGTGAACTTATTGA
TTTAGGTGTTGAAAACGACATCGTTGATAAATCAGGAGCATGGTATTCTTACAATGGCGAACGAATGGGTCAAGGTAAGGAAAATGTTAA
AATGTACTTGAAAGAAAATCCACAAATTAAAGAAGAAATTGATCGTAAATTGAGAGAAAAATTAGGTATATCTGATGGTGATGTTGAAGAA
ACAGAAGATGCACCAAAGTCATTATTTGACGAAGAATAG

Gene: rny (ribonuclease Y)

Position: 1263920 to 1265479, length: 1560 nt, orientation: FORWARD

Perfect match to: (11819-97-CP003194-[1314297:1315856], allele observed in CC80+CC1+CC88)

Sequence:

GTGAATTTATTAAGCCTCCTACTCATTTTGCTGGGGATCATTCTAGGAGTTGTTGGAGGGTATGTTGTTGCCCGAAATTTGTTGCTTCAAAA
GCAATCACAAGCTAGACAAACTGCCGAAGATATTGTAAATCAAGCACATAAAGAAGCTGACAATATCAAAAAAGAGAAATTACTTGAGGC
AAAAGAAGAAAACCAAATCCTAAGAGAACAAACTGAAGCAGAACTACGTGAAAGACGTAGTGAACTTCAAAGACAAGAAACCCGACTTCT
TCAAAAAGAAGAAAACTTAGAGCGTAAATCTGATCTATTAGATAAAAAAGATGAGATTTTAGAGCAAAAAGAATCAAAAATTGAAGAAAA
ACAACAACAAGTAGATGCAAAAGAGAGTAGTGTTCAAACGTTAATAATGAAGCATGAACAAGAATTAGAACGCATCTCCGGTCTCACTCAA
GAAGAAGCTATTAATGAGCAACTTCAAAGAGTTGAGGAAGAACTGTCACAAGATATTGCAGTACTTGTTAAAGAAAAAGAAAAAGAAGCT
AAAGAAAAAGTTGATAAAACAGCAAAAGAATTATTAGCTACAGCAGTACAAAGATTAGCAGCAGATCACACAAGTGAATCAACGGTATCA
GTAGTTAACTTACCTAATGATGAGATGAAAGGTCGAATCATTGGACGAGAAGGACGAAACATCCGCACACTTGAAACTTTAACTGGCATTG
ATTTAATTATTGATGACACACCAGAAGCGGTTATATTATCTGGTTTTGATCCAATAAGAAGAGAAATTGCTAGAACAGCACTTGTTAACTTA
GTATCTGATGGACGTATTCATCCAGGCAGAATTGAAGATATGGTCGAAAAAGCTAGAAAAGAAGTAGACGATATTATTAGAGAAGCAGGT
GAACAAGCTACATTTGAAGTGAACGCACATAATATGCATCCTGACTTAGTAAAAATTGTAGGGCGTTTAAACTATCGTACAAGTTACGGTCA
AAATGTACTTAAACATTCAATTGAAGTTGCGCATCTTGCTAGTATGTTAGCTGCTGAGCTAGGCGAAGATGAGACATTAGCGAAACGAGCT
GGACTTTTACATGATGTTGGTAAAGCAATTGATCATGAAGTAGAAGGTAGTCATGTTGAAATCGGTGTAGAATTAGCGAAAAAATATGGTG
AAAATGAAACAGTTATTAATGCAATCCATTCTCATCATGGTGATGTTGAACCTACATCTATTATATCTATCCTTGTTGCTGCTGCAGATGCAT
TGTCTGCGGCTCGTCCAGGTGCAAGAAAAGAAACATTAGAGAATTATATTCGTCGATTAGAACGTTTAGAAACGTTATCAGAAAGTTATGA
TGGTGTAGAAAAAGCATTTGCGATTCAGGCAGGTAGAGAAATCCGAGTGATTGTATCTCCTGAAGAAATTGATGATTTAAAATCTTATCGA
TTGGCTAGAGATATTAAAAATCAGATTGAAGATGAATTACAATATCCTGGTCATATCAAGGTGACAGTTGTTCGAGAGACTAGAGCAGTAG
AATATGCGAAATAA

Gene: Q5HGE4 (putative protein)

Position: 1265776 to 1265991, length: 216 nt, orientation: REVERSE

Perfect match to: (11819-97-CP003194-[1316153:1316368:r], allele observed in CC80+CC96)

Sequence:

TTATTTTAATTGTAAATCTGTTTTCTTTAATTCTTTTATAACTTCTGCAGTATCATAACAATTTGTTGCAATCGTTGAATATCTCTCTGCTAAAC
GATATGCATTAATGTAAAGCTTTAAACTTTCTTTAGCTATATCCTCTGCATCTTCGAATTTTGATGGGTTAGACATAACCACTAATTCTGCAAA
TTTTTCTGGATCAATATTAATAGACAT

Gene: ymdB (putative hydrolase)

Position: 1266165 to 1266962, length: 798 nt, orientation: FORWARD

Perfect match to: (N315-BA000018-[1284212:1285009], highly conserved allele)

Sequence:

ATGAGAATAATGTTTATAGGGGATATCGTAGGTAAAATTGGACGAGACGCAATTGAAACGTACATACCTCAACTGAAGCAAAAGTATAAA
CCAACAGTTACAATTGTAAATGCTGAAAATGCAGCACATGGTAAAGGTTTGACTGAAAAAATATATAAACAATTACTAAGAAATGGTGTAG
ATTTCATGACTATGGGTAATCACACATATGGTCAACGTGAAATTTATGATTTTATAGATGAAGCAAAACGACTAGTAAGACCAGCGAATTTT
CCGGATGAAGCGCCGGGAATTGGTATGAGATTTATACAAATTAATGATATTAAACTTGCAGTTATTAATCTGCAAGGAAGAGCGTTTATGC
CAGATATTGATGATCCTTTTAAAAAGGCAGATCAATTAGTCAAGGAAGCACAAGAACAAACTCCGTTTATATTTGTTGATTTTCATGCAGAA
ACAACTTCTGAAAAGTATGCAATGGGATGGCATTTAGATGGTAGAGCTAGCGCTGTTGTTGGAACGCATACACACATTCAAACAGCAGATG
AACGTATTTTACCAAAGGGGACAGGGTATATAACGGATGTTGGTATGACAGGTTTTTATGATGGCATTTTAGGAATAAATAAAACAGAGGT
AATTGAGCGTTTTATCACTAGTTTGCCACAAAGACATGTTGTTCCAAATGAAGGTAGAAGTGTATTATCTGGTGTTGTTATTGATTTAGACA
AAGAAGGTAAAACAAAGCACATCGAACGTATATTGATAAATGATGACCATCCATTTTCAACATTTTAA

Gene: porA (pyruvate ferredoxin oxidoreductase alpha chain)

Position: 1267102 to 1268862, length: 1761 nt, orientation: FORWARD

Perfect match to: (11819-97-CP003194-[1317479:1319239], allele observed in CC80)

Sequence:

ATGAAACCACAATTATCGTGGAAAGTTGGCGGTCAACAAGGCGAAGGTATTGAATCAACTGGGGAAATCTTCGCTACGGCTATGAATAGA
AAAGGATATTATTTATATGGATATAGACATTTTTCAAGTCGTATCAAAGGTGGACATACGAATAATAAAATTAGAGTTTCTACGACGCCTGT
TCATGCAATTAGTGATGATTTAGATATTTTGATTGCATTTGACCAAGAAACAATTGATGTTAACCATCATGAAATGAGAGAAGACAGTATTA
TTTTAGCTGATGCCAAGGCTAAACCTGTGAAACCAGAAGGATGTCATGCACAGCTTATTGAATTACCTTTTACAGCAACCGCTAAAGAATTA
GGTACAGCATTAATGAAAAACATGGTTGCAATAGGTGCTACTAGCGCATTGATGAATTTGAATACAAATACATTTGAAGAACTTATTACTAA
TATGTTTTCTAAAAAAGGTGACAAGGTAGTTGAAGTCAATATCCAAGCATTAAACGAAGGTTATCAATTAATGCAATCCCGCTTACCTGAAA
TCGACGGGGACTTTGAATTAGAGTCAACAGATGCACTACCACATCTATATATGATTGGTAACGATGCCATTGGATTAGGTGCAATTGCTGC
AGGTTCACAATTTATGGCGGCATATCCTATTACACCTGCGTCTGAAGTTATGGAATATATGATTGCCAATATATCTAAAGTAAACGGAGCGG
TTATTCAAACAGAAGATGAAATTGCTGCTGTAACTATGGCTATTGGTGCAAATTATGGTGGAGTCAGAGCGTTTACGGCTAGTGCTGGTCC
AGGTTTATCTTTAATGATGGAAGCAATTGGATTATCTGGTATGACTGAAACGCCATTAGTCATTATTAATACTCAACGAGGTGGACCTTCTA
CTGGATTACCTACGAAACAAGAACAGTCAGATTTAATGCAAATGATTTATGGTACACATGGTGATATTCCAAAAATAGTTGTAGCACCAACT
GATGCAGAAGATGCATTTTATTTAACTATGGAAGCCTTTAATTTAGCAGAACAATATCAATGCCCTGTTATAGTTTTAAGTGATTTGCAATTA
TCTTTAGGTAAACAAACTGTTGAAAAATTAGATTATAATCGCATTGAAATTAAACGTGGTGAAATCATTCAATCTGATATTGAACGTGAAGA
AGATGATAAAGGTTATTTCAAGCGTTATGCGTTAACATCCAATGGTGTTTCTCCTAGACCTATCCCCGGTGTTAAAGGAGGTATTCATCATA
TAACTGGTGTGGAACACAATGAAGAAGGTAAACCTAGTGAATCTGCGTCAAATAGACAACAACAAATGGAAAAACGAATGCGTAAAATTG
AGCAGTTACTAATTGAATCGCCAGTAGAAGCTAACTTACAACATGAGGATGCAGATATTCTTTATATCGGTTTTATTTCTACAAAAGGTGCA
ATTCAAGAAGGTAGTAACCGTTTGAATCAACAAGGTATAAAAGTTAACACTGTACAAATTAGACAATTGCATCCATTCCCAACAAGCGTTAT
TCAAGATGCAGTTAATAAAGCGAAGAAAGTCGTTGTAGTGGAGCACAATTATCAAGGACAATTGGCTAGTATTATAAAAATGAATGTCAAT
ATTCATGATAAGATTGAAAATTATACAAAGTATGATGGGACACCTTTCCTACCACATGAAATTGAAGAAAAAGGCAAAATAATTGCTACTG
AAATAAAGGAGATGGTATAG

Gene: porB (2-oxoglutarate ferredoxin oxidoreductase subunit beta)

Position: 1268863 to 1269729, length: 867 nt, orientation: FORWARD

Perfect match to: (ED133-CP001996-[1329773:1330639], highly conserved allele)

Sequence:

ATGGCGACATTTAAAGATTTTAGAAATAATGTTAAGCCTAACTGGTGCCCTGGATGTGGCGATTTCTCAGTACAAGCTGCAATTCAAAAAGC
AGCCGCAAATATAGGGTTAGAACCTGAAGAAGTAGCTATCATCACCGGTATAGGATGTTCTGGCCGTCTTTCAGGATATATTAATTCTTATG
GCGTTCATTCTATTCACGGACGTGCATTACCTTTAGCTCAAGGTGTAAAAATGGCGAATAAAGATTTAACTGTTATTGCATCTGGAGGAGAT
GGTGATGGTTATGCTATAGGTATGGGGCATACAATCCATGCTTTAAGAAGAAATATGAACATGACGTATATAGTCATGGATAATCAAATTT
ATGGTTTGACAAAGGGACAAACATCGCCGTCATCAGCAGTAGGATTTGTTACTAAAACAACGCCAAAAGGTAATATAGAAAAAAATGTTGC
GCCTTTAGAATTAGCATTATCATCTGGTGCCACATTTGTAGCCCAAGGTTTTTCAAGCGATATTAAAGGATTAACAAAACTAATTGAAGATG
CAATTAATCATGATGGATTTTCATTCGTTAATGTCTTTTCACCATGTGTGACTTATAATAAAATTAACACATACGATTGGTTTAAAGAACATTT
AACAAGTGTTGATGACATTGAAAATTATGATTCTACAGATAAACAATTAGCGACTAAAACTGTTATTGAACATGAATCTTTAGTAACTGGTA
TTGTTTATCAAGATAAAGAAACACCATCATATGAATCTCAAATTAAAGAGTTAGATGATACACCACTTGCTAAAAGAGATATCAAAATTACT
GAAGACACCTTCAATGCATTAACTGAACAATTTATTTAA

Gene: DUF77 (putative protein)

Position: 1269823 to 1270116, length: 294 nt, orientation: FORWARD

Perfect match to: (N315-BA000018-[1287870:1288163], highly conserved allele)

Sequence:

ATGAAAGACACATTAATGAGTATACAAATAATTCCTAAAACACCAAACAATGACAATGTTATACCTTACGTAGACGAGGCGATTAAAATAAT
TGACGAATCTGGTTTGCATTTTAGAGTAGGTCCGTTAGAAACGACAGTACAAGGAAATATGAATGAATGTTTAATTTTAATACAATCATTAA
ATGAACGAATGGTGGAACTTGAATGTCCAAGTATTATTAGCCAAGTTAAGTTTTATCATGTGCCAGATGGCATCACTATTGAAACTTTAACT
GAAAAATATGATGAATAA

Gene: miaB ((dimethylallyl)adenosine tRNA methylthiotransferase)

Position: 1270250 to 1271794, length: 1545 nt, orientation: FORWARD

Perfect match to: (Strain_132-ACOT01000026-[470289:471833], allele observed in CC8+CC97)

Sequence:

GTGAACGAAGAACAAAGAAAAGCAAGTTCTGTAGATGTTTTAGCTGAGAGAGATAAGAAAGCAGAAAAAGATTATAGTAAATATTTTGAA
CATGTTTATCAGCCGCCTAATTTAAAAGAAGCGAAAAAAAGAGGTAAACAAGAAGTTCGTTATAATAGAGATTTCCAAATTGATGAAAAAT
ATCGCGGTATGGGGAACGAGCGTACATTTTTAATTAAAACATATGGATGTCAAATGAATGCACATGACACTGAGGTCATTGCTGGTATACT
TGAAGCATTAGGCTATCAAGCAACGACTGATATTAACACTGCAGATGTTATTTTAATTAATACATGTGCGATTAGAGAAAATGCCGAGAAC
AAAGTGTTTAGTGAAATAGGTAATTTGAAGCATTTGAAAAAAGAACGACCTGATATTTTAATCGGTGTTTGTGGTTGTATGTCACAAGAAG
AGTCAGTAGTGAATAAAATTTTAAAATCGTATCAAAATGTAGATATGATATTTGGTACACATAATATTCATCATTTACCAGAAATTTTAGAAG
AAGCATACTTATCTAAAGCAATGGTTGTTGAAGTATGGTCTAAAGAAGGAGACGTTATTGAAAATCTTCCAAAAGTCCGTGAAGGCAACAT
TAAAGCATGGGTCAATATTATGTATGGTTGTGATAAGTTTTGTACATATTGTATTGTTCCATTTACAAGAGGTAAAGAACGAAGCCGTAGAC
CTGAAGACATTATAGATGAAGTACGTGAACTTGCTCGTGAAGGTTACAAAGAAATAACGCTTTTAGGTCAAAATGTAAATTCTTATGGTAA
AGATTTACAGGATATAGAATATGACTTAGGAGATCTTTTACAAGCAATTTCTAAAATAGCGATTCCAAGAGTTCGTTTCACAACAAGTCATC
CTTGGGACTTTACAGATCACATGATTGATGTTATTTCAGAGGGTGGTAATATCGTTCCGCATATCCACTTGCCAGTTCAATCTGGAAATAAT
GCAGTATTAAAAATAATGGGTAGAAAATATACACGAGAAAGTTATTTGGATTTAGTAAAACGAATCAAAGATAGAATTCCTAATGTAGCAT
TAACTACAGATATTATTGTAGGGTATCCAAATGAATCAGAGGAACAATTTGAAGAAACTTTAACTCTGTATGATGAAGTTGGTTTTGAACAT
GCATATACGTATTTGTATTCACAACGTGATGGTACGCCTGCTGCTAAAATGAAAGATAATGTACCTTTAAATGTCAAAAAGGAACGATTGCA
ACGTTTGAATAAAAAAGTTGGTCATTATTCACAAATAGCTATGAGTAAGTACGAAGGACAAACTGTAACAGTACTTTGTGAAGGTAGTAGT
AAAAAAGATGATCAGGTTCTTGCTGGCTACACTGATAAAAATAAGCTAGTTAATTTCAAAGCGCCTAAAGAAATGATTGGTAAACTAGTGG
AAGTACGAATAGATGAAGCTAAACAGTATTCATTAAATGGCAGTTTTGTAAAGGAAGTAGAGCCGGAAATGGTGATTCAATAA

Gene: ymcA (putative membrane protein)

Position: 1271795 to 1272160, length: 366 nt, orientation: FORWARD

Perfect match to: (MW2-BA000033-[1289979:1290344], highly conserved allele)

Sequence:

ATGTATAATAAAGATGACGTGTTGAAACAAGCGGATAATATTGCAAATAAAATTAAAAATTTGGATACTATCAAAACATATCAACAAATTG
AAGCACAGATTCATCAGAACCAAACGATAAAGACTAAAATGGATATGTTAAAAAAGCATCAAAAACAAGCAGTAAACTTTCAAAATTACGG
GAAACAAAATGCGCTAGAACAGTCGGAACATACCATTCAAAGTATAGAAGCAGAAATAAATACATTGCCCATAGTTGAACAGTTTCAAACT
TCACAATATGAAGCGAATCAATTATTGAAAATGTTTGTATCAACAATGGAAACACGTTTAAATGACCATAATAAAGCCAAGCATAGTGATTA
A

Gene: thiW (ThiW family protein)

Position: 1272187 to 1272678, length: 492 nt, orientation: FORWARD

Perfect match to: (Strain_21193-AFEG01000003-[97055:97546:r], allele observed in CC25+CC8+CC25+CC88)

Sequence:

ATGAAATCAAGAAAACTGGCTATAACTGCACTTTTAATTGCAATAAATGTTGTATTAAGCAGTATTATCATCATTCCTCTAGGACCAGTTAAG
GCAGCACCAGTACAGCATTTTGTAAATGTATTAAGTGCGGTCATAGTAGGTCCTTGGTATGGATTAGCTCAAGCGCTTATATCATCAATTTT
AAGAGTTCTTTTTGGTACTGGTACAGCTTTTGCATTTCCGGGTAGTATGATTGGAGTTTTATTGGCTAGTATGTTTTACATATATCGTAAACA
TATATTCATGGCCGCGGTCGGTGAAGTACTTGGAACTGGTGTCATCGGAAGTTTAATTTGTATACCATTAGCATATTTCCTCGGGCTTCAAG
ACTTCTTCATTAAACCGTTAATGATTACGTTCATAGTCTCAAGTGCTATCGGATCTATTATAAGTTATTTCTTATTAATTACTCTAAAAAAACG
TGGTATTCTTCAAAGGTTTATAAAATAA

Gene: mutS (DNA mismatch repair protein)

Position: 1272981 to 1275599, length: 2619 nt, orientation: FORWARD

Perfect match to: (M0294-AQFZ01000019-[186527:189145], allele observed in CC5)

Sequence:

ATGTCTAATGTTACACCAATGATGCAGCAATATTTAAAAATAAAATCAGAATACCAAGATTGCTTATTATTTTTTAGACTAGGTGATTTCTAT
GAAATGTTTTATGAAGATGCCAAGGAGGCATCACGTGTACTTGAAATTACTTTAACTAAAAGAGATGCTAAAAAAGAAAATCCAATTCCGA
TGTGTGGTGTTCCGTATCATTCTGCAGATAGTTATATAGATACACTTGTTAATAATGGATATAAAGTAGCTATTTGTGAACAGATGGAAGAT
CCGAAACAAACGAAAGGTATGGTTAGACGTGAGGTATTAAGAATTGTGACTCCAGGAACTGTGATGGAGCAAGGTGGTGTAGATGATAA
ACAAAATAACTATATTTTAAGTTTTGTTATGAATCAACCTGAAATTGCGCTTAGTTACTGTGATGTTTCTACTGGCGAATTAAAGGTTACACA
TTTTAATGATGAAGCGACTTTATTAAATGAAATTACGACGATAAACCCTAACGAAGTTGTTATCAATGACAATATTTCCGATAATTTAAAAA
GACAAATTAATATGGTGACAGAAACAATAACAGTCAGGGAAACGTTATCATCAGAAATCTATAGTGTGAATCAAACTGAACATAAATTAAT
GTATCAAGCGACACAATTATTGCTAGATTATATTCATCATACACAAAAACGTGATTTATCGCATATCGAGGATGTTGTTCAATATGCAGCTAT
AGATTATATGAAAATGGATTTTTATGCTAAGAGAAACCTTGAGTTAACGGAAAGCATTCGATTAAAATCAAAAAAAGGAACGCTACTTTGG
CTAATGGACGAAACGAAAACACCAATGGGAGCACGCCGCTTAAAACAATGGATAGATAGACCACTAATAAGTAAAGAACAAATTGAAGCA
CGATTAGATATCGTTGATGAATTTAGTGCTCATTTCATAGAAAGAGACACCTTAAGAACATATCTTAATCAAGTGTATGATATTGAACGTCTT
GTTGGGCGTGTTAGTTACGGAAATGTTAATGCGAGAGATTTAATTCAACTTAAACATTCCATTTCTGAAATACCGAATATTAAAGCATTACT
AAATTCTATGAATCAGAATACTCTTGTACAAGTTAATCAACTAGAACCCCTTGATGATTTACTTGATATATTAGAACAGAGTTTAGTAGAAG
AACCACCAATTTCAGTTAAAGATGGCGGACTATTCAAAGTTGGTTTTAATACGCAATTAGATGAATATCTTGAAGCTTCAAAAAACGGAAAA
ACATGGTTAGCAGAATTACAAGCCAAAGAAAGACAACGTACAGGAATAAAATCATTGAAAATAAGCTTTAATAAAGTGTTTGGTTATTTTA
TAGAAATAACACGTGCCAACTTGCAAAATTTTGAACCAAGTGAATTTGGTTATATGAGGAAGCAAACGTTATCGAATGCTGAACGTTTTATA
ACTGATGAACTTAAAGAAAAAGAAGATATCATTTTAGGTGCGGAAGACAAAGCCATCGAATTAGAATATCAATTATTTGTTCAGCTACGTG
AAGAAGTTAAAAAATATACTGAACGTTTACAACAACAAGCTAAAATTATTTCAGAGCTAGATTGTTTACAGAGCTTTGCAGAAATTGCTCAA
AAATATAATTACACTAGGCCTTCATTTAGTGAAAATAAAACATTAGAATTAGTGGAATCTAGGCACCCAGTAGTGGAAAGAGTAATGGATT
ATAATGACTATGTGCCTAATAATTGTCGATTAGATAATGAAACATTTATATATTTAATTACAGGTCCGAATATGTCTGGTAAATCGACATATA
TGAGACAAGTTGCCATAATTAGTATAATGGCCCAAATGGGAGCTTATGTCCCTTGTAAAGAGGCAGTGTTACCTATATTTGATCAAATATTC
ACTAGAATAGGTGCGGCAGATGATTTGGTTTCAGGTAAGAGTACGTTTATGGTAGAAATGCTAGAAGCACAAAAGGCATTAACTTATGCA
ACAGAGGATAGTTTGATTATTTTCGATGAAATTGGACGTGGTACTTCAACGTATGACGGTTTAGCTTTAGCGCAGGCAATGATAGAGTATG
TAGCTGAAACATCACATGCTAAAACGTTATTTTCAACACATTATCATGAATTGACAACATTAGATCAAGCATTACCAAGTCTAAAAAATGTTC
ACGTCGCTGCTAATGAATATAAAGGTGAACTTATATTCTTGCATAAAGTCAAAGATGGTGCAGTTGACGATAGTTATGGTATTCAAGTTGCG
AAATTAGCTGATTTACCTGAAAAAGTTATTAGCAGAGCACAAGTGATTCTAAGCGAGTTTGAAGCGTCTGCTGGTAAAAAAATCATCGATAT

CAAATTTAAAAATGGTCGAAAATGAACCTGAAATTAATCAAGAAAATTTAAACTTAAGTGTTGAAGAAACAACTGATACTTTATCTCAAAAA
GACTTTGAACAAGCATCATTTGATTTGTTTGAAAATGATCAAGAAAGCGAGATTGAACTACAAATTAAAAATTTGAATTTATCTAATATGAC
ACCAATTGAGGCATTGGTGAAGTTAAGTGAATTACAAAATCAATTAAAATAG

Gene: mutL (DNA mismatch repair protein)

Position: 1275612 to 1277621, length: 2010 nt, orientation: FORWARD

Perfect match to: (Strain_21193-AFEG01000003-[92112:94121:r], highly conserved allele)

Sequence:

ATGGGGAAAATTAAAGAACTCCAAACCTCATTAGCAAATAAAATCGCAGCAGGTGAAGTAGTTGAAAGACCGAGTTCTGTTGTGAAAGAA
CTGTTGGAAAATGCTATAGATGCAGGCGCTACAGAAATAAGCATTGAAGTAGAGGAATCTGGCGTCCAATCTATTCGCGTAGTCGATAATG
GAAGCGGAATTGAAGCGGAAGACTTAGGATTAGTATTTCATAGACATGCGACTAGTAAATTAGATCAAGATGAAGATTTATTTCATATTAG
GACATTAGGATTCCGTGGTGAAGCACTAGCCAGTATTTCATCAGTTGCTAAAGTAACATTGAAGACTTGCACGGATAATGCTAATGGAAAT
GAAATATATGTAGAAAATGGTGAAATATTAAATCATAAGCCTGCAAAAGCGAAAAAAGGAACAGATATACTTGTAGAATCATTATTTTATA
ATACACCAGCACGTTTAAAATATATTAAAAGTTTATACACTGAACTAGGTAAAATAACAGATATTGTCAACAGAATGGCAATGAGCCATCCG
GACATTCGAATAGCACTCATTTCAGATGGCAAAACAATGTTAAGTACAAATGGTTCAGGACGAACTAATGAAGTGATGGCAGAGATTTATG
GGATGAAAGTTGCACGAGATTTAGTACATATATCTGGAGATACAAGTGATTATCACATTGAAGGTTTTGTTGCAAAGCCTGAACATTCTAG
AAGTAATAAGCACTATATTTCTATTTTTATTAATGGACGATACATTAAAAACTTTATGCTAAATAAAGCGATTTTAGAAGGCTATCATACACT
CTTAACAATAGGTAGGTTCCCGATTTGTTATATTAATATTGAAATGGATCCAATCTTAGTAGACGTAAATGTTCATCCAACAAAACTAGAAG
TGCGTTTATCAAAAGAAGAGCAACTATATCAATTGATAGTGAGCAAAATACAAGAAGCATTTAAAGACCGTATATTAATTCCTAAAAATAAC
TTGGATTATGTGCCGAAAAAAAAATAAAGTGTTACATTCATTCGAACAACAAAAAATCGAATTTGAACAAAGACAAAACACAGAGAATAATC
AAGAGAAGACGTTTTCATCTGAAGAAAGTAACAGTAAGCCATTTATGGTAGAAAATCAAAACGATGAAATAGTTATAAGAGAAGATTCATA
TAATCCATTCGTAACGAAAACGTCTGAAAGTTTAATAGCTGATGATGAATCTTCCGGTTATAATAATACACGTGAAAAAGATGAAGACTACT
TCAAAAAGCAACAAGAAATTCTACAAGAAATGGATCAAACATTTGATTCGAATGACGATACATCTGTGCAAAATTATGAGAATAAAGCGTC
TGATGATTATTATGATGTAAACGATATTAAAGGAACAAAAAGTAAAGACCCTAAACGAAGAATTCCATATATGGAAATTGTTGGCCAAGTA
CATGGAACGTATATTATTGCTCAAAATGAATTTGGCATGTACATGATTGACCAGCATGCAGCTCAAGAAAGAATAAAATATGAATATTTTCG
AGATAAAATAGGTGAAGTTACCAATGAAGTACAAGATTTATTAATCCCGTTAACATTTCATTTTTCAAAAGATGAACAATTAGTCATTGATC
AATATAAAAATGAGCTTCAACAAGTAGGTATCATGTTAGAACATTTTGGTGGTCATGATTATATTGTAAGTAGCTATCCAGTTTGGTTCCCTA
AAGATGAAGTAGAAGAAATTATTAAAGATATGATTGAGCTAATTTTGGAAGAGAAAAAAGTAGATATCAAAAAATTACGTGAAGATGTAG
CAATCATGATGTCATGTAAAAAATCTATTAAAGCGAATCATTATTTACAAAAACATGAAATGTCTGATTTAATTGATCAATTAAGAGAAGCG
GAAGATCCATTTACATGTCCACATGGTCGTCCAATTATCATTAATTTTTCAAAATACGAATTAGAAAAATTATTTAAGCGTGTGATGTAG

Gene: glpP (glycerol-3-phosphate responsive antiterminator)

Position: 1277636 to 1278169, length: 534 nt, orientation: FORWARD

Perfect match to: (MW2-BA000033-[1295820:1296353], highly conserved allele)

Sequence:

GTGAATAACAACATATTGCCTGCCATAAGAAACATTAAAGATTTAGAGAAACTGATTAAAACAGATTATAAAATGTGTGTGCTTCTAGATAT
GCATATAGGACATATAAAAAGTATTATGGAATTGCTGAAGCAAAATCATATAGAGTGTTTTATTCATATAGATTTGATAAAAGGTTTAAGCC
ACGATGAATTTGCAAGTGAATTTATTATTCAGCAATACAAGCCAAAAGGTATCGTATCGACTAAATCTAAAGTAATAAAAAAAGCTAAATCA
TTAAATACTTTAACGATTTTTAGAGTATTTATTATTGATAGTCAAGCATTGAAACGCAGTATAGATTTGATAAAAAAAGTTGAACCTGATTTT
GTTGAAGTACTTCCAGGTGTTGCGAGTAAAGCGATTCATCATATTCAGAAAGAAACAAACACACAAGTCATTGCAGGTGGCCTAATTAATA
CAATAGATGAAGTCAATGAAGCTGTTAAAAATGGAGCGAAATATGTAACAACTAGTTATGATAAACTTTGGTAA

Gene: glpF (glycerol uptake facilitator)

Position: 1278634 to 1279452, length: 819 nt, orientation: FORWARD

Perfect match to: (N315-BA000018-[1296691:1297509], highly conserved allele)

Sequence:

ATGAATGTATATTTAGCAGAATTCCTAGGAACTGCAATCTTAATCCTTTTTGGTGGTGGCGTTTGTGCCAATGTCAATTTAAAGAGAAGTGC
TGCGAATGGTGCTGATTGGATTGTCATCACAGCTGGATGGGGATTAGCGGTTACAATGGGTGTGTTTGCTGTCGGTCAATTCTCAGGTGCA

CATTTAAACCCAGCGGTGTCTTTAGCTCTTGCATTAGACGGAAGTTTTGATTGGTCATTAGTTCCTGGTTATATTGTTGCTCAAATGTTAGGT
GCAATTGTCGGAGCAACAATTGTATGGTTAATGTACTTGCCACATTGGAAAGCGACAGAAGAAGCTGGCGCGAAATTAGGTGTTTTCTCTA
CAGCACCGGCTATTAAGAATTACTTTGCCAACTTTTTAAGTGAGATTATCGGAACAATGGCATTAACTTTAGGTATTTTATTTATCGGTGTAA
ACAAAATTGCCGATGGTTTAAATCCTTTAATTGTCGGAGCATTAATTGTTGCAATCGGATTAAGTTTAGGCGGTGCTACTGGTTATGCAATC
AACCCAGCACGTGATTTAGGTCCGAGAATTGCACATGCGATTTTACCAATAGCTGGTAAAGGTGGTTCAAATTGGTCATATGCAATCGTTCC
TATCTTAGGACCAATTGCCGGTGGTTTATTAGGTGCAGTGGTATACGCTGTATTTTATAAACATACATTTAATATTGGTTGTGCAATTGCAAT
TGTTGTAGTTATTATTACTTTGATTTTAGGTTACATTTTAAATAAATCATCAAAAAAAGGTGATATCGAATCAATTTACTAA

Gene: glpK (glycerol kinase)

Position: 1279581 to 1281077, length: 1497 nt, orientation: FORWARD

Perfect match to: (11819-97-CP003194-[1329958:1331454], allele observed in CC80+CC1+CC779)

Sequence:

ATGGAAAAATATATTTTATCTATAGACCAAGGAACAACAAGCTCAAGAGCGATTTTATTCAATCAAAAAGGGGAAATTGCAGGGGTAGCAC
AACGTGAGTTTAAGCAATATTTTCCACAATCAGGTTGGGTTGAACATGATGCAAATGAAATTTGGACATCTGTGTTAGCTGTAATGACGGA
AGTAATTAATGAAAATGATGTTAGAGCTGATCAAATTGCAGGTATCGGTATTACAAACCAACGCGAAACAACGGTGGTTTGGGACAAACAT
ACTGGCCGCCCAATTTATCACGCAATTGTTTGGCAATCACGTCAAACACAATCAATTTGTTCAGAATTAAAACAACAAGGATATGAACAAAC
ATTTAGAGATAAGACAGGATTACTTTTAGATCCGTATTTTGCAGGTACAAAAGTTAAATGGATTCTAGACAATGTTGAAGGTGCACGAGAA
AAAGCAGAAAATGGCGATCTATTATTTGGAACGATTGATACTTGGTTAGTATGGAAATTATCAGGAAAAGCTGCGCATATTACTGATTATTC
AAATGCGAGTCGTACATTAATGTTTAATATCCATGATTTAGAATGGGACGATGAGTTATTAGAACTACTTACAGTACCTAAAAATATGTTGC
CAGAAGTTAAACCTTCAAGTGAAGTATATGGTAAGACAATTGATTACCACTTCTATGGTCAAGAAGTACCAATCGCTGGTGTAGCTGGTGA
TCAACAAGCAGCATTATTTGGACAAGCTTGCTTCGAACGTGGTGACGTGAAAAACACATATGGAACTGGTGGCTTCATGTTAATGAATACA
GGTGACAAAGCGGTTAAATCTGAAAGTGGTTTATTAACAACAATTGCTTATGGTATTGATGGAAAAGTAAATTATGCGCTTGAAGGTTCCA
TCTTTGTTTCGGGTTCAGCAATCCAATGGTTACGTGATGGATTAAGAATGATTAATTCAGCACCACAATCAGAAAGTTATGCGACACGAGTT
GACTCTACTGAGGGTGTTTATGTTGTTCCAGCTTTTGTAGGTTTAGGAACACCATATTGGGATTCTGAAGCACGTGGTGCGATTTTCGGTTT
ATCACGTGGAACTGAAAAAGAGCACTTTATCCGTGCAACTTTAGAATCACTATGTTACCAAACTCGTGACGTTATGGAAGCAATGTCAAAA
GACTCTGGTATTGATGTCCAAAGTTTACGTGTCGATGGTGGTGCAGTTAAAAACAACTTTATTATGCAGTTCCAAGCAGACATTGTTAATAC
TTCTGTTGAAAGACCTGAAATTCAAGAAACTACAGCTTTAGGTGCTGCATTTTTAGCAGGATTAGCAGTTGGATTCTGGGAGAGTAAAGAT
GATATCGCTAAAAACTGGAAATTAGAAGAAAAATTCGATCCGAAAATGGATGAAGGCGAAAGAGAAAAATTATATAGAGGTTGGAAAAA
AGCTGTTGAAGCAACACAAGTTTTTAAAACAGAATAA

Gene: glpD (aerobic glycerol-3-phosphate dehydrogenase)

Position: 1281187 to 1282908, length: 1722 nt, orientation: FORWARD

Perfect match to: (Strain_21235-AFTQ01000042-[8906:10627:r], highly conserved allele)

Sequence:

ATGTGGGACAAGTCTCTCGTTTTTACATTTTTTAGGAGGCGTTTTGGAATGGCATTGTCTACTTTTAAGAGAGAACATATTAAAAAGAATTT
AAGAAATGATGAATATGATTTAGTAATTATTGGTGGCGGTATTACAGGTGCAGGTATTGCACTAGACGCGAGTGAAAGAGGAATGAAAGT
TGCATTAGTTGAAATGCAAGACTTTGCACAAGGAACAAGCTCAAGATCTACAAAATTAGTCCATGGTGGTTTACGTTACTTAAAACAATTCC
AAATTGGAGTAGTTGCCGAAACTGGTAAAGAACGTGCGATTGTTTATGAAAATGGACCTCATGTTACGACTCCAGAGTGGATGCTTTTACC
AATGCATAAAGGTGGAACATTTGGTAAATTCTCAACATCAATTGGTTTAGGAATGTATGATCGTTTAGCAGGTGTTAAGAAGTCTGAACGT
AAAAAAATGTTATCTAAAAAAGAAACTTTAGCTAAAGAACCATTAGTTAAAAAAGAAGGTCTAAAAGGCGGCGGTTACTATGTTGAATATC
GTACTGACGATGCGCGTTTAACTATTGAAGTTATGAAGCGTGCTGCTGAAAAAGGCGCAGAAATTATCAACTATACTAAATCTGAACACTTC
ACTTATGATAAAAATCAACAAGTAAATGGTGTTAAAGTTATAGATAAATTAACTAATGAAAATTATACAATTAAGGCTAAAAAAGTGGTTAA
TGCAGCAGGTCCATGGGTTGATGATGTTAGAAGTGGTGATTATGCACGCAATAATAAAAAAATTACGTTTAACTAAAGGTGTACATGTTGTT
ATTGATCAATCAAAATTCCCATTAGGTCAAGCAGTATACTTTGATACTGAAAAAGATGGAAGAATGATTTTTGCAATTCCACGTGAAGGAAA
AGCGTATGTAGGTACTACAGATACATTCTATGACAATATCAAATCTTCACCATTAACTACACAAGAAGACAGAGACTATTTAATCGATGCGA
TTAATTACATGTTCCCTAGTGTTAATGTTACAGATGAAGATATTGAATCAACATGGGCAGGAATTAGACCATTAATTTACGAAGAAGGCAAA
GACCCTTCTGAAATCTCTCGTAAGGATGAAATTTGGGAAGGTAAATCAGGTTTATTAACTATTGCAGGTGGTAAATTAACAGGCTATCGTCA
CATGGCTCAAGACATTGTTGATTTAGTATCTAAACGCTTGAAAAAAGACTACGGTTTAACATTTAGTCCATGTAATACAAAAGGTCTGGCAA
TTTCAGGTGGCGATGTAGGTGGTAGCAAGAACTTTGATGCGTTTGTAGAGCAAAAAGTAGATGTAGCTAAAGGATTCGGCATTGATGAAG
ATGTTGCAAGACGTTTAGCATCTAAATATGGTTCAAATGTTGATGAATTGTTCAACATTGCGCAAACATCTCAATACCATGATAGCAAGTTA
CCATTAGAAATTTATGTAGAACTTGTTTATAGTATTCAACAAGAAATGGTATACAAACCTAACGATTTCTTAGTTCGTCGTTCTGGTAAAATG

TATTTCAATATTAAAGATGTATTAGATTATAAAGATGCTGTCATCGATATTATGGCAGATATGCTTGATTACTCTCCAGCTCAAATTGAAGCA
TATACTGAAGAAGTTGAGCAAGCAATTAAAGAAGCGCAACATGGAAATAATCAACCAGCAGTTAAAGAATAA

Gene: pldB2 (putative lysophospholipase, locus 2)

Position: 1283058 to 1283972, length: 915 nt, orientation: FORWARD

Perfect match to: (N315-BA000018-[1301115:1302029], highly conserved allele)

Sequence:

ATGACTGAAAAACAATTTAAATTAACTGTACAAGATAATACGAATATTGAAGTTAAAGTGAATTTTACAGATGTAGATTCAAAAGGAATTAT
TCATATATTTCATGGTATGGCTGAACATATGGAACGTTACGATAAATTAGCACATGCACTTTCAAAGCATGGCTTCGATGTGATACGTCATA
ATCATCGAGGACATGGTATTAATATTGATGAATCAACAAGAGGGCATTACGATGATATGAAACGAGTTATCGGTGATGCCTTTGAAGTAGC
GCAAACAGTGAGAGGCAATGTTGATAAACCATACATTATAATCGGACATTCAATGGGATCCGTTATAGCTAGATTGTTTGTAGAAACATAT
CCGCAATATGTTGATGGTCTAATTTTAAGTGGTACTGGTATGTATTCATTATGGAAAGGTTTACCAACCGTTAAAGTGTTACAACTGATTAC
AAAAATTTATGGTGCTGAGAAACGAGTTGAATGGGTTAACCAGTTAGTATCAAATAGTTTTAATAAAAAAATACGTCCATTACGTACACAA
AGTGATTGGATTTCTAGTAATCCAATTGAAGTAGATAACTTTATTAAAGATCCATATAGTGGATTTAATGTGTCAAATCAATTATTATATCAA
ACAGCCTATTATATGCTACATACATCACAATTAAAAAATATGAAAATGTTAAATCATGCCATGCCTATATTATTAGTTTCAGGATATGACGAT
CCTTTAGGTGATTATGGTAAAGGGATTTTAAAATTGGCGAATATATATAGAAAAGCTGGCATTAAAAATGTTAAAGTGAATCTTTATCATCA
TAAACGTCATGAAGTGTTATTTGAAAAAGATCATGACAAAATTTGGGAAGACTTGTTTAAATGGTTGAATCAATTTTATAAAAAATAA

Gene: miaA (tRNA delta (2)-isopentenylpyrophosphate transferase)

Position: 1283990 to 1284925, length: 936 nt, orientation: FORWARD

Perfect match to: (N315-BA000018-[1302047:1302982], highly conserved allele)

Sequence:

ATGAATAAAAATAAGCCTTTTATTGTAGTAATTGTGGGGCCAACTGCTTCAGGTAAAACAGAGCTTAGCATAGAACTCGCGAAGCGTATCA
ATGGTGAAATCATAAGCGGTGATTCTATGCAAGTTTACAAACATATGAATATTGGAACTGCAAAAGTAACACCTGAAGAAATGGATGGTAT
TCCACATCATTTAATTGATATCTTGAATCCTGATGATACATTTTCAGCATATGAATTCAAGCGATTAGCAGAAGATTTAATTACTGATATAAC
GAATAGAGGTAAAGTTCCAATCATAGCAGGTGGAACAGGCTTATATATTCAATCATTAATATATAATTATGAATTAGAAGATGAAACAGTT
ACACCTGCACAATTATCCATAGTTAAACAAAAGTTATCTGCATTAGAACATTTAGATAATCAGCAACTACACGATTATTTAGCTCAATTTGAT
GCGGTTTCTGCAGAAAATATTCACCCTAACAACCGCCAAAGAGTGTTGCGCGCTATTGAATATTATTTAAAAACAAAAAAACTTTTGAGTAA
TCGCAAGAAAGTGCAACAATTTACTGAAAATTATGATACATTATTATTAGGGATTGAAATGTCGCGTAAAACATTATATTCAAGAATAAATA
AACGTGTTGATATTATGTTGGATCACGGATTATTTAGAGAAGTGCAACAACTTGTTGAACAAGGCTATGAATCTTGCCAAAGTATGCAAGCT
ATTGGATATAAAGAATTAATACCTGTGATTAACGGACAAATGATTTATGAAGATGCTGTCAATGATTTAAAGCAACATTCACGCCAATATGC
AAAACGACAAATGACATGGTTCAAGAATAAAATGAGTGTTCATTGGTTAGATAAAGAAAATATGTCACTTCAAATGATGTTAGATGAGATT
ACAACCCAGATTAAGTAA

Gene: hfq (RNA chaperone)

Position: 1284940 to 1285173, length: 234 nt, orientation: FORWARD

Perfect match to: (N315-BA000018-[1302997:1303230], highly conserved allele)

Sequence:

ATGATTGCAAACGAAAACATCCAAGACAAAGCACTAGAGAATTTTAAAGCAAACCAAACTGAAGTAACTGTATTCTTTCTAAACGGTTTCCA
AATGAAAGGTGTTATTGAAGAATACGACAAGTATGTCGTAAGCTTAAATTCTCAAGGCAAACAACACTTGATTTACAAACATGCGATCAGC
ACTTATACAGTAGAAACTGAAGGTCAAGCATCTACTGAAAGTGAAGAATAA

Gene: gpxA-L1 (glutathione peroxidase locus 1)

Position: 1285395 to 1285871, length: 477 nt, orientation: REVERSE

Perfect match to: (N315-BA000018-[1303452:1303928:r], highly conserved allele)

Sequence:

TTATAATAATTTTTCTATTTCTCTTTCGATTTGAACAGGTTTTTTTTGAGGTGCAAATCGTTTAACAACGTTACCTTCGCGATCCACTAAAAACT
TAGTGAAATTCCATTTGATTTTCTCATTAAAGAATCCGTGTTGTGCCGCAGTCAAATATCTAAATAAAGGTAATTGATGTTCCCCTTTTACGT
CTATTTTTTGATGCATAGGGAAGGTAACACCATAGTTTAATTTACAGTTTTGAGCTGCTTCTTCGCCTGATCCAGGTTCTTGACCACCAAATT
GATTACAAGGGAAACCTAGAATTACAAACCCTTGATCTTTGTATTTCTCGTATAATGATTGCAAACCTTCAAATTGTGAAGTAAAGCCACATT
CGCTAGCTGTATTAACAATTAGCATAACGTCACCCTTATATGCATCTAATTTGTAAGTAACACCTTTATTTGTTTCTACTACAAAATCATAAAT
TGTCTCCAT

Gene: ynbA (GTP-binding proteinase modulator)

Position: 1285983 to 1287221, length: 1239 nt, orientation: FORWARD

Perfect match to: (COL-CP000046-[1343457:1344695], highly conserved allele)

Sequence:

ATGGCTCAGCAACAAATTCATGATACTAAAAATAAACTAGAAAAAGCTGTCTTAGTCGGTGTACATGCTCAAGATGATAAGCAATTTAATTT
TGAGTCTACAATGGAGGAATTATCATCTTTATCAGAGACTTGCCAACTTGAAGTGTTGGGTCAAATTACTCAAAACAGAGATCGTGTAGATC
GCAAATATTATGTTGGTAAAGGTAAAATTGAAGAAATTCAAGCATTTATTGAGTTCAAAGATATTGATGTAGTCATCACAAATGATGAATTA
ACGACTGCACAATCCAAATCACTAAATGAAGCTTTAGGTGTAAAAATTATTGATAGAACTCAGTTGATTCTTGAAATATTTGCATTAAGAGC
AAGAAGTAAAGAAGGTAAATTGCAAGTAGAGCTAGCACAACTTGATTATTTATTACCTAGATTGCAAGGCCATGGTAAAAGCCTTTCTCGT
TTAGGTGGCGGTATTGGAACTAGAGGCCCTGGTGAAACGAAGTTAGAGATGGATCGCAGACATATTCGAACTCGTATGAATGAAATTAAA
CATCAATTGCGGACGGTAGAAGAACATCGCGAAAGATATCGAAATAAAAGAAATCAAAATCAGGTGTTTCAAGTAGCTTTAGTTGGTTATA
CAAATGCTGGTAAATCATCATGGTTTAATGTTTTAGCAAATGAAGAGACGTATGAAAAAGATCAATTATTTGCAACGTTAGATCCTAAAACA
CGACAAATTCAAATAAATGATGGATTTAATTTAATTATTTCAGATACTGTTGGTTTTATACAGAAACTACCTACGACGTTAATTGCAGCTTTT
AAATCAACTTTAGAAGAGGCTAAAGGTGCAGATTTATTAGTACATGTCGTAGATAGTAGCCATCCTGAATACCGTACGCAGTATGACACAG
TTAATGATTTAATCAAACAATTAGATATGAGTCATATTTCTCAAATAGTTATTTTTAATAAAAAGGACTTATGTGATCATGCATCAAATCGTC
CAGCAAGTGATTTGCCTAATGTTTTTGTTTCTTCTAAAAATGATGGTGATAAATTACTTGTTAAGACGTTATTTATTGATGAAATCAAAAGGC
AATTAACTTATTATGATGAGACAATTGCGACGAATAATGCAGATCGATTATATTTTCTAAAACAACATACATTAGTGACTGAACTTAAATAT
GATGAAATTGAAAATGTTTATCGTATAAAAGGATTTAAAAAATAA

Gene: ynbB (putative C-S lyase)

Position: 1287240 to 1288478, length: 1239 nt, orientation: FORWARD

Perfect match to: (COL-CP000046-[1344714:1345952], highly conserved allele)

Sequence:

ATGAAAGATATAAGTAAGATAGTAGCTGACGTCGAATCAACGTTAGCACCATATTTTAAAGAAATTGAAGAAACAGCATATATTAATCAAG
AAAAAGTATTAAATGCATTTCATCATGTCAAAGCAACCGAAAGTGATCTACAAGGATCAACAGGATACGGGTATGATGACTTTGGACGTGA
TCATTTAGAAGAAATATATGCGCAGGCATTTAAAGCAGAAGATGCAATTGTTCGTCCGCAAATTATTTCAGGTACGCATGCGATTACTATTG
CATTACAAAGTTTATTAAAACATGGTGATGAATTAATTTATATAACGGGTAGTCCATATGACACTTTACTTGAAGTCATTGGCGTAAACGGA
AATGGTATTGAAAGTTTAATGGAGCACGGCGTATCGTATAAAGATATTGCACTTAAAGAAGGTAAGATCGATATTGAAAGTGTGTTAGATG
GGGTTTCTGAGCGCACCAAAGTAATAGCGATTCAACGTTCGAAAGGCTATGATCAAAGACCTTCAATTCCGCTAGATGAAATTGAAAAGGT
AATTACTAGGTTGAAAAACGTGCATCCTAATATTTTAATATTTGTGGATAACTGTTATGGGGAATTTGTTGAAAGACGTGAACCTATAGAAT
GTGGTGCCGATTTAATAGCAGGATCATTAATTAAAAACCCTGGCGGTGGTTTAGCTAAGATTGGTGGATACATTGCTGGTAGAAAAGATTT
AATTGAACGATGTGGTTATAGATTGACAGCACCTGGTATTGGTAAAGAAGCGGGTGCATCATTAAATGCATTGCTTGAAATGTATCAAGGT
TTCTTTTTAGCACCACACGTTGTCAGTCAGAGTCTTAAAGGTGCATTGTTTACTAGTTTATTTTTAGAAAAAATGAATATGAACACAACGCCG
AAGTACTACGAAAAACGAACTGATTTAATTCAAACAGTTAAATTTGAAACGAAAGAACAAATGATTTCATTTTGTCAAAGTATTCAACACGC
ATCCCCAATTAATGCACATTTTAGTCCAGAACCTAGTTATATGCCTGGTTACGAAGATGATGTTATTATGGCAGCTGGTACGTTTATTCAAG
GTTCATCGATTGAATTATCTGCAGATGGACCTATTCGTCCTCCTTATGAAGCATATGTTCAAGGAGGATTAACATATGAACACGTTAAAATT
GCTGTGACAAGAGCTGTTAATCAGTTGAAAGAACAAGGACTTATATAA

Gene: glnR (glutamine synthase repressor)

Position: 1288721 to 1289089, length: 369 nt, orientation: FORWARD

Perfect match to: (RF122-AJ938182-[1273563:1273931], highly conserved allele)

Sequence:

ATGATATCGAATGATGCAATCAGACGAAATATGGCTGTCTTCTCTATGAGTGTAGTAAGTAAGTTAACGGATTTAACGCCAAGGCAAATAC
GTTACTATGAAACACATGAACTCATCAAACCTGAAAGAACAGAAGGTCAAAAACGTCTGTTCTCACTCAATGATTTGGAAAGATTACTAGA
AATTAAATCATTATTAGAAAAAGGATTTAATATCAAAGGGATTAAACAAATCATTTATGACTCACAAGAGCATTTAACAACAGATGAACAAG
AGATAAGAAAAAAGATGATTGTAGATGCCACGCAAAAGCCTATTGGAGAAACTTTGCCAATAAATCGTGGTGATTTATCCCGATTTATTAA
ATAA

Gene: glnA (glutamine synthase, type I)

Position: 1289108 to 1290448, length: 1341 nt, orientation: FORWARD

Perfect match to: (MRSA252-BX571856-[1349006:1350346], highly conserved allele)

Sequence:

ATGCCAAAACGTACTTTCACTAAAGACGACATTCGTAAATTTGCAGAAGAGGAAAATGTAAGATATTTAAGATTACAATTCACTGATATTTT
AGGAACAATTAAAAATGTTGAAGTGCCTGTAAGCCAATTAGAAAAAGTACTTGATAACGAAATGATGTTTGACGGTTCTTCTATCGAAGGT
TTCGTACGTATCGAAGAATCAGATATGTACTTACATCCAGATTTAGATACTTGGGTAATCTTCCCATGGACTGCTGGACAAGGTAAAGTTGC
ACGTTTAATTTGTGATGTATATAAAACAGATGGAACACCATTTGAAGGGGATCCTCGTGCAAACTTAAAACGTGTATTAAAAGAAATGGAA
GATTTAGGCTTCACAGACTTTAACCTAGGGCCTGAACCAGAATTCTTCTTGTTTAAGTTGGATGAAAAAGGGGAACCAACTTTAGAACTTAA
TGATGATGGTGGATATTTCGATTTAGCACCTACAGATTTAGGTGAAAACTGTCGTCGTGATATTGTTTTAGAATTAGAGGATATGGGCTTCG
ATATTGAAGCTAGTCACCATGAAGTTGCCCCTGGTCAACATGAAATTGACTTTAAATATGCAGATGCTGTTACAGCATGTGATAATATCCAA
ACATTTAAATTGGTTGTTAAAACAATCGCACGTAAACATAATTTACACGCAACATTTATGCCTAAACCATTATTCGGTGTGAATGGTAGCGG
TATGCACTTTAACGTTTCATTATTCAAAGGTAAAGAAAATGCATTCTTTGATCCAAATACTGAAATGGGCTTAACGGAAACTGCATATCAATT
TACAGCAGGTGTGCTTAAAAATGCACGCGGATTTACTGCTGTATGTAACCCGTTAGTAAACTCATATAAACGTTTAGTACCTGGTTATGAAG
CACCATGTTATATTGCATGGAGTGGTAAAAACCGTTCACCATTAATCCGTGTACCATCTTCAAGAGGATTATCTACTCGTATCGAAGTACGTT
CAGTAGATCCAGCTGCAAACCCATACATGGCGTTAGCTGCAATCTTAGAAGCTGGACTAGATGGTATTAAAAATAAATTAAAAGTTCCAGA
ACCAGTTAACCAAAATATTTACGAAATGAACCGTGAAGAACGTGAAGCAGTAGGCATTCAAGACTTACCTTCAACACTTTATACTGCATTAA
AAGCAATGCGTGAAAATGAAGTTATTAAAAAAGCTTTAGGAAATCATATCTATAATCAATTTATTAATTCAAAATCAATTGAATGGGATTAC
TACAGAACTCAAGTATCTGAATGGGAAAGAGATCAGTACATGAAGCAATATTAA

Gene: A6U1C8 (putative protein)

Position: 1290941 to 1291138, length: 198 nt, orientation: FORWARD

Perfect match to: (COL-CP000046-[1348415:1348612], highly conserved allele)

Sequence:

ATGAATTGCTATGATGAAATATTCAATACAATCAAAGAATTGATAGAAAACAAAGAGATATCGAGTTATCAAATTAATAAAGATACTGGGA
TAAGTTACGGTAATATTAATGCTATGCGCCGTAGAGAAAGAAGAATAGAAAATTTAAGCTTAAAGAATGCAAAGATCTTATATGAATATGC
GAAAAAGGTATTGTAA

Gene: Q2FYY4 (putative protein)

Position: 1291827 to 1292051, length: 225 nt, orientation: FORWARD

Perfect match to: (COL-CP000046-[1349301:1349525], highly conserved allele)

Sequence:

TTGACAAAAATTATGAACCAGTTTAAAGAAATTTATAATACAATAGAAAAATTACTAAATGATAAATCAATATCTAATTATAGAATTAATCAA
GACACTGATGTTTCTTATGGTGGTATAAGTGAATTAAGAAGCGGGAAAAGAAAAGTGAATAATTTAACTTTAGAAACAGCGGAAAAACTCT
ATAATTACCAAAAACAATTAGAAATAATGATTGAAGATTAA

Gene: A5ISJ3 (putative protein)

Position: 1292345 to 1292551, length: 207 nt, orientation: FORWARD

Perfect match to: (N315-BA000018-[1310400:1310606], highly conserved allele)

Sequence:

ATGAATGAGATTGAAACTATTATAAGTGAAATAGAAAAGTTATTAACTAACAATACACCATATAGTATTTCAAAAAATTCAGGTGTACCACG
TCAAACAGTTACAGATTTAAAGGTAGGTAAAACTAAAATAAAAGAAGCTAAATTTAAAACGATAATCAAGTTATATGAATATCAAAGAACA
TTAGAAAATAAAACAGAATGTTAA

Gene: Q2YXQ4 (putative protein)

Position: 1293791 to 1293976, length: 186 nt, orientation: FORWARD

Perfect match to: (COL-CP000046-[1351264:1351449], allele observed in CC8+CC22+CC80+CC239+CC4803)

Sequence:

ATGTCAGAATACAAGAAAAAAATAATTGAATTAATTGAAAGTAATTTAACAGGATATGAAATTTCTAAAAAAACTGGAGTTTCTCAATACGT
ACTTTCACAATTAAGACAGGGCAAACGCGAAGTAGATAATCTAACCCTGAATACAACAGAAAAATTATATGAATATGCCAATAAAGTTTTGT
AA

Gene: Q2FYX8 (putative protein)

Position: 1294721 to 1294972, length: 252 nt, orientation: FORWARD

Perfect match to: (N315-BA000018-[1312768:1313019], highly conserved allele)

Sequence:

ATGCTTACATTAATAAAATTGAAAGAAGATGAACAGGTTATAATATATGAATATATACCTGAAGATGATATAAGTAACGGTAAAGGTTCAG
TAACTTTTAATAAAAAAGATGCAGAGGTTATAGATTTCTCATTATCTGAAATAGAAAATGAAGAATATTTTATGTTATATCGTAATAAGTCTT
TTTCTGTAGTAAGAGACTTTATCGAGAAGCAAGAATTTCCCGAAAATTATAAAATAGCATGGTATTAA

Gene: Q8NWX1 (putative protein)

Position: 1295447 to 1295881, length: 435 nt, orientation: FORWARD

Perfect match to: (11819-97-CP003194-[1345824:1346258], highly conserved allele)

Sequence:

TTGCTTAAAGATGCCAAAGAAAACAATGATAGCAATGAAGTAGCTTATTTATTAAAAGATGGTAAAGTTACAAAAGTATATGGTGATCAAG
ATAGTGTATCTTTTGCACCAGGGGAAAAAGCAACAGAATTGTTATTTAACAGTAAACCGAATTCAATTGTTATGTTACATAACCATCCTGGG
CAGTCTAGTTTTTCTCTTACAGACTTGTATTTATTTATATTTAATAATTCTATTAAAACACTGACAATTGTTACAAATAAAGGTCAAACAAAGT
ACTTAACTAAGACAAAAGAATATTGCAAATCAACTTGTATTGATTGTATTAAAAAATATAATAAAAATAAGAATATAAAAAAATTCAATCAT
AAGGATATTGATATGATTCTAAAGAGATTATATAATAGTGGTAACATAATATATAAAGTTAGGTGA

Gene: A0A0H3JW92 (bacteriophage capsid protein)

Position: 1296169 to 1296381, length: 213 nt, orientation: FORWARD

Perfect match to: (MW2-BA000033-[1314367:1314579])

Sequence:

ATGGAGAAAAATGAAAGTAATATTACTGACGTAACTCAGAACGAAGAGCAACTAGACAACAGTGATGAACAATCACAACAGAATGAGAAA
ACATTTTCTCAAGAAGAAGTATCACAATTGATTAAAGAGCGTATAGCTAGAGAACGCAAAAAATCAGATGAACGTATTAAAGATGCGGTTC
AAGAAGCTGAGAAGTTAGCTAAAATGAAGTAG

Gene: Q8NWW7 (putative protein)

Position: 1297162 to 1297623, length: 462 nt, orientation: FORWARD

Perfect match to: (TCH70-ACHH02000011-[409438:409899:r], allele observed in CC1+CC80)

Sequence:

GTGCCACATGTTGGCAGTTGGCGAGACAAGTTCTTTAAAGAGCGTGAAGGTAAATATCAGGTAGAAGTAAAAGAAGCTAAATTACAAGAA
AAAGCTAAAAACCAGATGAAAGAAATGATTGAAAGTTGTAAAATGAAAATAGAAATAAATTGTGAAAAACAAAATGGACATATGTTAGGT
CATCATCTATATAATGAAAATAAAAAAAGAGCCATTTTAAATAATAAGAAATTGCCTAGCTATACAATACTTTCTATAGATTTATTGAATGAA
TTGTTAAGAGAAAAAATGTCAACAGGCAATCTAATATTAAGTGATGAGCTATTTGATATGAAAGAGATTATTAATTTTAATCAAATTATTGG
AAAAGCACATATCGATAATGTGTATATTGAAACCAGAAAAGGAAAAGTGCATTATTCGAAGACAGGTGCTCATATAGTACCTTATATTGAT
AAGTAG

Gene: A6QGL8 (aromatic amino acid beta-eliminating lyase/threonine aldolase)

Position: 1298385 to 1299410, length: 1026 nt, orientation: REVERSE

Perfect match to: (11819-97-CP003194-[1348762:1349787:r], highly conserved allele)

Sequence:

TTAGATTAATTCTAATAGTTTATTTAAATTTTCTTCGGTTGTCGCCCAGCTGGTTGCGAATCTAACAACACGATGTTGATCATCGTATTTTTCC
CAAATAGCAAATTTAACTTTTTGTTCTAACTCTGCTATTTTTTCGTTACTTAAAATAAAAAATTGTTGATTGGTTGGAGAATCAAAGTAAAGA
CGATAGCCTTTATTTATAAACCCGTCTTTCATCTTATTTGCCATTTCGATAGCATGTCTGCTTATATTAAAATATAAATTGTCCGTAAATAATTC
TAAAAATTGTATGCCTGTTAACCGTCCTTTTGCTAGAAGGGCACCGTGATGCTTGATTCGAGTGGTAAATTGTTTCGGTTCATTATTTTTCGT
AAAAACAATGGCTTCCCCGCATAATGCACCTATCTTCGTACCACCTATATAAAATACATCACAATATTTAGCGATGTCTTTAATAGTCATATCT
GATTGGTCACTCATCAATCCATACCCTAATCGTGCACCATCCATAAATAATGGAAGCTGATATTGCTTACATACTTTACATAACTCTTTCAATT
CTGATTTAGAGTATAATGTGCCATATTCTGTAGGATGAGAAATATATACCATTCCTGGGAATACCATATGGTCCTTTTTAAAATCACTTTTAA
ATGTCTCCATGTAAGTTTCAACATCTGAAGCACTAACTTTTCCTTCCTTAGAGGGTATAGTAATTACTTTATGTCCACTATATTCAATTGCACC
GCCCTCATGCACAGCAACATGACCAGTGTCTGCTGAAATAACCCCTTCGTAACTTTCTAACATTGAATTAATAACAACCTGATTGGTTTGCGT
TCCACCTACTAAAAAAACGGATAGTAGCATTTGGACAATCAATTGTATCTTTAATCTTTTCAATTGCCTGAGCTGTGAATTGATCAAAGCCATA
TCCCGAAGCTTGTACAAGATTTGTATCTACTAATCGTTTTAATACTTTTTCATGAGCACCTTCTAAATAATCATTTTCAAATGAGATCAC

Gene: A5ISJ9 (putative protein)

Position: 1299680 to 1299877, length: 198 nt, orientation: FORWARD

Perfect match to: (N315-BA000018-[1315775:1315972], highly conserved allele)

Sequence:

ATGGAACAAATTAAACTTAAAACTTTTACAGCTGAGACTTTAGAGTTATTAGAAAAAAACATTAATGCTTTTTTAAGTTCTGAAGAAGCTAC
AAATTTAAAATTAGTAAATATTACTATTAAAGAAATAGAAGAAAGAACATTCCCAAATAATGAAGAAGAATTCAATGCAATTTTAACTTTAT
CTGTGAATAAATAA

Gene: cls1 (cardiolipin synthases 1)

Position: 1299934 to 1301415, length: 1482 nt, orientation: FORWARD

Perfect match to: (COL-CP000046-[1357732:1359213], allele observed in CC8+CC80+CC96+CC239)

Sequence:

ATGCGATTTACATTTTCAAACGATTTAGGAACGTTATTTACTATTATTTTAGCCATTGGATTCATCATTAATTTAGTATTGGCTTTTATTATTAT
CTTTTTAGAAAGAAATAGGCGTACAGCGAGTTCAACTTGGGCATGGCTATTTGTACTTTTTGTCTTACCATTGATTGGTTTTATTCTTTACTTG
TTTTTTGGTAGAACCGTTTCGGCACGCAAATTGAATAAAAACAATGGTAACGTGTTAACGGATTTCGATGGACTTTTAAAACAACAAATAGA
AAGCTTTGATAAAGGTAATTATGGTACTGATAACAAACAAGTTCAAAAACATCATGATTTAGTACGTATGCTTTTGATGGATCAAGATGGTT
TTTTAACTGAAAATAATAAAGTTGATCATTTCATTGATGGAAATGATTTATATGATCAAGTTTTAAAAGATATTAAAAATGCAAAAGAATAT
ATCCATTTAGAGTACTATACTTTCGCTTTAGATGGTTTAGGTAAAAGAATTTTACATGCTTTAGAAGAAAAATTGAAACAAGGTCTAGAAGT

AAAAATATTATATGATGATGTTGGATCTAAAAATGTTAAGATGGCAAATTTTGATCATTTTAAATCGTTAGGTGGAGAAGTTGAAGCATTTT
TTGCTTCAAAATTACCGTTATTGAATTTCAGAATGAATAATAGAAATCATAGAAAAATCATCGTAATCGATGGTCAACTAGGTTATGTCGGA
GGATTTAACATTGGTGATGAATATCTAGGATTAGGAAAATTAGGATATTGGAGAGATACGCATTTACGTATACAAGGGGATGCGGTTGAT
GCACTGCAGTTGCGATTTATTTTAGACTGGAATTCGCAAGCGCACCGTCCACAATTTGAATATGATGTTAAGTATTTCCCTAAAAAGAACGG
ACCATTGGGCAATTCACCAATTCAAATAGCTGCAAGTGGCCCGGCTAGTGACTGGCATCAAATTGAATACGGTTATACAAAAATGATTATG
AGTGCAAAGAAATCTGTATATTTACAATCACCATATTTCATTCCGGATAATTCATATATAAATGCCATTAAAATTGCTGCTAAATCAGGTGTA
GATGTACATTTAATGATTCCATGTAAGCCAGATCATCCATTAGTATATTGGGCGACATTTTCAAATGCCTCTGACTTATTATCAAGTGGTGTT
AAAATTTATACGTATGAAAATGGATTTATACATTCTAAAATGTGCTTAATTGATGATGAAATCGTATCAGTGGGCACAGCAAATATGGACTT
TAGAAGTTTTGAATTAAATTTTGAAGTAAATGCCTTTGTATATGATGAAAATCTTGCTAAAGATTTAAGGGTGGCTTATGAACATGATATTA
CAAAATCAAAACAACTAACCAAAGAATCATATGCCAATAGACCGCTGTCTGTTAAATTCAAAGAATCGTTAGCAAAATTAGTTTCGCCAATT
TTATAA

Gene: Q99UF3 (putative protein)

Position: 1301802 to 1301990, length: 189 nt, orientation: REVERSE

Perfect match to: (N315-BA000018-[1321100:1321288:r], highly conserved allele)

Sequence:

CTAATTTTTTCGCTTATTATAAAACGAAACAATCAAGTAAACGATAAAGCCTACAAAGATACCCAATAAAATAGATAGTACTGCCGTCACTA
TTAATGGTAATTTGAAAAATATTTGTAGGAAAATACCAATGATAATTGCGATAATTACTGCAATTAATGTGACTGTATTTTCATTTGAAATGT
TCAT

Gene: nuc2 (thermostable extracellular nuclease locus 2)

Position: 1302129 to 1302662, length: 534 nt, orientation: FORWARD

Perfect match to: (N315-BA000018-[1321427:1321960], highly conserved allele)

Sequence:

ATGAAGTCAAATAAATCGCTTGCTATGATTGTGGTAGCCATCATTATTGTAGGTGTATTAGCATTTCAATTTATGAATCATACGGGTCCTTTC
AAAAAGGGGACGAATCATGAAACTGTACAAGATTTAAATGGTAAAGATAAAGTACATGTTCAAAGAGTTGTGGATGGTGATACATTTATT
GCAAATCAAAATGGTAAAGAAATTAAAGTTAGGCTTATAGGGGTTGATACGCCAGAAACGGTGAAACCGAATACGCCTGTACAACCATTT
GGCAAAGAAGCATCAAATTATAGTAAGAAGACATTAACAAATCAAGATGTTTATTTAGAATATGATAAAGAAAAACAAGATCGCTATGGTA
GAACATTGGCGTATGTATGGATAAGTAAAGATCGTATGTACAATAAGGAATTAGTGGAAAAGGGACTTGCTAGAGAGAAGTATTTTTCAC
CAAATGGCAAATATAGAAATGTATTTATAGAAGCACAAAATAAAGCTAAACAACAGAAATTAAATATTTGGAGTAAATAA

Gene: Q5HG96 (putative protein)

Position: 1302805 to 1303659, length: 855 nt, orientation: REVERSE

Perfect match to: (COL-CP000046-[1363806:1364660:r], highly conserved allele)

Sequence:

TTACTTGAATATAGCTGTGATTTGATCAACCTGTTCTTTATTTAAAGGGTGTTTAGATAGCTCTATGGCTTCGCTAATAATACGTCGTAACGA
AGATTTTGTTGAAACTGTATCAACATATTTATCTCTTTCTTCAGAACCTTGAATGATATTAACTGTACCATCACTAAAGTAAATAACACCGGTA
CTTTGGACTTTAAAGTTAAAGTTTTGTTCAATATGATCCTTTAATGCTTTCGCATTATTTGCTGCTAATTGATACGGATCATAATCATAAAAAT
CATAAATAACACGATTTGGTTGAACTGTTTCTGTAAAAGTATAGATAGACTTTCTTGATGAATTAAACTGATCATGATATTGCTGGCTAATGT
AATGACCGACAACTTTTTCAATATTACTATTGCTTATTTCTGTATCATGCTCATCAGGTACATCAAAGTGATAAAATGTTTTTCACCCCAGCT
TTTTACATCAACGTTTATTAAACCGATATCTGAAACAATGATAAAATCAAATGAACGTGCATACTCGAAAAACGGATGTTTAGTAGCTAAAT
TGCTTGTAACGATAATATCGTAATATTTTAATTTACCATTTTCGAGATAGCTATCTAAAATCTTGCGTAATTCTCTTTTAGCATTTTTGTATGCA
TGGTGGCCAACATCATTATTAGAACTCAGCATTTGACTTCTTAATTCAGCATTTTCTGCACTTAATGCTTTGTTCTTTTTAATAAGTTGCTTTCT
TGCATAAACTTCGGTATCTATTTTACTATTACTATACCTTTGATTTAAAACTAATATACCAATTAATGCTACAATGATAATGATAAGTACAACA
TAAAAAGACAT

Gene: Q5HG95 (putative protein)

Position: 1303975 to 1304565, length: 591 nt, orientation: FORWARD

Perfect match to: (MRSA252-BX571856-[1385422:1386012], highly conserved allele)

Sequence:

TTGAGAGTGAATGAAATGAATGCTAAAGAACAATTAGTGGACAATTTAATGAAAACATCATCGCAATTATTTAAATTTCACGGTGAAGTTG
CCATGCAGCTTTTCTTAAATGATGAATTAAAATTACCTTCTATTGTTGAAATATGCGTGGAACGTAAGCGTTTAAGTGATATTGTGAAAGTTA
TTCCGCAATCATATGCGTTACTATACATAGATAAGCAAGATCAAGCAATAGCTAAAGAAGATTTATCACTTTCAAAAATTGCAAAAGTTTAT
GTGCAATATGATGATACAACAATAATGAGTATTTTCGTTTATGATGTAGTAAACGATGAATGGATTTTTAGATTGGATCCGAATATACGTAT
ACCTAAGAGTAACATATACTTCCATAGTTTAAATTGGGATGTGGATTATATTAAACCGGAGATCGTTCTAATGTATGATCTAATGCAACACC
ATCAGTATCATCATTATTCCAATTATAAACGAGTCATAGATGCATTAAGCTACTATCAATTTTTTATTTTAAAATTTGTAGTAGGTGAGCAAC
GTATTAAGGATGCAATCCAGAGAACAATAAATAATTAA

Gene: thrA (aspartokinase)

Position: 1304619 to 1306001, length: 1383 nt, orientation: REVERSE

Perfect match to: (08-02119-CP015645-[846420:847802], allele observed in ST582+CC5+CC8+CC97+CC398)

Sequence:

TTAGTTTTTATCACACAAGTTTTTTAATGCAACCCCGTGATAGCAAAACTCATATGTAGATAATACAGCTTTTTCAGCATCATCTACATGAATT
CCAAACATCATCGAGATTTCCGAAGCACCTTGGTTAATCATTTTTAAGTTAATTTTTGATTCAGCTAAGGCATGTGTAATTTTATTTGCAGTAC
CGATGACTTTATTCATACCTTCGCCGACAATCATTAATATTGCTAAATCATGCTCAATACTTAGCTCATCAACATCACATTTTTGACGAATTTC
ATTTAATACTTTTGTTTCTTTATTTTGAATTTGTTTTGAACGCATAACGATACTGATAGTATCAATACCTGAAGGCATATGATCAAATGAAATA
TTATTATCCTCTAAGACACCTAATATCTTTCTAGTAAACCCGACTTGTCTATTCATTAAATACTTTTTGATATTAATAACGGTAAAATCTTTATC
ACAACTTATACCGCTAATCACATTTTTCGCATTTATTTCTCTATCATGCACTATAAATGTACCTTTATCTTGAGGGCGGTTCGTATTTTTAATCA
CCACAGGGATGCGATCTTTATAAAGTGGTTGTAAGGCTTCATCATGGAAAACACTAAAACCAGCATAAGATAATTCACGCATTTCTCGATAA
GTGATTTCTTCGATTAATTCAGGATCTTTGATGATATTTGGATTAGCTTTATAAATACCAGACACATCGGTGAAATTTTCATAAATTGTAGCT
CTAACACCACTTGATATGATGGCGCCAGTTATATCTGATCCGCCACGTGGAAATGTAACTATATATCCTTCATGAGATACGCCGAAAAATCC
TGGGATAATTAGTTTCTCATCATAATCTCTTAATTTTTTAATTTCAGAGTAAGCACTATCTAATATTTGTGCTTCTTGTGGGACGTCAGTAACA
AAAATACCCGCTTCCTTCGGTGATATATATTTTGTTGGTATACCTTGACTATTATTATATAAAGCTATCAATTGCGCATTAAAATCTTCACCAC
AAGAAAGTAATGCATCTAATAGTCTCTTTGGTTCATTTTTTAATTGATTAATATAATGTTCCAAAGTCACATCTATCGTCCGTAAAATACTTTC
ATCCATTTGCAATTCTTTTACAATATCATCATAACGCTGAATAATTTCTCTTTTTTTATCATGATAATCAAGATGATTAATGACCTTTTCATATA
ATCTGATTAACAAATCAGTTGTTTTAATATCATTATCATGTCTTTTACCTGGAGCAGAAACGATAACAATCTTTCGCTCTGGATCAGAATTAA
CAATATTTAAAACCTTTTTAATTTGAGTAGCATTGGAGACGGAGCTACCACCGAATTTGGAAACTTTCAT

Gene: hom (homoserine dehydrogenase)

Position: 1306192 to 1307472, length: 1281 nt, orientation: FORWARD

Perfect match to: (COL-CP000046-[1367193:1368473], highly conserved allele)

Sequence:

ATGAAAAAATTAAATATAGCATTATTAGGATTAGGTACTGTCGGATCTGGTGTTGTTAAAATCATCGAAGAGAACCGACAGCAAATTCAAG
ATACATTAAATAAAGATATTGTCATAAAGCATATTCTTGTTCGAGATAAATCTAAAAAGAGACCGCTAAATATTAGCCAATATCATTTAACTG
AAGATGTTAATGAAATTTTAAATGATGATTCATTAGATATTATCGTTGAAGTCATGGGAGGAATTGAACCAACTGTAGATTGGTTAAGAAC
AGCACTTAAAAATAAAAAACATGTTATTACCGCAAATAAAGATTTATTAGCAGTACATCTTAAACTTTTAGAAGATTTAGCAGAAGAAAATG
GTGTAGCTTTAAAGTTTGAAGCGAGTGTAGCAGGTGGTATTCCGATCGTAAATGCCATAAATAATGGTTTGAATGCGAATAATATTTCAAA
ATTTATGGGAATTTTAAATGGTACCTCTAATTTTATTTTATCTAAAATGACTAAAGAGCAAACGACATTTGAGGAAGCACTTGATGAAGCGA
AAAGACTTGGTTTTGCTGAAGCGGATCCAACTGATGATGTAGAAGGGGTAGATGCAGCGCGTAAAGTTGTCATTACATCATATTTATCATT
TAACCAAGTCATTAAATTAAACGACGTTAAACGAAGAGGAATTAGTGGCGTAACTTTAACTGATATTAATGTAGCCGATCAACTGGGGTAT
AAAATTAAATTGATTGGTAAGGGAATATATGAAAATGGCAAAGTTAATGCATCGGTAGAACCAACGTTAATTGATAAAAAGCATCAATTAG
CAGCTGTAGAGGATGAATATAACGCGATTTATGTCATTGGTGATGCTGTTGGTGACACGATGTTTTATGGAAAAGGAGCAGGCAGTTTAGC
AACAGGTAGTGCCGTTGTCAGTGATTTATTGAATGTAGCATTATTCTTTGAATCAGATTTACACACATTGCCACCACATTTTGAATTAAAGAC
AGATAAAACACGGGAAATGATGGATTCAGATGCAGAAATTAATATTAAAGAAAAATCCAATTTCTTTGTAGTAGTGAATCATGTCAAAGGT

TCAATTGAAAATTTTGAAAATGAGTTAAAGGCAATATTACCATTTCACCGATCATTAAGAGTTGCAAATTACGATAATCAATCATATGCCGCT
GTTATAGTTGGATTGGAATCATCACCGGAAGAATTAATCACTAAGCATGGATACGAAGTTGACAAAGTATACCCAGTAGAAGGAGTTTAA

Gene: thrC (threonine synthase)

Position: 1307478 to 1308539, length: 1062 nt, orientation: FORWARD

Perfect match to: (11819-97-CP003194-[1357855:1358916], highly conserved allele)

Sequence:

ATGAGAAGATGGCAAGGATTAGTAGAAGAGTTTAAAGCACATTTACCAGTAAATGAAAATACACCAAAATTAACATTGAACGAGGGAAAT
ACACCACTCATTCATTGTGAAAATATGTCTAAAATACTAGGCATAGATTTATATGTGAAGTATGAAGGTGCCAATCCGACAGGTTCATTTAA
AGATCGCGGTATGGTAATGGCTGTGACAAAAGCAAAAGAGCAAGGTAAGAAAATTGTAATATGCGCTTCGACTGGAAATACATCAGCGTC
TGCAGCAGCATATGCAGCGAGAGCAGGTTTAAAAGCTATCGTCGTAATACCAGAAGGTAAAATTGCATTAGGTAAATTGTCGCAAGCAGT
AATGTATGGTGCAGAAATCGTTTCTATTGAAGGAAACTTTGATGAAGCTTTAGAAATTGTAAAAGAAATTGCAAAAAGTGGCGAAATCGAG
CTTGTAAACTCTGTCAATCCATTTAGAATCGAAGGACAAAAGACAGGCTCATTTGAAATTGTACAACAATTAGACGGTGAAGCACCTGATAT
TTTAGCGATTCCTGTAGGTAATGCAGGTAATATTACTGCATATTGGAAAGGCTTTAAAGAATATCATGAAGCTAAAGGATCACAATTGCCG
AAAATGTTTGGCTTCCAAGCTGAAGGCGCATCACCAATTGTTCAAAATAAAGTCATTAAAAATCCTGAAACGATTGCAACTGCTATTCGAAT
TGGTAATCCTGCTAGTTGGGATAAGGCGACTAATGCTCTTAAAGAATCAAATGGATTAATAGATAGTGTTACTGATGATGAAATTCTAGAA
GCATATCAGTTAATGACAACTAAAGAAGGTGTCTTTAGTGAACCAGCGAGTAATGCTTCTATTGCAGGTTTAATTAAATTGCATAGACAAG
GTAAATTACCTCAAGGTAAAAAAGTAGTTGCTATTTTAACTGGTAATGGATTAAAAGATCCTGATACTGCTATTTCACTACTAGATAATCCG
ATAAAGCCATTGCCAAATGATAAAGATAGCATTATCGATTATATTAAAGGAGCTTTATAA

Gene: thrB (homoserine kinase)

Position: 1308541 to 1309455, length: 915 nt, orientation: FORWARD

Perfect match to: (MW2-BA000033-[1329946:1330860], highly conserved allele)

Sequence:

ATGTCGAATGTTTTGGAGTTAACAATTCCTGCATCAACAGCCAACCTTGGAGTTGGCTTTGATTCTATAGGTATGGCTTTAGATAAATTTTTG
CATCTGTCTGTAAAGGAAACATCAGGGACAAAATGGGAATATATTTTCCATGATGATGCATCTAAGCAATTGCCTACTGACGAAACAAACTT
TATTTATCATGTAGCACAACAAGTTGCTTCTAAATATAGTGTTGACTTGCCTAATTTATGTATCGAAATGAGAAGTGATATTCCATTGGCAAG
AGGGTTAGGTTCGTCAGCTTCTGCTTTAGTAGGAGCTATATATATCGCAAATTATTTTGGTGATATCCAACTGTCTAAACATGAGGTATTAC
AATTAGCGACTGAAATCGAAGGACATCCTGATAATGTTGCGCCGACCATTTATGGTGGTTTAATCGCTGGATATTATAATGATGTCTCGAAA
GAAACGTCAGTTGCACATATCGACATACCAGACGTGGATGTGATTGTAACGATACCAACTTATGAACTAAAAACAGAAGCATCAAGACGTG
CTTTACCACAAAAATTAACACATAGTGAAGCGGTTAAAAGTAGTGCAATTAGTAATACAATGATTTGTGCATTAGCACAGCACAATTATGAA
TTAGCAGGTAAACTCATGCAACAAGATGGCTTTCATGAACCGTATCGTCAGCATTTAATTGCTGAATTTGATGAAGTGAAAACAATTGCTAG
TCAACATAATGCCTATGCAACTGTAATTAGTGGTGCTGGACCAACTATTTTAATATTTAGTCGTAAAGAAAATAGTGGGGAATTGGTTCGCT
CTTTAAATAGTCAGGTAGTATCATGCCATTCTGAATTGGTCGATATTAATATCAGTGGTGTTAAAGAACGAATTGTATACCAATAG

Gene: yxeH (putative hydrolase)

Position: 1309513 to 1310316, length: 804 nt, orientation: FORWARD

Perfect match to: (N315-BA000018-[1328813:1329616], highly conserved allele)

Sequence:

ATGACAAATTATAAAGTTGTCGTTTTAGATATGGATGACACATTGCTAAATTCAGATAATGTGATATCAGAAGAAACTGCAAATTATTTAAC
AGCAATTCAAGATGAAGGTTATTATGTTGTTTTAGCATCTGGTAGACCTACTGAAGGTATGATTCCAACTGCTAGAGATTTAAAATTACCTG
AACATCATAGCTATATTATTAGTTATAACGGTAGTAAAACGATTAACATGACTAATGAAGAAGTAGAAGTAAGTAAATCGATTGGTAAGCA
AGATTTCGATGAAATTGTAGATTATTGTCGAGACAGAGGCTTTTTCGTTCTTACATATCATGATGGTCAAATTATTTACGACAGCGAACATG
AGTATATGAATATTGAAGCAGAATTAACAGGTTTACCGATGAAACGTGTTGATGATATCAAAGCGTATATTCAAGGCGATGTACCCAAGGT
CATGGGTGTAGATTATGTAGCGAATATTACAGAAGCTAGAATTGATTTGAATGGTGTGTTCAATGATAATGTAGATGCTACGACAAGTAAG
CCATTCTTCTTAGAATTTATGGCCAAAGATGTTTCAAAAGGTAATGCAATTAAAGCGTTATGTCACAAATTGGGATATTCGGTGGATCAAGT
CATTGCTTTTGGTGATAGTATGAATGATAAATCAATGTTTGAAGTCGCAGGTCTAGCTATTGCTATGGGGAATGCATCAGATGAACTTAAGC
AATATGCAGATGAAGTTACGTTGGACCATAATGAAAATGGTATTCCACATGCGCTCAAAAAAATTGTTATAA

Gene: Q5HG88 (putative protein)

Position: 1310609 to 1310923, length: 315 nt, orientation: REVERSE

Sequence:

TTATTTAGCTGTTTGTGTCCAGTCATGCTTACCTCTGAAACGTTTTACATGAGCATATATTTGGTCAGCAGTATAATCTAAATTTGTATAAACT
GTAATACTGAACGCTGATTGGTTTTCAGCAAATGATTCTTCGGAAAATAATTCCTTTAAGTGCATAAATAAATCATTCATTTGTTTATGGTTT
AAACCAGGATATTCCCTTAAAGTTCTCTTTAATAATTGTCCGTTCTTTTCTTCTAATGCTTGAACTACAATAACAAATCTTTCATCTTGCTTTAG
TACATCATCAAATAAATTTGTTTGTCCAATCAT

Gene: Q5HG87-lysP2 (putative amino acid permease)

Position: 1311141 to 1312595, length: 1455 nt, orientation: REVERSE

Perfect match to: (MW2-BA000033-[1332546:1334000:r], highly conserved allele)

Sequence:

TTATTTTTGATTGTCATAATCGTGTGGTTTTAAATTAATTGTTTCTAGCTTTACAAATTTTGTTTTGTGAATGATTTTATGAATAAAGTAAATCA
ACGCTAGAATGATTAAAGGTAAAAAGTTTTTAAAAGCATTTAACCATTGATCTTTTAAAATATATTCAACTGAGCCACCAAATAGCAAGAAT
AATAGTGTAGTGATGACAATGATTGGTCCTAATGGATAAAAAGGTGCTTTATATGGTAGGACCTTATTAGGGTCTTGACCTTGTTTTTTAAT
AGCTTGTCGCAATCGTATTTGTGACCAAATGCTTGATCCCCAAACAACTATAATCATTGAACCAATAATTTCAAGTAAATTAAAAACGGCATT
TGAATTAAAGTTTGCATAAATAATAACAATAACAACGACTGCATAAGTAGTTAATAATGCTCTTAAAGGTAACTTAGTTGTCTTGTTTAATTT
ACTTAAAAATTGGGGTGCTTTTTTGTCTGAACTTAAGGAATACAACATTCTGCCTGTTGTATAAACACCTGAATTTGCAGCGGATAATAATG
AAGTTAAAATAACCGCGTTGATTACTGATGCTGCAAAGGCTATGCCTACTCTATCGAATACAATTGTAAATGGGCTTTGACTTATTGAACTA
CTTGCTCTTAATAATGATGGATCTGTGTACGGAATAATTGCACCAATTACTGCAATTGATAAGACATAGAATAAAAGAATACGCCAAAATAC
TTGTTTAATTGCCTTAGGCATAGACTTTTTAGGGTCATCTGATTCACCAGCAGTTACTGCTACTACTTCTGTACCACCAACCGAAAATCCGGC
GACTAATAATACGCCTAAGAAACCAGAGATACCACCAACAAACGGTGCTTGGCCTTTTGTATAGTTTTCAAATCCATATGTATGACCACCTA
AGATACCGAAAATCATTAAAAAGCCAAAAATAACGAATACGATAATTGTTAACACTTTAATCAATGATAACCAAAACTCAGTTTCTCCAAAT
GATTTTACAGAAAAAATGTTTAATAATAGTAAAATTGTAATAAAGATTAAGCTCCAAGTAATGGGGTGGAAAAATTTAAATGTGTCCCAGA
AATAAAGCACATTTGACGCTACTATGACATCAACACTTGTAACTAATGACCACAATGCCCAATACAACCATCCCATGGTAAAGCCAAGAGAT
GAGTCAATAAAGCGTGTTGAATAAGAGCTGAATGAACCTGATACTGGATAAAATGTTGCCAACTCTCCAATTGATGACATTAAGAAATATA
GCATGACACCAATAACAAGATAAGCGAGTATAGCGCCTCCAGGACCAGCTTGAGAAATGATATTACCAGTAGCTACAAATAGACCAGTCCC
AATTGCACCACCTATAGCAATCATGGAAATGTGTCTTGAGTTAAGACTACGGTTCATTTTATTATCTTCCAT

Gene: katA (catalase A)

Position: 1312799 to 1314316, length: 1518 nt, orientation: FORWARD

Perfect match to: (VRS9-AHBS01000029-[48501:50018], allele observed in CC5+CC8+CC97+CC772)

Sequence:

ATGTCACAACAAGACAAAAAGTTAACTGGTGTTTTTGGGCATCCAGTATCAGACCGAGAAAATAGTATGACAGCAGGGCCTAGGGGACCT
CTTTTAATGCAAGATATTTACTTTTTAGAGCAAATGTCTCAATTTGATAGAGAAGTAATACCAGAACGTCGAATGCATGCCAAAGGTTCTGG
TGCATTTGGGACATTTACTGTAACTAAAGATATAACAAAATATACGAATGCTAAAATATTCTCTGAAATAGGTAAGCAAACCGAAATGTTTG
CCCGTTTCTCTACTGTAGCAGGAGAACGTGGTGCTGCTGATGCGGAGCGTGACATTCGAGGATTTGCGTTAAAGTTCTACACTGAAGAAGG
GAACTGGGATTTAGTAGGGAATAACACACCAGTATTCTTCTTTAGAGATCCAAAGTTATTTGTTAGTTTAAATCGTGCGGTGAAACGAGATC
CTAGAACAAATATGAGAGATGCACAAAATAACTGGGATTTCTGGACGGGTCTTCCAGAAGCATTGCACCAAGTAACGATCTTAATGTCAGA
TAGAGGGATTCCTAAAGATTTACGTCATATGCATGGGTTCGGTTCTCACACATACTCTATGTATAATGATTCTGGTGAACGTGTTTGGGTTA
AATTCCATTTTAGAACGCAACAAGGTATTGAAAACTTAACTGATGAAGAAGCTGCTGAAATTATAGCTACAGATCGTGATTCATCTCAACGC
GATTTATTCGAAGCCATTGAAAAAGGTGATTATCCAAAATGGACAATGTATATTCAAGTAATGACTGAGGAACAAGCTAAAAACCATAAAG
ATAATCCATTTGATTTAACAAAAGTATGGTATCACGATGAGTATCCTCTAATTGAAGTTGGAGAGTTTGAATTAAATAGAAATCCAGATAAT
TACTTTATGGATGTTGAACAAGCTGCGTTTGCACCAACTAATATTATTCCAGGATTAGATTTTTCTCCAGACAAAATGCTGCAAGGGCGTTTA
TTCTCATATGGCGATGCGCAAAGATATCGATTAGGAGTTAATCATTGGCAGATTCCTGTAAACCAACCTAAAGGTGTTGGTATTGAAAATAT
TTGTCCTTTTAGTAGAGATGGTCAAATGCGCGTAGTTGACAATAACCAAGGTGGAGGAACACATTATTATCCAAATAACCATGGTAAATTTG
ATTCTCAACCTGAATATAAAAAGCCACCATTCCCAACTGATGGATACGGCTATGAATATAATCAACGTCAAGATGATGATAATTATTTTGAA
CAACCAGGTAAATTGTTTAGATTACAATCAGAGGACGCTAAAGAAAGAATTTTTACAAATACAGCAAATGCAATGGAAGGCGTAACGGAT

AATGTTAAACGACGTCATATTCGTCATTGTTACAAAGCTGACCCAGAATATGGTAAAGGTGTTGCAAAAGCATTAGGTATTGATATAAATTC
TATTGATCTTGAAACTGAAAATGATGAAACATACGAAAACTTTGAAAAATAA

Gene: rpmG2 (50S ribosomal protein L33, locus 2)

Position: 1314407 to 1314556, length: 150 nt, orientation: FORWARD

Perfect match to: (RF122-AJ938182-[1299702:1299851], highly conserved allele)

Sequence:

GTGCGCGTAAACGTAACATTAGCATGCACAGAATGTGGCGATCGTAACTATATCACTACTAAAAATAAACGTAATAATCCTGAGCGTATTG
AAATGAAAAAATATTGCCCAAGATTAAACAAATATACGTTACATCGTGAAACTAAGTAA

Gene: rpsN (30S ribosomal protein S14)

Position: 1315010 to 1315279, length: 270 nt, orientation: FORWARD

Perfect match to: (RF122-AJ938182-[1300305:1300574], highly conserved allele)

Sequence:

ATGGCTAAGAAATCTAAAATAGCAAAAGAGAGAAAAAGAGAAGAGTTAGTAAATAAATATTACGAATTACGTAAAGAGTTAAAAGCAAAA
GGTGATTACGAAGCGTTAAGAAAATTACCAAGAGATTCATCACCTACACGTTTAACTAGAAGATGTAAAGTAACTGGAAGACCTAGAGGT
GTATTACGTAAATTTGAAATGTCTCGTATTGCGTTTAGAGAACATGCGCACAAAGGACAAATTCCAGGTGTTAAAAAATCAAGTTGGTAA

Gene: guaC (GMP reductase)

Position: 1315436 to 1316413, length: 978 nt, orientation: FORWARD

Perfect match to: (CIG1612-AHVW01000005-[819715:820692], allele observed in CC8+CC188)

Sequence:

GTGAAAATATTTGATTACGAAGATATTCAATTAATACCTAATAAATGCATAGTTGAAAGTAGGTCTGAATGTGATACAACTATCCAATTTGG
TCCGAAAAAAATTCAAGCTACCTGTAGTTCCTGCAAATATGCAAACAGTTATGAATGAGAAATTAGCGAAATGGTTTGCTGAAAATGATTACT
TTTATATCATGCATCGTTTTGATGAAGAAGCAAGAATACCTTTTATAAAACATATGCAAAATTCAGGCTTATTTGCATCTATTTCAGTTGGTG
TAAAGAAAGCGGAATTTGATTTTATTGAAAAGTTAGCTCAAGAAAAATTAATCCCCGAATATATTACAATAGATATTGCGCATGGTCACTCA
GATTCAGTGATAAACATGATTAAACATATAAAAACCCATATACCTGATAGTTTTGTTATTGCTGGTAATGTTGGCACGCCAGAAGGTGTTAG
AGAATTAGAAAATGCTGGTGCTGATGCTACCAAAGTCGGTATAGGTCCTGGTAGAGTTTGTATTACAAAGATTAAAACAGGTTTTGGTACT
GGTGGTTGGCAGTTAGCGGCATTAAACATATGTAGTAAAGCAGCTCGTAAACCTTTGATTGCCGATGGTGGTATAAGAACGCATGGCGAC
ATTGCTAAATCAATTAGATTTGGTGCATCAATGGTCATGATTGGTTCATTATTTGCTGCACACGAAGAATCACCTGGTGAAACTGTAGAACT
TGATGGTAAACAGTATAAAGAATATTTTGGTAGTGCATCTGAATTTCAAAAAGGCGAACATAAAAATGTAGAAGGTAAAAAAATGTTTGTA
GAACATAAGGGTTCATTAATGGATACCTTAAAAGAAATGCAACAAGATTTACAAAGCTCAATTTCATATGCCGGTGGAAAAGACTTGAAAT
CATTACGTACTGTAGATTATGTTATTGTTAGAAACTCTATTTTCAACGGTGATAGAGATTAA

Gene: ylbC1 (putative protein)

Position: 1316588 to 1317451, length: 864 nt, orientation: FORWARD

Perfect match to: (MW2-BA000033-[1337993:1338856], allele observed in CC1+CC5+CC80+CC239)

Sequence:

TTGGATACACCGAAGAAACAACAATTTGCCTTTAATAATATACAAATGAACATGTCGAAATCAGATGTTGAGAAAACATTAAATAAACCAA
AAAGAGTGACATTTAATGAATATGGTACGAAGTGGTATACGTATTATGATGACGATTACAATAATTTTATAATGATAAGTTACATGAAAGAT
AAAGTTAATGCGTTATATACAAATCAAAATATAATCACTTCAAAATCAAAAATTAAATACAATACACCTAAATCGGTTGTAAGGCAAAGATT
AGGCGAACCAGAAACAGAGATTGTTAAAGGTAGAGTGCGTTACGAACAAAATAATAAAGAATATGATGTTTTCCATAAAAATCACATTTAT
ACGACGGTATTTTATGATAAGCATCGACGTAATAATGTAACAGCTGTTTTACAAGTAAGTGATGCTATGGAAAATAGATTAAAAGAACAAT
ATGGAGCACCTTCGAAATCGCTTGCAGATAGTTTTGAACTACAAAATTTTGATTTAGTTAATGCTGAAAGAAAACAACATCAATTATTTACAT

TGAAGTATTCTAAACAGAATTCTGAAACTGCACGTAAGCATAGTAAAGATATGGCCAACAATCATTATTTTGATCATACAAATTTAAAAGGT
CAATCACCATTTGATCGATTGAAAAAAGATGGTATTACATTTAACTCAGCCGGAGAGAATTTAGCATATGGTCAAGTTAGTAGTATCTATGC
ACATCAAGGATTAATGAATTCTATTGGTCACAGAAAAAATATTTTAAATGATACGTTTAAAATATTAGGTGTTGGTGTTGATTTTAATGATG
AAAAACAACCTTTTTGGACAGAAAATTATACTGGTTAA

Gene: lexA (DNA damage-inducible LexA repressor)

Position: 1317830 to 1318453, length: 624 nt, orientation: REVERSE

Perfect match to: (N315-BA000018-[1337131:1337754:r], allele observed in CC5+CC80)

Sequence:

TTACATTTCGCGGTACAAACCAATTACTTTCCCAATTACAGCAACATTGTCGAGGTAAATTGGCTCCATTGTACTATTTTCAGGTTGTAATCG
ATAACGATTTTTTTCTTTATAGAAGCGTTTGACAGTTGCTTCATCTTCCTCAGTCATAGCAACAATAATGTCTCCATTTTCTGCTATGGTTTGA
CTGCGAACAATTACTTTGTCTCCGTCTAATATACCAGCCTCAATCATACTGTCGCCTACGACGTTTAATATGAATATGTCGCTATTATGTGTC
GATGTTAAGTGTTCAGGTAATGGAAAATACTCTTCAATATTTTCTACAGCGGTAATAGGAACACCTGCTGTGACTTTACCAATAACTGGCAC
ATGAATCGTTTCTTCCATATTAATATTATCATTTGTTTGATCACTTACAATTTCTATAGCACGTGGTTTCGTTGGATCTCTTCTTATATATCCTTT
TTCTTCAAGACGTGAAAGATGACCATGAACAGTTGAACTGGATGCTAAGCCAACTGCTTCACCAATTTCGCGAACACTAGGCGGATAACCT
TTCATTTGAACAACTTGTTTAATATAGTTATATATTTCGCTTTGTCGTTTTGTTAATTCTCTCAT

Gene: Q5HG79 (putative membrane protein)

Position: 1318596 to 1318829, length: 234 nt, orientation: FORWARD

Perfect match to: (N315-BA000018-[1337897:1338130], highly conserved allele)

Sequence:

ATGTTTTACAATAAATATAAAAACGTATCAACATATATCATCATATTTTTAGTTTCAAGTGCAGCCTTTGCAATATTCTTGTTAAGTGCGAACG
TTAGTGCTCACTCGGAACAAGTGTACGAAATGACTGACCATCAAATTAAGAACAATACGATAAATAAAGCATACGAACATAAAGACCCTAC
AAACAATGGCGAACAAAGAGATGGGAAAGTGTTCGCTTTAATAAATTGA

Gene: DUF896 (protein of unknown function)

Position: 1318966 to 1319205, length: 240 nt, orientation: FORWARD

Perfect match to: (RF122-AJ938182-[1304261:1304500], highly conserved allele)

Sequence:

TTGAGTAATTCTGATTTGAATATCGAAAGAATTAACGAGTTAGCTAAAAAGAAAAAAGAAGTAGGATTAACTCAAGAAGAAGCAAAGGAG
CAAACAGCCTTAAGAAAAGCTTATCTTGAGAGTTTTAGAAAAGGGTTTAAACAACAAATTGAAAATACTAAAGTAATTGATCCAGAAGGTA
ATGATGTAACACCTGAAAAAATTAAAGAGATACAACAAAAAAGAGATAATAAAAATTAA

Gene: tkt (transketolase)

Position: 1319326 to 1321314, length: 1989 nt, orientation: FORWARD

Perfect match to: (11819-97-CP003194-[1369703:1371691], allele observed in CC80+CC5+CC72)

Sequence:

ATGTTTAATGAAAAAGATCAATTAGCTGTTGATACGCTACGTGCACTAAGTATCGACACAATCGAAAAAGCGAATTCTGGTCATCCAGGATT
ACCTATGGGAGCTGCCCCAATGGCTTACACTTTGTGGACACGTCATCTGAATTTTAATCCACAATCTAAAGATTACTTCAATAGAGACCGTTT
CGTATTATCGGCAGGGCATGGTTCAGCATTATTGTATAGCTTGTTACATGTTTCTGGTAGTTTAGAATTAGAAGAATTAAAGCAATTTAGAC
AATGGGGTTCTAAAACACCAGGCCATCCTGAATACAGACATACTGATGGTGTAGAAGTTACTACTGGACCACTTGGACAAGGTTTTGCTAT
GTCAGTAGGATTAGCTTTAGCAGAAGATCACCTAGCAGGGAAATTTAATAAAGAAGGATATAATGTTGTAGATCATTACACATATGTATTA
GCTTCTGACGGTGATTTAATGGAAGGTATATCGCATGAAGCAGCTTCATTTGCTGGACATAATAAATTAAGTAAATTAGTTGTTTTATACGA
TTCAAATGATATTTCATTAGATGGCGAATTAAACAAAGCTTTTTCTGAAAACACAAAAGCTCGTTTTGAAGCATATGGTTGGAATTACTTACT

AGTTAAAGATGGTAATGATTTAGAAGAAATTGATAAAGCGATTACTACAGCTAAATCTCAAGAAGGACCAACGATTATTGAAGTTAAAACA
ACAATCGGATTTGGTTCACCGAATAAAGCAGGAACTAATGGTGTTCATGGGGCACCTTTAGGTGAAGTTGAAAGAAAATTAACATTCGAAA
ATTACGGTTTAGATTCTGAAAAACGTTTTAATGTTTCAGAAGAGGTATACGAAATTTTCCAAAATACTATGTTAAAACGTGCTAATGAAGAT
GAATCTCAATGGAATTCATTATTAGAAAAATATGCAGAAACATATCCTGAATTAGCAGAAGAATTTAAATTAGCGATTAGTGGTAAATTGCC
TAAAAATTATAAGGATGAATTACCACGTTTTGAACTGGGTCATAATGGTGCATCTCGTGCTGATTCTGGTACTGTTATTCAAGCAATCAGTA
AAACTGTCCCTTCATTCTTTGGTGGATCAGCAGACCTTGCTGGTTCAAACAAATCCAATGTAAATGATGCAACAGATTATAGTTCTGAAACA
CCTGAAGGTAAAAATGTGTGGTTTGGTGTACGTGAATTTGCTATGGGTGCTGCTGTAAATGGTATGGCTGCACATGGAGGTTTACATCCAT
ATGGTGCAACATTCTTCGTATTTAGTGATTATTTAAAACCAGCGTTACGTTTATCATCAATTATGGGATTAAATGCAACGTTCATCTTCACAC
ATGATTCAATTGCAGTAGGTGAAGATGGTCCTACTCATGAACCAATTGAACAATTAGCTGGATTAAGAGCTATTCCAAATATGAATGTTATC
CGTCCTGCTGATGGTAATGAAACAAGAGTAGCATGGGAAGTTGCCTTAGAATCTGAATCTACACCTACTTCATTAGTATTGACACGTCAAAA
CTTACCGGTTTTAGATGTACCAGAAGATGTAGTTGAAGAAGGCGTTCGAAAAGGTGCCTATACAGTTTATGGCTCTGAAGAGACACCAGAA
TTCCTATTATTAGCTTCAGGTTCAGAAGTTAGTCTTGCAGTTGAAGCTGCTAAAGATCTTGAAAAACAAGGTAAATCAGTGCGTGTTGTTTC
AATGCCTAACTGGAATGCATTTGAACAACAATCTGAAGAATATAAAGAATCAGTTATTCCATCAAGCGTAACAAAACGTGTTGCGATTGAA
ATGGCTTCACCGCTTGGATGGCATAAATATGTAGGTACTGCAGGTAAAGTTATTGCTATTGACGGCTTTGGCGCAAGTGCACCTGGCGATT
TAGTAGTTGAAAAATATGGATTTACTAAAGAAAATATCTTAAACCAAGTTATGAGCTTATAA

Gene: UPF0154 (protein of unknown function)

Position: 1321592 to 1321834, length: 243 nt, orientation: FORWARD

Perfect match to: (RF122-AJ938182-[1306887:1307129], highly conserved allele)

Sequence:

ATGGCAACTTGGTTAGCAATTATTTTTATAGTAGCTGCATTAATTTTAGGTTTAATTGGAGGTTTCCTTTTAGCTAGAAAATATATGATGGAC
TACTTGAAGAAAAACCCACCAATCAACGAAGAAATGCTTCGTATGATGATGATGCAAATGGGTCAAAAACCTTCTCAGAAGAAAATTAATC
AAATGATGACGATGATGAATAAAAATATGGATCAAAATATGAAGAGTGCGAAAAAGTAA

Gene: ccdC (membrane protein involved in cytochrome C biogenesis)

Position: 1322013 to 1322480, length: 468 nt, orientation: FORWARD

Perfect match to: (N315-BA000018-[1341314:1341781], highly conserved allele)

Sequence:

GTGCTCTATTTAATATTTTCAATCATTGTAGCTTTATTTATGGGAACTATAGTTATAGTTATTCGTATGAAAGCTCAAAATTATCCGGTAAATG
AGAAAAAAATAGTTTTGCCACCGTTTTTTATGGCGACCGGTGCATTGATGTACGTCGTTCCATATTTTAGGCTAACAGGATCGGAAATGCTA
GAAGCCTTTATAATTGGTTTGCTTTTTTCTACAGTTCTAATTTGGACTTCTCGATTTGAAGTCAAAGGTACAGAAATTTATATGAAACGATCT
AAAGCATTTCCAGTTATTTTGATTTCATTACTTATCATTCGTACTGTGATGAAAATATTCATTAGTAATGAAATAGATCCTGGAGAATTAGGC
GGCATGTTCTTTTTATTAGCATTCTGTATGATTGTTCCTTGGAGAGCAGCAATGCTATATAAATACAAAAAACTAAAGAAAACATTAATCAAT
TAA

Gene: sbcD (nuclease SbcCD subunit D)

Position: 1322604 to 1323725, length: 1122 nt, orientation: FORWARD

Perfect match to: (11819-97-CP003194-[1372981:1374102], allele observed in CC80)

Sequence:

ATGAAAATCATACATACAGCAGACTGGCACTTAGGGAAAATATTAAATGGCAAACAGCTTTTAGAAGATCAAGCGTATATTTTAGATATGT
TCGTAGAAAAAATGAAAGAAGAAGAACCCGATATCATTGTGATAGCTGGAGATTTATATGACACAACATATCCAAGTAAAGATGCAATCAT
GTTATTAGAACAAGCGATTGGAAAGTTAAATTTAGAACTGCGTATACCAATAATTATGATTAGTGGAAATCACGATGGTAAAGAGAGGTTA
AACTATGGGGCGAGTTGGTTTGAACATAATCAGTTATTTATAAGAACAGATTTTACATCGATTAATTCACCAATAGAGATAAATGGGGTTAA
TTTTTATACACTCCCTTATGCTACTGTGAGCGAAATGAAACACTATTTTGAAGATGACACCATTGAAACGCATCAACAGGGAATTACGCGCT
GTATTGAAACAATAGCACCGGCAATTGATGAAGATGCCGTCAATATTTTAATTAGTCATCTGACTGTTCAAGGTGGAAAGACATCTGATTCT
GAAAGACCATTAACTATTGGAACGGTTGAGTCAGTTCAGAAACGTGTTTTTGATATATTTGATTATGTCATGCTAGGTCACTTGCATCATCC
ATTTAGTATAGAAGACGACAAAATTAAATATAGTGGCTCCTTATTGCAGTATTCATTTTCGGAAGCGGGTCAAGCTAAAGGGTATAGACGT
GTAACAATTAATGATGGCATTATTAACGATGTATTTATTCCTCTTAAGCCACTTAGACAATTGGAAATTATCTCAGGCGAATATAATGATGTT

ATTAATGAAAAAGTTCATGTGAAAAATAAAGATAATTATTTACATTTTAAACTTAAAAATATGTCTCATATTACTGATCCAATGATGAGTTTA
AAACAAATTTATCCTAATACTTTAGCGCTGACGAATGAAACTTTTAATTATAATGAAGAAAATAATGCTATAGAAATAAGTGAAAAAGATGA
CATGTCAATTATCGAAATGTTTTATAAACATATAACTGATAAAGAATTATCGGATATCCAATCTAAAAAGATAAAAAAATATTTTAGAAAACG
AATTGAGAAAGGAGGATTAA

Gene: sbcC (nuclease SbcCD subunit C)

Position: 1323729 to 1326758, length: 3030 nt, orientation: FORWARD

Perfect match to: (IS-24-AHLM01000038-[49483:52512:r], allele observed in CC8+CC1+CC8+CC97)

Sequence:

ATGAAACCATTACATTTAAAGTTGAATAATTTCGGCCCCTTTTTAAAAGAAGAAATTGATTTTTCTAAAATTGATAATAATGAATTGTTTTTAA
TAAGTGGTAAGACTGGATCGGGTAAAACAATGATTTTTGATGCAATGACTTATGCCTTGTTTGGTAAAGCATCAACTGAACAAAGAGAAGA
AAATGATTTGAGAAGTCATTTCGCTGATGGTAAACAGCCGATGTCAGTAACATTTGAATTTCAATTAAATCAGCGAATTTATAAAGTGCATA
GACAAGGCCCTTATATCAAAGAAGGTAATACAACAAAAACGAACGCTAAATTTGATGTATTTGAGATGGTGGATGGCAAGTATGAAATTA
GAGAAAGTAAAGTAATTTCAGGTAACCAATTCATTATTGAATTATTAGGAGTAAATGCAGATCAATTCCGACAATTGTTTATTTTGCCTCAA
GGTGAATTCAAACGCTTTTTAATATCAAACAGTCGTGAAAAGCAAGGGATATTAAGAACACTGTTTGACAGTGAAAAATTTGAAGCTATAC
GAGAAATATTAAAAGAAGAAGTAAAAAAAGAAAAAGCTCAAATCGAGAATAGATATCAACAAATTGACCTTTTATGGCAAGAAATTGAAT
CATTTGATGATGACAATATAAAAGGCTTATTAGAGGTTGCCACTCAACAGATAGACAAATTGATTGAAAATATACCACTTTTACAAGCTAGG
TCGAAAGAAATACTAGCATCTGTAAATGAAAGTAAAGAAACTGCTATTAAAGAATTTGAAATAATAGAAAGAAAACATTAGAAAATAATA
TATTAAAAGATAATATTAATCAACTCAACAAAAATAAAATTGATTTCGTTCAATTGAAAGAACAACAACCTGAAATAGAGGGAATTGAAGCT
AAGTTAAAGTTGTTACAAGATATTACAAACCTATTGAATTATATTGAAAATAGAGAAAAAAATTGAAACTAAAATTGCTAATAGCAAAAAAG
ATATTTCTAAAACCAATAATAAAATATTGAATCTTGATTGTGATAAGCGAAACATAGACAAAGAGAAAAAAATGTTAGAAGAAAATGGAGA
TTTAATTGAAAGTAAAATCTCTTTTATTGATAAAACTAGAGTATTATTTAACGATATTAATAAGTATCAACAAAGTTATCTCAATATTGAACG
CTTGAGAACTGAGAGTGAACAATTAGGTGATGAATTAAATAATCTAATTAAAGGTTTAGAAAAGGTCGAAGATTCAATAGGTAATAACGAA
AGTGATTACGAGAAAATTATCGAACTAAATAATACGATAACGAACATAAATAATGAAATTAATATAATTAAAGAAAATGAAAAAGCTAAAG
CTGAATTAGATAAACTATTAGGTAGTAAGCAAGAGTTAGAGAATCAAATTAATGAAGAAACATCTATATTGAAGAATCTCGAAATAAAATT
AGATCGCTACGATAAAACAAAATTGGACTTAAATGATAAAGAAAGCTTTATAAGTGAAGTTAAATCTGCTGTAAATATTGGAGATCAATGT
CCGATATGTGGTAATGAAATTCAGGATTTAGGGCATCATATTGATTTTGAAAGTATTGCTAAACGTCAAAATGAAATTAAAGAAATTGAAG
CAAATATCCACGCTATAAAATCGAATATTGCTGTGCATAATTCTGAAATTAAATTTGTTAATGAAAAAATATCGAATATTAATATTAAAACGC
AAAGTGATTTTTCACTTGAAGTATTGAATAAGCGTCTGCTAGAAAATGAAAATGCATTGAATAATCAAAGAGATCTTAATAAATTTATAGAA
CAAATGAAAGAAGAAAAAGATAATCTAACGTTGCAAATTCATAATAAACAATTGCGTCTAAATAAAAATGAATCTGAGTTGAAATTATGTC
GAGATCTCATCACTGAATTTGAAACACTCTCGAAATATAATAATATCACTAATTTTGAGGTGGATTATAAGAAGTATGTTCAAGATGTGAAT
CAGCATCAAGAACTCTCAAAGGAGATTGAAGATAAGTTAATGCAATTGTCTCAAAGAAAGTTAATTGAGCAAAATAATCTAAATCACTATG
AAAATCAACTAGAAACTTACAATAATGACTTAGAATTGAATGAACAATCTATTGAAATGGAAATGTCGAGGCTGAATTTAACTGACGACAA
TGATATAAATGAAATAATAGCCTGGAGGGGCGAGCAAGAGGAATTAGAGCAGAAAAGGGATACTTATAAAAAACGTTATCATGAATTTGA
AATGGAAATAGCTAGGTTAGAATCATTAACCAAGGATAAAGAGTTATTGGACTCTGATAAATTAATAGATGAATATGAACTGAAAAAAGG
AAAGATGAATACACTGATAGATGAATACTCTGCTGTTCATTATCAATGTCAAAATAATATTAAAAAAACACAATCTATAGTTTCGCATATTAA
TTACTTAAATCAAGAATTAAAGGATCAACAAGAAATATTTCAATTGGCTGAGATTGTCAGTGGTAAGAACAACAAAAATCTTACATTGGAA
AACTTTGTATTAATTTACTATTTAGATCAAATTATTGCTCAAGCAAATCTGAGATTAGCAACAATGTCAGATAATCGATACCAACTAATTAGG
CGAGAAGCGGTTTCTCATGGTCTTAGTGGCCTAGAAATTGATGTATTTGATTTGCATTCAAATAAGTCTAGACATATTAGCTCGTTATCCGG
TGGAGAAACTTTCCAATCGTCGCTTGCATTAGCTTTAGGGTTAAGCGAAATTGTACAGCAGCAATCAGGAGGTATTTCACTAGAATCAATAT
TTATTGATGAAGGATTCGGTACATTAGATCAAGAAACGCTTGAAACAGCGTTAGACACTTTATTAAATCTTAAATCAACTGGTAGAATGGTT
GGGATTATTTCACATGTGAGCGAATTGAAAAATAGAATACCTTTAGTTTTAGAAGTGAAATCAGATCAATATCAGAGTTCAACAAGATTCAA
AAGAAATTAA

Gene: mscL (large-conductance mechanosensitive ion channel)

Position: 1326829 to 1327191, length: 363 nt, orientation: REVERSE

Perfect match to: (MW2-BA000033-[1348234:1348596:r], highly conserved allele)

Sequence:

TTATTTTTTCTCACGTAATAAATCTCTGATTTCAGTTAATAACACAACATTTTCTTCCACAACTGCTTCTTCTTCGGCTTCTTCTTTCTTCATTAA
TGTATTTGCAATCTTAACAAAGATGAATAAAGCAAACGCGATGATAATAAAGTCGATAACAGATTGGATAAATAAACCGTATTTAATACCCC
AGAATGACCATTCTTTAGCAAAATCAACTGATCCGAAAATTTTACCAATTAATGGCATAATGATATTTTCTACTAATGAAGATATAATCTTGT
TGAAAGCTGCACCCATCACAACAGCAATTGCTAAATCTAAGACGTTACCTTTTAAGGCGAACTCTTTGAATTCTTTTAACAT

Gene: opuD1 (glycine betaine transporter 1)

Position: 1327395 to 1329041, length: 1647 nt, orientation: FORWARD

Perfect match to: (Bmb9393-CP005288-[1395516:1397162:r], allele observed in CC239+CC8)

Sequence:

ATGAATTCTTCTTCACCAGAGAATCCAAATGGAAAGAAGTATTCACCAGTCTTCATCTATAGTGCAATTGTTGTTGCTATAGTCGTATTACTT
GGTGCATTTTTACCTGAACAATTCAACTATGTTACCAATAATATTAAAATGTGGATTACAGAAAAGTTAGGTTGGTATTATCTTATTCTTACT
ACGATTATCGTGTTCTTCTGTATATTCCTTATTTTTAGTCCTATTGGAAAACTTAAACTAGGTAAACCAAATGACAAACCTGAGTTTAATACAA
TTTCATGGTTTGCTATGTTGTTTAGTGCTGGTATGGGGATAGGTTTGGTGTTTTATGGTGCAGCAGAACCGATGGCGCACTTTGCTACGCCA
CCTACAGCAGATCCCAAAACTACTGAAGCTTATACTGAAGCTCTACGTTCAACATTTTTCCATTGGGGATTCCATGCTTGGGCTGTTTATGGT
GTTGTTGCGTTAGCGTTGGCATATTCGCAATTCCGTAAAGGTGAACCAGGTTTATTATCTAGAACTTTACGTCCTCTTTTAGGTGATAAAGTA
GAAGGTCCTATTGGAATTTTTATTGACGTTTTATCTGTATTTGCGACAATCGTTGGGGTAGCCGTTTCGTTAGGTATGGGTGCTCTACAAATT
AATGGTGGTTTACATTACTTGTTCAATGTTCCAAACAATACGTTTGTACAAGCGATTATCATCATTGTTGTTACTATCTTATTTATAGCAAGTG
CATGGTCTGGATTAAGTAAAGGTATTCAATACTTAAGTAACTTGAACATTGGTTTAGGTACTATTTTAATGGTAGCTGCTTTAATTGTTGGAC
CAACTGTTCTTATTTTAAATATGTTAACTAGCTCTACGGGTAGTTTACTAAACACATTCTTGTTTAATAGTTTTGATACAGCAGCTTTAAATCC
TCAAAAACGTGAATGGATGTCTTCATGGACACTTTATTACTGGGGTTGGTGGTTAAGTTGGAGTCCATTCGTTGGAGTGTTTATTGCACGAG
TTTCAAAAGGACGTTCAATTAGAGAGTTCATTTCTGGTGTCTTGCTAGTTCCAGCAATTGTTAGTTTTGTTTGGTTTAGTGTCTTTGGTGTATT
AGGCATCGAGACAGGTAAGAAACACAAAGAAATTTTTGATATGACTCCTGAAACACAGCTATTTGGAGTGTTTAATCATGTGCCATTTGGC
ATTGTTTTATCGTTGATTGCATTATTATTAATTGCATCATTCTTTATTACATCTGCTGACTCAGCAACATTTGTATTAGGAATGCAAACAACAT
TTGGTTCATTAAATCCATCTAGTATGGTAAAAGTTGTTTGGGGAATTTCACAGGCCTTAATAGCATTTGTACTTTTATTAGCTGGTGGCGGTA
ACGGCGCTGAAGCTTTAAATGCGATTCAAAGTGCTGCAATTATAAGTGCATTCCCATTCTCCTTTGTCGTCATACTCATGATGGTAAGTTTCT
ACAAGGATGCGAACCAGGAACGTAAATTCCTAGGTTTAACATTGACTCCGAATAAACATCGCTTACAAGAATATATCAAGAGTCAACAAGA
AGATTATGAATCTGACATTCTTGAAAAGCGTCAGTCACGTAGAAATATAGAGAAAAAAGATAACTAA

Gene: tx_universal2 (rho-independent terminator)

Position: 1329363 to 1329395, length: 33 nt

Perfect match to: (Strain_21194-AGTU01000149-[993:1025], allele observed in CC45I)

Sequence:

CTGAACGAAAATGCGCTTGTAACAAGCTTTTTT

Gene: tx_universal2 (rho-independent terminator)

Position: 1329365 to 1329403, length: 39 nt

Perfect match to: (Strain_21331-AGTV01000040-[104403:104441], allele observed in CC398)

Sequence:

GAACGAAAATGCGCTTGTAACAAGCTTTTTTCAATTCTA

Gene: acnA-citB (aconitate hydratase)

Position: 1329871 to 1332576, length: 2706 nt, orientation: FORWARD

Perfect match to: (11819-97-CP003194-[1380248:1382953], allele observed in CC80)

Sequence:

ATGGCTGCAAATTTTAAAGAGCAATCAAAAAAACATTTTGACTTGAATGGCCAAAGTTATACTTACTATGATTTAAAAGCTGTAGAAGAGCA
AGGCATTACTAAAGTTTCCAATTTACCTTATTCAATTCGTGTTTTGTTAGAATCTTTACTTCGTCAAGAAGATGATTTTGTAATTACAGACGAT
CATATTAAAGCTTTAAGTCAGTTTGGAAAAGATGGAAATGAAGGCGAGGTACCATTTAAACCTTCTCGTGTTATTTTACAAGATTTCACAGG
TGTACCAGCCGTAGTTGATTTAGCTTCTTTACGTAAAGCAATGGATGACGTTGGGGGAGATATTACTAAAATTAATCCAGAAGTACCGGTG

GATTTAGTTATTGACCACTCAGTTCAAGTGGATAGCTATGCAAATCCAGAAGCTCTTGAACGTAATATGAAATTAGAATTTGAACGTAACTA
TGAACGTTATCAGTTTTTAAATTGGGCAACGAAAGCATTTGATAATTACAATGCAGTTCCTCCTGCAACTGGAATAGTTCACCAAGTTAACTT
AGAATATTTAGCAAGTGTTGTACATGTTCGTGATGTAGATGGTGAAAAAACTGCATTTCCGGATACATTAGTTGGTACTGATTCACATACAA
CAATGATAAATGGTATTGGCGTACTAGGATGGGGTGTTGGTGGTATTGAAGCTGAAGCTGGAATGCTTGGACAACCTTCTTATTTCCCAAT
TCCAGAGGTTATTGGTGTACGACTAGTAAATTCATTACCACAAGGCGCAACAGCAACTGATTTAGCGTTAAGAGTAACTCAAGAGCTACGT
AAAAAAGGTGTTGTTGGTAAATTTGTAGAGTTCTTTGGTCCAGGTGTACAACATTTACCACTAGCAGACCGTGCTACAATTGCAAACATGGC
ACCAGAGTATGGAGCAACTTGCGGATTCTTCCCGGTTGATGATGAATCTCTTAAATATATGAAGTTAACTGGTAGATCAGATGAGCATATC
GCGCTAGTAAAAGAATATTTGAAACAAAACCATATGTTCTTTGATGTTGAGAAAGAAGATCCTAATTATACAGATGTTATCGAATTGGATTT
ATCAACAGTTGAAGCATCGCTTTCAGGACCAAAACGTCCTCAAGATTTAATTTTCTTAAGTGATATGAAATCATCATTTGAAAATTCTGTAAC
AGCTCCAGCGGGCAACCAAGGACACGGTTTAGATAAAAGTGAATTTGATAAGAAAGCTGAAATTAACTTCAAAGATGGATCAAAAGCTAC
AATGAAAACAGGTGATATTGCAATAGCAGCAATTACATCATGTACAAATACATCTAACCCTTATGTAATGTTAGGTGCAGGTTTAGTTGCTA
AAAAAGCAGTTGAAAAAGGCTTGAAAGTTCCTGAATACGTTAAAACTTCTCTAGCACCAGGATCAAAAGTTGTTACCGGATATTTAAGAGA
TGCTGGCTTGCAACCTTATTTAGATGATTTAGGCTTCAACTTGGTTGGTTATGGATGTACAACTTGTATCGGTAATTCAGGTCCTTTATTACC
AGAAATTGAAAAAGCGATTGCTGATGAGGACCTATTAGTGACATCTGTATTATCTGGTAACCGTAACTTTGAAGGTCGTATCCATCCTCTTG
TTAAAGCCAATTACCTAGCTTCACCACAGTTAGTTGTTGCTTATGCATTAGCTGGAACGGTTGATATTGATTTACAAAATGAACCTATTGGTA
AAGGTAATGACGGTGAAGATGTATATTTGAAAGATATTTGGCCATCAATTAAAGAAGTTTCAGATACCGTTGATAGTGTTGTAACACCTGA
ATTATTTATTGAAGAATATAATAACGTATACAATAACAACGAATTATGGAATGAGATTGATGTAACTGATCAACCTCTATATGACTTTGATCC
TAATTCAACATACATTCAAAATCCATCATTCTTCCAAGGATTATCTAAAGAACCGGGTACGATTGTTCCATTAAATGGTTTACGTGTTATGGG
TAAATTCGGTGATTCTGTGACAACTGACCACATCTCTCCAGCAGGTGCAATTGGTAAAGATACGCCAGCTGGTAAATATTTACAAGATCATC
AAGTGCCTATTCGTGAATTTAATTCATATGGTTCAAGACGTGGTAATCACGAAGTAATGGTTCGAGGTACGTTTGCTAATATACGTATTAAA
AACCAATTAGCGCCAGGTACTGAAGGTGGTTTTACAACTTATTGGCCAACAAATGAAGTAATGCCTATCTTTGATGCTGCAATGAAATATAA
AGAAGATGGTACAGGTTTAGTTGTATTAGCTGGTAACGATTATGGTATGGGTTCATCTCGTGACTGGGCAGCAAAAGGTACAAACTTATTA
GGTGTTAAAACAGTTATTGCACAAAGTTATGAACGTATCCATCGTTCAAATTTAGTTATGATGGGTGTATTACCATTAGAGTTTAAAAAAGG
TGAATCAGCTGATTCTCTTGGTCTAGATGGTACAGAAGAAATTTCTGTTAATATTGATGAAAATGTTCAACCACATGACTACGTCAAAGTTA
CTGCTAAGAAGCAAGATGGTGATTTGGTAGAATTTGACGCTATGGTTCGTTTTGACTCACTTGTTGAAATGGATTACTATCGTCACGGTGGA
ATTTTACAAATGGTTTTAAGAAATAAATTAGCGCAATAA

Gene: yneP (putative thioesterase)

Position: 1332756 to 1333223, length: 468 nt, orientation: FORWARD

Perfect match to: (11819-97-CP003194-[1383133:1383600], highly conserved allele)

Sequence:

ATGATATATAGTATTACAGAAATAGAAGCGCGTTATGCTGAAACTGATAAGATGGGTGTAATTTATCACGGGAATTATGCAACTTGGTTTG
AAGTTGCGCGGTTGGATTATATATCGAAGTTAGGTTTTAGTTATGCTGATATGGAAAAACAAGGAATCATTTCACCTGTGACTGACCTCAAT
GTCAATTATAAAAAGTCTATTTTTTATCCAGAAAAAGTTAAAGTTAAAACTTGGGTTGAAAAATATTCGAGATTACGTTCAGTGTATAAATAT
GAGATTTTTAATGAAAAAGGTGAACTTGCAACTACAGGTTCCACAGAATTGATTTGCATTAAAGAAGATACTTTTAAGCCTATACGGTTGGA
TCGTTATTTCCCAGATTGGCATGAAGCCTATAGTAAAGTGCAAACGCTCAATAATGAAGGGAAAACAGTAGAGATAATGGATGGTATTGAT
TCTTTATAA

Gene: yneR (putative iron-sulphur cluster biogenesis protein)

Position: 1333430 to 1333726, length: 297 nt, orientation: REVERSE

Perfect match to: (11819-97-CP003194-[1383807:1384103:r], allele observed in CC80+CC15+CC25+CC80+CC88)

Sequence:

TTATTTTGTGGAATAAGAAATTTCATCTTCGTGATCAACTACATTTACAATAATGTGGTCATCTTCAAAGTACCACAAATCTTTTTCCGCTACG
ACAACATTTAAATCGTCATATTGTTGTTCATAGCCAATATCAACATCTTCCTTTGGTTCAACTGTAAAAGCAGGACTAAATCCTTGCTTGAGTT
GGAATTCGCCACCATATCTTACAAAAAACACGAGCACTTTATTATTTTCAGGCAACTCTAGTTCATTTTTAAACCAAGTTACTGCTGCATCAG
TAAGTTCTATTTGCAT

Gene: plsY (glycerol-3-phosphate acyltransferase)

Position: 1334102 to 1334710, length: 609 nt, orientation: REVERSE

Perfect match to: (JKD6159-CP002114-[1340294:1340902:r], highly conserved allele)

Sequence:

TTACATCCATTTTATTTTAGGTTCTTCGCCTCTAAAAATCCTTGCGATATTAGAGCGATGTCTAATTATCAATATGATTGAAACTAAGAAACTA
ACGACTAATAAAATGTAGTCTTGAATGATAAGCGAGCCAATCACACAGCAAATTGCTGCAACGATACTTGCTAAAGAAACATATTTAAAAA
TCTTCAATACAATAAAGAAGATAATTGCAAGTATTAGTAAAAGTATCGGATTGACTCCCAAGACGACACCTGCACTAGTTGCAACCGCTTTG
CCACCTTGGAATTTTAAATAAACAGGATAAACGTGTCCAAGTATAGCGAATAAGCCAACAATTAAACCATTTGTAAAAAAAGTACTAATAG
GGCCATCTGCGTGAACTGGTAACCATAAAGGGAAGAAAACAGTTATGAACCCTTTGAAAATATCTAGAAATGTTACCAAGAATCCTGCAGG
ACGACCTAATACTCTAAAGCTATTAGTAGCGCCAGTATTACCACTACCAAATTGTCTAATATCTTTTTTGAAAAATAATTTTCCAATTACGAAT
CCACTTGGGAAAGCGCCGATAAGATAACTTAGTAGTAACATGACGATTATCATCAT

Gene: grlB (DNA topoisomerase IV, subunit B)

Position: 1334916 to 1336907, length: 1992 nt, orientation: FORWARD

Perfect match to: (IS-111-AHLS01000043-[2124:4114], allele observed in CC8+CC5+CC97)

Sequence:

ATGAATAAACAAAATAATTATTCAGATGATTCAATACAGGTTTTAGAGGGGTTAGAAGCAGTTCGTAAAAGACCTGGTATGTATATTGGAT
CAACTGATAAACGGGGATTACATCATCTAGTATATGAAATTGTCGATAACTCCGTCGATGAAGTATTGAATGGTTACGGTAACGAAATAGA
TGTAACAATTAATAAAGATGGTAGTATTTCTATAGAAGATAATGGACGTGGTATGCCAACAGGTATACATAAATCAGGTAAACCGACAGTC
GAAGTTATCTTTACTGTTTTACATGCAGGAGGTAAATTTGGACAAGGCGGCTATAAAACTTCAGGTGGTCTTCACGGTGTTGGTGCTTCAGT
TGTAAATGCATTGAGTGAATGGCTTGAAGTTGAAATCCATCGAGATGGTAATATATATCATCAAAGTTTTAAAAACGGTGGTTCGCCATCTT
CTGGTTTAGTGAAAAAAGGTAAAACTAAGAAAACAGGTACCAAAGTAACATTTAAACCTGATGACACAATTTTTAAAGCATCTACATCATTT
AATTTTGATGTTTTAAGCGAACGACTACAAGAGTCTGCGTTCTTATTGAAAAATTTAAAAATAACGCTTAATGATTTACGCAGTGGTAAAGA
GCGTCAAGAGCATTACCATTATGAAGAAGGAATCAAAGAGTTTGTTAGTTATGTCAATGAAGGAAAAGAAGTTTTGCATGACGTAGCTACA
TTTTCAGGTGAAGCAAATGGTATAGAGGTAGACGTAGCTTTCCAATATAATGATCAATATTCAGAAAGTATTTTAAGTTTTGTAAATAATGT
ACGTACTAAAGATGGTGGTACACATGAAGTTGGTTTTAAAACAGCAATGACACGTGTATTTAATGATTATGCACGTCGTATTAATGAACTTA
AAACAAAAGATAAAAACTTAGATGGTAATGATATTCGTGAAGGTTTAACAGCTGTTGTGTCTGTTCGTATTCCAGAAGAATTATTGCAATTT
GAAGGACAAACGAAATCTAAATTGGGTACTTCTGAAGCTAGAAGTGCTGTTGATTCAGTTGTTGCAGACAAATTGCCATTCTATTTAGAAG
AAAAAGGACAATTGTCTAAATCACTTGTGAAAAAAGCGATTAAAGCACAACAAGCAAGGGAAGCTGCACGTAAAGCTCGTGAAGATGCTC
GTTCAGGTAAGAAAAACAAGCGTAAAGACACTTTGCTATCTGGTAAATTAACACCTGCACAAAGTAAAAACACTGAAAAAAATGAATTGTA
TTTAGTCGAAGGTGATTCTGCGGGAGGTTCAGCAAAACTTGGACGAGACCGCAAATTCCAAGCGATATTACCATTACGTGGTAAGGTAATT
AATACAGAGAAAGCACGTCTAGAAGATATTTTTAAAAATGAAGAAATTAATACAATTATCCACACAATCGGGGCAGGCGTTGGTACTGACT
TTAAAATTGAAGATAGTAATTATAATCGTGTAATTATTATGACTGATGCTGATACTGATGGTGCGCATATTCAAGTGCTATTGTTAACATTCT
TCTTCAAATATATGAAACCGCTTGTTCAAGCAGGTCGTGTATTTATTGCTTTACCTCCACTTTATAAATTGGAAAAAGGTAAAGGCAAAACA
AAGCGAGTTGAATACGCTTGGACAGACGAAGAGCTTAATAAATTACAAAAAGAACTTGGTAAAGGCTTCACGTTACAACGTTACAAAGGTT
TGGGTGAGATGAACCCTGAACAATTATGGGAAACGACGATGAACCCAGAAACACGAACTTTAATTCGTGTACAAGTTGAAGATGAAGTGC
GTTCATCTAAACGTGTAACAACATTAATGGGTGACAAAGTACAACCTAGACGTGAATGGATTGAAAAGCATGTTGAGTTTGGTATGCAAGA
GGACCAAAGTATTTTAGATAATTCTGAAGTACAAGTGCTTGAAAATGATCAATTTGATGAGGAGGAAATCTAG

Gene: grlA (DNA topoisomerase IV, subunit A)

Position: 1336907 to 1339309, length: 2403 nt, orientation: FORWARD

Perfect match to: (CN1-CP003979-[1327667:1330069], allele observed in CC72+CC8+CC72+CC97)

Sequence:

GTGAGTGAAATAATTCAAGATTTATCACTTGAAGATGTTTTAGGTGATCGCTTTGGAAGATATAGTAAATATATTATTCAAGAGCGTGCATT
GCCAGATGTTCGTGATGGTTTAAAACCAGTACAACGTCGTATTTTATATGCAATGTATTCAAGTGGTAATACACACGATAAAAATTTCCGTA
AAAGTGCGAAAACAGTCGGTGATGTTATTGGTCAATATCATCCACATGGAGACTCCTCAGTGTACGAAGCAATGGTCCGTTTAAGTCAAGA
CTGGAAGTTACGACATGTCTTAATTGAAATGCATGGTAATAATGGTAGTATCGATAATGATCCGCCAGCGGCAATGCGTTACACTGAAGCT
AAGTTAAGCTTACTAGCTGAAGAGTTATTACGTGATATTAATAAAGAGACAGTTTCTTTCATTCCAAACTATGATGATACGACACTCGAACC
AATGGTATTGCCATCAAGATTTCCTAACTTACTAGTGAATGGTTCTACAGGTATATCTGCAGGTTACGCGACAGATATACCACCACATAATTT
AGCTGAAGTGATTCAAGCAACCCTTAAATATATTGATAATCCGGATATTACAGTCAATCAATTAATGAAATATATTAAAGGTCCTGATTTTCC
AACTGGTGGTATTATTCAAGGTATTGATGGTATTAAAAAAGCTTATGAATCAGGTAAAGGTAGAATTATAGTTCGTTCTAAAGTTGAAGAA
GAAACTTTACGCAATGGACGTAAACAGTTAATTATTACTGAAATTCCATATGAAGTGAACAAAAGTAGCTTAGTAAAACGTATCGATGAATT

ACGTGCTGACAAAAAAGTCGATGGTATCGTTGAAGTACGTGATGAAACTGATAGAACTGGTTTACGAATAGCAATTGAATTGAAAAAAGA
TGTGAACAGTGAATCAATCAAAAATTATCTTTATAAAAACTCTGATTTACAGATTTCATATAATTTCAACATGGTCGCTATTAGTGATGGTCG
TCCAAAATTGATGGGTATTCGTCAAATTATAGATAGTTATTTGAATCACCAAATTGAGGTTGTTGCAAATAGAACAAAGTTTGAATTAGATA
ATGCTGAAAAACGTATGCATATCGTTGAAGGTTTGATTAAAGCGTTGTCAATTTTAGATAAAGTAATCGAATTGATTCGTAGCTCTAAAAAC
AAACGTGACGCTAAAGAAAACCTTATCGAAGTATACGAGTTCACAGAAGAACAGGCTGAAGCAATTGTAATGTTACAGTTATATCGTTTAA
CAAATACTGACATAGTTGCGCTTGAAGGTGAACATAAAGAACTTGAAGCATTAATCAAACAATTACGTCATATTCTTGATAACCATGATGCA
TTATTGAATGTCATAAAAGAAGAATTGAATGAAATTAAAAAGAAATTCAAATCTGAACGACTGTCTTTAATTGAAGCAGAAATTGAAGAAA
TTAAAATTGACAAAGAAGTTATGGTGCCTAGTGAAGAAGTTATTTTAAGTATGACACGTCATGGATATATTAAACGTACTTCTATTCGTAGC
TTTAATGCTAGCGGTGTTGAAGATATTGGTTTAAAAGATGGTGACAGTTTACTTAAACATCAAGAAGTAAATACGCAAGATACCGTACTAG
TATTTACAAATAAAGGTCGTTATCTATTTATACCGGTTCATAAATTAGCAGATATTCGTTGGAAAGAATTGGGGCAACATGTATCACAAATA
GTTCCTATCGAAGAAGATGAAGTGGTTATTAATGTCTTTAATGAAAAGGACTTTAATACAGATGCATTTTATGTTTTTGCGACTCAAAATGG
CATGATTAAGAAAAGTACAGTGCCTCTATTTAAAACAACGCGTTTTAATAAACCTTTAATTGCTACTAAAGTTAAAGAAAATGATGATTTGA
TTAGTGTTATGCGCTTTGAAAAAGATCAATTAATTACCGTCATTACTAATAAAGGTATGTCATTAACGTATAATACAAGTGAACTATCAGATA
CCGGATTAAGGGCAGCTGGTGTTAAATCAATAAATCTTAAAGCTGAAGATTTCGTTGTTATGACAGAAGGTGTTTCTGAAAATAATACTATA
TTGATGGCCACACAACGCGGCTCGTTAAAACGTATTAGTTTTAAAATCTTACAAGTTGCTAAAAGAGCACAACGTGGAATAACTTTATTAAA
AGAATTAAAGAAAAATCCACATCGTATTGTAGCTGCACATGTAGTGACAGGTGAACATAGTCAATATACATTATATTCAAAATCAAATGAA
GAACATGGTTTAATTAATGATATTCATAAATCTGAACAATATACAAATGGCTCATTCATTGTAGATACAGATGATTTTGGTGAAGTAATAGA
CATGTATATTAGCTAA

Gene: alsT (sodium:alanine symporter family protein)

Position: 1339559 to 1341019, length: 1461 nt, orientation: FORWARD

Perfect match to: (11819-97-CP003194-[1389936:1391396], allele observed in CC80)

Sequence:

TTGAAAGATTTCGATAGTTTAATACCTGGATGGTTTAAAGAATTTGTCCATGTTGGTACCGATTTAATATGGTCTCAATATTTAATTGGTCTA
TTATTGACAGCTGGATTCTTCTTTACAATTAGTTCTAAATTCGTTCAATTACGAATGTTACCTGAAATGTTTAGAGCTTTAGTAGAACGTCCA
GAAACTTTAGAAGATGGTAAGAAGGGTATTTCGCCATTCCAAGCATTTGCGATTAGTGCTGGTTCGAGAGTTGGTACTGGTAATATTGCTG
GTGTTGCGACTGCGATTGTTTTAGGCGGTCCAGGTGCAGTATTTTGGATGTGGGTTATTGCATTTATAGGTGCAGCGAGTGCATTTATAGA
AGCGACTTTGGCTCAGGTTTATAAAGTACATGATAAAGATGGTGGATTCCGTGGTGGTCCAGCTTACTATATTACTAAAGGTTTAAATCAAA
AATGGCTAGGTATCGTATTTGCGATTTTAATTACAATTACATTTGCATTTGTATTTAACACAGTGCAATCTAATACAATTGCGGAGTCGTTAA
ATACGCAATATAATATTAGTCCAGTAATCACAGGTATTATTTTAGCAATCGTAACAGCTATTATTATATTTGGTGGTGTACGTAGTATTGCTA
CGTTATCTTCGTTAATTGTACCGATTATGGCTATCATTTACATTGGTATGGTTTTAGTAATATTGCTATTTAATTTAGATCAAATTGTTCCTATG
ATAGGTACGATTATTAAAAGTGCATTTGGTATCGAACAAGTAACTGGTGGCGCTGTAGGTGCTGCGGTTCTTCAAGGTATCAAACGTGGTT
TATTCTCTAACGAAGCTGGTATGGGTTCTGCGCCGAATGCAGCGGCAACTGCTGCCGTACCACACCCTGTTAAGCAAGGTTTAATCCAATCA
TTAGGTGTGTTCTTTGATACAATGTTGGTTTGTACAGCAACTGCAATCATGATTTTACTATATTCAGGACTGAAATTTGGTGATAACGCACCT
CAAGGTGTTGCAGTTACTCAATCAGCACTTAATGAGCATTTAGGTTCTGCTGGAGGTATTTTCTTAACAATAGCAGTTACACTGTTTGCATTT
TCATCTGTGGTAGGTAATTACTATTACGGTCAATCTAATATTGAATTTTTATCAACAAACCGTGTAATATTATTTATCTTTAGATGTCTTGTTG
TAGTACTTGTCTTTGTCGGTGCAGTTGTAAAAACAGAAACAGTATGGAATACGGCAGACTTATTTATGGGCTTAATGGCAATTGTAAACATT
ATTTCCATTATAGGACTGTCCAATGTAGCTTTTGCATTGATGAAAGATTATCAAAAGCAGAAAAAAGAAGGCAAGAACCCTGTCTTTAAACC
TGAAAACTTAGAAATTAACTTATTTGGAATTAGTGCTTGGGGCGCTAACAAATATAAGAACTCTGATAAATAA

Gene: glcT (transcriptional antiterminator)

Position: 1341519 to 1342370, length: 852 nt, orientation: FORWARD

Perfect match to: (NN50-BAEA01000026-[49232:50083:r], allele observed in CC4803+CC8+CC239)

Sequence:

ATGGGAGAATATATTGTTACTAAAACATTGAACAACAATGTCGTAGTATGTACTAATAATGATCAAGAAGTTATTTTAATCGGTAAAGGTAT
TGGTTTTAACAAAAAAGAGGGAATGGCGTTAAACGACCAAACTATTACAATAGAGAAAATTTATAAATTAGAAAGTGAGCAACAAAAAGC
ACATTATAAAAGTTTAGTTGAAATCGCTGATGATAATGTATTACAAGTAATTATTGATTCGTTGAATTTTATTTCTAATACTGCGATGAATGT
TGATTCAAAACAACTTGTAGTTTCATTAACGGATCATATTATATTTGCTTATAAACGCTTAAAACAAAATCAAGTTATTAGCAATCCATTTGTT
ATGGAAACTATGCAGTTATATAGTGATGCATATCATATTGCTAAACAGGTGATTGATCAGTTAAATGCAGCATTAGATGTACATTTTCCTGA
AGATGAGATAGGATTTATTGCATTACATATTGCATCTAATACAGAAGATTTATCTATGCATGAGATGACCTTGATCAATAATGTTATTAAAA
AAGGTATAGATATCATTGAATCAGACCTTGTGACAACTGTTGATAAGGAATCATTACAATACCAACGTTTTATAAGACACGTACAATTTTTA
ATTCGCCGATTAAGAAGAAAAGAATATATACATGCACAAGATGATTTTGTGTCTATGATTAAAAATCACTATCCGATTTGCTATAACACAGC

ATATAAAATTTTAACTATGATACAAAAACAATTTGATGTTAATATCAGTGAGTCTGAAATTATATATTTAACATTACACATTCATCATTTTGAA
GAAAGGATTAATCAATCCTAA

Gene: yubA (putative membrane protein)

Position: 1342505 to 1343713, length: 1209 nt, orientation: FORWARD

Perfect match to: (M0239-AIWE01000006-[149371:150579], allele observed in CC188+CC8+CC4803)

Sequence:

TTGAATGAAAATGAAAAGAATATAAGAAAGAATTTTTTAAAATTTACCGAATCACGGTATATGAAGTTTGTTGGTGGGAATGATTTAGTCTT
CTCATTAATAGCGCTAGTATTGTTGGGTATTGTTATTTTTATTTTCGAAAAAGTATCATATGTTTTTGATCCTTTTATCATCGTTTTTAAGACGA
TAGCAGCACCTATCATCGTCTCTTTAATTCTATTCTATCTATTTAACCCAATCGTAAATATGATGGAACGTTATAGAATACCAAGAGTTGCAG
GTATTTCTATTATTTATCTAGCTGTAGTAGGTGTTATTACGTTAATTGTTAATTTATTGATACCTATTATTGGTTCGCAAGTAGATAGTTTAGT
TAAAAATTCACCGCAATATCTAGAAAAATTAATTAATTCTATTGATAAAATAGCAAATAATACGTTTTTCTCTTCGTATTATAGTCAAATTAAT
GATTGGTTAAATTCTTTACCTAAGAAAATACCATCTATGTTAAGTGAATTTACAGATGGCTTTGGGTCTAAAATTGCAACGTTTGCAGAAAC
GATTGCTAATATTGGCGTTGTGATTGTCACAACACCATTTGTACTATTCTTTATGCTTAAAGATGGACATCACTTCAAAGAATTTTCAACGAA
TATTATGCCACCGAAATTCCGAAAAGATTTTCATGATCTACTTGAAAAAATGAGTGTTCAAGTTGGTTCATACATTCAAGGACAAATTATCGT
TTCATTCTGTATCGGTATACTGTTGTTTATCGGTTATTCGGTTATCGGGTTGAAATATAGCTTAGTATTAGCTAGTATTGCGGCAGTTACAAG
TGTTGTACCATATTTAGGGCCTACTATAGCGATTTCTCCAGCTATTGTAATAGCTGCTATAACATCGCCGTGGATGCTCTTAAAATTAGCAGT
AGTATGGACTTTAGTACAATTTGTTGAAGGGCACTTCATTTCACCAAATATTATGGGTAAAACACTTAAGATTCATCCACTTACAATCATTTT
CATTTTACTGTGTGCAGGCAAATTGCTTGGTATTGTAGGCGTTATTTTAGGTATTCCGGGATATGCTATTTTAAAAGTATTAGTTACTCATTT
ATTCCAATTATTTAAACGTCGATACAATCGTTTCTATGGTAATGATGTAGGTGAATATGATATTAAAGAAAGTAATAAAATAGTTGAATAA

Gene: mprF (lysylphosphatidylglycerol synthetase)

Position: 1344194 to 1346716, length: 2523 nt, orientation: FORWARD

Perfect match to: (NN50-BAEA01000026-[44886:47408:r], allele observed in CC4803+CC8+CC239)

Sequence:

ATGAATCAGGAAGTTAAAAACAAAATATTTTCAATCTTAAAAATTACGTTTGCTACAGCTTTATTTATTTTTGTAGCAATCACATTGTATCGG
GAGTTATCTGGTATTAACTTTAAAGATACGTTGGTTGAATTTAGTAAGATTAACCGTATGTCCTTAGTGTTACTATTTATTGGTGGTGGGGC
ATCGCTTGTTATTCTATCAATGTATGATGTGATTTTATCTAGAGCTTTAAAAATGGATATATCCTTAGGCAAAGTTTTAAGAGTAAGTTATAT
CATCAATGCATTGAATGCGATTGTAGGTTTCGGTGGCTTTATTGGTGCAGGCGTTAGAGCAATGGTTTATAAAAACTATACGCATGATAAA
AAGAAATTAGTTCACTTTATATCCTTAATACTTATTTCAATGTTGACAGGTTTAAGCTTATTATCATTGCTAATTGTATTCCATGTTTTCGATGC
ATCTTTAATCTTAGATAAGATTACATGGGTAAGATGGGTATTATATGTAGTGTCATTTTTCTTACCATTATTCATTATTTATTCAATGGTTAGA
CCACCCGATAAAAACAATCGTTTTGTAGGATTGTACTGCACTTTAGTGTCGTGTGTTGAATGGTTAGCAGCTGCAGTTGTATTATATTTCTGT
GGTGTAATTGTTGACGCTCATGTATCATTCATGTCCTTTATTGCAATATTTATCATTGCTGCATTATCAGGTTTAGTCAGCTTTATTCCTGGTG
GTTTCGGCGCTTTCGATTTAGTTGTATTACTAGGATTTAAAACTTTAGGTGTCCCTGAGGAAAAAGTATTATTAATGCTACTTCTATATCGTTT
TGCGTACTATTTTGTACCGGTAATTATTGCATTAATTTTATCATCATTTGAATTTGGTACATCAGCTAAGAAGTACATTGAGGGATCTAAATA
CTTTATTCCTGCTAAAGATGTTACGTCATTTTTAATGTCTTATCAAAAGGATATTATTGCTAAAATCCCATCATTGTCATTAGCAATTTTAGTA
TTCTTTACAAGTATGATCTTTTTTGTAAATAACTTAACGATTGTTTACGATGCTTTATATGATGGAAATCACTTAACGTATTATATTCTATTGG
CAATTCATACTAGTGCTTGTTTATTACTTTTACTGAATGTAGTTGGTATTTATAAGCAAAGTAGACGTGCCATTATCTTTGCTATGATTTCAAT
TTTATTAATCACAGTGGCGACATTCTTCACTTACGCTTCATATATTTTAATAACATGGTTAGCTATTATTTTTGTTCTGCTTATTGTAGCTTTCC
GTAGAGCACGTAGGTTGAAACGCCCAGTAAGAATGAGAAATATAATTGCAATGCTTTTATTCAGTTTATTTATTTTATATGTTAACCATATAT
TTATTGCTGGAACGTTATATGCATTAGATATTTATACGATTGAAATGCATACATCTGTATTGCGCTATTACTTCTGGCTTACGATTTTAATCAT
CGCTATCATCATAGGTATGATTGCATGGTTGTTTGATTATCAATTTAGCAAAGTACGTATTTCTTCTGAAATTGAAGATTGCGAGGAGATTAT
TAATCAGTACGGCGGTAATTATTTGAGTCACTTGATATATAGTGGTGACAAGCAGTTTTTCACTAATGAAAATAAAACAGCATTTTTAATGT
ATCGTTATAAAGCAAGTTCATTAGTGGTTCTTGGAGATCCGTTAGGTGATGAAAATGCCTTTGATGAATTGTTAGAAGCATTCTATAATTAC
GCTGAGTATTTAGGCTATGATGTTATATTCTATCAAGTTACAGATCAACACATGCCTTTATATCATAATTTCGGTAACCAATTTTTCAAATTAG
GTGAAGAAGCAATTATTGATTTAACGCAATTTTCAACTTCAGGTAAAAAACGCCGTGGATTTAGAGCGACTTTAAACAAATTCGATGAACTT
AATATTTCATTCGAAATTATTGAACCACCGTTTTCAACTGAATTTATAAATGAGCTTCAACATGTAAGTGATTTATGGCTAGATAATCGTCAG
GAAATGCATTTCTCTGTGGGTCAATTTAATGAAGAATACTTATCTAAAGCGCCAATTGGTGTAATGCGAAATGAAGAAAATGAAGTAATTG
CATTCTGTAGTTTAATGCCAACATACTTTAATGATGCCATTTCAGTCGATTTAATTAGATGGTTGCCAGAGTTAGATTTACCATTAATGGATG
GATTATACTTGCACATGTTACTTTGGAGTAAAGAACAAGGTTATACAAAATTTAATATGGGTATGGCAACGTTATCAAACGTTGGTCAATTG
CATTATTCATATTTAAGAGAACGACTTGCAGGCCGTGTCTTTGAACATTTCAACGGTCTATATCGTTTCCAAGGATTACGTCGTTATAAATCT

AAATATAATCCGAATTGGGAACCACGCTTTTTAGTTTATCGTAAAGATAATTCGCTTTGGGAATCACTTTCTAAAGTAATGCGTGTAATACGT
CACAAATAA

Gene: msrA1 (peptide methionine sulfoxide reductase A1)

Position: 1346919 to 1347428, length: 510 nt, orientation: REVERSE

Perfect match to: (D30-ABFB01000025-[27852:28361], allele observed in CC8+CC5+CC97)

Sequence:

TTATTGCTTATTTTTGTATTCTTGGCGTATTTTTTGTTCTTCTGCATAGCGCTCTGGATTTTTCTTATAAAAATCTTGGTGATAGTCTTCGGCTT
TGTAAAATTGTGACGCGGGTAATATTTTTGTTGCAATTGCCTTATCAGCATTAATCGTATTTTTAAGCTGCTCGATATAAGTCTCAGCGAGTT
CTTTTTGATGATCATTAGTGTAGAAAATAGCTGTTTGATATTGAGGACCACGGTCTTGATATTGACCACCTGTATCTAATGGGTCAATGACT
GAGAAAAATATTTCTAATAACTTATTGTATGAGAATAATGCAACATCATATTGAATTTCAACAGTTTCTAAATGACCACTCGTACCTGATTTT
ACTTGTTCGTAAGTAGGATTTTCAATATGTCCGCCCATATATCCAGAAGTTACTTTTTCTATGCCGTCAAAGGTGTCAAATGGTTTCGTCATA
CACCAAAAGCAACCTCCGGCAAAATAAGCTGTATTAATATTCAT

Gene: tagT-lcpA (phosphotransferase)

Position: 1347565 to 1348548, length: 984 nt, orientation: FORWARD

Sequence:

ATGGATAAAGAAACTAATGACAACGAATATAGACGTCAAAGTGAACATCGCACTTCGGCGCCTAAGCGAAAAAAGAAGAAGAAAATTAG
GAAATTACCTATCATTCTTCTGATTGTTGTAATTTTACTTATCACATTAGTTGTATATATTGTACATAGTTACAATAGCGGTGTAGAATATTCC
AAGAAACATGCGAAAGATGTTAAAGTACATCAATTTAATGGACCAGTAAAAAATGATGGTAAAATTTCTATTCTTGTACTCGGTGCAGATA
AAGCACAAGGTGGACAATCAAGAACAGATTCTATCATGGTTGTTCAATATGACTTTATCAATAAAAAAATGAAAATGATGTCTGTCATGCGT
GATATTTATGCAGATATTCCAGGATATGGAAAACACAAAATTAATTCAGCATACGCTTTAGGTGGTCCAGAGCTACTTAGAAAAACACTTGA
TAAAAATTTAGGAATTAATCCTGAATATTATGCAGTAGTTGATTTTACTGGATTTGAGAAAATGATTGATGAATTAATGCCTGAAGGTGTAC
CAATTAATGTCGAAAAAGATATGTCGAAAAATATTGGTGTATCTTTGAAAAAGGGTAACCATAGGTTGAATGGTAAAGAATTACTTGGTTA
TGCAAGATTCCGTCACGACCCTGAAGGTGACTTCGGACGTGTGCGACGTCAGCAACAAGTGATGCAAACATTGAAAAAAGAAATGGTTAA
TTTTAGAACAGTTGTTAAATTACCAAAAGTTGCAGGTATTTTAAGAGGCTATGTGAATACAAACATTCCTGATTCAGGGATTTTCCAAACAG
GTTTGAGTTTTGGTATCCGAGGTGAAAAAGATGTTAAGTCATTGACTGTGCCAATCAAGAACTCATACGAAGATGTCAATACAAATACTGA
TGGTAGTGCATTACAGATTAATAAAAACACAAATAAACAAGCTATTAAAGACTTTTTAGATGAAGATTAA

Gene: txbi_lcpA (bidirectional rho-independent terminator of lcpA)

Position: 1348541 to 1348559, length: 19 nt

Perfect match to: (MRSA252-BX571856-[1431394:1431412], allele observed in CC30)

Sequence:

AAGATTAAAAATAAACAAG

Gene: dmpI (4-oxalocrotonate tautomerase)

Position: 1348636 to 1348821, length: 186 nt, orientation: REVERSE

Perfect match to: (N315-BA000018-[1368054:1368239:r], highly conserved allele)

Sequence:

TTATTGATCTGACTTTCTTACGCCAGCCACACCATAATGGTTTGGTTTCATTTCTTCTATAACAACGTGAATTGCTTGTCTATTTGCCCCCGTT
GTTTTTTCTACGGCGTCAGTTACTTCGCTAACTAAATTTTTTAATTGTTCATCCGAACGACCTTCTAATAATTTTACATTGACGATTGGCAT

Gene: uvrX (DNA-damage repair protein)

Position: 1348969 to 1350231, length: 1263 nt, orientation: FORWARD

Perfect match to: (N315-BA000018-[1368387:1369649], highly conserved allele)

Sequence:

GTGTATAATTATCATTTATTAGAAGATAGGGATGTTCTATGTATTGACCAAAAAAGTTTTTTTGCGAGTGTTTCTTGTATTGAAAAGGGGCT
AGATCCATTAGAAACAAAGTTAGCTGTTGTTGCAGATACTAAGCGTCAGGGTTCTGTAATATTGGCTGCGACACCTAAATTAAAAGAATTA
GGCATCAAGACAGGGTCGCGATTGTTTGAAATACCACATAGAAATGATATTTACATTATCAATCCAAGTATGCGTAAATATCTTAATGTTTC
AGTTGCTATTTCTAAGATTGCATTGCGTTATATTCCACCTGAAGATTTACACCAATATAGTATTGACGAATTTTTTATGGATGTTACTGATAG
CTATCATAGATTTAGTTCTACAGTACATGCATTTTGCGAAAGACTTAAACGTGAAATTTATGAAGAAACAGGCATTTATTGTACTGTGGGCA
TTGGTTCTAATATGTTATTAAGTAAAATTGCTATGGATGTTGAAGCGAAGCATAGTCAAAATGGTATAGCTGAATGGCGATATCAAGATGT
ACCAACGAAATTATGGCCAATTCAGCCCTTGCGAGATTTTTGGGGTATTAATCGTCGAACAGAAGCCAAATTGAATAAAAGAGGAATTTTT
ACTATAGGAGATTTAGCGAAATATCCATATAAATTTTTAAAAAAAGAGTTCGGTATTTTAGGTGTTGATATGCATCTACATGCGAATGGGAT
AGATCAGAGTAAAGTACGTGAAAAGCACAAGATCAGCAATCCATCGATATGCAAAAGTCAAATATTAATGAGAGATTATCATTTTGATGAA
GCAAAAGTAGTAATGCAAGAGTTAATTGAAGATGTTGCTAGCAGAGTTCGAGCAAGAAAAAAAGTGGCAAGAACGATACATTTTGCCTTT
GGCTATAGTGATGAAGGCGGTGTACATAAGCAATATACTTTGAAAGATCCAACAAACTTAGAAAAAGATATTTATAAAGTAGTAATGCATT
TTGCAGATAAATTATGTAATAAACAAGCACTATATCGTACGCTAAGTATATCTTTGAGTCAATTTATTAATGAGGATGAGCGACAGTTAAGT
CTGTTTGAAGATGAATACCAACGCAAACGTGACGAATGCCTAGCTAAAACGATAGACCAATTACATTTGAAATACGGCAAAGGTATTGTGT
CCAAAGCAGTATCGTTTACAGAAGCAGGTACAAAACATGGTAGATTAGGTTTAATGGCTGGACATAAAATGTAA

Gene: tyrA (prephenate dehydrogenase)

Position: 1350368 to 1351459, length: 1092 nt, orientation: REVERSE

Perfect match to: (IS-111-AHLS01000043-[17575:18666:r], allele observed in CC8+CC1+CC8+CC97)

Sequence:

TTATTGGATATAACAATCAAAATCACTCAATGCTTGCATACCGCGTTCTCGGTCAGTAGGATTTTTGAAACTAATTTTTAAAGCACCGTATAT
ATCTTCGCGTACTTCTAAGATTCTTAAGTTGCTTATAGATATGTTATGTAAACTCAGGATATAAGTCACTTTACTTATCATACCTGATTCATCC
GGAATATCTACATATAGATCATACGCAGTATTTAGTCCACCTAGTTGTTTAGCGGGTAGTGCGTCGCGATACGATTTAGCTTGGGCAAAAAA
TGATAACAATTTTTCAGAATCATTGCTTTCAATTAGTCTTTCTAAATCTTGAAACTGACTTTTTAGCTGTCGAATCATTTCTAAAATATACGTTT
TATTACTCAAGGTGATATCTTTCCACATTTGTGCATTACTACTAGCTATACGAGTGATATCACGAAAACCACCAGCTGCAAGTTTATTAACTA
AATGATGTTCTTGACCGTTCTTTTGACTAACATGAACTAAACTAGATGCAACGATATGAGGTAAATGACTTACGACGCTTGTTACGTAGTCG
TGTTCTTCAGCAGTAGTTACAATAAATTTAGCAAGAGTAGGTGATAACAGTTCTTTTAACGTGTTTGCTGCTTGCTCATTTCTTGGCTCATTAT
AGACTAAAATATAATAAGCGTTTTCAAATAAGTGCTTTTTAGCATTTAGTACACCAGATTTATGACTACCAGCCATTGGATGACCACTGACTA
AATGAATATTATGCTTTAATAAATTGGATTCGTGTTGCTGTATCATTGCTTTAGTACTACCAGTATCAGAAACAATAACACCAGGTTTAGTTG
GCATATCTATAAGCTCGCTAAGATATTTATTTGTGATAGCAACAGGTGTTGCATAAATAATTACATCGGCTTTTTTAATAGCTTCACTATAAT
TTAAACATTTTTCATTAATAATGCCGATTGATTTAGCTTTATCTAACTGAGAAGTATCTGCATCGTATGCAATAATATTAGTATTAGGGTTAT
GGTATTTTATATTGCTAGCAAGACTTCCACCAATTAATCCAAGCCCAACAAATAAAACTGTTGTCAT

Gene: yhfE (putative endoglucanase)

Position: 1351624 to 1352655, length: 1032 nt, orientation: FORWARD

Perfect match to: (11819-97-CP003194-[1402001:1403032], allele observed in CC80)

Sequence:

ATGGAACCAATATTAGAAATGATTAAAACATTAACAGGTATTAATAGTCCTTCAGGAGACACAGAAGAAGCAATTCAATTTGTCGAAAAAT
ATGCAAATGACTTGGGTTATCAAACAACACTAACAAATAAAGGTGCGTTATTAATAACAGTGCCAGGCAAAAATGATGAAGTACAACGCTG
TATTACTGCTCATGTTGATACTTTAGGTGCAATGGTTAAAGAAATTAAAGAAGATGGTCGCTTAGCAATAGAATTAATTGGAGGATTCACGT
ATAACGCGATTGAGGGTGAATATTGCCAAATTAAAACTGATGCTGGTCAAATATATACAGGAACAATTTGTCTGCATGAAACAAGTGTTCA
TGTATATAGAAATAATCATGAAATACCTAGAGATCAAAAGCATATGGAAATAAGAATTGATGAAGTAACTACATCAGAAGAAGATACAAA
GAGTTTAGGTATTTCAGTAGGTGATTTTGTTAGCTTTGATCCACGTACAGTTATCACGTCATCAGGTTTTATTAAATCTCGTCATTTAGATGA
TAAAGCTAGCGTAGCGATGATACTACAATTACTAAAGAAATTAAAAGAAGAGCAAATAATATTACCACATACAACGCAATTTTATATTTCTA
ATAACGAAGAAATAGGTTACGGTGCAAATGCATCAATTGATTCGAAAATCAAAGAATATATTGCATTAGATATGGGCGCGTTGGGAGACG
GTCAAGCATCGGATGAATATACAGTTTCTATTTGTGCCAAAGATGCTTCAGGTCCATATCATAAGCAATTGAAATCGCACCTAGTTAATCTTT
GCAAAATAAATAACATTCCATATAAAGTAGACATATATCCATATTATGGTTCAGATGCTTCAGCAGCTTTACATGCTGGTGCGGATATCAGA

CATGGTTTATTTGGCGCTGGCATTGAATCATCTCATGCAATGGAACGAACACATATTGATTCTATTAAAGCGACAGAGAAATTACTATATGC
ATATTGCTTATCACCAATTGAGTAA

Gene: tbox05 (T-box leader element)

Position: 1352717 to 1352890, length: 174 nt

Sequence:

AATTTTCTAAAGAAATAGTAGCAGATATGAAACGTAGCAAATAGAAAGCTAATGGGTGATGGGAATTAGCACGCCATATCTTGTGAATTGG
ACTTTGGAAAACAATTGAATGAGTTTTGAAAGTGAACATGAATTATGTTAACTAAGGTGGCACCACGGTAACGCGTCCTTACA

Gene: tbox06 (T-box leader element)

Position: 1352918 to 1353104, length: 187 nt

Sequence:

ATTTAGACAAAATGTAGTAGTTAATTAAAGGTAGCAACAGAAAGTTAGTGGATGATGTGAACTAACACCGAGATTAATGAAATTGGGTTTT
GTCTGCAACAGAAAAATTATATATAGTAAAGAGTGAACTATGAATATTTCGAATATTCGGTTAATTTAGGTGGTACCACGCGTCAGCGTCCT
TTAT

Gene: trpE (anthranilate synthase component I)

Position: 1353147 to 1354553, length: 1407 nt, orientation: FORWARD

Sequence:

ATGGATATATTTTATAAAAAAATAAAAGCAAATGTAACGCCCGAAGTTTTAGCACAACTTCATTCCAAGAAGATCATTTTGGAAAGTACAAA
TCAACAACAAACTAAAGGTCGCTATTCAGTTGTTATTTTTGATATTTATGGCACTTTAACTTTAGATAATGATGTATTATCAGTAAGTACTTTA
AAAGAATCGTATCAAATCACTGAAAGACCGTACCATTATTTAACGACTAAAATAAATGAAGACTACCATAATATTCAAGATGAGCAACTTAA
GTCATTACCATTTATATCTGGATATGTTGGGACGTGTAGCTTTGATTTAGTAAGACATGAATTTCCTAAATTGCAATCAATACAATTAGAAGA
TCACAAGCAGCACGATGTAAGGTTATATATGGTTGAACAAGTTTATGTATTTGACCATTACAAAGATGAGTTATATATCATCGCGACGAATC
AATTTTCAAATTCAACAAAATCAGATCTTGAGAATCGAGTTATTAAGTCTATCGAAGACTTAACTAAAATCCAACCATTCATGCCTACACAAG
ATTTTGATTTTAAAACTAAAGAAATTCAATCAAACATTTCTGAAGAAAGATTTATCGAAATGATTCAGTATTTCAAAGAGAAAATAACAGAA
GGGGATATGTTCCAAGTTGTGCCATCAAGAATTTACAAATATGCGCATCATGCTAGTCAGCATTTAAATCAACTTTCGTTTCAACTGTATCAA
AATTTAAAACGACAAAACCCAAGTCCATATATGTATTATCTTAATATCGATCAACCATATATTGTCGGTAGTTCTCCCGAAAGTTTCGTAAGT
GTCAAAGATCAAATTGTAACAACTAATCCTATTGCAGGTACGATTCAACGTGGTGAGACGACACAAATAGATAATGAGAATATGAAACAAC
TACTTAATGATCCAAAAGAATGCAGCGAACATCGTATGCTAGTTGATTTAGGACGTAATGATATTCATAGAGTAAGTAAAATCGGTACCTCA
AAAATTACTAAATTAATGGTTATTGAAAAATATGAACATGTTATGCATATCGTAAGTGAAGTCACAGGTAAAATAAATCAAAATTTATCGCC
AATGAAAGTTATTGCGAATTTATTACCAACAGGTACCGTTTCAGGTGCACCAAAATTACGTGCAATTGAAAGAATATATGAACAATATCCAC
ATAAACGGGGCGTTTATAGTGGTGGTGTTGGATACATAAATTGTAATCATAACTTAGATTTTGCATTAGCAATTCGAACGATGATGATAGAT
GAGCAGTATATCAACGTAGAAGCTGGTTGTGGCGTTGTATATGATTCTATTCCTGAAAAAGAACTGAATGAAACGAAATTGAAAGCTAAAA
GCTTATTGGAGGTGAGCCCATGA

Gene: trpG (anthranilate synthase component II)

Position: 1354550 to 1355116, length: 567 nt, orientation: FORWARD

Perfect match to: (COL-CP000046-[1415613:1416179], highly conserved allele)

Sequence:

ATGATCTTAGTTGTAGATAATTATGATTCCTTTACATATAACCTAGTGGATATTGTTGCTCAACATACTGACGTCATTGTTCAATACCCTGATG
ATGATAATGTGCTGAATCAATCGGTGGACGCTGTTATTATATCTCCTGGTCCAGGGCATCCATTAGACGATCAACAGTTAATGAAAATCATA
TCAACCTATCAACACAAACCCATTTTAGGTATTTGTTTAGGGGGCTCAGGCACTGACTTGTTACTACGGTGGAGAAGTCATTAAAGGCGACAA
GGTTATGCACGGCAAAGTTGATACACTAAAGGTTATATCGCATCATCAACATCTGTTATATCAAGATATACCAGAACAGTTTTCAATTATGA
GATATCATTCATTAATAAGTAACCCTGACAATTTTCCAGAAGAATTGAAAATTACTGGACGTACCAAAGATTGTATACAGTCATTCGAGCAT

AAAGAAAGACCGCATTATGGTATTCAGTACCATCCTGAATCATTTGCTACAGACTATGGTGTCAAAATAATTACAAATTTCATTAATCTAGTG
AAGGAAGGATGA

Gene: trpD (anthranilate phosphoribosyltransferase)

Position: 1355122 to 1356120, length: 999 nt, orientation: FORWARD

Perfect match to: (MW2-BA000033-[1376529:1377527], allele observed in CC1+CC8+CC97)

Sequence:

ATGACATTACTAACAAGAATAAAAACTGAAACTATATTACTTGAAAGCGACATTAAAGAGCTAATCGATATACTTATTTCTCCTAGTATTGG
AACTGATATTAAATATGAATTACTTAGTTCCTATTCGGAGCGAGAAATCCAACAACAAGAATTAACATATATTGTACGTAGCTTAATTAATAC
AATGTATCCACATCAACCATGTTATGAAGGGGCTATGTGTGTGTGCGGCACAGGTGGTGACAAGTCAAATAGTTTCAACATTTCAACGACT
GTTGCTTTTGTTGTAGCAAGTGCTGGCGTAAAAGTTATAAAACATGGTAATAAAAGTATTACCTCAAATTCAGGTAGTACGGATTTGTTAAA
TCAAATGAACATACAAACAACAACTGTTGATGATACACCTAACCAATTAAATGAAAAAGACCTTGTATTCATTGGTGCAACTGAATCATATC
CAATCATGAAGTATATGCAACCAGTTAGAAAAATGATTGGAAAGCCTACAATATTAAACCTTGTGGGTCCATTAATTAATCCATATCACTTA
ACGTATCAAATGGTAGGCGTCTTTGATCCTACAAAGTTAAAGTTAGTTGCTAAAACGATTAAAGATTTAGGTAGAAAACGTGCAATCGTTTT
ACATGGTGCAAATGCTATGGATGAAGCAACACTATCTGGTGATAATTTGATATATGAATTGACTGAAGATGGAGAAATCAAAAATTACACA
TTAAATGCGACTGATTATGGTTTGAAACATGCGCCGAATAGTGATTTTAAAGGCGGTTCACCTGAAGAAAATTTAGCAATCTCCCTTAATAT
CTTGAATGGTAAAGATCAGTCAAGTCGACGTGATGTTGTCTTACTAAATGCGGGTTTAAGCCTTTATGTTGCAGAGAAAGTGGATACCATC
GCAGAAGGCATAGAACTTGCAACTACATTGATTGATAATGGTGAAGCATTGGAAAAATACCATCAAATGAGAGGTGAATAA

Gene: trpC (indole-3-glycerol phosphate synthase)

Position: 1356122 to 1356904, length: 783 nt, orientation: FORWARD

Perfect match to: (CIG1835-AIEQ01000005-[834702:835484], allele observed in CC1+CC8)

Sequence:

ATGACGATTTTATCAGAAATTGTTAAATATAAACAGTCACTTTTACAAAATGGCTATTATCAAGACAAACTTAATACCTTGAAAAGTGTGAA
GATTCAGAATAAAAAATCTTTTATAAACGCAATTGAGAAAGAACCAAAGCTAGCAATTATTGCAGAAATTAAATCGAAGAGTCCTACAGTT
AATGACTTACCTGAACGAGATTTATCGCAACAAATCTCAGATTATGACCAATATGGTGCAAATGCCGTGTCCATTTTAACTGATGAAAAGTA
CTTTGGTGGTAGTTTTGAAAGATTACAAGCATTGACGACAAAAACAACATTACCCGTATTATGCAAAGACTTTATTATAGACCCGCTTCAAA
TTGATGTTGCTAAACAAGCTGGTGCATCTATGATTTTATTGATCGTTAACATCTTATCTGATAAACAATTGAAAGATTTATATAACTACGCTA
TATCGCAAAATCTAGAAGTGTTAGTTGAAGTACATGATCGCCATGAATTAGAACGTGCCTATAAGGTTAATGCTAAATTGATTGGTGTAAAT
AACAGGGACTTAAAACGATTTGTTACAAATGTGGAACATACAAATACTATTTTAGAAAATAAAAAACCAAATCATTATTATATTTCTGAAAG
TGGTATTCACGATGCATCTGATGTAAGAAAAATCTTGCATAGTGGTATCGATGGCTTACTAATAGGTGAGGCGCTTATGCGTTGTGACAAT
CTATCTGAATTTTTACCACAACTGAAAATGCAAAAGGTGAAGTCATGA

Gene: trpF (N- (5'-phosphoribosyl)anthranilate isomerase)

Position: 1356904 to 1357536, length: 633 nt, orientation: FORWARD

Perfect match to: (CIG2018-AHVV01000008-[332593:333225], allele observed in CC8+CC15)

Sequence:

ATGAAATTGAAATTTTGTGGCTTTACATCAATAAAGGATGTTACAGCGGCCAGTCAATTACCTATTGATGCGATAGGTTTCATCCATTATGA
AAAAAGTAAAAGGCATCAAACAATTACCCAAATAAAAAAGTTAGCGTCTGCTGTTCCAAATCATATCGATAAAGTATGTGTCATGGTAAATC
CTGATTTAACAACAATTGAACACGTATTAAGCAATACGTCAATTAACACAATACAGTTACACGGCACAGAATCTATTGATTTTATACAGGAA
ATTAAAAAGAAATATTCAAGCATTAAAATCACTAAAGCTTTAGCTGCAGATGACAACATAATCCAAAACATAAATAAATATAAAGAGTTCGT
AGATTTATTTATTATCGACACACCCTCAGTGTCGTATGGTGGTACCGGTCAAACATATGACTGGACTATTTTGAAGCACATAAAAGACATAC
CTTACTTGATAGCAGGAGGCATTAACTCTGAAAATATTCAAACAGTTAATCAACTTAAATTATCACATCAAGGTTTTGATCTTGCATCAGGTA
TAGAAGTAAATGGGCGAAAAGATATAGAAAAAATGACAGCAATTGTAAATATTGTGAAAGGAGATAGAGATAATGAATAA

Gene: trpB (tryptophan synthase beta chain)

Position: 1357529 to 1358743, length: 1215 nt, orientation: FORWARD

Perfect match to: (11819-97-CP003194-[1407906:1409120], allele observed in CC80+CC12+CC772)

Sequence:

ATGAATAAACAAATACAAACGGAAGCAGATGAATTAGGTTTCTTTGGTGAATACGGAGGGCAATATGTTCCAGAAACATTAATGCCAGCAA
TTATTGAGTTGAAAAAAGCTTATAAAGAGGCAAAAGCAGACCCAGAGTTTCAAAGAGAACTGGAATACTATTTATCAGAGTATGTAGGAC
GCGCGACGCCACTTACATATGCTGCATCATATACTGAAAGCCTAGGTGGCGCTAAAATATATTTGAAACGAGAGGATCTAAATCATACAGG
CGCCCATAAAATTAATAATGCGTTAGGTCAAGCGTTGCTTGCTAAAAGAATGGGCAAGAAGAAGCTTGTTGCTGAAACTGGTGCGGGCCA
ACATGGTGTAGCTAGTGCTACGGTTGCTGCATTATTTGATATGGAACTTGTTGTCTTTATGGGAAGTGAAGATATTAAAAGACAACAACTTA
ATGTATTTAGAATGGAATTACTTGGTGCAAAGGTTGTGGCAGTTGAAGATGGTCAAGGGACTTTATCGGATGCAGTTAATAAAGCATTGCA
ATATTGGGTAAGTCATGTAGATGATACACATTATTTATTAGGTTCTGCATTAGGTCCAGACCCGTTCCCAACGATTGTTAGAGATTTTCAGA
GTGTGATTGGTAAAGAAATAAAATCACAGATATTGAAGAAAGAAGGTCGACTTCCGGATGCAATTGTAGCATGTATCGGTGGTGGCTCAA
ATGCAATCGGTACATTTTATCCATTTATTAAAGATGATGTTGCATTATACGGTGTTGAAGCCGCAGGTCAAGGCGAGGATACTGATAAACAT
GCACTTGCAATTGGCAAAGGATCACCTGGCGTATTACATGGTACTAAAATGTATTTAATTCAAGATGAAGATGGGCAAGTGCAACTAGCAC
ATTCTATTTCAGCAGGACTTGATTATCCTGGTATTGGACCAGAACATTCTTATTACCACGACATTGGTAGAGTAACTTTTGAAAATGCAAGTG
ATACACAAGCAATGAATGCTTTAATCAACTTTACAAAACATGAAGGTATTATACCTGCAATTGAAAGTGCACATGCACTGAGTTATGTTGAA
AGACTAGCGCCTACGATGTCGAAAGAAGATATTATTGTAGTAACTATTTCTGGACGTGGCGATAAAGATATGGAAACAATTAGACAATATA
TGGCAGAGCGAGGTCTTGCAAATGACTAA

Gene: trpA (tryptophan synthase alpha chain)

Position: 1358736 to 1359464, length: 729 nt, orientation: FORWARD

Perfect match to: (Strain_21282-AMPF01000005-[29185:29913], allele observed in CC8+CC1+CC8)

Sequence:

ATGACTAAATTATTTATACCTTATATTATGGGCAATAAAGATTTGATTGAAAATGCAACATTGTTGAGTGAAAATGGTGCAGATATAATTGA
AATTGGAGTACCTTTCTCTGATCCGGTTGCTGATGGTCCAGTTATCATGGAAGCAGGTCAACAAGCGATTAAACAAGGCATCACGATAGAT
TATATTTTCAATCAATTAGAAAAACATGGTGATCAAATTAAGTGTAACTATGTATTAATGACGTATTATAATATTATTTGTCATTATGGAGAA
CAAGCGTTTTTTGAAAAATGTCGAGATACTGGTGTCTACGGCTTAATTATTCCTGATTTACCATATGAATTATCGCAAAGTTTAAAACAACAA
TTTAGTCACTATGGCGTCAAAATCATATCGTTAGTTGCGATGACTACTGATGACAAACGTATAAAAGATATCGTATCCCATGCGGAAGGCTT
TATTTATACTGTGACGATGAATGCGACAACAGGGCAAAACGGTGCGTTTCATCCAGAATTAAAACGAAAAATTGAGTCAATTAAAGCGATA
GCCAATGTGCCAGTTGTCGCAGGATTTGGTATAAGAACACCACAACATGTTGCAGATATAAAAGAGGTTGCAGATGGCATTGTCATTGGTA
GCGAAATCGTTAAGCGATTTAAATCTAACACGCGTGAGGAAATCATTAAATATTTACAATCTATCCAACAAACATTGAATAATTAA

Gene: femA (aminoacyltransferase)

Position: 1359786 to 1361048, length: 1263 nt, orientation: FORWARD

Perfect match to: (08-02119-CP015645-[791309:792571:r], highly conserved allele)

Sequence:

ATGAAGTTTACAAATTTAACAGCTAAAGAGTTTGGTGCCTTTACAGATAGCATGCCATACAGTCATTTCACGCAAACTGTTGGCCACTATGA
GTTAAAACTTGCTGAAGGTTATGAAACACATTTAGTGGGAATAAAAAACAATAATAACGAGGTTATTGCAGCTTGCTTACTTACTGCTGTAC
CTGTTATGAAAGTGTTCAAGTATTTTTATTCAAATCGCGGTCCAGTGATCGATTATGAAAATCAAGAACTCGTACACTTTTTCTTTAATGAAT
TATCAAAATATGTTAAAAAACATCGTTGTCTATACCTACATATCGATCCATATTTACCATATCAATACTTGAATCATGATGGCGAGATTACAG
GTAATGCTGGTAATGATTGGTTCTTTGATAAAATGAGTAACTTAGGATTTGAACATACTGGATTCCATAAAGGGTTTGATCCTGTGCTACAA
ATTCGTTATCACTCAGTGTTAGATTTAAAAGATAAAACAGCAGATGACATCATTAAAAATATGGATGGACTTAGAAAAAGAAACACGAAAA
AAGTTAAAAAGAATGGTGTTAAAGTAAGATATTTATCTGAAGAAGAACTGCCAATTTTTAGATCATTTATGGAAGATACGTCAGAATCAAA
AGCTTTCGCTGATCGTGATGACAAATTTTACTACAATCGCTTAAAATACTACAAAGATCGTGTGTTAGTACCTTTAGCGTATATCAATTTTGA
TGAATATATTAAAGAACTAAATGAAGAGCGTGATATTTTAAATAAAGATTTAAATAAAGCATTAAAGGATATTGAAAAACGTCCTGAAAAC
AAAAAAGCGCATAACAAGCGAGATAACTTACAACAACAACTTGATGCAAATGAGCAAAAGATTGAAGAAGGTAAACGTCTACAAGAAGAA
CATGGTAATGAATTACCTATCTCTGCTGGTTTCTTCTTTATCAATCCATTTGAAGTTGTTTATTATGCTGGTGGTACATCAAATGCATTCCGTC
ATTTTGCCGGAAGTTATGCAGTGCAATGGGAAATGATTAATTATGCATTAAATCATGGCATTGACCGTTATAATTTCTATGGTGTTAGTGGT
AAATTTACAGAAGATGCTGAAGATGCTGGTGTAGTTAAATTCAAAAAAGGTTACAATGCTGAAATTATTGAATATGTTGGTGACTTTATTAA
ACCAATTAATAAACCTGTTTACGCAGCATATACCGCACTTAAAAAAGTTAAAGACAGAATTTTTTAG

Gene: femB (aminoacyltransferase)

Position: 1361067 to 1362326, length: 1260 nt, orientation: FORWARD

Perfect match to: (144_S7-CP010943-[1285676:1286935], allele observed in CC772+CC8+CC30+CC97+CC772)

Sequence:

ATGAAATTTACAGAGTTAACTGTTACCGAATTTGACAACTTTGTACAAAATCCATCATTGGAAAGTCATTATTTCCAAGTAAAAGAAAATAT
AGTTACCCGTGAGAATGATGGCTTTGAAGTAGTTTTATTAGGTATTAAAGACGACAATAACAAAGTAATTGCAGCAAGCCTTTTCTCTAAAA
TTCCTACTATGGGAAGTTATGTTTACTATTCGAATCGTGGTCCAGTAATGGATTTTTCAGATTTAGGATTAGTTGATTATTATTTAAAAGAGT
TAGATAAATATTTACAGCAACATCAATGTTTATATGTTAAATTAGATCCGTATTGGTTATATCATCTATATGATAAAGATATCGTGCCATTTG
AAGGTCGCGAGAAAAATGATGCCTTAGTAAACTTGTTTAAATCACATGGTTACGAGCATCATGGCTTTACAACTGAGTATGATACATCGAG
CCAAGTACGATGGATGGGCGTATTAAACCTTGAAGGTAAAACACCCGAAACATTGAAAAAGACATTTGATAGTCAACGTAAACGTAATATT
AATAAAGCGATAAACTATGGTGTTAAAGTCAGATTCCTTGAACGTGATGAGTTCAATCTTTTCTTAGATTTATATCGTGAAACTGAAGAGCG
TGCTGGATTTGTATCAAAAACAGATGATTATTTTTATAACTTTATTGACACATATGGAGATAAAGTATTAGTACCATTAGCATATATTGACCT
TGATGAATATGTGTTAAAGTTGCAACAGGAATTGAATGACAAAGAAAATCGTCGTGATCAAATGATGGCGAAAGAAAACAAATCAGATAA
ACAAATGAAGAAAATTGCAGAATTAGATAAGCAAATTGATCATGATCAGCATGAATTATTGAATGCAAGTGAATTGAGCAAAACGGACGG
CCCAATTCTAAACCTTGCTTCTGGCGTTTATTTTGCAAATGCATATGAAGTGAATTATTTCTCTGGTGGTTCATCAGAAAAATATAATCAATTT
ATGGGACCATACATGATGCATTGGTTTATGATTAACTATTGCTTCGATAATGGCTATGATCGTTATAATTTCTATGGTTTATCAGGTGATTTT
ACGGAAAACAGTGAAGATTATGGCGTATACCGCTTTAAACGTGGATTTAATGTACAAATCGAAGAATTAATAGGGGATTTCTATAAACCAA
TTCATAAAGTGAAATATTGGTTGTTCACAACATTGGATAAATTACGTAAAAAATTAAAGAAATAG

Gene: Q99UA6 (putative membrane protein)

Position: 1362615 to 1363361, length: 747 nt, orientation: FORWARD

Perfect match to: (N315-BA000018-[1382033:1382779], highly conserved allele)

Sequence:

ATGAATCGAATTGAAAAACATGCCAAAAACACTTTTATTATTCTGATGCTCATTATGTTATTTTGGATTTTTATGAGTTTTATTTTCCAGAAAT
TATTATTTCCACCTTCGAAAAATAATTTAACTACATATGAAGCATTAAAATATTATACACATTTAAAGGGATATTACGGTTTGGATCATATAT
CAAAAGGAATAGCATACATTGCCTGTGTGTTAATTCCATTCAATTTCTTTTTTAGATTCAATGACATCAAAAAAGATAATAACTATAATAATA
TTATAAGTACGTTATTCCTATTATTATACTTTTTAGTTAATGGAATTTCGTTGATTATTCAAGGATTCACTGCAGAATTTACAATTAGTTTAATT
AGTGAGTCAAATATTCATAATAATCATGAATTTGCTGTGAACTTATTTAGATATGTCATACAAGAAGGTGGTATTTCTTTTAGTACATATCTA
GTGTGTAACTTTTCTATAATAATGTGGTTATTCTTTTCGTGTTCTTTATTAAAAGAACGAAAACCAGTTGTCAGATGTTTACCATTAATTATAT
CTTGTTTAAAATTAATATTAATCCTACTATTTTTACTTTCAATACTTTTGGTCATATATCAAACTCAATCTGCACAAATCTTATTTATTTTTATAG
ATTTTCTAAATTTTGTCGCGTTAATTTTAGTTTATTTATGTACTAATCCTAATAATAGAGGTATTGATAAAATAGCATGTGTTAAATAA

Gene: Q5HG42 (putative protein)

Position: 1364408 to 1364881, length: 474 nt, orientation: REVERSE

Perfect match to: (COL-CP000046-[1424887:1425360:r], highly conserved allele)

Sequence:

TTAATAAAATTCGTCCTCGAACATTTCTTCCTCTTCATCTAATCCAAATAATTCTGCCATTTCTCCATGTTCAATTAACATGTTTAAATATGCAT
CGCGGAGTTCTTCTTCACTCATATCATTAATCATTTCTTTAAGACTATCAATCCACATATTTCTGCGTAATTGATAGTCTTCTTCAACTTCGTTT
AACATCATTATATGTTTATTTGCTGCTTCTGGACTAGCTGTAAAGAGTAATGCAATCATATGTTTACATATCACTCGTCTTCCATCAGCATGA
GGACAATTACATATGGATTTTCTAGGATGTTCCATATCAATATAACAACGATATACTTTGTTGCCACTGCCCTTTACTTCAGCCTCATGCTGC
GTTTCTGAAAATGATTTTAAGTTAATGACGCATTCACTTTGATAATAATTAAAGCCTCTTTCTATAGAACGAATACTTGCAATATCAAGTAAT
CCCAT

Gene: nikF (oligopeptide ABC transport, ATP-binding protein)

Position: 1365005 to 1365706, length: 702 nt, orientation: REVERSE

Perfect match to: (11819-97-CP003194-[1415381:1416082:r], allele observed in CC80+CC88+CC361)

Sequence:

CTAATATGAAAATGCTTGCACTAATTCTTTTGTATAAGGGTGTCTATCAACATTAAATAATTCCTCTATTGCAAAATCATCGACTATCATGCCA
TCCTTAAGAACGATAATTCTATTAACTAAGCGTTGTAACACGGATAAATCATGAGAAATAACGATAAAATGATTTAAGTTCGTAATCGTTTG
CGCTTTTAATATATTTATTACATTTTGTTCAGCTATAACATCTAAATTTGAAGTTATCTCATCACATATTAAAACGCGAGGCTGTGCTAATAAC
GAACGCATGACATTAAATCTTTGTAATTGTCCGCCACTCACTTCGCTTGGTAATTTAGTCAATAATTGCGCGTTCAACTCAAAAGTAGATAAA
TGTTGTAATAATAATTGATCCTGAGCAGTATTATCAGTTAGGCCTCTGTAATAATATAACGCTTCTTTTAATGAGGTCTCAATCGTCCAATCA
GGGTTAAAGCTAGTTAAAGGGTGTTGGAAAATCGGTAACACAGCATTGTCACTTAAGTAAATTTCTCCTTTAACAGGTTTAAACAAGCCAA
GAACCAATGAAGCGAGCGTACTTTTACCACAGCCACTTTCGCCTAAAATACCAACATTTTCTCCATCGGGTATAGTAATATTGATATCTTGTA
GCACCATCTGCTTTTTATTATAACCAAAAGTCACATGTTTTAACTCAATCAT

Gene: nikD (oligopeptide ABC transport, ATP-binding protein)

Position: 1365699 to 1366472, length: 774 nt, orientation: REVERSE

Perfect match to: (11819-97-CP003194-[1416075:1416848:r], allele observed in CC80)

Sequence:

TCAATCATAGTAATCCCTCTTTAATTGTGTTCTATATTTAATTAGACGTTCAGTATACGGATGCAAATGCTCATACTTGAAATGATTAATATTG
CCTCGTTCAATGATTTGACCTTCTTTTAAAACATAAATGTACTGACAATATTTCAATACATGACTTAAGTTATGTGTGATAATAAATAATGTTT
GACCATGTTCTAATACAATATGCTGTAATAAATCCATCACTTGATTACCGTTCAAAGCATCCAATGATGCAACTGGTTCGTCTGCAATGATTA
ATTTAGGCTCCAACATGAGAACGCTTGCTATGTATACGCGTTCAAGTTGGCCCCCAGAAAGTTGGAAACTATATTTATTTAATATATCTTTGC
TTTGTAAATTAACCCACGACAAAGCCTTATCAATTTTGGACAAAGCCTCTTCTTTACTACTTTTATAATGCTTACGATAAATCGCAGTTAACTG
TTTACCTAATTTAGTATGGTCGTTAAAACTTTCTGCATAATTTTGAGAAATATAGCCAATTGTATGACCATAATATTGACTCAATCTACTAACA
TTTTCTCCATCAAATTGGTACGAATCATACGTGCAGGTTAAATCAAATGGTAAATATTCAAGTAAGGCTTTAGCAATCAAACTTTTTCCAGCG
CCGCTCTCTCCAATCAAGGCATTAATCTGTTGACTAAAAATTTTCAAATCAATCCCTTTAATAAGAGATTTCTCACAAGTATTCTTTATTGTTA
AATTTTGTATATCAATGAGACTCAT

Gene: nikC (oligopeptide ABC transport, transmembrane permease subunit)

Position: 1366459 to 1367289, length: 831 nt, orientation: REVERSE

Perfect match to: (CIGC93-AHVD01000017-[36587:37417:r], allele observed in CC15+CC80)

Sequence:

TCAATGAGACTCATCATATTCACCCCGTTGTTTCAGCAATCTATCTCTTAGTGCATCACCGGTTAAATTAAAAATTAAAATAGTTATAGCAAT
GACTGAAGCAGGTGCAATCAACATAATTGGATGAGACGAAATAAAATCACGACCTTGTTGTAACATAGCGCCCCACTCTGGTGTTGGCGGT
TGTGCACCTAACCCAATAAATGATAGTGAACTTATATATAGAATGATTTTACCGAAATCAACGACCATCAAAACGATAATAGCCGGTATAAT
TTTAGGTGTTAAATGACGTATTAATATTGTTCTTGTAGGTACATGAAATAATTGTGCCATTTTTATATAAGGCTTATTCATTTCGCTATTAACT
ATACTTCTAGTCAACCTTGTGTAATTCATCCATTTTATTACTGTAATTGAGATAACTAAATTCCATAAAGATGGTTGAAAAAAGCTTGCTAAA
GCAATCATGATGATAAATTCTGGAATACTTAGACCAACATCAATAAACCTTAACACTAATCGTTCAATCCACCCTTTTTTATATCCGGCAAAT
AGACCTAGTGTAACACCTATGACAACGATAGCTATTAATGTTAAAACAGTAACAAACAATGTTGAACGTGCACCGATAATAATTCGGGTAA
ATAAATCTCTCCCATAATCATCAGTTCCTAATAAATGCAACCAACTAATAGGTTCAAAAGTTTGTGATAAATTGACTTTGGTTGCATTTTCACT
ACTGACAAAGAATTGCAGTACAATTACCACAAAAATAAATGCAACGAATACAAAAAATATCAGGTTATTCTTTGAATATATTTTATGCAT

Gene: nikB (oligopeptide ABC transport, transmembrane permease subunit)

Position: 1367282 to 1368268, length: 987 nt, orientation: REVERSE

Perfect match to: (11819-97-CP003194-[1417658:1418644:r], allele observed in CC80+CC5+CC8+CC239+CC4803)

Sequence:

TTATGCATGGCGGTCACTACTTTCTGATATCAATGGTGTATTGGTTTTGCTTTTTGGATTTCCTAATTGTAAACGCTGCTTCGGATCAAGTAAT
AACGTTAATAAATCAGCAATCGTATTGATAATAACAACGAAGAAGCCAATAAATAACACGCATCCTTGAATAACAGGATAATCTCGAGATTT
AATACTATCCATTAATAGATAACCAATACCAGGTATATCAAATAAATTTTCAATCACTACAGTACCACCTATTAGACTGCCAAGTGAAATCCC

TAGTAATGGGATAATCGGCAAAATTGTTGGTTTTAGTAAATCATGAATTAAAATATAACGTTCATTCATACCGCGTAATCTTGATGCTTGTAC
GATATTACTTTGCAATAACATCAATAAATTAGAACGCACTAAACGAATGATGTATGCACACATACCTAAAGATAGCGTGATTACAGGTAATA
TAAACTGACTTAGTATAACGCTATCTATATTCATTAAATTTGTGACAATAAATAATAAAATAATACCGATAAAGAACGCTGGTAAACTAATC
GATAGTGTTGAGATCACTCTAATCACTTTATCCGTCCACTTATGAAATCGTTTGGCTGCTATAATGCCGAGCGGTATAGATATGCATAACGA
CACTACTAATGTTGAAAATGATATGAGTAATGTTATGGGTGCATAGTTGAATAATATCTGTGTTACCGGTTCTTTTGATTCAAAACTTTTTCC
TAAATTAAAATGTAATAAATGATTCATCCAATGCCACCACTGTACCAATAAAGAATCATTTAATCCCAATTTATCTTTGGTTGCATTTATTTGT
TCCGTCGACACTTGTGCTACATCAAGATGTAATATTTTATCAACAGGATTGCCTGGTGATAATTTCATTAAAATGAATGTAAGTGTAGAAAT
AACAAATAAAACAACTATCATTTGCATCAGTCTATACAACATAGACTTTATTATGAACAT

Gene: Q5HG37 (putative protein)

Position: 1368572 to 1368916, length: 345 nt, orientation: REVERSE

Perfect match to: (N315-BA000018-[1387988:1388332:r], highly conserved allele)

Sequence:

TTATTTTTTATCTTTACGGCGAAGTTCAGCGCCCTCATAGCCGTATTTTTCAATTTGCTTTTCTAATTTACGCGCTTTTCTTTCTTTACGCCAATT
TCTAGTAAAATACCATAATAGAAAACTAATTAATAAACTCATAATCGCTAAAAATGCAGCGTATCCTAATAATGGTTGATATTTTATATCTTG
AAAATTTGGAATAAAAAATGCAAGCACACCTAATATAACAAATGTAATTACTGCAGATACAAACCATTTATTTAAAACTAAGCAACAGAATA
TTGTTAATAAAATCATTATTAATGTTGTGATCCATAAATAATTAGGCATATCGAATAATGTCAT

Gene: pepF2 (oligoendopeptidase F, M3 family)

Position: 1369121 to 1370935, length: 1815 nt, orientation: FORWARD

Perfect match to: (DSM_20231-AMYL01000002-[648936:650750], allele observed in CC8+CC15)

Sequence:

ATGTCTCAAGGTTTACCTTTAAGAGAAGATGTTCCTGTTTCAGAAACATGGGATTTAGTAGACTTATTTAAAGATGATCAACAATATTATGA
AAGTATTGACGCTCTAGTACAACAAGCAAATCAATTTCATCATACATATGCAACAACATTAAATTCAATCGAACAAATTAATACTGCTTTAGC
TGAATTAGAAAATATTTTAATTGCCTTAGATCGCTTAAGTAATTATGCAGAACTACGTTTAAGTGTAGATACTAGTAATATCGAGGCACAAG
TATTGAGCGCTAAATTATCTACTACATACGGTAAAATTGTTAGCCAATTATCATTTGTAGAGTCAGAAATACTTGAATTACCAGAAGAAATA
CTTCAACAATTAGAAGAATCATGTCCATATCAACACTATATTAAACAGTTAATAAAACAAAAGCCATTCCAATTATCTGCGTCGGTAGAACA
AGTATTAGCAACTTTATCACCTACGCTAAACAGTCCTTACGATTTATACGGCACGACAAAAATGCTAGATATTACATTCGATTCATTTGAACA
TGATGGTACAACGTACCCTGTCGACTATGCTACGTTTGAAAATGATTATGAAGATAATAAAGATCCTGAGTTTAGACGTAAAAGTTTCAAAT
CGTTTAGCGATGGGATTCGAAAATATCAGCATACTACCGCGGCTACATATAATATGCAAGTACAACAAGAAAAAATTGAAGCTGATTTACG
TGGATTTGAATCAGTCATCGATTATTTATTACATAGTCAAGAAGTAACGCGTGATATGTTTGACCGTCAAATCGATATGATTATGCGTGACT
TGGCACCAGTTATGCAGAAATATGCTAAACTTTTACAACGTATTCACGGATTAGATAACATGCGTTTTGAAGACTTGAAGATTTCTGTAGAC
CCTGATTATGAACCAGAGATTTCAATTGAAGACTCAAAAAATTATATTTTCGGTGCGTTAAGTGTTTTAGGTGATGACTATACAAACATGTT
ACGTGAAGCATACGATCAGCGATGGATTGATTTTGCACAAAATAAAGGTAAAGATACAGGCGCATTTTGTGCAAGTCCATACTTTACACAT
TCATATGTGTTTATTTCTTGGACTGGTAAAATGGCTGAAGCATTTGTCTTAGCACATGAATTAGGTCATGCAGGTCATTTTACATTAGCTCAA
AAACATCAACAATATCTTGAATCAGAAGCATCAATGTACTTTGTTGAAGCCCCTTCTACAATGAATGAAATGTTGATGGCCAATTATTTATTT
AACACAAGTGATAATCCAAGATTTAAGCGTTGGGTTATTGGCTCAATTTTATCTAGAACATATTATCATAATATGGTTACCCATTTATTAGAA
GCTGCTTATCAACGTGAAGTGTATCACAAAGTAGATCAAGGTGAATCTTTAAATGCGCCGACATTAAATGAAATAATGCTAAATGTTTATAA
ACAATTTTTCGGAGATGCAGTAGATATGACTGAGGGTGCTGAATTAACATGGATGCGTCAACCTCATTACTATATGGGATTATATTCGTATA
CGTATTCTGCTGGCTTAACAATCGGAACTGTCGTTTCTCAAAAGATTAAAAATGAAGGCCAACCAGCTGTTGATGCTTGGTTAGAAACATTG
AAAAAAGGTGGTAGTGTATCACCTGTCGAACTTGCAAACATTGCAGGTGTAGACATTACTACAGAACAGCCACTTAAATCTACAATTAAAT
ATATTTCTGATTTAGTCGATGAAGTTGAAAAATTAACAGATGAAATTGAGCAAGCAAATAACTAA

Gene: phoU (phosphate ABC transporter, regulatory protein)

Position: 1371075 to 1371716, length: 642 nt, orientation: REVERSE

Perfect match to: (JKD6159-CP002114-[1377244:1377885:r], highly conserved allele)

Sequence:

TTATTGTTCGTAATGTGTACCTGTTAAATAAAAATAAACACTTTCAGCGATGTTAATAATATGATCACCAATACGTTCTAAATGTCTTGCTGCT
AAATGAGCTTGTGCAGCGACAAATGGATCGTTATCAATAAGATACGTTGCGTTAATAATATGACTATATAAGTCATCGATATCTTCATCACG
CTCGATTATTTCTCTTATTAATACGGTATCTTTCTTTTTAAATGCTTGATCTAAATCCTTTAACATTAACATAGCTAATTTACCCATTGTCTTTAA
ACGGGTTAACACATAATCATCTGTAATCTTTGTACGCAATCGAATATTGGCAATACTCGAGGCATTATCTCCTATTCTTTCTAAATCGGAGGC
GATTTTTAATGAAGAAATCATCATACGCAAATCACTCGCAATGGGCTGTTGCTTTGTAATTAACATGATAACTCGCTCATTAATATCATAATT
TAATTGATTGATATGTTTATCGTTTTTAACTGTTTGTCGTGCAAAGCCTCTATCGTCAATACTTAATGATTTTATACCATTTTCAATACTCACAT
AGACATTTGCACCTAACCGACGTAATTCTTTTATTAAATCATCAAGTTGCTCCTGATATCGTTGTCTAATTATTGCCAT

Gene: pstB (phosphate ABC transporter, ATP-binding protein)

Position: 1371723 to 1372574, length: 852 nt, orientation: REVERSE

Perfect match to: (N315-BA000018-[1391139:1391990:r], highly conserved allele)

Sequence:

TCAACCAAACCTTCCTGAAATATAATCTTCTGTTTTCTTGTTTGATGGGTTAGAGAAAATTTTATCAGTATCATCATATTCATTGACATAACCA
TTTAAGAAAAATGCAGTTTTATCTGATACACGAGCTGCTTGTTGCATATTATGTGTAACCATAATAATTGTATACTTTTCTTTTAGTTCTTGAA
CCAACTCTTCTACTCTTAATGTTGAGATTGGATCTAATGCTGATGTCGGTTCATCCATTAAAATGACTTCAGGTTCAATTGCTAAACAACGCG
CGATACAAACACGTTGTTGTTGCCCACCGGATAAACTATATGCATTTGTGTGCAACCTATCCTTTAATTCATCCCAAATTGCAGCGCCACGTA
ATGATTTCTCAACGATTTCATCAAGAACTTTTTTATTTTTAATACCGTGAATCTTTGGACCGTAAGTAATATTATCGTATATTGATTTTGGAAA
TGGATTAGGTTGTTGAAAGACCATGCCCACATTTGTACGTAATTGTTCTTTAGAATATTTTTGATCAAAAATGTCTTGATCTCGATATAATAT
TTTACCAGCTGTTTTCACAGAAGGTACTAACTCAACCATTCGATTCAAAGTTTTAATATATGTTGATTTGCCACAACCAGATGGACCTATAAT
GGCAGTAATTTGGTTTTCATAAATATCTAAATTAATATTTTGTAATGCATGATTTTCGCCATACCATAAGTCTAAATTTTGTGTTGAATATATA
ACAGAATGTGAGTTTGTATCATCTGGTGTTTTATGATGACTTTGTGAGACATCAAACGTATGACTTTGAGATATTTGTTTAGTTTGTGCAAGT
GTTTGCGCCAT

Gene: pstA (phosphate ABC transporter, transmembrane permease subunit)

Position: 1372621 to 1373538, length: 918 nt, orientation: REVERSE

Perfect match to: (N315-BA000018-[1392037:1392954:r], highly conserved allele)

Sequence:

TTAGAATTTTTTACTAAATTTGTTACGTAAAATAATCGCAACGCCATTCATTAAGATTAAGATAACTAGTAAAACGATAATGCCTGCCGATGC
AACATTCTGGAATTCTTCTTGAGGCATTTTCGCCCAAGTAAATATTTGGATTGGTAATGCTGAAAATTGATCCAATATACTTCTAGGTGTTGC
CAATAATATAGTCGGTATACCGATTAGCACAAGTGGCGCTGTTTCTCCCAGTGCTCTTGAAAGAGACAAAATGAATCCAGTTAAAATACCA
GGTAACGCTGCTGGTAAGACAACACGTCTTATCGTTTGCCATTTATTAGCACCTAAACCATAAGAAGCTTCGCGTACTGAGTTAGGTACAGC
TCTAATTGCTTCCTGACTTGAAACAATAATAATTGGTAATATCAGTAAGGTCATTGTTAGCGCTGCTGCCAATATACTGTTACCCATTTTCAA
GGCTTCAATCCCCGCACCACCAACGAACAAAGTGTAACCTAATAACCCAAATACAACTGATGGTACACCAGCTAAATTGGAAATACTGATTT
TAACAAACTGAGTAAATTTGTTGTTTTTCGCATATTCTTCTAAGTATATAGCTGTACCTATTCCTAGGATGATTGATAATGGAATGATACTTA
ACATTAACCAAAGTGAACCGATTAACGCGCCTTTAACGCCAGCCATAGATGGTGTTGAAGAAGAAAAATTAGTGAAAAACTGTAAATTTAA
ATGACTTACCCCTTTAATCAATGTTTGAGTTAACAACGCAATAAGTACGACAAGTCCTAATAATGTACATGCTAAAAATATGAGTTTGAACA
CTTTATTTTTAACCGTTCTGGATGATAAATGTTTTTGGACAAGTTGTTGATCGACGAGTGATTGTCTATTATTATCTGTCGTTTCCAT

Gene: pstC (phosphate ABC transporter, transmembrane permease subunit)

Position: 1373540 to 1374466, length: 927 nt, orientation: REVERSE

Perfect match to: (11819-97-CP003194-[1423916:1424842:r], highly conserved allele)

Sequence:

TTAATACTCCTCCCTAAAACGCTTAGAAATCCACTGAGAAAGTAAATTCATGATTAAGGTAAAGATAAATAGTGTGAACCCTACAGCATAAA
TACTGTAATAAATATTTGATCCAAATGTTGCATCACCTGTCGCTATCTCAACAATATATCCAGTCATTGTTTGAATCGAACTTGTTAAACTTAA
TGAAGCTGTTGGCGAACTACCTGCCGCTAATGATACAATCATCGTTTCTCCAATTGCTCTTGAAATCGCGAGAACGATTGAAGCTACAATAC
CTGATGTTGCTGCGGGAAGTACGACTTTAGTTGCTACTTCTAATTTAGTTGCTCCAAGTCCATAGGCACCTTCTCGAATTTTATTTGGTACAG
ATGCCATTGCATCCTCACTCAAACTTGTGATGAGAGGGACAATCATAATACCGACAACTAAGCCGGGACTTATAGCATTAAACTCTCCAAGA
CCTGGTATGAAAGATCTTAATACTGGTGTAACAAAGGTTAATGCAAAGAAACCAAACACAATTGTTGGTATTCCTGCTAAAATTTCTAATAT

CGGTTTAATTATGCGTCGTGCACGGTCACTTGCATATTCACTTAAATAAATTGCTGCACCAAGCCCGACTGGAACTGCAAATATAGTCGCAA
TAACTGTGATTTTTAAAGTCCCTATTATCAATGCCCAGATACCAAACTTAGGGTCTGAACCGGTAGGATTCCAAGTAGTAGAAAATAGAAAT
TCAGTTATTGGAATTCTGGTGAAAAAAGTGATGGTTTCTAAAAGCAATGTGATTAATATACCTAGTGTTGTTAAAATGGAAATCGCTGAAAT
TGCGGCTAAAATAACTGGTATAATTTTGTCATTATGCTTCCCTTTTTTATTATTATTTTTTTCGATTAAAGCTTTAACATTAGTAGATGAAGTCA
T

Gene: pstS (phosphate ABC transporter, substrate-binding protein)

Position: 1374657 to 1375640, length: 984 nt, orientation: REVERSE

Perfect match to: (ATCC51811-ADVP01000009-[226:1209], allele observed in CC1+CC8+CC97+CC239+CC398)

Sequence:

TTATTTTTTGTCTTCAGACTTTTTATCATCAGATTTCTTGTCGTCTGATTTTTGATTTTTATCAATAAATGCTTTTAAATCATCTAATTGTGATTT
GTATGTTTTCTCTGGTGCTGCTACATATCCAGCTTCTTCAGCTGCTTTACCTTTATCTTCTAAGACGAATTTGATAAATTCTGACATTACTTTAT
TATCTTTCAATGCTTTTTCATTTACATAAATGAATAATGGTCTACTTAATGCATAAGAGTTATCTTGAATTGTTTTTTTCGTAGGCTCTGTTGCT
TTACCATTTTCATCTTTGATTTTAACTTCTTTTAATTTATCTTTATTTTGAACGTAGAAGTTATATCCAAAGTATCCGATTCCCTCTTTGTTTTTC
GTTACAGAAGAAACGATAGCATTTGTATCAGCATTTTTTTCTGCTTTAATATCTTCTTTATTCATTACTTCATTTTCAAAGAAGTCATAAGTAC
CATGACTTGAGTTTGGTGATACAGCATTTATTTTTTTATCTGGCCATTTACTATTAACATCTTTCCATGTTTTAGCTTTTCCAGAATAAATTGCT
TTTAATTGCTGTTTGTCTAATTCATCCACAAAATCATTTTCTTTATTTACAGCAACCGTTACACCATCTTGCGCAATTTTGAATTCTTTGTATTT
GATATTCTTATCTTGTAATTTTTGCTTCTCTTCATCTTTAATTGGTCTAGAAGCATCAGCGAAGTCGATATCTCCTGCAATGAATTTTTGGAAA
CCAGCACCTGTACCAGCTTGTCCTGCTGAGATTTTAGCATCCGAGTGATCTTGAGCCCATTTTTCATTTAATTTCTCCACAATTGGTGCTACTG
TTGATGAGCCATCACCTTTAGCTTCCCCTTTTAAATCACTATTACCACTGCCACCATTACCGCCACCACAAGCACCTAATAATAGTGTTGCACC
TAAAGCTGTAGTACCAACAAATTGCCATTTTTTCAT

Gene: cvfB (conserved virulence factor B)

Position: 1376610 to 1377512, length: 903 nt, orientation: REVERSE

Perfect match to: (st2344-FGWF01000001-[117611:118513], allele observed in CC779+CC1+CC80)

Sequence:

TTATTCTTTTGAGTCAATTCGACTCCAACCTTTTTTAGTTAAAGTGATTTTACCTGTTTCTATATTAATAATCTTCTGTTTATATAAGTGACCGA
TTGCACGTTTGAATGAACCTTTACTCATATTGAATACTTCTTTAATCGCTTCAGGGCTTGATTTGTCCCAGAATGGTAATTCACCATCATATTC
AACTAGTAAATCAAAGATGACTTGGCCGTCATCGTCTAAACGTTCATGTGCAAGTGGTAAAAATGAACCATTTAACTCACCTTTATCATTAT
GCCCGATAATTCTAACTTGAACAGATTCACCTAATCTTGGTTCAGCTTTACGTTCTGATTCATGTACGAAAATTTTGTAACCTGATTCGCTTAA
TAAAAAGCTACCAATTCGTAATACGCGGTAAGGTTTGGCTTCAATGACTTCGTTTTTTAAATTATCGTCGTGTACAGGTGTAAACATATTTTC
TACAACAGATTCACTCGCTAATCTTCCATACATATGATTCTCACGGTCAATTCGTAATGTGACTAGCAAATGATCACCAGGTTGTGGCCATAG
TGATTTCACTTTTGGTAAATCTTCCCATGGTACTAACACTTCACGGGGTAATCCAACATCTATACGTGCCCCATCGCGATCCGTTTTAAGTAC
TTTAGCAAAGTCATATTTATCTTTCGTAATATCAGGCATATTTTGAGTTGCAAATAATTCACCTGAACGGTTTGGATAAATGAAGAAACTATA
TTCTTCACCTACTTCTAATTCATCATCATCGTTCATTTCTGATTGGTTTAACTTTACGTTTTCACCGTTTGGTCCTTTTAAAAGGTAAGTTGAGC
CTTGTAACCCTACTACTTCAAGGAATTCTATAGAACCTACTATATCTTTGTCTAATGCCAT

Gene: ykpA (ABC transporter, ATP-binding protein)

Position: 1377659 to 1379260, length: 1602 nt, orientation: FORWARD

Perfect match to: (ATCC51811-ADVP01000008-[13034:14635:r], allele observed in CC1+CC5)

Sequence:

ATGTTACAAGTAACTGATGTGAGTTTACGTTTTGGAGATCGTAAACTATTTGAAGATGTAAATATTAAATTTACAGAAGGTAATTGTTATGG
ATTAATTGGTGCGAATGGTGCAGGTAAATCAACATTCTTAAAAATATTATCTGGTGAATTAGATTCTCAAACAGGACATGTTTCATTAGGTA
AAAATGAACGTCTAGCTGTTTTAAAACAGGACCACTATGCTTATGAAGATGAACGCGTGCTTGATGTTGTAATTAAAGGTCACGAACGTCTT
TATGAGGTTATGAAAGAAAAAGATGAAATCTATATGAAGCCAGATTTCAGTGATGAAGATGGTATCCGTGCTGCTGAACTTGAAGGTGAA
TTTGCAGAAATGAATGGTTGGAATGCTGAAGCTGATGCTGCTAACCTTTTATCTGGTTTAGGTATCGATCCAACTTTACACGATAAAAAAAT
GGCTGAATTAGAAAACAACCAAAAAAATTAAAGTATTATTAGCGCAAAGTTTATTCGGTGAACCAGACGTACTATTACTGGATGAGCCTACT
AACGGTCTCGATATTCCAGCAATCAGTTGGTTAGAAGATTTCTTAATTAACTTTGATAATACTGTTATCGTAGTATCGCATGACCGTCATTTC

TTAAATAATGTATGTACTCATATCGCTGATTTAGACTTCGGTAAAATTAAAGTTTATGTTGGTAACTATGATTTTTGGTATCAATCTAGTCAG
TTAGCTCAAAAGATGGCTCAAGAACAAAACAAGAAAAAAGAAGAAAAAATGAAAGAGTTACAGGACTTTATTGCTCGTTTCTCAGCTAACG
CTTCTAAATCTAAACAAGCAACAAGTCGTAAAAAACAACTTGAGAAAATTGAATTAGATGATATTCAACCATCATCAAGAAGATATCCTTTC
GTTAAATTCACGCCTGAGCGTGAGATTGGTAACGACTTATTAATCGTTCAAAATCTTTCTAAAACAATTGACGGCGAAAAAGTATTAGATAA
TGTATCATTCACAATGAATCCAAATGATAAAGCGATTTTAATTGGAGATAGTGAAATTGCAAAAACAACATTACTTAAAATATTAGCTGGCG
AAATGGAACCAGACGAAGGTTCATTTAAATGGGGTGTTACTACATCATTAAGTTACTTCCCTAAAGATAACTCAGAGTTCTTTGAAGGTGTA
AATATGAATCTCGTTGATTGGTTAAGACAATATGCTCCTGAAGATGAACAAACAGAAACATTTTTACGTGGTTTCTTAGGTCGTATGTTATTT
AGTGGTGAAGAAGTTAAGAAAAAAGCTAGTGTGCTTTCAGGTGGAGAAAAAGTACGTTGTATGCTAAGTAAAATGATGTTATCAAGTGCG
AATGTACTTTTACTTGACGAACCTACTAACCACTTAGACTTAGAAAGTATTACTGCTGTCAATGATGGTCTTAAATCATTTAAAGGTTCTATC
ATCTTTACTTCTTATGACTTCGAATTTATCAACACGATTGCAAACCGTGTTATCGATTTAAATAAACAAGGCGGCGTTTCAAAAGAAATTCCA
TATGAAGAATACTTGCAAGAAATCGGCGTTTTAAAATAA

Gene: RF00168 (lysine riboswitch)

Position: 1379864 to 1380039, length: 176 nt

Sequence:

ATATTTTGATGAGGCGCATCAATCATGAGTAAAGTTTAGATTACTGTCTGCTAACAGCTAAATTTGAAAGGGTGCGATGCCGAAGCGATTA
TAATAGCAGTTATAATTTGTTGGACTTTTTGGTTAAGAGCTGAGAGTTTGTCATTATTTAAAAATAATGGAGTGCATCACTTGTA

Gene: lysC (aspartate kinase)

Position: 1380121 to 1381326, length: 1206 nt, orientation: FORWARD

Perfect match to: (11819-97-CP003194-[1430499:1431704], allele observed in CC80+CC22+CC772)

Sequence:

ATGGTAACAAGAAGTGTGTTGAAATTTGGCGGATCATCCGTCAGTGATTTTACAAAAATAAAAAGGATCGCTGAAATGTTAAAGGAGCGA
GTCAATCAAGATGAACAATTAATTGTCGTTGTAAGTGCTATGGGTAACACAACAGATCAATTAATGACGAATGTATCGACCTTGACTAAAG
CACCAAAACAACAAGAACTGGCATTATTATTGACAACCGGAGAGCAACAAACTGTATCTTATTTATCAATGGTATTAAATGATATCGGTGTG
AATGCCAAAGCAATGACTGGCTATCAAGCGGGTATTAAAACCATTGGCCATCATTTAAAAAGTAAAATTGCTCAAATTAATCCTCAAACATT
TGAACAAGCCTTTCAAGAAAACGATATTTTAGTAGTTGCTGGATTTCAAGGCATCAATGAACATCAGGAATTAACAACTTTAGGCAGAGGC
GGTTCTGATACGACCGCTGTGGCACTTGCTGTTAGTAATCAAATACCTTGTGAAATTTATACCGACGTTGATGGTGTGTATGCCACTGACCC
AAGACTTTTACCAAAAGCTAAACGACTAGACATCGTCTCATATGAAGAAATGATGGAAATGAGCGCTTTAGGTGCTGGTGTACTTGAAACA
AGAAGTGTTGAATTAGCTAAAAACTATAATATCCCTTTATATTTAGGAAAAACTTTATCGAACGTGAAAGGAACATGGATTATGTCAAATGA
AGAAATATTAGAGAAAAAAGCAGTTGCTGGTGTGGCTTTGGATAAACATATGATGCATGTAACAATTAGTTATCCCCTACCTGACAATCAG
CTACTTACCCAACTATTTACGGAACTTGAAGAAGGTGCTGTAAATGTTGATATGATTTCACAAATCGTCAACTTGGATGGACTACAACTATC
CTTCACGATTAAAGATAGTGATTTTCATCAAATTTCTATGATTCTTGAAACATTAAAGAATCAATATGAAGCATTAGCTTATAAAATCAATGA
GCATTATGTCAAAATTTCATTAATTGGCTCAGGCATGCGTGATATGTCAGGTGTGGCATCAAAAGCATTTTTGACATTAATTGAAAATAATA
TACCTTTCTACCAAACAACAACTTCTGAAATAAGTATTTCATACGTCATTGATGATTTTAATGGGCAACAAGCGGTGGAAAAACTATATGAC
GCATTTAACATTTAA

Gene: asd (aspartate semialdehyde dehydrogenase)

Position: 1381390 to 1382379, length: 990 nt, orientation: FORWARD

Perfect match to: (MW2-BA000033-[1402758:1403747], allele observed in CC1+CC7+CC80+CC96)

Sequence:

ATGACAAAATTAGCAGTTGTGGGTTCAACAGGATTAGTAGGTACAAAAATGTTGGAGACATTAAATCGTAAAAATATTCCTTTCGATGAAT
TAGTATTATTTTCATCAGCACGTTCTGCAGGGAAAGAAGTTGAATTTCAAGGAAAAACATATACAGTTCAAGAATTAACTGATGCTCGTGCA
AGTGAACATTTCGATTATGTATTAATGAGTGCTGGTGGCGGTACAAGCGAACACTTTGCCCCACTCTTTGAAAAAGCTGGTGCAATCGTTAT
AGACAATTCAAGTCAATGGCGTATGGCAGAAGATATTGATTTAATCGTTCCGGAAGTCAATGAACCTACTTTTACAAGAGGTATCATTGCCA
ATCCAAACTGCTCTACGATTCAGTCTGTTGTGCCATTAAAAGTATTGCAAGATGCTTATGGTTTAAAACGAGTGGCATATACAACATATCAA
GCTGTATCAGGTTCAGGGATGAAAGGTAAGAAAGATTTAGCTGAAGGTGTAAATGGTAAAGCACCAGAAGCATATCCACATCCAATTTAT
AATAATGTGTTACCGCATATTGATGTGTTTTTAGAAAACGGATATACAAAAGAAGAACAAAAAATGATTGATGAGACGAGAAAAATTTTAA

ATGCGCCAGACTTAAAAGTAACAGCAACATGTGCACGTGTGCCTGTTCAAGATAGTCATAGTGTTGAAATTGATGTAACGCTTGAAAAAGA
AGCGACTGCAGAAGATATTAAAGCATTATTTGATCAAGATGACCGCGTTGTTTTAGTAGACAATCCAGAGAACAATGAATATCCAATGGCA
ATCAATTCTACTAATAAAGATGAAGTGTTTGTTGGCCGTATACGTAGAGATGATTCATTAGAAAATACTTTCCATGTATGGTGTACATCAGA
CAATTTATTAAAAGGTGCTGCATTAAATGCTGTACAAGTATTGGAACAAGTTATGCGTTTAAAAGGAGCGAATTAA

Gene: dapA (dihydrodipicolinate synthase)

Position: 1382381 to 1383268, length: 888 nt, orientation: FORWARD

Perfect match to: (11819-97-CP003194-[1432759:1433646], allele observed in CC80)

Sequence:

ATGACACATTTATTTGAGGGTGTTGGCGTTGCACTTACAACCCCTTTTACAAATAACAAAATTAATATTGAAGCTTTGAAAACACACGTTAAT
TTTTTACTAGAAAATAATGCCCAAGCAATCATCGTTAATGGAACTACTGCTGAGAGCCCTACTTTAACAACAGATGAAAAAGAACGCATTCT
AAAAACAGTTATTGATCTTGTAGATAAACGTGTTCCTGTCATAGCAGGAACTGGCACTAATGATACTGATAAGTCAATCCAAGCTTCAATCC
AAGCTAAAGCCTTAGGTGCTGATGCAATTATGTTAATTACGCCCTACTACAACAAAACGAATCAACGTGGTTTAGTCAAACACTTTGAAGCG
ATTGCAGATGCTGTGAAATTACCAGTCGTGCTGTACAATGTTCCTTCAAGAACGAACATGACAATTGAACCAGAAACTGTAGAAATATTAA
GTCAACATCCTTATATAGTTGCTTTAAAAGATGCTACGAATGATTTTGAGTATTTAGAAGAAGTGAAAAAGCGAATTGATACAAATTCATTT
GCATTATATAGTGGCAATGATGACAACGTCGTTGAATACTATCAACGTGGCGGTCAAGGGGTTATCTCTGTTATTGCCAATGTCATTCCTAA
AGAATTTCAAGCGTTATACGATGCTCAACAAAGTGGATTAGATATTCAAGATCAATTTAAACCAATCGGCACACTGTTATCAGCTTTATCAG
TTGATATTAACCCAATTCCTATTAAAGCTCTAACAAGTTATTTAGGATTTGGAAATTATGAATTACGTCTACCATTGGTTAGCCTAGAAGATA
CAGATACTAAAGTGCTTCGTGAAGCATATGACACATTTAAAGCGGGTGAAAATGAGTGA

Gene: dapB (dihydrodipicolinate reductase)

Position: 1383265 to 1383987, length: 723 nt, orientation: FORWARD

Perfect match to: (11819-97-CP003194-[1433643:1434365], allele observed in CC80)

Sequence:

GTGAAAATATTACTAATTGGCTATGGTGCAATGAATCAACGCGTTGCTAGATTAGCAGAAGAAAAAGGACATGAAATCGTTGGGGTCATT
GAAAATACACCGAAAGCAACAACGCCATATCAACAATATCAACATATTGCAGATGTTAAAGATGCCGATGTTGCAATAGATTTTTCAAATCC
AAATCTACTTTTCCCTTTATTAGATGAAGAGTTTCATTTGCCATTAGTTGTGGCAACAACTGGCGAGAAAGAAAAACTACTTAATAAGTTAG
ATGAATTGAGTCAAAATATGCCTGTATTTTTCAGCGCGAACATGAGTTATGGCGTTCATGCATTGACTAAAATTTTAGCAGCTGCTGTTCCCC
TACTTGATGATTTCGATATCGAATTGACTGAGGCACATCATAATAAAAAAGTAGATGCACCAAGTGGTACGTTAGAAAAATTGTATGATGT
GATCGTATCTTTGAAAGAAAATGTAACACCTGTGTATGATAGACATGAATTAAATGAAAAACGCCAGCCACAAGATATTGGTATACATTCTA
TTCGTGGAGGTACGATTGTCGGTGAACATGAAGTTCTATTTGCCGGCACTGATGAAACGATTCAAATCACGCATCGTGCACAATCAAAAGA
TATTTTTGCGAATGGTGCAATACAAGCAGCAGAACGCTTAGTTAATAAACCAAACGGCTTTTATACGTTTGACAACTTATAA

Gene: dapD (2,3,4,5-tetrahydropyridine-2,6-dicarboxylate N-acetyltransferase)

Position: 1384014 to 1384733, length: 720 nt, orientation: FORWARD

Perfect match to: (TCH130-ACHD01000018-[3638:4357:r], allele observed in CC72+CC1+CC72+CC97)

Sequence:

ATGGTACAACATTTAACAGCTGAAGAAATTATTCAATATATAAGTGATGCTAAAAAGTCTACACCAATAAAAGTATATTTAAATGGTAATTT
TGAAGGCATCACATATCCAGAAAGTTTTAAAGTATTTGGTTCAGAACAATCTAAAGTAATCTTTTGTGAAGCGGATGATTGGAAACCTTTTT
ACGAAGCATATGGTAGTCAATTCGAAGATATAGAAATTGAAATGGATCGTCGCAATTCTGCCATTCCATTAAAAGACTTAACAAATACGAA
TGCACGAATTGAACCAGGTGCGTTTATTAGAGAACAAGCCATTATTGAAGATGGTGCTGTCGTTATGATGGGCGCAACAATTAATATTGGC
GCAGTCGTTGGCGAAGGTACAATGATTGATATGAATGCTACTCTCGGTGGTCGTGCTACAACTGGTAAAAATGTACATGTAGGGGCTGGC
GCAGTATTAGCAGGTGTGATTGAACCCCCTAGTGCTTCACCAGTTATAATCGAGGATGATGTATTAATCGGTGCAAATGCAGTTATTTTAGA
AGGTGTACGTGTTGGTAAAGGTGCTATTGTTGCAGCTGGCGCGATTGTGACACAAGATGTACCAGCTGGTGCAGTTGTTGCTGGTACACCT
GCAAAAGTGATTAAGCAAGCTTCTGAAGTACAAGATACTAAAAAAGAGATTGTAGCAGCATTAAGAAAACTGAATGACTAG

Gene: hipO (hippurate hydrolase)

Position: 1384876 to 1386027, length: 1152 nt, orientation: FORWARD

Perfect match to: (11819-97-CP003194-[1435254:1436405], allele observed in CC80+CC22+CC80+CC772+CC4803)

Sequence:

ATGAATGAATTAGAATTTGTTACGAAACATCGCCGTCATTTACATCAACATCCTGAATTAAGCTTACATGAATTTGAAACAACTGCTTATATT
AAAGCGTTTTTAGATAGTTTAAATATTAAATACGATTGCCCATTGGAAACTGGCGTCATTGCATACTTAGAAGGTAATGGCTCACATACGAT
AGCGTATAGAGCTGATATTGATGCGTTACCTATTTTAGAGGAAAATGATGTGCCTTATCGCAGTCAATCTGATCATGTGATGCATGCTTGTG
GACATGATGGTCATACAACTGCATTAATGCTTTTTGTACAACGTTGCAAAGACATGCAAGACGCAGGTCAATTACCGCAAAATGTCGTTTTC
ATTTTCCAACCTGCAGAAGAAACTGGTGGCGGTGCAAATCGATTAATAAAAGCCGGTGCCTTTGATAAGTATCCAATTGAAGCGGTATTTG
GTATTCATGTTAACCCATTTGCTGATGAAGGCATTGCAGTGATAAGAGATGAAGAAATTACGGCCAGCGCAACAGAGTATCGCTTTTTCTTA
ACAGGCCTGTCAAGTCATGTTGCTGATAAAGAACAAGGTCATTCTTGTGGTGAAGCATTACAACATGTATTAACTCAAATATCACAAATTCA
ACAATTTCACCTTAACGGTTTGAAACGAAATATTGTTCATATTGGTCATTTTAAAGCTGGTGAAGCGATTAACACTGTACCAAGTAATGGTT
ATTTAGAAGGTACTATTCGTACATATGATATTGATGATTTAACAATCGTTAAAAATCAAATGCACAAGATAGCAGAAAGTGTCAAGCTTCTG
TTTAATGTAGATTGTGAAGTTAAATTTGCAGAAGGTTATCCCCCTACAATCAATAGTCCGAAATTACGTACTCAAATAGAGGACGCCTTAAT
AAAAGCTGATTTAAATGTCTATGACAAACCAACGCCATTCTTATTTGGGGAAGATTTTAGTTTTTATGGTCAACAACTAGCTCCAGCTTACTT
TGTTTTTATAGGAACACGAAATGAAGATAAAGGTTTTGTAACTGGTTTGCACACATCACATTTAAATTTTGATGAAAAAGTGTTAATAAACG
TGGTTAATTTTTACGAAAATTTATTAAATAATTACAAAGAGGTGTAA

Gene: alr2 (alanine racemase, locus 2)

Position: 1386032 to 1387117, length: 1086 nt, orientation: FORWARD

Perfect match to: (COL-CP000046-[1446512:1447597], allele observed in CC8+CC22+CC188)

Sequence:

TTGACAGCAACATGGTCTGTAAATAAGAAAATATTTTTACAAAATGCAATCACAGTCAAAAACAATCAGCCATTAATGGCAGTTGTTAAAAA
TAATGCATATCACTATGACCTAGAATTTGCTGTAACTCAGTTTATCCATGCAGGTATAGATACATTTAGCACAACATCACTACGAGAAGCAA
TTCAAATTAGACAACTTGCTCCAGATGCAACAATCTTTTTAATGAATGCAGTTTACGAGTTTGATTTAGTCCGTGAACATCAAATACACATGA
CTTTGCCGTCGTTGACATATTACTATAACCATAAAAATGATTTAGCAGGTATTCATGTTCACTTAGAATTTGAAAATTTATTACATCGGTCTG
GATTTAAAGATTTAAACGAAATTAAAGAAGTATTGAAAGATCACCATCATAATCAAAATGCAAAAATGATTATTAGTGGTTTATGGACCCAT
TTTGGATATGCTGATGAATTCGATGTGTCAGATTATAATGTTGAACGTTCACAATGGATGGAAATTGTTGAAGCACTTTTATCTGAAGGTTA
TCAGTTCGACCTAATCCATGCTCAAAATAGTGCGAGTTTTTATCGGGAAGGACAAATATTACTACCCCACCATACACATGCGCGTGTAGGTA
TTGCGTTATACGGTTCAAGACCATATAGTTCACTGAATCAACATGATATAGTTCAGTCATTAACTGTAAAAGCACATGTTATTCAAGTGCGC
GAAGTACAAGCTGGTGATTATTGCGGTTATAGCTTTGCCTTTGAAGTGACTAAAAACAATACAAAATTAGCTGTAGTTGATATCGGTTATGG
CGATGGAATTTTAAGAACTCGTGCTAAACATGAAGCACTTATCAATGGTAAACGCTACCCGATACGTGCATTAATGATGAGCCATATGTTTG
TTGAAGTTGATGGCAATGTACATGCACAAGATGAAGTTATTCTTTATAATAATGATATCCGCATCGATGAATATACCTTTAAAGGTGTTGGT
GCAAATTCTGAACAATTAAGTGCTATGAATCATGATTCTTTAAAAAAGGAGTACATTTCAAATGACTGTTAA

Gene: lysA (diaminopimelate decarboxylase)

Position: 1387107 to 1388372, length: 1266 nt, orientation: FORWARD

Perfect match to: (CN1-CP003979-[1376624:1377889], allele observed in CC72+CC80+CC101+CC1217)

Sequence:

ATGACTGTTAAATATAATCAAAATGGCGAATTAACAATGGATGGTATTAGTTTAAAAACGATTGCGCAAAGCTTTGGTACACCTACCATTGT
TTATGATGAACTACAAATTAGAGAACAGATGCGCCGTTACCATCGCGCATTTAAAGATAGTGGATTAAAATACAATATTTCATACGCCTCAA
AAGCGTTTACTTGCATTCAAATGGTCAAACTTGTAGCTGAGGAAGATTTACAGTTAGATGTTGTTTCTGAAGGTGAATTATATACAGCTTTA
GAAGCAGGTTTTGAACCGAGTCGCATCCATTTCCATGGTAACAATAAAACGAAACATGAAATTAGGTATGCTTTAGAAAATAATATCGGTT
ATTTTGTTATAGATTCATTAGAAGAAATTGAATTAATAGACCGCTATGCTAATGATACGGTTCAAGTTGTATTACGAGTTAATCCAGGTGTT
GAAGCACATACACACGAATTTATTCAAACTGGGCAAGAAGATAGTAAGTTTGGATTATCAATTCAATATGGCTTAGCTAAAAAAGCAATTG
ACAAAGTCCAACAATCTAAACACTTAAAATTAAAAGGTGTACATTGTCATATTGGTTCACAGATTGAAGGTACAGAAGCATTTATTGAAACT
GCTAAAATTGTTTTACGTTGGCTTAAAGAGCAAGGCATTCAAGTTGAATTATTAAACCTTGGTGGTGGCTTTGGTATTAAATATGTTGAAGG
TGACGAAAGTTTCCCTATCGAAAGTGGTATTAAAGATATTACAGACGCAATAAAATCCGAAATTAAAGTTCTAGGTATAGATGCACCAGAA
ATAGGTATTGAACCGGGACGATCAATTGTAGGTGAAGCTGGCGTTACTTTATATGAAGTTGGAACCATTAAAGAAATTCCAGAGATTAATA

AATATGTTTCAATCGATGGCGGTATGAGTGATCATATCAGAACTGCACTTTATGACGCAAAGTATCAAGCATTGCTTGTTAATAGAAATGAA
GAAGCAGATGACAGTGTAACTATAGCTGGAAAATTATGTGAGTCTGGTGATATCATTATTAAAGACGCTAAATTACCTTCATCAGTCAAAC
GTGGAGACTATCTTGCTATATTATCAACTGGTGCATATCATTACTCTATGGCATCCAATTACAATCAAATGCAAAAGCCTTCTGTGTTTTTCTT
AAAAGATGGCAAAGCACGTGAAGTTATAAAGCGACAATCGTTAAGACAACTCATTATTAATGATACAAAATAA

Gene: msaC (RNA modulating SarA production)

Position: 1388612 to 1389013, length: 402 nt

Sequence:

TTAGGTTTTATGCTTTACTTGCTTTTTCAAATAATATAAAACGATAAGAATTAACAACAAATATAATGTATAAAACAGAATTTCATCAAATCC
AAAATCTTTACTTGTAATTAATACATCAATATCTTCTAATTCCATCATCTCTGCTTTAATATAATTATTAAACTTTGGCAAAGTCATTAAATGTA
CATTGTAAATATAGTAATAAAAGTCGATAAATATGATGTTAAAAAATATGTATGTTTTATCAATATTAGTTTTGAAAAATAATATCCCAGAAA
TCATTATCGGAATCACTAATACAATCATCATTGCTGCTAAAATATTAATATTTAAACCAATTGCAGATAATACACCGAAAACTAATAAATTAG
CAACTAAACTAAGGATAAGATATTTCAT

Gene: msaB-cspC-L2 (RNA chaperone locus2)

Position: 1389210 to 1389410, length: 201 nt, orientation: REVERSE

Perfect match to: (RF122-AJ938182-[1373964:1374164:r], highly conserved allele)

Sequence:

TTATAGTTTAACAACGTTTGCAGCTTGTGGACCGCGGTCGCCTTCAACTACTTCAAACTCAACAGCTTGACCTTCTTCTAATGATTTGTAACC
ATCTTGGTTAATTGCTGAAAAATGTACGAATACGTCATTTTCTCCTTCAACTTCGATAAAGCCGAATCCTTTTTCAGCGTTAAACCATTTAACT
GTACCTTGTTTCAT

Gene: msaA (domain of unknown function 1033-containing protein)

Position: 1389581 to 1389889, length: 309 nt, orientation: REVERSE

Perfect match to: (N315-BA000018-[1409203:1409511:r], highly conserved allele)

Sequence:

TTAATCAATTATTGGTAGTTTATAATATACTTCATTATTTTGTAAAACAATGAAACTATAAAATATTTGTAAATCCTCTTCACAATCTTCGCAAT
AGTTCATGTCACAATTATTATAAAATGCATAAATATTATATTTGCCTGTGACATAATTATCGAACTGAACTTTACATTGATTAATCAATTGTTG
ATAGTGCTTAAACATGTCATCAAAATCTTGATATTGATATTTTTCAACAATGTTTTCTGGCCAGTCACTGAATAACCACCATCCCTCATAATCG
GCTCTAATTTTGGTAACTGTCCACAT

Gene: acyP (acylphosphate phosphohydrolase)

Position: 1390052 to 1390321, length: 270 nt, orientation: FORWARD

Perfect match to: (11819-97-CP003194-[1440430:1440699], allele observed in CC80)

Sequence:

ATGAGACATGTACATTTACAAGTATTCGGACGCGTTCAAGGCGTCGGATTTAGATATTTTACACAACGCATTGCAATGAACTATAACATTGT
CGGTACTGTTCAAAATGTAGATGACTATGTAGAGATATATGCACAAGGGGATGACGCAGATATAGAGAGATTTATTCAAGGTGTAATTGA
AGGTGCCTCACCAGCATCAAATGTAACAAGCCATCAACTTGAAGAGTTAGAACTAAATCAAAAATTATCGGATTTTCGATCAATATAA

Gene: xpaC (5-bromo-4-chloroindolyl phosphate hydrolysis protein)

Position: 1390340 to 1390969, length: 630 nt, orientation: FORWARD

Perfect match to: (RF122-AJ938182-[1375090:1375719], highly conserved allele)

Sequence:

ATGACAGTGAGATATAATATTTCTCATATATTTGGGGTGTTAGTGGGAATTCCTGTAGCGTTTTTAACAAGCATATTTGGGATGATTGCACT
TGATGTATCTTTTTTAATTGATATGTCTATTGGTATTGTTGGCTTTTTAATGACATACCTACCGATACAAAAACTCACTTCACGCAAATATTTA
AACGAAATTGGTTTGACTAGAAAAGACTATCGCTATATTCGAAATCAGTTAAATCATACACACCAAAAACTTAGAGGTATTTTAAAAACGTA
TGTCAATATAAGATCAATTAAAGATTTTAGGCAGATTAATGATATATACCAAATTTCACGTTCTATTTATACGACAGTTAGACAGAGACCTGC
ATCATTTTATAAAGTTGAAGGCTTTTTTTATTCTCATATTGATAATGCTTTAAATTTGGTTGATGCATATACACGTCTAGCAAAAATGCCCAAA
AAATCAATTAATGAACAGCAAAAGTTAGAACAAACACGAATTACTTTGGATGAGGTCAAACGAACATTAATCGCTGATTTAAAGCGTCTCA
ACGAAGATGATTATGAACGTTTAGATATTGAAATGGAATTAAATAAGTTACATCAAAAACATCATCAAGATTGA

Gene: terA (putative tellurite resistance protein)

Position: 1391001 to 1392137, length: 1137 nt, orientation: FORWARD

Perfect match to: (11819-97-CP003194-[1441379:1442515], allele observed in CC80)

Sequence:

ATGACTGAAAATAAAAGTTTCAAAGAAAGCTATCCACTAGATGATTTTATAAGCGATAAAGAATTATCGAATACTACTATTCAAAAAGAAAA
GTTAACAATTGAACAACAAAAACAGGTAGACACAATCAGTAAACAAATTAACCCTTTAGACAATGAAGGTTTATTAGCGTTTGGTTCTGATT
TACAGAAACAAATGTCTCAATTTTCACATCAAATGTTGGATGAAGTACAAAGTAAAGATGTTGGTCCTATTGGAGATACTTTGTCAGATCTA
ATGTCAAAACTAAAGTCAGTTAATCCAAATGAGTTAAATACTGATAAACCATCTATGTTAAAAAGAATTTTTAGCAGAGCAAAGTCGTCTAT
CAATGAAATCTTTTCAAGAATGCAATCAGTTAGTGCTCAAGTCGATCGCATAACGATTCAACTGCAGAAACATCAAACACATTTAACAAGAG
ATATTGAATTATTAGATACGCTATATGATAAAAACAAACAATACTTTGATGACTTATCATTGCATATCATTGCTGCACAGCAAAAAAAGTTGC
AATTAGAAAATGAAAAGCTACCACAATTGCAACAGCAAGCGCAGCAATCCACTAATCAAATGGATATTCAACAAGTTGCAGATATGCAGCA
ATTTATAGATAGACTAGATAAACGCATATATGACTTACAGCTTTCAAGACAAATAGCTTTGCAAACTGCGCCACAAATTCGTATGATTCAAA
ATGTTAATCAAGCACTTGCCGAGAAGATACAAAGTTCAATTTTGACAAGTATTCCACTATGGAAAAATCAAATGGCCATTGCGCTTACATTA
ATGAGACAGCGTAATGCAGTTGCTGCACAACGAGCTGTCACTGATACAACTAATGATTTATTAACAGCAAATGCTGAAATGTTGAAACAAA
ATGCGATTGAAACTGCAACAGAAAATGAGCGTGGCATTGTTGATCTTGATACATTGAAACGTACACAGCGTAACATTATTGAGACAATTGA
AGAAACATTAATTATTCAACAACACGGTCGCGAAGAACGACAATTAGCTGAAAAAGAATTACAACAATTAGAACAAGATTTAAAGTCACAT
TTAGTGAACATCAAAGGACCGAATAAACAATCATAA

Gene: brnQ3 (branched-chain amino acid transport system II carrier protein)

Position: 1392210 to 1393553, length: 1344 nt, orientation: REVERSE

Perfect match to: (11819-97-CP003194-[1442588:1443931:r], allele observed in CC80+CC8+CC80+CC239+CC4803)

Sequence:

TTATTCCTGTTGATATTTAATTGGATCTTGTTTTACAAATATGCCGACTAGATAGCCTAATATCGTTGCAATAATTGCTACTGGGAACCACTCT
AAAGAATACGCTCTTAGAGGCAATGATTCTATAAAGTTAATTTTCAGCCAACCTAACTTACTAATAACACTGAAAATCGACAATATAAATAC
GATAATAACTGGAATTTGTTGTGAAATGCGTTTTGTCGGTATGAATTTGGCAATTAAAATTAATAATACAACAGTTATTGCTACTGGGTATA
CAATGCTTAATACCGGAATTGACATTGAGATAACAGCATTTAAACCTTGGTTAGCAATAATAAAACTCATTAAAATGAAAACTAATACAAAT
GCTTTGTATGATACTTTAGGTACGATTCTATGGAAATATTCAGAAACTGCAACAATAAGCCCGCATGCTGTAGTTAGACATGCCAGCGCCAC
AATGATGCCCAATAAATATTTTCCGAATGAACCAAATCCTGTTGAAGCCATTGTCGTTAATAAATATGTCCCAATGTTTCGATCTTTGGATTT
CAATTGATCTAACGTCATGTCACTTACTGGCATATGATTACCAATATAACCTAATGAAATATATATGAAAATTAAAGCTACGGCTGCAATTAA
ACCAGCAGTCAAAGTTTGTTTGAATATTTGATTTGTTTTAGTAATGCCTGTTAGTTTTACTGCATTAACAACAATCATTGAAAAAGCAATTGC
TGCAATGGCATCCATTGTTAAATAGCCTTGTGTAAAGCCTTCAGCAAAACTTGAAAAATTAGAATGATATAGTGCTTCATTGCCCTTTCCAGC
ACTATTACCGCTAAAGTCTAAGTATCCTTTAATAATCATCGCTAAAATAGTAATCAATAATAATGGTGTTAATAATGAACCAATACGATCGAT
TAACTTAGATGGATTTAAACAAATATACAAAACGACTATGAAGTAGATAATCGTAAATATAAATAAAGCGATACTACTATTGTTATGTATAA
TTGGTGTAATTGTCATTTCAAAAGATGTAGATGCAGTTCTAGGTATTGCGAAAAGTGGACCAATAGTCAAATAAATGATGATTAAGAACAA
TATTGAAAATTTAGGTGAAATTTTATTTAATGCGCCAATATATCCTTCTTTATCAAGTGCACCTACAATCACACCTAATAATGGTAAACCAATC
CCAGTTAGAACAAACGCTAAAATGGCGGGCCAGAAAAATTGACCACTATCCAATCCGAGATTAGGAGGAAAAATTAGATTGCCTGCGCCA
AAAAACATAGCGAAGAGCGTAAACCCAATGACCCATGTATTTTTATTCAT

Gene: cobT (putative cobalamin biosynthesis protein)

Position: 1393778 to 1395664, length: 1887 nt, orientation: REVERSE

Perfect match to: (MRSA252-BX571856-[1477250:1479136:r], allele observed in CC30+CC80+CC772)

Sequence:

TTATAAAGATTTAAGTAGTAATTTTTTTAATAATGGAGAGAGATGTCCAGGTAAATGCGCAACACCTTCAACGAAAATAGCATATTGACCAT
AAATATTATGAATTGTTTGTTCAACATCTTCAGTTATTGGATCTTGACTCAAAAATACATTAAATACTTCAATACCAAATTTACGTGACATTTC
TACAGCTTCATACGTATCAATAATACCATCTTGACTATAATTAAATGCAGACGGTTCGCCGTCTGAAAATACGATTAAAAATCGTTGATGTTG
ATTTCGACGCATTAATCGTTCACTTGCAACTCTAATAGCAACACCATCACGATTATCATCTTGAGGTTCAAGTGCCATAATACGTGGGCCATC
TTTTTCAAAGGTTGAGTAATCATAGTTAATAATTTCATTAATGATATTTGGTTGTGCATGATCGTCTGAATCAAAGGCATCCTCACTGAATGA
TAAAATTTCATGTTTAATGTTCAATGCTTTTAACGTCTCGTGGAATAACACTACACCTTTCTTCGTTTCAGCCATTTTATCATGCATACTTGCTG
ACGCATCAATTAACAATGTAAACGTCGCATCAAATGATTTACTTAAATCTTGTTTTTTGTAAAACAATTTATATTGATCGTCGATAAACCAATT
AATTAAATCCTTTTGTAATCGTCCTTTTGTTAAATTAAAACGTGCATCTCGTTGTTCTCGTTCAATCGTTTTCTTTATAATTTGAATTAAGTCTT
TGATTTCATATTGTACGTCTTGTTTCGATTCTTGATATTCTAAAACATATTGTGGCTCAATTTCAGGAATTTGCCATTTTATTTCTACATTTTTG
TTCACACCATCAAGTTGAAATGCTTGGCTTTGACCAACAGCATCGCCTTCTTCACGATTTAGCGTATCGTTTGAACCTTTGCCCTTTTTGGTCA
TCATATCAGTCATATCATCTGTAGCATCGCCTTCACGTGCTTCATCATTACCCAATGTTTCACTATTTTGTCCTTCGTGAAGTTCCATTTCTAAA
TAGGCGCCACCTTTTGACTCACTATCTGCAGATTTAGAGTCAGCTTTTTCACTTTCGTTATCATCATCTTCTGAAGTATCATCTTGCCCATCGA
CTTGACTTGCATCAGTTCTCTTTAAATCATCAAACTCTGGACTAGCTAATGTATTATATAGTGTTTTAGGTAAGTAATAATATTCGTTTAGCAT
ATCTTCTTTTAAAATATCATCTACTTGATACATAATTCTTTGAGCCAGATACATATTATCTTCAGAATTTTGATTTTGGAAAAAAATTAGGTAAA
TATAAGAACATATTAACTACAATATCATCTAAATCTGAATGTATAGATGGTATATCAAAGAAATCTTGGCTTAAAAATGCATGTTCTAAATAC
AAAAATAATAAATCTGTATATTGTGTTTTAGTACGATACACTTTAATTTGAGATTCCGTATATGATATACGTGTATCTAGGCGAAGATCAATT
AATTTAGCAGTACTTGGGCGCTCAACTTTAATAGAATTTAATACGCGCATATCTTCTAATAATTTAAAAAGTTGTTGATAAAATTTAGGGTGT
TTAAAAGTTTTATCTTGTACTACTTCATTTACAATTTGTACATCCATCATATGATAACCGTAAGCAGCTAACATAACATCTGTTTTTAAACCAG
CCATTTCGATATGGCTTGGTCGATGTGACCAAAACCAACTCGTAATCAAAACATTTTGAACAGGATTATAGTAAGGGAATTTTTGGATTTTA
ACTTGTGTTTGTTCATTTTTTAATAAGAGTCGAGCTAAGTCTTGCAACATCATTACTTGCTTGGCATCTAACTGTTCGTCGTTAAATTTTATGA
AACGATCACTCAT

Gene: norQ (putative nitric-oxide reductase activation protein)

Position: 1395678 to 1396469, length: 792 nt, orientation: REVERSE

Perfect match to: (11819-97-CP003194-[1446056:1446847:r], allele observed in CC80+CC239)

Sequence:

TTAAAAGTTTAGTTCTACAGCATTATATATTGCTTGTTGTTCACGTTCATCTTCCAATTTATCAATAATTGTACGTTTAATTGCACGTTCAACTG
GCATTACAGTGATTAAATCACACAAGTCTAATAATGCACGGATACTAGCGGCTTCTTCAGAAATTTGTCCCTGCTTAGACATAGTACGTAAA
TCTTCGTTAAACTTAATAATTTGTTCGATTTGTTTATCATCTTGTAATAAACTTTGCTCTTTAATCACATTTTTTAAAATGTCCCCGTCAATATA
ATCAACGTGAATAACAACAAAGCGATTTTTTAGTGCTTCATTCATTGGCAAAGTACCAACATAACCTTCATTTATTGCTGCTATAACGTTAAA
TCCTGGTACAGCTTTGATTACTTCACCAGTGTATGGATTCGTAATTTGACGACGATAATCTAAGACCCCATTTAATACAGGCAATGTTTCAGG
TTTAGCCATATTTATTTCATCAATATATAAAATATGCCCCTCTTTCATAGCTTTAATAACTGGACCATCTACAAAGACAATTTCTTGTTGACCTT
CCGCATTTGTTTTAATTGTTTTAAAGCCTAATAAGCTTTCTGTATCTAAATCAACAGAACAATTGACTTGATGCATGGGTGTATCAACAACTT
CACTTAATGTTTCTGCCAACTTTGTTTTCCCTGAACCTGTTGGACCTTTAAGTAAAATATTTTTATTTAAATCAAATAATGCCTTCGCATCATTG
AAAACTGTTGAATCTGAATTCTTATAATGTTTTAGTGCCAT

Gene: yozC (protein of unknown function)

Position: 1396650 to 1396853, length: 204 nt, orientation: REVERSE

Perfect match to: (N315-BA000018-[1416273:1416476:r], highly conserved allele)

Sequence:

TTAAACTTCTTTGAAATATGTTTTATAGTAACCGCCAACTAATCCAGAATTATCTATTATTTGATAATATTCTTCAGTTTCGTTGATAACATCAT
ATTGTTTGCCAACCGTTAACATGTCAGAAACTGTAAATTTCTTTGCATCTGTATTAATGACTTCTACTTTCTTGATTGGTGTACGTTCTTTCCA
AGTTTCATGTAACAT

Gene: Q5HG08 (putative metalloenzyme)

Position: 1396882 to 1397691, length: 810 nt, orientation: REVERSE

Perfect match to: (DSM_20231_T-AMYL01000003-[4753:5562:r], allele observed in CC8+CC188)

Sequence:

TTATTTTTGTAAAGTGAATATAAACGATAATCCACTGTTTTCAGTAATTCTAATTGATTCAATATCATCTTGCTCAAGCGTTTGATATGGTATT
TCCACTTCATCTAAGCGCGATTTCGCTTGATTAAAGAAATCTGCATCTTTCGTTTCAAACTCAACTTGATCGACTGCGCCATATTCCGGCATT
GTCATTTCGGTTTCTACAGGCATTAAATGTATTTCGCCACCTAGACCACCCGTTCCAATTTTGAATACTTGGACATGATAGTCAGCATTGTCG
AAGGGTTGGTATTCTGCAAATACTTCAAGTCCGAATATATTTGTTAAAATTTGACCTGTAATATCTACATGATTCACTTTAAGAATCACTGGT
CCTAAACCTTGCACTTGATGTAACGGATTGACCGCACTCTCAAAAGAAGGCATACCTAAACCAACGCCATAATTATTCTCGTTTGAATATATT
GAGAAAATATGACCATTGTTATCTTCGAGACTGAAATATTTATTGCCATTTAATTCTTTAACTGTTGTAAATGGAATATCCTTATTCGATAATA
TTTCCGCATACTCCTCTAAACCTGAGTCAGTTGGTGTACGTAATCCAATACCCACAAAGTGAGATTCTTCTAATTGCTCACTTGGAACTTGTA
TAAATTGAAGTCTTGTTCCTGGGCTTATATCTGCATCGCCGAAACGAATTGAGTTTTCAAGAAGTTCTTCATAATTTAATCCTAATATGTCAA
CCATGAAATGTTTTGTCTGTTCTATATTTGTTGTACCTAATGTTATACTTCTAAGTCCACACAT

Gene: odhB (2-oxoglutarate dehydrogenase complex subunit E2)

Position: 1398271 to 1399539, length: 1269 nt, orientation: REVERSE

Perfect match to: (11819-97-CP003194-[1448649:1449917:r], allele observed in CC80+CC15+CC4803)

Sequence:

TTAAGATTCTAATAATAAGTCTTCTGGGTTTTCAATTAATTCTTTAATTGTTTTTAAGAATCCAACTGCTTCTTTACCGTCAATAATTCTATGAT
CATAGCTTAATGCAATATACATCATTGGACGATTTTCGATTGTATCTTGATCAATCGCAATTGGTCTTGTAATAATTGAATGCATGCCTAAGA
TTGCAGCTTGATTACCATTGATAATTGGCGTACTCATCATTGATCCAAAAATACCGCCATTTGTAATCGTAAATGAACCATTAACCATATCAT
CTAAGCCAAGTTTTTTCTCTCGTGCTTTAACAGCTAAATTAGCAATTTCTGCTTCGATTTCTGCAAAATTCTTTTTATCACAATCTCTTACAAAT
GGTACTAATAATCCATCATCTGTAGAAACAGCTACACCAATATCATAATATTGTTTCGTAATCATGTCGTCGCCGTCGATTTCTGCATTAACTT
CTGGATACTTTTTCAAAGCTGCTACAGAAGCTTTAGTAAAGAATGACATAAATCCTAATTTAGTACCATCATGATCTTTCATAAATTGTTCTTT
CTTACGTTTACGCAATTCCATAACATTTGTCATGTCAACTTCGTTAAATGTTGTTAACATAGCTGTATTATTAGATACCTCTAATAATTTTTTG
GCAGCTGTTTTCTTTCTGCGTGACATTTTTTCACGAATCACTGGTTTTGTAGGATATTGATTGTATTTTTTCTCTTCTTTTGCAGGTGCTTGTTG
TGTTGTTTGTGTTGATGCCGGTGCCTGTTGTTTCTTATCAATATCTTCTTTACGAACCACATCATTTGTTTTCGGACTTACTTCAGCAAGATTC
ACACCATTTTCACGAGCATATCGACGCGCAGAAGGCGTAGCATTAATACGTTGCTGATTGTCATCATTTGCTTGATTGACTTCAGCTTTATCT
ACCGAATTATTTGTTGTTTCTTCTTTTTTATTATTTGTTTCTTCATTTTGTTGTGGAGTATTGTCGTTACTATTTTCTTTAGAAGCATTGCCACTA
CCTTCGCCGATGATAGCAATTGCTTGTCCAACTTCTACAGTGTCGCCTTCACTTGCAAGTTGTTCAGATAATACACCTGCTTCTTCAGATACA
ACTTCGACATTAACTTTATCAGTTTCTAATTCAAGAATAGCTTCACCTTTTTCTACGCTATCCCCTACGTTTTTCAACCATTCTGCAATGGTACC
TTCTGTAATAGATTCTGCTAATTCTGGAACTTTAACCTCTGGCAT

Gene: odhA (2-oxoglutarate dehydrogenase complex subunit E1)

Position: 1399553 to 1402351, length: 2799 nt, orientation: REVERSE

Sequence:

CTAGTTATTTTTTAATGCATTTTCTATAATTTTATTTTGAACAAGTTTATGAATTTCTCCATCGCCTTCAGCTGGAGCAGCCCTTTGAATTCTGC
CATGATAACTTAAATCATATTTATCTGCAACTAGCACTTTAACATATGGATAGACATATAACCATGCACCTTGATTTTTAGGTTCTTCTTGTAC
CCATGACACTTCTTCAAGGTTTGGCAATTGTGCTAGTAATGCTTCAATCTCTTCCTCTGGGAATGGATACAATCTTTCAATCGCAACGAGTAA
CACTGATTCGTCTGGATTTTTAGCTAATGCTTCTTTTAAATCAATGAACATTTTACCAGTTGCTAAAATAACTTTTGTAACCTTATCCGCTTGA
TATGATTCTGTCAAAATTGGCTCAAATCCACCAGAAGTAAACTCATCAATTGGTTTGGCAACTGTTTTATTTCTCAATAAGCTTTTTGGTGAC
ATAACAACCAATGGTCGCATTTGTTCAGAATCTAAACTAGCTGCTTGTGCACGCAATAAGTGGAAATAATTACTTGAACTAGATAAGTTGAC
AACTGTGCAATTATTTTCAGCAGCTAATTGTAAAAATCGCTCTAATCTTGCTGATGAATGTTCAGGTCCTTGACCCTCATATGCATGAGGTAA
GAATAATGTTAATCCTGAACGTTCTCCCCATTTTGAGCGAGAACTGAATAAGAAGTTGTCAAAAATCATTTGTGACATATTTGCAAAATCAC
CATATTGCGCTTCCCAAATATTGAAGCTTTTTTTGTTTTCCACATTATAGCCGTATTCAAAACCAACTACTGCTGCTTCTGAAAGCGGAGAATT
GTGTATATCAAATGTCGCTTTTTGATCAGGAACATGATGTAAAGGTGTATATGTTTCACCTGTTTGCTCATCATGTAACACGGCATGCCTATG
ATTGAATGTACCACGTTCACTATCTTGACCAGTTAAGCGAATCGGTGTACCATCTTGTAAAATTGTCGCAAATGCAAGTTGTTCTGCTTGTGC
CCAATCAACTAAACCATCTTCTTTATTAAACGGCTCATGACGCTTCTCAAGAACTTTGTTTAACTTTTTCAAAATGTTAAAGCCATCCGGATAT
GTTAACAATGCATCATTTATTTCTTTCAAATGATCAAACGTAAATGATTGTTCGTCTGCTTGTAACGGTAATGCAAGTTCTGCAGGTTTTTCC
ATATCTGGATTATCCATTTTATCAGCTTTATTAATTTTATCATGTGCTTGTCTTAGTTCCTTTTGGACTTGTTCTATAAATGAATGCATTTCATC
TTCTGAAATGACACCTTCATTAACAAGCTTTTTACCAAACACATATTCAACAGAGTCATGTTTGCGAATATTCTGATAAGGAACTGGATTAGT
AATTGATGGTTCATCCATTTCGTTATGACCGAAACGACGATAACCTACTAAATCAATAACGACGTCTTTATGAAACTCTTTTCTAAATTCCATT
GCAATATCAATTGCTTCAATAGTAGCTTCAACGTCATCAGCATTGACATGGAATATTGGCACATCATAACCTTTGGCCACATCTGTAGAATA

AGTTGTTGAACGTGCATCAATTGGTTCTGTAGTAAATCCAATTCTATTGTTAGTAATAATATGCAATGAACCACCCGTAGAATAGCCTTTCAA
GTTTCCTAAGTTCATTGTTTCGAAGTTAATTCCTTGACCAGGATAAGCAGCATCGCCATGTATAATAATTGGCATTGCTTTATGATGATCAGT
CGTCGGAGCCCCAGCACGTTGTGTATCATCTTGTGCTGCTCTCGTACGCCCCTCAACAACAGGTGCAACAATTTCCAAGTGACTTGGATTGT
TAGCCAGTGCAATACGCTGCATTGTACCGTATGAATCAGTAGTTTTAATGCCACCAAGGTGATATTTCACATCACCAGTCCATCCAGCAGTT
AACTGCAAGCTACCATCTTCAGGTAAGAATTTCATTGGATCTGTATGCATAAATTCTGAAATCATCATTTCGTACGGTTTTTCTAAGACATGC
GTTAAAACGTTTAAACGTCCACGGTGAGCCATGCCTATTTGTATATTTTTAATACCTTCTTTCGCAGCAATCGTAATAGTACGTTGTAACATC
GGTACAAGTGCGTCTACCCCTTCAATTGAAAAACGCTTTGCACCAACGAAGTTTTTATGAAGATATTTTTCAAACCCTTCAACATACGCTAAT
TGTTTGAATAGTGCCCTTTTTTCGTTATTATTTAACGTTACTTTATATGGCGTTTCAATTCTTCTTTTTAACCAACCACGTTCGGTATTGTTATT
AATATGTGTATACTCAAATGCAATTGGTCCTTTGTAACGTTTTTCCATTCTTAAAATTGCTTCATAAGCATTATCATAAATGTCGGCAAAGTG
ATCTGAAACAATTCCTGCTGATATACCTTCCAAAGTCTGTTGATCTAAATCAAAGTCTTCAATCTCTAATTTAGGTACATGTTTCCTTTTTGGA
GGATTTACAGGATAAATATCGGCTTTAAGATGCCCGTATTGGCGAATATTATCAATTAAACGCATGACACGCTTAATTGTGCCGTCGCTATT
TTGACTACTTGTACTTTTTAAAGCTGGTACAATTGAGTCATCATTCTTAATTGTGCTGAATAAGACTTGTAAATCTTCTGGTACAGATGATGG
ATCTTGTAAAAAGTCATCATATAGATCTAACATTAGACCTAAATTCGCACCGAAGTTTACAGGAGCCTCTGAAACTTCTTTTCTTTCGTTAGT
CAT

Gene: arlS (two component sensor/regulator, sensor histidine kinase)

Position: 1402635 to 1403990, length: 1356 nt, orientation: REVERSE

Perfect match to: (ED133-CP001996-[1467518:1468873:r], allele observed in CC133+CC93)

Sequence:

TTAAAATATGATTTTAAACGTTGTTCCTTTGTTAATTTCACTTTTAATTTTAATCGATCCTCCGTTTAATTGAATGATTTTTTGAGCAATAGATA
ATCCGAGTCCATTACCGCCTTGACTTCTTGAACGAGATTTATCCACTCGATAAAAGCGATCAAAAATGAAATCTTGATCTTCCTCTGGAATAC
CAATTCCATGATCTGTAATTTCAATTATTTTTTGCTTATTTTTTAACCTTGTCTTAACTTTAATTTTCTTATTCTTCACATCATATTTGATTGCATT
ATCAATAAAGATTAAAAATAATTGTTCGAATTGATGAGGTTTCATTTTAATTTCTAGATTTTTAGATGTCAGATCCGTATCAAATTGATAATC
AGGATGCAATTGTTTTAATGAGTGTATTCGCGAGCGAATTTCATCATTAATATGCACGGTCTGCGCTTCAGAAGAAATGTCATTTACATCTC
CTTTAGTCAATTCAAGTAATTCTTCGACTAATTTTATGATACGATTCATTTCTTCAATAGAAATATTTAACGATTCTTCTAATACTGCTGGGTCT
TTTTTTCCCCATCGCTGAATCAAATTTAAATGACCTTGAATAATTTGTAATGGTGTTCGTAATTCATGTGACGCATCTTCAACAAATTGTCTTT
GTTGATTAAATGATTCTTCAATTTGGCTCATCATCTCATTAAACGTATTTGCTAAATTATCTATTTCTTCATAATTTGTATTTAATTGCAATTTA
TTTTGAAAACCATCTCGTCGAATCTCAATCATTTTATTTGATAAACTGACAAGCGGTTTAGTAATTTGTGTTGAAAATACATAACTGATTGTG
GCAGTTATAATTGTTGCAATCACTCCAAATGCGAGCGCAATGATATACAATGATTTTACGATGTTATCATAATTTTCTAGTGAATGAATTAAC
AAGCTATACCCTTTGAAATCTTGCGTTGTGATTGGTTCTTTAATAATTAAATATTCAATGCCTTTATAGCGTTTTTTTATTACGCGGTCAAAAT
ACCGGTGTTCATATCCAGGTTCAACTCTCACTGTGTTATCATTCGATGTCTCAAATAATTTATTATTATGCTCATCATAAATAATAATCTCTTG
AAAATTACCTAAAGATGCATTCAAGTCTAATGCAGATATATCTTTAACAGGCTTAGAATGAAATAAATTATTAATATCGCTTGAGCTTCGTTC
TGCATCATCAAGCTCACTATTATGCAGTGTATCTTTCAAGAAAAAGATAATAATTAAACAAAACAAAAATATCGTGACAAACGTAATCATCG
TGGTAACAATAATCCAGTTATTGCGCAATTTACGTTTTGTCAT

Gene: arlR (two component sensor/regulator, transcriptional regulator)

Position: 1403987 to 1404646, length: 660 nt, orientation: REVERSE

Perfect match to: (11819-97-CP003194-[1454365:1455024:r], allele observed in CC80+CC8+CC239+CC4803)

Sequence:

TCATCGTATCACATACCCAACGCCACGAACTGTTTCAATCATTTTGTCACGATCGTATGGTTTTAACTTGTTTCGTAAGTATCTTATATAAACA
TCTACGACATTTGTTTCTACTTCACTATTATAACCCCATACATGATTTAAAATTTGTTCCCGTTGCATAACATGGTTTTTATTTTCAGCTAGAAG
ATATAGTAAATCATACTCTGTTTTTGTTAATTCAATTTCTGCGCCATTTACCGTCACTTTAAAAGCGTTCTTATCAATTGTAATACCGTTGACAT
CGATAATATCCTTTTGTGGCTGACGACGTAAAATTGCACGAATTCTTGCTAAAAGTTCTTCAATATCAAATGGCTTAACTATATAATCGTCTG
CACCGTAATCAAGCCCAGCAACTTTGTCATACGTATCACTTTTCGCTGTAATTATAATGATAGGTGTAGATTGTTGTTGTCTAATTTTGCGAC
AAATTTCTAAGCCATTAATTGACGGCAACATTAAATCTAATATGATTAAATCATAGTAATGGCTAAGCGCTTTATCTAAACCGTCTTGTCCAT
CATACTCTGTGTCCACATTGTAATTTTCATGTGTGAGTTCCAATTCAAGAAATCTTGCTAAGTTTTGTTCATCTTCTACTATTAAAATTTGCGTC
AT

Gene: pgpB (putative phosphoesterase)

Position: 1405434 to 1406048, length: 615 nt, orientation: REVERSE

Perfect match to: (11819-97-CP003194-[1455812:1456426:r], allele observed in CC80+CC30+CC80)

Sequence:

TTAATTTATTAATTTATTTCTAAGTAATAACGTTGATAAAATAATGCAAAATACGCCACCAATAATGCCGGCAATAATATCTGTTGGATAATG
TACACCTAGATATACACGTGATATGGAAATCAATAAAATCATAGCTGCACATAACCCTATAAGAATACCTTTTGAATTACCTTGATTTAATCG
ATTTAATAGATAGATACCACTTCCAAAATATGCAGTTGATCCCATAGCATGACCGCTAGGAAAACTAAAGCCTGTTATATCAATTAAACGCA
GCAATGTAGGTCTTTCTCTATCGAATATATTTTTTAATGCTGGATTCAAAATTCCAGATAGTGCCATTGTTAATGCAAAAAATAATGCTTCAA
TTTTGTGGCGCTTTAACATGAGATATGCCACAAGAAGTAATGAAATACATAACATTGCCCAGACTTCACCTACTTTAGTAGCCCCAAGCATG
ATAGATGTCGTAATAAAGCTCTCTGATGAATATATAAATTCATAAACTTCATTATCAATCCATTTCCCTAGTCTTGATTCGTGGAAAAACGCG
ATAATTCCAAAAACCAATGTAAAAACGATGAGCAAAGAGATACGTTTCCATTGACTCAT

Gene: murG (undecaprenyldiphospho-muramoylpentapeptide beta-N-acetylglucosaminyltransferase)

Position: 1406065 to 1407135, length: 1071 nt, orientation: REVERSE

Perfect match to: (11819-97-CP003194-[1456443:1457513:r], allele observed in CC80+CC188)

Sequence:

TTAATTCAATGCATCTTTAATCATCTTATCAAATAAAGCTTCTTTCGTATAACTTTGTTCATACGATTTCATATTATTGATAATTCGAGTTCTTT
CCTGTTCCATTTCATTTAGTTCTTGTAATAAAATTTGTGCTGTTAATTGTTCTTCATCAATCGTTTTAGCATAACCTTTATCAGCAAAATGATTT
GCATTGTCAATTTGGTCGCCTCGGGATTGATCTAAACCTAATGGTACTAATAACATTGGTATACGTAATGTTAAGAACTCATAAATCGCATTT
GATCCAGCTCTACTTATTACTGTATCCGTAATTGCTAATAAATCTGTTAAATCCTCTTTAACAAATTCATATTGTATATATCCTGATTTTTTAAC
TTGAGCATCTTTTAATCCTTTACCAGTTAAATGTATCACTTGATATTGTTGTAATAATGCATCTAAGTTTTCGCGAATAATGCTATTTAATTTTT
TACTTCCTAAGCTTCCACCCATAACGAGTAAAACTTTTTTATTTTCATTAAAGCCTGTTAATTGATAACCATTATGTGCATTACCATTTTTTAAA
TCTTCTCGAATTGTTGCTCCAATAAAATCAGCTTTCTCTTTAGGTAGGTAGTTTAGCGTTTCTTCAAATGTTGTATATATTTTCTTGGCAAATTT
AAGTGCTATCTTATTCGCTAATCCTGGTGTTAAGTCAGATTCATGAATAATAGTTGGTATATTTAATGATTTGGCTGCAATAACAACAGGCAC
AGATACAAATCCACCTTTTGAAAATAATAGATCAGGTTTTTCTTTTTTCAAAACTTTACGAGCATCAAGAATACCTTTCAATACTTTAAATACG
TCTTTGGCATTTTCTAAAGAAATATATCTTCTTAATTTACCACTCGAAATAGGATAATACTTAATTTCTGGTAGTTGTGATTCAATCATTTCTCT
TTCAATACCATTTTTAGAACCAATATAAAGCGCTTCATAACCTTGTGATAATGCAGTTGGAATTAAACTTAAATTTACTGATACGTGTCCAAC
TGTTCCCCCTCCGGTAAATGCGATTTTCGTCAT

Gene: Q7A0W4 (putative acetyltransferase)

Position: 1407147 to 1407656, length: 510 nt, orientation: REVERSE

Perfect match to: (11819-97-CP003194-[1457525:1458034:r], highly conserved allele)

Sequence:

CTATTCTTTTAAATTTTTATAATATGCATAAAATGGTTCACCTTTATCATATGGCGGATATTCCATTAATTGTTCACCGACCTTATGAAATCCA
AATTTGGCAAATAAACCTTGTGCAGGTTTGTTTAACGCAAAGGTGTCCGTTAAAATAACTTCTGCACCACGTGCTTTAACTACATCAATAACA
TAATTGAATAATTCTGTAGCAGCTCCTTTATATTCTTTCGAACCAGTTAATCGATGAATAACAAAGGCGCCTTCTCTATTTACTGGCCAGTCA
ATGTCATCATACCATTCTGCTTGGTCTTGGTCGACAACAATAAAGCCATAAATTTTGTCATTTTCCTCTAATACGTACAAATAATCTTTAGCAA
TATCTTCTTCAAAATGTTCTAAAAGTGGGTACTGATCGTCCCATTGCTCGTTGTCGTGTTCTTTCATTAATTCTTTTGCTTCTTCTACTAGATTT
AAGATTTGATCTAAATCTGACATTTTACCTAGACGGATCAT

Gene: ctpA (carboxy-terminal processing protease)

Position: 1408843 to 1410333, length: 1491 nt, orientation: REVERSE

Perfect match to: (11819-97-CP003194-[1459221:1460711:r], highly conserved allele)

Sequence:

TTATTTTAAAATATTAATCAACTTATCGAGAACATCATCATGTTTATTAGCTTTTTCAACTAATAACTCAGTAAATTTATTATTCGTTTCTTTATT
AAATTCACCAGTTACCTCAAGTTTATTCGCTTGTTGGAAAGCTTTAACTTGATTTTCTAAAGCTTTATCAAATTGCGTTGATTCATTATCAACT
TTATAACCTAAAGCTGATAAACCAATTTTAATAGTTTTAATATTTTTATCATCGTCTCCAACTTTAAATGTTTTCGTATTAGGAATGACATTTA
AAGATTGATATTTAGGTGTGTCAATAGTAACGTCTGGTTTAATGCCTTTACCGTGAATATAATGACCATCTGGCGTTAACCATTTCATTTCAG

TATATTTTAACAATGAACCATCCTTAAACTCTCTTGTAGTTTGTACGACACCTTTGCCGAATGTTTTTGACCCATAAACTTTAGCTTTATTATA
GTCTTTTAGCGCACCAGTAAACACTTCAGAAGCGCTAGCTGAACCTTCATTCACTAAGATGGATATATCCATGTCTTTCGCTTCTTTTAACGC
ATCATTAGAAGTTTGAATTGCTTCAGTATCTTTACCTTTTTCTAGTTTAACAACAGTTTTTCCTTTATCGATAAAAATATTTGCCATTTTAACAG
CTTCATCTAGTAGTCCACCTGGATTATTTCTTAAATCTAAAACAATCTTTTTCAAACCATCTTTGTGAGCTTTTAGAACTGCATCTTTCAATTCA
CCTGATGTATCATTCTGGAATTTATTAATAGTAATAACTCCAACTTTACCTTTTTTCTTATACTCAACACTTTTAACATGAATTTTTTCACGTTTA
ATCTTAACGTCTTTTTCTTCACTACCTCGTTGAACAGTTAAAGTGACTTCAGTGTTTTCTTTACCACGAACATCTTTGACAACTTCATCTAATGC
TTTACCTTTAATTGATTTTCCATTTACTTTAGTAATGACATCTTTAGGACGAATGCCAGCACGTTCTGCTGGAGATCCCTTCATAGGACTAGTA
ACCATAATTTGATCATTTTTCTTTTGCATTTCTGCACCAATACCTACAAAATCACCTGAAACACCTTCATTAAAGGATTTCGTTTGTTCTTTTGT
TAAATATTCAGAATAAGGATCTTTTAATTCTTTGACCATGCCATCAATTGCAGCTTTACTTAACTTGTCAGAGTCCTGTTTTTTGTAATAATCA
CTATTTAAGATTTTATACACATTTTCAATTTTATTTAAGTTTGCTTGATCAGTTTTGTTTAAACCACTTATTTTTTGATTTATAAAAATATATGCA
ACAACTGTGATGACAGCTGTTATTAGGATTGTACCAATTAATATTGATATGAATTGCCAACGTTTTAAGTGAACGCGTTTCGATGAATTAGT
TTCTTGGTCTTGATTGCTTGTTGCAATTTCAGCGCGTTCATCATCGGATGAAGATGTGTGTTGCTTATCATCCAT

Gene: yozE (putative RNA binding protein)

Position: 1410525 to 1410746, length: 222 nt, orientation: REVERSE

Perfect match to: (N315-BA000018-[1429279:1429500:r], highly conserved allele)

Sequence:

TTAAAATTTTAGCCATTCCGTATATTCTTCATATAAATCATCAAATACAGACATTGGCAATGTGAAATCACCATGTGTCTCAATATAATCAGA
CAGTATGTTAAAATCATCATCGTGTTTTGGGAAAGCAAGATCGTCAAATATCTCTTCTGCTAGACGACCTTTATCGTCGTGTCGACCACGAA
CTGTCATGACAAATTGATAAAACGAATAGTTTTTCAT

Gene: crr (glucose-specific phosphotransferase system IIA component)

Position: 1410746 to 1411246, length: 501 nt, orientation: REVERSE

Perfect match to: (COL-CP000046-[1471380:1471880:r], highly conserved allele)

Sequence:

TTAGTTCATTGTCACATCAATCACTTTTGTTTCACCTTTAATCACAGCATTTTCATCATAAATATTAATTGAAGCTGCTTGATCAGTGTTAGTA
ATTATAATTGGTGAAATTACAGATTTAGCGTTATTATTAATATATTCAAGGTTGAATCTTACTAATGGATCTCCGACGTTAACTTCGTCACCA
CTAGACACTAACACTTCAAATCCTTCACCGTCTAATTGAACTGTGTCTAAACCGATATGAACTAATAATTCTAATCCGTTATCTGCTTTTAACC
CAATCGCATGCTTAGTTGGAAAGACATTGTCAACACGTCCTGCAATTGGAGACACAACTTCTCCTTCAGTTGGATTAATACCAAAACCTTCG
CCCATCATTTTTTGTGCGAATACAGGATCTGGAATATCTTCAATTTTCACGAATTCTCCAGTTAATGGTGCATAAATTGCGATATCTTTCTGAA
CTTCTTTGCCTTTTCCGAATAATTTTTTAAACAT

Gene: msrB (peptide methionine sulfoxide reductase B)

Position: 1411258 to 1411686, length: 429 nt, orientation: REVERSE

Perfect match to: (MW2-BA000033-[1433566:1433994:r], highly conserved allele)

Sequence:

CTACTTATCAAAATGTGATATTAAATCGCCATAACCCAATTCTTCTAACTTTTCATATGGAATAAATTGAATTGCAGCGGAATTGATACAGTA
TCTTAAGCCGCCACTTTCTTTAGGTCCATCATTAAAGACATGTCCTAAATGACTATTTGATTCTTCTGAACGCACTTCAGTTCTCAACATACCA
AATGATTTGTCGACTAATTCTATAATTTCATCGTCATCAAGCGCTTTGGAAAAGCTAGGCCATCCACATTCAGAATGAAACTTTTCTTCAGAT
GTAAATAAAGGTTTACCAGAAATTTTATCTACATAAATTCCTTTAGCAAAATGATTCCAATATTCATTCATAAATGGTGGTTCAGTGCCGTTT
TCTTGTGTAACAATATATTCTATATCTGTTAGTTCACTTTTATCTTTTTTAAGCAT

Gene: msrA2 (peptide methionine sulfoxide reductase A2)

Position: 1411679 to 1412212, length: 534 nt, orientation: REVERSE

Sequence:

TTAAGCATTTTGATTCCCCCAATGTGATTCTATAAACGCTTTTCTACCTGAACCACGTTGATATTGGTAATAATGTACCGGGTTCTTTTTGTAA
AAATCTTGATGGTAGTCTTCAGCTGGATAGAAATTTTTATATGGTTTAATAGGTGTAATCACTGGTTTCTTGAAAATACCTTGTTCATTTAATT
GTTGCTTTTTAAACTCAGCAGCCTTTTTCTGATGTTCATCATGATAGAAAATGACTGGTTGATAGCTTTCGCCTCTATCGAAAAATTGCCCTT
GATCATCAGTTGGGTCAAATGTTTTGAAATATATGTCTAATATATTTTCAAAGGAAGTAACCTCTGGATCAAACGTAATTTGTACTGCTTCGA
CATGGCCGGTTTGATTCGTACATACCTGTTCATAAGTTGGGTTGTCAACATGACCGCCACTATAACCAGATACGACTGACTTGATGCCTGGA
TATGATGTAAATGGTTTAACCATGCACCAGAAACATCCTCCTGCTAATGTTGCATATTCTTTTGTCAT

Gene: degV2 (DegV domain-containing fatty-acid-binding protein)

Position: 1412298 to 1413137, length: 840 nt, orientation: REVERSE

Perfect match to: (N315-BA000018-[1431052:1431891:r], highly conserved allele)

Sequence:

TTACTTCTTAAGGACTACGAGGCCAATCGCACCTTGACCAGTATGTGCAGAAATAACTGGTGTAGTTACATTTATATCGTAATTATTCACATG
AAAAGCTTCATTAAAAACTTTCTTCAATTTATCAACATATTCAATGACGTTAGCATGTGCGACACCAATGGATTTGATTTCATGATCTCCTATA
AATTCAGCAATTTCCTTTTTCAAGTATTGGATACTAGAATTTTGAGTTCTCGCATTGTGCACAAGCTCTAAGCGACCATCATCTAGTGTACCA
ATTGGTTTAATTTTCATAAGATTACCAATCAAACCTTTTGTTTTACTAATTCTGCCACCTTTAATTAATTGATTCAATTGCCCTATAACTACAAA
TAATTTAATGTTTTCTCTTAAATGATTTAACTTTTTAACTATTTCAGAAGTTGAGACACCTTCTTTTACAAGCTCTACTAGGTGTTGTATTTGAT
ACCCTAAACCAAAAGAAATAGATTTTGAATCAATAACAGTTACATTAGCATCTACCATTTGACTTGCTTGGTAAGCAGTGTTATATGTACCAC
TTAATCCTGAAGAAAGATGAATACTTATGATTTCAGAGCCATCTTTTCCTAGTTCTTCATAAGCAGATATAAATTCACCTATGGCTGGCTGAC
TTGTCTTTACATCTTCATCATTTTCAATATGATTAATAAATTCTTCTGATGTAATATCTACTTGGTCAACGTATGAAGCTCCTTCAATAGTTAAA
CTTAAAGGAATTACATGAATGTTGTTTGCTTCTAAGTATTCTTTAGATAAATCGGATGTTGAGTCTGTTACTATAATCTGTTTTGTCAT

Gene: folA (chromosomal dihydrofolate reductase)

Position: 1413152 to 1413631, length: 480 nt, orientation: REVERSE

Perfect match to: (11819-97-CP003194-[1463530:1464009:r], highly conserved allele)

Sequence:

TTATTTTTTACGAATTAAATGTAGAAAGGTATGTGGAATTGTATTTTTCTCATCTAGTTTACCTTCAACTGAAGAGGCAACTTCCCAGTCTTCA
AATGTATAAGGTGGAAAGAACGTATCACCACGGAATTTACCTTCAATAACAGTAATATACATGTCGTCCACTTTATCAATCATTTCTTCAAAT
AATGTTTGCCCTCCAAATATGAAAACATGGCCCGGTAGTTGGTAAATATCTTCAATAGAGTGAATTACATCAACGCCCTCTACGTTGAAACT
TGTATCTGAAGTAAGTACAACATTTCGACGATTCGGTAGTGGTTTACCAATCGATTCAAATGTCTTACGACCCATTACTAAAGTATGACCTGT
TGATAATTTTTTAACATGCTTCAAATCATTTGGTAGGTGCCAAGGTAATTGATTTTCAAAACCAATTACTCGTTGCAAGTCATGTGCAACTAG
AATGGATAAAGTCAT

Gene: thyA-chr (thymidylate synthase)

Position: 1413831 to 1414787, length: 957 nt, orientation: REVERSE

Sequence:

CTACACTGCTATTGGAGCTTTTATTGCTGGATGTGATTTATAGTCAACAATTTCCAAATCTTCATAATTTATGTCGAAAATAGACTTGTCACTG
TTAATTTTTAATGTTGGAGGATTGAAGCTTTCACGTGCTAATTGTGTTTGAATCGCATCAATATGATTTGAATAAATATGTGCATCTCCAAAT
GTATGCACAAATTCTCCTACTTCAAGTCCACATTCTTTGGCAATAAGGTGTGTCAATAAAGCGTAGCTTGCGATATTAAATGGCACACCTAA
AAAGATATCTGCGCTACGTTGGTATAACTGGCAACTTAACTTACCATCTTGGACATAAAACTGGAACATGGTATGACAAGGCGGAAGTGCC
ATTGTATCAATTTCTGTTGGATTCCATGCAGATACGATGTGTCGTCTTGAATCTGGATTATGCTTAATTTGTTCAATTACTGTTTTAAGTTGAT
CAAAATGATTACCATCTTTATCAACCCAATCTCGCCATTGTTTACCATAAACATTTCCTAAATCACCGAATTGCTTCGCAAAAGTATCATCTTC
AAGAATACGTTGCTTAAATTGTTTCATTTGTTCTTTATATTGTTCGTTAAATTCAGGATCACTCAATGCACGATGCCCGAAATCTGTCATATCT
GGACCGTTATACTCGTCTGATTTTATATAATTTTCAAAAGCCCATTCGTTCCAAATATTATTATTATATATTTTAATAAGTATTGGATGTTTGTATC
TCCTTTAATGAACCATAATAATTCGGTTGCTACTAATTTAAAAGAAACTTTCTTTGTCGTTAATAGTGGAAATCCTTTAGATAAGTCAAAGCG
AAGTTGATGACCAAATTTCGAAATCGTACCTGTATTTGTGCGATCATTTCGTGTATTTCCTATTTCTAAAACTTCTTCACAAAGACTGTGATAT
GCTGCATCAAATGAATTCAACAT

Gene: cvfC4 (conserved virulence factor C operon, protein 4)

Position: 1415211 to 1415648, length: 438 nt, orientation: REVERSE

Perfect match to: (N315-BA000018-[1433965:1434402:r], highly conserved allele)

Sequence:

CTATTTACAATTTTCGTCAAAGGCATCCTTTAAGTCCATTGCAATGTCATTAATATCTCTACCTTCGATAAATTCTCTAGGCATAAAATAAACT
AAATCTTGACCTTTGAATAAAGCATACGAAGGACTAGATGGTGCTTGCTGAATGAATTCTCGCATTGTAGCAGTTGCTTCTTTATCTTGCCCA
GCAAAAACTGTAACTGTATTTGTAGGTCTATGTTCATTTTGTGTTGCAACTGCTACTGCAGCTGGTCTTGCTAATCCAGCTGCACAGCCGCAT
GTAGAGTTAATAACTACAAAAGTAGTGTCATCAGCATTTACTTGGTTCATATACTCCGATACTGCTTCGCTCGTTTCTAAACTTGTAAAACCA
TTTTGAGTTAATTCGCCACGCATTTGTTGCGCAATTTCTTTCATATAAGCATCATATGCATTCAT

Gene: cvfC3 (conserved virulence factor C operon, protein 3)

Position: 1415664 to 1416788, length: 1125 nt, orientation: REVERSE

Perfect match to: (N315-BA000018-[1434418:1435542:r], highly conserved allele)

Sequence:

TTAAATTGTTCTGTTTGCCATTTGTTTCCATACTGAACCAAGTGCCTCATCTCCGTTTTCAATACGAGATATGGCCATTTCAATTTGTAATTTA
ACTTCAAACGCATTGTCATTAATATGGGCTTTTAGTGCGGGAAGCTGCTCTGCATTACCTTCATCAAAGATAAACATAGCAGCACGCCACCT
AACGATTTTCTGTGGATCATCTAATAGTAGCACCATTTCTGGTAGTGCCTCTGGATACCCTAAATCGCTTATGCAATCCCCTGCTGTTCTTCTT
ACAGCAGGACTTTTATCACGAAGCCCCTTATATAAATACGGTAAAATTTCTTTACTTTCAATCATACCTAATAATACAATCGCTTGACGTCTA
ACCGGTACTTTTTCATCAGATAAAGCTAAATCAAGTAGCGGTATATCTTCAAAAGTCGGCTTTGGAAAATGGTTTAACATTCGTAATCGAGT
CTTCCAATTATCAGTTGCATGATATTCATCCAAAGAGACATGTCGATAGAAATGATAATTATTAGTTGCGTGATTTTCTTCTAAAGCATGTTT
TACCAATACGGGTAACTGTGATTCTGGATAGGTAGCTAGCACTTCTTCTAGGACACCATCCATTACTTCTTCAATATTTCCATAGCGATTTCC
TAAATCTAGCCATTTACGCATAAAAACAATATTGTCATGTGCTGTTTGCGCTTGAGTCATATGATCAACATATGTTTGTGGTAATTGTTCTCTT
AATTCTTGGTCAGCAGAAGTTAGCTTAATTTGATACGGTATACCCTTAAAAGTTAATAATTCAGCTTTAATTTCACCAAAATGATTGTCAATT
TGAGGTTCATTTACAGATTCTAAAACCTTATTCGCATCAGAAAAAGCAGCTTTAATATCAGGTAATATGACTTCCCAATCAGCTTTTGGTGCC
TTATCAACAGCTAAGAAGTTCATGACATGAAAAATGGAAGTGATACCATCTATAGATAACAACTGATTTATAAATCTTGGTTGTGTTTCTTCT
ACTTTTTTATAAGTATTAGATAACTTGTCTTCTCTTGTATATGACAAAACAACTTTCATTGTATTTGGACTTGGTGTTGGCTCTATACGTAAAA
TTTCCAT

Gene: cvfC2 (conserved virulence factor C operon, protein 2)

Position: 1416826 to 1417077, length: 252 nt, orientation: REVERSE

Perfect match to: (N315-BA000018-[1435580:1435831:r], highly conserved allele)

Sequence:

TTATTCGAATACAGCCTCTACTTTTGGCAATACTGTTTCCCAATTTGCGTCATTTTCTTTATCTACTGAAATAAAGTCCATAACATGGAAAATT
GATTTAACGCCTTCAACCTTTAAGATGTCATTAATAAATGCTGGCTGTGAATCATCAACTTTAGTATACGTATCTGATGTCATACCTTCTCTGC
TTTCACTAAGTGTAATCTTCATTGTGTTGTGGTTCGGTGTTTCTGATATAGATATAATTTTCAT

Gene: cvfC1 (conserved virulence factor C operon, protein 1)

Position: 1417089 to 1417283, length: 195 nt, orientation: REVERSE

Perfect match to: (RF122-AJ938182-[1401344:1401538:r], highly conserved allele)

Sequence:

CTATTGAAGTTCATTTCCTAATTGTTTTATTTCTTTCCCTATTGAACACTCATTGATACAAAAATGATGCGCCTTCGTTTTCCCCTCTAATTTAC
GCAGACGAGTTTTGATTGGACATTGATTGCAATAAGTATTCATTAAATCATCTATTTTCGCGAGTGCATTTTGCTCTGAAAAAGTTAAAATAC
GCGACAC

Gene: Q5HFZ1 (putative membrane protein)

Position: 1417528 to 1418232, length: 705 nt, orientation: FORWARD

Sequence:

ATGTATAATGAAATTTTAGGACTAGTTACGTTTATTGCAACGTTCGTACTTATGGTACTTATGTATCGCTTTTTTGGTAAACAAGGTTTAATT
GCATGGGTTGCAATTGGCACAATCATTGCCAACATACAAGTGATTAAAACTGTAGAAATCTTTGGTATTTCAGCAACTTTAGGTAATGTCAT
GTTTGCTTCTATTTATTTAGCAACAGATATATTAAACGATATTTATGGGCGTAGAGTTGCAAAAAGAGCAGTTTGGTTAGGCTTTTCATCAAC
ATTAATTATGATTATTGTTATGCAATTGTCATTACATTTTATTCCTGCACCAGAAGATATGGCACAAAAAGCATTACACGCAATCTTTGATGTT
GTGCCACGTATTGCTTTAGGCTCAATCGTCGCATATATTATTGGTCAACATATTGATGTATTTATCTTTTCACTAATTAAAGAAGTATTTAGTT
CTGATAAAACGTTTTTCATCCGTGCATATGGTAGTACATTTTTAAGCTCAATAATTGATACAGCTTTATTTGTAGCTATCGCTTTTATCGGAAG
TTTACCTGGTACAGTTGTATTTGAAATATTTATTACAACTTACGTATTAAAATTAGCTTCAACAGTTTTCAATGTACCATTTGGATATATTGCT
AAATCATTTTATCGTAAAGGTAAGATTGATAAGTTAGATCAAGGCTATTAA

Gene: SIRU01 (staphylococcal interspersed repeat unit 1)

Position: 1418315 to 1418483, length: 169 nt

Sequence:

CCAACTTGCATTGTCTGTAGAAATTGGGAATCCAATTTCTCTTTGTTGGGGCCCATCCCCAACTTGCACATTATTGTAAGCTGACTTTTCGTC
ATCTTCTGTGTTGGGGCCCACACCCCAACTCGCATTGCCTGTAGAATTTCTTTTCGAAATTCTCTGTGTTGGGGCC

Gene: repeat_nySAgamma (repeat element)

Position: 1418373 to 1418543, length: 171 nt

Sequence:

CCAACTTGCACATTATTGTAAGCTGACTTTTCGTCATCTTCTGTGTTGGGGCCCACACCCCAACTCGCATTGCCTGTAGAATTTCTTTTCGAAA
TTCTCTGTGTTGGGGCCCACACCCCAACTCGCATTGCCTGTAGAATTTCTTTTCGAAATTCTCTGTGTTGGGGCCCC

Gene: tx_universal2 (rho-independent terminator)

Position: 1418548 to 1418586, length: 39 nt

Perfect match to: (Strain_21331-AGTV01000040-[104403:104441], allele observed in CC398)

Sequence:

TAGAATTGAAAAAAGCTTGTTACAAGCGCATTTTCGTTC

Gene: rnhA (ribonuclease HI)

Position: 1418831 to 1419232, length: 402 nt, orientation: FORWARD

Perfect match to: (11819-97-CP003194-[1469209:1469610], highly conserved allele)

Sequence:

ATGGCGAAAATAAATTTTGATGCTGCGACGAAAGGAAATCCAGGCATAAGTACATGTGCCATTGTAATCAAAGAAGATGAGCAGCATTAT
ACATATACACATGAGTTAGGCGAAATGGATAACCCACACTGCAGAATGGGCTGCTTGTATCTATGCATTGAAACACGCCCGTGAATTAAACG
TTAGCAATGCGTTGTTGTACACTGACTCTAAGTTAATTGCGGATAGTGTTAACGCTGGTTATGTAAAAAATGCAAAATTTAAACCTTATTTTG
ATCAATTGGAAATATTTGAACAAGATTTTGATTTATTATTTGTTAAATGGATTCCGAGAGAACAAAACAAAGAAGCGAATCAACACGCACA
GCAAGCATTGTACAAATTAATTAAAAAGAACAAATAA

Gene: ebh (cell wall associated fibronectin-binding protein)

Position: 1419562 to 1450934, length: 31373 nt, orientation: TRNC-RVRS (no stop codon)

Sequence:

ACATCTTCTTCATCTTCTTTACGACGACGTTTTGGTAAGAAGAACGGCGTATGTTTAACTTTATCGAGTGACTCGCCATTATTTAGCGAATCT
TTTTCTTCAACAGTAACATCTTCTTCATCTTCTTTTCTGCGACGTTTCGCAAATAAAAGTGGTAAATGTTTTGTATCGTCTAAAGTCTCTTTTAT
TGAATCTTTATTATTATCTCTTATTTCTAATTCTTCCTCTTCATCTTCTTTACGACGACGTTTGGCAATGAAGAACCAGAAACTAGCTAGTAAG
CCAGAGATACCCACCACACCAATAGCGTTTTTAATAACATTACCGAAATTATTACTAAAGTGACGCATATGAAGTGGATCAATGTCATCATC
TTCATCAATTTTCTTTTTATGTCCAATAGTCGAACTGTTAAATGGATGATTTGCAGTTCCATAACCAATTGTTAAATGGCTATTAGATTCATTT
CCAGTACTTGACGATTGTTTAGCACGATCAGATATTACAATTTGTACTGCTGAAATTCGAGCAATACCATTGTTCAATGCAGCCTCAACTTGC
TGTAATGTATGCGCATTATTAATATCTCTAATTGTTTCAAGCACAATTTCGTTAATTTGATTCATTGCTGCTTGTTTTTCATCAGTTGTGCCAAT
TTCACTATTTCGAATACTATTTATTTTTTCATTTGAAAAGTCTTTAATTCTACTAATTGCTTCTTGCTTGCGTTTAGCTAGTTCTTTTGCTGTTGG
ATTAGCTGCTTTCATTTGAGCTTTAAATTGTTCCAATTGCTCACTTATTTCATCGATGCTTTGCGCACGTTGAATCGCTTGAATTGCTTGTTCTT
TTAATTGATTTAACTTAGCAATAGCTTCTTGCTTCTCTTTATCTGTTAGATCAGTACGAGCTTTGATTTCATCAATCATATGTTGAATTGCATCT
TCAATCGATTTAATTGCATTTGATTTTGCTTGTTCAATCGTAAATTGTTCTGGATTAAATTGTTCAATATGCGCTTGTTCTTGTTGTTGAATTTC
TTCAACTGAATGAACATCAGGTGCATTGTTAATATAATCAATTGCTTGTTGTTTAAGCTTATTAACTTCTGCAATTGCAGCTTCTTTTTGTTCC
AGTGTTAATGTCACACTATTATTGATTTCATCAATCTTTTGTTGTGCCGCATTTTCGATTGATTCAATTGCCTGTTGTTTGACTACTGACAATTC
TTTTTCTACAACTTTTACAGGAACATTAACAATCACTTTTGATCCATCAAGCAATGTAACTTCAACTTTTGCTGTTAAGCTATCAGAAATCGTT
GCAGTTGATGGTGTATCGATAACTTCTGCTGAAAGCTGATCAATTAAGTTTATGTGTGCAAGAATATCTTGTTCTGTAATGATGTCATCACG
ATGTACAATGAGTTCACCATTAACTAGGATTTGCTCAGGTGTTGCTTCAAAAATTTCATTTACAGCAGGTTGTTCATCAACCTCCCATACATG
TGTATTTCTAATGTCATCTAAACCTAAGTTTAATCCTCGATTTAATTGATCTATAGTTTGAGCATTCTCAACGTTTTGTAGTGCTCGTTTTTCAG
CTTCGTCAATTTCTTTGAGCGCTTTTGCTTTTTGCTCAGGTGTTAAATCTGGATTTGAATTGATTTGATCACGCTTCGCATTAGCTAAGGCTTT
TATTGCATTTTTCGCATCTTCTTTAGCTTTCACTAAATCTTTGATGTCTTGTAATGCTTGCGCAAGTTGTGCTTTTGCCTGCTCAATTTCTTCTTT
AGTCAGCGCATTGTTAATGTCGTTATGACCTTGTTGAAGTATTTGATTAATTCGATCTTTAAGTGCTTGTTTTTCCTTATCTGTTAGATTTGGA
TTTTGATCGATTTCGTCAATTAAAGCTTGTACACGTTTATCAATATCTTGTTTCGCATTTTCTTTAGCTTTCACTAAATCTTTGATGTCTTGTAA
TGCTTGTGCAAGTTGTGCTTTGGCTTGTTCAATTTCTTCTTTTGTCATCGCATTGTTAATGCCGTTATGACCTTGTTGAAGTATTTGATTAATTC
GATCTTTAAGTGCTTGTTTTTCCTTATCTGTTAGATTTGGATTTTGATCGATTTCGTCAATTAATGATTGAACTTGCTTATCAACGTCTTTATTG
GCATCAATTTTAGCTTTTGGTATTTCATTGGCATGCACTTGTTCAATCGCGTGGTTGCCTGCTGTTTGAACTTGAGATACAGCCTGATTACTT
GTTGCTTTATTAATGTTGTTGATGATGCTGTTTGCCAATTCTTCTGCTTTATTTTTCGCAATATGCTTGTCTTGATCCGTCGCATTTGAAGCTTC
GATTTCTTTTAGCTTATTAGCTAAAGCTTGATTAATAGATTGAATTGCCTTGTCTTTAGCATCTTGTAGTCGTTGATCACCATTAAGATTATGG
ATTGCATCATTGACTGTTTGAATTGCGCCGTTAATATCATTCACATTTGTGTTATCACTATTTAGCAATGTATTTGCTAGACGTTTGGCATCAT
CGAAGTTTGTTTTAGCATTATCGTCAGCGTTTTGGTAATTGACAGTTTGCTCTGCATTTGGAATTTCATTGTCAACTAAATGTTTCAATGTTTC
CATTGCATCATTTAAGTCAATTTGATTATTAACAATATCTGTTACATCTGATACAGTATCGGCATTGTTAATTGCTTTATGTGCAAGATCTTGT
TGCTGTTGATTTAATCCATTTAACGAATTAACAAACGCATTTGCTTTATCTTTGGCATTTGCAAGGTTTTGGTCTCCATTTAATGCATTTTGAG
CATCGATAATATTTTGTTTCAATTGCTCTGCTTCAGCTTTTGCAATTGCATTACCTTGCACTTTATCTAACTCATGTGCTGCATTCGCAATTGCA
TTATCATAATTTGCTTTCAAATTGTCATCTGCATTGATGTAATTTGTGCTGCTGATAACACCAAGGTGCTGTTCGTCAATATATTGTTGTACTG
CACCCATTGCATTATCTAATTTAGTTGCTCTATTAATTGCTCGAGATGCTTTATTTAGCGTTTCAGCATTATTAATTTGTTGGATTGCTAATTGT
CTTTGAGCATTGTTTAGATGTGTTAATTGATCCAATCTTTGTAATGCTTCAGACTTACGATTATTAAGTCTTTCTTCACCATTTAATGCCTGTTT
CGCTGCCGTTACTGCATCATTTAATTTGATAACTTGTTCTGCAGTTAAGTTTTGACCAGTCGATTTATCAAGTGCTTGTTTCGCTTTATCAACC
GCTTCATCATATACTTGTTTGACTTGTGTATCTGCATTGACATAGTTCGTGCTACCTTTCGTGCGTCCTTCATTGCCAGTGATTTCTTCTGACA
ATTGATCCATTGCACGATTAAGTGCCTTAGCCTCATCTACAATTTGTTGGATTTGATTTAAATCGTTTGATTGATCGATGCGACCTTTAAATCC
ATCTTGTTGAGCATTGTTTAATGCATTCAATTGGTCGATGTCATGTTTACCATTTGTTTTAGCAAGTGCTACACGTTCATCACCATTTAATGCT
TGTTTTGCATTTAAAATATTTTGAAGTGCTTGATCCACTTGTTGCTTATTCGCATTTTGTTTCAATACATTTTCAGCATCAGCAATCGCTTGTTT
ATAAGCCTTTTGCTTATCTGAATCTGCTTGCGTGTAATCAACTGTTTGCTCAACATTTGTATGATCATTAACAGCTTGTTGTAATTGATCCATT
GATTGGTTCAATTGCGTTGCTTGATCTACTAATTCAGCGATTGGTTGAAGTTGCGTCGCTTGATCAATGTTTTGTTTAGCAGTTGCGATTTGA
TCAGCATTTAAATGTGTTAATTGGTCAATAGTTTGTTTCGCTTGTGTTTTAGCTTCAGCGACTCTCTCATTACCATTTAACTCATTCACTTTTTC
TTGAAGCTTAGTTAATGCTTGTTCTACAGCGTCTTTATTCGCATTTGAACCATTAGTTGGATCTGTAATGCTTTGTGCAGCTTGTAACGCTTG
ATCTACTGCTTCTTTTTTATCAGTTGACGCTTCAGTGTAATTTGGTTGAGCCTTATCTGTATTCACTTGATCAACTTTATTTTTCAATGTTTCCA
TCGCGTGATCAAGTTCAGTAGCAGTTTGAACATGTTGTGCAACCTCTGTTCTTGTAGGCGCTGCATTTATAGCATCAGTTAAAGCTTGTTGTT
GAGCATGATTTAAGTTTGGTAATGCATTAACAGTCGTTACTGCTTGTTGTTGATCACGAGCAAGTTTTTGATCACCATGTAGGTTATCTTTTG
CAGTTGTTACTGCTTGTGTTAATTGTTCAACTTGTGCTTTATCGAGTGTTGGATTACCTGTTTGGTTAATTAAATCTTTGGCTTGTTGTACTGC
TGCTTGGTATGCATCCTTTTGTGGCTTATCTTCATTGATAAACTTGCTACCAGATTCTGTTTGTTGTTGATCTTGAATACTATTACGTAATGCT
TCCATTGCTTGATTAAGTGCTTTTGCTTCAGCTAATTTTTGTGCTACTTGATCACGAGTTGCTGCGTTGTTTATTTGACTTTCAAGTGCTTGAC
GTTGCGGATTGTTCAAACCATTTAATTGATTTAAATCAGTAACCGCATGTTGTTTATCGTCTGCAAGTTTTTGATCACCATGTAAGTTATCTTT
AGCTTGGTTAACAGCTTGTGTCAATTGTTCAACTTGTGCTTTATCAAGCGTTGGATTGCTAGTTTGATTAATCAAATCTTTGGCTTGTTGTACT
GCTGCTTGGTAAGCATCTTTTTGCGGTTTATCTTCATTAATAAACTTGCTACCAGATTCTGTTTGTTGTTGATCTTGAATGCTATTACGTAATG
CTTCCATTGCTTGGTTAAGTGCTTGTGCCTCAGTTAATTTTTGTGCTACTTCGCCACGAGTTGTTGCATTATTGATTTGATTTTCTAGTGCTTG
ACGTTGTGGTGTATTCAAGTTAGACAAGTTATTTAACGTTTCTGTTGCACGTTGCTTATCTTGAGCTAATTTTTGATCACCATGTAAGTTGTTT
TTAGCATCAGTCACTGCCTGTGTCGCTTGATCAATGATTGATTTAGCTAATGTAGGATCAGTTGTTTTGTTAATCAAATCTTTCGCGTGTTGT
ACAGCTTGCGTATAAGCATCTTTTTGTGCTTGATCTTCATTAATAAATTTACTACTTGCTTCAGTTTGTGGTTGATCTTTAATACTTTCTTTTAA

TGCTTTCATCGCTTCATTTAATGCTTGCGCTTGTGCAATGATTTCAGCCACTTTATCACGAGTTGTTGCATTATTAATTTGATTTTCTAGTGCTT
GTTGTTGTGCTGGTGTTAATTGATCTAAATGATTTAGAGAATTTCCAGCAGTTTGCTTATCTTGCGCTAATCGTTCAACACCGTCTAATGCAT
TTTTAGATGATGTTACTGCTTGTGTCGCACTTGTTACATTACCTTTATTGATAGTTGGATTATTTAATCCTGCAATGATAGACTCAGCATTTTG
AACTGCTTCATCATATGCTTGTTTTTTATTCGGTTCTGCATTGACATATGCACTGCTCATACGTGTTGCATCTTTGTCAGCAATACTTTGTTGC
AATGCATCCATAAGTTGATCTAATGCTTGTGCTTCAGTCAAATCTTGCTTAACTGCTGTTCTAGTTGTTTCGCTATCAATTAACGTATCTTCCA
TATGTTTTTGTGCATTGTTTAGATGTGCTAATTGACTAACCGTTTGTTTAGCATGATCTTTATCATTTTGTAATTTCACATCACCATGTAATGCT
GCTTTCGTTGTATTCACAGTTGCCGCTGCTTGATTAATCGCATTATTATCTAATGTTGCAGTTTGTTCATTGATAATATTATTTGCGGCTTGAA
CAGCTTGATCATAGTTTTGTTGCTCTGGTTGATCTTCGTTGATATATTTGCTATTTGCTTTAGTTGCTGCGTTATCTTGAATGCTCTGTCTTAAA
CCATGCATTGCTTGGTTAAGCGTATTCGCATTTTGTTCAATTTGATGAACTGCAGTTACTAAAGTTGCAGCTGTAACTTGGTCTTTTAATGCT
GTTTTTTGAGGATCATTTAAGCTTGTTAAAGCATCAATTGCTTGCGTCGCATTTGTTTTAGCAACTTGTAAATTATGATCTCCATTTAACGCTT
GAAGTGTTGTTTGAACTTTAGTAATAGCTTGTTCTACTTCTATTTGGTTCGCATTTTGACCAGTTTGTTTGTTAATCATTGCTGCTGCTTCATTT
ACCGCTGTATTATAAGCAGTTTGTTTATCAGTATCAGCATTGATGAAGTTACCACGTTGTTCAACGGCTTGTTGATCTGCAATCGCATTAATT
AAGTTACCCATCGCAGTATTTAAAGATTCTGCTGTTTGTTTGTGACCATTAACTGCAGCAATATCAGATGATTGCGTAATTTGTGATTGTAAC
TGTTGCTTTTGCGCATTATTCAAATGATCTAATTGATCAAGTCTATTGTTCGCTTCTTGCTTAGCTGCTGCAAGGTTTTCATCACCATCTAATG
CAACTTTCGTATTATTCACTTGTGTCGTTGCACCATTAACATCATCTGGATTCATCGTAGGCGATGTAGTTTGATTAATGATATTATTAGCAG
CTGTTACAGCTTGGTTATAGTCATTTTGTCTTTGCGCAGTTGCATCATGATAATCTTCAGATGCTAATGTCGTTTGATTATCCGCAACACTATT
TCTTAATAATTCCATCGCATGATCTAACGTTTGTGCTTTTTGATTAATCGTAGTGACATCTGTAACAAGTGGCGCTTGTGTAATTTGACTTTCA
AATGATTGTTTTTGTGCATCATTTAAGTGTGTTAAGTTCGCCAATTGTTGTTTTGCTTGTTGTTTTGCATTTGCCAAATTGTCATTACCATTTAA
TGCTTGTTTCGCTGCGTTCACTTCATTAGTGATGCTTTCAACTTGTGCTTGTACTTCATTAGTACCACTTTGTTTATTCAATAATGCTTCAGCTT
TTGCTACTGCCTGTTTATACGCATTTTGTTTGTCAGGATCTGCATTTAAGTAATTACCGTCCGCTAATGTTTGAGCTTTATCATTAATAGCTTG
TGATAATTGATCCATTGCATGATCTAGGCTTGTTGCTGTTTGTTTAACTTGGTTCACTGTAGCAATTTCAGGCGATTGATCGATTTGACCTTTT
AATGCTTGTTTTTGAGCATCGTTTAAATGCGTCATCCCACTTACGGCATCTTTAGCATTTTGCTTTTCAGTTGCCAATTTAGCTTCACCGTTTA
AGCCATTTTTAGCATCAGTCACTTGAGTTAATGCACGTGTTATTTCATCTGGGTTAAGCGTTGGATTTGTCGTTTGATTGATAATACCTTCCG
CTTGAGACACTGCATTTGTATATGCTGTTTGCTTATCGGCATCAGCATCATGATAGTTCTCACTTGCTTTGACAGTATCTTTGTTTGCAATACC
TTGTTTCAAGTTACTCATTGCTGTATTCACATTTTGTGCATTTTGTTTAGTTTGTTCAACTGTAGCTAACGCTTGTGCTTGATTGATTTGGTTAC
GTAATGCATCACGTTGTGGTTGATTTAAATCGCGTAACGTATCAAGATTTGCTAAAGCATCCTGTTTCGCTTGTGCTAATTTTTCATCACCGT
TTAATGCATCTTTCGCTTGATTCATAGTTGTAACTGCTTGTGTTACTGTATCAGGCGTCAATACAGGTGTTGGTGTGCCATTTGCGATTTGTT
GCGCTTGGTTAGCTGCATTGTTATATGCTTGTTGTTTGTCTTGATCTGCTTGTGTAAAGTCAACTGATTGTGGTACTTGACTGTTTGCTTGAA
TTTGTTGTTTCAACGTACCCATTGCATTATTTAACGCATCAGCATTTTGCTTAATAGCATTAACACCTGTAACAAGATCTGCATGTGATACTTG
ATCTTTTAATGCTGATTTTTGTGGTTGATTTAAGTTTGGTAAATGATCGATTGCTGTATTCGCATTGTCTTTAGCAACTTGTAAGTTATGGTTA
CCGTTTAAATCACCCTTAGCTTGAGTCACTTGTTGTAACGCTTGAGCCACTTGTTGTGGATCCACGTTTGCATTTGGTGTACCACTAATGATT
TGTTCAGCATGTGCTACCGCATTGTTGTAAGCATTTTGCTTATCAGTATCAGCATTGACAAAGTTACCATTTGCTTTAGTTTGGTCTTTATCTG
CAATACCTTGTTTCAATTGTGTCATTGCTTGATTTAAATCTTGTGCTGTTTGCTTAATCGTGTTTACATTTGCAACAGTTTGCGCAGCATCGAC
TTGTTGTTTTAATGCATCTTTTTGCGCTTGATTTAAATCATTTGCATTTGTAATTACTTGTTTCGCTGCATCTTTTGCACGTTGTACATTGTCATT
ACCATTCAATGATTGTTTCGCTTCGTTCACACGTTGCATCGCTTGTTCCACTTCAGTTTGACTTGCATTGCTGCCATTTGCTTTAGATAAAATA
CCTTCTGCATGTGAAACCGCATTAGTATAATCATCTTTCTTAGCTTGATCTGCATCAGTAAAGTTAATGCTATTTAATGTCTCAGTTTTATCTT
GTAATGCATTGTTTAAGTTAGTCATAGCATTATTCAGATTTTGAGCATTTTGCTTAACATTATTAACTGTTGCAATATCTGGTGCTTGTTCAAC
TTGAGTTGTTAGCGCTTGTTTTTGAGCATCATTTAAATGATCTAGTGTACCAAGTGTTGTCTTAGCATCTTGTTTGGCATCAGCTAATTTTTGA
TTACCGTTAAGTGCTTGATTTGTGTCATTCACTTTAGTTAATGCTTGTTGAATTGTATTTGGATCCATTGTTGGATTTGTTGTTTGTTTTAACA
ATTCTTCTGCTTTTGTTACTGCATTATCATATGCTGTTTGCTTGTCAGTATCAGCATCGTGATAGTTCTCGCTACCTTTAATTTGTGCTTTATTC
GCAATACCTTGTTTCAATTGTGTCATCGCGTCATTAAGCGTTGTTGCCGCTTGTTGAATAGCATTGACGTTTGGTACAAGTGTTGCTTGCGTG
ATTTGTTTGCTGTATTCATCACGTTGCGCTTGGTTTAAGTTTGGCAATTGATCAATAGCATGTTGAACATTTTGTTTCGCCGTTGCTAAGTTTG
TATTACCATTTAAATCATTTTTAGCTTGCGTAACTTTATTTAACGCTGCAGTAATTTCGCTAGGTGTAACGACAACATCAGGCGTACCACTAA
TTAATGCTTCAGCTTTCGCTACTGCTTGATTATATGCATTTTGCTTGTCAGAATCTGCATTGACAAAGTTACCATCAGCTTTTGTTTGTTCTTTA
TCTGCAATGCCTTGTTTTAATTGTGTCATCGCATTGTTTAACTCTTGCGCCGTTTGTTTAACATTGTTTACACCAGCTACAGTAGTTGCATTTT
GTACTTGTTGTTTTAATGCATCTTTCTGTGCTTGGTTAAGATCATTAGAGTTATTAATTAATGCTGTTGCTTCGTCTTTTGCATGTTGAACGTT
GGCATTACCATTTAATGCTTGTTTTGCTGCATTTACTTGTTGGATTGCTTGTTCAACTTCAGTTTGTGTTGCATTACCACCATTAGCTTTTGAA
ATGATATTTTCAGCATTTGTAACTGCAGTGTTATATGCTTGTTGTTTTGCTTGATCTGCATCAGTGAAGTTAACTGTTGCTTTCGTAGCATCTT
TATCAGCTATCGCTTGTTTCAAGTTACCCATCGCAGTATTTAAGTTTTGCGCATTTTGCTTAATTTGATTTGCTTCATCAACAGTATGCGCGCC
ATTAATTTGATTAGTAACAGCTTGTTTTTGCGCATTATTTAAGTTGTCTAATGTACCTAAAGATTGCGTTGCTTGTTGCTTCGCTGCCTCTAAG
TTTTCATTACCATTTAAAGCATTTTTAGACGTGCTTACTTGTCCAGCAGCTTGATTGATAACAGTCGGATCTAATGAAGGGTTTGTAGTTTGA
TCAATAATACCTTGTGCAGTTGTGACAGCATTATTGTACGCATCCTTTTTATTCGGACTTGCATCAGTATAATTTTGGTTTTGTTTTGTTGTCG
CATTATCTGCAATACTTTGACGTAATTTGTCCATTGCTGCATCAACATTGTTTGCTTTTTGTTCATTACCTTGTGCTTCTGCAACAGTAGTCGAT
TGTTGTACCAATTGTTTTAATGCCTCTTTTTGTACATTTGTTAAATGGCTTAAACCGTCAATTGCTGTATTTGCGTGTTGTTTAGCTTTTTCAAG
GTTTTGAGTACCATTAAGCGCTGCTTTCGTTGTATTCACTTGATTCATAGCTGCTTCAACTTGATCTTTAGGCACGTTTGTACCTGTAGATTTA
TTTAAAATATTTTCAGCATTACGAACCGCTTCATTGTATGCATTTTTCTTCTCTGGATCTGCATCTGCAAAGTTTTGACTACTAAGTGTAGTGT
CTTTATCATTCAAGCTATTTTTCAAGTTACCCATCGCTGTATTCAGTTCCTGAGCTGTTTGAATGGCTTGCGTTACTTCAGCTACAGTGTGCGC
TTGATTGATGTTTCCAGTAACAGCACCTTTTTGTGCATTGTTTAAGTTATCTAACGTAGATAATGTTTGGTTTGCATTGTTTTTAGCATCTGTT
ACTTTTCTATCACCATTCAACGCTTGTTTAGTAGTTGTTACAGTTGAAAGTGCTGCTTCAACTTGTGATTGGATCGCATTTGTACCATTTGCTT
GATTAATAATATTTTCAGCAGCAGTTACCGCATTGTTGTATGCTGTTTGTTTTTCTGGATCTGCATCTGTAAAGTTAACACTTTGTTTCACTTG

ATCTTTGTCTTGGATTCCGTTGATTAAACGTTCCATTGCTTGATCTAATTGTTCAGCTTTAGTTTTCACTTGATTTACTGTGCTAATATTTGGTG
CATTTGTCACTTGTGTATTTAAATCTTGCTTTTGAGCATTGTTTAAATGACTTAATCGGTTGATGTTAGCAGTTGTTTCTTGCTTTTTATTTGCT
AAGTTTTGCGCACCATTAAGCGCAGTTTTGTTAGTGTTCACTTGATTAGCTGCTTGTGTTACTGCAGATGGATCAAGTGTTGGCTGACTCGTT
GCATTAATGATATTTTCAGCACTTGTAACTGCTGTATTGTATGCATTTTGTTTATCTGTATCTGCATCAACATATGGTTGACTCGCTTTTACTTC
ATCTTTATTCGCAATACTATTTCGAAGTTGATTCATTGCATTGTCAAGATTTGTTGCATTGTCTTTCACACTTTGTACACCAGTTACTTGTGTCG
CGCTATCAATTTGACCAGTAATACTTTGTTTTTGTGCATCAGATAAATGTGTCATTTGACGTAATGCATCTTTAGCTTGTTGCTTCGCAGTTGC
TAAGTTTGTATCACCATTTAAACTTGTTTCAGCATTATTGACACGTTGTGTTGCCGCAGTAACATCTGCTGCATTTGTAATCACATTAGGTGA
ACCATTTACGATATTCTTCGCAGCATTATATGCATCAGTGTAAGCACCTTGTTTATCAGGACTTGCGTTAGTGTAATTACCACCAGCTACAAT
TGTGTCATGATCACCAATTGCTTGTTTTAATTGATCCATCGCATTATTAAGTGATGTAGCACTATTTTTCAGATCAGTTACACCTGATACGCGT
TGTGCAGCATTCACTTGTTGTTTCAATGCTGTTTTTTGCGGTGTATTCAATTGTGTCAAGTTATCGATAGCTTGTGTAGCTTCAGTTTTAGCGC
GTTGTAAGTTCGCGTCACCGTTCAAAGCATTTTCAGTAGACGTTACACTTTGAATTGCTTGTTCAACAGCTTGCTTATCTTTATTCGTACCATG
ATCTTTATTTAAGTAAGATTCAGCTTGTGTGATGGCATTATCAAATGCTGTTTTCTTAGTTGGATCTGCGTCAATGTAGTTTTCACTCGCTTTA
GTTTGATCTTTATTTGCGATTGCTGACTCTAATCGTTGCATTGCACCATCTAAGTCTTGTGCTTTCGTTTTAACACTATTTACACCGTTGACTGT
TGTTGCACCTTCGATTTGTGATGTTAAATCTTGTTTTTGTGCATTATTTAAGTGTGTTAATGTACCTAAGTTTGCTTTCGCATCTGATTTTGCTT
GTCTTACATTTGCATCACCGTTTAAAGCATTCTTAGCAGTCGTTACTTGATTTAATGCAGCTTCAACTTGTGCTTTTGTCATATTTTGACCGTTT
GCTTTATCTAAAATACCTTGAGCATTCGTAACAGCATTTGTGTAAGCATCTTTCTGACCTTGGTCAGCATCTGTAAAGTTCACTTGTTGTTTAA
CAGTGTTTTGGTCATTAATACCATTTTGAAGTTGACCCATTGCTGTATTTAATTCATTAGCAGTATTTTGTGCAGCAGTTACGTCTGCTACTGT
CGTTGCATTGTCAATTTGACTAGTTGCTGCAGTTGATTGCGCGTTATTCAAGTTACTATATGAAGCTAATGCACGTTTTGCTGTTTCTTTCGCT
TGAGCAACTTTATTATCACCATTCAAGTCGTTTTTAGCTTGATTCAATTGGTTAAGCGCTGCTTGAACTTGTGTTTCATTCGCGTTACTGCCAT
TTGCATTAGTAATTGCTTCTGCTTGTGTAACTGCAGTATCATACGCTTGTTGTTTTGGTTGATCCGCATCTGTGTAATTGACACTACGTTTCGT
ATTATCCTTTTCAGCAAGTGCATGTTTCAAATTACCCATCGCTGTGTTTAACGATGTTGCTTTATTTTTAGCCGCTGTGACAGCTGCAAGATTT
GGTGCTTGATCCACTTCAGCATTTGCCGCAGTTCGTTGTGCATTGTTCAAGTCTGTCAAACGACCGATATCTGTTTTCGCAGTTTGTTTTGCA
GCTGCTAATTTTTCATCACCGTTCAATGCTGACTTCGCACTATTAACTTGGCTCGCTTTTTGATTAATCGTATCTGGATTCATTTCAGGATTATT
CGTTGCACTAATAATACCCTCAGCATCAGATACTGCATGGTTGTATGCATTTTGTAGATCTGCATTCGCGTCTTGATAATCTTCGCTTGATTTA
GTCGTATCTTTAGAAGCAATACTTTGTCTTAATTGATTCATTGCTTGATTTAAAGTACCAGCATTTGCTTGGATGCCTTGAACACCAGCAACA
GTTGTACCGCGTTCAATTTGTTCTGTTAAGTTTGCTTTTTGAGCATTAGTTAAATGTGACATTGTCGCTACTTGTTGTTTAGCTGTGTTCTTCG
CTTCATTTAATCGCGCGTCACCATTTAATGCTGTTTTAGCAGTATTCACACGTTGTAATGCTTGTTCAACGGCTACTTTATTGTCATTTGAACC
AGCTGTTTTATCTAATAACGTTTTAGCTGCTGTTACAGCATCATTGTATGCAGTTTGTTTATCTCTATCTGCATCAGTATATTTCTGAGCTGCT
TTTGTAGCTGCTTCGTCATTAATACCACGTTGTAAGTTGCTCATTGCAGTATTTAATTCAGATGCCATTGTAGACACTTGATTTACACCTGCAA
CTGTTGTCGCACCTTCAATTTGTGATTTCAATGCTGCTTTTTGTGCATTATTAATTGATGTAAGGTTATTCAATGCATTTTTCGCAGTTGTCTTA
GCGTTCGCAACATTTTGATTACCGTTCAATTCGTTTTTAGCACGTTGTACATTTTGTAACGCAGTTTCGACACCGTCTTTTGCAGTATTTGGAC
CTTGTGCTTTATTTAAAATTTGTTCAGCTTGCGTCACTGCATTTGTATATGCATTACGTTTCGCTTCATCTGCATCAGTGAAATTAACACCTTG
CTTAATCGTATTTTGATCTTGAATACCATTTTTCAAGTTCGTCATAGCTGTATTTAATGCGTCCGCATTATTTTGCGCTTGTGTTACTTCATTAA
CATGCGTTGCACCTTCGATTTGGCGTTTCGCTGCATCTTTTTGTGCATTAGTTAAGTCACTTAAGCCATTCAAATGTTGCTTCGCATTTGTTTG
CGCAGTTCTTAAGTTTTCTTGACCATTTAACGCTTGTTGTTTAGCTGTCACTTGGTCTTTCGCTTGATTAATTTCTTGCGCATTCATTGATGGA
CTAGTTGTTTGACCAATGATTGCTTTTGCTGCAGTGACTGCATTGTTGTAGTCATTTTGTTTGTTTTGACTTGCATCTGTGTAGTTTTGATCTG
CTTTAATCGTTGCTTCATTCGCAATGCTATCTCGTAGACCTTTCATTGCAGTGTTTAATGTTTGTGCATTATCACGAACAGTTTGAACATTTGG
CAACGTCGTCGCTTGACCCACTTGTTCTTTAAGTTTTGCTTTTTGCGGACCATTTAAAGAAGTTAAACCATCAATTGCTGTGTTAGCATTTTGT
TTCGCTACTTCTAAATTATGATTACCGTTTAACTGCGTCTTCGCATTCGTTACTTGCGTTGCTGCATTTGTAACATCTGATGGTGTTAACGTTG
GTGTTGGTGTACCATTAACGATACTTTCGGCAGCTGTCACTTTTTCATCATATGCTTGACGTTTATTCGCATCAGCATTGACATAGTTACCACT
AGTTTTTGTGTCAGCTTTATCAGCAATAGCACGTTTTAAAGCTGTCATCGCAGTATTTATTTCAGTCGCAGTATGTTCAACACCATTTGCTGC
AGATACGCGTCCTGCACTTGTTACTTGTGCTTTTAATGCTTCTTTTTGTTTTGTATTTAAGTCCGAAGCGTTTGTAATCGCTGTGTTAGCAGCC
TGTTTCGCACGTTCTAAGTTTTGAATACCGTTTAATAAAGTATTTGCTTGTGCAACAGCTTGCATTGCTCTTTCAACATCTGCTTTAGGTGTAT
TACCGCCAGCTGTTTTATTTAAAATAGTTGCTGCTGCATTTACTGCTTGAGAATAAGCAGTTCGTTTAGCATCATCAGCATCTTGATAATTTT
GACTTTGTAACGTCGTGTCTTTATCACGAATTGATGTTTCTAATTGACCCATAGCACCATCTAATTGTTGCGCTTTGGCTTTAACTGTATTAAC
ACCTTCAACATTTGTTGCTTGTGTAATTTCATTATCTAACGCTGTACGTTGTGCATTATTAATGTGTGTTAATGTACCTAACGTTTGTTTCGCA
GCGGCTTTAGCTTCATTTAATTTCGCATCACCGTTCAACGCCGTCTTCGTACTGTTCACATTTTGTAATGCTTGTTCAACTGCTGCTTTGTCTA
CATTTTGACCACTAGCTTTTGTTAAAATTGCTTTCGCTGCATTTACAGCTTGATCATAAGCTGATTTCTTATTTGGCTCTGCATCTAGGTATTTC
TGAGTTTGTTTAGTTTGTGCCTCATCATTGATACCGTTTTGTAAACTATGCATTGCGTTATTTAATTCTGTTGCTTTGGCAGTTTCTTGATTTAC
ACCAGCTACTGTTGTTGCACCATCAATGCTACGCGTTAACGCATCTTTTTGTGCATTGTTAATTGATGTTAAGTTATTTAAGTTGTTTTTCGCA
GTTGTCTTAGCTTGAGCTAAGTTTTGCGCACCATTTAATGCATGTTCTTTAGTTGTCACTTGTGATGTTGCTTGCGTAATAGTATTTGGTTCCA
TCGTTGGGTTCGATGTTTGATTAATGATTGCTTTTGCTGCAGTAACTGCGCTGTCGTACTCGTTACGATTAGTTGGACTTGCGTCAGTGTAGT
TTTGACCTGCTTTAATTGTTGCTTCATTCGCAATACTATCTCTTAAGCCTTTCATCGCTGTATTCAACGTTTGAGAACTATTTTTAACAGTTTGA
ACACCTTCTAATGTAGTTGCACTTTGAACTTGTTCTTTTAATTTTGCTTTTTGTGCATTATTCAATTGTGCTAAGCCATCAATTGTATTATTAGC
ATTTTCTTTAGCAACACGTAAGTTGTTATCGCCATTTAATGCTGTTTTAGCCGCATTGACTTGTGAAGTCGCACTATTTACATCATTGACTGTT
AATGTCGCATTCGGTGTACCATTAATGATATTTTCAGCGTTAGTCACTTTTGAATCATACGCTTGACGTTTTTCTTGATCAGCATTGACATAAT
TACCGCTTGCTTTTGTTCTTTCTTTATCAGCAATAGCTGCTTTAAGTGCTGTCATCGCACCGTTCAATTCAGTAGCTGTATGTTGAACGTTATT
TGCATCAGATACACGTTGTGCATTGTTAACTTGTGTTTTTAATGCATCTTTTTGTTTTTGGTTTAAGTCATGTGCGTTGTTAATTGTGTTCGTC
GCATTTGTTTTAGCTTGAGCTAAGTTTTGTACACCATTTAACGCTGCTTTTGTTGTATTGACTTGATTTGCCATGCCATTAATCGCATTAGCAT
CCATATTTGGATTGTTCGTTGCATTAATAACACCATTTGCATTATTTACAGCAGTATTGTAATTATTTTTGTTGCTATCAGATGCATCAAGATA

ATTTTGACTTGTTTTCGTCGTATTATCATTTTGGATACTTGTACGTAATGCACCCATGGCAGTATTTAATGTATTACCTTTATCTTTAACACCAT
TTACACCAGCTACATTTTGCGCTTGTTCAACTTGATGCTTCAAGTTGTCTTTTTGTAATTGTGTTAAGTAAGGTAAACCATCAATCGTATTTGT
TGCTGAAGTTTTCGCATTTCTTAAGTTGTCAGCACCATTTAAAGCCGCTTTCGCTCTTGTAACTGCATTTAATGCATTATCAACGTCTGCTTTA
GATGTGTTACCACCAGTTTGTTTATTTAAAATGCCTTCAGCCGCTGTGACAGCTTGCGTATATGCATTTCGTTTTGATTCATCCGCATCAAGA
TAATTTTGATTTCTTAATGTCGCATCTTTATCATTGATTGAACCTTGTAAGCTATTCATTGCGCCGTCTAATGTATTGGCATTTGTTTTTACAGT
ATTAACGCCATCAACAGTCGTCGCTTGATTGATTTGGCCTTCTAAGTCAGTACGTTGTGCATTCGTAATATGGTTTAAAGTGCCTAAGTTTTG
TTTAGCTGCCGCTTTCGCTTCTGCCAGTTTTGCATCACCATTCAATGCATCTTTCGTACTTGTTACTTGTTGTAATGCACGGTCAACTGCTGCT
TTATCTGAATTTGAACCTGTTTGTTTATTTAAAATTGCTTTCGCAGCAGCTTCAGCTTGATCATAAGCAGTTTTCTTACTTTGTTCAGCGTCAC
GATATTTTTGAGTTTGTTTTGTTGCATTTTCATCATCAATACCATGTTGTAATTGACCCATAGCTGTATTAAGTTCATTTGCAGTTTGTTGGAT
ACTTGTTACATTTGCAACACGTTGCGCACTTGTCACTTGTGCTTTCAACGCATCTTTTTGCGCTTTATTTAAGTTAGTAGCACCATCGATGGCA
TTTGTTGCTGTTTGTTTCGCTTGCGTTAAATTATGTGTACCATCTAATGCATTTTTCGTTGATGTCACTTGTGTAGCGATTTGGTTAATTGCAT
TAGCATCCATATTTGGATTGCTTGTTGCATTAATGACACCATTAGCACTATCAACAGCATTGTTATAGTTTGTTTTGTTACTTCCTGTAGCATC
AAGATAGTTTTGGCCATTTTTCGTAGCTGTGTTATCTTGAATGCTATTTCTTAACGTACCCATAGCACCATTTAATGTGTTGGCACTTGATTTA
ACAGTATCTACACCTGCAACATTTTGTGCTTGTTGCACTTGTTGTTTCAATTTATCTTTTTGAGCAGTTGTTAAATGTTGAAGTCCGTTTATAG
TAGTGTTTGCATTTGATTTAGCTTGCTCTAAATTATGGTTACCATTTAGGGCACCTTTCGCATTAGCAACTTGTGACAACGCGTTTTCAACGG
CAGCTTTATCTAAATTTGATCCACTATTTTTATTTAAAATATTTTCTGCGTTCGCAACGGCTTGAGTATAAGCAGTTTTCTTATCTGAATCAGC
ATCATGATAGTTTTCACTTGCTAGTGTATTTGCTTTATCATTAATACCATTTTGCAAGTTAGCCATCGCTGTATTCAATTGTGTACCTGTATTA
GATACAGCTTCAACACCAGCTACAGTTGTTGCTTGATCAATTTTAGTTGTTAAGTCACGTTTTTGTGCATTATTAATTGACGTTAAGTTATTTA
ATGCATTTTTAGCTGCATTCTTCGCATTTGTAACGTTTTGATCACCATTTAATGCATTTTTAGCACTTGAAACATTTTGAATAGCCGCTTCAAC
ATCTTGTTTAGACGTATTTGCACCTTGCGTTTTATTCAGAATTGCTTCAGCTCTGCTTACCGCATTTGTATACGCATCACGTTTAGCTTTATCT
GCATCAGTGAAGTTAACACCTTGTTTAACTGTGTTTTGATCATGGATAGCTTGTTCCAAGTTGCCCATTTGCGTGTTTAACTCAGTTGCTGCA
TTTTTAGCAGCTGTTACCTCACTCACATGACCTGCACGGTCAATTTGTGATGAGATGGCAGATTTTTGGTTATTTGTTAAGTGTGATAACGTA
TTTAAGTTTTGCTTAGCAGTGTTTTGTGCATTTCTTAAGTTTTCAGCACCGTTTAAACCATTTTTAGCATTATTCACTTGTGTTGCAGCTTGGG
TAATCGCACTAGCATCCATAGTCGGATTGTTAGTTTGATTAATGATACCTTTCGCATTTGACACAGCGCTATTATATGTTGATTGGTTATTCG
GACTTGCGTCTGTATAGTTTTGACTTGCTTTGACTGTTGTTTCGTTAGCAATACTATCTCTTAAGCCTTTCATTGCATTGTTCAATGCTTGTCCA
TTTGTTTGAACAGATTGTACGTCTTCTAATCTATTGGCTTGTCCCACTTGTTCTTTTAATTTAGCTTTTTGAGGAGTATTTAATTGCGTTAATGC
ATCAATAGCAGTATTGGCGTTTTGTTTTGCTACACGTAATCTTTCGTCACCATTTAATTCTTGTTTCGCGCTGTTTATTTGATTAGCTGCAGCT
GTTACTTCTGAAGGTGTCGTAACAACCGTTGGCGTACCGCTAATAATATGTTCAGCATTGGTAACTTTAGTTGTGTAAGCATTTTGTTTAGTG
CTATCGGCGTTGACATATTTACTGCTTGCTAACGTATTCGTCTTATCTGCGATGGCATGTTTTAATGTGCCCATCGCCGTGTTCAGTTCAGTC
GCATTACGTTGTACATCTTGTGCATTAGATACGCGTTGAGCACCATTAGCTTGTGCTTTTAATGCATCTTTTTGATGTTGATTTAAATCAGAT
GCGCCATTGATTGCTGTAATCGCTGCTTGTTTCGCATTGTTTAAGTTTTGCGTACCATTTAATGCATGTTTCGCATTATTAACATTATTAAGTG
CTTGTTCGACTGCTGTTTTCGCTGTATTCGGTCCAGTTTGTTTATTTAAAATGGTTTCGGCTGCTGATACAGCTTGATTGTATGCATTACGTTT
TTGCTCATCAGCATCCAAGAAGTTTTGGCTCGCTAATGTTCCTGACTTATCGTTGATAGCCGTTTGTAAGTTACCCATTGCGCCATCTAAACT
ATTTGCACTTTGTTTAACAGATTCAACACCAGCTAAGTTTGTAGCTTGTGAAATTTGATTTGTTAAATCATTACGTTGAGCTGTAGTGATATG
CGTTAAAGTACCTAAATGTTGTTTCGCTGCGTTTTGAGCTGCAATTAATTTTGCATCGCCATTCAATGCATCTTTCGCAGTATTCACACGTTGC
AATGCTGCTTCAACTGCATTTTGCGCAGTGTTTGGACCATGTTGTTTATTTAAAATCGCTTTCGCTGCAGTAATAGCATTATCATACTCTTGTT
GTTTATTTGTATCAGCATCACGATATTTCTCAGATGATTTAACTTGAGATTCGTTGTTAATACCACTTTGCAAGCTAGCCATAGCTTGATCTAG
ATGTTGTGCATTTTGTTTTACAGTATCAACACCACTAACTCTTGTCGCACTACTAATTTGACTAATCAAATTGTTCTTTTGAGCATCTGTAATA
CTCGTTAATGTGTTTAAGTACGTTTTTGCATTTTGTTTTGCCGTAGCTAAGTTTTCATCACCGTTAAGTGCCGTTTTAGAACTATTCACTTGCT
CTGCTTTTTGTGTAATCGTACTTGGATTCATTTCTGGATTACTATTTGCATTAATAATCGTTTCAGCAGCAGCTACTGCGTTGTTATATGCTGT
TTGCTTATCATTATTAGCATCTACGTAATCTTCACTTGCTTTAGTCGCATCTTTGTTGGCAATACTTTGTCTTAACGTATTCATGGCTTGATCTA
ATGTATTGGCATTTGATTGAACCGTATGAACACCAGCGACAGTAGTACCACTATTAATTTGGTTTGTTAAATTCGTTTTTTGTGCAGTTGTTA
AATGCGTCATATTATTTAACTGCTGTTTCGCTGTTTGCTTCGCTTGATCTAATAAACGCGTACCATCTAAGTTATTTTTAGCAGAATTCACTTG
ATTCATCGCTTCAGTAACTTGATCTTTCGTTTTATTTTGACCATTTGATTTATTTAAAATATCTTTCGCAGCTTGTACCGCATTTGTGTATGCTG
TTTTCTTACTTGGTGTCGCATCTTGATAATTTTGACTATTTAATGTCGTTTGTTCGTCATTGATTGCACCTTGCAAGTTACCCATCGCTGTATTT
AAATCTGTACCTTGTTGTTTAACAGTATTTACGCCAGCAATATTTGATGCAGCATTAATTTTAGATTTAACATCTGCTTTTTGTGCATTATTTA
AATGCGGTAATGCATCAATTGCTCTTGCAGCATCTGTTTTAGATTGTGCTAATTTTTCATCACCATTTAATGCATTTTTATTAGAAGTAACAGC
TGAAGTTGCACGATTAACATCATCAACAGACATCGTTGGATTTGTTGTTTGATTAATGATTGTTTCGGCACTTGAAACTGCACTGTTATATGC
ATTTTGTTTAGCTGTATCTGCATCCGCATAATCTTCTGTACGTTTCACTTGATCTTTATCTGCAACAGCTTGTCTTAAGTTACCCATTGCACTAT
TCAAGTTTGTTGCATTTTGCTTAATTGTATTAACTGCATCAATTTGATGCGCACCATTAATTTGCGATTGTAAGTTTTGACGTTGTGCATTATT
TAAATTGTTTAAATGACCTAATGCAGTGTTCGCTTCTTGTTTCGCAGTATTTAACTTAGCTTCACCATTCAATGCATGTTCTTTACTTGTGACA
TTTGATAAGGCATTGTTAACATCACTTGGTGTTATAACTGGATGTTGTGCATTACCATTAATAATGTCATTCGCATGGTTGTATGCATTGTTG
TAATCATTTTTCTTGTTAGTATCTGCGTTGACATAATTATCACTTTGTACGACTTGGTTATGATTAGCAACGCCACGTTTTAGACCTGTCATTG
CAGTGTTTAAGCTTTGAGTTGTTTGTTTAATATTATTTACTGCTTGAACATTTTGCGCACTATTCACTTGTTGTGTTAATGCATTCTTTTGCGCT
TGGTTTAAATCACTTGCATGCGTAATCGCATTTGTTGCTTCTGTTTTCGCACGTTGTAAGTTTTGTTGACCATCTAAAGCATCTTTCGTCGATT
TAACAGATGCTGCTTTTTGGTTCACTGTGTTAACATCCATCGTTGGATTAGTCGTTTCTCCAATGACACCTTTAGCCGCATTAACTGCATTATC
ATACGCTTGTTTATTAGCTGGTGTTGCGTCAGTGTAATTTTGACCTGATTTAATTGTATTATTATCCGCAACACTGTCTTTTAATTTCGTCATC
GCAGTATTTAAAGCCGTAATGTTAGACTTAATTGTTTCAAGCGCAGCATGATTTTGAGCAGCATTAATTTGTTGCGTGAAATTATTTTGTTGT
GCTTGGTTTAAGTTAGATGCACCATGTAATGTTGTTAACGCTGGTTGTCTATCTAATGTTAAACCTTGACGTGCTGTATTTAATTGATCCTTA
GCTTGGTTTGCCTCTGCCACTTTATCATTGATATTTTGGACCGTTGGATTGCCATTCAACACTTGATTAATTTCAGTTAACTTTTGACGCGCTG

CTTGTAATTTTTGGTTGTACGCATTTAACGAAGCGGTCGTCATACCTGTTGTATCTGTTGGCTGATTAATGCTTTGTTCTAATTGCGTTTTCGC
ATTTTGAAGCGGCGCTTTATCTGGTGTTAAAGCTTGACGTGCATTATCCAAATCAGATTTAGCTTGATTTGCTGCAGACGTATTTGTATTAAT
TTGATCTACAGTCGGTGAACCTGCTAATACTTGACTGATTTGTTGAACCTTATTACGTGCAGCCGTTAACTTCGCATTATAAGCATTTACAGA
GTCTTGTGTCATACCAGTTGTACTTGTTTGCGTGTCAATACTATGTTGTAGTTGATTTTTCGCATTTTCTAAAGGTGCTTTATCGACTGTTAAG
CCATTACGTGCTTGATCAAGTGCTGTTTTAGCAGCATTTGTTGCTGTCACGTTTTGACGAATCGTTGCAACATCTGGATGAGATGCCAGTAC
GCGATCAATTTCTTGTATTTTAGTTCTAGCTGCTGAAAGTTTTTCATTAAATGTAGCAACAGATGCGCTTGTCATACCAGTCGTACTCGTTGG
CTGATCAATATCATTTTGCAACTGAGTTTTTGCAGTTTGTAATGGTGCCAAGTCTGGTGTTAATCCAGCAATTGCTTGTTTTAAGCTATTATAT
TTTTCTTCTACTTTTGTTTTTTCTGCGGCAATTTGTTGGTCTGTCGTGTCACCATTGTTAATAACATTTTGTGCATTTGTTGATTCTGTTTGACCT
GCACGTTTAGCATTTTCATATGCTTGGATTGATGATTGTGTCATACCATCAGTAGTTACTGATTTATTGATTTCTTCATCAAGTTTCGTCTTAG
CAGTTCTTAAAGCACTATTATCAGCTAAAGGTACTAATTGATTAATTGCTTGCGTTAATCGCTCATTGACACGATTTACATTTGTTAACGCAG
ATTGTACCTCTTGCACTGTTCTTATTGGCTTCTGAATGATAGCATTAGCGCTATTTTTAGCACTTGTTAAGTCACTTTGAAGTGCACGAATCG
AATTATTGTAAGCAGTAATACTTGCCGGCTTCTTACCAGTCGTTGTACCTGTGCGATTCAATTGTTGCACTGCTTGCTCTAAGGCATGTGTAT
CTGCAGTTAAATCATGTTTCGCTTGGTTTAATGCTGTTAATGCGTTATCAACACGATGTTTTTCATCTGAAATTTGTTGTGCAGTTGCATCGCC
ATTGTCAATAACACGTTGAGCTGCAGTTATTTCAGTTTCTGCTTCACGCTTCTTCGCATTATAGTTATCAATACTTTGTTGCGTCATACCAGCA
GTTGATGGTACTTGGTTCACAGAACTTTGTAAGTTATTTTTAGACGTTACTAATTGGCTATTATCTTCTTTATTTTGAAGTAATGCTTTAGCTT
GATCAATCTTAGTTTGTGCTGCACGAACTTTAGTTAGTGCGTCAGAAACTTGTTGTGGTGTTGCACGCTCATTATTAATCACTTGTTGTGCTT
CTGTTTTCGCAGTATTGATTTGTTGTTGCGCATTATGCATTGCATTATTGTACTGCGTAATTGTACCTGGCTTTTTACCTTCAGTGCTTACTGG
ATCATCTAAATGATTTTTAGCTGTGATTAACTCACGTTTATCCGCTTTTGTGAAAATGGACTCTTGTACTTCTTCAGTACTACCATCATTGTAA
GTTACTGTCACAGGAATCGTCGTTGTGCTACCACCAGCTAAATTAGTAGGCATTGCTGTGCCATTTTTAATCGTTGCAGTACGTTTATTAGCA
ACTTGAACTGCATTGTTAATTTCAGCTGCTGTTACATTTGAACCATAATCTTTCACAATTTCAGTTGTGTTGACAGTATGTGCTGCCGGTGCA
GTTAATGTACTTGGATTACTACTTACTGAGTGACCTGTACCTGCTTTCGGAGTAATTGTGATTGATGAATTTGGTTTTATAGTATTGGCATTG
AACGTCACTTTACCAGTTTGTGCATCTAACGTTACATAGTCAGGCTTATTCGCAATTGTCCATTGATTATTCTGACCACGAACAACATTAATT
GTCTTACTATGTTCTGCACCATTACCCACTTTTTCAGTGTAAGCAATATCCATTGCTTGTGTTGGATTAATTAAATGTCCTGATGGATTATTAG
GCGTGATATCAATATGACCATTTTGCCAAATCTTAGTAGTCGCTTGGTTCGGTTGTGGTGCGACAACTGTGAAATCATCACTACGTTGCTCA
TCACTGATTGTTTCACCACTACCTTGTGTTGCAACAACTTGAATTGTATCAGCAGGATTGAAAGTACCTGCTGCAACAGTAATACCATTATTA
GTTCCAGCAATACCTGCTACAGTTGTTGCAGATGCTTCTTTCACCCATGGACTTGTATTATTGCGACGTGTAAATGTTGTCACGACACTGCCG
TTACGTTTAATAACTAATTTATCAGCATACGTCGTTACGTTACCGGCATGTGTATTCACTGTTTGGTTTGCTCCAGGTGCAATTGTAATCGCTC
CTGCCGCTGTTTCAGTCACAGTTGGTTTCGCTGGTTGCACATCTTTTACTACAAATTTCGCTGGTAAAGATGTTGCAAAAGTATGTCCGTTAT
AGATGACGTCATATTTTGCGTTAACGACTTTAGCCATATTAGGTTTATTCATAGCTGCCCAGTTTGCATCGTTTGTACCAGTTGCTGCATTATT
CCATTTATACGTAAATCCATCTGTTGGTAAACCATTAGCGTTTTGCATATGTGCATATCCTGATGCTTGCGTACCACTTGCTAAAGTGCCACC
AACTGTTGTTGTATAAGTAGTTTGAGGGAATTCAAATTGATATACGTTCACAGTTACAGGAACTCGTTTAGCAGCTGAAATACCTGGATATG
TGACATCGACATTTAAATGTTGAACGCCTGCTTGCTGGTTATTTGGTTGTTGTCTATTTGCCCATGCTGCTGTAATACCATTCGTATTAGTATT
TGGATCAAATGTAATGTAATCAATAGCGTTTGTACCATGTGTCAAATTTTGACCTTTCACATCACGTGATGGCGCCTTAGCATTAGCAACTG
GATAAACTTTGACTGGAACTTCAACATTACGCGTACCTTGACCACTAGGTAATGTTACAACCGCAGTTTTATGAGTGTTACCGACTGTATTCT
TCCATGTATCTGGACTATCATGCCATGCAACCGTTGCCCCATGTGGCGGGTTTTGAATAAATCTTTCTACGTGTCCGAAATCAAAACCGTCG
CCACCTTTAATAAATACAGCGCCTTCAGTAGTTGCTTGTAATTGTGGTGTCACTGTTACTGTTGCACTGTCATTTGAATCAACAGATTCATTTC
TTGTTACATCTATCGCTCGACCATGTTCATCTTGAGTAGTGTACGTCACATTATTCATTGTGATAGAAGACCTTGCCTTAATCACACCATTTGG
TAACGCATCACTTACCGTTACAACTGCAGTATTACCACTACTATAAGTCGTGTTTGTAATCGTTAATGGCGTATTATCTGCTTTAAATAATTTA
ATAGATGAACTACTTAACACATTATTAATTTTAATTTGTTGGTTAGTAAGACCTGCTTTATAAGTCACAGAGTTGCCATCAATTCGCGGTGGA
TCTGGTTTAACTTTTGCTAAAATCTTGATAATTTGCGTTTGGTGATTATCATAAGTAACTCGAATATCCCTTGTTGAAATACCAACCACATCTT
TACTTGGTCCATATGAATTTGTAAATTGGAAATTCATACGTTGCGCATTTTGTGTCCAAGAATCATTAACTGGTTTAACATATTGTTTCGCATC
ATACACATCAGATACACCTGGGAACACAGCGCCTCTATTCGTCTCAAAGACATGCTTCGGTACTGTACTTACTACTTTATATGTTGCTGTTTT
CGTAATCGGCGTTGTTTCACCATCAATTAAGATATGTGCAGTTACTGTTATATCTTCACCAATACGTGTATTATCTTTATTTGGCGCTTGACCA
CTTACCCAAGTAATCGTCGCATCTGGAATGGCACTACCATTAGATAACTTAAAGTAATCTGATGCACTAGAACCATTACCTGCTGGGAAGTC
TTGGCCTTGTACAGTGTAATGCGAATGTGCAACGATTTTTGGAATGACATGCTTTACAGGTACAGTCACTGTTGATGTTGTTCCATCTTGATA
AGTAGCAGTTACTGTAACATTGGCATTATTTCCAGTACGACTGACATTACTAACTGTTTTACTAGTGATTTCATTCGCACTTGCTCTTGCATAA
CTTCTATTTGGTACTGTTTCAGTAAACGTTAATGAATTAATAATTGCTGTTTGATCAGCTTGTGATACTGTCGTATTATTCGAAATATTGGCAA
TTCTCACAGGATTAGCAGCCGTTGATGAAGTACCAACTCGATATTTATCACGCAAAGGTTTCACTGTTACATTGAACGAAGTTGTAGCTGTA
TTGCCGCTTGTATCAGTTGCTAATAAATTGATTGTCTTATTAGTTGCTGATGTCACATTTGGTGCCGTTGCAGAAACATGTTGATGGTTATTA
TCAACAGTACCTGTAATTTGTGATGTATTTGGTACAGTTACAGATTGCACACCAAAGTTATCATTCGCTGTAATAGTATTTGTAAATGTTTCA
CCTGAGAACACTTGATGATTAGTGTAATTACCTACTGAAATTGTAGGATTCACTAAGTCACTTGGTACAAAATAAATGTTAATAGCGTCAGT
AGTATTTCCTGTATTTTGATTTAAATGTTCAACATATTGTTTTGGACCATATGGCGTTAAGTATAACTGTGCTTTATAAACTGCATCACTTGCA
TTATGTGTAGAATTACTTTTTACAACGTGGTCAATTGTAAATGCGCCAGCACCATTAGCTGCCGGTTCGTTTACGTTAACAACAGTTGAGTTA
CTATGGTTTGCACGAGTATTTGAATTTGTAACAGGTTGCCCACCATAACCGTTTGAAGGGTTGATGATGTCTAACACTTGAATCACTTGACC
ATTCAACGTAAATTGACGTTGACCATCAGTTGTCGCATTTGATTGTGAATAACCAGTCGTTCTAAAGTTAGGTCTTCCGCCAGCTTCTGCTTG
TGTTTTTTCATTACCAGTAATATTATTACGCAATCCAGGCACTGTAGTAGTCGCTTTTAGCATCGTTAAAATATCATTAGTTGTAATTTGTGTG
CCTAATGTAGCATCATAACGATAAATTAAAGATTTATGGTCATCAGACCATGATAAACCACCATCTGTTGGTCTGTTTGCAATTTTTGCATTC
GTCCATGTCACTGTATAATCTTGCGGGAAATTAACCCAACGCAAGAAATTCATATTAGCTAGGTTTGACGCGAATGATTTCGTTAATCGACC
TTTATTAATATTTACTGTAATAGTACTTACATTAGCACCATTAGGTGTATTTGTAACTGAAATATCACCTTCGGCAAGTGTAATTACATCTCTA
TTTGCATTAATAAAAGCTTGTTTTACTTTTGAGATTTCATCAGTAGTTAAATCATATGTTGACGTACGATAGACACGTTGATCGCCTTCAGGT

TGTTTCACATCAACGATTTTTAAACTAATATAGAAATCTTCAGTGTCTTTATGATATGCATTTGAAGCAACTATGTGATATAACTGCGTGCCT
GCATTCATTTGACTAACTTCTTGTGCAGTTGGAATAGTTCTATCAGATGTAATATTTGTAAATGTGCCACTTGGAATAGCTTGTCCATTACTT
AAAGTAAAATATTGTTTAATATCACCAATATTAAATGATTGTCCTTTAGCAATCGTTACCGTTGCTGTTTTAGCTGCATTGGCAGTTTGATATT
CAGATTTCACAACTGGTTCGTATGTCATCACATTTGTAGCATCAAGCGTTGTTGATGAGCCATCACGGTAATGTAATGTGACATTACCATTAT
TATCGACAGTTACTGGATCAGTTGATGCTAAATATCCTCTTATATTTTGGTTTTTATTCATAAAGGCAGTAATTATGCTTTGCTTTTCATCATT
AGATACAGCAGTCGGATTGACTACTACAACTTTCTCAGTATTTCCTAATACAATCGGATGAGCATCTTCACTAATTTTACCTACATGAATTGA
CACATGTTTAGATTGACTATCATTTGTCGTATTATTGACATTGTCTGTCGCTGATACTTTAAATGTAATATCACTGTTAAATGCCTGATTCATA
GAGACTCTACCTGTAATAGCTAATGAGCCGTTTTTGTTGTCGGATTTAGTTAAATTTGATGTCCAACCACTTGGCAATGTCGTAATAGTTGTA
CTTTTTAATCCAGAATGTCTATCTGAAACCCCCATAGTAAAGCTAACTTCATCACCACGATAATATTTGGCATTTATTCCAACAGGATTATTGA
TTGTTGGTGGAATGTCATCTGGCAAAATAGTAACAGCATTACTCATATTAGATGCAACTACTTTATTATAATAAACTACTGCTCTAATTTGTT
TACCTACTGGTGGTATGAATACAACATAATCTTTTGGTTGTATAGTAATTGTGTTATGGACCCCATCAGGTGTACCAGTCGCAAGTATCGTA
TAACCAGATGGTATTTGATCTGCCTCACTACCACCATTCGTAGTTTGATTTGTTGGTGCAACTAAATAAACCAATGCAGATGGATCCAACGG
TATTCCCGAAATATTAACAGGTACTTTTTGTAATGCTGTACCTCTTAATTGCTCAGCTGTCGTTGTAATTGTTGGTGTATGTGGTTTAACACG
AATTTCGAATAATTGTTCTTTACGTCCAAAACGTATCGTTCCAAAGTAATCCCCTGGTAATAAGTTACCAACAAGTTCAATATTGCGTTCTCT
GGCATTATTAGGTCTATTTGTCGAGTTTTCAATGTGTAATGTACGCGGAATACCTGTAATTTGCCAATCTTTATCTGTAAAATTCTCATTACTT
GTTAAATTAATACGTAATGGTTGAATAGATGTTACGCTACCACCTAAATTCACGGAAGCATTCCCTTGGTTACCTGCATATACATAGAAAAC
TGGATGCCCAGCTTGTTTTGAAAATGGTTTTTCAGCAATATTTTGAGTATCGTCATTTTTAAAGAATTTAATATTATGTTGGATTTCAGGTAAT
ACCGTTAAGTTAACAGGAACTAAAGTTTCACTATTACCATAACCATCAGGATAGTCGAAACCAATCATTAATCTTATTGGGTTTCTAGTTCTT
GCTGCTTCTTGTAATTTTTGATCAGTAAATAATTGACTTAATTGATATTCATTTAATTCCTGAACGCCCCCCGGTCTAAAAGCGTTAGTAGTTG
CATTAGTTGGGTAAAAACCTAACACTTTATTACTCGGCGTCTTAACATAACTTAATGGATCATCAACAAAATCATCTATATTTAGGTTTGTAC
TTTCTAATACCTTTTTCGTTTGTACATTTCCTTGACTCACTTCTACACGTTTAGTTGTACCTATGCGATATGTACGATTAACCACTTGTCTTAAA
CCATTAAGACGTTGTTGATGGTCTTGCGTTGATTCGACGTATAGTTGACTGTACATAAATAATTGATTGTACTCTAAAGCACGCCCACCTGCT
GCATAGTACAATTTATTAACAGTTGGACCTTGTGTTTTAAATGAAATTGTATAACTTGCATCACCTTGACCGATGAAAGTATAAACTTTAGGA
ACACCCGGTGATTCAGCAGGTTTTTGACCATTAATATATTCAAAATTTTGTCTACCAAATGCTTTAGTAGCAGCTGTTGCTCCAGCTTCACTA
AAATAATCACTCGCTCTAGATAAATCTTCTAAAGAATAAACATGGACAGTTGGCCAGCTATATATTACTTGGCCACTTCTATTTCTTATTTTAA
AGTCATGTGAACGATCAGGATCATTTACTCCATATTCCCACATTTGTTGAAGTGGTTTATTTGCACCTGCTCCTGCTCCATGACTCCATTGTAC
ATTTGTTCCATCTGAATTAACTGTTACAAAGTCAGTTCTTCCTACTGGCACTTGGTCTGCTGGTAATGCAAACCAGAAAATCATATTTTGATG
TGGAGCATGTCCTTTATTAAACTTAATAACCCATTGGTAGCCATCTCCAGTTTGTTTCGCTGTCATAAGAGCAATTTCCTTTGAGTCAGAGGC
CTTCAATGTATTCCATGGTGTAGTTGGTAAATTATATGGCGCCCCTTTACTAGAAGCATTGATATATGTACCTACAGGATATCCTGAACCATC
ATTAGCATTTTCAACATAATATGATGAATTTGCATTTTGATAGTCTGATACTGATCTAGGCTCTGCAGCTCTAGACTCAACAGAACGCGTTTG
AATTCTGTTTGATCGACGGCTAGTTTGTTGACGTTGAGGCATTGTACGAATTAAAGCAACAGCTTGTTCCAATTCTTTGTATGCAAGTTGCA
AGTCTTTATTGTTTATTGGATCATTACTGCCTAATAACGTATTAATCTTATCAAAAATGCCTTTTACATAGTTAAATGTGCCAGAATCTGCATT
TTCACGGTCAATAAAGCGATAATCATGATAATTTGCATCAAAAAATGCTTGTAATTCATTTCGGTCATGTTGTACGTTACCATTATCAGATGA
ATCTGAAGCTGTTACTACATTTGCTTCATGATTTTCTAAATGAGCCGCTCTATTGTCGTTTCCATTTTTATTAGCAGATTGACTTTCGTTTTGTT
TATGCTTACCTTCTTCTTTATCTGGTTGTGTTGTCGCAGCCGTTGCCGAATCTTTCTTTGTATTTACATTTTGAGATGCTGGTTGTTCATCATTA
GTAGTAGATGATTGCGCTACTTTTTGATCTACTAAATTAGCTTGACTAATATTTGGTTGCTCATTTTCATGAGTAGCAGATAATGATTGACTA
TTTTGTGAATTTTGAGAATTTTGTACTTGAGATTCTCGATTCTCAGTCTGTTCATTATTACTTTGTTGTTTCTGTTTAACCACGCTTGCTGGTTG
ATTTGTTTCAGCAGCATGTGCTTGTGATGTATTGAATCCTAAAAATACCAATGTCGCAATGACAGTTGAAAATGTACCAACTGTATATTTACG
AATACTAAACTTTTGAATTTTATCACGATAATTCAC

Gene: norB (multidrug efflux pump)

Position: 1451318 to 1452723, length: 1406 nt, orientation: REVERSE

Sequence:

TTAATTCTCAATTATCATAATTGAGTGTCGTTTTGTTTAGGCACAAGTAACAAAATGATAACGAATGATAATATTCCCATACCTGCATTTAAC
CATAATGCAATCATTGCACCTGTATAAATGTTTGTCATATTTGATACGATTGCATATACTGCACCACTCAATGCGACGCCAAATGCTCCACCT
AATGCAGAAGCCATTTTATAGATACCTGCAGCAACGCCTACTTTTTCTAACGGTGCATTTGCAATTGCTGTATCTGTTGATGGTGTAGCATAT
ATCCCTAGTCCTAAACCAAAGAATAAATAACCTATAATACAACAAATGACATACAATATTTCTGGCAAGAAAGTTAATGAAATGAGACATTC
TCCGACAATAAGAACTCCTGTTCCAATTAACATTGGTTTCTTGCATCCGAGTGTTTGAAGTAACTTTTCACCAACACGAATCATAATTAGTAC
CATTACTAAATAAGTGATTGATAAACTTCCTGCTTGCAATGAAGAATATCCTAAACCTCTTTGAACAAATGTGTTGGCTACTATTAATGTTCC
TGCAACACCATTTAACAAAAAGTTTGAAGCTGTTGCACCTGTGTAAGCTTTATTTTTAAATAATTTAAAATCGATTAAAGGATTTGTAGCACG
CTTTTCAAGAACTATAAATAAACTAAAAGATCCAATTGCAATTGCTAATAAAGTGATAAAAAGAAGTGAGGTTACACCTAATTCTGATCCTT
TAGTAATTAAAATATTTAAACTGAGGAGCATAATGACTAAAAGAACCAGACCTTTAATGTCAAATTTATTTAGAGAAATCGATTTAGATTTA
GTTTCAGGTGTGCCTTTAATAAGAAACAGTGCAATTAATGAAATTATAATTGATAGGATGAAAATCCAACGCCAACCTAAAAGCGTTGCAA
CTGCACCTCCAAAAAATGAACAAACACCAGAGCCGCCCCATGAGCCAATTGACCAATAACTTAAAGCGCGTTGTCTATCTTTCCCAATGTAA
TATGACTTAATAATAGACAAAGTTGCAGGCATAATACATGCTGCTGAAAGTCCTTGAATTAATCTTCCTATAATAAGTAATAAAGGAATATT
TGAAATAATGATTAATAATGAACCTAATATATTTAAGATAATACCAATGTTCGTGAGTTTAATTCTGCCATATTTATCAGCAAGACCACCTGC

TCCTACTACAAACATTCCTGAAAATAAAGCAGTTATACTAACGGCGATATTAACCGTTCCAATATCTGTATTGAAACTATCTTCAAGTATTGG
TACAACATTAACCAATGATTGTGCAAATAGCCAAAACGTTATTACACTTAAAACAATTCCTATTAACAACTTATTATTGCCTTCAAATGCCTCT
CTTGACGGCTTTTCCAT

Gene: steT (serine/ threonine exchanger transporter)

Position: 1452879 to 1454201, length: 1323 nt, orientation: REVERSE

Perfect match to: (CIGC345D-AHVO01000007-[163488:164810:r], allele observed in CC8+CC22)

Sequence:

TTATGCTGCTTTTTGTTTCTTCTTATAGTAATAAACTGGTATACCAAGTGCTGTTATTAGAATTCCAATGATTGCTAATATAAATTGTGTAAAC
AGTGTATTAATTAATACAAAAGATCCTGCCAAAATAGCAATTAAAGGTATGATCGGATATAACGGTACTTTATATGGACGCTCCATATTTGG
TTCACGTTTTCTTAAAATTATTACCGCAACAAATGACATACAATAGAACAACCAAATAACAAAGATTAACATGTTTGTAATTGTATCAAATGC
TCCCATTGACATCATGATGATAGCGATTATAAGTTGTATAATTGCGCCAAACCATGGTGCGCCAGATTTTGTTAATTTTGCGAATAAGTGGC
TAAATGGCAATAATTTTCTTTCAGCCATTGCATATGGTACGCGCATACCAGTCATAGTATAGCCATTGATCGTACCATAAACAGAAATTAATA
TACCGATTGTAATAATTTTACCGCCATTTTCACCAAATAATATTTTTGATGTATCTGAAGCTGCATTTAAGTTACCAGCAAGTAGTTCTATTGG
CAACGTTAATAAAAATGTAGCGTTAATTAATAAATATACAGCCATAATACAACCGATACCAACTGAAATCGCTAAAGGTAAATCGCGTTTAG
GATTTTTAAGTTCCCCCGCAACATTTCCTACATGAATCCAACCATCATATGCAAACATAGTTGCTAATAAACCACTACCAATTGCTGTAAAGA
AACCATTTCCTGAATTACCTGTAGTTGGAATTAATGAAAAAGTGATATCTCCAGATTGAAAAATACCAAAAATTACAATAACGATGATTGGA
ATCAGTTTAATTACTAAAGTAACTGATTGTAAAATTCCGCCTGCTTTTGAACCTAGGAAATTTATCAACACAATAGATAACGCAGATGCGATT
GCTATTGGTATTAACGAACCTATAGATAAATGGAATAAATTAATTAGCTGTGTCGCAAATACGATAGACAATGCTGCTACGTTAGCTGGAA
AATAAATAAATGATTGCGCCCAACCTGATAGGAAGCCCCAGAAATCACCGTATGTATATTCTATATACTTCGTTAAGCCACCTGTTTCAGGG
ATTGCAGCAGCAAGTTCTGCTGCTGTTAACCCCGCACAAATGGTAATGATGCCGCCTAGGAACCATACAAACAAGGCCATTCCTGCTGTTCC
TGTTACTTCTGTTACGTTTGATATTTTAAAGAATACTCCTGAACCAATAACTGTCCCCATAACAATAGCAAACGCTGAGAAGAAACCTATATT
TTTTTGTAATTCTTTACCATTTGACAT

Gene: ilvA1 (threonine dehydratase locus 1)

Position: 1454232 to 1455272, length: 1041 nt, orientation: REVERSE

Perfect match to: (MW2-BA000033-[1474553:1475593:r], allele observed in CC1+CC8)

Sequence:

TTAACCTACCACACCCTTGCTTGTATCTGCAATATTCAGTCCATGTTCAATGACACCTGAAACTCTAGTTAAGTCAACATTCCCGCCTGAAACT
AAAGCAACAACATTTTTATCTTCAAGCCATTTATTGTTTATTTTTCCACTTAAAATTGCAGCTGTTGGTAATGCGCCTGCACCTTCAGTAATAA
TTTTGGCACGCTGCATTAAATCTTTCATAGCATGTTCAATTTCTTCTTCAGTAACAAGAATAAATTCATCTACTAAATGTTTAACTACTTCATAT
GTTTGTTCACCAGGAACTTTTACATCACAACCATCTGCTATCGTGCTATCCACTCGATGTTCAGTTAAATCTCTCTTATAGAAAGACTCAGCCA
TACCATGAACATTCTCAGATTGAACACCGATAATATGAATTGAAGGGTTAAATGATTTTAATGCGGTGGCAATACCTGCAATTAATCCTCCA
CCGCCAACTGGTACGATGACTGTATTCACATTCCAAATATCATCTAAAATTTCTAAACCAATTGTTCCTTGGCCTGCCATTACAAACTTATCGT
CATATGGATGAACGATTGTCATGCCATTTTCTTTCGCTAATTCTTCCATATAAAGTCTAGTTTCGTTAAAGTTTTTACCTTTTAAAATAACCTTT
GCCCCATAGCCTTTTGTTGCTTGTTGTTTCGCTTGTGGTGCTGTTTCAGGCATTACGATAGTTGCATCAATGCCTAATAATTTAGCTGTTAAA
GCAACACCTTGTGCATGGTTCCCAGCAGATGCTGCGATAATGCCTTTTTCTTTTTGTTCATCTGTTAAGTGATTAATTTTATTGCTAGCGCCTC
TAAATTTAAAAGATCCTGTGAATTGCATATTTTCTAATTTTAGAAATACATTCCCTTTAGTTATACTTTGGCTTAAATACATTGATTTAATTAG
AGGTGTTCGACGAATAAATGGTTTAATGCTTGCTTTAGCTTCTTCGATATCTCCTAAACTTACAATATGTGCTGTTTGTAATGTAACTGTGTT
GGTTGTCAT

Gene: ald1 (alanine dehydrogenase 1)

Position: 1455367 to 1456485, length: 1119 nt, orientation: REVERSE

Perfect match to: (MRSA252-BX571856-[1539564:1540682:r], highly conserved allele)

Sequence:

CTATTCGATAACATTTAATATTTCTTTATAATCTAGGTCATGTGATGAAGCTAATCCTTGATTTGTCACTTGTCCTTGGTAAATGTTTACACCA
GTACTTAAGGCTTCATTATCTTTAATTGCTTGTTCTAAGCCTTTGTCACAAATTTCTAATATATAATCAATATTTCCTTGTGCTAATGCCATTGT
TGAAGTTCTTGGGACTGCTCCTGGTTGATTTGGTACACCATAATGAATCACACCTTCTTCTTCATACACTGGATCAGAAATTGTAGTTGGTCT

AATTGTTTCAATAGTTCCACCTTGGTCAATAGCTATATCGATTAATACTGAACCTTTTTTCATTGATTTAACCATCTCACGAGTAACCAATTTT
GGCGGTTTCGCACCTGGAATTAAAATTGTAGAAATAAATACATCTGCTTTCTTAATTTGTTCTGCTAAATTTTCTGGTGTTGATTTGACTACT
GTGACATCTTTTTCTGCATACATATCTTCAAGATATTTAATGCGGTCATCGTTTAACTCGATAATGATTACTTTAGCATTTAGTCCCAAGGCAA
CATTTGCTGCATTTGTTGCTGCTACGCCACCACCGAAAATCACATATGTACTACCAGGTATATCCACATTTTCATGTACACCAGTCACTAAAG
TACCTTGACCACCATGTTGTGCTTCAGAGTAGTAAGCTCCCATAATTGCTGAGCGTTGACCTGCTATAGCACTCATTGGCGCTAATAATTCTG
CTTTTCCATTTTTTATAATGGTTTCACCACTAATCGCAGTTACACCAACTTCTTGCATTTTTTCTACTATTTCTTTTGAAGATGCTAGATGTAAA
AATCCCCAGATAATTTGATTCTTTTTGAAATATTGATATTCGCTTTCATGAGGTTCTTTTACTTTGATAACAAGATCAGCTTCCCATGCTTGTTC
GTGAGTTACGATCTTAGCGCCTTCTTTTTCATACATATCGTTAGAAAATCCTGAACCAATGCCAGCATTTTTTTCAACAATTACTTTATGTCCT
GCATCCGTTAACTTACGCACATTTTCGGGTGTGCAAGCTACACGTCCTTCACCTTGTTTTAATTCTTTGACTACTGCAACTAACAT

Gene: exo53 (putative 5'-3' exonuclease)

Position: 1456961 to 1457839, length: 879 nt, orientation: REVERSE

Perfect match to: (11819-97-CP003194-[1507339:1508217:r], highly conserved allele)

Sequence:

TTAAAAATGGGATGAAATATATTTTCCTGAAACATGTAATTCATGTTCGTTACAAATTGATAAAATATGATTAAGCGTTGTTGCAAATGACAT
TTTCTCAAAAAGCGCTTCACTATCAATTGGTACTTGTGTGTGAATTTCAGCCAGTCGCTTCGATAAATATAGTTCATCTAAATTGTCATTAATC
TTATTACGTTGCCCAGCTGATAATGCATCAATGTTTTCAACCACATTTTCAACGCTTTGATATTGCTGAATTAACTTAATAGCCGTTTTCTCAC
CAATTCCTTTAACACCTGCATATCCATCTGCTGTATCACCCATAAACGCTTTAATATCGATTAGTTGTTGTGGTTCAAGGGCATATTCTTCGTT
AAAACGATGTAATGTATATCTATTATAAATGTTAAAACCTTTTTTAATTAGCCAGACTTCAACATTGTCATTAATACATTGCAGTAAATCTTTG
TCGCCCGTAATAATATAGACATCGTTATCAGTTGAATATTGTTGTGCTAATGTACCTATAACATCATCCGCTTCATAGTTTTTAACGCCAATAT
TTACAAAGCCAAATTGCTCTGAAATTTCTTTAACATAATCAAATTGTGGTATCAATTCTTCTGGTGGTGCAGAACGATTTTGCTTATAACCAT
CAAACATATCATTTCTAAAAGTTGATTGTCCCATATCCCAACATACAGCTACATGTGTAGGGCGTATTTCATGTATTGCCGAAAAGATATGAC
GCACAAACCCTTGTATTCCATTTGTAGGTACACCTTGTGAATTGTACATAAATTGTTTATGAAGACTTGTAGCGTAGAAATGTCTAAATAATA
GTGCCATACCATCTACAAGTAATATTTTATTAGGCAT

Gene: Q2FGZ8 (putative protein)

Position: 1457859 to 1461299, length: 3441 nt, orientation: REVERSE

Perfect match to: (11819-97-CP003194-[1508237:1511677:r], allele observed in CC80)

Sequence:

TTAATATAAAATAGTTTTCAGTTGTTGATGTTTATCTTTCAATTGATTGATAAGTGTTGTATCTAAATCAAATGCTAATAAATCGTTTATTTGC
GTTTGAATATTAGCTTCAAAACTTTCAAATTGCGATTCAGCTTCTACAGCCATTTGCTTAACATATAATTCAAGTTGTTGCCTCAATAATCCCA
TTTGTGGTTGTAATAATCCTAACGTACTTTGACAAATTGATTCATGTATATCTCTTTGCCCATTTGGATTCAAAATTTTACGTTTTGTTAATTGT
TTAGGCAATGCATTTAGCATATCATTGAAATCGATGTGCAATAATGGTTGCTCTATATTAGCTGATTCAAAGTTAAACTGAGGATTAACAAT
GACATGTAAATCTGCTAATTGTTGAACGATTGGTGCAATTTGCTCAGTGAGTTGCTTATTAAAGTATTTTTTTATACGTTCTGTAATTAAAGA
TTGCTCTAAAAACAATCGTTGATGAATTTGATCTAAGTATACTTTCGTAGACACTTTCTTTTCTTCATTAAAATCACTATTTTGCGTCATTTGA
GAATTAAACACTGATTTAACATCGTCAAGTAGTTGTAATTTTAAACGTGCATTTAAATGATAAACTTGTTCTTCAACTTCGTTGTCTGTATGTT
GTAACGTCGCATCAATTAATTGATGTTGTAAACGCTGTTTATCTTTATAGACTGTTAACCTTTGTTGGCGACGTGAAATATCAGCTTTATTTG
TTTCAAATTCTGTAATCATCTCTACATAAGAACGATCCATTTGTTTAAGCTGATGAATCATTTGTTGTTCTAAAATTGATTTAGATTCAACATC
AACAAATTGTTGTATGCTTTGTTTTAATTGATCAATGCCCTTATCTTCAGCTTGTAATGCATTTCGACTTGATACAGCAAAAATGTCTGATTGT
AAGTGTACTTGTCTTAATGCATCTGATACATATGTTTCAACCGCTTCAAGATCATCTTGACTTTCTGCTAAATCAGCAGCATTAATTACCATTT
TAAATGCTTGGTTTTCATTCAACTGGTTCATATCTTTCATGTGTTCTATAAACGCCTTGTCATTATCAGTAAATGAATGATTAAAATAACTTAC
ATACAATATTAAGTCTGAAGAAGTTAAAATTTGCTCGGTTTCATTTGTATGCCTTTGGTTATTTGAGTGTAGCCCTAATGAATCAACAATTAT
TTTACCTTTTAACCAATCATGCATTAATGCAATGTGTACTGTTTTAACAAATGTTGCGTATTCATCTTCTGCACTCCACTTTTTCAATTCTTGTT
GATTAATGGCATGTTTTTCACCATTTTCCAACATATTGACATACAAATTATAATGTTTTTCAACTGCATGTACAAAAGCGAGTTGATTTTTATT
TAAATGTGATTTTAACTTTTCTAAATCTGAATTAATAAAGTCTTCTATAGTTGAAAAAGACATATCTTGGTATTCAACTACTGCATTAATTTCA
TCTAATAATTGCGATTGTGATTTTAGCGTTATATAACTCTCGTCTCCATAAGATATTTCTGTAGTAGCTGCTGTAGTAGGATTTGGAGAACTG
ACTAAAATCTGCTCGCCTAATAATGCATTTATCAAACTACTTTTACCAGCACTAAATGTTCCAAATACACCTATTTTTATTAATTTATTATCTAT
ACGTGTTAATGTTTCGTGGATATCTTGCTTTGTACGCTTGAACAAAGGCACATCCGAAATTATATCAAGCGCTTTTTGAATATCTATAGACAT
ATTTGTTGTAGCTGTATTTTGATTTAGCTGCTGATTATCACGATTATCCTGAGTATTATCAGTAGCCACTTGATATGTTGTCTCTTGTCGACCT
ATTAATTTATCTAGAGATTCATCTAAATGAATATAGTAGTGACGATAATTCTTAGTCGTCAATGACTGACGCAGCTCATTTAATTCTGTATAA
CGTTGATATTCTTTTAAATCATCACTTTCTTCTGTTGGTAATTCATCTGCCTGCACATTTTCTATTATTTCTTTAAAAATTGGTGTTGATTGTTGT
TCAACATATTTCTTAATGGCTTTAACCACTTCGTCTGAAAATGTAAGTACATAAGTATTGCTGATTGATGTTTGTGGTTGATATAAATCTTCA

ATCATTTCCGGCTTAACGTCATAATGCTGATTTAATACTTTATCTGAAGCTTCTTTTTTATTGATAAAACGCGTTACAAATGACATATCTTCTC
GCATTGGTTGACGAATTTGTTGATTAACATGTTCTTGTAACGCATCTGTCGCTGTTAATAATCGCTGTTGTTGAATTTGTAGCTTTTTCTTCTT
TTTATTAAAAAACCCACCAACATTAAAGTCTTGAGACATACTTTCCAAATAACTTCGTAACATTTCTCTCATATTATGCGGCATAATGTATGCA
TTTTCTAAAATATTTTTACGCTTATTCTTTAAAAATGCCATTAATTCATCTGGATTATTTAAAAGTTGTGCCTCTTCACTAATTGCTTGATGTTG
TTGACTATTTAAAAATGCTTGTTCAAACTCCGCTTCTTCGATACCTAAATCTTCTAGTACTTCCTGAATTTCAGACTGAATGTAATCTAGCTGA
GCTTCGGTAATGTATTCAACCGTTCTTGATGTATAATCCTCTATTGTCTCTCTATGTTGATCTAATGAAATTAGATAACTTGATAATACTTCAA
GTTCATTTTCAGGGTGATCAAATTTAGATACATAAAAGGTGCGTTCTAATTTAATGCCCCAATCTGCAATTGATTTTTCAACTCGTGATTTAA
ACGTAGAGAATGACAATTCATCGTCTTGATGCTTGTCAATTTGATTAATGATAAACACAACAGGTATTCCAACATCATTTATATGCTTCATAA
ACTTAAAGTTAAGTTCAGATTGAACGTGGTTATAGTCAACCGTATAAAATATCATATTACTTGTATACATATATTGTTCTGTTATTGACTGAT
GTGATGCAACATTTGAATCAACACCTGGTGTATCTTGCAACGTAAACCCATTTTCAAATTTAGCTGATTGAAAATTAATTTCTACAGATTCAA
CGTCGACATTTTGGCGATTCATTTCCCTTACTTCATCATAATTAGATAATTTGGCATACGTTTGATTCGGCAAATTAGCAATAATATCGTGATT
GTCTGAAACTGACACAATAGCAGTATTACTTGTCGTTGGTACAGGTGAACTTGGTAAGATATCTTGTTCAATTAATAAATTTATCAGTGTCG
ATTTACCTGCAGAAAAATGTCCAACGAACGAACATGTATATTGCTGCAAATATACTTTCTTAATTACTTGGTTAATTGTATGTAAAAGTGCTT
CATTTCGCGACTTTTCAACTTCTTTTTTTAATTTATATAAAAGATCTAATTGTTCTTTATTAATCAT

Gene: piuB (iron-regulated membrane protein)

Position: 1461603 to 1462943, length: 1341 nt, orientation: FORWARD

Perfect match to: (KLT6-APFH01000004-[924930:926270], allele observed in CC12+CC8)

Sequence:

ATGAAAAATACATTTAATCCATTACAAAGATTACATTTCTATGCAGCAATATTTATTGCTCCACTGTTAATCACTTTAACCATTTCGGGCATTG
GTTACTTGTTCTTTCCAGAAGTTGAAAATAATATTTATAAGAATGAGTTTTTTGGTGACAGTGATGTAAAAACGCATCAAACATTAAATGAT
GCAGTACATCAAGTTGAACAACAATATGAAGGATTCTTTGTAAGTAAAGTTAGCATACTTGATGAACCATATAACAAACGAATTACACTGA
GTGATATGGCAGGAAATCAACGTTACGTCTTTCTAGATCATAACAATCAATTTGTTGCAGATCAAAATGCGAAACATACGTATTCTAATGTG
ATGCGAAGTATACATAGTTCTTTGTTTACTGAAAATACTATTATTAATTATTTAGTAGAGTTAACCGCATGTTGGACGATATTCATGATTTTAT
CTGGTACTTATTTACTCATTAAGAAGCATTTAATTTCTAACAAAGGTAAGGCACTTCGTTGGCAAAAGTGGCACGCAATGATTGGAGTTATC
ATTGCAATTCCAGTATTTGTATTAGTTTTAACTGGATTGCCATGGTCTGGTTTTATGGGCAGTAAAATTGCCGGTATGATGGACACAAACGG
TGACCTTGGTCAAGGTGAATTAGCGATTAATCCACCTAAATCAGATTTGAACGAATTACCTTGGGCTACACGTAAAAATAAACAGCCAGCTT
CATCCGAAAAAGGTTCAAGTGGTCATCATGGTAATGCAGCAATGCCTCAAACCAAATTAGATTATCAAATATCTATTGATAAGGTCGTTGTA
CAAGCGCAAAAAGCTGGTATTAAAAAGCCGTTTTCAATCGTATATCCAAGTGATAAAAATGGTACCTTTATTGTATCTAATACTAGTAATTC
AGGTGTTACTGGGCTAGATGTATCACCTTACAAGGAACAAACACTTTATTTCGACCAATATAGCGGTAAAAAGCTAGGTACGATTAAATAT
GATGACTACGGTATTATTGCTAAATGGTTTACATGGGGCATTCCGCTTCACGAAGGTCATTTATTCGGCATTTTAAATAAAATCATTAATTTA
TTTGTATGTATCGCTTTATTAGTAGCCATTGGCATGGGGTTTGTCTCTTGGATAAAGCGTACAAAAAATACTGCAGTAAAAGTACCACATCG
CGTAAAAAAACCAGCATCTATATCACTCATAATATGTTTAATTGTATTAGGATTATTAATGCCATTATTTGGATTATCACTTATCCTTGTATTT
ATAATTGAATTAATATTATATATTAAAGATCGTCGTGCTAAACAATAA

Gene: Q5HFY0 (putative protein)

Position: 1463149 to 1463481, length: 333 nt, orientation: REVERSE

Perfect match to: (11819-97-CP003194-[1513527:1513859:r], allele observed in CC80)

Sequence:

TTAATTCATATGCATGTCGTGAATTTTCATCATTAAACCGTGTGGGACGTCATCATGTTTAACAATCTCTTGTTTATAAAATGTCTTCCCATCA
TCTTTAGAAACTAATTTTACAAAGTCTCCTTTTTTCGGTTTAAAATCATTTGATGGTTCTACTTTGATATTATGTTTGACTAATCCATCTTTTTCA
CTGACAACTTTTTCTGCATTTGTTTTACTATTCATATAACCAAAATAAGTCGTTTTTTGACTAGAAAAAATCATTAAACATACAACGGCAATAC
CAATAACAATCAATGCAATAGATAAACCTAGACCCATTTTTTTACCCAT

Gene: ypsC (site-specific DNA-methyltransferase)

Position: 1463565 to 1464710, length: 1146 nt, orientation: REVERSE

Perfect match to: (11819-97-CP003194-[1513943:1515088:r], allele observed in CC80+CC361+CC772)

Sequence:

TTAGTTTTCAATAGTTTTACGTTCTGTCTTTTTACCCCAGTATTGATAATAAGTACATTCTATATATCCATTAAATAACTTACGTCGCTTCGTTG
CTTTACGATCTACTAAATATTCAAATTCTTTATTACTTGTTAAAATGTATGTAGATAAAAATGGATGTTGTTTCATTAGTTTACCAATATAACG
GTACATTTCTTCAACTTCTTCACGATCACCAATACGTTCACCATATGGAGGATTTCCAATTAACGCCACCGGTTCTTCTGTATCAATTGTTAAT
GTATTGACATCTTTTACACTAAATTTAATAATATCAGACAACCCAACTTCTTCAGCGTTACGCTTAGCAATCTCTACCATTTCTGGATCGATAT
CAGAAGCATATACTTCGATTTCTTTATCATAATCAGCCATCTTATCCGCTTCATCACGGTAATCATCATAAATATTTGCTGGCATGATGTTCCA
TTGCTCTGATACGAACTCGCGATTAAAACCAGGTGCGATATTTTGAGCAATTAAACAAGCTTCTATAGCTATTGTACCCGAACCGCAAAATG
GATCAATTAAAGGTGTATCACCTTTCCAGTTTGCAAGACGGATTAAACTTGCTGCCAACGTTTCTTTAATTGGTGCTTCACCTTGTGCTAATC
TATATCCACGTCTGTTCAAACCAGAACCTGATGTATCGATAGTCAATAATACATTATCTTTTAAAATGGCAACTTCAACGGGGTATTTCGCAC
CTGATTCATTTAACCAACCTTTTTCGTTATATGCGCGACGTAATCGTTCAACAATAGCTTTCTTAGTTATCGCCTGACAATCTGGCACACTATG
TAGTGTTGATTTAACGCTTCTACCTTGAACTGGGAAGTTACCCTCTTTATCAATTATAGATTCCCAAGGGAGCGCTTTGGTTTGTTCGAATAA
TTCGTCAAACGTTGTTGCGTTAAAACGTCCAACAACAATTTTGATTCGGTCTGCTGTGCGCAACCATAAATTTGCCTTTACAATTGCACTTGC
GTCTCCTTCAAAAAATATACGACCATTTTCAACATTTGTTTCATAGCCTAATTCTTGAATTTCCCTAGCAACAACAGCTTCTAATCCCATTGGA
CAAACTGCAAGTAATTGAAACAT

Gene: rnpB (ribonuclease P RNA component)

Position: 1464905 to 1465301, length: 397 nt

Sequence:

ACTAGTAGTGATATTTCTATAAGCCATGTTCTGTTCCATCGTACTCATCACGTGCACTAGTCACACTGGTACTCAGGTGATAACCATCTGTCT
ACACCACTTCATTTCGCGAAGTGTGTCTCGTTTATACGTTGAATTCCGTTAAACAAGTGCTCCTACCAAATTTGGATTGCTCACTCGAGGGGT
TTACCGCGTTCCACCTTTTATATTTCTATAAAAGCTACGTCACTGTGGCACTTTCAAATTACTCTATCCATATCGAAAGACTTAGGATATTTCA
TTGCCGTCAAATTAATGCCTTGATTTATTGTTTCATCAAGCACGAACACTACAATCATCTCAGACTGTGTGAGCATGGACTTTCCTCTATATA
ATATAGCGATTACCCAAAATATCA

Gene: gpsB (cell cycle protein)

Position: 1465361 to 1465705, length: 345 nt, orientation: REVERSE

Perfect match to: (N315-BA000018-[1484229:1484573:r], highly conserved allele)

Sequence:

TTATTTACCAAATACAGCTTTTTCTAAGTTTGAAATACGTTTTAAAATATCTACATTATTTGAAGATGTATTTGTTGTTGTATTATTCGAAGAA
AAACTTTTATTGTCCTGAGGTCTTGATGTTGCTACACGTAGTCTTAATTCTTCTAATTCTTTTTTAAGTTTATGATTCTCTTCTGATAATTTTAC
AACTTCATTATTCATATCGGCCATTTTTTGATAATCAGCAATAATGTCATCTAAAAATGCATCTACTTCTTCTCTTCTATAGCCACGAGCCATC
GTTTTTTCAAAATCTTTTTCATAAATATCTTTTGCTGATAATTTCAATGAAACATCTGACAT

Gene: ypsA (conserved hypothetical protein)

Position: 1465719 to 1466282, length: 564 nt, orientation: REVERSE

Perfect match to: (MW2-BA000033-[1486463:1487026:r], highly conserved allele)

Sequence:

TTAGAAACTTTGATCTTCAGACCACTGTAAGTCATTGATGAATGCTGTTAATTCATCGAACGTCACAATATCACAAGTATAGTTTGTTTTATC
CATAAAATCAACTAACATCTGCTTGAAGAACTTAGGACTTGCCTCTTGTTCCTCATCATAAATGAGCAATGTTTGATCCGAATGTTCAAGCAT
AAATTGATCTGCTTGTTTAAATTGAAAAGGACCCTGATACGACGTATGAAAAATACTATCAACATAATCTGCATGCTTAATTATGTTGGCAT
ACTTACTTTGATTATGTTCATTCCACTTTTCTGTATGTCCTTGGAACGGTGTAATTACAGCAAACTTTAACGAATCATATGTTCGTTGTAATTC
AATAACAACTTCTGCAGTCCATAATTCTATACCCATTTGCCCTTGTATTAACACCCATTCTAATCCTTCATCCAACAGTTGTTCAATTTTATGTT
TTATAAATTGTTTTAAATAATGTACTTCAGGTGCGTCATCTTTAAAAATGTTTAATTCGAATGATTTGTAACCTGTTACATAAACTGTTTTAAC
CAT

Gene: yppE (conserved hypothetical protein)

Position: 1466275 to 1466625, length: 351 nt, orientation: REVERSE

Sequence:

TTAACCATGAGTACCCTCTTTTGTTACGCCATCTAATATATTTGTAAATCATATTGAACACTTTTAAGCTTTTCTATAAATAACTTTCGACTTG
TTCGCTTAAAATGACATTCGACAGAAAGTTGTTCAATGTTAGCAATCAGTAGCTCAAATTTCCTTGAATTCATATAAGGTACTTCTATAATAA
ATTCACGATGTAATTTGATCTCATTGAGCATGCTATCAATATGTTCATTATATGGCTTTACAGTTTGGTAAAAATCATGATCTTGTTGTTGTGA
TTTCACATTTTCAAAATTTTGTTGCATGTTGTTAACTTCATAAATTAGTGATTCGACTACATCATTCAT

Gene: recU (Holliday junction resolvase)

Position: 1467165 to 1467791, length: 627 nt, orientation: FORWARD

Perfect match to: (N315-BA000018-[1486033:1486659], highly conserved allele)

Sequence:

ATGAATTATCCAAATGGTAAACCATATCGTAAAAATAGTGCTATAGACGGAGGGAAAAAGACCGCTGCCTTTAGTAATATTGAGTATGGTG
GACGTGGTATGTCACTTGAAAAAGATATCGAACATTCAAATACGTTTTATCTTAAAAGCGACATTGCAGTTATTCACAAAAAGCCTACGCCA
GTACAAATAGTTAATGTCAACTATCCTAAGCGGAGTAAAGCTGTGATTAACGAAGCTTATTTTCGTACACCTTCAACAACTGATTACAACGG
CGTTTATCAAGGTTATTATATTGATTTTGAAGCAAAGGAAACTAAAAACAAGACGTCCTTTCCTTTAAATAATATTCATGACCATCAAGTCGA
ACATATGAAAAATGCATATCAACAAAAAGGTATTGTGTTTTTAATGATTCGTTTTAAAACGCTAGATGAAGTTTATCTTTTACCCTATTCAAA
ATTCGAAGTATTTTGGAAGAGATATAAAGATAATATTAAAAAGTCTATAACAGTTGATGAAATACGAAAAAATGGTTACCATATTCCTTATC
AGTATCAACCAAGATTAGACTATCTAAAAGCAGTTGATAAGTTGATATTAGATGAAAGTGAGGACCGCGTATGA

Gene: pbpB (penicillin binding protein B)

Position: 1467788 to 1469971, length: 2184 nt, orientation: FORWARD

Perfect match to: (JKD6008-CP002120-[1536398:1538581], allele observed in CC239+CC8)

Sequence:

ATGACGGAAAACAAAGGATCTTCTCAGCCTAAGAAAAACGGTAATAATGGTGGGAAATCCAACTCAAAAAAGAATAGAAATGTGAAGAG
AACGATTATTAAGATTATTGGCTTCATGATTATTGCATTTTTCGTTGTTCTTTTACTAGGTATCTTATTGTTTGCTTATTATGCTTGGAAAGCAC
CTGCTTTTACCGAAGCTAAATTACAAGATCCGATTCCTGCAAAGATATATGACAAGAACGGAGAACTTGTTAAAACATTAGATAATGGCCA
AAGACATGAGCATGTAAATTTAAAAGACGTGCCGAAATCAATGAAAGACGCAGTACTTGCAACTGAAGACAATCGTTTCTACGAACATGGC
GCACTTGATTATAAACGTTTATTCGGTGCAATTGGTAAGAACTTGACTGGTGGATTTGGTTCTGAAGGTGCCTCAACATTAACACAACAAGT
TGTTAAAGATGCATTTTTATCACAACATAAATCTATTGGACGTAAAGCTCAAGAAGCATACTTATCATATCGTTTAGAACAAGAGTATAGTA
AAGATGATATCTTCCAAGTATATCTAAACAAAATTTACTATTCTGATGGCGTAACAGGTATTAAAGCTGCTGCTAAGTATTACTTTAATAAAG
ATTTAAAAGATTTAAACTTAGCGGAAGAAGCTTATTTAGCCGGTTTACCTCAGGTTCCAAACAACTATAATATTTATGATCATCCAAAAGCTG
CTGAAGATCGTAAAAACACTGTTTTATACTTAATGCATTATCATAAACGCATTACAGATAAACAGTGGGAAGATGCTAAGAAAATCGATTTA
AAAGCGAACTTAGTAAATCGTACTCCTGAAGAACGTCAAAACATTGATACAAATCAAGATTCTGAGTATAATTCATACGTTAACTTTGTGAA
ATCTGAATTAATGAATAATAAAGCATTCAAAGATGAAAATTTAGGTAATGTATTACAAAGTGGTATTAAAATTTATACAAACATGGATAAAG
ATGTTCAAAAAACATTACAAAATGATGTTGATAATGGTAGCTTCTACAAGAATAAAGACCAACAAGTTGGTGCAACGATTCTTGATAGTAA
AACTGGTGGTTTAGTTGCTATATCTGGTGGACGTGATTTCAAAGACGTCGTTAACAGAAACCAAGCAACAGATCCTCACCCTACTGGTTCAT
CTTTAAAACCTTTCTTAGCGTATGGACCTGCCATTGAAAATATGAAATGGGCAACAAACCATGCGATTCAAGATGAATCTTCATATCAAGTT
GACGGTTCTACATTTAGAAACTATGATACGAAGAGTCACGGTACTGTATCTATTTATGATGCTTTACGACAAAGTTTCAATATCCCAGCTTTA
AAAGCTTGGCAATCAGTTAAGCAAAATGCTGGTAATGATGCACCTAAGAAATTCGCTGCCAAACTTGGCTTAAACTACGAAGGCGATATTG
GTCCATCTGAAGTACTTGGTGGTTCTGCTTCAGAATTCTCACCAACACAATTAGCATCAGCATTTGCTGCAATCGCTAACGGTGGTACTTATA
ACAACGCGCATTCAATTCAAAAAGTAGTTACTCGTGATGGTGAAACAATCGAATACGATCATACTAGCCATAAAGCGATGAGTGATTACAC
TGCATACATGTTAGCTGAGATGCTAAAAGGTACATTTAAACCATATGGTTCTGCATATGGCCATGGTGTATCTGGAGTAAATATGGGTGCT
AAGACAGGTACTGGTACTTACGGTGCTGAAACTTATTCACAATATAATTTACCTGATAATGCAGCGAAAGACGTGTGGATTAACGGCTTTAC
ACCTCAATACACTATGTCAGTGTGGATGGGCTTCAGTAAAGTTAAACAATATGGTGAAAACTCATTTGTGGGACATAGCCAACAAGAATAT
CCACAGTTCTTATATGAAAATGTGATGTCAAAAATTTCATCTAGAGATGGCGAAGACTTTAAACGTCCTAGCTCAGTAAGTGGTAGTATCCC
ATCAATCAATGTTTCTGGTAGTCAAGATAACAACACTACAAATCGTAGTACACACGGTGGTAGTGACACATCAGCAAACAGCAGTGGTACT
GCACAATCAAATAACAATACTAGATCTCAACAATCTAGAAACAGCGGTGGATTAACAGGTATATTCAACTAA

Gene: Q1YBU7 (putative protein)

Position: 1470536 to 1470877, length: 342 nt, orientation: REVERSE

Perfect match to: (MW2-BA000033-[1491278:1491619:r], highly conserved allele)

Sequence:

CTATACCTGCTTATTCTTTAACCTTGTTTGATAACTAGCATTCATTTTAATCAATTCAGTTTTAAAGGTCTTCATTTGTCGATAGCCCATAAAAT
GATATTTACGTTCAATCATATAATCATATCTTTCTTTAAAATTCATTGGCACAACAGGATATTGATTTAATTTATCAAAATCAATATTGCCAGT
TATGTCATTAACCTGCTTAAAATAATCAATAATTTTACTATGGTATTCATCCAAGTAGGGCTTTGCTTCACTAGAACCTAATGTCTTATTTTTG
GCAAATACATCAATCTCTTGTTCTAAATTATCAAATACCTCTTTTGTAATATTTGTCAT

Gene: nth1 (putative endonuclease III, locus 1)

Position: 1470882 to 1471541, length: 660 nt, orientation: REVERSE

Perfect match to: (COL-CP000046-[1531312:1531971:r], highly conserved allele)

Sequence:

TCACGCTTCTTTCAAACTAGCTTTATAACGTTTTTGTCCTTCTCTACAATCTTCTAATAGTGGACAAATATCGCATTTAGGTTTTCGGGCTAAA
CAGTGGTATCTTCCAAAGAAAATGAGTTGATGATGGCTCCTATTCCATCTATCTCTAGGTATGACAGAACATAAACGGTCTTCTACCTGTCTC
ACATTATCTTTCCAACGATTAATACCTAAGCGTTTAGAAACACGTTCTACATGCGTATCAACAGCTAATGAAGGTTCATCAAATGCTACACTC
ATGACTACATTAGCAGTTTTACGTCCTACACCTGCTAAACTTTCTAATTCCTTATGTGTTTGTGGTATTTCTCCATTAAATTGATCAATCAAAG
ATTGACAAAGTTTCTTAATATTCTTAGCTTTGTTACGATACAGACCGATAGAACGAATATCATTCATAAGTTCTTCATCACTGACTGCCAAAT
AATCTTCAGGCGTTTTGTATTTTTTAAACAGCTCAGTTGTTACTCTATTTACTAGAACGTCTGTACATTGCGCTGACAATAATACAGCAATAG
TTAATTCGAACGGATTATCATGTTTTAATTCACATTCTGCATCCGGAAACATATTTGCTATAACATCAATCATTTCTAATGCTTTTTTCTTACTT
ACCAT

Gene: dnaD (primosome, DnaD subunit)

Position: 1471531 to 1472217, length: 687 nt, orientation: REVERSE

Perfect match to: (RF122-AJ938182-[1443496:1444182:r], highly conserved allele)

Sequence:

TTACTTACCATCAAGGTTCTCCCCGTTTAACCAATCAAATTTAGGTACCGTTTTAACTGTGTGCGTCATTTTCGGTTTATTAAATTTTTCTCTTA
TTTTTCTAGAATCGTCAATTGTTTTGACATTGTTTTTCTTCCAATTAAGTAAAATACGATCCATATATTTAAAGCTAAGTTTATTCAAACTATTC
GCCTCGTCTAATGCCGCTTGTATAATTGCACTATCGTGTTTATCAACATCAATCCATTGATTTAACGTTTCTATTTCATATGGAGATAACGGCC
TTGCAAATGTATCCTCTAAAACTCTAAATAATTGTTTAAATTTTTCTTTACTATTTTGCTCTTTCGTTTCCATACTTTGTTGCTTCAATATATGAC
TTAATTTTTCGAAAAAAGGATCTAGATTCATATATTCGGTAAATCTACCTTCTTCATCTTTTTGAACTTGTAATTCTAGCAATTCACGTTGTATC
AAATTTTGAATAACCATTGTAATATCGCGTGGTTGCATAGTTGAGCCCTTCTGAAGTAATTCAATTGAAGGCTGTTTATTTGATGTTTCGGAA
GCATAAATCAATTTAAGCAAAATGACTAAATCTTGCTCATCTAAACCTAAGTCACTGTAATGGTCTAATAATTCTCTTCGTATCACTACAGGT
CTTGCTTTTAATTGATATTTATCCAT

Gene: asnS (asparaginyl-tRNA synthase)

Position: 1472545 to 1473837, length: 1293 nt, orientation: REVERSE

Perfect match to: (11819-97-CP003194-[1522923:1524215:r], allele observed in CC80+CC1+CC88)

Sequence:

TTATGGATATAAACGGTTTAATAATCTTGGGAATGGCGCTGTTTCACGAACGTGTTCAACACCAGAAATCCATGCTACTGTACGCTCTAAAC
CTAGACCAAATCCACAGTGTGGCACACTACCATAACGACGTAAGTCTAAGTAGTAACTATAAGCTTCTTCGTCTAATCCATGTTCTTTAACGC
GTTGTTCTAACAATTCTAAGTCATCCACACGTTCAGATCCACCAATAATTTCACCGTATCCTTCAGGTGCAATTAAGTCTGCACATAATACAG
TTTCTTCATTTTCAGGATTTGGTTGCATATAGAAAGGCTTAATTTTAGTTGGATAATTAGTAATAAACACCGGTAAATCATAATGATTAGCAA
TGGCTGTTTCATGTGGCGCACCAAAATCTTCGCCCCATTCAATATCATCAAAGCCTTCTGCTTTTAAGAATTCAATTGCATCATCATATGAAAT
TCTAGGGAATGGTGTCGCAACTTTTTCAAGTTTTGATGTATCACGCTCTAAAATTTTCAACTCTAGTTTACAATTTTCTAAAACTGATTTTACA
ACATGTGTTACATATTGTTCTTGAATTTCTAAACTTTCAGCATGATTTGTGAAAGCCATTTCTCCTTCAATCATCCAGAACTCGATCAAGTGTC
TACGTGTTTTTGATTTTTCAGCTCTGAAAGTTGGACCAAATGAAAATACTTTTCCGTGTGCCATTGCTGCAGCTTCTAAGTATAACTGACCAC
TTTGAGATAAAAACGCATCTTGATCAAAGTATTTAGTATGGAATAATTCACTTGTACCTTCTGGCGCACTTGCTGTCAAAATTGGTGGATCA

ACCTTTGTAAATCCATCTTTGTTGAAAAAATTCATACGTTGCACGAATAACTTCATTTCTAATTTTCATTACAGCATGTTGTTTTTAGAACGTA
ACCATAAATGACGGTGATCCATTAAGAATTCTGTACCATGATTTTTAGGTGTAATCGGATAGTCATGCGCTTCTGAAATAACTTCAATTGATT
TCACTTGCATTTCGTATCCTAAGTCAGAACGATTATCTTCTGTAATTGTGCCTGTAACGTATAGAGATGATTCTTGAGTAATTTCTTTCGCAA
GTTTGAATACCTCTTCATCAACTTCTGATTTAACTACTACGCCTTGCATAAAGCCTGTTCCATCACGTAATTGTAAAAAGGCGATTTTACCACT
TGAACGTTTATTTGTTAGCCAAGCACCAATTGTAACGTCTTGGTTTAAATGATCTTTCGCTTGTTTAATCGTTGTTTTCAT

Gene: dinG1 (damage inducible ATP-dependent 3'->5' nuclease, locus 1)

Position: 1474159 to 1476852, length: 2694 nt, orientation: REVERSE

Perfect match to: (11819-97-CP003194-[1524537:1527230:r], allele observed in CC80+CC188+CC4803)

Sequence:

TCACTTTTTCTTTTTTTGAATTTGTCTTAATAATTTTCCAAACTGTTGAATGTCGCCTTTTTTCTGACGATAATTTTCAAGTGTTTGTTCAAAAAA
GTTTTTATAATTACTGTTTATGAGTCGATCATCAAATGAAACTATTATGCCGCGATCATTTTCATTTCTAATTAATCTTCCAAGTCCTTGTCTAA
AACGTGTAACTGCATCAGGTAATACATATTCCTTGAAAGTTGAAGTGAATTCAGAATCCATAAGCCAATATTTTGCATTATGCTTGTTCATAA
ACGGTAACTTCGCTATCATCACACATTTAATACCATTTGCTTGAAAATCAAAACCTTCAAAAAAATGTTGACGTACCAAGCAGTATGGCCTTAT
CAAAATTATTAAACTGTTGTACTATTTTATAATTTTGGTTCTGCTGTTGTGTTAATACAACATAATCTTCAAATTCTGGCAATTCATTTAGCAT
ATCTTGTACCATATGCATCATTTTATAACTCGTAAATAAGACTAAACATTTTGATGACGTTATAGTCGTATATTCAATAATATAGCTTACAATC
GATGCTACATACTCATCTATATTTTTATATTGATAAGATGCTACATCACTCGGTATAAATACACTTGTATTTTTTGCACTTTGTAACGACGTGT
TAACTTCAAATGTATTAAAGTGAACATCTTTGTTGAATAACTGTTTAAAAGCTTCAAACGAATGATTAAATTTTAAAGTACCAGATATAAAAA
TGAGTGATTTAAATTTTTCCAATACTTGTTTCGTTAATACATCTTTTACAGCGTAATCTTTCACATATAAACGTATTGTTGATTTTTGAGATAA
ATTTTTAATCGAAATGAAACTAGTATGTCCAGCTTTTAAACTTTGTTCGATATTTTTAAATTTATCTTTTAAATACAACAACTGTTTCCGTAATG
ATTTAACTGTTTTATGACTAATGCCATTGAATATTTCTAGCGTTTTATTTAACTTATCGATAATCGCATGTAAATCCTTCAAAATGTCTTTTGTT
TCAAAAGTAAATACATTATGGAAGCGATGAATATCATCATCATAAACATCAGAATCATTGATAATCGTAAATATCGTTGAGAACAATTGCTC
ATTTAACTCATGAATCTCATTCATACTAGCCTTCAAGCCAAAAATATCAATTGGTGCAATATCTAATTTTTCCAAAATTCGCTGCTTTTCCAGT
TGATCAATTGCCTTTAACAATTTTTCATTTTCGTTTTTACCAATCAAACCAAGCTGATATTTAATATCAGCATAACTCAACTCATTTGTCACTTG
ATTTAAGGCATAGTCTGGTAAGCGATGTGCTTCATCCACTATACAATCATCAAACAATTGATATATTGAATTTTCAACATCAGAATGAATTAA
ATGTGCATGATTTGTAATACCAATTTGAATGTTCTGTGCATTTCGCTTAATAAAATTATAATAATGAACATCGTGACGTGCCGGTACATATGT
TTCAATTTTCTGGTCAAAATACATCTTTTGACCACCTTTTAAATTTAATTCCTGTATATCTCCGGACGGCGTCTCTGTAATCCAAATCAGCAAT
TGCATTTTCAAGATATTCACTTCGTAATTACTTGTGTCATCTTTTAAAATTTGACTAATAAGCCCCAATGAAATGTAATCACTTTTACTTTTAAT
CAATAGTGCATTAATTTTAAAATTTAACGCTTCATTCATTGCTGGAATATCTTTTTCTAACAATTGACTTTGCAGTAATTTGGTATTGGTAGAA
ATCATAACATGCTTTCCAGTTTCAATATTATACATCAAGGCCGCAAGTAAATATGCTAACGATTTACCACTGCCTAGTGATGCTTCAATCATT
GCTTTTTCACTATGCATGAGCTGATCTAATATAGTTTCCGCTAAATATAATTGCTGCGGTCGATATGTTAAGCCAAGTTGATCTACAGCTTTG
CTATATAAAGACTTCAAGCTGCCATTATAATTTGTTGTCGGCTTTTTAAAATCAACTTGCTTACGATAGATAATCTGTTCGAACTTTTCGTACG
ATTTATCCAATGGCTTTGCATCATATTGCCTAACCATCTCAAAGAAAATATCATACAAATCGTATTTCAACTGTTTACTTAAATAATATAATTG
CTTCAAAGTATCTAACGGTAACTTTTCAAATTTTTCAAAAGCTAATATCATCAATTTAGCAGTAGTAGCGGCATCTTCGTCAGCTCGATGGGC
ATTTGCTAAGGTAATACCATGTGCCTCTGCTAATTCACTTAATTGATAGCTTTTATCTGTAGGAAAAGCTATTTTAAAGATTTCTAGTGTATCT
ATAACTTTTTTGGGACGATATTGAATATTACAATCTTTAAATGCCTTTTTAATAAAATTTAAATCAAAATCTACATTATGAGCGACAAAAATG
CAATCTTTTATCTTATCGTAGATTTCTTGTGCAACTTGATTAAAATATGGCGCTTGTTGTAGCATATTTTCTTCAATGGATGTTAACGCTTGAA
TGAACGGCGGAATCTCTAAATTTGTTCTAATCATAGAATGATATGTATCAATAATTTGGTTATTGCGCACAAACGTTATACCAATTTGAATGA
TATCGTCAAAATCTAATTGGTTGCCTGTTGTTTCCAAATCCACAACGGCATAGGTTGCCATACCCAT

Gene: birA (bifunctional protein BirA)

Position: 1476876 to 1477847, length: 972 nt, orientation: REVERSE

Perfect match to: (SA_ST125_MupR-ASTH01000001-[355340:356311:r], allele observed in CC5)

Sequence:

TTAAAAATCTATATCTGCACTAATTAAACGGTGTGATTCACCCGCTTCATCTCTAACAATTAAATAGCCATCGTAATCTAAATCAATTGCTTGT
CCTTTAAACTGTTTATCATTTTCTGTAAATAGCAACGTTCTATTCCAAATATTAGAAGCTGCAATATATTCTTCACGAATTTCAGAAAAAGGTA
ACGTTAAAAATTGATTATATCTTTTTTCAATTTCTTGAAGTAATATCTCTAAAAATTGATATCTATCTAATTTATTTTTATCATGTAATTGTATA
CTTGTTGCTCTATGTCTAATACTTTCATCAAAGTTTTCTAGTTGTTGCGTCAAATTAATACCTATACCACATATTATTGCTTCTATACCATCATT
ATTAGCAACCATTTCAGTTAAGAAACCACACACTTTACCATTATCAATATATATATCATTCGGCCATTTCACTTTGACTTCATCTTGACTAAAA
TGTTGAATCGCATCTCTTATCCCTAATGCAATAAATAAATTAAATTTAGATATCATTGAGAATGCAACGTTAGGTCTTAACACGACAGACATC
CAAAGTCCTTGCCCTTTTGAAGAACTCCAATGTCTATTAAATCGCCCACGACCTTTCGTTTGTTCATCACTCAAGATAAAAAATGAAGATTGA
TTTCCAACAAGTGACTTTTTCGCAGCAAGTTGTGTAGAATCTATTGAATCGTATACTTCACTAAAATCAAACAAAGCAGAACTTTTTGTATAT

TGGTCTATTATACCCTGATACCAAATATCTGGGAGTTGTTGTAATAAATGCCCTTTATGATTTACTGAATCAATTTTACATCCCTCTAACTTTA
ATTGGTCAATCACCTTTTTTACTGCAGTGCGTGAAATATTAAGTGATTCCGCAATGCTTTGTCCAGATATATAATTCGGTTTATTTTTATAGAG
TAATTGAAGTACATCTTGACTATATTTTGACAT

Gene: cca-papS (tRNA CCA-pyrophosphorylase)

Position: 1477834 to 1479036, length: 1203 nt, orientation: REVERSE

Perfect match to: (11819-97-CP003194-[1528212:1529414:r], allele observed in CC80)

Sequence:

CTATATTTTGACATGATTATCCACCCATTTCAAAATTTCAGTTTCTTCGTTGCTTACTTTACCTGTTACAATCGCTATCTCAATTTGTCTTAGCA
CATCTTTTAACCACGGACCACTTTTGGCATTTAAATGTGCCATAAGTACACCACCATTAACCATCATGTCTTTTCTATTATGCATAGGTAATCG
ATGTAATGTTTCATCAATCGTTTGAAGGTTAACGATTAATGGTTCATGTCCTTGAATATCATTTGCCTTTAACACGTCTGCTGCAACCATTACA
TTTTTAATGAGATTCGTATCATAATCATAAACAAACATTTTTAATTGTTCTTTTGTAATAATACTTGGTAATGCATTCATAATTTGAATATATTG
ATTGATATCTTTTACTTGTCGATTACTTAGCTTTAAAGGCTTCAATGAGTAATTAATATCAAATTTAACTGATGCTATAGCAATCAACAATTCT
AAATCAATTGCTTCAGTCACATTAATTTGATTCATATCAAGATGTTCGAAATATGGCATATAATTAAATGCTTTCAGCGATTTTAAATGATTA
AAACTCTTTTCAACATTAATACCTCGCATTAATTTAGTTAGTTCAATCACTATACGCTCAATTGATAAAAATTTAATATCTGCCATTTGTGTAC
GCATCGCTTCGAATGTTTCCGTTGCAATATCAAATGATAATTGTGACTGGAATCTTAAACATCGAATCATACGTAAAGCATCTTCTTGGAATC
GTTCCTCAGCTATACCTACAGTTCTTATTATTCGATTATTAATATCTTGTTGACCATCAAAATAATCATACAATTTGTATGCTGTATCCATTGCT
ATCGCATTCATCGTGAAATCTCTTCGTTGCAAATCTTCGTATAAATCACGAACAAATGTAACACCACTTGGTCTACGGTGATCGACATAATCT
TCTTCAGCCCGGAATGTTGTCACTTCATAATTTTCATCATTAAAAACTACATTTATAGTGCCATGTTCTTTACCTACAGGTATCGTATGACTAA
AGATGGATTCTATTTCATCTGGCGTTGCACTTGTTGTGATATCTATATCATGAATATTTCTTCCCATGACATAATCTCTGACAGAGCCACCTAC
ATAATATGCTTCAAAACCAGTGTCTTGAATTTGTTCTAATATAGGCCTTGCCTGTTCAAATAATGATTTATCCAT

Gene: bshA (N-acetyl-alpha-D-glucosaminyl L-malate synthase)

Position: 1479041 to 1480183, length: 1143 nt, orientation: REVERSE

Perfect match to: (VH60-ALWG01000026-[60523:61665:r], allele observed in CC772+CC8+CC772)

Sequence:

TTACTCGCCTTTACTTTTGTTATGCTCATTTAGCATTTTTTGATAATAATACTCATATTGATCTGTAATGAGTTCTGATCCAAAACGTTCAGCAA
TATCTGCTAGCATGTTTTTCTGAAGTTTGTTGTATAACACCTTATCTTCAAGTAATCGGATAGCATAGTCACTCGCTGAATCACAATCACCCA
CATCTACGACAAATCCAGTTTCACCATGTTTAATAACCTCTTTAATTCCACCGGCATTTGAACCAATTGGAACGACGCCTGTTTTCATAGCCTC
AAGTAAAGTTAGTCCAAAGCTTTCTTTTTCACTTAATAATAATACTAAGTCAGATAATTGGTAAAATTCACTTACGCAATCTTGTTTCCCTAAA
AATAAAACATCCTCTTCTACGTTTAACTCTTTCGTCAATTGACGCATTGGCACTAATTCAGGACCATCTCCAAGTAAAATTAATTTACTAGGTA
TCTTTTCACGTACTTTTGCAAATGTTTCTATAATAGTATCTATGCGTTTTACTTGTCTAAAATTCGATACATGTATTAACACTTTTTCATCTGGT
GCTATACCAAATTGTGATTTTAGTGCTGTGTTATGTTTAGTTGGAAACTCATTTTCACGTACAAAATTATAAATCGGTATAATTTCTTTGTTAG
TTTCGATAATTTCATGTGTTTCTTGCGCTAAAGATTTACTCACACTTGTCACAATATCACTTTTTTCAATGCCAAATTTAATTGCACCTTGGAGT
GAATGATCATAGCCCAAAACAGTAATATCAGTACCGTGTAGCGTTGTCATAATTTTTATATCTTTACCTGACATCTCACGAGCTAAAATCCCA
CAAATTGCATGAGGTACAGCATAGTGCATATGCAACAAATCAAGATCATATTCTTTAATAACTTCAGCGATTTTAGTACTTAACGTAATATCA
TACGGTGGATACTGAAATACTGCATATTGATTCACTTCAACTTGATGAAAAATCATATTCGGTAATGGTTTTCTTATTCTAAACGGGATATTT
GAAGTGATAAAATGTACTTCGTGACCTCGCTCTGCTAATTTAATTCCTAATTCTGTGGCAATAATTCCAGAACCACCCATGGACGGGTAACA
TGTTATACCTATCTTCAT

Gene: ypjD (MazG nucleotide pyrophosphohydrolase)

Position: 1480424 to 1480741, length: 318 nt, orientation: REVERSE

Perfect match to: (11819-97-CP003194-[1530802:1531119:r], highly conserved allele)

Sequence:

TCACTTTCTTTCGAATCGATTTTTATCTCTTGTATTAAACTTTTCCATTGTTTCATTAAAGCTCTCTGTCATATCTATTCCCATTGAATTCGCTAA
ACATAACAACACAAATAAATTATCACCTAATTCTGCTTTAATCGTATTTGCTTCCTCTGAATCTTTCTTCTTTTTTCACCATAGGTATGATTTA
TTTCACGTGCAAGTTCGCCCACTTCTTCAGTCAATCTAGCTAAGTTAGCTAATGGTGAAAAATATCCTGTTTTAAATTGTCCAATATATTTATC
AACTTCACGTTGCATTTCTACCATTGATTTCAT

Gene: yugP (putative metal-dependent protease/peptidase)

Position: 1481077 to 1481784, length: 708 nt, orientation: REVERSE

Perfect match to: (N315-BA000018-[1499949:1500656:r], highly conserved allele)

Sequence:

TTAATCACTTGAACGCGCAAGCAAAATGATACGTACAAGCTCTGCTACAGCGACAGCAGTTGCTGCAACATAAGTCATTGCTGCTGCAGAT
AATACTTTACGCGCATGCTTGTATTCTTTTTCATTTACAATGTTCAATGCCGTAATTTGTTTCATCGCTCTTGAACTCGCATCAAACTCAACTG
GTAACGTAACAATTGAGAATAATACCGCTAATGACATTAAACCAGCACCAATCCATAAAGCAGTTGAACCAAATGCACTACCTATCGCTGTT
AAGATAATACCTAACATGATGATCATATAACTTAATGAACTCCCTAGGTTTGCAACAGGTACTAATGCTGCTCTGAATCTTAAGAACCAATA
TCCTTGGTGATCTTGAATGGCATGACCAACTTCGTGGGCTGCAATTGCAGTTCCAGCAACTGATGGTCTGTCATAGTTTGCAGGAGATAGT
GAAACAACTTTCTTTTTAGGATCGTAATGATCTGTTAAGAATCCTTCACCTTTAACAACTTCGACATCATAAATACCGTTTGCATGTAAAATTT
CTAATGCAACTTCACGACCCGTTTTACCACTAGTTGATCTAACTTGTGAATATTTCTCATAGTTAGATTTAACTTTGTGTTGTGCCCATAAAGG
AAGCACCATTAATATTACGAAATAAATTATCATAGTAAAAATTGAAGACAATAAACTCAC

Gene: ypjA (putative integral inner membrane protein)

Position: 1481829 to 1482416, length: 588 nt, orientation: REVERSE

Sequence:

TCATTTACACTTTAATAATTTGTTTAATTCAATATAAAGCAAAAGTCCAAAAACACTCAGACAACATGATAATACACCAATTTGCCACACATG
CGTAGTTATAAAATCATAATATGGAAATTGAAGGTGAAAATAGTCAATATAATCATTCAAAAATACCCAAGTCATCGCTACACTGATTCCAA
TCATAGAACGTTTAAACCTAGGATAGAAGTAAATTGCCTGAACGGCCATTATACTGTGGGAAAACATTAATATCAAACCATTTACTGTAATA
TCACCTTGTTCAATAATAAATAATATATTCATAATAACTGCCCAAATCCCATATTTGAATAATGTTACAAATGCCAGTGCATCGATAATACTAT
TTTGTTTTTGAATTAATATCAATGTGATAGAAATAACTAAGTATAATATTGCAGTTGGGCTATCTGGAACAAAAATCTTAAAATGCCACGAC
GTATGACTTAATTGTTCACCATACCATATATAACCATAAATCATCCCTAATATATTACAAACGAGTAGCATCATTAACCAAGAACGTTGATAA
AGTGTATATTGCCAAAATGTATTAATCGTCAT

Gene: ypiB (conserved hypothetical protein)

Position: 1482406 to 1482981, length: 576 nt, orientation: REVERSE

Perfect match to: (11819-97-CP003194-[1532784:1533359:r], highly conserved allele)

Sequence:

TTAATCGTCATCTGCTAAGTCCTCAAATTGATTATGTTTATTTACTAGCTTGAGTGTATTTAAAATTTGCGTTAGTTGATAAAAACGTTGCTTT
TCATTCATCTGTAAACTTAAATCAATATTATGTAGCAAGTAATCTATTAATAACGCATGTTTATGCCGATCTATAGCCATACTATTTAAGTCAT
GAAGATAAGTTTGATAACTGGGCGATTCCGTTAGTTGAGCAACTATTAAATCATCTAGCCTTTGCTCACGCTTTGACACGTTTGCGAAGTGA
ATTTGAATATCAAAAGCACAGTTATGATTAGCGATATAATCAAATATTTCATTTGTATTCATTAACTTTATATTACGCTTAGTAAATTGAATTG
CAGAAGCGTGACTTCCCACTTCTGCAATTTCTAATGTTTCATGATGATTAATTTTTGTATCTACAAAATGAATGTTTGCCAATTTCGCCTCATT
CACTTTTATATAGTTAAGCACCCAAACTGCAATACGCGACTTAAATCGATATTGAAAAAGTAAATATTCAATAAAACTTTCTTTAATTTGATT
GAGTGTCTCTGACAT

Gene: ypiA (putative protein)

Position: 1482995 to 1484239, length: 1245 nt, orientation: REVERSE

Perfect match to: (11819-97-CP003194-[1533373:1534617:r], highly conserved allele)

Sequence:

TTAAGATTGCAATCTTGATAATTCGTCATGCCAATTTTCGTTACTTGGTTCTAGTTCCAACAATTGATTTAAAATAGTAATTGCTTGTTCCTTTT
GACCAATTTCAATTAAATAGAAATAATAATCACTCATAAAATCAATATTTGTTTTCATCGTTGGATATGCTAATTCAAAGAAATGTTGAGCTT
CTTTATCTCGCTCTTCTTGACCATAGGCGAACGCTAAATGCCACATGAATGTAGGATCCAAATCTTCTTCATCTACATATGTTAATAATTCAAT
GATTGCTTCATAATCTTCTTCATTACGATATAAATCGCTTAAAATCAATAAAGGTTCTTGGTAAGCATTATCAACCTCTAATGCTTGTTTTAAC

AATAATACACCTTCATTAGCATCGCCGTGTTCTATTTCCAAACATCCAGTTGTATACATTAACTCTTTATAAAATTGACTTAGTCGTAATCCTT
CTTTACCCGTCTCAATGGCATCTGGATAATTTTTTTCATTTTCATATAATGATTGTAAATACAAGTAGCCTTGAATATAATCAGGATCTTTAGA
AAGTAATGTAGTCATTATTTTAATTGCTTCTTGAGTGATGTCATTTTTATCGTAAGAAATGGCTTTTTTGAGATAATCTTCTGAAGTCATTTCA
TCTTCATTAATTTCATCGTATAAGCGAATCGCATCACTATAGTTACCACTTTGTAAACTACAATCTGCCATACGAGAGAATAAGTTTACACCA
TTAACTTGATATTCACCAGTTTCTAAAACGGTTTCGTATTCAGAGGTAGCACGTAAATATTGACCATCATAATATAACATTTCAGCCAATGCA
AAATGGATTATTGGATCATTTGGCTCTAGTTCAAGTGCTTCTTGTAATTTATCAATAGCAACTTCCATCATATTAATTTGTTGATATAAATCTG
CTTCTAACATCAACTTTTCAGGTGATGGTTCAACATAACTTAAATATTCTAACGCTTCGTCAGTTTGATTTTCAGACATTAAACCTTCAATAAA
ATAAATCAGCAATTCACTTTCGTCTGGATATTTTTGATATAACACGCGGAATACTTCCAAACCTTGTGGCATTAATCCAAAATTGTAAAGTGT
CTCTCCTAGAATAAATAATGCGTCATCGTTGTCAGTAGTTATTGCTTCATTAACACGAGAGTCTAAATTTTCTAGTTTTTGTAGATTGATATCG
TCTATTAATTTATAGATATCTTCCAT

Gene: aroA (5-enolpyruvylshikimate-3-phosphate synthase)

Position: 1484246 to 1485544, length: 1299 nt, orientation: REVERSE

Perfect match to: (IS-24-AHLM01000019-[62424:63722:r], allele observed in CC8)

Sequence:

TTATCCCTCATTTTCTAAAAGCTTTAGTTTTGGTAAAAATCCTGGAAATGATACATTTACAGCATCAAATTGTTTGATTTTGACAGGCTCGCTT
GAAAGTAGAGAAGCAACTGCAAGCATCATTCCTATTCGATGATCAGTTAAACTATCAACTGTTGCATTTGTTTTAAATTCTGACGGATGAAT
AATCAATCCATCATTAGTTGGTTGTAATTCAAACCCTAACAAGTTTAACATATCAGCCGTTGTATCAATTCTATTTGTTTCTTTTACTTTTAATT
CCTCGGCATCTTTAATTGTACTCGTGCCAACTGCTTGTGTACAAAGTAATGCTATTACAGGCAGTTCATCAATTGCTTTTGGCACTAATTCTCC
TTCGATTGTTATTGGTTGAAGCATTGGTGTGTATTGAATACGAATAGAAGCAGTAGGTTCAGCACCAGTTGTTTGATTGAAAAGTTGGATAT
TACCGCCCATTTTTTCAACAATATCAATAATACCTGAACGTGTTGGATTGATTCCAATATTATGAATCGTTACATCACTTCCTGGTGTGATAA
GTGCTGCAACAATAAAGAACGCTGCAGATGAAATATCGCCAGGAACATGAAAATCTGCAGGTTTAATGTATCGAATTGCTTCAGGGGTTGT
ATTAATTGATAACCCTTCTGCTTCAATTGGAATATTAAAATGTTTGAACATCGTCTCAGTATGATTTCGACTTACATCTAATTCTTTAATGATG
GTCGGTTCCTTAGAAAACAAACTTGCAAATAAAATGGCACTTTTTACTTGTGCACTTGCAACTTCCATTTGATAATTTATACCTTTTATGACAG
ATGGCTTAATAATTAATGGTGTATAATTATCTTCAATACCTTCAATATTCGCATCCATAAGTTTCAATGGTCTCAAGACACGATCCATTGGCCT
TTTACCAATTGAAACATCGCCAGACAAAACACTTTCAATACCTAAACCACTTAACAAACCTGCCAATAATCGTGTTGTCGTACCAGAATTACC
TGTATACAATACTTGATGTGGCGTGTTAAAAGATTGATATCCTGGGGAAGTCACAACTAATTTTTCATCATCTTCTTTGATTTCTACACCTAA
CAGTCGGAAAATGTCCATCGTACGACGACAATCTTCGCCAAGTAGTGGCTTATATATAGTAGATACACCTTCAGCTAGCGACGCCAACATG
ATTGCACGGTGTGTCATTGACTTATCGCCCGGCACTTCTATTTCGCCCTTTAACGGACCTGAAATATCAATGATTTGTTCATTTACCAT

Gene: aroB (3-dehydroquinate synthase)

Position: 1485554 to 1486618, length: 1065 nt, orientation: REVERSE

Perfect match to: (COL-CP000046-[1545985:1547049:r], allele observed in CC8+CC80+CC239)

Sequence:

CTACTTAAAATATGTTTTTAATTGTTCACATGCATGTTGTAATGTTAGTTGATCAACATGTTGTACAACGATATCTCCAAATTGTCTAATCAAG
ACCATTTGTACACCTTGCTTATCATTCTTTTTATCACTTAGCATATATTGGTATAACGTTTCAAAATCCAAGTCAGTTATCATGTCTAAAGGAT
AGCCGAGTTGTATTAAATATTGAATATAATGATTAATATCATGCTTAGAATCAAACAAAGCATTCGCAACTATAAATTGATAGATAATGCCA
ACCATCACTGCATGACCATGAGGTATTTTATGATAGTATTCAACAGCATGACCAAATGTATGACCTAAATTTAAAAATTTACGTACACCTTGT
TCTTTTTCATCTGCAATAACAATATCCAGCTTCGTTTCAATACCTTTAGCAATATATTTATCCATACCATTTAATGACTGTAATATCTCTCTATC
TTTAAAGTGCTGTTCGATATCTTGCGTCGCTGATTCACCATTCAATAACGCATGCTTATAAACTTCTGCATAGCCACTTAATATTTGCTCAAAT
GGTAACGTCTTTAAAAAGTCTAAATCATAAATCACAGCAGTTGGACGATAAAATGCACCGATAAGGTTTTTACCTTGCTTTGAGTTAATACC
CACTTTACCGCCAACACTAGAATCATGCGCTAGTATAGTCGTTGGCACTTGTATAAAGTGAACGCCTCGTAAAAGTGTCGCCGCAATAAACC
CAGCAAAATCACCAGTTGCACCACCACCAACAGCAATAATTGCTGTATTACGAGTTACATGATGGGATAAAATATACTCTAATGTTTCTTGA
TATTGCTCAAATGTTTTCGTCTTTTCACCAGCTGGAATAATAACTTTATGTACATTTTCATATGATAAAATATCATCAAATTTATCAGCAAAAT
ATTGATTTACATGCTCGTCAATTAATATAAAACTTTGATCAAACTGATCAATATACGTGCTAATATGGTCAATTGCACCGTGTTCAACATATA
TTGGATAATTATTTGAAGGGTATGTTGTTTGTAATTTCAT

Gene: aroC (chorismate synthase)

Position: 1486644 to 1487810, length: 1167 nt, orientation: REVERSE

Perfect match to: (11819-97-CP003194-[1537022:1538188:r], allele observed in CC80+CC188+CC772)

Sequence:

TTAAAACTCAATATTTAATTGTCTGCGGTCAGCAATTTGTTGTTGAAGTTGTTCAATATGGTTTGATTGAAACTCTTCAAGTAATGCTTTCGCT
ATTTCAAATGCAACAACGTGTTCACACACTATACTTGCTGCAGGTACAGCACAACTATCAGAACGTTCAATTGTTGCCTTAAAGTCTTCTTTA
GTATTTATATCAACTGAATTTAATGGTTTATATAACGTAGGAATAGGTTTCATTACACCATTTACAATAATTGGCATACCATTTGACATACCA
CCCTCTAAACCACCTAAATGATTAGATCCACGATAATAACCAATTTCACTATTATATAGAATTTCATCTTGAATCTCACTACCTGGCTTTTCAG
CTGCTTTAAATCCTTCTCCAAAGCTTACACCTTTAAAAGCATTTATGCTGACAACACCTTGTGCAATCTTACCATCTAACTTACGATCATAATG
CACATAACTACCAACACCAACAGGCATATTTTCAACTACAACTTGAACGACACCGCCAATTGAATCTCCTTCATTTTTAGCTTCGTCAATTTTA
TCTCGCATTGCTTGTGCGATACTGTCATCAATTACACGAACATCATTACGATCAAGATTTGCTTTAAATGTTTCTGAATCATAAAAATCTTTAT
CTTTAATGCCACCTATTTCAACAACACGACTGTATATATCGATATCTAACTGTTGTAATAACACTTTACATAAGGCACCGACTGCAACTCGAG
CTGCTGTTTCTCTAGCAGATGATCGCTCTAGCACATTTCGTAAATCACGATGATTATATTTCATACCTCCAACCAAATCTGCATGACCAGGTC
TTGGTTTTGTAATAGTACGTTTCATATTTTCACGTTCTTCTTCACTTATTGGAGCTGCTCCCATAATTTTTCTCCAATGCGTAAAGTCATCATTG
GTTACAACCATAGTAATTGGACTACCTAATGTATAACCATTTCTAACGCCTGATACTATTTCTACTGTATCTTTCTCAATTTGCATGCGTCGGC
CACGACCATAACCCCCTTGACGCTTGAACATTTCTTTATTAATATCTTCAACTTTAATTTCTAAATTTGCTGGTATACCTTCGACAATAACTGTT
AATTGAGGTCCATGTGATTCTCCTGAAGTTAGATATCTCAT

Gene: Q2FYG8 (putative membrane protein)

Position: 1488196 to 1488397, length: 202 nt, orientation: REVERSE

Perfect match to: (11819-97-CP003194-[1538574:1538775:r], allele observed in CC80)

Sequence:

TTAAGCGCTTTTGTTATACTTTGACTTCAAAAATATAATCAATTCTTTTACTAATACATAAAAAAATATAAATGAGAGTCAGTATAAAAATAA
TTTTTGTTATAGGATTAAGTGGTTTATCTAAAAATATAGCACCATATAGTAATTCCATTAATTTTAAGAACATTAAAATCCCCAAAATAATCG
AAATAATATTCTTCAT

Gene: ndk (nucleoside diphosphate kinase)

Position: 1488606 to 1489055, length: 450 nt, orientation: REVERSE

Perfect match to: (394_SAUR-JVIV01000066-[17346:17795], highly conserved allele)

Sequence:

TTATTCATATAACCATGCATCACGTGGTGAAGCATAGCTAGTAATTTCATTTTCATTAAACCATAGATTAATTTCACGTTCAGCAGACTCTAAT
GAATCTGAACCGTGAATGATATTTCTACCAACAGTTAAACCTAAATCACCTCTAATTGATCCTGGTGAAGCTTCTGAAGGATTTGTGCTGCC
AATAATATGTCTAGATACATTAACTGCATCTTCACCTTCAACTACCATTGCGAACACTGGTGCTGATGTAATAAATGAAATTAAATCATTATA
AAATGGTTTACCTTGGTGTTCACCATAATGTGTTTCAGCAAGTTCCATTGGTACTTGCATTAATTTACCACCGACAAGTTTAGTCCTTTTCTT
TCAATTCTTGAAATTACTTCACCAATTAGATTTCTTTGTACTGCATCTGGTTTAATCATTAAAAATGTACGTTCCAC

Gene: gerCC (heptaprenyl diphosphate syntase component II)

Position: 1489147 to 1490106, length: 960 nt, orientation: REVERSE

Perfect match to: (COL-CP000046-[1549577:1550536:r], allele observed in CC8+CC239+CC4803)

Sequence:

CTACGTGTTTCTTGAACCCATTTTTTTCGTCAAACTTAAAAGTAGTGATTTCGGATGTCCATCTGGTAACTCAGAAATCAAATCCAAAGCTTT
ACTTAAATACTTCGAACTTACTGCCTTAGCCTCATCGATGCTGTCAGATTTTCTAATGATTTGGATACATTCTTCAAATTCTTTACGTTCACTAT
CACGACGTAATTGTTCAATTTTCAATTTGAAGTCTGGGTTTTTACGCATTTCTAATAAAATCGGTAACGTAATATGACCATTAAGCAAATCAC
TTCCGACCGGCTTACCTAATTTCTTTTCGGTACTTGTGAAGTCTAATACATCATCAATGATTTGGAAGCTCATACCTATATAATGACCAATCAT
TTTCAATTTTCGTACAGTCTCTTTATCAGATTGAGATGTAATTGCACCAACTTCAGTTGATAATTGAATTAACAGTGCTGTTTTGCGATTGATA
CGTCGTAAATAATTGATAATTGTCTGTTGACTGTTAAATTGGTCTTGAAATTGGAAAAGTTCCCCTCTACAAACATCAACGATAGATTCAGAT
ATTAATTGATGTACACGATTATCTTTAACTGCCATTAAGTGTTCAAGTCCTAATGCCAATAAAAAATTCCCAGTTAAAATAGCAGTTGTCTGA
TCCCATTTCTTTGATATGGTTAACTTGCCTCGACGCTTGTCGCTTTTATCAATAACGTCATCATGAACAAGTGTTGCCATATGAATTAACTCTA

ATGCGACTGCAACTTGATACGTTTGTTCAGACGTTTGTTCATCTTTGCCAAATTGGCTACTCAGAATAACAAATGCTGGGCGTACTCTTTTAC
CACCAGAAGACAATAAGTGTAATGATGCCTGTTCTAATACAGAATCTTTACTTTTTATTGCCTTTTCAAGTCGTTGTTCCACTTTCTTAATTTC
ATTGTTCATGTTTAACTTTGCCAC

Gene: ubiE (menaquinone biosynthesis methyltransferase)

Position: 1490108 to 1490833, length: 726 nt, orientation: REVERSE

Perfect match to: (11819-97-CP003194-[1540486:1541211:r], highly conserved allele)

Sequence:

TTAATCACCTTTGGTATTATCTTTTTCTTTATAGCCAAGGTGCATTGCAGCAACGCCCCCTGTAAAACTACGTACTCTTACATTTATGAAACCC
GCTTCTTCAAACATGCGCTTCAACTCTTCTTTTCCAGGAAAATTAAACGTAGATTGCTGTAACCATTCATATTCTTCTTTTGATTTTGCAAATA
ATTTTCCAAAAATAGGCATAACAAATTTAAAGTATAATGCATACATTTGTTTAAAGACTGGCAAAGTTGGTTGGCTCGTTTCAAGACATACC
ACCATACCACCTGGTTTAAGTACTCTATTCATTTCTTTTAACGCGACTAAATAGTCTGGCACATTTCTTAATCCAAACCCAATTGTTACATAAT
CAAAAGAATTGTCTTCAAACGGCAATTCCATTGCATCACCATGAACAAGTTTAACATTTTCCATTGAAGCAGTTTTTTCTTTTCCTACTTCTAA
CATATTCTCACTAAAGTCAATACCAGTAACTTCACCTGTTGGTCCTACAGCTTTGCTTAATGCGATTGTCCAATCACCAGTACCACAACAAAC
ATCTAATGCTTTCGTCCCTTTTCTAACGCCCATGTCTTTCATGACGCGTTTTCTCCATACTTTATGCTGCTCAAAACTAATAATATTATTTAATC
TATCATATTTTTTTGAAATATTTTGAAAAACGCGATGTACTTGCTCTTTATTTGCTTTATTGTCAGCCAT

Gene: gerCA (putative protein)

Position: 1490836 to 1491408, length: 573 nt, orientation: REVERSE

Perfect match to: (N315-BA000018-[1509707:1510279:r], highly conserved allele)

Sequence:

TTAATTACCTCTACTTTTTAAATAACTTTTTTGGATATCGTGTAAGTAATGCTTTACTTCACTTTGATTATATTTCTTGAAGTATGATGGATAGT
AATCAGACATATCTTCAAATAAATAATTATATATTTCCGACTCATCAATATTGATACCGAAATGAGATAACGTAATATATGGGAAAAGTGTTT
CAATTTTTACTATTGCTTGAGAAATTTCATAATCATTTAAAGCTTGATGATGTAATGAAGATTTCAATTCATTAATTTCAACAATTGCTTTACT
AATTTCATTTTGAAATGATAAATCATTGATTTCTGCTAGTAGCGTATAAAAAATGTGCACTAATTAAATCTCCAATCAAAATGGAATGTTTAGA
CAAATGATTATATGTAATGTCATCAAGGTGTCTCATTGATGTGTCAATTGTGAGGCATGCTACTTTGGCAACATCTGGAATATCATATGAAT
CAAGTAACTTACCTAACCGATGATTAATATTAATGGATTCATATTCTGATACACCCTTTAATCTTTCTTCTATTTGTCTTTCCAATTTGCTAACA
GTTGTTTCCAT

Gene: hup (DNA-binding protein HU)

Position: 1491839 to 1492111, length: 273 nt, orientation: REVERSE

Perfect match to: (RF122-AJ938182-[1463493:1463765:r], highly conserved allele)

Sequence:

TTATTTTACAGCATCTTTTAATGCTTTACCAGCTTTGAATGCTGGAACTTTACTTGCTGGGATATCAATTTCTTTACCAGTTTGAGGGTTACGA
CCTTTACGTGCAGCACGTTCACGTACCTCAAAGTTACCGAAACCAATTAATTGTACTTTTTCACCTTTAGCAAGTGAGTTTTGGATTGATTCG
AATACAGCATCTACTGCTGAACCAGCTTCTTTTTTAGTTAAATCAGCTTGCTCTGCAACTGCATTGATTAAATCTGTTTTGTTCAT

Gene: gpdA (glycerol-3-phosphate dehydrogenase)

Position: 1492282 to 1493280, length: 999 nt, orientation: REVERSE

Perfect match to: (11819-97-CP003194-[1542660:1543658:r], allele observed in CC80+CC22+CC4803)

Sequence:

TTATTCAGATTTTTTATCGCGCTCCATTAAATCTTTTACGCATTCTTTTACTGAGATATTTTCAAATAATACTCTATATAATGCATTTGTAATTG
GCATATCCACATTTTTTTCTTTAGCTAAATGATAAACTGATTTAGTTGTATAAATACCTTCAACAACCATATTCATTTCAGATAATGCTTGATC
CATTGATTCACCTTGTCCAAGTTTATATCCTAATGTGAAATTTCTAGAATGTGTTGATGTGCAAGTAACGATTAAGTCACCGATACCACCTAA

ACCTAGAAATGTCATAGGATCGGCACCTAACTTTTCACCTAATCTACTAATTTCCGCTAAACCACGAGTCATTAATGCAGCTTTTGCATTATC
ACCGTAGCCAATTCCAGCTACGATACCACTTGCTACTGCGATGATATTTTTCAATGCACCACCAAGTTCAACACCAATCAAGTCATCATTCGT
GTACACACGCAAATAATCATTCATAAATAAATCTTGCGTTAATTTACTTACACTTTTATCTTTTGATGAAGCAGCAACTGTAGTTGGTTGCTT
GACTACAACTTCTTCCGCATGACTTGGCCCTGACAACACGCCAATACCTGCATTATATTCAGGTGAAATAGAATCTTCAATCATTTCTGACAC
ACGTTTAAACGTCCCATTTTCAATACCTTTAGCAACATGTATAAAAGTCTTTTTAGAGGTCAGCTTATCATTAATTTGAGAAGCAACTTCTCGC
ATTGCTTTAGTAGGTAAAGCCATTAAGTAAATATCTGCAAATTGAATTGCTTTGGTCATATCTGAAGTAGCGATGATGTTAACATCTAATTTC
GCGTATTTTAAATACTTTTTATTTGTATGACATGTATTTAATTCATCAACAGCATCTTGATTTTTACCCCACATCAAAACATCATGTCCATTTTC
TGCAAGAACATTGGCAAGGGCTGTCCCAAAACTTCCCATACCAAAAACGGTAATTTTAGTCAT

Gene: engA (GTP-binding protein)

Position: 1493297 to 1494607, length: 1311 nt, orientation: REVERSE

Perfect match to: (M0239-AIWE01000007-[70203:71513:r], allele observed in CC188+CC5+CC80+CC88+CC4803)

Sequence:

TTAATTTCTCTTTCGAGCTATAATATGAATTGGTGTACCTTCAAAACCAAAAGCGGCACGGATTTGATTCTCTAAATAGCGTTTATAAGAAAA
ATGCATTAATTCTACATCATTAACAAATACAACAAATGTCGGTGGTTCTATAGCAACTTGTGTTGCATAAAAGACATTCAAACGTCTACCTTT
GTCTGTTGGTGTAGGGTTCATGGAAATTGCATCAGTAACAACTTCATTTAAAGTTGAACTTTGAACACGTTTTTTATGGTTTTCACTTGCTTC
ATTAATGTAAGGGAATAATGTACGTAATCTTGTGCGTTCTTTAGCAGACACAAAAGCAATTTGTGCATAATCTAAAAATTGGAATTCTTTAC
GTACTTCATCTTCAAATTTCTTCATCGTTTTACTATCTTTTTCCACAGTATCCCATTTATTTACGACAATCACGACTGCTTTACCTTGTTCATGTG
CATATCCTGCAACGCGTTTATCTTGTTCAATGATGCCTTGTTCTGCATCAATGACCACTAAAACAACATTTGAACGTTCAATCGCTTTTAAAGC
TCTTAGTACTGAATATTTCTCAGTTGATTCATATACTTTTCCTTTTTTACGCATACCAGCAGTATCGATTAAAACATAATCTTGTCCATCATAAC
TATACTCTGTATCAATAGCGTCTCTCGTTGTCCCTGCAACATTAGAAACGATAACGCGATCTTCACCTAAAATAGCATTTACTAAACTTGATTT
ACCTACGTTTGGTCGTCCAATAATGGATAGTCGAATTGTATCTTCATCATAAGGATCTTCTTCCTCTTCACCAAAATGAGAAACAACTGCATC
TAACAAGTCACCAAGACCTAAACCATGTGACCCTGATATCGGATACGGTTCACCAAATCCTAATGAATAGAAATCATACACGTCTGTACGCA
TTTCCATATTATCTACTTTGTTAACCGCTAATACGACCGGTTTTTTAGATTTGTATAAAATTTGAGCGACCATTTCATCGCTTTGTGTCAATCCT
TCACGCACGTTAACCATAAAAATAATAACATCCGCTTCATCTATGGCGATTTCTGCCTGCGCTCTAATTTGTGTTTGGAATGGTGCATCACCA
ATTTCAATACCACCTGTATCAATAATATTGAAATCATGTGTTAACCATTCACCTGAAGAATAAATACGATCTCGTGTTACACCTGGCGTGTCT
TCCACAATCGAAACACGTTCTCCAACTATTCTATTAAAAATTGTAGATTTACCTACATTAGGCCTACCTACAATAGCTACTATAGGTTTAGTCA
T

Gene: rpsA (30S ribosomal protein S1)

Position: 1494829 to 1496004, length: 1176 nt, orientation: REVERSE

Perfect match to: (JKD6159-CP002114-[1500053:1501228:r], highly conserved allele)

Sequence:

TTATAGTTTAAGATTTTTAAGTTTATCACCAATCATATCGCCAATTGTTGGATTATCTTCTTCTTCGCTTTCTAAGTACGCCTTAGTCGTAGAA
GGATCACTTTCAACAACATCTTCGTTTGGTAATGTTGCTTTAATAGATAGTGATACTCTTTCATTCTCTTCATCAATACCTAATATTTTAACATT
TACTTGTTGACCAGGTTCTAACACTTCACCTGGCGTACCAATGTGTTTGTGTGCAATTTCAGAAATATGTACAAGTCCTTGTACACCTGGTGC
AATTTCAACAAATGCACCAAAGTTTGCCAATCTTACTACGACACCTTCAATGACATCATTTTCGTGGAATTGACCTTTAATATTTTCGAAAGG
TGTTGGTAACGTATCTTTGATTGATAATGAAATACGTTCTGTATCTCTATCAATAGATTTAATTTTAACTTTAACATCTTGACCAATTGAAACT
ACTTCTTCTGGTGTTTGAACATGTTCGTGAGAAAGTTCAGATACATGCACTAAACCATCAACACCGCCAATGTCTATAAATGCACCAAATTG
AGTTAAACGCGCTACTTTACCATCAATAACATCGCCTTCATTTAAAGATTGTAATAATTGATCTTTTTTAGCATCGTTTTCTTCTTGTTCAACTG
CTTTACGGCTTAAAATGACTCTATTATTTTCAGGATCCAATTCTTCAACTTTAATACGAATTGTTTGTCCATCAAACACAGAGAAATCCTCAAT
GAAGTCTGTTGAAATTAGTGAAGCCGGAACAAAACCTCTTTGTCCTACATCAACAACCAAACCACCTTTAACAACTTCTGTTACTTTCGCTTC
GATGATTTCATTATTATCTAATTTTTCTTGTAAATAACTATAAGACTTCTCAGTTTCAAGTTGTCTTCTAGATAAGATGTAAGCTCCAGTTTCA
TTTTCTTCATCAAACTCAACTTTAGTGACATATGCTTCAACTTCGTCGCCCTCTTTTACAACTTCACTTGGGCTATCAATATGATGCGTAGATA
GTTGACTAATAGGAATAATCCCATTAAATTTACCACCGTTGATATGAACAACAACTTGCTTGTCTTCAACTTGTTGTACCTCGCCAGTGACTT
TGTCACCTTCTTTAATATCGTTAATCATTGATTCATTGAATTCTTCAGTCAT

Gene: cmk (cytidine monophosphate kinase)

Position: 1496702 to 1497361, length: 660 nt, orientation: REVERSE

Perfect match to: (RF122-AJ938182-[1468371:1469030:r], highly conserved allele)

Sequence:

TTATTTAATTTGACTCACCATCGCTAAAATTTCGTCAGTAACTTCTTCAATCGACTTGCCTGTCGTATCTAATGTCACTGCATCATCTGCTTTTC
TTAATGGTGATATTTCACGGTTCATGTCATATTGATCACGAGCTTCAATATCACGTTTTAAATCTTCAAAATTTGATTCGATACCTCTTAATTG
ATTATCTTTATATCTTCTTTCTGCTCGCTCTTCAACTGATGCAATCATATATACTTTTAAATCTGCATCTGGTAGCACTACAGTTCCGATATCGC
GACCATCCATTACGATACCTTTTTCTGCAGCTAACTCTTTTTGTTTTTTAACGGCGAATGAACGTACTGGCTCTTTAGATGCAACGTATGAAA
CATGTTGCGTCACATCATTATTTCTTAAAAAGTCTGTTACATCTTCGTTATCTAAAATGACACATTGACCTTTATCTGCTTTATAAGTTAAATCT
AATGTTGTTTGGTCAACTAGTTTTGCAAAGTCCTCAGTTTTGTTTAATTTTAAATATTTGTATGTTAATGCACGATACATTGCTCCTGTATCGA
CATAAATCATTGATAGTTCGCTGGCTACACGTTTCGCAATTGTACTTTTTCCGGCAGCAGCTGGACCATCTAATGCAATATTAATGGCTTTCA
T

Gene: ansA (L-asparaginase)

Position: 1497438 to 1498406, length: 969 nt, orientation: FORWARD

Perfect match to: (T0131-CP002643-[1566344:1567312], highly conserved allele)

Sequence:

ATGAAACATCTACTTGTTATTCATACTGGTGGCACCATTAGTATGTCACAAGACCAATCTAATAAAGTAGTAACAAATGATATCAACCCTATT
TCAATGCATCAAGATGTCATAAATCAATATGCACAAATAGATGAATTAAATCCTTTTAATGTACCATCACCTCATATGACAATCCAACATGTT
AAACAATTAAAGGATATTATTTTAGAAGCAGTAACAAATAAATATTATGATGGTTTCGTTATCACGCATGGTACCGATACGTTAGAAGAAAC
TGCCTTTTTACTTGATTTAATATTAGGTATCGAGCAACCTGTTGTTATTACTGGCGCAATGCGCTCGTCTAATGAAATTGGTTCTGACGGATT
ATATAATTATATTTCCGCTATTCGCGTTGCCTCTGATGAAAAGGCCCGTCATAAAGGCGTGATGGTTGTATTTAATGATGAAATTCATACGG
CGCGTAATGTTACCAAAACACATACGTCTAATACAAACACATTTCAAAGTCCAAATCATGGTCCGCTAGGTGTATTGACAAAGGATCGTGTG
CAATTCCATCATATGCCATATCGCCAACAAGCATTGGAAAATGTCAATGACAAACTAAATGTACCATTAGTAAAAGCATATATGGGTATGCC
AGGTGACATTTTTAGTTTTTATAGTCGAGAAGGTATCGATGGTATGGTTATTGAAGCGTTAGGACAAGGCAACATACCTCCAAGCGCATTA
GAAGGCATTCAACAATTAGTATCTTTAAATATACCTATTGTGCTAGTTTCACGTTCCTTTAATGGTATTGTGAGTCCAACTTACGCATACGAT
GGTGGTGGTTACCAACTCGCACAACAAGGTTTTATTTTTTCTAACGGCTTGAATGGTCCAAAAGCAAGATTAAAATTATTAGTCGCGTTAAG
CAATAATTTAGATAAAGCTGAAATCAAATCATATTTTGAATTATAA

Gene: ypdA (FAD-dependent pyridine nucleotide-disulphide oxidoreductase)

Position: 1498521 to 1499507, length: 987 nt, orientation: REVERSE

Perfect match to: (ED133-CP001996-[1559947:1560933:r], highly conserved allele)

Sequence:

TTATGATTCTAAGGGCGTTTGTTTCTTAGCTAGCATGCTTTGAGCAATAATGCCCCCGTGGAATTTACCATTTTCAATAAAAATGGTATTCGC
ATCGTTCCCTGCAGCAATTACACCTGCAATATAGCAATTTTCGATATTTGTTTCGTATGTTTCTTTATTATACATAGGCGCTGTTCCAAATTCA
TTTGTATTAATTTGAATGCCTACAGATTTTAAAAATTCATAATCGGGATGATAACCAATCATCGCAAATACATAATCATTGTGTATCGTTTTAC
TTTCACCATTTACTTCATAAGTCACAGTATCTTCAGTTATTTGGGTAACATTAGCATTAAATTCCATGTCAATTTTTTCATGATTTACTAATGCT
GTGAAATTTGGAAGTATCCACGGTTTAATTGAAGGCGAATAATCTCCACCACGATATAGAACCGTCACGTTAGCACCAGCTTTTTCCAACTC
CAAAGCAGCATCGATAGCCGAATTCTTACCACCGATAATTACAACATCTTGATCAAAATACGGATGTGCCTCTTTAAAATAATGGAACACTT
TAGGTAAATCCGCACCTTCAACTTCTAATGTATTATGCTGACCATAATAGCCTGTCGCGATTGTTAAAAATCGACATTCATAAACATCTTTCG
TCGTAGTAATAGTAAATTTATTATTCATTTTTTTAACAGTTAATACTTCTTCAAATGCATTTACTTTTAATTGATGATGTTTTACAACTTCTCGG
TAATAAACTAGCGCTTGATTACGTCTTGGTTTACTTTCTTCAACGATAAACGGTACGTCCCCAATACTTAATTTATCACTTGATGAGAAAAAT
GTTTGGTGAGTAGGATAATTGTAGATTGATTCAACGACATTACCCTTTTCAATAATTAAGGTATCAATACCTTTTCTTTTTTGTTCAATAGCCG
CACTTAATCCGCATGGCCCTCCACCAATTATGATACTTTCAACTTTTTGCAT

Gene: ebpS (cell surface elastin binding protein)

Position: 1499909 to 1501369, length: 1461 nt, orientation: REVERSE

Sequence:

TTATGGAATAACGATTTGTTGACCGTTTCTAATATTGTTACCACTTAAACCATTGGCACGTCTAATTTTTTCAACATTTTCCGGTGAACCTGAA
CCGTAGTATTGAATTGCGATACGGTATAAGTTTTCTTGACCATTCACTGTATGTCTTTGGCCACCACCTTGACGTTGTTGTTGCTGTTGTTGA
TTTTGATTAGCTTGTTGTTGATTTTGATTATTTTGTGCTTGATTGTTCGCTTGATTAGCGTTGTTTTGATCATTATCAGATTCATCTTTAGTCGC
TTTGTCTTGATCCTCTTTTGATTTATCACTGTCTGTAGATTTTGATTTATCTTTAGAAGCGTCTTTAGATGTGTCTTTGTCTTTACTTTCATCAGC
ATTATTTTTATTTGTATTCGCGATTTTATTTTCTTTTGTACCATTATTATGATTGTTTAATGCCATGCCTCCAAATATCGCTAATGCACCGATAA
TTAGTACAGCTGCAATTAAAGGTAACAATACTTTGGCCATGCCACCTTTTTTACGTTCTTTATCTCTGTCATGATTGTCATGTTCATCATGGTT
TTGGCTTGCATTATTAGAGGCATGTGGTTTTGAAGCGGCAGAAGCACTTTTACTTGCTGCGCCTCCAGCCAAACCTGCTGTTCCAGCACCGA
TCGCTGCACCTTTTTTGCCATTATGATGATCTTTAGACTTATCTTGAGACGCTTTATCCTCAGTCGAGTTATTCGACTTGTCAGAATTACTTTT
GTTTTGAGCGTCATTTGAATGTTTCTTAGCTTTAGAAACACCCATTGCACCAGCTGCACCTGCAACACCCGCTGTTCCAGCACCAATTGCTGC
GCCTTTTTTACCACTATGATGATCTTTAGATTCATCTTTGTCTTGCTTAACAGTTACATCATCATGTTTATCTTTTGATGTATTTGCTTGGTTAG
CACCTGTTGCAAAATATGGTTTAGGTTGCTGAGATTGTTCAGCTTCACTCTTATCAGAAACTGTTGAATGCTCAGTGTTATTTTCTGCTTCTTT
AATAGTCTCGTGTTTATCATTGTCTTCGATTGGTTCTGGATGTGATTTATCCATTGCAAAAGCATTCTTATTATAATATTCCTCTTCATGTTGA
GGTGTACTGTCTTGATGGCTAGGTTCTTGACTTTCAGTACTGTGTGATGATTCGACTTGACGATCATCTATTGTGCCAGCCTCATTTTGAACA
TTGTCTTCAGATGTTTGTGATTCATTGTGAACTTGTTTATTATGATTCGTTGCTAAATCACGGCGTCTTTTTCTTCTTTGGGCATTTCTTGGCG
GAAACTGTTGCTCCGTATTCTCTATTGTATCCTGATGTTCTAATTCTGATTGGTCTTTTTCAACATCTTCCGTATGGTCTTGATGTGAATTTGT
GTCTATCGATTGACGATTTTTTTCAAAGTCATCTTTAAAATTATTAGACAT

Gene: recQ2 (ATP-dependent DNA helicase)

Position: 1501522 to 1502901, length: 1380 nt, orientation: REVERSE

Perfect match to: (IS-105-AHLR01000124-[11020:12399], allele observed in CC22+CC5)

Sequence:

CTATCTGAGAAATAAATTTTGCAACTTTTCATCAAATCCAATACTTCTAATTACCTTCTTCTTATTTAAAATTGCGATATCAGTTATATTAGAAT
CATTGTCACAACATCGATCTTGTGCCGGTGGATATTCACCGAAAAATTCTAATAAATACTTCCGTCTACATTGATCCAATTTGCAATAGCCAA
TCATGCGAAAGAATCCTAATTGCTTTCGTTTAAATGATTGCTTAAATATCTGTTTCAAGGCGCCGATACTATAGAATGATTGCAACGTTGTCA
AAACGGCTTGTTTATCGGGAGCTAAAAATTCTCCTATTTCGAAATTTTGTACATCTTCTTCTGTTATCATATCTGCAAATAATAACGTTTCTAA
AATATATTTATCGTCCGGTTGGAATAAACTAATTGCCTGACTTAGTTCACCATCGCGACCCGCACGGCCAATTTCTTGAATGTAGTTAGAAG
GACTTGTTGAAAGATGAAAGTGAATGATTGTGCGAATATCTTTTTTATTAATTCCCATACCAAAAGCACTCGTTGCGACTATAATCGGAATA
TCATTATTTAAAAATTGTTGTTGAACTGTGTGTCGCTCTTGATAATTCATATCACCATGATAAATACCTGTAAGAAAACCTGAATCATAAATA
AGTTGCGCTAAATTCAGACACATCTTTTTCGATGAGACATAAATAATCGTTGGTCCCGACTGTTGTAGAAACGGCAGCAACCATTCAATTTT
ATCTTCATCATCATGAAAATTAAGATGCTTAAAGCTTATATTTGGGCGATTCATTGTAGTTTTAATAACATTGAATTGAATCGCTAACATTTCC
GTCAAATCATCTTGTAAATGCGGTGGTGCAGTTGCTGTCAATGCTAAGACAACCGCTTCTTTAAAATGCTTTGTTACTTTTCCTATTAGAGCA
TAATGTGGTCTGAAATCATATCCCCATTCAGATAGGCAATGTGCTTCATCTAGAACAATCATGCCAAAGTCTATCATAGATATTAATTTAAAA
TTTGACGGTTGCAGGAGAAATTCTGGACTTAGAAAGATGAAGCGGCTATGTCGTAAACATTTAATATTATGCTTTTTCTCAATTTCATCCATA
CCAGAGTGAATACATGTTACACGTTTTTCTCCATTTATTTTCAACTGCATAACTTGGTCATCCATTAAAGATATTAACGGTGAGATAATTAAT
GTCGGCTTACCTGATAAATACGTAGGTATTTGATAACACAAACTCTTTCCACTTCCAGTTGGAAGAATACCTAGAGTGTGTTGTTGAGACAT
TATACTTTCTATAATTTCCTGTTGTCCCGGTTTAAAATTCTCGAAGCCAAATTTGTTTCGTAAAATATCATGCAACAT

Gene: A5IT10 (putative protein)

Position: 1502891 to 1503844, length: 954 nt, orientation: REVERSE

Sequence:

TCATGCAACATTCAGATCACCTCTTTCAAATCCAACGATTAATACTTTTAATTGAAAATATGATAACGTGTCAAATTGTTCTTTGTAAAATTTT
AATCGTTCACCACGATGTTGTTGATAAAAATTTAAAAATTGCAGTTGATCTTCTTGTTCAACATAATCATCGTAATTAGACATATAACCTTTG
ATTAAGATTTCAAGTACATGATCTTCGATAGTATTGATTTTAACTTGTTGTTGAGCCGCTATATCTTCCATCGTGAATTGTTCAAGCAATCTTG
TATAAGTAATATAAGTTTGGTTTAATAATGTAGGTTTCATTATTATTTTTGATAAAATAGTATATTCTCCACTTTCTAATTCAAACATCATTGTC
ACTAAATTATTCATTTCGTATTCAAACAATTGCTGTTGAGATAGCTTTTCTATTAAACTAACTTGCTGTCTCGTATACATTGGTTCATCATAGC
CTTGCAAATAATAATGTAAGTAACATGGCCCTTTTAAAGTTATTGCTTTAAACAAGTTATGTAGTTCATTTTCAAAATCAATTTGTAATTGGTT
TTCTTTAATGTAGTTATAGACAATCTTTACAGTTTCTTGTATCTTATTATTTTGAGAGATTGGTACGAAACGAAAAACATGTTGTTTGGTATTA
GACATGGTTTGCACTAATAGTTGAATTGCTTGAAATGTTTGCCCCATGCTTTCAAATGTATATCTAGGATGCAACATGATTTCCATTTCAGCA
TTAAATTCATTGATTTTTTCTAAAAATAGCTCAAAAGACGGATATTTTAATAGTGGTAAACTGTGATATAATGACAACTGTTGTTGACTACAA
GCGTCAAAAAAGGTTTGGTGAGATTTCTTACCAACTAAGATATTGTAAATACTTTTATTTGTTTTATAGTTAAATGTTTGTTGTAATGCTGTTT
TTATAATGTGTTGCAA

Gene: fer (ferredoxin)

Position: 1503952 to 1504200, length: 249 nt, orientation: FORWARD

Perfect match to: (11819-97-CP003194-[1554330:1554578], allele observed in CC80+CC72+CC80+CC772)

Sequence:

TTGGCAAAATATACAATCGTTGATATGGATACTTGTATTGCATGTGGTGCATGCGGTGCAGCAGCACCAGATATATATGATTACGACGACG
AAGGTATTGCTTTCGTAATCCTTGACGATAACCAAGGTACTGCAGAAGTACCTGAGGAATTATATGAAGATATGGAAGATGCAATTGATGG
ATGCCCTACAGATTCTATTAAAATTGCAGACGAATCATTTGATGGGGACGCTTTAAAATTTGAATAA

Gene: ribU (riboflavin ECF transporter, substrate-specific component)

Position: 1504306 to 1504851, length: 546 nt, orientation: REVERSE

Perfect match to: (11819-97-CP003194-[1554684:1555229:r], allele observed in CC80)

Sequence:

TTAAATTCTTTTCAAGAAATTCGCAAGCCTTCTATATAGTAAAATAAATACAATAGAAATAACGATACCTTTAATAATATTGAATGGTATAAT
TCCTGAAACAATGATTACTTTAAGATTATTTGCGATATCAGCTAAGTTAAATATCATACCGTACAAAGGTAATAGAACGAAATAGTTCAAGA
TACTCAACACGATAGTCATAACGATTGTTGCAATGATTAATCCAGTAATCAAAGATTTTGTTGAACGTTTATTTTTATAGATGGCGTAAGCAG
TTAATAAGAAACTTGCGCCTGCTAAAAAGTTAGCAAATGGTCCAACTGGATCGCCCATACTAAATAAGTAGTTCAATAAATTTTTAACCAGT
GCAACTACGATACCGGCAATTGGTCCAAACGTAAATGTAGCTAGCAATGACGGTACATCACTAAAATCTAAAGTTAAGTATGGTGGCAAAA
ATGGTATAGGAAACTTGATAAAAGTTAACACAAACGCAATCGCGCTCAACATACTTATTGTGATAAGACGTTTATTTTGTTGCAT

Gene: graD04 (putative lipoprotein)

Position: 1505460 to 1506374, length: 915 nt, orientation: REVERSE

Sequence:

TTATTCTTGATCAAACACACCATATTCTGAAGTGCTTTCACCGTTATAATTTGGTTTAATCGTATTTATTTTATTGTCTGAAAATTGTATACTAA
AATCTATATCAGTTGGTAAATCTTTTTCTTTTTTTATTTCTTCTGCCATTTTTATTACATTTTCACTGTTACTTTTTTTAGTATAGTTCTTATTTTTA
CTAAATAGAGTAGTAGTTACCGAAACTTTAGACTTATCTTTTAATTGCTTCTTAACATTTGACAATTCTTTTGAAAATTCACTGTCACTTTTGTT
TAACAATGGTTCAAAATAGTCTCTATACTCAGCTAAATTATAAGGTATGGCCGAAATATAAAAATATTCATTTTCATAACCACTATTTTGCGT
CTTATTAATTGCTTCTTTTGTAAATCCTGTATATTGATATTTCTCTTCATTATCTTTGAAAAATTTATATAATTTATCATATTTTTCTTTTTGTGCT
CGATATTCAAACCCACTGAGTACTGCACCAACTAAAGTACTCATATCATCGCCTTTATCCTTACTTCTTAATGAGCTATCACTGTCAATAATTG
ATTTATCAAATGGAATACTCGCATTAAATACGATGTCGTGGTCATCACAATGCACGAATACTTCTACACCATCGCCACTACCTATAACATTTG
TAGCTTTAACTTTTAGACCGAAGTTATCCATAAAAAAATTGTTCGCCTCGCTTAGCAATTTTATCTTTATGCTTCTTTGCAAATTCAATCGCATCT
TTTTCTGCAGGTGGTTGGAATCCTTGTCCTACATATTTTGAAGCATCCATTTCTTCTGGTACAGATTTTGTTTCTTTGTTGGATTCGTTATTGG
TAGTTGAACATCCTGATAGTAGTATCGTTGCCATTAAGATAAATTTTGCTTTTTTAAGCAT

Gene: A5IT17 (putative protein containing attachment site of PVL-phage)

Position: 1506389 to 1506595, length: 207 nt, orientation: REVERSE

Perfect match to: (TCH959-AASB02000242-[2061:2267:r])

Sequence:

TTATGCGCTTTTAAAAGTTTCATTTTGTAAATAATTATACTCTCTTAATTTATTTGTGTCTTATAGTTTCTCTCTATATAAATCTTCAAATACATC
TGCCTGCTTAAATCGTTTTTCGTTAACTGTAAAGTCATTATCTTTATTGATGTCTTGATAAACCCTATAGCTACTCAATTCTGTTAAATCTCTAT
CATTTATTTTATTCAT

Gene: graD03 (putative lipoprotein)

Position: 1506588 to 1507502, length: 915 nt, orientation: REVERSE

Perfect match to: (TCH959-AASB02000242-[2260:3174:r])

Sequence:

TTATTCATCTTCAAATGCTCCAAACGACACTACTTTCTTATCATCAAAACGAGCTTTTTTTGTGCCAATAAGTTTATTTCCTAATTGAGTAGTTA
TAGTATTCTTGATTGGCATATCTTTTGTTCTTTCAATTTTCTCGGATAAATCTATTACATTATTTATCTTTTCTTTTCTATTCCTTTCATCATTCGT
ACTAAACAACGTTGCTACAGTATTTGTATTAGCAGTATAGTTTAGCTCTTTTCTAGCTCGTTGCATACCCTCTTTAAATTCTTTATCATTTTTAT
GAATCAATGGTTCATAATATTTACGATATTCTTTTAAATTTCTTGATAAATATGTGATATAAAAATATTCATTTTGATACCCGACGTTTTGCGT
CTTGTTAATTGCTTCTTTAGTAAAACCTGTATATTGATATTTCTTTTCATTTTCTTTGAAAAATTTATATAAGTTATCATACTTTTCTTTTTGCGC
TCGATATTCAAAGCCACTCAGCACTGTACCCACCATCGTACTCATATCATCACCATTGTCATTACTACGCATTGATCCTTTTTGATGGATGGC
ATCTTTGTACAAAGGTAGACTTGCATTAAATACAATGCCATGATCATCGCAATGCACATAAACTTCTACACCATCATCTTTACCTACAACATT
GGTAGCCTTTACCTTCAGTCCAAAGTTGTCTTTAAAGAATTGTTCACCTACTTTTTCAAATTCTTTACGATGCTTCTTCGCAAATTCAATCGCA
TCTTTTTCTGCAGGTGGTTGGAAGCCTTGTCCAACATATTTTGAAGCTTCCATTTCTTCTGGTACTGATTTTGTTTCATTGTTGGATTCGTTATT
GGTAGTTGAACATCCTGATAGCAGTAATGTTGCTATTAAGATTAGTTTAGTTCTTTTAAACAT

Gene: graD02 (putative lipoprotein)

Position: 1507560 to 1508465, length: 906 nt, orientation: REVERSE

Sequence:

TCATTCGACCTCAATCCTTATAGACTCATTATCACTGTAATTAACTCGATTAGTACTAATAGTAGATTTTGCTAGTTGTAAAAATATTTTCGTA
TTTTCAAAATTAAGGTGTAACTTTTTCGTACTTTCAGACAAATCTATAACATCATCTAATTTTTTGTCCTTTGAAAAGTTACTACTTTTCGAAAA
CAATGTAGTATGTACTTCTATTTCAGCTTTATAGCCTACTCCTTTCCTTGCTTCTTTCATACCTTTTTTAAAATTCAGATTATTTTTATTTATTAG
GGGTTCGTAATATTTTCTATATTCTTGGAGCGTCGGTATATTAGCAACTACATAAAAATATTCATTTTCATATCCACTATTTTGCGTCTTATTA
ATTGCTTCTTTTGTAAATCCTGTATATTGATATTTCTCTTCATTATCTTTGAAAAATTTATATAATTTATCATATTTTTCTTTTTGTGCTCGATATT
CAAACCCACTGAGTACTGCACCAACTAAAGTACTCATATCATCGCCTTTGTCCTCACTTCTTAATGAGCTATTACTGTCAATAATTGATTTGTC
AAATGGAATACTCGCATTAAATACGATGTCGTGGTCATCACAATGCACGAATACTTCTACACCGTCGCCACTACCTACAACATTGGTGGCTT
TAACTTTTAGACCGAAGTTATCCATAAAAAATTGTTCGCCTCGTTTGGCAATTTTATCTTTATGCTTCTTCGCAAATTCAATCGCATCTTTTTCT
GCAGGCGGTTGGAAGCCTTGTCCTACATATTTTGAAGCTTCCATTTCTTCTGGTACTGATTTTGTTTCATTGTTGGATTCGTTATTGGTAGTTG
AACATCCTGATAGTAGTATCGTTGCTATTAAGATTAGTTTTGTCCTTTTAAACAT

Gene: lukF-PV (Pantone-Valentine leukocidin subunit F)

Position: 1508990 to 1509967, length: 978 nt, orientation: REVERSE

Perfect match to: (11819-97-CP003194-[1559368:1560345:r])

Sequence:

TTAGCTCATAGGATTTTTTTCCTTAGATTGAGTATCTATTAATTTAACTGTATGATTTTCCCAATCAACTTCATAAATTGATGTATGAGTTGCTC
TATTTTCATCTTTATAATTATTACCTATCCAGTGAAGTTGATTCCAAAAGTTTGTATATCTATCCATTTCTCTTTGATAAGTAACAGTAATTTTT
GATTTTTTTGCAGCGTTTTGTTTTCGAGATAGGACACCAATAAATTCTGGATTGAAGTTACCTCTGGATAACACTGGCATTTTGTGATATTCC
AAGAAGTTTTGTCCAGCATTTAAGTTGCTTTGTCTTGAGCCTAAAAACATTTCATTACCATAAGTTGAATGATAACTATCTCTGCCATATGGT
CCCCAACCATTATTCATAATTTTATGTGCTTCAACATCCCAACCAATTTTTTTGAAATTAGTTCTTTTATCTAAGCTAGTTCTATAGCTTTCTTGT
TTATAGTTAATTGTCTCTGAAAAAGATTTTGAACCATTACCTCCACCTGATAAGCCGTTAGAGATATTAATATCTCCACCATAAGAATAACCT
ACCGTTTGTTGTACTTGAAATTCTTCATTTTGATTTTTAGGTGCATAATCTACAACGTTTACTGAGTCATTAGAATCTGAATTAATTGAAATGT
TGTACTTAGAACCCCAATAAAATTGAGAACTAATAGTGTCTTTTGGATTTGGCTTTGTATAGCCAGAATAAATGTTTCCAGCAGCTTTGAGTA
TTAATGTATCTTTATCATAACTTTTATCTTTAATAAAATTAAAAGTTAAAATCTGAGAAATTTTTAACTTATCGGAATCTGATGTTGCAGTTGT
TTTGTACAAAGTAATTTTATCATCAACCTTTTTCTCACTTACAGGTGTGATATGTTGAGCTGCATCAACTGTATTGGATAGCAAAAGCAATGC
AATTGATGTAACAACTGATGATTTGACTATTTTTTTCAT

Gene: lukS-PV (Pantone-Valentine leukocidin subunit S)

Position: 1509969 to 1510907, length: 939 nt, orientation: REVERSE, SNP in start codon

Sequence:

TCAATTATGTCCTTTCACTTTAATTTCATGAGTTTTCCAGTTCACTTCATATTTAACTGTGTAATTTCTGTTTACAAATGCGTTGTGTATTCTAG
ATCCTTCTAAATAACTATTGCCATAGTGTGTTGTTCTTCTAGTAGCATGAGTAACATCCATATTTCTGCCATACGTTATTTCAAATTCACTTGT
ATCTCCTGAGCCTTTTTCATGAGAAACAGTTGCAATAAATGAAGGATTGAAACCACTGTGTACTAATGGGGGTAATTCATTGTCTGGCACAA
AATAGTCTCTCGGATTTTGACTATATGGTTTATATCCAACAAATAAATTTGGATCATGTCCAGACATTTTACCTAATGATGTGATAAATGAAT
TAGCTTTTATTCCCCATTGAACACTTTTTGAATTTTGATGTTCTACTTCACTGATATAGTTTTGTTGATTATAACTAATTGTTTTTGAATAATTA
AATGAACCATTACCTCCTGTTGATGGACCACTATTAAAATTACCACCTATGTTATAACCTAATGTTTGACTAACATTTACTGAATCTATTTTAT
TTTTAGGTAGATAATTTATTAAATCTACATTAGGGTCATTTGTTTTGAGACCAATATTGTATTGGAAAGGCCACCTCATTGCTTTTATATGATC
TGTGTTTTTGTAATTGTAATAAGTAGTCTTTGAATTGATAAAACCTTGCATTTTTAAAATCAAAGCGTCTTTGTTATACTTTTTATCTTTAACAA
AATCAAACTGAATATTTTGTGTGACCCCCCACTTATCGCTACTTGTATCTTCTGTTCTTTTGACTACCTCAGCGCCATCACCAATATTCTCAATA
TTGTTATCAGCTTTAGATTCATGAAACGAAGTAGCAATAGGAGTGATTATTCCTAACGATAATGTTGCAGCTAATAGTCTTTTTTTGACCTT

Gene: O80065 (putative protein)

Position: 1511047 to 1511139, length: 93 nt, orientation: TRNC-RVRS (no stop codon)

Perfect match to: (10S-AYXU01000292-[209:301])

Sequence:

GTTCATTACCAAACAAATTATAAATAATTTATATTTTGATTGATAATAAACATAAAACCACGATATAGTTGACCATAACAACTTTTTAATCAT

Gene: amidase-1

Position: 1511289 to 1512743, length: 1455 nt, orientation: REVERSE

Perfect match to: (11819-97-CP003194-[1561675:1563129:r])

Sequence:

TCAAACTGCACTAAACTTACCAAAACTGCTTATTCTATTACCTGCCTTGTCTACCTCTCCTGTAGCAATATAACGACGTTGTCCACTATTAGCA
ATATAAGTAATCCATCTATACCCATTGATGCAATATGCTCCGTCATATTTGATTGTTGCGTTATTAGGTAATACACCTGTAATTCTTGAATTAG
TTGAATAGCCATCCCTCACGTTATTACCTTTAACATTGGCAACTGTGTAATAACCAGTTTCTTTTTTATACGGTACATTATTTTTATCGGGTGT
ATAACCTGCTGGCACTGGCGGATTCTTTTGGTTTTTAGCTGATGTTTTAACATTACCAGCTACCAAACCACCTATAGGCTTACCATGAATCGC
ACCAGCTATTAATTTAGAATACAAGTCATAATTCTTCTTAATCCAATCCATATCTTTTTTATTAGTAATAAAACCTAATTCAGATAAACGATAA
TTGATATTTATTTCTGCTGATACATTAACGTTCAGTAAATCATTACGAGGTGTTACACCTCTTATTTGTCCTAAGTTATTTTTAATAACATCTTG
TATACTTTTATCAATAGTATCTGCATTGAATTGACTTGAAATAATAACATGCCCACCACTTGCACTTTCTCCTGCTGCGTCTAAATGAATCTCT
AGAACAATGTCATACCCCTGTGATTTAACCCAATATAAGCCATAATCTTTATTATTTCCTACATTAACACCGTAAGCAGTATCTTGATACATAT
CTTGTGATTGACTTGAGCCACCATATAATGCAACTTCGTGACCTGCATGTCTTAAATACTTAGCGATATTTGGTGTTATATATTTACGGATAA
AATCACGTTCATTTGTTCCGTTTCCGACTGCTCCAGGATCGTTATAACCATGACCGGCTACAAGCATAATTTTTTTAGGTTTAATTACTGCTTG
CTTTTTGGCAGTTGCTTGCTTAATAACGCTTTTAGCTTTATCTCCAACACTTACTTTATCTGGGAAATTTAATCTAATAAAATACATTGGGTCA
TCGTAATAATGAACATGTCTTGTAACGCTTTCGGGACCCCAACCAGGTTGCGCAACGCCATTTGTCCAACCTTTACCATTCCAATTTTGGCCA
AACGATGTGAAAGTGTTTAGATTAGCGCTCTCAACAATTTCAACATGTCCAGCTCCGCCACCATACTTTGACGGGAAAACGACAATGTCCAA
CTTTTGCGGTAAAAAGCTATCATAGTTTTTAATTATTTGCCCGTATTTTTCAATCCTTGCTTTATTATCAAATGGAATATTATAAGCGTATAAA
CCTTGTAACCTTTCGCCTGTTGCTATCATAAAAAACATATTTGCGTAATCGTAACACTGAAATCCATAAAACAAATCAGGATTGAACTGCTTC
CCTAATGAATTATCAAACCATTTTTCTGCTTGGTTTTTTGTTATCAACAT

Gene: holA-1 (holin)

Position: 1512754 to 1513056, length: 303 nt, orientation: REVERSE

Perfect match to: (MW2-BA000033-[1533153:1533455:r])

Sequence:

CTACCCTAAATCATTTGTGTCGTTCATATTCGTAGGTGTCATTACTTCTTTAATTGGCGCTTGCCCTGTTGCTTTTCTATACTTGTTTTCAGCTT
TATATTTCTTTAGCTTTTGATTTGCCCATTTACCTTCTTGAGATGTTGGATTGTCTTTATACGTAGTATATAAAGCAACAACAGTAAGTATTAT
TGATGATATAGTCTCATCGTCTACTGGAATCGGGCTAATACCTTTGTTCGCTAAGAATTGATTTACTAATGCTAAGATCAATACGATGTATCT
TGTTATTACTTTTGCATCCAT

Gene: Q9MBN6 (putative bacteriophagal protein)

Position: 1513192 to 1513491, length: 300 nt, orientation: REVERSE

Perfect match to: (MW2-BA000033-[1533591:1533890:r])

Sequence:

TTAAATGCCAAAAATAGTTTTTAACAAGGCTATAACAAATGTACTTAGAATCGTCCCTATTAATCCTAGAATCCACATCTTGATGTCTCTAAT
ATTTTTAGCATTTTTCTCTTTATTTTTTTCATCTTCTTCTTTGTCACGCCTTAGTTCTTCGAAATTTCTATCTAACTTGTCATAAATTTTTTCTTGC
GTTCTCAGACTGTCTTCTATTCTGTCGAATTTTTCAAACATAGTCTTATCATTTTCTTCTAATCGCGTTAAACGCCAATCTTGTTCGTGTCGTTT
GGTAAATCCAAACAT

Gene: hypothet_phage_protein

Position: 1513537 to 1513701, length: 165 nt, orientation: REVERSE

Perfect match to: (Phage-3A-ORF089-[AY954956.1-[[21287:21451]])

Sequence:

CTATGACTCTAGATTTTCTGGATACTTTTCTCCTGTAATAATTGCATATTCCTCTTTATCTATAACTTCCATATCTACATACCACGCTATATCTTC
TTTACTATATTCTTTCAATTGATACCATGTTTTAATATCTTCGAATGTTGGTGAAATTAATTTAAGCAT

Gene: DUF2479 (putative bacteriophagal protein)

Position: 1514083 to 1515549, length: 1467 nt, orientation: REVERSE

Sequence:

TTATTTTCTCACTCCTATAATTTTGTTGATTGTTCCTCTATTTGCATTGGCACCAGAACCTCTTTGACTTCCTAAGTCGAAATAAACATCGTTCG
ATATTGTTAAAGATGTACGACTAGATTTAGTTAACCCAAACTCATAAACGCCTCCACCGTTACCGTCATTATCCGGTAAATTTGATGGGTTCA
ATGAAATTTTCCCGCCACCAAAGGGGTTGCCAAACTCAGTAAAATCTCCCCCTGGAAAAGTTCCATAAAAAATTAACAAAATAAATTGATCT
AAACTTTCATTAAGATATAATGTTGAGCCAACGCCATTTGCCGTCCCATCAAAAATAACTGAATATCTTTTATTAAACTTGTCATCTGTGTATA
GTTTGGCGTTGCTTTCAGCCGTATTAGCTTTTGATTGCGCGTTTTGAACAGTTTCAAAAGGCGTATTGTAATCATTAAGGGCTAATTCTGACC
AATCAGACCAAGAACCTGCTTCTTTTCTCTTAACAAATACTTTATTTGTACCATTTGGACGATATGTCATACGTTTGTAGTCGGAAGTTACTAC
TAAATATTCGACAATACCATTAGTGCTTACGCCTCTTGGATAATTTATGGCTTGTGAGACGTAAATAAATTGGGTTGAATCTCCTACTCTTTG
TTCTGGATTATTAAAATCAAATCCAGTAATCTGTGTTATTTTACCGTCGTCTTTAGTAATCTTAGATTTTTGCCAATTTGAAGTTGAACCACTT
GTGACTAAACCGCCGCTATTCACTGACTGCTTGAAAGCCTCATGTTTCTCATCCATATATCGCTTTTGCTCATCGAATGTTCTTGAATAAGCTT
GCGCTTTATTCTCCAAATCGGTTATACGGCTATTAGCGAGTTGCTTTAATTCATCTATACTTGACGATTTTGCTATTTGAATATCTGATAGCCC
TTTTTCTTTAGCTTTTTCAATCAGACTCGCATAATCTTCACCATTTTTTATAGCCTCGTCCATTGCTTTCGCACGATCCACAATAGTTTTTTCTAA
TTCTTGAAACTCAACAATATAGTGCAGTTTTGTTTCAGAGGGAATCTTGCTAAACAAACTTTTTTCAACGTTAAATGTGATAGTTCTCTCGAC
AACTACCACGTCTGAATTACCTAATTCTGCAACCGAAACTTGAGCTTGATAACTTCCATCTCGTTTAATCACATCATTAGGTAATTGAAATTTT
AATATACCTTTAAATGGATCTAATATTTCTAGTGGAGCAACTACCATGACTCCTTTACCTCGAATCGCTATTCTTGCTTTGATATTTTCTTCACT
CAATAATAAAGGTTGATTATTTTTAGTGATATTAAAAAGAAGAACAGAAGAATCACTCTCTCCTGTTCTAAAAGTTATATCTAGATTTGAAAT
ATTTTCATAATGCGCTGTATTCTCTAAATTAATATTTACAGATTTTTCTAAATTACTCAT

Gene: minor-phi (bacteriophage minor structural protein)

Position: 1515549 to 1517459, length: 1911 nt, orientation: REVERSE

Sequence:

TTAACTTATAATTCTCCCTTCGTGTAAAGTCCATGGCCCTGAACTTGTTTTGCTATCATAGTTCTTCAATAGTATTTCACAAGATGCTGTAACA
CTATTACGAACTAGCCTATGAACAAAACCACCTGTGTTTGAAGCTTCTACATATAAGTTCCAACCAGCTACCCCTTTACGTTCAGTTGGAAAA
TCTGTAAAACGTTTTGTATCATCCGTAGTTAAATAAAACGACATACCTACTATGTTAATATCTGACATTTTTGTGATGAATGAAGGTACTCTC
TCCCATTTACCACTATTTTTAGGCACATAATTCCAGTCCGAAATGTCTCCAGTTCTCCCAGAAAGCACCCTTTCAAAAGTCATCATGTTTCTTG
CATAACTATTACGCGTTAATATCTGAATAACATCTCCACCAGTTTGTGGTGGTTTTACTTCCAAGAACCAACCTGCATCGCGCCATTCTCTTG
GTAGTGGGAAATCGTCAATTTGAACTGTATGATCTGTATATAAATAATAAAGACCAGGCTCTGTTAACATTCCAAGATTTGTTAGTTTATCA
GGTCTCATTGGTAAGGGCTTAACTCTACCGCCTGTGTCACTCATGATAAAAGGGACGCCTCTAGAGTGCAGAATTTCTAAAATCCCTCTTTG

GCCAATCATGAAAATACGATGTGTTCTATTTCCATCGCCACCAACAGTAACACCTAGCATCAAAGCCTTTTTACCGCTATCTTTATCATAGTAT
ATTTGTAGACCCTCTGCTTCCGCAAATTCACCAGGAAATGAATCAAGTGTTCCACCATAATCAGCATTAACTTGATATGCTTCTTCTCCTGTTT
CTAAATCGAAAGCCGTTAAATAGTTTCTATTATTCGGATTACTATCTCCTGTGTACCAATACAAGTATTTTTCATCAAAAGTCACACCTTGCAT
TGGCTGAGTTTCATTTGTTAGTCTCATAGGAATGCTAATTTTATACAAAACTTTATCAATGTTCTTATCAACATCGTCTAAACTTCTTATCTCTA
TATAATTCATAGAGTTTTCAAGTTCCCATTGACTTCTAGGTCTCTCAATTCTGTATAGAATTTTATTTTCTTTTTCATTTATGACTGGTGTGATA
TAAGGCTTTTCTGGATGTCCTGTAAATACGTCTTGCATACCATACTTGCCATAACTAATCTCTACATTAGGCGTGTACTTAAAGCGAACTAAT
GTGTTTTCATTATTACCATTTAAAATAAAACTATATATCCATAACTCATCATCAATATATCTATAGCCGTTGTGCGTACCATGACCTCCGCCTA
CAATTAATGAGCTATCTATAAATTGACCATTAGGTCTTAAACGACTTAGCATATAGCCGTTATTTCTAGCTTGTGTCATGTATACTATGCCTG
TTCTATTATCAAACCAGAAGGATTGCATCACTGCATTTGTAAGTGGTGCAAGTTCTGTTACAAATAAAAATTCTTGCTTATCAGGTTCAAAAC
GGTACTCGATATCAAGAATTTGTTGTTTAGCCTTATTTAGTTCTCTTATAGTTTCTTCTTTATTATTTTGAGTTTTGGTTTCCCAATCGTCTAAA
TGTTCTTTTAATGTATCAAAGGTTTCGCCATTTACATTAACTCGAGCTTGAACAATCTCATTAGCGCTATTATTACGCGGTGCCAAAACAAGT
GCGTTAATTTGACTTTGTAAAGATTTATTTACTGCTGCTTGCGATCTACCATTATAATAAATTTGCTCAGCGAAGTGTTGAATTGTTTTAGCTT
TCTGATGCAACTTAAACTCTGTTGTCAATCCAAGCGCAAATTGCTCTATTCTTTGTAAGTTTTGTATTTCCTTAGCTCTATAATCTCGACCTGCT
AAAGCTCCCAAATCCTTTATTAAATACAAATTTTCCAT

Gene: hypothet_phage_protein

Position: 1517475 to 1517765, length: 291 nt, orientation: REVERSE

Sequence:

CTAATAAAATAGCACTGTACCAAGTTTCCCACTATCGTCAACTGTTATTTTCCACAATTTACCGTTTGGGGATTTCTGTACAATGCTATTTTGA
ATAATTCCTGCTTCGCCTATTTTTAAATTATCTAATTTATTTTTATCATCTACCGAAATGATACCGTCTTGAGGTAGTCCATCAATATCACTACT
CCCTGCATAAGGTACCCCGTTTATAGCTTTCCAATGTGTAGCTGGAAAGTACTGTTTATCGTTTTCAAGTAGCGCTTTGATTTTAACTTCTTCT
GTTGCCAT

Gene: Q8SDP1 (phiSLT orf 527-like protein)

Position: 1517765 to 1519348, length: 1584 nt, orientation: REVERSE

Sequence:

TTATATTAATACACTCCCTATATCCATTGTCTCGAAAGGAGAATTCAAAGTACTAGTGTATAAATGATTTATACGATTTGCTTGATAGTTATAT
CTATTATCTTGTGCAATAACTCGTCTGTTAAGCGCTTGTTGAATTTGTACCATATCTTTTATTTCATTGCTGAAAGACACTTCATCTATTGCGTT
TACAAATGGGTGTGACCTATCAAGTTTAACAACCTTTAATTCAGTGTTATATCCCATTAATTCATGAACAAAGAATACGCTATCTCTTGGCTC
TATTTTTTCATAACCCATATAATTAACATCTAATTCAGTCTTAGGAGTATCATTTATTTGCTTTTTTGCAAATTCTAGCAACTTATCCTGTGTTT
CGATATCTTCATTTGTTTGCGTATTAGCATATCGAATCCCAAACTGCTTTGCACTATCTGCGACGTAGTCAACAATTGCTTTGTATTGATTGCG
ACCTGAATTGTCAGCAATTAAATTTAAGACTGTTGATTTTTCAGTTCCAACGTACATACAAGGCTTAGCTTTTTTATTTGAAGAGATATCAAT
TCTATTTTTGGGGTCTTCTCCTAAAAATATCATTTCTAAAACGTGCTTGCCTTTATCAATATTTTTTATTAAATCTATTGTTTCAGACTGAACCG
ACTTAGCAAAACAAGAAATTTGCTTAATTTGCTTGCCGTCTAAAATCAACTTATATATTCCACCTTGGGAACCTTTTTTGATTGTAAATCTAAC
TGTTTCATTACCATAGTTGCAATCAAAGTTAATAGTTGCTTTAGAACCAATTGTTTCTGTGCGATAAGTGCCTTCTTTTATGAAATCATTTGAG
TAATTAATGTCAGTTATTTTAATAGGATTATAATTTTTCTTTTCCTCGGCTGTGTACTTTTTCCCAAAAACTTTTATAGCTGTTCTTAATTCCAA
CGTACTGACAGTAGCAGACACAGTATCAGTATTATATTGATACCTTATTACTTTTTCACTTCTTTGATAGAATGTTTCAGGAGAATAAAAACA
TATCTCCGTATCATTTGGATAAATAATACAACCAAACAAATCTACTGCTTCTTTACAATATTCTAAGCCATTTTTGTTACCTAATTCATCAATTG
GTATTTTTCTTTTAAAATCTCCAATTATTTTATAGGTCATCTTGACTGACGTTTTTTGATTTGCAAATCCATATCTTAAGTACTCATCTAAAGAA
TATTCTGGTATTTTACCAGTTTCGCTAGTGTCGTCATCAAGCTTATTTGATTCCACTGAGTGATTTTGAAATTCATACATTATGTGATATGCCG
TAACTTCAATAAAAACTTTATCACCTTCAACCTTTGGCGCTGTCTGCTTAATTGTGTATTTTTCACCATGATAAATTATGAAGTTTTCACAAAT
CAATAAATCAAAAACAAAACTATTATGAGTAGTTCTATACACTGTAAAGGTGATATACCTAGCTTCATTCAGTTCATAATATTCTTTAAAAGA
ACCGTAATCTACGTCTAGTAAATTTTCACAAATCAATTCATTAAAATCCATTACTGATAAATGATCATGATAATCCAT

Gene: Q4ZCS6 (putative bacteriophagal protein)

Position: 1519357 to 1520181, length: 825 nt, orientation: REVERSE

Sequence:

CTACCTATAAATAAAAGGAAACTTAAATGTTGTTTTAATATCACTGACGTCTCCTTTAATCTTAAATTCATTTTTACCTGGCGCTAATGTTATA
ATGCCCCTATTTGTATCAATTCCGACTCTATTTATATCTCGATATGCATACACACCATCTAAAACAAAATCAGTGTTTTTATCTATGCTTTTCTT
GTATTTAAAAATATCACCTGTTGTATAGTTAACCAGTTCAAATCCTCCACTCGCATTTAAATTAATTAATATTTTTAAATCGTGCTTGAATCGT

GGATTTATCGTATCAGTAGAACCGTTCCAAATAGTGAATTGATTTGATGTATGAGTATATTTAGGTGTGAAATCAATAGGAATTCCATTTTC
AAACATCCAATTAGAGTCGAATAAGAACTCGCTATCAGTCCAATTAACTGATTCAGAATATCCTTTATAAACATTTAAACTTACTTCAATTTC
AGTTGAAGAACCATCTTTTAAATTAGATGTAACATTAGCTGTATTCACTGCGTATTTAACGCCAGGCATTTGAGAAGTAATAACATAATAAG
GATGTCTACGATTAAATACAGATCTAAACCAATGCTCAAATAAATTTAAATCTATAACATCTATACCATCATAGCCAAACCTTAATACTAATG
AAAAAGGCGCAAAACTAATTGCGCCCGGTAAAATACCATCTACTCCGTTAATAGTTACACTATTATCATTAGTGTTAGGACTTTCAGCCCTTG
CATCTAAAAATATAAGTTGATTAAAATCTGTTATTACTTCTTCCTTGTAACCATCTATGATTTTTACAAAAGATTGCAT

Gene: tailfiber-6200-phiSLT-2 (bacteriophage tail fiber protein)

Position: 1520181 to 1526357, length: 6177 nt, orientation: REVERSE

Perfect match to: (S1800-CAWB010000109-[8568:14744])

Sequence:

TTAATTAGTCAAACCTCCCATATAATTATTTGCATTTGCTCTATGTCCACTTTGTTTTGACAATACCTTTTCTAAATCTCTAATTGCATCACTAG
AGCTTAAGTTATTATCCTGAGAAGAAACAGTTTGAATCAATGCATCTGTTAATTTATTTCCTTTATCCGTTAGCATAACAATTTGTTTCAATAA
TTTTTCAACCGTAGAAGTATCATTATTTACAGTGATGTTATTTTGCTTGCCATCCATACCGATGATGCGCATAACCTGTTCAGTTAATTGCATT
GCTCTTTTGCGTCGTGTCAACGGAATAACCATTTCTGGTTTATCTCCTTCCCCCACTTCAGCGATTTGATGTTTAGTTATAAGACCTCCATTCG
CATAGGCATAATCTCCTGCGCGTTTAAAGCCTCCCCAACCGTACTTAGCAACAATGTATTTCATTGCTGAAATCGCCTCATCAGTAGGATTAT
AAATATTTCCATGTCCTGGCTTAGCAAAAGCTCTAAATGAAGGTTCAATCATTTGGAACATACCTTTAGACGGTGTACCTGCTCTTGCGTTG
GAATCCCAGTTGTTAATAGCATCTGCCTGATAGTTACTTTCACGCTTGGCTACTCTTAACATCTGAGTAGTGATATAGTCAGATCTATATCGT
CCACCTAAAATTGATTGTGCTCTTAGAATAGCTCTTTTTGCATTCTCGTATCCGCTTCCGCCGACCTTGCCACCGCCACCTTTAAGTGACTTCA
ACCATTTCATAGGATCTACTGCTGTATCATTACCTGGATGTGAACCTCTCATCAATTGGAAATGTAAGTGTGCTCCTCTAACGAAATTACCTG
TAGCACCGGATTTCCCTATCAGTTGACCAGCTTTAATACGTTGGCCTTGTCTTGCTAATTGCTTAGATAAATGCATATACCAATTCCATTCGTT
AGCACCGGTCTTAATTTGTATAGAATTACCGCCACCGTAATCAGTCCATACTTTATCAGCTATACCGCCTTTAACAGCATAAATGTTCGTTCCT
GTAGGCATACCAAAGTCGATACCATAGTGACGACCGCCATTAAAGTTAAGTCCACCTGTGTAACTCCCAAACCTTTGCCAAATTGGATGGTC
AAATAGATAGCTTCCATCGCCTCCGCCACCAAAATCTTCAAACCACGATTTTACTTTGTCTACTAATTTCTTTTTGAGCAATGAGTACGCGCCT
TTAGCAATTTTTACTGTAGCGTTAGCTCCGCCTCCAAAATTAATATTTAAACCTGACATTACTTTATTTACTAGTTTCCCTGGATGTTGTACAT
AATCCCACACATCGCCAATTTTATCGCCTAACCAAGATGCACCATCTTTGATTTTATCGCCTGCCGCTTCAACCATTTCTTCTGCACCTTTTTTG
ATATTATGAGCTGTGTTTTTAGCTTTAGAACCGAAGTCCCCTGCTTTTTTACCAAGATTTTCAGTAACTTGTTCCATCCATTTTTTCTTTTTCGT
ACCACCATGGAATTTTGGCAAAACACCCATCCGCTGTAACTTCAGAGTGTCATTGGCATTTATTACACTATCTCCAACTCCTAGTGGAACAAC
CACATCTCGTCCTTGGGGTGCATGGAATGTTCCGTCAGCTCTGTGAATTACTTCTTGAACTCCACCACCTGGGGCATTTCCAGAACCTCTATC
ATTTAATACAGCAAATGTCGGTTGCGTTAATGCTCCCGAATTATCGGTAGCTACACCTTTTCCTGCTAAAGTACCAGTAGACAATGTAGGTA
TTGGCTTGATGAGATTTTTATCAGTAATGGCTTTAGATATTTTATTAATACCGCCAATCATGCTATTCAAACCGCCAATAGCTTTATTAGCAAC
ATTTTTACCTAAATCAGCCGCAGCTCTGCCCATGTCTTTACCAATATCTCTAATCCAATCATATGTTCTTGATAGCCATTTTCTAAAACCATTAA
ATACTGATTTAGCGTTAGACCATGCCGAACTTGAAATTGCATCAAAACGATCGTGGGCTCTTGAATACATATCCCCAGTCCAACCTTTTAAA
GATTTGTATGAGTTACTAAACCATTTCGATGTTCCTTTCCAAACGGATTTTGCATTCGACCATGCTGTACTAGAAATATTATCCCATTTCGAGC
GCGATTTATTAGCCATATCCGTTAGCCAGCCCTTTGCACTTTTATATGCATTGCTAAACCATTTTGATGTGCCTCTCCAAATAGATTTTGAATG
CGCCCAAGCTTTATCTGAGGCATCTGAATACTTTTGCTTAGTTTGATTGTAAATACTTCCTGTAGTCGATTTAACAGATTGCCAAGCTTTTCCA
AACCATTTACCAGTACTATTAGCTATAGCCTTAGTGTGATATCCTACAGAACTTTTGGCTGAGCTCCAACTTGAACTTAATTTGTTTGGAATC
CCTTTGATTCCACTCCACATTTTTTTCATTTCGCCGCCAAAATGATTAGCATTTCTTCCCATTTTACTAAAGGCTTCGCCAGTTTTAGTTTTTAC
GCCGTCCCAAGCATTTCCAAACCATTTCTTTATATTTTCTCTGTTTCTATGAGCTGTTTCTTCTTGTTCTTTAGCGTATTTATCACTTTTCTTCTTT
TGGTCTTCTCTGAAGTTAGACCACCAACTTTTAAGACCATTCCACCACTTTTCAGTATTTTTATATACACGACCACTGGATAAATCCATCTCTT
TATCAATATCTTTATTTTGCTTTTTAACAACGTCTACTACAGCATCTTTTTTAGATTTTGCCTTTCTTACTTCATCCTTATGTCTTTGATCAGCAA
TAGCTAACAATTTATCTTTTTCAGACTTAGAAAGGTTGACGTTATTTTTTATAGCAATGACATCATCTTCATACTGCTTGTCTACTTCTTTTTTT
CTTGCCTTTCTTGCTTTTTCTGCTTCTTTAATTGCTTTGCTCGCTTCGTCTATTGAATAAGCATTTCTGTTTCTTTGCATTCTTACTAAAATACGC
TCTTGCTCTTTTTCAGTCTTACTCAATTCTTTAACAGTGATGTCACGTCTTTGATTTTCAAGCTTTTCAATTTCTTTTCTTTCATTTTCTGAAATCT
GACCATCACTCAAAGCTTTTTCTTTCAATTCTTTGATTTTCTGATTGAGTTCTTGCTCTTTTTTAATTCGCAAATCATTTTTTTCTTTAGTTCGAG
TTAAAATGTTTTGCTTTTCTTGTTCATCGAATGCACTATACTTATCAATAAGTTCTTGAGTTTTTTCGAGTTCCTTTTTATTTCTTTTTTTCTATTTC
AGCTATAAGGTTATTAGATAAATCCGTTTCAATTTTCAAAAGTTTTTTTGCTTTGTCTTCTGTTATTTGACCCGAGTTTAAACGTACTTTTTCCA
TGATTCTGTTGTTCTCTTCAGAATAGTGTACGTACTTTTCTAAAGCTTTTTCTGTTTCTTTTGAAACACCCTTTCCCTAACACTTTTACAGTATCA
GATGCTTTTTTAGAAGCTGTGCCCATGGTTTGCATAAACCCCTTTAAACTTGTTGACTCCTACTTTCAGAAGGTCATCATCACTTAATGATTTAT
AGCCATCTTTCATATCTTTTGAAAATTTTTCTTTGAAACTTCTCCCTATACTTCCAAGATAATTTTTAAACTCTCCGAGCTTTCTAACAGCACCG
CCGATGATTTTGCCACCAAAAAACTTTATAGTTTCTCCTAAACCGTTAATACCTTTTCTGAACCATTCCACATGATCATATGTGGTCTTAAAAA
CTTTATAAGCAATTGTAATAGCAGTTATTGTTGCACCAATAGGACCTGTTATAAAACGTAAAGCTATTCCTAAACCTCTAGCTCCACCACTAA
AGGCAAAAACACTTTTAGAAGCTAATCCTATTCCTTTACCTAAAAGTTTAAAAGGCAAGATTGCTAGATTTGCAGTATTTTTCAAAACATTTA
TAGGTTTTAAATTAAACAACATAGCTCCGGCTAATCCTTTAAAGCCTTTTGACGTTTTTCCTGTTGTAGAACCAAGAAATAAGGTTTGAAGAC
CTAAAGATTTCATTGCTTTTGAATTGGTATTAGAAAGTATTGTATTTTCAGCAATGCGTCTATTTAATGATGCATATCCTTTGGCCGCGCTTCC
AACTGCACGTATTAATAAGCCTCCAGCAAGAACAGCAGGACCAATAGATGCACCAAAAATTGCTAAGCCTACCGAAGCCTTTCTAACCCAA

CCAGGAAGATGTGTAAATCCATCAACTAATTTTGTTAATCCTTCCGCACCTGCTCTAATCATAGGCGTTAAATCTTTACCAACTTCAATTGCTA
ACGATTCAAAAGCGCCACCTAATTGTTCCAGAGCGCCTTTGAGATTATCTTTCATCAAATCTGCTGCTTTTTTACTTTCGCCATTGGAATTCTT
TAAGGATTTACTATAGTTATTAATTTTATCTGGCCCCGCTTCAATCAAGGCTAAAAATCCACTTGCTGCTTCAGTACCAACTATTGTAGCCACT
GTAGCTAGTTTTTGTTCTCGTGTCATGCCTTTCATGTTGTCTTGGAACTGTCTAATCAATTCGCCCATTCCAACAAACTCACCTTTAGCATCAG
ACAAATGAATACCTAATTTTTTCATTTCCTTAGCTGTACTTTTACTTGGATTAGCTAGCCTAATAAACGAAGCTCTTAATGCAGTACCTGCTTG
AGACCCCTCTAAACCTGAGTTAGATAAAACTTCAATTGCTGCAGAAGTGTCCTCTATTGAAACTCCTAATGCTTTTGCTGGAGTACCTGCATA
TTTTAATGCATCTCCCATATATTGAATATCTGCAGCACTATCATTTGCTGATCTCGCAAGTAAATCAGCAACATGATTTGCATCAGATGCTTTT
AAACCGAAAGAGTTAATCGCTGAAGCCATTACAGTTGCAGTTGTAGCCATTTCTGCACCACTTGCTTCTGCTGCGCTGATAACACCTGGCAT
AGCCTCCATTGTTTGTTTGGCATTAAAGCCTAAAGCTGCCAATTCTTCCATACCTTTAGCAACTTCGTTAGCACTTTTACTTGTTTTAGCTCCTA
AGTCAACCGCTTGATTAGACATGCTTTTTAAGTCTTTACTGCTTGCTTGTGCAATCGCTCCAACTCGAGACATTTGCCCTTCAAAGTCTGCACT
CGTTTTTAATGCTGCACCTAAACCTAAAGTAATCGGTGTAGATACGCCCATCGTCATTGTACGTCCCAGGGAAGTCATTTTGTTTCCAATAGA
ACTAAATTTCTTTGACATGACATCCGCTTGACTTGCAAGTTTACCGAAATGACTTTGAGCTATCATTTGTTCTTTGTTAAAAGTCTTCATTTCG
GATGAAGCTTTATCTATTGAACGCTCCAAATTATTTAAAGCAGCTTTTTCTTTATTAACAGCTGTTTCAACTTTTGCGACATTAGCGCTATGAT
TCTTAATAGTATTGTTTAAATCATTAAATTCTTTTTCTGTTTGCTTTAATTTAGTATTAGTTTTAGCGTAAGAACTTTCAATTTTATCATTTGATT
TTGAAAGATTGTCATTTTGCACTTTTAGTTTTTGAACTTGATTGCCTTCTTGTTTATATTGTTCAACAAGTGCTTTATGCTTAGCGGACTGCTTC
TGTACTGCGTCACTTGCTCTTTTTAGTTGTGCAGTAGTAGCTTGGTTACTATTCTTAAGCTTTTGTTCTGCATCTCTCAACTGTTTAAGTTTTTG
ATACGCATCTTGTTTACGTTGATTTGTACGTTTATATTGATTTTCAGCTTTTTTAAGTTCTGTATTCGATGATTTTAAGGCTTCTTTAGATTTAT
CAAGAGCTAATTTTTCTTTTTTATTGGCTTCTACTAACTTTAAATATGCTTTCTCAACATCTTTTACACTGGATTTAGCTTTTTGGTAATTAGCG
TTAACTTGTTTAAGCTCATCTTCTACTTGAGAATACATCTTTTTTTGAACTTTAAGCCTATCATTTAACCCCTTAATTCTCGCCTGATATTTTTCC
ATTGATTTTTCAGACTTATCAAATGCTGACAGATTAGCTTTCATTTCACTATTAACAACACCTAATTGTCGCTTTAAACCTTTCATGCCTTCTTG
GACACCTAAATGGTCTAATTTCAGCTCCAAGGTCATGCCTTCTACTTTTTCATTCAT

Gene: hypothet_phage_protein

Position: 1521176 to 1521319, length: 144 nt, orientation: REVERSE

Perfect match to: (Phage-42e-ORF106-[AY954955.1-[[13576:13719]])

Sequence:

CTATACCGCCTTTAACAGCATAAATGTTCGTTCCTGTAGGCATACCAAAGTCGATACCATAGTGACGACCGCCATTAAAGTTAAGTCCACCT
GTGTAACTCCCAAACCTTTGCCAAATTGGATGGTCAAATAGATAGCTTCCAT

Gene: Major-tail (major tail protein)

Position: 1526979 to 1527434, length: 456 nt, orientation: REVERSE

Perfect match to: (Phage-42e-ORF028-[AY954955.1-[[7461:7916]])

Sequence:

CTATCCGTTTGTTACTGTCACTGAAATTTGTCCTGACTTATCGCTTCCATCAGTAGACGTAGCAGTGATTACTGAAGTACCTTCAGCTACACC
GTGAATTGCTCCTGTATTTTCATCTACAGTAACAAATTCTGGATGTTCACTTGTATATTTCAATATTTTATTCGTTGCTGTGCTTGGTGCAATG
TTTGGCTCAACATTGTCATCGGTATTTACCATAATTGATTTAGTTTCTGGTGTAAATGATACGCCTGAGACTAGAATTGGATTGGTTTTGAAT
TGAGGTACATCAACTTTACTAGATTCTTTACCATTTTCTTCCCATGCCACTTGGTAAGTACCTTTTGGATAAGTTGTATCCGCTTCTAAATTAG
ATAAAGTTACTGACACTTTGCCTTCACCTTGTTCAGAAGCTACGACGTCGTCTCCTTTATAAACCTTTAAAGTTTTAGTCAT

Gene: hypothet_phage_protein

Position: 1527468 to 1527668, length: 201 nt, orientation: REVERSE

Perfect match to: (Phage-42e-ORF083-[AY954955.1-[[7227:7427]])

Sequence:

CTATTCTGCTGAAACTGTTGCAGATTTTGAATTAACTGCTACTTCAACATTTTGGGGATTAGCTGGGTAACGAACCTGCAGAATCCTCTGAAT
GATCTTCACTGTCCGTGTATCCAACGAATACTTTTTTGAAGAATTCTGCTTCTCCTTCTTTACCTTCATGATAACCGTATACAATACCTTGTGA
CGTTCCATCAACAT

Gene: Major-tail (major tail protein)

Position: 1527526 to 1528167, length: 642 nt, orientation: REVERSE

Perfect match to: (Phage-42e-ORF021-[AY954955.1-[[6728:7369]])

Sequence:

TTAGCTGGGTAACGAACCTGCAGAATCCTCTGAATGATCTTCACTGTCCGTGTATCCAACGAATACTTTTTTGAAGAATTCTGCTTCTCCTTCT
TTACCTTCATGATAACCGTATACAATACCTTGTGACGTTCCATCAACATCAACTTTTCTATTCATCCAGTCACCTGTTAATTTTGTAGGTTCTG
GGGCTTCTGCTTTTTCACCTCGTGTTTTAAATTCAATTGAATCTAAACTAAAAGTACCTTTAAGTAAGGCTACATATACCGGCTGACCTGTTA
AACCATCTTCCGATTCGCCAATTACTGTTACATACGGTGCTCTTGTATTCTCTCCTACCCAAGATGTACCATTTTTATCTTTAGTACGTCCAATA
ACTGTGTTTAAATCATCACTTGGAATATTGAAAATACTCATGTCAGACTTAACTTCATTAGTACCTTGTTTTTTCATCCATACACGTTTGTTAG
ATGCAAACATATCTACTAAATCTGGTGCTAAACCTGTGATATTTAGGTCAACTGTACCACCTTTTTCATCTTCCCATGTCATGCGTTTAACTAC
TTTTGTTGCTTCTGGGTTAAAAACTCCAACGTATAATCTTTTAAAACCTACTTTATAAGAACCTTGTCCTTCTGCCAT

Gene: hypothet_phage_protein

Position: 1528598 to 1528999, length: 402 nt, orientation: REVERSE

Sequence:

TTATCTGGCCAACTCCTTTTTTATAATTTCTCTATACTTCCTTTCACTAGCAGCTAATGTTTTTGCAATAACTCCAAAACCTCTTGGTGTATATTT
TTTACCATCTCTTGTATAACCATGTTCATTCAAGTGAATAATGTTTTTGCGATTCATAGGACCTACCCATTCAATTAAAACAGCTCTTTCTTGA
CTGCCAACTTTTGTATAAGGTTTAGATTTAGTCATTTCTTCTCTATACTAGCACCCGTATCTTTAAAACTCTCGAATTCTTTCTTTAAAGTCTTTAT
AAAAAATTCAGATGCTTTATTTAAAGCTCTATCACTCTTAGCTTGCATTGCTTGTTTACCGTATACCGATTCTAATTTCTTCAACACTTCAGGT
ATCCCTTTAATTTCTACACTCAT

Gene: DNA-packaging

Position: 1529340 to 1529618, length: 279 nt, orientation: REVERSE

Sequence:

CTACCACTTTTTTAATTTCAAAATCATTTTTTGCAATCCTTTTTCATTAAACACCTTGCTTCTAGATTGGTCATTTGAGTACCCACGACTTTCAT
AATCTCTTGCAATGATATATTTAATCGCTGTACAAAAAAGCGGGTATTCCAAGTCATCTTTGTCATAATCTGGAACCCCACTTAATAGTAACT
CAGACTTAGCCGATTGAATGAGACCCTCAATTAAATCATCTTCGAAACTATAGTCAATTCTCAACCACAATTTAATTTCTTCTAAACTCAT

Gene: hypothet_phage_protein

Position: 1529629 to 1529739, length: 111 nt, orientation: REVERSE

Perfect match to: (Phage-42e-ORF172-[AY954955.1-[[5156:5266]])

Sequence:

CTATTCGGCTGATATTACAGCTGATTTAGCCTTAGCTGTTACATTAACCTTTTGGGGCTTAGCTGGGTAATGGACCTGTATTTTCTTTTGCTTT
TGCAATTCTGAATGCAC

Gene: capsid-L (phage capsid protein)

Position: 1529687 to 1530850, length: 1164 nt, orientation: REVERSE

Perfect match to: (MRSA252-BX571856-[1610781:1611944:r])

Sequence:

TTAGCTGGGTAATGGACCTGTATTTTCTTTTGCTTTTGCAATTCTGAATGCACTGTCTAATGTACGTTGCTGATCATACCATGCTGTTAATACA
AACAAATATTCGCCTTTTTTAACATCTTTATCAGTGTCATAAGTTGTTCCATCATAGTTAATTCCAAAATAATTGAAATCTCCCACAATAGGTT
TAACTGCTGCATCTGTAAATACTACTGGTTTGCCAAATACTTTTTCTGCTGGTGTGTCAAAGAAATTTGTTGTTCCATTTGAAAGAACACTAA

TAATTTTGACATAATCTGCATATCGCATATAAATTGTTGCGTTATCACGATAATCTTCATGTAAATCTGCTAAAGCGTTAATAATAGCATCAT
ACATGTCTGCTCCCTCAACTTCTTTAACAGATCCATTATAAAATGACATGTGTTCTAATCCAGATTTAGGACTTACTGCTAAGGCATCTTTACG
CTCTTTAGCTGCTAATCCTGATTGTAGTGCGTTTTCAACCCAGTTTACTAAATCTACATCTGATCCATGAATTACAGTATCTGAAATTGCAGCA
AATACTTTGAATTTATTAGTAGTGAACTTGACTGTATCACCTTTTGCTTTTAATTCTTTTGCTGTTTCTACGTCTGTAATGAAATCATCATCGTC
TAAAGTGTATGAAACTCTTGGAATCTCTAAACCTTTAATGTTAGTTAGACGAGCTTTTTCACGTAATTGGTTTTTAGCAAATGGTTCTGAAAC
AATTTCTTTAGAAAGTGTTTTTGGTAAGAGCTTATCTCCACCTGAATCATTTCCTGTTGGTAAAGCGTGTAATAAACGTTGTGCCTCCATTGA
AGGTTTTTCAAATTCATTTGGTAAAATCGCGTGACGATAAAACTCTGCCTTAGCTTTAACCATCTTCTCATTATCACTTAAAGATTGATAAGCT
TCTCCTTTATCTTTAACTTTCGCTTTTTCTTTCTCTTCAATGTCTTGCACTTGTCTTTCAACAATGTTAAATCTTTGTTGTAAACCTGCTTTTTCTG
TTTCTAGTTGTTTGATGTCTTCCATATCAATATTTGGATCTGTTGCTTTCTGACTCAATTCATCATTTTTATTTTTTAATTGTTGTCCAATCATAC
CTAAGGATTGTTTTAATTCATATAATGTCGGCAT

Gene: Q5HIZ6 (bacteriophage prohead/Clp-protease)

Position: 1530862 to 1531635, length: 774 nt, orientation: REVERSE

Sequence:

CTAATAATTCATTGTCATTTTTAAAATTTCGCATTCGCGTTTAATTTTTTCTCTTTTTTCTTTTTCTTCTAGTGACATACTTTCTTTAGGTGTTTCA
ACCAATTCAGATGTATCTACATCATCAATTTTAGTGATTTTGTCTACATCTTTCTTTAAATCTTCTGGGACGTTCTCGAAACGCTTATATTGCTC
TTTAGAGATACTAGCAGCTATTTCATTAGCTCCTAAAATTTCATCTATCAAGCCGAAAGACAAGGCTTCTTCTGCAGTAAGCCAAGTTTCTGC
ATCTAACATCTGTTTTAAGTGTTCTTGACCTAAATCTTTTGCTTTATCTAAATAAGCTGAATTACTAACAGCATCTGTTTTTTCAAGTAAATCC
GCTGTCTTTCTTAATTCTTCTGCATTACCTACAGTCATAACCCATGAATTATGAATCATTAAAAAACTATTTTTGTGCATAAAAATAGTGTCAC
CACTCATAGCGATAACACTAGCAATTGATGCCGCTAAGGCATCGACATAGATATTAATTTTTGCAGGATGCATTTTTAGCATATTGTATATTG
CATGCCCTTCAAATACACTGCCTCCAGATGAATTTATATGAACATCTATTTCACTGATGTCTCCTAGTTCATCTAGTTTATTTTTGAAATCTGT
AGCAGTTACATCACTTTCAAACCATTTATCACTTACAATATCACCATAAATAAATATTTCACCTTTACTTTTTGATTTTCTTTTCATTTGAAAAT
ACTTAGCTTTCATTGACAT

Gene: portal2-phi2958PVL (bacteriophage portal protein type 2)

Position: 1531619 to 1532857, length: 1239 nt, orientation: REVERSE

Perfect match to: (AB045978-[18780:20018])

Sequence:

TTAGCTTTCATTGACATTTTTATCACCACCTTTCAAAGATTTTCTTAATTCAAGTGGCGTGTCAATTGGGTATAAATCACCGCTTATTAGCGGC
TTATCTCCACCTTCAACTGGTGGTAAATCTTCCCACTCTCTAATGTCATTTATAGTGTAGTAACCACTACGAACTGCTTTAAAGTACACTTCTG
CTTGTGTTGCACTATCAGCCCTTAAATAAGATTTAACGTTAAATTTAAAATACCTATTTTTTTCTCTGTCTGTTTTAGTAAGTAGTTTCCGATTA
AATTCTTCTTCATACTGTTTGACGATTGGCAATAAGGTATGCTGCAAGTAAAATCTGTTTAACTCTTCATTTTTCGCGAAATTTGTATTTGATC
TTGCATTTAAGAATACTGAGGGCAATTGAAAAACGTTAGCTACTCTTTCTCTTGTTAAATTCTCGCTTGCCACTATATCTTCAGAGACATATTT
TTTAGGTAACGGTTCGATTTCAACACCAGGCTCTTGGAATAATATTCCACCGTTTTCTTCATAGTACTGTTTGAAATCTTCTAACACTTGCTGC
CTTTTTTCTTTACCTACATTGGAACCATATTTAAGCATGAAAGAATCAGGTTTTTGCATTTCTGTAAGATTAAAGGTTCTTACTGCATTATCAA
AATCAGTTGTATTCTTCAACACATCAATCGGACTAATGCCTTGCACCATATTAGATGCCACGATGTGTTTAAAATGCAACATGTCCATATTAT
GAACAATCAATTTATTTCCAGTTGCAGCATGAATGGAATAATAAAGTTCACGTGATTGGTTTTCAATTAACATTTCAACAACATCTGGATTTA
ATAAGAAAAGCTTTGATGGTTGATGATAGATGTCTCGTTCAATTAGCACATATGCATTACCTTTTTCATTTCTGATTGTTTCAATTTGATTAAT
AAAATCAAAACTGCTCAGAGAATTATTCGGTGACACTGTAAGTAAATCAGATACTTCTGTATTAACTACTTTATAATCTTCATACATTTTCAA
GGGCAAACTAGCCATCGAATTAGATAACTTTGTAATAGCTGAAAATATCGTTTCATTAGTTTCAAGCGTATTATTAATTACACCCCAAAAAG
ATCTATTTTTCCATGGGCTAAAGTCATAAAGCTTAGAAGTTGACTGATCAATCCAATTGTCTATCAATTTTTTCTTTATGCGTGTGACAATATT
CTCTTTTGCGATAACATTCAC

Gene: terminaseL2 (bacteriophage terminase large subunit)

Position: 1532862 to 1534553, length: 1692 nt, orientation: REVERSE

Sequence:

TTAACGCATTATGTCTTTAATACTAATAAACTCTATGTTTCCTTCACCACTATCAGAAACAACTTTATTCATAATATCTGTATATGTGTTTAAAA
ATGCTGCAAAGCCATCTATTTTACGATATCTGCTTTGCTTAGACGGCAACCAGTTTCCGTTTCTGTCTAGTTTCAACTGAACATTATTGATATA
CCATTTCATTAAAGGATTATTATTAAATATTATTTTCCCATCTAAAAACATTTCTTTTAAATCCTTCAATGCAGGGCTCAAGGTCAAAGCTCCT

TGTCTTGTTTCTTCCGTTTCAAACCCGTAATTTTTTAACTCTTGATTTAGTTTGAATGCGTTCGCTCTATCATAAGTAATTTTTTCTACTACATA
ATGCTCATTCATCTTAATTATCCAATTTAAAACATCTTGGTAGTCAATATAAGGCTTATCTTGCACTGTTAATAAGCCATCTTCTTCCCATTCTC
TATAGGGTATTTTTTCGTTAGAATATTCAACTTTGTGCTTAGGAATCCATGAATGCGATAAAACTGCAACTTTACCATTATCTAACGCAAAAG
TAGCACACGCGGCTGTAAAGTCCTCTGTTTCTGATAAATCATAACCAATCGTGCACGGTCTGCCTTCCAGCTCTTCTAAAGAAACAATTTCAT
TATTTTTTTGGAGTGTTGGGTAATCAATAAAACTCATCTCGTCATTATTAGCAAAGATATTAAACCTTTTGGTTATAAAATCTCCACGTTCAGC
TGGTGTTCTCTTAGCTTTTTCCCACTCTTCTTTCATCTCATCTAAATTTATAGAGACACCTAAGTTTGGTTTTGCTTTTATCCAGTTCGACGAAT
CATTAATATCATCGTCATCATCCAAAGATGCTAAATAATAAAAAGTTCTTTCGTCTTCTATGATTTGATCTAAGGTGTCTCTTCCCGCTTCTAC
CATATCAACAAGTGGACCATCTAATTGATACCCTGCTGTCGTAATGTAGATGAGAAGAGGTTGTAACCTTGCAGCTCTTGAGTTTTTTATAA
CTGAAATCAATTTATAGTCTTTAAATTCATGAATTTCATCAAAAATCCCCATGTGTGTATTCAATCCATCTAACTTATCGCTATCTGATGCTTG
GGGCATAATTTTTGATATCGTTGCGTCATAATGGATTTCATCTCTTAATGTTCTGAAATTTTTATCAAGCTTTGGGCTAGCTTTTATCATCGCC
TTAGATTCATCGAATAATATTCTAGCTTGTTTCATTACGTTTGCTAAAAGATGGATTTCAGCGCCGTTTTCTCCATCTTGAGAAACAGCATAG
TTAGCAACACCAGATATAGTAGTTGTTTTACCATTTTTTCGCCCCATAAATATCAAAGCTTCTTTAAACCTGCGCAGTTTTGTTTCTTTATGAA
CCCAACCAAACAAACTGCCGATAATAAAATGTTGCCATGGCTGTAATACAAGTTGACGTTTAGATCCTTTGGAAGGTTTACAAAACTTTTCT
ATAAATCGAATAGGACGATGCGCTAATTCTTCATCAAATACCCATTTACCTCCATTTTCTAAATATCTAAGATGCCTATCACATTCTTTTCTAA
CATATTTGCTTGTTTTTATTTTCCCTTGAGTGACTTGCTCTGCATACCATGTTGTTAATAGTTTTGGTGAAGGTTCATTTAAAACTTTAATAGTC
AC

Gene: nuclease (HNH-endonuclease)

Position: 1534975 to 1535289, length: 315 nt, orientation: REVERSE

Sequence:

TTAAATTTTTAGAACTCTAATTTTCTTAAGATTACTTTTGTCATTATCATTTGCATGAATTTTGTTATGACAGCCATAACAAACTGACATTAGAT
TATCTAATTCTAAAGCTTTGTTAAAATCTTCATCAACATAAATAATGTGATGCACTATGTTTGCATCTGTTACAATATCTTCGCGTAAACACAT
TTGACAAAGATGATTATCTCTATCTAATGCTATCTCCCTTAACTTCTTCCATGCTTTTGAATGATAGAACCAATCGTATTGATATGACTTACGA
CCATGCTTATAAATGTTATTGTGCTTGGTCAT

Gene: helc-1350 (helicase 1 (ca1350nt))

Position: 1535896 to 1537254, length: 1359 nt, orientation: REVERSE

Perfect match to: (93b_S9-CP010952-[336391:337749])

Sequence:

TTACTTATGCTTAGCTATTCTTGCTTTAATAGCTTTCATTAATTCTTCTTGCGTTAGTTCTTTATTTTGTAAAGCTTTATATACTCTTTGATCTAT
TGTGTTATCGGTCATGATATGATGAATAATAGTCGTATGATTTTGTCCTTGTCTGTATAATCTAGCATTTGCTTGTTGGTATAATTCCAAGGA
CCATGTAAGTCCAAACCAAACAATAATGTGCCCACCTTGTTGTAAGTTTAATCCATGTCCTGCACTTGCTGGATGTGCTATAAGCAACTTAAT
GTCTCCACTGTTCCAACGTTCTTTATAGTTTGAATCCTCTAATGTGGTTGCTTCCTTAAACCTTTGAAGTATTCTTTCTTTATCGTGTTTGAAGT
TATAAAACAATAGTATTGGTTGGCCTTGAGACTCCTCTATAATTTCCTCTAACTTATCTAACTTCTTATCATGTATAAGTCTTACATCTTCCTCA
TCTGTATAAACTGCGCCGTTAGATAGTTGAAGTAGTTTCTGACTTAATGATGCCCCATTTTGAGCTACAACTGTTCCTTCTTCTTCCGATTCTA
AAATATAGTTTTTTTCTAATTCTTCATATACTTTTCTTTCTTTTTCTGATAAGACTACTGTTTGTTTAGTATCAACTCTGTCAGGCATATCCAGAT
AATCTTTCGCTTTCATGCTTAAACATATATCTTCTATTTGTTTATATATCTTTTCTTCAGATCCGTCTCTTAGCTCCCACTTAAAAATATGTTCGC
TAACTTGATGAGTTGGTTTAAAGTACCTTTCTCGATAACGACTGAATGAAGACTCAAGTCTTTCACCTCTGTCTATCAAATAAACTTGAGCCC
ATAAATCCTGTAAACTATTTGGACTAGGTGTTCCTGTTAATCCTATAAATCTATTAATGAGTGGTAATTTCTTTTTAATAGATTTAAACCTTTG
ACTCTTAGGACTTTTAAATGTAGACAGTTCATCAATCACAACCATGTCAAATGGCCATTCTTTTTTATATTGATCACATAACCATTTAGTATTT
TCTTTATTGGTTACATAGATATCAGCCTCTGTGTTTAATGCATCATTTCTTTCTTTAGGTGTTCCTAAAACTAAAGACACTTTCAGATGATTTA
AATGGTTCCACTTATCAACTTCATCAACCCATGTATCTTTAGCAACTTGTTTAGGTGCTATAACTAACATTTTTTTAGTGTCTAACAACTGCAA
TTCACTAAATGCTGTAAGTGTTGATACTGTTTTCCCTAGACCCATATCTAAAAACAGACCGTATTTCTCATTATCTATCACTTTATCTATTGCAT
ACTTTTGATAGCTATGTGGTTTGAAGTCAATCGCCAA

Gene: SNF-Helicase

Position: 1535896 to 1537263, length: 1368 nt, orientation: REVERSE

Sequence:

TTACTTATGCTTAGCTATTCTTGCTTTAATAGCTTTCATTAATTCTTCTTGCGTTAGTTCTTTATTTTGTAAAGCTTTATATACTCTTTGATCTAT
TGTGTTATCGGTCATGATATGATGAATAATAGTCGTATGATTTTGTCCTTGTCTGTATAATCTAGCATTTGCTTGTTGGTATAATTCCAAGGA
CCATGTAAGTCCAAACCAAACAATAATGTGCCCACCTTGTTGTAAGTTTAATCCATGTCCTGCACTTGCTGGATGTGCTATAAGCAACTTAAT
GTCTCCACTGTTCCAACGTTCTTTATAGTTTGAATCCTCTAATGTGGTTGCTTCCTTAAACCTTTGAAGTATTCTTTCTTTATCGTGTTTGAAGT
TATAAAACAATAGTATTGGTTGGCCTTGAGACTCCTCTATAATTTCCTCTAACTTATCTAACTTCTTATCATGTATAAGTCTTACATCTTCCTCA
TCTGTATAAACTGCGCCGTTAGATAGTTGAAGTAGTTTCTGACTTAATGATGCCCCATTTTGAGCTACAACTGTTCCTTCTTCTTCCGATTCTA
AAATATAGTTTTTTTCTAATTCTTCATATACTTTTCTTTCTTTTTCTGATAAGACTACTGTTTGTTTAGTATCAACTCTGTCAGGCATATCCAGAT
AATCTTTCGCTTTCATGCTTAAACATATATCTTCTATTTGTTTATATATCTTTTCTTCAGATCCGTCTCTTAGCTCCCACTTAAAAATATGTTCGC
TAACTTGATGAGTTGGTTTAAAGTACCTTTCTCGATAACGACTGAATGAAGACTCAAGTCTTTCACCTCTGTCTATCAAATAAACTTGAGCCC
ATAAATCCTGTAAACTATTTGGACTAGGTGTTCCTGTTAATCCTATAAATCTATTAATGAGTGGTAATTTCTTTTTAATAGATTTAAACCTTTG
ACTCTTAGGACTTTTAAATGTAGACAGTTCATCAATCACAACCATGTCAAATGGCCATTCTTTTTTATATTGATCACATAACCATTTAGTATTT
TCTTTATTGGTTACATAGATATCAGCCTCTGTGTTTAATGCATCATTTCTTTCTTTAGGTGTTCCTAAAACTAAAGACACTTTCAGATGATTTA
AATGGTTCCACTTATCAACTTCATCAACCCATGTATCTTTAGCAACTTGTTTAGGTGCTATAACTAACATTTTTTTAGTGTCTAACAACTGCAA
TTCACTAAATGCTGTAAGTGTTGATACTGTTTTCCCTAGACCCATATCTAAAAACAGACCGTATTTCTCATTATCTATCACTTTATCTATTGCAT
ACTTTTGATAGCTATGTGGTTTGAAGTCAATCGCCAAATGTTCCAC

Gene: Q4ZCF7 (putative bacteriophagal protein)

Position: 1537244 to 1537534, length: 291 nt, orientation: REVERSE

Perfect match to: (ED133-CP001996-[339907:340197])

Sequence:

TCAATCGCCAAATGTTCCACCTACCATTCTGATAAAAGTATTTACTTGTTCTTTATTCCATAACACATATACTTTATGATCTCTATTTTCAAATT
GTCTATGCACATATTTTTGTAAAGGATGCAACTTTCCTTTTTCTTGCTTCATTTCTACAAAATATGTTTTTCCTTCTGGCATAATAATAATTCTA
TCTGGCACACCTCTTGTTCCAGGTGCAACCCATTTTAAACATAAACCGTTTAGCTTTGTTATCTCTTTCACTAAATATTTTTCTAATGTCGATTC
TTTCAT

Gene: virE (bacteriophage virulence-associated protein E)

Position: 1537875 to 1540322, length: 2448 nt, orientation: REVERSE

Perfect match to: (11819-97-CP003194-[1588261:1590708:r])

Sequence:

TTATATTAAATCTTCTAAACTTTCATCTCTTACATAAGCTATCTGTACACCATAATCTTTTCCAAATCGAATTTTCCCACTTTTATTACCATCATA
TACAGACCAATTGTCTAATTGTCTTAAGATGTTTGAAATCTTTCTAATTTCCATAGATCCTCTACTATCTCCCTTATCTTTACCAAAACATTCAA
CAAACACTTCAAGCGCACAGACCTTATTTCTTTCAACGTAATCTACATTTCCTGTTGGTAACATATCAACATCACCTTGATAAAATCGTCTTCG
TTCAAAGATAGTTAAGTCATCCCAATTGCTAGGAATTGGTGTGTTAAGATATTCATCAATAATGCCTGTATATGGAGATTCCTCAGTATGTTT
GCTTTGTATTGAACGCATTTCTTCTTCTAGTTCAGGGTTGAGGAATAACTCTTCTCCTTGTTCATAATAATATTTAGCTTCTGCCCAAATTTGG
TCGATCTCTTCTTTGGTTAGTTTAGACCAGTTCACTTCAACTCTCTCTGGATTTACAGTCATTGGCCAAAAACGTCTTCCACCAGTTTCATCTCT
TAAGAAATCAACTTTATTAGTTGTACCAATGAAAATACATTGCCTTGGAAAATCTTCAATATAATGTCCATAAGCAACACGAAACCGGTCAA
CTTGTTTAGATATGAAATGCTTAATAGCTTCAACTTCAGCTTTTCTTGTAGCTGCAAGTTCTGCCATTTCCATTAGCCAAACGCCTTGTAAGGC
CTCATAGGCTTCTTTACCTGTAACAGAAACTAAACTGTCAGAAAACCATGCACCACCTAATTTTTTTAGCAAAGCAGATTTACCTACACCTTG
AGGACCATAAAGTGTAAGCATATAGTCAAATTTACATCCAGGCTCCATTACTCGAGCAATTCCAGCAGTCAATGCTTTTTTGGTAGTTGTTCT
ATTCACTTCAGTATCTTCAACACCTAAGTATTTGATAAATAACTTTTCAAGACGTTTATGTCCATCCCACGATATTTTATTTAGATAATCCCTTA
CTGGATGATAAGCATTTTGCATTGCTACGCTTATAATGGCATCTTTTGTTTTACCTGAATGGTGTATGTCATAAATCTTTTCGATATAACTTCT
TAAACTGCTATCATCACCGTCTTGCCATTGACGTGTCTTAAAATTAGTATTCCATGGCACTTTCCCTAAGCATTCAATTTGTTTTGTAAATTCA
TTAAATGCTATTTTTCCTTTTAAATTTGGATCATTACGCAATATAATTTCTATATTTGGGATACTAGCTTTGAAAGTACCTTTCGAAGTAATTTC
TAACGTCTCAGACCATGCATCATCGCTATTTACTATTTCATCGAAATCCTGCATTGCATCAGACATTTTGTCGTTAATTAATTGCTTTTTAACA
ACTTCATCATTTTGCGCTCTTTGCTGCATTGCTTTATAACTAGGTAGTCGATTAACCGGAGTATCTGTTTTAGCGTCTTCATCTTGAGCACCAT
ATAAGTGTATGCGTACTAAATCAAAACTGTTCACAAGCATACCGCTTACGGGATCCGTATTATGATGAGAATAGGCAAACTTGTTATTTTCG
TATAACACCAATCCACCTGCAGTTGAACCTTCATGATAGGTATAACGGTTAGTAGAATGTTTTTCGTATAAGTCAGGAATAAAAGTTGATAT
AGCTTCTTCTATCGTATAGGCTCTACAAAATGCGCCAACAATTCCCGGTTTTTCTTCTGGGTCACCTTGCTTATCTGCTAATCTTTTAGTCTTAC
TCTCTTCCTTTGAAGACGTTGGCCATTCTAATGTGTCAGTCCAATCAACATATTCATTTAATATTTTATCTGGATCTAACAAAGGTAAATCTTC
ATAGGTAAAGAAAAATTCTGCATCATTGCTAGTTGAAGGCCAATACATTAACCTATGTGGTTGATAAGTTGTATCATCGAAGTAATCCATGC
CAACGATATCTGCCACTTTACGTCCAATAGCCTCATACTCATCCGCATTTACATTCCGTTTTAAAGGAATCACTAAACGTAATCTTGGACTTAT
CTCTCTATGCTTATGTGTTGAATACAAACAATATGCAAAATCATAAAACATAGATAATATGTCGGTCATATCTTGAGCAGCATAATCGATATC

AAGTGTTAGCATTGAACGATTCATGACTTGACCAGCACGCCGTTTGCCTTCTTTTAAATATCCACCGACAAATCCGCCAACATCTTTTATATC
GGCTTGTTCAGACTTAGACATTTTATTGTACTCAGTTAAATCTTCTTTAGTTCTAACTGTTTGTGCTAGCTTCTGCATAAAGTCAGACCAAGCC
ATATTGTGATTAGTCCAATGTGTAGATAAACGACTAGCAGCATAAGAATATGAGACATCACGATCATATTTAATTGTTTCTATTTGAGTGAC
TTTGTCTAACAT

Gene: Q4ZCN3 (putative bacteriophagal protein)

Position: 1540374 to 1540574, length: 201 nt, orientation: REVERSE

Sequence:

TTAGCTTTTGAATCTTTTTCTAATTCGTTCAACTTCATTTTCATAATCTTCTATACCTTCAACACCATTATTTTTTACTAACTGCTTGAAAAGATA
AGCATTCATATACTCCAATGCTTCTATGGTTTTCATCTTATGAGAAATGCTACTTAACAAGATCAATAAAAATATAGATAAAACAATTGAAAT
GACAATCCACAT

Gene: hypothet_phage_protein

Position: 1540516 to 1540617, length: 102 nt, orientation: FORWARD

Perfect match to: (Phage-42e-ORF198-[AY954955.1-[[r:41798:41899]])

Sequence:

ATGCTACTTAACAAGATCAATAAAAATATAGATAAAACAATTGAAATGACAATCCACATATTTACAACACCTCCAGTGCTATTGCTAAACAC
ATTAATATAA

Gene: rinB (Transcriptional-activator)

Position: 1540642 to 1540794, length: 153 nt, orientation: REVERSE

Sequence:

TTACCATGAAACTTCAGCTCTGATTTTTTCAAAGTCACTTGGCGCCTCTACATCATCATTAGCCGTCATCATAATATATACTTGCTCAGTTACA
TACTTACCTAGCTCATACATCGCTAGTAGGAATAATAATCTCAAAATTTCTTTAACCAC

Gene: hypothet_phage_protein

Position: 1540797 to 1540997, length: 201 nt, orientation: REVERSE

Sequence:

CTAAACACCCCATGTTAATTTATCGATAATTTGTATAGCTTGTTTTAATGCGTCTCTTTTTTCTTCGATATCTCTATTATCGCCATCTTCATCAG
CTGACATTAACTCACTGTCATATTCATATAATAGTTCTGATATTTCATTACTAGCTACTACTAATAAGTTTTCATCTAATGTCTGAGTTACCGTT
TTCTTTGGCAT

Gene: dut-phi (dUTPase)

Position: 1541387 to 1541914, length: 528 nt, orientation: REVERSE

Sequence:

TTACACTCCGCTACTTCCGAAGCCTTTTGCTCCACGTTCTGAAACACTCTCAAATTCCTCCACTTGCTTTAGTTCAGGTGTCCATATAGGCACG
ATAACCAATTGAGCTAGTTTGTCGCCTTTATATATTTCGTAATAATCATTTATAGTAAAAGGTTTTTTCTTAGCATTATTTACAAAAGAATTTT
CCATTTCTCCTTGTATATCAAAACACTGTTCGTTAGTTGTTAAATATACTTGTGCATCATTCTTGATATTAATCCCTAAATTGCCATGATATCCC
GCGTCTATCTTGCCTGTTTCAATCACTAAATGCGTTTTACTACTTACACCACTACGGCTAGTTAATAGTCCGACATAGCCCTCTGGTATGCTCA
CAGCTACATCTGTTTTAATCACTGCCTTTTCTTGTGGCTCGAGTACGACAGTTTCAGCTGAGAATATGTCATAACCTGCATCCGTCTTATGATT
TCGTTCTGGCATTCTAGCGTTTTCTGATAATAGCTTTACTTGTAGGATGTTAGTCAT

Gene: hypothet_phage_protein

Position: 1542804 to 1543004, length: 201 nt, orientation: REVERSE

Perfect match to: (Phage-47-ORF068-[AY954957.1-[[35978:36178]])

Sequence:

TTAACAATTAGGCAGACCAAACGACATGCATTCGTCGTATAGCTCTTCATTACTTATGCTTGCCTTATAGTTTTCAATCACATTGCTAACTTCT
TTATGACTCATTGCTTTAACTTGTTCGTCTGTATATTTTTCGCAGTCTTCTAATTCCAGTTGCTCCTGTAATGACATCACATATTCAACTTGTCT
TTGGGTTGCCAT

Gene: DNA-binding_protein

Position: 1543264 to 1543665, length: 402 nt, orientation: REVERSE

Sequence:

TTATGCTTCACTCCATTTCTTGAACATTTGGTTATAAGTGACATCGAACCAGTACGGATCACGTGAATGTTTTTGTGGCGTTCCATCATAAAG
CCATGGTCTTAATCTTCTCTTTCTTTCCTGTTCATATTCCGCTCTCACATTTCGTTGGTATCGGTTCAAAATCGCTTTTTTTCTGATTTTTTCTCTC
CCTTTTTCTTCATCTTTTATTTGACTCTGCATATATTCAACTTCATCTTTAGATTTTGAGTCTTTTCTTCCACACAATAATTCATCGCCGCGCATT
TTATGTTTGTATCTATATCTAAGAAGTTCTGGAGATATGTGATATTTTTCTGAAACTTCTCTCAATGTCATTAGTTTTCCTTTGATACGCACTCT
TATAACTTTTCTTCTAGCCAT

Gene: hypothet_phage_protein

Position: 1543665 to 1543850, length: 186 nt, orientation: REVERSE

Perfect match to: (Phage-47-ORF081-[AY954957.1-[[35132:35317]])

Sequence:

TCATTCCACCTCTAAATCTAAAACCTTGATATTTATAACGTTATATTTTAATAGTTCACCTGGATTATTAAATAAATAGTCCGCCAAATTTTCTT
TTTCTTTATCAATCTGATTGTAATTAACACTTTCGACTTCTGTAGGAATTCTAATGTCAACAGAAGCATTGATATAAGCTTGATGTTGCAT

Gene: pol-phi12 (bacteriophage DNA polymerase)

Position: 1543863 to 1545824, length: 1962 nt, orientation: REVERSE

Sequence:

CTAATCCTTCATATAAAACGGAGAAGTAAACCCGTCACTATTCAAATTCAATCCTTTTGCCCAATCAACAGGCTTATTCATGATAGTTTCGAT
TTCCTTAAGTCCATTTGAACCTCTAGGTATTTCTACAATTACTTCATCATGGACATGGCCAACTATTTTAAAACCTAATGCTTCAAGCCTTGCT
ATAGAAATCGCAAGTAAATCCCTTGCAGTTGCTTGAACAATATTCTCGACTAACTTCCCACCATACGTTTTTAACTTTGACCATTTACGGTTAA
GATCTAACCCCATAAATTCAACAACTTGACTACCCCAACTATTTTCACCAACTAAAGCTTTTGGATAAGCTAAAGCTCTTCCACTAGGCAGTT
CAATCATTAGAAAACCTTTTTTCATATAAAATCTAAGTCCATGTGTATGATGCGTCTTTCGGGATTTTACAGTATTAATTGCAGCCTCTTGGC
AAGCCTTCCAAAAAATTAACTATGTTAGGATTTGCGTTACGCCAACTATCAACTAAACCTTGTAACTCGTTTTCTTCAATGCCCATTTCCAATGC
ACCCATTGCTTTTAAAGCTCCAGCGCCACCTTGATAGCCTAAAGCTAATTCGGACACTTTTCCTTTTTGTCTGAGAGGGTCGCCTTTAGTTAT
GCTTTCTACCGGTACATTAAACATTTGAGAAGCCGATGCTTCATATATCTTTCCGTGTGTGTTGAATACATCTAAACGCCATTGTTCTTTTGCA
TACCATGCTATGACTCTTGCCTCTATTGCAGAAAAATCACTTACTGCTAGTTCATTACCTTCTTCAGCAGTAAATGTCGTCCTAACTAATTGAC
TTAATAAGTCTTGAGGATGAACATTGAGTAATAAATCTAAATCATCAAAACGTTGTTCTTTAATAAGATCTCTTGCTATTTCTAATTCAGTATC
TGAAATATAATGCTTTGTTAAATTCTGAAGTTGTACACCTCTACCTGCCCATCTTCCAGTACCGGCACCGTAAAATTGAAACAGACCTCTTAC
CCGTTCATCACTGCACATCATGTCATGCATTTTGTTGTATTTTTTCACACTGGTTTTAGACATTTGCAATCTAATTTCTAGCATTTTTTTAGCTT
TTCCTGTTGCTTCTTTTAAGTACTCCTGAACCGTTTTCTTTTGTAAATTAGGTATATCTAATCCTTGTTCATCCTTTAACCAAGCCAATAACTGT
GTAGGACTATTAGGATTTTCTAAACCTGTTATATGTTTAGCTTGTTTAAGCAATTCTTCTTTACTCTGCTTATCGAGCACATTAGCTCCTAACA
TCAATGATTTAGAAAGCTTAATACCTCTGTCGTTTATATGTTGGTCAAAAACCCAATATGTTTGTTCAATTGCAGTTACTGGAAAGTCTTTAA
TTTTATTAGCAATCGCCATTTCTACTTCTACATCTCGAATACAGTAATCTATAAATTGTTGCCATTTTTCAAGATCATGTTCAGGTAGGTTTCTT
GTTCTTCCTCCATTAACTTTTGTTGGTTTACAAGGTATAGAGAAATAACGAATTAAATTTTTACCTGCTTTATCTTTTTGGCTTTGTAGTCTTAA
AACTTCTCCAACTTTATCAAGCGAAGCAGGTAAGCCAATACGCATTGAATTAACCATTGTGCAAATCCATTCTTCAGGTGGCATCTGTTTATT
AAAATGTTTAGCAAGACAAGTTCTTTCGAAATTAGCATTGAATGCATACTTTTTTACAGCAGGGTCAAATAGAGTAATTTTAAACGTCTCATA

ATCAGCGTGGAAAGGCTCATTATCTACTTTAGTCATGTCAATCGCACTAATCGCTCCACCATCTATCGAATAAGCTATAATTAAAATTTCGAA
ATCTTCAGCTTCTGTGTATTTATAGGCACCACATTTCGAAATATCGTTACTGCTGTATGTTTCAATATCTATATTCATAAATTTCAA

Gene: hypothet_phage_protein

Position: 1545884 to 1546441, length: 558 nt, orientation: REVERSE

Sequence:

TTATAAGAAATCCTCATCATCAGTGTCTAATTCATCAAAATCATCTTCTGCTGCACTTGCACCGCCAAGAGGTTCGCCTTTTTCTACAAGTTGA
ATGTTGTTCAATCCAACTGCGATACCCTTATTACCATTTGTGTTGAATGGAAATAAATTGATTGAAGCTCTAATATAGTCACCACTTACATTA
GTTCCAGAATCCGTTAATCTAATTTTGTTTTGGTCAATAATACCAGGTGCTTGTTTGCTTGATGCGTTAATAAAATAAGCGTCTTGATAATTC
ACATCATCTTCTCTTTCAGTATCTCCATCACGTAATGGAAGTTTCAGATTTGCAGGAACTTTGCCTCCAAACTTACTAACTTTTCCTTCTTCTTT
AGCAGCTTCTATAGCTTGTTCAATGGCTTTTATCGTACTTGTATCTGATTTAGGAATGATTAAACTGATTGAATACTTTGCTTCTTGCCCTTCT
TGCATACTGTGAGGTTCAAAAATATGTGCATATGATGCTCTTACTTTTCCTGTAATCACTTTAGTTTTATTTAATACTTTTGCTTTCAT

Gene: Q4ZCH8-phi12 (bacteriophagal protein)

Position: 1546467 to 1547633, length: 1167 nt, orientation: REVERSE

Perfect match to: (11819-97-CP003194-[1596853:1598019:r])

Sequence:

TTATAGTTTGTCAAAATCATCTTCAGCAGATTGCTTTATAGCTGGTCGTTTATCAGACTCGGTAGCAAGTGTTAATTTACCTTGTGGCTTTTCT
ATAAAGCCTTCTGCAATTTTAGAAAATGCTTTTTTGCCGATTAATTTTTCTAAATTCGTAATGCTAAGTAACTTGGTTTCTGTAATATCTTCAG
GTTTATAACCCGCTTCAACTAACTTTTCAAGCGTTGCATTTGTATCAGTTATCATTCTTCGCGAGCGACCTTCTACAAGCTTCCAACCAGGATA
GTTTTTATCATTTTCTTTCGCTTGATCTAGTGCATATTTTTCTACTTCATCAGCCCATTTTTTGATGTCAGGCAGTTTATATAAAAGTTCTGCAA
TCTCTTCATCACTCAACAAATGTGGTGGCTTTTGAGGCACATTTTGCATGTATTCTGCACGTGTTCTACATGAATGCTTTATCTTACAGAATCT
ACAATGACTACCTGCTTTAAACTCACCTTCACCGTTATAAGCAAGTCTGGCTAATGGTTTAACAAAATCGGTTCCCCATTGAAGTAATCTTGA
TATTGGTAACTCTTCAGTAGAAAAGTTATCTATTCGTGGTTGTATGATAGTCATGCGAACTGTATGAATGTCATACATTAAACTAAGCAGTTC
ATATGCGCCCAAGCCATATAATCTAAGTTGAGGATTATCTATAGCTGAAACTTCAATGCCTTTACCGTATTTAAGGTCAATAATTTCAAGTAC
ACCACCTGAAAATATAATGACATCACCAGTACCAAAAGATTCAGGGACGTATTTACCTAAATCCAATTTTGTTTCAAATAAAGCTATTACATC
GTCATCTCTACTCAAAGCCTCGTTATACTTTTCTTCTACATTAGCTACGTACTCTTCAACATATTCGCGCAACTCTTCACTGTAATATTGATTTC
GCTTATAATTTTGAAAAGCTTTATTAAACTCAAACTGTGTTAGGCCTTCATATTTAAGACTGAAATATAACTCACTTAACTCATGAGCGAATG
TACCTTTTTCAGCAAAAACTGAACTTTTATCTGCAATACCTTCACTTGCCTTAATACTCGGTGGACAGTTTAGCCATTGTTTTGCTCCACTTGC
ACTAAGCTTTGCATGAGCTCTATTTGAGTGATCTAGCTTCAT

Gene: hypothet_phage_protein

Position: 1548007 to 1548330, length: 324 nt, orientation: REVERSE

Sequence:

TTAAAATTGGTTAGCTAGACGAATCATTAACTTGATACGTTCTTCTATTTCTCTAGGGTCATCACTTTGTTCGTTTAATCTTGCCAATAACTCG
AATTGTTCTTCTAATATCTCTTTCTTACGTTCTACAACAGTTAAATGTAACTGCGGTTCAACAACACGCCAGATACCCCAACTTTCCAATTCAA
TCTTTCCTTTTTTCTTAAGTCTTGAAAGTGTGGATTTTGCATGTGTTTTAGATATCCCAAAAACTTCAACAACATCATCAGAATTGAAATTGTC
ATATGTTGCAAAATGTGATAGTATTTTTTGTTGTAAGGTCAT

Gene: Homeodomain-like

Position: 1548584 to 1548847, length: 264 nt, orientation: REVERSE

Perfect match to: (Phage-3A-ORF048-[AY954956.1-[[29235:29498]])

Sequence:

CTAATACCATTTTTTATGCTTTCTGATCAAATACTCTTCTAATTTAGAAATATTAATCAATGTTCCCGTTGCTGAATAATCAATGTATAAATTTT
CTACACCTAAATTATCTTCACGGTAATATTTCAACCAGTTGTATACTGTACTTCTACATACTCCAAACAATTGATGGATTTGTGTAGGTGTTGC
GTATAACTTTTTCACAAATTTTTCTTCGCCTCGATATGTGTTTTCTGGTGTTGGTGGTATTATGATTTTTGGCAT

Gene: Q9B0H0-phiSLT (putative protein of phage phiSLT)

Position: 1549142 to 1549507, length: 366 nt, orientation: FORWARD

Perfect match to: (MW2-BA000033-[1570113:1570478])

Sequence:

ATGCCCTTGATATCTGATGAATTTGATACACTTACTAAAGACCAACAATATATCTTGTCCGTACTCTACAAAGATTATTTAGAATGTGTAAAG
TTAGGTTCGGTTAAATTAACCTGCAATAATTTTGGAAGTGCTAAAGATATACATACAAAGTATTTTCAAAAACTACATTTCGAAGATGTAAA
ATACGATTTAAATAAACTTAAAAACTCTGGGTTCCTAAACGGCGTGTATGCTAGTAACACTATTTATCATGTAACAATTTCAGACAAGACTGT
TGTTTACTTTGAAAATGAGTTTAAAAACAATTTAAAAAGTATCATTGATAGCATTTCTAAAATTGCTTCAATAATTCCTGGTCTCTAG

Gene: hypothet_phage_protein

Position: 1549476 to 1549721, length: 246 nt, orientation: REVERSE

Perfect match to: (Phage-3A-ORF054-[AY954956.1-[[28361:28606]])

Sequence:

CTAAAATTGCTTCAATAATTCCTGGTCTCTAGTTGGGTTTATAACTTCCCAATCATTTGCCATGAGGTCATCGGCTGAAGGTTGCCAATATCT
GATAAGGTTTGTCCCATCGCTATTTGAAATGATGCATTGTAAAAAACTATCATTTGTTGGTAATATCTTAGTTCGATGACTTTCTTTCCAATCT
TTCCGTGTCATAGAGACAAGATTTTTTGTAGCTATCTTAGTTGCTTCTTGAATGTTCAT

Gene: Na-K-ATPase

Position: 1552054 to 1552668, length: 615 nt, orientation: REVERSE

Sequence:

TTAATATTCATTCATTAAATATAAATCCAATTTAATTTGTTGTTTAAGGTCTACAAGTGTATGTTTAATATACAATTCATCGTTTGACGGTAAA
TCAGATACTTTGAAATCTTGTCGCTCAACCTCTAGTAAATCGAAATCGCTACCAGCTGAATTATAGGTTTTAAGTTCACCCTCTTCAATGATTC
TGTTTTCAAAGTCTTTAATAACTATAAATACTGGTTTACCGTTGTTATTAAACAACTTGTCTCTTTTGTCTAATAAGCTTATACAATCCAAATTC
ATAAACTTTCTTGTTTCATTAATTAACCAGATAATGAATTTAACAATTAAAGGATTAAATACAAGCACTGTTAAAACAAAAATAATTAGAAAC
CAAATATTTGCTTTTAGACCTGTAAGCAACTGAAGTAAACTCAAATTTTTTAAATCAACATTATTAAAAATTATAAAAGTATAAAACCATATC
AAACATGTTTCAATAGAAAAAATCAATAATACAGGAGTATTGATAATCTTGTTTTTTTCACTAACTAAACCTATCATTGTTAGATATTTATATG
GTATGTAACCTAAAACTCCTGTAAGAAGAAGCGCCCCTAGAAATTGAGTCAT

Gene: int-1 (integrase)

Position: 1552794 to 1554000, length: 1207 nt, orientation: FORWARD

Perfect match to: (11819-97-CP003194-[1603180:1604386])

Sequence:

ATGTGGATTGAAAAATTTAAAAACAAAAATAACGAAACTAAATACAGATATTACGAGAAGTACAAAGATCCATACACAGATAAATGGAAG
CGCGTAAGTGTTGTGTTGAACAAGAATACAAAACAATCTCAAAAAGAAGCAATGTTTCGTTTAGAAGAAAAAATAAAAGAAAAACTGAAC
AACAAGTCGTCAAGCGAATTAAAAACTTTGACTTTTCACGCGCTATTAGATGAATGGCTTGAATATCATATAAAAACATCAGGTTCAAAGTT
GACTACTCTTAATAATATAAAAATAAGAATTAAAAACATTAAACGATACTGCTCTGAGAACTTGCTTTTAAACAAACTTGATACAAAATATAT
GCAGATATTTATTAATAAATTATCAGATATCTATTCTCAAAATCAAGTAACCCGTCAACTCGGAGATATGAAAGGAGCTATTAAATATGCAG
TTAAATTTTACAATTATCCAAATGAATATTTGTTAACTAATGTCAAAATTCCTAAAAGAAGAAAAACAATAGAGGATATCGAAAAAGATGAA
TCTAAAATGTACAACTATTTAGAAATGAACCAAGTCCTACAGATACGTGATCATATACTAAATGATAATAAGTTACACAAGCGAAATCGCAT
TTTAATTGCCAGCATCTTAGAAGTACAGGCTTTAACTGGTATGCGCATAGGAGAACTACAAGCACTGCAGGAAAAAGATAATAGATTTATT

AAACAAAACTATCAATATAACAGGTACAATTCACCGCATTAAATACGAGGAAGGATTCGGATACAAAGACACTACAAAGACTATAAGTTCA
AAAAGAAGTATCAGCATCAATTCTAGAACCGTAGAAATTTTTAAAAAGATAATACTGGAAAACAAAATGTTGAAAAGATGGAATTCGAGCT
ATGTTGACAGAGGGTTCATATTCACAACAAAAAAAGGGAATCCTTTATGTAATAATCAAATCGCCGGTGTGCTTAAGAAAACTACAAAAGC
TTTAAATATGAATAAGAAAGTTACCACGCACACATTTAGACATACACACATAACTTTATTAGTAGAAATGAATGTTTCTTTAAAAGCAATTAT
GAAAAGGGTAGGACATGTAGATGAAAAAACAACCATTCGCATATATACTCATGTAACTGAAAAAATGGATAGAGAACTAACTCAAAAACT
CGAAAACATTCCAAGTTAG

Gene: A5IT17 (putative protein containing attachment site of PVL-phage)

Position: 1554081 to 1556082, length: 2002 nt, orientation: TRNC-FRWD (no stop codon)

Sequence:

TTGAAATAAACATATCATCATAATGAGATGGTTCAAAAATCTGATTAACAATTAACGGTTTCATATTTCTAACTATATCATCTAAGTGATTTTC
TAAAATCGGCGAAGCTATTTTTAAATTATTTAAAAATTCGTCTAAGTTTTTAAGTTTACTTTTTAAAACTTCAAGCATTTTATCAATTTCGTCAA
CATTTATTCCCATTGCCTTTATCTTTATTTTCATTCCTGATTTATATATTAAACTGCTTATTTCATAAATATGTCTCACAGCTGTTTCTACCGAAT
GTATAACTTCAAGAATGTAGTTTAATACTGGTTCAAAATAATTATTGTAAGTTTCTTGAACATAATCACTAATTTTGTTAACTGCATTATCAAT
ATGGTTTTTTAAAATTGTCATTTTTCTAGTTAAATAATTAGAAGATTGTACTTGACCCGAAATCATATGTTCACCACTATAATTAATAGATAAG
TTTCCATCCATTACCGTTGCATCTTGATGAGACATTATATTTTTAATATCGGTTATCTGCTCTCCATATACATCATTTTGCTTTTTCACAAAATTT
ATATTATTATTCACAACATTTAAATGCTTCATCATTTCCTCTGTCACGCCATCTTCAAAATCATGATCTAAATTTTTAAACATTTCTTTAATTAC
GTTATCAAGTGAATCATGTAAACTTTCTATGCCACGTTGTAATTTTTTATCTAAATCTTCAAGAATTTCTTCAATAGTATTTAATTCAAGAATA
AGGACTCCAGCTGCTGGAGCTATTAAGATTATTACGACAAGATTTTTTTTGACATCATCAATTGCGCTAAATACATCCTGTTTAAAAGAAGTT
AATTCCCTACTCATTCCTTCAAGGATATCAACTTCGACAATCTTTTTAAAATCTTGTTTAAGTTTATGTTTTCTTTCTCCAAAATTAGAATTTTC
AACCTGTGAAATATTATAGCTCTCATTCAAATAATTTACACAATCGGTTAACATATTACTTGTTTCACCAGTTACCAAATTACTTAATGCACTA
AGATTTTCTAGATTTAATTTTATAGGAGTCCCTTTACCCGTTCTCGCTATTGAATCTCCTGTCCACACATTAATTGGAATTCTATCATCCATATT
TATATCGATTTCAATAGTTTTTTTGACTTTTTTACCATTTTTAATCATAGTATCTTTAACTGATTTAATTTTATAAACTGCTAAATCCGTATAAGT
ATTATATCTTGGATGTAATTTTCTTTTATAACCTAAATGACTATTTACTAATCCATCCATCGTTGGCACACCAGTTTCTAAATTCACTCGTTTTC
CTAGTGTCGGAGTGGCAAATGGATCCTGTAGCCAAGATAAAATATCAAACTTGCTATGATAATTAATCATATTTTTAAAATTTTGAGATTTG
AAATTTTCCCATATTGACTTTGGTAACATGGCAGGGTTTGTCGAAACTATATTTTTATCCGGATTAATCAAACCTTGATGGTTAGCACTTGCT
CCCCCTTGAGAATTGCCTCCATCAGACTTAATAGTTTTGTTTTTATACGTAGACGGATTTTGCTTATATTTCCTTAAAAAATCAGAGCTTTTAA
GTGTATCTGCATCTTTTAGTTTCTGTTCATACGATTTAATATAAGCATTATTTTGTTCTAATAAAGAAGTTGACTTATTTTTATCATTCATTAAC
TTTATATTTTCAATCCAATCATCAGCAACCTTCCCACGAAAAGGATTGTTGCTTCCGATACTCTTATTATTATTTGTACCTTGGAAGATAATAG
CTTGTTGTCCTGTTAATTCATTATTGCTATTTACCAATTCAAAAACTTTTGCATCTGTACCACCAAAACTTTTAGAATTAGGATTATCTGAATA
ATCTTCAATTAGTCTATATTTATTATTATTTACTCTAATTACATATCTTTTGTCCAAATCTTGATATGCCCAGTATCCACTTAATTCTGCTAAATC
TCTATCATTAATTTTTTT

Gene: graD04 (putative lipoprotein)

Position: 1556072 to 1557004, length: 933 nt, orientation: REVERSE

Sequence:

TTAATTTTTTTCATGATTAAATACTCCTACTTCTATTGAATCTTTCTTACTGTAACTAGGTCTTGCTGAATTTATATATTTATCAGTAATTTGAA
GAGAAACATTCAAATCATGAGGAATATTCTTTTTCTTTTTTAGTACTTCAGAAAAATCAATCATCTCGTCTATTGTATTATCTTTAGTGTAATT
GTCTTTAGTGCTAAATAAAGTTGTAACAACATATGGACGAGATTTATCTTTAAGTTGTTTTTTTGAGTTTTTCATACCGTCCCTAAAAGTCTTA
TCATCCTCTTTGATTAAGGGTTGAAAATACTTTTTATATTCATCTAAAGTATATGGAATTGCAGTCAAATAATAATATTCATTTCTAAAACCAC
TATTCTGCGTTTTCATAATTGCATTTTCTGTATAACCAGTATATTTATATTTACTTTTATATTCTTTCAAAACTTCTGTTAAACTATCCAACTCTT
GCTTATGTGCTCGATATTCAAATCCACTGAGTACTGTACCAACTAAAGTACTCATATCATCGCCCTTATCCTCACTTCTTAATGAGCTATCACT
GTCAATAATTGATTTATCAAATGGAATACTCGCATTAAATACGATATCGTGGTCATCGCAATGCACATAAACTTCTACACCGTCGCCACTACC
TACAACATTGGTAGCTTTAACTTTTAGACCGAAGTTATCCATAAAAAATTGTTCGCCTCGTTTGGCAATTTTATCTTTATGCTTCTTCGCAAAT
TCAATCGCATCTTTTTCTGCAGGTGGTTGGAAGCCTTGACCTACATATTTTGAAGCTTCCATTTCTTCTGGTACAGATTTTGTTTCTGTTTTTGT
GTCTTTTTTTGATTCATTTTCCATCGTGGAACATCCCCCTAAAATTAATGTCGTAGCTAAAACTGATCCAATGAATTTTTTCAT

Gene: srrB (two component sensor/regulator of respiratory response, sensor histidine kinase)

Position: 1557249 to 1559000, length: 1752 nt, orientation: REVERSE

Perfect match to: (FSA090-CCEO01000003-[88423:90174:r], allele observed in SCHW-ST206-303-253-142-196-202-197)+CC8+CC692)

Sequence:

TTATTCTGGTTTTGGTAGTTTAATAATAAATGTTGTGCCTTTTCCTAATTCGCTTTTAACATCTATGGAACCACCATGCTCTTCGATAATCATTT
TACAAATGAACAAACCTAAACCGGTACCTTGTTTACCTCGCGTTCTCGCTGCATCAACTTTATAAAAACGATCAAATACTTGTTGTAAATGTT
CTGGTGCAATGCCTGTGCCTGTATCTTTAATGTATAAAATATCTTCGCTTTCATTTTCATCACAAGTAATTGCAATTTCATCTCCAGGTTTCGT
ATAACGTGATGCATTATCAATTAAGTTCGTTAGTACTTGGTCCATGCGATCCATATCATAACTCCAAACACGCTTCTTACAATAATTAAAAGT
CATATTTAGACCTAAATCATCAGCTTGTTGGCGATACTTAATTTTCATCTTATCTAGTAACGCTGCAATAGGCTGAACTTCTTTATTTACGGAT
AACCCTTCAGCATCCATGCGTGCGACATTTAACAATTCATTAACTAAACGATTTAAACGTTTCGATTCATCAAGGACAATGGCAAGCGATTCT
TTTATTTCATCCGGTTCTGTAACAATACCATCTACAATTGATTCAGTATAACCTTGAAGTAATGATATCGGTGTACGTAATTCATGTGATACA
TTAGCAATGAAATCTTTCTTCATTTGATCTAGATTGTGCTCATTAGTCATATCACGAACTGTCACAACAACACCACTTTTACCTCCCTGTTCAA
TCTTGTCGATATAGCTTGTGGTCACAACAAAGAATCGTGCATTCATTTCTAAATCACGCATTTCCGTTTGTTTTGATTTAAAAGTATCTTCAAT
TTGTCTTAATAAGAAAGCTTTAGCATCTTCATCAATATTGTCCATAATATCATTCGCCATCTTATTAGATAAGATAATTTGTCGACTCTCATTA
ATACCTAGGACACCTTCTACCATAGAGTTAATTAAGCTGTCTCTAATATTTTTAGATGTGGATAATGCGTCGACATGCTCTTCGATTTCTGTA
CTCATCTGATTAAATGCCTGCGATAATTGACCAATTTCATCTTTCGTTGTGACAGAAGGTTTATAAGAGTAATCCCCTTCAGATACACGTGTA
GCTTGGTCTCTTAAACGTCTTAAAGGTTTTGTAATTCTTGATGATAAGAAAAACGCAAAGACTGTTGTAATTGTTAAGAAAATAACAGCCGT
AATTATAGTGATAATCGTAATAGCATTATTTGTATCTTCGATTGATTTCAAGTCTTTATATATAAAGACTCCACTATATTTGCTATGACTATTC
TTCTGTGCTTTTGTTGGATAGCCTAACAAAATATATGTTTGAGATGAGCCCTTTTCTTTAATCGTTACATTTCGAGTAACAGATTTACCTTTAT
CAAACACATCGTCAAAATGGTCGTTGTTGACTACTTCATTCAACATTTGCTTTTTAATATTAGAAAGTGAAGCCGTTGATTGACGATGTTTAT
TATTTATAATCATCAACCCACCAGGATTTTCAATTAATGTTTGACTATATTTTATTGCTTCTTCTTTATTATGTGATTGTTCGACCAGTGAACTT
ATACGTCTAGCATCTTCTCTTATGGCATTTTCGGTTTCTTGTGTGAAATAGTATTGCATAAAGGTAATTAAAGCAATACTTAATAAAATTAAA
ACTGTCGTCACTATTAAAATAATAGTTAACCACAGTTTAATTACGACACTATTTAGCCGGCTCATCAT

Gene: srrA (two component sensor/regulator of respiratory response, transcriptional regulator)

Position: 1558981 to 1559706, length: 726 nt, orientation: REVERSE

Perfect match to: (TW20-ST239-FN433596-[1624445:1625170:r], highly conserved allele)

Sequence:

CTATTTAGCCGGCTCATCATTAGATTTAACCTCAAATTTATACCCAACGCCCCAGACTGTTTGAATCATATGCGCAGCTTCACTAGACACACG
ATTTAACTTTTCTCTAAGTCGTTTAACATGAGTATCAACTGTTCTTAAATCACCATAGAATTCATAATGCCAAACTTCTTTTAATAATTGTTCAC
GGTCAAATACTTTATTTGGTGTTTTAGCTAAATATATTAATAATTCGTACTCTTTAGGAGTCAAATTAACTTCTTGATTATCAGCAAGTACGC
GATGTGCATCATTATCTATTTCTAAATGTTTAAATTCAATCACATCACGTGCGTGAGGTTCGCTTTGTTCTACAGTTGTAGATTGCGTTCTTCT
TAGAAGTGCTTTAACTCTTAAGACTACTTCTCTTGGTGAAAATGGTTTGACGATATAATCATCTGCACCAGATTCAAAACCTTCAACACGGTT
TGTTTCTTCACCTTTAGCAGTCAACATAATAATCGGTGTTTGTTTATGTTCACGCAATTTAGTTGCCACCTGGATACCATCCATTTCAGGCAAC
ATTAAATCTAGTAGTATGCAAGCATAATTATTCTCCATTGCAAGTTCATAAGCCTCTTGGCCATTACTTGCTTCATGGATTTCAAAAGATTCTC
TTTCTAAATACATTTTAAGTAATCTTCTGATTCTATCCTCATCATCTACGATAAGTATTTCGTTCGACAT

Gene: rluB (23S rRNA pseudouridine synthase)

Position: 1559839 to 1560576, length: 738 nt, orientation: REVERSE

Perfect match to: (11819-97-CP003194-[1610224:1610961:r], allele observed in CC80+CC239+CC4803)

Sequence:

TTATTTCCCATGTTCTGCTAAATGACGCATCACTTTTACTTCATGAGGCGTCAATACACGTCCTTCACCAGCATTCAAACCGACAACATTTAAA
GGCCCATATTCAATACGAGACAGTTTCGTCACTTGATGACCAAAATGTTCGAACATTCTTCTGACTTGGCGATTACGACCTTCTGTAATTGTA
ATTTCAACCAATGTTGTGTTTTTATCTTTATCTTGTTTCTTAACTTTCACTTCAGCCGGTTGCGTCATAACATCTTCTAATTCAATACCTTTTTCT
AGCGCTTTCACTTCTTCTCTCATTAAATAACCTTTTAATTTCGCAACATATTTTTTCTTAATTTGATATCTTGGATGTGTCATTAAATTAGTAAA
TTCACCATCATTTGTGAGTAATAACAATCCAGAAGTATCATAGTCTAAACGACCAACAGGATAAATACGTGCTTCTATATCTTTAAAGTAATC
TGTAACCACTGTACGTCCTCTATCATCAGATACACTTGTTATCACTTGAGTTGGTTTATGGAATAAAATGTAAATTTTGTCTTCTAGTTCTATT
TTAATACCTTCAACTTCAATCGTATCTGATGGCTTCACTTTTGTTCCTAATTCAGTGACAGTCGTACCATTCACTTTCACTTTTCCTTCAGAAAT
TAAAGTTTCTGCCTTACGTCTTGAAGTATAACCGCTATTAGCTATACGTTTTTGTAATCGTTCTAATTCTTTAGTCAT

Gene: scpB (chromosome segregation and condensation protein B)

Position: 1560569 to 1561111, length: 543 nt, orientation: REVERSE

Perfect match to: (N315-BA000018-[1534159:1534701:r], highly conserved allele)

Sequence:

TTAGTCATTATTTTCTCCTTTTTGATTGACTAGATTACTGAAAAAAGCATCCATTTCTTCATCGTCTTCTTCAGTTGTCGGCAAATCTTCTATAT
TGGAAATACCAAATACATTTAAAAATAAATCAGTAGTAATTAACTGTTGGCTACGCTGTTCATTAACCACTTTAGCCTCAACTAGTCCTTTGG
CAATCAATGTCTTAACTGCACCATCTGAATTGATACTACGAATTAATTCAATATCACTTCTTGATAATGGCTGGTTATAAGCAATAATTGATA
GTACTTCCATTGCTGCTTGTGATAATTTCATTTGTGACTTTTGTTCAATTAATTGTTCAATATACGTTGCCGCTTCTTTTTTAGTCGTTAAAACA
TACGTCGTTCCAAATCGTTGTATCATTAATCCATGTGATGAATAATTTTCAATTAATTCAACGAGTTGGTCTTTCGACATATCTAATATTTCTA
ATAGTTGTTTTTCATCTAAACCTTCATCGCCAGCTGTAAATAAAAGCGACTCTAATATACCATGATTATCCAA

Gene: scpA (chromosome segregation and condensation protein A)

Position: 1561104 to 1561835, length: 732 nt, orientation: REVERSE

Perfect match to: (TCH959-AASB02000244-[1963:2694:r], highly conserved allele)

Sequence:

TTATCCAAAATGGTAGTTCACTCCTCTAATAATGTTAATATCTTCAAAATTACGTTGTTGCTCAATATTAATTATTCCTGCTTTTGACATCTCTA
AAATAGCTAAAAAGTGAGTGACTACTTGTTCAATTGGCTCAGAAAACGTAAACAGACTAAAGAAGTTAAAATGATCTTTATCTTTCAATCTC
GATGTCACTTGTTCTGTAGCTTGTTGAATGGTAAATGTCTCTTTTCGGATTTCAACAGATTTAGGTGTATTTAACTCAACTCTATTTTTAACTC
TTTGATAAGCTACAATTAATTCAGTTAAATCAATCGTATGATTTGGATCCCAAGATTCATCTGTTTCCAAATGAGATAAATCTGTCGGTCTTTT
TGTAAAATAAAAATCTCTTTCTTCTTTCATGTCATTTAAAATAGCAGTATATTCTTTATAATTTTGATATTCTATTAAACGCCCAACTAAATCTT
CCCGTGGGTCATCATCAACATCCATATCTGATGTTGATTGTGGTAATAGCATCTTACTTTTAATCATTAAGAGTTCTGACGCTAATACTAGGT
ATTCACTTGCAATATTAATTTCAAGCTGTTTCATTGCATGAACGTACTGCATATACTGCTCTGTTAATGCTTGCATAGGAATATCATAAATATC
TATTTCAAATTTTTGGATAAGATGCAACAATAAATCTAATGGTCCATTGAAAGCATCTAATTTAACTTCATACAT

Gene: Q2FY75 (putative protein)

Position: 1561927 to 1562433, length: 507 nt, orientation: FORWARD

Perfect match to: (11819-97-CP003194-[1612312:1612818], allele observed in CC80+CC12+CC188)

Sequence:

ATGCAACAGGCACTGATAAATTTCTATTATCAATTTCATACTAAGCAACATTATTTCCTATGTCATGACATTTTAGAAGATGCATGGAAAGCT
GAAAATAACTACAGTAAGCAAGATGCAGTTGTTAGTTTAATCTTGTTTGCAACTGCTTGTTACCATTATCGCCGCAATAATTTAAAAGGCGC
CTATAAGTCTTTTAATAAATCTAAAGAAATAATACAAAATGCTAAAGACAGAGATACGTTATACCTTAATTTAAATGATTATCAGTTGTTAAT
TGAGCAACAGATTGCAAAATTGAATGCAGCAAAGCCTTTTTCATCTGTTATATTACCAATAACTCCAGTCTTTGAACGCATCATCAAGGCCA
ACTATCCAGACTATGATTATATAATCAGGAAACAGCAACAGATCCTTTTATTGTAGACCATCACATGCGCCGTGATCGTTCAGAAGTGATTGCA
GCAAAAGAAGAAGCGATTCAATTAAGAAAACATAGAAGTAATTAA

Gene: xerD (chromosomal tyrosine recombinase D)

Position: 1562511 to 1563398, length: 888 nt, orientation: REVERSE

Perfect match to: (CIGC93-AHVD01000017-[184897:185784:r], highly conserved allele)

Sequence:

TTATGCTCTAGGATGAAATTGGTTATACATTTTTCTAATTTGAGATTTCGAAACATGTGTATAGAGTTGGGTAGTAGATATGTCAGAGTGAC
CTAACATCTCTTGCACTGCTCTTAAATCTGCGCCATTTTCCAATAAATGTGTCGCAAAAGAGTGGCGTAACGTATGTGGCGTTAACGTCTTTT
TAATGTTTGCCTTTACACCATTTTGTTTAATCATTTTCCATATTGCTTGTCGCGATAAAGGTTTACCATGCATATTTAAAAATAAGACTTCGGT
AATAGTCTTTTTTAAAAGTTGCGGTCTAATCGTTTCAATATAAGTAGTTAAGTACTCAATGACTGCGTCGCCTAATGGTACAATTCTTTCTTTA
TCGCCTTTACCAAATACGCGTACAAATCCCATTATTAAGTTCACGTTCTCTAACTCTAAATGTATCAATTCAGATACACGCATTCCCGTTGCGT
ACAGAAGTTCTAACATCGTACGATCACGATAACCATTAATTTTATTTAGATCCGGCGTTTCTAATAAAGCCAACACTTCGTCAACATTTAAAA

CGTCAGGCAATTTTTTGTCATATTTTGGTGAATCTAATAATACCGTTGGATCTTTCGCCGCATATTTTTCTCTTATAGCAAATTGATGAAAACT
ACGGATTGTTGAAATAAATCGCGCAATAGATTTAGCAGATTGCCCTTGGTCTATTAAATGCCCCAAACACTCTTGAATTAATTGTCGATCTAT
AAAATCAATATGCGAGATATGATGTTCAGTCATATAATCTTGATACTTTTTCAAATCACGTCTATAAGCACCAATTGTGTTAGAACTTAGTCC
TTTTTCAATTTGTATAAAACGCAAATATTCTTCAATAATTGTTTCCAT

Gene: fur (ferric uptake regulator)

Position: 1563447 to 1563896, length: 450 nt, orientation: REVERSE

Perfect match to: (MW2-BA000033-[1584362:1584811:r], highly conserved allele)

Sequence:

CTATCCTTTACCTTTAGCTTGGCACGTTTCACACACACCATGGAAAGTTAAACGATGATCTAAAATTTTAAAATTGAACTCATTTTCAACTCGA
TTTTCAACTTCTGGTAACAAATCTTCATCGATTTCATCTACACGACCACATTCCATACATACTAAATGATGGTGGAAATGTTTTGCGCCTTCTT
TTCTTAAATCAAAACGAGCGACGCCATCACCAAAGTTAATTTTGTCGACAACTTTTAGTTCAGCTAACAACTCTAACGTTCTGTATACTGTCG
CCAAGCCAATTTCAGGCGCTTTATCTTTTACTTTCAGATATACGTCTTCAGCACTTAGATGATCTTTTTCATTTTCAATTAGAACTCTAACAGT
AGCTTCGCGTTGTGGCGTTAGCTTATATGATGATTGTTGTAATTGTTGCTTAACGCGATTTAATCGTTCTTCCAA

Gene: nudF (nucleoside diphosphate phosphohydrolase F)

Position: 1564001 to 1564543, length: 543 nt, orientation: REVERSE

Perfect match to: (Strain_21334-AGTW01000023-[7089:7631:r], highly conserved allele)

Sequence:

TTATTTAGAATGATTATAATTTAATAATAGGTGTTGCAATGCAATGATAGTCTTCGCATCTTCTATTTCTTTATTCATTAGCATCGATTTAACA
TTCTCAATCGGAACTTTAATGACTTCGACAAATTCATCTTCATCTAAGTGAACTGTACCTTCTTCTAAATTATCCGTAAAATATATTGATAATT
GTTCATCGCAAAAACCTGGTGAACCATACATATCTACAACATGTGTTAACTCTTTGGCAATATATCCTGTTTCTTCTTCTAATTCACGCTTTGC
CGCTTCGACTCTATCTTCATCATCTTCTAATTTACCAGCTGGAATTTCTAGCAATGGTTTTTCTACTGGTTTACGATACTGTTTCACTAATACGA
CTTCTTTTTTAGGTGTCACTGCACAAACAGCAACTGCACCATTATGATAAACTAATTCTCTTGTTGACGTTTCACCGTTTGGTAATGTCACTGT
ATGAATTTCTACATCTACAATTTTGCCATTATAAATAACTGTTCGATCAATTGTTTTTTCATTTAAATCCAT

Gene: yhdN (putative oxidoreductase)

Position: 1564625 to 1565533, length: 909 nt, orientation: FORWARD

Perfect match to: (MW2-BA000033-[1585540:1586448], allele observed in CC1+CC12+CC22+CC80+CC188)

Sequence:

ATGCAAAAAAATATATTAAAAAGTGGTATTTCATTATCTGAATTAGGTCTAGGTTGCATGAGTTTAGGTACAGATTTAAAAAAAGCCGAACA
AATTATAGATTGTGCTGTTGAAAATGGTATCACTTATTTTGATACAGCAGATATGTACGATAAAGGTATAAATGAATCAGTTGTTGGTAAGG
CACTTCTTAAATATCAACAACGCGATGATATTGTCATTGGTACAAAAGTAGGCAATCGTTTAACAAAAGATGGCAGTACAAAATGGGATCC
GAGTAAATCCTATATTAAAGAGGCAGTTAAAGGTTCACTAAAGCGTTTAGGTATCGATCATATCGATTTATATCAACTTCATGGCGGAACCA
TTGATGACCCATTAGACGAAACAATAAGCGCATTTGATGAATTGAAACAAGAAGGAATTATACGTGCTTACGGTATTTCTTCTATTCGCCCA
AATGTAATTGATTATTATTTAAAACATAGTCAAATCGAAACGATAATGTCTCAATTCAATTTGATTGATAATCGTCCAGAATCATTATTAGAT
GCAATTCACAACAATGATGTTAAAGTATTGGCAAGAGGACCTGTGTCTAAAGGATTATTAACTTCAAACAGTGTTAATGTGCTCGACAATAA
ATTTAAAGATGGTATTTTTGATTATTCTCATGATGAATTGGGTGAAACAATAGCCTCTATTAAAGAAATTGAAAGTAATTTATCTGCATTGAC
ATTTAGTTATTTAACATCACATGACGTGCTTGGTTCCATCATTGTAGGTGCAAGTAGCGTCGACCAATTAAAAGAAAATATTGAAAACTATC
ATACTAAAGTTAGTTTAGATCAGATTAAAACAGCAAGAGCTCGTGTAAAGGATTTGGAATATACCAATCATTTAGTGTAG

Gene: Q5HFS1 (putative protein)

Position: 1565749 to 1565997, length: 249 nt, orientation: REVERSE

Perfect match to: (N315-BA000018-[1539339:1539587:r], highly conserved allele)

Sequence:

TTAAAACATCTTTCTATATTTCACTTCGCATGTTGATTCATCATTATTAGTTATTATTTGTACACCCAGCACATTTCCTTGCAACACAAGTAGTT
TGAATTTTTCACAAGTATAATATAATGTACCGTCTGAAATTTGGTCTACAGAAATATCGCCTAAAATATCCAGCACTGTAAATTCTTCAAATA
CTGATAGTTGTTCCGCATATCGTACACAAAGTCTTACCACACTCTCCGATTGACAGTTCAT

Gene: yqjQ (oxidoreductase)

Position: 1566013 to 1566768, length: 756 nt, orientation: REVERSE

Perfect match to: (N315-BA000018-[1539603:1540358:r], allele observed in CC5+CC9+CC12+CC80+CC97)

Sequence:

TTATGCTTTATTTTTAAATAATTTAGGGAAACATCGTTCAAAAAATCTAGGCGCAATTTGATACATTTTCAACGCATGATGCATCCATTTAGG
CCGATTAATTTCCAATTGTTTTGTTTTAATGCCATAAATGATATCTTCTGCAAGCTGATTAGCATCAAGCATAATTTCCCCCATCTTTTTAGCAT
ACTTCATTGATGGGTCGGCTTTTTGATGAAAAGGTGTATCAATCGGGCCAACATTAACTGTCATGATATGTAAGTTTGGTGACTCTAGTCTT
AAAGCATTCATTAATGCATAAAACCCTGCTTTCGATGCCCCATAATGTGCAGCATTTGCTTGTGTGGAAAATGCAGCTTGACTTGAAATACC
TACAATATGTGCGTTAGATGTTAAATATGGTCTCAACACAGTATATAAAACATTAAAACTAATTAAATTAAGCTGATACGTTTCAATCATTTC
TGAAAAACTATGGTCTGAAATAGATTTGAAATAACCTAAACCTGCACTATAAATGAATCCATCGAATGATGTATTGTCTTCAAATTGCAGTG
CCTGTATCGACTTCAAATCATTTAAGTCACAAGGAATAACATTTATAGTTTTCCCCAATTCTTGTTCAAAGATTCTAGTTGCTTTATCAACATC
ACGCACCAACAACGTTACATGCACTTTATTTTCTAGTAACTTTCGGACAATCGATAAACCTAAACCACTCGTACCACCAGTCACTATAAAATG
TTGTCCTTTCAT

Gene: proC (pyrroline-5-carboxylate reductase)

Position: 1566910 to 1567725, length: 816 nt, orientation: FORWARD

Perfect match to: (RF122-AJ938182-[1491108:1491923], highly conserved allele)

Sequence:

ATGAAACTCGTATTTTATGGAGCTGGTAATATGGCACAAGCTATATTTACAGGAATTATTAACTCAAGCAACTTAGATGCCAATGATATATA
TTTAACAAATAAATCTAATGAACAAGCTTTAAAAGCATTCGCTGAAAAACTAGGTGTTAACTATAGTTATGATGATGCGACATTATTAAAAG
ATGCAGATTATGTATTTTTAGGTACCAAACCACATGACTTTGATGCTCTAGCAACACGCATCAAACCACATATTACAAAAGACAATTGCTTCA
TTTCAATTATGGCAGGTATTCCGATTGATTATATTAAACAACAATTAGAATGCCAAAATCCAGTTGCTAGAATTATGCCAAACACAAATGCG
CAAGTTGGACACTCTGTTACTGGCATTAGTTTTTCAAACAACTTTGACCCTAAATCTAAAGATGAAATTAACGATTTAGTTAAAGCATTTGGT
TCTGTAATTGAAGTATCAGAAGATCATTTACATCAAGTAACAGCTATCACCGGAAGCGGCCCAGCATTTTTATATCATGTATTCGAGCAATA
TGTTAAAGCTGGTACGAAACTTGGTCTAGAAAAAGAACAAGTTGAAGAATCTATACGCAACCTTATTATAGGTACAAGTAAGATGATTGAA
CGTTCAGATTTGAGCATGGCTCAATTAAGAAAAAATATTACCTCTAAAGGTGGTACGACACAAGCTGGCCTTGATACATTGTCACAATATGA
TTTAGTATCTATTTTCGAAGATTGTCTAAACGCTGCCGTCGACCGTAGTATTGAACTTTCTAATATAGAAGACCAATAA

Gene: rnz (ribonuclease Z)

Position: 1567830 to 1568750, length: 921 nt, orientation: REVERSE

Perfect match to: (Strain_21305-AFNO01000031-[150800:151720:r], allele observed in CC25+CC5)

Sequence:

TTAGATTTTAAAACTATCAAAATCTTTTACAAAATTAAAATTAGGTGTATCTTCATTTTGTATCAATGTTTGATAAATTTCATTTATATCTTCTG
TATTATAGCGATTGCTCAAATGTGTAATCAACGTACGTTTAACATTGGCTTCTTTTATCAATGCAAATACGTCTTCAATATGGCTATGATGAT
AATTGTTGGCTAAATGCTTTTCACCATCAATATAGGTCGCTTCATGTACCATCAAATCAGCATTTCTAGAAATCACATGTTCATTAGAACATG
GTTTTGTATCACCAAAAATTGCTACAACTGGACCCTGTTTGGACTCACCTCTAAAATCTTTTGATTGATAAACTTGACCATTATGTTCAAATGT
ATCATGAGATTTTACTTCTTGATATTTAGGACCTGGTTCAAGACCAATGTTTTTTAACGCTTCAACATTGATTGTACCTGTAGTTTCAGGTGCC
ATTACTCTATATCCATATGATGGAATACCATGATTAAGTAAATGCGCTTCTACAGTAAAACCATCATGATGATATGTTAGATGATCATCGATT
TCAATATATGTAATTGGATAGTTTAAATGTGACTCTGATAAATTCATAGACATTTCCACATATGCTTTAATTCCTTTTGGTCCAACTAATGTAA
GCGGCTTTTGTTCACCGCCCTGAAAAGAACGACTAGAAAGTAATCCTGGCAAACCAAAAATATGATCGCCATGCATATGAGTAATAAATAT
ATGTGTCACTTTTCCTAATTTGATTGCATGATGTAAAATTTGGTGCTGTGTACCTTCACCAACGTCGAAAAGCCATATGGAATTGGAATATG
GTTCTAAATTTAAGGCGATTGCTTGTGTATTTCTCTCTTTTGTAGGCAAACCTGCACTCGTTCCAAAAAATGTAACTTCCAT

Gene: zwf (glucose-6-phosphate 1-dehydrogenase)

Position: 1569063 to 1570547, length: 1485 nt, orientation: FORWARD

Perfect match to: (11819-97-CP003194-[1619448:1620932], highly conserved allele)

Sequence:

TTGAGTACTAAAAACAAACACATCCCTTGTTTAATCACAATCTTTGGTGCAACTGGTGACTTAAGCCATCGTAAGTTGTTTCCATCAATATTC
CATCTCTACCAACAAGACAATTTAGATGAACATATTGCCATCATCGGTATTGGACGTCGTGACATTACTAATGATGATTTCCGTAATCAAGTA
AAATCATCAATTCAAAAGCACGTAAAAGATACAAACAAAATTGACGCGTTTATGGAACATGTCTTCTATCATAGACATGATGTTAGTAATGA
AGAAAGCTATCAAGAATTACTAGATTTTAGTAATGAATTAGATAGCCAATTTGAATTAAAAGGTAATCGACTATTCTATTTAGCAATGGCAC
CACAATTCTTTGGCGTTATTTCTGATTATCTAAAATCTTCTGGTCTTACTGATACAAAAGGATTTAAACGCCTTGTTATCGAAAAACCATTCG
GTAGTGATTTAAAATCAGCCGAAGCATTAAACAATCAAATTCGTAAATCATTTAAAGAAGAAGAAATTTATCGTATTGACCACTATTTAGGA
AAAGACATGGTTCAAAATATCGAGGTATTACGTTTTGCGAATGCGATGTTTGAACCATTATGGAATAACAAATATATTTCAAACATCCAAGT
TACATCTTCTGAAATACTAGGTGTTGAAGATCGTGGTGGTTATTATGAATCAAGTGGTGCACTAAAAGATATGGTGCAAAATCACATGTTAC
AAATGGTTGCATTATTAGCTATGGAAGCACCTATTAGTTTAAATAGTGAAGATATCCGTGCTGAGAAAGTAAAAGTACTTAAATCACTACGT
CATTTCCAATCTGAAGATGTTAAAAAGAACTTTGTTCGTGGTCAATATGGCGAAGGCTATATCGATGGTAAACAAGTTAAAGCATACCGTG
ATGAAGATCGCGTTGCAGATGACTCTAACACACCTACCTTTGTTTCAGGTAAATTAACAATTGATAACTTTAGATGGGCTGGTGTACCATTC
TATATTCGTACTGGTAAACGTATGAAATCTAAAACAATTCAAGTTGTCGTTGAATTTAAAGAAGTACCAATGAACTTATACTATGAAACTGA
TAAACTATTAGATTCAAACCTATTAGTAATCAATATCCAACCTAATGAAGGTGTATCTTTACATCTAAATGCTAAGAAAAAATACACAAGGTAT
CGAAACTGAACCTGTTCAATTGTCTTATTCAATGAGCGCTCAAGATAAAATGAATACTGTAGATGCATATGAAAATCTATTATTCGATTGTCT
TAAAGGTGATGCCACTAACTTCACGCACTGGGAAGAATTAAAATCAACATGGAAATTTGTTGATGCAATTCAAGATGAATGGAATATGGTT
GATCCAGAATTCCCTAACTATGAATCAGGTACTAATGGTCCATTAGAAAGTGATTTACTACTTGCTCGTGATGGTAACCATTGGTGGGACGA
TATTCAATAA

Gene: graE (putative transcription regulator, AraC/XylS family)

Position: 1570777 to 1571643, length: 867 nt, orientation: FORWARD

Perfect match to: (11819-97-CP003194-[1621162:1622028], highly conserved allele)

Sequence:

TTGGACGTTATCAAGCAAATACAACAGGCAATTGTTTATATTGAAGATCGTTTATTAGAGCCTTTCAATTTGCAAGAATTAAGTGATTACGTT
GGTCTTTCGCCATACCATCTTGATCAATCATTTAAAATGATTGTCGGCTTATCTCCAGAAGCTTATGCACGCGCGCGTAAAATGACACTCGCT
GCAAATGATGTGATTAATGGTGCTACACGACTTGTAGATATCGCTAAAAAATATCACTATGCAAATTCAAATGATTTTGCAAATGATTTTAG
TGATTTTCACGGCGTATCACCTATTCAAGCTTCTACTAAAAAAGATGAATTACAAATTCAAGAGCGATTATATATCAAATTATCAACTACTGA
GAGAGCACCCTATCCATACAGATTAGAAGAGACAGATGATATTTCATTGGTTGGATATGCACGATTTATAGACACTAAGTATTTGTCACATC
CTTTTAATGTTCCGGATTTTTTAGAAGACTTGCTCATTGATGGTAAAATTAAAGAGTTACGACGATATAATGACGTTAGTCCATTTGAACTAT
TTGTTATTAGTTGTCCTCTTGAAAATGGTTTAGAAATATTTGTAGGTGTACCAAGTGAACGTTATCCTGCACACTTAGAAAGTCGATTTTTAC
CTGGCAAACATTGTGCGAAATTCAATTTACAAGGTGAAATTGATTATGCAACTAATGAAGCTTGGTACTATATTGAATCAAGTTTGCAGTTA
ACATTGCCATATGAACGAAATGATTTATATGTTGAAGTGTACCCTCTCGATATTTCATTTAATGACCCATTCACTAAAATTCAGCTTTGGATTC
CTGTTAAACAGAGTCCTTATGACGAAGATTAA

Gene: malA-yugT (alpha-D-1,4-glucosidase)

Position: 1571725 to 1573374, length: 1650 nt, orientation: REVERSE

Perfect match to: (CN1-CP003979-[1513896:1515545:r], allele observed in CC72+CC5+CC72+CC80)

Sequence:

TTAATTTAGTTCGATAACACATGCTTCATATGGACGTAACTGTTTTAAATTAACTTTGGCATCATAATTAAATAGCTTTACTTCTCCATGGCTT
AAATCAAATGGTACAGTTAATTCTGCTTCGTGGTTAGTAAGATTACCTACAATAAGAACTTGCTTTTCATTTAATGTTCTCGTGTACGCAAAA
ACTTGTGAATTTTCAGCATCTACTAAATCAAATTGACCATATACGTATACATCATTAGACTTTCTTAATTGAATTAAATCTTTATAAAATTGTA
ATACTGAATGCTCATCTTCTAATTGTTGTGCAACATTGATAGTTTTATAATTCGGATTCACTGGGAACCACGGTTCACCATTTGTAAATCCTCC
ATTTAACGTATCATCCCATTGCATTGGTGTGCGAGAATTATCTCGGTTCTCATCTTTATATTTCGCAAGTAAAGCGTCTACATCTCCACCTTGA
GCTTTCACTATTTGATAGTCATTTTTAACAGCAACATCGTTAAACGTTTCAATACTTTCAAATGGATAATTCGTCATACCAATTTCTTGACCTT

GATAAATGAATGGCGTACCTTGTTGCAAGAAATAAACAGCTGCATGACTTGTTGCTGATTCATACCAATACTTGTCATCGTCACCCCACGTC
GATACACGTCGTGGTTGGTCGTGATTTTCAATAAACAACGCATTCCAACCTTTATTTTCAAGTTGTTTTTGCCATCTATTTAATACAGATTTAT
ACGAATTTACATCAAAGTGAGAATCACCACTATTCCACAGTCCCAAATGTTCAAATTGGAATATCATATTAAATTTACCATTTTCTTCCCCGAC
CCAGTCATCAGCATCATCAGGGCTTACACCATTCGCTTCACCGACAGTCATAATGTCATACTTACTTAATGAGCGATCTTTCATCTCTTGTAA
CCAAGTTTGTATACCTGGCTGATTCATATCTACATCAAATGCTGGGGCATATGTTTTACCCTCAGGTACAGGTAAGTCACCCGCTTCAAACGT
CTTCTTAATATGCGTAATTGCATCTACTCTAAATCCATCAATGCCTTTATCAAACCACCAGTTCATCATTTCAAATACAGCATCTCTAACTTCTG
GATTACCCCAATTCAAATCAGGTTGTTTTTTACTGAATAAATGGAAATAATATTGCTCAGTATTAGCATCATATTCCCATGTAGATCCATTAA
AGATACTTTCCCAGTTGTTAGGTTCAGAGCCATCTGGCTTTGGATCTTGCCAAATATACCAATCACGTTTGGGATTGTCTTTACTAGATTTAG
ATTCTATAAACCAAGGATGTTCATCAGATGTATGATTTACAACTAAATCTAAAATAAGCTTCATGCCTCTATCATGAACACCTTTTAATAAAC
AATCAAAGTCTTCCATCGTTCCAAATTCATCCATAATTTCTTGATAGTCACTAATATCATAACCATTGTCATCATTAGGTGATTTAAACATTGG
ACTGAGCCAAATGACATCGATACCTAAATCTTTTAAGTAGTCCAATTTATCAATCATTCCAGGTAAATCCCCAATACCATCGTGATTACTATC
ATTAAAACTTCTTGGATATACTTGATATGCTACTGCTTCTTTCCACCATTGCTTATTCAT

Gene: malR-kdgR (transcriptional regulator of malA)

Position: 1573390 to 1574409, length: 1020 nt, orientation: REVERSE

Perfect match to: (11819-97-CP003194-[1623775:1624794:r], allele observed in CC80)

Sequence:

CTATCGCTGTGTTGATTTTCTTATTTTTAATTCTGTATCTATAATGACGAGTTCAATAACATCCTGTGCTTTGTTTTTCAATATATTTAAAATTG
CTGAACCAGCCTGTTGACCTAACATTCGAGGCTTGATGTCAATACAGGTTTGAGGTGGTGACGCAATTTCGGTTAAATAAGAATCATTGAA
CGTTGCTGTCATTACATCTTTCGGAATTTCAATATTAAGTTCATATAGGACACTTAAAATCGCTAAATGTAACATAGCATCTAACGAAATGAT
TGCCTGTTTAATATTTGGGTCCTTCAAACGCGTATGTAGATTTTGCATGTAATTAAAAATAACTTCTCTTTCATTACTAGTCTCAATAATTTGA
TAATCCAGATTAAATTGTGATGCAACTGTTTCAAATCCTTGAATCCTGTCTTTCGAAACTTCAAAATCGCCTTTTTCTGTAATAAATATTAATT
CATCTACACCTTGTTCAATAACATGTCGTGTCAAATTTTCAGAAGCTAATATATTATCATTATCTATATGTGTAAATTGATGATCTATATCCGA
TGTAGGCTTACCAATCACAATAAATGGCATGCTTTCATCAATTAACATTTGTTTAATCGGATCATTTTCTTTTGAATAGAGCAGTATAAACGC
ATCAACCATTCGTTGTTTAATCATTTTATAAACTTCATCCATTAAATCATTCATATTATTTGAGACTGTCGTTTGTGTACCATAGCCATGCTGG
TTACATGTTTCAGAAATCCCTAGCAATACATTGATGTAGAATGGATTCAGTCGAATAGGCTCCTCAGACCCTTTTAACACTAAACCAATTTTA
TATGTTTGGTTTGTAATTAAAGTTCTAGCAGCGGTATTAGGAAAATAATTCAATTCTTCCATAACTTTCTTCACTTTTGAAATTGTCGCTTCGC
TAATACGTTTATTTCCTTTTATAACTCTTGAAACTGTCGAAGGAGAAACACCGGCTTTTAGTGCAACATCTTTAATCGTAACCAT

Gene: Q5HFR3 (glyoxalase family protein)

Position: 1574603 to 1574974, length: 372 nt, orientation: REVERSE

Perfect match to: (ATCC51811-ST1-ADVP01000005-[229935:230306], allele observed in CC1+CC80)

Sequence:

TTAGTCACTAACTGCAAAATAGTTACCTTGGCCATCTTGAAAATTAAATACACGTTGACCATTCATTTCTACTATATCATGCACAGTTAAACCT
AAATCATTTAATTTTGAGTATAATGCATCAAAGTTTTTCTCTTTAAACATTAAAGATGGTGTTCCTAGGTTCACTTCCGGGCTATACTTTTCAA
TAAATTCTTTTGCCATAATCGTCAATGACGTTTCAGCATCTTTCGTAGGTGATACTTCAACTGCAACATAGTCCTCAGCTAACGGTGTTTCACT
TACAACAACAAATTCTAAAGTTTCTGTCCAAAATGCTTTCGCTTTGTCAACATCATCAACATATAACATAACTTGATTTAACTTTTCCAT

Gene: gnd (6-phosphogluconate dehydrogenase, decarboxylating)

Position: 1575193 to 1576599, length: 1407 nt, orientation: REVERSE

Perfect match to: (11819-97-CP003194-[1625578:1626984:r], highly conserved allele)

Sequence:

TTATTCTTCAATCCATTGTGTATGGAATACGCCTTCTTTATCTTTTCTTTCGTACGTGTGAGCACCGAAGTAGTCACGTTGTGCTTGAATTAAG
TTTGCAGGTAAATCAGCTGCACGGTAACTATCATAGTAATTAATACTTGATGAGAAACCAGGTGTTGGTACACCATTTTGAACACCAGTTGC
GACAACATCACGTAACGCATCTTGATATTCAGTAACGATGTTTTTAAAGTAAGGATCTAGCAATAAGTTTTGTAATCCTGGATTATTATCGTA
AGCATCTTTGATCTTTTGTAAGAATTGTGCACGGATAATGCAACCTTCTCTCCAAATCATAGCTAAATCACCAAGTTTTAAATTCCATTCATTA
TCTTCACTTGCTTTACGCATTTGCGCGAAACCTTGTGCATAAGAACAAATTTTACTCATATATAATGCTTTACGAATTTTTTCTAAAAAGTCTT
TCTTGTCACCATCAAATGATGCTTTTGGACCATTTAATTCTTTAGAAGCATTTACGCGCTCTTCTTTGATTGAAGAGATAAAACGTGCAAATA

CAGATTCAGTAATGATTGTTAATGGAATACCTAATTCTAATGCGTTAATTGAAGTCCATTTTCCTGTACCTTTTTGACCTGCAGTATCAAGAA
TTTTTTCAACTAATGCTTCTTTATTTTCATCTAATTTCATGAAAATATCACCAGTGATTTCAATTAAATAACTTTCTAATTCACCAGCATTCCAG
TCTTTGAACGTTTGAGCAATGTCTTCATGAGACATGCCTAATAATTCTTTCATCATAGCATAACTTTCTGCAATTAATTGCATGTCAGCATATT
CGATACCATTGTGTACCATTTTCACATAGTGTCCAGCACCATTAGGTCCAATATAAGTAACACATGAAGCACCGTCTTTTGCCTTTGCAGCAA
TTGCATCAAGAATATCTGCAACTTTGTTATAAGCTTCTTCTTGTCCACCCGGCATTAATGACGGACCAGTTAACGCTCCAATTTCACCACCAG
AAACGCCCATACCAATAAAGTTGATTGCACTTTGTGCTAATGCTTTATTACGTCTGATAGTATCTTGATAGTTTGTATTACCACCATCAATTAA
AATATCTCCATCATCTAATAAAGGTAACAAACTATCAATCGTTGCATCCGTAGCTTTACCTGCTTGAACCATTAATAAAATTTTACGTGGTTTT
TCTAAAGAATTAACAAATTCTTCCAATGAGTACGTTGGATGAATATTTTTCCCTTTTGATTCTTCAACCATTAAATCAGTTTTTTCACTTGAGC
GGTTAAATACAGATACACTATATCCGCGTGATTCAATATTCCAAGCTAGGTTTTTACCCATAACGGCTAAACCAATAACTCCAATTTGTTGTG
TCAT

Gene: Q5HFR1 (peptidase T-like protein)

Position: 1576667 to 1577800, length: 1134 nt, orientation: REVERSE

Perfect match to: (11819-97-CP003194-[1627052:1628185:r], allele observed in CC80+CC5+CC80)

Sequence:

TCATTTCAATTGTCGCGCAACTATTTTGATTATTTCTAACACTTGACTTGCAAGCAAGTTCAATGATTTAATCGGCATTCTCTCATTTGTTGTAT
GGATTTTTTCATAACCCACTCCTAAAATGACTGAAGGAATACCAAATGTATTAATAATACTGCCGTCTGAACCGCCACCAGAAATAATTGTA
TTTGCAGATAATCCTAAATTACGAGCACTTTCTTGTGCAATTTTAACAACCGTTTCATTATCATTAATTTTAAATCCTGGATAACTTTGCTCCA
CTGTTACTACTGCTTTCCCACCTAATTCTGATGCAGTAGTTTCAAACACATCAGTCATATGTTTGACTTGTGTTTTTATTCTTTCTGGATCGTGA
GAACGTGCCTCTGCTTCTAAAATGACTTCATCTGCAACAATATTCGTAGCTGAACCGCCATGAAACTTACCAATATTGGCAGTAGTTATTTCA
TCAACTTGTCCTAATTTCATTCGACTAATTGCTTTCGCCGCAATATTAATAGCACTAACACCCTCTTTTGGCGTACTTGCATGAGCCGTTTTGC
CAAAAATTTTAGCTGAAATTAACATTTGCGTCGGTGCACCTACAACCGTAGTGCCGACATCAGCACTTGCATCAATAGCATAACCAAAGTCC
GCGTCCAACAACTCTGAATTTAATTCTTTAGCACCAATTAAACCTGATTCTTCTCCAACAGTAATCACAAATTGAATTTGTCCATGTGGGATTT
GTTGTTCCTTTATCACTTGCAAAACTTCAAGCATCGCTGCTAATCCTGCTTTATCATCTGCACCTAGTATAGTCGTACCATCAGAGTATATGTA
GCCGTCATCTTTTACAATTGGCTTTACATTAATTGCGGGTACAACAGTATCCATATGGCTCGTCAAATATAATTTAGGTACTTCGCCTTCTTCG
ATAGTACTATTCATTGTACACACTAGATTATTGGCACCTAATTTAGGATGTTTAGCCGCTTCATCTTCTTTAACATCTAACCCTAATGCTATGA
ATTTTTCTTTTAAAATAGGTTGGATTGTTGATTCATTCCCTGTCTCAGAATCGATTTGTACAAGTTCTAAAAACGTATTAAGTAATCTTTGCTC
ATTAATCAT

Gene: yqjA (YqjA like protein)

Position: 1578369 to 1579349, length: 981 nt, orientation: REVERSE

Perfect match to: (11819-97-CP003194-[1628754:1629734:r], highly conserved allele)

Sequence:

TTAAGCTACTTCACTATGCATTTTCAATGAACCAAATTGCGATTTGATTTGTAAATATTCTTCTAATTCATTTAATATTTGAATAATACTTGCTC
TCGAGTTAAGCGCTTTGTGTGTTGTTGGCAATGGCAGTTCATCCAATTTCAAACGCGTCTCATACAAATTGTGTAAACGCATTGCTGTATAG
TCATTACTATTCACATTTAGACCAATTTCTTTCAGCAGTGACGCAACATCATTTAAAAGCGGATCTTTATGACAGATACTTTCGATGAGCGGT
TTCATTCTCATTAACAATTCCACTTGCTCTTCTCGCATATCAAAATAATGATAGTATGAATTTTCGTTTCTAACAAAATGATTTTTAACATCTCG
GAACGCGATAGACTTCGCCTTTTTAATATTTAAAAGTAACACTTCAAATTCAATCGCAATGGTATCTTCATATTTTTCACAAATATAACTATAT
TTACTAAAAATATCAGCAATTTGTTGCTCAATTTTACATTTGTATTCGTCTAGTTGTTTGTCTAAACTTGGCATCATTAAATTCATTGTAAATG
CAATGCTTAGTCCAATTAACAGTAATAATGTTTCATTAACAATTAAATGTGCATCAATTGATTTTGCATTAAAAACATGAAGTAATATAACGC
AACTCGTAATGACACCTTCTTGTACTTTTAATACGACAGTTAATGGTATAAATAACAATACGATAATACCGAGTACAATTGGACTCTGACCTA
ATAAACTAAATATTGCTGAACCTAAAAACAATACTAAAAAACATGATACTAATCTTGAAATAATCGCTTGTAGCGAATGTACTTTTGTATGTT
TAATACATAATACGACTAATATGGCGCTTGAAGCATAATTATCTAAACCTAACAGCTTACTAATAATTACACCTAAAGTCATACCCACTGCTG
TTTTTATTGTTCTAAATCCAATCTTGTAAGGATTTAACTTTAACAT

Gene: yqiW (putative protein)

Position: 1579363 to 1579800, length: 438 nt, orientation: REVERSE

Perfect match to: (RF122-AJ938182-[1509914:1510351:r], highly conserved allele)

Sequence:

TTATCTTTCTTCACAATATTTATTGAATAATGTTTGTAATTGATTAATTACGTTCATCACATCATGACCTTCGATTTGATGTCTTTCAATCATTTC
TGTAATCTTTCCATCTTTTACTAATGCAAATGACGGACTTGAAGGCGCATAACCTTCGAAGTATTCACGCGCTCTTTGTGTCGCTTCTTTATCT
TGTCCAGCAAATACTGTCACTAGACGATCAGGTAATACGTCATAATGTAAAGCATGTGATGCTGCTGGTCTTGCGATACCACCTGCACAACC
ACATACAGAATTGATCATAACTAGTGTTGTACCATCTTGTTTAAGAACTTTGTCAACATCTTCTGCAGTAGTTAATTGCTCATATCCCGCAGA
TTCAATTTCATTCCTTGCTTGTTCTACAACACCGTTCATGTATAAATCGAAATTCATGTCCAT

Gene: bfmBB (branched-chain alpha-keto acid dehydrogenase E2)

Position: 1580151 to 1581425, length: 1275 nt, orientation: REVERSE

Perfect match to: (11819-97-CP003194-[1630536:1631810:r], highly conserved allele)

Sequence:

CTAATATATATTTGTATTTTCTAAAGTATACTGTTCGATACGCTGTTTAATATGATTCATAAATTTACCTGTTTGTAAACCATCTAAAATACGA
TGATCAATTGAAATACATAAATTAACCATGTTACGAATTGCAATCATATCATTAATTACTACTGGCTTTTTAACGATTGATTCTACTTGTAAAA
TCGCTGCTTGTGGATGATTTATAATACCCATTGATGATACTGAACCAAATGTACCAGTATTATTTACCGTAAATGTACCGCCCTGCATATCTT
CAGCTGTCAATTGCTTATTACGCGCTTTCGTTGCTAAAGTATTAATTTCTCTAGCTATACCTTTGATTGACTTTTCGTCTGCATGCTTAATCACA
GGTACGTATAATTTATTTTCATCAGCAACAGCAATTGAAATATTAATGTCTTTATGTAAGACAATTTCATTTCCTTGCCAGCTACTATTTAATA
AAGGATATGCTTTTAAAGCATCTGCTACAGCTTTTACAAAGAAAGCAAAGAACGTTAGATTATATCCTTCTTTATTTTTAAAGCTGTTTTTATA
ATGATTTCTCGTATTCACAAGATTTGTAGCATCTACTTCAATCATCATCCATGCATGTGGAATCTCTGTTACACTATTAACCATATTTTGCGCA
ATTGCTTTACGCACACCATTTACTGGTATTGTGCTGTTTTCACTATTGTCTTCAGATGATTGGTTACTTGATGTATCTACTGATGTTGATTTTG
TTTGAACTTGTTTGTCAGATTGAGCTGTGGTACCACCATTTTCAATAACTGACATTATATCCTTCTTAGTTACACGACCTTCAAATCCACTACC
TACAACTTGTGATAAATCAATGTCATGCTCTGAAGCGAGTTTAAATACAACAGGTGAAAAGCGACCATTATTACGTGGTTGATTTTGTTTAG
CAGTAGATGTCTGTTCCACTGTTGCACTAGCTTTTTTAGTAGATTTCTGAGTATGCTCATCCACTTTTGCTTGTATCTCTTCAGTTGTTTCATTT
GTCTTTTCATCAGCAGTTTCAATTTTACAGATAATTGTATCAATAGCTACTGTCTGCCCCGCTTCAACTAAAATTTCTGTAATTGTTCCTGATA
TCGTGGAAGGGACTTCAGCTGTCACTTTATCTGTAATAACTTCACATAATGGTTCATATTCATCAATATGATCACCAACAGAAACTAACCATT
GTTCAATGGTGCCTTCATGAACACTCTCACCTAACTTAGGCATTGTTATTTCCAT

Gene: bfmBAB (2-oxoisovalerate dehydrogenase beta subunit)

Position: 1581438 to 1582421, length: 984 nt, orientation: REVERSE

Perfect match to: (COL-CP000046-[1596543:1597526:r], highly conserved allele)

Sequence:

CTAGAATTCTGCTAATTCACGCATTTTATTTAAGATTTTTTCTGGATTCATCATAATTTCATTTTCTAATACAGGAGAAAATGGCATAGATGGT
ACATCTGGAGCAGCTAAACGCATGATTGGTGCATCTAAATCGAACAAGCAATGCTCTGCAATAATCGCTGACACTTCTGACATAATACTACC
TTCTAAATTATCTTCAGTTACAAGTAAAACTTTACCTGTATGTTTAGCACGATCAATAATTGTTTCTTTATCTAATGGATAAACAGTTCGTAAA
TCAACGACTTCAACATTGATACCGTCTGCAGCTAAAATATCCGCTGCTTGTAAACAATAATTGACCATTAATCCATAACAAAATACTGTTAAA
TCTTCACCTTCACGTTTCACATCTGCTTTTCCTAAAGGTACAGTGTAATATTCTTCTGGCACTTCTTCCTTTAAGAAACGATAAGCTTTTTTATG
CTCAAAGTACAATACTGGATCATTTGATTCGATAGATGATAATAAAAGCCCTTTAGCATCATACGGTGTGGAAGGAATAACAATTGTTAAAC
CTGGCGATGAAGCAAATATACTTTCAATACTTTGTGAATGATATAGTCCTCCGTGAACACCGCCACCAAATGGTGCACGAATCGTTAATGGG
CATTGCCAATCATTATTTGAACGATAACGCATTTTCGCAGCTTCACTAATAATTTGATTTGTCGCAGGTAAAATAAAATCTGCAAATTGAATT
TCTGCAATTGGTCTTTTACCTACCATAGCTGCACCAATGGCAGTTCCAACAATATTTGACTCAGCTAATGGCGTATCGATAACTCTGTCTTCA
CCATATTTTTGTTGCAGTCCTTGAGTAGTACCAAATACGCCACCTTTTCTACCAACATCTTCACCAAGAATAAACACATCTTTATTTTGTTGTA
ATGCTAAGTCTTGTGCCTGGCGTATCGCCTCTAAATAAGATAATTTAGCCAT

Gene: bfmBAA (branched-chain alpha-keto acid dehydrogenase E1)

Position: 1582421 to 1583413, length: 993 nt, orientation: REVERSE

Perfect match to: (N315-BA000018-[1557790:1558782:r], highly conserved allele)

Sequence:

TTAGTTAAGACTCCCTTCTTCGTACACAAATGCATAGGCTTCTTCGACACTTGGATATGGCGCGTCTTCAGCAGCCTTTGTCGCTTTATTGAT
GATGTCTTTATGCTCCGCTTCTATTTCTGCCAACCAAGCATCATCGATAATGCCAGCTGAAAGCAACTCTTTTTTGAACTTTTCATTGCAGTCT
GCTTTTTTAAGCGCTTCACGCTCTTCTTTCGTACGATATTGGTCGTCATCATCTGATGAATGAGCTGTCATACGACTTGTTACTGCTTCAATCA
AAGTTGAACCTTGACCAGAAATAGCTCGATCTCTTGCTTCTTTCATCGCTTTATACATTGCTAATGGATCATTACCATCTACTTGTTCACCATG
TATACCGTAACCAAGTGCTCTATCCGATAATTTTTCAGCTGCGTATTGTAATGAATCAGGTACTGAAATTGCATATTTATTATTTATAATGAC
ACATACAAAAGGAAGTTTGTGTACACCCGCGAAGTTTAAACCTTCATGGAAGTCACCTTGGTTTGAGCTACCTTCACCAACAGTTGCTGTTG
CAATTTTCTTCTTACCATCCATTTTTAAAGCTAAAGCAGCACCAACAGCATGGGGTATTTGAGTTGCTACCGGTGAACTTTGAGACAAAATAT
TCTTAGCTCTACTACTAAAGTGTGATGGCATTTGTTTTCCACCAGAGTTAACATCGTCTTTCTTTCCAAACGCTGATAAAAACGTATCATACG
CTGAGATACCCATATAAGTAACGAAAGCTAGATCTCTATAATAAGGCGCTGTAATATCACCTTCTTCTAATGCGTATGCCATCCCAATCTGA
GTTGCTTCTTGTCCTTGACCACTTACAACAAATGGAATTTTACCTGCACGGTTCAATAACCACAGTCTTTCATCTATTTTTCTACCTAAATCCAT
CCATTTATATATTACTTTTAGGTCTTCTTCGCTAAGGCCTAATGATTTATAATCAATCAT

Gene: lpdA (dihydrolipoyl dehydrogenase)

Position: 1583429 to 1584850, length: 1422 nt, orientation: REVERSE

Perfect match to: (MW2-BA000033-[1604342:1605763:r], highly conserved allele)

Sequence:

TTATACGTGAATAGCTCTACTTTCTGCTTTCAATCCTAATTCCATCAACACTTCAGAGATGGAAGGATGTGCGTGTGTTGTTAGTCCTAATTC
TAATGCCGAGCCATTCATGAACTGTAACAGTGATGCCTCATTAATCAATTCTGTTACATGTGGACCAATCATATTAATACCCACAATTTCTTC
AGTTGATTGATCAATCACCATTTCGCTATACCCTTCGTTTGTGTCATGGCTATCAATCACTGCTTTACCAATTGCTTTAAATGGTACTTTAAAA
CTTTTAACTTTCATTCCCTCTGCCTTTGCTTGTTCAATGTTTAAACCGATAGAAGCAATTTCAGGTTGTGAATAAATACACTTAGGCATCATGT
TATAGTTTACTGGGATTGGGTTCCCCTCAAACATATGATCAACAGCCACAACACCTTCTTTTGATCCAACATGTGCCAATTGTAATTTTCCTAT
ACAATCACCAGCTGCATAAATATGTTTATCTTCAGTTTGTTGAAATTCGTTCGTTAAAATATGTCCTGATGTTGAAAGTTTTATTTTAGTGTTG
TTTAAACCAATATCTGATGTGTTAGGTTTTCTACCAATCGATAGCAACACTTTATCTACTTTAATTATGTCTGAGGAAATTTCAAACGTAACAC
CATCTTCGTTAACATTTATATCATTTTCAGAAAGTTTTATTCCCTCATAGAATTTAACACCACGTGCTGACAATGATTTTTTTAATAGTTGTGA
AGCTTGTTTACTTTCAGTTGGTAAAATTCTTTCACCTGCTTCTATAACTGTTACGTCAACACCTAAATCTATCATCAATGATGCAAATTCCATT
CCGATAACACCACCACCAATAATACCAATACTTGATGGTAACGTCTTTAATGATAATATATCATCGCTAGATAAAATTTTATCATGATCAAAT
GATAAGAATGGCAACTCTGCAGGCGAAGAACCAGTTGCAATTAATACAAATTGGTTGGGTAATAAGTCTGATTCACCATCTTCATATTCGAC
AGAAATTGTGCCACTTTGAGGTGAAAATATAGATGTACCTAGAATACGTCCCGTGCCATTATAAATGTCAATGTGATTGTGTTGCATTAAAT
GCTTTACACCTTGATACATTTGATTAATAATGTCTTCTTTTCGTGCCAACATATTTTCAAAATTAACATTAGCATCTTTGACATCAACGCCAAA
CATTGCTGCCTGTTTTACTGTTTGAAATACTTCAGCAGATTTAAGCAGCGATTTAGTAGGAATACAACCTTTATGGAGACAAGTACCTCCTAA
TAGTTGTCGTTCTACTATTGCCACTTTTTTACCTAATTGAGACGCACGTATCGCAGCAACATATCCTGCAGTACCTCCACCGAGAACGACTAA
ATCATATTGTTTCTCTGACAT

Gene: recN (DNA repair protein N)

Position: 1585001 to 1586680, length: 1680 nt, orientation: REVERSE

Perfect match to: (11819-97-CP003194-[1635386:1637065:r], highly conserved allele)

Sequence:

TTATCTACGTCTTTGATTGTGTTGTATCATTTCTCTTGCATTTTCTCGAGTTAAATCAGTAACACTTGCACCTGAAATCATTCGTGCAATTTCAT
CTACTTTATCATCGCTAATTAACTCTTGAACTTGTGTTGTTGTACGATCATCTTTTGATGATTTCGAAATTAATAAATGATGGTCGCTCATCGA
TGCAACTTGTGGTAAGTGAGAGATACAAATAACTTGTATATATTCTGCTATATCTCGCATTTTCTCTGCCATTTTTTGTGCAGCTTGTCCAGAT
ACACCTGAGTCAACCTCATCGAATAAAATTGCAGTTTGACCTCTCGATTTAACAAAAATACTTTTTAACGCTAACATAATTCTAGAAAGTTCT
CCACCTGACGCAATTTTATTTAAACTTTTTAATGGTTCCCCTTTATTTGGACTGATTAAAAATTCTACAAATTCGATTCCATCAATATTCGGTTC
TTCTAATTTTTTAAATGAAATCTCAAGATTTGCGTCTTTCATTTGTAAGTTTTGAATTTCCGATACAATGTGGTCTCTTAACTCCCTAGCGACT
ATACGACGCTGCTTTGATAACGCTTGTCCAACCTCTATAACTTGATTATACAATGCATTTATTTCTTCTCGTAACTGCGATGTACTTTGTTCAT
AGTTTTCAATTTTATTGATTTCATTATTAAGCTTTTCTTGATATGCGATTAATTCTGAAATATCTTTTCCATATTTACGTTTTAAATTATTCAGCA
AATTCATACGAGATTCATACTCGTTTAACACTTGTTCGTCGAATTCTGTATTAGCCATTTCATCATATAACTCATGTTTTGCATCTTCTAAAAT
GTAGTAAAATTGATCAATATCTTCTTTTAATTTGTCATATTTGTTTGGAACTATATCGTTTATTGTTAACAAATGGTTGCTTAGTTCATATAAA
CGATCAGTGATAGCATTTTCATCCGTTAATGTCATATGTGCGTTATTAAGCGCTAAGCTTAATTTTTCAGAGTTTTGAATGCGTTTAATATCTA
TTTCAAGTTGCTCTATTTCGCCTTCTTTTAGATGTGCTTCAGACAATTCTTCTAATTGGAATTTCATTAAATCTAAACGCTGTAGCAATGCTTG
GTCTGCTGATTCTAAATCTTCTAACTCTTGCTTTTTGGCTTTATAATTTTGAAAAGTTTGGTGATATTTATCCAACAAATCTTGATAACGTGAT
TCTGCGTAATTATCCAATAATGTTAAATGGTATTTTTGTTTCAACAAAGACTGCGTTTCATGTTGGCCATGAATATCTAATAATTCTTGCATAA
CTTTTCGTAAATCTTGTAAAGTAACTGTTTGATTATTAATTTTACAAAGACTTTTACCAGAGCTGAAAATTTCCCGTTTAACTAATAAAAAATC

TTCATCTACATCAATATCCATATTTTTCAATATATGTATAGCATCTTTACTCTCGTCAATATCAAATATACCTTCGATGACAGCCTTTTTTTCAC
CATGTCTTACAAAATCAGATGAAGCTCTCATTCCAATTAATTGTCCAATTGCATCTATAATAATTGACTTACCTGAACCCGTTTCACCACTTAA
AACAGTTAAACCATCAGAAAATTGAATTTCTAATTCTTCAATAATAGCAAATTGCTTGATTGATAAGGTTTGTAACAT

Gene: ahrC (arginine repressor)

Position: 1586696 to 1587148, length: 453 nt, orientation: REVERSE

Perfect match to: (MW2-BA000033-[1607609:1608061:r], highly conserved allele)

Sequence:

TTATAACAAATTGAAAATTCTTGACTTGATTTCATCACTTGCCTCTTTGCTTCGACAAATAATTAAACAAGTATCATCACCACAAATTGTGCCT
AGTACTTCTTCCCAATTGATTTGGTCTAATATAGCTCCAATAGATTGTGCATTACCAGGTAATGTTTTTAGAACAAGTAAATTATCAGTACCA
TCTATATTAACAAAGGAATCCATTAAATAACGTCCCAATTTTTCTAAAGGATGGAATTTTCTATCATTTGGTAAACTATAAACATATTGACCT
GAAGGTATAGGTACTTTAATAAGTTGTAGTTCTTTAATATCACGAGAAACAGTTGCTTGAGTGACATTTAAATCATAATCGTTTAATCGTTTA
ACTAATTCATCTTGTGTCTCTATCTGTTCATTTGAAATAATTTCTCTAATTTTTATATGCCTAACCGATTTTTTGGGCAC

Gene: ispA (geranyltranstransferase)

Position: 1587580 to 1588461, length: 882 nt, orientation: REVERSE

Perfect match to: (COL-CP000046-[1602685:1603566:r], highly conserved allele)

Sequence:

TTAGTGATCCCTGCTATAAAATAAATCAACGATTTCTAATAAGTGTTTTGTATTGAATTGTTCATCAATTTGCGTTAGTTCATCCACTGCTGCG
TCTCTATGATAAGTCAATTTATCTTCTGCGCCATCTTTCCCTAATAAACTCACGTACGTACTTTTATTATTTTCAAGATCGCTGCCCACTTTTTT
ACCTAACTTTGCTTCATCACCATAGCAGTCTAATAAATCATCTTTAATCTGGAACATCATACCTAAATGATAACTATAACTTTCTAAATGTTCT
TTAGTTGTATCATCGACATTAGCGATATCTGCTGCACTCATAACCGCAAAAGTTAATAATGCTCCTGTTTTTGTTTTGTGTATCATTTCCAAAG
TTTCAAGATCAATTGGTTGGCCTTCGCTTTGCATATCTAACATTTGACCGCCGACCATTCCAACATGACCACTTGCTATTGACAGCCGTTGTA
GAACTTTTATTTTTACTTCATCAGTTAATCTATCATCACTTGAAATAAGTTCAAATGCTTTAGTTAATAAAGCATCACCTGCTAATATCGCAGT
CCACTCACCATATACTTTATGATTTGTTAATTTTCCTCGTCGATAATCATCATTATCCATCGCTGGTAGGTCATCATGAATAAGTGAATATGTA
TGAATCATTTCTAGTGCAATTGCGCTCTTCATACCTAACTCATACTCGGTATTTAGTGAATCTAAAGTGAGTAATAACAGAACTGGTCGGAT
GCGTTTACCTCCAGCATTTAATGAATACAACATACTTTCTTCTAGCTGAGTATCCATTACTGATTTATTTATCGCAACCGATAATTCATTATTG
ACTTCATCTATTAATTTATTCATCGGTAGATTCGTCAT

Gene: xseB (exodeoxyribonuclease 7 small subunit)

Position: 1588439 to 1588669, length: 231 nt, orientation: REVERSE

Perfect match to: (N315-BA000018-[1563808:1564038:r], highly conserved allele)

Sequence:

TTATTCATCGGTAGATTCGTCATTTTTTACATCCTCAGCTTCTTCTTTTATTAAGTCATTCACCTTTTTTTCGGCATTTTTTAAAGTTGTGTCACA
AGCTGCTGATAGTTTCATACCACGTTGATATAAATCTAATGATTCCTCTAAAGATACTGTTTCATTATCTAATTTTTGAACAATTTGCTCTAAT
TCTTGCATCATTTCTTCAAAACTTTGCGTTTCTTTAGTCAT

Gene: xseA (exodeoxyribonuclease 7 large subunit)

Position: 1588662 to 1589999, length: 1338 nt, orientation: REVERSE

Perfect match to: (11819-97-CP003194-[1639047:1640384:r], highly conserved allele)

Sequence:

TTAGTCATTATTACACCTTACTTTCGTAACTTTTGCATCTACTAAGCCATCTTTCATTGTTAACGTCAATTGATCATTTTCTGTTAAATCTTTAGT
ACTCGTAATGACTTCGTCTTTTTTATTAACAATTGCATATCCACGCAACATTGTATTAGTTGGACTTAAATTGTTTAAGTTTTCTACTTTATTTT
TCAAATCATTTTTATAACTTAATATCTTAGAATTCAATAATTTAACAAGTTGGTTTGTCAATTGAAGATTATTTTGTTGTTCTTGATTAACACTA

CTTAGTAATGCTTTTAAATTATAACGTTGTTGCAACAGCATTAATCGATGTCTCTGTTGTTCAAAAGTTGCCTGAATTTGTTGTTTCAGTCTCT
TTTCTAAATCATCTCGACGTTGTATCTGTTGATCATACAATAAAGTTGGTTGTTTAAACTTGTAATACGATGACAAATGTTCAACATGTTTAC
GTTGTTGTTCTAAATGTTTCTTGATGAAACGAGTCAATGTAAACTGATATTGCTGTATTTGTTGCAGCAATTCATATTGGTCTGGTGTTGCAA
TAACAGCAGCTTGAGTTGGAGTCGCAGCTCTGATGTCTGCAGCAAAATCACTTAATGTAAAGTCTGTTTCATGACCTACTGCTGATATAATC
GGTGTCTTACAATTATATATTGCACGGACGACAGCTTCTTCGTTGAAATTCCATAAATCTTCTATGGATCCACCACCTCGACCTACAATAATG
GTATCTACACCTAAACTATCTGCATATTCAATTTTTTCAATAATGTCGTCTTTTGCTTTTTCACCTTGAACCAAAGTACTAATTTGTATTTGTTC
AGCTAATGGAAAACGACTATTTATCGTTGAATGGATATCTCGAATTGCGGCACCTGTACTCGCTGTTAAAACTGCAATTTTTTTAGGAAACTT
AGGTATTGATTTCTTATTCGCTTTATCAAAACAACCTTCTTCAGTTAATTTTTTCTTTAATGCTTCTAATTTTTGATATAAGTTCCCTATACCATC
TAATTGCATTTTATTTACATAAATTTGATAGTTTCCACGACGTTCAAAAACAGAAACACGTGCTTCTAATAAGACTTCATCTCCTTCTTTAGGT
TCGAAGTTTAATTTAGAAGCACTACCTTTGAACATCATGGCACTTATAACGCTCTCTTTATCTTTCACATTAAAGTATAAATGACCACTTGAAT
GCTTTTTAAAATTTGAAAGCTCACCTTTAATCAATACAGATTGGAGATGTGGATCTTGATCAAATTTATATTTAATATATTTCGTTAAAGCTG
AAACACTTAAATAATCTGACAT

Gene: nusB (transcription antitermination protein)

Position: 1590016 to 1590405, length: 390 nt, orientation: REVERSE

Perfect match to: (ED133-CP001996-[1600826:1601215:r], highly conserved allele)

Sequence:

TTATTTTTTTATATTACTCAATACACCATTTATAAATTTATAATGATCATCATCACTGAATTGTTTTGTTAATTCAACTGCTTCATTCATTACGAC
TTTAGCAGGTGTATCACTGTGTAATATTTCATATGTTGCCATTCTTAAAATAATACGATCCGTTTTTAATAAACGTGCAATAGTCCAATCTTTT
AAATAAGGACTAATTGTCTCGTCTAATACAGGTTCGTGATCTTTAACGCCAGAAACTAGCCAATGAATAAATTCGAAGTCTAAATCTGGATT
ATCGTCTTTAATAAAGCTTATCGCTTCATTTATCGTTAAATCACTGTCCTTCATTTCTAATTGAAATAAAGTTTGAAAAGCTTGCACTCGGGAT
TCTTTACGACTCAT

Gene: yqhY (putative protein)

Position: 1590465 to 1590827, length: 363 nt, orientation: REVERSE

Perfect match to: (RF122-AJ938182-[1521014:1521376:r], highly conserved allele)

Sequence:

TTACTTTTCAATAACGATTTGTGTAATGTGAATATTAATTTGCTTAGGTTCTATCGCTGTCATATTAGAAATTGAATTAAAAATTGACGTTTGA
ATTTTGTTTGCAGTTTTTGAAATATTAACACCATGTTTTAATGCACAATATACATCTATATATATGCCATCTTCTTTACTCTCGATTTTTAAATC
ACGGCTTAAATTTTTACGACTAACTTTTTCTAAATTTGTTTCTTTTAATTCAGCAAAATGGCCAGTGATGCCTTCGACTTCCGAAGTAGCTATA
CTTGCAATAACAGATAGCACTTCTGGCGCTATTTCTACTTTACCTAATTTTGAATTTGAATAATCAGTTACTTTGACCAT

Gene: accC (acetyl-CoA carboxylase, biotin carboxylase)

Position: 1590842 to 1592197, length: 1356 nt, orientation: REVERSE

Perfect match to: (T0131-CP002643-[1614409:1615764:r], allele observed in CC239+CC8)

Sequence:

TTAACCTTCATCATTCATAATGCTATTTTGCTCTAAAAAGTTTGTATTAAATTTACCGCTTCTAAATATATCGTTATTCAATAATTTAATATGGA
ATGGAATAGTTGTATCAATACCAAGAACCACAAATTCACTTAGTGCACGAATGCCAGCCATAATCGCTTCATCTCGTGTCGGTTCATGTATG
ATTAATTTCGCTACCATCGAATCATAATATGGCGGTATCGTATAATTAGTATAACATGCTGACTCTATTCGAACACCATATCCACCTGGTGCA
AGATATTGCTCAATTTTACCTGGTGATGGCATAAAGTTCTTGTAAGGATTTTCAGCATTAATTCTAAATTCAATTGCGTGTCCTGTTAATTTAA
TATCTTCTTGTTTATACGGTAACACGTCACCCATAGCAACTTGTAATTGTAATTTAACTAAATCAATTCCTGTTACCATTTCAGTTACAGGATG
TTCTACTTGAATACGTGTATTCATTTCCATAAAATAAAATTTATTATCATTTAAATCATATATAAACTCAATTGTTCCCGCATTTTCATAATTTA
CAGCTTTCGCTGCACGAACTGCGGCATTTCCCATTTCACGACGTGTTTCATCATCTAAAATTGGGGAAGGTGCTTCTTCCACTAATTTCTGCA
TACGTCTTTGAATTGTACAATCACGTTCTCCTAAATGAATTACATTACCATAGCTGTCCCCAACAATTTGGATTTCAATATGGCGGAAGTTTT
CGATGAATTTCTCCATATAAAGTCCACCATTACCAAATGCAGTTTGAGCTTCTTGTTCTGTCATTCGGAAGCCAGTTTCAAGTTCTTTTTCATC
ACGAGCAACACGGATACCTTTTCCGCCACCACCAGCAGTAGCTTTAATGATGACCGGATAGCCAATTTTCTTAGCGATTTTCTTAGCTTCTGA
GACGTCTTTCATTAAACCGTCACTACCAGGAACAACTGGAACATTGGCTTTGATCATTTCTGCCTTAGCAACATCTTTGATACCCATTTTTTG
GATAGATTGATAACTTGGTCCAATGAACTTCAATTGGCATGCTTCGCATAATTCTGCAAAATCAGCATTTTCAGCTAAAAAGCCATAACCCG

GATGAACGCCATCACAACCTGTAGAAGTTGCAATAGATAAGATGTTCGGAATATTTAAATATGAATCTTTAGACAAAGTGGGACCTACGCA
ATATGCTTCATCAGCAATTTGAGTATGTAGCGCATCTTTATCCCCTTCAGAATAGATTGCAACAGTTTGGATGCCTAAATCACGACAAGCGC
GAATAATCCTAACTGCGATTTCACCGCGGTTTGCAATTAAAACCTTTTTCAT

Gene: accB (acetyl-CoA carboxylase, biotin carboxyl carrier protein)

Position: 1592197 to 1592661, length: 465 nt, orientation: REVERSE

Perfect match to: (N315-BA000018-[1567566:1568030:r], allele observed in CC5+CC8+CC72+CC80+CC239)

Sequence:

TTATTTCACCTTAAATAACGGTTGGCCATACTCTACCATTTGTCCGTCTTCTACTAAGATTTCAACAATTTCACCTGAAATTTCTGCTTGAATTT
CATTAAATAGTTTCATTGCCTCTAAAATACACACTGTTGTTTCATTTGAAACAGTGTCCCCAACTTGCACATATGCTTCTTCGTCTGGAGATG
GCGATTTGTAAAATGTACCTACCATAGGTGCATTAATTGTTTTGTGATTATCTGAAGTTGGCTTTGGAGCTTCAGTTTTATTGCTATCAGTTG
ATTGTGCTTGAGGCATAGGCATTGCCGCAGCTTCAACTGGCATTTGTGAGATTTGTGGCGTGATAATCTCAGTTTCTTTTTCTTTCTTAAGCG
TCACTTTGCCTTTAGTATCTTCAATATTGATTTCCGTTAAAGTTGATTTATCCAGAATTTCAATTAATTCTTTGATTTCTTTAAAGTTCAT

Gene: efp (translation elongation factor P)

Position: 1593135 to 1593692, length: 558 nt, orientation: REVERSE

Perfect match to: (COL-CP000046-[1621582:1622139:r], highly conserved allele)

Sequence:

TTATCCTCTTGAAATGTAGCTTCCATCACCAGTGTTGATAATTAAAACGTCACCTTCGTTTACAAATAAAGGTACATTTAATGTATAACCAGTT
TCAACAGTTGCCGATTTAGTGGCACCAGTTGCAGTATCACCTTTAATACCAGGTTCTGTTTCAGTTACTGTTAATTCAACAGTTTTAGGTAAT
TCAACACCGATAGTTTCACCTTCGTATGTTTGAATTTGTACTTCCATACCTTCTTTTAAGTAATTCAATTCTTCTTTTAAGTAATCACTTGAAAG
TTCTGTTTGTTCAAAGCTTTCATTATCCATAAATACATGATTATCTCCGTCAGCATATAAATATTGCATGCGACGATTTTCAATCATTGCTGGT
TCAACTTTTTCACCAGCTCTAAACGTTTTCTCTTGAATTGCACCAGTTCTTAAATTACGTAATTTTGAACGAACGAATGCTGAACCTTTACCAG
GCTTTACATGTTGGAAGTCTATAACTTTCCAAATAGCGTTATCAACAGAAATTGTTAAACCTGTTTTAAAATCATTAACCGAAATCAT

Gene: yqhT (putative aminopeptidase)

Position: 1593718 to 1594779, length: 1062 nt, orientation: REVERSE

Perfect match to: (N315-BA000018-[1569087:1570148:r], highly conserved allele)

Sequence:

TTATGTTAAAACTATAAGGTCTTTTGTGCATTTAGTAAAGACTTGACAACCATTTTCTGTAATTAAAATATCATCTTCTATTCTTATACCGCCC
AAACCTTCTATATAAACACCAGGTTCTACTGTAACACAGTTGTTAACTTGAAGTTTATCTTGTATCGTACGAGCCAGCATTGGCCCTTCATGG
ATTTCTAAACCAATACCATGTCCTAGTGAATGTCCAAATTCTTTTCCATACCCTTTTGACTCTAAATAGTTTCTTGAAATGGCATCAGCTTCTG
CACCAGTCATGCCAGGTCTAATCTCATTAATTGCTTTCATTTGAGATTCAAGTACTATTTGATATATTTCTTTCAGTTTAGGATCTGGTTCTCC
AATAGCAAATGTTCTAGTAATATCTGAACAATAGCCGTTATAATACGCGCCAAAATCTAATGTAATCATGTCGCCTTTTTCAATAATTTTATC
ACTTGCAACACCATGTGGTAATGCACCTCTATGACCAGATGCTACAATCGTATCGAATGATGGTCCATCTGCTCCTAATTCTAGCATTTTGCT
TTCTAATATTGCCTTTAATTCTTTTTCAGTCATGCCTGCTTTTACAACAGTTAAAATATATTCATATGTTTCATCAACAATATTAGCTGCTTTTT
GAATTAAAGCAATTTCGTCAGCATCTTTGACGTCTCTAATTTTATCTACAGTATTAGAAATGCTTATTAATGATATACGGCTTTTATTTAATTC
AAGGTATGTATCATAACTTACATGATGCCCCTCAAAACCTACATTTTCAAAATTTTCTTGGTGTAGCAATTCTTTAATCTCACCAATAATAGTA
GATTTACGATTAATAATTTCATAATTTGGCGCCTGCTTAGTTGCTTGATCAATATATCTAAAGTCTGTTATCAAATATTGTTTATCTTTAGATA
TGATAAGTGCTCCACTGGTACCAGTAAAACCTGATAAATATCTTCTATTGTAATCCGAAAGAATGATAATCGCATCTAAATGTTTTTGTTCTA
AAATACGATGCACTTGTGTTATTCTGCTCAT

Gene: Q5HFM8 (putative lipoprotein)

Position: 1594884 to 1595465, length: 582 nt, orientation: FORWARD

Perfect match to: (11819-97-CP003194-[1645268:1645849], allele observed in CC80+CC22+CC80)

Sequence:

ATGAAAAAATTGGTTTCAATTGTTGGCGCAACATTATTGTTAGCTGGATGTGGATCACAAAATTTAGCACCATTAGAAGAAAAAACAACAG
ATTTAAGAGAAGATAATCATCAACTCAAACTAGATATTCAAGAACTTAATCAACAAATTAGTGATTCTAAATCTAAAATTAAAGGGCTTGAA
AAGGATAAAGAAAACAGTAAAAAAACTGCATCTAATAATACGAAAATTAAATTGATGAATGTTACATCAACATACTACGACAAAGTTGCTA
AAGCTTTGAAATCCTATAACGATATTGAGAAGGATGTAAGTAAAAACAAAGGCGATAAGAATGTTCAATCGAAATTAAATCAAATTTCTAA
TGATATTCAAAGTGCTCACACTTCATACAAAGATGCTATCGATGGTTTATCACTTAGTGATGATAATAAAAAAACGTCTAAAAATATCGATA
AATTAAACTCTGATTTGAATCATGCATTTGATGATATTAAAAATGGCTACCAAAATAAAGATAAAAAACAACTTACAAAAGGACAACAAGC
GTTGTCAAAATTAAACTTAAATGCAAAATCATGA

Gene: Q5HFM7 (putative protein)

Position: 1595479 to 1595697, length: 219 nt, orientation: FORWARD

Perfect match to: (N315-BA000018-[1570848:1571066], highly conserved allele)

Sequence:

ATGCGTAATATAATATTTTATCTTGTACTTATTATTGCTGCGATTGGATTAGTAATGAATCTAGATGCCTTTATTTTTTCAATCGTCAGAATGT
TAATCAGCTTTGCTGTAATAGCTGGTATTATTTATCTGATTTATTATTTCTTCATCTTAACTGAAGACCAACGCAAATATCGCAAAGCAATGC
GTAAGTATAAAAGAAATCAAAGAAGAAAATAG

Gene: lipM (octanoyltransferase)

Position: 1595761 to 1596591, length: 831 nt, orientation: REVERSE

Perfect match to: (MW2-BA000033-[1616677:1617507:r], highly conserved allele)

Sequence:

CTACTTTCTAAACATCCATTCATCTGAACGATATTTTTCAGTTAATTCTTCCACTTCTGCCAATTGAGCTTCTGTTAATTCAAGTGGCTTTAATT
CTATATTTAAACCTTTCTTAAAACCTTTCTCGAAAGCTTCTTCCATTTGACTAATAGTAATGTGTTTATCTGAAATATCATTGATGGCAACTGC
TTTTTCAACGAATGCCTCTTTCATTTTTAATTTTAATCTTTCATTTTTATAAATAAACATATCAAACAGTTCATCAATATCAATATCTTGTAAAAT
CGAACCGTGTTGGAGGATTACGCCCTTTTGTCTCGTTTGAGCACTCCCAGCAATCTTACGGCCTTCAACAACTAGCTCATACCAACTTGGTGC
ATCAAAACACACTGAACTTCGAGGTTGTTTTAATTTTTGACGCTCTTCAGGCGTTTTAGGTACCGCAAAATAAGTATTAAATCCTAAGTTTTT
AAATCCTTCTAATAATCCTTGTGAAATCACTCTGTACGCTTCTGTAACTGTAGAAGGCATATTCGGATGCGATTCAGGCACAATCACACTGTA
AGTTAACTCTTTATCATGTAGCACCCCACGGCCACCAGTTTGACGCCTTACGAGACCAAAACCTTTCTCTTTAACCTTATCAATATCAATTTCT
TTTTGTAGCCTTTGGAAATACCCTATTGATAATGTTGCAGGATTCCATGTGTAAAAACGTATAACTGGATCAATTTCACCTCTAGAGACAAAA
TTTAATAACGCTTCATCCATTGCCATATTATAATATGGGTCTTTACTTCCTGTATTAATAAAATTCCAAGTTTCAGTCAT

Gene: yqhL (putative sulfur transferase)

Position: 1596749 to 1597135, length: 387 nt, orientation: FORWARD

Perfect match to: (M0239-AIWE01000007-[130117:130503], allele observed in CC188+CC80)

Sequence:

ATGAGTGCTAGTTTGTACATCGCAATAATTTTAGTTATAGCAATTATTGCTTATATGATTGTTCAACAAATTCTTAACAAGCGAGCTGTTAAA
GAATTAGATCAAAATGAATTCCATAATGGGATTAGAAAAGCTCAAGTCATCGATGTTAGAGAGAAAGTTGACTATGACTACGGTCACATTA
ATGGGTCTCGCAATATTCCTATGACAATGTTCAGGCAACGATTCCAAGGATTAAGAAAAGACCAACCGGTATACTTATGTGATGCCAATGG
GATTGCTAGCTATAGAGCCGCTCGTATTTTGAAAAAAAATGGATATACAGATATCTATATGTTAAAAGGCGGCTATAAAAAAATGGACTGGA
AAAATAAAGTCTAAAAAAATAG

Gene: gcvPB (glycine cleavage system P protein subunit B)

Position: 1597499 to 1598971, length: 1473 nt, orientation: REVERSE

Perfect match to: (COL-CP000046-[1625947:1627419:r], allele observed in CC8+CC15+CC80+CC239+CC772)

Sequence:

TTATTTTTCCTGTTTAAGATTTTCAAACTTTAATATTGGTTTACGAGCAGCTGTAGCTTCGTCTAATCGATCAATCACAGTTGTATGTGGTGCT
TCTAGCACTTTATCAGGATCATTTTTAGCTTCTTCAGCAATACTAATTAATGTATCGATAAAATAATCAAGTGTTTCTTTAGACTCTGTCTCAG
TCGGTTCAATCATCATACCTTCTTCAACATTTAATGGGAAGTATATTGTTGGTGGATGTACACCGAAATCTAATAATCGCTTAGCCATGTCTA
AAGTACGTACACCAAATTCTTTTTGACGCACACCACTTAACACAAACTCGTGTTTACAATATTGTTTATAAGGTATTTCAAAGTGTTCAGATA
AACGTGCTTTAATATAATTCGCATTAAGAACCGCTGCTTCAGAAACCTCTTTAAGTCCAGTTGCTCCCATAGTTCGAATATACGTATAAGCTC
TTAAGTAAATACCAAAGTTACCATAAAATGGTTTTACACGTCCGATAGAATTTTTAATGTCATTATCATATTTAAATTTGTCGCCATCTTTAAT
AACCATTGGCTTTGGTAAGTAACTTGCTAGTTCTTTTACTACACCGACTGGACCTGAACCAGGACCGCCACCACCATGTGGACCAGTAAATG
TTTTATGCAAGTTTAAATGAACAGCATCAAATCCCATATCTCCTGGGCGAACTTTGTCCATAATAGCGTTTAAATTCGCACCATCATAATATA
ATAGACCACCAGCATTATGGACGATTTCACGGATTTCCATAATATTTTTTTCGAAAATACCTAAAGTGTTTGGATTAGTTAACATAATAGCTG
CTGTATTTTCATTTACAACACGTTTCAAGTCATCAATATCAACTTCGCCACGTTCGTTTGATTTTACAGTAACTGATTTAAATCCTGCAAATGA
AGCTGAGGCTGGATTCGTACCATGCGCAGAATCTGGCACAATGACTTCATCACGATGACCTTCACCATTATTCTCATGGTAAGCTTTAAATA
TCATCAATGCAGTCCATTCACCATGTGCGCCAGCAGCTGGTTGTAATGTCACCTCATCCATACCAGTAATTTCTTTTAATTCTTCTTGCAAACT
ATAAATAATTTCTAATGAACCTTGAACTTGATCTTCATCTTGTAATGGATGTGATTCACTAAATCCTGGTATTCTAGCAACCTTTTCATTAATT
TTAGGGTTATACTTCATCGTACATGAACCCAATGGATAAAATCCGTTGTCTACACCGAAATTTTTATTTGAAAGTTCAGTATAATGACGTACT
AAGTCTAGTTCAGCAACTTCAGGAAACTCCGCTTTGTTTTTACGAATAAATTTATCATCTAACAATGACTCAACAGAATTTGTTTTAATATCAC
TTTTTGGTAATGAATATGCATATCTGCCTTCACGAGATCTTTCAAAAATTAATGGACTTGATTTACTAGTCAT

Gene: gcvPA (glycine cleavage system P protein subunit A)

Position: 1598964 to 1600310, length: 1347 nt, orientation: REVERSE

Perfect match to: (MW2-BA000033-[1619881:1621227:r], highly conserved allele)

Sequence:

CTAGTCATTTAACTCACCAGCCTTTTCTACAAATGTATCGATTTCATCTTTTGTTCTTAATTCAGTTACAGCTATTAACATGTGATTTTTAAAGT
CGTCTGAAACAACACCTAAATCAAAACCACCGATAATATTGTACTTCACTAATTCCTCGTTAACTTGTTGAATTGGTTTGTCAAATTTGACTAC
AAACTCATTGAAAGATGTACCATCTAATACTTCAAAACCTTTTTTAATAAATTGCTGTTTAGCATAGTTAGCATGTTCTATATTTTGAACTGCA
ATATCATAGATACCTTGTTTACCAAGTGCTGACATTGCAATTGATGACGCTAGAGCATTTAATGCTTGGTTAGAACAAATATTAGATGTCGC
TTTATCACGTCGAATATGTTGTTCACGTGCTTGTAATGTTAATACAAAGCCACGATTACCTTCATCATCTTGTGTTTGACCGACTAATCTACCT
GGCACTTTACGCATTAACTTTTTCGTCGTTGCAAAATATCCACAATGTGGCCCACCGAATTGAGCAGGAATTCCGAATGGCTGAGTATCACC
TACAACAATATCTGCACCAAATGAACCTGGAGGTGTAAGTAATCCCAATGCTAATGGATTTGCATATACGATAAATAATGCTTTTTTATCTTC
AATAAAGCTATGAATCTTTTCAAGATCTTCAATTGAACCGTAGAAGTTTGGATATTGTACTGCAACAGCTGCTGTTTCATCATCCACTGCTGC
TTCTAATTTTTTCAAATCTGTAACAGTGCCATCTAAATCGATTTCCACTACTTCGAATTCCTTACGCGTCTTAGCATAAGTATGAAGTACTTGT
AATGCTTGATAATGTAAACCTTTTGAGACTACAATTTTATTTTTCTTTGTTTGACTAAATGCTAAGATACATGCTTCAGCAAAGCTAGTCATCC
CATCATACATAGAAGAATTTGCTACATCCATATCTGTTAATTCACAAATTAAAGTTTGGAACTCAAAAATGGCTTGTAATTCACCTTGAGAAA
TTTCCGGTTGATATGGCGTATATGCTGTGTAAAATTCTGATCTTGAAATCATAGCATCCACAACTGATGGCGCGTAATGATCATAAACACCA
GCACCCAAAAATGATGTATGCGTTTCTTTAGTGATATTCTTGCTTGCAATGCGATTTAATCTTCTAAGTAACGTTGTTTCCGCTTCGCCTTCAG
CAATATTTAAATCTCTATTTAATAAAATGTCACTTGGTACATCACCGAATAATTCTCCTATAGATTTTGCACCAATTGTTTGTAACATTTCTTGC
TTGTCTTTTTCAGTTAAAGGTATATAACGATGACTCAC

Gene: gcvT (glycine cleavage system T protein)

Position: 1600330 to 1601421, length: 1092 nt, orientation: REVERSE

Perfect match to: (MW2-BA000033-[1621247:1622338:r], highly conserved allele)

Sequence:

TTATTTATCAATTTGATTTTTCTTAACAATTTTCGCTTTTAATTGACGCTTACGAACTTGAACAAGCAACTCTCTACCCATTTCAAACTCATCTC
TTTTTATCATTGCAAGTGCAATTGATTTTCCTGATGATGGAGACTGTGTTCCTGAAGTTACTTCTCCAATAATATTTCCATCTAAATCCATAAC
TTCATAACCAGTTCTTGCAATTCCTTTTTCAAGTAATTCTAATCCCACTGTTCTTCTTGGTGCACCATTTTCTTTTTGATCTTTTAATACAGATTT
ACCAATAAAATCAGCATCAATTAATGGTTTACTTGCAAAAGCGATACCACCTTCATATGGTGTAATTGATTCAGTTAAATCTTGTCCATGTAA
TGGCAATCCAGCCTCTAATCTTAATGTATCACGAGCGCCTAACCCACATGGCATAACATTATACTCTAATAAACCATCCCAAATTTTTTCAGT
ATCATCGATATTACAATAAATTTCAAACCCATCTTCACCTGTGTAACCTGACTGAGATAAAATGACGTTTGCTCCAAATAATTTGACACCCTG
TTTAAATTCAAACATTTTCATTTCAGTTACATCTTCATCAACTAATTGATTAATTAAATCTCTAGCTTTTGGTCCTTGTATTGCTAATTGACCAT
ATTGGTTTGATACATTTTGTACTTCAACATCAAATTTCTCTTTGTGTTTTAAAATCCAATTAAAATCTTTTTCAGTATTAGCAGCATTAACAACT
AATAAATAATTGTCGTCAGCTAATTTATATATTACTAAATCATCAATAATACCGCCTTCTTCATTACATAAAGCAGTATATAATGCTTTTGAAG

TAGTTAAATTATCAGTATCATTTGATAATAAATATTGCACAAACTGACTAGCATCTTTACCTGTTACTTCAATTTCACCCATATGACTAACATC
AAACAGGCCAATTTCGTATCGAACAGCATTATGCTCCTCTTTAATACTTGAAAATTGAACAGGCATCGCCCATCCTCCGAATTCCACAATTTT
TGCACCTCTATCAACATAATTTTGATATAAAGGTGTTTGTTTTAAATCACTTGACAT

Gene: aroK (shikimate kinase)

Position: 1601580 to 1602104, length: 525 nt, orientation: REVERSE

Perfect match to: (11819-97-CP003194-[1651964:1652488:r], allele observed in CC80+CC10+CC15+CC30)

Sequence:

CTAATACTGATCACTCGCTTTTATTAAATTTAGCAATTCATAATATATTTCTGAAATTGATAGCAAATGACTATCAAATTTCTTGAATGCGATT
TCATTATATCTTAAAATCCGTGAGCAATACAAGTCATTTAACTGCGTGATTGTCTTATTATTTGCATTAGGTCGATGTGGGTCATCATTGATT
CGACTATATATAATATCAATATTACAATCTAACCAAATAATGTTTTTTTGATTTTTCAAAAAATTAAATGCCTCTTCACTTTCAATAATACCACC
ACCAGTAGCAATTATATCTGCAGTGTTAATACATTCTTGCAAACATGTGAACTCTAAATTCCTGAAATATTGTTCACCATGTTTACTAAATATT
TCTGGTATTGTTAACTTATACTTTTCTTCGATATATGAATCTATATCAATAAATGATAAATTTTGCTCATCTGCAACGTATTTACCAATCGTAG
ATTTACCGGTACCCATGAAACCAATTAAAATTATTGGTGATTTATCATGATTCAT

Gene: comGF (late competence protein F)

Position: 1602339 to 1602836, length: 498 nt, orientation: REVERSE

Perfect match to: (Strain_21333-AHKA01000045-[45493:45990:r], allele observed in CC15+CC80)

Sequence:

TTATACATAAATAGTTTTAGTCTGCACATTTGTACCGACTTTAACTGTTATCGTTATTTTAATAATGGATTTATAGTAGATATTTACAGTAAAT
GCTGTAACATTATTAATCATTGTTATATTTCCTCTGTCATTTACAACTTTAATAATTTTATTATTTATTAATTTGTATTCGATCATTTCTTCACCTT
TATGTAAAATGATACGCTGTTGCCTAATTTCAATATCGTTTCTATCTACTCCTTTAAAATCCTCTAGAATATCTCTTGAGAAAAATTCGAAATC
TACATTTGTTAATTCCCTACTTTCAATTAGAAAAGTTTTGCTAAGTCTAATTAAGTCTGGAACAATTAGTAAAGTTATACTTATAACCATCATC
GCTACTAACATTTCAATGAGCGAAAAAGCTTTGACATTAATACTGTATACATGTCTTTTGATAAGAAGTGGTATTTTTTGAAATAGCACAAAT
TTGTTGGTCACTTTGCTTAATATCAT

Gene: comGE (late competence protein E)

Position: 1602754 to 1603053, length: 300 nt, orientation: REVERSE

Perfect match to: (11819-97-CP003194-[1653136:1653435:r], highly conserved allele)

Sequence:

TTAATACTGTATACATGTCTTTTGATAAGAAGTGGTATTTTTTGAAATAGCACAAATTTGTTGGTCACTTTGCTTAATATCATACTTCCCTATA
GTTACCCCCTTCTTAAGTTCTTCTTTATTAATTTTAGATACAGTCGTCAAAATTACTTTAGAAGCATCAATTGTTTGTAGTTTATGGTTTATACT
CGCTTGCATTTGATTCATCATTGGTATCAATAGTAATGTAATCAATCCAATTAGCAAAAATCCAGCCATACTATCTATTAAGAATGATCCTTT
ACACTTATAGCTTTTCAT

Gene: comGD (late competence protein D)

Position: 1603040 to 1603486, length: 447 nt, orientation: REVERSE

Sequence:

TTATAGCTTTTCATAACGAATTCTTCCTTTTTCAATATGGAATATTATTCTATAAATTGAATTGTTATTGTCAATTGTTATACTACCAAATTTAT
TGATATTCCCTTTTTTATCAAAGGCAATAATATCAACTTTTGCAACATTAATTATTTTGCCTACTTTTAATTTTAGAAATCGTATTTTATTATTCT
CTATTACTTTAATAGTGTCACTGTTTTCATAAAATCTAACATTGATATATCCTTGATTTGCTATAGCTTGCGACTTAATATAATTCAATTCAGTA
ATAAAAGAAATGATATTTGCCTCATCATCTATTACTCTAAGATTGCTTAATCCTTTAGATGTCATTGTCAAAAGTAGAAATATACTGATTAAC
ATCATTACCACAAGCATCTCAATCATAGTAAATGCTGACTGCTTTCTAATTTGCAACTGCTTCTCCAT

Gene: comGC (late competence protein C)

Position: 1603464 to 1603775, length: 312 nt, orientation: REVERSE

Perfect match to: (RF122-AJ938182-[1534011:1534322:r], highly conserved allele)

Sequence:

CTAATTTGCAACTGCTTCTCCATTACTAATTGTTATTGTCTCTCCTGATTTACATGTCTTTTGTGCTTCTTTTATAAAACCATCTGCAATTAAGTC
TTCAATAGACGATGGATTTCTATTATGTTTCAATGCATACGCTTCAATTTGACTATTAACCATTTTTACCTGTGCATTACAACCTGTTGATTGT
ATGTGAGCAGTTTGTTTAGCAATATTTGGAATGATTAAAATTAATAATAAACTGATGATTAATAACACTAATAGCATCTCTATCAATGTAAAC
GCTTGAGTTTTCTTAAGAAATTTAAACAT

Gene: comGB (late competence protein B)

Position: 1603789 to 1604859, length: 1071 nt, orientation: REVERSE

Perfect match to: (Strain_21334-AGTW01000023-[48654:49724:r], highly conserved allele)

Sequence:

TTATTTTATACTTTGCATCATTTGAAACATTGGTAACATAATTACTAAATAAATTGCGACAATAAATAAACCTAAAATCAAAAATAAAATAGG
CTGTAAAAACTGAGTCTGTTTTATCGCTTTATCTTCTATTTGTTTTACTAATATTTGCGAATATAACTTTAGTTCTACTTCTAGCTTCCCTCTCTT
TTCACCTTGTAGCACAAACTTAATTAATTGAGGCTTAAAGCATTTTAGTTTTTCTAAAATTTGAGGCAAACCATATCCCATTTCTGAATAAGTT
AATAAGTATTTACCTAGAAACTGTCTAAATGGATCACTACTATGGTTAATATAAACGTCTACTATTGATTGAAGTGTAATACCATTTTTATAA
AACAACACTAATTCATTAGTTACAAAATAAGTTTTAAATAATTGGAAATAGCCTGATATTAGCGGTAGTTTCATCACAAAGTTTATCTTATTG
AGCATATTTAAATTGTTATAAATTAATTTCATAATAATAGCCAACATAGATACTATTATGAGCATTACTACAATTATAGTAGGTAAGCTGGTA
ATGAAAAAAGACAATGTTTTTTGAAAAGAAGATAGTTGAATATTCATAGAAGTATATAATTGTTGAAACTGTGGAATTACTGTGAGGTTTA
ATATAATAATCATAGCAATAAAGATAGAAACTAGTATTAAGGGGTATTGCAGTGTCTTTAACAATCGTTGTTCTGACTTTCTATTCACTTTCA
TATAATTTACGGTTTCTTCTAGAACGTCTATAATATTGCCAAATCTTTCTGCCAAATATACTTGCATGACGATAGTATCGCTATAACCTATCAG
TGATAATATTTGATTGCATGGTGCACCATTTGAAATTTCACTTAGAATGGTGGTACCTAATTGCTTATTTTTATATGTCATTTGAAGATTTAAA
AATTGAAAACTTTGATACAGAGTGAAACCATATTTCAACAAATTACATAAATTTGAAAGTAAGTCGATTTGTTGGGCCTTACTTAATTGTCGC
TTCTTAGAATGTAGTTTAAATGTATTTATCCATTGTAGTTTCAC

Gene: comGA (late competence protein A)

Position: 1604831 to 1605805, length: 975 nt, orientation: REVERSE

Perfect match to: (11819-97-CP003194-[1655213:1656187:r], allele observed in CC80+CC30+CC72)

Sequence:

TTAAATGTATTTATCCATTGTAGTTTCACAAATGACACCTGCTTTTGTCATATCATCAAGTTTATCTTCTAAGTTCTTAAATGATGATGGTAAT
GAATGATTATGGGAAAAGAAATATCGGAGTTGTTGCTGAGATAGAATTTCACATACTAATTGTCGCTGTTGCTTAATAGTAGTTACAAGTCG
TTGGTTTATAATTAAGTTAGTTGCCTGTATCAATTCTTGTACAGAAATGCCCATTTCTAATAGCCTTAAAATAGCACCTTTACAATCAGTTGCA
TGCAATGTAGTCAGAACAAGGTGACCACTTAAACTAGCCTGTATAACACACTTGGCAACATCTTTATCTCTGATTTCACCTATTAAAATAACA
TCAGGATCACATCTTAAAATAGCTTTAAACGAATTTACATAGTTAATGCCAGCTTTATCATTCACATTAATTTGGACGATACCAGGAATTTGC
ATCTCTACAGGATCCTCTATAGAAATTACATTTAAATTCAAGGCTTTATTCGCGTATGAGACCATTTGATACATTAATGTACTCTTTCCTGAAC
CAGTTGGCCCACTAAACAATAGTAATCCTTGTTTCTTATTCATGAGGTGTTTAAAATCATTGAATTTATAAGTTGATTTCTGTTGTTGAAAAA
ATTGAGGTACAATTCTGATAACACAACTTTCTTGGCCAAGTGACAATGGTAAAGTTGATATTCTCAAAAAATATATTTTATTGAAATGGTAA
CTATATCGACCGCTCTGTGCGACTTGCTGTGTAGAAACATCAAGCCCAGCTTGAAACTTCATATAAACTAATAACTTTTGATAAATGCTATTC
CCAATTTGTTCATACTGCTCCAAGTTATCATTAATTCTAAATTTAATACTTACTTCATTTTTAACTGGAATAAAATGTACATCACTCGCTTTCAT
TTCTATCGCTTTATTAATTATTTCTTGAAATAGAATCTTCAA

Gene: yqgX (putative metal-binding hydrolase)

Position: 1605857 to 1606480, length: 624 nt, orientation: REVERSE

Perfect match to: (N315-BA000018-[1581225:1581848:r], highly conserved allele)

Sequence:

TTAACCGTGTAAAAATGGATTTAATTGTTCATCATCAACCGTCGTATATGGACCATGTCCAGGGAATAAAGGTAAATCGCCTTCTAATTCAA
ATATTTTATCTTGAATAGAATCAACTAGCGTTTCATAATCACCTTTATATAAATCTGTACGTCCGATTCCATTATTAAATAATGTATCTCCAAC
AACTGCGAATTCATCGAACACATATGTTAAACTTCCTGGTGAATGTCCAGGTGTGTGTAACACATTAAACTTAAATCCTTCTATTTCTGTGCT
ACCTTCGTTTAACTTTTCAGGAGTTACCTTACTTGTAATAATTGGTAATCCATATTGCTTAAATTTATCTGCCCCATTTTTAACGGGATCTTTTA
GAAAATCAAACTCTGCTTCATGCATATAAACCGGGACATCGAATCGATCAACTATATCATCGACTGCTCCGATATGATCAAAGTGTGCATGT
GTTAATAAAATAGCTTTTAACGGTTTATTTATTTGGTTTAATTTTTTAATAATTTTTTCACTTTCACCTGAAGGGTCAATCAGAATAACAGCTTT
GTCATTTTCGATGAAATACGTATTAGTATCAACTAAGCCTAAAGTTAAGCTTGAAATCCTCAT

Gene: yqgV (putative protein)

Position: 1606477 to 1606806, length: 330 nt, orientation: REVERSE

Perfect match to: (N315-BA000018-[1581845:1582174:r], highly conserved allele)

Sequence:

TCATAGGTTTTCACCACTATTTTCTAAATGTTTTTGTACTGATGTTAGTTTATCATTCATTTTTCTAGATTTGTCTCGTCGGTCATCAATACGGA
TATTTGTACAAACTCTACTTAAACCTTTATCAAAAGGTAATTCATGTATCACTTGCACAACTTCTAATACATCGCTTAATTCACCTTCAATTAG
AGTATTCATTGGTGTTAATTGAAAATCAATTTTACCCATTGCTTTATATTCTTGAAGTTTTTTCTGAATATCTGCAATATATTTACTAACACTCG
GACCTTCCGTTCCAACTGGAATAACAACCACATCAACAATAGCCAT

Gene: glk (glucokinase)

Position: 1606806 to 1607792, length: 987 nt, orientation: REVERSE

Perfect match to: (A9765-ACSN01000002-[4390:5376:r], allele observed in CC8+CC1+CC8+CC97)

Sequence:

TTATTTTACCCCCTCTTTATCTAATACATAGGTCTTGATTAATCCTGCTGCTCCTGTAATACCTGCATCATTACCTAATTTCGCTTGTACAATTT
CAGTTTCAAATTGAGCAGGTGCAAATGTTAAATTATGATATTCTGTTTTAATATTTTCAATTAAAATAGGTCCTGCAGTAGACATTCCTCCAC
CTAGAACGATATATTTCGGATTACTTGTAATACTAATAATACTACATAAATATCCAATATAGTTTGCAACCTTTTCAGTAATGAAAATACAGA
ATTGGTCACCAGCTTTTGCCGCATCAAAAACAGCTTTTGCTGTAACCTTATTTTCTTTAATCAATTCTAATATAGAAGATCTAAACGTCAACTT
CGGATAGTAGAAGTTAACTAAGTTAACAACGCCTGTCGCTGAAGCAACTGTTTCAATACATCCAGAACGACCACAATTACATTTAAATCGTT
GATCGAAGTCTGCTCTAAAATGACCTATTTCTGCGCCAGAGCCATTATGACCATGTACGATTTCACCATTTGAAATAATTCCTCCACCTAGAC
CTGTACCAAGTGTGATGGCAACAACATCATCGGCACCTTCACCAGCACCTTTGTGTTTCTCCCCTAAAGCAGCTATGTTAGCATCATTATCTA
CATACACTGGACAATCAACGAATTGTTCAAAAATCTCACGTACATTAACTTTTTCTGGCCAATATAAGTTTACTGCTCCATTTACTGTACCTTT
TTCAAAGTCAACAGGACCTGGTACACCAATACCTACGCCAAGTACATTTGAAAAATTATAATTATTTTCATTTACTTTTTCAACAAACGAATC
ATAAATTCCTTTCAAAAGTGTATATCCTGTACTATCAGATGTATCAGTGTGAATAGACCATTTATGTAATTGTTCTAATTCAGGTGTGAAAAT
ACCTAATTTACAAGTCGTCCCGCCTACATCAGCTGCTAAAATAATTTTTGTCAT

Gene: yqgQ (putative single-stranded nucleic acid binding protein)

Position: 1607789 to 1607992, length: 204 nt, orientation: REVERSE

Perfect match to: (RF122-AJ938182-[1538336:1538539:r], highly conserved allele)

Sequence:

TCATTTCTGTTCATTCCTTCTCTGATTAATTATCAACGTACATTTCAAATATTCTTCCTTAGATAACAGTTCATATTGATACAATGATGAAATCT
CCTGTTGAATCATTTCGTACATATCTTCTGGATTTTTAAAATATATTAGAAATCCGTAACTTTTCAATAACTGTTGTACATCATAAAAGTTATT
TATTTTTCGACTCAT

Gene: gluP (transmembrane rhomboid domain peptidase)

Position: 1607973 to 1609436, length: 1464 nt, orientation: REVERSE

Perfect match to: (NN50-BAEA01000025-[170278:171741:r], allele observed in CC4803+CC8+CC30)

Sequence:

TTATTTATTTTTCGACTCATTTGATTTAGTCAACTCTTTTTCTAAGTTAATATAATCTGTATTTTTAGGGTCTGCATTTAATGCTTTACGCACATA
TTTTAATGCTTTTTCATCATCATTTAATGAACGATTTGCTATCGCTAACTCAAAATTTAATAAACCTGATTTAGGAAACATTCTAAGTCCTCGC
TCCCATTCTGTCATACCTTCAGACTTAGAATTGATAGTAGCCATAATCATACCACTTAAATAATATGTTTGATCATCGGCATAATTTTTATTTA
TTGTCTGCTTCACAATATTTTGAGCATTATCATAATTACCACTAGTCATATCATCTTTGATCAATTTATTATAAATATTATCTTCTTTAATTGTA
AAAATTCTAATTTGAAGTGCAATAAATATAACAAGCATACCAATTAGTAAAATCCAAAAAATATTACGATTCACTTTATAGTAATAGCCAATT
AAAGTTATTAATAAACCACCAATGAATCCTCCAATATGCGCCACAATATTTATATTTGACATAAACAGAGAAACACCAACTAATATCACTAAT
GCAATTAATAACTGTCCCAACATTTTTTTGTTAAATGTTTTTGAAACATACATCATCGCAAAAATTGATCCAATCAGACCAAATATAGCACCA
CTAGCCCCAACTGAAATTGTAGTCGTATTAAATGATAGTGATACAAAGTTTCCAAACAACCCTGCAATAAAGTATACAGTTAACATCCGCCA
TGAACCAATAATTGCTTCGACTATTTTACCAAAAATAAATAATGAAAGCATATTCATAAGTATATGTTCAAAACTAAAATGTAAAAACATCG
ATGTAACAATTCGATACCATTCACCATGTACGACATTAAAATGCACTAACCCGCCAACATCTAATAATTTTACATCCGAAAAATTATTTAAAT
ATAAAATCATACATAACCATATTAAGACATTTACAAATATTATTGTGTATGTTGCCGGTGAAAATTTCTGCATATATTTATCTAAAAAGTTATC
GGTTAATACTTTGCGTTTATAGAACATATATGTCTTTTTATCATTATCTTCTGAAATAAGTCTAGCCAAAAATATATTCGGCATATGCTTTATC
AAATCTTTTGTGTGTCTTATAACATTGAATTTAATTCTAATTGGCGAAACTTCATTTAATTGTTCTTCTGAAAATTCCGATTCTGTAAAATAAT
AAAATTCAAAACTTTGTGGTTCGAAAGAAATAAAATTTGCTATTTCATCTTTGTGTTCAAGCACTTTCGCTTTGTCAAAACGTATTTCTTGGGT
AGACTTGATATGTTGTTTAAAAATGACTACTTGTTTTTTTCTTTTATGGGCTAACCAAATTTCTTGGTCATCTTTTTCCCTACTGACAATATCAA
AATTCAAGTACCTAATCCAATAATATATTGTTTTCCAAAATTGTTTGTCTATGTTCAT

Gene: yqgN (putative 5-formyltetrahydrofolate cyclo-ligase)

Position: 1609448 to 1609987, length: 540 nt, orientation: REVERSE

Perfect match to: (MW2-BA000033-[1630408:1630947:r], highly conserved allele)

Sequence:

CTATGCTGATTGATATATAATCAATTTATCGACTGGTTGATCGAATGATTCCGGTTCAAATGATGTTATTTGAAAATCGTATAATAAGCTTAT
TGTCTTTGTCTGATAATTAGCTAAAAACCTGTCGTAATAGCCACCACCATACCCAATTCTATATCCATCGTCTTGAAATCCAACACCAGGAAC
AACAATTAAATCTAGGTTATTCGTTGTTTCACCTTTTGAAGTTGGATAGTAAATCCCCTTATTATCGACATCAATATCTTTGAGATTAAATATT
TCTTTAAAAGTCATTTGATGATTTAAATAATCCATTTTCGGTACAAAAATACGTTTATGATCCATTAAGGCTTGTTCAATAATAGAAAAAGTA
TCTACTTCATGATTAAAAGAAAGAACTAGCGCAATTGCGTTTGCTTCTTTGTATTCTTCAGTTGCAAAAAATTGATTTCTTAACCATGTGTCTG
CTTTTCGCTTTTCAGCTTTATTAAAATTCTTCATTTTATGTAAAATGTATTTTCTAATCTCATTTTTAGTCAC

Gene: rpmG1 (50S ribosomal protein L33, locus 1)

Position: 1610196 to 1610345, length: 150 nt, orientation: REVERSE

Perfect match to: (RF122-AJ938182-[1540743:1540892:r], highly conserved allele)

Sequence:

TTATTTTGTTTCACGGTGTAAAGTTTGTTTGTTTTCACGTGAACAGAATTTCTTCATTTCAACACGTTCTGGATTATTTCTTTTGTTCTTAGTTG
TAATGTAGTTTCTGTCACCACATTCCGTACAAGCTAAAGTTACGTTTACGCGCAT

Gene: pbpC (penicillin binding protein C)

Position: 1610458 to 1612533, length: 2076 nt, orientation: REVERSE

Perfect match to: (Strain_6850-CP006706-[1535383:1537458:r], highly conserved allele)

Sequence:

TTATTTGTCTTTGTCTTTATTTTTATCATCTTTACCTAACTGCTTAAAGTAGTAGTTAATTACATCTCTACCTAAGTCTCCACCTGTTAACCATG
GTGGTGGTACAGGCTGATTTGTATATACAATTGAAAACGCTAATTTTGGATCATCAATTGGCGCGTATCCTATATAAGTAGAGTTAACTCTT
GGCTCTCCGTTTTGGAACACTTCAGCGGTACCCGTTTTACCAGCAGTAGGTACTACTGTATCTTTAAAACTAACATATCCAGTACCATCTTTA
TCATTAAATGCCATTTTGAATCCTTCTTGAATTTGTTTGATTTCCTTTTCAGTATTATTAACCTTGTTCAAGACAGTGCCATTAATTTTCTTCTTG
AGTGGACCAACCTCATCTTTATTAGTTGATTCATGAATCGTTAATCCAATGTGTGGCTGTATTCTATAACCATCATTCGCTATAGTTGAAACA
TATTGTGATAATTGTAATGGTGTATAGGTATCATATTGACCAATTGATAAATCTAGATAATTACCTGGATTATTTGTTAATGGTTCGATTTGA
CCTCTTGTTTCATTTGGTAAATCTATCCCTGTTTTCACACCTAAGCCTACTTGATTTAATCCTCTTCTAAGCTTTTGGGCAGGTGAACTTATATC

TGAAGGTAAAGCCATACCAGAATAATAAGGGTCTCCCGCTAATTTTAATGCTGTTTTAAACATATATACGTTTGATGAATGCATCAAAGCTT
GCTTATCATTAATAGATACATGCCCGTTTTTATTGAAGTATGATCGTTTTGTCAAACCACCTTGGAAATGTAATGGTTCATCGACCATTGTTTC
TCCAACTTTGATAGCTTTATTCTGATAACCAGCTAATAATGTTCCACCTTTTACAGAAGATCCAACCGCAAATTGAGAAGTAAACGTACCAAT
GTCATAATCAGTCATTTTACCACTCTTATTAATCTGCTTTCCGGCAAGCGCAAGAATGTCTCCATTTTTAGGATTTTGTACAACCATCATAGCA
TTATCCATATCTTTTGCACCTTGGCTGCGAAGCTTCTTAATTTGTTTATCTAATAATGCTTCTACTTCTTTTTGAAGATCTATATCGATCGTTAA
TTTCAAATCTTGACCGCGAGCGCCAGGATTTAACACTTCTGAAGATGTAACTTTACCAGATTTGTCCGTTGTGTATTTCATTTCTTTCTTCTTA
CCACGCAATACATCTTCATATTGATATTCTAGGTAAGATTTTCCAACACGATCATTGCGTGAATATCCTTTGGATAAGTAATGTTCTGTCAAT
TCTTTTGGAATACCTTCAGCAGGTGTCGATACATCTCCGAATATACCTCTTAAAGTATCGCCATATGGATATTTTCTATCCCAATCCATAGAC
GTGTTAACACCTGGTAATTTGGAAAGTTGCTGAGAAACTGCTGCATACTCTTTTTCACTGACATCTTCATTTTTTATCATTTGTGGATCTAAAA
CTGTTCCTGCATTCATCTCTCGAAAAATAGCTAAAACTTGTAAATCTTTAGAAGACAATTCATCTAATTGTGATTTTCCGATTTTCGATAACAG
TTGTTTATCATATTGATCTTGTTTAATACTTCCATCTGCTAACATAGCTTGTTCTTTTGTCATCATTGCTTTTGCTTTTTTAGGATGCAACTGAA
TCCAGAAATCTTTCTTATCACGTTCTGTAATTTTCTTAGTATCCATCTTGATTAGCTTTGATAACTTTTCAGCCGTATCCAACATTTCCGATTGT
GTTGTTTTTCGACCCCTAGTATATGTAATAGCCATTTTAGAAGCATTATCAACTAAAACTTTCCCATTTCTGTCTAAAATACGACCTCTTGGCA
CAGACTCATTCACTGTAATGTTTTCATCATTTTTTATAATTTGTTTATAATGTGAGCCTTGTGCGATTTGTAAATAACCTAAACGTAGTACTAG
TACTGCAAAAATAAATACAATCACACCAAATATAAAGTTAATTCTCTTGTTAATTGTATTTTGAACGATTTCATCATTTGATTTTTCTTTTAGTC
TTTTTAACAA

Gene: sodA-L2 (superoxide dismutase, locus 2)

Position: 1612654 to 1613253, length: 600 nt, orientation: REVERSE

Perfect match to: (N315-BA000018-[1588022:1588621:r], highly conserved allele)

Sequence:

TTATTTTGTTGCATTATATAATTCGTCAACTTTTTCCCAGTTAACTACATTCCAAAATGCGCCAATGTAGTCAGGGCGTTTGTTTTGATATTTT
AGGTAATAAGCGTGTTCCCATACGTCTAAACCTAAAATAGGTGTTTTACCCTCAGTTAATGGATTATCTTGGTTTGGTGTAGTCACAATTTCT
AACTGGCCATTGTTTACGACTAACCAAGCCCAACCTGAACCAAAGCGTGCAGCTGCTTTGTCAGCAAATTCTTTTTTAAATTCTTCTAAAGAA
CCCCATTGTTCTTTAATTTTTTCTACTACAGTACCTTTTTCTTCTGAGTTTGGTGAAAGTAACTCCCAGAATAATGAATGGTTTAAATGTCCAC
CGCCATTATTACGTACAGCAGTTTGGATGTTAGCTGGTACACTGTCTAAATTAGCAACAATTTCTTCAATAGATTTAGATTCTAAATCTGTAC
CTTCTACTGCAGCATTTAATTTCGTAACATACGTGTTATGATGTCTGTCATGGTGAATTTCCATAGTTTCTTTGTCAAAATGTGGTTCTAATGC
ATCAAATGCGTATGGTAATTTTGGTAATTCAAAAGCCAT

Gene: zur (zinc uptake regulator)

Position: 1613529 to 1613939, length: 411 nt, orientation: REVERSE

Perfect match to: (N315-BA000018-[1588897:1589307:r], highly conserved allele)

Sequence:

TTAATCTTGGCAAGACTCACATACACCATAAACTTCAAGTTTGTGTTTGTGAATATTAACACCAGGTAGTGATAACTTTATCTGATCTATTGG
ACAATAATCTATTACCTTTGTATCTCCACACTTTTCACAGATAAAATGATGATGATGATGGTTTGTACAAGCGATTCTAAACTTCATTTCACCA
TCAAGTTCTGTATTTTCAATAATCCCTAAATCTTTAAATAAGTGCAGGTTTCTATATATTGTGTCGAATGAAATTCCAGGATAATTTTCATCCA
TAACTTGTTGTATATACTTTGCGTTTATATACTTATCTTCTTCGACAAAAATATCTAACATATCTTTACGTTTATCTGTATATTTTAAACCGTTCT
CTTTTAAAATTTTAATAGCATCATTTGTATTCAT

Gene: znuB (zinc ABC transporter, transmembrane permease)

Position: 1613926 to 1614759, length: 834 nt, orientation: REVERSE

Perfect match to: (N315-BA000018-[1589294:1590127:r], allele observed in CC5+CC72+CC80)

Sequence:

TCATTTGTATTCATTGATATTAGCTCCCTTTTTAAACTTCATTCGCATTTTCTGATAAGCCATTGTAATCATAAGTAAAATAACTAGTAGAACT
ACAATTACACCACCCGGAGAAATGTCCATATAGAAAGCTAGGACTAAGCCTAATATTACTGATAATTCACCTAAAAATACACTTAGTAATAT
CAATTGCTTAAAACTTTTTGTTATTCGCATACTTATTGCAATTGGTAACGTGATTAAAGCACTTACTAACAGTATCCCTACAACACGCATTGA
GGCAGAAATAACCATCGCTACAATAACAATAAATAAAAATTGAATCCATTTAGGAATGCCAATGACTTTACTATATTCCTCATCAAATGACA
ATATAAATAATTCTTTATAAAACAATGTAATAAACAGAACAACTATGATGGCAATGACAATAATCGTTGTTAAATCACTTATATTCACTGCGC

TTATTGAGCCAAATAGCAATCCAACAATTTCTTGATTGAACCCATCAGCTAATGAAATGAAGATTGCACTCAAGGCGATACCAGCACTCATT
ATAATTGGAATAGCAATTTCTTGGTAAGCAGTGTATGACGTTCTTAATTTTTCAATTAGAAGCGCACCTACTATTGCGAATAAGATTCCAAAC
CACATTGGATTAATAAATACTAGTGTTGGCATAATAGTAAGTAAAAACATACCGAAGGATATACCACCTAAAGTTACATGACTTAGAGCATC
AGCTATAAGTGATAGTCGTCTAACAACGATAAAAGCACCGATTAGAGGCGCAATAAAACCTATCAAGATACCACTAATTAAAGAGTACCTC
AT

Gene: znuC (zinc ABC transporter, ATP binding protein)

Position: 1614831 to 1615616, length: 786 nt, orientation: REVERSE

Perfect match to: (N315-BA000018-[1590199:1590984:r], allele observed in CC5+CC15+CC80+CC97+CC361)

Sequence:

TTAATTACAACATTCTCGATTATGCTGATGATCGACAAAACGTACAGGATGTCCATAAATTTTTGAAATTTCAACTTCATCAAGTGATTTAAA
CTCATCAGTTGTACCATGGAAATGCAAATGCTTATTTAAACATGCTACTTCAGTAGCAGTATCTGCTACAACACCGATATCATGAGTAACTAA
GATAATGGTGATACCTTCTTGTTTTAATTGATCTAAAGTATTATAAAATTCACTTACATGTTTTGCATCAATACCATTCGTTGGTTCATCAAGT
ACTAATACTGCAGGTTCTGAAATCAATGCTCGGGCAATCATTACACGTTGTTGTTGACCACCTGATAATTCTGCTATATTTTTATGAATTAAA
TCACTTATATTCAGTCTTTCTAGTACTTTAATCACTTTTTCATTATCTTTGCTATTAAATGTTTGGAAAAGACGTTTTGTCTTTGTTAATCCGCTT
AAAACAACTTCTTTAACACTTGCTGGGAAACCTGAATTAAAGGCATTTGCTTTTTGTGATACATAGCTTAATTTAATTGATGTTTTCTTATTTT
TAAAATCAATACCTTCAACAAAAATCTCACCACTTTGTAAAGGTAATAACCCTAGAATCAACTTCAATAATGTTGATTTACCAGCACCATTTG
GTCCAACAATCGCTAAAAATTCACCTTTATTTATTTTAATGTTTATATTTTCTAACACTTTTTTATGATCATAGTAGTAATTGACATTTTTCAATT
CAAAGACTGGTGTCATCGTATTCTCACCTCGCAT

Gene: nfo (endonuclease IV)

Position: 1615742 to 1616632, length: 891 nt, orientation: REVERSE

Perfect match to: (11819-97-CP003194-[1666124:1667014:r], allele observed in CC80+CC361)

Sequence:

TTATTGTTGCATAACCTTATTTTTTAATTCTGGGTCAAATTGCTGTTGTTTTAACATTTCAATTTCAAGTTTATATGGCGGTTTTTTATTTTTCTT
ATCTTCACCAACATAAGGTGTTTCTAAGATTTTCGGAATATCTTTAAAACTATCATGATGCACAATGTAATTTAATGCATCAAAACCAATGTA
ACCGAAGCCAATATTTTCATGTCGGTCTTTTTGAGCGCCACGGTCATTTTTAGAATCATTGACATGAACAACTTTGATTCTGTCGACTCCAAT
AATTTTATCAAATTCATTTAATACGCCATCAAAGTCCTCTTTCACATTATATCCAGCATCATGCGTATGACATGTATCAAAACATACTGATAAA
CGTTCGTTATTATGAACTCCATCAATAATACGTGCTAACTCTTCAAATGAGCGACCAATCTCTGTACCTTTACCTGCCATCGTTTCAAGCGCA
ATACGTACATTATTGTCATTCGTTAAAACTTCATTTAATCCTTCAATAATCTTATTAATTCCGGCATCAACACCAGCTCCAACATGCGCACCTG
GATGTAATACAATATCTTTAGCCCCTATAGCTTGCGTTCTTTCAATTTCTTGTTGCAAGAAATCTACACCAAGATTAAACGTTTCTGGTTTGGT
TGTATTTGCAATATTAATGATGTATGGTGCATGAACAACAATATTAGATAAGCCATATTTTTCCATCACTTCATGACCTTTAGTTATATTTAAA
TCTTCAATACTTTTACGGCGCGTGTTTTGAGGTGCACCAGTATAAATCATAAATGTCGTTTCACCATATTCATGCGCTTCTATAGCAGAACCT
TCTAACATCTTTTTACCACTCATTGAAACATGTGATCCTAATAACAT

Gene: cshB (DEAD-box ATP dependent RNA helicase)

Position: 1616642 to 1617988, length: 1347 nt, orientation: REVERSE

Perfect match to: (COL-CP000046-[1645088:1646434:r], highly conserved allele)

Sequence:

CTAACCTTTTTTGTTTTGCTTACGTTTTTGTCTATTTTGCTGCTTACTAAATTGCTTACGCTCTTGACGTTTCATTTTTTCAACTTCTTGTTTAAAT
TTCTTCTTATAACCTGGTTTAACTTTGTTTTTAATTTTACTTCGAACTTTGTTCTTCACTTGATTAGTTAAATGGTCATCTTTGCGCATTCTTGCT
TGACGCTGATTGTGCGCTTTAACTTCTTTTAACTCACCATCTTTAATATCAACAGTATTGAATACAAAACCGCGATCTTCTATTAATGAAATAT
TGTGTTCTTCATCAGGACTATAAAGCGTAATTGCTACACCTTTATAATTCCCACGACCAGTTCGTCCGACTCTATGCGTAAAGAAGTCAATAT
CATTTGGCACATCAAAATTGATGACATGACTAACACCTTCAATATCAATACCACGAGATGCTAAATCGCTGGCAATAACGTATTGGAATTCT
AAATTACGTATACGTTTCATTTGTTGTTTACGTTCACGTGGCGTTAAGCCACCATGAATCATACCAACTTTAATACCAGCTTCATTTAGTGAA
CGTGCTAAATCATTTGCATTATCTCTACTATTACAGAAAATAATACATAAGTATGGATTTAGTATATCAATTAAATTTAAAGTCTTTTCAACTT
TAGCTGCACCTTTAGTAGGTATTAAATAGAATTCGATGTTCTTTTTATTTTGTTTTTTACTGTCGACAGCTACATATTCTGGATGACTTAAATA
TTTATTTAAAAATGGTTGTAACTGTTGTGGGATTGTAGCACTAAACACCGCAATATTTGCATTATCTTCCAATCTTGCAGCAATGTAATCTAC

ATCTTCAATTAATCCTAAGTCAATCATAAGATCCGCTTCATCAATAACTAAATATGATGCTAAGTGCACATGTAAATGTCCCGTTTTAGCTAA
GTCATTAATTCTAGTAGGGGTGCCTATAATCAATTGTGGTTGTGCATTACAACGTTGTCTATCTTTCTCTATATCTGTACCACCAATAAAAACT
TTAACTGAAACACCAGCTTTAAATTGGCTTAAATGGTTCGCTGCATCGTATAGTTGTTGTGCAAGTTCTCTTGTTGGTGCAACTACGATTGCT
TGTGGTTCTTTTATTTCACTATCAATTAACTGCATTAATGGTAATAAAAATGCATGAGATTTCCCTGTACCCGTTTGAGATTGACCAATTAAAT
TTGTTCTCTTTAGTATTCTTGGAATAATTCGATTCTGAATTTCAGTTGGTTTTTCAAAATTAAGGTCTTTCACAGCGTCAATTAAACTAGATTC
TAGATTAAATTGTTCGAATGGATGTTTTGCCAT

Gene: ybgI (protein of unknown function)

Position: 1618102 to 1619202, length: 1101 nt, orientation: REVERSE

Perfect match to: (GR1-AJLX01000009-[185476:186576], allele observed in CC361+CC8+CC25+CC80+CC239)

Sequence:

TTAAATATATTGAAATGGATCTGTATTAATTGTAGATGCTTCAACATCTATATTTATTTTTTCTGTATTGAACCAATTCATTAGTAACGTTTTTA
AACCTTCTTTCATCACATATTCGCTGTAATGATTAATATCAATTAAATTCACACCATGAATTTTAGCATCTAAGGCATCATGATGTTTAATATC
ACCTGTAACAAAGACATCTGCGCCTTGTTGGACAGCTTGATATTCATATCCAATACCTGAACCACCAATAATTGCAATACGTTTAATTTTCTG
ATTAGACTCACCAACAAAACGGACACTTGGGATATTTAATTTAGATTTAATATCAGCTGCGAAATCTTCCAATGTCATTTGATTATCCACTTC
TGCCATAACGCCAAGTCCATAAAGGGATGTTTGTTTTATCTCAATAAAATCAAATACCGGTGTTTCATATGGATGGTATTGTTTAATTAATTG
CTCAGCCCTTGACTTTTGATATGCATCTATCATAAATTCAATTTTAACTTCATCTACATATTCAATTTTATCAATTTGTCCTATTGTTGGATTAG
CTTCACCAACTGGTTTGAATTGCCCTCTTCCTTCACTTTCAAAGAAACAATATTCATAATTACCTTCTTGCGCTAATCCATTTTCACTAAGCTTA
TCTTTAAATGGTCCAACATTATCCTTAGGTATATATGTTTGAACTTTATAGTATACATCTTGTTGATTATTTATTATTGAAATGTTCTTCAAACC
CATCGCCTTCGCCAACATCATATTGACACCATACGGATTTACATCTAAATTTGTATGCATCGCTATTAAATTAATGTCATGTTGAATTAGTTTT
CTAATGATCAAACCATAACCATTAGCTTTTAATGATGTTACGCCTTTAAAGATTAGAGGATGATGACTAATAATAGTATTATAACCTTTTTCG
ATTGCTTCATTTACTACTTCCAACGTACAGTCTAATGCTGTTAAAACACCAGTAACTTCAACATCTTCATCACCTATTAACAATCCTACATTATC
CCAAGATTCAGCAGTACTAAATGGCACATGATGATCTAACAATGTCATTAAATCAGCTATTTTCAT

Gene: trmK (tRNA (adenine (22)-N (1))-methyltransferase)

Position: 1619205 to 1619882, length: 678 nt, orientation: REVERSE

Perfect match to: (11819-97-CP003194-[1669587:1670264:r], allele observed in CC80+CC188+CC361)

Sequence:

CTATAACACCCTTTCAATTACAGCAATTTCGTCATTAATTTGAGCTAAACGTTGATGATGTTGTTCAGTATTGAGTTTCGATTTAATATGATAA
AGTGCTTCTAACTCTCTTTGCCATTTTTTTATAAAATATTCATTTTTGTTGTTCAGCAATTTTGGTCCGAATTTCAATTCATCAGATGATAGCTC
TATTAATTGTGTAGAATATTCTGCTACAACAATTTCATAAATATGGCCTTTTTCTTCCATTATTATTTCATCAATTATTTCATAATTCAATTGTT
GTAATGTTTGTCTTAAATTTTCAGTTTGTATATTACTTTGTAAAATCAACCTTGGATGTTGACTTAACTTATCTTGCCCATCTTTTAAAATTTTA
GCAATAAGTGGTCCGCCCATACCACAAATTGTGATATTATCGATTACGTCCTCAGGATGAATAACACTTAAGCCATCCCCTAAACGTACATC
AATTCTATCTACTAATTGGTTTGCAGCTACATTTTTCACAGCAGCTTGAAAAGGGCCTTGAATAACTTCTCCAGCAATACCGCATTCGCATAA
ATGGTTTTGAATTGCATAGATTGGCAAATAAGCATGATCTGAGCCAATATCCGCGATTGTACCTTGTTTTAAAAATCGACTTACCGTCGTTAA
TCGGTTATTTAACGAAATCAT

Gene: sigA (sigma factor A of RNA polymerase)

Position: 1620013 to 1621119, length: 1107 nt, orientation: REVERSE

Perfect match to: (NN50-BAEA01000025-[182318:183424:r], allele observed in CC4803+CC5+CC8+CC239)

Sequence:

TTAATCCATAAAGTCTTTCAAACGTTTACTACGACTTGGATGTCTTAATTTTCTAAGTGCTTTTGCTTCGATTTGTCGAATACGTTCACGTGTA
ACACCGAAAACTTTACCAACTTCTTCAAGTGTTCTTGTTCTGCCGTCATCAAGACCAAATCTTAATCGTAATACATTTTCTTCTCTATCAGTTA
ATGTATCAAGCACATCTTCTAATTGCTCTTTTAATAATTCATAAGCTGCATGATCTGAAGGACTTTGTGCTTCCTGATCCTCAATAAAGTCTCC
TAAATGACTATCATCTTCTTCACCAATTGGTGTTTCTAATGAAACAGGTTCTTGCGCAATTTTTAAAATTTCACGAACTTTTTCTGCTGGTAAA
TCCATTTCTTCACCAATTTCTTCTGGTGCTGGATCTCGACCTAAGTCCTGTAATAATTGACGTTGAACACGAATTAATTTATTAATTGTTTCTA
CCATATGCACAGGGATACGAATCGTACGTGCTTGGTCAGCAATTGCACGAGTGATTGCTTGTCTAATCCACCATGTTGCATATGTTGAAAAC
TTAAATCCTTTGTTAAAGTCAAATTTTTCAACAGCTTTAATAAGACCCATATTACCTTCTTGGATTAAATCAAGGAATAACATACCACGACCTA

CGTATCTTTTAGCAATACTTACAACTAAACGTAAGTTCGCTTCTGCAAGTCTTGATTTTGCTACTTCATCACCTTGTTCAATACGTTTGGCTAA
TTCGATTTCTTCTTGTGCACTTAATAAGTTAACACGCCCAATTTCTTTAAGGTACATACGAACTGGGTCATTTATTTTAACACCTGGAGGGGC
ACTAAGATCACTTGGATTCAGTTTCTCGTCAGTATCTGAACTATCTTTTTCATTAACTAGTGAAATATCATTATCATTTAATTGATCAAAGAAA
TCATCCATTTGATCAGAGTCGATATCAAAATTCTGAAGTTTTTCAGCAATTTCTTCATGACTTAAATGACCCTCTTTTTTACCTTTTTCAATTAA
TTGCTTCTTAACATCTTCTAATGTTAATGTCGGATCAATTGTTTGTTTTTTAATTTTAACTGTGTTATCAGACAT

Gene: dnaG (chromosomal DNA primase, variant 1)

Position: 1621343 to 1623160, length: 1818 nt, orientation: REVERSE

Perfect match to: (11819-97-CP003194-[1671725:1673542:r], allele observed in CC80+CC12+CC96+CC361)

Sequence:

CTACATGCGTTCTTTATTCTTAGCAACAATTTGCTGTAAATAGTATTTTTGTAATTCTACATCGCCAATCCTTGTAGCTTCCCTTAATTTATGAT
TCAATGACTCAATTGTTTCTTGTCCTTTTTCATTAATAACATTGACATAATCATCAATTTCATTTTCATATGGTTCGTCATTCAAATTATATTGTT
CTAAGCTAATTAGTGTTTCTCTCAACTCATTTGAATTAACATACTGTACAGCATCACTGATATTATATTGATCATTTTCCGCATAAAAATCATG
TAAGACTTCGAATACATATTTAAAATGCTGATTTGTGAAGTTATCCTTATCAACACTTTCATAATAGTTTAAAAATGTATCTTTATCTCTCATT
AAATGTTTTAAAAATGCTCGCTCCGCTTTTTCTTGACGGCTCAAATTGTCAAATTGTGCCATACCAATTGGCTCAGGTTCAATATAACCGCCA
TACTCATCATAACCGCCATACTCATCTTCTGGATAATAATTGGCTGGTGCTTGATTGAATTGTATTTCGTTAGCTAACTGCTCAGGACTGACA
TTGAAAAATGGCGCAACATCATTTATAGCCTTTTGTTGCAGAATTGATGACTTCATAAGTGAAATGTCATGACTCAGTTCTTTCAAATAACGT
TCATATGAAAGGTCATTATGTGCAATTTCATCTTTTAATATACTTACTTTATAATGTGCAAATGACTTTTTGTCATTTTTTACAAAAGTAGTAA
ATGCGTCGTTGCCATACTTACCAATGTATTCATCCGGATCCATGCCAGATGGCAATTGTATAACAAATACATTTAGCCCTTGCTGTAACAAAT
GTTGACCTGTTTTAAGTGTTGCTTCACTACCCGCAAAATCCCCATCAAACATTAATGTTATATTTGATGTTAACTTTCGTATAAAGGTAATATG
TTCATCTGACAACTGTGTACCCATTGTTGCAACAACGTTTTTCAAGCCAGCAGTATCAGATTTTATAACATCCATAAAACCTTCTAGTAATAC
AATTTCATCTAATTTTCTAATTGATTTACGTGCTTTATCTAAGTTATATAACAACTTTCTTTTTTGAAAGATAGGCGTTTCAGGACTATTTAGGT
ATTTTGGTTCTTGACCGGTATATGTTCGACCTGAATATCCAACAATTCTTCCTTGCGCATTTTTCAAAGGAAACATAATACGATTTCGAAATCT
ATCGTAATAACTGAAATTTTCTTCGTTACGTGATAATAATCCGGCTTCATATGCTAATTCAATATCGTAACCCTTTTTTTGAAGAAAATCATGA
CAAAAATGTGAGCTATCGGGTGCAAAGCCAATGCCTCGCTCTTTAATAAGCGCATCTGTAAAACCACGTTCTTGTAAGTATGTTAATGCTTG
TTCGCCTTCGACTGTCTTTGTTAAAGCGTAATAATAAAATTCTTGTATTAACTCATGCATTTCAATCATTTGTAAATCATCAGAAGCAATTTGA
ACATTTGAGTTAGATTGTGTTGCCTCAATATCTACAGCAACATTAACTCTATCACCTAATTCTTTAACCGCTTCAACAAATGATATGTCTTTAA
TTTCTTGAGTAAATTGAAAAACATTGCCACCTTTTTTACAACCAAAACAATGACAAATTTGTTTATCTTCAGAAACTGTAAATGAAGGTGTCT
TTTCATCATGAAAAGGACACAAACCTATATAATTGCGTCCTCTCTTTTCTAATTTTACATATTCACTTACCAAGTCTAAAATGTCGGTTTTATCT
TTTATTTCATTAATGATCGATTGATCTATTCGCAA

Gene: yqfL (positive regulator of gluconeogenesis)

Position: 1623221 to 1624039, length: 819 nt, orientation: REVERSE

Perfect match to: (930918-3-ABFA01000002-[84377:85195], highly conserved allele)

Sequence:

TCATTTCGATTTATTTTGTTCAATATAATGGATTATATCGTTTGCTGTTTCTTCGATTGCTTTTTGAGAAACATCAATGACAGGACATCCAATT
TCACTTACGATTTCTTCAAAGTAATTCAATTCTTCTTGAATTCGTGCTTCTGTTGCATATCGAGCTGTGTCACCTAGTCCTAATTGTTTTAGTCG
CTCTTTTCTAATGCGATTTAATTTTTCTTCACTTATTTTAAGTGCGATACATTTCTTTGGATCAATATCATATAAGCCATCTGGCGGTGTCACTT
CTGGTACAATCGGTACATTCATAACTTTGTAACTCTTATGCGCTAAATACTGAGATAATGGTGTCTTTGAAGTTCTCGAAATACCAAGTAAAA
CAATATCAGCTTTAGGTAATCCTTTAGGATCTTTACCATCATCATATTTAACTGCAAACTCTATCGCATCAATTTTCTTGAAATATGCATCATC
TAATCTATGAACGATACCTGGCTCATTATAAGGTTTTTCTTCAACCGAAGCTGATAATAAATCCATTAATGGCCCCATGATATCGACAGACTT
CAATTGGAATTCTGCTACTTTCTCACTCATATATTGCTTCATTTCAGGTTTAATAAGTGTATAAACAATGATAGCATTTGTATCTTTTGCAACT
TGAATCACTTCATCAACATCTTCAAAAGATTCAATATATGGATATCTTAATAATTCATTTTTACATTGCTTAGGATTGAATTGTGAAATACCTG
CCCTAGCAACTAACTCTGCCGTTTCACCTATAGAATCTGAAGCTACGATAATTTTAATTTTTTCCAT

Gene: ccpN (catabolite control protein N)

Position: 1624050 to 1624673, length: 624 nt, orientation: TRNC-RVRS (no start codon)

Perfect match to: (11819-97-CP003194-[1674432:1675055:r], highly conserved allele)

Sequence:

CTATTCTTTATATAATGCTACTAATAACTTAGCTATTGTTGTTTTGGAAATTCTTCCAATTACTTCATACTTTTGATTATCTTTTTTTCTTACAAT
TGGAATCGAATCAATTTCTTTTTCAATCATTCTATCTGCTGCGTATATGACTAATTCGCTTTCCTCTAAATAAGTGACATTAGGCATACGTGTC
ATATTTACACTGATAGGTACTGTATGAATATCTGCTCCAATCATTGAAGCTCTTAATAAATCTTTTCTTGAACACACACCAACAAAATCGTTAT
CTTCATTAATAATAAATAATGTACTTACATCTTCTAAAAAAATTGTACAAATAGCGTCATAAACTGTTGTATTCTCTCTTAGCACAACAGGTTG
AGACATATAGTCCTTAACCTCAAATTGTCGAAGTTTTTCATTAAAAAATTTACCTTTTGATTTACCTGAATAATAATATCCAACTCGGGGACG
CGCTTCTAAAAAACCTGACATTGTTAATATCGCTAAATCTGGTCTAAGCGTTGCTCTTGTTAAATTCAACTTATCTGCTATTTGTTCACCAGTA
ATGGGTCCTTTAGTTTTAACAATTTCGATGATTCGTTCTTGTCTTTGACTGAGTTCTAT

Gene: glyS (glycyl-tRNA synthetase)

Position: 1625008 to 1626399, length: 1392 nt, orientation: FORWARD

Perfect match to: (11819-97-CP003194-[1675390:1676781], allele observed in CC80+CC12+CC361)

Sequence:

ATGGCAAAAGATATGGATACAATTGTTTCATTAGCAAAGCACAGAGGTTTTGTGTTCCCTGGTAGTGATATTTACGGTGGTTTATCAAACAC
ATGGGATTATGGTCCTTTAGGTGTTGAATTAAAGAATAATGTTAAAAAAGCTTGGTGGCAAAAATTCATTACACAATCACCGTTTAACGTTG
GTATCGATGCTGCAATCTTAATGAATCCAAAAGTATGGGAAGCTTCAGGACACTTAAACAACTTCAACGACCCAATGATTGATAATAAAGA
TAGTAAAATTCGATATCGCGCTGATAAATTAATTGAAGATTATATGCAAGATGTTAAAGGTGATGAAAACTTCATTGCCGATGGTTTAAGTT
TTGAACAAATGAAAAAAATTATTGACGATGAAGGTATTGTTTGTCCTGTAAGTAAAACTGCTAACTGGACTGAAATTCGCCAATTCAATTTA
ATGTTTAAAACATTCCAAGGTGTAACTGAAGATTCTACAAATGAAATTTTCTTACGTCCTGAAACAGCACAAGGTATTTTTGTAAACTATAAA
AACGTGCAACGTTCAATGCGTAAAAAATTACCATTTGGTATCGGTCAAATTGGTAAATCATTCCGTAATGAAATCACTCCAGGTAACTTCAT
TTTCAGAACAAGAGAATTTGAACAAATGGAACTTGAATTCTTCTGTAAACCTGGTGAAGAAATCGAATGGCAAAATTATTGGAAAACTTTT
GCAAGTGACTGGTTAACAAGCTTAAATATGAGCAGTGAAAATATGCGTTTACGTGATCATGATGAAGATGAATTATCTCATTACTCAAATGC
AACAACTGATATTGAATATAAATTCCCATTTGGTTGGGGTGAGTTATGGGGTATCGCAAGTCGTACAGACTTCGACTTACGTAAACATGCTG
AACACTCTGGTGAAGATTTCAGATACCATGATCCAGAAACGAACGAAAAATATATTCCATATTGTATCGAGCCATCACTTGGTGCAGATCGT
GTAACATTAGCTTTCTTATGTGATGCATATGATGAAGAAGGCGTTGAAGGTAGTAAAGATGCACGTACAGTTTTACACTTCCATCCTGCATT
AGCACCATATAAAGCAGCGATTTTACCTTTAAGTAAGAAATTATCTGGCGAAGCGATTAAGATTTTTGAGCAATTAAGTTCTAAATTCTCAA
TCGATTTCGATGAATCACAATCTATCGGTAAAAGATACCGTCGTCAAGATGAAATCGGTACACCTTATTGTGTAACATTCGACTTTGATTCAT
TAGAAGATAATCAAGTTACAGTACGTGACAGAGATTCAATGGAACAAGTTCGTATGCCAATCTCAGAGTTAGAAGCTTTCTTAACTGAAAA
AACAAAATTCTAA

Gene: recO (DNA repair protein O)

Position: 1626550 to 1627302, length: 753 nt, orientation: REVERSE

Perfect match to: (Strain_21269-AFTU01000053-[96573:97325:r], allele observed in CC121+CC5+CC8+CC239+CC4803)

Sequence:

TTATTGTTCCAATCTTTTTAATTGGTTGATTAGTTTCTGACTTTTAAAAAACATACCTGCATATTCACGATATAACATTAAAATGATATCTGAC
ATTTCATCAATAATTTCTTGATGGATATTCAATGAATTCATTTTATCTATTGGTAATTTTTGTAATACATCTAATAAATATAGTGTTTTATTCGA
TAATATAACTGCATGTACATCTTTAGAAGCCTCTTGCCTTGAAATCGCACCGTCAAACTTAAAACTATAACCTATTAAATCTGCTTGTGTGTC
ATTACCACTCACAGCACAGCGATTAAATGATGCAGTAAAACCAAATCGTTTCATACACTTTAACATAACTACGACTGACATTAACTGTGCAG
ATGTACCTGATTCTATTTTTTCAAGAACAAATTGTAATAATTGATAGTTATATGGTGCAATGTCACCTTCATCCATTGAGCGCTCAATAGTTTC
AGCTGCCAGAGAGGCATAACTGCTTACGTAAAGGTCCATTTGTAATTTATAATGTTGACTAATAACATCTACAGAATTTAACGTTCCCATACC
TCGCCACTGATTGTAAATAAACAAACCATAAACAAACAATTGCGTTTGCGCTTGTAAACCCGTCTTAACTTTTTTAGCACGCCTTGCCATAAG
TGGTACTTTTGCACCATGCTCATTTAAAATCGTGATAATTTTATCAGATTCACCATAATCAACTGCTTTGATGATAATCCCTTTTTGGCGCATT
AACAA

Gene: era (GTP-binding protein)

Position: 1627324 to 1628223, length: 900 nt, orientation: REVERSE

Perfect match to: (COL-CP000046-[1655751:1656650:r], highly conserved allele)

Sequence:

TTAATCTTGGTCTTCAACATAACCAATTTGGCGAATAAAGTTAACTTTGTTTCGCCAGTCTCTTTGAACTTTGACCCATAATTCTAAGTAAACT
TTAGAGCCTAGAAGCATTTCTATATCACGTCTCGCACGTTTTCCTACTTCTTTTAACTTTTTACCGCCTTTTCCAATGACAATTCCTTTTTGCGA
ATCTCTTTCAACATATATAGTTGCTTCGATATGAACACGATCTTCGCTTTCTTTAACCATACGGTCCACATTAACACCAATCGCATGAGGGATT
TCTTCACTTGTAAGATGAAGGATTTTTTCACGAATGATTTCACCCACTACAAATTGTTCAGGATGGTCTGAAATTTGATCATCTGGATAATAT
TTAGGTCCTTCGGGTAAATACGTCTTTAAAACATCAATAAAATGATCGACATTTAGCCCTTCTAATGCTGAAATAGGTACAATCTCTGTAAAG
TCCATATAACTTTGATATTCTTCAATCTTTGGCATTAATTCATCTGGATGCACTAAATCTATTTTATTTAATACTAAAAATACTGGTGTCTTAAC
ATTTTTCAACATTTCTATAATATATTCATCGCCTCGTCCAATTTCCTCATTGGCATTAACCATAAACATGATTGCATCTATCTCAGATAATGTAT
TTTTAGCGACTTTCATCATATAGTCACCTAATTTGTGTTTAGGTTTATGAATACCTGGCGTATCAATGAATATAATTTGCGCGTCATCTCTTGT
CATAACACCTTGAATTTTATTTCTAGTTGTTTGAGCTTTATCGGACATGATTGCTATTTTATGGCCGATCACTCTATTAACAAATGTTGACTTT
CCTACATTTGGTCTACCTATAATTGAAACAAATCCTGATTTATGTTCTGTCAT

Gene: cdd (cytidine deaminase)

Position: 1628224 to 1628628, length: 405 nt, orientation: REVERSE

Perfect match to: (N315-BA000018-[1603591:1603995:r], allele observed in CC5+CC8+CC22+CC239)

Sequence:

TTATTCTAAATCCTTTCCTGAAAATCCAAATGGTAGTAACTCTGCGACTGTCATCATAACCATATCTCCTTTATGATTTGTCATATACACAGGC
ATATCATCATCACATAATTCCTTCAAAACTTGACGACATGCACCACAAGGTGATGACGGTTTATCTGCATCTACGGTTACAGTTATTGATTCA
AAATCACCTGGTCTGTATCCTTGAGAAATTGCCGATACCAAACTAGCTCGTTCAGCACATATCGATAATGGATAAGAAGCATTTTCTACATT
GGTACCATAAAAAGTTCTACCGTCTTTCGTTTTTAAATAAGCCCCTACTTTAAATTGACTGTATGGCGAATATGATTCTTGTTGTGCTTTTCTA
ACTTCTTGAAAATAATGAGGTTGATAACTCAT

Gene: dgkA (diacylglycerol kinase)

Position: 1628639 to 1628983, length: 345 nt, orientation: REVERSE

Perfect match to: (COL-CP000046-[1657066:1657410:r], highly conserved allele)

Sequence:

CTAAAATAACGCTATAAAATGTGGTAAAAATACTATTAAACCTATAATAAATGCTAATATTGAAACTATAAGTACACTAAAAGCCGCAATAT
CTTTAGCGTATTTAGCTAAATCATGATATTCAACGGTCACTAAATCGACAACATATTCAATAGCAGTGTTTAAAGCTTCAACAGTGAGAACT
AATGCAATAGCAATGAGTATAAATATCCACTCAATCCGATTAATATTTAGTACGAGACCAAAGACAATAGCAACAATCATTGCAAACACATG
TAAAAGAAATTTATAGTCTTTTTGAATTAAGATTTTCAGCCCATCAAGTGCATATTTAAACCTTTTCAT

Gene: ybeY (ribosomal RNA endonuclease)

Position: 1628986 to 1629453, length: 468 nt, orientation: REVERSE

Perfect match to: (COL-CP000046-[1657413:1657880:r], highly conserved allele)

Sequence:

TTAGTCTCGTGTTAATCCATATGCGTTTAATATTGTATCTTGTCGACCAAACATTTCCTTTTCATCCGCTTCAGTCATATGATCATAACCTAATA
GATGCAAAAATCCATGTAATGCTAAAAATCCTAATTCTCGTTCAAAAGAATGTCCGTAATTGTTTGCTTGTTCTTGCGCTACATCCGTACAGA
TAATTATATCCCCTAAAACACGTGGTATATCAAGACCACTAAAATCAATCTCTGGCTCATCTTCTTCTAAAGCAAATGAGATTACATCTGTAA
CTTTATCTTTATCTCTATATGTTCGATTAATTTCTTGTATTTCTTGTTTATCTACAAATGTAACAGAAAGCTCAGCATCGTCTTCTATATGCTCTT
CTTTTTTAGCAAATTCTAATAAATCTTCAATTTGTTTATACCAAGCATCTTTAACTAAGCCTGTGTGATCGCTAAAATCTATCGTAAACAT

Gene: phoH (phosphate starvation-induced protein)

Position: 1629454 to 1630374, length: 921 nt, orientation: REVERSE

Perfect match to: (COL-CP000046-[1657881:1658801:r], highly conserved allele)

Sequence:

TTAATTCTCTCCTTCATAATGTTCAATGATCTTACTTACCAATGGATGTCTTACTACATCGCTCTGATCTAATTTCAATATACTTATACCTTTAA
CGTTGTGTAACCTACTGACCGCTTCCTTAAGTCCACTTTTAACACCTTTAGGTAAATCGATTTGAGTTTGGTCACCAGTAACTACCATTTTTGA
GCCAAAACCTAGTCTTGTTAAAAACATTTTCATTTGCGCATGTGTCGTATTCTGCGCCTCATCAAGAATTACAAATGCATCTTCTAATGTTCGT
CCGCGCATATATGCAAGTGGCGCTATTTCGATAATGCCTCTTTCAATAAATCGCTCTGTTTGTTCACGCCCAAGAACAGTATATAGACCATCA
TATAAAGGTCTTAAATATGGATCTACCTTTTCTTTCAAATCTCCTGGTAAAAATCCAAGTGACTCTCCTGCTTCAACAGCAGGTCTTGTTAATA
CAATACGTTTAACAGCACCTTTACGGAGTTGCTTTGCTGCATAAACTACAGCTAAGAATGTCTTACCTGTACCAGCAGGACCTATACCAAAT
ACTAAATCATTATTTTTCATGGCATTAACATATATACGTTGCCCCATCGTTTTCGCACGAATCGTCTTACCAAATGCATCTTTAGTTATCTCTTC
ATCATATAAATCTAACAGATGTTGAATTGTGTTATTATGCGCCATTTTAATAGCTGCTTCAACATCTTTAATTGTAATATTATTACCTAAATCA
ATAACCTTCAGCAAATTGATTAATACTGATTCCGCTTTTTCTACGTTTTCTATTTTTGTACCTTTAACGGCAACTTCTTGTCCTCTTGCATGGAT
GACAACATCGAAACTCTCTTCAATTGCTTTTAAATGTTCATCATTATTTCCAATTAAAGCTTGAGATTGGTTCAT

Gene: Q5HFI8 (putative protein)

Position: 1630705 to 1631403, length: 699 nt, orientation: REVERSE

Perfect match to: (RF122-AJ938182-[1561230:1561928:r], highly conserved allele)

Sequence:

TTATAATTGTTTTGGTTTAGCTAAAATTTCTGACCATATCATACCATTGATTACTTCATCTTTATCAAATTGGAACGCTGATTTAGTCAAATCTT
TTTCGACATTTTGAGAATTAAGCAACTGCTTTAATTTCAAACGTTTTGTACGTTCAGATAAGTATTTATCCTCAATAATATCTCTAGCACGTTT
TTCCATTTTAGCAATTTGCTTTTCTTTTTCTCTATCAATGTCAGTACGAATCGTTTTAATATCATCTCTAAGTGATTTTTCTAATTGACGTCTAAT
TTCGTCAGAATTATCTTGTCTAGAAGGTCTATGTTTCTGCTCCACCGGTTCTGCTTTAGGCACTGTAATTGGTTTCTCTGTCATCGGTTTTGTT
TCTGGTTGTTTTTTAGGTGCCATAGGTTCTACAACAGGTTTCGATTTAGGCTCTTCCTTTGGAAGTTCATCGAATAAAGGTGGTAACGTATCA
TCATATTTTCGTTTCGATGATTTCTTTTCTTCTTCATTTAATTCTTCACTTATTTCTTTAAACGTTCGCTCAATTTCTTCAAAAAAGCCACCTTTTT
TTGGTTCATTATCGGTAGATGTTTTTTGAGGTGGCTTTTGATTTTGTCTATCTTTATGACTATTTTCGCGCATAGTAGTAATGATAGAAATGAT
CACTGATATGACAAAAATTAGAATACCGACACTCAT

Gene: floA (flotillin A)

Position: 1631420 to 1632409, length: 990 nt, orientation: REVERSE

Perfect match to: (MW2-BA000033-[1652380:1653369:r], allele observed in CC1+CC15+CC80+CC188+CC772)

Sequence:

TTAATGTTCAGGTGACTCATCATCACTTTGATCAGTTCGTTTATTAATTGCATTTCTCATGCCTGTATCAGCTTCGATATTTTTCAAATTATAAT
AATCTTTAACACTGATATTACCTGAACGTAATGCTTCAGCCATAGCTAATGGTACTTCAGATTCGGCTTCAACTACTTTAGCATGCATTTCTTG
TACACGCGCTTTCATTTCTTGCTCAGTTGCTACAGCCATAGCTCTACGTTCTTCAGCTTTTGCTTGTGCAATATTTTTGTCTGCTAATGCTTGTT
CAGTTTGTAAGTCTGCACCAATATTTTTACTAATATCAACGTCAGCAATATCAATTGATAAAATTTCAAATGCAGTACCTGAATCTAAACCTTT
GCTTAAAACTGTTTTAGAAATATTATCTGGGTTTTCAAGTACTTCTGTATGATGCTTACTAGAACCAATTGTTGAAACGATACCTTCACCAAC
ACGTGCGATGATTGTTTCTTCACCAGCACCACCAACAAGTCGAGCAATATTAGCTCTAACTGTGATACGAGCTTTGGCTTTCACTTCAATACC
GTTCATTGCTACACCTGCGATAAATGGTGTTTCAATGACTTTAGGATTAACAGACATTTGAACCGCTTCTAATACGTCACGTCCTGCAAGGTC
AATTGCAGCAGCACGTTCGAAAGGAAGATCAATGTCAGCACGTTGTGCAGCAATATTAGCGTCAACAACTCTGTCAACATTTCCTCCTGCTA
GATAATGCGATTCTAATTGGTTTGTTGTTAATGCTAGTCCTGCTTTATGCGCTTTAATTAATGGCGCTATAACTTTTCTTGGAGATACACGAC
GTAAACGCATACCAACCAATGTACCTATACCAACATGAACGCCAGCTGCTAACGCTGAAATCCATAAACCAATGGGTACAAATGAGAATAA
AATAAGTAATGCAACTACTATAATAACTGCTATTACGATAAAACTTAAACTAAACAT

Gene: yqeZ (putative membrane bound hydrolase)

Position: 1632427 to 1633134, length: 708 nt, orientation: REVERSE

Perfect match to: (11819-97-CP003194-[1682809:1683516:r], highly conserved allele)

Sequence:

TTAATCTACTTCCCTCACAACTACTCTTGTTCCTTCAACTTCAAGGATTTTTACCGTTTTATTGCGTAAAATAAAGTTGCCATCTGAAACAGCA
TCAATACGTTCATTTTCACAAAAAATAATCCCTGCAGGTCGAAGATCTGTAACTGTTTGAGCAGTCTTTCCTACGAGGTGCGAGCGGTTATC
ATGAGAATTGTAACCTGACTCAGAATTAGTTGAATCTTTTAAGATAACTTTATCCAAAAACGGAATCTTTCTGTTGAAAATCTTCACTAATAT
CACCCATTCTACAATCGTTAAAATCAAGGCAACGATAACATTCGCAAGCATAAATAGCAAATTATCACCGAGCGTTGTTATGCTTATAGTTA

TCAGTATCATGCCAATAATACCAATTACTGCACCAACTACAAATAATTCAATTACAACTAATATAACGCCAATTGAGAATATTAAGATAGAAT
GCATATTGACATTTCCTTGGATTAGAAATCCCAAAAATAAAATAAGTAATGATAATGTGGCGATAATACCAGCTGCATTGATTTTTTTAGAG
TAAAGTTGATATACAAATCCTAAGAATGTCAAACAGGTTAATATTAACGTAAAAATAGGTTGAACAATTATATTACTAACTTGTTCAACCCA
AGTATCTCCAGCCGTTGATTCCAAGATAGTTGTCATTTGTAAAAAATTATTATAACTCAC

Gene: rpsU (30S ribosomal protein S21)

Position: 1633354 to 1633530, length: 177 nt, orientation: REVERSE

Perfect match to: (RF122-AJ938182-[1563879:1564055:r], highly conserved allele)

Sequence:

TTATTTGAATTTACGTTTACGTGCAGCTTCTGATTTCTTTTTACGTTTTACGCTTGGTTTTTCGTAAAATTCACGTTTACGTACTTCTTGGATTG
TTCCACTTTTAGAAACTGAACGTTTAAATCTACGTAACGCATCTTCAAGTGATTCATTTTTACGTACTACTGTTTTAGACAT

Gene: mtaB (tRNA methylthiotransferase)

Position: 1633823 to 1635169, length: 1347 nt, orientation: REVERSE

Perfect match to: (11819-97-CP003194-[1684205:1685551:r], allele observed in CC80+CC10+CC72+CC80+CC772)

Sequence:

TTAAACTAAAACTTCTCTATCAGATTTATTTGTTGCGAAATCAACAACTTTAATTGCTTGCCCTTCATTTAATGGATAATTTGCTTGCGTAATTT
TAACTTTTACAATTTGACCTATGAGTGATTCGTCACCTTCAAATTGTACTTTCATATAATTATCTGCATATCCAACTAATGTGCCTTCTGTGTCA
CCCTGTTCCTCAGGAATTACTTCAAGCACATCTTGATCAAATTTAGACGCATATAACTTTCCGAGTTGATTGCTTAGCGTAATTAACTTATGC
ACCCGTTCATTTTTAATTTCTTCATCAATTTGGTCATCCATTCTTGCAGCTGGCGTGCCAATTCTAGGAGAATAAGGGAAAACATGCAGTTCA
GAGAACTTATGCTTTACGATAAAATCATATGTTTCTTGGAACTCTGCTTCAGTTTCACCTGGGAAACCAACAATTACATCACTCGTAACTGCC
AAGTCTGGTAAAGCTTTATGCAATTTTGTTAATCGTTCTGAAAATCTATCCATTGTATACTTACGTCTCATACGTTTTAATACTGTATCTGAAC
CAGATTGTAATGGAATATGCAAATGACGCACAACTTTTGTTGAACGTTCTAAAACGTCAATTACTTCATCTGTAAGTTGACTTGCTTCAATTG
AAGAAATTCGAATTCGTTCTAATCCATTAATCGTTTCAAGATCACGTAATAATTGGGCCAAGTTATAATCTTTTAAATCTTGACCATATCCACC
TGTATGAATTCCTGTCAATACAATTTCCTTATATCCTGAATTCACTAGTTGCGTCGCTTGTTCAACTACTTTTTCCGGATCTCTTGAACGCATTA
AGCCACGAGCCCATGGAATAATACAGAATGTGCAGAAGTTGTTACAACCTTCTTGAATTTTTAATGACGCACGTGTTCTATCTGTAAAATAT
GGGACATCTAATTCTTCATATTTACGATTTTTCATGATATTTCCAACACCATTAATTGGTTGGCGTTCTTTACGGAATTCATCAATGTAACCTA
ATAGTTTATGTCTATCTTGTGTACCAACTACTACATCGACACCAGGAATTTCCATAATTTCAGCTGATGAAGTTTGCGCATAACAACCTGTTA
CACAGATTACAGCATCAGGATTTTGTCTTATTGCACGTCTAATTATTTGACGACTTTTTTTATCACCTGTATTCGTTACTGTACAAGTATTAAT
AACAAATACATCAGCATTCGCTTCAAAGTCAACGCGCTCATAGTTTGCTTCTTTAAATAATTGCCAGATTGCTTCAGTTTCATAATGGTTTACT
TTACAACCTAATGTGTGAAACGCAACTGTTGACAT

Gene: rsmE (ribosomal RNA small subunit methyltransferase E)

Position: 1635176 to 1635928, length: 753 nt, orientation: REVERSE

Sequence:

TCACCCCATTAATTCTTTTTCATAACTTATTGCACTTAACGCATACAATGGCGCAGTTTCTGCCCGTAAAATTCTCGGTCCAAGACCAACAACT
GTACTAGTATTACTAAATAATGAAATTTCATTTTCTGACAAACCACCCTCAGGACCAAAAATCATCAACACTTTATCCTGAGCCTTGAATTGT
TGTAAAGTTTGCTTGAAATTGCTTAACTCACCATCTTTTGCTTCCTCTTCATATGCAATAAGAATATAGTCATAATTATCAATAGTATCACAAA
TTAATTTTAAATTCGACTCGAATTGAATAGATGGAATCACTAAACGATAGCTTTGTTCAGCAGCTTCTTTAATTATTTTTTGCCAACGCTCTAT
CTTTTTGGCAACTTTTGCCTCGTTTAATTTAACAATTGAACGTTCCATGCTCACAGCTATAAATGATGAAGCACCCAATTCAGTAGCTTTTTGT
AGCAACCACTCATATTTGTCAGCTTTGATTAGTCCACTGCAAATCGTAACATTAACTGGCAATTCTGTATTAATATTTTGTTTTTCTTTTAAATC
AACTGCAATTTTATCACTTGTTATGTCAGCAATTTCACATAAATAAACTGTTTGATCATTAAAAGTTAAAATAATTTTACTACCAACATCATAT
CTCATTACATTTGTTATATGATGAATATCTTCTTTTTTTGTAATAAAAAAACGCTGACTTACATCAGCGTTTTGGTCTATGAAATAACGTTGCA
C

Gene: prmA (ribosomal protein L11 methyltransferase)

Position: 1635930 to 1636868, length: 939 nt, orientation: REVERSE

Perfect match to: (K12S0375-JYGF01000011-[116067:117005:r], allele observed in CC692+CC5+CC8+CC97)

Sequence:

TTATTCACTCACTTTCTGGCCAACAAGACAAACCCAACCGTTGTCATGTTGTTCTGAAATAATTTTAAAACCTACACGCTCCATATGTGACTGT
ATACCTTCATACTTCTCTTTTATAATACCAGAAGTAATAAAATAACCGCCTTCATTTAGAGTATTATAAGCATCTTCAATCATTTCATCAATAA
TATGCGCTAAAATATTTGCTATTACAATATCAAATTTTTCTGTTTCGTCTTTCAATAAGTTACCTGGAACAGCTTCAATTAACGTTTCACAATG
ATTTCTTCTGAAGTTTTCTTTAGCTACATTCACTGCCATTTCATCAATATCCAACGCTTTAATACGTTTTACACCGATTAGATGACTTGCAATAC
TTAATATACCTGAGCCAGTACCAACATCAATTACTGAATGCTGTGGCAATACATATGTTTCTATTGCCTTCAAACACATACTTGTAGTCGGAT
GATCACCTGTTCCAAAAGCCATACCTGGGTCGAGCTCAATGCAAAGCTCTTCATCCGCTTCTTTAGCATATGTTTCCCAACTAGGAACTATTG
TGAACTTCTTCGACGCTCGGAATGGATGGAAATAGTTTTTCCATTCATTTTCCCAATCCGTCTCTGCAATAATTTGCTCACTGAATTGAACGTT
ATGTTGATCAAGTTCATCTAAATTTAATAACTCATCTTTAATTTGCTGTCGCAACTTATCATCATAAGTCATTTCATTAAAATAGGCTTTCAAT
CTTACTCCCTTATCTGGATAATCCTCTTTTTTCAAAGCGTAAATTTCACCGTATTTATCTTCTGGTTGGTTAATTAAATCATCTGAATCTTCTAT
CACGACACCATTTGATCCATGATTTTCAAGTATATTGGTAGCCAATTCTACTGCTTCATGATTAATAATAATTGAAAGCTCTGTCCAGTTCAT

Gene: dnaJ (chaperone)

Position: 1636872 to 1638011, length: 1140 nt, orientation: REVERSE

Perfect match to: (Tager_104-CP012409-[2739774:2740913], allele observed in CC49+CC34+CC49+CC72+CC96)

Sequence:

TTATTCTCCCTTAAAGAATCTTTTTGCTCTATCTTTAAAATTCGAAGGTTGTTCATTAATTTCTTCACCATTTAATTGGGCAAATTCTTTCATTA
GTTCTTTTTGTCTATCTGTTAATTTAGTAGGCGTTACTACTTTAATATCAACATATAAATCTCCGTATCCATAGCCATGAACATTTTTTATACCC
TTTTCTTTTAAGCGGAATTGCTTACCTGTTTGTGTACCAGCAGGGATTGTTAACATAACTTCATTATTTAATGTTGGTATTTTTATTTCATCGC
CTAAAGCTGCTTGTGGGAAGCTAACATTTAATTTGTAATAAATATCATCACCATCACGTTTAAATGTTTCAGATGGTTTAACTCTAAATACTA
CGTATAAATCACCAGCAGGTCCTCCATTCACGCCTGGAGAGCCTTCACCAGCTAATCTAATTTGTTGTTCATTGTCGACACCTTCAGGTACTT
TCACTTCTAATTTAACTGTTTTATTTTCAGTACCTTTTCCGTGACATGTTGGACAAGCTTCTTCAAATTCTTGACCACTTCCATTACATTTAGGA
CAAACTTGTTCAGTACGAACTCTACCTAAAATTGTGTTTTGTTCTACAGCTACATGACCAGCGCCATTACAGTAACTACAAGTCTTTTTACTTG
TTCCAGGCTTTGCACCATCACCATGACATGTTTCGCATGTTACATCTTTACGGATTGAAATTTCTTTTGTTGTACCAAATACCGCTTCTTCAAA
TGTCAATGTCATTGTATACTGAAGATCATCACCTTTTTGCGGTGCATTTGGATCTCTTTGTCTGCCGCCACCGAAGAAAGAGCTAAAGATATC
TTCAAAACCGCCGCCACCGAAGCCGCTAAAACCACCAAAGTCAGAGCCATTGAATCCTTGTCCACCAAAACCTTGTGGCCCGTCATGTCCAA
ATTGATCATAGCTTGCGCGTTTATTATCATCACTTAAAACTTCATAGGCTTCAGAAATTTCTTTAAACTTTTCATCTGCACCTTCTTCTTTGTTA
ATATCTGGATGATATTTTTTCGAAAGCTTTCGATACGCTTTTTTGATTTCATCTTTTGAAGCATCTTTACTAATGCCTAAAACTTCATAATAGTC
TCTTTTGGCCAC

Gene: dnaK (chaperone)

Position: 1638147 to 1639979, length: 1833 nt, orientation: REVERSE

Perfect match to: (Strain_3989-ALWH01000022-[260582:262414:r], allele observed in CC772+CC5+CC97)

Sequence:

TTATTTTTTGTCGTCGTCTTTTACTTCTTTAAATTCAGCATCTTCTACAGTACTATCGTTGTTTTGACCAGCATTTGCACCTTGTGCTTGTTGTTG
CTGTTGAGCCGCTTGCTCATATACTTTTGCTGATAATTCTTGAATCACTTTTTCAAGTTCTTCTTTTTTAGATTTAATATCTTCTATATCTTGACC
TTCTAAAGCAGTTTTAAGAGCGTCTTTTTTCTCTTCAGCAGATTTTTTATCTTCTTCACCAATATTTTCGCCTAAATCAGTTAAAGTTTTTTCAA
CTTGGAATACTAGACTGTCAGCTTCGTTTCTTAAGTCTACTTCTTCACGACGTTTTTTATCTGCTTCAGCGTTAACTTCAGCATCTTTTACCATA
CGGTCGATTTCTTCGTCTGATAATGAAGAACTTGATTGAATTGTAATTCTTTGTTCTTTATTTGTACCTAAGTCTTTTGCAGTTACATTTACAAT
ACCGTTTTTATCGATATCAAACGTTACTTCAATTTGAGGTTTACCACGTTCAGCTGGTGGAATATCAGTCAATTGGAATCTACCAAGTGTTTT
ATTATCCGCAGCCATTGGACGTTCACCTTGTAATACGTGTACATCTACTGATGGTTGATTATCTACTGCTGTTGAATAGATTTGAGATTTAGA
TGTAGGAATCGTAGTGTTACGTTCAATTAACGTATTCATACGTCCACCTAAAATTTCAATACCTAAAGATAGTGGTGTTACGTCTAATAATAC
TACGTCTTTAACGTCACCTGTGATAACGCCACCTTGGATTGCAGCTCCCATTGCCACTACTTCGTCCGGGTTTACTCCTTTGTTAGGTTCTTTA
CCGATTTCTTTTTTGACAGCTTCTTGTACTGCTGGAATACGAGTTGATCCACCAACTAAGATAACTTCATCGATATCTGAGTTTGTTAAGCCA
GCGTCTTTCATTGCTTGGCGTGTAGGTTCCATTGTTCTTCTAATTAATGAATCTGATAATTCTTCAAATTTAGAACGAGTTAAGTTTACTTCTA
AGTGTAATGGACCGTTTTCACCAGCTGAGATAAATGGTAATGAGATTTGAGTTTGTGATACACCTGATAAGTCTTTTTTAGCTTTTTCAGCA
GCATCTTTCAAACGTTGTAATGCCATTTTATCTTGAGATAAGTCTACGCCATTTTCTTTTTTGAATTCTGCAACTAGGTAGTCAATAATTACTT
GGTCAAAATCATCACCGCCAAGTTTGTTGTCACCGGCTGTTGATAGTACTTCGAATACACCGTCACCTAATTCTAGGATAGATACGTCAAAT

GTACCGCCACCTAAGTCAAAAACAAGAACTTTTTCATCTTTATCAGTTTTGTCTAAACCATATGCTAATGCTGCAGCTGTTGGTTCATTAATG
ATACGCTCAACTTCTAAACCAGCAATTTTACCAGCATCTTTAGTTGCTTGACGTTCAGCATCGTTAAAGTATGCAGGTACTGTAATTACAGCT
TTGTCAACTTTCTCACCTAAATAGCTTTCAGCTGTATTTTTTAAGTTTTGTAAAATCATAGCTGAGATTTCTTGTGGTGTGTATGATTTACCTTC
AATATCTACTTTATAATCAGTACCCATATGACGTTTAATAGATTGAACAGTGTTTGGGTTTGTAATAGCTTGACGTTTAGCTACTTCACCAAC
TTGAGTTTCTCCATTTTTGAAAGCTACAACAGATGGTGTTGTACGTGAACCTTCAGGGTTTTGAATTACTTTTGGCTCATCGCCTTCTAATACT
GTTACACATGAATTTGTTGTACCTAAGTCTATACCAATAATTTTACTCAT

Gene: grpE (cofactor of DnaK)

Position: 1640048 to 1640674, length: 627 nt, orientation: REVERSE

Perfect match to: (11819-97-CP003194-[1690430:1691056:r], allele observed in CC80)

Sequence:

TTATTGGTTTACTTTGACCATTGATGGTCTTAATACTCTATCTTTAAGCTTGTATCCTTTTTGTAGTTCTTGAGTGATTTCGCCAGATTCAAAAT
CAGGGTTATCATCTTGAACTACAGCTTGGTGAATATTTGGATCAAATGCTTCACCTTCAGTTTTAATAACTTCAAGACCATTATCTTTTAGTGC
GTTAATCAAACTTTCATGCACCATTTGTACACCTTTTTGAAGAGATTTAAAAGTCTCATCATCACCTTCAATTTGAAGTGCACGTTCTATATTG
TCTATTGCTGGTAAAATATCTGTTAACACACGTTGTGCTTGATATGTTTTATTTATTTCATTTTCTTTTTGAATTCTACGCTTATAATTTTCAAAC
TCAGCGTAGAGCCTTAAATATTTCTCTTCGTTTTCATCTGCTAATTGTTGAAGTTCATTAATTTTTTGATCTTTTGGATCTATTTCTTCAATAAC
ATTCTCGTCAGACGTTTCTTCTATTGCTTCATCTTGTAAATGACCTTTACTTTCTTCAGCTTGTTCAACTGAATCATCAATATTTTGTTTGATGT
TTGTTTCTTCAACTGTTGATTCAGTGTTTTTTTTAACTGATTCGTCTTTATTTGTCAT

Gene: hrcA (heat-inducible transcription repressor)

Position: 1640706 to 1641683, length: 978 nt, orientation: REVERSE

Perfect match to: (MRSA252-BX571856-[1726822:1727799:r], highly conserved allele)

Sequence:

TTACCAAATTCTATTTAATAATTGAATGACATTTTGATAATGCATAGCTGTAGGTCCAATCACAGCGATTTGACCTTTTAACGTTTCATCAAAA
TGATATTGACTTGTTACAATTGAAATATCACTTAAGCTGTCATCAATTTCATTACCAATTTTTACATTAATATTTGGTGAAGATATATCTTGTA
ATAATTCTGCAATTCTATTTGATTCTATATATTGTAGAATGGGCTGAATTGAAGATACATTACTTTCATTCAATGCATCAATAAGTTTAACCTT
TCCACCCATATAAATGCTATTACTTTGATTAGAAATATGATTATTCATCGTATTTATCAATTTATTGATAAATATTTCTTCCTGCTCTGATTGAA
CAAAAGAGACAATATCATCTTGTAAATTCTGATTAAACTCAGTTAGTTTGTTAGTAACAAAATTTGATATTGTATTTAGTTTATCATTACTAAA
CGGTATGTCTGAAGCAAGATGTACATGCTCAACATGACCTGATGAAAATACGATAACCATTATAACTAAATTAGGATTAGCACGAATCAAA
TGTACATTATTGATAATATCTTGTTTATGATTAGGATGAACAACTAAAGTTGTATATTGAGATATATTTGATAATTCATCTGCAAAATATGTC
AATGCTGATGATACATCATATTGATTCTCAACTAACAATTGATTTAATCGTCTTAATTTATTTGTTTTTTGATGAGATGTTTGTTCAAGTAAAC
GATTGACATAATACCTAAAACCTAATTGTGATGGCGAACGCCCTGAAGAACTATGTGTCTTTTCGATATAGTTTAAATCTTCAAGCTGTTTCA
TCTCATTTCTAATTGTAGCAGGACTAACATTCAAGTTATGTCGCTCAATTAGTGTTTTAGAACCAACGGGTTGTCCAAAATCAACATAATCCT
CAACAATTGCGTTTAATATACTCAATTGCCTATCTGTAATCAT

Gene: hemN (oxygen-independent coproporphyrinogen III oxidase)

Position: 1641784 to 1642941, length: 1158 nt, orientation: REVERSE

Perfect match to: (Strain_21340-AGTX01000034-[14107:15264], allele observed in CC188)

Sequence:

TTAATCATTTATTAGGAAAGCTTCAAAAACCTCATTACCTATGACTTTCCCTCTATTTGTAAGTGCAATCGCATCGTTCTTTTCTACAATTAATT
CCTTCTCTTTTAAATTATTTATTGTTTGACCAAAGACACTTTCAATAGATTGGTCAAACTTCTTTTTGAACCTACTACTACTCACACCTTCATTT
AAACGCAAACCAAGAAACATTTCTTCTTCCATTCTCTCAGTCAAAGAAGGTTTATTTGATACTAAAATTGCTTTACTTCTTTATTTATAGCTTT
GATATAATGATTCACTGGATTGATATTCGTATAACGCACACCATCTACATAACCACTTGCACCTGCTCCAAATCCATAATATTCCTCATTAAAC
CAGTAAACCTTATTATGTTCTGATTCATGGCCATCTAATGCAAAATTAGATATTTCGTATTGATGGAAAGGAGATTGTTCTATCTTAGACATC
AACAACTGATACATGTCAGCACCTAAATCCTCATTAGGGAGTTTAAGCAACCCTTTTCTATACATATTATAAAATTGGGTTTTAGGTTCAAGT
ATTAAGCCGTAACTCGAAATATGTTGAATATCCATATCTAAAGCTAGATCTAAACTTTGTTCAAAATCTTCAATCGTCTGTTTCGGTAAATGA
TACATTAAATCTAAACTGATTGATTTAATACCTGCGTTTTTAGCATTTAACACCGAAGTGTAAATATCTTCAGTATTGTGCGTTCTACCTAAAA
CAGACAATAACTCCGGCTTGAATGTTTGAACGCCCATTGAAATTCTGTTTACTCCATATTTTTCTAATAGTTGGACTTTCTCTTTAGTTAACTC

ATCAGGATTTGCTTCAAATGTATACTCGCCTGTGATTGTAAACGTATCACGTATTGCTTTAAGTAATCTTTCCAACTGATTAATAGAAAGGGC
CGTTGGTGTGCCGCCACCTACATACATGGTCTTTAAGTTCCTATATTTGCTGTAGACATTTCTGTTATTAGTGCATCTAAGTACTCATCTACA
GGTTGATTCTGTATAAAATATTTATTGAAATCACAATATGTACATATTCTTACACAAAATGGAATATGTATATATGCACTTTGTACCGTCATTT
TAATGCCCCGCTTCTCAGACTGTTTATCCAA

Gene: STAR (Staphylococcus aureus repeat element)

Position: 1643170 to 1643391, length: 222 nt

Sequence:

GGGAGTGGGACAGAAATGATATTTTCGCAAAATTTATTTCGTCGTCCCACCCCAACTTGCTTTGTCTGTAGAAATTGGCAATCCAATTTCTCT
GTGTTGGGTCCCCACCCCAACTTGCATATTATTGTAAGCTGACTTTTCGTCAGCTTCTATGTTGGGGCCCACACCCCAACCTGCATTGCCTGT
AGAATTTCTTTTCGAAATTCTCTGTGTTGGGGCCCC

Gene: lepA (elongation factor 4)

Position: 1643532 to 1645355, length: 1824 nt, orientation: REVERSE

Perfect match to: (11819-97-CP003194-[1693914:1695737:r], allele observed in CC80)

Sequence:

TTATTCGTCATCCATTTTCAATACAGCCAAGAAAGCATCCTGTGGAATTTCAACATTACCAACTGCTTTCATCTTAGCTTTACCTGCTTTTTGTT
TTTCAAGTAATTTACGTTTACGGCTTATGTCACCGCCATAACATTTAGCTAAAACGTTTTTACCCATTGATTTAATATTTGTACGTGCTACAAT
TTTTTGTCCTATTGCAGCCTGTACAGGTACTTCAAATTGCTGTCTTGGAATTAACGTTTTAAGTTTTTCAACTAATGCTTTACCACGTTCATAT
GCAAAATCTCTATGAACTATGAAGCTTAGAGCATCCACTTTATCACCGTTTAATAAAATATCCATCTTCACTAAATTACTTTCTTTATTTTCGAT
GAACTCATAATCAAATGATGCATATCCTTTGGTATTAGATTTAAGTTGATCGAAGAAATCAAATACAACTTCAGCTAAAGGTAATTCATAAA
CAATATTTACACGAATATCATCTAAATAGTCCATATTTATAAATTGTCCACGTTTACGTTGACATAATTCCATTACTGCACCTACATAGTCATT
TGGAACCATCATAGTTGCACGAACATATGGCTCAAATATTTTATCAATTTTATCACGATCTGGCATTTGTGCTGGGTTATCAACCGTCACTTC
TGAACCGTCCCTTAAAATACATTGATAAATTACAGATGGTGCTGTTGCAATTAATTCAATGCCAAATTCTCTTTCAATTCTTTCTTGAATTATT
TCCATGTGTAACATACCTAAGAAACCAGTTCTATAACCAAAACCTAATGCTTGTGATGATTCAGGCTCGAATTCTAATGATGCATCATTCAAT
TGTAATTTTTCTAATGCTTCTCTTAAATCATTATAATTTTTGTTATCTATTGGGAACAGTCCGCAATATACCATTGGATTCATTTTCTTATAACC
TTGCAGTGGTTCTGATGCAGGTCTACTAGCTAATGTGATTGTGTCACCAACCCTAGAATCATCAACATTTTTAATACTTGCAATAATATAACC
AACATCACCAACTGTTAATTCATCAACAGGAAGCTGCTTAGGTGTATTAATTCCAACTTCTGTTACTTCGAACTCTTTACCAGTGGCCATCATT
CGAATTTTATCTCCGGCTTTAACAACACCGTCTACAATTCTTATCGATGAAATTACCCCTCTATATGGATCATACTCAGAATCAAATATTAACG
CTTTTAGTGGTGCTTCTGGGTCGCCATCTGGAGCTGGCACAACTTCAACTATTTTCTCTAGTATCTCTTCAATTCCAATGTTAGATTTAGCACT
TGCTAAAACAACATCGTCTTGGTCTAAACCTATCATATCTTCAATTTCTTGTTTCACGCGTTCAGGTTCTGCAGCAGGTAAATCAATTTTGTTA
ATAACAGGCAATAACTCTAACTCATTATCTAATGCCAAATAAACATTTGCTAATGTTTGTGCTTCGATACCTTGAGCCGCATCTACTACTAAA
ATCGCACCCTCACAAGCTGCCAAAGAACGTGACACTTCATATGTAAAATCGACGTGTCCAGGCGTATCGATTAAATGGAATGTATAAGTATT
TCCATCTTTAGCTTCGTACTTTAAACGTACTGCGTTTAATTTGATTGTAATACCACGTTCTCTTTCTAAATCCATTGAATCTAGTAACTGATCTT
GCATATCTCTTGTTTCAACTGATTTGGTATTTTCTAAAATTCTATCAGCCAATGTAGATTTTCCGTGGTCAATATGTGCTATAATCGAGAAATT
CCTTATATTCTTTCTTCTTTTTAAGCGTTGCTCATTATCCAT

Gene: rpsT (30S ribosomal protein S20)

Position: 1645701 to 1645952, length: 252 nt, orientation: FORWARD

Perfect match to: (RF122-AJ938182-[1576162:1576413], highly conserved allele)

Sequence:

ATGGCAAATATCAAATCTGCAATTAAACGTGTAAAAACAACTGAAAAAGCTGAAGCACGCAACATTTCACAAAAGAGTGCAATGCGTACAG
CAGTTAAAAACGCTAAAACAGCTGTTTCAAATAACGCTGATAATAAAAATGAATTAGTAAGCTTAGCAGTTAAGTTAGTAGACAAAGCTGC
TCAAAGTAATTTAATACATTCAAACAAAGCTGACCGTATTAAATCACAATTAATGACTGCAAATAAATAA

Gene: holA (DNA polymerase III, delta subunit)

Position: 1645997 to 1646971, length: 975 nt, orientation: REVERSE

Perfect match to: (MW2-BA000033-[1666957:1667931:r], allele observed in CC1+CC80+CC188+CC361)

Sequence:

TTATAAAGATAGAATGAATAATTCCAGTATTAACTGTTTATCCATATATGATGATTTAAGTTTATAATCAGTTTCCGCACAAGCATCTATAAT
ATTCAATAATTCATCAAGTTGATAATGTCTTACTTGTCCTAACGCTAATTTTACTCTGTATGGATGTACGCCTATTGTTTTAGCAATTTGCTGT
CCACTATATCCTTTTTGACTCAAAATCTTACATTGATAAAATAATCGGTAATTACTTGTGATTAGTGCAAGTAATTTAATAGGTTCTTCTTTCA
TAGTTATTAAATCTTTTACTAAATGAATTGCTTGTTCTTTCTTTCTTTTCTGAATGTATTCAGTCAGTAAAAATACATTTTGCTCTAAACTTCTAT
TAATAATTTGGTTAACATCCTGCTTATTAATTGTTGGTCTATCGCCTAAAAATAAAATCAACTTTTCTATCTCTTGTGAGACAATATTAAAGTT
AATACCTGTCAACTCAATAAATAAATCTAATGCATCTCTTTTGATATCTTTGAAATTCTCATTTAATTTACTTGAATCCATTTTTTTATTTCTTC
TTCAGACATTTGCTCTATTTTTTTAAGCCTTGCATGCTTTTTTAGAGTTTTAGTTAACTTTTTTCTTTCATCAAGTTTATTTTGATATATCTCAAA
GACAATCAAATTTTCGCCATCATATTTTTCAATAAATTCTATTAATTGATTTACATTATGAGCCATATCTTTTGGCGCTTTTTCACCTGTAAATA
TATATGCATTTTTAACCAAAATTGCTTTTTTATCTGAAAAGAAAGGCAATGTTAATGTTTCTTCAACAATTGGTGCAATCTCTGTTTCGTATAA
ATTATATTTCACAAAGTTAAAGTCATCTCTATCACTTTTCAAAAATTGTGATATAATTTCTGCACTTTGTTTTTCAACCAATTCAGGCACATCTC
CATAAATAGCTACAATATTGTCGCTCAT

Gene: comEC (DNA internalization-related competence protein)

Position: 1647028 to 1649229, length: 2202 nt, orientation: REVERSE

Perfect match to: (Strain_118-AJGE01000022-[8560:10761], allele observed in CC772+CC15)

Sequence:

CTATAAACCACTTGCATTTCCATAAGAGTTTGAATCAACTTTTAAATTATCATCTAAGTCAATTGTAACTTGACCGTTTTGTTGACTATTGTAA
ATGCGACTGCGAATCCTTTGCAATCGTTTAACAACTTCTATATTAGGAAGATGATACATATTGTTCTTCCCAGAAGAAATCAAACTTATTTTA
GGCTTAATCATCTCTATAAATTCTTTAGAACTACTTGTCTTGCTCCCATGATGACCTACTTTTAAAATATCAATCTCCGGCAAGTTATATTTTTT
TAGTAGTAAAGATTCATTATTTTTACTAGCATCGCCCATTAATAAAACTTTTTTATTTTGATATGTAATCATAGTAATAATCGAATACTCATTTT
TATCTCGGCTATTTGGAATAAAACTATCAAAAAATAGAAAACTACTATCTCCAAGTTTAAAACTACTAACTTGTCTTACATCTATAAGTTTAAT
GTTGTACTTATGGCTTAATTTCGATAATAACATCAATGTATTACTACTATATCCCTTATTGTATATCACTATATGTTTAATTTTAATATGACTAA
TAATATATTCCACTTCACCAATATGGTCATTGTGTGGATGTGTTAAAATTAGATACTCTAATTCATTTATCCCTCTTTCATTTAGCGTTGGTAA
AATATGATATTTAGAAATTGAATAACTAGGTTGTTTAGTATCATCAATCACTTTCCCACCTGTATCAATCAAGACATTTTGGTTCTTACCGCCT
TCATATAAAATACTGTCTCCCTGCCCCACATTTAACATTGTAATTTTGTGATGTGAATTTGTTGGAAACGTTATTAATAATGTCAGAATAATTA
TAGTCCAAAACGTAACCAATATATATTTACGTTTAGCCAATAACCAAAATATGTAATAAACAGAAATTATAAATATTATAAATATCCAATCAT
TAAACTTGGGAACAGAAAAATGTGATTGCTTAATTCTTGTGAATAGGTCTAGTAACCAGTCATGAAAATTAAAACTTAGGTCAACCAAGTA
ATTTAGCGGCGTTAATCCCACAATAAAATGACTTGTAATAAAGAATAAAATAGATAGCGGAAACAATATAATCGAATAGTACGGTACAAAA
ATCAAATTAGATAAAAATCCCACCCATTGAAGTTGATGAAAGTTTGGAATGGCAACGATAAATGAAGCTAATTGTGCAATAAACGTAATTA
TGAATAATGATTGTAACTTTGACAATTGCTGTAAAAAAGGAAAAAGTAGCATAATAAAAAATGAAATGATGAATGAAAATTGAAATCCAAT
ATCATAAACAACTAGTGGATTTAAAATAAACATAATTATAAATGCAAATGCTAATAGCTGAATACCCTTTATTTTAATTTGCTTAGTAATAAG
CAGTACAAGAGTTGTCATTATTATAGCTCTTACAGCACTAGGTGCATAATTTGTGTATTGAGCAAATAAAGCTAATACAATGATTGTAATTCC
TTTAATGACAAATAAAGGTAAATTTAATCGTTTTAAAGGTTTGTAAATTAAGAATACAATTGCAGCTATATGCGAGCCACTAACTGCCAGCA
AATGATATATACCTATCTCTTTAACACGTTCCTTAAATTGCTCATTAATTTCTTTTACGTCACCAGTAATCAATGCCATAATACGATCCGGAAA
CTTAATACCCGAATCATAAATTCGATTCATTATAAACTGTTTATGTTTCTCAATTAAATTAGACCGGTTCGATTCTAGGCAGCTTTGTACAACT
ATACTTTGTAATTTAAGAGTTACAAACTTATTGTCATTAACTTTGAATTGTCCTTTAACAATACAATTACGACTTTCAATTTTCTTTAAATCAAA
ATTCTTTTTATTTGTTAAAAAGAAAGGATATATTTCATTTTTTAAACTCAACCTACCTTTATATGTGTCACTACCTTGACGTTGAATTTGGATTA
CTTGAGCACGCTCATTAAACTGAGAATTACGTTCAATATAATTGATATAATTAAATATTGCTTGTTGTGAATAATGTAAATACCATGAGGAG
AAAATGATTAAAAAGAGAGAAATAGGGGCATAAACGATTTTATTTTTACGATAAGTAATATACAAAAGTAAAATGAAAAGAAATGTAGAG
AGCACTTTGCTAGAATTCCAAAGCACTCCTACAATCATTGATAACGCGACATACAGCAA

Gene: comEB (competence protein EB)

Position: 1649234 to 1649665, length: 432 nt, orientation: REVERSE

Perfect match to: (11819-97-CP003194-[1699616:1700047:r], highly conserved allele)

Sequence:

TTAACCTTTAGTCAGATATTTAGCAACATATTCTGGTGAAAATGGAATTTTTTTAAATTCAATACCAGATTGTTTGAGTAATTTAGTTGCATAT
TCATGGTTATGATAATCTTCTGCATAGTAGATACGCTTTATACCTGCTTGAATAATTGACTTTGTACAATTTAGGCATGGAAAATGAGTAACA

TAGATTGTTGCACCTTCAGTAGATACACCTTGTTTTGCACATTGTAATAAAGCATTCATTTCTGCATGTATCGTTCTGATACAATGTCCATCTT
CAATTAAACATCCTTCATCTATACAATGCACCTCGCCAGCTACAGAGCCATTATACCCACCAGCAATAATACGATTATCCTTAACAATCGTTG
CACCTACAGATAATCTTTGACAAGTTGAACGTAATGCTAGCAAATGACTTTGTGCCAT

Gene: comEA (competence protein EA)

Position: 1649787 to 1650473, length: 687 nt, orientation: REVERSE

Perfect match to: (11819-97-CP003194-[1700169:1700855:r], allele observed in CC80)

Sequence:

TTATGTCGTGAAATAAGATTTCAGTTTATCAAAAGTTTTACTTCCAAAACCTTTTACTTTTTTCAAATCGTCAATTTCTTGAAATGCACCTTGTT
GGTTGCGATATTCAACAATTGCATTAGCTTTAGCTTGCCCTACTCCAGGAACAGACATCAATTCTGATACAGATGCCGTATTTAAATTTACTT
TAGTATTATTTGTGTTCCCATTTTTTACGTGCACACTGTTTACTTCAATTTGTGGTTCAACATTCTTTTGTCCTTTATGAGGTATGAAAATCATT
TTTTGATCTGTTAATTTTTCAGACAAATTAATTCGACTTACATCTGCATCATCCAATAATTGTGCTTTATCAAGTAAATCAACTACTCTATCCTT
AGATGTCATTTTATAAACATTAGGATGTTTAACAGCACCTTTTACATCGACATATACAGGACCTTTATTTTTGGAATTATCTCCATCTTTGACC
TGGACATCTTCTAATTTGGACAAACTATTATTTTCACTAGTGCTTTGTTTCAGAGCAGTATCTTTATTTTCAAAATTTCTTGAAGTATAATCATC
TTGTCTCCAGAATATAAAACCAATTAATACCATAATTAAAACAACAGCACTTATAATATATAACTTCCATTGAGTTAAAAAATCTTTATAGCG
TAATAAAAAATTGATACAATAAAACCAC

Gene: yqeM (putative protein)

Position: 1650513 to 1651229, length: 717 nt, orientation: REVERSE

Perfect match to: (MW2-BA000033-[1671473:1672189:r], highly conserved allele)

Sequence:

TTATTTTTTCGCAATGAAAAACAATCTTTCTGCATCTTCATTATGTTCATCTATATTAAAATCAGTAAATGTTTCAACATGTTTAAAACCAACTT
GAGCTAACCAAGACAAATATGTCTTTTCATCAAATGTTCTCTGAAAATGAGACTCATCAAATCTTGAATATGTTTCATCTTCATGTCGAATGA
AAAATGTCATATCATGATAAACACTTAAAGGTAAATCTCCTTGCACAGCATCCCATGCTAAAAAAATGTCCCCTTTATCATCAATATAACTTT
GATTATTAAACAAAGTCATCATTTTATAAACAGTATGTACATCAAAAATAAATACACCTGAATCAGTCAGATGATGATAAACATTGATGAAT
GTTTCAATCACTGCAGTTTCATCTTGCAAATAATTTAGAGAGTCACAAAAAAATAGTGATGATATCAAATTGTTGTTGCAAATCAAAAGATGT
CATATCTCCTTCAAGCCAATTTACATTTGCTGATTTTTGAGCTGCAACAGTCAACATATCAACACTTAAATCCATACCAGTAACATTACCTAAA
GCTTCTAATTGAACTGTTAAACTACCAGTACCGCATCCAATATCTAAAATATTTGATTCATCTTTGCAGTGATTTTTTACAATTTCAAACCATT
TTTCATATGGTTGATCTTGAGTCAATTGATCGTACACTAGGCTCATTTCTGCATATTGCGACAT

Gene: rsfS (ribosomal silencing factor)

Position: 1651232 to 1651585, length: 354 nt, orientation: REVERSE

Perfect match to: (MW2-BA000033-[1672192:1672545:r], highly conserved allele)

Sequence:

TTAATACGCAACCTGACTATATGATTCTAATGGTGCATCTTGATATAACTTTTCAATATTATAATAATTTCTTTCGTCTTTATGGAAAACATGT
ACCACAACATCAGCTAAGTCAATTAATATCCAACGCGCTTCATTGTATCCTTCCATACGTTTTACTTCTATATTTTGTTCATTGGCTACTTCTTT
CACCGCTCTAGCAATCGCTTGAACTTGTCGTTCATTATTTCCGTGCGTTACAACAAAATAATCTGTCATATCGCTGATACCTTTCATTTCTAAA
GAAATCGTATCTTCGCCTTTTTTATTGTCAATTGCATCCACAGCAATTGCTAATAATTCTTGTGAATTCAT

Gene: yqeK (putative hydrolase)

Position: 1651586 to 1652170, length: 585 nt, orientation: REVERSE

Perfect match to: (11819-97-CP003194-[1701968:1702552:r], highly conserved allele)

Sequence:

TTAATCATCCTTTATTCTTTCGTCACTATAGTTATAATAATTTAAACAGTCAATCGTCTTATTATATACCGTAATATCTTTCTGTATTAAAAATA
GTACTGTGCGTTTAGAAATTTCATAAATTGTCTTATCTAAACTACCTTGATTGTATGCCATATCTCGAATATCATCAACTCCTGGGATTGTTCT
TCCAGGTTCGATGTAATCTGCAATAAAAATCAGTTTTTCAGTTTTTGTCATTTGTTGACGTCCAGTAGTATGGTATTTGATAGCCATTAATACT
TCCTCATCATTGATACCATATTCATGTTCCATGATTGCTGCACACACAGGGCCATGCAATATTTCACTACCATAACTCAGTAGATCATTACCTA
ATTCGTATTGTCGAACAATTTGATACATTTTACCTAAATCATCATATTTACAGAAATCATGTAATACACCTGCTAATTCTACTTTACTAGTGTC
TCCATCATAAATTTCTGCCAATTTAATCGCTGTTTCTGCAACTCTTAAAGAATGATTATAACGTTTCTCTGGCAGTTTCTCTTTTGCAAGCCGT
TTTGCTTTTTCAATGTTCAT

Gene: nadD (nicotinate-nucleotide adenylyltransferase (Deamido-NAD (+) pyrophosphorylase) (Deamido-NAD (+) diphosphorylase))

Position: 1652160 to 1652729, length: 570 nt, orientation: REVERSE

Perfect match to: (COL-CP000046-[1680525:1681094:r], allele observed in CC8+CC80+CC97+CC239+CC4803)

Sequence:

TCAATGTTCATATAATCCTTCCCCCTTAATATAGTTTTCAACGGATTTAGGAACAAGAACTTGGATAGATTTCCCTTCACTAACTCTTTGTCGA
ATCATTGTCGAACTTATATCTACCCTAGGTATCTGAATTGCAATCATAGCATTTTCAACATTTTGACTATTTTTGTCTCGATTTACAACTACAA
AAGTAACCATTTCTTTTAAGTATTCAATTTGATACCATTTCTCTAGTTGGTTATACTGATCCGTCCCAATAACAAAGTACAACTCACTGTCTTT
GTGTTGCTCCTTGAATGCCTTGATCGTGTCATAGGTATAACTTTGACCACCACGTTTAATTTCATCGTCACAAATATCTCCAAAACCAAGCTC
GTCGATAATCATCTGTATCATTGTTAATCTGTGCTGAACGTCTATAAAATCATGGTGCTTTTTCAATGGAGACATAAAACTAGGTAAAAAAT
AAAATTCATCTGGCTGTAATTCATGAAATACTTCGCTAGCTACTATCATATGTGCAGTATGGATAGGGTTAAACTGACCGCCGTAAAGTACT
ATCTTTTTCAT

Gene: yhbY (RNA-binding protein)

Position: 1652732 to 1653022, length: 291 nt, orientation: REVERSE

Perfect match to: (11819-97-CP003194-[1703114:1703404:r], allele observed in CC80+CC1+CC772)

Sequence:

TTATGGCAATTCAATTTCTTTATTATCTTTAGATTCTCTATAAATCACTATCATAGATCCAATCACTTGCACTAATTCACTACGCGTAGCTTCGC
TTAATGTTTCAGCTAATTCTTTTTTATCATCAAAGTTATTTTGTAGTACATGTACTTTAATCAATTCTCTGTTTTCTAACGTATCATCTATTTGTT
TAATCATATTTTCGTTGATACCGCCTTTTCCAATTTGAAAAATCGGATCAATATTGTGTGCTAAACTTCTTAAGTATCTTTTTTGTTTGCCAGTA
AGCAT

Gene: aroE (shikimate 5-dehydrogenase)

Position: 1653026 to 1653832, length: 807 nt, orientation: REVERSE

Perfect match to: (11819-97-CP003194-[1703408:1704214:r], allele observed in CC80+CC12+CC361)

Sequence:

TTATTCTCCTTTTAATTGTTGTAAAACTGCTGTTTTCATAGAATTAATATCAGCATCTTTATTAGTCCAAATTTTAAAGCTTTCCGCACCCTGGT
AAACAAACATATCTAAGCCATTATAAATATGGTTTCCCTTGCGCTCTGCTTCCTCTAAAATAGGTGTTTTATACGGTATATAAACAATATCACT
CATTAAAGTATTGGGAGAAAGATGCTTTAAATTAATAATACTTTCGTTATTTCCAGCCATACCCGCTGGTGTTGTATTAATAACGATATCGAA
TTCAGCTAAATACTTTTCAGCATCTGCTAATGAAATTTGGTTTATATTTAAATTCCAAGATTCAAAACGAGCCATCGTTCTATTCGCAACAGTT
AATTTGGGCTTTACAAATTTTGCTAATTCATAAGCAATACCTTTACTTGCACCACCTGCGCCCAAAATTAAAATGTATGCATTTTCTAAATCTG
GATAAACGCTGTGCAATCCTTTAACATAACCAATACCATCTGTATTATACCCTATCCACTTGCCATCTTTTATCAAAACAGTGTTAACTGCACC
TGCATTAATCGCTTGTTCATCAACATAATCTAAATACGGTATGATACGTTCTTTATGAGGAATTGTGATATTAAAGCCTTCTAATTCTTTTTTC
GAAATAATTTCTTTAATTAAATGAAAATCTTCAATTGGAATATTTAAAGCTTCATAAGTATCATCTAATCCTAAAGAATTAAAATTTGCTCTAT
GCATAACGGGCGACAAGGAATGTGAAATAGGATTTCCGATAACTGCAAATTTCAT

Gene: yqeH (putative GTP-binding protein)

Position: 1653846 to 1654946, length: 1101 nt, orientation: REVERSE

Perfect match to: (11819-97-CP003194-[1704228:1705328:r], allele observed in CC80+CC239+CC4803)

Sequence:

TTATAAAATAGAATTTCTTAATACAACATCAACATTTTTAGGAACACGAACGATTACTTTAGCCCCTGGTCCTATAGTTATAAAGCCTAGACC
AGAGATCATAACATCGCGTTTCTCTTTGCCTGTTTCAAGTCTAACAGCCTTTACCTCATTAAGATCAAAATTTTGTGGATTTCCAGGTGGCGT
TAATAAATCACCAAGTTGATTACGCCATAAATCATTAGCCTTCTCCGTTTTAGTACGATGTATATTCAAGTCATTAGAAAAGAAACAAACTAA
CGGACGTTTACCACCTGATACATAATCTATGCGCGCTAGACCGCCGAAGAATAATGTCTGCGCCTCATTTAATTGATATACGCGTTGTTTTAT
TTCTTTCTTAGGCATAATAATTTTCAATTCTTTTTCACTAACTAAATGCGTCATTTGGTGATCTTGAATAATACCTGGTGTATCATACATAAAT
GATGTTTCATCTAAAGGAATATCTATCATATCTAAAGTTGTTCCAGGGAATCTTGAAGTTGTTACTACATCTTTTTCACCAACACTAGCTTCAA
TCAGTTTATTAATCAATGTAGATTTCCCAACATTCGTTGTCCCTACAATATACACATCTTCATTTTCTCGAATATTCGCAATTGATGATAATAA
GTCGTCTATGCCCCAGCCTTTTTCAGCTGAAATTAATACGACATCGTCAGCTTCCAAACCATATTTTCTTGCTGTTCGTTTTAACCATTCTTTAA
CTCGACGTTTATTAATTTGTTTCGGCAATAAATCCAATTTATTTGCTGCTAAAATGATTTTTTTGTTTCCGACAATACGTTTAACTGCATTAATA
AATGATCCTTCAAAGTCAAATACATCCACGACATTGACGACAATACCCTTTTTATCCGCAAGTCCTGATAATAATTTTAAAAAGTCTTCACTTT
CTAATCCTACATCTTGAACTTCGTTATAATTTTTCAAGCGGAAACAACGTCTGCAAATCACGTCATCACGAAACATATTATGCTCTGGTACAA
AACCAGGTTTATTTTTATCTTCAGATTGAAGTGGCGCACCACAACCGATACATTTTAAAATGTCAGACAA

Gene: yqeG (HAD-superfamily hydrolase)

Position: 1654947 to 1655474, length: 528 nt, orientation: REVERSE

Perfect match to: (N315-BA000018-[1630256:1630783:r], highly conserved allele)

Sequence:

TCAATTTTCCTCCCATGTGATATAACCTTTTTTACTGAAATGACGTAATAATCGTCTTTCAATTAATCTATTAAACTTAGTAATAAAGCCATCA
GTTCGTTTAACTGGAACAACCATAATTGTATATAGACCTCGACGATTACCACCAAATACATCAGTAAGCATTTGGTCACCTATAACAACAGTT
TGATCTGGTCTGATATTCATCTTAGTTATTGCTTTATCAAACGCTTTCCCCATTGGCTTTCTCGCTTTAAAAATAAAATCGATGTCTAAATGCT
GACTAAAACTAGCAACACGAGACTCATTATTATTAGACACGATTGTAATAGTGATTCCTTTTTCATTAGCTTCCTTAAACCATGCTTTAACAC
GTTCTGTAGGTTCTTTAACATCCCAACCTACTAGCGTATTATCTAAATCTGTAATAATACCTTTAACGCCTTTGTCCACTAACTTGTCTAAATC
AATTTGAAATATTGATTGAACATATGAATTCGGCATAAAAAACTTGCGAACTAAACCCAT

Gene: mtnN (5'-methylthioadenosine/S-adenosylhomocysteine nucleosidase)

Position: 1655494 to 1656180, length: 687 nt, orientation: REVERSE

Sequence:

TTATAATTGAGACACTAATGCTTTAACAGTTTGACTTGATGATACAGCTGCTTTTTCTAAAAATGCTTCGAAGCTCATTTCCGCTTCTCCATTT
GCTAAGTCTGAAACTGCACGAACTACAACAAATGGTACATTAAATTGATAACATATTTGTGCAATTGCAGTTGCTTCCATTTCAACCGCCATC
GCATTTGGAAATGCTTTTTTAATTTTTTGGCGTTGTTCAACACTACCGATAAAGCTATCACCACTTACAATTAAGCCTACTTTAGCTGTTAATT
GTTGTTGTTGTACAACTTGAGATACTTTTTCTATTAAAGGTTTACTTGATTGAAATGCTACCGGCATCTGTGGTATTTGTCCATATTCATAACC
AAATGCTGTTGCGTCTGCATCATGATATTTTACATCATCACTTATAAGAACGTCACCTACATTTAAACTTTCATCTAAAGCTCCAGCAGAACCT
GTATTAATAATGACGTCCGGTTTAAACTTATTAATTAATAATGTCGTAGAAATTGCAGCATTAACTTTTCCAATGCCACTTTGGGTAATCACT
ACTTCTCTATCTTTTAAAATGCCAGTATAAAATTTAACATGTGCAACTGAAATTTCGCTTAATTGTGTTAATTTATTTTTTAATATTGTTACTTC
TTCTTCCATGGCACCAATTATACCAATCAT

Gene: Q5HFG1 (putative protein)

Position: 1656494 to 1656763, length: 270 nt, orientation: FORWARD

Perfect match to: (MW2-BA000033-[1677454:1677723], highly conserved allele)

Sequence:

ATGGCTGAAAATAATCAAAATAGTCTCGTAACAAAGATAGCTACATACGGCAGTTTTATTGCAATTGCGTCGTTTGTCATTTTATTTATAAGT
ATATTTTTAAAGTTTTCATTTAATATCGAGTTTACAGCAACAATTATGAACATATGTCGCTACACTCTAATTCTAGGATTTATACTTATGTCCTT
ACCTGATGTTGTTGATAAAAACATCAAGAAAATTATATTCGATGCATTTATTATCATCGTCTTTATCTTCTTTTTACTATAG

Gene: entX (putative enterotoxin homolog)

Position: 1656965 to 1657717, length: 753 nt, orientation: REVERSE

Perfect match to: (11819-97-CP003194-[1707347:1708099:r], allele observed in CC80)

Sequence:

TTATGATTTGAATAAATAGATATCTAAATGCAATTTGTCACTATTGATAGTTTTATTATCAGCATACATTTTTAATACCTCACGACCATACTCA
CCATTTAAATTATAAAAATCATATTCTACATTTTGTTCATCATCATAATGATATTTAACATACCCTTTAACAATGTCGCCACCTTGTTCGTACAA
CTTATACTTCTCATTCAAATAATTTCTTAATTGAACATCTAATTCTTGAATGGTAACAATTTTCTTTTTCGTTTTTACTGCTGTTAATTCTATTTC
TGCTTGATGTCCGTCAATCCATAAATTACAAGGTATATTCTTATAATCATCGTACTTATTATTGTCACTTAAAGTAACACCACCATAACTACAT
TGCGTTTTATTGGTTGCTCCCCCATGACATCCGTATCCATAATTTCCAGCAAATATATCGATATCCTTATTTATAAATTTCTTTGAAAGTGCCT
CATTTTCAAATTCAACTTTAAAATCCTTTTTCCAATCATCATTTACGAACATGTCATGAAATAACAAATCATGCTTCAACAGTCTATCGTTTGT
GTTTTTATTTTCAAGCTGATTTGGATTGATGAATGACATCTTAGCATTACTTAGACGCTTTGAATCAAACTTACTTTTATGATGTAAGTCTGAA
TATTCGATTGCACTTGCTGAATTTGTAAAAATTCCTTCTAACAATAATATTAAAATAAATCCTGTTAAATATTTAACTTCAAACTCCCCCAA

Gene: Q5HFF9 (Fido domain protein)

Position: 1658131 to 1659351, length: 1221 nt, orientation: REVERSE

Perfect match to: (Strain_333-ALWF01000008-[95953:97173], allele observed in CC772+CC8+CC88)

Sequence:

TTATTTTATAATCGAATTTAGAAATTCATCACTTATCTCATAAATTGTTGGTCTACTTTTAATTTTAACTAAGTAGTTATCATATTTTTTAATTGT
ATTATTAATTTTTACTCTTCCTACTCCCAATATGTTTTCTAAATCAATTAATGTTAATCTATTTCTTTTATTTCCAAACAATTTATCCATTGAAAG
GAGATACAAAAATTCATAATCTATCTGTTTATAATGACTAGTTAAATAAAGCGTTAATTTTTCAGTCGCATCCATTTTTGGTATTAACTCATCA
AGTATTCTTTCTTGTCCTGCTATCAATAGCTCAAGCATTGTGTCAATAAATTCCGTCAAATCACCACAATTCAAATAATTTGAAGCAGTCATA
AACGCCTTGTAATATTTCGATTTATTTCTATTTATAACATATGAAAATGTCAAAGCAGTATAATTATCATAATAATCACTCAAGAGCTTGGCA
ATTATAAATCTTCCAACTCTACCATTGCCATCATAAAAAGGATGTATATATTCAAACAGATAATGACTAGCCATGATTTTGAACGGCTGAGG
CGCATCAAAATATTTTAAAAATGTTAGCATTTCACCTATATATTCAACAATTTTGGTTTCAGGTTGTAACCCAACATGTATATATTTATTCGTT
GACCCATCATGCACACCGACAAAATTTTTACGAAATAGCTCTCCATCTAACTTATCTTGTTCGTTAATTTCATTTGAAACTAATTTATCATAAA
TCGCTCTAATGTCTCTTACATTATCAACTTTAATTTTTTTATTAAGTTCTATCTCTTTATATTGATCCACGAGGCCTCTGAACTTAAGAAATTCT
GATGCTTGGTTATTTAACGCATGTGCAATCTCTTGTTTAGTACTAAATACATTTTCAATTTCATTAGTACTTTGTAATTCATCGATTAATAAAT
CATTAAAATATTGTTCTCTAGCTGCATATGGTAAAGAATTAAGTGCTCTATCAATTTTTCTACTATTAATTGATATTAATTCTTGTTTTTTTGAT
AGATTTTTAGTCACCATAAAGAATAGAGGATATTCCAAATCATTAACTTTTTTTCCATTTTCCATAGGTATGATATTAATATTAGTATTGAAAG
AAGCTAAAGAATTAAATCTTTTAGTATACTCTTCCTTCATTTTACTTTCATTGTGTTCATGAAAAATACTTTTTAAAGTTCTGTAACCCAT

Gene: ycsG (putative branched chain amino acids transporter)

Position: 1659640 to 1660869, length: 1230 nt, orientation: REVERSE

Perfect match to: (Z172-CP006838-[1727535:1728764:r], allele observed in CC239+CC80)

Sequence:

TTAAAATAACTGAAGTACTTCTTTGAATGAGAATATACCTGTCAATATTGTAACAAGTACTGCAACGATACCAAAGATGAACATCCAATTTG
GGTGTTTATAATCACCAACAATTGATTTCTTTTTACTTGCAATCAAAATTGCACCTAACGTAATAGGTAAAATCCATCCATTTATCGCGCCTGC
TATAATTAAAAGGCTGATTGGTTTTCCAATAAATAAGAAAATCATTGTTGAAATAACGATAAACACAATCACAATTAAATTACTTCTTTCGTT
AAGTGATTTATGAAGTGTTTTTAAAAATGTTGCGCTTGTGTATGCTGAGCCAATTACTGATGACATAGCTGCAGCAAATAACACAATACCAA
AAATATTCTTTCCAATTGGTCCAATTGCGTGTTCAAAAACTGACGCTGGTGGATTTTCAGAACTTAGTGTCACACCTGTTACAACAACTCCTA
ATACCGCTAGGAATAGTAACGTTCTCATAATACCTGTAGTTAAAATACCAGCAATTGCTGATTGATTTACAAATGGTAAATATTGCTTACCTT
TAATGCCAGAGTCTAATATACGATGTGCACCTGCAAAGGTAATATAACCACCTACAGTTCCACCAACTAACGTAATGATTGGCAAGACTAAT
TTCATTGGATGTTCTGGCGCAAATGTATGCACAAAAGCATCACCATAAGGTGGATTAGAAACAAACATCACATATGCCACAACTAAAATCAT
CACAATACCAAGAATCATTGAAACAACGTCCATAATTTTTTGGCCACTTTTACTTACAAAGATTAATATTGCAAAGATTGCAGTAATAGCTGC
GCCCCATTTTACATCTAATCCAAAAATTGCATTTAAACCTAAACCGGCACCAGCAATATTACCAATATTAAAAGCTAGACCACCAAATGCAAT
CAATATTGAGATAACAGTACCAAGCCCAGGAACAACTTTATTTGATATTTCTTGACCTCTTAAACCAGTTACAACTAATATGCGCCATATATT
AATTTGTGCACCAATGTCAATGATGATAGACAGTAATATGGCAAATGCGAAACTTGCAAAAAATTGTGATGTAAATACTGCTGTTTGCGTTA
AAAATGCTGGCCCAATTGCAGAAGTTGCCATCAAAAATACAGAACCTAATAATAACCTTTTATGATTTTTTGTGAATTCAAAGTCACTTTCTT
TTTTAAGCTTTAAATTTCTCCCCAT

Gene: ycsF (uncharacterized protein family UPF0271)

Position: 1660881 to 1661633, length: 753 nt, orientation: REVERSE

Perfect match to: (N315-BA000018-[1636542:1637294:r], highly conserved allele)

Sequence:

CTATAAGGATTGAATATCAATGCCTTCTTTCATTAAAATTTCTCTAATTTTCGAAACAAATAATAATGCATGTTCTCCATCACCATGCACACAA
ATTGTATCTGCTTGTAACGTTACTTCCTTATTGTTTTTTGAAATAACTTTATTTTCCTTCACCATCTTTAAAACCTGCTTAAGTGCTTCGTCAGT
ATCAGTAATCACAGCATCACTTTCTTTTCTACTAACGAGCTGCCCATCATCTTCGTATCGTCTATCAGCAAACACTTCAGAAGCTGTAATTAAT
CCGACATTCTTTGCTTCTGAAATTAGATATGAATTTGCTAATCCTACTAACACTAGTGATGGATCAAAGTCATAAACAGCTTGTGCTATAACG
CTTGCTATTTCTCTGTCTTTTGCACCCATCTGATACAACGCACCATGCGGTTTAACATGATTAATTTTAACTTGATGAATGCGACAAAACCCTT
GTAATGCACCTAATTGATAAATCATCAAATTATAAATCTCGTCGTTAGAGATATCTATATTTCGTCTGCCAAAGCCTTTCAAATCAGGTAAAC
CAGGATGTGCACCTACTGCAACATTATGTGCTTTGGCAAGTTTTACCGTTTCATTCATTACATTTTCATCACCAGCGTGAAAACCACAAGCAA
CATTCGCACTTGTAATTAACGGAATAATTTGATGATCACCACCAAAGGAATAATTTCCAAATGCTTCGCCTAAATCACAATTCAAATCAACTC
GCAT

Gene: accC2 (biotin carboxylase subunit)

Position: 1661633 to 1662994, length: 1362 nt, orientation: REVERSE

Perfect match to: (T0131-CP002643-[1685135:1686496:r], allele observed in CC239+CC8)

Sequence:

TTATAATTCCACCCCTTTAACAATTTGATGTTTTTCTAAAAATTTAATATCAACATCTTTTGCATCTCCATCACGATATAGTGGATAATTTAAAA
CTGCATATAAAAAAATCGGCAGTTGTAGAAAATCCATCTATCACCATTTCATCTAAGGTGGCTTTCAACTTATCAATTGCTGAAGCTCTATCAT
GAGATTTTACAATTACTTTAGCTACTAAAGAATCATAATATGGTGAAACTTGATAACCGTGATATAGTAAAGAATCGACTCGCACATTAAAG
CCTTGAGGTAAATGTAACGCTGTCACTTTACCTGGTGTTGGTTGAAATTTCTTTTCAGGATTTTCGGCATTTATTCTCGCTTCTATCACATGAC
CATTAAATTGAATATCGCTTTGTGAAAAAGGTAAATGATTATGTTCCAATAAATACAATTGTGCTGCAACCAAATCACGTTCTGCTCGCATCT
CTGTAACAGTATGTTCAACTTGTATTCGAGCATTCATTTCAATAAAGTAATGTGCGGTATCAGTTACTAAAAATTCAATCGTACCTGCACTTC
TATAATTTGCTGCACGTGCAACTTTAACAGCATCGTTACATATTTGTTGTCGTCTTTCTTCAGTTAATGCTGCACAAGGAGATTCTTCGATTAA
TTTTTGATTTTTACGTTGTACAGAACAATCACGTTCCCCTAAATGTACAAAATTATCCTGCCCATCTCCCATAACTTGAACTTCAACATGTTTT
GCAACAGGTATAAAAGCCTCAACATAAACACGATCATCATCAAAGTATTTTTTTCCTTCACTTTTAGCTTCTTTAAATGCCTTTTCTAAATCTTC
AGCTTTCTTTACAATACGTATACCTTTACCACCACCGCCACTGGCAGCTTTGATAACAACTGGATAACCGATGTCTTTGGCAAGCTTCTCAAT
TTCAGACACATGGTTCACAGCACCATTTGATCCTGGAATCACAGGAACACCTGCATGATGAACTGTTTGTCTTGCTGTTATTTTATCCCCCAT
CATTTCCATCGTTTTTTTAGTAGGCCCTATAAACGCTATGCCTTGTTCCTCAACGGTTTGAGCAAATTTTGTTGATTCTGATAAAAAGCCATAT
CCTGGGTGAATTGCATTAGCACCAGTGATTTGTGCAGCAGATATGATGCGGTCAATATTTAAATAACTATCTAAAGCATTAGCTTCCCCAAT
ACATATAGCTTGATCTGCTAAATGTACATGCAAGCTTTGCTCGTCCCCTTTTGCATAAACTGCTACAGTTTCAATCCCATATTCTCTGCAAGCT
CTTATAATCCTTACAGCAATTTCACCTCTGTTCGCAATTAAACAACGAAGCAT

Gene: accB2 (biotin carboxyl carrier protein of acetyl-CoA carboxylase)

Position: 1663008 to 1663457, length: 450 nt, orientation: REVERSE

Perfect match to: (H19-ST10-ACSS01000047-[169624:170073:r], allele observed in CC10+CC72+CC80)

Sequence:

TTACTTAATACGTACCAAAACTTGGTCGTATTCAACATTTGTGCCATGATCAGCTACTATTTCAGTAATTTCTCCAGCAACATCTGTTGTTACC
TCGTTTAATACTTTCATCGCTTCAACATATCCTATAATATCTCCCTTGTTAACTTTGTCACCGACATTCACAATTGGTTCATTTAATTCTTTACTA
TCTTGTAAAAAGAATGTACCTACCATTGGTGATTTAATGTCATGATAATCATTTGTCGAAACATCGGAGTTATCATTCGCTTTTGAAGCTGTC
AAATCATTATTGTTCATACTTTGATTTGATTGATTACTGTGTGCAGCCAAATGATTCGAGTCAGTGAAGTCAATTTCTATTTCATCTTCAAAAT
TTTTATATTTAAATTTCTTAACATCATTTTCCTTCACTAATTTGATTATTTGTTCGATTTTTTCAATATTCAT

Gene: kipA (allophanate hydrolase subunit 2)

Position: 1663459 to 1664469, length: 1011 nt, orientation: REVERSE

Perfect match to: (CN1-CP003979-[1604150:1605160:r], highly conserved allele)

Sequence:

TTACAAATCCCCTTTTAAAATTGTTGCTAATTTTTTCGAAGTATGTCGCAAGCTAGATGTTTCAAAAATTGGAGTCTTTTGATGACTCTTAAGA
ATTTCATTAAACAGAGACATTTGTTCCCGATTCTTATCTACAGCTTCTTGGAATGATATCCATTTAAATTGAATTGTATCTTGTGGTTTCATCT
GTGCTAACTTTGGCAGATCAAATTTGCATACAGTTGCAATTTTGGTATAACCACCTATCGTTTGTTTATCATTAAGCAGAATAATAGGTTGAC
CATCATTTGGTACTTGAACACTACCAAGAGCAACCGGTTCAGAAATGATATCTGCTTGATTAATTGGTGCAACGCTGTCACCTTCCAAACGA
TAGCCCATACGGTCTGATTGTTCAGTAATTAAATATGGATGATTTACAATTTTCGCTCTAGCCTCTTCAGAAAATGCCTCGAATTGAGGTCCT
TGAAGAATGTGTATAATATTATTTTCTGGCAATAAATCGTCCTGTAAATGAATAGTCTTTCCAATGTTTTCTTTAAAGTCATTATTTATTTTCA
CTGTTATTACATCATTAGCTAATAACTTTCTACCTTTGAATCCTCCTATACTGCTTCGGGTATGTGTTGCATAACTTTCAGCAATAGAAGGTAC
GTTGATAGAATGACCAAAAGTAAGATAACCGCGTGCACCTTTGGTTATAGCACCTATTTTTAAAATGTCACCTTTCTCAGCTAATATGACAG
AATTCATTGATATAGTTTTATTATTTAGCGAAGCATTAATACTACCACCGGTTATAACAAATGTATTTTGCGTATTAAATTGAATGGTAGGAC
CAATCAAAGTATATTCAATCGCTGGACCATCATTGTTAATTAATGACTGCGCAACCTTAAAACTAAATTGATCCATGGCACCTGCGCCTGAA
AATCCAATATGTTCATAACCTTTTCTTCCTAGATCTTGTACCGTTGAAAAGAGACCTGGTTGTAAAATCTTAATTGACAT

Gene: kipI (allophanate hydrolase subunit 1)

Position: 1664459 to 1665193, length: 735 nt, orientation: REVERSE

Perfect match to: (COL-CP000046-[1692825:1693559:r], highly conserved allele)

Sequence:

TTAATTGACATTTTCAATCACCACCCAGTCATCAACATTAAAGTTGCCATCTGATATATCTCTTTCGATTTGTATAAATTTCTGTTCATCTATTG
CATAAAATTGTATCCATTCTCCTGCTTCGTACATTGACATTGGTTCACGCTCGCTGCTAAATACTTTTAACGGTGTGCGTCCAATAATTTGCCA
TCCGCCAGGAGAATCTGATGGATATAGTCCTGTTTGATTATTCGCAATACCTACAGAACCTGCATGAATTTTTAACCTTGGCTGATTACGTCT
AGGTGTATGTAGTTGTTCATCAAGTCCGCCTAAGTATGGAAATCCTGGCATAAATCCTAGCATATATATTAAATAAGGTTTACTTGTATGTTT
TTCAATAACTTGCTCAACAGTTATTCGATTATGCTTTGCTACTTCTTCAATATCTGGTCCATATGTACCACCATATTGAACAGGTATTTTAATA
ATACGATTGGTTTGATTCACAGCATGAACATTTTTTTCATTAAATTTGTTAAGTTCTAAATTTTCAATTAATTTAGAAGATGTTATAGCTTGTT
CATCAAAATATATTAGAACTGCTCGATACGAAGGGACAATATCTTGAATTTCTAATATTTCTTTTTCTCGTATCCACCGTACCATTGCTGTGAC
ATTACGATATGTCTCTTCGGATATTTTATTTTCAAAATAAATCATAATTGTCTGCTCGTTAATAAATCTTACATCCAC

Gene: greA (transcription elongation factor)

Position: 1665519 to 1665995, length: 477 nt, orientation: REVERSE

Perfect match to: (11819-97-CP003194-[1715901:1716377:r], highly conserved allele)

Sequence:

TTATTGGATATTAACAATTTTTACGTTCATTTCGCCACCATTAGGTAGTGGAACACGAACTTCATCATCTAAACCTTTACCAATTAACGCTTTA
GCCATTGGTGATTCATTTGAAATCTTACCATTAAATGCATCTGATTCAGCTGAACCAACGATTTGATAACTTTCCTCTTCATCACCTGGTAATT
CTACAAACGTTACTGTTTTACCAATTTTAACAATGTTGTTATCTCCAGTATCTTCAATGATTAATGCATTTCTTAACATATGCTCAATTCTTTGA
ATATCTTGTTCGATGAATCCTTGTTCATCTTTTGCTGCATCATACTCAGAGTTCTCTGATAAGTCACCAAATGAACGTGCAACTTTAATTTTCT
CTACAACTTCAGGACGCTTAACTGTTTTTAATTCTTCAAGTTCACGCTCTAATTTTTCAAAACCTTCTTGAGTCATTGGATATTGCTTTTGATTT
TCCAT

Gene: udk (uridine and cytidine kinase)

Position: 1666023 to 1666646, length: 624 nt, orientation: REVERSE

Perfect match to: (RF122-AJ938182-[1596490:1597113:r], highly conserved allele)

Sequence:

CTATTGCTTGCTAACTAAAGACTGAATTTTTGTTGTCATAATATCTATTGCAACTTTATTGCTCCCACCTTCAGGAATAATTATATCAGCATAT
TTCTTAGTCGGTTCAATAAATTGGTCATGCATAGGTCTAACAACACTTAAATATTGATTGATAACAGAGTCCATTGAACGCCCACGCTCTTTA
GTATCTCGTGTTAAACGGCGTAATATTCTCAAGTCTGCATCTGTATCAACATATATTTTAACATCCATCATATCACGTAATACCTTATTTTCTA

AAGCGAAAATACCTTCTACGATAATAACATCTTTAGGTTTAAAATCAATGGTAATGTCACTTCTTGTATGACTAGCATAATCATATGTCGGTA
CTTCTACTGCTTTACCATTTTTCAAGTCTTTAAGATTTTCAATTAATAAATCATTATCGAATGCAAATGGATGGTCATAATTGGTTTCTAGGCG
CTCGTCGAAAGTCAAGTGCTTTTGATCTTTATAATAGTAATCTTGAGCAAGTAAAGCGACACTATGACCTTCTAAGTTTTTCATAATTTCGTT
AGTTACAGTTGTTTTTCCTGAGCCAGATCCACCAGCTATGCCAATGATTGTAGTAGCTTTCAT

Gene: yrrO (putative hydrolase)

Position: 1666646 to 1667914, length: 1269 nt, orientation: REVERSE

Perfect match to: (11819-97-CP003194-[1717028:1718296:r], allele observed in CC80+CC239+CC4803)

Sequence:

TTAGCCAATTTCCTTTCTCATCATGTTGTTTGGATATATCGGGCGATCCACTTTAATTTGAACGATTTGTAATGGATGGCGCGCCGCGTCTAA
GCTGTTACCTTCTTCATCATAAATTGCTTCTACTACTTGTGTAAATGTTTCAATTTCTGGACCAAAGAATTCTATTTCTTGACCTGGTTTAAAGT
TATTTCGTTGTTGAATAGTCGCAATTTTTGTATCTTCATTATAGTCTAATACCAAACCACAAAAATCAAATGGTGATTTTTTAGATTGTTGTTG
ACCAAACATCTGTTCTTCATAACCAGGTGTTCCCTCAAAGAATGCTGGTGCAGTGTCTCTATTTGCACATTTATCTAACTCTATTAACCATTCC
GGATTAATCTTAAAGTTGTCAGGATCTGCCGCATACGCATCAATGACTTTACGATATACTGAGACAACTGTTGCAATATAATGAATTGACTT
CATACGTCCTTCAATTTTTAATGAGTCCACACCAATATCCATCATTTGAGGAATTGATTCGATTAATTTTAAATCTTTAGGACTCATCGCAAAC
GGTGTAACTTCACCTTGATTATAAAATACATCAAGTTCACCATTATCATCAACTTCTAATAATTCATAATCCCAACGGCAACTTTGACAGCAA
CCGCCTCTGTTGGAATCCCTTGCAGTCATATGATTACTTAATGTACATCTACCTGAATAGGCGATACACATAGCACCATGAATAAATGCTTCG
ATTTCAATATCTACTTTTTCCTTCATTTCACGCATTTCCATCGCGCCGGTCTCACGTGCTAATACAACACGATCCAATCCTTCTTCTTTCCAATA
TTCTACAGCTTTGTAATTAGAAAGTGATTGTTGAGTAGATAAATGAATTTCAAGTTTTGGCGCAACTTCTTTACATGTTTCGATAATTAAAGG
ATCTGCAACAATGATACCTGTCGCACCAGTCTTTTCCAAATTACGCAAATATGATTCTAGACCTTCAATATTCTCATCATGTGCAATAATATTT
GTCGTAACATAAATTTTGGCACCGTAACGGTTCGCAAATTCAACACCTTCAGCTATTTCTTCCATCGTGAAATTATCAGCATTTGAACGTAAT
CCATATTCTTGACCACCTAAAAATACGGCATCAGCGCCATAATGTACTGCTATTTTTAACTTTTCTAAGTTTCCAGCAGGTGCTAATAATTCTG
GTTTCTTCATAACTGTTTTAGGAGTTGATTTAATCTCTTCTATTGTCTTCAT

Gene: yrrN (putative hydrolase)

Position: 1667926 to 1668849, length: 924 nt, orientation: REVERSE

Perfect match to: (COL-CP000046-[1696292:1697215:r], highly conserved allele)

Sequence:

TTAATATACTGTTTGTTTATATAAGAAACCTTCGTCAAATGGTCGATGATCAGGTTGAATTTCTTCAATTGGATCAATCAACATAAATTTCTCA
TCTTCATAGATTTCAGGATCTTCATTGTACAAATCTATCGCTTGACGATACTGTTCCGTTACCACATTAATATATTCTTCCGTTTGTAGAATAC
CATCGATTTTAAATGAATCTATACCCGCCTCAAAAAATGGTGCTAATTCTTCAATTAAACAAATGTCGTTTGGTGACATAATGTGCGTACCAT
TGTAATCTTCGTAAACTGGGTAATTATTTTGTCTTTCTTCATCATAAAGTAATAAAGATTGTTCATCATTGCGACGTTCAATTTTCATTTGGCG
ATCTTGGAACGTATAATAATTGCCTAGTAGCATACGCTTTGATTGGAACATACAAGTCATTCCTTGAACTTGCACCTCAATTTCCACATTTGA
ATTTTCTTTTATATTAATAATTTCATCCAAATTCAGCTCACGTGCTAAGACAGCTCTTGATGCGCCTCTTTTACCCCAGTAATTACATTGAAAA
TGATTAGTTACTAACGTCTCTGCATTCCAATGAAGTGGTATTGGATTTTCTTGCGCCTTCACATACATTACTACTGCTGGATCCCCGAAAATA
ATTCTGTCAACTCGTATTTCATGTAAAAAATTAATATAATCTTCTACAGCATCTAAATGATAATTATGAAATAATCCATTCACTGCCGCATATA
CTTTTTTATCGTTTTTGTGAGCTAATGCGACAGCCTCTGTCATTTGTTGTCTATTGAATTCCCCTGGAAGTCTTAAACCAAACTTTTGCTCGCC
AATTACAAAAGCATCTGCACCTAAATCAATAAGTGTTTCCATATGGCTTAATGACTTGGGTGTGACAAGTAATTCTGTCAT

Gene: yrrM (putative acyl-CoA O-methyltransferase)

Position: 1668852 to 1669490, length: 639 nt, orientation: REVERSE

Perfect match to: (N315-BA000018-[1644513:1645151:r], allele observed in CC5+CC80+CC772)

Sequence:

TCATTCTCCTTTAATTGAAATCGCTAATCCATCGTCTATATTTAAAAAATTCGTTGTATATCCTGGTTGCTTTATTAACCACTCATTATAATCTT
GAACCTTTTTAACCATTTGTCTTACATTTCTCGATCTAACAATCCCAATATCCGATACAAAACCGTGATATAAAACATTATCTGTAATTACGAG
ACCTTGGTGCTTTAAAAGTGGTGTATATATTTCAAAAAATTTCTTTGATTGCGCTTTTGCTGCATCAATAAATATCATATCATAAACTTTGTCA
TTTACATTTTCAAATTGCTCTAAAGCATTACCTTCAATAATTCGAACCTGGTTTTCAAAATGATAAGTAGCTAAATTTTGTTTAGCATATTGAA
TCATCGTTTCATTACGCTCTATCGTTGTGACATGAATGTCATCAGATATAGAAGCGAATTGCATAGAACTATAGCCGATTGCTGTACCAATTT

CTAAAATATTTTTAACATTATTCATACGAATTAATTGCTTAATTAAATCTAATGTTAAACGATCTACAATTGGCACTTCATTTCCCTCGGCAAA
TTCACGCAAAACTTCGATTGAACTATTTTGATGTTGATGTAAATCTATTAAATATTTTTTATTTAGGTCATCCAT

Gene: yrzB (putative anti-sigma factor)

Position: 1669775 to 1670083, length: 309 nt, orientation: REVERSE

Perfect match to: (N315-BA000018-[1645436:1645744:r], highly conserved allele)

Sequence:

TTATTCTTCCATTTCAGTATTTACAACTTCTTCAATCATGTCCCATTCTTCATCAGTTTCGATTGGTACTAACTTACCACCGTCACCTGACTCAT
CTGGTTCATTGATCATTGGTACAAGCTCAATCATATCGTCTTCATCTGATTGAGCACCTTCTTCAGCTAAGATAACATACTCTTTTTTGAATTC
AGGATGATAAAATTCTAAAACTTTTCGGTATAAAACTTCATTTCCCTCTTCATCGAATAAAGTTAATAATTCTTCTTCGTTATTAATTTCTAGTT
GTGAATCATGATTATGTTCAGTCAT

Gene: yrrK (putative Holliday junction resolvase)

Position: 1670098 to 1670526, length: 429 nt, orientation: REVERSE

Perfect match to: (RF122-AJ938182-[1600565:1600993:r], highly conserved allele)

Sequence:

TTAATGTAGTGAATCTAAATAGCCTTGTAAAATAAATACCGCTGCCATTTTATCAATCACTTGTTTTCTTTTTTGTCTTGAAACATCTGCTTCTA
ATAATGATCGTTCAGCAGCCATTGTGCTTAATCTTTCATCCCACATCACAATCTCAATAGAAGGATAAGCTTCTAATAATTTTTCTTTATATGT
TAACGAAGCTTCGCCTCGAAATCCTATTGAATTATTCATGTTTTTAGGTAGTCCTATTACGACTGTACCCACATTATGTTTTTTAATAATGTCT
ACTAATTGGTCAATACCTAATTCATTATTTTCTTCATTGATTCGGAGTGTGTCTAATCCTTGTGCCGTCCAACCCATTATATCACTAATTGCAA
TTCCTACCGTTCTACTACCGACATCGAGTCCTAAAATTTTATGTTGTAACAT

Gene: yrzL (putative protein)

Position: 1670530 to 1670790, length: 261 nt, orientation: REVERSE

Perfect match to: (COL-CP000046-[1698896:1699156:r], highly conserved allele)

Sequence:

TTATTTATTTTGCTCTTTTAAATAGTAAGAAACAAGCTCTTCCATAATAACATCTCTATCAATATGACGAATTTGATTTCTTGCTTCATTTTGGC
GTGGAATATACGCAGGGTCACCTGATAATAAATAACCTACAATTTGGTTTACGGCATTATATCCTCGTTCATCTAATGTTCGATAAACATTAT
TTAAAACATCTCTTACATCTTGCGTTGGAAGTTCTTCATAGTCGAATTTCATTGTTTTATCAAAGTTTTCCAT

Gene: alaS (alanyl-tRNA synthase)

Position: 1670853 to 1673483, length: 2631 nt, orientation: REVERSE

Sequence:

TTATAGATTTTTAATGTAATCTTTAATAAAGCTTAATGATTTTGAGATATTTTCAGGTTGTGTACCGCCACCTTGAGCCATATCTGGACGACC
GCCACCTTTACCACCAACGATTGGTGCCATTTGTTTGATAAGATCACCGGCTTTAACGTTATTTGTTAAAGATTTAGGGACAGTTGCAACCAT
CGATACTTTATCATCAACATTACTTGCAAGAATGATAATTGTATCTTGTAGTTTAGATTTAAAATCGTCCATTGTCGAGCGAATTGCTTTCGC
ATTTGGTACATCCACTTCAGTAACCAATACTTTATAGCCATTGATTTCTTCAACTTGATCTTCAATATTACCCATTTTAAGTGATGTGATTTCTT
TGTCACGTTGCTCTAATTGTTTTAATAATGCTTTTTCTTCATCTTGTAATTGTGTTAACTTATCGACTACTTGATCATCAGATTTCACTTTCATCT
GTGATTTCATCGTATTAAATTTCTCTTGAATATCTTCTAAATATAAGAAAGCTGCTTTACCTGTTAATGCTTCAATACGACGCACACCAGCTCC
TGTACCTGACTCACTTACTATTTTGAATAAGCCAATTTCAGAAGTATTGCGGACATGAATACCACCACATAATTCAATTGAAAATGGTGCCAT
ATTTACTACACGCACAACATCACCATATTTTTCACCGAATAATGCCATTGCGCCCATTTCTTTAGCTGAAGCAATATCCATTTCTTGAATGTTA
ACGTCAATACCTTTCCAAATTTCTTCATTTACTAAGCGTTCAACTTGATCAATTTCATCATTAGTCATTGGACCAAAATGAGAGAAATCAAAA
CGTAAACGATCTGCTTCTACTAGTGAACCAGCTTGGTTAACATGATCACCCAGTACTGATTTCAACGCTGCATGTAATAAATGTGTTGCACTA
TGGTTCTTTTGAATGTCACGTCGATCATTTTGGTTCACTTCAGCAGACATTGTAGCGCCAACATTTACTTGGCCAAATTGTACTACTCCTTTAT

GCAAGTTTTGACCATTTGGTGCTTTGGTTACTTCACTAACAGCAATTTCAAAATTGTCATTATAAACAATACCTGTATCCGCAACTTGTCCACC
ACTGACTGCATAAAATGGTGTTTCCGTTAACATGAAGTATACTGTTTCACCCGCTTCAACTTGTGAAACTTCTTCACCATTATATATCAAGTG
TGTTAGTGTTGTTTGAGCTGTCGCAGTATCATAACCAACAAAAGTACTTGCAGATGTAATATTTTTCAATACTTCACTTTGAACTTGCATTGA
TTGAGAATTTTGACGTGCTTGACGTGCACGATCACGTTGTTGTTGCATTTCTGACTCGAATGTTGTCATATCAACTTTCAATCCTGCTTGAAC
TGCTATTTCTTCAGTTAATTCAATTGGGAACCCGTACGTATCATACAATTTAAATGCATCTTTCCCATTAATTTCATTTGTTGTCGCTTTAGCTT
TTTTAATTAATTCATTTAAAATCGCTAAACCATCTTCTAATGTTTCATGGAATCGTTCTTCTTCAGACTTTATAACACGCTTAATGAAATCTGCT
TTTTCCTTAACATTTGGATAATATGGTTCCATAATGTCTGCAACAATATCAACAAGTTTGTACATAAATGGCTCATTGATTCCTAACGTTTGAC
TAAAACGAACGGCACGACGTAACAATCGACGTAATACATATCCTCTACCTTCATTGGCAGGTAATGCACCATCAGAAATTGCAAATGCAATC
GTACGAATGTGGTCAGCAATTACTTTAAATGCCACATCTTGTTCGTTGTTTACTAAATATTGTTTACCTGATACTTTTTCGATTTCATTCATTAT
AGGCATAAATAAATCTGTTTCATAGTTAGTACGTACATTTTGAGAAACTGAGGCCATACGCTCAAGCCCCATGCCAGTATCAATGTTTTTATT
AGGTAATGGTGTGTAACTATGATCTTTATTATGATTGAATTCACTAAATACTAAGTTCCATACTTCAAGATAGCGTTCATTTTCTCCACCTGG
ATACATTTCTTCTGCCGGATCGTCTTGTCCATATGCTTCTCCGCGATCATAGAAAATCTCAGTGTTTGGTCCTGAAGGCCCTTCACCAATATCC
CAGAAGTTACCTTCAATGCGAATAATACGACTTTCTTCAAGCCCAATATCTTTATGCCAAATGTTGTATGCTTCCATATCTTCCGGATGAATC
GTAACGTACAATTTATCTGGCTCCATACCCATCCATTTATCACTCGTTAAAAATTCCCAAGCAAATTCAATCGCTTCTTGTTTAAAATAATCAC
CAATTGAGAAGTTACCTAACATTTCAAAGAATGTATGGTGACGCGCTGTGAAACCAACATTTTCAATATCATTTGTACGAATAGCTTTTTGA
GAGTTTACAATTCTTGGCTTTTTAGGTGTTTCACGTCCATCAAAATATTTCTTTAATGTTGCTACACCTGAATTAATCCATAATAATGTATCAT
CATCAATTGGCACTAATGGTGCAGAAGGTTCAACCATATGTCCTTTTTCAACAAAGAAATCTAGATATTTTTGTCTAATTTCACTCGCTTTTAA
CTTTTTCAT

Gene: tbox07 (T-box leader element)

Position: 1673615 to 1673792, length: 178 nt

Sequence:

AAAAGGGACGAACGTTATCGCGGTACCACCCTAGTTATAAATGCAATTCAACACATTTATCACTTTAAATCGACTATACAGTTGTGCATAAA
GTAGCGTTCACTAATGTTTGTTGTACTTTTCACCAACCAGTACATCTCTGATAAACAAATCATTAACTACTCATCTTTATACGAAT

Gene: recD (ATP-dependent DNA helicase)

Position: 1673826 to 1676303, length: 2478 nt, orientation: REVERSE

Perfect match to: (11819-97-CP003194-[1724208:1726685:r], allele observed in CC80+CC5+CC22+CC4803)

Sequence:

TCAACGTTCTATAAAGTCATACGGCGTGATTTCTCCCATATTAATCATTGGGTCAATTTTAAACATTGTAGCTTCCGTTAATACATTTGTATCT
GTTTTTGTTGAATCAGACATAACTTCTTCACTATCATTCGATGACATTGGCGCTTCTACTTGATCATCTATTGTCGTTTGTGAAGCTCCTGTAT
CATTAGTTGCTGTGTTTTCCAGCATTTCTTCATCTTCTGAATTAAAATAATTTTTCAACAATGTACATAATTGTGTTAAACGCGCTTGACCATTT
GTTTTTAATCCAATATCAAATGCTTCTGGATCACCAAGTAGAACTAAACTCGTTTTCGCTCTAGTTAAACCAGTATATAATATCGGTCTTTGTA
ACATTCTAAAATACTGTTTAACAATAGGCATGATAACAATAGGAAATTCTGAACCTTGTGATTTATGGATTGATGTACAATAAGCATGTGTT
AATTCCATCATATCTTGTTTCGTAAATGTAATTTCATTACCTTCAAAATCCACAACAAGTACATCTTTATTAAGGGCATTTTCTTTCGCCCAAA
AAATACCAACAATAACTCCTATGTCACCATTGAATATATTATCATTTGGCCTATTAACAAGTTGTAATACTTTGTCACCTTTTCTAAAGACTAC
ATCACCAAACTCAATTTCTCGTGTGTCTTTCTTTTTAGGGTTTAAAATATCTTGTAAAACTTGATTTAAACGTTTAATACCGGCATTTCCTTTAT
ACATTGGTGCAAGCACTTGAATATCAGCCATAGTATACCCTTTATTAACAGCACTAGTAACTACCTTCTCTACAACTGTTGGTATTTGGTTTG
CCTGACAGTTAATAAAACTTCTATCATGAAAACGCTGTGTAATATCAATTTTCTGACCCAACTTCATTCGATGTGCTAATTCTATAATGCTTGA
ACCATCTTGTTGACGATATACTTCAGTCAGATTAACTCGTGGTATAGCTTTCGATTCAATTAAATCTTTAAATACTTGACCAGGACCTACAGA
AGGCAATTGGTCCTCATCACCTACAAATATCAATTGTGCATCTAAAGGAACTGCACTTAAAAATTGGTGGAACAACCAAGTATCTACCATAG
ACATCTCATCAATGATTATGAGTCGTGCGTTTATTTCATTTTCTAATATATCCTCTGGCTTTGTGTCTTGATTCCAACCTATTAAACGATGAAT
CGTCATAGCTTCTAATCCAGTTGACTCTTGTAGTCTCTTGGACGCTCTTCCTGTTGGCGCTGCTAATACAACTGGATAATCATCATTGACATA
ATCATCATAATCTAATGATAAGCCATGAATCTCAGCATATAATTCAACAATACCTTTAATTACTGTCGTTTTTCCTGTTCCCGGTCCACCGGTT
AATAGCATCACCTTAGAATTGATAGCCGTTTGCAAAGCTTCTTTTTGTGAAGCTGCATAGTTCACTTGATTCGCATCTTCTATTTCACCAATAT
GCATTTGTAAATCTGACTGTTCAATTTCTGTAAGTTTATTTGTATGCGTCTTTATTCTGAATAAGTTTTGAACACTTTTGATTTCAGAATAATA
CAAACTTGGAATTGCAACTTGTTCATTGTCAATAATTAGTCGTTTTTCCTCATTTAAGTATTGCAACATTTCGTCTAATTTTTCAGGTTCGATG
ACCTCTTCATCTTGATAATTTAATACATCAACCGTTAAATCTATAACAACATTGATAGGCAAATATGTATGTCCCTGTTTAATACATTCTTCTTC
TAACGTATAGAGCAACGCAGCTTTTAATCGTTCATTATCGTTATAAGCGATACCAATATTTCTAGCAAGTTGATCTGCTTTATTAAAACCAAT
ACCTTTAATATCATAAATCAATTGATATGGATTTCGATCTAAAATAGTCAGTGTATCGCCGAGATAAAACTGATAAATTGCCATTGAAAGTTT
AGGACCAAACCCTAAATCATGTAAACGAATCATTATTTTTTCAGATTCTTGATTTGCTGAAATTTGTTCTGCAATTTGTTTCTGTTTCTTTTTAG
ATAATCCCGAAACTTTTTCTAGCACTGAATGGTCATCTAATATATCATTTATCGCATTGTCACCTAATGTATTAACAATATTTTGAGCTGTCTT

TTTACCTACACCTTTAAACAAATCACTAGATAAATAACTTATAATTGCTTCTTTCGTTTGTGGCATTTCTTTTTCAAAAGTCTCTGCTTTTAATT
GTTTACCATAACGTGGATGATCAACAACTTGCCCTTTAAATGTGTAGACATCGCCTTCAACAATATTCGGAAGAAACCCTACAACAGTTGGC
ATTGTATCAAAGTCTTCATTTGTTTCAATAGTATCTACTTTAAGCACTGTATAAAAATTATCACTGTTTTGAAACAATATCGCTTCAACAGTAC
CTTTGATCATTGAATAATCAAATAGTGTAGGGTCTGACAT

Gene: yrrB (tetratricopeptide repeat family protein)

Position: 1676305 to 1676973, length: 669 nt, orientation: REVERSE

Perfect match to: (11819-97-CP003194-[1726687:1727355:r], allele observed in CC80)

Sequence:

TTACTCCTCCTCTTTCATTTTAGTGAATGTTTTCAGCGCATGCTGACTTAATAAGTGTTTAGGGTCGATAGTCACAGCTTCTCTTAAAATGAGTT
ATTGCTTCATCAATATCTTCATTTTTCATAAATAACGCTAAGCCCAAATTGTATCTTGCATCAACATGATTTTTATCAATCGTTAATACATGTTT
AAGTTGAGTTATGGCTTCATTAAACATTTCTAATTGACATAATACAAGACCATATTGAAATTGAACTTCTGCATCTTTGTCTTTATCTAGTTCC
GCAGCAGTCATTAAATACGGCAATGCCAGCTTAAATGATTCTAACTGATTAAACGCCATACCGATCATATAATTACAATCAACTTGTTCAATC
TCTGTTTGTAATGCTTGTTGATATAATTTAATAGCTTCTTGATAACGTTGCTGATTATAATATACATTTGCTAGATTATAAAATACGACGCCAT
TCTTCGGATCTATTGTTAAAGCTTTTTGGAAAAAACGCTCTGCCTTTTCAATCTCATTCGCATCAGCAAGTACAATACCAGCATTAATATAATT
TTCAATAATTGTAGGATCTTCTTCGATATTTCCGAACAATGCTTGTAACGCTTCTTCTATTTTTCCATTTTGTATGTATTGATAAATTGTTTGTT
GATCTATCAT

Gene: STAR (Staphylococcus aureus repeat element)

Position: 1677083 to 1677300, length: 218 nt

Sequence:

GGGAATGGAATAGAAATGCTAAAGAACCATTAACGGTTTATTATGTAATGGTTCTTCCACATTAGCCACCACTATTATGTATTTAAAAATAG
GAATACATAATTAGATTCATGCATAGGGAGTGGGACAGAAATGATATTTTAACAAAATTAAATTCGTTATCCCCAACTTGCATTGCCTGTAG
AATTTCTTTACGAAATTCTCTGTGTTGGGGCCCC

Gene: STAR (Staphylococcus aureus repeat element)

Position: 1677200 to 1677420, length: 221 nt

Sequence:

GGGAGTGGGACAGAAATGATATTTTAACAAAATTAAATTCGTTATCCCCAACTTGCATTGCCTGTAGAATTTCTTTACGAAATTCTCTGTGTT
GGGGCCCCATCCCCAACTTGCACATTATTGAAAGCTGACTTTTGGTCAGCTTCTGTGTTGGGGCCCCGTCCCCAACTTCCATTGCCTGTAGA
ATTTCTTTACGAAATTCTCTATGTTGGGGCCCCGCC

Gene: SIRU01 (staphylococcal interspersed repeat unit 1)

Position: 1677247 to 1677415, length: 169 nt

Sequence:

CCAACTTGCATTGCCTGTAGAATTTCTTTACGAAATTCTCTGTGTTGGGGCCCCATCCCCAACTTGCACATTATTGAAAGCTGACTTTTGGTC
AGCTTCTGTGTTGGGGCCCCGTCCCCAACTTCCATTGCCTGTAGAATTTCTTTACGAAATTCTCTATGTTGGGGCC

Gene: mnmA (tRNA-specific 2-thiouridylase)

Position: 1677552 to 1678670, length: 1119 nt, orientation: REVERSE

Perfect match to: (MRSA252-BX571856-[1763791:1764909:r], highly conserved allele)

Sequence:

TTATACAACATAATTTAATTGACCTTCATTTTTGAACACATCGTCAATTGTTGCACCACCAAGACACACATCACCTTGATAAAAAACAACTGC
TTGTCCAGGTGTGATTGCTCTTACTGGCTCAGCAAAAGTAACACGTAGTGCATGGTCGTTTTCACGTTTCACAAAAACTTTCGTATCTTTTTG
GCGATATCTAAATTTAGCTGTACATTCAAAACCTTGATCTAAGTCATTATCTTCTGGATTTACAAATGAATAGTCTGAAGCAATTAAGTAATC
ACTGTATAATGCATCGTGATGGAATCCTTGTTCTACATATAAAACATTATCTTTTAGGTTTTTACCGACAACAAACCAAGGATCGCCATCTCC
ACCTATACCTAATCCATGTCTTTGTCCTATTGTGTAATACATCAAACCACTATGTTTACCCATTTTCTTACCATCAAGTGTTATCATATCACCCG
GTTGTGCAGGTAAATATTGTGATAAAAATGTTTTAAAGTTTTTTTCGCCGATAAAACAAATGCCTGTAGAATCTTTTTTCTTAGCAGTAACAA
GTCCTTGTTCTTCAGCAATTCGACGCACTTCACTCTTTTCGATGTCGCCAATTGGGAACATCACTTTTGAAAGTTGTTGTTGAGATAATTGATT
CAAGAAGTATGTTTGATCTTTATTATTATCTACACCACGTAACATTTCAACATGACCATCTTCATGACGATGTATGCGTGCGTAATGTCCTGT
TGCTACATAATCTGCACCTAAATTCATCGCATGATCTAAAAAGGCTTTAAACTTAATTTCTTTATTACACATAACGTCTGGATTTGGAGTACG
ACCTTTTTTGTATTCATCTAAGAAATACGTAAAGACTTTATCCCAATATTCTTTTTCAAAATTAACAGCGTAATATGGAATGCCAATTTGATTA
CACACTTCAATAACATCGTTGTAATCTTCAGTTGCAGTACATACGCCATTTTCGTCAGTGTCATCCCAGTTTTTCATAAATATGCCAATGACAT
CATAACCTTGTTCTTTTAAGACGTGGGCTGTTACAGAACTATCTACACCGCCTGACATACCAACGACAACACGTATATCTTTATTTGACAA

Gene: csd1 (cysteine desulfurase 1)

Position: 1678671 to 1679813, length: 1143 nt, orientation: REVERSE

Perfect match to: (11819-97-CP003194-[1729053:1730195:r], allele observed in CC80+CC8+CC80+CC88+CC239)

Sequence:

TTATGACTCCTCCTTAAATTTAAAATATATTTTATGAATTTCAGCTACAATTGCATTAATTTCATTTTCAGTAGTCAATTCGTTAAAACTAAATC
GAATCGAATGATTTGATCGCTCCTCATCTTCGAACATTGCATCTAAAACATGCGACGGTTGTGTAGAGCCTGCTGTACATGCAGATCCAGAC
GACACATAGATTTGTGCCATATCCAACAATGTTAACATCGTTTCAACTTCAACAAACGGAAAATATAGATTTACAATATGGCCTGTAGCATC
CGTCATTGAACCATTTAATTCAAATGGAATCGCTCTTTCTTGTAATTTAACTAAAAATTGTTCTTTTAAATTCATTAAATGAATATTGTTATCGT
CTCGATTCTTTTCTGCTAATTGTAATGCTTTAGCCATCCCAACAATTTGCGCAAGATTTTCAGTGCCTGCACGGCGTTTCAATTCTTGTTCACC
GCCAAGTTGAGGATAATCTAGTGTAACATGGTCTTTAACTAGTAATGCACCGACACCTTTTGGTCCGCCAAACTTATGAGCAGTAATACTCA
TTGCGTCGATCTCAAATTCGTCAAACTTAACATCAAGATGTCCAATTGCTTGAACCGCATCAACATGGAAATATGCATTTGTCTCAGCAATAA
TATCTTGAATATCATAAATTTGTTGCACTGTGCCAACTTCATTATTTACAAACATAATAGATACTAAAATCGTCTTATCTGTAATTGTTTCTTCA
AGTTGGTCTAAATCAATAGCACCTGTATCATCAACATCTAGATATGTTACATCAAAACCTTCTCGCTCTAATTGTTCAAAAACATGTAACACA
GAATGATGTTCAATCTTCGATGTGATAATGTGATTACCCAATTGTTCATTTGCTTTTACTATGCCTTTAATTGCCGTATTATTCGATTCTGTTG
CGCCACTCGTAAATATAATTTCATGTGTATCTGCACCAAGTAATTGTGCAATTTGACGTCTTGACTCATCTAAATATTTACGCGCATCTCTTCC
CTTAGCATGTATTGATGATGGATTACCATAATGCGAATTGTAAATCGTCATCATCGCATCTACTACTTCAGGTTTTACTGGTGTGGTCGCAGC
ATAATCTGCATAAATTTCCAT

Gene: limB2 (putative monooxygenase locus 2)

Position: 1680125 to 1681138, length: 1014 nt, orientation: FORWARD

Perfect match to: (CIGC93-AHVD01000018-[73488:74501], allele observed in CC15+CC80)

Sequence:

ATGGCGATTAAATATTCAGCATTAAACCTTGTCCCTATTCGAGAAGGTGACGATGAACAAACAGCAATTAATGATATGGTTAATCTCGCACA
ACATTTAGACGAATTATCATATGAAAGATATTGGATTGCTGAACACCATAACGCTCCCAACCTAGTAAGTTCAGCAACTGCTTTATTAATTCA
ACATACGTTAGAACATACGAAACACATACGTGTAGGTTCTGGAGGCATCATGTTACCTAATCATGCTCCATTAATCGTTGCGGAACAATTTG
GCACGATGGCAACATTATTTCCAAATCGTGTCGATTTAGGATTAGGACGTGCACCTGGAACAGATATGATGACCGCAAGTGCATTAAGACG
AGATCAACATGATGGTGTTTATAAATTTCCAGAAGAGGTTTCATTATTACAACAATATTTCGGCCCTGCTCACCAACAAGCATATGTTCGTGC
TTATCCAGCAGTAGGTAAAAATGTGCCTTTATACATTCTTGGTTCTTCAACAGATTCTGCACATTTAGCTGCTCGCAAAGGGCTTCCATATGT
GTTCGCTGGACATTTTGCACCTCAACAAATGAAAGAAGCTATCGAAATTTACAAAACGTTATTTGAACCTTCTGATGTATTAGACGAACCTT
ACGTTATTGTATGTTTAAATACAATCGTTGCTGAAAATGATGACGAAGCACAATATTTAGCTTCATCTATGGCACAAGTAATGGTTAGTATC
ACTCGTGGCAGAATGCAGCCCGTTCAACCGCCAACACATGAACTACAAAATATATTAACGCCGAGAGAATACGCGATGGCTATGGAAAGA
CAGAAAATATCATTAATAGGTTCAGAAAATACTGTTCAACAAAAAATTCAAGATTTTATGGAAACTTATGGTGAAGTCAACGAAATTATGGC
AATAAGTTATATTTATGATAAAGATATGCAATTAGACTCTTATCGTCGGTTCAAGAATGTTATAAATCAGATAAATGAAAAAAACACTTTAT
AA

Gene: Q5HFD8 (putative small protein)

Position: 1681375 to 1681521, length: 147 nt, orientation: REVERSE

Perfect match to: (N315-BA000018-[1657152:1657298:r], highly conserved allele)

Sequence:

TTATTTCTCTTTATCTTCATTTTCTTTTTTCTCTTCGTTATTCGATCCTGTATATTCATTTATCTTATCTTTTACATTTTTAACTTGTTCATTATCGC
TATTTTTAAATTTTTCTACTGCGTCTTTAGCTTTATCCATAAAACTCAT

Gene: csbD-L2 (stress response protein, locus 2)

Position: 1681561 to 1681743, length: 183 nt, orientation: REVERSE

Perfect match to: (N315-BA000018-[1657338:1657520:r], highly conserved allele)

Sequence:

TTATTTTTTAAGTTTATCAATTGCATCAGTTATTTTGTTTTTAGCATTTTCAACAACTTCTTTTGCTTTACCAGTCGCTTTATCTTGCTGACCTTC
TTTTTCTAATTCTTTGTTATCAGTAACGTTACCTACTGTTTCTTTAACATTTCCTTTAAATTGATCGAACTTACTTTCGTCTGCCAT

Gene: cymR (oxidation-sensing transcriptional regulator)

Position: 1681843 to 1682265, length: 423 nt, orientation: REVERSE

Perfect match to: (N315-BA000018-[1657620:1658042:r], highly conserved allele)

Sequence:

TTAAATATAAAACATGTATCCGTCTAAATCTTCACTTGTATCTACATATTCCGCTAAATATTTCAATGTTGTATTATCTAAAACATCTCTCACTG
CATCTCTCATGCGAATCCATAGTTGTTTTTGCGCAGGTGGTTCTGATTCAATACTTTCAACAAATGTAATTGGACCTTCTAACAGTCTTATAAT
ATCCCCTGCTGAGATTTCTTCCGCTGGTACTCTTAATTGGTATCCACCTTTAGCACCGCGTACACTTCGAATTAACCCCGCATTTCTTAAAGGA
CCTACAAGCTGTTCTAAATATAAATCACTCAAATTATTTTCTTCAGCAATTGACTTTAATGATATACATCCTTGCCCCTCTTTTTTAGCAAGAG
AAATCATCAATGTAAGTCCATATCTCCCTTTAGTAGAAATTTTCAT

Gene: rarA (DNA-dependent ATPase)

Position: 1682350 to 1683624, length: 1275 nt, orientation: FORWARD

Perfect match to: (Strain_21343-AHKV01000003-[24731:26005], allele observed in CC88+CC22+CC80)

Sequence:

GTGAGTACAGAACCATTAGCATCGAGAATGCGCCCAAAAAATATAGATGAAATCATTTCCCAACAACATTTAGTTGGACCAAGAGGCATTA
TCAGAAGAATGGTTGATACAAAAAAATTAACTTCAATGATTTTTTATGGTCCACCTGGTATAGGCAAAACAAGTATTGCCAAAGCAATTTCG
GGCAGTACGCAATATAAATTCAGACAATTGAATGCTGTAACTAACACTAAAAAAGATATGCAACTTGTTGTTGAAGAAGCTAAAATGTCTG
GTCAAGTTATCTTGTTATTAGATGAAATACATCGACTAGATAAAGCTAAACAAGACTTTTTATTACCTCATTTAGAAAATGGCAAAATCGTCT
TGATCGGTGCTACAACTTCAAATCCTTATCATGCTATCAATCCAGCGATTCGTTCAAGAGCGCAAATTTTTGAGTTATATCCTTTAAATGATG
AAGATGTGCGCCAAGCTTTAACTCGTGCAATAGAAGATGAAGAGAATGGTTTGAAAACATATCAACCCAAAATTGATGAAGATGCCATGG
CCTACTTTTCTACACAAAGTCAAGGTGATGTTCGTAGTGCGTTAAATGCATTGGAATTAGCTGTATTAAGCGCAGATAATGACAAAGACGGT
TATCGACATGTTACATTGCAAGATGCTAAAGACTGTTTACAAAAAGGTGCATTTGTAAGTGATAAGGATGGTGACATGCATTACGATGTTAT
GAGCGCTTTCCAAAAATCTATCCGTGGTAGCGACGTCAATGCCGCTTTACATTATTTAGCACGATTAATTGAAGCTGGAGATTTACCTACAA
TAGTTCGACGATTACTTGTAATTAGCTATGAGGATATAGGCTTAGCCTCACCTAATGCTGGTCAGAGAACACTTGCTGCTATTGAATCAGCA
GAACGTCTAGGTTTACCAGAAGCTAGAATCCCACTAAGCCAAGCAGTAATCGAACTATGCTTATCACCTAAGTCAAATTCAGCAATGAGTG
CCATTGATAGTGCATTGTCCGATATTAGAAACGGTCATGTGGGTCAAATTCCAAACCATTTAAAAGATGGACATTATCAAGGTGCTAAAGAT
CTAGGCCGATCTATTGGTTACAAATATCCACACCAATATGTTAATGGCTATGTTTCACAGCAATATTTACCTGATAAACTAAAAAACAAAATT
TATTACGAACCAAAAACGACATCTAAAAGTGAACAACAACTCAAAGAAATATATAACAACTTACTTAAACAAAGGCCGTAA

Gene: yrvM (putative N6-threonylcarbamoyladenosine cyclase)

Position: 1683785 to 1684558, length: 774 nt, orientation: REVERSE

Perfect match to: (KLT6-APFH01000004-[1099317:1100090:r], allele observed in CC12+CC80)

Sequence:

TTATTGCCCTTTGTCTTTAATGCGACGAACTGGAATATCTTTTAATACGTCATTCACCACATAACTTGCACAAATTAATCCAACAACACTTGGT
ACAAAGGCATTTGAAGAAGGTGGCATTTGTCCTTTTCGATTGATAGCATTTTTATCTCCAACTATATCTTTTACATCTTCTCTTATGACAATTG
GACTTTCATCTGAAAATACAACTGGAATCCCTTTACGAATTCCTAGTTTTTTCAATTTTTGACGAATAATTTTGGCCATTGGATCGGTATGTGT
TTTAGAGATATCTGAAATTGTAAAACGTGTTGGATCTGTTTTATTTGCAGCACCCATACTGGAAATCACTTTAATCCCTCGGTCAAGACACTC
TTTCATTAAGTGTACTTTGTACATTATTGTATCACTTGCATCTACAAAATAATCTATATCGTAATTATCGAAAATTTCTTCATATGTCTCTTCTG
TATAAAACAAATGTAAAGGCGTGACTTTACAATCTGGATTAATTAATTTAATACGTTCTTCCATCAAAGAAACTTTACTTTGTCCTACTGTTGT
AGTTAAAGCGTGTAATTGTCTGTTTACATTTGTAATATCAACATCATCTTTATCTATTAATATAATATGACCAATATTCGTTCTTGCTAATGCTT
CAGCAGCAAATGAACCAACACCTCCAACGCCAAGTATGACAACAGTTTGTTGCTTCAATAAATCTAAACCTTGTTGTCCAATCGCTAGTTCAT
TTCTTGAAAATTGATGTTTCAT

Gene: ssrS (6S RNA)

Position: 1684718 to 1684949, length: 232 nt

Sequence:

ATAAAAATACGCAAGACAAAGTCTTGCGTATCGATAGAGTCCGTATTGCCGTAGTTATAATAGCTTGATCATTCGGCCTGTTATATACAGGT
GGGTGCCCTGTTTCTTGTTTTGTACGTCCTTCATATAAGGCGTGTACGCTGCAAGAAAACCCATTGGGCTCCCTTGATCAAAGAGTGTTAGG
CCCAAATTAAAAAGCAAACTTACGAACAACTCAGATGACTATCTTATG

Gene: aspS (aspartyl-tRNA synthase)

Position: 1685019 to 1686785, length: 1767 nt, orientation: REVERSE

Perfect match to: (Newbould_305-AKYW01000001-[265352:267118], allele observed in CC97+CC12+CC772)

Sequence:

TTAGTGACGAATTCGCAAAGAAAGTTCTTCTAATTGTTTATCAGAAACTTCACCAGGCGCATTCGTTAATAAACATGTAGCAGATGCTGTTTT
AGGGAATGCGATTGTATCTCTCAAGTTTGTTCTATTAGTCAATAACATGACTAATCGGTCTAATCCTAATGCAATACCGCCATGTGGTGGTG
CACCATATTTAAATGCATCTAGTAAGAAGCCGAACTGTTCTTGTGCTTGTTCTTTAGTAAATCCAAGAACTTCGAACATTTTTTCTTGTAACTC
ACCATCATGAATTCTGATTGAACCGCCACCTAATTCATAACCATTTAATACTATGTCATAAGCATTTGCCTCAGCTTCTTCTGGCGCAGTGCC
AAGCTTAGCAATATCAGCTTCTTTTGGAGATGTAAATGGATGATGTGCTGCAACGTAACGTTTCGCATCTTCATCATATTCTAATAATGGCCA
ATCTGTCACCCATAAGAAGTTTAATTTTGTTTCATCGATTAAACCTAATTCTTTAGCTAATTTGACACGTAATGCACCTAAACTTTGTGCAACG
ACATTTGGTTTGTCTGCAACAAACATTACTAAGTCACCAGCTTCAGCACCAGTTAATGTAAGTAATGTTTCAACATTTTCTGTTTCAAAGAAA
CGTCCAATTGGACCTGTCAAACCATCTTCCACAACTTTAACCCACGCTAATCCTTTAGCACCATAGATGTTTACAAATTCTGTTAAAGCATCCA
TATCTTTACGAGTATATTGTTCAGCTGCACCTTTAGCGACAATTGCTTTAATTTCACCATCATTTTCAACAGTATCTTTAAATACTTTAAAGTCC
ATATCACGTCCTAATTGAGAAACGTCAATTAATTCCATTTCAAAACGTGTATCTGGTTTATCAGAACCATAGCGACGCATCGCTTCTTTATAT
GTCATGCGTGGGAAAGCGCCATTAATTTCAACGCCTTTAACTTCTTTAACAACTTTTTTAAGCATTTCTTCACCCATTTGCATCACATCTTCTT
GATCTACAAAACTCATTTCAATATCGACTTGTGTAAATTCAGGTTGACGATCTGCACGTAAATCTTCGTCACGGAAGCATTTTACGATTTGGT
AGTATTTGTCAAATCCACTAATCATCAATAATTGCTTAAATAATTGTGGTGATTGTGGTAATGCATAAAATTCACCATCATGAACACGAGAT
GGTACTAAATAGTCACGTGCACCCTCAGGTGTTGACTTCGTTAGTACTGGTGTTTCGATGTCAAAGAACCCTTCATCATCCAAATATTGACG
AATAGAACGTGTAATTTGATGTCTCATTTTAAATGTTTGCGCTAACTCTTGACGACGTAAATCTAAATAACGGTATTTTAATCGAATATTTTC
ATCAACGTTAACATTTTCTTCATTTATAGAAAATGGTGGTGTCTCAGATTTATTAATCACTTTAATATTTGTAACTTGTACTTCAACTTGGCCA
GTTTTAATTTTAGGATTAACTGTTTCAGGGTCACGCTTCGTAACTGTACCTTGAACTTCTACAACATATTCAGAACGTACTGTTTCAGCAATTT
TCAATGCCTCTTCTGAAAATGCAGGATTAAACACGACTTGTACAATTCCTTCTCTATCTCTTAAATCAACGAAAATCAATCCACCTAGGTCAC
GACGATTGTTAACCCATCCTTTTAATGTAATTTCTTGTCCTAAAAATGCTTCAGTAACTAATCCACAATAAGTTGTTCTCTTACTCAT

Gene: hisS (histidyl-tRNA synthetase)

Position: 1686801 to 1688063, length: 1263 nt, orientation: REVERSE

Perfect match to: (930918-3-ABFA01000002-[20528:21790], allele observed in CC8+CC97)

Sequence:

CTACTTCTTAAAATATTCGACTAATGCGTCTAATTCAATTGTTTCAGATTCACCAGTTGTCATATTTTTAACATCGATTTTATTATTTTCTAATTC
TTGATCACCAATAACGATTGTAAACTTGGCACCTAAACGGTCTGCTTGTTTCATTTGTCCTTTAATTTTACGCTGTAAGTAGTCTTTATCTGCT

TTAATACCATTATGTCTCAAATGATTTAATAACTTCACAGCATATCGATCTGCTTGATCACCCATTGTAACAATGAATAAATCTAAGTTTTCTT
CAATATCTAATTCGATACCTTCTTCTTCAAGTGCAAGCAATAATCGTTCTATACTTAGCGCAAAACCAATACCTGTTTCACTTGGACCATCTAG
CAATTCTAATAAACCATTATAACGGCCACCACCACAAAGCGTTGTAATGGCACCATCATAGTTAGGGTTATCCATCATTAATTCAAATGCTGT
ATGTGTATAATAATCCAATCCACGAACTAAGTTAGGATCTTCAATATATGGAATACCTAAATCATCTAAATAAGCTTTTACTTGTTCATAATA
TGCCTTTGATTCCTCATTTAAGAAATCAGTGATTCTAGGTGCAGTCTTAATCGCTTCTTTATCACGGTCAACTTTACAATCCAAAATTCGCATC
GGATTTGTATGCAAACGTGATTGACAATCTGAACAAAATTCATGAATTACTGGTTCAAAGTGTTTCACTAACGCTTCGTTATATTCTTTTCGA
GACGCCATATCCCCTACACTATTAATAACAAGCTTTAAATGTTTTAATCCAAATGATTGATAAATATGCATAACCATAGCTAATACTTCTGCA
TCTACGCTAGGATTTTCAGCACCAATAGCTTCTACACCAAATTGATTAAATTGACGATAGCGCCCTTTTTGCTTACGTTCATATCTAAACATCG
GTCCATTGTAATAAAGTTTAATTGGTTGGTTTGGATTACCTTGCATTTTATGTTCAATATATGAACGCACAACTGCAGCTGTTCCCTCAGGTC
TTAATGTAATACTTCTATCGCCTTTATCTTTAAATGTATACATTTCTTTTTGTACGACATCGGTTGAATCACCAACACCTCTTGCAAAAAGATC
TGTACTTTCAAAAATTGGTGTTCTTATTTCTTTATAATTATAAAATGTCATTAATTCATCTAATTGATTTTCAATGTAACGCCATTTCTTTGAAT
CTTCAGGTAAAATATCCTGCGTCCCTCTAGGTATTTTAATCAT

Gene: lytH (N-acetylmuramoyl-L-alanine amidase)

Position: 1688524 to 1689399, length: 876 nt, orientation: REVERSE

Perfect match to: (COL-CP000046-[1716773:1717648:r], allele observed in CC8+CC80+CC239+CC630+CC4803)

Sequence:

CTACGCAGAAAAATAAATTTTAAGGCCATCAACAATTGCTTGTTCTAAAATTTGTCTATGTAATTGATCTTTAATCATCGTTTCATCAGTTGG
GTTACTAATATAACCTAATTCTAATAAAACAGCAGGAACTTTTGTTTGTCTTAACACTTGATAATTTTCTTGTCTTGAACCGCGATTAGAAAGT
AGACCTTTCTTCTGAATCGTAGCGTCTAACGTATCTGCTAAAGCTCTTTGATTATCATGATACCAATAAACTGTCATTCCATTTGCATTAGATG
ATTCTAACGCATCATTATGTATACTCAAATAGGCATCGCCTTTGATATCACGATTTTCTAGTGAAACATATGTATCGTCTGTTCTTGTCATCTT
AACAGTTGCGCCTTCTTTTTCTAAAGTACGCTGCAATTCTTTTGCTGTTTTCAACGTATAATCTTTTTCTAAACTTTTATATTTAGTATTGCTTG
AAGCACCCTGGTCACTACCTCCATGACCAGGATCAAGCACTATTGTTTTACCTTGCAAAGGATTTTTCTCCTTCGTATTATCCGCGACAATAT
CTAAATTTGTGTGCCATCCAGCTATCCAACCTTTTTCATTACTGGATGTATCTTCAACTTCAATCCATTTACCTACTTTACCAATCTTTTTAAAA
TGGTCACCTTTTTCAACTTTATATATGACTGGATACGCAGCGTTTGGACCTGTACGTAATTCAGCATTTTCAGTTATCGTGATGTTCCCACTAT
CTTCACTATTGCTATTCAGCAATAAAAATAAAAAGATGATAAATAAGACAAAGGCAATCACTACTATTAGAGTACGTTTATTTTTAAGACCCT
TTTTAGATAACCATGCCTCTATTTTTTTCAT

Gene: dtd (D-tyrosyl-tRNA (Tyr) deacylase)

Position: 1689396 to 1689848, length: 453 nt, orientation: REVERSE

Perfect match to: (N315-BA000018-[1665173:1665625:r], highly conserved allele)

Sequence:

TCATTGAATTTTGCCGTCCTGACTTTCATAAATAATAGTGACTGGACCATCATTATTTATGCTAACATTCATGTGTGTTCCAAATTCACCTGTT
TTCACAGTAAGACCATACGCTCGTAGCGCATCATTAAAATACTCATAAATTTTTACCGCTTGATCAGGATTTTTAGAATTTGAGAAACCTGGA
CGGTTACCTTTTTTTACATCTGCATAGAGAGTAAATTGTGAAACTGATAGTATTTCACCATTCATTTGTTGGATATTAAAGTTTAATTTATTAT
TGTCATCTTCAAATAATCTTGCATTAGCAATTTTCTTTGCAATTACATCTGCATCTTGCTCTGTAGAGTTCTGACCGATACCGACTAATAAACA
ATATCCTTTTTTGATTTGATTATTTAATGTATCATTCGTCACCGATGCTTCTTTAACTCTTTGTACAACTACTTTCAT

Gene: relA (ppGpp synthase I)

Position: 1689860 to 1692049, length: 2190 nt, orientation: REVERSE

Sequence:

CTAGTTCCAAACTCTTGTTACTGTATAAACATCACCAAGTTGTTTGATCTTTTCTACCACACGATAAACATCATTCACGTTTTTCACCATGACA
CTAATATTTATTATTGCATTTTTATCAATATCTGAACGTCCTGAAACTTTAATTAAATTGCCGGCTGTCGAGCTAACAGCTTGTAGTACTTCAT
TCAACAAGCCATTTCGGTCATACGCAGTTACCTCTAAATCAACCTGATATTTTTGAGTTGCGTCTTTTGATTTTACCCATTCAACATTAATTAG
TCGTTCAGTTTCGTTCTTAATATTTGGGCAATCAGTGCGATGTACTTTAATACCGTGACCTTTGGTGATATAACCTACAATATCATCACCTGG
TATAGGATTACAACATTTTGACAACTTGATAAGTACATTTTCTAAACCTTCTACATAGACACCACTATCAGTAATGATGTTGTCTTTAATAGG
CAATGATTTCGTAACTTCTTGTGCTTCATTTAAAGCACGTTGTTTATCTAAAATACGTTGTCTTTCAGTTAATTTATTAACAATCTGTAAGGAT
GTCACGCCGCCAAATCCTACAGCTGCGAATAAATCATCTTCATTTACAAAGTTATATTTTTCATTAACAACCTGAATATTTTTCTCTGTCAAAA

TATCTTCGACTCTAAATCCTTGCTCTTTTATTTCAGCTTCAACCATCATTCGGCCTTTTTCAATATTAGATGAACGATCTTGTTTTTTGAAGAAA
CTTTTAATTTTACCTTTGGCACTAGACGATTTAACAATTTTCAACCAATCACGACTTGGTCCATATGAATGTTTACTAGTACGTATTTCAACAA
TATCGCCTGTTTGTAAAATATAGTCAATTGGTACAATTTTGCCATTCACCTTGGCACCAATCATCTTATTACCTACTTCACTGTGAATCGCATA
AGCAAAATCAATCGGCACAGCACCATATGGCAACTCAATAACATCACTCGCTGGGGTAAATGCGTATACTTTGTCACTCTGTAAGTCATATT
TTAAGGTTTCCATAAATTCTTGAGCGTCAGACGATGTATGATCCGCTTCAGCTAATTCTTTTAACCAATTTAACTTATTTTGATAAGTTTGATC
TTTTTCACTTACTTTTTTACCTTCTTTGTAAGCCCAGTGTGCTGCAACACCATGCTCAGCAATTTCGTGCATATCAAACGTTCGTATTTGGATTT
CGAGCGGGTCTCCATTTGGGCCTACTACTGTAGTATGCAATGACTGATACAAATTTTGTTTAGGCATTGCAATATAATCTTTAAAACGTCCTG
GCATCGGTTTCCATAACGTATGCACCAACCCAAGTATCGCATAACAATCATTAATAGAATTGACAATAACACGTATCGCCAACAAATCAAAA
ATTTGATCAAATTGTTTTTTCTGCTTCATCATTTTCCGATAAATACTGTAAATATGTTTAGGTCTACCATTTATATCGCCTTCGATATTCATTCG
GTCCATTTCAGTACGTATTCTATCAATAGCCGTTTCGATATACGCTTCACGTTCACTACGTTTCTTCTTCATTAAATTGACTATTCTAAAATATT
GCACATTATCAATATAACGAAGAGCCGTATCTTCTAGTTCCCATTTGATTGTATTAATACCAAGACGATGTGCTAATGGTGCATAAATTTCTA
ATGTTTCTCGAGAAATTCTAATTTGTTTTTCGCGCGGCATGGCTTTCAAGGTACGCATATTATGTAATCTGTCTGCTAATTTCACCAAAATTAC
GCGTACATCTTTGGCAATCGCAATAAATAACTTGCGATGATTTTCAGCTTGTTGTTCTTCTTTTGAGCGGTATTTTACTTTTTTAAGCTTCGTC
ACACCATCAACAATTCGAGCAACTTCTTCATTGAACATTTCTTTTACATCTTCAAATGTATACGGTGTATCTTCAATTACATCATGCAAAAAAC
CTGCGACAATCGTCGGTCCGTCTAATCGCATTTCTGTTAAAATACCTGCAACTTGTATAGGATGCATAATGTATGGTAATCCGTTTTTTCGGA
ACTGACCTTTATGTGCTTCATAAGCAATATGATAGCTTTTTAAAACATACTCATATTCATCTGCTGACAAATATGATTTTGCTTTGTGAAGAAC
TTCGTCTGCACTATATGGATATTCGTTGTTCAT

Gene: apt (adenine phosphoribosyltransferase)

Position: 1692477 to 1692995, length: 519 nt, orientation: REVERSE

Sequence:

TTATTCGTCGTATGAGATTAAACTCATAACATCGTAATCTTTAATTTTTTCAATACCATTTAAATATTTCAATTCAATTATAAATGTAATACCTA
CTACGATACCGCCTAATTTTTCAACTAATTTTATTGCTGCTTCAATCGTACCACCAGTAGCTAATAAATCATCTGTAATTAACACACGTTGACC
TGGTTTAATTGCATCTTTGTGCATTGTTAAAACATTTGTACCATATTCTAGGTCATACTCATAACGAATGACTTCACGAGGTAATTTCCCTTCT
TTTCTAACAGGTGCAAAGCCAATCCCCATTGAATAAGCTACAGGACAGCCAATGATAAAGCCACGCGCTTCAGGTCCTACAACGATATCAA
CATCTCTGTCTTTTGCGTATTCTACAATTTTATCTGTTGCATAGCCATATGCTTCACCATTATCCATAATTGTAGTAATATCCTTGAAACTAACA
CCTGGTTTCGGCCAATCTTGAACTTCTGATACGTATTGCTTTAAATCCAT

Gene: recJ (single stranded DNA-specific exonuclease)

Position: 1693017 to 1695290, length: 2274 nt, orientation: REVERSE

Perfect match to: (Strain_21283-AHJZ01000115-[49570:51843:r], allele observed in CC8+CC97)

Sequence:

TCACGACAATTGTGACTTTATCCAATTTTTTATTTCTGAAAAATCTTGATATAATAATTGCTTTTCAACATCCATACGTTGTTGTCTTAATTGAT
ATACTTTGCTGGAATCAATCGATCTTTTATCAGGTTGTTGATTGATTCGAATTAAACCATCTTCTTGTGTTACAAATTTTAAGTCTAAGAAAAC
TTTCAACATGAATTTAAGTGTATCTGGTTTCACACTTAAATGTTGACACAATAACATACCCTCTTTCTGGATATTTGTTTCTTGTTTAGTTATTA
ATGCTTTATAACACTTTTTAAAAATATCCATATTAGGTATACCATCGAAGTAAATCGAATGATTATGTTGCAAAACTATATAAAGTTGAGAAA
ATTGCAGTTGTTGCAAGGAATTAGACAAGTCTTCCATTGACGTTGGTAAATCTCTTAATACTACTTTATCAGTTTGTTGTTTAATTTCTTCACC
ATAATAATATTCATTCGCATTTACTTTATCACTTTTAGGATGAATAAGCACGACAATATTTTCATCATTTTCTGTAAAAGGTAAACTTTTTCGC
TTACTTCTATAATCTAATATTTGCTGTTCATTCATCGCAATATCTTGAATAATTAATTGCGGTGATTGATTACCATTCCATTCGTTGATTTGAAC
AGATCCTAATATATTAATTGGCTGTTCATCTTGTAACTCAGGTTCTAAGTGACCATTTTGCCAAAATAGCGCGGCGATATTACTTTCACCAAG
TGTCAATTTTAGATGATTTTTTTGTTGACCTATCGCCTTAACTGAAGAAACTGATAAATCATCCATTTCAAAAATAGGTCTAGAAAAATCTGT
TCCGAAGGGTCTTAAACGATTCATATCACGAATATTTTTAATCGTTATATCATTTTCTGTTAATAATACATCTACTTGCTTTACGGGATCTAAC
GAAGTTGTTTTAGATAATTCTTTCATCCATTTATTTAAACCTTCAGCTAACGATTCTATATTTTCAATATCCATCGTCATACCTGCAGCCATATG
ATGGCCGCCAAATTTAGCGATTAACTCTTGATGTGCTGATAGTATTTCAAACATCGACACTTGATCAATTGATCTGGCGGAACCTTTTGCAT
GATTTTGCTCCCTATCAATATTTAAAATTAATGTTGGCAAAGCAAATGTTTCGACAATTTTCGAAGCAACAATACCTAAGACACCTTCATGCC
AATTTTCTTTTGCTAAAAGTAAAAATAAATCTCCCTTTTTAACTTTCGTTTCTGCCATAGCCATTGCTTCTTCTGTGATAGTTGCTACAATATCT
TTTCTTTCACGGTTAAAATGTTCAACTTGTTCTGCTAAAAATGCAGCTTCTTCTTCGTCGTCAGTCATCAACAATTCGCAAGCTAATGATGCGT
CATCTAAACGACCTACAGCATTAAGTCTAGGTCCAATAATAAAACCAATTGTTTCTTCATCAATATTGTCATTGTATCCCGCTTCTTTTAGCAA
TGCTTTAACAGAGGTCGGACATTGATCATTCAAGACTTTTAATCCTTGTTTCACTAATGATCGATTTTCATCAGTTAAGGATACTAAATCCGC
AATGGTACCTATCGCAACTAATGCTTTAAAATAATCAGGTACATTTTCAATCAATGCTTGTGCTAATTTATACGCAACACCTGCACCACACAA
TTGTTGGAACGGATAATTAAACGATGGATGCATTGGATGTACGATTGCATATGCTTCTGGTAATGTACTACCAATTTCATGATGATCAGTTA
CAATGACATCAACTCCTAAATCTTGAACCATTTTAATTTCATTATGACCTTGTATGCCATTATCAACAGTTATGATTAATGTTATGCCTTCATC

ATGAGCATTTCTAAATGCTAGTTCGTTTGGTCCATATCCTTCGGTAAAGCGGTTAGGAATATGCCATCCTACTTGTGCACCTAAAAGTTGTAA
TGTTGTCACTAAAATTGTAGTTGAGGTAACACCGTCGGCATCGTAATCACCATAAACTAGGATTTTCTCATCATTCGCTATCGCTCTTTTAATT
CTTTCAATAGTCTTAGTCATATCGCTCAATTGCAGTGCATCATGATTGATATCTGTATCTGAAATGATGGATTCTATTGCTTGTTCATCAATAA
TCGATTTACTTTCTAATATTTTTTTTACGATTGGCGTTAACTTTAATTTTGATGTTAATTCATCACTTATGTATTCAGCTGGTTTAGTTAATTTCC
ACTTATACTTCGGTTTAATCAT

Gene: secDF (preprotein translocase fused subunits D and F)

Position: 1695493 to 1697772, length: 2280 nt, orientation: REVERSE

Sequence:

TTAAACTAAAATCTTTTCATCGTTCGATTTCTTTTCTTTATATACAACTAATTTGTGTTTCGGCGATTTTTTCAACTGACGTTTTTTCATTATTCC
CCATAGCGGAACGGCAATGAAGATTGAAGAGAATACACCAGAAATCAATCCGATAAATAATGCTAAAGTAAAGTTGAATATCGTAGGAGC
ACCGAAGAATAGTATAGCAACTACTACTACAATAACTGTTAATACTGTATTAATTGAACGCGTCATTGTCTGTCTAATTGATCTATTAACGAT
ATCATCAATTTGTTCTGTTGTCGTAATCACTTTAACCTTTTGTAAGTTTTCACGTACACGGTCAAACGTTACGATTGTATCATTAATTGAATAA
CCGACAATTGTTAATACAGCGGCGATAAATGTTAAATCTACTTCAATTCTAAATAAACTGAAAATCGCTACTATAATGAATACATCATGTAAT
AATGCCAATACAGATGAAAGACCCATGCGCCATTCAAATCGTAATGATACATAGATGATGATACCTATCGATGCATAGATTAATGCAAGCA
TTGCATTTTTTGCTAATTCCTGTCCAATAATTGGTGATACAGTATTAATTTGAGGTGTGTCACCGAATTTCGATTTAATATTATCACTCAATTT
ATTATCTTGAGCACGCGTTAAATCGTCTTTAAATTGAACAGTTGCTACTTTATTATCTTTACCATTGATTTGAATTTGATCCGCTTTAAGTCCA
CTATCTTTTACAACTTGCTCAACCTTTTGTTGAGTAATTGCTTGTTTAGATTGGAAATCTACACGTGTACCACTTGAGAAATCAATTCCTAAGT
TTAACTTGAAGATATAAAGAATAACTAAACCGACAACTACAATTAAAATACTTACTCCAATTAATGGCTTAGCTAATTTAACAAAATTCCATT
TCTCGAATGAAGTTTTAAGGTCATGAACATCTACACCTTCATTAATATCATGTCGTTTATTCTTTTTAACACCAAATAACCAAAATTGATTTTT
GAATATATTTGATGAAACAAGTAATGATAATAAGAATCTTGATAAGAACACGGCTGTAACAAAGATCATTAGAATACCTAATAATAACATT
GTCGCGAAACCTTTAACTGAACTTTCACCGAAGAAGAATAATACTGCTGCGGCGATAACTGTTGTTAAGTTAGAATCAAAAATTGTTAGGA
ATGAACTTTTGTTTGCTTTAGAAAAGGCTTGCTTTATCGTTCTACCTATTCGAAGTTCATCCTTAATACGCTCATACATGATAATATTGGCATC
TACAGCCATACCTACACCTAATACCAACGCCGCTAATCCTGGTAAAGTTAGAACCCCGGAAATGAAATTAAATGCTACTAACGTTAGATAGA
TATAAGTTGTCAATGCAATAATCGCTACTAAACCAGGTAATCGGTAGAATCCAAGCATGAATAAATAAATCAATGCTACACCAATAAACGAT
GCAAACACAGTTTTATCTAATGCATCTTGACCAAATTGGGCACCTACTGAGTTTGAATAAATTTCTTTCAAGTCAACTGGTAAAGAACCTGCA
TTTAACAATTCGGCGATTTGTTTTGCTTTTTTAACGCCTTCTTGTCCTTTAAATCCACCCGAGATTTCTACGCTATCAGAATTGATTGGTTGATC
AACACTTGCTGCAGAAATAAATTTAGGGTTTTTCTTTTGTGCTTCTTTTTTATAGCTGTCACCTTTTTTGAAATCTAACCAAACAACCATGACA
TTATCACGTTTCTTAGAGATTTCTTCCGTTACTTTTTTAAATTTGTTTTTGTCTTTTACTTTAAAAGTAACTGTAGGCTGGTTTGTTTCCTGTTTA
AATTCTTGTTTGGCAGATCCCTGTTTAATATCAGAACCGCTTAATTTTACTTTATCTTCTGCATCGCGAATTGTTAAATTAGCTTGAGAAGATA
AAATTTTACGTGCTTCATTCTGGTCTGTTACACCAGCAAGTTGAACTCTAATTCTATTAGGTTCTTCAACTTGAATTTTAGGTTCCGAAACACC
TAAAACGTTAACACGATTTTCTAATGTTTGCGCTGTTGATTGTAAGGCTTTTTTATCTATTTTGTCGCCTTTATTTAAAGGATCGACTTGATAA
AGCACCTCAAATCCACCTTGCAAATCAAGTCCTAAATTGACATTCTTTATAACACTTTTATAAGTTGCAGCCATTCCGGCAAACAACAATACG
ACTAAAAGCAAGAACGCAATTATTCTACTACTTTTCTTCAC

Gene: yajC (preprotein translocase subunit yajC)

Position: 1698047 to 1698307, length: 261 nt, orientation: REVERSE

Perfect match to: (RF122-AJ938182-[1628361:1628621:r], highly conserved allele)

Sequence:

TTATGAAGGGTCAACTTGTTTAATAGCAGGTTTTTCGAAAGTTAATTCAGTACCATGACCATTAACTGTAATAACAACAGTTGTTTCATCTAC
TGCTTTAACAGTACCTTTAATACCACCAATAGTTGTAATTCTTTGACCAGATTGAATGTTATTAATCAACTCACGATGCTGTTTCGCACGTTTT
TGTTGTGGTCTGATCATCAAGAAATACATAACCGCAAAAATTACGACTATATATATTAGTAATGAAAATTGCAT

Gene: tgt (queuine tRNA-ribosyltransferase)

Position: 1698326 to 1699465, length: 1140 nt, orientation: REVERSE

Perfect match to: (CA347-CP006044-[1723189:1724328:r], highly conserved allele)

Sequence:

TTAAAAGTTTTTTGGGTTCTCAACATTTAATCCATATTGCTCGAAGAATTCTTCTTTGAAATCTAAAAGACGATCTTCTCGAATGGCTTGTCTT
ATATCTTCCATTAATTTTAGCAGAAAATGTAAATTATGAATAGTAGTAAGACGAATACCAAAAGTTTCCTCTGCCTTGATTAAATGACGTATA
TACGCTCTTGAATAGTTTTGACATGTATAACAGTCACAATTCTCATCTAACGGTCTTAAATCATCTGCAAATTTTGCATTTTTAATAACTAAAC
GACCTTGCGATGTCATACAAGTACCATTTCTGGCAATACGTGTCGGTAAGACACAATCAAACATATCCATGCCGCGAATACTACATTCGATT
AACGCATCTGGAGATCCTACACCCATTAAATATCTTGGTTTATCTTTAGGCATAAACTGCTCTGTATGTTCAACCATTTTATACATAACCGGTT
TAGGTTCACCAACTGACAAACCGCCGATTGCATAACCAGGAAAATCTAATTCTACTAAATCCTTTGCACTTTGTTCTCTTAAATCTTCATATTC
GCCACCTTGTATAATGCCGAACAATGCTTGATCTTCAGGTCTTTGGTGTGCATCTAGACATCGTTTCGCCCAACGTGTTGTACGTTCAATAGA
TTTTTTTACATAATCATATTCAGCCGGCATCGGTGGACATTCATCAAATGCCATCATAATATCAGAACCTAAATCATTTTGAATTTGCATTGAT
TTCTCAGGACTCAAAAATAATTTAGACCCATTAGTATGATGTCTAAATTCCACGCCTTCTTCTGTAATTTTACGTAAATTACTTAAACTAAACA
CTTGGAAACCGCCTGAATCTGTAAGAATCGGACCATCCCAATTCATGAATTTATGTAATCCCCCAGCGTGTTTGATAATATCATTTCCGGGTT
GTAACCACAAATGATATGTGTTGCCCAAAATGATTTTTGCTTCAATTTGTCTTAACTCTTCTGGACTCATTGTTTTAACGGTTGCTTTAGTACC
AACTGGCATAAACATAGGTGTTTCAAATGAACCGTGTGGTGTGTGCACGATACCTAAACGCGCACCTGATTGTTTACAAGTTTTAATGTGTT
CGTATGTTACTGCAGGCAT

Gene: queA (S-adenosylmethionine:tRNA ribosyltransferase-isomerase)

Position: 1699488 to 1700513, length: 1026 nt, orientation: REVERSE

Perfect match to: (11819-97-CP003194-[1749870:1750895:r], allele observed in CC80+CC188+CC772+CC4803)

Sequence:

TTATATAATTAACATTGCATCGCCAAAACTAAAGAATCTATATTCTAAATTTACTGCCGTTTTATAAGCATTCAGAACATTTTCACGAGTACTA
AACGCTGATACTAGCATAACTAATGTTGATTTTGGTAAATGAAAATTAGTAATCTGGCCATCAATTGCTTTAAAATCAAATCCTGGATAAAT
AAATATATTAGTCCAGCCACTCGTTTCAACAAATTTATCATGATCGCGTCGAATTGTTTCAAGTGTACGTGTTGAAGTTGTACCAACTGATAT
AATGCGATGTCCTTTGGACTTAGTATCATTTAATAAATCAGCTGTTTCTTGTGTCATTTGATAATATTCACTATGCATTTCGTGGTCATTCACA
TCATCGACGCTCACCGGTCTAAACGTACCTAACCCAACATGTAATGTAACAAATGCGATATTAACACCTTTATTTTTAATTTCAGTTAATAACT
CATCAGTAAAATGTAATCCTGCTGTTGGTGCTGCCGCTGAACCACTTTCTTTAGCGTAAACTGTTTGATAACGATCTGGATCATCTAAACGTT
CTTTGATGTATGGTGGCAGTGGCATTTCCCCTAATTCATCTAATCTTTCTTGTAAAATACCTTCATAATGTAAACGCATGATGCGTCCACCTTG
ATCCATTTCTTTTATGCATTCAGCTATAATTTTGCCATTACCAAAATTCAATTTATTACCAACTTTAATACGCTTAGCTGGTTTCAGTAAGACTT
CCCAATCATTACCTTCAATTTGAGTTAACATTAACATTTCAACTTTTGCACCAGTTTCTTCTTTTAAACCAAAAAGTCTAGCTGGCATTACTCG
CGTATCGTTAAGCACTAATGTATCACCAGGTCTAAAATACTCAATGATATCTTTGAAATGTAAATGTTTCATTTCACCAGTTTCTCTATCCATG
ACTAATAAACGACTATGATCACGATCTTTTAAAGGCGTTTGAGCAATTAATGATTCTGGTAAGTCATAGTCAAATTCTTCAATATTCAC

Gene: ruvB (Holliday junction ATP-dependent DNA helicase subunit B)

Position: 1700515 to 1701519, length: 1005 nt, orientation: REVERSE

Perfect match to: (11819-97-CP003194-[1750897:1751901:r], highly conserved allele)

Sequence:

TTATTCTCTCTCCTCATTCGACTTTGCAAAATGTTCATAAGCTAATGGTGTTGCTTTTCTGCCACGTGGCGTACGTTCTAAAAAGCCTTTCTGA
ATAAGAAATGGCTCATAAACGTCCTCAATTGTAATACGTTCTTCACCAATTGTTACGGCAATCGTATCTAAACCAACAGGTCCACCATTATAC
TGCTTAATAATACAGTTCATCATTTTATGATCAATGTAATCTAGTCCGTGTTGATCAACTTGAAGTAAACCTAATGCGTGCTTCGTTGTTTCAA
TGTATATTTGTTCATCTTCATTCACTTGCTGGAAGTCTCTTACCCGCTTCAATAGTCGATTTGCTACTCTTGGAGTCCCTCTAGAACGTTTAGC
AAGTTCAATGGCACTTTCTTCATCAATACCTGTGCCTAAAACCTCAGCTGTTCTAATAATGATTTCTTTTAAATCTGATTCATTATAATATTCTA
ATCTTAAGTGCACACCAAATCGATCCCTTAGTGGACCTGTTAAGCTGCCAGCTCGCGTTGTTGCACCTACCAAAGTGAATGGAGGTAAGTC
GATACGGATACTTCTAGCCTCATCGCCTTTACCAATGATAATATCTAAAAAGAAATCTTCCATTGCAGGGTATAACACTTCTTCAACAACACT
ACTCAGTCTGTGTATTTCATCAATAAACAAAACATCTCCAGGTTGAAGTCCTGATAAAATTGCAGCCAAATCACCAGGTCTTTCTAATGAAG
GCCCTGATACTGTACGTATATTAACTTCCATTTCATTGGCAATGATATTAGATAATGTTGTCTTACCTAATCCAGGGGGGGCCAAAAAGCAAT
ACATGATCTAATGGTTCATGACGAAGTTTAGCCGCTTTAATAAATACTTCTAAATTACTTTTTATTGAATTTTGACCAATATATTGTCGTAATC
TCGTAGGTCTAAGCGACAATTCGAAATCAGTTTCTTCACTATGCATTGATTGATCAACCATACGCTCATTCAT

Gene: ruvA (Holliday junction ATP-dependent DNA helicase subunit A)

Position: 1701551 to 1702153, length: 603 nt, orientation: REVERSE

Perfect match to: (11819-97-CP003194-[1751933:1752535:r], allele observed in CC80+CC20+CC80+CC445)

Sequence:

TTAAGATACAACTAATTGAAGACCTGCCTTAACAGCTTCATCAACTGAGTCATATTTATTTTTATTTAACGTTTTCTCAACTTTTGCAAGCTCT
CGTTTAGAATAACCTAATGCTTCTAACGCTAACATTGCTTCTTGCACGAATTGATCTTGCACCGTCGAAGTAGCGTCTACTTGTAATAATGAA
TCGCTATCTTCTTCAGTAATTTTCACTTTACCTTTTAAATCTAAGACAATCTGTCTTGCCGTTTTCTTACCAATTCCTGGGAATTTAGTTAAATA
CGTATCATTTTCATTTTCAATGGCACGTTTTACTTCATTAGGCGTACTTGTCGCTAAAATAGCTAAAGCTGATTTCGGACCAATACCAGTAAC
TTTAATTAAACTCAAGAACATATCTTTCTCTTCTTCACTACTAAATCCATACAATAATTGTGCATCTTCACGAACAATTAAAGATGTATGAATT
AAAACTTCATGATCTAAATGCTTTTGAAAACGATAAGAATTTGGTGTTTGAATTTCATAACCAACACCAGCAGTTTCAACAACTACGTGTGTA
GGATATAAATGTGTTAACTTACCTTTGACATACGCGTACAT

Gene: pheB (ACT domain-containing protein)

Position: 1702167 to 1702625, length: 459 nt, orientation: REVERSE

Perfect match to: (N315-BA000018-[1677944:1678402:r], highly conserved allele)

Sequence:

TTACATACTCATACTAATTAATTCTACTTTTGATACATAATCTAAATTTCTCAAAGCGCCAATAACATCTTCTACTGAAGTTTCTTTAGATTTAG
CATTCAGTGATAATGTTATTGTTGCTTTTTCTTCCATTGGAATACTTTGATGAATCGTTAATACAGATAGTTCTAACTTTGATATAACATCTAG
TACACGTGCCAACATACCCACAATATCAGTTACATATAAAATTAATGTAAATTCTCGATGGTCAAGCATTTTATCGTCTACTGGAAATATCGT
TTCTCTATATTTATAAAAAGCACTTCTAGATAGATCAAACTGTTTAACGGCATCATAAATGGACAATGTCGGATCACTTTTTAAGGCATCTTT
AATCTTCAATGTTTTAACCACGGATTCAGGCAAGACATCTTCTCTAATTAAATAAAACTTTTTATAATCTTTATTGTCCATCAT

Gene: obgE (GTPase obg)

Position: 1702635 to 1703927, length: 1293 nt, orientation: REVERSE

Perfect match to: (KLT6-APFH01000004-[1118167:1119459:r], allele observed in CC12+CC6+CC12+CC239+CC1217)

Sequence:

CTATTCAACGAATTCAAATTCTCCGCCAAGAATTCTAACGATATCACCATTTTTACAACCACGTTCTCTAAGCGCATCATCAATACCCATCGAA
CGCATTTGACGAGCAAATCGACGTACTGCTGGATCACTGTTAAAGTCAGTCATTTTAAACATTCTTTCAATAGCATTACCACTTACCACATAA
GCACCATCATCATCTCTTGAAATTGTAAATTTATCTTGTGACGGTGTATGTTTATATAATACTCGGTTAATGCCAACTGACTCCTCTTCTTCAA
CTGTGAAGTCAACATCTTTATATTCTTCTAATTTATCTGCTATTGCATATAATAATTGATCAATATTATCACGCGTTATTGTTGAAACTGGAAT
AACTGGCACATCTTCGCCAATTTCTTCTTTAAACAAGTTTAAATTATCTTGTGATTCAGGTAAATCCATCTTGTTAGCTACTACGATTTGAGGT
CTATCTTCTAAACGTTGCTCGTACGCGGCTAATTCTTGATTAATAACTTTATAATCTTCAATAGGTTCTCTACCTTCAGAACCACTCATATCAA
TCATGTGAACAATAACTTTTGTTCTCTCTACATGTCTTAAAAATTGATGTCCTAATCCAACGCCATCAGATGCACCTTCAATTAAACCTGGTAA
ATCTGCCATAACAAAACTACGTTGATCAGGTGTTGAAACAACACCTAGATTTGGTTTAATCGTTGTAAAATGATATGCCCCAATTTTAGGCTT
AGCTTTTGAAACGATAGATAATAAAGTCGATTTACCCACACTAGGGAAACCTACTAATCCTACATCAGCTAATAATTTCAATTCTAAAGATAC
ATCTAATTCCTCACCTGGTTCACCTTTTTCACTGAAGTCAGGTGCAGGGTTTCTAGGTGTTGCAAAACGTGAATTACCTCGGCCACCTCGACC
GCCCTTCGCTACTACAGCTCTTTGACCATCTTCAACAAGATCTGCTAACACTTCGTCTGTTTCAACATTTTTAATAATTGTACCAGGTGGAACT
TTTAATACTAAATCTTCCGCATTTTTACCATGCATATTACTACTTTGGCCATTTTCACCTTTGCTTGCTTTAAAATGACGTTGATATCTAAAATC
TAATAACGTTCTTAAACCTTCATCCACTTCAAATACGACTGAAGCACCTTTACCACCGTCACCGCCAGCTGGTCCACCAAATGGTACATATTT
TTCTCTTCTGTATGCGGTAATACCATTACCACCATCACCGGCTTTAAGAGATATTTTGACTTGATCGACAAACAT

Gene: SIRU15 (staphylococcal interspersed repeat unit 15)

Position: 1704006 to 1704292, length: 287 nt

Sequence:

ATTAGGGGCTCTTATGCAGTTGCTTTTAAATTTACTTTACTCAAATTGCTGATATTTCCGAATACCAAATATGATACTTTTTTTGCTTTTTATAC
TGCAACTGTATTCTTTTGTCCCCTTATAGTTTAGCGATTAGGGGCTCTTATGCAGTTGCTTTTAAGTTTACTTTACTCAAATTGCTGATATTTCC
AAGTACCAAACATGATACTTTTTTGCTTTTTATACTGCAACTGTAATCCTTTTAGCCCCTTATATATTGTAAATAATTAGGGGCTCTTATGCAG
TTG

Gene: SIRU15 (staphylococcal interspersed repeat unit 15)

Position: 1704406 to 1704692, length: 287 nt

Sequence:

ATTAGGGGCTCTTATGCAGTTGCTTTTAAATTTACTTTACTCAAATTGCTGATATTTCCGAGTACCAAATATGATACTTTTTTTGCTTTTTATAC
TGCAACTGTAATCTTTTGTCCCCTTATAGTTTAGCGATTAGGGGCTCTTATGCAGTTGCTTTTATAAATCTTTGCTTTAAAATACTTATATTTCT
CAGCACCAATTATGGTACAGTTTATTCTTTAAACGCTGCAACTGTATTCCTTAGCCCCCTTATATATTGTAAATAATTAGGGGCTCTTATGCA
GTTG

Gene: rpmA (50S ribosomal protein L27)

Position: 1704815 to 1705099, length: 285 nt, orientation: REVERSE

Perfect match to: (RF122-AJ938182-[1634464:1634748:r], highly conserved allele)

Sequence:

TTATTCAGCTACTGCATATACAGAAACTTGTTTTTTGTCGCGACCTTTACGTTCGAATTTAACAACGCCGTCGATTTTAGCGAATAATGTATC
ATCGCCACCACGACCTACATTTTCACCAGGGTAAATTTTAGTACCACGTTGGCGATATAAAATTGAACCACCTGTTACGAATTGACCGTCAG
CACGTTTAGCACCTAAGCGTTTTGATTCAGAGTCACGTCCGTTTTTTGTAGAACTTACCCCTTTTTTAGATGCGAAGAATTGTAAGTTTAATTT
TAACAT

Gene: DUF464 (putative protein)

Position: 1705111 to 1705431, length: 321 nt, orientation: REVERSE

Perfect match to: (N315-BA000018-[1680354:1680674:r], highly conserved allele)

Sequence:

TCACTTATAATTTAATCTAATATTCTCATTATATTCTTCTTCAATAGTTTGTAAAGACACAAGCATTGTTTGAAGAATTAGTTGCGCTTCATCG
TTATTTGTATCAACGCTTCTTATATGAAAATGACCACCATTGTCGTCATAATTGATATCTGGTCTCTCAGATGTCAATCCTATAATCGCATTAA
CACTACCAAACAATACAGCTGAAGCTCCAGCACAAACGATATCATGACCATATTCACCATGGTCAGCATGGCCATCCATAATAACGTCTGTT
ACTTTGCCTTCATCATTAACTGTAATATCAACAGTAATCAT

Gene: rplU (50S ribosomal protein L21)

Position: 1705437 to 1705745, length: 309 nt, orientation: REVERSE

Perfect match to: (11819-97-CP003194-[1755819:1756127:r], allele observed in CC80+CC445)

Sequence:

TTACGCGTTGATTTTATCGATTGTTAATTTAGTGTATGGTTGACGATGGCCTTTTTTACGTTTTGAATTTTTACGACGTTTGTATGTGAATACA
GTAATTTTTTTACCGCGACCTTGTTTATTAACAGTAGCAGTAACTGTTGCACCTTCAACTGTTGGCGCTCCAACTTTAACTGAATCTCCACCTA
CAAATAATACTTTATCAAATGTAAAAGTATCTCCTTCGTTTACGTCTAATTTTTCAACGAAAATTTCTTGACCTTCTTCTACTTTGATTTGTTTT
CCACCTGTTTCAATAATAGCAAACAT

Gene: L21_leader (ribosomal protein L21 leader RNA)

Position: 1705758 to 1705954, length: 197 nt

Sequence:

CTGTATAATAAGTCACGCCATACATAGGTGACATTTCGTTATTGAGTCAAGCGTTTGCACTCTATGTTGTTTCGGTCGCAACTCGAGTCATTT
ATAAACATAAACTCTTCTCGTTTCTTCCTTCAACGCCTTGATTGACAATCGCTTTCTTCATTAAAAATGTTCTTGAACCTATATTTGAGCGGTT
GTATGTAGCT

Gene: mreD (cell shape determinant D)

Position: 1705981 to 1706511, length: 531 nt, orientation: REVERSE

Perfect match to: (COL-CP000046-[1733628:1734158:r], highly conserved allele)

Sequence:

TTACCATTGACGACGTTTCATGTCAATGTCATTGTTTGTTTTTTTAAGGAACTTTATAATCAACGGATAAAGCATAATCAACAGTACAAAATT
CATAATTAATGTTGGCAATAATCTAAAGACTACAAAATGAATAATATCAAATTGAATGAATCCTAACATACCGTATATTAATGCCACATAGA
CTTCTAATAATAAGGTGCTGGCTAATATAATAATGAATAACATCGAATGATCTTTGTAAAAAATTTTAAAGAATCGATCTATAAGTGCTAAA
AACAATATATAGCCAAATAAGTACACTCCATAAATACTACCAAAGTATACATCAGTCATTACGCCTAAAAATATGCTGAGCAATAATGATAC
GCCAAAGCCACGATACACTACCATCATTAAAATATACATAAATGTAAGGTGTGGTACAAATACAAGTTCAAACTTACCTATGTGCATTGGAA
TAAGAAGCCCAATTGCAGTATCTATATAAAATAGTAAAATACCTATCAAAAAATAATACAGTGTACGCAT

Gene: mreC (cell shape determinant C)

Position: 1706511 to 1707353, length: 843 nt, orientation: REVERSE

Perfect match to: (st2344-FGWF01000005-[78452:79294], highly conserved allele)

Sequence:

TTATTTATCCCTGCTTTCATCATCAGGAATTGTTTTAGGATCTCTTTTTGCAACATAAACATGACTCAAATCTGTTAAGTCTGCACCAGTCTTA
ACCCTAACTTCTTTAGCTAAGCCGTATTGATCATTTTGAACCTTAGTCACTTCTCCTATATATAAATTACTTGGTAGTTGATCAGCTAATCCAC
TTGTAACGACTTTATCACCTTTTGAGATATTATCTCTATTATTAATGTCACTAATTACAAGTTCTGAGTTCTTTTCATCATAACGATCAATTAAA
CCAAATATATTTTTAGAACCGTGTTGTATATTTACAGATAATTTACCCGCACGTGTATTAGTTGAGATTAAATCAACTTGTGAAGAAAATTTA
TTAACTTTAGTAACTCTTCCAACAAAACCTTGTGATGTCATCACAGCCATATTTGAAGTTATACCTGATTTAGATCCCTTATCAATTACAATTG
TATTCATCCACTGATCCGGATTTCTTGCTAAAACTGTAGTAGAAATAGGATCAAATTTTGAAATATCTTTTAAATCAAGCTCTTTTTTTAATTT
TTCATTTTCCGCTTCTAATTGTTGGTTCTTAGATTCTAACTGGCTAATCTTATTTTTAGATTCTTTAGAATCTCCTTTTTTAAAAAAGTCCCCAAT
CGTACCAGCAACAAAATTAACTGGATAACTCACAACTCGTTGTCCAAAAGACACAGAATCACCTATATATTGTTCAGGAGGTGATTGAGATT
GTGAACGTATGGACAGCCCAATTAATGCAATAAAAACGATAATTGCACATAAAACAACAATTAATTTGTTATTTTTAAAAAACTTAAGCAC

Gene: Q5HFB3 (putative protein)

Position: 1707746 to 1708219, length: 474 nt, orientation: REVERSE

Perfect match to: (TCH1516-CP000730-[1758820:1759293:r], highly conserved allele)

Sequence:

CTATTGATAAGCATTTTCAGGTTTTAGTTGTAAATTTTGCCCTAATTCATTTAAGTCTTGTTGCATTTCAAATTCAGTATTGTAAACACGCATT
GACTCATCTCCAAATTTATAAAGAATAAATTCGTCTCCTCTTTGACCTATAATATATTGATCATTATAAGCCATGCGATTCATTCCAGACACAG
CCATAAACTCTTGTTTATCTATCATTTTAAATACATTTTTAATTTGGCTTAATGGTACATTCTTTATTAAATCATTTTCTTTTAATGAATATTTCT
CACCACTTACATAAACATGTTGGGCATCATTAGTGGGTTGCGTAACTTGATTCATTTGATCATTATCGTGATTTACAGGATTCCAATCTTGTT
CTTTTAAAAAAGGTGCGTATTTCAAGGCGATATAAACAATTATCACAATTGCTATTACTGCTATAATGTTTTTAATCATACTAAATATCAATCT
CAT

Gene: Q5HFB2 (putative protein)

Position: 1708693 to 1708977, length: 285 nt, orientation: REVERSE

Perfect match to: (COL-CP000046-[1736340:1736624:r], allele observed in CC8+CC25+CC80+CC239+CC4803)

Sequence:

TTACTTACGCCCAAATGCGTTAGATAAAAATCGTATCAGCGCGTAGCTACCACTTAGTAAAAGGCCCCAAACGATTCCAGTAAAAATTGAGC
CAATTGCTACACTTAATAAAGGAAAAGCTTGTTTCGTAACGATTACACCTGCTAACGCGGTTACTAAAATACCTAACGTAGATAAAGCTAAT
GTTATTAAAAGATTGCGCTTTGTTACAGGTTCATTTTTAATATTTAACTGCATGGTATAAACCATTGGCACGAGAACCCCGATAATAATAAAT
AGAGTCAC

Gene: radC (DNA repair protein C)

Position: 1709045 to 1709731, length: 687 nt, orientation: REVERSE

Perfect match to: (MW2-BA000033-[1730020:1730706:r], highly conserved allele)

Sequence:

TCAATCATTTTCATCAAAGTAACCCGCTTCTACAAGACTGGTAAATCTATTATCACCGATTATAATATGATCCAATAAATCTATCCCTAAAATC
AAACCACACTCCTTCAACCTCATTGTTGTTATGATATCTTCTTGTGAGGGCGTTACATCACCGGATGGATGATTATGAACTGCGATGATTGCA
TTGGCATTTTCTCTCACCGCAATACTAAAAATTTCACGTGGATGTACAATCGAACTATTTAATGTACCTTTAAAAACACAGGTTTCTTTAATCA
CTACATTTTTTGAATTTAACAATAAAATGACAAAATGTTCTTGTGTTAAATCTTTCATTGTTGGAATCATATAATCAGCAACATCACTTGGTTG
CGTTATTTTTATACGATTATTTTCAGCTCTTCTCCCCATCCTTTCCCCTAACTCAAATGCTGCTTTTAAAGTAATTGCTTTTTGTAATCCAATCCC
TTTAACTTTTATCAAATCGTTAATTGAAGATTTTTTCAATTCATTCAGATTCGAAGCAGATTTAAGCAGTTCATTACTAATGTCTATGCTCGAG
AATCCTTTTCTTCCGGTGTTAATTAATATAGCTAATAATTCTGTATTCGAAAGACTTTTTGCACCATGGCTTAACAAACGTTCTCTTGGCATTT
CTGAAGTTACCATTTCTTTAATTTTCAA

Gene: comC (pseudopilin signal peptidase)

Position: 1709728 to 1710435, length: 708 nt, orientation: REVERSE

Perfect match to: (Strain_21333-AHKA01000003-[5222:5929:r], allele observed in CC80)

Sequence:

TCAAAAATATACGCCTCCTAAAAATTGATGGATATCATTATAAAAAAGTGAATTGATAAAAAAGGAAATAAATATAAATGGAACAAGGGGT
AATAGTTTAATCGGCTTAAATATCATGGTAATTAAAGCAACTAAACCAGCAATGACAAATGTAAATAAAATGACATAAATAGTGAATTGGA
GAGGGAAAAACAAAGAAAGTGCAGATATTAGTAAAACGTCACCATAACCAATATATGCCCGAAATAAAAAGTAGAATATATGCGTGGTCA
TACTAATAATGATAAAAGCTACTGGATAAATCATACTTAACGAGAGAGAAACGATACAATAAATTATAATTAAGCGACAATCTAACATTAAA
GAAGTGATATCGGTCATAGTAAAAATAAGCAGAAAAACATATGTAGTTATAAATAGCGTAGCATTTACGTATGTGAAATCATACTTAATAA
AGACGATAGGTATTAAAGCAAAGGTTTCCCCTAAGAAATGTGTTAGGGAAATACGCTTTCGACAGTTTCGACATCGCCCTTTTAATAATAAA
AAACTAATAATCGGCATTAATTCATACCATTTGAGTGATGAATTACAATAATCACATTTCGATCTTCTATGTAAATAATCAAATGACGTTTCTT
CTATAGATATAAATTGATATAGAAAACTAAAGATACAACTGCAACTATAAGATAACAATACTACCAA

Gene: folC (folylpolyglutamate synthase/dihydrofolate synthase)

Position: 1710705 to 1711976, length: 1272 nt, orientation: REVERSE

Perfect match to: (RF122-AJ938182-[1640282:1641553:r], highly conserved allele)

Sequence:

TTATAATGCTTCAAAGTCTAATTTTGATTTAACTTCACTTATGAAATACAGACTACCGGTAATTACTAATGTATCACCTTGATAATTTTTTATA
AATTCAACGTAGTCATCTACTAATTGTATTTCATCATTTTCAATACTACCTACAATTTCTTCTTTGCGTAACGCTTTCGGAAAATCAAATTCAGT
TGCATAAAACGTATGCGCAATTAAACTTAAATGTTTGACCATCTCGTTAATCGGTTTTCCGTTTATTGCTGAGAACAAAATATCTACTTTTTCT
TTATCATGGTACTGTTTAATTGTATCAATTAGAGCATCTATACTCTCTGAATTATGTGCGCCATCCAAAATGATTAAAGGTTTGTCATGCACC
TGCTCAATACGTCCAGTCCAACGAACTGATTCAATACCGTCTATCATCTTATTGAAATCTAATTCAATTAATCCTTGTTCATTTAATTCAATAA
GAGCTGTTATGGCTAATGCAGCATTTTGTTTCTGATGTTCACCTAACATGCTTAAAATGATTGTTTCTAATTCATAATCTTTATAACGGTAAGT
AAATTCATCATTTTGCGATACAACAACAATTTCTCTATCTAATTCAATTGGCTTTGCATGTTGTTCAATTGCGCGTTCACGAACATATTTTAAT
GCATCTTCATTTTTAACAGCATATATCACTGGAACGTTAGGCTTTATAATCGCGCCTTTATCCCTAGCAATATCTAGATAAGTACCACCTAAA
ATATCTGTATGGTCTAGACCGATACTAGTTAAGATTGATAAAACCGGTGTAAAGACATTTGTCGAATCGTTCTTTATACCCAATCCAGCCTCA
ACAATGACAAAATCAACAGGATGTATTTCACCAAAATATAAAAACATCATCGCTGTGATTATTTCGAATTCAGTTGCAACACCTAAATCTGTT
TCACGTTCCATCATTTCACTTACTGGTTTAATACGTGATACTAATTCTACAATAGCGTCATTTGATATTGGCACACCATTTAGACTAATTCGTT
CATTAAATGTTTCAATAAACGGCGACGTAAATGTACCTACTTCATAACCATTTTCAACTAAAGCTGTTCTAAGGTAAGCAACTGTAGAGCCTT
TACCATTTGTGCCACCTACATGAATACCCTTAATGTTATTTTGAGGATTATTAAATTGTGCTAGCATCCATTCCATACGTTTAACACCTGGTTT
GATGCCAAATTTAGTTCTTTCGTGTATCCAATACAAGCTCTCTAGGTAATTCAT

Gene: valS (valyl-tRNA synthase)

Position: 1711989 to 1714619, length: 2631 nt, orientation: REVERSE

Perfect match to: (11819-97-CP003194-[1762371:1765001:r], allele observed in CC80)

Sequence:

CTATGCTTTTAATTGTTCAATTCTTGCCTTCACACCATCATATTTTTCTTGATAATCTTGTTTTTTACGTTTTTCTTCATTTATAACCTTTTCAGGT
GCTTTACTTACAAAGTTTTCATTAGAGAGCTTTTTATCTACTCTATCTAATTCGCTTTGAAGTTTAGCTAATTCTTTTTCCAAACGGCTGATTTC
CTTATCCATATCAATTAGCCCTTCTAATGGTAATACCACTTTACCTGCAATTACAACTGATGTCATTGCTTTCTCAGGAATTTCCACGTCAGTG
CTAATATTTAAGGTACTAGGATTACAGAATTTGATTAAATAATCTTTGTTTTGTGATAAAGTTGTTTCAATTTCTTTATCTTTAGCTTGAATTA
AAATAGGTATTTCTTTAGACAATGGCGTATTTACTTCTACACGTGATTGTCTTACAGATTTAATGATTTCAACAAGTTGTTGCATTGTTTGTTT
ACTTTCTTCAAAAATCAATGATTCACGCACTTCTGGCCATGAAGCTTTAACAATTGTGTCACCTTCATGTGGTAAACTTTGCCATATTTTCTCT
GTTACAAATGGCATGAATGGATGTAGCATTCTCATAATATTGTCTAAAGTATAACTCAATACTGAACGTGTAACTTGTTTTTGTTCTTCATCA
TTACTATTCATTGGAATTTTACTCATTTCAATGTACCAATCACAGAAATCATCCCAAATGAAATTATATAATGCACGTCCAACTTCGCCGAATT
CATATTTGTCACTTAAATCAGTAACTGTTGCAATCGTTTCATTTAAACGTGTTAGAATCCATTTATCTGCTAATGATAAGTTACCACTTAAATC
GATATCTTCAACTTTAAAGTCTTCACCGATATTCATTAAACTGAAACGTGCCCCATTCCAGATTTTATTGATAAAGTTCCACACTGACTCAACT
TTTTCAGTTGAGTATCTTAAATCATGTCCTGGAGATGAACCTGTTGCTAAGAAGTAACGCAAGCTATCAGCACCGTATTCGTCAATAACATC
CATTGGATCCACACCATTACCTAATGATTTACTCATCTTACGCCCGTCTTCAGCACGAACTAAACCGTGTAATAATACATCATTAAATGGACG
ACGATCTGTAAATTCTAAGCCTTGGAATATCATGCGTGCTACCCAGAAAAAGATAATATCGTAACCTGTAACTAAGGCATTTGTTGGGTAGT
ATCGTTTAAAGTCTTCACTTTCTAAATCAGGCCAACCTAACGTAGAGAAAGGCCATAAAGCACTTGAGAACCACGTATCTAATACATCTTCA
TCTTGTTGCCAATTTTCAATATCAGTTGGCGCTTCTTCTCCAACATATATTTCGCCTGTTTCTTTATGATACCAAGCCGGAATTTGATGACCCC
ACCATAATTGTCTTGAAATCGTCCAATCTCTAATATTTTCCATCCATTGGTTAAATGTATGTTCGAAACGTTGCGGATAAAAATCAATACGAT
CATCTGTTTTTTGGTTATCTAATGAACGTTTCGCTAAGTCTTCCATGCGCACAAACCATTGTGTTGATAAATATGGTTCAACAACAGCGCCAG
ATCGTTCTGAATGACCTACAGAATGAACATGATCTTCAATCTTGATAACTAAATCTTGTTCTTTTAAATCTTTAACTAGCTGTTTACGACAATC
AAAACGGTCCATACCTTCATATTTACCCGCTTTGTCGTTCATTTTACCATTTTCATCCATAACGATAATATTTTCTAATTGATGTCTTTGACCAA
TTTCAAAATCATTAGGGTCATGTGCTGGTGTCACTTTCATAGCACCAGAACCGAAGTCTATATCAACATACTCATCTGCTAAAATAGGCAGTT
CGCGTCCTACGATTGGTAATATAACAGTTTTACCGATTACATCTTTGTATCGTTCGTCATTAGGGTTAACAACAATCGCTGTATCACCTAACA
TCGTTTCTGGTCTTGTTGTTGCAATTTCAATAAAACCTTCACCATCAGCGTAAGGATATTTAAAATGATAAAACGCACCTTGAACATCTTCAT
GTATTACTTCAATATCAGATAAAGCTGTACGTGCTTTAGGATCCCAATTTATAATACGTTCGCCACGATAAATAATTCCTTTATTGTATAAATC
AACAAAAACTTTTTTAACTGCTTTACTTAAACCTTCATCTAAAGTAAAACGTTCTCTACTATAATCTAAACCTAGACCTAATTTAGCCCATTGC
GCACGAATAAATGACGCATACTCTTCTTTCCAATCCCATGCCTGTTCTAAAAACTTTTCACGACCAAGATCATATCTAGTTATTCCTTGTTCAT
TTAATTTAGCTTCTACCTTTGCCTGTGTCGCAATACCAGCATGATCCATACCTGGTAAGTATAACGTATCGTATCCTTGCATACGTTTCATACG
TGTAATGATATCTTGTAAAGTCGTATCCCATGCATGTCCTAAATGTAATTTACCAGTTACATTTGGTGGCGGGATAACAATTGTATATGTTTC
TTTTGATTTATCTTCTGACGGTTTAAAATAACCATTCTTTACCCATTCTTCATAACGTCCCGCTTCAACTTCACGAGGATCATATTTTGATTTCA
TTTCCAT

Gene: tbox08 (T-box leader element)

Position: 1714663 to 1714964, length: 302 nt

Sequence:

AAATAGGACGGATATTCCGTGGTACCACCTATATTCAAGAAGGATGATTAATATCAAATTCACTCTTTTAACATAATTGGAATAATCATACC
AATACTATCATCGTGAAATTTGAAATGCTTCATCTCTTCAAGCACTCTAGATTATGATTAACGCTCAAACACGTCTTAGCCTACTATTAATCAC
GTTCAGCTAAGATACTCTGTGGGCTACCTTCAGTAAAAATCATTTACATACTCACACCAAATCATATGCTCTCTTTAAAATAATTTGAACTTAC
TCTTCCCAAATCCTATATTAAA

Gene: tag (DNA-3-methyladenine glycosidase)

Position: 1715022 to 1715582, length: 561 nt, orientation: FORWARD

Perfect match to: (11819-97-CP003194-[1765404:1765964], highly conserved allele)

Sequence:

ATGAATGAATGCGCATTTGGTACTAAAGATCCAGTCTACTTAGACTATCATGATCATGTATGGGGACAACCGCTCTATGATAGCAAGGCATT
GTTTAAACTTTTAGCATTAGAATCACAACATGCTGGGCTATCTTGGTTAACTATTTTAAAAAAGAAAGAAGCCTATGAAGAAGCATTTTATG
ATTTCGAACCAGAAAAGGTAGCACAAATGACCGCTCAAGATATCGACCGCTTAATGACTTTTCCAAATATCGTTCATCATCGTAAAAAATTA
GAAGCAATTGTTAATCAAGCTCAAGGGTATTTAAAAATTGAACAAGCATATGGTAGTTTTAGTAAATTTTTATGGTCATATGTAAATGGTAA
GCCTAAAGATTTGCAGTATGAACATGCTTCTGATCGTATCACAGTTGATGATACTGCAACACAACTATCTAAAGATTTAAAACAATACGGGT

TTAAATTTTTAGGTCCAGTAACAGTATTTTCGTTTTTAGAAGCAGCCGGTTTATATGATGCACATTTAAAAGATTGTCCATCAAAGCCTAAAC
ACAATTAA

Gene: abrB (putative ammonia monooxygenase)

Position: 1715805 to 1716878, length: 1074 nt, orientation: REVERSE

Perfect match to: (MW2-BA000033-[1736780:1737853:r], highly conserved allele)

Sequence:

CTAATCTTCACGCTTATTCGAACGATATTTTAAAAAGTAATTAATTCCTGGTGCAATAATAAATAGGATGAAAAAGATTCGGAAAATATGAT
AACTCGTAATCATAGCAACATCGGCACCAGTAGCTAATGCAACTAAAACTATCTGATTAACCCCTCCTGGTGCTGCACCAAGAAACAATTCA
TTAATAGGATTATTATCAAAGAAATGTATGACATAAACCATGATTAGCGCACCAATTATCAACATAATATTTTGAATTGTAATTGCGATTGCT
AGTCTACCTTTTAAATCTGACAATAAATGCGCAATTTGAACTCCAATTCTAATCATATATATTAGTTGTGCCATGTTCAACAACCAATGATCTA
GTGTAAATGTTAAACCTGTAGAAAAAATTCCAAACAATTAATACAATGAGTGGTGCTAATAATTGAAATGTTGGAAACTTTATTTTAGACATA
ATTAGATAAACTATAAAGATAGCTATCGCTAAAATAACTATTTGCCCTATGTTTAATACTTGTGATAAAGGCAAGACTTTTGTTAACTTTCCA
TTCGCATGCATGTTACCATCATGAAAAAAATATGAAATGAACGGTACTAAAACAACAACAAATATAATTCGTGATGTTTGCGTTAAGCTAAC
AACTAACAAATTAGCACGTTTGTCTTGTTCAGCCATGACCAGCATTTGTGTTAGTGCTCCTGGTATAACACTTAAAATAGCTGTTTCTGTATT
AATACGTGCAATTTTTTTAAAAATAAATGCCATTACTATTGCAATTAATAATATCGAAATAGATACAACAATAATCGAAAGCCAATTGTCTTT
AATATCCATAACGACATTTTTCGTAAACGTTGATCCGATTTGCACACCTAATAGTACAATACCTAATTCACTAAGTAAGAATGGCCATTTAAT
ATCAAGTTTGAAAACTTTTACACAAATGATTGATGCGATAATAGGACCAAACATAAATGGGAGTAATACGTGCGATGAATACAGTAGAATA
CTAATAAAAAATGATAAAACGAACACAATGAAATTATTTCTATATATCATTGTCAT

Gene: gsaA (glutamate-1-semialdehyde 2,1-aminomutase, locus 1)

Position: 1717074 to 1718360, length: 1287 nt, orientation: REVERSE

Perfect match to: (MW2-BA000033-[1738049:1739335:r], highly conserved allele)

Sequence:

TTATTTTACAATACGACTTAAAGCCGTATCAAATGCTTGAATCGTTTTTTCAATATCTTCTTTCGTGTGTGCCGTAGATAGGAATGTACCTTCA
AATTGAGATGGTGGTAAAAACACACCTTCTTTTGCCATTTCTCGATACATTTCTGCAAATAATTTCAAATCACTTTTATTCGCTTGTTCAAAAT
TAGTTACAGGTCCTTCATTTAAGAAATAACCAATCATTGAACCTGCTCTATTTACAGTTATTGGTACATTGTGTTTAGCAAATACACGCTTTAA
ACCGTCTTCAAGTATATCGCCTAACATATTAAAATACTCATATGTCTCTGGCGTTAATTGGCTTAACGTTTCATAACCACTTGTCATTGCAAGA
GGATTTCCTGATAACGTACCCGCTTGATAAATATTTCCTAATGGTGCTATATGATCCATGATTTCTTTTTTACCACCAAAAGCACCTACAGGT
AGTCCTCCACCGATAACTTTTCCTAAGCAAGTTAAATCTGGTGTCACACCAAAGTAACCTTGTGCACAATGATAACCGACTCTGAAACCAGT
CATTACTTCATCGAAAATTAGCAATGCGCCGTATTCAGTCGTAATATTTCTTAATCCCTGTAAAAAACCTTCAATCGGCGGTACGACACCCAT
ATTACCAGCAACAGGTTCTACGATTACACCAGCAATATCGTCTCCAAATTTTTCGAAAGCGATTTTAAGTGCATCTAAATCATTGTATGGAAC
TGTAATTGTATTTTTAGCAATACCTTCAGGCACACCAGGAGAATCCGGCAATCCTAATGTTGCCACCCCAGAACCAGCTTTGATTAATAACG
AATCACTATGACCATGATAGCAACCTTCAAATTTCACAATTTTATTTCTTCCAGTATAACCACGTGCTAATCTTAAAGTATCCAATGTAGCTTC
TGTACCAGATGACACCATACGCACTTTTTCTATTGAAGGTACTCGGTCAATAACGAGCTGCGCCAATTTATTTTCAAGTAATGTTGATGCACC
AAAACTTGTACCTTTATCAATTGCTTCATGTAAATGACTAATAACTTGAGGGTCTCTATGTCCTAAAATAAGTGGCCCCCAACTTAGTACATA
GTCGATATACTCGTTACCATCGATATCATAAATTTTTGAACCTTTACCGTGATCCATAAAAATTGCTGGTGTATCTACTGATTTAAATGCGCG
TACTGGACTATTTACACCACCAGGCATTAAAGTTTCAGCAACCTTCATTGCTTCTTCTGATTTCGTATATCTCAT

Gene: hemB (porphobilinogen synthase)

Position: 1718408 to 1719382, length: 975 nt, orientation: REVERSE

Perfect match to: (NN50-BAEA01000025-[279907:280881:r], allele observed in CC4803+CC8+CC22+CC30+CC80)

Sequence:

TTATTTATCTAAATAGCGACAAATGTCCTTTGCAAAATACGTAATAATCATATCAGCACCTGCACGTTTCATTGAAACCATTTGTTCCATAAC
GACACGTTCTTCATCTATCCAACCATTTTGTGCCGCTGCTTTAGTCATACTATATTCTCCACTCACATTATATGCAACAACTGGAACATTCGTA
TGATTTTTAACATCTCGAACTATGTCTAAATAACTTAGAGCAGGTTTAACAATCATCATGTCGCACCCTTCTTTAAGATCACTTTCTAATTCAC
GAAGTGCTTCCAAACGGTTAGCAGGGTCCATCTGATACGTTTTTCTATCCCCAAATGATGGCGCTGAATCTGCTGCATCTCTAAAAGGTCCA
AAGAAACTTGATGCATACTTGACACCATAACTCATTATAGGAATATTGTAATAGCCGGCTTCATCTAATCCACGACGAATTTCAGCAACAAA

ACCATCCATCATATTACTTGGCGCAATAATATCAGCACCAGCTTCCACTTGAGAAATTGCTGTTTTAACAAGCAGTGGCAATGATTTATCATT
GTCAACGTCATGTGTATGGTCATCAATCACGCCACAATGACCATGATCAGTATATTCACATAAACAAGTGTCTGCAACAATTAATAAGTCAT
CATACATTTTTTTAGCAATACGTGTTGCCTGTTGAATAACACCATCGTGAATGTATGCACCAGTACCTATATCATCTTTTGAGTTTGGAACAC
CGAAAAACATAATGGCACGTATGCCTAAGTCATAAGCTTCTTTTAATTCACTTTCAAGTAAATTCAAACTGATTTGGTATACACCTGGCAATG
ACTTAATTTCTTTTTTCACATCGTCTTTTTCAACTACAAAAATTGGATATATTAAATCTTCTTTTCTTACATGATTCTCTCTAACCATATCTCTCA
TTGTCGCTGATGATCTCAATCTTCTATGTCTATCAAATTTCAT

Gene: hemD (uroporphyrinogen III synthase)

Position: 1719385 to 1720053, length: 669 nt, orientation: REVERSE

Perfect match to: (Strain_21343-AHKV01000009-[40724:41392], allele observed in CC88+CC25+CC80+CC361)

Sequence:

TTAGCCCCTACTTTCTAAAATCTTTTCAATTAGTGATTCGAGTGTTTGAATTTCTGCAATTGTTACTGGTTGTTGATATGATTTAATGGTCCGT
GCTGTTTGTTCTCCAATAGCAAAATACGACTTGAATTTTGGTACAAATCCTTCATTAAAATAATAACGTACTGCCGACGAACTTGAAAATGTT
AATGCATCGATTTGTTGATGTTCTATCATTTCTTTAACATCTTGTATATTTTGTTTGTTAGGCACTGAAGTATATAAATCTATTTTAACAACTTC
ATTATCTTTAGATAACGCTGCTAATAACAATGGTCTCGCCAATTCACTCGAAGGCAAAAGTATTTTTTGGTTAGTTTGATTAAATGATTTTAA
AAATCCTTCTTGAGAAAAGTCGTTTGGCATAAAATCAACTCGAATGCCAAGTGATTCACAATATTGCGCTGTCTTACTTCCTATCACAGCAAT
GTTATCAACATTAATTCCTTTTAGATATTTATAAAAGAATTTCACAGCATTTTTAGATGAAAAAATAAGCCAGTCATAGTGTTGATTTAACAA
ATGAATATCAAAATTTAGTGGCTTTATATCAATAAAGGGTTTGTGAATAATTGATACTAAATCACTTTGCATGTCATTTGTTTGTGTCATAAC
TACAACTGGCTTCAT

Gene: hemC (porphobilinogen deaminase)

Position: 1720075 to 1721001, length: 927 nt, orientation: REVERSE

Perfect match to: (11819-97-CP003194-[1770457:1771383:r], allele observed in CC80+CC8+CC80+CC88+CC239)

Sequence:

TTAATGTTGTTCATTTAAGCGTTTTATAATTTCATAAGCACCTTGCTCTTTTAATTTGTTACTCACTGTTTTGCCTAACTCAACCGGATCTGTTC
CGTTCATTGTATATTCAAATCGCTCTTTACCATCTGGGGTCATAATTAAACCTGTAAATTCGATTTCGTTTTGATCTGAGATTGTAGCATATCC
TGCAATTGGCACCTGACAACTACCATCCATTTCTGCTAAAAACGTTCGTTCAGCAGTCACACATTTTGCAACCTCATCATTATGTACTTTGCTT
AATAATGTTAATAGTTCTTCATCGTCACTACGACATTCTATCCCTAAAGCACCTTGTCCGATTGCAGGTAACAATGTATCTCTATCAAGATAA
GATGTTACAATATCATCTGACCAGCCCATTCTTCTTAAACCAGCTGCAGCTAAAATAATCGCATCATAATCTTCAGTTTGTAACTTTTCTAATC
GTGTATCTATATTACCTCTAATCCATTTAATCTCTAAATTAGGATACTTAGATAATATTTGTGCACCACGACGTAATGAACTAGTACCAATAAT
ACTGCCTTCTGGCAATTGGGATAGTGGTGTATGTGTTTTAGAAATATACGCATCAAAAGGTAATTCTCTATCAGGGATACAACCTAATGTTA
AACCTTCCGGAATTACACTTGGTACGTCTTTAAGCGAGTGTATTGCCATATCGATATTTTTTTCAAAAAGTTCATGTTGTATTTCTTTAACAAA
TAAGCCTTTGCCTCCGACTTTAGACAATTGTTTATCTACTATACGATCGCCTTTCGTGACAATTTCTTTAATTTCAATTTCTAGATTTGGCTCGA
CAGCTTTTAATTTATCAATAAATTGCTGGCTTTGTGTTAAAGCTAATTTACTTCTTCTGGAGCCAACGACTAATTTACGCAT

Gene: hemX (membrane uroporphyrinogen III methylase)

Position: 1721043 to 1721858, length: 816 nt, orientation: REVERSE

Perfect match to: (COL-CP000046-[1748690:1749505:r], highly conserved allele)

Sequence:

TCAATTCACAAAATGTGTTGCAAAAAATAAATTAATCATATTTAAGCAAAATAAAATAATGTTATAGTATATTAAATATCTTGAATTCAACCA
TTTGTTGATTCTAAGTAAAATATAACTTCCATATAATACTGTAATAATTGAAGAGAGTATTACCTTCGGGTCAATGAATATACGTTCACCAAC
TGAAATTACACCCCACTGTGTACCTAAAATAATACTAAATATGAGAATTATCCACCCACTTAACGTTGAGTAAAACACAATTGATTCAAGTGT
AGCAACGCTACCAATTCTAAAGTATTTTTGATCAAAACGTTTTTCCTTCAAATTACGGTATTGCATGATATACAGTAATGCATTGACAAAAGC
TAAGGCAAAGAAGACATAACTTAACACAGCTAGACCGATATGGACTAACAGTAACTCGTCTACAACAGCAATTTTCTGAACCTTATTAGTAT
AATGTGTCGGTTGAAATGTATTCATCCCTAAAAGTGTTAACCCTATTAAATTCAAGAAAAACACAGAGAAATTCAAAACTTTGATAAGGTTA
AGAATTAAAGAGATTGAAATAATCAGCCAACTTAAAGTATAGAATACATCTGAAATAGACCCTAATGGAATATGTCTAGTTTGTATAATAAA
AATAGATAAAGAGATTGTTTGTAAAACCCAAACAATCCCCAATAAATATATGCCTAAACTTCTAATCTTATGACTTTTTTGTACAAAATCATA
AAAATAGCAAATGATACTGATTAAGTATATTAATAATATAATTTCATTGAATCGAATAAACAGGTTTTCTTGCAT

Gene: hemA1 (glutamyl-tRNA reductase)

Position: 1721880 to 1723226, length: 1347 nt, orientation: REVERSE

Perfect match to: (11819-97-CP003194-[1772262:1773608:r], allele observed in CC80)

Sequence:

TTATTCAAAACTAAAGATACGTCGCGCTGAAATTTCCTTGACCTTGCTTTCTTTTTGTTGCTTCGCTTGCTCATGAGGACATTCAGCTTCGATA
TCAAATATATTTTGAAATAGCTCTAATTTTTCATTACTTTTCTTATCACTACTTAATTCTTTGGCCTGTTTAATAGGATCTTTCAACATTTGATTG
ATGATACTTTTTGTATGTTTAGATATAATTTTACGTTCTCTTTCGCTTAACCCTGGCAATTTACGATCAATACTGTCCATCGTTTCTGCTTGAAT
TGCCATAGCTTTTTCACGTAAAGCTCTAATCACTGGAACAACACCCAACATACTAATCCACTCATTGTGTGCATGTATTTCTGCAGGAATTTG
TTCCGAAATTGTTGCAGCCGCTAATTGTCGCTCACGTAAGTTTGCATCAACTAAACCTTTTAAGTCATCAACATCATAATTAAAGATGTTTGT
GATGGCACTAATACCAGGTTCAATATCTCGAGGAACTGCAATATCAATCAATACTAGTGAATCTTGCTTTCTATTTTCTGCAATTCTTTCTATC
ATTTCATTTGTAATGATATAAGATTGTGCACTCGTTGAACTAATCACAATATCTGCACTTTCAAGTAAATTTGGTAATGATGATAGTTCATCA
TATTTCACTTGATGCTTTGCTGCTAATTTCATAGCATTTTCAATTGTTCTATTTACTACTGTAATATCAGTAATTCCAGAACCAAGAAGATTTA
ATAGTGATAATTCACTCATTTCCCCTGCACCAATAATGATAGCTTGCTTACTTTTCAATTTGCCAAATACTTTTTTCGCCAACTCGACCGCAGC
ATAAGACACACTTACAGCATTATCAGCTATATCTGTTTCATTATGTGCTCTTTTTGCAAAAGTAATTGCCTGTTTAAATAGATGATTAAAAATT
GTTCCTGTCGTACCTGTGCTTTGCGCTAAGAAAAATGCATCTCTTATTTGACCTAAAATTTGAGTTTCTCCAAGTACGATTGAATCTAAACCA
GAAGTGACACGCAATAAATGTTCTACTGCTTCGTCCCCCACTTTTACTTCTGACATTGCTTTAATATCAGCTACTTCAAATCCAAATGCACGA
GCTAGAAATCGTTGAATATAGTAACGACCTGTGTGAATTTGATCAACAACAGCATATACTTCAGTTCGATTACATGTTGATAATATGACATTT
TCTAAAATAGATTTAGTTTCATATAAATCTTCATGGGCAATTCGTAAGGCATCATCTCTAAAAGCAACTTGCTCTCTTAGTGCAACATCAGCT
GTGCGATGATTTATACTAATTGCAATAAAATGCAT

Gene: engB (GTP-binding protein)

Position: 1723443 to 1724033, length: 591 nt, orientation: REVERSE

Sequence:

CTATGAAATATACGGTTCAATTAAATTCCATATTTGTTGTTGTTTATTATTTTGAATTGATGAATAACTTACAATTGTATCGTCTGGGTCCATA
TCTAATTGTGTCTTAATATTTTTAATATGCTTTTGAACTTTACCTTTTGGAATTTTGTCTTCTTTAGTGCATATAACTAAAGTAGGAATATCAAA
ATGTTTCAAATAATTGTACATTAAGATATCATCTTGTGTTGGATCATGTCTTAAATCAACTAATTGAATAACTAATTGCAAATTCTCTCTCTTA
GTTATATATCCCTCAATCATTTTCCCAAATTTTTCACGTTGTGTTTTACTTACTTTAGCATATCCATACCCTGGAACATCCACAAAAATAAGTTG
TTCATCTATATTATAAAAATTTAACGTTTGCGTTTTGCCGGGTTGCTGTGATGTACGTGCCATATTTTTTCTGCCAATCATACTATTGATAAAT
GTAGACTTACCTACATTAGATCGACCGCTCAGTGCAACTTCAGACAATTCTGTTTCTGGATATTGTTCTTCTTTTACTGCACTAATGATTAATT
CTATATTATTAGGATTAACTTTCAT

Gene: clpX (ATP-dependent chaperone protease, ATP-binding subunit)

Position: 1724187 to 1725449, length: 1263 nt, orientation: REVERSE

Perfect match to: (N315-BA000018-[1706141:1707403:r], highly conserved allele)

Sequence:

TTAAGCTGATGTTTTACTATTATTAATTAAATTGCCTTCTGCGTCGTATAGTTCTGGTTCAGTTTCTTCATTAATTGTTTGTGCTGTAATAACTA
CCTTCGTTACATTTTCGTTAGAAGGCACATCAAACATAATATCGATTAACGATTCTTCTATGATTGAACGTAAACCACGCGCACCTGTTTTTCT
TTCAATTGCTTTTTCACTAATTGCTGATAAAGCTTCTTCAGTGAACTCTAAATCCACATCATCTAATTCCAGCATTTTAGTATATTGTTTCACAA
GTGCATTTTTAGGTTGCGTTAAGATGTTTTTCAACGCAGTTACATCTAATGTTTCTAAATTAGCTACAATTGGCACACGTCCGATAAATTCAG
GAATCAAACCATAGGCTTGCAAATCTTCTGGGCGAATTTGTGCTAATAATGCTTGTTCGTCATATTTATCAGCTTCATTGCTTGAGAAACCAA
TAACTTTTTCACCAAGACGGCGCTTAATCACTTCTTCAATACCATCAAAGGCACCACCAAGAATAAATAAGATATTTGTTGTATCAATTTGAA
TCATTTCTTGGTTTGGATGTTTGCGTCCACCTTGTGGCGGAACACTTGCAGTCGTACCTTCTAAGATTTTAAGCAATGCTTGTTGAACACCTT
CACCTGAAACGTCACGTGTTATAGATGTGTTTTCAGATTTACGTGCAATTTTATCAATTTCATCTACATAAATAATACCTTTTTCGGCTTTATC
AATGTCAAAGTCAGCTGCTTGAATTAATCTCAACAAGATATTTTCAACATCATCGCCTACATAACCAGCTTCAGTTAAACTTGTCGCATCTGC
AATTGCAAATGGTACATTCAACGTCTTGGCTAATGTTTGAGCTAATAATGTTTTACCACTACCTGTTGGCCCAATTAATGCAATGTTACTTTTT
TGTAATTCAACATCATCTTCTTTTGGTCCTAATTGTTGAATACGCTTATAGTGGTTATAAACAGCTACAGCTAAAGATTTTTTAGCTTTTTCTT
GACCAATAACATATTCGTTTAAATGATCCATAATTTCTTTAGGAGTAGGTAATTCTGTCATCGCTTCAGAAGTGTTTTGAGCTAATTCTTCTTC

GACGATTTCTGAGCATAATTCAATACACTCATTACAAATATATACACCACTTCCTGCTACAAGTTTTTTTACTTGATCTTGGTCTTTTCCGCAG
AAAGAGCATTTCAAATTTTCTTCATCTTCATTGAATTTAAACAT

Gene: tig (trigger factor)

Position: 1725600 to 1726901, length: 1302 nt, orientation: REVERSE

Perfect match to: (MW2-BA000033-[1746575:1747876:r], highly conserved allele)

Sequence:

TTAATCTTCTTTAGTTCCTTCAACGAACTTTGCGTTATCTCTTAATAAATCGATAACTTTTTGGATACGAACATCATTTTTAATGATATCAGTAT
TACCTAAAGTATTTTTGATATCTTCAACTGAGATATTAAATTGTTTACTCATTTTTTCTAATTCTTTATCGATATCTTCATCAGTAGCTTCGATTT
TTTCAGCTTCAGCGATCGCAGTTAAAGTTAAGTTAGTTTTAACACGTTGTTCTGCATCGTCTTTCATTTGCTCTCTTAATTGAGTTTCATCTTG
ACCTGAGATTTGGAAGTACGTTTGTAAATCTAAACCTTGTTGTTGAATTCTTTGTGCAAATTCAGACACCATACGATCTAATTCAGTATTAAC
CATTGCTTCAGGAATATCGATTGTTGTATTATCAGTAGCTTTTGTAATCGCTTCTTCTTTTTCAACATTTTCAGCATCTGTAGCTTTTTGTTCAG
CTAAACGTTTACGTAAGTTTTCTTTGTACTCGTCTACTGTATTTGCTTCTGCATCTAATTCATTAGCAATTTCATCTGTTAATTCTGGAACTTCT
TTAAATTTAATTTCGTTAACTTTTGTTTTGAAAGTTGCTTCTTTACCAGCTAATTCTTCAGCATGGTATTCTTCTGGGAATGTTACGACAACAT
CTTTTTCTTCGTCAACTTTCATACCTTCTAATTGCTCTTCGAAACCAGGTATGAATGAACCTGAACCGATTTCTAAATCGTAACCTTCAGCTTG
TCCACCTTCGAATTCTTCTCCGTCAACTGAACCACTAAAGTCAATGTTAACTGTGTCGCCATTTTCAACAACACCATCTTCTTTAACTACCATTT
CAGCTAAATGTCCTAAGCTGTGGTCAATCGCTTCTTGTAACTCATCATCAGATAATTCAGTTTCTTGTTTTTCAATTTCAAGACCTTTATAGTC
TCCTAATTTAACTTCTGGCTCAACTGTAACTGTTGCTTCAAAAATGAAATCTTTACCTTTTTCAATTTGAGTAACACTTACTTCTGGTTGTGCA
ACTGGTTTAATATCAGTTTCGTCAATTGCTTCACCATAAGCATCTGGTAATAAAATGTCGATAGCATCTTGATATAATGCTTCTACACCAAAG
CGTTGTTCAAAAATTGGACGTGGCACTTTACCTTTACGGAATCCAGGTACGTTAATTTGTTTAACCACTTTTTTGAATGCTTGATCTAACGCTT
TGTTTACTTTTTCTGCAGGAACAGTAACAGTTAATAAACCTTCGTTACCTTCCTTTTTTTCCCAAGTTGCTGTCAT

Gene: ysoA (tetratricopeptide repeat domain protein)

Position: 1727064 to 1727993, length: 930 nt, orientation: REVERSE

Perfect match to: (N315-BA000018-[1709018:1709947:r], highly conserved allele)

Sequence:

TTAACTATTATTATTCAAATCTAATTGCTGAATAAAATTAAATGCATCGATACTTTGTAATTCACATTTAAGTCCTAACATATTTTTGAAATAA
CATTCGTATGCATTAATCCATTTATTCGTTTCAAACAAAGTTTCAATATCAATTGGATAAATCATGATAGAGTGATTATTCATAATATGGTGT
GCTTCTTCAGCGATATGTAATGCACCATCATTTAATGTTTCCATAACGTTAGGTATAACTTTTTCTTTCAGTGTTGTATGTTCTAGCCCATTTA
AATTAGCTGGTACAAAAGTTACATCCATACCATACTTTTCAATTGTCAGTTCTTGTGTACAATTTGCAAACCTTAAATACTCAATCATTAAACT
AATGAGGTTATAACTGTACGTATTAGATTTTAATATATATAATACCGTTTCTTGAAATTGAAAATGACCATTGTCTATTAACTTCAAAATCAA
GTGCGTCTGTTCCCTCATTGATAACGTATCAAAATCAGTTAATGACTGAGTCAATCGTTTTTCATCTTCAATTAATTTTGACTTAGCAAATTCT
TTAAGAGGATGTAATGCCATTCTTGTTTTGTGATCTTTGACTTCATCAATAATTTGATGAATTACTTCTACCGCTTCAAAATATTGTCCCAAAC
CAATCAAACTTTTCACGTAATAAATCATCAATGCATCATATTGTTGTATGCCAGTTTTCAATAAGACAATTGTTTCTTCTCTTAATTCAAGAAA
TGACCCCGTCTCATATAACATACGGCATTTCATCATTGCTATTTCTTCAGTGAGTTCAAATTGACGTTCATATTGCACAATATATTCATACATC
AGGTCATAGTTTTGGTCTTGTTCAGCACGTTTTATATCTTTATATAACTTTTTCGAAGAGTTAGGAAACTGAATAATATCTGACAT

Gene: ymaB (enzyme involved in deoxyribonucleotide synthesis)

Position: 1728012 to 1728620, length: 609 nt, orientation: REVERSE

Perfect match to: (11819-97-CP003194-[1778394:1779002:r], allele observed in CC80)

Sequence:

TTATAAATCTTGAAGGATTAATGCGCTCCACGTTTCGAAATCATCATATGACTCTATGTTGCCTTTTTCAACCCATTTTATTCGTAAAGTATCT
GTTTCTTTAGCTTCTACATCATTCGTACTTACAGTGATTTTAAATACAACACCAATATGTACCTTGCCCACTTCATTATTATCGTCATTAATAAA
ACCGATATATTCCATATTTTGTGAATCTTGCTCACTTAAACCTACTTCTTCTTCTAATTCTCTCTGTGCATTAACTCTCAATACTTCGTTAATAG
ATTCTGCTCCTGGAACATCATTCATATGACCGCCTACACCTATTGAAGATTGTCCATGCAATCGAGCTTCTCCACCGCCAGATAATCGTTCAT
ACACTAATATCTCGCCATGCTCATTTTCAAGTAAACAATAAGAAATGAGTTGTTTATATGATGGATCCTCTTCCATATCGCCGCGTCGCTTAA
CTTCATATTGACTTAGCGCATCAAAAATATTTTGACCTTCTGGTTTATTATTATTTAAAAACCCATTGAAAGTATTCTTTTCATTATTAAAAATA
ATTTCTCTAGGTACTACAATGATTTGTTCATCAAATTTAGACAT

Gene: rplT (50S ribosomal protein L20)

Position: 1728762 to 1729118, length: 357 nt, orientation: REVERSE

Perfect match to: (N315-BA000018-[1710716:1711072:r], highly conserved allele)

Sequence:

TTATTTTAAAGCATCTTTAGCTTTAGTTACTAATTGAGCAAATGCTTTTTCGTCAGAAATTGCGATTTCTGATAACATTTTACGGTTAATGTCG
ATACCAGCTTTTTTCAAACCGTTCATTAAACGTGAGTAGCTCATTTCATGTTGACGAGCTGCTGCGTTGATACGTGTAATCCATAATTTACGG
AAGTCACGTTTACGTTGACGACGGTCACGGAAAGCATATTGACCTGATTTCATTACTTGTTGCTTAGCTACTTTGTATAATGTATGTTTTGAA
CCGAAGTAACCTTTAGCTAATTTAATCGTTTTTTTACGACGCGCTCTTGTTACTGTTCCACCTTTAACTCGTGGCAT

Gene: rpmI (50S ribosomal protein L35)

Position: 1729165 to 1729365, length: 201 nt, orientation: REVERSE

Perfect match to: (N315-BA000018-[1711119:1711319:r], highly conserved allele)

Sequence:

TTATTTTTTGTATGCTAATAATTGTTTTACACGTTTCATATCGCTCTTAGACACTAATCTAGCTTTACGTAATTGACGTTTTTGTTTAGTGCTCT
TGTTTGCGAATAAGTGAGATGTGAAAGCTCTTGAACGTTTTAATTGACCTGAAGCAGTTCTTTTAACACGTTTAGCTGCTCCGCGGTGAGTT
TTCATTTTTGGCAT

Gene: infC (translation initiation factor 3)

Position: 1729394 to 1729921, length: 528 nt, orientation: REVERSE

Perfect match to: (MW2-BA000033-[1750369:1750896:r], highly conserved allele)

Sequence:

TTATTTTTCAGCTGTTGGCGCTAACATGATAAACATTTGACGCCCGTCCATTTTAGGTTTTTGTTCAACTGTTGCTATATCTTTGCATTCATCT
GCATATTTTTCTAGCACACGTTGACCAATTTCCTTATGCGTAATGGCACGCCCTCTGAAACGAATAGATACTTTACATTTATCGCCTTTAGTTA
AGAATTTACGTCCGTTTTTCAACTTAGTTTGGAAATCATGTTCCTCAATTGTTGGACTTAAACGAATTTCTTTAACATTGATAATTTTTTGTTTC
TTTTTCATTTCTTTTTCTTTTTTCTGTTGTTCGAATTTGAATTTACCGTAATCCATAATTCTTGCAACTGGTGGTTTCGCATTCGGTGCAACGAC
CACTAAGTCTAAATCTACACGTTCAGCCATTTCTAAAGCTTCACGCTTTGATTTAACACCAATTTGTTCACCATCTTGACCGATTAAACGTAAT
TCTTTTGCACGAATTTTGTCATTGATTTGAGTTTGATCTTTTGCTATGGTTGACAC

Gene: lysP1 (putative amino acid permease)

Position: 1730150 to 1731643, length: 1494 nt, orientation: REVERSE

Perfect match to: (08-02119-CP015645-[420140:421633], highly conserved allele)

Sequence:

TTAACCTTTTATTTCATCCATTGACACGTCTTGACGTAAATCTACTTGTTCTAATGGAATTTTTTTCGTTTTATATCGAAGCTTATGATAAATAA
AGAATGCTAAAAATACTGGGATTCCCATATACGTAATTAAGAAGCGACTAAAATTAAAATCTCCTGTTTTAATAAAGTCAACATCTTGACCA
ATAATTACTACAATACATAAAAAGCCAGCAAATAATGGTCCGAATGGGAATAATTTAGCAGTATATTTTAATTTAGATTTGTCATAATTTTGT
TTATCAAATGCTCTTCTAAATCGATAATGACTTACTGCTATACCTACCCAAGCAATAAAACCAGTTAAACCACTTGCTGCAACGATATATTCG
TATGCACCTTTTGTAAGGCTTTGTAATACGAAAATTATCACTACAATGATTGCTGTAACGAGTAACGACATATATGGCACACCGTTTTTATTT
GTTTTACCAAATGCTTCAAACGCTAATTTATCTTTACTCATTGAATATAGCATTCGAGTTGAAGCATACATACCTGAGTTACCTGCTGATAAC
ACAGACGTTAAAATGACTGCATTCATAAATGATGCTGCAAACGCAAATCCAGCATTTTTAAACACTAATGTGAATGGAGACGTTGCTACATT
ATCACTACCACCCATTAATGCACTACTATCATAAGGAATTAACATACCGATAACAAAAATGGCTAAAATGTAAAATAATAAAATTCTCCAGA
ATACTTGTTTAATTGCTTTCGGCACAGCACGTTCAGGATTTTCTGATTCACCAGCCGTAATACCAATTAACTCAGTACCTTGGAATGAGAAAC
CAGCGATTAAGAATACACCTAGAATTGATAATAAACTTCCTCCTAAGTTGCCACCAAGAATAGGACCTTCACCTTTATTAAATATTTCGAATC
CTACAACATGACCACCCATGATTCCGACAATCGTTAATAAACCAATTGCAATGAAAACAATAACTGTAACCACTTTTATCAATGCCAACCAGT

ATTCACTTTCACCATAGACGCGAACTGATAACGAATTCAGACTAAAAATTATAACTAAGAACAACGCACTCCATGCCCAAGCGGGTATGCCT
TGCAATGGTGTCCAATATTGAATGACTTGTGCTGCAATCGTAATATCTGCTGCTACAGTTACTACCCAGTTAAACCAATAGTTCCAACCAAGC
GCAAACCCTAAAGATGGATCAACAAATCTTGTAGCATATGTACTAAATGAACCTGATACTGGCAAATACGTAGCCATTTCGCCAAGTGACG
TCATTAAGAAAAATACCATTATTCCGATAATTGCGTATCCTATTAATGCACCCAAAGCACCTGCATCATGAATTGCTCCACCAGAAGTTACAA
ATAAACCTGTACCAATACAACCCCCAATCGCAATCATAGAAATATGACGATCTTTAAGTCCCCTTTTGACAACATTGTTACTTTCATTTTGAAC
TTTTGACAT

Gene: Lys_riboswitch (lysine riboswitch)

Position: 1731788 to 1731963, length: 176 nt

Sequence:

ATATAAGGTAGCACATCACATTCTGTGACAGTACAGTTCCTATTCGAAAACTGTCCCAATAGATATAATTTATGGTTTATATCTATTTCGGCA
TCTTTACCTTTCACTTGTTCAACTTATGTACCATAAATACTTCTGACAAGTTACTAATTAAACATGCAACCTCTAACTCAATT

Gene: thrS (threonine--tRNA ligase)

Position: 1732069 to 1734006, length: 1938 nt, orientation: REVERSE

Perfect match to: (TW20-ST239-FN433596-[1804756:1806693:r], allele observed in CC239+CC8+CC30)

Sequence:

CTATCTATGTTTTTTTAAACGAATTTCATCAACTAGATTCCAGATAAATTCATCTTTTTCAACTGTTTCTTGGTCTTGCGATCCATATTGACGCA
CATTCACTTGATTATTTTCAACTTCCTTATCCCCAACTACGATTTGATAAGGTATTTTTTGCATTTGAGCTTCTCTAATTTTATAACCCATTTTTT
CATTACGGTCATCAATACTTACACGAACGCCTTGAGATTTCAATTCATCTTGTAATTGGCGCGCATAATCATAATGTAAATCAACGTTAACTG
GAATGATTTGAACTTGTTTTGGCGCTAACCAAGTTGGGAATGCACCTTTAGTTTCTTCAGTTAAAAATGCTACAAATCGTTCCATTGTTGATA
CAACACCACGATGAATAACAACTGGACGATGATGTTCACCATCTTGACCAATATAAGTTAAATCAAAACGTTCTGGTAATAAGAAATCAAGT
TGTGCTGTTGATAATGTCTCTTCTTTACCCATCGCTGTTTTAACTTGAACATCTAGTTTCGGACCATAGAATGCCGCTTCACCAATCGCTTCTT
CGTACGATAAGCCAAGCTCATCCGCTGCCTCTTTAAGCATATTTTCAGCTTTATTCCACATATCATCATCATCAAAGTACTTTTCTTTATCTTCA
GGGTCTCTATAACTTAATCTAAAGCTATAATCCTCGAAACCAAAGTCTTTATACACATCAATAATCATGTTTACAACGCGTTTGAATTCTTCTT
TAATTTGATCAGGTCGAACAAAGATATGTGAATCATTTAAAGTCATACCACGAACACGTTGTAATCCTGATACAGCACCACTTGCTTCATATC
TATGCATCGTTCCTAGCTCAGCGATACGGATAGGTAATTCACGATATGAATGTGGTTTATTCGCATAAATCATCATATGATGTGGACAGTTC
ATTGGACGTAATACCATAGATTCAGTTTCATCTAACTGCATTGGTGGGAACATATCTTCTTGATAGTGATCCCAGTGACCAGATGTTTTGTAT
AAATCAACATTAGCAAGTACTGGTGTATAAACGTGGTCATACCCCATGCTCACCTCTTTATCAACAATGTAACGTTCAATTTCACGTCTAATT
GTTGCACCGTTAGGTAACCATAATGGCAAACCAGCACCAACTAATTGGCTATTTGTGAATAATTCTAACTCTTTACCAATTTTACGATGATCA
CGTTCTTTACGCTCTTCTAACATTTGTAAATGTGCTTTTAATTCTTTTTTATCAAAGAAAGCAGTACCGTATATACGTTGTAACATTTTGTTGTT
ACTATCTCCACGCCAGTATGCACCTGCTGTAGATAATAGTTTAAACTCTTTAATTTTAGCTGTTGATGGAACGTGAACTCCACGACATAAATC
AGTAAAATCACCTTGACTATATAATGTTACATTTTCATCTTCAGGAATCGCGTCGATTAATTCTAATTTGTATTCATCATTGCTGAATAACTCT
TTCGCTTCATCTCGTGAAACCACTTTTCGTTCGATTTTCATATTTTCGTTAACGATTTGTTTCATTGTTTTTTCAATTTGTTCAAAGTCATCAGAT
GAGATGTTTTGGTCAATGTCGAAGTCATAGTAGAATCCACCTTCTATTACAGGACCTACACCAAATTTAACATTACCATATAACCTTTTAATC
GCGTGTGCCATTAAATGTGCAGTAGAATGACGTAATACCTCTAACGCTTCTTCACTACCTGGTGTCACAATTTCAATTGATCCATCAGTTTCA
AGCGGTTTAGTTAAATCTACAAGTTGCCCGTTAAATTTGCCGGCAACAGCTTTTTTACGTAATCCAGGACTAATTGATTGTGCTATATCTTCA
GTAGTAGTACCTTTATCAAACGCCTTTTTATTACCATCTGGAAATTGAATATTAATTTGTTCCAT

Gene: tbox09 (T-box leader element)

Position: 1734167 to 1734378, length: 212 nt

Sequence:

ACAAGGGACGAGATCGTCGTGGTACCACCCTAGTTATCTAATACAATTTATGTATTAAATATCTCTGCTTAAGATAACGGTCTTGATCCGGG
TATTCATTACAAATACCATAAATGAAGTAGTAATCATCTAATTTATTAACCATATTCTCATCAAACAATGGCTTTCTGTGTAATATTGATTAGA
TCATCTTGTCTTCAATCATTTAAACG

Gene: dnaI (helicase loader)

Position: 1734419 to 1735339, length: 921 nt, orientation: REVERSE

Perfect match to: (SA-083-JXIE01000095-[292146:293066:r], allele observed in CC101+CC8+CC97)

Sequence:

TCAATTGTTTCTGAAATTTTCTCCTGATAAAAAGTATGGTGTTGACAAAGATTTGACACGTTCAATAATACGTGCTGCTTTAGTCTTCTCTTCA
CCATCACGAGTCATCGCTAAATGATGTTCCAATTCACTATAGTCAAAATTAGAACTAAAGAATGTTGGTAATTCATGAACCATTCGATAATG
TAGCAAAGGTCCAATTACCTCATCTCTCACCCATGGAGTCACTTCTTCAGCCCCAATATCATCAAGCATTAAAATGTTTGCTTCTCTTACGCGA
TGTAATTTCTTTTCAAAAGAACCATCTTTAAAGCCACCTTTTAATGTTCTAATAAATTCCGGTAAATAAATAATTGTCGAACGTACCTTCTTAG
ATTTGAGCTGATTCGCAATTGCACCTAGAATAAAAGATTTACCTGTCCCAAATGGACCATAAAGGTAAAGGCCTTTCACTTGTTCCCCATTA
GTTATTGCTGTACAAATATCATCTGCTGCCATAGCTACATCAAGACGGTCTCGATGATTCATATAAATATCTTTCAATTTGGCATTTAAAGTA
TCTCGTTGCATATGATGAGATGTAATTAGCTCAGCTTCAAAGCGTTCTTCGTCGTACTTGATTTTACACGGGCATTGTAAATAGCGTATTTTA
ATTCGGTTATTATCAACATATAACTCAGGCACATGCCCCTTTACGAAATTTGGACAATCAGCAAATTTATGACCGTCATAATGTTTTTGTTGA
TCTTTATACTCTTGTAACACATTTAAGTCTTCATCAATCATAGCATTCGTTAATTCAGCTCGATGCGCTTCCAAAAATTGCTTAACATCTGGGT
CATTGATTACTTCTTTTTTTATCTTTTCTATTCTTTTTTCAAAGTCCTGCGACGTGTTAATTATACTTTTAAATTGCTTCAT

Gene: dnaB (chromosomal replication initiation and membrane attachment protein)

Position: 1735339 to 1736739, length: 1401 nt, orientation: REVERSE

Perfect match to: (11819-97-CP003194-[1785721:1787121:r], allele observed in CC80)

Sequence:

TTATTGACTGTCCTCCTCCCATTTTTTAGATAATTTATCTAGAAATGCTTGTCGATCTTGCTCTAATTGTTGATCATCTACGCTATTATCTTTAG
CCGAATCTTCTTCACTAGGTTTATCTCTATTTTCTAACCATTTAGGTGTTTTTTCTTTTGAAATACGATTACGTTGCCCATAGTATGAACCACGC
TTTTGGTAATTTTCGCTAGAACCCTCATTTTTAAGTTGATTAACTTTTTTAGCGTAATTATATGCTTCTTTAGCTGTCTTAATACCTTTTTTCTTC
CAATTTGATGCTATTTCCAAAATATACGCTTTAGGAAGTTTCATATCTTCTTTTAACATGACAAATTGCAACAAAATATTAATGACGCCAAAA
GACATTTTTTCACGTTCAATTAATTCTTCAACCATTGTCTTTTGCGATATAGTTGGTTCTGATTCAGACCAAGAAGCTAACATATCAATTGGAC
TCGTTTGTTCAAGTAACTCAAACCATTCATCACTTTGTGGCTTTGGATTCACTTCTGAAGATTTGCCCGTCGAAGATGATGTAGCAGGAGATT
TCACCTGTAATTTAGGCATTTGATTTTCGTGTTCCATTAAGTAATACGAGCGTGCTTGTTTACGCATTTCTTCAAAGGATAACTGTTGTCCACT
TGTAATTGAATTTAAAATAACATGCTTCATGCCATCTGCTGTTAAACCATATAAAGTCGCGAGTTGTGTAATTAAACGCTTTGCATCTTTGGT
AACAATGTCTTGACTAATAAAATGTTTACCTAACATTTGTCTCAACATTTCAAAGTCAAAAGATTCATTTGATAAATCGATACCTTGGTACGG
TTCATTAATCGGAATATCACTTGTATCCATATCTATTTTTGTAGACGGCACTTTAAAAACATCAGTAAATTGTCTTGTTACCTGTTTAAATTCA
CTCAAATCAATTTGTTGATACTCAAAGTATTTCTTCAACTCATGAAATCGACGATGCTCGACTTCACTATATAAAAAGATTGACAACATTGGA
TCATTAAAAAATAAATGTGCTGAAGGCGGTTGAATTAATTGGTAAACAAATTGTGTTTCTTGTTCATCATGTTTGACAAACGCCTTTAACAAT
CCAATCGCTTCAAGTAAGTCCATTTGTTGTCTAAACTCTAGTAAATTAATTTTAAGTTCATTCATAAAAATATAATGAGAAAGAATCAATGTT
TCATTATGACTTTCTTTAACGAATTGAGTCATAAAATGATATAAACCCACTGCTTGCGTTCCAATTAGCGGTGTATACAGTCGATTCAATACC
TCTAAATGATTCGTATTTAAATCAAAGTGTTGCATAACTTTGAATTGATCCTTCGGTCTTAAGCCGAATTCGAAGGCTTGTCGTCCCAT

Gene: nrdR (transcriptional repressor of nrdD/nrdG operon)

Position: 1736740 to 1737210, length: 471 nt, orientation: REVERSE

Perfect match to: (MW2-BA000033-[1757714:1758184:r], highly conserved allele)

Sequence:

TTAAGCATCACTCCGTTTGTTTTCGCTTAAAATCCCTTGCATCGATGCTAACAATTGATCAACATCTTTAAATTCTTTATAGACTGATGCAAAT
CTAACATATGAAACTTGATCAACATGCATTAACAAGTTCATAACGTGTTCACCTATATCTCGTGAAGACACTTCCGTATGACCTTCATCTCGT
AATTGCCATTCAACCTTGTTAGTTATGTCTTCAAGTTGTTGATATCTAACTGGTCGTTTCTCACAAGAACGCACAAGTCCATTAAGTATCTTTT
CTCTTGAAAACTGCTCTCTTGTGCCATCTTTTTTCACAACTATAAGCTGACTAACTTCGATATGTTCAAATGTAGTGAAACGTGTTCCACAATT
TTCACATTCTCTTCGTCTTCGAATGGCATTTAATTCATCGGCATGCCTTGAATCTACAACTTTAGATTGTGTAGAATTACATTTCGGGCATTTC
AT

Gene: gapB (glyceraldehyde 3-phosphate dehydrogenase, locus B)

Position: 1737421 to 1738446, length: 1026 nt, orientation: REVERSE

Perfect match to: (394_SAUR-JVIV01000024-[173:1198], allele observed in CC6+CC692+CC772)

Sequence:

TTAACTTGCACTTACAGTTTCTTTTGATGTCAAAAGTGCTCCAATTTGCTCAGCAACATCTACAACTCTATTTGAATAACCCCATTCATTATCA
TACCAAGCAATAACTTTTACTTTATTCCCTGACATGACCATTGTTGATTTTGCATCAATAATAGCTGAATTTGGATTAGTATTAAAATCAACAG
ACACTAGTGGTTGATGTTCGACTTCTATGATACCTTCTAAACCTGCATTTTCAAAAGCTTGGTTTACTTCTTCTGCAGTTACTTCTTTTTCTAAA
TCAACAACTAAATCAACGAGCGATACATTCTTTGTTGGTACACGTAATGCCATGCCGTGTAATTTACCTTCTAATTCTGGTAATACTTCTTTTA
AAGCTTTCGCCGCACCAGTAGAAGTAGGAATAATGCTTTCATTACATGAGCGTGCACGTCTTAAATCTTTATGTGGATTATCAATATTTTTTT
GGTCATTTGTAATAGCGTGAACAGTAGTCATTAAACCATTAACTATTCCAAACTGATTATTTAAAACTTTTGCAACTGGACCAATGCAATTAG
TAGTACATGAAGCATTACTAAAAATGTCAAATGCTTCTATATCTAATTGGTAATCATTTACGCCTTTAACTACCATTTGAACATGTCCACCTTT
TGAAGGACCAGTTAACAAAACTTTTTTGGCACCTGCCTTAATATGTGCGATGGCTTTATCACCATGATTAAATTTACCAGTTGCATCTATAGC
AATATCGATATCTAATTCTTTCCATGGCAAGTTTTCAGGATTGCGATCAGCAACCAATTTAATTTTATGATCACCAACTTGCAATCCATTTTCA
ATCGGTTCAACTTTTAGATTATATTTTCCATGTGTTGTATCGTAATTGATTAAATGTGCAATTGTTTCGGGTGGATAACTAGCATTTATCGCTA
CTACATTTAAATTTTTATTTTGTAATGCAATACGTAATACCATTCTTCCAATTCTACCCATACCATTAATTGCAATATTCGTTGACAT

Gene: coaE (dephospho-CoA kinase)

Position: 1738616 to 1739239, length: 624 nt, orientation: REVERSE

Perfect match to: (Strain_103564-AHZI01000014-[34078:34701:r], allele observed in CC15+CC80)

Sequence:

TTAATCTTCTTCTCCGTAATTCGGCTTTTCAATATAACCTTCTTCTTCTAACAATCTCTCAAGGTTTTGTTTTAATTCAAGTTTATCCCCTAAATT
ATCGATAACATGATCGGCCATTCGGCTTTTTTTATCAATAGAAATTTGGCTATAGACACGTGCTTTCGCATCTTCTAATGACAAATTATTACG
TTGCATTAAACGATCCATTTGTATACTTTCAGAAGTGTATACAACCCACACTTCGTCTACTGTATTTTCCAATTCATTTTCAAATAATAATGGA
ATATCCATGATTACATTATATCCTTGTTTTAAATATTCTTGCTTTTCTTCTTCCATAATATCTCGCACGATAGGATGAATGATAGCATTTAATTC
TAAGCGTTTTTCTGGATGATTAAACACTAGATCACCCATATAACGACGATTCATCTCACCATTTTCATCAATTGCTTCATCACCAAAGACTTCT
CGTACTTGAGCTAAACCTTTACTCCCCTTTTTAACAGCTTCCCTGGCTGCTTTATCAGCATCTACTACTTTAAAACCGAATACGGATAAGAGTT
CTGATACTGTTGATTTTCCTGAGGCGATTCCACCTGTTAGACCAATAACTTTCGGCAT

Gene: mutM (DNA glycosylase)

Position: 1739255 to 1740127, length: 873 nt, orientation: REVERSE

Perfect match to: (11819-97-CP003194-[1789637:1790509:r], allele observed in CC80+CC5+CC80+CC239+CC4803)

Sequence:

TTATTTTTGACATACTGGACAATAATGACTATTTCTTGTCGCGATGATTTTTGTTTCAATTTGACTTCCACACACTTTGCATACCGGCTGCTTAT
ATACATTAAGATGCAATTGCATCTCACCAGTTTTTCCATCAGCATGACGATAATCTGAAATACTTGTACCGCCATATTTAATACCTTCTTCTAG
TACTTCTCTAACATAATAAAAAACCATTTCTTGTTGTTGGTGTGTTAAGTCTTTTACTTTTTTATCTGGTAAAACACCTGCACGAAACAACGCT
TCACATGCGTAAATATTTCCACAACCTGCGATTACTTTATGATCCAAAATCACTTGTTTGATTGGTTTATTCTTATTAGACTGTTGATGAATTC
GATTTAAATAATACGTCAATGCTTCATTTGAAAAAGGTTCAGGCGCTATTTCTAAAAATGAAGGATAAGATGCTACAGACGCAACATTTCTA
ATTTCTCCAAAACGACGTATATCTGAATAAATTAACTTTTTGTCATTTGACAACTCAAAAATAACATGCCAATGCTTACGATAATTAGGTATC
ATAATATCTTCAAGTTCATCTACAATGAAAAAACCACCCGCCATACCTAAATGACTAATTAATGTACGTTGTTCTCGTTTATTATCTAGCTGAA
AAACGATATATTTACTTCTTCGTTCTACATTTGTAATGGTATAGCCTTCCGATAAAGTTTTAAAAGTATCTAATTCAATTCCTTTTATAATTGTT
TCCTTGCCTTGAGCTTTACCTTCGATTACTTTATCCGAAAATATAACGTGTTCAATTTTTTGATTTATAACGTAGGGTTCAATTCCTCTTTTTAC
ATGTTCTACTTCTGGTAATTCGGGCAT

Gene: polA (DNA-directed DNA polymerase I)

Position: 1740143 to 1742773, length: 2631 nt, orientation: REVERSE

Perfect match to: (11819-97-CP003194-[1790525:1793155:r], allele observed in CC80+CC4803)

Sequence:

TTATTTTGCATCATACCAAGTTGCACCATAACTTGAGTCTACTTTTAATGGAACATCTAATTGCAATGCATTTTCCATTATCTCTTCTACAAATT
CACTAAATGAATCTACTTCTGACTTAGGTACTTCAAAAATTAATTCATCGTGTACTTGTAATAATAGTTTAGCTTGATATGTTGTCTCTTTCAT
TTTTTGTGCAAATTTAACCATTGCCAGTTTAATGATATCTGCAGCACTGCCTTGTATTGGCGTATTCATAGCAGTACGTTCAGCAAAGCCGCG
TAAATTAAAGTTACGACTCGTAATATCAGGAATATAGCGTCGACGATGTAGCAATGTTTCCACATAACCTAAAGCTTTGGCATCTTTTACAAT
ATCAGACATATATTGTTTTACACCTGGGAAACTAGCTAAATAATCATCAATGAATGCTTTTGCTTTTTTACGAGTAATACCTAAACTTTGACTT
AAACCATAATCACTTATCCCATAAACAATTCCAAAGTTAACCGCTTTTGCTTGACGACGCATTAAACTATCGACTTGATCAGCCTCTACACCA
AATACTTTCATAGCAGTTGCTGTATGAATATCATCGCCGTTGATAAATGCTTCTTTCATACTCTCATCTTGTGTAATGTGTGCTAATACACGCA
ATTCAATTTGAGAATAATCTGCTGATAATATAACGCTATCTTTTGAAGTTGGTTTAAAGGCTTTTCTTATTTTACGCCCTTCTTCAAGTCTAAC
CGGAATATTTTGTAAATTAGGATCTACACTTGATAAACGTCCAGTTTGCGCTAAAGTTTGGTTAAAACGTGTATGGATACGTTGATCATCAC
TAATTACCTTTTGCAATCCTTCAACATAAGTAGACTGTAACTTAGATAATTGACGATATTCTAAAATATAATCAATGATAGGATGTTCACCTT
GCAATTGCTCTAAGACATCTACAGCAGTAGAATATCCTGTTTTCGTCTTTTTAATAACAGGTAATTGTAATGTCTCAAACAATACAACACCTA
ATTGCTTAGGAGAATTTATATTAAAATCTTCACCAGCTGCATCATGGATATTTCGAATCAAGACGTCTAATTTTTCTTGAATTTCTTTTTCCAT
TTCTTCTAAATCATGAACATCTGTAAATATACCAATTTCTTCCATTTCACTTAAAATCTTAGCTAGCGGTAGCTCTAAATCAGCTAAGAGTTCT
ACCTGATTGTATTCTTCTAATTGTTTATCCATATTTGGTTTCGCAAAGTAAATTGCATCAGTAATAGAAGCAACATATGGATTTAAAACATCA
TCTTCAGGCACCTTAAATTTCTTACCTTTTCCATATATACTCACATCGTCTTTCACAAAACTTTGACCGTACAATGAAACAACTGATTGAACAT
CACTAATCGTACGCGATGGATCAATTATATAACTGGCCAACATAATATCGAAAGAAATATTTTGAATATCAATTCCCAATCTATGTGATGCTA
CATATGTTTTTTTAGCATCATATACTACTTTTTTCGTATTCGGATTTTCTAACCATGAAACTAGTTCGGCATAATTATTTATGTCATCCGCATTA
ATTACAATATGTTTCTCACCTGTAAATAAAGAGAATTTCAAAATATTATTTCGCAAATAGTTACCACCGTCTAATTCGAAATGGATGGCCGCT
TCTTTCAATGAAGTAAAATCAATATTATCAAAAGACGTTTCAATTTCAAATGTCTTTTCTATTGCATCTTCAACACTTGCTGATTGATCAATGT
CAGCCAACAATTGTTTGAATTCTAACTTCTTAAACAATTCGATTTTTTCTTGTTGTTCATCTTGATGAGTCATTAACGTATCTTCAAGTTTTACT
TCAATCGGACTATCTACATTAATCGTTGCTAATTCTTTACTCATTAATGCATCTTCTTTGCTATTTTGAAGTTTTTCTTTTAACTTTTTACCTGAA
ATTTCATCTAAATGTTCATAGACACCTTCTACTGTGTCAAATTGGTTTAGCAATTTTATTGCTGTTTTCTCTCCAACACCTGCAACACCTGGTA
TATTATCAGAAGTATCTCCCATTAATCCTTTCATATCAATAATTTGATTAGGTGTTAACCCGTTGTATTTTTCCGCAATAAAGTCAGGTGTATA
ATGATCAACATCAGTAACACCTTTTTTAGTGTAATAAATGGTTACATTATCCGTTGCAAGTTGTGTTAAATCTCGGTCTCCCGTAATAATAAT
TGTCTGAAAGCCCGCTTTATCTGCTTCTTTACTTAAAGTTCCGATAATATCATCTGCCTCATAGTTATCTAATTCATAACGTTTAATATGATAA
GCATCTAATAATTGGCGAATATAAGGAAATTGCTCACTTAGTTCAGGCGGCGTTTTCTGGCGTCCACCTTTATATTCACTATATTTTTCATGT
CTGAAAGTCGTTTTACCTGCATCAAACGCTACTAAAAAATGATTTGGCTTTTCTTCTTTTAAAATCTTCTCTAGTAACATTGCAAAACCATATA
CTGCATTGGTATGAATGCCTGCTTTGTTTGATAACAAAGGTAATGCATAAAAAGCTCTAAAACTTAAGCTATTACCATCGATTAATACTAATT
TATTCAC

Gene: Q2YTE9 (putative membrane protein)

Position: 1743067 to 1744554, length: 1488 nt, orientation: REVERSE

Perfect match to: (Strain_21333-AHKA01000003-[38560:40047:r], allele observed in CC80+CC1)

Sequence:

TTATGAATCGTCTGAACCAAATGGAATATATTTAACTTTGGTCATTTTCGGTAATTCTTCATAATTATTAAACCACTCTTGGTAATTTTTATTG
ATTGGTGACGGATGATGCATATAATGTTCAAACGGCAATGATTCCACTGTATAAACCTCTTGTCGTGGATGATCTTTAATTTGTTGTTTTAAT
AACTGAACTCTTTTTTCATGTTCATAATGAACATAAATAAATGCACTAAGATATATAACAGCTAAAACTAGTGATGCACCTTTAATAAAATTA
ACATTGATTGACTTATATTTCCGAAATTCTTTTAATAAAATAATTAAAATGATTACATGTATCGTATAAACAATCAAAAAATTACCTGGTTCAA
TTGGAGTAACAATGACTAGTGTCGACGCCGAAACACATATTGCAATAAGTAAGGAATATAAAGTAATTTGTGTTTTACGATCATTAATAGAC
AAATAAATACCTACAAATATCGAAAACGCAAAGTAACCACATACAATTACGTTCACAAAACCAACCAATCCAATATCTGTATTTTTATTTAAT
AAAAACTGATTGTAAAATAGTAAATAATAAAGCGGTAAAGTGATAAATCCTATCATAATGATACGACGCTTTAATATTGTTAAATGTACATA
CCGATCACTTCTAAGCAGTAGATAAATAATAAGCGCTGCAATCATACTTAAAATTATAATCTGACTGAAAATGACACCATATGGAAGTGACG
TTGATATCATTTCTGCAAATTTTGAAAAGATACCTTGATTATTTGAAACTTGTTGATATTCTGATCCTTCAAATAAAATTTTGCGATAATTTGG
ATTGGAAAACATAATGATTGTACCTATAGTAGCAATCATAAATGAAAACAATAATTTATAATTAAGTGTTCGATTTGCAGTAAATTCATATA
GAAAAGCTAAAATTAAAATGATACAGTGGAATAATGTCACATTCTCCATAAATAATTGACCGAAGAAGCATAAAACATAGAATAGTACAGT
AACACTTACCGGCTGCTTTTCTTTATATATAATTGCATTAATACAATAATAAATAATAAAGAGTGAAATTAGTGTTGATGTCGCATAATTATA
AAATCCTGCAAACCAGCCATATGTATCTGCATAAATAGCACTTGGTAAAATTAACATTAAAGAAAATGCTAACAAATAATAGCTTGTCCACG
CTTTACAACGTGTAATGTGCATAATCATCCAAATGATGCCCATACTAATGAGACCATATGAAAGCCAACGTAACCAGCTTACATGTACAGCT
ATAATTTCAAAGATATTTCCGATATAGCGACCATTAAGTGATGCAAATCCTACTTTTAAAATATCAGTATTATAATTGCTAAACCATTGTAAA
TCATCGTGCATGAGTGGTAGTAAGATACCCATAAAAGTATAAAACAATAATATCGCAATTAATATCAAAGTTGTCTTGTGTAATTGAATTGT
TTTCAC

Gene: phoR (two component sensor/regulator of alkaline phosphatase, sensor histidine kinase)

Position: 1745053 to 1746714, length: 1662 nt, orientation: REVERSE

Perfect match to: (DAR4145-CP010526-[1835468:1837129:r], highly conserved allele)

Sequence:

TTATTCTTTATAATCTTTTAGAATAACTTTGAACGTTGAGCCTTTGCCAACTTGACTATTCACTTCAATATTGCCTTGGTGGGCCTCTACTATAT
GCTTTGTAATTGATAATCCAAGACCTGTCCCACCAGAATCTCTACTTCGTGCTTTATCTACTCTATAAAATCTTTCAAAAATACGTTGTTGGTC
TTCTAATTTAATACCTATACCAAAATCTTGTACTTCGAAAATGACACGAAAGTCATCTCGATACACACGAACGTTAATATCTCCATCTTCATAA
GAATAGTTAATTGCATTCGTTAACAAATTCGTGATAACTTGAGCAATTTTACTTTCTTGTGCTTTAACAATGACATCTTTTTCAATATCAGTAT
GAATGGAAATATTTTTTTGATTGGCTTGAGTCATCATATTATCAATAATACGCCGCGTTAAATCTGATAAATTCATATAGTCTGTATCTAACT
CGGTATGTTGCTCAATATGCGATAAATCTAACAAATCTGTTACTAAAGATTCTATTCGATTTGATTCTTTTAAAATTATGTTTAAAAACATGTC
TAATGATTCCGCATCATTTTTTGCACCATCAATAAGCGTTTCAGCAAACCCCTTAATTGAAGTAATAGGTGTTTTTAATTCATGTGAAACATTT
GCTACAAATTCACGTCTTAGATTTTCAAGTTGTTTCAGATTTGTTATGTCATGCATCACAACTAAAATCCCTTGCAAACTTTTTTGAGACCTAG
TTAAAATCGGAACGCATGAAATATCAAAGTACTTGGCATGGACTTGGTTTATTGCAACTTCCAATTGTTCATAAATAGGTTTTTCAACTTTAA
AACTTTCTAAAATTAATTGCTCAATTTCAGTATTAACATAGCCGTGATAGCCTACTTGTTCAATATTATGCGAGATGTTGAACTGTTCATAATA
CGCTTTATTTGCAACAACGATTTTTCCATTTCGATCTATCATTAAAATAGCACTTGGAATATTTTCAATCGTTGTTTTTAAACGGTTGGATTGA
ATTTTTTGCTCATTATTAAGCTTTTGAAGGCGTCGTGCTAAATCATTGGTAGACACAAAAAGCGCTTTAGTTTCTACAACATTACTTTCAGGT
ACACGTATGTGATAATAACCATTTGCCAACAATTGTGTTGCATAAGTAACTTCTTGAATGGGACGGATTAATGTACGCTTAAAACTACGGCT
TGCAAAATACAGACAAATGAGTACAACTAAACATGTCAAAATAAGATATTTCCACAACGTCCAATGCATTTCTGTAATATCGTTATTGTAACC
TTTAATCCATACATGATAACCGTTAACCTTCTTATTAAAAATAAAAACGTCCCTTTTTTGAATATAGCTATCACTATTTGGGATAGCTTTCAGT
TTGCCACTACTAAACACCTTTTTATCATGTTTAGTTATCAATAAATCTATATTTTGCTGTTTTACAATTTCTTTAACTTTATCAATCTCATTATCT
TGGACTAAATAAATATATGATCTTGCATCTGTTGCTAGAGCTTGTTCGTGTTTTTCTGATAAAACATATGTGATGGAAGCGTGAATAATAAT
GCCTAATGTAACAAAACTGATAATTAATATACTGCTTATCAATAACATTAAGCGGTGGTGAAACTTCAT

Gene: phoP (two component sensor/regulator of alkaline phosphatase, transcriptional regulator)

Position: 1746714 to 1747418, length: 705 nt, orientation: REVERSE

Perfect match to: (930918-3-ABFA01000021-[4250:4954], allele observed in CC8+CC97)

Sequence:

TCATTGTTCTTTAGGTCTTTCCAATTTATAGCCTAAGCCACGCACAGTTTTAATAAGTTGTGGCTTCTTGGGATTATCTTCTAATTTATCTCTTA
AATGACTGATATGTACATCAACAATTCTTGAGTCTCCTGCAAATTCATAATTCCATACCGTATTTAACATATGTTCTCTCGTAATGACTCTGCC
TTGTCTTTCTATCAAATAAAGCAAGAGTTCAAATTCTTTCGGTGTTAGCTCCAATAACTCATTATGCTTATATACTTCAAAATAATCGGGTCTA
ATACGTATCGATCCGATGGTAATATCATCATCAATCTCTTCTACTTCTTTTACAAATTGAGAACGTCTTAAAATGGCTTTCACACGGGCAACA
ACTTCTCTAGGTGAAAAAGGCTTAGTCATATAGTCATCGGCACCTAATTCTAAACCTAATACCCGATCAAATTCATCATTTTTCGCCGTTAAC
ATTAATATAGGGACTAAATTTTTATTTGTTCTTACAGTCTTACATACGTCAATGCCATCTTTTTTAGGTAGCATAACATCTAAAATAATTAAAT
CTGGCTGTTCACTTTCTACCTTTTCTAAAGCCTCATCACCATCAAATGCGACAACAACTTCATAACCAGCTGTTTCTAAGTTATATTTAAGTAA
TGTTACGATTGAATGTTCGTCATCTACTACCAACACTTTTTGCGACAT

Gene: citC (NADP+-dependent isocitrate dehydrogenase)

Position: 1747808 to 1749076, length: 1269 nt, orientation: REVERSE

Perfect match to: (CN1-CP003979-[1688812:1690080:r], highly conserved allele)

Sequence:

TTATTTTAAATTTTTAATCAATTCATCTGCAAATGCTGATGTAGAAACTTCTTCAGCACCATCCATTAAACGGGCAAAGTCATAAGTAACAAC
TTTTGAAGCAATTGTATCTTCAATTGAATCTGTAATCTTATCTGCCGCTTCTTGCCATCCTAAATGTTCTAACATTAATACAGAACTTAAAATT
ACTGAAGATGGATTCACTTTATTTAAACCTGCATATTTTGGAGCTGTACCATGTGTTGCTTCAAAAATAGCATGACCTGTTTCATAATTAATG
TTTGCACCTGGCGCAATACCAATACCACCAACTTGTGCAGCTAAAGCATCTGAAATATAGTCACCATTCAAGTTCATAGTTGCTACAACATCA
TGCTCAGCTGGACGAGTTAAAATTTGTTGTAAGAAAATGTCAGCAATAGAATCTTTAATGATAATCTTGCCTTCTTTTTCAGCTTTTTCTTGA
GCAGCATTAGCAGCATCTCTGCCTTCTTTTTCAACAATTTCGTCATATTGTTGCCAAGTGAATACTTGATCACCAAATTCAGATAATGCTAAAT
CGTAACCCCACTGCTTAAATGAGCCTTCTGTAAATTTCATAATATTACCTTTATGAACTAAAGTAACTGATTTACGGTTATTATCGATAGCATA
TTGTATAGCTGCTCTAACTAATCGCTCAGTTCCTTCTTTAGAAACTGGTTTAATACCAATACCTGAAGTTTCTGGGAATCGAATGTTTGTCGC
ACCCATTTCGTTTTGTAAGAAGTCAATTACCTTTTTAACTTCTGTTGTACCTTCTTTAAATTCAATACCAGCATAAATGTCTTCAGTATTTTCAC
GGAAAATAACCATATCAACATCTTGTGGACGTTTAACAGGTGATGGTACTCCTTTAAACCAACGTACCGGTCTTAAACAAGTAAATAAATCT
AATTCTTGGCGTAAAGCCACATTTAATGATCTAATACCACCACCAATTGGTGTTGTTAAAGGTCCTTTAACAGCAATTAAATATTCTTTAATT
GTATCAAGTGTTTCTTGAGGTAACCATTCACCAGTTGTATCAAATGCTTTTTGGCCAGCTAGCACTTCTTTCCATTCAATGCGTTTTTCGCCAT

TATAGGCTTTCTCAACAGCAGCATCTATAACTCGGCTTGCTGCCTTCCAAATATCCGGTCCAATTCCATCACCGATAATAAATGGGATAATTG
GTTCATTAGGTACGTTTAATCCTTCAGTTCCTTGAGTAATTTTTTCTGCAGTCAT

Gene: citZ (citrate synthase)

Position: 1749125 to 1750246, length: 1122 nt, orientation: REVERSE

Perfect match to: (MW2-BA000033-[1770098:1771219:r], highly conserved allele)

Sequence:

TTATTTTCTTTCTTCAAGCGGGATATACTTACGATTCGTTTCGCCAATATATTTCGCTCTAGGACGCATAATTCTATTATCTTTATATTGTTCTA
AAATATGAGCAATCCATCCTGCAGAACGACTTACAGCAAAGATTGGCGTGAATAAGTCATGAGGTATTTCCATACAGTGATAAACACTCGC
ACTATAAAAATCAACATTAGGAATTAATCCTTTTTCTTCTGCCATACGTTTTTCCATTTTCACTGACATTTCAAATAATTCTTCACGACCAGCGT
CTTTCGTAATTTGACGGCTCATTTCTCTTAAATATTTCGCTCTAGGATCACCATCTTTATATACACGATGACCGAAGCCCATTACTTTATCTTTA
TTAGCAAATTTTTCATCTAAGTAAGCATCAACATTTTCAATTGACCCAATCTCAGATAACATCGTCATAACTTGTTCGTTTGCACCACCATGTA
ATGGCCCTTTCAGAGAACCTACAGCTGCTACAATACCTGAGTACATATCTGACAATGATGATACCGCACAACGTGCTGTAAATGCAGATGC
GTTCAACTCATGATCAGCGTGTAAAATAAGTGCTTTATTGAAGGCTTCTACTTCTATATCTGTTGGTAATTCCCCACGTAACATATATAGGAA
GTTTGCCGCATAACTTAAGTCAGGATTAGGCTTAAGTGGTTCTTTATCTTGTCTTACTCGAGCAAACGCTGTAACTAATGATGCTACTTTAGC
CTGTATACGCATTGCTCTTTCATAACGATTTTCATCTGATTCATTTTCAGCATCAGGATCGAAATGTGCAATATATGATAATGACGTACGTAA
TGCTGTCATTGGATGCACGTGATCTGTAACATACTCCTCAAAATGTGTATACACACGAGGATTTAATGTCATGTATTGATTTAATTTCCCTTTT
AGATGAGCAAGCTCTTCTTCGTTTGGCAATCTATAGTTCCATAATAGGAAAATAACTTCTTCAAATTGCGCATTTTCAGCTAGATCATCAATA
TCATAGCCGGCATAAGTCAATTGACTTTCAATAATTGAACTTATTTTAGTCTCCGCTGCGATAACCCCTTCTAAACCTCTTTGTAATTCTGCCA
T

Gene: aapA1 (amino acid permease locus 1)

Position: 1750594 to 1751955, length: 1362 nt, orientation: FORWARD

Perfect match to: (NN50-BAEA01000025-[312092:313453], allele observed in CC4803+CC8+CC188)

Sequence:

ATGGCTGAAAAATTACAAAGGGAACTGAGCAATCGCCACATACAATTAATTGCAATTGGCGGTGCAATTGGTACAGGCTTATTCTTAGGTG
CTGGTCAAACGATTGCATTAACCGGCCCTTCAATTCTATTAACATACATCATTATAGGATTTATGTTATTTATGTTTATGCGAGGTTTAGGAG
AAATCATTATACAGAATACTGAATTTAAATCTTTCGCAGATGTAACCAATACATATATTGGGCCTTTTGCAGGATTTGTTACCGGATGGACAT
ACTGGTTCTGTTGGATTATTACAGGTATGGCTGAAGTAACGGCTGTGGCAAAATATGTTAGCTTTTGGTTCCCAGAAATTCCAAACTGGATA
AGTGCACTATTTTGTGTACTGTTATTAATGTCATTCAACCTACTTAGCGCAAGACTTTTCGGAGAATTAGAATTTTGGTTCTCTATCATTAAAA
TAGCGACAATTATTGGTTTAATAGTAGTTGGTTTCGTCATGATTCTATTTGCATTTAAAACTCAATTCGGGCATGCCAGTTTCACAAATTTAT
ATGAACACGGCATATTCGCTAAAGGTGCTTCTGGATTCTTTATGTCTTTCCAAATGGCATTATTCTCATTTGTAGGAATTGAAATGATTGGTG
TTACAGCTGGGGAAACAAAAGATCCAGTTAAAACAATTCCAAAAGCAATTAACAGTGTACCCATTAGAATTTTAATATTTTACGTTGGTGCG
TTAGCGGTTATCATGTCTATTATCCCTTGGCAGCAAGTTGATCCTGATAACAGTCCATTCGTAAAATTATTCGCATTGATCGGAATTCCGTTT
GCTGCGGGCTTGATTAATTTTGTAGTATTAACCGCTGCTGCTTCATCATGTAACAGTGGTATATTCTCAAATAGCCGTATGCTTTTCGGTTTA
TCAAGTCAACAACAAGCACCTCCGAACTTTTCTAAGACGAATAAATATGGCGTTCCACATGTTGCAATCTTTGCTTCATCAGCATTATTACTT
GTGGCAGCATTACTAAACTATATTTTCCCAGATGCGACAAAAGTATTTACGTATGTGACTACCATCTCTACAGTGTTATTTTTAGTTGTATGG
GGTCTGATTATCATTGCATATATCAATTATAGTCGTAAAAACCCAGATCTACATAAAAATGCTACGTACAAACTATTAGGTGGCAAATATAT
GGGCTACTTAATATTTGTATTCTTCATTTTTGTGTTCGGGTTATTATTTATTAATGTTGATACAAGACGTGCAATTTATTTTATTCCGATTTGGT
TTATACTTTTAGCATTTATGTACTTAAGATATAAACGTATCGCTGCTAAATCAAATAAATAA

Gene: pykA (pyruvate kinase)

Position: 1752303 to 1754060, length: 1758 nt, orientation: REVERSE

Perfect match to: (COL-CP000046-[1781926:1783683:r], allele observed in CC8+CC97+CC239+CC772+CC4803)

Sequence:

TTATAGTACGTTTGCATATCCTTCAAAGATTTTACCTTGAGCAGCATCAATCGTAACTAACATGTTATTGCTTATGTTTTTAACAGCTTTTTCTA
CACCTACAACTGTTGGAATACCTTTTTCTAAACCAACAATTGCACTTGGTGATGTAATACCATTTTCTTCTGTAATTAAGCCTAAAGCTTTTTC
TACATAAGGTACAAACGTTTCATCGATTGAGTTAGTAACGATAACTTTGTCAGATAAATCTTTACCTTCTAAATCTTTAACAGTTTCAGCAAC

TAACGTAGTACCAACAACTGATCCACGTCCAATACCTTGACCATTAGCAATTTCGTCACCAACTAGGTGGATTTTCATCATATTAGTAGTTCC
AGTTTCACCAGTTGGTACACCAGCAGTAATAATGATTAAATCACCATTAGATACTCTACCAGTTTCAACAGCTGTTGCAACTGCATTGTTTAA
CAATGCATCTGTACTCTTACGTCCTTTTTTAACTACAGGTTGAACTCCCCAAACAATTGAACATTGACGTGCAGTTTCTTCACTTGGAGTCACC
GCAATAATGTCTGAATGTGGACGATATTTAGAGATAGTACGTGCCGTTGAACCACTTTCAGTAGCAGCTACAATTGCTTTAACATTTAAGTT
TAAAGCTGTATGTGCAACCGAAATACCGATAGCATTCACTAATGAAGTTTCAACTAATTTAGTACGATCTGACAATAACTTTTTGTAATCTTG
GGCTGCTTCAGCTGATACAGCAATATTCCTCATTGTTTTAACAGCTTCTTCAGGATATAAACCAGCAGCAGTTTCACCAGATAACATTACTGC
ATCTGTACCATCATAGATTGCGTTGGCAACGTCACTAGCTTCTGCACGTGTAGCACGTGGGTTACGTTGCATAGAATCTAACATTTGTGTAG
CTGTAATAACTGGTTTACCTAATTTGTTACATTGTCTGATTAAATCTTTTTGAACCATTGGTACTTTTTCAGGTGGAATTTCAACACCCATGTC
ACCACGTGCAACCATTAAACCATCAGACACTTCAAGAATTTCCGCAATATTATCAATACCTTCTTGGTTTTCAATTTTAGGGAATACTGAAAT
GTTAGCTTTTTGTTCTTCTAAAATTTCACGAATTTCTAAAACATCACTAGGACGACGTACGAAACTTGCTGCAATGAAGTCAACATTTTCTTTA
ATACCGAAACGGATATCTTCAGCATCTTTTTCTGTAATACCAGGTAAACTTACTCTTACGCCAGGTAAGTTAACACCTTTTTTGTTTTTAAGCT
CACCAGAGTTTAAAATATCACATTTAACTTCTTTTTTAGCATGGTCAATATCTTTAACTTGTAATTCAATTAAGCCATCATCAAGTAAAATGTA
TGAACCTACTTGAACATCGTTAATTAAGTTTTCATATGTTACTGAGAACTTTTCAGGTGTTCCTTCAACTTCATTCATGCTAACAATAACTTCG
TTACCACGTTCAAGTTCAATGATACCGTCTTTCATATTATGCGTACGAATTTCTGGACCTTTTGTATCTAATAAAATTGCTACAATTTTGTCTA
ATCTTTTAGCTACTTTACGAATTGTATCAATTCTACCTTTATGCTCTTCATGACTACCATGTGAAAAGTTTAATCGTGCAACGTTCATACCAGC
ATTGATTAATTTCTCAATCATTTCTTCTGATTCTGAAGCTGGTCCAATTGTACATACAATTTTAGTTTTTCTCAT

Gene: pfkA (6-phosphofructokinase)

Position: 1754082 to 1755050, length: 969 nt, orientation: REVERSE

Perfect match to: (11819-97-CP003194-[1804464:1805432:r], allele observed in CC80)

Sequence:

TTATATAGATAACTTGTTAGCAAGTTCATATAGACTATAATCAAATTTATGATCTTTACCATCAAAAATTTCATCAAAAGATGTTGCTACAATT
TTATTGTTCTTAATTCCAACACCCTTAGCTGTTTCACCTTGCATTAATAAGTCTACCGCATATCCACCTAAACGTGATGCTAAAACTCTATCCG
CACCTGTTGGGCTACCACCACGTTGAACGTGACCTAACACAGACACTCTATTATCAACATTGATGTATTGTGATAATTCTTTTTGACAATCTT
GCGCAGTCATACAACCTTCTGCTACAAGAACGATTGAGTGTTTCTTACCACGTTTAATACCTTGTTCAATTTTATCAGCTATTTCTTTAATATC
TGTTTTCACTTCTGGAACTACAATTGTCTCAGCACCAACTGATAATCCAGCCCATAATGCTAGATCTCCACAATCACGGCCCATTGCTTCAAT
GATAAATGTTCGTGCGTGACTTGACGCAGTGTCTCTAATTTTGTCGACTAAGCCAATAATCGTATTTAATGCTGTGTCAAATCCAATTGTAAA
ATCAGTACCATTGATATCATTGTCAATCGTACCAGGAATACCGATAGTTTGAATTTCTTTGCATTCCTCACTGATGCGTTGTGCACCGCGATA
ACTACCGTCACCACCAATAACTACAAGGCCCTCAATCCCTCTTTTACGTAAGTTTTCGATTGCAACTTTACGTACTTCTTGCTCCTTAAACTCT
GGACATCTTGCTGAATACAAGAATGTACCTCCACGCTGAATCGTATCCCCAACTGACCCTAATTCAAGTTTATGAATATCATCATTTAACAAT
CCTTGGTAACCATGATACACACCATAAACTTCAATTTCATTGTAAATTGCTGTACGAACAACTGCTCTTACGGCAGCATTCATTCCAGGTGAA
TCTCCACCACTAGTTAAAACTGCAATTTTCTTCAT

Gene: accA (acetyl-CoA carboxylase, carboxyl transferase, alpha subunit)

Position: 1755319 to 1756263, length: 945 nt, orientation: REVERSE

Perfect match to: (A5948-ACKD01000012-[44781:45725], allele observed in CC8+CC97+CC239)

Sequence:

TTATTCTATATAAGAACCGATATTTCTGAATTTTTCAAAGCGATCATTAGCAATTTCATCACGTGATAATGACTCAAGTGAATCTAACTGTGC
AACAAACGCTGATTTAATAGCTAAAGCTTGCTGTTCAACATCTTTATGTGCACCGCCAAGTGGTTCAGAAATGACATCATCTATAATACCTAA
TTGCTTAATATCATGAGCAGTAATTTTCATTGTTTCAGCTGCAATTTTAGCCAAATTACTGTCTTTCCATAATAATGCCGCTGCACCTTCAGGA
GATATAACAGAGTAAGTACTATTCTCTAACATCAATACTTTATTGGCAATACCAATACCTAGGGCACCTCCACTGCCACCTTCACCAATAACA
ATCGCAATAACTGGTACTTTTAATGAAGCCATCTCAATCAAATTTGTTGCGATAGATTCACTTTGTCCACGTTCTTCAGCAGCTTTACCAGGA
TATGCACCTTTTGTATCTATAAATGTAAAGATAGGACGATTGAATTTTTCAGCTTGTTTCATTAAACGTAATGCTTTTCGATAACCTTCTGGAT
GCGCCATACCAAAATTTCGATAAATATTATCTTTTGTATCTTTTCCACGTTGTTGTCCAATAACTGTAACAGCACGACCATTTAAAAAGCCAAT
ACCACCAATCATTGCTGGATCATCTCTAAAATTACGATCACCATGTAGTTCCATAAACGAATCAAAGATATATGGAATATAATCTAGGGTCG
TAGGTCTTTCTTGCAAACGCGCAATTTGCACACGATCCCATGGTTTTAGATTTGTATATATTTTTTTAGTTTCTCGTTCCAATGACGCTTCAAG
CATGTCAATTTCTTCTTGTAAATCCACATCATTTTTATCTTGAGATTCTTTTAAAGATTCAATTTTATTTCGAATTTCAAAAAGTGGTTTTTCAA
AATCTAACAT

Gene: accD (acetyl-CoA carboxylase, carboxyl transferase, beta subunit)

Position: 1756263 to 1757120, length: 858 nt, orientation: REVERSE

Perfect match to: (MW2-BA000033-[1777236:1778093:r], highly conserved allele)

Sequence:

TTATTTAGTCACCTCTTGATGGATTTTTAGAATTTCAGACAATGTTTGACGCATATCATTACGATGTACAACTTTATCCAATTGTCCATGCTCT
AATAAAAATTCTGCAGTTTGGAAATCATCTGGCAATTTTTCGTTTATTGTCTGTTCAATAACTCGACGACCTGCAAAACCTATCAACGCTTTT
GGCTCACTTAAATTTATATCACCAACTGATGCAAAACTTGCAGATACACCACCAGTAGTTGGATGTGTTAAATATGATATATATAATAGTCC
AGCGTCAGAATGACGTTTTAAAGATACACTGGTTTTACCCATTTGCATCAAGGAAATAATACCTTCTTGCATACGTGCACCACCACTTGCAG
AGAAAAGAATAAATGGTAAACGGTTCTCAGTGCAGTAATCAATGATGCGACATATCTTTTCACCGATAACCGATCCCATACTTCCCATTCTA
AAACGTGAATCCATGACAGCAACGCCAAATTTCATACCATCTAGTTGCGCTGTACCAGTCACAACTGCTTCTTTAAGACCTGTCTTTTGTTGG
TCCTTTTCAATTTTTTCTAAATAACTTGGAAAATCTAATGGATTCGCAGAGGTCATTCCCTTATCGAATTCTGTAAATGATCCTTCATCAGAAA
TTGCTTCTATACGTTTATACGCAGTTAAAGCAATATGATGATCACAATTAAAGCACACATTTAAATTTTCAGCTAATTCTTTTGTGTACATAAT
TTTCTTACACTTTGGACACTTAGTCATAATACCTGCAGGCACATCATTATTTTTAGAGTCTTGTACTGTAAGATATTTCTTTTTCTTGTTCGAT
TAAAAAAATCTTTAAACAT

Gene: maeB (malate dehydrogenase)

Position: 1757315 to 1758544, length: 1230 nt, orientation: REVERSE

Perfect match to: (N315-BA000018-[1739269:1740498:r], highly conserved allele)

Sequence:

TTATTGTAAATCTGTAAGTTTCATTGTTTTATCATACACATCTTGCGGATCAACTTCAATCCTAGCTACTCCAGATTCCATTGCCGCTTTAGCA
ACATTACGAGCAACTGATGGCGCTACACGTTTATCAAACGGTCCTGGGATACAGTAGTCTTCATTTAATTCAGAACTATCGATTAAATCAGC
AATCGCTTCTACAGCTGCCTTTTTCATTTCTTCATTTATATGTGTAGCTTCAACCTCTAATGCACCTCTAAAAATACCAGGGAAAGCTAATACA
TTATTAATTTGGTTAGGATAGTCTGAACGTCCTGTACCAACAACTCGTGCACCTGCCGCTTTGGCATCATCAGGTATTATTTCAGGATTTGGA
TTAGCCATTGCAAATATAATTGGATTATCTGCCATACTCTTAACCATATCTTGTGACAGCGCATTAGCTACAGAAACTCCGATAAATACATCT
GCGTCTTTTACGACTTCTTCTAAAGACCCTTCAATCTTATCTTTATTTGTCCATTTAGCTACAACATCTTTCGTAGGATTCATACCATATGAACG
TCCTTCAAAAATTGCGCCTCTTGAGTCACACATAACCATATTTCTTACACCATACGCGTATAGTAATTTAACAATGGCTATTCCTGCTGCACCA
GCACCATTTAGTACAACTTTTATTTTAGCAATATCTTTGTTAACAACTCTCAATGCATTTACCAAACCTGCCATTGTTACAATTGCTGTACCAT
GTTGATCGTCATGGAATACCGGAATATTAGTTTCTTTTTTCAATCGTTCTTCAATTTCAAAACAACGTGGTGCCGAAATATCCTCTAAATTAAT
ACCACCATAATTAGGTTCTAACAACTTAACTGTTTTAATGATTTCTTCGGTATCAGTTGTATTTAACGCAATAGGCACCCCATTGATACCAGC
GAAGCTTTTGAATAATACTGCTTTACCTTCCATTACAGGAATACTTGCTTCAGGTCCAATGTTACCTAAACCTAATACCGCTGTTCCATCAGTA
ATAACTGCAACTGTATTTCCTTTAATTGTGTAATCATATACTTTTCTTTTATCTTCATAAATATCTTTACACGGTTCAGCAACGCCAGGTGAGT
ATGCTAAACTTAATTCCTCTTTATTAGTAACTTTTACATTTGGTTTAACTTCTAATTTACCTTGATTACGTTTGTGCATTTCCAATGCTTCATCTC
TTAATGACAT

Gene: dnaE (DNA polymerase III subunit alpha)

Position: 1758994 to 1762191, length: 3198 nt, orientation: REVERSE

Perfect match to: (11819-97-CP003194-[1809376:1812573:r], allele observed in CC80)

Sequence:

TTATATAAGCCTAATATCACTAGGGTTAAAGGATTGTATAAAATTATTAAACATACTATCTTTTTGATTAATATAGCCTAAAGTAGTCATTTGT
TTAATCGTTTCATCATAAAAGGATAACATAACATCATTAGCATTCTCTTTCGTAGCTTTAATCATCTCTTCAAACATATCTATTTGTGATTTATT
TCTAATTATAATTTGTTTGGCAAATGCTAATTTTTGTTCTTCAAAAGTGGCTAATGTTTGAATCTCATTTATAATTAGTTGCCGTTGTTGCTTTC
TATGGTCAAATTTCCCGCTAACTATAAACAAGTCATTATGTGATAACAACTCTTCGTACTTTTTAAACTGATTAGGGAAAATCACACCATCTA
AAGTTTCAATGCCATCATTTAATGTGACGAATGCCATATTTTGACCATTTTTAGTTCGAATTTGTTTAACTTTATCAAACTGTACTAATATAGG
TTTATTATTCTGCGCGTTACTCAATTTAAATATCGTTAAATATTGTTTGGCAACAAACTTTTTATCTACTGGGTGTTGCGAAACATAAAATCCT
AAATATTCTTTTTCGTACTGACTAATAAGTGCATCAGGCAATTCTTCTTTATCTTCATACATCTGTTTTGGCGTTAAAATATCAAATAAAAAAC
CATCTTGTTCAATGTTTAAATCGCCATCCAACACTTGATCAATAGCTTGCAACAACGTTGAACGTGTTTTACCAAAAGCATCAAACGCTCCCA
CTAAAATCAGTGCTTCAAGTAACTTTCTCGTTTTGACTCTCTTCGGTATACGTCTAGCAAAATCAAAGAAATCTTTAAATTTGCCGTTCTGATA
ACGTTCATCAACAATCACTTTCACACTTTGATAACCAACACCTTTAATTGTACCAATTGATAAATAAATGCCTTCTTGGGAAGGTTTATAAAA
CCAATGACTTTCGTTAATGTTCGGTGGCAATATAGTGATACCTTGTTTTTTGCTTCTTCTATCATTTGAGCAGTTTTCTTCTCACTTCCAATAA
CATTACTTAAAATATTTGCGTAAAAATAATTTGGATAATGGACTTTTAAAAAGCTCATAATGTATGCAATTTTAGAATAGCTGACAGCATGT
GCTCTAGGAAAACCATAATCTGCAAATTTCAGAATCAAATCAAATATTTGCTTACTAATGTCTTCGTGATAACCATTTTGCTTTGCACCTTCTA

TAAAATGTTGACGCTCACTTTCAAGAACAGCTCTATTTTTTTTACTCATTGCTCTTCTTAAAATATCCGCTTCACCATAACTGAAGTTTGCAAA
TGTGCTCGCTATTTGCATAATTTGCTCTTGATAAATAATAACACCGTAAGTATTTTTAATATAGGTTCTAAATGTGGATGTAAATATTGAAC
TTTGCTTGGATCATGTCTTCTTGTAATGTAAGTTGGAATTTCTTCCATTGGACCTGGTCTATACAAAGAAGTTACAGCAACAATATCTTCAAA
GTGTTCCGGCTTTAATTTTTTTAATACACTTCTTACACCGTCAGACTCTAATTGGAATATGCCAGTCGTATCTCCTTGCGACAACAATTCAAAC
ACTTTTTGATCATCAAACGGAATCTTTTCGATATCAATATTAATACCTAAATCTTTTTTGACTTGTGTTAAGATTTGATGAATAATCGATAAGT
TTCTCAACCCTAGAAAATCTATTTTTAATAACCCAATACGTTCGGCTTCAGTCATTGTCCATTGCGTTAATAATCCTGTATCCCCTTTCGTTAA
AGGGGCATATTCATATAATGGATGGTCATTAATAATAATTCCTGCCGCATGTGTAGATGTATGTCTTGGTAAACCTTCTAACTTTTTACAAAT
ACTGAACCAGCGTTCATGTCGATGGTTTCGATGTACAAACTTTTTAAAATCGTCAATTTGATATGCTTCATCAAGTGTAATTCCTAATTTATGT
GGGATTAAACTTGAAATTTCATTTAATGTAACTTCATCAAACCCCATAATTCTTCCAACATCTCTAGCAACTGCTCTTGCAAGCAGATGACCG
AAAGTCACAATTCCAGATACATGTAGCTCGCCATATTTTTCTTGGACGTACTGAATGACCCTTTCTCGGCGTGTATCTTCAAAGTCAATATCA
ATATCAGGCATTGTTACACGTTCTGGGTTTAAAAAACGTTCAAATAATAGATTGAATTTAATAGGATCAATCGTTGTAATTCCCAATAAATAA
CTGACCAGTGAGCCAGCTGAAGAACCACGACCAGGACCTACCATCACATCATTCGTTTTCGCATAATGGATTAAATCACTTACTATTAAGAA
ATAATCTTCAAAACCCATATTAGTAATAACTTTATACTCATATTTCAATCGCTCTAAATAGACGTCATAATTAAGTTCTAATTTTTTTAATTGTG
TAACTAAGACACGCCACAAATATTTTTTAGCTGATTCATCATTAGGTGTCTCATATTGAGGAAGTAGAGATTGATGATATTTTAATTCTGCAT
TACACTTTTGAGCTATAACATCAACCTGCGTTAAATATTCTTGGTTAATATCTAATTGATTAACTTCCTTTTCAGTTAAAAAATGTGCACCAAA
ATCTTCTTGATCATGAATTAAGTCTAATTTTGTATTGTCTCTAATAGCTGCTAATGCAGAAATCGTATCGGCATCTTGACGTGTTTGGTAACA
AACATCTTGAATCCAAACATGTTTTCTACCTTGAATCGAAATACTAAGGTGGTCCATATATGTGTCATTATGGGTTTCAAACACTTGTACAAT
ATCACGATGTTGATCACCGACTTTTTTAAAAATGATAATCATATTGTTAGAAAATCGTTTTAATAATTCAAACGACACATGTTCTAATGCATTC
ATTTTTATTTCCGATGATAGTTGATACAAATCTTTTAATCCATCATTATTTTTAGCTAGAACAACTGTTTCGACTGTATTTAATCCATTTGTCAC
ATATATTGTCATACCAAAAATCGGTTTAATGTTATTTGCTATACATGCATCATAAAATTTAGGAAAGCCATACAATGCATTGGTGTCAGTTAT
GGCAAGTGCATCAACATTTTCAGACACAGCAAGTCTTACGGCATCTTCTATTTTTAAGCTTGAATTTAACAAATCATAAGCCGTATGAATATT
TAAATATGCCACCAT

Gene: nrnA (oligoribonuclease)

Position: 1762212 to 1763153, length: 942 nt, orientation: REVERSE

Perfect match to: (Strain_21343-AHKV01000046-[1637:2578:r], allele observed in CC88+CC22+CC80)

Sequence:

TTAGTTAAGTTTTGTGCGTAAAGCTGTAGCAAGTTGCTCAAATTCATCCCAGCTGTCCACTGAAACTCCTGACGCATTCGGATGACCACCGC
CACCAAAATCTTGCGCAATATCATTAATAATCAATTGCCCTTTAGAGCGTAAGCGACATCTTATTTCACTACCCTCATCAACTGCAAACACCC
ATATTTTCAAACCTTTGATGTCAGCAATTGTATTAACAAACTGAGATGCTTCATTTGGCTGAATACCGAATTGCTCCAATACATCTTCAGTTAT
TTTAACTTGGCAGAATCCATCATCCATAAGTTCGAAATGTTGTAAAACATAACCTTGAAACGGCAACATTTTTGGATCCTTCTCCATCATTTTA
TTTAAAAGCGCATTATGATCAATATCATGCCCAATTAACTTTCCAGCAATTTCCATAGTATGTTCTGAGGTATTGTTAAAAAGGAATCGCCCA
GTATCACCGACGATACCAAGATATAAAACGCTCGCGATATCTTTATTAACAATTGCTTCATCATTAAAATGTGAGATTAAATCGTAAATGATT
TCACTTGTAGATGACGCGTTCGTATTAACTAAATTAATATCACCATACTGATCAACTGCAGGATGATGATCTATTTTAATAAGTTTACGACCT
GTACTATAACGTTCATCGTCAATTCGTGGAGCATTGGCAGTATCACATACAATTACAAGCGCATCTTGATATGTTTTATCATCAATGTTATCT
AACTCTCCAATAAAACTTAATGATGATTCCGCTTCACCCACTGCAAATACTTGCTTTTGCGGAAATTTCTGCTGAATATAGTATTTTAAACCAA
GTTGTGAACCATATGCATCAGGATCTGGTCTAACATGTCTGTGTATAATAATTGTATCGTTGTCTTCGATACATTTCATAATTTCATTCAAAGT
ACTAATCAT

Gene: ytoI (putative protein)

Position: 1763414 to 1764712, length: 1299 nt, orientation: REVERSE

Perfect match to: (11819-97-CP003194-[1813796:1815094:r], highly conserved allele)

Sequence:

CTATACTTCGTCAAACATTTGGCATATCACGAGAGCGCTCGCTACTTTGTCGTTTTGACTATGCATGTTCACTTCTATTTTGGCGAAGTTTCTT
CCGACGTCTAGTATGCCAAAGCGCACTGTTATATGTGATTCAATAGGTACTGTTTTAATATACACGATATTTAAGTTCTCTATCATGACATTA
CCTTTTTTAAATTTACGCATTTCATATTGTATTGTTTCTTCTATAATACTTACAAATGCCGCTTTACTTACTGTTCCGTAATGATTGATTAAAAG
TGGTGAAACTTCTACTGTAATTCCATCTTGATTCATTGTTATATATTTGGCGATTTGATCGTTAATTGTTTCACCCATCTGAGGCTGTCTTCCT
AAAAGTTGCATAGACTTTAAAACATCTTGTCTATTAATCACACCCACTGTCTTTTTATTACTCGAAACGACAGGAATCAATTCAATACCTTCCC
AAATCATCATATGCGCACAACTTGCTACTGTACTCATAGCATTTACATAAATAGGATTTCGCGTCATCACTTTATCTATTTCGTCGTCGTCCTT
TGTATTAATCATCTCTCGACTTGTTACAATACCTACTAATTTATAAGACTCATTGACTACCGGAAATCTTGTATGGCCAGTTCGATTCGCCATA
CGCTTATAATCTGCTATTTTCATCGTATCAAACAGCACAGATAAATCATCTAATGGCGTCATTATATCTTGAACTATTAAGATATCTTTTCGTA
TTTTCTGATTAAAAAGTGCTTTGTTGATAATATTTGCAACTAGAAATGTATCATAACTTGATGATAGAACAGGTAAATCATGTTCATTCGCAA

AATTAATAACTTTATTAGATGGCTTAAATCCACCAGTAATTAATATAGCCGTACCTCTTTTTAAAGCTTCAATCTGCACATCTTCACGATTTCC
GACAATCAATAATGTCTTTGGACCAATATACTTTAAAATATCTTTGAGTTCCATTGCTCCAATTGCAAATTTAGATACCATCTTAGTGATACCT
TTGTTGCCACCTAACACTTGGCCATCAATAATATTGACAATTTCATTAAAAGTTAAATGTTCAATTTCATTACGATTACGTTTTTCGATTCGAA
CCGTACCAACACGATCTATCGTTGCGACCATGCCCATTTTATCAGCATCTTTAATTGCACGATATGCTGTCCCTTCAGATACGTTTAAAAATTT
AGCGATTTTACGCACCGAAATTTTAGAGCCTATAGATAACGATTCAATATAATCTAAAATTTGTTCATGTTTTGTCAT

Gene: uspA (universal stress regulator)

Position: 1764917 to 1765330, length: 414 nt, orientation: FORWARD

Perfect match to: (N315-BA000018-[1746922:1747335], highly conserved allele)

Sequence:

ATGTATAAAAATATATTACTTGGTGTAGACACTCAGTTAAAAAATGAAAAAGCACTAAAAGAAGTGTCTAAATTAGCTGGCGAAGGTACAG
TCGTAACAGTTTTAAACGCAATCAGCGAACAAGATGCTCAAGCATCAATTAAAGCAGGTGTTCATTTAAACAAACTTACTGAAGAACGAAG
CAAGCGATTGGAAAAAACACGCAAAGCTTTAGAAGATTATGGTATTGATTATGACCAAATAATTGTTCGTGGTAATGCAAAAGAAGAACTA
TTAAAACATGCTAATAGCGGTAAATACGAAATTGTTGTTTTAAGTAACCGTAAAGCAGAAGACAAAAAGAAATTTGTACTTGGAAGTGTCA
GCCACAAAGTAGCAAAACGTGCGACTATCCCTGTATTAATCGTTAAATAA

Gene: ytkL (metal ion-dependent hydrolase)

Position: 1765635 to 1766324, length: 690 nt, orientation: REVERSE

Perfect match to: (11819-97-CP003194-[1816017:1816706:r], allele observed in CC80)

Sequence:

TTAAAACTGAACAGATTCACCTGGTTTTAAAATTTGCACGTCCCCTACATTAACAGCATCTTTAAATTGTTGTGGATCTTGTTCGATTAATGG
GAATGTATCATAATGAATCGGTACAGAAATTTTTGGTTTAATAAATTCATTAATAGCATAACTTGCATCATCAATACCCATCGTAAAATTATC
TCCAATTGGTACAAAACATACATCAACTGGATGACGTTTCGCAATAAGTGACATATCACTAAACAGACCTGTATCACCAGTATGATAAATTG
TTTTTCCTTCAACTTCAAACACGATACCCATTGGCATACCTAAATAAACTGGAATACCATTTTCATGTGTAAAACTTGAACTATGAAATGCTT
GAACAAATTTAACGCTTCCGAAATCAAAGTTTGCTTTACCACCAATATTCATACCATGAACATTTTCAACACCGTGATATGAAGAAAGATAG
TCAGCCATTTCTGCACTTCCAATTACTGTTGCTCCTGTTTTCTTTGCTAGTTCCACAACATCACCAAAATGATCAAAATGACCGTGCGTTAAAA
CGATATAGTCTACCTGCACTGTTTCAATATTCAAATCACACTTAGGGTTATTTGAAATAAACGGATCTACGATAACCTTTTTGTTGTTCCCTTC
TAAATAAATCGTTGATTGACCTTGAAATGATAACTTCAT

Gene: pepQ (Xaa-Pro dipeptidase)

Position: 1766499 to 1767554, length: 1056 nt, orientation: FORWARD

Perfect match to: (11819-97-CP003194-[1816881:1817936], highly conserved allele)

Sequence:

ATGACAAAAATATCAAAAATAATAGACGAACTGAACAATCAACAAGCTGATGCAGCATGGATTACAACACCGTTGAATGTATATTATTTTAC
TGGATACCGTAGCGAACCCCATGAAAGATTATTTGCATTATTGATTAAGAAAGATGGTAAACAAGTACTATTTTGTCCAAAAATGGAAGTC
GAAGAAGTCAAAGCATCACCTTTCACAGGTGAAATCGTTGGATATTTAGACACTGAAAACCCTTTCTCACTTTATCCTCAAACAATCAATAA
ATTACTAATTGAAAGCGAGCACTTAACAGTAGCACGCCAAAAACAATTAACCTCTGGTTTCAATGTCAATTCATTCGGAGATGTTGATTTAA
CAATCAAACAATTGAGAAATATTAAATCCGAAGATGAAATTAGCAAAATACGTAAAGCTGCTGAGTTAGCAGATAAGTGTATCGAAATAGG
TGTTTCTTATTTAAAAGAAGGTGTGACTGAACGTGAAGTAGTCAACCATATTGAGCAAACTATCAAACAATATGGCGTCAATGAAATGAGT
TTTGATACGATGGTTTTATTTGGAGATCATGCCGCATCACCTCATGGCACACCAGGAAATCGCAGATTAAAAAGCAATGAATATGTACTATT
TGATTTAGGTGTAATTTATGAGCATTATTGTAGCGATATGACACGTACCATTAAATTTGGTGAACCTAGCAAAGAAGCACAAGAAATTTATA
ATATTGTATTAGAAGCAGAAACATCTGCAATCCAAGCAATTAAACCTGGAATACCTTTAAAAGATATCGATCATATCGCTAGAAATATTATT
TCAGAAAAAGGTTATGGTGAATATTTCCCTCATCGCTTAGGTCATGGCCTAGGATTACAAGAACATGAATATCAAGATGTTTCAAGTACTAA
TTCTAATTTGTTAGAAGCTGGCATGGTTATTACAATCGAACCAGGTATTTATGTACCAGGTGTTGCAGGTGTAAGAATTGAAGATGACATAC
TTGTCACTAATGAAGGATATGAAGTATTAACACATTACGAAAAATAA

Gene: ald2 (alanine dehydrogenase 2)

Position: 1767870 to 1768988, length: 1119 nt, orientation: REVERSE

Perfect match to: (MW2-BA000033-[1789036:1790154:r], highly conserved allele)

Sequence:

TTATAATTGTAATGCTTCTTCTACAGATTTATATTCCATTTCAAATGCCTCTGCAACGCCTTTATTGGTTACGTGACCTTTGTAAGTATTTAAAC
CTAATGATAATGGTTGATTTGATTTAAATGCTTCTCTATACCCTTTATTAGCTAGCATGAGCGCATAAGGTAGCGTAGCATTATTTAAAGCTA
ACGTCGAAGTACGCGGTACTGCACCTGGCATATTTGCAACTGCATAATGAACCACACCATGCTTAATATATGTAGGATCATCATGTGTCGTA
ATTTTATCAGTTGTTTCAAAAATACCGCCTTGATCAATAGCAATGTCAATAATAACTGACCCATTTTTCATTTGTTTAATCATGTCTTCTGTTAC
AAGTCTTGGCGCTTTAGCACCTGGAATTAAAACTGCACCTATTACTAAATCACTTTGTTTAACATACAACTCAATATTCAACGGATTTGACAT
AATTGTATGTACACGTCCACCGAATAAATCATCTAATTGTTGTAAACGCTTTGGATTAACATCTAAAATCGTAACATCTGCACCTAGTCCTAG
TGCAATTTTAGCTGCATTTGTTCCTGCTTGACCACCACCGATAATAGTTACTTTACCCTTAGGTACTCCTGGGACACCACCTAGTAGAATTCC
CATACCACCATTAAGTTTTTGTAGGAACTCTGCGCCAACTTGAGCTGACATTCTTCCTGCTACCTCACTCATTGGTGATAACAATGGTAAAGA
TCGGTCTGGTAACTGCACAGTCTCATATGCAATACTAATTACTTTTCTATCTATCAAAGCTTGTGTTAATTTTTCTTCATTTGCTAAATGAAGA
TAAGTGAATAATACAAGCCCTTCTTTAAAATATGGATATTCAGATTCAAGTGGTTCTTTAACTTTAATAACCATATCCACATCCCAAACTTTTG
CTTGTTCAGCAACAATCTCAGCACCTGCTTCTTTGTAATCTACATCTTCAAAGAATGATCCTGAACCCGCATTTGTTTCCACTAAAACAGTATG
CCCACTTTCTACTAAAGCGTGCACACCACTTGGTGATAAACCAACACGATTTTCATTATTTTTAATCTCCCTTGGTATACCAATTTTCAT

Gene: yxiE (phosphate starvation protein)

Position: 1769129 to 1769629, length: 501 nt, orientation: FORWARD

Perfect match to: (MW2-BA000033-[1790295:1790795], highly conserved allele)

Sequence:

ATGATTACTTACAAAAATATTTTAATCGCAGTTGACGGTTCACATGAAGCGGAATGGGCATTTAACAGAGCAGTTGGTGTTGCTAAACGTA
ACGATGCGAAGTTAACAATTGTGAATGTAATTGATTCAAGAACGTATTCTTCTTATGAAGTTTATGATGCTCAATTTACTGAAAAATCTAAGC
ATTTTGCAGAAGAATTATTAAATGGTTATAAAGAAGTAGCTACTAACGCTGGTGTTAAAGATGTAGAAACGCGTCTAGAGTTTGGCTCTCCT
AAATCTATCATTCCTAAAAAGCTTGCACATGAAATTAATGCAGACTTGATTATGAGTGGTACATCAGGCTTAAATGCCGTGGAAAGATTTAT
TGTTGGTTCTGTATCAGAATCTATCGTTCGTCATGCGCCATGTGACGTGTTAGTTGTTCGTACTGAAGAGTTACCAGCAGACTTCCAACCAC
AAGTTGCAACAACTCAATTACGTGAAAAATATCAAAATTAA

Gene: ackA (acetate kinase)

Position: 1769875 to 1771077, length: 1203 nt, orientation: REVERSE

Perfect match to: (11819-97-CP003194-[1820257:1821459:r], allele observed in CC80)

Sequence:

TTATTTTAAACCACCGAATGTCATAACATCACGGGCAATCATACTTTCTTCATCTGTTGGAATAACGACAACTTTAACTGGTGAATGAGGATA
GTTAATAAATCCTTCTTTACCACGTAGTAAGTTTTCATTTTTCTTAGGATCCCAGTAAACACCCATAAATTCTAAGCCTTCAAGAACTTTCGCA
CGAATTTCTACTGAGTTTTCACCGATACCTGCTGTAAATACGATAACATCAACACCATGCATTCTCGCAGCATATGATCCAATATATTTGTGA
ATTTTAGAAGCAAATACATCTAAAGCCATTTGTGAACGTGCTTTACCTGATTCAGCTTCTTCTGATAAGTCACGTAAATCACTAGATGTACCT
GATAATCCTAATAAACCTGATTCTTTGTTTAAGATTTCCAATACTTGTTCAGCAGTTTTACCTGTTTTTTCCATAATAAATGGAATTAAAGCAG
GGTCAATATTACCAGAACGAGTACCCATTGTTACACCAGCAAGTGGTGTGAAGCCCATTGATGTATCAATAGATTTACCGCCATCGATAGCT
GCAATTGATGCTCCATTACCAATGTGACATGAAATAATACGTAAATCTTCAATTGGCTTATCTAACATTTCTGCCGCTCTTTGTGATACAAATT
TATGGCTTGTACCATGGAAACCATACTTACGAATGCCATAATCTTTATAATAATGATATGGCAAGCTATATAGATATGCTTTTTCAGGCATTG
TTTGATGGAATGCTGTATCAAAAATTGCCACATGAGGGATATTTGGTAATAATTTACGGAAAGCACGAATACCCATCAAGTTAGCTGGGTT
GTGAAGCGGTGCTAATTCGCTTAATTCTTCAATTTCCTTTTCAACCTCATCAGTAATAGCTACTGATTCAGGGAATTTTTCACCACCATGTACA
ACACGGTGACCTGTTCCATCGATATCGTTAATATCATTAATAATATTGTGCGCTTTAAAAGCATCCAACATGATATCAACTGCCTCAACGTGA
TCCTTGATATCTTGTACTGATTTAACTTTTTCCCCGTTGACTTCGATTGTAAAAATTGAATCCTTCAATCCGATTCTTTCTATTAAACCTTTTGTT
ACTAATTCCTCTTCAGGCATTCTAATTAATTGAAATTTTAATGATGAACTACCAGCATTGATAGCCAAGATTAATTTTGACAT

Gene: ytxK (putative nucleic acid methyltransferase)

Position: 1771165 to 1772112, length: 948 nt, orientation: REVERSE

Perfect match to: (11819-97-CP003194-[1821547:1822494:r], highly conserved allele)

Sequence:

TTATTTTTTAGGACGATTTGTGTCCATCCACTGATTTAACTCTGTCATAAATCCTTGAAATTGTGAAGGATTTTTGAAATCAGGAATATTTGCC
AATAATACTTCAACTGGCTTTGTTTCACCCGATTTTTTCTTTTGTAAAATTAATATAGATTTTCGCGCTTTTTCATTTTTAAATAAAGTTGGTGG
TAAATTTAAAAATGCTTGCATCTCTGTCTCTGTTGCAATATATTTTTCAAGCTGTTTTACATGTTCACCTGTAAAAATATTACTTGGTACCACT
AGAAAGGCATATCCAGCATCTTTTAATGCATTTATTGCTTGTTCTATTAATAAATAATGTGAATAACTATGTCCTTCTTCAAAACCTAGCTTAA
ACTCCTTACTTCTTTCATCAATTGGATAATAGCCTACTGGAAAATCACCAATAACGATATCTGCTTCTTCTAATGGTAGTGGCATGATGGCAT
CTTGAGGATACACATCGAAAGGAATTTCTAAGAAGTTTGCTAAATGTACACTAACACGTGATAAAACTGGATCAACTTCAATTAAATGATGC
ATAACCGCAATTTCAGGTAACACTTCTTTTACAGTAGCACTTAAATGACCGGCACCACTTGCAATATCAACAATATGTAATTCTTCTTGGTTG
TTCATAAAACGCTCAACTAAAAATCCTAGTATCAATCCAATTGAATCTGGTGTAATTTGATGATTTGCTTGTATCTTTTCTTCCTGCATTAAAC
TTAAATATGCAAATTGGAATGCTTTACGTCGATCTTGTAACGTCGATTGTTCTAACAATCCTCTTTCATTGGTATATACTTGTTCCATTGCTAG
CCCAAGATTTTCAATAAAACTTTGGCCATTTTCATTATTTAATGTTTTAGCTTTTTCATCTAATGTATGAAACAAGCGTTCCATAATTGTTTGTT
GTTCTGCCAT

Gene: tpx (thiol peroxidase)

Position: 1772235 to 1772729, length: 495 nt, orientation: REVERSE

Perfect match to: (11819-97-CP003194-[1822617:1823111:r], highly conserved allele)

Sequence:

TTAAATATTTTTGTATGCAGCTAAAGCAGCATCAAAATCTGGGAAATCAGTACCTTCACTAACGATTTCTTTATAAACAACTTTATTATCTGCA
TCTAATACAAATACTGCACGAGCTAATAAGCGAAGTTCTTCCATAACAACGCCATAGTTTTCACCAAATGATAAGTCACGGTGGTCACTTAA
TGTAATGACATTGTCTAAACCTGCTGAAGCGCACCATCTTTTTTGTGCGAATGGTAAGTCTGCTGAAATTGTAAGCACAATCCCCTCTTCTTT
AGAAGCTTCAGAGTTGAATTTGCGAGTCTGCTGATCACAAACACCTGTATCAATTGATGGTACCACACTAATTAATTTCTTTTTACCAGCATA
ATCTGCTAATGTTACTTGATTTAAGTCATTATCTAACACTGTAAAATCAGGTGCAAAATCACCTTCATTAATTTGTTGACCTTTTAAGTGGATT
GGTCCACCTTTGAATGTTATTTCAGTCAT

Gene: ytnM (putative transporter)

Position: 1772827 to 1773597, length: 771 nt, orientation: REVERSE

Perfect match to: (TCH959-AASB02000248-[34283:35053:r], allele observed in CC7+CC9+CC96)

Sequence:

TTATACAAATTGTTGAATATAATCAAAAGCATTTTTTAAAATTAAAATAGCAGTAATAATAATAAATAATACTTTTACATAGCCAACACCTTG
TTTGATAGCAAAATGTGCCCCAGTATATGACCCAGCAATCATGCTTGTAGCCATTATTAAACCTATTACATAATCTACTTGTCCTAATACCAT
AAATAATACAAGCGCACCTATATTAGAAGCAAAGTTCAAAACTTTAGCATTTCCTGCTGCACTTAAAAAATCAAAACCAAAGACTAACAATA
CAAAAAGCATAAATGAACCTGTTCCCCCACCTACAAATCCATCATAAAAGCCGATTAATATAAAAAGTGCTGCAAATAGTATGGCTTTCTTA
AATGTAAATTGAGTAAACGTACGTGTATTGCCCCAATCTTTTTTAAGTAATGTGAATATAAACACCGACGAAAGTGCAATAATGATTAAAGG
TTTCAATATTTGTGACGGAACCATCGTTGCAATATATGCGCCACATGCAGATGCCAAAAATACAAAACCAAATAATTTGGCAACAACATATA
AGTCCACTTTACCGGACCTTATAAACTTTATCGTACTAGTTAAAGAACCAAATGAACTTGCCAATTTATTTGTACCTAAAGCCACAGATGGTG
GTAGACCGATTGCTAATAATGCTGGCGTAGAAATTAAACCACCACCCCCCACAACCGAATCTATAAACGCCGCGATAAAACCAAATAAAAT
TATGATTATAATCATCGTTAAGTTCAAATCCAT

Gene: thiI (thiamine biosynthesis protein)

Position: 1773641 to 1774864, length: 1224 nt, orientation: REVERSE

Perfect match to: (KPL1845-AZJA01000018-[161090:162313:r], allele observed in CC96+CC72+CC772)

Sequence:

TTATAAAAAGTCATTTATTAATTGGTTTGTTTGCTGTTCTTTAATAGTTTTATAATCACTAGTTATTTCAAGTGTTTCAATATTTTCAACAGCAC
GATTAATCATCTCTTCAAAATCAAAGACACTTTCATATTGGACTACCTTATCAAAGTTTGGTTCGGTTACTGGATTTTTAGGGGTGAAAATTG

TACAACAATCTTCAAATGGTTGAATAGATGTTTCAAATGTACCGATTTCTTTCGATTTAATAATAATTTCTTCTTTATCGTAAGTTAATAAAGG
ACGTAATACAGGAGTAGAAGTTACATTATTAATTGCATACATGCTATGAAGTGTTTGACTGGCTACCTGCCCTAGGTTTTCACCATTTACAAT
AGCTAAAGCCCCTATTTGATGTACTAATTTATCAGCAACACGCATCATCATACGTCTCGTTGAAGTCATTGTATATCTTGGATGTACAACTTT
ATTTACCTGTTTTTGCAATTCTGTAAATGGTACAATATGCAATTTAATTGGTCCAACACGTTCAGCTAAAATACGTGTCAATTCAATAACTTTT
TCTTTTGCTTGATCACTTGTAAATGGTGGACTATGGAAATGAATCGCTTCAATTGTTACGCCACGTCTCATCACTTCCATCCCAGCAACTGGT
GAGTCTATACCGCCTGAAAGCATTAGTAACGTCTTACCACCAGTACCAACTGGTAATCCACCTGAACCCGGAACAACTTCTTCATACATATA
AATTGCATCTAATCTAACTTCCACTCGAATTTCATGATCTGGACGTTTGACATTCACTGAAATATTGTCGAAGTGCTTCAATACTGCACCACC
CAATTCACGCTGTAATTCATACGTATCCATTGGGAAATTTTTATCGGCACGCTTAACATCAATTTTAAATGTGCTGTTTTCTTCAAATTGCTGC
GCAAATTTAATTGCCGCTGCACTCATTGCATCTATTGTTTTTTCTACTTTTAATACTGGACTAATAGATTTAATACCGAAAATTTTTGATAATC
GATATGTTATTTCATTTATATCTGCATGGTCTTCAAGTTCAATATACATACGATCTCGTTTGCCTTTAACGACAAACCCATCAAGTCCTTTTAA
TGACTTATTTACATTATTTCTTAATTGATTTACAAATTTTTTTCTATTTGAACCCTTTAATGTTAACTCCCCGTATCTAACAAGCAAGTGATCAT
ACTTCAT

Gene: csd2 (cysteine desulfurase 2)

Position: 1774864 to 1776003, length: 1140 nt, orientation: REVERSE

Perfect match to: (11819-97-CP003194-[1825245:1826384:r], allele observed in CC80+CC1+CC772)

Sequence:

TTATTTTAGCAACTCCTTAATTTCCTCATAAATGATGATAAATATTTCTTTAAACCTTGCTATATCTTCTTTAGTTGTAGTAGCCCCAAATGATA
ATCTTATACTACCTTCAATAGATTTGTCTGATAATCCCATTGCAGCCAATACTTCATTTAATTTATTACGTTTAGATGAACAAGCACTCGTCGT
AGATATCATAATGTCATATTTTGAAAAAGCATTAACTAATACTTCACCTTTTACGCCAGGAAAACTAATATTTAAAACGAATGGTGAACCTG
AAGTTGAAGAATTAATATAAACTCCATGATATTTATTTAAAAATTGACGGACGTCATTATTTAACTCAGTAACAAATGCATTCAATGCTTCAA
AGTTTTCATTAGCTATCTTCATCGCTTTAACCATTGCAATATCATTTGGCAAATTAACTGTTCCACTTCTAACACCATATTCTTGACCACCACCA
TGGACAGTTGGTTCAACATTTTGAATGTGATTTACAAGTAAGACGCCTTGGCCTTTTAAACCATTAAACTTGTGTCCACTTAAACTAATACTA
TCTATGTTATTGAGATCCATTGAAATTTTGCCGAATGCTTGAACTGCATCTACATGAAAATGTGCCTTAGGATAATTTTTTATAACTTTAGCC
ATTTGTGGAATAGGCTGTATTTGTCCAGTTACATTATTTACATACATACATGTTACTAAACCGACTTTGTCTGACATTAATTCTTTGAAGTGTT
CTAAGTTAATACTGCCATCTTTCTTTACATCAACATATTTAACTTTAAATCCTTCGTGTGCTTCCAAATATCTTACAACCTCTAATACGGACGG
ATGCTCTAACACGGATGTAATTATTTCCTTCGCTGTATCAAATTTACGATAGGCAATACCTTTTAAAGCAAGATTATTGGATTCAGTTGCACC
ACTAGTGAATACAACATCATAATTTGTTTTTGAATTAATCATTGCATTAATTTGGGCTTTTGCTTGTTGTAGTAATTGATTTGCCTGCAAACCA
GCTTTATGCGGACTATTCGGATTATAATACATTGATTGATTTACTTTTAAATAAGTATCTAACACTTCTTCAAATGCTTTCGTCGTTGCCGCAT
TATCTAGATATATCAA

Gene: ezrA (septation ring formation regulator)

Position: 1776375 to 1778069, length: 1695 nt, orientation: REVERSE

Sequence:

CTATTGCTTAATAACTTCTTCTTCAATATGTTTAGTAACACCTGGCTCAACACTTTCAAGAGCTTGCTCTGCAATTTCAATCGCACGCTTATAG
CGATTATTTTTAAATAATCGTTCAGCTTCATTTAAGCTCTTATCAACATTGCTATAGTCCTTACGATATCTATTTCCATATTGAATTAATTTCTC
TGCATAAACAGCATTAACAAGAACATCATTTGCTTCATCTTCAAATGTATTCATTTGAATCACAATTTTAGACACTTTATCTTTTAACTGTTTA
ACGTGTATTGGACGTTCACTAAATTGTTCGTTAACATCACGAACTTCATGATCAATTTCATTTTTCATGATGATAAACCCTTTCAGGAACGCTT
GTTAAGTTAGAAGCAAGTAATCGACGATACACTTCTTCTTTCTTCGATTGTACTCGTAGCAGATTGTCTTCTGCTTCTGCTTCATCTTCACGCA
ATTGAATCAGATGATTTTGTAGCTTTTCTTGTTTGTCATTAATAACTGTGACATGATCTTCTAAATATTGCAAATTATCCTGAACCTCGCTATA
TCGCACAGCAGATTTAGACATTTCTTTTAAAATATCATCATATACAGAAATTAAACTTTGAATTTCATTTTCAAATTGACGAACACTTTGAGCA
TCAGATTCATTTATATAGTAGTTTTCACGTACATATTCAATTTCTGTTTGCAATGTATAATTCATGTCTTTAGCTTTGAATAAGTTATCCGTAAT
GATATCTTTTGTTTCTTCGACATCATTTTTAGCTTTAACTTCATGTTCAATTAAATCATACATGTCATCTAACTTATCATTGATATTAGCTAGTTT
ATCATTAGCTTCTTCTAATTCTAAGCGGCTAATTAATGGTTCAACGAAACTAAGCTCTGTTTTTAAGCTTTGTAATGTACTGTCTACTTTTACG
TGATCCAGATCATACCCTTCAACTTTAAGATCACGGCAACCATATTTTAAATCTTGGAATTGACCAGGTAATTCTTTTTGAGTTTCTCTAATTA
ATTCTGGTATTTCTTCCATATAAGATCTTAGCTGTTTCATTTGTTCATTCAAGGCAGCTATATGGTTGTGCGCTTGTACATAATTACCATCAGC
TTTTAGTACTTCATATTGCTCTAACCTTGGCTCAAACTTTTCAATTTCAGTTTCAAGTAGACTTGCTGCCTCACCAAATTGATGACGATTTGCT
AAAACATCACGTTTCATTTCACGATAATCAACCTTACATTTGTCATATAATTCATCATTATCTTTGTATAACGCAATAATTTCATTTACATCTTC
TAATTGTTGCTGATAGCTTTGTTCGTAACTATCCATCAACTCATTTGCATCATCAATTTCACTTTGAGATGCGTTGAAACTAAATTTATCTAAT
AAAGCCTCAGCATTATGGATTTTTTCTTCCACAGGAGCTAGATACTTATTTGTACTTTCTACGTTGTCCTTTTTCATTGCATCGTATTTCGTTTT
TGTTTCACCTTTTAAATTCAACTTAGATAATTGTGCAAGGTTTTGATCAAAAGGTAACGTCTCAATTTCATTTTTACGTTCGATTGCTTTTTCA
ATAATTTGTCGTTTATTTGAACGTAAATAGAATAATACACCTACAGCAATCAATATAATCACAATTATTGCCAAAATGATATATAACACCAT

Gene: ytsP (GAF domain protein)

Position: 1778206 to 1778670, length: 465 nt, orientation: FORWARD

Perfect match to: (11819-97-CP003194-[1828587:1829051], highly conserved allele)

Sequence:

ATGACAACAATTAACCCAACAAACTACACATTATTAAAGAAACAAGCAGCAAGCCTTATTGAAGATGAACATCATATGATTGCTATTTTAAG
TAATATGTCTGCCTTATTAAATGATAATCTAGATCAAATTAATTGGGTCGGCTTTTACTTATTGGAACAAAATGAACTTATACTTGGACCTTT
CCAAGGACACCCCGCTTGTGTCCACATTCCAATTGGAAAAGGTGTATGTGGTACAGCCGTTTCAGAACGTCGTACACAAATTGTAGCTGAT
GTTCATCAATTCGAAGGACATATCGCTTGTGATGCTAATAGTAAGTCTGAGATTGTCGTTCCAATTTTCAAAGATGATAAAATTATCGGCGT
CTTAGATATCGATGCCCCTATAACGGATCGATTTGATGACAATGACAAAGAACATCTTGAAGCAATTGTTAAAATTATTGAAAAGCAACTCG
CATAA

Gene: rpsD (30S ribosomal protein S4)

Position: 1778914 to 1779516, length: 603 nt, orientation: FORWARD

Perfect match to: (MW2-BA000033-[1800080:1800682], highly conserved allele)

Sequence:

ATGGCTCGATTCAGAGGTTCAAACTGGAAAAAATCTCGTCGTTTAGGTATCTCTTTAAGCGGTACTGGTAAAGAATTAGAAAAACGTCCTTA
CGCACCAGGACAACATGGTCCAAACCAACGTAAAAAATTATCAGAATATGGTTTACAATTACGTGAAAAACAAAAATTACGTTACTTATATG
GAATGACTGAAAGACAATTCCGTAACACATTTGACATCGCTGGTAAAAAATTCGGTGTACACGGTGAAAACTTCATGATCTTATTAGCAAGT
CGTTTAGACGCTGTTGTTTATTCATTAGGTTTAGCTCGTACTCGTCGTCAAGCACGTCAATTAGTTAACCACGGTCATATCTTAGTAGATGGT
AAACGTGTTGATATTCCATCTTATTCTGTTAAACCTGGTCAAACAATTTCAGTTCGTGAAAAATCTCAAAAATTAAACATCATCGTTGAATCA
GTTGAAATCAACAATTTCGTACCTGAGTACTTAAACTTTGATGCTGACAGCTTAACTGGTACTTTCGTACGTTTACCAGAACGTAGCGAATTA
CCTGCTGAAATTAACGAACAATTAATCGTTGAGTACTACTCAAGATAA

Gene: ugpQ2 (chromosomal glycerophosphodiester phosphodiesterase)

Position: 1779732 to 1780475, length: 744 nt, orientation: REVERSE

Perfect match to: (MW2-BA000033-[1800898:1801641:r], highly conserved allele)

Sequence:

TCACTTAATAAATAATTTCGGATTATCTGTAATTAATCCATCTACACCCATTTGTCTTAATTTTTCTCCAGTTTTCAATTTATTAACTGTGTATG
GCATAACCTGTAATTGATGATGATGTGCTTTATCAACAAATTTTCTGGTCACTAATGCATAATTAGGATTAACATAACTAGCAATTTGAGCAA
TTCTTGAAAAGTTTGGCTTTTTATACCAATAATTACGTTTACTACAAAGCACACCTAATTCATATATACTGCCCAATGTGTTTAACTTTTCGAT
GCATTCGATATCAAAAGATTGTATAACTACTTGTGTCGCATCGACTTTCTTTTCTTCCAAAAATGCCAGCAATTTACATTCTATTTCTGGATAT
AAATTCGGACTTTTCAATTCAATCAATAGCTTTTTATCATACTTTAAGCATAATGAAAGTACTTCATCTAACGTGGGTATTCGTTCTCCCTTAA
AAGCAACATCTTTATAACTACCAAAATCAAATGATTTTAATTGCGATAATGTGTAATCAGAAATACGCCCCTTACCATCCGATGTTCTATCAA
TTGTTTCATCATGTATCACAACAAAATGTTGGTCTTTGGTCAAATGAACATCTATTTCTAACATAGCAACATTGAGCCCCATTACCTCTCGATA
ACCGACCATTGTATTTTCAGGAAAATCACTCGGCAATCCACGGTGCGAAACAATTTGTAATTCATCTTTCAGTTTATTCAGAGTCAT

Gene: osmC (peroxiredoxin-like)

Position: 1780555 to 1781001, length: 447 nt, orientation: FORWARD

Perfect match to: (Strain_21334-AGTW01000018-[29112:29558], allele observed in CC9+CC80+CC97+CC239)

Sequence:

TTGCATCAACATGACTTTAAAGTCCAAACTTCTTGGCAAGGTGGTCGTAACAATGTCGGAAACGTTCAAGGCGACATACTTTCAGAGAATAT
TTCTATACCTGCTTCTTTAGGTGGTGTTGGTATAGGAACAAATCCCGATGAAATGTTAGTATCAGCCGCTTCATCATGTTATATCATCTCATT
AGCAGCTACTCTTGAACGTGCAAAGTTCACAGATATTTCAATTGAACAACAATCGATTGGAACAGCTTGTTTAAATAACGGAAAATTCAGTA

TGTCAAAAATTGTGCACCATCCTCAAATTCAAATTCCAAGTGATCAAATAGCACAATTAGAAAAGCGATTACCAAAATTGATAACAATTGCA
GATAATAATTGCATGATTTCAAATGCTGTAAGAAATAATGTGGACATAAAAATCTATCCCAGCATTCAGGCCAAATAA

Gene: pucG (vitamin B6-dependent (S)-ureidoglycine glyoxylate aminotransferase)

Position: 1781115 to 1782275, length: 1161 nt, orientation: FORWARD

Perfect match to: (JKD6159-CP002114-[1791204:1792364], highly conserved allele)

Sequence:

ATGTATTATCATCAACCGTTGTTATTAACACCTGGCCCAACCCCTGTACCTGATGCCATTATGAGAGAAATTCAAGCACCTATGGTTGGTCAT
CGTTCTAAAGATTTTGAAGACATCGCACAACAAGCATTTCAAGGTCTAAAGCCAATATTTGGGAGTCAAAATGATGTACTTATTTTAACATC
TAGCGGTACAAGCGTCTTAGAAGCTAGTATGTTGAACATTGTAAACCCTGAAGATCACTTCGTTGTCATTGTTTCAGGTGCCTTTGGTAACC
GATTTAAACAAATTGCACAAACTTATTACAAAAATGTGCACATTTATGACGTAACATGGGGAGAAGCTGTAGATGTTAAAGATTTCATCAAT
TATCTTTCAACTTTAGATGTTGAAGTTAAAGCAGTATTTAGTCAATATTGCGAAACATCTACGACAGTGCTACACCCTATTCACGAGTTAGGA
AATGCCATCAATCAATTTAATAGTAATATTTATTTTGTAGTTGACGGCGTAAGTTGCATTGGTGCTGTTGATGTTGACATTAACAAAGATAAA
ATTGATGTACTTGTTTCTGGTAGTCAAAAAGCAATTATGTTACCTCCAGGATTAGCTTTTGTAGCTTATAGCCACCGTGCAAAAGAACGTTTC
AAAGAAGTAACTACGCCAAAATTTTATCTAGACTTAAATAAATACATTTCGTCACAAGCTGACAATTCTACACCGTTCACACCAAATGTGTCT
TTATTTAGAGGTGTAAATGCATACGTTGAAACCGTAAAAGCAGAAGGTTTCAATCACGTAATAGCACGACACTATGCAATTAGAAATGCAT
TAAGAAGCGCCTTAAAAGCATTAGATTTAACTTTATTAGTCAATGATAAAGATGCATCTCCAACGGTTACAGCATTCAAACCTAATACAAAT
GATGAAGTGAAAATAATCAAAGATGAACTTAAAAATCGGTTTAAAATAACAATTGCTGGTGGTCAAGGCCATCTTAAAGGTCAAATTTTAA
GAATTGGTCATATGGGGAAAATTAGTCCTTTCGATATTTTATCGGTAGTATCTGCTTTAGAAATTATTTTAACTGAACACCGTAAAGTTAACT
ATATCGGTAAAGGTATATCAAAATATATGGAGGTTATTCATGAAGCAATTTAA

Gene: serA (D-3-phosphoglycerate dehydrogenase)

Position: 1782262 to 1783866, length: 1605 nt, orientation: FORWARD

Sequence:

ATGAAGCAATTTAATGTACTCGTTGCAGATCCCATATCAAAAGATGGTATCAAAGCATTATTAGATCACGAACAATTTAATGTAGATATTCA
AACTGGCTTGTCCGAAGAAGCATTAATCAAAATTATACCTTCATACCATGCTTTAATCGTTCGTAGTCAAACTACGGTCACTGAAAATATCAT
AAATGCTGCTGATTCTTTAAAAGTAATCGCACGCGCCGGTGTTGGTGTAGATAATATTAATATTGATGCTGCAACATTAAAAGGTATTTTAG
TTATTAATGCCCCAGATGGTAATACGATTTCAGCTACTGAACATTCACTGGCAATGTTATTATCAATGGCTCGAAATATTCCGCAAGCACACC
AATCACTTACAAATAAAGAATGGAATCGAAATGCATTTAAAGGTACTGAGCTTTATCATAAAACATTAGGTGTCATTGGTGCTGGTAGAATT
GGTTTAGGTGTTGCTAAACGTGCGCAAAGTTTCGGAATGAAAATACTAGCTTTTGACCCTTACTTAACGGATGAAAAAGCAAAATCTTTAAG
CATTACGAAGGCAACAGTTGATGAGATTGCCCAACATTCTGATTTCGTTACATTACATACACCACTAACACCTAAAACAAAAGGCTTAATTA
ATGCTGACTTTTTTGCCAAAGCAAAACCTAGTTTGCAAATAATCAATGTGGCACGTGGTGGTATTATTGATGAAAAGGCGCTAATAAAAGC
ATTAGACGAAGGACAAATTAGTCGGGCAGCTATCGATGTGTTTGAACATGAACCTGCAACTGATTCGCCTCTTGTTGCACATGATAAAATTA
TTGTTACACCTCATTTGGGTGCATCAACAGTCGAAGCTCAAGAAAAAGTGGCAATTTCTGTTTCAAATGAAATCATCGAAATTTTAATTGAT
GGTACTGTAACGCATGCAGTGAATGCACCTAAAATGGACTTAAGCAATATAGATGATACTGTAAAATCATTCATCAATTTAAGCCAAACAGT
TAGTGAATTAGCTATTCAATTAATGTACAATGCACCAAGCTCTATTAAAATTACGTACGGTGGCGACTTAGCCTCTATTGATAGTAGTTTATT
AACACGTACAATTATTACTCATATTTTAAAAGATGATCTTGGTCCTGAAGTCAATATTATCAATGCTCTAATGTTGTTAAATCAACAACAAGT
GACATTAAATATTGAAAATAATAAAGCAGAGACAGGTTTTAGTAACTACTTAGAGGTAGAACTATCAAACGATAGCGATTCCGTTAAAGTT
GGCGCTTCTGTCTTTACAGGTTTCGGTCCAAGAATTGTTAGAATTAATAATTTTTCTGTAGACTTAAAGCCAAATCAATATCAAATTGTGTCA
TATCATAATGATACTCCAGGTATGGTAGGAAAAACTGGCGCATTGTTAGGTAAATACAATATCAACATTGCATCTATGACTTTAGGTAGAAC
TGAAGCGGGCGGAGATGCGCTAATGATTTTATCCGTTGATCAACCTGTTTCAAACAATATAATTGATGAACTTAAACAAGTTGGTGAATACA
ATCAAATTTTCACAACTGAATTGACGGTACAGTCATAA

Gene: Q5HF49 (haloacid dehalogenase-like hydrolase)

Position: 1784016 to 1785146, length: 1131 nt, orientation: REVERSE

Perfect match to: (11819-97-CP003194-[1834397:1835527:r], highly conserved allele)

Sequence:

TTACAAATTATCTAGTACACTTCTAAGTTCACCTAAATGATTAATAACATAGTCGGCATGATGCGCTTCTAACTCACCTGCAGCATCCTTACCT
TTTAAACCTGTTAATGTTCCAATAAACGTTGCACCTATTTTTTGAGCACTTAATAAGTCAGCTAACGAATCGCCTACTATAAATACGTCATCTT
TATTTACAATGTTATCTTGCTTATTGATATAAGATTCATATTTATCGCGATTATTACCATATAAAGCTGCGATATAACTAAAAGGATTCGGCTT
TCCTAATGGTCGTGCTTGCGGATACATATTCTCTGCTTCTAAAACATCACTTGCTGTTGCAATAAAATCAGCCTCAAAATATGGTAACAATCC
TAAATTTTCAAATGGCACAACAGTCTCAGTATAAGGACGACCTGTTGCAATACCTAATTCGAAACCAGCACCTTTTAAATCATTCAGAAGTA
CCTTAACTTCATCTACTGGTCTCAAAATAATTTCTTGATAAATATAACCTGTCTTAAAAGTAGTTCGTGCTATTTTCTTTTCAACATCTTCATAC
AACTTCGATCCTAAATACCATTCTTGATAAACTTCTTGCGCTAACGTCCATAATGCACCCTTCAAACTAAATAAAGTAGCATCCGAAACATGT
AACTCTGTTGTTGCAAACTCTTCCAAAGCAGCATAAATATTATTTTTACCAACTTTTACATTATCCAAAAATTGTAAAGGTAGTTGTTCATTTA
AATTAAAACAGTCTGCTAAGTTTGTGCTTATATTTTGCAACTTCAATTCGACAGGCTCATCTTGATACATGAATGCCTCAATTTCATCATGTG
ATAACTTTTTCAAAATATCAATTAAATGAATACTAAAAACGATAAATAACATATCCCAATTTGAATTCAGCCCTAGCGATTTTAATTTGTTTAA
AATCTTATCTTTTTGAAAAATTCGATTCCTAATGTCTTGTATATCGTTATCAGTCAAAGTTTCCCAATCTATATGTGAATGAAGACCTAAATAA
CACTTATCCATTAATAATTCATATACCGTTAATGCAGAGACATCGAAACAACGTTCTTCACTTAAAAAAACGCCATCAACATCAAATAAAATT
TTCTTCAC

Gene: nagE (PTS system, N-acetylglucosamine-specific IIBC subunit precursor)

Position: 1785253 to 1786719, length: 1467 nt, orientation: REVERSE

Perfect match to: (11819-97-CP003194-[1835634:1837100:r], allele observed in CC80+CC22+CC4803)

Sequence:

TTATAAATGTTTTTCAATTTCATCAGCAACCTGCTGTACGTGTGTACCGACAATAACTTGAGTTGAATGTTTACCATTAACAGTAACACCAAC
TGCACCGGCGCTTTTAATCTTCTGTTGATCAATAATAGATGTGTCTTTTAACTCTAGACGCAACCTTGTTGCACAATTAGTTAAATTAACAAT
ATTCTCTTGACCACCTAAACCTTCTAATATTTGTATAGCATGTTGATGATATTTACTTTGTTTAATATCATTTTCACCAGGAGCAATATTATCTT
TTACAACTGTTGGATCAACTAATTCATTTTCACCTCTACCAATCGTATTCAAGTTAAATACTTGGATTACTACACGGAAAATCACATAGTATA
AGATGAAAAATACAACACCTTGAACAAGCAACATCAATGGATGATTTGATACCGGATTAATTAGTGATAACACATAATCTATCAAACCTGCA
CTAAATGAAAATCCAGCTGTCCAATGGAATGTAGCTGCGATAAATAAAGATAATCCTGTTAATAACGCATGAACAACATATAAGATTGGCG
CAACAAACATAAATGCAAACTCAATCGGCTCTGTAACACCAACGAAAAATGCTGCAACTGAACTCGCTAGGAACCAACCGTAAACTTGTTTT
TTCTGAGTCGTTTTAGCTGTATGATACATTGCTAACGCAGCCGCTGGAATACCGAACATCATGATTGGGAAGAATCCCGCTTGATAGCGTCC
TGTAATACCTTTTATAGCATCTTTGCCACTTTGGAATTTACCAATATCATTAATACCAATCGTATCAAACCAGAACACACTATTCAGTGCATGA
TGTAATCCTGTAGGAATTAATAGTCTGTTAGCAACACCATATATGAAAGCACCAAACGATCCTAAACCAACTATAGATTCACCAAATTTTAC
AATCCATGAATAAAGTAGTGGCCATAAGAATAACAATATGACAACTAAAAATGTACAGTAAAATGCAGTCATAATTGGAACTAGACGTTTA
CCACTAAAAAATGATAATGCTAATGGTAATTCTGTTTCACTAAACTTATTGTATGCATAAGCTGCTATTAAACCTATTACAATACCAACAAAG
ACATTGCCATTATTCATCTTTTCAAAAGCTGAATTTATTTCCGAAGCTTTCATTCCTAATAAAGGCGCTAATTTCATTGGTGATAATACAACTG
TAACTAAAAAATATCCTAACGTAGCTGCAAGCGCGACTGCACCATCATTTTTCTTTGCCATTCCTATAGCTACACCAATTGCAAATAAAATAC
CTAATTGCTCTAAAATCGTAGTACCTACCGTAGTAAAGAACATTGCGATTTTCGGCGTCGCATGAAGTGCATTTAACGTATTACCAATTCCG
GCAATAATTGCTGCAGCCGGTAAAATGGCAACTGGTAACATTAACGAACGCCCTAAATTTTGGAAAAATTTATACAT

Gene: plsC (1-acylglycerol-phosphate (1-acyl-G3P))

Position: 1786847 to 1787464, length: 618 nt, orientation: REVERSE

Perfect match to: (MW2-BA000033-[1808013:1808630:r], highly conserved allele)

Sequence:

TTATAAACTTTTTACAATTTCATGCAATTCTTGTTGTAACTTTGCTGTTCGTGTTTCAATCTCTTTTGTAATATAATCGATACGCTCGTTTCGTTT
TAAATCTTTAGGTAAATCGTTAATATCGATTGGTTTACCAATATTTATGTATGCTTGTCCTGTTAAAAGACCGTGAATCTTAGTAGGACCAAC
ATAAGCAACAGGTAATATTGGTGACTTACTTAACATTGCAATTGTTGAAGCACCACGTTTCAAAGGTGCACCTTCTTGCGATGTGCGAGAAC
CTGTTGGGAAGATACCAACTGTCTTATTATCTTTCAACAAATTGATTGGGCGTTTTAAAGTACTAGGTCCTGGATTTTCACGATCTACAGGAA
ATGCATTTAAAGACGTTAAAAATTTACCAATCCATTTATTTTTGAATAATTCTTTTTTAGCCATATAATGAATTTGATTAGGATATAATGCCAT
ACCTAGCATAATGACTTCGTTATAACTTTCATGCGTACAAGTTACGACATATTTACTATCCTTAGGAATATTATCTTTACCGATTACGTATAAT
GATTTTGACATTTTAACTAAAATGAAATTCAAAATCTTACTAATCACTGAATACAT

Gene: htrC (putative serine protease)

Position: 1787636 to 1788910, length: 1275 nt, orientation: FORWARD

Perfect match to: (11819-97-CP003194-[1838017:1839291], allele observed in CC80+CC239)

Sequence:

ATGTCAGATTTTAATCATACAGATCATTCTACAACAAACCATAGCCAAACACCTAGATACAGAAGACCTAAATTTCCATGGTTTAAAACAGTC
ATCGTTGCATTGATTGCTGGAATTATTGGTGCACTTCTAGTACTTGGTATAGGCAAAGTATTAAATAGTACAATTTTAAATAAAGATGGTTC
AACTGTTCAGACAACAAATAATAAAGGTGGCAATCAATTAGACGGTCAAAGCAAGAAATTCGGTACCGTTCATGAAATGATAAAATCTGTC
TCCCCTACAATTGTTGGAGTTATTAACATGCAAAAAGCATCAAGTGTAGACGACTTATTAAAAGGTAAATCATCTAAACCATCTGAAGCTGG
AGTAGGTTCAGGTGTTATCTATCAAATAAACAACAATTCAGCTTATATCGTTACAAACAATCATGTTATTGATGGCGCAAATGAAATTAGAG
TCCAATTACATAATAAAAAACAAGTTAAAGCGAAATTAGTTGGTAAAGATGCAGTAACTGATATTGCTGTACTTAAAATTGAAAATACAAAA
GGTATTAAAGCGATTCAATTTGCCAACTCTTCAAAAGTACAAACTGGCGATAGCGTATTCGCAATGGGTAACCCATTAGGATTACAATTTGC
TAACTCTGTAACATCTGGTATCATTTCAGCAAGCGAACGTACGATTGACGCTGAGACAACTGGTGGCAATACAAAAGTTAGCGTTCTTCAAA
CAGATGCTGCTATTAACCCAGGTAACTCAGGTGGCGCATTAGTAGATATTAATGGTAATTTAGTTGGTATTAACTCAATGAAAATTGCTGCG
ACACAAGTTGAAGGTATCGGGTTTGCTATTCCAAGTAATGAAGTTAAAGTAACAATTGAACAACTTGTAAAACATGGTAAAATTGACCGCC
CTTCGATTGGTATTGGTTTAATTAATTTGAAAGATATTCCTGAAGAAGAGCGCGAGCAACTTCATACTGATAGAGAAGACGGTATTTATGTC
GCCAAAGCTGATAGTGATATTGATCTTAAAAAAGGTGATATTATTACAGAAATTGATGGCAAGAAAATTAAAGATGATGTTGATTTAAGAA
GCTATTTATATGAAAATAAAAAACCTGGTGAATCAGTCACTGTTACCGTTATCCGTGATGGTAAAACAAAAGAAGTTAAAGTGAAATTAAA
ACAACAAAAAGAACAACCAAAACGTCAAAGCCGATCAGAACGTCAATCACCTGGCCAAGGCGATAGAGATTTCTTTAGATAA

Gene: tyrS (tyrosyl-tRNA synthase)

Position: 1789004 to 1790266, length: 1263 nt, orientation: REVERSE

Perfect match to: (Strain_21340-AGTX01000041-[65734:66996], allele observed in CC188+CC8)

Sequence:

TTATTGATAGTTAACCATGAAGTATTTTTTCTTACCGCGACGAATAATCGTAAATTCGCCATCAATTTTATCTTCTGGTGCTAAAGCATAATTA
ACATCTTGTTGTCTCTCACCATTAATATAAATCGCACCATTGTTAACATCTTCACGTGCTTGTCGTTTAGAAGGAGAAATGCCTGTTTCAATA
AGGACTTCAACGATATTTGTTGTGTCATTTGATAATGTCACTTGAGGCACATCTTTAAATCCATCTTTTAATTCTTTCGCTGATAATGATTTTA
AATCACCACTAAATAATGCTTGTGAAATACGGATTGCATCATTTAATGCATCTTCACCATGAATAAATTTAGTTACTTCTTCAGCTAATGTTTT
TTGAGCTTCACGTAAATGCGGTGCTTCATTTTTAGATTGTTCTAAGCGATCAATTTCTTCTTTTCCTAAGAAAGTAAAGTATTTTAAGAATTTA
ATTACATCTTCGTCTGATTGATTAATCCAGAATTGATAAAATTCATAAGGACTTGTTTTTTCAGCATCTAACCAAACAGCACCTGACTCAGAC
TTACCAAATTTCTTACCATCTGATTTAGTTACAAGCGGAATAGTTAAACCGTATGCGTCTGTTTGACCATACATACGACGCATTAATTCAATA
CCACTTGTGATATTACCCCATTGATCTGATCCACCTACTTGAATCTTACAATTCAATTCTCTATTCAAATGACCGAAATCAATAGCTTGTAAAA
TCGTGTATGTGAATTCTGTATATGAAATACCATGTTCTAAACGACTTTGGATTGAATCTTTACCTAACATGTAATTAACGCCGACGTGTTTAC
CATAGTCACGTAAAAAACTAATTAATGAGATTTGTCCTAACCAGTCTCTATTATTAACAAGCACTGCACCATGGTCTGTTCCAAATTCAAAAA
TATTGTGCATTTGCTTACAAATACCTTCGATATTTTTATCTACTTGTTCTTCTGTTTGTAGCACACGTTCTTCTGATTTACCTGATGGATCACCA
ATCATACCTGTACCACCGCCAATTAAAACGATAGGACGATGTCCATGTTCTTGAAAACGTCTTAATGTTAAGAATGGTAGTAAGTGACCAAT
ATGTAAACTATCTGCCGTTGGATCGGCACCGCAGTATAACGTCACTTGTTCTTTATTTAATAAATCTTCAATACCTTGTTCATCAGTTTGTTGA
TAAATAAGACCTCTCCATTTTAAATCTTCAATTAATACATTCGTCAT

Gene: tbox10 (T-box leader element)

Position: 1790358 to 1790559, length: 202 nt

Sequence:

TGTAAGGGCGCGATTGCACGTTACCACCAAACTTAAACATAATCATAAGATAATGTTCACTCTATTAATGATACGTTCATTAATAAACGTAG
GACATGTTAGTTATAAAGGTGTATTCATATTATTAAAAACACTAGTTCACAGCGACCACTAGCTCTCTGATGATTTCAAATAATATTACTTGT
CCTTTTATCCTATTCTT

Gene: sgtA (monofunctional transglycosylase A)

Position: 1790648 to 1791553, length: 906 nt, orientation: FORWARD

Perfect match to: (930918-3-ABFA01000009-[29505:30410:r], allele observed in CC8+CC97)

Sequence:

ATGACGAATCAAGACAACAATCATCAATTGAATCATCGTATATATCATTTTGAAAAGATATATAAAGCTATCAAACATGTCATTGTTTTTATA
TTTATGATTTTCATTGCCATCGTTGCTATCGCTGTGATTGCGATGTCTTTATATTTTCATCATTTAACTAAAACGTCCGACTCATTATCAGATG
ATGCTTTAATAAAAAAAGTTCGACAAATACCTGGCGATGAATTATTAGATCATAATAACAAAAATTTATTATATGAGTATAACCATTCTCAAA
ACTCACTCATTATAGGCCCTAAAACATCAAGTCCAAATGTCATTAAAGCATTAACGTCATCTGAAGACACTTTATTTTATAAACATGATGGCA
TCTTACCAAAGGCGATTTTAAGAGCAATGATACAAGATATTTTTAATACTGATCAAAGTTCAGGTGGTAGCACAATTACACAACAACTTGTT
AAAAATCAAGTTCTTACCAACGAAAAAACATATAGTAGAAAAGCAAATGAACTTCGCCTAGCAATTAGATTAGAACACCTACTCTCAAAAG
ATGAAATTATATATACATATTTAAATATAGTTCCCTTCGGTAGAGATTATAATGGCGCTAATATTTCCGGAATTGCATCCGCTTCATATAGTT
TGTTTGGTATTCCACCAAAAGATTTATCAATTGCACAATCTGCATACCTTATCGGTTTGTTGCAAAGCCCATATGGCTATACACCCTACGAAA
AAGATGGAACATTAAAATCGGATAAAGATTTGAAATATAGTATCCAAAGACAACATTATGTATTAAAGCGTATGTTAATCGAAGATCAAAT
CACTGAAAAAGAATACAACGACGCATTAAAATATGATATTAAATCACATTTGTTAAATCGAAAAAAGCGTTAA

Gene: isdH (haptoglobin-binding surface anchored protein)

Position: 1791756 to 1794446, length: 2691 nt, orientation: REVERSE

Perfect match to: (M0239-AIWE01000008-[53320:56010:r], allele observed in CC188+CC80)

Sequence:

TTATTTAGATTCTTTTCTGAATTTAGGAATGAATAAAGCTAACATACCTAATAACGCATATAAGCCCCACCATGATTGGCTTGAAGTTGTTTC
TCCAGTTTTTGGTAGCATTTTAGATTGACTAAGTTTGTTTTCTTTCGATGGTGTGCCGGCTTTTTCTTTTGTTTTCACTGTTTATCTACAGTTT
TATGAATATCACTCGGCAGATGTTCAGTTATTTTTTTGTCAGTAACTTTGTCTGTATTATTATAATTTTGTTTCACTACGTCAAGCTTTGCTGCT
TTTCCAGTATGATTATTTTTATCGGCAATATGATTCAGCTGTATGACTTTGTCTTTATTTTTATTAGAGTCTTTATCAGTATCGACATTCGATGA
CATACCAACGCTATTATCGGCATCTTTATCCACATTTCTATCAGTATCTTTGGCAATTTGAGTATCCATTTCTTTTAAATCATACTTATCGAAGT
GATTGTTATTCATCATATTTGTTGATGAATGATCAATATTTAGATGCGCATCTTTGTCAACACTACTATCAGTATCTTTAACCATGTCAGACTC
TGGTTCTATCACATCTGCTTTATCAGACGCATCTTTAGGATTTGTTGCAGTGCTGCTATTTTCAGCTACATCTGTATCAGCAACCTTATCTTCTT
GTCCTGTTTGTACATTTAGCGGTTCACTCGTGTTATTTTGTGATGTATCATCATCTTTTGTATTGATATCCTGATTTATAATTCTGACATGATAT
TGACCTTCATAACCAATGTTTGCCACAACGACTTTAACAATCGCATTGTAAACTGCTTTGTCAGGTATATATGGGAAAATCAGCGTTCTAGA
ATTATTTTTAGGATCTTTAGAAACAGTAGTGACACGTTTACCTTCTACAATTAAATCTTTCCAGTAACTGTCATCCTTTGTTTTCATCACTACAT
ATTTTTGACCATTTAAAGTTGCTGTATAGAATGGATGTTCAACAAAGCCGTCCATAACTGACTCACTATTTTCTTCACTTTCAAAAACAACAA
AATGCGCTTCTTGTAAATCTGTTAATTGATCATTTGTAGGTGTAACATTTTCAAATTCCGTCACTGCTGATTTAACTTGATCAGCTAACTCTAC
TCTAGTTTGATCTAATTTCTTTTTATATTCCGCCTTATATTTTTCTGGCAATTTCTCTTGTAATTTTTCTAATTCATAAACTTGTCTTTCTAACGTT
TTAGCTTTGTGATACGGAGCTAATAATTTTTGTAAATTGTATGTTTCTTCATCCACATAGTCGTCTGGGTTATTAGTAATAGGCTGTGCAAAG
ACCATTAGCGTATAATCATAGTCTTCATGGATGTTCTCACCATATTCAATAGATGACACAATTTTAACTTCTCTCGTACCATTAGATACTGGG
AAACGAATATAGGCATAATCTTTATCAGAATCATATGATACTAATTCGACTGGTAACTTTTTGTCACCTTCATAAACTTCAAATTTCTTCCATG
TTGAAGCTGTCTTTAAACCTAATTCAATTATTGGTCCTGTTTTTGTAAAAATGACAGTTGCTGGTTCAACAGTACTAGCATAATGATAGAACT
GTCTTTCACCTTTATCATTTTTCATTTGAAAATCAATTGGTCGCCAATTATCAGCTGTATGTTCTTTATCGATGATAGCCGGGTTTTTAATTGC
ATCTTGTAGTGATTCATCTGCTGGTGGATACTGTTGATTAACATCCGACTGATTACTTGACTCATTCGTTTTATCGTTAGTATTACCATTAGAA
TTTGTTTCATGAGCTGGTTGGCTTGACGCTTGATCTGCATTCGTTGACGATTTTGGTTGTGCAGGTTGACTCATATTGGTCGTTGCCTGCGGT
TGATTATTAGCATTGTTGATCGTTGATGTATTTTGATTAGATGTATTCGTGTTTGTTTGATTACTTGCGACTGAACTTGATTGATCATTCGTTA
CTACTGCATCATTTGTATCTGATTTTACAAGTGAAGGATCGTTATAAATAGGTTTAGCAAATACTAATTTAGTATAATCATAATTTGTTTCTTC
TCCATCATCAATTTGAGTCGAAGAAACAATTTTCAATTCTTGTGTGCCATCTGAAACTGGGAATCGAATATAGGCATGGTCTTCTGGTACAG
GACTATATGATACAAGTCTCACTGGCAATTTTTGATTGTTTTCATAGACTTCAAACTTTTTCCATGTTGAAGCAGTATTGATGTCTAATTCAAC
TTCTGCTTTCTTTTTAGTGTAATACACATCTGCTGGATCTTTGATGCTGAAAAAGTGATAGTACTGCGTTTCATTGTTTTTATCTAATAATTGG
AAATTGACTTGTTCTCTTGGACCTATATCATGTTCTTTATTTTCTAATGCAGGATCTTTAATTGCATCTTTAAGTGATTCATCCGCTGCAGGAT
AGTTTTTCGCAGTGTTTGCTGGTTGCGTAGCAGGTTGTGTTTGATTTGTATCCTTTGGTGGCTGAGTTGTAGTTGCATTATTATTTTGATTTTC
CGAGATTTTATCTGAAGTATTTGTATTTTCTGCTGCTTGCGCTTGATGTTGAGAAGTAATTAAAAATAGTGTACTGACAATGACCGATGCAA
CGCCTAGAGTTGATTTTCTAATAGAATAGAAAGACCTTAATTTTGGGTGATGTTTGTTCAT

Gene: fhs (formyl-tetrahydrofolate synthase)

Position: 1794780 to 1796447, length: 1668 nt, orientation: REVERSE

Perfect match to: (11819-97-CP003194-[1845161:1846828:r], allele observed in CC80)

Sequence:

TTAGAATAACCCAATTGCATGACCATCATCAGTAACATCCATGTTTAATGCTGCTGGTTTTTTAGGTAAACCAGGCATAGTCATGATTGCACC
TGTCAACGCTACGATAAATCCTGCACCTGTTTTCGCTTCTAATTCACGAATTGTAATTTCAAATCCTGATGGTGCACCTAACAACGTTTGATC
ATCTGAGAATGAATATTGTGTTTTCGCCATACATACTGGGTAATTATCCCAACCATTTTCTTTAAATTGTTTTAATTGTTTTTGCGCTTTACTGC

TAAACGTTACTTTTGAACCGCCATAGATTTCAGTCACAATCTTTTCAATCTTTTGCTCTAATGGTAATTCTAATTCATATAAAGGTTTAAATGA
ATTAGGTTGATCAATGACTTCTAATACTTCATTTGCTAAGTCAACGCCACCTTTACCACCTTTTTCCCAAACTTCAGTTAAGGCAATTCGTACA
TTATTTTCTTTAGCCCAAGATTTTACATATTCTACTTCTGCATCAGTATCATGTATAAATGCATTAATTGCAACAACCGGTTCTACACCGAATT
TTTTAATATTATTAACATGACGCTCTAAATTAACAATTCCTGCTTTTACTGCTTCTACATTTTCTTCTTTTAAATTATCTTTCGCTACACCACCAT
GCATTTTTAACGCACGAATTGTCGCAACAACAACGACAGCTGCCGGATCAAATCCTGCTTCACGCGCTTTAATATCCATGAATTTTTCAGCGC
CTAAGTCTGAACCAAATCCAGCTTCCGTTACAACGATATCAGCTAAATCACGTGCTGTTTCAGTTGCTAAAATTGAGTTACAACCGTGTGCG
ATATTCGCAAATGGTCCACCATGAACTAATGCAGGTGTCCCTTCAATTGATTGTACTAAGTTTGGTTTAATTGCATCTTTTAAAATCATTGCA
AGTGCACCTTCCACTTTTAAATCTGCAACTGTAACTGGCTTGCGATCTCTAGTGTAACCAATAGTAATACGACTAATTTTATCTTTTAAGTCTT
TAATACTTCTACTTAAACATAAAATCGCCATAATTTCAGACGCTACTGTAATATTAAAGCCATCTTCACGTGGTACACCATTTGTAGGCCCAC
CTAACCCAACGTTTACATGTCTAAGTGCACGATCATTCATATCTAATACACGTTTCCATTCAATACGTCTTTGATCGATTCCTAATTCATTACC
TTGGTGAATATGATTATCGATAAACGCAGACAATGCATTATTTGCAGTTGTAATCGCATGGAAATCTCCGTTGAAATGTAAGTTGATATCTT
CCATAGGTAAGACTTGCGCATAACCACCACCAGTCGCACCACCTTTGATACCAAATGTTGGTCCTAAAGCAGGCTCTCTTAATGCAACCATA
ACGTTTTTATTTAACTCATGGAATGCATCAGCTAAACCAACTGTAACCGTTGATTTACCTTCACCAGCTGGTGTTGGGCTCATCGCAGTTACT
AAAACAACTTTCCCTTTGTTTTCTCTTGGCGTTATTTTATTAATGTCGATTTTAGCTTTGTAATGACCATAAGGTTCTAATGCATCCTCTGAAAT
ACCTACTGATGCAGCAATATCCTTAATTGGTTGTAGTGTTGATTGATTCGCAATATCTAAATCTGATAAATGAGTCAA

Gene: acsA1 (acetyl-coenzyme A synthase)

Position: 1796887 to 1798593, length: 1707 nt, orientation: REVERSE

Perfect match to: (TCH70-ACHH02000010-[81366:83072], allele observed in CC1+CC80)

Sequence:

TTATTCCATTGTACTTAAATCCCCAGCATCTAAATTTAATTCCCAAGCTTTTAATACACGTCTCATAATTTTACCTGACCGTGTTTTAGGTAATT
TATCTTTAAATTCGATTTCACGTGGTGCTGCATGTGCCGACAAACCTTCTTTAACAAATAAACGAATTTCTTCTTTTAATTCGTCTGTTGGTTC
ATATCCTTTTCTCAATGCAACAAACGCCTTAATTATTTCACCGCGAACCGGATCAGGTTTACCAATAATTCCCGCTTCGGCAACTGCTTCGTG
TTCAACCAATTTAGACTCAACCTCAAATGGTCCAACTCGTTCACCAGCTGTCATAATTACATCATCAACACGTCCTTGGAACCAGAAGTAACC
ATCTTCATCTTTATATGCCGAATCACCAGATACATACCAGTCTCCAATAAAATATGATTTATATTTTTCTGGATTCTTCCAGATACGATACATC
ATTGATGGCCAGCCTTTTTTTATAGCAAGGTTGCCCATTCGATTTGGTGGTAATTCATTACCTGCATCATCGATAATTGCAGCTTGAATACCA
GGTAATGGTTTGCCCATTGAGCCAAGCTTGACGTCCATCGTTGGATAGTTAACAATCATATGTCCACCTGTTTCTGTCATCCACCAAGTATCT
AACACCGTTAAACCGTATACTTTTTTCGCCCATTTCATAACTTCAGGATTTAAAGGCTCACCTACTGATAGAATCGAACGTAACGATGACAAG
TCATATTTCTCAACAATATCGTCACCAGCACTCATTAACATTCTTAAAGCTGTTGGTGCCGTATACCAAATCGTCACTTTAAAATCTTCAATCA
TACTATACCACTGTTCTGGTGAAAAGCGACCACCAGCTATACAATTTGTAGCGCCATTTAACCATGGTGCAAAAATACCATAAGATGTTCCT
GTAACCCAACCTGGATCTGCTGTACACCAATAAACATCATCTTCTTGTAAATCTAATACATATTTTCCAGAAATATAGTGCACTAACATTGCTT
GTTGAACATGCAATACACCTTTAGGTTGCCCAGTAGAACCTGATGTATAATGTAAAATCAAACCATCATCCGACTTTAACCATTCAATGTCAA
ATTCATCGCTAGCAGTTTCCATCAAACTAATGAAGTCTATGTAATTGTCTTCTACATCCTCATCTACGACAACAATTTTTTTCAAGTTCGGTAA
TTTATCTACAGGTACTCGAGGTAACAATGCCTTATTAGTAATTAACACTTTAGCTTCACTGTTCTCTAATCTATCCGCAACTGCCTTTTCCATA
AATGCTTCAAATAACGGCCCAACAATTGCACCAATTTTTAAAACACCTAACAACGCAAAATATAGTTCAGGTGTACGCGACATAAATATAAA
TACTCTGTCACCTTTGGCAACTTCTGCATGTTCAGACAAAACATTCGCTGCTTTATTAGATAACCGTTGCATATCTTTATAAGTATACGATTCT
TTTCTGTACTCATCTTTGTAATTTAACGCTATTTTATCCCCTAATCCTTGATCTACATGGCGATCTATACATTCATATGCCATGTTCATTTTTCCA
GTTTCACTCCAAGAAAATGCTTGTTCTACGTCTTTCCAATCAAAAGTATTATATATTTCTTCATAATCTTTAAGGTTATGTTTACCTTGCGCTCC
TTTATAAACTTCGACTTTCAT

Gene: acuA (acetoin utilization protein A)

Position: 1798762 to 1799394, length: 633 nt, orientation: FORWARD

Perfect match to: (TCH959-AASB02000248-[60210:60842], allele observed in CC7+CC1+CC12+CC80+CC361)

Sequence:

ATGAATCATTTAAAGACGTATCAATCCGAAGATTATTACATTCATGACAAGCAATTTGTTATTGAAGGTCCTTTAACATACGAAGATTTGAA
AGCGCTTACTTTCGATGCGCATTTAACCGCATTTAGAGATGCTGAAGATCAGTATGAAGCTTTGTTAGAAATTACAACATTACCAGAAGGTA
GAATTTATGTTGCTCGCCAAGATCAACTCATTGTGGGTTATGTCACTTTCCACTATCCTGATGAAATTGAGCGCTGGTCTACAGGTAACCTTC
CATATTTAATCGAATTGGGGGCAATTGAAGTCAGCATCAATTTTAGGCAATTACATCTTGCAGAAAAGCTAATACAACTTAGCCTTTCTACA
CCAGAATTCGAGGATTATATCGTTATAACTACTGAATATTACTGGCATTGGGATTTAAAAAATTCAAAGTTAGATGTATTTGACTATAAAAA
ATTAATGCAGCGGTTAATGGCAACTGGTGGACTTGAAATATTCGCTACAGATGATCCAGAAATAACAAGTCATCCAGCTAATTGTTTAATG
GCAAGAATTGGCAAAAATATTACATTAGAACAGCAACAAGCGTTTGATGATATTCGTTATATGAATCGGTTTTTCTTTTAA

Gene: acuC (acetoin utilization protein C)

Position: 1799419 to 1800588, length: 1170 nt, orientation: FORWARD

Perfect match to: (TCH70-ACHH02000010-[79371:80540:r], allele observed in CC1+CC80)

Sequence:

ATGCAACAACATTCATCAAAAACTGCATATGTTTATTCAGATAAGTTATTACAATATCGATTTCATGACCAACATCCCTTCAATCAAATGCGT
TTAAAATTAACAACAGAGCTACTTTTGAATGCAAATTTATTGTCTCCAGAACAAATAGTACAACCTAGAATTGCAACAGATGACGAATTAAT
GTTAATTCATAAATATGATTACGTCGAAGCAATTAAGCATGCTTCACATGGCATTATCAGTGAAGATGAGGCTAAGAAATATGGATTAAAT
GATGAAGAGAATGGTCAATTTAAGCATATGCACCGCCATAGTGCCACAATTGTTGGAGGCGCTTTAACTTTAGCAGATCTTATTATGTCAGG
CAAAGTATTAAATGGTTGTCACTTAGGTGGTGGATTGCATCACGCTCAACCTGGTCGAGCTAGTGGTTTTTGTATATACAATGATATTGCAA
TTACCGCACAATACTTAGCTAAAGAATACAATCAACGCGTTTTAATCATAGATACCGATGCACATCATGGAGATGGTACACAATGGAGTTTC
TATGCCGATAACCATGTTACTACTTATTCTATCCATGAAACCGGAAAATTTCTTTTCCCAGGCTCTGGTCACTATACTGAGCGCGGTGAAGAT
ATCGGCTATGGACACACTGTAAATGTCCCACTTGAACCGTATACAGAAGATGCATCATTTTTGGAGTGTTTTAAATTAACAGTTGAGCCTGT
CGTAAAGAGTTTTAAACCTGATATTATTCTAAGCGTAAATGGTGTCGATATACATTATCGTGATCCACTAACTCATCTAAATTGTACGTTACA
TTCATTATATGAAATTCCATATTTTGTAAAATATTTAGCTGATTCTTATACGAATGGAAAGGTAATTATGTTTGGTGGCGGAGGCTACAATAT
TTGGAGAGTCGTACCACGTGCATGGAGTCATGTATTCTTAAGTTTAATTGATCAACCAATTCAAAGTGGTTATTTACCGTTAGAATGGATTA
ATAAATGGAAACATTATTCATCTGAATTATTACCTAAAAGATGGGAAGATCGTTTAAATGATTATACCTATGTCCCTCGCACAAAAGAAATT
AGTGAAAAAAATAAAAAATTAGCTTTACATATAGCGAGTTGGTACGAATCTACTCGTCAATAA

Gene: ccpA (catabolite control protein A)

Position: 1800686 to 1801675, length: 990 nt, orientation: REVERSE

Sequence:

TTATTTTGTAGTTCCTCGGTATTCAATTCTGTGAGGTAAAACTACATTTGGTTCTTCTATCTTTTCATCATTCATATATTTTGTTAATAAGCGCA
TCCCTACTGCACCGATATCATATAATGGTTGAATAACACTAGAAAGTTGTGGTCTAACCATCTCAACTAAACGTGTATTATTGAAACTAATAA
TTTGTAATTCCTCTGGAACTTTAATACCAGCATCCATTGCACTATGCATAATACCAATAGCTTCTTCGTCACTAATACATAATATAGCATCTGG
AAGATTACCTTTCATTTTGGCAAAAGCTTTTACGCCTTCTTTATAACTTTCAGCACCAGAACAATTCAATGTATCACCTAATTGAAGACCATTT
TTATTTAACACTTCAGTTAAACCTTCTAAAACATCTTCTTGAGCTTTTTTAGAATGTTCTCCACCTACTAAAGCAAATGATTTAGCGCCTTTTTC
AATTAATTCTCCCGTAATTTCTTTCGCAGCTTCAGTAAAATCAATATTAACTGATGCTATATGTGCATCCTTACCATTTGTTCCTGATACTACTA
CAGGTACAGATGATTGATTTATCAATTCTTTCATTTCTTCAGTAATTGTACCACCAAGGAAAATAATACCATCAACTTGTTTACTTAATAAGTT
ATTAAAAATTTCTTTTTCCTTTTCAGGATCGTTATCTGAATTTGAAATAATTGAGTGATATTTATACATTGTTGCAATATCTTCAAGTCCACGA
GCAAGTTGTGAATAATAGATATTAGATATATCTGGAATGATCACACCTACTGTTGTTGTCTTTTTACTAGCTAAACCTCTAGCAACAGCATTT
GGACGATAATTCAAACGCTTAATGACTTCGTTAACTTTATTTTTAGTTTCTGCTTTAACATTTTGGTTCCCGTTAACAACACGCGACACTGTGG
CCATAGAGACACGCGCTTCTCTTGCTACATCATATATAGTAACTGTCAT

Gene: aroA2 (bifunctional chorismate mutase/phospho-2-dehydro-3-deoxyheptonate aldolase)

Position: 1802215 to 1803306, length: 1092 nt, orientation: REVERSE

Perfect match to: (MW2-BA000033-[1823378:1824469:r], allele observed in CC1+CC72+CC88)

Sequence:

TTATTTTAACTTTTTAGCGTTATATAAATCAGCTAAAGGCTTTAATTCATCATAAAATGCTTGGAATTCATCTAAATCCATTTGTTGACCCGCA
TCACTAAGTGCAACAGATGGATCTGGATGCACCTCAGCCATAACACCATCAGCACCTACTGCTAATGCTGCTTTCGCAGTTGGTAACATGAT
ATCTTTACGACCTGTACTATGCGTAACATCTACCATGACTGGTAAGTGTGTACCTTGTTTTAAAATTGGTACTGCTGAAATATCTAAAGTGTT
ACGTGTCGCCTTTTCATAAGTTCGGATTCCGCGTTCACATAAAATAATGTTTTGATTACCTTGTGAAGCAATGTATTCAGCTGCATAAACAAA
CTCTTCGATTGTAGCAGATAAACCACGTTTTAATAGAATAGGCTTTTTCGTACGCCCAGCTTCTTTTAATAACTCAAAGTTTTGCATATTACGT
GCACCAATTTGGAATACATCTAAATATTCATCGGCTACTTCAAAATCATTAGGATTTACAATTTCACTAACTACATTTAAATCGTATTTATCTT
TAATCTGTTTAAGTATTTTAAGTCCTTCAACACCTAGGCCTTGGAAGTCATAAGGCGATGTACGTGGTTTAAAAGCACCGCCACGAATAAAT
TTTTCACCTTTAGCATGTAAGTTTTTAGCAACAGCTTCAACTTGTTCAAATGATTCAACTGAACATGGCCCAAATACAAATGATTTATTGCCGT
CTCCAATAATGCCCCCATTATCAAATGTTACAATCGTATCTTCAGGTTTCAACTTACGTGATACATATAAATGTTTTTCATTTTCAGATTTTTGT
AAATCTGTAGAGGCTTTGAAAATTTCTTTAAATAATTGCTTAATAGTATTATCGTTGAATGGTCCTTTGTTACTATCGATTAAGTCGTTAAGC

ATTTCTTTTTCACGTTGTGGATCATAGATACGTGTACCTTGTTTTAATTTTTCTTCCCCAATTTTTTGTGCTAGTTCACCACGTTTAGATAATAA
GTCTAAAATTTGATGATTCAGTGATACAATCTCACTTCTGTATGATTCTAATTTATTACTCAT

Gene: Q99TC6 (putative protein)

Position: 1804006 to 1805364, length: 1359 nt, orientation: REVERSE

Sequence:

TTAGTCATTGAATGTACGTTTCTCTATTTTACTTTTAGCTTTCTCAACTTTAGCATTTGGCGTTTTTGATGCATTGGATGATTTAGCATTTGTTG
TTTTTGAATTCTTATTACTAGTTTGATTATTCTTTTGAGTTGACTTACTTGAACTACTTTGTTTCTTTGCACCTTGTGATGATTTCTTATTATGTT
GCTTTTTCTGACCTGAAGTTTTATTTGTAGATGCTTTTGATGCATTTCGTTTATTTGAAGGTGTTGTTTTTTTAGATTGTTTACCTGATTTCTTG
TCAACAGCTGTATTATTAGTTGTTTTTTCATCAGCTTTACGTGTAATAACACCATTTTCAAATTTAGCTTCTTTTTGTTTAACAGTGTCTTTATT
ATCTGTATTAACTGTCTCTGTCTCTGATACATCGTTCTTTTTAGTAACTAATTGAGGAATTTCTTTCAAGTCATTTTTAGCAACTGGTTTTTCTG
CAAATAACGCTTCAGTTAATTGACTCTCTTTTGAACCTGGTGTTAATTTAGCTTGCTTCTGTTTTGCAGCGTTTGCTAATCTTTCAGCTTGTGA
CTCTGGTTTTGATACTTCTACCTTTGCTGCACTTGCATCTTTAGTTAATGTGTCACTGTCTTTCTCTGCTTGTGCTTCTTTTTTAGCTTCTTGAAC
CTCTTGTGCCTCTTGTGATGTATCACTTAAATTATTTGCACTTGCTTCTTCTTTTATCGCTGCTTGTTGTGCTTTCAATGCCACTGCTTTTGGTTC
TTCATTTGATACAGCTGCACTTTTATCTGTTTCTGCTTGTGCTTCTTTTTTAGCTTCTTGAATCTCTTGTGCCTCTTGTGACGTGTCACTTAAATT
ATTTGCACTTGCTTCTTCTTTTATTGCTGCTTGTTGTGCTTTTAATGCCACTGCTTTTGATTCTTCATTTGATACTTCTGCACTTTTATCCGTTTCT
GTTTGTGCTTCTTTTTTAGCTTCTTGAATCTCTTGTGCTTCTTGTGATGTATCACTTAAATTATTTGCACTTGCTTCTTCTTTTATCGCTGCTTGT
TGTGCCTTTAATGCCGCTTGCTCATTTTTAGATTTGTTTAAAAATCCTTCAACACGTTCTTTTGTATAGGCAACCGTTTCTTCAAGTTGCGTTTT
TCTTTCTTCAAACTTTTGCGACAGTTCTTGTTCTTTGACTTTTAAATCATCTGCTTTTTGATAAACTTTATTTTTAAAATACAAACCTAGAGCTG
ATCCTACAAGGGCACCTGTTATAAAACTAACAACAAAATCTTTACGATTAGGTAAGGCTTCATTTTGGTAAGTATGTTTATTTTTTAATGTTC
TTTTATTATTTTGTTGTTGCGTCAT

Gene: yoxC (general stress protein)

Position: 1805438 to 1805929, length: 492 nt, orientation: REVERSE

Perfect match to: (N315-BA000018-[1788940:1789431:r], highly conserved allele)

Sequence:

TTATTTATCTACTCGAGAAGTATAGCTATGATTTGCATCAGTTGCTACATTATTAGCTTTGTAATTTGCACTTCCACGACGGTAGTGTCTATTT
TGCCATTTGTCTGCAATTTCCATTGCAACATTTGACCATTGAACAACTTGTGAGATTTTATCTTCATTTTGAGAAATATTATGTGTAATTGAAT
TTGTTACACGATCTACAGAGCTGTTTAACGTTTGTACTGAGTCACCGATACCTTTAACAGCATCTACAACTGAGTTTAAACGATCTACTTTAC
CTTGGATATCCTCAGTTAAACGGTTTACTTTATGAAGTAAATCTGTTGTTTCACGAGTAATACCTTGAACTTGACCTTCTACACCGTCAAGTG
TTTTTGCAACATAATCTAAGTTTTTCTTAACAGAATTTAATACAGCTACGATACCGATACATAAAATTAAGAATGCAATCGCAGCGATAATTC
CAGCAATTGGTAAAATCCAATCCAT

Gene: murC (UDP-N-acetylmuramate--L-alanine ligase)

Position: 1806003 to 1807316, length: 1314 nt, orientation: REVERSE

Perfect match to: (MW2-BA000033-[1827172:1828485:r], highly conserved allele)

Sequence:

TTAAAACGCATTTTTCATGCCTAATTTATCTAAATATGCATTTTGTAATTTTTGAATATCACCTGCACCCATAAATAAAACAACAGCATTATCA
AATTGTTCTAATACATTAATAGAATCTTCATTAATTAACGATGCACCTTCAATTTTATCAATTAAATCTTGTATCGTTAATGCGCCAGTATTTTC
TCTAATTGATCCAAAAATTTCACATAAGAATACACGATCTGCTTTACTTAAACTTTCTGCAAATTCATTTAAAAATGCTTGTGTTCTAGAGAAA
GTGTGTGGTTGAAATACTGCAACAACTTCTTTATGTGGATATTTCTTTCGTGCTGTTTCAATTGTAGCACTAATTTCTCTTGGATGGTGTGCA
TAATCATCTACAATAACTTGATTTGCAATTGTAGTTTCATTGAAACGACGTTTAACACCACCAAACGTTTCTAATGCTTCTTTAATATTTGTAA
CATCTAGCTTCTCTAAATAACTAATCGCAATTACAGCTAATGCATTTAAAACTGTATGGTCACCATATTGTGGAGACAGGAAGTGATCATAA
AACTCACCATCCACATACACATCAAAAGCAGTACCTTTATCCGTAATTTGAATATTTTGAGCATAAATGTCATCCGAATCTTTAAATCCATAG
TAATAAATTGGAACATCTGCTTCAATTTTACGTAGATGTTCATCATCACCCCAAGCAATAATACCTTTTTTAACATTATGTGCCATTTCTTGGA
ATGCATCAAAAACATCATTAATATCTTTAAAATAATCAGGATGATCGAAATCAATATTTGTCATAATTGCGTAATCAGGTTTATAACTTAAAA
AGTGACGTCTATATTCACATGCCTCAAAAGCGAAATAATCACTTTCAGGCAATCCCATACCTGTGCCATCACCAATTAAAAATGAAGTCTTTT
TATCACCATTCATAACATGTGATAATAAACCTGTTGTAGAAGTTTTACCATGTGCACCAGTTACAGCTACTGAAGTATATTGATCAATAATCT

GTCCTAAAAAAATCATTATAACTTACAACATCTAATTTCAATTGATGTGCACGTACTATTTCTTCATGGCTACTCGCGAATGCATTACCTTGTAT
AACTACCATATCTTCTTTTATGTTATTAGCATCAAATGGTAATATTTTTATCCCCTTATTTCTAAGAGCAACTTCTGTAAATACGTAGTTCTCAA
TATCCGATCCTTGAACTTCATGTCCTAAATCATGCATGATTTGTGCTAATGAACTCATGCCAGAACCTTTAATTCCGACAAAATGATAGTGTG
TCAT

Gene: Q5HF33 (putative cell division protein)

Position: 1807340 to 1811164, length: 3825 nt, orientation: REVERSE

Perfect match to: (Z172-CP006838-[1884508:1888332:r], allele observed in CC239+CC8)

Sequence:

TTATTCTTTATTTAAATCTGCTTCCGTAACATAAACATCCCTTGGTTTTGAACCATTAGCACTCGAAACATAACCGAGTTGCTCTAATTGATCG
ATAATTCTTGCTGCTCTATTATAGCCAATTTGGAAATGTCTTTGGATTAATGATGTTGAAATATGTCCTTCATTAACCATAAATGCACAAACAT
CATCAAATAATTCATCTTGTGATTGTGTTTGTGTTTTTTTCAACAATTCTTTTTCTTCAAATAGATAGTCCGGTTCTCTTTGTTGTTTGATAAAA
TCAACAACATCATCAATTTCGTCATCAGAAACAAATGTACCTTGAACTCTAATCGGTTTATTCATACCGCTACCAAGATATAACATATCGCCA
TATCCTAACAAGCGTTCTGCTCCACCACTGTCTAATATCGTTCTCGAATCTACACTTGATGATACCATAAATGCAATTCTTGTTGGTATGTTGG
CTTTAATTAAACCTGTAATTACATTGACAGATGGTCTTTGCGTAGCTACTAACATATGAATACCACATGCTCTCGCTTTTTGAGCAATTCTAG
CAATAGACTGCTCAACTTCTTGCGGAGCCATCATCATTAAATCAGCCAACTCATCAATTACAATGACAATTTTTGGCATTCTTTCATCATATG
GTGCTTTTTTGTTAAATGCTGTTATATTACGTACATGGTAATGTGCAAATAACTTATAACGTCGTTCCATTTCTTCTACGGCCCATTTTAAACT
CTGTGTAGCTGCTTTGACATCTGTAATTACCGGTGCAACTAAATGTGGCAAACCATTATAAGGAGCTAATTCAACCATTTTTGGATCGATAA
GTAATAATCTTAATTCCTCAGGGTGATTTTTATATAGTAAAGACATCAAAATACTATTGATACAAACTGATTTCCCTGATCCAGTTGCACCTG
CAATTAGTGCGTGTGGCGTTTTAGCAATATCCATAAGTAATGGTTCATTATTAATTCTATACCCCATCGCAACTGTTAATTTAGATTCAGCAT
TTTTAAAACTTGGAGATTCAATAATAGAACGTAAGTTGACCGTCGTTGGATTTTGGTTCGGAACTTCAATACCAACACGACTAGTTCCTGGA
ATTGGCGCTTCTATACGAATATCTTTCGCTGCCAATGCCATTTTAATGTCATCTTGTAATGCCGTAATTCTTGAAACTTTAACACCTTTTTCAA
CTGATAATTCAAATCTTGTAACACTTGGACCTTCAGTTACATCTTGTACTTCTGCAGGTACATTAAAGTAAAATAATGCGTCATTCAGTTCTTT
CTTTTTATCTGTAATCCAGTCCACGTCCGGCTCAATAACTTGTGGTTCTTCTAGTAATGAAACACTTGGCAATTTAATATTTGGGCCTTTACGA
ATCATCGGCTTAGATGTTTGTACAGCTTGATTTGTTGCAACATCTGATTGGTTCGTCCGAGGTCTATTTTCATTAGCTGTATCTTCATTTTGAT
TTGAAAATGATGACTGTAAATCTTTTTGTTGATCATCATTATCTTGTTGTCCACTAGTATTGTTTGGATGTGTTGTTTCTTCGCTTTCTTCAGTT
ATGTCGCTTACTTCTGAAACTGAAGACGAAGTTGATTGATCAACTTGCTCATTTTCCAATTGATTATCATCTTGACTTTCTTCAAATATTTCCG
TTTGAGTTGAATCAACAGAAGGTTTCTGCTCTGAATATTGTTGTGCATTTTGATAATCATTTTCTGTTTCTGCATGACCAATAAGTTGATTGTT
CTCAACATTGTTTGATGTCATATTATTTGTTTTATATGCATTTGTATTTGACTCTTGTGAATCAGTGCTTGATGATGGTGTGGCTTGACTCGCA
GGCATTCTTTCACTCACAGCTTGCTTACTTTGTACAGGCTTTAATTCAGGCACATTGACTTTTGAATGCTTTTTACGATCCATCATACGCTTTTT
ATCAGATGGCGTCATGACAACATTAAATGGTCTTTTACTTAAAGTTGAAACCTTTTCAGTCTGTTTCTTTGGATTTACGTTTTCAATAGTTTTC
TCTTCGACATTTTGTTCACTTTCAGCTGCTTCGTTTTGCACATTTTCTGTAATTTCTGCGTTATTCTTTTCTATTTCATTCATGCTTGAATCTTCC
ACAACATGACCATCATTCACATCATTCGTTTTCAATGAGTTGACACTTAAGTTTGTTTGGTCATCTTTTTTATGACGCGGTGCTAATTCAATTT
CATTATCAACCGTTTTATCGATATTATTAACTTGAGATTCAGTTACTGCGTGCTCTTGCGTTTTAGGAGCGTCTACATGCATTTCAGACGCGTT
TCTATTGGTATTATCTTCGATATTTGAAGTAGTATCCTCATCTGCTTCTGTATCATTTAATTCATAAAATTCTGCATCATCGATTTGACTTCCTG
AATATTCTTCTTTATTCGTTGCTCCATCTTCTTCTGAATCAGCAATTAATCTGGATTGATTTTTTAGTTCATCTTTATCATTTACTTCAACGTTAT
GTTGTAGTGCTGATTGATGTTGAGACGTTACATTCGAAACCGTAACTTCATCATCTGACAATTGTTTTGTTGTCGATACTTGATTCAAACTAA
CTTCCTCATAATTGTATGGACTTTCATCTACATGAGTGTCATTAGTGTCTTCATTTTGTATTGACGCTTGTTTATTTTCATCATTTGATACAGTT
TCATTCTCTATGCCATTTGTATGAAGCTGACTAGCATCTGTACTATTATCTGTTAAGTCACTATCATCATTTAATGAACTATCACCAACATAGC
GTTCCGCTTGTTTAGCATACATTTCATCAATTGCACGTTGTATTGCATCTTGCTCTTCGTTTTTATGTTCTTCACGCTTTTGTTGCAACGCTTTTT
TAAATCGACGCTTTTGAAGCACTTTACGTTCTCGTTCACGTCTAATTTCTTCAACAATTTGTGAAGCATAAATATTTTCAATTTTGATAGTATT
ATCAACTTTTGAATAATTAGGCATTGATTTTGTTGTTGATGATACATTTTCATCAGATGATTTAGATGCAGTTCCTTGTATGACAACACTATCA
TTTTGTTTTTCTTGTTCTAATTGTTTATTTTGAGACGTTTGCGTCTTAGCTACATATTTATCATATTTTTGTTTATCTGACTCAACTTTTTCTGAA
GGTTTACTTACAGGGATACGACCATTTTCTAACTTTTTAGGTTTCATTGTGCCAAAAATAGCTGACGGTACCTCTGAAGTCTTGAAACTTTCT
TTGTGATAATCTGGAGTTGAATCTTTAGCACGATGATTTGTATTATTAGAATACATATTATGTGTTTTTGACTTGTGCGTTTCCTTCTCAATAC
CATTAATTGCAGAAACATATGTACCTGGCTTATTCGTATGGTAATGTGAATGATCTTTATATTTTATACTTTGCTGTGATATTTTAGAATTCCC
ACGTTGTTCACTATAATTTTGTTCTTCAGTTGTTTGATTTCTTCTACGGCGATGTCGTTTTTGTGAACGAGAATCGTGGCTTTGTTTGCGATAG
TCTCGATGGTATTGTTCTTTTTCATCTGAAATAGTATCTGCAGATTGTTCAACATTTTCATTTTCATAAGCTACGCTCATAGGAAAACGGAATT
TTCCCCTCGGACGACTATAAATATCATTATTTTGAGGCAGTAATGAGTCATGATCGTTATCTATATTTTGTGATTCTTGACGTCTTTTTTTCTTT
CTATGAATCAAGTCATCATTTGAATCATTATCTTCGCCGAATAATTTATCAAACCAGCTCAT

Gene: pheT2 (tRNA-binding domain protein)

Position: 1811185 to 1811781, length: 597 nt, orientation: REVERSE

Sequence:

TTATTCAAAAAATGCTTGTCCAATTTCATAGCTGTCATTTAATACCATAATACCTTTTTCTTCAGGTGCATTAGGTAAATTCAATTCTTTCATTG
AACAAATCATACCGCTTGAGGCAACACCACGTAATTCAGCATCTTTAATTACCATACCGCTAGGCATCACTGCACCTACTTTAGCAACAACA
ACTTTCTGTCCAGCTTCAACGTTAGGCGCGCCACATACAATTTGTAATGTGTCATTTCCAACGTTTACATTTAGTACACTTAATTTATCTGCAT
CAGGATGTTTGTCTTTAGTTTCAACGTAACCAACTACAAATTTCGGTGATAAATCAGCATTTAATTTATAATCAAAACCAGCTTCTGAAATAC
GCTTTTGGAATACATTTACAAGTTCATCAGTTAATTTAATATGACCTTTTTCTTCAATTGTTATATCTTTTGAAATTTCAAAAATATTATAACCT
ACAACATTACCTTCATTAGTAATTTCAACAACATTACCTTTTTTATTGTAGTTTAATTCACCTTCAACTGGTTCAATTTGTAAAAATGCGACATC
TCCTACATATTTAGGATTGTAAAATAAATTCAT

Gene: Q5HF31 (putative protein)

Position: 1811810 to 1812667, length: 858 nt, orientation: REVERSE

Perfect match to: (COL-CP000046-[1841588:1842445:r], highly conserved allele)

Sequence:

CTATTTATCTTTATTAAATTTACGACGATTTGCTTCTAAACGCTGAATCACGTTTGGATCTCTTTTTTGTTTATTATTTTTACCTAAAATAAATAT
CGGTTCAAGATGACCCTGTTTATATCCAAAGGATAATGATGTAATTGGAACTAGACCTTTAGTGAAAAATTCCATTGTTAAATGTGCCATCA
CATCATATCCTGTTTTATTGCGTATATCTGCAATAATTAACACATCTTGGTGTGGCACTGCTACGAGCATTTCGCCTTGACATTGTGCCTCAAT
TTCATTTAAAAATGCAGTATTTAGTATCCTACTTGCATCATACCCGTCATTTGAGTTAATAAAATAAAAAATATTACCTTTTACTTCATCAGTC
GTATATGAATTTGACAATTTTCTAACATTAAACAGAGACATTTCTCTTATTTGTTGTTCAGTTAACTTCAAATCCTCTAACATGCTTTCGTCAAT
TAGACGATATGATTTCCCTAAATCGACTGCATAATAAACTGCTGTTTCTGCAGTATGCTCATCATAGATAAAAGGAACACCTTGTTTAGTTTT
TTTATCAAAGCTAGTCGCTCTAATGACAGGCATAATTTGACTAGATGATATACTCTCAAGGGTTTTATCTGCCATTTGTGCAATAGCTTCATC
AACGTAATAAACAATTTCATCTACAATTTTTTCTTTTTTATCTTCATATTTTGCGACTATAGCGTTAAGTTTAATCGTGATACCTTTGTTATTAT
CTGTTCGATAAATACGCAAAGTTTCTTCTTCACGATTAAATTTAAAATCAACGTCTAAATGGCTTAAACGTTCCTTTAATTTATCTCTCATTTG
AAAGGTATTCAT

Gene: ytpP (putative thiol-disulfide oxidoreductase)

Position: 1812767 to 1813078, length: 312 nt, orientation: REVERSE

Perfect match to: (11819-97-CP003194-[1863148:1863459:r], allele observed in CC80)

Sequence:

TTACACGTATTGAGCTAAAAATGCATCTATCTGTTCAATTGATTTTCGTTCTTTTCCAATATAACTTCCAAGCAGTTCTCCATTTTTATATACTA
GAAAACTTGGAATCCCCATAATACCATTTTCAATACAAATATCCATAAATTTATCACGGTCTACTGATACGAAGTCAAACATAGGATATCTCG
CTTCTAATTCCGGTAAATCTGGTTCTATCACTCTACAATCTGGACACCAGCCTGCAGTGAATTCAAATACTGTAGCACCTAGTTTTAAAGATT
CAAATTGTTGTTCTGATTCAAGTTGTTTCAT

Gene: pepA1 (glutamyl aminopeptidase, locus 1)

Position: 1813143 to 1814219, length: 1077 nt, orientation: REVERSE

Perfect match to: (MW2-BA000033-[1834312:1835388:r], highly conserved allele)

Sequence:

TTATTTATATTGTAATGTTTCTATTTGATTATTATCTAAATTACAAATGGCTTCTGAAAGTAAAGATCTAGCTGCAAAATAGTCTCTTATATCA
AATACTGAGTCTGTACTATGAATATATCGTGCACATACACCAATAACTGCAGTCGGAATACCAATATTAGCTTTATGAATTTCTCCACCATCT
GTTCCACCTGGTGACATATAGTATTGATGTTCAATGTCATGTGCTTCTACTAACTTTAATAAATAGTCTCTAAATACAGGCTTTAAAATCATT
GTACCGTCTTTTATGCGAATTAACGTCCCTTTACCAAGTTCACCAGATAATGGTTGGTTTCCTTTAACGTCATTGGCAGGTGAACAATCAACT
ACAAATGCAACGTCTGGGTCTATCATCTCTGCAGATGCTTTCGCACCTCGTAATCCAACCTCTTCTTGAACATTTGCGCCAACATACAAGTCT
ACATCTAATTCTATATCTTTTAATAATTCTAGTATTTCAATTGCCAAGACACAACCATAACGATTATCCCATGCTTTAGCACTATATCGATGTT
CAGATAACTGTGTGAATGGCGTGTGAGGTACAATTGTATCTCCTATATCTATTCCGCGCTCACGCACCTCATCTTCATTTTGAGCACCTATAT
CTAATGTTAAATCTTTAATTTCCGGTGCACCTTCACTACCAGTACGAAAATGTTTAGGTATATTAGAAACAACACCGATAATTTTATCGCCAT
TTCTATTTTTAATTACTAAGCGTTGTCCTTGCCAAATATCATTTGCAACACCACCTAAATTTGTGAATTGAATCATTCCATTTTTAGTGATATTT
GTAATCATAAATCCGATTTCATCCATATGTGCTGCAATCATTACACGTTTTGCATTTGGATTTTTAGATTTTTTCACACCAAAAAATCCACCCA

TACGATTTTCAATAAATTCATCTACGTACGGCGCCATTTGCTGAGTCATATAATTTTTTACTTCTTCTTCAAAACCTGGTGCCCCATGAAGCTC
AGTTAAAGTTTGAATTCGTTGTAATGTTACTTTTTTATTTATGTTCAT

Gene: ytzB (putative small protein)

Position: 1814306 to 1814617, length: 312 nt, orientation: FORWARD

Perfect match to: (MW2-BA000033-[1835475:1835786], highly conserved allele)

Sequence:

ATGACTAAACTGAAATATATAATTCCAACAATAATTGCAGTAGCCATTGTAATTATTTCTACCATTTCAATCATCCAATATATTAATCGTAAAC
GCTATAATCCCGTTAAAGTACTTAATGAAGTAAAATCATATTTTATGAATGTCAAAGGCTCATATATCGTTTATGAACCATTCGTTCATCCTG
AAACTGATAAATACCGTTTAGTTTATCAAGGTGGAATTACAACTATTAAAAATGGTCAAAATATTCATTATGATTTTTATGCAGATGCATATA
CTGGTGAAGTCATTAACATTGTAGAGCGTTAA

Gene: ytnP (metallo-beta-lactamase superfamily protein)

Position: 1814742 to 1815584, length: 843 nt, orientation: REVERSE

Perfect match to: (N315-BA000018-[1798244:1799086:r], highly conserved allele)

Sequence:

TTAGTTATTATCAACTAATGTTTCACGTAAAATATATGCATCTATGTTTTCACCATCATCGCTGTATTTTACAGCAAAGTAGTTTTCATCATGA
TAAAACAAGAACCAATATTGTTGCTGAATAAAATATGGTATCATGCGTTCTTTTTCACGAATCGATTGCATAGGATAATCATCATATGCCGTT
ACCCATAGAGGATTTTTATGTGCAGTAGTTGGGAATATATCACCCATATGAACTGCTTTATCTCCTTGACTTTCAATCGTAATAATCGTGTGG
CCAAAGCTATGACCTCCACTATGTTGCATCTTGATACCCGGAACCGGTTCAAAATGTTTTTCGAATAAAATCAACTTGTTACTATAATCGCCT
TTATTCTTATCCCAGTAAGTTGATTTACTTCTTATATTAGGTGCAATAAACTCATGCCACTCATCTTGTTGCACAACATGAATCGCATTTTCAA
AAATTGCATGTCCCGCTTGATCAGTCAAACCGGCAGCATGATCAAAATGCATATGTGTCATTAGCACATAATCAATATCCTTTGGCGTTAAA
TTATAATTTGCCAAATCAGCAATTATATGACTTTCTTCATCTACTCCAAAATTACGTAATTGCTTTTCAGATAATTTACCATTACCAATACCCGC
ATCTATAATCAAATTATATTGAGCCGTTTGAATCAAAATTGGATGTGTCGGTAAATTGATTTGATTTCGTTCATTTGCATTGTATTGCTTTGAC
CACAACGGCTTCGGAACAACACCAAACATTGCACCGCCATCCATTTTTGTATTGCCACCATTTAGATAATGAATAGATATATCCCCGATCTTC
AT

Gene: trmB (tRNA (guanine-N (7)-)-methyltransferase)

Position: 1816054 to 1816698, length: 645 nt, orientation: REVERSE

Perfect match to: (11819-97-CP003194-[1866435:1867079:r], highly conserved allele)

Sequence:

CTATTTTTGTGAATGGAATTTCGCTTCCATACGATAAATACGTGACCCTTTATCCGAAAATTTCTTTTCATATTCTGTTAAAATATTACTGCCAT
CGTCTTCTTGATGTAAATTTAGATTTATTTTTGTAAAATACATTCCAAATTGAGACATACTTTCTAAACTGTAGGCAAATAGTCCTCTGTTATC
AGTTTTAAAATGTAAATCTCCTTCATCATTTAAGATTTGTTGATACAACGCTAAAAACGTATGATACGTTAAACGTCGTTTTGCATGACGATT
TTTTGGCCATGGATCTGAAAAGTTCAAATAAATACGCGAAACTTCGCCGTCTTTAAAATATTCATTTAATTCAATGGCGTCATTACAAATAAT
CTTTAAATTTGTTAAACCCATCTCTTTAACTTTATCCAATACTTTATAAACGATACTTTTCTCACGTTCCATTGAAATATAGTTAATATGAGGAT
TTTGAGCAGCTAATGTTGTAATAAACTGCCCCATACCCGAACCAATTTCAATGTGTATCGGTTGCGTTTTATCAAACCATTCAGTCATTTTCCC
TGCATGTTGACCGTCCATGTCAACCAATTCAGGATGATCTTTTAAATAATCTTCAGCCCATGGTTTGTATCGAACTCTCAT

Gene: ytmP (putative phosphotransferase)

Position: 1816713 to 1817504, length: 792 nt, orientation: REVERSE

Perfect match to: (N315-BA000018-[1800216:1801007:r], highly conserved allele)

Sequence:

TTAAATAAACATGTTACTATTCATAACTTCATTTAGGAATTTAAGCCAAGTGTTCATATCCTTATATCTTTTTTGCTCTTCATACCATTGAACAA
GACCTATAGATTGAATTACCGTATACCATTTCATACGTTTATTTAAATTCAAGCTCTCTTGAACACCATATGTTTCAAGCCATTCAGACCATTG
TTGTTGTGGAACATAGTTGTAAAGCAGCATTCCGATATCAATTGCCGGGTCTGCAATCATTGCACCTTCCCAATCAACTAAAAATAGTTCATC
TCGATCGGATAATAACCAATTATTATGATTCACATCACCATGTACAACAGTGAAAAAACGCGAATCTAAACTCGGTATATGCTCTTCTAAAT
AGGTTAATGATTTTCTCACAATATGATGTGTTAAAACTTCTCTTGATAAAGAGGCATTAATTTTATTAAGCATAATCTCAGGAGTAATAGGTT
CCATTTCCATACGCTTTAACATACTTAATAAAGGTCTAGAATTGTGTATCTTCTTTAATAAATGTGCAACTCTTGTTTGCTTCATTTCGTTTGAA
GATAGTTCACGCCCATTTTTCCAATGTTGTGCTGTAACAACCTCGCCTGTTTCTATGCGTTTCGTCCATACTAATTTGGGCACAATACCTTCTG
CTGATAATGCCGCAATAAATGGATTTGAATTTCGTTTTAAAAACAACTTTTGTCCATCTTGTTCAGCCATATATGCTTCACCAGATGCACCAC
CTGCTGAATCAAGTGTCCACCCTAATTGATAAAACTGCTCCAA

Gene: daaA (D-alanine aminotransferase)

Position: 1818054 to 1818902, length: 849 nt, orientation: REVERSE

Perfect match to: (N315-BA000018-[1801557:1802405:r], highly conserved allele)

Sequence:

TTAAATACTGTGTGACTCTATATACTTTTCAAATCCTTCTTGTAGTTGACGTGTAATTGGGCCAACTTTACCATCATTAACTGGTTCACCATCT
AATTTAATAACAGGTGTAACCTCAGCTGAAGTACTTGAAACAATAACTTCATCTGCGTTTTTCAAGAAATCTACAGTAAACGTTTCTTCTTTA
AATGGGATGTTATAGTCTTCGGCAATTTTTTTAATTACAATTCGTGTAATACCATTAAGAATATAGTTGTTAATCGGATGTGTATAAATCACA
CCGTCTTTAATTGCATAAGCATTACTTGAAGATCCTTCAGTTACAGTTTCACCTCGATGTTGAATTGCTTCAACTGCATTATATTTCACAGCAT
ATTCTTTTGCTAATACATTTCCTAATAAGTTCAAGCTTTTAATGTCGCAACGTAACCATCGGATATCTTCAACGGTAACACCATTCACACCATT
TTCTAAATGATCATAAGGACGATCATAACTCTTTGTATAAGCAACAATTGCTGGTTCTACTTCAGGTGTCGGGAAGCTATGATTCCTTTCAGC
TACACCACGCGTTGCTTGAATATAAATTGCCCCAGTTTCAATTTGATTCATATCAACTAATTTACGAGATAGTTCAATTAATTCTTCTACAGAA
TAATTTAAATCTAAACCAATCTCATTGGCACTACGTAAAAATCTTTCATAATGTTCTGTTACTGTAAATAACTTACCATTATATACTCGAATGT
ATTCATAAATACCATCGCCAAATACGTATCCTCTGTCGTTGTATGAAACCTTTGCTTCACTTGGACTTACAAACTCACCATTTAAAAAAATTTT
TTCCAT

Gene: ytjP (Mn (2+)-dependent dipeptidase)

Position: 1818906 to 1820315, length: 1410 nt, orientation: REVERSE

Perfect match to: (Strain_21333-AHKA01000003-[114398:115807:r], allele observed in CC80)

Sequence:

TTATTCCTCCACGCATAATGAATAAATTGCTTCTAAGTAAATACTAGTTGCGTTAAATAACTGTTTTTTAGTGATATATTCATTTTTCTGATGC
ATTAAATCTTCAGAATCACTAAACATTGCGCCAAATGCTACACCCTTGTCTAAGTTTCTCGCATAAGTACCGCCACCTATAGTATAAGGTTCA
GTCATATCATTTGTTTGATTTCTATATGCAGTAACTAACTTTTGTACAAAAGGATCATTTTTATCAACATAATGTGGTGGTTGGACTTTACCTA
ATTTCACTTCAAAGCCATATTGTTGAATCTCATTTGCAAAACGATCCATAGCTTTTTCAAATTCAAATCCTTCTGGGTAGCGTAAGTTGATACC
GAAAAGACCTGCGTTTTCATTATCATATGTAATAACACCAATGTTAGTTGTCACGTCACCCATGACATCTGTATGGAATTTCATTCCCATCTTT
TCACCAAAATCTGAATTAAATAAGTAGCGATTACTAAATGCTACAAACGCTTGTGCATTATTATCAAGATTTAATGATGCTAAGAATTTTAGT
AAGTAAAGACCCGCATTCACACCGATAGATGGATCCATACCATGAACCGCTTTACCTTCAACTGTTAAAACTAGAATGCCACTATCAACAGT
ACTATCACCTTGTAAATGATTTTGTTCTAAAAAGTACTCAAAGTCTTGAATAACATCTGTCATATTTTCTTTAACAAGCACTCTTGCTTCTGCA
TGATCAGGCACCATGTTGTAACGTTCACCAGATTTAAAAGTTATTAATTCATAATCAGGTTCATCTTGATCTTCAGCAAGCTTATTTTGAACT
AAGTCAAATGTTGTAATGCCTTTTTCACCATGAATACACGGAAATTCTGCATCTGGTGCAAAACCTAATGTTGGCATTTCTTCTGTTTTAAAA
TAGCGATCCGTACATTTCCAATCAGATTCTTCATCCGTACCAATAATCATATGAATACGTTTCTTCCAATCCACATTCATATCTTCTAATATCTT
AATTGCATAATAAGCAGCAATTGTTGGACCTTTGTCATCAAGTGTACCTCTAGCTATGATAGCATCTTCTGTTACAACCGGCTCGAACGGAT
TACTATCCCATCCATCACCAGCAGGAACAACGTCAACATGACATAAGATACCTAATACGTCACTTCCTTTACCTGCCTCAATTCTTCCTGCAAT
ATGATCCACATCATGTGTTGTAAATCCATCTCTATGTGCAATTTCATACATGTAGTCTAATGCCTTACGAGGACCTGGACCAACTGGTGCGTC
TTCTGATGCTTTTGCATCATCTCTCACACTTTCAATTGCTAATAATCCTTTTAAGTCATTAATGATTTGATCTTCGTATTGTTGAACTTTTTCTTT
CCACAT

Gene: Q5HF22 (putative protein)

Position: 1820520 to 1820942, length: 423 nt, orientation: REVERSE

Perfect match to: (COL-CP000046-[1851013:1851435:r], highly conserved allele)

Sequence:

TTATTCTTTATCAGATAATGCATTTTTATTCTTTTTTAAATCTTCTTCAGTGACGATACGTAAATTATTATTTGGTGCTGCGCCACCTTCATCAT
CAAATTTACCTTTTTCAATACTTTCGTCAGTCTTATTGTCATATTCGGTAAATTTTGATTTTTCTTCTTCGAAAAATGCTTTTGGATTATTTTTA
ATCTATTAGCATATTCTTTCGGATTTGTTTTTACTTCTTTAATTGTTTCATTAGCAATTGTTCCTAATTGCGTCGCTTTATCCTTAGCATTATCTT
TATAGCTTTGAGGATCTTGTTTATATTTATTATATTCTGCTTTCAGCTTGTCACGACTATCTTTACGTGTAACAAGTACAGCTGCTACAGCGCC
ACCTATACCTAAAATCGCTTTAAATAAATTACCTTTTGCCAT

Gene: rsuA (pseudouridine synthase)

Position: 1820959 to 1821654, length: 696 nt, orientation: REVERSE

Perfect match to: (11819-97-CP003194-[1871340:1872035:r], allele observed in CC80+CC12+CC15+CC80)

Sequence:

TTATTTATAATTTAATTTGTCAAAATCATTTTCAGTTAATAAACGATATTCTCCTGAATCTAGATTGCTGTCCAATTCTAAATCAGCAATTTTGA
TACGTCTTAAATGTAATACCTCATTTTGAATGCTATGAAACATTCGTTTAACTTGATGATATTTTCCTTCATAAATTGTTACGTGTGACGTTTG
ATTATCAATATAAGTTAAAATTGCAGGCTTAACCTTGCCATCAGACAGTGTTACACCCTCTTTAAAAGCTTGAATGTCGTCTTCAGTGATAGG
ATTTGCTGAAATAACTTCATATTTTTTAGAAACATGTTTGTTTGGACTCATTAATTCATGATTAAAATCACCATCATTCGTTATCAATAAAAGC
CCTTCTGTATCTTTATCAAGACGACCAACCGGAAAAATATTTAGATGTTGGTATTCAGGTATTAAATCAATAACGGTTTTTGAATGATGATCT
TCAGTTGCTGATATATAACCTTTTGGCTTATTTAACATAATATAGACATTTTCAATGTATTCTATTAATTCTCCACGAACTGTTATCTTATCGTT
TTCTGGTTCTATATGTGTTTTTGGTGATTTAATTACTTGTTCGTTGACATTTACAAGGCCTTTTTTAAGTAACTGTTTGACCTCATTACGTGTAC
CAACGCCCATATTTGCTAAAAATTTATCTATTCTCAT

Gene: ytgP (putative membrane protein)

Position: 1821651 to 1823312, length: 1662 nt, orientation: REVERSE

Perfect match to: (COL-CP000046-[1852144:1853805:r], highly conserved allele)

Sequence:

TCATCGTAAAAACCTAACTCTACGTCTTAATTTTTCAGGAATTTCACCTAAGAATTCGTCCGCAAGACGCGTTTTAATTGTGATTGTACCGTA
AATTAGAATACCTACTGTAACACCTAAAATAATAATGATTAAGTAACCAAGTTTAGTAGGTTCTAAGAATAGATTTGCAAGGAAGAATACTA
ATTCTACACCTAGCATCATAATAAATGAATACAAGAATATTTTTGCAAAATGAATCCAACTATAGCTGAATTTAAACTTCGCATATTTTTTAA
GAATATAGAAATTACATCCAATTGCAAATAATAATGCGATACTAGTACTTAAAATTGCACCAGGTGTATGGAATAACATAATTAATGGATAG
TTTAACGCTAACTTGATAACTACAGAAGCTAAAATAACATAAACTGTTAATTTCTGTTTATCTATACCTTGTAACATTGATGCCGTTACACTTA
ATAGTGAAATTAGTATTGCTACAGGCGCATAATAGAATAATAAGCGACTACCATCATGGTTAGGGTCATGACCTAAAACAATTGGATCGTA
ACCATAGAAAACTGTGAATAATGGTTGTGCCAAGGCCATAATTCCAATACTAGCTGGAACAGTTATAAACATTAATACACCAATAGATGTTC
TAATTTGATGATGCATTTCATGTAAGCGACCTTCTGCAAATGTTTTTGTAATATAAGGAATTAAACTCACTGCAAAACCAGCACTTAATGATG
TCGGAATCATTACAATTTTATTAGTTGACATATTTAGCATATTAAAGAATATATCTTGTAACTGTGAAGGTATACCAACTAAAGATAAAGCAC
CGTTATGTGTAAATTGATCTACTAAGTTAAATAATGGATAATTCAAACTTACAATAACGAACGGTATACTATAAGCAATAATTTCTTTATACA
TCTTGCCATATGACACATCTATATCTGTGTAATCAGATTCGACCATACGATCAATATTATGCTTACGCTTTCTCCAGTAATACCAGAGTGTGA
ATATACCAATAATCGCACCAACTGCTGCTGCAAAAGTAGCAATACCATTGGCTAATAAAATAGAGCCATCAAAGACATTTAGTACTAAATAA
CTTCCGATTAATATGAAAATCACGCGTGCAATTTGCTCAGTTACTTCTGACACTGCTGTTGGCCCCATAGATTTATAACCTTGGAATATCCCT
CTCCATGTCGCTAATACAGGAATAAAGATAACAACCATACTAATGATTCTTATAATCCAAGTAATATCATCGACTGACCAACCGTTTTTATCA
TGAATGTTTCTAGCTAATGTTAATTCAGAAATATAAGGTGCTAAGAAATACAGTACCAAGAAACCTAAAACACCGGTAATACTCATTACAAT
AAAACTCGATTTATAAAATTTCTGACTTACTTTATATGCCCCAATAGCATTATATTTCGCAACATATTTCGAAGCTGCTAATGGTACACCTGCT
GTCGCAACTGCAATTGCAATATTATATGGTGCATAAGCGTATGTGAACGGCGCCATATTTTCTTGTCCACCAATTAAATAGTTGAATGGAAT
GATAAAAAGTACGCCCAATACCTTGGTAATTAATATACTAATGGTAATTAAAAAGGTTCCACGCACCATTTCTTTACTTTCACTCAT

Gene: Q1Y9Y5 (NAD (FAD)-utilizing dehydrogenase)

Position: 1823719 to 1824987, length: 1269 nt, orientation: FORWARD

Perfect match to: (N315-BA000018-[1807575:1808843], highly conserved allele)

Sequence:

ATGTATCAAACAATTATTATCGGAGGCGGACCTAGCGGCTTAATGGCGGCAGTAGCTGCAAGCGAACAAAGTAGCAGTGTGTTACTCATTG
AAAAAAAGAAAGGTCTAGGTCGTAAACTCAAAATATCTGGTGGCGGTAGATGTAACGTAACTAATCGATTACCATATGCTGAAATTATTAA
GAACATTCCTGGAAATGGGAAATTTTTATATAGTCCCTTTTCAATTTTTGATAATGAATCCATCATAGATTTTTTTGAGTCTAGGGGTGTTAA
ATTAAAAGAAGAAGATCACGGGCGTATGTTTCCAGTTTCCAACAAAGCACAAGACGTGGTTGATACATTAGTGACAACTATCGAACGCCAA
CATGTAACGATTAAAGAAGAAGAAGCTGTTAGTAGAATCGAAGTTAATACAGACCAAACTTTCACTGTACATACTCAAAATAATAGTTATG
AAAGCCATTCGCTAGTGATTGCTACAGGTGGTACAAGTGTCCCTCAAACTGGTTCAACTGGTGATGGTTATAAGTTCGCACAAGATTTAGG
TCATACCATTACTGAGTTATTCCCGACCGAAGTTCCAATTACATCAGCTGAACCTTTCATCAAATCCAATCGTCTAAAAGGTTTAAGTTTAAA
AGATGTTGAATTGTCAGTACTTAAGAAAAATGGTAAAAAACGCATCAGTCATCAAATGGATATGTTATTTACTCATTTTGGTATCAGTGGTC
CAGCTGCATTAAGATGTAGTCAGTTTGTTTATAAAGAACAAAAAAATCAAAAGACACAGCACATTTCTATGGCAATCGATGCATTTCCTGAA
TTAAACCATGAACAATTAAAACAACACATCACATCATTATTATCGGACACACCAGATAAAATCATTAAAAACAGTTTGCATGGTCTAATTGA
AGAGCGCTACTTACTGTTCATGCTGGAACAAGCAGGAATCGATGAAAATACCACATCACATCACTTATCAAATCAACAATTGAACGACTTAG
TAAATATGTTTAAAGGGTTTGTATTTAAGGTGAACGGGACATTACCTATAGATAAGGCATTTGTCACAGGTGGTGGTGTGTCACTTAAAGA
AATTCAACCTAAAACAATGATGTCTAAATTAGTTCCGGGATTATTTTTATGTGGTGAAGTATTAGATATACATGGTTATACTGGTGGTTATAA
TATTACAAGTGCACTCGTAACAGGACATGTCGCTGGATTATATGCCGGACATTACTCACATGCATCAATGGAATAA

Gene: sasC (LPXTG-sorted surface protein C)

Position: 1825104 to 1831664, length: 6561 nt, orientation: REVERSE

Perfect match to: (AH2-CVOS01000014-[60291:66851:r], allele observed in CC630+CC8)

Sequence:

TTATGATTCTTTTTCGTTTTTAGTACGTCTTCTAGCTAACAAAGCCGCACCTGTAATCAGTGCAAATTCTTTCAATGGTAAATCCATTCCTTCA
GAACCTGTATTTGGAAGTTCTTTTTCAACTTTGCGCGATTCATGTGTCTCTTCTTTTTTAATAGGCGTACAAACTTTTGGAGCTGGCTGAATTT
CTTTTGGTGATACTTTCGTCGCTTCAGCTGGTAATTTAATTGCTAAAATTTCATCAACAATGAATTGCGTGTGTTGTTTGATGTCATTTAATGT
CGCATCTTCATCAATCATTCTATTGCCATCTGCAACATATTGATCAATTAATACTTTTACTTTAGCTAATTGTTCTGGTGTTGCGATCGCTTTGA
ATTTCGCATATGTTTGTTGAGCAATGTTATCAATGCGCAGTAAGCTATTTTCTTTTTCAGTAATTACTGCTTCTATATCGCTTAATGCAACATT
AAATCGTTTTAAAACTGCATCAACATCAGCATTAGTGCGTGCTGTTTTTAATTCTTCATCCATTTGTAATTTTAAAGCAGTTATAGCTTTTAAT
GCATCAGCCTTATTACGATCACTTACTTTTCGATAATTTTGCACTAAAGCAGTGACGCGTGCAAGATCATCATTAATCGTTTTTTCAGCATCT
GGCTTTTTAATAGGATGTACATCTAAATCATGTATTGTTTGTAGATTTAATGATGCTGTTTTATCAACTTGTGCATTGCTACGATCTTGATCAA
TTTGTCCAATAGCAGTGTCATAAATATTTTGTAACTGTGCTAATATACTATTTCTTTCTTCTACCGTTGCTTGAATATTCGCTTCAATTGCTTGT
TTTTTATCGTTGAATAATGTTGTCAATTGTTCTCGAGCAGACGCCTTTCTGTTAATAACAGGTTCGATTTCACGAATTTCGTTTTTTTCATTAT
GCAATAAATATGCCACATCAGCATTAGTCACTGCACTAGCAATTTGTTGTTTAGCTTTAATTAACTCTTTTTCAACTTGTGCAATTGCAATATT
TTGTTCTTCATCTGTCGCTTCGTTATTTGCTTTAATTAAATTAATTTTATTTGTAGCGATATTTTGAATTTGTTGTAATGCTGTTGTTTTAACTGT
TGTCGCTGGTTTAATTTTTGAAATAATATTTTGAGCATTTATACTATCTTGATTAACTTGGGCAGTCTTATCTGCATGATTGATCTGATCAATA
GCCTGATTAAGTGCTTGTTCTACTAAATGTTTAGCAGCTAGTCTTTCTTCTTCAGTTGATAAATCGCTTTGATCGATTAGTGCATTTTGAGCTT
CGGCTTTTACACCAACAGATTGACGCGCTGCTGGTTTAACTTGAACTTTAGGTAAAATCACTTTGATGTTGTCGTTGCCATCAGTCTCAGTTC
GATCCACTTCTGCATTCGTTTTGTTTTGTGCAATGTCATTTTTAATTGTATTTACAATTTTATTTAAAGTATCAATAGCAACATCTCTTTCATCTT
GAGTAGTATCCAACGTATTTCGGATTGCATCGAGTTGATTTTTATTATTTTCTTCAATGCTATCAAGCGCAGCTCGTTTACGTCTTACTTTAGG
TTTTATTTGCTCTATTGCCTTGATTGTTTGATTTCTAACATCAGTAACAGCAGCATCTTGATTTGTATTTTCTATTTCTTGTTGCGCTTGTTTGA
GTGTGTCGCTAATTAATTGATTCGCTTCATCTAATTCATCAACTGTTGCATGTGGTGTATCTTTTATTGATTCTACTTGATTTTCTGCACTTGCT
TTTATTGCTTGTTGTGCTTCAGGCTTAATTACAATATGAGGTTGTACACCTTTTAGTGTAGCAATGCCATTTGTTTCAACACGTTTCACATCAT
TATTCGTTACTGCTTGATTGATGTTTTGTAATGCAAGTTTTTCATTATTCGCTAATTGATTTAAAGCAACTTGTTTTTCTTCATCAGTCGCATGT
TCAGCTTGCTCTATTTCTTGCTTTTTAGTCTCATATTGTTGCTTTACTGCATCTCTAGCAGCTGCTCTAACAATATGTTCAGGCGCTACTAAAG
CAATGCTATCAAGCGCTTGACTTGTTGTATCATCAACTTGTTGATTTGTTCTATTATTCGTAATATCTGTCATGGCTTGATTTACAAATTCATT
GATTTTATCTAGTGCTACTTGTCTTTCTTCTGCTGTTGCTTCTTTATCCTGATTAATCTTAGCACGTAATTCATTCGCTTTTTGATTGATTTTTTC
ACGTGCAGCTGGTTTAACTTTTGTTTCAGGTTGAATAATTTTAATCGCTGATACACCATTTGTTGCTGCTTGATTCACCTGACTATTCGTTTGA
GCTTGGTCAATAGCTGCAAGTGCTTTTTCTTTTTCTTTTGCTAATGCTTGTGAAGCAACTTCTTTCTCATTATCTGTTGAATCAAGACTATTATC
AATTTGCTGTTGCTTTTCTTTAACAGCTTTTTCAATATCTGCAATTGCCTTTGGTTTAATTACTACTTCAGCTTCAACATTATCTATAGCATTTAC
CGCTTGATTTGTAGTTGCGTCTACCTGATCATTTGTTTGGTTTTGATTAATTTGATTAAATGCTTGATCTTTAAGTTGATTGATTTGATTAACA
GCAGCCTGCTTTTCTTCGTCAGTTGCATTAGGTGTTTGTTTAACCGCTTCAATACGCTTCGCCACTTCAGCAGTGATTTTATCTCGCGCTGCTT
GTTTTTTTACTACGCCAACTTGAACAGCATCGATATTATTCTCTGCTACTGTCGCAGCTTGGTCTACTTCTGCATTTGTGTTAGCTTGTTTAAT
ACTTTCAATAGCTTGTTGTCTGTCTTGATTTAAAGTATTGATTGCAGCATTTTTCTCATCATTCGTTGCATCTGGTGTAGCATTGATTTCAGCT
AATTTAGCATTATAATGCTGATTGATTTGTGCTAATGCTGCAGGTTTTTTAACAATATTTGGCTGAATCGCATTAATTGCTTTTGTACCTAATT
GTTGCGCTTGATCTACTTCCGCATTTGTATCAGCTTGATTTATATTATTAATTGCCGTTGCTAACTCTTGATCCACTTGATTTAAAGCCACTTG
CTTTTCTTCAGTTGTTGCATTTGTGTTTTGATTAATTTCTTGCTTTTTAGCAGTTGCTAAATCATTTAATACACCTGTAGCAGTTTGTTTCTTCGT
TACATGCGGTTGAACTGCGCCGATTTGATTGACTGCATCGTCTCTAATACTATTGACCATCGCAGTAGTAGACGTCACACCAATATCAGTTA
ATGCTCTATTTTTAAGTGTATTGACACGATTTATCGCTTCTTGTTTCTCTTCTCGAGTCGCGCCAGGTGTTGCATTGATATTTGTTATCGCCTCT
CGCGCTTTATCATTTACAACATTGCGAGCATCCGTTTTAACTTGTGTTGCCGGTTGAATCACAACTATATTTTGTGTGCCCTGATCTTTTGCTT

GTGCAACTTCTTGATTCGTATCTGCTGCATTAATATTTTGCTTCGCTGTGGCTACAGCTTGATCAAGTAATTGTTGTGCTGCTTGTTGTTCTTC
GAGCGTCGCATCATTATTATTAAATATCGTATTATGTTGCGTTTCCGCACTTTTATCAATGGCATTTTTTGCATCTGTTTTTACCTTTGTAGCTG
GTGTAATTGCTTGTATTCCTTGTATCGCATTTGTCTTCACTTGTTCCACTTCAGCATTGGTATTAGCGTTTAAAATGTTTGTGTTTGCTGCAGT
TACAGCAGCATTCACTTTGTCAATTGCTGCTTGTCTTTCTTCTTGAGTCGCATCAGGATTAGCATTGATCTCTGCGATATGTTGTTGTGCATCA
TGTGATACAGCATCCCTTGCAGCTTGCTTAACAACAGTTACAGGAGCAATTGGATTAATGGCATTTAGACCATCTCCTTTGGCGTTATCAAC
ATCAGCATTTGTTGTTGCTTGATTGATTTGTTCTAAAGCATGGTTGGTTGCTTGAGTTAATTCGTTCAATGCTGCATTTTTCTCTTCCTGAGTT
GCTTCTCTATTACTATTTATTTGTTGTCTTTTCGTTGCTGTTGCTTGGTTAATTGCATCTCTTGCAGCTTTTTTATGAGTTACTTGAGGAACAAC
TGCTCCAATAGTATTGATGCCATTATTTTTAGCTGTTTCAACGTCAGCATTTGTAGTAGCATTCGTAACATTATCAATAGCATCTGTTTCATCC
GTAGCTAATTGATTTATTGCATCTTGAATCTCGTCTTCAGTAGCATCTGGTGTTGCATTGATAATTTCCCTTTGTTTCGTTGCTTTATCACGTAT
TGCTTTTTTAGCATTTGGTTTAACAACCGGTGTTGCAGTATCCCCACTTAAGGTCTGTATACCTTGATCTTTGATTCTAGTAACGCCATCATCA
GTCGTTTGGTCACCAATATTACCAATTATTTCATTTTTATGTTCCTCGATAACTTGTATTGCTGCTTGTTTTTCTTCATCTGTCAATTCATCATTT
TGATTAACTAAATCTTCCATTTGGTCAACTTTTTTATTAACTGCATTTTCAGCATCAACACTTCGAATTAACGTATGTTGCATTTGATTAGTTAA
TGAATCAATATCTGCTTGAGAAACTCTTTTATTTAATGGTACATTGTTACGATTTTCATCTAAAATCGTTTGTGCGCGTCGTTTCAGACCATTA
AAGATATCTAATGACGCAAATGTATAATCAGCTTGTTGAATACGTCTGTCAACTTCGGCTTGTAATGCATCTTTATTCATGATGATATCGATA
GTATATGGATTTGTATTTATAGTATGACTTTCTGCAGCTGAATTAATGAAATCTTGTGTATATGTTTTATACGTTAATGTCTCGTTAAATGTTA
CTGTTCTTGGTGTCGGCACATTATTTACACGTAATTTATATCTTAAATCGAGTATTTTATCAGGCATAAGTCGTGCCGGAGAGTTTGCTGTAC
CTCCTCCAGTACTTTTAATTGTTATCACACGATTTGCTGCATCATATGTAACATTCATATCATTAACATCAACGCCTGAATTGTTACTTGGAAA
ATCTTTAGTCAATGAATTGTTCACATATTCAACACCTTCAGGTAATTGAACTTGATATACAAAGTCATTTGTATCTAGAGAAGCACCAGAATT
ACCATTATTCTTTAATGATGTTGTTACAGTAAACTCTTTATTATTTGTTGCTGTTGGATCCATTGTTCGTCTTTCAACATAAACATGTGACCCA
GAATGAAGTCCGATAGAGTCAACAAAGCTATAGTATTTGTAACCATCTTTTAGTTGATAAATGCCACGCGCATCTGTTATTGCGTCATTTTTA
GGTACAAATTGAATTTTGAGATTTCTCACATTATCAGGTACTTTAAATAAACGCAAAGTTGGACCGCCTTCAACAGTCTTTTCAGCAATCGTA
TCATTAGTATCAGCATTTTTGATAATAACATTTGTTGCGCCTTGACCGTTTTTAGTAGTCATTGTATTAAATTCAAAGATTAATTCAGAATTCG
GATTTACTGTTAATGCTTTCTCGATACCATTAAAATCGCCATGGTCATTCGTATCAGTTCCATGTATACGACCTAATGCAATTACATTTCCTTG
TGCTTGATAGTTCTTAGCATCAGCTGAATCAAACATACTTGTTCTAACCATCGCATGACTAAACACACCTACTTTACCACCATTGATAAGTGT
AAAGCCTGGTAAATTATCAACAACTGTTACTGATGGTACAGAACGGTTGGTACTTGGTCTAATACCATTGTCATCAAATGACAGCACTTCGT
TAGGTGCATTTTGTCCTGCATTATTGGCATTAGGATCAGTTGTAGGCGTGTATGGCGCTGTAATTGCAACTGGTGCACCACCGTTTCCTGCC
GCTGCAGCCGCTGGATCTGCTGGAGTTGCATTAGGATCTGCCGGTGCCGCACGTCTACTTCTCTTTTTCGGTCTATTAGATGCTGGTTCAGC
AATTGCAACTAGCTCTGGTTTATCTGAAGAATGACGAACATCTTCTTGAATTTCCTTTAAAGTTAGATGTCCTCCTGAACCATTTTCATTTGTT
TTATTTGGTACATTTAATGTGGTATTATTCGACACTGCATCATTATTATTAGCGTTTGACACTGTCTCAGTTGCTGTTGTATTGCCATCTGTAT
GATTATTGTTTTGTGCTGAAGGCGTACTTGATTGCACTGATGTTGGCGTAGCTTGATTTACTATACTACCATTATTATGATTAACCAATGCTT
GATTCGTTGCTTGATTGGTTGTTGCAGATTGATTAGGTGTATTCTGCGCACTATTTGCTAAACCTCTATTATTAGCAACATCTTTATCTTGTGA
ATTTACAGGTGTTGCTTGATTAGTATCGCTTTGTACATTATTATCCGTAGTTAAGGCTTGTGCACCATTTGGGTTTGAAAGTAATAAAACTGT
TCCGATTAAAGTAGAGAATATGCCTACTTTATACTTCCTAATACTATATTTATTTTTCTTTAACAAATTCAT

Gene: ytwF (putative sulfur transferase)

Position: 1831990 to 1832301, length: 312 nt, orientation: REVERSE

Perfect match to: (11819-97-CP003194-[1882371:1882682:r], allele observed in CC80+CC12+CC88)

Sequence:

TTAAATACTTTTTATTTCCAAACCTTCATCGCCCCATGCGTGCATGCCGCCTTCGACATTTACAGCATCAATGCCATTTGCCTCTAAATATTCT
ACAACTTTAGCGCTTCGAACTCCACCAGCACATACAATATAATATATTTCATTTTTATTAAATGAATTTAAATTATCCGGAATGGTATCCATTG
GAATCAACTTTGCATTAGGAATATATCCCATTGCTGTTTCTTCGTCAGTACGAACATCAACAATTTGAACTGGTTTAGATTCTAAAAGTTTGT
TTTTTAATTCATCTGTAGTAATTGACTTCAT

Gene: leuS (leucyl-tRNA synthetase)

Position: 1832323 to 1834737, length: 2415 nt, orientation: REVERSE

Perfect match to: (Strain_21310-AFNP01000056-[181318:183732:r], allele observed in CC22+CC80)

Sequence:

TTATTTAGCTACAATATTGACTAATTTTTGAGGAACAGCGATGACTTTCATGATGTCTTTACCTTCAATACTCGCTTTAACATTGTCATTAGAT
AAGGCAATTTCTTGCATTTCTTCTTTTGATGTATCTTTAGCAATTTTAATTTTAGCTCTCAATTTACCATTCACTTGAACAACGATTTCTACTTC
ATCATCTACAAGTAGTGCTTCGTCATAAGTTGGCCAAGGTTGGTACGTAATAGACTCTTCATGTCCTAATTTTGACCATAATTCTTCACCGAT
ATGTGGTGCAATAGGTGCTAACATTTTAACGAAGCCTTCAATGTAAGGTTTATAAACTTCATCAACTTTATAACACTCATTAATAAATACCAT
TAATTGACTAATAGCAGTATTAAATCCTAATGTTTCAAAGTCTTCTGTTACCTTTTTAACAGTTTGGTTATAAACTTTATCTAAAGATTTATTAT

TTGTAGTTACAATTTTTGAACTCAATGTCCCATCTTCATTTACCATTAAACGCCATACGCGATCTAAGAATCGACGAGACCCATCTAATCCTTT
TTCACTCCATGCAATTGCAGCATCTAAAGGTCCCATAAACATTTCGTAAAGACGTAAAGTATCTGCACCATGAGACTGTACTATATCATCAG
GATTGATTACATTTCCTTTAGATTTACTCATCTTCTCATTACCTTCTCCTAAAATCATACCTTGGTTAAATAATTTTTGGAAAGGTTCTTTAGTA
GGTACGATACCCAAATCATAAAGGACTTTATGCCAAAATCTTGCATATAATAAGTGAAGAACCGCATGTTCTACTCCACCGATATATAAATC
AACAGGTAACCAATGTTTTAATTTTTCAGGATCTGCTAACATATTTTCATTTTTAGGATCGATGTAACGTAAATAATACCAACAACTACCTGC
CCATTGTGGCATTGTATTTGTTTCACGACGTCCTTTCATACCTGTTTTTTCATCTACAACATTTACAAATGAATCAATATTAGCTAGTGGAGAC
TCACCAGTCCCTGATGGCTTGATTTCATCTGTTTCAGGTAACAACAATGGTAGCTCTTCTTCAGGAACAGTTGTCATTGTTCCATCTTCCCAAT
GAATGACAGGAATTGGTTCGCCCCAATAACGCTGACGACTGAATAACCAATCTCTTAATTTGTAATTAACTTTCTTTTCGCCAGCACCTTTTT
GCTCTAATAATTGAATAGCTTTAGTAATTGCCGCTTCATTTTCTAAACCATCAAGTTCACCAGAATTAATATGTTTACCTTCACCAGTGTATGC
TGCTTCTTCAACATTTCCACCTTCGATGACTTCAATGATTGGCAAATCAAACTTTTTAGCAAATTCATAATCTCTGTCATCATGCGCTGGTACT
GCCATAATTGCTCCAGTACCATATGTTGATAATACATAATCAGCAATCCAAATTTGTACTTTTTCACCAGATAAAGGATTAATTGCATATGCA
CCAGTAAACACACCTGATTTATCTTTTGCTAAATCTGTACGTTCTAAATCCGACTTTTTAGAAGCTTCTGTTTGATAAGCTTTTACTTTTTCTTT
ATATTCATCAGTTGTAATTGAATTAACTAATGCATGTTCAGGACTTAAGACTAAGAATGATGCACCATAGATTGTATCTGGTCTAGTCGTAA
ATACTTCTACTTTTCCTTCCGTATTATCTACATCAAATGAAACTTTGGCCCCTTCAGAACGTCCAATCCAATTGCGCTGCATATCTTTTAAAGA
TTCAGGCCAATCTAAATCATCTAAATCTGCTAATAATTGATCTGCATATTCTGTGATTTTAAGTACCCATTGTTTCATCGGCTTACGATAAACT
GGATGTCCACCACGTTCAGAGACACCATCAATCACTTCTTCGTTAGATAAAACAGTGCCTAATGCTGGACACCAGTTAACTGCAACTTCATC
AACGTATGCTAAACCTTTGTTATATAACTGTATGAAAATCCACTGTGTCCATTTATAGTATTCTGGATCTGTTGTATTAACTTCACGATCCCAA
TCATAACTGAACCCTAATTCTTTAATTTGTCGTTTAAAAGTTTGGATATTTTTCTTTGTAAATTCACGTGGGTCGTTGCCAGTGTCTAAAGCAT
ATTGCTCTGCTGGTAATCCGAATGCATCCCACCCCATCGGATGTAATACATTATATCCTTGCATTCTTTTATATCTTGAAATGATATCTGTTGC
TGTATAGCCCTCAGGATGTCCAACATGTAAACCAGCACCTGATGGATATGGAAACATGTCTAAAGCATAAAATTTCTTTTGACCTAAGTTAT
CATTTGTTTTAAATGTTTTATTTTCGTCCCAATAGTCTTGCCATTTCTTTTCAATTTGATTGTGGTTGTAATTCAA

Gene: yttB (major facilitator superfamily transporter)

Position: 1835028 to 1836209, length: 1182 nt, orientation: REVERSE

Perfect match to: (11819-97-CP003194-[1885409:1886590:r], highly conserved allele)

Sequence:

TTATGCATCTATTTTTTTAGGTTGCGTATTATTCTCCTTGAAAACCATTAATAATATTAATGCAAATACAAGTAGTAGCATCATACCGATAAAC
ATCATGCGCATATTAAACGCATCAACTAATACACCACCAAGAAATGGACCAAATGCTTTTCCTACTGTAGCAGCTGAATTCACAAAACCTTG
GTACTGTCCTTGCTTACCATCTGGCGCTAACTGATTGGCTATAGTTGGAACTGCTGGCCATACAAACATTTCTCCAAAAGTTAAAATAATCAT
ACCGACAACAAATATTGTAAAGTTTTCGGCAAAACTCGTGACAAAGAACGACAACATAAAAATGATGATGCCGACAAACATTTGCTTCTTTA
AGTTTCCTTTTAACAGATAGAGAATCGGTTTAATTAATGGTTGTGCTACTAAAATCATTATTCCGTTAATTGTCCATAAAACACTATATTGTG
CCATTGAAATATTAATAGATTGTGTAAATGAAGCGATTGTAGACTCCCATTGAATATATGCAACCCAACAAATTGCAAACATTGCACAAATT
AGTACTAATGAAATAAATCTTGCTTTATTCTTTTTACCAGTAATATCTAAATGAGTTGGATATTTAACTTTCGCATTAATTTCAATATTAAATT
GCGTTACCGCGACAAGCGCAAACACAACATACATAATAAGATTGGCTAAAAAGATATAGTTAAAGCTAAATTCTGCGACAAAGCCGCCCAT
TGCAGCACCGACAGCCACACCAATATTTTGCGCTAAGTATATCGCATTAAACGTTTGTCTTCCGCCATTTGGCCACACTGCTCCAGCCATAGC
GTATATCGCAGGAATAATCATTCCGCCACCAAACCCTAACATTACAAGCCATACAGCATACCAAGGCCACCCGTGAAAGAAATTAAGTAGC
GTTGTACTACAAAGACAAGTGAAAGTTCCAATTAAAATCGTCTTGTATCCACCTAATTTATCAAATAGTGAACCACCTAATAAGTTTCCAATA
ACCATGCCAAATGAATTTATCATTAGCACTAAACCAGCAACAGTTAAACTTTTTCCAAGTTCTTGTTTCATATAAATTGTATTTAAAGGCCAC
AAAAAACTGGAACCAGTAATATTTAACGCCATGCCAATTACTAGCCACCAGACTGATTTAGGTATATTCAT

Gene: ytqA (putative Fe-S oxidoreductase)

Position: 1836319 to 1837272, length: 954 nt, orientation: FORWARD

Perfect match to: (COL-CP000046-[1866812:1867765], highly conserved allele)

Sequence:

ATGGGCAATCATTTCCAATACGCTTTTGAAAACAAACGTTATCACACATGGAATTACCATTTAAAAAATAAATTTGGACAAAAAATATTTAA
AGTTGCATTGGATGGCGGGTTTGACTGTCCTAACCGCGATGGCACTGTAGCACATGGTGGATGTACATTTTGTTCTGCTGCAGGTAGCGGA
GACTTTGCAGGTAATCGTGCAGATTCAATCGCAGTACAATTTAAAGAAATTAAAGAAAAGATGCATGAGAAATGGCACGAAGGAAAATAT
ATTGCTTATTTTCAGGCATTTACAAATACACATGCACCGGTTGAAGTATTAAAAGAAAAATTCGAACCTGTACTTAAAGAACCGGGTGTTGT
GGGATTATCTATTGGTACGCGTCCTGACTGTCTACCAGACGATGTTGTCGAATATTTAGCAGATTTGAATCAACGAACATACTTATGGGTTG
AATTAGGACTACAAACAATCCATCAGTCAACATCTGATTTAATCAATCGTGCCCATGATATGAAAACTTATTATGATGGTGTGGCAAAATTA
CGTAAGCATAATATCAATGTATGTACACACATCATTAATGGCTTACCTGGCGAAGACTATGACATGATGATGGCTACTGCCAAAGAAGTTG
CACAAATGGATGTACAAGGTATTAAAATTCATTTACTTCATTTGTTAAAAGGTACACCGATGGTAAAACAATACGATAAAGGTTTATTAACT

TTTATGACTCAAGAAGAGTACACAAACCTAGTTGTGGACCAATTAGAAGTGATTCCCCCTGAAATGATCGTTCACCGAATTACCGGTGATG
GTCCAATAGATATCATGGTAGGTCCAATGTGGAGTGTTAATAAATGGGAAGTATTAAATGGCATCGATGCTGAATTAGCACGTAGAAATTC
TTATCAAGGCTTGCGTTACAAGTCTAAGGTGAAGCAATGA

Gene: ytqB (rRNA methylase)

Position: 1837269 to 1837832, length: 564 nt, orientation: FORWARD

Perfect match to: (RF122-AJ938182-[1767931:1768494], highly conserved allele)

Sequence:

ATGAAATTAGAACGTATACTCCCTTTTTCAAAAACACTTATTAAACAACATATAACACCAGAAAGTATTGTTGTAGACGCAACTTGCGGTAA
CGGCAATGACACTTTATTTTTAGCCGAACAAGTACCAGAAGGACATGTTTATGGTTTCGACATTCAAGATTTAGCTTTGGAAAATACACGTG
ATAAAGTTAAGGATTTCAATCATGTTTCTTTAATAAAAGATGGACATGAAAATATTGAACATCATATAAATGATGCACATAAAGGTCATATT
GATGCAGCCATCTTTAACCTAGGTTATTTGCCTAAAGGTGATAAATCTATCGTGACAAAGCCTGACACGACAATCCAAGCTATTAATTCATT
GCTATCATTAATGTCAATTGAAGGTATTATTGTACTTGTTATATATCATGGTCATAGCGAAGGACAAATTGAGAAGCATGCATTGCTTGATT
ACTTGAGTACTTTGGATCAAAAGCATGCGCAAGTTTTGCAATATCAATTTTTAAACCAACGTAATCATGCTCCATTCATTTGTGCCATAGAAA
AAATTTCTTAA

Gene: rot (repressor of toxins)

Position: 1837952 to 1838353, length: 402 nt, orientation: REVERSE

Perfect match to: (RF122-AJ938182-[1768614:1769015:r], highly conserved allele)

Sequence:

TTACACAGCAATAATTGCGTTTAAACTATTTTGCATTGCTGTTGCTCTACTTGCAATCGCATCACTGATGAAATTCAACAACTCTACTTTCTCT
TGTTGTAACTTTTCATTGAAATGAATAATAACTGTTCTTTCATCGTCAACAGGACGCTCTTTGTAAATCCATTCTAATTCAACTAAATTATTAT
ACGTTCTCGTACGCTTATACGGTTTAACTTCAACAAATCTGTCCATTTCTTTAAGCGTCATAGAACCTTTTTGCCATAAAGTTAGTAAAATTAA
AATTTCTTCTCTAGACATTTTGTATTCGCTTTCAATCTCGCTGAAAATTGAGTTAATGTCACCCAAAAGTGTTTCTAATTGCAAAATCCCAAAT
ACAGTGTCGTTATTTACTTTTTTCAT

Gene: pldB1 (putative lysophospholipase, locus 1)

Position: 1838925 to 1839752, length: 828 nt, orientation: REVERSE

Perfect match to: (RF122-AJ938182-[1769587:1770414:r], highly conserved allele)

Sequence:

TTAAATTTCTACAATTTCGTCATCTTCAACAATAAAGCCCATTGTATTGACGCTGTTATTTAAGAAAGTCAGAATATAACGCATTACTTCATCA
CGTTCTGGCTCATTGTGAACCTCGTGGTAAAAACCTTGCCAAGCTTTAAAATATAATTCAGGTGTTTGATATTTTTCTTTAAACTCATCAATTG
CCCTAGTATCAACAATTAAATCCTTCGTTCCATACATTAATAGCGTTGGCATTGGTTGAATGTCATGAATATGAGCCATCGTATCTTTCATCG
TCTCATTAATTGTATTATACCAATGATACGTTGCTTTTTTTAACATTAAACCATCGTTAACTGTTTCTTCAACAATTTCTAAATTACGTGTTAAA
TCTTTTGGTTCTACACCAACATTAAAACGTGTGTCTTTTGAAATTTTACCTATATTTGAAACAAGTTTATCTTTACGATTTTTTCCATTCTTTTG
AAGTTCTAGCATAGGAGAAATTAACATCATCCCCTCGATTGGCAATTCTACTTTTTCAAGTAAATTTAATAAAATCAAACCGCCAAGTCCTAC
CCCTAATACATAAGTAGGAATTTTATATTCATTAGCTATCTTTAACCAGTCTAGCAAACTTTCGTGATACGTTTGAAAGTTTTCAATTTGTCCT
TTATTAGCTCTTGAAGTTTGACCTTGACCAGGCAAATCTCCCATAATCACATGATAGCCATTTCTTCTTAACATCGTAATAACATATGCATATC
TTCCCGTATGTTCTAATATATTATGAGCAATAACAACGACGCCTTTCGCATCATTTTCAGCTTCCCACTTCCACAT

Gene: putA (proline dehydrogenase)

Position: 1839986 to 1840987, length: 1002 nt, orientation: FORWARD

Sequence:

ATGGCACTATTAAAGAATTTTTTTATCGGATTATCTAATAATAGTTTTTTAAACAACGCAGCAAAAAAAGTGGGCCCACGTTTGGGCGCCAA
TAAAGTCGTTGCCGGAAATACAATTCCAGAGTTAATTAATACAATCGAATACTTAAATGACAAGAATATCGCTGTTACGGTAGACAATTTAG
GGGAATTTGTCGGTACAGTTGAAGAAAGTAATCATGCTAAAGAACAAATTTTAACAATTATGGACGCGCTTCATCAACATGGCGTAAAGGC
ACATATGTCTGTTAAATTGAGTCAGTTAGGTGCAGAATTCGACTTAGAATTAGCTTACCAAAATTTAAGAGAGATTTTACTTAAAGCAAATA
CTTACAACAATATGCATATAAATATTGATACTGAAAAATATGCTAGCCTACAACAAATTGTTCAAGTTTTAGATCGCTTAAAAGGCGAATTTA
GAAATGTTGGTACTGTAATTCAAGCATATTTATACGATAGCCACGAATTAGTTGATAAGTACCAAGATTTACGATTACGTTTGGTTAAAGGT
GCATATAAAGAAAACGAATCAATTGCATTTCAATCTAAGGAAGACGTAGATGCAAATTACATCAAAATAATTGAACAACGTTTGTTAAACG
CACGCAATTTCACTTCAATTGCAACACATGACCATCGCATCATTAATCATGTAAAACAATTTATGAAAGAAAATCACATTGAAAAAGATCGT
ATGGAATTCCAAATGCTCTATGGTTTTAGATCAGAGTTAGCAGAAGAAATCGCAAATGAAGGCTATAATTTCACTATTTATGTACCTTATGG
CGATGATTGGTTTGCGTATTTTATGAGAAGATTAGCAGAACGCCCACAAAACCTATCTCTTGCTGTAAAAGAATTTGTGAAACCTGCTGGCT
TAAAACGTGTTGGCATAATTGCAGCTTTAGGAGCTACAGTTATGTTAGGTTTAAGTACAATTAAAAAATTATGCCGTAAATAG

Gene: ribH (6,7-dimethyl-8-ribityllumazine synthase)

Position: 1841109 to 1841573, length: 465 nt, orientation: REVERSE

Perfect match to: (COL-CP000046-[1872609:1873073:r], highly conserved allele)

Sequence:

CTATGCTTTTATAGATTTTAATAAATTAGCCATTTCAATTGCACTTACTGCTGCTTCAGCACCTTTATTGCCAGCTTTCGTACCTGCTCTTTCCA
CAGCTTGTTCAATACTTTCAGTCGTTAAAATACCAAATATGACTGGTACATTAGTTTGATCATTCACTTTAGAAACACCTTTCGCGACTTCATT
ACAAACATAATCATAATGAGACGTAGCACCGCGAATTACGCATCCTAATGTAATTACTGCATCATAATTTCCTGATGAGGCTAATTTTTTAGC
TACTAAAGGAATTTCAAACGCACCTGGCACAAATGCTACATCAATATTGTCTTCATTAACATCATGTCGAATCAAAGTATCTTTTGCACCTTC
AAGTAATCTTCCAGTGATAAAATCATTAAATCGACTAACTACGATTGCAACTTTCAAATCTTTTCCAATTAATTTACCTTCAAAATTCAT

Gene: ribA (bifunctional 3,4-dihydroxy-2-butanone-4-phosphate synthase/GTP cyclohydrolase II)

Position: 1841586 to 1842767, length: 1182 nt, orientation: REVERSE

Perfect match to: (GR1-AJLX01000042-[2706:3887:r], highly conserved allele)

Sequence:

CTATATTAAATGACCCATTTTTATTTTTTTCGTTTCCATATAATCATGATTATGTACCGTTTCTGGTACGATAACTTCAATTCTTTCTGCAATATC
AATGCCATATTGTTTTAATCCCTCAAATTTACTTGGATTATTACTTAATAAATTGATATGTTCGATGTTAAAATATTTTAAAATCTGTGCAGCA
ATATGATAATCTCGTAAATCTTCATCAAAACCTAATGCTAAATTTGCAGTTACTGTATCATATCCTTGCTCAATTAATTCATATGCGCGTAATT
TGTTTAACAATCCTATGCCACGACCTTCTTGAGGTAGATAAATAATCATGCCACCATGTTCATTGATATACTTCATAGACGATTCAAGTTGAG
CACCACAATCACAACGTTGACTATGGAAAATATCGCCTGTAAGGCACGCAGAATGTAAGCGTACATTTTCATGTTGTCGAATTGCACCTTTT
GTCAGTACAACTATCTCTTCATCTGTGTATGTCGCTTTAAAACCATACATATCAAATGTTCCGAAATCTGTAGGCATTTTCACTTTTGCCTTAA
ATTCAATTTCTGGTTCTAATTTTTTACGATATTCAATTAAATCATCAATCGTAATCATCTTTAATTGATGTTTTTCTTTAAACTTTTGTAAATCTT
GTCCTTTCGCCATCGTGCCGTCATCATTCATAATCTCACAAATGACACCAGCGGGCTTGGCACCAGTAAGTTTAGCTAAATCAACAGCCGCT
TCTGTGTGTCCATTTCTAGCTAATACGCCTTTATCTTGTGCTACTAATGGAAATAAATGACCAGGACGATTAAAATCTTTAGCTTCACTACTA
GGATCAATGAGCTTTTTGGCAGTCAATGTACGTTCATAAGCACTAATTCCTGTTGTTGTATCTACATGATCAATACTCACTGTAAATTGCGTA
CCAAAGATGTCGGAGTTATCATCAACCATTTGTACCAAATCCAAACGTTGTGCAATATCTTTAGACACTGGTGCGCATATTAATCCCCTTGCT
TCTTTCGCCATAAAATTAATGGTATTATCGTTCATCCATTCAGTAACCGCTACTAAATCACCTTCATTTTCACGATTCTCATCATCTACTACAAT
AATTGGTTCTCCATTTTTTAAAGCCATTAAAGCACTGTCAATATTATCGAATTGCAT

Gene: ribB (riboflavin synthase subunit alpha)

Position: 1842778 to 1843410, length: 633 nt, orientation: REVERSE

Perfect match to: (COL-CP000046-[1874278:1874910:r], highly conserved allele)

Sequence:

CTAAAAACCAAATGCTCTTAATTTATCTACAGATAATTGGTCTTTATCTTTATTTAAAATATTTTCAACATATTTAAACAAAACGTCTGTTTCTA
AATGTACTTTATCTCCTAATTTTTTGGATGATAAAATCGTTGAACGCCTCGTTTCTGGAATAAGATGAATGTCAAAACTGTTATCATGCTTAT
CAAATACCGTTAGACTAACACCATCCACAGTAATAGACCCTTGCTTAACTAACTGATTATTAATATGTTGGCTACATTGAATCGTAATAATTT
TTGCATTGGCTGTTTCATTTATTTTTGAAACTGTTCCTAGTTCATCTACATGACCGAGGACAAAATGTCCACCAAACCTACCGTTACCACTCAT

GGCACGCTCTAAATTTACTTCTGATTGTCGCTTAACATCTGCTAAATAGGTTTTATTTTCAGTGCCTTTAATTACTTGAACAGTAAAAGATGTC
TGATTAAAATCAATCACTGTTAAACATGCACCATTAACACTGATGGAATCACCAATATGCATATCTGCCGTAATCTTATGTGCTTCAATTTCA
ATCGTCCTGACTGATTGACGAATTTGAACACTTTTAACGACACCTATTTCTTCAACGATGCCAGTAAACAT

Gene: ribD (riboflavin specific deaminase)

Position: 1843417 to 1844460, length: 1044 nt, orientation: REVERSE

Perfect match to: (N315-BA000018-[1827275:1828318:r], highly conserved allele)

Sequence:

TCACTTCTTTCGTAAAGTTAATTTAACATTTTGATTTAATAACTCGGAATGAACAATTTCAAATTGGTTCGCATCTGGTATCTCAATCACATCA
TTTGTTTGATAAAATTGATAATTTCCAGATCCGCCAATTAATTTCGGGGCATAATAGAGAATAAATTCATCTATATAATTAGATTGGAGAAAT
TCTGAAGTAGTGGTTGGACCTGCCTCGACTAGCAAAGTTCCAACTCCTCTTTTATATAAATTGTGAAGAATTGTTGTTAAATCACAAGACTTC
AAGTAAATAATTTCAATATGTGTTTGATTGCTTGTTAAATTTGGATTTTCAGTATATATCCAAATTGGTGTTGATTCATCTTGATAAATTTGCT
GATTAAAATGAATATTCCCAGACTTAGACAATATTACTTTTATAGGGTTTTTTCCATCTTGAATACGTGTAGTATATTGTGGATCATCTAATTC
AACTGTACGTCTTCCAGTTAACACTGCGTCGTGTCGATGTCTTAACTTATAGACATCTTGTTTAACCTCTTTGTTAGTAATCCATTGACTTTGT
CCATTATCATTCGCTTGTTTACCATCTAAACTTGCAGATACTTTCACTGTAATTTGTGGCAGTTGCTTTGCTTTTGCTTTAAAAAAGTCTTGGT
ATAATTGTGATGCCCGTTCATCATCAACGCATTCAACCTCAATACCGTGAGCCCGTAACGTCTCATCACCATGTGTGTCTAACGAATTGTCTT
TTGTTGCGTATACTACTTTTGCTATCTTACAATCAATAATTTTGTTAACACAGGGTGGTGTTGAACCAAAATGACTACATGGCTCTAACGTAA
TATAAATCGTCGCACCTTCAGCATTTTGTTGTGCCATATCAAGTGCTTGAACCTCCGCATGCTTGTCACCTTTTCTCAAGTGTGCACCAATACC
AACAATCCTACCTTCATTAACTACAACAGCGCCAACGGGTGGATTAACACCTGTTTGACCTTGTACCATATTTGCAAGTTGAATCGCATAATC
CATAAATTGACTCAA

Gene: Q5HF04 (putative pyridine nucleotide-disulfide oxidoreductase)

Position: 1844941 to 1846443, length: 1503 nt, orientation: REVERSE

Sequence:

TTATTGATTGTAACTGCGCTGTTGTTGCTCATTCGATTCTAAAGCATCATATAATTGAGATACTGTATGCGCAACTTGTTCTACTATCATTTTC
ACACCATTTCGTAGTTTATTAACACCGTTTGTCATTTGACCTATCGCAATCATATTTGTTAATGTTCCAAACCTTGGACTAATAACTTGATTGG
TTTCCGGTATGATTTGTATGCCTCTCATTGGATGTGCTTGTACAATTTGTCTATTTTCAAGATTTTTAATTAATTGATCATCTTGATCCAATTCA
TTTAAATGACTTTTTGCACCTGTCGCGTTAATGACAACATTATACATGTCTACTGATTCTTGGTTTTTGTATGAAAAATAATACAACTTGCCAT
CATGTTTCACATCTTCTAAATCTTTTTTCAAAATTAAAGACTTATTTTCTATTAATTCAATAATTAGTTCAGCAGTTCTTGGAGGCATTGGATTT
GAATTTAATTGAATCATCTTTGAGTATTTTTGATTAAATTGATGTTGATCTTCAATACTTAAGCTATTCCATATCCAATTTAAATTCTCTTTCAA
ATGTTCAATCATACTTTGGAAAATGCCCATTTCTGTTGGACGCGCTAAATCATACTTCAAATCTGCAATATGATTTCCTGTACGTCTATGTACT
AATTTTTTAAAATCAATGTCATATTCAGCACATTCTTTTAAAAATAAAGAAACTATAGTATCAAGCGGTGCATTGCCGAAATGATGTTTTTTA
ATGTCATTTAATTTGTCTTTAGTTAAGTACTTGAATGTCACGTCTATCATTGTACCTCTTACACTTGGTAAATGAGCAGAACGACTCGTCATA
GTAATTGGTAATTTCGGATGATGAGCAGCAACATAACGGACAACATCTAAACTGGCAAGGCCTGTACCAATAATCGCAATATCGTCCAGTT
CATTTACTTCGTCTAACGTATTATATGTTGGATAAGGCGTAGCGATATATCCTTTTTTACCCTTTAAGTTATATGGATCATGGTAGGCAAATG
TACCACATGTTAAAAATACATAATCGTACGCTTGCCATGATTGTTCTGAATTTGTAGTACATATGTAATAAGTTAAATTCGTTTCATCGATATT
AGAATTTGTATAAATCTCTTGAACTTTATTATAATTAGTTGATATATTTGGATATTTTTTCGTGAACATAGATAAATAAGATTTCATATAATGT
CCGAATACAAATCTCGGTAAATATGCAGGTTCATCAAAATTAAAATCAGTTTGTTGTTTATACCACTTCCAAAATTCAGTCTCATCATCTAAA
TTTAAACTCATCTTTTTCGAAGGCATATTAATTAGCAGCTCAGAACTATCATTTTGAAATGGTACGCCCTGTCCCATATTTACTTTATCATCGT
ATAAATCTATATCTAATTGATTAAACTTCGGGTGCTTAACTAACTCTCTCAATACACTTACACCAGCAGTTCCCATGCCTATTATTGCTACACG
CAT

Gene: arsR (repressor of arsenic resistance operon)

Position: 1846966 to 1847280, length: 315 nt, orientation: FORWARD

Perfect match to: (N315-BA000018-[1830824:1831138], highly conserved allele)

Sequence:

ATGACGTATAAAGAACTAGCAACATTTTTAAAAGTTTTATCAGATTCAAGCAGATTAGAAATACTAGATTTACTTCTTGTGGAGAGTTATG
CGCTTGTGATTTGTTAGCACATTTTCAATTCTCTCAACCCACACTTAGCTATCATTTGAAAGCATTAGTAAAAACCAACTTAGTTACGACACG

AAAAATCGGAAATAAACATTTATACCAGCTTAATCATAATATTTTTGAGTCCGTAATTAATAACTTGTCTAAGGTTCATACCTCTAACCAACG
ATGTTTTTGTCATAACCTTAAGACTGGTGAATGCTAA

Gene: arsB (arsenical pump membrane protein)

Position: 1847280 to 1848572, length: 1293 nt, orientation: FORWARD

Perfect match to: (N315-BA000018-[1831138:1832430], highly conserved allele)

Sequence:

ATGATGACAACTTTAGCGACACTCATTTTTCTAGTAACTTTATTATTTGTATTATGGCAACCTAAAGGCCTAGATATTGGCATTACCGCATTA
ACTGGTGCCTTTATTGCTGTTATTACTGGTGTTGTAAGTTTTTCCGATGTTTTCGAAGTAACAGGTATTGTTTGGAATGCTACTTTGACTTTTG
TCTCAGTCATTCTTATTTCATTAATATTAGATAAAGTTGGATTATTCGAATGGTCAGCTATTCACATGCTTCATGCTTCAAAAGGAAATGGTTT
AAAAATGTTCGTTTATATCATATTATTGGGTGCCATTGTTGCTGCATTTTTCGCAAATGATGGCGCAGCGTTAATCTTAACGCCTATTGTATT
AGCGATGGTTAAAAATATAGGTTTTAGTAAGCGGGCCATATTCCCTTTTATTATTGCGAGTGGTTTTATAGCTGACACAACTTCTTTACCTTT
GATCGTGAGCAATCTAGTGAATATTATATCTGCTGATTATTTTCATGTAGGATTCGTTCGATATTTTAGTAGAATGATTATACCTAATTTATTC
TCGCTTTTAGCAAGTATTATAGTATTGTGGTTATATTTTAGAAAGGCGATACCTAAAACGTTTGATGATAATAATATAAAGCATCCTAAAGAT
GCCATTAATGATTTAAAGCTATTTAAAATTTCATGGATTGTTCTAGTTATATTACTTTTCGGCTATCTAATCAGTGAATTTACTAAAATTCCGG
TATCAATTTTCACTGGAATCATTGCTTTTATTTTTCTAATGTTGGCTCGTAAATCAAATGCTGTAAATATTAAGCAAGTCATTAAGGGCGCAC
CTTGGAATATAGTATTATTTTCAATTGGTATGTATATCGTCGTATTCGGCTTAAGAAATGCTGGCATTACTTTAATATTGGCTAAAATATTAG
AATATATTTCCAATTACGGTCTATTTAGCACCATTTTGGGAATGGGCTTCATTTCAGCGTTTTTATCATCAATAATGAATAATATGCCTACAGT
TTTAATAGATGCGATTGCTATTGGTCAATCAAATGTCCATGGCATGTTAAAAGAAGGCCTAATTTATGCGAATGTTATCGGTTCTGATTTAG
GTCCAAAAATTACACCGATAGGCTCTTTAGCTACATTACTGTGGTTACACGTCTTAACACAAAAAGATGTTAAGATTTCTTGGGGCACATAC
TTTAAAACTGGTATCATCATTACAATTCCAGTACTATTTATAACCCTCATAGGGTTGTATCTAACACTTATCATATTTTAA

Gene: gad-sagB (glucosaminidase B)

Position: 1848659 to 1849513, length: 855 nt, orientation: REVERSE

Perfect match to: (MW2-BA000033-[1870324:1871178:r], highly conserved allele)

Sequence:

TTACTTATTCAAATGTTTACTGTCATCTTTATACACAAAGTATTTGAAGTATTTTCCTTCAGTCTTCATATTCTTATAAAAGTCAGCGATAATTG
TTGCATTACTTTCTGCCCACTTAATATCAGTAGCATATTGATGTTCTCCTGGATTTTTTGGATTCCATCTCATACTATACAATGTATTTTGATCT
GTGCTTGATAAGAAGTGCTTATGAATGAAATCAGCACCGCCTGAAATAGCTTTTTCAGGTGTATCCCAACCATGCTTTTTAGCATATTCTGCA
CCTGTTTTAATTGGGTCTTTATCAAGGGCTCCTACTCCATAGAAATTGTAGTATTTTTTGCCATCAATTTCGACTCCATTAGCTAATTCACTTTT
AACTGCGCCAGTTTCTAATAATGCATGTGAAATTAAATAAACTTCGTTAACGTGCTTATCTTTAGCAGCTTTTAAGAAATCATCCGTATGTTT
CAATAACGTTGGTCTATCTACTAACATACGTTTAATTCTATTTTTATCAATCCCTTGATACTTTGATAAATCTAAAAATTGATATTTTTGCTTTT
CATTATCGATAAAAGTACCGCTATCCATTGCACTTTTAATTTCAGTTGCAGATGCATCTCTCCATGCATCATTCTTTTTATTTGATACCTGTTGA
CTCGTATAATTATTTATTTGTTTCTTTGCTGCATCGTTTAATGTAACATTTAACTTTTCAATCTTAATGTCGGATTTAACATGTTTGAAAAATAT
CTGATCGGATATCATTGAGAAAAATAAAAATGAGACAACAGCAAATATGACAACAAGTCCTATTATTCCAAAAATAGAACCTTTCTTGTGTT
TATTCAT

Gene: Q5HEZ9 (putative protein)

Position: 1849789 to 1850013, length: 225 nt, orientation: REVERSE

Perfect match to: (N315-BA000018-[1833647:1833871:r], highly conserved allele)

Sequence:

TTACTTAGTTTCTTTTTTATAATCCATCATAATAAAACTTGCTGCATTTGATCTATTTTTATAATAGTACTGATTTAAATCAATCGTGCCTTCAA
TTTGACCATCACGATACATCATTTCATTAGTGAAGTTATTTATCATCACGAAATCAGCGCGATCTCTTATAACATCTAAATCGTCGTTTCGTGC
AAAAAAATGTTCTAAATTTAAATGTGCGTAATCCAT

Gene: sigS (RNA polymerase sigma factor S)

Position: 1850212 to 1850682, length: 471 nt, orientation: FORWARD

Perfect match to: (MW2-BA000033-[1871877:1872347], highly conserved allele)

Sequence:

TTGAAATTTAATGACGTATACAACAAACACCACAAAATCATACACCATCTTTTAAAAAAATATAATATTAGCTATAATTATGATGAGTATTAT
CAACTACTCTTGATAAAAATGTGGCAATTGAGTCAGATATATAAACCCTCAAGCAAGCAATCTTTATCCTCTTTTTTATTCACTCGATTAAATT
TTTACCTTATCGATTTATTCAGACAACAAAATCAATTAAAAGATGTCATTTTATGTGAGAATAATTCACCAACATTAACTGAACAACCAACAT
ACTTTAATGAACATGACCTTCGTTTACAAGATATCTTCAAGCTTTTAAATCAAAGAGAAAGACTATGGCTCAAACTATACCTTGAAGGATAC
AAGCAATTTGAAATTGCTGAAATCATGTCATTATCGCTTTCAACGATTAAATTAATTAAGATGTCCGTTAAGCGTAAATGCCAACATAATTTT
AATTAG

Gene: Q5HEZ7 (putative protein)

Position: 1850795 to 1851238, length: 444 nt, orientation: FORWARD

Perfect match to: (11819-97-CP003194-[1901176:1901619], allele observed in CC80+CC239+CC4803)

Sequence:

TTGCAAGACAATTCTACTAAATATCTACTTTATATCCAAACTGCTACTTCAAACCATCTCGAAACAAATTGTGTCTTTTTACATTGCGATTACA
TTCTTAAAGTTCCAATTAACAAACTCGTTTCATATTATGCGAAATTGCATTTATCATCACAAAGTGTGCTAATTGAGACTGCAAAAAACATAC
TAAATATTAATAAACTGGTTCCTATTTACATCAACCCAAAAACTATACTTTTTCCGTTAAAACATAAACGTGCACCGATACAAATTTATATCAA
TGCACATTATATTGTTGGTATGACTGCCATAGAGAATTCAACATTAATACATTTTCAAGAAGGCATTCAGCTAGAAGTTGATGAGCCATTTTC
ACTAGTTTCAAAAAAAATGTCATGAAAGTTTAGCTTTGAAGCACTTCATTGAAAATACTATTTCGAATTAA

Gene: Q5HEZ6 (putative membrane protein)

Position: 1851225 to 1851668, length: 444 nt, orientation: REVERSE

Perfect match to: (MW2-BA000033-[1872890:1873333:r], highly conserved allele)

Sequence:

CTATTTCGAATTAAATTTAATTTCTAAAAGTTTCTTAGTAAAGTATGATGTCACAATTTTTATAAAATATATGCTAACAATCATTAATAAAATC
GCTCCAATAATTGTTAAAATAACAACAGTTGTTGATAAATCTACCTTAGGACTAACGAAAAATTGTACGCCAGATAGACTTGTAATTAGCCC
AAATAAAAACAAGCCTAATAAAGCAACATAAGCAAACATCATCACTACTAATGTAACAAACATACTATAACTTTTTGTTTCTTTATTTCGAAA
TACATTTTGTCTTAAGTAAGCATTAGCAAAGGTAACAGGCTTATTTAAGTTAACGCTGTCATCATCAGATATAGAAGACAATCGTTGATCTA
ATGCTTGTTTCTCCTTCTTGTTCAAAAACCATAATTGCTTTTTAACTTTTTTATCAAATGATGCTTTATTCAT

Gene: Q5HEZ5 (putative CAAX protease)

Position: 1851964 to 1852599, length: 636 nt, orientation: FORWARD

Perfect match to: (11819-97-CP003194-[1902345:1902980], highly conserved allele)

Sequence:

ATGCAAAAAATTCAAAGACTTTTTTTACGATGATTTATCGGTTACACGAGGAAATTATTTTTTAACTTTAATGGCAGCATTTTTTATTACTATCA
TTTTATTTATCGGCATAGTTGTCAGTGAAGTACATTTACTTTATAGCATGCTAATTGTATTAGTAGGTTTAATTCTATTGAGGCTATTCAAAAT
CAATTTATTCTCTTTTAAAAAAATTAACATTGTCTCAAGTTATTTATATTATAGGCGGTGCACTATTAATTTATGGGTTAGATAATCTTTATTTAT
ATTTTCATGACGTACCGGCAAATGAACAACAATTAGAGCAAGAAATACGAAATACACCATTCTATATTTCTATTTTCACTGTTACCATCATCC
CCGCTATTGTGGAAGAAATTGTTTTTCGCGGTATGATAATAAGGGTTATCTTCAGAAAACACTTGTTTTTAGGGTTAATTGTGTCTAGTTTAG
TTTTTGCATCATTACACGAATCTGACACTTGGATTGGTTATTTACCTTACTTATATTCTGGTTTGATTTTTGGTATAATTTATATAAAAACAAA
ACGATTAGAAGTGGTAATATTCATGCACTTCTTAAATAACTTGTTAGCTCTGCTCTTTATAATATGGGGATAA

Gene: tal (transaldolase)

Position: 1853040 to 1853753, length: 714 nt, orientation: REVERSE

Perfect match to: (MW2-BA000033-[1874704:1875417:r], highly conserved allele)

Sequence:

TTATAGAATAGAAAGACCTGAAGATTGAATATCTTTCGCAAAGCCTTTAACTGTATCTACTGATAATTCGTTAATATCGCGACCTAAGTTTGT
ATTCACTTTTTTCACAACATCTGCAGGGCATGTAATAATATCTGCACCAATTTCATCAGCTTGAATCACATTGAATAATTCGCGGCAACTTGC
CCATAATAATTTAACGCCGTCTTTACTATGCGTAACTTTTACAGCCTCTTTCATTAATGGTAATGGATCTACGCCTGTATCTGCAATACGTCCT
GCAAATACTGAAACATATGTTGGCACACCTTCAGTTACTGCTTCAGTTATTTCTTTAACTTGTTCAATTGTGTAAACAGCCGTAACGTTTAATC
TCACATTGTCAGCTGAAAGCTTTTTAATTAAAGGAATCGTTGATTCACCTTTTGTATTTACAATAGGAATTTTAACAAATACATTTTCGCCATA
TTGTTTTAGAATTGCTGCTTCTTTTTCCATAGTTTCTAAATCGTCTGCAAATACTTCAAATGAAATTGAAGCATCTGGAATTTCTTTCACAGCT
TCTTCAGCAAAAGCTTTGTAATCTGTTACGCCCGCTTTCGCCATTAAACTAGGATTTGTTGTAAAACCATCCACTTGTTTGTTTTTATAAGCTG
CTTTCATTTCTTCAATATCTGCACCGTCCGCAAATACTTCTACATTTAGTTTAGCCAT

Gene: Q7A500 (putative protein)

Position: 1854013 to 1854315, length: 303 nt, orientation: FORWARD

Perfect match to: (MW2-BA000033-[1875677:1875979], highly conserved allele)

Sequence:

ATGTCTCGTTCAAAAAAATACTTTTACTTATCTAGCTTAATGATTATTTTAAGCTTTTTCTTTAATACAAATAACGTTTTCCTAAGTGGACTTTT
TAATTCTTTTATTAAATTAATACTTTTCTGCAGTGTTATTAACTCAATTGTACTAATTTTGTCTATAATTTTTGCAGATCGTTCAATTAAATCACT
AAAGCCTGATGCAGATTGGATTAGAATTGCGAGTAAAAGTTTGCCTTGGATTATTCTAATTGTTATTTTAGTACATATCTTTTCAATTGTTCG
TACATTCGGTTTTATTTAA

Gene: crcB1 (protein CrcB homolog 1)

Position: 1854493 to 1854936, length: 444 nt, orientation: FORWARD

Perfect match to: (MW2-BA000033-[1876157:1876600], allele observed in CC1+CC8)

Sequence:

ATGCATCGACAATTTTTGTCGTCGCGTTGCCAAAACCTCTTTTTTAAATTCAAACTACTTCTTTTCGAGGTGAACCAAATGCAATATGTATATA
TTTTTATCGGTGGTGCTTTAGGCGCTTTATTACGTTACCTCATTTCTTTTCTGAATACTGACGGAGGTTTTCCAATCGGAACACTGATAGCCA
ATTTGACTGGTGCCTTTGTAATGGGATTGCTAACAGCCTTAACAATTGCATTTTTTTCAAACCATCCGACGCTAAAAAAAGCTATTACGACTG
GTTTTCTTGGTGCTTTAACGACTTTTTCAACATTTCAATTAGAATTAATACATATGTTTGATCATCAACAATTTATAACTTTACTACTATATGCT
GTAACAAGTTATGTCTTTGGTATTTTGTTATGTTACGTCGGTATAAAACTAGGTGGTGGTTTATCATGA

Gene: crcB2 (protein CrcB homolog 2)

Position: 1854933 to 1855286, length: 354 nt, orientation: FORWARD

Perfect match to: (11819-97-CP003194-[1905314:1905667], allele observed in CC80+CC7+CC80+CC97)

Sequence:

ATGATATCAATCATTTTAGTCATGATTGGCGGCGGTTTCGGTGCAATTACTAGAAGTGCCATTACTGATTATTTTAATCATAAATTTACTTCA
AAGTTACCTATCGCAACATTGATAGTAAATCTAGTTGGTAGTTTTTTAATTGGATTAACTATAGGCTTATCAATTTCAATCTCATGGTTCCCTG
CGTTCTTTGTTACCGGTTTTTTAGGTGGCTTAACAACTTTCTCAACGTTAGCCAAGGAACTTACACTAATGATGACGCCAAAATTTAATATTA
ACCTTTTTCTCAATTATTCACTTTTACAATTCATCATTGGATTTATAGCTTGTTATATTGGCTATCATATTTAA

Gene: tx_universal2 (rho-independent terminator)

Position: 1855528 to 1855566, length: 39 nt

Perfect match to: (Strain_21331-AGTV01000040-[104403:104441], allele observed in CC398)

Sequence:

TAGAATTGAAAAAAGCTTGTTACAAGCGCATTTTCGTTC

Gene: ytbE (glyoxal/methylglyoxal reductase)

Position: 1855669 to 1856502, length: 834 nt, orientation: REVERSE

Perfect match to: (11819-97-CP003194-[1906050:1906883:r], allele observed in CC80+CC188+CC4803)

Sequence:

CTATCCTTCAAAAGTTTTTGGATCAGGTCCAATTCTCTTATCTTGATTTAAACCATCAATTCGCGTCATTTGTTCATCTGATAATTCGAAATCA
AATATTTGGAAGTTTTCAGAGATTCTGTTTGGTGTTACCGATTTAGGGATTGTAACCACACCATGCTGCACATTCCATCTTAAAACAACTTGG
GCAGGTGACTTTCCTAATTCTTGAGCAATGTCTTTAATTGTCTCATCATTTAAAATTTGTGCATTCATCAATGGTGACCAAGATTCCATCACG
ATATGTTGTGCTGCCAAATATAATTTCAATTTATGTTGCGTTAAATATGGATGATATTCAACTTGATTAATTACAGGTTTAATTGACACTTGT
GCCAACAAAGCTTCCAAATGTTCAGGTTCAAAATTGCTGACACCTATATTTTTAACTTTATTATTTTTATATAAATCTTCCATACCTTTCCATGT
ATCAACCATTACGGCTTCGTTCGTACCTGGCCAATGTACTAGATACAAATCTAAGTATTTTAAACCTAATCTAGATAAACTAGCTTCGTAAGC
AACTGCTACATTTTCACGACCGAAATCCTCAAAATATAATTTTGAAGTAATAAATAAGTCTTCTCTAGCAATACCAGTTGACTCCAATCCGGC
ACGAATGCCAGCACCTACTTGTTCTTCATTCCCATAAACTTTTGCGGTATCAATACTACGATATCCTTGTTCAATGGCATACTTAACACTTTCC
ATGCAATTTTCATCATTTTCCACACGAAATGTCCCTAAACCAATTTGTGGCATCGTGTTTCCATTATAAAATGTTTTAACCTCCAT

Gene: nrd (nuclease-related domain (NERD))

Position: 1856714 to 1857622, length: 909 nt, orientation: REVERSE

Perfect match to: (11819-97-CP003194-[1907095:1908003:r], highly conserved allele)

Sequence:

TTAGTTGAATGATTCAATTTTATCCATCATTTGTTGTAAGTCTTCCACGTTGTATTGAATACGACCATGGAATACAAATTTGTTAAAGAACTC
GTCTAATTGTTCAGCACCGACAAGCACTTTGACAGCACTATTTTGATTATAATTTGAAATCGTTACATCGCCTTCATTTTTAGGATTAAAGTAT
AAAATTGAAGTTGGCGTATATTTGGCACCTAATTCTTTTTGTAAGTCTTCAGCCAATTGTTTAATCGCCTCAATTTGATCTGAATAATTTACAA
AAGATAATGAACGTTTGTCATCATTTTGATCCATCACAATAGTTTGTGGTCTAGATTTATCTAAATCCAATGTATCAAATACTTGTTCCATTGG
TGGTAAATCTTTAAATTGACCGCCACTAATACCATTATAAACATGACCTTTTAACAATTGAGAATCAATAATATAAAGACCAGTTCTTGTTAA
TACTAAATGACTAATTCGTTCAATATTATTAAAGCCATCCTTTGGTAAAAAGATATTTGCCATAATGTGCATATCTTCTGGTCGAATTCGTTTT
TCTTTAACTAATCTTTCACGAATACCAATTAATCTCATGTCCGTTACATATTCACTATGATTTTTCGAGAACAATTTTAATGCGTCAATCTCAC
GATCTTTTGTACTAACCATGTGATTATAATCTTCTTGTTGTTTTGTAATTGTCTTTTTATTTTGAATACGCTCTTTCTCTAAAGCTTCTTCATGAG
ACTTTTTAATGTTTTGTTCTTGTTGTTCATACTTTTCTTCTGTTTGTCGCTTAACTTTTTTCTTACTACCTAAGGCAACTAAGAAAAGGACAAAA
AAGATTAATGCAATGACTACTGCAATAATGAGTCCAATGACTACCGGTGAAGATAAATCCAT

Gene: metK (S-adenosylmethionine synthetase)

Position: 1857747 to 1858940, length: 1194 nt, orientation: REVERSE

Perfect match to: (11819-97-CP003194-[1908128:1909321:r], allele observed in CC80+CC1+CC772)

Sequence:

TTATTTTACTGCGTCTTTTAATTCTTCCACTTTGTCTAATTTTTCCCATGGGAATAAGACATCTGTACGTCCAAAATGACCATAAGCAGCAGTT
TGTTTGTAAATTGGTTGTTTCAAATCAAGCATTTTAATAATACCAGCAGGTCTTAGGTCAAAGTGTTTTCTAACTGCTTCAACAAGTTGCCCT
TCAGAAACTTTACCTGTTCCAAATGTATCAATTGCAATTGACACTGGTTCTGCAACACCAATCGCATATGCCAATTGTACTTCACATTGATCT
GCTAAACCTGCTGCAACAATATTTTTAGCCACATAACGTGCAGCGTATGCAGCTGAACGGTCTACTTTTGTAGGATCCTTACCACTGAAGCA
TCCGCCACCATGACGTGCATAGCCACCGTACGTATCAACAATGATTTTACGTCCTGTTAATCCTGCATCACCTTGAGGTCCACCGATTACAAA
GCGTCCTGTAGGATTGATGTAGAATTTAGTTTGTTCATTAATCAAGTTTTCTGGAACAGTTGGATAAATGACATGTGCTTTAATGTCTTCTTG
AATTTGTTCAAGTGTCACATCCTCAGCATGTTGTGTTGATACGACAATCGTATCAATACGTACTGGGTTATCATTTTCATCATATTCAACAGT
GACCTGAACTTTACCGTCTGGTCGTAAATAATTTAACGTACCATCTTTACGCACATCTGATAAACGTTTTGCCAATTGATGTGATAAATAAAT
TGCTAGAGGCATATATGTCTCTGTTTCATTCGTTGCGTAACCAAACATTAAACCTTGGTCACCTGCACCTGTTGCTTCAATTTCTTCTTCGCTA
TCTTTATCACGATACTCTAATGCTTTATCCACGCCTTGTGCAATGTCAGGTGATTGTTCATCAATCGCAGTTAAAATTGCCATTGTTTCATAAT
CATAACCATATTTTGCTCTTGTGTATCCAATTTCTTTAATTGTTTCTCTAACAACTTTCGGAATATCAACATATGTTGTTGTAGAAATTTCGCCG
GCGATCAATGCCATACCTGTTGTAACAGTTGTTTCACAAGCTACACGTGCATTTGGGTCGTCTTTTAAAATAGCATCTAATATTGCATCTGAC
ACTTGGTCAGCGATTTTATCTGGGTGTCCTTCTGTAACAGACTCTGAAGTAAATAATCGTTTGTTATTTAACAT

Gene: pckA (phosphoenolpyruvate carboxykinase [ATP])

Position: 1859312 to 1860904, length: 1593 nt, orientation: FORWARD

Perfect match to: (NN50-BAEA01000011-[58320:59912], allele observed in CC4803+CC8+CC239)

Sequence:

ATGTCAGTAGACACATACACTGAAACAACTAAAATTGACAAATTACTGAAAAAACCAACGTCACATTTTCAACTTTCGACGACACAACTTTA
TAATAAAATCTTAGACAATAACGAAGGGGTATTAACAGAACTTGGTGCTGTTAATGCAAGTACTGGAAAATATACTGGTCGTTCGCCTAAA
GACAAATTTTTTGTCTCTGAACCTTCATATAGAGATAACATTGATTGGGGAGAAATTAATCAACCTATCGATGAAGAAACTTTCTTGAAGTT
ATACCATAAAGTACTAGACTATTTAGATAAAAAAGATGAACTATACGTATTTAAAGGCTACGCTGGTAGCGATAAAGATACAATGTTAAAA
CTTACAGTCATCAATGAATTAGCATGGCATAATTTATTTGCTAAAAATATGTTTATTAGACCTGAATCAAAAGAAGAAGCTACAAAGATTAA
ACCTAACTTCACTATCGTTTCTGCACCACATTTTAAAGCAGATCCAGAAGTTGATGGTACTAAATCTGAAACCTTTGTCATTATTTCATTTAAA
CACAAAGTCATTTTAATCGGCGGTACTGAATACGCTGGTGAAATGAAAAAAGGTATCTTCTCTGTAATGAATTATCTCTTACCGATGCAAGA
TATTATGAGCATGCATTGCTCAGCAAACGTTGGTGAAAAAGGCGATGTTGCATTATTCTTTGGTCTATCTGGCACTGGTAAAACAACCTTAT
CGGCTGACCCACACCGTAAACTAATCGGTGATGATGAACACGGATGGAATAAAAACGGGGTCTTTAATATCGAAGGTGGCTGCTATGCAA
AAGCAATTAATCTTTCCAAAGAAAAAGAACCACAGATTTTTGACGCAATCAAATATGGTGCAATTTTAGAGAACACTGTAGTTGCAGAAGA
TGGTTCAGTGGACTTTGAAGACAATCGTTATACAGAAAACACGCGTGCCGCTTATCCAATTAATCACATTGACAATATTGTAGTACCATCAA
AAGCAGCACATCCAAATACAATTATTTTCTTAACTGCGGATGCATTTGGTGTTATTCCACCGATTTCAAAGTTAAATAAAGACCAAGCAATGT
ATCATTTCTTGAGTGGTTTCACTTCTAAATTAGCTGGTACAGAGCGTGGTGTGACAGAACCTGAACCATCATTCTCAACATGTTTCGGAGCA
CCGTTCTTCCCGTTACACCCTACTGTTTACGCTGATCTATTAGGTGAACTTATCGATTTACATGATGTTGATGTTTATCTTGTTAATACTGGAT
GGACTGGCGGAAAATATGGTGTAGGACGTAGAATCAGCTTACATTACACACGTCAAATGGTAAACCAAGCGATTTCTGGCAAATTGAAAA
ATGCAGAATATACAAAAGATAGTACGTTTGGTTTAAGCATTCCTGTAGAAATTGAAGATGTACCGAAAACAATTTTAAATCCAATTAATGCT
TGGAGCGACAAAGAGAAATATAAAGCACAAGCAGAAGATTTAATTCAACGTTTTGAAAAGAACTTCGAAAAATTTGGTGAAAAAGTTGAA
CATATTGCTGAAAAAGGTAGCTTCAACAAATAA

Gene: ytmA (putative peptide hydrolase)

Position: 1861157 to 1861927, length: 771 nt, orientation: REVERSE

Perfect match to: (Strain_21266-AFTT01000016-[264029:264799:r], allele observed in CC12+CC80)

Sequence:

CTAGCTATGCAACTCTACTTGGTTCATAAACTCTTTAATATAAGTCAATGTTTCAACCATCGCTGGTGGTCTTGGCACATGTCCTTCTGCCATT
TGATAAAATGTTTCATGCGTGGCACCTTTTAACTCTAGTTGGTCCGCTAAATAATACGCATGATGAATACCAACTTGCTGGTCTTTCCCTCCA
TGTACAATTAATATTGGCGGACTGTTTTCATTAATGTTTGGAATCGCTTGGCGTGCCTCATATGCCGCTCGATCTTTTTTCGGATGACCAATC
ATTCTTCGTAGCATGCCTCTTAAATCGACACGTTCTTCATACATTAAATCAATATCTGAGACACCACCCCAGATTGTATAACTTGTTACTGGTA
AGTCTTGAAATGTCAACAATCCTTGTAAACCACCTCGCGAAAAACCAACCATGTGGATAAATGCATGTGGATATTTATCATGTAGCAATCTT
AATAGTTGCGTCACATCATTTAAATCGCCACGATAAAATTCGTCTTTACCTTCACTCCCATTGTTACCTCGGTAGTATGGCCCAATCACTAAA
GTTTGACTATCTGAAAATTGCATTAATCTACCTGCGCGCACACGTCCTACTTGACCTTTGCCACCTCGCAAATATACTACAATGCGATTTACTT
CATGATGTGGTGTCATCATTAAAGCTTTTACTTGTAAGTCATCTGACAAATATGTAATTTCTTCGAATTGATGCGTAAAAGATTCAATTGGCA
TTCGTTTACGTTTGATAAAATCCAA

Gene: rppH (8-oxo-dGTPase (antimutator))

Position: 1861908 to 1862387, length: 480 nt, orientation: REVERSE

Perfect match to: (11819-97-CP003194-[1912289:1912768:r], allele observed in CC80)

Sequence:

TTACGTTTGATAAAATCCAAGTGATTGCACCCTCTCTACGCATTTTAAAATGGTACTATCTTGCAGTAAGAAACTCCGTTGTGCGAGTTCAAT
ATCATTGATACAGTTAAACAACACTGGCCCTGCTGTTTCTAAATAATCGTTCTTGCTTACCAATGATTCAACTTCGATAAAATATACATCTTTT
ACAAAATCAGTTTGATCATGTGTTTCAATGGTATATTGTGCTATGTAATGAATATTTTTAACTTTGGCGCCTGTTTCTTCATATAATTCACGTG
TAACTGCTTCAGCACTACTTTCCCCGCGTTCCCTTTTACCACCAGGAAATTCAATCCCCCGTAAATTATGTTTGGTAAAAAGCAATTGATTTTT
AAACGTTGGAATAGCTAGCACATGATTGCCATCTGCTATCTCATTATCCTTTTTAAATGTCAAATTAACTTGACGATTATCTTTATCCCTAAAC
TTCACGCGCAT

Gene: ytjA (membrane protein insertion efficiency factor)

Position: 1862447 to 1862704, length: 258 nt, orientation: FORWARD

Perfect match to: (RF122-AJ938182-[1793726:1793983], highly conserved allele)

Sequence:

ATGAAAAAGATATTCTTGGCGATGATTCATTTTTATCAACGTTTCATTTCGCCACTCACTCCACCAACTTGTCGTTTTTATCCAACATGTTCAG
AGTACACTAGAGAAGCGATTCAATACCACGGTGCTTTCAAAGGCCTTTATTTAGGCATCCGTCGTATTTTAAAATGTCATCCGCTTCATAAA
GGTGGCTTTGACCCTGTTCCGTTAAAAAAAGACAAGTCAGCAAGCAAGCATTCACATAAACATAACCATTAA

Gene: menC (O-succinylbenzoic acid synthetase)

Position: 1862701 to 1863702, length: 1002 nt, orientation: REVERSE

Perfect match to: (11819-97-CP003194-[1913082:1914083:r], allele observed in CC80+CC1)

Sequence:

TTAATATGGTTGTAATTGAGTTATATCCACTAAAGGGGGGCGAAATTCGAGTCGCCCCTCTTTTAATATGCCTGAATGCGCCACCACATCTT
GATCAAAATAATAACCTGCTGGTGTAACATCTCCTGGATAATCACCTTTACGAGCAAGCATCGCTGTAAAATAACGGCTTAAACCATATTCG
TACATGCCGCCAATAACCACTTTTACACCATGACTTTTCAAAGTATCAATTGCCGTTTGCACTTTATCAATGCCACCTAGACGAAATGGTTTTA
ATACAACAACTTTCACATTGTATAATTCTATCAAATTAATTATGTCCAACAACGATGTTGCCTTTTCATCAAGGGCTATTGGAGGTATTGTTCC
ATCCACTACGTCATCAAGCATGGAGATATCTTTAAATGGCTCTTCGATATAAAGAACCTGTTCACGCGCTAATAACTGTAACTGTGTGAAAT
CTTGACGATCCAAGGACTCATTTGCATCTATAACCAATTGAAAGTGAAAGTCTAATTCCCGTAACACTCTAATTTGATGCATGATTTGAGGC
GTCCATTTTAATTTAATTCTGGTCGGCTTTGTTGCTTTCAATGACTCTAGTTGTTTATTTGATAAGCCGCTCGCTGTCGCTCCATATGCTACTG
AAAATGAAGGCAGTACATGAAACATTTGATACAATGCCATGACAATAGTTGCCCCTTGCAGCAGGCGTATTTTCCAATGAATCTACTAATTTT
AGTGCTGCTTCATACGTTTCAAATGATTTATTTCTATTATCTTCGAACCATTGCTCAATTACATGTTTCACTGAAGCAATTGTTTCATGATCAT
ACCAATCTGTTTGAAAAGCGTTACATTCCCCGAAATATGCATTTCCTTTATCATCAATCAATTCGATAAACAAACAATCACGATGTGTTAAAG
TGACTTTCGGTGTTACAATTTGTGACTTAAATGGCTCACTATATTTATAAAAATGCAAAGCTGTCAACTTCAT

Gene: menE (O-succinylbenzoic acid CoA ligase)

Position: 1863707 to 1865185, length: 1479 nt, orientation: REVERSE

Perfect match to: (11819-97-CP003194-[1914088:1915566:r], allele observed in CC80+CC1+CC479)

Sequence:

TCATCCTCTATACAACTTATTTCTTTGTAATTTACCTGTTGATGTATAAGGTAAAGTATCAACCTTTTCAAAGTGTTTCGGTACTTTATATTTCG
CTAAATGTTGTGATAAATATGCAATCAATTGTGCCTTTGAAATGTCACTTTCACTGACAAAATATAATTTAGGCACTTGTCCCCAAGTATCAT
CAGGATGCCCTACACATACTGCGTCACTGATACCTGGAAATTGCTTCGCTACCGTTTCAATTTGATATGGATAAATATTTTCACCGCCACTAA
TAATTAAATCTTTACGTCGGTCATAAATCATGACATAACCTTCATGATCTATTTCAGCAATGTCACCCGTATTAAAATAACCATTTTCAAACGT
ACCCGTTAAATCTGTTGGATACAAATATCCATTCATCACATTGGCACCTTTAATCATTAATTCTCCATGACCTTCTTTATTAGGATTTTTAATTT
TAACGTCTACATTGGCACTTGGCATCCCTACAGTGTCAGGACGTGCATGCAACATTTCCGGTGTTGCTGTTAAAAATTGTGAACATGTCTCA
GTCATACCAAATGAATTATAAATTGGCAGGTTATATTGTAATGCCGTCTCTATCAAAGTGGCAGATAATTTAGCACCGCCAAGTAATATTTTT
TGCAAATTATAAGGTTCATGTAAACCTTGTTGCATAAGCCAATTTAAAGTTTGTGGCACAAGCGAAATGTGCGTGATGCGTTCATTTTTAAT
TATCGTTAAAATTTGTTCGGCATTGAATTTATCAACAATGCGCACAGTAAACCCTTCAATAACAGCTCTTAAAAGTACACTGAGACCCGAAA
TATGATAAATCGGCAAGACAGATAGCCAATTAGTATCACGATCAAATCCCAAGCTCTCTTTACATCCGATTGCACTGGCATAATGATTACGA
AACGTTTGCGGCACCGCTTTTTGAGGGCCAGTTGTCCCTGATGTAAACATAATCGATGCAATGTCATCTAAATTAAATGAAGTATTTAATAT
GTTGGACGGCGACTCTTTCGGCACCACAGTTTCATTCGATGTATCATATTGGATATCCATTGTGTTGTCCAACAAACCGTTCATTGTAATATC
CGTTCCAGCGAATTCAATATCATCCAGCGATACAATTTGAAACCCTCGCAATTCCAGTGGCAAGGTACAAAAAATCAATTGTACATCGATTG
ACCTCATCTGATTCTTCATCTCATTAGGTGTCAACCTTGTATTAATCATCGCAATTTCAATATTTGCCAACCAACAAGCATGTATTAAAATGAT
CGATTGAATCGAATTATCTATGTATAGCCCGACACGAGATTGTTGATAAGCCTTGAGTCTTTTAGCCAATAGACTCGCTTCACAGTATAAATT
TTGATAAGTATAAGATTCTTGACCGTCTGTTATCGCAATATGATGTCCATTTTGTTGTGCTTGTTTATATAACCAAAAGTCCAT

Gene: Q5HEY1 (lipoprotein)

Position: 1865344 to 1865826, length: 483 nt, orientation: REVERSE

Perfect match to: (MW2-BA000033-[1887133:1887615:r], allele observed in CC1+CC9+CC80)

Sequence:

TCAGTTATTTTTATTTAATTTTAGTGTCATTCTGTCATTTTGATGTGGTGATTTACCCATTGTTGCCACATCATCTGCAATGTCAATTGGTATAC
GATTCATGTCTTGTAATACACTTAAATGGAATACTTCATCATCTAAATTTTCAATGAGATATACATAATATGTTATCTTATCCTTTTTATATTTT
AACGTTTTCCAAAAGTCCGACTTGCAATTCAATACATTATCCGGAATATATTCAATAAATAAGTAACGTTTGCTGCCTACTTTGTCTACGAAA
TATTTTACAGTGCCTTTTTCTATACCTCTTATATGTGCATAGTCTGCTGAAAAGTAAATACTACCTATTGTTTCATTATGTTGTTGTATTTCAAA
TCGTTGGCCTACTATTTTATTATTTGTGCCACAACTACTTAAAAAAATCAGTAGATATAGCATTAAACATATTTTCATCCCCTTGAATTTTAAA
GACTTTTTCAA

Gene: Q5HEY0 (putative calcium binding protein)

Position: 1866102 to 1866752, length: 651 nt, orientation: FORWARD

Perfect match to: (MW2-BA000033-[1887891:1888541], allele observed in CC1+CC9+CC80)

Sequence:

ATGAAAATAACATATAAATATAGAGGAGATTTACCTTTGAATACAGAGAACAACAAGAATCAAAACCGATCTGTTAAAAATTCTGAAAGAC
GTGGCATGTTAAAAGGATGCGGCGGTTGCCTTATTTCTTTCATTTTATTAATAATCTTATTATCAGCCTGTTCAATGATGTTTAGTAATAATG
ACAATTCCACTAGTAATCAATCATCAAAAACGCAATTAACTCAAAAAGATGAAAATAAAAATGAAGATAAGCCTGAGGAAAAATCAGAAAC
AGCAACAGATGAGGATTTACAATCAACCGAAGAAGTACCCGCAAATGAAAATACTGAAAATAATCAACATGAAATTGATGAAATAACAAC
AAAAGATCAATCAGATGATGAAATTAACACACCAAACGTTGCAGAAGATAAATCACAAGACGACTTGAAAGATGATTTAAAAGAAAAGCA
ACAATCAAGTAACCATCATCAATCCACGCAACCTAAGACCTCACCATCAACTGAAACAAACACGCAACAATCATTTGCTAATTGTAAGCAAC
TTAGACAAGTATATCCGAATGGTGTCACTGCCGATCATCCAGCATATCGACCACATTTAGATAGAGATAAAGATAAACGTGCATGTGAACC
TGATAAATATTAA

Gene: DUF4352 (immunoprotective extracellular protein)

Position: 1866833 to 1867828, length: 996 nt, orientation: FORWARD

Sequence:

ATGAGCAATCAATTCAAAAGCGAAGAAGAGCGAAGACAATGGGAACAATTCCAAGCTTTCCAAAATCAACAAAACCAACAGAACCAGCAA
TACGGACAAAAGAAATCTAAAAAAGGATGGTTCTGGGGCTGTGGTGGTTGTCTAGTATTATTTATTTTAATTATCATCGGTATTTCAGCTTG
TACAGCTGGTATTACAGGTAACTTTGGCGGAAATAGTTCTAAAGAAACGAACAAAACCCATAAAATTGGAGAGACTGTAAAAAATGGTGA
TCTTGAAGTCACAGTTAATTCAGTTGAAACTATGAAATCAGTTGGTCCATCTATCGCGCCAACAAATGCTAAAGGTACATTTGTCGTTGCTG
ATGTGACGATTAAAAACAAAGGTAAAGAAGCGTTAACAATTGATAGTTCAATGTTTAAACTGAAATCTGGTGATAAAACATTTGAAGCAGA
TAATACAGGCTCAATGTCTGCTAATCAAAATGACAACGGTAGTATAGAAAATTCATTTTTCTTACAGCGTATAAATCCTGATAGCACTGCTCA
AGGTAAAATTGTTTTCGATGTGTCAGAAAACATAGCCAACGCAAAAGATAAAAAATTAGAAGTTATTTCTAGTTTATTTAGCGTCAAGAAG
ATTACATTTGATTTATCCGATGCTAAAAAAACATCAAAAGCTAAAAAAGACAAGCAAGATACAGAAGTAGCTGCTGCGAGTTCAAATAGCG
ATAATGTAAGTTATGAAGCTTCGGCTACTACACCTGCTACAACTTCTAGTGCAAATAATGATTCTGAAGAAAATGAACAGTCTAGTAAAGAT
GAAGATAAGCAGAATGCGTCTAAAAGTGATAAATCTAGTGTAGAAAAAAGTGAATCTAATGAAGAAACTGCTACTGTAGAATCAGCACCT
CAAAGCAAACCTGTTACAAGTGAAGCACCACCTAACCAAAATAATCACAACGAAGATAGCATGTACGACGCTTCAACAGAATAA

Gene: Q5HEX8 (putative lipoprotein)

Position: 1867902 to 1868528, length: 627 nt, orientation: FORWARD

Perfect match to: (MW2-BA000033-[1889691:1890317], allele observed in CC1+CC80)

Sequence:

ATGAAATTCAAAGCTATCGTTGCCATTGCATTATCATTGTCACTATTAACTGCTTGCGGTGCTAATCAACATAAAGAAAATAATAGTAAATCA
AATGACACTAATAAAAAGACGCAACAAACTGACAACACTACACAGTCAAATACAGACAAGCAAATGACACCACAAGAAGCCGAAGATATC
GTTCGAAACGATTACAAAGCAAGAGGTGCTAACGAAAATCAAACATTAAATTATAAAACAAATCTTGAACGAAGTAATGAACATGAATATT
ATGTTGAACATCTAGTCCGCGATGCAGTTGGCACACCTTTAAAACGTTGCGCTATTGTTAATCGACACAATGGTACGATTATTAATATTTTTG

ATGATATGTCAGAAAAAGATAAAGAAGAATTTGAAGCATTTAAAAAGAGAAGCCCTAAATACAACCCAGGTATGAGTGATCATGATGAAA
CAGATGGTGAGTCAGAAGACATTCAACATCATGACAATGATAATAACAAAGCAATTCAAAATGACATACCAGATCAAAAAGTCGATGATAA
AAGTGATAAAAATGCTGTTAATAAAGAAGAAAAACACGATAACGGTACAAATAATTCTGAAGAAACTAAAGTTAAATAA

Gene: Q5HEX7 (putative protein)

Position: 1868569 to 1868913, length: 345 nt, orientation: FORWARD

Perfect match to: (MW2-BA000033-[1890358:1890702], allele observed in CC1+CC80)

Sequence:

ATGATAACATTTGAAAATATACAACAACTTGAAAAATACACCTTAATGACTATGCATGGTCTTTTTAATCAACTTAAACTCGGTATTATTTCA
ATCGACAATGCAGAGCATACGCTCTTTACACCTTATATGATGGAAACACTCTCTTCCCTAGGCATGAAAGACAGCATTGTCGATTTAATTCAT
AAAGGGACTGAATTAGAAGACTTTGCAGCATTTAATTTATCAATTGAAGATACAGTTACAGTCTGTTTACAAAGAACTGAAGAACTATTAAA
TCAATACAAAAATGTGGAATTCAATGACAAAATATTAATCAATTGGCGTATTATGCAAAAGTAATAG

Gene: A5ITW8 (putative protein)

Position: 1869011 to 1869583, length: 573 nt, orientation: FORWARD

Perfect match to: (MW2-BA000033-[1890800:1891372], allele observed in CC1)

Sequence:

GTGGAGAAAAATGAATATATAGCTAAATATAATGAATATAGTCAATTATTAGACGCTACATACTCGCAAGCCGTAGCATACCTTTTAAATAA
ATATGGCGCTGTAACCGATGACTATTATAAGGAAAAATCATACACGCGATTTTTAAATGGAGAAATCAAAAGTATTACAAAAGGAAAATAC
ACTAGAGCTGGTGAAGGATTATATTGTCATCACATAAGCGAAGACAAATTCCAAAACTTATCTGACCTAAGATTCATTTCCGAATTTAAGTA
CTCATACAACTATCAAAAGAAAGAAAACTTAGTTTACTGCGATTTAATCGAGCATTTAATTTTACATGCAATTATTACAAAAGAATCCAATGG
CCAATTTGGTGTGGCTGGATTATGTCAAATGATCAAACCAACAGTCATTGATTGGTACATTAGCGAATATAATCCAAAACCAGCATGGATGC
AAGCCACCAAAGCACGTGCCTATTTGCCTAGAATATTAGTAGAGAAACTACTCATTAAAATTGACGATATGTTAAAAGAAATAGAAATATAT
GATTTCCTTGAGTCTAGATAA

Gene: Q6GFQ3 (putative protein)

Position: 1869732 to 1869850, length: 119 nt, orientation: TRNC-RVRS (no start codon)

Perfect match to: (MW2-BA000033-[1891521:1891559:r], highly conserved allele)

Sequence:

TTAGTTTTCTTTTGGTTTTAATAAATCGACTAGATTTTCACAATATTTATCAAATATGTATTCCTAAATTATACAGCCTTAATCCAGCAGCTACT
TTCGAAACTTCCAACTTAGTTGAT

Gene: DUF955-L1 (putative bacteriophagal protein)

Position: 1869781 to 1870338, length: 558 nt, orientation: REVERSE

Perfect match to: (11819-97-CP003194-[1920162:1920719:r], allele observed in CC80+CC1+CC6+CC80)

Sequence:

TCAAATATGTATTCCTAAATTATACAGCCTTAATCCAGCAGCTACTTTCGAAACTTCCAACTTAGTTGATATAAGGTTCAATAGTTTGTTTCAT
TCTTTTTCAGATAAACCAGAACTTAAATTGATATTATTGACTTCATAAAAATTATAGACTAATGCCTCTATTTGCTTTTTAGGCATAAGTAAGT
CGACTGAAAACTGATTTACGTCGCTTTCATAAATCATTTCATGTAAATTCTTTAGACTATTATCGTTGTTATCTTTAATTTTGTATAAATAACG
GCCGAGTTCACGAGCTATTGCAAATCTTGTATTATTAATCGAGTGATTATTATTGATATAAATCGTTCTTCCACTTAAATAACCCGAAGTATT
ACCCTCCATTTTAATATATTTAACTTTTAAATTAAGTTGAAATAATAGCTTGTCTATGTCAATAGCAAAGTGTTCAGAAGTAATAAAAAGTTG
ATCCATTTTGTCCTTAACTAATGGCTGAATTAATTGAACTATTTCTGGTTCTAAAATATCTTCATAATGAACTTTCTCAATAACTTTCAA

Gene: DUF955 (putative bacteriophagal protein)

Position: 1869781 to 1870338, length: 558 nt, orientation: REVERSE

Sequence:

TCAAATATGTATTCCTAAATTATACAGCCTTAATCCAGCAGCTACTTTCGAAACTTCCAACTTAGTTGATATAAGGTTCAATAGTTTGTTTCAT
TCTTTTTCAGATAAACCAGAACTTAAATTGATATTATTGACTTCATAAAAATTATAGACTAATGCCTCTATTTGCTTTTTAGGCATAAGTAAGT
CGACTGAAAACTGATTTACGTCGCTTTCATAAATCATTTCATGTAAATTCTTTAGACTATTATCGTTGTTATCTTTAATTTTGTATAAATAACG
GCCGAGTTCACGAGCTATTGCAAATCTTGTATTATTAATCGAGTGATTATTATTGATATAAATCGTTCTTCCACTTAAATAACCCGAAGTATT
ACCCTCCATTTTAATATATTTAACTTTTAAATTAAGTTGAAATAATAGCTTGTCTATGTCAATAGCAAAGTGTTCAGAAGTAATAAAAAGTTG
ATCCATTTTGTCCTTAACTAATGGCTGAATTAATTGAACTATTTCTGGTTCTAAAATATCTTCATAATGAACTTTCTCAATAACTTTCAA

Gene: DUF1433-var4 (DUF1433 family protein)

Position: 1870524 to 1870970, length: 447 nt, orientation: REVERSE

Perfect match to: (11819-97-CP003194-[1920905:1921351:r], allele observed in CC80+CC361)

Sequence:

CTAATCCAACGATCCCGAAAAGAAGAACAACGGCGGTTCTGCTTCATATTCACTTTTATCCAAGTGTTCATTTTTAATTATTTGATCGACGGT
TTTCCAATTTTTTACTTCTTGCTCTTTTAATAGTTTCCCAAGTTTTTCTTCGTCATACCCTATATCTCTTTGAAATTGCTTTCTACCACCTACATTA
ATAAAAGCTTGAAATTCATAGCCTTTATTGTTATTTATATAACCACCTATAAAATAACTATCCATTGGCGACTTTTTAAATCTCGTTACTTTAA
GTGAGTTATAATGATTCAGGTTATACTTAAAAAATAAATCTATTCTGTTTTCTTGCGTCTCAATGTAATGGTCTTTTTTTTGTTTATTATGCGC
ATATGCAATAACTATTATAGCTATTAAAATTACACCAACTATTATTACGATATATTTCTTTTTCATCAC

Gene: DUF1433-var17 (DUF1433 family protein)

Position: 1870967 to 1871410, length: 444 nt, orientation: REVERSE

Perfect match to: (11819-97-CP003194-[1921348:1921791:r], allele observed in CC80+CC88+CC361+CC2970)

Sequence:

TCACTCTAATCTCCCAGAAAAGAAGAATAACGGTGGATCGGCTTCATAATCACTTTTGTCCAAGTGTTCTTTTTTTATAATGTCTGTAGATTTT
AATTCATTTTTATGGTCTTTTTCTTTAAATAATTTACCAAATGTCTTATCATCGTATCCTATGCTATCTTCAAATTGATGATTGTCAGTTGCAGA
GATGAGAACTTTAAAATCATAATTTTTGTTGTGATTAACATATCCATCCACAATATAGCCACCCATAGGCGTCTTTTTAAAACTAGTAATTTTC
ATTGAATGATAATTATTAAGGTTATATTTAAAATATAAATCTATTCTCTTTTCTTGTATTTCAATGTAATGGTCTTTTTTGTTTTTATTGATTAA
ATAGCCAGTACTTATTAAGAGTGCTATAATTAAGGAAATTATAAATATTATTTTTTTTCTTTCCAT

Gene: Q6GFP5 (bontoxilysin)

Position: 1871453 to 1873063, length: 1611 nt, orientation: FORWARD

Perfect match to: (11819-97-CP003194-[1921834:1923444], allele observed in CC80+CC88+CC2970)

Sequence:

ATGAAATATACAGACAAAATCTATCATAAAGTATCTGACTTTTCTTATTCAAAGTTAAAAAAAGATAACACTTATAAAATTTTAGGCCAAGAT
TACAAAGTCATCGAAAAACGCGACGACACCCAGAATGGGTTTAGAGCATATGTTTTCGCTCCAGTAATCAAAGGTAAAGTTGATAAGAGTC
AACTTTTAATAGGTTACGCTGGCACTGACCCATATAGTCTAAATGATATTCGTACTGATTTACAACTTCCCATTCATCATAACACAAATAATCT
AAAAATTAATAATCATTATAAAGATTTAAAAAACTCAACTATTCACAAAAAATACAATACAATAAAAGATAAAAATGACCTTTTTAGTTTTTC
TTCTAATTTAAATAATAAAGCACTTTTCGGTGATCTATGGGCAGCTAAGTTTGCACCGACTCAAATGGACGAAAGTGATGCTTTTACTGAAT
CAGTTAAAAAGAAATATCCAGATAGTGAAATTCACCCATTAGGACATAGTTTAGGTGCGTTTTTAGCTCAATATAATTTAATAAAACATAAT
CTGGATAACGGTACAACGTTTGCAGCACCTAACGTATACCATTCATTTACTGGAGACTTTAAAAAAGACATTGATAATGGTGTCTATGATTC
AAAAATACGCAATATTGGCCATTTTGATGATCCTATTAATAATTTGAATTTTTTTAATCCTCGTATTGGCAAAAATATTACATCAATGCCACAT
TATGATGGATTTATAACTAACCTTCCTTTGATTGGTCAACACACACTTAAATCTTACAATGATTTCGATGCAGATGGTAATGTGAAGGAAAT
GCCAGAACTTGAACAATTTAATGATGACCAATCTCTTAATAATAGATTTCAATTATTTGACCCATTTGGTATAAAAAGCAGATTTTTCGACTC
TATTAAAATCGATAGTTCTATATTAAATAGAACAAGTGAAAATGATTTTGATCCTTTTGCTATTCTCAATAAAAAAGATAAATTATTCGAATC
AAATGACCTTGATATAGAAGGATTTAATAATAAACAAAAAGAAGATTTAAGCATTGATTTAAATCCTTTTGATGATGAATTACATGATGAGG

ACGAACCATTAATTCCCAAAAGAGGCGATAGTAAAAAATTAAAAGCAGAAAAGAAAATCTCTGTAAAAAAAGGTAAAGGTGGTTCCAAAA
ATACATGTAAAAAAGTAAAAATCCAGCCTGATGAAGTGCGTAATATCGCCAACACAATTCGTGATAGACTATTTTTATATAATCAAATCACT
AATGCATTGGATGAATATAAAGAAGAAACAATATCAGGGGCTAATCGCATTTTAGATAAATATCAATCGGAGCTTCTAAGCGGTTCACATG
AATTTATAACACCTTACGATCTTGAACAATATATGGAGATTTTAGCAGTTGAGGGAAGTTCAGGAAACTTCAAGTTTTATAATAGTGGTTTA
ATGGATGACTTAATTCAGGAATTAGATCAAAACAAAAAAGACTTGCTACAATTTGCTGAAAAACTAGAATTTGCTGCAAATAAATTCGAAG
AAAAAGATTTAGAAGAAAGCGATGTTTTTGGACTATTTAGTTAA

Gene: A0A0E1X646 (putative protein)

Position: 1873078 to 1873377, length: 300 nt, orientation: FORWARD

Perfect match to: (GR1-AJLX01000047-[17617:17916], allele observed in CC361+CC80+CC361)

Sequence:

ATGAGCTCAAAAGATGCAAAAAAAAGTCAAGAAGAAGCTAGATTACAACAATTAAAGCAAGAAATGCGTAATAGTACTGATAATTTGGTT
AGCAATGCTAAAAATAATGTAGCATTAAAATCTCAAACATTAAATGAAATCATTGAAACAATTAATAATTCTTCGCAAAATTTAGAAGGTAC
ATTTGAAGGAAAAGCAAGCGAAGCAGCCCAACAAAGTATAAATAAACTCAAAAACAATAATGAAAATCTTAAACAAAAATTTGAGACCCTA
TTAAATACAATGAAGATAAATGGATAA

Gene: hsdS (type I restriction-modification system site-specificity determinate)

Position: 1873504 to 1874712, length: 1209 nt, orientation: REVERSE

Perfect match to: (GR1-AJLX01000047-[18043:19251:r], allele observed in CC361+CC80)

Sequence:

TTATATAAACATTTTTTGAAGTAAAACCTGCTTACGTCGCTTAAGATACATTATTTTTTCTTGCTGCAATTCAATTAGTTTTTCATTTCTATTAA
AAAACTGCCCCACTTTTTCTTGTTCGTGATTGCTTGGCATATTCAGTGTTTGAGATTTGACAATTTCTGTATTTAAATTAACTTGACTACCTGG
TTGACCGTATTTACTCCATTTATCCTTGAAATATTCAAGCCAATAGTACATAAAGAATAAATTAAACTTAGGATTTAAAAATATTAAAAATCC
ATCATGAACTCCCGTTTTCACAAAATTCATTACAGGTTTTCCAATACTTGCCGCAATACTTAATAGTAAATGTGTTGTTACTAAAACCCTTGTT
TTTTCTTGACCTTCAATTGATAATTTTTGTTCCAAATGATAAATTTTCCCGTTTTGATTTGTAACATCAGAAATTCTTAGCCATCCTATGTCTGA
TTCTTTATTAAACCATTTAGGATCTTTAATAGGTCTAGGAGAAGCCCCTCGTACAATTTGTGATAATTCCCCTAATTGCTTCTCTTCCCAATCT
GGATAATCATTACCATTCTCATCTTTGAATCGCAATTCCTGTGAGAAAATTTTCTGCATATAGCCTTTTTTCTGTTGTTGAAGTAATTCGAGTT
TTTGTTCTTCTAATTCAATTTGTCGGTCGAGTTTGCTGAAGAATTGTCCTATTTTTTGTTGTTCACATAATTCTGGAAAATATACTCTTATATTT
TCTATTAACCCCCAATCAGCCCTTGGCATCTTAGAACCAGCCGATTTACTAGCAACATCAGAATATCGTTTTGTTTGTATAAAATAATATAGA
AATAAATTCAATAATTTATCTTCTTTCGTTGATTTCAAAACCCATATTTCTGATGAACACACTCCACTTTTTTTTGTAAAATAATACTTATTCAA
ATATGGTCTGAGCTTTCCATACAAAACATTTTGTGGATTGAATTTATTTTTTTGACTTGAAAATTCTTTTGAATTATAAATTTTAATTAATCGA
CCCGTATTTTGTTCAATACAATCCAATTCTATATCAATACTTGCATTCTCATTTTGAGGGTTAAATTTTTCACTCTTATTCGTTGCTAGATTTCC
AAAAATGTCTAAAGAATATTCGCCTTCAAACCCTGGGAACCTCAATTCTGGCACATTTTTCGTTTGTGTATTACTCAT

Gene: hsdM (type I restriction-modification system DNA methylase)

Position: 1874705 to 1876261, length: 1557 nt, orientation: REVERSE

Perfect match to: (HST-105-AZTH01000020-[652:2208], allele observed in CC88)

Sequence:

TTACTCATCTTTCAACACCCCAAGTTCTTTCAGGTATGCATTGATTTCTTGTTCAATTTCTGCGATTTCTTTGTCGATATTTTTCAAATCTTGTTG
GACTTGATCTAAATCAATTGGTGCTTCTTCTTCGAATGTATCGACATATCTCGGTATGTTTAGGTTGTAATCGTTATCGGCGATCTCTTGTAAT
GTCCCGCTGTAGCTATATTTATCAATCGTTTCTTTACGCTTATATGTGTCTATAATACGTTCGACTTGGGCATCGCTTAAATGGTTTTGATTTT
TTCCTTTTTCAAAATCATTGGATGCATCGATAAATAATACGTTATCGTCTTGTTGGCGACATTTTTTAAATACAAGGATACATGTTGGAATAC
TTGTCCCATAGAAAATATTGGCTGGCAAACCAATCACGGCTTCTAAGTAGTTCTTTTCTTCTATTAAATAGCGACGAATCACACCTTCTGCGG
CACCACGGAATAATACACCATGTGGGAGTACGACTGCCATGGTACCTTCATCGTCTAGGTAATGTACCATGTGTTGAATAAAGGCAAAGTC
TGCTTTGGATTTTGGCGCAAGCTTGCCGTAACCACTGAATCGTTCATCATTTTCAAATTTTGAATCTGCTGTCCATTTCGCACTGTATGGTGG
GTTCGCAATAACCGCATCAAATGTATTGCCTAAAAAGGCTGGATTTTCCAATGTGTCATCATTACGGATCTCGAAGTTCTCATAACGCACATC
ATGTAATAACATATTCATGCGTGCTAAGTTGTATGTAGTATTGTTACGTTCTTGTCCGAAATAACGATACACTTGCGTTTCTTTACCAACACG
TAACAGTAATGAACCTGAACCACATGTTGGATCATATACGTGACGTAATTTATCTTTACCGTCTGTGACAATCTTCGCCAGTATCTTAGATAC

TTGTTGTGGTGTATAAAACTCGCCTGCTTTTTACCCGCTGTCGCCGCAAAGCGCCCGATAAGGAATTCGTATGCATCACCTAACATATCAAT
TTCCATGTCACTGTGAACGAATGGTAAGTCGTCAAGATTAACCATAACTTTGGAAATTAACGCAGTACGTTCTTTGACATTGTTACCTAAAC
GTGTTGAACTCAAATCCATATCGCTGAACAGTCCGATAAAGTCATTTTCACTTTCTTCACCTAGTGTTGATGTTTCAACTTTACGAATTGCCGT
CGTCAGGTGTTCGATATCGAAATCTTGCGTTTCAATTTCACGAATCATCGCGCTGAATAAATCTTGTGGCTCAATGAAGTAACCGACTTGAT
CAATTAATTCTGCTTTTAAATCTTCACGATATTCTTCATCTGCCCATGCTTCTTGATACGTAATATCTTCACCTGCCAAGGCATCTGCATATTCT
TGTTCAGCTTTTTCAGATAAGAAGCGATAGAAAATCAAGCCTAAAATGTAATTACGGAATTCACTCGCATCCATGTTCCCTCTTAAATCATTC
GCAATCGACCATAATTTTTTATGTAATTCAGCTTGTTGCTGACGTTGTTTTTCAGTAATAGACAT

Gene: splF (serine protease F)

Position: 1876628 to 1877347, length: 720 nt, orientation: REVERSE

Perfect match to: (IS-157-AICH01000006-[5022:5742], allele observed in CC239+CC8+CC97)

Sequence:

TTATTTATCTAAATTATCTGTAATGAATTTCTTAATTTCAGGAGAGAAATAAACAGCGAATCCTCTTGTGCTTTCACCTGATGGCTTATTACCT
GCATAGATTACACCAATAGCTTCGTGTTTACTATTTAATATAGGTGAACCAGAGCTACCAGGCTGAATAATTGCATCCGATGTCACTATATTC
CCATTCACTGATAGTACTTTACCAGTTGATTCATACATTTGTAGTTTATTTCCATTAGGATTTGGATAACCAATGACTGATATAGGTTCATTTT
CTTTAGCTTCTGATGCTATATTAAATTTACTAGTGAAATCTTTGAATTTTCTACCTTTTGGTTGTGTTGATTTTTCTTCAACTTGTACAACCGCA
ATATCTTCTTTACCAGGATAATCTACAATCTTAGTAACTTTATAAAGTCCACCACCGTTATTATAAAAACCATTAGGATGTGCTTTGATTTCAT
CACCGACTTTCATGTGATAGGTAACATGTTTATTGGTAATGATTGTATGATTTCCAACTACAAATCCTGTTCCAGCGCCCATCCATGTAACAC
CACTGTATGGTGCAACATTTGTATTTGTAATTTGTTTAACAGTATTTTCGGCTTTGGCTGTTTGTTGAATACCTTCAACCATTGTTGTGCCGAC
ACCAGTTATTGATGTTAAAATCGTCAATGCTGCAATACTTTTGATGATTATATTTTTATTCAT

Gene: splD1 (serine protease D)

Position: 1877505 to 1878224, length: 720 nt, orientation: REVERSE

Perfect match to: (11819-97-CP003194-[1927886:1928605:r], allele observed in CC80)

Sequence:

TTATTTATCTAAATTATCTGCAATAAATTTCTTAATTTCAGGAGAGAAATAAACAGCAAATGACCTTGTACTTTCACCTGTTGGTTTATCACTA
GCATACATAACACCAATTGTTTCTCGCTTACTATTTAATATAGGTGAACCAGAGCTGCCAGGTTGGACAACCGCATCAGATGTCACTATATTT
CCATTCACTGATAGTACTTTACCAGTTGATTCATACATTTGTAGTTTATTTCCATTAGGATTTGGATAACCAATGACTGATATAGGTTCATTTT
CTTTAGCTTCTGATGCTATTTTAAATTTGCTAGTGAAATCTTTGAATTTTCTACCTTTTGGTTGTGTTGATTTTTCTTCAACTTGTACAACCGCA
ATATCTTCTTTTCCAGGATAATCTACAATTTTAGTAACTTTATAGAGTCCACCACCGTTATTATAAAAACCATTAGGATGTGCTTTGATTTCAT
CACCGACTTTCATGTGATAAGTAACATGTTTATTGGTAATGATTGTATGATTTCCAACTACAAAGCCTGTTCCTGCGCCCATCCATGTAACAC
CACTGTATGGTGCAACATTCGTATTGGTAATTAATTTCACACTATTTTCGGCTTTAGCCGTTTGTTGAATACCCTCAACCACTGTTGTGCCGAC
GCCAGTCACTGATGTTAAAATCGTCAATGCCGCAATACTTTTGATGATTATATTTTTATTCAT

Gene: splC (serine protease C)

Position: 1879222 to 1879941, length: 720 nt, orientation: REVERSE

Perfect match to: (CN1-CP003979-[1823521:1824240:r], allele observed in CC72+CC8+CC72+CC96)

Sequence:

TTAATGTTCAATGTGTTTTTGAATAAAATCTTTGATTTGAGGCGTAAAGTATACGGCACCATTATATTCAGAACCAATTTTTCCAATACCGCC
ATACACCACACCTATGACCTCATTGTTAGAATTTAGAACTGGTGATCCTGAATTCCCGGGTTCAATGTATGCATCAAAATTTAAAATATTGTC
TTTGATTCTTTTTATAGTTCCTGTAGATTCAAACTGTTTAAAACTATTTTGAGCAGGTAATGAGTAACCAATAACTTTAATTTTGTCATCAACT
TTAGCATCTTTCGCAAAATTGAATGCTTGGACATTTTCATTAAAATTAAAGCCTTTTGGTCCACGTTCGACTGCTTGTTCTTCAATATTCATGA
CAGAGATGTCTTCATCACCCGGATAATCAGAAATGCTTTTAATTTTATATATACCACCATTTCCTTTGTCACCGTTTGGATGGGCAGTAATTCT
ATCGCCAACTTTATAATCTTTTGATACATGTTTATTGGTGATAATTGTATTTTTTTCCAATTACAAAACCTGTCGCATCTTTAAATGAAACGACG
CCATTATATGGAAAAATATTTGTATCTTTAACTTGCGTAACATTCTTCTCTGCATTTGCTATTTGTTGTGTCTCTTCAACGACTGCAGCATTTAT
TCCAGTTACTGAGGTTAGAATGGCTAATGCTGCCATGCTTTTAATGACTATATTTTTATTCAT

Gene: splB (serine protease B)

Position: 1879999 to 1880721, length: 723 nt, orientation: REVERSE

Perfect match to: (11819-97-CP003194-[1930380:1931102:r], allele observed in CC80+CC96+CC1153)

Sequence:

TTATTTATCTATGTTTTCTGCAATGAATTTTTTAATTTCTGGTGTAAAGTAGACGCCATATGCATTTCTGTTATCATCATTTTTTACATCAGAAG
CAAAATGAATACCAACTAATTCGTTGTTGCTGTTTAATACAGGTGATCCAGAGTTTCCGCTTTCAGTATGCGCTGAATATACAATACTGCTAC
CTTCTACTGACATCACAGGGCCAGTTGACTCATATAAAACATATTTATTTTTGTATGGGTGTGGATAACCAATCACTTTAATTCGCTCACCAG
CTTTAGCCCCTGCCGCATATTTGAATGGCGTTACATTATCATTAAAATTAAAGCCTTTTGGTCCACGTTCTATTGCACGCTCTTCAACTTGAAT
GACTGATACATCTTCTTTACCTGGATAATTAATAATCTTTTTAATCGAATAAATACCACCATTACCTTTATCACTATTTGGATGTGCAGTAATA
CGATCGCCCACTTTGTAATTTTTCGACACATGTTTATTTGTTAAAATAGTATTCTTTCCAACTACAAATCCAGTTGCACTTTTAAAAGCAACTA
CACCAGTATATGGAAAAATATTAGTATCTTGAATTTTTGTGACATTATTTTCTGCTTTGGCAGTTTGTTGAACTTCCTCAACCAATGTTGTTCC
AATACCTGTTACAGATGTTAAAATTGTTAATGCTGCTAAACTCTTGATGACTACGTTTTTGTTCAT

Gene: splA (serine protease A)

Position: 1880846 to 1881553, length: 708 nt, orientation: REVERSE

Perfect match to: (KPL1845-AZJA01000018-[275219:275926:r], allele observed in CC96+CC5)

Sequence:

TTATTTTTCAATATTATTTTGAATAAATTCTTTTAATTGTGGTGTGAAATAAACACCGAAATTCTTTTCAGATTCATCTTTTCCACTACCTGCAT
ATAAAATACCAATCAGTTCATGTTTAGAATTCAATACAGGAGATCCTGAATTACCTGGTTGTGCATACGCATCAAATTCCATAAACGTTCCAC
TGATATGGTTAATCGTTCCTGTCGATTCAAACATTTTATATTTTGTTTGTGCACCCTTTGGATAACCAATAACAGAAATTCTATCTTTCGCTTTT
GCTCCGTCTGCAAATTTTGTATAACTAACGTTCTTATTAAAATTCAAACCTTCTGTACTTGTTTCATGAACATGAACTATCGCAAGGTCTTCTT
TTCCGGGATATTCTACAATGTCTTTAACGTCGTAGTTTCCTCCGCCTTTACCTTTACTCGAATGATGTGCTGATACTCTATTTTTAAAAATATC
ATTACTTTTAGCGATATGTTTGTTAGTTACGATTGTATTTTTACCAACAACTACACCAGTACCACCCGCAAATGCTACCACTGAATTGTATGG
TGCCTTAGTTGCATCGGTAATTTCTTTGACATTCTTTTCTGCTTTGGCAATTGAATGAGGCTGATTAGAAATATTTTCAGCGAAACCAAGAGA
TGTTAAAATAGTTAAAGCAGTTAAACCTTTAACCATTACATTTTTATTCAT

Gene: A6QHZ7 (putative protein)

Position: 1882013 to 1882204, length: 192 nt, orientation: FORWARD

Perfect match to: (N315-BA000018-[1865577:1865768], highly conserved allele)

Sequence:

ATGGAATTTTTTTCGAGATATGTTATTTCGGGGATCAGATTTTGCGATTTCGCCTTGGATAGAGTAGTTGCGATTTGTAAATACGTTTTTCAG
GTTCGATTCAATTTCGTTGTTAAGATAATATGGATTAGTTTCACTGACATGTTGATGCATGTCTTTTTTGTTCTGATAAAGTATAAAATGTTTA
CTTAA

Gene: Q2FXC0-ear2 (putative protein)

Position: 1882517 to 1883080, length: 564 nt, orientation: FORWARD

Perfect match to: (11819-97-CP003194-[1932898:1933461], allele observed in CC80)

Sequence:

ATGAATACAAAATTTTTAGGTAAAACATTAGTAGCAAGTGCTTTAGTATTAACAACATTAGGAACAGGCTTACATTCTTCATACTTAGGATTA
GATACAAATAAAGTTGTTAAAACAGCTAAAGCAGAAGAAAAAACAAATACAGTGATTTGGCAAGGAGTTAAAAAGTCACTAATAGATTCA
AATATAATTAGTGGCAACCAAGGAGAAACCGTAAAAATATCTTTTTTACTAAATAATGGACAATCCACAAGTATAGAATCTACTGCAAATCA
TGACAGTTTTACTAATACTTCAAATAATTTTTCTAAACTCACTCAAATTGATATAAAAAAAGAAAACATTCCTGGTGATGATTTTAATACGAC
TGTAGACGCAAATAATACATGGAAAAACTTAACTTCCAGCCTAGTATCTAATGGATTAGCAGAGAAAGGTCAAACAGTTAGTATTTACAGC
AAAGATACAAGTACAGTAATTACCGCTAAAATTGGTGAAGACTTCAATGATCATAAAGGCTTTTACTTAAATAAAAGAGACATCAATAAAAT
CACAATTGAATAA

Gene: sprB (small pathogenicity island RNA B)

Position: 1883082 to 1883552, length: 471 nt

Sequence:

CCTTAAATTCAAATTTCCTTAATTTATAAAACATTAATAACTAACCCTTTCTTACAAACCACCTAGCCCATAATATATTAAAACGTCATTTTTAT
TTCAAATAGCGGAAAATAAAAAAACCATTCCGCATTAACTTTGGCAAGTTGGAATGGTTAAAAAAATATTTTAGAAGCCAGCGTTTGGCTT
GCTTTGACGACATGTTCGCGCATGTCGTCTTGTTTTTACATTTATATAATAACACGCAATTCAATAAAATGTCTAATCAATAAACCTCAATTAC
ATTCAAATTCAAAAACTTTTCACCTAATATTCATCGCTTAAATAAACATTACGTTTAATTAACCAGACATTGTCAATCGCGTCTAATACCCCTG
CTGTCCGAAAACAAAATTATTTTGTATTCATATTTAACTGTTCACTTTCCTCCCTTTTCAACGCAACTAAATTTCTTTTACCGTTAGCGCCCCAA
TC

Gene: epiG (lantibiotic ABC transporter, transmembrane permease)

Position: 1883568 to 1884266, length: 699 nt, orientation: REVERSE

Perfect match to: (11819-97-CP003194-[1933949:1934647:r], allele observed in CC80)

Sequence:

TTAATCTTTAATTGTTCTACCGCTCCATTTATTGAATCCTTTAAAGAGTAAAACTGCCAATAGCAACGTGATAATAATATAGATTACCATTGTC
ATCATCACTGGTATATTTCCTTCAATATACATATATACATAACGCGTAGCATATGTTATTGGTAAATAGAACCACGAATGATCTCCAAGCACT
TCTAATCCAAAATAAACGTTAAATATAAACATTAAAACGCCGACAACAATAGCCAATACATCTTTAATGAAAATACTAAAAATAAAAAGTAG
CAGTAATATAATTACATTGAAAAACAATGATACGCCTATAAACATAAGTGTTATTTTCATATCATGTGAATGCCACAATAAATTAATTGATAC
TAATAGAATACATACGGCTGTAATAGTATAAGTAAAAATCATTGATGCAATTAACCAATTTAACCTATTAGCTTTTCCTAATATATGATTAAA
GTGACCAATATTTTCTTCAAAATTGATGACTTGATAGACGTTTATAGAAATTAATAGTGATGTAATTGCATTAAAACTCGCTGTAAACAAACT
TATTTGTCGACCATTCCATAAATTTACGTTTAAATACCAATTTATAAATAATATAAACAATATGGTTACAATAATGGGTACAAATGTGAGCGC
TTGCTTAGAAAATTTCAATTTACATGACTTTAATTCGTTAATTATCAT

Gene: epiE (lantibiotic ABC transporter, transmembrane permease)

Position: 1884263 to 1885024, length: 762 nt, orientation: REVERSE

Perfect match to: (ATCC51811-ADVP01000003-[52077:52838], allele observed in CC1+CC8+CC25)

Sequence:

TCATTTTAATCGCCAACTTTTCTTATTATTTAAAAATGTTACTATCGCGAAAATAATGATGCTAACAATAATCGAAATATATAAGGCATTGAG
ATCATTAAAGTATTTTGAATTATGGTTTACAACTAAACCATTAGGTAGTATACCAAGCGTAATAAGCGGGATTCTCTGCATTATCCCCCACGG
TAACACCCAAAATAAACTCTTCAATGTTAAAAACAAGACACAAATGACTGATAATACTAAGTTTATTAATATTGATACGAACACACCAAAGT
ATCGAGTTAATAAAAAGTTGAGCGGTATCAATGGTAGAGATACTACATACATCAACAATATTGTCACCAATAACAACATAGCATTAACCGG
ATGTGGATTAATAATTAGGTCACCTATATAAGCAATAATAAATACTAAAAAGCAATGTACCAAAAATGCTATTGATAAAATGAAAATCTTTG
CTCTTATTTCTTTTGTAATCGACCAATTATTACTTAAGTAATAATTAAATGATTTATTTCTCATTTCAATTTTAAATAACGAATTACAAGCCATA
CATAATACAATCGGGATGAAAGCAATTGGCCAAATATTAAATAGTAAAGTTATATATGGTGACACACTATTCGCTGTTCCCGTATTACTTTT
GGCGAATAAGACTGTGAAAATAGCAAAACAAAGAAATACCAGCGGACTTATTAATAAAACATACATACTGAAAGAATTTTTAAATTTGATA
CCTTCTATCTTTAAATGTATCAT

Gene: epiF (lantibiotic ABC transporter, ATP-binding protein)

Position: 1885021 to 1885713, length: 693 nt, orientation: REVERSE

Perfect match to: (394_SAUR-JVIV01000076-[6428:7120:r], allele observed in CC6+CC8)

Sequence:

TCATTTGTAATCTCCTTTCGTTATTTTGAAGAATATCTCTTCTAAGTTTTCATCTTTGTTATTTCTTTGCTGATATTTTAGCTCACCCTCATGAAT
AATACCGATATGATCTGCTAAAACTTGGATTTCAGATAAAATGTGACTTGAAATAATAATACTAGTACCTTGTTCTGTTAATAATTTTAGAAG
TTCTCTAAGTTCTTGGATTCCATATGGGTCTAAACCATTAGATGGTTCGTCTAATACTAAAATTTCTGGCTTTTTAATTAATGCCATTGCAATT

CCAAGTCTTTGTTTCATACCTAAAGAAAAGTCCTTAACCTTTTTCTTTTTATCGACATTTAAATTGACTAAGCTTAAAACACTATTAATTTCGCT
GAAATCAACGCTTTCATTTAAACACACAATTTTCAAATTATCTTGTGCACTTAAATGATTATATGTCGCAGGTGTTTCAATAAGCGAACCAAC
TTTGTGCAAATCATTTCGTGTCATTGGTCTATTATCTAATTTAATTTCCCCTGAATCTTGTTGAAGTATGCCACATATAATTTTCATAAGTGTC
GTTTTACCGGCTCCATTAATACCAAGTAATCCATAAACTTCGTTATTTTCTAAAGTGATATTAATATGCTTTAATACATCTTGATTTTTAAACC
GCTTCGAAATGTTTTCAGTAACTAATTTTGCCAT

Gene: epiP (autocleaving serine protease)

Position: 1885736 to 1887109, length: 1374 nt, orientation: REVERSE

Perfect match to: (CIG2018-AHVV01000018-[44597:45970], allele observed in CC8+CC25)

Sequence:

TTAACTTGCTTTTTGATTTGCTACATTTAATGCTTTATACACATCAAGCTCACCATGCCCATATCTACTAAATGGTTTATTATTCTTAGATGTCC
CATGCTGATATAACAATTCAATCGCTTTATCTGGATGTTTTTCAAGATGATATTTATCAATGATTAAAGCTAGTGCTCCCGAAACTTTAGGTG
TGGCTAATGAAGTTCCAGCTTGATAAATATATCTTCCGTTATTGGCAGTAGTTAAAATGTTCTCCTTATGCATATACCCTTCATTCATCCATTT
ATCCACACCGAATTGATTTAAATAAGCAAATGATCCTCCGGGCGCAGCAATATCTGTATAATTCATACCAAAATTGGAAAACTCAGATAGAT
TACTCTTTTGATCTGTAGATCCTACTGTAACGACATTGTCCATAGATGCAGGAACATCTTTCACTTCGCCATTACCTTGATATTCACGCTGTAA
TTTTAGTTTCTGTTTGTCATTGACATCAATACCATCATTACCAGCTGCAGCAACAACGATAGATTTTTTCTTCTTGGCGTAATTGATTGCTTTCT
GTAACGCATCGTATTCTACTTTTTCATCTTTTCTAAATGTTTGATGGTCATTTTTGTCCAAAATAGTATAACTACCAACACTAATATTAATGACT
TGATTTCCATCATTTGCAGCTTGAACAATCGCTTTTGATACCCAAAGCAGTTCTGTTTTTTTACTACCAAACACGCGATACATTGTAAATTTGT
TATTCGGTGCAACACCTATTAACTTACCATTAGCACTCGTTTGACCCGACACCATCGTGCCATGTCCTTTCCTATCATTGACATCGTGAACATC
ACCTGTTTCCTCCGGTTCAGTACCTCTAAAACCGTTTAAAGGTACTAAATTTTTAGAATCAGTCGAGAAATTATTTTTCAAATCGTCATGGTTT
TTCATCACACCTGTATCTATGATTGCTATTTTTGTGTTAGCCTGTTTTGGCAAATCATCATACGATGCACCATTATTGGTTATTTTATTCATATC
CCATTGTCTTGAAAATAATGACTCATTTGATGTTCTGTCTATTGTTTTCTCGCTAGTAATACAAGTTGAACATGTGGCATTGATATATTTAATA
TCATTTTTATAGTTTGCTAAAGCATTAGCATGCATTTTCGTCATTTTAATCTGTGCCACATGTAATTCCGGTATATTATAGACAACGTTTAAGG
ACTTCTTTTTAACTAACTTGTTAAATGTTGCTGTATTTTTGTATTCAACACTGTAATATAGTTCTTCTGACGCTGATGCATTACTCATTGTAATA
GATATTAAAAGACTCAATATTATTAAACTAATAATAGCGCGCTTTATGATTTTCAT

Gene: epiD (lantibiotic epidermin biosynthesis protein EpiD)

Position: 1887119 to 1887637, length: 519 nt, orientation: REVERSE

Perfect match to: (MW2-BA000033-[1912385:1912903:r], highly conserved allele)

Sequence:

TCAAATGAATTCCAGAACTTTATATGGTTCAGGTGCGACAACATTCTTTTTAAATGTTTTTGACGCTAATTCATAACTTTCTGAAATATTTGCT
GGATATATTGATACACCATAATCTTTTAATAATCGAATATTATTTTGAGTAACTGGATTTTCCCACATTCGTAAATTCATATTTGGAAATATAG
AAAGTTTTTCAAAAGCTGTATGACAAATAGTTAATAATAAATTGTCACATATACCATTTGCAATTTTATTAATCGTATTAGAAGTCGCAGGTA
AAATAATAATCTTGTCATGTTTATTTGCTATATCTACATGATTTAAAAAAGGGTCTTCAAATTCGTCATAATAGTTATCGCAAAATTGCTTTAA
TATTTCACCATTAATAAACTTTCTACCGTTTGTTGACGCGATTACATTTACTTCATCAAATTTTGATTTTAATTCAATAATATAGTGGCTAATAT
TGATACTATTAACTGAGCCACACAAACAAATCAATACATTTTCTCCCAT

Gene: epiC (lantibiotic epidermin biosynthesis protein C)

Position: 1887653 to 1888898, length: 1246 nt, orientation: REVERSE

Sequence:

TCAGCTAATTAAAAACGCATTGGTCCAATTTTTTTTCATTGAGAATTTTTGTATTTTTCAGTGTCATCAATGTTAGATAGACCCCTAATTCACC
TTCTAAAAGGCCAACTTTCCTACTTTTAAATACATCTTGCTTATTTTCATTAATGTCTATAAAATTCACCAGATAATCTTCCTTATAATGAGAAA
TCAGTTTATTTATTAATACAGTGATATAATCAGACACTTCATTTAATTCAAAATTCAAATTCATAATTGTTAACATTAATATTTGTGATGACAA
TCCATGACAAATAGTTGGACTTATTAAATTTTCATCTTTATCATTTACTACTTGTAGCATCACTTTTTTAGACATTTTAATTATTTCATCATCTTG
TAATGCTTGGCCGATTAAAAACAACGTCGTCATTACACCCGTATTGCCATAACACCAACCATTCCGAATAAAATTGAAATGATTACGTTCAG
AAATTAAATCGTAACGCTGCAACCATCTTTCGTGGTTACAAAATTTTTCGTCCATGATAAATTTGTACATGTCTTTTAATATGTGCTGATGATT
TTCAATCGTAATTCCTTTAATCACGCATAATGCAAATAAAGACATCGGTCCTAGCACTCCATGTGCAAGGCCAAGATTGATATTACCTTCAGT
AAAATAATTTTTATCAGACTCTAAAAATTGACTTTCTTGTGGGACTACCCAGCTATTTTTAGAATATTGAATATCTTTAAAGTATACTAATATG

CTTTTTAATGCTTTAACATTAAATTCATTCTCATCTACTCTATTTAGCAAATATCTCCCTATCCCAGCTAATCCACTGACAGTATCATAATTTAA
TGGATTTAATGGTTCTTGTAATATTTGATCAAATTTATTTTCTATTTCATTTACAATGTGACCATCAATATTATTTAAAATATTTTGATAATTTTT
GCCATTTTGCGATGCTAAATCCATTGAAAATGCAATACCTGAAAGTCCACCAAACAAAGAATTGTTATATTGACCACTCTGTAAATATGGTG
CTAATTTCATGATGTACTTATGCACAATTTGCTCAGTATTAATATCGAAAACTTTTTGATAAGCATCTAAAAACAAAATGATACCTGGTATAC
CATGTGATAAAGTTGAAGGTTCAAAATAATCGGTTTCAGTAGATGCTTTTGAAATGAAATCATCTACTTCTGAAATCTCTTGAAACTTCTTCT
TCAATATTAGATTCATATCAATCAT

Gene: epiB (lantibiotic epidermin biosynthesis protein EpiB)

Position: 1888891 to 1891884, length: 2994 nt, orientation: REVERSE

Perfect match to: (RN4220-VC40-CP003033-[1812317:1815310:r], allele observed in CC8)

Sequence:

TCAATCATTTTTGTCACCACACCAATGCTTTTGAGTTTTAACAATTTCTTTTACAATAGATAAAACAAATGTTTCTTGATCCTTATCAATGCCAA
ATATTCGATTGCAGCGCATATGAATTAAGCTGCCAATAATCCTTGACCTTTTAGTAGTTAAAGTTTTTTTAAGCCCTTCATTTAATTTGCTCAA
AATTTGGTTGCCATCTTTTAAAATTCGATAAATACCACTATAGTGTTTAGCCATATATTCAAAATTATTAGCAGGATTACATATAGTTACAAG
TTCATTTTTAAAAGGTCTAATATCTTTACTTTTAAATGATGTAGGTACATAGTTATTAACAATTTCCATACGCTCTTCATTAGAAATTGAAAAC
ATCTCAAATAAATACATTATTGAAATAGCAACAATATATGGTCGATCAAACTTAAAATCATTTTCAATAATATTGACAGCTAAATTCGTGTCA
TATTCAAAGAAGGCTTCTATTTCATTAATCGTGTTCTTTCCACCATATCTAAAAAATTCTGGCTCGTATGATACAAATTCATAATCTGAAACTT
CTGTCGTTTGTCGAACATGTGGTAACCAATTCTTCAAGATGCTGTATATTTCTGCGTAATTTTCGTCATTCCTGAATAACCTCAACTTTAAAAT
GTCACCTTGTTTGATATAACGCATTAAAAAATATTGATCTATATCTCCCTTATCTTTAAGATGTTTTACAAATGGATACAAATTGTCGATGATA
AATGTATCTTGCGATGGCTTATCGATATGAATATGTAATGCAAACCAGTCTTTGTCATATTCAATATCTGTATTTTTATATTTTGGAATTTCGA
TTTCGGGTCCACGATAAGATGATTTTTTATAAATTGGTGTAACAATTTCATACACATGATCATTCTTTGATTGAGGTAAAAATGATTCTACTA
AACGTACACGTTTATGCTTCTTATATTCTTTCATCAATAAATATCTATGATTAGCTAATGATAAATTTAGTAGAAGCTTGTTATCTCCATAAAC
TAAATTCACGATATTAGGTATATTGAATTGTTCTTGATATTTCAAAAATTGTTGATCCCATTCTTCATTTTTAGGTAAAGGTAATACCATCTCG
TTAATTTTCCAATATGCTGGTTTAAGAATGACATTTTTATATCTGATTCGTGGTGAATAACTAAATGAATCAATACTCACATCATTCATAGGTT
CAATGCATTTCACAGATTCCATTGAAATTTCTCGTAACAATCGATATAAATTACATTCTTTTAAAAAGTTATACATATTGTTTGATTCAAATAG
TACCCTTTTATTTAGTTGGCTAGAATATAAATATAATTTGTTAAAGGTAGCACCAACATAAATATCATCTAAGGTAAGCTCATACTTTGAATA
ACTATTACTACTTCCTAAATTCAATGAATATTCGTATGAGTCATGATTAGTTAATACATTATGATTTCTCGGATATTTCGGTATGTTATTTATA
GAAATCATTTCTACATTGTCATCACATATCATCTTCTGATAATAATGTCCTTTTTCATGTTCTAATTTTGCTAAAGTTTCAGTATCAATTAAATG
ATGAAATCGCCCAAATGTAGCGCCAGCATTAAAAGACGCCGTTAAAGGACTAATAACAATGAGCTCATTGTACTGATTGTAAAAACGTCCT
AAATAAAGTTCAGCATAAACATCTGCACTCATTGGCGCATGATAATGATTGATTTCATTATCGTTAATCAATGACTCTACATCTTTTTCATTTA
TGACTATCTCATCGTTATTTCTCAAGGCATGTAAAAATTTTTGCTTAAGCATTACAATATTATTTCCATCGGTTTCTTCTTCTTGAAAAATTGTT
GTCCCGAAACCTGTTATATCTGAAATCAAATCTTTAATATTCACGAGTTGTTCAAATCCATACTTTTCAATAAAACGGTTGTGAAGAACTTTC
AAATCTGTGAATCCAATATTATTTCTTGATAAAAGCCACAATAAGTACGCTGCTTCAGATATATTTGTTGCTATATCTTGATGTAAATAATTGT
TAATCATGTCAATTTTGGTATCAATTTGAAGATAGTTTTTACATTTGAATAGTGCCTTCATATGATGAATAATGTCTTTATATAATTCCTCGCC
AAATCCAATTTCAGTTTTTTCATAAGCTAATATCAATTTCTGGATTTCTCTAATTTTTTTCACAAAATCATCATTATGTAAAGAAAGTTTATTTA
AAATATAATTTAAATTATCGCTATATGATAACGGTGGTCTGATTGTTGAATATATAATTTCCTTTGAGACAAGATTATGGATATATACTTTCA
CTTTAGTAATATCATTAATTTCAAATTCTTGATTTATCTTTTCAGCTAAATTTGAAAAAGTAATATTATTATTCGTCACAGTGGTTTTGATGAAT
ACGAGTAATTCGCTATTTTTAATGGAAAAAGATGTATCTTTATTGTTGTTTAAATAAATAGCACTTTGTTCATTTAAATATATTCGATCATTGA
TAATATGTGCTTTACTATTCCAAATAACTTTCAAATTTTGATAATATTCATCAATACTCTCAATATAACTAACCAGTTTATACAACCATTCGCCA
TCGACTTTCACATATTTTTGAATACTATTTCCAACTTTAAGACGTGTTGGTTCATTTACAAAATGCCCCAAATTAATACCACTTAATAATCCAA
ATGGAGTTGGACGTGTTGACATTCTAATTAAATATTTTAATAAACTTTCTTTAGCATCTCTAACCTTCTTGTTATCGCCATCAAAATTTATTTCT
TGTAAAGTACAATATAAGTTAAATGTTGTTGTTAGGATTGATTCTTTCATCTGAGCATTTAATTGTAAGTCGCTATATTTAATTTGTTCAGTAT
TTAAAAATAAATTGAAAAATTCAACTGATAATAAAGGTGTTCTAATCATACAAATACTTGACGGTTCGTAAATAGTGTTCAT

Gene: epiA (lantibiotic epidermin)

Position: 1891949 to 1892092, length: 144 nt, orientation: REVERSE

Perfect match to: (MW2-BA000033-[1917214:1917357:r], highly conserved allele)

Sequence:

TTAACAGCAGAAGCTATTAAAACTACCAGTCTTAGCACAACCAGGAGTACATAAACTATGACTTGTAATACGTTCGTCACCTGCTGAATCAT
TTGAGTTATTGTTTGCTTTAACTTGCACGTCTAAATCAAGAACTTTTTCCAT

Gene: bsaX (putative bacteriocin of Staphylococcus aureus)

Position: 1892536 to 1892727, length: 192 nt, orientation: FORWARD

Perfect match to: (MW2-BA000033-[1917801:1917992], allele observed in CC1+CC8+CC80+CC239+CC4803)

Sequence:

ATGACATGTTCTAAGAGGGTCAATATAATAACCAAAGCTTGTCATTACTTATATGTAAAGATAGAGGCTAATTACATAATCCTAACTTATTTA
ACAGGAAGTCATGAAAACTATATTACAACTAAAAGTATAACTTTCCATCACCAAAGTCAGAATTTATTGAAACACTCAACTGATAATATAGA
AGGCTAA

Gene: epiA (lantibiotic epidermin)

Position: 1892829 to 1892972, length: 144 nt, orientation: REVERSE

Perfect match to: (MW2-BA000033-[1918094:1918237:r], allele observed in CC1+CC8+CC80+CC239+CC4803)

Sequence:

TTAACAACAGAAGCTGTTAAAACTACCCGTCTTTTCACAACCAAAGCTACAAAAAAGATGGCTAGTTATTCTTTCGTCACCCGCTGAATCATT
AGTGTTGTTATTTCCTTTAACTTGCACGTCTAAATCAAGAACTTTTTCCAA

Gene: lukD (leukocidin D)

Position: 1893214 to 1894197, length: 984 nt, orientation: REVERSE

Perfect match to: (NN50-BAEA01000023-[39835:40818], allele observed in CC4803+CC1+CC8)

Sequence:

TTATACTCCAGGATTAGTTTCTTTAGAATCCGTACCGATTAATTTAACAGTATGGTTTTGCCAGTCAACTTCATAAGTAGATGTAAACGTTAC
TGTATTTTGATTTTTGTAGTTATTACCAACCCAGTGTAGTCGATTCCATTGATTAGTATATCTATCCATTTCTCTTTGGTAAGTTACTTTGATTT
TAGATTTTTTTGTATCATTTTGTTTATGAGAAAGTACGCTTATAAATTCTGGGTTAAAGTTACCACGCGCCAATAAAGGCATTTGATGTGTTG
GCAAGAAATTTTGTCCAGCATTTGAACTACTTTGTCTACCACCTAAAAACAGTTCATTACCATATGTTGGGTCATAACTATCTCTACCATATG
GTCCCCAACCATTATTCATAATTTTGTGCGCCTCAACACCCCAGCCAATTGATTTATGATTTGTTTTTCTATCAATCGTAGTTCTGTAACTTTCT
TGTTTATAATTTATCGTTTCTGAAAATGATTTTGATCCATTTAATCCACCTGATAAGCCATTAGATATATTAATATCTCCGCCATAAGAATAAC
CTAATGTTTGTTGAACTTGGAATTCTTCATTTTGATTTTTAGGTGCATAGTCAACAACATTTACAGCATCATTTGATTCTGAACTAACCGAAAC
ATTATACTTACCGCCCCAATAAAACTGTGAGTAATTGTAATCTTTTGGATTAGGCTTTTTATAACCTGAATTAATGTTTCCGGCTGCCTTAAGT
ACTAACGTATCTTTGTCATAACTTTTATCCTTAATGAAATTAAACGTTAAAATTTGAGAAATATTCAATTTATCATTATCAGATGTTGCTGTTG
TTTTGTATAAAGTGATTTTGTCATCTACTTTTTTCTCGCTTACAGGTGTGATATGTTGAGCTGCATCAACTGTATTCGATAGCAAAAGCAGTG
CAATTGATGAAGCAACTGATGATTTGACTAATTTTTTCATTTTCAT

Gene: lukE (leukocidin E)

Position: 1894199 to 1895134, length: 936 nt, orientation: REVERSE

Perfect match to: (11819-97-CP003194-[1944579:1945514:r], allele observed in CC80)

Sequence:

TTAATTATGTCCTTTCACTTTAATTTCGTGTGTTTTCCAATTAACTTCATATCTAACTACAAAGTTTCTATTTACAAATGCATTATGCTTTCTTTC
TGCGTAAATACCAGTTCTAGGGAATAAAGTCGCATATGTAATATCTAAGTTTCTACCATATGAAATTTCAAATTCACTCGTATCACTTGAACC
TTTTTCATGTGATAGTGTAGTGATAAATGATGGATTAAAGCCACTTTGAACTAAAGGTGGCAATTGATTATCAGGAGCAAAATATTCTCTTG
CTGAACCTGTTGGACCATTTGGACTTTGTACGAATAAATATCTATCATGCGCAGATTTTTTACCATCAGGCGTAACAAATTCGTTTGCTTTAA
CACCCCATTTAACAGATTTTGAGTTTTGCTTGTCTACTTCACTGACATAACTCTTTTGGGTATAACTAATTGTTTTAGAATAATTAAATGAGCC
ATTGCCACCTATAGATGGTGCTGACTGGAAATTACCTCCAATGTTATATCCTAATGTTTGACCAACATCAGTAGTTTCTATTTTGTTTTTAGGA
AGGTAATTGATTAAGCTAACATTTGGATCTTTAGTCGTCAGTCCTATATTATATTGGAATGGCCAAATCATTCGTTTAGTTAATTCATATCCA
CTACCCTTCACATCTGAAAATGAAGTTCTGGAATTAATAAAACCTTGCATTTTAACAATTAAAGCGTCTTTGTTATATTTTTTATCTTTTACAA
AGTCGAATTGAACATTTTGAGTAACGCCCCATTTCTTACTACTTACATCCTCCGTACGTTTGATTACTTCAGCACCATCACCAATATTCTCTAT
ATTAGTATTTGCTCTAGATTCTTGAATCGGAGATGCTAAAGGTGCAATCAGTCCTACTGACAAAGTTGCAGCTAACATTTTTTTCTTAAACAT

Gene: sprC1 (small pathogenicity island RNA C)

Position: 1895252 to 1895959, length: 708 nt

Sequence:

ACAATTTATTCATTTATGCTGCTGAAAACCGGTCATTTTGGCGCAACCTTCATTACCTTTAAATAGTTCCCTTTATATTCGAACAATATGATAA
AATTACCGATACTCAATCTTTTCTCTGTAAAAATAAAAACGAATTTTAATCAAATCTTCAAAAATAATGTTTGTATTATCACAAATAATCTATA
ATTGAATTTAAAATTAACTGATTATTAACTATTGTCCGTTAAACGAAGATGATTACTTCGTATACTGTTATAACTGAAATGTTCTATATATCAT
TTCGTAATATCGCTTACTTCATCTAAAAAAATTTAATGCTATGATTTAGTCTAATTAAAAACATATATATATTTACTATGAACTAAAATTTCAC
TTTAAAAATAAAAAACCATGATTTCGAAGTCTTCATAAACTGAAGCTTCTACTCTCATGGCAATTTATACTATGATATTCAATTATAATGCCTA
GTCTCATAGTAAACGGCTACTACATTCGCATGTACTTCGTTTTACTGTCCACGCATGGTCGTTGACTTATTTATATTATAATATAAATATTTTA
TTTTTCAATACTTAATTTTAAAAATCCCCTATGAAAATGTTGATGAGCTACATCTTTAAATCTTATTATACATTGTATAAAATTATATTGCGAG
GTAGTAAATTGATATTATACACTTTTGGTTGTGTATGCATCTCACCTATC

Gene: Q2FXA9 (putative bacteriophagal protein)

Position: 1896018 to 1896473, length: 456 nt, orientation: FORWARD

Perfect match to: (COL-CP000046-[1937475:1937930])

Sequence:

ATGGCTAAACTTGATTTAAATAGTCTTGACGACGAGCACGTAAAATTATTAATAAATGAATTAAAATATCCAGAAACTCATATCGATGTAAA
TGAATTAAAAACAATAGTTGCTAGTCGAATAAATGAAAGGCAAGAAATAATAAGTTTTAAGTTAGGAATAAAGTACTTATTAACAATAAAA
AGAGGGAACATAGAAAAAGATAGGTTTTCAATTTCAATCATTTTCAAAGATACCTATCACACCCTAGTTAGAATAGATATTAACGGTGGTAC
TCACGATAATCCAGATGGAACAATCGCTCCGAAAAGTCATATTCACATATATAATGATAAGTATGATAAAAAGGATAGGTTTGCTTATGAA
ATTAACTTGAAAGATTTCCCCGACATCTATAACTTGTATAATGTGTATATGTCGTTTTTAGAGTATAATAATATAAAAGACCTTGAATAA

Gene: Q2FXA8 (putative protein)

Position: 1896486 to 1897274, length: 789 nt, orientation: FORWARD

Sequence:

ATGACTACATTTGATGCTAAAAAATTAAAAAAAGAATATCTTGATTGGTATAATCAGACCTTAGAGTTTTCTAATTTATCAAACAATGTAGTA
AGAATAGATACTCCTTTTAAAGATAATTCTTTAGATAATTTAATAATTTACGCTTTATACGATCAGTCCAGAGACATGATTACATTGACAGAT
GACGGCTATACTATATTTGATTTAGAAAATAATGGTATTTCTTTAAATAAATCAAAAAAACGTAAAAAGATTTTTGAAGAGCACCTTTCAGCT
TACGGTATTAAATATAACGATAAAACTCACGAAATTTTTGTTCAAACTAACTTTAAAAATTTTAATAAATCGAAACATAATTTATTACAGTGC
CTTATATTTGTTAATGATATGTACTTACTTTCTAATCCTAAGTCACAGAACATATTTACAGAAGATGTTGCAAACAAATTGGATGAACATAAC
ATTTATTATGGAAGAGATTTACCTATTATAGGAAGCAGTGGTGTTGTTCATAATTTCGACTTTTTTATTAGCGCTAAGAAAAATCAAAAAGA
AAAATTTATCAATGCTATTTCTAATCCTAATAATTCTATGATTATTAAGTCGAAAATAACGGATGCTATGCAAGCAAAAAAAATAAAAAGAC
ACAGGCAAAATGAGTTTATTTTTATTTTAAATGACTCAAAAAAAGAAATAAATGAACATAATAAAAATCTTCTTCATGAAAACTATATTAGTA
CAATAGATTATAGCGAATTAGATGAAAAGATAGGTTTATTGATTTAA

Gene: ydeN (esterase with alpha/beta hydrolase fold)

Position: 1899580 to 1900134, length: 555 nt, orientation: REVERSE

Perfect match to: (AH2-CVOS01000018-[3171:3725:r], highly conserved allele)

Sequence:

CTATCGTGTCATGTAATCTTGCATCCGATCTTGCAACGCTGTAAATGTTTCGAAGCCATCCTCTTCTAAGAAGTGCCCTCCATCTTCCACGATT
CGCAAGTTCCCCTCTAATGCATTCATTAAACGCTGGGTCTCTTTATATGAAACGTATTTGTCATTTTTAGAACTCAATCCATAAAAATTGTCAA
CTTTCTTTTTAATATTATCGTAATCAATGGTTACATTACTTAAATCAATATCTAAATCTATATTTTCTGCATCTTCTTTAAAGCCCGCTATACTA
AAAAAGCCTTCAATCGGCTGATCAATCATTTCAATATATTTTAAAGCTGTGATTGAACCTAAACCATGTGTTACAAAATATGTATCCTTTTTG
CGTACATTAATTTGTTTCGTCATAGCTTCAATCCACTGATCCACTGTCTTCGCTTCAGGGGATTCAAAATTAAATAATGTTACGTCATATCCTT
CTAAAGTTAAGTTATGCTCCAACCACTGATACCAATGATTTCTACTATTTCCATGCATAGAATGTACAATAATTACATCTGTCAT

Gene: hemY (coproporphyrin synthase)

Position: 1900455 to 1901855, length: 1401 nt, orientation: REVERSE

Perfect match to: (JKD6159-CP002114-[1917375:1918775:r], allele observed in CC93+CC80+CC707+CC834+CC1290)

Sequence:

TTACAACTCTGCGATTACTTCTTCAGCAGCAACTTTACCTTGCGTAATACAATCAGGTAGTCCAACCGCTTCAAAAGATGCACCAGTTACTCT
AAGTCGTGGATATGTTTGTTTAATATGTGCTTGAATCTGTCTAATTTGTTGAATATGACCGACATGGTACTGTGGCATACTTTTCGGCAAACG
ATTGACAATTGTAAATTCAGGATCACCTTTAAATGTCATCATTTGACTTAAATCTCTACGTACAATCGATACTAATTCATTATCTGTATGATCA
TCAACCACAGTATCACCTGGTTTACCTACATACGCACGAATCAAAACCTTACCTTCTGGTGTAGTAAATGGCCATTTTTTCGATGTCCAAGTA
CATGCGGTAATGTCTGTATCACTTGTTCTCGCAATCACGAAGCCAGTACCATCATAAGTATTTTCAATGTCTTTTTCATCAAATGCCAATACA
ACAGTTGCAACAGTCGTACTATCCATCGTTTTAAAGTAATCAAATGCTGGATCTTGCCCGAACCAATTCAAAAAGACTTGATGTGGTGTTGT
CACTAATATTCCATCGAATACATCTTCTTGTTGATTACTGTAGACAATTTTATATTGCTTTTGAGATGTAATAATATCATCCACTGACGTATTG
TAGCGTATTGTCACACCTTTATTTTTAACATCTTGTTCTAATGCTTCAATAAATGAGCTTAAACCATGCTTAAATTGTTTGAATTGTCCTTTTGG
TGCGCCAGGATATAATTGTCTTTGTTTCAGACGCTTATTTTTCTCATCCTTCATACCTTTTATCAGACTTCCGAATGCCTCTTCTTTTTCTTTAAA
ATTAGGAAACGTACTCATCAAACTTAATTTATCAATATCGGTACCATAAATACCACCCATTAAAGGCTCTATTAAATTCTCAAGTACCTCATT
ACCTAATCTTGCTCTGAAAAATGCACCAACAGAAATGTCACCATTCTGCATTTGAGTAGGTTTTTTTAATAAATCAAACCCTGCTCTTAACTTT
CCAAATGGTGAAATTAATTTAGTTGTCACAAACGGTTTGATATCTGTCGGAATTCCCATAATTGATCCACCTGGAATAGGATACAATTTGTTT
TTCGCAAAAATATAAGATTGTCCAGTCGTATTTGTAACAATATCTTGTTCTAATCCAATATCTTTCGCTAATTCTGTCATGATCGTTTTTCTACC
TAAATAAGATTCAGGCCCTAGTTCAATCATATAACCATCTTTACGATACGATTGAATCTTTCCCCCCGGACGATTCGATGCTTCAAAGATGGT
TACATCAATATTAGGATCTTGCTGTTTTAAAAAATATGCACTTGATAAACCTGTTATCCCCGCTCCTATAATAGCCACTGATTTAGTCAC

Gene: hemH (ferrochelatase)

Position: 1901879 to 1902802, length: 924 nt, orientation: REVERSE

Perfect match to: (11819-97-CP003194-[1952259:1953182:r], allele observed in CC80)

Sequence:

TTAAAATATAGACTTGATTTCATCAACAATTGCACCGATAAATAATGGATGTGTATTCGGCATTTTTGGACGATAATAATTCGCACCAATATC
ATCGCAAACAACTTTACATTCATAATCATTGTCATAAAGCACCTCTAAATGCTCACATACAAAACCTACTGGCGTATATATAAAGTTTTTATA
CTGATGTTTTTCATATAAATCACGTGTTAAATCTTGTACATCTGGCCCTAACCAAGGTGTACCTGTATTACCTTCAGATTGCCAACCAATCGC
GATATGTTCAATATTAGATTGTTCTTTAATTAAAAGCGCAGTATGTTCTAGTTCTTGTGGATATGGATCATTATTCTTTTCGATTAAACCTTTT
GGCAAACTATGTGCCGAAACAACTAATACCGTGTCTTTATGTTCCTCTTCCGGTATTTGAGCTAATGTTTCGTTGACTTTATTCGTCCAATATT
CAATAAATTTAGGTTGTTCATAATAATGTTTCACATGTGTAAGTTGAATACCATATTTTGCAGCTTCTTCATCAGCACGTTTGTCATATGATCC
TACTGAAAATGAAGAATAATGTGGTGCTAGTACTACAGTGATTGCTTCAGTAATACCATCATTGTGCATTTGTTCAACCGCATCTTCGATAA
ATGGTGAAATGTGTTTTAATCCTAAGTATAGTTTAAATTCAACATCTGCATATGCTTTATTTAATGCTGAAGCTAGCGCATCAGCTTGGTCAT
CTGTTGTACCTGCTAATGGTGATAAACCACCTATAAATTCATATCTATCTTTCAAATCTTGAAGTTCTTCTTCAGATGGACGTTTACCATGTCT
AATATCTGTATAATATGGCTCTATGTCACTTTCTTTATAAGGTGTGCCATAAGCCATAACTAATAATCCCATTTTTTTAGTCAT

Gene: hemE (uroporphyrinogen decarboxylase)

Position: 1902860 to 1903897, length: 1038 nt, orientation: REVERSE

Perfect match to: (CIG1612-AHVW01000005-[1422639:1423676:r], allele observed in CC8+CC5+CC97)

Sequence:

TTATCTTTGTGTATATGTGTGTACGAATTCGCTTACTTTACGTAACGTCTCTGGTTGCACTTCTGGGAATACACCGTGTCCTAAATTAAAGAT
GTGTTTACCGTTCTCCATACCTTGATCTAATATTGGTTTCAATCTCTCTTCAATGACATTCCATGGTGCTAATAAAATTGATGGATCTAAATTC
CCTTGTAATGTTTTAGTAACGCCTAATTGTTGAGCCTGATTAATAGACGTTCTCCAATCTAGGCCTAATACATCAATCGGTAAATCATTCCAT
TCATTGATTAAATGACTGGCACCTACACCGAATAAAATTACCGGCACATCATGTTTTTCTTTAACCTCACTGATTAATCGAATCATATGTGGT
TTAATGTAACGTCTGTAATCCTCGACATTTAATGCACCTACCCATGAATCGAAAATTTGAATCAATTCGGCACCTGCTTCGACTTGTGCTGTT
ACATATTTAACAGATACATCAACTAAATGATTCATTAAAGCAAACCATGTTGCTTCATCTCTATACATCATCGCTTTTGTAAAATTGTAATTTT
TCGATGGTCCGCCTTCAATCATATATGACGCTAATGTAAATGGTGCCCCAGTAAATCCTATTAGCGGCACATTTAACTTTTCTTCTGTTAAAA
GTTTAATTGTATCTAATACATATGGTACATCTCGTTCGGGGTCTATTTGAGAAAGTTTCTCAACATCTTGAATTGTTTTGATTGGATTATGAAT

CACTGGACCAATACCCGATTTAATTTCTACATCGACACCAATTGGCTTTAATGGTGTCATAATATCTTTGTATAAAATTGCTGCATCTGTATG
ATAATTATCAACTGGTAAATGTGTTACATAAGCGCACAACTCCGGCTGATGTGTAATATCGAATAGTGAATATTTTTCTTTCAATTTTCGATA
TTCTGGTTGCGAACGGCCAGCTTGTCGCATAAACCAAACAGGTGTATGTGATGTTTCTTCACCTTTGATCATTTTTAAAATTGTATTGTTTTTA
TTATGCACCAT

Gene: traP (signal transduction protein)

Position: 1904160 to 1904663, length: 504 nt, orientation: FORWARD

Perfect match to: (Strain_21333-AHKA01000031-[5147:5650], allele observed in CC15+CC80)

Sequence:

ATGAAGAAACTATATACATCTTATGGCACTTATGGATTTTTACATCAAATAAAAATCAATAACCCGACCCATCAACTATTCCAATTTTCAGCA
TCAGATACTTCAGTTATTTTTGAAGAAACTGATGGTGAGACTGTTTTAAAATCACCTACAAAATATGATGTTATCAAAGAAATTGGTGAATT
CAGTGAACATCATTTCTATTGTGCAATCTTCATTCCATCAACAGAAGATCATGCATATCAACTTGAAAAGAAACTGATTAGTGTAGACGATA
ATTTCAGAAACTTTGGTGGCTTTAAAAGCTATCGTTTGTTAAGACCTGCTAAAGGTACAACATACAAAATTTATTTTGGATTTGCTGATCGAC
ATGCATACGAAGACTTTAAGCAATCTGATGCCTTTAATGACCATTTTTCAAAAGACGCATTAAGTCATTACTTTGGTTCAAGCGGACAACATT
CAAGTTATTTTGAAAGATATCTATACCCAATAAAAGAATAG

Gene: ecsB (ABC transporter, transmembrane permease)

Position: 1904787 to 1906010, length: 1224 nt, orientation: REVERSE

Perfect match to: (MW2-BA000033-[1930217:1931440:r], allele observed in CC1+CC12+CC72+CC80+CC88)

Sequence:

TTAGTCTCGTAATAATGTTTCCTGATATTTCAACTTTTTAATAATACTTCTAATCGTTAATAAACCTACAATATAGAAAATGAGTACAACATAG
AATAACGTCATATGTTTTATAATAAATGTCACTGCAAACACAGTACAAATAACAAACATTAATCTATATAAAAACTGTTCATACCCTTTGATT
ACCTTCTCTTCAGGTACAGGCCACACTTGAGGCCATAAGCCATATGCTTGCTGTGAATAAAATTGTGCCATTTGTAACAATATAATATATACA
AATAAACACCCAATAATTGCTGTCACTAATGGATATGATAACCAAACCATTAATAAAACTGCAATAATTACTAACCTAAAGATAATATTAAAT
GCGTCTCTCCCTCTTATAAAGCTTCTAATAAATAAGAATAAATACATCGCATTAGAGTTAAATTTACTACCCTTTGGAACTGGTAAAAGTATA
TCTAGATAACTTCTTCTGACTGCAGATTCTTTCAAATGTTTTACATCGGTGAACATATTAACAAATTTATAATAATTCATATGATGTCGATGTT
CGATTGCAATCATTTTCTCCCAAGGATACAAAAAGCCTGGTTTATATTTTTTAACTAAAAATTCTATTAACACAGGCAAAGCAACCATCACAA
ATGCGATGTACCATTTTGGAGCTAATAGTAAGTAATATGTTAGAGCAAAGGTGATGAATGATATTAAATTAACTTGCCATGTTTTAAGTCCC
GATTGATACCATTGCCATCTTAAGCGTAAACCAACATATGGAAAAATTAATGCACTGACTCCAAAACAAATATAAAATGCCACATTATGTTG
ATTAATATTGTAAAACAACGGGAACATTACAATAACAATAATGAGTTGGATTAATATGCGCGCAAAGTAACTATATAAAATCGCATGACGC
ATAAATTGAGACATGTGTTTTTCAAATGGTAATAAAAAGATTTTATCCGCTTCTTTTAGGAGTGGTCGCATTGGAAAAATGGATGTCAATGC
TACAATCACTGCTGCTATTAATGAAAAATTGATATTCGTTGGAATATGTTTTAACCATTCACCATATCCAAAAATAAATGCACCCAGCAAAAT
AAGTAAAAAGACCATGAAATGACCATTAAATATAAACTTATTATAATAATTTTTCTCTTTACGAAGGGCATGTAATCTTTTATTAAATAATGT
GGTCGCTTGGTTATGCAT

Gene: ecsA (ABC transporter, ATP-binding protein)

Position: 1906003 to 1906743, length: 741 nt, orientation: REVERSE

Perfect match to: (11819-97-CP003194-[1956383:1957123:r], allele observed in CC80+CC12)

Sequence:

TTATGCATGTACATCTCCACCTTGCGTCACATGAATATATATATCGTCTAATGTTTGTTTATGTAAGCCAGTTTGTTGTCTCAATGCTTCTAAA
TCTCCAAATGCAACGGCTTCACCTTCGTCTAGTATGATAAAACGATCACAATAACGTTCAGCTGTTGCTAAAATATGTGTACTCATTAGAACG
GTTCTACCTTCGTTTTTCTTTTCAACCATTAAATCTAACATGGATTGAATTCCTAATGGATCTAGGCCAAGGAATGGTTCGTCTATAATATACA
ATTCGGGATTAACGATAAACGCACAAATAATCATGACTTTTTGTTTCATCCCCTTAGAAAAATGACTCGGAAAAACTTTCAATTCATTTTCTA
AACGGAATGTCTTTAATAATGGCATTGCTCGATTCATCGCTTCATCACGATCAATATCATATGCCATTGCTGTCATCTCAATGTGTTCCTCTAA
TGTGAGTTCTTCATAAATAACCGGTGATTCCGGAATATAAGATAACTTTCTTCTATAAGCCTCTATGTCATCATTAATGTTGATATCTGAAATT
GATAGAGATCCTTCCATAGGTGTAAGCAATCCTAGCATATGTTTAATCGTTGTACTCTTACCAGCGCCATTAAGGCCAATAAGTCCAACGAT
TTCGCCTTTGTTTAATTCAAAATTTATATCTTTAATTACAGGGCGTTTTCCATATCCACCTGTAAGCTGTTCTACTTTAACTGTCAT

Gene: hit (histidine triad cell-cycle regulator)

Position: 1906877 to 1907299, length: 423 nt, orientation: FORWARD

Perfect match to: (N315-BA000018-[1890292:1890714], highly conserved allele)

Sequence:

ATGTCAGAAACAATTTTCGGCAAAATTTTAACTGGAGAAATTCCTAGCTTTAAAGTATATGAAGACGATTATGTCTATGCCTTTTTAGATATA
TCACAAGTTACTAAAGGACATACGTTATTAATTCCTAAAAAAGCTTCTGCTAATATCTTTGAAACTGATGAAGAAACAATGAAACATATCGG
TGCAGCATTACCTAAAGTAGCAAATGCTATTAAGCGTGCATTTAATCCTGATGGTTTAAACATTATTCAAAATAATGGTGAGTTTGCAGATC
AATCTGTATTTCATATTCATTTCCACTTAATTCCTCGATATGAAAATGATATTGATGGATTTGGTTATAAGTGGGAAACACATGAAGACATTT
TAGATAACGATGCAAAACAACAAATTGCTGAACAAATTCAAGCACAATTTTAA

Gene: gvpP (gas vesicle protein-like protein)

Position: 1907441 to 1907806, length: 366 nt, orientation: FORWARD

Perfect match to: (N315-BA000018-[1890856:1891221], highly conserved allele)

Sequence:

ATGAAAGCATCACGCATTCTATTCGGTATCGGTGTTGGCGTAGCAGCTGGTTTTGTAGTTGCACTTCAAGGACGAGACGACAAAAGTGTCA
AGAACAACACGATCGATCGTACTGCCCCTACTGGTTCAAAATCAGAACTACAACGTGAATTTGAAACGATTAAACAAAGTTTTAATGACATT
TTAAACTATGGTGTTCAAATTAAAAACGAAAGTGCGGAATTTGGTAGTTCAATTGGTGGTGAAATTAAGTCATTACTTGGAAACTTCAAATC
TGACATTAATCCTAATATTGAACGTTTACAGTCACACATCGAAAATTTACAAAATCGTGGCGAGGATATTGGAAACGAAATTTCTAAGTAG

Gene: yhaJ (putative bacteriocin)

Position: 1908539 to 1909096, length: 558 nt, orientation: FORWARD

Perfect match to: (MW2-BA000033-[1933969:1934526], allele observed in CC1+CC80)

Sequence:

ATGTTTTTATGTAAAAGACAAATTGATATCAATGCACGATTTGGTTTGCCTAGAATTGCATTTATGAGTGCAGTTGCAACCATCATTATGTTT
TTAGTTAGTTATGAAGTAATGTATTTTTTATCTAATACGCCATTATCAGATAGACATTTTCTCATCTTTTTATTACTTGTATTTATGACGTATCC
ATTACATAAAAGTATACATTTGTTATTTTTCTTACCATATAGAAAATCGTTTAAAGTTCATAAGTTGACTAAAAGAAAATGGCTTATATTCTAT
AATACCTACGTCAATCAACCTGTACACAAATTTTATTTTTGCATTAACTTAATATTGCCGTTAATTATCTTATCTGCAATGTTCGTTTATCTAAC
AATTTTATTCCCGCAATATGGACATTATTTTATGTTCTTATTGGCATTGAATTTCGGTATTTCCATTACAGATTTATTATATTTAAAAATAATTA
TATTTTCTAATTATGGACAATATATAGAAGAACATAGTACAGGTATTAATATTTTGAAAAAAATTAAAAATCCATATCATTTATAA

Gene: prsA2 (extracellular chaperone)

Position: 1909301 to 1910263, length: 963 nt, orientation: FORWARD

Perfect match to: (DAR4145-CP010526-[2002319:2003281], highly conserved allele)

Sequence:

ATGAAGATGATAAACAAATTAATCGTTCCGGTAACAGCTAGTGCTTTATTATTAGGCGCTTGTGGCGCTAGTGCCACAGACTCTAAAGAAA
ATACATTAATTTCTTCTAAAGCTGGAGACGTAACAGTTGCAGATACAATGAAAAAAATCGGTAAAGATCAAATTGCAAATGCATCATTTACT
GAAATGTTAAATAAAATTTTAGCTGATAAATATAAAAATAAAGTTAATGATAAGAAGATTGACGAACAAATTGAAAAAATGCAAAAGCAAT
ACGGCGGTAAAGATAAATTTGAAAAGGCCCTTCAACAGCAAGGTTTAACAGCCGATAAATATAAAGAAAATTTACGTACTGCTGCTTATCA
TAAAGAATTACTATCAGATAAAATTAAAATCTCTGATTCTGAAATTAAAGAAGACAGCAAGAAAGCTTCACATATTTTAATTAAAGTTAAAT
CTAAGAAAAGCGACAAAGAAGGCTTAGATGATAAAGAAGCGAAACAAAAAGCTGAAGAAATTCAAAAAGAAGTTTCAAAAGATCCAAGT
AAATTTGGTGAAATCGCTAAAAAAGAATCAATGGATACTGGTTCAGCTAAAAAAGATGGCGAATTAGGTTATGTTCTTAAAGGACAAACTG
ATAAAGATTTTGAAAAAGCACTATTTAAGCTTAAAGATGGTGAAGTATCAGAGGTTGTTAAATCAAGCTTTGGATATCATATTATTAAAGCT
GATAAACCAACAGACTTTAACAGTGAAAAACAAAGCCTGAAAGAAAAATTAGTCGATCAGAAAGTACAAAAAAATCCAAAATTATTAACTG

ATGCATACAAAGATCTATTAAAAGAATACGATGTTGACTTTAAAGATCGTGATATTAAATCAGTTGTCGAAGATAAAATCTTAAACCCTGAA
AAACTTAAACAAGGTGGCGCACAAGGCGGACAATCCGGCATGAGCCAATAA

Gene: cbf (replication enhancer)

Position: 1910384 to 1911325, length: 942 nt, orientation: REVERSE

Perfect match to: (FPR3757-CP000255-[1972640:1973581:r], allele observed in CC8+CC5+CC30+CC239)

Sequence:

TTAATCGAGTGATTCAGGATTGTAGAATCTACGATTTTCAAGACCAAATATTTTATCTGTAAACTGACCCTTGTCAGTTTTTTTATATGCCTTT
TCAAACATATTCATTCTAGCATCGATATTATCGATATAGCATAAAATTTCTGCTTCTTTTAAGTATGGCAGTTTTGGCGAACCATACTCTAACT
TACCATGATGAGATAAAATCATATGTCTTAACAACATGATTTCTTCTCCTTCAATGTTCAATTCACGAGCTGCTTCAACTACTTCATCACTCGC
AATCGAGATGTGTCCTAATAAGTTACCTTCGACTGTATACGACGTCGCAACAGGACCACTCAATTCTCTAACTTTACCAATATCATGCAAAAT
AATACCACTATATAACAAACTTTTGTTTAACAATGGATAAATGTCACAAATTGATTTTGCAATACGTAACATCGTTAATACATGATAGCTTAA
GCCACTCGCAAAGTTATGATGATGAGAACTAGCAGCTGGATATGTGTAAAATCGTTCTTGATATTTTTTCAATAAATGACGTGTGATACGTT
GTAAATTAGCATTTTCAATATCTAGCAAATAATGAGAAATCTCTTCTTGTATTTCTGCCGGTGATAAAGGTGCACCATCTACAAATTGTTCTG
TTTTTAATTGATCTTCAGTTGTCGCTAGTCTAATTTGGTTGACTTTCATCTGTTTATTTCCGCGATAGTTTATGATGTCACCTTTAACATGTACA
ATTTCTTCAGGCTTGATTGTTGCCATATCATTTTTTGTAGCCGTCCAAAATTTCGCTTCAATTTCACCACTTTTATCTTGCAAATGTAATGTCAT
ATAATCTTTACCTTGTGCTGTTACACCCTGTGTAGCTTTATGCACTAAGAAAAAGTGATCAACTGAATCTCCGGGATTTAGATTCTCTATATTT
CTCAT

Gene: Q5HET2 (DNA double-strand break repair ATPase)

Position: 1911322 to 1914258, length: 2937 nt, orientation: REVERSE

Sequence:

TCATCGTTTCCCGCCTTCCTCTATTTTGTTTAATGTAATCACTTCTTTTGATGGAACAATATTATCTTTTACACATGTAAAGTATAGTACTTGAT
AGTGTTCTGATAATGATCGTAAATAATTCAACATTTTTTCAGTACGCTTTTTATCAAAATGAACAAATGCATCATCAACAATTAATGGGAACG
GATAATATGGTCTTAGTACCTTAATTAAACTGATACGTAAAGCTACATAAAGTAATTCTTTTGTAGATTGACTTAGTTCAACAGGATCATATA
ATTGACCATTAACATGTTTAACCGTAATTGAATCTTCATTATAGTTAATCATCGTATATCTGCCATCTGTTAAATGCTTCAATATTTCTACCGCT
TCATTAATAACTTGAGGCAAACGTTTATCTTTAATTTGTTTAATGTGTTCATCAACTAAACTTTGTAAATAACTTAAACTTGCCCAATCTTTTG
CGATATCATTAAGTTGATTTTTAAGACTGTGATATTCATGTCTTAAATTAGCAAGCGTTGTATCAGTTTCCATGTGATTGATTTGTGCACTTAA
ATCACTGACTTGTGCTTGCATTTCAAGATATTGCTCATTATATTCGTCAACTTGAGTAGCCAATAAATGATCTTCTTCTTCAAGTTGTGCAGTT
GTTTTTTCACTTAAACTAGAACTTAATTCATAAGAATAGTTTTGGTTCTCAAGATATTTAGTTAAATCATTAAAACGACTCAAATTACTAGTAT
AAGTTTGGTAATCTTCATGATGTTGGTAAAAATCTTCTTCAGTACCAACATTGATAAAATCGAATAGTGCTGTAATTTCTTTATTATTTTCTTC
TAATTGAGCATTTAAATGATTTAATTCATTTGTAACAAGTTTGGTATTTTCAGCATTAATACGCCATTTTTCATTCGTGTCTTCAGCTGATTTCA
ACCATTGTTGCACATCGTGGAATAAAGATAATTTGTTGAAATAAACAAATTGTGATTTTGTAACAGCTTCAGCATGATTGTAGAATGTATCT
AATTCTTGAACCAATTGCTGGCGTTGTTGATTTAAATCACTGATATGTTGATCTAATGCTTTAATATTCGCCATTGTAGAAATACTATCAACA
ATTAAATCATTTGAAATTTTAGATGATAAGTATAATTCATCCTTAACGTTTTCAACTGTCGATTGTAATTCATCATGACGCCCTTTCGCATCAT
TTAAACGACCTTCAATATACTGACGTTTCTCTTCTAAAATATCTTTATTTTTCAATGCCTGTTGCCAGTGATCACGAATGCGATATTGCTCATC
AAGATCAAAATCTAAGTCATAGTTTTCATCTAAAATGGCTAGTTGTGCTTTAATTTCTTCGATTTCATCTGTGATGGCCTCGCTATAATCTACT
TCTTTTGATTTAGACATGATGATACCGATAACAAATACTAAAGTTAATACTGCGAAAATAATACCAAACAGCATGTTGTTTGAAATAAATGA
GAAGGCAGTTAAACCAATACCTACTAATGTTAAAAGAATAAACGTTGTTCGTAACAATTTTTGACGTTTTTGTTTTTCTTGTTGCTCAATTTCG
AAACGTTCTTTCAATTTGCTATACAAGTTCTCTTTTTCGTTTAATTCAATGACTTGTTGTGAGTATTCTTTTTTCTTTTCAAAAGTCTCTTCAGG
AACTATTTTTTCTTCAACAGAATCTAGTTCGCTATGAACCGCATTATCTTCGATTTTATTTTCTTCTAAACTACGTTCTAATTGTTTAATGTGTG
CAGCTTGTTCTTGTTTATTCTTGATTTGCTCACTGACATAACTTTTCATTGCCTCTGAACTATCTACGTCATGATGCGTTTCAGACCAACCAAT
ATTTGCTTGCAATTCATCTTTATCACGTTGTTTATTCGCAATATCCTTTTCGATTGCAGTAAGTTCAAATTCTTTATTTTTAATTTCATTTTCTTG
TTGATTCAAACTAATAAAGGCGTCAATATCAGATTGCTTAACTGGCTCTAATTGAGTCGCTTCTTCTTTAAGTTGAGCTAAACGCTCATTTCTT
AAACCAATATCTCTTTCTAACGATTGCTTATGCGCTCGTGCTTTTTCGTAACGATCCACACCTTTTTCTGGGAATGTGATTGGCTCAATATTTA
ACTGTTGTTCTAGAGACTTCCATTCTTGTGAATGATCATGTAAAGCAACCTCTTTTTGTTTTTCTTCATGCATTTTTGATAATTGATTTAAATTA
TGCTTTAAATTCTCTAATCGACGTGATGATTTATCACGATCATCTACTAAGCGATGATATGTTTCTAGCTTAGCTTCTTCTTCACGAATTTGAC
TTTCTAGTTGTTTTAATTGCTCAATTTGTTGATTAATGATCGGATTTTTACCTGATTTTTTATATAATTCATCTTTTTTACGATTAATCACTTCGC
GCATTGACGTGAATTCAGTTGATCCTAAAGCCCCTGCTTGTAATAAATAATCTTGCAATTGTTTTTCATTTAGATTTCTATGAATGTCTTGAAG
CCCTAGTACATCAAATGAAAAGATACCTTGATATGTCTTTTTAGAAATATAATTAAGTTTCTTTTGTAACCAAGCATCATCACGCACAGCACC
ATTAGGTAAATATACTTTCACATCACCTTGAGCACTGCCTTTAATTCGTTCAACTTCAATCTCTAAGCCATCATCAAGAATAAGTACTAATTTA
CCACCGTATTGGTTACCTAGACGTGGTTCTAGTCTTGGCTCTTTAGACTTTTTAGTTGGAAAACCAAATAATATCGAATGGATGAATGCTTG

AATCGTCGATTTACCCGCTTCATTTTCACCAAAAATTTCAGTGAAGTTTTTATTAAATTCAATTTTACGTTGAACAAATTGACCGTAACCATAA
ATTTCAAGTGATTTAATTATCAT

Gene: Q5HET1 (DNA repair exonuclease)

Position: 1914248 to 1915444, length: 1197 nt, orientation: REVERSE

Perfect match to: (11819-97-CP003194-[1964628:1965824:r], allele observed in CC80+CC239+CC4803)

Sequence:

TTAATTATCATTTTGTTCACCTCTCATTTCAGCTTTTAATATTTCTTCAGCACGATTAACTAATGCTGTATGGTCGAATGTTCCATAATCGTCTA
GGAACTTTGATGCCCTTGGATTTAAATATAAATCTGACATCGCTTTATCAAAAACAGTTTGATCGACTAATAATTCCGCTGAAAATTCATTAA
CTAAAGGTGACTCATCATTTTGTGCATATTGTATTTTTAACTCATCAATATATACAAATTGATTTTCGTTTTCTTCATAATCTGTAATCATTTCT
TCAACTTGTAATAAATCTTGAGGTGAAATTAATGTCTCACTATTAATAACAAGCGTTAAACGATAAAAGGCTTTTCCTTCTTCTCTCACTTGTT
CTTTAAAGTTTTGAATGACCTCGTATAAACCTTGCTTAGATGTCTTATCCGTTTCAATAGTTGCTTCTTCAAATCTAATATACTGTGTAGGATA
AAATTTAGTCTTTAATTTTAAGTGGTCACCCTCGATTAATAAGCAACCTTTTTCACCTTGCTCATTAAAATGTCTACCTTGAATATTACCTGAA
TAGTTAATTACAGGCATATCTCTTAATTGTTGACGTTCATGTATATGACCTAAAGCCCAATAATGATACAATTTACTGTTTAAATCTTCTAAAA
TGAACTCGGTATATCTTTCGTTAACTGAAGATTTACTATACGTACCATGCAAGACACCAATATGTATGCCTTTTTGGCCTTGACTTGATGGAT
ATTCATCAATCTTGTTCTCATAACTTGCTCTATTTTCATAACTAAATCCGTGAATATAAATTGTTTCACCAGATTTAGTAATTGCTTCATACGTC
TCAACTTTATTTGAAAATACAGAAACATTATCTGGCCAGTTTGATGAAATCTTTGATGATAATGGGTCGTGATTACCATGACAAACATAAAC
AAAGATTTGTTCATTTTGTAAACGTTCAAATTGCTGTTTTAAGAAAATTTCAGCACGTAATGTTCTGTTTTCACTATCAAATAAATCACCCGCG
ATAATTACAAAATCAACATCTTGTTGTAATGCAATATCTACAATATTTTTAAAACTTTCATAAGCACTTTTTTGAACATCCTCAAAAATTTTAG
GGCTTATATGACTCTTAGATTTGAAAGGACTATCTAAATGTAAATCCGAACAATGAATAAATTTAACCAT

Gene: UPF0342 (putative protein)

Position: 1916326 to 1916670, length: 345 nt, orientation: REVERSE

Perfect match to: (RF122-AJ938182-[1905520:1905864:r], highly conserved allele)

Sequence:

TTAGTCAGCGTAAATTTCGTCTAATGGTTTAACGATAATTTGGTTGATTTCTTGGAATACTTGACTCATTTTTTGTTCAGCATTCATTAATGCA
GAGATGTTTTCATCTTTTTCAATTGCTTGCGCTTGTTCTTGCGCTTTTTGTAAATCTTCTTCAGCAATTTCTTCACCTTGCATTTGTTTTTGTTGG
AAGTTAATTTGAGTTTCACGGAACTCGTCGAATAACTTTTTAGATTCTTCGTTAGCTTTTACATTAGCGAATGCTTCTTTGATTGCTTTGTATT
CTTCGCTTTCTCTTAAAGCTTGTTCTAATTGATTTGCATAATCATATAAATTTACTGCCAT

Gene: UPF0754 (putative membrane protein)

Position: 1916739 to 1917863, length: 1125 nt, orientation: REVERSE

Perfect match to: (Strain_21334-AGTW01000033-[106201:107325:r], allele observed in CC9+CC1+CC80+CC772)

Sequence:

TTAGACAAAGATTGCAACCAAACCTTGGAAAAATCCAATAATACCACCTAAAATGAAACCTAATGACATAATCAACTTTAGTTCTTTGTTAGC
AATTTCTATAATTAATTTTTCAATATAATCTAAGTCAAACGTATTAATTTGTTCTTCAATTAATCCTCGTAAATCCACTTTTTTCATAATTGTAG
ATAAGTGGATCGATAATTTTTCAATGATTAAATTTGCTAATTTGCTAGAGAGTTGACCTTCTAAATAATCAACGAATTGAGGCATTAACGTA
ACCACTGGCTTATTCGCTTGTTTGCTTGCATATGTTGTTACATACACTGATAGATTTTCAGCGATTTCATTAAACTGCGATGCATCTAACAGTT
CATTCAATGGTTTATCTTTAAAAGTTTGATATTCATTGGTAATTAACGATGTCACAATTGTTCTTGCTTTAGGATGAGATGTTAAACGTATAA
GTTCTTGTTGAATGCGATCTGCAATGCTCTCTTTTGTCATAAACATTTGCAACATACCAATTAACTTACCTTTCTCATTGAAAAAAGTATCCAA
CATATCATTAATATCTTGTGTGCCTTTTGCAGATGATAAATAATTCCTTGCACGATCACATAATAAGTCTGTTGCGTTATCTACATGCTGATCT
AAAAATGTTACAAGTTGATTTGGCAATAAAGATGCTATTGTTTGGTTTTGATGCTTTGTATAATAATTATTCAATTGTGATTCAATATATTGA
TTTCCATTAGTTTGCAATACTTGTTCTAAATCAATATCGATTTGAGAAGTAATTTGTTTTATTGACAATTGATCTTTCGTCAACTTTTGTAACTG
CTGTTGAATCATAGATTCTATTGCTTGCTGTGATTGCTCGCTTTTTAATTTTTCATTAATTAAAGTTTCTGTAAGCAAATGCTCTTCAATCACTT
GGCCAATTTTAGTTGCAATTTCTTCGCGTCTTTTCGGTATTAAACCTGGTGTAAATGGGACTCTAAATTTAAATATATAGTATGGTTTAAAGG
GGTGAAACAGCATTCTAATTGCAATTACATTAGTAATGCCCCCAATTATCGCCCCTACTACAATCATAAATATGATAATAAATAGTGCATTCA
T

Gene: xdrA (DNA binding helix-turn-helix protein)

Position: 1918042 to 1918506, length: 465 nt, orientation: REVERSE

Perfect match to: (JKD6159-CP002114-[1934849:1935313:r], highly conserved allele)

Sequence:

CTAGATATGTACTAATTCTTCTTTAGCATTTCTGTTAAAGTATGTTTCAGCTTCTCTCATTTTAGAAGTACCGAAAATTGGTTGGTTATCTGGA
TTTAATACACGATAAATATTGCTTACTTTGTTACTTTGTAAAATGTACCCATTACGAGTCTCAATGTTATTCCAATAAATATCACTTGTTGGTG
CATGGTTTGGATAAGCACAGTGATTTCTTGAAATTGTTTGAACGATTTCTAATGGATCACAGCCAAATGTACTGTTTAATACTTCAGAGTCTC
TGAATAATGCACAAATTGAAATACAAGTTGTCCAGTTTGGTAATACTCTCTCTTTTTCGATTTGTACTAAAGTCTTTTTAGAAAGTCCAATTGT
TTGCGCCATAGTATCTTGCGTATAACCAGCCTCTATACGAACCATTTTAAATTTTGTTTGAATTAAATCTGTAAAACTCTGTCTATCCAT

Gene: airR (anaerobic iron-sulfur cluster-containing redox sensor response regulator)

Position: 1918862 to 1919485, length: 624 nt, orientation: REVERSE

Perfect match to: (JKD6159-CP002114-[1935668:1936291:r], highly conserved allele)

Sequence:

CTAAATCAACTTATTTTCCATTGCATAAATTGCTGCTTGTGTACGATCGCTAACTTGTAATTTACTAAATATATGACTGACATGTGTTTTAATT
GTTTTTTCAGATACAAATAAAGTTTCTGCAATCTCTTTATTTGTTTTACCTTTAACCATTTCACGTAACACTTCAATTTCTCTCTTTGACAACTTA
TTCGTGTAGTGTGGTTTTTGGCTAACTGTTTCGAATACATCTTGTGCCTTAGGATGTATCATTTTTTCACCGTTCATAACTCGTCTAATAGTTT
CAATTAATTGCTGAGGCTCAACGTCTTTCATTTCATAACCATCAGCACCTTTATTGATTGCTGAAATTACATGTTCATCATCAACATAACTTGT
TAATACCAAAACTTTAATATCCGGATAATGTGCCTTAATATATTCCGTAATTTCAATACCATTCATGCCAGGCATCACTAAATCTAATAGCAC
AATATCAGGGTGCTCATGCTCTTTTAAATATTCTAAAAATGTTTCTCCATCTGCAAAGTCTTGTAAAACTTCTATGTTTTCAATCGTGGATAAT
AAAAATCGCAATCCTTGTCGCACAATATAATGGTCATCTACTAATATTACTTTGTTCAT

Gene: airS (anaerobic iron-sulfur cluster-containing redox sensor histidine kinase)

Position: 1919507 to 1920619, length: 1113 nt, orientation: REVERSE

Perfect match to: (11819-97-CP003194-[1969887:1970999:r], allele observed in CC80)

Sequence:

CTATTTTATAGGAATTGTGAATTGTATTTGTGTACCCTTTGTTGGCTGAGAATGAAAGGTCACTTTACCTCTTAATAATTTAACTCTTTGTTTT
ATGTTATTAATACCGTGTGATGAAGCTATCTGAACATTATCGATCTCAAATCCTTGACCATAATCAATCACGTCAATATATAGTATATCGTTC
ATTTGTTTTAATGTAAGATCCATTTTATTCGTATCAGCATGTTTCTTAACATTATTAATACACTCTTGTAATGCTCTATATATGTTTTCTTCGATT
TCATTAGATAAATCGATTAAACCTTCTACATTTACATTTAATTGTATATGCATTAATTTACTATATGCTGTCAAAGCATGAATTAAACCTTGCT
CAAGTCCAACTGGCTTAAGTTGCCAAATCAATGCACGCATTTCATTAACGGCATTTTGACTCGTTTCCTCAATCGTCTTGAATGCTTGTTTAG
CGATGGATTCGCTTGACATGCCATACGCAGCATGTGCTGTTAGTTTTACAGAAAATAACATTTGATTTACTGAATCATGTAAATCTCTAGCTA
AACGATTACGTTCATTAATTTTTGCTGCTTCTTTTTCACGGTCTGTTAAATAAATACGTTTGATGGCTGACCCTAATTGAAATGCGACAGACT
CTAGCAACTCTAAATCTTCATCACTATATATTTCAGTATTTGGGGACGCTACATTTAAAATGCCGAATTGTTCTTGACCCGATTTAAGTGGTA
CCGTTGCATGATGTGTAATATTGTCATTTTGGCTAGGAAATGCTTTAGAGGCTAAGTTAATACGAGAACAATTGACGATATTCGACGCTTTC
ATTAGCCTACGTTGATTAAATGCTTTCACACACCAACAAGACCCATCTTTAATATAGTGACAGTGGTCTGCTGTCAAAGATTGTGGTAGAGC
CACATGTGATACAAGTTCATGTTCACCTACGCTATTGATGAAAAAATATCCAGCCTGTCGTGAAATTACTGCCCTCAATTAAATATTTTAACGC
ACCTTGGGTCATGCTATACATTTCAGTTTCTTCGTTTAAAAATTCGGCAATCTTTTTTAATAAAGCTAGTCGCGTCCTTTGTTCCAT

Gene: rluA2 (RNA pseudouridylate synthase)

Position: 1920782 to 1921603, length: 822 nt, orientation: FORWARD

Perfect match to: (COL-CP000046-[1962282:1963103], highly conserved allele)

Sequence:

ATGAAATTTAAAATACCAGAAAACTTTAATGACTTAAGTTTACGAGATATTTTCCAACAACTTAAGGTACCTAAAAAAGATTTACATCATTTA
AATATGTCTAAAGATATTACTATTAATGATAAACCTGCGCGATTAATGGATAAAGTGCATACTGGCGACGATGTATTTGTTCCAACCATCGA
TGAAAAAAGTAATTATGTTCCAAGTTATCGTTATGCACAAATTAAATACGAAGACGATGATATGGCAATCGTAATGAAACCTAAAGGTGTT
AAGACTCACCCTAATGATTTAAAAGAAAGCAATACTTTAATGAATCATGTGATTTACACTATTGATAGTGACTATGTCGAACCAATTCATCG
ACTGGACCAGGAAACAGTAGGATTATTAATTGTTGCTAAAAATCCTTTAATGAAAAAAAATTCTTGATCGCATGTTAGAAGACAATGATATTA
CGCGGATATACAAAGCAAATGTTAAGGCACTTTTACCTTTAAAACCACAAACGATTGATATGCCAATTGGTAAAGATAAATTCCATTCGAAT
AAACGACGTGTGTCTCCTACTGGACAGCGTGCAATTACACACATTTTAACTTCAAAAATGATAAAAGAAGCTGTGTGCCAACTTGAAATCAA
GTTGGATACTGGACGTACTCATCAAATTCGTGTGCATTTAGCTGAAATTGGTCACCCTGTTATTGGTGATCCTTTATATGGTGATTCAACGTT
AAGACAATTAGAACTTGAAAGTTACAAAATAGAGTTTGTGCATCCCTTGACTAAGGAAGTCATTTCCGTTTCTTTGGATGACTAA

Gene: fumC-citG (fumarate hydratase)

Position: 1922066 to 1923451, length: 1386 nt, orientation: REVERSE

Perfect match to: (11819-97-CP003194-[1972446:1973831:r], allele observed in CC80+CC88)

Sequence:

TTAATGAGGATCTACCATATCTTCTGGTTTAATCCATGCTTCAAATTGTTCTTCTGTAACATATCCAGTTTGAATTGCAGATTCTTTTAAAGTT
AAACCTTCTTTATGGGCTTTCTTAGCAATTTGAGCAGCTTTTTCATAACCAATATGTGGATTTAATGCAGTAACTAACATTAATGATTGATTTA
AATAATTATCAATATTCTCTTCGATTGGTTCAATGCCCACTGCACAATTGTTATTAAATGTTTCCATACCGTCAGCTAAAAGGTAAATTGATTG
TAGTGTATTATGCATAATAACTGGTTTATAAACATTCAATTCAAAGTTACCTTGTGAACTTGCGAAGCCAACAACTGTATCATTACCCATTAC
TTGGACTGCAACCATTGTTAACATTTCACATTGTGTAGGATTAACTTTACCAGGCATAATTGATGAACCTGGTTCATTTTCAGGGATAGAAAT
TTCTGCCAAACCAGCTCGTGGCCCTGAAGCCAACCATCTCACATCATTAGCAATTTTCATTAAGTCTCCTGCTAGCGCCTTCAATGTTCCATG
TAGTTGTACTACTTCATCATGTGCTGTTAATGCATGGAATTTATTTTCAGAAGATACAAATGGATAACCCGTATTTTCTGAAATATAATGTGC
CACTTTATCACCAAATTCAGGATGCGCATTAATACCAGTACCAACAGCCGTACCACCGATGGCAAGATTTAAAATGTGCTTCTTAGATTCAG
ATAACATTATTTCGCAACGGTCAAGCATATAACGCCAGCCACTAATCTCTTGTCCTAGTTTGATCGGCGTTGCATCTTGTAAATGTGTACGAC
CAATTTTAATAATTGAATCAAATTTGTCTTCTTTTTCTTTCAAAGTATTTCTTAAAAGTTTTAATGCAGGTTCTAATTTTGTTTCAACCTCTTGAT
ATAATGCAACGTGCATAGCAGTTGGGAATGTATCATTCGAACTTTGTGATTTATTTACATCATCATTTGGGTGGATACTTTCATCACTTTGAT
GATCTTTTAAATACATATTAGCAACATAACTTACTACTTCGTTCACATTCATATTACTTTGTGTACCGCTTCCTGTTTGCCATACAACTAGTGG
GAAGTGTTCATCTAATTCACCTGATAAAATTTGATCACATGCGTATACAATGGCATCTTTTTTTGCCTCGCTTAATTTTCCTAAATCAAAATTA
GCTAATGCTGCTGCACGCTTTAGTTGTGCAAAACCATAAACTACTTCGATTGGCATACGCTCTTTACCAACTGGGAAATTACGTTTACTTCTT
TCTGTTTGAGCACCCCAATATTTATCTGCAGGTACTTCTATTTCTCCAAAAGTATCATGTTCAATTCTTACTGACAT

Gene: Q5HES3 (putative protein)

Position: 1923647 to 1924042, length: 396 nt, orientation: REVERSE

Perfect match to: (MW2-BA000033-[1949075:1949470:r], highly conserved allele)

Sequence:

TTAATTACGATACTTTGTTTTAGTAGCTTCAACCGTAGCAATAGCTGTAAGTATATATAATACAGCACTAACAATTGTCGTATATGGATTTAG
AGCAACAAGCGTACCTAATACTCCTGTTAAACTCGCATAAAACCCATCAAGTGTATAATACAATATTCCTGTTATTAATAAACAACAAATTAC
ACTTATTACAATACCTGATTGATTACTTTTAATGAATGTTTGCGCATTAACATCATCGATTAATCCTTTTGATAAATTGAGTTGTAATTTTATTA
CTTTGAAAATAACAGGTAAATATAATGCCCCAATTGCCAATGGAAAAGCTTTAATTGATATTAAACTTATAATAACTGTTGCTATCAATAATT
GAATCCAGTATTTTCCTAACAT

Gene: Q5HES1 (putative protein)

Position: 1925056 to 1925208, length: 153 nt, orientation: REVERSE

Perfect match to: (11819-97-CP003194-[1975436:1975588:r], allele observed in CC80)

Sequence:

TTACTGACTGTCTTCTTCAGAATTTTTTTCTTGATCATTTTGATCAGAAATTTGTTCCATTTCTTTACCTAATTCTTTTAAATCTTCAAAATCCGT
TACCATACTGTTTTCTTCTTCATGATAATTTAATTTTGGATCTTTGTCTTTAGACAT

Gene: Q5HES0 (putative glucosamine-6-phosphate deaminase)

Position: 1925233 to 1925832, length: 600 nt, orientation: REVERSE

Perfect match to: (11819-97-CP003194-[1975613:1976212:r], allele observed in CC80)

Sequence:

TTAAGCAAAGCGTGACGTAAAGTAAGCTTTAACATCTTCAGGTAAACCTGCAGCCGCTTCTTTATCAAGAATAACATTTACCATTCTATGTGC
TTTTAAATCGGCTGGTTCGAAGCTTGTTTTACCATTTTCTTGATATAATTTTTCAACTACATCTCGTTTATTAGCACCTGTCACTACTAAGAAA
ATTTCTCTTGCTTCCATTAGTCCTTGACGAATACTAACATTTAACTTACCTTGCTCATCGATAGAAACAACTTGTAATGTTAATTTCCCTTTATT
TTCTTTAGTTTTAATCTTATCAGCGATTAATTCGATTGCATCTTTTTCATAAGCAATTGGATAAACTTGACCTGCTGGTACACCTAACGCTTCG
AAATATGATTTTTTATCGTCATAATCTAAAATATTTATTTGGCTAAAATCAACAGCATGTTTTTCAACATTTTTCTTTAATTCATTTAGAACTGG
CGCTTGATCTGTATCTAAATGAAAACCTGCAATTGTAGTAGGATTATTGTTAAATTGCTTTCTAATAATATCAGCAGCATATTCTGCTACAAG
TTGACTATTGTCAAAGACTTTAAAGTTCATTGCCAT

Gene: cspR (tRNA (cytidine/uridine-2'-O-)-methyltransferase)

Position: 1925992 to 1926462, length: 471 nt, orientation: REVERSE

Perfect match to: (N315-BA000018-[1908978:1909448:r], highly conserved allele)

Sequence:

TTAATTCAATCCTGGAAAATCTTGTTGACGTAACGCTTCATAAATTAACAACGCAGCAGTATTTGATAAATTTAATGAACGAATATGTTCACT
CATAGGAATTCTTAACGCTGTGTCTTGATATTTCTCTTTCACCCAGTCTGGTAATCCTGTCGTTTCTTTTCCAAAAATGAAGTAAAAATCTTTG
TCATGATTTGAAAAATCAAAATCACTATAAGTCTTTTTACCAAATTTTGTTAATAAGTAATACTCGCCATTTGTGGCTTCAAAAAATGCTTCAA
TACTATCATGATACGTAATATTCACAAATTCCCAATAATCTAAACCGGCTCTTTTTAACATTTTATCATCAGTTCTAAATCCAAGAGGTTTAAT
TAAATGTAAATGTGTGTTTGTACCTGCACACGTACGTGCAATGTTACCAGTATTAGCTGGGATTTCTGGTTGATATAAAACGATATGATTTG
TCAT

Gene: queG (epoxyqueuosine reductase)

Position: 1926467 to 1927594, length: 1128 nt, orientation: REVERSE

Perfect match to: (MRSA252-BX571856-[2028550:2029677:r], allele observed in CC30+CC80)

Sequence:

CTATTCTCTCCTTGTGTCTAATCCTTTTATCATTTCATTCTGAACTTCTGCATCCTCTTGATCATAATTAGCATTGATAAAATCTCTTGCTTCTTC
CCCAAGAATTTGACCAATGGCCCAATAAGCAGTTGCTCGAATCATCGGTCTTTCATCTGTTGTTGCAACTTTTTTCAATTCTGGAATTGCATC
CACTTCATTAAAATGCGCTAATGCTAAAATAGCATTTCGTTGTATCGGCTTTTTACCACGCCAAGCACCTGCAAGGTGACCATATGTTTGTTT
GAATTCTTTATTAGACATACGTAGTAAAGGTACTAATCTTGGCTTTAAAATTTCTGGTTCCAAAATAATGTCATCTTGTTCGGTATTAATACCT
CTATTTTTCGGACAAACTTGTTGACACGTATCGCAACCATATAATCTATTCCCAATTTTATAACGATATTGGTCAGGCATATAGCCTTTTGTTT
GCGTTAAAAAACTAATGCATTTCTGACTATTTAATTGGCCATTTCCAACTAATGCACTTGTTGGACAACGATCAACACAAATTGTACAATCAC
CACAGCTATCTAATAATGGATCATCAGGTTCAAAAGGTATACTGACTAACATTTCACCGAGGTATGTCCATGTTCCTAGTTTAGGATTGATG
ACAAAGCCATTACGACCAACAAATCCTAAACCTGCACGTTCTGCTACTGCCCTATCTGATAATACACCCGTATCTACCATAGATTTTATTTCA
ACATCTGGAACTTTAGATTCAATAAATGAAGCTAACATGTCTAATCGTTTACGCATAATTGTATGATAATCTTGACCCCACGATGCTCTAGCA
AATAAGCCTCTGCGATCACCTCTAACACTCTTAGGTGCACCTTTCAGTTTGTTAGGATAACCAACTGCAATTGCTATGATTGACCTTGCTGTT
GGTAAGGATAATTTAGGCTCCGTTCGTAAAGCAATATCAGATTCTTCAAATCCTGAGGCATAACCATTTGCATGATATGCTTCTAGCTTTTGC
TTCAATTCATCAAAGGGATCGGCAGTAGTAAATCCAATACTGTCGATACCAATTGTATATGCATAGTCAATGATATCTTGCTTTAACTGCTTT
GTATCCAA

Gene: artR (high affinity arginine ABC transporter, ATP-binding protein)

Position: 1927745 to 1928473, length: 729 nt, orientation: REVERSE

Perfect match to: (N315-BA000018-[1910731:1911459:r], highly conserved allele)

Sequence:

TTATAATACTCTTGCTAAGAAATTTTGTGTTCTTTCATGTTGCGGTTGTTCAAATATTTCGACTGGTGTGCCTGACTCTACGACAACGCCATCT
GCCATAAATATGACTTTGTCACTTACATCTTTGGCAAATCCCATTTCATGTGTCACAACCACCATGGTCATACCTTCTTTGGCTAGGTCTTTCA
TTACTTTTAATACATCACCAACTACCTCAGGATCTAATGCTGAAGTTGGTTCATCGAATAAAATAACATCTGGATGCATTGCTAAAGCTCTTG
CAATTGCTACCCTTTGCTTTTGACCACCTGATAATTGATTCGGATATACATCTGCTTTTTCTTTTAATCCCACTTTATCTAATAACGACAATGCT
TCCTTATGTAATTCATCGTTATTATCTTTCTTTAATAATTTAGGAGCTAAAATAATATTATCGACAACTTTTTTATGTGGAAATAGGTTGAAGT
TTTGAAATACCATACCCATTTTTTGACGTAGTTTATCTACTTGTGTCCCTTTTTCCGTTAAGTCATTGCCTTCAAAAATCACTTGACCTTTAGTG
GGTACTTCTAATAAATTCATACATCTTAACAATGTACTTTTACCACTACCAGATGGACCTATTATTGCTACTACTTCCCCTTGATTGATTTCAA
GATTGATATCTTTTAAAACTTCATTATCTCCAAAAACTTTATTAAGATTGTTTATTTTAATCACTGGCAT

Gene: artQ (high affinity arginine ABC transporter, transmembrane permease)

Position: 1928460 to 1929917, length: 1458 nt, orientation: REVERSE

Sequence:

TTAATCACTGGCATTCAATCTCCCTTCAATCATGTTCATAATGCGTGTAAGTACAAATGTTAAGACAAAGTATAATGCTGCTGCCACTATTAA
TGGTGTGAATGGGTCAAATGAAATACCTTGAACCACTTGTGCATTAAACATAATTTCGCCAACTCCAATTGTTGACACAATAGATGATTCTTT
AATTAAAGTGACAAATTCATTACCTAAAGCTGGTAAAATATTTTTAATTGCTTGTGGCATGATTACACTTTTCATCGTTTGTCTATAATTCAAA
CCTAGACTACGTGCGGCTTCCATTTGGCCTTTATCAACAGCATTTATACCTGCACGAATAATTTCAGCAATGTAAGCTGAGGAATTAATAACT
AATGCAATTGTTCCACAAACAAGAGCTGAAATGTCTAGTCCTAATGCAGCAGTTATACCAAAGAATACGATAAATACTTGAACTAACATTGG
TGTTCCTCTTAATATTTCGATATAGATAGAAGCAATCCATGAAATAATTTTTATTTTACTTAATTTCATTAACGCAACGAATGCACCTAAAATA
GAACCTAATGCAACACCGATAAGTGAAATTAATATTGTAATCTTAATTCCTTTCAAGAAAAAACTACCATACTTAGAAATAAAACCACTGTCA
TCATTCATCGCATTTGCAGCATTAGTCATATATTTATCGATTAATCCTTTATCTTTAACCTCTTTAATCGTTTTATTAATTTGTGACAATAATTTT
GGTGAATCTTTTGGCACTGCTATCACTGTATCTTTTTCTTCTTCATTAAATTTCACATTGGAAATTCCTAATTTAGGATTTTGTTTTAAATATGC
TTCTGCTACAGGTTTTTCAACTACAGCACCTTCAACCTTTCCACTTTTAAGTGCTAATATAACGTCTGGTAAACGGCTTAATGAAGTAATAGA
TGCATTTTCAATTTCGGTTTGAGCGATTTTTTCTTGTTCAGTTCCCTTTTGTGCCCCTACTTTTTTATTATTAAAGTCTTTGATATCTTTATATTC
ATTAACTTTATCTTTCTTTACAAGCATGATATTTTTAGTCATCATATATGAATCTGAAAAATCAACTTGCTTCTTACGTTCAGGCGTTGAAGTC
ATTCCGGAAATAATAATATCAATTTTTCCAGTTTTAAGAGCTCCTAACAAACTATCAAATGACATATTGACGATTTTTAATTTTAAATTATTAT
CTTTCGCAATTTTTTTAGCTAAATCAATATCTACACCTGCATACTCAGTCTTACCATTAACTGTATGCTCAAATTCCATTGGTGCATAATCTGC
AGAAAGACCCACTCTAAGTTCACCGCGTTCTTTAATCTTCTCCCATGTTTGATCTTGTTCAGCATGTGCTGTGGGATTGATATACACCATAGC
ACTGCTAATTAACAACCCTAATACTAATATAAACCTAATTAAACACTTCAT

Gene: Q1Y9N4 (phosphotransferase system (PTS), EIIC domain protein)

Position: 1930175 to 1931236, length: 1062 nt, orientation: REVERSE

Perfect match to: (11819-97-CP003194-[1980555:1981616:r], highly conserved allele)

Sequence:

TTATTGTTTTGGAACTTCAAATTTATAAATATCATTAGAATATATAGGAATGAATTTTTTACACAATTCATGAACAATAAATGCCGATACAAA
TGGTATGACAAAATAACCAAGTGCTAATAAAATAATATTCATTGTTGGATCACCTGACATTCGGTTAAATGCATTTATTGGGCCAACTAATC
CTGTATATCCAAATCCAGCAGACAATGGTGTTCCTTTAACCTGAAGAACGTAAGCAATGATTCCAGTAATTATTCCATTTATAGTCAATGGTA
TCGAAATAATTAAATTTTTCAAGTAAACTGGGATCATCATTTTTGCAGCTCCTATGAGTAAAACTGCATTTACACCAATAGAATTGACACGCA
ATGAGCCAAATAAGAAAGTAACACAGGCAGCCACTATACCTAGGTTTGCTGCTCCACTTCCTAGTCCGTTTAAACTAATCGCAGTTGCAATC
GCTACTAACGATATTGGTGTTACCATTAATAATGAAAATGCCACACTAATAAGTATAGACATTAACAACGGATTTAAGTCTGTAAAAGAATG
AATTACATTTCCAATTGCTTGAGTAATTTTTCGAATGTAAGGTAATGTGATTAGACCGATACCCCCACTAACGATAGGTACTAAAACTGGTA
ATATAATTAATTCAAAAGATCCAAGTTTGTTTTGTAATACCATATATATAAGACATGCAATAATAACAACCAAACTCGTATTTATAATGTCAC
CTATACCTTTTAACATAAAACTATTATTACTATATACAACAGCACCTGAACCAATCATAGCTGATGTACCTACTATAGCAGCACCTGCGCCAT
TAAATTTAAATTGATGAGCAGCTAAAACCCCAATAATAAATGCCATAAATGATTGAATTAGTATCACTAACTGATACGTTAATTCTAAAATTT
CATTACCACTTTTAAATATTTTTAATACTTCACCTAATAGAGCATTCGGAACAAGTGCAATAACAACACCAGCACCAATAGAATTTAAAATCT
TACTGAAAAACTGTTTATTATCAGCATTATTTGCGTTACTCAT

Gene: IGR_rrl_rrf (intergenic spacer between rrl and rrf)

Position: 1934343 to 1934637, length: 295 nt

Sequence:

CATATGAATGTAATTTATACATTCAAAACTAGATAGTAAGTAAAAGTGATTTTGCTTCGCAAAACATTTATTTTGATTAAGTCTTCGATCGAT
TAGTATTCGTCAGCTCCACATGTCACCATGCTTCCACCTCGAACCTATTAACCTCATCATCTTTGAGGGATCTTATAACCGAAGTTGGGAAAT
CTCATCTTGAGGGGGGCTTCATGCTTAGATGCTTTCAGCACTTATCCCGTCCACACATAGCTACCCAGCTATGCCGTTGGCACGACAACTGG
TACACCAGAGGTATGTC

Gene: rrl (23S ribosomal RNA)

Position: 1934638 to 1937337, length: 2700 nt

Sequence:

CATCCCGGTCCTCTCGTACTAAGGACAGCTCCTCTCAAATTTCCTACGCCCACGACGGATAGGGACCGAACTGTCTCACGACGTTCTGAACC
CAGCTCGCGTACCGCTTTAATGGGCGAACAGCCCAACCCTTGGGACCGACTACAGCCCCAGGATGCGATGAGCCGACATCGAGGTGCCAA
ACCTCCCCGTCGATGTGAACTCTTGGGGGAGATAAGCCTGTTATCCCCGGGGTAGCTTTTATCCGTTGAGCGATGGCCCTTCCATGCGGAA
CCACCGGATCACTAAGTCCGTCTTTCGACCCTGCTCGACTTGTAGGTCTCGCAGTCAAGCTCCCTTATGCCTTTACACTCTATGAATGATTTCC
AACCATTCTGAGGGAACCTTTGAGCGCCTCCGTTACCTTTTAGGAGGCGACCGCCCCAGTCAAACTGCCCGCCTGACACTGTCTCCCACCAC
GATAAGTGGTGCGGGTTAGAAAGCCAACACAGCTAGGGTAGTATCCCACCAGCGCCTCCACGTAAGCTAGCGCTCACGTTTCAAAGGCTCC
TACCTATCCTGTACAAGCTGTGCCGAATTTCAATATCAGGCTACAGTAAAGCTCCACGGGGTCTTTCCGTCCTGTCGCGGGTAACCTGCATC
TTCACAGGTACTATGATTTCACCGAGTCTCTCGTTGAGACAGTGCCCAAATCGTTACGCCTTTCGTGCGGGTCGGAACTTACCCGACAAGGA
ATTTCGCTACCTTAGGACCGTTATAGTTACGGCCGCCGTTTACTGGGGCTTCGATTCGTAGCTTCGCAGAAGCTAACCACTCCTCTTAACCTT
CCAGCACCGGGCAGGCGTCAGCCCCTATACATCACCTTACGGTTTAGCAGAGACCTGTGTTTTTGATAAACAGTCGCTTGGGCCTATTCACT
GCGGCTCTTCTGGGCGTTAACCCTAAAGAGCACCCCTTCTCCCGAAGTTACGGGGTCATTTTGCCGAGTTCCTTAACGAGAGTTCGCTCGCT
CACCTTAGAATTCTCATCTTGACTACCTGTGTCGGTTTGCGGTACGGGCACCTATTTTCTATCTAGAGGCTTTTCTCGGCAGTGTGAAATCAA
CGACTCGAAGACACAATGTCTTCTCCCCATCACAGCTCAGCCTTAACGAGTACCGGATTTGCCTAATACTCAGCCTTACTGCTTAGACGTGCA
ATCCAATCGCACGCTTCGCCTATCCTACTGCGTCCCCCCATCGATTAAAACGATTATAGGTGGTACAGGAATATCAACCTGTTATCCATCGCC
TACGCCTGTCGGCCTCAGCTTAGGACCCGACTAACCCAGAGCGGACGAGCCTTCCTCTGGAAACCTTAGTCAATCGGTGGACGGGATTCTC
ACCCGTCTTTCGCTACTCACACCGGCATTCTCACTTCTAAGCGCTCCACATGTCCTTACGATCATGCTTCAACGCCCTTAGAACGCTCTCCTAC
CATTGTCCAAAGGACAATCCACAGCTTCGGTAATATGTTTAGCCCCGGTACATTTTCGGCGCAGTGTCACTCGACTAGTGAGCTATTACGCA
CTCTTTAAATGATGGCTGCTTCTAAGCCAACATCCTAGTTGTCTGGGCAACGCCACATCCTTTTCCACTTAACATATATTTGGGACCTTAGCT
GGTGGTCTGGGCTGTTTCCCTTTCGAACACGGACCTTATCACCCATGTTCTGACTCCCAAGTTAAATTAATTGGCATTCGGAGTTTGTCTGAA
TTCGGTAACCCGAGAGGGGCCCCTCGTCCAAACAGTGCTCTACCTCCAATAATCATCACTTGAGGCTAGCCCTAAAGCTATTTCGGAGAGA
ACCAGCTATCTCCAGGTTCGATTGGAATTTCTCCGCTACCCTCAGTTCATCCGCTCACTTTTCAACGTAAGTCGGTTCGGTCCTCCATTCAGTG
TTACCTGAACTTCAACCTGACCAAGGGTAGATCACCTGGTTTCGGGTCTACGACCAAATACTAAACGCCCTATTCAGACTCGCTTTCGCTAC
GGCTCCACATTTACTGCTTAACCTTGCATCAAATCGTAACTCGCCGGTTCATTCTACAAAAGGCACGCCATCACCCATTAACGGGCTCTGACT
ACTTGTAAGCACACGGTTTCAGGTTCTATTTCACTCCCCTTCCGGGGTGCTTTTCACCTTTCCCTCACGGTACTGGTTCACTATCGGTCACTAG
AGAGTATTTAGCCTTAGGAGATGGTCCTCCCAGATTCCGACGGAATTTCACGTGCTCCGTCGTACTCAGGATCCACTCAAGAGAGACAACA
TTTTCGACTACAGGATTATTACCTTCTTTGATTCATCTTTCCAGATGATTCGTCTAATGTCGTCCTTTGTAACTCCGTATAGAGTGTCCTACAA
CCCCAACAAGCAAGCTTGTTGGTTTGGGCTCTTCCCGTTTCGCTCGCCGCTACTAAGGGAATCGAATTTTCTTTCTCTTCCTCCGGGTACTAA
GATGTTTCAGTTCTCCGGGTGTGCCTTCTGATATGCTATGTATTCACATATCGATAACATGACATAACTCATGCTGGGTTTCCCCATTCGGAA
ATCTCTGGATCAAAGCTTACTTACAGCTCCCCAAAGCATATCGTCGTTAGTAACGTCCTTCATCGGCTTCTAGTGCCAAGGCATCCACCGTGC
GCCCTTAATAACTTAATC

Gene: rrs (16S ribosomal RNA)

Position: 1937765 to 1939317, length: 1553 nt

Sequence:

AGAAAGGAGGTGATCCAGCCGCACCTTCCGATACGGCTACCTTGTTACGACTTCACCCCAATCATTTGTCCCACCTTCGACGGCTAGCTCCT
AAAAGGTTACTCCACCGGCTTCGGGTGTTACAAACTCTCGTGGTGTGACGGGCGGTGTGTACAAGACCCGGGAACGTATTCACCGTAGCAT
GCTGATCTACGATTACTAGCGATTCCAGCTTCATGTAGTCGAGTTGCAGACTACAATCCGAACTGAGAACAACTTTATGGGATTTGCTTGAC
[truncated: 875,105 more chars]
