# Supplementary material for: Molecular investigations on a chimeric strain of Staphylococcus aureus sequence type 80
Source: PLoS One. 2020 Oct 14;15(10):e0232071. doi: 10.1371/journal.pone.0232071 (PMC7556507; doi:10.1371/journal.pone.0232071)
Supplement: S4 File — (PDF) [file pone.0232071.s004.pdf]

Gene: dnaA (chromosomal replication initiator protein)

Contig: 01_NODE_8 (fragment from pos. 242580 on), position: 517 to 1878, length: 1362 nt, orientation: FORWARD

Sequence:

ATGTCGGAAAAAGAAATTTGGGAAAAAGTGCTTGAAATTGCTCAAGAAAAATTATCAGCTGTAAGTTACTCAACTTTCCTAAAAGATACT
GAGCTTTACACGATCAAAGATGGTGAAGCTATCGTATTATCGAGTATTCCTTTTAATGCAAATTGGTTAAATCAACAATATGCTGAAATTA
TCCAAGCAATCTTATTTGATGTTGTAGGCTATGAAGTAAAACCTCACTTTATTACTACTGAAGAATTAGCAAATTATAGTAATAATGAAAC
TGCTACTCCAAAAGAAGCAACAAAACCTTCTACTGAAACAACTGAGGATAATCATGTGCTTGGTAGAGAGCAATTCAATGCCCATAACAC
ATTTGACACTTTTGTAATCGGACCTGGTAACCGCTTTCCACATGCAGCGAGTTTAGCTGTGGCCGAAGCACCAGCCAAAGCGTACAATCC
ATTATTTATCTATGGAGGTGTTGGTTTAGGAAAAACCCATTTAATGCATGCCATTGGTCATCATGTTTTAGATAATAATCCAGATGCCAAA
GTGATTTACACATCAAGTGAAAAATTCACAAATGAATTTATTAAATCAATTCGTGATAACGAAGGTGAAGCTTTCAGAGAAAGATATCGT
AATATCGACGTCTTATTAATCGATGATATTCAGTTCATACAAAATAAAGTACAAACACAAGAAGAATTTTTCTATACTTTTAATGAATTGCA
TCAGAATAACAAGCAAATAGTTATTTCGAGTGATCGACCGCCAAAGGAAATTGCACAATTAGAAGATCGATTACGTTCGCGCTTTGAATG
GGGGCTAATTGTTGATATTACGCCACCAGATTATGAAACTCGAATGGCAATTTTGCAGAAGAAAATTGAAGAAGAAAAATTAGATATTCC
ACTAGAAGCTTTAAATTATATAGCAAATCAAATTCAATCTAATATTCGTGAATTAGAAGGTGCATTAACACGTTTACTTGCATATTCACAAT
TATTAGGAAAACCAATTACAACTGAATTAACTGCTGAAGCTTTAAAAGATATCATTCAAGCACCAAAATCTAAAAAAATTACCATCCAAGA
TATTCAAAAAATTGTAGGCCAGTACTATAATGTTAGAATTGAAGATTTCAGTGCAAAAAAACGTACAAAGTCAATTGCATATCCACGTCA
AATAGCTATGTACTTGTCTAGAGAGCTTACAGATTTCTCATTACCTAAAATTGGTGAAGAATTTGGTGGGCGTGATCATACGACCGTCATT
CATGCTCATGAAAAAATATCTAAAGATTTAAAAGAAGATCCTATTTTTAAACAAGAAGTAGAGAATCTTGAAAAAGAAATAAGAAATGTA
TAA

Gene: dnaN (DNA polymerase III subunit beta)

Contig: 01_NODE_8 (fragment from pos. 242580 on), position: 2156 to 3289, length: 1134 nt, orientation: FORWARD

Sequence:

ATGATGGAATTCACTATTAAAAGAGATTATTTTATTACACAATTAAATGACACATTAAAAGCTATTTCACCAAGAACAACATTACCTATATT
AACTGGTATCAAAATCGATGCGAAAGAATATGAAGTTATATTAACTGGTTCAGACTCTGAAATTTCAATAGAAATCACTATTCCTAAAACT
GTAGATGGCGAAGATATTGTCAATATTTCAGAAACAGGCTCAGTAATACTTCCCGGACGATTCTTTGTTGATATTATAAAAAAATTACCTG
GTAAAGATGTTAAATTATCTACAAATGAACAATTCCAGACATTAATTACATCAGGTCATTCTGAATTTAATTTAAGTGGCTTAGATCCAGA
TCAATATCCTTTATTACCTCAAGTTTCTAGAGATGACGCAATTCAATTGTCGGTAAAAGTACTTAAAAACGTGATTGCACAAACGAATTTT
GCAGTGTCCACCTCAGAAACACGCCCAGTACTAACTGGTGTGAACTGGCTTATACAAGAAAATGAATTAATATGCACAGCGACTGACTCA
CACCGCTTGGCTGTAAGAAAGTTGCAGTTAGAAGATGTTTCTGAAAACAAAAATGTCATCATTCCAGGTAAGGCTTTAGCTGAATTAAAT
AAAATTATGTCTGACAATGAAGAAGACATTGATATCTTCTTTGCTTCAAACCAAGTTTTATTTAAAGTTGGAAATGTGAACTTTATTTCTCG
ATTATTAGAAGGACATTATCCTGATACAACACGTTTATTCCCTGAAAACTATGAAATTAAATTAAGTATAGACAATGGGGAGTTTTATCAT
GCAATTGATCGTGCATCTTTATTAGCACGTGAAGGTGGTAATAACGTTATTAAATTAAGTACAGGTGATGACGTTGTTGAATTGTCTTCTA
CATCACCAGAAATTGGTACTGTAAAAGAAGAAGTTGATGCAAACGATGTTGAAGGTGGTAGCCTGAAAATTTCATTCAACTCTAAATATA
TGATGGATGCTTTAAAAGCAATCGATAATGATGAGGTTGAAGTTGAATTCTTCGGTACAATGAAACCATTTATTCTAAAACCAAAAGGTG
ACGACTCGGTAACGCAATTAATTTTACCAATCAGAACTTACTAA

Gene: yaaA (RNA-binding S4 domain protein)

Contig: 01_NODE_8 (fragment from pos. 242580 on), position: 3671 to 3916, length: 246 nt, orientation: FORWARD

Perfect match to: (RF122-AJ938182-[3670:3915], highly conserved allele)

Sequence:

GTGATTATTTTGGTTCAAGAAGTTGTAGTAGAAGGAGACATTAATTTAGGTCAATTTCTAAAAACAGAAGGGATTATTGAATCTGGTGGT
CAAGCAAAATGGTTCTTGCAAGACGTTGAAGTATTAATTAATGGAGTGCGTGAAACACGTCGCGGTAAAAAGTTAGAACATCAAGATCG
TATAGATATCCCAGAATTACCTGAAGATGCTGGTTCTTTCTTAATCATTCATCAAGGTGAACAATGA

Gene: recF (DNA replication and repair protein F)

Contig: 01_NODE_8 (fragment from pos. 242580 on), position: 3913 to 5025, length: 1113 nt, orientation: FORWARD

Sequence:

ATGAAGTTAAATACACTCCAATTAGAAAATTATCGTAACTATGATGAGGTTACGTTGAAATGTCATCCTGACGTGAATATCCTCATTGGAG
AAAATGCACAAGGAAAGACAAATTTACTTGAATCAATTTATACCTTAGCTTTAGCAAAAAGTCATAGAACGAGTAATGATAAGGAACTCA
TACGTTTTAATGCTGATTATGCTAAAATAGAAGGTGAGCTTAGTTATAGACACGGCACGATGCCATTAACAATGTTTATCACTAAAAAAG
GTAAACAAGTCAAAGTGAATCACTTAGAGCAAAGTCGTCTAACTCAATATATTGGACACCTCAATGTGGTTCTATTTGCGCCAGAAGATTT
GAATATTGTAAAAGGCTCTCCTCAAATAAGACGACGCTTTATAGATATGGAGTTGGGCCAAATTTCTGCTGTTTACTTAAATGATTTAGCT
CAATACCAACGTATTTTAAAGCAAAAGAATAATTACTTAAAGCAGTTACAATTAGGCCAAAAAAAGGACTTAACAATGTTGGAAGTATTA
AATCAGCAGTTTGCTGAATATGCAATGAAAGTAACTGATAAACGTGCACATTTTATTCAAGAGCTAGAGTCGTTAGCGAAACCGATTCAT
GCTGGTATCACAAATGATAAAGAAGTGTTGTCGCTGAATTATTTACCTAGTCTTAAATTTGATTATGCTCAAAATGAAGCGACACGACTTG
AAGAAATTATGTCTATTCTTAGCGATAATATGCAAAGAGAAAAAGAACGAGGCATTAGCTTATTCGGACCACATCGAGATGATATAAGTT
TTGATGTGAATGGCATGGATGCTCAAACATATGGTTCTCAAGGACAGCAACGTACAACAGCTTTGTCCATTAAATTAGCTGAAATTGAGT
TAATGAATATCGAAGTTGGGGAATATCCCATCTTATTATTAGACGATGTACTCAGTGAATTAGATGATTCGCGTCAAACGCATTTATTAAG
TACGATTCAGCATAAAGTACAAACATTTGTCACTACGACATCTGTAGATGGTATTGATCATGAAATCATGAATAACGCTAAATTGTATCGT
ATTAATCAAGGTGAAATTATAAAGTAA

Gene: gyrB (DNA topoisomerase II, subunit B)

Contig: 01_NODE_8 (fragment from pos. 242580 on), position: 5035 to 6969, length: 1935 nt, orientation: FORWARD

Perfect match to: (Strain_21252-AHJV01000015-[25179:27113], allele observed in CC45IV8+CC395)

Sequence:

ATGGTGACTGCATTGTCAGATGTAAACAACACGGATAATTATGGTGCTGGGCAAATACAAGTATTAGAAGGTTTAGAAGCAGTACGTAA
AAGACCAGGTATGTATATAGGATCGACTTCAGAGAGAGGTTTGCACCATTTAGTGTGGGAAATTGTCGATAATAGTATCGATGAAGCAT
TAGCTGGTTATGCAAATCAAATTGAAGTTGTTATTGAAAAAGATAACTGGATAAAAGTAACGGATAACGGACGTGGTATCCCAGTTGAT
ATTCAAGAAAAAATGGGACGTCCAGCTGTCGAAGTTATTTTAACTGTTTTACATGCTGGTGGTAAATTTGGCGGTGGCGGATACAAAGTA
TCTGGTGGTTTACATGGTGTTGGTTCATCAGTTGTAAACGCATTGTCACAAGACTTAGAAGTATATGTACACAGAAATGAGACTATATATC
ATCAAGCATATAAAAAAGGTGTACCTCAATTTGACTTAAAAGAAGTTGGCACAACTGATAAGACAGGTACTGTCATTCGTTTTAAAGCAG
ATGGAGAAATCTTCACAGAGACAACTGTATACAACTATGAAACATTACAGCAACGTATTAGAGAGCTTGCTTTCTTAAACAAAGGAATTC
AAATCACATTAAGAGATGAACGTGATGAAGAAAACGTTAGAGAAGACTCCTATCACTATGAGGGCGGTATTAAATCTTATGTTGAGTTAT
TGAACGAAAATAAAGAACCTATTCATGATGAGCCGATTTATATTCATCAATCTAAAGATGATATTGAAGTAGAAATTGCGATTCAATATA
ACTCAGGATATGCCACAAATCTTTTAACTTACGCAAATAACATTCATACGTACGAAGGTGGTACGCATGAAGACGGATTCAAACGTGCAT
TAACGCGTGTCTTAAATAGTTATGGTTTAAGTAGCAAGATTATGAAAGAAGACAAAGATAGACTTTCTGGTGAAGATACACGTGAAGGT
ATGACTGCAATTATATCTATCAAACATGGTGATCCTCAATTCGAAGGTCAAACGAAGACAAAATTAGGTAATTCTGAAGTGCGTCAAGTT
GTAGATAAATTATTCTCAGAGCACTTTGAACGATTTTTATATGAAAATCCACAAGTCGCACGTACAGTGGTTGAAAAAGGTATTATGGCG
GCACGTGCACGTGTTGCTGCGAAAAAAGCGCGTGAAGTAACACGTCGTAAATCAGCGTTAGATGTAGCAAGTCTTCCAGGTAAATTAGC
TGATTGCTCTAGTAAAAGTCCTGAAGAATGTGAGATTTTCTTAGTCGAAGGGGACTCTGCCGGGGGGTCTACAAAATCTGGTCGTGACTC
TAGAACGCAGGCGATTTTACCATTACGAGGTAAGATATTAAATGTTGAAAAAGCACGATTAGATAGAATTTTGAATAACAATGAAATTCG
TCAAATGATCACAGCATTTGGTACAGGAATCGGTGGCGACTTTGATCTAGCGAAAGCAAGATATCACAAAATCGTCATTATGACTGATGC
CGATGTGGATGGAGCGCATATTAGAACATTGTTATTAACATTCTTCTATCGATTTATGAGACCGTTAATTGAAGCAGGCTATGTGTATATT
GCACAGCCACCGTTGTATAAACTGACACAAGGTAAACAAAAGTATTATGTATACAATGATAGGGAACTTGATAAACTTAAATCTGAATTG
AATCCAACACCAAAATGGTCTATTGCACGATACAAAGGTCTTGGAGAAATGAATGCAGATCAATTATGGGAAACAACAATGAACCCTGA
GCACCGCGCTCTTTTACAAGTAAAACTTGAAGATGCGATTGAAGCGGACCAAACATTTGAAATGTTAATGGGTGACGTTGTAGAAAACC
GTAGACAATTTATAGAAGATAATGCAGTTTATGCAAACTTAGACTTCTAA

Gene: gyrA (DNA topoisomerase II, subunit A)

Contig: 01_NODE_8 (fragment from pos. 242580 on), position: 7006 to 9669, length: 2664 nt, orientation: FORWARD

Sequence:

ATGGCTGAATTACCTCAATCAAGAATAAATGAACGAAATATTACCAGTGAAATGCGTGAATCATTTTTAGATTATGCGATGAGTGTTATC
GTTGCGCGTGCATTGCCAGATGTTCGTGACGGTTTAAAACCAGTACATCGTCGTATACTATATGGATTAAATGAACAAGGTATGACACCG
GATAAATCATATAAAAAATCAGCACGTATCGTTGGTGACGTAATGGGTAAATATCACCCTCATGGTGACTCATCTATTTATGAAGCAATG
GTACGTATGGCTCAAGATTTCAGTTATCGTTATCCGCTTGTTGATGGCCAAGGTAACTTCGGTTCAATGGATGGAGATGGCGCAGCAGCA
ATGCGTTATACTGAAGCGCGTATGACTAAAATCACACTTGAACTGTTACGTGATATTAATAAAGATACAATAGATTTTATCGATAACTATG
ATGGTAATGAAAGAGAGCCGTCAGTCTTACCTGCTCGATTCCCTAACTTATTAGCCAATGGTGCGTCAGGTATTGCTGTAGGTATGGCAA
CCAATATTCCACCACATAACTTAACTGAATTAATCAATGGAGTGCTTAGTTTAAGTAAGAATCCTGATATTTCAATTGCTGAGTTGATGGA
GGATATTGAAGGTCCTGATTTCCCAACTGCTGGACTTATTTTAGGTAAAAGTGGAATTAGACGTGCTTATGAAACAGGTCGTGGTTCAAT

TCAAATGCGCTCTCGTGCGGAAATTGAAGAACGTGGCGGTGGTCGTCAACGTATTGTTGTCACTGAAATTCCTTTCCAAGTGAATAAAGC
ACGTATGATTGAAAAAATTGCAGAGCTCGTACGCGACAAGAAAATTGACGGTATCACTGATTTACGTGATGAAACAAGTTTACGTACAG
GTGTGCGTGTCGTTATTGATGTGCGTAAGGATGCAAATGCTAGTGTCATTTTAAATAACTTATACAAACAAACACCTCTTCAAACATCATT
TGGTGTGAATATGATTGCACTTGTAAATGGTAGACCGAAGCTTATTAATTTAAAAGAAGCGTTGGTACATTATTTAGAGCATCAAAAGAC
AGTTGTTAGAAGACGTACGCAATATAACTTACGTAAAGCTAAAGATCGTGCCCACATTTTAGAAGGATTACGTATCGCACTTGACCATAT
CGATGAAATTATTTCAACGATTCGCGAGTCAGATACAGATAAAGTTGCAATGGAAAGCTTGCAACAACGCTTCAAACTTTCTGAAAAACA
AGCTCAAGCTATTTTAGACATGCGTTTAAGACGTCTAACAGGTTTAGAGAGAGACAAAATTGAAGCTGAATATAATGAGTTATTAAATTA
TATTAGTGAATTAGAAGCCATTTTAGCTGACGAAGAAGTGTTATTACAATTAGTTAGAGACGAATTGACTGAAATTAGAGATCATTTTGG
TGATGATCGTCGTACTGAAATTCAATTAGGTGGATTTGAAGATTTAGAAGATGAAGACCTCATTCCAGAAGAACAAATTGTAATTACACT
AAGCCATAATAACTACATTAAACGTTTGCCGGTATCTACATATCGCGCTCAAAACCGTGGTGGCCGTGGTGTTCAAGGTATGAATACATT
AGAGGAAGACTTCGTTAGTCAATTAGTAACGTTAAGTACACATGATCATGTATTGTTCTTTACTAACAAAGGTCGTGTTTATAAACTTAAA
GGTTATGAAGTGCCTGAGTTATCAAGGCAGTCTAAAGGTATTCCTGTAGTGAATGCTATTGAACTTGAAAATGATGAAATCATTAGTACA
ATGATTGCTGTTAAAGACCTTGAAAGTGAAGATAACTTCTTAGTGTTTGCTACTAAACGTGGTGTCGTTAAACGTTCAGCATTAAGTAATT
TCTCAAGAATTAATAGAAATGGTAAGATTGCGATTTCGTTTAGAGAAGATGACGAGCTAATTGCAGTTCGCTTAACAAGTGGCCAAGAA
GATATCTTAATCGGTACAGCGCATGCATCATTAATTCGATTCCCTGAATCAACGTTACGTCCTTTAGGTCGTACAGCAACAGGTGTGAAAG
GTATTACACTTCGTGAAGGTGACGAAGTTGTAGGGCTTGATGTAGCTCATGCAAACAGTGTTGATGAAGTATTAGTAGTTACTGAAAATG
GTTATGGTAAACGTACGCCAGTTAATGACTATCGTTTATCAAATCGTGGTGGTAAAGGTATTAAAACAGCTACGATTACTGAGCGTAATG
GTAATGTTGTATGTATCACTACAGTAACTGGTGAAGAAGATTTAATGATTGTTACTAATGCAGGTGTCATTATTCGACTAGATGTTGCAGA
TATTTCTCAAAATGGTCGTGCAGCACAAGGTGTTCGCTTAATTCGCTTAGGCGATGATCAATTTGTTTCAACGGTTGCTAAAGTAAAAGA
GGATGCAGAAGACGAAACGAATGAAGACGAACAATCTACTGTATCTGAAGATGGTACTGAACAACAACATGAAGCGGTTGTAAATGAT
GAAACACCAGGAAATGCAATTCATACGGAAGTAATTGAATCAGAAGAAACTGACGAAGATGGACGTATTGAAGTAAGACAAGATTTCAT
GGACCGTGTTGAAGAAGACATACAACAATCATCAGATGATGATGAAGAATAA

Gene: nnrD (ADP-dependent (S)-NAD (P)H-hydrate dehydratase)

Contig: 01_NODE_8 (fragment from pos. 242580 on), position: 9757 to 10587, length: 831 nt, orientation: REVERSE

Sequence:

CTAACTTTCTAATTGTTTCATTGCGTAAGGTATTTCATTGATAAGTCTTGATGGTGGCACCACATACATATCTTTTGCAAGATTTTCGCCAA
TAAAACTATGTGTATATGTGGCACTCATAACCGCTTCTTTTAAGTTATCAAATTGACCAACAAAACTTGTAATCATACCAGCAAGTGTATC
GCCCATACCACCAGTCGCCATTGCTGGGCTACCGATTGTCAATTTAAAGTCTTCATCTTTAAAGAAAATTTCAGTGCCATGTTTTTTAAGTA
CAACAGTTGCGCCTAAACGATCAACTGCTTCACGATTACGCTCATATGTCTGTTCCTCAATCGGAATACCACTTAATCGCTCCCATTCTTTG
AGATGCGGAGTAAAAATCACGCGACATGTAGGTAATTGCGGTTTCAGTTTACTAAATATTGTAATCGCATCGCCGTCTACGATTAAATTTT
GATGCGGTTGTATATTTTGTAGTAGGAATGTAATGGCATTATTTCCTTTGAAATCAACACCAAGACCTGGACCAATTAATATACTGTCAGT
CATTTCAATCATTTTCGTCAACATTTTCGTATCATTAATATCAATAACCATCGCTTCTGGGCAACGAGAATGTAATGCTGAATGATTTGTTG
GATGTGTAGCTACAGTGATTAAACCACTACCGCTAAATACACATGCACGAGCCGCTAACATAATGGCGCCACCTAAGTTAGCAGATCCAC
CAATTAATAAAATTTTGCCATAATCACCTTTATGTGAATCTTCTTTACGCTTAGGAATGTTAATAGAATTTAACGTTTCCATAGTGATATAA
CCTCCCAT

Gene: hutH (histidine ammonia lyase)

Contig: 01_NODE_8 (fragment from pos. 242580 on), position: 10895 to 12409, length: 1515 nt, orientation: FORWARD

Sequence:

ATGACTTTATATTTAGATGGTGAAACACTAACAATTGAGGATATTAAATCATTTTTACAACAACAATCAAAGATTGAAATTATTGATGATG
CGTTAGAACGTGTCAAAAAAAGTAGAGCGGTAGTTGAACGTATTATTGAAAATGAAGAAACAGTTTATGGCATTACAACAGGATTCGGA
TTATTTAGTGATGTGCGTATAGACCCAACACAATACAATGAATTACAAGTGAATCTGATACGCTCACATGCCTGTGGATTAGGTGAGCCA
TTTTCAAAAGAAGTAGCACTCGTTATGATGGTATTAAGATTAAATACATTATTAAAAGGGCATTCAGGTGCTACCTTAGATCTCGTAAAAC
AATTACAATTCTTTATAAATGAGCTTATTATACCAGTGATACCGCAACAAGGTTCACTAGGTGCATCAGGAGATTTAGCACCACTATCACA
TTTAGCATTAGCATTAATTGGTGAAGGGAAAGTATTGTACAGAGGGGAAGAAAAGGATAGTGACGATGTATTAAGAGAATTAAATAGA
CAACCTTTGAACCTTCAGGCTAAAGAAGGTTTAGCATTGATTAATGGTACGCAAGCTATGACAGCTCAAGGTGTCATTAGTTATATAGAA
GCAGAAGATTTAGGTTACCAATCTGAATGGATTGCTGCATTAACGCATCAGTCTCTTAATGGCATTATAGATGCATATCGACATGATGTG
CACGCTGTTCGTAATTTTCAAGAACAGATTAATGTGGCAGCGCGTATGCGTGATTGGTTAGAAGGATCAACATTAACGACGCGACAAGC
AGAAATACGTGTACAAGATGCATATACGTTGCGTTGTATACCACAAATCCATGGCGCGAGTTTTCAAGTATTCAATTATGTAAAACAGCA
ATTAGAATTTGAAATGAATGCGGCTAATGATAATCCACTTATATTTGAGGAAGCAGATGAAACGTTTGTTATTTCAGGTGGGAACTTCCA
TGGACAACCGATTGCTTTTGCATTAGATCATCTTAAATTAGGTGTAAGTGAATTAGCAAACGTATCGGAACGTCGTCTAGAGCGACTAGT
AAATCCTCAATTAAATGGTGATTTACCAGCATTTCTTAGTCCAGAGCCAGGATTGCAAAGTGGCGCGATGATTATGCAATATGCTGCTGC

AAGTCTCGTTTCTGAAAATAAAACTTTAGCGCATCCAGCGAGTGTTGATTCTATCACTTCATCTGCGAACCAAGAAGATCACGTATCTATG
GGAACTACAGCTGCTAGACATGGTTATCAAATTATTGAAAATGCAAGACGTGTGTTGGCAATCGAATGTGTTATTGCATTACAAGCAGCA
GAGTTGAAAGGTGTCGAAGGATTATCACCAAAAACACGTCGCAAGTATGATGAGTTTCGAAGTATCGTGCCATCCATTACACATGACCGT
CAATTTCATAAAGATATTGAAGCGGTTGCACAGTATTTAAAGCAATCAATTTATCAAACGACTGCATGTCACTAA

Gene: tbox01 (T-box leader element)

Contig: 01_NODE_8 (fragment from pos. 242580 on), position: 12487 to 12697, length: 211 nt

Sequence:

CATTGTTCGTAGGACAAGTAATATATAGTGTTCGATATCAGAGAGCTTGTGGTTAGTGTGAACAAGAATCAACATATATATGAATCTACC
TACTTATTTAAAAGAACAATCGGTGATAACCGTTATTTTAGTGAAGTGCAATTTAGGTTTAGTGTATCTTTATAACTTAAATTGTTAAATAG
GGTGGCAACGCGTAGACCACGTCCCTTGT

Gene: serS (seryl-tRNA synthase)

Contig: 01_NODE_8 (fragment from pos. 242580 on), position: 12787 to 14073, length: 1287 nt, orientation: FORWARD

Perfect match to: (Strain_18341-HE579069-[12793:14079], allele observed in CC5)

Sequence:

ATGTTAGACATTAGATTATTCAGAAATGAGCCTGACACAGTTAAGAGCAAAATTGAATTACGTGGAGATGATCCAAAAGTTGTAGATGA
AATTTTAGAATTGGATGAGCAACGACGTAAATTAATTAGTGCAACAGAAGAAATGAAAGCACGTCGTAATAAAGTAAGCGAAGAAATCG
CATTAAAAAAACGTAATAAAGAAAATGCTGATGATGTGATTGCTGAAATGCGCACATTAGGTGACGATATTAAAGAAAAAGATAGTCAA
TTAAATGAAATTGATAATAAAATGACAGGTATCCTTTGTCGTATTCCAAATTTAATAAGTGATGATGTACCTCAAGGTGAATCTGATGAAG
ATAACGTTGAAGTTAAAAAGTGGGGTACACCACGTGAGTTTTCATTTGAACCAAAAGCACATTGGGATATTGTAGAAGAATTGAAAATG
GCTGATTTTGATCGTGCAGCAAAAGTTTCAGGTGCGCGTTTTGTATATTTAACAAATGAAGGTGCGCAATTAGAGCGTGCTTTAATGAAC
TATATGATTACAAAACATACAACACAACATGGTTATACAGAAATGATGGTACCACAGCTTGTGAACGCAGATACAATGTATGGTACAGGT
CAATTACCTAAATTTGAAGAAGATTTATTTAAAGTAGAAAAAGAAGGATTATATACAATTCCAACTGCTGAAGTACCATTAACGAATTTCT
ATCGTAATGAAATTATTCAACCAGGTGTACTTCCTGAAAAATTCACTGGTCAATCTGCATGTTTCCGTAGTGAAGCAGGATCAGCAGGTA
GAGATACAAGAGGATTAATTCGTTTACATCAATTCGATAAAGTGGAAATGGTACGTTTTGAACAACCTGAAGATTCATGGAATGCTTTAG
AAGAAATGACAACAAACGCAGAAGCAATTCTAGAAGAGTTAGGTTTACCATACCGTCGTGTTATTTTATGTACAGGTGATATTGGATTTA
GTGCAAGTAAAACATATGATTTAGAAGTTTGGTTACCAAGCTACAATGATTATAAAGAAATTAGTTCATGCTCAAACTGTACGGATTTCCA
AGCGCGTCGTGCTAACATCCGCTTCAAGCGTGACAAAGCAGCTAAACCAGAATTAGCACATACATTAAATGGTAGTGGTTTAGCAGTTG
GACGTACATTTGCTGCTATTGTTGAAAATTACCAAAATGAAGATGGAACAGTAACAATTCCAGAAGCATTAGTACCATTTATGGGTGGTA
AAACACAAATTTCAAAACCAGTTAAATAA

Gene: azlC (branched-chain amino acid ABC transporter, transmembrane permease subunit)

Contig: 01_NODE_8 (fragment from pos. 242580 on), position: 14721 to 15416, length: 696 nt, orientation: FORWARD

Sequence:

ATGACAACACATTTAAGTTTTAGACAAGGCGTGCAAGAGTGTATCCCAACATTATTGGGTTATGCCGGTGTTGGTATTTCATTTGGTATTG
TGGCTTCGTCTCAAAACTTTAGTATTTTAGAAATTATTTTGTTATGCCTTGTGATATATGCCGGTGCTGCGCAATTTATTATGTGCGCGCTG
TTTATAGCAGGTACACCGATATCAGCGATTGTACTAACTGTATTTATCGTAAATTCAAGAATGTTCCTTTTAAGTATGTCGCTTGCACCAAA
CTTCAAGACATATGGGTTTTGGAACCGTGTTGGATTAGGTTCATTAGTAACTGACGAAACGTTTGGTGTTGCCATTACACCTTATTTAAAA
GGAGAAGCTATCAGTGATCGCTGGATGCATGGTCTTAACATTACAGCATATTTATTTTGGGCAATTTCATGTGTAGCTGGGGCTTTATTTG
GCGAATATATCTAAAATCCACAAGCATTAGGATTAGACTTTGCTATCACGGCTATGTTTATCTTTTTGGCCATTGCGCAATTTGAATCAATT
ACTAAATCGCGATTAAGAATTTACATAGTACTCATTATTGCCGTCATAGTAATGATGTTATCGCTAAGTATGTTTATGCCTTCATATCTAGC
AATATTAATTGCAGCCACAATTTCAGCAGCGTTAGGAGTGATGATGGAACGATGA

Gene: metX (homoserine O-acetyltransferase)

Contig: 01_NODE_8 (fragment from pos. 242580 on), position: 16105 to 17073, length: 969 nt, orientation: FORWARD

Sequence:

ATGACAAATTACACAGTAGATACTTTAAATCTAGGGAAATTTATTACAGAATCTGGGGAAGTCATAGATAACTTGCGTTTGAGATATGAG
CATGTCGGTTATCATGGACAACCATTAGTTGTAGTTTGTCATGCATTAACTGGCAATCATTTAACATATGGAACAGATGATTATCCGGGTT
GGTGGCGAGAAATTATTGATGGGGGATATATACCCATTCACGATTATCAATTTTTAACATTTGATGTTATTGGTAGTCCTTTCGGTTCAAG
TTCACCTTTAAACGACCCTCATTTTCCTAAAAAATTAACATTAAGAGATATTGTTAGAGCGAATGAACGAGGTATACAAGCGCTTGGTTAT
GACAAAATTAATATTTTAATAGGGGGAAGTCTTGGAGGTATGCAAGCGATGGAACTACTTTACAATCAACAGTTTGAAGTAGATAAAGC
CATTATTCTTGCTGCAACAAGCCGAACATCATCTTATAGTAGAGCTTTCAATGAAATTGCGCGTCAGGCCATTCATCTTGGTGGTAAGGAA
GGTCTAAGTATTGCACGTCAATTAGGTTTTTTGACATATCGATCATCAAAAAGTTATGATGAACGTTTCACGCCGGATGAAGTAGTCGCAT
ACCAACAACATCAAGGTAATAAATTTAAAGAACGATTCGATTTGAATTGTTATCTGACACTGCTAGATGTATTGGATAGTCACAACATTGA
CCGAGGTCGCACAGACGTAACGCATGTTTTTAAAAATTTAGAAACGAAAGTCTTAACAATGGGCTTTATAGATGATTTGCTATATCCGGA
TGATCAAGTTCGTGCTTTAGGTGAACGTTTTAAATATCATCGTCATTTCTTTGTACCTGATAACGTTGGTCATGATGGATTCCTACTAAACT
TTAGTACCTGGGCACCTAACTTATATCATTTCTTAAATTTAAAGCATTTTAAGCGCAAGTAA

Gene: yybS (putative transmembrane helix protein)

Contig: 01_NODE_8 (fragment from pos. 242580 on), position: 17381 to 18304, length: 924 nt, orientation: FORWARD

Perfect match to: (CA347-CP006044-[17407:18330], highly conserved allele)

Sequence:

GTGTTTTCAAAAATACAACCTAAAGCAACAATAATTGCAACGATTACGTTGGTATTTGTCGCTTTAGCTTTATATCTAGTGCCTGGTTTAG
GACTAATATTTGCATTATTTGCAACCATACCAGGTATCGTTTTATGGAATAAATCAATACAATCTTTCGGGATTAGTGCACTTATTACAGTA
ATTATAACAACTGTTTTAGGTAATACTTTCGTTTTAAGTGCCATCATATTAGTCTTAATTGCAAGTTTAATTATTGGTCAATTGCTCAAAGA
AAGAACGTCTAAAGAAAGAATATTATACGTAACAACAGTAGCGATGAGCTTAATTTCATTAATCGCTTTTATGTTACTACAAACATTCGGA
AGAATTCCACCATCAGCGAGCATAGTAAAACCTTTCAAGCAAACATTACATGAAGCGATTACGATGAGCGGTGCCGATGCGAATATGAC
CCAAATATTAGAAGAAGGGTTTAGACAAGCGACCGTTCAATTACCAGGTTTCATCATTATCATTACATTTTTAATCGTCTTAATTAACTTAA
TCGTTACATTTCCGATTTTACGAAAATTTAAAATCGCTACACCTGTATTTAAGCCACTTTTCGCGTGGCAAATGAGCGGTATTTTTATTATGG
ATATACATTATTGTTATCATATGTTTATTATTTACAGGTCAACCGAGTGTGTTCCAGAGCATTCTTTTAAACTTCCAACTTGTGTTATCATTA
GTAATGTATATTCAAGGTTTAAGTGTTATTCATTTCTTTGGTAAAGCGAAAGGTTTGCCGAATGCAGTAACGATTTTACTATTGATTATCG
GTACAATACTGACACCTACGACACATATTGTAGGACTACTTGGTGTTATCGATTTAAGTTTGAATTTGAAGCGAATCATGAAAAATAATTC
TAAAAAGTGA

Gene: gdpP (cyclic di-AMP phosphodiesterase)

Contig: 01_NODE_8 (fragment from pos. 242580 on), position: 18319 to 20286, length: 1968 nt, orientation: FORWARD

Sequence:

ATGAATCGGCAGTCCACTAAGAAAGCTTTACTAATACCATTTGTCATCATGATCATCACAGCAATTGTTTTAATGGGTGTATGGTTTATCTT
TAATAGTCTTATAGCATTAATTGCATCTATCGTTCTTGTCGTGATGATTATTGTTAGCATCGTTTTATTCAGACAAGCTTTAATGAAAATGG
ATAGTTATGTAGATGGTTTGAGTGCTCAAATTTCAACAACAAATAATAAAGCAATCAAACATTTACCAATTGGTATCATTGTTTTAGATGA
AAATGATCACATCGAATGGGTTAACCAATTTATGACAGATCATATGGAAGCAAATGTCATTTCTGAATCTGTAAATGAAGTATTTCCAAAC
ATTTTAAAGCAATTAGATAGAGTGAAATCCGTTGAAATAGAATATAATCAGTATCATTTCCAAGTACGTTATTCTGAGAATGATCACTGCC
TCTATTTCTTTGATATAACTGAACAAGTACAAACAAATGAACTATATGAAAATTCTAAACCAATCATTGCGACATTATTTTTAGATAACTAC
GATGAGATTACGCAAAATATGAATGATACGCAGCGTTCGGAAATCAATTCAATGGTAACGCGTGTTATTAGTCGATGGGCAACTGAGTA
TAATATATTCTTTAAAAGGTATAGTTCCGATCAATTCGTAGCCTATTTAAATCAAAAAATATTAGCTGACTTAGAAGAATCTAAATTTGATA
TCTTGAGTCAATTACGTGAAAAAAGTGTTGGTTATCGTGCCCAATTAACATTAAGTATCGGTGTTGGTGAAGGCACTGAAAATTTAATCG
ACTTAGGTGAATTATCACAATCAGGCCTAGACTTAGCATTAGGACGCGGTGGCGACCAAGTTGCAATTAAAAGTATTAATGGTAATGTGC
GCTTCTATGGCGGTAAGACTGACCCGATGGAGAAACGTACTCGTGTAAGAGCGCGTGTTATCTCACATGCGTTAAAAGATATCCTTGCAG
AGGGTGACAAAGTCATTATCATGGGACATAAACGTCCTGACTTAGATGCAATTGGTGCAGCAATCGGTGTGTCTAGATTTGCAATGATG
AATAATTTAGAAGCATACATCGTATTAAATGAGACTGACATTGATCCAACATTACGACGCGTGATGAACGAAATAGATAAAAAGCCAGA
GTTAAGAGAGCGATTTATTACATCAGATGATGCTTGGGATATGATGACATCTAAGACAACCGTAGTGATTGTTGATACGCATAAACCGGA
ACTGGTTTTAGATGAAAATGTCTTAAATAAAGCAAACCGTAAAGTTGTTATCGATCATCATAGACGTGGTGAAAGCTTCATCTCTAATCCA
TTGTTGGTATATATGGAACCATATGCAAGTTCGACAGCTGAATTGGTAACAGAGTTACTGGAATATCAACCAACAGAACAACGTTTAACA
CGTCTTGAATCAACAGTGATGTATGCAGGTATTATTGTAGATACAAGAAACTTTACATTACGAACAGGATCAAGAACATTCGATGCAGCG
AGTTATTTACGTGCACATGGTGCAGATACGATTTTAACGCAACATTTCTTAAAAGATGATGTGGATACTTACATTAATCGATCTGAATTAA
TTCGAACTGTAAAAGTCGAAGATAATGGCATAGCCATTGCGCATGGTTCAGACGATAAAATTTATCATCCAGTAACAGTTGCACAAGCAG
CAGATGAACTGTTAAGTTTAGAAGGTATTGAAGCATCATATGTTGTTGCGAGACGTGAAGATAATCTGATTGGTATATCTGCGCGTTCAC

TCGGTTCAGTAAATGTCCAGTTAACAATGGAAGCACTTGGTGGCGGTGGACATTTAACCAATGCTGCAACACAACTTAAAGGTGTGACA
GTCGAAGAGGCGATAGCACAATTACAACAAGCAATTACAGAACAATTAAGTAGGAGTGAAGATGCATGA

Gene: rplI (50S ribosomal protein L9)

Contig: 01_NODE_8 (fragment from pos. 242580 on), position: 20283 to 20729, length: 447 nt, orientation: FORWARD

Sequence:

ATGAAAGTAATTTTTACACAAGATGTTAAAGGTAAAGGTAAAAAAGGTGAAGTTAAAGAAGTACCAGTAGGTTATGCAAATAACTTCTT
ATTGAAAAAGAATTATGCTGTAGAAGCAACACCAGGTAACCTTAAACAATTAGAGTTACAGAAAAAACGTGCAAAACAAGAACGCCAAC
AAGAAATTGAAGATGCTAAAGCATTAAAAGAAACTTTATCAAACATTGAAGTTGAAGTATCAGCAAAAACTGGTGAAGGTGGTAAATTG
TTTGGATCAGTAAGCACAAAACAAATTGCCGAAGCACTAAAAACGCAACATGATATTAAAATTGATAAACGTAAAATGGATTTACCAAAT
GGAATTCATTCCCTAGGATATACGAATGTACCTGTTAAATTAGATAAAGAAGTTGAAGGTACAATTCGCGTACACACAGTTGAACAATAA

Gene: dnaC (replicative DNA helicase)

Contig: 01_NODE_8 (fragment from pos. 242580 on), position: 20761 to 22161, length: 1401 nt, orientation: FORWARD

Sequence:

ATGGATAGAATGTATGAGCAAAATCAAATGCCACATAACAATGAAGCTGAACAGTCTGTCTTAGGTTCAATTATTATAGATCCAGAATTG
ATTAATACTACTCAGGAAGTGTTGCTTCCTGAGTCATTTTATAGGGGTGCCCATCAACATATTTTCCGTGCAATGATGCACTTAAATGAAG
ATAATAAAGAAATTGATGTTGTAACATTGATGGATCAATTATCGACGGAAGGTACGTTGAATGAAGCGGGTGGCCCGCAATATCTTGCA
GAGTTATCTACAAATGTACCAACGACGCGAAATGTTCAGTATTATACTGATATCGTTTCTAAGCATGCATTAAAACGTAGATTGATTCAAA
CTGCAGATAGTATTGCCAATGATGGATATAATGATGAACTTGAACTAGATGCGATTTTAAGTGATGCAGAACGTCGAATTTTAGAGCTAT
CATCTTCTCGTGAAAGCGATGGCTTTAAAGACATTCGAGACGTCTTAGGACAAGTATATGAAACAGCTGAAGAGCTTGATCAAAATAGTG
GTCAAACACCAGGTATTCCTACAGGATATCGAGATTTAGACCAAATGACAGCAGGGTTTAACCGAAATGATTTAATTATCCTTGCAGCGC
GTCCATCTGTAGGTAAGACTGCGTTCGCACTTAATATTGCACAAAAAGTTGCAACGCATGAAGATATGTATACAGTTGGTATTTTCTCACT
AGAGATGGGTGCTGATCAGTTAGCCACACGTATGATTTGTAGTTCTGGTAATGTTGACTCAAACCGCTTAAGAACGGGTACTATGACTGA
GGAAGATTGGAGTCGTTTTACTATAGCGGTAGGTAAATTATCACGTACGAAGATTTTTATTGATGATACACCGGGTATTCGAATTAATGA
TCTACGTTCTAAATGTCGTCGATTAAAGCAAGAACATGGCTTAGACATGATTGTGATTGACTACTTACAGTTGATTCAAGGTAGTGGTTCA
CGTGCGTCTGATAACAGACAACAGGAAGTTTCTGAAATCTCTCGTACATTAAAAGCATTAGCCCGTGAATTAGAATGTCCAGTTATCGCA
TTAAGTCAGTTATCTCGTGGTGTTGAACAACGACAAGATAAACGTCCAATGATGAGTGATATTCGTGAATCTGGTTCGATTGAGCAAGAT
GCCGATATCGTTGCATTCTTATACCGTGATGATTACTATAACCGTGGTGGCGATGAAGATGATGACGATGATGGTGGTTTCGAGCCGCAA
ACGAATGATGAAAACGGCGAAATTGAAATCATCATTGCTAAGCAACGTAATGGCCCAACAGGCACAGTTAAGTTACACTTTATGAAACA
ATATAATAAATTTACAGATATCGATTATGCACATGCTGATATGATGTAA

Gene: purA (adenylosuccinate synthase)

Contig: 01_NODE_8 (fragment from pos. 242580 on), position: 22439 to 23722, length: 1284 nt, orientation: FORWARD

Sequence:

ATGTCATCAATCGTAGTAGTTGGGACACAATGGGGAGACGAAGGAAAAGGAAAAATAACGGATTTCTTGGCAGAACAGTCAGATGTTA
TCGCGCGTTTTTCAGGTGGTAATAATGCAGGCCATACCATTCAATTTGGCGGAGAAACATATAAATTACATTTAGTACCATCTGGTATCTT
TTACAAAGACAAATTAGCGGTAATCGGTAACGGAGTCGTTGTTGATCCAGTTGCACTATTGAAAGAATTAGACGGATTAAATGAACGTG
GCATTCCTACAAGTAATTTACGTATATCTAATCGTGCGCAAGTGATTTTACCATATCACTTAGCACAAGATGAATATGAAGAACGTTTACG
CGGTGATAATAAGATTGGTACAACTAAAAAAGGTATCGGTCCAGCATATGTAGACAAAGTTCAACGTATCGGTATTCGTATGGCAGATTT
ACTTGAAAAAGAAACATTCGAAAGATTATTAAAATCAAACATTGAATATAAACAAGCATATTTTAAAGGTATGTTTAACGAAACATGTCC
ATCATTTGATGATATCTTTGAAGAATATTATGCAGCAGGTCAACGTCTAAAAGAATTTGTAACAGACACATCAAAAATCTTAGACGATGC
ATTTGTAGCAGATGAAAAGGTACTTTTCGAAGGTGCGCAAGGTGTAATGTTAGATATCGACCATGGTACATATCCATTCGTTACATCAAG
TAATCCAATTGCAGGTAACGTTACTGTTGGTACAGGTGTAGGTCCTACATTCGTTTCAAAGGTAATTGGTGTATGTAAAGCTTATACATCA
CGTGTTGGTGATGGTCCATTCCCTACTGAATTATTCGATGAAGATGGACATCATATTAGAGAAGTTGGTCGTGAATACGGTACAACAACA
GGACGTCCACGTCGTGTAGGTTGGTTTGATTCAGTTGTATTACGTCACTCTCGTCGTGTAAGTGGTATTACAGATTTATCTATTAACTCAA
TTGATGTTTTAACAGGCCTAGACACAGTGAAAATTTGTACAGCTTATGAATTAGACGGTAAAGAAATTACTGAGTACCCAGCAAACTTAG
ATCAATTAAAACGTTGTAAACCAATCTTTGAAGAGTTACCAGGTTGGACAGAAGACGTAACAAGTGTGCGTACTTTAGAAGAATTACCTG
AAAATGCACGTAAATATTTAGAGCGTATTTCAGAATTATGTAATGTACAAATTTCTATCTTCTCAGTTGGTCCAGATAGAGAACAAACAAA
CCTATTAAAAGAATTGTGGTAG

Gene: walR (two component sensor/regulator of autolysis, transcriptional regulator)

Contig: 01_NODE_8 (fragment from pos. 242580 on), position: 24919 to 25620, length: 702 nt, orientation: FORWARD

Sequence:

ATGGCTAGAAAAGTTGTTGTAGTTGATGATGAAAAACCGATTGCTGATATTTTAGAATTTAACTTAAAAAAAGAAGGATACGATGTGTAC
TGTGCATACGATGGTAATGATGCAGTCGACTTAATTTATGAAGAAGAACCAGACATCGTATTATTAGATATCATGTTACCTGGTCGTGAT
GGTATGGAAGTATGTCGTGAAGTGCGCAAAACATACGAAATGCCAATTATAATGCTTACTGCTAAAGATTCAGAAATTGATAAAGTGCTT
GGTTTAGAACTAGGTGCAGATGACTATGTAACGAAACCGTTTAGTACGCGTGAATTAATCGCACGTGTGAAAGCGAACTTACGTCGTCAT
TACTCACAACCAGCACAAGACACTGGAAATGTAACGAATGAAATCACAATTAAAGATATTGTGATTTATCCAGACGCATATTCTATTAAA
AAACGTGGCGAAGATATTGAATTAACACATCGTGAATTTGAATTGTTCCATTATTTATCAAAACATATGGGACAAGTAATGACACGTGAA
CATTTATTACAAACAGTATGGGGCTATGATTACTTTGGCGATGTACGTACGGTCGATGTAACGATTCGTCGTTTACGTGAAAAGATTGAA
GATGATCCGTCACATCCTGAATATATTGTGACGCGTAGAGGCGTTGGATATTTCCTCCAACAACATGAGTAG

Gene: walK (two component sensor/regulator of autolysis, sensor histidine kinase)

Contig: 01_NODE_8 (fragment from pos. 242580 on), position: 25633 to 27459, length: 1827 nt, orientation: FORWARD

Sequence:

ATGAAGTGGCTAAAACAACTACAATCCCTTCATACTAAACTTGTAATTGTTTATGTATTACTGATTATCATTGGTATGCAAATTATCGGGCT
GTATTTTTACAAATAACCTTGAAAAAGAGCTGCTTGATAATTTTAAGAAGAATATTACGCAGTACGCTAAACAATTAGAAATTAGTATTGAA
AAAGTATATGACGAAAAGGGCTCCGTAAATGCACAAAAAGATATTCAAAATTTATTAAGTGAGTATGCCAACCGTCAAGAAATTGGAGA
AATTCGTTTTATAGATAAAGACCAAATTATTATTGCGACGACGAAGCAGTCTAACCGTAGTCTAATCAATCAAAAAGCGAATGATAGTTCT
GTCCAAAAAGCACTATCACTGGGACAATCAAACGATCATTTAATTTTAAAAGATTATGGCGGTGGTAAGGACCGTGTCTGGGTATATAAT
ATCCCCGTTAAAGTCGATAAAAAGGTAATTGGTAATATTTATATCGAATCAAAAATTAATGACGTTTATAACCAATTAAATAATATAAATC
AAATATTCATTGTTGGTACAGCTATTTCATTATTAATCACAGTCATCCTAGGATTCTTTATAGCGCGAACGATTACCAAACCAATCACCGAT
ATGCGTAACCAGACGGTTGAAATGTCCAGAGGTAACTATACGCAACGTGTGAAGATTTATGGTAATGATGAAATTGGCGAATTAGCTTT
AGCATTTAATAACTTGTCTAAACGTGTACAAGAAGCGCAGGCTAATACTGAAAGTGAGAAACGTAGACTGGACTCAGTTATCACCCATAT
GAGTGATGGTATTATCGCAACAGACCGCCGTGGACGTATTCGTATTGTCAATGATATGGCACTTAAGATGCTTGGTATGGCGAAAGAAG
ACATCATCGGTTATTACATGTTAAGTGTATTAAGTCTTGAAGATGAATTTAAACTGGAAGAAATTCAAGAGAATAATGATAGTTTCTTATT
AGATTTAAATGAAGAAGAAGGTCTAATCGCACGTGTTAACTTTAGTACGATTGTGCAGGAAACAGGATTTGTAACGGGTTATATCGCTGT
GTTACATGACGTTACTGAACAACAACAAGTTGAACGTGAGCGTCGTGAATTTGTTGCCAATGTATCACATGAGTTACGTACACCTTTAACT
TCCATGAATAGTTACATTGAAGCACTTGAAGAAGGTGCATGGAAAGATGAGGAACTTGCGCCACAATTTTTATCTGTTACCCGTGAAGAA
ACAGAACGAATGATTCGACTGGTCAATGACTTGCTACAGTTATCTAAAATGGATAATGAGTCTGATCAAATCAACAAAGAAATTATCGAC
TTTAACATGTTCATTAATAAGATTATTAATCGACATGAAATGTCTGCGAAAGATACAACATTTATTCGAGATATTCCGAAAAAGACGATTT
TCACAGAATTTGATCCTGATAAAATGACGCAAGTATTTGATAATGTCATTACAAATGCGATGAAATATTCTAGAGGCGATAAACGTGTCG
AGTTCCACGTGAAACAAAATCCACTTTATAATCGAATGACGATTCGTATTAAAGATAATGGCATCGGTATTCCTATCAATAAAGTCGATAA
GATATTCGACCGATTCTATCGTGTAGATAAGGCACGTACGCGTAAAATGGGTGGTACTGGATTAGGACTAGCCATTTCGAAAGAGATTG
TGGAAGCTCACAATGGTCGTATTTGGGCAAACAGTGTAGAAGGTCAAGGTACATCTATCTTTATCACACTTCCTTGTGAAGTCATTGAAG
ACGGTGATTGGGATGAATAA

Gene: walH (extracellular negative effector of the regulator of autolysis)

Contig: 01_NODE_8 (fragment from pos. 242580 on), position: 27452 to 28786, length: 1335 nt, orientation: FORWARD

Sequence:

ATGAATAATAAGGAACATATTAAATCTGTCATTTTAGCGCTACTCGTCTTGATGAGTGTCGTATTGACATATATGGTATGGAACTTTTCGC
CTGATATTGCAAATGTCGACAATACAGATAGTAAGAAGAGTGAAACGAAACCTTTAACGACACCTATGACAGCCAAAATGGATACAACT
ATTACGCCATTTCAGATTATTCATTCGAAAAATGATCATCCAGAAGGTACGATTGCGACGGTATCTAATGTGAATAAACTGACGAAACCTT
TGAAAAATAAAGAAGTGAAGTCCGTAGAACATGTTCGTCGTGATCATAACTTGATGATTCCTGATTTGAGCAGTGATTTTACATTATTCGA
TTTTACGTATGATTTACCGTTATCATCTTATCTTGGTCAAGTACTGAACATGAATGCGAAAGTACCAAATCATTTCAATTTCAATCGTTTAG
TCATAGATCATGATGCTGATGATAATATCGTGCTTTATGCTATAAGCAAAGATCGCCACGATTACGTAAAATTAACAACTACAACGAAAA
ACGATCATTTTTTAGATGCATTAGCAGCAGTGAAAAAAGATATGCAACCATATACAGATATCATCACAAACAAAGATACGATTGATCGTA
CGACGCATGTTTTTGCACCAAGTAAACCTGAGAAGTTAAAAACTTATCGCATGGTATTTAACACGATTAGTGTTGAGAAAATGAATGCTA
TACTATTTGACGATTCAACCATCGTTCGTAGTTCTAAGAGTGGTGTTACAACCTACAACAATAATACAGGTGTCGCAAACTATAACGATAA

AAATGAAAAATACCATTATAAAAACTTGTCCGAAGATGAAGCAAGTTCCAGCAAAATGGAAGAAACGATTCCAGGAACCTTTGATTTTAT
TAATGGTCATGGTGGTTTCTTAAACGAAGACTTCAGATTGTTTAGTACGAATAATCAGTCAGGCGAGCTAACATATCAACGTTTCCTTAAT
GGTTATCCAACGTTTAATAAAGAAGGCTCTAATCAAATTCAAGTCACTTGGGGTGAAAAGGGCGTCTTTGATTATCGTCGTTCGTTATTGC
GCACCGATGTTGTGTTAAATAGTGAGGATAATAAATCGTTGCCGAAATTAGAGTCTGTGCGTTCAAGCTTAGCGAACAATAGTGATATTA
ATTTTGAAAAAGTAACGAATATCGCTATCGGTTACGAAATGCAAGATAATCCGGATCATAATCACATTGAAGTGCAGATTAATAGTGAAC
TCGTACCACGCTGGTATGTAGAATATGATGGCGAATGGTATGTATATAACGATGGGAGGCTTGAATAA

Gene: walI (extracellular negative effector of the regulator of autolysis)

Contig: 01_NODE_8 (fragment from pos. 242580 on), position: 28787 to 29575, length: 789 nt, orientation: FORWARD

Sequence:

ATGAACTGGAAACTGACGAAGACACTTTTCATTTTCGTGTTTATTCTTGTCAACATCGTGTTAGTATCGATTTATGTTAATAAAGTCAATCG
CTCACACATTAATGAAGTTGAGAGTAATAATGAAGTTAATTTTCAACAAGAAGAAATCAAAGTACCGGCTAGTATTTTAAATAAATCAGT
TAAAGGTATACAATTAGAACAAATTACGGGGCGTTCAAAAGACTTTAGTTCTAAAGCTAAGGGCGATTCGGATTTGACCACATCAGATG
GTGGAAAATTATTAAATGCAAACATTAGCCAATCGGTAAAGGTCAGTGACAATAACTTAAAAGATTTGAAAGATTATGTTAACAAACGTG
TGTTCAAAGGTTCAGAATATCAATTAAGTGAGATTAGTTCTGGTTCTGTAAAATACGAACAAACGTATGATAATTTCCCGATTTTGAACAA
TAGTAAAGCGATGTTGAACTTTAATATAGAAGATAACAAAGCGACTAGTTATAAACAATCAATGATGGATGACATTAAGCCCACAGATG
GTGCAGATAAGAAGCATCAAGTGATTGGTGTGAGAAAGGCAATCGAGGCATTATATTATAATCGTTACTTGAAAAAAGGTGATGAAGTC
ATTAATGCTAGACTCGGTTACTACTCAGTCGTGAACGAGACGAATGTTCAATTGTTACAACCAAACTGGGAAATTAAAGTGAAGCATGAC
GGTAAGGACAAAACGAATACTTACTATGTCGAAGCGACAAATAATAACCCTAAAATTATTAATCATTAA

Gene: walJ (exonuclease associated with regulator of cell wall autolysis)

Contig: 01_NODE_8 (fragment from pos. 242580 on), position: 29963 to 30763, length: 801 nt, orientation: FORWARD

Sequence:

ATGAGCCGCTTGATACGCATGAGTGTATTAGCAAGTGGTAGTACAGGTAACGCCACTTTTGTAGAAAATGAAAAAGGTAGTCTATTAGTT
GATGTTGGTTTGACTGGCAAGAAAATGGAAGAATTGTTTAGTCAAATTGACCGTAATATTCAAGATTTAAATGGTATTTTAGTAACCCAT
GAACATATTGATCATATTAAAGGATTAGGTGTTTTGGCGCGTAAATATCAATTGCCAATTTATGCGAATGAAAAGACTTGGCAGGCAATT
GAAAAGAAAGATAGTCGCATCCCTATGGATCAGAAATTCATTTTTAATCCTTATGAAACGAAATCTATTGCAGGTTTCGATGTTGAATCGT
TTAACGTGTCACATGATGCGATAGATCCGCAATTTTATATTTCCATAATAACTATAAAAAGTTTACGATTTTAACGGATACGGGTTACGT
GTCTGATCGTATGAAAGGTATGATACGTGGCAGTGATGCGTTTATTTTTGAGAGTAATCATGACGTCGATATGTTGAGAATGTGTCGTTA
TCCATGGAAGACGAAACAACGTATTTTAGGCGATATGGGTCATGTATCTAATGAGGATGCGGGTCATGCGATGACAGACGTGATTACAG
GTAACACGAAACGTATTTACTTATCACATTTATCACAAGATAATAACATGAAAGATTTGGCGCGTATGAGTGTTGGCCAAGTATTGAACG
AACACGATATTGATACGGAAAAAGAAGTATTGCTATGTGATACGGATAAAGCTATTCCAACGCCAATATATACAATATAA

Gene: sasH (cell-wall attached adenosine synthase A)

Contig: 01_NODE_8 (fragment from pos. 242580 on), position: 30990 to 33308, length: 2319 nt, orientation: FORWARD

Sequence:

ATGAAAGCTTTATTACTTAAAACAAGTGTATGGCTCGTTTTGCTTTTTAGTGTGATGGGATTATGGCATGTCTCGAACGCGGCTGAGCAG
CATACACCAATGAAAGCACATGCAGTAACAACGATAGACAAAGCAACAACAGATAAGCAACAAGTAACGCCAACAAAGGAAGCGGCTC
ATCATTATGGTGAAGAAGCGGCAACCAACGTATCAGCATCAGCACAGGGAACAGCTGATGAAATAAACAATAAAGTAACATCCAACGCA
CCATCTAACAAACCATCTACAGCAGTTTCAACAACAGTAAACGAAACGCGCGATGTAGATACACAACAAGCCTCAACACAAAAACCAACT
CGCACAGCAACATTCAAATTATCAAATGCTAAAACAGCATCACTTTCACCACGAATGTTTGCTGCCAATGTACCACAAACAACAACACATA
AAATATTACATACAAATGATATCCATGGCCGACTAGCCGAAGAAAAAGGGCGTGTCATCGGTATGGCTAAATTAAAAACAGTAAAAGAA
CAAGAAAAGCCTGATTTAATGTTAGACGCAGGAGACGCATTCCAAGGTTTACCACTTTCAAATCAATCTAAAGGTGAAGAAATGGCTAAA
GCGATGAATGCAGTAGGTTATGATGCTATGGCAGTCGGTAACCACGAATTTGACTTTGGATACGACCAGTTGAAAAAGTTAGAGGGTAT
GTTAGACTTCCCGATGTTAAGTACTAACGTTTATAAAGATGGAAAACGCGCATTTAAACCATCAACGATTGTAACGAAAAATGGTATTCG
TTATGGAATTATTGGCGTAACGACACCAGAAACAAAGACGAAAACAAGACCTGAAGGCATTAAAGGTGTTGAATTTAGAGATCCATTAC
AAAGTGTGACAGCAGAAATGATGCGTATTTATAAAGACGTAGATACATTTGTTGTTATATCACATTTAGGGATTGATCCTTCAACACAAA
AAACATGGCGTGGTGATTACTTAGTGAAACAATTAAGTCAAAATCCACAATTGAAGAAACGTATTACAGTCATTGATGGTCATTCACATA
CCGTACTTCAAAATGGTCAAATTTATAACAATGATGCATTAGCACAAACAGGTACAGCACTTGCGAATATCGGTAAGGTTACATTTAATTA
CCGCAATGGAGCGGTATCGAATATTAAGCCGTCATTGATTAATGTTAAAGACGTTGAAAATGTAACACCGAACAAAGCATTAGCTGAAC

AAATTAATCAAGCTGATCAAACATTTAGAGCACAAACAGCAGAGGTTATTATTCTAAATAATACGATTGATTTCAAAGGAGAAAGAGATG
ACGTTAGAACGCGTGAAACAAATTTAGGAAACGCGATTGCAGATGCTATGGAAGCATATGGCGTTAAGAATTTCTCTAAAAGACTGAC
TTTGCCGTGACAAATGGTGGAGGTATTCGCGCCTCTATCGCAAAAGGTAAGGTGACACGCTATGATTTAATTTCAGTATTACCATTTGGA
AATACGATTGCGCAAATTGATGTAAAAGGTTCAGACGTCTGGACGGCTTTTGAACATAGTTTAGGCGCTCCAACAACACAAAAAGATGG
TAAGACAGTATTAACAGCGAATGGCGGTTTACTACATATCTCTGATTCAATCCGTGTTTACTATGATATGAATAAACCGTCTGGCAAACGA
ATTAACGCTATTCAAATTTTAAATAAAGAGACAGGTAAGTTTGAAAATATTGATTTAAAACGTGTATATCACGTAACGATGAATGACTTCA
CAGCATCAGGTGGCGACGGATATAGTATGTTCGGTGGCCCTAGAGAAGAAGGTATTTCATTAGATCAAGTACTAGCAAGTTATTTAAAA
ACAGCTAACTTAGCTAAGTATGATACGACAGAACCACAACGTATGTTATTAGGTAAACCAGCAGTAAGTGAACAACCAGCTAAAGGACA
ACAAGGTAGCAAAGGTAGTGAGTCTGGTAAAGATACACAACCAATTGGTAAAGACAAAGTGATGAATCCAGCGAAACAACCAGCGCCA
AGTAAAGTTGTGTTGTTGCCAGCGCATAGAGGAACTGTTAGTAGTGGTAGAGAAGGTTCTGATCGCGCATTGGAAGGAACTGCTGTATC
AAGTAAGAGTGGGAAACAATTGGCTAGCATGTCAGCGCCTAAAGGTAGCGCACATGAGAAACAGTTACCAAAAACTGGAACTGATCAA
AGTTCAAGCCCAGCAGCGATGTTTGTATTAGTAGCAGGTATAGGTTTAATCGCTACTGTACGACGTAGAAAAGCTAGCTAA

Gene: orfX (23S rRNA methyltransferase)

Contig: 01_NODE_8 (fragment from pos. 242580 on), position: 33675 to 34154, length: 480 nt, orientation: FORWARD

Sequence:

ATGAAAATCACCATTTTAGCTGTAGGGAAACTAAAAGAGAAATATTGGAAGCTAGCCATAGCAGAATATGAAAAACGTTTAGGCCCATA
CACCAAGATAGACATCATAGAAGTTCCAGACGAAAAAGCACCAGAAAATATGAGCGACAAAGAAATTGAGCAAGTAAAAGAAAAAGAA
GGCCAACGAATACTAGCCAAAATCAAACCACAATCCATAGTCATTACATTAGAAATACAAGGAAAGATGCTATCTTCCGAAGGATTGGCC
CAAGAGTTGAACCAACGCATGACCCAAGGGCAAAGCGACTTTGTATTCGTCATTGGCGGATCAAACGGCCTGCACAAGGACGTCTTACA
ACGCAGTAACTACGCGCTATCATTCAGTAAAATGACATTCCCACATCAAATGATGCGGGTTGTGTTAATTGAGCAAGTGTATAGAGCGTT
TAAGATTATGCGTGGAGAAGCATATCATAAGTGA

Gene: sRNA6 (antisense RNA associated with orfX)

Contig: 01_NODE_8 (fragment from pos. 242580 on), position: 33855 to 34138, length: 284 nt

Sequence:

CAACGAATACTAGCCAAAATCAAACCACAATCCATAGTCATTACATTAGAAATACAAGGAAAGATGCTATCTTCCGAAGGATTGGCCCAA
GAGTTGAACCAACGCATGACCCAAGGGCAAAGCGACTTTGTATTCGTCATTGGCGGATCAAACGGCCTGCACAAGGACGTCTTACAACG
CAGTAACTACGCGCTATCATTCAGTAAAATGACATTCCCACATCAAATGATGCGGGTTGTGTTAATTGAGCAAGTGTATAGAGCGTTTAA
GATTATGCGTGGAGA

Gene: DR_SCC (direct repeat of SCC)

Contig: 01_NODE_8 (fragment from pos. 242580 on), position: 34136 to 34154, length: 19 nt

Perfect match to: (JS395-CP012756-[1418304:1418322])

Sequence:

AGAAGCATATCATAAGTGA

Gene: dam5 (type II restriction-modification system, endonuclease and methyltransferase)

Contig: 01_NODE_8 (fragment from pos. 242580 on), position: 34449 to 37196, length: 2748 nt, orientation: FORWARD

Sequence:

ATGGGAAAAAAAAAAGAACTTAAGTATTACAGAGATAGAGAATTCTGTAAATACTTTGATTAATCATTTGAAAGAGAAAGATTTTATAACT
GAATTTTTAAGTTTCTATGATATACCAAAAACTTCAATTACACGAGCAAAAGTAAAATTTGATAAAGGTGAACCATTTATAATTAAAAATA
AAGTATACTATACAGAAATTCAAGGTGAAGTTATTACAGCAATAGATGCTATTGAGCATGAAATATTAAATCAGAAATCCAAACCGAGAT
ATCTTATTGCAAATAATTATTCTGAAATTGCAGCTTTAGATATTAAAACTCGAGACACACTTAACATTCCATTAAGTGAGTTACCATCAAAA
GCTGATTTCTTTTTAGCGTGGAATGGTATCGAAAAGTCAGATTATCAATCTGAACATCCAGCCGATAGAAAAGCAGCTGAACGGTTTGCA
AAACTTTACGATGTGCTTGAAAAGGATAATCCTAATGTTAAAGAACACTCATTTAACACATTTTTAATTCGTATATTATTTTTATTGTTTGCA

GAAGATACGGGAATTATGAAGAAAGGTATATTTACTAATACACTTAAAATAAGAACAAAAGAAGATGGCAGTAATTTTAATGAAGTTAT
TGAAGAGTTATTTGAAATTTTAAATACTAATGAACTAAATCGAGATAAAAAATCAGATTGGTTAAAAATATTTCCTTATGTTAATGGTAAA
CTTTTTAGTGAGCCTCACGTATCCTTAATTTTTACAAAGAAATCTAGAAAGTTACTGATTGAAGCTGGCGAGCTTCTAAATTGGAACGAAA
TTAATCCTGATATTTTGGGTTCAATGATTCAAACAGTATCCTCTTCTAAAAAAAAGGCAAGTATCAGGAATGCACTACACTAGTGTTCCTAA
TATTATGAAAGTAATAAAGCCTCTTTTTTTAGATGAACTTTATAAAATATTTAATGATTTATCTGCTCAATATGAAGAAAATAAAATTAAAA
ATATTACAGAGAAAACAAAAAAAAACCTATAATAAGAAAATAATTAACTCTTTATCGGATTTGTTAGAAAGAATTTCAAGGATTAATTTTTT
AGATCCTGCATGTGGTTCAGGAAATTTTTTAATTATAGCATATAAAGAAATCAGACGTCTTGAAATAAAAATACTTGTTTTATTAGATGAA
ATACAGCAATCAGACACTATGCCAATGACAGCGATACATTTAGAAAATTATAATGGTATTGAAGTTGATGACTTTGCGCATGAAGTAGCG
AAATTATCTCTCTGGATTGCTGAACATCAAATGAATGAAGAAATGGAAAAAGCTTTACCTGGTTATATTTCGGCTCTACTACCCTTAAAAG
ATTCTGGAAATATAGTTTTAGGAAATGCGTTGAGGATTGATTGGAATAATATTATACCTCAAAATAAAAATGAGGAAATTTATATATTTG
GTAACCCACCCTATATTGGAGCAGCTAATAAAAACGAAAACCAAAAAAACGATTTAGCTTTTGTGTTTCATGATACAGATATTTCATTTGG
AAAATTGGATTATATCACGGGATGGTTTTATAAATCTGTTAAATTTATGGAAAAAAGAAAATCTGTCTTTGCATTTGTTTCAACAAATTCA
ATTGTCCAAGGAGAACAAGTTTCTTTAATTTGGCCTGAATTATTTAAAACTGCACAAATAAGTTTTGCTTATCCATCTTTTAAATGGTCTAA
TAATGCAAAATCAAATGCTGGTGTTACAGTAGTGATAATTGGTTTCGAATATAAAGATTATTTAGGACCAAAAAGTATATATTCTTCAAAT
GGAACAGTAAAAAAGGTGGATAATATTACACCTTATTTAGTTGAAGGGGTAAATATAATTGTTGAAAAAGAAAATAAATCAATAAATGG
CTTTACAGAGATGGTTAAAGGAAGTTCTCCAACTGATGATGGTGGATTAATATTTTCAGAGTATGAATATCAACAAGCTATAGAACTTTAT
CCAAATTTGAAAGATATTTTAAAAAAATATCAAGGATCAAGAGAATATATTAATGATATAAGCAGATATGTTCTTTGGATGTCTGATGAT
GACGCTAAATTATTTAAAAATAATCCTATAATTTCAAAAAGACTTGAACACGTACGTCAATTTAGATTAAATAAAAAGGGAAACACCTTGA
AAAAAGCTGAAACACCTTGGGAATTCGTTTCTAATGGTAAAAGAAAAGCAGCTTTGAAAAAAAAATAAAAATATGAAACAAATCTTAATAC
CACGAGTTTCTTCAGAAAATAGATATTACGTTCCAATGGGATATGTTAATAAAGATACTATAATATCTGATTCATCTATGGCAATTTATGA
TGCTCCATTATGGCTTTTAGGATTACTTCAATCACGAATGCATATGGTTTGGCTTAGAGCAATTGGTGGAAAATTAAAAACTGATTATAGA
TACTCCTCAGGGCTAGTTTATAATACTTTTCCAATACGAAGATTATCTCCTCAACGATTAAAGGAAATTGAACGTGTGATAACGGATATTT
TAGATTTAAGGGAGTATGAAGGTGGTAGTTTAGCTTACCTTTATAATAGTAAAACTATGCCAATTAGTTTAAAAGAAAAACATCAAGAAC
TTGATGGTATAGTGGAACGCGCATACCGACAAAAACCTTTTAATAGTGATGAAGAACGTTTGAGTACCTTACTAACATTATATAAAGAAA
AGAAGGTAATAGTAGATGACAACTAA

Gene: helicase5 (DNA helicase, associated with dam)

Contig: 01_NODE_8 (fragment from pos. 242580 on), position: 37186 to 39151, length: 1966 nt, orientation: FORWARD

Sequence:

ATGACAACTAATTTATTCGAAGTTAATTTTGAAGAAAAAAATGAAAAAAATACTAATAATCTTGGTATGAGAGAAATGCAAGAAAAAGTT
TATAAAAAACGCTTTGCACGTTATTTATTAGTTAAAGCACCTCCAGCCTCTGGTAAATCGCGTGCTTTAATGTTTGTGGGTTTAGATAAATT
AAATACTCAAGGTCTAAAAAAAATAGTTATTGCAGTTCCAGAAAAATCAATCGGAAAATCGTTTCGTAATACTAAACTAACAAATTATGG
TTTTTATTGGGATTGGAATGTAAAACCTTGTAATAATCTAACTATTGGAGTAGGTGAAACTTCAAAAGTTAAAAGATTTGTTGAATTTATG
AAATCTAATGATAAAGACGACAATATTCTCATTGCAACACATGCCACTTTGCGTTATGCATTCGAAGAATTAGATGATTCAGCTTTTGATA
ATTCATTACTTGCAATTGATGAGTTTCATCATGTCAGTCGAGATGACAGTTCAGTGCTTGGTAATGCTTTGCGCAGTATTATGAGTAATTC
AACTGCACACATTTTAGCCATGACCGGATCCTATTTTCGAGGTGATTCTGTGCAAATTTTAGAGCCGAAAGATGAAGATAAGTTTGAAAA
AGTAACTTACACATATTATGAGCAATTAAATGGCTATCAATATTTAAAATCTTTTGCTATGGGTTATAGTTTTTATCGAGGACAATATACAG
AAGCTTTAGATGAAGTTATAGATGTGTCGAAGAAATCGATTATTCACATACCTAATATTAATTCTAGTGAATCAACTAAAGATAAATATGA
CGAAGTTGATCGAATTATGGATTTAATTTCTGATGGTGGAAATATTTATCAAAATAGTGATGGTATATATGAAGTTAATCGGCCTGATGG
AAAAGAATTATTAGTTGCCGATTTAGTCAATGAAGATTCAAGAGAAAAGGTTACATCTTATTTAGCTAATATAACTGATGATATAAAGGA
TTTAGATAAATTAGATATTATCATTGCACTTGGTATGGCTAAAGAGGGTTTTGATTGGCCCTTTGCAGAATACGCTTTGACGATTGGTTAT
CGTAATTCTTTGACAGAAATTATTCAAATTATCGGACGAGTAACTCGAGATAGTGCTAATAAAAATCATGCTCAATTCACAAATTTAATTG
CACAGCCTGATGCTCAAGATGATGAAGTATTATATGCAGTTAATAGTCTTATGAAAGCAATAACTGCGTCATTACTTATGGAGCAAGTTTT
AGCACCTGTTTATAATTTTAAACCTAAGGATAAAAAAATAGACAATCCTGATGATATATCTATAAAAGGATTGAAACCTTCTAATACAACA
GAACGAACTAAAAAAATAATTAAAGATGATATGGCTGACTTGAAGTCAAGTATATTACAAAGTGAGCATATTCAAAATGCTATTGTATCT
GGTTCTGATGCTCAAATGATTAACAAGACTTTAGTCCCGCGTGTGATTATTGAACGTTATCCTGATTTAACAGAGGAAGAATTGGAAACA
GTTCGTCAACATACAGTTGCTAACGTTAATTTTTCAGCAAGTTCTAAAGTGATTTCAAATGATGGTAGTCGTGAAATTATAAAAATGGCTG
ATAGATTTATTAATATAGATGAATTAAATATAGATTTAATTGATTCTATAAATCCATTTCAGCGCGCGTATGAAATGATAAGTCGTGATAT
TAATGCTCCAACGTTACAATTTATTCAAGATTATATGTCATCGAAAAAATATGAATTTACCAATGAACAACTAGTGAATGCTTATCAACGT
GCAAAACAATTTCGAATTGAAAATGGGCGTAATCCAGAAAGAAATAGTAAAGATGAGGGAGAAAGATATTTAGCTTTTGCACTTTTAAG
ATTAGCTGAAATGAAACGTGAACGAGAGGCTGATAAAAAATAATAATGAGTTACTTAGATGA

Gene: YeeC-like (putative protein from SCC/ACME elements)

Contig: 01_NODE_8 (fragment from pos. 242580 on), position: 39154 to 40594, length: 1441 nt, orientation: TRUNCATED

Sequence:

TTTTTTCAGATGAACTTTTTGAAGATTTAACAAAACAAGAAAAAGAGAAAAAAATTGAAAAGTTAGATCCAGAAATCGGTAAATTTCAAG
AAATAATAAATTTTGTAAAAAATAAAAATAGAGAACCTGAAAAGACAAATCGATGGTCAGAAGAACGTGCTTTGTGGCAAGACTTCAA
GGATTTCGTAATAAAAAAGAACGTTCAGATAAAGTAAAACATATTGATGAATTAAATCTATTAAATAAGCAAGGAGATTTTTTAGATGAA
TTGAACTTGACTGTTAATAGAGAATCAGTAAGTAATATTGATGAAGTACTAAATGATAACCAATTACTTGATAACTTTTCGGATTTACTTG
ATACTTCAAGATATAAAAAGACTGTTAATGCATTGAACAAAAAAAGTTCACGTAAACGAGCTGATAATTTTAATGAATATAAAGAATTAT
TTAAAAAAGTACATGAAGAAATAGCAAGTGGTAAAAGAAAAATTTTACCATTTAAGAAATATGATATTGAAGAAGGTAGATTTTATATAC
AAAATGGTGTAATGCTGTATATTGTATCAATCTCTGATGAAAAATTTATCGGAGACAATGGTAAAGAGAATGCCCGCATGCATGTTGTAT
ATGAAAATGGAACAGAGAACAAACAGCTATTACTGCAATCGTTAGCGTCTTCATTATACTCTAATGAACGTCATGGTAGAATGGTAACAG
AAATTATTGATGAAAATTCATTATCTGAAAGTTTTGGCGCAGAATTTACGACAGGATACATTTACGTTTTAAAATCGTTAAACACGAATCC
AGAAATTAGTCAATTAACTCATTTATATAAAATTGGCTTTACTAAAAACAGTATAGAGTCTAGAATTGTTAATGCAGAAAATGAAGTTACT
TATTTAAATGCTCCAGTTCGTATAGTATTGTCTGTAGAGGTAAAAAATCTTAATGCACAGTTGTTAGAGCGTACGTTACATCATACATTTC
ACGATAAACAAGTTATATTTCAAAATAGTAAATATAAAAAAGCAACAGAATGGTATATTTTACCACTTGAAGAAATAGAGAGCAGAATTA
ATGAAATATTATCTCATATTCAATTTTAGTTTAGAAGGTTTTTGATTGAATAAAAAATAAACTAATAAGAGCATCCCTCACCGCAAAGTGA
AGGATGCTCTTTTTTTATTAGAATATAGTTTTCATTTTGATAGATAATGGTTAATAATTATCTGTAAATCATTATTAAGTACCGTTGTTATAG
AGTCATCATTCATAAAAGCTTCATAGATTTTATCAAGTATTTCCTCATCTTCAATCGCTGTAAAATGATGTACTAAACCTTTTACTATTGATA
CATAATTTTGAGATGCTAAGCAACTAGCAACCGTTCGCCAGTCTGATTGTGTAGTTTCATGGTTCATAGATAATCCTCCTT

Gene: A9UFT0 (LPXTG protein homologue)

Contig: 01_NODE_8 (fragment from pos. 242580 on), position: 40359 to 40580, length: 222 nt, orientation: REVERSE

Sequence:

TCATTTTGATAGATAATGGTTAATAATTATCTGTAAATCATTATTAAGTACCGTTGTTATAGAGTCATCATTCATAAAAGCTTCATAGATTT
TATCAAGTATTTCCTCATCTTCAATCGCTGTAAAATGATGTACTAAACCTTTTACTATTGATACATAATTTTGAGATGCTAAGCAACTAGCA
ACCGTTCGCCAGTCTGATTGTGTAGTTTCATGGTTCAT

Gene: Q9KX75 (putative protein)

Contig: 01_NODE_8 (fragment from pos. 242580 on), position: 40595 to 41097, length: 503 nt, orientation: REVERSE

Sequence:

TTATTTCAATGTCCATTTTTGACGTGCTTTAGGATTGAGTGGATGCATGATTTCATTTGTTGCGGGGTTCATGAGTTGCTTCACTTTCTTTTT
ATGTGGTTTCAACATTTCTATTACTTCATTGACACGTGCTTCAACAACGGGTTTTTAGCGTAAGCGCCCCAAGCTAATATAACTTCATCCGA
TTCTTTCACAGCCTTCATAATTTGAATATTTGTATGATTATCAAAAGCATTTTTTGAATGTTTAATATTAATAGGTGTTGTAATATTAGAATA
TAGATTCACAAAATTGATTGAACCAAATGCATCCATTTCTGAAACTTTGTTCATAATGAGCTGAGTTGTTAGGTCGATGTTGAGAATGCCA
TCATAATGTGGATACATTGTAATGATTGTAATTGTTTGTTTTTCACTATCCCATTTTTTCTTAAGTAGATAGCGATGTTCTTGATCATCACTG
AATATAGCTTCGGTTTCTAATGTACTTTTGATTGTTTTCAT

Gene: Q7A207 (putative protein)

Contig: 01_NODE_8 (fragment from pos. 242580 on), position: 41113 to 41424, length: 312 nt, orientation: REVERSE

Sequence:

TTAGTATTCTTCTGGTAAAAGCATCACATAATAAAGAAAGTCTACATCATCTTCACGAATGACGTAGACTTTTTTAGGTAATGCATTTTGTT
TTTTTACATAGTTTGTATAGTGATATTCCAATTTGTATGCGGGTTGTTCTTGTTCATGTGTGATTGACAGTATATTCTCATCTTCTTGTAGTC
TAAAAATGTGTAGGTAATCTGTATGAGGTTTATTATCTCTTTCTTTCACCATATTCCAAAGTAAGATTTGAAGGTCTAGAGATAGGTGTTC
ACTAATGCCTCTTGTGATGTATCGATTGATTTTCAT

Gene: Q7A206 (putative protein)

Contig: 01_NODE_8 (fragment from pos. 242580 on), position: 41426 to 41512, length: 87 nt, orientation: TRNC-RVRS (no start codon)

Perfect match to: (G265-LCL_10074-[66452:66538:r])

Sequence:

CTATTTTCCTCCATTTTGCTTTTCTTTCATGATGTCAATCACTTCGTTAATGACTGTAACAGATATTTGTGCCACTTTGATCCAATT

Gene: ccrB-1 (cassette chromosome recombinase B, type 1)

Contig: 01_NODE_8 (fragment from pos. 242580 on), position: 42328 to 43956, length: 1629 nt, orientation: REVERSE

Perfect match to: (isolate 275757-[41820-43448])

Sequence:

TTAGATTTGAAAATAGAGTGTTTGAGGCAAAAACAAAGACGAAGTGCTGAGGAGCACTTCGTCTAGATTATTATTATTGAAAAGTTGTTT
AATAATTTCATTATTAAGTTTTAGTGTAACGTAGAATTGCTTTTTATGATTCTCATCTTTACGAATGTCAATACGGTCAATAACCGTTAGGT
ACAAAGCTTTGAGCTGTGATTTATCCATGGATTCTATATTTTGAAATATTCGTTGTAATAGGGCAGCGATTTGTTTCGTATCATAAGATGG
TTTCTCTTGATTTTGTTGCTGCTTGAGTTGATTAATTTGGTTTGTAATGTCATTGAGTTGTGTTTCATATTGATGAATGGTTGGTTTGAGTG
CAGATGTTAAGTCTGGATTGTCTTCGAGGGTTTGAATTAGATTTTTTAGTTTAGTGTTTATTTCATCAAATTGTTGTTGCTTATATGCGATA
TCATGGTTAAGTGCAGCCATATCAATTTGACTATCTTGATTAACACGTTCGACAACTTGTTTGAGAACTTTATCGCTTTTGACAATTTCAAG
TATTTGATCCATGACGTATTTCTCAATCACATCAGCTCTAACACTATTGGCTGAACATACCTTTGATCCTTTGTTGCGAAAGTTACTACAAG
AATAATAGCGAATACGTTTCTTAGTTCCATCTTTGAGTGTATTAGTAGTATTACTCGCGGCCATAGGTGCTCCGCATTGGGGACAATGAAC
GATACCGGTTAATAGATTAGTTCCTTTACCGTGAACTTGGGGTTTTTGACTGACTTGCTTTTTACGTGCTTGCACTTTATCCCATAATGCTT
GGCTAATAATAGGGGCGTGCTTACCCTCAGCTATGATCGGTTTATCATTCAGTCCTTTACGACGTTTTTCATTCCAATCTTTGTACTTCGCA
AATTGAATTTTACCCATATAGAAGGGATTGGATAGAATATAAGTAATAGAACTTATACTGAAAGGCTTCCCTTTTTTAGTGACATAGCCTT
TATGATTGAGTGTATTGGCAATTTTACGATAGCCATGCCCTTTGGCATAAGATTCGAAAATATATTTTACAATATTAGCTTCATGTTGATTA
ATCATCAATTCGTGTTTACTGTCGGGTATTTTGTCATAGCCTAGCGGTAAATTTCCTTGATAATAGCCTTCTTGGGCACGTCTGGTTTGGCC
CATGAATACGTTCTCGACAATGTTATTACGTTCAAATTCTGAGAAACTCGCAAGTATTTGTAACATGAGTTTTCCAGAAGAAGTATTGACT
TCCATACGTTCAGACAAACTGAAAAATTCGACATTTTGCTTATGCAAATCTTCGACAATTTTGAGTAAGTCTGATGTATTACGTGCCAGTC
GGTTTGTTTTGTACACCATGACACAATCGATATGACCTTCATTCGCATCCTTCAACATACGTTGGAGCTCAGGACGGTTCATAGATTTTCC
GGAAATACCACGATCTGCGTACACATCTACCACTTTGAAGTTATTAAATTGACAGTATTCTTCAATCTGATTGATCTGGCCTGTTATCGAGT
AGCCTTCAGTACTTTGGATTTCCGTTGATACACGAACATAGATAGCGACACGTTTTTGTTTGAGTTGTTGCAT

Gene: ccrA-1 (cassette chromosome recombinase A, type 1)

Contig: 01_NODE_8 (fragment from pos. 242580 on), position: 43977 to 45326, length: 1350 nt, orientation: REVERSE

Sequence:

TTAAGCAATCGATGATTGCGCCGTTTGATTGACGATGTTTAAGGGCTCATTTTTGAAATAGATACCCGTAAGTGTTTTATTTTTGGTAATAT
GAATTTCATCAATGTAAGGGTACAACATGTTTAACGTGAAACGTTGTTGTATGATATTTTGAAAAACCTTTCGAATTTGATGTGCATTGAT
TGATGTTATAGGGTTTGACTGTTGTCGAAATGATTGCGATTGTTCTCTGAACGTATCTGCATCAATTTTGCCTTGGGCTAATTTTTCTATCA
GCTGCTCGTGATTGAGTGTAGTTTTAGTTTCTATATCTCTTTGTCTTTTGAGTCGTTGTTGAATAGTATGATTTATTTTTGAATAGAGCTGTT
GATTTTGAAAGAAGTCCTGACAAGTCGCTAAAACACTTGTTTCTAATTCTTGTGCGTTGATTCCTTTAAATTCACAAACAAAGCGAGCGTT
ATTCATATTTTTCGGACAGACATAGTAACGCAATGAATGATTTGGTTTTCGGACGGTCATGTTTGTGAGTGTCGAATGGCAATAGGGACA
TTTGATTTTTTGTTTGAGTTGATTTTCTGACGGCTTACGTTTGACTTGTTTCTGAGTTCGGGTAACTTGAGCTTCTTCGTATATCGTTGTACT
GACAATAGCTGGGAACATGTTTTCATATTGTCCGTATTGATTGATAACACGGCCACAGTAATTAGGGTTAAGGATAATATTACGCACTTG
ATAGGGCTTGCGATTAATGAATTTATCATCAGCTTCTAAGTATTGCGCAATTTTTTTATAACCATAACCTTGAAGGTAATAATTGAACACA
GCTTTTACTGTTGGTGATTTTACTGTGTCTATTGTGAAAGTACCATTATGATAGTGATACCCAAAGGGTGCATGTGTTGTAATTAGTTTAC
CTTGTCTCGCTTTTTCTTTGATTCCATTTTTGACTTGTTCGCCTATATTATCAGATTCTAGTTCGGCCAAGCTGATGAAAATATTGAGTTTGA
GCCGATCGAATGCTTTATCCATGTCGAAGTAGCCATCATGAACGCTTAAGATATGGATATGGTATTTTTGACACAATTTCATGAGTTTTAA
TGCATTTTTAAGATTGCGATGAAGTCGGTTTAATCTGTAACAACATAATACGTTACATTGTCCTTGTTGAATCAGTTCAGTAATTTGTTGAT
AACCATTCCGCTTATCAGTACGTCCTGATTGCTTATCGCTATAAAAGGTAATGTATGTAATATTATGTTTTTTGGCTAATGCCTCTATGGTT
TGTTTTTGTGCTGCTAGGGATTGTTGCTTTGTAGTGCTCTGTCGTAAGTAACCTATTGCTTGTTTCAT

Gene: ORF-NoKK12 (undescribed ORF from SCC/ACME elements)

Contig: 01_NODE_8 (fragment from pos. 242580 on), position: 45329 to 45859, length: 531 nt, orientation: TRUNCATED

Sequence:

TATTTCCTCCTTCCACATTGATAATATATATTTATGAACGAATTTATGCATATGCCCAACGCCATCAGGCGTTGGGCATTAACTTTAGTCAT
CAGCGTGATTGATTTCTTCAATCACTAAATCAGCGAGTAATGTGATTAATTCGTCCATATTCTTCCTCCTAATTGACTTTGATAGTAGTAAA
AGTGTTATACCAACTTTGGAAATATTGTAATGAACTCAATTTTTCGCTTTGAGCAGATTTGCTCCAACTCAGGATAATCTTCATAAATGCCC
CAAAAGACGATACGTTTAGGTTTGACGTGCTTCAGTTTAATATCATTTATTAATTGGTTATCGTCGTTATAATCTTTTGTGATTTTATAACCA
TGTTCTTTACAATATGCTTTTACAGTATCAATAGGTTCGTCTAAGTAGACTTCTCTTTCATCTGTAATATTATGTGCTAATGCAACTACATTC
ATAGTTAATACCTCCGGTTGATTTAATGAGCTACAACGCATAGAGCGTTGTAGCGGTATAATATTCAAA

Gene: cch (cassette chromosome helicase)

Contig: 01_NODE_8 (fragment from pos. 242580 on), position: 45861 to 47648, length: 1788 nt, orientation: REVERSE

Sequence:

TTATAGTTCTAAATCGTCACTTGCTTCTTTTTCTTTCGCTTGTTTAATAAAATTATTAAGCAAATCTGGATTTGTATTATCATCAAATTTCGGT
GGTGTGAAAGTTTCTGTATTGGAACTTAAGCCAAAAATAGATGCAAGTTCTTTATCCAATTTAAGATGATAGAATACTATCGTTTTTGATT
TGCCCGTTGCATCTTTGACACTTCGTTTTGTTGTCTTACGATCTCGGTCCGATTCCATATAACCTTTTTCTATCAAAGCTTCAACTACATTGT
TGGTGTCCTGAAATTGATTATTTTTAAGCATCTCTTTGAAAACTTTGGCAATTATCTTAACTTGTATATGGTCATCTTTAAGTTCTATGAGAC
CGAACCCCTCCAGCATATTTGATAATCGACCATCATCTGAAAATTTATTTCTGTACTGTGCTACAAATTGGACAATTAGTTCGATTGCTTTA
TCGGACAGAGACCTTTCGCTCACCGAATCTGAGTGGTAATTCACTAGGTATTCTCTAACTGCATTCAAATCGATAGGGGTTGCGATAACA
CGTTCTAATATTCGTGCTGATGTCGTAATGGTGGCGTAGCGCTTGAACATACGTATGCCTACATTGTTTGTTTCATTGTTAAGTTGTGTTTT
GAACCAATCGTGTTCTCTATGAAACCAATTGATTACTTCAGATTCACGATTTAGAAGATATTCAGCTACTAACGGCATTATGTGCCCATAG
TTTTTTGATGTTCCTTTTTTAATGGCGTCTGCATTGTCAGCACTTGTTGTAAAGACATCAGAAATTTCTATACATCTTGCTCTGAGACCATCA
TTCGCACTAGCATCCCTGAAAATCGTGTGTTCTGCTGTACTAATAACACTAGATCCAAAGTGGTGAGGATTTTTTACATTTCCTTCTCGATT
AGCACGTAGACGACCTTGCCCCTCTGCTAGGGAATAAAGTAAACCATTTGTATCTTTAAAGGTAGCTGCTGATAATTCATCTAAGGCAAT
AGGAACACCAAAGTTGTTACTTAAGTATCCTTCAATTGCATTACGGGTGCCGTTCCAACCTCTGAATAATGTTTGATCACCTTTAGATGGA
TTACCTGCTATTGAAACTGCTAATGCCGCTGCAGTCGATTTACCCGTACTAGAGTTCCCCATAAATGAAAATATCGTGCCAAAATATTCGA
CTTCATGCTTTGTTTTTAAGAACGCAGTCACTAATGAAGAAACACCAAATATCACAGCAAGCTCCAGTAACAAGTTTCCTTTTACTTCATCA
AGATACATTTGCCACCAGCTTTCAAATGTACCTTTAGGTTGTAAGTCATACTTTGTTTCACAAATGATTTCATCAGCCTGAGACTGCTCGAT
GTCTTTTGAAAGATATGGTTCATCTAATGAAATGACAGTACCTTCATCAGTACTTAAGACACCTACACCTGTATACAATGTAGAAATGGGC
AGTGATTGACGCATTAATTGTAGTGCATTACTTAGCGATTTAATGTACGTTTCATTGATGCTGAAGCTAAACATAACCAGTCTAGGTAACT
TTTGTGTCGTTAGAATATCTGACGTTTCAATACGTTTAATATTTTTGCCATCCGTGATAGTCAATTTCTCAACCCCTGTAGTAGGGTCGAGA
AATTTGTTTTCTATGATGATTGGACTAGATAAACGAATTATCTTATCTTCGTCGTTTTTGTTTTTTGCTCGAATAAGTTCATACCATGCAGTT
GAATCTAACCAGTATGGATATTGCTCGAAGATGTTGTTAGCCAT

Gene: orf7795 (putative protein)

Contig: 01_NODE_8 (fragment from pos. 242580 on), position: 48072 to 49631, length: 1560 nt, orientation: REVERSE

Sequence:

TTATTTTTTAAAATATTTTTGTCTCAAGTTTAGATTATCAATTACATTCATAGAATTAATTAGAGATAGATGAGTTTCACTAGAAATTTTAAA
GTCATAAATGAATAAGTTTAGGTCGTACTTATTTGATTTACTATGAGTTAGGTCATTTTTAGCATGGAGAGGTGTTATTGTATTACTCCATT
TTTTTGTCGTTTTTATAATTTGATTACTCAAATTAAGAACTTCTATAGGCGCATTTGTATGAAAGAAATTCATGTTCTCGCAAATATGAGCG
TAAACACCTGATAAAGTACTAAGCAAAGTTTCCATAAATGGTAGCTGATCCATATAAAATAATTTATGTATAAACAAATTTGATGCATTTA
TAAGTTCATTATCGATGATATGAAAAGATGAAAAATCACCTTCATCATCTTCATTTCCTAATTTGTCATCTGTGAAGACAATGTGTAAGTG
ATTTTCAGTTAATTCATCTATGTCGTTATGTAGTTGTTGAAAATCAGCGTTGAATTTTAATTTTTGTTCCTTAAAATCTTGGAGTTCTGAATA
ATTAACTTTTTGCTTTTTTAATATATTTAATTCTTGCTTGTTAAATAGTAAAGCAAAATAGCTATTGTTGCCAGAGGCATTTTTGTGTTTAGC
ATGTGTATTACTATCTATGTACAAATATCTAAAATAAAAAACAGACAAAATTGTTAGTACATCACTAAAAGGGAACCTTCCACGAATGTTG
TCTTTTGATAATTTACCAATTAATGAATTTAGATTTGCAGTTATCTGTTTTTCTTCATCTGTGTTGATATCAGGGAACCTATTTAATAGTGTC
TGTATTATAGATGAAATAAGTTGTTCTTTTGTGAAATATCTATGGAATATCTCATTATTTTTGTCATTTAAATCTATATTGATTTGTGTACTT
ATATCAGCAATCTTATTTAAGTTTTCCCATTCTTCATGTGAATATGATGAGGAGTAGTTATTTGAATAATTATAGTTTTTACGTTCGAGACC
ATTTTTAATTGTAAAAGCAGTGTTTAGCATATTATCCAGTGATGTGATATTCGGATTATTTTCATCGTAAAATCTAAAAGGTATATCATTTT
TATAACCAAAAGTAGCATTTACAGTAGCTCTTTTAAAAGTAGTGTCGTCAAGAGCTATAGATCCATCATTATGTAATTTTAATAGTACATG
TTCCTTTAGTTTGCCTTTAGATGTGAACAAATAAGTGAAAATTGCATTTTTATATTCTTCAAATTGATCATCTAAGTTTGAAGCATCCATGA
AACTTGTTAAGACTTCATAAAATCCTTCGTTGAATCCACGCATAAAAGTATCTACACCATATTCAATACCTAACAAATCTATTAAATTTGAT
AAAATATTTTTTATATAAAGTTTATTATTTTGAAATTGATAACGTGTGTAATTGCCTTTATTATCGAATTCGACTTCAATTGATGATGAACCT
TCAATACCTTCTAACGATATGTTTATTTGTTTGATTATATCCTGCATGAATTCAGAGTTATACTTTGAAATAAGTTTTAGTGTCAT

Gene: IR_IS431 (inverted repeat of IS431)

Contig: 01_NODE_8 (fragment from pos. 242580 on), position: 49898 to 49913, length: 16 nt

Perfect match to: (Plasmid_pHKK701-L38972.1-[631:646])

Sequence:

GGTTCTGTTGCAAAGT

Gene: tnpIS431-06 (transposase for IS431)

Contig: 01_NODE_8 (fragment from pos. 242580 on), position: 49958 to 50632, length: 675 nt, orientation: REVERSE

Perfect match to: (Strain_21342-AHKU01000078-[56395:57069:r])

Sequence:

TTAACTTGTTAGCATGATGCTAATTTCATGGCATGGCGAAAATCCGTAGATCTGAAGAGACCTGCGGGTCTTTTTATATAGACCGTAAATA
CATTCAACACCTTTTAAAGTATTCTTTGCCGTATTGATACTTTGATATCTTGTCTTTCTTACTTTAATATGACGATGATCTTGCTCAATGAGG
TTATTCAGATATTTCGATGTACAATGACAGTCGGGATTCAGTTTAAATGCTTTAATTACTTTAGCCATTGCTACCTTCGTTGAAGGTGCCTG
ATCTGTAATCACCTTTTGAGGTTTACCAAATTGTTTAATGAGACGTTTGATAAACGCATATGCTGCATGATTATCTCGTTGCTTACGCAACC
AAATATCTAATGTATGTCCCTCTGCATCAATGGCACGATATAAATAGCTCCATTTTCCTTTTATTTTGATGTATGTCTCATCAATACGCCACT
TATAATAGGCTTTTTTATGCTTTTTCTTCCAAATCTGATACAAAATTGGGGCATATTCTTGAACCCAACGGTAGAGCGTTGAATGATGAAC
GTTTACACCACGTTCCCTTAATATTTCAGATATATCACGATAACTCAATGCATATCTTAGATAGTAGCCAACGGCTACAGTGATAACACCTT
TACTGAATTGTTTATATCTGAAATAGTTCAT

Gene: putative surface protein with similarity to DLJ55-14705 (Unidentified ORF from SCC/ACME elements)

Contig: 01_NODE_8 (fragment from pos. 242580 on), position: 50766 to 52318, length: 1553 nt, orientation: FORWARD

NOTE: *Assembly might be faulty due to repetitive elements. In isolate 275757, there were discrepancies between Nanopore and Illumina affecting this gene´s sequence.*

Sequence:

ATGTATTTAGGAGGGAGTATTATGAAAAAAAATCTCAAATGAAAGTAATTAATACATATTCTATAAGGAAATGTAATATTGGAGTTGCTT
CTTTTATTATTGGTTCATTTTTATTTTTAGGAGTGAACGGTAATATAGCCGAAGCCAATGATGAGAGACAAGAAGTTGACAATGGTCAAC
AACAAAAGTTAAATAAAGCTGATAATGATAGACAAGAAGTTGACAATGGTCAACAACAAAAGTTAAATGAAGCTGATAATGATAGACAA
GAAGTTGACAATGGTCAACAACAAAAGTTAAATAAAGCTGATAATGAGAGACAAAAAGTTGACAATAGTCAACAACAAAAGTTAAATAA
AGCTGATAATGAGAGACAAGAAGTTGACAATAGTCAACAACAAAAGTTAAATAAAGCTGATAATGATAGACAAGAAGTTGACAATAGTC
AACAACAAAAGTTAAATAAAGCTGATAATGATAGACAAGAAGTTGACAATAGTCAACAACAAAAGTTAAATAAAGCTGATAATGAGAGA
CAAGAAGTTGACAATAGTCAACAAGAGTTGTTAAAAAAAAATAAATGAATCAACAAATAGAGAATCAACGGTGAAGAGACTTTTAGAAGA
TGTTTATTCTAAAGAGAATGCTCGAGGTATTCTTAATAAAATAAAACCAGAAGATTATAATTTAAGTTCTAAAGAATTAATAAATAAAATA
ATTAAAGCCGGAATAGATTATTCTGAAGAAAATAATAGTAAGTACAAAGTCTTTGCTTCTGTTCCTCAAGCTAGTATTACTTTGAAAAGTG
AGTATAATATTGGTAATTCTTTAGTGGTTAGAAGCGATAAAGTTATAAAACCAGAAGATTATGTTAATTATCCAGAAGGGTATCACGTTC
AAAGTTATGTTTCCAAAGGAAAAGAATATCCTAGTTTAGATGATATAGTTAAAAAAAGAGTCACAGGTAGTTATAAAATTACGTATCGAT
TATTAGATAGTAATGATGAACCTGTTCACGGAGAAGATGGTCAAGCGATTGTAAGATCATTAAATATTTATTTCTATAATCCAGAACCAGC
TTTAGATATAATTAAAGAAATCGAAGAAGATATAGATGAAAATAATTATCAAAATGGCGAGGAGATAAAAGAAGATTTAGAAAAAAATAA
AAAATGAACTAGAAAAACCAACTAAAATACCAGATGTACCTGCTTTGATTGATGAAATAAATAATAAGAAAAACCAGCTTGAAAATTTAG
ATACGACGGCACCAGACGCACCAAAAGTTAAGGATACAGAGTCAGGCAGCAAGAAAATCACTGGTGAAGGATCAGAACCAGGCAATGA
CATTACCGTAACGTTCCCTAGCGGAAAAACAAGTCAAGGTAAAGTTGGTCAAGATGACAGCGATTCAGACTCCGGCAAAGAAGTATTAC
CAGAAACTGGTGAACAATCAAATTCAAATAATGCGACTTTATTCGGATCATTATTTGCTGGCTTAGGATCTTTATTCTTATTCGGTAGACG
CCGTAAAAAAAGATGCAGATAACAAATAA

Gene: IR_IS431 (inverted repeat of IS431)

Contig: 01_NODE_8 (fragment from pos. 242580 on), position: 52496 to 52511, length: 16 nt

Perfect match to: (Plasmid_pHKK701-L38972.1-[631:646])

Sequence:

GGTTCTGTTGCAAAGT

Gene: F8WKF9 (putative membrane protein)

Contig: 01_NODE_8 (fragment from pos. 242580 on), position: 52714 to 53066, length: 353 nt, orientation: FORWARD

Sequence:

ATGTGATAGCGAATAGACATCAATATACGATGTATTTGAATGGTGAAGAAGTAGGCACACTTGAGATGAAACAGTTCTTCAAAAGAGGG
GGAAAGCAACAAATTCCTTATATGTTTAATTACAAATTTGAAGTGTTTGATGTAAGCAATCCGTTTTTTAGTAATGAAACGAAAATCACAT
TTTAGAATGACGTATTATTAGCCGCAAAGCGTAGTTTTTTAGATATTTTAAAAAGCAAACGAACTAAAAAATGAGGCGAAAAACATACTA
TACACATTTACAGCACTAGAGTAGAGAAAGAAATATTAATAACTATTTACTTACAATGCATAATCAATAAGCAAACACAATAA

Gene: EHQ67276 (putative protein)

Contig: 01_NODE_8 (fragment from pos. 242580 on), position: 53324 to 53749, length: 426 nt, orientation: FORWARD

Perfect match to: (C427_ST42-ACSQ01000050-[1158:1583])

Sequence:

TTGATATATTGGAATTTATATAAAATTTTTAAATATAAAATAAGTCATATATTACTCGCTTGTATCTTCACTAATGTCGGATTAACTAAAATT
ATTATGTATTCAACGCCAGTATTAATGTTTATCTATCCGTTAGCGATTACACTGATTTTATTAACGCTTGTCAGTCCATTAATTAATCATTCG
ACAATTGTACATAAATTTACAACGTTCTTTACAATGTTTGCAGCGTTCTTTGATGGTTTAAACGCATGCCCAGAATTCTTTGCAAAGACATC
ATTTGCACAAGCGTTAATAATTTTCGCTGAACGTTATTTACCGTTCTTCACAATTGGAATGGGTTGGATTGTTCCGGCAATCATAGGATTT
GTAGTAGGACTTATCGTTTATTTAATTCGTTCTCGTAGACAAACTCAAACACAATAA

Gene: opp3A-A8YYZ6 (putative S-adenosyl-L-methionine-dependent methyltransferase)

Contig: 01_NODE_8 (fragment from pos. 242580 on), position: 54158 to 54796, length: 639 nt, orientation: FORWARD

Perfect match to: (C427_ST42-ACSQ01000050-[1992:2630])

Sequence:

ATGGCGTGTGACCATCAAGAAGCTTGGGAGAGAACTATAGAAATGCTTGATACATCGGATATCAAAGGGAAGACAATCTTAGATGTGG
GATGTAATCAAGGCGGATTTTTAAGAAAATTATATGACACAACGCCATTTAAAGAAGGTGTCGGCATAGATTTAGCACGTTTATCTTTAG
AAAAGGCAGAAACTTTAAAAGGTGAACGACCACTTACATATTATTTAACGGATAAACCACAAGAGACAAATAGAACATTTGATACTGCTG
TAAGTACATCTGTTTTGTACTTAATCGAAGACATTCCACAACATGCACAAGATTTAAAAGAAGTGTTGAAACCGGGAGGCGTTTACTATG
CTTCATTCGCCGATTTAACCAATAATCCTAGTCGTCAATTTATGGATGATACGATTAATCAATACGGCGCAACACCTTCTCAGAACCATTCT
TTAAAACATATCGTTGATAGCTTTGTTGATGCAGGGTTTGAAGTTGCAGTAATGAAAGAACCTGTGCCTGTCGTGATTGATTTAACACATT
ATAGTGATTTTTATCTATCACCAAATGATTATCTACAAACACTTTATGAGGAATCATTTTTAATTAAAGCAAGCGTGAAAGAAGGTATCGG
GAAATGA

Gene: opp3B (oligopeptide permease, channel-forming protein)

Contig: 01_NODE_8 (fragment from pos. 242580 on), position: 56349 to 57305, length: 957 nt, orientation: FORWARD

Sequence:

ATGCTTAAACGAACAATAAAATTAATACTTTACTTAATCGTGAGTTCGTTTATTATATTCGTTTTAGTCGAGAAGACATCAGGTAATCCAG
CGATACTATACTTACAACGTCATGGTTATACGTCGATTACCCAGGAAAATATTGAAGCAGCACAACATAAACTTGGTTTGGGCCAGCATTT
TTTATTAAGATATATCGATTGGGTTGGACATGCACTTACTGGTAACTTGGGATACAGTTTTAGTACAAATGAGCCAGTAACAACTATGATT
ATGGAGGCCGTCATTCCAACATTAATATTAATCGTTGTTTCAAGTTGTATCATGTTGCCATTGGGTTATATGGTTGGTTATTTTATTGGGAC
GCGCCCACATACTCGATACGCGAATGGTATTCGTGGATTCGCTCAAGTGATGACTTCTATGCCGGAATATTGGTTAGCCATTTTGTTTATA
TATTATTTAGGAGTACGTTGGCAGTTGCTACCTTTTGTAGGTAGCGATTCCTGGCAACATTTCGTGTTACCCATCTTCACCATTGTTGTTAT

AGAAGGATGTCATATCTTATTGATGACATCGCACCTTATTGCGCCAACGTTAGATAACGATGCGTATCAACTTGCTCAGTTAAGACATTAC
TCATTAAAGGCACGTATCATTGTCCAGATTAAAGAGATATTTGCACCACTCATGACCATTTCAATTAACAGTGTGATTCATTTAATTGGAA
AAGTAGTCATACTTGAAGTAATCTTCAGCATGTCTGGTATAGGGAAATTATTAATTAATGCTATCAATCAACGAGATTATCCACTTATTCA
AGGCATTGTGGTCTTTATCATTGTCTTAATTATGTTCATTAATTATTTAGGCGACATTATTATTTTAAACAATGAACCTAGACTTCGACGAC
GACATACCAAACGCCAAGCCCATGAGAAAAGAGGTGTGTCGTGA

Gene: opp3C (oligopeptide permease, channel-forming protein)

Contig: 01_NODE_8 (fragment from pos. 242580 on), position: 57305 to 58072, length: 768 nt, orientation: FORWARD

Sequence:

ATGAAAAAAATATCAAACTTACATCGCCATAAGTGCAATATTAAGTGTGATGTTTGTGCTAATTGTATATTGCTTTATGCAAGACACACAAA
ACTTGGACCCACTTCAATCACCTAATAGCCAACATTGGTTAGGCACGGATCAATTAGGCAGAGATTTCTTAGTCAGACTGATTGTAGGTA
GCCTAGTCACATTAAGCTTAACTAGCGTGGTAATTCTGTTAAGTGTTTGTATTGGACTGGTCTTTGGGTTAATTGCAGGTATAGAAAGAA
AATGGTTAGACCAAATCATCATGTTTATTGCTGATATGCTATTGGCAATTCCTTCGTTTATTATTGCATTAGTCATCTTAAGCTTAGTAAGT
AATTCAATGCTAGGTTTGATACTTGCTTTAACGATTGGATGGATAGGCCGTTATTTACGTTATTTCAGAAATTTAACACGAGATATTCAAA
AACGTCCCTTTGTCAGTTATGCAAGACTAAGTGGTAACTCAACGTTTAAAACGACAGTGACGCACATGATTCCGCATTTACTAAGTAATAT
ATTTGCTTTGGTCACGGCTGACTTTGGCAAAATGATGCTAAGTATATCTGGACTTGCTTTTTTAGGCCTAGGTATTAAACCACCGACGCCC
GAGTTAGGAACCATTCTCTTTGACGGGAAAAGTTATTTCAATGGAGCACCTTGGCTCTTCTTCTTCCCTGGTGTATTGTTAGGAGGTTTCG
CCTTATTATGTCAAATGATCAACAAAAAAATAACGCAATGA

Gene: opp3D-A8YZ00 (nickel/peptide ABC superfamily ATP binding cassette transporter, ABC protein)

Contig: 01_NODE_8 (fragment from pos. 242580 on), position: 58039 to 58806, length: 768 nt, orientation: FORWARD

Sequence:

ATGTCAAATGATCAACAAAAAAATAACGCAATGAATACTGTAGTTAAAGTCAATCAATTATCCATCTTAGATCATAATCAGTCATTGTTAA
ACGATGTGAATTTGACAATAACTAAAGGTGCATTTCATTGCATTATAGGTGAGAGTGGTAGTGGGAAGTCATTATTGACGAGAACTATTC
TTGGAATGAAGCCTTCACAACTAAGCTATCAAGGAGATATCAAGATTGATTTAAATAAGGTGGATGCCGTTTTTCAAGATGTTCAAAGTA
ATATGTTTCAAAACGTGAACTTGGCTAAACATTTCCAATATATTTATGAAGCTAACCATTCACAACTATCTAAACAAGATATCAAGGAAGA
CGTCCTAGATAAAATGCAATTACTCGGTTTAAATCAAGGTGAACAATTACTTAAACGATATCCCTTTGAACTTAGTGGAGGTATGGCACA
ACGTATAGCCTTTATAATGTCATTAATTAGACGCCCGGACTACTTATTTTTAGATGAACCGACGAGTGCGCTAGATCAAGAGAATGTTAA
AAAGTTTATGCACTATTTGATTAAGGCACAGGAACATTATCAAATGACAATTGTCTTTATTACACATGATATTAACTTAGTTAAGGATTAT
GCCACTCATATTAGTATTATGCAGCAAGGTCAATTGATTGAGAGTGGTGAGGCATCATCCATTTTAGCTAACCCCATACATAGCTATACGA
AAAATTTAATTGCTATCGCACATCGGAGACAGGCCTATGCTTAA

Gene: opp3E-A8YZ01 (nickel/peptide ABC superfamily ATP binding cassette transporter, ABC protein)

Contig: 01_NODE_8 (fragment from pos. 242580 on), position: 58799 to 59433, length: 635 nt, orientation: TRNC-FRWD (no stop codon)

Sequence:

ATGCTTAAAATTGAAAGATTAACTAAATACATTGATGGGAAACTTATCTTCAAAGATATATCATGTGCAATTAATGACCAACATTTACTTA
TAAGTGGTGAAAGTGGCTGCGGTAAGTCTACGTTAGCCAAGATTATCGCCGGATTAGATATGAATTATCAAGGTAACTTATACTTTAATG
GCCAATTACGTAAGTCATATACGGCAAAAGAATGGATGAAACACATCCAATATGTGCCCCAGTATCAACGTGACACATTAAATAAGCGTA
AAACCGTATTATCTACGTTATTAGAACCACTTAAGAATTACAAATTTGATAAGCAGAGTTACACATCAAGAATAGAAGCGGTACTTAGAC
AATGCAAGTTACCACAAAGCATACTTAATCAAAGCATTTCGACGCTAAGTGGTGGACAATTTCAACGTGTATGGATAGCAAAGGCACTAA
TTCTAGAGCCGGAGATATTGATATTAGATGAAGCAACGACAAACTTAGATGTGATTAACGAGGAAGAAATACTTCAAATGTTAATTACGT
TAAAGCAAACGCAATTAATCATTATTTCACATGATGCGTACGTCTTGAGTCGCTTTAAAGGCGTTCAGTTACAACTCAAACAATTGAATAA
TTA

Gene: tnp_A8YYY6 (transposase)

Contig: 01_NODE_8 (fragment from pos. 242580 on), position: 59520 to 60186, length: 667 nt, orientation: TRNC-RVRS (no start codon)

Sequence:

CTAGGCTATTAATTCTCTGTATTTTACAGGGGATAAGTAGCCTAGTTTTTGTTGAATTCGATTATTATTATAGTTTTTAATATTCTTTTCGAC
AATATCTATTACAAATATGATTAGAGCGATTAAGCTCATTATTGATGTAAAAAGTTTCAGACTTTAGGGAGGAATGGAAACTTTCTATCG
GGGCGTTATCGGCAGGTGTTCCCTTTCGGGACATACTTCTGATAATGCCTTTTTCTTCGCACAATTGATAATAAGCATAAGATGTATAAAC
GTTGCCTTGATCACTATGTAATATACAGCCCTCAGGTATATCGATTTGATTTAATGTATCATTAACTAAACTTTGGTCTTGTTTATCATCTAT
TTTATACGCCACAATTTCTCCGTTATAAATATCCATTATCGAAGATAAATACAACATAGAATGACCAAATGGTAAATAAGTAATATCGGTT
GTTAATACTTCCATTGGACAACTCGCTTTAAATTGTCTTTGTAATAAATTGTCCGTTTTATAATACGTTTTACCTATTCTTGTCGTCTTTTTAG
GTCTAACTCGGCAGTTCAAATGATGCTTCTGCATCATTCTCTGTACTTTCTTATGATTAATTGGTGATGTATAACATTGATTAATCAATGCT
GTAATCTTACGATAACCGTAGGT

Gene: DR_SCC (direct repeat of SCC)

Contig: 01_NODE_8 (fragment from pos. 242580 on), position: 61576 to 61594, length: 19 nt

Perfect match to: (JS395-CP012756-[1418304:1418322])

Sequence:

AGAAGCATATCATAAGTGA

Gene: Q6GKL1 (HTH transcriptional regulator)

Contig: 02_NODE_3, position: 2252 to 2827, length: 576 nt, orientation: FORWARD

Sequence:

ATGGAAAAAAATCAACGAAAAAAACGCAGTGATGCCGAATATAACCAACAGATTATCTTAACTACAATGGAAGATTTACTAGAACAGGG
TGAAGATATAAGTGCTAAGAAAATGTCAGATATTGCTAAAATATCAGGTGTTGGAGTTGGTACATTATATCGTCATTTTGAAAGTAAAAC
ATTGTTATGTCAGGCTATTATGGATAAAAAAGTCGATCAAATGTTTATAGAAATAGAAGATGTTTTAGCGGAAAAATACACAGTGGCCCGT
GCGAGATAAAATCAATATTATATTAACAAAGTATCTAGATTTAAAAGAAGCAAATTTCACAACATTAAATTTTATAGAAAAATCCAGTTCA
CACTCTAGTTCTGTAATTAATATTCCATTCTTTGAGCGACTAAAAAACTTATTAATTCAACAATTCGAAAATGTTAATTCCATAGCAGACTT
GGATTTTAAACTTAATCTAATGCTCAATGCATTTTCTTCAGATTTCTATTATTTTGTAAAACATAACCAAAAATTAACAAAACAACAATTTTT
AGACAAATTACTAGATTTATTTCTAAAATAA

Gene: ORF CM14 (enterotoxin-like protein)

Contig: 02_NODE_3, position: 3980 to 4759, length: 780 nt, orientation: FORWARD

Sequence:

ATGAGAAAAGTACTTATACTAATCACTTTGTTATTTGGTTACAGTTGCTATTTGTTGTTGGAAGCTAAAGCAGAGACACAAAATGATCCAA
ATATAAGTGAATTAAACAAAGCTAGTCAATATACGGGTTCATGGCATAATATATGGTATTTATATAATAGCGATCCAGTCAATGCTAAAA
AAATTAAGCTGAGTGACAAATTTTTGAGTCATGATTTCATTGTTCCAATAAATAGCCCTGGTCATTACGACTATGTTAAAACTGAGTTAAA
AGACAGTACGATGGCGAGTTCATTTGATGGGAAAGAAGTCGATATTTTTGGCGTCAATTATTTTCATCAATGCTATTTTTCAAATGAAAAT
ATACAGTGTGATAGTAATCAAGGAGCTGGAAGTAAAAAAACTTGTATGTATGGTGGTATAACATTAAATGAGAACAATACCAATAATAG
AATTCAGCCAATTGTTGTAAAAGTTTACGAGAATGACAGCGTTACGCTTTCTTTTGATATCAATATTGATAAAGAGACTGTAACTATTCAA
GAGTTAGATTATAAAGTGAGAAATAAATTAATTTCTAAAATCAATTTATACCATTTAGGTGGCACTTCATATGAAACGGGATACATTAAAT
TTATAGAAAATGCTAATCGTTATTATTGGTATGATATGATGCCAGACCCTAGCTTTACTCAGTCTAAATATTTGATGATATATCGAGGTAA
TGAAACAGTTGAATCAGCCAAAACGGAAATAGAAGTGCACTTAACTAAAAAGTAG

Gene: dusC (tRNA-dihydrouridine synthase C)

Contig: 02_NODE_3, position: 4826 to 5812, length: 987 nt, orientation: REVERSE

Sequence:

CTATAATTCTATTTTAACGTCTTCGTCCATTTGGGCTTCAAATTCATCTAGTAGTGCTCGTGCTTCTGCAATTGATTGTGTGTTCATCAATTG
ATGGCGAAGTTCGCTAGCGCCTCTTATGCCACGCACATAGATTTTAAAGAATCTACGCAAGCTCTTGAATTGTCGTATTTCATCTTTTTCAT

ATTTGTTAAACAATGATAAATGCAATCTCAACAGATCTAATAGTTCTTTGCTTGTATGTTCGCGTGGTTCTTTTTCAAAAGCGAATGGATTA
TGGAAAATGCCTCTACCAATCATGACGCCATCAATGCCATATTTTTCTGCAAGCTCAAGTCCTGTTTTTCTATCGGGAATATCACCGTTAAT
TGTTAACAATGTGTTTGGTGCAATTTCGTCACGTAAATTTTTAATAGCTTCGATTAATTCCCAATGCGCATCTACTTTACTCATTTCTTTACG
TGTACGAAGATGAATAGATAAATTTGCAATGTCTTGTTCGAAGACATGCTTCAACCAATCTTTCCATTCATCGATTTCATAGTAGCCAAGG
CGTGTTTTAACACTTACCGGAAGTCCACCTGCTTTAGTTGCTTGAATAATTTCGGCAGCGACGTCAGGTCTTAAGATTAAGCCGGAACCCT
TACCTTTTTTAGCAACATTTGCTACAGGACATCCCATATTTAAGTCAATGCCTTTAAAGCCCATTTTAGCTAGTTGAATACTCGTTTCACGG
AACTGTTCTGGCTTATCTCCCCATATGTGAGCGACCATCGGCTGTTCATCTTCGCTAAAAGTTAAGCGTCCGCGTACACTATGTATGCCTTC
AGGGTGACAAAAGCTCTCGGTATTTGTAAATTCAGTGAAAAACACATCCGGTCTAGCTGCTTCACTTACAACGTGTCGAAAGACGATATC
TGTAACGTCTTCCATTGGCGCCAAAATAAAAAATGGACGTGGTAATTCACTCCAAAAATTTTCCTTCAT

Gene: A6TXM6 (putative protein)

Contig: 02_NODE_3, position: 6121 to 6324, length: 204 nt, orientation: FORWARD

Sequence:

ATGGAAGATTTAAAACACTCTTTAAAAGATTTAGGTTGGTATGACTTATTTTTTACAGTACCTATGTTTCTACTATTCGTGTATCTGCCGAA
TTATAATTTTATAACTATATTTATTAACATTGTTATCATTATTTTCTTTTCCATAGGTTTGATTTTAACTACTCATATAATTATAGATAACATTA
AGAACAACTCTAAATGA

Gene: A6QD71 (putative protein)

Contig: 02_NODE_3, position: 6374 to 6670, length: 297 nt, orientation: TRNC-FRWD (no stop codon)

Sequence:

ATGGCGACTGAGAAAGATGTAAATGATTTATTTTTAAATCATGTGAATTCAAATGCTGTTAAAACTAGAAAGATGATGGGAGAATATATT
GTTTATTATGATGGTGTGGTTATAGGTGGTTTGTATGATAATAGATTATTGGTCAAGGCGACTAAAAGTGCACATCATCAATTTCAAGAT
AATACATTAGTATCGCCATATCCTGGTGCCAAGGAAATGGTTTTAATTCCACACTTTGCCGAAGTAACAAATCTAAATGATTTATTTGAGC
TCATAAAAAATGATTTGAAAAACACA

Gene: Q6GKK6 (putative protein)

Contig: 02_NODE_3, position: 6870 to 7481, length: 612 nt, orientation: FORWARD

Sequence:

GTGAACGATATGTTAATTAGTCTTGTAATCCCAGTTTTTGCTTTGTTAGTTATTGGTGGTATTATTTGGATGATTATAGAAGGTATAGTACA
TATTTCAAAAAAGAATAAAGCAATTGATAACTTTTTTAACAAAGTTAATAAAGTAAGTGAGACATATAAATTCGCTACTACTTTTTTATTTC
TAATCTTGGCTACGGCTGGTATTTCTCAATTTTATCTATATTATATAGTATCAGAGTTTCTTTTTTTGCTGGTTATATTTACCTTTGGTATTGC
AGGTATCATTTTTTTAATGCCATATGGCTTATGTTTCCTACCACTTTATAAACAAAAAAAGAAAAAACAGACATTTAAAAAATACATGGCTT
ACACTACGATTGGTTTGTCAATTTATCTAGGCTTATCTCTAGTTTTGGTTCACACTACGAAAATTTATATGGACGAAGGTGGCGTAAGATA
CTATTACGGTAGTTTTGTAATGAAACAAGCGGGCGGTTATGCTTATTTAGCTTTAGCAGTACTTTCAACGTTGTTAATTGTTGCGAAAAAA
GCTACAAATAAAAATAAAAAAATCGAAACCGTCGACAATACAAATATAACGGAAAGATAA

Gene: Q7A890 (putative NTPase)

Contig: 02_NODE_3, position: 7749 to 10901, length: 3153 nt, orientation: FORWARD

Sequence:

ATGAGTCAATTGCTAAATGATACGTTATCGGCTTGGTTGTTAATTGAATCTTTAAGTCCAGGAGAAGTAAATTTTACAGCGGAAGATATA
CTCTCAGCTGAAAATTTTAAAAATGGTGCAAAGCAAGCGCAACTTCAAAGTTTTGATGAATATTTTGAAATATGGAATGATGAACGCTTT
ATTATATCAGAAGAAAAATCAGAGATTGGCGAAATCATTTTTAAATTTTACAGACACTGTTTCCGCTATAATGAAATTAATTTGAAAATTC
AGGATATTTTTGATGATTATTCAGATATTCATAATCCAAATGGGACACACTGTTATGGTTACACATTTAATATAGATAAACACGGTCAAGT
GATAGTTGATTCTATACATATTCCGATGATTATGAGTGCATTGAAAGAAATTGAAAAGAATAAAAATGCTAATATAGAAGAAAAATTTAA
TGATTCTGTTGAAAAATTTGTTCAAAAAGTAAAAGAAATTTTAGTGGATGAACCAATTAATGAATTTAAATTGAAGAAGATGGACATAGC
TTATGATGAGTATTTTTCTGTATTAAATTCAAAGAAAGATGGATTATTTGCACATTATGTAGCAATAGAATATGTGAAAGATAGTGATTTA

CCACAGCCAGAATTTAATAGCTTCTTCATAAGTGATATTGAGAAAGCAAGAAAATCTCCCAATCAAACTTTAATTGATTACATTGAGGGTG
TAGAAGAAAGTAAGCGCATAGAAGTGGATGAAAATAAAGAAATGTTCGACAAATTTTTACATCCTTCACGTTTGCCTGATGGACGCTGG
CCATCACAGACTGAGTTTAGATTGTCATTAATGCAACAACTTGCTGTAAACCAAATTACGAGTGGTAATGAAAGGATAAGTTCAGTTAAT
GGGCCACCAGGGACAGGTAAGACTACTTTATTAAAAGATATATTTGCGCATCTAGTAGTTGAAAGAGGTAAAGAGTTAGCCAAACTAAA
TAATCCTAAAGATACATTTGTCAAAACAAAAACTCATGAAACAGATGATAAATATGTCTATTTACTTAAGGAATCTATTGCTAAATATAAG
ATGGTAGTCGCATCTAGTAATAATGGAGCTGTTGAAAATATATCTAAAGATTTACCGAAAATTGAAGAAATTATAAGAAATCCCGAAAAA
TGTAAATTCCCTAAATATGAACAGAATTATGCAAATTTAGCACATGAATTAAAAGATTTTGCTGAAATAGCTGAAGATTTGATTGGTGAA
AGTGCCTGGGGCTTATTTTCTGGAGTATTTGGTAAAAGTACTAATATTAACCAAGTATTGAGTCATATGTTAAAACAAGATGCGAATGAT
ATTGGCTTTGCTAAATTACTACAAAATGAGAATAATCGTATGAGTTATAACGAGTTAATGAGTGAATGGCAATCACATCAACGTGCATTTT
TAGAAGAGTTGAGGCATGTTGAAATGTTAAAAGAAGAATCTATTAGAGCCTATGATGTTTATAAAAATTGTGAGTCTTACTCTAAGGTTG
AACATGAACTAAATAGCAAAAAAATGAATGTTAAGGAAAAGTTGAATCATTTAGAAATTCAAATATCTTGTGACAATAAAGAAATTGAAG
ATTTGGATGATCGAATTAATTATAATACAAAGCAACTCGAAACTTTAAATGAGTTAATTAAATCCATCAGAGATAGTAACAAAGGGTTTG
TTAACAAGCTGAAGGCGATATTTAATTCAGAAGAAGATGAAAGATATAAAAAACATAATGCAGAGAAGCAACAATTATTAGGACAACAG
ATAGAGTTAGAGAAATGTAAAAAAATTAAAAATGAAGACCTTGTTAGCAAACTAAAAGAAAAAGAGAAATTAATTAAACAATTAACTAA
AGTACAGTTGCAATTAGACGAGTTAAATTCACAGTTACAAGAGTTAGAAGCATATCGTATTGAGTCAAAAATTACAATTCCAGAAAAAGA
TTTTTGGAGTGACAACAATTATGATGAGCGCCAAGTTACTAATCTGTGGACGAGTGACGAACTTCAATACAGACGTGCCATGCTCTTTTTA
AGAGCAATGATATTGCATAAATTATTATTGATTGCTAATAATACAACTATTTATTATGCGATTAATGATTTTAAAGATAGAAGGAAATTAA
TTGATGCAAATCCAGATAAAGTACACAACGCATGGAATGTGATGCATTTAATATTTCCAGTAGTTAGTACGACGTTTGCAAGCTTTAAATC
TATGTATGGGGGCATACCAAAAGATTTCATAGACTACTTATTTATTGATGAAGCAGGACAAGCAATACCTCAAGCAGCTGTGGGAGCATT
ATATCGTTCAAAAAAAGTTGTAGCTGTAGGTGATCCGATTCAAATAGAACCGGTTGTGACTTTAGAAAGTCATTTAATTGATAACATTCGT
AAAAATTATCATGTTCCGGAATATCTAGTTTCTAAAGAAGCTTCTGTGCAGTCTGTTGCAGACAACGCCAATCAATATGGTTTTTGGAAAT
CTGATGCTACTGATAGTAATCAAAAAACCTGGATAGGCATACCTTTATGGGTGCACAGACGATGTTTAAAACCTATGTTCACGATAGCTA
ACCAAATCGCTTATAATAATAAAATGGTGTTGCCAAGTAATATTACAAAAGTAGGTAAAACAGGTTGGTATGACGTTAAAGGAAACGCA
GTTCAAAAACAATTTGTGAAAGAGCATGGTGAAAAAGTAGTGGGATTATTAGCTGATGATTGGATTGAAGCAATTAAGGAAGGTAAAA
ATGAACCGAGCTCATTTGTAATATCGCCTTTTTCAGCAGTACAGCAACAGATTAAACGTATGTTAAAGCAACAACTACCGACTAGAATTGA
TATTGAACGTACAAAAATTAATCAATGGGTCGATAAATCCATTGGTACTGTTCATACTTTTCAAGGTAAAGAGGCTCAGAAGGTGTATTTT
GTAATAGGTACTGATAATACCCAAGATGGTGCTGTGAACTGGTCATGCGAAAAACCAAACTTGTTAAACGTTGCAGTGACAAGAGCTAA
GAAAGAGTTTTATGTAATTGGCGACATGCAAAGAATACAGATGAAACCATTTTATGAGACGATTTTTAAAGAAAGAAATGTAAAATAA

Gene: Q2YUT2 (putative protein)

Contig: 02_NODE_3, position: 10958 to 11440, length: 483 nt, orientation: FORWARD

Sequence:

ATGGAGAAATTTAACAACTGGATATTAAATGCAATAAGTGGATCTCAAACAGACAAGAATGAAACAACTGAAGAATTAAAAGGGGCAA
AATTTATCATTTTATATGCATATTCAATGCTCGTTTTGCTTGCGTTAGTAATTTCTAACATATTCATTCACATTTTGGAGCCTAAACTATCAA
TCACCACTCAAATCATCATCGTTTTGATTTTAATTGAAGCACTAATTGGACTGCGTTTCTTGAAAGCGTACGATGTTAAGCGTGGCAAAGA
TAAAGAAAATAAGAAAAATAGTAAGGATTTCGTTAAACTAAAATCAATTTTAGTAGCAATTTTATTTACATCATTGGCGCTGACAGCAGG
TACTGTAGCTGATATATACGGTTTCACTGACTTAGGAAATACTAGAAGTGATTTAATCGTTTGGAGCATAGGTGGTATTATATTTGGCCTC
GTATTTTACACAATGGAAGATAAAAGATAA

Gene: plc (phosphatidylinositol-specific phospholipase C)

Contig: 02_NODE_3, position: 11645 to 12631, length: 987 nt, orientation: FORWARD

Sequence:

ATGAAAAAGTGTATTAAGACTTTGTTTTTAAGTATCATTTTAGTAGTGATGAGTGGTTGGTATCATTCAGCACATGCGTCAGATTCGTTGA
GTAAAAGTCCAGAAAATTGGATGAGTAAACTTGATGATGGAAAACATTTAACTGAGATTAATATACCGGGTTCACATGATAGTGGCTCAT
TCACTTTAAAGGATCCAGTAAAATCAGTTTGGGCAAAGACTCAAGATAAAGATTACCTTACCCAAATGAAGTCGGGAGTCAGGTTTTTTG
ATATTAGAGGTAGAGCAAGTGCTGATAATATGATTTCAGTTCATCACGGCATGGTTTATTTGCATCATGAATTAGGAAAATTTCTCGATGA
TGCTAAATATTACTTGAGTGCTTATCCAAACGAAACAATTGTGATGTCTATGAAAAAGGACTACGATAGCGATTCTAAAGTTACGAAGAC
ATTTGAAGAAATTTTTAGAGAATATTATTATAATAACCCGCAATATCAGAATCTTTTTTACACAGGAAGTAATGCGAATCCTACTTTAAAA
GAAACGAAAGGTAAAATTGTCCTATTCAATAGAATGGGGGGTACGTACATAAAAAGTGGTTATGGTGCTGACACGTCAGGTATTCAATG
GGCAGACAATGCGACATTTGAAACGAAAATTAATAATGGTAGCTTAAATTTAAAAGTACAAGATGAGTATAAAGATTACTATGATAAAA
AAGTTGAAGCTGTTAAAAATTTATTGGCTAAAGCTAAAACGGATAGTAACAAAGACAATGTATATTTGAATTTCTTGAGTGTAGCGTCTG
GAGGCAGCGCATTTAATAGTACTTATAACTATGCATCACATATAAATCCTGAAATTGCAAAAACGATTAAAGCAAATGGGAAAGCTAGAA
CGGGTTGGCTGATTGTTGACTATGCAGGATATACGTGGCCTGGATATGATGATATCGTAAGTGAAATTATAGATAGTAATAAATAA

Gene: lpl-locus0080-SAOUHSC_00052 (tandem lipoprotein-like protein, first locus)

Contig: 02_NODE_3, position: 12852 to 13622, length: 771 nt, orientation: FORWARD

Perfect match to: (COL-CP000046-[89394:90164], highly conserved allele)

Sequence:

ATGATGAAACGATTAAACAAATTAGTGTTAGGCATTATTTTTCTGTTTTTAGTCATTAGTATCACTGCTGGTTGTGGCATAGGTAAAGAAG
CGGAAGTTAAGAAAAGCTTTGAAAAAACATTGAGTATGTACCCTATTAAAAATCTAGAGGATTTATACGATAAGGAAGGCTATCGTGAT
GATCAGTTTGATAAAAATGATAAAGGTACATGGATTATAAATTCTGAAATGGTTATTCAACCTAATAATGAAGATATGGTAGCTAAAGGC
ATGGTTCTATATATGAATAGAAATACCAAAACAACAAATGGTTACTACTATGTCGATGTGACTAAGGACGAGGATGAAGGAAAACCGCA
CGACAATGAAAAAAGATATCCGGTTAAAATGGTCGATAATAAAATCATTCCAACAAAAGAAATTAAAGATGAAAAAATAAAAAAAGAAA
TCGAAAACTTTAAGTTCTTTGTTCAATATGGCGACTTTAAAAATTTGAAAAATTATAAAGACGGAGATATTTCATATAATCCAGAGGTGCC
GAGTTATTCGGCTAAATATCAATTAACTAATGATGATTATAATGTAAAACAATTACGCAAAAGATATGATATACCGACGAGTAAAGCTCC
AAAGTTATTGTTAAAAGGTTCAGGGAATTTAAAAGGCTCATCAGTTGGATATAAAGATATTGAATTTACGTTTGTAGAGAAAAAAGAAG
AAAATATATACTTTAGTGATAGCTTAGATTATAAAAAAAGCGGAGATGTATAA

Gene: lpl-locus0080-SAOUHSC_00053 (tandem lipoprotein-like protein, first locus)

Contig: 02_NODE_3, position: 13674 to 14444, length: 771 nt, orientation: FORWARD

Perfect match to: (NN50-BAEA01000031-[49388:50158:r], allele observed in CC4803+CC8)

Sequence:

ATGATAAAACGTGTAAATAAATTAGTGCTTGGTATTAGTCTTCTGTTTTTAGTCATTAGTATCACTGCTGGTTGTGGCATGGGTAAAGAAG
CGGAAATAAAGAAAAGTTTTGAAAAAACATTGAGTATGTATCCGATTAAAAATCTAGAGGATTTATACGATAAAGAAGGATATCGTGAT
GATCAATTTGATAAAAATGATAAGGGAACATGGATTGTAAATTCTCAAATGGCAATTCAAAATAAAGGAGAAGCTCTAAAAATAAAAGG
CATGCTTTTGAAGATAGATAGAAATACAAGAAGTGCAAAAGGATTTTACTATACTAATGAAATAAAGACGGAGAAATACGAAGTAGCTC
AGGATAATCAAAAAAAAATATCCAGTTAAAATGATTAATAATAAATTCATTTCTACTGAGGAAGTTAAAGAAGAAAACATAAAAAAAGAA
ATCGAAAACTTTAAGTTTTTTGCGCAATATAGCAATTTTAAAGATTTAATGAATTATAAAGATGGAGATATATCATATAATCCAGAGGTGC
CGAGTTATTCAGCTCAATATCAATTAACTAATGATGATTATAATGTAAAACAATTACGTAAAAGATATGACATACCAACAAATAAAGCGCC
GAAGCTGTTGTTGAAAGGTACAGGGAATTTAAAAGGTTCATCAGTTGGATATAAAAAAATTGAATTTACTTTTTTAGAGAATAAAAATGA
AAATATTTACTTTACTGATAGTCTACATCTTAAACCGAGCGAGGATAAATAA

Gene: lpl-locus0080-MW0073 (tandem lipoprotein-like protein, first locus)

Contig: 02_NODE_3, position: 14795 to 15487, length: 693 nt, orientation: FORWARD

Perfect match to: (MW2-BA000033-[84355:85047], allele observed in CC1)

Sequence:

ATGGGTAAAGAAGCGGAAATTAAAAAAAGTTTTGAAAAAACGTTGAGTATGTATCCGATTAAAAATCTAGAAGATTTATACGATAAAGA
AGGATATCGTGATGATCAATTTGATAAAAAAGATAAAGGCACATGGATTATTAATTCTGAAATGGCGACTCAAAATAAGGGAGAAGCTT
TGAAAATTAAAGGTATGGTCTTATACATGAATAGAAATACAAAAACAACAAAAGGATATTATTATGTAAATGCAATAAAGAATGATAAA
GACGGAAGACCCCAAGAGAATGAAAAAAGGTACCCAGTTAAAATGATTGATAATAAAGTTATTCCAACAAAAGAAATTAAAGATGAAAA
CATAAAAACAGAAATCAAAAACTTTAAGTTCTTTGTTCAATATGGCAACTTTAAAGATTTGAAAAATTATAAAGACGGAGATATTTCATAT
AATCCAGAAGCCCCGATTTATTCAGCGAAATATCAACTAACTAATGATGATTATAATGTAAAACAATTACGTGAAAGATATGATATACCG
ACGAATAAAGCGCCAAAGTTATTGTTAAAAGGGTCAGGGAACTTAAAAGGTTCATCAGTTGGATATAAAAATATAGAATTTACTTTTGTA
GAGGAAAAAGGTAAAAATATATACTTTAGTGATAGCTTAGATTATAAAAAAAGTGGAGAAGTATAA

Gene: lipC3-MW0074 (putative lipase class 3)

Contig: 02_NODE_3, position: 15490 to 16866, length: 1377 nt, orientation: FORWARD

Perfect match to: (MW2-BA000033-[85050:86426], allele observed in CC1+CC1290)

Sequence:

ATGGCTAAAGCAGAATATGAAATTGATCCTGGAAAAATAACAAGTAATTCGGAAGAAACTAGCGCAATATCTAATATTAGTTATGAAATA
GAAAATGCAAATGATAATAATTTAGATAATGAAAAAATTAGAGGGCAAATTACTAAATTAAAAAAGGGTGGGAATTTTCCTAAAAACTTA
GACTACATAGATAGTTATACAGACCCCAGTACGGGGACCACGGCAACGGCATTTTTAAATAAAGATACAGGTAAAGTTACTGTTGGAAT
GGCTGGTACAAACTTTCACGGCGATCAACTTAAAAGAGTAGCACTTAGTTCGATGTCTCCGTTATTATTCCCACCCTCTAAGCAAGATATG
AAAGATGTACGAGGCACTATGAAGGATGGTGTAGCGGATTTAGCCATTGGAGTAGGAATGGTCAATTATAAAGGAAAACATTTTGCAAA
TACACAGCAGTTTATTGAGAATTTACAAAAAAAGTACGAAATTGATACCGTTACTGGTCATTCTTTAGGAGGGAGAGATGCTATCTTTCTT
GGGCTTCGTTATAACATCAAAAATGTTGTGGCATACAATCCTGCTCCGTTAGAAGTAAAAAGCATTCGTAATAAATTTGGTGGTCAATTGT
TTAGAAATACGACATTTCCAGATGAAAAATATTTAAAAGAGTTAATGGATAATTATGATGGAGATATTACTAAGGTTATAACTCAAAAGG
ATGGATTAGACTATTTAGTGAAGCGTACAGATCATTTAACTTGTGGTGATGTGTTACGTATAAACAATGGTCAGGGCCATGCGATGGAG
AATTTTTTAGGAGAAAAAGAACAACGTGAAATAATCGAAGAACTAATGTTAGTAAAAGGATATCGCGATGCAAATGACAAAGCATTTAA
GGCTTTGAAAAAAAAATACTGAGAAAAAACTCGGTAAAATAGATGAAATCAAATCGAAGTTACTGCAAACGAACGGTGGCGCCCTCTCGT
CTTCTCAACAAAAATTGCTTGAAACTTTAGTGGCATTTTCGGTAGCTGAAGGACTAAGTAAAATGGTTGACCAAGAACTTCAGCAGTTGA
AAAATATGTTCAATCTTATGGATGAGAAATTTGAAGCAAATTGGAAAGACGCACAAGAAGCTAGTGATATTGTAGGTAAACATCTTTCAT
ACCCAGAAAAGGTAAGTGCATTAGATAACGGTGGTGTCAATGAAAGTAAACTTGCTACCGAACCACATAATGAAATAAAAGACAAACTA
AATAAAATTACGGACTTATCTAAGAAATATAATTCGTATTTACAGCAAATCGAAAAAAGTATTAATGAAATAGTTGCTAAGGATCAACAA
TTGGCAGGGCAAATAGGTGATTTAATATGA

Gene: Q8NYT6 (HTH-type transcriptional regulator)

Contig: 02_NODE_3, position: 17759 to 19996, length: 2238 nt, orientation: FORWARD

Sequence:

ATGCAACGTGATTATTTAATTCGAGTAGAAACTGAAAGCATGCCAGATTTCAAAAGGCTCAATGGTTTAATGATTGGTTTTGTTATTAAAG
GTGAGGCACATATTTATGATGAAAATAACATGACGCAATGCAACAGTGGCGACATTTTCATCATTAACCACCGTGACTTGTATCGATTTCA
ACTTCAACAAGATGGCATCATATGTTATATCCAATTCCAAATGAAATATTTGGCAGACAAGTTTGATGATGCGCATTGTCTATATTTTCACT
TAACAGATGCGACCACAACCAAGAATATACATCAACTGAGAAATATAATGGCAAGACTGGTTTCAACACACATTCGACATAATGAGTTGT
CTAAATTGACTGAGCAACAACTTGTGATTCAGTTGCTTATGCATATGATTCATTATGTCCCGCGTACATATCATTCGAACCAAAGTATCTTA
AATGATGATAAAGTGAATCAAGTATGCGACTATATCGAGTTACATTTTCATGAAGATTTAAGCCTTTCAGAATTAAGCGAATACGTTGGG
TGGTCAGAGAGCCATCTGTCTAAAAAGTTTACAGAATCGCTAGGTGTAGGATTCCAACATTTCTTAAATACGACGCGAATTGAGCATGCG
AAACTCGATTTAACATACACAGATGAAACGATTACTGATATTGCATTGCAAAATGGCTTTTCAAGTGCAGCGAGCTTTGCGAGAACATTT
AAACACTTTACGCATCAAACGCCTAAACAATATCGAGGTGATCGTCCAGCAATCACTGAAAATCAACAATCGGCACAACATAATTATCAC
GACCGTGAATTGATATTACTTTTAAATGACTACATTGAAGAAATGAATCATTTCATTGAAGATATTGAAAAGATGAACTATAAAGAGATT
GCCTTTAAACCAACTAATCAACAACTAAATCAATTTAATCATATTATTCAAGTGGGCTATTTGAGGAATTTGCTCAATACACAGTATCAATC
ACAGTTGCTTACATGTCATCATGATTTTCAAGTCAATGAAGTATTAGCATATGATGTGATGCCATATATTATGAAAAAGCTCAATGCGCCA
TTCACGTATGATGCAGAGATTTCGAATATATTTTATGATATCGATTTGTGTTTAGACTTTTTATTAGATCATAATTTTAGTCTAACCATGCAT
TTGAATCAGTATGACTCACGAGATTATATCGATGCATTCAAAGTATTTATCCATCACGTTGCCCTGCATGTCAGTCATAGAAAAGATTTGA
AGTTCAACTTGTATGTGACGACATTGCACAATGCTTTGATTGAAATGATTGATTATTTTAAAGCGTTATTCCCTAATGGTGGCTTGTACATT
CACTTAGATCAAGCTACGGAAAGACATCTACCATTGTTGAAACGACTTGAGCCACACATCGACCATTTTGTATTTGATGCCAATTCAAATG
ATGCTGTTGATTTTAATAAAATGAATGATGATGAATTTAAAACCGCGAGTCAAATGATTATTAATAAAACGAATTACCTTATCGACTTAAT
ACATCGTCATAACCTAAAGCGTCCACTCATTTTACTCAATTGGAATACATTGACGGGTGATACATTTATAACCAACGGCGAATATTTTAGA
GGTGGTATCATCATTGAGCAGTTATTAAAGTTAAGCTCTAAAGTAGAGGGTATCGGGTATTGGTTGAATTATGATTTGCACGTTAGTCAT
TGTAAAAATGAACGGGATTATATGAATTCTATTGAACTGTTTCATCAATATAATGGAAAACGTCCGGTCTATTTCACGGCATTGCTATTTA
ATAAATTAACAAGCAATATTTTGTATTCTGATGATACATGTATTGTCACGGGAACTGATTCAAATTTTCAAATATTGTTATATGATGCAAA
GCATTTTAATCCGTACTTAGCGTTGGACAATCAAATGAATATGCGTGCAACGGAAATGATCCATTTGAACATTAATGCCTTGGAAGAAGG
TATGTATAAGATTAAACATTTTACCTTAGATAAAGAAAATGGTGCGTTATTTAATCTTTGGCGCAAACATCATACGATACATGGCATGGAC
AAGGACTCTATAGATTACGTTAATCGAATGAGTTTTCCGAAATTAGAAGTATATGATATAGATATCACGGACACACTGGCATTAAACATT
AAAATGATTACGAATGGGATTCACTTAATTGAAGTAAAACGTTACCCAAGTTCATAA

Gene: Teg15as (antisense RNA)

Contig: 02_NODE_3, position: 19787 to 20030, length: 244 nt

Sequence:

GATAAAGAAAATGGTGCGTTATTTAATCTTTGGCGCAAACATCATACGATACATGGCATGGACAAGGACTCTATAGATTACGTTAATCGA
ATGAGTTTTCCGAAATTAGAAGTATATGATATAGATATCACGGACACACTGGCATTAAACATTAAAATGATTACGAATGGGATTCACTTA
ATTGAAGTAAAACGTTACCCAAGTTCATAAAATGATCACAAATCACAAATTTTGATATACATAA

Gene: Q8NYT5 (Mn (2+) binding amidohydrolase)

Contig: 02_NODE_3, position: 20147 to 21325, length: 1179 nt, orientation: FORWARD

Perfect match to: (DAR4145-CP010526-[95066:96244], highly conserved allele)

Sequence:

ATGAATCAACAATTAATTGAAACTTTAAAATCTAAAGAAGGCAAAATGATTGAGATCAGACGTTATTTACATCAGCATCCAGAATTATCTT
TTCATGAAGATGAAACGGCGAAATACATCGCTGAATTTTACAAAGGTAAAGATGTGGAAGTAGAAACGAATATCGGACCACGTGGAATT
AAAGTAACGATTGATTCAGGGAAACCTGGTAAAACATTAGCAATCCGTGCAGACTTTGATGCATTACCTATTACTGAAGATACAGGATTA
TCTTTTGCATCACAAAATAAAGGTGTTATGCACGCATGTGGTCACGATGCACATACAGCATACATGCTTGTATTAGCAGAGACGCTTGCT
GAAATGAAAGATAGTTTTACAGGAAAAGTCGTTGTGATACATCAACCAGCTGAAGAAGTACCACCAGGTGGTGCTAAAGCAATGATTGA
AAATGGTGTATTAGACGGTGTTGATCATGTATTAGGTGTACACGTCATGAGCACAATGAAAACAGGTAATGTGTATTACAGACCTGGTTA
TGTTCAAACAGGACGCGCATTTTTCAAATTGAAAGTTCAAGGTAAAGGTGGTCATGGTTCATCACCCCATATGGCCAATGATGCCATTGT
TGCAGGTAGCTACTTCGTCACAGCGTTACAAACAGTTGTATCTAGACGACTAAGTCCATTTGAAACCGGTGTTGTCACAATCGGTTCATTT
GACGGTAAAGGTCAATTCAATGTCATTAAAGATGTTGTTGAAATTGAAGGTGATGTACGTGGATTAACAGATGCTACAAAAGCAACAAT
TGAAACAGAAATTAAACGTTTATCAAAAGGATTAGAAGCATTGTATGGTGTAACTTGCACATTAGAATATAACGATGATTATCCTGCATT
ATATAATGATCCAGAGTTTACTGAGTACGTGGCTAAGACGTTAAAAGAAGCAAACCTTGATTTTGGTGTCGAAATATGTGAACCACAACC
ACCTTCAGAAGACTTTGCATACTATGCTAAAGAACGTCCAAGTGCCTTTATTTATACAGGTGCAGCTGTGGAAGATGGTGAAATTTACCC
ACATCATCATCCTAAATTTAACATTTCAGAAAAATCATTACTTATTTCGGCAGAAGCTGTAGGGACAGTTGTTTTAGATTACCTTAAAGGA
GATAACTAA

Gene: norC (multidrug efflux pump)

Contig: 02_NODE_3, position: 21327 to 22715, length: 1389 nt, orientation: FORWARD

Perfect match to: (MW2-BA000033-[90887:92275], allele observed in CC1+CC772+CC1290)

Sequence:

ATGAATGAAACGTATCGCGGGGGCAACAAGTTAATCTTAGGTATTGTATTAGGTGTTATTACATTTTGGTTGTTTGCACAATCACTTGTAA
ATGTTGTACCAAATTTACAACAAAGTTTTGGTGCAGACATGGGAACAATTAGTATTGCGGTAAGTCTAACCGCACTATTTTCAGGCATGTT
TGTTGTTGGAGCAGGCGGCCTAGCAGATAAAATTGGGCGCGTGAAAATGACGAATATCGGTTTATTGTTAAGTATTATTGGTTCAGCATT
AATTATTATTACGAATTTACCGGCATTATTAATTTTAGGTCGTATTATACAAGGCGTATCAGCAGCGTGTATTATGCCTTCCACATTGGCCA
TTATGAAAACTTATTATGAGGGTGCTGAACGTCAGCGCGCCTTAAGCTACTGGTCTATCGGTTCTTGGGGTGGAAGTGGTATCTGTTCAC
TCTTCGGTGGGGCAGTTGCGACAACTATGGGTTGGAGATGGATTTTCATCTTCTCAATTATCGTTGCCGTACTTTCAATGTTACTCATCAA
AGGGACGCCTGAAACGAAATCAGAAGTTACCAATACACATAAATTTGACGTTGCAGGGCTAATTGTTCTAGTAGTTATGTTGCTAAGTTT
AAACGTTGTCATTACTAAAGGTGCAGCACTTGGTTACACATCATTATGGTTCTTTGGTTTAATTGCAATCGTAATTGTAGCATTCTTTATTT
TCTTAAAAGTTGAGAAAAAAGTAGATAATCCGCTTATTGATTTTAAATTATTTGAAAATAAACCATATACAGGTGCAACGATTTCGAACTT
CTTATTAAACGGTGTTGCAGGTACATTAATTGTAGCGAATACATTCGTGCAACAAGGTTTAGGTTATACAGCATTGCAGGCAGGATACTT
ATCAATTACTTATTTAATCATGGTGTTATTGATGATTCGAGTTGGTGAAAAATTATTACAAAAAATGGGTTCTAAGCGACCAATGTTATTA
GGTACATTCATTGTGGTCATTGGTATTGCACTTATTTCATTAGTATTCTTACCAGGCATATTTTATGTTATTAGTTGTGTCGTAGGATATTTA
TGTTTCGGACTAGGCTTAGGTATTTATGCAACACCTTCTACAGATACAGCTATTTCGAATGCACCGTTAGATAAAGTTGGCGTTGCTTCAG
GTATTTATAAAATGGCTTCATCACTTGGTGGTGCATTCGGTGTCGCAATTAGTGGTGCAGTATATGCCGGTGCAGTTGCTGCAACTAGCA
TTCATACAGGTGCGATGATTGCACTTTGGGTTAACGTATTAATGGGAATCATGGCATTTATCGCAATTTTATTCGCGATTCCTAATGATGA
TAAACGTGTCAAAGATGCGAAATAA

Gene: nptA (putative sodium:phosphate symporter)

Contig: 02_NODE_3, position: 23202 to 24863, length: 1662 nt, orientation: REVERSE

Sequence:

TTAAATTTCAGTTGTTGCAATTTCTTCATCTGTAGGTACATCATCGTTAAGGCCAACAAGTGCTTCAGAAACATTTCGTGAATGATAACCG
ATACGTTCAAGAACACCAATCATATCGATATATAGTAATCCGCCTTTTGTTGTACATTCACCACGATTAAGGCGTTTAATATGACCTTTGCG
TAGTTTATGTTCAATATTAAATGATTCTCTACTACGTTCTACAATTTCATCTTTTTTCGTTTTGTCATAAACATCTAACATGTCGATGGCTTTA
TCAAATGACTCAGCAACATGGTTGAATAATTTATCCATACCGCGTTGTGCATCTTCTGTAATGCGAATATCTTCATCATGTTGGCGTTTTAA
TTGAGCGACATACTCTTCTGTTAGCTCTGCTACTTTTAAAATAGAGCGATTGACATCAAACATAACTGCTAAACGCTCAACGTCAGCCTTC
GTTATGGCTTTTGTAGAAATTCTAACTAAATAATTTCGAATGCTATCATTGATTGTTTCAACAGCTTGGTGCTTTTGTTCAAGCTTTTTGATC
AATTTTTTATCGTCTTTTGTAATTTCGCGAATGTCTTCAAACATTGATAAGACAATCTGACCAACATTTTGTAATTCTTTTTGAGTTTCTTGT
AATGCAACACCAGGTGCGTGATAAACAAGATCTTTGTTTAAGTGCTGAGGTTTATAGTCATCAGCAATATCTTTACCTGGGACAAGCTTT
GTAACAATCCATGCTAAACCTGCTACAAATGGTAATTGAATCAAAGTATTTGTTATGTTGAAGATACCATGTGATACTGCAATCGTCATCG
CTGGTTTTAAGTGCCATACATCTTGTAACAAACTAATCAAATGAATCACAACTGGCAAGAAAATTGTGAAGATAATTACCCCGATTAAGTT
AAAGATGACGTGTACAAGCGCCGCACGTTTTGCAGCGATTGAGCCGGCTAAACTAGCTAAGATAGCTGTAATCGTTGTACCAATATTATC
GCCTAGTAACACAGGGATTGCTGCGTTTAAGCTAATTAAATCTTGTTGATAAAATTCTTGTAAAATACCAATCGTCGCACTTGAACTTTGA
ACTAGTGCTGTTAACCCTGCGCCGACAATAACAGCAAGTATTGGATTTGATGACATATCAAGCATTAATTGCTTAAATCCATCTAATGATG
CTAAAGGTTTAACGGCATCACCCATAAATTCTAGACCGAAGAATAGAGAACCGAAACCGAATAGTATGCGGCCAATGTTATTAATTTTAG
AGCGTTTAAAGAAAAAGATTAAAAATGCACCTAATGCTAAAATTGGCATTGCATATTCGCCTAAATCTATACCGATAATAAATGCAGTTAC
CGTTGTTCCGATATTAGCACCCATTATCACTCCAATGGCTTGTTTCAATGTCATAAATCCAGCTGTTACCAGTCCGATTGTGATAACTGTCG
TACCTGAACTACTTTGTATTAAAATAGTTACAACGATACCTGCAATAACACCTAATACTGGATTTGATGTAAATTTGTTTAAAATATCTCGT
AGCCTGTCTCCTGCTGATGCTTGAAGCCCGTCTCCCATGATTTTTAAGCCGTAAAGGAAAATACCTAAACCACCTAAAAAGGAGAAAATG
ACTTCTGTAACCGACAT

Gene: Q2YUS5 (67 kDa myosin-crossreactive streptococcal antigen homologue)

Contig: 02_NODE_3, position: 25187 to 26962, length: 1776 nt, orientation: REVERSE

Sequence:

TTATAACAATTTGTGTTCTTTTAATAATGACTCAATGTACGTACCTTTTATCTTTTTAAGGAATCCTGCTAATGCGAGTTTTTGCATTTTCGA
ATCTTTAGTAATCTCGCGCAAATCTTGATGGTCATTCAGTTCGTATATGGCATCCATTAAGACGCGAAGATCAAATGTACTATTGATGACC
TCTGGAATACCACGATCTATATTTAGTAATTGATAAACAGCTTCCATGGCAGTACGAACCGAATATTCTGTTGTAAATACAGTATCTCGCT
CTGTTTCTGCAAAGTTACCAATAAATGCTAAATTTTGTGATTGATGCGGGACGACTAAAGGTCTGTCGCCGATAGCACGCGTCATGAAAT
AAGAGGTAATATATGGCATATAAACAGGAATCGTATTAGATGCATGTTTTGCTAAGTCTTCAATTTTGTCAGTTGGTACACCTAAGTGATA
CAGCCATTCTTGACATATTTCATTACCACTACATTCTGTGATTGGCTTTTTAATATAATCGCCGTTTACATCTGAATATAAGGCATAAATCCA
TGTAGATATTTCATTTTTAGGTTGGTCTTTAAACTGTTGCTGACGATTGATTGTAAAACTGATTTGCCATGCAGAATCATTGATTGTAATAA
TTCCGCCAGTAACCGTTTTGCCTGCAAGAGGGTCACATTTACAAATGCTTTCTATTGTATCGATAATATCTTTATTGTTTGTTGTCGATGTT
GCAGAAACAAACCAACTCTTTTGAGGAATATTTTGGCAAAACTTATCAGGATTACCAAATTCAGGACTTTGTCGCGCTAAATTTTTCCATA
GTGTCCAACTACCACCTAATTCGTCAGTTGGTGGTGCTGGTGTATCATTATCACCATACGTAGAGCTTTCCGTAATACTACCGTTTGTCACA
AAGACAAGATCATCTACAGTCAGTTTAATTGACTCTGCTTTGCCATGTCGGTGAATTAATATTTCTCGGGCAATTTTTTGACTTGTCGTAAC
ATCTACTTTAATATCTTCTACTTTTACACCGTATTCAAATTGAACCCCATGCGATTTTAAATATTCAACCATAGGTAATACTAAAGATTCATA
TTGATTATATTTAGTGAATTTTAAAGCTGAAAAGTCTGCGAGACCACCAATATGATGAACGAATCGCATTAGATAGCGACGCATTTCCATA
GCAGAATGCCACGGTTCAAATGCAAACATCGTTTTCCAGTAAATCCAAAAGTTTGAATTAAAGAAGTCATCGGAAAATACATCTGTTATTT
TGACATCATCTAAATCTTCTTCATTCGTTAAGCATAAATCTAAAATTTCTTTAATCGCTTTTTTGGTCAAAGTGAAGTCTCCATCGGTAACTA
AACGTTGACCCTGTTTTTCAATAACTCGACAGCGAGAATAGTTAGGGTCTTCTTTGTTTAGCCAATAGAACTCATCTAATACAGACGCGTT
ATCGATTTCTAATGAAGGGATAGATCTGAATAAGTCCCACAAACATTCAAAGTGGTTCTCCATTTCACGACCACCTCGGACAACATAGCCT
TTTAAAGGCATATTTTCACCATCAAGACTACCACCTGCTTTAGGTAACTCTTCTAAAATATGAATCTTCGAACCTTCCATTTGACCATCCCTT
ATTAAAAAACAAGCTGCAGCAAGTGAAGCTAGACCAGATCCGATTAAGTAAGCGGATTTGTTTTCTACATTTTCAGGTTTTTTAGGGCGC
GCAAATGCTTCATAATTTCCATAACTGTAATACAT

Gene: DUF1648 (putative protein)

Contig: 02_NODE_3, position: 27313 to 27786, length: 474 nt, orientation: FORWARD

Perfect match to: (MW2-BA000033-[96873:97346], allele observed in CC1+CC80+CC445)

Sequence:

ATGTCTAAAATCAGGTCTTTTACAATATTAAGTCTACTTATTTACTTAGCTATGATGTGCTATACAGTAGTGACCTATTCAAAATTACCAAC
CAAAGTACCTATTCATTATAATTTAGCAGGGGATGCTGATAACTTTGCTGATAAATGGGTGCTGCTTTTGATTAATAGCGCATTTATAGTG
ATTTGGCTTATATTTTTCATTGCAGGTAGATACTATGAACGATTTGCCAAATGGTCACATTATAATCATACACCACGTGAAATTCGAGCGA
TTAAATTATTTTTAAGTACGTTAAATTTAGAGATTATGAGCTATATGTCTATCTTCACAGTATTAGAAATTTGGCAAATACAACACCATCAT

CAATTCAATTTACTATGGTTTAATATGATATTTATCATTATCATTGGTTTGACGCTTGTCATATTTTGTTTACTTCCTACAATTCATAAAATGA
GAGATTCTCAATAA

Gene: lctP-locus1 (L-lactate permease)

Contig: 02_NODE_3, position: 28048 to 29640, length: 1593 nt, orientation: FORWARD

Perfect match to: (MW2-BA000033-[97608:99200], allele observed in CC1)

Sequence:

ATGACACTACTTACTGTAAATCCATTCGATAATGTTGGATTATCAGCCTTAGTTGCAGCAGTACCTATTATTTTATTTTTATTATGCTTAACC
GTTTTTAAAATGAAAGGCATTTATGCAGCATTGACAACTTTGGTTGTTACATTGATTGTGGCTTTATTTGTATTTGAATTACCAGTGCGTGT
ATCAGCAGGTGCGATTACAGAAGGCGTTGTTGCCGGTATTTTCCCAATAGGATATATCGTTTTAATGGCAGTTTGGTTATATAAAGTTTCT
ATTAAAACAGGACAATTTTCTATTATTCAAGATAGTATTGCAAGTATTTCAGAGGACCAAAGAATCCAACTATTATTAATTGGATTTTGTTT
CAACGCATTTTTAGAAGGTGCAGCAGGATTTGGTGTGCCAATTGCGATTTGTGCAGTATTATTAATTCAACTTGGATTTGAACCATTAAAA
GCAGCGATGTTATGCTTAATTGCTAATGGTGCGGCGGGTGCCTTTGGTGCAATTGGTTTACCAGTTAGTATTATTGATACGTTTAACTTAA
GTGGAGGCGTTACAACATTAGATGTTGCGAGATATTCAGCATTAACACTTCCAATTTTAAACTTTATTATCCCATTTGTTTTAGTATTCGTT
ATAGATGGCATGAAAGGTATTAAAGAAATTTTACCTGTCATTTTAACAGTGAGTGGTACATATACTGGATTACAATTATTATTAACAATAT
TCCATGGTCCAGAACTAGCAGACATTATCCCATCACTAGCAACAATGGTGGTGTTAGCATTTGTTTGTCGTAAATTTAAACCGAAAAACAT
TTTCAGGTTGAAAGAATCAGAACATAAAATTCAAAAACGAACGCCTAAAGAAATTGTCTTTGCTTGGAGTCCGTTCGTCATTTTAACTGCC
TTTGTTTTAGTATGGAGTGCACCATTCTTCAAAAAATTATTCCAACCTGGAGGTGCACTTGAAAGTTTAGTAATAAAATTGCCAATTCCAA
ATACTGTGAGTGATTTATCGCCTAAAGGAATTGCGTTGCGTCTCGATTTAATTGGTGCAACTGGGACAGCGATTTTATTAACAGTAATTAT
TACAATTTTAATTACGAAGCTAAAATGGAAAAGTGCAGGTGCTTTATTGGTCGAAGCAATTAAAGAATTATGGTTACCGATCCTTACAATT
TCAGCTATCCTAGCTATTGCTAAAGTTATGACATACGGTGGTTTGACTGTAGCAATTGGACAAGGTATTGCTAAAGCTGGAGCAATTTTCC
CATTATTCTCTCCAGTATTAGGTTGGATTGGTGTGTTTATGACTGGTTCAGTTGTAAATAACAATACTTTATTCGCACCTATTCAAGCGACA
GTAGCACAACAAATTTCAACAAGCGGTTCATTACTTGTGGCAGCTAACACTGCAGGTGGTGTAGCAGCGAAACTTATTTCACCACAATCA
ATTGCCATTGCGACTGCAGCTGTTAAAAAAGTTGGTGAAGAATCTGCATTATTAAAAATGACGCTAAAATACAGTATTATATTTGTTGCTT
TTATTTGTGTTTGGACGTTTATACTAACGTTAATATTCTAA

Gene: spa (immunoglobin G binding protein A)

Contig: 02_NODE_3, position: 29969 to 31333, length: 1365 nt, orientation: REVERSE

Sequence:

TTATAGTTCGCGACGACGTCCAGCTAATAACGCTGCACCTAACGCTAATGATAATCCACCAAATACAGTTGTACCAATGAATGGATTTTCT
TCACCAGTTTCTGGTAATGCTTGAGCTTTGTTAGCATCTGCATGGTTTGCTGGTTGCTTCTTATCAACAACAAGTTCTTGACCAGGTTTGAT
CATGTTTTTATCAGCTAATTTGTTATCTGCAGCAATTTTGTCAGCAGTAGTGCCGTTTGCTTTTGCAATGTCATTTACTGTATCACCAGGTTT
AACGACATGTACTCCGTTGCCGTCTTCTTTACCAGGTTTGTTGTTGTCTTCTTTGCCAGGCTTGTTGCCATCTTCTTTACCAGGTTTTTTGTT
GTCTTCTTTACCAGGTTTGTTGCCGTCTTCTTTACCAGGTTTTTTGTTGTCTTCTTTACCAGGTTTTTTGTTGTCTTCTTTACCAGGCTTGTTG
TTGTCTTCTTTGCCAGGTTTGTTGCCGTCTTCTTTACCAGGTTTGTTGTTGTCTTCCTCTTTTGGTGCTTGAGCATCGTTTAGCTTTTTAGCTT
CTGCTAAAATTTCTTTGCTCACTGAAGGATCGTCTTTAAGGCTTTGGATGAAGCCGTTACGTTGTTCTTCAGTTAAGTTAGGTAAATGTAA
AATTTCATAGAAAGCATTTTGTTGTTCTTTGTTGAATTTGTTGTCAGCTTTTGGTGCTTGTGCATCATTTAGCTTTTTAGCTTCTGCTAAAAG
GTTAGCGCTTTGGCTTGGGTCATCTTTTAAGCTTTGGATGAAACCATTGCGTTGTTCTTCGTTTAAGTTAGGTAAATGTAAGATTTCATAG
AAAGCATTTTGTTGTTCTTTGTTGAAATTGTTGTCAGCTTTCGGTGCTTGAGATTCGTTTAATTTTTTAGCTTCACCTAAAACGTTAGTGCTT
TGGCTTGGATCGTCTTTAAGACTTTGAATGAAACCATTGCGTTGCGCTTCGTTTAAGTTAGGCATGTTCAAGATTTCATAGAAGGCGCTTT
GTTGATCTTTGTTGAAGTTATTTTGTTGCGCATCAGCTTTTGGAGCTTGAGAGTCATTAAGTTTTTGAGCTTCACCTAAAACGTTAGCACTT
TGGCTTGGATCATCTTTAAGGCTTTGGATAAAACCATTACGTTGATCAGCGTTTAAGTTAGGCATATTTAACACTTGATAAAAAGCATTTT
GTTGAGCTTCATCGTGTTGCGCAGCATTTGCAGCAGGTGTTACGCCACCAGATATAAGTAATGTACCTAAAGTTACAGATGCAATACCTA
CACCTAGTTTACGAATTGAATAAATGTTTTTCTTTTTCAAATTAATACCCCCTGTATGTATTTGTAAAGTCATCAT

Gene: sarS (HTH-type transcriptional regulator)

Contig: 02_NODE_3, position: 31718 to 32470, length: 753 nt, orientation: REVERSE

Perfect match to: (RF122-AJ938182-[67420:68172:r], highly conserved allele)

Sequence:

TTATTCAAAAACAAGATGTAAATGATCTTTATCTGCTAATAATTGATTCACTTGAGCTAATAATTGTTCAGCATGGTCTTGCTGCGCGTCAT
CCATATGAATTAAAATTTTTCTTTCATCTTCAGTTGAGCGTTCTTTTATTAGATAGCCTTGCTTTTTTAAATTATTGAGAGCTCTAACAGTTT
GAGGGTATTTATGGTGGATTGTTTCAATTAAATCTTTAAGAAGAACGATGTTTTTATTTTGAGAAGTGATAATAGCTAGAATTGTGAATTC
TACAAAACTTAATGTTAGATGTTTTTTGATAATATTCTTGAAATACATTGTATACATCATCAAGTTTAGAAATTCTTTACTATCTTTTGGTAT
CATCTGTGATTCACTTTGATCTGCAAGGTTAAATTGTTTAATGATTTGATCAAACAATGTAACACGTTCTGCAATTTTCTCTCGTTGTTCTTC
AGATATTGAAATGTAAGTATTACGCTCATCAATTTTACTTCGAACTTTACTAATATATGAATGTTTCACAAGTACTTTTATATGCTGTACTA
AATCCGATTGTTTATAACATAAATCTGAAACAATCTTCTTAAATGGAAGTGTGTTTTCTTGCTGATGAAATAAATAAGTCAGTAATATAAA
TTCTTTTATAGTCATATCGACTTCAGGCTTGACTTTTTTCTTAAAACGAAACATATATGCTTCAATGATTATAAAATCTCTAATTTTGTCATG
GTTATTATATTTCAT

Gene: sirC (siderophore 'staphylobactin' ABC transporter, transmembrane permease subunit)

Contig: 02_NODE_3, position: 32839 to 33837, length: 999 nt, orientation: REVERSE

Perfect match to: (MW2-BA000033-[102549:103547:r], allele observed in CC1+CC6+CC20)

Sequence:

TTATAACTTTTTCACGGTTAATAATAAGTATATGAAGAATGGGGCACCAAAAGCAGCAATAAATACACCTGCTGGCACTTCTTTAGGCAA
GAATAAGGTACGCCCAATTAAGTCTGCAATAACAATTGATATGGCACCAATCATTGCTGACATTAGTAACTTTTTAGCATAACTTCCGCGA
ACGATTGTTTTCGCGATATGTGGTGCGATTAAACCGACAAACCCAATGTTACCTACTAAACTGATTGCCATAGATACGAGTATAGTAGAA
GTGATTAATTGGATTAGTTTCATACGTTGTACATGTAAGCCTAAGCCAATCGCTACAGGGTCATCAAGTATAGATATTTTCATTTTTGGTAT
AACAAGAAATAACAACGGCACAACAGCTAAAATAACCATACCCAAGATGATTGTATCTTTAAACGTAGCACCGTAAAGACTTCCGACTAG
CCATGTATAAGCTTTGGCAGCAGATAATTGCTTCGTTGTAATGAGTAATCCTTGGACAAGCGCAATAAACAACGTTTGCATCGAAATACC
GATGATTATGAGTGTTGTCGGGCGTATTTGTCCTTTCGTTTGAAACACTAATAGTATCATCATTGCAATTGCGCCACCTAATACAGCGAAT
AGTGGAAGTAAATGTATCGTTAAATGGCTGAAAAATGCAATAAAGACAACGGCACTTAAGCTAGCACCACCTGTGATACCGATAATATC
AGGTGAGGCAATTGGATTTTTTAATACATTTTGCAACATTAAACCACTCATTCCTAGTGCGGCACCTGCTAAAATCGCAAGTGTAATGCGA
GGTAAGCGTAATACTTCTAAAGTGAATTGATCCATACTGTCATTTGGATTTATAAAGTACATCAGTACGCGTTGTAATGGTATAAAGCTTG
AACCACTCATCATACTTACCACTGAAACGATGGCTAAAAAGATTAACGCGAAGATGAGATGGTAATTGTCTTTTTTATTAATCTTTTCGGT
CAT

Gene: sirB (siderophore 'staphylobactin' ABC transporter, transmembrane permease subunit)

Contig: 02_NODE_3, position: 33834 to 34829, length: 996 nt, orientation: REVERSE

Perfect match to: (MW2-BA000033-[103544:104539:r], allele observed in CC1+CC5+CC130-ST582)

Sequence:

TCATAAGCGTTGACGTCCTTTCTTCATAATATAGATTAAGACAATAGCGCCAATGACAGCGGTAACGACACCGATAGGCAACTCTAGTGG
CTTAATTATTATACGAGCAACAATGTCTGAAATGATCATTAGGATTGCTCCAGCTAATGCAGTAAAAGGAATTAAATACTTATAGTTTGGT
GGTAATAATCGTTTGCTAATATTCGGTACGATAAGACCCACAAAGACGATTGCTCCAGCTACGGCTACCGAAATACCGGCTAACATACTG
ATGAGCATAATAATCATCCATTTGATTAATTTTATGTTTTGACCGAGGCCGATTGCAATGTCGTCACTTGTCATCAAGATGTTGATGTGTG
CAGCCATGCTAAATGCAATTAAAATAAGTATCAATACAAGCGGAATAATCCATGGGATATCCCAAATATTACGTAATGAAACGGAGCCAC
TTAACCAAAATAATAGGCCTTGTAAGTCTGTTTCGTTCATAATAAGTATGCCTTGAGTAAAGGCTGTAAATAGCATCGCAATCGCAGCACC
TGCCAAAATGACACGGTGAGGTGAGAATAGTGTTTGTCTAAACATACCTAGCGCAACAACTAATACAGTAACAACAATGGCACCTAAAA
ATGCAATAACTACAATCATTTTAAAAGATTGAATTTGGATAAATGTAATACTAAAAATGACAAAAAATACTGCGCCTGCATTGACACCGA
AAAGCCCTGGTGATGCTATTGGGTTTCGTGTAAGTGCTTGCATCAACAAACCTGAGACAGCAAGGGCAGCACCAGTCAATAACGCAATG
ATTGTTCTCGACGCCCGTGCACCAGTGACAACATCATGTAAATCGTTTTCACTATCAAAGTTGAATAACGCCTGTATCACCGTACCTGGTG
ACACAAGCGTATTTCCAATCATTAAACTTAAGATAGCTACTATTGCAAGACATAAACCAGCAATAACGATTTGGTATTTTGGTTTAAGTAG
CAT

Gene: sirA (siderophore 'staphylobactin' ABC transporter, substrate-binding protein)

Contig: 02_NODE_3, position: 34845 to 35837, length: 993 nt, orientation: REVERSE

Sequence:

TTATTTTGATTGTTTTTCAATATTTAACTTTTCATATAAATCGTCAATCAATTTTAATGATGATTTATATCCGCCAGCTAAGTTCCAAGTGATT
TCATCTAAATCATCAGATACTTGGTTGTTTTTAACTGCGTCTAAATTTTTCCATTCTTTACTTGAAGTCCATTCGCTTTCAGTCTTTTTAACTA
ATGCAGCATCTTTCGCATTTGGATCTGATTTTACTACAAAAATATGATCAGCGTTCATTAATGGAATGCTTTCTTTGGATGTAAGTTGGATA
ATATCTTTACCATTATCAACTTGTTTTTGTAAGTCTTTATTACGTTTGAATCCTAAATCATTTAAGATTTCACCAGCATATCCACCAGCATAA
ATTCTTGTATGATCAGCACGGAAGTTAACAACTGAAGCTTTCAATGGCCATGCATCTTTATACTTTGCTTTTGCATCTTTTTGGAATGCAGC
TACTTTATCATCGTACTTTTTAAGTAAATCTTCAGCTTCTTTTTCTTTCCCTAAAGCTTTCCCCATTAACTTAGTTGTATCTTTGAATTTGAAA
ACTGTATCAGTAGAAACTGTTGGTGCGATTTTAGATAATTGATCGTAAACTTTTTCATTTCTAACTTTTGACGCGACAATTAAGTCCGGTTT
TAATTTAGAGATTTCCTCTAAATTAGGTGCAGGTTCTTGACCTACAATCTTAGTATCTTTTAAATCATTTTTTATGTATTCGAATTTCGGTTT
TTGTGTCCATGATTCTACAGCACCTACAGGTTTAACACCTAAAGATACAGCGACGTCAGTGGCACCTTGATATAGCGTAACAACACGCTTT
GGTTTCCCTTTAATTTCAGTTGTACCCATTGCATGTTTAATTGAAGTTGTTTCCTTATCTTTGTTATCAGATGATTGTTTATTTGAATTCCCAC
TACATCCTGCTAAAACAAGTAGGAAAGCAAGCGTAACAACAAGCATTTTAATTACTTTATTCAT

Gene: sbnA (siderophore biosynthesis protein A)

Contig: 02_NODE_3, position: 36068 to 37048, length: 981 nt, orientation: FORWARD

Perfect match to: (RF122-AJ938182-[71767:72747], highly conserved allele)

Sequence:

TTGATTGAAAAAAGTCAAGCATGTCACGATTCATTGTTAGATTCTGTAGGGCAAACACCTATGGTTCAACTTCATCAACTATTTCCGAAAC
ATGAAGTGTTTGCAAAGTTAGAGTATATGAATCCTGGAGGCAGCATGAAAGATCGACCTGCCAAGTACATCATTGAACATGGTATTAAA
CATGGTTTAATCACTGAGAATACACATTTAATTGAAAGTACTTCTGGTAATTTAGGCATTGCGTTGGCAATGATAGCTAAAATCAAGGGA
TTAAAACTCACGTGTGTTGTTGATCCTAAAATATCACCAACAAATTTGAAAATTATTAAAAGTTATGGTGCCAATGTAGAAATGGTTGAAG
AACCTGATGCACATGGGGGTTATTTAATGACTCGTATTGCAAAGGTGCAAGAACTGTTAGCCACTATTGACGATGCATATTGGATTAATC
AATATGCGAATGAGTTAAATTGGCAATCCCATTATCATGGTGCAGGCACAGAGATTGTTGAAACAATTAAGCAACCTATAGATTATTTTG
TCGCGCCAGTCAGCACGACAGGTAGCATTATGGGTATGAGTAGAAAAATAAAAGAAGTGCATCCAAACGCACAAATTGTTGCTGTTGAT
GCGAAAGGGTCAGTCATTTTTGGTGACAAACCTATTAATAGAGAATTACCTGGTATCGGTGCTAGTCGTGTACCCGAAATATTGAATAGA
TCAGAAATTAATCAAGTGATCCATGTAGATGATTATCAATCTGCTTTGGGCTGTCGAAAACTGATTGATTATGAAGGCATATTTGCCGGA
GGTTCAACAGGTTCGATTATTGCAGCGATTGAGCAGTTGATAACGTCAATTGAAGAAGGTGCAACAATTGTCACGATTTTACCAGATCGA
GGCGATCGTTACTTAGATTTAGTTTATTCAGATACATGGTTAGAAAAAATGAAATCAAGACAAGGAGTTAAATCAGAATGA

Gene: sbnB (siderophore biosynthesis protein B)

Contig: 02_NODE_3, position: 37045 to 38055, length: 1011 nt, orientation: FORWARD

Perfect match to: (08-02119-CP015645-[2121450:2122460:r], highly conserved allele)

Sequence:

ATGAATAGAGAGATGTTGTATTTAAATAGATCAGATATTGAACAAGCGGGAGGTAATCATTCACAAGTTTATGTGGACGCATTAACAGA
AGCATTAACAGCCCATGCGCACAATGATTTTGTACAACCGCTTAAGCCGTATTTAAGACAGGATCCTGAAAATGGACACATCGCAGATCG
AATAATTGCAATGCCAAGTCATATCGGTGGTGAACACGCAATTTCAGGTATTAAGTGGATAGGTAGTAAGCACGACAATCCATCGAAAC
GTAATATGGAGCGTGCAAGTGGTGTCATTATTTTGAATGATCCAGAAACGAATTATCCAATTGCAGTTATGGAAGCAAGTTTAATTAGTA
GTATGCGTACTGCAGCAGTTTCAGTGATTGCAGCTAAGCATTTGGCTAAAAAAGGATTTAAAGACTTAACAATCATTGGATGCGGGCTAA
TCGGAGACAAGCAATTACAAAGTATGTTAGAGCAATTCGATCATATTGAACGCGTGTTTGTTTACGATCAATTCTCTGAAGCATGTGCAC
GCTTTGTTGATAGATGGCAACAACAGCGTCCGGAAATTAATTTTATTGCGACAGAAAATGCTAAAGAAGCAGTATCAAATGGTGAAGTA
GTCATTACATGTACCGTAACGGATCAACCATACATTGAATATGATTGGTTACAAAAGGGTGCATTTATTAGCAACATTTCTATCATGGATG
TGCATAAAGAAGTCTTTATTAAAGCTGACAAAGTCGTAGTAGACGACTGGTCACAATGTAATCGAGAAAAGAAAACTATTAACCAATTG
GTGTTAGAAGGTAAATTCAGCAAAGAAGCACTTCATGCTGAACTAGGACAACTTGTGACAGGTGACATACCAGGACGTGAAGACGATG
ATGAAATCATATTACTTAATCCGATGGGTATGGCTATCGAAGATATTTCAAGTGCTTACTTTATTTATCAACAGGCACAACAACAAAATAT
TGGGACAACATTGAACCTATATTAA

Gene: sbnC (siderophore biosynthesis protein C)

Contig: 02_NODE_3, position: 38076 to 39830, length: 1755 nt, orientation: FORWARD

Perfect match to: (MW2-BA000033-[107786:109540], allele observed in CC1+CC6+CC20-ST582)

Sequence:

TTGCAGAATCATACAGCAGTCAATACAGCACAAACGATAATATTAAGAGATTTAGTTGATGCATTATTATTTGAAGATATAGCCGGTATT
GTATCGAATAGTGATATTACTAAAGAAAATGGACAAACGCTTTTGATATACGAACGTGAAACACAACAAATAAAGATACCTGTTTATTTT
AGTGCTTTAAATATGTTTCGTTATGAAAGCTCACAACCAATTACGATAGAGGGAAGGGCGTCTAAGCAACCTTTAACAGCAGCTGAATTT
TGGCAAACAATTGCTAATATGAATTGTGATTTAAGTCATGAATGGGAAGTGGCTCGAGTTGAAGAAGGACTGACTACTGCTGCCACACA
GCTTGCTAAACAATTATCAGAATTAGATTTAGCGTCACATCCTTTTGTGATGTCAGAGCAGTTTGCAAGTTTAAAAGATCGTCCATTTCATC
CATTAGCTAAAGAAAAAAGAGGATTAAGAGAAGCGGATTATCAAGTGTATCAAGCTGAATTAAATCAATCATTTCCTTTAATGGTTGCAG
CAGTTAAAAAGACACATATGATTCATGGCGATACTGCAAATATCGATGAATTAGAAAATTTGACAGCACCTATAAAAGAACAAGCGACA
GACATGTTAAATGATCAAGGGTTATCAATAGATGACTATGTACTATTTCCGGTACATCCTTGGCAATATCAGCATATTTTGCCGAACGTCT
TTGCGAAAGAGATTAGTGAAAAGTTGGTTGTACTATTACCGTTAAAATTTGGAGATTATCTGTCGTCTTCAAGTATGCGTTCATTAATTGA
TATTGGCGCACCGTATAACCATGTCAAAGTACCATTTGCAATGCAGTCATTAGGGGCATTAAGGCTAACGCCTACGCGTTACATGAAAAA
CGGAGAACAAGCAGAACAATTATTACGTCAGCTTATAGAAAAAGATGAAGCACTAGCTAAGTATGTCATGGTTTGTGATGAAACAGCTT
GGTGGTCATATATGGGTCAAGATAATGATATTTTCAAAGATCAATTAGGTCATCTAACTGTTCAGCTAAGAAAATATCCCGAAGTGCTAG
CCAAAAATGATACGCAACAGCTAGTGTCAATGGCAGCACTCGCGGCAAATGATCGCACTTTATATCAAATGATTTGTGGAAAAGATAATA
TTTCTAAAAATGATGTCATGACGTTATTTGAAGATATCGCGCAAGTCTTTTTAAAGGTAACACTATCATTTATGCAATACGGCGCATTACC
AGAGTTGCATGGTCAAAATATATTGTTGTCATTTGAAGATGGACGTGTACAAAAATGCGTGTTACGTGATCATGATACTGTCAGAATTTA
TAAACCATGGCTAACAGCACATCAGCTTTCATTGCCGAAGTATGTCGTCAGAGAAGATACACCTAATACGCTAATTAATGAAGATTTGGA
AACATTCTTTGCTTATTTTCAAACATTAGCTGTATCGGTAAATCTATATGCCATTATTGATGCAATTCAAGATTTATTTGGTGTAAGTGAGC
ATGAACTTATGTCGTTGTTAAAACAAATTTTAAAGAATGAAGTGGCAACTATTTCCTGGGTTACAACTGATCAGCTAGCTGTCAGACACAT
TTTATTTGATAAACAGACGTGGCCATTCAAACAAATTTTATTACCATTGCTATATCAACGTGATAGTGGTGGAGGTAGTATGCCTTCAGGT
TTAACTACCGTACCAAATCCAATGGTGACATATGATTAA

Gene: sbnD (siderophore biosynthesis protein D)

Contig: 02_NODE_3, position: 39823 to 41079, length: 1257 nt, orientation: FORWARD

Perfect match to: (ATCC51811-ADVP01000042-[20093:21349:r], allele observed in CC1)

Sequence:

ATGATTAATCAGTCTATATGGCGCAGTAACTTTCGCATTTTATGGCTCAGTCAGTTTATAGCGATTGCTGGACTGACAGTACTTGTGCCAT
TATTGCCAATTTATATGGCATCACTACAAAATCTATCAGTTGTAGAAATACAGTTGTGGAGTGGTATAGCGATTGCTGCTCCAGCTGTAAC
GACGATGATAGCTTCGCCGATATGGGGGAAGCTAGGTGATAAGATTAGCCGGAAATGGATGGTGTTAAGAGCGTTACTTGGTTTGGCG
GTATGCTTATTTTTAATGGCATTGTGTACGACACCATTACAGTTTGTACTTGTGAGGTTATTGCAGGGACTATTTGGTGGTGTTGTTGATG
CATCAAGTGCGTTTGCGAGTGCAGAGGCGCCAGCTGAAGATCGTGGAAAGGTATTAGGAAGACTGCAAAGTTCAGTCAGCGCAGGGTC
TCTTGTGGGGCCATTAATTGGCGGTGTTACAGCTTCGATATTAGGTTTTAGTGCGTTACTGATGAGTATTGCCGTTATTACTTTTATTGTCT
GTATTTTCGGTGCATTAAAATTGATTGAAACGACACATATGCCAAAATCACAAACACCAAATATTAATAAAGGTATTCGCCGTTCATTTCA
ATGTCTATTATGCACACAACAAACATGTCGATTTATTATCGTTGGCGTTTTAGCAAACTTTGCTATGTATGGCATGCTAACTGCATTATCAC
CACTTGCTTCATCAGTGAATCATACAGCGATAGATGACCGTAGTGTGATTGGATTTTTACAGTCCGCATTTTGGACGGCTTCGATATTAAG
CGCGCCTTTATGGGGACGCTTTAATGATAAATCATATGTTAAATCAGTATATATATTTGCCACGATTGCATGTGGTTGTAGTGCGATACTG
CAAGGTTTAGCGACGAATATAGAGTTTTTAATGGCTGCAAGAATACTTCAAGGATTAACATATAGTGCATTGATTCAAAGTGTCATGTTT
GTTGTCGTGAATGCGTGTCATCAACAACTTAAAGGCACATTTGTTGGAACGACGAACAGTATGTTAGTTGTTGGTCAAATTATTGGCAGT
CTTAGTGGCGCTGCCATTACAAGTTATACTACACCAGCTACTACGTTTATCGTTATGGGCGTAGTATTTGCAGTAAGTAGTTTATTTTTAAT
TTGTTCAACCATCACTAATCAAATCAACGATCACACATTAATGAAATTATGGGAGTTGAAACAAAAAAGTGCAAAATAA

Gene: sbnE (siderophore biosynthesis protein E)

Contig: 02_NODE_3, position: 41069 to 42805, length: 1737 nt, orientation: FORWARD

Perfect match to: (08-02119-CP015645-[2116700:2118436:r], allele observed in ST582+CC5+CC8+CC101+CC1290)

Sequence:

GTGCAAAATAAAGAATTAATACAACATGCAGCGTATGCGGCTATCGAACGCATTTTAAATGAATATTTTAGAGAAGAAAATTTATATCAA
GTACCACCTCAAAATCATCAATGGTCTATACAATTATCAGAGCTCGAAACTTTAACGGGTGAATTTCGCTATTGGTCTGCGATGGGGCATC
ATATGTATCATCCAGAGGTATGGCTTATCGATGGAAAAAGTAAAAAAATAACAACTTATAAAGAAGCAATTGCGCGTATTTTGCAACATA
TGGCTCAAAGTGCAGATAATCAAACGGCAGTGCAACAACATATGGCGCAAATTATGTCTGACATCGATAATAGCATTCATCGCACGGCG
CGTTATTTGCAAAGTAACACAATAGACTACGTAGAGGATCGTTATATCGTTTCAGAACAATCTTTATACTTAGGTCATCCATTTCATCCGAC
TCCTAAGAGTGCAAGTGGGTTTTCAGAAGCAGATTTAGAGAAATATGCACCCGAATGTCATACATCATTCCAATTGCATTATTTAGCTGTG

CATCAAGATGTTCTGCTCACGCGCTATGTAGAAGGTAAAGAAGATCAGGTTGAGAAAGTGTTGTATCAATTAGCAGACATAGATATATCA
GAGATACCCAAAGATTTTATTTTATTACCAACACATCCTTATCAAATCAATGTGTTGCGACAGCATCCACAGTATATGCAATATAGTGAAC
AAGGTTTAATAAAAGACCTTGGCGTTTCCGGTGATTCAGTGTACCCGACGTCTTCGGTTAGAACTGTATTTTCAAAAGCATTAAACATTTA
TTTAAAATTACCGATACACGTTAAAATCACTAATTTTATACGTACGAATGACCTTGAACAGATTGAACGGACAATTGATGCCGCGCAAGTT
ATCGCATCAGTCAAAGATGAGGTTGAAACACCCCATTTTAAATTGATGTTTGAAGAAGGATATCGTGCATTGTTACCGAATCCATTAGGG
CAAACAGTTGAACCTGAAATGGATTTATTAACAAATAGTGCCATGATTGTTCGTGAAGGGATACCGAATTACCATGCTGATAAAGATATT
CATGTATTGGCGTCATTATTTGAAACGATGCCTGATTCACCGATGTCTAAGTTATCACAAGTGATTGAGCAAAGTGGTTTAGCACCAGAA
GCATGGCTTGAATGTTATTTGAATCGTACATTATTGCCGATATTAAAGCTGTTTAGTAACACAGGCATTAGTCTAGAAGCACATGTACAAA
ATACATTAATTGAATTAAAAGATGGCATACCCGACGTATGCTTTGTCAGAGATCTTGAAGGCATTTGTCTATCTAGAACGATTGCTACTGA
AAAACAACTTGTGCCAAATGTTGTGGCAGCATCAAGCCCTGTTGTATATGCACATGATGAAGCATGGCATCGTCTTAAATATTACGTTGT
AGTAAATCACTTAGGACATTTAGTATCAACTATTGGTAAAGCGACTAGAAATGAAGTTGTGTTATGGCAACTTGTAGCGCATCGTCTTAT
GACTTGGAAAAAAAGAATACGCGAATAACGCAGTGTTTGTTGACTGTGTAGAAGATTTATATCAAACGCCGACCATTGCGGCTAAAGCGA
ATTTGATGAGTAAATTGAATGATTGTGGTGCAAACCCTATTTATACACATATACCAAATCCAATTTGTCATAACAAGGAGGTATCGTATTG
TGAATCAAACAATTCTTAA

Gene: sbnF (siderophore biosynthesis protein F)

Contig: 02_NODE_3, position: 42825 to 44564, length: 1740 nt, orientation: FORWARD

Perfect match to: (CO-23-AJKF01000006-[16811:18550:r], allele observed in CC8)

Sequence:

ATGCACCAACTGGTATCATCACTTATTTATGAGAATATTGTTGTGTATAAAGCGTCATATCAAGACGGTGTCGGTCATTTTACAATAGAAG
GACATGATTCAGAGTATCGTTTTACTGCTGAAAAGACACATAGCTTTGATCGTATACGTATCACATCACCAATTGAGCGTGTCGTAGGAG
ATGAGGCAGATACAACAACAGACTATACACAATTATTGAGAGAGGTTGTATTTACATTTCCTAAAAATGATGAAAAGCTAGAACAATTTA
TTGTTGAGTTATTACAGACAGAATTAAAAGATACACAAAGTATGCAGTATCGAGAATCAAACCCACCAGCAACACCTGAGACATTTAACG
ACTATGAATTTTATGCGATGGAAGGGCATCAGTATCATCCAAGTTACAAATCACGTTTAGGATTTACGTTGAGTGATAATTTGAAATTTGG
TCCTGATTTTGTACCAAACGTTAAACTGCAGTGGTTAGCTATCGACAAAGATAAAGTAGAAACGACGGTATCAAGAAATGTTGTAGTTAA
CGAAATGTTACGTCAACAAGTTGGCGATAAGACTTATGAACATTTTGTACAGCAAATTGAAGCGTCTGGCAAACATGTAAATGATGTTGA
GATGATACCTGTACACCCATGGCAGTTTGAACATGTCATCCAAGTTGATTTGGCTGAAGAAAGGCTTAATGGCACAGTACTATGGTTAGG
GGAAAGTGATGAGCTATATCATCCTCAACAATCGATTCGTACGATGTCGCCAATAGACACGACAAAATATTATTTAAAGGTACCAATAAG
TATAACGAACACTTCAACGAAACGAGTGTTGGCGCCTCATACAATTGAAAATGCAGCGCAAATTACGGATTGGTTAAAGCAGATACAGC
AACAAGATACGTATTTAAAAGATGAATTAAAGACAGCTTTTCTAGGGGAAGTCTTAGGACAGTCTTATTTAAATACACAACTTTCGCCTTA
TAAACAAACTCAAGTTTATGGTGCGTTAGGTGTTATATGGCGTGAAAATATATATCACATGTTAATCGATGAAGAGGATGCGATACCATT
TAATGCACTTTATGCAAGTGATAAGGATGGTGTACCATTCATTGAAAATTGGATTAAACAATATGGTTCTGAAGCTTGGACAAAGCAATT
TTTAGCTGTAGCGATTCGTCCAATGATTCATATGCTTTATTATCACGGTATTGCCTTTGAATCGCATGCACAAAATATGATGCTCATTCATG
AAAATGGTTGGCCTACACGTATTGCCTTAAAAGATTTCCATGATGGTGTTCGTTTTAAGCGTGAGCATTTAAGTGAAGCTGCTTCACACCT
GACATTAAAGCCAATGCCAGAAGCACATAAAAAAGTGAATAGTAATTCATTTATTGAAACAGATGACGAACGTTTAGTACGCGACTTTTT
ACATGATGCATTTTTCTTTATTAATATCGCCGAAATCATCTTATTTATTGAAAAGCAATATGGTATCGATGAGCAGCGACAATGGCAATGG
GTTAAAGACATTATCGAGGCGTATCAAGAAGCATTTCCAGAGTTGAATAACTATCAACATTTCGATTTGTTTGAACCTACGATTCAAGTTG
AAAAGTTAACGACACGTCGATTATTAAGTGACTCCGAGTTAAGAATTCATCATGTTACAAATCCATTAGGTGTAGGAGGTATCAATGATG
CAACAACTATCTCTGAAACATAG

Gene: sbnG (siderophore biosynthesis protein G)

Contig: 02_NODE_3, position: 44539 to 45315, length: 777 nt, orientation: FORWARD

Perfect match to: (11819-97-CP003194-[130453:131229], allele observed in CC80+CC1)

Sequence:

ATGCAACAACTATCTCTGAAACATAGATTAAACAATGGTGATTCAGTTTATGGCATTTTTAATTCTATACCGGACCCATTGATGATCGAGG
TTATCGCAGCAAGCGGGTATGACTTTGTTGTGATTGATACAGAACACGTGGCGATTAATGATGAGACACTAGCGCATTTAATTCGTGCAG
CTGAAGCAGCGCATATTATACCAATTGTACGTGTCACTGCAGTGATAGATAGAGATATCATTAAAGTGTTAGATATGGGTGCGAGAGGT
ATTATTGTGCCACACGTTAAAGATCGTGAGACAGTTGAGCATATTGTGAAATTAAGTCGTTATTACCCGCAAGGATTAAGAAGTTTGAAT
GGTGGTCGCATGGCAAGATTTGGACGTACACCATTACTTGATGCAATGGAGATGGCTAATGAGCATATTATGGTGATTGCCATGATAGA
AGATGTTGAAGGGGTTATGGCCATTGACGATATAGCACAAGTCGAAGGTTTAGACATGATAGTCGAAGGTGCCGCAGATTTATCGCAGT
CACTTGGCATACCATGGCAAACGCGTGATGATCAAGTAACATCACATGTTCAACATATTTTTGAGGTTGTGAATGCACATGGTAAACATTT

TTGTGCATTACCACGTGAAGATGAAGATATTGCAAAATGGCAGGCACAAGGTGTACAAACATTTATTTTAGGTGATGATCGCGGAAAAA
TATATCGCCATTTAAGTGCATCTCTAGCGACGTCTAAACAGAAAGGGGATGATGGCTAA

Gene: sbnH (siderophore biosynthesis protein H)

Contig: 02_NODE_3, position: 45315 to 46517, length: 1203 nt, orientation: FORWARD

Perfect match to: (ATCC51811-ADVP01000042-[14655:15857:r], allele observed in CC1)

Sequence:

ATGCGTATAGTTCAACCTGTTATTGAACAATTAAAAGCACAATCTCATCCAGTTTGTCATTATATCTATGATTTAGTCGGACTGGAACATCA
TTTGCAACATATTACATTGTCATTGCCGAGTAATTGTCAAATGTACTATGCAATGAAAGCAAATAGTGAACGAACAATCCTAGATACAATT
AGTCAGTATGTTGAAGGATTCGAAGTTGCATCTCAAGGTGAAATAGCAAAAGGTCTTGCTTTTAAACCAGCAAATCATATTATTTTTGGT
GGCCCTGGTAAGACAGACGAGGAACTAAGATATGCAGTAAGTGAAGGTGTTCAGCGTATTCATGTTGAAAGTATGCATGAATTACAACG
GCTAAATGCCATCTTAGAAGATGAAGATAAGACACAACACATTTTATTGCGTGTTAATTTAGCAGGACCATTTCCCAATGCAACCTTGCAT
ATGGCAGGACGCCCAACACAATTTGGTATTTCTGAAGACGAAGTTGATGATGTCATTGAAGCTGCGCTAGCAATGCCAAATATTCATCTA
GATGGCTTTCATTTTCATTCTATTTCTAACAATTTAGACTCGAATTTACACGTCGATGTAGTGAAACTTTATTTAAAAAAGCAAAATCATG
GTCTGAAAAACATCGATTTCCACTCAAACATATCAATCTTGGTGGTGGTATAGGGGTTAACTATGCAGATTTAACTAGCCAATTTGAGTG
GGATAATTTTGTAGAAAATTTTAAAACACTTATCGTTGAGCAAGAAATGGAAGATGTGACATTGAACTTTGAATGTGGGCGCTTTATTGT
GGCACATATTGGTTACTATGTGACAGAAGTGCTAGATATTAAGAAAGTGCATGGTGCTTGGTATGCCATTTTAAGAGGAGGTACGCAAC
AATTTAGACTGCCGGTATCTTGGCAGCATAACCATCCTTTTGAAATTTATCGCTATAAGGACAATCCATATTCATTTGAAAAAGTTTCAATT
TCGAGACAGGACACAACGTTAGTCGGTCAATTATGTACACCGAAAGATGTCTTTGCTAGAGAAGTACAGATAGACGCAATCAGTACAGG
CGACGTTATTGTTTTCAAATATGCAGGTGCATACGGATGGTCTATTTCACATCACGATTTCTTAAGCCATCCACATCCTGAATTTATTTATTT
AACGCAAACAAAGGAGGATGAATAA

Gene: sbnI (siderophore biosynthesis protein I)

Contig: 02_NODE_3, position: 46521 to 47285, length: 765 nt, orientation: FORWARD

Perfect match to: (08-02119-CP015645-[2112220:2112984:r], highly conserved allele)

Sequence:

TTGAATCATATTCATGAACATTTAAAATTGGTACCAGTAGATAAGATTGATCTTCACGAAACATTCGAACCTTTAAGATTGGAAAAGACGA
AAAGTAGTATTGAAGCAGATGATTTTATACGTCATCCTATTTTAGTGACAGCGATGCAACATGGTAGATATATGGTTATAGATGGTGTGC
ATCGGTATACAAGTTTGAAAGCGTTAGGATGTAAGAAAGTTCCAGTGCAAGAAATCCATGAAACACAATATTCAATTAGTACGTGGCAA
CATAAAGTTCCATTTGGTGTGTGGTGGGAAACGTTACAACAAGAACATCGCTTGCCATGGACTACTGAGACAAGACAAGAAGCGCCATT
TATTACGATGTGTCATGGTGATACAGAACAATATTTGTATACGAAAGATTTAGGCGAAGCACATTTTCAAGTATGGGAAAAGGTTGTCGC
AAGTTATAGTGGTTGTTGTTCTGTAGAGAGAATTGCACAAGGTACATATCCTTGTCTTTCTCAACAAGATGTACTCATGAAGTATCAGCCA
TTGAGTTATAAGGAAATTGAAGCGGTTGTTCATAAAGGGGAAACTGTGCCAGCAGGTGTGACACGCTTTAATATTTCAGGACGATGTCTT
AATCTTCAAGTACCACTGGCATTACTTAAACAAGATGATGATGTTGAACAACTGCGCAATTGGAAGCAGTTTTTAGCAGATAAGTTTGCC
AATATGAGATGCTATACTGAAAAAGTATACTTGGTGGAGCAATAG

Gene: Q5HJP4 (putative membrane protein)

Contig: 02_NODE_3, position: 47481 to 47888, length: 408 nt, orientation: FORWARD

Sequence:

ATGAGCTCAATTATAGGAAAAATAGCAATTTGGATAGGCATCGTAGCTCAAATATATTTTAGTGTCGTTTTTGTTAGGATGATATCTATTA
ATATTGCTGGAGGATCTGATTACGAAACAATTTTTTTATTAGGATTAATATTGGCTCTTTTCACTGTTTTACCAACCATCTTTACTGCGATTT
ATATGGAAAGTTACTCTGTAATCGGAGGTGCACTTTTTATTGTTTATGCTATTATTGCACTGTGTTTATATAATTTCTTTTCGTCAATTTTAT
GGCTGATTGGTGGTATTTTGCTGATTTGGAATAAATACTCAAAAGATGAATCGACAGACGAAAATGAAAAAGTTGATATTGAAAGTACA
GAGAATCAATTTGAATCTAAAGATAAAATCACTAAAGAATAA

Gene: Q5HJP3 (putative protein)

Contig: 02_NODE_3, position: 47938 to 48564, length: 627 nt, orientation: FORWARD

Sequence:

ATGGAAAAAAATGTAGAAAAATCATTCATAAAGATAGGTTTATATTTTCAAATAGCTTATATAGTACTCATGGCTATAACTTTATGTGGGT
TTGTAATTTGCTATGGACTAATTTTCGGCCTTTTCTATTTATTATCAGGTAGCACAGCTGATTATTCAATAGTAACAATAGTTATATCGGCA
ATAATTTCTATATTTGTAATTATACTTTCAATCGTACCTGTCATCGTATTGGCATCTGACTTATTTAAAGAAAGGATTTCAAAAGGTGTCAT
ATTAATTGTATTGGCTATTATCGCTTTAGTATTATGCAACTTTGTATCTGCAATACTCTGGTTTGTTTCAGCCATATCTATTTTAGGTAGAAA
AAAATTAGTATCTACAGCAGATACTACCACTATTCAAAAAAGTAAAGGGAACGAAAATCAAGCATCACATAAAGACACGTGTAAAAAGG
AACTTGATAGTCAAGACATGATGGAACATCCTGAGTTTAAAAATCCCACGACTAAAAACCTAGAAGGATTAAACGAAGAAATACATAAA
GATGAAGCTACAACTAAAGTTGACAGTGATAACACGCAACCGCCGATTGAATCAAAAGACCATGTCTCGAAAAAAGATTGA

Gene: butA (acetoin (diacetyl) reductase)

Contig: 02_NODE_3, position: 48776 to 49552, length: 777 nt, orientation: FORWARD

Perfect match to: (MW2-BA000033-[118486:119262], allele observed in CC1)

Sequence:

ATGACAAACAACAAAGTAGCGTTAGTAACTGGCGGAGCACAAGGGATTGGTTTTAAAATTGCAGAACGTTTAGTGGAAGATGGTTTCAA
AGTAGCAGTTGTTGATTTCAATGAAGAAGGGGCAAAAGCAGCTGCACTTAAGTTATCAAGTGATGGTACAAAAGCTATTGCTATCAAAG
CAGATGTATCAAACCGTGATGATGTATTTAATGCAGTAAGACAAACTGCAGCTCAATTTGGCGATTTCCATGTCATGGTTAACAATGCCG
GCCTTGGACCAACAACACCAATCGATACAATTACTGAAGAACAGTTTAAAACAGTATATGGCGTGAACGTTGCAGGTGTGCTATGGGGT
ATTCAAGCCGCACATGAACAATTTAAAAAATTCAATCATGGCGGTAAAATTATCAATGCAACATCTCAAGCAGGCGTTGAGGGTAACCCA
GGCTTGTCTTTATATTGCAGTACAAAATTCGCAGTGCGAGGTTTAACACAAGTAGCCGCACAAGATTTAGCGTCTGAAGGTATTACTGTG
AATGCATTCGCACCTGGTATCGTTCAGACACCAATGATGGAAAGTATCGCAGTGGCAACAGCCGAAGAAGCAGGTAAACCTGAAGCATG
GGGTTGGGAACAATTTACAAGTCAGATTGCTTTGGGCAGAGTTTCTCAACCAGAAGATGTTTCAAATGTAGTGAGCTTCTTAGCTGGTAA
AGACTCTGATTACATTACTGGACAAACAATTATTGTAGATGGTGGTATGAGATTCCGTTAA

Gene: galE (UDP-glucose 4-epimerase)

Contig: 02_NODE_3, position: 49897 to 50868, length: 972 nt, orientation: FORWARD

Perfect match to: (COL-CP000046-[127490:128461], allele observed in CC8+CC5+CC12+CC121+CC772)

Sequence:

TTGGAAAGAGTTTTGATAACTGGTGGGGCTGGTTTTATTGGGTCGCATTTAGTAGATGATTTACAACAAGATTATGATGTTTATGTTCTAG
ATAACTATAGAACAGGTAAACGAGAAAATATTAAAAGTTTGGCTGACGATCATGTGTTTGAATTAGATATTCGTGAATATGATGCAGTTG
AACAAATCATGAAGACATATCAATTTGATTATGTTATTCATTTAGCAGCATTAGTTAGTGTTGCTGAGTCGGTTGAGAAACCTATCTTATCT
CAAGAAATAAACGTCGTAGCAACATTAAGATTGTTAGAAATCATTAAAAAATATAATAGTCATATAAAACGTTTTATCTTTGCTTCGTCAG
CAGCTGTTTATGGTGATCTTCCTGATTTGCCTAAAAGTGATCAATCATTAATCTTACCATTATCACCATATGCAATAGATAAATATTACGGC
GAACGGACGACATTAAATTATTGTTCGTTATATAACATACCAACAGCGGTTGTTAAATTTTTTAATGTATTTGGGCCAAGACAGGATCCTA
AGTCACAATATTCAGGTGTGATTTCAAAGATGTTCGATTCATTTGAGCATAACAAGCCATTTACATTTTTTGGTGACGGACTGCAAACTAG
AGATTTTGTATATGTATATGATGTTGTTCAATCTGTACGCTTAATTATGGAACACAAAGATGCAATTGGACACGGTTATAACATTGGTACA
GGCACTTTTACTAATTTATTAGAGGTTTATCGTATTATTGGTGAATTATATGGAAAATCAGTCGAGCATGAATTTAAAGAAGCACGAAAA
GGAGATATTAAGCATTCTTATGCAGATATTTCTAACTTAAAGGCATTAGGATTTGTTCCTAAATATACAGTAGAAACAGGTTTAAAGGATT
ACTTTAATTTTGAGGTAGATAATATTGAAGAAGTTACAGCTAAAGAAGTGGAAATGTCGTGA

Gene: wcaJ-tuaA (capsular polysaccharide biosynthesis glycosyltransferase)

Contig: 02_NODE_3, position: 50831 to 51523, length: 693 nt, orientation: FORWARD

Perfect match to: (MW2-BA000033-[120541:121233], highly conserved allele)

Sequence:

TTGAAGAAGTTACAGCTAAAGAAGTGGAAATGTCGTGAAAATGACATTGAAGCTGTCCATAATAATAAGGGTTATGCCTATCAAAGAAA
ATTAGACAAACTAGAAGAAGTGAGAAAAAGCTATTACCCAATTAAACGTGCGATTGACTTAATTTTAAGCATTGTTTTATTATTTTTAACTT

TACCGATTATGGTTATATTCGCCATTGCTATCGTCATAGATTCGCCAGGAAACCCTATTTATAGTCAGGTTAGAGTTGGGAAGATGGGTA
AATTAATTAAAATATACAAATTACGTTCGATGTGCAAAAACGCAGAGAAAAACGGTGCGCAATGGGCTGATAAAGATGATGATCGTATA
ACAAATGTCGGGAAGTTTATTCGTAAAACACGCATTGATGAATTACCACAACTAATTAATGTTGTTAAAGGGGAAATGAGTTTTATTGGA
CCACGCCCGGAACGTCCGGAATTTGTAGAATTATTTAGTTCAGAAGTGATAGGTTTCGAGCAAAGATGTCTTGTTACACCAGGGTTAACA
GGACTTGCGCAAATTCAAGGTGGATATGACTTAACACCGCAACAAAAACTGAAATATGACATGAAATATATACATAAAGGTAGTTTAATG
ATGGAACTATATATATCAATTAGAACATTGATGGTTGTTATTACAGGGGAAGGCTCAAGGTAG

Gene: epsF (capsular polysaccharide biosynthesis glycosyltransferase)

Contig: 02_NODE_3, position: 51733 to 52899, length: 1167 nt, orientation: FORWARD

Sequence:

TTGAAAATTATATATTGTATTACTAAAGCAGACAATGGTGGTGCACAAACACATCTCATTCAACTTGCCAACCATTTTTGCGTACACCATG
ATGTTTATGTCATTGTAGGCAATCATGGACCAATGATTGAACAACTAGATGCAAGAGTTAATGTAATTATTATCGAACATTTAGTAGGAC
CAATTGACTTTAAACAAGATATTTTAGCTGTCAAAGTGTTAGCGCAGTTATTCTCGAAAATTAAACCTGATGTTATCCATTTACATTCTTCC
AAAGCTGGAACGGTCGGACGAATTGCGAAGTTCATTTCGAAATCGAAAGACACACGTATAGTTTTTACTGCACATGGATGGGCTTTTACA
GAGGGTGTTAAACCAGCTAAAAAAATTTCTATATTTAGTTATCGAAAAATTAATGTCACTTATTACAGATAGCATTATTTGTGTTTCAGATTT
CGATAAACAGTTAGCGTTAAAATATCGATTTAATCGATTGAAATTAACCACAATACATAATGGTATTGCAGATGTTCCCGCTGTTAAGCAA
ACGCTAAAAAGCCAATCACATAACAATATTGGCAAAGTAGTTGGAATGTTGCCTAATAAACAAGATTTACAGATTAATGCCCCGACAAAG
CATCAATTTGTTATGATTGCAAGATTTGCTTATCCAAAATTGCCACAAAATCTAATCGCGGCAATAGAGATATTGAAATTACATAACAGTA
ATCATGCGCATTTTACATTTATAGGCGATGGACCTACATTAAATGATTGTCAGCAACAAGTTGCACAAGCTGGGTTAGAAAATGATGTCA
CATTTTTGGGCAATGTCATTAATGCGAGTCATTTATTATCACAATACGATACGTTTATTTTAATAAGTAAGCATGAAGGTTTGCCAATTAGC
ATTATAGAAGCTATGGCTACAGGTTTGCCTGTTATAGCCAGTCATGTTGGCGGTATTTCAGAATTAGTAGCTGATAATGGTATATGTATG
ATGAACAATCAACCAGAAACTATTGCCAAAGTCTTGGAAAAATATTTAATAGACAGTGATTACATCAAAATGAGTAATCAATCTAGAAAA
CGTTATTTAGAATGTTTTACTGAGGAGAAAATGATTAAAGAAGTGGAAGACGTTTATAATGGAAAATCAACACAATAG

Gene: wzy (capsular polysaccharide polymerase)

Contig: 02_NODE_3, position: 52880 to 54118, length: 1239 nt, orientation: FORWARD

Sequence:

ATGGAAAATCAACACAATAGTAAATTACTAACATTGTTACTTATCGGTTTAGCGGTTTTTATTCAGCAATCTTCGGTTATTGCCGGTGTGA
ATGTTTCTATAGCTGACTTTATCACATTACTAATATTAGTTTATTTACTGTTTTTCGCTAACCATTTATTAAAGGCAAATCATTTTTTACAGTT
TTTCATTATTTTGTATACATATCGTATGATTATTACGCTTTGTTTGCTATTTTTTGATGATTTGATATTTATTACGGTTAAGGAAGTTCTTGCA
TCTACAGTTAAATATGCATTTGTAGTCATTTATTTCTATTTAGGGATGATCATCTTTAAGTTAGGTAATAGCAAAAAAGTGATCGTTACCTC
TTATATTATAAGCAGTGTGACTATAGGTCTATTTTGTATTATAGCTGGTTTGAACAAGTCCCCTTTACTAATGAAATTGTTATATTTTGATG
AAATACGTTCAAAAGGATTAATGAATGACCCTAACTATTTCGCGATGACACAGATTATTACATTGGTACTTGCTTACAAGTATATTCATAA
TTACATATTCAAGGTCCTTGCATGTGGTATTTTACTATGGTCTTTAACTACAACGGGGTCTAAGACTGCGTTTATCATATTAATCGTCTTAG
CCATTTATTTCTTTATTAAAAAGTTATTTAGCAGAAATGCGGTAAGTGTTGTGAGTATGTTAGTGATTATGCTGATATTACTTTGTTTTACT
TTTTATAATATTAACTACTATTTATTCCAATTAAGCGACCTTGATGCCTTACCGTCATTAGATCGAATGGCGTCTATTTTTGAAGAGGGGTT
TGCATCATTAAATGATAGTGGATCTGAGCGAAGTGTTGTATGGATAAATGCCATTTCAGTAATTAAATATACATTAGGTTTTGGTGTTGGC
TTAGTGGATTATGTACATATTGGCTCGCAAATTAATGGTATTTTACTTGTTGCCCATAATACATATTTGCAAATTTTTGCGGAATGGGGCAT
TTTATTCGGTGCGTTATTTATCATATTTATGCTTTATTTACTGTTTGAATTATTTAGATTTAACATTTCTGGGAAAAATGTAACAGCAATTGT
TGTAATGTTGACGATGCTGATTTACTTTTTAACAGTATCATTTAATAACTCAAGATATGTCGCTTTTATTTTAGGAATTATCGTCTTTATTGT
TCAATATGAAAAGATGGAAAGGGATCGTAATGAAGAGTGA

Gene: wzx (capsular polysaccharide extrusion protein)

Contig: 02_NODE_3, position: 54108 to 55538, length: 1431 nt, orientation: FORWARD

Perfect match to: (144_S7-CP010943-[67185:68615], allele observed in CC772+CC72+CC772)

Sequence:

ATGAAGAGTGATTCACTAAAAGAAAATATTATTTATCAAGGGCTATACCAATTGATTAGAACGATGACACCACTGATTACAATACCCATTA
TTTCACGTGCATTTGGTCCCAGTGGTGTGGGTATTGTTTCATTTTCTTTCAATATCGTGCAATACTTTTTGATGATTGCAAGTGTTGGCGTT
CAGTTATATTTTAATAGAGTTATCGCGAAGTCCGTTAACGACAAACGGCAATTGTCACAGCAGTTTTGGGATATCTTTGTCAGTAAATTAT

TTTTAGCGTTAACAGTTTTTGCGATGTATATGGTCGTAATTACTATATTTATTGATGATTACTATCTTATTTTCCTACTACAAGGAATCTATA
TTATAGGTGCAGCACTCGATATTTCATGGTTTTATGCTGGAACTGAAAAGTTTAAAATTCCTAGCCTCAGTAATATTGTTGCGTCTGGTATT
GTATTAAGTGTAGTTGTTATCTTTGTCAAAGATCAATCAGATTTATCATTGTATGTATTTACTATTGCTATTGTGACGGTATTAAACCAATT
ACCTTTGTTTATCTATTTAAAACGATACATTAGCTTTGTTTCGGTTAATTGGATACACGTCTGGCAATTGTTTCGTTCGTCATTAGCATACTT
ATTACCAAATGGACAGCTCAACTTATATACTAGTATTTCTTGCGTTGTTCTTGGTTTAGTAGGCACATACCAACAAGTTGGTATCTTTTCTA
ACGCATTTAATATTTTAACGGTCGCAATCATAATGATTAATACATTTGATCTTGTAATGATTCCGCGTATTACCAAAATGTCTATCCAGCAA
TCACATAGTTTAACTAAAACGTTAGCTAATAATATGAATATTCAATTGATATTAACAATACCTATGGTCTTTGGTTTAATTGCAATTATGCC
ATCATTTTATTTATGGTTCTTTGGTGAGGAATTCGCATCAACTGTCCCATTGATGACCATTTTAGCGATACTTGTATTAATCATTCCTTTAAA
TATGTTGATAAGCAGGCAATATTTATTAATAGTGAATAAAATAAGGTTATATAATGCGTCAATTACTATTGGTGCGGTAATGAATTTAGTA
TTATGTCTTGTTTTGATATATTTTTATGGAATTTACGGTGCGGCTATTGCACGTTTAATTACAGAGTTTATCTTACTTATTTGGCGATTTGTT
GATATTACTAAAATCAATGTGAAGTTGAATATTGTAAGTACGATTCAATGTGTCATTGCTGCCGTTATGATGTTTATTGTGCTTGGTGTGG
TCAATCATTATTTGCCCCCTACAATGTACGCTACGCTGCTATTAATTGCGATTGGTATAGTAGTTTATCTTTTATTAATGATGACTATGAAA
AATCAATACGTATGGCAAATATTGAGGCTTCTTCGACATAAAACAATTTAA

Gene: sodA-L1 (superoxide dismutase, locus 1)

Contig: 02_NODE_3, position: 55806 to 56405, length: 600 nt, orientation: FORWARD

Perfect match to: (11819-97-CP003194-[141261:141860], highly conserved allele)

Sequence:

ATGGCATTTAAATTACCAAATTTACCATATGCATATGATGCATTGGAACCATATATAGATCAAAGAACAATGGAGTTTCATCACGACAAAC
ATCACAATACGTACGTGACGAAATTAAACGCAACAGTTGAAGGAACAGAGTTAGAGCATCAATCATTAGCGGATATGATTGCTAACTTA
GACAAGGTACCGGAAGCGATGAGGATGTCAGTCCGTAATAATGGCGGTGGTCATTTTAACCATTCATTATTCTGGGAAATACTATCACCT
AATTCTGAAGAAAAAGGTGGCGTAATAGATGACATCAAAGCGCAGTGGGGCACTTTAGATGAATTTAAAAATGAATTTGCAAATAAAGC
AACAACATTATTTGGATCAGGTTGGACTTGGTTAGTTGTTAATGATGGCAAATTAGAAATTGTGACAACGCCAAACCAAGATAATCCATT
AACAGAAGGCAAAACACCAATCTTACTATTTGATGTTTGGGAGCATGCCTACTATCTGAAATATCAAAATAAACGTCCAGACTATATGACT
GCATTTTGGAATATTGTTAACTGGAAAAAAGTTGATGAATTATACCAAGCAGCAAAATAA

Gene: sasD (Staphylococcus aureus surface protein D)

Contig: 02_NODE_3, position: 56773 to 57498, length: 726 nt, orientation: FORWARD

Perfect match to: (MW2-BA000033-[126483:127208], allele observed in CC1+CC12+CC20+CC72)

Sequence:

ATGAAAAAATTAGCAACAGTAGGTTCTTTAATTGTAACAAGCACTTTAGTATTCTCAAGTATGCCTTTTCAAAATGCGCATGCCGACACAA
CTTCAATGAATGTGTCGAATAAACAAAGCCAAAATGTACAAAATCATCGTCCTTATGGCGGAGTAGTACCACAAGGAATGACGCAAGCA
CAATATACTGAATTAGAGAAAGCTTTATCCCAATTAAGCGCTGGCAGTAATATGCAAGACTATAATATGAAATTGTATGATGCGACGCAA
AATATTGCTGATAAATACAATGTGATAATTACAACTAATGTAGGGGTATTTAAACCACATGCTGTTAGAGATATGAATGGCCATGCGTTA
CCTTTAACAAAAGATGGCAATTTTTATCAAACGAATGTAGATGCAAATGGTATTAATCATGGTGGTAGTGAAATGGTGCAAAATAAAACA
GGTCATATGAGTCAACAAGGCCATATGAATCAGAACACACACATGAACCAACAGCCACACATGCAACAAGGTCATATGCAATCATCAAA
CCATCAAATGATGAGTCCAAAAGCAAATATGCATTCATCAAATCATCAAATGAACCAAAGTAACAAAAAAGTTTTACCAGCTGCTGGTGA
AAGTATGACATCAAGTATTCTTACTGCAAGTATTGCCGCACTACTATTAGTATCTGGGTTATTCTTAGCATTTAGACGACGTTCAACAAAT
AAATAA

Gene: Q5HJN3 (transcriptional regulator, GntR family)

Contig: 02_NODE_3, position: 57689 to 58444, length: 756 nt, orientation: REVERSE

Perfect match to: (MRSA252-BX571856-[151160:151915:r], allele observed in CC30+CC1+CC5+CC8+CC239)

Sequence:

TTAATAATCCGATTGTCTTATACGTGTCAGTGTTAATTCAGAGATTTCCTGTGGAATATACCACTTATTAATCATAATTGGATAAGGTGTTT
GTGCGTACAATGTTTCAATAATCAGCCAACAATGTGTATCACCATCAAACACGTGACTATGATTTTTGAAGTGGGGCGCTTTGGTAATAG
ACATTTTTAAATCTGATTGATATGCATTGCTATAAATCGTTTGCTCAACGAATGTCTTCATGTCGTCTTCGTTTTGTGTATTCACTTTAAATG
TGTCAATGACATTTAACGGTATAAAGGTAAAGCAAAATGCATCAGCTTGCTTAGAATGATTGTCCTTTTTTTGATAATAGCGTTCCATTGC

AATGACGGCAGAAGGATGGTTTGCAAACAAATGATTTGTATATTCACTTTCTAAATCAACACGATAATTAATTGATGACATAGATACGCG
AGCTAGCAATATTTGATCAAGTGGATGCTTAAATTGATCCATACTTGAAGCGTGTTGGGCATTTGTTTGTGGAATAACAAAGTGTCCCTTC
CCTCTTGTACTCTCTACGATGCCATCTTCGGCTAACAATTTTATAGCTTGGCGCAAAGTCATACGACTGACATCAAAGCGCGCACAAAGTT
CCTTTTCAGTAGGTAATGCATGGCCACTCGGATATTTTCCTATTTGAATTTCTTTATATAACGTATTATAAATCGTTAAAAATTTTGGTTGTG
TTTGCGTCACGTAGACAACCTCCAT

Gene: deoD-L1 (purine nucleoside phosphorylase locus 1)

Contig: 02_NODE_3, position: 58680 to 59387, length: 708 nt, orientation: FORWARD

Perfect match to: (ED98-CP001781-[113469:114176], allele observed in CC5+CC8+CC72)

Sequence:

ATGAAATCAACACCACACATTAAACCAATGAATGACGTCGAAATTGCAGAAACGGTTCTATTGCCAGGAGATCCGTTAAGAGCTAAGTTC
ATTGCAGAAACTTATTTGGATGATGTGGAACAGTTCAATACAGTGCGAAACATGTTTGGTTTTACCGGAACATATAAAGGTAAAAAAGTT
TCTGTCATGGGTTCAGGTATGGGTATGCCATCTATTGGCATTTACTCTTATGAATTAATTCATACATTTGGTTGTAAAAAATTAATTCGCGT
TGGTTCTTGTGGCGCGATGCAAGAAAACATTGATTTATATGATGTGATTATTGCACAAGGTGCCTCTACTGATTCAAATTACGTTCAACAA
TATCAATTACCAGGTCATTTTGCGCCAATTGCTTCTTATCAATTATTAGAAAAAGCAGTTGGAACAGCACGTGACAAAGGTGTACGTCATC
ATGTAGGTAATGTGTTATCAAGTGATATTTTCTATAACGCGGATACAACAGCGAGTGAACGTTGGATGCGTATGGGTATTTTAGGTGTAG
AAATGGAATCAGCTGCATTATACATGAATGCAATTTACGCTGGTGTCGAAGCATTAGGTGTGTTCACAGTGAGCGATCATTTAATTCATG
AAACGTCAACAACACCTGAGGAAAGGGAACGTGCATTTACAGATATGATTGAAATTGCACTGTCATTGGTTTAG

Gene: tet38 (major facilitator superfamily permease)

Contig: 02_NODE_3, position: 59394 to 60746, length: 1353 nt, orientation: FORWARD

Perfect match to: (MW2-BA000033-[129104:130456], allele observed in CC1+CC25+CC45I+CC72)

Sequence:

ATGAATGTTGAATATTCTAAAATAAAGAAAGCAGTACCTATTTTATTATTCTTATTTGTATTCAGTTTGGTTATAGACAACTCATTTAAATT
GATTTCTGTAGCCATTGCTGATGACTTAAACATTTCTGTAACGACAGTAAGTTGGCAAGCGACATTAGCCGGTTTAGTAATTGGTATTGGC
GCTGTAGTATACGCTTCATTATCTGATGCCATTAGTATACGCACACTATTTATTTATGGCGTGATATTAATCATTATCGGATCAATTATTGG
TTACATTTTCCAACATCAATTCGCATTACTTTTAGTTGGACGTATTATTCAAACTGCCGGTTTAGCCGCTGCAGAGACATTATATGTGATAT
ATGTTGCAAAGTATCTTTCTAAAGAGGACCAGAAGACTTACCTTGGCTTAAGTACGAGCAGTTATTCCTTGTCATTAGTTATCGGTACATT
ATCAGGTGGATTTATTTCTACGTATTTACACTGGACAAATATGTTTTTAATTGCATTAATCGTAGTATTTACGTTGCCATTCCTATTTAAATT
ATTACCAAAAGAAAATAATACGAATAAAGCTCATTTAGATTTTGTTGGCTTAATTCTAGTGGCAACTATTGCTACAACAGTCATGCTGTTT
ATTACGAACTTTAATTGGTTATATATGATTGGTGCCTTAATTGCGATTATCGTTTTTGCGCTATATATTAAAAATGCGCAACGTCCATTAGT
AAATAAATCATTTTTCCAAAATAAACGTTATGCTTCATTTTTATTTATAGTATTTGTAATGTATGCTATCCAATTGGGTTATATTTTTACGTTC
CCATTCATAATGGAGCAAATTTATCATCTGCAACTAGACACAACATCACTGTTATTAGTACCGGGTTATATAGTAGCAGTCATCGTTGGTG
CACTAAGTGGTAAAATCGGCGAATATCTGAATTCAAAACAAGCGATTATCACAGCAATTATTTTAATCGCACTGAGCTTGATTTTACCTGC
ATTTGCAGTAGGTAATCACATTTCAATCTTCGTCATTTCTATGATATTCTTTGCAGGTAGCTTTGCTTTAATGTATGCACCTTTACTTAACGA
AGCCATTAAAACAATAGATCTTAATATGACAGGTGTGGCTATTGGTTTTTATAATTTAATTATTAATGTGGCGGTATCTGTAGGTATTGCG
ATTGCTGCGGCTCTAATCGATTTTAAAGCATTAAATTTCCCAGGCAATGATGCATTAAGTTCACATTTCGGTATTATTTTAATTATTTTAGG
TTTAATGAGTATTGTCGGATTAGTTTTATTCGTCAGCTTAAATCGTTGGACACAATCTGAAAAATAA

Gene: deoC-L1 (deoxyribose-phosphate aldolase locus 1)

Contig: 02_NODE_3, position: 60827 to 61489, length: 663 nt, orientation: FORWARD

Perfect match to: (CN1-CP003979-[120453:121115], allele observed in CC72+CC8+CC72+CC361)

Sequence:

ATGAAATTTGAGAAATATATAGATCACACTTTATTGAAGCCTGAGTCAACACGTACGCAAATCGATCAAATCATCGATGAAGCGAAAGCA
TACAATTTTAAATCTGTATGTGTGAATCCAACACATGTTAAATATGCAGCAGAGCGACTAGCTGATTCAGAGGTGCTCGTTTGTACGGTA
ATAGGATTCCCATTAGGTGCGTCGACAACTGCAACGAAAGCATTTGAAACAGAAGATGCAATTCAAAATGGTGCAGATGAAATTGACAT
GGTCATCAACATCGGCGCATTAAAAGATGGACGTTTTGATGATGTACAACAAGACATTGAAGCAGTGGTTAAAGTTGCGAAAGGTCACA
CAGTAAAAGTGATTATTGAGACGGTATTGTTGGACCATGACGAAATTGTAAAAGCGAGTGAATTAACAAAAGCGGCTGGTGCGGACTTC

GTTAAAACTTCAACAGGTTTTGCAGGTGGCGGTGCGACTGCAGAAGACGTTAAATTAATGAAAGATACAGTAGGTGCTGATGTAGAAGT
AAAAGCATCAGGTGGCGTACGTAATTTAGAAGATTTCAATAAAATGGTTGAAGCAGGTGCGACACGTATTGGTGCGAGCGCAGGTGTTC
AAATTATGCAAGGTTTAGAAGCAGATTCAGATTACTAA

Gene: deoB (phosphopentomutase)

Contig: 02_NODE_3, position: 61517 to 62695, length: 1179 nt, orientation: FORWARD

Perfect match to: (JH9-CP000703-[147606:148784], allele observed in CC5)

Sequence:

ATGACAAGACCATTTAATCGTGTACATTTAATCGTAATGGATTCAGTAGGTATTGGTGAAGCGCCAGACGCAGCTGATTTTAAAGATGAA
GGTTCACATACTTTAAGACATACCTTAGAAGGTTTCGATCAAACTTTACCAAACCTTGAAAAGTTAGGTCTAGGGAACATCGATAAATTAC
CAGTAGTAAATGCAGTTGAACAACCAGAAGCATACTATACTAAATTGAGTGAAGCTTCAGTTGGTAAAGATACAATGACTGGTCACTGG
GAAATTATGGGTTTAAATATTATGCAACCTTTTAAAGTATACCCTAATGGATTCCCTGAAGAGTTAATTCAACAAATTGAAGAAATGACAG
GTCGTAAAGTTGTTGCTAACAAACCGGCATCGGGTACGCAAATTATCGATGAGTGGGGCGAGCACCAAATGAAAACTGGTGACTTAATT
GTTTATACAAGTGCAGACCCAGTATTGCAAATTGCTGCACATGAAGACATTATCCCATTAGAAGAGTTATATGATATTTGTGAAAAGGTT
CGTGAGTTGACAAAAGACCCTAAATATTTAATTGGTCGTATTATCGCACGTCCATATGTTGGTGAACCAGGAAACTTTACACGTACATCTA
ATCGACATGACTATGCGTTAAAACCTTTTGGTAAAACTGTCTTAGATCATTTGAAAGACGGTGGTTATGATGTTATTGCCATCGGTAAAAT
TAATGACATTTATGATGGTGAAGGTGTAACAGAAGCGGTTCGTACGAAGAGTAACATGGACGGTATGGATCAATTGATGAAAATTGTTA
AGAAAGATTTCACAGGTATTAGCTTCTTAAACTTAGTAGACTTTGATGCATTATACGGTCATCGTCGTGATAAACCAGGTTATGCACAAGC
AATTAAAGATTTCGATGATCGCTTGCCAGAACTGTTTAGCAACTTAAAAGAAGACGATTTAGTAATTATTACAGCAGACCATGGTAATGA
CCCGACAGCGCCAGGTACGGACCATACGAGAGAATATATCCCAGTAATTATGTACAGTCCGAAATTTAAAGGTGGTCATGCACTAGAAA
GTGATACTACATTCAGTTCTATCGGTGCAACTATAGCAGATAATTTCAACGTAACATTACCAGAGTTCGGTAAAAGTTATTTAAAGGAATT
GAAATAG

Gene: phnE2 (phosphonate ABC transporter, transmembrane permease)

Contig: 02_NODE_3, position: 62826 to 63641, length: 816 nt, orientation: REVERSE

Sequence:

TTAAACAATACGTTTTCGGATTGAACCGGAAATTAAATCTACAATTGCGACCATTAGTACTAAACCGATTAATATAATACCTACACGGTCC
CAAGAACGTGTTTGAATGGCAAATATGAGTGGTGTCCCGATACCACCAGCCCCAATTAGCCCCAGTATAGAAGCTGAACGTAAGTTTAGT
TCAAAGCGATAAAGTATGAGAGATAGAAAGGCAGGCATAATTTGTGGTATGACTGCAAATACGAGTGTTTTAATCTTATTCGCACCACTG
GCCTTTAATGATTCTATAGCACTGAAATCTAGACCTTCAATATCTTCAGCTAAAAGTTTCCCAAGCATACCTACAGAATGGATACCTAAAG
CTAATACACCTGAAAATGAACCTGGGCCAACAGCTTTGATAAATATAAGTGCCATTACAATTTCTGGGAAGACACGTATAACACTTAAAA
TAAATTTGCTAACACCTGAAACCGGGCGTAACTTTACCATATTATTTGTACCTAGAAATGCTAATGGAATACAGATAATTGCGGCGATGA
AAGTACCTACAACGGCTATCGCAAAGGTTTCAAGTAAACCACGTAATAAGTCTTCGCCATCTGGTATATAGATATAGCTGATGTCAGGAT
GGAATAATCCGCTGAATATGGATTTTAAGATTTCTAATGATTTACTTTTAAGTTCTAAACTTGGTACACCTGCAAATGCCCAGATGATAAT
AGCTAAGACAACAATTGCAATAAGCCATCTTTTAATCAATTTTCGTTTGTGTGCTTTTGTGTGAACATTATATTTTGCTATTTCCTGTGTCAT

Gene: phnE1 (phosphonate ABC transporter, transmembrane permease)

Contig: 02_NODE_3, position: 63638 to 64438, length: 801 nt, orientation: REVERSE

Perfect match to: (MW2-BA000033-[133348:134148:r], allele observed in CC1+CC6)

Sequence:

TCATGCGAGATGTGCCCTCACTTTCGTACTGATGTAATCAATGACGACGACAATAACTAAAGTAAATAAAATAATCGTTGCTGTTTTTGGA
TATTGAAATAAACCAAGTGTTTGATCATAAAACAATCCAATACCGCCAGCGCCGACTAATCCAAGCACAGCTGAAGCACGTATATTTACTT
CAAATGCATATAATACGTATGACATAAATGACGATATGGCTTGTGGTACAACACCGAAAACAATCCATTTTATTTTATTAGCGCCAACAGC
CGTCATTGCTTCCATTGGACCTGGATCTATCGTTTCCAATGATTCATATAATAATTTTCCAATAATACAGATAGTTAAAATAAACAGTGCTA
ATATCCCTGGAATTTGACCGATTCCAAATACAGCCACAAAGATTGCTGCTAATAGCAAATCCGGTATAGTACGAACTATATTTAAAATAAA
ACGCGAGGGTATTGAAATCCACTTTTGATGAACGATATTGCTAGCACATAATAACGCAATTGGTATTGAAACGATGCTACCTAATACTGT
ACTTACGATAGCCATTCGAATAGTATCTAACATTGGCGTTGTAATTTGTTGTAAATACTCGAAATCAGGTGGAATCATTTGTTTGAATAGA

TCACCTATTTGAGGTATTCCTATCATTAAATCTCCAAAATTAAACCCCGTATAAATGAAGCTCCAAATGATAAGCACAATGATTAACATGA
AGGTAAAACTCGTTTTTAAAGAAACCTTTTTCTTTAAAAGGGAGTCATACTTTGTAGGTGTTTCTAAAGGCAT

Gene: phnC (phosphonate ABC transporter, ATP-binding protein)

Contig: 02_NODE_3, position: 64440 to 65213, length: 774 nt, orientation: REVERSE

Perfect match to: (MW2-BA000033-[134150:134923:r], allele observed in CC1)

Sequence:

TTAGTTCACTCCTAGCTTTTCATCTTCTTTAATTGTACGTCCATATATTTCACTAAATACGTCATCTGTTGCTTCAGATGCAGGACCATCATA
GACAACCTCACCATCACGTAAACCAATGATGCGTGTGCCATATTCTTTTGCTAAGTCAACAAAATGTAAGTTAATTAAAATTGTGATGCCT
AATTCTTGGTTGATTTTTCTTAAATCATCCATAACCTGTTTCGTAGTTAATGGGTCTAATGAAGCAACTGGCTCATCTGCAAGAATAATTTC
AGATTCTTGGCATAGTGCACGTGCAATAGATATACGTTGTTGTTGACCTCCTGATAATTCATCAGAGCGTTGATTATATTTATCTAAGATG
TTGACCCGTTCTAGTGCATCCATTGCTTTAATTTTGTCTTCTTTTGGGAATAAACCTAATACCATTTTCCAAGTAGGGTGATAACCTACACG
TCCACTTAGTACATTTCGTAATACACTTGACCGTTTAACTAAATTAAAATGTTGGAAAATCATACCTATATTTCGGCGCATTTCTAATAGTG
CTTTACCATGGGCTTTAGTGATTGATTTACCTTGGATGAAAATTTCACCTGACGTGATATCATGCAAACGATTTACAGATCTTAATAACGT
GGATTTCCCAGCACCAGATAGTCCGACAATGACTGCAAATTCACCTTTTTCAATATTTAAGTTAATATTTTTCAAGCCTACATGACCGTTAG
GATAGACTTTACTGACGTTTTTAAATTCGATTTGACTCAT

Gene: phnB (phosphonate ABC transporter, substrate-binding protein)

Contig: 02_NODE_3, position: 65427 to 66383, length: 957 nt, orientation: REVERSE

Perfect match to: (TCH70-ACHH02000005-[353692:354648], allele observed in CC1)

Sequence:

TTATTTCATATCTTTAACTAATTTTTCGTACTCTCTTACAATGTCGAAATTTGAATCTTTCGTTTCTGTGTATCCTTCATGTGAATAAACTTCG
CTAATAATTTTGTGACCTTCTTTTGATTTAGCAATGTCTATAAAAGCTTTTTTCAATTTTTCTTGGAAATCTTTATCCATATCTGGTCTTACAG
AAATTGTGTCATTCGGAATAGCTTGTGTTAATTTTAAAATTCGTGTGTCTTTAAATACATTTGGTTGGTCTTTTTTCACAGTATTACGTGCAT
CGTTAAATACAGCCGCAGCATCTACATCTCCATTTAATAATGAGATAACTGCTTGGTCATGACCTTTAACATTCACAATTTTCATATCTTTA
GTTGCATTAATACCTGCTTCGTTTTTTAACATCGCAAGTGGGAATGTATATCCAGCAGTTGATGTTACATCTTGTAAGGCAATTTTCTTACC
TTTTAAATCTTTCAAGCTTTTAATTTTTGAGTCTTTTTTAACAAGAATTTCTGACTTATAACTATCTACAAGTTCTTTACTTGCTGAACCATCT
TCTTTTACACCGAAACGTTGTGCTTGTAATAATAAATCAGCTGCTTTTTGATCATGTGCTAATGTGTATGCCGTTGGTGGTAAGAAACCAA
CATCAACTTTTTTAGACTTCATAGCTTCAACAATTGTATTGTAGTTAGTTGATACAGACACTTTAACTGGAATCCCTAATTCTTTAGATAGT
AATTTTTCTAATGGTTTTGCTTTAGCCTCTAATGTTCCAGCATTTTGCGAAGGTACAAATTGTACTGTTAATTCTTTAGGTTTGTATCCTCCT
GATTTAGAATCCGAATCATTACTAGCGTTCTTTTGATTATCTAAAGAACTTGAATTTCCACATGCTGCTGCAAAAACAATGACTGCTAACAT
TAATACAAATAAACACTTAAATTTTTTCAT

Gene: Q5HJM4 (putative exported protein)

Contig: 02_NODE_3, position: 66612 to 68156, length: 1545 nt, orientation: FORWARD

Perfect match to: (Mu50-BA000017-[158798:160342], allele observed in CC5+CC8)

Sequence:

ATGAAAAAAAATATATAAGTCATTAACTGTCTCTGCAATTGTTGCAACGGTATCATTAAGTGCTTTACCGCAATCTTTAGCTATAACGCATG
AATCGCAACCTACAAAGCAACAGCGAACGGTATTATTCGATCGTTCTCATGGTCAAACAGCTGGTGCTGCAGATTGGGTTAGTGATGGT
GCATTTTCAGATTATGCGGATTCAATACAAAAACAAGGTTATGACGTTAAAGCTATTGATGGTCATTCGAACATAACAGAAGCAAGTTTG
AAAAGTTCTAAAATATTTGTAATTCCTGAGGCTAATATTCCTTTCAAAGAATCAGAACAGGCAGCAATTGTTAACTATGTGAAACAAGGT
GGCAATGTTGTCTTTATTTCAGATCATTACAATGCTGACCGAAATTTAAATCGTATTGATTCATCAGAGGCAATGAATGGTTATCGACGTG
GAGCATATGAAGATATGTCGAAAGGTATGAATGCAGAAGAAAAAAGTTCTACTGCAATGCAAGGTGTGAAAAGTTCAGATTGGTTATCT
ACAAACTTTGGCGTACGTTTTCGATATAATGCACTAGGTGATTTAAATACGAGCAATATTGTTTCTTCAAAAGAGAGTTTCGGTATTACTG
AAGGTGTGAAATCTGTCTCTATGCATGCCGGATCAACATTAGCAATTACTAATCCAGAGAAAGCAAAAGGTATTGTGTATACACCAGAAC
AATTGCCAGCGAAAAGTAAATGGTCACATGCTGTAGATCAAGGTATTTATAATGGGGGCGGTAAAGCAGAAGGCCCCTATGTAGCAATT
TCTAAAGTTGGAAAAGGTAAAGCAGCATTTATCGGTGATTCATCACTTGTGGAAGATAGTTCGCCCAAATATGTAAGAGAAGATAATGG
AGAAAAGAAGAAAACATATGATGGTTTTAAAGAACAAGACAACGGTAAGCTATTAAATAATATAACGGATTGGATGTCTAAAGATAGTG

ATGGGAAATCACTTAAGGCGAGTGGACTAACATTAGATACAAAGACTAAGTTGCTTGATTTTGAACGACCAGAGCGTTCAACTGAGCCT
GAAAAAGAGCCATGGTCACAACCGCCGAGTGGTTATAAATGGTATGATCCAACAACATTTAAAGCAGGTAGTTATGGCAGCGAAAAAG
GCGCAGATCCTCAGCCAAACACACCAGATGATCATACGCCACCAAATCAGAACGAAAAAGTAACATTTGATATCCCGCAAAATGTTTCTG
TAAATGAGCCATTTGAAATGACAATACATTTAAAAGGATTTGAAGCAAATCAAACACTTGAAAATCTTAGAGTTGGTATTTACAAAGAAG
GCGGACGTCAAATCGGACAATTTTCAAGTAAAGATAACGATTATAACCCACCAGGTTACAGTACTTTGCCAACAGTTAAAGCAGATGAAA
ACGGAAATGTCACAATTAAGGTCAATGCTAAAGTACTTGAAAGTATGGAAGGTTCAAAGATTCGTTTAAAACTCGGTGACAAAACCTTGA
TTACAACAGACTTCAAATAA

Gene: cpdB (putative 5' nucleotidase)

Contig: 02_NODE_3, position: 68207 to 69742, length: 1536 nt, orientation: FORWARD

Perfect match to: (Strain_21193-AFEG01000009-[314024:315559:r], allele observed in CC25+CC20+CC25+CC188+CC361)

Sequence:

ATGTCAAACATAGCATTTTATGTCGTGAGTGACGTACATGGTTATATTTTCCCAACAGATTTTACGAGTAGAAATCAATATCAACCTATGG
GATTGTTACTAGCGAATCATGTTATAGAACAAGACAGAAGGCAGTATGACCAAAGTTTTAAAATAGATAATGGTGATTTTTTGCAAGGGT
CACCATTTTGTAATTACTTAATCGCGCATAGCGGCAGTAGCCAGCCTTTAGTTGATTTTTATAATCGAATGGCATTCGACTTTGGTACGCTT
GGTAATCATGAATTTAATTATGGATTGCCATACTTAAAAGACACTTTACGCAGACTCAATTATCCAGTTTTGTGCGCTAATATATATGAAA
ATGATAGTACATTGACTGATAACGGTGTGAAGTATTTTCAGGTTGGAGATCAAACTGTTGGTGTGATAGGTTTAACGACACAATTTATTC
CCCATTGGGAACAACCAGAGCATATTCAGTCACTTACGTTTCATAGTGCTTTTGAAATACTTCAACAATACTTACCTGAAATGAAGCGACA
TGCAGATATCATTGTGGTTTGTTACCATGGTGGATTTGAAAAGGATTTAGAAAGTGGTACGCCGACCGAAGTATTAACGGGTGAAAATG
AGGGATATGCCATGTTAGAAGCGTTTTCTAAAGATATAGATATCTTTATTACGGGTCACCAACATCGACAAATTGCTGAAAGGTTTAAGC
AAACGGCTGTGATTCAACCTGGTACGAGAGGTACAACTGTAGGCAGAGTAGTCTTGAGTACTGATGAATATGAAAATCTATCCGTTGAA
TCATGTGAATTACTTCCTGTTATAGATGATTCCACATTTACTATTGATGAAGATGACCAACATTTACGAAAGCAGTTAGAGGACTGGTTAG
ATTACGAAATTACTACATTGCCATATGATATGACGATTAATCATGCATTTGAGGCACGTGTGGCACCGCATCCTTTTACAAATTTTATGAA
TTACGCTTTATTAGAAAAAAGTGACGCAGATGTTGCCTGTACAGCTTTGTTTGATTCTGCTAGTGGTTTCAAGCAAGTCGTGACGATGCG
AGATGTTATTAACAATTACCCATTTCCAAATACATTTAAAGTTTTAGCTGTAAGTGGTGCCAAACTTAAAGAAGCCATTGAACGATCAGCA
GAATATTTTGACGTGAAAAATGATGAAGTTAGTGTGAGCGCAGACTTCCTTGGACCCAAACCACAACACTTTAATTATGATATATATGGT
GGCGTAAGTTATACCATTCATGTTGGAAGACCAAAGGGACAACGTGTGAGCAATATGATGATTCAAGGTCACGCAGTTGATTTAAAGCA
GACATATACAATTTGTGTAAATAATTATCGTGCAGTTGGCGGTGGTCAGTATGATATGTTTATCGACGCGCCAGTTGTGAAAGATATTCA
AGTTGAAGGCGCACAATTACTTATTGATTTTTATCAAATAATAATTTGATGCGTATCCCGCAAGTTGTTGATTTTAAAGTTGAAAAGTGA

Gene: Q1Y4B9 (putative DNA-binding protein)

Contig: 02_NODE_3, position: 69809 to 70023, length: 215 nt, orientation: TRNC-FRWD (no start codon)

Sequence:

CTTTAGACGACGTGATTGAAATGTCACGTCCTGTCAAAATGAATAGTTTATTAAAGTTGTATTTTGATAGAAATTTACTGACACCACAAAA
ATTATTGAATTATTTAAAAGTTGATGAAACATTTTTGAATCATCTAGCAGGTATTAATTTAAAACTCTTTAAGGATTATGTTAATGAAAATA
GAGAATATAATATAACGAATCTATATAAATAA

Gene: repD-chr (pseudogene similar to plasmid replication initiation protein)

Contig: 02_NODE_3, position: 70282 to 71174, length: 893 nt, orientation: TRUNCATED

Sequence:

TGTGTAAAACTGGATACTCTAATATCCGGTAATAGCCGGTTAAATTGACATAGGATGTCACTAGCTATTCCAAAATTGTCTTTTGACGCTA
TAACGATTGTTGGGAATCTTAGCATGTCTAACGCAGAGAGACTTTCACATTTTATGAGTACTAATCCTGAAGTTCGGCTTTGGGATATTTT
ACAAACAAACTTTAAAGCTAAAGCTCTTAAAGAAAAAGTTTATATTGAATATGACAAAATAAAAGCAACTCTTTGGAATAGACGTAGTAT
GCGCGTTGAATTTAATCCTAATAAGCTTTCGCATGATGAAGTGCTTTGGTTAAAACAAAATATCATCAGTTATTTGGACGATGTTAGTTTT
ACGAGATTAGATTTGGCTTTTGATTTTGAATTTGATTTAAATGACTATTATGCATTGTCAGATAAGTCGGCAAAGAAAACTATATTTTATG
GACGTAATGTAAAACCAGAAACAAAATATTTTGGTGTGCGTAATAGTGATAGGTTTATTCGGATTTATAATAAAAACAAGAACGTAAAGA
TAATGCAGATGTTGAAATTGATTCAACATTTCTATGGCGTGTGGAAATTGAATTAAAACGAGATATGGTTGATTGTTGGAAAGATTGTTT
TGATGATTTGCATATTTTAAAACCGAATTTAAAAATGATTGAAAATATACAAGAACGAGCAATGCTTCATTTATTAACTCACGAAGAAGA

GGAATGGGGAAATTTAGAAAGACGTACTAAAAATAAATATAGAGATAAGTTGAAAAATATAGCGTCTATTGATTTGACAGATTTAATGA
AAATATCTTTAAGAGGAAATGAAAACCAATTGCAAAAACAAATCGACTTTTGGTTGAATTAATTATTATAACTAAAGAAC

Gene: Q2G1L1 (putative protein)

Contig: 02_NODE_3, position: 71345 to 71632, length: 288 nt, orientation: FORWARD

Perfect match to: (MW2-BA000033-[141055:141342], allele observed in CC1+CC8+CC25+CC72+CC80)

Sequence:

ATGTTTATAATAATATTGAAAGATATTTCTGTTTTCCTTTTGAATAATAAACTTAAAAGAGAATATAAAATAATGCTTATTAAAATTAGGGG
TGGAAATATGAAAAGAGATTTTTTTGAAAAGTGGTCGTTTATTTTAAATTTAATTTTATTTTGTGCTTTTTTGTTTTTAGTGGTTCTGATGTT
TTATAAGGATGTCGATTTGATTTATATTTTTTGTACATTATTAATATCATTATTACCTTTGCTAAATATTATAAAAAGATATAAGAAAAAGA
CGGATAATTAA

Gene: tnpIS200 (transposase of IS200)

Contig: 02_NODE_3, position: 72021 to 72505, length: 485 nt, orientation: FORWARD

Perfect match to: (MW2-BA000033-[141731:142215])

Sequence:

ATGTCATCTGACACAAACAGTTTAGCACATACGAAATGGAATTGTAAGTAACATATTGTCTTTGCACCTAAATACAGAAGACAAGCGATA
TATGGAAAAATAAAAAAAGATATAGGGATTATATTACGTCAATTATATGAAAGAAAAGGTGTAGAGATAATTGAAGCAGAGGTATGTAA
AGATCATATCCATATGTTAGTAAGTATACCACCCAAACTTGGGGTATCATCATTTGTTGGCTATTTAAAAGGAAAAGTAATTTAATGATAT
TTGATAGACATGCTAACTTAAAGTATAGATATGGAAATAGAAAGTTTTGGTGTAAAGGATTTTATGTGGATACAGTAGGTAGAAATAAA
AAAGTGATTGAAAATTATATTCGTAATCAATTACAAGAGGATATCGTTGCAGATGAAATCTCAATGGAAGAATATTTAGATCCTTTCACTG
GAGAGAAAAATAAAAAAAGAAAGAAAAAAGAGTAA

Gene: adhE (alcohol-acetaldehyde dehydrogenase)

Contig: 02_NODE_3, position: 72955 to 75564, length: 2610 nt, orientation: FORWARD

Sequence:

ATGTTAACTATACCTGAAAAAGAAAATCGTGGATCGAAAGAACAAGAAGTGGCAATTATGATTGATGCTCTAGCTGACAAAGGGAAAAA
AGCATTAGAAGCATTATCTAAAAAGTCACAAGAAGAAATTGATCATATTGTTCATCAAATGAGCTTAGCAGCTGTTGATCAACATATGGT
GCTAGCAAAATTAGCGCATGAAGAAACTGGAAGAGGTATATACGAAGATAAAGCGATTAAAAATTTATACGCTTCTGAATATATATGGA
ATTCAATAAAAGACAATAAGACAGTAGGGATTATTGGTGAAGATAAAGAAAAAGGATTAACGTATGTAGCGGAACCAATTGGTGTTATT
TGTGGTGTTACGCCAACAACAAATCCTACGTCGACAACTATTTTTAAAGCGATGATTGCAATTAAGACAGGAAATCCAATCATTTTTGCAT
TCCATCCAAGTGCACAAGAATCGTCGAAGCGTGCAGCAGAAGTTGTATTAGAAGCGGCAATGAAGGCAGGTGCACCTAAAGATATTATT
CAGTGGATTGAAGTGCCTTCTATCGAAGCAACAAAACAATTAATGAATCACAAAGGTATTGCATTAGTTCTAGCAACAGGTGGTTCGGGC
ATGGTTAAGTCTGCATATTCAACTGGCAAACCGGCATTAGGTGTGGGACCAGGTAACGTGCCGTCTTACATTGAAAAAACAGCACACATT
AAACGTGCAGTAAATGATATCATTGGTTCAAAAACATTTGATAATGGTATGATTTGTGCTTCTGAACAAGTTGTAGTCATTGATAAAGAA
ATTTATAAAGATGTTACTAATGAATTTAAAGCACATCAAGCATACTTTGTTAAAAAAGATGAATTACAACGCTTAGAAAATGCAATTATGA
ATGAACAAAAAACAGGTATTAAGCCTGATATTGTCGGTAAATCTGCAGTTGAAATAGCTGAATTAGCAGGTATACCTGTCCCCGAAAATA
CAAAACTTATCATAGCCGAAATTAGCGGTGTAGGTTCAGACTATCCGTTATCTCGTGAAAAATTATCTCCAGTATTAGCCTTAGTAAAAGC
CCAATCTACAAAACAAGCATTTCAAATTTGTGAAGACACACTACATTTTGGTGGATTAGGACACACAGCCGTTATCCATACAGAAGATGA
AACATTACAAAAAGATTTTGGACTAAGAATGAAAGCTTGTCGTGTACTTGTAAATACACCATCAGCGGTTGGAGGTATTGGTGATATGTA
TAACGAATTGATTCCGTCTTTAACATTAGGTTGTGGTTCGTACGGTAGAAACTCAATTTCACATAATGTTAGTGCGACAGATTTATTAAAC
ATTAAAACGATTGCTAAACGACGTAATAATACTCAAATTTTCAAGGTGCCTGCTCAAATTTATTTTGAAGAAAATGCAATCATGAGTCTAA
CAACAATGGACAAGATTGAAAAAGTGATGATTGTCTGTGACCCTGGTATGGTAGAATTCGGTTATACAAAAACAGTTGAGAATGTATTA
AGACAAAGAACGGAACAGCCTCAAATTAAAATATTTAGCGAAGTCGAACCGAACCCATCAACTAATACAGTATATAAAGGTCTGGAAAT
GATGGTTGATTTCCAACCGGATACAATCATTGCACTTGGTGGTGGTTCAGCGATGGATGCTGCAAAAGCAATGTGGATGTTCTTTGAACA
CCCTGAGACATCATTCTTCGGTGCTAAACAAAAGTTCCTAGACATCGGTAAACGTACTTATAAAATAGGCATGCCTGAAAATGCGACGTT
CATTTGTATCCCTACGACATCAGGTACAGGTTCAGAAGTAACACCATTTGCAGTTATCACAGATAGTGAAACAAATGTAAAATATCCGTTG
GCTGATTTTGCTTTAACACCTGACGTTGCAATTATTGACCCTCAATTTGTGATGAGTGTGCCAAAAAGCGTTACAGCAGATACAGGAATG

GATGTACTAACGCATGCAATGGAATCATATGTATCTGTAATGGCTTCAGACTACACAAGAGGTTTGAGTCTACAAGCGATTAAATTGACG
TTCGAATATTTAAAATCATCTGTTGAAAAGGGTGATAAAGTTTCAAGAGAGAAAATGCATAACGCATCAACTTTGGCTGGTATGGCATTT
GCAAATGCATTCTTAGGCATTGCACACTCAATTGCGCATAAAATTGGTGGCGAATATGGTATTCCGCATGGTAGAGCGAATGCGATATTA
CTACCGCATATTATCCGTTATAATGCCAAAGACCCGCAAAAACATGCGTTATTCCCTAAATATGAGTTCTTCAGAGCAGATACAGATTATG
CAGATATTGCCAAATTCTTAGGATTAAAAGGTAATACGACAGAAGCACTTGTAGAATCATTAGCTAAAGCTGTCTACGAATTAGGTCAAT
CAGTCGGAATTGAAATGAATTTGAAATCACAAGGTGTGTCTGAAGAAGAATTAAATGAGTCAATTGATAGAATGGCAGAGCTCGCATTT
GAAGATCAATGTACAACTGCTAATCCTAAAGAAGCACTAATCAGTGAAATCAAAGATATCATTCAAACATCATATGATTATAAGCAATAA

Gene: capA-L1 (capsular polysaccharide biosynthesis protein A, locus 1)

Contig: 02_NODE_3, position: 75909 to 76577, length: 669 nt, orientation: FORWARD

Perfect match to: (MW2-BA000033-[145619:146286], highly conserved allele)

Sequence:

ATGGAAAGTACATTAGAATTAACAAAAATTAAAGAAGTATTACAAAAAAACTTGAAGATTTTAATTATTTTACCGCTATTATTTTTAATTAT
TAGCGCTATTGTTACATTTTTCGTCTTATCACCTAAATATCAAGCTAATACTCAAATCTTAGTGAATCAAACTAAGGGTGACAATCCTCAGT
TTATGGCACAAGAGGTTCAAAGTAATATTCAACTTGTAAATACGTATAAAGAAATTGTTAAAAGTCCTAGAATTTTAGATGAGGTGTCAA
AGGACTTAAATGATAAGTATTCACCATCTAAATTGTCGAGTATGTTGACAATTACAAACCAAGAAAATACGCAACTTATCAACATCCAAGT
TAAAAGTGGTCATAAACAAGATTCGGAAAAAATTGCGAATAGCTTCGCTAAAGTTACAAGTAAACAAATTCCGAAGATTATGAGTGTGG
ATAACGTATCAATTTTATCTAAAGCAGACGGTACAGCAGTTAAAGTCGCACCAAAAACTGTAGTGAATCTAATCGGTGCATTCTTTTTAGG
ATTAGTTGTCGCGCTTATATATATCTTCTTCAAAGTAATTTTCGATAAGCGAATTAAAGATGAAGAAGATGTAGAGAAAGAATTAGGATT
GCCTGTATTGGGTTCAATTCAAAAATTTAATTAA

Gene: capB-L1 (capsular polysaccharide biosynthesis protein B, locus 1)

Contig: 02_NODE_3, position: 76593 to 77279, length: 687 nt, orientation: FORWARD

Perfect match to: (M1216-AIYW01000015-[45514:46200], highly conserved allele)

Sequence:

ATGTCAAAAAAGGAAAATACGACAACAACACTATTTGTGTATGAAAAACCAAAATCAACAATTAGTGAAAAGTTTCGAGGTATACGTTCA
AACATCATGTTTTCAAAAGCAAATGGTGAAGTAAAGCGCTTATTGGTTACTTCTGAAAAGCCTGGTGCAGGTAAAAGTACAGTTGTATCG
AATGTAGCGATTACTTATGCACAAGCAGGCTATAAGACATTAGTTATTGATGGCGATATGCGTAAGCCAACACAAAACTATATTTTTAAT
GAGCAAAATAATAATGGACTATCAAGCTTAATCATTGGTCGAACGACTATGTCAGAAGCAATTACGTCGACAGAAATTGAAAATTTAGAT
TTGCTAACAGCTGGCCCTGTACCTCCAAATCCATCTGAGTTAATAGCTTCAGAAAGGTTTAAAGAATTAGTTGATCTGTTTAATAAACGTT
ACGACATTATTATTGTCGATACACCGCCAGTTAATACTGTGACTGATGCACAACTATATGCGCGTGCTATTAAAGATAGTTTGTTAGTAAT
TGATAGTGAAAAAAATGATAAAAATGAAGTTAAAAAAGCAAAAGCACTTATGGAAAAAGCAGGCAGTAACATTCTAGGTGTCATTTTGA
ACAAGACAAAGGTCGATAAATCTTCTAGTTATTATCACTATTATGGAGATGAATAA

Gene: capC-L1 (capsular polysaccharide biosynthesis protein C, locus 1)

Contig: 02_NODE_3, position: 77282 to 78046, length: 765 nt, orientation: FORWARD

Perfect match to: (RF122-AJ938182-[113726:114490], highly conserved allele)

Sequence:

ATGATTGATATTCATAACCATATATTGCCTAATATCGATGACGGTCCGACAAATGAAACAGAGATGCTGGATCTTTTAAAACAAGCGACA
ACACAAGGTGTTACAGAAATCATTGTAACATCACATCACTTACATCCTCGATATACCACACCTATAGAAAAAGTGAAATCATGTTTAAACC
ATATTGAAAGCTTAGAGGAAGTACAAGCACTAAATCTAAAGTTTTATTATGGTCAGGAAATAAGAATTACCGATCAAATCCTTAATGATA
TTGATCGAAAAGTTATTACCGGTATTAATGATTCACGCTATTTACTAATAGAATTTCCATCAAATGAAGTTCCACACTATACTGATCAATTA
TTTTTCGAATTACAGAGTAAAGGCTTTGTACCGATTATTGCACATCCAGAGCGGAATAAAGCAATAAGTCAAAACCTTGACATACTATAC
GATTTAATTAACAAAGGTGCTTTAAGTCAAGTGACAACGGCGTCATTAGCGGGTATTTCCGGTAGAAAAATTAGAAAATTAGCAATTCAA
ATGATTGAAAACAATCTGACACATTTCATCGGTTCAGATGCGCATAACACAGAAATCAGACCGTTCTTAATGAAAGACTTATTTAATGATA
AGAAATTACGTGATTATTATGAAGATATGAACGGATTTATTAGTAATGCGAAGTTAGTTGTTGATGATAAAAAAATTCCTAAACGAATGC
CACAACAAGATTATAAACAGAAAAGATGGTTTGGGTTATAA

Gene: capD (capsular polysaccharide biosynthesis protein D)

Contig: 02_NODE_3, position: 78066 to 79889, length: 1824 nt, orientation: FORWARD

Sequence:

ATGGCACATTTATCTGTGAAATTGCGGCTTTTAATACTAGCATTAATCGATTCACTGATAGTGACATTTTCAGTATTCGTAAGTTATTACAT
TTTAGAACCGTATTTCAAAACATATTCTGTCAAATTATTAATATTGGCAGCTATATCACTATTCATATCGCATCATATTTCAGCATTTATTTT
TAATATGTATCATCGAGCGTGGGAATATGCCAGTGTGAGTGAATTGATTTTAATTGTTAAAGCTGTGACGACATCTATCGTTATTACGATG
GTGGTCGTGACAATTGTTACAGGCAATAGACCGTTTTTTAGATTGTATTTAATTACTTGGATGATGCACTTGATTTTAATAGGTGGCTCAA
GGTTATTTTGGCGTATTTATCGGAAATACCTTGGAGGTAAGTCATTTAATAAGAAGCCAACTTTAGTTGTTGGTGCTGGTCAAGCAGGTT
CAATGCTGATTAGACAAATGTTGAAAAGTGACGAAATGAAACTTGAACCGGTATTAGCAGTCGATGATGACGAACATAAACGCAATATC
ACAATTACTGAGGGTGTAAAAGTCCAAGGTGAAATTGCGGATATTCCAGAACTAGTGAGGAAATATAAGATTAAAAAAATCATCATTGC
AATTCCAACTATTGGTCAAGAGCGTTTGAAAGAAATTAATAATATTTGCCATATGGATGGCGTTGAGTTATTGAAAATGCCAAATATAGA
AGACGTCATGTCTGGTGAGTTAGAAGTGAACCAACTTAAAAAAGTTGAAGTAGAAGATTTACTAGGCAGAGATCCTGTTGAATTAGATA
TGGATATGATATCAAATGAATTGACGAATAAAACTATTTTAGTTACGGGTGCAGGTGGTTCAATAGGATCAGAAATTTGTAGACAAGTTT
GTAATTTCTATCCAGAACGTATTATTCTACTTGGCCATGGTGAAAACAGTATTTATTTAATCAATCGTGAATTGCGAAATCGCTTCGGAAA
AAATGTTGATATCGTTCCTATTATAGCGGATGTGCAAAATAGAGCGCGTATGTTTGAAATTATGGAAACGTATAAACCATACGCAGTTTA
TCATGCAGCAGCACACAAGCACGTGCCGTTAATGGAAGAGAACCCTGAAGAAGCAGTACGTAATAATATTTTAGGTACGAAAAATACTG
CTGAAGCTGCTAAAAATGCAGAGGTGAAGAAATTCGTTAGGATTTCTACGGATAAAGCCGTTAATCCGCCTAATGTCATGGGAGCTTCAA
AGCGAATTGCAGAAATGATTATTCAAAGTTTAAATGATGAAACGCATCGAACAAATTTTGTTGCAGTGAGATTTGGTAATGTACTTGGAT
CGAGAGGATCTGTGATTCCACTTTTCAAAAGTCAAATTGAAGAAGGTGGGCCAGTTACTGTGACACATCCTGAAATGACACGTTACTTTA
TGACAATTCCTGAAGCTTCTAGACTAGTTTTGCAGGCAGGGGCATTAGCAGAAGGTGGCGAAGTATTTGTGCTAGATATGGGAGAACCA
GTGAAAATTGTAGATTTGGCACGTAATTTAATTAAGCTAAGTGGTAAAAAAGAAGACGACATACGCATTACTTATACAGGGATTAGACCC
GGCGAAAAAATGTTTGAAGAGCTTATGAATAAAGATGAGGTTCATCCTGAACAAGTATTTGAAAAAATTTATCGTGGCAAAGTACAACA
TATGAAATGTAATGAAGTTGAAGCGATTATTCAAGACATCGTCAATGACTTTAGTAAAGAAAAAATTATTAACTATGCCAATGGCAAAAA
GGGAGATAATTATGTTCGATGA

Gene: capE (capsular polysaccharide biosynthesis protein E)

Contig: 02_NODE_3, position: 79879 to 80907, length: 1029 nt, orientation: FORWARD

Perfect match to: (MW2-BA000033-[149588:150616], allele observed in CC1+CC9)

Sequence:

ATGTTCGATGACAAAATTTTATTAATTACTGGGGGCACAGGATCATTCGGTAATGCTGTTATGAAACGGTTTTTAGATTCTAATATTAAAG
AAATTCGTATTTTTTCACGCGATGAGAAAAAACAAGATGACATTCGAAAAAAATATAATAATTCAAAATTAAAGTTCTACATTGGTGATGT
GCGTGATAGTCAAAGTGTAGAAACAGCAATGCGAGATGTTGATTATGTATTCCATGCAGCAGCTTTAAAACAAGTGCCGTCATGTGAATT
CTTTCCAGTTGAGGCAGTGAAGACAAATATTATTGGTACAGAAAATGTCTTACAAAGTGCTATTCATCAAAATGTTAAAAAAGTCATATGT
TTATCTACAGATAAGGCAGCGTATCCTATTAATGCTATGGGTATTTCAAAAGCAATGATGGAAAAAGTATTCGTAGCCAAATCAAGAAAT
ATTCGTAGTGAACAAACGCTGATTTGTGGTACAAGATACGGTAATGTGATGGCTTCAAGAGGATCAGTAATTCCTTTGTTTATCGACAAA
ATCAAAGCTGGAGAACCTTTAACGATTACAGATCCTGATATGACAAGATTTTTAATGAGTTTAGAAGATGCCGTAGAACTAGTTGTTCAT
GCATTTAAGCATGCAGAAACAGGCGATATTATGGTTCAAAAAGCACCAAGCTCAACGGTAGGGGATCTTGCGACCGCATTATTAGAATT
GTTTGAAGCTGATAATGCAATTGAAATCATTGGTACACGACATGGAGAGAAAAAAGCAGAAACATTGTTGACGAGAGAAGAATACGCA
CAATGTGAAGATATGGGTGATTATTTTAGAGTGCCGGCAGACTCCAGAGATTTAAATTATAGTAATTATGTTGAAACCGGTAACGAAAAG
ATTACGCAATCTTATGAATATAACTCCGATAATACACATATTTTAACGGTGGAAGAGATAAAAGAAAAACTTTTAACACTAGAATATGTTA
GAAACGAATTGAATGATTATAAAGCTTCAATGAGATAG

Gene: capF (capsular polysaccharide biosynthesis protein F)

Contig: 02_NODE_3, position: 80920 to 82029, length: 1110 nt, orientation: FORWARD

Perfect match to: (MW2-BA000033-[150629:151738], highly conserved allele)

Sequence:

TTGAATATTGTAATTACAGGAGCAAAAGGTTTTGTAGGAAAAAACTTGAAAGCAGATTTAACATCAACGACAGATCATCATATTTTCGAA
GTACATCGACAAACTAAAGAGGAAGAATTAGAGTCAGCATTGTTGAAAGCAGACTTTGTCGTGCATTTAGCGGGTGTTAATCGACCTGA

ACATGACAAAGAATTCAGCTTAGGAAACGTGAGTTATTTAGATCATGTACTTGATATATTAACTAGAAATACGAAAAAGCCAGCGATATT
ATTATCGTCTTCAATACAAGCAACACAAGATAATCCTTATGGTGAGAGTAAGTTGCAAGGGGAACAGCTATTAAGAGAGTATGCCGAAG
AGTATGGCAATACGGTTTATATTTATCGCTGGCCAAATTTATTCGGCAAGTGGTGTAAGCCGAATTATAACTCAGTGATAGCAACATTTTG
TTACAAAATTGCACGTAACGAAGAGATTCAAGTTAATGATCGGAATGTTGAACTAACGCTAAACTACGTGGATGATATCGTCGCTGAAAT
AAAGCGTGCTATTGAAGGAACTCCAACGATTGAAAATGGTGTACCTACAGTACCAAACGTATTTAAAGTGACATTGGGAGAAATTGTAG
ATTTATTATACAAGTTCAAACAGTCACGTCTCGATCGAACATTGCCGAAATTAGATAACTTGTTTGAAAAAGATTTGTATAGTACGTATTT
AAGCTATCTACCTAGTACAGACTTTAGTTATCCCTTACTTATGAATGTGGATGATAGGGGTTCTTTTACAGAATTTATAAAAACACCGGAT
CGTGGTCAAGTTTCTGTAAATATTTCTAAGCCAGGTATTACTAAAGGTAATCACTGGCATCATACTAAAAACGAAAAATTTCTAGTCGTAT
CAGGTAAAGGGGTAATTCGTTTTAGACATGTTAATGATGATGAAATCATTGAGTATTACGTTTCTGGCGACAAATTAGAAGTTGTAGACA
TACCAGTAGGATACACACATAATATTGAAAATTTAGGTGACACAGATATGGTAACTATTATGTGGGTGAATGAAATGTTTGATCCAAATC
AGCCAGATACGTATTTCTTGGAGGTATAG

Gene: capG (capsular polysaccharide biosynthesis protein G)

Contig: 02_NODE_3, position: 82033 to 83157, length: 1125 nt, orientation: FORWARD

Perfect match to: (MW2-BA000033-[151742:152866], allele observed in CC1+CC188+CC361)

Sequence:

ATGGAAAAACTAAAATTAATGACAATAGTTGGTACAAGGCCTGAAATCATTCGTTTATCATCAACGATTAAAGCATGTGATCAATATTTTA
ATCAGATATTAGTACACACTGGTCAAAATTATGATTATACATTGAATCAAATTTTCTTTGATGATTTGGAATTGAGACAACCGGACCACTA
CTTAGAGGCAGTTGGAAGTAACCTTGGAGAAACGATGGGGAATATTATTGCGAAGACATATGACGTTTTATTACGCGAACAACCAGATG
CACTTTTAATTCTTGGTGATACAAATAGTTGTTTAGCAGCAGTATCTGCTAAACGATTAAAGATTCCTGTGTTCCACATGGAAGCGGGTAA
TAGATGCTTTGATCAGAATGTACCTGAAGAAATCAATCGTAAAATTGTTGACCATGTCAGTGATGTGAATCTACCTTATACGGAACATAG
CAGACGTTATTTATTAGATGAAGGCTTCAATAAAGCGAATATCTTTGTGACAGGATCACCGATGACAGAAGTGATAGAAGCGCATCGAG
ATAAAATTAATCACAGTGACGTTTTAAATAAACTAGGATTAGAACCGCAACAATACATTTTAGTATCTGCGCATAGAGAAGAGAATATCG
ATAATGAAAAGAATTTTAAATCATTAATGAATGCGATAAATGATATTGCCAAAAAGTATAAAATGCCTGTGATTTATTCAACGCATCCAAG
AAGTTGGAAGAAAATTGAAGAAAGTAAATTTGAATTTGATCCATTAGTTAAAAAGTTAAAGCCATTTGGTTTCTTTGATTATAATGCATTG
CAAAAAGATGCATTTGTTGTGCTATCAGATAGTGGAACATTGTCAGAAGAGTCGTCTATTTTGAAGTTCCCTGGTGTCCTTATTCGAACTT
CCACAGAAAGACCGGAAGTACTAGATAAAGGTACGGTTATTGTAGGTGGTATTACCTATAACAATCTAATCCAATCCGTTGAACTAGCAA
GAGAGATGCAAAACAATAACGAACCGATGATTGATGCTATTGATTATAAAGACACTAACGTTTCGACAAAGGTAGTTAAAATTATTCAAA
GCTATAAAGATATTATCAATCGAAATACTTGGAGGAAATGA

Gene: capH8 (capsular polysaccharide synthesis enzyme Cap8H)

Contig: 02_NODE_3, position: 83160 to 84239, length: 1080 nt, orientation: FORWARD

Perfect match to: (MW2-BA000033-[152869:153948], highly conserved allele)

Sequence:

ATGAGGATAGCGATTCTTGGCGCTACTAACATTAAGCATATGTCATTATTATCACATTATTTAAACCACATTGATTTGAATATCAATGAGG
TGGACATTATATACACTGACAAATATGATATCGAAGAACATATCCAAGGCATCAATAATTACTATAAATATAAAGTAGATATTAAAGAAG
ATTGGACATTTATCAAAAAAGCTATTGCTTACTATCGATTTAGGCCATACGCTATGAAAATTCTTAAAGAAAATCGTTATGATTTTGTCATA
GTATGGGGAAGTTATACAGGACACTTATTTAAAAGTTTTTTAGAAAAACACTATAAAAATAAATTCATTTTAAATATAAGGGACTACTTTT
TTGAAAATAATAAACTTATTAAGTATAGAATGAAAAAAATCGTTGATGCTAGCAGGGTGACAACATTATCTTCAGAAGGTTTTCTTAAATT
TTTACCTAAATCTGAAAAATATAGAATTATTTATAGTTATAACATGAGTATTATTAGAGAAAGTAATGTAACCGATGGGTTTAAAAAAAGA
TGGCCAATTAATATAGGTTTTATTGGTAATGTTAGATTTAATGAGATAAATCAAAAATTGATAAAGGAACTGGCAAATGATTCAAGGTTC
CATATGCAATATTTTGGAACAGGGTCGGAAAAATTAGAAGTCTTTGCTCGAGAAAATTTTATTAATAATATTACATTTTCTGGCGGCTTTG
ACTTGAAAGAAACACCGAAATATTTAAATGAAATTGATATACTTAATAATTTGTTTGGTAATCAAAATATTGCTTTAGATACTGCTTTATCC
ATAAGAATGTATTATGCGTTGTTTTTAAACAAACCTATTATTACAACAGACGATACATTCACCGCTACAGAAGCTAATAAATTCGGACTGG
GTTTTAGTATTAATCCAGAAAATTTAAAAGGTATTGGTGATGAATTGATGGATTGGTATAACAATTTGGATGTAATGGACATTAATCATA
AAAGAGAAGCTTATAGAAACGATGTAATTGAAAATAATAAACAGTTCTATCAAGAGATAGGCAGGATATTTAATGAATAA

Gene: capI8 (capsular polysaccharide synthesis enzyme Cap8I)

Contig: 02_NODE_3, position: 84232 to 85626, length: 1395 nt, orientation: FORWARD

Perfect match to: (MW2-BA000033-[153941:155335], allele observed in CC1+CC12+CC101+CC121+CC130)

Sequence:

ATGAATAAGATATATAATGTCACTTCATATGTTATTGCCATTTTAATGTTTCCTTGCCTTATGTTAGGTGATAAACCTTTATTATTTTTAGCA
CCTATAAGTTATGGAGTAGGAAAGCTCTTTATAAGCTTCTCGAATAATCCGAATTTTAAATTTTCGAAAATTGTATACGATGTTTTAGGTTT
TCTTAGATTAGTATTTATACCTGCTATGATAGTGTTTTTCCAGGATTCAACTATAGATAATTTACCATTAGGACAAGCTTATTTTAATCAAG
CGGTTATTTATATGAGTGTGGAGTTTATCATAGGCTCGCTATTTATATTGATACTATCTAAATTATTCAAACATGAAGTGGTATCAAGAAA
TAGCTTTACACTTTCTGGATCATCAATTTATTACATTGTGTTTGGTCTTGTTATTTGTGGGATTTTTGTAGCTTTTCCCGAAGTGCGCAAAAA
CATATCATTTTTAATTATTAAAACAGATGCAATGGGAAGAGGAACCGAAGCAACAAGTGGTTTAAATGTTCTTTTTGTAATGCTATTTCAA
CTTGCCTTAGCGTTATTATTCTTAATAATCGCATATGCTTCATATAAAAAGTATAAAGAGAATCCTAAAATTATTTATGTTGTATTACCGCT
AGCTATAGGAATTTTAAATATTAGTTTAATTGTTGGTGAAAGAAGAAGTTATCAACTTTATACAATGGTTGCTGTTTTAACAGTGGTGTCA
ATCTTGTTTTCTAAGCATAAAAGACGTATCAATATCATTATTATATCTGTAGGTATTTTCGTATTAGCATTGATGACACTATATAAAGAATT
GTATGTGTTTAATTATAGTTCATATAGCGAGGCGTTAAATAGTACAAGTGTAAGTAATCTTAAAATAGTTGACACGTTGCAGTCATATTTT
TATGGACCTAGCAATATTGCAGCTTCTATAGACTATTTGAATTACTATAATGGTTCGTTCAAACAATATTTATTTGATAATACCAGAGCTGT
TTTTGGGTTTAATTTCTTTTTAGATAAAAAGCAGTTGATTACTAGCCAACTTTTTAACCAATTGATATATGGTAGTAAACAATTGACTGGTC
ATCTAATTTCGAGTGCTGGATATGGAATTATTTATTTTGGACCCTTATTCTTCTATTTGAATTTAATTGCAAATATCTTTTTTGCATTTCTGA
GTGAGTATATTATTCGGAAGAGTCATTCGTTAGAAGTGATATTTATTGGTACATATATTTATATGAGACTTATTACGAGTATTTTTAGTCAC
CCGACACCATTAATCACATTGATTTCTATGATTTTAGTTGTATATGTTATAGCTATTATCCCAGGCATTATAATAAAAAAATTTACTAAAAA
AGTAGGGATAGAATGA

Gene: capJ8 (capsular polysaccharide synthesis enzyme Cap8J)

Contig: 02_NODE_3, position: 85623 to 86180, length: 558 nt, orientation: FORWARD

Perfect match to: (MW2-BA000033-[155332:155889], allele observed in CC1+CC7+CC12+CC15+CC96)

Sequence:

ATGATTGTAAAAACATTTATGAAATCGAAAATATTTAGATTAATGAATACACCACTATTATTATTTTATAAGAAAGAATATTTAACTGGAT
ATTATTTTGAAAATAAAGTGGCTGGATGGTTATGGGCGTGGAAAGCTGTTCCGTTCAAGTTGTTAGGAATAAATACAAGTTTGCCATTTC
CTGCAGATATAACTGTTAGAATGCATAACCCTAATAACATTGTTTTTGATAAAAATGATATTCATATTTTTCAATCGCCCGGGACGTATTTT
AATAATTTTTCAGCAGTTATATATATATAGGTAGAGGTGTTTATATAGCGCCTAACGTAGGTATTATTACAGCTAATCATAATATTAAAAATTT
GAAGTCACATGCACCAGGTGAAGATGTCAAAATAGGGAATTATAGTTGGATTGGAATGAACTCAGTTATATTACCAGGAGTAGAATTGG
GGGAACATACAATTGTAGGGGCTGGGTCGGTTGTAACAAAAAGTTTTCCAGAAGGTAACGTTGTTATAGGTGGCAATCCAGCGAAAATC
ATCAAGAAAATCTGA

Gene: capK8 (capsular polysaccharide synthesis enzyme Cap8K)

Contig: 02_NODE_3, position: 86189 to 87427, length: 1239 nt, orientation: FORWARD

Perfect match to: (MW2-BA000033-[155898:157136], allele observed in CC1+CC80+CC188+CC361)

Sequence:

ATGAGATTAAATAAATTTATTGGCGATTCGTTTTTAATGATTTTAAGCAGTGGCATCGCTCAAGTCATATTAATCATCACTACCCCAATTAT
TACAAGACTATATTCACCTACAGAATTTGGTGAGTTTACAATTTTTTCAAATATCGCAATGATTTTAATACCAATAATAAATGCAAGATACG
ATTTGTTGATTGTGAATACCAAAAATGACCGTAGTGCTAATATACTTTCACAAATCAGTTTTTTGATATCATTGCTTATTTTATTAATACTGA
TACCAATATTTGCGATTAGTGCATGTTTATACCCAAACTTTATATTAGATTTTATTTTCATTATTATTATGTTGTTTTTGGTAAGTTTAACAA
ACATTTTTACAAATTATCTAAATAAGGAAAGAAAGTATAAAGTGTTAAGTTTGATTAATGTGTTTAGAGCTGGATCAATGGCTTTACTTCA
AATCATTTTCGGACTTTTAGCATTAGGAAGTTTAGGATTAATTATTGGTTTTTCATTATCCTATATCGCAGGCATTACACTAGGATATAAAA
CGTTTAAAAAGCACTTTAATATTGTGAGAGATAAAGAAGAAACTAAAGCATTATTTTTAGAAAATAAAAATCAGTTAGTTTATTCAACACC
ATCAATATTATTAAATAGTTTGTCTTTCTCGGTTGTTGTGTTCTTTATAGGTATTTTGTATACCAATACAGAAGTGGGTATTTATGGTATGG
CCATAAGAGTACTAGGCATACCAGTGACAATTATTTCATTAGGGTTATCAAAAATATTTATGCAACAAGCCAATGACTATTATATTGAACA
TGGTAACTTCCGAAATTTATTACTTAAATTTAGTTCCATACTGGTTATAGTTTCTATAATTCTTTATGTGCCACTTTATTTGTTCAGTGAAGA
ATTAGTCAATATATTATTAGGACATAGCTGGGTTGACGCAATTACAGTTATAAAAAATTGTTATCCCATTATTTGTTATAAGGCTGATTGTAT
CAACGGTATCACTTTCTGTGATTGTATTACAAAAACAACAGTTAGAATTAATACTACAAGCGTTATTTTTAATAGGTACTACTGCAACATTT
GTTATATCAAAAATGCTTAATTTAACTTTTTTAAACTTTGTATCTATTAATACAATTGTTTTAATCGTATCGTACATGATATTTTTCATAGCAC
TCTATTATTTTGCTAAAAATAAACAGTTCAAAAAATTCTTAG

Gene: capL (capsular polysaccharide synthesis protein L)

Contig: 02_NODE_3, position: 87461 to 88666, length: 1206 nt, orientation: FORWARD

Perfect match to: (MW2-BA000033-[157170:158375], allele observed in CC1)

Sequence:

ATGAGTGAAAAAAAGATTTTGATTTTATGTCAGTATTTTTATCCGGAATATGTATCTTCTGCGACGTTACCAACTCAATTGGCGGAAGATT
TAATTGCGAATCACATTAATGTCGATGTCATGTGTGGATGGCCATATGAATATAGTAATCATAAACAGGTTTCTAAAACCGAGATGCATC
GTGGTATTTGCATTCGACGTCTCAAGTATTCGAGGTTTAATAACAAAAGTAAGGTTGGAAGGATCATCAATTTCTTTAGTTTATTTTCAAA
ATTCGTGATTAATATACCTAAAATGTTGAAATATGATCAGATTCTTGTTTACTCTAATCCACCAATCTTGCCATTAATACCAGACGTTTTAC
ACAGACTGCTTAAGAAAAAATATTCTTTTGTGGTGTATGATATAGCACCTGATAATGCGATTAAGACAGGTGCAACTCGTCCAGGTAGCA
TGATTGATAAACTGATGCGTTACATTAATAGACATGTCTACAAGAATGCTGAAAATGTCATTGTCCTTGGTACGGAAATGAAAAACTACTT
ACTAAATCATCAAATTTCTAAAAATGCTGACAATATCCATGTGATTCCTAACTGGTATGACATGCGTCAATTACAAGACAATCGTATCTAT
AATGACACATTTAAAGCTTACCGTGAGCAATACGACAAAATTTTATTGTATAGCGGTAATATGGGGCAGTTACAGGATATGGAGACACTT
ATCTCATTTTTAAAATTAAATAAGGATCAGCCTCAAACGTTAACAATACTTTGTGGTCATGGTAAGAAATTTGCAGATGTCAAAACGGCAA
TAGAAGACCATCGTATTGAAAATGTTAAAATGTTTGAGTTTTTAACAGGTACAGACTATGCTGACGTATTAAAAATTGCGGATGTATGTA
TTGCATCGCTGATTAAAGAAGGCGTCGGTTTAGGCGTGCCGAGCAAGAATTATGGCTATCTTGCAGCTAAGAAAGCGTTGGTACTCATC
ATGGATAAGCAATCTGATATCGTTCAACATGTTGAACAATATGATGCGGGTATCCAAATTGATAATGGCGATGCACATGCCATTTATAAC
TTCATCAACACTCACTCGAGTAAGGAATTGCACGAGATGGGTGAGCGCGCACATCAACTGTTTAAAGATAAATATACGAGAGAAATTAA
TACTATGAAGTATTACAATCTGTTGAAGTGA

Gene: capM (capsular polysaccharide synthesis protein M)

Contig: 02_NODE_3, position: 88677 to 89234, length: 558 nt, orientation: FORWARD

Perfect match to: (N315-BA000018-[179995:180552], allele observed in CC5)

Sequence:

ATGAAGCGATTATTCGATGTAGTGAGTTCAATATATGGTTTAGTAGTTTTAAGTCCGATTCTGTTAATTACAGCATTACTAATTAAAATGG
AATCACCTGGACCAGCCATTTTCAAACAAAAAAGACCGACGATTAATAATGAATTGTTTAATATTTATAAGTTTAGATCAATGAAAATAGA
CACACCTAATGTTGCAACTGATTTAATGGATTCAACATCGTATATAACAAAGACAGGGAAGGTCATTCGTAAGACCTCTATTGATGAATT
GCCACAATTATTGAATGTTTTAAAAGGAGAAATGTCAATTGTAGGTCCTAGACCAGCGCTTTATAATCAATACGAATTAATCGAAAAACG
TACAAAAGTGAACGTGCATACGATTAGACCAGGTGTGACAGGACTAGCTCAAGTGATGGGGAGAGATGATATCACTGATGATCAAAAA
GTAGCGTATGATCATTATTACTTAACACATCAATCTATGATGCTTGATATGTATATCATATATAAAACAATTAAAAATATCGTTACTTCAGA
AGGTGTGCATCACTAA

Gene: capN (capsular polysaccharide synthesis protein N)

Contig: 02_NODE_3, position: 89234 to 90121, length: 888 nt, orientation: FORWARD

Perfect match to: (TCH130-ACHD01000025-[6853:7740:r], allele observed in CC72)

Sequence:

ATGAGAAAAAATATTTTAATTACAGGCGTACATGGATATATCGGTAATGCTTTAAAAGATAAGCTTATTGAACAAGGACATCAAGTAGAT
CAAATTAATGTTAGGAATCAATTATGGAAGTCGACCTCGTTCAAAGATTATGATGTTTTAATTCATACAGCAGCTTTGGTTCACAACAATT
CACCTCAAGCAAGGCTATCTGATTATATGCAAGTGAATATGTTGCTGACGAAACAATTGGCACAAAAGGCTAAATCTGAAGACGTTAAAC
AATTTATTTTTATGAGTACTATGGCAGTTTATGGAAAAGAAGGTCAGGTTGGTAAATCAGATCAAATTGATACACAAACACCAATGAACC
CTACGACCAACTATGGTATTTCCAAAAAGTTCGCTGAACAAGCATTACAAGAGTTGATTAGTGATTCGTTTAAAGTAGCAATTGTGAGAC
CACCAATGATTTATGGTGCACATTGCCCAGGAAATTTCCAACGGTTAATGCAATTGTCAAAGCGACTGCCAATCATTCCCAATATTAACAA
TCAGCGCAGTGCATTATATATTAAACATCTGACAGCATTTATTGATCAATTAATATCATTAGAAGTGACAGGCGTGTATCATCCTCAAGAT
AGCTTTTACTTTGATACATCGTCAGTAATGTATGAAATACGTCGCCAATCACATCGTAAAACGGTATTGATCAACATGCCTTCAGTGTTAA
ATAAGTATTTTAATAAGTTGTCGGTCTTTAGAAAATTATTCGGCAATTTAATATACAGCAATACGTTATATGAAAATAATAATGCACTTGA
AGTTATTCCTGGAAAAATGTCACTTGTTATTGCGGACATCATGGATGAAACGACAACCAAAGATAAGGCATAA

Gene: capO (capsular polysaccharide synthesis protein O)

Contig: 02_NODE_3, position: 90175 to 91437, length: 1263 nt, orientation: FORWARD

Perfect match to: (MW2-BA000033-[159884:161146], allele observed in CC1)

Sequence:

ATGAAGTTAACAGTAGTTGGCTTAGGTTATATTGGTTTACCAACATCAATTATGTTTGCAAAGCATGGCGTCGATGTGCTTGGTGTTGATA
TTAATCAGCAAACGATTGATAAGTTACAAAGTGGTCAAATTAGTATTGAAGAACCTGGATTACAAGAGATTTATGAAGAGGTACTGTCAT
CGGGAAAATTGAAGGTATCTACAACGCCAGAAGCATCTGATGTTTTTATCATTGCCGTTCCGACGCCGAATAATGATGATCAGTACCGGT
CATGTGACATTTCGCTAGTTATGCGTGCATTAGATAGTATTTTACCATTTTTAAAAAAAGGGAATACTATTATTGTAGAGTCGACAATTGC
GCCTAAAACGATGGATGATTTTGTAAAACCAGTCATTGAAAATTTAGGGTTTACAATAGGTGAAGATATTTATTTAGTGCATTGTCCAGA
ACGTGTACTGCCAGGAAAAATTTTAGAAGAATTAGTTCATAACAATCGCATCATTGGCGGTGTGACTAAAGCTTGTATTGAAGCGGGTAA
ACGTGTCTATCGCACATTTGTTCAGGGAGAAATGATTGAAACAGATGCACGTACTGCTGAAATGAGTAAGCTAATGGAAAACACATATA
GAGACGTGAACATTGCTTTAGCTAATGAATTAACAAAAATTTGCAACAACTTAAATATTAATGTATTAGATGTGATTGAAATGGCAAACA
AACATCCGCGTGTTAATATCCATCAACCTGGTCCAGGTGTAGGCGGTCATTGTTTAGCTGTTGATCCGTACTTTATTATTGCTAAAGACCC
TGAAAATGCAAAGTTAATTCAAACTGGACGTGAAATTAATAATTCAATGCCGGCCTATGTTGTTGATACAACGAAGCAAATCATCAAAGC
ATTGAGCGGGAATAAAGTCACAGTATTTGGTTTAACTTATAAAGGTGATGTTGATGATATAAGAGAATCGCCAGCATTTGATATTTATGA
GCTATTAAATCAAGAACCAGACATAGAAGTATGTGCTTATGATCCACATGTTGAATTAGATTTTGTGGAACATGATATGTCACATGCTGTC
AAAGACGCATCGCTAGTATTGATTTTAAGTGACCACTCAGAATTTAAAAATTTATCGGACAGTCATTTTGATAAAATGAAGCATAAAGTG
ATTTTTGATACAAAAAATGTTGTGAAATCATCATTTGAAGATGTATCGTATTATAATTATGGCAATATATTTAATTTTATCGACAAATAA

Gene: capP (capsular polysaccharide synthesis protein P)

Contig: 02_NODE_3, position: 91484 to 92659, length: 1176 nt, orientation: FORWARD

Perfect match to: (MW2-BA000033-[161193:162368], allele observed in CC1+CC361)

Sequence:

ATGTGTTTGAACTTCAGAGAGGATAATGTTATGAAAAAAATTATGGTTATTTTCGGTACGAGACCCGAAGCAATAAAAATGGCACCATTA
GTAAAAGAAATTGATCATAATGGGAACTTTGAAGCGAACATTGTGATTACAGCACAACATAGAGATATGTTAGATAGTGTGTTAAGTATA
TTTGATATTCAAGCTGATCATGATTTAAATATTATGCAAGATCAACAAACGTTAGCGGGCCTTACGGCTAATGCGCTTGCTAAACTTGATA
GCATCATTAATGAGGAACAGCCGGATATGATTTTAGTACATGGTGATACTACAACGACTTTTGTAGGAAGTTTGGCAGCATTTTATCATC
AAATTCCGGTCGGACATGTAGAAGCTGGACTTCGAACACATCAGAAATACTCACCATTTCCTGAAGAGTTAAATCGAGTCATGGTAAGTA
ATATTGCTGAATTGAATTTTGCGCCAACAGTAATTGCAGCTAAAAATTTACTTTTTGAAAACAAAGACAAAGAGCGTATCTTTATTACTGG
AAATACAGTTATTGACGCATTGTCAACAACAGTTCAAAATGATTTTGTTTCAACGATTATTAATAAACATAAAGGCAAGAAAGTTATTTTA
CTAACAGCGCATCGTCGTGAAAATATTGGGGAACCGATGCATCAGATTTTTAAAGCAGTAAGAGATTTGGCAGATGAATATAAAGATGT
TGTCTTCATTTATCCAATGCATCGTAATCCAAAGGTAAGAGCGATTGCCGAAAAATATTTATCTGGGAGAAATCGGATTGAATTAATTGA
GCCATTAGATGCGATTGAGTTCCATAATTTTACAAATCAATCGTACCTCGTGCTGACAGATTCTGGTGGTATTCAAGAGGAGGCTCCTACA
TTTGGAAAACCTGTGTTGGTATTAAGGAATCATACAGAGCGTCCCGAAGGCGTTGAGGCGGGAACATCGAGAGTAATTGGCACAGATTA
TGACAATATTGTTCGAAATGTGAAACAATTGATTGAGGATGATGAAGCATATCAACGTATGAGTCAAGCGAATAATCCATATGGTGATG
GACAAGCATCACGACGTATTTGTGAAGCAATAGAATATTATTTTGGATTGCGCACAGACAAGCCGGATGAATTCGTACCTTTACGTCACA
AATAA

Gene: isdI (heme oxygenase)

Contig: 02_NODE_3, position: 92724 to 93050, length: 327 nt, orientation: REVERSE

Perfect match to: (MW2-BA000033-[162433:162759:r], allele observed in CC1+CC72+CC88)

Sequence:

TTATTTTTGATAGTGGTAGCCAATATCATATTTGAATACTTTATTTGATAATATTGGACTTTGCTGTCCATCGTCATCACTTTTTAAACGTAC
ATTTTTATGAGCTTCTTTAAATACATCGGAATTCAACCAATTATTAAAGCTATCTTCAGATTCCCAAATAGTTAAGATTTTAACTTCGTCTGT
ATCCTCAGTATTTAATGTTTTAGTGACAAACATTTGTTGGAAGCCTTCAATAGTTTCAATGCCTTGTCTATTGTAAAAACGTTCAATCGTTT
CTTCCGCACTGCCTTTTTGTAATTGTAATCTATTTTCTGCCATAAACAT

Gene: ybaN (putative membrane spanning protein)

Contig: 02_NODE_3, position: 93057 to 93440, length: 384 nt, orientation: REVERSE

Perfect match to: (MW2-BA000033-[162766:163149:r], allele observed in CC1+CC8+CC22+CC239)

Sequence:

TCACTCCTCTATTTTATGATTTGATTTGGGTAATGTTTTTACAAATGTAAAGAGTACAGCGGTTTGTATGATAACCATTATGATTAATCCTA
TACGTACTGCAAGAACATCCACCATATAAATTGAAAAACCTATTACAATGTATAAGCTAATTAAAATTTTAATTTTCTGTTGTAGCGTGTAG
CCTCGATGTAAATAAAAGTTTTCTACATATTCTTTATAAATTTTTTGATTAATAAGCCAATTGTAAAAGCGATCTGAACTTCGAGCAAAACA
AAAAACTGCTACGAGTAAAAAAGGGGTCGTTGGCAGTAAAGGTAATACGGCACCTGCAATACCAAGCGCTGTAAATATTAAGCCAATGA
CGATTAAAATAAGTCGCAT

Gene: aldA1 (aldehyde dehydrogenase, locus 1)

Contig: 02_NODE_3, position: 93867 to 95354, length: 1488 nt, orientation: FORWARD

Perfect match to: (MW2-BA000033-[163576:165063], allele observed in CC1)

Sequence:

ATGGCAGTAAACGTTCGAGATTATATAGCAGAGAATTATGGTTTATTTATCAATGGGGAATTTGTTAAAGGTAGCAGTGACGAAACAATC
GAAGTGACTAATCCAGCAACTGGAGAAACACTATCACATATTACAAGAGCAAAAGATAAAGATGTCGATCATGCAGTCAAAGTGGCGCA
AGAGGCATTTGAATCATGGTCATTAACTTCTAAATCAGAACGTGCACAAATGTTGCGTGATATTGGTGATAAATTAATGGCACAAAAAGA
TAAAATTGCAATGATTGAAACATTAAATAATGGTAAACCGATTCGTGAGACAACAGCAATTGATATTCCATTTGCTGCAAGACATTTCCAT
TATTTCGCAAGTGTTATTGAAACAGAAGAAGGTACAGTAAATGATATCGATAAAGACACAATGAGTATCGTACGACATGAGCCGATTGG
CGTCGTAGGTGCTGTTGTTGCTTGGAACTTCCCAATGCTATTAGCTGCATGGAAGATTGCGCCAGCCATTGCTGCAGGTAATACAATTGT
GATTCAACCTTCGTCTTCAACACCATTAAGTTTATTGGAAGTTGCTAAAATTTTCCAAGAGGTATTACCTAAAGGTGTTGTCAATATACTAA
CGGGTAAAGGTTCAGAATCAGGTAATGCAATTTTCAATCATGATGGTGTAGATAAATTATCATTTACGGGCTCAACTGATGTAGGTTATC
AAGTTGCCGAAGCTGCAGCAAAACATCTAGTACCCGCTACATTAGAGCTTGGTGGTAAAAGCGCCAATATCATATTAGATGATGCTAATT
TAGACCTTGCAGTTGAAGGTATTCAGTTAGGTATTTTATTCAACCAAGGTGAAGTATGTAGTGCAGGTTCTCGATTATTAGTTCATGAAAA
AATTTATGATCAATTGGTGCCACGTTTACAAGAGGCATTTTCAAATATTAAAGTTGGAGATCCACAAGATGAAGCTACACAAATGGGTAG
TCAAACTGGTAAGGATCAATTAGATAAAATTCAATCATATATTGATGCAGCAAAAGAATCAGATGCACAAATTTTAGCAGGTGGTCATCG
CTTAACTGAAAATGGATTAGATAAAGGGTTCTTCTTTGAGCCGACATTAATTGCTGTGCCAGACAATCATCACAAATTAGCACAAGAAGA
AATATTTGGACCAGTGTTAACAGTGATTAAAGTGAAGGACGATCAAGAAGCAATTGATATAGCTAATGATTCTGAGTATGGTTTAGCAG
GCGGTGTATTTTCTCAAAATATCACACGTGCATTAAATATTGCTAAAGCTGTACGTACAGGACGTATTTGGATTAACACTTACAACCAAGT
ACCAGAAGGCGCACCATTTGGTGGTTATAAAAAATCAGGTATCGGTCGAGAAACTTATAAAGGTGCGTTAAGTAACTATCAACAAGTTA
AAAATATTTATATTGATACAAGCAATGCTTTAAAAGGTTTGTACTAG

Gene: czcD (Co/Zn/Cd efflux potassium/proton antiporter)

Contig: 02_NODE_3, position: 96000 to 96959, length: 960 nt, orientation: FORWARD

Perfect match to: (MW2-BA000033-[165709:166668], highly conserved allele)

Sequence:

ATGAATGGGAAAAAGGCGAATACGATAAACAGATACAAATATTTTCATCATGTCAATCATCAAAAAATTCAACAAAGTTCTAAAAAGACG
CTGTGGGCATCACTTATCATCACATTGTTATTTACAGTGATTGAATTTGTCGGAGGTTTAGTATCTAATTCATTGGCATTACTGTCAGATTC
ATTTCATATGCTTAGTGATGTATTAGCACTTGGTTTATCTATGTTGGCCATTTATTTTGCAAGTAAAAAGCCGACTGCACGATACACATTTG
GATATTTAAGATTTGAGATATTAGCTGCATTTTTAAATGGTTTAGCATTAATTGTAATTTCAATCTGGATTTTATATGAAGCTATTGTACGT
ATTATTTATCCGCAACCAATTGAAAGTGGCATTATGTTTATGATTGCTAGTATTGGTTTACTCGTTAATATTATTTTGACAATTATCCTTGTA
AGGTCTTTAAAACAAGAAGACAATATCAATATTCAAAGTGCATTATGGCATTTCATGGGAGACTTATTGAACTCTATTGGTGTCATCGTTG
CAGTTGTATTGATTTACTTTACAGGATGGCGCATCATCGACCCAATCATTAGTATTGTAATTTCACTCATCATTTTACGTGGTGGTTATAAA
ATTACGCGTAATGCGTGGTTAATTTTAATGGAAAGTGTGCCTCAACATTTGGATACTGATCAAATTATGGCAGATATTAAAAACATAGAT
GGCATATTAGATGTACATGAATTTCATTTGTGGAGTATTACAACAGAGCATTATTCATTAAGTGCCCATGTTGTGTTAGATAAAAAATATG
AGGGTGATGATTATCAAGCGATTGATCAAGTATCATCATTGTTGAAAGAAAAATATGGCATTGCACATTCAACGTTGCAAATTGAAAACT
TGCAATTGAATCCATTAGATGAGCCATACTTCGACAAATTAACATAA

Gene: tx_universal2 (rho-independent terminator)

Contig: 02_NODE_3, position: 97031 to 97063, length: 33 nt

Perfect match to: (Strain_21194-AGTU01000149-[993:1025], allele observed in CC45I)

Sequence:

CTGAACGAAAATGCGCTTGTAACAAGCTTTTTT

Gene: tx_universal2 (rho-independent terminator)

Contig: 02_NODE_3, position: 97033 to 97071, length: 39 nt

Perfect match to: (Strain_21331-AGTV01000040-[104403:104441], allele observed in CC398)

Sequence:

GAACGAAAATGCGCTTGTAACAAGCTTTTTTCAATTCTA

Gene: Q5HJK0-srpF (alpha-helical coiled-coil protein)

Contig: 02_NODE_3, position: 97634 to 98146, length: 513 nt, orientation: FORWARD

Perfect match to: (N315-BA000018-[188955:189467], highly conserved allele)

Sequence:

ATGACATTATTTTTATTAGAAGCTAACAATCTTGATTTTGCATCAACGAAAGAAGAACTAGAAGCAAAGGCAGCATCACTATCTACGAAG
ACAATTCCAACATTAATTGAAGTACAAGCTACTGAAAATTTAACTCATGGTTATTTTATTGTGGAAGCAAATGACGAAGCAGAAGCTAAA
CAATTTTTAACAGAAGCAGATATTAGTATTCAATTAGTCAAAGAAGTACGCTTAGTTGGTAAAGATTTAGATGAAGTTAAAAATGGTGAT
GCACATGTTGATTACCTTGTAACTTGGAACATTCCGGAAGGCATTACGATGGATCAATATTTAGCACGTAAAAAGAAAAATTCTGTTCATT
ATGAAGAAGTGCCAGAAGTTGAATTTAAACGCACATATGTATGTGAAGATATGTCTAAATGTATTTGTTTATACAACGCACCTGATGAAG
AAGCGGTACGTCGCGCGCGCAAAGCAGTTGATACACCGATTGATGGCATCGAAAAACTTTAA

Gene: tauB (taurine ABC transporter, ATP-binding protein)

Contig: 02_NODE_3, position: 98488 to 99228, length: 741 nt, orientation: FORWARD

Sequence:

ATGATTAAAATACAACAATTACAACATCACTTTGGATCACATAAAGTAATTCATAACTTTAATTTGGACATTAGCAAAGGAGAAATAGTCA
CTTTCATAGGAAAAAGTGGTTGCGGAAAGTCTACTTTACTCAATATCATCGGTGGATTTATTCATCCATCATCTGGACGTGTCATTATTGA
TAACGAAATTAAACAACAGCCGTCTCCAGATTGTTTAATGCTATTTCAACATCATAATTTGCTGCCATGGAAAACGATTAATGACAACATT
AGGATTGGATTTCGACAGAAAATTAGTGATGAAGAGATTAACGCACAGCTTAAATTAGTTGATTTAGAAGGCAGGGGAAAGCATTTTCC
CGAGCAACTGTCCGGGGGTATGAAACAACGTGTGGCACTATGTCGAGCGCATGTGCATAAGCCTAACGTTATATTGATGGATGAGCCAT
TAGGTGCATTAGATGCATTTACACGTTATAAACTTCAGGACCAACTAGTGCAACTAAAACATAAAACGCAATCAACTATTATTTTAGTGAC
GCATGACATTGATGAAGCTATTTATCTTTCCGACCGCATTGTTCTGTTAGGTGAAGGGTGCAATATTATTTCTCAATATGAAATTACAGCA
TCACATCCACGCAGTCGTAATGATAGCCACCTACTTAAGATTCGTAATGAAATTATGGAAACATTTGCATTGAATCATCATCAAGTTGAAC
CTGAATATTATTTATAA

Gene: tauA (taurine ABC transporter, substrate-binding protein)

Contig: 02_NODE_3, position: 99242 to 100216, length: 975 nt, orientation: FORWARD

Perfect match to: (N315-BA000018-[190563:191534], allele observed in CC5+CC1+CC72+CC80)

Sequence:

ATGAAAAGGTTAAGCATAATCGTCATCATTGGAATCTTTATAATTACAGGATGTGATTGGCAAAGGACGTCTAAAGAACCGTCTAAAAAT
GCCCAAAATCAGCAAGTGATTAAAATTGGATATTTGCCGATTACACATTCAGCTAATTTGATGATGACTAAAAAATTATTATCACAATACA
ATCATCCGAAATATAAACTAGAATTAGTTAAATTCAATAATTGGCCAGATTTAATGGACGCATTAAACAGTGGTCGTATTGATGGTGCATC
AACTTTAATAGAGCTAGCGATGAAATCAAAACAGAAGGGCTCAAATATAAAGGCTGTGGCATTGGGCCATCATGAAGGCAATGTCATTA
TGGGACAAAAAGGTATGCACTTAAATGAATTTAATAATAATGGCGATGATTACCATTTTGGTATACCACATCGTTATTCAACACATTATCT

TTTACTTGAGGAATTACGTAAACAATTAAAGATTAAACCGGGGCATTTTAGCTATCATGAAATGTCGCCAGCAGAAATGCCAGCCGCATT
GAGTGAACACAGAATTACAGGGTATTCTGTAGCCGAACCATTCGGTGCACTGGGTGAAAAGTTAGGCAAAGGTAAGACTTTGAAACATG
GTGATGACGTTATACCTGATGCGTATTGCTGTGTGCTAGTACTGAGAGGGGAATTGCTTGATCAACACAAGGATGTAGCGCAAGCATTT
GTACAAGATTATAAAAAGTCTGGCTTTAAAATGAATGATCGCAAGCAAAGTGTAGACATTATGACGCATCATTTTAAACAAAGTCGTGAC
GTTTTAACACAGTCAGCGGCATGGACATCCTATGGTGATTTAACAATTAAGCCATCCGGCTATCAAGAAATTACGACATTGGTAAAACAA
CATCATTTGTTTAATCCACCTGCATATGATGACTTTGTTGAACCGTCATTGTATAAGGAGGCATCGCGTTCATGA

Gene: tauC (taurine ABC transporter, transmembrane permease)

Contig: 02_NODE_3, position: 100213 to 100974, length: 762 nt, orientation: FORWARD

Perfect match to: (MW2-BA000033-[169923:170684], allele observed in CC1+CC5)

Sequence:

ATGACACGTCCCACAAATAACAAATTTATATTACCTATTATCACATTTATTATTTTCTTAGGCATTTGGGAAATGGTCATTATTATTGGGCA
TTACCAACCTGTATTGTTACCTGGTCCTGCTCTTGTAGGAAAAAGTATATGGACTTTCATTGTTACTGGAGAAATTTTCCAACATTTAGCAA
TTAGTTTATGGAGATTTGTAGCGGGCTTTGTTGTCGCATTGTTGGTTGCTATTCCATTGGGCTTCTTGCTTGGAAGGAATCGTTGGCTATA
CAACGCTATCGAACCGCTATTTCAATTGATTAGACCGATATCTCCGATAGCATGGGCACCATTTGTTGTTCTATGGTTTGGTATTGGTAGT
TTGCCAGCGATTGCGATTATTTTTATCGCCGCTTTTTTCCCAATTGTGTTCAATACTATTAAAGGCGTTAGAGACATTGAACCTCAATATTT
AAAAATAGCGGCAAATTTAAATTTAACTGGGTGGTCATTGTATCGCAATATATTATTTCCCGGGGCATTTAAACAAATCATGGCTGGGAT
ACATATGGCGGTAGGAACAAGTTGGATATTTTTAGTTTCTGGTGAAATGATTGGTGCACAATCGGGATTAGGTTTTTTAATCGTTGATGC
ACGAAATATGTTGAACTTAGAAGATGTTTTAGCAGCAATATTCTTTATCGGATTATTTGGTTTTATTATTGATCGATTCATTAGTTATATTG
AGCAGTTTATACTTAGAAGATTTGGTGAATAA

Gene: Q5HJJ8 (putative acyl-CoA dehydrogenase)

Contig: 02_NODE_3, position: 100987 to 102018, length: 1032 nt, orientation: FORWARD

Sequence:

ATGACTTTAGAAACGCTTATCAAAGAACAATTAGATCCTCATTTAGTAGAAGTTGATGAAGGGACGTATTATCCGAGAACATTTATTCAAC
AATTATTTGTAGATGGTTATTTCGGTGAGGCGGCATTGAGAAAAAATGCTGAAGTAATCGAAGCTGTATCGCAGTCTTGTTTGACAACAG
GATTTTGTTTATGGTGCCAATTAGCTTTTTCAACGTATTTAGAAAATGCCACGCAGCCACATTTAAATAATGACTTACAACAGCAATTGTTA
TCTGGAGAAATATTAGGTGCTACCGGATTGTCTAATCCGATGAAGTCATTTAATGATTTAGAAAAGTTGAACCTTGAACACACTTATGTTG
ATGGACAATTGGTTGTCAGTGGACGTATGCCAGCTGTAAGTAATATTCAAGAAGACCATTATTTTGGTGCGATTTCGAAACATGAATCAT
CAGATGAATTTGTCATGTTCATTCTACGTGCCAATCAAGATGGTATCACTCTCGTTGAAAAAACAAATTTTTTAGGGGTAAATGGCTCGGC
TACGTATCAAATTACTTTGAATCAAGTCGTAGTGCCACAATCACAAATTATCACGCATGATGCGAAGCAGTTTGCGGCAACTATTCGCCCA
CAATTTATTGCTTATCAAATTCCAATAGGATTAGGCTCAATTAAAAGTTCTTTAGAGTTAATTGATGCATTTTCAAATGCGCAAAACGGAA
TAAATCAATATTTAGAGTATGATGTTGAAGCTTTTAAAAAACGTTATCGTCAACTTAGAGAAGAATATTATGCAATATTAGATGACGGTAA
CTTAACTTCACATTTAAATGAATTAATATCATTGAAGAAGGACATCGGCTATTTATTGCTAGATGTAAATCAAGCTTCTGTTGTCAATGGT
GGTTCAAGAGCGTACACACCATATTCGCCACAAGTTCGCAAGTTAAAAGAAGGAGTCTTCTTTGCAGCATTGACACCAACATTAAGACAT
TTAGGTAAACTTGAAGCAGAGTTGAAGGGGTAA

Gene: yrhF (putative regulator protein)

Contig: 02_NODE_3, position: 102235 to 102594, length: 360 nt, orientation: FORWARD

Perfect match to: (MW2-BA000033-[171945:172304], highly conserved allele)

Sequence:

GTGAATACTATAGATACGCATACTAAAGAACAACAATTCTCGAATCTAGTAAGATCTTATCGTAAAGAATACGTGGGTAAAGGACCCAAT
AGTATTCGAGTGTCGTTTAAAGATAATTGGGCGATTGCACATATGACAGGTGTTTTGAGTAAAGTTGAGAGTTTTTACCTAAACGACAAA
CGCAATGAATCGATGCTCCATTATACACGCACAGAGAAGATTAAACAGATGTATAAAGAAATAGATGTAAATGAGATGGAAAGTCTTGT
AGGCGCTAAGTTTGTAAAATTATTTACAGATATTGATTTGAATGATGATGAAGTCATTTCAATATTTGTTTTCGATAAGTCAATAGAATAA

Gene: fdh (NAD-dependent formate dehydrogenase)

Contig: 02_NODE_3, position: 102768 to 103793, length: 1026 nt, orientation: FORWARD

Perfect match to: (MW2-BA000033-[172478:173503], highly conserved allele)

Sequence:

ATGAAAATCGTAGCATTATTTCCAGAAGCAGTAGAAGGTCAAGAAAATCAATTACTTAATACTAAAAAAGCATTAGGATTAAAAACATTT
TTAGAGGAAAGAGGACATGAGTTCATTATATTAGCAGATAATGGTGAAGACTTAGATAAACATTTACCAGATATGGATGTGATTATTAGT
GCGCCATTTTATCCTGCATATATGACTCGTGAACGTATTGAAAAAGCACCGAACTTGAAATTAGCAATTACAGCAGGTGTAGGATCTGAC
CATGTAGATTTAGCGGCAGCAAGTGAACACAATATTGGTGTCGTTGAAGTTACAGGAAGTAATACAGTTAGTGTGGCAGAACATGCGGT
TATGGATTTATTAATACTTCTTAGAAACTATGAAGAAGGTCATCGTCAATCAGTAGAAGGTGAATGGAACTTGTCTCAAGTAGGTAATCA
TGCGCATGAATTACAACACAAAACAATTGGTATTTTTGGATTTGGTCGAATTGGACAACTTGTTGCTGAAAGATTAGCGCCATTTAATGTA
ACATTACAACACTATGATCCAATCAATCAACAAGACCATAAATTGTCTAAATTTGTAAGCTTTGATGAACTTGTTTCAACAAGTGATGCGA
TTACAATTCATGCACCATTAACACCAGAAACTGATAACTTATTTGATAAAGATGTTTTAAGTCGTATGAAAAAACACAGTTATTTAGTGAA
TACTGCACGTGGTAAAATTGTAAATCGCGATGCGTTAGTTGAAGCGTTAGCATCCGAGCATTTACAAGGATATGCTGGTGATGTTTGGTA
TCCACAACCTGCACCTGCTGATCATCCATGGAGAACAATGCCTAGAAATGCTATGACGGTTCACTATTCAGGTATGACTTTAGAAGCACA
AAAACGTATTGAAGATGGAGTTAAAGATATTTTAGAGCGTTTCTTCAATCATGAACCTTTCCAAGATAAAGATATTATTGTTGCAAGTGGT
CGTATTGCTAGTAAAAGTTATACAGCTAAATAG

Gene: lmrP (integral membrane transporter)

Contig: 02_NODE_3, position: 104179 to 105429, length: 1251 nt, orientation: FORWARD

Perfect match to: (ED98-CP001781-[158098:159348], allele observed in CC5)

Sequence:

ATGAAACGCTTAAGTACGACTTTGAAAGTACGATTGATTAGCAATTTTTTACAGCTAATTATTACGACAGCATTTATACCGTTTATAGCACT
ATATTTAACAGATATGTTAAGTCAATCAATTGTCGGTATATATCTTGTTGGTTTAGTGGTTCTAAAATTTCCATTGTCCATTATATCTGGTTA
CCTTATTGAGATTTTTCCGAAAAAGTTGCTAGTACTTATTTATCAAGCGACGATGGTGATAATGCTTGTGTTCATGGGCGTATTTGGGTCA
CATCAATTGTGGCAAATTATTGGTTTTTGTGTTGCATATGCCATATTTACAATCGTTTGGGGATTACAATTTCCAGTTATGGACACATTAAT
TATGGATGCAATTACCGAAGACGTGGAACATTATATTTACAAGATTAGCTATTGGATGACGAACTTATCGGTAGCTATTGGGGCATTGTT
AGGTGGCTTGATGTATGGCTACAGTATGTTACTACTTTTCTTAATAGCAGCTTGTATATTTTTAATTGTACTCTTTATTTTATATATTTGGTT
ACCTCAAGACCGAAATCAAGTAAAGCAAAGTGATGACAAGAGGCATGCAAGTCGTTATCAAAAATTACAAATAATGAATATATTTCGCA
GTTATAAATTAGTTTTGAAAGACCGTAATTATATGTTATTGATTTCGGGGTTCAGTATCATCATGATGGGTGAATTTTCAATCTCCTCATAT
ATTGCTATTAGACTAAAGGATCAGTTTGAAACAATAAGTATAGGTTCATATGATATTACAGGTGCTAAGATGTTAGCAATCTTGCTAATG
ATTAATACGGTCGTCGTCATTTTACTCACGTATTCAATCTCGAAAGTTGTATTGAAAATAGATTTTAAAAAAGCTTTAATCACTGGTTTGCT
GATTTATATTGTTGGCTATAGTGGTCTAACCTATCTTAATCAGTTTGGCTTATTAGTTGTTTTTATGATAATTGCGACTGTAGGTGAAATTA
TTTATTCGCCTATAGTTTCAGAACAACGCTTTAAAATTATTCCTAAAGCTAAAAGAGGAACATATAGTGCAGTTAATGCATTAGGTATTCA
TTTTTCAGAAACACTAGCTAGGTTAGGGATTGTGTTGGGTGTTTTCTTAACGTCATTACAAATGGGACTGTATATGTTTATCGTTTTAACAA
TTGGTGCTAGCATGCTTGTTGCTGGTGTATTTGGGGGACAAAAACAAGTGAATACAAATTGA

Gene: ausA (nonribosomal peptide synthase)

Contig: 02_NODE_3, position: 105876 to 113051, length: 7176 nt, orientation: FORWARD

Sequence:

ATGATTATGGGTAATTTGAGATTTCAACAGGAATATTTTCGTATATACAAAAATAATACAGAATCAACGACACACCGTAATGCGTATTGG
GTTAAACTCGCTAAAAATGTTGAAGCTACTAAAATGATGTATGCATTATCGACAATTGTGCAACAACATGCATCTATAAGACATTTTTTTG
ATGTTACTACCGATGACAATTTAACAATGATACTTCATGAATTTCTGCCTTTTATTGAGATAAAACAAGTTCCATCTTCTTCCGCAAACTAT
GATTTAGAAGCTTTTTTTAAGCAAGAATTAAGTACTTACCATTTTAATGATTCACCTTTATTCAAAGTTAAATTGTTTCAGTTCGCTGATGCT
GCATATATACTATTAGATTTTCATGTGTCCATTTTCGATGATAGTCAAATTGATATTTTTCTTGATGATTTATGCAATGCATATCGTGGCAAT
ACTGTTATTAACAATACTCGACAGCATGCACATATAAATAGAAATGATGATAAAGACAATCAAGATGCATCGCATATAGCATTAGACTCA
AACTATTTTCGGTTAGAGAATAACTCTGACATCCATATTGATAGTTATTTTCCAATTAAGCATCCATTTGAACAAGCTTTATATCAAACGTA
TTTGATTGATGATATGACATCAATAGATATGGCATCGTTGGCTGTTAGTGTGTATTTAGCTAATCATATAATGAGTCAACAACATGATGTC
ACATTAGGTATACATGTACCATCACATTTACCAAATGATTTACACGGAAATATTGTGCCGTTAACGTTAACAATCGATGCAAAAGATGTAT
GTCAACGTTTTACAACAGATTTTAATAAATGTGTGTTGCAAAATATGTCGCAATTACAGTGCGCGAAGTCTTCGCTTTCACTAGAGACTAT
TTTTCATTGTTATCATCATATGATGTCTTGTTGTAATGATGTTATTGAGGATGTACATCAAATACATGATGCACATACATCTTTAGCGGATA
TTGAAATTTTTCCACATCAACACGGGTTCAAAATTATATATAACAGTGCAGCATATGATTTGCTCTCAATCGAGACGCTGAGTGACTTAGT

TCGAAATATTTATTTGCAAATTACTGAAGAAAATGGAAATAAACGAACAACTGTAGATGAACTTAATTTGATGACAGAACGTGATATTCA
ATTATATGACGATATCAATTTAAGTTTGCCTGAGATAGATGATGCGCAAACAGTTGTTACCTTATTTGAGCAACAAGTTGAAGCAACGCC
GAATCATGTCGCTGTGCAATTTGACGGAGTGTTTATAACATATCAAACATTGAATGCACGCGCGAATGATTTAGCACACCGTTTGAGAAA
CCAGTATGGTGTTGAACCTAATGATCGTGTCGCTGTCATAGCTGAAAAAAGTATTGAGATGATAATAGCGATGATAGGTGTGTTGAAAG
CTGGTGGGGCTTACGTGCCAATTGATCTGAACTATCCAAGTGATCGTCAGGAGTACATTTTAAAAGATGTAACGCCTAAAGTTGTAATAA
CGTACCAAGCTTTATATGAAAATGGTAAACAAAATATTAATCACATTGATTTGAATAAGATAGCGTGGAAAAATATTGATAATCTTTCTAA
ATGTAACACGTTAGAAGATCATGCTTATGTTATTTACACGTCGGGGACAACTGGTAACCCTAAAGGGACACTAATTCCGCACCGAGGTAT
TGTTCGCTTGGTCCATCAAAATCATTATGTACCATTAAATGAAGAGACGACGATTTTGTTATCAGGAACTATAGCCTTTGATGCTGCAACA
TTTGAAATATATGGTGCATTGCTCAATGGTGGAAAGCTGATTGTTGCTAAAAAAGAACAATTATTAAATCCAATAGCGGTAGAACAATTA
ATCAATGAAAATGACGTTAATACTATGTGGTTAACCTCCTCATTATTTAATCAGATTGCTAGTGAACGAATAGAAGTATTGGTACCGTTAA
AGTATTTATTAATTGGTGGAGAAGTATTGAATGCTAAGTGGGTGGATTTGCTTAATCAAAAACCGAAGCATCCTCAAATTATTAATGGTT
ATGGACCAACTGAAAATACAACATTTACAACGACGTATAATATACCTAACAAAGTTCCAAATCGTATTCCTATTGGTAAACCGATTCTGGG
TACTCATGTTTATATCATGCAAGGCGAGCGTCGGTGTGGCGTTGGTATTCCTGGAGAATTATGTACAAGTGGCTTTGGGTTAGCTGCAGG
TTATTTAAATCAGCCAGAATTGACAGCAGATAAATTTATCAAAGATTCAAATATAAATCAGCTGATGTATAGAAGTGGTGATATCGTTCGT
TTGTTACCCGATGGCAACATAGATTATTTATATCGAAAGGACAAACAAGTTAAGATTCGAGGGTTTAGGATTGAGTTGTCAGAGGTTGA
GCATGCGCTCGAGCGTATACAAGGTATTAATAAAGCAGTTGTTATTGTTCAAAATCATGATCAAGATCAGTATATCGTTGCTTATTATGAA
GCGATGCATACATTATCACATAATAAGATTAAATCACAATTACGTATGACCTTACCGGAGTACATGATACCAGTTAATTTCATGCATATTG
AGCAAATTCCTATTACTATTAATGGGAAATTAGATAAGAAGGCATTGCCTATCATGGACTATGTCGATACGGATGCCTATGTAGCACCGA
GTACAGATACCGAACACTTGCTATGCCAAATTTTTGCAGATATTTTACATGTGAATCAAGTAGGTATTCATGATAATTTCTTTGAATTAGGT
GGCCATTCATTAAAAGCAACGTTAGTGGTGAATCGGATAGAGGCATCTACTGGGAAACGATTACAAATTGGTGATTTATTACAAAAGCC
AACTGTATTTGAACTAGCACAAGCGATTGCTAAGGTTCAAGAACAAAACTATGAAGTGATTCCAGAAACTATAGTTAAAGATGATTATGT
GCTGAGCTCTGCACAAAAGCGTATGTATTTATTATGGAAATCAAACCATAAAGATACGGTGTATAACGTACCTTTTTTATGGCGGTTATCA
TCAGAACTTAATGTAGCTCAATTGCGACAAGCAGTGCAGCGTTTGATAGCGCGACATGAGATTTTACGAACACAATATATTGTTGTAGAT
GATGAGGTTCGACAACGTATTGTGGCAGATGTTGCAGTTGACTTTGAAGAAGTTAACACGCATTTTACGGATGAACAAGAAATCATGCG
CCAATTTGTAGCACCTTTTAATTTGGAAAAGCCAAGTCAAATTAGAGTGAGATACATTAGAAGTCCCTTACATGCATACCTCTTTATAGAT
ACGCATCATATCATTAATGACGGTATGAGTAATATACAATTAATGAATGATCTTAACGCACTTTATCAACATAAATTATTGTTACCACTTAA
ATTGCAATATAAAGACTATAGTGAGTGGATGTCGCATCGTGATATGACGAAACATAGACAATATTGGTTATCTCAATTCAAAGATGAAGT
ACCTATTTTAAGCTTACCGACAGACTATGTTAGACCAAATATTAAAACGACAAATGGAGCAATGATGTCATTTACAATGAATCAACAAAT
GAGACAGCTACTTCAAAAGTATGTAGAAAAGCATCAAATTACTGATTTTATGTTCTTTATGAGTGTGGTCATGACGTTGTTAAGTAGATAT
GCTCGAAAAGATGATGTTGTTGTCGGTAGTGTGATGAGTGCGCGTATGCATAAAGGCACGGAGCAAATGCTAGGCATGTTTGCTAATAC
GTTGGTATATAGAGGGCAACCGTCACCTGATAAAATGTGGACACAGTTTTTACAAGAGGTTAAGGAAATGAGTTTGGAGGCATACGAGC
ATCAAGAATACCCATTCGAATGTTTAGTAAATGACTTAGATCAATCACATGATGCCTCACGGAATCCATTATTTGATGTCATGTTAGTACT
ACAAAACAATGAAACGAATCATGCTCATTTTGGGCATAGTAAATTAACACACATTCAACCCAAATCAGTGACGGCGAAATTTGATTTATCT
TTCATCATTGAAGAAGATCGCGATGACTATACAATCAATATCGAGTATAATACCGATTTATATCACTCAGAAACAGTTCGTCACATGGGTA
ATCAATGTATGATTATGATTGATTATATTTTGAAGCATCAAGATACACTACAAATTTGTGATATACCAAACGGCACGGAGGAACTTCTAAA
TTGGGTCAATACGCATGTTAACGATCGAATGCTTAATGTCCCGGGAAATAAATCTATCATAAGTTACTTTAATGAAGTTGTCTCACGACAA
GGTAATCATGTTGCGCTAGTCATGAATGATTTGACAATGACGTATGAAACATTACGCAACTATGTGGATGCCATTGCGCACATGCTCCTA
TCAAATGGTGTGGGCAATGGTCAACGGGTTGCCTTGTTTACAGAACGTAGTTTTGAAATGATTGCGGCGATGTTGGCGACAGTTAAAGT
AGGTGCATCTTATATACCTATCGATATTGATTTTCCGAATAAACGACAAGGTGCAATTTTGGAGGATGCTAAAGTAACTGCAGTCATGTCT
TACGGCGTTGAAATTGAAACGACATTACCAGTCATTCAATTGGAAAATGCTAAAGGCTTTGTTGAATCAAAGGAAAATGAACAATATGAT
GATTTACATGGCAATCAACTTGAAAACACAGCGATGTTAGATAATGAGATGTATGCTATTTACACATCTGGTACGACCGGGATGCCTAAA
GGGGTTGCCATACGACAACGAAATTTGTTGAATTTAGTGCATGCATGGTCAACTGAATTGCAATTAGGCGACAATGAAGTATTTTTGCAA
CATGCAAATATTGTTTTTGATGCATCAGTTATGGAGATTTATTGTTGTTTGTTAAATGGTCATACGCTTGTGATTCCAGATAGAGAGGAAC
GTGTTAATCCAGAACAGTTACAACAACTCATTAATAAGCATCGTGTGACGGTTGCGTCGATTCCGTTACAGATGTGTAGTGTTATGGAAG
ACTTTTATATTGAAAAGTTGATTACAGGCGGGGCAACTAGTACGGCATCCTTTGTTAAATATATTGAGAAGCATTGTGGCACGTATTTCAA
TGCCTATGGACCATCTGAGTCAACAGTCATCACATCGTATTGGTCACATCAATGTGGTGATTTGATACCTGAGACGATTCCAATTGGCAAA
CCCTTATCTAACATCCAAGTGTATATTATGTCAGATGGTTTGTTATGCGGTATTGGTATGCCAGGCGAGTTGTGTATTGCAGGTGATAGTT
TAGCGATAGGATATATTAACCGTCCAGAATTAATGGCTGATAAATGGCAAAATAATCCATTTGGTAAAGGAAAGTTGTATCATAGTGGTG
ATTTAGCACGTTATACATCTGATGGTCAAATTGAATTTTTAGGAAGAATAGATAAACAAGTGAAAGTTAACGGGTACCGTATTGAACTTG
ATGAAATTGAAAATGCAATATTAGCTATTCGTGGTATATCTGATTGTGTTGTTACAGTAAGTCACTTTGATACGCATGATATATTGAATGC
TTATTATGTCGGAGAGCAACAAATGGAGCAGGATTTGAAGCAATATTTAAATGATCATCTGCCTAAGTATATGATTCCTAAGACTATAAC
GCATATCGATTATATGCCATTAACCACGAATGACAAGGTGGATACTACGCGTTTGCCAAATCCATCAACTATACAACAGTCTAATAAAGT
GTATAGCGAACCATCTAATGAAATTGAGCAGACATTTGTTGATGTATTCGGAGAGGTATTGAAACAAAATGATGTCGGTGTTGACGATG
ATTTCTTTGAACTTGGTGGTAACTCATTAGAGGCGATGTTAGTTGTCTCGCATTTAAAACGATTTGGCCATCATATTTCAATGCAGACATTA
TACCAATATAAAACCGTGCGACAGATTGTTAATTATATGCACCAAAATCAACAATCATTAGTTGCATTACCGGATAATCTTTCGGAATTAC
AAAAGATTGTTATGTCTCGTTATAATTTGGGTGTTTTAGAGGATAGTCTAAGTCATCGACCTATAGGAAATACACTATTGACTGGCGCGA
CAGGGTTTTTAGGTGCTTATCTGATTGAAGCACTACAAGGATACAGTCATCGCATTTATTGTTTCATACGTGCTGATAATGAGGAAATAGC
AAGGTATAAGTTGATGACGAATTTAAATGATTATTTTTCAGAAGAGACGGTTGAAATGATGTTATCAAACATTGAAGTCATTGTTGGTGA
TTTCGAGTGTATGGATGATGTTGTTTTACCAGAAAACATGGATACGATTATTCATGCAGGTGCTCGTACAGATCACTTTGGTGATGATGAT
GAATTTGAAAAAGTAAATGTTCAAGGTACTGTTGATGTCATACGTTTGGCACAACAACATCATGCAAGGTTAATATATGTGTCTACGATA

AGTGTGGGAACTTATTTTGATATAGACACAGAAGATGTGACATTTTCAGAAGCGGATGTTTATAAAGGGCAACTACTAACATCACCATAT
ACACGGAGCAAATTTTATAGTGAATTAAAAGTATTAGAAGCTGTAAATAATGGCTTAGATGGTCGGATTGTACGTGTTGGTAATTTGACG
AGTCCTTACAATGGAAGATGGCATATGAGAAATATAAAGACTAACCGTTTTTCAATGGTAATGAATGATTTGTTACAACTGGATTGTATC
GGGGTTAGCATGGCTGAAATGCCTGTAGATTTTTCTTTTGTGGATACGACTGCAAGACAAATTGTCGCATTAGCACAGGTCAACACACCA
CAAATCATTTACCATGTGCTATCACCTAATAAAATGCCGGTGAAATCTTTGTTAGAATGCGTTAAGCGCAAAGAAATTGAACTCGTCAGC
GATGAATCATTTAATGAAATTTTACAGAAACAAGACATGTACGAAACGATTGGATTAACTAGTGTTGACCGTGAACAACAACTAGCAATG
ATAGATACAACATTAACATTAAAAATAATGAATCACATCAGTGAAAAATGGCCAACGATAACTAACAATTGGCTGTATCATTGGGCACAA
TATATCAAAACAATATTCAATAAGTAA

Gene: ausB (holo-[acyl-carrier-protein] synthase)

Contig: 02_NODE_3, position: 113064 to 113708, length: 645 nt, orientation: FORWARD

Perfect match to: (08-02119-CP015645-[2046917:2047561:r], allele observed in ST582+CC25+CC361)

Sequence:

ATGACAGTATTTGTAATGCAATTACAGAGTAACTTGAAAAGTATTGAAGAATTAATATCACAAAGTCGTTGGTCATATAAAAAACCGCGT
ACAGTCAACTATAGATACAATCAAGATAAACTCATGCACAGATTGGGAGATATTTTAGTGCAATATGGAATTCAACATGACACAGGTTTA
TTACCACATGAATGGCATTATCACATTTCGCCACGAGGTAAGGCAGATATTGTTCAACACAATCGTGATGGACAGCCCATCTATGTGAGC
TTATCATATAGTTATCCTTATATCGTGTGTGTTGTCGATAAAGAACCAGTTGGTATTGATATCGAAAAGATATCACAACGTTTAGACTGGC
GTACGTTAGTGACGTGTTTCTCTACAAACGAAGCACATCAAATATGTAGTTTAAATGATTTTTATCAAATATGGACACAAAAAGAAAGTTT
TACAAAATTGATTGGTGAAGGTTTAATCAAAGGATTGGACATTTATGATATGACACAATCACACTTTTATCAATCACGTGAAGTGAAGTTC
AAACAATTTATTTTTGATCAGTTTATGGTACAGGTATGTTTCTTAGGACAGGCACCCTGGGGGTATAAAAAAGTGTCTGTATTTCAGTTAT
TGAGTAGTTAA

Gene: Q5HJJ2 (putative protein)

Contig: 02_NODE_3, position: 114036 to 114530, length: 495 nt, orientation: REVERSE

Perfect match to: (MW2-BA000033-[183746:184240:r], highly conserved allele)

Sequence:

TTAATTTAATTTATAGCGATAGACAAAATGTTGTCGCACTAATTCAATTGTCATCATCCAGACGATATGGCCAAAGAATTCTGACAGATGC
TCTTGGAATGGTTGATCCCACACAGCAGGTACAGTATGCATGATTGGCATAATGATAAGGTGGAATAATACCCAAATAGCAATACCAAA
AACAGCACCTTGTCCCATTGCTAAGTAAGCGTATTTTTTAACTAATATGCAGTAAATAATTGCAATGACGATAGAAAAACTAAAGTGGAC
AATAAAGCTTACCCAAGGCAATTCCATATTTGAAAATGTATATGTTTGATGCGTAAACTCACTACTAAATCCTAATTGTTGCAATAACTCTT
GAGGTGGGTTCGTTGCATTACGTTCTGGTGTGCGAGGTGGAAACATGACCTCCCAACCTAATTTTACAATTCCAGATAACAAGCCACCGA
TAATTCCAGCATAAATGAATATACCCATTTGTCGTTTCGTCAA

Gene: argB (acetylglutamate kinase)

Contig: 02_NODE_3, position: 114779 to 115549, length: 771 nt, orientation: REVERSE

Sequence:

TCATAGTTATGAATTAAGGATTGTTGTGCCAACAAAATCATTATTGTAAATAGATTCAATGATATTTGGCTTGTTTCCTGATGCAATGATA
ACTTTAGGACAGCCATTTTCAATCGCATTTTTGGCATCTAGCACTTTGGGAATCATACCTCCATAAATATCACCATGTTCAATATATTGATG
AATATCGACTAATGGCAATTGAGGTATAACAACATCATTGATGAGTACACCTGCAATATTACTTAATACATAAATAGGCGCTTTTAATGAT
GATGCAATAAAATAGGCAAGCGTGTCAGCATTAATATTGTAAAATTCTCCATCATGGTTATTGAAACCAATCGAATTGATGATAGGTACA
AATTTAGTACATAAATACTGTAAAGCATCCTTATTTAAAGCGGTCGGAACACCGACATATCCATATTGTTGATCAAAAGATGTAATTTCAA
ACAGCTGTGCATCCAAACCACATAAGCCTATTGCAGAACATTGGTGCTGGTTAAATTGAGCTACTAATGCAGTGTTAACGTCTGCAATGA
GCGTGTGTTTAGTAATGGTCATGGTTGCTTTATCAGTCACTCTTAGGCCATTAACAAAGTGTGGCTCGATTTGCTGGTTTGATAATGCTTC
ATTAATAAATGGGCCACCGCCATGAACGATAATGGGGTAGATGTTGTTTGATCGTAAATGCTTAATGTTGTTAATAATTGATGGATGCAT
GTCACTAAGTGTACTGCCACCAATTTTAATGACAATAAATTTCAT

Gene: argJ (arginine biosynthesis bifunctional protein)

Contig: 02_NODE_3, position: 115565 to 116806, length: 1242 nt, orientation: REVERSE

Perfect match to: (MW2-BA000033-[185275:186516:r], highly conserved allele)

Sequence:

TTATGTTCGATATGATGCGTTGATACGCACATAATCATAGGATAAATCACAACCGTATGCAGTCGCTGCAGCGTTACCTAAACCAAGCTG
AACGTCAATTGTGACATTTTCATGAGTTAATGTATTCGACATAGCTTGCTCATCAAATAGTACAGCCATACCTTTATCAACGACAGGTATTT
GGTTCAGTTGAACATATGTGCAGTTAGGATCAATTTCACATCCGCTGTAGCCAATAGCTGTAATGATTCGACCAAAATTGGCATCTTCGCC
AAAAATAGCTGATTTTACTAGATTTGAACTTACGATAGTTTTACCGATTTTTCTTGCATCTGATATTGATTTAGCGCCTGACACATTGACGC
TGATTAACTTTGTTGCGCCTTCGCCATCTCTGGCTATAGCTTTAGCTAAAAATGTACAGACAAAATTGAATGCATCAACAAATGTTTCCCAT
TGTGGATGGTCTTGACTAAGTATTTGGTGTTCAACTTGGTGATTTGCCATGACTAATACCATGTCATTTGTACTTGTATCGCCATCAACAGT
AATCATATTAAATGTATGGTCAGTCGAAGATTTTAATAATTGATGAAGTGTATTCGATTCAATCGATGCATCGGTTGTTATAAAAGCAAGC
ATGGTAGCCATATTTGGGTGAATCATACCTGAACCTTTGGTGCTACCACCAATTGTAACGGTTTTACCATCGATTTTTAGTGATACAGCGA
TATGTTTTGTACAGGTATCAGTTGTTAAAATTGCCTCGTTAAACGCACCTGGCGTTGCAAAATTAGCATCCTTAATATGTTCGGTCCCAGTC
TTAATTTTATCCATAGGCAAATATTCACCAATGACCCCAGTTGAAGCAACAGCAACATGCTCAGATGGTATTTGAAGTTGTTGAGCAACCC
ATGTTTGTGTTTGTCGTGCATCATCTATGCCTTGTTGACCGGTACAAGAATTTGCATTAGCTGAATTAACAACAAGTGCTTGTAATTTTCCT
TTAGACTTTTGTAAAGTGTCTTCAGTGACAATAAGTGGTGCAGCTTTAAACTGATTTAAAGTATATACGGCAGCTGCACTTGCCAAAGAC
GATGAGTAAATCCACCCAAAGTCTTTTTTGTTAGCGCGTAAACCGATGTGCATACCACCAGCCGTGAAGCCTTGAGGTGTACTGATATCG
CCATGTTTAATAATTGAAAAGTTATATTGTTGTGATGTCGTTTCTTGATGTTTCAT

Gene: argC (N-acetyl-gamma-glutamyl-phosphate reductase)

Contig: 02_NODE_3, position: 116818 to 117852, length: 1035 nt, orientation: REVERSE

Perfect match to: (MW2-BA000033-[186528:187562:r], highly conserved allele)

Sequence:

TTATGGATAAACTGGTGATTGATTTAGGCCAGTCGTCACTTCAAAATCATATAATATATTTAAATTTTGAATGGCTTGCCCACTTGCGCCTT
TGACAAGGTTATCAATCACTGATACTAAAATTGCTGTTTGCGTTGTTTCATCTACATAGATGCCGATATCGCAGTAGTTACTACCGAGTAC
TTCTTTTGTGGTTGGAAAAGTCCCAATATCTCTAATTCTGACAAATGGCTGATTAGCATAATAAGAGGTCATTAATTTATGTAATGATTCA
GTCGTATATTCAGATGATAATTTGACATATATTGTTGATAAAATACCTCGTGTCATTGGTACGAGATGTGGTGTAAATATGACTGATACAT
CTTGACCCGCAATGATAGATAAATATTGCTCGATTTCCGGTTTGTGTTTATGGTTTCCGATTGCATAAGCGCTTAGATTTTCATTCATTTCT
GAAAAATGAACACGTTGTGATAATGAACGACCAGCACCTGACACGCCGGTCTTAGCATCAATAATAATAGATGACAAATCTACTATTTTT
TCGCTAATAAGTGGATGTAATGCTAATAATGTTGCTGTAGGGAAACAGCCAGGATTAGAAATGAGCTTCGTTCCATTGTTATCAAACGAT
TGCCATTCTGAAATGCTGTAAATAGCATGATTCAAATCATCTTGTGCTGCAGCAGTTTCTTTGTAATATGCTTCATATATTTCACGATTCTTA
ATTCTAAATGCGCCAGATAAATCGATAACATGAATACCTTTTTCTACTAAGGGAGGGATACATGTTTTACTTACGGGTGCTGGTGTCGCA
AAGAAAATTACATCACAGTCATTATTGTCCACTGTAAGTGCTTCGAAATGTTGCATAATATGTTGTAAATGTGGAAATGTTAATTTCAACG
GTTCATCTACTTTTGAATGTGAGTAGATGTGTGCAATCGTTACATGAGGATGTGTTTGTAACAATCGAATTAATTCAATTGCGCCATAACC
GCTACCGCCAACGATACCTACTTTAATCATCAT

Gene: rocD1 (ornithine aminotransferase 1)

Contig: 02_NODE_3, position: 117888 to 119072, length: 1185 nt, orientation: REVERSE

Perfect match to: (COL-CP000046-[194970:196154:r], highly conserved allele)

Sequence:

TTAATTTTTAAAAACGTCTTGAAAAGCTGCAACAATTTGATGGATTTCCTCTTTATCAATGACTAGAGGTGGAGACAATCGAATGATAGTA
CGATGCGTGTCTTTGCATAAGATTCCACGTTGAATCAGTTGATCCACAAAAGGTGCAGCATCTGTGTTAAGCTCTATGCCTATAAATAAAC
CACGACCTCTAATTTCTTTAATACTAGGATGTTTAAGTTGTAGCAACGCTTTTAATAAAAATGAACCTAAGCGTTCTGATCGTTCAACCAGT
TGTTCATCTTTAAGTACATCAAGCGCTGCCGTCGATATTGCAATGGCTAAAGGGTTACCACCAAATGTTGAACCATGTGTACCTGGTGTTA
GAACACGCATGACATCATTATTTGCAAGTACAGCAGATACAGGGTATAAGCCGCCACCCAATGCCTTACCTAAAATATAAATGTCTGGAA
CGACTTGCTCCCATTCCATAGCAAACCATTTCCCAGTTCTACCAAGACCAACTTGAATTTCATCTGCAATCAATAATATTTGATGTTTATCAC
ATAGTTGACGCACAGCTTGAATATATCCTTTCGGTGGTATATTAACGCCACCTTCACCTTGAATTGGTTCCAAAATAATTGCTGCTGTATTC
GGTGAAATAGCTTGTGTTAATTGTTCAATGTCTCCAAAATCTACTGTTGTAGTGCCTTGAAGTAGGGGGTGAAATCCTGCTTTATATGCGT
CGTGGTTAGATAGTGATAATGAGCCAAGTGTACGACCGTGAAAATTGTTATTCATAGCGATGATTTCAACTTGTCCGTCAGTAATGCCTTT
AACTTCAGAGCCCCATTTTCTAGCAATTTTAATGGCTGCTTCAACAGCTTCAGTACCAGAGTTAAGGGGGAGTACTTTGTCTTTCTTAGCA
AGATGACAAATTTTTTCTTCCCATTTCCCGAGATTGTCACTATAAAGGACACGTGAAATGATAGACAACTTTGAAGCTTGTTCTGTCATCG

CTTTAACAATTGTTGGATGACAATGGCCTTGGTTTGCAACTGAAAAACCCGAAATGCAATCTATATATTGTTTGCCATCAGTATCCCAAAC
TTTGACACCTTTACCTTTAGAAATGACAAGCTTAAGTGGTGCATAATTATTAGAGCTATAATAATCAGTTAATTCAATGATTGAATTCAT

Gene: brnQ1 (branched-chain amino acid transport system II carrier protein)

Contig: 02_NODE_3, position: 119325 to 120680, length: 1356 nt, orientation: REVERSE

Perfect match to: (MW2-BA000033-[189035:190390:r], allele observed in CC1+CC5)

Sequence:

TTATGCTTGTGGAATTTTACGATGCTTAATTTTATAAATAATGAAGCCGATAATGAAACCAATCAAACTGAGAACAACCCAGCCCATACCA
ATGTCTGATAATGGTAAATATTTTTGGCTGAAATTAATCAAAGTTTGTGAGAATGATGTGCTTGAAATGAACTCTGGACTAGCTTTTAATC
CATCTACTAATGCAGCAATCATTGTAAAGAAAATGGTACATTGATAAATAAGTTTTGAATGATGGAATTTGCTACTAAATAATGTTAGTAC
AATCAAGGCAATTGCTAATGGATATAAGAACATTAACACTGGGACTGAGTACATAATAATCTTAGTTAAACCAACATTCGCGAATAAGAA
CGAAATAAAGCTTACAACTGTTGCAATCGCTAGGTAATTCATTTTAGGGAAAAGGTGTTCGAATGTTTCTGAAAATGCCGTAATCAAACC
GATGGCTGTTTTTAAACAAGCAACCATAACGATAAGTGACAACAGGACGATACCGTAGTTACCTAAGTAGTATTGAGTAATTTGCGCTAA
GGCAATACCACCATTTTCACTAAGTTTGAAATGACCAATACTTAATGTACCCATGATTGCTAGTAGGGTATAAATGATCCCCATCATAATG
ATACTGATAGTACCAGACTTAATTGTTTCTTTAGCGATATCAGTTGGATTTTCGATACCTAACTTTTTAATCGTTGCAACAATGATAATACC
AAATGCCAATGACGCTAGCGCATCTAAGGTATTGTATCCATCTAAAAAGCCGTTAAATAAGGCATTTGATTGATATTGTTTACTAATAGGT
GCATCAGATATGCCACCTAATGGATGGATAAAAGCAAATAATAAAATAATTGCTAATAATACTAAGAATACCGGATTTAAAAATTTACCG
ATATATTCTAAAATTCTTGATGGCTTTCTCGCAAAAAACCATGCAATCACAAAGAAGACGAAGCTAAAAATAAATAAATATAAAGTGATTT
GCTTTGGTGATAAAAATGGCGAAAATGCAATTTCAAATGATGTCGTTGCCAGTCTAGGTAAGGCGAAAAATGGTCCGATAACTAGATAT
AAGGCAATCGTGAAAATGTAAGCATATGTTTTATTAACACGCGATGCAATTTCAAATAAACCAGATGTCTTTGAAATGCCAATAGCAATG
ATACCTAGAAATGGTAAGCCAATTGCTGTAATTAAAAATCCTAAGTTAGCGATAAAAACGTTAGAACCAGCAGCTTGACCCAAGTGTATT
GGGAAGATAAGATTGCCGGCACCAAAGAATAAACCAAATAACATAGAACCTATAAACATGTTTTCTTTAAATGTTAGTTTCTTCTTCAT

Gene: ywoC2 (isochorismatase)

Contig: 02_NODE_3, position: 120956 to 121453, length: 498 nt, orientation: REVERSE

Perfect match to: (MW2-BA000033-[190666:191163:r], allele observed in CC1+CC8+CC4803)

Sequence:

TTAGTTCAATATATTTAGAAAGTCGTTTGTAGTTATTGTTTGTCCCATCAATGGGAATACATTATCTATTGGAAATTGATGTAGCGTTTCGT
TTTGTGCACTCATCATATCTGTAACAAAAAACTGATTGTAGTTTAATTGATAGGCATCTCGCGCTGTCGTATCTACGCCAATATGCGTTGC
GACACCACCAAGAACAATCGTATCAATTCCTCGACGTCGCAATTGTAAGTCCAAATCTGTTCCTACAAATGCACTAAAATGTCGTTTGTCT
ATGACAAAATCGTCATCTCTCTTGTCTAATAAATGATGGAAACGACTGTAGTCGTCGCCTTCTTTTGGTGGTAATGAGATCATTGCATTTG
GTTGCAATACATCTTTACCATCATAGAAATTCACGCGAACAAAAGCGATAAAGCCATTGTTTTTTCTAAAAACATCTATTAATTTATTAGCG
TTTTGAACGACATTTTCAGCTGTATATGGGGCATAATCCAT

Gene: ipdC (indole-3-pyruvate decarboxylase)

Contig: 02_NODE_3, position: 121580 to 123220, length: 1641 nt, orientation: REVERSE

Sequence:

TTATTTATTTTGTTGTGAAAAAGCTTTAGCGATATCGATGAGTTTCTTCGGTGCGTCTTCGACAGACATTTTGACTTCGACAAAATGCATCA
CATCGGGATGACCATTAATTGCATTAAACGTGTCTTGTAAATCTTTTGATGATTCAACGTCATGAATTTCAACATTTTTACCACCAAATACA
GCTGGTAAAGCTTTATAATCCCACATGTGAATTTCATTATAAGGTTCATACATGCCGTGAATAAGTCGTTCTACCGTATAGCCGTCATTATT
AATCACAAATAATACCGGTTTAATATGCTGTCTAATCATAGTTGAAATAGCTTGAACAGTTAGTTGCAATGAGCCATCACCAATTAATAAT
AAGTTACGACGATCTTTGTCTGCTAATTGTGAACCTAATGTTGCAGGTAATGTATAGCCGATAGAACCCCATAACGGTTGCCCTATAAAA
GTATTGTTTTTGTATAATGCTAAATCATAAGCACCAAAGAATGATGTACCTTGATCAGCAATGATGACATCATTTGGTTTTAAGAAATTTT
GCATCATTTTAAAATAAGTTTGTTGTGTTAATGGTTCTGTGCCAACAGTATAATCGGGTGATGTTGGACGATGATACGCAGGGAACGTTG
CGTTATTCGTATATGAAATATTGGATAATTGTTTTAACAATGATGGTAGAGATATTTCATCATTTGTAACATCGTCAATTTTGATATTGTGA
TGATTTAACATAACGACATCATCGATATTGAATTGGTATGAAAAACCTGCTGTTGCTGAATCTGTTAATTTGGCTCCAATATTTAAAATTAA
ATCGCTGTTGTCCACATAATCTCGTATTTTATCTTCGGCAATTTTCCCATCGTAAATACCCATATAATATGGATTTTCCTCATTAAAAGCACC
TTTTCCTAATGAAAGTTGTGCTACTGGTATCTGTGTTTGATTTACAAAATCTTCTAATTCTTGATGAAGGTGAAAACTGTTGATTTCATGTC

CAGTAATGATGATAGGCTGCTTCGATTGATGCAATTTAGATGTTAATAACTCTATATATGTTGATGCATCCGTATCTTTGGCTGCCGTTACT
TCAAATGGTGTCGGTATCTCAATTTCAGAGATTGCGACATCGATTGGTAAATGTAAATGAACTGGGCGTCTTTCGGCGATTGCTGTATTA
ATTAAACGTGGTATTTCGGTTGTTGCATTTTCAGGTGTGATATAACCTTGTGCAACGGTTATATGTGCAAACATTTTTCGGTAGTCGTCAA
ATGTACCTTCACCAAGTGAGTGATGTACATATTTACCGGCATGTTCAACAGCACGTGTCGGCGCACCTGTAATCGCAATGACAGGTATGC
GTTCAGCATATGAACCTGCGATACCGTTGACGGCACTTAATTCGCCAACACCAAATGTAGTAACTAATGCAGCGAGTCCATTAAGACGGG
CATAACCGTCCGCTGCGTAACTTGCGTTTAATTCATTTGTATTTCCTACCCAATCTACATTGGGATTGCTGATAATATCGTCTAGAAAAGCG
AGATTAAAATCACCAGGAACACCAAAAATTTTATCGACGCCTGCTCGATGAATAGCGTCAATTAAGTAAGCTCCAATGCGTTGTTTCAT

Gene: glcA (glucose-specific PTS transporter protein II, ABC component)

Contig: 02_NODE_3, position: 123493 to 125538, length: 2046 nt, orientation: REVERSE

Perfect match to: (MW2-BA000033-[193203:195248:r], allele observed in CC1)

Sequence:

TTATTTAGCTTCAAATAATTGATCGCCAAATGAAATGTTGCCATGTTCACCTTGTTTAAAATCAAGGTTTGTAATGTTTCCTTGTGTCACGA
TAATAGGCGTAATATCACTCTTTGCATGATTGCGGATGTAGTCTAAATCAAAGTTGATTAATAAATCACCTTGTTTAACTTCTTGACCTTCC
TCAACATGTAAAGTAAAGCCTTCTCCGTTTAATTTAACAGTGTCTAAACCGATGTGGATTAATAATTCTAAACCACTATCTGATACAAGAC
CAATTGCATGTTTTGTTGGGAAAATCATTTGTACTTTACCGTTGAATGGTGCACGAACTTCACCTTGTGAAGGTTTGATAGCGATACCGTC
ACCCATCATTTTTTCGCTGAACACTTGATCAGGCACTTCTGATAATGGTGTTACTTCACCAGTTAATGGTGCATGCACGATATGGCTCAATT
CGCTTGTTGCAGATTTATCTTCTGCAACAACAACAGTTTCGTCTTTATCGTCTTCCATAGTAGTAGGATTTTCTACTACTTGACCATTCATAA
TCTGTTGCATTTCATGTTTGATTTGGTCAGATTTAGGACCAAAAATTGCTTGCATATTATTGCCGACTTCTAATACACCAGATGCGCCTAAA
TCTTTCAAACCAGGAACATCAACTTTAGATTTGTCGTTAACTTCAACACGTAGACGTGTGATACAAGCGTCTAAATGTTTAATGTTTGCTTT
GCCACCCATAGCTTCTAATACTGCATATGGTAATTCAGTTGCTGAAGCAGTAGCCGCTTGTGATTGTTTATCTTCACGACCTGGTGTTTTGT
ATTTTAATTTTACAATTAAGAATCGGAATACGAAGTAGTAAATAACTGCGTATACAAGACCTACAGGAATAACTAACCACCATTGTGTCTT
ATTAGGTAGTATACCGAGTAAGAAGTAGTCGATGAAACCACCTGAGAATGTATAACCTAGATGAAGATCTAATAAGTACAATGTTAAGA
ATGATAAACCATCAAGTACTGCGTGAATAAAGAATAATAATGGTGCTACAAATAAGAATGAGAATTCTAATGGTTCTGTAATACCAGTTA
AGAATGATGTTAAAGCAGCAGAACCCATTAAACCTGCTACTACTTTCTTATTTTCAGGTTTAGCTGTGTGATAAATTGCTAAAGCTGCTGC
AGGTAAACCGAACATCATAACAGGGAATTCACCTTGCATGAATTTACCAGCTGTCAAATGTGCGCCTTCACGAATTTGTTCGATAAAGAT
ACGTTGGTCACCGTGAATAATTTCACCAGCTGCATTTTTCCATGAACCAAACTCGAACCAGAACGGTGCGTGGAAAATGTGATGTAGACC
GAATGGAATTAATAAACGCTTGATGAAACCAAATAAGAATACGGCAACACCAGTATTTGAATCTAATAATCCTGTACTGAATGCATTTAA
TCCTGATTGAATCGTTGGCCAAATTAATGCCATTGGGAATGCTAAAATAAATGATGTTGTAGCCATCATAATAGGTACGAAACGCTTACC
AGCGAAGAAACCTAAATAAGATGGTAAGTTAATGTTATAGAACTTGTTATAACACCAAGCTGCCAGGGCCCCGATTATAATACCGCCGAA
CACACCTGTTTGTAATGTTGGGATACCTAAAATGCTAGCGTAACCACTCGCTGGATCACCAATATTCTTAGGTGTAACTTGTAAAAAGTCG
CCCATTGTTTTGTTCATGATTATGTAACCGACGAATGCTGCGATAGCTGCTACGCCATCACCGCCAGCTAATCCGATTGCGACACCTAATG
CGAAAATCATAGGCAAGTTATCAAAAATGATACCACCAGCACCTGTCATTAATTTAGCGACAGTTTGTACGCCACCATTTTGTATAAACGG
CAAGTAGTGTTGTAATGATTCACCTTGCATAGCTGTACCGATAGCTAATAACAGACCCGCTGCTGGTAAAATTGCAACAGGTAACATTAG
CGCTTTACCAATACGTTGCAATTGACCGAAAAGTTTCTTCCTCAC

Gene: rsaK (ncRNA of Staphylococcus aureus K)

Contig: 02_NODE_3, position: 125597 to 125808, length: 212 nt

Sequence:

AATAGACTGTGTTGGTAACCATCACAGCTTAATTTAACTCATGCCTAATCTTACTTAGTAACACGTTGGTGTATGTAATTAATGTAAAAGA
AGCAATTGACTAAGTAGTATATAAAACTGATACGTTAGTTTATCTAGCTTACCATCACATCTTATTGAATCTTATTTGTTTGCGACTCCTATT
TTAGCATGGTGAAGTATGCGTTTTCAAT

Gene: DUF871 (putative protein)

Contig: 02_NODE_3, position: 126123 to 127178, length: 1056 nt, orientation: FORWARD

Perfect match to: (MW2-BA000033-[195833:196888], allele observed in CC1+CC25)

Sequence:

TTGACAGGCTTTTCAGTGTATTTAGGACAACCTTTAGATGAAGCGTATATTAAGCGAATGATTAAACAAGGTTACCAAATGATTTTTACAT
CTGTACAAATACCAGAAGAAGATGACGAGACAAAATATCATTATTTCACAAAACTACTCAATTTATTAAAACATGAACAAGTGACTTACCT
CATAGATGCTAATCCATCTATATTAACACCATCTTTTTATGAGCATCTTCGACAATATGATGCGCAATTTATGATTCGTATCGATCATAGTA
CATCAATTGAGGCAATCGAAGCGATAATGGCACAGGGTTTAAAGTGCTGTTTGAATGCAAGTATTATTTCCCGGGAATTGTTAACAAGCT
TACATCAACAATTGAATGATTTTACATTACTTTCATTTTGTCATAACTATTATCCAAGACCAGATACGGGATTATCTGTTGACTTGGTCAAT
AAGAAAAATGAACTCATTTATCAATTTAATCCAAAGGCACAAATATATGGTTTTATTGTAGGGAGTGATTTGCGAGGTCCTTTGCATAAA
GGCTTGCCAACAATTGAAGCAACGAGACATAGTCATCCTGTCGTTGCAGCTAAATTATTACAAGAAACTGGTGTATCTGAAGTGTTAGTT
GGAGACTCATTGATTGAAATGAGGCAGGCAAAACAACTTATAGATTTTTGCAAGCATAGGCATTTCACGTTATGTATTGAAGAAGTGTTT
GATACGACAGTGACTTACCTTTTCGATATGTGTCATAAAGTACGCCCGGATAATCCGGAAAATGTCATTCGTTCGGAAACGTCAAGACAA
ATATGTCCACATTCGATTCAACCACAGTTTACGACGCAACGACGCATTGGTTCAGTAACCGTTGATAATTTGAATAACGGACGTTATCAAG
GCGAAATGCAAATTGTGAGACAAACGCTTAGTGCACATGACAATGTGAATGTTGTTGCACAAATTATTAAAGAAGACTTACCACTGTTAA
GTTGTATCGAGCCGAATGATACATTTGATTTTCAAAAAACTAGGGAGTGTAAGAAGTGA

Gene: murQ (N-acetylmuramic acid 6-phosphate etherase)

Contig: 02_NODE_3, position: 127175 to 128074, length: 900 nt, orientation: FORWARD

Sequence:

GTGATGGAAAATAGTACGACCGAAGCGCGTAATGAAGCGACGATGCATCTTGATGAAATGACTGTGGAAGAGGCTTTAATTACGATGA
ATAAAGAAGATCAGCAAGTCCCGTTAGCAGTTCGAAAGGCAATACCACAATTGACAAAAGTAATTAAAAAAACAATTGCACAGTATAAA
AAGGGTGGACGATTGATTTATATCGGTGCAGGTACAAGTGGAAGGTTGGGTGTCTTAGATGCAGCGGAGTGTGTACCTACATTCAATAC
TGACCCTCATGAAATTATAGGTATTATTGCTGGTGGACAACATGCTATGACGATGGCTGTAAAAGGTGCGGAAGATCACAAAAAATTAG
CGGAAGAAGATTTGAAAAATATAGATTTAACATCAAAAGATGTCGTTATAGGAATTGCCGCGAGTGGCAAAACGCCATATGTTATAGGC
GGTTTAACATTTGCTAACACAATCGGTGCTACAACAGTATCTATTTCATGCAATGAACATGCAGTTATAAGTGAAATTGCGCAGTATCCAG
TAGAAGTTAAAGTTGGTCCAGAAGTATTAACTGGTTCAACACGTTTAAAGTCTGGTACAGCACAAAAGTTAATTTTAAATATGATTTCAAC
CATCACAATGGTTGGTGTCGGAAAAGTTTACGATAACCTCATGATTGATGTTAAAGCAACCAATCAAAAACTGATCGACCGTTCAGTGCG
TATTATTCAAGAAATATGTGCTATCACATATGATGAAGCAATGGCGTTATATCAGGTATCTGAGCATGATGTGAAAGTTGCGACAGTTAT
GGGTATGTGTGGCATTTCTAAGGAAGAAGCAACAAGACGGTTATTAAACAATGGTGACATTGTTAAACGAGCAATCAGAGATAGACAAC
CTTAG

Gene: Q5HJI0 (phosphotransferase system EIIC component)

Contig: 02_NODE_3, position: 128086 to 129540, length: 1455 nt, orientation: FORWARD

Perfect match to: (MW2-BA000033-[197796:199250], highly conserved allele)

Sequence:

ATGACCAAAGAACAACAACTTGCAGAACGAATTATTGCTGCAGTAGGTGGTATGGATAATATAGATAGTGTCATGAACTGTATGACACG
TGTGCGTATTAAAGTATTAGATGAGAATAAAGTAGATGACCAAGAACTAAGGCATATTGATGGTGTCATGGGTGTTATACACGATGAAC
GCATTCAAGTTGTGGTTGGACCTGGTACAGTCAATAAAGTGGCTAATCATATGGCGGAATTAAGTGGTGTTAAACTAGGTGACCCAATA
CCACACCATCACAATGATAGTGAAAAAATGGACTATAAATCATATGCAGCTGATAAAGCAAAGGCGAATAAGGAAGCGCATAAAGCAAA
ACAAAAGAATGGTAAGTTGAATAAAGTATTGAAATCAATTGCCAATATCTTTATACCGTTGATTCCTGCATTTATTGGAGCTGGATTAATT
GGTGGTATTGCAGCAGTACTGAGTAACTTAATGGTGGCAGGCTATATTTCAGGTGCTTGGATTACGCAACTTATAACAGTATTTAATGTC
ATTAAAGACGGTATGTTAGCATACTTAGCTATTTTCACTGGTATTAATGCGGCTAAAGAATTTGGTGCGACACCAGGACTTGGTGGCGTG
ATTGGTGGTACAACGTTATTAACGGGTATTGCTGGTAAAAATATTTTAATGAATGTCTTCACTGGAGAACCATTGCAACCTGGACAAGGT
GGGATTATTGGCGTTATTTTTGCCGTTTGGATTTTAAGTATTGTCGAAAAGAGATTACATAAAATTGTGCCAAATGCGATTGATATTATTG
TAACGCCGACTATTGCATTGTTGATTGTAGGACTATTAACTATCTTTATCTTTATGCCATTAGCAGGTTTTGTTTCAGACAGTTTAGTTTCA
GTAGTTAACGGAATTATTAGTATTGGTGGCGTATTTAGTGGATTTATCATTGGTGCAAGCTTCCTACCGTTAGTTATGTTAGGGCTTCATC
ATATTTTTACGCCAATTCATATAGAAATGATTAACCAATCAGGTGCTACTTACTTATTGCCAATTGCAGCGATGGCTGGTGCTGGACAAGT
AGGTGCCGCATTAGCACTTTGGGTAAGATGTAAACGCAACACAACATTACGTAATACTTTAAAAGGTGCATTGCCAGTTGGTTTCCTAGG
TATCGGAGAACCATTAATCTATGGTGTGACTTTGCCATTAGGTCGACCTTTCTTAACTGCTTGTATTGGTGGTGGTATTGGTGGCGCTGTA
ATAGGTGGAATTGGACATATTGGTGCCAAAGCAATAGGCCCAAGTGGTGTGTCACTATTACCATTAATCTCAGATAATATGTATTTAGGT
TATATTGCAGGATTACTTGCTGCGTATGCTGGTGGATTCGTTTGTACATATTTATTTGGAACGACAAAGGCGATGCGACAGACAGATTTG
TTGGGTGATTAA

Gene: rpiRB (putative phosphosugar-binding transcriptional regulator)

Contig: 02_NODE_3, position: 129540 to 130418, length: 879 nt, orientation: FORWARD

Perfect match to: (COL-CP000046-[206624:207502], highly conserved allele)

Sequence:

ATGATGACAAATATTTTATATCGCATTGATAAGCAGTTGAGTGATTTTACGAAGACAGAAAAGATAATCGCTGATTACATTTTAAAGAATC
CACATAAAATCATTGATATGACTGTGAATGATTTGGCAGATGTTACGAATGTTAGTACAGCATCAATTGTTAGATTTAGTCGGAAAATGA
CACATCAAGGTTTTCAAGAGCTAAAGATTGCGATATCTCGATACTTACCCGAAGATATTGCAACCAATCCACATTTAGAATTGATTGAAAA
TGAATCTGTAGAAACTTTGAAAAATAAAATGATTGCTAGAGCAACGAATACGATGCGATTTGTAGCTACTAATATTATGGATGCGCAAAT
TGATGCAATTTGTGATGTGTTGAAAAATGCCAGGACAATATTTTTATTTGGATTTGGCGCATCGAGTTTGACTATTGGTGATCTTTTTCAA
AAGTTATCTCGTATTGGCTTAAATGTCAGGTTATTACATGAAACGCATTTACTTGTGTCAACATTTGCGACGCATGATGATAGAGATTGCA
TGATTTTTGTGACGAATCAAGGTAGTCATAGTGAATTGCAGTCAATTGCACAGGTGGCCACACATTACAGTATTCCCATCATAACTATATC
TAGTACAGCTAATAATCCAGTGGCTCAAATTGCAGACTATGCATTGATTTATGGCAGAACTGATGAAAATGAAATGCGTATGGCGGCTAC
AACGTCACTATTTGCACAGTTATTCACGGTAGATATATTGTACTATCGATTTGTAGCATTAAATTATCATGCGATTCTAGATTGTATAACCC
AATCGAAAATGGCACTTGATAATTACAGGAAGCATCTTGCGACGATAGATTTTAAACATTAG

Gene: A5IP63 (putative protein)

Contig: 02_NODE_3, position: 130612 to 130893, length: 282 nt, orientation: REVERSE

Perfect match to: (N315-BA000018-[221933:222214:r], highly conserved allele)

Sequence:

TTAAATGCTGAAGAATAATTTTAATGCTAAATGAACTAATACGAAGGTGCTAAAAGCAATTATTATCAAATTAGTGATATGAATGAAATG
TTTAAATCGTTTGCGTTGATTGTATTCGGGACTTTTATAGAGTAGGTAGTAGTACATAAAGAGTATGGCTGCGAACATGCCGAATATTGA
AGTGAAGTTACCTACAGTGACTGCCATGTAATGATTTAAAGTTAAATAGTAACTTAGACTAATAATAAAAAATAAAATGATTTGATAGGT
AATTAACACCAT

Gene: hsdR (type I restriction-modification system endonuclease)

Contig: 02_NODE_3, position: 131106 to 133895, length: 2790 nt, orientation: FORWARD

Sequence:

ATGGCATACCAAAGTGAATACGCATTAGAAAATGAAATGATGAATCAACTTGAACAATTGGGTTACGAAAGAGTAACGATACGTGATAA
TAAGCAATTGCTTGATAATTTTAGAACGATTTTAAATGAGCGTCATGCGGACAAATTAGAAGGCAATCCCTTAACAGATAAAGAATTTCA
ACGTCTGTTAACGATGATTGATGGAAAAAGTATTTTCGAGAGTGCCCGTATTTTACGTGATAAATTACCACTTAGACGTGATGATGAGTC
TGAGGTTTATTTGTCGTTTTTAGATACGAAAAGTTGGTGTAAAAATAAGTTTCAAGTGACGAATCAAGTATCTGTCGAGGATACATATAA
AGCACGTTATGATGTAACGATATTAATCAACGGACTACCCCTTGTCCAAGTTGAATTGAAACGTCGAGGTATTGATATTAATGAGGCGTT
TAACCAAGTAAAACGTTACCGCAAACAAAATTACACAGGCTTATTCCGCTACATACAAATGTTTATCATTAGTAATGGTGTTGAAACGCGA
TACTTTTCTAATAATGATAGCGAACTATTGAAGAGTCACATGTTTTATTGGAGTGATAAACAGAATAACCGTATCAATACATTGCAATCGT
TTGCTGAGTCATTTATGAGACCTTGTCAATTAGCTAAGATGATATCGCGCTATATGATTATTAATGAAACAGATAGAATACTGATGGCAAT
GCGTCCGTATCAAGTGTATGCGGTAGAAGCACTTATTCAACAAGCGACTGAGACAGGGAATAATGGATATGTATGGCATACAACTGGAA
GTGGTAAGACGTTGACTTCTTTTAAAGCGAGTCAGATTTTATCACAGCAAGATGACATTAAGAAAGTTATCTTTTTGGTTGACCGTAAAG
ACTTGGATAGTCAAACAGAAGAGGAATTTAATAAATTTGCTAAGGGTGCTGTAGACAAAACTTTTAATACCTCGCAACTGGTACGCCAAC
TAAATGATAAAAGTTTGCCACTTATTGTAACGACGATTCAAAAAATGGCTAAAGCGATTCAAGGGAATGCCCATTTATTAGAACAGTATA
AAACGAATAAAGTTGTATTTATTATTGATGAGTGTCATCGCAGTCAATTTGGTGACATGCATCGTCTAGTTAAACAACATTTCAAAAATGC
CCAATACTTTGGATTCACTGGTACGCCACGTTTTCCAGAAAATAGTAGTCAAGATGGTAGAACAACTGCAGATATTTTCGGTAGATGCTT
ACATACGTATTTAATTAGAGATGCCATTCATGATGGTAATGTACTTGGTTTCTCAGTTGACTATATTAATACTTTTAAAAATAAAGCTTTAA
AAGCAGAAGATAACAGCATGGTTGAAGCAATTGATACGGAAGAAGTATGGTTAGCGGATAAACGTGTGGAATTAGTAACACGACATAT
CATCAATAATCATGATAAATATACACGTAATCGTCAATATTCAAGTATATTTACAGTCCAAAGTATTCACGCGCTTATTAAATATTATGAGA
CATTTAAGCGACTTAACAAAAAGTTGGAACAACCGTTAACGATAGCTGGTATATTTACGTTTAAACCTAATGAAGATGATCGTGATGGTG
AAGTGCCATATCATTCACGTGAAAAATTAGAGATAATGATTAGTGATTATAATAAAAAGTTCGAGACGAATTTTTCAACAGACACAACTA
ATGAGTATTTTAATCATATTTCAAAAAACGTTAAAAAGGGCGTTAAAGATAGTAAAATTGATATCTTAATCGTTGTTAATATGTTCTTAACT
GGTTTTGATAGTAAAGTACTGAACACTTTATATGTTGATAAGAATTTAATGTATCATGATTTAATTCAAGCGTATTCACGTACAAATAGGG
TTGAAAAAGAATCAAAGCCATTTGGTAAAATTGTAAACTATCGTGACTTGAAAAAAGAGACAGACGATGCACTGAGAGTATTCTCACAA
ACAAATGATACGGATACAATTTTAATGCGCAGTTATGAAGAGTATAAAAAAGAATTTATGGACGCTTATCGTGAGCTTAAAATGATTGTG
CCGACACCACACATGGTTGATGACATTCAAGATGAAGAAGAGCTAAAGCGCTTTGTTGAAGCTTATCGTTTATTAGCTAAAATAATATTA

CGTTTAAAAGCATTTGACGAGTTTGAGTTTACAATTGATGAAATTGGAATGGATGAACAAGAGAATGAAGACTATAAAAGTAAATATTTA
GCTGTGTACGATCAAGTAAAAAGAGCGACGGCTGAGAAAAATAAAGTATCCATTTTAAATGATATTGATTTCGAAATAGAAATGATGCG
TAATGATACGATTAATGTGAATTATATTATGAATATATTGAGACAAATTGATCTTGAAGACAAAGCGGAACAACGTCGTAACCAAGAACA
AATTAGACGCATTTTAGATCATGCAGATGATCCGACATTGAGGTTAAAACGAGATCTAATTAGAGAATTCATCGACAATGTTGTACCTTCT
TTAAATAAGGATGATGATATCGATCAAGAATATGTTAATTTCGAAAGTATTAAAAAAGAAGCGGAGTTCAAAGGATTTGCTGGAGAGAG
ATCTATCGATGAACAAGCCCTAAAAACAATTTCAAATGACTACCAGTATAGTGGTGTTGTAAACCCACATCACCTTAAAAAAATGATTGGT
GATTTGCCATTGAAAGAAAAGCGTAAAGCAAGAAAAGCCATTGAATCTTTCGTGGCAGAAACAACTGAAAAATACGGTGTGTAA

Gene: A5IP65 (putative protein)

Contig: 02_NODE_3, position: 134042 to 134641, length: 600 nt, orientation: REVERSE

Perfect match to: (MW2-BA000033-[203752:204351:r], allele observed in CC1)

Sequence:

TCACTGAATGCTAATACTAATCAAAGAAAAAGTGATAATTAAAATAAGATATATAATACCCCAAATAGTGGATGCTCGTATAGAAAGTTT
CGACGTACCAATTAGCATGACTGTAAGTATGTATGCTTGTACAAAAAATTGTAAGTCGAAAGTACCCAGAATGCGATTTCCTTTAGCAAA
AATATTTAAACTAGTAATAGAATAGTCAATTAATGATAAACCGGCACTTATTTGGATGGTAAGTATAATTAAACTAATAGCATTTGTAAAC
AGTATGTAGCTTAACGTTGCAGAGAATATAGATTTTGTATTAGCATCTGAGCGCATAATTCTAGAGATAACTAGAATGATTAAAAATGTA
AATAATACGCTGAAAACTCTTGAGAAAAAAATACTAATTTGACTAAAAATTTTTATTTGTGCTAATGATGTCTCTGAGAGTCCTGCTTCACT
CAATAACTTTTTATAGTCCACAGATAGTCCACTAACAAATGTATATGTAACAATTATTATCAAAGTCAGAAACAGCTTAATGATTAACTTTG
GTTGAATGCGGATATTTGCAAAGTGATTAAGCAAAGGTAGTTTTGATAATGACAT

Gene: A5IP66 (putative RND transporter component)

Contig: 02_NODE_3, position: 134767 to 135522, length: 756 nt, orientation: FORWARD

Sequence:

TTGAGGCGTAAAAGGAAATGGCTAATTGTTATTTGTGTATTATTAGTATTGATTACGATATCACAAATTTTGAAACATTTTAACTATGGAA
CTGAGAGTAAAGAAGCATACAATACATATACGGTTGAAAAAGAAAGACAATTAGATTTACAAGGTAAGGCATATCCACGTGAAGTTAAA
ACATATTATAAAAATAATCAGGTTGGAACATATTTGGGTGTGCAAGTAGCGGACGGGCAAACAGTCAAAAAAGGAGACAAAGTTAACTT
AAGTATTACGAGTACGGGTAAAACAGGAGCGGGGATTATAAAACAAATCTCTGAGTTGCCTATTAGTTATGAAGAGAATTTAAGTCTTCA
CGAAAATGATGCCCTGCATTTACCAGAAAGTAACAACGATGGGGAATTAGCAAATTCAAAATCGATTTCGGCATCTCCAATATTTAAGAG
TAATAACAACTCTGAATTATCAAAGTATGGAGTTATAATTGATGATCTTAGTCTACCAATACGTGCAGGTTATTCGTTGGAAATTAAAGTC
CCATTAAATGCGATTAAAATACCTAAATCAGTATTAACAAAAGGCAATAATGTTTTTATTGTTAATAAAAATAGTATTGTGGAAAAACGGA
ATATTCGTATAAAAAGAATCAATGGTGACATTATTGTAGAAAAGGGGTTAAAACCTGGAGACAAGTTAATTAAACAACCTAAAAGTACA
ATGAATGATGGTGATAAGGTTGAGATTTCGTCATGA

Gene: A5IP67 (putative ABC transporter, ATP-binding protein)

Contig: 02_NODE_3, position: 135519 to 136199, length: 681 nt, orientation: FORWARD

Perfect match to: (N315-BA000018-[227197:227877], allele observed in CC5+CC1+CC97)

Sequence:

ATGATAGAATTAAAGAATGTTTATAGGACTTTTAAAAACGGTGATAAAAATATTCGAGTTTTAAAAGACATTAATATACATATCAATGCA
GGTGAATTCATAGCGATTATGGGACCATCAGGCTCAGGGAAGAGCACACTGATTAATATCTTAGGATTTATAGATCGAGAGTATCATGG
TGAATATTTGTTTGAAGGAAGTAATTATCAGACACAATCTGATAATCAATTGGCAAACATAAGAAATAAAACAGTTGGATTTGTTTTTCAA
AATTTTAAACTAATTCATAATAATACTATTTTAGAAAATATAAGTATGCCACTGGTATATACAAATATGAATACGATAGAACGAAAGAATA
AAGTTATGACTGTGCTCAGTGCTGTAGGCCTAGATGGCAAAGGTGATTTTACTCCTAGTAAACTATCAGGCGGTCAACAACAACGTGTTG
CAATTGCGCGAGCCATTATAAATAATCCAAAGTTTATTATTGCAGATGAGCCAACGGGTGCTTTGGATTCAGATACATCTAAAATTATTAT
GGACATATTTACATCATTAAACCGAGATAATCATACTACAATTATTTTAGTCACACATGATCGTAAAGTAGCAGAAAAAGCTGATCGAATA
ATTCATATTTTGGATGGTCGTATACAACATGAAGAGGTGATTAAATGA

Gene: A5IP68 (putative ABC transporter, transmembrane permease)

Contig: 02_NODE_3, position: 136196 to 137374, length: 1179 nt, orientation: FORWARD

Perfect match to: (N315-BA000018-[227874:229052], allele observed in CC5+CC1)

Sequence:

ATGAGGAAAGTTTTAAATATTTTTACAATAGCTTTTAAATCAATATTGAAAAATAAAGGTAGAAATATATTTACAATGATAGGTATAATCA
TCGGTATTTCTTCTGTTATTACGATAATGTCTTTGGGAAATGGCTTTAAAAAAACTGCTGCTGATCAATTTTCAGATGCTGGCGCCGGAAA
ACAAGAAGCGTTGATTAGCTTTACTTTTAAAGTTGATGAGAAAATCAAAAAGTATCCTTTTAATCAAAGAGATATAGAGTTGGTAAATCA
AGTTGATGGCGTACTAGATGCGAAGCTAAAGGAAAATAAAGAAGAAGGAATTGAAGCCACAATAACTAATGTCCAGAAAAAAAGTGAC
ATCTTCATTATAAAAAAACAAAATTTACACTCTTTTAAAGTAGGTAGAGGGTTTGATAAAGAGGATAATGAATTACGTAAGAAAATAGTG
GTTATAAATGATCAAGTAGCAAAAACTGTATTCAATAATAATGCTATTGGTAAATCGCTATATATTGAGGGACAGGGATTTGAAGTCATA
GGAATTACGGATAAACTATATTCTGACTCATCTACCGTTATAATGCCTGAAAATACATTCAACTACTATATGGGACACTTACATCAAGGTC
TGCCTACCTTACAGATAATTATTGAAGATGGTTACAATAAAAAAACTGTAGTTAAAAAAGTAGAATCACTATTAAATAAAAAAGGATCGG
GCTCTGTTTTAGGTGAGTATACATATACAGACACTGAAGAAATTATCAAAAGTATTGATAAAATTTTTGATAGTATTACTTATTTTGTAGC
AGCTGTTGCCGGTATTTCACTTTTTATTGCTGGTATTGGAGTTATGAATGTCATGTATATATCTGTAGCTGAAAGAACAGAAGAAATTGCG
ATACGACGTGCATTTGGTGCAAAAAGTCGAGATATTGAACTACAATTTTTAATAGAGAGCATTTTAATATGTGTGACAAGTGGTTTTATTG
GACTTATTTTAGGTGTTGTGTTTGCAACAATAATTGATGTATTAACACCAGATTATATTAAAAGTGTAGTAAGTTTGAGTTCTGTCATAATT
GCGGTTAGTGTGTCGATATTAATTGGACTTCTTTTTGGATGGATACCAGCTCGCGCAGCATCTAAGAAAGAGCTCATAGATATTATAAAA
TAG

Gene: A5IP69 (putative protein)

Contig: 02_NODE_3, position: 137581 to 137802, length: 222 nt, orientation: FORWARD

Perfect match to: (RF122-AJ938182-[174161:174382], allele observed in CC705+CC1+CC5+CC9+CC97)

Sequence:

ATGCTAAAATTACATAAAAAAATAGCTTGGACAGGAATCAAAGGTTCAGCTATAACTACATTTATTACTGCCTTATATAATGGAAGTAGT
GTTTGGAACGCGCTAGCTGTTGCTGGTATTGCTTTTGGTGGTGGCGCTGGTACTGCGGTTGCAGCTCTTGGTCGTGCAACAGTAATGAGA
TTTATCAAACGTTGGGGCGTACGAAAAACAGCTGCTTGGTAA

Gene: A5IP70 (putative transmembrane protein)

Contig: 02_NODE_3, position: 137840 to 139558, length: 1719 nt, orientation: FORWARD

Perfect match to: (N315-BA000018-[229518:231236], allele observed in CC5+CC1+CC25+CC97)

Sequence:

TTGGGAAAGCCTCTTTTTAAAAAAGGAGATTCAATGAATAATTCAATATTTTATACCTATTTAAAATTTAATCTTAGATATTTTATCCGTAG
AAAATTTGAAATTGTAAAAAGGTCGCGACTTAATGTTACTACAAAAATACTGTTTTTAATTAAATTGATTTTTTCGTCACTATTATTATTTAT
TTCAACTATTATGTTAGCTGACTATTTCAGTGATTATATTGATTTTATAGAATATTATTTCTATGCGAGTATGTTTATTTACATTGTATTTATA
CCATTAATACCTAGAACTAAATTATTGATCAATCCAATGGATAAACAGCTGTTATATAGATCTAGTTTGAGAGAAGTTGACATTTTTAACTT
GATATATGCTTCAGACGTTTTAAAAAAGTTTGTTGGATATTTTAATTTGTTGATAGTTGGTATGGCTATTAGTTTATATCATATTAGTCCTTT
TATATTTAACCTCAAGGTTTTTTGTGCACTAATATACTTTTCAACAATCTCTTATGTAATTCAAATTATTTTTATAAATATTAAAGTCATAAG
CACAAAAAAATTATTTTCTTATCACTATTGTGTTTTTAATTTTATGATGGGAAGCATTGTTTCTGTTATTGGATTTGTCGTCTCATTTCTTTTA
GTAAATTTATTAATAAAGCCCTTTTTAGTTTTTTCGACATATGTAATAAAAAGTAAGAAAACTTTTGAATGGTATAGTTATTTTCAGGATAT
AAAGAACGAAGTCATTATTATTAATGAAAAAGTTATGCAATTATTAGATTATATATCACCTCACACTATTTTTATGAGATTAAACTTTGTGA
ATATATCATTTGTGATTATTACAATAGCTTTATTTATAGCGTTATATTATTTTAATAAAATAGGGTTTTGGTATAGAAAAGAAGATATCTAT
CTATCTTCAACAAATATTAATTTTTCGTTGCCAATTTCAATAAAAGCTATAACAACAATACAGCTAAAACACTGGATAAGTAACAAAGAAG
AAGTTAATTTACATAAACCTTTCTTCTATATTAGTTATTCAATATGGTTCTTTTTAGGGTGTTTAATCTATTTTTCACAGATGGCATATAATC
AGTTAGCTAATGCAGTTATTTTTATTTTGATATTAAACACTATAACTCGTGATAGTTTTTCTGCGGGGACGGATTTTTTTACTAAATCATTAC
GTTTTGATTCAGATAGAAAAAGTATCGGTTTGTATAGGATGTCAAACACTGATTTTAAACAAATTTACGATTCTAAATTATCTCTAATTCGC
TTTATTGGTTTTAAAGAAACAATACTTGCTATTATTTTATTAGCAATATTTTTAAATCAAGACATATATTTATATATAATTGGATTTGAAATA
ATAATCATCAATACAGTAATAATTCCTAATTTATCATTATTACCTTCGTATTTATCTCCACATTTCAATCATCAACATTACAGTGAATTAGAG
TCTTTTGAAGAACAAATTTTTCTTGAAGATACTGTTTTTGATAAAGTTAAGAATTTTTTATCGTCATCATATTTTTTAATTCTATTTGTAGGTT
ACTTATCTCAACGTAATTATATTGACATAATGATTTTTATAATTATTTTCAACATTTTAGCCTTAATACTTGTTTCGTTGTTTATTAGGTTTTC
TAAACAAAAAAATAACAAAGTCTTGGAGAAAGAGGGATTTATATTTATAG

Gene: A5IP71 (putative transmembrane protein)

Contig: 02_NODE_3, position: 139617 to 140060, length: 444 nt, orientation: FORWARD

Perfect match to: (N315-BA000018-[231295:231738], allele observed in CC5+CC1+CC97)

Sequence:

TTGGGATGCTTGGTTTATTACTTATTAAATATTGATTTAGGTAAAATGATAGTTGCTCAAGATTTAAATTTACAAGAGATTTTCTTTCATAA
TTTAATTTATTTCTTCTCTGCAATATTTGGTTTTCTGACACTTGGTCTGTTAAATATAGGTATGTTACTCATAAATTCTGGGATGTTAGGCTT
TTTTATAGCACATGGAATCTATAGCAATCAATTAAAATCTATTCTATTAGTATTAGCGCCACACGCTATATTTGAGATTTTAGCACTTTTAA
TTGCTTCAACATTCTCATTAGGTTTTTTATCATACCTTTATCAAAGAATGGTAAAGAAAAATAAAATAAAAAAATAAGATGTTCCATAATTTT
GTAACAACAATAATAACTGTTATTGTATTAACAATAATCGCATCATTATTAGAAGTTTATGTAACCATAAATTAA

Gene: A5IP72 (putative ABC transporter, ATP-binding protein)

Contig: 02_NODE_3, position: 140072 to 140794, length: 723 nt, orientation: FORWARD

Sequence:

ATGCTAGTTTTAAATAATCTTCAAAAAAAATTTACTAATTCAAAAATGGTTTTGAATAATGTGAATGCTAAGTTTAGAAGTGGGAGCATCA
ATTGCATAGTGGGAGTAAATGGGGCAGGTAAAACGACTTTGTTAAATATTATTAGTAGTATATTGATGCCAACAAAAGGTGATGTATATT
TAAATAGTGAAAGTATTTTTGAAAATAGCACACTAAAAAAAGAGATTTTTTACACACCAGTAAATCCCTTTTTTTATGAAAATTTAAGTGCT
AAAGATAATTTGTACTTAATATGTAGCCTATATAATCGGAAAATAGATCAAATAACTATTGAGAAAACTATTAAAGATGTAGGTTTGAAT
ATAGAAGATCTAAATATACCTGTATATAATTTTTCAAGTGGTATGAAGCAAAAACTAAATTTTGCTTCTATGTTATTAGTAGATTCAAATGT
TTTATTATTAGATGAACCATTTAATGCCCTTGACCATGTTGCTCAAAAAAAATTTCACACGAATTTTAAAGGGATTAGTTTCTAAAGAAAAG
ATAATAATTTTCACTAGTCATTTGCCTAATACAATTTTAGAACTCTCAGAAAATATTTATTTGTTAAAAGATGGATATTTTACAGATAAACG
AAAAGCACAGTCATTTAATCAAACCACGTTAGAAACATGGTTGTTAGAAAGTCAAAAAGAGGTGATACCTTATGAAGAAAAATGA

Gene: A6TXY8 (putative protein)

Contig: 02_NODE_3, position: 140781 to 140957, length: 177 nt, orientation: FORWARD

Perfect match to: (N315-BA000018-[232459:232635], allele observed in CC5+CC1+CC25)

Sequence:

ATGAAGAAAAATGATTTGTTAGTAATGAGTGTAGTATATATTTTAACTTCAGTCATAATGTTCTACATTAATGGATTAAGTAAACAATTTTT
TACATATGTAGTTGGTTTCCCAATTATTTATTTTGGTTATATATGTCTAGTTAATCTTATAAAAAAAAGAAGCCGGAATAATTAA

Gene: A5IP73 (putative protein)

Contig: 02_NODE_3, position: 141004 to 141192, length: 189 nt, orientation: REVERSE

Perfect match to: (RF122-AJ938182-[177582:177770:r], allele observed in CC705+CC1+CC5+CC25+CC97)

Sequence:

TCAAAAGGAGCTAGCAAATAAAGTTGGAGTAACTAGACAAACAATATCTCTTATAGAAAAGGGTGTACATAACCCCTCTCTATCACTATG
TAAAAATATTTGTTCGGTGTTGAATAAAAATTTGGATGAGATATTTGGAGAGAAACCACAATAAAAGAGCTTATTGAAAGATAATCTAAA
AGTCGACAA

Gene: oppF2 (oligopeptide ABC transporter 2, ATP-binding protein)

Contig: 02_NODE_3, position: 141667 to 143259, length: 1593 nt, orientation: REVERSE

Perfect match to: (MW2-BA000033-[211376:212968:r], allele observed in CC1+CC5)

Sequence:

TTATTTAGCAATAACTGCTACTTCTGAAATAAGTTGCTTTGCATAGTCTGATTGCGGATGTTTGATAATATCTTCTGTGTTATTCAGTTCAA
CGATTTCGCCATTTTTCATAACTGCAACGCGATCACATATTTCATTGATAACACCCATGTCATGTGTGATGAATAAATAAGTGATGCCGAA
GTCTAACTGTAATTGTTTTAATAACTCGATGATATCTTTTTGAATTGAAACGTCTAAAGCGGACACTGCCTCGTCGCAAACAATCACTTTAG
GTTCTACAGCAAGTGCTCTCGCGATACTTACACGCTGACGTTGCCCACCAGATAATTCGTGTGGATAGCGATATAAGAAACTTTGATCTA
GGCCAACCTTTTCTAACAACGATACGACAGTTTTAATAATGTCATCATTATCTTTGACTTTCCCATGAATGATTAGTGGTCGTTTAATCACA
TCAATGACTTTAAATCTTGGATTAATAGATGCGAATGGATCTTGAAAAATCATTTGTATCTCTTGTCGTAAAGATTTCAATTCATCATCTTT
AAATAAACTTAATGGTAATTCGTTATACCAAATAAAGCCTTCTGACACTTCCTTTAGACCGACGACCGTCTTAGCTAATGTCGATTTCCCTG
ACCCTGATTCACCGACAATGCCTAATGTTTCGCCTTTTCTAATACCCAAGTTAATATCATTAACTGCTCGGTATAGGCTGCCACTCGGTGAT
GTGTAATCCACGCTCACGCGATCGAATTTTAATAAAATATCATTGTTTAACGGTCTTGGCGGACGCGTTTGATGAATATCAGGAATCGCAT
CTATTAAGCGTTTTGTATAGGTATGTTGTGGCGATTTAAAAATACTTTCAACCGTGCCACTTTCAACGACACTTCCATCTTTCATTACAATC
ACATCGTCGCAAAATTGATACACAGCGCCTAAATCGTGAGTGATAAAAATAATAGATGTTTCTGTGTACTCATAAAGGGACTTCATTAACT
GCAGTAATTGATTTTGTGTACTGGCATCTAATGCCGTTGTTGGTTCATCTGCGATTAAAATTTGTGGCTTTAAAATCAATGCCATTGCTATC
ATGACACGTTGACGCATACCACCAGAAAGTTCATGTGGATAAGCATCAAATTGTCGAGTTGCATGTTTTATACCTACTTTTTCTAAAATGT
CTATTGTCATCGACTTTGCTTCAGATTTAGATACACGTTTATGTTGAAATAGTACTTCTGTAATTTGTTTGCCAATCGTTAATCTTGGATTCA
ACGAAGAGAGTGGATCTTGAAAAATCATTGAAATATCCTTACCTCGAATTTGTTGTAACGCTGAAGTTGATAAATTATTTAACGATTGCCC
ATTAAAAATAATTTCTCCTGTTAATGTGTGATCTGGATAATCTGGTAGTAGCCCTAAAATAGATTTAGCAGTAATACTTTTTCCTGATCCTG
ATTCACCAACAATACCTAGGATATGTTTTTTTCGTAATTCGAAAGAGACATTTTTTACCGCTTGAACTGTAGTTTCATCATAATTGAATTGT
ACATTCAGACTGTTGACTTCTAATAAATTTGACAT

Gene: oppB2 (oligopeptide ABC transporter 2, transmembrane permease)

Contig: 02_NODE_3, position: 143387 to 144694, length: 1308 nt, orientation: FORWARD

Perfect match to: (502A-CP007454-[933161:934468:r], allele observed in CC5)

Sequence:

TTGTATCACAAGGCATTTTACAAGTTTGTATTATCGGTATTAAGCTTGCCAATATTTTTATATGCAGTGATAAAGTTTTTCTTTTCTGCTAAG
AGAAAAAATTTTTACGCTAATAATTCTGAAATTTCAGAAATTGAACGAGCGTTACATCAAAAATATAAATATTTATCGCAGCAAAAGTCAT
CCACACAAATACATAAAGAAGCATTAAAAATATTCAAGGCACAAAGTTCTAATACGAGTTCAAAGAATATTGAACAAGCACATTTTTCAA
CATACTTTGAAAATTTATTATTTCATAAGTTCATCATGATCAAAGTAATATTGGCCTTGCCGATGTTCATCTTATTGACTATTTATTTACAGC
CATTAGTTAGATATATTTTTGAGAGAATTGTCATGGCTGTGATTGTCATCATTGGTGTTATTGTCAGTGTGTTTACCATTCTGTATTTTTCAC
CGCTTGATGCGGCTTATAGCATACTGGGACAAAATGCAACAAAGGCACAGATACATCAATTCAATGTATTACATCATCTTAACGAACCTT
ATTTTATTCAATTGTGGGATACCATCAAGGGTGTTTTTACCTTTGACTTAGGTACGACTTACAAAGGGAATGAGGTTGTGACTAAAGCAG
TTGGCGAAAGAATTCCAATTACAATAATTGTCGCAGTATTAGCGCTAATTGTGGCATTAATTATTGCAATACCAATTGGTATTATCAGTGC
GATGAAGCGAAATAGTTGGCTTGATATCACGTTAATGATAATTGCATTAATTGGTTTATCTATTCCAAGTTTCTGGCAAGGGCTATTATTC
ATTTTAGCGTTCTCATTGAAATTGGATATTTTGCCACCATCTTATATGCCAGAACATCCAATATCGTTGATTTTACCTGTACTTGTCATTGGA
ACAAGTATTGCTGCTTCTATCACGCGTATGACAAGGTCTTCTGTACTTGAAGTAATGCGCAGCGATTATGTTTTAACTGCTTATGCAAAAG
GATTATCGACGACACAAGTTGTTATTAAACATATTTTGAAAAATGCCATTATTCCAATTGTAACGTTAGTTGGTCTTCTAGTGGCAGAGTT
ACTAGGCGGTTCAGCAGTGACGGAACAAGTATTTAACATTAATGGTATCGGGCGTTATATCGTCCAAAAACAACTAATACCTGATATCCC
AGCAGTCATGGGTGGGGTCGTATATATATCAATTGTAATATCTTTAGCAAACTTAATTATTGATATATTTTATGCTTTAATCGATCCAAAAT
TACGTAGTGAAATTAACGAAAGGAAGTGA

Gene: oppC2 (oligopeptide ABC transporter 2, transmembrane permease)

Contig: 02_NODE_3, position: 144700 to 145863, length: 1164 nt, orientation: FORWARD

Perfect match to: (MW2-BA000033-[214409:215572], allele observed in CC1+CC25+CC772)

Sequence:

ATGGTAAAACTTACAACAAAGATAGCTTCCTTAAAACTATTCGCAAGTTATGCTATAGCAACTTATATTTTAGTTATATTAACGAGTGCATT
AAATCTTTTTAAAGGTTATGTGGCCGATACGTTCTATATTGCTGAAACATTGCTAATCGTTTTAACCATCATTTTAATTATCATTTTAACAAC
GGAACAAACATGGAAGCATCATGACTTATGGCGACGTATCGTCGAAGTGTTGTTATTGTTGATGACATTAACAGGCAACGTATTTACATT
ATTAATGTTTGTAAGTATTAGACGTTACCAACGTACATCGCAAATACATAGTTATAACGGGTGGGAATCGTTTATACGAAAAACTACTAG
ACATCGTATTGCGATTATCGGGTTACTTATTTTAGTCTACATGCTGACATTATCAATTGTGTCACAATTTACATTTGATACGACATTGGCTA
CTAAAAATCAGTTCAATGCACTGTTACATGGACCGAGTCTAGCCTATCCGTTTGGTACTGATGATTTCGGTAGAGACTTATTTACACGCGT
AGTTGTAGGAACGAAGCTGACATTTTCAATTTCAATTATTTCAGTAGTTATTGCAGTTATTTTTGGTGTGTTACTAGGCACTATCGCAGGTT
ATTTTAATCATATTGATAATTTAATAATGCGAATTTTAGATGTAGTGTTTGCAATTCCATCATTATTGTTAGCGGTGGCAATTATTGCATCA
TTTGGAGCAAGTATTCCAAATTTAATTATTGCTTTAAGTATCGGTAATATACCATCATTTGCACGGACAATGCGTGCCAGTGTTTTAGAAA

TTAAACGCATGGAATATGTAGATGCAGCACGTATCACTGGTGAAAACACTTGGAATATCATATGGCGTTATATTTTACCGAATGCGATTG
CGCCTATGATTGTACGTTTTTCATTAAATATAGGTGTGGTTGTATTAACAACAAGTAGTTTAAGTTTCCTAGGACTTGGTGTTGCACCTGAT
GTAGCTGAATGGGGCAACATTTTACGTACCGGTAGTAACTACTTGGAAACGCACAGTAATTTAGCTATTGTACCTGGTGTTTGTATTATGT
TCGTCGTTTTAGCATTTAATTTTATAGGTGATGCAGTGCGTGATGCACTAGATCCAAGAATTCATTAA

Gene: oppA2 (oligopeptide ABC transporter 2, substrate-binding protein)

Contig: 02_NODE_3, position: 145880 to 147655, length: 1776 nt, orientation: FORWARD

Perfect match to: (Strain_18341-HE579069-[213404:215179], allele observed in CC5)

Sequence:

GTGAAGAAAATCATTAGTATCGCAATTATAGTTTTAGCGTTGGTATTAAGTGGTTGTGGTGTCCCTACGAAATCAGAAGTGGCTCAAAAG
TCATCGAAAGTTGAAGTGAAAGGCGAGCGACCAACAATACATTTCCTAGGACAAGCAAGTTATGAAAATGATATGAATATCGTTAAAGA
TCAATTGGAAAATGCAGGATTTAACGTGAAGATGAATATCCAACCAGATTATGGTAGCTATCGTACACAACGTCAAGCCGGCAATTATGA
TATCCAAATTGATGACTGGATGACAGTGTTTGGTGACCCGAACTATGCTATGACGGCATTATTTAGTTCTACAGGATCAAATAGTTTATTG
AAAGATAAACATGTAGACCAGTTGTTAAATAAAGCTTCTACTCAAAATGAAGCAGATGTTAAACAAACATATAAGCAAATTGAAGATGAA
GTTGTATTTGATAAAGGGTATATGGCGCCTTTATATGGATCAAAAAAGAATTTAGTATATGACAATAAAGTGTTAGATAAAAATAGTGTT
GGATTGCCAAATTCACGTGCATTAATATGGCAACAATTTGATTACAACAATAGTAGAGAACGAGATACGCGGCCACTTGTGATGACACAA
CAAGATGGTGAAATTCCTACATTGGATCCAATACGTTCAATTGCGCCGTCAGTATATTCAATTAATATGAATATGTACACAAGGTTATTAT
TATTAGATGAAAATGATCATTTAACAACGAAAGGTTCGTTAAGTCATGATTATGCTGTGAATAAGGACAATAAAGCATTTTATTTCTTGTT
AAGAGATGATGATTATTTTGCGAAAGTGGTCAATGGACAAGCACGTAATACTGGAGAGCGTGTATCGGCTGAAGATGTTAAGTTTTCTTT
AGATAGAGCACGTGATAAAAAGTCTGTGCCTAACAATAATACTTACAATATGCACAAACATATAAATGACATCAAGATATTAAAAGATGA
GGACATCGATCAGTTGCGTAAAGAGAAAGACAAGGACGATAAATCAATCTATGATAAGTTGATTAAAGCTTATAACGTCAAATCGTTAA
CGACAGATGGTCAAAAAGTAAATAATAAAGACGGTATTTATCAAATTGTTAAAATTACGACAGATCAATCGATGCCTCGAGAGGTAAATT
ACTTAACACACTCTTCGGCAGGCATTTTATCTAAAAAATTTGTTAATCAAGTAAATCAAGAATATCCAAAAGGATATGGGGATAGCAGTA
CAATTCCTGCAAATTCAGATGGGAAAAATGCGCTGTATGCAAGTGGCGCATACATTATGACACAGAAAAATGCATATCAAGCAACGTTTC
AACGTAATCCAGGATTCAACGAAACAGAAAAAGGTAGTTATGGACCAGCTAAAATTAAAAATATTACATTGAAGTTTAATGGTGACCCG
AATAATGCATTGTCAGAACTTAGAAATCATTCAATTGATATGTTGGCAGATGTGAATCAAAAACATTTTGATTTAATTAAGTCGGATAAAA
ATTTAAGCATTATTCGCAAAAATGGACGCAAGTCAGTCTTTTTAATGCTAAATATTAAAAAAGGTATATTTAAGACGCATCCAAACTTGAG
ACAAGCAGTAGTTAATGCGATAGATCAGGATCAATTTATTAAGTTTTATCGTGGCGATAAATTTAAAATTGCATCACCGATTACACCACTT
GTCGATACTGGTAACGAGCAACGTCAAGATTTAGAAAAAGTAGAAAAAGCCATCAATCAATAA

Gene: ggt (gamma-glutamyltranspeptidase)

Contig: 02_NODE_3, position: 147693 to 149699, length: 2007 nt, orientation: FORWARD

Perfect match to: (MW2-BA000033-[217402:219408], allele observed in CC1)

Sequence:

ATGGTCATTAACTTAAATGACAAACAGACAAAAACATCTAAAGAAGGGTTAATTTCCGTATCACATCCTCTTGCGGCTAAAATTGGTAAG
GATGTATTAGATCAAGGTGGCAACGCCATGGATGCAGTGATTGCAATTCAACTGGCGTTGAATGTGGTAGAACCATTTGCATCAGGTATT
GGTGGTGGCGGGTATTTGCTATATTATGAGCAAAGTACTGGCAGTATAACTGCGTTTGATGCGCGTGAGACAGCCCCTGCACATGTAGA
TAAACAATTTTATCTAGATGATTCAGGCGAATATAAATCATTTTTTGATATGACTACACATGGTAAAACTGTCGCTGTGCCAGCAATTCCA
AAGCTGTTTGATTATATTCACAAGCGTTATGCTAAGTTGTCATTGGAAGATTTAATTAATCCTGCAATTGAACTAGCTATTGAAGGTCATG
CAGCCAATTGGGCTACTGAAAAATATTCGCGCCAGCAACACGCACGATTGACAAAGTATTATGAAACGGCACAAGTATTTACGCATGAA
AATCAATATTGGCGTGAAGGTGATTGGATTGTACAACCCGAATTAGGTAAGACATTTCAAATATTAAGAGAACAAGGGTTTAATGCATTT
TATAAAGGTGACATTGCGAAACAATTAGTCAATGTTGTCAAAGCATGTGGTGGGACAATCACTTTAGAGGATCTAGCCAAATATGACATT
CAGATTAAAGCGCCAATCAGTGCAACATTTAAAGACTATGACATTTATTCAATGGGACCATCTAGTTCTGGCGGTATCACGGTAATTCAA
ATATTGAAGTTATTAGAACATGTCGATTTACCATCTATGGGTCCAAGATCTGTTGATTACTTGCATCATTTGATACAAGCGATGCATTTAG
CATATAGTGATCGTGCGCAATATTTGGCGGATGATAATTTTCATGAGGTGCCTGTACAGTCATTAATTGATGACAATTATTTAAAAGCACG
CAGTACGCTCATTGATAGCAATAAAGCAAATATTGATATAGAGCATGGTGTTGTGTCTGATTGCATTAGTCATACAGATGTTGAAGAAAA
TCATACCGAAACAACTCATTTTTGTGTGATTGATAAGGAAGGTAATATTGCTTCATTTACGACATCAATCGGTATGATTTACGGATCGGGT
ATCACGATTCCAGGCTACGGCGTGTTATTGAATACGACAATGGATGGCTTTGATGTAGTAGATGGTGGTATTAACGAAATTGCACCATAT
AAACGACCACTAAGTAACATGGCTCCAACGATTGTGATGTATCACGGGAAGCCAATATTAACAGTAGGTGCACCTGGTGCCATAAGTATC
ATTGCTAGTGTTGCGCAAACATTAATCAATGTATTAGTGTTTGGCATGGATATTCAGCAGGCTATAGATGAACCTAGAGTTTATAGTAGC
CATCCTAATCGCATTGAATGGGAGCCTCAATTTTCACAATCTACAATATTAGCATTGATTGCACGTGGACATGCAATGGAACATAAACCAG
ATGCCTATATTGGAGATGTACATGGACTACATGTTGACTTGAATACACGTGACGCATCGGGAGGTGCTGATGATACGAGGGAAGGTACA

GTGATGGGTGGTGAGGTATTATCAATTAGAAAACAACCATTACTTAGTCCCGAAATATACGATAATGACACCCATCGTGTATATTTCAAT
GATGTGCAGTTACCTTTATTGGCAGATCAAGTGCGATGGATGCATGACAAATATTGGGTTGATGAAAGTGTCGTTAGAATCATCTTCTCT
GAAGTTAGTGCACATATTGAAGATTTAAGAAGTTATGAAAACGCAGGAGAAAATTATATAGATATTGCCTGGTTAGCACGAAAGAAAGG
TTATCAAGTGGCATTGAAAGATGATGGTTTATACTTAACTGATGATACATATACTTCAGTGAAACGGAACACAAATGCATACTATAGATAT
GATCGAGATAGTATCACAAGATAG

Gene: DUF576_Saur (putative tandem lipoprotein)

Contig: 02_NODE_3, position: 150012 to 150785, length: 774 nt, orientation: REVERSE

Perfect match to: (MW2-BA000033-[219721:220494:r], highly conserved allele)

Sequence:

TTAATGTTCACTTAATTTATTATAAGTAAGGCTTTCTTTGTATATAATTTGGTCTTTTCCAGGACTTGAAAAATAAAAATAAAGTTTCTGATC
TTGATGATTATTGCCTTTTAAATCACTCATTCCCTTTAATTCCACCTTTGTTGATGCATTTCTGGGAATATTATATCTACGTTTTAATTGTTTA
ACGTTTGGATTATCATTCGACAACTTACCAGATAAATTATAATAATCAGTGGTAGGATCATGTGTGACTTTCAAATCATTGCTATTTAAAAT
GCTTGTTAAATCACCACTTTGAATCAAAAATTGATTGTTTTCGATTTTTTGTTTCAGCGCGGGATCTTTTACGTCTTTTGTGAAAACGATTTT
ATTATTAACTACTTTTACTGGATAACTTTTGTATGTCGAGTCAGTAGCATTTTTTCTATCGTTTGTAGTTGTGTCATATTCACCAGTTATTTT
ATGTGTGTTCTTATCTACCTTTAACAACATACGGTCTTCTTTTAAAAGCTCATCTGATCCAACAACTGAATAAGAGGATTCTATATACCATG
TGTCTTGATCATTATTTTCATAATGGGGATTATCGTGACCATCAATTTCATAAAGCGTTTCTAAGTTTTTAATAGGATACGTACTTAGTACT
TTTTTAAGACCATCTTTCAAATGAATTTGTTCCCACTTCATTGCCAAAAACATACCGCCACTGACTACAATTGAAATAATAATAATTGCTGC
TAAGTTTAACCAGAAAATTTTATGTGCTTTCAT

Gene: acpD (FMN-dependent NADH-azoreductase)

Contig: 02_NODE_3, position: 150975 to 151601, length: 627 nt, orientation: REVERSE

Perfect match to: (MW2-BA000033-[220684:221310:r], allele observed in CC1+CC5)

Sequence:

TTAAAAAATTGTTGCTAATTTTTCAGCATTATTAATACTAGTTGCTTTAATTTCTTCAGTCTTATGAGGTTCAGCATTGTGTCCTTCAATAAT
GATTGTTTCATATGATGGCACACCTAAGAATGTCATAATTGTTCTTAAATAACGGTCACCCATTTCAAAATCAGCAGCAGGTCCTTCAGTA
TAATATCCACCACGTGATTGAATGTGTAATACTTTTTTGTCAGTTAGTAAACCTTGTGGTCCTTCAGCAGAATATTTAAAAGTTTTACCTGC
AATTGAAATAGCATCAATATATGCTTTAACTACAGGTGGGAAAGAAAGGTTCCACATAGGCGTTACAAATACATATTTATCTGCACTTAA
AAATTCTTCTAAAATGTCACTCAATCTTGAAACTTTCATTTGTTCATCATCAGTTAACGTTTCGCCATTACTCATTTTTCCCCAACCAGTTAAT
ACATCTTTGTCAATAACTGGAATATAAGTTTCAAATAAATCAATATGTTTCACTTCATCATCAGGATGTTGTTGTTGATATGTTTCGATAAA
TGCTTTACCAGCCGCCATAGAATTTGATACCAGTTCATTAAAAGGGTGTGCTGTAATATATAATACTTTTGCCAT

Gene: Q5HJG7 (M23/M37 family peptidase)

Contig: 02_NODE_3, position: 151810 to 152388, length: 579 nt, orientation: FORWARD

Perfect match to: (MW2-BA000033-[221519:222097], allele observed in CC1+CC7+CC8+CC22+CC80)

Sequence:

ATGACAAAGCGACCAAAACGTATTTTGGCAACAATTATCATTTTTCTTTCACTATTATTTACGATTATTTATATAGATGACATTCAAAAATG
GTTTAACCAATATACCGATAAATTGACACAAAATCATAAAGGACAAGGACACTCAAAATGGGAAGACTTTTTTAGAGGGAGTCGGATTA
CTGAGACTTTTGGTAAATATCAACATTCACCATTTGATGGTAAGCATTATGGCATTGATTTTGCATTGCCAAAAGGTACACCAATTAAAGC
GCCGACGAATGGTAAAGTAACACGTATCTTTAATAATGAATTGGGCGGCAAGGTATTACAGATTGCCGAAGACAATGGAGAATATCACC
AGTGGTATCTACACTTAGACAAATATAATGTCAAAGTAGGTGATCGAGTCAAAGCAGGTGATATTATTGCATATTCAGGCAATACAGGTA
AACAAACGACAGGCGCACATTTACATTTTCAAAGAATGAAGGGTGGCGTAGGTAATGCATATGCAGAAGATCCAAAACCGTTTATCGAT
CAGTTACCTGATGGGGAACGTAGCCTATATGATTTGTAG

Gene: malK (maltose ABC transporter, ATP-binding protein)

Contig: 02_NODE_3, position: 152771 to 153868, length: 1098 nt, orientation: FORWARD

Perfect match to: (COL-CP000046-[225404:226501], highly conserved allele)

Sequence:

ATGGCAGAACTAAAGTTAGAGCATATTAAAAAGACGTATGATAACAACAATACTGTAGTGAAAGATTTTAATCTACATATTACTGACAAA
GAATTCATTGTATTTGTTGGACCATCGGGATGTGGTAAATCAACAACATTACGAATGGTTGCTGGACTAGAGTCTATCACATCTGGAGAT
TTTTATATTGATGGGGAACGCATGAACGATGTTGAACCAAAGAATAGAGATATTGCGATGGTATTTCAAAACTATGCATTATATCCACAT
ATGACTGTTTTTGAAAATATGGCATTTGGGCTAAAGCTACGTAAAGTAAATAAAAAAGAGATTGAACAAAAAGTTAATGAAGCAGCTGA
AATATTAGGATTAACTGAGTATCTTGGTCGTAAACCAAAAGCGTTATCTGGCGGACAGCGTCAACGTGTTGCTTTGGGCAGAGCTATTGT
TAGGGATGCGAAAGTCTTTTTAATGGATGAACCATTATCGAATCTTGATGCGAAGCTTCGAGTACAAATGCGCACAGAAATATTGAAATT
ACATAAGCGACTTAATACTACGACAATTTATGTTACACATGATCAAACTGAAGCATTGACGATGGCTAGTCGAATTGTTGTTTTGAAAGAT
GGCGACATTATGCAAGTCGGCACACCTAGAGAAATATATGATGCCCCTAATTGCATATTTGTGGCGCAATTTATCGGCTCACCAGCAATG
AATATGTTGAATGCTACAGTTGAAATGGACGGATTGAAGGTAGGAACACACCATTTTAAATTACATAATAAAAAATTTGAAAAGTTAAAA
GCTGCTGGCTACTTAGACAAGGAAATTATTTTAGGTATTCGAGCTGAAGACATTCATGAAGAACCAATATTTATTCAAACTTCTCCAGAGA
CACAATTTGAATCTGAAGTAGTTGTATCCGAACTGTTAGGTTCAGAAATTATGGTACATAGCACATTCCAAGGAATGGAATTGATTTCTAA
ATTAGATTCAAGAACTCAAGTGATGGCGAACGACAAGATTACACTAGCATTTGATATGAATAAGTGTCACTTTTTTGATGAAAAAACAGG
AAATCGTATCGTCTAA

Gene: malE (maltose ABC transporter, substrate-binding protein)

Contig: 02_NODE_3, position: 153881 to 155152, length: 1272 nt, orientation: FORWARD

Perfect match to: (Strain_21334-AGTW01000044-[49722:50993:r], highly conserved allele)

Sequence:

ATGTCTAAAATTTTAAAATGTATCACGTTAGCCGTGGTAATGTTATTAATCGTAACTGCATGTGGCCCTAATCGTTCGAAAGAAGATATTG
ATAAAGCATTGAATAAAGATAATTCTAAAGACAAGCCTAACCAACTTACGATGTGGGTGGATGGCGACAAGCAAATGGCGTTTTATAAA
AAAATTACGGATCAATATACTAAAAAAACTGGCATCAAAGTAAAGCTTGTAAATATTGGTCAAAATGATCAACTAGAAAATATTTCGCTA
GACGCTCCTGCAGGAAAAGGTCCAGATATCTTTTTCTTAGCACATGATAATACTGGAAGTGCCTATCTACAAGGCTTAGCTGCTGAAATC
AAATTATCAAAAGATGAGTTGAAAGGTTTCAATAAGCAAGCACTTAAAGCGATGAATTATGACAATAAGCAACTAGCATTGCCAGCTATC
GTTGAAACAACCGCACTTTTTTATAATAAAAAATTAGTGAAAAATGCACCGCAAACGTTAGAAGAAGTTGAAGCTAATGCTGCCAAACTA
ACTGATAGTAAAAAGAAACAATACGGTATGTTATTTGATGCTAAAAATTTCTATTTTAATTATCCGTTTTTATTCGGCAATGATGATTATAT
TTTCAAGAAAAATGGCAGTGAATATGATATTCATCAGCTAGGACTAAATTCAAAACATGTCGTCAAGAATGCTGAACGATTACAAAAATG
GTACGACAAAGGGTATCTTCCTAAGGCAGCAACACATGATGTCATGATTGGTCTTTTTAAAGAAGGAAAAGTAGGACAATTTGTCACTGG
ACCGTGGAACATTAATGAATATCAAGAAACGTTTGGTAAAGATTTAGGAGTAACAACATTACCTACAGATGGTGGCAAACCTATGAAAC
CATTTCTAGGTGTACGTGGTTGGTATTTATCTGAATATAGTAAACATAAGTATTGGGCTAAAGATTTAATGCTGTATATCACTAGTAAAGA
TACATTACAAAAATATACAGATGAAATGAGCGAAATTACTGGACGTGTTGACGTGAAATCATCTAATCCAAATTTAAAAGTGTTTGAAAA
GCAAGCACGTCATGCTGAACCGATGCCTAATATTCCTGAAATGCGACAAGTTTGGGAACCGATGGGCAATGCAAGCATATTTATTTCAAA
TGGTAAGAATCCTAAACAAGCGTTAGATGAGGCGACGAATGATATAACGCAAAATATTAAGATTCTTCATCCATCACAAAATGATAAGAA
AGGAGATTAG

Gene: malC (maltose ABC transporter, transmembrane permease)

Contig: 02_NODE_3, position: 155155 to 156423, length: 1269 nt, orientation: FORWARD

Perfect match to: (MW2-BA000033-[224864:226132], allele observed in CC1+CC8+CC72+CC188+CC4803)

Sequence:

ATGACGAAACGTAACCCTAAATTAGCGGCATTATTATCTGTTATACCTGGTTTGGGACAGTTTTATAATAAAAGACCCATTAAAGGGACG
ATATTTTTTATCTTTTTCATCAGTTTTATTTCTGTTTTTTATAGCTTTTTAAATATTGGTTTTTGGGGATTGTTCACATTAGGGACAGTACCTA
AGTTAGACGATTCTCGTGTCTTACTTGCACAAGGTATTATTTCTATCTTACTCGTTGCTTTCGCAATCATGCTATATATCATTAATATTTTAG
ATGCATATCGTAATGCTGAACGATTTAATCGCAATGAGGAAATAAAGGATCCGAAGGCGCGTATGGTGGCAACATGGGACAAGACGTTC
CCATACTTACTAATCTCACCAGGTACATTCTTATTGATATTTGTAGTTGTATTTCCATTAATATTTATGTTTGGAGTAGCATTTACAAATTAC
AATTTATACAACGCGCCTCCGAGACACACATTAGAATGGGTTGGTTTAGATAACTTTAAAACGTTATTCACAATTGGCGTTTGGCGTAAA
ACATTTTTCAGTGTTATTACTTGGACATTAGTATGGACGCTTGTTGCAACGACACTTCAAATTGCATTAGGGCTGTTTTTGGCAATTATTGT
AAATCACCCTGTCGTCAAAGGTAAGAAATTTATCCGTACTGTGTTAATCCTACCTTGGGCTGTACCATCATTTGTGACAATTTTAATATTTG
TAGCGTTATTTAATGATGAATTTGGTGCGATAAATAATGATATTTTGCAACCTTTATTAGGTGTAGCACCAGCATGGTTAAGTGATCCGTT
TTGGGCAAAAGTGGCATTAATCGGCATTCAAGTATGGCTTGGATTCCCATTTGTCTTTGCACTGTTCACTGGAGTACTGCAAAGTATTTCA

TCAGATTGGTACGAAGCAGCAGATATGGATGGTGCGTCTAGTTGGCAAAAGTTTAGAAACATCACATTCCCGCATGTCATTTACGCCACA
GCGCCATTGTTAATTATGCAATATGCAGGTAATTTCAATAATTTTAATCTTATTTATCTATTTAATAAAGGCGGTCCACCAGTGTCAGGGCA
GAATGCTGGTAGTACAGATATCTTGATATCTTGGGTGTATAATCTGACATTTGAGTTTAACAACTTCAACATGGGTGCAGTTGTGTCATTA
ATTATTGGATTTATTGTTGCTATTGTCGCATTTATTCAATTCAGACGTACAAGTACGTTTAAAGATGAGGGAGGTTTATAA

Gene: malD (maltose ABC transporter, transmembrane permease)

Contig: 02_NODE_3, position: 156425 to 157264, length: 840 nt, orientation: FORWARD

Perfect match to: (MRSA252-BX571856-[241712:242551], highly conserved allele)

Sequence:

ATGACAAAGAAGAAAAACATATTAAAAGCAATCGGTATTTACAGTTTTATAGCGATGATGTTTGTCATCATTTTATATCCACTACTGTGGA
CATTTGGCATTTCCCTTAATCCAGGTACGAACTTGTATGGTGCCAAAATGATACCAGACAATGCAACATTTAAAAATTATGCATTCTTACT
ATTCGATGACAGTAGTCAATACCTGACTTGGTATAAAAATACGCTTATCGTAGCATCTGCAAATGCACTGTTTAGTGTGATATTTGTCACG
TTAACAGCATATGCTTTTTCTAGATATCGCTTTGTTGGTCGTAAATACGGGCTGATTACATTTTTGATTTTACAAATGTTCCCTGTATTAATG
GCAATGGTCGCAATCTATATTTTGCTAAATACAATTGGATTATTAGATTCTTTATTTGGACTAACACTGGTATATATTGGTGGATCAATACC
GATGAATGCCTTTTTAGTGAAAGGTTACTTCGATACGATTCCAAAAGAACTTGATGAATCTGCCAAAATTGATGGTGCAGGGCATATGCG
TATTTTCTTACAAATTATGCTTCCATTAGCTAAGCCGATTTTAGCAGTTGTTGCTTTGTTCAATTTTATGGGGCCATTTATGGACTTTATATT
ACCTAAAATACTATTAAGAAGTCCTGAAAAATTCACATTAGCAGTTGGATTGTTCAACTTTATTAATGATAAGTATGCAAATAATTTCACA
GTGTTTGCAGCAGGGGCAATTATGATTGCAGTACCTATAGCAATCGTATTCTTGTTCTTGCAACGCTATTTAGTATCAGGTTTAACAACAG
GTGCGACAAAAGGTTAG

Gene: yrbE (putative NAD-binding oxidoreductase)

Contig: 02_NODE_3, position: 157338 to 158414, length: 1077 nt, orientation: FORWARD

Perfect match to: (Strain_10497-HE579061-[224860:225936], allele observed in CC5+CC8+CC22+CC188)

Sequence:

ATGACGATTAAAGTTGGAATCATTGGGTGTGGTGGTATTGCGAATGGCAAGCACATGCCAAGTTTACAAAAAGTTGAAAATGTTGAAAT
GATCGCATTTTGTGACGTAGACATTTCGAAAGCAGCGAGTGCGGCAGAAGCATACGGAACTGACAATGCAAAGGTTTATGATGATTACA
AAGCATTGTTAAAAGATGACACGATTGATGTTATCCATGTTTGTACGCCAAATGACTCGCATTGTGAAATTACTGTAGCAGGGTTGCATG
CTGGTAAACATGTGATGTGTGAAAAACCAATGGCTAAAACGACAGCAGAAGCTCAAAAAATGATAGATACAGCTAAATCAACAGGTAAA
AAATTAACAATAGGTTATCAAAATCGTTTCCGAGCAGATAGTCAATTTTTACATCAAGCAGCGCAACGTGGCGACTTAGGAGACATTTAC
TTCGGAAAGGCACATGCCATTCGTCGTCGAGCAGTACCAACATGGGGTGTCTTTCTAGACGAAGAAGCTCAAGGTGGAGGACCATTAAT
CGATATCGGTACACACGCTTTAGATTTAACGTTATGGATGATGGATAATTATGAACCAGAATCAGTGATGGGTTCAACATTCCATAAATT
AAATAAACAGCATCATGCGGCAAACGCTTGGGGTTCATGGAATCCAGATGAATTTACAGTTGAAGATTCTGCGTTTGGATTTATTAAAAT
GAAGAATGGAGCGACGATCATTTTAGAATCCGCTTGGGCGATTAATTCTTTAGAAGTGGATGAGGCAAAATGTTCATTATCAGGAACTA
AAGCAGGTGCTGATATGAAAGATGGTCTACGTATTCATGGTGAAGACATGGGTACACTTTATACCAAACACGTTGAATTGGAAAACAAA
GGCGTCGACTTTTATGAAGGTAATGAAGTGGATGAAGCTGAAGAAGAAGCAAAAGCTTGGATTGATGCAGTTGTAAATGATACTGAACC
AGTTGTGAAACCGGAACAAGCAATGGTAGTTACAAAAAATTCTTGAAGCGATTTATCAGTCTGCAAAATCAGGCAAAGCAATTTACTTTGA
ATAA

Gene: mviM (NADH-dependent dehydrogenase)

Contig: 02_NODE_3, position: 158439 to 159479, length: 1041 nt, orientation: FORWARD

Perfect match to: (MW2-BA000033-[228148:229188], highly conserved allele)

Sequence:

ATGACAAAATTAAAAGTTGGTGTGATAGGTGTTGGTGGTATTGCACAAGACCGTCATATTCCAGCATTGCTGAAACTCAAAGACACAGTC
TCATTAGTTGCAGTACAAGATATTAATACAGTGCAGATGATTGATGTTGCGAAGCGCTTTAATATACCTCATGCAGTTGAGACACCTAGC
GAGCTGTTTAAACTTGTTGATGCGGTGGTCATTTGTACACCTAATAAATTCCATGCTGATCTTTCTATAGAAGCATTGAACCATGGTGTCC
ATGTATTGTGTGAAAAGCCAATGGCGATGACGACGGAAGAGTGTGATCGCATGATTGAAGCGGCTAATAAAAATCACAAATTATTAACT
GTCGCATATCATTATCGTCACACAGATGTGGCAATTACTGCTAAAAAAGCAATTGAATCAGGTGTGGTTGGTAAACCTTTAGTAGCACGT
GTACAAGCGATGCGTAGGCGTAAAGTGCCTGGCTGGGGTGTTTTTACCAATAAAGCGTTGCAAGGTGGCGGTAGTTTAATCGATTATGG

TTGCCACTTGTTAGACTTATCTTTGTGGCTACTAGGTAAAGATATGGTGCCGCATGAAGTGCTAGGAAAAACATATAATCAATTGAGCAA
ACAACCGAATCAAATTAATGATTGGGGAACATTTGATCATACTAAATTTGATGTCGATGATCATGTTACTAGTTATATGACATTTGCCAAT
CGAGCAAGCATGCAGTTTGAATGTTCGTGGTCTGCAAATATCAAAGAAGATAAGGTTCACGTTAGTTTATCAGGAGAAGATGGCGGTAT
CAATTTATTTCCATTTGAAATATATGAGCCCCGCTTTGGAACTATTTTTGAAAGCAAAGCTAATGTTGAGCATAACGAAGACATTGCTGGT
GAGAGACAGGCGCGTAACTTTGTCAATGCGTGTTTAGGGATAGAAGAGATTGTGGTGAAACCGGAAGAAGCACGCAATGTAAATGCCC
TTATAGAAGCGATTTATCGTAGCGATCTTGATAACAAGAGCATACAACTTTAA

Gene: iolE (sugar phosphate isomerase/epimerase)

Contig: 02_NODE_3, position: 159534 to 160502, length: 969 nt, orientation: FORWARD

Sequence:

ATGAAAATAGGTGTATTTTCAGTATTATTTTACGATAAAAATTTTGAAGATATGTTAGATTATGTCTCAGAATCTGGATTGGATATGATTG
AAGTTGGAACAGGTGGTAACCCAGGAGATAAATTTTGTAAGTTAGATGAGTTGTTAGAAAATGAAGACAAGCGCCAAGCATTTATGAAG
TCAATCACAGACAGAGGCTTACAAATAAGTGGTTTCAGTTGTCATAACAATCCAATTTCTCCAGATCCGATAGAAGCGAAAGAAGCCGAT
GAAACGTTACGTAAAACAATCCGTTTAGCAAATCTATTAGACGTGCCAGTTGTTAATACATTTTCTGGCATTGCAGGATCAGATGATACCG
CTAAAAAGCCTAATTGGCCTGTTACACCTTGGCCAACAGCCTACTCTGAAATTTATGATTATCAGTGGAATGAAAAGTTGATACCATATTG
GCAAGATTTAGCTGAGTTTGCAAAAGAGCAAGATGTAAAAATTGCCATAGAGTTGCATGCAGGATTTTTAGTGCATACACCATATACAAT
GTTGAAGTTACGTGAGGCTACAAATGAATATGTCGGTGCTAACTTAGATCCTAGTCATCTATGGTGGCAAGGTATTGACCCAATTGCTGC
GATTCGCATATTAGGCCAAGCAAATGCAATTCATCACTTCCATGCTAAAGATACGTATATTAATCAAGAAAATGTAAATATGTATGGTCTA
ACTGATATGCAACCATATGGTAACGTTGCGACAAGAGCATGGACATTCCGTACAGTTGGTTATGGACATAGTCCATATGTATGGGCAGAT
ATCATAAGTCAACTTATTATTAATGGATATGATTATGTATTAAGTATTGAACATGAAGATCCTATTATGTCAGTAGAAGAAGGTTTCCAAA
AAGCTTGTCAAACTTTGAAATCTGTTAATATTTACGACAAGCCAGCAGACATGTGGTGGGCATAA

Gene: Q7A1X1 (isoprenylcysteine carboxyl methyltransferase)

Contig: 02_NODE_3, position: 160863 to 161357, length: 495 nt, orientation: REVERSE

Perfect match to: (N315-BA000018-[252541:253035:r], highly conserved allele)

Sequence:

TTAAATATTCATTAATTTCTCTTCTTGTTTAATACGTACATATAAGAAATACGCATACGGTACTAATAAAATAGTTGTATATGTTGCGTGTG
TTAATAATAATACACCGATTAATTCAGGAATGATGTTTAAGAAGTAATTTGGGTGTTTTGTAATTTTATATAATCCAGATTTAATAATAGG
ATGGTTAGGTAAAATGAATAATTTTAATGTCCAAATACCACCTAAAGTTTTAATAACCATAAATAACATGATATAAGCAAAGATTAATATA
ACTAAGCCAATACCATTTGCAAAGCTAAATGTATCTTTATTAATAAATGCCTCTACACCAGCCAATACATAAATTAAAACGTGTGTTATTGC
TAAAAACTTCGAATTTTTAACGCCATATTCAACTGCACCGTCTGCTTTTAATTGTTTTGAGTGATTAATAGATATCTTTAAGCTGACAAGTC
TGATACAGAAAAAGATAAGTAATATAGATAGAATCAT

Gene: uhpT (hexose phosphate transporter, major facilitator superfamily)

Contig: 02_NODE_3, position: 161590 to 162969, length: 1380 nt, orientation: FORWARD

Perfect match to: (N315-BA000018-[253268:254647], allele observed in CC5+CC1+CC188+CC772)

Sequence:

ATGAACTTTTTTGATATCCATAAGATTCCGAACAAAGGCATTCCATTATCGGTACAACGTAAATTATGGCTTAGAAACTTCATGCAAGCTT
TCTTTGTAGTGTTCTTTGTTTATATGGCTATGTATTTAATTCGAAACAACTTTAAGGCGGCCCAACCGTTTTTAAAAGAGGAAATTGGATTA
TCTACATTAGAACTTGGTTATATCGGATTAGCATTTAGTATCACGTACGGTTTAGGGAAAACATTACTTGGATATTTTGTCGATGGACGTA
ACACAAAACGTATTATCTCGTTCTTACTTATCTTATCTGCGATTACAGTTTTAATTATGGGATTTGTTTTAAGTTACTTTGGTTCTGTAATGG
GATTATTAATTGTACTTTGGGGACTTAACGGGGTGTTCCAATCAGTTGGTGGACCTGCAAGTTATTCAACGATTTCAAGATGGGCGCCAA
GAACGAAACGTGGCCGATACTTAGGATTTTGGAATACATCACATAATATCGGTGGTGCCATTGCAGGTGGTGTTGCACTTTGGGGTGCT
AATGTATTCTTCCATGGAAATGTTATAGGGATGTTCATTTTCCCATCGGTGATTGCATTACTTATTGGTATCGCAACATTATTTATCGGAAA
AGATGATCCAGAAGAATTAGGATGGAATCGTGCTGAAGAAATTTGGGAAGAGCCGGTCGATAAAGAAAATATTGATTCTCAAGGTATG
ACGAAATGGGAGATCTTTAAAAAATATATCCTGGGAAATCCTGTTATATGGATTCTATGTGTTTCAAACGTCTTTGTATACATTGTACGAA
TCGGTATTGATAACTGGGCACCGTTATATGTGTCAGAGCATTTACACTTTAGTAAAGGCGATGCAGTTAATACGATATTCTACTTTGAAAT
TGGTGCATTAGTTGCAAGTTTATTATGGGGCTACGTATCAGACTTATTAAAAGGTCGTCGTGCAATTGTAGCTATTGGCTGTATGTTTATG

ATTACATTTGTTGTCTTATTCTACACAAATGCTACAAGTGTCATGATGGTTAACATTTCATTGTTTGCATTAGGTGCGTTAATCTTTGGTCC
GCAATTATTAATTGGTGTATCATTGACTGGTTTTGTTCCTAAAAATGCCATCAGTGTAGCAAACGGAATGACAGGTTCATTCGCGTATCTA
TTCGGTGACTCAATGGCGAAAGTTGGTTTGGCGGCTATTGCTGATCCAACACGTAACGGTTTAAACATCTTTGGATATACATTAAGTGGA
TGGACAGATGTTTTCATCGTCTTCTATGTTGCATTATTCCTAGGCATGATTCTATTAGGAATCGTTGCTTTCTATGAAGAAAAGAAAATTAG
AAGTTTAAAAATTTAA

Gene: rsaG (ncRNA of Staphylococcus aureus G)

Contig: 02_NODE_3, position: 163024 to 163217, length: 194 nt

Sequence:

CCCCGACGGCATGTGCGTGAAGAGATGAAAGATACTGCTTCTACCCTTGCAAATATATCATCTCTATGTCTCGGGGCAGATCATAATTCCC
TGTTATGAAGTATCCTTATTTGCCCGACTTAGGGTGACTCAATGAATTTACTCCTTACAATAAAGACATATAGCGGTGTCAATATTGTAGG
GAGTATTGTTTT

Gene: yesN (two component sensor/regulator, transcriptional regulator)

Contig: 02_NODE_3, position: 163328 to 164086, length: 759 nt, orientation: REVERSE

Perfect match to: (MW2-BA000033-[233037:233795:r], allele observed in CC1+CC22+CC188)

Sequence:

CTATTTTGCTTGCTTACAATAATCACTTGGCGACATTTGTAAATATTTTTTAAAATGATAGCTAAACATTTTATACTCTGAAAAGCCTACTTT
GTCTGCAATTTCATAGTGTTTGTAATGTCGATCTAACAATTGCAGAGATTGTAAAATACGATAGCGATTTAAATAATCGACAATTGTAATA
CCAACATGATCTTTAAATGTTCGCATCGCATACGATTCACTAACATCGATATGTTGAATTAAATCTGAAACAGTCACTTTCGTTTGATAAGA
TTGCTTAATTTGATCAACAATTTGATTTACATAATAATCATCGTATTCTACTTTTAATAATGGTTGGAAGGCATCATGACAAGATGCTAAGC
TACGGCCGTTCTGTGATTGTTGCTCTAATAAGGTACGGACAAGTCTTCCTAAAATAACTTCTAATTGTGCATGGTCCACTGGTTTTAATAA
ATAATCAAGAACATGATGTTGAATACCGGCTTTCATATATTCAAAGTCATCGTAACTCGATAATATGATGACATTACAATCTAGATGCGCA
ATATCATTGAGTAAATCGACGCCATTTTTACGTGGCATACGAATATCAGTAATTACTAATTCTGGCTGATGTTGTTGAATTAGTGATAATG
CTTCAACACCATCTTTAGCAGTGTATATTGTATTGAAATGATAGTCTCCCCAAGGAATGATTTGCTTTAAACCTTCTCGAATAATTCGTTCA
TCATCACAAATAACTACCTTAAACAT

Gene: yesM (two component sensor/regulator, sensor histidine kinase)

Contig: 02_NODE_3, position: 164079 to 165635, length: 1557 nt, orientation: REVERSE

Perfect match to: (ATCC51811-ST1-ADVP01000041-[205131:206687], allele observed in CC1)

Sequence:

TTAAACATCTACATTCCCCCTTGAAAGTGGTATTTTATAACAAATTAACGTACCTTGATTACGCTTTGAAAAAATATGGAGTCGTGCATGT
GAACCATATTGAATCATTGCTTTATTGTGTAAATGATTTAATCCCAAATGCTTAGTATCAAATACATCATTATTAAGAGATTGGCGTACATA
TTGCAGGCGAGATGACGACATCCCAATACCATTGTCGCAAACTAAAACATGTAAATTCTGACGTGCCAATGTCAGGCGTATAGTAATATC
CAATGACTCAGTATCTCTACCATGTTTAATAGCATTTTCTATGAGTGGTTGAAGCATCATTTTACCAATTGTCTGGTGACGCGCTTCTTCAG
AACTTTCGATATGGAGCTTAATCATGTCATCAAAACGGATGTTTTGTATCGCAACATACTGTTCAATGTAGTTCAACTCTTCGTTTAATTCC
ACTGTATGTGAGTTTGTACGTAATGAGTAACGTAACATTTGCGATAATTGTTGGACCACAGTTTGTGCTAATTTCGGAGATAACGTAATTA
AATATTGTATTGTTTGCATCGTATTGAATAGGAAATGAGGTTGGAATTGGCGTTCTATTTCCTTTAACTGAATATCACGCAAGCGACGTTC
TGTATGCTCGATAGAATGGATCAGTTGCTCATTTGATTCAAATAAATCGTAAATATAATTATTAATTTCTTCTAGTTCACTGTTGTTTTTTAA
AGGTGTATATGTACCTAGATGACGATTTTTGGCATAGTAAATTTTTTGAATAATCGTTTCGATATCTTTTGTTTGTCGTTTAGCCATATTATC
TGCGCTAATGAAACCAAATATTACTAGTAAAACAAGAACTACGGCCATAACAATTAACAACGTGATACCATCTTCAATGTTTTCATGTATA
TCTTTATAAATAATGAGACGATGGTCAGCATGGTTTAATTTTACAGATTCATTCATAAATCCGAATTGTTGTGGTCTATACTTTTCACCTAT
AGTAAAACGGTCATCGTTGGCGTATAAAATATTGTCATATTGATCAACGATAAGTGCGAATTGTCGGCTATCTTTCTTAATTTCACTTAAA
CGTGGGGTGTTAGCCATATAAATTTTAAGCATATATGTACTATTTTTGAATTTAAGCTGATGCGTTGAAAATAAATACATATTTTTAGTGTT
TAAATGTTCATAATTATTGGTTATAAACTGATTTGGTCCAGATAATTCATAATAAAGTGTTGCGGGCTGTTGGTGTATTAATTTTAATAATC
CACGTTTTGTAGCGGTAACATCATGATGATTTGTTAAATCGAGCTCTTGAAACGAATTATTATGCTGTGTAATAAATGTCTGAATCTGCTTT
TCAGTGTGATGTAAAGATGACTGACTTTCATCAACATGTTGATGAATCGTACGATGCTCAATCCAAATATAGATGGCATAGAAGCTTACT

AGTCCAATAATAATGACTAAAAATACCGGAAAAATAGTAGACGCAAATAACGATCGTCTCAATTGATGTCTATAAGGTTTGTATGCCGTC
AT

Gene: hptA (phosphate sensing protein)

Contig: 02_NODE_3, position: 165632 to 166600, length: 969 nt, orientation: REVERSE

Perfect match to: (MW2-BA000033-[235341:236309:r], allele observed in CC1+CC80+CC772)

Sequence:

TCATTGAATCATCTCCAAAAATTTATGATGTGGAATGTCCGGTAATTTAGATTTCGGTATTAAAGGTATGTTCTTAAGATTTTCGATAGACT
GATCGCTTTGTTCACTAACATCCTTTCGAATTGACTTGGCATCGAACTCTGCAACTAATCGTTGTTGTACTGAGCGGCTTGTTAAATATTGC
ACTAACTTTTTACGCTTAGGATGAGGGTGTGCATTTTTAACTAAAGCAATACCATCAACATTTAACATTGTTCCTTCAATTGGATAAACGAT
TGATACAGGATAACCTTTGTTTTTCCATGTGCGTGCATCTTGTTCGTAGCTTAGACCTGCGTAATATTTACCTTTTGCAACATCTTCAATGA
CTTTAGACGTCTTTGACAGTTGCATCGCATGGTTTTGGAATTGATGCACATCACTTACTCGATGATGCATGCTATAAATAGCGCGCATATG
TTGATAGCCTGTCGTTGTTGTATTTGGATTTGAGTACGCAATTTTACCTTTAAGTATAGGTTGTAATAAATCTTGATAACCTCGAATCTTAA
TATCTCCTTGTAAATCTGAATTCACTACTATAACTGTTGGCATTAATAGAAAACTAGTAACATATTTATTGTTCGAGCGATAATCCTCTAAT
TGCTGTGTTACAGATGTATCTTGATAGGGAACAAAATCTTCTGGATGATCAATTGTTTCTGATAACACACCACCCATAAAGACGTCACCAC
GCTCCGAAAAATCTTCGTTATGCAAGTTTGAAAGCAGTACTTGAGTAGATCCATGTTTGATTTCAATTTTGACATGCTCTTGTTTTTCAAAT
TCATTTAAAATTGGACGAATCAAGTTTGATTGATACGGAGAATAAACTGTTAATACATTTTTATCTGATTCAGAGTGACGCGTATTAGCGC
ATGCTGATAAAAAAATGAGAAATAATAGCAAGATATAAATTTTTGATTTCAT

Gene: pflB (formate acetyltransferase)

Contig: 02_NODE_3, position: 167188 to 169437, length: 2250 nt, orientation: FORWARD

Sequence:

ATGTTAGAAACAAATAAAAATCATGCAACAGCTTGGCAAGGATTTAAAAATGGAAGATGGAACAGACACGTAGATGTAAGAGAGTTTAT
CCAATTAAACTACACTCTTTATGAAGATAATGATTCATTTTTAGCAGGACCAACAGAAGCAACTTCTAAACTTTGGGAACAAGTAATGCAG
TTATCGAAAGAAGAACGTGAACGTGGCGGCATGTGGGATATGGACACGAAAGTAGCTTCAACAATCACATCTCATGATGCTGGTTATTT
AGACAAAGATTTAGAAACAATTGTAGGTGTACAAACTGAAAAGCCATTCAAACGTTCAATGCAACCATTCGGTGGTATTCGTATGGCGAA
AGCAGCTTGTGAAGCTTACGGTTACGAATTAGACGAAGAAACTGAAAAAATCTTTACAGATTATCGTAAAACACATAACCAAGGTGTATT
CGATGCATATTCTAGAGAAATGTTGAACTGCCGTAAAGCAGGTGTAATCACTGGTTTACCTGATGCATACGGACGTGGACGTATTATCGG
TGACTATCGTCGTGTAGCTTTATATGGTGTAGATTTCTTAATGGAAGAAAAAATACACGACTTCAACACGATGTCTACAGAAATGTCAGA
AGATGTAATTCGTTTACGTGAAGAATTATCAGAACAATATCGTGCATTAAAAGAATTAAAAGAACTTGGACAAAAATATGGTTTCGATTT
AAGCCGTCCAGCAGAAAACTTCAAAGAAGCAGTTCAATGGTTATACTTAGCATACCTTGCTGCAATTAAAGAACAAAACGGTGCAGCAAT
GAGTTTAGGTCGTACATCAACATTCTTAGATATCTATGCTGAACGTGACCTTAAAGCAGGCGTTATTACTGAAAGCGAAGTTCAAGAAAT
TATTGACCACTTCATCATGAAATTACGTATTGTTAAATTTGCTCGTACACCTGATTACAATGAATTATTCTCTGGAGACCCAACTTGGGTAA
CTGAATCTATCGGTGGTGTAGGTATTGACGGACGTCCACTTGTTACGAAAAACTCATTCCGTTTCTTACACTCATTAGATAACTTAGGTCC
AGCTCCAGAACCAAACTTAACAGTATTATGGTCAGTACGTTTACCTGACAACTTCAAAACATACTGTGCAAAAATGAGTATTAAAACAAG
TTCTATCCAATATGAAAATGATGACATTATGCGTGAAAGCTATGGCGATGACTATGGTATCGCATGTTGTGTATCAGCGATGACAATTGG
TAAACAAATGCAATTCTTCGGTGCACGTGCGAACTTAGCTAAAACATTACTTTACGCTATCAATGGTGGTAAAGATGAAAAATCTGGTGC
ACAAGTTGGTCCAAACTTCGAAGGTATTAACAGCGAAGTATTAGAATATGACGAAGTATTCAAGAAATTTGATCAAATGATGGATTGGCT
AGCAGGTGTTTACATTAACTCATTAAATGTTATTCACTACATGCACGATAAATACAGCTATGAACGTATTGAAATGGCATTACATGATACA
GAAATTGTACGTACAATGGCAACAGGTATCGCTGGTTTATCAGTAGCAGCTGACTCATTATCTGCAATTAAATATGCACAAGTTAAACCA
ATTCGTAACGAAGAAGGTCTTGTAGTAGACTTTGAAATCGAAGGCGACTTCCCTAAATACGGTAACAATGACGACCGTGTAGATGATATC
GCAGTTGATTTAGTAGAACGCTTTATGACTAAATTACGTAGTCATAAAACATATCGTGATTCAGAACATACAATGAGTGTATTAACAATTA
CTTCAAACGTTGTATACGGTAAGAAAACTGGTAACACACCAGACGGACGTAAAGCTGGCGAACCATTTGCACCAGGTGCAAACCCAATG
CATGGCCGTGACCAAAAAGGTGCATTATCTTCATTAAGTTCTGTAGCTAAGATCCCTTACGATTGCTGTAAAGATGGTATTTCAAATACAT
TCAGTATCGTACCAAAATCATTAGGTAAAGAACCAGAAGATCAAAACCGTAACTTAACTAGTATGTTAGATGGTTACGCAATGCAATGTG
GTCACCACTTAAATATTAACGTATTTAACCGTGAAACATTAATAGATGCAATGGAACATCCAGAAGAATATCCACAGTTAACAATCCGTGT
ATCTGGTTACGCTGTTAACTTCATTAAATTAACACGTGAACAACAATTAGATGTAATTTCTCGTACATTCCATGAAAGTATGTAA

Gene: pflA (pyruvate formate-lyase-activating enzyme)

Contig: 02_NODE_3, position: 169460 to 170215, length: 756 nt, orientation: FORWARD

Perfect match to: (RF122-AJ938182-[205576:206331], highly conserved allele)

Sequence:

ATGCTTAAGGGACACTTACATTCTGTCGAAAGTTTAGGTACTGTCGATGGACCGGGATTAAGATATATATTATTTACACAAGGATGCTTA
CTTAGATGCTTGTATTGCCACAATCCAGATACTTGGAAAATTAGTGAGCCATCAAGAGAAGTCACAGTTGATGAAATGGTGAATGAAATA
TTACCATACAAACCATACTTTGATGCATCGGGTGGCGGTGTAACAGTCAGTGGTGGCGAACCATTGTTACAAATGCCATTCTTAGAAAAA
TTATTTGCGGAATTAAAAGAAAATGGTGTGCACACTTGCTTAGACACATCGGCTGGATGTGCTAATGATACAAAAGCATTTCAAAGGCAT
TTTGAAGAATTACAAAAACATACAGACTTGATATTATTAGATATAAAACATATTGATAATGACAAACATATTAGATTGACAGGAAAGCCT
AATACACACATCCTTAACTTCGCGCGCAAACTGTCAGATATGAAACAACCTGTATGGATTCGACATGTCCTTGTGCCTGGTTATTCTGATG
ATAAAGACGATTTAATTAAACTAGGGGAATTCATTAATTCTCTTGATAACGTCGAAAAGTTTGAAATTCTGCCATATCATCAGTTAGGTGT
TCATAAGTGGAAAACATTGGGCATTGCATATGAATTAGAAGATGTCGAAGCGCCCGATGATGAAGCTGTTAAAGCAGCCTACCGTTATG
TTAACTTCAAAGGGAAAATTCCCGTTGAATTATAA

Gene: Q2YV51 (putative protein)

Contig: 02_NODE_3, position: 170205 to 170513, length: 309 nt, orientation: FORWARD

Perfect match to: (MW2-BA000033-[239914:240222], allele observed in CC1)

Sequence:

TTGAATTATAAATACAATTCAGACCGAAAAGAAAGCATATGCAACTTCAAGAGTGAAGGGGCATATGCTTCTTTTTCAATTGAGTATAGA
GTATTAGCAAGACGTAGTAAGTATATGAGACAACTTCTACAATGGTTGAAGGAAGACGTTTTTGTAAGTAGCTATGCTGATAAAGAATGT
GATGTCTTGTTAAAGGTGGGGTTCCAATATCATCATTTAGCTGATGTTGAATGGGTTATTATTTGCTACTTGCATATGAATATGAGTCTTTT
CAAATTTTTATTGACCCTGAGTAATGAAAAATATTAA

Gene: glpQ1 (glycerophosphoryldiester phosphodiesterase)

Contig: 02_NODE_3, position: 170551 to 172314, length: 1764 nt, orientation: FORWARD

Perfect match to: (CN1-CP003979-[225413:227176], allele observed in CC72+CC630+CC1290)

Sequence:

ATGAAGAGAATTAGTAAAGATATATGGGCAGTATTTAAATTACTGTATCAAAATAAAGGGCGTTTTAGCATTAATGCCTTACTATTGCAG
TTAATCATGATTTTTATTAGTAGTACATACTTAATTTTACTATTTAATATGATGTTAAAAGTAGCTGGGCAAAGCCAACTTACGATTAACAA
TTGGACGGAAATCGTTAGTCATCCCGCCAGTGTGATACTTCTTATTATATTCATATTAAGTGTTGCCTTTCTGATTTATGTAGAGTTTTCATT
GTTAGTTTATATGGTTTATGCCGGCTTTGATCGACAGATTATTACATTTAAATCCATTTTTAAAAATGCCTTTGTAAATGTGCGTAAACTCA
TAGGTGTACCAGTTATTTTCTTTGTTATTTATTTAATGTTAATGATACCCATTGCCAACCTAGGACTAAGTTCAGTATTAACAAAAAATATTT
ACATACCTAAATTTTTAACGGAAGAACTTATGAAAACGACGAAAGGTATAATCATTTACGGTACCTTTATGATTGCTGTATTTATATTAAA
CTTTAAATTAATATTTACCTTACCGTTAACGATTTTAAACCGTCAGTCGTTATTTAAAAAATATGAGACTAAGTTGGCAAATTACGAAGCGA
AATAAGTTTCGACTTGTTATAGAAATAGTTATATTGGAACTCATCATTGGTGCGATTTTAACATTAATTATTTCAGGAGCAACATATCTTGC
TATTTGTGTAGATGAAGAAGGAGATAAGTTTTTAGTCTCATCAATTTTATTTGTTGTATTGAAAAGCGCATTGTTCTTCTATTATTTATTTAC
GAAATTATCATTAATCAGTGTGTTAGTACTGCACTTAAAACAAGAGAATGTATTAGACCAACCGGGCTTAGAATTTAAATACCCAAAACC
GAAACGGAAGTCTAGGTTCTTTATAATTTCAATGGTACTTGCAGTGACATGTTTTATCGGTTATAACATGTACTTACTTTACAATAATACTA
TCAATACAAATATCTCCATTATTGGCCATCGTGGTTTCGAAGATAAAGGCGTTGAAAATTCTATTCCGTCATTGAAAGCTGCTGCAAAAGC
GAATGTCGAATACGTTGAGTTAGATACAATTATGACGAAAGATAAACAATTTGTTGTTAGTCATGATAACAATTTGAAACGTTTAACAGG
TGTTAATAAAAATATTTCTGAATCTAATTTCAAAGATGTCGTCGGTTTGAAAATGCGTCAAAATGGACATGAAGCAAAACTTGTATCCTTA
GACGAATTTATTGAAACGGCTAAACAATCAAATGTGAAGCTACTAGTAGAGTTAAAGCCACATGGTAAAGAACCAGCAGATTATACACA
ACGTGTTATTGATATTTTGAAAAAGCATGGTGTTGAACATCAATATCGTGTGATGTCATTGGATTATGATGTGATGACTAAGTTGAAAAA
AGAAGCGCCATATCTCAAGTGTGGTTATATCATTCCGTTGCAGTTTGGTCATTTTAAAGAAACATCATTAGATTTCTTTGTCATCGAAGATT
TTTCTTATTCGCCAAGACTTGTTAATCAAGCGCACTTGGAAAATAAAGAAGTCTATACTTGGACCATTAACGGCGAAGAAGATTTAACGA
AATACTTACAAACCAATGTTGATGGTATTATCACAGATGACCCAGCATTAGCTGATCAGATTAAAGAAGAAAAGAAAGACGAAACATACT
TCGATCGTTCTATAAGAATTTTGTTTGAATAA

Gene: Q2YV49 (putative protein)

Contig: 02_NODE_3, position: 172478 to 172822, length: 345 nt, orientation: REVERSE

Perfect match to: (CGS03-ABWY01000005-[110346:110690:r], allele observed in CC5+CC8)

Sequence:

CTAATTGTTTTGTGAATGCAAAGGGTTAGAAATTGAATCGTAAATACTTTCTAATCTATGTTTCGCTTTAGTCATTTGATCCAAATTTTTAG
TGCGTATAGCGGATTTTGCAATATAGTGCGCAGCTAAAATATCGCGTTTTTGAAACGCATCTAAATTTAGGTACGATAATTTATTTAAGTC
AGTGTTTGCTATTAATTCATGTAATTGATCTACAAGCGCTTGATGTTGATACGTATGTGATGTAGTTTCAGATTTGCTTGCTAATTTAATAC
CAGTCGTATCAAGGAGCGCCGCTTTAATACCAGCAACTAAATATGTTTTGATTTTCATTTGTGTTGTCAT

Gene: coa (staphylococcal coagulase)

Contig: 02_NODE_3, position: 173012 to 174913, length: 1902 nt, orientation: FORWARD

Perfect match to: (MW2-BA000033-[242721:244622], allele observed in CC1)

Sequence:

ATGAAAAAGCAAATAATTTCGCTAGGCGCATTAGCAGTTGCATCTAGCTTATTTACATGGGATAACAAAGCAGATGCGATAGTAACAAA
GGATTATAGTGGGAAATCACAAGTTAATGCTGGGAGTAAAAATGGGAAACAAATTGCAGATGGATATTATTGGGGAATAATTGAAAATC
TAGAAAACCAGTTTTACAATATTTTTCATTTACTGGATCAGCATAAATATGCAGAAAAAGAATATAAAGATGCAGTAGATAAATTAAAAA
CTAGAGTTTTAGAGGAAGACCAATACCTGCTAGAAAGAAAAAAAGAAAAATACGAAATTTATAAAGAACTATATAAAAAATACAAAAAA
GAGAATCCTAATACTCAAGTTAAAATGAAAGCATTTGATAAATACGATCTTGGCGATTTAACTATGGAAGAATACAATGACTTATCAAAA
TTATTAACAAAAGCATTGGATAACTTTAAGTTAGAAGTAAAGAAAATTGAATCAGAGAATCCAGATTTAAAACCATATTCTGAAAGCGAA
GAAAGAACAGCATATGGTAAAATAGATTCACTTGTTGATCAAGCATATAGTGTATATTTTGCCTACGTTACAGATGCACAACATAAAACA
GAAGCATTAAATCTTAGGGCGAAAATTGATTTGATTTTAGGTGATGAAAAAGATCCAATTAGAGTTACGAATCAACGTACTGAAAAAGA
AATGATTAAAGATTTAGAATCTATTATTGATGATTTCTTCATTGAAACCAAGTTGAATAGACCTAAACACATTACTAGGTATGATGGAACT
AAACATGATTACCATAAACATAAAGATGGATTTGATGCTCTAGTTAAAGAAACAAGAGAAGCGGTTGCAAAGGCTGACGAATCTTGGAA
AAATAAAACTGTCAAAAAATACGAGGAAACTGTAACAAAATCTCCAGTTGTAAAAGAAGAGAAGAAAGTTGAAGAACCTCAATCACCTA
AATTTGATAACCAACAAGAGGTTAAAATTACAGTTGATAAAGCTGAAGAAACAACACAACCAGTGGCACAGCCATTAGTTAAAATTCCAC
AGGGCACAATTACAGGTGAAATTGTAAAAGGTCCGGAATATCCAACGATGGAAAATAAAACGTTACAAGGTGAAATCGTTCAAGGTCCA
GATTTCCCAACAATGGAACAAAACAGACCATCTTTAAGCGATAATTATACTCAACCGACGACACCGAACCCTATTTTAGAAGGTCTTGAA
GGTAGCTCATCTAAACTTGAAATAAAACCACAAGGTACTGAATCAACGTTAAAAGGTACTCAAGGAGAATCAAGTGATATTGAAGTTAA
ACCTCAAGCATCTGAAACAACAGAAGCATCACATTATCCAGCAAGACCTCAATTTAACAAAACACCTAAATATGTTAAATATAGAGATGCT
GGTACAGGTATCCGTGAATACAACGATGGAACATTTGGATATGAAGCGAGACCAAGATTCAATAAGCCATCAGAAACAAACGCATACAA
CGTAACGACAAATCAAGATGGCACAGTAACATATGGCGCTCGCCCAACACAAAACAAACCAAGCAAAACAAATGCATACAACGTAACAA
CACATGCAAATGGTCAAGTATCATATGGCGCTCGCCCGACACAAAACAAGCCAAGCAAAACAAATGCATATAACGTAACAACACATGCA
AATGGTCAAGTATCATACGGAGCTCGCCCGACACAAAACAAGCCAAGCAAAACAAATGCATATAACGTAACAACACACGCAAACGGTCA
AGTGTCATACGGAGCTCGCCCGACATACAAGAAGCCAAGTAAAACAAATGCATACAATGTAACAACACATGCAGATGGTACTGCGACAT
ATGGGCCTAGAGTAACAAAATAA

Gene: fadA (3-ketoacyl-CoA transferase)

Contig: 02_NODE_3, position: 175516 to 176700, length: 1185 nt, orientation: REVERSE

Perfect match to: (MW2-BA000033-[245225:246409:r], allele observed in CC1+CC15+CC188-ST582+CC834+CC2885)

Sequence:

CTAACGCACATATTCAAATATAGCAGCTGCACCCATACCGACACCAATACACATCGTAACCATGCCGTAACGGCTATCGGGACGTCTACC
CATTTCATTAAGTAAACGCGCGGTTAACATTGCGCCTGTAGCACCTAATGGATGACCTAAAGCAATAGCGCCACCATTCACATTCGTACGT
GATATATCTAGACCTACTTCTTTAATAGATGCAATTGTTTGAGAAGCAAATGCTTCGTTCAATTCGATCAAATCAATGTCTTCAACAGATA
GATTGCTGAGTGACAATACTTCAGGAATCGCATATGCAGGCCCAATACCCATAATTTTCGGGTCAACGCCTACTGCCTTATAACCAACGA
ATCGTGCAATAGGTGTCACGCCTAGTTCTTTCACTTTATCTCCAGACATTAAAACTACAAATCCTGCACCATCAGAAAGTGGGGCAGATGT
TCCCGCAGTCACAGTGCCGTCAGCTTTAAATACTGTACGTAATTTGGCTAATGCCTCCATCGTGGTGTCAGGGCGTATAAATTCATCTTGG
TCAAAGATATTTGTGTGTACTTTTGGTCCTGCGTTTGTATATTCAACTGAGTTTACTTGTATTGGGATAATTTCATCGTTGAACCGACCATC
ACGTTGTGCTTCATAGGCACGTTGATGACTTCTGACAGCATAAGCATCTTGATCTTCGCGTGATACGTCAAATTGGGATGCTACATTTTCA
GCAGTTAAACCCATAGGATATGACGCACCTATATCATCATATTGTAAGGTAGGATTGTTTGTGGGCTCGTTGCCACCCATTGGTACGGCA
CTCATCAATTCAACGCCACCAGCTACAAGTATATCTCCTTGACCAGCCATAATTTGATTGGCTGCAATCGCGATGGTTTGTAATCCTGATG
AGCAGTAGCGATTCACTGTTTGACCCGGTACCGTGTCAGATAATCCCGTAAGCAATGCAATCGTTCGTGCAATGTTTTGTCCTTGTAATCC
TTCTGGAAAAGCCGTACCAACAATGACATCTTCAATCATATTCTTATTGAATTTTCCGTCAATACGTTTCAATACGCCTTGTAATACTTTGG

CTGCGACATCATCAGGTCTTTCGTGGAATAATGCGCCTTGCTTTGCTTTCGCTGCGGCTGAACGCCCATAAGCTACAATGTATGCTTCTTG
CAT

Gene: fadB (NAD binding 3-hydroxyacyl-CoA dehydrogenase)

Contig: 02_NODE_3, position: 176730 to 178991, length: 2262 nt, orientation: REVERSE

Perfect match to: (TCH70-ACHH02000005-[241027:243288], allele observed in CC1)

Sequence:

TTAATTACGTAATGGCTTACCAGTTTTTAACATATGTGCAATTCTTTCATATGATTTTTTAGATTTTAGTAAGTCAATAAAGCCAATTTTCTC
CAACGATTGAATGTAACGTTGATTGATAAATGTATTTCTTGGTAAATCACCACCCGCTAAAATTGTGGCGATATTTAAGGCGATATGATAA
TCATGGTCGCTAATAAAATGACCCCGTCTTTGCGCATCTAATTGTCCTTGGATCAATGCTTTGAAGTCTTCACCTAAAGCGATATATTGATG
TCTAGGATTCGGAATATAGTTTGTTTCTGCTTCATATTTCGCACGTTTGAGCGCAACTTCGACACGTTGTGCTGTATTGAAAATAATCGTAT
CTGTATCACGTAAATAACCATAACGACGTGCCTCAAAGGCATTTGTAGAGACTTTCGCAAATGCGATATTCGTCAGTACTTTTGTCATGGA
AGCTTGTTTGTCATCAAACTTATGCGATGTGCGTAATATGCGATCAGCCATTTCTGCAAGGCCACCGCCACTCGGTAATAAGCCAACGCCT
GCTTCAACAAGACCGATATACGTTTCACTTGCAGCGACAACAATAGGTGAGTAAAGTACAAGCTCACAGCCACCGCCTAAGGCACGACCT
TGAACAGCTGTGACTACTGGTTTCAAACTATACTTCAAACGATTAAAGCTATAATGTAATTTATCAATTGATTGTGCAACGACATCATCTA
CAAGACCGTCTTCATGCGCCTTTTTCATTAAGAAAAGGTTAGCACCCACACTGAAATTGTTACCATCTGCATAAATAACCATACTTGTGTA
ATGGTCATTTTCCAGTAAATCAATCGCATCAACTAACGCATCGTTGAATTCATCGGTAATGACATTATTTTTACTTTGTAATTTCAGTAACA
GTTGATCATCATGAGTTACGGAAAGTTTGGCATCACCTTTATCCCAAAGTTCATCTTTTACGAAGTGAGAAACAGGTGTTGCATATTCGAT
GGTCTCATCTTGTTTATAAAAGCCACCATCTAAATCACTAATCCATTGTGGTAAGTCTCCAATTTCGTCTTCCATACGTGTTTTAACACGTTC
ATATCCCATTGCATCCCATAATTGGAATGGACCAAGTTTCCAGTTGAACCCCCAGACAAGCGCACGGTCTATGTCTCGGAAATCATCGGT
AGCTTTAGGTACATTGATAGCAGAGTAATAGAAATTATTACGTAATGTCTCCCATAAAAATAGTCCCGCTTCGTCTTGCGCATTGAATATG
ACATCAAGGTTATGCACTAAGTCTTTATTAAATTCATTTAAAATTGGTAATTGTGGTTGCGATACAGGTACATAATCTTGTTTTTCAACATC
GTAAACAAGTCGAGCTTTAGTTTCTTTATCCTTTTTGTAAAATCCTTGTTTCGTTTTACGTCCGAGTGCGCCATTGTCAAACAACGTATTTAC
AATTTTGACATCATGAAAATAAGGCGTTTCTTCAGGTACTTGTTGCATGCCTTTAATTACAGACACTGCAATATCTAAACCGACTAGGTCA
GATAGGGCATATGTACCTGTTTTAGGACGACCAATCGCTTGCCCAGTTAAAGCATCCACATCTACAATGCTTAACTTGTGTTGCTCGGCGC
GATACATAATATCATTCATTGTTTGCGTGCCGACTCTATTTGCGACAAAGCCAGGCACATCATTGACGACAATGACACCTTTACCTAACAC
ATTTTGCGCGAAATTTTTTACATCTAATATGATAGATTCCTTCGTGTGTGACGTAGGTATTAACTCCACTAATTTCATAATACGTGGTGGGT
TAAAGAAATGTAGACCAAAGAATCGTTCTTGATCCTTCTCGTTAAATGTTTGAGCAATCGCATTAATTGGAATACCTGATGTATTTGTAGC
GAATAAAGCATCTTCTTTAGCATGTTGTAGAACTTGTTGCCAAACAGCATGCTTAATTTCAATATCTTCTTTAACTGCTTCGATATATAAAT
CAGCATCATCATTTACCAAGTCATCATCAAAATTACCATATGTTAAATGACTCGCTAGATTTAAGTCGAATAGTAGCGGCCGTTTCTTATCT
GTAATTTTATCGTAAGATTTTTTCGCAATGAGATTTGGATCGTTTTTGTCCACTACAATATCTAATAGTTTTACTTTAAGTCCAGCATTCACA
AAGAGTGCTGCCAGTTGAGCGCCCATCGTGCCTGCGCCAAGAACGGTTACTTTATTAATTGTCAT

Gene: fadD (putative acyl-CoA dehydrogenase)

Contig: 02_NODE_3, position: 179178 to 180389, length: 1212 nt, orientation: REVERSE

Perfect match to: (COL-CP000046-[251819:253030:r], allele observed in CC8+CC5+CC772)

Sequence:

TTATACGAAAGCAGAATCTCCAGTCAAAGCGCGTCCAATTACTAAGGCATTAATTTCATGTGTACCTTCGTACGTGTAAATCGCTTCTGCA
TCAGAGAAGAAACGTGCAATATCATAATCGTCAGCTAGTATGCCATTACCACCTGTAATACCACGGCCCATAGCTACTGTCTCACGCAAA
CGTAAGGCATTCATCATCTTCGCCGTTGAAGTTGCAACCTCGTCATATTCACCATGTGCTTGCATATTAGCTAATTGAGCACATGTTGCCAT
TGCTTGAGCTAAATTACCTTGCATCATTGCTAGCTTTTCTTGTATTAACTGATATTTACTAATTGGTTTGCCGAATTGCTTACGCTCAGTGAC
ATAATCTAATGTGGCACGTAAAGCGCCAGCCATACCACCTGTAGCCATATAAGCAACGCCTGCTCTCGTTGAATAAAGAATTTTGGCAAT
ATCTTTAAAGCTTGTTATGTTTTGTAAGCGATCCGCTTCATCTACTTTGACATTAGTTAATTTAATTAGGGCGTTAGGAACAATGCGAAGT
GCGATTTTATTATCAATGACTTCAATATCGACGCCATCTTGTTCTGGTCTGACTACAAAGCAATGGGGTTTGCCAGTTTCTTTATTTACTGC
GAATACTGGAATGACATCAGATACATGTGCACCACCAATCCATTTCTTTTCACCATTGATAACCCAAGTATCGCCCTGGCGTTCAGCGACT
GTTTCAAGACCTCCCGCAACGTCCGAACCGTGTTCTGGTTCAGTTAAAGCAAAGCATGTACGCAGTTCATGTGACTGTAATTTAGGTACAT
ATTTCGCAATTTGTTCTTTGCTACCTCCGAAATAGAAAGTGTTATGCCCTAAACCTTGGTGAACACCGAGTAGGGTAGCTAAGGAAATATC
AAATCGCGCGAGTAGGTAAGACATGAAAAACTGAAATAGTTGACTAGGCATTTTGGCGTTTGGACGATCCTTGTAAAGTAATGGATTGT
TAAAATAATTTAATTCTCCCAGATCTTTAAAATAGTCCTCGGGTACAGTAGCGTCTATCCAATGTTGATTAATATTTTCACGGTACTTACTTT
CTAGCAATGAATCTACTTGTTGTAAAAATTCGACTTCACCGTCTGTTAAACCTTTAGCAATACTAAGTACATCTTCAGGAAATAATGTTTTT
AAGACCGTTTCTTTTTCAAATGTCAT

Gene: fadE (putative long-chain-fatty-acid--CoA ligase)

Contig: 02_NODE_3, position: 180501 to 182006, length: 1506 nt, orientation: REVERSE

Perfect match to: (COL-CP000046-[253142:254647:r], allele observed in CC8+CC5+CC772)

Sequence:

TTAAAGTGTTTGTGAGACTTTGTCATTCATCATTTGTCGAATCGCAAGTTTATCTGGTTTCTGCGTACTGTTTAACGGCATATGTGTCACTG
GTACATACATTCTTGGGACTTTATAACCTGCTAAACGACTTCGCATATGTTGATCTAAAATTTCAGCGTAATGAGGTTCATCTTCGCGAAG
TATAATGGCTGCAGCAATTGATTCACCATATTTTGGATGATCATAGCCAACGACCACACACCGGTCTACTAGTGGATGCTCAGCTAAAGC
ATTTTCGACTTCGGATGGTAAGACATTTTCGCCACCAGTTATGATTAATTCTTTTTTGCGGTCAATAATAAATATATCGCCATCGTCGTCCA
TCTTCGCTAAGTCACCAGTTAATAAATAACGACCATGAAATGCTTTGGCAGTCTCTGCTGGTTTATTCCAATATCCTGGCGTGACATTTTTA
GCCTTAATTGCAAGTTCGCCAATCTCACCAGTAGGTACTTCCTCACCGTTATCATCAAGGATACGTGCATCAACGAACATGACTGCTTTAC
CAATACTCATTGGCTTACGTTTTGAATTTTCCGGTGTATTAACAAGTACAAGAGGTGCTTCAGTTAAACCATAGCCGTTAATAATGTTTAT
GCCATATTGTTTAAAAGCTGCTTGGATACTTGGTAATGGTTGTGAACCACCTTGGATGATATAATCCATAGCTTTAAAATTTTCAGGATTA
AAATTACTAGCACGTAGCGTACTATAATACATTGTCGGAATCATGATAATAAATGTAGGGTGATATTGTGCAATCATGTCATTCAATTCTT
CGCCGTTAAAGTAACGTTGAAGAATAAGTGTGCCACCTGACATCAATACTGGTAATACAGTATCGTTAAACCCTAAAACATGGAACATTG
GTGTTGATACAATCGTAATATAGTTTGAATTGAACTTATACGTCAGCTCTAAGTTTGCACCGTTATGAACAAATGATTCATATGAGAACAT
CACACCTTTAGGTGATCCGGTTGTACCACTTGTATAAATTAATGCTGCAAGATCTTGTGGTTCGACAGGTGTTGCTTGAAAAGGTTGGTG
ATAATCTGGATTTACGATTTCATCATATTGCGCTACATCAATATCCATATGCAATAAGTTTTGGTCAATATCGGTGAGTGAACTTAAATGTT
TTTCAGCATAGAAGAGCAGTTTTAATTGTGCATCTTCCACAATGGCTGCAATTTCTTTTGGGTTAAGCCGCCAATTCAATGGTAAAAAAAC
CGCACCTGTTTTAAAACAAGCAAACAATAAATCTAATATTGCAATATCATTTGGCGCAAAAATACCGATAACATCGCCTTTTTTAACACCTT
GAGATGTTAAATAATGTGCCATATTATCAGCGCGTGCATTGAGTTGTTGGTATGTCCAAGATGTTTGTTTTGCGTGATCAATAACGGCAG
GCTTGTCATCATCGAAGTCTGAACGCGTTTTTATCCAATCGAAATTCAT

Gene: fadX (putative acetyl-CoA/acetoacetyl-CoA transferase)

Contig: 02_NODE_3, position: 182032 to 183594, length: 1563 nt, orientation: REVERSE

Perfect match to: (N315-BA000018-[273754:275316:r], allele observed in CC5)

Sequence:

TCATACTTTATGAATTGATTGTTTAAGTTGTCCCCATTTTTCTTTGTAAATGCTGGTATCAATTAATTTTAAATGATCAGCAATAATTGGTTT
AAAAGCCATTTGATTCAAAATATCTTTATGCAAATCAAGACCTGGTGCAATTTCAATTAGTTTCAAGCCTTGATTGGTGAGTTCGAATACT
GCACGCTCAGTAACAAAATAGACTTCTTGCTCGAGTGATTGTGAATATTGTGCATTAAAGTCGATATGGCTCACATCTGATACAAATTTCT
GGTTTTGTCCTTCAGTTTCAATGTTTAATCGTTGATTATGGCATGAGACATGACTGCCAGCTACAAAAGTACCTGAAAAGATAATTTTATTT
ACAGATTGCGTAATGTCTATAAAGCCACCACATCCATTTAGTCGGTCATTGAAGTAAGACACGTTGACATTGCCGTATTGATCAACCTCAG
CAAAGCTAAGATAGGCAACTGATACACCATTGTTATAAATAAAATCCCATGCTCGATCATGAGGCATGCGCACATCTGCATTGTAATTCAT
ACCAAAATGTTCACGACTCCCAACGAATCCACCGAAAATGCCAACATCTAAAATCGGTTGCACATCATGTTCAACACATTCTTCGTGCAAT
AAATTAGAGAGTTCATTATTGATGCCATAACCGATGCTAATTGTATCGCCATAAGTTAAAAACTGAGCAGCACGTCGGAGAATCAATTTG
CGACTATTAAAAGGTAATGCGGGTTCAGGTATTCCATCAATTCGTTCTTCTCCAGACAAGGCTGGTAAATAATGACTCTGAATTACTTGGC
GGTGATTCTTTTCATCTTCTGTGACGTATACATAATCGACAAGATTTCCTGGGATAACAACTTCATTCGGTTTTAGTTGATATCCGTCAACT
AAAGCTTTAACTTGTACAATAACTTTCCCATGATTGGCTTTCGCGTTTAATGCGACATGATAACACTCGCTCAAGTACGCTTCTTGAGTTAA
ATAAATGTTACCTTGTTGATCTGCGTATGTTCCTCTCAGTAGTGCCACATCAACGCTAGGGAATGTGTAATGTAAGTATGTTTCATCGTTG
ATGGTTACTAATGAAACTAAATCATCAGTTGTTCGTGTATTTACTTTACCGCCACCGTATCTAGGATCAACAGCTGTGTTTAATCCGATTTT
AGTAATAACTCCAGGTAATAATTGATTACTCTGACGATAATGAGTTGCAATGATACCTTGTGGTAAAAAATAAGCTTCAATGTCATTATTT
TTCATTGCTTGTGCCGTTTTGGAAGAAGCCGTTAAAATGCTCATAATGACACGTTTAATCATGCGACGTTCTATAAAATCATCTAAATCCG
GTGCGGCACCTAAACTATGGATATCATTCGCTAATATAAACGTTAAATCTTTGGGCGTATGATATGTGTCATGTTGCGCTAACACAGCAC
GTAGAACTTCGGCGGGTAAGTTGGCTACAGCTAATGCTGGTAAACCAATCACATCACCATCTTTAATGATATGTTGTAAGTCGTGCCATG
TGATTTGTTTCAA

Gene: prsW-prsS (protease responsible for activating sigma-S)

Contig: 02_NODE_3, position: 184050 to 185192, length: 1143 nt, orientation: FORWARD

Perfect match to: (N315-BA000018-[275772:276914], allele observed in CC5+CC72+CC772)

Sequence:

ATGTCATCAGCAAGTACACAATCAACTAAGACGTCCGACATACATAATGAATCTATCAATAAACAAATGGAAGCCAAAGCGCATGAAACA
GCGCAAAATGCAGATTTAAAAACCGAAGCAAGAAGTTTATTTGATAATGCAACCAAATCAATCGGTAGACTAGCCGGCAATGATGAAAG
CTTAAATCTTAATTTAAAAGATATGTTTTCTGAAGTATTTAAGCCGCATACTAAAAACGAAGCAGATGAAATATTTATAGCGGGTACTGCT
AAAACTACGCCAGCAATTTGTGACATATCAGAAGAATGGGGGAAGCCATGGCTCTTTTCTCGAGTATTCATCGCTTTCACAGTAACATTTA
TTGGATTATGGGTCATGGCAGCGATTTTTAATAACACTAACGCGATTCCGGGTCTCATTTTTATAGGGGCTTTAACAGTACCATTATCGGG
TTTGTTCTTCTTTTATGAATCAAATGCGTTTAAAAATATTAGCATTTTTGAAGTTATTATCATGTTCTTTATTGGCGGCGTATTTTCATTACTA
AGTACGATGTTATTATATAGATTTGTCGTTTTTAGTGATCAATTCGAAAGGTTTGGTTCTTTAACATTTTTCGATGCATTTTTAGTAGGATT
AGTTGAAGAAACTGGAAAAGCACTCATTATTGTTTATTTCGTCAATAAATTGAAAACAAATAAGATTTTGAATGGATTATTAATCGGTGCT
GCTATTGGTGCAGGGTTCGCAGTTTTTGAATCAGCAGGTTATATTTTGAATTTCGCTTTAGGGGAAAATGTCCCATTATTAGATATTGTCT
TCACACGTGCGTGGACTGCGATTGGTGGTCATTTAGTTTGGTCAGCGATTGTTGGTGCTGCAATAGTTATTGCGAAAGAACAGCATGGCT
TTGAATTCAAAGATATTTTTGATAAACGCTTTTTAATATTCTTTTTATCAGCCGTTGGTTTACATGGCATTTGGGATACATCTTTAACTGTAC
TTGGCAGTGATACGTTAAAAATATTTATTTTAATCGTTATTGTGTGGATACTTGTATTCATTTTAATGGGGGCAGGTTTAAAACAAGTGAA
TTTACTGCAGAAAGAATTTAAAGAACAACAGAAAAAAGTAGACGAATAA

Gene: nikA (nickel  ABC transporter, substrate-binding protein)

Contig: 02_NODE_3, position: 185506 to 186981, length: 1476 nt, orientation: REVERSE

Perfect match to: (MW2-BA000033-[255215:256690:r], allele observed in CC1+CC5)

Sequence:

TTATCGTTCAATCGTTGTTCGATAATCGATTAAATAGATACCTTCAGGTGTTACTTTATAATTTTTAACCTTAGCGTTAGCAGCGACTATTT
GATCGTTGTAAGCAATATAACTGTTTGGTACATCTCGACTTGATAATTTAATAATATCATTAGAAATATTGTGACGTTCCTTAACATCTACA
GTATGATTCAATTGATTAATTAAATCATCGACGTTGCTATTATTGTAGTCTCCTTTATTAATAGCACCATCTTTTTTATATGCTTGATTAAAG
AAATAACCTGTATCACCGCGAGGGATTGTCCCAAAACTATACATCGTTGCATCCCATGCAGAACGGTCTTTTAAGTAACCTTCTATGTCAT
CAACACTTTTAATGTCGATTTCAATATTTGCTTTTTTAGCATCTGATTGTAATACTTGCGCAATTTTCGATAGCTCTGGACGACCGTCATAC
GTAATTAACTTAATTTTTAAAGGGTGTTCTTTTGTATAACCATCTTTAGCTAATAACGTTTTTGCTTGTTCGATATTTTGTTTGGTTAACTTA
GGTTCTTTAATATATGGAATTTTATCATTAAATGGACTCGTTGCAGGTTTCGCATAACCTTGATAAATATGATCTGCAATACCTTGTCTATC
AATGATATGATCTAATGCTTCACGAACGGATTTAGTCATTTTTTTATTAGTATGATTATACATAAGTAAAGAAGTTCTAAATCCAGATTCTT
TTGACACTTTTAAATTTTGATTATTTTCTATGTCTTGAACTTTATTAACTGGGACATCAGTTATTAAATCATCTTTTTGAGATTCTAAATTTCT
GACGCGATTATTGCCGTCTTCTTGGTACGTCACAGTAATATGATCAAGTTTGGGTTTACCTTGCCAATAGTCCTTAAAATTCGACAATGAT
ATTTTTCGAGATTGCTTATAATCTTTTATTTGGTAAGGGCCTGTACCAACAGGAGTTTGATTAACATCTGATTTAGCATCTGTATCATAAAT
TGCCATAAAAGGATTAGCTAATTCAGATACAAGTTCAGGGTAAGCGGAGTTGGTTTTAATTGTCAGTTTTTGACCTTTAGCGGTAATTGAT
GATATTGGTAATGAATATTTGACCAAGTCGCTTTTTTTCATGCTATTTTCAAGGCTAGATTTCACTTTTTCTGCAGTCAATTTTTGACCGTTT
TGAAATTTAATATTATCTTTTAATTCTATATCTAACGTTGTATCATTTGGTTGATGATACGATTTCACTAATGCTTTTTCTATTTTTCCTTGAT
CATTTGTTTTAAATAATGATTCTGCAGCACCAATCTTAACTGGTACATCTGTTTCATAAGGTGCAATAGACTTTGTTTTTAACGGTAACGAA
ATATTTAAGTCTTTGCCAGATGAATGCATTGAGCCACATCCTGATAACACTAATACTGCTGAAAATATAGTTGCTAGTCTTTTAAACTTCAT

Gene: DUF488 (putative protein)

Contig: 02_NODE_3, position: 187179 to 187535, length: 357 nt, orientation: REVERSE

Perfect match to: (MW2-BA000033-[256888:257244:r], allele observed in CC1+CC7+CC12+CC72)

Sequence:

CTAAGTATTGAGCAACTGCTGTAGTACTACAGCTTGGTTATGTTTAGTATCTTTTGCTGCATATAACAATAGAACATGATTATGCTGATTTA
CAATATCCTTTAATTTTTCAAAAGCATCTTTTTGTGCATCCTGATCACGTAATTCTTTTTCATATTTTTCTTTAAAAGCTCCAAAAAGTTTAGG
ATCATGTTGGAACCATTGTCGCAACTCAGTAGAAGGGGCAATGTCTTTTAACCAATAATCTAGGTTAGCAGTTCTTTTCGAAATACCTCTC
GGCCAGACTCTATCGACTAGGATACGAATAGCGTCGGTATTATCTTTATTGTCATAAATCCGTCCAATATCTACGGTCAT

Gene: Q5HJD9 (putative protein)

Contig: 02_NODE_3, position: 187692 to 187865, length: 174 nt, orientation: REVERSE

Perfect match to: (MW2-BA000033-[257401:257574:r], allele observed in CC1)

Sequence:

TTATTTATTTTTACGTTTAGTTAAATACTTTAAACCAACTGCAAAGACCGTGTACCCGGCTATGGTTTGTATCAATGTTTTAATTAAATTATT
ATTTTTAACAATAATATTTGCAGTAACAATACTTACGAAATATAATGCAAATACTTTTACGTAACGTTGATTAAGTTTCAT

Gene: hmp (radical nitric oxide-detoxifying flavohemoglobin)

Contig: 02_NODE_3, position: 187891 to 189036, length: 1146 nt, orientation: REVERSE

Perfect match to: (MW2-BA000033-[257600:258745:r], allele observed in CC1)

Sequence:

TTATACTGCAACGCTTAGTCTTGGAATAAATGTTTCGTAGTGTACGCGATCCATATCGTAATTTAAAGATTTAAGTGCTTCGATCATAGATT
GTAAGAATTTTGTACCACCACAGATATAAATTTCAGGTTTATTTGCTAAAAATGCTTGTAATTCTTCAGCGCCAATATAGCCTTGTTTATCT
TTTAAGTGTGTATATAATTTAGCGTTGTCATGATGGCTTGCGATACTGTTGAAGTTGTCTTTGAAAGGTAAATGTTGTTCATTTTCAGCAA
CTTGAACCATCTGTGTATCTAAACCTTTGGCAGAGGCAGCTTCATACATAGCTACTAAAGGTGTAACACCAATACCTGAACCTAAGAAAA
GTTGTGGTTCAGTCGTATTCTCTAATACGAATCCACCTACAGGCGCAGCTAAATTAATCATATCGCCTTCTTTAATCTCATCGTGTAAAATT
GTTGAAACTTCGCCTTCATGTTCTGTTGTGACATCACGTTTAACGCCAAAAGTTAAATGGTTTTTTTCACCTGATACGATAGAATAGTGAC
GTTTAGCTCTATATGGAAGTTTTTCACTAGAAACATCAACTGTGATGTATTGGCCTGGTGTAAATTCACTAAAGTCATATTCTTCAGTTTCA
ACTGTAAATGATTTAATGTCTTCAGATTCTTGTTTAATATTGGTAATTTTGAATGGTTTAAAACCTATCCACATCATTTGATCATAAATTTCT
TTTTCAATTTGGATGAACACATCCGCAATAACGCCATATGCTTTTGCCCAAGCTTGAATGACAGGGTCATTTTCTTCTAATCCTGTCACGTC
TTGAATGGCTTTTAATAAATTTTCCCCCACAATTGGATAATGTTCAGCATAAACTTGTAGTGCGCAGTGTTTATATGCGACTGGCATAATG
ACTGGTTTAATAACACTTAAGTTATCGATATTAACCGCTGCGGCCATTACAGCTTGTGCTAATGCTGAAGATTGCATGCCTCGTTTTTGGTT
CGTTTGATTAAACATGTTTAAAAGTTCAGGATGCGCTTTAAACATTTTTGGATAAAAGATTGACGTAATTTCTGTCCCTTTCTCTTTAAGTA
AAGGCACTGTTTGTTTGATAATGTCTTTCTCTTGTTCTGTAAGCAT

Gene: lctE (L-lactate dehydrogenase 1)

Contig: 02_NODE_3, position: 189609 to 190562, length: 954 nt, orientation: FORWARD

Perfect match to: (ED133-CP001996-[224313:225266], highly conserved allele)

Sequence:

ATGAACAAATTTAAAGGGAACAAAGTTGTATTAATAGGTAATGGTGCAGTAGGTTCAAGCTACGCATTTTCATTAGTGAACCAAAGCATT
GTTGATGAATTAGTCATCATTGATTTAGACACTGAAAAAGTTCGAGGAGATGTTATGGATTTAAAACATGCCACACCATATTCTCCAACAA
CAGTTCGTGTGAAAGCTGGCGAATACAGTGATTGTCATGATGCGGATCTAGTTGTCATCTGTGCTGGTGCTGCACAAAAACCTGGAGAA
ACACGTTTAGATTTAGTATCTAAAAACTTGAAAATATTCAAATCAATTGTTGGTGAAGTAATGGCATCAAAATTTGATGGTATTTTCTTGG
TAGCTACAAATCCTGTTGATATTTTAGCGTATGCAACATGGAAATTCTCTGGTTTACCTAAAGAACGTGTTATAGGTTCTGGTACAATTTTA
GACTCTGCACGCTTTAGATTATTGTTAAGCGAAGCGTTCGATGTTGCGCCACGTAGCGTCGATGCTCAAATTATTGGTGAACATGGTGAC
ACTGAATTACCAGTATGGTCACACGCTAATATTGCGGGTCAACCTTTGAAGACATTACTTGAACAACGTCCTGAGGGCAAAGCGCAAATT
GAACAAATTTTTGTTCAAACACGTGATGCAGCATATGACATTATTCAAGCTAAAGGTGCCACTTATTATGGTGTTGCAATGGGATTAGCTA
GAATTACTGAAGCGATTTTCAGAAATGAAGATGCCGTATTGACTGTATCAGCATTATTAGAAGGCGAATATGATGAAGAAGATGTTTATA
TTGGTGTTCCAGCAGTCATCAATAGAAACGGTATTCGCAACGTCGTAGAAATCCCATTAAACGACGAAGAACAAAGCAAGTTCGCACATT
CAGCTAAAACATTAAAAGATATTATGGCTGAAGCAGAAGAACTTAAATAA

Gene: ptsIIBC (putative PTS transport system, IIBC component)

Contig: 02_NODE_3, position: 190882 to 192411, length: 1530 nt, orientation: REVERSE

Perfect match to: (FPR3757-CP000255-[284200:285729:r], allele observed in CC8+CC1+CC5+CC8)

Sequence:

TTAATCCCCGAGCAATTCTTCAATTTCATTTTTGATAACTGTAACGTGAGGCCCATAAATTACTTGCACACCAGTGCCTTGCTGGATTACAC
CTTTGGCACCAGTACTTTCGAGTAATACTTTATCGACTTTGTCATTTTGATGAAGTGTGACGCGTAGTCTCGTTGCACAACAGTCAACGAT
TTCAATGTTATCTTTGCCTCCCAAACCAGCAACAATAGTTTGTGCTCTTTCAGTAGCCTCAACTTGTTGTGCTGCAGCTTTATCTTCTCGACC
AGGTGTTTTGAAATTAAATTTCGTAATTAAGAATCTGAAAACGATGTAATACAAACAGAACCACACAATTCCAATAGGTATGACGTATAG
GTAGTTTGTTTTACTATTACCTTGTAGCACACCAAAGAGTAAGAAATCGATAAAGCCTCCACTGAAGGTTTGACCAATTGTAATGTTGAAA

ATGTCTGCCATCATAAATGCTAATCCATCAAAGAAGGCATGGATTACATAAAGAATAGGTGCGACAAACAAGAAACTAAACTCTAAAGG
TTCGGTAATACCTGTTAAAAATGAAGTGAGTGCAGCGGATAACATTAAACCGCCGACAACTTTTTTATGTTCAGGTTTAGCTGTGTGATA
AATTGCAAGTGCGGCACCACATAAGCCGAACATCATCGTAATAAAACGGCCTGACATAAAGCGTGACACACCTGAATAATACTTCGTCAC
ATCTGGATCACCAAGTTGAGCAAAGAAGATGTTCTGCGTACCTTGAACTAAGTGCCCTTTGACTTCTAAAGTACCACCAAGTGCCGTCTG
CCAAAACGGTAAGTAAAAAATATGGTGTAAACCGAGTGGACCTAACAATCTTAAGATGAAGCCATAAACAAAAGTACCGATGGCACCTG
TTTTCGTTACAAATCCACCAACATGATAAATGCCGGCTTGTATGCTTGGCCAAATGAAAAACATCAATACACCTAAAAAGATTGCGGCAA
ATGCTGTGACAATAGGGACAAATCTAGAGCCACCAAAGAAACCTAAATACGGTGGTAATACCACTTTGTGATATTTGTTGTGAAGTATTG
CGGTCATAATACCTGTGATAATCCCGCCAAAAACACCGGTTTCAACCGTTTGTATACCGAGCACCATGCCTTGTCCATTTTGTGCAAGCTG
ATCTTTTGCCAATGTGCCCGTGATAGTTAATAAGCCATTCATAGTTGCGTTCATAATTAAGAAACCGAGCAGCGCAGCTAAACCTGCAGT
ACCTTTATCGCTTCTAGATAATCCGATTGCGACACCAATTGCAAAGATGACCGGTAAATTTTGGAAAACAATACTACCTGCAGCTGACATT
AATGTAAAAATATTTTGTAATAAGGTAATATCTAAAATAGGGTATGCTTTAACGGTGTTTGGATTACTTAATGCACCACCGATACCCAACA
ATAGACCTGCAGCTGGTAAGATTGCGATAGGTAACATAAAGGACTTGCCGAACTGCTGTGCTTTTTCAAATAAAGATTTCAT

Gene: rihA (inosine-uridine preferring nucleoside hydrolase)

Contig: 02_NODE_3, position: 192773 to 193708, length: 936 nt, orientation: FORWARD

Perfect match to: (COL-CP000046-[265414:266349], allele observed in CC8+CC1+CC5+CC8)

Sequence:

ATGAAAAGAAAGATTATTATGGATTGTGATCCAGGACACGATGATGCAATAGCATTAATTTTAGCGGGGGCAATTGACAGTCCACTAGA
GATATTAGCTGTAACAACAGTCGCAGGTAATCAATCAGTTGACAAGAATACGACAAACGCCTTGAACGTATTGGATATTATGGGACGCC
AAGATATAGCAGTAGCGAAAGGTGCGGATAGGCCGTTAATTAAACCAGCTGCCTTTGCTTCTGAAATACATGGGGAATCTGGATTAGAT
GGTCCGAAACTACCGTCGACACCATCACGTCAAGCAGTTGCAATGCCAGCATCAGATGTGATTATAAACAAAGTGATGACGAGTGATAC
ACCTGTAACAATTGTAGCGACAGGTCCTCTTACGAATGTAGCAACGGCATTGATTCGTGAGCCAAGAATCGCTGAGCATATTGAATCTAT
TACTTTGATGGGTGGTGGTACATTTGGAAATTGGACGCCTACAGCAGAATTCAATATTTGGGTAGATGCTGAAGCAGCGAAGCGTGTTTT
TGAAAGTGGGATTACTATAAATGTGTTTGGTTTAGATGTAACACATCAAGTTTTAGCCGACGATCACGTGATTGAACGCTTTGAAAGTAT
CAATAATCCTGTTGCACAGTTCGTCGTAGAATTATTGCAATTCTTTAAGAAGACATACAAGACTCACTTTAATATGGATGGTGGTCCAATA
CATGATGCTTGTACAATTTTGTATTTGTTACAACCAGAATTGTTTACAATGGTACCCGTTAATATCGACATTGAACATCAAAGTCCACTAAC
TTATGGCACTATGGCTGTCGATTTAAATCATGTTACAGGTAAGCCTGCCAATGCTTATTTTGCTACAGCAGTTGATGTTGAAGAAGTGTGG
AACTTGATAGACCATAAGTTACGTACATACGAATAA

Gene: bglG (transcriptional antiterminator, PTS regulator)

Contig: 02_NODE_3, position: 194045 to 196141, length: 2097 nt, orientation: FORWARD

Perfect match to: (MW2-BA000033-[263754:265850], allele observed in CC1+CC445)

Sequence:

ATGAACGGGGATAATCAGCAAATACTCAGAGAAATTGTATTGAATCCTACTATTCATGGTAAAGAACTTGAATCGATATTTGGTTTGTCTC
GTAGACAACTAGGATATCGCATTCAAAAAATCAATTTGTGGCTTGAACAAGAGGGTTATCCAAAACTTGAAAGAACAAGCCAAGGAAAT
TTTATTGTAAGTTCTGAAATCATGACGTTATTCAAACGAGATGTATCAGAGCAGCAAATGTTAAACGGCAACAATGTCATTTTTAGCATAG
AAACACGTCGTTATTATTTAATGCTCATGCTTTTTAGTAAGGAAAACGCAATGTCTCTAAACCATTTTTCAATTGATTTACAAGTCAGTAAA
AATACTGTCATTCACGATATAAATCATGTGAAAGAGCAATTGGAAAATCATGGTTTGTCACTTAAGTATTCTCGAAAACATGGTTATGAAA
TTGTTGGTGATGAATTTGAAGTTCGCCGTTTCTTCATTAAGTTGATTGATCAAAGGTTGAATCATGATATTACTAAAAGTGAAGTTTTAAA
GGCGCTCAACTTAACATTCGAAGATATCGCATATCAAAAAGACAAAATCAAACAGGTAGAACAATTTTTGAAGAGTCGCTTTATAGACAA
ATCACTTAGTTCATTGCCTTATGTCCTTTGTGTGATTCGTAGACGAATTCAAAGTGGTCATGTGATGAATCCATTAAATATTAATTATCAGT
ATTTGAGGGATACGAAAGAATATCAAGCAACGGAGATTATGACGCAACTTGAGCCGGATTTGCCAGAAGCGGAAAAGTTATATTTGACA
TTACACTTACTTTCAACAAGTGTGCAATGGACTGATTTGCAGGAATCAGATAACATATCGAATTTAACGATGGCTATCGCTCAAATGATTC
ACCATTTTGAACAAATCACTTTTATTAACATTGAAGATAAGGAGAAATTATCACAGCAACTCTTGTTACATTTAACGCCTGCTTTTTATAGG
ATTAAATATAACTTAACGGATCGTGATGAATTAATAAATCCTTTACAAGGAAATTATCAATCCTTATTTCATATGGTGAAACAATCATGTCA
ATCGTTAACTGAATATTTCGGAAAATCGTTGCCTGATAATGAAATAGCATATTTAACCATGTTGTTCGGAGGTAGTTTGAGACGTCAAGA
TGAAAACTTCGATGGCAAGATAAAAGCTATTATCGTGTGTACACAAGGCACGTCAGTATCACAAATGATGTTATACGAGTTGCGAAACTT
ATTTCCAGAAATTATTTTCTTAGATGCGATTTCACTTAGAACATTTGAAAATTACACATTAGATTATGACATCGTCTTTTCACCAATGTTTGT
CCTAACACATAAAAAAATTATTTATCACAAAAGTAGCTTTATCTGAAAATGAGCAACGAAAGTTACGTAAAGAAGTGATGAAGTACATTAA
TAAGGAATCGGCTGACATTGATAAGGAAATAAACAAGTTAATGGCATTAATTGAACGCACTACGACAGTTAATGACATTACAGAACTAC
GTGATGGTTTAGAAGATTTTATTGCGAATTATAATTCAATTTCAACCATTAATGGATCGATTGTCACACAAAATAAGACATTAGATTTAGC
TGACTTGATACCGGCAAGGCACGTGAAAAGAATACATCATGTTGAAAATATTGATGAAGCTATTGCTAAAGCAAGTGATGTGTTAGTTGC

TAATCATTTTATTGATATTAAATATATTCATGAGATGCAACAGGTATTTGATGATTCGTATATGGTTATCATGCAAAATATTGCTATTCCAC
ATGCATACTCTGAAAAGCATGTACATAAAACAGCGATGAGTATGTTGATATTACAAGAACCAATATACATGTCAGATGGCACAGCAATCC
ATATTATTGTACCTATTGCTGCTGTTGATAAAGTGACACACTTAAGAGCGTTACTACAATTGAGAGATGTGGCGCAAGACAATGACGCAA
TTAAGCGCATCATACAAAGTCGCAAAAAATTCTGATGTAAATGAGATTTTAAAAAATTATTCAAATAAAGAAGCGAGGGAAAATGGATGG
GACAGCAATTAG

Gene: Q5HJD2 (phosphotransferase system sugar-specific component IIA)

Contig: 02_NODE_3, position: 196126 to 196593, length: 468 nt, orientation: FORWARD

Perfect match to: (N315-BA000018-[287848:288315], allele observed in CC5+CC1)

Sequence:

ATGGGACAGCAATTAGTGCATAAAGAAAATATAATGCTCAATTTGTCGGCAACTGATAAAGAATCCGTATTGTCACAAATGTCAGATGTG
TTATTTCAAAATGGGTTCGTGAAGTCAACGTTTAAAGATGCAGTCATCGACAGAGAAAAAGAATTTGCTACTGGTTTACCAACACATCTAT
GTTCGGTCGCTATACCGCATACAGATGTCGAACATATTAACCATAGAACGATAGGTGTGGCTGTTCTAGAAAAAGAGGTGCCGTTTATTG
AAATGGGAACACTTGATCAACAGACAGAAGTGAAAATCGTTTTTATGTTAGCGATGGATAAAGTAGATGATCAACTTAAGTTGTTACAAC
AGTTGATGCAAATTTTTCAAAGTGAAGAAAAATTGGAGCAGATTCTGCGAACGAAAGATGAAACGATTTTAGCAACACTAATCAATGATT
ATTTGGAATATAACTAA

Gene: Q5HJD1 (phosphotransferase system sugar-specific component IIB)

Contig: 02_NODE_3, position: 196616 to 196894, length: 279 nt, orientation: FORWARD

Perfect match to: (N315-BA000018-[288338:288616], highly conserved allele)

Sequence:

ATGAAACAAGTATTAGTAGCGTGTGGTGCAGGTATTGCAACGTCAACAGTAGTAAATAATGCAATTGAGGAAATGGCAAAGGAACACA
ATATTAAAGTAGATATTAAACAAATCAAAATTACAGAAGTTGGACCTTATGAAGACACTGCAGATTTATTAGTTACAACTGCAATGACAA
AGAAAGAATATAAATTCCCAGTTATCAACGCACGTAATTTCTTAACTGGTATTGGTATTGAAGAAACAAAACAACAAATCTTAACAGAGT
TACAAAAATAA

Gene: gatC1-Q2G2C8 (PTS system transmembrane permease)

Contig: 02_NODE_3, position: 197121 to 198380, length: 1260 nt, orientation: FORWARD

Sequence:

ATGAGTTACTTCACTGATTTTGTAAGGGGATTTTTAGATTTAGGTGCAACTGTTATTTTACCGGTTGTCATATTCTTGCTTGGCCTATTCTTT
AGGCAGAAAATTGGAGCGGCATTTAGGTCTGGTTTAACAATAGGTGTGGCTTTTGTAGGGATTTTCTTAGTCATCGATTTATTAGTTAAA
AATTTAGGGCCAGCAGCACAAGCGATGGTTAAAAATTTAGGCGTCAGTCTGAATGTGATTGATGTAGGTTGGCCAGCAACATCATCTATC
GCTTGGGCATCATCTGTCGCAGCATTTATTATTCCACTCGGAATCATAGTTAACGTTGTATTGCTAGTAACTAAAGTGACAAAGACGATGA
ATGTAGATATTTGGAATTTTTGGCATTATACGTTTACATCAGCAATGGTTTATGCCGTATCAGGCAGTATTTGGCAAGCGTTATTAGCAGC
AGTTATTTTCCAAGTTATCTGTTTGAAAGTAGCAGATTGGACAGCACCGATGATGAGTGAGTTCTTTGATTTACCAGGTGTATCGATTGCT
ACAGGAAGTACAATTTCTTATGCACCAGGTATTTACTTAGTTAAATTGTTACAAAAAGTACCCGGTCTGAATAAGTTAGATGCTGATCCTG
AAACAATTCAAAAACGTTTTGGCGCATTTGGAGAGTCTATCTTTGTCGGATTAATTTTAGGTTTAGGTATTGGTGTGTTAGCAGGTTACAA
ACCTGGAGACATCATTAATTTAGGAATGTCAATGGCTGCAGTAATGGTATTAATGCCTAGAATGGTAAAAATCTTAATGGAAGGTTTAAT
GCCAGTTTCAGAGTCTGCAAGAACATGGCTAAATAAACGTTTTGGCGAACGTGAAATTTATATTGGATTGGATGCGGCTGTAGCATTAG
GTCATCCAGCGGTTATTTCGACAGCATTAATTTTAGTACCTATCACTGTTTTATTAGCCGTTATTTTACCAGGAAACCAAGTACTACCTTTT
GGTGACTTAGCAACTATACCATTTGTTGTCGCGTTTATTGTTGGTGCAGCAAGAGGAAACATTATTCATTCTGTCATTGTGGGCACGATTA
TGATTGCAATTTCATTATATATATTGCAACAGACGTAGCACCCATTTTCACAGATATGGCGAAAGGTACGAATGTACAAATGCCAAAAGGTT
CATCTGAAATTTCAAGTATTGATCAAGGTGGTAATATCGTTAACTATCTTATCTTTAAACTATTTAGTCTATTCAATTAA

Gene: gutB (L-iditol 2-dehydrogenase)

Contig: 02_NODE_3, position: 198398 to 199453, length: 1056 nt, orientation: FORWARD

Sequence:

GTGAAAGCTTTAGTAAAAACAAGAGAAGGGCATGGCAACTTAGAACTTCTTGATAAAGAAGTTGCAACACCGCTAGATGATAAAGTAAA
GATTAAAGTACATTATGCAGGAATTTGTGGCACAGACATTCATACTTATGAAGGGCATTATAAAGTTAATTTTCCAGTGACATTAGGTCAT
GAATTTTCTGGTGAAATCGTTGAAGTTGGAGCAGACGTTAAAGATTTTAAAGTTGGTGACCGTGTCACTTCTGAAACGACATTCTATGTTT
GTAATGAGTGTGAATACTGTAAATCAAAAGACTATAATTTATGCAACCATCGAAAAGGTATTGGAACACAAGTTGATGGCGCATTTACTA
ATTATGTCATTGCACGTGAAGAAAGTTTGCATCATATTCCAGATGAAGTATCGTATCAGTCTGCAGCTATGACAGAACCATTAGCATGTG
CACATCATGGCGTTTCTAAGATTCAAGTCAATTCAGGCGATGTAGCAGTTGTAATGGGACCTGGGCCAATCGGATTACTTGTAGCACAAG
TGTTAAAAAGTAAAGGCGCAACTGTTGTGGTAACTGGATTGGACAATGACAAAGTCAGATTAGATAAAGCAGAAGCATTGCACATGGAT
TATGTAGTCAATTTACAACAAACAGACTTAAAAACGTATATCAATGGAATTACAGACGGTTACGGTGCAGATGTTGTTGTTGAATGTTCA
GGTGCAGTTCCAGCAGCACGACAAGGTTTGGATATTTTACGCAAAAAAGGTTTCTACAGTCAAATAGGTATTTTTAAGGATGCTGAAATT
CCATTTGATATGGAAAAAGTGATTCAAAAAGAAATAACAGTTGTTGGTAGTAGAAGTCAAAAGCCAGCAGATTGGGAACCTTCATTGCA
ACTTATGGCGGATGGTTTAGTAAATGCTGAAGCTTTGGTGACAAAAATATATGATATTTCGAAATGGGACGAGGCGTATCAACATTTAAA
ATCTGGCGAAGGTATTAAAGCATTACTTAAGCCGCTCGATTTAGATGAAAATGAAGGAGAGAATTAA

Gene: Q5HJC7 (putative protein)

Contig: 02_NODE_3, position: 199455 to 199601, length: 147 nt, orientation: FORWARD

Perfect match to: (MW2-BA000033-[269164:269310], highly conserved allele)

Sequence:

ATGGTAGAATCAATGCTAACTTTTATGCTTGGGCCATTAAGACAAATCACTGATTTTTATATGGAACATTTACTCGTAAGTAATTCCATTGT
CATTGCAGGTTATTTTGCGACAGGTATTTTTAAAAAGAAAAAAGTTGTGAATTAA

Gene: Q5HJC6 (putative sugar-phosphate dehydrogenase)

Contig: 02_NODE_3, position: 199625 to 200668, length: 1044 nt, orientation: FORWARD

Perfect match to: (MW2-BA000033-[269334:270377], allele observed in CC1+CC188)

Sequence:

GTGAAAGCATTGAAATTATATGGCGTGGAAGATTTACGGTATGAGGATAATGAAAAGCCAGTCATTGAAAGTGCGAATGACGTTATTGT
TAAAGTACGAGCGACTGGCATATGTGGTTCAGACACGTCACGATACAAAAAAATGGGGCCATACATTAAAGGTATGCCATTTGGTCATG
AATTTTCAGGTGTTGTAGATGCCATTGGAAGTGATGTTACGCATGTTAATGTAGGCGACAAAGTGACAGGTTGCCCAGCAATACCTTGTT
ATCAATGCGAGTATTGTTTAAAAGGTGAATATGCACGATGTGAAAAGTTATTCGTTATTGGCTCATATGAACCTGGATCGTTCGCGGAAT
ATGTCAAATTGCCAGCGCAAAATGTTTTAAAGGTTCCAGACAATGTTGATTACATTGAAGCAGCAATGGTTGAGCCATCAGCCGTTGTTG
CGCATGGGTTTTATAAATCGAATATACAACCTGGTATGACTGTTGCAGTAATGGGGTGTGGCAGTATAGGTTTGTTAGCTATTCAATGGG
CACGAATATTTGGTGCTGCACATATCATCGCTATAGATATAGATGCGCATAAACTAGATATTGCAACATCATTGGGCGCACATCAAACAA
TCAATTCAAAAGAAGAAAATCTTGAGAAATTCATCGAAAATCATTACGCCAATCAAATCGATTTAGCTATAGAATCATCAGGTGCTAAAG
TTACGATTGGTCAAATATTGACGCTACCTAAAAAAGGTGGCGAGGTGGTATTACTCGGAATACCATATGATGATATTGAGATTGATCGCG
TTCATTTTGAAAAAATTCTGCGTAACGAGTTGACAGTATGTGGCTCTTGGAACTGTTTGTCCAGTAATTTTCCGGGCAAAGAGTGGACGG
CAACCTTACATTATATGAAGACGAAAGATATTAATGTAAAGCCTATTATTTCTCATTTTTTACCGTTAGAAAAAGGCCCAGAGACATTTGA
TAAATTAGTTAACAAGAAAGAACGATTTGATAAAGTCATGTTTACGATTTATTAG

Gene: tarI1 (2-C-methyl-D-erythritol 4-phosphate cytidylyltransferase 1)

Contig: 02_NODE_3, position: 201196 to 201912, length: 717 nt, orientation: FORWARD

Perfect match to: (Strain_16035-HE579065-[268768:269484], allele observed in CC5+CC8+CC772)

Sequence:

ATGATTTATGCAGGTATTTTAGCAGGAGGTATTGGTTCGAGAATGGGGAACGTGCCATTACCAAAACAATTTTTAGATATTGATAATAAA
CCGATTTTAATCCATACAATTGAGAAGTTCATTTTAGTGAGTGAATTTAATGAGATTATTATCGCAACGCCAGCACAGTGGATTTCCCATA
CACAGGATATTTTAAAAAAATATAACATTACAGATCAACGTGTCAAAGTAGTTGCAGGTGGTACGGATCGAAACGAAACAATTATGAAC
ATTATCGACTATATTCGCAATGTAAATGGAATTAATAATGATGATGTGATTGTAACTCATGATGCCGTAAGACCATTTTTAACTCAACGTA
TTATTAAAGAGAATATTGAAGTAGCAGAAAAATATGGTGCAGTAGATACAGTCATTGAAGCAATTGATACGATTGTAATGTCTAAAGATA

AACAGAACATACACAGTATCCCTGTAAGGAATGAAATGTATCAAGGCCAAACACCACAATCATTTAATATTAAATTATTACAAGATAGTT
ATCGCGCCTTAAGTAGTGCACAAAAAGAAATCTTATCAGATGCATGTAAAATCATTGTCGAATCTGGACATCCAGTTAAATTGGTACGTG
GAGAACTATACAACATTAAAGTGACAACACCGTATGATTTAAAAGTAGCAAATGCCATTATTCAAGGTGATATTGCCGATGATTAA

Gene: tarJ1 (CDP-ribitol synthase)

Contig: 02_NODE_3, position: 201905 to 202930, length: 1026 nt, orientation: FORWARD

Perfect match to: (MW2-BA000033-[271614:272639], allele observed in CC1)

Sequence:

ATGATTAATCAAGTATATCAACTCGTTGCACCGAGACAGTTCGACGTCACATATAATAATGTTGATATTTATGGTAATCATGTCATTGTAA
GACCTTTATACTTGTCTATTTGTGCAGCTGATCAAAGGTATTACACAGGTCGAAGAGATGAAAATGTACTGCGCAAAAAATTACCAATGT
CACTAGTTCATGAAGCTGTTGGTGAAGTTGTATTCGATAGTAAAGGCGTATTTGAAAAAGGTACGAAAGTAGTAATGGTGCCGAATACA
CCTACAGAAAAACACGACGTGATTGCCGAGAATTACCTGCCCTCTAGTTATTTTAGATCTAGTGGTTATGATGGTTTTATGCAAGACTACG
TTGTGATGGCACATGATCGTATCGTTCCGCTGCCTAATGCCATTGATTTGAGTACGATTTCATACACAGAGTTAGTGTCAGTAAGTTATCA
TGCTATACAACGATTTGAACGTAAATCTATACCTTTGAAAACCAGCTTTGGTATTTGGGGTGATGGTAACTTAGGTTATATTACTGCTATTT
TGCTACGTAAGTTGTACCCAGAAGCTAAAATTTATGTATTTGGTAAGACAGACTATAAATTAAGTCATTTTTCATTTGTAGATGACATCTTT
ACAGTAAATCAAATACCAGATGATCTTAAAATTAATCATGCATTTGAATGTGTTGGAGGTAAAGGAAGTCAAGTTGCACTTCAACAAATA
GTTGAACATATTTCACCAGAAGGCAGTATTGCTTTGTTAGGCGTAAGTGAATTACCCGTGGAAGTGAATACACGATTAGTACTTGAAAAA
GGATTAACATTGATTGGTAGTAGTCGAAGCGGCTCTAAAGATTTTGAGCAAGTTGTTGATTTATATCGTAAGTACCCAGACATAGTTGAA
AAGTTAGCCTTATTAAAAGGACATGAAATTAATGTATGTACGATGCAAGATATCGTCCAAGCGTTTGAAATGGATTTATCGACATCTTGG
GGAAAAACAGTATTGAAATGGACGATTTAA

Gene: tarL1 (glycosyl/glycerophosphate transferase)

Contig: 02_NODE_3, position: 202952 to 204646, length: 1695 nt, orientation: FORWARD

Perfect match to: (MW2-BA000033-[272661:274355], allele observed in CC1+CC5)

Sequence:

ATGACAAAAACGAAACAAGCAATACATATTGATAACATATACTGGGAACGTGTTCAGTTATATATTGAAGGACATAGTGAAGGTGTCGA
TTTAACATCAGGACAATTTGTTCTGAGGAATTTAACCGAAACAAAAACATTAGAAGCAAATGAAATGAAAATAGACGGTAATACATTTAT
ATGTAGATTCAACGTCGCAATATTAGACGATGGGTATTATTTACCAATGGATAAATATTTATTTGTTTATCATGACCAGTTAGAGTATATT
GGACAACTTAATCCAAATATTATTGATCAAGCTTATGCGGCATTAAATGAAGAGCAAATTGAAGAATACAATGAGCTGACTACACAAAAT
GGAAAAGTGAACTATTTATTAGCGTATGATGCTAAAGTTTTCCGTAAAGGTGGCGTATCACAACATACGGTCTATACCATTACTCCGGAA
ATAGCAAGTGACGTTAACGAATTTGTATTTGATATTGAAATCACCTTACCTCAAGAGAAATCAGGGGTCATTGCGACAAGTGCACACTGG
ATTCATAAACAAGGTCATAAAGCTTCATTTGAAAGTAGAAGTTTCTTATTTAAAGCTATTTTTAATATTACAAAGTTACTACATATTAAAAG
AAGCAAAACAATATTATTCACATCAGATTCGCGTCCGAATTTATCAGGGAATTTCAAGTATGTATATGATGAGTTACTACGCCAAAAAGTA
GATTTTGATTATGATATTAAAACGGTATTTAAGGCGAATATTACGGATAGACGTAAATGGAGAGACAAGTTTAGATTGCCATATTTACTT
GGTAAGGCAGATTATATTTTTGTTGATGATTTCCATCCATTAATTTATACGGTTCGCTTTAGACCATCACAAGAAATTATTCAAGTGTGGCA
TGCCGTTGGTGCTTTTAAAACAGTTGGCTTTAGTCGTACAGGTAAAAAAGGTGGTCCATTTATCGATTCATTAAACCATCGTAGTTACACG
AAAGCATATGTTTCATCAGAAACCGATATTCCATTTTATGCTGAAGCATTTGGAATTAGAGAAGAAAATGTTGTACCAACAGGTGTACCA
CGTACTGATGTACTATTTGATGAAGCTTATGCAACACAAATTAAACAAGAGATGGAAGATGAATTGCCAATTATAAAAGGTAAGAAAGTT
ATTCTATTCGCACCGACATTTAGAGGTAATGGTCACGGTACGGCACATTATCCATTTTTTAAAATTGATTTTGAACGTTTAGCAAGATACT
GCGAGAAGCATAATGCAGTTGTGTTATTCAAAATGCATCCGTTCGTAAAAAATAGACTTAATATTTCACGTGAACATAGACAATACTTTAT
CGATGTGTCAGATCATCGTGAAGTTAACGATATTCTCTTTGTTACAGACTTGTTGATTAGTGATTATTCATCTTTAATATATGAATATGCAG
TATTTAAAAAGCCGATGATTTTCTATGCATTTGACTTAGAAGATTACATTACGACGCGTGATTTCTATGAACCATATGAATCATTTGTTCCA
GGTAAAATTGTACAATCCTTTGATGCATTAATGGATGCTTTGGACAATGAAGATTATGAGGTTGAAAAAGTTGTACCATTCTTAGATAAA
CATTTTAAATATCAAGATGGTCGCTCAAGTGAACGTTTAGTCAAAGATTTGTTTAGACGCTAA

Gene: tx_universal2 (rho-independent terminator)

Contig: 02_NODE_3, position: 205253 to 205291, length: 39 nt

Perfect match to: (Strain_21331-AGTV01000040-[104403:104441], allele observed in CC398)

Sequence:

TAGAATTGAAAAAAGCTTGTTACAAGCGCATTTTCGTTC

Gene: tarF (CDP-glycerol:poly (Glycerophosphate) glycerophosphotransferase)

Contig: 02_NODE_3, position: 205614 to 206783, length: 1170 nt, orientation: FORWARD

Perfect match to: (Strain_21193-AFEG01000009-[176975:178144:r], allele observed in CC25+CC5+CC25+CC361+CC445)

Sequence:

ATGATTAAAAATACAATTAAAAAATTGATAGAACATAGTATATATACGACTTTTAAATTACTATCAAAATTGCCAAACAAGAATCTAATTT
ATTTTGAAAGCTTTCATGGTAAACAATACAGCGACAACCCCAAAGCATTATATGAATACTTAACTGAACATAGCGATGCCCAATTAATATG
GGGTGTGAAAAAAGGATATGAACACATATTCCAACAGCACAATGTACCATATGTTACAAAGTTTTCAATGAAATGGTTTTTAGCGATGCC
AAGAGCGAAAGCGTGGATGATTAACACACGTACACCAGATTGGTTATATAAATCACCGCGAACGACGTACTTACAAACATGGCATGGCA
CGCCATTGAAAAAGATTGGTTTGGATATTAGTAACGTTAAAATGCTAGGAACAAATACTCAAAATTACCAAGATGGCTTTAAAAAAGAAA
GCCAACGGTGGGATTATCTAGTGTCACCTAATCCATATTCGACATCGATATTTCAACATGCATTTCATGTTAGTCGAGATAGGATTTTGGA
AACAGGTTATCCAAGAAATGATAAATTATCACATAAACGCAATGATACTGAATATATTAATGGTATTAAGACAAGATTAAATATTCCATTA
GATAAAAAAGTGATTATGTACGCGCCAACTTGGCGTGACGATGAAGCGATTCGAGAAGGTTCATATCAATTTAATGTTAACTTTGATATA
GAAGCTTTGCGTCAAGCGCTGGATGATGATTATGTTATTTTATTACGCATGCATTATTTAGTTGTGACACGTATTGATGAACATGATGATT
TTGTGAAAGACGTTTCAGATTATGAAGATATTTCGGATTTATACTTAATCAGCGATGCGTTAGTTACCGACTACTCATCTGTCATGTTCGA
CTTCGGTGTATTAAAGCGTCCGCAAATTTTCTATGCGTATGACTTAGATAAATATGGCGATGAGCTTAGAGGTTTTTACATGGATTATAAA
AAAGAGTTGCCAGGTCCAATTGTTGAAAATCAAACAGCACTCATTGATGCATTAAAACAAATCGATGAGACTGCAAATGAGTATATTGAA
GCACGAACGGTATTTTATCAAAAATTCTGTTCATTAGAAGATGGACACGCGTCACAACGAATTTGCCAAACGATTTTTAAGTGA

Gene: tarI2 (2-C-methyl-D-erythritol 4-phosphate cytidylyltransferase 2)

Contig: 02_NODE_3, position: 207059 to 207775, length: 717 nt, orientation: FORWARD

Perfect match to: (MRSA252-BX571856-[293159:293875], highly conserved allele)

Sequence:

ATGAAATACGCTGGTATTCTAGCTGGAGGTATAGGCTCAAGAATGGGTAACGTACCTTTACCTAAACAATTTTTAGATTTAGACAACAAA
CCGATTTTAATCCATACATTAGAAAAATTTATTTTAATTAATGATTTTGAAAAAATTATTATCGCGACGCCACAACAATGGATGACGCATAC
GAAAGATACACTTAGAAAATTCAAAATTTCTGATGAAAGAATTGAAGTCATTCAAGGTGGTAGCGATCGTAACGATACAATTATGAATAT
CGTTAAACATATTGAATCAACAAATGGTATTAACGATGACGATGTCATTGTGACACATGATGCAGTTAGACCATTTTTAACGCATCGTATT
ATTAAAGAAAATATTCAAGCTGCTTTAGAGTACGGTGCAGTAGATACAGTGATTGATGCTATAGATACGATTGTTACATCTAAAGATAAT
CAAACGATTGATGCAATTCCAGTGCGTAATGAAATGTACCAAGGTCAAACACCTCAATCGTTTAATATTAATTTATTAAAAGAAAGCTATG
CACAGTTGAGTGATGAGCAAAAGAGTATTTTATCTGATGCTTGTAAGATTATTGTAGAAACAAACAAACCGGTTCGACTTGTAAAAGGTG
AGTTATATAACATTAAAGTAACAACACCTTACGATTTAAAAGTAGCGAATGCTATTATTCGAGGTGGTATTGCCGATGATTAA

Gene: tarJ2 (CDP-ribitol synthase)

Contig: 02_NODE_3, position: 207768 to 208793, length: 1026 nt, orientation: FORWARD

Perfect match to: (MW2-BA000033-[277535:278560], highly conserved allele)

Sequence:

ATGATTAATCAAGTATATCAATTAGTTGCACCTAGACAATTTGAAGTTACGTATAACAACGTAGATATTTACAGTGACTATGTCATTGTAC
GTCCTTTATATATGTCAATTTGTGCTGCCGATCAAAGATATTATACTGGTAGCCGTGATGAGAATGTCTTATCTCAGAAATTGCCAATGTC
TTTAATTCATGAAGGTGTTGGTGAGGTCGTATTTGACAGTAAAGGTGTGTTTAATAAAGGTACAAAAGTAGTTATGGTACCGAATACGCC
GACAGAAAAAGACGATGTCATTGCTGAAAACTATTTAAAATCGAGCTACTTCAGATCAAGTGGACATGATGGGTTTATGCAAGATTTTGT
GTTGCTAAATCATGATAGAGCTGTACCACTACCTGATGATATTGATTTAAGTATTATTTCATATACAGAGCTTGTAACAGTAAGTTTGCAT
GCTATTCGTCGTTTTGAAAAGAAATCTATTTCAAATAAAAAATACATTTGGTATTTGGGGTGATGGTAACTTAGGTTACATTACAGCCATTT
TATTACGTAAATTATATCCAGAGTCTAAAATATATGTCTTTGGTAAAACAGATTATAAATTGAGTCACTTCTCATTTGTTGATGATGTCTTC
TTTATTAATAAAATACCTGAAGGCTTAACATTTGATCATGCATTTGAGTGTGTGGGTGGTCGCGGTAGTCAATCAGCCATAAATCAAATG
ATCGATTACATTTCACCAGAAGGAAGCATTGCACTGTTAGGTGTAAGTGAGTTCCCAGTAGAAGTTAATACACGTCTAGTATTGGAAAAA

GGACTAACGTTGATTGGTAGTAGTCGAAGTGGTTCAAAAGATTTCCAAGATGTTGTAGACTTATACATTCAATACCCAGATATTGTAGAT
AAATTAGCGTTGTTAAAAGGTCAAGAATTTGAAATTGCAACAATTAATGATCTTACAGAAGCTTTTGAAGCAGACCTGTCTACATCTTGG
GGTAAAACAGTATTAAAATGGATTATGTAA

Gene: tarL2 (glycosyl/glycerophosphate transferase)

Contig: 02_NODE_3, position: 208815 to 210503, length: 1689 nt, orientation: FORWARD

Perfect match to: (A5948-ACKD01000026-[141970:143658], allele observed in CC8+CC45I+CC398)

Sequence:

TTGGTTAAAAGTAAGATATATATAGATAAAATCTATTGGGAACGTGTTCAGTTATTCGTTGAAGGACATAGTGAAAACCTAGATTTAGAA
GATAGTAATTTTGTATTAAGAAATTTAACTGAGACACGTACAATGAAGGCGAATGATGTCAAAATAGATGGGAATCAATTCGTTTGTCGT
TTCAATGTAGCTATCTTAGATAATGGTTATTACTTACCTGAAGATAAGTACTTATTAGTGAATGAGCAAGAACTTGATTATATTGCACAGT
TAAACCCAGATGTGATTAATGATGCATATCAAAATCTAAAGCCAGAACAAGAAGAAGAATACAACGAATTAGAAACACAAAATGGTAAA
ATCAATTTCTTATTGCAGACTTACCTAAAAGAATTTAGAAAAGGCGGCATTTCGAAGAAAACGGTTTATACTGTTACACCTGAAATTTCTA
GCGATGTTAATGAATTTGTCCTTGATGTTGTTGTAACGACTCCGGAAGTTAAAAGTATTTATATCGTTCGTAAATATAAAGAATTACGTAA
GTATTTCCGCAAACAATCATTTAATACAAGACAATTTATTTTTAAAGCGATATTTAATACGACGAAATTTTTCCACTTGAAAAAAGGGAAT
ACGGTGTTGTTTACATCAGACTCTAGACCAACGATGTCTGGAAACTTTGAATACATCTATAACGAAATGTTACGTCAAAATTTAGATAAAA
AGTATGATATTCACACTGTTTTTAAAGCGAATATTACAGATAGACGTGGCATCATCGACAAGTTTAGATTGCCATATTTACTTGGGAAGGC
AGACTACATCTTTGTTGATGACTTTCACCCATTGATTTATACAGTGCGTTTTAGACGTTCTCAAGAAGTTATTCAAGTATGGCATGCCGTTG
GTGCCTTTAAAACAGTTGGCTTTAGTCGTACTGGTAAAAAGGGTGGACCATTTATTGATTCATTAAATCATCGTAGCTATACAAAAGCTTA
TGTATCATCTGAAACCGATATTCCATTCTACGCTGAAGCATTTGGTATTAAAGAGAAAAATGTAGTGCCTACAGGTGTTCCACGTACTGAT
GTACTATTTGATGAAGCTTATGCGACACAGATCAAACAAGAGATGGAAGATGAATTACCAATTATTAAAGGTAAGAAAGTCATTCTTTTC
GCACCAACATTTAGAGGTAGTGGTCATGGTACAGCACATTACCCATTTTTCAAAATTGATTTTGAACGTTTAGCAAGATATTGCGAAAAAA
ATAACGCGGTTGTATTATTTAAAATGCATCCATTTGTGAAAAATAGACTTAATATTGCAGACAAACATAAACAATATTTTGTTGACGTTTCT
GACTTTAGAGAAGTTAATGATATACTGTTCATAACAGATTTATTAATTAGTGACTATTCATCTTTAATATATGAATATGCAGTATTTAAAAA
GCCAATGATTTTCTATGCATTTGATTTAGAAGATTATATTACGACGCGTGATTTTTATGAACCATATGAATCATTTGTTCCAGGTAAAATTG
TGCAATCATTTGACGCATTAATGGACGCCTTGGACAATGAAGATTATGAAGGAGAAAAAGTCATTCCATTCTTAGATAAACATTTTAAAT
ATCAAGATGGCCGATCAAGTGAGCGTTTAGTCAGAAATTTATTTGGTAGCTAA

Gene: tarS (beta-GlcNAc appending glycosyltransferase)

Contig: 02_NODE_3, position: 210536 to 212261, length: 1726 nt, orientation: FORWARD

Perfect match to: (N315-BA000018-[301718:303443], allele observed in CC5+CC1+CC8)

Sequence:

ATGATGAAATTTTCAGTAATAGTTCCAACATACAATTCAGAAAAGTATATAACAGAATTACTTAATAGCCTTGCGAAACAAGATTTTCCGA
AAACTGAATTTGAAGTGGTTGTAGTTGATGACTGTTCAACAGATCAAACGTTACAAATAGTTGAAAAGTATCGCAATAAATTGAACTTGA
AAGTAAGTCAACTCGAAACAAATTCTGGTGGTCCAGGTAAACCTAGAAATGTGGCGTTAAAACAAGCAGAAGGTGAATTTGTATTATTT
GTGGACTCCGATGACTATATAAACAAAGAGACTTTAAAAGATGCAGCAGCATTTATTGATGAACATCACTCCGATGTCTTATTGATTAAA
ATGAAAGGTGTTAATGGTCGTGGTGTACCACAATCTATGTTTAAAGAAACAGCACCTGAAGTTACTTTGTTAAATTCAAGAATTATCTATA
CTTTAAGCCCGACTAAAATCTATAGAACAACATTACTAAAAGATAATGACATTTATTTTCCAGAAGAATTAAAGAGTGCAGAAGATCAATT
ATTTACAATGAAAGCATATTTGAATGCAAATCGAATCAGTGTGTTAAGTGATAAAGCGTATTATTATGCTACAAAGCGTGAAGGTGAACA
TATGAGTAGTGCGTATGTTTCACCTGAAGACTTTTACGAAGTCATGAGATTGATTGCTGTAGAAATATTAAATGCAGATTTAGAAGAAGC
TCATAAAGATCAAATCTTAGCAGAATTTTTAAATCGTCATTTTAGTTTTTCTCGTACGAATGGCTTCTCACTTAAAGTTAAACTAGAAGATC
AACCGCAATGGATTAATGCTCTAGGAGACTTTATACAAGCAGTTCCAGAACGTGTAGATGCATTGGTGATGAGTAAATTACGACCATTGT
TGCACTACGCGAGAGCGAAAGATATAGACAACTATAGAACTGTAGAAGAAAGTTACCGTCAAGGTCAATACTACCGTTTTGATATTGTA
GATGGTAAATTAAACATTCAATTCAATGAAGGCGAACCATACTTTGAAGGCATTGATATCGCTAAGCCAAAAGTGAAAATGACAGCATTT
AAATTTGATAATCATAAAATTGTTACAGAGCTAACGTTAAATGAATTTATGATTGGCGAAGGACATTATGATGTCAGACTTAAATTACATT
CACGAAACAAGAAGCACACAATGTATGTACCTTTAAGTGTCAATGCGAATAAACAATATCGTTTTAACATTATGTTAGAAGATATTAAAG
CGTATTTACCTAAAGAAAAAATTTGGGATGTTTTCTTAGAAGTCCAAATAGGTACGGAAGTATTTGAAGTGCGTGTTGGTAATCAACGTA
ATAAATATGCATATACTGCAGAAACAAGTGCATTAATTCATTTGAATAATGATTTTTATAGATTAACACCGTATTTCACAAAAGACTTTAAT
AACATTTCGTTATACTTTACAGCTATTACATTAACGGATTCAATCTCATTGAAGTTAAAAGGTAAAAACAAAATCATTTTAACTGGTCTGGA
TCGTGGTTATGTATTTGAAGAAGGTATGGCTAGTGTCGTGCTAAAAGACGACATGATTATGGGAATGTTAAGCCAAACGTCAGAAAACG
AAGTGGAAATCTTACTTAGTAAAGATATTAAGAAGCGAGACTTCAAAAATATTGTTAAGTTAAACACTGCACATATGACTTACTCGCTAA
AATAAATAA

Gene: scdA (iron-sulfur cluster repair protein)

Contig: 02_NODE_3, position: 212401 to 213075, length: 675 nt, orientation: FORWARD

Perfect match to: (MW2-BA000033-[282168:282842], highly conserved allele)

Sequence:

ATGATAAATAAAAATGACATAGTAGCAGATGTAGTAACAGATTATCCGAAAGCAGCAGATATTTTTAGAAGCGTAGGTATAGACTTTTGT
TGCGGTGGACAAGTAAGTATAGAAGCAGCATCCTTAGAAAAGAAAAATGTAGATTTGAACGAATTATTACAGCGTCTCAATGACGTTGA
ACAAACGAATACACCAGGTTCGCTTAACCCTAAATTTTTAAATGTTTCGTCACTTATTCAATATATTCAAGCAGCATATCATGAACCTCTTA
GAGAAGAATTTAAAAATTTAACACCTTATGTGACGAAATTATCGAAAGTACATGGACCTAACCATCCATATTTAGTCGAGTTAAAAGAAA
CATATGATACATTTAAAAATGGCATGTTAGAGCATATGCAAAAAGAAGATGATGTTGATTTTCCAAAACTAATTAAATATGAACAAGGTG
AAGTAGTAGACGATATTAATACAGTGATTGATGATTTAGTATCTGATCACATTGCAACGGGACAATTGTTAGTGAAAATGAGCGATTTAA
CATCTAGCTATGAACCACCGATAGAGGCATGTGGTACGTGGCGACTCGTTTATCAGAGATTAAAAGCACTTGAAGTGTTAACACATGAGC
ATGTTCATTTAGAGAATCATGTTTTATTTAAAAAAGTATCATAA

Gene: lytS (two component sensor/regulator, sensor histidine kinase)

Contig: 02_NODE_3, position: 213319 to 215073, length: 1755 nt, orientation: FORWARD

Perfect match to: (MRSA252-BX571856-[299425:301179], allele observed in CC30+CC25+CC30+CC239)

Sequence:

GTGCTATCGCTAACAATGTTATTACTTGAGCGTGTAGGTTTAATTATTATTTTGGCCTATGTGTTGATGAATATTCCATATTTTAAAAACTT
AATGAATCGTCGACGTACATGGAAAGCACGTTGGCAATTATGTATTATTTTCAGTTTGTTTGCCTTAATGTCTAATTTAACTGGTATCGTCA
TCGATCATCAACATAGTTTGTCAGGAAGTGTGTACTTCCGTTTAGATGATGATGTATCTTTAGCTAACACACGTGTATTAACGATAGGTGT
CGCAGGATTAGTTGGTGGCCCTTTTGTAGGTCTATTTGTTGGCGTTATTTCAGGTATTTTCAGAGTGTATATGGGTGGGGCGGATGCACA
AGTTTATCTTATCTCATCTATATTTATCGGTATAATTGCTGGTTATTTTGGCTTACAAGCTCAAAGACGCAAGCGTTACCCGAGTATTGCGA
AAAGTGCCATGATTGGAATTGTTATGGAAATGATTCAAATGTTGAGCATTTTAACATTTTCCCACGACAAAGCATATGCGGTTGACCTCAT
ATCATTAATTGCACTACCAATGATTATTGTTAATAGCGTCGGTACGGCGATTTTTATGTCTATTATCATTTCAACATTAAAGCAAGAGGAG
CAAATGAAGGCTGTTCAAACACATGATGTACTGCAATTGATGAACCAGACATTGCCGTATTTTAAAGAAGGATTGAATAGAGAATCGGC
ACAGCAAATTGCGATGATTATTAAAAATTTAATGAAAGTATCTGCCGTAGCAATTACAAGCAAAAATGAAATCTTATCGCATGTAGGTGC
AGGTAGTGATCATCACATACCAACAAATGAAATATTAACAAGTCTGTCTAAAGATGTATTGAAATCAGGAAAGTTGAAAGAAGTTCATAC
TAAAGAAGAGATTGGTTGTAGTCATCCGAATTGCCCGCTTAGAGCAGCTATCGTGATACCACTTGAGATGCATGGTTCTATCGTCGGTAC
ATTGAAGATGTATTTTACAAACCCTAATGATTTAACTTTTGTGGAACGTCAACTTGCAGAAGGATTGGCAAATATTTTTAGTAGCCAAATT
GAACTTGGTGAAGCCGAAACGCAAAGTAAGTTATTGAAAGATGCTGAGATTAAGTCATTACAGGCACAAGTGAGTCCACATTTTTTCTTC
AATTCAATTAACACGATTTCAGCTTTAGTTAGAATAAATAGCGAAAAGGCACGAGAGTTACTATTAGAATTGAGTTATTTTTTCAGAGCGA
ATTTACAAGGCTCTAAGCAACATACGATTACTTTAGATAAAGAGTTAAGTCAAGTGCGTGCATACTTATCACTCGAACAAGCACGTTATCC
AGGAAGATTTAATATCAATATTAATGTTGAAGACAAATATCGCGATGTGCTTGTACCACCATTTTTAATTCAAATTTTAGTTGAAAATGCC
ATCAAACATGCGTTTACGAATCGAAAGCAAGGTAACGATATTGACGTGTCAGTGATTAAAGAAACTGCAACACATGTACGTATTATTGTA
CAAGATAATGGTCAGGGTATTTCTAAAGATAAAATGCATTTGTTGGGAGAAACATCTGTAGAATCAGAGTCTGGAACTGGTAGTGCTTTA
GAAAATTTAAACTTACGCCTAAAAGGATTATTTGGAAAATCCGCAGCATTACAATTTGAATCGACATCGAGCGGTACCACTTTTTGGTGT
GTACTTCCTTATGAAAGACAAGAGGAGGAATAA

Gene: lytR (two component sensor/regulator, transcriptional regulator)

Contig: 02_NODE_3, position: 215076 to 215816, length: 741 nt, orientation: FORWARD

Perfect match to: (04-02981-CP001844-[305384:306124], allele observed in CC5+CC772)

Sequence:

ATGAAAGCATTAATCATAGATGATGAGCCATTAGCACGTAATGAATTAACATATTTATTAAATGAAATTGGTGGTTTTGAAGAAATTAAT
GAGGCAGAAAATGTAAAAGAAACATTGGAAGCACTACTGATCAATCAATATGACATTATATTTTTAGATGTCAATTTAATGGATGAAAAT
GGGATCGAATTAGGAGCTAAGATTCAAAAGATGAAAGAGCCACCTGCGATTATTTTTGCAACTGCACATGACCAATACGCAGTACAGGC
ATTTGAATTAAATGCGACAGACTATATTTTGAAACCGTTTGGTCAAAAACGTATTGAACAAGCAGTCAATAAAGTGCGTGCGACTAAAGC
CAAAGATGATAATAGCGCAAGTGCAATTGCGAATGATATGTCGGCGAATTTTGATCAAAGTTTACCTGTTGAAATAGACGATAAAATTCA

CATGTTAAAGCAACAAAATATTATTGGGATTGGCACACATAATGGTATTACAACCATACATACAACGAATCATAAATACGAAACAACAGA
GCCATTGAATCGTTATGAAAAACGATTGAATCCCGCTTATTTCATACGTATTCATCGTTCATATATTATTAATACGAAACACATTAAAGAAG
TGCAACAATGGTTTAACTACACTTATATGGTAATATTGACAAATGGTGTCAAGATGCAAGTTGGACGTTCATTTATGAAAGATTTTAAAGC
GTCGATAGGGTTACTTTAA

Gene: lrgA (antiholin-like protein)

Contig: 02_NODE_3, position: 215929 to 216372, length: 444 nt, orientation: FORWARD

Perfect match to: (RF122-AJ938182-[251476:251919], highly conserved allele)

Sequence:

ATGGTCGTGAAACAACAAAAAGACGCATCAAAACCAGCACACTTTTTTCACCAAGTCATTGTAATTGCTTTAGTACTCTTTGTATCGAAAA
TAATTGAATCATTTATGCCAATTCCTATGCCTGCATCAGTAATCGGTTTAGTATTATTATTTGTATTATTATGTACTGGTGCTGTTAAGTTAG
GCGAAGTCGAAAAAGTAGGAACGACACTAACAAATAACATTGGCTTACTCTTCGTACCAGCCGGTATCTCAGTTGTTAACTCTTTAGGTG
TCATTAGCCAAGCACCATTTTTAATCATTGGACTAATAATCGTCTCAACAATACTATTACTTATTTGTACTGGCTATGTCACACAAATTATTA
TGAAAGTTACTTCGAGATCTAAAGGTGACAAAGTCACAAAAAAGATCAAAATAGAGGAGGCACAAGCTCATGATTAA

Gene: lrgB (antiholin-like protein)

Contig: 02_NODE_3, position: 216365 to 217066, length: 702 nt, orientation: FORWARD

Perfect match to: (MW2-BA000033-[286132:286833], allele observed in CC1)

Sequence:

ATGATTAACCACTTAGCACTAAATACACCTTACTTCGGAATACTGTTATCCGTTATACCATTTTTCTTAGCGACCATATTATTTGAAAAAACT
AATCGTTTCTTCTTATTCGCACCGCTATTTGTCAGTATGGTATTTGGTGTGGCCTTCCTCTATTTAACAGGCATTCCGTATAAGACTTACAA
AATAGGTGGAGACATTATTTACTTCTTCTTAGAACCGGCAACAATCTGTTTTGCGATTCCGTTATATAAAAAGCGTGAAGTGCTTGTTAAA
CATTGGCATCGTATCATCGGAGGTATTGGTATCGGTACAGTTGTAGCGTTATTAATTATTTTAACTTTTGCGAAGTTAGCACAATTTGCCA
ATGATGTTATTTTATCAATGTTACCTCAAGCAGCAACTACAGCGATTGCGTTACCAGTATCAGCTGGTATCGGTGGTATAAAAGAATTAAC
ATCATTAGCAGTTATTTTAAATGGTGTCATTATTTATGCCCTAGGTAATAAATTCTTGAAGCTTTTCCGAATTACTAACCCTATTGCCCGAG
GATTAGCACTTGGAACAAGTGGTCACACATTAGGTGTAGCACCAGCCAAAGAATTAGGACCTGTAGAAGAATCAATGGCAAGTATAGCT
TTAGTGTTAGTTGGTGTAGTTGTTGTAGCAGTTGTGCCTGTCTTTGTAGCAATATTCTTCTAA

Gene: Q5HJB2 (putative transcriptional regulator, GntR family)

Contig: 02_NODE_3, position: 217174 to 217878, length: 705 nt, orientation: REVERSE

Perfect match to: (MW2-BA000033-[286941:287645:r], allele observed in CC1)

Sequence:

TTACTTTTTACTAGGAATATAAAACTGTGCATGACGATAATGAAATACGATGTCAGACGAATCAAAGGGTTTGCCAGTCATTGTATAAAA
AGTCTGGTGGTAACGTAAACATGGTTCACCTGTAGACAATTGTAGTAATGAAGCTTCACTTGAAGTGAGTTTATCTACATTAAAGAAAAT
ATCTGAAAAACCAATACGAAGTTTCATGTTTGATTCTAAATAATCGAAGATAGAGCCCTTAGCAATATCATCATTTAAATATTTCACGATTT
CTTTATGATAATAAGAATATTCGATACATAAAACATCATCGTCCACGAATCTTAATCGCTCTAAATAGTAGACGGTATCATCTGCATTTAAT
TGGAGTTCATCTTGTACAGATTTAGGTGGCGTTGCCATCTCCTTAAAAACAAGTACCTTACTTGTCATTCGATGTTCACCTAAACTTTTAGA
GAAACCATTAGTCTTAAAGACGTTGATACGATTGGCATCTGCAATATTTCTCACATAAATACCACTGCCTTGTGCTTGATAGATCAAACCA
TCTTGTTCCAATAAGCCTAATGCTTTAATGATAGTACTCTTACTTACTTGATAACGTTCTTTTAATTGCGTCACGCTTGGCAATTTATCACCG
GGTTTGAATTTAGATTGATGTATAAACGCATTAAGTTGCTTAGCAATATGTTCATACTTTAACAA

Gene: Q1YAD5 (PTS system, IIA component)

Contig: 02_NODE_3, position: 218027 to 218818, length: 792 nt, orientation: FORWARD

Perfect match to: (ATCC51811-ST1-ADVP01000041-[151890:152681:r], allele observed in CC1)

Sequence:

ATGAGCAATAAATATAAAGAACAAGCCCAAGACATTCTTACAGCTGTAGGTGGTGTCGAAAACATTGTTGATGCAACGTATGACACGAA
GTGCATTACAATTCATATGCAACATACAATTCCTTCTACAGCAAATGAAGTGAAACAAATAGTTGATGTGACATCTGTAGCAGAAAATGA
TGCGCAGTTAGTCATAAAATTAAATGGAAATGTCGATGAAGTGTATCAGCAATTACAGCGATTAATTAAGAATGCTAATGTCGAAGAGA
GTGAGAATACTGACAATATTAATAGCCAAGATACAAGTTATATACCTCAAGTAAAAGTAACAACACCAATTTTAGTGAAAGCACCAATCG
CTGGTCGTCGTATTTTACTTAAAGAAGTAAGAGATTCAATTTTTAGAGAGAAAATGGTAGGCGAAGGCTTGGCAATCAAAGCTCATGAA
GAATCCAAAGTAATCGCACCGTTCAATGGTTTAATATCTATGATTGTACCAACTAAGCATGCAGTTGGTATTCAATCAGAAGACGGTGTG
GACATAGTCATTCATATTGGCGTGAATACAGTTGACTTGGAAGGTAAAGGGTTCAAGTGCTTTGTAAAGCAAAATGATCGTGTTGAAGC
AGGGCAAACGTTGTTACAATTCGACCAGCAATATATACAACAACAAGGCTACAATGCTGACGTTATTGTCGTTATTAGCAACTCTGCCGA
TTTAGGAAAAGTAGAACTGACAATGAATGAAATCATTACGACTGAAGATGTTATTTTTAAAATATTTAAAAACTAG

Gene: bglA (putative 6-phospho-beta-glucosidase)

Contig: 02_NODE_3, position: 218834 to 220270, length: 1437 nt, orientation: FORWARD

Perfect match to: (MW2-BA000033-[288601:290037], allele observed in CC1)

Sequence:

ATGACCAAATTACCGCGAAATTTTATGTGGGGTGGCGCTCTTGCCGCAAATCAATTTGAAGGTGGATATGATAAAGGGGGTAAAGGGTT
AAGTGTAATTGATGTTATGACGAGTGGTGCACATGGCAAAGCACGTCAGATTACAAAGTCTATAGATCAAAATCACTATTATCCAAATCA
TGAAGGTATTGATTTTTATCATCGTTATAAGGAAGATATTGCCTTGTTTAATGAAATGGGATTGAAATGTTTACGTACGTCGATTGCGTGG
ACACGTATCTTTCCGAATGGGGATGAAGATGTGCCAAACGAAGAAGGACTCGCCTTTTATGATCGTATCTTTGATGAATTAATTGCACAA
GATATTGAACCTGTTGTGACGTTATCACATTTTGAGATGCCACTTCATTTAGCGAAACATTATGGTGGATTTAGAAATAGAGAAGTTGTCG
ATTATTTTGTGCATTTTGCGCGTGTTGTATTTGAAAGATATAAAGATAAAGTGACATATTGGATGACGTTTAATGAAATTAATAATCAGAT
GGACACATCAAATCCTATCTTTTTATGGACGAATTCTGGGGTAGCATTGACAGAAAATGATAATCCTGAAGAAGTCTTGTATCAAGTAGC
ACATCATGAACTTTTAGCCAGTGCTTTAGCAGTTCGTCTTGGTAAAGAGATTAATCCGAAGTTTAAGATTGGAACAATGATTTCACATGTA
CCCATTTATCCATATTCGTGTCATCCGAAAGATATGATGGAAGCACAAATTGCGAATCGCTTACGTTTCTTTTTCCCGGATGTCCAAGTGA
GAGGTTATTATCCAAGCTATGCTAAAAAAATGTTGGCACGAAAAGGATATGATGTTGGATGGCAAGAAGGGGACGACAGTATTTTACAG
CAGGGCACGGTTGATTATATTGGCTTTAGTTATTACATGTCTACGGCTGTAAAACATGATGTTGATACTACAGTTGAAAACAACATCGTCA
ACGGTGGTTTGAATCATTCTGTGGAGAATCCGCATATCGCAACGAGTGATTGGGGTTGGGCGATTGATCCAGATGGCTTAAGATATACA
TTGAATGTGTTATATGATCGTTATCAGTTACCACTTTTTATTGTGGAAAATGGTTTTGGTGCAGTTGATGAAGTGGTAGATGGACATATTC
ATGATGATTATCGCATTGAATATTTAAAAGCACATATTACAGCAGCGATAGAAGCAGTTGATCAAGATGGTGTAGATTTAATCGGTTATA
CACCGTGGGGAATCATTGATATTGTTTCATTTACAACCGGTGAAATGAAGAAACGCTATGGTTTAATATATGTTGATCGAGATAATGATG
GTCATGGCACGATGGAACGCTTGAAAAAAGATTCGTTCTATTGGTATCAACAAGTGATAGCATCAAATGGAGATAAATTATAA

Gene: SIRU01 (staphylococcal interspersed repeat unit 1)

Contig: 02_NODE_3, position: 220348 to 220511, length: 164 nt

Sequence:

GGCCCCAACATAGAAGCTGGCGGAAAGTCAGCATACAATAATGTGCAAGTTGGCGGGGCCCCAACACAGAGAATTTCGAAAAGAAATT
CTACAGGCAATGCAAGTTGGCGGGGCCCCAACATAGAAGCTGGCGGAAAGTCAGCTTACAATAATGTGCAAGTTGG

Gene: repeat_nySAgamma (repeat element)

Contig: 02_NODE_3, position: 220402 to 220567, length: 166 nt

Sequence:

GGGGCCCCAACACAGAGAATTTCGAAAAGAAATTCTACAGGCAATGCAAGTTGGCGGGGCCCCAACATAGAAGCTGGCGGAAAGTCAG
CTTACAATAATGTGCAAGTTGGCGGGGCCCCAACACAGAAGCTGGCGGAAAGTCAGCTTACAATAATGTGCAAGTTGG

Gene: Q5HJA9 (putative methyltransferase)

Contig: 02_NODE_3, position: 220818 to 221579, length: 762 nt, orientation: REVERSE

Perfect match to: (11819-97-CP003194-[301808:302569:r], highly conserved allele)

Sequence:

TTAGTCCTCTTTTTTAGCGACAAAAGTAATATAATTCATATCTTTACGCAATTTAGTCATCGTTTTAAACATTTTACAAAACATTGGTCGATT
TTCTTTTTTTAAAGCATTGTTGATAATCTTTATAGTTCCAACAATACCTTCGTCATAAATTAAACCTTTTGGTGTCATTAAACTCATTGGACC
AGTATGATAATGCACATGATTAAAACCAGCTTGATTATATAAATCTAACCAGCCAAGTTTCGTCTGCGGTGAGACATTGACATTAATTGCT
GCAGATAATGATTTAACTACATGTGTGGCATGTGATTCATTAACGATGACAATATCATGTGTTAACAAGATACCCCCAGGCTTTAAGACTC
GGTAGTACTCGCGTAATGCTTTTTCCTTTATGGTGATGGGTAACATTGTTAACATTGCTTCATTTAAAACGATATCGAATTGATTGTCATCA
AAGGGCAATTTAACAGCATTCGCTTGTTGAACTTGAATATATGATTCAAGACCTGCTGCTGAAATATTTTCCTGTGCTTTTTCTAATGCTTT
CTTATTTATATCAACGCCTTGAATGTGACAGCCATATGTATGAGCTAGATAAATAGATGTTGTGCACATATTACATGCCACTTCTAACACTT
GTTTATCTTGTGAAAATGCCCCTTGTTGTATTAACCAATCTGTTGCTTCTTTACCACCGGGGCGTAGACGAGTTTTTCCTAATTTAGCTAAA
AATGTATGACCAGCTTCTTTAGACAT

Gene: rbsK (ribokinase)

Contig: 02_NODE_3, position: 221830 to 222744, length: 915 nt, orientation: REVERSE

Perfect match to: (JKD6159-CP002114-[295903:296817:r], allele observed in CC93+CC1-ST582)

Sequence:

TTAAACTTGATTTACTTCTTCTAGTAGAGGAATAGATGCTTGCGCGCCGTGTTTTTGTACAGTGAGTGAGCTCGCTTTATTACCAAAATCA
ATAGCATCTGCTAAGTTATCTTGCGACTTGTTTAAGCGACTGACAAATGCACCAATAAATGTGTCGCCTGCAGCAGTTGTATCAATCGCAT
TTACTTTATAAGCTTCGATGTGTTGGCTTTGATTTTTAGTAGCAAAATATGTACCTTGCTTACCTAGCGTAATCAAAACAGTCTTAATGCCT
AAAGATAAAAAGTAATTGGCATTGTCTTTCATAGATTGTTCATTAGTTACTTTAATCCCAGATAACAATTCGGCTTCTGTTTCGTTTGGCAC
AATAATATCGATTAATGATAATAATTCATTAGGTAATGCTTTCGCTGGTGCAGGATTTAATACTGTCGTCACACCATGTGCCTTGGCAATT
TCAAATGCAGATATAATAGCCGGGATGGGTACTTCTAATTGTGCAACGACAAAGTCTGCATTGATTATAGCGTCTTTTGCGTTAATAACAT
CTTCAGGTGTCATCGTCATATTCGCACCACCATAAACATAGATGGTGTTTTGTCCTTCTGCATTCACAGTGATAAAGGCTTGGCCTGTTTTT
GCTTCAGTTGATTTGATAATATATGATGTATCAATATGAGCCGCTTTAAAATCTTCTAAGATGAAATCAGCAACGCCATCAGTGCCAATTT
TAGTAATAAATGTTGTGTCTGCTTGCATACGTGCAGTGGCAATAGCCTGGTTGGCACCTTTACCTCCGCCGAATGCTTTTTGTGCTTCTTCA
ACATGTAATGTTTCGCCTGGTTGTGCATATCTTTCAACTGTTAAAAATTGATCGACATTCGTTGAACCTAAAATAACAACTTTGTTGGTCAT

Gene: rbsD (D-ribose pyranase)

Contig: 02_NODE_3, position: 222772 to 223176, length: 405 nt, orientation: REVERSE

Perfect match to: (MW2-BA000033-[292539:292943:r], highly conserved allele)

Sequence:

TTAAAAAGTAACATTCGATTCTAATGCAATATTAGAGTAGGGCGTTGTTTCACCAGTACGAATATTACCTTTATTTAATGGGTGAGCTAAG
TTACTTTTCATTTCTTCGTGAGGAATGAAAATGATTTCGATTTCCGATGAAATCAATTGTTTAATTTGTTGCAATTGTGTAGGGTTATGTTC
TTTTATTTCTTCTGCTAAGTATATTTTTTGGATTTCCATTTCTTCTAACACTGTAGCTAAGACATCAATAAAGCGTGGTAAGTTTTTAGTTAC
AGCTAGGTCGATACGACGATGATCATTTGGAATTGGCATGCCAGCGTCATTAATCGTTAATAAATCAAAATGACCAATTGTCGCGATTGC
TTTTGAAATATGTTCATTTAAAACAGCTGATTTTTTCAT

Gene: rbsU (ribose uptake protein)

Contig: 02_NODE_3, position: 223191 to 224072, length: 882 nt, orientation: REVERSE

Perfect match to: (N315-BA000018-[314313:315194:r], highly conserved allele)

Sequence:

TTATTTTATAAATACTGTAACAGAAGCGGCTACTAAAATGAGTACTAAGCCGATGATTGTAATAACCATTTCTTTTGACGTTTTATGTTGTT
TTAAGAAATAAATACCAGTTAATGTAGCAAGCACAACGGATGTTTGAGAAAGAATAAATCCAGTTGCTAAACCATTCATATTAGGTTGTG
CTGAAATAAGATATGTTAAAGCACCAAATGCAAAGAAGAAACCTGAAATAATTTGTAACCACGTAATTTTATTACGGAATGGATTCTCTG
CTTTCATATTCATAAAGCCATAAATGACTGCAACAATTACCATACCCATTGCTTGAGGTAAAAAGGCAGTTAGGCCATCAATAGAAGTTGC
TTGCGGTGCAGCTGAATATAACCAGTATCCAAATTCACCAATTAACAGAAGTACCACTGCACGACGTAAATTTTTGGCGTTACTTGCTTCT

TTGCGTTCACTCCAAACTGTCATACGCGCTCCAATTAGAATAACGACTAAAGCTGTAAATCCAATGATTTTATGACCAATGCCTGGCCAAT
TTCCTAATGCAAAGACACCCCATAAAGATGCGCCTAATAATTGGAATGCTGTTGTGACTGGCATGGCACGAGATGAGCCGACTAATTCGA
ACGCTTTAAATGTAATGATTTGTCCGAATCCCCATCCTGCACCTGATAATAAGGCGAATAGCAAATTGGTTCCAGTAGGGAAGCCACTTG
ATGTGACTACGGCTAATATAATAGCGAAGATTAACGTACCTACAGTAGCACCGATAATTTGATGTACAGGTTTACCACCAAACTTTGAAG
CGACTGTTGGGAAGAAGCCCCAGCCAATTAAGGGGCCTAACCCGATAAGTAATGCAACAATGCTCAT

Gene: rbsR (ribose operon transcriptional repressor)

Contig: 02_NODE_3, position: 224304 to 225302, length: 999 nt, orientation: REVERSE

Perfect match to: (COL-CP000046-[296295:297293:r], allele observed in CC8+CC772)

Sequence:

TTAGTTTGAAAGATGATAGCCAGTTGTTGCACGAATTTTTAAAGTCGTTGGTAATTCAATCATATCAATGGATTTATCTAAGTGCTGTAAT
CGTTGAAGTAATAAGGTTAAAGATGTTTTGCCAATATCAGTTATAGGTTGTGCCACAGTAGTTAAAGGTGGCGAGACGTACGCTGCATA
ATCAATGTCGTCATAACCTATTAATGAGATATCTTTCGGAATACTGATGCCATGTTCAATTAGTCCTCGTAAAATGCCAATAGCGAGTTCA
TCGTTAATAGCGAAGATTGCAGTGGCAGATTGAACCATGATGTCATCAACAATGGTTAGCCCACCGCGCTTAGATAATTCAGTATGGACG
ATTTGTGGTTCTGGCAATTGATTCGCGCGCAAAGTATCAACAAATCCAGCGACACGAGTCGACATATTCGCCATCATGTCATATGGTGCA
ACAATCATCATATTGTTGTGACCGAGTTCTATTAAATGTTGTGCTGCAAGTTGTCCGCCTTGATATTCATTTGTCCGAACAAAATCTGTATA
GCCTTGATGGTCATTTTGATCCAGTACGACATAAGGTACATGATGTTTCTTTAGATAGTTATTTAGGGCGTCCGGGGATGAAATGTATTGT
GCGATAATTAATCCGTCAATACCTCGATCAATTAAATGTTTAATATTGTCATACAAATCAGTTGCTGTAGATGTTAAAAAGCATAAATCAA
CATCAGATGGTTTATGGTCATGAATACTTTGCATCAGTGCTGAGAAAAACGGATTTGTTAAGCTAGGCAAAATGACGCCAATAGTTTGAA
TTTTACTGCCGCGCAATTGTTTTGCATGTTTATTAGGGGCATAGCCTAAACGTTCTGAAACAGCATGTACGTTTTTTATCGTTGTTGCGGA
AAAACGACTATCATTATGATTTAAAATATGTGACACAGTTGTAACTGATACACCAGCTTCTCTAGCAACATCTTTAATTGACACTTTTTTCA
T

Gene: Q5HJA4 (putative protein)

Contig: 02_NODE_3, position: 225453 to 225634, length: 182 nt, orientation: FORWARD

Sequence:

TTGAGGCGAGAGAATATATTATTAAGGTTAAATAACTTTTAATGATTATGATAACAAGAATTTTAAAATGTTTATTGTGATGAAAAATACA
AAAGTCATTATTTTAAATAATAAAATATTTGTTTTTGAAAAAAAGTATTAAAAAACATAACGAAATGATGTATATTGAACTATGTGATTGA

Gene: Q5HJA3 (putative protein)

Contig: 02_NODE_3, position: 225684 to 226076, length: 393 nt, orientation: FORWARD

Perfect match to: (MW2-BA000033-[295450:295842], highly conserved allele)

Sequence:

ATGCAATTCAAATTAAAAGAAGAAGAGATTATTAGTTTTTTAGAATTGAAATATCCAGAAAAAGAGTTCGAATATGGTCGTTTGTTAGTT
GGACAACATAAACGTGATGATTTAGATGTTTATTACTTTGGTGATACGTTTTTAATGTGCACGATTATTTCATTCAAGACATTTGAAATTAA
AGAAACAGTAGAATTATCATATGATGCTGTTAATCGTATTGTGTTAAAAGATGGATGGTTATTTAGAAAAATGAGAATAGAAACAATGCA
AAAAGTGTTAAAATACGGTACATCTAAATTAATGTTAACTGATTTTCAAAAAGAGAATTATAATAAATATATTCAAGGTCAGAAACAACG
CGTGATATTTGAAAATGGCCATTTTGTCTAA

Gene: yusP (major facilitator superfamily transporter)

Contig: 02_NODE_3, position: 226204 to 227580, length: 1377 nt, orientation: REVERSE

Perfect match to: (N315-BA000018-[317324:318700:r], allele observed in CC5)

Sequence:

TTAAGATTTAACCGTTTTTGGAATGATCATGTATGCAGACAATGAGCCAAGGATCATCAATACAATGCTGACGATAAATGTTACGGTTGC
AGCTACACTTGGTGCATAGTTTAGTTGTAACATACTGAAAACTGTAGTACTTAGTGCTATACCAAAGGCGCCACCTAATGTACCACTCATT
TTATATAATCCTGTAGCTAAACCAACTTTTTCATTTGGCATACTGAAAATTGCAATTGTAAGGCCGGGTGTTGCGACTAAACCATTTCCAAT
TGCACAAATGACGAAACCAATGATAACTGCAATGACATATTGTGATGCCGATAATTGAGTCATGCTAATAATAGTGATGCCGATGACAG
GGAACAACGGACCAATGATGAGCATCAATTTGCCACCGAAACGTAATGTTGCTTTTTCACCTAAACGAATCATCGCAACTGCCACAATGG
CATATGGCAATGTAACAAGTCCAGATTGCGCAGCTGATAAACCAAGGTGTGTTTGAGCATATATGAAAAAGACCACTGTTACGCCTAGG
CCACTATTTAAAACAAAGTTATTTAAAAATGCACCAATGAACGGACGGTTGCGTAATACTGAGAAATCAATAAAAGGTACTTCATGTCGA
CGTTCGATGATGATGAATATCAACGTAGTGATGATAAAAATGCTCAGACAAATGAATGAAAATGTACTAAACCAACCTTGTTCGAATCCT
TGTGTTAACAATAATGTAAAGCTACCAATCATAACAGCGAAAATCGACATACCTTTGTAATCGAATGGATGACGATGGCTATGTTGACTT
ACTTTTTCAGGTGTGCCTTTTAGAAGTAATATGGCAATGAAAGCAATGACTATACTAATGATGAAATTCGTTTGCCATCCGAAATTTGTGG
CAATTAAACCGCCGATAACACCAGCTAGGCCGATGCCCCCAACAGTACTAATCATTAGATAACTAATCGCTCGTCTTAAATGTTCTCCTTT
AAATTGATTATTTAAAACGCCAACTGTTGAAGGTAACAAGATAGCTGCTGATAGACCTTGTAAAATTCTACCGATGATGAGCAGTGCAGT
GATGTCCGATATAATTAATAGAAGAGATGCAAACATACTGATTATGAGACCCATGTATGTCATTCTCAGTTGTCCTATTTTATCAGCAATA
TCACCTGCAGCCACCATGAAGATACCTGTGGCGAAGGAAGTTAAACTAATAGATAAATTTAACACGGCAGGAGAGGTTTGATACGTTTG
ACCAACGAGAGGTCCTATATTAATAAATGATTGTGCAAACAACCAATATGTTAATGCAGACAACATAATCGCAATAATAATATTACTGCG
TGGTGAAGATTGTGTGTTATTCAC

Gene: yxeI (choloylglycine hydrolase family protein)

Contig: 02_NODE_3, position: 227814 to 228806, length: 993 nt, orientation: FORWARD

Perfect match to: (MW2-BA000033-[297580:298572], highly conserved allele)

Sequence:

ATGTGCACAGGATTCACAATACAAACTTTAAATAATCAAGTACTTCTTGGACGCACGATGGATTATGATTATCCATTAGATGGTTCGCCAG
CAGTGACGCCTAGAAATTATCGTTGGACATCTCGCACTGGCACGACAGGCCAAACGCAATATGGCTTTATTGGTACAGGAACAGATATG
GAAGGTTTTATTTATGGTGATGGTGTTAATGAACATGGCGTTGCCATTTCAACACAATATTTCCGAGGTTATAGTTCATATGGATCAACAC
ACAAAGCGGGCGCGATGAATATTACGCAAAATGAAATTGTGACATGGATTTTGGGATATACAACAAGCATTGAAGATATGAAACAACAA
GCATCCCAAATACATGTTGTAGCGGTATATTTAAATGACATCGGTGAAGTTCCGCCATTGCATTATCATGTTTCCGATGCAACTGGACATT
CAGTCGAAGTTTCATTTAAAGAGGGTGAAGTGGTTATAAAAGATAACCCTATTGGTGTCTTAACAAATCATCCAGACTTAGATTGGCATT
ATAGTAATTTAAGACAATATATCAATATTTCTCCTTATCCAGCAACAGCAAAGTTATTGGAAGGTGTAACGATTGAACCTTTAGGCAATGA
AGCAGGTACATTTGGATTGCCAGGTGGATTTACTTCAACTGAGCGCTTTGTGAGAATGGCATTTATGAAAGCAAACATTGCTCAAAACAA
TGATAAAGAAATGGATTTAATGAATGCATTTTATTTATTAGATGCGGTAAATATACCGATTGGAATTGTACGTCCGCATGATGCTGACAAT
CACTATACGATGTATCAGACCGTAATAAATTTAACTACAAGAACGTTATATATTAAGTATTATGGCAGCAATGAATTAGTAGCATTAAAGC
TCACAGATGATTTAATTAATAGAAAAGATATGACGATTTTTAAGCCTGAGAAGCATATCACTATTAGAAAGTTGAATGACAATCAATAG

Gene: lytM (lysostaphin)

Contig: 02_NODE_3, position: 229132 to 230082, length: 951 nt, orientation: FORWARD

Perfect match to: (Strain_21193-AFEG01000009-[153788:154738:r], allele observed in CC25+CC188+CC692)

Sequence:

ATGAAAAAATTAACAGCAGCAGCGATTGCAACGATGGGCTTCGCTACATTTACAATGGCGCATCAAGCAGATGCAGCAGAAACGACAAA
CACCCAACAAGCACATACACAAATGTCAACACAATCACAAGACGTATCTTATGGTACTTATTATACAATTGATTCTAATGGGGATTATCAT
CACACACCTGATGGTAACTGGAATCAAGCAATGTTTGATAATAAAGAATATAGCTATACATTCGTAGATGCTCAAGGACATACGCATTAT
TTTTATAACTGTTATCCAAAAAATGCAAATGCCAATGGAAGCGGCCAAACATATGTGAATCCAGCAATAGCAGGAGATAACAATGACTAC
ACAGCGAGTCAAAGCCAACAGCATATTAATCAATATGGTTATCAATCAAATGTAGGTCCAGACGCGAGCTATTATTCACATAGTAACAAC
AACCAAGCGTATAACAGCCATGATGGTAATGGAAAGGTCAATTACCCTAATGGCACATCGAATCAAAATGGCGGATTAGCAAGTAAAGC
GACAGCTAGTGGTCATGCGAAAGACGCAAGCTGGTTAACAAGTCGTAAACAACTACAACCATATGGACAATATCACGGTGGTGGTGCGC
ATTACGGTGTCGACTATGCAATGCCTGAAAATTCACCAGTTTACTCATTAACTGATGGCACAGTAGTACAAGCAGGTTGGAGTAACTATG
GTGGCGGCAATCAAGTAACTATTAAAGAAGCGAACAGTAATAACTACCAATGGTATATGCATAATAATCGTTTAACTGTTTCAGCTGGTG
ATAAAGTCAAAGCTGGTGACCAAATTGCATATTCAGGTAGTACGGGTAATTCAACAGCGCCTCACGTACACTTCCAACGTATGTCTGGTG
GCATCGGTAATCAATATGCAGTAGACCCAACGTCATACTTGCAAAGTAGATAA

Gene: Q5HJ98 (putative ABC transporter, ATP-binding protein)

Contig: 02_NODE_3, position: 230134 to 230793, length: 660 nt, orientation: REVERSE

Perfect match to: (MW2-BA000033-[299900:300559:r], allele observed in CC1+CC188)

Sequence:

TTAATAATAGGTATAAGCCCATTGCTTGTCCTCTAAAACATGTTGATAAAAAGGATCTTGGCCAATTAACTTGATATCATCTGCAAGTGCT
TCAACTTCATCTAAATGATGGGTAGTTAATATAATTAAACATTTAGATTTCATAATGTTAAGCAGTTGGTGGATGTCATGTCTAGATTTCA
AATCAATACCAACTGTCGGTTCATCTAAAATTAGAATTCGAGGTTGACCTAGTAAACCTACTAATATATTAATTTTACGTTTATTCCCACCG
GACAATGTAGATACTTTGGCAGACGTATCATCAAAATTTAATTGCTGTAAATATTCGTTGATAGTTGTATCGTTAATTGGATTTTTACAAA
GTGATTTAAAAAAATTAATGTTTTCAGCCACTGTCATGTGTTCAAATAACGCAATGTCTTGTGGCACATAACCGATGTGATTTTGGATTTG
TCTTTGATTCCATTTTTCGCCGAAATAGTTGATAGTTCCATCATTAGCTTTTTCAATACCAGCGATCATACGAAGTAATGTTGATTTTCCAGC
GCCATTATCACCAAGTAATACGGTTAAACGATTACTATCAAAGGACATGGTTAAATGATTGAAAATCTGTTTGTTACGGTAACGCTTTGAA
AGGTTATTAATTTCTATCAT

Gene: Q5HJ97 (putative ABC transporter, transmembrane permease)

Contig: 02_NODE_3, position: 230807 to 231727, length: 921 nt, orientation: REVERSE

Perfect match to: (MW2-BA000033-[300573:301493:r], allele observed in CC1+CC72+CC361)

Sequence:

TTACATGAAAATAATCAAGTATACGATACCCATAGCAAGTGCATATATAAATGTCATGAATAATCGATGACTTATTGTTTGAATATGGAAT
AAGATAAAGACGATACCTATCTCATAAATCAATATAAGTAACAGTGATTTTAAGTAAAATATTAAGCTGAGTGGTTGAGACAAATATAGA
CTAACTGCCAATAGTACCAACAATAACAAGATCGTATGTGTCATTACATAAGTACTATATAGTTTGAAACGGCTTAAATGATATTGTGATA
ATCGTTGCAATGCTGCTTGTTGGTTTAAACGATAATGAAGTACTACTTGAACAGCGCTAACAAATAAAATCACAGCAAAGATTAAGCTAA
TTGAAATAGATTGTTGTGCTTGTTTAGTAAGCGACACAAATTTGATTTTAGATTCAGGTGTATGTTTATGATAGGACTTGTTGATAGCATC
GATGGATTGATGTTGTTTCATATCCTCAAGGTGTTCATAAATAATGTTAGGAATTTGCTGTTCATATAATGAACTACTAACAATTTCTACAG
CAATACCACCTATAAAGTCATCTCTACCATATAACTGTATCGTTTCTTTTAAACGGTTCTCTTTTAATTTTTGAGAGAAACCTTTAGGAATTT
GCATACTTAAAATAGCTTCCTTTTTAGTAACATCATCTTCAATATAGCTTTCATCTTCATCGACTTTTTTAATAGTTACATAGTCAGATTGTTT
AATTTTATTGACGAATGATTTTGATGCAGTGGTTTGGTCTAAATCTTGAATGGTAATCGGTATTTTGAAGTTGTCATGTGCTACACGGTAA
CCGATACCAATAAGTACGAGTGCGATGACAATGGTTGTTACGAGCAAGATGTATTGTAACCATTGCTTGAACACAACAAGTTGTATATAA
GGCTTCAT

Gene: Q5HJ96 (putative ABC transporter, ATP-binding protein)

Contig: 02_NODE_3, position: 231724 to 232890, length: 1167 nt, orientation: REVERSE

Perfect match to: (MW2-BA000033-[301490:302656:r], allele observed in CC1+CC15+CC25+CC188)

Sequence:

TCATTGACGATACCTCCAAACCAATACAGCTAAATTAATTATCAAAAGTGCGATGAAACTAAGATAGAAACTAGGGTGCAGTTCTAAAAT
GTAGTTGTTTAAAATAATTTCTAACAATTGATTTGTTACAACTGCGAACGGTTGAATATTGAAAACGCCATTTGCTATATGTTGTAAAAAA
ATCGTAGGTATTGTTAAACCAGATAACACCAGGATGACAATAGCTAATATGACTTTACTAATACTATTCAACAAGCCTGTTGTTAAAAGTT
CGATGAGTAATAACCACAGTATTAAAAAGGTAACATAATAGCTTAAATGAATGGCTAACGTTGGCCAATTATATAATTCAAAGGTATTCG
GAATACTGAACACAATCCAAACTACACCAACGATACTCCATAACATAGTATAAAACCATGTAATCAACACACGAATGATTAATAAACGCTC
TTTAGAAAAATGAAACATTTTCAATCGCGCTTTCAATACAGTATCTTGATTCATTTTCAAAACTGTAAATAAAGATAGTGCAAAGATGAAT
ACCGTTGTTAAAAAACCTGTAATTGCATAATAACTGCCCGTATCGTATAAATGAATCGGTTCTAAGTTAAATGCACCTGAACGGTTTAATC
CTGTAATCAGCAAATCAGTCATAACATTGATACTGTCAGAATGTGATGCTTTCGGTGCTAAGTCTTGAAAAGCTAAGATGCCACCCATTG
ATCGCATAAGACGTTGGTAAACAGAATCTGTTAGCTGAGATAGCACGACACTTTTCATGGATTGTTGATCATATGTATATACTGAAATTG
GTAGTTCGCCTTGTTTATAAAATGCCTTGGTCATACCTTTATCAAAAACAAAATAGCCTTGAAGTTTATGTTTTTTTAACAAAGTATGTGCT
TGCTTATCATCATATGCCTTAATGCTCACGTTTTTTCCTAGGTTACTCCCTTTACCAATAGAGTTTAAGATTAATTTCGTTTCACTTGATTGA
TCTTTATCTACGACACCTATATTAAAATGATTGTCATCTTCTGTTACATGTTGGATCGTCGTTAATGTAATAACGAGTGCCGCTAATATAAA
TAGTAAATAGATAATCAAATACCACTTTTTCAATAAAAAAGAGTGGTAGATGCGAAACAAATGTATTGTTTTCAT

Gene: Q5HJ95 (putative membrane protein)

Contig: 02_NODE_3, position: 232958 to 234481, length: 1524 nt, orientation: REVERSE

Perfect match to: (MW2-BA000033-[302724:304247:r], allele observed in CC1+CC25+CC188)

Sequence:

TTATTTAGATGCCTTTTTTAAAATTGATTCAAACATTTTGCCGCCATTTTTTTCGATTTCTTTTTCAAGTTTTTCACGGTCTTTTGAAGATAGA
CTATTGAAATCTTTCGCACCACTATCATCAAAATCAATATCTGCTTTCAATTTTGTGCTAGATTTTAAAATGAAATTAATTGGTTCTTCAGCA
TATTTGATGCCGATATTTAACGTAGATTTCTGAGTGTTATTTTTGACGTCAGAATCTATATTATTTTCAAAAGTGAATTCATTTTCGTCGCTA
TATTTATCTAATGCGACAGTAATTTTACCTTTATCTTGACGTTTTGTGCCATCTACTTTTTCTTGGTTATCTAATTTGATTTTTGATTCATCAT
ATTCTGTCTTTTTACCAAATTCGTATTTATCACTGTATTTATTGTCTTTTTCCTTAGAAGATACGCCTTTAATTGTATATTTCGCTTCAGCATA
CGTGTATTTATCTTGATCGAAATCAAGTGCGTAATCTAGTTTTAACTTATCGTCTTCTAAAGTATTAGTACCTTTGATTTTAGTTTTATTATT
TTCTTTGTCTGTAATAGTAATTTCTCGTTTTACAATCGTATGTTTTTCGGTATAAATTTTAGATTGAATTTTAGCAAATTCATCCTTTTTAGTT
TCTTTGACATCGTCAATTGCTTTTTTGATGTCTTTTTCAAAGTCTTTTGTAGCACCTTGTTCTTCCATTAATTTTTTAAGGTCTTTATCCTTTTT
AGCTTCTTCTAATACAGCTAATGTAATTTTTTTAGTGTCAGCTCTGCTAAGTGTTAACGTGACAGGTCTAACTTTGTACTTTTCACCATTAAC
CTTAATTTCTTCTTTTTTACCTTTATCAAAATTATCGTCATCTAATTTGTCGACAATAAGTTCGGAATATTTTTCGGCAATTTTGCTGTAGTCA
CTTTGTTGTGCTTGAGCATTACTAAAAAGAGTATTTAAATTTAGTTGTTGGTTTGTAATACCATTTTCTTTTGCTGTTTCTTCATCTTCACCT
GTAAGTTTTGAATAAGTTGATAATAAATCAGAATTATTAACACTATATTTCCCTTTAAATAATGGTGATTCGAAATAATGCTTATCTTTATC
TGCAGCTAACTGGAATTTCCCTAATGCAGAGTCTGCTATTGTTGGTTCAAGATTAATCATTGATTTCTCTTTTTTAGGATCATGTCCATATG
ACATTTTAATTTTCGAAGCATTAACAACAGATTTAGGAATACCAAGCCCCTTAACAATTTCATCTGATGCATCTGCGCTTAATTCTAATGAA
GATAAAAATGAATTATCTTTCATCTTTTCTTGGAACTTCACTTCATTTTCAAAACGGTCATTAAAATAATCTTTATACATTTTTGCTGTTTGTT
GTTCACTTTTTAGGTATGTATTTTTCGGTGTATTAGCAAAAAATGCATAAACTCCCCATGCGATTCCACCTATTAATAATAAGACAACAATA
ATAGGAATTATAATTTTTAGTTTTTTAGACAT

Gene: ssaA (staphylococcal secretory antigen A)

Contig: 02_NODE_3, position: 234812 to 235714, length: 903 nt, orientation: REVERSE

Perfect match to: (08-02119-CP015645-[1928452:1929354], highly conserved allele)

Sequence:

TTAATGGATGTAATTATATGATGAAACTTCTGAAGCAGAGATGGTTCTTGATGAAACGATATATTCACCAATCCAGTTCATTTCTGAAATT
AGAATACTTCCATCAATATTAACTTTTTCAACGTAGGCTACATGACCAAATGGACCATTTACTGTTTGTAAAATTGATCCTCGTGTTGGGTG
TCTATCTACTTTGAAGCCATTGCTTGAAGCTTGGCCTGCCCAGTTTTTAGCATCTCCCCAAAATGTACTAATCGTGTGTCCATCTTTGGCAC
GTTTATCAAAGACATACCATGTACATTGTCCAGCAGTATATAAGTTGTTCTTACTTGTGATAAGAGGCTGATCAATGATTTTACCGTTACCT
AATGCTAAAGGTTTACCGTCAGCGGTCTTTGCTTTGTCATTAAATTCGGCGATTTGTAATTCGTCATACAATTCGTCTAAATCGATGCCTGT
AATAAGCCCTTTGTTATCTTTTGAAAAAGCGTTATTTAATTCATCGTCATTGTCTTCGACATTCGGTATTGCTGGTGTCAAAGGATTGCTTG
GTGACGTTTGAGGCGGTGTGTGTGAATCAATTGCGTCATTAATGTGCGTATACTGACCACTTAATGAAGAATGGTACTGATTGTTGTTAA
GATCACGTTGATTTGCGTGTTGATGATTGTCGTTTGTACGTGACTGGTTTTGATGATTGTTGTTTGGCGTGTTGTTTTTGTCATATGTATAA
GTATACGCGCCGGTGTCTTTATTCACTTTGAACTGTGCGTTTGGGTGTGCTTTCTTTGCTTCTTCTAATGTTTTGCTATCATTCGTATATGCT
TGAGCCGAGTTAGGCGACATACTAAATAAAGTAAGAGTTGTCATCGTCAGTAAAATTGTTTTCTTCATAATAACCAT

Gene: esxA (virulence factor esxA)

Contig: 02_NODE_3, position: 235953 to 236246, length: 294 nt, orientation: FORWARD

Perfect match to: (N315-BA000018-[327073:327366], highly conserved allele)

Sequence:

ATGGCAATGATTAAGATGAGTCCAGAGGAAATCAGAGCAAAATCGCAATCTTACGGGCAAGGTTCAGACCAAATCCGTCAAATTTTATCT
GATTTAACACGTGCACAAGGTGAAATTGCAGCGAACTGGGAAGGTCAAGCTTTCAGCCGTTTCGAAGAGCAATTCCAACAACTTAGTCCT
AAAGTAGAAAAATTTGCACAATTATTAGAAGAAATTAAACAACAATTGAATAGCACTGCTGATGCCGTTCAAGAACAAGACCAACAACTT
TCTAATAATTTCGGTTTGCAATAA

Gene: esaA (protein esaA)

Contig: 02_NODE_3, position: 236329 to 239358, length: 3030 nt, orientation: FORWARD

Perfect match to: (MW2-BA000033-[306095:309124], allele observed in CC1)

Sequence:

ATGAAAAAGAAAAATTGGATTTATGCATTAATTGTCACTTTAATTATTATAATTGCCATAGTTAGTATGATATTTTTTGTTCAAACAAAATA
TGGAGATCAATCAGAAAAAGGATCCCGAAGTGTAAGTAATAAAAATAATAAAATACATATCGCAATTGTTAACGAGGATCAACCAACGA
CATATAACGGTAAAAAAGTTGAGCTGGGTCAAGCATTTATTAAAAGGTTAGCAAATGAGAAAAACTATAAATTTGAAACAGTAACACGA
AACGTTGCTGAGTCTGGTTTGAAAAATGGTGGATACCAAGTCATGATTGTTATCCCAGAAAACTTTTCAAAATTGGCAATGCAATTAGAC
GCTAAAACACCATCGAAAATATCGCTACAGTATAAAACAGCTGTAGGACAAAAAGAAGAAGTAGCTAAAAACACAGAAAAAGTTGTAA
GTAATGTACTTAACGACTTTAACAAAAACTTAGTCGAAATTTATTTAACAAGTATCATTGATAATTTACATAATGCACAAAAAAATGTTGG
CGCTATTATGACGCGTGAACATGGTGTGAATAGTAAATTCTCGAATTACTTATTAAATCCAATTAACGACTTCCCGGAATTATTTACAGAT
ACGCTTGTAAATTCAATTTCTGCAAACAAAGATATTACAAAATGGTTCCAAACATACAATAAATCATTATTGAGTGCGAATTCAGATACGT
TCAGAGTGAACACAGATTATAATGTTTCGACTTTAATTGAAAAACAAAATTCATTATTTGACGAGCACAATACAGCGATGGATAAAATGT
TACAAGATTATAAATCGCAAAAAGATAGCGTGGAACTTGATAACTATATCAATGCATTAAAACAGATGGACAGCCAAATTGATCAACAAT
CAAGTATGCAAGATACAGGTAAAGAAGAATATAAACAAACTGTTAAAGAAAACTTAGATAAATTAAGAGAAATCATTCAATCACAAGAG
TCACCATTTTCAAAAGGTATGATTGAAGACTATCGTAAGCAATTAACAGAATCGCTGCAAGATGAGCTTGCAAATAACAAAGACTTACAA
GATGCGCTAAATAGCATTAAAATGAACAATGCTCAATTCGCTGAAAACTTAGAGAAACAACTTCATGATGATATTGTCAAAGAACCTGAT
ACAGATACAACATTTATCTATAACATGTCTAAACAAGACTTTATAGCTGCAGGTTTAAATGAGGATGAAGCTAATAAATACGAAGCAATT
GTCAAAGAAGCAAAACGTTATAAAAACGAATATAATTTGAAAAAACCGTTAGCAGAACACATTAATTTAACAGATTACGATAACCAAGTT
GCGCAAGACACAAGTAGTTTGATTAATGATGGTGTGAAAGTGCAACGTACTGAAACGATTAAAAGTAATGATATTAATCAATTAACTGTT
GCAACAGATCCTCATTTTAATTTTGAAGGCGACATTAAAATTAATGGTAAAAAATATGACATTAAGGATCAAAGTGTTCAACTCGATACAT
CTAACAAGGAATATAAAGTTGAAGTCAATGGCGTTGCTAAATTGAAAAAGGATGCTGAGAAAGATTTCTTAAAAGATAAAACAATGCAT
TTACAATTGTTATTTGGACAAGCAAATCGTCAAGATGAACCAAATGATAAGAAAACAACGAGTGTTGTGGATGTAACATTGAATCATAAC
CTTGATGGTCGCTTATCGAAAGATGCATTAAGCCAGCAATTGAGTGCATTATCTAGGTTTGATGCGCATTATAAAATGTACACAGATACA
AAAGGCAGAGAAGATAAACCATTCGACAACAAACGTTTAATTGATATGATGGTTGACCAAGTTATCAATGACATGGAAAGTTTCAAAGA
CGATAAAGTAGCTGTGTTACATCAAATTGATTCAATGGAAGAAAACTCAGACAAACTGATTGATGACATTTTAAATAACAAAAAGAATAC
AACAAAAAATAAAGAAGATATTTCTAAGCTGATTGATCAGTTAGAAAACGTTAAAAAGACTTTTGCTGAAGAGCCACAAGAACCAAAAA
TTGATAAAGGCAAAAATGATGAATTTAATACGATGTCTTCAAATTTAGATAAAGAAATTAGTAGAATTTCTGAGAAAAGTACGCAATTGC
TATCAGATACACAAGAATCAAAAACAATTGCAGATTCAGTTAGTGGACAATTAAATCAATTAGATAATAATGTGAATAAACTACATGCGA
CAGGTCGAGCATTAGGCGTAAGAGCGAATGATTTGAACCGTCAAATGGCTAAAAACGATAAAGATAATGAGTTATTCGCTAAAGAGTTT
AAAAAAGTATTACAAAATTCTAAAGATGGCGACAGACAAAACCAAGCATTAAAAGCATTTATGAGTAATCCGGTTCAAAAGAAAAACTT
AGAAAATGTTTTAGCTAATAATGGTAATACAGACGTGATTTCACCGACATTATTCGTATTATTGATGTATTTACTATCAATGATTACAGCAT
ATATTTTCTATAGTTATGAACGTGCCAAAGGACAAATGAATTTCATTAAAGATGATTATAGTAGTAAAAACCATCTTTGGAATAATGTCAT
TACGTCAGGTGTTATTGGTACAACTGGTTTGGTAGAAGGATTAATTGTCGGTTTAATTGCAATGAATAAGTTCCATGTATTAGCTGGCTAT
AGAGCGAAATTCATCTTAATGGTGATTTTAACTATGATGGTCTTCGTACTTATTAATACGTATTTACTAAGACAGGTAAAATCTATCGGTA
TGTTCTTAATGATTGCTGCATTGGGTCTATACTTTGTAGCTATGAATAATTTGAAAGCGGCTGGACAAGGTGTGACTAATAAAATTTCACC
ATTGTCTTATATCGATAACATGTTCTTCAATTATTTAAATGCAGAGCATCCTATAGGCTTGGCGCTAGTAATATTAACAGTACTTGTGATTA
TTGGCTTTGTACTGAACATGTTTATAAAACACTTTAAGAAAGAGAGATTAATCTAA

Gene: essA (protein essA)

Contig: 02_NODE_3, position: 239358 to 239816, length: 459 nt, orientation: FORWARD

Sequence:

ATGTTGATGAATAGCGTGATTGCTTTAACTTTTTTAACAGCATCTAGCAATAATGGCGGACTTAATATTGACGTGCAACAAGAAGAAGAA
AAGCGAATCAATAATGATTTAAATCAATATGATACAACGCTATTTAATAAAGACAGCAAAGCGGTTAATGATGCGATTGCTAAGCAGAAA
AAAGAACGACAACAACAAATAAAAAATGATATGTTTCAAAATCAAGCGAGTCACTCGACTCGCTTGAATGAAACTAAAAAAGTGTTATTT
TCCAAATCTAACTTAGAAAAGACTTCGGAGAGTGATAAAAGCCCCTATATTCAAAACAAGCAGGAGAAAAAAATATTCCCGTACATTTTG
ATGTCTGTAGGGGCTTTTTTGACTTTAGGATTTGTCATTTTTTCAATTCATAAAGGGAGACGAACGAAAAATGAATCAGCACGTAAAAGT
AACATTTGA

Gene: esaB (protein esaB)

Contig: 02_NODE_3, position: 239788 to 240030, length: 243 nt, orientation: FORWARD

Perfect match to: (RF122-AJ938182-[276651:276893], highly conserved allele)

Sequence:

ATGAATCAGCACGTAAAAGTAACATTTGATTTTACTAATTATAATTACGGCACATATGACTTAGCAGTACCAGCATATTTACCGATAAAAA
ACTTAATAGCTTTAGTATTGGATAGTTTGGACATTTCAATATTTGATGTCAATACACAAATTAAAGTGATGACGAAAGGTCAATTACTTGT
TGAAAATGATCGACTCATTGATTATCAAATCGCTGATGGAGATATTTTGAAGTTACTATAG

Gene: essB (protein essB)

Contig: 02_NODE_3, position: 240043 to 241377, length: 1335 nt, orientation: FORWARD

Perfect match to: (08-02119-CP015645-[1922789:1924123:r], allele observed in ST582+CC1)

Sequence:

ATGGTTAAAAATCATAACCCTAAAAATGAAATGCAAGATATGTTAACGCCTTTAGATGCTGAAGAAGCAGCTAAAACAAAATTACGCTTA
GATATGAGAGAGATTCCTAAGTCTTCAATTAAACCAGAACATTTTCATTTAATGTACTTATTAGAACAACATTCTCCATATTTTATAGATGC
TGAATTAACTGAACTACGTGACAGTTTCCAAATACATTATGACATTAATGACAATCATACACCTTTTGATAATATTAAATCATTTACTAAAA
ATGAAAAATTACGTTACTTACTCAATATCAAAAATTTAGAAGAAGTAAATCGTACACGCTACACATTTGTGTTGGCACCAGATGAATTATT
TTTCACAAGAGATGGATTACCCATTGCTAAAACAAGAGGGTTACAAAATGTTGTTGATCCATTACCTGTGTCAGAAGCTGAATTTTTAACA
AGATATAAAGCACTGGTTATCTGTGCATTCAATGAGAAACAATCATTTGATGCTTTAGTTGAAGGAAACTTAGAACTACATAAAGGAACG
CCATTTGAAACTAAAGTTATTGAAGCGGCAACGTTAGATTTACTAACGGCATTTTTAGATGAACAGTATCAGAAACAAGAACAAGATTAT
AGTCAAAATTATGCATATGTACGCAAAGTAGGACATACCGTTTTCAAATGGGTTGCTATCGGTATGACAACGTTAAGTGTTTTATTAATTG
CATTCTTAGCCTTTTTATATTTTTCAGTAATGAAGCATAATGAGCGCATTGAAAAAGGATACCAAGCATTTGTAAAGGATGATTATACGCA
AGTACTAAATACGTATGATGATTTAGATGGTAAAAAATTAGATAAAGAGGCACTTTACATTTATGCCAAAAGTTATATCCAAACAAATAA
ACAAGGTTTAGAAAAAGATAAGAAAGAAAATTTACTTAATAATGTGACACCAAATTCAAACAAAGACTACTTATTATATTGGATGGAATT
AGGACAAGGACATCTTGATGAAGCGATTAATATTGCCACTTATTTAGATGATAACGATATTACAAAGTTAGCGTTGATTAATAAATTAAAT
GAGATTAAAAATAACGGAGATTTATCGAATGATAAACGTTCTGAAGAAACGAAAAAGTATAACGATAAATTGCAAGATATTTTAGACAA
AGAAAAACAAGTTAAAGATGAAAAAGCGAAATCTGAAGAAGAGAAAGCAAAAGCGAAAGATGAGAAATTAAAGCAACAAGAAGAGAA
CGAAAAGAAACAAAAAGAACAAGCACAAAAAGATAAAGAAAAACGCCAAGAAGCTGAAAGAAAAAAATAG

Gene: essC (protein essC)

Contig: 02_NODE_3, position: 241399 to 245838, length: 4440 nt, orientation: FORWARD

Sequence:

ATGCATAAATTGATTATAAAATATAACAAACAATTGAAGATGCTCAATTTGCGAGATGGTAAGACATATACTATTAGCGAAGACGAGCGT
GCAGATATTACGTTGAAATCGTTAGGCGAAGTCATTCATTTAGAACAAAATAATCAAGGTACTTGGCAAGCGAATCATACTTCTATTAATA
AGGTGCTTGTTAGAAAAGGTGACCTTGATGACATTACATTACAGCTTTATACAGAAGCTGATTATGCATCATTTGCGTATCCTTCAATTCA
AGATACGATGACAATTGGACCAAATGCGTATGATGATATGGTTATTCAAAGCTTGATGAATGCCATCATTATTAAAGATTTTCAATCAATA
CAAGAATCACAATACGTACGCATTGTGCACGATAAAAATACAGATGTGTATATTAACTATGAACTACAAGAGCAACTAACGAACAAAGCT
TACATTGGTGATCATATTTATGTTGAAGGGATATGGCTCGAAGTACAAGCTGATGGTTTAAATGTATTGAGTCAGAATACAGTGGCATCG
TCATTAATTCGCTTAACACAAGAGATGCCACATGCACAGGCAGATGATTACAATACGTACCATCGTTCGCCAAGGATTATTCACCGTGAA
CCGACGGATGATATTAAGATTGAAAGACCGCCACAGCCAATACAGAAGAACAATACAGTGATATGGCGTTCCATTATACCGCCATTAGTA
ATGATTGCTTTAACTGTTGTCATCTTTTTAGTGAGACCAATTGGTATTTATATTTTAATGATGATTGGTATGAGTTCAGTAACGATAGTATT
TGGTATTACAACGTATTTCTCTGAAAAGAAAAAGTATAACAAAGATGTTGAAAAACGAGAGAAAGATTACAAAGATTATTTGGATAATA
AATCTAAAGAAATTAATAAAGCGATTAAAGCACAACGTTTTAGTTTGAATTACCATTATCCAACGGTTGCTGAAATTAAAGATATCGTTGA
AACGAAAGCACCAAGAATATATGAAAAAACATCGCATCATCACGATTTCTTACATTATAAGTTAGGTATTGCGAATGTAGAAAAGTCATT
CAAATTAGATTACCAAGAAGAAGAATTTAACCAACGTCGTGATGAATTATTCGACGATGCTAAAGAATTGTATGAATTTTACACAGATGT
AGAACAAGCACCATTAATCAATGATTTAAATCATGGACCGATTGCATATATTGGTGCACGACATCTCATTTTAGAAGAATTGGAAAAAAT
GTTAATCCAATTGTCAATATTCCATAGTTATCATGATTTAGAGTTTCTATTTGTGACACGTGAAGATGAAGTTGAAACATTGAAATGGGCA
CGTTGGTTGCCACATATGACATTGAGAGGTCAAAACATTAGAGGATTTGTTTACAATCAACGAACACGTGACCAAATTTTAACGTCAATTT
ACAGCATGATTAAAGAACGTATCCAAGCTGTGCGTGAACGCAGTAAAAGTAATGAGCAAATTATTTTTACACCGCAATTAGTGTTTGTCA
TTACAGATATGTCATTAATTATTGATCATGTCATTTTAGAATATGTAAACCAAGATTTATCAGAATATGGTATTTCATTAATCTTTGTTGAA
GATGTGATTGAAAGTTTGCCAGAGCATGTAGATACCATTATTGATATCAAGTCTCGTACTGAAGGAGAACTGATTACGAAAGAAAAAGA
ATTAGTTCAATTGAAATTTACACCTGAAAATATTGATAACGTCGATAAAGAATATATCGCGCGACGTTTGGCGAATTTGATACACGTCGA
ACATTTGAAAAATGCAATTCCTGATAGTATTACATTTTTAGAGATGTATAACGTGAAAGAAGTAGATCAGCTTGATGTGGTTAATCGATG
GAGACAAAACGAAACATACAAAACGATGGCAGTACCTTTAGGTGTAAGAGGTAAAGATGACATTTTATCATTGAACTTACATGAAAAAG
CACACGGACCACATGGTTTAGTTGCTGGTACCACTGGGTCAGGGAAATCTGAGATTATCCAATCATACATTTTATCTTTAGCTATTAATTTT
CACCCACATGAAGTTGCGTTCCTATTGATTGACTATAAAGGTGGGGGTATGGCGAACTTATTTAAAGATTTAGTCCATTTAGTTGGTACG
ATTACAAACTTAGATGGTGATGAAGCGATGCGTGCCTTAACATCAATCAAAGCCGAATTGAGAAAACGTCAACGTTTATTCGGAGAGCAT

GATGTTAACCATATTAATCAATACCATAAGTTATTTAAAGAAGGTATTGCGACAGAACCAATGCCACATTTATTCATTATTTCCGATGAGTT
TGCCGAATTAAAATCAGAACAACCTGATTTTATGAAAGAACTTGTATCAACGGCACGTATTGGACGTTCGTTAGGTATTCATTTAATACTT
GCGACACAAAAACCATCGGGTGTTGTTGATGACCAAATTTGGTCTAACTCTAAATTTAAATTGGCATTAAAAGTACAAGATAGACAAGAC
AGTAATGAAATTTTAAAAACACTAGATGCAGCAGACATTACATTACCAGGTCGTGCGTATTTACAAGTTGGTAATAATGAAATTTATGAA
TTGTTCCAATCTGCATGGAGTGGAGCAACATATGACATCGAAGGCGATAAATTAGAAGTTGAAGATAAGACGATTTACATGATTAATGAT
TATGGTCAACTTCAAGCAATCAACAAAGACTTGAGTGGACTTGAAGATGAAGAAACGAAAGAAAATCAAACTGAGTTAGAAGCGGTTAT
CGATCATATAGAATCTATTACAACACGATTAGAAATTGAAGAAGTTAAGCGTCCATGGTTACCACCATTACCAGAAAATGTATATCAAGA
AGATTTAGTAGAAACAGATTTCAGAAAATTATGGTCAGATGATGCAAAAGAAGTGGAATTAACATTAGGACTTAAAGACGTACCAGAAG
AACAATATCAAGGGCCGATGGTATTACAATTGAAAAAAGCTGGTCACATCGCGTTAATCGGAAGTCCAGGATATGGTAGAACAACGTTC
TTACACAACATTATTTTCGATGTTGCAAGACACCATCGTCCTGATCAAGCACACATGTACTTGTTCGATTTCGGTACCAATGGTTTGATGCC
AGTCACAGACATACCACATGTCGCTGATTACTTTACAGTAGATCAAGAAGACAAGATTGCGAAGGCGATACGTATATTTAATGATGAAAT
CGATCGCCGTAAGAAGATTTTAAGTCAGTATCGTGTTACTAGTATTTCTGAATATCGAAAATTAACTGGTGAAACAATTCCGCATGTCTTT
ATTCTTATTGATAACTTTGACGCAGTAAAAGATTCACCTTTTCAAGAAGTTTTTGAAAATATGATGATTAAAATGACACGTGAAGGGCTAG
CATTAGACATGCAAGTAACCTTAACTGCCTCAAGAGCTAACGCTATGAAAACACCAATGTACATTAATATGAAAACGCGTATCGCCATGT
TTTTATATGATAAATCAGAAGTGTCGAACGTAGTAGGACAGCAAAAATTTGCGGTTAAAGATGTAGTGGGTCGAGCATTGTTAAGTAGC
GATGACAACGTATCATTCCATATTGGCCAACCATTTAAACATGATGAGACCAAATCATATAATGATCAAATTAATGATGAAGTATCGGCG
ATGACAGAATTTTATAAAGGTGAAACACCAAGTGATATTCCTATGATGCCAGATGAAATTAAATATGAAGATTACAGAGAATCATTAAGC
TTACCGGATATAGTTGCAAATGGTGCTTTACCAATTGGATTAGATTATGAAGGTGTTACACTACAAAAAATTAAATTAACTGAACCAGCA
ATGATTTCATCAGAAAATCCGAGAGAAATTGCGCATATTGCTGAAATTATGATGAAAGAAATTGACATATTAAATGAAAAATATGCGATT
TGTATCGCAGATTCAAGTGGAGAGTTTAAAGCTTATAGACATCAAGTGGCTAACTTTGCCGAAGAAAGAGAAGACATTAAAGCGATTCA
TCAACTAATGATTGAAGACTTAAAACAAAGAGAAATGGACGGTCCGTTTGAAAAAGATTCACTTTACATTATCAATGATTTTAAAACATTT
ATTGATTGCACGTATATTCCGGAAGATGATGTTAAAAAACTTATTACAAAAGGACCAGAACTTGGCTTGAACATTTTATTTGTCGGCATTC
ATAAAGAATTAATAGATGCTTATGATAAACAGATTGATGTTGCACGTAAAATGATTAACCAATTTAGTATAGGTATTCGTATTTCAGACCA
ACAATTCTTTAAATTTAGATTTATTCAACGAGAACCTGTTATTAAAGAAAATGAAGCATATATGGTCGCAAACCAAGCTTATCAAAAGATT
AGATGGTTTAAATAG

Gene: esxC (protein esaC)

Contig: 02_NODE_3, position: 245868 to 246260, length: 393 nt, orientation: FORWARD

Perfect match to: (MW2-BA000033-[315634:316026], allele observed in CC1+CC6+CC97+CC1153)

Sequence:

ATGAATTTTAATGATATTGAAACAATGGTTAAGTCGAAATTTAAAGATATTAAAAAGCATGCTGAAGAGATTGCGCATGAAATTGAAGTT
CGTTCTGGATATTTAAGAAAAGCTGAACAATATAAGCGATTAGAATTTAATTTGAGTTTTGCACTAGATGATATTGAAAGCACAGCAAAG
GACGTACAAACTGCAAAAACTAGTGCTAATAAGGACAGTGTAACTGTTAAAGGAAAAGCGCCCAATACGTTATATATCGAAAAAAGAAA
TTTGATGAAACAAAAGCTTGAAATGTTGGGTGAAGATATCGATAAAAATAAAGAATCCCTCCAAAAAGCTAAGGAAATTGCTGGAGAAA
AGGCAAGTGAATATTTTAATAAAGCAATGAATTAA

Gene: esxB (virulence factor esxB)

Contig: 02_NODE_3, position: 246276 to 246590, length: 315 nt, orientation: FORWARD

Perfect match to: (MW2-BA000033-[316042:316356], allele observed in CC1+CC6)

Sequence:

ATGGGTGGATATAAAGGTATTAAAGCAGATGGTGGCAAGGTTGATCAAGCAAAACAATTAGCGGCAAAAACAGCTAAAGATATTGAAG
CATGTCAAAAGCAAACGCAACAGCTCGCTGAGTATATCGAAGGTAGTGATTGGGAAGGACAGTTCGCCAATAAGGTGAAAGATGTGTT
ACTCATTATGGCAAAGTTTCAAGAAGAATTAGTACAACCGATTGCTGACCATCAAAAAGCAATTGATAACTTAAGTCAAAATCTAGCGAA
ATACGATACATTATCAATTAAGCAAGGGCTTGATAGGGTGAACCCATGA

Gene: esaE (putative protein)

Contig: 02_NODE_3, position: 246587 to 247264, length: 678 nt, orientation: FORWARD

Perfect match to: (394_SAUR-JVIV01000045-[22830:23507], allele observed in CC6+CC1)

Sequence:

ATGATGAAAGATGTTAAGCGAATAGATTATTTTTCTTACGAAGAATTAACAATTTTAGGTGGTAGTAAATTACCTCTCGTAAATTTTGAAT
TGTTTGATCCATCAAATTTTGAAGAAGCTAAAGCGGCTTTAATTGAAAAGGAATTAGTAACAGAGAATGACAAGTTAACTGATGCAGGTT
TTAAAGTGGCTACATTAGTCAGAGAGTATATTAGCGCAATTGTAAATATTCGAATTAATGATATGTATTTTGCACCATTTAGCTATGAAAA
AGATGAATATATTTTGTTAAGCCGGTTTAAAAATAATGGATTTCAAATACGAATTATAAATAAAGACATTGCATGGTGGTCGATTGTACA
ATCATATCCTTTATTAATGAGACAAGAAAAGTCCAATGATTGGGACTTTAAACAAATTGACGATGAAACATTGGAGAACTTAAATAATGA
AAGTATCGATACGATTGGGCGTGTTTTAGAAATTGAAATATACAATCATCAAGGTGACCCTCAACAAAGTTTATATAACATTTATGAACAA
AATGATTTGCTACTCATTCGATACCCATTAAAAGATAAAGTACTGAATGTTCATATTGGCGTCATTAATACATTTATACGAGAATTATTTGG
ATTCAATACTGATGAAAATCATATTAATAAAGCAGAGGAGTAA

Gene: esxD (putative protein)

Contig: 02_NODE_3, position: 247264 to 247581, length: 318 nt, orientation: FORWARD

Perfect match to: (MW2-BA000033-[317030:317347], allele observed in CC1+CC6)

Sequence:

ATGACGTTGAGTGGAAAAATTAGTGTTAAAGCTGAAACGATTGCACATGTTGTAAAAGAATTGGAAAGCATTAGTCAAAAGTATGATGA
AATAGCTCAAAACTTTGGAAAAATTGCGCAATTAAATTACTACAGTAGTGAAAAAGCTTCACATTCTATGGAAAACGGCTATAGTAGTGC
TGCAACAGTCATTAGTGGTCTCAAAGGTCCATTGAGTACACTCGGTGGTGGTGTCATGAATTCAGCACAAAAGTTCTTTGAAGCAGATGA
ACATTGGGGTACGGAATTTGCCAAGCTTTACTATAATATTGAGGGATAG

Gene: essD (putative protein)

Contig: 02_NODE_3, position: 247591 to 249435, length: 1845 nt, orientation: FORWARD

Perfect match to: (MW2-BA000033-[317357:319201], allele observed in CC1)

Sequence:

ATGACAAAAGATATTGAATATTTAACAGCTGATTATGACAATGAAAAGTCATCTATCCAAAGTGTAATAGATGCAATAGAGGGGCAAGA
CTTCTTAGATGTAGATACAACAATGGATGATGCGGTAAGCGATGTCAGTTCTTTAGACGAAGATGGCGCAATATCATTAACAAGTAGTGT
AGTAGGTCCACAAGGATCTAAATTAATGGGCTATTATCAAAATGAGTTATATGATTATGCATCTCAATTAGATTCGAAAATGAAAGAAAT
TATTGACACGCCATTTATAGAAGATATAGATAAAGCATTCAAAGGTATAACGAATGTTAAATTGGAAAATATATTAATTAAAAATGGCGG
TGGTCATGGTAGAGATACCTATGGGGCTTCTGGGAAAATTGCAAAGGGAGATGCCAAGAAAAGTGACAGCGATGTTTATAGCATCGAT
GAAATATTAAAATCGGATCAAGAATTTGTAAAAGTAATTGATCAGCATTACAAAGAAATGAAAAAAGAAGATAAGAAATTATCTAAGAG
TGATTTTGAAAAAATGATGACTCAGGGCGCTTCTTGTGATTACATGACAGTAGCTGAAGCGGAAGAGCTAGAGGAGCAAAAGAAAAAA
GAAGAAGCTATAGAGATTGCAGCACTAGCTGGTATGGTAGTTTTATCTTGTATTAATCCTGTTGCTGGAGCAGTAGCTATTGGTGCTTATT
CCGCCTATTCAGCAGCAAATGCAGCCACAGGAAAAAATATTGTAACTGGAAGAAAGCTATCTAAAGAAGAACGAATCATGGAAGGACTT
TCGCTTATTCCATTGCCAGGTATGGGCTTCCTCAAAGGTGCTGGGAAAAGTTTAATGAAATTAGGCTTCAAAGGCGGAGAAAAATTTGCA
GTTAAAACAGGATTGCAAAAGACAATGCAACAAGCGGTTAGTCGTATTTCACCTAAAATGGGAATGATGAAAAACAGTGTGTTGAATCA
ATCTCGTAACTTTGCTCAAAATACTCATGTTGGACAAATGCTGAGTAACATGCGTGGTCAAGCAACTCATACTGTTCAACAAAGTAGAAAT
TGGATTGGACAACAAGCACAAAATGTCAAACGAATAGTGAATAATGGACTTGATAAAGAAATAGCACATCCATTTAAACAACAACTTGC
ACCAGCGGGAATGGGTGGTATAAAATTTGCTGAAACAACTACTTTGAGAAACATGGGTCAAAACATGAAACGTGCTGTTACACCACAAA
ATCACGTGACACATGGTCCGAAAGATAGTATGGTGAGAAGTGAAGGTAAACATAGTGTAAGTAGCCATGAAATAAATTCATCAAAATAT
GTTGAATCACCAAACTACACCAAGGTTGAATTCGGAGAACACTATGCAAGACTTAGACCTAAGAAACTAAAAGCAAATATAGAATATACA
ACACCAACTGGTCACATATATCGGACCGATCATAAAGGGCGCATAAAAGAAGTTTATGTAGACAATCTTTCTCTAAAAGATGGCGGTCGT
AATAACCATGCACAAAGAACTGTGGGGGGAGAGGATAGATTACCAGACGATGATGGAGGTCATTTAATCGCTAGAATGTTTGGTGGGT
CAAAAGACATAGATAACCTTGTGGCACAAAGTAAATTTATCAACCGTTCATTTAAGGAAAATGGACAATGGTATAAGTTGGAAAAACTAT
GGGAAAAAGCAATCAAGTCTGGGAAGTCAATAGAAAATGTTAAAGTTGAAGTTAAATATAAAGGTAATAGTCAAAGGCCAACCGAATTT
TCAGTTAGATATAATATTAATGGTGAGCAGTTTTTAAAAAATATAATTAATAAATAG

Gene: DUF600-locus2 (putative protein)

Contig: 02_NODE_3, position: 249446 to 249940, length: 495 nt, orientation: FORWARD

Perfect match to: (MW2-BA000033-[319212:319706], allele observed in CC1)

Sequence:

ATGACTTTCGAAGAAAAACTAAGTCAAATGTATAACGAGATTGCGAATAAGATTAGCAGTATGATACCGGTAGAATGGGAAAAGGTATA
TGCAATGGCATATATAGATGATGAAGGAGGCGAAGTGTTCTACTATTACACAGAACCTGGAAGTAATGAATTATACTATTATACTAGTGT
ATTAAATAAATATGATATATCGGAATCAGAATTTATGGATTCAGCGTATGAGTTGTATAAACAATTTCAAAAGTTAAGAAATATATTTAAA
AAAGAAGGACATGAACCATGGACATCATGCGAATTTGATTTTACAAATGAAGGTAAATTAAAAGTTTCATTTGATTATATTGATTGGATA
AATACAGAGTTTGATCAATTGGGCCGTCAAAATTATTATATGTACAAAAAATTTGGGGTTATACCAGAAATGGAATATGAAATGGAGGA
GGTTAAACAAATCGAGCAATATATTAAAGAGCAAGAAGAAGAATAG

Gene: Q1Y4R2 (putative protein)

Contig: 02_NODE_3, position: 250151 to 250834, length: 684 nt, orientation: FORWARD

Perfect match to: (MW2-BA000033-[319917:320600], allele observed in CC1)

Sequence:

ATGACTACTAAAGAAAATATTGATACTCTTCGAAAGCCAGGTGCACAAGCCTTAAGTTTAATATCATTGTTTTTGATACTTTTTTCATGTCT
AACTTTCTTTTTTGGTTTAGATTATGAAAGGTTTCCAAACTATTTAAAGATAACGACAATTATAGAATTAATAATTATTGTAATTAGTTTACT
TCAATGGATTAGATTTATAGACTTCGAAAAAGAAAGTACACAGAAATATAAAAAAATATATGCTCGATTTTTAGTTATTATAAATGTGTTA
ACTACTATCACTGTAGTATTTGCACTGTGTAACCTTTATTATTTTGCAGCTGTACAAAACCATTACGATTTATTCAATTATTGGTTAATGGGT
ACGATTTCAATCATAATTAGCTATTTATTATTAGTAATTGGCGGAATGTTTACGTTGTTAAAATTACCTAAAGTAACAAAACGCTGGGGGG
GTAAAACTAAAACACATTTCGGTTTATTATTAACTGCGTTGAGCTCATTTATATATATTGAAAAAATTATCGAATATATATTGATTCCTAAT
GTCGTAGAATCCAAGTTTATAATTATTGTGAGCATGATGGTTATCGCCGGAGCACAATTTGTGGCATTTCAATTTATTATGCAATACAGTA
GATTCTATATTTTTGAATTAAACACTGAAGATGATGACTAA

Gene: Q1Y4R3 (putative protein)

Contig: 02_NODE_3, position: 250981 to 251592, length: 612 nt, orientation: FORWARD

Perfect match to: (MW2-BA000033-[320747:321358], allele observed in CC1+CC121+CC2059)

Sequence:

ATGGAGTTCTTATTATTAATTGTCGTAGCCGGACTGTATTATATTATATATTTAACTGCTGTGATGTATTCTGAAAAAATAGTAGTATTGCC
TATAATCATCTATGCCATTGTGTTTGTAGTAATTGGTATCACTTATATTTTTATAGGTGACAGCTATGATCAGTTAACAAATTTCAATGTGA
TTTTGTATATGGGGAGTTTGTTTTATGCATGGATGGCTTTTAGAAATCTTTGGAACAGACCATTATTATTAAAATATAAGAACATTACAGA
TAGTTCAAGTGGAATAGTTAATAAATCTGAATATAATTCAGTTGAAAGCTTACGTATAAACATTGAAATAGCTAAGTATAAAGGGATTAT
TTCTTTGATAGTAGCTATAGTACTAACGGTATTAATGACATTAAAGTCAACGCCTCAAATTACTGCGGAAACACGTGACTTAAGTATCTCA
TTTTTCATACTCAGCTTATTTATCATTATTATATTTGCTGTTTGGGATTTAATTATTAGAGTTAGAAAAGGAGCGTTTGCTTTTGTTGTAATA
AGGCCAATATTATTCAGTTGTTGGTTATTTATTCTGAATATGATTTTATCAAGATTAGTATAA

Gene: DUF600-locus6 (putative protein)

Contig: 02_NODE_3, position: 251791 to 252291, length: 501 nt, orientation: FORWARD

Perfect match to: (MW2-BA000033-[321557:322057], allele observed in CC1)

Sequence:

ATGACTTTCGAAGAAAAACTAAATGAAATGTACAACGAGATTGCGAATAAGATTAGCAGCATGATACCGGTAGAGTGGGCAAAGGTAT
ATGCAATGGCATATATCGATGAAGAATCTGGAGAAGTGTTTTTCAATTATACTGAACCAAGAAGTGATGAATTATTTTACTATACAAGCG
TGATAAAAAAGTATAATATTTCGAGATCAGGATTTATGAATTCAGTATATGAGTTGCATGATCAATTTGAAAAACTAAGAGAATTGTTTAT
AGAAGAAGGACATGAACCATGGACATCATGTGAGTTTGATTTTACAAAAGAAGGTAAATTGAAAGTATCTTTTGATTATATAGATTGGTT
AAATACAGAATTTGATCAATTGGGTCGTGAAAATTATTATATGTACAAAAAATTTGGGGTTATACCAGAAATGGAATATGAAATGGAAG
AAATTAAAGAAATCGAGCAATATATTAAAGAGCAAGATGAAGCTGAACAATAG

Gene: DUF600-locus7 (putative protein)

Contig: 02_NODE_3, position: 252302 to 252802, length: 501 nt, orientation: FORWARD

Perfect match to: (MW2-BA000033-[322068:322568], allele observed in CC1)

Sequence:

ATGACTTTCGAAGAAAAACAAAGTGAAATGTACAATAAAATTGCAAATGAGATTAGTGGAATGATACCGGTTGAGTGGGAAAAGGTAT
ATACAATTGCTTATGTAGATAATCAAGGTGGAGAGGTCATTTTTAATTATACTAAACCTGGAAGTGAAGAATTGAATTATTATTCAGATAT
TCCTAAAGATTGCAATGTCTCAAATGATATTTTTATGGATTTATGGATGAAAGTTTATCGAATGTTTGATGAGTTAAGAGAAACTTTTAAA
AAAGAAGGACATGAACCATGGACATCATGTGAATTTGATTTTACAAGAGATGGTAAATTGAAAGTATCTTTTGATTATATAGATTGGTTA
AATACAGAATTTGATCAATTGAGTCTTGAAAATTATTATATGTACAAAAAATTCGAGGTTATACCAGAAATGGAATATGAAATGGAAGAA
ATTAAAGAAATCGAACAATATATTAAAGAGCAAGAAGAAGCTGAAATATAG

Gene: DUF600-other (putative protein)

Contig: 02_NODE_3, position: 252813 to 253313, length: 501 nt, orientation: FORWARD

Perfect match to: (MW2-BA000033-[322579:323079], allele observed in CC1+CC6)

Sequence:

ATGACTTTCGAAGAAAAACTAAATGAAATGTACAACGAGATTTCGAATAAAATTAGTAGCATGATACCAGTAGAATGGGAAAAGGTATA
TACAATGGCTTATATAGATGATGGAGGAGGTGAAGTATTCTTTAATTATACTAAACCAAACAGCGATGAATTGAATTATTACACCGATAT
ACCTAAGGAGTATAACATTTCTGTGCAAGTATTTGATGATTTATGGATGGATTTATATGATTTGTTTGAGGAATTAAGAAATTTATTTAAA
GAAGAAGGACTAGAACCATGGACATCATGCGAATTTGATTTTACAAGAGAAGGTACATTGAATGTATCTTTTGATTATATTGATTGGGTT
AATACGGAGTTTGATCAATTAGGCCGTGAAAATTATTATATGTACAAAAAATTTGGGGTTATACCAGAAATGGAATATGAAATGAAAGA
AATTAAAGAAATCGAACAATATATTAAAGAGCAAGATGAAGCTGAAATATAG

Gene: DUF600-locus9 (putative protein)

Contig: 02_NODE_3, position: 253324 to 253824, length: 501 nt, orientation: FORWARD

Perfect match to: (MW2-BA000033-[323090:323590], allele observed in CC1+CC6)

Sequence:

ATGGCTTTCGAAGAAAAACTAAATGAAATGTACAATGAGATTGCGAATAAAATTAGCAGTATGATACCGGTAGAATGGGAAACGGTATA
TGCAATGGCTTATGTAAATGAAAGAAGTGGAGAAGTGTTTTACAATTATACTGAACCAAGCAGTGATGAATTATTTTACTATACGAGCGT
GTTAAATAAATATAATATACCAAGATCAGAATTTATGGACTCAGTATATGAATTATATAAGCAATTTGATAATTTAAGAGAATTGTTTATA
GAAGAAGGACTCGAACCATGGACATCATGTGAATTTGACTTTACAAGAGATGGCAAATTGAATGTATCTTTTGATTATATTGATTGGGTG
AATTCAGAATTTGGACCAATGGGAAGAGAACATTATTATATGTATAAAAAATTTGGAATTTGGCCTGAAAAAGAATATGCCATAAATTGG
GTTAAAAAAATAAAAGATTATGTTAAAGAGCAAGATGAAGCTGAACTATAG

Gene: DUF600-other (putative protein)

Contig: 02_NODE_3, position: 253835 to 254335, length: 501 nt, orientation: FORWARD

Perfect match to: (MW2-BA000033-[324112:324612], allele observed in CC1+CC6+CC395)

Sequence:

ATGACTTTCGAAGAAAAACTAAGTCAAATGTACAACGAGATTGCGAATAAGATTAGCAGTATGATACCGGTAGAGTGGGAAAAAGTATA
TACAATTGCTTATGTAGATGATCAAGGTGGAGAGGTCGTTTTTAATTATACTAAACCAGGTAGCGAGGACTTGAATTATTACACATATATC
CCTAGAGAGTATAATGTCTCTGAAAAAGTATTTTATGATTTGTGGACGGATTTATATAGATTGTTTAAGAAGTTAAGAAACGCATTTAAA
GAAGAAGATTTAGAACCATGGACATCATGTGAATTTGACTTTACAAGAGATGGTAAATTGAATGTTGTATTTGATTATGTTGATTGGATG
AATTCAGAATTTGGTCCATCAGGAAAGGAAAACTACTATATGTATAAAAAGTTTGGTGTTTTACCAGAAACGGAATATGAAATTAATAAA
GTTAAAGAAATCGAGCAATATATTAAAGAGCAAGATGAAGCTGAACTATAG

Gene: DUF600 (putative protein)

Contig: 02_NODE_3, position: 254857 to 255357, length: 501 nt, orientation: FORWARD

Sequence:

ATGACTTTCGAAGAAAAACTAAGTCAAATGTACAACGAGATTGCGAATAAGATTAGCAGTATGATACCGGTAGAGTGGGAAAAAGTATA
TACAATTGNTTATNTANATGATCAAGGTGGAGAGGTCATTTTTAATTATACAAAACCTGAAAGTGAAGATTTGAATTATTACACGGATAT
ATCTAGAGATTATAATATTTCAGAAGAAATATTTGATGATTTATGGATGAATCTTTATTACTTGTTTATGAATTTGAGGGATTTATTTAAAA
AAGAAGGTCTTGAACCATGGACATCATGTGAATTTGATTTTACAAGCGAAGGTAAATTAAAAGTTTCATTTGATTATATTGATTGGATAA
ATACAGAGTTTGATCAATTAGGCCGTGAAAACTATTATATGTATAAAAAGTTTGGTGTTTTACCAGAAATGGAATACGAAATGGAAGAAG
TTAAAGAAATCGAGCAATATATTAAAGAGCAAGATGAAGCTGAACTATAG

Gene: Q1Y4S0 (putative protein)

Contig: 02_NODE_3, position: 256478 to 256852, length: 375 nt, orientation: FORWARD

Perfect match to: (MW2-BA000033-[326247:326621], highly conserved allele)

Sequence:

ATGAAAAGAATATTGGTAGTATTTTTAATGTTAGCAATTATATTGGCAGGTTGTTCTAATAAAGGTGAAAAGTATCAAAAAGATATTGAT
AAAGTGTACAAAGAACAGAATCAAATGAATAAAATTGCCTCGAAAGTACAAAACACTATTAAAACAGACATTAAACAAGAAGACAGTAA
TACACATGTTTATAAAGATGGTAAAGTCATTGTTATTGGTATTCAATTATATAAAGATCGTGAAAAAATGTATTATTTCGCATATGAAATA
AAAGATGGTAAGGCAGAGATTAATAGAGAAATAGACCCAATTAAGTATATGAAAGACCATAAAGCAGATTATGAAGATGAAAATGTAG
AAGTGGAAAAAGATTAA

Gene: Q2G173 (putative membrane protein)

Contig: 02_NODE_3, position: 257002 to 257400, length: 399 nt, orientation: FORWARD

Perfect match to: (MW2-BA000033-[326771:327169], allele observed in CC1+CC7+CC445)

Sequence:

ATGGAAAAATCGATCAAAATAATGACAATAATAGGAATTGTTATTCAGGGTTTAGCAACGGTATTTAGTTTACTATTGATGGTTTTAGCA
GCATCAGGTGTAATGACTACAGATGTGTCAACAACAGTTAATGGTGAGGTTGACCCAGTTGATGCAGAAACAGCAGCAGCAATTTTCAC
TGTATTATTCTTATTCCTATTCATATTTGGAATCATTTCAATTATTTTAGGTGCAATCGGTATGTTTAAAGCATCTAAAAACAAAAAAATGA
GTGGTATATTGTTGATTATTGGAGCTGTAATAAGTGGTAACATAATTACATTTGCTTTATGGTTAGTCAGTGGTATTAAACTACTTACTAAT
AACAAGCCTAAAGATGAAATAAGCGACTTATCATAA

Gene: nirC-focA-L1 (formate/nitrite transporter, locus 1)

Contig: 02_NODE_3, position: 257650 to 258474, length: 825 nt, orientation: REVERSE

Perfect match to: (MW2-BA000033-[327419:328243:r], allele observed in CC1+CC445)

Sequence:

TTAATTATTTTTATAAAATTGATGATGATCATTCAAGTAAGCATAGAATAAACCTATAATGAGTCCTCCTCCAATATAGTTACCGATAAAAG
CCGCAGCGATATTCGAAATAGCTGGTATGAAGTGCAATGTATCAACTTGATAAATTAAACCACCCATAAATAAGCAACTGTTGTAAACGA
CGTGTTCATAACCCATAAAGGCGAATATGGTAACACCGAACATCATGACAAACATTTTTGCGAGTACATCGTCAATTTGCATGGCAATAA
CTAATGAAATATTGATAAAGAAATTGGCGAATATCGCTTTCATTAATATACTTACAAAACCAGTAGACAACGTTTTGTGCTCAATAACTGC
TGATAACTGATTTAACATATCTGGCGTCATTACATTTGAAAAACGCATGAAACTAAATAAAATGGCAGCACCTAAAATATTTCCTGCAAAG
CACAATAAAAATATTTTCAATACTCTAGTCGGTTTAATTACTTTATAATACAGGCCTACAGTAAAGTACATGAAGTTACTGGTTAGTAGTTC
GGAGTTTGTAAATAAAATGAGTACTAACGCAAAGCTGAATGTAATGGCACTGGCCATATTCACAATGCCTGGCGGTAAATCTGGTTCGT
GTGTTGCTTTAACTGATAATACGAAGACCGTAATAATCCCGATAATAAATCCTGCCATCATCGCGCGTAATAAATAACGTTTTAAATAAAC
GCTTTGTAATATATCTTTTGTTCTTATTGTTTCGACTACGTTATTTACCCAGTCGTCCCCATAAAATATTTTATCCCATTTAATATGTTTCTCC
TTCAC

Gene: brnQ2 (branched-chain amino acid transport system II carrier protein)

Contig: 02_NODE_3, position: 258713 to 260020, length: 1308 nt, orientation: REVERSE

Perfect match to: (MW2-BA000033-[328482:329789:r], allele observed in CC1+CC188)

Sequence:

TTAAGTTGTAGCTTGTTTCGGACGGCGTATAAATACATCGATTATGAAACCGATAATAGCAAAGAGCATGAATGGTACAAGCCAAGCTAA
ATCGATATCTGCTAAAGGTAACATCATAAACGATTTCAAAATAACACCGTGTAATAAGTTAAAACTATTTAGTATTTGTAAAATTGAAATA
ATCAATGTAATAACAGTTGCGAGTCGATAGGCCCAACTGAATCTGAATGTGCTAAACATGTTAGCAAATGATATCAGTACAAGTGCAATA
GACACGGGATATATTAATGTCAATAATGGGACAGCAATTTTTAAAATCATTTCTAAACCAAGTGTTGTAAATAAGAACCCTATGATAGAG
AAAATAAGTGCGAATATTTTATAAGAAAACTTAGGTACGTGTTTCTTAGTAAATGTGGCGCAAGCATTGACGAGTCCTATACATGTTGTT
AGGCATGCAAGGATAACCGTCATTCCAAATACGAGGTTACCGAACGAACCAAATACTCGTAATGAGTTGTACGTCAATATATCTGTACCA
TCTTTAAAGTTTCCTGGAGCTGTTGATGCCCCAACGTATGCAAGTGCAAAGTAAATCATTCCAAGTAATATGGCTGCAATAAGACCTGAA
AAGCAGACATATTTTAAAATTTTCATGCGATCTGTGAGGCCTTTAAACTTATAGCCATTGACAATGACAACGGAAAAAGCTAACGCAGCA
ACAAGATCCATTGTAAAATAGCCTTCCAAACTTCCTGAAATGAAAGGATGTGTTATATATTTATCCTTAGGTGCACTTAGTGCAGATTCAG
GGTTGAAAATGACAGCAATACTTAATAGAGAGACCATTAATAGTAATAACGGTGTTAATAATTTACCTAAATTATCAACGATTTTCGATG
GATTTAAACTAATCCAGTAAACGATGGCAAAAAAGATTGCTGCGAATATAATTAAAGTCCATTGGTTGTGCACAGGTAAAATGTGTCTTG
TACCAATTTCGTACGCGACATTTGCAGCACGTGGAATACCGTAAAATGCTCCGATAGACATGTAAATCACGACAGCAAAAATAAACCCGA
ACCATGGATGTATACGATTGCCTACACTTTCAACACCTTCATCATAAAATGCAACAACAATAACAGTAATAAAGGGGAGTAATATGCCTG
TAAGAGCAAAGCCTAGCATACCAATCCACATATTTTGACCCGCTGTATGGCCAAGCATGGGCGGGAATATTAAATTTCCGGCTCCAAAAA
ATAGTGAAAATAACATGAGGCCCGAAATAATAACTTGTTTTTCAA

Gene: Q99WS0-sapS (secreted acid phosphatase)

Contig: 02_NODE_3, position: 260604 to 261494, length: 891 nt, orientation: FORWARD

Perfect match to: (MW2-BA000033-[330373:331263], allele observed in CC1+CC50)

Sequence:

ATGAATAAAATTTCAAAGTATATTGCAATAGCATCATTATCGGTAGCGGTTACAGTTTCAGCACCACAAACGACAAATTCTACAGCGTTTG
CCAAAAGTTCTGCTGAAGTTCAACAAACGCAACAAGCTTCTATACCAGCATCACAAAAGGTGAATCTTGGTAATCAAAATATTATGGCAG
TGGCTTGGTATCAAAATTCAGCTGAAGCAAAAGCATTATATTTACAAGGTTATAACAGTGCGAAAGTTCAACTAGATAAAGAGATTAAAA
AGAATAAAGGTAAACATAAGTTAGCTATTGCTTTGGATTTAGATGAAACAGTTTTAGATAATTCTCCATATCAAGGCTATGCATCAATACA
TAATAAACCTTTCCCAGAAGGTTGGCATGAATGGGTACAAGCTGCTAAAGCTAAACCTGTCTATGGCGCAAAAGAATTCTTGAAATATGC
TGACAAAAAAGATGTCGATATCTACTATATTTCTGATAGAGATAAAGAAAAAGATTTAAAGGCAACACAAAAGAACTTAAAACAACAAG
GTATCCCTCAAGCTAAGAAGAGTCATATTTTACTAAAAGGTAAAGATGATAAGAGTAAAGAATCACGCAGACAAATGGTTCAAAAGGAT
CATAAACTTGTCATGCTATTTGGAGATAATTTATTAGACTTTACAGATCCAAAAGAAGCTACAGCTGAATCTCGTGAAGCATTAATTGAAA
AACATAAAGACGATTTCGGTAAGAAATATATCATTTTCCCTAACCCAATGTATGGTAGTTGGGAAGCTACAATTTACAACAATAACTATAA
AGCAAGTGACAAAGCAAAAGATAAATTACGTAAAAATGCTATTAAGCAATTCGATCCTAAAACAGGCGAAGTTAAATAA

Gene: Q2G168 (ABC transporter, transmembrane permease)

Contig: 02_NODE_3, position: 261744 to 262793, length: 1050 nt, orientation: FORWARD

Perfect match to: (M1216-AIYW01000019-[6032:7081], allele observed in CC445+CC1-ST291)

Sequence:

ATGTTTTTAGCTTGGAATGAAATACGGCGCAACAAATTGAAGTTTGGACTAATTATTGGTGTGTTAACGATGATTAGTTACTTGCTATTTT
TATTATCTGGATTGGCGAATGGTCTTATCAATATGAATAAAGAAGGTATTGATAAGTGGCAAGCAGATGCCATTGTTCTAAATAAAGATG
CCAATCAAACTGTGCAACAATCTGTTTTTAACAAGAAAGATATTGAAAATAAATACAAGAAGCAAGCTACTTTGAAGCAAACAGGGGAA
ATTGTGTCTAATGGCCATCAAAAAGACAATGTTTTAGTGTTCGGTGTTGAAAAGTCATCATTTTTAGTTCCGAGTTTAATAGAAGGGCATA
AAGCGACTAAAGATAATGAAGTGTTAGCTGATGAAACACTTAAAAATAAAGGATTTAAAATTGGCGACACATTATCACTATCTCAATCAG
ATGAAAAATTGCATATCGTAGGTTTTACAGAAAGTGCAAAATATAATGCGTCACCAGTCATTTTCACGAATGACGCTACCATTGCCAAGA
TCAATCCTAGATTGACTGGAGATAAAATTAATGCAGTTGTTGTACGTGATACAAATTGGAAAGACAAAAAATTAAACCAAGAGCTTGAA
GCGGTAAGTATTAATGACTTTATTGAAAATTTACCAGGTTATAAACCACAGAACTTAACATTAAACTTTATGATTTCATTCTTATTTGTCAT
TTCAGCTACAGTTATAGGCATTTTCTTATATGTCATGACATTACAAAAAACAAGTTTATTTGGCATATTAAAGGCTCAAGGATTTACGAAT
GGCTATTTAGCGAACGTAGTTATTTCGCAGACGCTCATATTAGCATTATTTGGTACGGCATTTGGCTTACTGTTAACAGGCGTTACAGGTG
CATTTTTACCTGATGCAGTACCTGTCAAATTCGATGTACTAACATTACTCGTATTTGCAATTGTGTTAATGATTGTCTCTGTATTAGGAAGT
TTATTCTCCATTTTAACAATTAGAAAAATAGATCCGTTAAAGGCGATTGGGTAG

Gene: A5IPH6 (ABC transporter, ATP binding protein)

Contig: 02_NODE_3, position: 262806 to 263483, length: 678 nt, orientation: FORWARD

Perfect match to: (MW2-BA000033-[332575:333252], allele observed in CC1+CC6+CC22+CC72-ST582)

Sequence:

ATGTTGAAATTTGAAAATGTAACAAAGTCATTTAAAGATGGGAATCGTAACATTGAAGCGGTTAAAGATACAAATTTTGAGATAAATAAA
GGTGATATTATAGCATTGGTTGGACCTTCTGGCTCTGGTAAAAGTACATTTCTAACTATGGCAGGTGCTTTACAAACGCCGACATCTGGG
CACATTTTAATCAATAACCAAGATATTACGACAATGAAGCAAAAAGCATTGGCAAAAGTTAGAATGTCTGAAATAGGTTTTATTTTACAA
GCTACAAACCTTGTGCCATTTTTAACGGTAAAGCAACAATTTACATTATTGAAAAAGAAAAATAAGAATGTTATGTCTAATGAAGACTATC
AGCAACTTATGTCACAATTAGGGCTAACTTCATTGCTTAATAAGTTACCTTCAGAAATTTCAGGTGGTCAGAAACAACGTGTGGCGATAG
CTAAAGCGTTATATACGAATCCGTCGATTATTTTAGCGGATGAACCTACCGCGGCGTTAGATACTGAAAATGCGATTGAAGTCATTAAAA
TTCTACGTGATCAAGCCAAACAAAGAAAGAAAGCATGTATTATTGTTACACATGATGAACGACTTAAAGCATATTGTGATCGTTCATATCA
TATGAAAGATGGCGTCCTTAATCTTGAAAATGAAACAGTAGAATAG

Gene: pfoR (putative

Perfringolysin O regulator protein)

Contig: 02_NODE_3, position: 263691 to 264722, length: 1032 nt, orientation: FORWARD

Perfect match to: (Strain_21269-AFTU01000045-[71492:72523:r], allele observed in CC121+CC1+CC121+CC398)

Sequence:

ATGGATTTATTGATAGGTACTTTATTTTTATTTTTGGTCTTAGTGATTTTTACATTATTTACATATAAAGCACCTAATGGTATGCGTGCCATG
GGAGCATTAGCTAATGCAGCAATCGCAACATTTTTAGTAGAAGCATTTAATAAATATGTTGGTGGCGAAGTATTCGGTATTAAATTTTTA
GAAGAGCTAGGAGACGCTGCGGGAGGTCTAGGTGGTGTCGCTGCCGCTGGATTAACAGCATTAGCTATCGGTGTGTCACCAGTATATGC
ATTAGTTATAGCAGCCGCGTGCGGTGGTATGGATTTATTACCAGGTTTCTTTGCGGGTTATATGATTGGATATGTGATGAAATATACAGA
GAAATATGTGCCGGATGGTGTCGACTTAATTGGATCGATTGTCATCTTAGCGCCATTAGCTCGTCTTATTGCAGTATTATTAACGCCAGTA
GTGAATAGTACATTGATTCGAATAGGTGATATTATCCAAAGTAGTACGAATACGAATCCAATTATCATGGGTATCATTTTAGGTGGTATTA
TCACGGTTGTCGGTACAGCGCCATTGAGTTCAATGGCATTGACAGCATTATTAGGTTTAACGGGTGTACCTATGGCTATTGGTGCCATGG
CAGCATTTAGTTCGGCATTTATGAATGGGACGCTATTCCATCGCTTAAAATTAGGTGATCGTAAGTCTACGATTGCAGTAAGTATTGAACC
TTTATCACAAGCAGATATTGTATCAGCCAATCCAATTCCAATCTATATTACAAATTTCTTTGGTGGTGCGATTGCTGGTTTAATTATTGCTA
TGTCAGGTTTAATTAACGATGCGACAGGTACAGCTACACCGATTGCAGGATTTTTAGTTATGTTTGGATTTAATCATCCGACGACAATTGT
GATTTATGGTGTAGTAATGGCGATTGTAGGTGCGCTTGCAGGTTATCTTGGTTCAATTGTATTTAAAAAATATCCAATTGTTACTAAGCAA
GACATGATTAATCGAGGTGCAGTAGACGCATAG

Gene: Q5HJ57 (PfkB family carbohydrate kinase)

Contig: 02_NODE_3, position: 265065 to 266183, length: 1119 nt, orientation: FORWARD

Perfect match to: (MW2-BA000033-[334834:335952], allele observed in CC1+CC772)

Sequence:

ATGAGCGATTCTGAGAAAGAAATTTTAAAAAGAATTAAAGATAATCCGTTTATTTCACAACGTGAACTTGCTGAGGCAATTGGATTATCT
AGACCCAGCGTAGCAAACATTATTTCAGGATTAATACAAAAGGAATATGTTATGGGAAAGGCATATGTTTTAAATGAAGATTATCCTATT
GTTTGTATTGGCGCAGCGAATGTAGATCGTAAGTTTTATGTGCATAAAAATTTAGTTGCAGAAACATCAAATCCTGTAACGTCAACACGC
TCTATTGGTGGCGTAGCAAGAAATATTGCTGAGAACTTAGGTAGGCTTGGCGAAACGGTCGCTTTTTTATCTGCTAGTGGACAAGATAGT
GAATGGGAAATGATTAAACGATTGTCCACACCATTTATGAATTTGGATCATGTTCAACAATTTGAAAATGCGAGTACAGGTTCATATACA
GCTTTAATTAGTAAAGAAGGCGACATGACATATGGCTTAGCAGATATGGAAGTGTTTGACTACATTACGCCTGAATTTTTAATTAAGCGT
TCACACTTATTGAAAAAGGCTAAGTGCATTATTGTCGATTTGAATTTAGGCAAAGAGGCATTAAACTTCTTATGTGCCTATACCACGAAAC
ATCAAATCAAATTAGTTATCACCACGGTTTCTTCTCCAAAAATGAAAAATATGCCTGATTCATTACATGCTATTGATTGGATTATCACGAAT
AAAGATGAAACAGAAACATACTTAAATTTAAAAATAGAATCTACTGATGATTTAAAAATAGCTGCTAAACGCTGGAATGATTTAGGTGTT
AAAAATGTTATTGTGACAAATGGCGTGAAAGAACTCATTTATCGAAGTGGTGAGGAAGAAATCATTAAGTCAGTTATGCCATCAAATAGT
GTGAAAGATGTTACAGGTGCAGGCGATTCATTCTGTGCTGCAGTAGTGTATAGCTGGTTAAATGGGATGTCTACTGAAGATATATTAATT

GCTGGTATGGTTAACGCAAAGAAAACGATAGAAACGAAATATACAGTTAGGCAAAACCTAGATCAACAGCAACTTTATCACGATATGGA
GGATTATAAAAATGGCAAATTTACAAAAGTATATTGA

Gene: psuG (pseudouridine-5'-phosphate glycosidase)

Contig: 02_NODE_3, position: 266158 to 267081, length: 924 nt, orientation: FORWARD

Perfect match to: (MW2-BA000033-[335927:336850], allele observed in CC1)

Sequence:

ATGGCAAATTTACAAAAGTATATTGAGTATTCTCGAGAAGTTCAGCAAGCACTGGAGAACAATCAACCGATTGTAGCATTAGAATCAACA
ATTATATCGCATGGTATGCCGTACCCACAAAATGTTGAAATGGCAACAACAGTAGAGCAAATTATCAGGAATAATGGTGCCATTCCAGCA
ACCATAGCCATTATAGATGGCAAAATTAAAATTGGTTTAGAAAGCGAAGATTTAGAAATACTGGCAACTAGTAAAGACGTTGCTAAAGT
ATCTAGAAGGGATTTAGCAGAAGTTGTTGCGATGAAGTGTGTTGGTGCTACTACTGTAGCGACGACGATGATATGTGCTGCAATGGCTG
GTATTCAATTTTTTGTTACAGGAGGTATTGGGGGCGTCCATAAAGGTGCAGAACATACGATGGACATTTCAGCAGACTTAGAAGAACTGT
CTAAAACAAATGTCACTGTTATCTGTGCAGGTGCCAAATCAATTTTAGACTTACCTAAGACGATGGAGTATTTAGAAACAAAAGGCGTTC
CAGTTATTGGATATCAAACGAATGAATTGCCAGCATTCTTCACTCGCGAAAGCGGTGTTAAGTTAACAAGTTCGGTTGAAACGCCAGAAC
GACTTGCTGACATTCATTTAACAAAACAGCAGTTAAATCTTGAAGGTGGCATTGTTGTTGCTAATCCAATTCCATATGAGCATGCCTTATC
AAAAGCATATATTGAGGCAATCATAAATGAAGCTGTTGTTGAAGCGGAAAATCAAGGTATTAAAGGTAAGGACGCCACACCGTTCTTGT
TAGGGAAAATTGTAGAAAAAACGAATGGTAAAAGTTTAGCAGCAAATATAAAACTTGTTGAAAACAATGCGGCGTTGGGTGCTAAAATT
GCTGTCGCTGTTAATAAATTATTGTAG

Gene: nupC2 (nucleoside permease C, locus 2)

Contig: 02_NODE_3, position: 267092 to 268312, length: 1221 nt, orientation: FORWARD

Perfect match to: (N315-BA000018-[356915:358135], allele observed in CC5+CC1)

Sequence:

ATGAATATTTTATTCGCTATCACAGGGATAGCATTTGCACTATTTGTTGCGTTTTTATTCAGTTTTGATCGTAAAAACATAGACTTCAAAAA
GACGTTAATAATGATATTTATTCAAGTGTTGATTGTGTTATTTATGATGAACACAACGATTGGTTTAACAATCTTAACTGCATTAGGCTCAT
TTTTTGAAGGGTTAATAAATGTTAGTAAAGCAGGTATAAACTTTGTTTTTGGAGATATACAAAATAAAAATGGCTTTACGTTCTTTTTAAA
TGTGTTGCTGCCATTAGTGTTCATTTCTGTATTAATAGGCATTTTTAATTATATTAAGGTATTACCATTTATTATCAAATATGTAGGTATCGC
TATTAATAAAATAACGAGAATGGGGCGCTTAGAAAGTTACTTTGCTATTTCAACAGCAATGTTTGGACAACCAGAAGTATATTTAACAAT
AAAAGATATTATTCCGAGATTATCTAGAGCGAAATTATATACAATTGCGACGTCTGGTATGAGTGCTGTTAGTATGGCAATGTTGGGTTC
ATATATGCAGATGATTGAGCCCAAGTTCGTAGTTACAGCTGTAATGTTAAATATTTTTAGTGCGCTTATCATCGCCAGTGTAATCAATCCC
TATAAATCTGATGATAGTGATGTTGAAATTGATAACTTAACTAAATCAACGGAAACGAAATCAGTGAATGGAAAAACAGGAAAACCTAA
GAAAGTTGCCTTTTTCCAAATGATTGGTGATAGTGCGATGGATGGGTTTAAAATCGCTGTTGTAGTAGCCGTAATGTTGTTAGCATTTATT
TCATTAATGGAAGCAATTAATATCATGTTTGGTAGTGTTGGTTTGAACTTTAAACAGCTTATTGGCTATGTGTTTGCACCAATCGCATTCTT
AATGGGGATTCCATGGAGCGAAGCTGTTCCAGCTGGCTCTTTAATGGCGACTAAATTAATTACAAATGAGTTTGTAGCAATGCTTGATTT
TAAAAATGTCCTGGGTGATGTATCAGCTCGAACACAAGGTATCATTTCAGTTTACTTAGTAAGCTTCGCTAATTTTGGTACGGTTGGTATC
ATCGTAGGTTCAATTAAAGGCATTAGTGATAAACAAGGAGAAAAAGTTGCATCCTTTGCAATGAGGTTGCTACTTGGTTCAACTCTAGCT
TCTATCATTTCAGGATCAATCATTGGCCTAGTATTATAA

Gene: nanT (N-acetylneuraminic acid transporter)

Contig: 02_NODE_3, position: 268418 to 269950, length: 1533 nt, orientation: REVERSE

Sequence:

CTACTTTTTCGTAGCCGTTTTTGAAATGTATGTTGATGGTTTATCTTTTTCAAAAATTGTTAATCCCGTTATATCTTTTTTATGTTTTGAAGG
GACAATGAAGCTAAGTATATAAGCAAAGACAAAAGCAACTGTAAATGAAATGGTAGATACATAGAAAGGTGAGTTACCTTTGCCAACAC
CATTATAGACATAAGCAAAGATGATACCCAATATTAATCCACAAATAACACCGAATGTATTCGTACGTTTAGTGAAAATACCAACTGCAA
ATACACCAGCCAATGGAACGCCGAATAATCCAGTCACAAACAAGAATAAATCCCATAAGTCATTTGAATTAGAAGCAATTAAGTATAGTG
ACATTCCAAAACCGAAAATACCTGCAATGATGATAATGAAACGTGCAAAGTTAACTTCGTGTCGCTCGCTACCTTTTCCGAAGAAGCGTT
GCTTAATGTCGATTGAAATACAAGCAGATATAGAATTTAAACTAGATGAAATGGTAGACTGTGCAGCGGCGAAAATGGCTGCAATAAGT
AATCCTGCTACAAATGGTGGCATCTCAGTCAAAATGAAATATGGCACTACAGATGATGTATTGAAGCCTTTTGGTAAAACAGCTTCATGT

GTATAAAATGAATACAGCATTGTACCCATACCATAAAATAAGGGGGCTGAAATTAAAGCTAGGATACCATTTGTCCATAACGATTTATTT
GTTTCTTTTAAACTATCAGAAGCTTGATAACGCTGCACGACGTCTTGACTCGCTGTGTATTGATACAAGTTGTTGAAAATATTTCCTAGGA
AAATAATTGGAATGGCAGCTGCCGCAGTATTTAGTTTCCAATTGTCTGCACTAATTAATTTTTTGTGCTCAATCGCATCTGCAAAGACAGT
GCCAAAACCGCCTTTAATGTTCATAACACCTAGAATAATGATAACTAAAGCGCCGCCTAATAAAATGACGCCTTGAATGAAATCACTCCA
AACCACACCTTCGAATCCACCTAAAAATGTATATAAAATACATAGTAAACCAACGAGTGATGCAACGATATAAGGGTTCATGTCTGATAC
AGATGTGATTGCTAATGTTGGTAAGTAGATAACGATTGCAACACGCCCTAAATGGTAAACGACAAATAATAATGAGCCAATAACACGTAT
GCTAGGGCCAAATCTAGCTTCTAAATATTCATATGCTGATGTTACTTTTAACTTTTTAAAGAAAGGGACATAGAAATAAATGAGTAATGGA
ATAATTGCGACGATAGCAATGTTACCAGCGATATATGACCAATCTGTTAAAAATGCTTTCTCTGGTGTCGACATAAATGTAATCGCACTTA
ACGTAGTAGCATAAATTGAAAAGCCAACTACCCAAGATGGCAAGCGACCACTTGCGGTAAAGAAACTATTGGTACTTTGGCTCGCGCGC
TTGGTAAAATAAATGCCAATGAACAACATAGCTAGTAGATAAATGATAACGGCAACCCAGTTTAGTGTGCCAAATCCAATTTCTTTCAT

Gene: nanK (N-acetylmannosamine kinase)

Contig: 02_NODE_3, position: 271029 to 271889, length: 861 nt, orientation: FORWARD

Sequence:

GTGTATTACATCGCAATCGATATTGGAGGCACTCAAATTAAATCGGCAGTTATTGATAAGCAATTGAATATGTTTGACTATCAACAAATAT
CAACGCCGGACAACAAAAGTGAGCTTATTACTGACAAAGTATATGAGATTGTAACAGGATATATGAAGCAATATCAGTTGATCCAACCTG
TCATAGGTATTTCATCAGCAGGCGTTGTTGATGAACAAAAAGGCGAAATTGTATACGCAGGGCCAACCATTCCGAATTATAAAGGTACTA
ATTTTAAGCGATTATTAAAATCACTGTCTCCTTATGTCAAAGTAAAAAATGATGTAAACGCTGCATTACTAGGCGAATTGAAATTACATCA
ATATCAAGCAGAACGGATCTTTTGTATGACGCTTGGTACAGGCATTGGGGGTGCGTACAAGAATAATCAAGGTCATATTGATAATGGTG
AGCGTCATAAGGCAAATGAAGTTGGGTATTTATTGTATCGTCCAACTGAAAATACAACGTTTGAGCAACGTGCTGCAACGAGTGCATTGA
AAAAGCGCATGATTGCCGGAGGATTTACGAGAAGCACACATGTGCCAGTATTGTTTGAAGCAGCTGAAGAAGGTGATGATATTGCAAAA
CAAATATTGAATGAGTGGGCAGAAGATGTAGCAGAAGGGATTGCCCAAATACAGGTCATGTATGATCCAGGGCTTATATTAATTGGGGG
CGGTATATCTGAACAAGGAGATAATCTCATTAAATATATCGAGCCGAAAGTTGCACACTATTTACCAAAAGACTATGTTTATGCACCAATA
CAAACGACTAAGAGTAAAAATGATGCAGCATTATATGGCTGTTTGCAATGA

Gene: nanR (repressor of nan gene cluster)

Contig: 02_NODE_3, position: 272166 to 272966, length: 801 nt, orientation: REVERSE

Perfect match to: (MW2-BA000033-[341935:342735:r], allele observed in CC1)

Sequence:

TTATTTGTTGTCTAGGATAATAGATTTAGTATGTTGATAAGTTTGACTCAGATTCGTATTTTCTAATAAATGATAGCTCACGATATCGATTA
AAAAGAGTGTCGCAATTTGTGTGTTGATAAATTGATGGTCGGTATTACGCGATTGATCCGTTGTTAAAAGTACTAAATCTGCACAATCTGT
AAGTTTGCTACCTTCGAAATTTGTGATGGCAACGACATATGCACCATGAGATTTGGCGACTTCCGCTGCAGAAATTAATTCCGAAGTATTA
CCACTATTTGACATAGCAATAAACATATCCGAATGAGATAGTAGGGATGCCGATATTTTCATTAAATGTGAATCGGTAGTAACATTACCTT
TTAGCCCCATACGAATCATACGATAATAAAATTCAGTCGCTGATAAACCAGAGCTACCTAGTCCAGCAAAGAGTATATGTCGACTTGATT
GAAGTTTGTCGATAAAGGTTTGGATAATGTCGTTATCAATAAATTCACCAGTTTGTTGAATGATTTGTTGATGATATTTATGAATTCTTTGA
ATAATTGGGCTATTTTCAATAACTGTCTCTGTCATTTCTTGTTGAATATTAAATTTTAAATCTTGGAAATTCTCATAATCCAGCTTATGACTA
AAGCGTGTCATCGTTGCTGGTGATGTACCAATCGCATGGGTTAAGGAGTTAATCGTTGAAAAGGCATCGCTATAACCATTTTGTCTTATAT
AATTGACGATGCGTTTATCAGTTTTTGTAAATAAATGTTGATAACGTTGAACACGATTCTCAAATTTCAT

Gene: nanE (N-acetylmannosamine-6-phosphate 2-epimerase)

Contig: 02_NODE_3, position: 273107 to 273775, length: 669 nt, orientation: FORWARD

Perfect match to: (MW2-BA000033-[342876:343544], allele observed in CC1)

Sequence:

ATGTTACCACATGGATTAATAGTATCTTGTCAGGCACTACCAGATGAACCATTGCATTCATCTTTTATTATGTCGAAAATGGCATTAGCTG
CGTATGAAGGTGGTGCTGTTGGTATTCGCGCAAATACTAAGGAAGACATTTTAGCAATTAAAGAAACGGTAGATTTACCAGTTATTGGCA
TTGTGAAACGTGACTATAATCACTCAGATGTTTTCATTACAGCAACGTCAAAAGAAGTTGATGAACTGATAGAAAGCCAATGTGAAGTCA
TTGCATTGGATGCAACGTTACAGCAACGTCCAAAAGAAACGTTAGACGAATTAGTATCATATATTAGAACACATGCACCGAACGTTGAAA
TCATGGCTGATATCGCGACCGTTGAAGAAGCTAAAAATGCCGCACGACTTGGCTTTGATTATATTGGCACGACGTTACATGGCTATACTA

GTTATACGCAAGGACAATTACTTTATCAAAATGACTTCCAATTTCTAAAAGATGTACTACAAAGTGTTGATGCAAAAGTTATTGCGGAAG
GTAATGTCATTACACCGGATATGTATAAACGCGTGATGGACTTAGGCGTTCATTGTTCAGTCGTTGGTGGTGCGATAACACGACCAAAAG
AAATTACGAAACGTTTTGTTCAAGTTATGGAAGATTAA

Gene: Q5HJ49 (nucleoside recognition membrane gate protein)

Contig: 02_NODE_3, position: 273906 to 275219, length: 1314 nt, orientation: REVERSE

Perfect match to: (CGS00-ABWS01000003-[271518:272831:r], allele observed in CC30)

Sequence:

TTATCCAAAAATAAGTAAAGCGACGGGGATGGTGATTAATAGCGACAATGCCACGCGTAAAAACCAAATGATGATGAGTTTCCAGACAG
GTATTTTAATTTCAGTTGCTAGTATACATGGCACTAATGCTGAGAAAAAGATAATGGCTGATACGCTTACTACACCGACGACAAATTTAGT
ACTCATTGCAGCTTTAGTTACTAACAAAGATGGTAGAAACATCTCTACAATAGAAATCGCTGACGCTTTTGCAAGTAAAGCCTGATCAGC
AATTGGGAAAATATAAATAAATGGATAGAAGATATAGCCAAGCCAATCAATGAATGGTGTATAGTTCGCTACAATCAGTCCTAAAAAAC
CAATCGATAATATAGAAGGCAAAATACCAACAGTCATTTCTAAACCGTCTTTCAAATTGTCCCAAACGTTCTTCACGAGGGATGGTGTTAA
TGCATTTTGTTTCATCGCCTCTGCATATGCAGTTTTCAGTCTGCTTCCTTCAATAGCAACTTCTTGTTCTCCTTCTTGTCCGTTATAATATTCT
GTTGATTCATTGGTGATTGGCGGTAGCCATGCAGTAATTGCAGTCACGACAAATGTGATGACTAAAGTTATCCAAAAGTATAAATTCCAA
TGCGGCATTAATCCTAAAGTTTTAGCAACGATAATCATAAAAGTTGCTGAAACTGTTGAAAAGCCAGTCGCAATAATCGTGGCTTCTCGTT
TGTTGTACATCCCTTGCTTATAGACACGATTAGTAATCAATAATCCTAAGGAATAACTGCCGACAAACGAAGCTACTGCATCGACAGCGG
ATTTTCCTGGTGTTTTAAAAATAGGTCTCATAATAGGCTCCATATAAACACCGACAAATTCTAATAAGCCATAGCCTACTAATAAAGAAAG
CGCAATTGCACCTACTGGAATTAAGATACTTAATGGCATCATTAATTTTTCAAACAAGAACGGACCATAGTTAGCTTTAAATAGTATTGAT
GGACCGATTTTAAATACATACATTATACCAATCATTGCACCTGCAACTTTAAATAATGTAATGACCAAGTTTGTGATTGAAGTCATAAAAG
TACGTCTCACTATTGGTAACGCTGTACCAATTAAAATCATAATCAGTGCAACATAGGGCATAAGTGGACCTATGATTGAGCGAATGGCTA
GATGAACATGATCGACGAAAATAGTGTTGTTACCATTAATCGTAAAAGGAATAAAGAAACATAGTATGCCCACTAAACTATAGACAAAA
AAACGCCATGCACTTGGTTGTTGTGCATTAGAATGATATTGATTCAT

Gene: lip2 (lipase 2)

Contig: 02_NODE_3, position: 275637 to 277709, length: 2073 nt, orientation: FORWARD

Perfect match to: (MW2-BA000033-[345406:347478], allele observed in CC1+CC20)

Sequence:

ATGTTAAGAGGACAAGAAGAAAGAAAGTATAGTATTAGAAAGTATTCAATAGGCGTGGTGTCAGTGTTAGCGGCTACAATGTTTGTTGT
GTCATCACATGAAGCACAAGCCTCGGAAAAAACACCAACTAATGCAGCGGTACAAAAAGAAACACTAAATCAACCGGGAGAACAAGGG
AATGCGATAACGTCACATCAAATGCAGTCAGGAAAGCAATTAGACGATATGCATAAAGAGAATGGTAAAAGTGGAACAGTGACAGAAG
GTAAAGATACGCTTCAATTATCGAAGTATCAATCAACACAAAATAGTAAAACAATCAGAACGCAAAATGATAATCAAGTAAAGCAAGATT
CTGAACGACAAGGTTCTAAACAGTCACACCAAAATAATGCGACTAATAATACTGAACGTCAAAATGATCAGGTTCAAAATACCCATCATG
CTGAACGTAATGGATCACAATCGACAACGTCACAATCGAATGATGTTGATAAATCACAACCATCCATTCCGGCACAAAAGGTATTACCCA
ATCATGATAAAGCAGCACCAACTTCAACTACACCCCCGTCTAATGATAAAACTGCACCTAAATCAACAAAAGCACAAGATGCAACCACGG
ACAAACATCCAAATCAACAAGATACACATCAACCCGCGCATCAAATCATAGATGCAAAGCAAGATGATACTGTTCGCCAAAGTGAACAG
AAACCACAAGTTGGCGATTTAAGTAAACATATCGATGGTCAAAATTCCCCAGAGAAACCGACAGATAAAAATACTGATAATAAACAACTA
ATCAAAGATGCGCTTCAAGCGCCTAAAACACGTTCGACTACAAATGCAGCAGCAGATGCTAAAAAGGTTCGACCACTTAAAGCGAATCA
AGTACAACCACTTAACAAATATCCAGTTGTTTTTGTACATGGATTTTTAGGATTAGTAGGCGATAATGCACCTGCTTTATATCCAAATTATT
GGGGTGGAAATAAATTTAAAGTTATCGAAGAATTGAGAAAGCAAGGCTATAATGTACATCAAGCAAGTGTAAGTGCATTTGGTAGTAAC
TATGATCGCGCTGTAGAACTTTATTATTACATTAAAGGTGGTCGCGTAGATTATGGCGCAGCACATGCAGCTAAATACGGACATGAGCGC
TATGGTAAGACTTATAAAGGAATCATGCCTAATTGGGAACCTGGTAAAAAGGTACATCTTGTAGGGCATAGTATGGGTGGTCAAACAAT
TCGTTTAATGGAAGAGTTTTTAAGAAATGGTAACAAAGAAGAAATTGCCTATCATAAAGCGCATGGTGGAGAAATATCACCATTATTCAC
TGGTGGTCATAACAATATGGTTGCATCAATCACAACATTAGCAACACCACATAATGGTTCACAAGCAGCTGATAAGTTTGGAAATACAGA
AGCTGTTAGAAAAATCATGTTCGCTTTAAATCGATTTATGGGTAACAAGTATTCGAATATCGATTTAGGATTAACGCAATGGGGCTTTAA
ACAATTACCAAATGAGAGTTACATTGACTATATAAAACGCGTTAGTAAAAGCAAAATTTGGACATCAGACGACAATGCTGCCTATGATTT
AACGTTAGATGGCTCTGCAAAATTGAACAACATGACAAGTATGAATCCTAATATTACGTATACGACTTATACAGGTGTATCATCTCATACT
GGTCCATTAGGTTATGAAAATCCTGATTTAGGTACATTTTTTCTTAATGGATACAACGAGTAGAATTATTGGTCATGATGCAAGAGAAGAA
TGGCGTAAAAATGATGGTGTCGTACCAGTGATTTCGTCATTACATCCGTCCAATCAACCATTTGTTAATGTTACGAATGATGAACCTGCCA
CACGCAGAGGTATCTGGCAAGTTAAACCAATCATACAAGGATGGGATCATGTCGATTTTATCGGTGTGGACTTCCTGGATTTCAAACGTA
AAGGTGCAGAACTTGCCAACTTCTATACAGGTATTATAAATGACTTGTTGCGTGTTGAAGCGACTGAAAGTAAAGGAACACAATTGAAA
GCAAGTTAA

Gene: A5IPQ2 (esterase/lipase-like protein)

Contig: 02_NODE_3, position: 277951 to 278778, length: 828 nt, orientation: REVERSE

Perfect match to: (MW2-BA000033-[347720:348547:r], highly conserved allele)

Sequence:

TTACATCATTGTTATAGCGTTTAAGAAATCAACAACTTTACGATAAATAGTGATTGCTTCGTCATTAGGTCTACGATCAAAATCATGCTCGT
TTTTATTCACGCGTTCAAATGTTGAATGTGGAACATGATTCATGATATGTTCGCTTTCCTCAACGGGAACATCATAATCGCCATTACAATGC
GCAATGAAAACAGGTGGAAGTGTTTTAAGTTCATCTGGTGCAATATTATATTTTGAATCAGTATAATCAGCAATGTTAATCATATTTATCC
ATTTACCTGTGCCACGTGCATAAACGTAGATTAAAAAACGTTGTGCGATTTGATCTTGAACAACCGGTGTTGGTGAAGTGAGTTGTGCAA
TCATTGTTTCGTTTACGCTTTGAGCTATTTTTGCGTAATAACTATTAGTTGTTTTAAAAGGTTCAGTGTTGATGCGACTATAACCATAAAAA
TCAATAACACCATCAATATCTCTGTCTCGTGCAATTAATAGACTTAAATATGCACCTGATGATCTGCCAAAGGTAAAAATAGGGCAATTAG
AATATTGTGATTGAATCGCATCGAATGATGCGTAGACATCCTCAATAATGCAATCGAGACTTACTTCTGGTAATAAACGATAACTTAGTTG
AATTAAATCGTAATGTTCCGTAAGGATATCGATATACTGTGGGGATAAATCGTTAGCTTTACCGAACATTAATCCACCACCGTGGATGTA
GACAATAGCGCCTTTTGTTGGTTGATTTTTTGCTTTAATAATTGTGTAAGGTAATGCAAATGCATCTTTAGTAATTACTTTATCTTTAATTTC
AGTCAC

Gene: Q6GJZ4 (putative NADH:flavin oxidoreductase)

Contig: 02_NODE_3, position: 278851 to 280050, length: 1200 nt, orientation: REVERSE

Perfect match to: (MW2-BA000033-[348620:349819:r], allele observed in CC1+CC8)

Sequence:

TTAAGACGATGAGTCATGATAATTCTGTTCCAATTGACGTAAAGCGTCACGGGTATGCTTCTTTAGACCTTCCCCATAATCCATCATTTTAA
CAATATCTTTAAAAGCAGCATGTGGAATGGCTAAATCTTCTAAATCTGCCATAGAAAATTCAAGATTGATATCATGTGGTCGCTGTTCAGC
AAGTTTATGCACAAAGTCAGGTTCTGTGACAAAAGGCGAAGACATGCCGACCATATCTGCATGTTGTAAAGCATCTAAAGCAGACTCTG
GAGAATTAATCCCGCCACTTGCAATTAAAGGGATACGACCTGCTAAATGTTCATAGACAATTTGGTTAACTGGTCGACCGAAATGATCAC
CTGGTGTACGAGACGTATTTTGATAAATATGTCGACCCCAGCTAGCGATTGCTAAGTATTGGATGTTTGAAACGTCCATGACCCAATCGA
TTAATTGGTTGAACTCGTCAATGGTATATCCTAAATCACTGCCTCTGGTTTCTTCTGGCGTTGCTCGAAATCCTAAAATAAAATTGTCAGGT
GCTTCTTTATCAATCACTTCTTGTACCGCACGCATAACTTCTAAACATAATCTTGCACGATTTTTTAATGAGTCGGCACCGTAATGGTCTGT
ACGTTTATTCGAAAAAGTTGAGAAAAATGTTTGAATCAGCAAACGTTGTGCAATCGAAATTTCCACACCATCAAAACCTGCTTTAATCGCG
CGTAATGTAGCATCGCGATACTGCTGAATGATGCTATTGATTTTCTCATGAGACATGGCGATAACATCGTGTTCAATCGGTGAATGCAAT
GTCATAGGGCTTGGTCCATACACCTTTCCAAAATTTAAAATGGCTTGATTTGAAAAACGACCAGCATGCGCTAGCTGGATAATAGCGAGG
CTACCATGTTGTTTCATCGTAGATGCCATGTTAGTTAATCCAGGGATACAAGCATCATGATCAATATTAAAGCCATATTCAAACAATTGAC
CATAAGGTTCAATGTAAGCAGCGCCGGTGACTTGCATTCCAGCTGAATTAGAGCGACGTGCAGCATAAGCCAAGTCTTCTTTTGTAATAT
AGCCTTCTTTTGTTGATGTGTTTACGGTCATTGGTGATAATACAAAGCGATTCGAAATTTTGATGCCATTAGGTAAGTGGATTGATTGTAA
AAGTGGTTTGTATCGGTACAT

Gene: limB1 (putative monooxygenase locus 1)

Contig: 02_NODE_3, position: 280352 to 281353, length: 1002 nt, orientation: FORWARD

Perfect match to: (COL-CP000046-[401051:402052], highly conserved allele)

Sequence:

TTGGTTAAATTAAGCGTATTAGACTATGCCTTAATAGATGAAGGTAAGGATGCACAAAAGGCATTGCAAGATTCAGTGACACTTGCAAAA
TTAGCAGATCGACTTGGCTTTAAGCGAATTTGGTTTACGGAACATCATAATGTACCAGCGTTTGCGTGTAGTAGTCCAGAACTTTTGATGA
TGCATACATTGGCGCAGACAAATCACATACGAGTTGGCTCTGGTGGTGTGATGCTGCCGCACTATCGACCTTATAAAATTGCTGAGCATT
TTAGAATGATGGCAGCGTTATATCCAAATCGTATTGATTTAGGTATTGGTAATAATCCAGGTACTACTATGGTAAAGCAAGCTTTAGATG
GAATAAATCCTACATATGATAGTTACGATGAATCGATTTCGTTATTACGTGATTATCTTACAATAAAGGATAAACCAAGTGCGCATACGTT
AGGTGTCCAACCACACATTGATCATTTTCCAGAAATGTGGTTATTAAGTAGTAGCGCAACATCTGCCAAAATAGCTGCCGAACTAGGTAT
AGGGCTTTCTGTTGGAACATTTTTGCTACCAGATATAAATGCGATACATGCAGCGAAGGATAACATTGATATTTACAAAAAACATTTCCAA
GCATCAACGATTAAAATGGACGCAAAGGTGATGGCATCTGTATTTGTCATTGTAGCTGATAACGAAGCGGAAGTAGCAGCATTACAACA
TGCCTTAGATGTTTGGTTATTAGGTAAATTACAATTTGCAGAATTTGAAGATTTTCCTTCAGTAGACACAGCACAAAAGTATAAGCTTAAT

GATCGAGACAAAGAGATGATTCAAGCACATCAAGCACGCATCATTGCAGGTACACAAGAAAAGGTTAAAGCACAATTAGATGATTTCAT
TGCTACGTTTGAAGTTGATGAGGTGTTAGTAGCACCGCTTATTCCAGGTATTGAACAGCGTTGTAAAACATTAAAATTACTCGCGGAAAT
TTATTTGTAG

Gene: gcvH1 (glycine cleavage system H protein, locus 1)

Contig: 02_NODE_3, position: 281387 to 281719, length: 333 nt, orientation: FORWARD

Perfect match to: (N315-BA000018-[371211:371543], highly conserved allele)

Sequence:

ATGAAAAAGTTAGCCAATTATTTATGGGTAGAAAAAGTAGGAGATTTGTATGTGTTTAGTATGACACCTGAATTGCAAGATGATATTGGG
ACAGTAGGTTATGTTGAATTCGTAAGTCCAGATGAAGTTAAAGTGGATGATGAAATTGTGAGTATCGAAGCATCGAAAACGGTCATTGA
TGTGCAAACGCCATTGTCAGGAACGATTATTGAGCGAAATACAAAAGCGGAAGAAGAACCGACAATTTTAAACTCTGAAAAACCAGAAG
AAAATTGGTTGTTCAAATTGGATGATGTCGATAAAGAAGCATTCCTAGCATTACCGGAGGCTTAA

Gene: UPF0189 (macro domain protein)

Contig: 02_NODE_3, position: 281720 to 282520, length: 801 nt, orientation: FORWARD

Perfect match to: (MW2-BA000033-[351489:352289], allele observed in CC1+CC8+CC4803)

Sequence:

ATGGAAACGTTAAAATCAAATAAAGCGAGACTTGAATATTTAATCAATGATATGCATCGAGAGAGAAATGACAATGACGTATTGGTAAT
GCCATCTTCATTTGAAGATTTGTGGGAATTATATCGAGGCTTAGCAAATGTCAGACCGGCATTACCTGTAAGTGATGAATATTTAGCTGTA
CAAGATGCTATGTTAAGTGATTTGAATCGTCAACATGTTACGGATTTGAAGGATTTGAAGCCGATAAAAGGTGACAATATCTTTGTTTGG
CAAGGTGATATCACGACGTTAAAAATCGATGCTATTGTTAATGCTGCAAATAGTCGTTTTCTAGGATGTATGCAAGCTAATCATGACTGC
ATTGATAATATTATTCATACAAAAGCGGGTGTTCAAGTTCGACTTGATTGTGCAGAGATCATTCGACAACAAGGGCGCAATGAAGGTGTA
GGTAAAGCCAAAATAACACGTGGATATAATTTGCCAGCAAAGTATATAATTCATACGGTTGGTCCGCAAATACGTCGATTGCCTGTTTCA
AAGATGAATCAGGACTTGTTAGCTAAATGTTATCTTAGCTGTCTTAAATTGGCTGATCAACATAGTTTAAATCATGTCGCTTTTTGCTGTAT
ATCTACAGGTGTATTTGCTTTTCCTCAAGATGAAGCAGCAGAAATTGCTGTTCGAACAGTAGAAAGCTATCTCAAAGAAACAAATTCAAC
ATTGAAAGTCGTGTTCAATGTATTTACAGATAAGGATTTACAACTGTATAAGGAGGCATTTAACCGTGATGCAGAGTAG

Gene: sir2 (NAD-dependent protein deacetylases, SIR2 family)

Contig: 02_NODE_3, position: 282510 to 283454, length: 945 nt, orientation: FORWARD

Perfect match to: (MW2-BA000033-[352279:353223], allele observed in CC1+CC8)

Sequence:

ATGCAGAGTAGTAAGTGGAATGCAATGTCTCTGTTAATGGATGACAAGACAAAGCAGGCTGAAGTATTGCGTACTGCGATTGATGAAGC
AGATGCGATAGTGATTGGAATTGGTGCAGGCATGTCTGCATCTGACGGATTTACATATGTAGGAGAGCGTTTTACGGAAAATTTCCCAG
ATTTTATTGAAAAATATCGCTTCTTTGATATGTTGCAAGCGAGTTTACATCCTTATGGCAGTTGGCAAGAGTATTGGGCATTTGAGAGTCG
TTTTATTACATTAAACTATTTAGATCAACCTGTAGGTCAGTCTTACCTCGCTTTAAAATCCTTGGTGGAAGGTAAACAGTACCACATTATAA
CTACGAATGCAGATAATGCTTTCGATGTAGCTGATTATGATATGACTCATGTATTTCATATACAAGGGGAGTATATACTGCAACAGTGTA
GTCAGCATTGTCATGCTCAAACGTATCGCAATGATGATTTAATTCGTAAAATGGTTGTTGCGCAACAAGATATGCTTATACCTTGGGAGAT
GATTCCAAGATGTCCAAAATGTGATGCCCCAATGGAAGTGAATAAACGTAAAGCGGAAGTTGGGATGGTTGAAGATGCTGAATTTCATG
CGCAACTACATCGTTATAATGCTTTTCTAGAGCAACATCAAGATGATAAAGTGTTGTATTTGGAAATTGGAATTGGTTATACTACACCACA
ATTTGTGAAGCATCCTTTTCAGCGTATGACACGTAAAAATGAAAATGCACTTTATATGACGATGAATAAAAAGGCATATCGCATTCCGAA
TTCAATTCAAGAACGTACCATACATTTAACTGAGGATATCTCAACATTGATTACAACAGCACTCCGGAACGATAGCACAACGCAAAATAA
CAACATTGGAGAGACAAAAGATGTACTTAATAGAACCGATTAG

Gene: lplA1 (lipoate-protein ligase A, locus 1)

Contig: 02_NODE_3, position: 283432 to 284454, length: 1023 nt, orientation: FORWARD

Perfect match to: (MW2-BA000033-[353201:354223], allele observed in CC1)

Sequence:

ATGTACTTAATAGAACCGATTAGGAATGGAGAATATATTACTGATGGTGCGATTGCACTCGCTATGCAAGTTTATGTTAATCAGCATATCT
TTTTAGATGAAGATATTTTATTCCCTTATTATTGTGATCCAAAAGTGGAAATTGGACGTTTTCAAAATACTGCTATAGAAGTGAATCAAGA
TTATATAGATAAACACAGTATTCAAGTAGTTCGCCGAGATACTGGTGGTGGCGCTGTGTATGTTGATAAAGGTGCCGTTAATATGTGTTG
TATTTTAGAACAAGACACTTCAATTTATGGTGATTTTCAACGATTTTATCAGCCAGCTATAAAGGCGTTGCATACATTAGGTGCAACAGAT
GTGATACAAAGCGGTAGAAATGATTTAACATTGAATGGTAAAAAAGTGTCAGGCGCCGCAATGACATTAATGAACAATCGTATTTATGG
CGGTTATTCGCTATTACTTGATGTTAATTATGAAGCAATGGATAAAGTGTTAAAGCCTAATCGCAAAAAGATTGCATCGAAAGGGATTAA
ATCTGTGCGCGCACGTGTTGGTCATCTTAGAGAAGCACTGGATGAAAAGTATCGTGGTATAACCATTGAAGAATTTAAAAATTTAATGGT
GACACAGATTTTGGGAATCGATGACATTAAAGAGGCAAAACGATACGAATTAACGGATGCAGATTGGGAAGCGATTGATGAATTAGTT
GATAAAAAGTATAAAAATTGGGATTGGAATTATGGCAAGTCACCCAAATATGAATATAATCGAAGTGAAAGATTATCATCAGGTACTGT
AGATATTACCATTTCTGTTGAACAAAATCGTATTGCAGATTGTAGAATTTTTGGTGATTTCTTTGGACAAGGTGATATAAAAGATGTGGAA
GAAGCATTACAAGGAACAAAAATGACAAGAGAAGATTTAACGCATCAGTTAAAGCAATTAGACATCGTTTATTATTTTGGTAATGTTACG
GTTGGATCATTGGTTGAGATGATTTTAAGTTAA

Gene: Q5HIW6-v1 (putative NAD-dependend oxidoreductase)

Contig: 02_NODE_3, position: 284770 to 285795, length: 1026 nt, orientation: FORWARD

Perfect match to: (MW2-BA000033-[354539:355564], allele observed in CC1+CC5+CC7)

Sequence:

ATGAATAATAAAGTATTAGTAACCGGTGGTACAGGGTTTGTTGGCATGCGAATTATTTCACGATTATTAGAACAAGGTTATGAGGTACAA
ACAACGATACGTGATTTAAGTAAAGCTGATAAAGTAATTAAAACAATGCAAGACAATGGCATTTCCACAGAGCGATTAATGTTTGTCGAA
GCGGATTTATCACAAGATGAACATTGGGATGAAGCAATGAAAGATTGTAAATATGTCTTGAGTGTAGCATCTCCGGTGTTTTTCGGTAAA
ACAGACGATGCAGAAGTGATGGCGAAGCCTGCCATTGAAGGCATACAACGTATTTTAAGAGCTGCAGAACATGCTGGCGTGAAGCGTG
TGGTGATGACTGCAAACTTTGGTGCAGTTGGTTTTAGCAATAAAGATAAAAATTCAATCACAAATGAAAGTCATTGGACAAATGAAGATG
AACCAGGCTTATCAGTATATGAAAAATCAAAATTGTTAGCTGAAAAGGCAGCGTGGGATTTTGTTGAGAATGAAAATACAACAGTAGAA
TTTGCCACAATCAATCCAGTTGCAATTTTTGGGCCATCATTAGATGCACACGTTTCAGGAAGCTTTCATTTATTAGAAAATTTATTGAATGG
TTCAATGAAACGTGTACCGCAAATTCCATTGAATGTTGTTGATGTGAGAGACGTAGCTGAACTACACATTTTGGCAATGACAAATGAACA
GGCTAATGGCAAGCGATTTATTGCTACAGCTGATGGACAAATTAATTTGTTGGAAATTGCAAAATTAATTAAAGAAAAGAGACCTGAAAT
AGCTCAAAAAGTTTCTACTAAAAAATTACCAGACTTTGTTTTGAGTCTAGGTGCTAAATTTAATCATCAAGCTAAAGAAGGTAAACTTTTA
TTAGATATGAATCGAAATGTAAGTAACGAACGTGCAAAAATACTTCTTGGTTGGGAACCGATTGCGACACAAAAAGAAGCAATTTTAGC
AGCTGTCGATAGTATGGCTAAGTATCATTTAATATAA

Gene: ulaA (putative sugar-specific permease, SgaT/UlaA)

Contig: 02_NODE_3, position: 285888 to 287234, length: 1347 nt, orientation: REVERSE

Perfect match to: (JH1-CP000736-[412562:413908:r], allele observed in CC5)

Sequence:

CTATTGCTGTGGCTTTTGACGGCCTTTTAATAAAATTGCTGTCGCACCTACGATAATAATAAATAGAATCGCACCAAATAATCCCATATATT
TTACTGCGTTACCGAACACGATACCGACAGTTAAAAAGTCTGTATCTGAGAATGTTGTTGCAGCACCACCTAATTCGCCTAAAAATGGCA
AGAATAATAATGGTAAAAACGTGATTAGGATACCATTTAGAGCGGCGCCAGCAACAGCACCTTTAATACCGCCTCTTGCATTACCGAATA
CAGCAGCCGTTGCACCTAAGAAGAAGTGTGCAACTACGCCAGGTAAAATGACGACGCCACCAAATAAGAATAAGATAAACATACCGATG
ACACCTGTAATAAAGCTGACAAAGAATCCAATTAATACTGCATTTTGTGCATAAGGGAACACAATAGGGCAGTCTAATGCAGGTTTAGAA
TTTGGTACAAGCTTTTCAGAAATTCCTTTAAATGCTGGGACGATTTCAGCTAAGATTAAGCGAACGCCCGTTAAAATAATAAATACACCAG
CAGCAAATGTCACACCTTGAATTAATGAAAAGACAATAAAGTTTTGACCATTACTAATAGACTCGTGTACATAACTAACGCCCGCAAATA
AGCATGCGATGAAGTAAAGTAATGCCATCGTAATCGAGATACTAATTGTACTTTCTCGTAAGAAACTTAAGCCTTTTGGAAATTTAATCTC
TTCCGTTGATTTAGACTTACCTTTGAATAATTGACCTACAGCACCTGCGGCAAAGTAACTGATTGAGCCAAAATGACCTAAAGCTACTTGG
TCATTCCCTGTAATTTTTCGCATCGTAGGTTGGAGTAATGCAGGTAATACTGCCATGATTAATCCTAATACGAGTGCGCCGATAACAATCG
TTAGCCAGCCTTTAATATGACTGACTGTTAAAATGATTGCTAAAAACGCAGCCATGTAAAATGTATGATGACCTGTTAAAAAGATATATTT
TAAATTAGTGAAGCGGGCAATTAAAATATTAACAATCATGCCACAGACCATGATGAGTGCAGCTGTTGTTCCAAAATCTTTTAAGGCTAG
TGAGACGATAGCTTCGTTGTTAGGTACGATACCTTGCACACCAAATGCGTGTTGGAATATTTTGCCGAATGGTTCAAGAGATCGAACGAC

GACATCAGCACCTGCACTTAAAATTAAGAAGCCTAATATCGTTTTAATGGTTCCTGAAGTGATCGTTGCGGCAGGTTTTTTCTGAACGATT
AAACCTATAAAGGCAATCAGTGCAACAAGAATGGCTGGTTGACTTAAAATATCGACTATAAAATTAAGGATTGCTTGCAT

Gene: Q5HIW4 (putative phosphotransferase system protein)

Contig: 02_NODE_3, position: 287249 to 287533, length: 285 nt, orientation: REVERSE

Perfect match to: (MW2-BA000033-[357018:357302:r], highly conserved allele)

Sequence:

TTAAATCATGTTAAGTTGTTGTAATTTTTCTGAGAGCTTTTGTTGTAATTCAGCTTTATCTAAAATATTATCAAGAACTAAGACATCCCCTA
GACGTTCGGCATTTTCAGCTAAATCTCTACCACAAATAAACAAGTCAGCCATCTCTGGACTTGCTGTCATAATGTCACTATGTTCAACTTCG
ATATCAGATGGTGCATTAAGTTGCCTAAGTGCTTCTTGTGCGTTCATTTCTACCATAAAACTACTTCCTAAACCGTGGCCACATACTACTAA
AATTTTCAT

Gene: Q5HIW3 (putative phosphotransferase system protein)

Contig: 02_NODE_3, position: 287535 to 287978, length: 444 nt, orientation: REVERSE

Perfect match to: (MW2-BA000033-[357304:357747:r], highly conserved allele)

Sequence:

TTAATCATGCTCCTTTAAAATGTTTTTAATGTCTTGTGCATTTGTTGCAGTTAATAGTTGCTGGACTGTTTGGTTATCGCCCAGTACGGTTG
CTAAATTTTGTAATACAGATAAGTGTGAATGATTGTCGATGGCACTCAATACAAAAATGAGAGATGCGTAGTGATCTTCATCACAAAATG
CCACATGTTGATTCAACTTTAATAGACTTAAACCAACTTGATGTACGTCATTGTTCGGTCTTGCATGTGCAATTGCAATTTCAGGTGCGATA
ACGATATAAGGTCCAAGTTCATTAACGCTATCAATCATTGCTTGAACATAGCCTTGTTCAATAATTTGTTCTTGTAGTAATGGCTGAGAAG
CTATAGTTATAGCTTCAGTCCAATCATTTACTTGTTCTTTTACAATGATGCGTGTTGTTGACAAAATGTCTAATGACAC

Gene: A8YZF7 (transcriptional antiterminator, BglG family)

Contig: 02_NODE_3, position: 287983 to 289938, length: 1956 nt, orientation: REVERSE

Perfect match to: (N315-BA000018-[377808:379763:r], highly conserved allele)

Sequence:

TTAAGCCTCCTTTGTCATAGTTAAAGCAATGTGTTGTTTAATTTTAAAAATATTCCCATCTAAGAAATCTTGTCGATATAAGTCGTTGCTTA
AGCATTCGCTTAACTGTCCCAATGCCTTTAAATGTGCATTGGGGTGGTCCGTTGCTAATGTAATTACAAGGTGAACGGGATCGTTAGCTTT
ACTACCAAAGATAATCCCTTCAGTGAAATATGTTAGTGCGAAACCTACACCATTCTGTACATAATCAGTACCAGCGTGAATAAGTGCAATA
TGTGGACTAATGACCATATATGACCCGAATTGTTCAAATTGTTTTAAAATTGCAGCTGTATAATTTGAATAGACAATGCCATCATTGATTA
AAGGTTGCACAGCCACTGCAATTGCGGATTCAATTGATAATGGTTGTTTATTTATAATGATGCGATGTTCAGGCAATAAATCTGCGAGTG
ACTTGCCATCAGTTGCCATTTTCATGACTCGTTGTTCTCTTGAGTCATTGATAATTTGATTCAATTTTTGACGAGATTGTTGATTGATAAAT
GGATCGACATGAATAACTGGTACAGCTGATATTTCACAAGGTACTGTTGAAATGACATAATCAATGTTATCTTGCAATAATCGACTTTCTT
CCAATTGATAAATGGAATAGGCATCCCAAATGTGAAACTCAGGATACAGGTGATTTAGTTTTGATTTTAAAAGTTGTGACGTGCCTATAC
CAGAACCACATAGTAAGACAACCTTAATCATTGATTGTTTATGTGTTGCAACACGCTCTATACTTGATGCGAAGTGAATTGTAATGTATGT
TAATTCATCTTCGTTGAAGCGAATAGCAGCATCTTGTTCAATTGGACTAATATGCTTGCTAACGGCTTCAATGATTTGAGGATAGCGACGC
ATAACTTCTTGCCTCAAAGGATTAGGTTGTAGCATATCGTATTTAATACGATGTATAGCTGGTTTGATATGTGTGATCAGACTGGTATGTA
ACTTGTTGTCTTTTGACATATCAATGCCTAATTCTTGGCTAACACAAGTGATCAATTCATGTATATTTTGCGATAAATCATGGTATTCAAAG
GTAATTGAAGATGCTGTATGTTCAGTCATTTTAGAGCCTAGTAAATGTAACGTGATAAAGATAATTTCAGACTCTGGAAATGTGACATTA
CAACTGCGTTCTAAGTTTTCTATCATTTTTGAAGCAATAGCATACTGATTAGTATGTCGCCATTTATCAATTTCATTGATAGGTATATCGAA
CGAAAAATTTTCATTTAAACGCTGAATGGCAATGAGTATATGATAGATTAAGCCATCGATAGCCGACTGAACTAAATGATAATTTTCACTA
TTTAATGTCTTAATAATGGCACGGCGAACCAATGCGATTGATTCTGAATTAAAGATATCCGCCTCTATAAAAGGTGCAGCTTGTTTCATAT
ATTGATGTATAAAGTGTGCATACGCTTTACGATAATGATCTTCCTCACCAATAATATTGAATCCTTTATTGTGGACATAATTTAACTTTAAA
TGGTATTGATCTAGTTGGGCTTGAATCATTTTAATATCATCTGCAATTGTCCGACGCGAAACATTAACATCTTGCGCAAGTTGCTTTGTTGA
AACAGGATCGGTTGTTTCGAATAACTTTAAAGCGATATGTGTGAGTCGTTCATCTTTTGAAAAATGAATTTGATTTGTTAGATTGTGCTCT
AATTCATTTAATAGTGTCGCGTGAGCTGTTGTTACTTTGATGCCTGCAGCTTTATTACGGCTGACTTGGTAATGATAAGTTTCAGCATATT
GCTCAATATATGCTATATCATATTGAATGGTACGAGGTGATACACCAAGTTGATTAGCAATGGTATTGATTGGAATAAACGTTTGCTCAT
GAATTAAAAGATACAAAATTTCGATTTGTCTATAACTTAACAA

Gene: mepR (MATE regulatory protein)

Contig: 02_NODE_3, position: 290150 to 290569, length: 420 nt, orientation: FORWARD

Perfect match to: (N315-BA000018-[379975:380394], highly conserved allele)

Sequence:

ATGGAATTCACTTATTCGTATTTATTTAGAATGATTAGTCATGAGATGAAACAAAAGGCTGATCAAAAGTTAGAGCAATTTGATATTACAA
ATGAGCAAGGTCATACGTTAGGTTATCTTTATGCACATCAACAAGATGGACTGACACAAAATGATATTGCTAAAGCATTACAACGAACAG
GTCCAACTGTCAGTAATTTATTAAGGAACCTTGAACGTAAAAAGCTGATCTATCGCTATGTCGATGCACAAGATACGAGAAGAAAGAATA
TAGGGCTGACTACCTCTGGGATTAAACTCGTAGAAGCATTCACTTCGATATTTGATGAAATGGAACAAACACTCGTATCGCAGTTATCTG
AAGAAGAAAATGAACAAATGAAAGCAAACTTAACTAAAATGTTATCTAGTTTACAATAA

Gene: mepA (multidrug and toxin extrusion protein)

Contig: 02_NODE_3, position: 290676 to 292031, length: 1356 nt, orientation: FORWARD

Sequence:

ATGAAAGACGAACAATTATATTATTTTGAGAAATCGCCAGTATTTAAAGCGATGATGCATTTCTCATTGCCAATGATGATAGGGACTTTAT
TAAGCGTTATTTATGGCATATTAAATATTTACTTTATAGGATTTTTAGAAGATAGCCACATGATTTCTGCTATCTCTCTAACACTGCCAGTA
TTTGCTATCTTAATGGGGTTAGGTAATTTATTTGGCGTTGGTGCAGGAACTTATATTTCACGTTTATTAGGTGCGAAAGACTATAGTAAGA
GTAAATTTGTAAGTAGTTTCTCTATTTATGGTGGTATTGCACTAGGACTTATCGTGATTTTAGTTACTTTACCATTCAGTGATCAAATCGCA
GCAATTTTAGGGGCGAGAGGTGAAACGTTAGCTTTAACAAGTAATTATTTGAAAGTAATGTTTTTAAGTGCACCTTTTGTAATTTTGTTCT
TCATATTAGAACAATTTGCACGTGCAATTGGAGCACCAATGATTTCTATGATTGGTATGTTAGCTAGTGTAGGCTTAAATATTATTTTAGA
TCCAATTTTAATTTTTGGTTTTGATTTAAACGTTGTTGGTGCAGCTTTGGGTACTGCAATCAGTAATGTTGCTGCTGCTCTGTTCTTTATCGT
TTATTTTATGAAAAATAGTGACGTTGTGTCAGTTAATATTAAACTTGCGAAACCTAATAAAGAAATGCTTTCTGAAATCTTTAAAATCGGT
ATTCCTGCATTTTTAATGAGTATCTTAATGGGATTCACAGGATTAGTTTTAAATTTATTTTTAGCACATTATGGAAACTTCGCGATTGCAAG
TTATGGTATCTCATTTAGACTTGTGCAATTTCCAGAACTTATTATCATGGGATTATGTGAAGGTGTTGTACCACTAATTGCATATAACTTTA
TGGCAAATAAAGGTCGTATGAAAGACGTTATCAAAGCAGTTATCATGTCTATCGGTGTTATCTTTGTTGTATGTATGATTGCTGTATTTAC
AATTGGATATCATATGGTCGGACTATTTACTACTGATCAAGACATCGTTGAGATGGCGACATTTATTTTGAAAGTAACAATGACATCATTG
TTATTAAATGGTATAGGTTTCTTGTTTACTGGTATGCTTCAAGCGACTGGGCAAGGTCGTGGTGCTACAATTATGGCCATTTTACAAGGTG
CGATTATCATTCCTGTATTATTTATTATGAATGCTTTGTTCGGCCTCACAGGTGTCATATGGTCATTATTAATTGCTGAGTCACTTTGTGCTT
TGGCAGCAATGTTAATCGTCTATTTATTACGTGATCGTTTGACAGTTGATACATCTGAATTAATAGAAGGTTAA

Gene: mepB (putative protein)

Contig: 02_NODE_3, position: 292138 to 292578, length: 441 nt, orientation: FORWARD

Perfect match to: (MW2-BA000033-[361907:362347], allele observed in CC1+CC12+CC25+CC188)

Sequence:

ATGTATAAATCTAAAATACTGTTGAAATATATTTTTAGTGAAGAACCAGAAGTTAAAGATTTAACTGAAGAAAAATATAATCAAGATTAC
GAAGCATTAACATTTAGCTTTAAAGAGGAAACATATCAAAGTAGGTTAGCTAAGAAAACACCGACTAAAGCAGGGTATTTTGTTACATGT
TGGACAAAAGACGAGAACAATTGTAATCAACCATACTCAAAAGAGGCATTTGCAGATTACTTAATGATTATAGTTATTGATGAAGAATTA
AGCGGTTATTTTTTATTTCCTAGGGAATTATTGGTAGAAAAAGGTATCTTAACTACATTTGAACATAAAGGTAAGATGGCTTTTAGAGTTT
ATCCTAAGTGGTGTAATCAATTGAATAAAACAGCAGGGCAAACACAAAAGTGGCAATGTAAATATTTTTTTGAATACTAA

Gene: glpT (glycerol-3-phosphate transporter)

Contig: 02_NODE_3, position: 292661 to 294019, length: 1359 nt, orientation: REVERSE

Perfect match to: (MW2-BA000033-[362430:363788:r], allele observed in CC1+CC12)

Sequence:

TTAATGATGAACGGTTTCTTGTCCTACTTTATTCCAAGTGAGGATAAAGCTCAACATTGCAAACACACTGATTGCTGTTAATAAAATAAAA
CCGACATCCCATCCGAATTTATCAACTACAGCACCTAAGACGATGTTGGCCATTACAGCACCAAACAGATAACCAAATAATCCTGTTAATC
CAGCTGCTGTGCCAGCTGCTTTTTTAGGTACATAATCTAATGCTTGTAAACCAATTAACATAACTGGTCCATATATTAAGAAACCAATGGC
AATTAATGAGACATTGTCTAACCAAGCATTGCCTGGAGGATTTAACCAATAAATTAATACAAATACTGTGACACCTAACATAAAGAAGAA
ACCTGCAGGTCCTCGACGACCTTTGAATAATTTATCAGAAATGTAACCACATAATAATGTACCAGGAATTCCAGCCCATTCGTATAAGAAG
TATGCCCAACCTGATGCTTTTAAGTCGAAATGCTTTTCTTCACTTAAGTAGACTGGCGCCCAATCAAGTACACCATAACGCACGAAATAAA
CAAATATATTTGCAAAGGCAATTGCCCATACCCATTTATTGTTTAGTACATATTTAAATAAAATTTCTTTTGTAGTTAATTCTGTTTCTAATG
TTTTCTTATCGCTTGTAGCAAAGTCGTTTTTATAAATTTCGATTGGAGGTAAACCTTGAGATTGAGGTGTGTCTCTAATCAATACGTATGAA
ATTGCGGCAATGATAAGTGCTAAGAGTGCAGGGTAAATGAATACACCTTCGAACCCTTTTAAATAACCAAAGTTGATAAATGCTGTTGTT
GTAATACCCCAAGCAGCAATAGGTGCCATAATACCTCCACCAACATTATGCGCAACGTTCCAAAGGGCAGTCTTACTTCCGCGTTCACTTA
CACTAAACCAGTGAACGAGAACACGGCCTGAAGGTGGCCAGCCCATACCTTGAAACCATCCATTTAAGAATAATAGGACAAACATAATA
CCGATACCTGATGTAAAGAACGGTACAAATCCCATTAACAAATTGACGATAGCAGTGAGTGCTAATCCAAGAACTAAGAATATCCGAGC
ATTGCTCCGATCACTTACAGTACCCATAAAGAACTTACTAAATCCATATGCGATGGAAACAGCAGAAAGTGCAAAACCTAGTTCCGCTTTT
GTAAAACCTTGCTCTTGCAATGCTGGCATCGCTAATGAAAAGTTTTTACGTAATAAATAGTACCCAGCGTAACCGATGAAAATACCAAGA
AATACTTGGAGACGTAATCGTTTATAGGTATCATCTATCTGATTTTCTGGCAAAGGCTTAATATGCTTTGCAGGTTTAAGAAAATTCAT

Gene: mhqA (putative hydroquinone-specific extradiol dioxygenase)

Contig: 02_NODE_3, position: 294331 to 295191, length: 861 nt, orientation: TRNC-FRWD (no stop codon)

Perfect match to: (MW2-BA000033-[364100:364960], allele observed in CC1+CC188)

Sequence:

ATGAATATAGTAGGGCATCATCACATATCCATGTATACAAAAGATGCAAAACGTAATAAGGATTTTTACACAAATGTCCTTGGATTACGA
TTAGTTGAAAAGTCGGTTAATCAAGACAATCCTTCAATGTATCATTTGTTTTATGGGGACGAAGTAGGTACAGCCGGAACAATTTTAAGC
TTTTTTGAAATTCCCAATGCGGGTCATAAGCAGCCAGGTACTGAAACGATTTATCGATTTTCTTTATTAGTACCAAATCAAGCGGCACTTC
ATTATTTTGAAAAACGCCTTGATAATAATGGTATTACGTCTGAACGTTTGTACTATCTTGGACAAGAAGGTGTTGTCTTTAAAGATGAAGA
CGACTTAGAAATCATATTGCTTGTTAATGATAGTTTTGAAGTACCACATCAATGGCAACATAACGTTTATAGTGAAATACCTCAAGCATAT
CAAATTTTAGGAATAGGGCCAGTCGAATTAAGAGTTAGAAATGCAGCGCGTACGGTAGAATTTTTGGAAAATGTCTTAGGTTATCGCAA
AAGAGATAATAAATCATTCGATGTGCTGACATTAGCACCACAAGGTTTATATTCGGATTTTGTAGTTATTGAGCAACAGGGACAACGTGA
AAGACCTGGACGAGGTTATATCCATCATATTGCAATTAATACACCACAAATGAGTGACTTAGATGCAATTTACAAGAAATTACAACAACA
AACACAAAGTAATTCAGGTATAATTGATCGCTATTTCTTTAAATCATTATACTATCGCCATAATTCAATTATGTATGAATTTGCGACTGAAG
CGCCTGGATTTACTATTGATACACCTCTTGAACAATTAGGAAGTCAA

Gene: ssuD (flavin utilizing monoxygenases, luciferase family)

Contig: 02_NODE_3, position: 295271 to 296332, length: 1062 nt, orientation: FORWARD

Perfect match to: (MW2-BA000033-[365040:366101], allele observed in CC1+CC188)

Sequence:

ATGGCAAAATTAGAAATGAATAAAAATACACCTCTTGAGTTTGGTTTGTATTCCTTAGGTGATCATTTATTGAATCCATTGAAAGGTGAAA
AAGTTAGTTATGAGCAACGTATTAATGAAATTATTGAAGCAAGTAAATTAGCAGATGAAGCAGGTATTGATGTTTTTGCAGTTGGTGAAA
GTCATCAGGAGCATTTTACAACACAGGCACCTACGGTTGTGTTAGGTGCAATTGCCCAAGCGACAAAGCATATCAAAGTTTCAAGTTCTT
CAACGATTATTAGTGCAACAGATCCTGTAAGAGTATTTGAAGACTTCGCGACATTAGATTTGATTTCTCATGGTAGAGCCGAAATTGTAG
CTGGCAGAGCATCAAGAACAGGTATTTTTGACTTGTTTGGCTATGATTTAAAAGACTATGATGAATTGTTTGAAGAAAAATTAGGTTTACT
TTTAGAGTTAAATAAAACTGAGCGTATTACTTGGTCTGGAAAATATCGTCCAGAACTTAGAAATATGAAAATATTCCCAAGACCAATCGA
TAATACATTGCCAATATGGCGTGCTGTTGGTGGTCCACCTGCAAGTGCTATTAAAGCGGGAAAACAAGGTGTGCCAATGATGATTACAA
CCCTTGGTGGCCCAGCAATGAACTTTAAAGGTTCTATAGATGCTTATCGTCAAGCAGCAACTGAAGCAGGTTTCGATGCTTCGCCTAAGT
CTTTACCAGTAAGTATAGCGAGTCTGTTTTATACAGCTGAAACAACTCAGGATGCTATGAGAGAATTTTATCCACATTTGAATACAGGGAT
GTCATTTATTCGTGGCGTTGGTTATCCGAAACAGCAATTTGCTAATTCACCAGATTATAGAGAAGCGCTAATGGTTGGAAGCCCGCAACA
AATTATTGAAAAGATATTGTATCAACACGAGTTGTATGGTCATCAACGTTTTATGGCACAGCTTGATTTTGGCGGTGTGCCATTTGAAAAT
GTTATGAAGAATATAGAGTTAATTGGCAACGACATTATACCGGCGATTAAAAAGCATTTATCAAAATAG

Gene: ssuE (flavin mononucleotide reductase)

Contig: 02_NODE_3, position: 296346 to 296912, length: 567 nt, orientation: FORWARD

Perfect match to: (N315-BA000018-[386167:386733], highly conserved allele)

Sequence:

ATGAATATTGTATTATTGTCAGGTTCCACAGTAGGTTCTAAAACGAGAATTGCTATGGATGATTTAAAAAATGAACTAGAAGTCATCAAT
GAGGGACATCAAATAGAGTTGATGGATTTACGAGAACTTGAATTAGAATTTAGCGTTGGAAAGAATTATCTAGATACTACAGGAGATGT
ATATAAATTAACGACGTCGTTAATGCAGGCTGATGTGATTTTTATTGGTTTTCCAATTTTTCAAGCTTCCATCCCTGGTGCTTTGAAAAATG
TGTTTGATCTACTTCCAGTCAATGCGTTTCGTGACAAGGTAATAGGACTTGTAGCGACAGCAGGTTCTAGTAAACATTATTTAATTCCTGA
AATGCATTTAAAACCAATATTGAGTTACATGAAAGCACATACGATGCAAACGTATGTATTTATTGAAGAGAAAGATTTTTCAAATCAACA
AATTGTCAATGATGATGTTGTATTTCGGTTAAAAGCGTTGGCACAATCCACAATGCGAACTGCCAAAGTACAACAACAAGTGTTTGAAGA
AGAAAACAACCAATACGACTTTTAA

Gene: yeiH (UPF0324 membrane protein)

Contig: 02_NODE_3, position: 296972 to 297967, length: 996 nt, orientation: REVERSE

Perfect match to: (MW2-BA000033-[366741:367736:r], allele observed in CC1)

Sequence:

TTAACTATATAGCCAATGAACGACGATAAAGGCAAGTGATGACAAGCATATTGAGGTAATAATGATTGTCATAAGCGGTTTAAGTGCGC
GATTTTTAAGATCTTTAAATGCAACATTTAACCCTAAAGCAACCATGGCCATTAATAAGCAAATTGTTGATACAGTATTTAAAATATTTAGC
AATGCTGACGGAATAGTTACATATGTATTCACTAAGGCCATAATGACAAATCCAATTAAAAAGTATGGAATGCTTATTCGACCATTGCTA
GATGATTCTGATGAACGGAAACGCATAATTAAAATAAGTACGATGGTTAATGGAATCAGTAAGAATACTCTGCCAAGTTTACCAAGAAG
TGCAATTTTAAGTGCATCACTACCACCAAAGCCACCAGCTAGGACAACGTGTGCAATTTCATGAAGACTAACGCCAGACCAAGCGCCATA
AACATTTGTCGTCATTGAAAAGATAGCATAGATAGCTGTATATATAAGTGAAAATATCGTACCAATCAATGCGATGATACCGATACTAAT
AGCTGTATCCTTTTCACGTGATTTGAATATTGGAGCGACTGCAGCAATAGCGGCAGCACCACAAACGCCTGTACCTACACCTAGTAATAA
TGCGATGTTTTTGTCACCATGCAACAGTTTGTTGACAAAGAGCATCATTACAATACTAAAAATAACGACACCTACATCGATGGCTAATAGT
CTACTACCTTGACCGATAATATCGAATATATTGAGTTTAAGTCCATATAGGATGATTGCAAATCTTAATAAATATTTAGATGAAAACGTAA
TACCTGAGCTATATTGTTCAGGATATCCTCTAAAGTGACGATATAGAATAGCGATTAATATCGCGATAGTTAATGCGCCAACCTTATCTAA
GATTGGCAATTTAGCAGCTAAAAAGCTAAATAATGCGACTATAAATGTTAATGTTAGTCCAATCATAAAATGCTTATTTTTCAATGATGCC
AT

Gene: rimL (ribosomal protein N-acetyltransferase)

Contig: 02_NODE_3, position: 298272 to 298817, length: 546 nt, orientation: FORWARD

Perfect match to: (MW2-BA000033-[368041:368586], allele observed in CC1+CC188)

Sequence:

ATGTTTGGAATGAAAGTGAATGAACAAATAATATTAAAAATTTTAGAAGCCCATGACACAGAAGCGCTTTTCAATTTAGTCAATCGTTCA
AGAAATTCACTTAGGGAATGGTTACCTTGGGTAGATGCAACTGAGCAACCATCAGATACGCGTGCGTTTATTAAAAGAGGACTTTTGCAA
TTTTCTGATGGTAATGGATTTCAGTGTGGCATTTGGTATGAAGGAACGCTAGTTGGTGTCATCGGTTTACATGAAATTAATCGCATGCAC
AGAAAAACTTCATTAGGATATTATTTAGATAAACAATTTGAGGGTCATGGGATTATGACACAAGCAGTTGAGGCATTGATAAAGTATTGT
TTCGAAGAGCTTGACTTAAACCGAATTGAGATTAGTGCCGCAGTTAATAATGAAAAAAGCCGAGCTATTCCTGAAAGGCTGGGATTTACT
AGAGAAGGTATGTTACGTGACAATGAATTACTAAATGGTATTTATTCATCGAGTTACATCTATAGTTTATTAAAATCAGAATACGACCAAA
AATGA

Gene: fepA (lipoprotein)

Contig: 02_NODE_3, position: 299084 to 299938, length: 855 nt, orientation: FORWARD

Perfect match to: (MW2-BA000033-[368853:369707], allele observed in CC1+CC30)

Sequence:

ATGAAAAAGTTAACAACGCTATTATTAGCATCAACGTTATTAATTGCTGCATGTGGGAACGACGATAGTAAGAAGGATGATTCAAAGAC
ATCGAAAAAAGATGATGGTGTTAAAGCAGAATTAAAACAAGCAACAAAAGCATATGATAAATATACTGATGAACAGTTAAATGAATTTTT
AAAAGGTACAGAAAAATTTGTTAAAGCGATTGAAAATAATGATATGGCCCAAGCAAAAGCGTTATATCCAAAAGTTCGTATGTATTATGA

ACGCTCTGAACCAGTTGCAGAAGCATTTGGAGATTTAGATCCTAAAATTGATGCACGTCTTGCAGATATGAAAGAAGAGAAAAAAGAAA
AAGAATGGTCAGGATATCATAAGATTGAAAAAGCATTATACGAAGATAAGAAAATTGATGATGTGACTAAAAAAGATGCACAACAATTA
TTGAAAGATGCAAAAGAATTGCATGCCAAAGCTGATACATTAGATATCACACCAAAATTAATGTTACAAGGTTCTGTTGACCTATTAAAT
GAAGTTGCAACTTCTAAAATCACAGGTGAAGAAGAAATTTATTCACATACAGATTTATATGATTTTAAAGCGAACATTGAAGGTGCACAA
AAAATTTATGACTTATTTAAACCTATTTTAGAGAAAAAAGATAAAAAATTAAGTGATGATATTCAAATGAACTTCGATAAAGTGAATCAAT
TATTGGATAAATATAAAGATAACAACGGCGGTTATGAGTCATTTGAAAAAGTATCTAAGAAAGACCGTAAAGCATTTGCGGATGCTGTTA
ATGCATTAGGAGAGCCACTAAGTAAAATGGCTGTGATTACTGAATGA

Gene: fepB (iron-dependent Dyp-type peroxidase)

Contig: 02_NODE_3, position: 299935 to 301164, length: 1230 nt, orientation: FORWARD

Perfect match to: (Tager_104-CP012409-[1234530:1235759:r], allele observed in CC49+CC5+CC49+CC361+CC2249)

Sequence:

ATGACAAATTATGAACAAGTTAACGATAGTACGCAATTTTCAAGACGTACATTTTTGAAAATGTTAGGTATTGGCGGTGCCGGTGTTGCA
ATTGGCGCAAGTGGTGTTGGTAGCATGTGGTCTTTCAAATCAATGTTCAATACACCAGAAGATCCGGAAAAAGATGCGTATGAATTTTAT
GGTAAAGTGCAACCAGGCATTACCACACCCACGCAAAAAACATGCAATTTCGTTGCGTTAGATTTGAAGTCAAAAGATAGAGATGCAATT
AAGGCAATGTTTAAAAAGTGGACGGTTATGGCTGATCGTATGATGGATGGTGATAAAGTTGGCAAGCCGAGTAACAATCCTTTAATGCC
ACCAGTAGATACCGGTGAATCGATAGGATTAGGTGCAAGCAAGTTAACGATTACCTTTGGGATTAGTAAGTCTTTGATGAAGAAAATTG
GGTTATCTAGTAAAATTCCCGATGCCTTTAAAGATTTACCGCATTTTCCGAATGATCAGTTAATAGACGATTACAGCGATGGTGATATTAT
GATTCAAGCATGCTCAAATGATTCGCAAGTATCCTTTCATGCGGTTCATAATTTAGTTCGTCCATTTCGAGATATTGTTAAGGTACGTTGG
GCGCAATCTGGTTTTATCTCTGCTAAAGGTAAGGAAACACCTAGAAATTTAATGGCATTTAAAGATGGAACAATTAATCCTAGAAAGAGT
AATCAACTTAAAGATTATGTGTTTATTGATGACGGATGGGCGAAACATGGAACTTATTGTGTTGTCAGACGTATTCAAATACACATTGAA
ACGTGGGATCGTACTGCACTGGAAGAACAAGAGGCTACATTTGGTCGGAAACGACATAGTGGTGCGCCGTTAACAGGTGGGAAAGAGT
TTGATGAAATTGACTTAAAAGCGAAAGATAGTCATGGCGAGTATATTATTGATAAAGATGCCCATACGAGACTAGCGAAAGAAGCAAAT
ACGTCAATTTTACGTAGAGCCTTTAATTATGTTGATGGTACGGATGACCGCACAGGTAACTTCGAAACAGGCTTGTTGTTCATTGCTTTTC
AAAAAGCGACAAAACAATTTATCGATATACAAAATAATTTAGGTAGTAATGATAAATTAAATGAATATATTACACATAGAGGTTCTGCTTC
ATTTTTAGTATTACCAGGTGTTAGTAAGGGAGGATACCTTGGTGAAACATTATTTGATTAA

Gene: fepC (high-affinity iron transporter)

Contig: 02_NODE_3, position: 301145 to 302857, length: 1713 nt, orientation: FORWARD

Perfect match to: (MW2-BA000033-[370914:372626], allele observed in CC1+CC188)

Sequence:

GTGAAACATTATTTGATTAAATTTGTAGCAATGCTAATAACTGCTGCTATGGTGTGTAGCTTTGGGTTACTGAAAAGTCAGGCAGCAAAA
CAACAAAGTATTAGTGATGTATATAGTGTGATAACGGATGCGAAATCTGCACTTTCTAATAATTCGATATCGAATGACAATAAGCAGAAA
GCAATTGAGCAAGTGGTAAGTGCAGTTAAGAAATTATCGCTTGAAGATAATAGCGAAGGTAATGCTGTCAAATCAGATGTGAGAAAGCT
TGAAGATGCAAAAGCGAATGATAATCAAAAAGATACACTTTCGCAATTAACGAAGTCATTAATTGCTTATGAAGAGAAATTGGCTAGTAA
AGATGCGGGTTCTAAAATTAAACTATTGCAACAGCAAGTCGATGCCAAAGATGCAGTAATGACAAAAGCGATTAAAGATAAAAATAAAG
CAGAGCTAGAATCGCTGAACAATAGTTTGAATCAGATTTGGACAAGTAATGAAACGGTAATTCGCAATTATGACGCAAATCAATATGGA
CAAATTGAAGTCGCATTACTGCAACTTAGAATCGCAATTCATAAGTCGCCATTAGATACAGCGAAAGTGTCACATGCTTGGACAACTTTTA
AATCAAATATTGATCATGTCGATAAGAAAAGTGATACGTCTGCAAAAGATCAATACCATGTATCACAATTAAATGATGAGTTAGAGAAGG
CGATTAAAGCTATCGACGACAATCAATTGTCGGATGCTGATGCTGCGCTTACACATTTTATAGAAATTTGGCCGTATGTTGAAGGTCAAA
TTCAAACTAAAGACGGTGCTTTGTATACGAAAATTGAAGATAAAATACCATATTATCAAAGTGTATTAGACGAACATAATAAAGCACATG
TGAAAGATGGTTTAGTAGATTTAAATAACCAAATTAAAGAGGTTGTTGGCCATAGTTATAGCTTTGTTGATGTGATGATTATCTTTTTACG
TGAAGGTCTAGAAGTGTTATTAATTGTAATGACATTGACTACTATGACGCGTAATGTCAAAGATAAGAAGGGTACTGCAAGTGTGATTG
GTGGTGCAATTGCCGGACTTGTACTGAGTATTATCTTAGCAATTACGTTTGTAGAAACTTTAGGTAATAGTGGCATTCTTCGTGAAAGTAT
GGAAGCGGGATTAGGTATTGTTGCGGTCATATTAATGTTTATCGTTGGTGTTTGGATGCACAAACGTTCAAATGCGAAACGCTGGAATG
ATATGATTAAAAATATGTATGCCAATGCGATTAGTAATGGTAATTTGGTATTGTTAGCGACGATTGGTTTAATATCTGTGTTGCGTGAAG
GTGTTGAGGTTATCATTTTCTATATGGGGATGATAGGTGAGCTAGCGACAAAAGATTTTATTATTGGTATTGCTTTAGCTATCGTTATATT
AATTATCTTTGCGTTATTATTTAGATTTATCGTCAGATTGATACCTATATTCTATATATTTAGAGTGTTGTCGATTTTTATTTTTATTATGGGA
TTCAAAATGCTTGGCGTAAGTATTCAAAAGTTACAATTATTAGGTGCTATGCCGAGACATGTCATTGAAGGATTCCCAACGATTAACTGG
TTAGGATTCTATCCAACTTATGAACCATTGATAGCACAAGCTGCCTATATTATGGTAGTTGCTATCTTAATCTTTAAATTTAAAAAATAA

Gene: tatC (twin-arginine translocation protein C)

Contig: 02_NODE_3, position: 302949 to 303650, length: 702 nt, orientation: REVERSE

Perfect match to: (MW2-BA000033-[372718:373419:r], allele observed in CC1+CC12+CC188)

Sequence:

TTAATGTGTTGGCGGTTTGCCTCGGTATGTAAATTTAACGATGAACGTACTGAACTCAAAGAGCAATATGAGTGGCAATGTGAGTAATAT
ATTTAATGTTAAATCGGGTGGTGCAATGATACTTGCTAATACAAAGCAAGCGAAATAAATATATTTTCGATAATGCTTCAATGATGTGGT
ATCTATAAGACCGAATTTTGCAAGTCCTATGAATAATATTGGTAATTGAAATAGAATGCCAAATGTGAATAACCAACGTATAAGTTCAATC
AAATATGCTTTAAAGCCAATGACAGGCGAAATGTTTAAAGTTAATGATAATTTTAACGCGAATTGAATGATCATTGGAAAGCCAACATAA
AATGCAAAAGCGACACCAAAAATAAATAAAAGTACGCTAAAAAAGCTATATTTATAAATAAATTGGCGCTCATTATTATGCAAACCAGGC
GCAACAAACGCCCACAATTGATAAAACATAACCGGTGAAATGAGACAAAACGCGATGAAAAATATAATCATCACGTATATTTGGATCATT
TCTGTGAATGAAAATGCATGTAAGGACACATGTGCACGGGTAATATACGTTATGAATGGTGTCATCCACCAAAATGATGAAACATATACG
ACGATGACCGTAATGACGAACGACAATAAAATTTTTACTAACCGATGGCGTAGTTCGCTAAAGTGAACCAT

Gene: tatA (twin-arginine translocation protein A)

Contig: 02_NODE_3, position: 303667 to 303882, length: 216 nt, orientation: REVERSE

Perfect match to: (MW2-BA000033-[373436:373651:r], allele observed in CC1+CC398+CC425)

Sequence:

CTATTGCTCTCGCTGTTGCTTCGATTCCTTACTGGGTGTATCGTGAGACTCTTTATCTAAATCTTCTGTTGCAGATTTAAATTCTTTTAAAGT
AGAACCGATAGCACGACCAAATTGTGGTAATTTTTTCGGACCAAAAATAATTAAAGCGATAATGCTAATGACGACAAGACTTGTTGGACC
TGTGATGCCTAAAATAAAAGTGTTAGTTATCAT

Gene: DUF1398 (putative protein)

Contig: 02_NODE_3, position: 303990 to 304379, length: 390 nt, orientation: REVERSE

Perfect match to: (MW2-BA000033-[373759:374148:r], allele observed in CC1+CC188)

Sequence:

TTATTGAGGGATTAATTCTGAAATAACAGCTTGGTCTTGCAAGTCGATATAAGTACAAGTGCCCGCTTGAATATCGATATGCCATTTATAA
ATGCCAGCTTCAGCCATTTCATCACAAAATGTTTCAAAATCTGTTTGTCCTTGTTGATGTCTAGTTAATACATCTTGAACGATAGATTGATT
TGATTTTTGAGCAACAGGAATCGTACTTTTCACAGATGACGTAACGATATCATCTTCTGATTGATGTACGTATGTTGCAGTGCCATCTTGA
ATGTTGACGATATTGTAAGTCATCCCCATATCTTTAAAAGCTTTGAATAGTTTTGGAAAATCAACACCAGTAAATTGTTGATGTGCTTGTT
GAATTGCAGATAATGTAAATGCCAT

Gene: Q5HIU5 (putative transcriptional regulator)

Contig: 02_NODE_3, position: 304619 to 304822, length: 204 nt, orientation: FORWARD

Sequence:

GTGCGTAATCGATTGAAAGAATTACGAGCACGAGATGGCTTAAACCAAACGCAGCTTGCCAAACTAGCGGGTGTTTCAAGACAAACCAT
TTCGCTAATTGAGCGAAATGACTTTATGCCATCGGTATTAACTGCAATAAAGATTGCTCGTATCTTCAATGAAACGGTGGAAACTGTTTTT
ATTATTGAGGAGGATGAGGTATGA

Gene: DUF3169 (ABC transporter, transmembrane permease)

Contig: 02_NODE_3, position: 304819 to 305532, length: 714 nt, orientation: FORWARD

Perfect match to: (MW2-BA000033-[374588:375301], allele observed in CC1+CC12+CC80+CC188)

Sequence:

ATGAAAATACTAAGATATATCGGATATCTTTTACTAGGTGGACTTGTAGGGGGTATCATTGGTGGAATTTTAGGTAATTTTGATGGATTG
GGTATTGAGAACTTGACGTTTGCGACATATACCAATGTCGTTGTAATATCGATTGTTGCGACGATTATTATCATATTGGTAGAAGCCATTG
TTTTGATGAATCAAAGACGTGCATTGAAGTATAAGCGACTTGTAGATGAAGAGGTAGATATCGATGCAACAGATCAATATGAATTGCTTG
CGAATCGTTATGTTTTAAATGGAAGTATATTAAGCGTTATACAGACAATTATTGCCTTTGTAGTGTTACTAATTTTTGTGGTAGGGCAAGC
TGAAGCAAATGCAATGCTATTCTTTTTAATACCATTTTTTGCTAGTGCTATTTTCAATACACAATTTACACTGTTTAATAGAAAATTTGATGA
CAGAATGCCAAAAATTGCAGATAAGAATTACACTGAAAAGCGATTGGAAATATTGGATGAAGGTGAACGCCATATAGAATTAATTGCAT
TATTTAAAACATATGCGATCAACTTATCAATATTGATACTAGCCATTATTTTTATAGGGTCTTATTCAATTGCTACAGGAATTAATCAAAGC
TTTAGTTTGCTACTTATCATTGCTATTTTCATATATAACGCCTTTAGCTATTTATTGAAGAGAAGACGTTTTTATTAA

Gene: Q5HIU3 (ABC transporter, ATPase)

Contig: 02_NODE_3, position: 305557 to 306399, length: 843 nt, orientation: FORWARD

Perfect match to: (ECT-R2-FR714927-[361391:362233], allele observed in CC5)

Sequence:

ATGACAACATTGTTAAACGTAGATCGTGTGAACAAACAATACAAAGATTCGGATTTTAAATTGCAAGATGCATCTTTAACGATTTCTACTA
ATGAGACAGTTGGATTAATTGGGAAAAATGGCTCAGGTAAATCGACATTAATTAATATTCTAGTAGGCAATCGACATAAAGATAACGGT
AGTATTGCATTTTTTGGAGAAGAACATGCTGCGGATGATGTCGAATATAAAGAACACATAGGTGTTGTGTTTGATGATCTGAGAGTACCT
AATAAATTGACTATTAAAGATATTGATAAAGTATTTCAATCTATTTATACGACTTGGAATAGTCAAAAATTCTTTGATTTAATCAAATATTT
CGAGTTACCACGACAAACTAAAATTAAAACTTTTTCAAGAGGGATGCGAATGAAGATAGCTTTAACGATTGCGCTTTCTCATGATGTGAA
GCTATTAATCTTAGATGAAGCAACTGCAGGTATGGATGTTTCTGGACGAGAAGAAGTAATGGAACTATTAGAAGATTTTGTCGCTCAAG
GTGGAGGCATCTTAATATCATCGCATATTTCTGAAGATATAGAACAATTAGCGGATAAATTAGTGTTTATGAAAGATGGACGAATGATTT
TAACTGAACAGAAAGATATACTTTTAGCACAATATGGAATTGTTACGACAGGAGATAAAGATGTTGAAATTACTAAGCATTTAATCATTG
CTTCTAGATTGTCAAAGGGGAAATATCAAATTTTAGTTAAAGATTATGCAGAAATTGAAAATGCAGAGCCTTTAAAACACATTGATGACG
CTACGAAAATCATAATGCGAGGTGAAGTATAA

Gene: Q5HIU2 (ABC transporter, transmembrane permease)

Contig: 02_NODE_3, position: 306399 to 307028, length: 630 nt, orientation: FORWARD

Perfect match to: (N315-BA000018-[396428:397057], allele observed in CC5+CC1+CC12+CC188)

Sequence:

ATGAAAGGTATGTTCCTAAGTAGTTTTTATGCAACGAGAAAGCAAACATATATTTATTTTATAGTCGCTATCATAGCTGCGGGATACTTTG
CAGTATTTAATCCGTTGATGAGTTCGGCAATGGCTGGGGTTATGTTAATCACACCCATTACTGATAATATTAAACATGAAAAAGACTCAA
GATGGATGTATTATGTATCTACTTTACCGGTTAAACGTAGTGATTATATTAAGTCATACTTTGCCTTTTATTTAATCTTATTCGGTGCAAGTT
TAATGATTGGATTAGTTGTGACTACGATCGTGACCCAAAGTGTGATGATTGGTATTATGTCAGGTTTAATGAGTTTTGGTATCATAGGGG
CATACTCTATCATTTTCCCATTGACATTTAAATTTGGCGCTGAAAACTCTAATGTCATTATGATAGTTGCATCTATCCTACTACTTTTTTCTTT
CGTGGTCTTTTTCTTTATATATGGTATGGTTAGTGGTGCATCGGCATTAGAATTTGAAAAAATTAGCACTGAAGGATGGTTAGTTGTCATA
GTATATGCGGTCATTGGTATCGTTATAACAAGTGTTTCTTATATATTGTCTATTAAAATTTTTAACAAACAAGAACTATAA

Gene: ltrA-L1 (low temperature requirement A, locus 1)

Contig: 02_NODE_3, position: 307418 to 308551, length: 1134 nt, orientation: REVERSE

Perfect match to: (COL-CP000046-[428258:429391:r], highly conserved allele)

Sequence:

TTATTCATGTTTATTACGTAGACGTTGATTTTTAAAATAAATCAGCATGATGCAGAAAGTCGCAACGGATACCGAGATAATCATTACTTGG
TCATGCCCCTTAAAGATAAGGCTGATGATATATGATACTACAAGTATGCCTAGTGAAGAAGAAATATATTTTGCGTTTGTCAGTTCATTAT
GGAAATAAGGCGTGATTAACCATAATCCAATATAGAATATTAAAACACTGATATACATCATATTAATTTCAAACAAGTCATTTAGTTTATT
GTTATTACTAAAAACAATTGCAGCATTAATCACACCTAAAGCGATATTGATTAATAGATGCGTATACGATAAACGGAAACCGATAGATGA
CAATTTATGATTAATGTAATTTTCAGTAATGATCCAATATACACCGAAAAGACTAATTAAAATCATAAATTGGAATATATAAATGTAACTA
AAATGATCAATGCTAAATGATGACGAAGCTAAACCAACCAGTACCTCGCCAAAGATAATAATTGTTAGTAACGAAAAACGTTCTACTAAA
TGCATCATATTAACAGGTGATAATACAAGATATTTCTGAAATGGAATAAGTCCTGTCGCTGCAATGAATACGCCTAAAAATCCAGGGATG
TAATGGATACTTTGTGGTAGTACTAATGATAGAAATGATAAAAATGAAATCACAAAGGCTACGCTCGCAAAAGCTTGACATGTACGCTTA

TCGCCATAATCTAACCCTGTACGTATATGTAATAAATACTGTAATCCGATACTTAAATACATAATTGCCACGCATAAGAAGAATGGGAAG
AATGTCTTTTCAAAGTCCGGATATAGGCTGTTAGATAGGAAGACCATGATGAACATATTAAACATCATAAACGAGACGTCTTTGAATGTA
ACTTGACCAAATCGATTTGTAAAAAATGTTTGATGAGACCACATTAACCATAAGAACAAACTCATGACGATGTATTTGAAAAATAAATCA
GCTGAAATGGAACCGTTTTGTGTTGTTAAAATCACATGTGCAATTTTTTGAATGGCATAGACGAAAATTAAATCAAAGAACAACTCATGG
AATCCTGCACGCTTTTCAGCTAAATGTTTTGGTGTTAATGCATTAACCAT

Gene: thlA-yqiI (acetyl-CoA acetyltransferase)

Contig: 02_NODE_3, position: 309090 to 310271, length: 1182 nt, orientation: FORWARD

Sequence:

ATGACGAGAGTCGTATTAGCAGCAGCATACAGGACACCTATTGGCGTTTTTGGAGGTGCGTTTAAAGACGTGCCAGCCTATGATTTAGG
TGCGACTTTAATAGAACATATTATTAAAGAGACGGGTTTGAATCCAAGTGAGATTGATGAAGTTATCATCGGTAACGTACTACAAGCAGG
ACAAGGACAAAATCCAGCACGAATTGCTGCTATGAAAGGTGGCTTGCCAGAAACAGTACCTGCATTTACAGTGAATAAAGTATGTGGTT
CTGGGTTAAAGTCGATTCAATTAGCATATCAATCTATTGTGACTGGTGAAAATGACATCGTGCTAGCTGGCGGTATGGAGAATATGTCTC
AGTCACCAATGCTTGTCAACAACAGTCGCTTCGGTTTTAAAATGGGACATCAATCAATGGTTGATAGCATGGTATATGATGGTTTAACAG
ATGTATTTAATCAATATCATATGGGTATTACTGCTGAAAATTTAGTGGAGCAATATGGTATTTCAAGAGAAGAACAAGATACATTTGCTGT
AAACTCACAACAAAAAGCAGTACGTGCACAGCAAAATGGTGAATTTGATAGTGAAATAGTTCCAGTATCGATTCCTCAACGTAAAGGTG
AACCAATCTTAGTCACTAAGGATGAAGGTGTACGTGAAAATGTATCAGTCGAAAAATTAAGTCGCTTAAGACCAGCTTTCAAAAAAGAC
GGGACAGTTACAGCAGGTAATGCATCAGGAATCAATGATGGTGCTGCGATGATGTTAGTCATGTCAGAAGACAAAGCTAAAGAATTAAA
TATCGAACCATTGGCAGTGCTTGATGGCTTTGGAAGTCATGGTGTAGATCCTTCTATTATGGGTATTGCACCAGTTGGCGCTGTAGAAAA
GGCTTTGAAACGTAGTAAAAAAGAATTAAGCGACATTGATGTATTTGAATTAAATGAAGCATTTGCAGCACAATCATTAGCTGTTGATCG
TGAATTAAAATTACCTCCTGAAAAGGTGAATGTTAAAGGTGGCGCTATTGCATTAGGACACCCTATTGGTGCATCTGGTGCTAGAGTTTT
AGTGACATTATTGCATCAACTGAATGATGAAGTTGAAACTGGTTTAACATCATTGTGTATTGGTGGCGGTCAAGCTATCGCTGCAGTTGT
ATCAAAGTATAAATAA

Gene: mdh (cyclase family protein)

Contig: 02_NODE_3, position: 310354 to 311106, length: 753 nt, orientation: REVERSE

Perfect match to: (MW2-BA000033-[380123:380875:r], allele observed in CC1+CC8+CC80+CC239)

Sequence:

TTAGTCATTTGATGGTTTAATTGCAAATGCTCTAACAGGGAACCCAGGTGCATCTTTTGGTTTAGGGCTGATAGCGTAAATGATGGCGCC
ACGAGTTGGTAATTGATCTAAATTAGTTAATAACTCGACTTGGTATTTATCCTGACCAAGAATATAACGTTCGCCAACTAAATCACCATTTT
TTACAACGTCCACAGATGCATCGGTATCGAATGTTTCATGACCAACAGCTTCAACACGACGTTCTTCAATTAAGTACTTCAAAGCATCTAA
TCCCCAACCCGGTGCATGTTGTTGTCCGTTCGCATCTTTGTTTTCAAACTTTTCAATATTAGGCCAACGTTTTGACCAATCGGTACGAAGTG
CAACAAAAGTGCCAGGTTCAATAGTACCATGCTCTTTTTCCCATGCTTCTATATGCGCACGTGTTACAATGAAATCATTGTTGTTCGCTACT
TCTGTTGAAAAGTCTAATACAATTAACGGCAATACCAATTCTTTTAAATCAATGTCTTCTAAATAACGTTTATTCTCGACAAAGTGAATTGG
TGCATCAATGTGAGTACCATATTGCGTTACAATATTCCAACGTTGCACATAGAAACCATGATCTTTAACCGTGAATAAAGTTGAAACTTCG
CCTTTTTCAAACTCACTAAAACGTGGTATTTCCGGATCAAATGTATGCGTTAAATCAACCCAAGTTGCTTGTTTTAAAGTATTTAATTGTTG
CCATAAAGGATATTGTGTCAT

Gene: metE (5-methyltetrahydropteroyltriglutamate--homocysteine methyltransferase)

Contig: 02_NODE_3, position: 311149 to 313377, length: 2229 nt, orientation: REVERSE

Perfect match to: (MW2-BA000033-[380918:383146:r], allele observed in CC1+CC88+CC188)

Sequence:

TTATTCTTGGCGTTTAGCTTTAACAGCATTCACAAGCACAGTCAATGCATCTTTAACTTCTTCTTCTTTTCGCGTTTTCAAACCACAGTCAGG
GTTTACCCAGAATAATGAGCGGTCGATTTGTTGTAGTGAACGATTGATTGCTGTAGTAATTTCTTCTTTTGTTGGAATACGTGGACTATGA
ATATCATACACACCTAGACCAATACCTAAATCATAATTAATATCTTCAAAGTCTTTAATTAAATCACCATGGCTACGAGATGTTTCAATTGA
AATAACATCAGCATCTAAGTCATGAATAGCATGAATGATTTGACCGAATTGAGAATAACACATATGTGTATGGATTTGAGTTTCATCACG
AACTGAAGACGTTGCAAGTTTAAATGATAAAACAGCATCTTTAAGATATTGTTCGTGATATTCAGAGCGTAATGGTAAGCCTTCACGTAA
TGCAGGTTCGTCAACTTGGATAACTTTGATTCCTGCAGCTTCAAGTGCTAATACTTCTTCGTTGATTGCTAAAGCAATTTGATCTTGAACGA

CTTTACGTGGTAAATCAACACGTTCAAATGACCAGTTTAGAATTGTTACAGGTCCAGTTAACATACCTTTAACTGGTTTATCTGTTAAGCTT
TGTGCATAAACTGTTTCATCAACAGTTAAAGGCGCTGTCCATTTTACATCACCATAAATGATTGGTGGTTTTACGGCACGTGAACCATATG
ATTGCACCCAACCGAATTTAGTTACTAAGAAACCTTGTAATTTTTCTCCGAAGAATTCAACCATGTCATTACGTTCAAATTCACCGTGAACT
AATACATCTAAGCCAATGTCTTCTTGAATTTTAATCCATCGAGCAATTTCATTTTTTAAGAATGTTTCATATGCTTCGTCTGTAATGCGTTTG
TTCTTCCAATCTGCACGGTATTTTCGAACTTCTCGGCTTTGTGGGAATGATCCAATAGTTGTTGTTGGTAAATCCGGTAAGTTCAAACGTTT
TTGTTGTTGTTCAATACGTTGCGCGAATGGTGATTGTCTTGAAGTACGCACGCTTTCGAAATCATAATCTAAGTTTTTGAATGATTGATTTT
GGAAACGCTCATAACGTGCTTTTAATTTATCATATTTAACACTATCGTTTTGATTAAATAGGCGACGCAATGCATCTAATTCGTCTAATTTT
TCAGTTGCAAAGCTTAAGCCTTCGCCAACACTTGTATCTAATGTTTCATCATCTAAAGATACTGGAACATGTAATAATGAAGATGATGGTT
GAATGACAAGTTCATTAGTGTGTGCTAACAATTTATCGATTAAGACTTTTTTAGCTTCAATGTCACTTGCCCACACATTACGACCATCAATA
ATTCCAGCGTATAATGTTTTTGATTTATCAAAATCTCCAGCTTCAATTTGTTTAAGGTTATAGCCATTATCATGGACAAAGTCTAAACCTAA
ACCACCAACAGGTAAAGAACTTAAGAATTTAAGATGTGCACGTTCAAAGTATGTTTGAATGACTAATTTTTTAGCAACACCAGCTTTTTCG
AAATAGTCATAAGCTTCACGTGTAATATTTTCATAGCTTTCGCTGTCGTCTGTAACTAAGATTGGCTCATCAACTTGAATGTACTCAGCACC
TGCATCAATTAATGATTCAAACACTTCTTTATAAAGTGGTAATAACGTTTTAACTTTTTCTTCAAAAGTTTGGTGACCGCCTTTTGATAATTT
AACAAAAGTAATCGGACCAACAATGACAGGGTGAGCGTTAACGTTTAAAGATTGGGCGTATTTAAAGCGATCTAATAATACATTGCGAC
TCACTTTAGGCTCAACATTGTCCCATTCAGGTACGATGTAATGATAGTTAGTGTTAAACCATTTTATAAGTGCACTTGCAACATGGTCTTTA
TTACCGCGAGCAATATCAAATAATAAGTCATCATCGATAGTTCTACCTTGGAAACGTTCTGGGATGATGTTGAATAATAATGACGTATCTA
AAATATGGTCATATAAAGAGAAATCACCAACTGGGATGCTATCTAAGTGATAGTACTTTTGTAATAATAAATTTTCTTTATGTAGATCAGT
TAATGTTTGATCTAATTCTTCTTTAGAAATCTTTTTTGCCCAATAACTTTCGATGGCTTTTTTCCATTCTCTTTTTCTACCTAATCTTGGGAAT
CCTAAGTTTGATGTTTTAATTGTTGTCAT

Gene: metF (methylenetetrahydrofolate reductase)

Contig: 02_NODE_3, position: 313374 to 315215, length: 1842 nt, orientation: REVERSE

Perfect match to: (08-02119-CP015645-[1844751:1846592], highly conserved allele)

Sequence:

TCATAATATTGCCTCCTTGTGAGCAGTAATAGATTTTGAGTATGCTGCAAGTTCTAATGAATCTTCGACATTTTGAAACGGTGTGATAATG
TATAAACCATTAAAATATTCATGAACAGTATCGATTAAATCCTTTGAAAGCTTAAGACTTAGCTCTCGTGTTTTGGCGTTATCATCTTTAAC
TGCTTCAAATTGTTGTAAAATTTCATCTGACATCTTGATTCCTGGCACTTCATTATGCAAAAAGAGTGCGTTTTTGTAACTTGCGATAGGCA
TAATGCCTATGAAAAATGGTTTGTTCAAGTGCTTAGTGGCATGGTAAATTTCAATGATTTTCTCTTTGCTGTACACGGGTTGTGTTATAAA
ATAAGACATTCCGCTTTCTATCTTTTTCTCTAATCTTTTGACGGCACCATCTAATTTTCGAACATTAGGATTAAAGGCGCCAGCGATGTTGA
AGTGTGTACGTTTCTTCAGCGCATCACCGTCAGTGTTAATACCTTGATTAAATCTTAGAGCGAGTTCAGTTAATCCTTTAGAATTAACATCA
TAGACATTGGTTGCACCTGGTAAGTGACCAACTTTTGAAGGATCACCAGTTATGGCTAATATTTCGTTAACGCCAATGAGCGATAATCCA
AGTAAATGGGACTGCAAGCCGATTAAGTTTCGGTCTCGACATGTAATATGTACGAGTGGTTCAATATTGTAATATTGTTTAATTAAGCTTG
CAGCAGCAATGTTGCTAATTCTGACAGTTGCCAATGAATTATCTGCGAGTGTTACCGCATCTACATTAGCTTTGTCAAGTTTAGCGATATT
TTCAAAAAATCTATCCGTGTCTAAATGTTTCGGTGTATCCAATTCGATAATAACGGTTGGACGTTCTTGAACCTTAGATGTTAATGATTGTC
TAACTTTATTTTGAGATGGATTGAAAAGTGCTTTCGTTGGTATCGGAATCACTTTTTTGTCATTAACAGGTTTAAGTGTCTGAATAGATTCT
TTAATAAATTTGATGTGCTCTGGCGTTGTACCACAGCAACCACCAATTAAACGAACACCTTCGTGAATTAAATTTTGAGCAACTTGGCCGA
AATATTGTGCATTGTCACTATACTTAAATTCACTATTTTCAATATCTAATAAGCTGGCATTTGGATAGCAAGATAAGTATGCGTGCTCTGGT
AATTCAATATGTGTGAAAGACTCTTGCATATGGTGCGGGCCATGATGACAATTGAGTCCCACGATGTTGGCACCACATTGAACGAGTTGT
TTTAATCCTTCATTGATTGCCTGACCATTAACTAAGTAATTTGTGTTTGAAGCGGTTAATTGAGCAATGATTGGAATGTCGTATTTCTTTCT
CGTTCGTGAAATGACATTTGTTAACTCTTCTAGGTCGTAATACGTTTCGAAAAGTAGCGCGTCAACGCCTTCTTCAATTAAGGTGTCTATTT
GAATTTCAGTATGATAAAGAATAGTTTGTAAGCTGATATCCTCTTGTTTGATACCTCTAAACCCACCAACTGTGCCTAATATATACGTATCT
TTATTTGCTGCTTTTTTTGCGATGCGTACGGCGGCTTGATGTATAGCTTTAACTTTATCTTCAAGACCGAATCGTTTTAACTTTTCAAAATTT
GCACCATAAGTATTGGTTTGAATGACATCAGCACCAGCTTCGATATATGAACGATGGATACGTTCAACTTTATCTGGATGGCTAAGATTAT
ATGCTTCTGGACAGGTGTCTAATCCTTCAGAGTATAAAATGGTTCCAATAGCGCCATCAGCTACTAAAACATTATCTTTCAATTGTGTGAG
GAATTGACTCAT

Gene: metC (cystathionine beta-lyase)

Contig: 02_NODE_3, position: 315184 to 316344, length: 1161 nt, orientation: REVERSE

Perfect match to: (Strain_21202-AGRO01000067-[91670:92830], allele observed in CC395+CC398)

Sequence:

TTATCTTTCAATTGTGTGAGGAATTGACTCATTGAATGCCTCCTTTAATGCGTATTTGATGTCTGCAATGAGTTCATCAGGATCTTCGAGAC
CAACACTTAATCGGAATAGACCGAAAGTGATACCACGTTCTTGTCTCACTTCTTCAGGTAGTGCAGCGTGAGACATTGTTGCTGGATGTG

AAAGGATCGTTTCAACACCGCCCAGACTCACTGAAACGAGTGGTAATGTCAGTGCATCGACAAATTGTTGTGCTTTAGACTCATCAGCTA
AACGAAAGCCAATAACGGCACCGCCATTTTTAGCTTGTTCTAAATGAGCAGTAGTGAGTCCCGGATAATAAACTTCTGAAATTTCATCTTG
CTTTATTAAAAATGACACGATTTTTTGCGCGTTTTCGACAGATTGTTTAAATCTGATTGGAAATGTTTTTAAATGTTTAGCAAGTGTCCAGC
TATCTTGAGCAGATAACATATTGCCTGTACCATTTTGTATTAAATAAAGAGCTTCACTAATTGCCTCATTATTAGTAATGACAGCACCAGCA
ATTAAATCGCTATGTCCACTTAAAAATTTTGTAGCACTATGAATGACAATATCAGCGCCAAGTAATAAAGGTGATTGGCCTAACGGTGTC
ATAAATGTATTGTCCACAGCTACCAGTAGTTCATGCTTTTCGGCTATTTTAGAAACAGCTTTGATATCAGTAATTTTAAAACAGGGATTCG
ATGGTGTTTCGATATAAATTAATTTTGTGTTTGATTGAATGGCACCCTCGATTTGTTCGAGCTTGGTAGTATCTACGGTTGTAAATTCAATA
TTAAATCGATTCAAAATTTGCTCAGTGAGGCGAAAAGTACCGCCATATACATCATCGGGTAAGATGACATGATCACCAGATTTGAAAGTC
AAAAGTACTGCTGAAATAGCAGCAATACCTGATGCAAAAGCAAAAGCGAATTTTCCCTGTTCTAATCGTGCTAACTTCTCTTCTAAAAGTT
CACGGTTAGGGTTGCCACTTCGTGCATAATCATATTTAACATCGCCACCAAGACTTGTTTGATGGAATGTTGAAGAATCATAGAGTGGTG
GGTTAGCTGAATGATATTCCACACCTCTACGCCAATCGAATATCACTTCTGTCTCTTTTGAAAGTGTCAT

Gene: metI (cystathionine gamma-synthase/cysteine synthase)

Contig: 02_NODE_3, position: 316341 to 317444, length: 1104 nt, orientation: REVERSE

Perfect match to: (MW2-BA000033-[386110:387213:r], allele observed in CC1+CC15+CC188+CC361)

Sequence:

TCATACAATCTCTCCAATCTGAGCTTTATCTAATGCTTGGATGATATCGCGTTCGATGTCTTCATAATTTTCAACACCTAGTGATAAGCGGA
TTAAATACTCATCAATGCCACGTTTATCTTTTTCAGCATCTGGCATATCAACATGTGTTTGGGTGTAAGGGAAGGTCACTAATGTTTCAGT
ACCTCCTAAACTTTCTGCAAAAATGCAAATGTCTAAATTTTCTAATAATTTAGCGACGCTATAGGCCTTGTTAAGTCTTAAACTAAGCATGC
CAGTTTGCCCGCTATATAGTACTTCGTCAATTGCTTGAAGTGACTGACATTTTTTAGCAAGTTTTCTAGCGTTTGATTGCGCACGCTCAATG
CGTAAATGCAAAGTTTTAAGTCCACGTAACAACAAATAACTATCTATTGGTGAAAGTGTTGCGCCAGTCATGTTGTGAAAATCAAACAAC
TGTTGCGCGAGTGATTCATCTTTGACGGTTACGACACCTGCTAGTACATCGTTATGTCCGCCAATATATTTCGTGGCTGAATGTAAGACTA
TATCAGCACCTTCTGCTAGTGGTGTTGAAAGATAAGGTGTTAAAAAAGTATTGTCGATAATTGACAATAAGCCTTTAGCTTTACAAAGTTG
ATAGTATGGCTTTACATCAATAGCAATCATTTGTGGGTTAGATATTGGTTCAATGAATAATGCAACTGTTTTATCAGTGATTTCTTTTTCAA
CTTGTTCATAATCTGTAAAATCAACGTACTTAAATTTGATATTGTATTGTTGCTCATAAAATTCAAATAATCTAAATGTGCCGCCGTATAAA
TCGAATGAAACTAAAATTTCATCGTGTGGTTTAAATAAATTACATATTAATTGAATGGCTGACATTCCACTTGATGTAGCGAATGATGCAA
TACCATGTTCGAGTTTGGCAAAACAGGTTTCAAATGTTGAGCGTGTAGGATTTTTAGTACGTGTATAATCAAAACCTGTCGATTGTCCTAG
TTTTGGATGCTTGTAGGCAGTAGATAAATGGATTGGATTCGCTATAGCACCGGTTGAATCATCGGTTAATGTGATTTGGGCTAACTGTGT
ATCCTTCAT

Gene: tbox02 (T-box leader element)

Contig: 02_NODE_3, position: 317510 to 317879, length: 370 nt

Sequence:

AAAAAGGACGAAAGCTTATGTTTCGCGGTACCACCTTTATTTGTTATTCCATCGCTGAAATAACCTTATTCAGTACGCATTAAAAGTAAAT
ATGCTTACTGAACAATTATCACAATTAAAGTCAGTAAGTAAGGATATAGTAATGTGCTATCCCATACTTATTAACAAAAAATCGTGCGTAA
AGAATCCAGTACGCCATTTAACATCAATGTTAATACTGTATCGCTATAACGGGCGAACCCGTAGACACCTCATATTGGCATCAACACTCCA
AGGCCATTTTCAAACACGCTTTCAAAATCTTCTCTCAGCTACTAAAGACTCTCTGTATAAGCAGGGTGTGTTTTACTTTCCTCTTTATTGTGT
TTAC

Gene: spo0J (chromosome partitioning protein)

Contig: 02_NODE_3, position: 318108 to 318953, length: 846 nt, orientation: FORWARD

Perfect match to: (CN1-CP003979-[380118:380963], highly conserved allele)

Sequence:

GTGAGTGAATTGTCAAAAAGTGAAGATCAACGTATTACTAAAACAAAAGATGAACAAATTAAGCAAATAGATATATCGGATATCAAACC
GAATCCGTATCAGCCCCGAAAAACTTTCGATGAAAATCATTTAAATGATTTGGCAGATTCAATTAAGCAATATGGAATTTTGCAACCAATT
GTGCTTAGAAAAACAGTTCAAGGTTATTACATTGTAGTTGGTGAAAGAAGGTTTAGAGCTTCGAAAATTGCTGGTCTAAAATACGTATCA
GCGATTATCAAAGATTTAACAGATGAAGATATGATGGAACTGGCGGTCATCGAAAATTTACAACGAGAAGACTTAAATGCGATTGAAGA
AGCTGAAAGTTATCAACGTTTGATGACAGATTTGAAAATTACACAACAAGAAGTAGCGAAGCGATTGAGTAAGTCGCGCCCGTATATAG

CGAATATGTTGAGGTTATTACATTTGCCGAAAAAGATTGCTGACATGGTAAAAGATGGGCGACTGACAAGTGCACATGGACGAACGTTA
TTGGCAATTAAAGATGAACAACAAATGCTTAGGTTAGCGAAACGGGTTGTTAAAGAAAAGTGGAGTGTTAGATATTTAGAAAACCATGT
TAATGAATTAAAAAATGTTTCGTCAAAGTCGGAAACAGACAAAGTAGATATAACTAAGCCTAAATTTATAAAGCAACAAGAACGACAGTT
GCGAGAACAGTATGGTACCAAAGTAGATATATCAATAAAAAAATCGGTTGGTAAAATCTCATTTGAGTTTGATTCACAAGAAGATTTTCT
AAGAATAATTGAACAATTAAATCGTAGGTATGGTAAATAG

Gene: ykuT (putative small-conductance mechanosensitive channel)

Contig: 02_NODE_3, position: 319110 to 319991, length: 882 nt, orientation: FORWARD

Perfect match to: (MW2-BA000033-[388879:389760], highly conserved allele)

Sequence:

ATGAATCAAGTCATGAATATTATTTCATCTCTATTTGAGCCATTAACAAAAATAGAAACATATGAAAACATTGCAACTAAAATCGCTATGA
TTGTTATTTATATTATCGTAGCCCTCATAGTTATTAAAATACTGAATAAAATGATTGAACAGGGATTTAAGATTCAAAATAAGAGTAAAAA
GAGTAACAAAAAGCGCTCTAAAACTTTAATATCTCTTGTTCAAAATGTAGTGAAGTATATCGTTTGGTTTATAGTTATTACGACGATTTTA
AGTAAATTTGGCATTAGTGTTGAAGGTGTTATTGCAAGTGCTGGTGTCGTAGGTTTAGCAGTAGGTTTTGGTGCTCAAACCATTGTTAAA
GACGTTATTACTGGATTTTTCATTATATTTGAAAGTCAATTTGATGTAGGTGATTATGTTAAAATAAACAATGGTGGTACGACTGTGGCAG
AGGGAACGGTTAAATCAATAGGACTTCGTTCAACACGAATCAATACAATTTCAGGAGAATTAACAATTTTACCAAATAGTAGTATGGGTG
AAATAACGAACTACTCAATTACAAATGGTACAGCTATCGTTAAAATTCCAGTGTCTGTCGAAGAAAACATTGATAATGTTGATAAAAAAC
TAAACAAACTATTTACTTCTTTACGTAGTAAATATTACTTATTTGTTAGTGATCCGGTTGTTATTGGTATTGATGCTATTGAAGATACAAGA
GTAATATTGAGAATATCTGCAGAAACAATTCCAGGTGAAGGATTTGCTGGAGCTCGAATTATTCGCAAAGAAGTACAAAAAATGTTTTTA
CAAGAAGGTATTAAAACACCTCAACCAATTATGACTGCTTATAATCATAGTGAAAACGGTGTTTAG

Gene: yyzM (putative DNA binding protein)

Contig: 02_NODE_3, position: 320021 to 320224, length: 204 nt, orientation: FORWARD

Perfect match to: (MW2-BA000033-[389790:389993], highly conserved allele)

Sequence:

ATGGCGTCAAAATATGGAATAAATGATATAGTAGAAATGAAAAAACAACATGCGTGTGGAACAAACCGTTTTAAGATTATTAGAATGGG
TGCAGACATAAGAATTAAATGTGAAAATTGTCAAAGAAGTATTATGATTCCACGTCAAACGTTTGATAAAAAACTTAAAAAAATCATCGA
ATCTCATGATGATACACAAAGATAG

Gene: yyaF (ribosome-binding ATPase)

Contig: 02_NODE_3, position: 320236 to 321333, length: 1098 nt, orientation: FORWARD

Perfect match to: (MW2-BA000033-[390005:391102], allele observed in CC1+CC239)

Sequence:

ATGGCTTTAACAGCAGGTATCGTTGGATTGCCAAACGTTGGTAAATCAACATTATTTAATGCAATAACAAAAGCAGGTGCTTTAGCAGCG
AACTATCCATTCGCTACGATTGATCCTAATGTAGGGATAGTAGAAGTGCCAGATGCTAGATTACTTAAATTAGAAGAAATGGTTCAACCT
AAAAAGACATTGCCGACTACATTTGAATTTACAGATATCGCTGGTATTGTGAAAGGTGCTTCAAAGGGAGAAGGGTTAGGTAATAAATT
CTTATCACATATTAGAGAAGTAGATGCGATTTGTCAGGTCGTTCGTGCATTTGATGATGATAACGTAACTCATGTTGCTGGTCGAGTAGA
CCCTATTGATGATATTGAAGTTATTAATATGGAATTAGTACTAGCGGACTTAGAATCTGTTGAGAAACGTTTGCCTAGAATTGAAAAATTA
GCACGTCAAAAAGATAAGACTGCTGAAATGGAAGTACGTATTTTAACAACTATTAAAGAAGCTTTAGAAAATGGTAAACCCGCTCGTAGT
ATTGACTTTAATGAAGAAGATCAAAAATGGGTGAATCAAGCGCAATTACTGACTTCTAAAAAAATGCTTTATATCGCTAATGTTGGTGAA
GATGAAATTGGTGATGATGATAATGATAAAGTAAAAGCGATTCGTGAATATGCAGCGCAAGAAGACTCTGAAGTGATTGTTATTAGTGC
AAAAATTGAAGAAGAAATTGCTACATTAGATGATGAAGATAAAGAAATGTTCTTAGAAGATTTAGGTATCGAAGAACCAGGATTAGATC
GATTAATTAGAACAACTTATGAATTATTAGGATTATCAACATATTTTACTGCTGGTGTGCAAGAAGTACGTGCTTGGACATTTAAACAAGG
TATGACTGCACCTCAATGTGCTGGTATCATTCATACTGATTTTGAACGTGGATTTATCCGTGCCGAAGTAACAAGTTATGACGACTATGTA
CAATATGGTGGCGAAAGTGGCGCTAAAGAAGCGGGCAGACAACGATTAGAAGGTAAAGAATATATTATGCAAGATGGTGATATCGTTC
ATTTCAGATTTAATGTATAA

Gene: Q5HIT0 (putative bacteriophagal protein)

Contig: 02_NODE_3, position: 321419 to 321610, length: 192 nt, orientation: REVERSE

Perfect match to: (RF122-AJ938182-[365662:365853:r], highly conserved allele)

Sequence:

TTAAAAGAATGGAATAATTTTACTCGCGTTAATAATATCTTGAGTGCTGAAAAATTGTTTGCCTTCGCCAGTATAAGCAGGCTCTAAAACA
AGATTAGCCTTTGCACAATAAAGCCATTCAGGATGAATGCCACTATTAAGTATCTCTTGGAATTCTTGAAAATCTTTAGACCAATCAATAT
TTAAATTCAT

Gene: rpsF (30S ribosomal protein S6)

Contig: 02_NODE_3, position: 321872 to 322150, length: 279 nt, orientation: FORWARD

Perfect match to: (MW2-BA000033-[391641:391919], highly conserved allele)

Sequence:

ATGTACATCGTACGCCCAAACATTGAGGAAGATGCTAAAAAAGCGTTAGTTGAACGTTTCAACGGCATCTTAGCTACTGAAGGTGCAGA
AGTTTTAGAAGCAAAAGACTGGGGTAAACGTCGCCTAGCTTATGAAATCAATGATTTCAAAGATGGCTTCTACAACATCGTACGTGTTAA
ATCTGATAACAACAAAGCTACTGACGAATTCCAACGTCTAGCTAAAATCAGTGACGATATCATTCGTTACATGGTTATTCGTGAAGACGA
AGACAAGTAA

Gene: ssb (single-stranded DNA-binding protein, chromosomal)

Contig: 02_NODE_3, position: 322171 to 322674, length: 504 nt, orientation: FORWARD

Perfect match to: (MW2-BA000033-[391940:392443], highly conserved allele)

Sequence:

ATGCTAAATAGAGTTGTATTAGTAGGTCGTTTAACGAAAGATCCGGAATACAGAACCACTCCCTCAGGTGTGAGTGTAGCGACATTCACT
CTTGCAGTAAATCGTACGTTCACGAATGCTCAAGGGGAGCGCGAAGCAGATTTTATTAACTGTGTTGTTTTTAGAAGACAAGCAGATAAT
GTAAATAACTATTTATCTAAAGGTAGTTTAGCTGGTGTAGATGGTCGCTTACAATCCCGTAATTATGAAAATCAAGAAGGTCGTCGTGTG
TTTGTTACTGAAGTTGTGTGTGATAGTGTTCAATTCCTTGAACCTAAAAATGCGCAACAAAATGGTGGCCAACGTCAACAAAATGAATTC
CAAGATTACGGTCAAGGATTCGGTGGTCAACAATCAGGACAAAACAATTCGTACAATAATTCATCAAACACGAAACAATCTGATAATCCA
TTTGCAAATGCAAACGGACCGATTGATATAAGTGATGATGACTTACCATTCTAA

Gene: rpsR (30S ribosomal protein S18)

Contig: 02_NODE_3, position: 322726 to 322968, length: 243 nt, orientation: FORWARD

Perfect match to: (RF122-AJ938182-[366969:367211], highly conserved allele)

Sequence:

ATGGCAGGTGGACCAAGAAGAGGCGGACGTCGTCGTAAAAAAGTATGCTATTTCACAGCAAATGGTATTACACATATCGACTACAAAGA
CACTGAATTATTAAAACGTTTTATCTCAGAACGCGGTAAAATTTTACCACGTCGTGTAACTGGTACTTCAGCTAAATATCAACGTATGTTG
ACTACAGCTATCAAACGTTCTCGTCATATGGCATTATTACCATATGTTAAAGAAGAACAATAA

Gene: Q1XZL6 (Abi-like protein)

Contig: 02_NODE_3, position: 323234 to 324172, length: 939 nt, orientation: REVERSE

Perfect match to: (MW2-BA000033-[393003:393941:r], allele observed in CC1+CC188)

Sequence:

TTATACTTTTGGTCTGTTGAAGTCAATAATTTTATCTAAAGCTATAAAAAATCTTTTGATAGCTAATGCATTATTATAATAGCTTTCGTTTCT
TTTATATCGCTTTTGAAGTTGGTCCAAATCGTGATATCTTGCTTGGATAATTGCATTACTACAAACTTGATTATGTAATTCTAGCGTAGCGA
AAGTATCTATGAAATTTTTTATTCCGAACATGTTTCTAGATATGCCTATATTATTCCCTTTTTCAAATAAATATTGAGGAGATCTACTGTCAT
AATTTAGATTTGCTATGATGGGTTGGTTATGAGCTGATTTGTTTCTTATATTTTTAACTAAAGGCATTAAAATATTAGCAACTCTCAATTCTT
CGTCATTGTACTTCTTGTAATAGAAGTTGAGAAACGAAACGAATTGACCTAGTTGCATGAATTCAATGCAAACCCATGCGGGTGGATTTT
GATAGTATTTATTCAACTTCTCGGGTAGTTGTCCTCGTTTATTCATATGCTTGAATATTTCGTTTTTATTTTTGATTTTGGTTTCCATAACTTC
TTCTGGTGTTCTTGAATTTGTGTCAAAATTTGAATTGCTATATGATTTATCAATACATAAGAACTCATCTATTATTTTATAACCATCTTCTTG
GTTATTTTCTGTTATTAGTTTTAAGACTAGACACTTTAAACTATGTTCAATATCTAAAGTTAAATGCAACATTGTGTATCTTAATTTCATATC
TATAGTTGCTAAATCTGATAAATAAGCAAATTCTATGAAATAGCCGCCATTCTTTTTTTCGAAATTTTTTCGGAAATAAGCTAGTTTGAAGA
AGTGATTATTTTTTCTAAGAATTTCATTTGCTTTTTCGGTGTCAATAATATTAAAAAATATATTCATCTGTTTTAATTTCGCTATTTGCTCATC
AAAATTGAGCAT

Gene: int5-setC (truncated integrase, associated with setC)

Contig: 02_NODE_3, position: 324211 to 324925, length: 715 nt, orientation: TRNC-RVRS (no start codon)

Sequence:

TTAACTCCCCAATCATTCAAATTTATTCATCATATCTTTCGCCATCTGATTAGTAACATGTGTGTATATCTCTAGATTTTTTTATAATCTGAAT
GACCTACATGCTCTTGCATTGCTTTTAAGTTAATTCCTAATTGAGCAAGTGTAGATATATGCGAATGATGTAATGTATGCGTCGTTACAGG
TTTCTTAATAGAACTAATCTCAGTTGTCTCTTTGATAGTGCCCCCTTTAATAATGTTGCTAATTTTGTTCGAGTCGATAGGGCCACCAGCTG
CATTTGTGAATATGTACCCTATATTAATAAACTTATCATTACAAGCATAAGTGTTCTTAGTAAGTCGATGCTATGGATAGTGAGGCCTATA
GCCCTATAGCTGTTACTTGTTCTAGTTGTCTCTTTTACTCCGGATGCTCCCGTCATTTTTCAGTTATCCAATTAACTTTACCGTCGATATCTA
GCGTTTTATCTTCATAGTTTATATTTACTCTCTTTATTGCAAGTAGCTCACCGATACGCATGTCATTAGCAATTTGAAACTGTACCATAGCTT
TTACCATTTTATAATTACGTTTTGTCGTTGGATATTTTTATACTTAATTACATAGTCGAAACAATCCAGTAACTCCTTATCTTCATTATCTTCT
AAAGTGTTATTACGTTTAGCTAGTAACGCATCACTATAGGGATATCTATTTTATCTATTACACATAT

Gene: setC (staphylococcal enterotoxin-like toxin X)

Contig: 02_NODE_3, position: 325131 to 325742, length: 612 nt, orientation: FORWARD

Perfect match to: (MW2-BA000033-[394900:395511], allele observed in CC1+CC72+CC188)

Sequence:

ATGTTCAAAAAAAAATGACTCGAAAAATTCAATTCTATTAAAATCTATTCTATCGCTAGGTATCATCTATGGGGGAACATTTGGAATATATC
CAAAAGCAGACGCGTCAACACAAAATTCCCCAAGTGTACAAGATAAACAATTCCAAAAAGTTGAAGAAGTACCAAATAATTCAGAAAAA
GCTTTGGTTAAAAAACTTTACGATAGATACAGCCAAAATACAATAAACGGAAAATCTAATAAATCTAGGAATTGGGTTTATTCAGAGAGA
CCTTTAAATGAAAACCAAGTTCGTATAAATTTAGAAGGAACATACAGAGTTGCTGATAGAGTATATACACCTAAGAGAAATATTACTCTT
AATAAAGAAGTTGTCACTTTAAAGGAATTGGATCATATCATAAGATTTGCTCATATTTCTTATGGCTTATATATGGGAGAACATTTGCCTA
AAGGTAACATCGTCATAAATACAAAAGATGGCGGTAAATATACATTAGAGTCGCATAAAGAGCTACAAAAAGATAGGGAAAATGTAAA
AATTAATACAGCCGATATAAAAAATGTAACTTTCAAACTTGTGAAAAGTGTTAATGACATTGAACAAGTTTGA

Gene: Q2G106 (putative exported protein)

Contig: 02_NODE_3, position: 326111 to 326512, length: 402 nt, orientation: FORWARD

Perfect match to: (MW2-BA000033-[395880:396281], allele observed in CC1)

Sequence:

TTGAAAACGATTTTAAAAACAATAACATATCTAGCACTTACTATCATTGGCGCTTATGCTGCTTTATTCATTTTAAAAACAATAGACTCTCA
TGGTATAACAGATCAATTTAACCCATTAGTAAAGGAAGATGTTTCTTATGTTAAAACGACTGAGGTGTCTACTAGAATGGATGATCAACT
CCGAAGTTATAGTCAAAGTGCTTTTAATAAAGAAGGGAAAGAGACGCAATTAATGTATACTGCTACATTTGATGTTAAACCGCATAGATA
CTTGAAAATTACACATAAAGGTCATCACGTAGAAACTTTTGAAGAAGTTGAAAAGGGGCAAGTACCTAAAAAAAGCATTAGACAAATTG
AGTCGATTATAGCGTGCTTATATTACATGGTTCAATTATAA

Gene: Q1Y9M1 (putative protein)

Contig: 02_NODE_3, position: 326660 to 327232, length: 573 nt, orientation: FORWARD

Perfect match to: (MW2-BA000033-[396429:397001], allele observed in CC1+CC9+CC188)

Sequence:

ATGAAATTAAAATCATTAGCAGTGTTATCAATGTCAGCGGTGGTGCTTACTGCATGTGGCAATGATACTCCAAAAGATGAAACAAAATCA
ACAGAGTCAAATACTAATCAAGACACTAATACAACAAAAGATGTTATTGCATTAAAAGATGTTAAAACAAGCCCAGAAGATGCTGTGAA
AAAAGCTGAAGAAACCTACAAAGGCCAAAAGTTGAAAGGAATTTCATTTGAAAATTCTAATGGTGAATGGGCTTATAAAGTGACGCAAC
AAAAATCTGGTGAAGAGTCAGAAGTACTTGTTGCTGATAAAAATAAAAAAGTGATTAACAAAAAGACTGAAAAAGAAGATACAGTGAAT
GAAAATGATAACTTTAAATATAGCGATGCTATAGATTACAAAAAAGCCATTAAAGAAGGACAAAAAGAATTTGATGGTGATATTAAAGA
ATGGTCACTTGAAAAAGATGATGGCAAACTTGTTTACAATATCGATTTGAAAAAAGGTAATAAAAAACAAGAAGTTACTGTTGATGCTAA
GAACGGTAAAGTATTAAAGAGTGAGCAAGATCACTAA

Gene: Q5HIS2 (putative protein)

Contig: 02_NODE_3, position: 327370 to 327633, length: 264 nt, orientation: REVERSE

Perfect match to: (MW2-BA000033-[397139:397402:r], highly conserved allele)

Sequence:

CTATTTATCCATGCGAATATCGACTTCTTCTAAATGTTTCTGATATTCTTTAACCTTACTTTCTAAAAACATTTCATATGGTGCATCAAAGAA
ATCAGCTAAATGCATGGCGTCTTTAAATTTAGGTTCATGGTGATGATTCTCCCATTCCCAAATTTGATGTGCTTCATATTTAGTACCATATT
TCTCATTTAATTGTTGTGCTAATTCGTCAATTTCTAAATTATGTTTAGTTCGTAAGTTATATAAAATATGCATATTCAT

Gene: Q5HIS1 (putative protein)

Contig: 02_NODE_3, position: 327927 to 328178, length: 252 nt, orientation: FORWARD

Perfect match to: (MW2-BA000033-[397696:397947], allele observed in CC1+CC9+CC188)

Sequence:

ATGTTTGGATTTATTGGAATGTTAATTATCGGTGGCTTAATTGGATGGGCTGCTGGTGCTATTATGGGTAAAGATATCCCAGGTGGTATT
TTAGGCAATATTATCGCAGGTATTATTGGATCATGGGTAGGTGGCAAACTATTCGGACAATGGGGTCCTGAATTAGGAAGTATTTACATC
TTGCCAGCATTAATTGGTTCAATTATCTTAATTGCAATCGTAACGTTAATTTTAAGAGCTATGCGTAAATAA

Gene: Q7A7J4 (putative protein)

Contig: 02_NODE_3, position: 328604 to 329185, length: 582 nt, orientation: FORWARD

Perfect match to: (MW2-BA000033-[398373:398954], allele observed in CC1+CC188)

Sequence:

ATGACGATTTATTTAGTTAGACATGGCGAATCAAAATCGAATTATGATAATAAACATTTTCGATCTTATTTTTGTGGACAATTAGATGTGC
CGTTAACGGATACTGGCACAAAAAGTGCAGACGATTTATGTGATTATTTTAAAGAGAAACAGATTAAACATGTATATGTTTCAGACTTATT
AAGAACACAGCAAACGTTTGAACATATTTTTCCATATGACATTGCATCAACGACTACGCCTCTATTAAGAGAACGTTCACTTGGCGTATTT
GAGGGTGAATATAAAGATGAAATCAGTGCGAATCCGAAATATGAAAAATATTTCAATGATCCAAACTTTAAAGACTTTCGTCATAGTTTT
TCACAAAAAGCGCCTGAAGGAGAAAGTTATGAAGATGTATATCAACGCGTCGAACATTTTATGAATCATGTTGTCAATGAAGATACACAA
AAAGATGATATCGTCATTGTTGCACATCAAGTTGTCATTCGTTGTTTGATGGTTTATTTTAACAAAGTTTCAAGGGAAGAAGCTGTGGATT
TAAAAGTTGAAAATTGCAAACCATATATCATTGAATAG

Gene: Q5HIR9 (putative membrane protein)

Contig: 02_NODE_3, position: 329251 to 329634, length: 384 nt, orientation: REVERSE

Perfect match to: (MW2-BA000033-[399020:399403:r], allele observed in CC1+CC9+CC188)

Sequence:

TTAATGGAAGTCATTCTTATCAACAGACTTACCACCAATAGCTAATGTGATAATTGCGCTTACGAACAAAATAATTGAAGTTATAAATGAC
GCTAATGTAAAAGGTGTAAATTGTGTGAGCGATAATATCAATGATAAAAAGAACCAAGAGATAGCCCCTAGAAATTGTAAAATAGCAGA
AGAAATTCTAAAACTCATATAATCATATATAGCTAAAACAAGTGAAGGTAATGCGACTAATACCATTATAATGATGTTGAGGGTGAATAA
ATATGGCTGTTCAAAAGTTACTGTGTTTGGTCCTGTTGGATGCATTGCTGTTAAGAACAAGCAAACAATCGTCGATAAAATTGCTAAAAT
AATAAAAGTAATTCGAACTTTCAT

Gene: A5IPV8 (putative lipoprotein)

Contig: 02_NODE_3, position: 329889 to 330515, length: 627 nt, orientation: REVERSE

Perfect match to: (MW2-BA000033-[399658:400284:r], allele observed in CC1+CC9)

Sequence:

TTATTTATCGATAACATCACTCTTGATACCTTTAGATTTTAAGAAATCTTTAATTTTATCTTGTTGCTTTTTATTAACATCACCGGCATATTTT
GTTGGCACGTCGACTACATTGATTTTATTTTGCGGTTGATAGCTAAGCTTTTCAATATCTTCATCAACATTGGCGATTGTACTATTTAAAGC
TTTGAAGTAATTCATCATTAATTCAACGGGTTTCTTATATTCTTTAGGAATATTGTTTTCAGTGACAAATTTCTTGAAATGCAAATCGTTTTT
AACAGCTAAGTTAGATAAGTGGCTAAGTGTTTCTGCTTGTTTTTCAGTCACTTTTGTTTGACTGTCAATTTGTTTATCTAGTTTATGTTGCAT
AATATATTTGTTCTCAAGTATATCGCTATTTACAGACAAATACTTTTCTATAGCTTGCTTCATCTCTGCATCACTAATATCACTATTTTTCTTA
TCTGAGTTAAAGATATCTTTTGTTTCTAATTTTTTAGCGCTTTTAGGTGCATGGATGCCAGTACTTGTGTGATGATCTTCGTTATCAGATTG
ATCGGACGCGCAACCTGTAAGAATTAATGCAGATGCTAAAAATGTACTTAGTAGTAATCTCTTTTTCAT

Gene: Q6GJR9 (putative protein)

Contig: 02_NODE_3, position: 330588 to 332198, length: 1611 nt, orientation: REVERSE

Perfect match to: (MW2-BA000033-[400357:401967:r], allele observed in CC1)

Sequence:

TTAGTTTGGAATGTCACGATGACCTTGCAATGACCATAGACGTAAATGATTACGTGCATGAGTTGCTTTTTCAATCAATAATGCGTCGTTT
TGAACGTTGTTAAGGATAGCATTATCTATAAATAACTGCATAATTGGTTGTATCAATTTAGACGTAGGTATCGTACGTAAAAGCATAATG
ATTTCGTTCACATACTTTTCCTTCTCAATATCATTTTTCATATTGATTTGTTTGCGTAGATAACTGGTGATCTCTTTAATTGCTTGATCAGATT
TCAAATAGTGATGTCTATCATAATAGATTAATTCTTCAACCGAAGCTGGTACATAATATCCATCATCAGAAATATCAATGCCAGACGCCTC
AGACTCGACAACATCAAATAACACAGGATGAAACACGAAGTCATCTTCAACATAATATAGCTCAGTACCAGCTAAAGTATCTAAGCTATA
TTGTAACTCTTCTTTACTAAAAGTAGCGTCATAAAATCTTTGTAAAAGATATAGTAGATGATTAGTTGATAAAATACCATACAAGTTTACA
GAACCAAGTATAATACGAAGAGGTAAAATATCTTTTTTATCTATTAATCCTTTCTTAATCTCAGAAATCAATTGAGTGATTGCATCACTTCT
TAAGCTATCGGTTGTACCAAAATCTTCATCTTCAAGTAACATATCTAAGTCAACTTCGTCTTCCATAAATGATAATTCTAACGCGTTTTTAA
ACTCTGCTAAACTATTCTCAATTGTTTGCTCATTAACAAATTGATTTACAGCTTTCTCAAAATCACCATTGCCGACTTGATTCACGAAATTTT
CAATATGTTTACGCACATCATTTGGCAAAATAAGACAATCATATTCAAAGTCGTAATAATAATAAAAATGAATAGTTAAAAAGTTATCAAA
ATTTATTAATTGCAGCTTTTCAAATTTAGTTAGTTCATTGTTTAATCGGTAGTTATCATCACTTTCAAGTATGCTAGAAAGAATATCAAACTC
AAATAGAGTAGCATTAGAAAACACTTGTTCAATGATGTGATTATTATTGAAAAATGCTGTCATAATTAATTCAACAAGATTTTGTTTGTTTT
TATTTGAGAATCCTTTGATACCATACTGCCTGCATATTGTTTTTAGCTCGTCATTAGTTTTTAACAGTAAAGCATGTTCTAATGTGACATCGT
TTAAGCGCGTATGTTGTCTTACACGTAACACTTCTTCATTTAAATTCATATGGTTTATTGTATCTAACGATTCGAATTCACCATATGCATTAT
CTAGTACCTCTGAATTGATATATCTTAAAAAGAAATTATTTACAAAGAACTCATCTTCGTTAAGTTGATAAACAAAACCTAGATGATTTGTT
AGCTTTAAAATATTAACATCGCGTTTATTTATAATTGTGACAGGATTACCATTAGATATAAGTGTTTCTATAAATTTTTGTTCTGTCTTATCC
ATATCATGATAAATTTTATGTATATTTTCAGGGCGTAAAGCATGCATCGTGACTTCAAATCCTTCTTTTTGGGTCGGTAAATTAAAATATTC
GCATGCGTTTTGTAATTTTCTTGGGTATATAAAATGAACATCAAGCAT

Gene: ahpF (alkyl hydroperoxide reductase subunit F)

Contig: 02_NODE_3, position: 332313 to 333836, length: 1524 nt, orientation: REVERSE

Perfect match to: (MW2-BA000033-[402082:403605:r], allele observed in CC1)

Sequence:

TTAGTTTCTGATAATATAGTCAAAGGCATTTAATGCTGCATTTGCACCAGCGCCCATTGAAATAATAATTTGTTTGTTCTTCTGATCTGTGA
CATCGCCAGCAGCAAATATTCCAGGTACGTTAGTATTATTGTTACGATCAATAACAATTTCACCACGTTCGTTTAATTCAACAGCATCTTTT
AACCATGATGTGTTTGGAAGTAAACCAATTTGAACAAAGATACCATCTAAGTTAAGTAGATGTTCTTCGCCGGTGCTCATGTCTTCGTAAC
GTATACCTGTAACATGGTCTTCTCCGACAACTTCAGTAGTTTTGGCATTTGTTTTGATATCAACATTTGATAAAGAACGTAAACGATCTTGT
AACACGTTGTCTGCTTTTAATTCGCTAGCGAATTCGAATAATGTAACATGATTAACGATACCAGCAAGGTCAATTGCTGCTTCAACCCCAG
AGTTACCGCCACCGATAACTGCTACGTCTTTATTTTCAAATAGAGGTCCGTCACAGTGAGGGCAGAATGCAACACCTTTATTAATCAATTG
CTCTTCACCTGGAATGTTTAGCTTACGCCAACCTGCACCAGTAGCAATAATGACTGTTTTACTTTCTAAGACAGCACCGTTTTCTAACGTAA
CTTTAATTGCTTCGTCAGTCTTTTCGATATCTGTAGCACGTATACCTGTCATTGCATCAATGTCATATTGATCAATGTGCGCTGCTAAGTTA
GAAGAAAATTCAGAACCAGTTGTTTCTTTAACAGTAATGAAGTTCTCAATACCAGCAGTATCATTAACTTGGCCACCGATACGATCAGCAA
CTATACCAGTACGTAAACCTTTACGTGCTGTGTAAATCGCTGCACTACCACTAGCAGGACCACCACCAACGATTAAGACATCATAAGGTTC
TTTATTTTCAAACTCAGATGCATCTGCCGTACTGCCTAGTTTCGAAAGAATATCTTGGATTGTCATACGACCATTGCCAAATTCTTCGCCAT
TTAAAAAGACAGCAGGGACTGCCATGATGTTTTCAGATTCTTCACGGAACACTGCACCATCAATCATAGAATGCGTGATGTTAGGGTTGA
TCACACTCATTAAGTTAAGTGCTTGAACGACATCAGGACATTTTTGACACGTTAAACTAATGAATGTTTCAAAATGGAATGAACCTTCTAA
GTTTTTAATTTGGTCAATGATTGACTGTTTTTCTTTAGGTGCACGACCACTAACCTGTAAAATTGCTAAAACAAGTGAGTTAAACTCGTGA
CCTAATGGAATACCTGCAAATGTTACACCTGTTTCTTCGCCAGGACGATTGACTGAGAAACTTGGTGTACGCTTTAAAGACTTTTCAGAAA
GTGAAAGTCTAGGTGACATATCAGAAATTTCTGTTAATAATTCTTTGAGTTCCTTAGATTTTTCATCTGAACCAAGGCTGGCAACGAATTC
AACGTTGCCCTCCATTAGTTCTAATAATTGTTTAAGTTGTTGTTTTAAATCAGCATTAAGCAT

Gene: ahpC (alkyl hydroperoxide reductase subunit C)

Contig: 02_NODE_3, position: 333852 to 334421, length: 570 nt, orientation: REVERSE

Perfect match to: (N315-BA000018-[422549:423118:r], highly conserved allele)

Sequence:

TTAGATTTTACCTACTAAATCTAAACCAGGTTGCAATGTTTTAGCGCCTTCTTCCCATTTAGCTGGGCATACTTCGCCAGGGTTTTTACGAA
CATATTGAGCTGCTTTGATTTTGTGAGCTAATGTACTAGCGTCACGGCCAATTCCGTCAGCGTTAATTTCAGATGCTTGTACAACACCGTC
TGGGTCGATAATGAATGTACCACGTTGAGCTAAACCAGTAGCTTCATCTAATACATCAAAATTACGAGTGATTGTTTGTGATGGGTCACC
AATCATAGTGTAAGTGATTTTGCTAATTGCATCTGAATGGTCATGCCATGCTTTGTGTACGAAGTGAGTATCAGTTGATACTGAGAATACA
TTTACGCCTAATTTTTGTAATTCTTCATATTGGTTTTGTAAGTCTTCTAATTCAGTTGGACAAACGAATGAGAAGTCAGCAGGATAGAAGC
ATACTACGCTCCAAGAACCTTTTAAATCTTCTTGTGTAACTTCTTTAAATTGATCTTTTTTTGGATCGAAAGCTTGCGCTGTAAATGGTAAG
ATTTCTTTGTTAATTAATGACAT

Gene: nfrA (NADPH-dependent oxidoreductase)

Contig: 02_NODE_3, position: 334913 to 335668, length: 756 nt, orientation: FORWARD

Perfect match to: (TW20-FN433596-[475823:476578], allele observed in CC239+CC1+CC239)

Sequence:

GTGTCAGAACATGTATATAATCTTGTGAAAAAGCATCATTCTGTTAGAAAATTTAAGAATAAACCTTTAAGTGAAGACGTTGTTAAGAAA
TTGGTAGAAGCTGGACAAAGCGCTTCGACGTCAAGTTTCCTGCAAGCATACTCAATTATTGGTATCGACGATGAGAAGATTAAAGAAAAT
TTACGAGAAGTTTCTGGACAACCTTATGTTGTAGAAAATGGCTATTTATTCGTCTTTGTTATTGATTATTATCGTCATCATTTAGTTGATCA
ACATGCTGAAACTGATATGGAAAATGCATATGGTTCAACGGAAGGTTTGCTAGTAGGTGCAATCGATGCAGCATTAGTTGCCGAAAATA
TTGCGGTAACTGCTGAAGATATGGGGTATGGCATTGTCTTTTTAGGATCATTAAGAAATGATGTTGAACGAGTTCGAGAAATTTTAGACT
TACCTGACTATGTCTTCCCGGTATTTGGTATGGCAGTAGGGGAACCCGCAGATGACGAAAATGGTGCAGCCAAGCCACGCTTACCATTTG
ACCATGTCTTCCATCATAATAAGTATCATGCTGATAAGGAAACACAGTATGCACAAATGGCAGATTACGACCAGACAATCAGCGAGTACT
ATAATCAACGTACAAACGGGAATCGCAAAGAAACATGGTCGCAGCAAATTGAGATGTTCCTAGGAAACAAAGCAAGATTAGATATGTTA
GAACAATTGCAAAAATCAGGCTTAATACAGCGATAG

Gene: tcyP (L-cysteine uptake protein)

Contig: 02_NODE_3, position: 335749 to 337137, length: 1389 nt, orientation: REVERSE

Sequence:

TTAGTGTGAAGTTAATGCAGCATTATCATTTGAATCGAAAGTATCTTTATCCCAATGTTTAGTTAACTTGGCGGTACCTGTACCAGCTAGC
ATTGAATCGTTCACGTTTAATGCTGTTCTACCCATGTCAATCAATGGTTCAACGGAGATGAGCACGCCGGCTAAAGCGACTGGCAAGTTT
AACGTTGACAACACCAATATGGATGCAAATGTAGCCCCGCCACCGACGCCAGCAACGCCGAATGAACTAATAATCACGACAGCGATTAA
CGTTACAATAAATTGTAAATCAATTTCTACATTAGCGACGGGTGCGACCATAATTGCAAGCATGGCAGGGTAAATGCCTGCACAACCATT
TTGTCCAATCGACAATCCAAATGTCGCAGCGAAATTGGCAATACCTTCTGGCACGCCTAGACGTCTTGTTTGTGTTTGTACATTCAATGGT
AAGGCACCCGCGCTTGAGCGTGATGTGAATGCAAAGATTAATACTTCCAAAGTCTTTTTAACATAGCGAATTGGGCTAATACCTAACAGG
CTTAAAATAATTAAGTGAATGATATACATCGTAATTAATGCAGCGTACGATGCGATTAAGAATTTTCCTAAAGTCCAAATGGCGCCAAAG
TCACTTGTCGATAATGTGTTGGCCATAATTGCTAATACACCGTATGGCGTTAAACGTAAGACGAACGTCACAATCGCCATTACTAGTGAAT
AGATAGCGTCAATCGCACGCTTAAGCAATTCACCATGATCAGGTTGTTTGCGTGCTACGCGTAAATAAGCAAATCCTATAAACGAAGCAA
ATATCACGACAGCAATCGTGGAAGTTGCACGTTGTCCAGTGAAATCTAAGAATGGATTTTTAGGCAATAATTCCAAAATTTGTTGTGGTA
ACGTATGTGCTGTTAAATCTTTCGCTTGTTTAGCAATTTCGCTTCCACGTGCTTGTTCAACGTTACCAAGGTTAATTGTTGATGCATCTAAA
CCAAACACCAAGGCATAAACAACACCAACAATTGCAGCAATGGTGACAGTGCCAATTAAAAAGATAAAAATGAGACTACCAATTTTAGC
AAACTTTTCTCCGATTTGAATTTTAGTGAATGCAGCGACAATAGAAATGAAAATTAAAGGCATCACAATCATTTGCAACAATGCAACGTA
ACCTTGTCCGACAATGTTGAACCAGTCACTTGTTGATGTAATAACATTCGAATGTGTTCCATAGACAAGATGTAATAACACGCCAAATACA
ATGCCGATACCTAAAGCTGTAAACACACGTTTCGCAAAAGATATATGTTTGCGAGCCATCATGTGCAATATTACGATGAAAATCACCAAT
ACAATAATATTAATCAGTGTAAGAAAAGCATTCAT

Gene: tx_universal2 (rho-independent terminator)

Contig: 02_NODE_3, position: 338150 to 338182, length: 33 nt

Perfect match to: (Strain_21194-AGTU01000149-[993:1025], allele observed in CC45I)

Sequence:

CTGAACGAAAATGCGCTTGTAACAAGCTTTTTT

Gene: tx_universal2 (rho-independent terminator)

Contig: 02_NODE_3, position: 338152 to 338190, length: 39 nt

Perfect match to: (Strain_21331-AGTV01000040-[104403:104441], allele observed in CC398)

Sequence:

GAACGAAAATGCGCTTGTAACAAGCTTTTTTCAATTCTA

Gene: Q5HIR2 (putative protein)

Contig: 02_NODE_3, position: 338311 to 339267, length: 957 nt, orientation: REVERSE

Perfect match to: (MW2-BA000033-[408080:409036:r], allele observed in CC1+CC5+CC49-ST291+CC398+CC425)

Sequence:

CTACAAGTTGAATAAACGTGCGAAGAAACCTTTTTTCTCTTCTTGAGGTTTTGGTTCTGACATTTCAGACTGTGAAGCTGTGCTGTCATTTT
CTGCTTTCGCTTCCAATGAATCGTTCGAAGTAGCTTGTTTAGAAGCTTCTGCATGGGCTTCTGCTACTTGTTGTTCAGCATCATGTTGCGCT
TCTGCATCTATTGTAGCTTCTTTATTTGAAGTCTCAGCTGCATTGCCATTTTTAGACTGCTCAACGCTGGCTTTTTCAATTGCTTCAGTTGCA
GAAATTTGATCCTCTTTATCTACTTTAGTAGTAGTGCCATCGCCTGCATTGTCTGCTTTAGTATCCGTCTTAGCGTCTGTTGACGTATTCACT
CCAGCAACCTTCGAATCTTTACTGTCAGCTTTATCTTCAGTAACAGCATTCTTTTCAACTTTATTTGTTGATTCAGCCAATACTTTAGCTTGA
GATGCTTGTGCTTCTTGGATAGCCTCTTGTGATGTTTGGATAGCTTTTAAGGATTCGCTATTTGAATCGATTTTAGATGTAAGTTGATTTTG
AAGTTCTTTTAATTCTTGTTGTTGCTGATGTACTTGATTCATCATTTGACCAAGTAGGTGACGTTCTTCGCGCATTTTCTGGATTTCTAGGC
GTAAATCTTCTACTAATTGAGCGACATTTTGATTAGTAGGTAAGTTTTTGTCATCGTTTTTGACAATGACTTGCAGGAAGTCTTTTTCTTTTT
CTAATTCATCAAACGCTAGATCATAACTATTTGTTTGTTTTACTTTGTCGGCAATGTCTTTGAAAAGCTCGATATCTTCTTCTTTGAAATCAG
TTGCTTCACGACCACGATATTCAGTCTTGCTTAGTTGGTAACCACGTTCTTCTAAATGTTGAACAATTTTACGGACTTGCTTCTCACTTAGTT
CTACGCGTTGTGCAAATTCTTTAGTTAACAT

Gene: Q5HIR1 (putative protein)

Contig: 02_NODE_3, position: 339385 to 340047, length: 663 nt, orientation: REVERSE

Perfect match to: (JH9-CP000703-[464666:465328:r], allele observed in CC5)

Sequence:

TTAAAATGGTTTACGTAAATCCATTTCTTCTAATAAAGCATAAATAATTTCACGTGCGATACCATTTGCAAATACATACTTCAAAATATAAT
TTGATTTAACACTTGTACCGTAGATATCCTTTGTCAGTTTAATATTTTTCTTTAAAAATAAGTCCACTTTTTTAGTGTGACCATCATTAGTTG
GATGAATGTAATCAATTTCAGTTTGTCTTTCAAATTGATGTTCGTATAAAAACTGTCTACGGTGTTCAATAATGAGTCCATTATCTTGTGTT
AAATCTGCAGGTTCTTTAGGGACTTCCATCATTAAAAAGTTAATACTTCTAGAATGAACAAGGTTTGATAATAAGTTTAGTTCATGTTCAA
TTTGATGTAATGTTTGCGTCATTAACTCATCATGGTTGGGTAGGTCTTTTGCGAATAAATAAATTAAAAATGCTGAATTGGTTGTGGCTTC
ATAATGTGCAGTTGCTAGACTGACAACAACATCGTTTTCTAAGCCTACGATAAAGACATAATCGTTTTCGGTTTTGTTGTTTTCTAATGAGC
GTTTGATGATACGTTTATCTTCAGTTAAAGATAAATTTAACTTAGTATCGTAAAGTTTAAGTGCTTCGTTGTAATGTGGATCTTTGACAGAT
TGAATGGTTTTAAATTCCAT

Gene: yflT (heat induced stress protein)

Contig: 02_NODE_3, position: 340190 to 340597, length: 408 nt, orientation: REVERSE

Perfect match to: (MW2-BA000033-[409959:410366:r], allele observed in CC1+CC188)

Sequence:

CTAAGCTTTGGGACCTTTAGATGCTTCAGCAAAATGTGTAATATCAATCTCTTCATAAGCTGAATTATTTTCATGCACTTCTTGATGTGATG
ATTTGTCACGAACCGCTACAACTAACATTTTATCGTCTAAAATAAGTTGTTTATATTTTTCTAATTCATCAGGCGCTAAGTTGTAACGTGAT
AAAACTGCATGTTCACCATCTTCTCCTGTTAACAGTTTAGTCATTCTATCACTAAATGTTCCACTTGTTGAGATAAGGGAGATTTCAGAGTC
GTGTAAGTCATTTAGGTGTAATTTACTTTTACTAATAATTGTTAGCTCTGATTCTAAATAACCTTCAGATTTCTTTTGATTGATTACGTTGTA
TAATTCGCCAGTGTCATTTACTACAGTAATATCTGCCAT

Gene: xpt (xanthine phosphoribosyltransferase)

Contig: 02_NODE_3, position: 341110 to 341688, length: 579 nt, orientation: FORWARD

Perfect match to: (N315-BA000018-[431130:431708], highly conserved allele)

Sequence:

GTGGAGTTACTAGGACAAAAAGTAAAGGAAGACGGCGTTGTCATTGATGAGAAGATTTTAAAAGTCGATGGATTTTTAAATCATCAAAT
TGATGCAAAGTTAATGAATGAAGTTGGTCGCACTTTTTACGAGCAATTTAAAGATAAAGGGATTACTAAAATCTTAACCATTGAAGCTTCC
GGTATCGCACCTGCAATCATGGCTGCACTGCATTTTGATGTGCCATGTTTATTTGCGAAAAAAGCAAAACCTAGCACTTTGACGGATGGT
TATTATGAAACATCTATTCATTCATTTACTAAAAATAAAACAAGTACGGTCATTGTTTCAAAAGAATTTTTATCAGAAGAAGATACTGTACT
CATCATCGATGACTTTTTAGCAAATGGTGATGCTTCATTAGGATTATACGATATCGCACAGCAAGCGAATGCTAAGACAGCTGGTATTGG
TATTGTTGTTGAAAAGAGTTTCCAAAATGGGCATCAACGTTTAGAAGAAGCAGGTTTAACAGTTTCTTCTCTCTGCAAGGTTGCTTCACTA
GAAGGAAACAAAGTGACATTGGTGGGAGAAGAATAA

Gene: pbuX (xanthine permease)

Contig: 02_NODE_3, position: 341688 to 342956, length: 1269 nt, orientation: FORWARD

Perfect match to: (MW2-BA000033-[411457:412725], highly conserved allele)

Sequence:

ATGAAAAAATTTAATCCTAAGTGTTCAACATCTTTTAGCTATGTACGCAGGTGCCATCTTAGTTCCAATCATTGTTGGTACAAGTTTGAAGTT
TACACCTGAACAAATCGCTTACTTAGTTACAGTAGATATATTTATGTGTGGGGTTGCCACATTTTTACAAGCCAATAAAGTAACAGGAACA
GGATTACCAATCGTTCTTGGATGTACATTCACGGCTGTTGCGCCCATGATTTTAATTGGTCAAACGAAAGGAATAGATGTACTTTATGGTT
CGCTATTTTTATCAGGGATATTAGTTATTATCATCGCGCCTTTCTTTTCACATCTTGTAAAATTCTTCCCACCAGTAGTAACGGGTAGTGTT
GTTACTATCATTGGTATCAATTTAATGCCAGTAGCAATGAATTACTTAGCTGGAGGTCAAGGTGCAAAGGACTATGGAGATGTTAAGAAC
ATTTTGTTAGGTTTAATGACATTAATCATTATTCTTGTTTTACAAAGATTCACAACTGGATTTATTAAGAGTATTGCCATATTAATTGGACTC
GTTTTAGGAACGATAGGTGCTGGCTTACTTGGGATGGTCGATATTAATCAAGTCAATCATGCCGGTTGGTTAGGCATCCCAGTGCCGTTT
AGATTCTCTGGATTTAGCTTTGATGTGACATCGACGTTAGTGTTCTTTATTGTAGCTATCGTTAGTTTAATTGAGTCGACAGGTGTCTATCA

TGCGTTAAGTGAAATTACCGGTAAGAAGTTAGAAAGAAAAGATTTTCGTAAAGGTTATACTGCGGAAGGTCTAGCGATAGTGTTAGGTT
CTATATTCAATTCATTTCCGTATACAGCCTATTCGCAAAATGTAGGACTTGTTTCTTTATCCGGCGCTAAGAAAAATAATGTTATATACGGC
ATGGTCGTGTTATTACTTATATGTGGTTGTATACCTAAGCTTGGTGCATTAGCAAATATCATACCGCTACCTGTGTTAGGTGGTGCGATGA
TAGCTATGTTTGGCATGGTAATGGCATATGGTGTTAGTATATTAGGACATATCGATTTTAAAAATCAAAACAATTTATTAATTATCGCTGT
ATCAGTAGGATTAGGTACTGGTATAAGCGCTGTACCACAAGCATTTAAAGGTTTAGGTGAACAATTTGCATGGTTGACTCAAAACGGAAT
TGTTTTAGGCGCAATCTCTGCAATTATTCTTAATTTCTTTTTTAATGGAATAAAGTATAAACAAACGGAAGAAAATGTGAAATAA

Gene: guaB (inosine-5'-monophosphate dehydrogenase)

Contig: 02_NODE_3, position: 342994 to 344460, length: 1467 nt, orientation: FORWARD

Perfect match to: (65-1322-ACJS01000018-[4683:6149], allele observed in CC30+CC239)

Sequence:

ATGTGGGAAAGTAAATTTGCAAAAGAATCATTAACGTTTGATGATGTGTTATTAATTCCAGCACAATCTGATATTTTACCGAAAGACGTTG
ATTTAAGCGTACAATTATCAGACAAAGTTAAATTAAATATTCCAGTTATTTCTGCTGGTATGGATACTGTAACTGAATCTAAAATGGCGAT
TGCTATGGCTCGTCAAGGTGGTTTAGGTGTTATTCATAAAAATATGGGCGTTGAAGAACAAGCGGACGAAGTTCAAAAAGTAAAACGCT
CAGAAAATGGTGTCATTTCAAACCCATTTTTCTTAACGCCAGAAGAAAGCGTTTATGAAGCAGAAGCATTAATGGGTAAATACCGTATTT
CAGGTGTACCAATTGTTGATAATAAAGAAGATCGCAACTTAGTAGGTATTTTAACAAACCGTGACTTACGTTTTATTGAAGACTTCTCGAT
TAAAATTGTAGATGTAATGACGCAAGAAAATTTAATTACAGCTCCAGTGAATACAACACTTGAAGAAGCAGAAAAAATTCTCCAAAAACA
TAAGATTGAAAAGTTACCATTAGTTAAAGACGGACGTCTAGAAGGTCTTATTACTATTAAAGATATTGAAAAAGTAATCGAATTCCCTAAT
GCAGCAAAAGATGAACATGGTCGTCTACTTGTAGCCGCAGCAATCGGTATTTCAAAAGACACTGATATTCGTGCTCAAAAATTAGTCGAA
GCAGGTGTGGATGTCTTAGTTATCGATACAGCACATGGTCACTCTAAAGGCGTTATCGATCAAGTGAAACATATTAAGAAGACTTACCCA
GAAATCACATTAGTAGCTGGTAACGTAGCAACTGCAGAAGCAACAAAAGATTTATTTGAAGCGGGTGCAGATATTGTTAAAGTTGGTAT
TGGCCCAGGTTCAATTTGTACGACGCGTGTTGTAGCAGGTGTTGGTGTACCACAAATTACAGCAATTTATGATTGTGCAACTGAAGCACG
CAAACATGGTAAAGCTATCATTGCTGATGGTGGTATTAAATTCTCAGGAGATATCATTAAAGCATTAGCTGCTGGTGGACATGCGGTTAT
GTTAGGTAGCTTATTAGCAGGTACTGAAGAAAGCCCAGGCGCAACAGAAATTTTCCAAGGTAGACAATATAAAGTATACCGCGGTATGG
GCTCTTTAGGTGCGATGGAAAAAGGTTCAAACGACCGTTACTTCCAAGAAGACAAAGCGCCTAAGAAATTTGTTCCTGAAGGTATCGAA
GGACGTACGGCATATAAAGGTGCGTTACAAGATACAATTTACCAATTAATGGGCGGTGTGCGTGCTGGTATGGGTTATACTGGTTCACA
CGATTTAAGAGAATTACGCGAAGAAGCACAATTCACACGTATGGGCCCTGCTGGTTTAGCAGAAAGTCACCCACATAATATTCAAATTAC
GAAAGAATCACCGAACTACTCATTCTAA

Gene: guaA (GMP synthase [glutamine-hydrolyzing])

Contig: 02_NODE_3, position: 344485 to 346026, length: 1542 nt, orientation: FORWARD

Perfect match to: (JKD6008-CP002120-[425291:426832], allele observed in CC239)

Sequence:

ATGGAAATGGCAAAAGAACAAGAGTTAATCCTTGTCTTAGACTTTGGTAGCCAATACAACCAATTAATTACACGCCGAATTCGTGAAATG
GGCGTTTATAGTGAATTACACGATCATGAAATTTCAATTGAAGAAATTAAGAAAATGAATCCAAAAGGTATTATCTTATCAGGTGGTCCA
AATTCAGTTTATGAAGAAGGTTCATTTACAATTGATCCGGAAATATATAATTTAGGAATTCCAGTACTTGGTATTTGTTACGGCATGCAAT
TAACTACTAAATTACTAGGTGGTAAAGTTGAGCGTGCCAATGAACGTGAATACGGTAAAGCAATCATTAATGCGAAGTCAGATGAGTTA
TTCGCTGGCTTACCAGCAGAACAAACTGTTTGGATGAGTCATTCTGATAAAGTTATTGAAATTCCAGAAGGCTTTGAAGTTATCGCTGATA
GCCCAAGTACAGACTATGCAGCAATCGAAGATAAGAAACGTCGCATTTATGGTGTTCAATTCCATCCAGAAGTACGTCATACAGAATATG
GTAATGATTTGTTAAACAACTTCGTACGTCGTGTTTGTGAGTGTAAAGGTCAATGGACAATGGAAAACTTTATCGAAATTGAAATTGAAA
AGATTCGTCAACGCGTAGGCGACCGTCGTGTATTATGTGCGATGAGTGGTGGCGTAGATTCATCTGTTGTTGCTGTATTATTGCATAAAG
CAATTGGGGATCAACTAACATGTATCTTTGTAGACCATGGCTTACTTCGTAAAGGTGAAGGCGACATGGTTATGGAACAATTCGGTGAA
GGGTTCAACATGAATATTATTCGTGTTAATGCGAAAGATCGTTTTATGAATAAATTAAAAGGTGTTTCAGATCCTGAACAAAAACGTAAA
ATCATTGGTAATGAATTTGTATATGTATTTGATGATGAAGCATCAAAACTGAAAGGTGTAGACTTCCTTGCGCAAGGAACACTTTATACA
GACGTTATCGAATCAGGTACCAAGACAGCACAAACAATCAAATCACATCACAATGTTGGTGGATTACCAGAAGACATGGAATTCGAATT
AATCGAACCAATCAATACATTGTTTAAAGATGAAGTACGTAAATTAGGTATTGAATTAGGTATTCCAGAACATTTAGTGTGGAGACAACC
ATTCCCAGGACCTGGTCTTGGTATTCGTGTACTTGGAGAAATTACTGAAGATAAACTAGAAATCGTTAGAGAATCAGACGCGATTTTACG
CCAAGTGATTAGAGAAGAAGGTCTTGAAAGAGAAATTTGGCAATACTTCACAGTGTTACCAAACATTCAATCAGTAGGTGTTATGGGAG
ACTACCGTACGTATGATCACACAGTAGGTATTCGTGCAGTAACATCTATCGACGGTATGACAAGTGACTTCGCACGCATCGATTGGGAAG
TCTTACAAAAGATTTCTAGTCGTATCGTAAACGAAGTAGATCACGTCAACCGCGTAGTCTATGACATTACATCAAAACCACCAAGCACAAT
TGAGTGGGAATAA

Gene: Q8NY68 (putative protein)

Contig: 02_NODE_3, position: 346538 to 347644, length: 1107 nt, orientation: FORWARD

Perfect match to: (MW2-BA000033-[416307:417413], allele observed in CC1+CC188)

Sequence:

ATGGATAAATCACTTGAAATTAACTTAAAAGAATTAGAAGGCATAATGTATAAAGAAAAAGTAATCGACGTTAAAGAGTATGATAATTTA
AAAGTAACTTTTGAGACACTAAACGATGCTCCTAAAAGTGAAATACCCACTCCCTTTACAATAGAACAAATGTTATATAAATTAGAAACTA
AAAATTTATATTTTCCTGAAATTAAGTCAAAAGAAAAATGTGTGGAGCTTTTACATAGTGTTGGCTATTATAATTTGAAGCATTTTATGTAT
AATGAATTTGGTAAAGATGAGCCGAAAAATTTTGATGAAGTATATATACTTTATAAATTTGATAGATATTTATTGAAACAATTATTTAGTT
TAGTAAATATGTTGGAAACTCATATAAGAAATATTATTTTAGAAGTATATATGTTAGAAATTGAGTATAATAACAAGCCTTCGACACTGTT
CTATTTAGATAAAGATTTATATTTTGAGAAAGTTAATGATGAGTATAAATATTCTGCCAAAAAGCTAAAAGAGTTCAATAGGTTACAAAAT
GTTTTTTGGAGAGCTATAGAAAAAAAGAAAAGTAACGATAACGTTAAACATAATATTAAAAAATACAATATTATACCAGCTTGGGTTTTA
TTCCAAAATTTTAGTTTTGGAGATTTGTCTACTTTTTATAGAACTACATTACCAACTTATAGAAATAAAGTAAGTAAGCGAATTGAAAATTT
AATAGAAAAAAATACTGGTATAAGCATAAAACTTCCAGAAAAGTTATTGTGTGCATGGTTGAATAGTATCAGGTTTTTGAGAAATAGAAT
AGCTCACACAGATATAATTTATGGAATTAATTTTACAAACACCTGTGCAAAACATCATAGTGATGAGGAAATGTATGTAAATATAGAAAA
GTATAAATATCAGCAAAGGTTAGTTACATTTTTATTAGCTATGAAGAAAATATTTATGAGTATGCCAGAAAATAATATTATTGAATGGAAT
GAGACTCTAACTAAGATAGAGAATAAATGTTCAGAACATAACTTTATTAAACTTTCTAGGTTAGGAGTTATTGAAAATAACCTTTCTTACT
TTAAAATAACTAAATAA

Gene: Q6GC78 (putative protein)

Contig: 02_NODE_3, position: 348270 to 348662, length: 393 nt, orientation: REVERSE

Perfect match to: (MW2-BA000033-[418039:418431:r], allele observed in CC1+CC22+CC88+CC188)

Sequence:

TTAAAAATCTATACTATCTATATTGAAATTTTTATTTATGGTTATGCCATAACTACTTTGTATTTCCGGTACTAAACTTTGCAATATTGGGTA
ATAGTTATTAGCGAAACCAATAATAATGGAATTAATCACATTGATACTAAGTTCTTTATCTTCAGGTAATGCATTTTCTATTATTTTGATTA
AGTCTATATATTTTATTTCATCAGAATTTTTATGTGCTAAATACTTCATAAAATCAGTTTGCACTTTTTCGAAAACACTATATCTTAAGTCCA
TTTGTACAAATATTAAGCTGACAGTTTTAGTTTCGAATTGACTTTGACCCAAAACTGAAGCAAGTTCTTCTATTGATTGATATAGCAATCTG
ATATCGCCGTTATCTTCAAGCAT

Gene: Q8NY63 (putative protein)

Contig: 02_NODE_3, position: 350089 to 350421, length: 333 nt, orientation: FORWARD

Perfect match to: (MW2-BA000033-[419858:420190], allele observed in CC1+CC9+CC25+CC80+CC188)

Sequence:

GTGGCAACACTAAAAGATGTAAAGACTGGTAAAAAGGAAATTTTGCCATCAACTGCAAAAGATAAAAATGGTAAAAATGTTACGTTAAT
TTATTTTGAAAAAGACGGTAAATTAGGTTTTTATGTGCAGAAAACACAAAAAGAACGTGGAGTAGGGAAATGCGTTTCTGGTATAGCGG
GTGGCGCAGTGACAGGAGGCACTACTTTAGGTCTTGCAGGTGCAGGAGTAGGAACAGTTACTATTCCAGTAATTGGGACAGTTAGTGG
AGGCGTAGTTGGAGCTGTTGGTGGTGCTGTCGGCGGTGGTCTAACCGGTGGAGCCACATTCTGCTAA

Gene: Q2YVN4 (putative protein)

Contig: 02_NODE_3, position: 351115 to 351297, length: 183 nt, orientation: TRUNCATED

Sequence:

ATCTATAATTAAATACGCACGTAAGTAATTTTGATCGTTCATTGTTATTGTTTTATGTTTTAGGAAATGTGCTATTGAAAGGTTTAAAGATG
ATTGGAATTTCTTGGTTTCTTGACTTATATAGTTATGAAAGAGGGCGTTGTCCTTAATGGCATTTCTGAGATCAGAAATGCTATTAATAAA

Gene: Q6GC74 (putative protein)

Contig: 02_NODE_3, position: 351526 to 353352, length: 1827 nt, orientation: FORWARD

Perfect match to: (MW2-BA000033-[421295:423121], allele observed in CC1+CC12+CC188)

Sequence:

ATGGGATATAGAATAGAAACAAGAACGTTTGAAGAGTTAGAAGGAAAATTAAAGTTACCAACTTTTCAAAGAGCACTAGTTTGGTCTAA
TAATCAAAAGCAAGAATTTTTCTCAACGCTGAAAGATGGTTTTCCTTTTGGTTCTATTCTGTTATACGAATATGAAAATGATAATAAATACA
GTCTTATTGATGGCCTGCAAAGGTATTCTACAATGGTAGATTTCATGGAGAATCCAACAAACTATATTGAATTTGAAGATTTTACATTGAA
AATTGCTGAATTGTATAAAGGGGCATCAGAGAGTACAAAAGCAGAAGTAGTTAATTATGCCGAAGATATTCTGCTGAAAATTATTGAAA
AATATATTGATTCTACAAAAGAAAATGTAAAAGTAAATGAACTAGCTAAAAAAATGGTGGATAAATTTCCAGTATTAGGTGAAACAGATA
TTAGAGATGAAATCACTCAAATACAGTCAGATGTTATTACTTATTTTAATAATCAACTAGATATTAGTAATGTCAAGATTCCGTGTATCTTC
TTTGAAGGAGAAGAGACTGAATTGGCAGAAGTTTTTCAAAGGTTAAACAGTGGAGGTAAAAAGTTATCGAAGTATCAAGTTTTTGCTGC
ACATTGGGACAGATATGAAGTTGAATTGGGAGATTTAAAGTATAGTGATGAGATACTTAAAAATGTAATAGAAAGGTACGAAAGATTAA
ATAATGATAGAGGTATCTTAATTGAAAATTTTGACCCTAAAGAAATGGAGACGTCAAGAGTAATTAATTTATCTGAATTATGTTATGGACT
TGGTAAAGTGATTTCTGATGAATTACAAGCATTTTTTAAAGAGTCAACAGAAGATATATGCAATGAATTAGGATTTCAAACAATGCTAATT
GTATTTGGCATTCCTACAAATAAGATGAATGAGTTACCATATAAATATCAATATATGAAGAATAAAAATAATATAGAAATCTTGTTTGAAA
AAATTGTGAAGATATATAAAATAATAAATAACAAGTTTAAAGAAGCTTTCTCTATGGTAGTATCGTCAAGAGAAGATAAATATGAAACTA
AATCTTTTACTAGATTACAAATAATGTCATTCTTTTCATCTTTATGGATCTCATCGTATAGTATTGAATTAGATGAAAATAAAAACACATTTT
CTATTAAACAAACTAAATCTTTGAAAAAAAATCAACAAGTGATTTTGTCTAATATCATAATTTATGCAATTTATGATGTGATAAGAAAGTA
CTGGTCAGGAACAGGTGATCGAAAATTAATGGATATATACATATCTCAAAACAATAGATATTTAAAGCCGTTAGCAAAAGAAGTGTTTAG
AAATGAGCTACTTAGATGGAATGAAGAGAATATAAATAAGACAAGTATTAATATTCAAACGGATGAGAAGATGATCATTACTTTTATTGC
TAATAAATTTAGAAGCTTCTATTCATCTTTGAATGATAATTTAGATTATGAACATATTTTTTCAAGAAAATTATACAAGCTGCATAAAGGTA
ATGCAATAATCCCTGCTGGATCTTTAGGTAACATCATGTTACTTGACTCTGGTATTAATAGAAGAAAAAAAGAAAAATTTCTTTATTCAGC
AATAGATATTGATAAGGCATCTATTACGGGAACTATAGAAAATCAGTATGTTAACAACAGTTTTTATCCAGATAAAAGAACAATAGATAC
TATTGAAATAGATATAATTGATGAAAAATATGATAGTTTAATAAAAATGATCAAAAATCGTGGTGATAAATTAATAGAAGAAGTTGTTAG
AGAATTATATATAAATAA

Gene: Q6GC73 (putative protein)

Contig: 02_NODE_3, position: 353890 to 354312, length: 423 nt, orientation: REVERSE

Perfect match to: (MW2-BA000033-[423659:424081:r], allele observed in CC1+CC12+CC25+CC188)

Sequence:

TTAGTTAATATATTTAATTGCTAACTTATTATTAGGGTCTCTTTCAAGAATTTTATTTGCGACCAATTGAATATTCTCATGTGAAATCATATT
AAAGTCTGGTTCTCCAAGGATTTCTAAAAATTCGTATAATAAATTAGCATTATTAAATTGCGAAATCTCTAACGATTGCCAATAATGATAG
TATGCTAAATCATAGGCATTATCTATGTAATTGAAATGAAAGTGATAGATGCTTGCGACCATTTCATGATATTCAATTGTATTTATTTGATT
CGCAAGATTTGATAAATATGATACTAATTTTTCATCTGAAATTTCGTTCGACATAAAGTATGTTAGAATATTCTCCATATCTTTATTCTCTAA
CATATTTTTTAATTCAGTTGGTAATAAATTTAATAGAATGTTAACGCTTTTCAT

Gene: Q2YVN4 (putative protein)

Contig: 02_NODE_3, position: 354894 to 355185, length: 292 nt, orientation: TRNC-RVRS (no stop codon)

Sequence:

TTTTCGTTTTTCCACTAACGTCTTTTTCAAGCTTTATATTTACAATTTTTAAGCAGAAGTATCCCATTATTTCAAGGTCTGATTTATCTATAAT
TAAATACGTACGTATGGAATTTTGATCGTTCATTTTTATTGCTTTATGTTTTAGGAAATGTGCTATTGAAGGGTTTAAAGATGTTTGGAATT
TCTTGGTTTCTTGACTTATATAGTTATGAAAGAGGGCGTTGTCCTTTATTAAATTTCTGAGATCAGAAATGCTATAAATAAAGTGATTAAG
ATTTTTTGTTTTCAT

Gene: Q6GC70 (putative protein)

Contig: 02_NODE_3, position: 355681 to 356088, length: 408 nt, orientation: FORWARD

Perfect match to: (MW2-BA000033-[425450:425857], allele observed in CC1+CC188)

Sequence:

ATGGAGCTATCTATTTTTTACAATGGGCAATTTTTTGTAGCATTGGTAGAATATAAAATGGAAAATAAATCTAAATTTATCCAATACACATT
TGGGAATGAACCTGATGATATAGAGGTATTGGATTTTATTCATCATCAATTAATGAAAATGATTGATGATGTGCAAACTATTGTTTATACG
AAAAATATTTCTAGAAAAGTAAACCCGAAAAAACTACAACGACAAATTGCTAAGGAGCAAAAGAAACCTAAATATTCTACCCAAGCACA
AATAGCTATTAAGAAAGAATTAGAATTAAAGAAAAAGCAAAAGCGGAAGCGCTATAAAGAAAAACGTGATGCATTTCAAAAAAGAAAA
AGAGAAATTAAAAAGGTTAAAGCAAAAGAGAAGCATAAGGGGCATTAG

Gene: DUF523 (putative protein)

Contig: 02_NODE_3, position: 356135 to 356617, length: 483 nt, orientation: FORWARD

Perfect match to: (MW2-BA000033-[425904:426386], allele observed in CC1+CC188)

Sequence:

ATGATTTTAATTAGTTCGTGTTTGATAGGCGATAATGTAAGATACGATGGTGGTAATCAATTGAATGTTAGATTGAAAAAATTAATAGAC
AGCGGAAAAGCTATTCACGCATGTCCAGAATTACTTGGTGGATTATTAATTCCTAGAGAACCTGCAGAAATTATTGGTGGCGATGGTTTT
GATGTATGGAATGACGCTGCAAAGGTTGTTACTATTTCGAACAAAGACGTAACTGACGATTATAAACATGGAGCAATAGTTACATTAAAA
ATTTTGAAAAAAATATCAATGTGATACAGTTATTTTAAAAGCAAATAGCCCATCATGTGGATCACAAGAGATATATGATGGGAATTTTACA
GGAAATAAGAAAAAGGGTGTAGGTGTGGCAACTGCTTTACTCATTAATGAAGGTATAAAAGTTTATGATGAAAATACGTTTTTTGACCAA
AACATGAATGAAACGATAGTACATGAAAAGTAA

Gene: Q6GC68 (putative protein)

Contig: 02_NODE_3, position: 356817 to 357506, length: 690 nt, orientation: FORWARD

Perfect match to: (11819-97-CP003194-[436634:437323], allele observed in CC80+CC1+CC12+CC188)

Sequence:

ATGGAAAAAGTAGCCATTTTAGTAGATGGTGGATACTATAGAAAGATAAGCGCAAAAGTCTACGGAAAAGTTACAGCGAAAGAAAGAG
CTGACGAGTTATATAGTTATTGCAATAGACACTTAAAAGAGACACATTTCAAAGAAGAAATATATAATAAATTATACAGAATATTTTACTA
TGATTGTCCTCCTATTGATAAGATAGTTTATCACCCCTTATTAAAAAAGAATGTAAATTTTTCCAATACTGATACGAAAAAGTGGACAGAA
GACTTTTTCAAAGAAATGAGCAAAAAAAGAAAGGTAGCATTGAGGTTAGGAGAGTTGAGTGAGTATTCAGTTGAATACAACTTAAAATA
CTCTATTACAAAAAAATTATTAAATGGAAGTATCGATTTAAATGATTTGAAAGAAAAAGATTTTTCCCTATCATTACAACAAAAAGGTGTT
GATATGAAAATAGGTTTAGACATAGCTTCATTATCATTTAAACATCAAGTAGATAAAATTATTTTAATAGCTGGTGATAGCGATTTTGTAC
CTGCTGCTAAACTAGCAAGAACAGAAGGAATTGATTTTGTATTAGATTCTTTAGGGGCAGATATTAGAAACAACTTGTCATTACACATTG
ATGGTAGGCGTACTTGTGATGAAAAATTTAAATTTTATTTAAATCAACTATTTAAATAA

Gene: Q5HIQ1 (putative membrane protein)

Contig: 02_NODE_3, position: 357765 to 358124, length: 360 nt, orientation: REVERSE

Perfect match to: (MW2-BA000033-[427534:427893:r], allele observed in CC1+CC15+CC188)

Sequence:

TTACCAAAGCATTGATATGAGTGCCAATACTGGCAATGTACCTTGTTTGAAAAAGATACTAATATTGCTTGAAAGGCCACCATAAATAGC
AACGCCAATGATATACACTAAAATAGCTGCGCATATTTCTTTTGGATTACTGCTGATAAACAAACCGTATATTAGCAAAATTCCGATTAAA
CCGTTATATACGCCTTGGTTCTTCAAAAGTAGGTTAATATTTTTGTCTTTCAATTTATCGACGCTTATATTAAATGTCTCGCTAGTCTTTTTG
GAAGTTGTAGCAATCGTTTCAAGGTACATAATATAGAAAAACTCTAATGCCACAAATATGATTAAAATTGTTGAGATGATATTCAC

Gene: Q2G0Y0 (putative protein)

Contig: 02_NODE_3, position: 358143 to 358988, length: 846 nt, orientation: REVERSE

Perfect match to: (ATCC51811-ST1-ADVP01000041-[11829:12674], allele observed in CC1)

Sequence:

TTAAATATTTTCTTGTAAAAATGATTGCAGTGTCTGTGGTTGATCATTGACTAATTGTTGGAAATCATTGGATTCTTGGTCTAATAGTCCTC
TTGCTCCTGCGTCGTACATTGATGCCAATAATGCACCAAAGCCTTTAGGTTCATCGTACATTTCTGCAAATGTCTCTAATGAAACGGGCTC
ATATTTAATTTCTGTGCCTGATGCCTCAGATAAAATTGCAGCAAGTTCTTTCATATCATAACTGTAGCCTGATAATAAATAGCGTTTGCCCC
AAGTATCTGGATTTTTAATAATAGCAATGACACCTCTAGCAATATCATTTCTAGTAATATAATTAATACGACCATCACCAGCTGGATAAATC
AGTTTATGCATATTCATCAATTCTGGTAAATATGGTTTAAGTGGATCCATGTACATTGCCATTCTTACATAGGTATAGTCAATACCACTTGT
TGCCAATAGACGTGCTGCATAACCAAAATAAGGACTCATATGGAATGGATTATTATGCTGATCTGCGTAATAACCTATGAAAATGATATG
AGCAACGCCACTTTGCTTTGCCGCATATACTAAATTTTCCACTTCAGGAATACGTTTGAATGATGGATGGATAATACTTGGAATAAACACA
ACGGTATCCATTCCTTTAAATACCTCTACCATGCTTTCGGGATTAAAATAATCTAATTGTCGAACAGGAACTTTTCCGCGCCAATCTTCTGG
AACTTTCTCAACATTTCTAACACCAATGTGAAAATGATCTATGTGATTTGCAATGGCTTGATTTGTAATATGTGTGCCTAAATGACCTGTAG
CACCTGTTAACATAATATTCAT

Gene: ssl01 (staphylococcal superantigene like protein locus 1)

Contig: 02_NODE_3, position: 359460 to 360140, length: 681 nt, orientation: FORWARD

Perfect match to: (MW2-BA000033-[429229:429909], allele observed in CC1+CC188+CC361)

Sequence:

ATGAAATTTAAAGCGATAGCAAAAGCAAGTTTAGCATTGGGAATGTTAGCAACAGGTGTAATTACATCGAATGTACAATCAGTACAAGC
GAAAACAGAAGTTAAACAACAAAGTGAGGCTGATTTAAAACTTTATTATAATGGACCAAGTTTTGAATATAAAAAAGTAACTGGATATG
GATTTATTGAAGGTAAAGATAGATTTATTGATTTTATATACAATGGACAATATAATAAAATATCTTTAGTTGGTTCTGATAAAGATAAATA
TAATGAAGAAGTTAACCCAGATATAGATGTGTTTGTCGTTAGAGAAGGAAACGGTAGACAAGCTGATAATCATTCGATTGGTGGCATAA
CAAAAACTAATAGAGGAGTGTATTATGACTATATACACACACCAATCCTTGAAATCAAGAAAGGTAAAGAAGAACCACAAAGTAGTCTAT
ACCAAATTTATAAAGAAGACATCTCACTAAAAGAACTTGATTTTAAATTAAGAAAGCAATTAATTAGTCAAAGTGGCTTGTATTCAAATGG
TCTTAAACAAGGTCAAATTACAATTACAATGAATGATGGCACAACACATACAATCGATTTAAGTCAAAAACTTGAAAAAGAACGTATGGG
CGAGTCTATCGATGGCAGACAAATACAAAAAATTCTAGTAGAAATGAAATAA

Gene: ssl02 (staphylococcal superantigene like protein locus 2)

Contig: 02_NODE_3, position: 360426 to 361121, length: 696 nt, orientation: FORWARD

Perfect match to: (MW2-BA000033-[430195:430890], allele observed in CC1+CC188)

Sequence:

ATGAAAATGAAATCAATTGTAAAAATAAGTTTGTTATTAGGAATATTAGCAACAGGTGTAAACACTACAACGGAAAAACCAGTTCATGCC
GAAAAGAAACCTATTGTAATAAGTGAAAATAGCAAAAAATTAAAAGCTTATTATACTCAACCTAGTATTGAATATAAAAATGTGACAGGT
TATATCAGTTTCATTCAACCAAGTATTAAATTTATGAATATCATAGATGGTAATTCTGTTAATAATATTGCTTTAATTGGCAAAGATAAGCA
ACATTATCATACGGGTGTACATCGTAATCTTAATATATTTTACGTTAATGAGGATAAGAGATTTGAAGGTGCAAAGTACTCCATTGGCGG
TATCACGAGTGCAAACGATAAAGCTGTCGACCTAATAGCAGAAGCAAGAGTTATTAAAGCAGATCATATTGGTGAATATGATTATGACTT
TTTCCCATTTAAAATAGATAAAGAAGCAATGTCATTGAAAGAGATTGATTTTAAATTAAGAAAATACCTTATTGATAATTATGGTCTTTAC
GGTGAAATGAGTACAGGGAAAATTACCGTCAAAAAGAAATACTACGGAAAGTATACATTTGAATTGGATAAAAAGTTACAAGAAGACC
GGATGTCCGATGTTATCAATGTCACAGATATTGATAGAATTGAAATCAAAGTTAGAAAAGCATAA

Gene: ssl03 (staphylococcal superantigene like protein locus 3)

Contig: 02_NODE_3, position: 361412 to 362482, length: 1071 nt, orientation: FORWARD

Perfect match to: (MW2-BA000033-[431181:432251], allele observed in CC1+CC239)

Sequence:

ATGAAAATGAGAACAATTGCTAAAACCAGTTTAGCACTAGGGCTTTTAACAACAGGCGCAATTACAGTAACGACGCAATCGGTCAAAGC
AGAAAAAATACAATCAACTAAAGTTGACAAAGTACCAACGCTTAAAGCAGAGCGATTAGCAATGATAAACATAACAGCAGGTGCAAATT
CAGCGACAACACAAGCAGCTAACACAAGACAAGAACGCACGCCTAAACTCGAAAAGGCACCAAATACTAATGAGGAAAAAACCTCAGC
TTCCAAAATAGAAAAAATATCACAACCTAAACAAGAAGAGCAGAAAACGCTTAATATATCAGCAACGCCAGCGCCTAAACAAGAACAAT
CACAAACGACAACCGAATCTACAACGCAGCAAACTAAAATGACAACACCTCCATCAACAAACACGCCACAACCAATGCAATCTACTAAAT
CAGACACACCACAATCTCCAACCATAAAACAAGCACAAACAGATATGACTCCTAAATATGAAGATTTAAGAGCGTATTACACGAAACCGA
GTTTTGAATTTGAAAAGCAGTTTGGATTTTTGCTCAAACCATGGACGACGGTTAGGTTTATGAATGTTATTCCAAATAGGTTCATCTATAA

AATAGCTTTAGTTGGAAAAGATGAGAAAAAATATAAAGATGGACCTTACGATAATATCGATGTATTTATCGTTTTAGAAGACAATAAATA
TCAATTGAAAAAATATTCTGTCGGTGGCATCACGAAGACTAATAGTAAAAAAGTTAATCACAAAGTAGAATTAAGCATTACTAAAAAAGA
TAATCAAGGTATGATTTCACGCGATGTTTCAGAATACATGATTACTAAGGAAGAGATTTCCTTGAAAGAGCTTGATTTTAAATTGAGAAA
ACAACTTATTGAAAAACATAATCTTTACGGTAACATGGGTTCAGGAACAATCGTTATTAAAATGAAAAACGGTGGGAAATATACGTTTGA
ATTACACAAAAAACTGCAAGAGCATCGTATGGCAGACGTCATAGATGGCACTAATATTGATAACATTGAAGTGAATATAAAATAA

Gene: ssl04 (staphylococcal superantigene-like protein locus 4)

Contig: 02_NODE_3, position: 362847 to 363692, length: 846 nt, orientation: FORWARD

Sequence:

ATGAAAATAACAACAATTGCTAAAACAAGTTTAGCACTAGGCCTTTTAACAACAGGTGTAATCACAACGACAACGCAAGCAGCAAATGC
GACAACACCGCCTTCAACTAAAGTGGAAACACCGCAATCAAAACCAAACGCGACAACACCATCTTCAACTAAAGTGGAAGCACCGCAAC
AAGCAGCAAACGCGACAACACCACCTTCGTCTAATGTAGACACATCACCACCACAATCGCCAACCACAAAACAAGTACCAACAGAAATAA
ATCCTAAATTTAAAGATTTAAGAGCGTATTATACGAAACCAAGTTTAGAATTTAAAAATGAGATTGGTATTATTTTAAAAAAATGGACGAC
AATAAGATTTATGAATGTTGTCCCAGATTATTTCATATATAAAATTGCTTTAGTTGGTAAAGATGATAAAAAATATGGTGAAGGAGTACAT
AGGAATGTCGATGTATTTGTCGTTTTAGAAGAAAATAATTACAATCTCGAAAAATATTCTGTCGGTGGTATCACAAAGAGTAATAGTAAA
AAAGTTGATCACAAAGCAGGAGTAAGAATTACTAAGGAAGATAATAAAGGTACAATCTCTCATGATGTTTCAGAATTCAAGATTACTAAA
GAACAGATTTCCTTGAAAGAACTCGATTTTAAATTGAGAAAACAACTTATTGAAAAAAATAATCTGTACGGTAACGTTGGTTCAGGTAAA
ATTGTTATTAAAATGAAAAACGGTGGAAAGTACACGTTTGAATTGCACAAAAAATTACAAGAAAATCGCATGGCAGATGTCATAGATGG
CACTAATATTGATAACATTGAAGTGAATATAAAATAA

Gene: ssl05 (staphylococcal superantigene like protein locus 5)

Contig: 02_NODE_3, position: 364056 to 364760, length: 705 nt, orientation: FORWARD

Sequence:

ATGAAAATGGCAGCAATTGCGAAAGCAAGTTTAGCATTAGGTATTTTAGCAACAGGAACAATAACGTCATTGCATCAAACTGTAAATGC
GAGTGAACATGAAGCAAAATATGAAAATGTGACAAAAGATATCTTTGACTTAAGAGATTACTATAGTGGCGCAAGTAAGGAACTTAAAA
ATGTTACTGGTTATCGTTATAGCAAAGGTGGCAAGCATTACCTTATCTTTGATAAACATCAAAAGTTCACTAGAATACAAATTTTTGGTAA
AGATATAGAAAGATTTAAAGCACGCAAAAATCCGGGATTAGACATATTTGTTGTTAAAGAAGCGGAAAACCGTAATGGCACAGTGTTTT
CATATGGTGGTGTCACTAAGAAAAATCAAGACGCTTATTATGATTATATAAACGCACCAAGATTTCAAATCAAGAGAGATGAAGGTGAC
GGTATTGCTACGTACGGTAGAGTACACTACATTTATAAAGAAGAGATTTCACTTAAAGAACTCGACTTTAAATTGATACAGTATTTAATTC
AAAATTTTGATCTGTATAAAAAGTTTCCTAAAGATAGTAAGATAAAAGTGATAATGAAAGATGGCGGCTATTATACGTTTGAACTTAATA
AAAAATTACAAACAAATCGCATGAGTGACGTCATTGACGGTAGAAATATTGAAAAAATAGAAGCCAACATTAGATAA

Gene: ssl06 (staphylococcal superantigene like protein locus 6)

Contig: 02_NODE_3, position: 365206 to 365904, length: 699 nt, orientation: FORWARD

Perfect match to: (MW2-BA000033-[435077:435775], allele observed in CC1+CC188)

Sequence:

ATGAAATTAAAAGCGTTAGCTAAAGCAACATTAGTATTGGGATTGTTAGCTACTGGTGTAATAACAACAGAAAGTCAAACAGTAAAAGC
GGCAGAATCAACTCAAGGTCAACACAATTATAAATCATTAAAATACTACTATAGCAAGCCAAGTATAGAGTTAATAAATGTAGATGGTCT
GTATAGACAACATTTAACTGATAAAGGTGCATATGTATGGAAAAATCTTAAAGATTATTATATTGGGCTACTAGGTGAAGATAGTAAGAA
ATTCAAATCAGATGTATACGGGGACCTAGATGCATTTTTAGTCATAGAAGAAGAACCTGTTAAAGGAAGACAATATTCAATTGGCGGTAT
AAGTAAGACAAATAGTAAAGAATTTAAAGAAAGAGAAGTCGATGTTAAAGTAACAAGAAAAGCAGACAGAGATACTACATCAACTAAA
GATAGTAAATTTAAAATTACAAAAGAAGAAATCTCGTTAAAAGAGTTAGATTTTAAATTAAGACAAAAATTGATGAAAGAAGAGAATTTA
TACGATGCAATTAACCATAGAAAAGGTAAAATTGTAGTTAAAATGGAAGATGATAAGTTTTATACTTTCGAACTTACAAAAAAATTACAA
CCGCATCGCATGGGTGACACGATAGATGGTACCAAAATCAAAGAAATTAATGTTGAGCTAGAATATAAATAA

Gene: ssl07 (staphylococcal superantigene like protein locus 7)

Contig: 02_NODE_3, position: 366326 to 367021, length: 696 nt, orientation: FORWARD

Perfect match to: (MW2-BA000033-[436197:436892], allele observed in CC1)

Sequence:

GTGAAATTAAAAACGTTAGCTAAAGCAACATTGGCATTAGGCTTATTAACTACTGGTGTGATTACATCAGAAGGCCAAGCAGTGCAAGC
AAAAGAAAAGCAAGAGAGAGTACAACATTTATATGATATTAAAGACTTACATCGATACTACTCATCAGAAAGTTTTGAATTCAGTAATAT
TAGTGGTAAGGTTGAAAATTATAACGGTTCTAACGTTGTACGCTTTAACCAAGAAAATCAAAATCACCAATTATTCTTATCAGGAAAAGAT
AAAGATAAATATAAAGAAGGCCTTGAAGGCCAGAATGTCTTTGTGGTAAAAGAATTAATTGATCCAAACGGTAGACTATCTACTGTTGGT
GGTGTAACGAAGAAAAATAACCAATCTTCTGAAACTAATACACCTTTATTTATAAAAAAAGTGTATGGCGGAAATTTAGATGCATCAATT
GAATCATTTTTAATTAATAAAGAAGAAGTTTCACTGAAAGAACTTGATTTCAAAATTAGACAACATTTAGTTAAAAATTATGGTTTATATA
AAGGTACGACTAAATACGGTAAGATCACTTTCAATTTGAAAGATGGAGAAAAGCAAGAAATTGATTTAGGTGATAAATTGCAATTCGAG
CACATGGGCGATGTGTTGAATAGTAAGGATATTCAAAATATAGCAGTGACTATTAATCAAATTTAA

Gene: ssl08 (staphylococcal superantigene like protein locus 8)

Contig: 02_NODE_3, position: 367360 to 368058, length: 699 nt, orientation: FORWARD

Perfect match to: (MW2-BA000033-[437231:437929], allele observed in CC1+CC59+CC188)

Sequence:

ATGAAATTTACAGCGATAGCTAAAGCGATATTTGTATTAGGAATATTAACAACAAGTGTAATGATAACAGAAAATCAATCGGTTAATGCA
AAAGGAAAGTATGAAAAAATGAACCGTTTATATGATACAAACAAGTTACATCAATACTATTCAGGACCTAGTTATGAGTTAACAAATGTT
AGTGGCCAAAGTCAAGGTTATTATGACTCTAACGTTTTGCTTTTTAACCAACAAAATCAAAAGTTCCAAGTGTTTTTATTGGGAAAAGATG
AAAATAAATACAAAGAAAAAACACATGGTTTAGATGTCTTTGCGGTACCGGAATTAGTAGATTTAGATGGAAGAATATTTAGTGTTAGTG
GTGTAACAAAGAAAAATGTAAAATCAATATTTGAGTCTCTAAGAACGCCGAACTTACTAGTTAAAAAAATAGACGATAAAGACGGTTTTT
CGTATGATGAATTTTTCTTTATTCAAAAGGAAGAAGTATCATTGAAGGAACTTGATTTCAAAATAAGAAAACTGTTAATTAAAAAATACAA
ATTGTATGAAGGGGCAGCTGATAAAGGTAGAATTGTTATTAATATGAAAGATGAAAATAAGTATGAAATTGATTTAAGTGATAAATTAG
GTTTCGAGCGTATGGCAGATGTCATTAATAGTGAACAAATTAAAAACATCGAAGTGAATTTGAAATAA

Gene: ssl09 (staphylococcal superantigene like protein locus 9)

Contig: 02_NODE_3, position: 368437 to 369135, length: 699 nt, orientation: FORWARD

Perfect match to: (MW2-BA000033-[438308:439006], allele observed in CC1+CC80+CC188)

Sequence:

ATGAAATTTACAGCATTAGCAAAAGCAACATTAGCATTAGGAATATTAACTACAGGTGTGTTTACAACAGAAAGTAAAGCTGTTCACGCG
AAAGTAGAACTTGATGAGACACAACGCAAATATTATATCAATATGCTACATCAATACTATTCTGAAGAAAGTTTTGAACCAACAAATATTA
GTGTTAAAAGCGAAGATTACTATGGCTCTAACGTTTTAAACTTTAAACAACGAAATAAAGCTTTTAAAGTATTTTTACTTGGTGACGATAA
AAATAAATATAAAGAAAAAACACATGGCCTTGATGTCTTTGCAGTACCTGAATTAATAGATATAAAAGGTGGCATATATAGCGTTGGCGG
TATAACAAAGAAAAATGTGAGATCAGTGTTTGGATTTGTAAGTAATCCAAGTCTACAAGTTAAAAAAATCGATCCTAAACATGGCTTTTC
GATAAATGAGTTGTTCTTTATTCAAAAGGAAGAAGTATCGTTGAAGGAACTGGATTTTAAAATAAGAAAAATGTTAGTCGAAAAATATAG
ATTGTATAAAGGCGCGTCAGATAAAGGTAGAATCGTTATTAATATGAAAGACGAAAAGAAATATGTAATTGATTTAAGTGAAAAATTAA
GTTTTGATCGTATGTTTGATGTAATGGATAGTAAGCAAATTAAAAATATTGAAGTGAATTTGAATTAA

Gene: ssl10 (staphylococcal superantigene like protein locus 10)

Contig: 02_NODE_3, position: 369494 to 370177, length: 684 nt, orientation: FORWARD

Perfect match to: (MW2-BA000033-[439365:440048], allele observed in CC1+CC72+CC188)

Sequence:

ATGAAATTAACAGCGATAGCTAAAGCTGCATTAGCTTTAGGAATTTTAACAACAGGAACTTTAACAACAGAAGTTCATTCAGGTCATGCA
AAACAAAATCAAAAGTCAGTAAATAAACATGACAAGGAAGCATTATACCGATACTACACTGGAAAGACTATGGAAATGAAAAATATTAG
TGCTTTGAAACATGGTAAAAATAACTTGCGTTTTAAGTTTAGAGGTATTAAGATTCAAGTTTTACTGCCTGGAAATGATAAAAGTAAATTT
CAACAGCGTAGTTATGAGGGGTTAGATGTGTTTTTTGTTCAAGAAAAAAGAGATAAGCACGATATATTTTATACTGTTGGTGGTGTAATA
CAGAATAATAAAACATCTGGAGTTGTCAGTGCACCAATATTAAATATTTCAAAAGAAAAGGGTGAAGATGCTTTTGTGAAAGGTTACCCT

TATTACATTAAAAAAGAAAAAATAACACTAAAAGAGCTGGATTATAAGTTGAGAAAGCATCTAATCGAAAAATATGGACTTTATAAAACA
ATCTCAAAAGATGGTAGGGTCAAAATTAGCTTGAAAGATGGCAGTTTTTATAACCTTGATTTAAGATCTAAATTAAAATTCAAATATATGG
GGGAAGTCATAGAAAGCAAACAAATTAAAGATATTGAAGTTAACTTAAAGTAA

Gene: hsdM (type I restriction-modification system DNA methylase)

Contig: 02_NODE_3, position: 370439 to 371995, length: 1557 nt, orientation: FORWARD

Perfect match to: (MW2-BA000033-[440310:441866], allele observed in CC1+CC188)

Sequence:

ATGTCTATTACTGAAAAACAACGTCAGCAACAAGCTGAATTACATAAAAAATTATGGTCGATTGCGAATGATTTAAGAGGGAACATGGAT
GCGAGTGAATTCCGTAATTACATTTTAGGCTTGATTTTCTATCGCTTCTTATCCGAAAAAGCAGAACAAGAATATGCAGATGCGTTGGCA
GGTGAAGATATCACGTATCAAGAGGCATGGGCAGATGAAGAATATCGTGAAGACTTAAAAGCTGAATTAATTGATCAAGTCGGTTACTT
CATTGAACCACAAGATTTATTCAGTGCGATGATTCGTGAAATTGAAACGCAAGATTTCGATATCGAACATCTGGCGACGGCAATTCGTAA
AGTTGAAACATCAACATTAGGTGAAGAAAGTGAAAATGACTTTATCGGACTGTTCAGCGATATGGACTTAAGTTCAACGCGACTAGGTA
ACAATGTCAAAGAACGTACTGCTTTAATCTCTAAAGTCATGGTTAATCTTGACGATTTACCATTTGTTCACAGTGATATGGAAATTGATAT
GTTAGGTGATGCATATGAATTCCTAATTGGGCGCTTTGCGGCGACAGCAGGTAAAAAAGCAGGCGAGTTCTATACACCACAACAAGTAT
CTAAGATACTGGCGAAGATTGTCACAGACGGTAAAGATAAATTACGTCATGTGTACGACCCAACATGTGGTTCCGGTTCATTATTGTTAC
GTGTTGGTAAAGAAACGCAAGTGTATCGTTATTTCGGTCAAGAACGTAACAATACCACTTACAACTTAGCACGCATGAACATGTTATTAC
ATGATGTACGTTATGAAAATTTCGATATCCGTAATGATGACACGTTGGAAAATCCAGCCTTTTTAGGACATACATTTGATGCGGTTATTGC
GAACCCACCATACAGTGCGAAATGGACAGCAGATTCAAAATTTGAAAATGACGAACGATTCAGCGGATACGGCAAACTTGCGCCAAAGT
CCAAAGCAGACTTTGCCTTTATTCAACACATGGTACATTACTTAGACGATGAAGGTACCATGGCCGTTGTACTCCCACATGGTGTCTTATT
CCGTGGTGCTGCAGAAGGTGTCATTCGTCGTTATTTAATTGAAGAAAAGAACTACTTAGAAGCCGTGATTGGCTTACCAGCCAATATTTT
CTATGGGACAAGTATTCCAACATGTATTTTAGTATTTAAAAAATGTCGCCAACAAGAAGACTATGTATTATTTATCGATGCATCCAATGAT
TTTGAAAAAGGAAAAAATCAAAACCATTTAACCGATGCCCAAGTCGAACGCATTATTAACACATATAAGCGTAAGGAAACAATTGATAAA
TATAGCTACAGTGCGACATTACAAGAGATCGCCGATAACGATTACAACTTAAACATACCGAGATATGTCGATACATTCGAAGAAGAAGC
ACCGATTGATTTAGATCAAGTCCAACAAGATTTGAAAAATATCGACAAAGAAATCGCAGAAGTTGAACAAGAAATCAATGCATACCTGA
AAGAACTTGGGGTGTTGAAAGATGAGTAA

Gene: hsdS (type I restriction-modification system site-specificity determinate)

Contig: 03_NODE_10, position: 871 to 1080, length: 210 nt, orientation: TRNC-FRWD (no start codon)

Sequence:

AAAATAAAAGGTATGATGCAAGGAGCAACCCAAGTTTATATAAATTATTCATCTATTAAATTGATATCTATACAATTGCCACTTCTTGAAG
AACAACAGAAAATAAGAGGGTTTCTAGAAGTTTTATCTGGAATAACTACTAAACAATTGCACAAGATAGACCAATTAAAAGAGAGGAAA
AAGGCGTTTTTACAGAAAATGTTTATTTGA

Gene: ssl11 (staphylococcal superantigene like protein locus 11)

Contig: 03_NODE_10, position: 1486 to 2178, length: 693 nt, orientation: FORWARD

Perfect match to: (MW2-BA000033-[443524:444216], allele observed in CC1+CC188)

Sequence:

ATGAAATTAAAAAATATTGCTAAAGCAAGTTTAGCACTAGGGATTTTAACAACAGGGATGATTACAACTACTGCTCAGCCAGTAAAAGCA
ATTGAGCAAAGCAGATTATCAGTTACTTCAAAAGATACACAAGAATTAAAAAAATACTACAGTGGAACAGGATATAATTTTCAAAATGTG
AGTGGTTATAGAGAAGGTAATAAAATGAACATTATTGATGGACCACAACTTAATGTAGTTACTTTACTTGGCACAGACAAAGAAAGGTTT
AAGGACGATGAAGATTATGAAGGACTTGATGTATTTGTTGTAAGAGAAGGGTCAGGTAAACACGCAGATAATATATCAATTGGTGGAAT
TACAAAAACAAATAAGAATCAATATAAAGACCCTGTACAAAACGTTAATTTATTGACTTCTAAGAGTAACGGTCAAAATACTGCTTCTGTG
ACTTCAGAATACTATAGCATCAATAAAGAAGAAATTTCATTAAAAGAACTTGATTTCAAACTAAGAAAGCAATTAATTGATAAACATGATC
TTTATAAGACAGAGCCTAAAGACAGCAAAATTAAAGTTTCTATGAAAAATGGCGGCTACTATACGTTTGAATTAAATAAAAAATTACAGC
CTCATCGCATGGGTGATACGATTGATAGTAGAAATATAAAGAAAATTGAAGTGAATTTATAA

Gene: slaP (exported SSL-associated protein)

Contig: 03_NODE_10, position: 2200 to 3702, length: 1503 nt, orientation: FORWARD

Perfect match to: (MW2-BA000033-[444238:445740], allele observed in CC1+CC188+CC425)

Sequence:

ATGAGAGAAAATTTTAAATTACGTAAAATGAAAGTCGGTTTAGTATCAGTTGCAATTACTATGTTATATATCATGACAAATGGCGAAGCA
GAAGCATCTGAAGGTAGCCAAACTGTTAAGAACCCAAAGGTGAATGCAACTGAAGAAATCAAAGTTGGATCACAACCAGTTCAAAATAA
TCAAGAAGTAAGTTCGGAACAAACAAAAAAGAATTTTGTTAATTTAGACCCCATTAAACCTGGTGCTCAAAAGGTAACAGGGACTACTTT
ACCCAGCCATATTATTCTAATGAATATAGATGGTAAAAGTGCTGATTCAGTAGATGGAGGAAATAGTGATTTAGTATTTGCTGATGAAAA
CGGAAGATTCGAGTATCCACTACACAATAGAAAAATTGTTCATAATCAAGAAATTGAGGTTTCGTCATCCAGTCCTGATTTAGGTGATGA
TGAAGAAGATGAAGAAGTAGAAGAAGATTCAACTGAAAAAGCTGGTACTGAGGAAGAAAACACAGATGCTAAAGCTACATACACAACA
CCACGATATGAAAAAGCGTATGAAATACCGAAAGAACAACTAAAAGAAAAAGATGGACATCACCAAGTTTTCATCGAACCTATTACTGA
AGGTTCAGGTATTATTAAAGGTCATACGTCTGTAAAAGGTAAAGTTGCTCTATCTATTAATAATAAATTTATTAATTTTGAAGAAAGAGCT
AAAGATGGAATTAGTAAAGAAGATACTAAAGCTAGTTCCGATGGTGTTTGGATGCCTATTAATGAAAAAGGATATTTTGATTTTGATTTC
AAAAAAAATCCTTTTGATAACTTAGAGTTAAAGAAAAATGATGAAATCTCATTAACATTTGCACCTGATGATGAAGATGAAGCATTGAAG
TCATTAATTTTCAAAACTAAAGTAACGAGTTTAGAAGATATTGATAAAGCAGAAACTAAATATGACCATACTAAAGTGGAAAAAGTAAAA
GTATTGAAAGATGTTAAAGAAGATTTACATGTAGATGAAATTTATGGAAGTTTGTACCATACAGAACAAGGTAAAGGTATTCTCGATAAA
CAGGGAACTAAAGAAATTACAGGTAAGACTAAATTCGCGAATGCAGTAGTGAAAGTATATTCTGACTTAGGTGATGCGCAACTGTTTCCT
GATATTCAAGTAGATGAAAATGGTAAATTTAGCTTTGATGCTGAAAAAGCTGGTTTCAGATTACAAAATGGAGAAACATTGAATTTTGCA
GTAGTTAAACCTATTACTGGTGAGCTATTACATCAAGGATTCGTTTCTAAGTATATCGATGTTTATGAATCTCCGGAAGAAAAGAAAGAA
CGTGAATTTGAAGAGAAACTTGAAAACACGCCTGCATATCATAAATTACATGGTGATAAAATTGTCGGCTATGATGTTCAAGGTAATCCA
TCAACTTGGTTCTATCCATTAGGTGAAAAGAAAGTTGAACGTAAGGCACCAAAATTAGAAAAATAA

Gene: Q2G0X2 (putative protein)

Contig: 03_NODE_10, position: 3809 to 4117, length: 309 nt, orientation: REVERSE

Perfect match to: (MW2-BA000033-[445847:446155:r], allele observed in CC1+CC188)

Sequence:

TTATTTTACGTGTTCATATTTTGAAACATCAAAGCCGTCTTGTTTAGCTTTGTTGATAATGTCTTTGATTGAATGTAGTCCTTTATCGGCGAA
GTATGATCTTAAGTTGTCTTTTGTAGCTTGGTCAGCATTCTTATCTAATAACACATCGATATAGCTTAATTCATGTTCTAAGAAGTTTGCAT
CATCATGTAGTACAAGTCCATTTTGAGAATAAACTTTCGCATCTGCTTGATTACTATATCCAACAACGCCAGTTGCTAAAACACCTACCATT
GCCGTAGCTACTAAAACCTTTTTAAATTTCAT

Gene: lpl-locus0450-MW0397 (tandem lipoprotein-like protein, second locus)

Contig: 03_NODE_10, position: 4453 to 5265, length: 813 nt, orientation: FORWARD

Perfect match to: (MW2-BA000033-[446491:447303], allele observed in CC1)

Sequence:

ATGAAGTATAAAACAGAGAGACGTGAAACGATGGGATATTTAAAAGGGTTTGCATTGTACATAAGTATCTTAATTTTAATAGTTTTTATA
GCAGGTTGTGGCAAAAGTGATAAAACAAAAGAAGATTCAAAAGAAGCACAAATTAAAAAGAGCTTTGAGAAAACTTTAGATATGTATCC
AATCAAAAATCTCGAGGATTTATACGACAAAGAAGGCTATAGAGATGGTGAATTTAAAAAGGGTGATAAGGGTACATGGACTTTACTCA
CAAGTTTTGCTAAAAGTAACAAACCAGGTGAGATAGATGATGAAGGCATGGTTTTATTTTTAAATAGAAATATAAAGAAGGCAACAGGA
TATTATTACACGAGCAAAGTTCATGATGAATTTAATGAAAAAGAGCATCAAAAAAAATATCATGTTGAACTTAAAAATAATAAAATAGTT
CTTTTGGATAATGTAGAAGATTCAAAGCTCAAAAATAAAATAGAAAATTTTAAATTCTTTAGTCAGTACGCTGATTTTAGAGATTTTAAAA
ACTATAAAAATGGAAATATATCAAGTGCAGATAATGTACCAAGTTTCGATGCAGAATATCAAATAAGTAATACGGATAAAAATGTAAAAA
AACTTAGAGAGGTTTATCCAATCACAACAAAAAAATCTCCAGTATTAAAATTACATATAGATGGTGACATAAAAGGAAGTTCAATTGGAT
ATAAAAATATAGAATTTAATTTTTCAAAAGTAAAAGACGAAGAAACAGCTGTTAGAGATTTTGTGAATTTTGGACCATCTGATGGAGTTA
GCTAA

Gene: lpl-locus0450-13 (tandem lipoprotein-like protein, second locus)

Contig: 03_NODE_10, position: 5313 to 6083, length: 771 nt, orientation: FORWARD

Sequence:

ATGGGATATTTAAAAAGGATTGGAATGTGTATAAGCCTGTTGATTGTAATCATTTTTGTAACATCTTGCGGTGGTGGTAATAAAATCACT
GGAGATTCAAAAGAAACACAAATCAAAAAGAGTTTTGCGAAAACGTTAGATATGTACCCAATCAAAAATCTTGAAGATTTATACGACAAA
GAAGGATATCGAGATGGCGAATTTAAAAAAGGTGACAAAGGGATGTGGACGATATACACAGATTTCGCTAAAAGTAATAAACCGGGTG
AATTGGATGATGAAGGTATGGTTTTAAATCTGGATAGAAATACTCGAACGGCTAAGGGATATTATTTTGTTAAGAAATTTTATGAAAAGG
ATAAATTACCTGATAGAAAAAAATTATAAAGTTGAAATGAAAAATAATAAAATTATCTTATTAGACAAGGTAGAAGATCCAAATCTAAAAA
AGAGAATAGAAAACTTTAAATTTTTCGGACAATATGCAAATTTTAAGGATTTGGAAAATTACAACAATGGCGACGTGTCAATAAATTGGA
ATGTTCCAAGTTATGACGTGGAATATAAAATGAGCAATAAAGATGAAAATGTTAAACAATTAAGAAGTCGTTATAACATTCCTACTGATA
AAGCTCCAATGTTAAAAATGCATATTGACGGGGACTTAAAAGGTAGTTCTGTTGGATATAAAAGGTTAGAAATAGATTTTTCAAAAGAAG
ATAGGGATATTTCAGTCATTGATTATTTAAGTTATAAGCCAGCGAAAAAATAG

Gene: lpl-locus0450-MW0399 (tandem lipoprotein-like protein, second locus)

Contig: 03_NODE_10, position: 6115 to 6906, length: 792 nt, orientation: FORWARD

Perfect match to: (MW2-BA000033-[448153:448944], allele observed in CC1)

Sequence:

ATGGAATATCTAAAAAGGCTTGCATTGTTCATAAGTGTTATTATTTTGACCATTTTTATAATGGGTTGTGATAGTCAAAGCGATACTGCAG
AAAATCCAAAAGAAGGTTCAAAAGAAGCACAAATTAAAAAGAGTTTTTCGAAAACGTTAGATATGTATCCAATTAAGAATCTCGAGGATT
TATATGACAAAGAAGGATATCGTGATGGCGAATTTAAAAAAGGTGATAAAGGGACGTGGACAATATCAACAGATTTTGCTAAAAGCAAC
AAACCAGGTGAAATGGATAGTGAAGGTATGGTGCTACATTTGAATAGAAATATGAGGACGGCAACTGGGCATTATACTATAAGAACAAC
TTATGATGAATTGGGTAAGATGACACGCGAAAAAAATTATCGTGTTGAACTTAAAAATAATAAGATAGTAGTTTTAGATAAAGTAGAAG
ATGTAAACCTTAAACATAAAATTGAAAATTTTAAATTTTTTAGTCAGTATGCAGATTTTAAAGATTTGAAAAATTATAAAAATGGAAGAAT
ATCTATCAATGAAAATGTTCCGTATTATGAAGCAGAGTACAAAAGGAATAATAGTGATGGAAATGTAAAAAAACTTAGAGAAAAGTACC
CAATTACAACCAAGCAGTCTCCAATATTAAAACTGCATATAGACGGAGATATTAAAGGTAGCTCAGTTGGATATAAGCAGATAGAGTACA
TGTTTTCTAAGGAGAAAGAGGATGAGACTTTTATGAGTGATTTTTTGAACTTCGGACCATCACATAGTAATTAG

Gene: lpl-locus0450-MW0400 (tandem lipoprotein-like protein, second locus)

Contig: 03_NODE_10, position: 6958 to 7755, length: 798 nt, orientation: FORWARD

Perfect match to: (MW2-BA000033-[448996:449793], allele observed in CC1)

Sequence:

ATGGGATATTTTAAAAGAGTTTTACTTTATATAATTGTTATGGTTTTGAGTGTTTTTATAATAGGTTGTGATAAATCAAGCGATACTTCAGA
AAAATCAAAAGGAGATTCAAAAGAAGCACAAATTAAAAAGAGTTTTGCAAAAACGTTAGACATGTATCCTACTGAAAATCTAGAAGACT
TTTATGACAAAGAGGGATATCGAGATGGAAAATTTAAAAAAGGCGACAAAGGTACTTGGGTTATTAGATCTGAAATGACAACAGAACTG
AAAAATGAAAATATGGTATCTAAAGGTATGGTCATACGTTTAAATAGAAATAGTAGAACATGCACTGGTGAATATTTTGTCAGGATAGTT
AAAGAAGACAGTGAGGGCAAGGTATATAGTGATGAACGAAAATATCCAGTGAAAATGGAAAATAATAAAATCATTCCATTAAAACCAAT
CGATGATGAAAAAGTAAAAAAAGAAATTGAAGAATTTAAATTCTTTGTACAATACGGGAATTTCAAAGAATTGGAAAACTATAAAGACG
GAGAAGTGACATATAACCCAGAAGCACCAATATACTCTGCACAATATCAATTGAAAAACAGTGATTACAATGTAGAACAATTACGTAAGC
GATATAATATCCCGACGCAAAAAGCGCCTAAATTATTATTGAAAGGCTCAGGTAATTTAAAAGGTTCATCAGTCGGATATAAAAATATTG
AATTTACCTTTGTTGAAAACAAGGAAGAAAATATTTACTTCACAGATAGTGTCTACTTTAATCCAAGCGAGGATAAATAA

Gene: lpl-locus0450-14 (tandem lipoprotein-like protein, second locus)

Contig: 03_NODE_10, position: 7934 to 8749, length: 816 nt, orientation: FORWARD

Perfect match to: (MW2-BA000033-[449972:450787], allele observed in CC1)

Sequence:

ATGATGGAGTATATAAAAAAAATTGCTTTGTACATGAGTGTATTACTTTTAATCATTTTTATTGGGGGATGTGGAAATATGAAAGATGAA
CAGAAAAAAGAGGAACAAACGAATAAAACAGATTCAAAAGAAGAACAAATCAAAAAGAGTTTTGCGAAAACGTTAGATATGTATCCAA

TTAAGAATCTCGAGGATCTATATGACAAAGAAGGATATCGAGATGGTGAGTTTAAAAAAGGTGATAAAGGTATGTGGACGATATATACA
GATTTTGCCAAAAGTAATAAAGCAGACGAATTAAGTAATGAAGGTATGGTCTTATACTTAGATAGAAATACACGGACAGCAAAGGGACA
TTATTTTGTTAAGACATTTTATGAAAAGGATAAATTCCCAGATAGAAAAAAATATAAAGTTGAAATGAAAAATAATAAAATTATCTTATTA
GACAAGGTAGAAGATCCAAAACTAAAAAAGAGAATAGAAAACTTTAAATTTTTCGGACAATATGCAAACCTTAAAGAATTGAAAAATTA
CAACAATGGTGATGTCTCAATTAATGAGAATGTTCCAAGTTATGACGCAAAATTTAAAATGAGCAATAAAGATGAAAATGTTAAGCAATT
AAGAAGTCGTTATAATATTCCTACTGATAAAGCACCGGTATTAAAAATGCATATTGATGGTAATTTGAAAGGAAGTTCTGTGGGTTATAA
AAAGTTGGAAATTGACTTTTCAAAAGGTGAAAAAAGCGATTTGTCAGTAATAGATTCATTAAATTTCCAGCCGGCGAAGGTGGATGAAG
ATGAAAGATAA

Gene: lipC3-MW0402 (putative lipase class 3)

Contig: 03_NODE_10, position: 8739 to 10064, length: 1326 nt, orientation: FORWARD

Perfect match to: (MW2-BA000033-[450777:452102], allele observed in CC1+CC188)

Sequence:

ATGAAAGATAAATACAAAATAGATCCAGGACCTATTAAATATAATACTGAGGAAACTACAGCTATTTCTAAAATAAGTTATGAAATTGAA
AATGCGAAATTATATGGAATTAAAAGTAAAACAATAGGTAGACAAATAGATCAATTAAAAGAAGCGAAAAAATTCCCTTCTAATCTTGAA
TATGTTGATAGTTATACTGATTCTCTTAATGGAGTAACAACTTCTGCTTTTTTAAATAAAGATACAGGCAAAGTAACTCTTGGGATGACTG
GGACTAATATACAAGGCGAAGCCTTTAAAAAGTTAAAAGAAGGTGAATTTTCAAGACAAAATTTTACCAATGCTTTGGAAACAGTTAAAG
ATGGATATGCAGATCTTAAAATATTATATTCTCCTGCATCTGATCAAAACTATAGATATGCGAATACGCAAGAATTTATAAATAAAATAAA
AAGTAAGTATGATATTGATTTTATTACTGGACATTCACTAGGTGGAAGAGATGCGGTAGTTCTAGGAATGAGTAATGGTATTCCGAACAT
TGTGGTTTATAATCCAGCTCCTATTTCTATAACTAGTTTGAATCCTAATTCCCCAGATGGAAAACGATTGTTAGAGTTATATAAAAATTATA
AAGGTAATATTACTAGGTTTGTTGCAGAAAATGATGCATTGACAGAAAATCTGAAGAAATATAAGCATTATGTTTTTTTCGGTAATGATA
AAGTCTTTAAAAATGGTAAAGGTCATGAAATGGAAGGCTTTCTGACCGAAGAAGAACAAAAAGCTATAAAAAAAGAACTTAAAAAACTA
CAAGGTTATGCAGAAGAAAATAATAAGTCATTTGTAAAGCATTCAAATAATGCTATCTCTAAATTAGCTAGTATAGAATTACTTAGAGCA
AATATGATGACTACAAATGGAGGAGGATTATCTTCTTCGCAGCAAAAAGTTTTAGAAAGTTTAACAGCTTTAACAATTGCGCAGTCATTC
AGTCAACTGATAGACGATGAAATTAATCAAATCAAAAAAAATGTATAATGAAAAGAAAAAGAAATTTGGAAAAAAATTGGGAAGACGCTCA
AAAAGCTGGAAAAGCTGTAGGTGAAGATTTGAGTGTAAATGGAGTTCTCAATGCTTTAGATGAAGGTCAAGTGAATGAAAGTAGTATG
GTAAGAGAACCTGAACAAATGATATCTGCAAAAGAAAGACAACTTTCAACGATAGGGTCTTCTGTATCAAATTATATTATGAGAGTTAGA
CTCAGTATTAATGAAATCGTTGATAAAGATCAAGTGCTTGCATCACAAATAGGTGGATTACTATGA

Gene: Q5HIM7 (putative protein)

Contig: 03_NODE_10, position: 11001 to 11195, length: 195 nt, orientation: REVERSE

Perfect match to: (MW2-BA000033-[453039:453233:r], allele observed in CC1+CC188)

Sequence:

TTACAAACCATAAAGCGCTGCAGTTAAGTATTGAATGGCATAGCCAATTGAAATGAAAATAGCAATCGTTATAATAGCACTGCCAATGGT
ATATAAAGTATGTTTCGTAGATAATCCTTCAGTCTTTGAACCAAAAACACTGAGTAGAATAAATGTAATACCAATGATATATAAAACTAAA
ATGACAAATAGCAT

Gene: cobW1 (cobalamin biosynthesis protein)

Contig: 03_NODE_10, position: 11393 to 12595, length: 1203 nt, orientation: FORWARD

Perfect match to: (MW2-BA000033-[453431:454633], allele observed in CC1+CC188)

Sequence:

ATGGCTAAAATTCCAGTTACGGTATTAAGTGGTTATTTAGGCTCGGGGAAAACAACGTTGTTAAATCATATTTTACAAAATCGAGAAGGT
CGACGTATTGCGGTAATTGTAAATGATATGAGTGAAGTCAATATCGATAAAGATCTTGTCGCAGATGGTGGGGGACTATCGCGCACAGA
CGAAAAATTAGTCGAACTTTCTAATGGTTGTATCTGTTGTACACTTAGAGACGATTTATTAAAAGAAGTTGAGCGTTTAGTGAAAAAAGG
TGGCATCGATCAAATTGTTATTGAGTCAACAGGGATTTCAGAGCCAGTACCTGTTGCACAAACTTTCTCATATATTGATGATGAACTTGGC
ATTGATCTTACAGCGATTTGCCGTTTAGATACAATGGTTACAGTTGTGGATGCTAACCGCTTCGTACATGACATCAACTCAGAAGATTTAT
TGATGGATCGTGATCAAAGCGTTGATGAAACAGATGAGCGTTCGATTGCTGATTTATTAATTGACCAAGTTGAATTTTGTGATGTATTGA
TTATTAATAAAATTGATTTAATTAGTGAAGAAGAACTTGCGAAGTTAGAAAAAGTGTTAAGTGCATTGCAACCTACTGCTAAAATTATTAA

GACAATAAATTCTGAAGTGGATTTAAAAGAAGTCTTAAATACGCAGCGTTTTGATTTTGAAAAAGCGAGCGAGTCAGCAGGATGGATCA
AAGAACTTGAGTCTGGTGGGCATGCATCGCATACACCTGAAACAGAAGAATATGGTATATCATCGTTTGTATATAAACGTCGACTTCCTT
TCCATGCTAAAAGGTTCAATGATTGGTTAGAAACCATGCCAAATAATGTCGTTCGATCAAAAGGTATCGTATGGCTAGCACAATACAATC
ACGTAGCATGTTTATTATCTCAAGCAGGGTCATCTTGCAATATTCATCCAGTTACATATTGGGTGGCTAGTATGTCTGAAGCACAACAAAC
GCAAATATTAGCAGAACGCCAAGACGTCGCAGCTGAATGGGATCCAGAATATGGCGATCGTCATACACAATTTGTCATTATTGGTACCGA
CTTAGATGAAGAAAAATTAACAAAAGAACTCGATGCATGCTTAGTCAATGCGCAAGAAATTGATGCAGATTGGCAACAATTTGAAGATC
CATATCAATGGCAAATTAGACCAGCACGATAA

Gene: psmA4 (phenol-soluble modulin alpha 4)

Contig: 03_NODE_10, position: 13252 to 13314, length: 63 nt, orientation: REVERSE

Perfect match to: (N315-BA000018-[469998:470060:r], highly conserved allele)

Sequence:

TTATTTTGCGAAAATGTCGATAATTGCTTTGATGATTTTAATGATAGTACCTACAATAGCCAT

Gene: psmA3 (phenol-soluble modulin alpha 3)

Contig: 03_NODE_10, position: 13378 to 13446, length: 69 nt, orientation: REVERSE

Perfect match to: (RF122-AJ938182-[437079:437147:r], highly conserved allele)

Sequence:

TTAGTTGTTACCTAAAAATTTACCAAGTAAATCTTTAAAGAATTTGAATAATTTTGCTACGAATTCCAT

Gene: psmA2 (phenol-soluble modulin alpha 2)

Contig: 03_NODE_10, position: 13498 to 13563, length: 66 nt, orientation: REVERSE

Perfect match to: (RF122-AJ938182-[437199:437264:r], highly conserved allele)

Sequence:

TTACTTACCAGTGAATTTCTCAATTAATCCTTTAATGAATTTAATGATTCCTGCAATGATACCCAT

Gene: psmA1 (phenol-soluble modulin alpha 1)

Contig: 03_NODE_10, position: 13595 to 13660, length: 66 nt, orientation: REVERSE

Perfect match to: (RF122-AJ938182-[437296:437361:r], highly conserved allele)

Sequence:

TTATTTACCAGTGAATTGTTCGATTAAGCTTTTGATAACTTTAATGATGCCAGCGATGATACCCAT

Gene: nuoL-mpsA (membrane potential-generating system subunit A)

Contig: 03_NODE_10, position: 14180 to 15664, length: 1485 nt, orientation: FORWARD

Sequence:

GTGTTAAGTTTTCAATTGCTATTTTCACTGTTTGTTATTGCGCTTATCATTGCATTGATAAGTGGCTTGTTGTTTTTAGCACCAGTTATGCCA
ATGAGATATATTAAATTACATTTATACATACTAGTCATGCCAGTATTATTTGCAGTCATTGGCTTTTTCGGTATTCATGGTCAACATGTCTT
AGGTCCATTTAAAATAGATCGTTTATCTTGGTTATTAGCTGGCTTTGTAATGGCGCTTGGTTTTATTATTCAAAAGTTTTCAATGCGATATT
TACTAGGTGATCATCATTATAGACATTACTTTCCATTGTTCACTGCGATTACGTCGTTTGCATCTTTAGCATGGATGTCTGAAGACTTAAGA
CTGATGGCACTCTGCTGGGGTATGACATTATTATGTTTAACATTGCTGATGAACGTTAATCGTTTTTGGAAAGTGCCACGTGAGTCTGCGA

AATTATCAAGTATGACATTTTTATGTGGTTGGCTTGCATTCGTTGGAGCAATTGTAACTATTTATATTGCGACTGGCGAGTGGCGCGTGCC
TCAACATATGTCTAATTCGACATGGTCATTGTTGACGAATGTACTACTTGTATTAGCTGTCATGATACCAGCAGCACAATTTCCATTTCATC
GATGGTTGATTGAATCTGTAACAGCACCAACGCCAGTATCGGCAATTATGCATGCAGGAATTGTGAATGCAGGTGGTGTTATTCTAACTC
GTTTTGCGCCGATATTTGATAATGGATTTGCGTTATCATTATTACTTATCCTTTCTAGTATTTCTGTATTGTTAGGATCGGGTATTAGCTTGG
TTCAAGTGGATTACAAAAGACAGTTAGTCGGCTCTACGATGAGTCAAATGGGCTTTATGTTAGTTCAATGTGCATTGGGTGTATATTCAG
CAGCGATTATTCATTTAACATTGCACGGTATTTTTAAAGCAACATTATTTTTACAATCAGGTTCTATCGTGAAGCGATTCAATATTCCAAAA
CAAGCATCTGCTAAAGACGCTTATGGCTGGATTGTCATGGGACGTGTATTAGCTATTATCGTGGCATTTCTATTTTGGATGAGTAGTGAC
AGAAGTGCATATGAAGTGTTAAGTGCACTCATTCTAGCATGGTCATTACTTGTATCTTGGAATCAGATGGTAGCCTTTAGTAAAGGGCGC
ATGGCACGTCTGGTTGGCATGATTTTGATTGCAATTGTGACATTTATCTACGTCATCACACACAATTATTTTTACGATGTATTACAAAATAT
AACAACACATGCGACAACGCCGCCTACAGTGAGTGTCATCATTAGTGTTGTCATTTTAATATTTGGTAGTTTATTAAGTATTTGGGTGGCG
CGTCATCGATACTCTAAGGGTTTTGCGGTATTGTACGTGTGGTTAGTTAATCTAGGTGAAGCACGCTCGAAAGCGATAGAAAGTCATCCG
AATTATTTGAAGAAGTATTTATAG

Gene: ybcC-mpsB (membrane potential-generating system subunit B)

Contig: 03_NODE_10, position: 15677 to 18382, length: 2706 nt, orientation: FORWARD

Perfect match to: (MW2-BA000033-[457715:460420], allele observed in CC1+CC188)

Sequence:

ATGACAACACAGTTAAATATCAATTCAGTCATTGAAAATGCGAAACGTGTTATTACACCATTATCACCGATTTCGATTTTTGCAGCACGAA
ATCCATGGGAAGGATTAGAAGCGGATACGTTTGAAGATGTTGCAAAATGGTTACGTGATGTTCGAGATGTGGATATTTTTCCAAATAAA
GCATTAATAGAAAGTGCTGTGGCACGTGGTGAATTAGACGAAAGTGTCTTTAATCAACTTGTTACTGATATGTTACTTGAACATCACTACA
ATATCCCGCAACACTACATCAATCTTTATATTGATAACATTAAAACATTAAAAGACGTACCTGCATCATATATGAATCATTCAAATGTTGAT
GTTGTTGCTGATCTACTATTAGAAAAATCAAAACGTGATATGGCTGATTCATATCATCACTATGATGTACGTCCGATGAGTGATGCAATAA
TAGATGAACAAGGTGAGCCACTTAGCGAACAAGTGAATCGTCAAATGATTAAATGGACGAAACTTTATATCGATCAATTTCTATCGAGTT
GGACAATGCCGAAGCGTGAGCAAAGTTTTTACCATGCATGGTTGCATTTAGCGCAACATGATCATAGTTTTACTAAAGCACAGCGCCAAG
TGATTAAAGGCTTGCCCAATGATCCTAAAATGACGATAGAGTCAGTATTAACTCATTTTTCAATAGATCAGGAAGACTACCAAGCTTATGT
TGAAGGACATCTTTTGGCGTTACCGGGTTGGGCAGGTATGTTGTATTACCGTTCACAACAGCATCACTTTGAACAACATTTGTTAACGGAT
TATTTGGCAATTCGGTTAGTTGTCGAACAATTGCTAGTTGGTGATGAGTTTAAGTCAGTCACTAAAGATTGTGAAAGTAGATCGGAAAAT
TGGTTTAAGCAAACTGTTGCATCATGGTGTTACTACAGTGATATGCCTAGCGATGTATTACTACAACATGACGTCAATGAAATTCAAACGT
TTATTCATTTTGCAGCAACTATGAATAAAAATGTATTTAAAAATTTATGGCTAATTGCCTGGGAAATGACATACGAATCTCAGTTAAAACA
AAAAATTAAAGCAGGTCATGAAAGTGTGGCGGGCGCATTAGATGTAAACCAAGTAAATGTCTCAGAAAATGATAACGCTAATCAGCCAC
ATTCAGTATTATTAAATGACACACAAGCAGTTGATGAAAATAATAGCGAGCTAAATCAGGTGGGCACATCAACGAAAGCGCAAATTGCA
TTTTGTATAGATGTTCGTTCAGAACCATTTCGTAGACATATCGAAGCAGCAGGGCCCTTTGAAACGATTGGTATTGCAGGTTTCTTTGGAT
TACCTATTCAAAAAGATGCCGTAGACGAACAATTCAAACATGATTCATTACCTGTCATGGTACCGCCGGCATATCGCATTAAAGAATTTGC
AGACCGCTACGATATGAATGTTTATCGACAACAGCAACAGACAATGTCATCGATGTTTTACACATTTAAATTGATGAAAAATAATGTTATG
CCTAGTCTGTTATTGCCTGAATTAAGTGGGCCATTTTTAAGCTTGAGTACCATTGTCAATTCGATTATGCCTAGAAAAAGTCGCGCGTCTTT
ACAAAAAATAACACAAAAATGGTTGAAAAAGCCTGAAACAAAGTTAACGATTGATCGTGAGTTTGACCGAACATCAGACTTACCTGTTG
GATTTACTGAGCAAGAGCAAATTGATTTCGCGTTACAAGCGTTGAAATTGATGGATTTAACCGAAGCATTTGCGCCGTTCGTTGTGTTAG
CAGGTCATGCTAGTCATTCTCACAATAATCCACATCATGCATCACTTGAATGTGGGGCTTGTGGTGGCGCATCAAGCGGTTTTAATGCTAA
GTTATTAGCGATGATATGTAATCGTCCAAATGTCAGACAAGGATTAAAACAATCAGGTGTGTATATTCCAGAGACAACTGTTTTTGCGGC
AGCAGAACATCATACGTCTACTGATACGTTGGCATGGGTATATGTGCCAGACACATTATCTTCTATTGCTTTAGATGCATATGAATCATTG
AATGACGCGATGCCGATGATTTCTGAACACGCGAATCGCGAACGTTTGGACAAACTGCCAACGATTGGTCGTGTGAATCATCCAGTGGA
AGAAGCGCAGCGGTTTGCGAGTGATTGGAGTGAGGTACGTCCAGAATGGGGCTTGGCTAAAAATGCATCATTTATAATTGGACGACGC
CAATTAACAAAAGGCATTGATTTAGAAGGGCGGACATTTTTACACAATTATGATTGGCGTAAAGATAAAGATGGCAAATTGTTAAATACT
ATCATTTCTGGTCCGGCGCTTGTGGCACAATGGATTAATTTACAATATTATGCGTCGACAGTTGCGCCGCATTTTTACGGAAGTGGGAAT
AAAGCGACACAAACCGTCACGTCAGGTGTTGGTGTCATGCAAGGTAATGCGAGTGATCTGATGTATGGCTTATCATGGCAATCTGTTATG
GCTGCTGATCGAACGATGTATCATTCGCCAATTCGTTTGCTTGTCGTTGTTCAGGCACCCGACTATGTTGTAGCAAGACTACTCGCGAATA
ATGACCATTTCGCTAGGAAGGTGTCTAATCATTGGCTGCGTTTAATGAGCGTTAATGAGGAAGGGCGTTTTAAAAGTTGGATTTAA

Gene: ybcI-mpsC (membrane potential-generating system subunit C)

Contig: 03_NODE_10, position: 18544 to 18906, length: 363 nt, orientation: FORWARD

Perfect match to: (MW2-BA000033-[460582:460944], allele observed in CC1+CC188)

Sequence:

ATGAAAAGAACGAAAGGTGAAATCGAAGCTGAAATCAGTAAAGCCATTACGCAATGGGAAAAAGATTTCCTTGGCAGAGGTTCCTTGTC
AGTTAAATCAGACATTTTAAGAGATATGGTGATTATTAGTTTACAAGGTATCTTAACGCCAGCAGAATATCGTGTGTGTAGTACGAATGA
AGGATTACTAAATATTAAACGAACACGTTCTGAATTAGTTGAATCCGGTGAGCAAGATTTGAATGATATCATTTTTAAAATTACAGGTATC
AAAGTGATGAGCTTCCATAGTGATTTAAGTACAGTTACAGGTGAACGTATTATCGTATTCAAACTTGAGGATAATTTGGAAAAGCATATT
TAA

Gene: Q5HIM2 (putative protein)

Contig: 03_NODE_10, position: 19135 to 19482, length: 348 nt, orientation: REVERSE

Perfect match to: (MW2-BA000033-[461173:461520:r], highly conserved allele)

Sequence:

TTACATAAAGAGTATATGTGTGACGTAGGCATATAATCGATAAAGTATTCCTAAAAAATTAGGTATAATGACCATTGTTGCAATAAGTTTT
TCGGCCTTGAAACCGATAACGCATAGTATAATAGGAATAAAATATAATACAAATATCCAGTAAATCAAATTTGAAAACGATGGATGAATA
GGGGTAAAGAATCTAACTAATGTAATGATTAACGCTATATAGACAAAAATAACTCTATTGATACGCCTTACCCCCTCTGTATAATAAATAT
AGATCGTACTAAATTGAAAATAGCAAAATATTGTTATTTCAATTATACAATGTTTTATTTGCAATATACATAACAA

Gene: Q5HIM1 (lipid phosphate phosphohydrolase 2 family protein)

Contig: 03_NODE_10, position: 19589 to 20263, length: 675 nt, orientation: FORWARD

Perfect match to: (MW2-BA000033-[461627:462301], highly conserved allele)

Sequence:

ATGATAGATAAAAAAATTAACATCACCGAAAATGACAGTGCCTTTATTTTTAATCGCGCTGATTGTATTTATAGGTATGTTTTACAGTGTAG
TGACAAATCAAGAATGGCTTAAAAATATAGATATGGGATCATTAACATGGTTTACAGATTATTTCGGTGAGCCACAACGTCAGTATGTTA
ACAATTTATTTAATTACTATATGACGTTTAGTGCGGAAATTGGAGATGTCAAAGGTGTCGTGTTGATTTCCATTATCGTCACAATCATACT
GTTTATTAAACAGAGGCATTTAGCGGTTTGGTTTGTGACATATTTGGTTTCAGGTGTCATCATGAACAAATTAATTAAAGATACTGTTTTA
CGTCCAAGACCATATAATCATTTAGCCGTTGATACAGGCTTTTCATTTCCAAGTGGACATTCCAACGCCAGCACATTATTATATTTCGCTTT
AATGATCATAATTATTTCACTTGCTGCTAAGACAATAACAAAAGTGTTGAGTGCGCTAGTTATGGGAATATTATGGCTTAGCATATTATTT
TGTCGCCTTTATTTTCATGCGCATTACTTTTCAGATGTCATTGGCGGCACGTCACTAGCAATCATTTGGGTAGCGTTATTCTTAATGGTATA
CCCATACTTTATTAATCATCGACGACAACGCGTTTAG

Gene: Q5HIM0 (putative esterase)

Contig: 03_NODE_10, position: 20300 to 21034, length: 735 nt, orientation: FORWARD

Perfect match to: (TCH959-AASB02000043-[20470:21204], highly conserved allele)

Sequence:

ATGAGAATTAAAACACCGAGTCCATCTTATTTAAAAGGCACAAATGGACATGCGATATTATTATTACATTCATTTACAGGTACAAATCGGG
ATGTGAAGCATCTTGCAGCTGAGTTAAATGACCAAGGATTTAGTTGTTATGCACCGAATTATCCAGGTCATGGCTTACTGTTGAAAGATTT
CATGACATATAATGTAGATGATTGGTGGGAAGAAGTTGAGAAAGCTTACCAATTTTTAGTCAATGAAGGTTATGAATCTATCAGTGCAAC
GGGTGTGTCTTTAGGTGGATTAATGACATTAAAATTGGCGCAACACTATCCTTTGAAACGTATCGCTGTCATGTCAGCACCAAAGGAAAA
GAGTGACGATGGTTTAATAGAACATTTAGTTTATTATAGTCAACGCATGTCGGATATTTTAAATTTAGATCAGCAAGCATCGAGTGCGCA
ATTAGCAGCAATTGATGATTATGAAGGTGAAATTACGAAGTTTCAACATTTTATTGATGATATCATGACAAATTTAAATGTTATTAAAATG
CCAGCTAATATATTATTTGGTGGTAAAGATGCGCCATCCTATGAAACAAGTGCACATTTTATTTATGAACATTTAGGATCAGTAGACAAAG
AATTAAATGGTCTGAAGGATTCGCATCATTTAATGACGCATGGAGAAGGCAGAGATATTTTAGAAGAAAATGTTATTCGCTTTTTCAATG
CTTTAACATAA

Gene: Q5HIL8 (transporter)

Contig: 03_NODE_10, position: 21490 to 22827, length: 1338 nt, orientation: FORWARD

Perfect match to: (MW2-BA000033-[463528:464865], allele observed in CC1+CC12+CC15+CC72+CC361)

Sequence:

ATGAAAAGACAACAATCACAATGGAAGTCATCAACTGGATTTATTTTAGCTAGTGCGGGTTCTGCAATCGGTCTTGGTGCCATGTGGAAA
TTCCCATATATGGCAGGGATTTATGGCGGCGGTGCCTTTCTAGCTATGTTCTTAATATTCACCATTTTTGTTGGGTTACCATTACTCATTAT
GGAATTCACTGTTGGGAAAATGGGACGGACATATACAACACAAATATATAGTAAATTAACTGGTAAAAAATGGCTCAATATCATTGGCT
GGAACGGTAATTTGGCCGTGTTTATTTTATTTGGCTTCTATAGTGTTATCGGTGGTTGGATTGTCATTTACATCGGACAAGTATTATGGCA
ATTAGTTATATTTCAACGCATCAATCATCTCCAAGAAATGAATTTTGAAGCGGTAATATCAAATCCTTGGTTAACTGTTCTAGGGCAAGGT
ATATTCATATTCGCTACGATGATTATTGTCATGTTAGGTGTTGAAAAAGGATTAGAAAAGGCATCGAAAGTTATGATGCCATTGCTGTTTG
TCTTTTTAATCATCATTGTGATTAAGTCTTTAACATTAGATGGCGCCTTAGAAGGTGTGAAATTTATTTTACAACCAAGAGTGTCAGAGATT
ACTGCTGACGGTATCTTGTTTGCGCTAGGCCAATCATTCTTTACGTTATCATTAGGAACTACAGGTATGATTACTTATGCGAGTTATGCCTC
TAAAGACATGACGATTAAGTCATCAGCTATTTCTATCGTTGTTATGAATATCTTTGTATCTGTATTGGCAGGTCTAGCTATATTTCCGGCGT
TACATAGTTTTGGCTATGAACCACAAGAAGGGCCTGGATTATTATTTAAAGTACTGCCAATGGTCTTTAGTCAAATGCATCTAGGCACATT
ATTCTATTTGGGATTCTTAGTGCTGTTCTTATTTGCGGCTTTAACGTCATCTATTTCTTTATTAGAATTAAATGTTTCTAACTTCACGAAGAA
TGACAATACAAAACGTAAAAAAGTCGCAGTGATCGGTAGTATTTTAGTATTTATCATTAGTATTCCAGCAACCTTATCTTTTGGTATCTTAA
AAGATGTAAGATTCGGTGCGGGAACGATTTTTGATAATATGGATTTCATCGTTTCGAATGTATTGATGCCATTAGGCGCATTAGGTACTA
CGCTTGTCGTAGGACAATTATTAGATAAAAAATTATTACAACAATATTTTGGTAAAGATCGATTTAGATTATTCAGTGGTTGGTATTACTT
AATTAAGTATGCGATGCCTGTCGTTATTATTTTAGTCTTTATCGTGCAATTATTTAGTTAA

Gene: mccA (O-acetylserine-thiol-lyase)

Contig: 03_NODE_10, position: 23044 to 23949, length: 906 nt, orientation: FORWARD

Sequence:

ATGATTACTTATGATTTAATTGGCAATACACCATTAGTACTGTTAGAACATTATAGTGATGATAAAGTTAAAATTTATGCCAAGCTTGAAC
AATGGAATCCTGGAGGCAGTGTTAAAGACAGACTCGGGAAATATTTAGTAGAGAAGGCAATTCAAGAAGGGCGTGTGCGTGCAGGTCA
AACTATTGTTGAAGCGACTGCTGGTAATACAGGCATAGGGTTAGCTATTGCAGCGAATAGACATCATTTAAAATGTAAGATCTTTGCGCC
GTATGGTTTTTCAGAAGAAAAGATTAATATTATGATAGCGCTTGGTGCAGATGTTTCAAGGACGAGTCAGTCTGAAGGTATGCATGGGG
CACAATTAGCTGCACGTTCCTATGCTGAAAAATATGGTGCCGTTTATATGAATCAATTTGAATCCGAACATAATCCGGATACATATTTTCA
TACATTGGGACCCGAATTGACTTCAGCATTACAGCAAATTGATTATTTTGTGGCTGGTATTGGCTCTGGCGGTACATTTACAGGTACCGCA
CGTTATTTAAAGCAACATCACGTGCAATGTTATGCCGTTGAGCCAGAAGGGTCCGTGTTAAATGGAGGGCCAGCTCATGCACATGACACT
GAAGGTACCGGTTCTGAGAAATGGCCGATATTTTTAGAGAGACGTCTTGTAGATGGGATATTTACGATTAAAGATCAAGATGCCTTTCGA
AATGTCAAAAGTTTGGCTATAAATGAAGGGTTGTTAGTAGGCAGTTCTTCAGGTGCAGCATTACAAGGTGCATTGAATTTAAAAGCGCA
ATTATCTGAAGGTACGATTGTTGTCGTATTTCCAGATGGTAGCGATCGATATATGTCTAAGCAAATATTTAATTATGAGGAGAATGATTAT
GAATAA

Gene: mccB (cystathionine gamma-synthase)

Contig: 03_NODE_10, position: 23942 to 25084, length: 1143 nt, orientation: FORWARD

Perfect match to: (MW2-BA000033-[465980:467122], allele observed in CC1)

Sequence:

ATGAATAAGAAAACTAAATTAATTCACGGTGGACACACAACGGACGACTATACAGGTGCAGTTACTACACCAATTTATCAAACAAGTACA
TATTTACAAGATGATATTGGTGATTTACGTCAAGGATATGAATATTCTCGTACTGCGAATCCAACAAGAAGTTCTGTAGAAAGCGTTATTA
CGGCATTAGAAAATGGCAAACATGGCTTTGCGTTTAGTTCAGGTGTTGCAGCTATCAGTGCAGTTGTTATGCTGTTGGACAAAGGTGATC
ATATTATTTTAAATTCAGATGTATACGGCGGTACTTATCGCGCATTGACAAAAGTATTTACACGATTTGGCATTGAAGTGGATTTTGTAGA
TACAACGCATACAGATTCAATTGTACAAGCGATACGCCCAACAACAAAGATGTTGTTTATTGAAACACCTTCTAATCCATTATTACGTGTT
ACTGACATTAAAAAGTCTGCTGAAATTGCGAAAGAACACGGTTTGATTTCAGTCGTTGATAACACATTTATGACACCTTATTATCAGAATC
CATTAGATTTAGGCATCGATATTGTCTTGCATTCTGCAACGAAATATTTAGGTGGACATAGTGATGTCGTTGCTGGTTTAGTTGCAACATC
GGATGACAAGCTTGCAGAACGTTTAGCATTTATTTCAAATTCAACAGGTGGCATTTTAGGACCTCAAGATAGCTATTTACTTGTGAGGGG
TATTAAAACATTAGGTTTACGTATGGAACAAATTAATCGCAGTGTTATTGAAATTATTAAAATGTTACAAGCACATCCAGCTGTGCAACAA
GTGTTCCATCCAAGTATTGAAAGTCATTTAAATCATGATGTCCATATGGCTCAAGCGGATGGCCATACAGGTGTGATTGCATTTGAAGTG
AAAAATACAGAAAGTGCCAAACAATTGATTAAAGCAACATCGTATTACACATTAGCTGAAAGTTTAGGTGCAGTGGAAAGTTTAATTTCA
GTACCTGCATTGATGACACATGCATCCATTCCAGCAGATATTCGAGCTAAAGAAGGTATTACAGACGGACTTGTAAGAATTTCTGTAGGT
ATTGAAGATACTGAAGATTTAGTCGATGATTTAAAACAAGCACTAGATACGTTATAA

Gene: metN2 (methionine ABC transporter locus 2, ATP-binding protein)

Contig: 03_NODE_10, position: 25381 to 26406, length: 1026 nt, orientation: FORWARD

Sequence:

ATGATTGAGTTTCGACAGGTAAGTAAGACCTTTAATAAAAAGAAGCAAAAAATAGATGCTTTGAAGGACGTATCATTTACGGTCAATCGC
AATGATATTTTTGGTGTGATTGGATATAGTGGTGCAGGAAAAAGTACGTTGGTAAGACTCGTGAATCATCTTGAAGCTGCCTCGAATGG
ACAAGTGATTGTAGATGGACATGATATTACGAATTATAGCGATAAAATGATGAGGGATATTAAGAAAGATATCGGTATGATATTTCAGC
ATTTCAATTTATTAAATTCAGCTACCGTATTTAAAAATGTAGCAATGCCACTCATTTTAAGTAAGAAAAGCAAAACAGAAATTAAGCAACG
AGTAACGGAAATGCTTGAATTTGTAGGATTTAGTGATAAAAAAGACCAATTTCCTGAAGAATTATCTGGTGGGCAGAAGCAAAGGGTGG
CTATTGCAAGAGCGCTTGTTACTAATCCGAAAATACTCCTATGCGATGAAGCAACAAGCGCATTGGATCCAGCAACGACTGCTTCGATAT
TGACGTTATTAAAGAATGTCAATCAAACCTTTGGCATTACAATTATGATGATTACACATGAAATGCGCGTTATTAAAGACATTTGTAATCG
TGTTGCTGTAATGGAAAAGGGTCAAGTGGTTGAAACAGGAACTGTTAAAGAGGTGTTTAGTCATCCTAAAACGACGATTGCTCAAAATT
TTGTGTCTACAGTTATACAGACTGAGCCAAGTACATCATTGATTCGTCGATTGAATGACGAACAAGTTGGCGATTTTAAAGATTATAAAAT
CTTCGTCGAGGAAACTCAGGTGACACAACCGATTATAAATGACTTGATTCAAATTTGTGGCAGAGAGGTTAAAATTTTATTTTCATCTATG
TCAGAAATACAAGGTAACACCGTATGTTATATGTGGCTTCGATTTAATATGGATCAACAATTTGATGACACGGCAATAAATCAATATTTCA
AAGAGAAAAATATTCAATTTGAGGAGGTGCATTAA

Gene: metP2 (methionine ABC transporter locus 2, transmembrane permease)

Contig: 03_NODE_10, position: 26410 to 27069, length: 660 nt, orientation: FORWARD

Perfect match to: (MW2-BA000033-[468448:469107], allele observed in CC1+CC9+CC188)

Sequence:

ATGTTTGGTTCTGATTTAGACAGTGCACAGTTATTACAAGCATTGTACGAAACGCTATATATGGTATCTATTGCTTTATTTTTAGGAGCAGT
GATTGGTATTCCATTAGGTGTCTTATTGGTAATTACTCGAAAACAAGGCATATGGCCGAATATAGTGATACATCAAGTTTTAAATCCTATA
ATCAATATTTTAAGGTCACTACCATTTATTATTTTGTTAATTGCGATTGTACCATTCACAAAATTAGTAGTAGGTACTTCAATTGGTACGAC
TGCTGCCATCGTGCCTTTAACAGTATATGTGGCACCTTACATTGCAAGACTTGTTGAAAACTCATTATTAGAAGTAGACGAGGGGATTATT
GAAGCGGCGAAAGCGATGGGCGCTTCACCACTACAAATCATTAGATATTTTTTAATTCCTGAAGCGTTAGGTTCATTAGTATTAGCAATTA
CCACTGCGATTATTGGACTTATTGGAAGTACGGCGATGGCAGGAGCTGTTGGCGGTGGTGGTATAGGAGACTTAGCTTTAGTGTATGGT
TATCAAAGATTTGATACGACGGTCATTATTATTACCGTTATTGTATTAGTCATTATTGTCCAAGTGATTCAAACGCTAGGGAATGTCCTAG
CTAGATTCATACGTAGACATTAA

Gene: metQ2 (methionine ABC transporter locus 2, substrate-binding protein)

Contig: 03_NODE_10, position: 27106 to 27948, length: 843 nt, orientation: FORWARD

Perfect match to: (MW2-BA000033-[469144:469986], allele observed in CC1+CC188+CC361)

Sequence:

ATGAAAAGATTGATTGGGTTAGTTATCGTAGCACTTGTATTATTAGCAGCGTGTGGTAGTAACAATGATAAAAAAGTAACAATTGGTGTC
GCATCAAATGACACTAAGGCTTGGGAGAAGGTTAAAGAATTAGCTAAAAAAGATGATATTGATGTGGAGATTAAGCACTTTTCCGATTA
CAATTTACCGAATAAAGCATTAAACGATGGTGATATTGATATGAATGCATTCCAACATTTTGCATTTTTAGATCAGTATAAGAAGGCACAT
AAAGGAACAAAGATTTCAGCATTGAGTACAACAGTTTTAGCACCGTTGGGCATTTACTCAGATAAAATTAAAGATGTCAAAAAGGTTAAA
GATGGTGCTAAAGTTGTCATTCCAAATGATGTATCAAACCAAGCACGTGCACTTAAACTATTAGAAGCAGCTGGTTTAATAAAACTGAAA
AAAGATTTCGGATTAGCAGGTACAGTGAAAGCTATAACGTCAAATCCAAAACATTTAAAAATTACTGCAGTAGATGCACAGCAAACTGC
GCGTGCTTTATCTGATGTCGATATTGCAGTTATTAATAACGGTGTAGCAACTAAAGCGGGGAAAGACCCTAAAAATGATCCGATATTTTT
AGAAAAATCAAATTCAGATGCTGTAAAGCCATATATTAATATTGTTGCAGTTAATGACAAAGACTTGGATAACAAAACATATGCTAAAAT
CGTAGAATTGTATCATTCAAAAGAAGCTCAAAAAGCGTTGCAGGAAGATGTCAAAGATGGAGAGAAACCTGTTAATTTATCTAAAGATG
AGATTAAGGCAATAGAAACGTCATTAGCAAAATAA

Gene: aaa (N-acetylmuramoyl-L-alanine amidase)

Contig: 03_NODE_10, position: 28280 to 29284, length: 1005 nt, orientation: FORWARD

Perfect match to: (SA40-CP003604-[455571:456575], highly conserved allele)

Sequence:

GTGCAAAAAAAAGTAATTGCAGCTATTATTGGGACAAGCGCGATTAGCGCTGTTGCGGCAACTCAAGCAAATGCGGCTACAACTCACAC
AGTAAAACCGGGTGAATCAGTGTGGGCAATTTCAAATAAGTATGGGATTTCGATTGCTAAATTAAAGTCATTAAACAATTTAACATCTAA
TCTAATTTTCCCAAACCAAGTACTAAAAGTATCTGGCTCAAGTAATTCTACGAGTAATAGTAGCCGTCCATCAACGAACTCAGGTGGCGG
ATCATACTACACAGTACAAGCAGGCGACTCATTATCATTAATCGCATCAAAATATGGTACAACTTACCAAAACATTATGCGACTTAATGGT
TTAAATAATTTCTTTATTTATCCAGGTCAAAAATTAAAAGTATCAGGTACTGCTAGCTCAAGTAACGCTGCGAGCAATAGTAGCCGTCCAT
CAACGAACTCAGGTGGCGGATCATACTATACAGTACAAGCAGGTGACTCATTGTCATTAATCGCATCAAAATATGGTACAACTTATCAAA
AAATTATGAGCTTAAATGGCTTAAATAATTTCTTTATATATCCGGGTCAAAAATTGAAAGTAACTGGTAATGCATCTACGAACTCAGGATC
TGCAACAACGACAAATAGAGGTTACAATACACCAGTATTCAGTCACCAAAACTTATATACATGGGGTCAATGTACATATCATGTATTTAAT
CGTCGTGCTGAAATTGGTAAAGGTATTAGTACTTATTGGTGGAATGCTAATAACTGGGATAACGCAGCGGCAGCAGATGGTTACACTAT
CGACAATAGACCTACTGTAGGTTCTATCGCTCAAACAGATGTAGGTTATTATGGTCATGTTATGTTTGTAGAACGTGTAAATAACGATGG
TAGTATTTTAGTTTCAGAAATGAACTATTCAGCTGCACCAGGTATTTTAACTTACAGAACGGTACCAGCTTACCAAGTAAATAATTATAGA
TATATTCACTAA

Gene: Q5HIL1 (putative protein)

Contig: 03_NODE_10, position: 29469 to 29738, length: 270 nt, orientation: REVERSE

Perfect match to: (N315-BA000018-[486130:486399:r], highly conserved allele)

Sequence:

TTAGTATTCATGCTTTACTTTGCGAATGCTTGTAAATAATCTAGCACCGTTTGTTATTAAAGTAACAACTGCCACTGCTTTTTGAACTTACG
TGGTGACTTAATTGAAATGTAAAAGTCTAACACGGTTCCTACAGTGCTTAAACCTAATGAAAGATATACTAATTTTTTATTTTTAGCATGAT
ATTTATAGCCATTGTAGCCGTCGACTATGAAACCTGCGACATTTAGTAAACTTGATAAACGTTGTGATTTGGAACGTTTTGCCAT

Gene: nudG (nucleoside diphosphate phosphohydrolase G)

Contig: 03_NODE_10, position: 29887 to 30282, length: 396 nt, orientation: FORWARD

Perfect match to: (MW2-BA000033-[471925:472320], highly conserved allele)

Sequence:

ATGATTAAATGTGTCTGTTTAGTTGAAGAAACAGCTGATAAAATATTACTTGTTCAAGTAAGGAATCGCGAAAAGTATTATTTCCCAGGT
GGTAAAATAGAAGAAGGGGAATCACGAGTACAAGCGCTGTTAAGAGAAGTAAAAGAAGAATTAAATTTAACATTAACAATGGATGAAA
TTGAATATATCGGGACAATTGTAGGTCCTGCATATCCACAACAGGATATGTTAACTGAGTTAAATGGATTTCGCGCATTAACCAAAATCG
ATTGGGAAAACGTAACTATCAATAATGAAATTACGGATATACGCTGGATTGATAAAGATAATGATGCGTTGATTGCGCCTGCTGTCAAAG
TTTGGATTGAAACGTATGGTGGTAAACATGACAAATAA

Gene: bltD (acetyltransferase, GNAT family)

Contig: 03_NODE_10, position: 30272 to 30757, length: 486 nt, orientation: FORWARD

Perfect match to: (N315-BA000018-[486933:487418], highly conserved allele)

Sequence:

ATGACAAATAATGACACCATCATGTTACGACATTATGTCCCACAAGATTATTCGATGTTAGAAGCTTTTCAATTAAGTGAAAGTGATTTGA
AGTTTGTTAAAACGCCAGAGGAAAATATTACAGCTGCAATGTCTGATAATGAAAGGTATCCCATCGTTGTAATGGATGGCAGGCAATGT
GTGGCCTTTTTTACATTACATCGTGGAAAAGGGGTCGCACCATTTAGCGATAACCAAGATGCAGTATTTTTCAGGTCATTTAGTGTTGATC
AACGTTATCGTAATAGAGGAATAGGTAAAGTGGTAATGGAAAAATTGGCGTCATTTATCACTTCAACATTTCAGGATATTAATGAGATTG
TGTTAACGGTTAATACTGACAATCCACATGCCATGGCACTTTATCGCCAACAAGGATATCAATATATGGGAGATAGTATGTTCATCGGAA
GACCTGTTCATATTATGGCGTTAACTATAAAATAA

Gene: yibF (putative membrane protein)

Contig: 03_NODE_10, position: 30926 to 31708, length: 783 nt, orientation: REVERSE

Perfect match to: (MW2-BA000033-[472964:473746:r], allele observed in CC1+CC7+CC9+CC188)

Sequence:

TTAATTGATAGACTCATCATTTTTGCGCTGTCGAGATGGTCTTTTTATTAAAAATGCCGTAATCCAAGCCGTAATCGGAATACTGATTGCA
ACGGCAATACCGCCTAAAATAATAGAAATAAATTCTTGGGCAAATATTTTCGAGTTTATAATATGACCAAATGAATATTTAAGTTTGAAAA
ACCAAATAAATAAAGCAAGTTGGCCACCAAAAAAGGCAAGGTAAATCGTGTTCGCAGATGTCGCTAAAATTTCTCTACCAACACGCATGC
CAGATTGGAATAATTCGTATTGCGTAAGCGTTGGATTCACTTGATGCAATTCATAAATGGGTGAACTAATGGTAATTGTTAAATCTATCAC
AGCTGCAATAACAGCAAGAATAATAGTGAACACCATAAATTGAACCATATCAATGCCAATATTCATTGAATACACATATGTTTCATCTTGT
TGTTCGGTTGAAAAGCCTTGTAGATGACCGAAGTAGACCGATAAATAAATGAGTGTAATCAACAATATTGTTGTAACGATAGTGCTGATA
AATGCAGCTTGTGTTTTAACATTGTAACTATTGAGTACGAATAAATTACAAGCGCCAATAATAATGCAGAAAAAGAATGTGACGACATAA
ATCGGTACGCCAAAAATAATCAACACAATACTAATAATTAAAATAGCAAAATTTAAAAATAGGGTTAAATAAGAGATGAATCCCTTTCTA
CCTCCGAAAATTATCATCAGAAAGAGGAGCAATAACGCCAATATAAATACAGCATTCAT

Gene: yibE (putative membrane protein)

Contig: 03_NODE_10, position: 31705 to 32817, length: 1113 nt, orientation: REVERSE

Perfect match to: (N315-BA000018-[488366:489478:r], allele observed in CC5+CC1+CC361+CC772)

Sequence:

TCATTGTTTCGCCCTCCTTAATGTTTCAAATATTTCCATAAACAATATTGTGATAGGAATTGTAAGTACGATACCTATACCACCTGTTAGTG
CGCGCGCAATTTCTAACGACCAATTCATCGAAATAGTATAAGTCACTGTATTGGCATTTTTTAAAAAGATTAAAAACATAGGTAGTGCACC
GGATAAATATGAGAATAATAAGATGTTAGTCATTGTTCCCATAATATCTTGGCCGATGTTTCGGCCAGCAAGCGCCCATCTCCTCATTGAA
ATGTGTGGCGTACGCTGTAAAATTTCATGCATACCACTAGCAATTGTAATTGCAACATCCATAATAGCGCCAAGTGAACCTATTAACACTG
AGGCTAGGAAGATATCTTTCGGTGGTAATGATAAAAAGTTCATCGTTTCATATTTAATGCCTTTACCGTCTGTCATATATATGATTAATTCT
GTTAAACCTATACTCAAAAAAGTTCCGATAATTGTACTGGCTATGGTAATGAGTGTGCGCATATGCCAGCCTGTAACGAGCAATAAAGTG
AGTATTGTTGAACAGATCATGGCAATGGTCATGAGTAAGAATAAATTAATATTGCTATGTTGAATATGAATGTAAATTGCGATTAATATG
GCAATAGAATTCAAGATTAACGATAAAATCGATTGCAGTCCGACTTTGCGACCAACCAATAATACAGTTAATAAGAACAAACCAGTGATG
ATAACCGTTAAGGTATCACGCTTCTTTTCTATAATATAAGCATCACTCGGCTTGTTAGAAATATGTAATAATACTTTTTCGTGTGTGCGAAA
TGCCTCAGAATCTGCTTGCGATTTGACGTACTGATGATTAATCGTCGTCGTTTCTCCAGCAAATTGACCATTTAATATTTTGACTTTTAATT
GATTTTTATATTTAATATCACGATTATTTTGTGCATCTTTTGTAGGTGTCGAAGAAACATGTTTGACATCTATAATTTGACCAATTGGTTTGT
TGTAAAAGTTCTCATTATTGAATGTAAATAAAATAGCACCAATGAATGCGATGCAGAACAAACCTAAAATTATATTAAATGGCTTTGTAAA
TAAATTTCTATATTTCAA

Gene: gltC (transcription activator of glutamate synthase operon)

Contig: 03_NODE_10, position: 32938 to 33822, length: 885 nt, orientation: REVERSE

Perfect match to: (MW2-BA000033-[474976:475860:r], allele observed in CC1+CC72+CC188+CC361)

Sequence:

TTAATGGAATGTAGACGTTTTAGTCATTAATTGCTGAATGAGTGTTAATAAGATACCAATATCACTCTTCGTATAAGGTTCTTTCGTAATA
GCACATATCGTTCTTTTTAATTCAGTATGATCTAATTTTATATCTATCCATGATTTAGATTCTGGTAAATGTATATTTTGTGATGAAATGATG
TAACCTTCTTTTTGACGAAGGAGATACTGCGCAAGTGGTTGGCTACTGATTGTGTATACATCTGATTTAGTAATCTTGCGCAATTGTTTTTT
TACAGTTTCGGCAAATGGTGCCAAGCAATAAATATGACTATGCTCAAACTGAATTAATGGTGGGTGTGTCGCCATCGTAATTGGATCGTC
TGAAGGCGCATATAAATGATAGTGCTCTTCGAATAAAGGTAGCATATGTAATTGTTTGTGTTTACGTATTTCTGGTGTAAGTTCCGTGAAA
CCAATGTCTATATTCCCATTTAATACGCTATTTATAATCGTGTCATGATCTAATAAGTTCGGTATGACATGTGTATCATTTTGTAAATGAAA
CGTTTGGATAAGTGGTAGTAACATGTGGGATACGTCACTCTCATCATAGCCAATGTAGATACTTTTATTTTTAGTTAATCCATGGCTTTGA
AATTGTTCAATCGTGCTATCTAAATGTTCAATAATGCGCAGAGCTTCATTAAATAATAATTTCCCTTCAGAAGTGAGCGTAATATTGCGTC
CTTGCTTTTTAAATAAAGACACATTAAGTTCTTGTTCTAATAATGTAATTTGACGGCTTATCGCTGATTGAGCAATGTTTAGTTCAAGTGCT
GTTTCGGAGATATGTTCTCTTTTAGCGACCTCGATAAAATATCTTAATTGTTTAATTTCCAT

Gene: gltB (NADH-glutamate synthase large subunit)

Contig: 03_NODE_10, position: 34004 to 38503, length: 4500 nt, orientation: FORWARD

Sequence:

ATGCACAATGAGAAATTAATTAAAGGCTTATATGACTATCGTGAGGAACATGATGCGTGTGGTATTGGTTTTTATGCGAATATGGATAAT
AAAAGGTCTCACGACATCATTGATAAATCGCTTGAAATGTTGCGACGCTTAGATCACAGGGGCGGGGTCGGCGCAGATGGCATCACTGG
TGATGGCGCAGGTATTATGACTGAAATACCTTTTGCATTTTTCAAACAACATGTAACGGACTTTGATATCCCAGGTGAAGGTGAATATGC
CGTGGGGTTATTTTTTTCCAAAGAACGCATTTTAGGTTCTGAACATGAAGCAGTTTTTAAAAAATATTTTGAAGGTGAAGGGTTATCAATT
CTTGGTTATCGTAATGTACCAGTTAATAAAGATGCCATTGCTAAACATGTAGCAGATACGATGCCAGTCATTCAACAAGTGTTTATTGATA
TTAGGGACATTGAAGATGTTGAAAAGCGTTTGTTTTTAGCGAGAAAACAATTAGAGTTCTATTCGACTCAGTGCGATTTAGAATTGTATTT
TACGAGCTTATCACGCAAAACAATTGTATATAAAGGTTGGTTACGATCAGACCAAATTAAAAAACTCTATACAGATTTATCGGATGATTTA
TATCAATCAAAGCTAGGGTTAGTGCATTCGAGATTTAGTACGAATACATTCCCGAGTTGGAAAAGGGCGCATCCTAACCGTATGTTAATG
CATAATGGTGAGATTAACACGATTAAAGGTAATGTGAACTGGATGCGAGCACGCCAACATAAATTAATCGAAACATTATTTGGCGAGGA
TCAACATAAAGTGTTTCAAATTGTCGATGAGGATGGTAGTGACTCTGCCATTGTAGATAATGCGCTAGAGTTCTTATCGTTAGCCATGGA
GCCAGAAAAGGCAGCGATGTTACTCATACCTGAACCTTGGTTATACAATGAAGCGAATGATGCAAATGTACGTGCATTTTATGAATTTTA
TAGTTATTTAATGGAACCGTGGGATGGTCCTACAATGATTTCGTTCTGTAACGGTGACAAACTTGGCGCGCTTACAGATAGAAATGGATT
ACGTCCAGGTCGTTATACGATTACTAAAGATAACTTTATTGTCTTTTCATCTGAAGTGGGTGTTGTGGACGTACCTGAAAGTAATGTTGCT
TTTAAAGGTCAATTGAATCCTGGAAAGTTATTGCTTGTTGATTTTAAACAGAATAAAGTCATTGAAAATAATGATTTGAAAGGTGCGATT
GCTGGAGAATTACCATATAAAGCGTGGATTGATAACCATAAAGTTGACTTTGATTTTGAAAATATACAATATCAAGATTCGCAATGGAAA
GATGAGACGTTATTTAAATTACAACGTCAGTTTGCATACACGAAAGAAGAGATTCATAAGTATATTCAGGAACTTGTAGAAGGTAAGAA
GGATCCTATCGGTGCAATGGGATATGATGCGCCAATTGCAGTGTTGAACGAGCGACCAGAATCACTATTTAATTACTTTAAACAGCTGTT
TGCACAAGTTACGAATCCACCAATTGATGCGTATCGTGAAAAAATCGTAACGAGTGAACTTTCTTATTTAGGTGGCGAAGGTAACTTACT
AGCACCTGACGAAACGGTTTTAGATCGTATTCAATTGAAAAGGCCGGTATTGAATGAATCACACATAGCAGCGATTGATCAGGAACATTT
TAAATTAACTTATTTATCAACGGTATATGAAGGGGATTTGGAAGATGCGTTAGAAGCATTAGGCCGAGAAGCAGTGAATGCTGTAAAGC
AAGGCGCTCAAATTCTAGTGTTAGATGATAGTGGATTAGTTGATAGCAATGGCTTTGCAATGCCGATGTTACTCGCAATAAGTCATGTGC
ATCAATTACTTATTAAAGCAGATTTACGTATGTCTACAAGTTTAGTCGCTAAATCTGGTGAGACACGAGAAGTGCATCATGTTGCTTGTTT
ACTCGCATATGGCGCGAATGCAATTGTGCCATACCTAGCGCAACGTACAGTTGAACAACTGACATTGACAGAAGGGTTACAAGGCACCG
TTGTCGATAATGTTAAGACATATACGGATGTATTGTCCGAAGGTGTCATTAAAGTAATGGCTAAGATGGGAATTTCGACAGTGCAAAGTT
ATCAAGGTGCACAAATATTTGAAGCGATTGGCTTGTCTCATGATGTGATTGATCGTTATTTTACTGGGACACAGTCTAAGTTATCGGGTAT
TTCGATTGATCAAATTGATGCTGAAAATAAAGCACGTCAACAAAGTGATGATAATTATCTTGCATCAGGTAGTACATTCCAATGGAGACA
ACAAGGTCAACATCATGCTTTTAATCCGGAATCTATTTTCTTATTGCAGCACGCATGTAAAGAAAATGACTATGCGCAATTTAAAGCATAC
TCTGAAGCGGTGAACAAAAATAGAACAGATCACATTAGACATTTACTTGAATTTAAAGCATGTACACCGATTGACATCGACCAAGTTGAA
CCGGTAAGTGACATTGTCAAACGCTTTAATACAGGGGCGATGAGTTATGGATCGATTTCAGCGGAAGCACATGAAACGTTAGCACAAGC
CATGAACCAATTAGGTGGAAAGAGTAATAGTGGTGAAGGTGGCGAAGATGCAAAACGTTATGAAGTACAAGTTGATGGAAGCAACAAA
GTAAGTGTGATTAAACAAGTTGCTTCTGGGCGTTTTGGTGTAACTAGTGATTATTTACAACATGCCAAAGAAATTCAAATTAAAGTTGCG
CAAGGTGCAAAGCCTGGTGAAGGTGGTCAATTACCTGGTACTAAGGTATATCCGTGGATTGCGAAGACAAGAGGGTCAACGCCAGGTA
TCGGTCTGATTTCACCACCGCCACATCATGATATTTATTCAATAGAAGATTTAGCGCAACTGATACATGATTTAAAAAATGCGAATAAAGA
TGCAGATATCGCGGTAAAATTAGTTTCGAAAACAGGTGTTGGTACCATTGCATCTGGGGTGGCAAAAGCATTTGCAGATAAAATTGTCAT
CAGTGGTTACGATGGTGGTACAGGGGCTTCACCTAAAACGAGTATTCAGCATGCCGGTGTTCCTTGGGAGATTGGTTTAGCAGAAACAC
ATCAAACATTAAAACTAAACGACTTAAGAAGTCGTGTTAAGTTAGAAACAGACGGTAAGTTATTAACTGGTAAAGATGTAGCGTACGCAT
GTGCGCTTGGAGCGGAAGAATTTGGATTTGCAACTGCACCATTAGTGGTGTTGGGCTGTATTATGATGCGTGTATGCCATAAAGATACAT
GTCCAGTAGGAGTTGCAACTCAAAACAAAGATTTACGTGCTTTATATAGAGGTAAAGCACATCATGTTGTTAATTTTATGCATTTTATTGC
ACAAGAATTAAGAGAAATTTTAGCATCTTTAGGTTTGAAACGTGTAGAAGACTTAGTTGGAAGAACTGATTTATTACAACGATCATCAAC
ATTAAAAGTGAATAGCAAAGCGGCTAGTATTGATGTTGAAAAACTGTTATGTCCTTTCGATGGGCCAAACACAAAAGAAATTCAACAAAA
TCATAATCTTGAGCATGGATTTGATTTAACAAATTTATATGAAGTAACGAAGCCGTATATTGCTGAAGGGCGTCGCTATACAGGTAGCTTT
ACAGTAAATAATGAACAACGTGATGTAGGAGTTATTACAGGTAGTGAGATTTCGAAACAATATGGAGAAGCAGGACTTCCTGAAAATAC
AATTAATGTTTATACGAATGGTCATGCTGGTCAAAGTCTTGCAGTATATGCACCGAAAGGCTTAATGATTCATCATACTGGAGATGCGAA
TGACTATGTTGGTAAAGGATTATCTGGTGGTACGGTCATTGTCAAAGCACCTTTTGAAGAACGCCAAAATGAAATTATTGCTGGTAACGT
CTCATTCTATGGTGCGACAGGTGGTAAGGCATTTATTAACGGTAGTGCAGGAGAAAGATTCTGTATTAGAAATAGTGGTGTAGATGTTG
TCGTTGAAGGTATCGGTGACCATGGATTAGAGTACATGACTGGTGGACATGTCATTAATTTAGGTGATGTAGGTAAGAACTTCGGTCAA
GGTATGAGTGGTGGTATTGCTTACGTTATCCCGTCTGATGTAGAAGCTTTTGTTGAAAATAATCAACTAGATACGCTTTCGTTTACAAAGA
TTAAACACCAAGAAGAAAAAGCATTCATTAAGCAAATGCTGGAAGAACATGTGTCACACACGAATAGTACGAGAGCGATTCATGTGTTA
AAACATTTTGATCGCATTGAAGATGTCGTCGTTAAAGTTATTCCTAAAGATTATCAATTAATGATGCAAAAAATTCATTTGCACAAATCATT
ACATGACAATGAAGATGAAGCGATGTTAGCTGCATTTTACGATGACAGTAAAACAATCGACGCTAAACATAAACCAGCCGTTGTGTATTA
A

Gene: gltD (NADH-glutamate synthase small subunit)

Contig: 03_NODE_10, position: 38521 to 39984, length: 1464 nt, orientation: FORWARD

Sequence:

ATGGGTGAATTTAAAGGATTTATGAAGTATGACAAACAGTACTTAGGTGAATTATCACTGGTAGACCGTTTGAAGCATCATAAAGCATAT
CAACAACGATTTACTAAAGAAGATGCCTCTATCCAAGGTGCACGATGTATGGATTGTGGAACGCCGTTTTGTCAAACCGGACAACAGTAT
GGTAGGGAAACAATAGGTTGTCCAATTGGAAACTACATTCCTGAATGGAACGACTTAGTGTATCATCAAGATTTTAAAACTGCTTATGAA
CGCTTAAGCGAAACAAATAACTTTCCTGACTTTACAGGGCGTGTATGTCCTGCACCATGCGAAAGTGCTTGTGTGATGAAGATTAATAGA
GAATCGATTGCGATTAAAGGTATTGAATGCACAATTATTGATGAAGCTTTTGAAAATGGTTGGGTAGCGCCGAAAGTTCCGAGTCGCCG
TAGAGATGAAAAAGTGGCAATCGTTGGAAGCGGTCCAGCAGGATTAGCTGCTGCTGAAGAACTTAATCTACTAGGATATCAAGTAACTA
TTTATGAACGTGCTAGAGAATCAGGCGGTTTATTAATGTATGGTATTCCGAATATGAAACTTGATAAAGATGTGGTTCGACGTCGTATTA
AGTTAATGGAAGAAGCGGGCATTACTTTCATTAATGGTGTTGAAGTAGGCGTTGATATTGATAAAGCAACGTTAGAATCTGAGTATGAT
GCCATTATATTATGTACTGGTGCACAAAAAGGTAGAGATTTACCTTTAGAAGGACGCATGGGTGATGGTATACATTTCGCTATGGATTAT
TTAACTGAACAAACGCAGTTGTTAAATGGAGAAATTGATGATATAACAATAACTGCAAAAGATAAGAATGTCATTATCATTGGTGCTGGT
GATACAGGGGCAGACTGTGTAGCGACAGCATTAAGAGAAAATTGTAAATCGATTGTTCAATTTAATAAATATACGAAATTGCCAGAAGC
AATTACATTTACAGAAAATGCATCATGGCCTTTAGCAATGCCGGTGTTTAAAATGGACTATGCGCACCAAGAGTACGAAGCTAAGTTTGG
TAAGGAACCACGTGCATATGGTGTTCAAACAATGCGTTACGATGTTGACGATAAAGGACACATACGTGGTTTGTATACTCAAATTTTAGA
GCAAGGCGAAAATGGTATGGTCATGAAAGAAGGACCTGAAAGATTTTGGCCTGCTGACCTTGTATTATTATCAATCGGCTTCGAAGGTA
CAGAACCAACAGTACCGAATGCTTTTAACATTAAAACGGATAGAAATCGAATCGTGGCGGATGATACAAACTATCAAACTAATAATGAAA
AGGTATTTGCTGCTGGAGATGCTAGACGTGGTCAAAGTTTAGTTGTATGGGCGATTAAAGAAGGTAGAGGCGTAGCGAAAGCAGTAGA
TCAGTATTTAGCGAGTAAAGTTTGTGTATAA

Gene: treP (PTS system, trehalose-specific IIBC component)

Contig: 03_NODE_10, position: 40815 to 42242, length: 1428 nt, orientation: FORWARD

Perfect match to: (N315-BA000018-[497474:498901], allele observed in CC5+CC188)

Sequence:

ATGGCTGTAAAAAGAGAAGATGTAAAAGCCATCGTAAGCGCTATTGGGGGAAAAGAAAATCTTGAAGCTGCAACGCATTGTGTAACAC
GATTACGTTTAGTGCTTAAAGATGAAAGCAAAGTTGATAAAGACGCATTAAGTAATAACGCGTTGGTCAAGGGGCAGTTCAAAGCAGAC
CATCAATATCAAATTGTCATTGGTCCAGGAACAGTCGATGAAGTGTATAAGCAGTTTATTGATGAAACAGGTGCTCAAGAAGCTTCGAAA
GATGAAGCGAAACAAGCAGCTGCGCAAAAAGGGAATCCAGTACAACGTTTGATCAAATTGTTAGGGGATATTTTTATACCAATATTACCT
GCGATTGTGACAGCTGGTTTGTTAATGGGGATCAATAATTTACTTACAATGAAAGGTTTATTTGGTCCAAAAGCACTTATTGAGATGTATC
CACAAATTGCTGATATTTCAAACATCATTAATGTGATTGCGAGTACGGCATTTATTTTCTTACCAGCATTAATTGGTTGGAGTAGTATGCG
TGTATTTGGTGGTAGTCCGATTCTAGGCATAGTCTTAGGTTTGATTTTAATGCATCCGCAATTAGTATCTCAGTATGATTTGGCAAAAGGG
AATATTCCGACGTGGAACTTATTTGGCTTAGAGATTAAGCAGTTGAATTACCAAGGTCAAGTGTTGCCAGTTTTAATTGCAGCTTATGTTC
TAGCTAAAATTGAAAAAGGATTAAATAAAGTCGTTCACGATTCGATAAAAATGTTGGTCGTTGGACCCGTAGCGCTTTTAGTTACTGGAT
TTTTAGCATTTATTATCATTGGACCAGTTGCGTTATTGATTGGTACAGGTATTACATCTGGTGTTACATTTATATTCCAACATGCAGGATGG
CTTGGCGGAGCAATATATGGATTGTTATATGCACCACTTGTAATTACAGGACTACACCATATGTTTTTAGCAGTAGATTTCCAATTGATGG
GTAGCAGCTTAGGTGGTACGTACTTATGGCCAATCGTTGCGATTTCCAATATTTGTCAGGGCTCTGCAGCATTTGGAGCATGGTTTGTCTA
TAAACGTCGTAAAATGGTTAAAGAAGAAGGCTTGGCATTAACATCTTGTATTTCTGGTATGTTAGGTGTTACTGAACCAGCCATGTTTGG
TGTGAACTTACCTCTGAAATATCCATTTATCGCTGCGATATCAACATCTTGTGTATTGGGGGCAATCGTTGGTATGAATAACGTACTTGGA
AAAGTTGGTGTTGGTGGCGTGCCAGCATTCATTTCAATTCAAAAAGAATTTTGGCCAGTATATCTCATTGTGACAGCTATTGCTATTGTTG
TACCATGTATACTAACAATTGTGATGTCTCATTTTAGTAAACAAAAAGCGAAAGAAATTGTTGAAGATTAA

Gene: treC (alpha,alpha-phosphotrehalase)

Contig: 03_NODE_10, position: 42306 to 43946, length: 1641 nt, orientation: FORWARD

Perfect match to: (M0239-AIWE01000023-[173242:174882], allele observed in CC188+CC239)

Sequence:

GTGTCGAAAGAAATAGATTGGAGAAAATCCGTTGTATATCAAATTTATCCTAAGTCGTTTAATGATACGACGGGGAATGGTATAGGAGA
TATCAATGGAATTATAGAAAAATTGGATTATATCAAGTTATTGGGTGTTGATTATATTTGGTTAACACCAGTGTATGAATCACCGATGAAT
GATAATGGCTATGATATCAGCAATTATTTAGAAATCAATGAAGACTTTGGAACGATGGATGATTTTGAAAAGTTAATCAAAGTTGCTCAT
CAAAAAGACTTGAAAGTGATGTTAGATATTGTCATTAATCATACGTCGACGGAGCATGAATGGTTTAAAGAAGCCCGTAAATCTAAAGAC
AACCCTTATAGAGATTATTACTTTTTCAGATCATCTGAAGACGGGCCGCCAACAAATTGGCATTCTAAATTCGGTGGTAATGCATGGAAG
TTTGATTCTGAGACAGATGAATATTATTTACATTTATTTGATGTCAGTCAAGCTGATTTAAATTGGGATAATCCGGAAGTACGTCAATCGT
TATATCGCATAGTCAATCATTGGATAGACTTCGGCGTTGATGGTTTTCGATTTGATGTCATTAACTTAATTTCTAAAGGTGAATTTAAGGA
CTCTGACAAAATAGGTAAAGAATTTTATACGGATGGTCCTAGAGTGCATGAGTTTCTGCATGAATTAAATCGTCAAACGTTTGGTAACAC
TGACATGATGACTGTAGGAGAAATGTCTTCGACGACGATTGAAAATTGTATTAAGTATACACAGCCAGAACGCCAAGAATTGAATAGTG

TTTTTAATTTTCATCATCTAAAGGTTGATTATGTTGATGGTGAAAAGTGGACAAATGCGAAGCTTGATTTCCATAAGTTAAAGAAAATTCT
GATGCAATGGCAACGAGGTATTTATGACGGTGGCGGATGGAACGCGATTTTCTGGTGTAATCATGATCAGCCACGGGTAGTGTCTAGAT
TTGGTGATGATACGTCGGAAGAGATGAGGATACAAAGTGCTAAAATGTTAGCTATCGCACTGCATATGTTGCAAGGGACGCCATATATT
TACCAAGGTGAAGAAATTGGTATGACGGACCCACATTTTACATCAATAGCACAATATCGCGATGTTGAATCGATTAATGCCTACCATCAG
TTGTTAAGTGAAGGGCATGCTGAAGCGGATGTGTTAACGATTTTAGGACAGAAGTCACGAGACAATTCGAGAACGCCTATGCAATGGAG
TGATGATGTTAATGCTGGATTTACAGCTGGTAAGCCTTGGATTGATATTTCGGAAAATTATCATCAGGTCAACGTTAGACAAGCACTTCA
GAATAAAGAGTCTATTTTCTATACGTATCAAAAATTAATACAATTAAGACATACGCATGATATTATTACGTATGGAGACATTGTGCCACGT
TTTATGGATCATGATCATTTATTTGTTTATGAACGTCATTATAAGAATCAACAATGGCTAGTAATTGCGAATTTCTCAGCATCGGCTGTTGA
TTTGCCAGAAGGATTGGCTAGAGAAGGTCGTGTTGTGATTCAAACAGGCACAGTGGAAAATAATACGATAAGCGGGTTTGGTGCAATTG
TAATCGAAACAAACGCGTAA

Gene: treR (trehalose operon repressor)

Contig: 03_NODE_10, position: 43971 to 44699, length: 729 nt, orientation: FORWARD

Perfect match to: (MW2-BA000033-[486009:486737], allele observed in CC1+CC80+CC96)

Sequence:

ATGGCGAAACAAAAAAAGTTTATGAAGATTTATGAGGCGTTGAAAGAAGATATATTAAACGGGCAGATTCAATATGGTGAACAAATTCC
GTCTGAACATGATTTGGTGCAATTGTACCAGTCATCTCGAGAGACCGTGCGTAAGGCATTAGATTTGTTGGCATTAGACGGCATGATTCA
AAAGATTCATGGTAAAGGGTCACTTGTCATTTATCAGGAGGTTACAGAGTTTCCATTTTCTGAACTGGTTAGTTTTAAAGAAATGCAAGA
AGAAATGGGCGTCGCATATTTAACTGAAGTTGTTGTGAATGAGGTTGTTGAAGCGCATGAAGTTCCAGAAGTTCAACATGCTTTAAACAT
CAATTCTAGTGAATCACTCATTCATATTGTTAGAACTCGTCGGCTTAACCAACATGTGAAGATTGTTGATGAAGATTATTTTCTAAAGTCG
ATTGTTTCAGACATAGGTAATGATGTTGCGAGTGATTCTATTTATGATTATTTGGAAAAGGTATTAAATCTTAATATTAGTTATTCAAGTAA
GTCTATTACTTTTGAACCGTTTGATGAACAAGCATATCAATTGTTTGGTGATGTATCGGTGGCTTATTCAGCAACAGTTCGAAGTATTGTG
TATTTAGAAAATACAATGCCGTTTCAATATAATATTTCAAAACATCTTGCAAATGAATTTAAATTTAACGACTTCTCAAGACGTCGTATAAA
GTAA

Gene: ffs (signal recognition particle RNA subunit)

Contig: 03_NODE_10, position: 44761 to 45030, length: 270 nt

Sequence:

CCGTGCTAGGTGGGGAGGTAGCGGTTCCCTGTACTCGAAATCCGCTTTATGCGAGGCTTAATTCCTTTGTTGAGGCCGTATTTTTGCGAA
GTCTGCCCAAAGCACGTAGTGTTTGAAGATTTCGGTCCTATGCAATATGAACCCATGAACCATGTCAGGTCCTGACGGAAGCAGCATTAA
GTGGATCATCATATGTGCCGTAGGGTAGCCGAGATTTAGCTAACGACTTTGGTTACGTTCGTGAATTACGTTCGATGCTTAGGTGCACGG

Gene: Q5HIK0 (acetyltransferase, GNAT family)

Contig: 03_NODE_10, position: 45342 to 45866, length: 525 nt, orientation: FORWARD

Perfect match to: (MW2-BA000033-[487380:487904], allele observed in CC1+CC80)

Sequence:

ATGCAAATATATTTAAGTACTTTAACAGAGTTAGATTATGATAAATCTTTAAATAGTATTGAAGAAAGTTTTGATGATAATCCTGAAACGA
GTTGGCAAGCACTTGCGAAAGTAAAACATTTAAGAAAATCTCCTTGCTATAATTTTGAATTAGAAGTAATAGCGAAAAATGAAAATAACG
ATGTCGTTGGACACGTTTTATTAATTGAAGTAGAAATTAATAGTGATGATAAGACGTATTATGGTTTGGCGATTGCCTCTTTATCAGTTCA
TCCTGAATTACGTGGACAAAAATTAGGTCGTGGCTTGGTTCAAGCAGTAGAAGAGCGTGCCAAAGCACAAGAGTATAGTACGGTTGTTG
TAGACCATTGTTTTGACTACTTTGAAAAGTTGGGTTATCAAAATGCTGCTGAGCATGACATTAAATTAGAATCTGGTGATGCACCGTTACT
TGTAAAATATTTATGGGATAATTTGACGGATGCACCACACGGAATCGTAAAATTTCCAGAACATTTTTATTAA

Gene: dnaX (DNA polymerase III gamma and tau subunits)

Contig: 03_NODE_10, position: 45935 to 47632, length: 1698 nt, orientation: FORWARD

Perfect match to: (11819-97-CP003194-[500143:501840], allele observed in CC80+CC1+CC7+CC97+CC101)

Sequence:

TTGAATTATCAAGCCTTATATCGTATGTACAGACCCCAAAGTTTCGAGGATGTCGTCGGACAAGAACATGTCACGAAGACATTGCGCAAT
GCGATTTCTAAAGAAAAACAGTCGCATGCTTATATTTTTAGTGGTCCGAGAGGCACGGGGAAAACGAGTATTGCCAAAGTGTTTGCTAA
AGCAATCAACTGTTTAAATAGCACTGATGGAGAACCTTGTAATGAATGTCATATTTGTAAAGGCATTACGCAGGGGACTAATTCAGATGT
GATAGAAATTGATGCTGCTAGTAATAATGGCGTTGATGAAATAAGAAATATTAGAGACAAAGTTAAATATGCACCAAGTGAATCGAAAT
ATAAAGTTTATATTATAGATGAGGTGCACATGCTAACAACAGGTGCTTTTAATGCCCTTTTAAAGACGTTAGAAGAACCTCCAGCACACG
CTATTTTTATATTGGCAACGACAGAACCACATAAAATCCCTCCAACAATCATTTCTAGGGCACAACGTTTTGATTTTAAAGCAATTAGCCTA
AATCAAATTGTTGAACGTTTAAAATTTGTAGCAGATGCACAACAAATTGAATGTGAAGATGAAGCCTTGGCATTTATCGCTAAAGCGTCT
GAAGGGGGTATGCGTGATGCATTAAGTATTATGGATCAGGCTATTGCATTTGGTGATGGTACGTTAACATTGCAAGATGCGTTGAATGT
CACAGGTAGCGTACATGATGAAGCGTTGGATCACTTGTTTGATGATATTGTACAAGGTGACGTACAAGCATCTTTTAAAAAATACCATCA
GTTTATAACAGAGGGTAAAGAAGTGAATCGCCTAATAAATGATATGATTTATTTTGTCAGAGATACGATTATGAATAAAACATCTGAGAA
AGATACTGAGTATCGAGCACTGATGAACTTAGAATTAGATATGTTATATCAAATGATTGATCTTATTAATGATACATTAGTGTCGATTCGT
TTTAGTGTGAATCAAAACGTTCATTTTGAAGTGTTGTTAGTAAAATTAGCTGAGCAGATTAAGGGTCAACCACAAGTGATTGCGAATGTA
GCTGAACCAGCACAAATTGCTTCATCGCCAAACACAGATGTATTGTTGCAACGTATGGAACAGTTAGAGCAAGAACTAAAAACACTAAA
AGCACAAGGAGTGAGTGTCGCTCCTGTTCAAAAATCTTCGAAAAAGCCTGCGAGAGGCATACAAAAATCTAAAAATGCATTTTCAATGCA
ACAAATTGCAAAAGTGCTAGATAAAGCGAATAAGGCAGATATCAAATTGTTGAAAGATCATTGGCAAGAAGTGATTGATCATGCCAAAA
ACAATGATAAAAAATCACTCGTTAGTTTATTGCAAAATTCGGAACCTGTGGCGGCAAGTGAAGATCACGTACTTGTGAAATTTGAGGAAG
AGATCCATTGTGAAATCGTCAATAAAGACGACGAGAAACGTAGTAGTATAGAAAGTGTTGTATGTAATATCGTTAATAAAAACGTTAAA
GTTGTTGGTGTACCATCAGATCAATGGCAAAGAGTTCGAACGGAGTATTTACAAAATCGTAAAAACGAAGGCGATGATATGCCAAAGCA
ACAAGCACAACAAACAGATATTGCTCAAAAAGCAAAAGATCTTTTCGGTGAAGAAACTGTACATGTGATAGATGAAGAGTGA

Gene: yaaK (DNA binding protein)

Contig: 03_NODE_10, position: 47722 to 48039, length: 318 nt, orientation: FORWARD

Perfect match to: (RF122-AJ938182-[471380:471697], highly conserved allele)

Sequence:

ATGCGCGGTGGCGGAAACATGCAACAAATGATGAAACAAATGCAAAAAATGCAAAAGAAAATGGCTCAAGAACAAGAAAAACTTAAAG
AAGAGCGTATTGTAGGAACAGCTGGCGGTGGCATGGTTGCAGTTACTGTAACTGGTCATAAAGAAGTTGTCGACGTTGAAATCAAAGAA
GAAGCTGTAGACCCAGATGATATTGAAATGCTACAAGACTTAGTGTTAGCAGCTACTAATGAAGCGATGAATAAAGCTGATGAGCTTAC
TCAAGAACGTTTAGGTAAACATACTCAAGGCTTAAACATCCCTGGAATGTGA

Gene: recR (recombination protein R)

Contig: 03_NODE_10, position: 48046 to 48642, length: 597 nt, orientation: FORWARD

Perfect match to: (MW2-BA000033-[490084:490680], highly conserved allele)

Sequence:

ATGCATTATCCAGAACCTATATCAAAACTTATTGATAGCTTTATGAAATTGCCAGGCATTGGTCCAAAGACAGCCCAACGTCTGGCTTTTC
ATACCTTAGATATGAAAGAAGACGATGTTGTTCAGTTTGCCAAAGCATTAGTAGATGTTAAGAGAGAATTAACATATTGTAGCGTATGTG
GTCACATTACTGAAAATGATCCATGTTATATTTGTGAAGATAAGCAAAGAGATCGTTCAGTTATTTGTGTTGTGGAAGATGACAAAGATG
TCATAGCTATGGAAAAAATGAGAGAATACAAAGGTTTATATCACGTTTTACATGGGTCTATTTCGCCTATGGATGGCATTGGACCAGAAG
ATATTAATATTCCTTCATTGATTGAACGCTTGAAAAACGATGAAGTTAGCGAATTAATCTTAGCTATGAACCCGAACTTAGAGGGGGAAT
CTACAGCCATGTATATTTCTAGATTAGTTAAGCCTATAGGTATCAAAGTGACGAGATTAGCACAAGGGTTATCGGTAGGTGGCGATTTAG
AGTATGCTGACGAAGTAACATTATCTAAAGCAATCGCAGGTAGAACAGAAATGTAA

Gene: yaaO (putative Orn/Lys/Arg decarboxylase)

Contig: 04_NODE_11, position: 1003 to 2340, length: 1338 nt, orientation: FORWARD

Perfect match to: (GR1-AJLX01000005-[36086:37423:r], allele observed in CC361)

Sequence:

ATGAAGCAACCTATTTTAAATAAATTAGAAAGTTTAAATCAAGAAGAAGCGATTTCTTTGCATGTTCCGGGTCATAAAAATATGACTATCG
GTCATTTATCTCAATTATCAATGACAATGGATAAAACTGAAATACCTGGATTAGATGATTTACATCATCCTGAAGAAGTCATTTTGGAAAG
TATGAAGCAGGTGGAGAAACATTCAGATTATGATGCTTATTTCTTAGTGAATGGCACCACTTCAGGAATATTATCTGTCATCCAGTCTTTT
TCACAGAAAAAAGGCGATATCTTAATGGCAAGAAATGTACATAAATCTGTATTACATGCGCTCGATATTAGCCAACAAGAAGGGCATTTT
ATTGAAACGCATCAAAGTCCGTTAACGAATCATTATAATAAAGTTAATTTAAGCCGTTTGAATAATGACGGTCACAAACTTGCTGTGTTGA
CTTATCCTAACTATTACGGTGAAACATTTAATGTAGAAGAGGTTATCAAATCTTTGCACCAATTAAATATTCCTGTACTCATTGACGAAGC
ACACGGCGCGCACTTTGGATTGCAAGGATTTCCAGATTCTACATTAAATTATCAAGCTGACTATGTTGTTCAATCTTTTCATAAAACGTTAC
CAGCTTTAACGATGGGCTCGGTACTTTATATTCATAAAAATGCACCTTATAGAGAAACTATTATAGAATATCTAAGCTACTTCCAAACATCT
AGTCCTTCGTATTTGATTATGGCTAGTTTAGAGTCAGCTGCCGAGTTCTATAAAACATATGATAGTACCGTGTTTTTTGATAAGAGAGCGC
AATTAATCGAATGTTTGGAGAAGAAGGGTTTTGAAATGCTTCAAGTTGATGATCCGTTGAAGTTGCTGATAAAATATGAAGGTTTTACAG
GTCATGATATTCAAAATTGGTTTATGAATGCACATATCTATTTAGAATTAGCGGACGACTATCAAGTATTAGCGATATTGCCGTTATGGCA
TCATGATGATACGTATTTATTTGATTCGCTTTTACGTAAAATTGAAGATATGATTTTACCGAAAAAATCAGTTTCTAAAGTTAAACAAACAC
AACTTTTAACAACTGAAGGTAACTATAAACCAAAACGCTTTGAATATGTTACTTGGTGTGATTTGAAAAAGGCAAAAGGTAAAGTTCTGG
CGCGACATATTGTCCCGTATCCGCCAGGGATTCCTATTATTTTCAAAGGAGAAACAATAACTGAAAATATGATAGAATTAGTAAATGAAT
ATCTGGAAACTGGAATGATAGTTGAAGGAATTAAAAATAATAAAATTTTAGTTGAGGATGAATAA

Gene: tmk (thymidylate kinase)

Contig: 04_NODE_11, position: 2342 to 2959, length: 618 nt, orientation: FORWARD

Perfect match to: (MW2-BA000033-[498743:499360], allele observed in CC1+CC80+CC101)

Sequence:

ATGTCAGCTTTTATAACTTTTGAGGGCCCAGAAGGCTCTGGAAAAACAACTGTAATTAATAAAGTTTACCATAGATTAGTAAAAGATTAT
GATGTCATTATGACAAGAGAACCGGGCGGCGTTCCTACTGGTGAAGAAATACGTAAAATTGTATTAGAAGGCAATGATATGGACATTAG
AACTGAAGCAATGTTATTTGCTGCATCTAGAAGAGAACATCTTGTATTAAAGGTCATACCAGCTTTAAAAGAAGGTAAGGTTGTGTTGTG
TGATCGCTATATCGATAGTTCATTAGCTTATCAAGGTTATGCTAGAGGGATTGGCGTTGAAGAAGTAAGAGCATTAAACGAATTTGCAAT
AAATGGATTATATCCAGACTTGACGATTTATTTGAATGTTAGTGCTGAAGTAGGCCGCGAACGTATTATTAAAAATTCAAGAGATCAAAA
TAGATTAGATCAAGAAGATTTAAAGTTTCACGAAAAAGTAATTGAAGGTTACCAAGAAATCATTCATAATGAATCACAACGGTTCAAAAG
CGTTAATGCAGATCAACCTCTTGAAAATGTTGTTGAAGACACGTATCAAACTATCATCAAATATTTAGAAAAGATATGA

Gene: darA (cyclic-di-AMP receptor)

Contig: 04_NODE_11, position: 2987 to 3316, length: 330 nt, orientation: FORWARD

Perfect match to: (RF122-AJ938182-[480866:481195], highly conserved allele)

Sequence:

ATGAAAATGATTATAGCGATCGTACAAGATCAAGATAGTCAGGAACTTGCAGATCAACTTGTTAAAAATAACTTTAGAGCAACAAAATTG
GCAACAACAGGTGGGTTTTTAAGAGCAGGTAATACAACATTCTTATGTGGTGTCAATGATGACCGTGTAGATGAAATATTGTCTGTGATT
AATCAAACGTGTGGTAATAGAGAACAGTTGGTTTCACCTATTACACCTATGGGAGGCAGTGCGGATTCGTACATTCCATATCCAGTTGAA
GTTGAAGTTGGCGGTGCTACTGTATTTGTTATGCCAGTTGATGCATTCCATCAATTTTAA

Gene: holB (DNA polymerase III, delta' subunit)

Contig: 04_NODE_11, position: 3530 to 4456, length: 927 nt, orientation: FORWARD

Perfect match to: (MW2-BA000033-[499931:500857], allele observed in CC1+CC8+CC30+CC80)

Sequence:

ATGGATGAACAGCAACAATTGACGAATGCATATCATTCAAATAAATTATCGCATGCCTATTTATTTGAAGGTGATGATGCACAAACGATG
AAACAAGTTGCGATTAATTTTGCAAAGCTTATTTTATGTCAAACAGATAGTCAATGTGAAACAAAGGTTAGTACATATAATCATCCAGACT
TTATGTATATATCAACAACTGAGAATGCAATTAAGAAAGAACAAGTTGAACAACTTGTGCGTCATATGAATCAACTTCCTATAGAAAGCA
CAAATAAAGTGTACATCATTGAAGACTTTGAAAAGTTAACTGTTCAAGGGGAAAACAGTATCTTGAAATTTCTTGAAGAACCACCGGACA
ATACGATTGCTATTTTATTGTCTACAAAACCTGAGCAAATTTTAGACACAATCCATTCAAGGTGTCAGCATGTATATTTCAAGCCTATTGAT
AAAGAAAAGTTTATAAATAGATTAGTTGAACAAGACATGTCTAAGCCAGTAGCTGAAATGATTAGTACTTATACTACGCAAATAGATAAT
GCAATGGCTTTAAATGAAGAATTTGATTTATTAGCATTAAGGAAATCAGTTATACGTTGGTGTGAATTGTTGCTTACTAATAAGCCAATGG

CACTTATAGGTATTATTGATTTATTGAAACAGGCTAAAAATAAAAAACTGCAATCTTTAACTATTGCAGCTGTGAATGGTTTCTTCGAAGA
TATCATACATACAAAGGTAAATGTAGAGGATAAACAAATATATAGTGATTTAAAAAATGATATTGATCAATATGCGCAAAAGTTGTCGTT
TAATCAATTAATTTTGATGTTTGATCAACTGACGGAAGCACATAAGAAATTGAATCAAAATGTAAATCCAACGCTTGTATTTGAACAAATC
GTAATTAAGGGTGTGAGTTAG

Gene: yaaT (protein related to DNA replication)

Contig: 04_NODE_11, position: 4457 to 5260, length: 804 nt, orientation: FORWARD

Perfect match to: (MW2-BA000033-[500858:501661], highly conserved allele)

Sequence:

ATGCCAAATGTAATAGGTGTTCAGTTTCAAAAAGCGGGAAAATTAGAATATTATACACCTAATGATATACAAGTAGATATAGATGACTGG
GTAGTTGTCGAATCTAAAAGAGGCATAGAGATAGGTATTGTTAAAAATCCATTAATGGATATTGCTGAAGAGGATGTTGTGTTACCTCTT
AAAAATATTATTCGCGTTGCTGATGACAAAGATATTGATAAATTTAATTGTAATGAACGAGATGCTGAAAATGCATTAATACTATGTAAA
GACATTGTAAGAGAACAAGGTTTGGACATGCGTTTAGTCAATTGCGAATATACATTAGATAAATCGAAAGTTATTTTTAATTTTACGGCG
GATGATCGTATTGATTTTAGAAAATTAGTAAAAATATTAGCGCAACATTTAAAAACACGTATCGAGTTGAGACAAATTGGTGTAAGGGAT
GAAGCCAAATTGCTTGGCGGTATCGGACCTTGTGGTAGGTCGTTATGTTGTTCTACATTTTTAGGAGATTTTGAACCAGTATCGATTAAGA
TGGCTAAGGATCAAAATTTATCATTAAATCCAACTAAAATTTCTGGTGCATGTGGTCGTTTGATGTGTTGTTTAAAATATGAAAATGACTA
TTATGAGGAAGTACGTGCACAATTACCTGATATTGGTGAAGCAATTGAAACGCCTGATGGTAACGGGAAAGTAGTTGCTTTAAATATATT
AGACATTTCTATGCAGGTGAAGCTTGAGGGACATGAACAGCCACTTGAATATAAATTAGAAGAAATAGAAACTATGCATTAA

Gene: yabA (initiation-control protein)

Contig: 04_NODE_11, position: 5277 to 5624, length: 348 nt, orientation: FORWARD

Perfect match to: (MW2-BA000033-[501678:502025], allele observed in CC1)

Sequence:

TTGGATCGCAATGAAATATTTGAAAAAATAATGCGTTTAGAAATGAATGTCAATCAACTTTCAAAGGAAACATCAGAATTAAAGGCACTT
GCAGTTGAATTAGTAGAAGAAAATGTAGCGCTTCAACTTGAAAATGATAATTTGAAAAAGGTGTTGGGCAATGATGAACCAACTACTATT
GATACTGCGAATTCAAAACCAGCAAAAGCTGTGAAAAAGCCATTACCAAGTAAAGATAATTTGGCTATATTGTATGGAGAAGGATTTCAT
ATTTGTAAAGGCGAATTATTTGGAAAACATCGACATGGTGAAGATTGTCTGTTCTGTTTAGAAGTTTTAAGTGATTAA

Gene: yabB (putative O-methyltransferase)

Contig: 04_NODE_11, position: 5898 to 6623, length: 726 nt, orientation: FORWARD

Perfect match to: (MW2-BA000033-[502299:503024], highly conserved allele)

Sequence:

ATGTTAAAAGAGAATGAACGATTTGATCAACTAATCAAAGAAGATTTTAGTATTATTCAAAATGATGATGTTTTTTCGTTTTCAACGGATG
CTTTGTTGTTAGGGCATTTTACAAAACCTAGAACAAAAGATATTGTGTTGGACTTATGTTCAGGCAATGGGGTGATACCGTTGTTATTGTT
TGCGAAACATCCACGACATATAGAAGGTGTTGAGATTCAAAAAACACTTGTCGATATGGCGCGACGCACATTTCAATTCAATGATGTTGA
TGAATATATAACAATGCATCACATGGATTTGAAAAACGTTACTAAAGTATTTAAACCTTCACAATATACTTTAGTAACGTGTAATCCACCTT
ATTTTAAAGAGAATCAGCAACACCAACATCAAAAAGAAGCACATAAGATAGCGAGACATGAGATTATGTGTACACTTGAAGATTGCATG
ATTGCAGCCCGTCATTTATTAAAAGAAGGTGGCAGGCTAAACATGGTACATCGTGCAGAGAGACTAATGGATGTCTTGTTTGAAATGAG
AAAAGTGAATATTGAACCTAAGAAAGTCGTTTTTATATATAGTAAAGTAGGGAAATCAGCACAAACGATAGTAGTAGAAGGTCGAAAAG
GTGGAAATCAAGGTTTAGAAATCATGCCCCCATTTTATATTTATAATGAAGATGGTAATTATAGCGAAGAAATGAAGGAAGTATATTATG
GATAG

Gene: yazA (putative UvrC-type endonuclease)

Contig: 04_NODE_11, position: 6616 to 6864, length: 249 nt, orientation: FORWARD

Perfect match to: (RF122-AJ938182-[484495:484743], highly conserved allele)

Sequence:

ATGGATAGTCATTTTGTATATATTGTAAAATGTAGTGATGGAAGTTTATATACAGGATACGCTAAAGACGTTAATGCACGTGTTGAAAAA
CATAACCGAGGTCAAGGAGCCAAATATACGAAAGTAAGACGTCCGGTGCATTTAGTTTATCAAGAAATGTATGAGACAAAGTCTGAAGC
ATTGAAGCGTGAATATGAAATTAAAACTTATACCAGACAAAAGAAATTGCGATTAATTAAGGAGCGATAG

Gene: rsmI (ribosomal RNA small subunit methyltransferase I)

Contig: 04_NODE_11, position: 6866 to 7705, length: 840 nt, orientation: FORWARD

Perfect match to: (N315-BA000018-[518064:518903], allele observed in CC5+CC1+CC45IV8)

Sequence:

ATGGCTGTATTATATTTAGTGGGCACACCAATTGGTAATTTAGCAGATATTACTTATAGAGCAGTTGATGTATTGAAACGTGTTGATATGA
TTGCTTGTGAAGACACTAGAGTAACTAGTAAGCTGTGTAATCATTATGATATTCCAACTCCATTAAAGTCATATCACGAACATAACAAGGA
TAAGCAGACTGCTTTTATCATTGAACAGTTAGAATTAGGTCTTGACGTTGCGCTCGTATCTGATGCTGGATTGCCCTTAATTAGTGATCCT
GGATACGAATTAGTAGTGGCAGCCAGAGAAGCTAATATTAAAGTAGAGACTGTGCCTGGACCTAATGCTGGGCTGACGGCTTTGATGGC
TAGTGGATTACCTTCATATGTATATACATTTTTAGGATTTTTGCCACGAAAAGAGAAAGAAAAAAGTGCTGTATTAGAGCAACGTATGCA
TGAAAATAGCACATTAATTATATACGAATCACCGCATCGTGTGACAGATACATTAAAAACAATTGCAAAGATAGATGCAACACGACAAGT
ATCACTAGGGCGTGAATTGACTAAGAAGTTCGAACAAATTGTAACTGATGATGTAACACAATTACAAGCATTGATTCAGCAAGGCGATGT
ACCATTGAAAGGCGAATTCGTTATCTTGATTGAGGGTGCTAAAGCGAACAATGAGATATCGTGGTTTGATGATTTATCTATCAATGAGCA
TGTTGATCATTATATTCAAACTTCACAGATGAAACCAAAACAAGCTATTAAAAAAGTTGCTGAAGAACGACAACTTAAAACGAATGAAGT
ATATAATATTTATCATCAAATAAGTTAA

Gene: metS (methionyl-tRNA synthetase)

Contig: 04_NODE_11, position: 7990 to 9963, length: 1974 nt, orientation: FORWARD

Perfect match to: (Strain_21193-AFEG01000016-[29305:31278:r], allele observed in CC25+CC22+CC25)

Sequence:

ATGGCTAAAGAAACATTTTATATAACAACCCCAATATACTATCCTAGTGGGAATTTACATATAGGACATGCATATTCTACAGTGGCTGGAG
ATGTTATTGCAAGATATAAGAGAATGCAAGGATATGATGTTCGTTATTTGACTGGAACGGATGAACACGGTCAAAAAATTCAAGAAAAA
GCTCAAAAAGCTGGTAAGACAGAAATTGAATATTTGGATGAGATGATTGCTGGAATTAAACAATTGTGGGCTAAGCTTGAAATTTCAAAT
GATGATTTTATCAGAACAACTGAAGAACGTCATAAACATGTCGTTGAGCAAGTGTTTGAACGTTTATTAAAGCAAGGTGATATCTATTTA
GGTGAATATGAAGGTTGGTATTCTGTTCCGGATGAAACATACTATACAGAGTCACAATTAGTAGACCCACAATACGAAAACGGTAAAATT
ATTGGTGGCAAAAGTCCAGATTCTGGACACGAAGTTGAACTAGTTAAAGAAGAAAGTTATTTCTTTAATATTAGTAAATATACAGACCGT
CTATTAGAATTCTATGACCAAAACCCAGATTTTATACAACCACCATCAAGAAAAAATGAAATGATTAACAACTTCATTAAACCAGGACTTG
CTGATTTGGCTGTTTCTCGTACATCATTTAATTGGGGTGTCCATGTTCCGTCTAATCCAAAACATGTTGTTTATGTTTGGATTGATGCGTTA
GTTAACTATATTTCAGCATTAGGCTATTTATCAGATGATGAGTCACTATTTAACAAATACTGGCCAGCAGATATTCATTTAATGGCTAAGG
AAATTGTGCGATTCCACTCAATTATTTGGCCTATTTTATTGATGGCATTAGACTTACCGTTACCTAAAAAAGTCTTTGCACATGGTTGGATT
TTGATGAAAGATGGAAAAATGAGTAAATCTAAAGGTAATGTCGTAGACCCTAATATTTTAATTGATCGCTATGGTTTAGATGCTACACGT
TATTATCTAATGCGTGAATTACCATTTGGTTCAGATGGCGTATTTACACCTGAAGCATTTGTTGAGCGTACAAATTTCGATCTAGCAAATG
ACTTAGGTAACTTAGTAAACCGTACGATTTCTATGGTTAATAAGTACTTTGATGGCGAATTACCAGCGTATCAAGGTCCACTTCATGAATT
AGATGAAGAAATGGAAGCTATGGCTTTAGAAACAGTGAAAAGCTACACTGAAAGCATGGAAAGTTTGCAATTTTCTGTGGCATTATCTA
CGGTATGGAAGTTTATAAGTAGAACGAATAAGTATATTGACGAAACAACGCCTTGGGTATTAGCTAAGGACGATAGCCAAAAAGATATG
TTAGGCAATGTAATGGCTCACTTAGTTGAAAATATTCGTTATGCAGCTGTATTATTACGTCCATTCTTAACACATGCGCCGAAAGAGATTT
TTGAACAATTGAACATTAACAATCCTCAATTTATGGAATTTAGTAGTTTAGAGCAATATGGTGTGCTTACTGAGTCAATTATGGTTACTGG
GCAACCTAAACCTATTTTCCCAAGATTGGATAGCGAAGCGGAAATTGCATATATCAAAGAATCAATGCAACCGCCTGCTACTGAAGAGGA
AAAAGAAGAGATTCCTAGCAAACCTCAAATTGATATTAAAGACTTTGATAAAGTTGAAATTAAGGCAGCAACGATTATTAATGCTGAACA
TGTTAAGAAGTCAGATAAGCTTTTAAAAATTCAAGTAGACTTAGATTCTGAACAAAGACAAATTGTATCAGGAATTGCCAAATTCTATACA
CCAGATGATATTATTGGTAAAAAAGTAGCAGTTGTTACTAACCTGAAACCAGCTAAATTAATGGGACAAAAATCTGAAGGTATGATATTA
TCTGCTGAAAAAGATGGTGTATTAACCTTAGTAAGTTTACCAAGTGCAATTCCAAATGGTGCAGTGATTAAATAA

Gene: tatP (deoxyribonuclease)

Contig: 04_NODE_11, position: 9994 to 10767, length: 774 nt, orientation: FORWARD

Perfect match to: (MW2-BA000033-[506395:507168], allele observed in CC1+CC96+CC779)

Sequence:

ATGTTAATCGATACACATGTCCATTTAAATGATGAGCAATACGATGATGATTTGAGTGAAGTGATTACACGTGCTAGAGAAGCAGGTGTT
GATCGTATGTTTGTAGTTGGTTTTAACAAATCGACAATTGAACGCGCGATGAAATTAATCGATGAGTATGATTTTTTATATGGCATTATCG
GTTGGCATCCAGTTGATGCAATTGATTTTACAGAAGAACACCTGGAATGGATTGAATCTTTGGCTCAGCATCCAAAAGTGATTGGTATTG
GTGAAATGGGATTAGATTATCACTGGGATAAATCTCCTGCAGATGTTCAAAAGGAAGTTTTTAGAAAGCAAATTGCTTTAGCTAAGCGTT
TGAAGTTACCAATTATCATTCATAACCGTGAAGCAACTCAAGACTGTATCGATATCTTATTGGAGGAGCATGCTGAAGAGGTAGGCGGG
ATTATGCATAGCTTTAGTGGTTCTCCAGAAATTGCAGATATTGTAACTAATAAGCTGAATTTTTATATTTCATTAGGAGGACCTGTGACATT
TAAAAATGCTAAACAGCCTAAAGAAGTTGCTAAGCATGTGTCAATGGAGCGTTTGCTAGTTGAAACCGATGCACCGTATCTTTCGCCACA
TCCGTATAGAGGGAAGCGAAATGAACCGGCGAGAGTAACTTTAGTAGCTGAACAAATTGCTGAATTAAAAGGCTTATCTTATGAAGAAG
TGTGCGAACAAACAACTAAAAATGCAGAGAAATTGTTTAATTTAAATTCATAA

Gene: rnmV (ribonuclease M5)

Contig: 04_NODE_11, position: 10934 to 11470, length: 537 nt, orientation: FORWARD

Perfect match to: (N315-BA000018-[522132:522668], highly conserved allele)

Sequence:

ATGAAAATCAATGAGTTTATAGTTGTAGAAGGACGAGATGATACTGAGCGTGTTAAACGAGCTGTTGAATGTGATACGATTGAAACGAA
TGGTAGTGCCATCAACGAACAAACTTTAGAAGTAATTAGAAATGCTCAACAAAGTCGAGGCGTTATTGTATTAACAGATCCAGATTTCCC
AGGAGATAAAATTAGAAGTACAATTACTGAACATGTCAAAGGTGTTAAACATGCGTATATTGATAGAGAAAAAGCTAAAAATAAAAAAG
GGAAAATTGGTGTTGAACATGCCGACTTAATTGATATTAAAGAAGCGTTAATGCATGTTAGTTCACCCTTTGATGAAGCTTATGAATCAAT
TGATAAATCTGTGCTAATAGAGTTGGGGTTAATCGTTGGGAAAGATGCAAGGCGCCGTAGAGAAATTTTAAGTAGAAAATTGCGAATCG
GCCATTCCAATGGTAAGCAGTTATTGAAAAAGTTAAATGCATTTGGTTATACCGAAGCGGATGTAAGGCAAGCTTTAGAAGATGAATGA

Gene: ksgA (ribosomal RNA small subunit methyltransferase A)

Contig: 04_NODE_11, position: 11484 to 12374, length: 891 nt, orientation: FORWARD

Perfect match to: (MW2-BA000033-[507885:508775], allele observed in CC1)

Sequence:

TTGGATAATAAAGATATTGCAACACCATCAAGAACGCGAGCGTTGTTAGATAAATATGGCTTTAATTTTAAAAAAAGTTTAGGACAGAAC
TTTTTGATAGATGTGAATATCATTAATAATATCATTGATGCAAGTGATATTGATGCACAAACTGGGGTGATTGAAATTGGTCCAGGCATG
GGGTCATTGACAGAACAATTGGCTAGACATGCTAAAAGAGTATTGGCATTTGAAATTGATCAACGTTTAATACCTGTATTAAATGATACA
CTATCACCTTATGATAATGTGACGGTGATTAATGAAGATATTTTAAAAGCGAATATTAAAGAAGCTGTTGAAAATCATTTACAAGATTGC
GAAAAAATAATGGTTGTTGCAAACCTGCCGTACTATATTACGACGCCAATTTTATTAAATTTAATGCAACAAGATATACCAATTGATGGCT
ACGTGGTGATGATGCAAAAAGAAGTGGGCGAACGCTTAAATGCTGAAGTAGGTTCAAAAGCATATGGTTCGTTATCAATTGTCGTACAA
TACTATACAGAGACTAGTAAAGTATTAACGGTACCTAAATCTGTATTTATGCCACCACCTAATGTTGATTCAATAGTTGTAAAACTGATGC
AGAGAACTGAACCGTTAGTAACAGTAGATAACGAGGAAGCATTCTTTAAGTTAGCAAAAGCAGCATTTGCACAAAGAAGAAAGACAATT
AACAATAACTATCAAAATTATTTTAAAGATGGTAAACAACACAAAGAAGTGATTTTACAATGGTTGGAACAAGCAGGTATTGATCCAAGA
CGTCGCGGTGAAACGCTATCTATTCAAGATTTTGCTAAATTGTATGAAGAAAAGAAAAAATTCCCTCAATTAGAAAATTAA

Gene: veg (conserved hypothetical protein)

Contig: 04_NODE_11, position: 12474 to 12737, length: 264 nt, orientation: FORWARD

Perfect match to: (RF122-AJ938182-[490353:490616], highly conserved allele)

Sequence:

ATGCCAAAATCAATTTTGGACATCAAAAATTCTATTGATTGTCATGTAGGAAATCGTATTGTACTGAAAGCCAATGGAGGCCGTAAGAAA
ACAATAAAACGTTCTGGAATTTTAAAAGAAACATATCCGTCAGTTTTCATTGTTGAGTTAGATCAAGACAAACACAACTTTGAGAGAGTAT
CTTATACATACACTGATGTGTTAACTGAAAATGTTCAAGTTTCATTTGAAGAGGATAATCATCACGAATCAATTGCACACTAA

Gene: ispE (4-diphosphocytidyl-2-C-methyl-D-erythritol kinase)

Contig: 04_NODE_11, position: 13047 to 13895, length: 849 nt, orientation: FORWARD

Sequence:

ATGATATATGAAACGGCACCAGCCAAAATTAATTTTACGCTCGATACACTTTTTAAAAGAAATGATGGCTATCATGAGATTGAAATGATA
ATGACAACAGTTGATTTAAATGATCGTTTAACTTTTCATAAAAGAAAAGATCGAAAGATAGTTGTTGAGATTGAACATAATTATGTGCCTT
CTAATCATAAAAATCTCGCATATCGTGCAGCGCAACTATTTATTGAGCAATATCAACTAAAGCAAGGTGTAACAATTTCTATCGATAAAGA
AATACCTGTTTCTGCTGGCTTAGCTGGAGGTTCGGCTGATGCAGCAGCAACGTTAAGAGGATTGAATCGACTTTTTGATATAGGGGCGA
GTTTGGAAGAATTGGCTCTGCTAGGCAGTAAAATCGGGACAGATATTCCGTTTTGTATTTATAATAAAACTGCACTATGTACTGGAAGAG
GAGAGAAAATCGAGTTTTTAAATAAACCACCTTCAGCTTGGGTGATTCTTGCTAAACCAAACTTAGGCATATCATCACCAGATATATTTAA
GTTGATCAATTTAGATAAGCGTTACGACGTACATACGAAAATGTGTTATGAGGCCTTAGAAAATCGAGATTATCAACAATTATGTCAAAG
TTTGTCTAATCGATTAGAGCCAATTTCTGTTTCAAAACACCCACAAATCGATAAATTAAAAAATAATATGTTGAAAAGTGGTGCAGATGGT
GCGTTAATGAGTGGAAGCGGACCGACTGTGTATGGGCTAGCACGAAAAGAAAGCCAAGCAAAAAATATTTATAATGCAGTTAACGGTT
GTTGTAATGAAGTGTACTTAGTTAGACTATTAGGATAG

Gene: purR (purine operon repressor)

Contig: 04_NODE_11, position: 13909 to 14733, length: 825 nt, orientation: FORWARD

Perfect match to: (MW2-BA000033-[510310:511134], highly conserved allele)

Sequence:

ATGAGATATAAACGAAGCGAGAGAATTGTTTTTATGACGCAATATTTGATGAACCATCCGAATAAATTGATTCCATTAACTTTTTTTGTGA
AAAAATTTAAACAGGCGAAGTCTTCAATAAGTGAAGATGTCCAAATTATAAAAAAATACATTCCAAAAAGAAAAGTTAGGTACAGTAATTA
CTACTGCTGGCGCAAGTGGTGGTGTTACGTATAAACCAATGATGAGTAAAGAAGAGGCGACTGAAGTTGTTAATGAGGTCATTACTCTA
TTAGAAGAGAAAGAACGTTTGTTACCTGGCGGGTATTTATTTTTATCAGATTTGGTAGGTAATCCATCGCTACTAAACAAAGTTGGTAAG
TTAATTGCCAGTATTTACATGGAAGAAAAATTAGATGCTGTTGTTACCATTGCGACAAAAGGTATTTCATTGGCAAATGCGGTTGCTAATA
TTTTAAATTTACCAGTAGTAGTGATTAGAAAAGACAACAAGGTGACTGAAGGTTCTACAGTTTCAATTAATTACGTTTCAGGATCTTCAAG
AAAAATAGAGACAATGGTACTTTCGAAGAGAACTTTAGCAGAAAATTCAAATGTTTTAGTTGTCGATGATTTTATGAGGGCTGGTGGCTC
TATTAATGGTGTTATGAATTTAATGAATGAGTTTAAAGCCCATGTAAAAGGGGTATCAGTACTTGTAGAATCAAAAGAAGTTAAACAAAG
ATTGATTGAAGATTATACTTCCTTAGTGAAATTATCTGATGTAGATGAATATAATCAAGAGTTTAACGTAGAACCTGGCAACAGTTTATCT
AAGTTTTCATAA

Gene: yabJ (translation initiation inhibitor)

Contig: 04_NODE_11, position: 14750 to 15130, length: 381 nt, orientation: FORWARD

Perfect match to: (N315-BA000018-[525948:526328], allele observed in CC5+CC7+CC12)

Sequence:

ATGAAAATCATTAACACAACAAGATTACCGGAAGCACTTGGACCATATTCGCATGCAACAGTTGTGAATGGTATGGTTTATACTTCTGGT
CAGATTCCATTGAATGTTGATGGGAAAATCGTAAGCGCTGATGTTCAAGCACAGACAAAACAAGTTTTAGAAAATTTAAAGGTTGTTTTG
GAAGAAGCAGGATCTGATTTGAATTCTGTTGCGAAAGCGACCATTTTCATTAAAGATATGAATGATTTCCAAAAAATAAATGAAGTGTAT
GGTCAATATTTTAATGAACACAAGCCAGCGCGTAGTTGTGTAGAGGTTGCGCGTTTGCCAAAAGATGTGAAAGTAGAAATTGAATTAGT
AAGTAAAATTAAGGAATTATAA

Gene: spoVG (septation protein spoVG)

Contig: 04_NODE_11, position: 15202 to 15504, length: 303 nt, orientation: FORWARD

Perfect match to: (RF122-AJ938182-[493081:493383], highly conserved allele)

Sequence:

ATGAAAGTGACAGATGTAAGACTTAGAAAAATACAAACAGATGGACGAATGAAAGCACTCGTTTCCATTACATTAGATGAAGCTTTCGT
AATTCATGATTTACGTGTAATTGAAGGAAACTCTGGCTTGTTCGTTGCAATGCCAAGTAAACGTACACCAGATGGTGAATTCCGCGACAT

CGCGCATCCTATTAATTCAGATATGAGACAAGAAATTCAAGATGCAGTGATGAAAGTATATGATGAAACAGATGAAGTAGTACCAGATA
AAAACGCTACATCAGAAGATTCAGAAGAAGCTTAA

Gene: glmU (UDP-N-acetylglucosamine pyrophosphorylase)

Contig: 04_NODE_11, position: 15848 to 17200, length: 1353 nt, orientation: FORWARD

Perfect match to: (08-02119-CP015645-[1721922:1723274:r], allele observed in ST582+CC361+CC398)

Sequence:

ATGCGAAGACACGCGATAATTTTGGCAGCAGGTAAAGGCACAAGAATGAAATCTAAAAAGTATAAAGTGCTACACGAGGTTGCTGGGA
AACCTATGGTCGAACATGTATTGGAAAGTGTGAAAGGCTCTGGTGTCGATCAAGTTGTAACCATCGTAGGACATGGTGCTGAAAGTGTA
AAAGGACATTTAGGCGAGCGTTCTTTATACAGTTTTCAAGAGGAACAACTCGGTACTGCGCATGCAGTGCAAATGGCGAAATCACACTTA
GAAGACAAGGAAGGTACGACGATCGTTGTATGTGGTGACACACCGCTCATCACAAAGGAAACATTAGAAACATTGATTGCGCATCATGA
GGATGCTAATGCTCAAGCAACTGTATTATCTGCATCGATTCAACAACCATATGGATACGGAAGAATCGTTCGAAATGCGTCAGGTCGTTT
AGAACGCATAGTTGAAGAGAAAGATGCAACGCAAGCTGAAAAGGATATTAATGAAATTAGTTCAGGTATTTTTGCGTTTAATAATAAAA
CGTTGTTTGAAAAATTAACACAAGTGAAAAATGATAATGCGCAAGGTGAATATTACCTCCCTGATGTATTGTCGTTAATTTTAAATGATGG
CGGCATCGTAGAAGTCTATCGTACCAATGATGTTGAAGAAATCATGGGTGTAAATGATCGTGTAATGCTTAGTCAGGCTGAGAAGGCGA
TGCAACGTCGTACGAATCATTATCACATGCTAAATGGTGTGACAATCATCGATCCTGACAGCACTTATATTGGTCCAGACGTTACAATTGG
TAGTGATACAGTCATTGAACCAGGCGTACGAATTAATGGTCGTACAGAAATTGGCGAAGATGTTGTTATTGGTCAGTACTCTGAAATTAA
CAATAGTACGATTGAAAATGGTGCATGTATTCAACAGTCTGTTGTTAATGATGCTAGCGTAGGAGCGAATACTAAGGTCGGACCGTTTGC
GCAATTGAGACCAGGCGCGCAATTAGGTGCAGATGTTAAGGTTGGAAATTTTGTAGAAATTAAAAAAGCAGATCTTAAAGATGGTGCCA
AGGTTTCACATTTAAGTTATATTGGCGATGCTGTAATTGGTGAACGTACTAATATTGGTTGCGGAACGATTACAGTTAACTATGATGGTG
AAAATAAATTTAAAACTATCGTCGGCAAAGATTCATTTGTAGGTTGCAATGTTAATTTAGTAGCACCTGTAACAATTGGTGATGATGTATT
GGTGGCAGCTGGTTCCACAATCACAGATGACGTACCAAATGACAGTTTAGCTGTGGCAAGAGCAAGACAAACAACAAAAGAAGGATAT
AGGAAATAA

Gene: prs (ribose-phosphate pyrophosphokinase)

Contig: 04_NODE_11, position: 17347 to 18312, length: 966 nt, orientation: FORWARD

Perfect match to: (MW2-BA000033-[513748:514713], allele observed in CC1+CC15+CC80+CC96+CC772)

Sequence:

ATGTTAAATAATGAATATAAGAATTCGTCATTAAAGATTTTTTCATTGAAAGGAAACGAAGCATTAGCGCAAGAAGTTGCTGACCAAGTA
GGAATTGAACTAGGTAAATGTTCAGTTAAACGTTTTAGTGATGGAGAAATTCAAATTAATATCGAAGAGAGTATTCGTGGTTGTGACGTA
TTTATTATTCAACCAACATCATATCCTGTGAATCTACATTTAATGGAATTATTAATTATGATTGACGCTTGTAAACGTGCTTCTGCAGCAAC
AATCAATATTGTAGTGCCATATTATGGATATGCAAGACAAGATAGAAAAGCCCGTAGCCGTGAGCCAATCACAGCTAAATTAGTTGCAAA
CTTAATCGAAACAGCTGGCGCAACTCGTATGATTGCGTTAGACTTACATGCACCACAAATTCAAGGATTCTTTGATATTCCAATTGACCAC
TTAATGGGTGTGCCAATTCTTGCTAAACATTTCAAAGATGATCCGAATATTAACCCAGAAGAATGTGTCGTTGTTTCACCAGACCATGGCG
GTGTTACACGTGCACGTAAATTAGCTGACATTTTAAAAACTCCAATTGCAATTATAGATAAACGTCGTCCTAGACCAAATGTTGCTGAAGT
GATGAACATTGTTGGTGAGATTGAAGGACGTACGGCAATTATTATTGACGATATTATTGATACAGCAGGTACAATCACTTTAGCTGCACA
AGCATTAAAAGATAAAGGTGCTAAAGAAGTATATGCTTGTTGTACACACCCTGTTTTATCAGGACCGGCTAAAGAACGTATCGAAAATTC
TGCTATAAAAGAATTAATCGTAACAAACTCAATTCATTTAGATGAAGACCGCAAACCATCTAACACTAAAGAATTATCTGTTGCTGGTTTA
ATCGCACAAGCTATCATTCGTGTATACGAAAGAGAATCAGTTAGCGTATTATTTGACTAA

Gene: rplY (50S ribosomal protein L25)

Contig: 04_NODE_11, position: 18462 to 19115, length: 654 nt, orientation: FORWARD

Perfect match to: (N315-BA000018-[529681:530334], highly conserved allele)

Sequence:

ATGGCTTCATTAAAGTCAATCATCCGTCAAGGTAAACAAACACGTTCAGATCTTAAACAATTAAGAAAATCTGGTAAAGTACCAGCAGTA
GTATACGGTTACGGTACTAAAAACGTGTCAGTTAAAGTTGATGAAGTAGAATTCATCAAAGTTATCCGTGAAGTAGGTCGTAACGGTGTT
ATCGAATTAGGCGTTGGTTCTAAAACTATCAAAGTTATGGTTGCAGACTACCAATTCGATCCACTTAAAAACCAAATTACTCACATTGACT
TCTTAGCAATCAATATGAGTGAAGAACGTACTGTTGAAGTACCAGTTCAATTAGTTGGTGAAGCAGTAGGCGCTAAAGAAGGCGGCGTA

GTTGAACAACCATTATTCAACTTAGAAGTAACTGCTACTCCAGACAATATTCCAGAAGCAATCGAAGTAGACATTACTGAATTAAACATTA
ACGACAGCTTAACTGTTGCTGATGTTAAAGTAACTGGCGACTTCAAAATCGAAAACGATTCAGCTGAATCAGTAGTAACAGTAGTTGCTC
CAACTGAAGAACCAACTGAAGAAGAAATCGAAGCTATGGAAGGCGAACAACAAACTGAAGAACCAGAAGTTGTTGGCGAAAGCAAAG
AAGACGAAGAAAAAACTGAAGAGTAA

Gene: pth (peptidyl-tRNA hydrolase)

Contig: 04_NODE_11, position: 19426 to 19998, length: 573 nt, orientation: FORWARD

Perfect match to: (MW2-BA000033-[515827:516399], allele observed in CC1+CC15)

Sequence:

ATGAAATGTATTGTAGGTCTAGGTAATATAGGTAAACGTTTTGAACTTACAAGACATAATATCGGCTTTGAAGTCGTTGATTATATTTTAG
AGAAAAATAATTTTTCATTAGATAAACAAAAGTTTAAAGGTGCATATACAATTGAACGAATGAACGGCGATAAAGTGTTATTTATCGAAC
CAATGACAATGATGAATTTGTCAGGAGAAGCAGTTGCACCGATTATGGATTATTACAATGTTAATCCAGAAGATTTAATTGTCTTATATGA
TGATTTAGATTTAGAACAAGGACAAGTTCGCTTAAGACAAAAAGGAAGTGCGGGCGGTCACAATGGTATGAAATCAATTATTAAAATGC
TTGGTACAGACCAATTTAAACGTATTCGTATTGGTGTGGGAAGACCAACGAATGGTATGACGGTACCTGATTATGTTTTACAACGCTTTTC
AAATGATGAAATGGTAACGATGGAAAAAGTTATCGAACACGCAGCACGCGCAATTGAAAAGTTTGTTGAAACATCACGATTTGACCATG
TTATGAATGAATTTAATGGTGAAGTGAAATAA

Gene: mfd (transcription-repair-coupling factor)

Contig: 04_NODE_11, position: 19998 to 23504, length: 3507 nt, orientation: FORWARD

Perfect match to: (CA347-CP006044-[541727:545233], allele observed in CC45I)

Sequence:

ATGACAATATTGACAACGCTTATAAAAGAAGATAATCATTTTCAAGACCTTAATCAGGTATTTGGACAAGCAAACACACTAGTAACTGGT
CTTTCCCCGTCAGCTAAAGTGACGATGATTGCTGAAAAATATGCACAAAGTAATCAACAGTTATTATTAATTACCAATAATTTATACCAAG
CAGATAAATTAGAAACAGATTTACTTCAATTTATAGATGCTGAAGAATTGTATAAGTATCCTGTGCAAGATATTATGACCGAAGAGTTTTC
AACACAAAGCCCTCAACTGATGAGTGAACGTATTAGAACTTTAACTGCGTTAGCTCAAGGTAAGAAAGGGTTATTTATCGTTCCTTTAAAT
GGTTTGAAAAAGTGGTTAACTCCTGTTGAAATGTGGCAAAATCACCAAATGACATTGCGTGTTGGTGAGGATATCGATGTGGACCAATTT
CTTAACAAATTAGTTAATATGGGGTACAAACGGGAATCCGTGGTATCGCATATTGGTGAATTCTCATTGCGAGGAGGTATTATCGATATC
TTTCCGCTAATTGGGGAACCAATCAGAATTGAGCTATTTGATACCAAAATTGATTCTATTCGGGATTTTGATGTTGAAACGCAGCGTTCCA
AAGATAATGTTGAAGAAGTCGATATCACAACTGCAAGTGATTATATCATTACTGAAGAAGTGATCAGCCATCTTAAAGAAGAGTTAAAAA
CTGCATATGAAAATACAAGACCCAAAATAGATAAATCAGTGCGCAATGATTTGAAAGAAACGTATGAAAGCTTTAAATTATTCGAAAGTA
CATACTTTGATCATCAAATACTACGTCGCTTAGTAGCGTTTATGTATGAAACACCTTCGACAATTATTGAGTATTTCCAAAAAGATGCAATC
ATTGCAGTTGATGAATTTAATCGTATTAAAGAAACTGAAGAAAGTTTAACAGTAGAGTCTGATTCGTTTATTAGCAATATTATTGAAAGTG
GTAATGGATTTATAGGACAAAGTTTTATAAAATATGATGATTTTGAAACATTGATTGAAGGCTATCCTGTCACTTATTTTTCATTATTCGCT
ACAACAATGCCGATAAAACTAAATCATATTATTAAATTTTCATGTAAACCTGTCCAACAATTTTATGGGCAATATGACATTATGCGTTCTGA
ATTTCAACGATATGTTAATCAAAACTATCATATCGTGGTTTTGGTCGAAACCGAAACTAAAGTTGAACGTATGCAAGCGATGTTAAGTGA
AATGCATATTCCATCAATAACAAAATTGCATCGCTCAATGTCATCGGGACAAGCAGTGATTATTGAGGGCAGTTTATCTGAAGGATTTGA
ACTACCTGATATGGGATTAGTTGTCATTACTGAGCGTGAGCTTTTTAAATCAAAACAGAAAAAGCAACGAAAACGTACGAAAGCTATCTC
AAATGCTGAAAAAATTAAGTCTTACCAAGATTTAAATGTGGGAGATTATATTGTTCATGTGCATCATGGTGTTGGTAGATATTTAGGTGTT
GAGACGCTCGAAGTGGGGCAAACGCATCGTGATTATATTAAATTGCAATATAAAGGTACGGATCAACTATTTGTTCCAGTAGATCAAATG
GATCAAGTTCAAAAATATGTAGCTTCGGAAGATAAGACGCCAAAATTAAATAAACTCGGTGGCAGTGAATGGAAAAAAACAAAAGCTAA
AGTTCAACAAAGTGTTGAAGATATTGCTGAAGAGTTGATTGATTTATATAAAGAAAGAGAAATGGCAGAAGGTTATCAATATGGGGAAG
ACACAGCTGAGCAAACAACATTTGAATTAGATTTTCCATATGAACTTACGCCTGACCAAGCTAAATCTATCGATGAAATTAAAGATGACAT
GCAAAAATCGCGTCCAATGGATCGCTTGCTATGTGGTGATGTTGGTTATGGTAAAACTGAAGTTGCAGTGAGAGCAGCATTCAAAGCTG
TAATGGAAGGAAAGCAGGTTGCATTTTTAGTTCCTACAACTATTTTAGCTCAGCAACATTATGAGACGTTAATTGAGCGTATGCAAGATTT
TCCTGTTGAAATTCAATTAATGAGTCGTTTTAGAACGCCTAAAGAGATAAAACAAACTAAGGAAGGACTTAAAACTGGATTTGTTGACAT
AGTTGTTGGTACACACAAATTACTTAGTAAAGATATACAGTATAAAGATTTAGGGCTGTTGATTGTAGATGAAGAACAACGATTTGGTGT
ACGCCATAAAGAGCGTATTAAAACATTAAAACATAATGTAGATGTACTAACATTGACTGCAACCCCAATACCTAGAACATTGCATATGAG
TATGCTAGGTGTGCGCGATTTGTCAGTGATTGAAACGCCGCCAGAAAATCGTTTCCCAGTTCAAACATATGTATTAGAACAGAACATGAG
TTTTATCAAAGAAGCTTTAGAAAGAGAACTATCCCGTGATGGCCAAGTGTTTTATCTTTATAATAAAGTGCAATCCATTTATGAAAAACGA
GAACAACTCCAGATGTTAATGCCAGATGCTAACATTGCAGTTGCTCATGGACAAATGACAGAGCGCGATTTAGAAGAAACGATGTTAAG
TTTTATCAATAATGAATATGATATTTTAGTAACGACGACGATTATTGAAACAGGTGTCGATGTCCCAAATGCAAATACTTTGATCATTGAA
GATGCAGATCGCTTTGGATTGAGTCAGTTGTATCAATTAAGAGGTCGTGTTGGTCGTTCAAGTCGTATTGGTTATGCATACTTCTTACATC

CAGCAAATAAGGTACTAACTGAGACTGCAGAAGATCGATTACAAGCGATTAAAGAATTTACGGAGTTAGGCTCAGGATTTAAGATTGCG
ATGCGTGATTTGAACATTCGTGGTGCTGGTAATTTGTTAGGTAAACAACAGCACGGCTTTATTGATACAGTTGGATTTGATTTGTACAGTC
AAATGTTAGAAGAAGCTGTAAATGAAAAACGTGGTATTAAGGAACCAGAATCTGAGGTGCCAGAAGTCGAAGTTGATTTAAACTTGGAT
GCATATTTGCCGACAGAATATATTGCAAATGAACAAGCTAAAATTGAAATTTATAAAAAGCTACGAAAAACTGAAACATTTGATCAAATT
ATCGACATTAAAGATGAATTAATTGATCGTTTTAATGATTATCCTGTTGAAGTAGCACGTTTGCTTGATATAGTGGAAATAAAAGTACACG
CATTACATTCAGGTATCACGTTGATTAAAGATAAAGGGAAAATAATTGATATTCATTTATCTGTAAAAGCCACTGAAAATATTGATGGCG
AAGTGCTGTTCAAAGCAACACAACCTTTAGGTAGAACAATGAAGGTTGGTGTTCAAAATAATGCAATGACAATTACTTTAACGAAACAAA
ATCAATGGCTTGATAGTTTGAAGTTTTTAGTTAAGTGCATTGAAGAAAGTATGAGAATCAGTGATGAAGCATAA

Gene: yabM (putative teichoic acid exporter)

Contig: 04_NODE_11, position: 23494 to 25020, length: 1527 nt, orientation: FORWARD

Perfect match to: (N315-BA000018-[534713:536239], allele observed in CC5+CC1+CC772)

Sequence:

ATGAAGCATAAAGAAGCATTTAATGGCGTTGTCGTGTTAACTGCTGCATTAATTGTCATTAAAATTCTGAGTGCTGTATATCGAATTCCAT
ATCAAAATATATTAGGTGATACAGGTTTGTATGCATATCAACAAGTGTATCCAATTGTAGCATTAGGAATGATATTATCGATGAATGCCAT
TCCTAGTGCAATTACACAAAATATAGGGAAGTATCATAGTGACGAAGCATATGCAAAAGCGGTCGCTTATATACAATTAGTTGGTATATT
ATTATTTATTGCTATTTTTGTGTTTGCGAACAATATTGCACATATGATGGGTGATAGCCATTTAACACCAATGATTCAAGCAGCAAGTTTAA
GCTTTATATTTATAGGTATGCTTGGCGTGTTAAGAGGTTATTATCAATCTGCAAATAATATGACAGTTCCGGCTATTTCCCAGGTTATAGA
ACAAGTTATACGAGTAGGTATTATCATTGTTACTATTGTTATTTTTGTAGACAGAGGTTGGACGATATATGAAGCGGGAACAATTGCTATT
TTAGCATCAACGATAGGTTTTTTAGGTTCTTCAATTTATTTAGTAGCGCACCGACCTTTTAAGTTTAAAATGGTAAATAACACTGCAAAGAT
TGTTTGGAAACAGTTCGCACTTTCGGTTTTGATTTTCGCTATCAGTCAATTAATCGTAATTTTATGGCAAGTGATTGATAGTGTTACTATTA
TTAAGTCACTTCAAGCGATACGCGTGCCATTCGATGTTGCCATAACTGAAAAAGGAGTCTATGACCGTGGTGCATCATTTATTCAGATGG
GATTGATTGTAACTACAACATTTAGTTTTGCGCTCATTCCTCTGTTAAGTGACGCAATCAAAATGAATAATCAGGTACTTATGAATCGTTAT
GCAAATGCGTCATTAAAGATTACGATTTTAATAAGTACAGCAGCGGGAATAGGATTAATTAATTTATTGCCTTTAATGAACGGTGTGTTTT
TTAAGACGAATGATTTAACCTTAACGTTAAGTGTTTATATGATTACGGTCATTTGTGTATCGTTAATTATGATGGATATGGCTTTATTACAA
GCGCAACATGCTGTGAGACCTATTTTTGTTGGTATGACGGCAGGATTGGTTATTAAATTTATACTTAATATCATTTTGATTCGTTTAAGTG
GCATTATTGGTGCGAGCATTAGTACTGTTGTATCATTAATTATATTCGGTACGATTATCCATATTGCTGTCACGAGAAAATACCACTTACAT
GCGATGAGACGATTTTTTATCAATGTTGTTTTAGGTATGGTATTTATGTCGATTGTTGTTCAATGCGTGTTAAACATAGTGACAACACACG
GTAGATTCACTGGACTCATTGAATTATTATGTGCAGCAGTATTAGGTATCATTGCATTGTTTTTCTATATTTTTAGATTTAATGTTTTGACAT
ATAAAGAGTTAACTTATTTACCATTTGGTTCAAAGTTGTATCAAATTAAGAAAGGAAGACGTTGA

Gene: mazG (tetrapyrrole methylase)

Contig: 04_NODE_11, position: 25020 to 26213, length: 1194 nt, orientation: FORWARD

Perfect match to: (MW2-BA000033-[521421:522614], allele observed in CC1+CC15+CC22+CC25+CC59)

Sequence:

ATGGCACATACCATTACGATTGTTGGCTTAGGAAACTATGGCATTGATGATTTGCCGCTAGGGATATATAAATTTTTAAAGACACAAGAT
AAAGTTTATGCAAGAACGTTAGATCATCCAGTTATAGAATCATTGCAAGATGAATTAACATTTCAGAGTTTTGACCATGTTTATGAAGCAC
ATGACCAATTTGAAGATGTCTATAATGATATTGTGGCGCAATTGGTTGAAGCTGCTAATGAAAAAGATATTGTCTATGCGGTTCCGGGTC
ATCCTAGAGTTGCTGAGACAACTACAGTGAAATTACTGGCTTTAGCAAAGGACAATACTGATATAGATGTGAAAGTTTTAGGTGGTAAAA
GCTTTATTGATGATGTGTTTGAAGCAGTTAATGTAGATCCAAATGATGGCTTCACACTGTTAGATGCGACATCATTACAAGAAGTAACACT
TAATGTTAGAACGCATACATTGATTACACAAGTTTATAGTGCAATGGTTGCTGCTAATTTGAAAATCACTTTAATGGAACGATATCCTGAT
GATTACCCTGTTCAAATTGTCACTGGTGCACGAAGCGATGGTGCGGATAACGTTGTGACATGCCCATTATATGAATTGGATCATGATGAA
AATGCATTCAATAATTTGACGAGTGTATTCGTACCAAAAATCATAACATCGACATATTTGTATCATGACTTTGATTTTGCAACGGAAGTGA
TTGATACTTTAGTTGATGAAGATAAAGGTTGTCCATGGGATAAAGTGCAAACGCATGAAACGCTTAAGCGTTATTTACTTGAAGAAACAT
TTGAATTGTTCGAAGCTATTGACAATGAAGATGATTGGCATATGATTGAAGAGCTAGGAGATATTTTATTACAAGTGTTATTGCATACTA
GTATTGGTAAAAAAGAAGGGTATATCGACATTAAAGAAGTGATTACAAGTCTTAATGCTAAAATGATTCGTAGACACCCACACATATTTG
GTGATGCCAATGCTGAAACTATCGATGACTTAAAAGAAATTTGGTCTAAGGCGAAAGATGCTGAAGGTAAACAGCCAAGAGTTAAATTT
GAAAAAGTATTTGCAGAGCATTTTTTAAATTTATATGAGAAGACGAAGGATAAGTCATTTGATGAGGCCTCGTTAAAGCAGTGGCTAGA
AAAAGGGGAGAGTAATACATGA

Gene: hslR (ribosome-associated heat shock protein Hsp15)

Contig: 04_NODE_11, position: 26210 to 26473, length: 264 nt, orientation: FORWARD

Perfect match to: (N315-BA000018-[537429:537692], highly conserved allele)

Sequence:

ATGAGATTAGATAAATATTTAAAAGTATCACGGTTAATAAAGCGACGTACGCTAGCAAAAGAAGTAAGTGATCAAGGTAGAATTACAAT
AAATGGTAATGTTGCTAAAGCTGGATCGGATGTTAAAGTTGAAGATGTGCTGACGATTCGCTTTGGTCAAAAATTAGTAACAGTTAAAGT
AACTGCATTAAATGAACATGCATCTAAAGATAACGCGAAGGGCATGTATGAAATCATTGAAGAGCGTCGACTTGAAGAAGCGTAA

Gene: divIC (cell-division protein)

Contig: 04_NODE_11, position: 26491 to 26883, length: 393 nt, orientation: FORWARD

Perfect match to: (MW2-BA000033-[522892:523284], allele observed in CC1+CC15)

Sequence:

ATGAAAAATAAAGTAGAACATATAGAAAATCAGTACACGTCGCAAGAGAATAAGAAAAAACAACGTCAAAAAATGAAAATGCGTGTTG
TTCGTAGGCGTATTACAGTATTTGCGGGCGTATTACTTGCGATAATTGTTGTTTTATCAATCTTGCTTGTTGTCCAAAAACATCGCAATGAT
ATCGATGCACAGGAGCGAAAAGCGAAAGAAGCACAGTTTCAAAAGCAACAAAATGAAGAAATTGCGTTAAAAGAAAAGTTGAATAATC
TGAATGACAAAGATTATATTGAAAAAATTGCGCGTGATGATTATTACTTAAGCAACAAAGGTGAAGTGATTTTTAGGTTGCCAGAAGACA
AAGATTCGTCTAGCTCAAAATCTTCGAAAAAATAA

Gene: yabR (putative RNA degradation protein)

Contig: 04_NODE_11, position: 26988 to 27389, length: 402 nt, orientation: FORWARD

Perfect match to: (MW2-BA000033-[523389:523790], highly conserved allele)

Sequence:

ATGTCAATCGAAGTTGGAAATAAGCTTAAAGGTAAAGTCACTGGTATTAAAAAGTTTGGTGCATTCGTAGAATTACCTGAAGGAAAAAG
TGGTTTAGTTCACATTAGTGAAGTCGCAGATAATTATGTTGAAAACGTAGAAGAGCACCTTTCTGTTGGTGATGAAGTAGACGTAAAAGT
ATTATCTATTGCTGATGATGGAAAAATTAGTCTTTCAATTAAGAAAGCTAAAGACCGTCCACGTAGACAACATACGAGTAAACCAAGTCA
TCAAAAACCAGTGCAAAAAGCCGAAGATTTTGAAAAGAAATTAAGCAATTTCTTAAAAGATAGTGAAGATAAATTAACTTCAATCAAACG
TCAAACAGAATCTAGACGCGGTGGCAAAGGTTCAAGACGTTAA

Gene: tilS (tRNA (Ile)-lysidine synthase)

Contig: 04_NODE_11, position: 27569 to 28864, length: 1296 nt, orientation: FORWARD

Perfect match to: (MW2-BA000033-[523970:525265], allele observed in CC1+CC15)

Sequence:

ATGCAGTTAAATAGTAATGGTTGGCATGTTGATGACCATATTGTTGTCGCTGTTTCTACAGGTATTGATAGTATGTGTTTATTGTATCAAC
TACTAAATGATTATAAAGATAGTTATAGAAAACTAACATGCTTACATGTCAATCATGGCGTTAGGTCAGCTTCAATCGAGGAAGCCAGAT
TTTTAGAAGTATACTGCGAACGTCATCACATCGATTTACATATCAAAAAGTTAGATTTGTCGCATAGTCTCGACCGAAATAACAGCATTCA
GAATGAAGCTCGAATTAAACGTTACGAATGGTTTGATGAAATGATGAATGTATTAGAAGCGGATGTATTGCTAACGGCGCATCATTTGG
ACGATCAATTAGAAACTATTATGTATCGTATTTTTAATGGGAAATCAACGCGTAATAAACTAGGATTTGATGAGTTATCGAAGCGAAAAG
GTTATCAGATTTATCGACCACTTTTAGCTGTCTCTAAAAAAGAAATAAAACAATTCCAAGAGAGATATCATATTCCATATTTTGAAGATGA
ATCTAATAAAGATAACAAATATGTTAGAAATGATATTCGTAATAGAATTATTCCAGCTATTGATGAAAATAATCAACTTAAAGCATCGCAT
TTATTAAAATTAAAACAATGGCATGATGAACAATATGATATTTTGCAATATTCAGCTAAACAATTTATTCAAGAATTTGTGAAGTTTGATG
AACAGTCAAAATATTTAGAGGTTTCTAGACAAGCTTTTAATAACTTACCAAACTCATTAAAGATGGTTGTGTTGGATTGCCTACTATCAAA
GTATTATGAGTTGTTTAATATTAGTGCTAAAACATACGAAGAGTGGTTTAAACAATTTAGTAGTAAGAAAGCACAATTCAGTATTAATCTC
ACGGATAAATGGATAATTCAAATTGCATATGGTAAATTAATAATAATGGCTAAAAATAATGGCGATACATATTTTAGAGTTCAAACTATT
GAAAAGCCAGGTAATTATATTTTTAACAAATATCGATTAGAGATACATTCTAATTTACCAAAATGTTTATTTCCGCTTACAGTGAGAACAC
GACAAAGTGGCGATACATTTAAACTGAATGGGCGCGATGGTTATAAGAAAGTGAATCGCCTGTTTATAGATTGTAAAGTGCAACAGTGG

GTTCGGGATCAAATGCCAATCGTATTGGATAAACAACAGCGCATTATTGCGGTAGGAGATTTATATCAACAACAAACAATAAAACAATG
GATTATAATTAGTAAAAATGGAGATGAATAG

Gene: hpt (hypoxanthine-guanine phosphoribosyltransferase)

Contig: 04_NODE_11, position: 28869 to 29408, length: 540 nt, orientation: FORWARD

Perfect match to: (RF122-AJ938182-[506747:507286], highly conserved allele)

Sequence:

ATGCATAATGATTTGAAAGAAGTATTGTTAACTGAAGAAGATATTCAAAATATCTGTAAGGAATTGGGAGCACAATTAACAAAGGATTAT
CAAGGTAAACCATTAGTATGCGTGGGTATCTTAAAAGGCTCAGCAATGTTTATGTCAGATTTAATTAAACGAATTGATACCCATTTATCAA
TTGATTTCATGGATGTTTCTAGTTATCACGGAGGCACTGAGTCAACTGGTGAAGTTCAAATCATTAAAGATTTAGGTTCTTCTATTGAAAA
TAAAGACGTATTAATTATTGAAGATATCTTAGAGACTGGTACTACACTTAAGTCAATTACTGAATTATTACAATCTAGAAAAGTTAATTCA
TTAGAAATAGTTACTTTATTAGATAAACCAAACCGTCGTAAAGCGGACATTGAAGCTAAGTATGTAGGTAAAAAAATACCAGATGAATTT
GTTGTTGGTTACGGTTTAGATTATCGTGAATTATACCGAAACTTACCATATATCGGTACGTTAAAACCTGAAGTGTATTCAAATTAA

Gene: ftsH (cell-division protein H)

Contig: 04_NODE_11, position: 29665 to 31758, length: 2094 nt, orientation: FORWARD

Perfect match to: (RF122-AJ938182-[507544:509637], highly conserved allele)

Sequence:

ATGCAGAAAGCTTTTCGCAATGTGCTAGTTATCGTAATAATAGGCGTTATTATTTTTGGTCTATTTTCATATTTAAACGGTAATGGAAATAT
GCCGAAACAGCTTACATATAATCAATTTACTGAGAAGTTGGAAAAAGGTGACCTTAAAACTTTAGAAATCCAACCACAACAAAATGTCTA
TATGGTAAGTGGTAAAACGAAAAATGATGAAGACTATTCATCAACTATTTTATATAACAACGAAAAAGAATTACAAAAAATTACTGATGC
TGCTAAAAAGCAAAACGGTGTAAAATTAACGATTAAAGAAGAAGAAAAACAAAGTGTCTTTGTGAGTATACTTTCAACATTAATTCCAGT
TGTAGTCATAGCGTTATTATTTATTTTCTTCCTAAGCCAAGCACAAGGTGGCGGTAGTGGCGGTCGTATGATGAACTTTGGTAAATCTAAA
GCAAAAATGTACGATAATAATAAACGTCGTGTTCGTTTCTCTGATGTAGCAGGGGCAGATGAAGAAAAACAAGAATTAATTGAAATTGTT
GATTTCTTGAAAGATAATAAAAAATTCAAAGAAATGGGATCTAGGATTCCTAAAGGTGTCTTACTTGTTGGACCTCCAGGTACTGGTAAA
ACATTACTTGCTAGAGCGGTTGCAGGTGAAGCTGGCGCACCATTCTTCTCTATTAGTGGTTCAGACTTTGTAGAGATGTTTGTTGGTGTTG
GTGCGAGCCGTGTTCGTGACTTATTCGATAATGCTAAGAAAAACGCGCCTTGTATCATCTTTATCGATGAGATTGATGCTGTTGGTCGTCA
ACGTGGTGCAGGTGTTGGTGGCGGTCATGATGAACGTGAACAAACCCTAAACCAATTATTAGTTGAAATGGATGGTTTCGGTGAAAATG
AAGGTATCATTATGATAGCTGCTACAAACCGTCCTGATATCCTTGACCCAGCCTTATTACGTCCAGGTCGTTTTGATAGACAAATTCAAGT
TGGTCGTCCAGATGTGAAAGGCCGTGAAGCAATTCTTCATGTTCATGCTAAAAACAAACCACTTGATGAAACGGTTGATTTAAAAGCAAT
TTCACAACGTACACCTGGTTTCTCAGGTGCTGATTTAGAGAACTTATTAAATGAAGCATCTTTAATTGCTGTACGTGAAGGTAAAAAGAA
AATTGACATGAGAGATATCGAAGAGGCAACGGATAGAGTTATAGCCGGACCTGCTAAGAAATCTCGAGTTATTTCTAAGAAAGAACGTA
ATATTGTTGCTCATCACGAAGCTGGTCATACAATTATCGGTATGGTACTTGATGAGGCAGAAGTAGTGCATAAAGTTACTATTGTTCCAC
GTGGACAAGCAGGTGGTTATGCAATGATGCTACCTAAACAAGATCGTTTCTTAATGACTGAACAAGAGTTATTAGATAAAATCTGTGGTT
TACTTGGTGGACGTGTATCAGAAGATATTAACTTTAACGAAGTATCAACAGGTGCTTCAAATGACTTCGAACGTGCAACACAAATCGCAC
GCTCAATGGTTACGCAATATGGTATGAGTAAAAAATTAGGACCATTACAGTTCGGTCATAGCAATGGTCAAGTATTCTTAGGTAAAGATA
TGCAAGGTGAGCCTAATTATTCAAGCCAAATCGCATATGAAATTGATAAAGAAGTTCAACGAATCGTTAAAGAACAATACGAACGTTGTA
AACAAATTTTATTAGAGCACAAAGAACAATTAATTTTAATTGCTGAAACATTATTAACAGAAGAAACATTAGTTGCTGAACAAATTCAATC
ATTATTCTACGAAGGTAAATTACCTGAAATTGATTATGATGCAGCTAAAGTTGTTAAAGATGAAGATTCTGAATTTAATGATGGTAAATTC
GGTAAATCTTATGAAGAGATTCGTAAAGAGCAATTAGAAGATGGACAACGTGACGAAAGTGAAGATCGTAAAGAAGAAAAAGATATTG
CTGAGGATAAAAAAGAAGCTGATAAATCTGATGAAAAAGATGAACCAGCACATCGACAAGCCCCAAATATCGAAAAACCTTACGATCCA
AATCACCCAGACAATAAATAA

Gene: hslO (33 kDa chaperonin)

Contig: 04_NODE_11, position: 31987 to 32868, length: 882 nt, orientation: FORWARD

Perfect match to: (MW2-BA000033-[528388:529269], highly conserved allele)

Sequence:

ATGACACACGATTATATTGTTAAAGCATTAGCATTTGATGGAGAGATTAGGGCTTATGCTGCTTTGACAACTGAAACTGTTCAAGAAGCA
CAAACGAGACATTATACATGGCCGACAGCATCTGCTGCAATGGGAAGAACAATGACAGCAACAGCTATGATGGGCGCAATGTTGAAAG
GTGATCAAAAATTAACTGTCACTGTAGATGGCCAAGGACCTATTGGACGAATTATTGCCGATGCAAATGCTAAAGGCGAGGTGCGTGCT
TATGTAGACCATCCACAAACTCATTTTCCATTAAATGAGCAAGGTAAACTTGATGTAAGACGAGCAGTAGGGACAAATGGATCTATTATG
GTTGTTAAAGACGTTGGAATGAAAGACTATTTCTCTGGAGCAAGTCCAATTGTTTCAGGAGAACTTGGTGAAGATTTTACTTATTATTATG
CTACAAGTGAACAAACACCTTCATCGGTAGGTCTTGGTGTATTGGTAAATCCTGATAATACGATTAAAGCAGCAGGAGGATTTATCATTC
AAGTTATGCCAGGTGCCAAAGATGAAACAATTTCAAAATTAGAAAAAGCAATTAGTGAAATGACACCAGTTTCTAAATTAATTGAACAAG
GATTAACGCCAGAAGGATTACTAAACGAAATCTTAGGTGAAGACCATGTGCAAATTTTAGAGAAAATGCCTGTTCAATTTGAATGTAATT
GTAGTCATGAGAAATTTTTAAATGCTATTAAAGGATTGGGCGAGGCTGAGATTCAAAATATGATTAAAGAAGATCATGGTGCTGAAGCA
GTATGTCATTTCTGTGGAAATAAATATAAATATACTGAAGAAGAATTAAACGTGTTGCTAGAAAGTTTAGCGTAA

Gene: cysK (cysteine synthase)

Contig: 04_NODE_11, position: 33047 to 33979, length: 933 nt, orientation: FORWARD

Sequence:

ATGGCACAAAAACCAGTAGATAATATTACTCAAATTATTGGCGGTACACCGGTAGTCAAATTGAGAAATGTAGTAGATGACAATGCAGC
AGATGTTTATGTAAAATTGGAATATCAAAATCCAGGTGGTTCTGTAAAGGATAGAATTGCTTTAGCAATGATTGAAAAAGCAGAGCGAG
AAGGCAAAATTAAACCTGGCGATACAATTGTAGAACCAACAAGTGGTAATACAGGTATCGGTTTAGCATTTGTATGTGCTGCTAAAGGAT
ATAAAGCAGTATTTACTATGCCCGAAACAATGAGCCAAGAGCGTCGTAATTTATTAAAAGCATACGGTGCGGAATTAGTTTTAACGCCTG
GATCAGAAGCGATGAAAGGTGCAATTAAAAAAGCTAAAGAATTGAAAGAAGAACATGGTTACTTCGAGCCACAACAATTTGAAAACCCT
GCGAACCCTGAAGTTCATGAGTTAACTACAGGTCCTGAGTTATTACAACAATTTGAAGGGAAAACTATCGATGCGTTCCTAGCTGGTGTT
GGTACTGGTGGTACGTTATCTGGTGTAGGTAAAGTTCTGAAAAAAGAATATCCTAACATCGAAATTGTTGCTATAGAGCCTGAGGCTTCT
CCAGTATTGAGCGGTGGTGAGCCAGGTCCACATAAATTACAAGGTTTAGGTGCTGGATTTATTCCAGGCACTTTGAATACAGAAATCTAT
GACAGTATTATTAAAGTAGGAAATGATACAGCGATGGAAATGTCTCGTCGAGTTGCTAAAGAGGAAGGTATTTTAGCAGGTATTTCATC
AGGTGCTGCGATTTATGCTGCCATTCAAAAAGCAAGAGAATTAGGAAAAGGTAAAACAGTAGTAACAGTATTGCCGAGTAATGGTGAAC
GCTACTTATCAACACCTTTATATTCATTCGATGACTAA

Gene: folP (dihydropteroate synthase)

Contig: 04_NODE_11, position: 34195 to 34998, length: 804 nt, orientation: FORWARD

Perfect match to: (Strain_21193-AFEG01000016-[4271:5074:r], highly conserved allele)

Sequence:

ATGACTAAAACAAAAATTATGGGCATATTAAACGTCACACCTGATTCATTCTCAGATGGTGGAAAATTTAATAATGTTGAATCAGCTATAA
ATAGAGTGAAAGCCATGATAGATGAAGGTGCTGACATTATAGATGTTGGAGGTGTTTCAACGAGACCAGGTCATGAAATGGTTTCATTA
GAAGAAGAGATGAACAGAGTATTACCTGTTGTTGAAGCTATTGTCGGTTTTGATGTAAAAATTTCAGTCGATACATTTCGAAGTGAGGTT
GCTGAAGCATGTTTAAAATTAGGCGTTGATATGATTAATGATCAATGGGCGGGTCTGTATGATCATCGTATGTTCCAAATTGTAGCTAAA
TATGACGCGGAAATTATTTTAATGCATAATGGAAATGGTAATCGTGATGAACCGGTTGTCGAAGAAATGTTAACATCTTTGTTAGCACAA
GCACATCAAGCTAAAATAGCTGGTATACCTTCAAATAAAATTTGGCTAGATCCAGGTATAGGTTTCGCTAAAACTAGAAATGAAGAAGCC
GAAGTTATGGCAAGACTGGATGAACTTGTTGCAACAGAATATCCAGTTTTATTAGCGACAAGCCGGAAACGTTTCACTAAAGAGATGAT
GGGTTATGATACAACACCGGTTGAAAGAGATGAAGTAACTGCAGCTACGACTGCATATGGTATTATGAAAGGCGTTAGAGCAGTACGCG
TTCATAATGTCGAGTTGAATGCTAAATTAGCTAAAGGTATAGATTTTTTAAAGGAGAATGAAAATGCAAGACACAATCTTTCTTAA

Gene: folB (dihydroneopterin aldolase)

Contig: 04_NODE_11, position: 34976 to 35341, length: 366 nt, orientation: FORWARD

Perfect match to: (MW2-BA000033-[531377:531742], highly conserved allele)

Sequence:

ATGCAAGACACAATCTTTCTTAAAGGTATGCGCTTTTATGGATATCATGGTGCTTTATCAGCTGAAAATGAAATAGGGCAAATTTTCAAAG
TGGATGTAACTTTGAAAGTAGACTTAGCTGAAGCTGGGCGTACTGATAATGTTATTGATACAGTTCATTATGGTGAAGTGTTCGAAGAGG
TTAAATCAATTATGGAAGGTAAGGCCGTTAATTTACTTGAGCATCTAGCTGAACGTATTGCAAATCGTATAAATTCACAATATAATCGTGT

AATGGAAACGAAAGTGAGAATCACTAAAGAAAACCCACCGATTCCGGGTCATTATGATGGAGTAGGTATCGAAATAGTGAGGGAGAAT
AAATGA

Gene: folK (2-amino-4-hydroxy-6-hydroxymethyldihydropteridine pyrophosphokinase)

Contig: 04_NODE_11, position: 35338 to 35814, length: 477 nt, orientation: FORWARD

Perfect match to: (MW2-BA000033-[531739:532215], allele observed in CC1+CC239)

Sequence:

ATGATTCAAGCATACTTAGGATTAGGTAGTAATATTGGTGATAGAGAAAGCCAGTTAAACGATGCTATAAAGATTTTGAATGAATATGAT
GGTATTAACGTATCTAATATTTCTCCGATTTATGAAACAGCACCAGTTGGGTATACTGAGCAACCTAACTTTTTAAATTTGTGTGTTGAAAT
TCAAACAACACTCACAGTATTACAACTGTTGGAATGTTGTTTGAAGACAGAAGAATGTTTACACCGTATTAGAAAGGAACGATGGGGTCC
TAGAACTTTAGATGTGGATATTTTGTTGTATGGAGAAGAAATGATAGATTTACCAAAACTGTCGGTGCCACATCCGAGAATGAATGAACG
TGCATTTGTTTTAATCCCATTAAATGATATAGCAGCAAATGTCGTAGAACCACGTTCGAAATTGAAAGTGAAAGATTTAGTTTTTGTAGAT
GACAGTGTAAAGAGATATAAATAA

Gene: lysS_leader (lysyl-tRNA synthetase leader peptide)

Contig: 04_NODE_11, position: 36208 to 36300, length: 93 nt, orientation: FORWARD

Perfect match to: (N315-BA000018-[547426:547518], highly conserved allele)

Sequence:

ATGGTTAATGATAAAGTATTAGAAACATCGAAAGAGATGTATGTTGAGCAAAAATGTCTGATATTTTATAAAACTTTAAAGGAAAATGTT
TGA

Gene: lysS (lysyl-tRNA synthase)

Contig: 04_NODE_11, position: 36353 to 37840, length: 1488 nt, orientation: FORWARD

Perfect match to: (MRSA252-BX571856-[556960:558447], allele observed in CC30+CC8+CC25+CC30+CC239)

Sequence:

ATGTCAGAAGAAATGAATGACCAAATGTTGGTTCGACGTCAAAAATTACAAGAATTATATGATCTTGGTATAGACCCGTTTGGTTCTAAA
TTTGACCGTTCAGGTTTATCTAGTGATTTGAAAGAAGAGTGGGACCAGTATTCTAAAGAAGAATTGGTAGAAAAAGAAGCGGATAGTCA
TGTCGCTATAGCTGGACGATTAATGACTAAGCGTGGTAAAGGTAAAGCAGGATTTGCACACGTTCAGGACTTAGCTGGACAAATTCAAA
TTTACGTTCGTAAAGATCAAGTTGGCGATGACGAATTTGATTTATGGAAAAATGCTGATTTAGGCGATATCGTTGGTGTTGAAGGTGTAA
TGTTCAAAACAAATACTGGCGAATTATCGGTTAAAGCGAAGAAATTCACGCTACTAACTAAATCATTGCGACCATTACCGGATAAATTCC
ACGGTTTACAGGATATTGAACAGAGATATCGTCAAAGATATTTAGATTTAATTACGAACGAAGATAGCACTCGTACATTTATTAATCGTA
GTAAAATCATTCAAGAAATGCGTAATTATTTAAATAATAAAGGTTTCTTGGAAGTAGAAACACCTATGATGCACCAAATTGCTGGTGGAG
CAGCTGCTAGACCATTTGTAACACATCATAATGCATTAGATGCAACGTTATACATGCGTATTGCTATTGAGTTGCATTTAAAACGTTTAATT
GTCGGTGGACTTGAAAAAGTATATGAAATTGGTAGAGTATTCCGTAATGAAGGTGTATCAACTAGACATAACCCTGAATTCACAATGATT
GAATTATATGAAGCATATGCAGATTATCATGACATTATGGATTTAACAGAATCTATGGTGAGACATATTGCCAATGAAGTGTTAGGTTCT
GCAAAAGTACAATACAATGGGGAAACGATTGATTTAGAATCTGCTTGGACTCGTTTGCATATTGTTGATGCTGTAAAAGAAGCTACTGGT
GTAGATTTTTATGAAGTTAAAAGTGATGATGAAGCTAAAGCTTTAGCTAAAGAACATGGTATTGAAATTAAAGATACAATGAAATATGGT
CATATTTTAAATGAATTCTTTGAGCAAAAAGTTGAAGAAACACTTATTCAGCCAACGTTTATCTATGGTCATCCGACTGAAATTTCACCTTT
AGCGAAGAAAAATCCTGAAGATCCTAGATTTACTGATCGTTTCGAATTGTTCATTGTAGGTAGAGAGCATGCAAATGCATTTACTGAATT
AAATGATCCTATTGATCAAAAAGGTCGTTTTGAAGCGCAACTTGTTGAAAAAGCGCAAGGTAATGATGAAGCGCATGAAATGGATGAAG
ATTACATTGAAGCGTTAGAATATGGTATGCCTCCGACAGGTGGTCTTGGTATCGGTATTGACAGATTGGTTATGTTATTAACTGACTCTCC
ATCAATCAGAGACGTATTATTATTCCCTTATATGAGACAAAAATAA

Gene: pdxR (transcriptional regulator of pyridoxine metabolism)

Contig: 05_NODE_4, position: 801 to 2183, length: 1383 nt, orientation: REVERSE

Perfect match to: (MW2-BA000033-[546677:548059:r], highly conserved allele)

Sequence:

TTACTTTATAATTAATGATTTTATTAGAGCGTCTACATGCGGTTTTAAAGCATCATCGTCTATACCGCCAAAGCCTAATATAAATTTAGGGG
TTTTCTTATAGTCTTGATCATCATCAAAATTATAAACTTGTAATTTTAACTTTACTTTGTTTGCTCTATCAAGACACTCTTGTAATGTTAATCC
ATTTTTTACTGTAATTGTAAAATGCATACCCGTTTCAGCACCTTGAATATCAAGCTGCTCTTTGTAAGGTTTCAATCTTTTTAAAATATAGGT
TAGTTTTCTACGATAAATTCGTCTCATTTTATTTAAATGCCTTTCAAAACCACCGGAAGATATAAACGTTGCAATAAGGTTTTGCATATGAA
CAGGTACAGTGTTGCCTTCAATGTGATTTTGAGAATGATATTTTTTCATTATAGAATAGGGTAACACCATATATGCAACTCGACAGCTAGG
AAAAATAGACTTTGAAAATGTACTGATATAAATCACTTTTTCTCCTCTTGAATATAGACCTTGAATTGCTGGAATGGGTTTGCCGAAATAT
CTAAACTCGGAATCATAATCATCTTCTATAATAAATCGTTCTTCTTTTTCTTGAGCCCATTGTATTAATTGAGTTCGCTTTTTTAAGTCCATCA
CATATCCAGTTGGAAATTGATGGGAAGGCGTTATATATACTATATTTTTTTGTGATTTAATAACTTCATCTACGTTTATTCCATTATCTTCAA
CTTCAATTTGTTCATATTCAACTTGTTTTTTATCTAAAATATTTTTGATTGGTGGATAACTAGGTTTTTCGATAATAAATGTTGAAGTATAAA
GTAAATCGACTAATTGATTTACTAATTGTTCGGTAGATGAGCCAATTATAATTTGATTAGGATCACAAATTACACCACGATTAGTAAATAA
ATAAAATGCCAGTTGAAATCTTAAATGTAATTCTCCTTGAAAATGTCCTCTACGTAATTGATTTAAATGATTTGTATCATAAAGATCTTTGG
AATACTTTCTGAAAAGTTCTATAGGGAAATGTTTCGTATCTATTTCATCCAAATTAAAAGCATAATCATAAGCTTCATCACTCGCTTTTGGT
TTATATGAATCATCATCAAAAAGAGAGGGGATAGGTTGATTGTTTAAAATTGTTAAAGATTCAATTTCGGACACAAAATATCCAGAGCGA
GGTCTTGAATAAATGTAACCTTCGTCTAATAGAAGTTGATATGCATGCTCTACGGTTGTTTGGCTGATAGATAAATGTTTGCTTAATTGTC
TTTTAGAATAAAATTTATCGCCTTCTTTAAATTGACCTTCAATTATTTGTTTTTTTAATTTTTCATAAAGTTGATGGTATAAAGTGTTTTTCAA

Gene: pdxS (pyridoxal synthase subunit)

Contig: 05_NODE_4, position: 2287 to 3174, length: 888 nt, orientation: FORWARD

Perfect match to: (04-02981-CP001844-[555203:556090], allele observed in CC5)

Sequence:

ATGAGTAAAATTATTGGATCAGACAGAGTCAAAAGAGGTATGGCTGAAATGCAAAAAGGCGGCGTTATTATGGATGTCGTTAATGCTGA
GCAAGCAAGAATTGCAGAAGAAGCAGGCGCGGTAGCAGTTATGGCATTAGAACGAGTACCTTCTGATATTAGAGCTGCTGGTGGCGTT
GCACGTATGGCAAACCCTAAAATTGTAGAAGAAGTAATGAATGCTGTTTCTATTCCAGTCATGGCTAAAGCACGTATTGGTCATATCACT
GAAGCAAGAGTATTAGAGGCGATGGGTGTTGACTATATTGATGAATCAGAAGTGTTAACACCAGCAGATGAGGAATATCACTTAAGAAA
AGATCAATTTACAGTACCATTTGTATGTGGATGTCGTAATTTAGGTGAAGCTGCGCGTAGAATTGGTGAAGGTGCTGCTATGTTACGTAC
TAAAGGTGAACCAGGTACAGGTAATATTGTTGAAGCTGTAAGACATATGAGACAAGTTAATTCAGAAGTTAGTCGATTGACTGTAATGA
ATGATGATGAGATTATGACTTTTGCGAAAGATATCGGTGCGCCTTATGAAATTTTAAAACAAATTAAAGACAATGGTCGTTTACCGGTAG
TTAACTTTGCAGCTGGTGGCGTTGCGACTCCTCAAGATGCTGCTTTAATGATGGAATTAGGTGCCGACGGTGTATTCGTTGGATCAGGTA
TTTTTAAATCAGAAGATCCAGAAAAATTTGCTAAAGCAATTGTTCAAGCAACAACACATTACCAAGACTATGAGCTAATTGGAAGATTAG
CAAGTGAACTTGGCACTGCTATGAAAGGTTTAGATATCAATCAATTATCATTAGAAGAACGTATGCAAGAGCGTGGTTGGTAA

Gene: pdxT (pyridoxal synthase subunit)

Contig: 05_NODE_4, position: 3178 to 3738, length: 561 nt, orientation: FORWARD

Perfect match to: (C427_ST42-ACSQ01000009-[2712:3272], allele observed in CC34+CC5+CC8+CC30+CC772)

Sequence:

ATGAAAATAGGTGTATTAGCATTACAAGGTGCAGTACGTGAACATATTAGACATATTGAATTAAGTGGTCATGAAGGTATTGCAGTTAAA
AAAGTTGAACAATTAGAAGAAATCGAGGGCTTAATATTACCTGGTGGCGAGTCTACAACGTTACGTCGATTAATGAATTTATATGGATTT
AAAGAGGCTTTACAAAATTCAACTTTACCTATGTTTGGTACATGCGCAGGATTAATAGTTCTAGCGCAAGATATAGTTGGTGAAGAAGGA
TACCTTAACAAGTTGAATATTACTGTACAACGAAACTCATTCGGTAGACAAGTTGACAGCTTTGAAACAGAATTAGATATTAAAGGTATC
GCTACAGATATTGAAGGTGTCTTTATAAGAGCGCCACATATTGAAAAAGTAGGCCAAGGCGTAGATATCCTATGTAAGGTTAATGAGAA
AATTGTAGCCGTCCAGCAAGGTAAATATTTAGGCGTATCATTCCATCCTGAATTAACAGATGACTATAGAGTAACTGATTACTTTATTAAT
CATATTGTAAAAAAAGCATAG

Gene: nupC1 (nucleoside permease C, locus 1)

Contig: 05_NODE_4, position: 3944 to 5158, length: 1215 nt, orientation: REVERSE

Perfect match to: (MW2-BA000033-[549820:551034:r], highly conserved allele)

Sequence:

TTAAATAAAGAATCCAGCGATTGCAGCTGAAATGAAAGATACTAGTGTTGCACCGAATAATAGTTTCAAACCAAAGCGGGCAACTGTATC
TCCTTTTTTGTCATTAAGTGATTTAATCGCACCTGAAATAATACCAATAGAGCTAAAGTTAGCAAATGATACTAAGAATACAGATGTAACA
CCTTTTGCGTGTTCAGATAAATCACTAAGTTTACCAAGTGCTTGCATTGCTACAAATTCGTTAGATAATAGTTTTGTCGCCATAACTGAACC
GGCTTGAACTGCATCTTGCCATGGCACACCGACTAAGAATGCAAATGGTGCAAAGACAAAACCAATTAATGTTTGGAAATCCCAAGAAA
TAGCGCCACCTGAAACTGTACTAAAGATATTGCTTACAATTCCATTTAATAGAGCGATAATAGCAATGTATCCGATTAACATTGCGCCTAC
AATGACAGCTACTTTAAATCCATCTAAAATATATTCTCCTAGCATTTCGAAGAATGATTGTTGTCTTTCTTCAGTTTCTTCAACTAATAATTT
GTCATCTTCTTCATTAACTTTATAAGGGTTAATAATTGAAGCGATGATGAAACCACCAAATAAGTTTAAGACAACAGCCGTTACAACATAT
TTAGGTTCAATTAAGGTAAAGTATGCACCGATAATTGAAGCAGAAACAGTCGACATTGCTGAAGCTGTTAATGTGTATAAACGTTGCTTA
GGTATGTATGGTAATTGTTTTTTAATTGAAATAAATACTTCAGATTGTCCCAAAATTGCTGCAGCAACTGCATTGTATGATTCTAAACGTCC
CATACCATTAATTTTAGAAATTAAGAATCCTAAAACATTAATGATTAAAGGTAAAATCTTTGTGTATTGAAGGATACCGATAATCGCTGAA
ATAAATACGATAGGTAATAATACACTGAAGAAGAATGGTGGTTGCTTAGGATCGATATATTGAATACCACCGAATACAAAGTTAACACCA
TCTGCTGCTTTTAATAATAAGTAGTTAAAACCGTTTGAAATACCACCAATAACCTTGATTCCCATTGTAGTTTTAAGCAAGATAAATGCAAA
GATAAGCTGAATTGCAAGTAAAATTCCTACATATTTCCAGCGAATATTTTTCCTGTCTGAGCTAAATAGAAACGCAAGTGCTAAAAAGAA
GATAATTCCGATAATCCCAATTAGAATATGCAT

Gene: ctsR (transcriptional regulator)

Contig: 05_NODE_4, position: 5316 to 5777, length: 462 nt, orientation: FORWARD

Perfect match to: (N315-BA000018-[560629:561090], highly conserved allele)

Sequence:

ATGCACAATATGTCTGACATCATAGAACAATACATCAAACGTTTATTTGAAGAGTCGAATGAAGATGTCGTTGAAATTCAGAGAGCGAAT
ATCGCACAGCGTTTTGATTGTGTACCATCACAATTAAATTATGTAATCAAAACACGATTCACTAATGAACATGGTTATGAAATCGAAAGTA
AACGTGGTGGTGGTGGTTACATCCGAATCACTAAAATTGAAAATAAAGATGCAACAGGTTATATTAATCATTTGCTTCAGCTGATTGGAC
CTTCTATTTCTCAACAACAAGCTTATTATATTATTGATGGGCTTTTAGATAAAATGTTAATAAATGAACGTGAAGCTAAAATGATTCAAGC
AGTTATTGATAGAGAAACGCTATCAATGGATATGGTTTCTAGAGATATTATTAGAGCAAATATTTTAAAACGTTTGTTACCAGTTATAAAT
TATTACTAA

Gene: mcsA (modulator of CtsR-dependent repression)

Contig: 05_NODE_4, position: 5796 to 6362, length: 567 nt, orientation: FORWARD

Perfect match to: (MW2-BA000033-[551672:552238], highly conserved allele)

Sequence:

GTGCTTTGTGAAAATTGTCAACTTAATGAAGCGGAATTAAAAGTTAAAGTTACAAGTAAAAATAAAACAGAAGAAAAAATGGTGTGTCA
AACTTGTGCTGAGGGGCACCATCCGTGGAATCAAGCTAATGAACAACCTGAATATCAAGAACATCAAGATAATTTCGAAGAAGCATTTGT
TGTTAAGCAAATTTTACAACATTTAGCTACGAAACATGGCATTAATTTTCAAGAAGTAGCGTTTAAAGAAGAAAAACGTTGCCCATCATGT
CATATGACTTTGAAAGATATTGCACATGTTGGTAAATTTGGGTGTGCTAATTGTTATGCAACATTTAAAGATGACATCATTGATATCGTCC
GCAGAGTTCAAGGTGGACAATTTGAGCACGTTGGAAAGACACCACATTCTTCACATAAAAAGATAGCTTTAAAGCGAAAAATCGAAGAA
AAGAATGAATATTTGAAAAAACTTATTGAAATCCAAGATTTTGAGGAAGCAGCCATTGTTAGAGATGAAATTAAAGCACTAAAAGCTGA
GAGTGAGGTGCAACATGATGACGCATAA

Gene: mcsB (protein arginine kinase)

Contig: 05_NODE_4, position: 6352 to 7359, length: 1008 nt, orientation: FORWARD

Perfect match to: (11819-97-CP003194-[559737:560744], highly conserved allele)

Sequence:

ATGACGCATAATATTCATGATAATATCAGCCAATGGATGAAAAGTAATGAAGAAACACCAATTGTTATGTCTTCTAGAATTCGGTTAGCG
CGTAATTTAGAAAATCATGTGCATCCACTAATGTATGCTACTGAAAATGATGGATTTAGAGTTATAAATGAGGTACAAGATGCCTTGCCA
AATTTTGAATTAATGCGTCTTGATCAAATGGATCAACAAAGTAAAATGAAAATGGTTGCAAAGCATTTGATTAGTCCTGAACTAATAAAA
CAACCAGCAGCCGCAGTATTAGTGAATGATGATGAATCTTTAAGTGTCATGATAAATGAAGAGGACCATATTCGTATTCAAGCTATGGGA

ACTGACACGACATTACAGGCTTTATATAATCAAGCTTCATCAATTGATGATGAATTAGATCGAAGCCTTGATATAAGTTATGATGAACAAC
TTGGTTATTTAACTACATGTCCTACCAATATAGGTACTGGTATGAGAGCAAGCGTGATGCTACATTTACCAGGTCTATCTATTATGAAAAG
AATGACACGGATTGCTCAAACCATTAATCGTTTTGGATATACAATCAGAGGTATTTACGGTGAAGGTTCGCAAGTTTATGGACATACTTAT
CAAGTATCCAACCAACTTACACTTGGTAAATCTGAGTTAGAAATCATAGAAACATTAACAGAAGTTGTTAATCAAATCATTCATGAAGAA
AAACAAATACGACAAAAGTTAGACACTTATAATCAATTAGAAACACAAGACCGTGTTTTTCGCTCGCTAGGTATTTTACAAAACTGTAGA
ATGATAACTATGGAAGAGGCTTCTTATAGATTAAGCGAAGTTAAACTTGGTATAGATTTAAATTACATTGAATTACAAAACTTTAAATTTA
ATGAATTGATGGTAGCTATACAGTCACCATTTTTATTAGATGAAGAAGATGACAAATCTGTAAAAGAAAAACGAGCAGATATACTAAGA
GAACATATAAAGTAG

Gene: clpC (chaperone-like protein C)

Contig: 05_NODE_4, position: 7373 to 9829, length: 2457 nt, orientation: FORWARD

Perfect match to: (Strain_21334-AGTW01000019-[6906:9362], allele observed in CC9+CC1+CC12+CC22+CC188)

Sequence:

ATGTTATTTGGTAGATTAACTGAGCGTGCACAGCGCGTATTAGCACATGCACAAGAAGAAGCAATTCGTTTAAATCATTCAAATATAGGA
ACAGAACACCTATTATTGGGATTAATGAAAGAACCTGAAGGAATTGCTGCAAAAGTATTAGAAAGTTTTAATATCACTGAAGATAAAGTA
ATTGAAGAAGTTGAAAAAATTAATCGGACATGGTCAAGATCATGTTGGTACATTGCATTATACACCTAGAGCTAAAAAAGTTATTGAATTA
TCGATGGATGAAGCTAGAAAATTACATCACAATTTTGTTGGAACGGAACATATTTTATTAGGCTTGATTCGTGAAAATGAAGGTGTTGCA
GCAAGAGTTTTTGCAAATCTAGATTTAAATATTACTAAAGCACGTGCACAAGTTGTGAAAGCTTTAGGAAACCCTGAAATGAGTAATAAA
AATGCACAAGCTAGTAAGTCAAATAATACTCCAACTTTAGATAGTTTAGCTCGTGACTTAACAGTCATTGCCAAAGACGGTACATTAGATC
CTGTTATAGGACGTGATAAAGAAATTACACGTGTAATTGAAGTATTAAGTAGACGTACGAAAAACAATCCTGTACTTATTGGAGAGCCAG
GTGTTGGTAAAACTGCTATTGCTGAAGGTTTAGCGCAAGCCATAGTGAATAATGAGGTACCAGAGACATTAAAAGATAAGCGTGTTATG
TCTTTAGATATGGGAACAGTAGTTGCAGGTACTAAATATCGTGGTGAATTTGAAGAGCGTCTGAAAAAGGTTATGGAAGAAATCCAACA
AGCAGGTAATGTCATCCTATTTATTGATGAGTTGCATACTTTAGTTGGTGCTGGTGGTGCTGAAGGTGCTATCGATGCTTCGAATATTTTG
AAACCGGCATTAGCACGTGGTGAATTACAATGTATTGGTGCTACTACATTAGATGAATATCGCAAAAATATTGAAAAAGACGCGGCTTTA
GAACGTCGTTTCCAACCTGTACAAGTTGATGAACCTTCAGTAGTAGATACAGTTGCTATTTTAAAAGGATTAAGAGATCGTTACGAAGCA
CACCATCGTATTAATATTTCAGACGAAGCTATTGAAGCAGCTGTTAAATTAAGTAACAGATACGTTTCAGATCGTTTCTTACCAGATAAAG
CAATTGATTTAATTGATGAAGCAAGTTCTAAAGTAAGACTTAAGAGTCATACGACACCTAATAATTTAAAAGAAATTGAACAAGAAATTG
AAAAAGTTAAAAATGAAAAAGATGCTGCAGTACATGCTCAAGAGTTTGAAAATGCTGCTAACCTGCGTGATAAACAAACAAAACTTGAA
AAGCAATATGAAGAAGCTAAAAATGAATGGAAGAATGCACAAAATGGCATGTCAACTTCATTGTCAGAAGAAGATATTGCTGAAGTTAT
TGCAGGATGGACAGGTATCCCATTAACTAAAATCAATGAAACAGAATCTGAAAAACTTCTTAGTCTAGAAGATACATTACATGAGAGAGT
TATTGGGCAAAAAGATGCTGTTAATTCAATCAGTAAAGCGGTTAGACGTGCCCGTGCAGGGTTAAAAGATCCTAAACGACCAATTGGTA
GCTTTATCTTCCTTGGACCAACTGGTGTTGGTAAAACTGAATTAGCTAGAGCTTTAGCTGAATCAATGTTTGGCGATGATGATGCGATGAT
CCGTGTAGACATGAGTGAATTTATGGAAAAACACGCAGTGAGCCGATTAGTTGGTGCTCCTCCAGGATATGTTGGTCATGATGATGGTG
GACAATTAACTGAAAAAGTTAGACGTAAACCATATTCTGTAATTTTATTTGATGAAATTGAAAAAGCTCATCCAGATGTATTTAATATTCT
ATTACAAGTTTTAGATGATGGACATTTGACAGATACAAAAGGACGTACAGTTGATTTCAGAAATACAATTATCATAATGACATCAAACGT
TGGGGCACAAGAATTACAAGATCAACGATTTGCTGGATTCGGTGGTTCAAGTGATGGACAAGATTATGAAACAATTCGAAAAACGATGT
TAAAAGAATTAAAAAATTCATTCCGTCCAGAATTTTTAAACCGTGTAGATGATATCATTGTATTCCATAAACTAACAAAAGAAGAATTAAA
AGAAATTGTAACAATGATGGTTAATAAATTAACAAATCGATTATCTGAACAAAACATAAATATTATTGTTACTGATAAAGCGAAAGACAA
AATCGCAGAAGAAGGATATGATCCAGAATATGGTGCAAGACCATTAATTAGAGCGATACAAAAAACTATCGAAGATAATTTAAGTGAAT
TAATATTAGATGGTAATCAAATTGAAGGTAAGAAAGTTACAGTAGATCATGATGGTAAAGAGTTTAAATATGACATTGCTGAACAAACTT
CAGAAACTAAAACACCATCGCAAGCATAA

Gene: radA (DNA repair protein A)

Contig: 05_NODE_4, position: 10313 to 11677, length: 1365 nt, orientation: FORWARD

Perfect match to: (N315-BA000018-[565627:566991], highly conserved allele)

Sequence:

TTGGCCAAGAAAAAAAGTGATTTTTGAATGTATGGCTTGTGGTTATCAATCTCCTAAATGGATGGGGAAATGTCCTAATTGTGGCGCTTGG
AATCAAATGGAGGAAATTGTTGAAAAAGCAGCCAATCCTAAACATGGAGTTAAAACCAAGGAATTAGCAGGTAAAGTACAAAAATTAAA
TAGTATTAAACATGAAACAACGCCGAGAGTGTTAACAGATTCAGCAGAATTCAACCGTGTATTAGGTGGAGGTATTGTGAGCGGATCGT
TAGTACTTATTGGTGGGGATCCAGGTATTGGTAAGTCAACGTTACTTTTACAAATTTGTGCATCGTTATCTCAAAAGAAAAAAGTACTATA
TATTACTGGAGAAGAATCGCTTAGTCAGACTAAATTACGTGCAGAGCGATTAGATGAAGATTCAAGTGAATTGCAAGTATTAGCTGAAA
CAGATCTTGAAGTTATTTATCAAACAGTAAAAGAAGAACAACCTGATTTATTAGTAGTGGATTCGATTCAAACAATATATCATCCTGAAAT

CAGCTCTGCGCCAGGTTCTGTTTCACAAGTTCGTGAAAGTACACAAAGTTTAATGAATATTGCTAAACAAATGAACATTGCAACTTTTATA
GTGGGTCATGTAACGAAAGAAGGTCAAATTGCTGGCCCAAGATTGCTAGAACACATGGTTGATACTGTGCTTTATTTTGAAGGCGATGA
ACACCACGCATATCGAATTTTGCGAGCTGTTAAAAACCGTTTTGGTTCAACGAATGAAATGGGAATCTTCGAAATGAAGCAAAGTGGATT
AAAAGGTGTAAATAATCCATCTGAAATGTTTTTAGAAGAACGTTCAACAAATGTTCCAGGTTCAACAATTGTTGCAACCATGGAGGGAAC
CAGACCACTTTTAATAGAAGTTCAAGCGCTGGTAACTCCAACGACTTTTAACAATCCGAGACGAATGGCAACAGGGATTGATCATAATCG
ATTAAGTTTGTTGATGGCTGTTTTGGAAAAGAAAGAAAATTATCTATTACAACAACAAGATGCTTATATCAAAGTAGCTGGCGGTGTAAA
GTTAACGGAGCCAGCAGTTGATTTAAGTGTAATTGTAGCAACTGCATCTAGCTTTAAAGATAAAGCTGTCGACGGATTAGATTGCTATAT
TGGAGAAGTTGGTTTAACGGGTGAGGTACGTCGTGTATCTCGGATAGAACAACGCGTGCAAGAGGCTGCAAAACTAGGTTTCAAACGT
GTAATTATTCCTAAAAATAATATAGGCGGATGGACATATCCTGAAGGTATACAAGTAATAGGTGTAACTACTGTACATGAAGCATTGTCA
TTTGCTCTTCATTCATAA

Gene: pilT (PIN/TRAM domain protein)

Contig: 05_NODE_4, position: 11702 to 12775, length: 1074 nt, orientation: FORWARD

Sequence:

GTGAATATCGTTAAACTAATGGTTATTATTATTTACTTAATTATTGGGAGCGCATTAGGAATAATTATTATTCCTGAAATTGCAAATGATCT
TGGATTACAAAACTCCAGCTTTTTAAAAAATCACTATGTAGATGGCATTATCGGTAGTATTTTTATGTTCTTAATTTTTGGTGTATTTATTAG
ACGAGTTACTAACGCTATAAAAGGTTTAGAACATTTTATTATGCGTAGAAGTGCTGTTGAAATACTATTCGCAACAATAGGTTTAATAATC
GGATTACTTATTTCTGTTATGGTGTCGTTTATATTAGAATCAATTGGTAACTCTATTTTTAATCATTTCATTCCTGTCATAATTACGATATTA
CTATGTTATTTCGGTTTCCAATTTGGCCTTAAAAAACGAGATGAAATGTTAATGTTTTTACCTGAGAATATAGCGCGTTCCATGTCACAAC
ATACTAAAAGTGCTACGCCAAAAATTATCGACACAAGCGCAATTATTGATGGTCGTATTTTAGAAGTCATTCGTTGCGGTTTTATCGATGG
CAATATTTTAATTCCACAAGGTGTTATTAATGAATTACAAATTGTTGCAGATTCAAATGACAGTGTTAAACGTGAAAAGGGTAAAAGAGG
CTTAGATATTTTAAATGAATTGTATGATTTAGACTATCCTACAAAGGTTATACATCCAACTAAAACACATAGTGATATTGATACGATGTTAT
TAAAACTAGCAAAACAATATCATGCAAGTATTATAACGACAGATTTCAACCTAAATAAAGTTTGTCATGTACATGGTATTAAAGCATTAAA
TGTTAATGATTTATCAGAAGCAATCAAACCTAACGTACATCAAGGTGATCAACTGCATATTTTACTGACAAAAATGGGTAAGGAGCCTGG
TCAGGCAGTAGGATATCTAGATGATGGTACGATGGTGGTTGTTGATAATGCTAAAAATCTTATTGGCAGTCATGTCAATTTAGAAGTAGT
CAGCTTATTGCAAACATCTTCAGGAAGAATTGTTTTTGCTAAAAAAATCGAAGATACAGTATCATTATAA

Gene: gltX (glutamyl-tRNA synthetase)

Contig: 05_NODE_4, position: 13332 to 14786, length: 1455 nt, orientation: FORWARD

Sequence:

ATGAGCGATCGTATAAGAGTAAGATATGCACCAAGTCCAACTGGTTATCTTCATATTGGTAATGCAAGAACAGCATTATTCAATTACTTGT
ATGCTAAACATTACAACGGAGATTTTGTGATTCGAATTGAAGATACTGATAAAAAACGTAATTTAGAAGATGGAGAAACATCACAATTTG
ATAATCTTAAATGGTTAGGATTAGATTGGGATGAGTCTGTAGATAAAGACAATGGCTACGGACCATATCGTCAATCTGAACGTCAACATA
TCTACCAACCATTAATAGATCAGTTACTAGCAGAAGATAAAGCATATAAATGCTATATGACAGAAGAAGAATTAGAAGCTGAACGTGAA
GCACAAATCGCTCGTGGTGAAATGCCTCGCTATGGTGGACAACATGCGCATTTGACTGAAGAACAACGTCAACAATTTGAAGCAGAAGG
ACGCCAACCATCAATTCGTTTCCGAGTACCTCAAAACCAAACGTATTCATTTGATGATATGGTAAAAGGAAATATTTCATTTGATTCAAAT
GGTATTGGTGACTGGGTTATCGTAAAAAAAGATGGCATTCCAACGTACAATTTTGCAGTAGCTATAGATGATCATTACATGCAAATTTCA
GATGTAATTCGTGGTGATGATCATATTTCAAACACGCCTAAACAAATTATGATTTATGAAGCATTTGGCTGGGAGCCACCTCGTTTTGGTC
ATATGTCATTAATTGTTAATGAAGAACGTAAAAAGTTAAGTAAACGTGATGGGCAAATTTTACAATTTATTGAGCAATATCGTGACTTAG
GTTATTTACCTGAAGCGTTATTTAATTTTATTGCGTTATTAGGTTGGTCTCCTGAAGGTGAAGAAGAAATCTTTTCTAAAGAAGAATTTATC
AAAATCTTTGATGAAAAGCGTTTGTCAAAATCACCAGCATTTTTCGATAAGCAAAAATTAGCATGGGTTAATAACCAATATATGAAACAA
AAAGATACTGAAACAGTATTCCAATTAGCATTACCTCATTTAATTAAAGCAAATTTGATTCCTGAGATGCCGTCAGAAGAGGATTTATCTT
GGGGACGCAAATTAATTGCGCTTTATCAAAAAGAAATGAGTTATGCCGGTGAAATTGTACCTTTATCAGAAATGTTCTTTAAAGAAATGC
CAGCTCTTGGTGAAGAAGAACAACAAGTGATTAATGGAGAGCAAGTACCAGAGTTAATGACGCACTTATTCAGTAAATTAGAAGCACTT
GAACCATTTGAAGCGGCTGAAATTAAAAAGACAATTAAAGAAGTTCAAAAAGAAACAGGAATAAAAGGCAAGCAATTATTTATGCCTAT
TCGTGTTGCTGTAACAGGCCAAATGCATGGTCCTGAATTACCAAATACAATTGAAGTACTTGGTAAAGAAAAAGTGCTAAACCGTTTAAA
ACAATATAAGTAA

Gene: tbox03 (T-box leader element)

Contig: 05_NODE_4, position: 14891 to 15116, length: 226 nt

Sequence:

ATAAAACGAAGGATTAGTAATTAAATTTATACGATGCAGAGAGTGTACGGTTGCTGTGAGTACAACGTAGAAATTAATGAATGCACCTTC
GTAAAATGAATTAAATATATAATGAGAGTGATGAGCATTAAGTTGACTTAGTTTCCTTGATAATTTGGAAGCGCCCGCAATATTATTAATG
TTATTCGCTAAATTCAGAGTGGAACCGTGCGGAAGCGCCTCTAAC

Gene: cysE (serine acetyltransferase)

Contig: 05_NODE_4, position: 15215 to 15856, length: 642 nt, orientation: FORWARD

Sequence:

TTGTTAAAAAGAATGAGAGACGATATAAAAATGGTATTTGAGCAGGATCCAGCGGCACGTTCAACATTAGAAGTCATTACAACGTATGC
AGGTTTACATGCAGTTTGGAGTCATTTGATTGCACATAAGTTATACAACCAAAAAAAATATGTTGCAGCACGCGCGATATCTCAAATTTCA
AGATTTTTCACAGGTATAGAAATCCATCCAGGTGCTAAAATTGGAAAGCGTCTATTTATAGATCATGGTATGGGCGTTGTAATAGGAGAA
ACATGTACAATTGGTGATAATGTGACAATCTATCAAGGCGTGACACTTGGTGGGACAGGGAAAGAAAGAGGGAAAAGACACCCAGATA
TAGGAGACAATGTTTTAATAGCAGCCGGTGCGAAAGTTTTAGGAAATATTAAAATAAATTCAAATGTAAATATTGGTACAAATTCAGTTG
TTTTACAATCAGTTCCAAGTTATTCAACGGTTGTTGGTATACCAGGACATATTGTTAAGCAAGATGGTGTTCGAGTTGGAAAAACATTTGA
TCATCGCCATCTACCTGATCCAATTTATGAACAAATTAAGCATTTAGAACGACAACTTGAAAAGACTAGGAATGGAGAGATTCAAGATGA
TTACATTATATAA

Gene: cysS (cysteinyl-tRNA synthase)

Contig: 05_NODE_4, position: 15840 to 17240, length: 1401 nt, orientation: FORWARD

Perfect match to: (Strain_18341-HE579069-[547242:548642], allele observed in CC5+CC239)

Sequence:

ATGATTACATTATATAATACGCTTACACGTCAAAAAGAAGTGTTCAAGCCTATAGAACCAGGGAAAGTAAAAATGTATGTATGTGGTCCT
ACTGTATATAACTACATTCATATTGGTAACGCAAGACCAGCAATTAATTATGACGTAGTGAGACGTTACTTTGAATACCAAGGATATAATG
TAGAATATGTATCAAATTTTACAGACGTAGATGATAAATTAATTAAACGTTCTCAAGAATTAAATCAGTCTGTTCCCGAAATTGCAGAAAA
ATATATCGCTGCTTTTCATGAAGATGTTGGTGCGTTAAATGTTAGAAAAGCGACTTCAAATCCAAGGGTAATGGACCATATGGATGACAT
TATTCAATTTATTAAAGATTTGGTGGATCGAGGTTATGCATATGAAAGTGGTGGCGATGTTTACTTTAGAACACGTAAATTTGAAGGTTAT
GGTAAATTAAGTCATCAATCCATAGATGACTTAAAAGTGGGTGCTCGTATAGATGCAGGAGAGCATAAAGAAGATGCACTTGATTTTAC
ATTGTGGAAAAAAGCGAAGCCTGGCGAGATTAGTTGGGATAGCCCATTTGGTGAAGGTAGACCAGGATGGCATATAGAATGTTCTGTA
ATGGCATTTCATGAGCTAGGACCTACAATTGATATACATGCGGGTGGTTCAGATTTACAATTTCCACATCATGAAAATGAAATAGCACAA
TCAGAAGCACATAATCATGCGCCATTTGCTAATTATTGGATGCATAATGGTTTCATTAATATTGATAATGAAAAAATGAGTAAATCACTAG
GCAACTTTATTTTAGTTCACGATATTATTAAAGAAGTTGATCCAGATGTACTAAGATTCTTTATGATTAGCGTACATTATAGAAGCCCAATT
AACTATAATCTAGAATTGGTAGAATCAGCACGTAGTGGACTAGAGCGTATTCGCAATAGTTATCAATTAATTGAAGAGCGCGCACAAATT
GCTACTAATATTGAAAATCAACAGACATATATTGATCAAATTGATGCGATTTTAAATCGTTTTGAAACAGTTATGAATGATGATTTTAATA
CAGCTAATGCAATTACAGCTTGGTATGATTTAGCAAAACTTGCGAATAAATATGTACTAGAGAACACAACATCAACAGAAGTAATTGATA
AATTTAAAGCAGTTTATCAAATTTTCAGCGATGTTTTAGGTGTACCGTTAAAATCTAAAAATGCAGATGAATTATTGGATGAAGATGTTGA
AAAATTAATCGAAGAGCGTAATGAAGCAAGGAAAAACAAAGATTTTGCACGAGCAGATGAAATTCGAGACATGCTGAAATCACAAAAC
ATTATATTAGAAGACACACCTCAAGGGGTTAGATTTAAACGTGGATAA

Gene: mrnC (minimal RNase III)

Contig: 05_NODE_4, position: 17233 to 17637, length: 405 nt, orientation: FORWARD

Perfect match to: (N315-BA000018-[572547:572951], highly conserved allele)

Sequence:

GTGGATAATCAACAAGATAATCACATTAAATTATTGAATCCATTGACCTTAGCATATATGGGAGACGCAGTCTTAGATCAATATGTACGTA
CCTATATCGTTTTAAAGCTTAAAAGTAAGCCTAATAAACTACATCAAATGTCTAAAAAATATGTATCTGCCAAAAGTCAGGCGCAAACGTT
AGAATATTTAATGGAGCAAGAATGGTTTACAGACGAAGAAATGGATATTTTGAAGCGAGGGCGTAACGCGAAAAGTCATACTAAAGCT
AAAAACACTGATGTTCAAACATATCGTAAAAGTTCAGCGATAGAAGCAGTGATAGGTTTTCTTTATTTAGAAAAAAGAGAAGAACGATTA
GAGGCATTATTAAATAAAATAATAACAATAGTAAACGAAGGTAG

Gene: yacO (tRNA/rRNA methyltransferase)

Contig: 05_NODE_4, position: 17645 to 18391, length: 747 nt, orientation: FORWARD

Perfect match to: (DAR4145-CP010526-[569661:570407], highly conserved allele)

Sequence:

GTGGAAGATACGGTTATTGTTGGTAGGCATGCTGTTAGAGAAGCGATTATTACTGGGCATCCGATAAATAAGATATTGATTCAAGAAGG
TATTAAAAAGCAACAAATTAATGAAATTTTAAAAAATGCAAAAGATCAAAAAATCATTGTTCAAACTGTACCAAAATCTAAATTAGATTTT
TTAGCAAATGCACCACATCAGGGTGTTGCAGCGCTTATTGCACCATATGAATATGCTGACTTCGATCAATTTTTAAAACAGCAAAAAGAA
AAAGAAGGTTTATCGACAGTACTTATATTAGACGGCTTAGAAGACCCGCATAACTTGGGATCAATTTTAAGAACAGCCGATGCAACGGG
AGTTGATGGTGTTATTATTCCTAAACGTCGTTCAGTTACACTAACGCAAACAGTTGCAAAAGCCTCAACAGGTGCAATTGAACATGTACCA
GTTATTCGAGTGACAAATTTAGCTAAAACTATCGATGAACTAAAAGATAATGGCTTTTGGGTAGCTGGCACTGAAGCTAATAATGCAACA
GATTATAGAAATCTAGAAGCGGACATGTCATTGGCTATTGTAATTGGTAGCGAAGGACAGGGTATGAGTCGCCTAGTAAGTGATAAATG
CGATTTTTATATTAAGATTCCAATGGTTGGACATGTAAACAGTTTGAATGCTTCGGTTGCAGCAAGTTTAATGATGTACGAAGTATTTCGA
AAAAGACATGATGTTGGAGAAATATAA

Gene: yacP (putative protein)

Contig: 05_NODE_4, position: 18391 to 18915, length: 525 nt, orientation: FORWARD

Perfect match to: (N315-BA000018-[573705:574229], highly conserved allele)

Sequence:

ATGAAAGAACGTTACTTAATCATTGATGGATACAATATGATAGGACAATCACCAACGCTAAGCGCCATTGCAAAAGAGAATTTAGAAGA
AGCTAGAATGCAATTAATAGATGCAATTGCAAATTATAATGCAGTTATTTCAGATGAAATTATTTGTGTTTTCGATGCTTATGACCAATCG
GGTGTTGAAAGAGAATACATGTATCATGGCGTTAAAACGATTTTTACCAAGGAAAAAGAAACAGCTGATAGTTTCATAGAACGTTATGTT
TATGAACTTTATGACAAGCATACTAAGCATATTACAGTTGTAACAAGTGATATGAGTGAGCAACATGCTATCTTTGGATCAGGTGCATAT
AGAATATCATCTCGCGAAATGTGGAGAGATTTAAAAGAAAATGAAATTGATGTGAGTAAATCATTAGATGATATAAGTGAAAACAAGCC
AAGAACTCGAATTCCGTTATCTTCTGAAATCCTTGCAGAATTTGAAAAAAATACGAAGAGGACATCATAAGAAATGA

Gene: sigH (alternative sigma subunit of RNA polymerase)

Contig: 05_NODE_4, position: 18996 to 19565, length: 570 nt, orientation: FORWARD

Perfect match to: (08-02119-CP015645-[1669578:1670147:r], allele observed in ST582+CC8+CC4803)

Sequence:

TTGAAATACGATTTGACAACTCAAGACAGTACAATCAAACGTAACAATGCAATAAATGATAAAGACTTCGAAAAGTTAGTAATGGATCTA
CAACCATTAATTATTCGACGCATCAAAACATTTGGATTTAATCATTATGATTTAGAAGACTTATATCAAGAAATACTTATACGGATGTATA
GGTCGGTCCAAACATTTGATTTTAGTGGAGAGCAGCCTTTCACAAATTATGTTCAATGTTTAATTACGTCTGTAAAGTATGATTATTTGAG
AAAATATTTAGCTACAAATAAAAGAATGGATAATTTGATTAATGAATATAGAGTTACGTATCCATGTGCAATAAAGCGTTATGATGTTGA
AAACAATTATTTGAATAAATTAGCAATTAAAGAGTTGATTCGTCAGTTTAAGTATTTGAGTGTATTTGAAAAAGATGTCATGTATTTAATG
TGTGAACAATATAAGCCGAGAGAAATTGCTCAACTGATGCATGTAAAAGAGAAAGTGATTTATAATGCCATACAACGATGTAAAAATAA
AATAAAACGTTATTTCAAAATGATTTGA

Gene: rpmG3 (50S ribosomal protein L33, locus 3)

Contig: 05_NODE_4, position: 19680 to 19823, length: 144 nt, orientation: FORWARD

Perfect match to: (N315-BA000018-[574994:575137], highly conserved allele)

Sequence:

GTGAGAAAAAATACCTTTAAATTGTGAAGCTTGTGGCAATAGAAATTATAATGTTCCTAAGCAAGAAGGCTCGGCAACAAGATTAACCTTA
AAGAAATATTGTCCAAAATGTAACGCGCACACAATTCATAAAGAATCGAAATAA

Gene: secE (preprotein translocase subunit E)

Contig: 05_NODE_4, position: 19879 to 20061, length: 183 nt, orientation: FORWARD

Perfect match to: (RF122-AJ938182-[543743:543925], highly conserved allele)

Sequence:

ATGGCTAAAAAAGAAAGTTTCTTTAAAGGCGTTAAGTCTGAAATGGAAAAAACAAGTTGGCCGACGAAAGAAGAGCTATTTAAATATAC
TGTAATTGTAGTTTCTACTGTTATATTCTTCTTAGTCTTTTTCTATGCCTTAGATTTAGGAATTACAGCATTGAAAAATTTATTATTTGGTTA
G

Gene: nusG (transcription antitermination protein)

Contig: 05_NODE_4, position: 20074 to 20622, length: 549 nt, orientation: FORWARD

Sequence:

ATGTCTGAAGAAGTTGGCGCAAAGCGTTGGTATGCAGTGCATACATATTCTGGGTATGAAAATAAAGTTAAAAAGAATTTAGAAAAAAG
AGTAGAATCTATGAATATGACTGAACAAATCTTTAGAGTAGTCATACCGGAAGAAGAAGAAACTCAAGTAAAAGATGGCAAAGCTAAAA
CGACTGTTAAAAAAACATTCCCTGGATATGTTTTAGTGGAATTAATCATGACAGATGAATCATGGTATGTGGTAAGAAATACACCAGGTG
TTACTGGTTTTGTAGGTTCTGCAGGTGCAGGGTCTAAGCCAAATCCATTGTTACCAGAAGAAGTTCGCTTCATCTTAAAACAAATGGGTCT
TAAAGAAAAGACTATCGATGTTGAACTCGAAGTTGGCGAGCAAGTTCGTATTAAATCAGGTCCATTTGCGAATCAAGTTGGTGAAGTTCA
AGAAATTGAAACAGATAAGTTTAAGCTAACAGTATTAGTAGATATGTTTGGCCGAGAAACACCAGTAGAAGTTGAATTCGATCAAATAG
AAAAGCTTTAA

Gene: rplK (50S ribosomal protein L11)

Contig: 05_NODE_4, position: 20803 to 21225, length: 423 nt, orientation: FORWARD

Perfect match to: (N315-BA000018-[576117:576539], highly conserved allele)

Sequence:

GTGGCTAAAAAAGTAGATAAAGTTGTTAAATTACAAATTCCTGCAGGTAAAGCGAATCCAGCACCACCAGTTGGTCCAGCATTAGGTCAA
GCAGGTGTGAACATCATGGGATTCTGTAAAGAGTTCAATGCACGTACTCAAGATCAAGCAGGTTTAATTATTCCGGTAGAAATCAGTGTT
TATGAAGATCGTTCATTTACATTTATTACAAAAACTCCACCGGCTCCAGTATTACTTAAAAAAGCAGCTGGTATTGAAAAAGGTTCAGGCG
AACCAAACAAAACTAAAGTTGCTACAGTAACTAAAGATCAAGTACGCGAAATTGCTAACAGCAAAATGCAAGACTTAAACGCTGCTGAC
GAAGAAGCAGCTATGCGTATTATCGAAGGTACTGCACGTAGTATGGGTATCGTTGTAGAATAA

Gene: rplA (50S ribosomal protein L1)

Contig: 05_NODE_4, position: 21433 to 22125, length: 693 nt, orientation: FORWARD

Perfect match to: (RF122-AJ938182-[545297:545989], highly conserved allele)

Sequence:

ATGGCTAAAAAAGGTAAAAAGTATCAAGAAGCAGCTAGTAAAGTTGACCGTACTCAGCACTACAGTGTTGAAGAAGCAATTAAATTAGC
TAAAGAAACAAGCATTGCTAACTTTGACGCTTCTGTTGAAGTTGCATTCCGTTTAGGAATTGATACACGTAAAAATGACCAACAAATCCGT
GGTGCAGTTGTATTACCAAACGGAACTGGTAAATCACAAAGTGTATTAGTATTCGCTAAAGGTGACAAAATTGCTGAAGCTGAAGCAGC
AGGTGCTGACTATGTAGGTGAAGCAGAATACGTTCAAAAAATCCAACAAGGTTGGTTCGACTTCGATGTAGTAGTTGCTACACCAGACAT
GATGGGTGAAGTTGGTAAATTAGGTCGTGTATTAGGACCAAAAGGTTTAATGCCAAACCCTAAAACTGGAACTGTAACAATGGATGTTA
AAAAAGCTGTTGAAGAAATCAAAGCTGGTAAAGTAGAATATCGTGCTGAAAAAGCTGGTATCGTACATGCATCAATTGGTAAAGTTTCA
TTTACTGATGAACAATTAATTGAAAACTTCAATACTTTACAAGATGTATTAGCTAAAGCTAAACCATCATCTGCTAAAGGTACATACTTCAA
ATCTGTTGCTGTAACTACAACAATGGGTCCTGGAGTTAAAATTGATACTGCAAGTTTCAAATAA

Gene: rplJ (50S ribosomal protein L10)

Contig: 05_NODE_4, position: 22397 to 22897, length: 501 nt, orientation: FORWARD

Perfect match to: (ED133-CP001996-[589915:590415], highly conserved allele)

Sequence:

ATGTCTGCTATCATTGAAGCTAAAAAACAACTAGTTGATGAAATTGCTGAGGTACTATCAAATTCAGTTTCAACAGTAATCGTTGACTACC
GTGGATTAACAGTAGCTGAAGTTACTGACTTACGTTCACAATTACGTGAAGCTGGTGTTGAGTATAAAGTATACAAAAACACTATGGTAC
GTCGTGCAGCTGAAAAAGCTGGTATCGAAGGCTTAGATGAATTCTTAACAGGTCCTACTGCTATTGCAACTTCAAGTGAAGATGCTGTAG
CTGCAGCGAAAGTAATTTCTGGATTTGCTAAAGATCATGAAGCATTAGAAATTAAATCAGGCGTTATGGAAGGCAATGTTATTACAGCAG
AAGAAGTTAAAACTGTTGGTTCATTACCTTCACACGATGGTCTTGTATCTATGCTTTTATCAGTATTACAAGCTCCTGTACGCAACTTCGCT
TATGCGGTTAAAGCTATTGGAGAACAAAAAGAAGAAAGCGCTGAATAA

Gene: rplL (50S ribosomal protein L7/L12)

Contig: 05_NODE_4, position: 22940 to 23308, length: 369 nt, orientation: FORWARD

Perfect match to: (N315-BA000018-[578254:578622], highly conserved allele)

Sequence:

ATGGCTAATCATGAACAAATCATTGAAGCGATTAAAGAAATGTCAGTATTAGAATTAAACGACTTAGTAAAAGCAATTGAAGAAGAATTT
GGTGTAACTGCAGCTGCTCCAGTAGCAGTAGCAGGTGCAGCTGGTGGCGCTGACGCTGCAGCAGAAAAAACTGAATTTGACGTTGAGTT
AACTTCAGCTGGTTCATCTAAAATCAAAGTTGTTAAAGCTGTTAAAGAAGCAACTGGTTTAGGATTAAAAGATGCTAAAGAATTAGTAGA
CGGAGCTCCTAAAGTAATCAAAGAAGCTTTACCTAAAGAAGAAGCTGAAAAACTTAAAGAACAATTAGAAGAAGTTGGAGCTACTGTAG
AATTAAAATAA

Gene: ybxB (16S rRNA methyltransferase C)

Contig: 05_NODE_4, position: 23483 to 24091, length: 609 nt, orientation: FORWARD

Perfect match to: (MW2-BA000033-[569359:569967], highly conserved allele)

Sequence:

ATGAGTCATTATTACGATGAAGATCCAAGTGTAATTAGCAATGAACAACGTATTCAATATCAATTAAACCATCATAAAATTGATTTAATAA
CTGATAATGGAGTGTTTTCGAAAGATAAAGTAGATTATGGTTCAGATGTTCTTGTTCAAACTTTTTTAAAAGCGCATCCACCTGGTCCAAG
TAAGCGAATTGCCGATGTTGGTTGTGGTTACGGACCAATTGGTTTGATGATTGCTAAAGTATCACCACATCATTCAATTACAATGCTAGAT
GTTAATCACAGAGCGCTAGCCTTAGTTGAAAAAAACAAAAAAATAAATGGTATTGATAATGCGATCGTAAAGGAAAGTGATGCTTTGTCT
GCTGTGGAAGACAAAAGTTTTGATTTTATTTTAACCAATCCACCAATAAGAGCAGGGAAAGAAACCGTGCATCGTATATTCGAGCAAGCA
TTACATAGATTAGACTCGAACGGTGAACTATTCGTTGTAATTCAGAAGAAGCAAGGTATGCCATCTGCAAAGAAAAGAATGAATGAACTT
TTTGGAAATGTAGAAGTGGTAAATAAAGATAAAGGATATTACATTCTGAGAAGTATAAAAGCTTGA

Gene: rpoB (DNA-directed RNA polymerase beta subunit)

Contig: 05_NODE_4, position: 24306 to 27857, length: 3552 nt, orientation: FORWARD

Perfect match to: (08-02119-CP015645-[1661286:1664837:r], highly conserved allele)

Sequence:

TTGGCAGGTCAAGTTGTCCAATATGGAAGACATCGTAAACGTAGAAACTACGCGAGAATTTCAGAAGTATTAGAATTACCAAACTTAATA
GAAATTCAAACTAAATCTTACGAGTGGTTCCTAAGAGAAGGTTTAATCGAAATGTTTAGAGACATTTCTCCAATTGAAGATTTTACTGGTA
ATTTGTCATTAGAGTTTGTGGATTACCGTTTAGGAGAACCAAAATATGATTTAGAAGAATCTAAAAACCGTGACGCTACTTATGCTGCACC
TCTTCGTGTAAAAGTGCGTCTAATCATTAAAGAAACAGGAGAAGTTAAAGAACAAGAAGTCTTTATGGGTGATTTCCCATTAATGACTGA
TACAGGTACGTTCGTTATCAATGGTGCAGAACGTGTAATCGTATCTCAATTAGTTCGTTCACCATCCGTTTATTTCAATGAAAAAATCGAC
AAAAATGGTCGTGAAAACTATGATGCAACAATTATTCCAAACCGTGGTGCATGGTTAGAATATGAAACAGATGCTAAAGATGTTGTATAC
GTACGTATTGATAGAACACGTAAACTACCATTAACAGTATTGTTACGTGCATTAGGTTTCTCAAGCGACCAAGAAATTGTTGACCTTTTAG
GTGACAATGAATATTTACGTAATACTTTAGAGAAAGACGGCACTGAAAACACTGAACAAGCGTTATTAGAAATCTATGAACGTTTACGTC
CAGGTGAACCACCAACTGTTGAAAATGCTAAAAGTCTATTGTATTCACGTTTCTTTGATCCAAAACGCTATGACTTAGCAAGCGTGGGTC
GTTATAAAACAAACAAAAAATTACATTTAAAACATCGTTTATTTAATCAAAAATTAGCTGAGCCAATTGTAAATACTGAAACTGGTGAAAT

TGTAGTTGAAGAAGGTACAGTGCTTGATCGTCGTAAAATCGACGAAATCATGGATGTACTTGAATCAAATGCAAACAGCGAAGTGTTTG
AATTGCATGGTAGCGTTATAGACGAGCCAGTAGAAATTCAATCAATTAAAGTATATGTTCCTAACGATGATGAAGGTCGTACGACAACTG
TAATTGGTAATGCTTTCCCTGACTCAGAAGTTAAATGCATTACACCAGCAGATATCATTGCTTCAATGAGTTACTTCTTTAACTTATTAAGC
GGTATTGGATATACAGATGATATTGACCATTTAGGTAACCGTCGTTTACGTTCTGTAGGTGAATTACTACAAAACCAATTCCGTATCGGTT
TATCAAGAATGGAAAGAGTTGTACGTGAAAGAATGTCAATTCAAGATACTGAGTCTATCACACCTCAACAATTAATTAATATTCGACCTGT
TATTGCATCTATTAAAGAATTCTTTGGTAGCTCTCAATTATCACAATTCATGGACCAAGCAAATCCATTAGCTGAGTTAACGCATAAACGT
CGTCTATCAGCATTAGGACCTGGTGGTTTAACACGTGAACGTGCTCAAATGGAAGTACGTGACGTTCACTACTCTCACTATGGCCGTATG
TGTCCAATTGAAACACCTGAGGGACCAAACATTGGATTGATTAACTCATTATCAAGTTATGCACGTGTAAATGAATTCGGCTTTATTGAAA
CACCATATCGTAAAGTTGATTTAGATACACATGCTATCACTGATCAAATTGACTATTTAACAGCTGACGAAGAAGATAGCTATGTTGTAGC
ACAAGCAAACTCTAAATTAGATGAAAATGGTCGTTTCATGGATGATGAAGTTGTATGTCGTTTCCGTGGTAACAACACAGTTATGGCTAA
AGAAAAAATGGATTATATGGATGTATCGCCGAAGCAAGTTGTTTCAGCAGCGACAGCATGTATTCCATTCTTAGAAAATGATGACTCAAA
CCGTGCATTGATGGGTGCGAACATGCAACGTCAAGCAGTGCCTTTGATGAATCCAGAAGCACCATTTGTTGGTACAGGTATGGAACACG
TTGCAGCACGTGATTCTGGTGCAGCTATTACAGCTAAGCACAGAGGTCGTGTTGAACATGTTGAATCTAATGAAATTCTTGTACGTCGTCT
AGTTGAAGAGAACGGCGTTGAGCATGAAGGTGAATTAGATCGCTATCCATTAGCTAAATTTAAACGTTCAAACTCAGGTACATGTTACAA
CCAACGTCCAATCGTTGCAGTTGGAGATGTTGTTGAGTATAACGAGATTTTAGCAGATGGACCATCTATGGAATTAGGAGAAATGGCATT
AGGTAGAAACGTAGTAGTTGGTTTCATGACTTGGGACGGTTACAACTATGAGGATGCCGTTATCATGAGTGAAAGACTTGTGAAAGATG
ACGTGTATACTTCTATTCATATTGAAGAGTATGAATCAGAAGCACGTGATACTAAGTTAGGACCTGAAGAAATCACAAGAGATATTCCTA
ATGTTTCTGAAAGTGCACTTAAGAACTTAGACGATCGTGGTATCGTTTATATTGGTGCAGAAGTAAAAGATGGAGATATTTTAGTTGGTA
AAGTAACGCCTAAAGGTGTAACTGAGTTAACTGCCGAAGAAAGATTGTTACATGCAATCTTTGGTGAAAAAGCACGTGAAGTTAGAGAT
ACTTCATTACGTGTACCTCACGGCGCTGGCGGTATCGTTCTTGATGTAAAAGTATTCAATCGCGAAGAAGGCGACGATACATTATCACCT
GGTGTAAACCAATTAGTACGTGTATACATCGTTCAAAAACGTAAAATTCATGTTGGTGATAAGATGTGTGGTCGACATGGTAACAAAGGT
GTCATTTCTAAGATTGTTCCTGAAGAAGATATGCCTTACTTACCAGACGGACGTCCGATCGATATCATGTTAAACCCTCTTGGTGTACCAT
CTCGTATGAACATCGGACAAGTATTAGAGCTACACTTAGGTATGGCTGCTAAAAATCTTGGTATTCACGTTGCATCACCAGTATTTGACG
GCGCAAACGATGACGATGTATGGTCAACAATTGAAGAAGCTGGTATGGCTCGTGATGGTAAAACTGTACTTTATGATGGACGTACAGGT
GAACCATTCGATAACCGTATTTCAGTAGGTGTAATGTACATGTTGAAACTTGCGCACATGGTTGATGATAAATTACATGCGCGTTCAACA
GGACCATATTCACTTGTTACACAACAACCACTTGGAGGTAAAGCGCAATTCGGTGGACAACGTTTCGGTGAGATGGAGGTATGGGCACT
TGAAGCATATGGTGCTGCATACACATTACAAGAAATCTTAACTTACAAATCCGATGATACAGTAGGACGTGTGAAAACATACGAGGCTAT
TGTTAAAGGTGAAAACATCTCTAGACCAAGTGTTCCAGAATCATTCCGAGTATTGATGAAAGAATTACAAAGTTTAGGTTTAGATGTAAA
AGTTATGGATGAGCAAGATAATGAAATCGAAATGACAGACGTTGATGACGATGATGTTGTAGAACGTAAAGTAGATTTACAACAAAATG
ATGCTCCTGAAACACAAAAAGAAGTTACTGATTAA

Gene: rpoC (DNA-directed RNA polymerase beta' subunit)

Contig: 05_NODE_4, position: 27994 to 31617, length: 3624 nt, orientation: FORWARD

Perfect match to: (TCH70-ACHH02000012-[97967:101590:r], allele observed in CC1+CC80+CC97+CC188)

Sequence:

TTGATTGATGTAAATAATTTCCATTATATGAAAATAGGATTGGCTTCACCTGAAAAAATCCGTTCTTGGTCTTTTGGTGAAGTTAAAAAAC
CTGAAACAATCAACTACCGTACATTAAAACCTGAAAAAGATGGTCTATTCTGTGAAAGGATTTTCGGACCTACAAAAGACTGGGAATGTA
GTTGTGGTAAATACAAACGTGTTCGCTACAAAGGCATGGTCTGTGACAGATGTGGAGTTGAAGTAACTAAATCTAAAGTACGTCGTGAA
AGAATGGGTCACATTGAACTTGCTGCTCCAGTTTCTCACATTTGGTATTTCAAAGGTATACCAAGCCGTATGGGATTATTACTTGACATGT
CACCAAGAGCATTAGAAGAAGTTATTTACTTTGCTTCTTATGTTGTTGTAGATCCAGGTCCAACTGGTTTAGAAAAGAAAACTTTATTATC
TGAAGCTGAATTCAGAGATTATTATGATAAATACCCAGGTCAATTCGTTGCAAAAATGGGTGCAGAAGGTATTAAAGATTTACTTGAAGA
GATTGATCTTGACGAAGAACTTAAATTGTTACGCGATGAGTTGGAATCAGCTACTGGTCAAAGACTTACTCGTGCAATTAAACGTTTAGA
AGTTGTTGAATCATTCCGTAATTCAGGTAACAAACCTTCATGGATGATTTTAGATGTACTTCCAATCATCCCACCAGAAATTCGTCCAATG
GTTCAATTAGATGGTGGACGATTTGCAACAAGTGACTTAAACGATTTATACCGTCGTGTAATTAATCGAAATAATCGTTTGAAACGTTTAT
TAGATTTAGGTGCACCTGGTATCATCGTTCAAAACGAAAAACGTATGTTACAAGAAGCCGTTGACGCTTTAATTGATAATGGTCGTCGTG
GTCGTCCAGTTACTGGCCCAGGTAACCGTCCATTAAAATCTTTATCTCATATGTTAAAAGGTAAACAAGGTCGTTTCCGTCAAAACTTACT
TGGTAAACGTGTTGACTATTCAGGACGTTCAGTTATTGCAGTAGGTCCAAGCTTGAAAATGTACCAATGTGGTTTACCAAAAGAAATGGC
ACTTGAACTATTTAAACCATTCGTAATGAAAGAATTAGTTCAACGTGAAATTGCAACTAACATTAAAAATGCGAAGAGTAAAATCGAACG
CATGGATGATGAAGTTTGGGACGTATTGGAAGAAGTAATTAGAGAACATCCTGTATTACTTAACCGTGCACCAACACTTCATAGACTTGG
TATTCAAGCATTTGAACCAACTTTAGTTGAAGGTCGTGCGATTCGTCTACATCCACTTGTAACAACAGCTTATAACGCTGACTTTGACGGT
GACCAAATGGCGGTTCACGTTCCTTTATCAAAAGAGGCACAAGCTGAAGCAAGAATGTTGATGTTAGCAGCACAAAACATCTTGAACCCT
AAAGATGGTAAACCTGTAGTTACACCATCACAAGATATGGTACTTGGTAACTATTACCTTACTTTAGAAAGAAAAGATGCAGTAAATACA
GGCGCAATCTTTAATAATACAAATGAAGTATTAAAAGCATATGCAAATGGCTTTGTACATTTACACACTAGAATTGGTGTACATGCAAGTT
CGTTCAATAATCCAACATTTACTGAAGAACAAAACAAAAAGATTCTTGCTACGTCAGTAGGTAAAATTATATTCAATGAAATCATTCCAGA
TTCATTTGCTTATATTAATGAACCTACGCAAGAAAACTTAGAAAGAAAGACACCAAACAGATATTTCATCGATCCTACAACTTTAGGTGAA
GGTGGATTAAAAGAATACTTTGAAAATGAAGAATTAATTGAACCTTTCAACAAAAAATTCTTAGGTAATATTATTGCAGAAGTATTCAAC

AGATTTAGCATCACTGATACATCAATGATGTTAGACCGTATGAAAGACTTAGGATTCAAATTCTCATCTAAAGCTGGTATTACAGTAGGT
GTTGCTGATATCGTAGTATTACCTGATAAGCAACAAATACTTGATGAGCATGAAAAATTAGTCGACAGAATTACAAAACAATTCAACCGT
GGTTTAATCACTGAAGAAGAAAGATATAATGCAGTTGTTGAAATTTGGACAGATGCAAAAGATCAAATTCAAGGTGAATTGATGCAATC
ACTTGATAAAACTAACCCAATCTTCATGATGAGTGATTCAGGTGCCCGTGGTAACGCATCTAACTTTACACAGTTAGCAGGTATGCGTGG
ATTGATGGCCGCACCATCTGGTAAGATTATCGAATTACCAATCACATCTTCATTCCGTGAAGGTTTAACAGTACTTGAATACTTCATCTCAA
CTCACGGTGCACGTAAAGGTCTTGCCGATACAGCACTTAAAACAGCTGACTCAGGATATCTTACTCGTCGTCTTGTTGACGTGGCACAAG
ATGTTATTGTTCGTGAAGAAGACTGTGGTACTGATAGAGGTTTATTAGTTTCTGATATTAAAGAAGGTACAGAAATGATTGAACCATTTA
TCGAACGTATTGAAGGTCGTTATTCTAAAGAAACAATTCGTCATCCTGAAACTGATGAAATAATCATTCGTCCTGATGAATTAATTACACC
TGAAATTGCTAAGAAAATTACAGATGCTGGTATTGAACAAATGTATATTCGCTCAGCATTTACTTGTAACGCACGACATGGTGTTTGTGA
AAAATGTTACGGTAAAAACCTTGCTACTGGTGAAAAAGTTGAAGTTGGTGAAGCAGTTGGTACAATTGCAGCCCAATCTATCGGTGAAC
CAGGTACACAGCTTACAATGCGTACATTCCATACAGGTGGGGTAGCAGGTAGCGATATCACACAAGGTCTTCCTCGTATTCAAGAGATTT
TCGAAGCACGTAACCCTAAAGGTCAAGCGGTAATTACGGAAATCGAAGGTGTCGTAGAAGATATTAAATTAGCAAAAGATAGACAACAA
GAAATTGTTGTTAAAGGTGCTAATGAAACAAGATCATACCTTGCTTCAGGTACTTCAAGAATTATTGTAGAAATCGGTCAACCAGTTCAAC
GTGGTGAAGTATTAACTGAAGGTTCTATTGAACCTAAGAATTACTTATCTGTTGCTGGATTAAACGCGACTGAAAGCTACTTATTAAAAG
AAGTACAAAAAGTTTACCGTATGCAAGGTGTAGAAATCGACGATAAACACGTTGAGGTTATGGTTCGACAAATGTTACGTAAAGTTAGA
ATTATCGAAGCAGGTGATACGAAGTTATTACCAGGTTCATTAGTTGATATTCATAACTTTACAGATGCAAATAGAGAAGCATTTAAACACC
GTAAGCGTCCTGCAACAGCTAAACCAGTATTACTTGGTATTACTAAAGCATCACTTGAAACAGAAAGTTTCTTATCTGCAGCATCATTCCA
AGAAACAACAAGAGTTCTTACAGATGCAGCAATTAAAGGTAAGCGTGATGACTTATTAGGTCTTAAAGAAAACGTAATTATTGGTAAGTT
AATTCCAGCTGGTACTGGTATGAGACGTTATAGCGACGTAAAATACGAAAAAACAGCTAAACCAGTTGCAGAAGTTGAATCTCAAACTG
AAGTAACGGAATAA

Gene: ybxF (RNA binding protein)

Contig: 05_NODE_4, position: 31754 to 32008, length: 255 nt, orientation: FORWARD

Perfect match to: (RF122-AJ938182-[555615:555869], highly conserved allele)

Sequence:

TTGTCTAAGGAAAAAGTTGCACGCTTTAACAAACAACATTTTGTAGTTGGTCTTAAAGAAACGCTTAAAGCGTTAAAGAAAGATCAAGTT
ACATCTTTGATTATTGCTGAAGACGTTGAAGTATATTTAATGACTCGCGTGTTAAGCCAAATCAATCAGAAAAATATACCTGTATCTTTTTT
CAAAAGCAAACATGCTTTGGGTAAACATGTAGGTATTAACGTCAATGCGACAATAGTAGCATTGATTAAATGA

Gene: rpsL (30S ribosomal protein S12)

Contig: 05_NODE_4, position: 32106 to 32519, length: 414 nt, orientation: FORWARD

Perfect match to: (MW2-BA000033-[577982:578395], highly conserved allele)

Sequence:

ATGCCAACTATTAACCAATTAGTACGTAAACCAAGACAAAGCAAAATCAAAAAATCAGATTCTCCAGCTTTAAATAAAGGTTTCAACAGT
AAAAAGAAAAAATTTACTGACTTAAACTCACCACAAAAACGTGGTGTATGTACTCGTGTAGGTACAATGACACCTAAAAAACCTAACTCA
GCGTTACGTAAATATGCACGTGTGCGTTTATCAAACAACATCGAAATTAACGCATACATCCCTGGTATCGGCCATAACTTACAAGAACAC
AGTGTTGTACTTGTACGTGGTGGACGTGTAAAAGACTTACCAGGTGTGCGTTACCATATTGTACGTGGAGCACTTGATACTTCAGGTGTT
GACGGACGTAGACAAGGTCGTTCATTATACGGAACTAAGAAACCTAAAAACTAA

Gene: rpsG (30S ribosomal protein S7)

Contig: 05_NODE_4, position: 32585 to 33055, length: 471 nt, orientation: FORWARD

Perfect match to: (N315-BA000018-[587899:588369], highly conserved allele)

Sequence:

ATGCCTCGTAAAGGATCAGTACCTAAAAGAGACGTATTACCAGATCCAATTCATAACTCTAAGTTAGTAACTAAATTAATTAACAAAATTA
TGTTAGATGGTAAACGTGGAACAGCACAAAGAATTCTTTATTCAGCATTCGACCTAGTTGAACAACGCAGTGGTCGTGATGCATTAGAAG
TATTCGAAGAAGCAATCAACAACATTATGCCAGTATTAGAAGTTAAAGCTCGTCGTGTAGGTGGTTCTAACTATCAAGTACCAGTAGAAG
TTCGTCCAGAGCGTCGTACTACTTTAGGTTTACGTTGGTTAGTTAACTATGCACGTCTTCGTGGTGAAAAAACGATGGAAGATCGTTTAGC

TAACGAAATTTTAGATGCAGCAAATAATACAGGTGGTGCCGTTAAGAAACGTGAGGACACTCACAAAATGGCTGAAGCAAACAAAGCAT
TTGCTCACTACCGTTGGTAA

Gene: efg (translation elongation factor G)

Contig: 05_NODE_4, position: 33178 to 35259, length: 2082 nt, orientation: FORWARD

Perfect match to: (Strain_21202-AGRO01000041-[17310:19391:r], allele observed in CC395+CC361+CC395+CC398)

Sequence:

ATGGCTAGAGAATTTTCATTAGAAAAAACTCGTAATATCGGTATCATGGCTCACATTGATGCTGGTAAAACGACTACGACTGAACGTATT
CTTTATTACACTGGCCGTATCCACAAAATTGGTGAAACACACGAAGGTGCTTCACAAATGGACTGGATGGAGCAAGAACAAGACCGTGG
TATTACTATCACATCTGCTGCAACAACAGCAGCTTGGGAAGGTCACCGTGTAAACATTATCGATACACCTGGACACGTAGACTTCACTGTA
GAAGTTGAACGTTCATTACGTGTACTTGACGGAGCAGTTACAGTACTTGATGCACAATCAGGTGTTGAACCTCAAACTGAAACAGTTTGG
CGTCAGGCTACAACTTATGGTGTTCCACGTATCGTATTTGTAAACAAAATGGACAAATTAGGTGCTAACTTCGAATACTCTGTAAGTACAT
TACATGATCGTTTACAAGCTAACGCTGCTCCAATCCAATTACCAATTGGTGCGGAAGACGAATTCGAAGCAATCATTGACTTAGTTGAAAT
GAAATGTTTCAAATATACAAATGATTTAGGTACTGAAATTGAAGAAATTGAAATTCCTGAAGACCACTTAGATAGAGCTGAAGAAGCTCG
TGCTAGCTTAATCGAAGCAGTTGCAGAAACTAGCGACGAATTAATGGAAAAATATCTTGGTGACGAAGAAATTTCAGTTTCTGAATTAAA
AGAAGCTATCCGCCAAGCTACTACTAACGTAGAATTCTACCCAGTACTTTGTGGTACAGCTTTCAAAAACAAAGGTGTTCAATTAATGCTT
GACGCTGTAATTGATTACTTACCTTCACCACTAGACGTTAAACCAATTATTGGTCACCGTGCTAGCAACCCTGAAGAAGAAGTAATCGCG
AAAGCAGACGATTCAGCTGAATTCGCTGCATTAGCGTTCAAAGTTATGACTGACCCTTATGTTGGTAAATTGACATTCTTCCGTGTGTATT
CAGGTACAATGACATCTGGTTCATACGTTAAGAACTCTACTAAAGGTAAACGTGAACGTGTAGGTCGTTTATTACAAATGCACGCTAACT
CACGTCAAGAAATCGATACTGTATACTCTGGAGATATCGCTGCTGCGGTAGGTCTTAAAGATACAGGTACTGGTGATACTTTATGTGGTG
AGAAAAATGACATTATCTTGGAATCAATGGAATTCCCTGAGCCAGTTATTCACTTATCAGTAGAGCCAAAATCTAAAGCTGACCAAGATA
AAATGACTCAAGCTTTAGTTAAATTACAAGAAGAAGACCCAACATTCCATGCACACACTGACGAAGAAACTGGACAAGTTATCATCGGTG
GTATGGGTGAGCTTCACTTAGACATCTTAGTAGACCGTATGAAGAAAGAATTCAACGTTGAATGTAACGTAGGTGCTCCAATGGTTTCAT
ATCGTGAAACATTCAAATCATCTGCACAAGTTCAAGGTAAATTCTCTCGTCAATCTGGTGGTCGTGGTCAATACGGTGATGTTCACATTGA
ATTCACACCAAACGAAACAGGCGCAGGTTTCGAATTCGAAAACGCTATCGTTGGTGGTGTAGTTCCTCGTGAATACATTCCATCAGTAGA
AGCTGGTCTTAAAGATGCTATGGAAAATGGTGTCTTAGCAGGTTATCCTTTAATTGATGTTAAAGCTAAATTATATGATGGTTCATACCAT
GATGTCGATTCATCTGAAATGGCCTTCAAAATTGCTGCATCATTAGCACTTAAAGAAGCTGCTAAAAAATGTGATCCTGTAATCTTAGAAC
CAATGATGAAAGTAACTATTGAAATGCCTGAAGAGTACATGGGTGATATCATGGGTGACGTAACATCTCGTCGTGGACGTGTTGATGGT
ATGGAACCTCGTGGTAATGCACAAGTTGTTAATGCTTATGTACCACTTTCAGAAATGTTCGGTTATGCAACATCATTACGTTCAAACACTC
AAGGTCGCGGTACTTACACTATGTACTTCGATCACTATGCTGAAGTTCCAAAATCAATCGCTGAAGATATTATCAAGAAAAATAAAGGTG
AATAA

Gene: tuf (translation elongation factor Tu)

Contig: 05_NODE_4, position: 35476 to 36660, length: 1185 nt, orientation: FORWARD

Perfect match to: (MW2-BA000033-[581352:582536], highly conserved allele)

Sequence:

ATGGCAAAAGAAAAATTCGATCGTTCTAAAGAACATGCCAATATCGGTACTATCGGTCACGTTGACCATGGTAAAACAACATTAACAGCA
GCAATCGCTACTGTATTAGCAAAAAATGGTGACTCAGTTGCACAATCATATGACATGATTGACAACGCTCCAGAAGAAAAAGAACGTGG
TATCACAATCAATACTTCTCACATTGAGTACCAAACTGACAAACGTCACTACGCTCACGTTGACTGCCCAGGACACGCTGACTACGTTAAA
AACATGATCACTGGTGCTGCTCAAATGGACGGCGGTATCTTAGTAGTATCTGCTGCTGACGGTCCAATGCCACAAACTCGTGAACACATT
CTTTTATCACGTAACGTTGGTGTACCAGCATTAGTAGTATTCTTAAACAAAGTTGACATGGTTGACGATGAAGAATTATTAGAATTAGTAG
AAATGGAAGTTCGTGACTTATTAAGCGAATATGACTTCCCAGGTGACGATGTACCTGTAATCGCTGGTTCAGCATTAAAAGCTTTAGAAG
GCGATGCTCAATACGAAGAAAAAATCTTAGAATTAATGGAAGCTGTAGATACTTACATTCCAACTCCAGAACGTGATTCTGACAAACCAT
TCATGATGCCAGTTGAGGACGTATTCTCAATCACTGGTCGTGGTACTGTTGCTACAGGCCGTGTTGAACGTGGTCAAATCAAAGTTGGTG
AAGAAGTTGAAATCATCGGTTTACATGACACATCTAAAACAACTGTTACAGGTGTTGAAATGTTCCGTAAATTATTAGACTACGCTGAAG
CTGGTGACAACATTGGTGCATTATTACGTGGTGTTGCTCGTGAAGACGTACAACGTGGTCAAGTATTAGCTGCTCCTGGTTCAATTACAC
CACATACTGAATTCAAAGCAGAAGTATACGTATTATCAAAAGACGAAGGTGGACGTCACACTCCATTCTTCTCAAACTATCGTCCACAATT
CTATTTCCGTACTACTGACGTAACTGGTGTTGTTCACTTACCAGAAGGTACTGAAATGGTAATGCCTGGTGATAACGTTGAAATGACAGT
AGAATTAATCGCTCCAATCGCGATTGAAGACGGTACTCGTTTCTCAATCCGTGAAGGTGGACGTACTGTAGGATCAGGCGTTGTTACTGA
AATCATTAAATAA

Gene: yhaA (M20 family peptidase)

Contig: 05_NODE_4, position: 36942 to 38117, length: 1176 nt, orientation: REVERSE

Perfect match to: (CIGC128-AHVY01000003-[39858:41033:r], allele observed in CC1)

Sequence:

TTAATTTGTTTTAAATACTTGCTCTAATTCAATGATTTTTAAAAATACAGCTACAGCGTATTTTAATGATTTTTCATCAATATCAAATTTGGG
ATTATGGTGTGGCGCTGTAATACCTTTACTTTCATTACCACAACCAGTCAGAAAGAATGCACCTGGTCGTACTTTCAAATAATGTGAAAAA
TCTTCTCCAATCATCATTAAATCTGATTCATTAAAGCGTACATGTAAGTCATTTGTTGCTTCTTTAATAACTTGATATGCTTTCTCGTTATTAT
GGACAGGCAAATACCCTTTAATATAATTCAAATCATAGTTAATATCATTTGCTATTGCTAAACCTTGTAAAAGCTTATCCATTTTGTCCATT
ACATGATTCTGTATATCTGAATCGAAAGTTCTAACTGTACCTTTACAAAATGCTTGATCAGGAATAACGCTATCTGTGGTGCCTGCTTGAA
TCATTCCAAATGAAAGTACAGCTTGTTTAACTGGATCGATCGTACGTGAAATTATTTTTTGTGCACTTAAAATGAACTCTGCCATGATTACT
ATTGGGTCAATGGTTTCATGAGGTTTGGCACCATGACCGCCACGACCTTTAATTGTGACACTAAATTCATCTGGAGAGGCCATGATTGCC
CCCGCACGTGAATGAATAGTTCCAGTAGGATAACCACTCCATAAATGTGTACCGTAAATTCTATCTACATTTTCCAGACATCCAGCATCTA
TCATTTCTTGAGAACCACCTGGCATGATTTCTTCACCGTACTGGAATATTAATACAACATTACCTTCTAATAAATGTTTATGTTCATCTAAAA
TCTCTGCTACAGTAAGTAAAATTGCTGTATGACCATCATGCCCACACGCATGCATACATCCTGGATTTTTAGACTTATAAGGCACATCGTTT
AATTCCTCGACAGGTAACGCATCAAAGTCAGCTCTTAATGCAATGGTAGGTCCTGTGCCCAAGCCTTTAAATGTGGCTTTGATACCATTGC
GGCCGATAGGAGTTTCAATATCACAAGATAACTGGCTTAATTGGTTAACAATATAATCATGTGTTTGAAATTCTTCAAAAGATAACTCAGG
GTATTGGTGTAAATAACGTCTGAGTTGAATTGTTTTATTTTCTTTATTATTTGCTAGTTGGAACCAATCTAACAC

Gene: kbl (2-amino-3-ketobutyrate coenzyme A ligase)

Contig: 05_NODE_4, position: 38288 to 39475, length: 1188 nt, orientation: FORWARD

Perfect match to: (MW2-BA000033-[584164:585351], allele observed in CC1)

Sequence:

GTGGTTCAATCATTACATGAGTTTTTAGAGGAAAATATAAATTATCTAAAAGAAAATGGTTTGTATAATGAAATAGATACAATTGAAGGT
GCAAACGGACCAGAAATCAAAATCAATGGGAAATCATACATTAACTTATCTTCAAATAATTATTTAGGACTAGCAACAAATGAAGATTTG
AAATCAGCTGCAAAAGCAGCTATTGATACACATGGAGTAGGTGCAGGCGCTGTTCGTACAATCAATGGTACATTAGATTTACACGACGA
ATTAGAAGAAACACTAGCAAAATTTAAAGGAACAGAAGCTGCAATAGCTTATCAATCAGGATTTAATTGTAATATGGCTGCTATTTCAGC
TGTCATGAATAAAAATGATGCTATTTTATCAGATGAGCTTAATCATGCATCAATTATTGATGGATGTCGCTTATCTAAAGCTAAAATTATTC
GAGTTAACCATTCAGACATGGATGATTTACGTGCGAAAGCAAAAGAAGCAGTTGAATCAGGTCAATACAATAAAGTGATGTATATCACT
GATGGCGTTTTTAGTATGGATGGTGATGTGGCTAAATTACCTGAAATTGTAGAAATTGCAGAAGAATTTGGTTTATTAACTTATGTAGAC
GACGCTCATGGTTCAGGTGTTATGGGTAAAGGCGCTGGTACGGTTAAACATTTTGGTTTACAAGATAAAATCGATTTCCAAATAGGTACG
CTTTCTAAAGCAATTGGTGTCGTTGGCGGTTATGTAGCAGGTACAAAAGAGTTAATAGATTGGTTAAAAGCACAATCACGACCATTCTTA
TTCTCTACATCATTAGCACCTGGGGATACCAAAGCAATAACTGAAGCAGTTAAAAAGTTAATGGATTCAACTGAATTACATGATAAATTAT
GGGACAATGCACAATATTTAAAAAATGGATTGTCAAAATTAGGATATGATACAGGTGAGTCAGAAACTCCAATTACACCAGTAATTATTG
GTGATGAAAAAACAACTCAAGAATTTAGTAAGCGTTTAAAAGACGAAGGTGTCTATGTGAAATCTATCGTTTTCCCAACAGTACCAAGAG
GTACAGGACGTGTAAGAAATATGCCTACAGCTGCACATACAAAAGACATGTTAGATGAAGCAATTGCGGCTTATGAAAAAGTAGGAAAA
GAAATGAAGTTGATTTAA

Gene: hchA (chaperone Hsp31 and glyoxalase 3)

Contig: 05_NODE_4, position: 39743 to 40621, length: 879 nt, orientation: FORWARD

Perfect match to: (MW2-BA000033-[585618:586496], allele observed in CC1+CC5)

Sequence:

ATGTCACAAGATGTAAATGAATTAAGTAAGCAACCAACGCCAGATAAAGCAGAAGATAACGCATTTTTCCCATCACCATATTCCCTTAGTC
AATATACAGCACCTAAAACAGATTTTGATGGTGTTGAACACAAAGGTGCCTATAAAGATGGTAAATGGAAAGTATTGATGATTGCTGCTG
AAGAGAGATATGTATTATTGGAAAATGGAAAAATGTTCTCTACGGGTAATCATCCTGTTGAAATGTTATTACCTTTACATCATTTAATGGA
AGCAGGTTTTGACGTTGATGTTGCGACATTATCTGGTTATCCAGTTAAATTAGAATTATGGGCTATGCCAACTGAAGACGAGGCAGTTAT
AAGTACTTATAATAAATTGAAAGAAAAATTAAAACAGCCAAAAAAAATTAGCAGATGTGATTAAAAATGAATTAGGACCTGATTCAGATTA
TTTATCTGTCTTTATCCCAGGCGGACATGCTGCAGTTGTTGGTATTTCTGAAAGTGAGGATGTTCAACAAACATTAGATTGGGCATTAGAC
AATGACCGCTTTATAGTTACATTATGTCATGGACCAGCAGCACTACTTTCAGCAGGGCTTAACAGAGAAAAATCTACATTAGAAGGATAC
TCTGTTTGTGTCTTCCCTGACTCATTAGATGAAGGTGCAAATATTGAAATAGGTTATTTACCTGGACGCTTGAAATGGTTAGTTGCTGATT

TATTAACTAAACAAGGATTAAAAGTAGTTAACGACGATATGACAGGAAGAACGTTAAAAGATCGTAAATTATTAACAGGTGACAGTCCTT
TAGCTTCAAATGAGTTAGGAAAATTAGCAGTTAATGAAATGTTAAATGCAATACAAAATAAATAA

Gene: araB (ribulokinase)

Contig: 05_NODE_4, position: 40780 to 42417, length: 1638 nt, orientation: FORWARD

Perfect match to: (MW2-BA000033-[586655:588292], allele observed in CC1)

Sequence:

ATGTCTTATAGCATTGGAATTGATTATGGAACAGCTTCAGGCCGTGTGTTTTTAATTAATACAACTAACGGTCAAGTAGTATCAAAATTTG
TGAAACCATATACACATGGTGTCATTGAAAGTGAATTAAATGGTTTGAAAATACCACATACATATGCACTTCAAAATAGTAATGATTATTT
AGAAATTATGGAAGAAGGAATATCATATATAGTACGTGAATCAAAAATAGATCCAGACAATATAGTAGGTATTGGTATAGACTTTACTTC
ATCTACTATTATTTTTACTGACGAAAATCTTAACCCGGTACATAATTTAAAACAATTTAAAAACAATCCACATGCGTATGTGAAACTTTGGA
AACATCATGGTGCATATAAAGAAGCAGAGAAATTATATCAAACTGCTATTGAAAATAATAATAAGTGGTTAGGCCATTATGGATATAATG
TTAGTAGTGAATGGATGATTCCCAAAATAATGGAAGTCATGAATCGAGCACCAGAAATTATGGAAAAAACGGCTTATATTATGGAAGCG
GGCGATTGGATTGTAAATAAATTAACTAATAAAAATATACGCTCGAATTGTGGATTAGGTTTCAAAGCATTTTGGGAAGAAGAAACAGG
GTTTCATTATGATTTATTTGATAAAATAGACCCCAAATTATCAAAAGTAATTCAAGATAAAGTATCTGCACCGGTTGTTAATATTGGTGAA
GCAGTAGGGAAACTGGATGATAAAATGGCACAGAAATTAGGATTATCAAAAGAGACTATGGTAAGTCCTTTTATTATTGATGCCCATGCT
AGTTTATTAGGTATTGGGTCTGAAAAAGATAAAGAAATGACTATGGTGATGGGAACAAGCACATGCCATCTTATGTTAAATGAAAAGCA
ACATCAAGTGCCAGGTATATCAGGTTCTGTAAAAGGAGCAATTATTCCAGAATTATTTGCTTATGAAGCGGGGCAATCAGCAGTAGGTG
ATTTGTTTGAGTATGTCGCTAAGCAAGCACCAAAGTCATATGTAGATGAAGCAGCAAATAGAAATATGACTGTATTTGAATTAATGAATG
AAAAGATAAAACATCAAATGCCAGGTGAAAGTGGGCTCATTGCTCTTGATTGGCATAATGGAAATCGAAGTGTATTAAGTGATAGCAAT
TTAACAGGTTGTATCTTTGGATTAACTTTACAAACTAAGCATGAAGATATTTATAGAGCATATTTAGAAGCTACAGCATTTGGTACTAAGA
TGATTATGCAACAGTATCAAGATTGGCATATGGAAGTAGAAAAGGTATTTGCATGTGGCGGTATACCTAAAAAGAATGCTGTTATGATG
GATATCTATGCGAATGTACTGAATAAAAAACTAATTGTTATGGATAGTGAGTATGCACCAGCAATAGGCGCAGCAATATTAGGTGCAGTC
AGTGGTGGCGCACATAATTCAATTAATGATGCAGTTGATGCTATGAAAGAGCCAATTTTATACGAAATTAATCCAGAAGCGGAAAAAGT
ACAAAGGTATGAAACATTATTTAAAGCTTATAAGGCTTTACATGATATCCATGGTTATAAAAAAGCTAATATAATGAAAGATATCCAGAG
TTTAAGAGTTGAGGGATAA

Gene: Q5HIC2 (uncharacterized epimerase/dehydratase)

Contig: 05_NODE_4, position: 42631 to 43596, length: 966 nt, orientation: FORWARD

Sequence:

ATGAAAAAAATTATGATTACTGGTGCATTAGGACAAATTGGTACAGAATTAGTTGTTAAGTGCAGAGAAATTTATGGGACAGATAATGTT
CTTGCTACAGATATTAGGGAACCTGAAGCAGACTCACCTGTACAAAATGGACCATTTGAAATCTTAGACGTAACAGATCGTGACCGTATG
TTTGAGTTAGTTAGGGACTTTGAAGCGGATAGTCTAATGCATATGGCAGCATTATTATCAGCAACTGCTGAGAAAAATCCAATTCTAGCT
TGGGATTTAAATATGGGTGGATTAATGAATGCATTAGAAGCTGCAAGAACTTATAATTTGCACTTTTTCACACCAAGTTCAATTGGTGCAT
TTGGAGACTCAACTCCTAAAGTTAATACGCCACAAGTAACGATTCAGCAACCTACGACAATGTATGGTGTAAATAAAGTAGCTGGAGAAT
TATTGTGTCAATACTATTTCAAACGTTTTGGTGTAGATACAAGAAGTGTTAGATTCCCAGGTTTAATCTCGCATGTTAAAGAGCCAGGTGG
CGGTACTACAGACTATGCTGTTGAAATATACTTCAAAGCAGTAAGAGAGGGTCATTATACAAGCTTCATAGATAAAGGCACGTATATGGA
TATGATGTATATGGATGATGCAATTGAAGCAATTATTAAACTTATGGAAGCAGAGGACGCTAAATTAGAAACTAGAAATGGTTATAATTT
GAGCGCAATGAGTTTTGATCCAGAGATGGTAAAAGAAGCAATTCAAGAATACTATCCCAATTTTACATTAGATTACGATGTTGATCCTATT
AGACAAGGTATCGCTAATAGTTGGCCGGATTCTATTGATACAAGCTGTTCACGTGGCGAATGGGGATTTGATCCTAAATATGATTTAGCG
AGCATGACTAAATTAATGTTAGAAGCTATTGAACAAAAAGATACTGTTAAAAATAATAACTAA

Gene: ilvE (branched-chain-amino-acid aminotransferase)

Contig: 05_NODE_4, position: 43931 to 45007, length: 1077 nt, orientation: FORWARD

Perfect match to: (COL-CP000046-[626612:627688], highly conserved allele)

Sequence:

ATGTCACAAGCAGTTAAAGTTGAACGACGAGAAACATTAAAACAAAAACCAAATACATCTCAACTAGGTTTTGGTAAATATTTTACTGAT
TATATGTTGAGTTATGATTATGATGCAGATAAAGGATGGCATGATTTGAAGATAGTACCTTATGGTCCTATTGAAATTTCACCTGCTGCAC

AAGGTGTTCATTATGGTCAATCGGTATTCGAAGGATTAAAAGCATATAAAAGAGATGGGGAAGTTGCACTTTTCCGTCCTGAAGAAAATT
TTAAGCGTCTTAATAACTCGTTAGCACGATTAGAAATGCCTCAAGTAGACGAAGCAGAATTGTTAGAGGGGCTAAAACAATTAGTTGATA
TAGAAAGAGATTGGATTCCTGAAGGGGAAGGTCAATCATTATATATTCGTCCATTTGTTTTTGCAACAGAAGGGGCACTTGGCGTTGGTG
CATCACATCAGTATAAATTATTAATTATTTTATCTCCTTCAGGTGCATATTATGGTGGTGAGACTTTAAAACCAACTAAAATCTATGTAGAA
GATGAATATGTGCGTGCTGTTCGTGGCGGTGTAGGCTTTGCAAAAGTTGCAGGTAACTATGCGGCAAGTTTATTAGCACAAACAAATGC
AAATAAATTAGGTTATGACCAAGTATTATGGCTTGATGGTGTTGAACAGAAATATATCGAAGAAGTTGGTAGCATGAATATTTTCTTCGT
TGAAAATGGAAAAGTAATTACACCAGAGTTGAATGGCAGTATTTTACCTGGTATTACACGTAAATCTATTATCGAATTAGCTAAAAACTTA
GGATATGAAGTCGAAGAGCGCCGCGTTTCAATCGATGAATTATTCGAATCATATGATAAAGGTGAGTTAACAGAAGTATTTGGTAGTGG
TACTGCAGCAGTTATTTCACCTGTGGGTACATTGAGATACGAAGATCGTGAAATCGTTATTAATAATAATGAGACTGGTGAAATTACTCA
AAAATTATACGACGTCTATACTGGTATTCAAAATGGTACTTTAGAAGATAAAAATGGTTGGAGAGTCGTTGTACCAAAATATTAA

Gene: ppaX1 (P-Ser-HPr phosphatase)

Contig: 05_NODE_4, position: 45258 to 45941, length: 684 nt, orientation: FORWARD

Perfect match to: (MW2-BA000033-[591133:591816], allele observed in CC1)

Sequence:

ATGGAATGGATATTATTTGATAAAGATGGCACGTTAATTGAATTTGATAGAAGTTGGGAAAAAATAGGGGTACGATTTGTACAATCATT
GCTTGAGACTTTCCCAGTACATAATAAAGAAGCTGCTTTAAGACAACTCGGTGTCATTAAAGAATCTATTGATCCAAAATCAGTGATGGG
TTCAGGATCTTTACAACAAATTATCCAGGCATTTAATGATGTGACGGGACAAGATACAACCGACTGGTCCAAGTCAACAAGTCAAAAGCT
GGTAGATGAACGTATTCCTGAAATTAATTGGGTAGAAGGTGTTAAAGAAGCACTTATCGATTTGAAAGCAAAAGGCTATCAACTTGGTAT
TGTTACGAGTGATACTAAAAAAGGTGTAGAACAATTTTTAGCACATACCAATGCTACCTCGTTGTTCGATTTGATCATTTCTACCGAAGCG
GATGCCTATGAGAAGCCAAATCCTAAAGTATTATCGCCTTTATTTGAGCAATATAATGTAGATCCTCAGAAAGTAGCTATAGTAGGAGAC
ACTGCTAATGATATGAAGACAGCAAGTAATGCAAATTTAGGTATGGCAATAGGTGTATTAACAGGTATTGCAACAAAAGAAGAATTACA
TGAAGCTGATATTATTTTAAATAGTGCGGCAGATATTTTAGAAGCTTTAAATTAA

Gene: dck (deoxyadenosine/deoxycytidine kinase)

Contig: 05_NODE_4, position: 46057 to 46719, length: 663 nt, orientation: REVERSE

Perfect match to: (MW2-BA000033-[591932:592594:r], allele observed in CC1+CC22+CC96+CC239+CC361)

Sequence:

TTATCGTGTATCTACTTGTCGATATGTTTGAATAATTCGAGCAATTTTGTCTATCATAGGATTTAAAGATTCGGGGTCCTTATGGATATCAT
ATTCATTAATATTGATACGTACAACTGGACATGCATTAAAGCTATTAATCCAATCGTCATAGCGTTTAAATAGCTTTTTCCAGTATTCAGGG
TCTGTATTAATTTCCATTTCGCGACCACGTTCAATAATACGATCAATGACCTCATCATAGTTACATTCTAAATAAATCATTACATCAGGTTTA
GGAAAATAAGGTGTCATGACCATGGCATTAAATAAGTCTGAATATGTTTTGAAATCTTCTTTACTCATTGTGCCTTCTTCTTCATGCATTTT
TGCAAAAATATCAACATCTTCATAAATTGATCGATCTTGGACAAAGCCACCACCATATTCAAACATACGCTTTTGTTCTTTAAAACGTTCAG
CTAAGAAGTAAATTTGCAAATGGAAACTCCATCGTTCAAAATCGCTGTAAAATTTATCTAAATATGGATTATGTTCGACATTTTCAAAAGA
CGTTTTAAAGTTTAATTTATCTGCAAGTGCTTGCGTTAGTGTTGATTTTCCAACACCAACTGTACCTGCAATGGTTATAATGGCATTTTGTG
GAATACCGTAATTATTCAT

Gene: dgk (deoxyguanosine kinase)

Contig: 05_NODE_4, position: 46712 to 47329, length: 618 nt, orientation: REVERSE

Perfect match to: (MW2-BA000033-[592587:593204:r], highly conserved allele)

Sequence:

TTATTCATTGGTAATATCTCCTATCATAGGTAATATAATATGTAATATATCTTCGTAATCTTGTTCATTTTTAAGAAAATCAATAGAAGTTGT
ATCGATTAAAACTACATTTGAACCATTACTTTGTAAGGACTCATAATACTCACGATAATCTTTTTTTAACTTTAACAGATATTCATCTTCTAT
TTGATGCTCAAAACTACGGTTACGTTTAGCAATTCTAGATTTTAACACATCAAGGTCTGCATCTAAAAAGATAATCATATTCGGCATAATC
ATATCTTCAGTTAAAATATCATAAATTTTACTGAATTTCTGAAATTCAACAGAACTCAAAGTATTTTTAGCAAATATCTTATTTTTATGTATA
TGATAATCACTAACTACACCTTGATTTAGTTGTGTTACATCTTGAAATTGCTTATATCTATTGCATAAAAAGAACATTTCAGTTTGAAAACT
CCATTTAGAGATATCTTCATAAAAGTCTGATAAAAATGGATTTTCTGTGATGATTTCTTTTTCTTCATAAAAATCTAAAGTTTGACTTAATTT
GTGTGCAAGTGAAGATTTACCTACGCCAATAGGACCTTCAATTGCTATAAAAGGTTTGTTCAT

Gene: tadA (nucleoside deaminase)

Contig: 05_NODE_4, position: 47396 to 47866, length: 471 nt, orientation: FORWARD

Perfect match to: (N315-BA000018-[602712:603182], highly conserved allele)

Sequence:

ATGACAAATGATATATATTTTATGACATTAGCGATTGAAGAAGCTAAAAAAGCAGCTCAACTAGGCGAAGTACCTATAGGTGCTATCATC
ACTAAAGATGATGAAGTTATCGCTAGAGCACATAATTTAAGAGAAACACTACAACAACCAACGGCGCATGCTGAACATATTGCAATTGA
ACGTGCAGCCAAAGTGTTAGGTAGTTGGCGTTTAGAAGGTTGCACATTATATGTAACCTTAGAACCATGTGTCATGTGCGCAGGAACAAT
TGTAATGAGTCGCATTCCAAGAGTCGTCTATGGCGCAGATGATCCTAAAGGTGGTTGTAGTGGCAGTTTAATGAATTTATTGCAACAATC
TAATTTTAATCATCGTGCAATTGTTGATAAAGGTGTACTTAAAGAAGCATGTAGCACATTATTAACAACATTTTTTAAAAACTTAAGAGCC
AATAAGAAATCCACCAATTAG

Gene: A5IQB3 (putative hydrolase, haloacid dehalogenase-like family)

Contig: 05_NODE_4, position: 48013 to 48882, length: 870 nt, orientation: FORWARD

Sequence:

ATGATAAAACTAATAGCCACTGATATGGATGGCACGCTACTTAATGCAGCACATGAAATTTCTCAACCTAATATTGATGCGATTAAATACG
CTCAAGAACAAGGGATAACGGTTGTTATCGCGACAGGTCGAGCATTTTATGAAGCACAAGCACCAGTTGCTGACACAGATTTAACAGTA
CCATATATTTGTTTGAATGGTGCTGAAGTACGTGATGAAACTTTCAATGTAATGAGCACTTCACACCTTAATAAATCGCTAGTACACAAAA
TTACAAATGTTTTAAAAGATGCAGGTATTTATTATCAAGTATACACGAGTCGTGCGATTTATACTGAAGATCCACAAAGAGATTTAGACAT
TTACATAGATATTGCTGAGCGTGCAGGTCAACATGCAAACGTTGAGCGTATTAAAAATGGTATTCAAAGACGCATAGATAATGGTACGTT
GAAAGTTGTTGATAATTATGATGCTATTGAAAACATACCTGGTGAATTAATTATGAAAATATTAGCATTTGATGAAAATTTAGAAAAAATT
GACAAAGCTAGTAAAATTTTAGCTGAATCTCCGAATTTAGCTATATCATCATCTTCGAGAGGAAATATAGAAATAACGCATTCAGATGCA
CAAAAAGGTATTGCGCTAGAAACAATTGCCGAAAGATTAGGGATTGAAATGAAAGAAGTCATGGCAATAGGTGACAATTTAAATGACTT
ATCAATGTTAGAGAAAGTTGGCTATCCAGTTGCGATGGAAAATGGTGCAGAAGAAGTTAAAAAAATAGCGAAATATGTCACAGATACGA
ATGAAAATAGTGGTGTTGGAAAAGCTATTATGAAATTATTACGTGAACAACAAGTTTAA

Gene: azo1 (FMN-dependent NADPH-azoreductase)

Contig: 05_NODE_4, position: 48903 to 49469, length: 567 nt, orientation: FORWARD

Perfect match to: (RF122-AJ938182-[572763:573329], highly conserved allele)

Sequence:

ATGAAAGGATTAATTATTATTGGCAGTGCACAAGTGAATTCACATACAAGTGCACTAGCAAGATACTTAACTGAGCATTTTAAAACACAT
GATATTGAAGCGGAAATATTCGATTTAGCAGAAAAACCGTTAAATCAATTAGATTTTTCAGGAACAACACCGTCTATTGATGAAATCAAA
CAAAATATGAAAGATTTAAAAGAGAAAGCAATGGCGGCGGACTTTTTAATATTAGGAACGCCAAACTATCATGGTTCATATTCTGGAATA
TTGAAAAATGCATTAGATCATCTAAATATGGATTATTTTAAAATGAAACCTGTAGGCTTAATAGGAAATAGTGGTGGTATTGTTAGTTCA
GAGCCATTGTCACATTTAAGAGTAATCGTCAGAAGTTTACTAGGCATTGCTGTACCAACTCAAATAGCAACACATGATTCTGATTTTGCTA
AAAATGAAGATGGTTCATATTACTTAAATGATAGTGAATTCCAATTACGAGCAAGATTATTTGTCGATCAAATTGTATCTTTTGTGAATAA
TAGTCCATATGAACATTTAAAATAA

Gene: sdrC (serine aspartate repeat protein C)

Contig: 05_NODE_4, position: 49900 to 52785, length: 2886 nt, orientation: FORWARD

Perfect match to: (ATCC51811-ST1-ADVP01000012-[76998:79883:r], allele observed in CC1)

Sequence:

ATGAATAATAAAAAGACAGTAACAAATAGAAAAGGCATGATACCAAATCGATTAAACAAATTTTCGATAAGAAAGTATTCTGTAGGTACT
GCTTCAATTTTAGTAGGGACAACATTGATTTTTGGGTTAAGTGGTCATGAAGCTAAAGCGGCAGAACATACGAATGGAGAATTAAATCA
ATCAAAAAATGAAACGACAGCCCCAAGTGAGAATAAAACAACTGAAAAAGTTGATAGTCGTCAACTAAAAGACAATACGCAAACTGCAA

CTGCAGATCAGCCTAAAGTGACAATGAGTGATAGTGCAACAGTTAAAGAAACTAGTAGTAACATGCAATCACCACAAAACGCTACAGCT
AGTCAATCTACTACACAAACTAGCAATGTAACAACAAATGATAAATCATCAACTACATATAGTAATGAAACTGATAAAAGTAATTTAACAC
AGGCTAAAGATGTTTCAGCTACTCCTAAAACAACGACTATTAAACCAAGAACATTAAACCGTATGGCTGTGAACACAGTAGCAGCACCAC
AACAAGGTACAAATGTTAATGATAAAGTTCATTTTTCAAATATTGATATTGCTATTGATAAAGGACACTTAAATAAAGATACTGGCAAAAC
AGAATTTTGGGCAACTTCAAGTGATGTTTTAAAATTAAAAGCGAATTACACAATCGATGATTCTGTTAAAGAGGGCGATACATTTACTTTT
AAATATGGTCAATATTTCCGTCCAGGATCAGTAAGATTACCTTCACAAACTCAAAATTTATATAATGCCCAAGGTAATATTATTGCAAAAG
GTATTTATGACAGTACAACAAACACAACAACGTATACTTTTACGAATTATGTAGATCAATATACAAATGTTAGCGGTAGCTTTGAACAAGT
CGCATTTGCGAAACGTGAAAATGCAACAACTGATAAAACTGCTTATAAAATGGAAGTAAGTTTAGGTAATGATACATATAGCGAAGAAA
TCATTGTCGATTATGGTAATAAAAAAGCACAACCGCTTATTTCAAGTACAAACTATATCAACAATGAAGATTTATCGCGTAATATGACTGC
ATATGTAAATCAACCTAAAAATACATATACTAAACAAACGTTTGTTACTAATTTAACTGGATATAAATTTAATCCAAATGCAAAAAACTTCA
AAATTTACGAAGTGACAGATCAAAATCAATTTGTCGACAGTTTCACCCCAGATACTTCAAAACTTAAAGATGTTACTAATCAATTTAATATT
ACTTACAGCAATGATAATAAGACTGCAACAGTTGATTTAATGAATGGTCAAACAAGTAGTAATAAACAATACATCATTCAACAAGTTGCG
TATCCAGATAATACATCAACAGATAATGGAAAAATTGATTATACTTTAGACACTGACAAAACTAAATATAGTTGGTCAAATAGTTATTCAA
ATGTGAATGGCTCATCAACTGCTAATGGCGACCAAAAGAAATATAATCTAGGTGACTATGTATGGGAAGATACAAATAAAGATGGTAAA
CAAGATGCCAATGAAAAAGGGATTAAAGGTGTTTATGTCATTCTTAAAGATAGTAACGGTAAAGAATTAGATCGTACGACAACAGATGA
AAATGGTAAATATCAGTTCACTGGTTTAAGCAATGGAACTTATAGTGTAGAGTTTTCAACACCAGCCGGTTATACACCGACAACTGCAAA
TGCAGGTACAGATGATGCTGTAGATTCTGATGGACTAACTACAACAGGTGTCATTAAAGACGCTGACAATATGACATTAGATAGTGGATT
CTACAAAACGCCAAAATATAGTTTAGGTGATTATGTTTGGTACGACAGTAATAAAGATGGTAAACAAGATTCGACTGAAAAAGGAATTA
AAGGTGTTAAAGTTACTTTGCAAAACGAAAAAGGCGAAGTAATTGGTACAACTGAAACAGATGAAAATGGTAAATACCGCTTTGATAAT
TTAGATAGTGGTAAATACAAAGTTATCTTTGAAAAGCCTGCTGGTTTAACTCAAACAGGTACAAATACAACTGAAGATGATAAAGATGCC
GATGGTGGCGAAGTTGATGTAACAATTACGGATCATGATGATTTCACACTTGATAATGGCTACTACGAAGAAGAAACATCAGATAGTGA
CTCAGATTCAGACAGCGACTCAGACTCAGATAGCGACTCAGATTCAGACAGCGACTCAGACTCAGATAGCGACTCAGATTCGGACAGCG
ACTCAGATTCAGATAGCGACTCAGATTCGGACAGCGATTCAGACTCAGACAGCGACTCAGACTCAGACAGTGATTCAGATTCAGACAGC
GATTCAGACTCAGATAGCGACTCAGATTCGGACAGCGATTCAGACTCAGATAGCGACTCAGATTCAGACAGCGACTCAGACTCAGACAG
TGATTCAGATTCAGACAGCGACTCAGATTCAGATAGCGACTCAGACTCAGACAGTGATTCAGATTCAGACAGCGATTCAGATACAGATA
GCGATTCAGACTCGGATAGCGACTCAGACTCGGATAGCGATTCAGACTCAGATAGCGATTCAGACTCAGACTCGGATAGCGATTCAGAT
TCAGACAGCGACTCAGATTCGGACAGCGATTCAGACTCAGATAGCGACTCAGACTCAGAAAGTGATGCCGATTCAGATACAGATAGCGA
TTCAGATGCAGGTAAACATACTCCGGCTAAACCAATGAGTACGGTTAAAGATCAGCATAAAACAGCTAAAGCATTACCAGAAACAGGTA
GTGAAAATAATAATTCAAATAATGGCACATTATTCGGTGGATTATTCGCGGCATTAGGATCATTATTGTTATTCGGTCGTCGTAAAAAACA
AAATAAATAA

Gene: sdrD (serine aspartate repeat protein D)

Contig: 05_NODE_4, position: 53152 to 57249, length: 4098 nt, orientation: FORWARD

Sequence:

ATGCTAAACAGAGAAAATAAAACGGCAATAACAAGAAAAGGCATGGTATCCAATCGATTAAATAAATTTTCGATTAGAAAGTACACAGT
GGGAACAGCATCAATTTTAGTAGGTACAACATTAATTTTTGGTCTGGGGAACCAAGAAGCAAAGGCTGCAGAAAGTACTAATAAAGAAT
TGAACGAAGCGACAACTTCAGCAAGTGATAATCAATCGAGTGATAAAGTTGATATGCAGCAACTAAATCAAGAAGACAATACTAAAAAT
GATAATCAAAAAGAAATGGTATCATCTCAAGGTAATGAAACGACTTCAAATGGGAATAAATCAATAGAAAAAGAAAGTGTACAATCTAC
CACTGGAAATAAAGTTGAAGTTTCAACTGCCAAATCAGATGAGCAAGCTTCACCAAAATCTACGAATGAAGATTTAAACACTAAACAAAC
TATAAGTAATCAAGAAGCGTTACAACCTGATTTGCAAGAGAATAAATCAGTGGTAAATGCTCAACCAACTAATGAGGAAAACAAAAAGG
TAGATGCCAAAACTGAATCAACTACATTAAATGTTAAAAGTGATGCTATCAAGAGTAATGCTGAAACTCTTGTTGATAACAATAGTAATTC
AAATAATGAAAATAATGCAGATATCATTTTGCCAAAAAGTACAGCACCTAAACGTTTGAATACAAGAATGCGTATAGCAGCAGTACAGCC
ATCATCAACAGAGGCTAAAAATGTTAATGATTTAATTACATCAAATACAACATTAACTGTCGTTGATGCAGATAAAAACAATAAAATCGTA
CCAGCACAAGATTATCTAGAATTAAAATCACAAATTAAAGTTGATGACAAAGTTAAATCAGGTGATTATTTCACAATAAAATACTCAGATA
CAGTACAAGTATATGGATTGAATCCGGAAGATATTAAAAATATTGGAGATATTAAAGATCCAAATAATGGTGAAACAATTGCGACTGCA
AAACATGATACTGCAAATAATTTAATTACATATACATTTACAGATTATGTCGATAGATTTAATTCTGTACAAATGGGAATTAATTATTCAAT
TTATATGGATGCTGATACAATTCCTGTTAGTAAAAACGATGTTGAGTTTAATGTTACGATAGGTAATACTACAACAAAAACAACTGCTAAC
ATTCAATATCCAGATTATGTTGTAAATGAGAAAAATTCAATTGGATCAGCGTTCACTGAAACAGTTTCACATGTTGGAAATAAAGAAAAT
CCAGGGTACTATAAACAAACGATTTATGTAAATCCATCGGAAAATTCTTTAACAAATGCCAAACTAAAAGTTCAAGCTTACCACTCAAGTT
ATCCTAATAATATCGGGCAAATAAATAAAGAGGTAACAGATATAAAAATATATCAAGTTCCTAAAGGTTATACATTAAATAAAGGATACG
ATGTGAATACTAAAGAGCTTACAGATGTAACAAATCAATACTTGCAGAAAATTACATATGGCGACAACAATAGCGCTGTTATTGATTTTG
GAAATGCAGATTCTGCTTATGTTGTAATGGTTAATACAAAATTCCAATATACAACTAGCGAAAGCCCAACACTTGTTCAAATGGTTACATT
AAGTTCAGACAATAGTAAATCTGCCTCAATGGGTAATGCTTTAGGATTCACTAATAACCAAAGTGGCGGAGCTGGTCAAGAAGTATATAA
AATTGGTAACTACGTATGGGAAGATACTAATAAAAACGGTGTTCAAGAATTAGGAGAAAAAGGCGTTGGCAATGTAACTGTAACTGTAT
TTGATAATAATACAAATACAAAAGTAGGAGAAGCAGTTACTAAAGAAGATGGGTCATACTTGATTCCAAACTTACCTAATGGAGATTACC
GTGTAGAATTTTCAAACTTACCAAAAGGTTATGAAGTAACCCCTTCAAAACAAGGTAATAACGAAGAATTAGATTCAAACGGCTTATCTTC

AGTTATTACAGTTAATGGCAAAGATAACTTATCTGCAGACTTAGGTATTTACAAACCTAAATACAACTTAGGTGACTATGTCTGGGAAGAT
ACAAATAAAAATGGTATCCAAGACCAAGATGAAAAAGGTATATCTGGCGTAACGGTAACATTAAAAGATGAAAACGGTAACGTGTTAAA
AACAGTTACAACAGACGCAGATGGCAAATATAAATTTACTGATTTAGATAATGGTAATTATAAAGTTGAATTTACTACACCAGAAGGCTA
TACACCGACTACAGTAACATCTGGTAGCGACATTGAAAAAGACTCTAATGGTTTAACAACAACAGGTGTTATTAATGGTGCTGATAACAT
GACATTAGATAGTGGATTCTACAAAACACCAAAATATAATTTAGGTAATTATGTATGGGAAGATACAAATAAAGATGGTAAGCAGGATT
CAACTGAAAAAGGTATTTCAGGCGTAACAGTTACATTGAAAAATGAAAACGGTGAAGTTTTACAAACAACTAAAACAGATAAAGATGGT
AAATATCAATTTACTGGATTAGAAAATGGAACTTATAAAGTTGAATTCGAAACACCATCAGGTTACACACCAACACAAGTAGGTTCAGGA
ACTGATGAAGGTATAGATTCAAATGGTACATCAACAACAGGTGTCATTAAAGATAAAGATAACGATACTATTGACTCTGGTTTCTACAAA
CCGACTTACAACTTAGGTGACTATGTATGGGAAGATACAAATAAAAACGGTGTTCAAGATAAAGATGAAAAGGGTATTTCAGGTGTAAC
AGTTACGTTAAAAGATGAAAACGACAAAGTTTTAAAAACAGTTACAACAGATGAAAATGGTAAATATCAATTCACTGATTTAAACAATGG
AACTTATAAAGTTGAATTCGAGACACCATCAGGTTATACACCAACTTCAGTAACTTCTGGAAATGATACTGAAAAAGATTCTAATGGTTTA
ACAACAACAGGTGTCATTAAAGATGCAGATAACATGACATTAGACAGTGGTTTCTATAAAACACCAAAATATAGTTTAGGTGATTATGTT
TGGTACGACAGTAATAAAGACGGCAAACAAGATTCAACTGAAAAAGGTATCAAAGATGTTAAAGTTACTTTATTAAATGAAAAAGGCGA
AGTAATTGGAACAACTAAAACAGATGAAAATGGTAAATATCGTTTCGATAATTTAGATAGCGGTAAATACAAAGTTATTTTTGAAAAGCC
TGCTGGCTTAACACAAACAGGTACAAATACAACTGAAGATGATAAAGATGCAGATGGTGGCGAAGTTGACGTAACAATTACGGATCATG
ATGATTTCACACTTGATAACGGATACTTCGAAGAAGATACATCAGATAGCGACTCAGATTCAGATAGCGATTCAGATTCAGATAGCGACT
CAGACTCAGACAGCGACTCAGATTCAGACAGCGACTCAGACTCGGATAGCGATTCAGATTCAGACAGTGATTCAGATTCAGACAGCGAT
TCGGATTCAGACAGCGACTCAGACTCGGATAGCGACTCAGACTCAGATAGCGATTCAGATTCAGATAGCGATTCGGACTCAGACAGTGA
TTCAGATTCAGACAGCGACTCAGATTCAGATAGCGATTCAGACTCAGACAGTGATTCAGATTCAGATAGCGATTCAGAATCAGACAGCG
ATTCAGACAGCGACTCAGACTCGGATAGCGACTCAGACTCAGACAGCGATTCAGACTCGGATAGCGATTCAGATTCGGACAGCGACTCA
GACTCAGACAGTGATTCAGATTCAGATAGCGACTCAGACTCAGATAGCGACTCAGATTCAGACAGCGATTCAGACTCAGATAGTGACTC
AGATTCGGACAGCGATTCAGACTCAGATAGCGACTCAGATTCAGACTCAGATGCAGGTAAGCACACACCTGTTAAACCAATGAGTACTA
CTAAAGACCATCACAATAAAGCAAAAGCATTACCAGAAACAGGTAATGAAAATAGCGGCTCAAATAACGCAACGTTATTTGGCGGATTA
TTCGCAGCATTAGGATCATTATTGTTATTCGGTCGTCGTAAAAAACAAAATAAATAA

Gene: bbp (bone sialoprotein-binding protein)

Contig: 05_NODE_4, position: 57643 to 61068, length: 3426 nt, orientation: FORWARD

Perfect match to: (MW2-BA000033-[603446:606871], allele observed in CC1)

Sequence:

ATGATTAACAGGGATAATAAAAAGGCAATAACAAAAAAGGGTATGATTTCAAATCGCTTAAACAAATTTTCGATTAGAAAGTATACTGTA
GGAACTGCATCGATTTTAGTAGGTACGACATTGATTTTTGGTCTAGGGAACCAAGAAGCTAAAGCTGCTGAAAACACTAGTACAGAAAA
TGCAAAACAAGATGATGCAACGACTAGTGATAATAAAGAAGTAGTGTCAGAAGCTGAAAATAATTCGACAACAGAAAATGATTCAACAA
ATCCAATTAAGAAAGAAACAAATACTGATTCACAACCAGAAGCTAAAGAAGAATCAACTAAATCAAGTACTCAACAACAGCAAAATAAC
GTTACAGCTACAACTGAAACTAAGCCTCAAAACATTGAAAAAGAAAATGTTAAACCTTCAACTGATAAAACCGCGACAGAAGATACATCT
GTTATTTTAGAAGAGAAGAAAGCACCAAATAATACAAATAACGATGTAACTACAAAACCATCTACAAGTGAAATTCAAACAAAACCAACT
ACACCTCAAGAATCTACAAATATTGAAAATTCACAACCGCAACCAACGCCTTCAAAAGTAGACAATCAAGTTACAGATGCAACTAATCCA
AAAGAACCAGTAAATGTGTCAAAAGAAGAACTTAAAAATAATCCTGAGAAATTAAAAGAATTGGTTAGAAATGATAGCAATACAGATCA
TTCAACTAAACCAGTTGCTACAGCTCCAACAAGTGTTGCACCAAAACGTGTAAACGCAAAAATGCGCTTTGCAGTTGCACAACCAGCAGC
AGTTGCTTCAAACAATGTAAATGATTTAATTAAAGTGACGAAGCAAACAATCAAAGTTGGCGATGGTAAAGATAATGTGGCAGCAGCGC
ATGACGGTAAAGATATTGAATATGATACAGAGTTTACAATTGACAATAAAGTCAAAAAAGGCGATACAATGACGATTAATTATGATAAG
AATGTAATTCCTTCGGATTTAACAGATAAAAATGATCCTATCGATATTACTGATCCATCAGGAGAGGTCATTGCTAAAGGAACATTTGATA
AAGCAACTAAGCAAATCACATATACATTTACAGACTATGTAGATAAATATGAAGATATAAAATCACGCTTAACTCTATATTCGTATATTGA
TAAAAAAACAGTTCCAAATGAGACAAGTTTGAATTTAACATTTGCTACAGCAGGTAAAGAAACAAGCCAAAATGTCACTGTTGATTATCA
AGATCCAATGGTCCATGGTGATTCAAACATTCAATCTATCTTTACAAAATTAGATGAAGATAAGCAAACTATTGAACAACAAATTTATGTT
AACCCATTGAAAAAATCAGCAACCAACACTAAAGTTGATATAGCTGGTAGTCAAGTAGATGATTATGGAAATATTAAACTAGGAAATGG
TAGCACCATTATTGACCAAAATACAGAAATAAAGGTTTATAAAGTTAACTCTGATCAACAATTGCCTCAAAGTAATAGAATCTATGATTTT
AGTCAATACGAAGATGTAACAAGTCAATTTGATAATAAAAAATCATTTAGTAATAATGTAGCAACATTGGATTTTGGTGATATTAATTCAG
CCTATATTATCAAAGTTGTTAGTAAATATACACCTACATCAGATGGCGAACTAGATATTGCCCAAGGTACTAGTATGAGAACAACTGATA
AATATGGTTATTATAATTATGCAGGATATTCAAACTTCATCGTAACTTCTAATGACTCTGGTGGTGGCGACGGTACTGTTAAACCTGAAGA
AAAGTTATACAAAATTGGTGACTATGTATGGGAAGACGTTGATAAAGACGGTGTTCAAGGTACAGATTCAAAAGAAAAACCAATGGCAA
ACGTTTTAGTTACATTAACTTACCCGGACGGTACTACAAAATCAGTAAGAACAGATGCTAAAGGTCATTACGAATTCGGTGGTTTGAAAG
ACGGAGAAACTTATACAGTTAAATTCGAAACGCCAACTGGATATCTTCCAACAAAAGTAAATGGAACAACTGATGGTGAAAAAGACTCA
AATGGTAGTTCTGTAACTGTTAAAATTAATGGTAAAGATGATATGTCTTTAGACACTGGTTTTTATAAAGAACCTAAATATAATCTTGGTG
ACTATGTATGGGAAGATACTAATAAAGATGGTATCCAAGATGCAAATGAGCCAGGAATCAAAGATGTTAAGGTTACATTAAAAGATAGT
ACTGGAAAAGTTATTGGTACAACTACTACTGATGCCTCGGGTAAATATAAATTTACAGATTTAGATAATGGTAATTATACTGTTGAATTCG
AAACACCAGCAGGTTACACGCCAACGGTTAAAAACACTACAGCTGAAGATAAAGATTCCAATGGTTTAACAACAACAGGTGTCATTAAA

GATGCAGATAATATGACATTAGACAGTGGTTTCTATAAAACACCAAAATACAGTTTAGGTGATTATGTTTGGTACGACAGTAATAAAGAT
GGCAAACAAGACTCAACTGAAAAAGGTATTAAAGATGTGACAGTTACATTGCAAAACGAAAAAGGCGAAGTAATTGGAACAACTAAAA
CAGATGAAAATGGTAAATATCGTTTCGATAATTTAGATAGCGGTAAATACAAAGTTATTTTTGAAAAGCCTGCTGGCTTAACACAAACAG
TTACAAATACAACTGAAGATGATAAAGATGCAGATGGTGGCGAAGTTGACGTAACAATTACGGATCATGATGATTTCACACTTGATAAC
GGATACTTCGAAGAAGATACATCAGACAGTGATTCAGACTCAGACAGCGATTCAGACTCAGATAGCGACTCAGATTCAGACAGTGACTC
AGACTCAGATAGCGATTCAGACTCAGAAAGCGATTCAGATTCAGACAGCGACTCAGACTCAGATAGTGATTCAGATTCAGATAGCGACT
CAGACTCAGATAGCGACTCAGATTCGGACAGCGATTCAGACTCAGATAGCGACTCAGATTCAGACAGCGATTCAGACTCAGATAGCGAC
TCAGATTCAGACAGTGACTCAGACTCAGATAGCGACTCAGACTCAGACAGTGACTCAGACTCAGACAGCGATTCAGATTCAGATAGCGA
CTCAGACTCAGATAGCGACTCAGATTCGGACAGCGACTCAGACTCAGATAGCGACTCAGACTCAGACAGTGATTCAGACTCAGATAGCG
ATTCGGACTCGGATGCAGGAAAACATACACCTGTTAAACCAATGAGTACTACTAAAGACCATCACAATAAAGCAAAAGCATTACCAGAA
ACAGGTAGTGAAAATAACGGCTCAAATAACGCAACGTTATTTGGTGGATTATTTGCAGCATTAGGTTCATTATTGTTATTCGGTCGTCGCA
AAAAACAAAACAAATAA

Gene: sdgA (serine-aspartate repeat glucosyltransferase A)

Contig: 05_NODE_4, position: 61187 to 62659, length: 1473 nt, orientation: FORWARD

Perfect match to: (08-02119-CP015645-[1626533:1628005:r], allele observed in ST582+CC1+CC8+CC239)

Sequence:

ATGAATTACATTTTAGGAACAATTTTAGAAAGTAAAATTACAGGTGTAGAAAAAGCGCAAATAAATAGATTGAAGTTGTTCAAACAACAC
GGCATATCTTCAAAATGTGTATATGTTAAATGGAATCCTTATTCATACACATATGCGAAGCAACATCAGATTGAAAATGATGTATTTACAA
TGTATGACTATTTTCAAAAAGCAATCAATTATAAAAAGACAAAGCAAGTTAACTGGATACAGTATTGGGAAAAGTCATGTAGGTACACAT
TGAAATTTGTGGAAAATTCAAATGATGTCAGAATATATGATGAAGAGCAATTTATAATGTATGCTCATTTTTTAGATAAACAGTATCATCA
TTTAAACTATGTGAATTATTTTGATCATAAAAGAAGAAAAGTAAAACGCGAATTGTATGATGGAAGAGGCTTTTTAAGTTGTTCTCGAATT
TTAGGTGAAGGACAACGGATTGTACTCGAAAATTACTATACACCTAATGGGGAAATCGTCATCCAAAAATATTTCGACGATATAAAAGG
GAAAAACACGCTCACAAAGGTTATCTTAAATGAAGATCAGCATCAACAATTTTTTGATACAGAAGATGAATTAGTTCAATATTTTCTCCAT
CAATTATGTAAAAATAATGATCAAATCATATTAGATCGTCCTCATGAATTAGGAAATGTTATAGCGGGATTAAATCAAAGTATTCCAGTTA
TTGTTGTGCTCCACAGTACACATTTAGCCGACACCGGTAATGGTATAAAAAGTTTTTATAAAACAGTGTTTAATAATTTAACACGTTATAA
AGCGATTGTTGTATCAACAGAACAGCAATGCCAAGATATTTCACAATATATTGAAAATAAAATACCAGTTATCAATATTCCGGTTGGCTAC
GTGGCAAATTTAAAGTATCAATTTGACATCAATCAAAAGGAGAAAAATCATATCATATCAATTGCTCGCCTCGTTGAAAATAAACAAATTA
AACATCAAGTTGAAGTGATCAAGCAATTAGTAACAAAACATCCCAATATTCAATTGAATATTTATGGACATGGAAATGGTTTGTCAGAAT
ATCGACAACTTGTAGAAGATTATCATTTATCGGAACATGTAAAATTTCATGGTTTTAAGACGCATATTAATGAAGAGATTGCTAAAGCAG
AACTGATGTTATCGACAAGTAAAATGGAAGGTTTTGGCTTAGCAATTTTAGAGTCGCTTTCAGTAGGTACACCAGTGATCAGTTATGATG
TAGATTATGGGCCATCAGAACTGATTCAAGATGGATTTAATGGCTATTTAGTACCTCAAGGTGACATCAATCAAATGGTTGAAAAGGTCG
ACCAATTACTAAATAATACTCAAAAATTGCAACAGTTTTCGATTAATAGCATAGAATCTGCACAACAGTACAATGCAACTACTATCAGTAC
AAAGTGGCAAAATATTTTAAACTAA

Gene: sdgB (serine-aspartate repeat glucosyltransferase B)

Contig: 05_NODE_4, position: 62783 to 64273, length: 1491 nt, orientation: REVERSE

Sequence:

TTATATAAGACGAACAAATTGGTCCCATTGTTTAATTAACGACGCTTTACTATATTGTTGCGCTTTTGCTAAACTACCTTTTGACAGTCGTT
GTTGTACTTCAGGATGATCAATCACATATTTTACTTTATCAAATAGGGCATCTTCATCATTTTTAGTAATTAAATAACCATTGAAATCAGGC
GTAATCAATTCGTTAGGTCCATATTTAATATCATAACTAATAACTGGAACACCATGTGCTAAAGATTCAAGTAGCGCTAAAGAGAAACCTT
CCATGTTACTTGTTATTAAACTCAAATAGGCATCGCTATATTCTTGGTCTAGATTGCTTAAAAAGCCGCGTAAGTAAACATGATTTTCCAAT
CCATATTTTTGTATCAATTCATTTAATTTTTTACTTTCAGAACCAAAACCATACATATGAAGCTCTATTTTTGGGACGTACGATACTAAGCGT
TTAATTAATTCAATTTGTTGATGTAATTGTTTTTCAGGTGAATAACGAGCAACGGAAATTAATTTAACACTGCGCTGATCTAATGTTTGGA
CTGGTGTATCAATTGTTTCACTATAGCCGACAGGAATATTAACAACTGGAATAGTATGGTTAATACGTTTTTCAACATCTAATTTTTGCTGC
TCAGTAGAAACGATAATTGCACGATATCGAGATAAATTTTCAAACATCGCTTTATATACATTTTTAAATGGCGATGAATCTAATGCATCAA
TATTTTTAATGTGTGTACTGTGAAGCACAGCTACTACTGGGATTGACTCAGGCGTTAAGTTGAAAATAGGTGCTGTGTACACATTACGAT
CACTGAAAAATAAATCCCCATGTTGATATAGTTGTTTAATGAAAAATGCGCCTAATTCCGTTTCATTATTAAAGAAATATTGTTTGTTAGCA
TAGTAAACAATAATTTTTTGTACTTCTGGTTTGCCATCCTTGTAAGAAAAATACTTTTCTAATTTTGTGTCACCTTCTGGATTATAGAAAAAT
TCACATAATGTTTGTTGTTTATCAACAAGAATCCTACTACAACTTAAAAAGCCACGCACATCATAAAAATCACGTTTTACTTTTCGTCTTTG
ACTATCAAAATGATTCACATAATCTAATATACGATATTTAGGATCTTGAAAATGGGCATACATTAAGAAACGCTCTTGATCATATATTCTA
AAGTCATGACTATTTTCAACATGTTTTAAAGTATAATGACATTCATCAGTCCAATACGACAACCAGTCAAATGGTTCATTGCGTTCTAAATA

TGTTGCTTCTTGGAAGAAATCATACATATTAATATAGTCAGAACTAGTAATATAATTTTGGGCATTTCTATATAAATATCTATTCCATGACA
GAAATACACATTGCGCTGGTCTTCCCATTTCTTTAAATAAATTTAAACGATTAATAATTGCTTTCTCTATCCCAGTTAAATTAACACCTAAAC
TATTACCTACAAAATAATTCAT

Gene: folE2 (GTP cyclohydrolase)

Contig: 05_NODE_4, position: 64555 to 65433, length: 879 nt, orientation: REVERSE

Perfect match to: (MW2-BA000033-[610358:611236:r], allele observed in CC1+CC22)

Sequence:

TTATTTGCGATACTTTAATTTAGCGAAAGCATCATGTTGATGGATAGACTCTTCATTACGACATTCGATATCGAAACCGTCTAACCAATCA
AATTCAACTAAGTCCGCGGCAATTAAACGAATTAAGTCTTCGACAAAACGTGGATTTTCATATGCACGCTCTGTCACACGTTTTTCATCAG
GACGTTTTAAAATAGGGTATAGAATTGAACTTGCATTAGCTTCCATTGCATCTAAAATTTTATTTTTATAGTCATCAACTATGTCTTGATCTT
TATTAATATATGTTTTAACAGTGACAACACCACGTTGGTTGTGCGCTGAATACTCACTTATTTCTTTTGAACAAGGGCATAGCGTTGTGAC
AGTTGCTTCAATAGTAAGTTCTTTACGTGTAACTTTATCACCGTCAATTGCTAATCCATAAGTGACATCGGCATTACCAACTGCTTTAATAT
TTGTGGTTGGACTATAGCGATCAAAGAACCATTTCCCAGAAACATCAACGCCTGCCGCATTTTGTTTCATATTCGTTTGTAAAGTGCGTAA
CACCTGATAAAGTGTATTAAATTCAAGTTCAATACCATTATCATAGTGCTTTTCAACACTTTCAATTATACGGCTCATATTAATACCTTTTTC
GTCTTTTGTTAAACTTGTTGAAAAACTAAATGTGCCAGCTGTTTGATACTGGTCAACAAGTACAGGGTACACTAAGTTTTTAATACCAACT
TCTTCTATTTCAAATAAAAAATCTTTATGTGTACTTTGTAAGTCTGTCATTTCGTTCTTAGTAGTAGGTTTCGTGCCTTCAATAGGATCTACG
GAACCAAAGTGTTTCCAACGACCTTCTCGTGTCGATAAATCAAATTCAGTCAT

Gene: bshB2 (bacillithiol biosynthesis deacetylase)

Contig: 05_NODE_4, position: 65446 to 66111, length: 666 nt, orientation: REVERSE

Perfect match to: (N315-BA000018-[620800:621465:r], highly conserved allele)

Sequence:

TTAAGATTTAAAGTGATATGTCCAATATGGTTCGACTGTTAAAAAGCTGTGTTGTTTACCATCGATTTCAGGACTTGCTAATTGTTTTAAAA
ATGGACCTGTTTGAGAAGCATGTGCTTCAAATGCCTTAATTTTAAGTTCTTTAAAATCTGTAATATCATTTTGAATATCAGGTTCTCCAAGA
GCTTCGGTTGCATCATTACTGAACGCAACTAAAGTTAAACGAGGGCGTTCTTCTTTAGGCATGCGTTCAACCGTTCGAATTACAGCGTCTG
CTGTTGCTTCGTGATCAGGATGTACTGCATATCCAGGATAAAATGAAATAATCAATGATGGATTTGTATCATCGATTAAAGATTTAATCAT
ACCATCTATATGTTCGTAGGGTTCAAATTCGACAGTTTTGTCACGTAAACCCATTTTTCTTAAATCAGTAATACCAATAACTTTACAAGCTT
CTTCTAGTTCACGCTCACGAATACTTGGTAATGATTCGCGTGTTGCAAATGGGGGATTACCTAAATTTCTGCCCATTTGTCCTAGGGTTAA
ACATGCATATGTTACAGGTATGCCTTTTTGGATATAACTTGCTAATGTGCCTGCAGATGAGAAGGTTTCATCATCAGGATGTGGAAATATT
ACTAATACATGTCTTTCGTCAGTCAT

Gene: yojF (protein of unknown function, DUF1806)

Contig: 05_NODE_4, position: 66125 to 66487, length: 363 nt, orientation: REVERSE

Perfect match to: (N315-BA000018-[621479:621841:r], highly conserved allele)

Sequence:

CTATAAATTAAATGGTCGCTCACTAATTTGAAGTGCTGCAGCGAGTTGACCTTCGTAATTAAAACCTGCAATTAAAAATTCATCATGTTCA
TTGACCTCAAAATGCGTTAGACCTTGTACATAAACCCAACCACCATTTGATAGTTTAAGACCAATGCGATAAGGTTCTTTATTACCACCTTT
TAGTTGTGCATGCGTATATGTTACTTGTATGTTTCTTAAAAATGTACCAGCATTAAAAACACGTTGATCGAAATGGTTCGCATAGGCCCCA
TTTGTCGTTTCAACATGCAGATACACAGGTTTATGTTCAAAAGAAGCAAGTAAATCTATAACTTCTTGTTCTTTAATTGGTTCCAACAC

Gene: nagB (glucosamine-6-phosphate deaminase)

Contig: 05_NODE_4, position: 66763 to 67521, length: 759 nt, orientation: FORWARD

Perfect match to: (MW2-BA000033-[612566:613324], allele observed in CC1+CC361)

Sequence:

ATGAAAGTATTAAACTTAGGATCGAAAAAACAAGCATCATTCTATGTTGCATGTGAGTTATATAAAGAGATGGCATTTAATCAGCACTGT
AAACTTGGTTTAGCAACTGGTGGTACAATGACAGATTTGTATGAGCAACTTGTTAAGTTATTAAATAAAAATCAGTTAAACGTAGACAAT
GTATCCACGTTTAATTTAGACGAATATGTAGGTTTAACCGCATCACATCCGCAAAGTTATCACTATTATATGGATGACATGCTTTTCAAAC
AATATCCTTATTTTAATAGAAAGAACATTCATATTCCAAATGGAGATGCCGATGATATGAATGCGGAAGCGTCAAAATATAATGACGTTTT
AGAACAACAAGGTCAACGTGATATTCAAATTTTAGGTATTGGAGAAAATGGTCATATTGGATTTAATGAACCTGGTACGCCGTTTGACAG
CGTTACTCATATCGTTGATTTGACTGAAAGTACTATTAAGGCTAATAGTCGATATTTTAAAAACGAAGATGATGTTCCAAAGCAAGCCATT
TCGATGGGACTTGCTAATATTCTTCAAGCCAAACGTATCATTTTACTCGCATTTGGTGAAAAGAAACGTGCTGCTATTACACATTTATTAA
ATCAGGAAATTTCTGTTGATGTTCCAGCCACATTACTTCACAAACACCCGAATGTTGAGATATATTTAGACGACGAAGCTTGCCCGAAAAA
TGTTGCGAAAATTCATGTCGATGAAATGGATTGA

Gene: hxlA (3-hexulose-6-phosphate synthase)

Contig: 05_NODE_4, position: 67598 to 68230, length: 633 nt, orientation: FORWARD

Perfect match to: (MW2-BA000033-[613401:614033], highly conserved allele)

Sequence:

GTGGAATTACAATTAGCAATTGATTTATTAAACAAAGAAGACGCGGCTGAGTTAGCAAATAAAGTAAAAGATTATGTAGATATCGTAGA
AATCGGTACGCCAATCATTTACAACGAAGGTTTACCAGCAGTTAAACATATGGCAGACAACATTAGTAATGTAAAAGTATTAGCAGACAT
GAAAATTATGGATGCAGCTGATTATGAAGTTAGCCAAGCAATTAAATTTGGCGCGGATGTAATTACAATACTAGGTGTTGCAGAAGATG
CATCAATTAAAGCAGCTATTGAAGAAGCTCATAAAAATAATAAACAATTACTAGTTGATATGATTGCTGTTCAAGATTTAGAAAAACGTG
CAAAAGAACTAGATGAAATGGGTGCGGATTATATTGCAGTACACACTGGTTATGATTTACAAGCAGAAGGGCAATCACCATTAGAAAGT
TTAAGAACCGTTAAATCTGTTATTAAAAATTCTAAAGTTGCAGTAGCAGGTGGAATTAAACCAGATACAATTAAAGAAATTGTCGCTGAA
AGTCCTGATCTTGTTATTGTTGGTGGCGGAATCGCAAATGCAGATGATCCAGTAGAAGCTGCAAAACAATGTCGCGCTGCAATCGAAGG
TAAGTAA

Gene: sis (sugar isomerase)

Contig: 05_NODE_4, position: 68232 to 68780, length: 549 nt, orientation: FORWARD

Perfect match to: (MW2-BA000033-[614035:614583], allele observed in CC1+CC80+CC361)

Sequence:

ATGGCTGAATTTAGTGACTATCAATTAATTCTAGATGAATTAAAGATGACTTTGTCACATGTTGAAGCGGATGAGTTTTCAACGTTTGCAT
CCAAAATACTACATGCTGAACATATATTTGTAGCTGGTAAAGGACGTTCAGGATTCGTGGCGAATAGTTTTGCAATGCGCTTAAATCAGC
TCGGCAAACAGGCACATGTTGTTGGAGAATCAACGACACCTGCGATTAAGTCGAATGATGTATTTGTAATTATCTCTGGTTCAGGTTCCA
CGGAACATTTAAGATTATTAGCAGACAAAGCAAAATCAGTAGGTGCTGACATCGTATTAATTACTACAAATAAAGATTCTGCAATAGGCA
ATCTAGCTGGGACGAACATCGTTTTGCCTGCAGGTACAAAATATGATGAACAAGGCTCGGCACAACCATTAGGAAGTTTGTTTGAACAA
GCATCTCAATTATTTTTAGATAGTGTTGTAATGGGATTGATGACTGAAATGAATGTTACGGAACAAACGATGCAACAAAATCATGCTAAT
TTAGAATAA

Gene: Q2FJ68 (putative haloacid dehalogenase-like hydrolase)

Contig: 05_NODE_4, position: 68893 to 69540, length: 648 nt, orientation: FORWARD

Perfect match to: (MW2-BA000033-[614696:615343], allele observed in CC1+CC5)

Sequence:

TTGAAGTTTGACAATTATATTTTTGATTTTGATGGTACGTTGGCAGACACGAAAAAATGTGGTGAAGTAGCAACACAAAGTGCATTTAAA
GCATGTGGCTTAACGGAACCATCATCTAAAGAAATAACGCATTATATGGGAATACCTATTGAAGAATCATTTTTAAAATTAGCAGACCGA
CCATTAGATGAAGCAGCATTAGCAAAGTTAATCGATACATTTAGACATACATATCAATCTATTGAAAAGGACTATATTTATGAATTTGCGG
GTATAACTGAAGCCATTACAAGTTTGTATAACCAAGGGAAAAAACTTTTCGTGGTGTCTAGTAAGAAGAGTGATGTATTAGAAAGAAATT
TATCGGCTATTGGATTAAATCACTTGATTACCGAAGCTGTTGGATCCGATCAAGTAAGTGCATATAAACCAAATCCTGAAGGCATACACA
CAATTGTGCAACGCTACAATTTAAATAGCCAACAAACGGTTTATATTGGTGATTCAACATTTGATGTTGAGATGGCGCAACATGCTGGTG

TACCATCTGCAGCAGTAACATGGGGAGCACATGATGCAAGGTCATTACTTCATTCAAATCCGGATTTTATAATCAATGATCCATCAGAAAT
TAATACAGTATTATAA

Gene: proP (putative proline/betaine symporter)

Contig: 05_NODE_4, position: 70042 to 71442, length: 1401 nt, orientation: FORWARD

Perfect match to: (MW2-BA000033-[615845:617245], allele observed in CC1+CC239)

Sequence:

ATGGATTTTAATAAAGAGAATATTAACATGGTGGATGCAAAGAAAGCTAAAAAAACCGTTGTTGCAACCGGTATCGGTAATGCAATGGA
ATGGTTCGATTTTGGTGTCTATGCATATACAACTGCGTACATTGGAGCGAACTTCTTCTCTCCAGTAGAGAATGCAGACATTCGACAAATG
TTGACTTTCGCAGCATTAGCCATTGCGTTTTTATTAAGACCAATTGGTGGTGTCGTATTTGGTATTATTGGTGACAAATATGGACGTAAAG
TTGTATTAACATCTACAATTATTTTAATGGCATTTTCAACATTAACCATTGGATTATTGCCAAGCTATGATCAAATTGGACTTTGGGCACCA
ATACTATTATTGCTTGCAAGAGTACTACAAGGGTTTTCAACAGGTGGAGAGTATGCGGGGGCAATGACATATGTTGCCGAATCATCTCCA
GATAAGCGTCGTAACTCATTAGGTAGTGGACTAGAAATTGGGACATTATCAGGTTACATAGCTGCTTCAATTATGATTGCTGTATTAACAT
TCTTTTTAACAGATGAACAAATGGCATCATTTGGTTGGAGAATCCCATTCTTACTCGGTTTATTCCTAGGATTATTCGGCTTATATTTACGT
CGTAAGCTGGAAGAATCACCAGTTTTCGAAAATGATGTTGCAACACAACCAGAAAGAGATAACATTAACTTTTTACAAATCATCAGATTTT
ATTACATAGATATATTTGTATGTTTTGTAGCTGTTGTATTCTTCAATGTTACAAACTATATGGTAACTGCATATTTACCAACCTATTTAGAAC
AAGTTATTAAATTAGATGCAACGACAACAAGTGTATTAATTACTTGTGTCATGGCAATAATGATTCCATTAGCATTAATGTTTGGTAAGTT
AGCGGATAAAATAGGTGAAAAGAAAGTATTTCTAATTGGTACTGGTGGGCTAACATTATTCAGTATCATCGCATTTATGTTATTACATTCA
CAATCATTTGTTGTAATAGTAATCGGTATATTTATATTAGGATTTTTCTTATCAACTTACGAAGCGACAATGCCAGGGTCGTTACCAACGAT
GTTTTACAGTCATATAAGATATCGAACTTTATCAGTAACATTTAATATCTCTGTTTCGATATTTGGTGGTACGACGCCATTAGTTGCAACAT
GGTTAGTTACGAAAACTGGAGATCCATTAGCACCTGCGTATTATTTAACAGCAATCAGTGTTATTGGCTTTTTAGTTATTACATTCTTACAT
TTAAGTACAGCAGGAAAATCTCTAAAAGGTTCGTATCCAAATGTAGATAACGAGCAAGATAGAGCTTATTATGCAGAACATCCAAAAGA
AGCATTATGGTGGGTTAAAGAACGTAAGAATTAG

Gene: rsaOI (structural RNA)

Contig: 05_NODE_4, position: 71443 to 71776, length: 334 nt

Sequence:

AGATTTTAATAAAAAGTATAAATCAATCGTATATAAGCACTTTAAAGCTAGTAGGTTCTGCTAACTTTAAAGTGCTTTTTAAATTGAGAAC
TGTAATTAGCCGTAATAAAGTTTTTGTATATACATAAACCCCCACTGCAATGATTATCGCAATGGGGGAAAGAGGGGACTTAAAGCATAT
GTTTAGCTTTGAATACTTAAAATTCTCTTGCTATTGAAATGTTAGGATGTAAATATGTCTTAGAGTATTTTGTCCAACGCAATTAATATTGA
GACTCTAACCTTCAATATTATTATAGAGAACACAAACTTAAATAGATTGGGTGACTTATTT

Gene: vraA (long chain fatty acid CoA ligase)

Contig: 05_NODE_4, position: 71986 to 73362, length: 1377 nt, orientation: FORWARD

Sequence:

ATGAACGTAATTTTAGAACAGTTGAAAACACATACTCAAAATAAACCTAATGACATAGCATTACATATCGATGATGAAACAATTACATATA
GTCAACTAAATGCCCGCATCACTAGCGCAGTTGAATCTTTGCAGAAATATTCACTTAACCCTGTCGTTGCTATTAATATGAAATCACCGGT
GCAAAGTATTATTTGTTATTTAGCTTTGCATCGTTTACATAAAGTGCCTATGATGATGGAAGGTAAATGGCAAAGTACTATACATCGTCAA
TTGATTGAAAAATATGGTATTAAAGATGTAATTGGAGATACAGGTCTCATGCAGAATATAGACTCACCGATGTTTATTGATTCAACGCAA
TTACAGCACTACCCCAATTTATTACATATTGGTTTTACTTCAGGGACAACTGGACTGCCAAAAGCATATTATCGTGATGAAGATTCATGGT
TGGCTTCTTTTGAAGTTAATGAAATGTTGATGTTAAAAAATGAAAATGCAATAGCAGCCCCTGGACCACTATCGCACTCGTTAACATTATA
TGCGTTATTGTTTGCTTTAAGTTCCGGTCGTACTTTTATAGGACAGACCACTTTTCATCCTGAAAAGTTACTTAATCAATGTCATAAAATAT
CATCATACAAAGTTGCTATGTTTCTTGTTCCAACGATGATTAAATCATTATTGTTAGTTTACAACAATGAACATACAATCCAATCATTTTTTA
GCAGTGGAGATAAGCTGCATTCTTCTATTTTTAAAAAGATAAAAAATCAAGCAAATGACATAAATTTGATTGAATTTTTTGGTACATCGGA
AACCAGTTTTATCAGCTATAACTTGAATCAGCAAGCACCAGTTGAATCAGTAGGTGTGCTATTTCCAAATGTGGAATTGAAAACAACGAA
TCACGATCACAATGGTATAGGAACTATTTGTATAAAAAGTAATATGATGTTTAGTGGCTATGTAAGTGAACAATGTATAAATAATGATGA
ATGGTTTGTTACTAATGATAATGGCTATGTAAAAGAGCAGTATTTATATTTAACGGGACGTCAACAGGATATGTTAATTATTGGTGGTCA
AAATATATATCCAGCACATGTTGAACGCCTTTTAACGCAATCTTCGAGCATTGATGATGCAATTATCATCGGTATTCCAAATGAGCGTTTT
GGTCAAATAGGCGTATTGCTTTATTCTGGTGATGTGACACTTACACATAAAAATGTAAAACAATTTTTAAAAAAGAAAGTGAAACGCTAT

GAAATTCCATCGATGATTCATCATGTAGAAAAGATGTATTACACTGCAAGTGGTAAAATTGCTAGAGAAAAAATGATGTCGATGTATTTG
AGAGGTGAATTATAA

Gene: vraB (acetyl-CoA c-acetyltransferase)

Contig: 05_NODE_4, position: 73364 to 74503, length: 1140 nt, orientation: FORWARD

Perfect match to: (N315-BA000018-[628718:629857], highly conserved allele)

Sequence:

ATGAATCAAGCAGTCATAGTTGCAGCTAAACGAACTGCATTTGGGAAATATGGTGGCACTTTAAAACATTTAGAGCCAGAACAATTGCTT
AAACCTTTATTCCAACATTTTAAAGAGAAGTATCCAGAGGTAATATCTAAAATAGATGATGTAGTTTTAGGTAATGTTGTTGGGAATGGT
GGCAATATTGCAAGAAAAGCATTGCTTGAAGCGGGGCTTAAAGATTCAATACCTGGCGTCACAATCGATCGGCAATGTGGGTCTGGACT
TGAAAGTGTTCAATATGCATGTCGCATGATCCAAGCCGGAGCTGGCAAGGTATATATTGCAGGTGGTGTTGAAAGTACAAGTCGAGCAC
CTTGGAAAATCAAACGACCGCATTCTGTGTACGAAACAGCATTACCTGAGTTTTATGAGCGTGCATCATTTGCACCTGAAATGAGCGACC
CATCAATGATTCAAGGTGCTGAAAATGTGGCCAAGATGTATGATGTTTCAAGAGAATTACAAGATGAATTTGCTTATCGAAGTCATCAAT
TGACAGCGGAAAATGTAAAGAATGGAAATATTTCTCAGGAAATATTACCTATAACCGTTAAAGGAGAAATATTCAACACTGATGAAAGT
CTAAAATCACATATTCCGAAAGATAACTTTGGCCGATTTAAGCCCGTGATCAAAGGTGGGACCGTTACCGCTGCGAATAGTTGTATGAAA
AATGATGGTGCAGTTTTATTGCTTATTATGGAAAAAGATATGGCATACGAATTAGGTTTCGAGCATGGTTTATTATTTAAAGATGGTGTTA
CGGTAGGTGTTGATTCTAATTTTCCTGGCATTGGTCCAGTACCAGCCATTTCCAACTTACTAAAAAGAAATCAATTAACGATAGAAAATAT
TGAAGTCATTGAAATTAACGAAGCGTTCAGTGCACAGGTAGTTGCCTGCCAACAAGCTTTAAATATTTCAAATACGCAATTAAATATATG
GGGTGGTGCATTAGCATCAGGTCATCCATACGGTGCAAGCGGTGCCCAATTAGTGACTCGATTATTTTATATGTTTGACAAAGAGACTAT
GATTGCATCTATGGGGATAGGGGGAGGTCTAGGAAATGCAGCATTATTTACTCGATTCTAA

Gene: vraC (putative protein)

Contig: 05_NODE_4, position: 74478 to 74843, length: 366 nt, orientation: FORWARD

Perfect match to: (RF122-AJ938182-[593815:594180], highly conserved allele)

Sequence:

ATGCAGCATTATTTACTCGATTCTAACCAGCGATTAAATGTGTCATTTTCTAAGGATAGTGTGGCTGCATATTATCAGTGTTTTAACCAACC
TTATAGAAAAGAAGTACCACCATTAATGTGTGCGTCATTATGGCCAAAATTTGATTTATTTAAAAAATATGCAAATAGCGAACTGATTTTA
ACAAAATCAGCAATTAATCAAACTCAAAAGATAGAAGTAGACACAATATATGTAGGGCATTTAGAAGATATTGAATGCCGACAGACTCG
CAATATCACACGTTATACAATGGCTTTAACATTAACTAAAAATGATCAACATGTCATAACGGTTACACAAACTTTTATTAAGGCGATGAAG
TAG

Gene: Q5HI98 (putative protein)

Contig: 05_NODE_4, position: 74846 to 75115, length: 270 nt, orientation: FORWARD

Perfect match to: (N315-BA000018-[630200:630469], highly conserved allele)

Sequence:

ATGAAGTTTAATGAGATATGGATAAATGAATATTTGGCGCTCGTAAATGATGATAATCCAATACATAATGAGATTGTGCCAGGACAATTA
GTGAGTCAAATGATGCTGATGGCTATGTCATTAGAGACAAACCAGTGTCAAATTAACTACGTTAAACCTATTTTAATAAATGAAAATATC
GAATTCATTGAACAACACGAACACGAAATTATAGCAATTAATGACGATGGAGAGATTAAAATAAAAATTTCTTTGAGCACAAAAAAATAA

Gene: ssr128 (small stable RNA 128)

Contig: 05_NODE_4, position: 75110 to 75502, length: 393 nt

Sequence:

AAATAACCGATATTAGCTGCATGAACGCATATTAATTAGGAGATGAAAGGACAGCTAATATCAGTTATGTATTGTTATTATTATTGGGAA
CAGAGATGAATATAGGTTACGTTTCTTTCTTTGCACGGGGATGCATTAATCTAAAATAATAATAACAACTATATCAATGTTTAATAAATTCT

GGATTATTGGAACGATTAGTCAATTTAACTAACTTTCATATGATCTATATCGTCTTGTAATAAAGAGAGCAATTTGAATATTTCAGTATCAC
TAAATGAATCGTCACATTTAATTGAAACATGCTGAAACGTTTTGGTTATAATTTCATAAACTGGTGCGCCTTCATGGTGATACTGTCGATA
AATAATCATAACCTATATTACCTCCTTT

Gene: thiD1 (phosphomethylpyrimidine kinase)

Contig: 05_NODE_4, position: 75990 to 76820, length: 831 nt, orientation: REVERSE

Perfect match to: (MRSA252-BX571856-[637913:638743:r], highly conserved allele)

Sequence:

TTAAACCTCTGTTACTTCAACATCGATATGTTCAATACGGTTGTATGCACCGTGATCCACAGGACCAACAAAATCATTCATTTTCCAACCGT
TTTTAATAGCAGAAGCGACGAAAGCTTTCGCGCTAATCACAGCTTCTTTCGGTGACTTACCGTTAGCTAAATATGCAGTTGTTGCCGCAGC
AAATGTACAACCAGCACCATGGTTATAACTTTGTTGGAACATGTCTGTTGTTAGTTGATAAAATGTTTGACCATCATAGTATAAGTCATAC
GATTTATCTTGATCTAAAGCTTTGCCACCTTTAATGATGACATGCTGTGCGCCTTTATCAAAGATAATTGTTGCAGCCTTTTTCATATCTTCA
ATTGAATTTAATTTACCTAATCCTGATAATTGACCCGCTTCAAATAAGTTTGGTGTCACTACCGTTGCTTTAGGTAGTAAATATTTAATCAT
CGCCTCAGTATTTCCAGGATTAAGCACTTCATCTTCGCCTTTACAAACCATGACAGGATCTACTACAAAATATTGTGCATTAGATGCCTCAT
ATACTTCTCCAGCACGTTTGATTATCTCCTCAGTACCTAACATACCTGTTTTAATAGCATCAGGTCCGATTGATAAAGCCGTTTCAAGTTGC
TTTTCAAATACGTCCATAGGTAATGGTGTGACATCGTGTGACCATGTATCTTTATCCATAGTAACGATGGCAGTTAAAGCGACCATGCCAT
ACGTATCTAATTCTTGGAACGTTTTCAAATCTGCTTGCATACCTGCGCCAGCACTTGTGTCAGAACCGGCAATTGTTAAAACTTTCTTTAAA
GCCAT

Gene: ung (uracil-DNA glycosylase)

Contig: 05_NODE_4, position: 77004 to 77660, length: 657 nt, orientation: FORWARD

Perfect match to: (COL-CP000046-[659764:660420], highly conserved allele)

Sequence:

ATGGAATGGTCGCAAATTTTTCATGACATAACAACGAAACATGACTTTAAAGCTATGCATGATTTTTTAGAAAAAGAATATTCGACTGCAA
TCGTATACCCTGATAGGGAAAATATATATCAAGCGTTTGATTTAACACCGTTTGAAAATATCAAAGTTGTTATATTAGGACAAGACCCGTA
TCATGGTCCAAACCAAGCACATGGATTAGCATTTTCAGTGCAACCTAACGCAAAATTCCCTCCATCTTTACGTAATATGTATAAAGAATTA
GCAGATGATATTGGATGCGTTAGACAAACACCGCATTTACAAGATTGGGCAAGAGAAGGCGTCTTGTTATTGAATACAGTTTTAACCGTA
AGACAGGGTGAAGCAAATTCTCATCGTGATATTGGTTGGGAAACATTTACTGATGAAATTATTAAAGCAGTGTCTGATTATAAAGAACAT
GTTGTCTTTATTTTGTGGGGGAAACCTGCACAGCAAAAAATAAAGCTTATCGATACATCTAAACATTGTATTATAAAATCAGTGCATCCTA
GTCCACTGTCTGCATATAGAGGATTCTTTGGATCAAAACCGTATTCCAAAGCGAATGCCTATTTAGAGTCAGTAGGAAAATCACCAATTA
ATTGGTGTGAAAGTGAGGCGTAG

Gene: ywdI (putative protein)

Contig: 05_NODE_4, position: 77661 to 78041, length: 381 nt, orientation: FORWARD

Perfect match to: (MW2-BA000033-[623464:623844], highly conserved allele)

Sequence:

ATGTTGAATAGAGAAACTTTAATAGCACGAATTGAGCAAGAATTAGTACAAGCAGAGCAGGCACAGCATGACCATGACTTTGAAAAACA
TATGTATGCCATACATATATTAACATCTTTATATGCTTCAACATCAAATACACCACATATTGGTGAACAACAAATGAATCGTCGTATTGCTA
ACCATAATCAAATGCCACAATCACAAATAACGCAGCCAACTCATCAAGTGACAGCTGCTGAAATTGAAGCGATGGGTGGTAAAGTAAAT
ACGCATTCAGCACATCATCATAATAAGTCATATTCACAACCTTCAAACCAACAACAAAGATTAGCGACAGATGATGACATTGGCAATGGT
GAATCCATATTTGATTTTTAA

Gene: ywdK (putative small membrane protein)

Contig: 05_NODE_4, position: 78174 to 78542, length: 369 nt, orientation: FORWARD

Perfect match to: (RF122-AJ938182-[597536:597904], highly conserved allele)

Sequence:

ATGAAATTATTTATTATTTTAGGTGCATTAAACGCGATGATGGCTGTCGGAACAGGTGCATTTGGTGCGCATGGTTTACAAGGAAAAATA
AGTGATCACTATTTATCAGTATGGGAAAAAGCAACGACGTATCAAATGTACCATGGCTTAGCATTATTAATTATAGGTGTAATTAGTGGT
ACAACTTCAATCAATGTTAACTGGGCTGGCTGGTTAATATTTGCTGGTATTATTTTCTTTAGTGGATCATTATATATTTTAGTATTAACTCAA
ATTAAAGTTTTAGGTGCGATTACGCCAATTGGTGGCGTATTGTTCATCATTGGATGGATAATGTTAATCATTGCGACATTCAAATTTGCTG
GTTAA

Gene: yfnA2 (cationic amino acid/polyamine transporter, locus 2)

Contig: 05_NODE_4, position: 78663 to 80147, length: 1485 nt, orientation: FORWARD

Perfect match to: (Strain_21334-AGTW01000032-[22275:23759], highly conserved allele)

Sequence:

ATGGAAAAAAAGAATAAGCAAATAGATAGAGGCGATTTAAAACAAAACCTATCTGAAAAGTTTGTATGGGCGATTGCATATGGTTCATG
TATCGGATGGGGCGCATTCATCTTACCAGGAGACTGGATTAAGCAGTCAGGTCCGATTGCAGCATCAATTGGTATAGTTATTGGTGCATT
ATTAATGATATTAATTGCGGTTAGTTATGGCGCATTAGTAGAGAGATTTCCAGTATCAGGGGGCGCGTTTGCCTTTAGTTTCTTAAGTTTC
GGCAGATATGTGAGTTTCTTCTCATCATGGTTTTTAACTTTTGGTTATGTCTGTGTCGTTGCTTTAAATGCGACCGCATTCAGTTTACTAGT
TAAATTCTTATTGCCAGATGTCTTAAATAATGGGAAACTATACACCATTGCGGGCTGGGACGTTTATATTACGGAAATCATTATTGCGACC
GTATTACTACTTGTATTCATGCTAGTAACGATTCGTGGCGCAAGTGTATCTGGATCATTACAATATTATTTCTGTGTGGCGATGGTAATCG
TCGTATTATTGATGTTCTTTGGTTCATTCTTTGGTAATAATTTTGCACTTGAAAATTTACAACCGTTAGCTGAACCTAGCAAAGGATGGTTA
GTGTCTATTGTGGTTATTGTATCCGTGGCACCATGGGCATATGTTGGATTTGATAATATTCCACAAACAGCAGAAGAGTTTAACTTTGCAC
CAAACAAGACATTTAAGCTTATCGTGTACAGTTTATTAGCAGCATCATTAACTTATGTTGTCATGATTTTATACACTGGTTGGTTATCAACA
AGTCATCAAAGTTTAAATGGGCAGTTGTGGTTAACAGGTGCTGTTACACAAACAGCATTTGGTTATATTGGATTAGGTGTATTAGCAATT
GCAATTATGATGGGTATATTTACTGGTTTAAATGGATTCTTGATGAGTTCAAGTCGCTTGTTATTTTCTATGGGACGTTCAGGTATTATGC
CAACAATGTTTAGTAAATTACATAGTAAATACAAAACACCATATGTCGCAATCATATTCCTAGTAGGTGTGTCGTTAATTGCACCTTGGCT
AGGAAGAACTGCATTGACTTGGATTGTAGATATGTCATCTACAGGTGTATCCATTGCCTACTTTATTACATGTTTGTCTGCAGCGAAATTA
TTCAGTTATAACAAACAAAGTAATACGTATGCACCGGTTTACAAAACGTTTGCTATTATCGGCTCATTTGTATCATTCATTTTCTTAGCGTT
GTTATTAGTGCCAGGTTCTCCTGCAGCACTGACTGCACCGTCTTATATTGCATTACTTGGATGGTTAATCATCGGTTTAATATTCTTTGTGA
TTCGATATCCTAAATTGAAAAATATGGATAATGATGAATTAAGTCGCTTGATTTTAAATAGAAGTGAAAATGAAGTTGATGATATGATTG
AAGAACCTGAAAAAGAAAAAACTAAATAA

Gene: DUF3815 (protein of unknown function)

Contig: 05_NODE_4, position: 80288 to 80740, length: 453 nt, orientation: REVERSE

Perfect match to: (N315-BA000018-[635642:636094:r], highly conserved allele)

Sequence:

TTATAATCGTTTAAAAGATTTCACGAACCCAGAAACAATTAATTTAGAAATTTGGTCGGCGAATAATAAACCTAATGCGATGGCTCCTGCA
ATAAGTGTTACTTCTAACATTGTATTTATTGCTGTGCTGAAATTTAATAAGACTAAATTTTTTGTAGCATCGTATGCTAAGCCACCAGGTAC
TAATGGAATGATACCCGTTACCATAAAAATGATGGCAGGTTCTTTTTGTTTACGAGCCATATAATGACTTAACAAGCCTAATGCTAAACTA
CCAAAGAAACTAGAGTATATAGTGTGCACATTAAAGCCGTTGAAGAATAAGGTGTAAACCATCCATCCACATGTACCAACGAAACCACAT
GATAGATATAATTTTCTAGGTGCATCAAAAATGACGCAGAAGAACATTGAAGCTAAAAAGCTAAAGATAAAGTTTAAGATCCAAAACAT

Gene: DUF1212_L1 (protein of unknown function DUF1212, locus 1)

Contig: 05_NODE_4, position: 80753 to 81517, length: 765 nt, orientation: REVERSE

Perfect match to: (MW2-BA000033-[626556:627320:r], allele observed in CC1+CC25+CC361)

Sequence:

CTATACTAAAATTAATACGCTACCAACGCCAGCACCGATGCCAAACGCAGTAACCAATGCTTCTAATGATTTCGTTGTGAACATCAACATG
TGTCCACCAAATAAATCTTGTATTGCGTTTGTTATTAATACACCAGGAACAATAGGCATGACTGCTGCAATGATAATAGTTGCCAAGTCAC
CTGTTGGGATAAGTGTATGTCCAATAACGGCGATAATCCCAATAACTAATGAACCAATGAATTCTGGGATAAACTGTGCATGTAACTTGC
GATCTAAAATCTCAGTGACTAGGTATCCTAGACTACCTGCTAATATCGCAGTTAAAACATCAATCAATCTACCACCTTGTAAATATAAGAA

ACTCATTGCAATCATTGCTGCAGCAAAACCTTTAAAGGGAAGACTGCTGTCACGCTTAGCAACATATATTTTTTCAAGTTGCGTTTTTGCTT
CGGCTAAAGAAATTTCATTGTTTGTAATTTGACGCGAAATTTTATTAGCTTGCGAAATTTTTATTAAGTTTGTATCTCGAGAGGTAATTCTA
AATATTCTAGGAAACGATTCCGAATGTAACGTAAACTGGATGACAGTGTTTGTAACAAAGCTGTTACTTTCACTGTAACCAAGTTTTTTTG
CAATACGTGTCATGGTATCTTCTACACGCGTACCTTCTGCACCAGATTCTAATAGTATGCGAGCAGCAAGCATGACAACGTCTTTGATAAG
TACCTCTTGTTTGTATTCTTCTGAATTTATGTCCAT

Gene: hemQ (Fe-coproporphyrin oxidase)

Contig: 05_NODE_4, position: 82066 to 82818, length: 753 nt, orientation: REVERSE

Perfect match to: (MW2-BA000033-[627869:628621:r], highly conserved allele)

Sequence:

TTAAGAAATCGCAAAGAATTGATCGAATTCGTTTGTGTTAATAAGATGTCCTACAAAGAAACTACCGAATTCACCGTATCGTGCTGTTGTT
TCATCAAAGCGCATTTCGTATACAATTTTTTTGAATTGTAATACGTCATCTGAGAACAATGTTACGCCCCATTCGAAATCATCAAACCCTAC
AGAACCAGTAATAAATTGTTTGATTTTGCCAGCATATTTTCTACCAATCATACCATGGTCATACATTAATTTTTGGCGTTCTTCCATAGTTAA
CATGTACCAGTTATAAGTTTCATTACGACGTTTGTTCATTGGATAGAAACAAATATAATCAGAATGTGGTAATTCTGGGTATAATCTTGCT
TTGATATGAGGGTTCTCATAAGGATCTTCATCAGATTTACCAGCTAAATAATTGCTCAATTCAATGACTGATACATATGAATATGTAGGGA
TTAAGAAGTCAGCAATGCGCAATTTGTTAAATTCATTTTCAATATGATTTAAAGACTTCATTTCAGGACGTAAGAACCATAATAACAAATC
TGCTTTTTGACCAGTTATATTATAAATAGCTTGATCACCAGATTTTGATGATCTTACAGTTGCTGTATTTTCTAAAAATGATTGAAATTCAG
TGACAAGTGCATCGCGTTCGTCCTTTGGAACTAGACGTAATGATGCCCAATCAACTGCATAAAATAAATGTAGACTATACCAACCATCTAA
TGTTTCGGCTGCTTGACTCAT

Gene: pta (phosphotransacetylase)

Contig: 05_NODE_4, position: 82986 to 83972, length: 987 nt, orientation: FORWARD

Perfect match to: (08-02119-CP015645-[1605222:1606208:r], allele observed in ST582+CC8)

Sequence:

ATGGCTGATTTATTAAATGTATTAAAAGACAAACTTTCTGGTAAAAACGTTAAAATCGTATTACCTGAAGGAGAGGACGAACGTGTTCTA
ACAGCTGCAACACAATTACAAGCAACAGATTATGTTACACCAATCGTGTTAGGTGATGAGACTAAGGTTCAATCTTTAGCGCAAAAACTT
GATCTTGATATTTCTAATATTGAATTAATTAATCCTGCGACAAGTGAATTGAAAGCTGAATTAGTTCAATCATTTGTTGAACGACGTAAAG
GTAAAGCGACTGAAGAACAAGCACAAGAATTATTAAACAATGTGAACTACTTCGGTACAATGCTTGTTTATGCTGGTAAAGCAGATGGTT
TAGTTAGTGGTGCAGCACATTCAACAGGAGACACTGTGCGTCCAGCTTTACAAATCATCAAAACGAAACCAGGTGTATCAAGAACATCA
GGTATCTTCTTTATGATTAAAGGTGATGTACAATACATCTTTGGTGATTGTGCAATCAATCCAGAACTTGATTCACAAGGACTTGCAGAAA
TTGCAGTAGAAAGTGCAAAATCAGCATTAAGCTTTGGCATGGATCCAAAAGTTGCAATGTTAAGCTTTTCAACAAAAGGGTCTGCTAAAT
CAGACGACGTGACAAAAGTTCAAGAAGCTGTCAAATTAGCACAACAAAAAGCTGAAGAAGAAAAATTAGAAGCAATCATTGATGGCGA
ATTCCAATTTGATGCTGCGATTGTACCAGGTGTTGCTGAGAAAAAAGCGCCAGGTGCTAAATTACAAGGTGATGCAAATGTCTTTGTATT
CCCAAGTTTAGAAGCTGGTAATATTGGTTACAAAATTGCACAACGTTTAGGTGGATATGATGCAGTTGGTCCAGTATTACAAGGTTTAAA
TTCTCCAGTAAATGACTTGTCACGTGGCTGCTCAATTGAAGATGTATACAATCTTTCAATTATCACAGCAGCGCAAGCCTTACAATAA

Gene: lipL (octanoyl-[GcvH]:protein N-octanoyltransferase)

Contig: 05_NODE_4, position: 83975 to 84811, length: 837 nt, orientation: FORWARD

Perfect match to: (MW2-BA000033-[629778:630614], highly conserved allele)

Sequence:

ATGGATTTAGCGAGTAAATATTTTAATGGCGTCAACTGGCGATATATCGATCATTCTTCTGGATTAGAACCTATGCAATCTTTCGCATTCG
ATGATACATTTTGCGAAAGTGTGGGCAAAGATATATCAGATAATGTTGTGCGTACTTGGATTCATCAACATACTGTTATTCTTGGTATTCA
TGATTCAAGATTGCCGTTTTTAAAAGATGGCATTGATTATTTAACGAATGAGATTGGTTATAATGCCATTGTTAGAAATTCTGGTGGCTTA
GGTGTCGTTCTAGATCAAGGTGTATTAAATATATCGCTGATGTTCAAAGGACAAACAGAAACAACGATTGATGAAGCGTTTACTGTGATG
TACCTCTTAATTAGCAAAATGTTCGAAAATGAGAATGTTGATATTGATACGATGGAAATTGAACATTCTTATTGCCCAGGAAAATTTGACT
TAAGTATCGATGGTAAGAAATTTGCAGGCATATCGCAACGAAGAGTTAGAGGCGGTATTGCTGTACAAATTTATCTTTGTGTTGAAGGCT
CTGGTTCAGAACGTGCATTGATGATGCAAACATTTTATGAACATGCTTTAAAAGGTGAAGTGACTAAATTTAAATATCCTGAAATTGAACC
ATCTTGTATGGCCTCATTAGAGACATTGCTTAACAAAACGATTACTGTTCAAGATGTAATGTTTTTACTATTATATGCAATCAAAGATCTTG

GCGGTGTATTAAATATGACGCCAATTACTCAAGAAGAATGGCAAAGATACGATACGTATTTTGATAAAATGATTGAAAGAAACAAGAAA
ATGATAGATCAAATGCAATAG

Gene: mvaK1 (mevalonate kinase 1)

Contig: 05_NODE_4, position: 85398 to 86318, length: 921 nt, orientation: FORWARD

Sequence:

ATGACAAGAAAAGGATATGGGGAATCGACAGGTAAGATTATTTTAATAGGAGAACATGCTGTTACATTTGGAGAGCCTGCTATTGCAGT
ACCGTTTAACGCAGGTAAAATCAAAGTTTTAATAGAAGCCTTAGAGAGCGGGAACTATTCGTCTATTAAAAGCGATGTTTACGATGGTAT
GTTATATGATGCGCCTGACCATCTTAAGTCTTTGGTGAACCGTTTTGTAGAATTAAATAATATTACAGAGCCGCTAGCAGTAACGATCCAA
ACGAATTTACCACCATCACGTGGATTAGGATCGAGTGCAGCTGTCGCGGTTGCTTTTGTTCGTGCAAGTTATGATTTTTTAGGGAAATCAT
TAACGAAAGAAGAACTCATTGAAAAGGCTAATTGGGCAGAGCAAATTGCACATGGTAAACCAAGTGGTATTGATACGCAAACGATTGTA
TCAGGCAAACCAGTTTGTTTCCAAAAAGGTCATGCTGAAACGTTGAAAACGTTAAGTTTAGACGGCTATATGGTTGTTATAGATACTGGT
GTGAAAGGTTCAACAAGACAAGCAGTAGAAGATGTTCATAAACTTTGTGAGGACCCTCAGTACATGTCACATGTAAAACATATCGGTAA
GTTAGTTTTACGTGCGAGTGATGTGATTGAACATCATAACTTTGAAGCCTTAGCGGATATTTTTAATGAATGTCATGCGGATTTAAAGGC
GTTGACAGTTAGTCATGATAAAATAGAACAATTAATGAAAATTGGTAAAGAAAATGGTGCGATTGCTGGAAAACTTACTGGCGCTGGTC
GTGGTGGAAGTATGTTATTGCTTGCCAAAGATTTACCAACAGCGAAAAATATTGTAAAAGCTGTAGAAAAAGCTGGTGCAGCACATACTT
GGATTGAGAATTTAGGAGGTTAA

Gene: mvaD (mevalonate diphosphate decarboxylase)

Contig: 05_NODE_4, position: 86323 to 87306, length: 984 nt, orientation: FORWARD

Perfect match to: (MW2-BA000033-[632126:633109], highly conserved allele)

Sequence:

TTGATTAAAAGTGGCAAAGCACGTGCACATACGAATATTGCACTTATAAAATATTGGGGTAAAAAAGATGAAGCACTAATCATTCCAATG
AATAATAGCATATCTGTTACATTAGAAAAATTTTACACTGAAACGAAAGTCACTTTTAACGACCAGTTAACACAGGATCAATTTTGGTTGA
ATGGTGAAAAGGTTAGTGGCAAAGAATTAGAGAAAATTTCAAAATATATGGATATTGTCAGAAATAGAGCTGGCATCGATTGGTATGCA
GAAATTGAAAGCGACAATTTTGTACCAACAGCAGCAGGGTTGGCTTCATCGGCAAGCGCATATGCAGCTTTAGCAGCAGCTTGTAATCA
AGCGCTAGACATGCAGCTGTCAGATAAGGATTTATCGAGATTGGCGCGAATTGGTTCGGGTTCTGCGTCGCGTAGTATTTATGGTGGATT
TGCAGAATGGGAAAAAGGGTATAGTGATGAGACGTCATATGCCGTTCCACTTGAATCGAATCATTTTGAAGATGACCTTGCCATGATATT
TGTTGTGATTAATCAACATTCTAAAAAGGTACCTAGTCGATATGGTATGTCATTGACACGAAACACATCAAGGTTTTATCAATATTGGTTA
GATCATATTGATGAAGATTTAGCTGAAGCAAAAGCAGCGATTCAAGACAAAGATTTTAAACGCCTTGGTGAAGTAATTGAAGAAAATGG
TTTGCGTATGCATGCCACGAATCTAGGATCAACACCGCCGTTCACATATCTTGTGCAAGAAAGTTATGATGTCATGGCGCTTGTTCACGAA
TGCCGAGAAGCGGGGTATCCGTGTTATTTTACAATGGATGCGGGACCTAATGTGAAAATACTTGTAGAAAAGAAAAACAAGCAACAGAT
TATAGATAAATTATTAACACAGTTTGATAATAACCAAATTATTGATAGTGACATTATTGCCACAGGAATTGAAATAATTGAGTAA

Gene: mvaK2 (phosphomevalonate kinase 2)

Contig: 05_NODE_4, position: 87319 to 88395, length: 1077 nt, orientation: FORWARD

Perfect match to: (TCH60-CP002110-[2709064:2710140:r], allele observed in CC30+CC239)

Sequence:

ATGATTCAGGTCAAAGCACCCGGAAAACTTTATATTGCTGGAGAATATGCTGTAACAGAACCAGGATATAAATCTGTACTTATTGCGTTA
GATCGTTTTGTAACTGCTACTATTGAAGAAGCAGACCAATATAAAGGTACCATTCATTCAAAAGCATTACATCATAACCCAGTTACATTTA
GTAGAGATGAAGATAGTATTGTCATTTCAGATCCACATGCAGCAAAACAATTAAATTATGTGGTCACAGCTATTGAAATATTTGAACAAT
ACGCGAAAAGTTGCGATATAGCGATGAAGCATTTTCATCTGACTATTGATAGTAATTTAGATGATTCAAATGGTCATAAATATGGATTAG
GTTCAAGTGCAGCAGTACTTGTGTCAGTTATAAAAGTATTAAATGAATTTTATGATATGAAGTTATCTAATTTATACATTTATAAACTAGCA
GTGATTGCAAATATGAAGTTACAAAGTTTAAGTTCATGCGGAGATATTGCTGTGAGTGTATATAGTGGATGGTTAGCGTATAGTACTTTT
GATCATGAATGGGTTAAGCATCAAATTGAAGATACTACGGTTGAAGAAGTTTTAATCAAAAACTGGCCTGGATTGCACATCGAACCATTG
CAAGCACCTGAAAATATGGAAGTACTTATCGGTTGGACTGGCTCACCGGCGTCATCACCACACTTTGTTAGCGAAGTGAAACGTTTGAAA
TCAGATCCTTCATTTTACGGTGACTTCTTAGAAGATTCACATCATTGTGTTGAAAAACTTATTCATGCTTTTAAAACAAATAACATTAAAGG
TGTGCAAAAGATGGTGCGTCAGAATCGTACAATTATTCAACGTATGGATAAAGAAGCTACAGTTGATATAGAAACTGAAAAGCTAAAAT

ATTTGTGTGATATTGCTGAAAAGTATCACGGCGCATCTAAAACATCAGGCGCTGGTGGTGGAGACTGTGGTATTACAATTATCAATAAAG
ATGTAGATAAAGAAAAAATTTATGATGAATGGACAAAACATGGTATTAAACCATTAAAATTTAATATTTATCATGGGCAATAA

Gene: DUF1450 (protein of unknown function)

Contig: 05_NODE_4, position: 88572 to 88913, length: 342 nt, orientation: FORWARD

Perfect match to: (N315-BA000018-[643926:644267], highly conserved allele)

Sequence:

ATGAAAAAATACATTCCTTATTTGTGATGAATGTCAGGCAGTCAATATAAGAACGTTACAAAAGAAGTTGGAAAAATTAGATCCCGATGCT
GAAATCGTGATAGGTTGTCAATCTTATTGTGGACCTGGACGCCGAAAAACATTCACTTTTGTTAATAACCGCCCACTGGCTGCGCTTACTG
AAGAAGAATTAATCGAAAAAGTTTCTCAACAATTAAAGAAACCACGTGATCCTGAAGAAGAAGAGCGTTTAAGAAAACGACATGAAGAA
CGTAAACGTCGTAAAGAAGAACAAGATAGAAAGCTTAAAGAAAAATTAGAAAAGCGAAAAGCACAACAATAA

Gene: ykgC (FAD-dependent pyridine nucleotide-disulphide oxidoreductase)

Contig: 05_NODE_4, position: 88965 to 90287, length: 1323 nt, orientation: REVERSE

Perfect match to: (ATCC51811-ST1-ADVP01000012-[39477:40799], allele observed in CC1)

Sequence:

CTAGAAATTAAATAAATCATTAAATGATTCGGCCATCGTAGGATGCGTATAAATATTATCTCGTAATACGGTATATGGAATGTTTTGATCA
ATCGCAAGTTTAATTATATTAATTAATTCTTCAGATTGCTTACCATATAATGTAGCACCTAAAATCATATTATTTTCATTATTAATGACTACT
TTAAATAAACCTCTTGGATCATTGTTAATTTTGTGACGAGGTATAGCACTTACTAAAAGTTGATGTTCAGTGTAATCATAATGTTGAGCGG
CAGCTTCTTTACTAGTTAATCCAACACGTGATAATGGTGGATCTATAAATACTGTATAAGGCACGCTACCTCTATTGTCAGTCGTACGTGA
CTGATTACCATATAACGCTGATTTGATAATTCGATAATCGTCTAAAGATATATACGTAAATTGAAGTCCGCCTTTAACATCACCTGCAGCA
TAAATATGCGGTACAGTTGTTTGAAGATGAGCATTGACTTTAATTTCGCCTCTGTCGCCTAATTCGATATCAGTATTTTCTAAAGCTAAATC
CGTATTCGGTTTGCGCCCGATAGCCAAAAGTACTGCATCAGCCTCAAAGTTACCAACGTTGGTATGGACTGTTGTATGATGATTGTCAGA
TGACAATTCAGTCGTTTCAACATTTGTATGCAATGTAATGCCTTTATTTTCTAAGTCAGTAATAGCATGTGCAACGACATCTTGATCTTCAC
GTGGCATAAATGATTCACCACGTTCTAATACTGTAACCTTACTACCTAAATTCGCAAACATTGAAGCAAATTCTAAGGCGATATAACCGCC
ACCTACAATAACGAGGTGCTTAGGTTGATAGTTAATGTTTAATAAACCTGTTGAATCGAAGACGTGTTTAGCTTGATCAAGGCCTTTAATG
TTAGGAATAACAGAGGTAGCACCAGTATTAATAATGATATGAGGTGCAGTAATACTATCGGCGATATCGTCATGCTGATCTAATAAATTC
ACTTCAGTATTAGATTTAAACTGCGCTTTAAAATCCAGTACATCAATGTTGTTATCGTCTGCTAATAAATGGTAATTTTTATTGTTTAGCGC
ATTGACAACATCGTTTTTACGGTTATAACTTGCTTCAAAAGATTTGCCTTCTAATCCATCATGTACAAGTGTCTTTGAAGCAATACATCCTA
TGTTTATACAAGTGCCTCCATACATTTTCGGAGATTGTTCGATAACTGCGACGTGTTGACCTGTTGATGCAGCATATTTGGCTAAAGTTTT
ACCAGCTTCCCCAAATCCTATTACAATTAAATCATATGTTTTCAT

Gene: Q5HI81 (transcriptional regulator)

Contig: 05_NODE_4, position: 90274 to 90714, length: 441 nt, orientation: REVERSE

Sequence:

TCATATGTTTTCATGACATAAATCCTCCTTTTGAATGTCTTCAATGACATCTTTGATTGTTTTTCCTTTATAAAAATTAATAATGATATTCTGT
TCGTCTTGCTCATAATGTGACATAGTAGTTGCAATATTACGAGCAATTTGACAGTGACTGCCTTCGTCGCCAGTAAATAGTCGTGTGTGAT
GTTCTTTCTCTAAGACAAAATGTTTATATAATGTTGCTAGAGAGACATCAGCGCTACGATCATTTGATAAATAACCACCATCTTTACCCCGT
ATTGTGTTAATCATTTTTAAATCGACAAGTTGAGTCGTCACGCGTCGTAATTGAACAGGATTTAAACAAGTTAATTCTGCTAATGAACTAC
TATTGAATTTTTTTGAATGATGCTTAGTTAAAAAAGCTAATACATGCACGGCAATGTTAAATTCTAAATTCAA

Gene: Q5HI79 (putative protein)

Contig: 05_NODE_4, position: 91339 to 92748, length: 1410 nt, orientation: FORWARD

Perfect match to: (MW2-BA000033-[637142:638551], allele observed in CC1)

Sequence:

ATGAGTATTGACATGTATTTAGACAGATCTCGAAACCAAGCTTCAAGTGTGGGGAATTTGAGTCAAACAATGAATTCAAATTATGATGCG
TTGGAAAAAGCAATTACTCAATTTATCAATGATGATGTGCTTAAAGGGAAAGCGTATACGTCAGCTAAGCAATTTTTTAGTACGGTGTTA
ATTCCATTATCAACAAGTATGAAAACATTGAGTGATTTAACGAAGCAAGCTTGCGATAATTTTGTGTCACGTTATACGAGTGAGGTTGAT
AGCATATCTTTAAAAGAATCAGAGCTTGAAGAAGATATCAGATCATTAAGTCAACAAATTACGCGATATGAAAATTTGAATAACAATTTG
AAAAAGCATGCTTCCGATAATCAGCAAGCCATTTCATCGAACCAACAAATAATACAAACATTAGGTCAACAAAAACATGAATTAGAAGAG
AAGCTACGCAAATTGCGTGAGTTTAATCAAAAATCACCAGAAATATTTAAAGAAGTTGAAGAATTTCAAAAAATTGTCCAACAAGGACTT
ACCCAAGCGCAAAATTTTTGGAACTTTTCAACAAATCAATTTAATATTCCTTCAGGTAAAGAACTTGATTGGGCCAAAGCAAGTCATGAAA
AATATTTGAAAGTTGCTATGGGGAAAATTGAACATAAAGCAGAGAAAGAAACTTTAAATAAAGCAGACTTTGCTGTTATAAAGGCATAT
GCCAAAGAACATCCAGAAGACGATATCCCGAAAAGTATATTGAAATATATAAATGACAATAAAGACAGTATTAAAAGAGATATAGGATT
AGATATTACGTCAACACTTTTAGAGCAAGGCGGTATAAATGCAAGTAAATTCGGTGTATTTATCAATACAGCAGGTGGAGTGAAAGGCC
CAGCAGGTCCAAATTCATTTGTGGAAGTCAAACGTACATCAGGTAATGTGTTTATAGAAAATGGTAGTAAATTTGCAAAAGGCGGAAAA
TACCTAGGTAAAGGTGTTGCTGGTGTAGGATTTGGTATAGGTATGTATGATGACCTTGCAAATGATGATAAAACATTTGGAGAGGCGTT
GTCGCATAATGGTATGACGCTTGCAGCTGGATCTGCAGGGACAGCAGTTGGAGCTGGATTAGCGACTTTTGTTTTAGGAAGTAATCCAG
TAGGATGGGTGATTTTAGCAGGTTTGGCTATGAGTACAGTATTTGCATTAGGAACAGATTTAATTTATCAAAATAATATTTTTGGATTAAA
AGATAAAGTAGACTGGGTAGGGCATAAAATCGATAATAGTATAGATGTTGTTAAAAAAACTACAGAAAAATCCATGGATAGTGTTGGGA
ATGCTGTTAGTGAAGCTAAAAATATTATTAGCAATCATATAAATCCAATGAAATGGGCGTGGTGA

Gene: DUF443 (putative protein)

Contig: 05_NODE_4, position: 93514 to 94146, length: 633 nt, orientation: FORWARD

Sequence:

TTGCTTTACGAATCTAATATTATTAACAAAAATCCCAGATATAGAATTATTAAATATAAAAACGACTATCTGATGATTGATTTAGTAAGCAC
ATGGTTAGTTTTGTTCTTTCCGTTTATTAATTGGTTAATCCCAAAAAAATATGTCCAAATCAGCAGAGAAGAGTTTGATAATTTAAATATTG
TTAAACCAGTTAAAAATAAAGCTTTATGGCCAGTTATAGGTAGAATACTTTTATTTGGCACTATGTTTAGAGATAAAATATATATACCTGA
TTCTCATTTAGAAAAAAATTGCGTTATTATCATTTGTTCGGTTTTATTATTAAGTATTCTAGTGTTTTATATATATTTAAATCAGAAAGTAAA
GTTATCTATCTATAATAATCGGAGTAGCAATGGAAAAATTATGATTTTTCCATCATTCAAGAATCTTTGCTTTGTACTATTTTCTTATTTTTT
CTGTGGTGGATTATCAATCATGTTCTTAGATGTTTTGATTAGTTTATCTATCCAAAATATTATAGTATTTATTGCTTGGGTTATTATGACAAT
GCTATTTTTCTTTATAAATATGTCTTCAATAATAGACAAAAAGATTCATGTTATATATTTAAGGTCATATAAATATTAA

Gene: DUF443-var9 (putative protein)

Contig: 05_NODE_4, position: 94926 to 95555, length: 630 nt, orientation: FORWARD

Perfect match to: (MW2-BA000033-[640729:641358], allele observed in CC1+CC8+CC239)

Sequence:

GTGCTTTGCGAATCTAGAGTCATTAATCAAAACCCTAAATATAGAATTATTAAATACAATAATGAATATTTCATGGTTGATTTAGTAAGTA
CTTGGATTGCTTATTTTTTGCCCATGATTAATTGGTTTATTCCCAAAAAGTACGCGAAAATTAGTAGAGAAGAATTTGAAAGTTTAAATAT
TGTCAAACCCGCTAAAAATAATACTTTCTGGCCTGTTGCAGGATTTGCAGTGTTATTAACAACCTTAACAAGAAAATATATCTATTTGCTTA
ACATCCATTTAGAAAAAGAAATAGTTATATTAACATGCTGTATGATACTTCTAGGTGTTTTCGCATTGTTTATATATATAAATACAAAATTG
AAGTTACATATTTTTGATAAAAATAAAAGTAATAACGAAAAGATCATATTAATACCTACATTTAAAAATATTTGTTTATCCTTATTTGCTTAT
ATATTATTTGGTGGATTGTCAACAATGGCTCTGAGTATGTTAGTAACTTCATCCCCTCAAAATATAATAGAATTTCTTGCTTTAATTGGCAT
GACTGCATGCTTCTTTCTACTGAATATGTCATCGGTTCTAGATAAAAAAATTCATGTTATTTTAAAAACAAATAAGTAG

Gene: DUF443-var1 (putative protein)

Contig: 05_NODE_4, position: 96502 to 97143, length: 642 nt, orientation: FORWARD

Perfect match to: (MW2-BA000033-[642305:642946], allele observed in CC1+CC8+CC239)

Sequence:

GTGGAGACATTGCTTTGCGAATCTAAAGTTATTAATAAAAACCCTAAATATCGAATAATCAAATACGATAGTGAATATTTAATGATTGATT
TGGCAAGTAATTGGATTGTCTTCTTCTTTCCATTTATTAACTGGCTCATACCGAAAACATATGTCAAAATCACTAAGAATGATTATGAAAAA
TTAAATATTGTCAAACCAGTTAAAAATAAATCGATAGGATGGACCATATTCGCGGGTATTGTGTTACTTGGTGGTACTGTAAGAAGAAAT
ACTTATTTATTTGATTTTCAATTAGAAGAACTAATTGTTTGGAGCAGCTGTTTCATTGGGTTTTTAGAGATTATATTTTTTTATTGTTATCTA

AATAAGAAATTAACATTAAATATTTATAATGAAAGTAAAAATAATGAACTTAAATTAAGATTATTACCCTCCTTTAAAAATATTTGTTTCAC
AATTTTTTATTACCTATTTACTGGTTTCATGTCTTATGGGGCATTTTACTTGTTGGTATTTGAAAATGTGCAAAATTTAATCTTATATGTTTCT
TGGCTTTTCATGACTATGCTATTTATGTTTATGAATATGCATTCAATTATAGATAAAAAAGTACATATATTCTTAAAGTCTAATAAATAG

Gene: DUF443-var7 (putative protein)

Contig: 05_NODE_4, position: 98271 to 98900, length: 630 nt, orientation: FORWARD

Perfect match to: (MW2-BA000033-[644075:644704], allele observed in CC1+CC8+CC239)

Sequence:

ATGTTGCTTTGCGATGTCAGAGTCATTTATAAAAATCCGAAATACAAAGTCATTCAACATAACGGTGAATACTTATTAGTCGATTTAGTAA
GCACTTGGTTCGTGTACTTTTTTCCTTTCATTAATTGGTTCATTCCAAAAAAGTACGCGATAATTAGCGAAGAAGAATTTGAAAATTTAAAT
GTTGTTAAACCAAATAAAAATAATGTTTTCTGGTCAGTTATAGGAAGTTCGGTTTTGTTTGGAGTTACTTTAAGGAAATACATACATGTTT
TTGATGTTCAATTAGATAAGCTAGTTGTAATGATATTGTGTGCTCTCGCTTTAATTTGTGTTATAGTTTTTTATTTTAACTTAAATAGAAAGC
TTAAGTTAAAAGTGTTTGATACAAATATTGAAAAAAATAAGAGAGTTATATTAATACCAACGTTTAAACTTGGCTGTTTTTTAGTTTTCGG
ATATATTTTCGCTGGAAGTTTTTCAATATTTTCATTAATTGCCCTTATGACAATCGAACCTCAAAATATAATAATATTTATTTATTGGATTAT
GATGACAATGCTTTTCTTTTTGTTAAATATGACTTCGATAGGTAATGAAAAAGTTCGCGTTATAATGAAAAATAATTGA

Gene: DUF443 (putative protein)

Contig: 05_NODE_4, position: 99015 to 99665, length: 651 nt, orientation: FORWARD

Sequence:

GTGGAGACATTGCTTTGTGAAACTGAAAATATTAATAAGAATCCCAAATATAGAATTATCAAATACAAAGATGAATATTTGATGATTGAT
TTAGTAAGTACATGGTTAGCACTCTTTTTCCCAATGATTAATTGGCTGATTCCAAAAAAGTACGTCAAAATCAGCGAAAAAGATTTTGAAA
CTTTAAACATTGTGAAGACAGCTAAAATCAATTCTTTTTGGCCAGTGGCAGGAAGTACGGTCTTATTTGGTGTTATGTTAAGAAGGTATTC
CCATTTATTTATCGTTAAATATGAATATAGTATAGTAATTTTAATTTGTTGCATCATAATACTAGGTATTTTTCTGTTTTTTTAATATTTAAAT
CAAAAGTTAAAGTTACAAATCTATAATGAAAACAAAAATAAAAGCAATAAGATAATCATATTTCCCACTTTAAAGAGTCTTTGTTTATCAA
TAGTTTTATATATTTATTTAGGTGGTGGTTCATTCTTTACTATTTATATGTTATTGACGATTGAAGTTCAAAATATAATATTATTTATAACTTT
GTTTGTAATATTTTTGTTTTTCTTATTTTTAAATATGTGCTCACTATATGACAATAAAGTTCATGTATTATTTAAATCAAATGGAATTGAAAA
GTTTTAA

Gene: DUF443-var11 (putative protein)

Contig: 05_NODE_4, position: 99702 to 100337, length: 636 nt, orientation: FORWARD

Perfect match to: (MW2-BA000033-[645506:646141], allele observed in CC1+CC8+CC30+CC239)

Sequence:

TTGCTATGTGAATCTAAAGTTATCAACAAAAATCCTAAGTATAGAGTTATTAAATATGGTGATGAATATTTAATGATTGATTTAGTAAGTA
CCTGGTTAACTTTATTTCTTCCTATGATTAATTGGTTAATTCCAAAAAAATATGTCAAAATCAGTAAAAAAGAATTTGACGATTTAAACATT
GTCAAACCTGTTAAAAATAAAGCTTTTTGGCCAGTTGCAGGTAGTACTATTTTGTTCGGAGTTACGTTTAGAAAATATATTCCTTCACTTAA
TATTCAATTAGAGAAAAACATGGTGATTGTAATATGTTGTGCAATATTTCTGGGTGTTTTAATACTTTTTTTATTTCTGAATCGTAAGCTAA
GGTTGGAAATTTATAATAATAACTCTAGTAAAGGGAAAATAATTTTATTTCCTTCATTAAAAAACTTTTGTTTCACAATATTTTATTATTTTT
TATTTGGCGGTCTTTCAATAATGGCTCTAAGTATGTTATTAACTTTAAATCCTCAAAATATAATAGGCTTTATTGGTTGGTTGGTAATGACT
GCAGGTTTCTTTCTGTTAAACATGTCATCGATTATTGACAAAAAAATTTATGTATTATCTAAAACTAACACGGTGGAAAAATGA

Gene: DUF443-var5 (putative protein)

Contig: 05_NODE_4, position: 100572 to 101198, length: 627 nt, orientation: FORWARD

Perfect match to: (MW2-BA000033-[646376:647002], allele observed in CC1+CC8+CC239)

Sequence:

ATGTTGCTATGCGAATCTAAAATCATCAATAAAAACCCAAAATATAGAATTATTAAATATAATGATGAATACTTAATGGTCGATATAATAA
GCACTTGGATTAGTTTATTTTTTCCTTTTATTAATTGGTTCATCCCAAAAGAATACGTCAAAATTAGTAGAGAAGAGTTTGAAAACTTAAAT
ATTGTTAAACCTGCTAAAAAGAATGTTTTTTGGCCAGTTGCAGGTAGCTCTGCTTTGCTGGGAGTTGCATTAAGAAAATATACACATTTAC
TTGACATTCAACTTGATAAAAAAATTAGTTATTGCCATATGTTGCATCACATTTATAGGGATTTTAATATTTTATGTACGCCTAATTAAAAAA
TCATCTTTAAATATTTATAATACTAAAAATAAAAGGTCAAAAATTTTTTTAATACCTACACTAAAAAATGTTTGTTTCACATTATTTGGATAT
ATTTTATTTGGCGGATTGACTATGCTATTCCTAGATGCACTATTATCAATGAGTTATCAAAACATAATAGTATATTTTGTTTGGATTGCAGT
TATAATGGGTTTTTTTCTAGTTAATATAGCTTTAATTATAGATAAAAACATTCATGTCATACTTAAAAACCAATAG

Gene: Q5HI66 (NADPH-dependent FMN reductase)

Contig: 05_NODE_4, position: 102617 to 103153, length: 537 nt, orientation: FORWARD

Perfect match to: (RF122-AJ938182-[617538:618074], highly conserved allele)

Sequence:

ATGATTACTGTTTTGTTTGGTGGGAGTAGACCAAACGGTAATACTGCACAATTAACAAAATTCGCTTTGCAAGATTTAGAGTATCAATGG
ATTGACGTGACACAACATCAGTTTAAACCGATACGTGACGTGAGACATACAGCAGAGACTATTACTTCATATGACGATGACTATTTGCCG
ATTCTAGATAAAATATTGGCTAGTGATACAATTATTTTTGCATCACCAGTGTATTGGTATAGCATTTCAGCACCATTGAAAGCATTTATCGA
ACATTGGTCAGAAACATTACAAGATAAACGATATCCTAATTTTAAGGCACAAATGGCCGAAAAGGATTTTAGAGTTATTTTAGTTGGTGG
AGATTGTCCAAAAATAAAAGCGAAGCCAGCAATTACGCAAATGAAATATAGTTTAGACTTTTTAGGTGCCACTTTAAATGGTTATATTATT
GGAACTGCTGAAAAGCCTGGTGACATCATGAAAGACAACTATGCCTTAGCACGTGCAACTGAGTGGAATAGTATATTGCAATAA

Gene: Q8NXU4 (GNAT family acetyltransferase)

Contig: 05_NODE_4, position: 103322 to 103798, length: 477 nt, orientation: FORWARD

Perfect match to: (N315-BA000018-[652698:653174], highly conserved allele)

Sequence:

ATGTTCAAGGTAAGACAAGCAACTGAAAAAGATGTTGTTCAAATTAGAGATGTCGCAACTAAAGCTTGGTTTAATACATACTTAAATATA
TACGCTGCGACAACAGTTAATCACTTGTTAGAAGCTTCATATAATGAACATCATTTAAAGAAAAGACTTCAAGAACAATTATTCTTAGTCG
TTGAAGAAGGTAATGACATCGTTGGCTTTGCTAACTTTATTTACGGTGAAGAATTATATTTATCAGCTCATTATGTTAAACCAGAATCGCA
ACATACAGGTTATGGTACAGCATTGTTAAATGAAGGATTATCACGTTTTGAAGATAAATTTGAAGGTGTTTACTTAGAAGTAGATAATAA
AAATGAAGAAGCAGTAGCTTACTATAAAGAGCAAGGTTTTACAATCTTACGCTCTTATGAGCCAGAAATGTATGGCGAAAAGTTAGACTT
AGCACTTATGTACAAAGCATTTTAA

Gene: Q5HI65 (phosphohydrolase)

Contig: 05_NODE_4, position: 103838 to 105133, length: 1296 nt, orientation: FORWARD

Perfect match to: (MW2-BA000033-[649642:650937], allele observed in CC1+CC239)

Sequence:

ATGACTAACGCATATGTAGATTTAAAATTAGTAGAAGAAAAAGTTTTTAAAGACCCGATACATCGATATATTCATGTTGAAGATCAATTG
ATATGGGATTTAATTAAAACTAAGGAATTCCAAAGGTTACGTCGAATTAGACAACTAGGAACACTGTACCTATCTTTTCACACAGCAGAA
CATAGTCGCTTTGGACATTCTTTAGGTGTGTATGAAATAGTTAGACGATTAATTGATGAGTCATTTATTGGTCATGATGCATGGGACAATA
AAGATAGACCGTTGGCATTATGTGCTGCATTATTACATGATTTAGGACATGGTCCATTTTCACATAGTTTTGAAAAAATATTTAATACAGA
CCATGAAGCATACACACAAGCGATTATTACTGGAGATACTGAGGTGAATGCTGTATTACGTAAAGTGGCGCCTGAGTTTCCAAGAGAAG
TTGCGGAAGTAATTAATAAAACGCATCATAATAAATTGGTCATTTCGATGATTTCGTCACAAATCGATGCGGATAGAATGGATTATTTACA
ACGTGATGCGTATTTCACAGGTGTATCATATGGTGCTTTTGATATGGAGCGTATTTTAAGATTAATGCGACCTTCTAAAGATGAAGTACTA
ATCAAAGAAAGTGGTATGCATGCAGTTGAAAACTTTATTATGAGTCGTTATCAAATGTATTGGCAAATTTACTTCCACCCAGTTAGTCGTG
GTGGAGAAGTGCTGCTTAATAATTGTTTGAAACGCGCAAAACAGCTTTATAATGAAGGCTATGAATTTAAGTTGCATCCACATGATTTTAT
TCCATTTTTTGAAGAGACAGTTACGATTGAACAATATGTTGAACTCGATGAAGCGGTAGTTACGTATTATTTGGAAAAATGGACAAAAGA
AGATGATGCCATTTTAAGTGATTTAGCAAGTCGATTTATTAATCGAGACTTATTTAAATATATTCCATTTGATGGCTCAATTATTACAATAT
CAGAACTGCAAGAACTGTTTGAAGCAGGTGGTATTAATCCAGATTATTATTTTGTGAGTGAAGCATTTTCGGATTTGCCATATGACTATGA
TCGACCGGGGTCAAATCGCAAACCGATTCATTTATTAAGACAAGATGGTACGATTAGAGAAATAAGCAATCAATCATTAGTCATTCATAG

TATTACAGGCATTAATCGCCAAGACTATAAATTATATTATCCTAGAGAAATGGTTGCAAAGATTAAAGATAAGACAATTAGAGAAGCTAT
TGAAAATTTGATTAATGAGCTTAATTAA

Gene: ywhD (putative protein)

Contig: 05_NODE_4, position: 105161 to 105682, length: 522 nt, orientation: FORWARD

Perfect match to: (MW2-BA000033-[650965:651486], allele observed in CC1+CC5)

Sequence:

ATGGAGGTTATATCATTGTCTGAGAAAAAAGGCTTTAATTTTAATATCATAAAAAATGACCCTCTAGATGGTCATAAAGGTACAAATATTG
GTTCAATTAGCTTAGACAATATTGCACCAGTTTTTATCGATGTTGCTAACAAAGAAGCATTTATTGATATTGGAGGCATGCATGCTCGTGC
CAAAGTTGAAAAAGGTGTGAAATGGATTACTGATAAAGCTGCTGTTGAAGGCGATGAAGCTAAAGAATATTGGTTGTGTTGGGTAACAA
CAGAACGTAATGAACAAGGACCATATTACGCTGGTTTAACAGCGTGCTATTTATTAGTGAATAAAGCAATTCGTCGTGGTTATAAAAGTA
TGCCTGAACATGTTAATATGATGGATAAATCAATGAAACATCATATTATCATTGATCAAATTGGTGACGAGAATAAAGCTATTTTAAAAG
ACTTTTTAATGAACCATGATGAAGGTATGTGGAAGCATTCTTCTGATGCTTTACATCAAGCATTTAATTAA

Gene: adhA (alcohol dehydrogenase I)

Contig: 05_NODE_4, position: 106181 to 107191, length: 1011 nt, orientation: FORWARD

Perfect match to: (MW2-BA000033-[651985:652995], allele observed in CC1+CC239)

Sequence:

ATGAGAGCAGCAGTTGTAACGAAAGATCACAAAGTAAGTATTGAGGACAAAAAGTTAAGAGCTTTAAAACCTGGTGAAGCGTTGGTAC
AAACGGAATATTGTGGCGTTTGTCATACCGATTTACATGTTAAGAATGCTGATTTTGGTGATGTTACAGGCGTTACTTTAGGTCATGAAG
GTATTGGTAAAGTCATCGAAGTTGCGGAAGATGTAGAATCATTAAAAATTGGAGACCGTGTGTCTATCGCTTGGATGTTCGAAAGCTGT
GGAAGATGTGAATATTGTACAACAGGTCGTGAAACACTTTGCCGTAGTGTGAAAAATGCTGGTTATACAGTAGATGGTGCAATGGCTGA
ACAAGTTATTGTTACTGCAGACTATGCTGTGAAAGTACCTGAAAAATTAGATCCAGCAGCAGCGTCTTCTATTACATGCGCAGGTGTGAC
AACTTATAAAGCTGTAAAAGTAAGTAATGTAAAACCTGGTCAATGGTTAGGTGTTTTTGGTATAGGTGGTTTAGGTAACCTAGCTTTACA
ATATGCTAAAAACGTTATGGGGGCTAAAATTGTTGCCTTCGACATTAATGATGATAAATTAGCATTCGCGAAAGAATTAGGTGCAGATGC
TATTATTAATTCTAAAGATGTTGATCCAGTTGCAGAAGTTATGAAATTAACTGATAACAAAGGATTAGATGCAACAGTGGTAACTTCAGTT
GCTAAGACGCCATTTAACCAAGCGGTTGATGTTGTAAAAGCTGGTGCAAGAGTTGTTGCTGTTGGTTTACCTGTTGATAAAATGAACTTA
GATATCCCAAGATTAGTGCTTGACGGTATTGAAGTAGTAGGTTCACTTGTTGGTACAAGACAAGACTTACGTGAAGCGTTTGAATTTGCT
GCTGAAAATAAAGTAACACCTAAAGTTCAATTAAGAAAATTAGAAGAAATCAATGATATTTTTGAAGAAATGGAAAATGGTACTATAACT
GGTAGAATGGTTATTAAATTTTAA

Gene: ywiB (putative protein)

Contig: 05_NODE_4, position: 107456 to 107884, length: 429 nt, orientation: FORWARD

Perfect match to: (MW2-BA000033-[653260:653688], allele observed in CC1+CC45I)

Sequence:

GTGAGTAGTAAATTGGATAAAAAAGTAAGTATTCAAACAAAGCAAGTGTTGAAACAGCACAACGAAAAAGAAAAATTTGAATTTACTAC
TGAAGGAACTTGGCAACAAAGGCAATCTAACTTTATTCGGTATGTAGAACAAATTGAGGATGCAACAGTTAATGTTACAATAAAAGTGG
ATGATGATAGCGTTAAGTTGATTCGTAAAGGCGACATTAATATGAATTTGCATTTTGTTGAAGGACAAACGACAACAACTTTTTACGATAT
ATCGGCTGGACGAATTCCACTAGATGTTAAAACATTACGCATTTTACATTTCGTAAGTGGAGACGGTGGCAAGCTAAAGATTCATTATGA
ATTATATCAAGATAATGAAAAAATGGGTTCTTATCAATATGAAATTAACTATAAGGAGATAGGCGAATGA

Gene: argS (arginyl-tRNA synthase)

Contig: 05_NODE_4, position: 107881 to 109542, length: 1662 nt, orientation: FORWARD

Perfect match to: (MRSA252-BX571856-[663784:665445], allele observed in CC30+CC25+CC30)

Sequence:

ATGAATATTATTGATCAAGTGAAACAAACATTAGTAGAAGAAATTGCAGCAAGTATTAACAAAGCAGGATTAGCAGATGAGATTCCTGA
TATTAAAATTGAAGTTCCTAAAGATACAAAAAATGGAGATTATGCTACTAATATTGCGATGGTACTGACTAAGATTGCAAAGCGTAATCC
TCGTGAAATTGCTCAAGCGATTGTTGATAACTTAGATACTGAAAAAGCACATGTAAAACAAATTGACATTGCTGGTCCAGGATTCATTAA
TTTTTACTTAGATAATCAGTATTTAACAGCAATTATTCCTGAAGCAATTGAAAAAGGTGATCAATTTGGACATGTAAATGAATCAAAAGGT
CAAAATGTATTGCTTGAGTATGTTTCGGCTAACCCTACAGGAGATTTACATATTGGTCATGCTAGAAATGCAGCAGTTGGTGATGCTTTA
GCTAATATTTTAACTGCAGCTGGCTATAATGTAACACGTGAATATTATATTAATGATGCTGGTAATCAAATTACTAACTTAGCGCGTTCGA
TTGAAACACGTTTCTTTGAAGCTTTAGGTGACAATAGTTATTCAATGCCAGAAGATGGCTATAATGGAAAAGATATTATTGAAATAGGTA
AAGATTTAGCAGAGAAACACCCTGAAATTAAAGATTATTCTGAAGAAGCACGTTTGAAAGAATTTAGAAAATTAGGCGTAGAATACGAA
ATGGCTAAATTGAAAAATGATTTAGCAGAGTTCAATACGCATTTTGATAATTGGTTTAGTGAAACATCTTTATATGAAAAAGGCGAAATTC
TTGAAGTTTTAGCAAAAATGAAAGAATTAGGTTATACGTATGAAGCTGATGGCGCTACATGGTTACGTACAACTGATTTTAAAGACGACA
AAGACAGAGTCTTAATTAAAAATGACGGTACATATACGTATTTCTTACCAGATATTGCGTACCACTTCGATAAAGTTAAACGTGGTAATGA
CATTTTAATCGATTTATTTGGTGCTGATCATCATGGTTATATTAATCGTTTGAAAGCATCTCTTGAAACGTTTGGTGTAGATAGTAATCGTT
TAGAAATTCAAATCATGCAAATGGTTCGTTTAATGGAAAATGGTAAAGAAGTGAAGATGAGTAAACGTACTGGTAATGCGATTACATTA
AGAGAAATTATGGACGAAGTTGGCGTTGACGCTGCACGTTATTTCTTAACTATGCGTAGTCCTGATAGTCACTTTGATTTTGATATGGAAT
TAGCGAAAGAGCAATCTCAAGACAATCCAGTTTACTATGCTCAATATGCACATGCGCGTATTTGTTCAATTTTAAAACAAGCGAAAGAGC
AAGGTATTGAAGTGACTGCTGCGAATGATTTTACAACAATTACTAATGAAAAAGCGATTGAATTGTTGAAAAAAGTAGCTGACTTTGAGC
CTACAATTGAAAGTGCTGCAGAACATAGATCAGCACATAGAATTACTAATTATATTCAAGATTTAGCTTCTCATTTCCATAAATTCTACAAT
GCTGAAAAAGTGTTAACAGATGATATTGAAAAAACAAAAGCACATGTTGCTATGATTGAAGCGGTCAGAATTACATTGAAAAATGCATT
GGCAATGGTCGGTGTAAGCGCACCTGAATCAATGTAA

Gene: tx_universal2 (rho-independent terminator)

Contig: 05_NODE_4, position: 109753 to 109792, length: 40 nt

Perfect match to: (LGA251-FR821779-[1071185:1071224:r], allele observed in CC425)

Sequence:

TGAACGAAAATTCGCTTGTAACAAGCTTTTTTCAATTCTA

Gene: nth2 (putative endonuclease III, locus 2)

Contig: 05_NODE_4, position: 109933 to 110568, length: 636 nt, orientation: FORWARD

Sequence:

ATGTTAGGAACTGATGAATTATATAAAGTTTTATATGAACATCTCGGACCACAATTTTGGTGGCCTGCTGATAATGACATTGAAATGATGT
TAGGTGCAATTTTAGTTCAAAATACTAGATGGCGAAATGCAGAAATTGCATTGAATCAAGTTAAAGAACACACGCATTTTAATCCAAATC
ATATATTAGAACTACCTATTGAAACGTTACAATCATTGATACATTCAAGTGGCTTTTATAAAAGTAAATCACTGACGATTAAAACATTATTA
ACATGGTTAGCACGACATCATTTCAATTATCAAGAGATTAATGAGCGATATAAAGGTGGATTAAGAAAAGAATTATTATCTTTGAAAGGT
ATTGGAAGTGAAACAGCAGATGTCTTACTTGTTTATATATTCGGACGTATTGAATTTATTCCAGATAGCTATACAAGAAAAATATATGATA
AATTAGGATATGAAAACACTAAAAATTATGATCAATTAAAAAAAGTAGTCACATTACCAAATCATTTTACAAATCAAGATGCTAATGAATT
TCATGCTCTGTTAGATGTATTTGATAAACATTACTTTAGAGACAAAGATATAAAGAATTATGATTTTTTAGAACCTTACTTTAAAAAGTAA

Gene: A5IQG3 (similar to iron-binding transport protein)

Contig: 05_NODE_4, position: 110879 to 111766, length: 888 nt, orientation: FORWARD

Perfect match to: (MW2-BA000033-[656683:657570], allele observed in CC1+CC1156)

Sequence:

GTGAAGAAATCGTTAATTGCTTTTATTTTGATTTTTATGCTTGTCCTGAGTGGCTGTGGTATGAAAGATAATGATAAACAAGGTAGCAATG
ATAATGGCTCGTCTAAATCGCCGTACCATAGAATTGTTTCGTTAATGCCTAGTAATACTGAAATTTTATATGAATTAGGATTAGGTAAATA
CATAGTTGGTGTTTCAACGGTTGATGATTATCCAAAAGATGTGAAAGAGGGTAAGAAACAATTTGATGCTTTGAATCTAAATAAAGAGG
AACTTTTAAAGGAAAAGCCAGATCTAATTCTTGCGCATGAGTCGCAAAAGGCAACTGCTAATAAAGTATTGTCATCATTAGAGAAACAAG
GCATCAAAGTAGTGTATGTTAAAGATGCACAATCAATTGATGAAACATACAACACATTTAAGCAAATTGGGAAATTAACGCATCATGATA
AGCAGGCTGAACAACTTGTTGAGGAAACTAAAGATAATATCGATAAAGTCATAGATTCAATTCCTGCTCATCATAAAAAATCAAAAGTAT

TTATTGAGGTTTCATCAAAGCCTGAAATATATACAGCAGGAAAGCATACATTTTTCAATGATATGTTAGAAAAATTAGAAGCCCAAAATG
TTTATAGTGACATTAATGGTTGGAACCCTGTAACGAAGGAAAGTATTATTAAAAAGAACCCAGATATATTAATTTCGACGGAAGCTAAGA
CAAGATCAGATTATATGGATATCATCAAAAAAAGAGGTGGATTCAATAAAATTAATGCTGTCAAGAATACACGTATTGAAGTTGTAAATG
GTGATGAAGTATCAAGACCCGGTCCACGTATTGATGAAGGATTAAAAGAATTAAGAGATGCAATTTATAGAAAATAA

Gene: yvrB (putative vitamin B12 permease)

Contig: 05_NODE_4, position: 111915 to 112865, length: 951 nt, orientation: FORWARD

Perfect match to: (Strain_10497-HE579061-[638703:639653], allele observed in CC5)

Sequence:

ATGACATTTAATAAAGTATTATTGAGCTGGATAGTCATATTGATTATAACAACTAGCATATATCTATTTTGGCAGTTGGGGGATATCAATG
ATGTATTTAACCAGTCTATTTTAATCAATGTTAGATTACCGAGATTATTAGAAGCATTGTTGACAGGTATGATATTAACAGTTGCAGGTCT
TATATTTCAAACAGTTTTAAATAATGCATTGGCAGATAGCTTTACATTAGGATTGGCAAGCGGTGCTACATTTGGTTCAGGATTAGCATTA
TTTTTAGGTTTAACAACGTTATGGATTCCTGTATTTTCAATAACATTTAGTTTGATAACATTAATAACTGTATTAGTCATTACGTCGGTATTG
AGCCAAGGCTATCCAGTTAGAATCTTAATATTAAGTGGTTTAATGATTGGTGCATTATTCAATTCACTTCTATATTTTTTGATTTTATTAAAA
CCTCGCAAATTAAATACAATTGCCAATTATTTGTTTGGTGGCTTTGGTGATGCAGAATACTCAAATGTATCCATAATAGCAATCACATTTAT
CATTGCACTGTTTGGTATATTTATCATTCTTAATCAACTAAAGTTATTGCAATTAGGAGAACTAAAAAGTCAGTCACTAGGCTTAAATGTTC
AATTGATTACATATATCGCGTTATGTATAGCTTCTATGATAACGGCGATAAATGTCGCATATGTTGGCATCATTGGATTCATTGGTATGGT
AATACCGCAACTCATTAGAAAATGGCAGTGGAAACAATCATTAGGAAGACAATTGGCTTTGAATATTGTAATTGGAGGACAAATAATGG
TTATGGCAGATTTTATTGGTAGCCATATATTGTCACCAGTACAAATACCGGCAAGTATTATCATTGCATTAATTGGTATACCAGTGTTATTT
TACATGCTAATATCTCAGTCGAAACGGTTACACTAG

Gene: ppaX2 (haloacid dehalogenase-like hydrolase)

Contig: 05_NODE_4, position: 112920 to 113639, length: 720 nt, orientation: FORWARD

Perfect match to: (N315-BA000018-[662115:662834], highly conserved allele)

Sequence:

ATGGATTTGAATCAAATTAAAGCAGTTGTATTTGATTTAGAAGGTACGTTGTTGGACAGAGTTAAATCTCGAGAGAAATTTATCGAAGAG
CAATATGAACGATTTCATGACTACTTAATTCATGTTCAACTGGCAGATTTTAAAAAAGCATTTATTGAGCTAGATGACGATGAAGATAATG
ATAAACCTGATTTATATAAAGAAATCATTAAACGTTTCCATGTAGATAGGTTAACTTGGAAAGACTTATTTAATGATTTTGAAATGCATTTT
TATCGTTATGTATTTCCTTATTACGATACTTTGTATACACTAGAAAAGCTATCGCAAAAAGGCTTTCAAATTGGTGTTATCGCAAATGGTAA
ATCTAAGATTAAACAATTTCGATTACATTCACTTGGTTTGATGCATGTTATTAATTATTTATCAACATCAGAAACAGTTGGTTTTCGTAAAC
CACATCCTAAAATTTTTGAAGATATGATTGATCAACTAGGGGTATTACCTGAGCAAATTATGTATGTTGGCGATGATGCGTTAAATGATGT
AGCTCCAGCACGAGCTATGGGCATGGTTAGTGTATGGTATAAACAAGAAGATGCTGAAATTGAACCACTCGAAGAAGAAGTTGATTTTA
CAATTACAACAGTGGAAGAATTATTAACCATTTTACCAATAAAAAATGATAATAAAGGAGAAAATTATGGATCTATTTACTAG

Gene: ydjP (alpha/beta fold hydrolase)

Contig: 05_NODE_4, position: 113623 to 114423, length: 801 nt, orientation: FORWARD

Perfect match to: (MW2-BA000033-[659427:660227], allele observed in CC1+CC101)

Sequence:

ATGGATCTATTTACTAGAAAAGATGGAACATCGATTCATTACAGTACATTAGGTGAAGGCTATCCTATCGTATTGATTCATACTGTACTTG
ATAATTATTCTGTGTTTAATAAATTAGCAGCACAATTAGCAAAATCATTTCAAGTTGTGTTAATTGATTTACGTGGACATGGCTATTCTGAT
AAACCTCGTCACATTGAAATAAAAGATTTTTCTGATGACATTGTTGAATTACTTAAATATTTATACATTGAAGAAGTTGCATTTGTATGCCA
TGAAATGGGTGGAATCATTGGTGCGGATATTTCAGTACGTTATCCTGAATTTACATCATCACTTATGTTGGTAAATCCAACATCTATTGAA
GGTGAATTACCGGAAGAACGTTTATTTAGAAAATATGCCCATATTATTCGAAACTGGGATCCTGAAAAACAAGATAAATTTTTAAATAAG
CGTAAGTATTATCGTCCGAGAAAAATGAATCGATTCCTTAAACATGTCGTAGATACAAATGAAATATCAACTAAAGAAGAAATTCAAGCA
GTTAAAGAGGTATTCAAAAACGCTGATATTTCTCAAACTTATAGAAATGTCGTAGTACCGACAAAAATTATTGCAGGAGAATTCGGTGAA
AGAACAACAAGATTGGAAGCTAAAGAAGTAGCTGATTTAATCCAAAATGCGGACTTTGAAGTATATCAAGAATCAAGTGCATTCCCATTT
GTTGAAGAGCAAGAAAGATTCGTCGAAGATACAGCTTCATTTATCAACAAACATCACGATGAAAAGCATGTTTAA

Gene: Q5HI54 (putative protein)

Contig: 05_NODE_4, position: 114559 to 115065, length: 507 nt, orientation: FORWARD

Perfect match to: (N315-BA000018-[663754:664260], allele observed in CC5+CC1)

Sequence:

ATGAAAAAGCTACTAACGGCAAGTATAATTGCATGTTCTGTTGTAATGGGAGTAGGCTTAGTGAACACTAGTGCCGAAGCAGCAAGTGG
CAACTCTATTGATACTGTTAAACAATTAATTAAGGGTGATCAGTCATTAGAAAATGTGAAAATTGGCGAATCTATTAAAGATGTTTTAACT
AAGTACAAAAATCCTATGTATTCTTACAATGAAGATGGAACTGAACATTATTACGAATTCCATACTAAAAAAGGTATGTTATTAGTAACTA
CTGATGGTAAGAAAAACAATGGTAAAGTAACTCATATTTCAATGATGTATAATGATGCTAATGGTCCAACATATCAAGCTGTTAAAAATT
ATGTTGGCAAAGCAGTAACACATACGGAATATAGCAAAGTTGCTGGTAATTTTGGATATATTGAAAAAGGCAAAACGACTTATCAATTTG
CCTCAGCACCAAAAGATAAAAACATAAAATTATATCGTATTGATTTGGAAAAATAA

Gene: Q5HI53 (putative protein)

Contig: 05_NODE_4, position: 115227 to 115943, length: 717 nt, orientation: FORWARD

Perfect match to: (MW2-BA000033-[661031:661747], allele observed in CC1+CC239)

Sequence:

ATGCGTATTAACGATAAAATTTTACTAGAAAATATAGAAGATTACTTTAATCATAAAGGTTTATCACCGCATTTGATTGATGATATTAAAG
AGAAAGTAATTACTGATATAAAAAATTCTGAAAAGAAAGATCAAGATTATATTGAATATAAAAGAAAATCTCCAGCACAAATCATATTAA
TGATTCAAAGGAATTTGTTTGCTTTACAAATGAATCCAGTTATTTTCTTTATTATAAACTTCATTCTCATATCCTATTTATATGATAAACAGT
ATGTTCAGTTTCAAGCTATTACTGGAATGAGTCTATTTTATTGTTTAGTGATTTTTCCAATGACTATTGTTGTATACCTAAGGGTGTCACAA
AAGAATTACTTGCGTAGTAATAAAATAGAAATGATTATGGGTACAATTATCGCTATCATATCCTTGTTATTAATTATATTACAAGCATTTAA
TATTACTTGGGGCGTTATACCAATTACAAATTTTGGACATCAATTTTTCTTTTTCATTGGTATTATTTTAGTAATTGCCGGCATATTTTATAA
GCGACTTGAGTTTTCGGGAATCGGCTTATTATTTTGTCAAAAAACCGTCGATGCAATGATTCATAATCCACAATCAGCCCAGATTTTTTCAT
TAATTATATGGATATTATTAGTAGTTCTAGTTATATATTTCACAATTAGATTATCTTCACGTACAAGATTATAA

Gene: Q5HI52 (alpha/beta hydrolase fold protein)

Contig: 05_NODE_4, position: 116112 to 116900, length: 789 nt, orientation: FORWARD

Perfect match to: (RF122-AJ938182-[632288:633076], highly conserved allele)

Sequence:

ATGAATAAAGTCACAATTAATCCTCAAATCCAATTAACTTATCAAATTGAAGGTAAAGGGGATCCTATAATATTACTTCATGGATTGGATG
GTAATTTAGCTGGATTTGAAGATTTGCAACATCAACTAGCATCATCATATAAAGTACTTACTTACGATTTAAGAGGTCATGGCAAGTCTTC
TAAAAGTGAATCATACGATTTAAACGATCACGTTGAGGATTTAAAAATTCTAATGGAGAAGTTAAATATTCATGAGGCACATATTCTAGG
ACATGATTTAGGTGGGGTAGTTGCTAAGTTATTTACAGATAAATATGCTTATCGTGTAAAATCATTAACTACCATTGCATCGAAGAAAGAT
GACTTAATACACAGCTTTACTCAATTGTTAATACAATATCAAGATGATATAGCGGGTTTTAATAAGTCTGAAGCGTATATTCTTTTATTTTC
TAAATTGTTTAGAAATCAAGAGAAGACGATGAAATGGTATCAAAAACAAAGAATATATAGCATTAAGTCTGAGGATGATAGTGCGGTGG
CAATTCGTTCATTAATTTTGCATAAAGATGAACCTATGTATTTAAAAAAACGTACATGTGTACCTACTTTGTTAATTAATGGGGAACATGA
TCCTTTGATTAAAGATAAGAATCATTTTAAATTGGAAGCGCATTTTTTAAATGTTACGAAAAAAATCTTCGAACATTCAGGACATGCACCG
CATATTGAAGAACCAGAAGCATTTATGAATTATTATTTAAATTTTTTAAAAAGCGTATCATAA

Gene: sarA (staphylococcal accessory regulator A)

Contig: 05_NODE_4, position: 117150 to 117524, length: 375 nt, orientation: REVERSE

Perfect match to: (RF122-AJ938182-[633329:633703:r], highly conserved allele)

Sequence:

TTATAGTTCAATTTCGTTGTTTGCTTCAGTGATTCGTTTATTTACTCGACTCAATAATGATTCGATTTTTTTACGTTGTTGTGCATTAACAAG
AATTAATACAGTTCTTTCATCATGCTCATTACGTTTTTTATCGAAGTAATCTTCTTGAGATAAAATTTTAACTGCTTTAACAACTTGTGGTTG
TTTGTAGTTTAAATGATTAATAATATCTTTAAGATAGTATTCTTTCTCTTTGTTTTCGCTGATGTATGTCAATACAGCGAATTCTTCAAAGCT
AATTGAAAATTCCTTTTTAATTAAACTTTTTAATTTGTCAGCATAAGTGACCATTGATAACAACTCAAAGCAATCATTGATTTTTGTAATTGC
CAT

Gene: teg49 (small RNA upstream of sarA)

Contig: 05_NODE_4, position: 117688 to 117883, length: 196 nt

Sequence:

AAAAATGAGAAGTAAACAAAAAAGTGTTTATAAAACAAATTATTCATTAGACACAGTGATTGTATTTCTGGGTTAGCATTTGGTTTAGTCA
AAAATATCAGCATTTTTTATAATTTACCTTAATTTAATCGCCAATACAAGGTAATTTATTTAATGAAACGATTTAGCGCAATTAAATGTTTC
GATATTCAATGTG

Gene: Q2YSV8 (putative protein)

Contig: 05_NODE_4, position: 118472 to 119401, length: 930 nt, orientation: REVERSE

Perfect match to: (N315-BA000018-[667669:668598:r], allele observed in CC5+CC1+CC361)

Sequence:

TTAAAATAATCTTAAAAGTATAATGCCTGTCAAAATACATAATAGACCAATAGTTTTTCTGGATGTCATTGCTATTTTAGGTGAACCAAATA
ATCCAAAGTGATCTATCAATATGCCCATTAGAATCTGGCCAAACATCCCAATAAGTGTTGTTAATGCTGCACCCATATGAGGCATTAAGAT
AATGTTAGCTGTTACAAAAGCCATACCAAGTATACCGCCAGTAAAATAGATAGGCTTTAATTTACCGAATTTTAAATGACTTGTTTTTAGT
TTTAAAGAACGATTAAAAATAGCGGTTAAAATCAATAGCGTTATTGACCCAATTGTAAATGATACTAATGATGCAAAGGCTGGTGAATGA
GTATGACTTGCTAAAGCACTATTAATTGTCGTTTGAATAGGTGGAAAGAAACCAAAAATAAATCCTAAGAGAAGCCAAAACAGTAAATA
CTTTTGATCAGTTAGTAATAAATTATTCTTGTTAAATTGATTCATTATGACGATGCCGACAATGAGTAACAATACTCCAATTGCTTTAATTA
AATTAAAATCATGAATTGTAGCGCCAAATAAGCCAAATGTATCAATAATGACACCCATAATAATTTGACCCGCAACTGTTGCAATTACAGT
TAATGTTGCACCTAATTTTGGCAATAACAATAAATTGCCAGTTAAAAAGCTAACCCCAAGCAAACCACCGACTACCCATGTGTAGTTAAAT
GATTGATTATTGTAAAAGTGAATAGTAAATACTTCTGGATTGATAATGATATTTAAAATAATTAAACAAATTGTTCCAACTGAAAATGAAA
TGAATGAAGTATAGAAAGGCGATTTAGTATACAGTGATAGTCTTGAATTGACAGATGTTTGGATAGGAATAAGCATTCCAACTAGGACA
CCAATGATATAGAAAAGAAGCAT

Gene: A6QET1 (putative protein)

Contig: 05_NODE_4, position: 119596 to 119820, length: 225 nt, orientation: REVERSE

Perfect match to: (RF122-AJ938182-[635775:635999:r], highly conserved allele)

Sequence:

CTATTTCTTATCTTCAAATAAAACAGTTGTTGTTTTATCAATAATTTGAGCAGAATGTACTTTGGTGATAGTACCACTATTTGACTTTATAAT
ATTTAAATCTACTAATGCATTCATACTATCACGTGCAACTTGCTCGGTCACTACGTTGTTTAATTGTGGTAATTGCAGTTTTACAGGCTTGT
CTAGTGAAGATTTAAAACTTAATTCAAGTGTTTTAGTCAT

Gene: Q7A1N3 (putative protein)

Contig: 05_NODE_4, position: 119837 to 120040, length: 204 nt, orientation: REVERSE

Perfect match to: (N315-BA000018-[669034:669237:r], allele observed in CC5+CC1)

Sequence:

TTAAATTGATAAAGATTTGATGAGTTCGATATTGTTATATGTTTCTCCAGTAAGTCGCTCAATAAGTTTGCTGAATGTTTTAATTTGGTCGT
TTGATGCATCAGGGTTAATGTTAGCGAATCGACGCTTAAATTCTGTTTGTTTGCCGTTAGCGTCTACTTTAGTAAATGATAATACAATAGT
GATGTGGTTTATTTTACTCAT

Gene: A5IQH4 (putative phage integrase family protein ??)

Contig: 05_NODE_4, position: 120199 to 120759, length: 561 nt, orientation: FORWARD

Perfect match to: (N315-BA000018-[669396:669956], highly conserved allele)

Sequence:

ATGAATAAAGTAGAAGCGATTAAATTTAATGATGATATTGTTAAAATGTATGAAGCGCTCAAGATAAAATCTGAACGTGACTATTTATTCT
TTAAGTTAGCTATACATAGTGGATTGAAAGTATCAGAATTATTAACAATTACAGTCTCTCAAGTTAAGAGACTAATTGAAAAGTGTACGTT
ATCAGAAATGTGTAAAGCACATTTTCATTCGTTGATTAAAATTAGGTTACCAGAAACATTATCGAAAGAACTACTTCAATATATAGAGGAC
AGGAGTCTTTCGAATGAAGACGTTCTTTTTCAATCACTACGAACAAATCAAGTATTATCTAGACAGCAAGCATATCGAATAATTCACCAAG
CATCAATTGAAGCTGGTATAGATAATGTAGGACTAACGACATTGCGTAAGACATTTGCATATCATGCTTATCAAAAAGGTATACCTATACC
AGTCATTCAAAAGTATTTAGGGCATCAATCTGCTATTGAAACACTAAATTTTATCGGTTTAGAAAATGAGTGTGAACATAGTATTTATATT
TCATTACAATTATAG

Gene: mrpA (monovalent cation/proton antiporter protein A)

Contig: 05_NODE_4, position: 120778 to 123180, length: 2403 nt, orientation: FORWARD

Perfect match to: (JKD6008-CP002120-[696775:699177], allele observed in CC239)

Sequence:

ATGAGTTTGGTTTATTTATTAATTGCTATACTTGTGATTATGGCGATGATACTTCTAATGTCTAAACGTAGAGCATTGGCTAAATATGCCG
GGTACATAGCGTTGGTTGCACCTGTAATTTCATCTATCTATTTTTTGATTCAAATACCATCAGTAGCTAAACTGCAATATCTTTCTACCTCTA
TTCCATGGATTAAGACATTAGATATTAATTTAGATTTACGTTTAGATGGTTTAAGTTTAATGTTTTCTCTTATTATTTCACTTATTGGAATTG
CAGTATTCTTCTATGCAACTCAATATTTATCCTCTCGAAAAGACAATTTACCAAGGTTTTATTTTTATTTAACGTTATTTATGTTCAGTATGA
TTGGTATTGTATTATCAGACAATACGATATTGATGTACATTTTTTGGGAATTAACGAGTGTATCATCATTTTTATTGATTTCATATTGGTAT
AACAACGGAGATAGTCAATTTGGTGCGATGCAATCATTTATGATTACAGTATTTGGTGGTTTGGCATTATTAGTTGGTTTTATCATGTTGT
ATATTATGACAGGAACGAATAACATCACAGAGATATTAGGACAAGCAGATCATATTAAGAATCATGGATTGTTTATCCCTATGATTTTTAT
GTTTTTATTAGGTGCATTTACAAAATCAGCACAATTTCCATTTCATATTTGGCTACCTAGAGCAATGGCTGCACCTACACCTGTAAGTGCTT
ATTTACATTCAGCCACGATGGTAAAAGCTGGTATCTTTTTATTACTTCGATTTACACCATTATTAGGTCTTAGCAATATGTACGTATATATC
GTTACGTTTGTTGGTTTAATAACAATGTTATTTGGTTCAATTACAGCTTTAAAACAATGGGATTTAAAAGGTATCCTAGCGTACTCTACAAT
CAGTCAACTTGGGATGATTATGGCTATGGTGGGTATAGGTGGCGGATATGCTCAACACCAACAAGACGCAATAGCATCTATTTATGTATT
TGTATTATTTGGTGCGCTATTTCATCTAATGAATCATGCCATCTTTAAATGTGCGCTTTTCATGGGAGTAGGTATTTTAGATCATGAAGCAG
GTTCAAGGGATATACGAATTTTAAGTGGAATGCGTCAACTATTTCCTAAAATGAATCTAGTCATGACGATAGCGGCTCTATCTATGGCTG
GAGTACCATTTTTAAATGGATTTTTAAGTAAAGAAATGTTTTTAGATGCATTAACACAAACTGGACAATTATCCCAATTTAGTTTGATTTCA
ATGATAGCTATCGTGTTTGTTGGTGTTATTGCGAGTGTTTTTACATTCACATATGCACTATACATGGTAAAAGAAGTATTTTGGACAAAAT
ATGATTCTAAGGTTTTTACTAAAAAAAATATCCACGAACCATGGTTGTTTAGTTTACCATCTCTTATATTAATGGTGCTAGTACCTGTAATC
TTTTTTGTACCAAATATATTTGGGAAGGGGATTATCGTTCTAGCATTAAGAGCTGTATCAGGTGGTAATCATCAAATTGATCAATTGGCAC
CACATGTTTCGCAATGGCATGGATTTAACATACCGCTTCTTTTAACCATCATCATTATTTTATTGGGTAGTGTACTAGCAATCAAAGTAGAT
TGGAAAAAAGTGTTCACAGGTAAAATTAGACAGATTTCAGTTTCAAAAAGCTATGAGATGGTATATCGACATTTTGAAAAGTTTGCTACG
AAGCGATTTAAACGTGTTATGCAAGATCGTTTAAACCAATACATTATTATGACCTTAGGCATATTTATGATTATCATTGGATATGGTTATAT
TCGAATTGGACTTCCTAAAGTACATCAGTTACATGTTTCTGAATTTGGGGCATTAGAAATTATATTAGCAATCGTAACTGTCACAATTGGT
ATTTCTTTAATTTTTATACGTCAACGACTGACAATGGTCATTTTAAATGGAGTCATCGGATTTGTTGTGACCTTATTCTTTATAGCAATGAA
AGCCCCTGATCTAGCATTGACTCAGCTAGTAGTTGAAACAATAACGACGATACTATTTATTGTCAGTTTTTCAAGATTACCAAACGTGCCA
AGATCTAACGCTAACAAAAAAAGAGAAATAATTAAAATTTCTGTATCACTCTTGATGGCACTTATTGTTGTATCATTAATTTTTATTACACA
ACAAACAGATGGTTTATCATCAATATCAGACTTTTATTTAAAAGCTGACAAACTAACAGGTGGTAAAAATATTGTAAATGCGATACTTGGT
GACTTTAGAGCATTAGATACATTATTTGAAGGATTAGTGTTAATTATTACTGGGCTAGGTATTTACACATTATTAAATTATCAAGATCGGA
GGGGACAAGATGAAAGAGAATGA

Gene: mrpB (monovalent cation/proton antiporter protein B)

Contig: 05_NODE_4, position: 123167 to 123592, length: 426 nt, orientation: FORWARD

Perfect match to: (MW2-BA000033-[668971:669396], highly conserved allele)

Sequence:

ATGAAAGAGAATGATGTCGTGTTAAGAACGGTCACGAAACTTGTTGTATTTATTTTATTGACTTTCGGATTCTATGTCTTCTTCGCAGGTC
ATAATAATCCTGGTGGTGGGTTTATTGGTGGTTTAATATTTAGTTCAGCGTTTATTTTAATGTTTCTGGCTTTTAATGTTGAAGAGGTTTTA
GAAAGTTTACCGATTGATTTTAGAATTTTAATGATTATTGGAGCATTGGTATCATCTATTACTGCGATAATACCTATGTTTTTTGGAAAACC
ATTTTTGTCTCAATATGAAACAACTTGGATACTTCCAATTTTAGGACAAATTCATGTAAGTACAATAACACTTTTTGAATTAGGTATTTTATT
CTCAGTTGTTGGTGTTATTGTCACAGTGATGTTGTCGCTTAGCGGAGGTCGATCATGA

Gene: mrpC (monovalent cation/proton antiporter protein C)

Contig: 05_NODE_4, position: 123589 to 123933, length: 345 nt, orientation: FORWARD

Perfect match to: (MW2-BA000033-[669393:669737], highly conserved allele)

Sequence:

ATGAATTTAATATTATTACTAGTTATAGGATTTTTAGTGTTTATAGGAACATATATGATTTTATCAATCAATTTAATTCGTATTGTAATCGGA
ATTTCAATATATACTCATGCTGGTAATCTCATTATTATGAGTATGGGAACGTATGGTTCTAGTAGATCAGAACCACTAATAACTGGTGGAA
ACCAATTGTTTGTTGATCCCTTGTTACAAGCTATTGTACTAACTGCAATAGTTATAGGGTTTGGGATGACTGCGTTTTTACTTGTACTTGTT
TATAGAACTTATAAAGTAACAAAAGAAGATGAAATTGAAGGCCTAAGGGGGGAAGATGATGCTAAGTAA

Gene: mrpD (monovalent cation/proton antiporter protein D)

Contig: 05_NODE_4, position: 123923 to 125419, length: 1497 nt, orientation: FORWARD

Perfect match to: (COL-CP000046-[706749:708245], highly conserved allele)

Sequence:

ATGCTAAGTAACTTATTGATTTTACCAATGTTATTACCATTCCTTTGTGCCTTAATCCTTGTATTTTTAAAAAATAATGATCGTATTTCTAAAT
ATTTATACTTAGGTACAATGACTATCACCACAATTATTTCATTAATGCTATTAATTTATGTTCAGCGTCACCGTCCAATTACGCTAGACTTTG
GAGGATGGTCAGCGCCCTTTGGTATACAGTTTTTAGGAGATTCTTTAAGTTTAATTATGGTTACAACCGCTTCGTTTGTGATTACTTTAATT
ATGGCATACGGATTTGGGCGTGGCGAACATAAAGCAAATCGTTATCACTTGCCATCGTTCATATTATTTTTAAGTGTTGGCGTGATAGGC
TCTTTTCTAACATCAGATTTATTTAATTTATACGTCATGTTTGAAATTATGTTACTAGCGTCATTTGTACTCATTACACTTGGACAATCTGTA
GAACAATTACGTGCTGCAATTATTTATGTTGTCTTGAATATTATTGGTTCATGGCTATTCTTATTAGGTATAGGTTTACTTTATAAAACAGT
AGGTACATTAAACTTTTCACATATTGCAATGCGTTTGAATGACATGGGAGATAATCGCACTGTTACAATGATTTCATTAATCTTCTTAGTCG
CATTTAGTGCGAAAGCAGCGCTGGTCCTTTTTATGTGGCTACCCAAAGCCTACGCTGTGTTAAATACTGAGCTTGCAGCATTATTTGCAGC
GTTAATGACCAAAGTAGGGGCCTATGCATTAATTCGATTCTTCACTTTACTATTTGATCAACATAATGATCTCATACATCCATTGCTAGCAA
CTATGGCTGCTATAACTATGGTCATCGGCGCTATAGGTGTCATTGCTTATAAAGATATTAAAAAGATTGCAGCTTACCAAGTCATAATCTC
AATAGGATTTATCATTTTAGGTTTAGGAACAAACACGTTTGCAGGTATTAATGGTGCAATATTTTATTTGGTAAATGACATTGTTGTAAAA
ACATTGCTATTTTTTATTATTGGTAGTTTAGTTTACATTACAGGCTATCGACAATATCAATATTTGAATGGCTTAGCTAAAAAAGAACCTTT
ATTTGGAGTTGCGTTTATTATAATGATTTTTGCTATTGGCGGCGTGCCTCCATTTAGTGGCTTTCCGGGGAAAGTACTTATTTTCCAAGGT
GCATTGCAAAATGGCAATTATATTGGACTAGCGTTAATGATTATTACTAGTCTAATTGCAATGTACAGTTTATTTAGGATACTTTTTTATAT
GTATTTTGGAGATAAAGATGGGGAGGAAGTTAATTTTAAGAAAATCCCGCTATATCGAAAAAGAATTTTAAGTATTTTAGTAGTTGTGGT
TATCGCAATCGGAATTGCTGCACCTGTTGTGTTAAATGTTACAAGTGATGCAACTGAGTTGAACACGAGTGATCAATTATATCAAAAACTT
GTAAATCCGCATTTGAAAGGAGAGGACTAA

Gene: mrpE (monovalent cation/proton antiporter protein E)

Contig: 05_NODE_4, position: 125420 to 125902, length: 483 nt, orientation: FORWARD

Perfect match to: (N315-BA000018-[674617:675099], highly conserved allele)

Sequence:

ATGAATCAAATAGTTTTAAATATTATCATTGCATTCTTATGGGTATTATTTCAAGATGAAGATCATTTTAAATTCTCGACTTTCTTTTCTGGA
TATCTAATTGGTTTAATTGTCATTTATATATTACACAGGTTTTTCAGCGATGATTTTTATGTTAGAAAAATATGGGTAGCTATTAAATTTTTA
GGTGTTTATTTATATCAATTAATAACATCTAGCATTAGCACGATTAATTATATTCTTTTTAAAACAAAAGATATGAACCCTGGATTACTTTC
ATATGAAACAAGACTAACAAGTGATTGGTCAATAACATTTTTAACAATTTTAATTATTATAACTCCAGGGTCTACAGTAATACGAATTTCTC
AAGACTCTAAAAAGTTTTTTATTCATAGTATCGACGTGTCAGAAAAAGAAAAAGATAGTTTGTTAAGAAGTATTAAGCATTATGAAGACT
TAATATTGGAGGTGTCGCGATGA

Gene: mrpF (monovalent cation/proton antiporter protein F)

Contig: 05_NODE_4, position: 125899 to 126201, length: 303 nt, orientation: FORWARD

Perfect match to: (RF122-AJ938182-[642078:642380], highly conserved allele)

Sequence:

ATGATACAAACAATAACACATATTATGATTATTAGTTCACTCATTATTTTTGGAATTGCATTAATCATCTGTTTATTTAGATTAATCAAGGG
ACCTACAACAGCAGATCGTGTCGTTACATTTGATACAACAAGTGCTGTCGTAATGTCAATTGTGGGTGTGTTAAGTGTACTTATGGGCAC
CGTTTCTTTCTTAGATTCAATCATGCTCATTGCCATTATATCTTTTGTAAGTTCTGTTTCAATATCACGCTTTATTGGTGGGGGGCATGTGTT
TAATGGAAATAACAAAAGAAATCTTTAG

Gene: mrpG (monovalent cation/proton antiporter protein G)

Contig: 05_NODE_4, position: 126176 to 126613, length: 438 nt, orientation: FORWARD

Perfect match to: (COL-CP000046-[709002:709439], highly conserved allele)

Sequence:

ATGGAAATAACAAAAGAAATCTTTAGTCTTATTGCTGCTGTGATGTTGTTGTTAGGTAGTTTTATTGCTCTTATTAGTGCAATAGGTATCGT
GAAATTCCAAGATGTTTTCTTAAGAAGTCACGCTGCGACAAAAAGTTCAACTTTATCCGTGTTATTAACTTTAATCGGTGTATTAATTTATT
TTATTGTGAATACAGGATTTTTCAGTGTGCGTTTATTACTGTCACTTGTTTTTATTAATTTAACTTCACCAGTCGGCATGCACTTAGTCGCTC
GCGCTGCTTATCGCAACGGCGCTTATATGTATCGAAAAAATGATGCTCACACACATGCATCAATATTATTAAGTTCAAATGAACAAAACTC
TACAGAAGCATTACAATTACGTGCTGAAAAACGAGAAGAGCATCGTAAGAAATGGTATCAAAACGATTGA

Gene: nhaK1 (Na+/H+ antiporter locus 1)

Contig: 05_NODE_4, position: 126953 to 128995, length: 2043 nt, orientation: FORWARD

Perfect match to: (DR10-AIDT01000015-[44610:46652:r], allele observed in CC398)

Sequence:

TTGGAAATATTTGAAACAATTCTTATATTTATAGCTGTTGTGATACTAAGTTCGTTTGTCCATACTTTCATACCTAAAGTACCCCTAGCATTT
ATACAAATTTTCTTGGGCATGTTACTATTTATTACCCCAATCCCTGTTCAATTTAATTTTGATTCTGAATTGTTTATGGTAACAATGATTGCG
CCTTTGTTATTTGTAGAAGGTGTTAATGTTTCTAGAGTCCATTTAAGGAAATATATTAAGCCAGTGATGATGATGGCATTAGGATTAGTCA
TTACTACTGTGATAGGTGTAGGTTTATTTATTCATTGGATTTGGCCAGATTTACCTATTGGAGCAGCATTTGCAATTGCTGCCATTCTTTGT
CCTACTGATGCAGTAGCAGTGCAAGCAATCACTAAAGGAAAGGTCTTGCCAAAAGGAGCAATGACAATTCTTGAAGGTGAGTCATTATT
GAATGATGCTGCTGGTATTATTTCATTTAAAATAGCTGTTGGAGTATTAGTTACAGGTGCTTTTTCACTTGTTGATGCTGTTCAGTTGTTTT
TAATTGCATCAATTGGTGGCGCAGTGGTTGGTTTACTTATAGGTATGGCATTAGTAAGGTTCCGATTAACATTGATGCGTCGAGGATATG
AAAACATTAATATGTTTACAATTATTCAATTGTTAACACCATTTGTTACGTATTTAATTGCTGAATTGTTTCACGCATCAGGAATCATTGCA
GCAGTAGTTGCAGGACTTGTACATGGTTTCGAACGTGACAGAATTATGCAAGTACGTACACAACTGCAAATTAGTTACAATCATACATGG
AATATACTAGGTTATGTTTTAAATGGCTTTGTTTTTTCAATATTAGGATTTTTAGTACCTGAAGTTATTATTAAAATTATCAAAACAGAACC
GCACAATTTAATCTTTTTAATAGGCATCACTATTGTTGTTGCTTTAGCTGTCTATCTATTTAGATTTGTTTGGGTTTATGTCTTATATCCTTAT
TTTTATTTAGCCATCAGTCCATTCCAAAAAATGATGACTAAAAATGATGATGATAATCCAACGACTGAGAAACCACCAAAGCGAAGTTTAT
ACGCTTTAATTATGACGTTATGTGGTGTGCATGGAACAATTTCTTTAGCAATCGCATTAACGTTACCGTATTTTTTAGCAGGGCATCATGCT
TTTACGTATAGAAACGACTTATTATTTATTGCATCTGGTATGGTTATTATTAGTTTGGTAGTTGCGCAAGTATTATTGCCATTATTAACGAA
ACCTGCACCTAAAACAGTAATTGGCAATATGTCGTTTAAAGTTGCTAGAATTTATATATTAGAACAAGTTATTGATTATCTAAATCAAAAA
TCTACTTTCGAAACAAGTTTTAAATATGGTAACGTGATTAAAGAATATCATGATAAATTAGCATTTTTAAAAACTGTAGAGAAAGATGATG
AAAACTCTAAAGAATTAGAACGTCTACAAAAAATTGCTTTTAATGTAGAAACAAAAACATTAGAGTCTTTAGTAGATGAAGGACAAATAA
CGAATAGTGTACTTGAAAACTATATGCGTTATGCTGAAAGAACACAGGTATATAGACAAGCATCATTAATAAGAAGAATGATTGTATTAT
TACGAGGTGCTTTATTAAAACGAAGAGTACAAACGAGAGTGAACTCCGCATCTTCACTTAGTGTTACGGATAACTTAATGGAATTAAATA
AAATTAATAAATTAGTCCATTATAATGTGGTTAGTCGTTTGTCTAAGGAAACAACAAAAGATAATACACTTGAAGTTGGAATGGTTTGTG
ACGGTTATTTAATGCGAATTGAAAACTTAACACCATCAAATTTCTTCAACTCAGCAAGTGAAGATACGATTACTAAAATTAAATTAAATGC
ATTGAGAGAACAACGTCGCATTTTACGTGAGTTGATTGATACAGATGAAGTATCAGAAGGTACAGCGTTAAAACTAAGAGAAGCCATCA
ATTACGATGAAATGGTTATTGTAGATAGTATGACGTAG

Gene: txbi_nhaK1 (bifunctional rho-independent terminator of nhaK1)

Contig: 05_NODE_4, position: 128999 to 129029, length: 31 nt

Perfect match to: (N315-BA000018-[678196:678226:r], allele observed in CC5+CC8)

Sequence:

CTAATTATGCTAAAAGGGATTGATGAAAAAC

Gene: rsaC (ncRNA of Staphylococcus aureus C)

Contig: 05_NODE_4, position: 129194 to 129787, length: 594 nt

Sequence:

AATAGCCACACTCATATGACATCGGATGAGTGTGGCTTAAGGATCTATGGGGGGAGGAAACCATAGATGTTTACTTTGATAGGCCAGAT
TAAATATCAAAGTATGCGATTATTTATAGCTTGATGCAAAAGTGGTATGCCTATTAAAAGTTACTGCACATAGCTTTTAATATTCCGTTCAA
AGGAAAGGGGCATACAATTGAACAATCTGTAATAGTACTTTTAACCAGCTATGCTAAAAGTCTAGTAGGGAGAACAGTTGTCCAATCACA
TAAGAACCTCTAACTTCGTTAGTACGATTAAGAAAAGCTTTTTAGTTAGTATGTAATACAATTTATTGACGCGCGTGAATCTCTTTTATAAG
AGTGTGTAGGGAATGGCGTTGTATAAATTGTATTAGAAGAACTTCTAACGCATCTCTGTGGTTAAAAGAGATGAAGGGAACGACAGTTT
AATTAAAACTGCATAAGAACTTCTAGCTTTTCTCTCTCGTTCAAAGAGAAGCAGCTGTTCGCAGTTTAATCAAAACCACATAAAGCTTTTA
ACTTTACTCTTTGATTTAAAGAGTGATAAATGTTTACAGTTTAATTAAAAC

Gene: mntC (manganese ABC transporter, substrate-binding protein)

Contig: 05_NODE_4, position: 130155 to 131084, length: 930 nt, orientation: REVERSE

Perfect match to: (RF122-AJ938182-[646068:646997:r], highly conserved allele)

Sequence:

TTATTTCATGCTTCCGTGTACAGTTTCAATATTTGATTTCATCATTTTGTAGTAAGAGTCACCTTTAGTGCCTTCTTTACCGATTGAATCTGT
GTACACTTCACCAAAGATATCTTTCTTCGTTTCTTCAGATAAACTTTCCATTGCTTTCTTATCAACACTTGTTTCTACTAATAAGTGTTTTAAT
TTGTGCTTTTTAACAAACTCAATAGCTTGTCTCATTTGTTCAGGTGTACCTTGTTTTTCAGTGTTAATTTCCCAAATATAACCTGGTGTAATA
CCGTATTGTTTTGAGAAGTACTTGAAGGCACCTTCACTTGTAATCATGGCACGTTGTTCTTTTGGAATGTCATTAAATTTGTCTTTACTGTC
ATTATTTAATTTTTCCAATTGAGCAATGTATTTGTTACCTTGCTTTTCATAATCTGCTTTATGTTTTTTGTCGTTATCGATAAATGTTTGTTGA
ATTGTTTTTACGTATTTAATACCGTTATCTAAACTTAACCATGCGTGTGGATCTTGTTTATCTTTGTTGCCTTCTTCACCGTTTAAATAGATA
GGTTTAACATCTTTTGATACTGCGATAACTTTTTTATCTTTTAATGATTTACCAGCCTGTTCTAAGGCTTTTTCAAACCAACCGTTACCAGTC
TCTAAATTTAATCCGTTGTATAAAATAACGTCAGCGTCAGTTAACTTTTTAATATCTTTAGGTTTAACTTCATATTCATGAGGATCTTGACCA
ACAGGTACAATACTATGAATATCGACGTTGTCTCCACCAACATTTTTAGCCATATCATATAAAATTGAATTCGTCGTTACTACTTTTAATTT
GCCATTTGACTTATCACTGCTTTGTTTACCACCAGTACCACATGCAGCAACTAGAAGTAATAAGGCTAATAATAAAGGTACTAATTTTTTC
AT

Gene: mntB (manganese ABC transporter, transmembrane permease)

Contig: 05_NODE_4, position: 131081 to 131917, length: 837 nt, orientation: REVERSE

Perfect match to: (N315-BA000018-[681665:682501:r], highly conserved allele)

Sequence:

TCATGTTAAACTTCCTCGTTTCTTTCTATTCGTAAATTTTGTGAAAAATAATGTGATGATATAAATTACAAACGTACAAAGTACGATTGTCG
CACCACTAGGAATGTTGTAAATATAGCTGTAATAAAGTCCGACAATTGAACTTATGACACTTATTAAACTTGCTATAATCATCATTGAGTA
TAGTTTTTTACTAATTAAAAATGCTGTAGATGCAGGTGTAATTAATAATGCAACTACAAGAATAATACCTACCGTTTGAATACTTGCTACT
GTTACTAATGAGAGTAACAACATCACAAAGTAATGTAATAACGTCGTATTTAGACCACTCATTCTACTAAACGTTGGATCGAATGTAGAA
ATCATTAATGGACGATAGAAAATAATGATTAGAATAAGGACGATTGAACCAATCACAATAGTTGTTAAAAATGCACTATTTGTGATTGCC
AGTAAATTACCAAACAGAATATGGTACAAATCTGTCGTAGTGTTTATTAAGCTAATAATAATAATCCCCGAAGCTAAGAAAGCGGTAAAA
CTAATTCCAATAGCGGCGTCAGGTTTCGTTTTACTACTAGATGTGATATAACCGATAAAAATACTTGCGATCATACCAGTTATAAGTGCGC
CTACAAACATTGGAATACCAAATAAGAATGATAGGGCAACACCAGGTAATACTGCGTGACTCATTGCATCTCCCATTAATGAAAGACCAC

GTAATACAATTAAACTACCAACTGTACCACAAACTATCCCTACAATAATTGAAGTTATCAATGCTCGATTCAAGAATTGATATGTAAATAA
ATGTTCGACAAACTCTAACAT

Gene: mntA (manganese ABC transporter, ATP-binding protein)

Contig: 05_NODE_4, position: 131911 to 132654, length: 744 nt, orientation: REVERSE

Perfect match to: (RF122-AJ938182-[647824:648567:r], highly conserved allele)

Sequence:

CTAACATGTTATATTGCTCCTTTGACTAGGGTCACTACAGTCAGTGCTACTCATAAATGTTTCGTTTAAGCGAGTGACACTCATAGCCTCTT
CACTATCACCAAAGTATCGTAATGTTTGATTTAATAGAATAATGCGATCAAAGTATTGCTTTGCTTTTGATAGATCATGGTGGATGATAAG
AATAAGTTTTCCTTGTTGTTTTAAGTTCTCGATTTTTGTCATGATTAATTTTTCGCTACTAAAATCAATTCCGACAAACGGCTCATCTAGAAA
ATAAACTTCACTTTCGGACATCAATGCTCTTGCTACTAGCACACGTTGTAATTGTCCACCACTTAATTCTGAAATTTGTCGATGACGTAAAG
ATTCTAATTCTAAATCGCTTAATAACTGTTTGAGTTTATCCCTTGCTGATTTATTAGGTCGTCTAAACCATCCAATTTCTTTGTAGCAACCTG
ATAAAATCACTTGTTCCACACTTATAGGAAAATCTAAATCAATATGTGCTTTTTGTGGAATATATGTAATATGTTGCAGTTGTTGTTGTATA
GGTTTGTTATATAACAATTTAGTACCGGTAGCATTAAATTCACCAATTAAAGACTTGATAAGGGAAGATTTACCAGCACCATTCGGGCCCA
TGATACCAATTATTTCGCCGCGTACTGGTATCGATAAGGAAATGTTTTTAAGTACATGCTTATTACCTAAAAACAGATTTAAATCTTTTGTT
TCTAACAA

Gene: mntR (manganese-dependent transcriptional repressor)

Contig: 05_NODE_4, position: 132776 to 133420, length: 645 nt, orientation: FORWARD

Perfect match to: (N315-BA000018-[683360:684004], highly conserved allele)

Sequence:

ATGTTAACTGAAGAAAAAGAGGACTATTTAAAGGCAATCCTTACGAATAATGGCGATAAAAACTTTGTGACAAATAAAATCTTATCTCAA
TTTTTAAATATTAAGCCTCCATCTGTAAGTGAAATGGTAGGACGTCTTGAAAAAGCAGGCTATGTTGAAACAAAACCATACAAAGGTGTT
AGATTAACAGAGGATGGTTTAACGCATACGCTTGATATCATTAAGAGACATCGACTATTAGAATTATTTTTAATAGAAATATTGAAATATA
ATTGGGAAGAAGTACATCAAGAAGCAGAAATTTTAGAACATCGAATTTCAGATTTATTTGTTGAAAGGCTGGATAGCCTGTTAAATTTCC
CAGAAACTTGCCCGCACGGCGGTGTGATTCCTAGAAATAATGAATATAAAGAGAAATATATAACAACGATTTTGAATTATGAACCTGGTG
ATATCGTTACAATCAAACGTGTGAGAGATAAGACCGATTTGCTAATATATTTGTCTAGTAAAGATATTTCTATTGGTAATGAAGTGGAAAT
TGTATCGAAAGATGAAATGAATAAAGTAATTATCATTAAACGTAATGATAATGTAATTATTGTCAGTTACGAAAATGCAATGAACATGTTT
GCTGAAAAATAA

Gene: Q5HI33 (putative membrane protein)

Contig: 05_NODE_4, position: 133500 to 134252, length: 753 nt, orientation: REVERSE

Perfect match to: (SA40-CP003604-[657945:658697:r], highly conserved allele)

Sequence:

TTAATTTATCAAGTGAGTATATTTGAGTAAAATATTCACTGCATAAAGATTGAAGATAATCCAGATTGTACTATAAATGAAGATAGGTACA
TGACTGAGTTCTTTAAGTGCACTACCATCCCACTGTGGACTCGGACGCTGGAAAGTCAATTTAGCAATCGTCCAACTAGATTGTAGAACTT
CGCCTAATAATACACCTAAAATATATTGATAACTCATTGTGACAAGTAGTTGAATTTCTACTATATTTTCATCTTTTAATATAAAATACAACA
TGATAGAAATTAAAGTTATAACAACAATGGGTGAGCCTTTTCTAGATGTTAAAATTAAAAAATAAATAAATATCAATAAATAGGTAAATA
TAAAGAAACTAGGTATCTGATAATGGCTCGACGCTAAACCTATCAATAACATAATAGGTGGCATTAAATAACCACCAATCGTTGTAAGCC
ATTGGCCTGCTAGATGTCTAGATTGTGTAATTGCGAATCCTTGTTGTAATGTCTGTTGTCGCTCTCGTGGACTTGTTACAATGACTAAATCT
TTTGCACGGCCACCAGCGAGTTTATTAAACAGTACATGACCAAATTCATGTGTTAAAACAGGGATATAGTTTAAAATGACATCTAAATAG
TTCAAAACAGGCTTATGTCTATATTGATGAATAGCAATATAACAAGCTGCAACAATAACGATAATGTATATATTAAGTTGAATTGTCGTAT
TAAAAAAGTTTGATAAATAATTCAT

Gene: tarA (N-acetylmannosamine transferase)

Contig: 05_NODE_4, position: 134488 to 135252, length: 765 nt, orientation: FORWARD

Perfect match to: (ATCC51811-ADVP01000011-[180639:181403:r], allele observed in CC1)

Sequence:

ATGACTGTTGAAGAAAGATCCAATACAGCCAAAGTTGACATTTTAGGAGTCGATTTTGATAATACAACAATGTTGCAAATGGTTGAAAAT
ATTAAAACCTTTTTTGCAAATCAATCAACGAATAATCTTTTTATAGTAACAGCCAACCCTGAAATAGTGAATTACGCGACGACACATCAAG
CGTATTTAGAGTTAATAAATCAAGCGAGCTATATTGTTGCTGATGGGACAGGAGTAGTCAAAGCTTCGCATCGTTTAAAGCAACCTCTAG
CGCATCGTATACCTGGTATTGAGTTGATGGATGAATGTTTGAAAATTGCTCATGTAAATCATCAAAAAGTATTTTTGCTAGGGGCAACTA
ATGAAGTTGTAGAAGCGGCACAATATGCATTGCAACAAAGATATCCAAACATATCGTTTGCACATCATCACGGTTATATTGATTTAGAAG
ATGAGACAGTAGTGAAACGAATTAAACTGTTTAAACCTGATTACATATTTGTAGGTATGGGATTCCCTAAACAAGAAGAATGGATTATGA
CACATGAAAACCAATTTGAATCTACAGTGATGATGGGCGTAGGTGGTTCTCTTGAAGTATTTGCTGGGGCTAAAAAGAGAGCGCCTTAT
ATCTTTAGAAAATTAAACATTGAATGGATATATAGAGCATTAATAGATTGGAAACGTATTGGTAGATTAAAGAGTATTCCAATATTTATGT
ATAAAATAGCCAAAGCAAAAAGAAAAATAAAAAAGGCGAAATAA

Gene: tarH (teichoic acid ABC transporter, ATP-binding protein)

Contig: 05_NODE_4, position: 135313 to 136107, length: 795 nt, orientation: REVERSE

Perfect match to: (MW2-BA000033-[681251:682045:r], highly conserved allele)

Sequence:

TTATTTAATAACGAAGCGGGACTCATCGAGTTTGTTTCTAAATTCTTTTTGTTCGGCTTTGGATTTCTTTTTAAAATCGTTAAGGAAAGCTT
CATATTTAGGTAATACATCATCAAGTTCACCGTAATCTTTTAACTTTCCGCCTTCAATCCAAGCAATCTTAGTACAAAATTGTCTCACTTGTC
CTAAGTTATGACTAACGAAAAAGATGGTTTTGTTTTGCTCTTTAAACTCGTAAATTTTATCTAAACATTTTTGTGCAAAAGTTTGGTCACCT
ACAGATAAAGCTTCGTCAATGACTAAGATATCTGGATTAACTGTGATATTAATTGAAAAACCAAGTTTTGCACGCATACCACTTGAATACT
TTTTAACTGGTTGATAAATAAACTCACCAAGTTCACTAAATTCAATAATCTTAGGTGTCATCGCTTTAATTTCTTTTCGCTTAAAGCCCATAC
ATAACATTTTAAATTCGATATTTTCAATCCCTGTAAGTTGTCCACTCAAGCCAGCACTAATTGCGATAACGCTGACTTCACCATTACGATCC
ACTTTGCCAACAGTAGGCGACAAAGAACCGCCAATGATATTGCTCAACGTTGATTTGCCGGAACCATTGATGCCAACAAGCCCTATGACG
TCGCCTTCATATGCTTTTAAACTAATGTCATCTAAAGCGAAAAATGTTTTGTTTTTATGTTTGGGAATGAGCGCATCTTTCATACGTTCTTTA
TTTGTACGATAAATACGATATTCTTTTGTTACATTTTTAATGTTTACCGAAACGTTCAT

Gene: tarG (teichoic acid ABC transporter, transmembrane permease)

Contig: 05_NODE_4, position: 136433 to 137266, length: 834 nt, orientation: FORWARD

Perfect match to: (MW2-BA000033-[682371:683204], allele observed in CC1)

Sequence:

TTGAAAGTGTGGTTTAATGGAATGTCAGCAATAGGAACAGTTTTTAAAGAACATGTAAAGAACTTTTATTTAATTCAAAGACTGGCTCAG
TTTCAAGTTAAAATTATCAATCATAGTAACTATTTAGGTGTGGCTTGGGAATTAATTAACCCTGTTATGCAAATAATGGTTTACTGGATGG
TTTTTGGATTAGGAATAAGAAGTAATGCACCAATTCATGGTGTACCTTTTGTTTATTGGTTATTGGTTGGTATCAGTATGTGGTTCTTCATC
AACCAAGGTATTTTAGAAGGTACTAAAGCAATTACACAAAAGTTTAATCAAGTATCGAAAATGAACTTCCCGTTATCGATAATACCGACA
TATATTGTGACAAGTAGATTTTATGGACATTTAGGCTTACTTTTACTTGTGATAATTGCATGTATGTTTACTGGTATTTATCCATCAATACAT
ATCATTCAATTATTGATATATGTACCGTTTTGTTTTTTCTTAACTGCCTCGGTGACGTTATTAACATCAACACTCGGTGTGTTAGTTAGAGA
TACACAAATGTTAATGCAAGCAATATTAAGAATATTATTTTACTTTTCACCAATTTTGTGGCTACCAAAGAACCATGGTATCAGTGGTTTAA
TTCATGAAATGATGAAATATAATCCAGTTTACTTTATTGCTGAATCATACCGTGCAGCAATTTTATATCACGAATGGTATTTCATGGATCAT
TGGAAATTAATGTTATACAATTTCGGTATTGTTGCCATTTTCTTTGCAATTGGTGCGTACCTACACATGAAATATAGAGATCAATTTGCAG
ACTTCTTGTAA

Gene: tarB (teichoic acid biosynthesis protein B)

Contig: 05_NODE_4, position: 137365 to 138468, length: 1104 nt, orientation: FORWARD

Sequence:

ATGAACGTTTTAATAAAGAAATTTTATCATTTGGTAGTTCGAATACTTTCTAAAATGATTACGCCTCAAGTGATTGATAAACCGCATATCGT
ATTTATGATGACTTTTCCAGAAGATATTAAGCCTATCATCAAAGCATTAAATAATTCGTCGTATCAGAAAACTGTTTTAACAACACCAAAA
CAAGCGCCTTATTTATCTGAACTTAGCGACGATGTTGATGTGATAGAAATGACTAATCGAACATTGGTAAAACAAATTAAGGCTTTGAAA

AGCGCGCAGATGATTATTATCGATAATTATTACCTATTACTAGGTGGATATAATAAGACTTCTAATCAACACATTGTTCAAACGTGGCATG
CAAGTGGTGCATTAAAAAACTTTGGATTAACAGATCATCAAGTCGATGTGTCTGACAAGACAATGGTTCAGCAGTACCGTAAAGTTTATC
AAGCGACGGATTTTTACTTAGTGGGTTGTGAACAAATGTCACAATGTTTTAAACAGTCTTTAGGTGCAACAGAAGAGCAAATGTTGTATT
TTGGCCTTCCGAGAATTAATAAATATTACACAGCTGATAGAGCAACGGTTAAGGCAGAGTTAAAGGATAAATATGGAATTACAAATAAG
TTGGCATTATATGTACCAACATATAGAGAAGATAAAGCAGATAATAGGGCTATTGATAAAGCTTATTTTGAAAAATGTTTACCAGGATAT
ACACTGATTAATAAATTACATCCATCAATTGAACATTCAGACATTGATGACGTATCTTCAATCGACACGTCTACATTAATGCTAATGTCAGA
TATAATTATTAGCGACTATAGTTCGCTGCCAATAGAAGCTAGCTTGTTAGATATTCCAACTATATTTTATGTGTATGATGAAGGAACATAT
GATAAAGTGAGAGGCCTGAATCAATTTTACAAAGCAATACCGGATAGCTACAAAGTGTATACTGAAGAAGATTTAATAATGACGATACA
AGAAAAAGAACATCTATTAAATCCGTTATTTAAAGATTGGCATAAGTATAATACTGATAAAAGTTTACATCAGCTCACAGAATATATAGAT
AAGATGGTGACAAAATGA

Gene: tarX (teichoic acid biosynthesis protein X)

Contig: 05_NODE_4, position: 138465 to 139526, length: 1062 nt, orientation: FORWARD

Sequence:

ATGAGGCTTACGATAATCATACCTACATGTAATAATGAGGCAACCATTCGACAATTGTTAATATCTATTGAGAGTAAAGAACACTATAGA
ATCCTTTGTATTGATGGTGGTTCTACTGATCAAACAATTCCTATGATTGAACGGTTACAAAGAGAACTCAAGCATATTTCATTAATACAATT
ACAAAATGCTTCGATAGCTACGTGTATTAATAAAGGTTTGATGGATATCAAAATGACAGATCCACATGATAGTGACGCATTTATGGTCAT
AAATCCAACATCAATCGTATTGCCAGGTAAATTAGATAGGTTAACTGCAGCTTTCAAAAATAATGATAATATTGATATGGTAATAGGGCA
GCGAGCTTACAATTACCATGGTGAATGGAAATTGAAAAGTGCTGATGAGTTTATTAAAGATAATCGAATCGTTACATTAACGGAACAACC
AGATTTGTTATCAATGATGTCTTTTGACGGAAAGTTATTCAGTGCTAAATTTGCTGAATTACAGTGTGACGAAACTTTAGCTAACACATAC
AATCACACAATACTTGTCAAGGCGATGCAAAAAGCTACGGATATACATTTAGTTTCACAGATGATTGTCGGAGATAACGATATAGATACA
CATGCTACAAGTAACGATGAAGATTTTAATAGATATATCACAGAAATTATGAAAATAAGACAACGAGTCATGGAAATGTTACTATTACCT
GAACAAAGGCTATTATATAGTGATATGGTTGATCGTATTTTATTCAATAATTCATTAAAATATTATATGAACGAACACCCAGCAGTAACGC
ACACGACAATTCAACTCGTAAAAGACTATATTATGTCTATGCAGCATTCTGATTATGTATCGCAAAACATGTTTGACATTATAAATACAGTT
GAATTTATTGGTGAGAATTGGGATAGAGAAATATACGAATTGTGGCGACAAACATTAATTCAAGTGGGCATTAATAGGCCGACTTATAA
AAAATTCTTGATACAACTTAAAGGGAGAAAGTTTGCACATCGAACAAAATCAATGTTAAAACGATAA

Gene: tarD (glycerol-3-phosphate cytidylyltransferase)

Contig: 05_NODE_4, position: 139589 to 139987, length: 399 nt, orientation: FORWARD

Perfect match to: (N315-BA000018-[690173:690571], highly conserved allele)

Sequence:

ATGAAACGTGTAATAACATATGGCACATATGACTTACTTCACTATGGTCATATCGAATTGCTTCGTCGTGCAAGAGAGATGGGCGATTAT
TTAATAGTAGCATTATCAACAGATGAATTTAATCAAATTAAACATAAAAAATCTTATTATGATTATGAACAACGAAAAATGATGCTTGAAT
CAATACGCTATGTCGATTTAGTCATTCCAGAAAAGGGCTGGGGACAAAAAGAAGACGATGTCGAAAAATTTGATGTAGATGTTTTTGTTA
TGGGACATGACTGGGAAGGTGAATTCGACTTCTTAAAGGATAAATGTGAAGTCATTTATTTAAAACGTACAGAAGGCATTTCGACGACTA
AAATCAAACAAGAATTATATGGTAAAGATGCTAAATAA

Gene: pbpD (penicillin binding protein D)

Contig: 05_NODE_4, position: 140104 to 141399, length: 1296 nt, orientation: REVERSE

Perfect match to: (MW2-BA000033-[686042:687337:r], allele observed in CC1+CC188)

Sequence:

TTATTTTCTTTTTCTAAATAAACGATTGATTATCATATGAACAATTAGTGCTAATCCAGCGACAAGGCATGCACCACCAATGATAGTGAAT
AATGGATGTTCTTCCCACATACTTTTAGCAACAGTATTTGCCTTTTGAATAATTGGCTGATGAACTTCTACAGTTGGAGGTCCATAATCTTT
ATTAATAAATTCTCTTGGATAGTCCGCGTGTACTTTACCATCTTCGACTACAAGTTTATAATCTTTTTTACTAAAATCACTTGGTAAAACATC
GTAAAGATCGTTTTCAACATAATATTTCTTACCATTTATCCTTTGCTCACCTTTAGACAATATTTTTACATATTTATACTGATCAAATGAGCG
TTCCATTAATGCATTCCCCATCATATTACGTTGCTTCTCGCCACCAAGGTTTTTATAGTCTCCCGCACCCATGATAACTTGATTAATTCTAAA
TTTACCTCGTTTGGTAGTAATCGTATGGTTGTAATTTGCTGTATCACTTGATCCAGTTTTTAAACCATCTGTACCCGGCAAACTCATTTTTGC
ACCTTCCAATGAAAAGTTGAATGTGTAATACGTAACTGCATGCGTTGTTGGTGCTAACTGCTTTGTAAAGTCTAATATTTTAGGTGTCTCTT

TAATCACGTGTAAATCTAAAATGGCATAGTCTCTAGCAGTCGTTACAGTACGTTCTTGGTCTTTATACTTTGTTGGTGCAAATGAACGTAA
TCTTGAATTTTCAGCACCCGTTGGATTGACGAAATGTGTATTTTTCATTCCGATAGCTTTAGCTTTGTTATTCATTAAATCAACGAAATCGC
TGGTGTTTTTTGATACCTTCTTAGCTAAAATTAATGCCGCGGCATTACTAGAATTAGATACTGTAATTTGTAATAGGTCTGCGATTGTCCAT
ACTTGTCCAGGATATAGTTTCGTATTACTCAACTCAGGTAGTGTAGACATAATATATTCTTTGTTCGTCATTGTGACAGTGTCATCAAGTGA
AAGCTGCCCCTTATTTACAGCTTCCAATGTTAAGTACATTGTCATTAATTTGGTCATAGACGCTGGATTCCACTTAGTATCGATATTGTATT
GATACAGTAATTGTCCAGTTTGACTTACATTAACAGCACTCGTCGGTTCGTATGCAGCTGACAAACCTGCATAACCATATTGATTTGCTGC
TTGTACAGGGGTTACGTCACTGTTAGCAGCTTGTGCATATGGTGTCATAATACTTAATGTTAAACATAAAATGATGATAATAGATATTAAA
TTTTTCAT

Gene: msbA1 (lipid A ABC transporter, fused ATPase and transmembrane permease)

Contig: 05_NODE_4, position: 141820 to 143547, length: 1728 nt, orientation: FORWARD

Perfect match to: (MW2-BA000033-[687758:689485], allele observed in CC1+CC188)

Sequence:

ATGAAACGAGAAAATCCATTGTTTTTCTTATTTAAAAAACTATCATGGCCAGTGGGTCTTATCGTTGCAGCTATCACTATTTCATCACTAGG
GAGCTTAAGTGGACTATTAGTGCCACTGTTTACTGGACGAATTGTAGATAAATTTTCCGTGAGCCATATCAATTGGAATCTAATCGCATTA
TTTGGTGGTATCTTTGTTATCAATGCTTTATTAAGCGGATTAGGTTTATATTTATTAAGTAAAATTGGTGAAAAGATTATTTATGCGATACG
CTCAGTTTTATGGGAGCATATCATACAATTAAAAATGCCATTCTTTGACAAAAATGAAAGTGGTCAATTAATGAGTCGATTAACTGACGAT
ACGAAAGTGATAAATGAATTTATTTCACAAAAGCTACCTAACTTATTACCATCAATCGTTACATTAGTTGGGTCACTAATCATGTTATTTAT
TTTAGATTGGAAAATGACATTATTAACATTTATAACGATACCGATATTCGTTTTAATTATGATTCCTCTAGGTCGTATTATGCAAAAGATAT
CGACAAGTACACAATCTGAAATTGCAAACTTCAGTGGTTTGTTAGGGCGTGTCCTAACTGAAATGCGTCTTGTTAAAATATCAAATACAG
AGCGTCTTGAATTAGATAATGCACATAAAAATTTGAATGAAATATATAAATTAGGTTTAAAACAGGCTAAAATTGCGGCAGTTGTACAAC
CAATTTCAGGTATAGTTATGTTGCTAACAATTGCAATTATTTTAGGTTTTGGTGCATTAGAAATTGCGACTGGTGCAATCACTGCAGGTAC
ATTAATTGCAATGATATTTTATGTTATTCAGTTATCTATGCCTTTAATCAATCTTTCAACGTTAGTTACAGATTATAAAAAGGCAGTCGGTG
CAAGTAGTAGAATATACGAAATCATGCAAGAACCTATTGAACCGACAGAAGCTCTTGAAGATTCTGAAAATGTATTAATTGATGACGGTG
TATTGTCATTTGAACATGTAGACTTTAAATATGATGTGAAGAAAATATTAGATGATGTGTCGTTCCAAATCCCACAAGGTCAAGTGAGTG
CTTTTGTAGGTCCTTCTGGGTCTGGTAAAAGTACGATATTTAATCTGATAGAACGTATGTATGAAATTGAGTCAGGTGATATTAAATATGG
CCTTGAAAGTGTCTATAATATCCCGTTATCTAAGTGGCGACGCAAAATTGGATATGTTATGCAATCAAATTCGATGATGAGTGGTACAATT
AGAGACAATATTTTATACGGAATTAATCGTCATGTTTCAGATGAAGAACTTATTAATTATGCTAAATTAGCGAACTGTCATGATTTTATCAT
GCAATTTGATGAAGGATATGACACGCTTGTAGGTGAACGAGGATTGAAACTGTCTGGCGGACAACGTCAACGTATTGATATTGCTAGAA
GTTTTGTTAAAAATCCTGATATTTTGTTACTTGATGAAGCAACAGCTAATCTCGATAGTGAAAGTGAATTGAAAATTCAAGAAGCTTTAGA
AACATTGATGGAAGGTAGAACAACGATTGTCATTGCGCATCGTTTGTCTACAATTAAAAAAGCCGGTCAAATTATATTCTTAGACAAAGG
ACAGGTAACAGGTAAAGGTACGCATTCAGAACTGATGGCATCACATGCGAAGTATAAAAACTTTGTAGTGTCTCAAAAATTAACAGATTA
A

Gene: nupG (purine nucleoside transporter)

Contig: 05_NODE_4, position: 143904 to 145133, length: 1230 nt, orientation: FORWARD

Perfect match to: (CIG290-AIES01000014-[68137:69366], allele observed in CC45I+CC45IV8+CC49)

Sequence:

ATGTTTTTATTAATCAACATTATTGGTCTAATTGTATTTCTTGGTATTGCGGTATTATTTTCAAGAGATCGCAAAAATATCCAATGGCAATC
AATTGGGATCTTAGTTGTTTTAAACCTGTTTTTAGCATGGTTCTTTATTTATTTTGATTGGGGTCAAAAAGCAGTAAGAGGAGCAGCCAAT
GGTATCGCTTGGGTAGTTCAGTCAGCGCATGCTGGTACAGGTTTTGCATTTGCAAGTTTGACAAATGTTAAAATGATGGATATGGCTGTT
GCAGCCTTATTCCCAATATTATTAATAGTGCCATTATTTGATATCTTAATGTACTTTAATATTTTACCGAAAATTATTGGAGGTATTGGTTG
GTTACTAGCTAAAGTAACAAGACAACCTAAATTCGAGTCATTCTTTGGGATAGAAATGATGTTCTTAGGAAATACTGAAGCATTAGCCGT
ATCAAGTGAGCAACTAAAACGTATGAATGAAATGCGTGTATTAACAATCGCAATGATGTCAATGAGCTCTGTATCGGGAGCTATTGTAG
GTGCGTATGTACAAATGGTACCAGGAGAACTGGTACTAACGGCAATTCCACTAAATATCGTTAACGCGATTATTGTGTCATGCTTGTTGA
ATCCAGTAAGTGTTGAAGAGAAAGAAGATATTATTTACAGTCTTAAAAACAATGAAGTTGAACGTCAACCATTCTTCTCATTCCTTGGAGA
TTCTGTATTAGCAGCAGGTAAATTAGTATTAATCATCATCGCATTTGTTATTAGTTTTGTAGCGTTAGCTGATCTATTTGATCGTTTTATCAA
TTTGATTACAGGAATGATAGCAGGATGGATAGGCATAAAAGGTAGTTTCGGTTTAAACCAAATTTTAGGTGTGTTTATGTATCCATTTGC
GCTATTACTCGGTTTACCTTATGATGAAGCGTGGTTGGTAGCACAACAAATGGCTAAGAAAATTGTTACAAATGAATTTGTTGTTATGGG
TGAAATTTCTAAAGATATTGCATCTTATACACCACACCATCGTGCGGTTATTACAACATTCTTAATTTCATTTGCAAACTTCTCAACGATTG
GTATGATTATCGGTACATTGAAAGGCATTGTTGATAAAAAGACATCAGACTTTGTATCTAAATATGTGCCTATGATGCTATTATCAGGTAT
CCTAGTTTCATTATTAACAGCAGCTTTCGTTGGTTTATTTGCATGGTAA

Gene: rsaD (ncRNA of Staphylococcus aureus D)

Contig: 05_NODE_4, position: 145283 to 145459, length: 177 nt

Sequence:

AAAAACCAAGTGCACATGGTAATACACTTGGCTTTTATGGGAAATGAATATTATTGTACATATGACAGTAAGGACTAGGTACAGTCATAG
TACTTCGAGCAAAATTTGTTTTGTTATTATAAACAACACAAAGGAGATAACTTCTCTATTGAAGAAGTTAAAAACATTATAGCAGAC

Gene: yxkD (putative membrane protein)

Contig: 05_NODE_4, position: 145658 to 146491, length: 834 nt, orientation: FORWARD

Perfect match to: (COL-CP000046-[728486:729319], highly conserved allele)

Sequence:

GTGAATAAAACGGTTAAAGATTTAATACTAGTTGTCTTAGGTTCATTTATCTTTGCTGCAGGTGTAAATGCATTTATTATTTCTGGTAACTT
AGGTGAAGGCGGGGTTACAGGTTTAGCAATTATTTTATATTATGCGTTTCATATTTCACCAGCCATCACTAACTTCTTGGTCAACGCAGTA
TTGATTGCCATAGGTTATAAATTTTTGAGTAAGAGAAGTATGTACTTAACTATTCTTGTAACAATTCTTATTTCAATATTTTTGAGTTTAACA
GAATCATGGCAAGTAGAAACTGGAAACAGCATTGTGAATGCCATTTTTGGTGGTGTAAGCGTTGGACTAGGAATCGGAGTAATTATCCT
TGCAGGCGGTACAACAGCAGGTACAACAATTTTGGCGAGAATTGCAACGAAATACCTCGATGTAAGCACGCCATATGCTTTGCTTTTCTT
CGATATGATCGTTGTTGCAATTTCACTTACAGTTATTCCACTTGATAAAGTATTAGTAACAGTAATATCACTTTATATAGGAACAAAAGTG
ATGGAATATGTCATAGAAGGTTTAAACACTAAAAAAGCTATGACGATTATTTCAACTAATCCCGACAAACTTGCCAAAGCAATAGACGAG
CAAATTGGAAGAGGTTTAACCATTTTAAACGGACATGGCTATTATACGCGTGAAGAAAAAGATGTCTTATACGTTGTTATTTCTAAAACAC
AAGTTTCAAAAGCAAAGCGATTAATTAAACAAATCGATAAAGATGCATTCCTCGTAATTCATGATGTAAGAGATGTCTATGGTAATGGCT
TTCTTGCAGATGAATAA

Gene: fhuC (iron-III-hydroxamate ABC transporter, ATPase)

Contig: 05_NODE_4, position: 146773 to 147570, length: 798 nt, orientation: FORWARD

Perfect match to: (N315-BA000018-[697357:698154], highly conserved allele)

Sequence:

ATGAATCGTTTGCATGGACAACAAGTTAAAATTGGTTACGGGGATAACACGATTATAAATAAATTAGATGTTGAAATACCAGATGGCAA
AGTGACGTCAATCATTGGTCCTAACGGCTGCGGGAAATCTACTTTGCTAAAGGCATTGTCACGTTTATTGGCAGTTAAAGAAGGCGAAGT
ATTTTTAGATGGTGAAAATATTCATACACAATCTACGAAAGAGATTGCAAAAAAAATAGCCATTTTACCTCAATCACCTGAAGTAGCAGAT
GGCTTAACTGTTGGGGAATTAGTTTCATATGGTCGTTTTCCACATCAAAAAGGATTTGGTAGATTAACTGCTGAGGATAAGAAAGAAATT
GATTGGGCAATGGAAGTTACAGGAACTGATACATTCCGACACCGTTCAATCAATGATTTAAGTGGTGGTCAAAGACAACGTGTTTGGATT
GCAATGGCATTAGCACAAAGAACTGATATTATCTTTTTAGACGAACCAACAACATATTTAGATATCTGTCATCAATTAGAAATACTAGAAT
TAGTTCAGAAGCTAAATCAGGAACAAGGTTGTACAATTGTCATGGTTCTTCATGATATCAACCAAGCGATTCGTTTCTCAGATCATCTTAT
TGCGATGAAAGAAGGGGATATCATCGCTACAGGTTCAACAGAAGACGTATTAACACAGGAAATTTTAGAAAAAGTTTTCAATATTGATG
TTGTTTTAAGTAAAGATCCTAAAACTGGAAAACCTTTATTGGTAACTTATGACTTATGTCGCAGAGCTTATTCTTAA

Gene: fhuB (iron-III-hydroxamate ABC transporter, transmembrane permease subunit)

Contig: 05_NODE_4, position: 147606 to 148610, length: 1005 nt, orientation: FORWARD

Perfect match to: (MW2-BA000033-[693545:694549], highly conserved allele)

Sequence:

ATGACAAATAGAGAGAATTCAACGCCATTGAAGTTTTTAGCCTATATTATAGGTTTAAGTATGATACTACTAATCACACTATTTATTTCTAC
ATTAATAGGTGACGCCAAAATTCAAGCCTCTACAATTATAGAGGCTATTTTTAATTATAATCCTAGCAATCAACAGCAAAACATCATCAAT
GAGATTAGGATTCCCAGAAATATAGCAGCAGTAATTGTGGGTATGGCGCTTGCAGTTTCTGGTGCGATTATACAAGGTGTTACCCGTAAT
AGTCTTGCTGATCCGGCGCTCATAGGTTTAAATTCAGGTGCTTCATTTGCTTTAGCATTAACATATGCAGTTTTACCAAACACTTCATTTTT
AATATTGATGTTTGCTGGATTTTTAGGTGCTATTCTAGGAGGTGCTATTGTATTAATGATAGGCCGATCTAGACGTGATGGATTTAATCCG

ATGCGTATTATTTTAGCGGGTGCAGCAGTAAGTGCTATGTTAACAGCGCTAAGTCAAGGTATTGCATTAGCTTTTAGACTAAATCAAACA
GTAACATTTTGGACTGCTGGAGGCGTTTCAGGCACAACATGGTCACACCTTAAGTGGGCAATTCCATTAATTGGTATTGCGTTATTCATTA
TATTAACAATTAGTAAACAACTTACCATTTTAAATCTTGGTGAATCATTAGCTAAAGGTTTAGGTCAAAATGTAACAATGATCAGAGGCAT
ATGTTTAATTATTGCTATGATTCTAGCAGGTATTGCAGTTGCTATCGCTGGACAAGTTGCATTTGTAGGTTTGATGGTACCTCATATAGCA
AGATTTTTAATTGGAACTGATTATGCTAAAATTCTACCATTAACAGCCTTGTTAGGTGGGATACTCGTGCTTGTTGCCGATGTGATAGCAC
GATATTTAGGAGAAGCGCCTGTTGGTGCAATCATTTCATTTATCGGTGTTCCTTACTTTTTATATTTAGTTAAAAAAGGAGGACGCTCAAT
ATGA

Gene: fhuG (iron-III-hydroxamate ABC transporter, transmembrane permease subunit)

Contig: 05_NODE_4, position: 148607 to 149623, length: 1017 nt, orientation: FORWARD

Perfect match to: (MW2-BA000033-[694546:695562], allele observed in CC1+CC96+CC188+CC772)

Sequence:

ATGATTAGTTCAAATAATAAACGCAGACAATGGATTGCACTGGCTGTTTTTAGCATTCTACTATTTCTAGGTTGTACTTGGAGTATTACCTC
AGGTGAATACAACATACCTGTTGAAAGATTTTTCAAAACTTTAATTGGACAAGGTGATGCCATTGATGAGTTAATCTTATTAGATTTCAGA
TTACCTCGGATGATGATTACTATTTTGGCTGGCGCAGCGCTTAGTATTAGTGGTGCAATAGTGCAAAGTGTCACGAAAAATCCAATAGCT
GAACCCGGTATATTAGGTATTAACGCAGGTGGCGGATTTGCAATCGCATTATTTATTGCAATTGGTAAAATTAATGCTGACAACTTTGTTT
ATGTACTGCCGTTAATAAGTATACTAGGTGGTATCGCCACTGCATTGATTATTTTTATTTTCAGTTTTAATAAAAATGAAGGTGTTACACCT
GCGAGTATGGTATTAATAGGTGTAGGTTTACAAACAGCATTATATGGTGGCTCAATTACAATTATGTCAAAATTTGATGATAAGCAATCT
GATTTCATCGCTGCTTGGTTTGCAGGTAATATTTGGGGTGACGAATGGCCATTTGTCATTGCATTTTTACCGTGGGTGTTGATTATTATTCC
TTACTTACTATTTAAATCGAATACACTAAATATTATTCATACGGGTGATAATATTGCACGAGGTCTAGGTGTAAGGTTAAGCAGAGAACG
TTTAATATTATTCTTTATCGCAGTGATGTTATCATCTGCTGCTGTAGCAGTAGCAGGTTCGATTTCGTTTATCGGATTAATGGGTCCGCATA
TTGCCAAACGTATCGTTGGACCACGTCACCAGTTGTTTTTACCAATTGCCATTTTAGTAGGGGCATGTTTACTTGTTATAGCTGATACAATT
GGCAAAATTGTATTACAACCAGGTGGGGTTCCAGCAGGTATTGTCGTAGCAATTATTGGTGCACCGTATTTCTTATATTTAATGTACAAAA
CGAAAAATGTATAG

Gene: dakK (putative dihydroxyacetone kinase, K subunit)

Contig: 05_NODE_4, position: 149857 to 150825, length: 969 nt, orientation: FORWARD

Perfect match to: (N315-BA000018-[700441:701409], highly conserved allele)

Sequence:

ATGATGAAAAAGTTAATCAATAAAAAAGAAACATTTTTAACTGATATGCTTGAAGGATTGTTAATTGCGCACCCAGAGTTAGATCTGATT
GCTAATACAGTTATTGTAAAAAAAGCTAAGAAAGAACATGGTGTAGCAATAGTCTCTGGAGGTGGAAGCGGACATGAACCTGCGCATGC
CGGTTTTGTTGCAGAAGGTATGCTAGATGCAGCGGTTTGTGGCGAAGTATTTACATCACCTACACCTGATAAAATATTAGAAGCTATTAA
AGCAGTAGATACTGGTGATGGTGTATTACTAGTTGTAAAAAACTATGCAGGTGACGTGATGAATTTCGAAATGGCACAAGAGCTTGCAG
AAATGGAAGGTATAAATGTTCAAACTGTTATTGTTCGTGACGACATTGCTGTGACAAACGAAGTACAACGTCGTGGTGTTGCAGGAACA
GTGTTTGTTCATAAGCTTGCCGGTTATCTTGCTGAAAAAGGTTATTCATTAACAGAGATAAAATCGCGTGTAGAAGCGTTGTTACCTGAA
ATTAAAAGTATTGGTATGGCAATTGAGCCACCGCTTGTTCCAACTACTGGAAAATATGGCTTTGATATTGAAGACGACAAAATGGAAATC
GGTATTGGTATACATGGTGAAAAAGGTATTCATAGGGAAGAAGTAAAGGATATTGATCATATTGTTGGAACATTGTTAGACGAATTGTA
TAAAGAAGTTACTGCCAATGATGTCATATTAATGGTAAATGGTATGGGTGGTACGCCGTTATCTGAATTAAATATCGTAACTAAATATATT
CAACAAAATTTAGCTGCAAGAACGGTTAATGTTGCTAAATGGTTTGTTGGTGATTATATGACATCTTTAGACATGCAAGGTTTTTCTATAA
CTATCGTGCCTAATAAACCAGAATATTTGGAAGCATTTTTAGCACCAACAACAAGTCAATACTTTAAATAA

Gene: dakL (putative dihydroxyacetone kinase, L subunit)

Contig: 05_NODE_4, position: 150867 to 151451, length: 585 nt, orientation: FORWARD

Perfect match to: (N315-BA000018-[701451:702035], highly conserved allele)

Sequence:

ATGAAAGTGAATGATATGAAAGCACGTTTATTAAATTTAGAAGAAACGTTTAAAAAACATGAATCTGAATTAACTGAATTAGATCGAGCA
ATTGGTGATGGTGACCACGGGGTTAACATGGTTCGTGGGTTTAGTAGTCTTAAAGACAAACTTGATGATAGCTCAATGCAATCATTGTTC
AAATCGACTGGTATGGCATTGATGTCAAATGTTGGGGGTGCATCAGGACCACTGTATGGCTTTAGCTTTGTTAAAATGTCTGCAGTCACC

AAAGATGATATGGATAATCAAGATTTCATTACACTAATTCAGGCATTTGCCGAAGCGGTTGAATCACGTGGTAAAGTTACTTTAAATGAA
AAGACAATGTATGATGTAGTAGCGCGAGCAGCAGAGAAGCTTGAAAATGGTGAAACTTTAACATTCAATGATTTACAGCAATTAGCAGA
TAATACAAAAGATATGGTAGCAACGAAAGGTAGAGCTGCATATTTTGGAGAAGAATCAAAAGGTTATATTGATCCAGGTGCTCAAAGTA
TGGTTTATATTTTAAACGCTTTGATTGGAGATGAAGATAATGCCTAA

Gene: dakP (putative dihydroxyacetone kinase, phosphotransferase subunit)

Contig: 05_NODE_4, position: 151444 to 151806, length: 363 nt, orientation: FORWARD

Perfect match to: (MW2-BA000033-[697383:697745], allele observed in CC1+CC361+CC692)

Sequence:

ATGCCTAAAATTATACTTGTTAGCCACAGTAAAGAAATTGCAAGTGGTACAAAATCTTTGTTAAAGCAAATGGCAGGTGACGTTGATATT
ATACCAATCGGGGGATTACCAGATGGTTCAATTGGAACTTCATTTGATATCATCCAAGAAGTTTTGACTAAATTAGAGGATGATGCATTG
TGTTTTTACGATATTGGATCTTCAGAAATGAATGTAGATATGGCTATTGAAATGTATGATGGTAATCATCGTGTGTTAAAAGTTGATGCAC
CAATTGTTGAAGGCAGTTTTATCGCAGCAGTAAAGCTATCAATCGGCGGTTCAATTGATGATGCATTAGCAGAAATCAAACAATCATTTT
AG

Gene: Q5HI15 (putative protein)

Contig: 05_NODE_4, position: 151922 to 152419, length: 498 nt, orientation: FORWARD

Perfect match to: (RF122-AJ938182-[667831:668328], highly conserved allele)

Sequence:

ATGCAACACCTTATAAAAAAACATGTATTGAATGGCGAGTTTGATTTAGTACGACAATTGATGTCCGAAACAGATTTTATGGAATTTGAA
GAAGCATATATTTCAAGTGCGCATGAAGTAGAAAGTATGATGTTTTATACATGTATTTTAGATATGATTAAGTACGAAGAATCATCTGAA
ATGCATGACTTAGCATTTTTATTGCTTGTGTATCCACTAAGTGAATATGAAGGTGCTTTGGATTCTGCTTATTATCATGCAGACGCTTCCAT
AAAACTTACTGACGGCAAAGAAGTTAAAAGTTTGTTACAAATGTTATTATTGCATGCGATACCAACACCTGTTATTTCAGATAAGAAGGCT
TTTGATATCGCCAAGCAAATTTTAAAATTAGATCCTAATAATAATGTTGCTCGTAACGTCTTAAAAGACACTGCCAAACGTATGGACAACG
TTGTTGTTGATATAAATGAATTACACCAACGTAATGCACGTTAA

Gene: Q5HI14 (putative membrane protein)

Contig: 05_NODE_4, position: 152876 to 153943, length: 1068 nt, orientation: FORWARD

Perfect match to: (MW2-BA000033-[698815:699882], highly conserved allele)

Sequence:

ATGATGATGAATAAAGAAGCAACAAAAATTGGATTTGCCTACGTCGGCATTGTAGTGGGCGCAGGATTTTCAACTGGACAAGAAGTTAT
GCAATTTTTCACTAAATATGGCTTGTGGGCTTATTTAGGTGTTATTATATCTGGTTTTATTTTAGCTTTTATTGGGCGCCAAGTAGCAAAAA
TTGGTACTGCCTTTGAAGCGACAAATCATGAATCAACATTACAATACGTATTCGGTGAAAAGTTTAGTAAAGTCTTTGATTATATTTTAAT
CTTCTTCTTATTTGGTATAGCTGTAACCATGATAGCTGGTGCAGGCGCAACATTTGAAGAAAGTTATAACATACCTACATGGCTAGGTGCT
TTAATTATGACATTAGCGATTTATATTACGTTGCTATTAGACTTTAATAAAATAGTACGTGCACTAGGTATCGTTACACCATTTTTAATTGTT
TTAGTTGTATTAATCGCTGGCGTTTATTTATTTAAAGGTCATGTTTCATTAGCAGAAGTTAACCAAGTAGTGCCTGAAGCAAGTATTTGGA
AGGGAATCTGGTTTGGTACAATATATGGTGGATTAGCTTTTTCTGTAGGTTTTAGTACCATCGTAGCAATCGGTGGGGATACTGAAAAGC
GTACAGTGTCAGGTGCAGGCGCGATGTATGGTGGTATTATCTATACTGTATTACTAGCATTGATCAACTTTGCATTGCAAAGTGAATATCC
AACTATTAAAAATGCCTCAATTCCTACATTGACGTTAGCAAATAATATCCATCCTTTAATAGCAACAGTGTTATCTGTTATTATGCTGGCGG
TTATGTATAATACTATTCTAGGACTAATGTATTCATTTGCAGCACGTTTTACAGAACCATACAGTAAAAATTATCATATCTTTATTATTATAA
TGATGGTAGCAGGTTATTTATTAAGTTTCGTAGGATTTGCTGAATTAATTAATAAGTTATATACAATTATGGGATATGTAGGCTTATTTATT
GTAGTAGCTGTAATTATTAAATATTTCAAACGTAAAAATGCGGATAAAAAACATATTGCTTAA

Gene: lip4 (putative lipase)

Contig: 05_NODE_4, position: 154094 to 155137, length: 1044 nt, orientation: FORWARD

Perfect match to: (N315-BA000018-[704678:705721], allele observed in CC5+CC1+CC25+CC361)

Sequence:

ATGAATAAAGATAATAAATGGACGATGATAACTGCGCTTTTTATAACTGTAATCAGTGTATTGTTAGCATTTCATCTGAAACAACATTATG
ACCAAATTACAAATGAGAACCATGCTAATAAAGACAAAATTAATATTAAAAATAAAAATGTGCGCATTTATCAAAACCTTACATACAATA
GAGTTTTCCCTAACAGTAAATTAGATATTATTACACCTGTTGATATGTCTTCTAATGCCAAACTGCCAGTTATTTTTTGGATGCACGGTGGT
GGTTATATTGCGGGTGATAAGCAGTATAAAAACCCATTATTAGCGAAAATTGCTGAACAAGGGTACATTGTTGTGAATGTAAATTATGCA
TTGGCGCCACAATATAAATATCCCACACCATTAATTCAAATGAATCAAGCAACTCAATTCATTAAAGAAAATAAAATGAATTTACCTATTG
ATTTTAATCAAGTAATTATTGGCGGTGATTCTGCAGGTGCTCAATTAGCTAGCCAATTTACGGCAATACAGACGAATGATCGCTTAAGAG
AAGCCATGAAATTTGATCAGTCATTCAAACCATCGCAAATTAAAGGTGCTATACTATTTGGTGGTTTTTATAATATGCAAACAGTTAGAGA
AACTGAGTTTCCAAGAATACAGTTATTTATGAAAAGTTATACTGGCGAAGAAGATTGGGAAAAGAGTTTTAAAAACATTTCACAAATGTC
GACAGTAAAACAATCGACAAAAAATTATCCACCAACATTTTTATCTGTTGGAGATAGCGATCCATTCGAAAGTCAAAATATAGAATTCAG
TAAGAAATTACAAGAATTGAATGTACCAGTAGATACTTTGTTTTATGATGGTACGCATCATTTACATCATCAGTATCAATTTCACCTTAATA
AACCTGAATCGATAGATAATATCAAAAAAGTGTTACTTTTCTTAAGTCGTAATACATCTTCTAGTGGTATTCAAACTGAAGAGAAACCACA
AATAGAAAATCCGAGTAATGAATTACCGTTAAATCCTTTAAACTAA

Gene: Q5HI12 (putative protein)

Contig: 05_NODE_4, position: 155466 to 155894, length: 429 nt, orientation: FORWARD

Perfect match to: (N315-BA000018-[706050:706478], highly conserved allele)

Sequence:

ATGACAAGGTCAGCATTAAAACCATTTAAAAATAAACGCGTTATGGTTACTGGACGTATACAACGTGTTTTGTTTAAAAATTATTTAGATA
GACATAGCACATTTAAGCCGAATGTAAGGATATTATTAAAAGATGTATTTGTTTCAGGTGTATCAATAGATCATTTATGGTTATATGAGAC
AAATAAATACTATGCATTGGCAATGGAACTTATTCATCAACGAGTAAAATTTAGTGCGAATGTTGTACCATATTACAAAATAAATAGAAAT
AATAATTTATTCGTACAAGATTATGGAATTAAGCGTAAAGGTAGGTTAATTACTGAAGAAGCTTACAATCAAAACAATCAGTATCAGGAT
AAGATATATGAAAAATTACCGGATATAGATTTTAGACTCGAAGATTTTTATAGTAAGGAAAACTAA

Gene: Q5HI11 (N-acetyltransferase)

Contig: 05_NODE_4, position: 156103 to 156609, length: 507 nt, orientation: REVERSE

Perfect match to: (N315-BA000018-[706687:707193:r], allele observed in CC5+CC1+CC361)

Sequence:

TTAATTAAGAATTTTAGCCATCATATAGTCATCGTAATATTTACCATCGATAAATAACTTATCTTTTAAAACGCCTTCGATTTGAAAATCGG
CACTTTTAAAAAGCTCGAGGGCAGGTTGGTTATTGAGTGGTACATTTGCTTCAATTCGGTGTATTTGATTGTTTAAACACCAAGCCATAAT
GGCATCAAGAAGTGCTTGGCCAATTCCACGATGTTGATATAATTTCTTTACACCTAAATCAATTTTAGCAACATGTTTAATGCGTTGAAAT
GGTGTCGTATTAACAAAGGCAAAGCCAACGAGTTGTTCATCACTTTCAGCAACAAAGATGACTTTATGTGGAGAAGTGATATATTCTTCT
AATTGTTTACTAGCCGACGTGACGCTAGGATCATATTCTCCTGGTGTGTAGAACATATACGGAGATTCGTCGTATATTTTCGCTAACATTG
AAATGAAATTTTCTACATCTTTGATACTAACTCTACGTATAATATGGGCCAT

Gene: graX (cofactor of GraRS)

Contig: 05_NODE_4, position: 156722 to 157645, length: 924 nt, orientation: FORWARD

Perfect match to: (N315-BA000018-[707306:708229], highly conserved allele)

Sequence:

ATGAAACCTAAAGTTTTATTAGCAGGTGGAACAGGATATATTGGTAAGTATTTAAGTGAAGTGATTGAAAATGATGCTGAACTTTTTACT
ATATCAAAATATCCAGACAATAAAAAAACAGATGATGTTGAAATGACTTGGATTCAGTGTGATATATTTCATTACGAACAGGTTGTTGCA
GCAATGAATCAAATAGATATTGCTGTATTCTTTATCGACCCAACAAAGAATTCTGCCAAAATAACACAATCATCAGCAAGAGATTTAACAT
TAATCGCAGCAGATAATTTTGGTCGAGCAGCGGCTATCAATCAAGTAAAAAAAGTAATCTACATACCTGGGAGTCGTTATGATAATGAAA
CAATTGAACGCCTAGGTGCATATGGCACACCTGTAGAAACAACAAATTTAGTTTTTAAACGTTCTTTAGTTAATGTAGAATTACAAGTTTC
AAAGTATGATGATGTTAGATCAACGATGAAGGTAGTTTTACCAAAGGGATGGACATTAAAGAACGTTGTAAACCATTTTATTGCATGGAT

GGGTTACACTAAAGGAACTTTTGTGAAAACAGAAAAATCACATGATCAATTTAAGATATATATTAAGAATAAGGTGCGACCGCTCGCAGT
ATTTAAAATAGAAGAAACAGCTGACGGAATAATAACTTTAATTTTATTGAGTGGAAGTTTAGTGAAAAAATACACAGTTAATCAAGGGAA
GTTAGAATTTAGATTAATCAAAGAGTCGGCAGTCGTTTATATACATCTATACGATTATATCCCTCGATTATTTTGGCCGATTTATTACTTTA
TACAAGCACCAATGCAAAAAATGATGATTCATGGCTTTGAAGTTGACTGCCGGATTAAAGATTTTCAAAGTCGATTAAAATCAGGAGAAA
ATATGAAATATACTAAATGA

Gene: graR (two component sensor/regulator, transciptional regulator)

Contig: 05_NODE_4, position: 157661 to 158335, length: 675 nt, orientation: FORWARD

Perfect match to: (RF122-AJ938182-[673568:674242], highly conserved allele)

Sequence:

ATGCAAATACTACTAGTAGAAGATGACAATACTTTGTTTCAAGAATTGAAAAAAGAATTAGAACAATGGGATTTTAATGTTGCTGGTATT
GAAGATTTCGGCAAAGTAATGGATACATTTGAAAGTTTTAATCCTGAAATTGTTATATTGGATGTTCAATTACCTAAATATGATGGGTTTT
ATTGGTGCAGAAAAAATGAGAGAAGTTTCCAACGTACCAATATTATTTTTATCATCTCGTGATAATCCAATGGATCAAGTGATGAGTATGG
AACTTGGCGCAGATGATTATATGCAAAAACCTTTCTATACCAATGTATTAATTGCTAAATTACAAGCGATTTATCGTCGTGTCTATGAGTTT
ACAGCTGAAGAAAAACGTACATTGACTTGGCAAGATGCTGTCGTTGATCTATCAAAAGATAGTATACAAAAAGGTGACGATACGATTTTT
CTATCCAAAACAGAAATGATTATATTAGAAATTCTTATTACCAAAAAAAATCAAATCGTTTCGAGAGATACAATTATCACTGCATTATGGG
ATGATGAAGCATTTGTTAGTGATAATACGTTAACAGTAAATGTGAATCGTTTACGAAAAAAATTATCTGAAATTAGTATGGATAGTGCAA
TCGAAACAAAAGTAGGAAAAGGATATATGGCTCATGAATAA

Gene: graS (two component sensor/regulator, sensor histidine kinase)

Contig: 05_NODE_4, position: 158328 to 159368, length: 1041 nt, orientation: FORWARD

Perfect match to: (ED133-CP001996-[719991:721031], highly conserved allele)

Sequence:

ATGAATAATTTGAAATGGGTAGCTTATTTTTTGAAATCTCGCATGAACTGGATATTTTGGATATTGTTTTTAAACTTGCTTATGTTAGGCAT
TAGTCTAATCGATTATGATTTTCCAATAGACAGTTTATTTTATATTGTTTCTTTGAATTTAAGTTTAACAATGATTTTTCTTATATTGACATAT
TTTAAAGAAGTAAAATTATATAAGCATTTTGACAAAGATAAAGAAATAGAAGAAATTAAACATAAAGATTTAGCGGAAACGCCATTTCAA
CGTCATACAGTTGATTATTTATATCGTCAAATCTCAGCGCACAAAGAAAAGGTTGTTGAGCAACAGTTACAATTGAACATGCATGAACAA
ACCATTACAGAATTTGTGCACGACATAAAAACACCTGTGACAGCCATGAAATTATTAATTGATCAAGAAAAAAATCAAGAAAGAAAACA
GGCATTACTATATGAATGGTCTCGTATAAACTCGATGCTGGATACACAGCTGTATATTACTAGATTAGAATCTCAACGCAAAGATATGTAT
TTTGATTACGTGTCACTTAAACGCATGGTCATTGACGAAATACAATTAACAAGACATATTAGTCAGGTTAAAGGTATTGGTTTTGATGTTG
ACTTTAAAGTGGATGATTATGTTTATACAGATATAAAATGGTGTCGTATGATTATTAGACAAATTTTGTCAAACGCATTGAAATATAGTGA
GAATTTTAATATTGAAATTGGGACAGAATTAAATGATCAACATGTTTCGTTATATATTAAAGACTATGGCAGAGGTATTAGTAAAAAAGA
TATGCCGCGAATATTTGAACGAGGATTTACGTCAACGGCTAACAGAAATGAAACGACGTCTTCAGGTATGGGTCTATATTTAGTAAATAG
TGTAAAGGATCAATTAGGTATTCACCTGCAAGTCACGTCGACTGTTGGTAAGGGGACAACTGTCAGATTGATTTTCCCATTACAAAATGA
AATTGTTGAACGCATGTCGGAAGTGACAAATTTGTCATTTTAA

Gene: vraF (ABC transporter, ATP-binding protein)

Contig: 05_NODE_4, position: 159512 to 160273, length: 762 nt, orientation: FORWARD

Perfect match to: (RF122-AJ938182-[675419:676180], highly conserved allele)

Sequence:

GTGGCAATTTTAGAAGTAAAACAATTAACAAAAATATATGGAACTAAAAAAATGGCACAAGAAGTGTTGCGAGATATCAATATGTCTATT
GAAGAAGGCGAGTTTATTGCTATTATGGGTCCCTCTGGATCTGGGAAAACGACATTATTAAATGTTTTAAGTTCAATTGATTATATTTCAC
AAGGTTCTATTACATTAAAAGGAAAAAAATTAGAAAAGCTTTCAAACAAGGAATTATCTGATATACGCAAGCATGATATTGGTTTTATTTT
TCAAGAGTATAATTTACTGCATACATTGACTGTTAAAGAAAACATAATGTTACCACTAACGGTTCAGAAGTTAGATAAAGAACATATGTTA
AATCGTTATGAAAAAGTAGCAGAAGCATTAAATATATTGGATATTAGTGATAAATACCCTTCTGAATTGTCTGGTGGACAAAGACAACGA
ACATCTGCTGCAAGAGCGTTTATTACATTACCTTCTATTATATTTGCTGACGAACCAACAGGTGCACTGGATTCTAAAAGTACTCAAGATT
TATTAAAACGATTAACAAGAATGAATGAAGCATTTAAGTCTACAATTATTATGGTAACGCATGATCCTGTTGCAGCAAGTTATGCCAATCG

AGTAGTGATGCTAAAAGATGGTCAAATTTTCACTGAATTATACCAAGGGGATGACGATAAACATACCTTTTTCAAAGAAATAATACGTGT
ACAAAGTGTTTTAGGTGGCGTTAATTATGACCTTTAA

Gene: vraG (ABC transporter, transmembrane permease)

Contig: 05_NODE_4, position: 160263 to 162152, length: 1890 nt, orientation: FORWARD

Sequence:

ATGACCTTTAACGAGATAATATTTAAAAATTTCCGTCAAAATTTATCACATTATGCCATCTATCTTTTTTCATTAATTACGAGTGTAGTATTG
TATTTTAGCTTTGTAGCATTAAAATACGCGCATAAACTAAACATGACAGAGTCATATCCAATTATTAAGGAAGGCTCACAAGTCGGAAGC
TACTTTCTATTTTTCATCATAATTGCATTTTTGTTATATGCCAATGTGTTATTTATTAAACGACGAAGTTATGAGCTTGCATTATATCAAACA
TTAGGTTTATCTAAATTCAACATTATTTATATACTAATGCTCGAACAATTACTAATATTTATAATTACGGCAATATTAGGTATTATTATTGGT
ATTTTTGGTTCAAAACTGTTATTAATGATTGTCTTTACATTATTAGGAATTAAAGAAAAGGTTCCAATTATTTTTAGTTTGAGGGCGGTATT
TGAAACATTAATGTTAATCGGTGTCGCTTATTTTTTAACCTCTGCTCAAAATTTTATATTAGTGTTCAAACAATCTATTTCACAGATGTCAAA
GAATAACCAGGTTAAAGAAACAAATCATAATAAAATTACATTTGAAGAGGTTGTTTTAGGCATCTTAGGTATAGTATTGATTACCACAGG
ATACTATCTATCTTTGAACATTGTTCAATATTATGATTCTATCGGTATACTTATGTTTATTTTATTGTCAACTGTGATTGGGGCATACTTATT
TTTTAAAAGCTCTGTTTCTCTAGTTTTTAAAATGGTGAAGAAGTTTAGAAAAGGTGTTATAAGTGTAAATGATGTCATGTTCTCATCATCTA
TTATGTATCGTATTAAGAAAAATGCTTTTTCACTTACGGTCATGGCAATCATTTCAGCGATTACTGTTTCAGTTCTTTGCTTTGCTGCTATAA
GTAGAGCGTCCTTATCAAGTGAAATAAAATATACTGCACCACACGACGTTACAATTAAAGACCAACAAAAAGCTAATCAATTAGCAAGTG
AATTAAACAATCAAAAAATTCCTCATTTTTATAATTATAAAGAAGTAATTCATACGAAATTGTATAAAGATAATTTATTTGATGTAAAAGC
GAAAGAACCATACAATGTAACAATTACTAGTGATAAATATATCCCTAATACTGATTTGAAACGTGGACAAGCTGATTTGTTTGTAGCGGA
AGGTTCTATCAAAGATTTAGTGAAACATAAGAAGCATGGTAAAGCAGTTATAGGAACGAAAAAACATCATGTTAATATTAAGTTGCGGA
AAGATATTAATAAAATCTATTTTATGACAGATGTTGATTTAGGTGGACCAACGTTTGTCTTAAATGACAAAGACTATCAAGAAATAAGAA
AGTATACAAAAGCAAAGCATATCGTCTCTCAATTTGGATTCGATTTGAAACATAAAAAAGATGATTTAGCATTAGAAAAAGTGAAAAATA
AAGTTGATAAATCTATTAAAACAAGAAGTGAAGCGATAAGCTCAATATCAAGTTTAACCGGAATATTATTATTTGTAACATCATTTTTAGG
TATTACATTCTTGATTGCTGTATGTTGCATTATATACATAAAGCAAATAGATGAAACCGAAGATGAGTTAGAGAATTATAGTATTTTGAGA
AAGCTTGGATTTACACAAAAAGATATGGCAAGGGGACTAAAGTTTAAAATTATGTTTAATTTTGGGTTACCTTTAGTTATTGCACTATCAC
ATGCATATTTTACATCATTAGCATATATGAAATTAATGGGTACAACGAATCAAATACCGGTTTTCATAGTAATGGGATTATACATTTGTAT
GTATGCTGTTTTTGCAGTGACGGCTTATAATCATTCCAAGCGAACAATTAGACATTCCATATAA

Gene: ykaA-pitR (regulator of inorganic phosphate transporter)

Contig: 05_NODE_4, position: 162877 to 163494, length: 618 nt, orientation: FORWARD

Perfect match to: (N315-BA000018-[713459:714076], highly conserved allele)

Sequence:

ATGTTTAGTAAGAAAAAAGATAAGTTTATGGTTCAATTAGAAGAGATGGTTTTCAATCTGGATCGTGCTGCTATTGAATTCGGTAAAATG
GATTTCAATACACATTTAGATTTAAAAGCATACTCAGACAACATTAAAACTTATGAGTCACATGGTGACGAATTAGTACATCAAGTAATTA
CTGATTTAAATCAAACATTTATCACACCAATTGAACGTGAAGATATTTTATCATTATGTGATGCAATTGATGATGTTTTAGATGCAATTGAA
GAAACGGCAGCTATGTTTGAAATGTATTCAATCGAATACACAGATGAATATATGGCTGAGTTTGTTGATAACATTCAAAAAGCAGTTGCA
GAAATGAAACTTGCTGTCGGCTTATTAGTCGATAAAAAATTATCACATATGCGTATTCATTCAATTAATATTAAAGAATTTGAAACAAACT
GTGATGGTATTTTAAGACAGTCAATTAAACATATTTTCAATAGCGAAACAGATCCAATCACTTTAATTAAAATAAAAGATATTTATGAAAG
CATGGAAGAAATCGCTGATAAATGTCAAATCGTAGCAAATAATTTTGAAACTATTATTATGAAAAATAGCTAA

Gene: pitA (inorganic phosphate transporter)

Contig: 05_NODE_4, position: 163510 to 164517, length: 1008 nt, orientation: FORWARD

Perfect match to: (MRSA252-BX571856-[721027:722034], allele observed in CC30+CC25+CC30+CC45I)

Sequence:

ATGTCATATATAATCATCGTCACTATAGCTGTAGTTATTTTCTCGCTGATATTTGACTTTATCAATGGATTCCATGATACAGCCAATGCAGT
AGCTACTGCTGTATCTACTAGAGCGTTAACGCCTAAAACGGCAATTTTAATGGCAGCAGTGATGAACTTTATAGGTGCTTTAACATTTACG
GGCGTTGCAGGCACCATTACTAAAGACATTGTCGATCCATTTAAATTGGAAAATGGATTAGTTGTTGTGTTAGCTGCAATACTTGCGGCT
ATTATTTGGAATTTAGCTACTTGGTTTTACGGAATTCCAAGTTCGTCTTCACATGCACTTATAGGTTCAATTGCGGGTGCAGCAATCGCATC

TGAAGGCTCATTTGGAGTGTTACATTACCAAGGTTTCACAAAAATTATTATTGTATTAATCGTTTCACCGATAATCGCATTTTGTGTTGGTT
TCTTGATGTATTCAATTTTTAAAGTTATCTTTAAAAATGCAAATTTAACAAGAGCGAATCGTAACTTTAGATTTTTCCAAATTTTCACAGCA
GCGTTACAATCATTCTCTCACGGTACGAATGATGCGCAAAAGTCAATGGGTATTATTACGTTGGCATTAATTGTTGCTAATGTACAGAATG
ATGGCAGCGTTGAACCACAGTTATGGGTAAAATTTGCCTGTGCGACAGCAATGGGGCTTGGTACTGCAATTGGTGGCTGGAAAATTATC
AAAACTGTAGGTGGTAATATTATGAAAATACGTCCAGCAAATGGTGCTGCGGCCGATTTATCATCTGCATTAACAATTTTTGTTGCATCAT
CGCTACATTTCCCATTATCAACAACTCACGTTGTGTCATCATCAATCTTAGGTGTTGGTGCTTCTAACCGAGCTAAAGGTGTAAAATGGAG
CACTGCGCAACGAATGATCATTACATGGGTGATTACATTACCTATTTCAGCATTGTTAGCAGGTTTACTATTCTATATACTTAACTTATTTTT
CTAA

Gene: ssaA5 (staphylococcal secretory antigen A5)

Contig: 05_NODE_4, position: 165105 to 165902, length: 798 nt, orientation: REVERSE

Perfect match to: (MW2-BA000033-[711044:711841:r], allele observed in CC1+CC188+CC2970)

Sequence:

TTAATGGATGAATGCATAGCTAGAAACTTCTGAAGCTGGAATTGTACGGTAGTTCATATTGTATGGACCATATGTGTAATTCATTTCAGAA
ATCAAGATACTACCATCACCATTGACACGTTCAACATAAGCAACATGACCATATGGGCCAGGTGTGCTTTGCATAATTGAACCAACTGAT
GGTGTGTTGTTTACTTGGTAACCATCATTAGCTGCGTTACCAGCCCAATACTTAGCGTCTGACCAATATGTGCTAATTGGACTACCAGCTT
GAGCACGACGGTCAAATACGTACCATGTACATTGACCAGCAGTGTATAAATTTTGGTGATTAAAAGATGATGCATTGCCATTGCTACCTG
TTGTAGCTGTTGGTGTTGTACCACCTGATCCACCATTAGGAATTTGTAATGTTTGGTTAGGCATAATTAAATAACCACGTAAGTTATTGGC
TGCCATTAATTGATCAACTGAAACACCATATCTGCTAGCAATGATATTTAATGATTCACCAGCTTGTACAGTATGAGATGATGCTGAACCA
GCTTGTGGAGAAGTGTTTGACGAATTTTGTGCATCACTTCCACCTACTGAGATAACTTGACCAGGGAATACCAAGTTGTTATCTAATTGGT
TATTTTGTTTAATACTCTCTACTGAAGTGTTGTATTTTTGAGCAATACTCCATAATGATTCACCAGATTGTACTGTATGTTGTGTAGAAGCT
TGTGCATCATGATGCGTTAAAAATGCAGCTGCACCAGATGTTGCTGTTATTGCAAATGCTAATTTTTTCAA

Gene: yetJ (pH-dependend calcium efflux protein)

Contig: 05_NODE_4, position: 166263 to 166907, length: 645 nt, orientation: FORWARD

Perfect match to: (MW2-BA000033-[712202:712846], allele observed in CC1+CC25+CC188)

Sequence:

TTGTCGCAAAATACAAATCATTCATATTATCATCAAAACCAGCATGCTCAATCAATAAGTAAAGTGTGGCTTTATTTTATGTATTATTGGAT
TATATTTGGCATAGGATGCTATCTAGGTCAGTTTTTACCATTAAGTTGGCGACAACCCTTGTCATTTGGATTACTGATTATTATTTTAGCAA
CACTTGTTTTTGAAAGAGCGAGACGGTTCGGTTTAATTATTTCACATATTTACGCTGTAGTGATCGGCTTATTGTCATACGCAACGTTTACC
ACGTATTTACAAAATTTAGGACCAGATATTTTCTATAAAAATATCGCATTAGCAATTTTTGCATTTATAGCATTTGGTATTATTGGTTATTTC
TTCGTTGGAGATGCATCGAGTATAGGCAAATATTTATTCGTTACATTAATAACATTAATTATTGCGAGTCTAATTGGTATTTTTCTTCAAAA
TCCTATTTTTTACACTATTATTACCGTCGTTAGTTTGTTGTTATTTCTACTTTATACTTTGTATGATTTTAATCGTTTAAAAAGAGGTGACTAT
TCACCAAGAGAAATGGGATTTAATCTATTTATTAATTTGTTGAATATTATTAAGGATATACTTTATCTTGCTAATATGTTCAGAAGATAA

Gene: rbf (regulator of biofilm formation)

Contig: 05_NODE_4, position: 167604 to 169754, length: 2151 nt, orientation: FORWARD

Perfect match to: (N315-BA000018-[718186:720336], allele observed in CC5+CC1)

Sequence:

ATGGCAAAATCATGCTTGCATATACTTACTAATAATGAATATGCGACAACGCGTTGCCAAGATGGCATAGTCTTATTTTGGCCAATTGACG
GGGAAATCGAACTACAAAAATTTCGTAAAAGTAAAATAATTGAAGATGATATATATATTATTAATCATCTGGATGTATTTAGTATTAAGAA
TAATAAAAAAACGATCATGTTGTATTTGAGTAGCGATTGGTTTGCGGAATTAGGCTTTACTTTCTTTAATTACCACTATACAGCAAAGTTG
ATTAAATCATCCTATAATTTGAAATGTCTACTATTAAAATTGACATATCGATACCTTGATAATCAGCCTCTTAATGACGCTGATATTAGAAA
ATTACAGGATATTATTAAAATCATTGCAAAAGAAGCAAGTATGGATAAAAAGATTGCACAAAATCAATATCGATATGCTTATTATGGTGA
TTTGCGTGATGAGCTCGAATATATTTATCAAAATGTAAATCAACGATTGACATTAAAAAGTGTCGCTGATAAATTATTTGTCTCAAAGTCA
AATTTGTCATCACAATTCCACTTACTTATGGGCATGGGTTTTAAAAAATATATTGATACTTTGAAAATTGGTAAATCGATTGAAATTCTACT
TACTACTGATAGTACTATTAGCAACATAAGTGAACATTTAGGTTTTAGTAGTAGCTCCACTTACTCTAAAATGTTTAAAAGTTATATGGAT
ATAACACCGAATGAATATCGTAATTTATCAAAATATAATAAATGTTTAATGCTAAAGCCAGAACCACTAGTAGGCAAAATGGTGCAAGAA

GTAAAAGAAATCATATTGAATTATATTGAACATTATAAAAACCACCTAACTGATGTTATACATATTGATGAAGACAAATTTGAAACACCTA
AATTGTTTCAAACGGTTATTCAAATAAATACTTATACAGAAATGAAATTAGTTTTCTTAGAAGGAATCTTTAAAACCTTATTGAATAAGAA
CAGTCAAGTTGTCTTTTTCATCATGCCATCGATTCTAAAAAGTAAAAATACCATGTCCGAAGAAGAAAAATTCACAATCATTAAAACAATA
ATTGAAAGTGATCTAAAGATAGCATTTAATATAAATGATATTGAAACAACTTATTTTGTTGAAGAAGCTTTTATGAGTGTTTTCAGACAAA
TATCTCCAAACGAATTAAGTAATCATAATAATTACGAAGTGCATTTTGTTTTTGATTTATCATTGATGGAAATTAGAACAATTTATCGAATG
ATATTAAAATTACATAACATCATGTTGAATGTGAAATTAGGATTGAACATTACCTGTTTATTTGAAAAACCTTCAGTTTTTAAATCACTAGT
ATCACAAATAAAGCGACTTAAATTCGATTCGTTAATAATAGATAATGCAAATTTAAGTAGCCCTTATTTGATGGGGGAAAGTGATGAGTT
ACTATTGAAAAATATTTTGCATTTTAAAAATTTAAAACAAGTAATTAATGAATTGGATATTGAACAAGAAAAGCTTATTTTTCTAAATGTTG
AAAATCATAAACTGCTTAATAATAAAGAACGAGATTTAAGTAATAGTGCTCCATTAATTTATAAGACATTAAGTGCGCTGTATCACAACTT
TGATGGCTTTGGATTAAACATTTTTGATAATCATCATACATTTAATGCGATGCATCTATATGATAAAAATGGATTTAAAACAACACTAGGT
CTTATATTGGAAAAATTTATCGAATATGTCTCGAAACCAAAATACGAAAACAGTTATTATTCTATTTTTGATATAGAGAATTATTATTGTCT
TGTTATTTATGATTGGCGAGTGATAGAGAGCGAGACAATTATGAGTAATTTTGAGGATAGTCAAGTTTATATAAATTTTAAAAACAATGT
TTTAAACGATAAATATCTAATTGTAATAGAAACATTGGACGAAAATAGTGGCAACATTAATCATTTGATTTCTAAAGAATTAAGAGATAA
ATATGAATGGAACCCTAGTTTACTATCTAAAATTGACAACTACCTTAAACCAGCAATAGAGATTAAAGAGCATAATTTTAGTGATAATTCC
TTGAATATTAACGTTACTTTTAATGCGTTATACATAATTAAAATAGGAAAAAAATAA

Gene: sarX (HTH-type transcriptional regulator)

Contig: 05_NODE_4, position: 169834 to 170259, length: 426 nt, orientation: FORWARD

Perfect match to: (N315-BA000018-[720416:720841], highly conserved allele)

Sequence:

ATGGCAAAAAAATTTAAGATAATAATGACAGAAGCATTGTCTTTATATATTTGGGGGTGCAACATTTTGAATACTGAGAAATTAGAAACA
TTGCTTGGCTTCTATAAACAATATAAAGCATTATCTGAATATATTGATAAAAAATATAAGTTGTCGCTAAATGATTTAGCAGTCTTAGATTT
AACGATGAAGCATTGCAAAGATGAAAAAGTACTTATGCAATCATTTTTAAAAACTGCAATGGATGAGCTAGATTTAAGTAGGACAAAATT
ATTAGTTTCTATAAGAAGACTAATTGAAAAAGAAAGACTTAGTAAAGTTAGATCATCTAAAGATGAGCGTAAAATTTATATTTATTTAAAT
AATGATGATATATCTAAATTTAATGCTTTATTTGAAGATGTAGAACAATTTTTAAATATTTAA

Gene: yeeI (putative transcriptional regulator)

Contig: 05_NODE_4, position: 170449 to 171165, length: 717 nt, orientation: FORWARD

Perfect match to: (N315-BA000018-[721031:721747], allele observed in CC5+CC1+CC49+CC80+CC239)

Sequence:

ATGGGACGTAAATGGAATAACATTAAAGAAAAAAAGGCCCAAAAAGATAAAAACACAAGTAGAATATATGCGAAATTTGGTAAGGAGA
TTTATGTTGCAGCAAAATCTGGTGAACCCAATCCAGAATCTAACCAAGCTTTAAGGTTGGTGCTTGAACGCGCTAAGACATATTCAGTGC
CGAATCATATTATTGAAAAAGCAATAGATAAAGCTAAGGGTGCTGGAGACGAAAACTTTGATCACCTAAGATATGAAGGATTTGGCCCA
AGCGGATCAATGCTAATTGTTGATGCGTTAACAAATAATGTAAATCGTACTGCCTCTGATGTGCGAGCTGCTTTTGGTAAAAATGGCGGT
AATATGGGTGTATCTGGATCAGTTGCTTATATGTTTGATCATGTGGCAACATTTGGTATTGAAGGAAAGTCTGTTGACGAAATACTTGAA
ACATTAATGGAACAAGATGTAGATGTAAATGATGTGATTGACGATAATGGATTGACAATAGTCTATGCTGAACCAGATCAATTTGCAGTC
GTTCAAGATGCGCTTCGTGCAGCAGGTGTTGAAGAATTTAAAGTTGCTGAATTTGAAATGTTACCTCAAACAGATATTGAACTTTCTGAA
GCGGACCAAGTAACATTTGAAAAATTAATCGATGCATTAGAAGATTTAGAAGATGTACAAAACGTATTCCATAATGTGGATTTGAAATAA

Gene: DUF985 (protein of unknown function DUF985)

Contig: 05_NODE_4, position: 171165 to 171638, length: 474 nt, orientation: FORWARD

Perfect match to: (MW2-BA000033-[717104:717577], allele observed in CC1+CC80+CC239)

Sequence:

ATGAAATCAGCAGAACAATGGATTGATGAATTGCAACTTGAATCACATCCTGAAGGTGGTTTCTATAGAGAGACAATTCGAGAAGTATT
GAAAGATGGACGCAGAGCGCCGTTTAGTAGTATTTATTTTTTACTTACAGATGACAATATTTCGCATTTTCATCGAATTGATGCTGATGAA
GTATGGTACTATCATGCTGGCGATTCTCTAACAATTCATATGATAAATCCGGATGGGGAATATACGACTGCAACATTGGGTACTGATATC
CAAAATGGAGATGTATTGCAATATGTAGTGCCTAAAGGAACAATTTTTGCTTCTTCAATCGAATTTTCAAATACTTATAGTTTAGTAGGTT

GTATGTGTCAACCGGCATTTGAGTTTAAGCAGTTTGAATTGTTTAAGCAATCTGAATTAATTACACAATATCCGCATCTTAAATCAGTGAT
TGAAAAATATGCTTTAAAATAA

Gene: A5IQM5 (putative protein)

Contig: 05_NODE_4, position: 171991 to 172635, length: 645 nt, orientation: FORWARD

Perfect match to: (N315-BA000018-[722573:723217], allele observed in CC5+CC1+CC239+CC4803)

Sequence:

GTGAAAGTAAAGTATATAGATAAACGTCACTGGCGTCGCCTAATTGATAGGGAATACACAGAGGTAAAAGTTAATAATAATAGGTTTAA
GGGTATTATAGGCTTAGTCACGATGAAAAAGGTTCGTGATCCTTTAGAGGTGACGGTAGTTGGACAAAATATCATTGTCGCAGATGACA
ATTATAAATGGTTGCAAATACTACCTGAAAAGAAACGTTATAGTATAACTGTAATGTTTGATAATAAAGGCAATCCATTAGAATATTATTT
TGATATAAATATCAAAAATATAACGCAAAAAGGTAATGCGCGTACAGTAGATTTATGTTTAGATGTTTTAGCGTTACCAAGTGGTGAATA
TGAGTTGGTAGATGAAGATGACTTAATGTTTGCATTAGAAAGTGAGCAAATTACAAAAAAGCAATTTCATGAAGCATATATGATTGCACA
TCAAATTATGGCAGAGTTAGAAAATGATTTTAAAGGATTCCAAAAGAAAATCATGTACTGCTTTAATAAAATTAATGCAAAGGCTCAAAA
AAATCATCAAAAGCCACAAAATAAAACTAATATTGAAAAAAGCAAACAAATAAAGCCTAAGCAATATAATCAAACTAAAAATCACCAACA
ACAAAAGAAAAACTAA

Gene: ccpE (catabolite control protein E)

Contig: 05_NODE_4, position: 172751 to 173617, length: 867 nt, orientation: FORWARD

Perfect match to: (Strain_21334-AGTW01000032-[115204:116070], allele observed in CC9+CC30+CC59+CC772+CC1464)

Sequence:

ATGAAGATTGAAGACTATCGTTTACTAATAACATTAGACGAAACGAAAACGTTACGTAAAGCGGCTGAAATTTTATATATATCTCAACCT
GCTGTTACACAAAGACTAAAAGCTATTGAAAATGCTTTTGGAGTAGATATTTTTATCAGAACAAAAAAACAATTGATTACAACAACTGAA
GGAACAATGATTATTGAGCATGCCCGTGACATGTTGAAAAGAGAGCGATTATTTTTTGACAAAATGCAGGCACATATTGGTGAAGTGAA
TGGAACAATATCAATCGGGTGTTCTTCTTTGATTGGACAAACCTTACTTCCTGAAGTTTTGAGCCTATATAATGCCCAATTTCCTAATGTTG
AAATACAAGTGCAAGTTGGTTCAACTGAACAAATTAAAGCAAATCATAGAGATTATCATGTTATGATAACTCGTGGAAATAAAGTAATGA
ATTTAGCTAACACACATTTATTTAATGATGATCATTATTTTATTTTTCCAAAAAATAGACGAGATGATGTTACAAAGTTACCATTTATAGAG
TTTCAAGCTGATCCGATTTATATAAATCAAATAAAACAATGGTATAACGATAATTTAGAACAAGATTACCATGCAACTATTACAGTGGATC
AAGTAGCAACTTGCAAAGAAATGTTGATTAGTGGTGTAGGTGTTACAATTTTGCCGGAAATTATGATGAAAAATATCAGCAAAGAACAA
TTTGAGTTTGAAAAAGTAGAAATTGATAATGAACCGCTGATTCGTTCGACATTTATGAGTTATGATCCGAGCATGTTGCAATTGCCACAA
GTTGATTCTTTTGTAAATCTCATGGCGAGCTTTGTTGAACAACCAAAGGCGTAG

Gene: A8Z196 (putative sugar:cation symporter)

Contig: 05_NODE_4, position: 174026 to 175246, length: 1221 nt, orientation: FORWARD

Perfect match to: (MW2-BA000033-[719965:721185], allele observed in CC1+CC5)

Sequence:

ATGGATATGTTTGCAGCGTTATTACAAATAAAGAATTATAAACTCTTTGTTGCTAATATGTTTCTACTAGGTATGGGTATTGCGGTTACGG
TCCCATATCTTGTTCTTTTTGCAACTAAAGATTTAGGTATGACAACAAATCAGTATGGATTACTTCTAGCATCTGCAGCGATTAGCCAGTTT
ACAGTAAATTCAATTATTGCTAGATTTTCGGATACGCATCACTTTAATAGAAAAATTATTATTATTCTCGCATTATTAATGGGTGCGCTTGG
TTTTTCAATATACTTTTTTGTAGATACAATCTGGTTATTCATATTACTATATGCGATTTTCCAAGGATTATTTGCACCAGCAATGCCCCAACT
TTACGCATCTGCTAGAGAATCTATCAATGTTTCAAGCTCTAAAGATAGAGCTCAATTTGCCAACACAGTATTACGTTCAATGTTCTCATTG
GGCTTTTTATTTGGTCCATTTATTGGTGCCCAATTAATCGGTTTAAAAGGCTATGCTGGATTGTTTGGTGGAACAATAAGTATCATTTTATT
TACTTTAGTACTTCAAGTGTTTTTCTATAAGGATTTAAACATTAAACACCCTATTAGTACGCAACAACATGTTGAAAAAATTGCTCCTAATA
TGTTTAAAGACAAAACGCTTTTATTACCATTTATTGCATTTATTTTATTACACATTGGACAATGGATGTATACGATGAATATGCCTTTATTTG
TTACTGATTATTTAAAAGAAAATGAACAACATGTCGGTTATTTAGCTAGTTTATGTGCTGGTTTAGAAGTGCCATTTATGATCATTCTTGGC
GTTTTATCATCTAGATTACAGACTCGAACATTGTTGATTTATGGAGCGATTTTTGGTGGTTTATTCTACTTCAGCATTGGGGTATTTAAAAA
CTTCTATATGATGTTAGCAGGACAGGTGTTTTTAGCTATTTTCTTAGCGGTTCTTTTAGGAATTGGTATTAGTTATTTCCAAGATATCTTAC
CAGATTTTCCAGGATACGCCTCAACACTATTTTCTAATGCAATGGTTATTGGACAGTTAGGCGGTAACCTATTAGGTGGTGCTATGAGTCA

CTGGGTAGGTTTGGAAAATGTATTTTTTGTATCAGCAGCATCAATCATGTTAGGTATGATACTTATATTCTTTACTAAAAATCAAAAAATTA
CAAAAGAGGATGTGATATCAACATGA

Gene: Q2YSN2 (putative membrane protein)

Contig: 05_NODE_4, position: 175243 to 175731, length: 489 nt, orientation: FORWARD

Perfect match to: (MW2-BA000033-[721182:721670], highly conserved allele)

Sequence:

ATGACAATTATTTTATGGCTACTTATCATCGCTGCCTTCATGTTAGCATTTGTTGGGTTGATTAAGCCGATTATTCCTTCTGTTTTAGTATTA
TGGGTTGGCTTTTTAATCTATCAATTTGGCTTTCATAATCAGCATTTATCATGGGTGTTTTATGTATCTATGGCATTGCTAACAATATTAATT
TTATGTGCCGACTTTTTAGCTAATAAATATTTTGTGAATCGCTTCGGTGGTTCTAAGTTTGGAGAGTATGCAGCTTTAATTGGTGTGGTTA
TTGGATGTTTTGTTTTACCGCCATTTGGAATTATTATTATACCTTTTATTTTGGTATTCATAGTTGAATTAATACAAGGCTATTCATTTGAAA
GAGCAGTTAAAGTAAGTATAGGTTCAATCGTAGCATTTTTAACAAGTAGTATAGCTCAAGCAATCATTATGTTTATAATGATTGTATGGTT
CTTTATAGATGCTTTATTGATTAATTAA

Gene: Q2YSQ6 (putative uncharacterized protein)

Contig: 05_NODE_4, position: 175830 to 176519, length: 690 nt, orientation: REVERSE

Perfect match to: (N315-BA000018-[726135:726824:r], allele observed in CC5+CC1+CC8+CC239)

Sequence:

TTATAAAAATGTATGAGAATGTTTTTTCGAAATATTTCTTTCAATGCGTAATCCAATAGGCATAACTATAAATGAAAATATAATAAATACCC
AATTGCTTACCTGAATATATGGTCCAAAGGCAAGTAATGATTGTGGAATAAAGAATACATAGAAAAATCCTACAATTAGTAGAATGATTG
CTAAATAGGTGAATATCCATATCCAATTTGAATTGTTAAAACACTGCAGTTGTATTGTTTTAAAACTAAACCATATAAATAAAAAGACAAT
AAAGAATCCAACGACTACTGAAAACGGGAATGAAACAAAATATAAATTACTTCCATTTTTTTCCATGAAAAATCCTAAAAATCCTTTGAGA
AAACTAACAATCCCAATTAATAGAACGATGTGTTGATAGATATATTTAAAAATATTTTTAAATGTTTCATTAGGCATCGCTTTTAGTTCTTT
TATTGCATGTGCTTTTGGGTCGTGATTGAAAAAATCTAAGGCTAATAAACCATGTTGTTCTGCGCTTAATAATTGTTTGAGTATACGGTTA
ATAATTAACTCTGTATCATGAGGGTTGACGCGAAAGTCAGAGCGCATATAAGTCATATAATTCTCGAAGATTTCTCTATCAGTATTGCTTA
ATCTTAATGATTTAACATTATTTTCTTTTGTTAATTGCGCAGTACTTTTCAT

Gene: A5IQN0 (putative acetyltransferase)

Contig: 05_NODE_4, position: 176646 to 177089, length: 444 nt, orientation: FORWARD

Perfect match to: (MW2-BA000033-[722585:723028], allele observed in CC1+CC8+CC239+CC425)

Sequence:

ATGAGGACACTTAATAAAGATGAACATAATTATATCAAGCAAATAGCTAATATACATGAGACATTATTGTCGCAAGTAGAATCCAACTAT
AAATGTACTAAACTGAGTATTGCTCTTAGGTACGAGATGATATGTTCAAGATTAGAACATACAAATGATAAAATTTATATATATGAAAAT
GAAGGTCAATTAATAGCGTTTATTTGGGGACATTTTAGTAATGAAAAAAGTATGGTTAACATTGAACTGCTATATGTTGAACCACAATTTC
GCAAACTGGGAATAGCTACGCAACTGAAGATTGCGCTTGAAAAATGGGCAAAAACTATGAATGCAAAGCGAATAAGCAATACAATTCAT
AAAAATAATTTGCCAATGATATCTTTGAATAAAGATTTAGGTTATCAAGTGAGTCATGTGAAAATGTATAAAGATATTGATTAG

Gene: A8YZU8 (putative lipoprotein)

Contig: 05_NODE_4, position: 177156 to 177551, length: 396 nt, orientation: FORWARD

Perfect match to: (N315-BA000018-[727461:727856], allele observed in CC5+CC1+CC8+CC239)

Sequence:

ATGAAGAAATTAATCATCAGTATTATGGCGGTCATGCTATTTTTAACAGGTTGTGGTAAAAGTCAAGAGAAAGCCACTCTGGAAAAGGAT
ATCGATAATTTACAAAAAGAAAATAAAGAATTAAAAGACAAAAAAGAAAAGCTTCAACAAGAAAAAGAAAAATTAGCAGATAAGCAAA
AAGACCTTGAAAAAGAAGTGAAAGATTTAAAACCTTCAAAAGAAGATAACAAGGATGATAAAAAAGACGAAGACAAAAATAAAGACAA

AGATAAAGATAAAGAGGCATCACAAGATAAGCAATCAAAAGATCAAACTAAGTCATCGGATAAAGATAATCACAAAAAGCCTACATCAG
CAGATAAAGATCAAAAAGCTAATGACAAACACCAATCATAA

Gene: Q5HHZ0 (putative protein)

Contig: 05_NODE_4, position: 177689 to 177988, length: 300 nt, orientation: FORWARD

Perfect match to: (N315-BA000018-[727994:728293], highly conserved allele)

Sequence:

ATGCATGAACAAGATTTTAGAATTTTAGAGGGTCAAGATATTACTTTGCCAGAATTAGGTAGAGAATTAGAAAATATTACAGGACATACG
ATTGCTGATTCTACTGGCGAAATTAAGCGTGTAATTGCACATTTACCAAACTTTGAGTCCGATACAGATACTTTTGTTGCTACATATCGTTT
AAACCATCAACAAGATTTTATAGATGCAACTTTTACTGCGCTGAAATCAGATAGAGCACGTTTAAAAGAAGTGCCAGTTCATGTTGAACTT
ATAAGTTATATTTCTAAATCAAAATAA

Gene: ykkB (putative acetyltransferase, GNAT family)

Contig: 05_NODE_4, position: 178068 to 178610, length: 543 nt, orientation: FORWARD

Perfect match to: (RF122-AJ938182-[693696:694238], highly conserved allele)

Sequence:

ATGATTTATTGTGAAACAGAGCGTTTAATATTAAGAGACTGGCATGAAGATGATCTGTTACCTTTTCAAAAAATGAATGCGAATTATGAC
GTACGTAAATATTTTCCAAGTTTATTGAGTTATCGTCGTTCAGAATTAGATATGAGAACTATGGATGCGGTTATTAAAGATTATGGCATTG
GATTATTTGCTGTAGAAGATAAAGAGTCCCATCAATGGATAGGCTTTATAGGTTTGAATTATATTCCAGAAACAAGCGATTATCCATTTAA
AGAATTACCGCTTTATGAAATAGGTTGGCGCTTGTTGCCAGAATTTTGGGGAAAAGGATTAGCAACTGAAGGCGCAAAGGCAACATTGA
AGTTAGCAGAAGAACATCAAATATACGATGTCTATAGTTTTACAGCAGAAGCAAATAAAGCTTCACAACGTGTAATGGAAAAAATTGGC
ATGACAGTGTATGATCATTTCGAATTACCCAATCTAAGTAAGTATCATTTATTAAAAAGGCAAGTGCGCTATTACATTAATCTTCGAAAGT
GA

Gene: yvdD (decarboxylase family protein)

Contig: 05_NODE_4, position: 178706 to 179272, length: 567 nt, orientation: REVERSE

Perfect match to: (MW2-BA000033-[724645:725211:r], highly conserved allele)

Sequence:

TTAATCGTATGAACGAGTACCCAGAGGTTTGAAATTTAATATTGATTCAATTAATGATTCCTTAGTGTCGCATAACGGTGCAAGAGCACG
ATACTTAGGATCAATAAAACCTTCCTCAATCATATGGTCAATCATTGTTTGTAGTGGATTGAAAAAGCCATTAATATTATAAATGGCAATA
GGCTTTTCATGGATACCTATTTGAGCCCAACTATACATTTCGAAAAATTCTTCTAGTGAACCTGCGCCACCAGGAGCCATGACAAATGCAT
CTGCAAGTTCTGCCATTTTATTTTTACGTTCATGCATAGAATCAACTAAAATTAATTCAGTTAAACGTTGGCTTGTGATTTCATGTTCATCTA
ACATTTTAGGCATGACGCCAATAGCTTTGCCGCCATGATCTAATACACCATCTTGAATGGCACCCATAATGCCAATTGACCCTGCACCAAA
TACTAATTCATAACCTTGTTCAGCAAAATATTTACCTAAATCGTATGCTTTTTGTACATATGAAGGGTCATGACCTTTGCTTGCACCACAAT
AAACTGCGATTCGTTTCAT

Gene: yqxD (conserved hypothetical protein)

Contig: 05_NODE_4, position: 179274 to 179732, length: 459 nt, orientation: REVERSE

Perfect match to: (N315-BA000018-[729579:730037:r], highly conserved allele)

Sequence:

TTAATCCAGCTCCTTAATTCGATGAATGACTTTTAATAGTGATTGTTCAAACACTTTTTGATCTTGCTTTGTAAAAGGTGGGGGACCTTTGT
GGCGACCACCTTGTTTTCTAATTTGTGCATTCATATATCGTTTATCTAATAGTTGTTGAATATTTTTGGAATTGTATATCTTCCCATTATGAT
GCATGACAATTAAGACTTTGTCGACTAATAAACTTGCGAGTCCATAATCTTGAGTGACTACGATATCATCCTTCGTTGATAATTGAACAAT
TTTGTAATCAACTGCATCTGGTCCATCATCAACATATAATGTTGATACATGTGGAGGATATAATTGGTTCGAAAAATGGCTGAAGCTCCG

AATAATTGTCACAAAAATGCCTGTCTCAGTTGTTAAATCTATAATAGAATCAACAACAGGACAAGCATCTCCATCAATAATAATATGTGTC
AC

Gene: Q5HHY6 (putative protein)

Contig: 05_NODE_4, position: 179735 to 180418, length: 684 nt, orientation: REVERSE

Perfect match to: (N315-BA000018-[730040:730723:r], highly conserved allele)

Sequence:

TTATGCCTCTGTATTGTTTTCTTTATTTTGTTGAGAGGCGCTTTTGGCAACATAATCTTTATATTTTTTAAATGACTTGATGCGTGCTTTATC
AGCTTCTTGTTGGCGTTTTTGTTCTTCTTTGTGTCGTTTTTCAATATTTTTTTGTAACTTTTTATTCATTTTAGCGATTTCTTTGCGATTTTTTTC
AGCTAGTTTATCGCCTTTTTTCTCAGTTTTCTCATCTAATTTATTAGGTGTTAAGCCTGCTTTTTCTTCGTATTTTTGTGATTTTTTCATATCTT
TAATACGTTGTATTTCATTCTTTTCGCGGGCTTTTTGCTCTTCTTTATGACGCTTTTCGATATTTTTTTGAAGTATTTTATTCATTTTATCAGC
GTCTTTACGATTTTGTTTAGCTAATTTTTCGCCTTTTTTCTCAATATAGGCAGGATCATGTTCTCTAGCAAACTTTTTAAGTTCACGTTTATTT
TCAAAATCTTGTTTTTTATCGCCGACATATTCTTTAACATCACTCGCTGTGTTACTGATTGCTGCAGATGTTTTTGAAGCAACTTTACTTGTA
GCATCTGTAACTTTTTGTACGTCCGGATGTTGTTTGATACGTTTACGTTCAACAATTAACGGTACCAATACAATTGGTAATACATTAATCAT
AAATTTGATGACTTTTTTCTTATCCAT

Gene: uppP (undecaprenyl-diphosphatase)

Contig: 05_NODE_4, position: 180590 to 181465, length: 876 nt, orientation: REVERSE

Perfect match to: (MW2-BA000033-[726529:727404:r], allele observed in CC1+CC121+CC133)

Sequence:

TTAAATACCTTTACCAATGCCAAATCCGAAGTAAAGTATAGCAATAAAGATTACTAATACAATTCTGTAAATGGCAAATGGAATTAATTTG
ATTTTGTTAATTAGATGCAAGAATGTTTTGATTGCAATTAGTCCAACAGTAAATGCAGCTAAAAAGCCTAAAATATAAAAAGGTATATCA
GTAATCTGAATATCTTGATAATGTTTTAATAAAGATAAACCACTAGCTGCTAACATAATTGGAACAGCCATAATAAATGTGAAGTCCGATG
CTGCTTTATGATTTAATTTCATTAATACCCCAGTTGAAATTGTTGAGCCTGAACGGCTGAAACCAGGCCACATAGCTACTGCTTGAGAGAT
ACCAATTACAAACGCTTGGAAATAATTGATTTGATCCACTGTTTGTGGGTTTTTAACTTTAGCTGAGTATTTATCAGCAATAATCATATAGA
TAGCACCTACGAATAAGCCAATCATAACAGTTGGCACACTAAATAAATGTTCTTCGATGAAATCATCAAATAGTAAGCCTAAAATACCTGC
TGGAACCATACCCACTAATACATGTAATAAATTTAAACGTCTTGGCTTTGAACGTCTTTGTTGATTGTTATCTCCTTCAACATGTTTGTGTTT
ACCAATATGTAAAATCTCTAAGAAGCGTTCGCGGAACACCCATGCTGCTGCAAAGACGGATCCTAATTGGATGACGATTTTAAATGTAAA
TGCTGATTGAGAACCTAAAAATTCAGATGATTTTAACCACATATCATCAACTAGAATCATATGTCCAGTAGAGGAAACAGGTGCAAATTC
TGTTAATCCTTCGACGACCCCTAAGATAATACCTTTTATTAATTCAATGATAAACAT

Gene: cydD (cysteine ABC transporter, ATPase subunit 2)

Contig: 05_NODE_4, position: 181684 to 183315, length: 1632 nt, orientation: FORWARD

Perfect match to: (MW2-BA000033-[727623:729254], allele observed in CC1+CC30)

Sequence:

GTGAAAAAAATTAACAACAATACTGTTTCAATATAAAATTTTTCCGGTACTCATGTTCTTGGTCAGTACTGGTCTCGGCATACTCGTTATAAC
GCAAAATATTTTAATAGCAGATTTTTTAGCTAAAATTATAAGACATCAATTTCAAGGTTTATGGATTGTATTATTTATTTTATTAGGTGTTTT
ACTTTTAAGAGCAACTGTGCAATTTCTAAATCAATGGTTAGGTGATACATTAGCATTTAAAGTTAAGCATATGCTTAGACAGCGGGTTATT
TATAAAAATAATGGTCATCCAATCGGTGAACAAATGACTATACTCACAGAAAACATTGATGGTTTAGCACCTTTTTATAAGAGTTATTTGC
CTCAAGTGTTCAAATCAATGATGGTTCCGCTCATCATAATCATTGCAATGTTTTTCATCCATTTCAATACCGCATTAATTATGTTAATAACTG
CACCATTTATTCCTTTGTTTTATATTATTTTCGGTTTGAAAACGCGAGATGAGTCAAAAGATCAAATGACTTATTTGAATCAATTTAGTCAA
CGGTTTTTAAATATTGCTAAAGGTTTAGTGACGTTAAAGCTATTTAATCGTACAGAGCAAACAGAGAAGCATATTTACGACGATAGTACT
CAGTTTAGAACTTTAACAATGCGCATTTTACGCAGTGCTTTTTTATCGGGATTAATGCTCGAATTTATAAGTATGTTAGGTATTGGATTGGT
TGCATTGGAAGCAACGCTAAGCTTAGTAGTATTTCATAATATTGATTTTAAAACTGCGGCAATTGCGATTATTTTAGCGCCTGAATTTTTAT
AATGCAATTAAGGACTTAGGGCAAGCGTTCCATACTGGAAAACAAAGTGAAGGTGCCAGTGACGTTGTGTTTGAGTTTTTAGAACAACC
GAACAATAATAATGAATTTCTATTAAAGTATGAGGAAAATCAAAAGCCATTTATTCAGTTAACAGACATATCATTTCGATATGATAATTCT
GATAGATTGGTATTAAATGATTTAAATTTGGAAATATATAATGGTGATCAAATTGCACTTGTAGGTCCAAGCGGGGCAGGTAAATCCACT
TTGACACATCTAATTGCAGGTGTTTATCAGCCAACAATAGGTACTATAAGTACAAACCAGCGTGATTTAAATATAGGAATACTTAGTCAAC

AGCCATATATTTTCAGTGCTTCTATAAAAGAGAATATTACGATGTTTAAAGATATAGAAAATAATACTATTGAAGAAGTGCTAGACGAAG
TAGGTTTATTAGACAAAGTGCAATCTTTCACAAAAGGCATTAACACAATAATAGGTGAAGGAGGCGAAATGTTATCTGGTGGACAGATG
AGACGCATAGAACTTTGCCGTCTTTTAGTTATGAAGCCAGATCTCGTTATATTTGATGAGCCTGCAACTGGTTTAGATATTCAAACAGAAC
ACATGATTCAGAACGTTCTGTTTCAACATTTTAAAGATACAACGATGATTGTCATTGCACATAGAGATAATACAATTCGCCATTTACAACG
ACGCTTGTATATAGAAAATGGAAGACTGATTGCTGATGATCGCAATATTTCAGTAAATATAACAGAAAATGGTGATGACTTATGA

Gene: cydC (cysteine ABC transporter, ATPase subunit 1)

Contig: 05_NODE_4, position: 183312 to 184985, length: 1674 nt, orientation: FORWARD

Perfect match to: (08-02119-CP015645-[1500268:1501941:r], allele observed in ST582+CC1+CC5+CC8)

Sequence:

ATGAAAACACGACTAAAATTTCAAGTAGATAAGGATTTATTGTTAGCTATAGTTGTTGGTGTTTGTGGAAGTTTAGTTGCGCTCGCCATGT
TTTTCTTAAGTGGTTATATGGTGACACAAAGTGCACTTGGTGCGCCACTATACGCTCTGATGATTTTAGTCGTTACAGTAAAATTGTTTGG
GTTTTTAAGAGCTATTACTCGATACGTAGAGCGCCTTATTTCTCATAAAGCTACATTTACAATGCTACGTGATATTCGGGTACAGTTTTTCG
GTAAATTAGTAAATGTCATTCCTAATGTTTACCGTAAACTGAGTTCTAGTGATTTAATTTCACGTATGATTAGTCGTGTTGAGGCATTACAA
AATATATATTTACGTGTTTATTATCCACCAGTCGTCATCGGTTTGACAGCGCTAGTTACAGTCATAGTTTTGGCGTTCATTTCAATCGGCCA
TGCGCTATTGATTATGGTTAGCATGTTGTTTACTTTACTCGTTGTTCCTTGGTTAAGCTCAAAAAAAGCACGTACTTTAAAGAAACATGCA
GCTAATGAACAGGCCCGATTTTTAAATCATTTTTATGATTATAAAGCTGGTATGGATGAACTACGTCGATTTAATCAAATTAATCATTATC
GAGATAATTTGATGGCTAAATTAAATCATTTTGATAAATTACAACTTAAAGAGCAACGCTTTTTAACGATTTATGATTTTATATTAAATATT
ATTGCTATGCTTTCGATTTTTGGTAGTTTAGTTCTAGGATTAATTCAAATTAATGCAGGCCAACTAAATATTATTTATATGACGAGTATAGT
TTTAATGGTCTTAACTTTATTTGAACAAGCTGTACCAATGACAAATGTCGCGTATTATAAAGCGGATACTGACCAAGCATTGCATGATATT
AATGAAGTGATATCTGTACCTTCTACTAATGGAAAAAAACCGTCTTAATGATAAGTATGATGCAACGAACATTTATGAAGTTAAGGACGCT
AGTTTTAAGTATTGGAACCAGCAAACGTATGTGTTGTCGGATATTAATTTTAATGTTAATAGAGGCGAAAAGATTGCGATTGTGGGGCCT
TCTGGTTCAGGAAAAAGTACATTACTACAAATTATGGCTGGGTTATATCAATTAGATAGTGGCTCTATTCGTTTCGAAAATATGGATATGT
TTGAAATAGATGACAAAGATAAGTTTGAATCGTTAAATGTCTTGCTACAATCTCAACAATTATTTGATGGTACAATACGTCAAAATTTATTT
ACCGATGAAAAAGATGAAGCGGTGCAAGCAATATTTAAGCAATTAGATTTAGAACATTTGGCACTAGAACGTCAAATTGACTTAGATGG
TCATACATTATCTGGCGGAGAAATTCAGCGTTTAGCGATTGCAAGGATGTTATTAAAAGATACTGCATCAACATGGATTTTAGATGAACC
AACAACTGCATTAGATAAACAAAATAGTTTAAAAGTTATGGATTTAATTGAAGCACATGCAGAAACATTAATTGTTGCTACACACGATTTA
ACTTTATTGTCACGTTTTGAGACCATCATTGTGATGATAAATGGTAAAATAGTTGAAAAGGGAAACTATCAACAATTACTCGCTAATCAAG
GTGCTTTATGGAATATGATTCAATATAATGCATAA

Gene: mgrA (regulator of autolytic activity)

Contig: 05_NODE_4, position: 185112 to 185555, length: 444 nt, orientation: REVERSE

Perfect match to: (RF122-AJ938182-[700739:701182:r], highly conserved allele)

Sequence:

TTATTTTTCCTTTGTTTCATCAAATGCATGAATGACTTTACCTAATAAGCGATTAAGTTCTTTAACTTCATCTTGAGATAAAGAAGAAGCTG
AAGCGACTTTGTCAGATGCATTACTTAATTCTGGTCTAATAGTTTCACTTTTGTCAGTCAAGTGAATAAATACTTCACGTTGATCGACTTCG
GAACGTTCACGCTTAATTAAGTCTACTTGTTCCATTCGTTTTAATAATGGTGATACTGTACCAGTATCGAGTGCTAATTCAGTTACGACTTT
CTTGACGTTTACAGGAGATTCATCCCATAAAATTGTTAAGACAAGAAATTGTGGGTATGTTAGATTGTACTTCTTAAAAACTTTGTTAGAG
TAGTAGCGATTAACTTGTCTTTGAGCATTGTACAAACTAAAGCATAGCTGTTCTTTTAAATTATGTTGATCAGACAT

Gene: cobW2 (cobalamin  (vitamin B12) biosynthesis protein)

Contig: 05_NODE_4, position: 185782 to 186708, length: 927 nt, orientation: FORWARD

Perfect match to: (MW2-BA000033-[731721:732647], allele observed in CC1+CC25+CC361)

Sequence:

ATGAAAAATAATAAAGATGAAAAAAATAAGAATATCCATAATTAACGGATTTTTGGGTAGTGGTAAAACCACGTTACTGACACATTATATT
AGTGAATTATTAAAAAATGATGAGAAAATTAAAATCATCATGAATGAATTCGGTACTTTTGATATTGATAGCAATAGTATTTCAAATGAAA
TTGAAGTCCATTCATTGATTAATGGTTGTGTTTGTTGCGATCTTAAACAAGAACTTGTCTATGAACTAAAAGCCATTGCTTTAAAAGGGGA
CGTTAATCATGTCATCATAGAAACGACAGGCATTGCGCATCCTTTGGAATTACTAGTTGCATGTCAAGATCCGCAAATCGTTAATTTCTTT

GAAAAGCCGATTATTTATGGTGTATTAGATGCGACTCGATTTTTAGAACGTCATCAATATACCGAAAATACAGTTTCGCTGATGGAAGAT
CAGTTGAAACTAAGTGACATGATTATTATTAATAAAATTGATCTTATAACTGATGACCGTCTTGAGAGAATTGATAAGCAATTAGGTATGA
TTTGTGCAAGTATTCCAACATATAAAACAACCTATGGAAAAGTTTCGTTGGAAGAATTGGACTTAACTGTTAAAGACAGAGAGATATCGT
CTCATCATCACCATCATCATGGGATTAAAAGTATGACTTACACGTTTACAGGTCCGATTGATCGTCAATTGTTTTATCAATTTATAATGAAA
TTACCGGAATCTGTTCTACGTTTGAAAGGTTATGTGTCATTTAGAGATCAACCAAATGCAATTTATGAATTTCAATATGCATATGGTTTACC
AGACTATGGAATAATTGGCATGCAATTACCATTAACGATTGTTATTATTGGTGAAACTTTAGATACAAATCACATACGTAATCAATTGGAT
ATGCTACAATTTACGTAA

Gene: ycsN (putative oxidoreductase)

Contig: 05_NODE_4, position: 186811 to 187719, length: 909 nt, orientation: FORWARD

Perfect match to: (JH9-CP000703-[779163:780071], allele observed in CC5)

Sequence:

ATGGAACAAATAATGATTAATCACTATGTTCATTTTTCTAGGCTTGTACAAGGTTTTTGGCGTGCAAATGAATGGAAGATGACTGCGAAA
GAGTTAAATTATTTTATAAATGAATTAGTTGAACGTGGAATTACAACGATGGATCATGCTGATATTTATGGGGATTATCAATGTGAATCAC
TGTTTGGTAATGCTTTGGATTTATCACCCGAATTAAGAAATAAAATTCAAATTGTTACGAAATGTGGTATCATTTTGCCTTCTAAGCAATTT
GATTTTACAAATGGACATCGTTATGATTTGAGTAGTAAGCACATCGTGAAATCTGTTGAACAGTCATTAATCAATTTGAATGTAGATTATT
TAGATAGTCTACTCATTCATCGTTCTTCACCATTGATGGATCCAGAACAAGTTGCTGATGCATTAACTAAACTTGTTAAACAAGGTAAGTT
GAAGTCATTCGGGGTGTCGAATTTTAATCATTCACAATACCAATTGTTAAATCAATATATTATGAAAGAAAGACTACATATTAGCATCAAT
CAATTAGAATTATCGCCATATCACGTTGATAGTTTACAAGATGGAACAATGGATTCAATGTATCAAAACCATGTTCAAATTATGGCTTGGA
GTCCTTTTGCAGGCGGTAAAATTTTCGACAAGGAAGATATTAAAGCGCAACGTATTATGAAAGTTGTTCAATCAATAGCTGACAAATATG
GTGTGAGTGACACAGCTGTGATGATAGCATGGTTAGTAAAAATACCGCATCGTATCATGCCGATACTTGGAACAAGTCAGTTAAAGCGT
ATTGATCAAGCAATCGAAGGGCTACAACTTAATTTAGATGATCAGTCGTGGTTTGACATTTACACCGCTATTATCGGACAAGATATTCCGT
AA

Gene: Q5HHX9 (putative protein)

Contig: 05_NODE_4, position: 187754 to 188041, length: 288 nt, orientation: FORWARD

Perfect match to: (N315-BA000018-[738059:738346], highly conserved allele)

Sequence:

ATGACAAACGAAGATAAACGTTTCGAACAATTAAGATTTGAACGCAAATTTATAGTTATTCCGTATTTAATTTATGCAGTCATTGTATTACT
ATTAAATATTTTCTATTCTGATTTGAAAATAACAATGACATTATTCGGACTTTTCTTTGCGTATAATGTAGTCATTTTGTTCATAGCATTTAT
TAAACATTATAAACGCACATTGTTACTAAGTCTTATATTAACAGTGCTTAGTGGCGCGGCATTCTTTGGAATTATTTATGTTTATGGCATTA
ATCATTTTTAA

Gene: yflS (putative malate transporter)

Contig: 05_NODE_4, position: 188324 to 189877, length: 1554 nt, orientation: FORWARD

Perfect match to: (04-02981-CP001844-[736270:737823], allele observed in CC5)

Sequence:

ATGTCAGAAGAAAAACATGTAGTGGAACATGAACAACAAAAGAAAGAAAAGACAAAAAAGCAATACAAGCCATTTTGGATTGTCATGA
GTTTTATAATACTTATAGTTGTACTATTACTCCCGGCACCTTCAAGTCTGCCGATAATGGCTAAGGCAGTACTAGCTATTTTAGCTTTTGCA
GTTATTATGTGGGTAACGGAAGCTGTATCATATCCGGTGTCAGCAACTTTAATTATTGGCTTAATGATATTACTTTTAGGATTTAGCCCGG
TTCAAAATTTAGGGGAGAAGCTAGGTAATCCGAAAAGTGGCAGTGCTATTTTAGCTGGAAGTGACCTTCTAGGAACTAATCATGCATTAT
CATTAGCGTTTAGTGGATATGCAACTTCAGCTGTAGCTCTCGTTGCAGCTGCATTATTTTTGGCTGCTGCTATGCAAGAAACGAATTTGCA
TAAAAGACTAGCTCTTTTAGTGTTATCAATTGTTGGTAATAAAACTAGAAATATAGTTATTGGAGCAATTATCGTTTCAATTGTACTTGCAT
TTTTCGTTCCTTCTGCAACAGCTAGAGCAGGGGCAGTTGTACCAATCTTGCTGGGTATGATTGCGGCATTTAAAGTTTCCAAAGATAGCA
AGTTAGCGTCTTTATTAATAATTACTTCAGTACAAGCTGTGTCAATTTGGAATATTGGTATCAAAACGGCGGCAGCACAAAATATCGTAGC
GATTAATTTTATAAACCATCAATTAGGATTTGATGTTTCATGGGGCGAGTGGTTCTTATATGCAGCGCCTTGGTCCATAGTTATGTCCGTA
GCTTTATATTTCATCATGATTAAAGTGATGCCTCCAGAAATTAATACAATAGAAGGTGGTAAAGATTTAATAAAAGAAGAATTGCATAAA
CTTGGCCCCGTTAGCCCACGTGAATGGCGTTTAATTGTTATATCGATGTTATTATTACTGTTTTGGTCAACTGAAAAAGTATTACATCCGAT

TGACTCTGCATCCATTACTATTATTGCTTTAGGTGTTATGTTAATGCCGAAAATTGGTGTCATGACATGGAAACATGTTGAAAATAAAATA
CCATGGGGAACAATTATCGTGTTTGGTGTAGGTATTTCACTAGGGAACGTTCTTTTGAAAACAGGTGCAGCTCAATGGTTAAGTGATCAA
ACTTTTGGTGTTTTAGGTTTAAAACATTTACCTATTATCGCGACAATTGCACTTATCACGCTTTTTAATATATTGATTCATTTGGGCTTTGCG
AGTGCAACAAGTTTATCATCAGCGTTAATACCTGTTTTTATTTCGCTAACCTCTACGTTACACTTAGGAGACCAGTCTATAGGATTTGTTTT
AATTCAACAATTTGTTATTAGTTTTGGTTTCTTATTACCTGTTAGTGCACCTCAAAATATGTTGGCTTATGGCACTGGTACTTTTACGGTTAA
AGATTTCTTGAAGGCAGGTATACCATTGACAATTGTAGGGTATATTCTAGTGATAGTTTTTAGCATGACTTATTGGAAATGGTTAGGTTTG
CTTTAA

Gene: phrB (deoxyribodipyrimidine photo-lyase)

Contig: 05_NODE_4, position: 189963 to 191336, length: 1374 nt, orientation: FORWARD

Perfect match to: (TCH959-AASB02000218-[128612:129985], allele observed in CC7+CC1+CC12+CC15+CC25)

Sequence:

ATGGCAATTGCTGTGTTATTAAATCGAATGTTTCGAATGGAACACAATCCATTATTTGAATATATTTATCAACAAAAAGAAGACATTGATG
CATGTTATTTTATCATTCCGGAAGAGGACATGTCTTCAGCTTCTGATTTGAAAGCACAGTTTTATCGCGGTACTTTGCAGCGCTTTTACCAA
TCGTTGCACGCAGAAAAGCTCACACCTTATGTCATGTCTTATGACGATATCATTTCATTTTGTAAAGAAAACAATATCTCTGAAGTAGTGA
CTGCGGGTGATATTATGAGTTATCATCTTGAAGAATATGATATTTTACATCAACGTTCTTTATTCAATGAAGCACGCATTGCCGTTACTTTG
ATACGTGGGAATCATTACTTTAAAGCGAGTAAAACAATGAATCAACAAGGGGAGCCATACAAAGTTTTTACTAGTTTCTATAAAAAATGG
CGACCTTACTTGAGGCATAGAGACGTATATCACTATGATTTAAAATCATTCGAAGACTTTGTCATTGCATCACCTGATGATTTAGTGTTTG
ATGACATAGCATTTGGATCCTCACAAATAATTGAACAGAATAAATGGCAACATTTTTTAGATCAAGATATACAGAATTACGAAAGCGGAA
GAGACTATTTACCTGAAGTATTAACAAGTCAGCTAAGTGTTGCTTTAGCATATGGATTATTAGATATTATTGAAATTTTTAATGATTTATTG
GCGCGTTATGATGAAGATGAGGCAAACTATGAAGCATTTATACGTGAACTCATTTTTAGAGAATTTTATTATGTGTTAATGACACAGTATC
CTGAAACCTCATACCAAGCTTTCAAACCTAAATATCGACAGATAAAATGGTCGCAAAATGAAGCGGATTTTAATGCATGGTGCGAAGGG
CAAACAGGATTTCCAATCATTGATGCAGCAATAATGGAATTGACACAAACTGGTTTTATGCATAATCGAATGAGAATGGTTGTGTCGCAA
TTTTTAACCAAAGATTTATTTATAGATTGGACATGGGGAGAAAAATTCTTTAGAAAGCACCTTATTGACTATGATGCAGCATCAAATATTC
ATGGATGGCAATGGTCTGCTTCTACAGGTACGGATGCAGTGCCGTATTTTAGAATGTTTAATCCAATAAGACAGAGTGAACGCTTTGATG
CTAAAGCTTTGTATATCAAAACATATCTTCCGATTTTTAATCAAATTGATGCAAAATATTTGCATGATACACAACGCAATGAGTCCAACCTT
TTTGAACAGGGGATTGAATTAGGTAGTCATTATCCAAGACAAATGGTAGATCATCAAGAAAAACGTACACAAGTTTTAGCTACATTTAAA
GCGCTAGACTAA

Gene: Q2G0A5 (putative membrane protein)

Contig: 05_NODE_4, position: 191491 to 191775, length: 285 nt, orientation: REVERSE

Perfect match to: (N315-BA000018-[741796:742080:r], allele observed in CC5+CC1+CC8+CC239)

Sequence:

TTATCTTAGTATGTCCGTAAATAAAGTGAGGTATAGTACGACATACTCTAAAAACGTAGCGAGATAAATATATTTCAATCTAACTTTTATG
TTTTGAGGCACTTGCCATTTAGGATATTGTCGTTCGTAATACGACACTTGTTGTATAAATACACCTAGTCCAAATGGCAGCATCATGAGTA
AGATACTTCTTAAATAACTTAAACCAATATCATGCCATATGTGTCCAATAATCAATTGAAAGACAATGATAGATACTATTAAAACGATTAT
ATTTATTGTCAC

Gene: Q5HHX6 (putative protein)

Contig: 05_NODE_4, position: 191772 to 192389, length: 618 nt, orientation: REVERSE

Perfect match to: (N315-BA000018-[742077:742694:r], highly conserved allele)

Sequence:

TCACTTGTTCAAACGCACTCCTTTTCCAAATAATAGAATTGCTGCTTGCATGACAACCATAAAACATACAAACATAGCAGTTTTAAGCGTTA
GACTTTCTAGAATGTGATTTAGAACATGTAAGGGCTCATTAAAGAAATAAACGGAATGTAAGCGTAAGAAACGACCAATATAAATTCCG
AATCCATTTAAAAACATTAGCACGACAACAATTAATCTATTAAGCCAACGGTGAGAAGTCAATGTTAGTATTTCAAAATAGATTAAAATCA
TCACATAAACCGCTAAGAAGACACCAAGCAGTAAATAGGTAAAGTATTTCCACTCACTTAAATTTAGTCCTGCGTAAAAGTTGAATTGAA
ATTGGTTCAAATGGATTAAATCAGTTACCATATAAAATGTATTTGGTAAAAGTAACACAAATATAAAACTATATATGATAAAGAGTGGCC

ATTCATACTTTTTATTCGGTTTGAATAATCGTAATAATAGACTAAGCTCAAAAGGTATATATGCTAAAAACAAATTTAAAGTCATAAATTG
AAAAATTTTAGTCTCAAAAAGGGAGACGATAAATAAAATTAAAAAGTAAATTCTAGCGATGTATCGAGATTGCAT

Gene: Q5HHX5 (putative protein)

Contig: 05_NODE_4, position: 192575 to 192997, length: 423 nt, orientation: REVERSE

Perfect match to: (N315-BA000018-[742880:743302:r], highly conserved allele)

Sequence:

CTATTGAGGATCAAGTAAATTTAAACCGACTGCATCTTTGAATGAACGTTGAATGTCCAAATCAGTTACAGTAAGAATGATTTCAGCAGTT
GCAGAAACAATTGCTTTAGCGAGATGTCCGCTAAAAGTTTCATATGATTGAGTACCGAAAGTAGCATGCAAATGTGCAAAATGACCATTG
TCTAGACGAGAAATATTACCTAATAAGCTCGTCAATTCCAGTGGCTCAGTAATATGTTTTTCTTCGTATTGTTTCGTTGTTAAATTGAAAAA
TTTTAATACAACGTCATCACATGCACCAATGCCGCTGACAGATGTAAATGTTAAGTCTTGGTCATCTGCAAAGGTTGTTATACATTCAACG
ATATCTTCTCCTTTTTCCAACACTAGTAGTATAGTATGATTACTTTTTTGCAATTTCAT

Gene: norA (multidrug efflux pump)

Contig: 05_NODE_4, position: 193206 to 194372, length: 1167 nt, orientation: FORWARD

Perfect match to: (COL-CP000046-[775963:777129], highly conserved allele)

Sequence:

ATGAATAAACAGATTTTTGTCTTATATTTTAATATTTTCTTGATTTTTTTAGGTATCGGTTTAGTAATACCAGTCTTGCCTGTTTATTTAAAA
GATTTGGGATTAACTGGTAGTGATTTAGGATTACTAGTTGCTGCTTTTGCGTTATCTCAAATGATTATATCGCCGTTTGGTGGTACGCTAG
CTGACAAATTAGGGAAGAAATTAATTATATGTATAGGATTAATTTTGTTTTCAGTGTCAGAATTTATGTTTGCAGTTGGCCACAATTTTTCG
GTATTGATGTTATCGAGAGTGATTGGTGGTATGAGTGCTGGTATGGTAATGCCTGGTGTGACAGGTTTAATAGCTGACATTTCACCAAGC
CATCAAAAAGCAAAAAACTTTGGCTACATGTCAGCGATTATCAATTCTGGATTCATTTTAGGACCAGGGATTGGTGGATTTATGGCAGAA
GTTTCACATCGTATGCCATTTTACTTTGCAGGAGCATTAGGTATTCTAGCATTTATAATGTCAATTGTATTGATTCACGATCCGAAAAAGTC
TACGACAAGTGGTTTCCAAAAGTTAGAGCCACAATTGCTAACGAAAATTAACTGGAAAGTGTTTATTACACCAGTTATTTTAACACTTGTA
TTATCGTTTGGTTTATCTGCATTTGAAACATTGTATTCACTATACACAGCTGACAAGGTAAATTATTCACCTAAAGATATTTCGATTGCTAT
TACGGGTGGCGGTATATTTGGGGCACTTTTCCAAATCTATTTCTTCGATAAATTTATGAAGTATTTCTCAGAGTTAACATTTATAGCTTGGT
CATTATTATATTCAGTTGTTGTCTTAATATTATTAGTTTTTGCTAATGGCTATTGGTCAATAATGTTAATCAGTTTTGTTGTCTTCATAGGTTT
TGATATGATACGACCAGCCATTACAAATTATTTTTCTAATATTGCTGGAGAAAGGCAAGGCTTTGCAGGCGGATTGAACTCGACATTCAC
TAGTATGGGTAATTTCATAGGTCCTTTAATCGCAGGTGCGTTATTTGATGTACACATTGAAGCACCAATTTATATGGCTATAGGTGTTTCA
TTAGCAGGTGTTGTTATTGTTTTAATTGAAAAGCAACATAGAGCAAAATTGAAAGAACAAAATATGTAG

Gene: yedL (putative N-acetyltransferase)

Contig: 05_NODE_4, position: 194665 to 195117, length: 453 nt, orientation: FORWARD

Perfect match to: (MW2-BA000033-[740604:741056], allele observed in CC1+CC7+CC188)

Sequence:

ATGACAGTAAAAAATTTATTTTTAGGCTTTGTTGCTGTAATATTAACCGTTTGTTTAATTGGTTTATTAATATTAGCAACAAATGAAGATGC
GCTTGCTAAGGTACATAAAACAATTAATACGCTTAACGCGATAAATGTATCAACTGAAGATACTTATAAAAAGAAAATGGATATTCTCAA
TATTCATACTGCTAAAGCATCTGAAGTGAATGAAAATATGAAAAAGCAAAATCATTTTAAACATCGTGTGAATGCAAATAAATCAAATTCT
TTTAACGAACAAGAGTGCCAAGTTATTGCTGATCGTTATGCAGATAAGCATATCAATGATAATTATGGTTTAGAAAGAATTTCTAAGACA
AATCATGGATATAATTATGTTTATTCCAATGATAATTCAACTAGTAAGCAACATGTAAGTATTTCAAATCAAGGCATAATAACGAAATAA

Gene: ybaK (transcriptional regulator)

Contig: 05_NODE_4, position: 195290 to 195772, length: 483 nt, orientation: FORWARD

Perfect match to: (MW2-BA000033-[741229:741711], highly conserved allele)

Sequence:

ATGGCTAAAAATAAGAAAACGAACGCGATGCGTATGCTTGATCGTGCAAAAATTAAATACGAAGTTCATAGCTTTGAGGTACCAGAAGA
ACATTTATCTGGTCAAGAAGTCGCAGAACTCATACAAGCAAATGTTAAAACAGTATTTAAAACGCTTGTTCTAGAAAATACAAAACATGA
ACATTTTGTATTTGTTATCCCAGTAAGTGAAACTTTAGATATGAAAAAGGCAGCTGCTTTGGTTGGAGAGAAGAAATTGCAGCTTATGCC
TTTAGATAATTTGAAAAATGTAACGGGATACATTCGTGGTGGGTGTTCGCCTGTTGGTATGAAAACATTGTTTCCAACAGTCGTTGACAA
ATCGTGTGAAAATTATAGTCATATCAGTGTGAGTGGTGGGCTTCGAACAATGCAAATCACAATAGCTGTTGAGGATTTGATTACAATAAC
TAAAGGCAAAATTGGAGCAGTTATCCATGAATGA

Gene: fruR (transcriptional regulator of fructose operon)

Contig: 05_NODE_4, position: 196025 to 196786, length: 762 nt, orientation: FORWARD

Sequence:

ATGATAATTACAGAAAAAAGACACGAGTTAATATTAGAAGAACTTTCGCACAAAGATTTTTTGACTTTACAAGAACTAATAGATCGAACT
GGATGCAGTGCTTCAACAATACGAAGAGATTTATCTAAACTACAACAATTAGGGAAATTGCAACGTGTGCATGGTGGTGCAATGTTAAA
AGAAAATCGTATGGTTGAGGCGAATTTAACTGAAAAATTAGCAACGAATCTTGATGAAAAGAAAATGATTGCTAAAATAGCAGCTAATC
AAATCAACGATAATGAATGCTTATTTATCGATGCTGGTTCATCTACATTGGAGCTAATTAAATATATTCAAGCGAAAGATATCATTGTGGT
AACCAATGGTTTAACACATGTAGAAGCTTTACTTAAAAAAGGTATTAAAACAATTATGCTAGGTGGTCAAGTTAAAGAAAATACACTCGC
TACGATTGGTTCTAGTGCTATGGAGATATTAAGACGATATTGTTTCGATAAAGCTTTTATCGGGATGAATGGATTAGATATTGAACTTGG
ATTAACTACTCCCGATGAGCAAGAGGCATTAGTTAAACAAACAGCAATGTCATTAGCCAATCAATCATTTGTACTTATAGATCATTCTAAG
TTTAATAAAGTATATTTTGCTCGTGTACCTTTGCTAGAAAGTACGACAATCATCACATCTGAAAAAGCATTAAATCAAGAATCGTTAAAAG
AATACCAACAAAAGTATCACTTTATAGGAGGGACTTTATGA

Gene: fruB (fructose 1-phosphate kinase)

Contig: 05_NODE_4, position: 196783 to 197703, length: 921 nt, orientation: FORWARD

Perfect match to: (MW2-BA000033-[742722:743642], highly conserved allele)

Sequence:

ATGATTTATACAGTGACTTTCAATCCTTCAATTGACTATGTCATTTTTACGAATGATTTTAAAATTGATGGTTTGAACAGAGCAACAGCAAC
ATATAAATTCGCTGGGGGGAAAGGTATTAATGTCTCGCGCGTCTTAAAGACATTGGATGTTGAGTCAACTGCCTTGGGATTTGCAGGTG
GATTTCCTGGGAAATTCATTATAGATACATTAAATAACAGTGCAATTCAATCGAATTTTATTGAAGTTGATGAAGATACACGTATTAATGT
GAAATTAAAAACAGGACAAGAAACAGAAATCAATGCACCGGGTCCTCATATAACGTCAACACAATTTGAACAACTGTTACAACAAATTAA
AAATACAACAAGCGAAGATATAGTTATTGTTGCTGGAAGTGTACCAAGTAGTATTCCAAGCGATGCGTATGCGCAAATTGCACAAATTAC
AGCACAGACAGGTGCTAAATTAGTAGTCGACGCTGAAAAAGAATTGGCTGAAAGCGTTTTACCATATCATCCACTATTTATTAAACCTAA
TAAAGATGAATTAGAAGTGATGTTTAATACAACAGTGAACTCAGACACAGATGTTATTAAATATGGTCGTTTGTTAGTTGATAAAGGTGC
GCAATCTGTTATTGTCTCGCTTGGCGGTGATGGTGCTATTTATATTGATAAAGAAATCAGTATTAAAGCAGTTAATCCACAAGGGAAAGT
GGTTAATACAGTTGGCTCTGGTGATAGTACAGTTGCAGGCATGGTGGCTGGAATTGCTTCAGGTTTAACGATTGAAAAAGCATTCCAACA
AGCAGTCGCATGCGGTACTGCCACGGCATTTGATGAGGACTTAGCAACACGGGACGCTATAGAAAAAATAAAATCACAAGTTACGATTA
GCGTACTTGATGGGGAGTGA

Gene: fruA (fructose specific permease)

Contig: 05_NODE_4, position: 197709 to 199667, length: 1959 nt, orientation: FORWARD

Perfect match to: (MW2-BA000033-[743648:745606], allele observed in CC1+CC239)

Sequence:

ATGAGAGTAACAGAGTTATTAACAAAAGATACAATAGCAATGGATTTAATGGCAAATGACAAAAATGGTGTTATTGATGAGTTAGTAAA
TCAATTAGACAAAGCAGGTAAATTAAGTGATGTCGCGTCATTTAAGGAAGCGATTCACAATCGAGAATCACAAAGTACAACTGGTATCG
GCGAAGGTATTGCCATTCCACATGCCAAAGTGGCCGCAGTTAAGTCACCAGCTATTGCGTTTGGTAAATCTAAAGCAGGCGTAGATTATC
AAAGTTTGGATATGCAACCAGCACACTTATTCTTTATGATTGCAGCGCCAGAAGGTGGCGCCCAAACACATCTAGATGCTTTAGCTAAGT
TGTCTGGTATTTTAATGGATGAAAATGTACGTGAGAAATTATTACATGCTTCATCACCTGAAGAAGTACTAGCGATCATAGATGAGGCTG
ATGATGAAGTGACAAAAGAAGAAGAGGCAGAAGCTGAAGCACAACAAGTTGCAACTGCAGAACAATCATCTAAACAATCTAATGAGCC
ATATGTGTTAGCAGTAACTGCTTGTCCAACAGGTATTGCACATACGTACATGGCACGTGACGCATTGAAAAAACAAGCGGATAAGATGG

GTATTAAAATTAAAGTAGAAACGAATGGTTCAAGCGGCATTAAAAACCATTTAACTGAACAAGATATTGAAAATGCAACAGGTATCATTG
TTGCTGCTGATGTTCATGTTGAGACGGATCGCTTCGATGGTAAAAATGTCGTAGAAGTACCAGTAGCAGATGGTATTAAACGCCCAGAA
GAATTAATTAATAAAGCATTAGATACAAGTCGTAAACCTTTTGTTGCCCGTGATGGTCAAAGAAAAGGTAACTCAAATGACAGTCAAGAA
AAATTAAGCCCAGGTAAAGCATTCTATAAACACTTAATGAACGGTGTTTCTAACATGTTGCCACTTGTAATATCTGGTGGTATTTTAATGG
CAATCGTATTTTTATTTGGAGTAGACTCATTTAAACCAAAAAGCTCAGAGTACAATGCGTTTGCAGAGCAGCTATGGAACATTGGTAGTA
AAAGTGCATTCGCGTTAATCATTCCAATTTTATCTGGATTCATTGCACGTAGTATTGCGGATAAACCTGGTTTTGCAGCTGGTCTTGTAGG
TGGTATGTTAGCAATTTCAGGTGGTTCAGGATTTATTGGTGGTATTATTGCAGGTTTCTTAGCAGGTTACTTAACACAAGGTGTTAAAGC
GATGACACGTAAGTTACCACAAGCATTAGAGGGATTAAAACCAACATTAATTTACCCACTATTGACAGTGACAGCTACAGGCTTATTGAT
GATTTATGTCTTTAATCCACCAGCATCTTGGTTAAATCATTTGTTATTAGATGGATTAAACAATTTATCAGGTTCTAATATTGTATTATTAGG
TTTAGTTATTGGCGCTATGATGGCAATTGATATGGGCGGTCCATTCAACAAAGCGGCATATGTTTTTGCAACAGGTGCGTTGATTGAAGG
TAATGCAGCACCAATTACAGCTGCAATGATTGGTGGTATGATTCCACCGTTAGCAATTGCGACAGCGATGTTAATTTTCAGACGTAAATTT
ACAAAAGAACAACGTGGTTCAATTATCCCTAACTATGTGATGGGTATGTCCTTTATTACAGAAGGTGCGATTCCATTTGCAGCTGCCGATC
CATTACGTGTTATTCCTTCAATGATGATTGGTTCAGGTATAGGTGGCGCAATTGCTTTAGGCTTAGGTTCACGAATTACTGCGCCACATGG
TGGTATTATTGTAATTGTTGGTACGGATGGTGCACACTTACTTCAAACTCTTATTGCACTTCTAGTTGGTACATTAGTTTCAGCATTAATTT
ACGGTTTAATCAAACCCAAGTTAACTGAAACAGAAATCGAAGCTTCAAAATCAATGGACGAGTAG

Gene: nagA (N-acetylglucosamine-6-phosphate deacetylase)

Contig: 05_NODE_4, position: 199975 to 201156, length: 1182 nt, orientation: FORWARD

Sequence:

GTGTCAGAATTAATTATATATAACGGCAAAGTTTATACTGAAGATGGCAAAATCGATAATGGTTACATTCATGTGAAAGATGGACAGATT
GTTGCAATTGGAGAAGGGGATGATAAAGCAGCAATTGATAATGATACGACAAATAAAATTCAAGTGATTGATGCTAAAGGTCATCATGT
ATTACCAGGTTTTATTGATATACATATTCATGGTGGTTATGGTCAAGATGCGATGGATGGGTCATACGATGGCTTAAAATATCTATCCGAA
AATTTGTTATCTGAAGGGACGACATCATACTTGGCCACTACAATGACGCAATCTACTGATAAAATAGATAAAGCACTTACAAATATTGCTA
AATATGAAGCGGAGCAAGATGTTCACAATGCAGCGGAAATTGTAGGTATACATTTAGAAGGACCATTTATATCTGAAAATAAAGTTGGT
GCTCAACATCCGCAATACGTTGTACGCCCATTTATCGATAAAATTAAACATTTTCAAGAGACTGCTAACAGATTAATAAAGATTATGACGT
TTGCACCTGAAGTTGAAGGTGCAAAAGAAGCGCTTGAAACGTATAAAGATGACATTATTTTTTCAATTGGTCATACAGTGGCAACATACG
AAGAAGCAGTCGAAGCTGTTGAGCGAGGAGCTAAACATGTCACGCATTTATATAATGCAGCGACGCCATTCCAGCATAGAGAACCAGGT
GTTTTTGGAGCAGCATGGTTGAATGATGCTCTACATACCGAAATGATTGTTGATGGCACACATTCTCATCCGGCATCGGTTGCTATTGCTT
ACCGTATGAAAGGTAATGAACGTTTTTATTTAATTACCGATGCAATGCGTGCAAAAGGTATGCCTGAAGGAGAATATGATTTGGGTGGA
CAAAAAGTAACTGTTCAATCGCAACAAGCACGTCTTGCAAATGGTGCGCTTGCTGGTAGTATTTTAAAAATGAATCATGGGTTACGTAAC
TTAATATCATTTACAGGTGATACATTAGATCATTTATGGCGAGTAACAAGTTTAAATCAAGCCATTGCATTAGGTATCGATGATAGAAAAG
GTAGTATTAAAGTAAATAAGGATGCAGATCTTGTTATTCTAGATGATGATATGAATGTAAAATCTACAATAAAACAAGGTAAGGTTCACA
CATTTAGCTAA

Gene: corC-mpfA (magnesium protection factor A)

Contig: 05_NODE_4, position: 201377 to 202726, length: 1350 nt, orientation: FORWARD

Sequence:

TTGGAAACTTCGACCATAATTAGTTTGATTATATTTATTCTATTAATTGCATTAACCACTGTATTTGTTGGTTCAGAATTTGCATTAGTAAAA
ATTAGAGCAACAAGAATTGAACAGCTAGCAGATGAAGGAAATAAACCTGCTAAAATAGTAAAAAAGATGATTGCTAATCTAGATTATTA
TCTTTCTGCTTGTCAGTTAGGTATAACAGTAACATCTTTAGGGTTAGGTTGGCTTGGTGAACCAACGTTTGAAAAGCTATTACACCCAATA
TTTGAAGCAATCAATTTACCAACTGCATTAACGACGACGATTTCGTTTGCAGTGTCATTTATAATCGTTACGTATTTGCATGTAGTACTTGG
TGAATTAGCGCCTAAATCTATAGCGATTCAACATACTGAAAAGCTTGCTTTAGTATATGCAAGACCATTGTTCTATTTCGGTAACATTATG
AAACCACTGATTTGGCTGATGAATGGTTCTGCACGTGTTATTATTAGAATGTTTGGTGTAAATCCTGATGCCCAAACTGATGCAATGTCAG
AAGAAGAAATCAAAATTATTATTAACAATAGTTATAATGGTGGAGAAATCAACCAAACTGAATTGGCATATATGCAAAATATCTTTTCATT
CGATGAAAGACATGCAAAAGATATAATGGTACCTAGAACTCAAATGATTACACTAAATGAACCTTTTAATGTAGACGAATTACTAGAAAC
AATAAAAGAACATCAATTTACGCGTTATCCAATTACTGATGATGGTGATAAAGACCACATTAAAGGATTTATTAACGTCAAAGAATTTTTA
ACTGAATACGCTTCTGGAAAAACGATTAAAATAGCAAACTATATACATGAGTTGCCAATGATTTCAGAGACAACACGTATCAGTGATGCA
TTAATTAGAATGCAACGTGAACATGTACATATGAGTCTTATTATAGATGAATATGGTGGAACGGCAGGTATTTTAACGATGGAAGATATT
TTAGAAGAAATCGTTGGAGAAATTCGTGATGAATTTGATGATGATGAAGTGAATGATATCGTTAAAATTGATAATAAGACATTCCAAGTA
AATGGCAGAGTACTATTGGATGATTTAACTGAAGAGTTCGGTATAGAATTTGATGACTCTGAGGATATTGATACGATAGGTGGATGGTT
ACAATCTCGTAATACCAATTTACAAAAAGATGATTACGTGGATACAACTTATGATCGCTGGGTTGTTTCAGAAATCGATAACCACCAAATT
ATTTGGGTGATATTAAACTATGAATTTAATGAAGCGAGACCTACTATCGGACAGTCTGATGAAGATGAAAAATCAGAATAG

Gene: yvgN1 (glyoxal/methylglyoxal reductase, locus 1)

Contig: 05_NODE_4, position: 202948 to 203787, length: 840 nt, orientation: FORWARD

Perfect match to: (MW2-BA000033-[748887:749726], highly conserved allele)

Sequence:

ATGTTGAATGAGATACAAATATTAAATAATGGATACCCGATGCCTTCAGTTGGATTAGGTGTTTATAAAATCTCTGACGAAGATATGACT
AAAGTTGTAAATGCTGCAATTGACGCAGGCTATAGAGCGTTTGATACAGCATACTTTTATGATAATGAGGCTTCACTAGGACGAGCATTA
AAGGATAATGGCGTCGATAGAGAAGATTTGTTTATAACAACGAAGTTATGGAATGACTATCAAGGTTATGAGAAAACATTCGAATATTTC
AACAAATCGATTGAAAATTTACAAACTGATTATCTTGATTTATTTCTAATACATTGGCCTTGTGAAGCAGATGGTCTATTTTTAGAAACATA
TAAAGCTATGGAAGAACTTTACGAGCAAGGTAAGGTAAAAGCAATAGGTGTATGTAATTTTAATGTTCATCATCTAGAAAAATTAATGGC
TCAATCAAGTATCAAACCAATGGTGAATCAAATTGAGGTACATCCATATTTTAACCAACAAGAATTACAAGAATTTTGTGATCGTCACGAT
ATTAAAGTGACTGCATGGATGCCTTTGATGAGAAATAGAGGACTACTAGACAACCCTGTCATTGTTAAAATTGCTGAAAAATATCATAAA
ACACCAGCACAAGTTGTATTACGTTGGCATTTAGCACACAATAGAATTATTATTCCAAAATCTCAGACACCTAAACGCATTCAAGAAAATA
TAGATATTTTAGATTTTAATTTAGAATTAACAGAAGTAGCTGAAATTGATGCTTTAAATAGAAATGCAAGACAAGGTAAAAATCCAGATG
ATGTGAAAATTGGGGATTTAAAATAA

Gene: csbB (putative glycosyl transferase)

Contig: 05_NODE_4, position: 203919 to 204902, length: 984 nt, orientation: FORWARD

Perfect match to: (MW2-BA000033-[749858:750841], allele observed in CC1+CC12+CC25+CC239+CC772)

Sequence:

ATGAAAATTAGAGTCGTCATTCCTTGTTTTAATGAAGGGGAAGTCATTACACAAACACATCAACAATTAACTGAAATACTTTCACAAGATA
GTAGTGTGAAAGGCTATGATTATAATATGCTTTTCATAGATGATGGTAGTACGGATACCACTATAGATGAAATGCAACATCTTGCCACAA
TAGATAGGCATGTCAGCTTTATTTCTTTTAGTAGAAATTTTGGAAAAGAAGCAGCTATGATTGCAGGTTACCAGCATAGTACTGAATTTGA
TGCAGTCATCATGATAGATTGTGATTTGCAACATCCACCTGAATATATTCCGAAAATGGTTGAAGGTTTTATGGAAGGCTATGATCAAGT
GATTGCAAAGCGTGATAGAAGTGGTGAAAATTTTAGTCGCAAAACATTAAGCCATTTGTATTATAAGTTAGTTAATTGCTTTGTAGAAGA
AGTACAATTTGATGATGGTGTTGGTGATTTTAGACTTTTAAGCCAAAGAGCTGTTAAATCCATTGCATCACTTGAAGAATATAATCGATTT
TCAAAAGGGTTATTTGAATGGATAGGCTATAATACTAAAGTGTTTACGTATCAAAATGTTGAGAGACAAAAAGGGGAATCTAAGTGGTC
CTTTAAAAAGTTATTTAATTATGGTATTGATGGATTGATTTCCTTTAATAGTAAACCTTTGAGAATGATGATTTATCTTGGCTTGTTTATCTT
TTCAATAAGCGTGCTATATATTATCTATTTATTCATCAATATTATGATATCTGGTGTTAATATTCCGGGATATTTTTCAACGATTGCAGCTAT
TTTATTATTAGGCGGCATACAGTTAATTTCAATTGGTGTTGTAGGTGAATATATTGGCAGGATATATTATGAAGTTAAGGCACGTCCTAAA
TATATTATTCAAGCTACAAATCTTTCAAGTATTGAAAATGATGAGAAGGATACCCATAAAGTTTATTCTAAATAA

Gene: saeS (histidine protein kinase, sae locus)

Contig: 05_NODE_4, position: 204974 to 206029, length: 1056 nt, orientation: REVERSE

Perfect match to: (RF122-AJ938182-[720527:721582:r], highly conserved allele)

Sequence:

TTATGACGTAATGTCTAATTTGTGTAATGTTACAGTCATCGTAGTTCCTACATCTATATCACTGCTTACACTGATTTTTGCGTTATTTTGTTG
CGCGAGTTCATTAGCTATATATAAGCCTAATCCAGAACCACCCGTTTTTGTATTACGAGAGTTTTCTACTCTGAATGTACGTTCGAATATAC
GTTCTTGTAGTTCTGGTATAATGCCAATACCTTCATCGCTAATAGCAATGTCGATAGTATCTTGATCTTTGTTTTCACTAATATTAATATCAA
TGCGACTACCAACATTTGAAAATTTTAGCGCATTATCAAGTAAGTTTGTTAAAATACGCTCAAGTGGCGTTCGATATTGATAAAATGCATC
AATTTCGCTACAGAAATTCACTTCTAATGTGCGGTTTTCATGTTTGATACGTTGCTCATATGGTTGCAATATTGATACAAGTAATTGGTCTA
GTTGTATTAATTCTGGGGGATATGTTTTACCTGTATTTAAAGTGATAATATGAGTCATATCATCAAATAATGTTGATAATCTGTTTGCTTGT
TTAATTAATATGTCGTATGACTCTTTAATCTCATGATCCTTTGTGATTATACCATCACGTAGTCCTTCAGAATATGAAATAATGCTTGCTAA
AGGTGTTTTTAAATCATGGGCTAAGTTTTGAATCAGTTCTGTTTTTTCTTGTTGTTCGGATTTAATTTGATTCATTTGTTGCGTAATTTCAGA
AGCCATTTTATTAAAAGATTGATTTAATTCATAAATTTCTTTTGGTGAATTAAACGTTTTATCATTGCTTGCGTAATTTCCGTTAGCAAATTG
CTTAGTTTTTATATTAAACTGCTTAATTTTTTGTATAAGTGGATTAATAAAAATACTACATATTAATAAGGTTAAACAGCTTGTAATTATTGT
CGTTAAGGTCAAAGTTAGTGTCATATGGCCGTTAAACCACATTAAAATATATGCAATTGCTAAAATAGTTGAAGTTAATAGTATACTCGAT
ACGACGCCAATAATGATTTGACTTCTAATTGATAACACCAT

Gene: saeR (response regulator, sae locus)

Contig: 05_NODE_4, position: 206029 to 206715, length: 687 nt, orientation: REVERSE

Perfect match to: (RF122-AJ938182-[721582:722268:r], highly conserved allele)

Sequence:

TTATCGGCTCCTTTCAAATTTATATCCTAATCCCCATACAGTTGTGATGGTATATGTTGTAAAGCTCTCTTTTTCTAATTTTTCTCTAATACG
GTGTATATGGACATTCACGGTATTAGCATCTTCGTAATAGTCATATCCCCAAACTTTTTCAAGTAATTCTGATTTAGAAATAACTTCATTTTC
TCTAGAAGCTAAATACCACAATAACTCAAATTCCTTAATACGCATAGGGACTTCGTGACCATTTACAGTCACAACTTTACTTAAGTTAATAA
GTGTTAATTCATCAAACGACAGTTGTTCAACTGGTTGATGATGGTATTTCTTCATTCTTGTAAGTAAATTATTAATACGTAAAACGAGTTCC
CTTGGACTAAATGGTTTTTTGACATAGTCATCTGCACCTAAAGTTAAGGCGTAAATGGTATCATGTTCTTGTGTTTTGGCAGTTAAATAGA
TAAAGGGGATATCTAATTTTTGCCTTTTCATTTCTTTGACAATGTCGTAACCATTAACTTCTGGCATCATGATATCAAGTACCATGATATCA
ATATCATTTGATAGTAAAGAAATTGCTTCTTTACCGCTAGTTGTCGTTGTTACTTTGTAACCTTCATATTCAAAATAGGTTTGACAAATGTC
TACAATGTCTTGTTCATCATCCACGATCAGTAAGTGGGTCAT

Gene: saeQ (putative protein)

Contig: 05_NODE_4, position: 206690 to 207163, length: 474 nt, orientation: REVERSE

Perfect match to: (MW2-BA000033-[752629:753102:r], highly conserved allele)

Sequence:

TCATCCACGATCAGTAAGTGGGTCATCTATTTTTTCACCTCTGTTCTTACGACCTCTAAAGTAATTAATGATTTCTTTAAGTGAAATCTGTTT
TAACAATGAGTGACTCATTAGTAAAAGGATAAAGAAAGTTAATTGAAGAGGATACGTAAATATCATATCTGCTAAGATATAATTTATCAT
AACAAAGGCTCCAAAGAAACTAGCAGCATATGCAAAAACTCCAAAAATTAAACCTAATCCAATTGCAATCTCTCCGAGTGGGACAACAAT
ATCAAATAATGACGTCGTATGTGCAACTATATTTGCGAAAAACCACTTATACCACTCTGGTGAATCAGTATTGTTAGCGATGACTGGTACT
AAACCTTTCAGCGTAAATCCGCCCGTTAATTTTTCGTAGCCTTGCATTAACATAACAATACCTGAACCCACACGAATGATAAATGTAACGA
GTAGCAATAATTTATTCAT

Gene: saeP (putative lipoprotein)

Contig: 05_NODE_4, position: 207506 to 207946, length: 441 nt, orientation: REVERSE

Perfect match to: (N315-BA000018-[757812:758252:r], highly conserved allele)

Sequence:

TTATTTTAATTTAGCGCCGCCGAAGATGACGTGAGCTTTTTTACAATAGATTGTTACTTCATCATATTTGCTTAAATCTACATTTTTAAGATC
AAATGTTTGTTTTTCTTTATCGTAGTCAACCATTGCGATTTCTTTACCGTTTTTAATGTCGCCATTTTTTGTTAGGTAGACGTATAAATCTGG
ACCTTTTGATGATTTGTAGTTAGTAAGCATTAATTTACCATTTTTAATCTCAGCTTTACCTTCAACAGTTTCACCGTTTTTAGAACTGAATGT
ACCTGTTAGGTGTTTTGTTTTATCAGTTTTAACATTGCTATCTTCTGACTTTGTTTTTTGTTCAGTTTTGTTACCTTGATCTTGTGAATTAGAA
TTACCACAAGCGCCTAAAGTTAATACAGAGGCAACAGCACCAACTGCTAAAAAATATTTTGTATTCAT

Gene: Q5HHW1 (putative membrane protein)

Contig: 05_NODE_4, position: 208226 to 208810, length: 585 nt, orientation: REVERSE

Perfect match to: (N315-BA000018-[758532:759116:r], highly conserved allele)

Sequence:

TTAAAATTTATAAACCTTTTGCCACAATAAAAACAACAAGAAAATAAGATAGACGATTAAATACAAGTACCAATATGGTGTTAAAACCATC
ATAACGATAAATGAAATGTTAATAATGATAAGTCCTTTAATCATTAGTTTCTTTATTTTGTTATTATTTTCAGCAGTTTCATCTAAGCTATTA
AATGTTGAAATAAACAACATAATACCACCAATGATGAAAATTGCAGGGGCTAAAAATGGAATAGACATATATATTGATTGTGTACTTGAA
AAGTCGGTAACACGTTGCGTTAAACTGACAACAATCGTTAATATGATAGCAATAAATTTTTGGTCTGTAATGTATACAGGTTTTAATTTTTT
ATCAATGTAAAACTGTAGTATCGTCCAAATGATAAACATCGTTATAGTACACAGAGTAATAAAGTTATATTTATCAATTATGAATGTATTT

ATAAAGGCGTTTATCGTAATAGATAACAACATTGCACTTAATGATAAATGTTGCATTTGATGCCTATATAAATTGAATCCAGTTATAAGTA
CGACGATAGCAATATTAATTAATATATATATAAAATCAT

Gene: queE (7-cyano-7-deazaguanosine biosynthesis protein E)

Contig: 05_NODE_4, position: 208909 to 209622, length: 714 nt, orientation: REVERSE

Perfect match to: (N315-BA000018-[759215:759928:r], highly conserved allele)

Sequence:

TTATACACCTTTTTTATTACTCCAAAGTAATGTATGAAGTTGTGGTAACACATAAACGTGATTCATATCATTACTTTGCATAACTAAATCCA
CCAACTGCTCGTAGCGTTCTAACAACTTTTCGGTATGATTATCTACGCTGTCTGATAAATATGGGTTACCAACTTGTAAATAGAAGGGAAT
ATCTGGATAACGGTGGTGTATCATTTTGGCAAAATCATAATCTTTATCGTCGAATACAACTACTTTTAAGTTTAATGAGGAAGGTACGCAT
TGTGTAATCACTTCATCTAACTTTTTTAAATCAGGTGTCATAGTTGAACTTGGTGGTTTTGGACTAATCGTTAAATCATCAATTTGTGTCAT
CCAAGGTTGGAATTTACTGCCTTGTGTCTCCAGTGCGCTGAAAATACCTTTATCTTGAAATAAGTCAACTAACTCTTGGATACCTTTAATTA
ATGCTGGGTTACCACCAGAAATTGTAACGTGATTAAATAAATCGCCACCAATTCGTTTTAATTCATCATAAATTTCTTCAGCGGTCATGAG
TTTTATATCGCCTTTAGCACTACCATCCCAAGTAAATGCAGAATCACACCAGCTACAGCGATAATCACATCCAGCTGTTCTCACAAACATC
GTTTTTCTACCGATTACTCGACCTTCACCCTGAATGGTTGGACCGAATATTTCGAGTACAGGAATTTTAGCCAT

Gene: queD (queuosine biosynthesis protein D)

Contig: 05_NODE_4, position: 209626 to 210045, length: 420 nt, orientation: REVERSE

Perfect match to: (MW2-BA000033-[755565:755984:r], allele observed in CC1+CC188)

Sequence:

TTACACCTGTTCCTTTGGTCTAAATACAACATAACTTGTTGGTGTTTCTCTTACAAATACTTGAATACATTTTGGTTGGTGTTCGAGCGATG
CCAAATTTTCTTTAACAATTTGATAAATTGTTTCCGCTACGATTTCAGTTGAAGGGATTTTGTTTTTAAAAGCAGGTAAGTTATTTAACAGT
TGATGGTCAAATTTACCGTGTATCATCTTTTTCAAATGGCTAAAGTTCACTAAGAAGCCAGTGTCATCTAGTTTATCACCGACAATTGTTAA
ATTAACAAAGTAAGTATGACCATGGACATTTTGACAAATACCTGCTTCTTCACAAGGAATGTGATGTGCAGCCGAAAAATTAAAGTCTTT
ATTTAATTCGAATTGATATGGATGCGTTGTACTAGGATAGATTTGTTGTAACAT

Gene: queC (7-cyano-7-deazaguanine synthase)

Contig: 05_NODE_4, position: 210047 to 210715, length: 669 nt, orientation: REVERSE

Perfect match to: (MW2-BA000033-[755986:756654:r], highly conserved allele)

Sequence:

TTAAAGCGCTCCTTTACTTTCAAGATATTGATTTAGTCCACGTTGACGTAAATGACAAGCTGGACATTCACCACAGCCATCCCCAATGATA
CCGTTATAGCATGTTAATGTTTTTGTACGAATATAATCTAAAACTTCGAGTTCATCACTTAATTTCCACGTTTCTGCTTTGTTTAACCACATT
AAAGGAGTATGAATGACAAAATCTTTGTCCATAGCTAGGCTTAATGTTACGTTCATTGATTTTATAAAACTATCGCGACAGTCTGGGTAGC
CTGAAAAGTCTGTTTCACATACGCCTGTAATAATATGCTTAGCCCCAATTTGATAAGCTAGAGCGCCTGCAAACGACAAGAAAAGTAAAT
TTCTAGCTGGAACAAATGTATTAGGTATACCATCTTCATTATTAGTAATTTCCATATCATGTTGTGTTAATGCGTTTGGAGTAAGTTGTGAT
AATAATGACATATCTAAAACGTGATGTTTCATTCCTTGATCTTGTGCAATTTGTTTTGCGACTTCAATTTCAGTATCATGTCTTTGGCCATAA
TTAAACGTTACGAGTTCAACTTCTTTGAAATGTTTTTTTGCATAAAAGAGACATGTTGTACTGTCTTGACCACCACTAAAGACAACGATGG
CTTTTTCATTATTTAATACACTTTCCAT

Gene: pabA (putative para-aminobenzoate synthase, glutamine amidotransferase, component II)

Contig: 05_NODE_4, position: 211066 to 211659, length: 594 nt, orientation: FORWARD

Perfect match to: (MW2-BA000033-[757005:757598], allele observed in CC1+CC80+CC96+CC188)

Sequence:

ATGATTCTAGTCATAGATAATAATGATTCATTTACATATAATTTAATAGACTATATTAAGACTCAAACGAAACTAACAGTTCAAGTTGTTG
GTATTGATAATCTGCTGATAGAAGACGTCATTAATATGAAGCCAAAAGCAATTGTTATTTCACCTGGGCCGGGTAATCCGGATGATTATC
CTATATTGAATGAAGTGTTAGAACAATTTTATCAGCGTGTACCTATACTAGGTGTATGCTTAGGATTTCAATGTATCGTGTCTTATTTTGGT
GGAAATATCATTCACGGCTATCATCCTGTACACGGACATACTACACAGTTACGCCATACCAATGAAGGTATTTTTCAAGGACTGCCTCAAA
ATTTCAATGTAATGCGTTATCATTCATTAATTGCTGACGGAGCGACTTTTCCAAATTGCTTAAAGATTACAGCCAAAAACGATGAAGCGAT
TATTATGGCATTTGAGCATATTACATTTCCGGTTTTTGGTGTGCAATATCATCCTGAATCTATTTTGAGTGAATACGGTTATCGACAAGTTG
AATTATTTTTATCGAAGGTAGGTGATTACTGTGAGAATAGAATATAA

Gene: pabB (anthranilate/para-aminobenzoate synthase component I)

Contig: 05_NODE_4, position: 211643 to 212794, length: 1152 nt, orientation: FORWARD

Perfect match to: (11819-97-CP003194-[802860:804011], allele observed in CC80+CC1+CC5+CC361+CC772)

Sequence:

GTGAGAATAGAATATAATTATCGCTACTATTTAACTGAAAATGAATATAAGCAATACCATATTCAATTAAAGGGATTTATAAAGAAGTAT
GTTGCTACTAAGTTGGCTGATGTGGGAGAAGTGATACACTTTGCACAAGCGCAGCAACGACAAGGTAGATATGTCTCGTTATATTTAAGT
TACGAAGCGGCAAAGTATTTTAATCATGTTATGTGTACACATTCATTAGCTAAAGATGATATTTATGCAGTAGCTTATAGTTTTGAAAAAG
CGGAAAGCATAAATTCAACATATGAACATCAAACTTCTTATGTATCAAAGCATCATTTTTCATTTGTTGAATCTTCTGAGGTTATGATGACT
AATATTAAACGTGTCCAACAAGCAATTGTTGAAGGCGAAACGTATCAAGTGAACTATACGGCGCGCTTAACGGATAACATTTATTATCCT
ATTAGTACTTTATATGAACGATTAACTCAATTTAGTAATGGTAATTATACTGCGTTATTACAAACAGATGAAATCCAAGTAGCGTCTATCTC
ACCAGAATTATTTTTTCAAAAAGGACAGTTTAACAATGTCGATAACGTTATCATAAGCAAACCGATGAAAGGGACAATGCCTAGAGGTAA
AACGGAAGCTGAAGATCAACAGTATTATAAAACATTGCAAACTTCTTCGAAAGATCGTGCAGAAAATGTCATGATTGTTGATTTACTAAG
AAACGATATAGGGAGAATATCACAGAGTGGCTCAATTAAGGTGTATAAACTATTTTTTATTGAGGCATATAAAACTGTATTTCAAATGAC
TTCGATGGTAAGTGGTACTTTAAAAACTAATACAGACCTAACTCAAATTTTAACATCGTTATTTCCTTGTGGTTCGATTACAGGTGCACCG
AAACTGAATACAATGAAATATATTAAACAATTAGAAAGTTCACCTCGTGGTATATACTGCGGAGCAATTGGACTATTACTTCCAACTGAA
GATGATAAAATGATTTTTAATATTCCGATTCGTACTATTGAGTATAAATATGGACAAGCGATTTATGGAGTCGGAGCAGGTATTACAATT
GATTCTAAGCCAAAAGATGAAGTGAATGAATTTTACGCAAAAACCAAGATTTTGGAGATGTTATAA

Gene: pabC (4-amino-4-deoxychorismate lyase)

Contig: 05_NODE_4, position: 212794 to 213402, length: 609 nt, orientation: FORWARD

Perfect match to: (MW2-BA000033-[758733:759341], allele observed in CC1+CC188)

Sequence:

ATGCAATTATTTGAAACAATGAAAATTGAAAATGGACATATCCCTAGACTTACTTATCATACTAATCGCATAAAATGTTCTTCTGAGCGAT
TAAACTTTAAATTTGATGAACATGCATGGCGAAATGAATTAAACGATGTAACAACAAAGTATCACAGTGGCCAATATAGACTTAAAATCG
TATTAAATGCTGAAAGCGAATTTGAAACGATAGTGTCACCTTTACCTGAGAAAAGTAGTTTTACAGCAAAATTTCAAGTGTTGCCCAAAG
TAGTTAATCCAACTTTTATAAATAATAAAACGACAGAACGAAAGCATTTAGCTCACAATCATGAAACAGATTTAATATTGCTAACTTCAGA
GGACGGCAAGGTCCTTGAATTTGATATTGGCAACATTGTCATTGAAGAGGATGGAAAATGGTACACACCAAGTTATAAAGATGATTTCTT
AAAAGGATGCATGCGTGATTATTTAATAGATAGTGACAAACTTGTTGAAAAAGACTTTAATAAAAACGAATTGATTTATAAATATCATAA
CAATGAGATACGTTTATTTTTGATAAATAGTTTACGAGAGGTTGCCGATGTCCACCTTTGCCTTTAA

Gene: Q6GIS6 (putative membrane protein)

Contig: 05_NODE_4, position: 213468 to 213674, length: 207 nt, orientation: FORWARD

Perfect match to: (MW2-BA000033-[759407:759613], highly conserved allele)

Sequence:

ATGATTATTGTTTATATTGTGCTGTTGTTAATTCTTGTATACGTAAATTATCGATTAGTGAATCGATTGCTATCTGAAAATAGAATATATGT
TGTTCGTTTGATAGCAACAATTACTACTGTTATAAGCTTTATCCTTGTATACGCATTAATTCACGAACTCATGCCTTTTGTTGTGCGGGCAA
TGGATTTAATGTATCACCAGTAA

Gene: ahs1 (allophanate hydrolase subunit 1)

Contig: 05_NODE_4, position: 213762 to 214472, length: 711 nt, orientation: FORWARD

Sequence:

ATGAAAATATATAGTCAAGGTGACCAAGCCATTGTAGTCGCAATTGAAAAAGAAGTATCTAAAAGTTTAACCGAAGATTTATTAACACTT
CGCTCATATTTAATTGAACAAAATTATCCATTTATTATAGAAATTGTGCCATCAGAATCAGACATGATGATTGTCTATGACGCAAGGGATA
TGATTAAACACCATAATATACAATCACCTTTTTTATACATGAAAGCACTAATAGAATCGATTCATTTAAACATAAAACATGATTTTAACCAG
CAAGATTTGATTGAAATACCAATTGTGTATGGTTCGAAATATGGTCCGGATTTAGAATCACTTTTAAAACATTACAAAGTCAAGCTAGAAA
CTTTTATTGAATTACATTCTAAGGCGCAATATTTTGTTTCGATGATGGGATATTCACCTGGGTTTCCTTATTTAACTGGATTAAATAAGAAA
TTGTATATTAATCACACGAGTAAACAGAAAAAATTCATTCCAGCTGGTTCTGTAGTACTTGAAGGGAAAAAATGCGGTATTGTAACTACG
GATACAATTAATGATTGGTTAGTTATTGGTTATACACCATTATCACTTTTTAATCCGAAAGAATCAGATTTCGCACGCTTAAAGTTAGGCG
ATAATATTAAATTTAGACCTATCAATGAAAATGAATTAGAAGTAGGAGCGTTTAAAGATGTCAATCATAATTGA

Gene: ahs2 (allophanate hydrolase subunit 2)

Contig: 05_NODE_4, position: 214456 to 215460, length: 1005 nt, orientation: FORWARD

Perfect match to: (MW2-BA000033-[760395:761399], allele observed in CC1+CC239)

Sequence:

ATGTCAATCATAATTGAAAAAAGTGGCTTATTCAGTAGCTTTCAGGACTTTGGCAGAAGGGGATATGAACATGATGGTGTAATTCCATGT
GGTGCACTTGATACTTTAGCACATGAAATTGCTAATCGATTAGTTGCAAATGACAAGAATGAAGCAACTTTGGAAATGACTAATAAAATG
GCAACGATTCGTTTTACAGAACCTACGCTGATTGCATTAGCAGGGGGTAATGTCAAAGCTTACACTGAGCATATGACTATATCTCCATATA
AATTGTATTTGTTAGATAAAGGCGATGTTTTAAAGTTTAGAGAAACAAGTTATACATCGCGAGTGTATTTAGCTGTGGGAGGCGGATTTG
AATTAGATGCATGGTTAGGATCTAACTCAACCGACTTTAATGTAAAAATTGGTGGTTTTAAAGGTAGAACATTACAAGATGGCGATGAAA
TAAAGCTTAAGAGAGATTATACAGCTCGTCATCATAAGTTATTTGAAAACCTTGCTCACACGAAACAAACAGATTGGGGTATTGATGGAT
ACGCCTTGTCATTTAATTATATGTCTGATGTATTTCATGTCGTTAAAAATAAAGGTACGGAAGATTTTAAAGAAGATGCCATTCAAAGATT
TGTGAAACATGATTATAAAGTAACGAGCAAAGCAAATCGCATGGGGATGATGCTTGAAGGTGAAAAAATCAAAGCCTTTTATGAAGATA
TGCCACCGTATCAGACTGTCAAAAAAGGAACGATACAAATTAAGCGTGATGGCACACCTATTATCTTATTAAATGATCATTATACGCTAG
GTAGCTACCCGCAAATCGGTACAATCGCAAGTTATCATTTAACGAAATTAGCACAAAAACCGCAAGGATCACGTTTGAAATTTCAATTTAT
AGATATTTTAACGGCTGAAAAGAACCTTGTTAAGTATAGTAACTGGTTAAACCAATTATTCCATGGAATAGAATATAGAATGCAATTAGA
AATGATGAAATAA

Gene: ltaS (glycerol phosphate lipoteichoic acid synthase)

Contig: 05_NODE_4, position: 215934 to 217874, length: 1941 nt, orientation: FORWARD

Perfect match to: (08-02119-CP015645-[1467379:1469319:r], allele observed in ST582+CC8+CC25+CC239)

Sequence:

ATGAGTTCACAAAAAAAGAAAATTAGTCTTTTTGCGTTCTTCTTATTAACCGTAATAACGATTACCTTGAAGACGTATTTTTCTTATTATGTT
GATTTTTCTTTAGGTGTTAAAGGTTTAGTACAAAACTTAATATTATTGATGAATCCTTATAGTTTAGTAGCACTGGTTTTAAGTGTGTTCCT
ATTCTTTAAAGGCAAAAAAGCATTTTGGTTCATGTTCATAGGCGGCTTCTTATTGACGTTCCTATTATATGCCAATGTTGTGTACTTTAGAT
TCTTCTCGGATTTTTTAACGTTTAGTACTTTAAACCAAGTAGGTAACGTAGAATCTATGGGTGGTGCGGTTAGTGCATCATTCAAATGGTA
TGACTTTGTTTATTTCATTGATACGTTAGTTTACTTATTCATTTTAATATTTAAAACAAAATGGTTAGACACAAAAGCATTTAGTAAGAAAT
TTGTTCCTGTCGTAATGGCGGCTTCAGTAGCATTATTCTTCTTAAACTTAGCTTTTGCTGAAACTGACAGACCAGAATTATTAACACGTACA
TTTGACCATAAATATTTAGTGAAATATTTAGGACCGTATAACTTTACAGTATACGATGGTGTTAAAACTATCGAAAATAATCAACAAAAAG
CGTTAGCATCTGAAGATGACTTAACAAAAGTATTAAATTATACGAAACAACGTCAAACAGAGCCTAACCCAGAATATTATGGGGTGGCA
AAGAAGAAAAATATTATTAAGATTCATTTAGAAAGTTTCCAAACCTTCTTAATTAATAAAAAGGTTAATGGTAAAGAAGTAACACCGTTTT
TAAACAAATTATCAAGTGGGAAAGAGCAATTCACATACTTCCCTAACTTTTTCCATCAAACAGGTCAAGGTAAAACATCTGACTCTGAATT
TACAATGGATAACAGTTTATACGGTTTACCGCAAGGTTCTGCCTTTTCATTAAAAGGAGATAATACGTATCAGTCACTACCAGCAATTTTA
GATCAAAAGCAAGGCTACAAATCTGATGTCATGCACGGTGACTATAAAACATTCTGGAACAGAGACCAAGTATATAAACACTTTGGTATC
GATAAATTCTATGATGCAACATACTATGACATGTCTGATAAAAACGTTGTAAACTTAGGCTTGAAAGACAAAATTTTCTTTAAAGATTCTG
CTAATTATCAAGCTAAGATGAAATCACCATTCTATTCTCATTTAATTACTTTGACTAACCACTATCCATTCACATTAGATGAAAAGGATGCG
ACTATTGAGAAGTCAAACACAGGTGATGCAACAGTTGATGGTTATATTCAAACAGCGCGTTATTTAGACGAAGCATTAGAAGAATATATT
AATGACTTGAAGAAAAAAGGATTATATGACAATTCAGTGATTATGATTTATGGTGACCACTATGGTATCTCTGAAAACCATAACAATGCC
ATGGAAAAACTATTAGGTGAAAAAATCACACCAGCTAAATTTACAGATTTAAACAGAACTGGTTTCTGGATTAAAATCCCTGGTAAATCT

GGTGGTATCAATAATGAATATGCTGGTCAAGTCGATGTAATGCCAACAATTTTACATTTGGCTGGTATAGATACGAAGAATTATTTAATG
TTCGGTACTGATTTATTCTCTAAAGGTCATAATCAAGTAGTTCCATTCAGAAATGGTGACTTTATAACAAAAGATTATAAATATGTTAATG
GTAAGATTTATTCTAATAAAAATAATGAACTCATAACTACTCAACCAGCTGATTTCGAAAAGAATAAAAAGCAAGTTGAAAAGGATCTCG
AAATGAGTGACAACGTGCTTAATGGTGATTTGTTTAGATTCTACAAAAATCCAGACTTCAAAAAGGTAAATCCTTCGAAGTATAAATATG
AAACAGGACCTAAAGCAAACTCTAAAAAATAA

Gene: uup (putative ABC protein involved in excision of transposons)

Contig: 05_NODE_4, position: 218151 to 220028, length: 1878 nt, orientation: FORWARD

Sequence:

ATGGAAGCATATAAAATTGAACATTTAAATAAATCTTATGCCGATAAGACTATATTCGATAACCTAGATTTATCAATTTCAGAAGGTGAAA
AAATAGGTTTAGTAGGCATAAATGGTACAGGGAAAAGTACGTTGTTAAAAGTAATTGGTGGTATTGATGATGATTTTACAGCCAATGTTA
TGCATCCAAATCAATATCGAATTCGATATTCGTCTCAGAAACAGGACCTTAATGAAGATATGACAGTTTTTGATGCAGTATTAAGTTCTGA
TACAACAACTTTACGCATCATCAAGCAATATGAGCAGACAGTACAAGCTTATGCGGATGACCAAAGTGATAAATTGTTCAAGCGAATGAT
GGATGCGCAAGATGCTATGGATCAACATGATGCTTGGGACTATAACGCTGAAATTAAAACAATCCTCTCAAAACTAGGTATACATGATAC
TACTAAATACATTAAAGAATTATCCGGCGGACAACAAAAACGTGTTGTACTTGCTAAAACATTAATAGAACAACCAGATTTATTGTTATTA
GATGAACCTACGAACCATTTAGACTTCGAATCAATCAGCTGGTTGATCAATTATGTGAAGCAATATCCTCATACTGTTTTATTCGTAACCC
ATGATCGATATTTTTTAAATGAAGTTTCCACTAGAATTATTGAACTAAACAGAGGTAAGTTAGCGTCATATCCTGGTAACTATGAATCTTA
TATTGAAATGCGCGCTGAAAGAGAAGTATCACTTCAAAAGCAACAACAAAAGCAACGAGCTTTATATAAGGAAGAACTTGCTTGGATGA
GGGCTGGAGCTAAGGCTCGTACTACAAAGCAACAAGCTAGAATTAATCGATTTAATGACCTAGAAAATGAAGTTAACCAGCAATATAAA
GACGATAAAGGTGAATTGAATCTTGCTTATTCAAGATTAGGTAAGCAAGTGTTCGAATTAGAAGACTTATCAAAGGCTATTAATGATAAA
GTATTATTTGAACATCTGACGGAAATTATTCAAAAAGGTGAGCGTATTGGTGTTGTTGGGCCAAATGGAGCTGGTAAAACAACACTCTTA
AATATTTTGAGTGGAGAAGACCAACAATTCGAAGGTAAATTGAAGACTGGACAGACGGTTAAAGTAGCTTATTTTAAGCAAACAGATGA
GACCCTGGATAGAGATATTCGTATGATTGATTATTTAAGAGAAGAAAGTGAGATCGCAAAAGAAAAAGATGGAACCTCGGTATCTATTA
CACAACTTCTTGAACGATTTTTATTTCCAAGTGCAACTCATGGTAAAAAAGTTTATAAATTATCTGGTGGAGAGCAAAAGCGTTTGTATTT
ATTACGTCTACTCGTACACCAGCCAAATGTTCTGTTGTTAGATGAACCGACAAATGATTTAGATACTGAGACTTTAACAATACTTGAAGAT
TATATTCATACTTTCGGTGGTACAGTGATTACCGTAAGCCATGATCGCTACTTTTTAAATAAAGTTGCACAGTCATATTGGTTTATTCATGA
TGGTCAGATGGAAAAGATTATCGGAACTTTTGAAGATTATGAAAGTTATAAAAAATCATTAGATAAAAATAAATCCACATTGAAGCAACA
ATCTAAATCTTCTACAACTGTACGTAAGAAAAATGGTTTATCATATAAAGAAAAGTTAGAATATGAACAATTGATGAAACGCATAGAACA
AGCGGAAGTAAGAATGGAAGAAATAGATGTGCTCATGATTGAGGCAAGTGCAGATTATGGGAAAATTAAAGAATTAAACGAAGAAAAA
GAACAACTTGAAATTCAATATGATTTAGACATCACAAGATGGAGTGAGTTAGAAGAAATTAAAGAACAACAATAA

Gene: recQ1 (ATP-dependent DNA helicase)

Contig: 05_NODE_4, position: 220040 to 221821, length: 1782 nt, orientation: FORWARD

Perfect match to: (ATCC51811-ST1-ADVP01000011-[94070:95851:r], allele observed in CC1)

Sequence:

ATGATGCAACAAACATTATCGCATTACTTTGGGTATGAAACGTTTCGACCAGGACAAGAAGAAATTATTAGCAAAGTATTAGACCATCGT
AATGTGCTTGGTGTCTTACCAACTGGTGGAGGTAAGTCTATATGCTATCAAGTACCAGGTTTATTGTTAGGTGGTACAACAATTGTAATA
AGTCCACTAATATCATTAATGAAAGATCAAGTGGATCAATTAAAAGCGATGGGAATTCAAGCTGCTTTTTTAAATAGTAGTTTGACTCAAA
AAGAGCAACAACGTATTGAAAAAGCATTATCAAATGGAGAAATTCAATTTTTGTATGTTGCACCAGAACGATTTGAAAACCGATATTTTTT
AAATATGCTTCAGCGTATAAAGATTCACTTAGTCGCGTTTGATGAAGCGCATTGTATTTCTAAATGGGGTCATGATTTCAGGCCGAGTTAC
CAAAATGTTATTTCAAAAGTATTTACGTTACCTCAAGATTTTACAATAATAGCGTTGACAGCAACTGCCACGGTTGAAGTACAGCAAGATA
TTAGAGAAAAGTTAAATATCGCTCAAACTGATCAAATTAAAACGAGTACTAAGCGTAGAAACTTAATTTTTAAAGTAAATCCTACTTATCA
ACGTCAAAAATTTATATTGGATTATATTAAAACACATGATGAAGATGCAGGTATTATTTATTGTTCTACACGTAAGCAAGTTGAAGAGCTT
CAAGAAGCCTTAGAAAGCCAGAAAATTGAAAGTGTTATATATCATGCAGGTTTGAGCAATAAAGAAAGAGAAGAAGCGCAGAATGATT
TCTTATTTGATCGTGTTAAAGTAGTCGTTGCTACAAATGCTTTTGGTATGGGTATTGATAAATCCAATGTACGTTTTGTTATTCATTATAAT
ATGCCTGGAGATTTAGAATCTTATTATCAAGAAGCCGGTCGTGCAGGTCGTGACGGGTTGAAAAGTGAATGTATTTTGTTATTTAGCGAA
CGCGATATCAATTTACACGAGTATTTTATAACAGTCTCTCAAGCTGATGATGACTATAAAGATAAAATGGGCGAAAAGTTAACTAAAATG
ATTCAATATACAAAAACAAAAAAAATGTTTAGAAGCAACAATTGTCCATTATTTTGAACCGAATGAAAAATTAGAAGAATGTGAACAATGT
AGTAATTGTGTTCAACAAGATAAATCATATAATATGACACAAGAAGCTAAGATGATTATTAGTTGCATCGCTCGTATGAAACAACAAGAG
AGTTATAGTGTTATCATTCAAGTGCTAAGAGGAGAGTCAACAGATTATATTAAGTACAAAGGTTATGATCAAATTTCAACCCATGGTTTAA
TGAAAGGTTACACAACATCAGAATTAAGTCACTTAATAGATGAATTAAGATTCAAAGGGTTCTTAAATGAAAATGATGAAATATTAATGT
GTGATACTTCAATTAAAAAATTACTCAGTAATGAAGTAGAAGTATTCACAACACCATTTAAGCAAAAAGCGACTGAAAAAGTATTTATAA

ATACGGTTGAAGGGGTTGACCGAGTATTATTCAGTCAGTTGGTAGAAGTTCGTAAAAAGTTAAGTGACAAATTAACGATAGCACCTGTA
AGTATATTTTCTGATTACACGTTGGAGGAATTTGCTAAACGTAAGCCTGCTTCGAAACAAGATATGATTAATATTGATGGCGTAGGTAGT
TACAAATTAAAACATTATTGTCCAGCATTTTTAGAAACGATTCAAAATTATAAAGCCAAAGTATAG

Gene: opuBA (osmoprotectant ABC transporter, ATP-binding protein)

Contig: 05_NODE_4, position: 222043 to 223020, length: 978 nt, orientation: FORWARD

Perfect match to: (Strain_21331-AGTV01000004-[78673:79650:r], allele observed in CC398)

Sequence:

GTGATTAAGTTTAAAAATGTAACTAAGCGTTATGGCAAACATGTTGCTGTCGATAACATTAGTTTCAATATTAATGAGGGTGAATTTTTTG
TGCTAATTGGACCTTCAGGTTGTGGAAAAACTACGACATTAAAAATGATTAATCGACTCATTCACTTAAGTGAAGGTTATATTTATTTTAA
AGATAAACCAATAAGTGATTATCCAGTATACGAAATGCGTTGGGATATTGGATACGTATTGCAGCAGATTGCATTATTCCCACATATGAC
AATCAAAGAAAATATTGCACAAGTGCCACAAATGAAAAAGTGGAAAGAAAAAGATATAGATAAAAGAGTAGATGAATTACTTGAAATG
GTTGGATTAGAACCTGAAAAATATAAAAACAGAAAACCTGATGAATTGTCAGGGGGGCAACGACAACGTGTAGGAGTTATACGTGCGTT
AGCAGCTGATCCACCAGTTATTTTAATGGATGAACCGTTTAGTGCATTAGACCCAATCAGCCGAGAAAAACTTCAAGATGATTTAATTGA
ATTACAAACTAAAATTAAGAAGACAATCATATTTGTTACACATGATATTCAAGAGGCGATGAAACTTGGTGATAAGATTTGTCTTTTGAAT
GAAGGGCATATTGAACAAATTGACACACCAGAAGGATTTAAAAATAATCCTCAAAGTGAATTTGTTAAACAATTTATGGGTAGTCATTTA
GAAGATGTTGCGCCATGTGTTGAAGAGAACGCAATTATCCGTGACTTGGATATTATGAAACCAATCGATGAGGTTACATCTATGAGCGCT
TATCCAATTGTTTATGACAATCAACCAATTGAAGTATTGTATCAACTTTTATCAGAGAGCGAGCGTGTCATTGTCATGCAAGAAGATAGCG
TAGGTCAATATGTTATTGATAGGAAAGATATCTTCAAATATTTGTCCCAGAAAAAGGAGGTAGCTCAACATGACTAA

Gene: opuBB (osmoprotectant ABC transporter, transmembrane permease)

Contig: 05_NODE_4, position: 223013 to 224527, length: 1515 nt, orientation: FORWARD

Sequence:

ATGACTAACTTTTTCGACATATTGAGTGAACGTAAGGGGCAACTCTTTTCGACAATGATAGAACATATTCAAATATCATTTATCGCATTATT
GATTGCAACTGCTATTGCGGTACCATTAGGTATTTTATTAACGAAGACTAAAACGATATCTGAAATCGTAATGAATATTGCGGCAATTCTT
CAAACCATACCATCGTTGGCATTATTAGGTTTAATGATTCCTTTATTTGGTATCGGTCGTGTGCCAGCAATTATTGCACTTGTAGTGTATGC
GTTGTTACCAATTTTAAGGAATACGTATACTGGAATTAAAGAAGTTGATCCATCACTCATTGAAGCGGCTAAAGGTATAGGTATGAAACC
ATTTAGACGTTTAACTAAAGTCGAACTTCCGATAGCAATGCCTGTTATAATGGCTGGTGTAAGAACGGCTATGGTATTAATTATAGGTAC
AGCAACACTAGCAGCATTAATTGGTGCAGGCGGACTAGGAGATTTAATTTTATTAGGTATAGACCGTAACAATGCATCGTTGATATTATT
AGGTGCAATTCCAGCAGCCTTATTGGCAATTATATTTGATTTAATTTTAAGATTTATGGCTAAATTATCTTATAAAAAGTTATTGATGACGT
TAGGTGTTATAGTGATGATTATTATACTGGCTATCGCTATTCCTATGTTTGCACAAAAAGGTGATAAAATTACGTTAGCTGGAAAGCTTGG
CTCCGAGCCATCGATTATTACAAATATGTATAAAATTTTAATAGAAGAAGAGACCAAAAATACTGTAGAAGTGAAAGATGGTATGGGCA
AAACAGCATTTTTATTTAATGCTTTAAAATCTGACGATATAGATGGGTATTTAGAATTTACTGGAACAGTTTTAGGCGAATTAACAAAAGA
ACCATTGAAGTCAAAAGAAGAGAAAAAAGTTTATGAAGAAGCTAAGCAAAGTCTTGAAAAGAAATATCAAATGACTATGTTAAAACCAA
TGAAGTATAACAATACGTATGCTTTAGCTGTAAAACGTGATTTTGCTAAACAACATAATATACGTACAATTGGTGATTTAAATAAGGTTAA
AGATCAACTTAAACCAGGATTTACATTGGAATTTAATGATCGTCCAGATGGTTACAAAGCTGTTCAAAAGGCTTATAATTTAAATTTAGAT
AACATACGTACAATGGAACCTAAGTTGAGATATCAAGCGATCAATAAAGGTAATATTAATTTAATAGATGCATATTCAACTGACGCTGAA
TTAAAACAATATGATATGGTTGTGTTAAAAGATGATAAGCACGTATTTCCACCATATCAAGGAGCACCATTATTTAAAGAAAGCTTTTTAA
AGAAACATCCAGAAATTAAGAAACCGTTAAACAAACTAGAAAACAAAATATCTGATGAAGATATGCAAATGATGAACTATAAAGTAACA
GTTAAAAATGAAGACCCATATACAGTTGCGAAAGATTATTTAAAAGCAAAAGGGTTAATCAAATAA

Gene: hisC2 (histidinol-phosphate aminotransferase locus 2)

Contig: 05_NODE_4, position: 224767 to 225825, length: 1059 nt, orientation: FORWARD

Perfect match to: (08-02119-CP015645-[1459429:1460487:r], allele observed in ST582+CC20+CC239)

Sequence:

ATGAAAGAACAACTTAATCAACTATCAGCATATCAGCCTGGTTTATCTCCAAGGGCATTGAAAGAAAAGTATGGCATTGAAGGAGATTTA
TATAAACTTGCATCAAATGAAAATTTGTATGGACCATCGCCTAAAGTTAAAGAAGCGATATCAGCACACTTAGATGAGTTATATTATTATC
CTGAAACAGGATCACCGACATTAAAAGCGGCGATTAGTAAACATTTAAATGTAGATCAATCACGCATTTTATTTGGTGCGGGATTAGATG

AAGTTATATTAATGATTTCTAGAGCTGTATTAACGCCAGGGGATACTATTGTTACAAGTGAAGCGACATTCGGTCAATATTATCACAATGC
GATTGTTGAATCAGCTAATGTGATACAAGTACCTTTAAAAGATGGTGGCTTCGATTTAGAAGGTATTTTAAAAGAAGTTAATGAAGATAC
GTCATTGGTATGGTTATGTAATCCAAATAATCCTACAGGTACATATTTTAATCATGAGAGCTTAGATTCGTTTTTATCTCAAGTACCTCCAC
ATGTACCAGTAATTATAGATGAAGCTTATTTTGAATTTGTGACAGAAGAGGACTACCCGGATACACTTGCTTTGCAACAAAAATATGACA
ATGCTTTCTTATTACGTACATTTTCAAAGGCGTATGGATTAGCGGGTTTACGTGTAGGATATGTGGTAGCAAGTGAACATGCGATTGAAA
AATGGAACATCATTAGACCACCATTTAATGTGACACGTATATCTGAATACGCAGCAGTTGCAGCACTTGAAGATCAACAATATTTAAAAG
AGGTAACACATAAAAATAGTGTTGAACGCGAAAGATTTTATCAATTACCTCAAAGTGAGTATTTCTTGCCAAGTCAAACGAATTTTATATT
TGTAAAAACAAAGCGGGTAAATGAACTTTATGAAGCACTTTTAAATGTAGGGTGTATTACGCGACCATTTCCAACTGGTGTTAGAATTAC
AATTGGTTTTAAAGAACAAAATGATAAAATGTTAGAAGTTTTATCAAACTTTAAATACGAATAG

Gene: tx_universal2 (rho-independent terminator)

Contig: 05_NODE_4, position: 225890 to 225922, length: 33 nt

Perfect match to: (Strain_21194-AGTU01000149-[993:1025], allele observed in CC45I)

Sequence:

CTGAACGAAAATGCGCTTGTAACAAGCTTTTTT

Gene: tx_universal2 (rho-independent terminator)

Contig: 05_NODE_4, position: 225892 to 225930, length: 39 nt

Perfect match to: (Strain_21331-AGTV01000040-[104403:104441], allele observed in CC398)

Sequence:

GAACGAAAATGCGCTTGTAACAAGCTTTTTTCAATTCTA

Gene: yorS (5' (3')-deoxyribonucleotidase)

Contig: 05_NODE_4, position: 226120 to 226662, length: 543 nt, orientation: FORWARD

Perfect match to: (MW2-BA000033-[772059:772601], highly conserved allele)

Sequence:

ATGACCCGTAAATCAATCGCGATTGATATGGATGAAGTATTGGCAGATACATTAGGAGAAATCATTGATGCTGTCAATTTTAGAGCGGAT
TTAGGTATTAAAATGGAAGCTTTGAATGGTCAAAAACTTAAACATGTTATTCCTGAACATGATGGATTAATTACAGAAGTATTGAGAGAA
CCAGGCTTCTTCAGACATCTTAAAGTGATGCCGTATGCACAAGAAGTTGTGAAAAAATTAACTGAACATTATGATGTATATATTGCTACAG
CAGCAATGGATGTACCAACATCATTTAGTGATAAATATGAATGGTTACTAGAGTTCTTTCCATTTTTAGATCCTCAGCATTTTGTTTTTTGT
GGTAGAAAAAACATCGTTAAAGCTGATTATTTAATAGATGACAATCCTAGACAGCTTGAAATTTTTACTGGTACACCGATTATGTTTACAG
CAGTGCATAATATTAATGATGATCGATTTGAACGCGTAAATAGCTGGAAAGATGTAGAACAGTATTTTTTAGATAATATTGAGAAATAA

Gene: dgkA1-bmrU (putative lipid kinase)

Contig: 05_NODE_4, position: 227287 to 228204, length: 918 nt, orientation: REVERSE

Perfect match to: (CN1-CP003979-[751792:752709:r], allele observed in CC72+CC8+CC72+CC239)

Sequence:

TTACAAATCATTGACAGTAAGTAACTGAATGGCATTTGGTATAACCTCAATATCAATAGGTGTTTCTAATGAAATTTCGCCATCAATATCA
ACTTTCATTGCTGGATCTGTTGTAAGTGAAATCTTTTTACCAGGTATATGCTCAATACCTTGAGTAATTTCATTCCAATTCATGCTATCACGC
TTTTTAAAAATATCATTTAAAATACTGAAACTTTGTTCATTAAAAATGAAAGTGTTCAGTTCACCATCTTGAGGAGACAAATCAGTCAATG
GTATACGACTACCACCAATGAATGGACCATTTGCTGTTAGTATCATGGTCGTTTCGCCAGAATATGTCTTATCATCTATTGATAATTGATAA
TTAAATTGTGTTGGATTTAGCAGTGTTTTGACAGTTGATCCAATATAACTCAATTTACCAAATATATCTTTTGAACCATCTTGTACGTTTTCA
GCGTTTTGAACAATGAGACCTAAGCCAACAAAGTTGAGTGCATATTGATTATTTATTTTAATTACATCGTATTTACCAACTTGTGCAGAAA
TCATTTGTTCACTAGCTTGTTTATGATTAGGTGCTATATTTAGCGTTTTTGTAAAATCATTAAAAGTACCGCCTGGTAAAATGCCAATAGGG

AGTTGAAGGTCATGTGTCATAACACCGTTTATAAGTTCGTTAACCGTGCCATCACCGCCAAGAATAAATAATATATCTACATCTTTTGCAT
AGTTTTTAGTTTTGATTTCTTGGCAATATTTAATAATGTCACCTTCGTTTTCACTCAATTGAATAGAAAGATGCTTACAAATTGAACTTAATG
CTGTTGTAACTTCCCCAATACCTTGATTAATATTTTTTAATCCACTGTGTTCATGGTAAAAGAGGACACCATGTGTATATTTATTTTCCAT

Gene: dtpT (proton-dependent di- and tripeptide transporter)

Contig: 05_NODE_4, position: 228473 to 229978, length: 1506 nt, orientation: REVERSE

Perfect match to: (08-02119-CP015645-[1455164:1456669], allele observed in ST582+CC239)

Sequence:

TTAACGTATACCTTTCATCGCTTTGATGATTAAAGGTGAGAATGCTAATACAATTGTTGTAACAATAATTGCAACAACACCTAGGAAAATA
AAGTAATTTGTTTGACCTAGTGGTTCTATTAACTTAACTAAAGTACCATTGATTGCTTGTGCAGAAGCGTTAGTTAAGTACCAAATACTCA
TCATTTGGGCATTAAATGCTTTAGGTGCTAACTTAACAGCAGCACTATTACCCGTTGGTGATAAGCATAGCTCACCGATAACACAAATAAT
GTACGATAAAATAACCCAGTTAACTGAAAAGTTTGATGAACCTGATGCATAACCTACAATACCAATTAGTATGTATGACGCACCTGCTAA
GAACGTACCAATTGCAAATTTTACTGGCAGGCTAGGTTGTTTAGTTCCAAGCTTTTGCCATAAAAGTGAAATAATTGGAGCTAGTAATAA
AATAAATAATGGGTTAATTGATTGGAAGATCGCTTCACCAAAGTTTGTTTTCCAACCAAATAAGTTTAATTTCATATCTGAATGTTCAATTC
CATATATGTTTAATACATTAGACCCTTGTTCTTGAATAGCCCAGAACACCATTCCAAGAATAAATAATGGAATAAATGCTTTAACACGAGA
ACGTTCAGTATCAGTGACATCTTTACTTCTAATAATTAAAGTGAAGTAAATGATTGGTAATGCAATACCTAATACTAAAACAGTATTACTA
ACTAAGTTAAATGATAATGAGTTAGTTAATGCACCAATAACGATAATTAATACAATTGCTAAAACAACACTTCCGATAATAAGACCATACT
TTTTCTTTTCAGCTGGTGTCAATGGGTTAGTAGGTTTCATACCAACGCTACCTAAGTTTTTGCGGTTGAAAAGTACATACCATACTAAACCT
AATGCCATACCAACTGCTGCAATCAAGAATCCGCCGTGGAAGTTTTTAACATTAACAAAGTGTTGCAAAATAATAGGTGATAATAATGCA
CCCATATTAACTGACATATAGAAAATAACAAAACCTGCATCCATACGTCTATCATTTTCAGGATATAAACGGCCAACGATATTTGAAATGT
TTGGCTTCATTAAACCTGAACCAATAATGATGAAGAACATTGATGTGAATAAGCCGATTAATGCAAATGGTAAGCTTAAACAAATATGTC
CGATAATAATAAAGACTGCACCTAATAAAGTAGCGCCTCTAGTACCTGTAATTCTGTCAGCAATCCATCCGCCTGGTATTGATGTCATATA
GATTAATGAACCATAAACTGACATAATTGACATAGCTGTTGTTTTATCAATTCCAAGGCCATTATCTGTTACGGCAAAGTACATGTAGAAA
ATGAGTAGGGCACGCATGCCATAATAACTAAACCTTTCCCAGAACTCTACAAAGAAGAGTACGCCTAGTCCTCGAGGATGCCCGAAAAA
TCCTGTTTGAGGTATGTCTTGAATTTGATTTCCATGGGAGTTTTGTTGTGTCAT

Gene: queF (NADPH-dependent 7-cyano-7-deazaguanine reductase)

Contig: 05_NODE_4, position: 230319 to 230819, length: 501 nt, orientation: REVERSE

Perfect match to: (N315-BA000018-[780923:781423:r], highly conserved allele)

Sequence:

TTAACGATTATCTATTTTTTCGGGATATAAATCATGATTCATCAAACGATGCTCAGCCATTTTTTCATATTTAGAATTTGGACGTCCATAGTT
TGTATAAGGATCAATAGAAATTCCACCACGTGGTGTGAACTTGCCCCAGACTTCAATATAATGTGGGTCCATAAGCTCTATCAAATCATTC
ATAATAATATTCATACAATCTTCGTGAAAATCACCGTGATTTCTGAAACTAAATAAGTATAATTTCAAAGATTTTGATTCAACCATTTTAAC
ATTTGGAATATATGAAATATAGATAGTTGCAAAATCTGGTTGCCCAGTAATTGGACATAATGATGTAAATTCTGGACAGTTGAATTTTAC
GAAATAGTCACGACCTTGATGCTTATTATCAAACGATTCTAATACATCAGGACGATAGTCAAAATTGTAAGTATTGTCTTGATTTCCTAAT
AAAGTTATATCTTGTAATTCATCTTGTTGACGGCCATGTGCCAT

Gene: yxxF (putative transporter)

Contig: 05_NODE_4, position: 230839 to 231705, length: 867 nt, orientation: REVERSE

Perfect match to: (MW2-BA000033-[776834:777700:r], highly conserved allele)

Sequence:

TTATTTTTTTATTATTTTGGCGTCTCGGCGTGCTTTTTCAAACATGTAATAACTTGCACCGATAATAACGACGTAACCTAATGTTGCATAGA
AATCTGGAGATTCTCCGAATAGAATAAATCCAAGTATTGCTGTGAAAATTATAGATGCATACGTGAAAATAGAAATATCTTTTGCTGCTGC
AAAACTATATGCTAATGTAACACCAATTTGACCCACAGCGGCAGCTAAGCCAGCCCCTAATAGATAAAGTATTTGCATCTGACTCATTGGT
TCATAGGTATATGCAGTGAAAGGTATTAAAACGATGACAGAAAATAAGGAGAAGTAAAATACTATAGTATATGGTGCTTCTCTTGTACTA
AGTGCTCGAACACATGTATATGCTGATGCTGCAAAAATACCTGAGAATAAGCCAGCTAATGATGGAATCATAGATGATGAAAATTCAGG
TTTCACTATTAAAAGCATACCTAAAATAGCAATTATCATTGCTGTAATTTGATACTTCCTTACCTTTTCATGTAAGAAAACAATGCTTAATAA
AATCGTCCAGAAAGGATTGAGTTTCATTAATGAATCGGCATCACTAAGTACCATATGATCAATGGCATAAATATTTAACAATACACCAATA

AGTCCAAGTGTTGATCGTGTTATTAATAAGGGTTGACTTGAAAGTCTGCCAAACATTGGCTGATGGTATTTATATATAAAAAATAATGGA
ATAAACATTGCTACTAAGTTTCGTGCTAATGATTTTTGAAAAACAGGAAGGTCACCTGCAAGTCTGAAAAACACTGACATAAAACTGAAA
CCAATAGCCGAAATTAAAATGGCAATGATACCTTTTACTTTAGGATTCAA

Gene: rnaIV (regulatory RNA IV)

Contig: 05_NODE_4, position: 232116 to 232345, length: 230 nt

Sequence:

TAATATAGTGTTGGTGAAACGAAAAAGACACAATATCTTGTGTTTTGTATGCAAATGCTTTATTTATGAAGAAATTACATTTAAAAGTAAT
TTAACACAGAAATTTAATAGTTATTATCAATTAATAGTCATATTTTTAGAAAATGTACTGAGCAAATGGAAGATATCCAATGATGTAAATA
CTACATATAGTGATTTTTATACATTCAACCCATATAAGCTACTATTTT

Gene: nrdI (stimulator of ribonucleotide reduction)

Contig: 05_NODE_4, position: 232506 to 232904, length: 399 nt, orientation: FORWARD

Perfect match to: (RF122-AJ938182-[747913:748311], highly conserved allele)

Sequence:

ATGAAAATAATATATTTTTCATTTACTGGAAATGTCCGTCGTTTTATTAAGAGAACAGAACTTGAAAATACGCTTGAGATTACAGCAGAAA
ATTGTATGGAACCAGTTCATGAACCGTTTATTATCGTTACTGGCACTATTGGATTTGGAGAAGTACCAGAACCCGTTCAATCTTTTTTAGA
AGTTAATCATCAATACATCAGAGGTGTGGCAGCTAGCGGTAATCGAAATTGGGGACTAAATTTCGCAAAAGCGGGTCGCACGATATCAG
AAGAGTATAATGTCCCTTTATTAATGAAGTTTGAGTTACATGGAAAAAACAAAGACGTTATTGAATTTAAGAACAAGGTGGGTAATTTTA
ATGAAAACCATGGAAGAGAAAAAGTACAATCATATTGA

Gene: nrdE (ribonucleotide-diphosphate reductase alpha chain)

Contig: 05_NODE_4, position: 232867 to 234972, length: 2106 nt, orientation: FORWARD

Perfect match to: (MW2-BA000033-[778862:780967], allele observed in CC1+CC9+CC239)

Sequence:

ATGAAAACCATGGAAGAGAAAAAGTACAATCATATTGAATTAAATAATGAGGTCACTAAACGAAGAGAAGATGGATTCTTTAGTTTAGA
AAAAGACCAAGAAGCTTTAGTAGCTTATTTAGAAGAAGTAAAAGACAAAACAATCTTCTTCGACACTGAAATCGAGCGTTTACGTTATTT
AGTAGACAACGATTTTTATTTCAATGTGTTTGATATTTATAGTGAAGCGGATCTAATTGAAATCACTGATTATGCAAAATCAATCCCGTTTA
ATTTTGCAAGTTATATGTCAGCTAGTAAATTTTTCAAAGATTACGCTTTGAAAACAAATGATAAAAGTCAATACTTAGAAGACTATAATCA
ACACGTTGCCATTGTTGCTTTATACCTAGCAAATGGTAATAAAGCACAAGCTAAACAATTTATTTCTGCTATGGTTGAACAAAGATATCAA
CCAGCGACACCAACATTTTTAAACGCAGGCCGTGCGCGTCGTGGTGAGCTAGTGTCATGTTTCTTATTAGAAGTGGATGACAGCTTAAAT
TCAATTAACTTTATTGATTCAACTGCAAAACAATTAAGTAAAATTGGGGGCGGCGTTGCAATTAACTTATCTAAATTGCGTGCACGTGGTG
AAGCAATTAAAGGAATTAAAGGCGTAGCGAAAGGCGTTTTACCTATTGCTAAGTCACTTGAAGGTGGCTTTAGCTATGCAGATCAACTTG
GTCAACGCCCTGGTGCTGGTGCTGTGTACTTAAATATCTTCCATTATGATGTAGAAGAATTTTTAGATACTAAAAAAGTAAATGCGGATG
AAGATTTACGTTTATCTACAATATCAACTGGTTTAATTGTTCCATCTAAATTCTTCGATTTAGCTAAAGAAGGTAAGGACTTTTATATGTTT
GCACCTCATACAGTTAAAGAAGAATATGGTGTGACATTAGACGATATCGATTTAGAAAAATATTATGATGACATGGTTGCAAACCCAAAT
GTTGAGAAAAAGAAAAAGAATGCGCGTGAAATGTTGAATTTAATTGCGCAAACACAATTACAATCAGGTTATCCATATTTAATGTTTAAA
GATAATGCTAACAGAGTGCATCCGAATTCAAACATTGGACAAATTAAAATGAGTAACTTATGTACGGAAATTTTCCAACTACAAGAAACT
TCAATTATTAATGACTATGGTATTGAAGACGAAATTAAACGTGATATTTCTTGTAACTTGGGCTCATTAAATATTGTTAATGTAATGGAAA
GCGGAAAATTCAGAGATTCAGTTCACTCTGGTATGGACGCATTAACTGTTGTGAGTGATGTAGCAAATATTCAAAATGCACCAGGAGTTA
GAAAAGCTAACAGTGAATTACATTCAGTTGGTCTTGGTGTCATGAATTTACACGGTTACCTAGCAAAAAATAAAATTGGTTATGAGTCAG
AAGAAGCAAAAGATTTTGCAAATATCTTCTTTATGATGATGAATTTCTACTCAATCGAACGTTCAATGGAAATCGCTAAAGAGCGTGGTAT
CAAATATCAAGACTTTGAAAAGTCTGATTATGCTAATGGCAAATATTTCGAGTTCTATACAACTCAAGAATTTGAACCTCAATTCGAAAAA
GTACGTGAATTATTCGATGGTATGGCTATTCCTACTTCTGAGGATTGGAAGAAACTACAACAAGATGTTGAACAATATGGTTTATATCAT
GCATATAGATTAGCAATTGCTCCAACACAAAGTATTTCTTATGTTCAAAATGCAACAAGTTCTGTAATGCCAATCGTTGACCAAATTGAAC
GTCGTACTTATGGTAATGCGGAAACATTTTACCCTATGCCATTCTTATCACCACAAACAATGTGGTACTACAAATCAGCATTCAATACTGA
TCAGATGAAATTAATCGATTTAATTGCGACAATTCAAACGCATATTGACCAAGGTATCTCAACGATCCTTTATGTTAATTCTGAAATTTCTA

CACGTGAGTTAGCAAGATTATATGTATATGCGCACTATAAAGGATTAAAATCACTTTACTATACTAGAAATAAATTATTAAGTGTAGAAG
AATGTACAAGTTGTTCTATCTAA

Gene: nrdF (ribonucleotide-diphosphate reductase beta chain)

Contig: 05_NODE_4, position: 235090 to 236061, length: 972 nt, orientation: FORWARD

Perfect match to: (N315-BA000018-[785694:786665], highly conserved allele)

Sequence:

ATGATAGCTGTTAATTGGAACACACAAGAAGATATGACGAATATGTTTTGGAGACAAAATATATCTCAAATGTGGGTTGAAACAGAATTT
AAAGTATCAAAAGACATTGCAAGTTGGAAGACTTTATCTGAAGCTGAACAAGACACATTTAAAAAAGCATTAGCTGGTTTAACAGGCTTA
GATACACATCAAGCAGATGATGGCATGCCTTTAGTTATGCTACATACGACTGACTTAAGGAAAAAAGCAGTTTATTCATTTATGGCGATG
ATGGAGCAAATACACGCGAAAAGCTATTCACATATTTTCACAACACTATTACCATCTAGTGAAACAAACTACCTATTAGATGAATGGGTTT
TAGAGGAACCCCATTTAAAATATAAATCTGATAAAATTGTTGCTAATTATCACAAACTTTGGGGTAAAGAAGCTTCGATATACGACCAATA
TATGGCCAGAGTTACGAGTGTATTTTTAGAAACATTCTTATTCTTCTCAGGTTTCTATTATCCACTATATCTTGCTGGTCAAGGGAAAATGA
CGACATCAGGTGAAATCATTCGTAAAATTCTTTTAGATGAATCTATTCATGGTGTATTTACCGGTTTAGATGCACAGCATTTACGAAATGA
ACTATCTGAAAGTGAGAAACAAAAAGCAGATCAAGAAATGTATAAATTGCTAAATGACTTGTATTTAAATGAAGAGTCATACACAAAAAT
GTTATACGATGATCTTGGAATCACTGAAGATGTGCTAAACTATGTTAAATATAATGGAAACAAAGCACTTTCAAACTTAGGCTTTGAACCT
TATTTTGAGGAACGTGAATTTAACCCAATCATTGAGAATGCCTTAGATACAACAACTAAAAACCATGACTTCTTCTCAGTAAAAGGTGATG
GTTATGTATTAGCATTAAACGTAGAAGCATTACAAGATGATGACTTTGTATTTGACAACAAATAA

Gene: sstA (iron compound ABC transporter, transmembrane permease subunit)

Contig: 05_NODE_4, position: 236769 to 237740, length: 972 nt, orientation: FORWARD

Perfect match to: (MW2-BA000033-[782764:783735], highly conserved allele)

Sequence:

ATGAAGTATTTATTAAAGGGAAATATTTTGCTTCTATTACTAATATTGTTGACAATTATTTCGTTGTTCATAGGTGTGAGTGAACTATCAAT
TAAAGATTTACTACATTTAACTGAGTCACAGCGGAATATTTTATTCTCAAGCCGAATACCAAGGACGATGAGTATTTTAATTGCTGGAAGT
TCGTTGGCTTTAGCAGGCTTGATAATGCAACAAATGATGCAAAATAAGTTTGTTAGTCCGACTACAGCTGGAACGATGGAATGGGCTAA
ACTAGGTATTTTAATTGCTTTATTGTTCTTTCCAACCGGTCATATTTTATTAAAACTAGTATTTGCTGTTATTTGCAGTATTTGCGGTACGTT
TTTATTTGTTAAAATCATTGATTTTATAAAAGTGAAAGATGTCATTTTTGTACCGCTTCTAGGAATTATGATGGGTGGGATTGTTGCAAGTT
TCACAACCTTCATCTCATTGCGCACGAATGCTGTTCAAAGCATTGGTAACTGGCTTAACGGGAACTTTGCCATTATCACAAGTGGACGCTA
TGAAATTTTATATTTAAGTATTCCTCTTTTAGCATTGACATATCTTTTTGCTAATCATTTCACGATTGTAGGAATGGGTAAAGACTTTACTAA
TAATTTAGGTTTGAGTTACGAAAAATTAATTAACATCGCATTGTTTATTACTGCAACTATTACAGCATTGGTAGTGGTGACTGTTGGAACA
TTACCGTTCTTAGGACTAGTAATACCAAATATTATTTCAATTTATCGAGGTGATCATTTGAAAAATGCTATCCCTCATACGATGATGTTAGG
TGCCATCTTTGTATTATTTTCTGATATAGTTGGCAGAATTGTTGTTTATCCATATGAAATAAATATTGGTTTAACAATAGGTGTATTTGGAA
CAATCATTTTCCTTATCTTGCTTATGAAAGGTAGGAAAAATTATGCGCAACAATAA

Gene: sstB (iron compound ABC transporter, transmembrane permease subunit)

Contig: 05_NODE_4, position: 237727 to 238683, length: 957 nt, orientation: FORWARD

Perfect match to: (MW2-BA000033-[783722:784678], highly conserved allele)

Sequence:

ATGCGCAACAATAATAAAAAAATAATGCTTTTAATTGCAGTAACGTTATTAATTAGTATGCTGTACTTATTTGTAGGTATTGATTTTGAAAT
ATTTGAATATCAATTTTCAAGTCGTTTAAGAAAGTTCATATTAATTATTTTAGTAGGTGCTGCCATTGCAACTTCAGTGGTGATTTTTCAAG
CGATTACAAATAACCGTCTATTGACACCATCAATAATGGGGTTAGATGCAGTTTATTTATTTATCAAAGTATTGCCAGTCTTTTTATTTGGA
ATTCAATCGGTATGGGTTACTAATGTATATTTGAACTTTATATTAACACTTATAACGATGGTGTTATTCGCACTAATCCTATTCCAAGGTAT
CTTTAAAATTGGACATTTTTCAATTTATTTTATCTTACTTATTGGTGTCCTTTTAGGAACATTTTTTAGAAGCATAACAGGTTTTATTCAACT
GATTATGGATCCTGAGTCATTTTTAGCAATACAAAGTAGTATGTTTGCTAATTTTAATGCTTCTAATTCGAATTTAGTTATTTTCTCAGCAGT
GCTATTAGTAATCTTATTAGTCATTACAATTTTACTATTGCCTTATTTAGATGTATTGCTTTTAGGTCGTGCTGAAGCAATTAATCTTGGGAT
ATCGTATGAAAAATTAACGCGAATTCTACTTGTAATAGTCTCAGTTTTAGTTTCTGTGTCAACTGCATTAGTAGGACCAATTACATTTTTAG
GTTTATTAACTGTAAATCTAGCGCATGAACTAATGAAGACGTATGAACATAAGTATATTTTAATTGCGACAATTTGCTTGAGTTGGATTAG

TTTATTTAGTGCGCAATGGGTAGTTGAAAATGTGTTTGAAGCTACGACAGAAATGAGTATACTTATTGATTTGATTGGTGGAAGTTATTTC
ATTTATCTATTAGTTAGAAGGAGAAATGCGCAATGA

Gene: sstC (iron compound ABC transporter, ATP-binding protein)

Contig: 05_NODE_4, position: 238680 to 239441, length: 762 nt, orientation: FORWARD

Perfect match to: (N315-BA000018-[788951:789712], highly conserved allele)

Sequence:

ATGATTCAAGTTGAAAATTTAACTAAAACTATAAATAATCAAATGATATTGGAAGATATTAGCATAGATATCGAAAAAGGTAAATTGACT
TCTTTAATTGGACCTAATGGTGCGGGTAAGAGTACTTTACTTTCAGCGATATGTAGGTTAATTCGTTTTGATAACGGTGAAGTGAAAATA
GATGGACAGCTCATGTCTGATTATAAAAATAATGACTTGTCGAAAAAAATATCTATATTAAAACAAACAAACCATACTGAAATGAATATT
ACGGTAGAGCAGTTGGTAAACTTTGGACGATTCCCTTATTCTAAAGGTCGTTTGACGAAAGAGGATCATGATATTGTCAATGATGCGCTA
GATTTGTTGCAACTACAAGATATCAGAAATCGTAATATTAAGTCATTATCTGGTGGACAACGTCAGCGTGCATACATTGCAATGACAATA
GCACAAGATACTGAATATATTTTGCTAGATGAACCATTAAATAATTTAGATATGAAGCATGCTGTTCAAATTATGCAAACGTTAAAAATGT
TAGCGCATAAAATGAATAAAGCGATTGTCATTGTGTTACATGATATTAACTTTGCGTCCTGTTATTCAGATCAGATTGTAGCATTGAAAAA
CGGACAACTAGTTAAGTCAGATTTGAAAGATAATGTCATTCAAAGTAGTGTTTTAAGTGATTTATATGACATGAATATTCAAATTGAACAT
ATAAGAAATCAAAGGATTTGTTTATATTTTAAGGATTGA

Gene: sstD (iron compound ABC transporter, substrate-binding protein)

Contig: 05_NODE_4, position: 239560 to 240588, length: 1029 nt, orientation: FORWARD

Perfect match to: (COL-CP000046-[822428:823456], highly conserved allele)

Sequence:

ATGAAGAAAACAGTCTTATATTTAGTATTAGCAGTAATGTTTTTATTAGCGGCATGCGGTAACAATTCTGATAAAGAACAATCAAAATCA
GAAACTAAAGGTTCTAAAGATACAGTAAAAATTGAAAATAACTATAAAATGCGTGGCGAGAAAAAAGATGGTAGTGACGCTAAAAAAG
TTAAAGAAACTGTTGAAGTACCAAAAAATCCTAAAAATGCAGTTGTGTTAGACTATGGCGCATTAGATGTAATGAAAGAAATGGGCTTAT
CAGATAAAGTAAAAGCATTACCTAAAGGGGAAGGCGGTAAGTCATTACCGAATTTCTTAGAATCATTTAAAGATGATAAATATACAAAC
GTTGGTAATTTAAAAGAAGTGAATTTTGATAAAATTGCTGCGACGAAACCCGAAGTAATCTTTATCTCTGGACGTACAGCTAATCAAAAG
AATTTAGATGAATTCAAAAAAGCTGCACCTAAAGCGAAAATTGTTTATGTTGGTGCAGATGAAAAGAACTTAATTGGTTCAATGAAACAA
AACACTGAAAATATCGGTAAAATTTACGATAAAGAAGATAAAGCTAAAGAATTAAATAAAGATTTAGATAACAAAATTGCTTCAATGAAA
GATAAAACGAAAAACTTCAATAAAACTGTTATGTATTTACTAGTTAACGAAGGTGAATTATCAACATTTGGACCTAAAGGTCGTTTTGGTG
GATTAGTTTACGATACATTAGGATTCAATGCAGTTGATAAAAAAGTAAGTAATAGCAATCATGGACAAAATGTTTCTAACGAATATGTTA
ATAAAGAAAATCCAGATGTTATTTTAGCGATGGATAGAGGTCAAGCGATAAGTGGTAAATCAACTGCGAAACAAGCATTAAATAATCCT
GTATTAAAAAATGTTAAAGCAATTAAAGAAGACAAAGTATATAATTTAGATCCTAAATTATGGTACTTTGCAGCTGGATCAACTACAACTA
CAATTAAACAAATTGAGGAACTTGATAAAGTTGTAAAATAA

Gene: Q5HHT3 (putative protein)

Contig: 05_NODE_4, position: 240905 to 241219, length: 315 nt, orientation: REVERSE

Perfect match to: (N315-BA000018-[791176:791490:r], highly conserved allele)

Sequence:

TTAAATTTCAAAATAAATATGATAGTGATATTTACAGCGATTGTTAAACCGAGATTGGCAATTTGGACAACGCTCTACCATCATATATTCA
TTGATTGTTAATTCGTGTTTGCATACACCGCATAAGATTGCTTTTTCGTTAAATGAAGGCTCAGACCAACGCTTAATGGCGTGCTTTTCAAA
CTCATTATGGCACTTATAGCATGGATAGTATTTATTACAACATTTAAATTTAATAGCAATAATATCTTCTTCGGTAAAATAATGGCGACAGC
GTGTTTCAGTATCGATTAATGAACCATAAACTTTAGGCAT

Gene: murB (UDP-N-acetylenolpyruvylglucosamine reductase)

Contig: 05_NODE_4, position: 241237 to 242160, length: 924 nt, orientation: REVERSE

Perfect match to: (ED133-CP001996-[802255:803178:r], highly conserved allele)

Sequence:

TTACGATTCCTTTGGATGTTCACCAATAATGCGAACTTCACGATTTAATTCAATGCCAAATTTTTCTTTGACGGTCTTTTGTACATAATGAAT
AAGGTTTTCATAATCTGTAGCAGTTCCATTGTCTACATTTACCATAAAACCAGCGTGTTTAGTTGAAACTTCAACGCCGCCAATACGGTGA
CCTTGCAAATTAGAATCTTGTATCAATTTACCTGCAAAATGACCAGGCGGTCTTTGGAATACACTACCACATGAAGGATACTCTAAAGGTT
GTTTAGATTCTCTACGTTCTGTTAAATCATCCATTTTAGCTTGTATTTCAGTCATTTTACCAGGAGCTAAAGTAAATGCAGCTTCTAATACA
ACTAAGTGTTCTTTTTGAATAATGCTATTACGATAATCTAACTCTAATTCTTTTGTTGTAAGTTTAATTAACGAGCCTTGTTCGTTTACGCAA
AGCGCATAGTCTATACAATCTTTAACTTCGCCACCATAAGCGCCAGCATTCATATACACTGCACCACCGATTGAACCTGGAATACCACATG
CAAATTCAAGGCCAGTAAGTGCGTAATCACGAGCAACACGTGAGACATCAATAATTGCAGCGCCGCTACCGGCTATTATCGCATCATCAG
ATACTTCGATATGATCTAGTGATAATAAACTAATTACAATGCCACGAATACCACCTTCACGGATAATAATATTTGAGCCATTTCCTAAATAT
GTAACAGGAATCTCATTTTGATAGGCATATTTAACAACTGCTTGTACTTCTTCATTTTTAGTAGGGGTAATATAAAAGTCGGCATTACCAC
CTGTTTTAGTATAAGTGTATCGTTTTAAAGGTTCATCAACTTTAATTTTTTCATTTGGGATAAGTTGTTGTAAAGCTTGATAGATGTCTTTAT
TTATCAC

Gene: grpB (putative nucleotidyltransferase)

Contig: 05_NODE_4, position: 242287 to 242805, length: 519 nt, orientation: REVERSE

Perfect match to: (MW2-BA000033-[788282:788800:r], allele observed in CC1+CC80)

Sequence:

TTATAAGTATTTTTTACATAGTTTTTCAAAGTATTGTTGCTTTTGCATCTCATATTGTCTAATTGTTAAGCTATGTTGCAATATTTGGTGCTTT
TTTGTATGGAATTGCAAAGCAATATCATCATTAGTTGATAAGAGGTAATCAAGTGCAAGATAAGATTCAAATGTTTGGGTATTCATTTGA
ATGATATGTAGACGCACCTGTTGTTTTAGTTCATGAAAATTGTTAAACTTCGCCATCATAACTTTCTTATTATATTTATGATGCAAGCGATA
AAACCCTACATAATTTAAGCGTTTTTCATCTAAGGATGTAATATCATGCAAATTTTCTACACCTACTAAAATATCTAAAATTGGCTCTGTTG
AATATTTAAAATGATGCGTACCGCCAATATGTTTTGTATATTTTACTGGGCTGTCTAAGAGGTTGAATAATAATGATTCAATTTCAGTGTAT
TGTGATTGAAAACAATTAGTTAAATCACTATTAATGAATGGTTGAACATTTGAATACAT

Gene: Q5HHT0 (putative lipoprotein)

Contig: 05_NODE_4, position: 242925 to 243803, length: 879 nt, orientation: FORWARD

Perfect match to: (MRSA252-BX571856-[831583:832461], highly conserved allele)

Sequence:

ATGAAAAAAATTGTTATTATCGCTGTTTTAGCGATTTTATTTGTAGTAATAAGTGCTTGTGGTAATAAAGAAAAAGAGGCACAACATCAAT
TTACTAAGCAATTTAAAGATGTTGAGCAAAAACAAAAAGAATTACAACATGTCATGGATAATATACATTTGAAAGAAATTGATCATCTAA
GTAAAACTGATACAACTGATAAAAATAGTAAAGAATTTAAGGCACTACAAGAAGATGTTAAAAACCATCTCATACCTAAATTTGAAGCAT
ATTATAAGTCAGCAAAAAATTTGCCTGATGATACAATGAAAGTTAAGAAATTAAAAAAAGAATATATGACGCTTGCAAATGAGAAGAAG
GATGCGATATATCAATTAAAAAAATTCATAGGTTTATGTAATCAATCTATCAAGTATAACGAAGACATTTTAGATTATACGAAACAATTTG
AAAAAAATAGATACAAAGTTGAATCAGAAATTAAATTAGCTGATAATAAAAGTGAAGCAACTAATCTTACGACAAAATTAGAACATAATA
ATAAAGCGTTAAGAGATACTGCGAAGAAGAACCTAGATGATAGTAAAGAAAATGAAGTAAAAGGCGCGATTAAAAATCACATTATGCC
AATGATTGAAAAGCAAATTACCGATATTAACCAAACTAATATTAGTGATAAGCATGTTAATAATGCAAGGAAAAACGCAATAGAAATGTA
TTACAGTCTGCAGAACTATTATAATACACGTATTGAAACAATAAAGGTTAGTGAGAAGTTATCAAAAGTCGATGTAGATAAGTTGCCGAA
AAAGGGTATAGATATAACTCACGGCGATAAAGCCTTTGAAAAAAAGCTTGAAAAATTAGAAGAAAAATAA

Gene: ytxJ (thioredoxin-like protein)

Contig: 05_NODE_4, position: 243957 to 244277, length: 321 nt, orientation: FORWARD

Perfect match to: (RF122-AJ938182-[759206:759526], highly conserved allele)

Sequence:

GTGGCTATAAAGCTAAGTTCAATTGACCAATTTGAACAGGTTATTGAGGAAAATAAATATGTTTTTGTATTAAAACATAGTGAAACTTGTC
CAATATCGGCAAATGCGTACGATCAATTTAATAAATTTTTATATGAACGCGATATGGACGGTTATTATTTGATTGTCCAACAAGAACGCGA

TTTGTCAGATTATATTGCTAAAAAAACGAACGTTAAACATGAATCACCTCAAGCATTTTATTTTGTAAATGGTGAAATGGTTTGGAATCGA
GACCACGGTGATATCAATGTGTCGTCATTAGCACAAGCAGAAGAATAA

Gene: glxK2 (glycerate kinase, locus 2)

Contig: 05_NODE_4, position: 244702 to 245826, length: 1125 nt, orientation: FORWARD

Perfect match to: (MW2-BA000033-[790697:791821], allele observed in CC1+CC30)

Sequence:

ATGAAAGTATTAGTAGCCATGGATGAGTTTCATGGAATTATTTCGAGTTATCAAGCTAATAGATATGTTGAAGAGGCAGTTGCAAGCCAA
ATTGAAACTGCAGATGTAGTTCAAGTACCATTGTTTAATGGAAGACATGAATTATTAGATTCTGTATTTTTATGGCAATCTGGGCAAAAGT
ATCGTATACCAGTACATGATGCAGATATGAATGAAGTTGAAGGTGTTTACGGACAAACTGATACAGGGATGACCGTTATCGAGGGGAAT
TTATTTTTAAAAGGTAAAAAACCAATTGTTGAACGAACAAGTTATGGTTTAGGAGAAATGATTAAACATGCATTAGATAACGACGCAAAA
CATGTTGTAATTTCACTAGGTGGGATTGATAGTTTTGATGCTGGTGCAGGTATGTTACAAGCATTAGGTGCTCAATTCTATGATGACGAA
GGGCGTGTCGTAGATATGAGACAAGGTGCTGGTGTAATTAAATATATTCGTCGTATGGATATGTCGAACTTACACCCTAAAATGGAAAC
AGCAAGAATTCAAGTAATGTCGGATTTTTCAAGTCGATTATATGGTAAGCAAAGTGAAATCATGCAAACTTATGATGCGCATCAGTTGAA
TCATAATCAAGCAGCAGAAATCGATAATTTAATTTGGTATTTTAGTGAGTTATTTAAAAGTGAATTGAAAATTGCAATTGGTCCAGTTGAA
CGTGGTGGTGCTGGTGGTGGAATTGCAGCAGTCTTGAATGGACTGTATCAAGCTGAAATATTAACCAGTCATGCATTAGTAGACCAACT
AACACATTTAGAAAATTTAGTTGAACAAGCGGATTTAATTATTTTTGGAGAAGGGTTAAATGAAAATGATCAGTTGCTAGAAACGACAAC
ATTGCGTATTGCAGAACTTTGTCATAAACATCAAAAGGTTGCCATTGCAATTTGTGCAACTGCTGAAAAGTTTGATTTATTTGAATCACAA
GGGGTTACAGCAATGTTTAATACATTTATCGATATGCCAGAAACTTATACTGACTTTAAAATGGGGTTACAAATTAGGCATTATACGGTTC
AGTCTTTAAAACTGTTGAAAACACATTTTAATGTTGAGGTTTAG

Gene: pepT (peptidase T)

Contig: 05_NODE_4, position: 246008 to 247234, length: 1227 nt, orientation: REVERSE

Perfect match to: (MW2-BA000033-[792003:793229:r], allele observed in CC1)

Sequence:

TTAGTGATTTTCAGCGATATCTTCTACAATTCCAATGATTACTTGTACTGCTTTTTCCATAACATCAATGGATGCATATTCATATGGGCCGT
GGAAGTTACCGCAACCTGTAAAGATGTTTGGAGTTGGTAACCCCATAAATGACAATTGTGAACCATCTGTACCACCGCGAATAGGTTCAG
TGTTTGCTGGAATATCTAATTTGGCAAAGACACGTTTAGGTATATCAATAATATGAGGCAATGGTAATATTTTTTCTGCCATATTGAAATA
TTGATCCGATATATCAACTTTAACTGGATAATTTTCAAAATGGGCATTGATATCGTCACGTATTTCTAAAATACGTTTCTTACGCAATTCGA
ATTGTTTTTTATCATGATCACGAATAATGTATTGCAAAGTTGCTTTTTCAACAGTTCCTTCAAAGTTCATTAAGTGATAAAAGCCTTCGTAT
CCTTCTGTTCGCTCCGGAACTTCACTATCAGGTAGCAAACTATCGAATTGTTCACCTAAACGTATTGCGTTTACCATTGCATTTTTAGCTGA
ACCAGGATGAACATTTACACCGTGGCATGTAATAACCGCTTCAGCAGCGTTAAAGCTTTCATATTGTAATTCTCCATATTGACTACCATCC
ATAGTATAAGCAAAATCAGCATTGAAGCGGTCAACATCAAATTTATGTGGACCACGACCGATTTCTTCGTCTGGTGTAAATCCAATGCGA
ATGGTACCATGTTTAATTTCTGGATGTTCTTGTAAATAACAAATAGCTTCCATAATTTCCACAATACCCGCTTTATCGTCTGCACCTAGTAA
CGATGTACCATCAGTTACCATTAATGTATGACCAACTAAACTGTTAAGTTCTGGAAATACTTTAGGATCTAAGACACGTTTAGTATTGCCT
AGTTTGTATGGCTTACCATCATAGTTTTCAATAATTTGCGGTTTAACATTTGAAGCATTGAAATCAGGTGATGTATCAACATGCGCCAAAA
ATCCAACTGTTGGGACATCGACATCGATGTTACTTTCTAATGTAGCAAATAAGTAGCCATTTTCATCTAAATCAGTTGGCAATCCTAATTGT
TGTAATTCTTTTTCTAATAAATGTAACAAATCCCATTGCTTTTCAGTTGAAGGTGTTGTTGTAGATTTTGGATCAGATTGCGTATCAATTGT
CGTATATCTTGTTAATCTATCTATCAATTGGTTCTTCAT

Gene: Q5HHS6 (putative membrane protein)

Contig: 05_NODE_4, position: 247248 to 247742, length: 495 nt, orientation: REVERSE

Perfect match to: (MW2-BA000033-[793243:793737:r], allele observed in CC1+CC12+CC88)

Sequence:

TTAAACTCTATTATTCATGTTGTAAGATTTTTTATATGTCTTACCTTTGATTTTACCATACAGTTGTTTGATACGTGTGTATAGGTAATATAG
AATTTCAGAAACTAATATACCGAAAGCAATCGCACCTGAAATCAGTGTAACTTCTAAAAATGTATTTACAGCACTTGTATAATCATTTGAT
ACTAAAAAACGAGTCGCTTGATAAGCTGCACCACCAGGTACTAATGGTATAATGCCTGGCACTATGAATATAATTACCGGTCGTTTATATC
TGCGGCTCATAGTATGACTCATTAAGCCTAAAATTAAGCTTCCCAAAAATGAAGCGCCAACTTTTCCAAACTCTAAATCTACCGTTAATTG

GTAAATCGTCCATGCAATGGCACCCACAAATCCACATGCTACTAAGAGGCGTTTGGGTGCATTGAAAATGATAGAGAAAAGTACTGTTG
ATATAAAGCTGATTGTAAAATGAAATAAATAAAATAGCAT

Gene: DUF1212_L2 (protein of unknown function DUF1212, locus 2)

Contig: 05_NODE_4, position: 247760 to 248521, length: 762 nt, orientation: REVERSE

Perfect match to: (MW2-BA000033-[793755:794516:r], allele observed in CC1+CC5+CC45I+CC49)

Sequence:

TTAAATGATTAATAAAACGATTGCGACACCAGCACCGATTGCGAATGCTGTTAATGCAGCTTCAACACCACGAGACATACCTGCAAGTAA
TTCACCCGCTAATAAATCTCGAATGGCATTGGTAATTAATATACCAGGGACAAGTGGCATGACACTGGCTATAGTAATGATATCTTGATT
GGTTGCAATGCCTAATTTAGTAAATGTGGCTGCAATGGATATGACCACAGCGGCTGCAACAAACTCTGAGAAAAATTTAATTTGTATATA
GCGTTGCACAAAGCTGAATGTTAAAAATGCGGATCCGCCAGCAATGACTGCAATCCAACAATCTGATGCGACACCACCAAACATAAATA
GGAAGAAGCCACATGCAATGGCAGCTGCAAAGAAATTCGTTAAAAAAGAATATTGTAATGATGCATGCTGTAAATGAATAAATTCAGAT
TTAGCTTCATCAATTGTGAGTTCTTTATTTGATATTTTACGTGAAAGACTATTCGTTAAAGCGATTTTCTCTAAATCTGTTGTACGCTCTTGT
ACACGAATTAATCTTGTACTTGTTCGATCGTTTAATGAAAAAATAATTGCAGTTGAACTGACAAAACTATATGTATTATGAAGACCATAAC
TATGTGCGATACGGTTCATTGTATCTTCAACTCGATATGTTTCAGCACCTGATTCAAGTAAAATTCTACCTGCAATTAATACAACATCAATC
ACTTTGTTTTCATCTATAATTGTGATTGAATCTGGCAT

Gene: gdpS (membrane bound diguanylate cyclase GGDEF domain-containing protein)

Contig: 05_NODE_4, position: 248717 to 249787, length: 1071 nt, orientation: REVERSE

Perfect match to: (Strain_21193-AFEG01000023-[44819:45889], allele observed in CC25+CC361)

Sequence:

TTATAAATTGATAATAGGGTTAAACATTACTTTGTTTCGCCCTTGATTTTTGGCTACATGCACCATATCGTCTGCATCTTTAAACACTTTACG
CTGTGATTTTGGATCGTCATCTGTTAAATATCCAACACCGATAGACACTGACAATTTAATAACTTCTTTGTTTGGTAAATGGAATGATGATT
TTTCAACACCCGAACGAATATTTTCAGCCAATTTAACACTTTGATCAAGTGAATAATTGTGAATGACAACTGAGAACTCTTCGCCACCATTT
CTAAAAATTTTAAATTGATTCGGCACATAGTTTTTAAGTAATTGAGACATTTGTTTTAATACAGCATCACCTGATTTGTGTGAGTAGGTATC
ATTGACATCTTTAAATCCATCGATATCGATTAATAATAAAGCGATACTTTGATGTTCTTTTTCAGCTTTTCGTGAAATTTCATTTAAATGTCT
ATCAAATTCTTTTACATTACCTAAGCCTGTTAAGTAATCATATTTATCTTCGTTTTCATAACGATTTACGAGTGAGAAGAAATGCCAAATAT
CGACAAATGTTATCGCTGAAGCTAAAGTGATAATTAATGAAATTGGTATTAAAATGATAACTTCCGATAGTGTGTAAATAGGACTCACTA
ACGCGACACCAAATAAAATGATTATTGTAACAACATTAAGTATTAATAATGATAGCACATCATTTTGTTTTAAAAATGGTCCAATAGCACT
TGTTACTGCAGCAATAACAATCAACGTAACACCGTACATAATCGAGTTGTTAAATACTACAATTTCAACAATTGCTACAATTACTGTGGCA
GATAATGTATAGACCATATTTGTAAATCTACCTAAAAACAATAAAGGAACGAATGTTAAGTGAATTAAATAATCTTCACGATAAGGGATA
GGGTAGACAGATAATAATAATGATACGATTGTCATTAAAACAGTGACATAAGCCTTAGAAAAAACCATACGTTTGTTTTCTGAATACTGT
AAGCGATGGAATAAATAGATTCCAGCGACTATAACAGATATATTGTATATAAATGCTTCGAACAT

Gene: tarO (N-acetylglucosamine-1-phosphate transferase)

Contig: 05_NODE_4, position: 250105 to 251160, length: 1056 nt, orientation: FORWARD

Perfect match to: (CA347-CP006044-[810751:811806], highly conserved allele)

Sequence:

ATGGTTACATTATTACTAGTTGCAGTAACAATGATTGTCAGTTTGACGATAACACCAATTGTTATTGCAATATCGAAAAGATTAAATTTAG
TTGATAAACCAAATTTTAGAAAAGTACACACTAAACCTATTTCAGTTATGGGTGGTACAGTGATTCTCTTTTCATTTTTAATAGGTATTTGG
ATTGGTCATCCTATTGAAACAGAAATCAAACCACTTATTATTGGTGCGATTATTATGTACGTACTTGGGCTTGTAGATGATATCTACGATTT
GAAACCGTATATAAAATTGGCTGGTCAAATTGCCGCTGCCTTAGTAGTTGCTTTTTATGGTGTGACTATTGATTTTATTTCGTTGCCAATG
GGTACAACGATTCATTTTGGATTTCTTAGTATTCCAATTACTGTGATTTGGATTGTTGCTATTACAAATGCAATTAACTTAATTGATGGACT
CGATGGTTTGGCGTCGGGTGTTTCTGCAATCGGACTCATTACAATAGGATTCATTGCAATTTTACAAGCTAATATTTTCATAACGATGATT
TGTTGTGTTTTATTAGGCTCTTTAATTGGGTTTTTATTTTACAATTTCCATCCTGCCAAAATATTTTTAGGTGATAGTGGGGCTTTAATGATT
GGATTTATCATCGGATTCCTTTCTTTACTCGGATTCAAAAATATTACAATTATTGCATTGTTCTTCCCAATTGTTATCTTAGCAGTTCCATTC
ATTGATACTTTGTTCGCAATGATTCGACGTGTGAAAAAAGGGCAGCATATAATGCAAGCTGATAAATCGCATTTGCATCATAAACTATTA
GCTTTAGGCTACACACATAGACAAACAGTATTATTAATCTATTCAATCTCTATTTTATTTAGTCTTTCGAGCATTATTTTGTATGTATCGCCA

CCATTAGGTGTTGTATTAATGTTTGTATTAATCATATTTAGTATTGAATTAATTGTTGAATTTACAGGATTAATAGATAACAACTACCGACC
AATATTAAATTTAATTAGTCGGAAATCGTCACACAAAGAGGAATAG

Gene: Q5HHS2 (putative protein)

Contig: 05_NODE_4, position: 251332 to 251973, length: 642 nt, orientation: REVERSE

Perfect match to: (MW2-BA000033-[797327:797968:r], highly conserved allele)

Sequence:

TTATTCAGTTGGTATATCGAAAGGTAACTGCTTTGGAGTTTCTTCAGTCAAATCGAAATTTCCTGCAGTCATTTGATTTAAAAAGTTAATAA
ACGCTTCATAGTCACTTTTAACGACATCGATATAGTAGCTTACCTTATCAGTGTAAGTTTGGTTTCTTAACATAAAATGAGTTGAAGCTAAT
TCATATTCAAATTTACCAGTTTGATCATAATTCAGTGTTACTATACATGGTACTGCTTCTCGTAGTTCGACACGTCCGACATCATAAATGAC
GTCTCTAACAGCACCGCTATAGGCGCGAATTAAACCGCCACCACCTAATTTAATACCACCAAAATATCTTGTTACTACGACACACGCATTA
TGAACATCGAGCTTTTTTAATATGTCTAACATTGGGACACCGGCAGTTCCTGTCGGTTCACCGTCATCATTCGCTTTTTGAATATTCATTTC
AGGTCCAATAGTATATGCAGAACAATTATGAGTGGCATCTTTATGTTCTTTTTTTATTGCAGCAATAAATGCTTTAGCTTCATCTTCATTTTG
AACAGGTTTGATATGAGCAATGAATCTTGATTTACTAATCACATTTTCAATAATGTGTTCTTTTTTAACAGTAATGATATTTTGTGTCAT

Gene: degV1 (DegV domain-containing fatty-acid-binding protein)

Contig: 05_NODE_4, position: 252117 to 252983, length: 867 nt, orientation: FORWARD

Perfect match to: (MW2-BA000033-[798112:798978], allele observed in CC1+CC12+CC398)

Sequence:

ATGAAAATTGCTGTGATGACCGATTCTACAAGTTATCTGTCGCAGGACTTAATCGATAAATATAATATTCAAATAGCGCCATTAAGTGTGA
CTTTTGATGATGGGAAGAACTTTACAGAAAGTAATGAAATAGCAATTGAAGAATTTTATAATAAAATGGCATCGTCTCAAACGATTCCAA
CAACAAGCCAACCAGCAATTGGCGAATGGATTACTAAATATGAAATGCTAAGAGATCAGGGTTACACAGATATCATTGTCATTTGCTTAT
CCAGTGGGATTAGTGGAAGTTATCAATCTAGTTATCAAGCAGGGGAAATGGTTGAAGGTGTTAATGTACATGCATTTGATAGTAAGCTT
GCAGCAATGATTGAAGGATGTTATGTATTACGTGCTATTGAAATGGTTGAAGAAGGATACGAACCACAGCAAATTATTGATGATTTAACT
AATATGCGTGAACACACAGGTGCATATTTAATTGTTGATGACTTAAAGAATTTACAAAAAAGTGGTCGAATTACTGGTGCTCAAGCATGG
GTTGGAACATTATTGAAAATGAAGCCAGTTCTTAAGTTTGAAGATGGCAAGATTATACCAGAAGAAAAAGTTCGTACTAAAAAGCGTGC
CATTCAAACATTAGAAAAGAAAGTATTAGATATTGTAAAAGACTTTGAAGAAGTAACTTTATTTGTCATAAATGGAGATCATTTCGAAGA
TGGTCAAGCGTTATACAAAAAGTTACAAGATGATTGTCCTTCAGCTTATCAAGTAGCATACTCTGAGTTTGGTCCAGTTGTTGCAGCACAT
TTAGGTTCTGGTGGATTAGGTTTAGGCTATGTTGGCAGAAAAATAAGATTAACATAA

Gene: comFA (type II DNA/RNA helicase)

Contig: 05_NODE_4, position: 253347 to 254429, length: 1083 nt, orientation: FORWARD

Perfect match to: (MW2-BA000033-[799342:800424], allele observed in CC1+CC88+CC239+CC4803)

Sequence:

ATGGATAATGTAACAAGATATAAAATAACAGAGAGTTCGCAAAGTTCATCACAAGCATATTATCATCTCTCATTTGAATTGTCGGAACAG
CAGTCTTATGCCTCAGAACATATTGTTCGAGCCATTAGAAAGAGACAAACGATTTTGTTATATGCCGTAACAGGTGCAGGTAAGACAGAA
ATGATGTTTCAAGGCATTCAATATGCAAGAATACAGGGAGATAATATAGCTATTGTGTCACCACGTGTAGATGTTGTTGTAGAAATTAGT
AAACGTATTAAAGACGCATTTCTTAATGAAGATATAGACATACTACACCAGCAATCAAGACAACAATTTGAAGGGCATTTTGTTGTATGC
ACAGTGCATCAACTTTACCGATTCAAACAGCACTTTGATACTATTTTTATTGATGAAGTCGATGCCTTTCCTTTATCAATGGATAAAAATCT
ACAACAAGCATTGAAGTCATCTTCTAAAGTTGAACATGCAACAATTTATATGACAGCAACACCACCGAAACAACTTCTGTCAGAGATTCCC
CACGAAAATATAATTAAATTGCCAGCTCGCTTTCATAAAAAATCACTTCCAGTTCCTAAATATCGTTATTTCAAACTTAATAATAATAAGAT
TCAGAAAATGTTATACCGAATTTTACAAGATCAAATTAATAATCAACGTTATACACTGGTGTTTTTAACAATATAGAAACAATGATTAAA
ACATTTTCGGTTTATAAGCAGAAAATTACTAAATTAACATACGTCCATAGCGAGGATGTTTTTCGCTTTGAAAAAGTTGAACAATTAAGGA
ATGGACATTTCGATGTCATTTTTACTACGACAATATTAGAACGTGGATTTACAATGGCAAATTTGGATGTTGTTGTTATCGATGCACATCA
ATATACTCAAGAGGCTTTAATACAAATTGCTGGACGTGTTGGACGAAAATTAGAATGTCCTACTGGAAAAGTATTGTTTTTCATGAAGG
AGTAAGTATGAATATGATTCAAGCTAAAAAAGAGATTCAAAGGATGAACAAATTAGCATTAAAAAGAGGTTGGATTGATGAATAA

Gene: comFC (type II DNA/RNA helicase)

Contig: 05_NODE_4, position: 254419 to 255096, length: 678 nt, orientation: FORWARD

Perfect match to: (N315-BA000018-[804687:805364], highly conserved allele)

Sequence:

TTGATGAATAATTGTTTGAGTTGTGGTGCTAAGTTATATGAAAATATAACCATTTATAATTTGTTCAAGAAACCTAATAGATTATGTGACA
GATGCAAAGAGAATTGGGACAATATTAAACTTGATATTAAAGCAAGGCGATGTTCAAGGTGCTTAAAACACTTAAATCAAGATGAAGCG
TATTGTTTAGACTGCAAGTTTCTATCGGCACACTTTAATTTAATGGAACAATTATATTGTCAATTTCAATATGACGGTTTAATGAAAGAGAT
GATACATCAGTATAAATTTTTGAAAGACTATTATTTATGTGAATTATTGGCACATTTGATTGAAATACCACAAACATCTTATGACTATATTG
TGCCAATTCCTTCTTCGCCGGCACATGATTTATCTAGAACATTTAACCCGGTAGAAGCAGTACTAAAAGCTAAAGGGATTCGCTTTGATAA
GATTTTAAAGATGTCAAATAGACCAAAACAGTCTCATTTAACTAAGAAAGAGCGTCTGGCAGATGAAAATCCATTTATTATTGATACGGA
ATTAGATTTAAATGGCAAGGAAATATTACTCGTTGACGATATTTATACAACTGGATTAACAATTCATCGTGCAGGGTGTAAATTATATGCT
AAAAATATCAGAAAATTCAAAGTGTTTGCGTTTGCACGATAG

Gene: yfiA (sigma-54 modulation protein)

Contig: 05_NODE_4, position: 255157 to 255729, length: 573 nt, orientation: FORWARD

Perfect match to: (N315-BA000018-[805425:805997], highly conserved allele)

Sequence:

ATGATTAGATTTGAAATTCATGGAGATAACCTCACTATCACAGATGCTATTCGCAACTATATTGAGGAAAAAATTGGTAAGTTGGAACGT
TATTTTAATGACGTACCAAATGCAGTGGCGCATGTTAAAGTTAAAACTTATTCAAATTCAGCTACTAAAATTGAAGTAACAATTCCATTGA
AAAATGTTACGTTAAGAGCTGAAGAGCGAAACGATGATTTATACGCAGGTATTGATTTAATTAATAATAAACTTGAAAGACAAGTTCGAA
AATATAAAACACGTATTAATCGTAAGAGCCGTGATCGAGGAGATCAAGAAGTGTTTGTTGCCGAATTACAAGAAATGCAAGAAACACAA
GTTGATAATGACGCTTACGATGATAACGAGATAGAAATTATTCGTTCAAAAGAATTCAGCTTAAAACCAATGGATTCAGAAGAAGCGGT
ATTACAAATGAATCTATTAGGTCATGACTTCTTTGTATTCACAGACAGAGAAACTGATGGAACAAGTATCGTTTACCGCCGTAAAGACGG
TAAATATGGCTTGATTCAAACTAGTGAACAATAA

Gene: secA1 (preprotein translocase subunit A)

Contig: 05_NODE_4, position: 256143 to 258674, length: 2532 nt, orientation: FORWARD

Perfect match to: (11819-97-CP003194-[847014:849545], allele observed in CC80+CC361+CC425)

Sequence:

ATGGGATTTTTATCAAAAATTCTTGATGGCAATAATAAAGAAATTAAACAGTTAGGTAAACTTGCTGATAAAGTAATCGCTTTAGAAGAA
AAAACGGCAATTTTAACTGATGAAGAAATTCGTAATAAAACGAAACAATTCCAAACAGAATTAGCTGACATTGATAATGTCAAAAAGCAA
AATGATTATTTAGATAAAATTTTACCAGAAGCATATGCACTTGTTAGAGAAGGCTCTAAACGTGTATTCAATATGACACCATATAAAGTTC
AAATTATGGGTGGTATTGCAATTCATAAAGGTGATATCGCTGAGATGAGAACAGGTGAAGGTAAAACATTAACAGCGACAATGCCAACA
TACTTAAATGCATTAGCTGGTAGAGGTGTTCACGTTATTACAGTCAATGAATACTTATCAAGTGTTCAAAGTGAAGAAATGGCTGAGTTA
TATAACTTCTTAGGTTTGACTGTCGGATTAAACTTAAACAGTAAGACGACAGAAGAAAAACGTGAAGCATACGCACAAGACATTACTTAC
AGTACTAATAATGAGCTAGGTTTTGATTACTTACGAGATAACATGGTGAATTATTCTGAAGATAGAGTAATGCGTCCATTACATTTTGCAA
TCATTGATGAGGTTGACTCAATTTTAATCGACGAGGCACGTACGCCATTAATTATTTCTGGTGAAGCTGAAAAGTCAACGTCACTTTATAC
ACAAGCAAATGTTTTTGCGAAAATGTTAAAACAGGACGAAGATTATAAATACGATGAAAAAACGAAAGCTGTACATTTAACAGAACAAG
GTGCGGATAAAGCTGAACGTATGTTCAAAGTTGAAAACTTATATGATGTACAAAATGTTGATGTTATTAGTCATATCAACACAGCTTTAC
GTGCGCACGTTACATTACAACGTGACGTAGACTATATGGTTGTTGATGGCGAAGTATTAATTGTCGATCAATTTACAGGACGTACAATGC
CAGGCCGTCGTTTCTCGGAAGGTTTACACCAAGCTATTGAAGCGAAGGAAGGCGTTCAAATTCAAAATGAATCTAAAACTATGGCGTCTA
TTACATTCCAAAACTATTTCAGAATGTACAATAAACTTGCAGGTATGACAGGTACAGCTAAAACTGAAGAAGAAGAATTTAGAAATATTT
ATAACATGACAGTAACTCAAATTCCGACAAATAAACCTGTGCAACGTAACGATAAGTCTGATTTAATTTACATTAGCCAAAAAGGTAAATT
TGATGCAGTAGTAGAAGATGTTGTTGAAAAACACAAGGCAGGGCAACCAGTGCTATTAGGTACTGTTGCAGTTGAGACTTCTGAATATA
TTTCAAATTTACTTAAAAAACGTGGTATCCGTCATGATGTGTTAAATGCGAAAAATCATGAACGTGAAGCTGAAATTGTTGCAGGCGCTG
GACAAAAAGGTGCCGTTACTATTGCCACTAACATGGCTGGTCGTGGTACAGATATCAAATTAGGTGAAGGCGTAGAGGAATTAGGCGGT
TTAGCAGTAATAGGTACAGAACGACATGAATCTCGTCGTATTGATGACCAGTTACGTGGTCGTTCTGGACGTCAAGGTGATAAAGGGGA
TAGTCGCTTCTATTTATCATTACAAGATGAATTAATGATTCGTTTTGGTTCTGAACGTTTACAGAAAATGATGAGCCGACTAGGTTTAGAT
GACTCTACACCAATTGAATCAAAAATGGTATCAAGAGCTGTAGAATCAGCACAAAAACGTGTAGAAGGTAATAACTTCGACGCGCGTAA

ACGTATCTTAGAATACGATGAAGTATTACGTAAACAACGTGAAATTATCTATAACGAAAGAAATAGTATTATTGATGAAGAAGACAGCTC
TCAAGTTGTAGATGCAATGCTACGTTCAACGTTACAACGTAGTATCAATTACTATATTAATACAGCAGATGACGAGCCTGAATATCAACCA
TTCATCGACTACATTAATGACATCTTCTTACAAGAAGGTGACATTACAGAGGATGATATCAAAGGTAAAGATGCTGAAGATATTTTCGAA
GTCGTTTGGGCTAAGATTGAAGCAGCATATCAAAGTCAAAAAGATATCTTAGAAGAACAAATGAATGAGTTTGAGCGTATGATTTTACTT
CGTTCTATTGATAGCCATTGGACTGATCATATCGACACAATGGATCAATTACGTCAAGGTATTCACTTACGTTCTTATGCACAGCAAAATC
CATTACGTGACTATCAAAATGAAGGTCATGAATTATTTGATATCATGATGCAAAATATTGAAGAAGATACTTGTAAATTCATTTTAAAATC
TGTAGTACAAGTTGAAGATAATATTGAACGTGAAAAAACAACAGAGTTTGGTGAAGCGAAGCACGTTTCAGCTGAAGATGGTAAAGAA
AAAGTGAAACCGAAACCAATCGTTAAAGGCGATCAAGTTGGTCGTAACGATGATTGTCCATGTGGTAGTGGTAAAAAATTCAAAAATTG
CCATGGAAAATAA

Gene: prfB (peptide chain release factor 2, programmed frameshift)

Contig: 05_NODE_4, position: 258989 to 260099, length: 1111 nt, orientation: FORWARD

Perfect match to: (MW2-BA000033-[804984:806094], allele observed in CC1+CC22+CC80+CC239+CC4803)

Sequence:

ATGGAATTATCAGAAATCAAACGAAATATAGATAAGTATAATCAAGATTTAACACAAATTAGGGGGTCTCTTTGACTTAGAGAACAAAGA
AACTAATATTCAAGAATATGAAGAAATGATGGCAGAACCTAATTTTTGGGATAACCAAACGAAAGCGCAAGATATTATAGATAAAAATA
ATGCGTTAAAAGCAATAGTTAATGGTTATAAAACACTACAAGCAGAAGTAGATGACATGGATGCTACTTGGGATTTATTACAAGAAGAA
TTTGATGAAGAAATGAAAGAAGACTTAGAGCAAGAGGTCATTAATTTTAAGGCTAAAGTGGATGAATACGAATTGCAATTATTATTAGAT
GGGCCTCACGATGCCAATAACGCAATTCTAGAGTTACATCCTGGTGCAGGTGGCACGGAGTCTCAAGATTGGGCTAATATGCTATTTAGA
ATGTATCAACGTTATTGTGAGAAGAAAGGCTTTAAAGTTGAAACTGTTGATTATCTACCTGGGGATGAAGCAGGGATTAAAAGTGTAAC
ATTGCTCATCAAAGGGCATAATGCTTATGGTTATTTAAAAGCTGAAAAAGGTGTACACCGACTAGTACGAATTTCTCCATTTGATTCATCA
GGACGTCGTCATACATCATTTGCATCATGCGACGTTATTCCAGATTTTAATAATGATGAAATAGAGATTGAAATCAATCCGGATGATATTA
CAGTTGATACATTCAGAGCTTCTGGTGCAGGTGGTCAGCATATTAACAAAACTGAATCGGCAATACGAATTACCCACCACCCCTCAGGTA
TAGTTGTTAATAACCAAAATGAACGTTCTCAAATTAAAAACCGTGAAGCAGCTATGAAAATGTTAAAGTCTAAATTATATCAATTAAAATT
GGAAGAGCAGGCACGTGAAATGGCTGAAATTCGTGGCGAACAAAAAGAAATCGGCTGGGGAAGCCAAATTAGATCATATGTTTTCCAT
CCATACTCAATGGTGAAAGATCATCGTACGAACGAAGAAACAGGTAAGGTTGATGCAGTGATGGATGGAGACATTGGACCATTTATCGA
ATCATATTTAAGACAGACAATGTCGCACGATTAA

Gene: lysM (LysM domain protein)

Contig: 05_NODE_4, position: 260508 to 261347, length: 840 nt, orientation: FORWARD

Perfect match to: (MW2-BA000033-[806503:807342], highly conserved allele)

Sequence:

ATGAAAAAAACTCTTACAGTGACGGTTTCGTCAGTGTTAGCTTTTTTAGCTTTAAATAATGCAGCACATGCACAACAACATGGCACACAAG
TAAAAACACCTGTTCAACATAATTATGTATCAAATGTTCAAGCACAAACGCAATCACCGACAACTTATACAGTAGTTGCTGGCGATTCATT
ATATAAGATTGCATTAGAGCATCACTTAACGTTGAATCAATTATATTCATACAATCCTGGTGTAACACCTTTAATTTTTCCTGGTGACGTGA
TTTCACTTGTGCCTCAAAATAAAGTGAAACAAACTAAAGCGGTTAAAACACCAGTAAGAAAAGCAAGCCAAGCTAAAAAGGTAGTAAAA
CAACCTGTACAACAAGCGTCTAAAAAAGTAGTAGTTAAGCAAGCACCTAAGCAAGCAGTAGCTAAGACAGTTAATGTAGCATACAAACC
TGCTCAAGTACAAAAATCAGTACCAACTGTACCTGTTGCACATAACTACAATAAATCAGTTGCTAACAGAGGGAACTTATATGCTTATGG
AAACTGCACATATTATGCTTTCGATCGTCGTGCACAATTAGGTAGAAGTATAGGAAGTTTATGGGGCAATGCAAATAACTGGAATTACGC
AGCAAAAGTTGCAGGATTTAAAGTAGATAAAACACCAGAAGTTGGCGCTATTTTCCAAACAGCTGCTGGCCCATATGGACATGTTGGTGT
TGTTGAATCTGTAAACCCTAATGGAACAATTACTGTTTCTGAAATGAACTATGCTGGATTTAATGTTAAATCTTCAAGAACAATTTTAAATC
CAGGAAAATATAATTACATCCACTAA

Gene: yfbR (nucleotidase)

Contig: 05_NODE_4, position: 261521 to 262171, length: 651 nt, orientation: FORWARD

Perfect match to: (MW2-BA000033-[807516:808166], highly conserved allele)

Sequence:

ATGGGTGTACATCAATATTTTAAAAGATTATCAGATATGGAAAGACTTATAAGATTACCTGGAAAATTTAAATATTTTGAACACAATGTTG
CAGCACACTCCTTTAAAGTAACTAAAATTGCTCAATATCTAGCAACAGTTGAAGAATATCATGGACGAAAGATTAATTGGAAAAGCTTAT
ATGAAAAAGCATTAAATCATGATTTCGCCGAAGTGTTTACTGGTGATATAAAAACACCTGTTAAATATGCGAGTAGTGAATTAAAAAAAT
TATTTTCGCAAGTTGAAGAAGAAATGGTAGAGACCTTTATTGAAGAAGAAATTCCATTACAATATAGAGATGTTTATAAGCAACGACTGC
AAGAAGGTAAAGATGATTCATTAGAAGGCCAAATACTTTCAGTTGCTGATAAAATTGATTTGCTTTATGAAACATTTGGAGAAATACAAA
AACGTAATCCCGAAGAATTATTTTTCGAAATTTATGAAATGAGTCTAGAAACAATTATTCAATTTGACCATTTAGCATCTGTACAAGATTTT
ATTAATAATATCATTCCAGAAATGTTGACTGAAAACTTTATACCTAGAACAGAATTAAGAGAAACAACCATGAACATTTTAAATAAAAGA
AAAGAGGAAAATGAATGA

Gene: DUF2198 (putative membrane protein)

Contig: 05_NODE_4, position: 262168 to 262404, length: 237 nt, orientation: FORWARD

Perfect match to: (N315-BA000018-[812436:812672], highly conserved allele)

Sequence:

ATGATATGGTATTTTAGCGCAGCATTCTTTCCATGTGTCTTGGTAGTATTATTTAGTGTAATAACAAGAAGTAAATGGGTCGGTACTATTC
TGACATTAATTTTAATTGGTGCCTCAATCTATAAAGAGTATTTCCATAACGAGTGGATTATTTTTATTGATGTAGTGTCATTATTAGCTGGT
TATTTAATTATAGATCAACTCGAATTTCATAAACATCAAGATGAAGATCGCTAA

Gene: uvrB (ultraviolet response system subunit B)

Contig: 05_NODE_4, position: 262667 to 264658, length: 1992 nt, orientation: FORWARD

Perfect match to: (MW2-BA000033-[808662:810653], allele observed in CC1+CC15)

Sequence:

GTGACAATGGTTGAACATTATCCTTTTAAAATACATTCTGATTTTGAGCCTCAAGGTGACCAACCGCAAGCAATTGAAGAAATCGTGGAA
GGTATTAAAGCGGGGAAAAGACATCAAACTTTATTAGGTGCTACTGGCACAGGGAAAACATTTACGATGAGTAATGTTATTAAAGAAGT
TGGGAAACCAACGTTAATTATCGCGCATAACAAAACATTAGCAGGACAATTATATAGTGAGTTTAAAGAATTTTTTCCTGAAAACAGGGT
GGAATACTTTGTAAGTTACTATGATTATTATCAACCAGAGGCATACGTACCGTCTACTGACACTTTTATTGAAAAAGATGCCTCAATCAAT
GATGAAATTGATCAACTACGACATTCTGCTACAAGTGCATTATTTGAACGCGATGATGTAATTATTATTGCTAGTGTAAGTTGTATATATG
GTTTAGGTAATCCTGAAGAATATAAAGATTTAGTAGTAAGTGTTCGAGTTGGTATGGAAATGGATAGAAGTGAATTACTTAGAAAACTT
GTAGATGTGCAATATACACGAAATGACATCGATTTCCAACGAGGAACGTTTCGAGTGCGTGGTGATGTAGTTGAAATATTCCCAGCCTCT
AAAGAAGAACTTTGTATAAGGGTTGAGTTTTTCGGCGATGAGATTGACCGTATCCGAGAAGTTAACTACCTAACAGGTGAAGTGTTGAA
AGAAAGAGAACATTTTGCGATATTCCCAGCTTCTCACTTCGTAACACGTGAAGAAAAGTTGAAAGTTGCGATTGAACGTATTGAAAAAGA
ATTGGAAGAACGATTGAAAGAATTACGAGATGAGAATAAATTACTAGAAGCGCAAAGGTTAGAACAGCGTACCAACTATGATTTAGAAA
TGATGCGAGAGATGGGATTCTGTTCAGGAATTGAAAACTATTCCGTACATTTAACTTTGCGGCCACTGGGTTCGACACCATATACTTTATT
GGATTACTTTGGCGATGATTGGTTAGTAATGATTGATGAATCACATGTGACATTACCGCAAGTTCGAGGCATGTATAACGGAGACAGAG
CGCGTAAACAAGTTTTGGTGGATCATGGATTTAGATTACCGAGTGCATTAGATAACCGTCCACTTAAATTTGAAGAATTTGAAGAAAAGA
CAAAACAACTTGTGTATGTATCTGCAACGCCTGGACCATACGAAATTGAACATACGGATAAGATGGTTGAACAAATTATTCGTCCTACTG
GTTTACTGGATCCTAAGATTGAGGTTAGACCTACTGAAAATCAAATTGACGATTTATTAAGTGAAATTCAAACAAGAGTTGAGCGTAATG
AACGCGTACTTGTTACAACGCTCACTAAAAAGATGAGTGAAGATTTAACCACATACATGAAAGAAGCGGGTATTAAAGTTAATTATCTGC
ATTCAGAAATCAAGACATTAGAACGAATTGAAATAATTAGAGACTTACGAATGGGTACATATGATGTTATCGTAGGTATTAATTTATTAA
GAGAGGGTATTGATATACCAGAAGTTTCTCTAGTTGTCATATTAGATGCAGATAAAGAAGGGTTTTTACGTTCTAACCGCTCATTAATTCA
AACAATAGGTAGAGCTGCGCGTAACGATAAAGGTGAAGTCATTATGTATGCCGATAAAATGACTGATTCGATGAAGTATGCAATTGATG
AGACACAACGTCGTCGAGAAATACAGATGAAACATAATGAAAAACATGGTATTACACCTAAAACAATTAATAAAAAAATACATGATTTAA
TTAGTGCTACTGTTGAAAATGACGAAAATAATGACAAAGCACAAACTGTGATACCTAAGAAGATGACGAAAAAAGAACGTCAAAAGACA
ATCGACAATATAGAAAAAGAAATGAAACAAGCAGCGAAAGATTTAGATTTCGAGAAAGCTACAGAATTAAGAGATATGTTATTTGAATT
AAAAGCAGAAGGGTGA

Gene: uvrA (ultraviolet response system subunit A)

Contig: 05_NODE_4, position: 264666 to 267512, length: 2847 nt, orientation: FORWARD

Perfect match to: (MW2-BA000033-[810661:813507], highly conserved allele)

Sequence:

ATGAAAGAACCATCCATAGTAGTAAAAGGTGCTCGTGCGCATAACTTGAAAGATATTGATATCGAACTACCTAAAAATAAATTAATTGTT
ATGACAGGTTTATCTGGGTCAGGTAAATCGTCATTAGCATTCGATACTATATATGCTGAAGGACAACGACGTTATGTTGAATCATTAAGT
GCCTATGCGCGTCAATTTTTAGGCCAAATGGACAAACCAGATGTTGATACAATTGAAGGATTATCGCCAGCAATTTCAATAGATCAAAAA
ACAACAAGTAAAAATCCAAGATCAACTGTAGCAACAGTAACAGAAATATATGATTATATACGTTTGTTATATGCACGTGTTGGTAAACCTT
ACTGTCCAAATCACAATATAGAAATTGAATCGCAAACAGTACAACAAATGGTTGACCGCATTATGGAATTAGAGGCACGTACAAAGATTC
AATTATTAGCACCTGTCATCGCTCATCGTAAAGGTAGTCATGAAAAGCTAATCGAAGATATTGGTAAAAAAGGTTATGTACGTTTAAGAA
TCGATGGCGAAATTGTTGATGTAAATGATGTACCTACTTTAGATAAGAACAAGAATCATACAATAGAAGTTGTTGTAGACCGATTAGTTG
TTAAAGATGGAATTGAAACACGACTAGCTGACTCTATAGAAACTGCCTTAGAGCTTTCAGAAGGACAATTAACAGTCGATGTCATTGACG
GGGAAGACCTTAAGTTTTCAGAAAGCCATGCTTGTCCTATATGTGGATTTTCAATCGGAGAGTTAGAACCAAGAATGTTTAGCTTTAACA
GTCCTTTTGGTGCTTGTCCGACATGTGATGGCTTAGGCCAAAAGTTAACAGTCGATGTAGACTTGGTTGTTCCCGACAAAGATAAGACGC
TAAACGAAGGTGCAATAGAACCTTGGATACCGACGAGTTCTGATTTTTATCCAACATTGTTAAAACGTGTTTGTGAAGTTTATAAAATCAA
TATGGATAAACCTTTTAAAAAGTTAACAGAACGTCAACGTGATATTTTATTGTATGGTTCTGGTGACAAAGAAATTGAATTTACATTTACA
CAACGTCAAGGTGGTACTAGAAAACGAACAATGGTTTTCGAGGGTGTAGTTCCTAATATAAGTAGACGATTCCATGAATCTCCTTCAGAA
TATACACGTGAAATGATGAGTAAATATATGACTGAACTACCTTGCGAAACTTGTCATGGAAAGCGATTGAGTCGTGAAGCGTTATCTGTT
TATGTAGGTGGTTTAAATATTGGTGAAGTAGTCGAATATTCAATCAGTCAAGCGCTGAACTATTATAAAAACATTGATTTGTCAGAACAA
GATCAAGCGATTGCAAATCAAATATTGAAAGAAATTATTTCCCGACTCACTTTTTTAAATAATGTGGGACTTGAATATTTAACGTTAAACA
GAGCTTCAGGTACACTTTCAGGTGGTGAAGCACAACGTATTCGATTAGCAACGCAAATTGGGTCGCGTTTGACTGGTGTCTTATATGTAT
TAGATGAGCCATCAATTGGACTGCATCAAAGAGATAATGATCGATTAATTAATACACTTAAAGAAATGAGAGATTTAGGAAATACTTTAA
TTGTAGTTGAACACGATGATGATACAATGCGTGCGGCTGATTACTTAGTGGATATAGGTCCTGGTGCTGGTGAACATGGAGGGCAGATT
GTGTCTAGTGGTACTCCTCAAAAGGTAATGAAAGATAAAAAATCATTAACAGGACAATACTTGAGTGGTAAGAAACGTATTGAAGTACC
TGAATATCGCAGACCGGCTTCAGATCGTAAAATTTCTATACGTGGAGCTAGAAGCAACAATCTTAAAGGGGTTGATGTGGACATACCACT
ATCAATCATGACGGTTGTTACAGGTGTATCAGGTTCTGGTAAAAGCTCATTAGTAAATGAAGTATTATACAAATCATTAGCTCAAAAAATT
AATAAATCTAAAGTAAAGCCAGGATTGTACGATAAGATTGAAGGTATTGATCAACTTGATAAAATTATTGATATTGATCAATCACCAATA
GGTAGAACGCCACGCTCTAATCCAGCAACATATACTGGTGTGTTTGATGATATACGTGATGTGTTTGCGCAAACAAATGAAGCTAAAATT
CGAGGATATCAAAAAGGGCGTTTTAGTTTTAATGTAAAAGGTGGACGCTGTGAAGCTTGTAAAGGTGACGGTATTATTAAAATTGAAAT
GCATTTTTTACCTGATGTTTATGTTCCTTGTGAAGTGTGTGATGGTAAACGATATAATCGTGAGACACTAGAGGTTACTTACAAAGGTAAA
AATATTGCTGACATTTTAGAAATGACTGTTGAAGAAGCAACACAATTTTTTGAAAATATTCCTAAGATTAAGCGCAAGTTACAAACACTAG
TTGATGTTGGTCTTGGATACGTCACATTAGGTCAACAAGCTACAACGTTATCAGGTGGTGAGGCTCAACGTGTGAAACTTGCATCTGAAC
TTCATAAACGTTCAACTGGTAAATCTATTTATATCCTAGATGAACCGACAACAGGGTTACATGTTGACGATATTAGTAGATTATTAAAAGT
ATTAAACCGATTAGTTGAAAATGGTGATACTGTTGTAATTATTGAACATAACCTAGATGTTATCAAAACAGCAGACTATATTATAGACTTA
GGTCCTGAAGGTGGTAGTGGCGGTGGTACTATTGTTGCGACTGGCACACCCGAAGATATTGCTCAGACAAAGTCATCATATACAGGAAA
GTATTTAAAAGAAGTACTTGAACGAGATAAACAAAATACTGAAGATAAATAA

Gene: STAR (Staphylococcus aureus repeat element)

Contig: 05_NODE_4, position: 267655 to 267878, length: 224 nt

Sequence:

GGGAGTGGGACAGAAATGATAAAGAATCACTAATGATTTATTATGTAGTGGTTCTTTGTCATTAGCCACAGCTATTGTGTACTTAAAAAT
AGGAATGCATGAGTGCAACTCATGCATAAGAAATACTAATTTCTAAAGAAAAAGTATTTCTTTATGTTGGGGCCCCGCCAACTTGCATTG
TTTGTAGAATTTCTTTTCGAAATTCTTTATGTTGGGGCCCCGCC

Gene: hpr (bifunctional kinase and phosphorylase)

Contig: 05_NODE_4, position: 268119 to 269051, length: 933 nt, orientation: FORWARD

Perfect match to: (RF122-AJ938182-[785133:786065], highly conserved allele)

Sequence:

ATGTTAACGACAGAAAAACTAGTTGAAACATTAAAGTTAGATTTAATCGCTGGTGAAGAAGGACTATCGAAGCCAATTAAAAATGCTGA
TATATCAAGACCGGGCTTAGAGATGGCAGGTTATTTTTCACATTATGCGTCAGATAGAATACAACTATTAGGAACAACGGAACTATCGTT
TTACAATTTATTACCAGATAAGGATCGCGCAGGTCGTATGCGTAAACTATGCAGACCAGAAACGCCTGCAATTATTGTGACGCGTGGATT
GCAGCCACCAGAAGAATTAGTTGAAGCTGCAAAAGAATTAAATACCCCACTTATAGTTGCTAAAGATGCGACTACAAGTTTAATGAGTCG
CTTAACAACGTTTTTAGAGCATGCACTTGCAAAGACGACATCTTTACATGGTGTTTTAGTAGATGTTTACGGTGTTGGTGTACTAATTACC
GGTGATTCAGGAATAGGTAAAAGTGAGACTGCGTTGGAATTAGTTAAACGTGGGCATAGATTAGTAGCAGATGATAATGTAGAAATAC
GTCAAATTAATAAAGATGAACTAATAGGGAAACCACCAAAGTTAATAGAACATCTATTAGAAATACGTGGACTAGGTATTATCAATGTTA

TGACTTTATTTGGCGCGGGTTCAATATTAACTGAAAAACGAATTAGATTAAATATTAATTTGGAAAACTGGAACAAGCAAAAGTTATATG
ACCGCGTAGGTCTTAATGAAGAGACGCTAAGTATTTTAGATACTGAAATCACTAAAAAAACAATACCTGTAAGACCTGGTAGAAATGTTG
CGGTAATTATTGAGGTCGCTGCAATGAACTATCGATTAAATATCATGGGCATTAACACTGCCGAAGAATTTAGTGAAAGATTAAATGAAG
AAATTATCAAGAACAGTCATAAGAGTGAGGAGTAG

Gene: lgt (prolipoprotein diacylglyceryl transferase)

Contig: 05_NODE_4, position: 269057 to 269896, length: 840 nt, orientation: FORWARD

Perfect match to: (CN1-CP003979-[793234:794073], highly conserved allele)

Sequence:

ATGGGTATTGTATTTAACTATATAGATCCTGTGGCATTTAACTTAGGACCACTGAGTGTACGATGGTATGGAATTATCATTGCTGTCGGAA
TATTACTTGGTTACTTTGTTGCACAACGTGCACTAGTTAAAGCAGGATTACATAAAGATACTTTAGTAGATATTATTTTTTATAGTGCACTA
TTTGGATTTATCGCGGCACGAATCTATTTTGTGATTTTCCAATGGCCATATTACGTGGAAAATCCAAGTGAAATTATTAAAATATGGCATG
GTGGAATAGCAATACATGGTGGTTTAATAGGTGGCTTTATTGCTGGTGTTATTGTATGTAAAGTGAAAAATTTAAACCCATTTCAAATTG
GTGATATCGTTGCGCCAAGTATAATTTTAGCGCAAGGAATTGGACGCTGGGGTAACTTTATGAATCACGAGGCACATGGTGGACCTGTG
TCACGCGCTTTTTTAGAAAAATTACATTTGCCAAATTTTATAATAGAAAATATGTATATTAACGGCCAATATTATCATCCAACATTCTTATAT
GAATCCATTTGGGATGTCGCTGGATTTATTATCTTAGTTAATATTCGTAAACATTTAAAATTAGGAGAAACATTCTTTTTATATTTAACTTG
GTATTCAATTGGTCGATTCTTTATAGAAGGATTACGTACAGATAGCTTAATGCTCACAAGTAATATTAGAGTTGCACAATTAGTATCAATT
CTTTTAATTTTAATAAGTATAAGTTTAATTGTATATAGAAGGATTAAGTATAATCCACCGTTGTATAGCAAAGTTGGGGCGCTTCCATGGC
CAACAAAAAAAGTGAAGTAG

Gene: yvoF (putative acetyltransferase)

Contig: 05_NODE_4, position: 269904 to 270389, length: 486 nt, orientation: FORWARD

Perfect match to: (MW2-BA000033-[815899:816384], allele observed in CC1+CC7+CC97)

Sequence:

TTGAGAAAATTTTTATCAAAAACACATCATCATACAAACCCTTTATGGCGTGTATACCGTCTTGTTAAATTTTCGAAAGTTTTTAAGAATGT
AATTATCATTGAATTTTCGAAATTTATTCCAAGTATGGTACTGAAAAGACATATATATAAACAACTTTTAAATATTAATATCGGTAATCAAT
CGTCGATAGCTTATAAAGTAATGTTAGATATTTTTTACCCAGAACTGATTACGATTGGTAGTAACAGTGTTATTGGTTACAATGTAACAAT
TTTGACGCATGAAGCATTAGTTGATGAATTTCGTTATGGACCAGTGACGATAGGATCTAACACTTTGATTGGTGCAAATGCTACCATTTTA
CCCGGTATAACGATTGGTGACAATGTAAAAGTTGCAGCTGGTACGGTTGTTTCAAAAGATATACCGGATAATGGATTTGCATATGGCAAC
CCTATGTATATAAAAATGATTAGGAGGTGA

Gene: yvcD (tetratricopeptide repeat protein)

Contig: 05_NODE_4, position: 270397 to 271836, length: 1440 nt, orientation: FORWARD

Perfect match to: (N315-BA000018-[820722:822161], highly conserved allele)

Sequence:

ATGGCGCAAAAGAATAATAATGTAATTCCAATGACTTTTGATGATGCATTTTATCGTAAAATGGCTAAACAGAAGTTTAAACAAAGAGAA
TATAAACGAGCTGCTGAATACTTTGAAAAAGTGTTAGAATTGTCACCTGATGATCTGGAAATTCAAATTGATTATGCACAATGTCTAGTGC
AACTTGGTATTGCTAAAAAAGCAGAACATTTATTTTATGACAATATTATTTATAATAGGCATCTAGAAGATAGCTTTTATGAATTGAGTCA
GCTCAACATTGAAGTTAACGAACCAAACAAGGCATTCTTGTTTGGTATTAATTATGTTATTGTTAGCGACGACCAAGATTATAGAGATGA
ATTAGATCAAATGTTTGATGTGAAATATCAAAGCGAAGAACAAATTGAACTTGAAGCTCAATTGTTTGTAGTTCAAATACTATTCCAATAT
CTTTTTTCTCAAGGTCGATTAAAAGATGCAAAGAATTATGTCTTACATCAACCACAAGAAGTTCAAGATCATCGTGTCGTACGTAATTTAT
TGGCAATGTGTTATTTATATCTCGGTGAATATGATACGGCTAAAGCATTGTACGAAGCACTATTACAAGAGGATAGTACAGATATATATG
CATTATGCCATTATACTTTGCTACTTTATAACACTAAGGAAAATGAACAATATCAAAAATATTTAAAAATATTAAACAAAGTTGTACCTATG
AATGACGATGAAAGTTTTAAATTAGGTATTGTATTAAGTTATTTAAAGCAGTATCGTGCATCACAACAATTGTTGTACCCTTTATATAAAA
AAGGGAAATTTTTATCAATTCAAATGTACAATGCTTTAGCATATAATTATTATTATTTAGGTGAAGAAGACGAAAGTCATTACTACTGGGA
TAAATTGAAGCAAATTTCTAAAGTGGAAATTGGACATGCGCCTTGGGTAATTGAAAATAGCAAAGAAGTTTTTGACCAACATATTTTGCC
ATTACTTCAAAGTGATGACAGTCATTATCGTTTATATGGTATTTTTTTATTGGATCAATTAAATGGTAAAGAAATTGTGATGACGGAAAGT
ATTTGGCAGGTTCTGGAAAATCTAAATAATTATGAGAAATTGTATTTAACGTATTTAGTTCAAGGTTTAACGCTCAATAAATTAGACTTCA

TTCATCGCGGCTTGTTAACGCTTTACCAAAATGAATTATTTGTAAGTGAAAATGATTTAATGGTTGCATGGATTAATCAAGGTGAACTCAT
AATTGCTGAAAAAGTAGATTTAACTGATGTTGAGCCATATATCGGTGCGTTTATTTATTTGTATTTTAAAAATCAACCTCGAAACGTTACA
AAGAAGCAAATTACAACATGGTTAGGCATAACACAATATAAACTGAACAAAATGATTGAATTTCTCTTGAGCATATAG

Gene: trxB (thioredoxin reductase)

Contig: 05_NODE_4, position: 271903 to 272838, length: 936 nt, orientation: FORWARD

Perfect match to: (M1015-ACST01000009-[96018:96953], allele observed in CC30)

Sequence:

ATGACTGAAATAGATTTTGATATAGCAATTATCGGTGCAGGTCCAGCTGGTATGACTGCTGCAGTATACGCATCACGTGCTAATTTAAAA
ACAGTTATGATTGAAAGAGGTATTCCAGGCGGTCAAATGGCTAATACAGAAGAAGTAGAGAACTTCCCTGGTTTCGAAATGATTACAGG
TCCAGATTTATCTACAAAAATGTTTGAACACGCTAAAAAGTTTGGTGCAGTTTATCAATATGGAGATATTAAATCTGTAGAAGATAAAGG
CGAATATAAAGTGATTAACTTTGGTAATAAAGAATTAACAGCGAAAGCGGTCATTATTGCTACAGGTGCAGAATACAAGAAAATTGGTG
TTCCGGGTGAACAAGAACTTGGTGGACGCGGTGTAAGTTATTGTGCAGTATGTGATGGTGCATTCTTTAAAAATAAACGCCTATTCGTTA
TCGGTGGTGGTGACTCAGCAGTAGAAGAGGGAACATTCTTAACTAAATTTGCTGACAAAGTAACAATCGTTCACCGTCGTGATGAGTTAC
GTGCACAACGTATTTTACAAGATAGAGCATTCAAAAATGATAAAATCGACTTTATTTGGAGTCATACTTTGAAATCAATTAATGAAAAAG
ACGGCAAAGTGGGTTCTGTGACATTAACGTCTACAAAAGATGGTTCAGAAGAAACACACGAGGCTGATGGTGTATTCATCTATATTGGT
ATGAAACCATTAACAGCGCCATTTAAAGACTTAGGTATTACAAATGATGTTGGTTATATTGTGACAAAAGATGATATGACAACATCAGTA
CCAGGTATTTTTGCAGCAGGAGATGTTCGTGACAAAGGTTTACGCCAAATTGTCACTGCTACTGGCGATGGTAGTATTGCAGCGCAAAGT
GCAGCGGAATATATTGAACATTTAAACGATCAAGCTTAA

Gene: STAR (Staphylococcus aureus repeat element)

Contig: 05_NODE_4, position: 273033 to 273257, length: 225 nt

Sequence:

GGGAGTGAGACAGAAATGATAAAGAGCCACTAATGATTTATTATGTAGTGGTTCTTACACATTAGCCACAGCTAATGTGTACTTAAAAAT
AGGAATACATGAGTAAAACTCATGCATAAGAAATACTAATTTCTATAGAAAAAGTATTTCTTTATCGTTGTCCCACCCCAACTCGCACATT
ATCGTAAGCTGACTTTTCGTAAGCTTCTGTGTTGGGGCCCCGCC

Gene: repeat_nySAgamma (repeat element)

Contig: 05_NODE_4, position: 273200 to 273366, length: 167 nt

Sequence:

CCAACTCGCACATTATCGTAAGCTGACTTTTCGTAAGCTTCTGTGTTGGGGCCCCGCCAACTTGCATTGTCTGTAGAAATTCTTTTCGAAAT
TCTCTGTGTTGGGGCCCACACCCCAACTCGCATTGCCTGTAGAATTTCTTTTCGAAATTCTCTGTGTTGGGGCCC

Gene: yvcJ (P-loop NTPase family protein)

Contig: 05_NODE_4, position: 273603 to 274514, length: 912 nt, orientation: FORWARD

Perfect match to: (MW2-BA000033-[819598:820509], highly conserved allele)

Sequence:

ATGGATAATAATGAAAAAGAAAAAAGTAAAAGTGAACTATTAGTTGTAACAGGTTTATCTGGCGCAGGTAAATCTTTGGTTATTCAATGT
TTAGAAGACATGGGATATTTTTGTGTAGATAATCTACCACCAGTGTTATTACCTAAATTTGTAGAGTTGATGGAACAAGGAAATCCATCCT
TAAGAAAAGTGGCAATTGCAATTGATTTAAGAGGTAAGGAACTATTTAATTCATTAGTTGCAGTAGTGGATAAAGTTAAAAGTGAAAGT
GACGTCATCATTGATGTTATGTTTTTAGAAGCAAGTACTGAAAAAATTAATTTCAAGATATAAGGAAACGCGTCGTGCACATCCTTTGATG
GAACAAGGTAAAAGATCCTTAATTAATGCAATTAATGATGAGCGAGAGCATTTGTCTCAAATTAGAAGTATAGCTAATTTTGTTATAGAT
ACTACAAAGTTATCACCTAAAGAATTAAAAGAACGCATTCGTCGATACTATGAAGATGAAGAGTTTGAAACTTTTACAATTAATGTCACA
AGTTTCGGTTTTAAACATGGGATTCAGATGGATGCAGATTTAGTATTTGATGTACGATTTTTACCAAATCCATATTATGTAGTAGATTTAA
GACCTTTAACAGGATTAGATAAAGACGTTTATAATTATGTTATGAAATGGAAAGAGACGGAGATTTTCTTTGAAAAATTAACTGATTTGTT

AGATTTTATGATACCCGGGTATAAAAAAGAAGGGAAATCTCAATTAGTAATTGCCATCGGTTGTACGGGTGGACAACATCGATCTGTAGC
ATTAGCAGAACGACTAGGTAATTATCTAAATGAAGTATTTGAATATAATGTTTATGTGCATCATAGGGACGCACATATTGAAAGTGGCGA
GAAAAAATGA

Gene: mgfK (putative gluconeogenesis factor)

Contig: 05_NODE_4, position: 274511 to 275506, length: 996 nt, orientation: FORWARD

Perfect match to: (11819-97-CP003194-[865327:866322], highly conserved allele)

Sequence:

ATGAGACAAATAAAAGTTGTACTTATCGGTGGTGGCACTGGCTTATCAGTTATGGCTAGGGGATTAAGAGAATTCCCAATTGATATTACG
GCGATTGTAACAGTTGCTGATAATGGTGGGAGTACAGGGAAAATCAGAGATGAAATGGATATACCAGCACCAGGAGACATCAGAAATG
TGATTGCAGCTTTAAGTGATTCTGAGTCAGTTTTAAGCCAACTTTTTCAGTATCGCTTTGAAGAAAATCAAATTAGCGGTCACTCATTAGG
TAATTTATTAATCGCAGGTATGACTAATATTACGAATGATTTCGGACATGCCATTAAAGCATTAAGTAAAATTTTAAATATTAAAGGTAGA
GTCATTCCATCTACAAATACAAGTGTGCAATTAAATGCTGTTATGGAAGATGGAGAAATTGTTTTTGGAGAAACAAATATTCCTAAAAAA
CATAAAAAAATTGATCGTGTGTTTTTAGAACCTAACGATGTGCAACCAATGGAAGAAGCAATCGATGCTTTAAGGGAAGCAGATTTAATC
GTTCTTGGACCAGGGTCATTATATACGAGCGTTATTTCTAACTTATGTGTCAATGGTATTTCAGATGCGTTAATTCATTCTGATGCGCCTAA
GCTATATGTTTCTAATGTGATGACGCAACCTGGGGAAACAGATGGTTATAGCGTGAAAGATCATATCGATGCGATTCATAGACAAGCTG
GACAACCGTTTATTGATTATGTCATTTGTAGTACACAAACTTTCAATGCTCAAGTTTTGAAAAAATATGAAGAAAAACATTCTAAACCAGT
TGAAGTTAATAAGGCTGAACTTGAAAAAGAAAGCATAAATGTAAAAACATCTTCAAATTTAGTTGAAATTTCTGAAAATCATTTAGTAAG
ACATAATACTAAAGTGTTATCGACAATGATTTATGACATAGCTTTAGAATTAATTAGTACTATTCCTTTCGTACCAAGTGATAAACGTAAAT
AA

Gene: whiA (transcription regulator)

Contig: 05_NODE_4, position: 275617 to 276561, length: 945 nt, orientation: FORWARD

Perfect match to: (MW2-BA000033-[821612:822556], allele observed in CC1+CC15)

Sequence:

ATGAGCTTTGCATCAGAAATGAAAAATGAATTAACTAGAATAGACGTCGATGAAATGAATGCAAAAGCAGAGCTCAGTGCACTGATTCG
AATGAATGGTGCACTTAGTCTTTCAAATCAACAATTTGTTATAAATGTTCAAACGGAAAATGCAACAACGGCAAGACGTATTTATTCGTTG
ATTAAACGTGTCTTTAATGTGGAAGTTGAAATATTAGTCCGTAAAAAAATGAAGCTTAAAAAAAATAATATTTATATTTGTCGTACAAAGA
TGAAAGCGAAAGAAATTCTTGATGAATTAGGAATTTTAAAAGACGGCATTTTTACGCATGAAATTGATCATTCAATGATTCAAGATGATG
AAATGAGACGCAGTTACTTGAGAGGAGCTTTTCTGGCAGGTGGCTCAGTGAATAACCCTGAAACATCTTCGTACCATTTGGAAATTTTTT
CTCAAAATGAGAGTCATGCAGAAGGCTTAACGAAACTAATGAATAGTTATGAATTGAATGCCAAACATTTAGAGCGAAAAAAAGGAAGT
ATTACGTATTTAAAAGAAGCGGAAAAGATTTCGGATTTTCTTAGTTTGATAGGTGGCTATCAAGCGTTATTAAAATTTGAAGACGTACGT
ATTGTAAGAGATATGCGTAATTCTGTTAACCGACTCGTTAATTGTGAAACGGCAAATCTAAATAAAACAGTTAGTGCTGCGATGAAACAA
GTTGAGAGCATTAAATTGATTGATAAAGAAATTGGTATTGAAAATTTACCAGACAGGTTGAGAGAGATTGCTAGAATTCGAGTAGAACA
TCAAGAAATTTCGTTGAAAGAGCTTGGAGAAATGGTATCAACTGGTCCAATTTCAAAATCAGGTGTAAATCACCGATTAAGAAAACTTAA
TGATTTAGCCGATAAGATTAGAAATGGTGAACAAATAGAATTATAA

Gene: clpP (ATP-dependent chaperone protease, proteolytic subunit)

Contig: 05_NODE_4, position: 277134 to 277721, length: 588 nt, orientation: FORWARD

Perfect match to: (N315-BA000018-[827630:828217], highly conserved allele)

Sequence:

ATGAATTTAATTCCTACAGTTATTGAAACAACAAACCGCGGTGAACGTGCATATGATATATACTCACGTTTATTAAAAGACCGTATTATTA
TGTTAGGTTCACAAATTGATGACAACGTAGCAAATTCAATCGTATCACAGTTATTATTCTTACAAGCGCAAGACTCAGAGAAAGATATTTA
TTTATACATTAATTCACCAGGTGGAAGTGTAACAGCTGGTTTTGCGATTTATGATACAATTCAACACATTAAACCTGATGTTCAAACAATTT
GTATCGGTATGGCTGCATCAATGGGATCATTCTTATTAGCAGCTGGTGCAAAAGGTAAACGTTTCGCGTTACCAAATGCAGAAGTAATGA
TTCACCAACCATTAGGTGGTGCTCAAGGACAAGCAACTGAAATCGAAATTGCTGCAAATCACATTTTAAAAACACGTGAAAAATTAAACC
GCATTTTATCAGAGCGTACTGGTCAAAGTATTGAAAAAATACAAAAAGACACAGATCGTGATAACTTCTTAACTGCAGAAGAAGCTAAA
GAATATGGCTTAATTGATGAAGTGATGGTACCTGAAACAAAATAA

Gene: yfcH (cell-division inhibitor)

Contig: 05_NODE_4, position: 277964 to 278866, length: 903 nt, orientation: REVERSE

Perfect match to: (N315-BA000018-[828460:829362:r], allele observed in CC5+CC1)

Sequence:

TTATTCTTTAATTAAATCTTCAAGTGCCATTTTTAAATTACTATATTTAAATTGGAATCCCAATGCTTGAATTTTATTAGGTAATACTTTTTGA
GTATCCAATACTACTGTTGACATTTGACCAAGTATGAGACGCATTGCAAGACTTGGTGCCCAAGTTTCATGGGGCTTATGCATAGCTCTTG
CTAAAGTGTAGCCAAATAAATTTTGACGCTCAGGTATAGGTGCAGTTAAATTAAACGGACCACTAGCTGACTCGTTATTTATTAAAAATAA
AATAGCTTGAATTAAATCATTGATATGAATCCATGAATACCATTGTTGACCAGAACCTAATCTACCACCAATGTAATATTTGTATGGTAGTT
TCATTGTTTGTAACGCACCGCCTTCATTCGATAAAATCATACCGAAACGACCGATGACAACTCGCGTACCTAATTGTTCAAATTGTTGTGC
GAAACGTTCCCATTGATACACAATATCTGATAAGAAATCAAATGGTAAAGTTTTATAAACTTCTGTGTAACTCATAAATAAATCAGGAGG
ATAGTAACCAGTGGCGCTAGCATTAAATAAAACTTTAGGTGCTTTATTACGTGATTTAAACAATTCATATAAAGCTTGCGTAGATTGAATT
CTACTTAGCATTAGCGTTTGTTTATATTCCGGTGTCCATCGTTTATTCAGTGTAGCACCTGCTAAGTTGATGACCACATCGATATTTTGAGG
AACTTTGTGTTCCCACCCAGATTTAGCCCAGTTGACATATGAAATTTTCTTATCATTTGAAATTTGGTCGTGTCGCGTTAATATCGTGATAT
GTGAATCTGATTTTTTAATTTCATTAACTAATTGAGATCCAACCATACCAGTCCCACCAGTAATTAAGTATTGTTTCAT

Gene: Q5HHP8 (putative exported protein)

Contig: 05_NODE_4, position: 279569 to 280198, length: 630 nt, orientation: FORWARD

Perfect match to: (MW2-BA000033-[825564:826193], allele observed in CC1+CC30)

Sequence:

ATGTCGAATCAAAATTACGACTACAATAAAAATGAAGATGGAAGTAAGAAGAAAATGAGTACAACAGCGAAAGTAGTTAGCATTGCGA
CGGTATTGCTATTACTCGGAGGATTAGTATTTGCAATTTTTGCATATGTAGATCATTCGAATAAAGCTAAAGAACGTATGTTGAACGAACA
AAAGCAGGAACAAAAAGAAAAGCGTCAAAAAGAAAATGCAGAAAAAGAGAGAAAGAAAAAGCAACAAGAGGAAAAAGAGCAGAATG
AGCTAGATTCACAAGCAAACCAATATCAGCAATTGCCACAGCAGAATCAATATCAATATGTGCCACCTCAGCAACAAGCACCTACAAAGC
AACGTCCTGCTAAAGAAGAGAATGATGATAAAGCATCAAAGGATGAGTCGAAAGATAAGGATGACAATGCATCTCAAGATAAACCAGA
TGATAATCAGAAGAAAACTGATGATAATAAACAACCAGCTCAGCCTAAACCACAGCCGCAACAACCAACACCAAAGCCAAATAATAATC
AACAAAATAATCAATCAAATCAGCAAGCAAAACCACAAGCACCACAACAAAATAGCCAATCAACAACAAATAAACAAAATAATGCTAAT
GATAAGTAG

Gene: gapR (regulator of glyceraldehyde 3-phosphate dehydrogenase)

Contig: 05_NODE_4, position: 280896 to 281909, length: 1014 nt, orientation: FORWARD

Perfect match to: (MRSA252-BX571856-[871492:872505], highly conserved allele)

Sequence:

GTGAAAGACTTATTGCAAGCACAGCAAAAGCTTATACCGGATCTCATAGATAAAATGTATAAACGTTTTTCTATTCTTACTACTATCTCAA
AAAATCAGCCTGTCGGACGTCGAAGTTTAAGCGAACATATGGATATGACTGAACGTGTACTGCGTTCTGAAACAGATATGCTTAAGAAA
CAAGATTTGATAAAAGTTAAGCCTACCGGAATGGAAATTACAGCTGAAGGTGAGCAACTGATTTCGCAATTGAAAGGTTACTTTGATATC
TATGCAGATGATAATCGTCTGTCAGAAGGTATTAAGAATAAATTTCAAATTAAGGAAGTTCATGTTGTTCCTGGTGATGCTGATAATAGT
CAATCTGTTAAAACAGAATTAGGTAGACAAGCAGGTCAATTACTTGAAGGCATATTACAAGAAGACGCGATAGTTGCTGTAACTGGCGG
ATCCACGATGGCATGTGTTAGTGAAGCAATTCATTTATTACCATATAATGTATTCTTCGTACCAGCCAGAGGTGGACTAGGCGAAAATGT
TGTCTTTCAGGCAAACACAATTGCAGCCAGTATGGCACAACAAGCTGGCGGTTATTATACGACGATGTATGTACCTGATAATGTCAGTGA
AACAACATATAACACATTGTTGTTAGAGCCATCAGTCATAAACACTTTAGACAAAATTAAACAAGCAAACGTTATATTACACGGCATTGGT
GATGCGCTGAAGATGGCGCATCGACGTCAATCACCTGAAAAGGTCATTGAACAACTTCAACATCATCAAGCTGTCGGAGAGGCATTTGG
TTATTATTTTGATACACAAGGTCAAATTGTCCATAAGGTTAAAACAATTGGACTTCAATTAGAAGACCTTGAATCAAAAGACTTTATTTTTG
CAGTTGCAGGAGGCAAATCGAAAGGTGAAGCAATTAAAGCATACTTGACGATTGCACCCAAGAATACAGTGTTAATCACTGATGAAGCC
GCAGCAAAGATAATACTTGAATAA

Gene: gapA (glyceraldehyde 3-phosphate dehydrogenase, locus A)

Contig: 05_NODE_4, position: 281962 to 282972, length: 1011 nt, orientation: FORWARD

Perfect match to: (MRSA252-BX571856-[872558:873568], highly conserved allele)

Sequence:

ATGGCAGTAAAAGTAGCAATTAATGGTTTTGGTAGAATTGGTCGTTTAGCATTCAGAAGAATTCAAGAAGTAGAAGGTCTTGAAGTTGT
AGCAGTAAACGACTTAACAGATGACGACATGTTAGCGCATTTATTAAAATATGACACTATGCAAGGTCGTTTCACAGGTGAAGTAGAGG
TAGTTGATGGTGGTTTCCGCGTAAATGGTAAAGAAGTTAAATCATTCAGTGAACCAGATGCAAGCAAATTACCTTGGAAAGACTTAAATA
TCGATGTAGTGTTAGAATGTACTGGTTTCTACACTGATAAAGATAAAGCACAAGCTCATATTGAAGCAGGCGCTAAAAAAGTATTAATCT
CAGCACCAGCTACTGGTGACTTAAAAACAATCGTATTCAACACTAACCACCAAGAGTTAGACGGTTCTGAAACAGTTGTTTCAGGTGCTT
CATGTACTACAAACTCATTAGCACCAGTTGCTAAAGTTTTAAACGATGACTTTGGTTTAGTTGAAGGTTTAATGACTACAATTCACGCTTA
CACAGGTGATCAAAATACACAAGACGCACCTCACAGAAAAGGTGACAAACGTCGTGCTCGTGCAGCGGCAGAAAACATCATCCCTAACT
CAACAGGTGCTGCTAAAGCTATCGGTAAAGTTATTCCTGAAATCGATGGTAAATTAGATGGTGGTGCACAACGTGTTCCTGTAGCTACAG
GTTCATTAACTGAATTAACAGTAGTATTAGAAAAACAAGACGTAACAGTTGAACAAGTTAACGAAGCTATGAAAAATGCTTCAAACGAAT
CATTCGGTTACACTGAAGACGAAATCGTTTCTTCAGACGTTGTAGGTATGACTTACGGTTCATTATTCGACGCTACACAAACTCGTGTAAT
GTCAGTTGGCGACCGTCAATTAGTTAAAGTTGCAGCTTGGTATGATAACGAAATGTCATATACTGCACAATTAGTTCGTACATTAGCATAC
TTAGCTGAACTTTCTAAATAA

Gene: pgk (phosphoglycerate kinase)

Contig: 05_NODE_4, position: 283111 to 284301, length: 1191 nt, orientation: FORWARD

Perfect match to: (MW2-BA000033-[829050:830240], highly conserved allele)

Sequence:

ATGGCTAAAAAAATTGTTTCTGATTTAGATCTTAAAGGTAAAACAGTCCTAGTACGTGCTGATTTTAACGTACCTTTAAAAGACGGTGAAA
TTACTAATGACAACCGTATCGTTCAAGCTTTACCTACAATTCAATACATCATCGAACAAGGTGGTAAAATCGTACTATTTTCACATTTAGGT
AAAGTGAAAGAAGAAAGTGATAAAGCAAAATTAACTTTACGTCCAGTTGCTGAAGACTTATCTAAGAAATTAGATAAAGAAGTTGTTTTC
GTACCAGAAACACGCGGCGAAAAACTTGAAGCTGCTATTAAAGACCTTAAAGAAGGCGACGTATTATTAGTTGAAAATACACGTTATGA
AGATTTAGACGGTAAAAAAGAATCTAAAAATGATCCAGAATTAGGTAAATACTGGGCATCTTTAGGTGATGTGTTTGTAAATGATGCTTT
TGGTACTGCGCATCGTGAGCATGCATCTAATGTTGGTATTTCTACACATTTAGAAACTGCAGCTGGATTCTTAATGGATAAAGAAATTAA
GTTTATTGGTGGCGTAGTTAACGATCCACATAAACCAGTTGTTGCTATTTTAGGTGGAGCAAAAGTATCTGACAAAATTAATGTCATCAA
AAACTTAGTTAACATAGCTGATAAAATTATCATCGGCGGAGGTATGGCTTATACTTTCTTAAAAGCGCAAGGTAAAGAAATTGGTATTTC
ATTATTAGAAGAAGATAAAATCGACTTCGCAAAAGATTTATTAGAAAAACATGGTGATAAAATTGTATTACCAGTAGACACTAAAGTTGC
TAAAGAATTTTCTAATGATGCCAAAATCACTGTAGTACCATCTGATTCAATTCCAGCAGACCAAGAAGGTATGGATATTGGACCAAACAC
TGTAAAATTATTTGCAGATGAATTAGAAGGTGCGCACACTGTTGTATGGAATGGACCTATGGGTGTATTCGAGTTCAGTAACTTTGCACA
AGGTACAATTGGTGTATGTAAAGCAATTGCAAACCTTAAAGATGCAATTACGATTATCGGTGGCGGTGATTCAGCTGCAGCAGCAATCTC
TTTAGGTTTTGAAAATGACTTCACTCATATTTCAACTGGTGGCGGCGCGTCATTAGAGTACCTAGAAGGTAAAGAATTGCCTGGTATCAA
AGCAATCAATAATAAATAA

Gene: tpi (triosephosphate isomerase)

Contig: 05_NODE_4, position: 284423 to 285184, length: 762 nt, orientation: FORWARD

Perfect match to: (N315-BA000018-[835146:835907], highly conserved allele)

Sequence:

ATGAGAACACCAATTATAGCTGGTAACTGGAAAATGAACAAAACAGTACAAGAAGCAAAAGACTTCGTCAATGCATTACCAACATTACC
AGATTCAAAAGAAGTAGAATCAGTAATTTGTGCACCAGCAATTCAATTAGATGCATTAACTACTGCAGTTAAAGAAGGAAAAGCACAAG
GTTTAGAAATCGGTGCTCAAAATACGTATTTCGAAGATAATGGTGCGTTCACAGGTGAAACGTCTCCAGTTGCATTAGCAGATTTAGGCG
TTAAATACGTTGTTATCGGTCATTCTGAACGTCGTGAATTATTCCACGAAACAGATGAAGAAATTAACAAAAAAGCGCACGCTATTTTCAA
ACATGGAATGACTCCAATTATTTGTGTTGGTGAAACAGACGAAGAGCGTGAAAGTGGTAAAGCTAACGATGTTGTAGGTGAGCAAGTTA
AGAAAGCTGTTGCAGGTTTATCTGAAGATCAACTTAAATCAGTTGTAATTGCTTATGAGCCAATCTGGGCAATCGGAACTGGTAAATCAT
CAACATCTGAAGATGCAAATGAAATGTGTGCATTTGTACGTCAAACTATTGCTGACTTATCAAGCAAAGAAGTATCAGAAGCAACTCGTA
TTCAATATGGTGGTAGTGTTAAACCTAACAACATTAAAGAATACATGGCACAAACTGATATTGATGGGGCATTAGTAGGTGGCGCATCAC
TTAAAGTTGAAGATTTCGTACAATTGTTAGAAGGTGCAAAATAA

Gene: gpmI (2,3-bisphosphoglycerate-independent phosphoglycerate mutase)

Contig: 05_NODE_4, position: 285187 to 286704, length: 1518 nt, orientation: FORWARD

Perfect match to: (RF122-AJ938182-[803823:805340], highly conserved allele)

Sequence:

ATGGCTAAGAAACCAACTGCGTTAATTATTTTAGATGGTTTTGCGAACCGCGAAAGCGAACATGGTAATGCGGTAAAATTAGCAAACAA
GCCTAATTTTGATCGTTATTACAACAAATATCCAACGACTCAAATCGAAGCGAGTGGCTTAGATGTTGGACTACCTGAAGGACAAATGGG
TAACTCAGAAGTTGGTCATATGAATATCGGTGCAGGACGTATCGTTTATCAAAGTTTAACTCGAATCAATAAATCAATTGAAGACGGTGA
TTTCTTTGAAAATGATGTTTTAAATAATGCAATTGCACACGTGAATTCACATGATTCAGCGTTACACATCTTTGGTTTATTGTCTGACGGTG
GTGTGCACAGTCATTACAAACATTTATTTGCTTTGTTAGAACTTGCTAAAAAACAAGGTGTTGAAAAAGTTTACGTACACGCATTTTTAGA
TGGTCGTGACGTAGATCAAAAATCCGCTTTGAAATACATCGAAGAGACTGAAGCTAAATTCAATGAATTAGGCATTGGTCAATTTGCATC
TGTGTCTGGTCGTTATTATGCAATGGACCGTGACAAACGTTGGGAACGTGAAGAAAAAGCTTACAATGCTATTCGTAATTTTGATGCCCC
AACTTATGCAACTGCCAAAGAAGGTGTAGAAGCAAGCTATAATGAGGGCTTAACTGACGAATTCGTAGTACCATTCATCGTTGAGAATCA
AAATGACGGTGTTAATGATGGAGATGCAGTGATCTTCTATAATTTCCGACCTGATAGAGCAGCACAATTATCGGAAATTTTTGCGAACAG
AGCATTCGAAGGCTTTAAAGTTGAACAAGTTAAAGACTTATTCTATGCAACATTCACTAAGTATAACGACAATATCGATGCGGCTATCGTT
TTCGAAAAAGTTGATTTAAATAATACAATTGGTGAAATTGCACAAAATAACAATTTAACACAATTACGTATTGCAGAAACTGAAAAATATC
CTCACGTTACTTACTTTATGAGTGGTGGACGTAACGAGGAATTTAAAGGTGAACGCCGTCGTTTAATTGATTCACCTAAAGTTGCAACGT
ATGACTTGAAACCAGAAATGAGTGCTTATGAAGTTAAAGATGCATTATTAGAAGAGTTAAATAAAGGTGACTTGGACTTAATTATTTTAA
ACTTTGCTAACCCTGATATGGTTGGACATAGTGGTATGCTTGAGCCGACAATCAAAGCAATCGAAGCGGTTGATGAATGTTTAGGTGAA
GTCGTTGATAAGATTTTAGACATGGACGGTTATGCAATTATTACTGCTGACCATGGTAACTCTGATCAAGTATTGACGGATGATGATCAA
CCAATGACTACGCATACAACGAACCCAGTACCAGTGATTGTAACAAAAGAAGGCGTTACACTTCGAGAAACTGGTCGCTTAGGTGACTTA
GCACCTACATTATTAGATTTATTAAATGTAGAACAACCTGAAGACATGACAGGTGAATCTTTAATTAAACACTAA

Gene: eno (enolase)

Contig: 05_NODE_4, position: 286834 to 288138, length: 1305 nt, orientation: FORWARD

Perfect match to: (RF122-AJ938182-[805470:806774], highly conserved allele)

Sequence:

ATGCCAATTATTACAGATGTTTACGCTCGCGAAGTCTTAGACTCTCGTGGTAACCCAACTGTTGAAGTAGAAGTATTAACTGAAAGTGGC
GCATTTGGTCGTGCATTAGTACCATCAGGTGCTTCAACTGGTGAACACGAAGCTGTTGAATTACGTGATGGAGACAAATCACGTTATTTA
GGTAAAGGTGTTACTAAAGCAGTTGAAAACGTTAATGAAATCATCGCACCAGAAATTATTGAAGGTGAATTTTCAGTATTAGATCAAGTA
TCTATTGATAAAATGATGATCGCATTAGACGGTACTCCAAACAAAGGTAAATTAGGTGCAAATGCTATTTTAGGTGTATCTATCGCAGTA
GCACGTGCAGCAGCTGACTTATTAGGTCAACCACTTTACAAATATTTAGGTGGATTTAATGGTAAGCAGTTACCAGTACCAATGATGAAC
ATCGTTAATGGTGGTTCTCACTCAGATGCTCCAATTGCATTCCAAGAATTCATGATTTTACCTGTAGGTGCTACAACGTTCAAAGAATCATT
ACGTTGGGGTACTGAAATTTTCCACAACTTAAAATCAATTTTAAGCAAACGTGGTTTAGAAACTGCAGTAGGTGACGAAGGTGGTTTCGC
TCCTAAATTTGAAGGTACTGAAGATGCTGTTGAAACAATTATCCAAGCAATCGAAGCAGCTGGTTACAAACCAGGTGAAGAAGTATTCTT
AGGATTTGACTGTGCATCATCAGAATTCTATGAAAATGGTGTATATGACTACAGTAAGTTCGAAGGCGAACACGGTGCAAAACGTACAG
CTGCAGAACAAGTTGACTACTTAGAACAATTAGTAGACAAATATCCTATCATTACAATTGAAGACGGTATGGACGAAAACGACTGGGAT
GGTTGGAAACAACTTACAGAACGTATCGGTGACCGTGTACAATTAGTAGGTGACGATTTATTCGTAACAAACACTGAAATTTTAGCAAAA
GGTATTGAAAACGGAATTGGTAACTCAATCTTAATTAAAGTTAACCAAATCGGTACATTAACTGAAACATTTGATGCAATCGAAATGGCT
CAAAAAGCTGGTTACACAGCAGTAGTTTCTCACCGTTCAGGTGAAACAGAAGATACAACAATTGCTGATATTGCTGTTGCTACAAACGCT
GGTCAAATTAAAACTGGTTCATTATCACGTACTGACCGTATTGCTAAATACAATCAATTATTACGTATCGAAGATGAATTATTTGAAACTG
CTAAATATGACGGTATCAAATCATTCTATAACTTAGATAAATAA

Gene: Q2YSE7 (putative membrane protein)

Contig: 05_NODE_4, position: 288477 to 288935, length: 459 nt, orientation: FORWARD

Perfect match to: (N315-BA000018-[839200:839658], highly conserved allele)

Sequence:

ATGGCTGATAGAACGAATAAAGAAATTAAAACAGGACGCTTTATTGCAACTGCATCAATCGTATTCTCAATATTATTGATTATTCATTACT
TTGTTTCGTTGGATAATGCGACTGCCAAAGCATTACTTAATTTAACGAATCAAAACACTTCAGATAAAGCGATTGATTACATTTTAAACAG
CTTTAGATTCACTGGTATTATGTATATTTTGGCTTATCTAGCAGGCTTCATCACTTTTTGGAATCGACATACTTATGTGTGGTGGTTTATGTT

TGCAGTTTATGTATCAAATAGTTTGTTTACGTTGATTAATTTATCAATCACAATTCAAGCAATAAAAGCTGCACACGGTGCGTACTTAACAT
TGCCAATTTTAATCGTTATTATAGGTTCGGTTGCATTAGCGATTTATATGCTTGTTGTTTCTATCAAACGTAAAAGTACATTTAATCGCTAG

Gene: secG (preprotein translocase subunit G)

Contig: 05_NODE_4, position: 289002 to 289235, length: 234 nt, orientation: FORWARD

Perfect match to: (N315-BA000018-[839725:839958], highly conserved allele)

Sequence:

ATGCATACATTTTTAATCGTATTATTAATCATTGATTGTATTGCATTAATAACTGTTGTACTACTCCAAGAAGGTAAAAGCAGTGGACTTTC
AGGTGCCATCAGTGGTGGTGCTGAGCAGTTATTCGGTAAACAAAAACAACGTGGCGTCGATTTATTCTTAAATAGATTAACAATTATTTT
ATCAATATTATTTTTTGTACTTATGATTTGCATAAGTTATCTTGGTATGTAA

Gene: est (putative carboxylesterase)

Contig: 05_NODE_4, position: 289351 to 290091, length: 741 nt, orientation: FORWARD

Perfect match to: (N315-BA000018-[840074:840814], allele observed in CC5+CC8+CC25)

Sequence:

ATGCAGATAAAATTACCAAAACCTTTCTTTTTTGAAGAAGGCAAACGTGCCGTGTTATTATTACATGGATTTACAGGCAATTCGTCTGATG
TACGTCAATTAGGTCGATTTTTACAAAAAAAGGGATATACTTCGTATGCACCACAATATGAAGGTCACGCGGCACCACCAGAGGAAATAC
TGAAATCTAGTCCTTTCGTTTGGTTTAAAGATGCGTTAGATGGTTATGATTATCTTGTTGAACAAGGTTATGATGAAATTGTTGTTGCTGG
TCTATCATTAGGTGGGGATTTTGCTTTAAAATTAAGCTTAAATAGAGATGTAAAGGGTATTGTAACGATGTGTGCACCAATGGGTGGCAA
AACTGAAGGTGCCATTTATGAAGGCTTTTTAGAGTATGCACGTAATTTTAAAAAGTATGAAGGTAAAAATCAAGAGACTATTGATAATGA
AATGGATCATTTTAAACCAACTGAAACTTTAAAAGAACTAAGTGAAGCATTAGATACGATTAAAGATCAAGTTGATGAGGTATTTGACCC
AATTTTAGTGATTCAAGCAGAAAACGACAATATGATTGATCCACAATCCGCAAATTATATATATGACCATGTAGATTCTGATGACAAAGA
CATCAAGTGGTATAGTCAATCAGGACATGTTATTACGATTGATAAAGAAAAAGAACAAGTATTTGAAGATATTTATCAATTTTTAGAGTC
ATTAGACTGGTCAGAATAA

Gene: rnr (ribonuclease R)

Contig: 05_NODE_4, position: 290125 to 292497, length: 2373 nt, orientation: FORWARD

Perfect match to: (M0075-AQFY01000015-[17119:19491], allele observed in CC5)

Sequence:

ATGAATTTAAAGCAATCAATAGAAGAGATTATTAATCAACCTGAATATGAACCTATGTCAGTGTCAGATTTTCAAGATGCATTAGGTTTAA
GTAGTGCCGACTCATTTAGAGATTTAATTAAGGTGCTCGTAGAATTAGAACAATCAGGATTAATTGAACGTACAAAAACAGACAGATATC
AAAAAAAGCATAGTTCAAAAGGTCATTCAAAATTGATAAAGGGAACGTTAAGTCAAAATAAAAAAGGCTTTGCATTCTTAAGACCTGAA
GATGAGGATATGGAAGATATATTTATTCCCCCAACAAAAATTAATCGTGCCTTAGATGGAGATACTGTAATTGTAGAAATACATCAATCT
AAAGGTGAACATAAAGGTAAAATTGAAGGGGAAGTTAAGTCGATTGAGAAGCATTCAGTAACTCAAGTTGTTGGTACGTATAGTGAAG
CTAGACATTTCGGATTTGTTATTCCAGATGATAAACGTATTATGCAAGATATTTTCATTCCTAAAGGACAAAGTTTAGGTGCAGTAGATGG
GCATAAGGTACTTGTTCAAATTACTAAATATGCTGATGGTTCAGATAATCCAGAAGGACATATTTCTGCTATTTTAGGACATAAAAATGAT
CCTGGCGTAGATATTTTATCTATTATCTATCAACATGGCATAGAAATTGAATTTCCTGATGAAGTGTTACAAGAAGCTGAAGCAGTACCTG
ATCATATTGAAAATACTGAAATTAAAGGCCGTCATGATTTACGTGATGAATTGACAATCACAATTGATGGTGCTGATGCTAAAGACTTAG
ATGACGCAATTAGTGTTAAAAAGTTAGCGAACGGTAATACGCAATTAACTGTAAGTATTGCTGATGTCAGCTATTATGTAACAGAAGGTT
CTGCATTGGATAAAGAGGCATATGATAGAGCGACAAGTGTATATCTTGTTGACCGTGTAATTCCAATGATTCCACATCGATTAAGTAATG
GTATTTGTTCATTGAATCCTAATGTTGATCGTTTAACTCTAAGCTGTCGCATGGAAATCGATGCTAGTGGTCGCGTTGTTAAACATGAAAT
TTTTGATAGTGTTATACATTCTGATTATCGAATGACGTATGATGCGGTAAATCAGATTATTACTGAAAAGGATCCTAACATTCGCGAACAA
TATAAAGAAATTACGCCTATGTTAGATTTAGCACAAGATTTATCTAATCGTTTGATTCAAATGAGAAAACGACGTGGTGAAATCGATTTTG
ATATTAATGAAGCAAAAGTATTAGTTAACGAAGACGGTATACCAACAGATGTTCAATTAAGACAACGTGGCGAGGGTGAACGTCTAATT
GAATCATTTATGTTAATTGCAAATGAAACAGTTGCTGAACATTTTAGTAAGTTAGATGTACCTTTTATTTACCGAGTGCATGAGCAACCTA
AATCAGATCGCTTAAGACAATTCTTTGATTTTATTACAAACTTTGGCATCATGATTAAGGGTACTGGCGAAGATATTCATCCAACAACACT
TCAAAAGGTTCAAGAAGAAGTAGAAGGTCGACCTGAACAAATGGTCATTTCAACAATGATGTTGCGTTCAATGCAACAAGCGCATTATG
ATGATGTGAACTTGGGACATTTTGGCTTATCAGCTGAATATTATACGCATTTTACATCACCAATTAGACGTTATCCTGATTTAACAGTTCAT

CGTTTAATCCGTAAGTATTTAATTGAGAAATCAATGGATAACAAAGAAGTGAAGCGTTGGGAAGACAAATTGCCTGAGTTAGCTGAACA
TACTTCTAAACGTGAACGTCGTGCTATTGAGGCAGAACGTGATACTGATGAATTGAAAAAAGCAGAATATATGATTCAACATATTGGTGA
TGAATTTGAAGGTATTGTCAGCTCAGTAGCTAACTTCGGTATGTTCATTGAATTGCCAAATACGATAGAAGGTATGGTTCATATTGCGAAT
ATGACTGATGATTATTACCGTTTTGAAGAGCGTCAAATGGCATTAATTGGTGAGCGTCAAGCTAAAGTATTTAGAATTGGTGACACAGTT
AAGGTTAAAGTGACGCATGTTGATGTAGATGAACGATTAATTGATTTTCAAATTGTAGGTATGCCTTTACCGAAAAATGATCGATCACAG
CGCCCAGCGCGAGGTAAGACAATTCAAGCCAAAACGCGTGGTAAATCATTAGATAAATCAAAATCTGATGATAAAGGACGTAAGAAAAA
GGGTAGTCAACGTAAAGGTAAAAATGAACGCAAAAATGACAAATCAGGAAATAGTAAACATAAGCCATTTTATAAAGATAAAAGTGTGA
AAAAGAAAGCACGTCGTAAGAAAAAATAA

Gene: ssrP (tmRNA binding protein)

Contig: 05_NODE_4, position: 292519 to 292983, length: 465 nt, orientation: FORWARD

Perfect match to: (MW2-BA000033-[838458:838922], allele observed in CC1+CC9)

Sequence:

ATGGCTAAGAAGAAATCACCAGGTACATTAGCGGAAAATCGTAAAGCAAGACATGATTATAATATTGAAGACACGATTGAAGCGGGAA
TCGTATTACAAGGTACAGAAATAAAATCGATTCGCCGAGGTAGTGCTAACCTTAAAGATAGTTACGCGCAAGTTAAAAACGGTGAAATG
TATTTGAATAATATGCATATAGCACCATACGAAGAAGGGAATCGTTTTAATCACGATCCTCTTCGTTCTCGAAAATTATTATTGCATAAAC
GTGAAATCATTAAATTGGGTGATCAAACACGTGAAATTGGTTATTCGATTGTGCCATTAAAGCTTTATTTGAAGCATGGGCATTGTAAAG
TATTACTTGGTGTCGCACGAGGTAAGAAAAAATATGATAAACGTCAAGCTTTGAAAGAAAAAGCAGTCAAACGAGATGTTGCGCGCGAT
ATGAAAGCCCGTTATTAA

Gene: ssrA (tmRNA, regulatory RNA)

Contig: 05_NODE_4, position: 293074 to 293435, length: 362 nt

Sequence:

GGGGACGTTCATGGATTCGACAGGGGTCCCCCGAGCTCATTAAGCGTGTCGGAGGGTTGTCTTCGTCATCAACACACACAGTTTATAATA
ACTGGCAAATCAAACAATAATTTCGCAGTAGCTGCCTAATCGCACTCTGCATCGCCTAACAGCATTTCCTATATGCTGTTAACGCGATTCA
ACCTTAATAGGATATGCTAAACACTGCCGTTTGAAGTCTGTTTAGAAGAAACTTAATCAAGCTAGCATCATGTTGGTTGTTTATCACTTTTC
ATGATGCGAAACCTTTCGATAAACTACACACGTAGAAAGATGTGTATCAGGACCTCTGGACGCGGGTTCAAATCCCGCCGTCTCCATAT

Gene: Q1YB79 (putative lipoprotein)

Contig: 05_NODE_4, position: 293848 to 294576, length: 729 nt, orientation: REVERSE

Perfect match to: (08-02119-CP015645-[1389440:1390168], allele observed in ST582+CC8+CC30)

Sequence:

TTATACTTCATCTAAACTACTGTGGTCGTCATCTTTTTGCTTTTCTTTTTCTTTCTCTCGTTCTTGTTCTTTTTTGTACTCTTCTTCAAATTCTTT
TTCTTTCTTTTCTACTTCTTCTCTTGTTTCCGCTCTATGAGAAAAATCCTCGGTTTTAAGTTTACTAAATTTGAATGATTTAGAATCAACTGTT
TTATCTTCTGAGTATTTATGGACATTTAAATTAATATTTCCATCACCTCTTAACTCATAGATAAACATGGCTTGTGCAGTTTTGCCTTTTTTA
ATTTGATCTTGGTTATGTTCTGTCCAATCTTTATATTTTTTATCACTTAAAAGATAACCATCTCTTAATTTATTTACTGTATTTTTATCATCTTG
AGTGATATTAATATAGTCATGAGAAATAGAAGATGGATTTAAATCTTTATCGTCTTTTTTAGCAGTAATTTCCATTTTAAAAGCGATATATT
TCTTTTTCTCATCTTTTTCATTGATGATAAACGGTTCTTTTATTTTAGCTTCAAATTTGTCACTAACAATAGTATCGCCTTTAATTTTTATATCC
ATATTTTTTTTGCTTTTAAATTCTTTAAGTTCTTCATTTAATTCTTCATTGTCATTTTCTTTCTTTTTGTGACTAGTGCTCTCTTTTTTTGCACTA
TCTTGATGATGTCCACAAGCACCTAAGATAAGTGTACTTGCTAATAATATCCCCATTACTTTTTTCAT

Gene: Q5HHN0 (putative membrane protein)

Contig: 05_NODE_4, position: 295174 to 295695, length: 522 nt, orientation: FORWARD

Perfect match to: (MW2-BA000033-[855549:856070], allele observed in CC1+CC80)

Sequence:

ATGATTAATATTATTTCAGCTATAGGATCTATTGGAACATTTATTATGGCTTTATTTTATTTTGTATCAGTTTCAGTTCAACTTTATCAAATG
AAAATTAGCTTTCTGCCAGCTTTAGGTTTTAACCAAATTTTATTAGAAAGGGAGGAGGATCAACTTAATATAATGAATTCGGCAACAGAA
GAGCATCATCATAAAGATTATATTAAACTATATAATTTAGGTGGCGGTGCTGCTAAAAAAAATTGCAATAGAGGTTTTATTGGGTAATGAT
AAAGTCATTCAGAAAAAAATACGTGAATATTTTACCTAGTAAAGAAGGGTACATGTTACCAATTAATAAAAATGTGTACGAAGAATTAGAA
AGAACGATTGAGAACAATGGTTATGAAGCTGATTTGAATGTACGTATGACTTATTATCATAATGTAAGTCGCAAACAACAGGAAGTTATA
TTAAAAGGTCAAATCGACTGTTTTAATACTTATAATAATAAAGAAATTTATGATTTGCAGTTTATCTAA

Gene: Q1XZI1 (putative acetyltransferase, GNAT family)

Contig: 05_NODE_4, position: 295834 to 296364, length: 531 nt, orientation: FORWARD

Perfect match to: (MW2-BA000033-[856209:856739], highly conserved allele)

Sequence:

ATGCAAATTAGACAAATACATCAACATGACTTTGCTCAAGTTGACCAGTTAATTAGAACGGCATTTGAAAATAGTGAACATGGTTATGGT
AATGAATCAGAACTAGTAGACCAAATTCGTCTAAGTGATACGTATGACAATAACTTAGAATTAGTAGCTGTTCTTCAAAATGAAGTTGTA
GGGCACGGTTTACTAAGTGAAGTTTATCTTGATAACGAGGCACAACGGGAAATTGGATTAGTGTTAGCACCTGTATCTGTTGATATTCAT
CATCAAAATAAAGGTATTGGGAAGCGATTGATTCAAGCATTAGAACGAGAAGCAATATTAAAAGGATATAATTTTATCAGTGTATTAGG
ATGGCCGACGTATTATGCCAATCTAGGATATCAACGCGCAAGTATGTACGACATTTATCCACCATATGATGGTATACCAGACGAAGCGTT
TTTAATTAAAGAATTAAAAGTGAACAGTTTAGCGGGAAAAACAGGTACCATAAATTACACATCTGCTTTTGAAAAAATATGA

Gene: clfA (clumping factor A)

Contig: 05_NODE_4, position: 296627 to 299467, length: 2841 nt, orientation: FORWARD

Perfect match to: (MW2-BA000033-[857002:859842], allele observed in CC1)

Sequence:

ATGAATATGAAGAAAAAAGAAAAACACGCAATTCGGAAAAAATCGATTGGCGTGGCTTCAGTGCTTGTAGGTACGTTAATCGGTTTTGG
ACTACTCAGCAGTAAAGAAGCAGATGCAAGTGAAAATAGTGTTACGCAATCTGATAGCGCAAGTAACGAAAGCAAAAGTAATGATTCAA
GTAGCGTTAGTGCTGCACCTAAAACAGACGACACAAACGTGAGTGATACTAAAACATCGTCAAACACTAATAATGGCGAAACGAGTGTG
GCGCAAAATCCAGCACAACAGGAAACGACACAATCAGCATTAACAAATGCAACTACGGAAGAAACTCCGGTAACTGGTGAAGCTACTAC
GGCAACGAATCAAGCTAATACACCGGCAACAACTCAATCAAGCAATACAAATGCGGAGGAATTAGTGAATCAAACAAGTAATGAAACGA
CTTCTAATGATACTAATACAGTATCATCTGTAAATTCACCTCAAAATTCTACAAATGCGGAAAATGTTTCAACAACGCAAGATACTTCAACT
GAAGCAACACCTTCAAACAATGAATCAGCTCCACAGAGTACAGATGCAAGTAATAAAGATGTAGTTAATCAAGCGGTTAATACAAGTGC
GCCTAGAATGAGAGCATTTAGTTTATCGGCAGTAGCTGCAGATGCACCGGCAGCTGGCAAAGATATTACGAATCAGTTGACGAATGTGA
CAGTTGGTATTGACTCTGGAGATACAGTTTATCCGCACCAAGCAGGCTATGTCAAACTGAATTATGGTTTTTCAGTGCCTAATTCTGCTGT
TAAAGGTGACACATTCAAAATAACTGTACCTAAAGAATTAAACTTAAATGGTGTAACTTCAACTGCTAAAGTGCCTCCAATTATGGCCGG
AGATCAAGTATTGGCAAATGGTGTAATCGATAGTGATGGTAATGTTATTTATACATTTACAGACTATGTTGATACTAAAGAAAATGTAAC
AGCTAATATTACTATGCCAGCTTATATTGACCCTGAAAATGTTACAAAGACAGGTAATGTAACATTGACAACTGGCATAGGTAGTACAAC
AGCAAACAAAACAGTATTAGTAGATTATGAAAAATATGGTAAGTTTTATAACTTATCTATTAAAGGTACAATTGACCAAATCGATAAAAC
AAATAATACGTATCGTCAGACAATTTATGTCAATCCAAGTGGAGATAATGTTATTGCGCCGGTTTTAACAGGTAATTTAAAACCAAATACG
GATAGTAATGCATTAATAGATCAGCAAAATACAAGTATTAAAGTATATAAAGTAGATAATGCAGCTGATTTATCTGAAAGTTACTTTGTG
AATCCAGAAAACTTTGAGGATGTCACTAATAGTGTGAATATTACATTCCCAAATCCAAATCAATATAAAGTAGAGTTTAATACGCCTGATG
ATCAAATTACAACACCGTATATTGTAGTTGTTAATGGTCATATTGATCCGAATAGCAAAGGTGATTTAGCTTTACGTTCAACTTTATATGG
ATATGACTCAAGGTTTGTATGGAGATCTATGTCATGGGACAACGAAGTAGCATTTAATAACGGATCAGGTTCTGGTGACGGTATCGATA
AACCAGTTGTTCCTGAACAACCTGATGAGCCTGGTGAAATTGAACCAATTCCAGAGGATTCAGATTCTGACCCAGGTTCAGATTCTGGCA
GCGATTCTAATTCAGATAGCGGTTCAGATTCGGGTAGTGATTCTACATCAGATAGTGGTTCAGATTCAGCGAGTGATTCAGATTCAGCGA
GTGATTCAGATTCAGCAAGTGATTCAGACTCAGCGAGTGATTCAGATTCAGCAAGCGATTCAGATTCAGCGAGCGATTCAGATTCAGCG
AGTGATTCAGATTCAGCGAGTGATTCAGATTCAGCAAGCGATTCCGACTCAGACAATGACTCGGATTCAGATAGCGATTCTGACTCAGAC
AGTGACTCAGATTCCGATAGCGATTCTGACTCAGACAGTGACTCAGATTCAGATAGCGATTCAGATTCAGATAGCGATTCAGATTCCGAC
AGTGATTCCGACTCAGACAGCGATTCTGACTCCGACAGTGATTCCGACTCAGACAGCGATTCAGATTCCGACAGTGATTCCGACTCAGAT
AGCGATTCCGACTCAGATAGCGACTCAGATTCAGACAGCGATTCAGATTCAGACAGCGATTCAGATTCAGATAGCGATTCAGATTCCAAC
AGTGACTCAGATTCCGACAGTGACTCGGATTCAGATAGCGATTCAGATTCCGACAGTGACTCAGATTCCGACAGTGACTCAGACTCAGAC
AGTGATTCGGATTCAGCGAGTGATTCGGATTCAGATAGTGATTCCGACTCCGACAGTGACTCGGATTCAGATAGCGACTCAGACTCGGA
TAGCGACTCGGATTCAGATAGCGATTCGGACTCAGATAGCGATTCAGAATCAGACAGCGATTCAGAATCAGACAGCGATTCAGATTCAG
ACAGCGACTCAGATTCAGATAGTGACTCGGATTCAGCGAGTGATTCAGACTCAGGTAGTGACTCCGATTCATCAAGTGATTCCGACTCAG
AAAGTGATTCAAATAGCGATTCCGAGTCAGGTTCTAACAATAATGTAGTTCCGCCTAATTCACCTAAAAATGGTACTAATGCTTCTAATAA

AAATGAGGCTAAAGATAGTAAAGAACCATTACCAGATACAGGTTCTGAAGATGAAGCAAATACGTCACTAATTTGGGGATTATTAGCAT
CAATAGGTTCATTACTACTTTTCAGAAGAAAAAAAGAAAATAAAGATAAGAAATAA

Gene: vwb ("van Willebrand factor" binding protein)

Contig: 05_NODE_4, position: 299688 to 301214, length: 1527 nt, orientation: FORWARD

Perfect match to: (TCH1516-CP000730-[876118:877644], allele observed in CC8)

Sequence:

TTGAAAAATAAATTGCTAGTTTTATCATTGGGAGCATTATGTGTATCACAAATTTGGGAAAGTAATCGTGCGAGTGCAGTGGTTTCTGGG
GAGAAGAATCCATATGTATCTGAGTCGTTGAAACTGACTAATAATAAAAATAAATCTAGAACAGTAGAAGAGTATAAGAAAAGCTTGGA
TGATTTAATATGGTCCTTTCCAAACTTAGATAATGAAAGATTTGATAATCCTGAATATAAAGAAGCTATGAAAAAATATCAACAGAGATTT
ATGGCTGAAGATGAGGCTTTGAAGAAATTTTTTAGTGAAGAGAAAAAAATAAAAAATGGAAATACTGATAATTTAGATTATCTAGGATT
ATCTCATGAAAGATATGAAAGTGTATTTAATACTTTGAAAAAACAAAGTGAGGAGTTCTTAAAAGAAATTGAAGATATAAAAAAAGATA
ACCCTGAATTGAAAGACTTTAATGAAGAGGAGCAATTAAAGTGCGACTTAGAATTAAACAAATTAGAAAATCAGATATTAATGTTAGGTA
AAACATTTTATCAAAACTATAGAGATGATGTTGAAAGTTTATATAGTAAGTTAGATTTAATTATGGGATATAAAGATGAAGAAAGAGCAA
ATAAAAAAGCAGTTAACAAAAGGATGTTAGAAAATAAAAAAGAAGACTTAGAAACCATAATTGATGAATTTTTTAGTGATATAGATAAA
ACAAGACCTAATAATATTCCTGTTTTAGAAGATGAAAAACAAGAAGAGAAAAATCATAAAAATATGGCTCAATTAAAATCTGACACTGAA
GCAGCAAAAAGTGATGAATCAAAAAGAAGCAAGAGAAGTAAAAGAAGTTTAAATACTCAAAATCACAAACCTGCATCTCAAGAAGTTTC
TGAACAACAAAAAGCTGAATATGATAAAAGAGCAGAAGAAAGAAAAGCGAGATTTTTGGATAATCAAAAAATTAAGAAAACACCTGTA
GTGTCATTAGAATATGATTTTGAGCATAAACAACGTATTGACAACGAAAACGACAAGAAACTTGTGGTTTCTGCACCAACAAAGAAACCA
ACATCACCGACTACATATACTGAAACAACGACACAGGTACCAATGCCTACAGTTGAGCGTCAAACTCAGCAACAAATTATTTATAATGCA
CCAAAACAATTGGCTGGATTAAATGGTGAAAGTCATGATTTCACAACAACGCATCAATCACCAACAACTTCAAATCACACGCATAATAAT
GTTGTTGAATTTGAAGAAACGTCTGCTTTACCTGGTAGAAAATCAGGATCACTGGTTGGTATAAGTCAAATTGATTCTTCTCATCTAACTG
AACGTGAGAAGCGTGTAATTAAGCGTGAACACGTTAGAGAAGCTCAAAAGTTAGTTGATAATTATAAAGATACACATAGTTATAAAGAC
CGATTAAATGCACAACAAAAAGTAAATACTTTAAGTGAAGGTCATCAAAAACGTTTTAATAATCAAATCAATAAAGTATATAATGGCAAA
TAA

Gene: emp (extracellular matrix protein-binding protein)

Contig: 05_NODE_4, position: 301565 to 302587, length: 1023 nt, orientation: FORWARD

Perfect match to: (MW2-BA000033-[861940:862962], allele observed in CC1+CC12)

Sequence:

ATGAAAAAGAAATTATTAGTTTTAACTATGAGCACGCTATTTGCTACACAAATTATGAATTCAAATCACGCTAAAGCATCAGTGACAGAG
AGTGTTGACAAAAAATTTGTAGTTCCAGAATCAGGAATTAATAAAATTATTCCAACTTACAATGAATTTAAAAAAGCACCAAAAGTAAAT
GTTGGTAATTTAGCTGACAATAAAAACTTTGTAGCTTCTGAAGATAAATTGAATAAGATTGTAGATTCATCGGCAGCTAGTAAAATTGTA
GATAAAAACTTTGCCGTACCAGAGTCAAAGTTAGGAAACATTGTACCAGAGTACAAAGAAATCAATAATCGCGTGAATGTAGCAACAAA
CAATCCAGCTTCACAACAAGTTGATAAGCATTTTGTTGCTAAAGGCCCAGAAGTAAATAGATTTATTACGCAAAACAAAGTAAACCACCA
CTTCATTACTACGCAAACCCACTACAAGAAAGTTATTACTTCATACAAATCAACACATGTACATAAACATGTAAATCATGCAAAGGATTCT
ATTAATAAACACTTTATTGTTAAACCATCAGAATCGCCTAGATATACACATCCATCTCAATCTTTAATTATCAAGCATCATTTTGCAGTTCCT
GGATATCACGCGCATAAATTTGTTACACCAGGGCATGCTAGCATTAAAATTAATCACTTTTGTGTTGTGCCACAAATAAATAGTTTCAAGG
TAATTCCACCATATGGTCACAATTCACATCGTATGCATGTACCAAGTTTCCAAAATAACACAACAGCAACACATCAAAATGCTAAAGTAAA
TAAAGCATATGACTATAAATACTTCTATTCTTATAAAGTAGTTAAAGGTGTGAAGAAATATTTCTCATTTTCACAATCAAATGGTTATAAAA
TTGGGAAACCATCATTAAATATCAAAAATGTAAATTATCAATATGCTGTTCCAAGTTATAGCCCTACACACTACGTTCCTGAATTTAAGGG
TAGCTTACCAGCACCACGAGTATAA

Gene: vwb2 (truncated "van Willebrand factor" binding protein)

Contig: 05_NODE_4, position: 302922 to 303443, length: 522 nt, orientation: FORWARD

Perfect match to: (MW2-BA000033-[863297:863818], allele observed in CC1)

Sequence:

ATGAAAAGGAAAGTATTAGTACTAACAATGGGTGTAATTTGTGCAACTCAATTATGGCATTCTAATCACGCAAACGCATTAGTATCAGAG
AGTGTTGAAACTAATTTTGTTGTAAAAGATTCGGATAATAAAAATATTTTACAAACCCATACTGAAATTACTACTGAGGAGAAATTTTCAG
TAGTAGAAAAGAGTCAATTAAATACACTAAAATCACTATCTAATGACAACTACATAGAATATGATTTACATACTAATCAAACAGGCATAAA
AAAAGGTTGGTTATATGGTTATAGTGAAATTGACTCATCACATTTCACAGACCGTGACAAACGCGTTATTAGACGTGATCATGTTAAAGA
AGCACAAAGCTTAATTAATGATTATAAATATACGCAAAAATATGAAGATCTCGCTAAGGCAACTGCAAAAGTAAGTACACTTAGTCAGTC
TCACCAAAATTATTTGAATAAACAAATTGATAAAGTGAATAATAAGATAGAGAAAACTGAAAAACGCTAA

Gene: nuc1 (thermostable extracellular nuclease locus 1)

Contig: 05_NODE_4, position: 303784 to 304470, length: 687 nt, orientation: FORWARD

Sequence:

ATGACAGAATACTTATTAAGTGCTGGCATATGTATGGCAATTGTTTCAATATTACTTATAGGGATGGCTATCAGTAATGTTTCGAAAGGG
CAATACGCAAAGAGGTTTTTCTTTTTCGCTACTAGTTGCTTAGTGTTAACTTTAGTTGTAGTTTCAAGTCTAAGTAGCTCAGCAAATGCATC
ACAAACAGATAACGGCGTAAATAGAAGTGGTTCTGAACATCCAACAGTATATAGTGCAACTTCAACTAAAAAATTACATAAAGAACCTGC
GACATTAATTAAAGCGATTGATGGTGATACGGTTAAATTAATGTACAAAGGTCAACCAATGACATTTAGACTATTATTGGTGGATACACC
TGAAACAAAGCATCCTAAAAAAGGTGTAGAGAAATATGGTCCTGAAGCAAGTGCATTTACGAAAAAAATGGTAGAAAATGCAAAGAAA
ATTGAAGTCGAGTTTGACAAAGGCCAAATAACTGATAAATATGGACGTGGCTTAGCGTATATTTATGCTGATGGAAAAATGGTAAACGA
AGCTTTAGTTCGTCAAGGCTTGGCTAAAGTTGCTTATGTTTATAAACCTAACAATACACATGAACAACTTTTAAGAAAAAGTGAAGCACAA
GCGAAAAAAGAGAAATTAAATATTTGGAGCGAAGACAACGCTGATTCAGGTCAATAA

Gene: cspC-L1 (RNA chaperone locus1)

Contig: 05_NODE_4, position: 304827 to 305027, length: 201 nt, orientation: FORWARD

Perfect match to: (RF122-AJ938182-[825949:826149], highly conserved allele)

Sequence:

ATGAATAACGGTACAGTTAAATGGTTTAATGCAGAAAAAGGTTTTGGTTTCATCGAAAGAGAAGATGGTAGCGACGTATTCGTACACTTC
TCAGCAATCGCTGAAGATGGATACAAATCATTAGAAGAAGGCCAAAAAGTTGAATTCGACATCGTTGAAGGCGACCGTGGCGAGCAAG
CTGCAAACGTAGTTAAAATGTAA

Gene: Q1Y1Z8 (putative protein)

Contig: 05_NODE_4, position: 305523 to 305741, length: 219 nt, orientation: REVERSE

Perfect match to: (MW2-BA000033-[865898:866116:r], highly conserved allele)

Sequence:

TTAATTTACAGCGTCAAATATACTTATTTCTAATGCTTTGGGGTCTACTGAAACAAGTAAAGAATGATCGATGTTACTAATATTGCCATTCT
CCAAATTTATTTCTGTGAGTATTTGGAAGCTACCATTAGGCAACGGTTTAACAATAGACAATTGCTTTTCCGCTTGTTGTATTAAAAAAGG
TTTTGTAGATTGATTATTAATATGCCATTCACTCAT

Gene: Q2G007 (putative protein)

Contig: 05_NODE_4, position: 305803 to 306087, length: 285 nt, orientation: REVERSE

Perfect match to: (MW2-BA000033-[866178:866462:r], allele observed in CC1+CC15)

Sequence:

TCAACCAAAATTTTGTTCGAATTCAATAAATGTCTTGTTTAAAATAGAAATATTGTAAATGTTATCGTCCAAAACTTCACCAGTTAAGTATT
TGTTTTGAATTAAAATTTGGCAGTTAGTTAAGAAGTCTTGATAATCACGATCACAAAAATAGTTTTCACGTGCATCTTTAGCATCGCCAAA
AAAGTTAGCGACTGTTTCTGTTTCTCCATTATTCGAACGTTCAATATATAATTTGTAAAATTTAGCTATTGTATACTTTTGTTCTTTAGTTAG
TTCATTCAA

Gene: Q1YB69 (putative exported protein)

Contig: 05_NODE_4, position: 306175 to 306744, length: 570 nt, orientation: REVERSE

Perfect match to: (COL-CP000046-[890101:890670:r], highly conserved allele)

Sequence:

TTAATAAGCTTTAACAAGCCTTAGTTTGTATGGATCTATAAAATTATCTTTAATTGCATAGGGTGAAATAATATGTAGTCCATAACTTTTAA
CTGATTTTTCACTTACACCAAATTTATAAGCTTGGTAGATAATTTTAGTACAATACGTAAATTTTTTGCTGTTCAAATTTAATGTAACTAGAT
AACGATGATTTGTATTCTCATAGTTTTTCTTAACCCATTCAGCCGCTTTTTTACCTGCACCAGGATAGCTGCAACGATAAACTTTCATCCAAT
CATTTTTGCCACTTGCATAATTATATTTAAAAGATTCGAAGGATTGTGTAGTTGGTTTGTCGCCAGGCCCCTCAATTTGTAAAATCGTTTTA
TCATCAATCGCGATACTACAATGACCAAAAAATCCCCACATGACAGGGCCTTTTGTAACAATAATATCACCAGGTTGTAATTGGAATTTGT
CATCTTGAATTTCTGAATACTTATTATCTGCAATTGTTTTTGGTGAGTTTATTGGGGATACGACAACGAATAATATAAGTAAAATTATCGTT
CGTTTAATATAGTTCAC

Gene: Q6GIJ6 (putative protein)

Contig: 05_NODE_4, position: 306932 to 307120, length: 189 nt, orientation: REVERSE

Perfect match to: (N315-BA000018-[858892:859080:r], highly conserved allele)

Sequence:

TTATAGCACACCAAATATAAATGTGTATATTAATATAGCGAATCCAAAAATATAGAGAATGACAGTGAAACTTAAATAGGACTCTTTTTA
GATTCTTTGCCAGTTTTTTTCATTAATACGAGTATAAACGTAGCGGCAACTAAGAAAATTAATGCTAGCCAAAACAGAATTGCAAAATGTA
AAGACAT

Gene: Q6GIJ5 (putative membrane protein)

Contig: 05_NODE_4, position: 307149 to 307409, length: 261 nt, orientation: REVERSE

Perfect match to: (N315-BA000018-[859109:859369:r], highly conserved allele)

Sequence:

TTAACCTATAAAACTATAAAAAAATAGGATAAATGTAATAGAGAACGAGTACGATATTTATAAAAAATAATATTTCACTTAACCAGTTTTTA
GTTATCATTGCAATGGTAAAGGATACGATGAGTATCACACCACAAATGATAATACCAGGCAGGAGCCAACATAAATCATCTAAATCTTTA
TTATATGTGATTAAAATATTAAAGATAACAAAAGTGGTAGTAATAACTATATTGATAGCATTTAACAAAATGTTATTCAT

Gene: DUF1250 (PF06855 family protein)

Contig: 05_NODE_4, position: 307749 to 307952, length: 204 nt, orientation: FORWARD

Perfect match to: (RF122-AJ938182-[828871:829074], highly conserved allele)

Sequence:

ATGACGTTTTACAATTTCATCATGGGTTTTCAAAATGATAACACACCATTTGGTATATTGGCCGAACACGTTAGTGAAGATAAAGCATTCC
CTCGATTAGAAGAAAGACACCAAGTAATTAGAGCATATGTGATGTCTAATTACACAGATCATCAATTAATTGAAACTACAAATAGAGCTA
TTAGCTTATATATGGCAAATTAA

Gene: Q6GIJ3 (putative protein)

Contig: 05_NODE_4, position: 307949 to 308185, length: 237 nt, orientation: REVERSE

Sequence:

TTAATTTGAGTAGTACCAATTATGATGTATTAGTGCATCCCAAATATCTTTTGTCTTAAAGTTTATTTCATCATTTCTTATCGAAAATGGTGT
AATAATGTCTTTATCTAACCAAGTGTTGATAAGTTCATTTGGTACACCATCTAACAACATTTCACTTTTACTAATTATAAAACATTCCCAGTC
AAGTGAAACATTTTGTGGATTCACATAATTACATTGATTATGATTATCCAT

Gene: gpmA1 (putative phosphoglycerate mutase)

Contig: 05_NODE_4, position: 308406 to 308990, length: 585 nt, orientation: FORWARD

Perfect match to: (N315-BA000018-[860368:860952], allele observed in CC5+CC97)

Sequence:

TTGTTGGCTAAAACGTTATATTTAATGCGCCACGGACAAACTTTGTTTAATTTTAAGGGACTAATTCAGGGATTTGGAGATTCGCCGCTAA
CAGAACTTGGAATTGCTCAAGCTCAAAAGGCACGTAGTTATTATGAAACTAAGGGGATAAACTTCGATTTATATGCATCATCAACGCAAG
AACGCGCAAGTGACACACTTGAAAATGTTGCACCTAACCAATCGTATCAACGTTTTAAGGGACTGAAGGAATGGCATTTTGGATTATTTG
AAGGTGAGTCAGTCTATCTATTTGATAATCTATACAAGCCTGAAGACTTATTCGGAGATCGAATTGTTCCTTTCAAAGGAGAGGCAAGGC
AACAAGTTGAAGATCGCATTGTGAAAACTTTACATGACATTATGTCTCAAACAAAGAATAATGCATTAGTCGTGAGTCATGGAACAATAA
TGGGAGTATTTTTAAGATATTGCCTTAAACTAGATGAAGCATTAAAGCATAATATCGGTAATTGTAATATCCTGAAATTTGAATATGACAA
TGGAACATTTAAATTTGTTGAGTTAATTGATCCAAATTTATAA

Gene: yisU1 (putative aminoacid related metabolite efflux transporter)

Contig: 05_NODE_4, position: 309063 to 309680, length: 618 nt, orientation: REVERSE

Perfect match to: (MW2-BA000033-[869438:870055:r], allele observed in CC1)

Sequence:

TTAGAATAATAATTGAATAAGTTTTTGTAATATCATCAGAGCAACAATAATAATTATTATGCTTGATAATTTATTGATTATTGTTAATAATTT
TCCGGTTTTATCAATTGATCCAACCATTTCTCCTAAAATTGCGAGTAAAAAGAACCATAACCATGAAACGCTAATACAAGCGATTGTAAAC
GCAATTTTATTGCTGCCACTATATAATGCAGCACTACTACCAATTACACCAATTGTATCTAAAATAGCATGTGGATTGAGTAATGAAACTG
ATAAAGCAAAACTTACTTGTTTCATTGGAGACATAATTTGAGCTTCTCCATCTGTTGAGGGTTTATCATGCCAAATGGTCCAAGCCATATA
CATCAAGAAAATTAAACCAACTATATAAATAATTGCTTGAAGTACAGGTAAAGACATAATAATGATAGATACTCCTACCACTGCAATAATA
ATAAGTAAGCTGTCTGACAACCCGGCTGCAATTATTGCAGGCAATACATATCTATATTTTGGTTGATTAGCTCCTTGGTTAAAAATAAATA
CATTTTGTGCACCTAAAGGTAAAATTAGACCAATCGCTAAGATAAATCCATGAATAATTGCGGTTACCAT

Gene: Q5HHL4 (putative acetyltransferase)

Contig: 05_NODE_4, position: 309835 to 310329, length: 495 nt, orientation: REVERSE

Perfect match to: (MW2-BA000033-[870210:870704:r], allele observed in CC1)

Sequence:

TTACTTATTAATCAAATCATAAAAAAGCCAATGTTCATCGAAATAGGTATTTCCGATTTTAATTGCATTTTTCTCAAAACCAAGAATGTCGA
ATCCTAGTGCACTATAGAAAACTTTAGCACCTATGTTGTTTGACACAATTGATGTAAGTAAAGATTCGTAATTGTTCTGCCGTGCATAATT
AATAATGAAATTAATGAGCTCGCTATTAATCGATTTATCGTTATTAGTAACAAAGTTGTATTTAATTAATGATTTATGTTCTTTTCCAACGT
ATCGAATTTGTTCTAAGGCAGCTGTTGCGATAAGTGTTTCGTTTTCATAACAGCCAAATACAATACATTTCGGTGAACCTTGATTTAAAAT
GTCATGTATTAAGGTGTCAGTCATTGTTTGACTATAATGTGAATCTTGAGTGAATTCTTCATTGACTGTCGAAACAAGTCTCTTATACTCAT
TAAAATCATTATTCGTTAATGTTTTAATTTCTATCAT

Gene: ohrB (peroxiredoxin)

Contig: 05_NODE_4, position: 310728 to 311150, length: 423 nt, orientation: REVERSE

Perfect match to: (N315-BA000018-[862690:863112:r], allele observed in CC5+CC1+CC15+CC88)

Sequence:

CTAATCTACAACATTTACATTTAAATCGACATTAATATTTCCTTGAGTCGCTTTTGAATATGGACAAAATTCATGAGCCATTTGTAAATATTT
TTCAGCTTCTTCTTGAGATATAACATTTTTAATTGTCGCATCAATTGAAACACTTAATTTAGGACTTTCTGAGTCTGAATCATCTTCTAGTCT
CACTGTTAGTGTTACTTCTGGATGAGCATCACGCACTTTGTTTTGCTTTAAAATTAGGTCGAAAGCACCGTTGAAGCAAGATGCATAACCT

GCTGCAAATAATTGTTCGGGGTTAGTAGCTTTACCATCTGCTTGAGCAGGCGGAACGATATCAATATCTAACGCTCGATCATCAGTATAA
ACATGTCCTTTACGTCCGCCAACATTCGTCGCTTTAGTTTCATAATGTATTGCCAT

Gene: aroD (3-dehydroquinate dehydratase type I)

Contig: 05_NODE_4, position: 311298 to 312014, length: 717 nt, orientation: FORWARD

Perfect match to: (MW2-BA000033-[871673:872389], allele observed in CC1+CC5)

Sequence:

ATGACACATGTGGAAGTAGTAGCGACTATCGCGCCACAATTATCTATCGAAGAAACTTTAATTCAAAAAATTAATCATCGTATTGATGCA
ATAGACGTATTAGAATTACGAATTGATCAAATTGAAAATGTCACAGTTGATCAAGTGGCAGAAATGATTACAAAGCTGAAGGTTATGCA
AGATTCATTCAAATTATTAGTTACGTATCGTACAAAGTTACAAGGTGGCTATGGGCAATTTATAAATGACTTGTATCTTAATTTAATATCA
GACTTAGCAAATATCAATGGCATAGATATGATTGATATAGAATGGCAAGCAGATATTGACATTGAAAAACATCAACGAATCATTACACAC
TTGCAACAGTATAATAAAGAGGTGGTTATATCACATCATAATTTCGAAAGTACGCCTCCATTAGATGAATTGCAATTTATATTTTTTAAAAT
GCAAAAATTCAACCCAGAATACGTTAAATTAGCAGTAATGCCACATAATAAAAATGATGTGTTAAATTTATTGCAGGCAATGTCTACATTT
TCAGATACTATGGACTGCAAAGTTGTTGGTATTTCAATGTCTAAACTTGGACTAATAAGTAGAACGGCTCAAGGCGTTTTTGGTGGTGCA
TTGACTTATGGTTGTATCGGAGAACCACAAGCTCCAGGACAGATTGATGTTACTGATTTAAAAGCACAAGTGACTTTATACTAA

Gene: ntrA (iron-regulated nitroreductase)

Contig: 05_NODE_4, position: 312097 to 312636, length: 540 nt, orientation: FORWARD

Perfect match to: (T0131-CP002643-[897861:898400], allele observed in CC239)

Sequence:

ATGGAATTACAACAAGCAATAGCTAATAGAAGAAGTGTGAAAAAATTTAAAAGAGATATGCACATAGATGACGCATTGCTATATCAAGC
AATTGAGAAAGCTGCTGATGCTCCAAATCACGGAATGAGGGAACCATGGAGAGTTGTGCATGTTCCGAAAGACAGATTAGGAGATATG
AGTAAGGATATTTCTAAATTTGCATTTCCTAATGAATTAGATAAGCAACAATGTCATTATGATGCAGTTACGAAACTAGGTGGCATGTTAT
TGCTTATTTTAAAAACAGATCCAAGACAACGTCAAAATGATGAAAACTACTTTGCATTTGGTGCATATGCACAAAATCTTATGTTGTTACTT
TATGAAGCGGGAATAGGTACATGTTGGAAATCGCCATTATATATCTATGATCCTAAAGTAAGAAAAACACTTGGTATAAAGAAAGATGA
AGTTCTTTCTGGATTCTTATATTTAACGGATTTAGAAGAAGATATGCCTAAAGCACCACGTAAAAATAGAAACTTAATTACATTATATTAA

Gene: trxA2 (thioredoxin)

Contig: 05_NODE_4, position: 312786 to 313106, length: 321 nt, orientation: REVERSE

Perfect match to: (MW2-BA000033-[873161:873481:r], allele observed in CC1+CC188)

Sequence:

TTATTTAAAAGTTTCTGCTAAAAATGATTCAACTTGCTCAGGTGACTTAGCATTTGCTGAATGAAGGTGTGCAATTTTGTCGCCGTTTTTAA
ATACTAGCAAGCTAGGGATACCCATAACTTCATTTTCAACAACTACATCTTCTAATTCATCACGATTAACAGTATACCATTGGTAATCTTTA
TATTGTTCTACGATTGGGTCAATCCATAAATCCATAGCACGACAGTCTGGGCACCATCCTGCCTCAAATTTAACAATTACAGGTGTATCGC
TATTAATCACAGATTTAAATGATTCATTACTTTTGATTGCTTGCAT

Gene: yusI (putative oxidoreductase)

Contig: 05_NODE_4, position: 313250 to 313606, length: 357 nt, orientation: FORWARD

Perfect match to: (MW2-BA000033-[873625:873981], allele observed in CC1+CC25+CC49+CC50+CC239)

Sequence:

ATGATTAAATTTTACCAATATAAGAATTGTACAACTTGTAAAAAGGCAGCAAAGTTTTTAGATGAATATGGCGTAAGTTATGAACCAATT
GATATCGTTCAACATACACCTACAATAAATGAATTTAAAACAATAATTGCAAATACAGGCGTAGAAATTAATAAATTGTTTAATACACACG
GCGCGAAATATCGTGAGCTTGATTTGAAAAATAAATTACAAACTTTATCAGATGATGAAAAGTTAGAGTTGTTATCATCTGATGGTATGT
TAGTAAAGCGTCCTCTAGCAGTAATGGGCGATAAGATAACGTTAGGATTTAAAGAAGATCAATATAAAGAGACTTGGTTAGCGTAA

Gene: gcvH2 (glycine cleavage system protein H, locus 2)

Contig: 05_NODE_4, position: 313764 to 314144, length: 381 nt, orientation: FORWARD

Perfect match to: (N315-BA000018-[865727:866107], highly conserved allele)

Sequence:

TTGGCAGTACCAAATGAATTGAAATATTCAAAAGAGCATGAATGGGTTAAAGTTGAAGGTAATGTAGCAACAATTGGAATCACAGAATA
CGCACAAAGCGAGTTAGGTGATATTGTTTTCGTTGAATTACCAGAAACAGATGATGAAATTAATGAAGGGGATACGTTTGGTAGCGTAG
AATCAGTTAAAACTGTATCAGAATTATATGCACCAATCTCTGGTAAAGTAGTTGAAGTCAACGAAGAACTAGAAGATAGTCCCGAATTTG
TAAATGAATCTCCATACGAAAAAGCATGGATGGTAAAAGTAGAAATTAGTGATGAAAGTCAGATTGAAGCTTTATTAACAGCTGAAAAA
TATTCAGAAATGATTGGTGAATAA

Gene: ywqG (putative protein)

Contig: 05_NODE_4, position: 314323 to 315201, length: 879 nt, orientation: FORWARD

Perfect match to: (MW2-BA000033-[874698:875576], allele observed in CC1)

Sequence:

ATGCTAAACATTCAAGACGTTAGTCATCTTTCTAAAAAGGAGCAAAAAGCATATAACCGTTTCGTAGAATCTGTAGAAAACGGTAATTTA
CCAGTACTACCATGTATTGAAATGGTTCTAAAAGAGATGAAAGAAGAAACATTAAACCAGAGTAAGATTGGTGGAATGCCATTTTTAAA
ATCTTTTAAAGATATACCATTAGATGAAAATAATGTACCAATGGTATTGTTAGCACAGATTAATTTGGATAATCTTCCAGAACAACAAGAA
TTATTTCCTGTAAAAGAAGGGATATTGCAGTTTTGGATTAGTTCAGAAGATCAAATGTATGGTATGTCTGAAAATTTAAAGGGAAACAAT
ATAAACTCAAGGCTTGTTTATATAAAAGAGCCAATTACAGATTTATCACTCGAAAATATTCAAGCGCATTTGAAGTCATTAGATGCTGATA
ATGAGGATATCCCGTTCAGTGGAGCATTTTCTATAGAATTTAGATTGTCGAAACAAACTATTACATGTACTGATTATAAGTACGATGAGG
ACGTGCTTGCATTGTGGAATAAAGTCAATCCATCCTTCGCGCTAAAATCAATGTTTGGTGGTTATGATGAATTGATGGAACCTGTGTGTA
ACACATTTACTGCTAAGGAACCATTTAATCAACTTGGTGGTTATCCATATTTTGACCAAATAGATCCAAGAACGAACGATCAAGAACTGAA
AATGTATGATAGAGTCTTACTGCAAATTGATTCTACAAGAGATGGTAATTCTTCGATTATATGGGGTGATTTAGGTATTGCCAATATCTTA
GTAAAATCTACTGACCTTGAGGCTATGAAGTTTGATGATTACATGTATTCATGGGATTGCAGCTAA

Gene: STAR (Staphylococcus aureus repeat element)

Contig: 05_NODE_4, position: 315256 to 315475, length: 220 nt

Sequence:

GGGAGTGGGACAGAAATGATATTTTCGCAAAATTTATTTCGTCGTCCCACCCCAACTTGCATTGTCTGTAGAAATTGAGAATCCAATTTCT
CTTTGTTGGGGCCCATCCCCAACTTGCATTGCCTGTAGAATTTCTTTTCGAAATTCTCTGTGTTGGGGCCCCTCCCCAACTTGCATTGCCTG
TAGAATTTCTTTTCGAAATTCTCTGTGTTGGGGCCCC

Gene: SIRU01 (staphylococcal interspersed repeat unit 1)

Contig: 05_NODE_4, position: 315307 to 315473, length: 167 nt

Sequence:

CCAACTTGCATTGTCTGTAGAAATTGAGAATCCAATTTCTCTTTGTTGGGGCCCATCCCCAACTTGCATTGCCTGTAGAATTTCTTTTCGAA
ATTCTCTGTGTTGGGGCCCCTCCCCAACTTGCATTGCCTGTAGAATTTCTTTTCGAAATTCTCTGTGTTGGGGCC

Gene: tx_universal2 (rho-independent terminator)

Contig: 05_NODE_4, position: 315537 to 315575, length: 39 nt

Perfect match to: (Strain_21331-AGTV01000040-[104403:104441], allele observed in CC398)

Sequence:

TAGAATTGAAAAAAGCTTGTTACAAGCGCATTTTCGTTC

Gene: SIRU01 (staphylococcal interspersed repeat unit 1)

Contig: 05_NODE_4, position: 315747 to 315911, length: 165 nt

Sequence:

CCAACTTGCACATTATTGTAAGCTGACTTTCCGCCAGCTTCTGTGTTGGGGCCCCGCCAACTTGCACATTATTGTAAGCTGACTTTCCGCCA
GCTTCTGTGTTGGGGCCCCGCCAACTTGCACATTATTGTAAGCTGACTTACCATCAGCTTCTGTGTTGGGGCC

Gene: yusF (small primase-like protein  (toprim domain))

Contig: 05_NODE_4, position: 316168 to 316554, length: 387 nt, orientation: FORWARD

Perfect match to: (N315-BA000018-[874673:875059], highly conserved allele)

Sequence:

ATGGCTATTGTAAATAAAGTGATAATTGTTGAAGGAAAATCTGATAAAAAAAGGGTGCAACAGGTTATTGCAGAACCAGTCAATATTATT
TGTACTCATGGAACAATGAGTATAGATAAGCTTGATGATATGATAGAATCACTGTATGATAAACAAGTTTTTGTATTAGCCGATTCTGATG
ACGAAGGAGATCGAATTAGAAATTGGTTTAAACGTTATTTGAGTGAAAGTGAACATATATTTATTGATAAAACTTACTGTCAAGTTGCGA
ATTGCCCCAAACAATATTTGGCGCATGTACTTTCAAAACATGGCTTTACTTGTAAGAAAGAAACACCTCTTTTACCGAATATAAATAATGA
AAGGTTAGTTTTAGTAAATGAATAA

Gene: yusE (putative thioredoxin)

Contig: 05_NODE_4, position: 316547 to 316843, length: 297 nt, orientation: FORWARD

Perfect match to: (N315-BA000018-[875052:875348], highly conserved allele)

Sequence:

ATGAATAATTCATTAGACATCAAAGATGTAACTACATTTTATGAGGAAGACAAACATTTAATCTTTGGTTATACACCAACGTGTGGTACTT
GTAAGGTTTCAGAAAGAATGTTAGACATTGCTAATGAAATATTGCAGTTACCATTATTGAAAATAGATTTAAACTTTTATCCTCAGTTTTGT
AAAGATATGCAAATCATGTCTACGCCGATTTTATTGTTGATGAATAAAGATAAAGAAGTAAAACGAATTTATGCATTTAAATCGGTGACT
GATTTGTTAGAAAATTTAAAATAG

Gene: metN1 (methionine ABC transporter locus 1, ATP-binding protein)

Contig: 05_NODE_4, position: 317093 to 318118, length: 1026 nt, orientation: FORWARD

Perfect match to: (COL-CP000046-[900796:901821], highly conserved allele)

Sequence:

GTGATTGAATTAAAAGAAGTTGTTAAAGAATATCGGACTAAAAATAAAGAAGTCCTTGCTGTAGATCACGTTAATTTATCGATTCGAGCA
GGATCGATTTATGGCGTCATTGGTTTTTCTGGAGCAGGAAAAAGTACTTTGATTCGAATGTTTAATCATTTAGAAGCGCCTACATCAGGT
GAAGTTATTATAGATGGAGACCATATAGGTCAATTGTCCAAAAATGGATTAAGAGCAAAAAGACAAAAAGTAAGTATGATCTTCCAACA
TTTTAATTTGTTATGGTCAAGGACTGTGTTAAAAAATATTATGTTTCCGCTTGAAATTGCAGGTGTCCCTAGAAGGAGAGCTAAGCAAAA
AGCATTAGAACTTGTCGAACTCGTCGGTTTAAAAGGTAGAGAAAAGGCTTATCCATCAGAGTTATCAGGTGGACAAAAGCAACGTGTTG
GGATTGCACGAGCGTTAGCTAATGATCCAACGGTCTTGCTTTGTGATGAGGCAACAAGTGCACTTGATCCGCAAACAACAGATGAAATTT
TAGATCTACTACTAAAAATTAGAGAACAACAAAATTTAACAATTGTACTAATTACGCATGAAATGCATGTCATTCGTCGTATTTGTGATGA
AGTTGCAGTTATGGAAAGTGGTAAAGTGATAGAACAAGGACCGGTGACACAGGTTTTTGAAAATCCGCAACACACTGTGACAAAACGAT
TTGTGAAAGACGATTTAAATGATGATTTCGAAACATCTTTAACAGAATTAGAGCCATTAGAAAAAGATGCATATATCGTTAGATTAGTTTT
CGCTGGTTCAACAACAACCGAGCCTATTGTATCGAGTCTATCAACTGCCTATGATATTAAAATTAATATTTTAGAAGCAAATATTAAAAAT
ACAAAAAAATGGAACAGTCGGCTTTTTAGTTCTGCATATTCCATATATTTCAAGTGTAGATTTCGGAAAATTCGAAAAAGAGTTAATTGAGC
GACAAGTTAAAATGGAGGTGTTAAGACATGGGTAA

Gene: metP1 (methionine ABC transporter locus 1, transmembrane permease)

Contig: 05_NODE_4, position: 318111 to 318806, length: 696 nt, orientation: FORWARD

Perfect match to: (COL-CP000046-[901814:902509], highly conserved allele)

Sequence:

ATGGGTAAATCATTTAGTGAAATTATAAATGAAATGATTACAATGCCTAATATTCAGTGGCCAGAAGTTTGGACTGCAATAGTCGAAACA
CTATACATGACAGTCGTCTCAACTATATTTGCATTTATACTTGGTCTTATTTTAGGTGTGTTATTATTCTTGTCTGCTAAAGGTAAGTCTATC
GGTGCAAGGTTATTTTATTCTATCGTTTCTTTCATTGTTAACTTATTTAGAGCGATACCATTTATTATTTTAATTTTATTATTAATTCCATTTA
CAAGTTTGATACTTGGAACGATAAGTGGTCCGACAGGTGCGTTACCAGCCTTGATCATTGGCGCAGCACCGTTTTATGCAAGGCTCGTAG
AAATTGCTTTTAAAGAAATTGATAAAGGTGTCATCGAAGCGGCTTGGTCAATGGGCGCTAATACTTGGACAGTAATTCGTAAAGTCCTTT
TACCTGAAGCTATGCCAGCGCTAGTGTCTGGCATTACAGTTACAGCAATCGCTTTAGTTGGTTCAACAGCAGTTGCAGGTGTAATTGGTG
CCGGTGGTTTAGGAAATTTAGCATACTTAACAGGTTTCACTCGAAATCAAAATGATGTCATTTTAGTATCAACAGTTTTTATTTTAATTATT
GTATTTATAATCCAATTCATTGGGGATTGGCTTACAAATAAACTTGATAAACGATAA

Gene: metQ1 (methionine ABC transporter locus 1, substrate-binding protein)

Contig: 05_NODE_4, position: 318824 to 319645, length: 822 nt, orientation: FORWARD

Perfect match to: (MW2-BA000033-[878973:879794], highly conserved allele)

Sequence:

ATGAAAAAATTATTTGGTCTTATTTTAGTATTAACATTTGCAGTTGTATTAGCAGCTTGCGGTAATGGAAACAAAAGTGGCAGTGACGAC
AAGAAAATAACAGTAGGTGCTTCACCAGCACCACATGCTGAAATTTTAGAAAAAGCAAAACCATTATTAGAGAAAAAAGGTTATGAACT
AGATATTAAAACAATTAACGATTACACTACACCTAATAAATTACTAGACAAAGGTGAAATTGACGCAAACTATTTCCAACATACACCATAT
TTAAACACAGAGAAAAAGGATAAAGGTTACAAAATCGTAAGTGCCGGTGATGTTCACTTAGAACCTATGGCTGTATACTCTAAAAAGTAT
AAAAGTTTAAAAGAATTACCAAAAGGTGCAACAGTCTATGTGTCTAATAATCCAGCTGAACAAGGACGTTTCTTAAAATTCTTCGTTGATG
CAGGTTTAATTAAAATCAAAAAAGGCGTAAAAATTGAAGATGCTAAGTTTAGTGATATTACAGAGAATAAAAAAGATATTAAGTTTAATA
ATAAACAATCAGCAGAATTCTTACCTAAAATTTATCAAAATGAAGACGCTGATGCTGTTATCATTAATTCGAACTTTGCAATCGAACAAAA
ACTAAATCCTAAAAAAGATTCTATTGCTGTAGAAAGTGCGAAAGATAATCCTTATGCAAACTTAATTGCTGTTAAAGAAGGACATCAAGA
TGATAAGAAAATCAAAGCATTAATTGAAGTATTACAATCTAAAGATATTCAAGACTTCATTAATGAAAAATACAATGGTGCAGTTATTCCT
GCTAAATAA

Gene: int1-SaPI1 (integrase)

Contig: 05_NODE_4, position: 319788 to 321008, length: 1221 nt, orientation: REVERSE

Sequence:

TTATCCAAGCTTTACCTGTTCAAGTTTGTTCATCATATCCTTATCCATCTGTTCAGTAACGTGTGAATAAATAGAAAGTGTTGTGCGGTGGT
CGGAATGGCCTACACGATCCATAATAGCTTTAAGTGACACTCCTTGTTGAGAGAGTAATGATATGTGGCTATGTCTTAATATATGTGAAG
AAATTTCTTTTTCAATACCCACATCTTTAGCTGATTCCCTAAGGATTTTATTGAATCTTTCAGTCTGCATTGGGTTGCCTTTATGATTAGTAA
ATACAAAATTTCTATTTAGATATCCATCATTCCATTTTGAATCCTTTTTGTTCTCCAGTATTGCTTTCTTTAATATCTCGCAACTTCTGCTACT
CAATCCAATTGTTCTATAACTAGACTCTGTTTTAGTGGTATCTTTTACACCGAATCCACCAGATTCATCGTGAAACCAATGGATGGTTCCGT
TAATATTTAAACTCTTATTATCAAAGTCTATATCTTCATTTTTAATGGCTAACATTTCACCAATACGCATACCATTTAAGGCTTGGAATTCTG
TCATGAGTGCAACGAATAAATAAAACCGCTTATGGATACCTGAGTGCATCTTCTGAGCCTTCATATTAATATCTTTGATGATAGATTGTAT
TTCATTTAATTCGAGGTATTTATTACGTTTAGCTTTCACTTCATCATAAGACTTAATCCTTTTATTTAACGTTATATCTTTTAAAAATTCTAAA
TCTTGCAGTTTGTAGATGCGTCTAGTGTATTCAAATACATTTTTGAATATGCTTAACGCATCTTTGTTTACTTGATATACATAACCTTTACTA
TCCATTTCATCGAAAACTTGTTGTGCATATGAAAGTGTTATTTTATTGATTAAAATATCTTCATCTACAAATTTCTTCAAAGTGTTTAGCTTA
CTCAATTTAGTTTTAATAGTTGTTCTTTTTGACCCAGACGTTTTGATATAATTCTGAAACCACTCATCACATGCGACATGAAAAGTTAATGA
TTTTAACGTTGTCGGTGTATTATCATTCAGTTTTGCCTCTATACGCTCATTTAAGCGTCTCTGAGCCTCTTTCTGCGACTGCTTACCATTCTT
ATTGAGAACCACGCTAACACGTCGCCATTTATTTGTGAGAGGGTCTTTGTACTTCTCATAATAGCGATATTTAGTTTCACCATGTTTATTAG
TAAATTTCTCGTGCCACAT

Gene: D0K396 (putative DNA binding protein)

Contig: 06_NODE_54, position: 300 to 872, length: 573 nt, orientation: REVERSE

Perfect match to: (JS395-CP012756-[565971:566543])

Sequence:

TTATTTTTGATTTTCATCATTATATATTCTTTGAATTACTGAATTAACAATCTCGCTTAACATTTTTTTATCGCTTTCTGATAAAACTTCATTAA
AACCAAGAGAATTTTTGTTATCTATGCCACTATAAAACAGCTTATATTCAGACTGGTTTAATAGCCATTTTAAATCAAAGTAAGGCTCGTCT
ATTTCCTCAATTTGGTATTGATTAAATTCGTTCATAGTAAGTTTAATTTCTTTTCTTTGGATTTTACTTGAATGCTTTTTTTGTTTTTCTCGGA
ATTTTTTAACAAACTCGTCATATTCTGTTCTCAATTGTTCATAGCTTATTTTTTTGTTTTCACTAAATATAGTTAGTAATTTTTCAATATCATC
ATGCATTTGAGATTGATCCATAAACAATACATACCAAAATAAAATATTTTTAGATGGAAAGCGCTTACCATTTTCTAATTTACTAACATACA
CATCTGATACACCTAAGTACTTTGAAAGAGTATCTAAAGTTAAATCTTCTTCTTTTCTAAAATTTTTAAGAATGTTTGAAAAATCTTCAAGA
CTATTTTGTTGCAT

Gene: E5QRR7 (putative protein)

Contig: 06_NODE_54, position: 1044 to 1265, length: 222 nt, orientation: FORWARD

Perfect match to: (O11-CP024649-[896625:896846])

Sequence:

ATGATTGCTAAAGTGAATTATTTGAAACGTTTGATTGCATTTGAAGGATTGAGTTTAAAAGATTTTGCAAGCGAAATAGAAGTGAATTCT
AACTTTTTGAATTCCATTGTTAATGGTAAAAGAACGACATCCCCAAAAACAGCTAATAAAATAGCGAAAAGACTCAATGTTGATATAAAA
GATATTTTCATCTTTATAGAAGAAAAAGAGGAGGTCAAATAA

Gene: D0K398-ED98 (putative DNA binding protein)

Contig: 06_NODE_54, position: 1266 to 1538, length: 273 nt, orientation: FORWARD

Perfect match to: (ED98-CP001781-[834326:834598])

Sequence:

ATGGCTAGAACAAAGTTGAAAGATATACCGACTAAGGAAAATACAATCAGTGAACCAAAACAAGTTGTAGTAAAACCACTGTTTGCTAA
ACCAAACGCGCTAGCAAGTATTTTTGGTATTTCGTATAGTTCGACAAATCGTATTTTAAAAGAGTGGGAGAAAGATCCTAAAGGTGTTGA
CGATTTGTATTATTCATTGTCATCAACTATGACGGTTATCAGTATTCCTAGATTTGAGGAGTACATGAAGAAACGTCATAAAAAATGGATG
TAG

Gene: Q6GJU3-var1 (putative protein)

Contig: 06_NODE_54, position: 1895 to 2278, length: 384 nt, orientation: FORWARD

Perfect match to: (IS-125-AHVC01000051-[4803:5186])

Sequence:

ATGAAAATCAAACAAAAATATCAATTATCAAAAGTGGTTAAAGTATTAGAAGTAGTATTATACGAGAAAAGTAAAACTTATGATGATATT
AGTTATCTCAATGAGGATACAGCATTTTATGAATATGCTTTAAAGTTAGTTCATAATGGATTGTTCAATATTCTTGCTGAATTAGATTTTGA
AGATGAAGCATTTTTAATTCTTGATGAAGTAACGATGACGCTAAGTGATGTCATGAAAGAAACACAACACGTTTATCGTTATAGTGTCAT
AGATGAAAAAGGTGAACATAAACATACAACAGATCGCAAAGGACACGTGATTGGGATATTAGAGTGGGCATTAGATTACATTGTGGGA
AATATTGAAGTGGAGGAATTATAA

Gene: Q6GJU3 (putative protein)

Contig: 06_NODE_54, position: 1895 to 2278, length: 384 nt, orientation: FORWARD

Sequence:

ATGAAAATCAAACAAAAATATCAATTATCAAAAGTGGTTAAAGTATTAGAAGTAGTATTATACGAGAAAAGTAAAACTTATGATGATATT
AGTTATCTCAATGAGGATACAGCATTTTATGAATATGCTTTAAAGTTAGTTCATAATGGATTGTTCAATATTCTTGCTGAATTAGATTTTGA
AGATGAAGCATTTTTAATTCTTGATGAAGTAACGATGACGCTAAGTGATGTCATGAAAGAAACACAACACGTTTATCGTTATAGTGTCAT
AGATGAAAAAGGTGAACATAAACATACAACAGATCGCAAAGGACACGTGATTGGGATATTAGAGTGGGCATTAGATTACATTGTGGGA
AATATTGAAGTGGAGGAATTATAA

Gene: Q5HHJ1-RN3984 (putative protein)

Contig: 06_NODE_54, position: 2279 to 2596, length: 318 nt, orientation: FORWARD

Perfect match to: (Strain_21266-AFTT01000017-[701309:701626:r])

Sequence:

ATGAATTGGGAAATTAAAGATGTAATGTGTGATATTGAAGTGATAAAAGAAAAAATCAATGATGTAGCTATCAAACATGGTTGGTTTGTT
GAAGATAAATTTGTCAAAAATGAATTAGAAACAAAACGGGAACATATTAATTTTTCTGCTAGCTATTTAGAACATCGTATACAAAATGAA
CATACAGTTGAGTTGTTACAGGTTTATTTAAAAGAGTTCGATGAACTTATACAAAAATTTCATGAAATAGAAAAAGCATCATCTGAGAACT
TTGGCGAGGTATCAGATGACGCAAAGAAATTAAAAATTACAGAGTAA

Gene: Q5HHJ1 (putative protein)

Contig: 06_NODE_54, position: 2279 to 2596, length: 318 nt, orientation: FORWARD

Sequence:

ATGAATTGGGAAATTAAAGATGTAATGTGTGATATTGAAGTGATAAAAGAAAAAATCAATGATGTAGCTATCAAACATGGTTGGTTTGTT
GAAGATAAATTTGTCAAAAATGAATTAGAAACAAAACGGGAACATATTAATTTTTCTGCTAGCTATTTAGAACATCGTATACAAAATGAA
CATACAGTTGAGTTGTTACAGGTTTATTTAAAAGAGTTCGATGAACTTATACAAAAATTTCATGAAATAGAAAAAGCATCATCTGAGAACT
TTGGCGAGGTATCAGATGACGCAAAGAAATTAAAAATTACAGAGTAA

Gene: O54473-phi (phage DNA-replication protein)

Contig: 06_NODE_54, position: 2663 to 3532, length: 870 nt, orientation: FORWARD

Perfect match to: (Strain_21266-AFTT01000017-[700373:701242:r])

Sequence:

ATGAATGAAATTAAATTAGAATACGACACACATGTTTCAGTGGTACATTATGAAAGTTTAGACTCACGTTCATTTAAGAGCTTTTCAATGT
CTAAATGGAGTAAGTTAATTAATAAACTGTCTGTGCCTATAGAAGCAAATTATAAGTATGCACGTGGTGTTGCTGTATATGGTGATATTA
AAAACGGTGCAAATGATCATGGTGAAATTATCAAAAAGCATCGCAATGACGTTAATGTCGTATACAGAGATGTGATTGTACTTGATTACG
ATGAAATAAATGATTTAAAGCAATTACATGAAGCAATCAGCTCAGCTTTAAGCAATGTTGCATGGTTTTGGCACACAAGTTACTCGCACA
GAACTGAACAAGCTAGAATACGCCTGTATATCCCTCTAAATGAGCGGATAAGTGCAGATGATTATCGTAAATATTCAAAGGTATTAGCAA
ACAAAATTGGTCATAAAGTTGATGAAGGTTCATATCAGCCAAGTAGATGTTTTGCATTACCAGTTATTCAAAAAGGGCACATATTTATTAA
ACGAGTGAATGACTGTCCAATTATCGATGTTGATATGCTCGAACAGTGGTCGAAGGAACTTGAACAATCAAATGCTAGTCCTAATGTTAT
AGGGTACACGCGACGTGATAGTGCGTACTGGCGTGAGTTAAGCTTTGGAACAACCGAAGGCAATCGTAACAATGCACTAGCTAGCTTAG
TTGGGCATTTATTAAGATGTCGCGTCAATGATTATATTGTGTATTCATATGCTTTATTATGGGGGAAATTCGCATGTAAACCACCTATGAA
AGAACAAGAAATCAACGCCACTTTTCAATCGATATTAAATAAACACTATAACAATTAG

Gene: hypothet. phage protein

Contig: 06_NODE_54, position: 4328 to 4624, length: 297 nt, orientation: REVERSE

Sequence:

TCAAAATAGTATTGAATCGATGGCGTCATTTTTGAACTCATTTCCAATGTTTAAAGATGATACACGTAACACACATCCTAAGTTTGTTACCA
GTGCCACATATAACTTTTCTAGTGGCGAAAGTAAATCAAGAAGTAATATTAATTTAACACTAAACGCTAAAAAAGAATGGCGAAATATTT
TAATTTCTACTGGTGAATCATCTATCGCAAATATGGCTGATGAAAAAGCAGGTGTATCAGCACGTGTAGTTACACTACAAGATCCACCAT
ATCCAGATAATTTTGATTTTACCAC

Gene: pif (phage interference protein)

Contig: 06_NODE_54, position: 5639 to 6019, length: 381 nt, orientation: FORWARD

Perfect match to: (KLT6-APFH01000004-[316314:316694])

Sequence:

ATGGATAAAGAACAACTTAAAAAGACTATATACGATTATGTAAAAGAATATAAGGAGATACCGATATATCAGTTAGAAGATTTGTTTGAA
GAAATAAATCACGACTATATAGGGAGAACTAGTATCACACACGATAAGGATGAGAATATTGTGTTTTGGAGTGGATGGAACAAAATTAC
AATGTTTGCGTTGATTGAATTAGTTAAAAGTGAACAACTTGATTTAGTGTATAGAGGTAGGTTTGTAATGCGTTATTTGTTGGATGGTAG
AGTTCCTAATTTACCATTAGCTATTTGTTATCCAGAAGATGGACAACAAACGGACGTGCCCTCATGGGTGCCTATGGTATTAAGAATAAAT
AAAGAGGAGAAAATCAAATGA

Gene: Q5HHI6 (putative protein)

Contig: 06_NODE_54, position: 6016 to 6657, length: 642 nt, orientation: FORWARD

Perfect match to: (Strain_21266-AFTT01000017-[697302:697943:r])

Sequence:

ATGAACATAGAAACTATTGTAAACCAATTTGAAACACGAGCAGGCACGTTACTAAGGTACTACACAGGATTATTAGAACATAGTAAAGT
GCAACCATGTTGCTTTAAGTTATATAATGATCCATTTGATATGGTTTACGTGGTAATGAACAGTAAGTTATTCGGTCATGTATATATTAAA
GATTGTAAAGTAAGGCAATCATTTGAATTAGCGTCACCTAAGCACACTGAGGGGCTTATAAGAAGCATAGAGGGGCATTATGTAGGTTA
TGAATTACATGACGGTAAACAGCTTTCTATTAGCGATATGATGGCCAGTCAATTGTTTGAAGATGAGTATTTTATGTATGGATTACAAACA
TATGCAGAATCAAATAATAGTGATGTGTTTAAGTACTTAGAAAATGGATTTGATACAGATACACTTGAGGGCATTCAATCGAGTAATACT
GATGTGATAGCGAATATTGAAATGTTGTATCAGTTAGCTACGGGAATCAATGAACCAGTACCAGAGTTAGTTGAGGGGTTAAAATTAGT
AACTGAGTTTGTACAAGATGAGAATGCGACACAAGAGGATTACAAGGCGTTAGAACGTAAATTGAATGATCTAAAAGCGTCTTACTATA
GCTTGAGTAAATAA

Gene: Q93CE0 (putative bacteriophagal protein)

Contig: 06_NODE_54, position: 7527 to 8105, length: 579 nt, orientation: FORWARD

Perfect match to: (IS-125-AHVC01000051-[10398:10976])

Sequence:

ATGAAAACTGAATCGTACTTTAAAGAATACAACCAATTTGTATTAGATCAACACAAGGCTATACAAGAATTGGAACAAGAGCGTAATGCA
TTGGAGAGTAAAATAAAGTTAGATAAGTCCACATACAAACAGTTAATCATGGATGGACAAGATGATAAAGCAGATAACCTATATCAAGC
AACAGATGCTGATGAAAAGAAACTAAAAGCACTTAATAAACGCTTAGAGACAAAGAAAAGTGTGTCGAAAGAAGTTAAATATCAAAAG
ACAATTGAATTATTAAAACATCAAAGCGAGTTGTCATCATTATATGAATCAGAAAAGCAATCAGCTTTAGGTAAATTAAAAAAGGTAGTC
GATGCATATAATGAGATCATTGATGAAATAGAAGATGTTAATGATAGATATGAAGATGAGCATCAGCAATATGCGAGTATTTATAGTCA
AGAACAATTATATGATGATAAAGAGGCTAGGGAAGCATTGAATGGCTACTTTAGAGAAAATATATTTACATCATATATTAATGGTAATGA
TTTGCCATACGAACACAATAACAAGTTGTTTTTAAAACGTTAA

Gene: hypothet. phage protein

Contig: 06_NODE_54, position: 8392 to 8919, length: 528 nt, orientation: FORWARD

Sequence:

ATGAAACTGCTTAAAACGAAGAATTGTTTATATTATCGTAATGGCGACAATAAACTATCTGAGTATCAACTATTAACGCAATTTAACCCAG
CATTTATTAATAAAAAAATTAAGATGTGTGAATTCCAAATTGAAAGTATGTACCATATGAGTGCGTCGACCACAACATGTGATGAAATAA
TGGGGGTCGTGTCTGTCTCATATCCAATTGAAAAACTAGTTATCAAAATTATTGAAACAAAGGCAAGATTACAAAACTATAAAAATCGAT
CTATAAGTAATATGGTGTTGTTGAAAATGGTACTAAATCATTATACAGAAAAAGAGCAGAAGAAAGTTGTAAAATATATGCGTTCAAATG
GACGATATAAGCCCTACAACGTCATTGAACGCTTACAGGTTGATTTGTATCAAGCAAGTATTAAACAACGTTCAGAACGTCAAAAACAAA
GAAATATAGCAATTGAAAATAGCAAGATTGCACGAGTAAATGCTTATCACCAATCTTCACATGTAAAAGTGGTGTAA

Gene: Q5HHI1 (putative protein)

Contig: 06_NODE_54, position: 8922 to 9263, length: 342 nt, orientation: FORWARD

Perfect match to: (IS-125-AHVC01000051-[11792:12133])

Sequence:

ATGGATAAAAAGCAAATAAAAGGCTTCGTGTGTGATTATCATAAGCGAACTAGAAGTGATGTATTAATAGATGATGATATAAATACTGAT
GAATTCTTTTCAATAGATGATGAAAACTCTAATGAATGGATGACAGACGATAATGTCGATGATCATATTATAAAGAATCACTTAGAAATG
ATTGTTGACCGATTAGCAACTGATAAAGAGTTTTATATTTTTGACTCCCTTATACAAGGACGTAATTATAAAGATATTAGTAGTATCTTAG
AGTGTTCAGAACAATCTGTAAGATTATGGTATGACACCTTATTAGACAAAATTGTGGAGGTGATAGAATGA

Gene: ear-nySa1 (enterotoxin-linked ampicillin resistance protein)

Contig: 06_NODE_54, position: 11646 to 12200, length: 555 nt, orientation: REVERSE

Perfect match to: (IS-125-AHVC01000051-[14515:15069:r])

Sequence:

CTAGTTTATAGTGATTTTATCTATATCCTTTTTAAACAACATCAATCGTTTGTTATCATTATAGACTTCACCGACTTTAGCTGAGATTTTAGG
GTCTTTTTCATCTTTACTATGAATAGTTACTGTATCACCGTCTTTCACAATCTGCTTTTCTTTTAATTTATCAGTTAATTTTTTCCATGCATCAT
TAGCGTCAAATCTATCTCCATTGGGATTTTTCCTTGACAAATCAATGCTTTTTACACTATCGCTATTGATCGGTTTGTTATTAGAATTGCTAA
GTTCATTTAAATTGGCGGTACCTTCAATACTGTTTTCACCGCCATTTGCTAATTTATAAGTCACTTTGACATTTTCATTATCTGTTTTATCGAT
AATATTCGCTTCTTTTAAAGCGTCTCTTACATTTTTCCACAATTCGTTATCTGTCGTTTGCTCAGCTTTTGCAACGTTATTAATACCATTATAA
TTTGAAGAAGAATGAAAACCTGAACCTACTGTTGTTAAAACTAAAGCACTTGCTATCAATGTTTTTGTTAATAGTTTTTTATTCAT

Gene: hypothet. phage protein

Contig: 06_NODE_54, position: 12798 to 12908, length: 111 nt, orientation: FORWARD

Perfect match to: (Phage-55-ORF187-[AY954963.1-[[25518:25628]])

Sequence:

ATGACAGAACAAATGTATTTAATATTGTTTTTATTAAGCCTACCATTGTTATTATTTATCGGGAGAAAAACACATTTTTATTGTTTAGATAA
AAAGAATGGACGTAGATAA

Gene: Q2YXQ4-phi (putative phage protein)

Contig: 06_NODE_54, position: 12895 to 13095, length: 201 nt, orientation: FORWARD

Perfect match to: (SA40-CP003604-[862157:862357])

Sequence:

ATGGACGTAGATAATATGAGTGATTATAAATTAAAAATAATTGAATTGATCAAAAGTGATATAACAGGTTACCAAATTCACAAACAAACT
GGCGTAGCGCAATATGTAATTTCACAATTAAGGCAAGGAAAGCGCGAAGTAGATAACTTAACTTTAAATACAACTGAAAAACTATACAG
TTACGCACGACAAGTGTTATAA

Gene: terminase-S-phiPT1028

Contig: 06_NODE_54, position: 12910 to 13095, length: 186 nt, orientation: FORWARD

Perfect match to: (Phage-55-ORF073-[AY954963.1-[[25630:25815]])

Sequence:

ATGAGTGATTATAAATTAAAAATAATTGAATTGATCAAAAGTGATATAACAGGTTACCAAATTCACAAACAAACTGGCGTAGCGCAATAT
GTAATTTCACAATTAAGGCAAGGAAAGCGCGAAGTAGATAACTTAACTTTAAATACAACTGAAAAACTATACAGTTACGCACGACAAGT
GTTATAA

Gene: csbD-L1 (stress response protein, locus 1)

Contig: 06_NODE_54, position: 13921 to 14115, length: 195 nt, orientation: FORWARD

Perfect match to: (N315-BA000018-[878504:878698], highly conserved allele)

Sequence:

ATGGCAGACGAAAGTAAATTTGAACAAGCAAAAGGTAATGTTAAAGAAACAGTAGGTAATGTTACTGATAATAAAAATTTAGAAAACGA
AGGTAAAGAAGATAAAGCTTCTGGTAAAGCGAAAGAATTCGTTGAAAATGCAAAAGAAAAAGCAACTGATTTTATTGATAAAGTAAAAG
GTAACAAAGGCGAGTAA

Gene: DUF368 (putative integral membrane protein)

Contig: 06_NODE_54, position: 14348 to 15199, length: 852 nt, orientation: REVERSE

Perfect match to: (MW2-BA000033-[880575:881426:r], highly conserved allele)

Sequence:

TTATTCATTTTCAGCGGTAATTCGACCTAAAGTCAAACTTACAATAAAACCGATGATAAATACTACTAATGAAACGAACCACATCACGATA
TTAGTTGGTAAACCTGGAAATACTGCAAAGAGGGAGCCAACAACAAAACCAATGATTAATGCAAAAGTCATTAGTTTATGATGTGTTAG
GAAATACTGGATAATTTTGCTTGAAATAATGAATCCAGCAAGCACGCCAAATCCGACTGCAAGTAATATAGGAAGACCTGCAAAGTTAA
GTTTAACAACTTCAGATATTGCTAGCATGACCGTACCATAGACGCCAAATACTAATAACATAAATGACCCTGAAATACCTGGGAGTAACA
TAGCACTAGATGCACACATACCTGCAATAAAATATTTAATAATAAGACTAGTTGATAGAGTAAGTGTTTCTCCAGCATGTTTATCACCATT
ATTCATTAATGTAATAACAATTAAGATAGCGATACCAGCTATAACCATCATGTAATGTTTAGTTGTAAATGACGTTTTATAGTTAGAAATTT
TCAATAAATATGGAACGATACCAATGATTAATCCACCAAAGAAAAACATAGTTGGAATATGGTGTTGGCTTAATAAATAATTAAAAAGAT
TACTTAGTGATCCCATTGCCAGTAACATTCCAATTATAATGGGGATTAAAAATGTAAAACTTGGCCAAAAACGTCGTGAGAATATGCCGC
TAATTGAAGCGATAAATTGATTGTAAATACCTAACAATAATGCGATAGTCCCACCGCTAACACCAGGTACCAAGTCACTCGTTCCCATAGC
AAAACCTTTTAGAATATTAATCCATTTAAATTGTTGCAT

Gene: sufC (iron-sulfur cluster assembly ATPase)

Contig: 06_NODE_54, position: 15527 to 16288, length: 762 nt, orientation: FORWARD

Perfect match to: (N315-BA000018-[880111:880872], highly conserved allele)

Sequence:

ATGGCATCAACATTAGAAATCAAAGACCTACATGTGTCTATTGAGGATAAAGAAATCTTAAAAGGTGTTAACTTGACAATTAACACTGAT
GAAATACATGCGATTATGGGACCAAACGGGACAGGTAAATCAACTTTATCATCTGCAATTATGGGACACCCAAGCTATGAAGTAACTAA
AGGAGAAGTACTTTTAGACGGTGTAAATATTTTAGAATTAGAAGTTGATGAAAGAGCAAAAGCAGGATTATTCTTGGCAATGCAATATCC
ATCAGAAATTACAGGTGTTACAAATGCTGATTTCATGCGTTCAGCAATCAATGCGAAACGTGAAGAAGGACAAGAAATCAACTTAATGC
AATTTATTAAGAAATTAGATAAAAACATGGATTTTCTAGACATAGATAAAGACATGGCACAACGTTATTTAAATGAAGGTTTCTCAGGTG
GAGAGAAGAAACGTAACGAAATCTTACAATTAATGATGTTAGAACCTAAGTTTGCAATCTTAGATGAAATCGATTCAGGGTTAGACATCG
ATGCATTAAAAGTTGTATCTAAAGGTATTAACCAAATGCGTGGGGAAAACTTTGGTGCATTAATGATTACACACTATCAACGATTATTAAA
TTACATTACTCCTGATAAAGTACATGTAATGTATGCTGGTAAAGTCGTTAAATCTGGTGGTCCAGAATTAGCAAAACGTCTTGAAGAAGA
AGGATATGAATGGGTTAAAGAAGAGTTCGGTTCAGCTGAATAA

Gene: sufD (iron-sulfur cluster assembly protein D)

Contig: 06_NODE_54, position: 16386 to 17693, length: 1308 nt, orientation: FORWARD

Perfect match to: (MRSA252-BX571856-[914431:915738], allele observed in CC30+CC25+CC30)

Sequence:

ATGACAACTGATATTTTGAACATTTCTGAAGAACAACTTGTTGATTATTCTAAAGCCCACAATGAACCTTCTTGGATGACAGAATTACGTA
AAAAAGCTTTGAAATTAACAGAAACTTTAGAAATGCCAAAACCTGATAAAACAAAATTAAGAAAATGGGATTTTGATTCTTTTAAACAAC
ACGATGTAAAAGGTGATGTTTATCAATCTTTATCACAATTACCTGAGTCAGTAAGAGAAATTATTGACGTAGATCATTCTAAAAACTTAGT
AATTCAACATAATAATACGATTGCGTACACACAAGTTGATGATAATGCATCGAAAGATGGCGTTATCGTTGAAGGTTTAGCAGACGCTCT
TATGAACCATAGTGATTTAGTACAAAAGTACTTTATGAAAGATGCAGTAACAGTAGATGAACATCGTATCACAGCGCTACACACGGCATT
AGTTAATGGTGGCGTATTTGTTTATGTTCCTAAAAATGTAGTTGTAGAACATCCAGTACAATACGTTGTGTTGCACGACGACGAAAATGC
AAGCTTTTATAACCATGTTATCATCGTTACTGAAGAAAGCGCCGAAGTCACATATGTTGAAAATTACTTATCAAATGCATCTGGTGAAGGA
AATCAATTAAATATTATTTCTGAAGTGATTGCTGGTGCAAATTCAAATATCACATATGGCTCAGTGGACTATATGGATAAAGGCTTTACAG
GTCATATCATTCGACGTGGTATTACTGAAGCGGATGCCTCAATTAATTGGGCACTAGGTTTAATGAATGAGGGTAGCCAAATTATTGATA
ATACAACAAATTTATTTGGTGATCGTTCAACAAGTTCACTTAAATCAGTAGTTATAGGTACAGGCGAACAAAAAATTAATCTAACATCTAA
AATCGTACAATATGGTAAAGAAACAGATGGTTATATCCTTAAACATGGTGTTATGAAAGAACATGCATCGTCTGTATTTAATGGTATCGG
CTACATTAAGCATGGTGGAACTAAATCAATTGCTAATCAGGAATCACGTGTATTAATGTTATCTGAACATGCTCGTGGTGACGCGAATCC
TATTTTATTAATTGATGAAGATGATGTACAAGCTGGTCATGCTGCATCAGTAGGTCGTGTTGATCCAGATCAACTTTACTATTTAATGAGT
CGTGGTATTTCTCAAAGAGAAGCGGAACGTCTTGTTATACATGGTTTCTTAGATCCAGTAGTACGTGAATTACCTATCGAAGACGTTAAA
CGTCAATTGAGAGAAGTAATTGAACGCAAAGTTTCTAAATAA

Gene: sufS (iron-sulphur cluster assembly cysteine desulfurase)

Contig: 06_NODE_54, position: 17808 to 19049, length: 1242 nt, orientation: FORWARD

Perfect match to: (MW2-BA000033-[884035:885276], highly conserved allele)

Sequence:

GTGGCCGAACACTCATTTGACGTTAATGAAGTAATCAAGGATTTTCCGATATTAGATCAAAAAGTCAATGGCAAACGTTTAGCATATCTT
GATTCAACAGCGACAAGTCAAACGCCTGTGCAAGTGTTAAATGTTTTAGAAGATTACTACAAGCGTTATAATTCAAACGTTCATCGTGGT
GTTCATACATTAGGATCATTGGCAACTGATGGTTATGAAAATGCCCGTGAAACCGTTCGTCGTTTTATTAATGCGAAGTATTTTGAAGAAA
TCATTTTTACACGCGGAACAACTGCGTCGATTAACCTTGTAGCACATAGCTATGGTGATGCAAATGTTGAAGAGGGCGATGAAATTGTTG
TCACTGAAATGGAACATCATGCCAATATTGTTCCTTGGCAACAGTTAGCAAAGCGTAAAAATGCGACATTGAAATTTATACCAATGACAG
CTGACGGTGAATTAAACATCGAGGATATTAAGCAAACGATTAATGATAAAACAAAGATCGTTGCTATTGCACATATATCTAATGTGCTCG
GTACAATTAATGATGTTAAAACCATTGCAGAAATAGCTCATCAACATGGTGCAATTATCAGTGTTGATGGGGCGCAAGCAGCACCACATA
TGAAACTTGATATGCAAGAAATGAATGCTGATTTTTATAGTTTTAGTGGTCATAAAATGCTTGGACCAACAGGTATTGGCGTATTATTTGG
TAAACGTGAGTTACTACAAAAAATGGAACCGATTGAGTTCGGTGGCGACATGATTGATTTTGTAAGTAAGTATGATGCAACATGGGCTG
ATTTACCTACTAAATTTGAGGCGGGTACTCCATTAATTGCTCAAGCAATTGGGCTTGCAGAAGCTATTCGCTATTTAGAACGCATAGGTTT
TGATGCAATTCATAAATATGAACAAGAATTAACGATATATGCTTATGAGCAAATGTCTGCAATTGAAGGAATTGAAATTTATGGCCCGCC
AAAGGATCGTCGTGCAGGTGTAATAACGTTTAATTTACAAGATGTACATCCACACGATGTTGCTACAGCCGTAGATACAGAAGGTGTAGC
GGTTAGAGCTGGGCATCATTGTGCGCAACCGTTAATGAAATGGTTAAATGTGTCTTCAACAGCTAGAGCGAGTTTTTATATATACAACAC
GAAAGAAGACGTTGATCAGTTAATAAATGCCTTGAAACAAACGAAGGAGTTTTTCTCTTATGAATTTTAA

Gene: sufN (iron-sulfur cluster assembly protein N)

Contig: 06_NODE_54, position: 19039 to 19503, length: 465 nt, orientation: FORWARD

Perfect match to: (RF122-AJ938182-[846053:846517], highly conserved allele)

Sequence:

ATGAATTTTAATAATCTAGATCAATTATATAGATCTGTCATTATGGATCATTATAAAAATCCTAGAAATAAAGGTGTATTAGATAACGGGT
CTATGACAGTAGATATGAATAACCCGACATGCGGTGACCGTATACGACTAACATTTGATATAGAAGACGGCATTATAAAAGATGCTAAGT
TTGAAGGTGAAGGTTGTTCGATTTCAATGGCAAGTGCATCGATGATGACACAAGCTGTTAAAGGTCATTCACTTGGAGAAGCAATGCAA
ATGAGCCAAGAATTTACGAAAATGATGCTTGGTGAAGACTATGTGATTACAGAAGAAATGGGAGATATTGAAGCATTGCAAGGTGTATC
TCAATTCCCAGCTCGTATTAAATGTGCCACATTAGCTTGGAAAGCATTGGAAAAAGGTACTGTTGCTAAAGAAGGTAAAGCAGAAGGTA
CGACTGAAGAAGAATAG

Gene: sufB (iron-sulfur cluster assembly protein B)

Contig: 06_NODE_54, position: 19654 to 21051, length: 1398 nt, orientation: FORWARD

Sequence:

ATGGCTAAAAAAGCACCTGATGTTGGGGATTATAAATATGGATTCCACGACGATGATGTGTCCATTTTCAGATCAGAACGTGGTTTAACT
GAGAATATCGTTAGAGAAATTTCTAACATGAAAAATGAGCCGGAATGGATGTTAGATTTCCGTCTTAAATCATTAAAATTGTTTTATAAAA
TGCCAATGCCTCAATGGGGTGGCGACTTATCAGAATTGAATTTCGATGACATTACTTACTATGTAAAGCCTTCAGAACAAGCTGAACGTT
CATGGGATGAAGTGCCAGAAGAAATTAAAAGAACTTTCGATAAATTAGGAATTCCTGAAGCTGAACAAAAATATTTAGCTGGTGTTTCTG
CTCAATATGAATCTGAAGTTGTTTACCATAATATGGAAAAAGAACTTGAAGAAAAAGGTATTATCTTTAAAGATACAGATAGTGCTTTAC
AAGAAAATGAAGAATTATTCAAAAAATACTTTGCTTCTGTAGTACCTGCAGCAGATAACAAATTTGCGGCGTTAAACTCAGCAGTATGGT
CAGGTGGTTCGTTCATTTATGTACCTAAAAATATCAAACTAGATACGCCACTACAAGCTTATTTCCGTATTAACTCTGAGAACATGGGTCA
ATTTGAACGTACATTAATCATTGCTGATGAAGGTGCTTCTGTACATTACGTAGAAGGTTGTACTGCACCAGTTTATACAACTAGTTCTTTAC
ACTCTGCTGTTGTGGAAATCATTGTGCATAAAGATGCGCACGTTCGTTATACTACGATTCAAAACTGGGCGAACAATGTATACAATTTAGT
TACAAAACGTACTTTTGTTTATGAAAACGGAAATATGGAATGGGTAGATGGTAACTTAGGTTCTAAGTTAACGATGAAATATCCAAACTG
TGTTCTTTTAGGTGAAGGTGCAAAAGGTAGTACATTATCTATTGCATTTGCTGGTAAAGGACAAGTTCAAGATGCCGGTGCTAAAATGAT
TCATAAAGCACCTAATACATCTTCTACAATTGTTTCTAAATCTATTTCAAAAAATGGTGGTAAAGTTATTTATCGCGGTATTGTTCATTTTG
GACGTAAAGCAAAAGGTGCTCGTTCAAATATTGAATGTGATACATTAATCTTAGATAACGAATCAACATCAGATACAATTCCATATAACG
AAGTATTCAACGATCAAATATCATTAGAACATGAAGCCAAGGTTTCAAAAGTTTCTGAAGAACAATTATTCTATCTAATGAGTCGTGGTAT
TTCTGAAGAAGAAGCGACAGAAATGATTGTTATGGGATTCATCGAACCATTTACAAAAGAACTTCCAATGGAATACGCGGTCGAAATGA
ACCGTTTAATCAAGTTCGAAATGGAAGGTAGTATTGGATAG

Gene: Q2YWM5 (putative protein)

Contig: 06_NODE_54, position: 21381 to 21695, length: 315 nt, orientation: REVERSE

Sequence:

TTAAAATAAGAAAACTATTAATAATGTTAAGTTCACTACAGATGTTGCTAATGGACCATAAGTTTTAAAGACATCTTCACTTTTATAACCAA
CAATCGCATCTAAAAATTGAACTAAGATCATTGCAATGGATATAGTTATCAAAAATATAGCACTATGAATGACTAAAGAAAAAATAGCTA
ATAAAAATAAAGGTAAGCTTCGACTAAGTGCATAATATGCATTTATATTATGGCTAGATGCACATGCTTGAATTGAATAACCTAAACTTAC
ACTGGCACTGATTATTGTAAATATTGCTAAAACAAAATACAT

Gene: corB (CBS domain comprising Mg2+ and Co2+ transporter)

Contig: 06_NODE_54, position: 22060 to 23100, length: 1041 nt, orientation: FORWARD

Perfect match to: (N315-BA000018-[886646:887686], allele observed in CC5+CC1+CC361)

Sequence:

GTGATCATTGCCATAATTATATTGATATTTATTTCGTTTTTCTTTTCAGGAAGCGAGACGGCATTAACGGCTGCCAATAAAACAAAATTTAA
AACTGAAGCTGACAAAGGTGATAAAAAAGCAAAAGGCATTGTAAAGTTACTTGAAAAACCAAGTGAGTTTATTACAACGATTCTAATTG
GGAATAATGTCGCGAATATTTTATTACCAACACTTGTTACAATTATGGCTTTACGTTGGGGGATTAGCGTTGGTATTGCATCAGCTGTTTT
AACAGTTGTTATCATTTTGATTTCCGAAGTGATTCCCAAGTCTGTCGCTGCAACATTTCCAGATAAAATAACAAGGCTTGTATATCCAATTA
TTAATATTTGTGTCATTGTGTTTCGCCCTATCACATTACTTTTAAATAAGTTGACGGACAGTATTAATCGAAGTTTATCTAAGGGTCAACCT
CAAGAACATCAATTTTCAAAAGAAGAATTTAAAACAATGTTAGCAATTGCTGGACATGAAGGTGCTTTAAATGAAATTGAGACGAGTAG
GTTGGAAGGTGTCATTAATTTTGAAAATTTAAAAGTAAAAGATGTAGATACAACACCTAGAATTAATGTGACGGCATTTGCTTCAAATGC
GACATACGAAGAAGTTTATGAAACGGTTATGAATAAGCCATACACTAGATATCCAGTGTACGAGGGAGATATTGATAACATTATTGGAG
TGTTTCATTCTAAATATCTGTTGGCTTGGAGTAATAAAAAAGAAAATCAAATTACAAACTATTCAGCTAAGCCATTATTTGTGAATGAACA
CAATAAAGCTGAATGGGTATTACGTAAGATGACTATTTCTAGAAAACATTTAGCAATTGTGTTGGACGAATTTGGTGGTACTGAAGCGAT
AGTGTCACATGAAGACTTAATTGAAGAATTATTAGGTATGGAAATTGAAGATGAGATGGATAAAAAGGAAAAAGAAAAACTTTCTCAAC
AGCAAATTCAATTTCAACAACGGAAAAATCGCAACGTATCTATATAA

Gene: npd (putative 2-nitropropane dioxygenase)

Contig: 06_NODE_54, position: 23114 to 24181, length: 1068 nt, orientation: FORWARD

Perfect match to: (M0239-AIWE01000002-[29601:30668], allele observed in CC188+CC1+CC188)

Sequence:

ATGTGGAATAAGAATCGACTTACTCAAATGTTAAGTATTGAATATCCAATTATACAAGCAGGTATGGCAGGAAGTACGACACCGAAATTA
GTTGCATCAGTAAGTAACAGTGGTGGGTTAGGCACAATAGGCGCAGGTTACTTTAATACGCAGCAATTGGAAGATGAAATAGATTATGT
ACGCCAATTAACGTCAAATTCTTTTGGCGTAAATGTCTTTGTACCAAGTCAACAATCATATACTAGTAGTCAAATTGAAAATATGAATGCA
TGGTTAAAACCTTATCGACGCGCATTACATTTAGAAGAGCCGGTTGTAAAAATTACCGAAGAACAACAATTTAAGTGTCATATTGATACG
ATAATTAAAAAGCAAGTGCCTGTATGTTGTTTTACTTTTGGAATTCCAAGCGAATCTATTATAAAAAGATTGAAAGAAGCAAACATTAAGC
TAATAGGTACAGCAACAAGTGTTGATGAAGCTATTGCGAATGAAAAAGCGGGTATGGATGCTATCGTTGCTCAAGGTAGTGAAGCAGGT
GGACATCGTGGTTCATTTTTAAAACCTAAAAATCAATTACCTATGGTTGGAACAATATCTTTAGTGCCACAAATTGTAGATGTCGTTTCAA
TTCCGGTCATTGCCGCTGGTGGAATTATGGACGGTAGAGGAGTTTTGGCAAGTATTGTCTTAGGTGCAGAAGGGGTACAAATGGGCACT
GCATTTTTAACATCACAAGATAGTAATGCATCAGAACTACTACGAGACGCAATTATAAATAGTAAAGAAACAGATACAGTCGTTACAAAA
GCGTTTAGTGGAAAGCTTGCACGCGGTATCAACAATAGGTTTATCGAAGAAATGTCCCAATACGAAGGCGACATCCCAGATTATCCAATA
CAAAATGAGCTAACAAGTAGCATAAGAAAAGCCGCGGCAAACATCGGCGACAAAGAGTTAACACATATGTGGAGTGGACAAAGCCCGC
GACTAGCAACAACGCATCCCGCCAACACCATCATGTCCAATATAATCAATCAAATTAATCAAATCATGCAATATAAATAA

Gene: yunF (putative protein)

Contig: 06_NODE_54, position: 24357 to 25205, length: 849 nt, orientation: FORWARD

Perfect match to: (MW2-BA000033-[890584:891432], highly conserved allele)

Sequence:

ATGATAAACATCGGATTAACAGGTTGGGGTGATCACTATTCATTATATGAAGATTTAGAACGCCAAACCGATAAACTTAAAACATATGCT
GGACATTTTCCGGTTGTCGAATTAGATGCGACATACTATGCGATACAACCGGAAAGAAATATATTGAAATGGATAAAAGAAACGCCTGA
TACATTTGAATTTGTGGTCAAAATTCATCAAGCACTCACATTGCATGCAGACTACAAAACATTTGCAGATACAAGGCAAGAACTATTTGAT
CAATTTAAGAATATGTTAGAGCCCTTACATACACAGAAAAAATTAGCAATGGTATTGGTTCAATTTCCGCCATGGTTTGACTGCAATGCAC
AAAATATCAAATATATTTTGTATGTAAGACAGCAATTACAAGCATTTCCAATGTGTGTAGAATTTAGGCATCAATCATGGTTTAGTGATGC
ATTTAAAGAACAAACATTGGCATTTTTAACAGAACATCAAATCATTCATGCAGTAGTTGATGAACCACAAGTGAAAGATGGCAGTGTACC
TTTAGTCAATCGAATCACAAATGAAATTGCGTTTGTACGTTATCATGGACGTAATCATTACGGTTGGACTAAGAAAGATATGTCAGATCA
AGAATGGCGCGATGTACGCTATTTATATGATTATAATGAGCAAGAATTAATAGACTTGGCACAAAAGGCACAAATATTAGCACAAAAAG
CTAAGAAAGTTTACGTCATATTTAACAATAATTCTGGTGGTCATGCAGCAAATAATGCCAAAACATATCAGCGATTATTGAATATAGAATA
TGAAGGGTTAGCACCACAACAATTAAAATTATTTTAA

Gene: yunE (putative membrane protein)

Contig: 06_NODE_54, position: 25218 to 26045, length: 828 nt, orientation: FORWARD

Perfect match to: (N315-BA000018-[890013:890840], highly conserved allele)

Sequence:

ATGTTATTAACAATTACATTATTAGTTTTAATCGGAGGTTTGTCAGCGATTATAGGGTCTATCGTAGGCATTGGAGGCGGTATTATTATCG
TTCCAACAATGGTTTACCTCGGTGTTGAACATGGATTACTACATAATATTACAACACAAGTAGCGATAGGGACGTCTTCAGTCATTCTAAT
TGTGACAGGACTTTCTTCATCACTTGGATATTTAAAAACAAAACAAGTTGATATTAAAAATGGTTCCATCTTTTTATTTGGACTATTACCAG
GTTCATTGCTTGGGTCCTTCATTAGTAGATATTTAACATTTGAGTCATTTAATTTATATTTTGGTATCTTTTTAATTTTCGTAGCCATTTTATT
AATGGTAAGAAATAAGATTAAACCGTTTAAAATTTTCGATAAACCCAAGTATGAAAAGACTTATGTAGACGCTAAAGGTAAAACATATCA
TTATAGTGTTCCACCATTGTTTGCTTTTATTACAACGTTTTTAATTGGTATATTGACAGGTTTATTTGGTATTGGAGGTGGCGCACTAATGA
CGCCACTAATGCTTATTGTATTTAGATTTCCACCTCATGTAGCTGTTGGAACAAGTATGATGATGATTTTCTTTTCAAGTGTCATGAGTTCT
ATAGGGCACATTGCTCAAGGTCACGTAGCTTGGGGTTATGCAATCATTTTAATTATTTCTAGTTATTTTGGTGCGAAAATCGGTGTCAAAG
TGAATCAATCAATTAAGTCAGATACGGTAGTAACATTATTGAGAACAGTAATGTTGTTAATGGGTATATATTTAATTATTCGTGCGTTGAT
TTAA

Gene: yunD (putative 5'-nucleotidase)

Contig: 06_NODE_54, position: 26072 to 27391, length: 1320 nt, orientation: FORWARD

Perfect match to: (BAA-39-ST239-AEEK01000071-[64480:65799:r], allele observed in CC239+CC1+CC239)

Sequence:

TTGAGGCTTACAATTTATCATACGAACGATATTCATAGTCATTTACATGAATACGAACGCATTAAAGCATATATGGCAGAACATCGGCCAC
GACTTAATCATCCTTCTTTATATGTTGATCTAGGTGATCATGTAGATTTATCCGCACCTATAACTGAAGCAACTTTAGGTAAAAAGAATGT
GGCATTACTAAATGAAGCAAAATGTGATGTTGCAACAATCGGTAATAATGAAGGGATGACCATTTCACACGAAGCTTTAAATCACCTTTA
CGACGAAGCAAAATTTATAGTGACATGTAGCAATGTTATAGATGAATCAGGTCATTTACCAAATAATATCGTTTCTTCTTATATTAAGGAC
ATAGACGGTGTGAAAATACTATTCGTTGCAGCGACAGCACCTTTTACCCCATTTTATCGTGCACTAAATTGGATTGTTACCGATCCACTTG
AATCTATAAAAGAAGAAATTGAACTTCAACGAGGTAAATTTGATGTATTAATCGTGCTAAGTCATTGTGGCATTTTCTTCGATGAAACATT
ATGCCAAGAATTGCCTGAAATTGATGTCATTTTTGGTAGTCATACGCATCATTATTTTGAACATGGTGAAATCAATAATGGTGTACTGATG
GCGGCAGCTGGAAAGTATGGTAATTATCTTGGAGAGGTTAATTTAACTTTTGAGGCACATAAAGTAGTACATAAAACTGCAAAGATTATT
CCTTTAAAAACATTACCTGAAGTTGAAACTTCATTTGAAGAAGAAGGAAAAACGTTAATGTCCAATTCAGTAATTCAACATCCAGTAGTGC
TTAAGCGTAGTATGAATCACATAACTGAAGCTGCATACTTATTAGCTCAAAGTGTTTGTGAGTATACACATGCACAATGTGCCATCATCAA
TGCTGGCTTACTCGTTAAAGATATTGTAAAAGATGAAGTGACAGAATATGACATTCATCAAATGTTACCGCATCCGATTAATATGGTAAG
GGTTAGACTTTTTGGTGTGAAATTAAAAGAGATTATAGCTAAAAGTAATAAACAAGAATATATGTATGAACATGCACAAGGTTTGGGTTT
CAGAGGGAATATATTTGGAGGATATATTCTTTATAATTTAGGGTACATTCATTCTACAGGGCGTTACTATCTGAATGGAGAAGAAATCGA
AGACGACAAAGAATATGTACTAGGTACGATAGATATGTATACGTTCGGTCGTTATTTCCCAACATTGAAAGAATTACCAAAAGAGTATTT
AATGCCAGAGTTTTTAAGAGATATATTTAAAGAAAAATTATTGGAATATTAA

Gene: lipA (iron-sulfur cluster comprising lipoyl synthase)

Contig: 06_NODE_54, position: 27475 to 28392, length: 918 nt, orientation: FORWARD

Perfect match to: (MW2-BA000033-[893702:894619], highly conserved allele)

Sequence:

ATGGCGACAAAAAACGAGGAAATATTACGTAAACCGGATTGGTTGAAAATAAAATTAAATACCAACGAAAACTATACAGGACTTAAGAA
GATGATGAGGGAAAAAAATCTTAATACTGTATGTGAAGAAGCTAAATGTCCTAATATACATGAATGTTGGGGTGCACGTCGTACAGCGA
CATTTATGATTTTAGGTGCCGTATGTACAAGAGCTTGTCGTTTTTGTGCGGTTAAGACAGGTTTACCTAATGAACTTGATTTAAATGAGCC
TGAACGTGTAGCTGAATCAGTTGAATTAATGAATTTGAAACACGTTGTTATCACTGCTGTTGCGCGTGATGATTTAAGAGATGCTGGTTC
AAATGTTTATGCTGAGACAGTACGTAAAGTTAGAGAAAGAAATCCATTTACAACGATTGAAATTTTACCATCAGATATGGGCGGGGACTA
TGATGCGTTAGAAACATTAATGGCGTCAAGACCTGACATTTTAAACCATAATATTGAAACTGTTCGTCGCTTAACACCGAGAGTTCGTGC
GCGTGCGACTTACGACAGAACATTAGAGTTTTTACGTCGTTCAAAAGAATTACAACCGGATATCCCAACTAAATCAAGTATTATGGTTGG
ATTAGGTGAAACTATAGAAGAAATTTATGAAACGATGGATGATTTACGTGCGAATGATGTAGATATTTTAACGATTGGTCAATATTTACA
ACCTTCACGTAAACATTTAAAGGTTCAAAAATATTACACGCCTTTAGAGTTTGGTAAATTAAGAAAAGTGGCAATGGATAAAGGGTTTAA
ACATTGCCAAGCTGGACCTTTAGTACGTAGTTCTTATCATGCGGATGAGCAAGTAAATGAAGCTGCTAAAGAAAAGCAACGCCAAGGTG
AGGCACAGTTAAATAGTTAA

Gene: DUF1027 (protein of unknown function)

Contig: 06_NODE_54, position: 28513 to 28905, length: 393 nt, orientation: FORWARD

Perfect match to: (MW2-BA000033-[894740:895132], highly conserved allele)

Sequence:

GTGAAGAATTTGATAAAAGTAGATCAACATTACTTTGAATTAATAGAAAATTATCGCGAATGTTTTAATGAAGAACAATTTATTGCTAGGT
ATTCAGATATTTTAGATAAATATGATTACATAGTTGGTGACTATGGTTACGATCAATTACGATTAAAAGGTTTTTACAAAGATTCTAATAA
AAAAGCAGAGATGAGTAAACGTTTTTCAAATATTCAAGATTACATATTTGAATATTGTAACTTTGGTTGTCCTTACTTTGTATTAAGACATT
TGTCTAAACAAGAGGTTAAAAAGTTAATCGAAGAAGTTCATCCGTCTGATGTGATAGATGACGACAATAAACTTCAAGATGTGAAGATTA
AGCCAACCATTCAAGATACTGAACATTAA

Gene: DUF3055 (protein of unknown function)

Contig: 06_NODE_54, position: 28984 to 29241, length: 258 nt, orientation: REVERSE

Perfect match to: (N315-BA000018-[895299:895556:r], highly conserved allele)

Sequence:

TTAATGAATGACTTCATTTAAATACTCAGTAATTTCATCGCCTTCTTCAGCATTTACACCTAAAATATGAGCGATATAGCCTTCTTCTTTTAA
ATCATCAGTACCGATAATACCGAATTTATTTGTTTGCATATTAAGTACGAGTGTCTTACCATAATGTCTATTTGTATGGACTAACATCAAAT
CATATCGACTATGCTCGCCAACAAAACCAACAAACTGAACTTGACTCTCTTCGTTGTCATCATATAAATACAT

Gene: yutE (conserved hypothetical protein)

Contig: 06_NODE_54, position: 29348 to 29782, length: 435 nt, orientation: FORWARD

Perfect match to: (MW2-BA000033-[895575:896009], highly conserved allele)

Sequence:

ATGTATTTTGTAGACAAAGATAAACTAACTCAGAAATTAGCCTATTTACAAGCATTAACTGATGATTATCATGAGAGCAAGCACAATCATT
ATGCATTTGAACGCATTGCTCAAATGTTGATAGAATCATCGGTAGATATAGGGAATATGATTATCGATGCATTTATTTTAAGGGATCCTG
GTAATTATAAAGATGTGATTGATATATTAGAACTAGAAAATGTTATTACTAAAGAAACACAGCAGGCGATTAATAAAACTGTCGGTATTC
GTAAACAATTTACATATGATTACACAGCCTTAGATATTAAGATTATCATGCCAATGTTTGATGACGCATTACCTTATTACAAACAATTTATT
ACAGAAGTAACGACATTTTTACATCAAGAAAATGTACCAGTAACAGCTTTTGGTAAAGGAGAAAATCAATAA

Gene: yutF (putative p-nitrophenyl phosphatase)

Contig: 06_NODE_54, position: 29782 to 30561, length: 780 nt, orientation: FORWARD

Perfect match to: (N315-BA000018-[896097:896876], highly conserved allele)

Sequence:

ATGAAACAGTATAAAGCGTATTTAATCGATTTAGATGGCACAATGTATATGGGAACAGATGAGATTGATGGAGCAAAACAATTCATCGA
TTATTTAAATGTAAAAGGCATTCCTCATTTATACGTAACTAATAATTCAACAAAAACACCTGAGCAAGTAACTGAAAAATTACGTGAAATG
CACATTGATGCTAAACCAGAAGAGGTTGTAACGTCAGCGTTAGCCACTGCTGATTATATTTCAGAACAATCACCAGGAGCATCAGTATAT
ATGTTAGGTGGGAGTGGTTTAAATACTGCGTTAACCGAAGCGGGACTTGTCATTAAAAATGACGAGCATGTTGATTATGTAGTTATTGGA
CTTGACGAACAAGTTACATATGAAAAGCTTGCGATTGCAACGTTAGGTGTAAGAAATGGTGCAACATTTATTTCTACAAATCCTGATGTA
TCAATTCCTAAAGAGCGTGGTTTATTACCTGGTAATGGTGCTATTACAAGTGTTGTAAGTGTATCGACAGGTGTATCGCCACAATTTATTG
GTAAACCAGAACCGATTATTATGGTTAAAGCATTAGAAATTTTAGGATTAGATAAATCCGAAGTTGCTATGGTAGGCGATTTGTACGATA
CCGATATTATGTCTGGTATTAACGTAGGTATGGATACGATTCATGTACAAACAGGTGTATCTACGTTAGAAGATGTGCAAAATAAAAATG
TGCCACCAACGTATTCTTTTAAAGATTTAAATGAAGCAATAGCTGAATTAGAAAAATAG

Gene: gyaR (2-ketogluconate reductase)

Contig: 06_NODE_54, position: 30594 to 31553, length: 960 nt, orientation: FORWARD

Perfect match to: (CO-23-AJKF01000089-[26877:27836:r], allele observed in CC8)

Sequence:

TTGGTAAAAATAGTTGTTTCGAGGAAAATTCCAGATAAATTTTATCAACAATTAAGTAAACTTGGTGACGTTGTTATGTGGCAAAAATCAT
TAGTGCCTATGCCTAAAGATCAATTTGTGACAGCACTTCGTGACGCAGATGCTTGTTTTATTACATTAAGTGAACAGATCGATGCAGAAAT
TTTAGCGCAATCACCAAATTTAAAAGTAATTGCGAATATGGCTGTAGGATATGACAACATCGATGTTGAAAGTGCAACAGCGAATAACGT
GGTTGTCACGAATACACCAAATGTACTTACTGAAACAACTGCAGAATTAGGATTTACATTAATGCTTGCTATAGCACGCCGTATTGTAGAA
GCTGAAAAATATGTAGAAGCAGATGCATGGCAAAGCTGGGGTCCTTATTTATTGTCAGGTAAAGATGTCTTCAATTCAACTATTGGAATA
TATGGTATGGGAGATATTGGTAAAGCTTTTGCAAGAAGGTTGCAAGGGTTTAATACTAATATTCTTTATCATAATCAATCAAGACATAAA
GATGCAGAGGCGGACTTTAATGCAACATATGTTTCTTTTGAAACGTTGTTAGCAGAAAGTGATTTTATCATCTGTACAGCGCCACTTACAA
AAGAAACACATCATAAATTTAATGCTGAAGCATTTGAACAAATGAAAAATGATGCAATTTTTATTAATATCGGTAGAGGACAAATTGTAG
ATGAAACAGCATTAATCGATGCACTAGACAATAAAGAAATTTTAGCATGTGGTTTAGATGTATTAGCAAATGAACCGATTGATCATACAC
ATCCATTAATGGGACGTGATAATGTTCTGATTACACCACACATTGGTAGCGCATCAGTAACAACACGGGACAATATGATTCAATTATGTAT
TAATAATATAGAAGCGGTTATGACAAATCAGGTACCACATACTCCAGTAAATTGA

Gene: dltX (putative protein located in teichoic acid biosynthesis gene cluster)

Contig: 06_NODE_54, position: 31965 to 32117, length: 153 nt, orientation: FORWARD

Perfect match to: (RF122-AJ938182-[864187:864339], highly conserved allele)

Sequence:

ATGAAATCTAAAAGTAAACAGCCACCTAATAAATATGTTGAAGCATTCAAACCATATTTATTAACACTATTGTATTTGGCAATATTTATTAC
TTTATATTTAATTTATGGCAGTGGCGACACACACAATAACTTCATTTATAATGAGTTCTAA

Gene: dltA (D-alanine-D-alanyl carrier protein ligase)

Contig: 06_NODE_54, position: 32133 to 33590, length: 1458 nt, orientation: FORWARD

Perfect match to: (JKD6159-CP002114-[876396:877853], highly conserved allele)

Sequence:

ATGACAGATATTATTAACAAGCTGCAAGCGTTTGCGGATGCAAATCCACAAAGCATTGCTGTTAGACACACAACTGATGAATTAACTTAT
CAACAGTTAATGGATGAGTCTAGTAAATTAGCACATCGATTACAAGGTAGTAAGAAACCGATGATTTTATTCGGTCACATGTCACCATAT
ATGATTGTTGGGATGATTGGTGCCATTAAAGCAGGATGTGGATATGTACCTGTAGACACTTCAATTCCTGAAGACCGTATTAAAATGATT
ATTAACAAGGTTCAACCAGAGTTTGTATTTAATACGACTGATGAATCATTTGAAAGTTTAGAAGGCGAAGTATTTACAATAGAAGATATT
AAAACATCTCAAGACCCAGTAATTTTTGATAGTCAGATTAAAGATAACGACACAGTATACACAATCTTTACATCTGGTTCTACTGGGGAAC
CTAAAGGTGTTCAAATTGAATATGCAAGTTTAGTTCAATTTACTGAGTGGATGTTAGAACTTAATAAATCAGGAAATGAACAACAATGGC
TTAACCAAGCGCCATTTTCATTTGATTTATCTGTAATGGCTATTTATCCATGTTTAGCATCAGGCGGTACATTAAATCTTGTAGATAAAAAC
ATGATTAATAAACCTAAATTATTAAATGAAATGCTAACAGCAACACCGATTAACATTTGGGTATCAACACCATCATTTATGGAAATGTGTT
TATTATTACCAACGCTTAATGAAGAACAATATGGTAGTCTTAACGAATTCTTCTTCTGTGGTGAAATTCTACCTCACAGAGCAGCAAAAGC
GTTAGTAAACCGTTTCCCAAGTGCGACGATTTACAACACATATGGTCCAACTGAAGCTACGGTAGCAGTTACAAGTATTCAAATTACACA
AGAAATCTTAGATCAATATCCGACATTACCTGTTGGCGTTGAAAGACCAGGCGCAAGATTATCTACTACAGATGAAGGTGAACTTGTTAT
CGAAGGTCAAAGTGTAAGTTTAGGATACTTAAAAAATGACCAAAAAACAGCTGAAGTATTTAATTTCGATGACGGTATTCGTACATATCA
CACTGGTGATAAAGCGAAGTTTGAAAATGGTCAATGGTTCATTCAAGGTCGTATTGATTTCCAAATCAAATTGAATGGCTACAGAATGGA
ATTAGAAGAAATTGAAACACAATTACGCCAGTCTGAGTTCGTAAAAGAAGCGATTGTTGTACCTGTATATAAAAATGATAAAGTTATTCA
TTTAATTGGTGCAATTGTGCCAACGACTGAAGTTACGGACAATGCAGAAATGACTAAAAATATTAAAAATGACTTGAAATCACGCTTACC
AGAGTATATGATTCCTAGAAAGTTTGAATGGATGGAACAATTGCCATTGACTTCAAATGGTAAAATTGACAGAAAGAAAATTGCAGAGG
TAATTAACGGATGA

Gene: dltB (putative membrane transporter)

Contig: 06_NODE_54, position: 33587 to 34801, length: 1215 nt, orientation: FORWARD

Perfect match to: (MRSA252-BX571856-[931760:932974], highly conserved allele)

Sequence:

ATGATTCCATATGGTGATTTTACATTCTTCTTAATTGCTTTAATTGCATTATTACCAGTCATTATACTTGGATTTTTAGGTAAGCGAAGTTAC
ATTTATAATGGCGTAGTTACAGCATTTATGATTGTGTTAATCTTTTCTTCTGATAAACATAATCTGTTTGACCAAAAGTATTTAAGTGTTCA
ATTAATTAGTTTTATTATTTACGTCGTATGGCAAGTTTTATTGATAATGTTTTATTATCATTCAAAACCAAAAAATAATTCATTTTCAAAATT
TGTAACTGTAATGGTTTTATCAATATTGCCATTAGCACTTGTGAAAGTGTTACAAAGTACATGGTTAGGTGGACATCAGATTCACTTCCAT
GAAAGTAAATTAATTGAATTTGTTGGTTTCTTAGGAATTTCTTATGTTACATTCAAAAGTGTGCAGTTAATTATGGAAATTCGTGATGGTT
CTATCAAAGAAATTAAAGTATGGAAATTAATTCAATTTATTTCATTCTTCCCAACGATTTCATCTGGACCAATCGATCGTTACAAACGTTTC
GTTAAAGACGATAAAAAAGTACCAACAGGCAATGAATATCGTGAATTAGTATTAAAAGCAATTCACATGATTATGCTTGGTTTCTTGTAT
AAATATATTGTTGCTTACTTTATTAACACATATGCAATCATGCCGTTACAATTAGACTTACATGGCTTTGTCAATTTGTGGTTATATATGTAC
GCATACAGCTTATATTTATTCTTTGACTTTGCAGGTTATAGTTTATTTGCGATAGCATTTAGTTATTTATTCGGTATTAAAACACCACCAAAC
TTCGATAAACCTTTCAAAGCGAAAAATATTAAAGATTTCTGGAATAGATGGCATATGACATTATCATTCTGGTTCAGAGATTGTATTTACA
TGAGATCTTTATTCTACATGTCTCGTAAAAAATTATTGAAGAGTCAATTTGCAATGTCTAACGTGGCATTCTTAATCAACTTCTTCATAATG
GGAATTTGGCATGGTATCGAAGTGTATTACATTGTTTATGGTTTATACCATGCAGCATTGTTTATAGGTTATGGCTATTATGAACGTTGGC
GTAAGAAACATCCGCCACGTTGGCAAAATGGTTTCACAACAGCACTTAGCATTGTGATTACATTCCACTTTGTAACATTTGGCTTTTTAATC
TTCTCAGGTAAACTTATATAA

Gene: dltC (D-alanine--poly (phosphoribitol) ligase subunit 2)

Contig: 06_NODE_54, position: 34819 to 35055, length: 237 nt, orientation: FORWARD

Perfect match to: (RF122-AJ938182-[867041:867277], highly conserved allele)

Sequence:

ATGGAATTTAGAGAACAAGTATTAAATTTATTAGCAGAAGTAGCAGAAAATGATATTGTAAAAGAAAATCCAGACGTAGAAATTTTTGA
AGAAGGTATTATTGATTCTTTCCAAACAGTTGGATTATTATTAGAGATTCAAAATAAACTTGATATCGAAGTATCTATTATGGACTTTGAT
AGAGATGAGTGGGCAACACCAAATAAAATCGTTGAAGCATTAGAAGAGTTACGATGA

Gene: dltD (putative exoprotein)

Contig: 06_NODE_54, position: 35052 to 36227, length: 1176 nt, orientation: FORWARD

Perfect match to: (N315-BA000018-[901367:902542], highly conserved allele)

Sequence:

ATGAAATTAAAACCTTTTTTACCCATTTTAATTAGTGGAGCGGTATTCATTGTCTTTCTATTATTACCTGCTAGTTGGTTTACAGGATTAGTA
AATGAAAAGACTGTAGAAGATAATAGAACTTCATTGACAGATCAAGTACTAAAAGGCACACTCATTCAAGATAAGTTATACGAATCAAAC
AAGTATTATCCTATATACGGCTCTAGTGAATTAGGTAAAGATGACCCATTTAATCCTGCAATTGCATTAAATAAGCATAACGCCAACAAAA
AAGCATTCTTATTAGGTGCTGGTGGTTCTACAGACTTAATTAACGCAGTTGAACTTGCATCACAGTATGATAAATTAAAAGGTAAGAAAT
TAACATTTATTATTTCACCACAATGGTTTACAAACCATGGTTTAACGAATCAAAACTTTGATGCTCGTATGTCTCAAACTCAAATTAATCAA
ATGTTCCAGCAGAAAAACATGTCTACTGAATTAAAACGTCGTTATGCACAACGTTTATTACAGTTTCCACATGTACACAATAAAGAATACT
TGAAATCTTATGCTAAAAACCCTAAAGAAACTAAAGATAGTTATATTTCTGGTTTTAAAGAGAATCAATTGATTAAAATAGAAGCGATTAA
ATCATTGTTTGCAATGGATAAATCTCCATTAGAACATGTTAAACCTGCTACAAAACCAGACGCTTCTTGGGATGAGATGAAACAAAAAGC
AGTTGAAATTGGTAAAGCTGATACTACATCGAATAAATTTGGTATTAGAGATCAATACTGGAAATTAATTCAAGAAAGTAAGCGTAAAGT
TAGACGTGACTACGAATTCAATGTTAATTCTCCAGAATTCCAAGATTTAGAATTACTTGTAAAAACAATGCGTGCTGCTGGTGCAGATGTT
CAATATGTAAGTATTCCATCAAACGGTGTATGGTATGACCACATTGGTATCGATAAAGAACGTCGTCAAGCAGTTTATAAAAAAATCCAT
TCTACTGTTGTAGATAATGGTGGTAAAATTTACGATATGACTGATAAAGATTATGAAAAATATGTTATCAGTGATGCCGTACACATCGGTT
GGAAAGGTTGGGTTTATATGGATGAGCAAATTGCGAAACATATGAAAGGTGAACCACAACCTGAAGTAGATAAACCTAAAAATTAA

Gene: nfuA (Fe-S cluster carrier protein)

Contig: 06_NODE_54, position: 36490 to 36711, length: 222 nt, orientation: REVERSE

Perfect match to: (MW2-BA000033-[902717:902938:r], highly conserved allele)

Sequence:

TTAGAATACTTGTTCTACTTCTATTACACCAGGCACTTCTTCGTGTAATGCACGCTCAATACCAGCTTTAAGAGTGATTGTAGAACTTGGGC
ATGTACCACATGCACCATGTAATTGTAATTTAACAATACCGTCTTCCACGTCAATCAATGAGCAGTCGCCACCATCACGTAATAAAAATGG
ACGAAGACGTTCAATAACTTCTGCTACTTGATCAAACAT

Gene: yuzD (putative sulfur oxido-reduction management enzyme)

Contig: 06_NODE_54, position: 36833 to 37156, length: 324 nt, orientation: FORWARD

Perfect match to: (RF122-AJ938182-[869055:869378], highly conserved allele)

Sequence:

ATGGAGCACGTGAGTGTGGTAGTATATGGGGCAGATGTTATATGTGCAAGTTGCGTTAATGCGCCAACATCGAAAGATATTTATGACTG
GCTACAGCCGCTATTAAAAAGAAAATACCCAAATATATCATTTAAATATACGTATATAGATATTACAAAAGATAATGACAACTTAACAGAT
CATGATTTACAATTTATTGAAAGAATAGAACAAGATGAACTATTTTATCCATTAATTACAATGAACGATGAATATGTAGCAGATGGTTATA
TACAAACAAAGCAAATCACTCGATTTATAGACCAAAAGCTTGTGAATGAATAA

Gene: yutJ-ndhF (type-II NADH:quinone oxidoreductase)

Contig: 06_NODE_54, position: 37215 to 38279, length: 1065 nt, orientation: REVERSE

Perfect match to: (TW20-FN433596-[990375:991439:r], highly conserved allele)

Sequence:

TTAACCATTATGATATTTATATAACCAAAGTACGCCGGATTTTAAAATAGAAGCGAGTCGTCCTGTTACAGTGCGGTCCATGATATATGCA
AAACCTTGCTTATCTCCTAATGACCCAACGATACCTTGTACCTTTAGTTCCGGCATTTTGTCAGGTAATGGTTCATTTAGCCATTGCTTTTTA
AGCACATCGGCAATTTGATCACCTTGAACTTCGGCTAACTGAGCACTTGGCGCATGTGGTAAATCAGCACAATCACCAACTACATAGACG
TTACGATATGTTGGTACTTGATGATACTGGTTAACTATCACGCGTCCATTACTATTTATATCAATCGGCAAGTTACGAACAACTTCAACAG
GTTGAATTCCTGCTGTCCATACAACTAAATCAATATCTTTAGGTTCATCACAGTTATATATTTTACCAGGTTCAACTTTATTAATATTTGAAT
TTGGAACAACGGTAACATTATTTTTGGCGAACCATTTCGCAACATACTTACTTAATTTTTCTGGAAAATTTCTTAAAATTCGCGGCCCACGG
TCATAAAGATATATTTCCAAGTCTGATCTACTTTCTCTTAATTCGCTGGCAAGTTCTATGCCGCTTAATCCAGCACCAACGATACCGACTTT
AGCACCTTCTGGTAGTTCACTAATACTATGGAAAGTATCCCGAGCCTTTGAGAGTGTTTGAATACTATGTGTATATTCTTCGGCTCCTGGA
ACGTTATGATATTTATCTTCACATCCTAAACCAATGATTAGCTCATCATAATCAATTTTAGAATTACCGACTGAGACAATTTGAGCATCTAA
ATCTATGTCGTTAATTTCACCATAAACTGTATTCACTTGTGGATGATTAGGGAATTTCATACGAACATCTTTATCTGATTTCGTGCCCGCAG
CTAAAGCATAAAATTCTGGTTTCAATCCATGAAATGGCATACGATCAACTAATGTGACTGTATAATCTTGTGGTAAAGAAGTAGTTAAAAT
GCGTGACATGATACGCATATTACCATATCCGCCGCCTAACAAAACTAAGTTTTTCAT

Gene: yuzB (conserved hypothetical protein, UPF0349 family)

Contig: 06_NODE_54, position: 38596 to 38832, length: 237 nt, orientation: FORWARD

Perfect match to: (MW2-BA000033-[904823:905059], highly conserved allele)

Sequence:

ATGAATCCGATCGTAGAATTTTGTCTCTCTAACATGGCAAAAGGTGGAGATTATGTTTTTAATCAACTGGAAAATGACCCAGATGTCGAT
GTGTTAGAGTATGGTTGCCTGACACATTGTGGTATATGTTCAGCCGGGTTGTATGCTTTAGTAAATGGTGATATTGTTGAAGGTGATTCG
CCGGAAGAATTATTACAAAATATATATGCACATATAAAAGAAACTTGGATTTTTTAA

Gene: sufA (chaperone involved in Fe-S cluster assembly)

Contig: 06_NODE_54, position: 38845 to 39204, length: 360 nt, orientation: FORWARD

Perfect match to: (MW2-BA000033-[905072:905431], highly conserved allele)

Sequence:

ATGCCAACAGTTATATTAACAGAAGCAGCTGCTTACGAAGTAAAAGATATGCTTAAAGCAAATGAAATGCCAGATGGCTATTTAAAAATT
AAAGTGAATGGTGGCGGGTGCACTGGTTTAACATACGGTATGGGTGCAGAAGAAGCGCCTGGTGAAAATGATGAAGTCTTAGAATACT
TTGGATTAAAAGTATTAGTAGACAAAAAAGATGCACCCGTATTAAATGGTACGACTATTGATTTTAAGCAATCATTAATGGGTGGCGGTT
TCCAAATCGACAATCCTAATGCAATTGCTTCATGTGGCTGTGGTAGTTCATTTAGAACTGCAAAAGTTGCAGGTAATCCTGAAAATTGCTA
A

Gene: yumB-ndhC (type-II NADH:quinone oxidoreductase)

Contig: 06_NODE_54, position: 39658 to 40866, length: 1209 nt, orientation: FORWARD

Perfect match to: (MRSA252-BX571856-[937831:939039], highly conserved allele)

Sequence:

ATGGCTCAAGATCGTAAAAAAGTACTTGTACTTGGTGCTGGTTATGCAGGTTTACAAACTGTAACTAAATTGCAAAAAGCGATATCAACA
GAAGAAGCAGAAATTACGCTTATTAATAAAAATGAATATCACTATGAAGCAACATGGTTACATGAAGCATCAGCAGGTACACTAAACTAT
GAAGATGTATTATATCCTGTGGAAAGTGTCTTGAAGAAAGACAAAGTGAACTTTGTTCAAGCAGAAGTAACAAAAATTGACCGTGATGC
TAAAAAGGTAGAAACAAATCAAGGTATTTATGACTTTGATATTTTAGTAGTAGCATTAGGTTTCGTTAGTGAAACATTCGGCATCGAAGG
TATGAAAGATCATGCTTTCCAAATTGAAAATGTTATCACAGCACGTGAATTATCACGTCATATCGAAGACAAATTTGCTAACTATGCAGCA
TCAAAAGAAAAAGATGATAACGATTTATCTATCTTAGTTGGTGGTGCTGGATTCACTGGTGTTGAATTCTTAGGTGAATTAACAGACAGA
ATTCCTGAATTATGTAGCAAATATGGTGTGGATCAAAATAAAGTTAAAATCACTTGTGTTGAAGCAGCACCTAAAATGTTACCAATGTTCT
CAGAAGAATTAGTTAACCACGCAGTTAGCTACTTAGAAGACCGCGGTGTTGAATTTAAAATTGCTACACCAATCGTTGCTTGTAACGAAA
AAGGTTTTGTAGTTGAAGTAGATGGTGAAAAACAACAATTAAATGCAGGTACTTCAGTATGGGCAGCTGGTGTACGTGGTAGTAAATTA
ATGGAAGAATCATTTGAAGGCGTTAAACGTGGACGTATCGTTACAAAGCAAGATTTAACAATCAATGGTTACGACAACATTTTTGTTATT
GGTGACTGTTCAGCGTTTATCCCAGCTGGAGAAGAACGTCCATTACCAACTACAGCACAAATTGCAATGCAACAAGGTGAAAGTGTTGCT

AAAAACATTAAACGCATCTTAAACGGTGAATCAACTGAAGAATTCGAATACGTTGATCGTGGAACTGTTTGTTCTTTAGGTTCACATGAC
GGTGTAGGTATGGTATTTGGTAAACCTATCGCTGGTAAAAAAGCAGCATTCATGAAAAAAGTGATTGATACACGTGCGGTATTCAAAATC
GGTGGTATCGGTTTAGCATTCAAAAAAGGTAAATTCTAG

Gene: pepZ (cytosolic leucine aminopeptidase)

Contig: 06_NODE_54, position: 40997 to 42472, length: 1476 nt, orientation: FORWARD

Perfect match to: (MW2-BA000033-[907224:908699], highly conserved allele)

Sequence:

ATGAATTTTAAATTAAATAACACACTAAGCAACGAAATAAATACATTGATTATTGGTATACCAGAACATTTAAATCAGTTAGAGCGCATTA
GTTTTAATCATATCGATATTACAGAATCACTTGAAAGACTAAAACATCAACATATTATTGGTAGTAAAGTTGGGAAGATTTATACAACTGC
ATTTGATGTACAAGATCAAACATATCGTTTAATTACAGTTGGTTTAGGAAACTTAAAGACACGTAGTTATCAAGATATGTTGAAAATATGG
GGACATCTTTTCCAATACATAAAGTCAGAACACATTGAAGATACGTATTTACTTATGGATTCATTTATTTCAAAATATGATCAGTTATCAGA
TGTATTAATGGCATGCGGTATTCAAAGTGAGCGTGCAACATATGAATTCGATCATTATAAATCAAGTAAGAAGGCACCGTTTAAGACGAA
TTTAAACCTTATTAGTGAATCATTAATTGAATTAGATTTTATTCATGAGGGTATCAGTATTGGCCAATCCATTAATTTGGCAAGAGACTTTA
GTAATATGCCACCGAATGTATTAACACCACAAACATTTGCAGAAGATATTGTTAATCATTTTAAAAATACAAAGGTCAAAGTAGATGTTAA
AGATTATGACACTTTAGTTTCTGAAGGATTCGGACTTTTACAAGCAGTAGGTAAAGGTAGTAAGCATAAACCGAGATTAGTAACCATCAC
ATATAATGGCAAAGACAAAGATGAAGCACCAATTGCCTTAGTTGGTAAAGGTATAACGTATGATTCTGGTGGTTATAGTATTAAAACGAA
GAATGGCATGGCTACAATGAAGTTTGACATGTGTGGCGCTGCGAATGTCGTTGGTATCATTGAAGCGGCTAGTCGTTTACAACTGCCTGT
AAATATTGTCGGAGTGCTTGCGTGTGCTGAAAATATGATAAATGAAGCATCAATGAAGCCAGATGATGTATTTACAGCATTAAGTGGTG
AAACTGTAGAAGTAATGAATACAGACGCTGAAGGTAGATTAGTCCTTGCAGATGCTGTGTTTTATGCAAATCAATATCAGCCTAGTGTGA
TTATGGACTTTGCTACATTAACGGGTGCAGCAATTGTTGCACTAGGCGATGATAAAGCTGCTGCATTTGAATCGAATAGTAAAGTGATAT
TAAACGATATATTACAAATAAGTTCTGAAGTCGATGAAATGGTATTTGAATTACCGATTACTGCAACCGAACGTGCAAGTATTAAACACA
GTGATATCGCTGATTTAGTTAACCATACGAATGGACAAGGTAAAGCGCTATTTGCGGCAAGTTTTGTAACACATTTTAGTGGTCAAACAC
CTCACATTCATTTCGATATTGCAGGTCCAGCAACGACTAATAAAGCTTCATATAATGGTCCAAAAGGGCCAACAGGATTTATGATTCCGAC
GATAGTACAATGGTTAAAACAACAATAA

Gene: nhaC3 (Na+/H+ antiporter)

Contig: 06_NODE_54, position: 42883 to 44199, length: 1317 nt, orientation: FORWARD

Sequence:

ATGATAAATGCAGTAGTAATAGCAGTAATTTTAATGATTGTGCTATGTTTATGTCGATTAAACGTAGTTATAAGCTTATTTATCAGTGCGC
TAGTTGGTGGCTTAATTTCAGGCATGAGCATTGAAAAAGTTATAAATGTATTTGGGAAAAATATAGTCGATGGTGCTGAGGTAGCATTAA
GCTATGCTTTATTAGGTGGATTTGCAGCATTAATTTCATACAGTGGTATCACAGACTATTTAGTAGGAAAAATTATAAATGCAATTCACGC
TGAAAATAGTCGATGGTCAAGAGTTAAAGTCAAAGTGACAATAATCATTGCATTATTAGCTATGAGTATCATGAGTCAAAACTTAATTCCT
GTACATATTGCATTCATTCCAATTGTCATCCCACCATTGTTAAGTCTGTTTAATGACTTAAAAATAGATAGACGTTTAATCGGTTTGATTAT
CGGTTTTGGTTTATGTTTCCCGTATGTGTTATTACCATATGGATTCGGTCAAATTTTCCAGCAAATTATTCAAAGTGGCTTTGCAAAGGCG
AATCACCCAATTGAGTTTAATATGATTTGGAAAGCAATGCTTATTCCTTCAATGGGGTATATTGTTGGCTTACTTATCGGTTTATATGTATA
TCGTAAACCACGTGAATATGAAACACGTAAAATTTCAGATAGTGACAATGTTACAGAGTTAAAACCATATATCTTAATAGTAACAATTGTA
GCAATACTAGCTACATTTTTAGTACAAACATTTACAGATTCAATGATTTTTGGTGCACTGGCAGGGGTACTCGTATTCTTTATTTCACGTGC
ATATAATTGGTATGAATTAGATGCTAAGTTTGTTGAAGGTATTAAAATTATGGCTTATATTGGTGTAGTTATTTTAACAGCAAATGGATTT
GCTGGTGTAATGAATGCTACTGGTGATATAGATGAATTAGTTAAAACTTTAACAAGTATTACTGGTGATAATAAATTATTTAGCATTATCA
TGATGTATGTGATAGGTTTAATTGTCACTTTAGGTATTGGATCATCATTTGCAACAATTCCTATTATTGCATCATTATTCATTCCTTTTGGAG
CGTCAATTGGACTAGATACAATGGCATTAATCGCATTGATTGGAACAGCGAGTGCATTAGGTGACTCAGGTTCACCTGCAAGTGATTCAA
CATTAGGACCAACTGCGGGATTAAATGTTGATGGCCAACATGATCATATACGCGATACATGTGTACCAAACTTCTTGTTTTATAATATTCC
TTTAATGATTTTCGGTACTATTGCTGCTATGGTACTATAA

Gene: yuxO (putative esterase)

Contig: 06_NODE_54, position: 44218 to 44592, length: 375 nt, orientation: FORWARD

Perfect match to: (MW2-BA000033-[910445:910819], highly conserved allele)

Sequence:

ATGACTCATTTATTAGAGACATTTGAGATGTCAATAGATCACCAGGAAGATGGTTTAGTTGTTATTTCTATGCCTGTTACTGATAAAGTAA
AACAACCATTTGGATATTTACATGGTGGTGCTTCGATTGCTTTAGGTGAAACAGCATGTTCATTAGGATCTGCTAATCTAATTGATACAAC
CAAATTTATTCCATTAGGTTTAGAGATGAATGCCAATCATATTCATTCTGCTAAAGATGGTCATGTTACTGCGACAGCTGAAATTATTCATC
AAGGTAAGTCGACACATGTATGGGATATAAAAATTAAGAATGACAAAGAACAATTAATTACAGTTATGCGTGGTACAGTTGCTATTAAG
CCTTTAAAATAA

Gene: Q5HHE0 (putative pyridine nucleotide-disulfide oxidoreductase family protein)

Contig: 06_NODE_54, position: 44648 to 45802, length: 1155 nt, orientation: REVERSE

Perfect match to: (MW2-BA000033-[910875:912029:r], allele observed in CC1+CC9+CC15+CC22)

Sequence:

TTATGCGCTATATTGTTGTAATTTTAGAAATGCTTGTTCAATGCGTTCGGCAGCTTTACGGCCACCCATAACATTTCTACCAAATGGTCCTA
ATTCTAAGTCTGCAAAGCATCCTGCGACAAATAGATTTGGTATCCATTCTAATTTTTCGGAAATAACAGGGTAATTACATTCGTTGATAGG
TGCATCATAATTTTGTATTAATTGCTTAATAAGTGGTTGTGACATAAAATCTTGTTCAAAACCAGTTGCAACCATAATCTGTTGATATGGAA
CAGAATTATTTTCAGTGTTAATTAAACCATCACTAATTTGAGTGATAGGTGTTTTATGCACATTTATACGACCATTTTTAATATGTTTTTAA
GGCGTAAGTACAGTTCGTGAGGCATTGATCCTTTATGACGTTCACGTTGTACAATGGCATTTCTTTCAGGCATGCTTTTAGTACTTAAAAA
TGTAGACATATTCTTCGGACCTAACCAACCAGGATCAGCATCAAAGTCATGTATTTCAATATCTTTATTTAGCCATAAATGAATCTTTTTAT
CGTTATCATGATTTAACAATTTAAGTGCAAGATGTGCAGCCGTAATGCCACTACCAACGATATGATCGGTCTTATCATATACTACTTGATC
ATGTTCTTTCTCGAAGATATGATTTACATTCTGTTTGTCTTTTAAAATGTCAGGCATAAACGGAATATTTGTACTACCTATTGCAATAACGA
CGCAATCTGTAGTGATAATTTGTCCATCTTCTAACTTGATATGCCATTTGTCTTCTTGTTTATCTAAAGTTTGAACTAAACCTTGAACCAAGC
AATCCTCTAATTGATATTGTTTAGAAGCATGTGCAATATGATCCATAAACATTGTCAATTCAGGTCGTTGATAAGGACCATAAAAAGCATT
TGTATATTGGTGCTGTTTAGCGAATTGTTTTAGATGGAACGGTTGTGGATGTACGTGATGTACAATCGGTGATCTTAAATAAGGCATTTCT
ATTCGATTTGTAAATGAGTTAAACCTTTGGCAAAAAGTTTCGTGTGGGTCAATGATTGTTAATCGGTCTGTTGTTAATCCGCTTGATAATA
GTTTTTGTGCGATTGCAGTTCCCTGTATGCCACCGCCGATAATTGTCCAATGCAT

Gene: mnhG (Na+/H+ antiporter, subunit G)

Contig: 06_NODE_54, position: 46051 to 46407, length: 357 nt, orientation: REVERSE

Perfect match to: (MW2-BA000033-[912278:912634:r], allele observed in CC1+CC9+CC15+CC72+CC188)

Sequence:

CTACAATTTTGTGTCTTTTAAGTCTTCTGAAATTTCATCGACTTTAGTCTTTTTAGTATAAGGCGTTTTAATATTATATGCTGCTTTCATAATC
ATATGACTTGAAAGAGGACCTGTAATTAATACAAAGATAATCGCAACGATTAATTGCATATTTACAAAACCTTGAGTAGCAATAAAATAT
AAAAACGTACCAAATAGTAATGACATTGCACCTAATGTTGATGCTTTTCCGGCAGCATGTGCACGTGAATATACATCTTCAAGTCTCAATA
ATCCTATAGCTGCCAGGGCGCTAATTAAAGCACCGATGATAACAAAGATAAGTGCAAGACTAATCAGTATGATTTTGATCAT

Gene: mnhF (Na+/H+ antiporter, subunit F)

Contig: 06_NODE_54, position: 46385 to 46678, length: 294 nt, orientation: REVERSE

Perfect match to: (MW2-BA000033-[912612:912905:r], highly conserved allele)

Sequence:

CTAATCAGTATGATTTTGATCATGTTCAATCACCTTACCTTTGTCCATAAATTTAGAGAATACTGCAGTACCTAAAAAAGCTAATATACCAA
TCATCATAATAACGACAATCATGTATTTAATATTTAATAAAATACTGAATAATGCTATAACTGCCATTAATTGAAGACCAATCGCATCTAAT
GCGACAACACGATCGGCAAGTGATGGGCCTAGCACAACGCGAATGAGCATAGCTAACATAGAAATGACAACTATGATTAATGCAATAAC
GATAATAACATTATGATTCAT

Gene: mnhE (Na+/H+ antiporter, subunit E)

Contig: 06_NODE_54, position: 46678 to 47157, length: 480 nt, orientation: REVERSE

Perfect match to: (MW2-BA000033-[912905:913384:r], highly conserved allele)

Sequence:

TTATATTTCGCCCACCTCTCTTACAATTTTCTCTAATGATGTTTTAATACTTTCTACTTCTTGCTCTTTAGTTGAAAAATCTATGGCATGAATA
TAAATTTTTGTACGATCGTCACTTACACCAAGCACTACAGTACCAGGTGTTAATGTAATTAAATTAGACAGCAAGACAATTTGCCAATCTT
TTTTTAAATCTGTGTGATAAACAAAGAATCCTGGTTCATTTTTAATCGAAGGTTTAATAATAATTTTCAAAACATCAAAATTAGCTTTAATC
AGTTCGATTAAGAAAATAATAACTAATTTAATAATACGATATAGCGTGATGACATAAAATCTACCTGGTAACACTCTGTGTAAGAGGTAA
ACAAGAACTAGGCCAAAGATGAAACCTAACACAAAGTTATTTGTTGTGTAACTATTTGTCACAAACAACCAAAACACTGCGATAATAAAG
TTTAATACTAATTGTACAGCCAT

Gene: mnhD (Na+/H+ antiporter, subunit D)

Contig: 06_NODE_54, position: 47159 to 48655, length: 1497 nt, orientation: REVERSE

Perfect match to: (MW2-BA000033-[913386:914882:r], allele observed in CC1+CC5+CC8+CC12+CC188)

Sequence:

TTATTTACCTCCTAATACAGCTTTAACGTAGGTTGATGGATTGTAGAATGTTTCTGCACCAGCTTTTACCATTGGATATAAGTAATCTGCTG
ACAATCCATATAAAACAGTTATCACAACTGCAACGATTGCAATCGTAGTTAAATATTTGACGTCGACTTTGTTATTAAGATCATATCCTTTT
GGTTGACCGAAAAAGCCTTGTAGGAATATGCGAATGACAGAATATAATACGACTAAACTTGATAATAAGACGATGACACCACTTAAATA
AAATCCTCTTTCAAATGTTGATTGGACAATAAAAAATTTTCCATAAAAGCCACTGAGTGGGGGAATGCCAGCTAAACTTAATGCTGCGAT
AAAGAATGACCAACCAAGTACAGGATATCGTTTAATTAAGCCACCAAATTGTCTTAAATCAGCAGTGCCTGTAATTTTAATCATAATTCCG
ATAAGCAAGAATAATGCAAGTTTTACTAACATGTCGTGCAATGTATAGTAAATAGCCCCAATCATACCTGACTCTGTCATCATTGCAACGC
CGACTAAGATCACACCTACAGCAATCATGACATTGTATAGGATGATTTTTTTAATGTTGGCATATGCAACAGCACCGACACAACCAAAGA
TGATCGTTAATAGTGCTAAGAATAAAATGACATAATGTGAAAAGCTTACATTATCACTAAAGAATAGGCTCAATGTTCTAGCGATTGCAT
AAACACCAACTTTTGTTAACAAAGCACCAAAGAATGCAATGATTGGAATTGGTGGTGCATAGTATGCACTAGGTAACCAAACAAACATTG
GGAATACGCCAGCTTTTGTAGCAAAAACAAAGATAAATAGTATGAAAACGATATTGACTAAGCCACTGTCATGCGCTGAAAGGTTAGCT
AATTTATTGCTTATATCTGCTAGATTCAATGTTCCTACTACTGAATATAAAATCGCTACACCCATTACGAAGAAGGATGACGATACAACGT
TAACAAGAACATATTTTATTGTTTCTTGTAGTTGAATTTTTGTAGAACCAATTACTAATAAGAAATAAGATGACATTAAAAATACTTCGAAA
AATACGAATAGGTTGAAAATGTCACCAGTTGTGAATGCACCAATGATACCTATTAACATAAATAGTACTGAAAAATAATAATAATATCTTT
CACGTTCAATACCAATTGTTTGGTATGAATATAAAATCACAATAGCTGTAATAATAATACTAGTAATTATTAGTAGGGCACTGAATATGTC
TAATACAAAGACAATACTGTATGGTGCTTTCCATGAACCTAGCTCTACGCGTATTGGTCCATGTTTAACAACATTTGCTAAATTGATAATT
GCCGCGACCAAGGTTAATAATGTACCGCCTAGTGCGACATAACGCTTTATAATAGGACGCTTTCCAATAAAGACAAGTAATATGGCTGTA
ATTACTGGAATAACTAGCGTTAACACAAGCATATTACTTTCAATCAT

Gene: mnhC (Na+/H+ antiporter, subunit C)

Contig: 06_NODE_54, position: 48648 to 48989, length: 342 nt, orientation: REVERSE

Perfect match to: (MW2-BA000033-[914875:915216:r], highly conserved allele)

Sequence:

TCAATCATCTTCTGGAACTCCTTTCATACTCTCAACGTTATCTGTGCCTAATTCTTTATATGTTCTAAATGCTAATACTAAGAAAAAGGCTGT
TGTCGCAAAGGCGATAACGATTGCTGTTAAAATAAGTGCTTGCGGGATAGGATCAACATAGCTTTTTACGTTCGCTTCATAAATTGGAAC
AGTACCATGTTTAAGTCCGCCCATAGTTATTAAAAATAAATTTGCTGCATGTGTTAATAGTGTAGTTCCCATAACAATTCGTATCAGACTTT
TAGACAAAACGAGATAGACACTAATTGCTGTGAGAATACCACTAACAAAAATCATAATAATTTCCAC

Gene: mnhB (Na+/H+ antiporter, subunit B)

Contig: 06_NODE_54, position: 48989 to 49417, length: 429 nt, orientation: REVERSE

Perfect match to: (MW2-BA000033-[915216:915644:r], highly conserved allele)

Sequence:

CTATTCGTTCTCTCCAATCGAAATAATAATTGTCATGACAGTACCAACTACTGCACATAAAACACCGAAATCAAAGAATACTGCTGTTGTC
ATATGAACAGGTTCTAATATAAATAACGGTATATCAAATGTGACATGCGTAAAGAAATTTTTGCCTAAAAACCAACTTGCGATAGGCGTC
GCAATACAAAAAACTAATCCGATACCTATCAAGATTTTAAAATCTAATGGGAAAATTTTACGCATTGTTTCTATATCAAATGCAATCGTAA

TGATAACAAGTGAACTTGCGAATAATAATCCGCCGACGAAACCGCCACCAGGTGTATAATGTCCTGCTAAGAAAAGTGAAAAACCAAAG
ACCATTACCATGAAAAAGATAATAACTGCAGCAAATTGCAAAATTAGATCATTTTGTTGTCTATTCAT

Gene: mnhA (Na+/H+ antiporter, subunit A)

Contig: 06_NODE_54, position: 49410 to 51815, length: 2406 nt, orientation: REVERSE

Perfect match to: (MW2-BA000033-[915637:918042:r], highly conserved allele)

Sequence:

CTATTCATGATTTTTCACCTCGTTACCTTGCGTTTGACGCTTTTTACGTAATTTAATCATTGTATATACAGCTAATCCTGCGATACCAAGCAC
AGATGACTCGAATAAAGTATCCATACCACGGAAATCAACAAGTATGACGTTTACCATGTTTTTACCGTGAGCTAAATCATAAACGTGCTCT
TGATAAAACTTAGATATCGATTCAAAATGTCTATTTCCGTATGCAATTAAACCGATAATAATGACGGACAAACCAACACCACCAGCAATTA
AAGCATTAGTAAGCTGGAATGAGCGCTTTTCATTATAACGATTTAAATTTGGTAAGTGGTAGAAGCATAATAAGAACAATGCTGTTGAAA
TAGATTCAACGACAAACTGTGTCAATGCTAAGTCGGGTGCTTTAAAGAATATAAACAATACAGACACAGCATATCCAACTGCACTTAACA
TAATGATGCTAAATAATCTTGATTTAGCGAAAAGAATTAAAAAGGCAGCACTTAATAATAAAATTACGATACAAACTTCGAAAATTCTAAT
CGGACTAACGTCTTTAAAATTAATGTTGAAAGGTACTGAGAATATAGTGACAAATGTTAATAAAATTAATGCACCAAAAATGATAACTAA
ATTATTACGTGAATAATCGGTAACATAGCTATTCGTCATCTTTTCAGAGTAGTTTGGAATAACATTTGCACTTCTGTTGTACCAATAATTGA
ATGTTAGTTTACCAGGTTGTCGTTGCAACAATTTCACCCAATAACTAAATGTCACAATTAGTAAGATACCTAAAATATAAATCACTAATGTT
GATAAAAAGGCAGGCGTTAATCCATGGAACATATGGAATTCAACATCATCAATTACCGTATGATTAATCGAAGATGTAGCTGGTTCAATA
ATCGAATTAGTTAAAATGCCAGGGAATAAACCAAATACAATTACTAATGTAGCTAAAATAGCTGGTGATAAAAGCATTAATATTGATACT
TCGTGTGCTTTTTTAGGTAATTGTTCAGGTTTATATTGTCCGAAAAATATATGCATTATAAATTTAATTGAATATACAAATGTGAAGACACT
GCCCACTATACCAATGATTGGGAATAGGTAGCCTAATGTATCAACACTGAATAAATTTGCTTGGCTTGCTGTAAATGTTGTTTCTAAAAAT
GATTCTTTTGATAAGAAACCATTGAACGGTGGTACACCAGCCATACTTAATGCTGTAATAACAGTGATTGTAAATGAAATAGGCATAATT
GTTAGTAAGCCACCTAATTTCTTAACATCACGTGTACCAGTAGAATGATCCACTGCACCTGTAATCATAAATAGGGCACCTTTAAATGTTG
CATGGTTGATTAAATGGAATATTGCAGCCGTAAATGCAGCAGCATATATTTTGCTATCATCGCCTTGATAGTGATAACTAATGGCACCGAT
TCCAAGCATCGCCATAATCATACCTAATTGGGATACTGTTGAAAATGCCAGTATACCTTTCAAGTCTTGTTGTTTTGTTGCGTTTAGCGAA
GCCCAGAATAATGTAATTAAACCAACGAGTGTGACAGTCCATACCCAACCTTGCGATGCTGCGAAGATTGGTGTCATTCGAGCGATTAAA
TATAACCCTGCTTTAACCATTGTTGCTGAATGAAGATAAGCACTGACTGGTGTAGGTGCTTCCATTGCATCTGGTAGCCAAATATAAAATG
GAAACTGAGCAGATTTTGTAAAAGCACCAATCATGATTAAAATCATCGCAAAAATGAAGAATGGGCTATTTTGAATTTCAGAAGCATGTT
GAATCATGTACTGAATGCTAAATGATTGTGTTGGTATAGCGAGTAAGATGATACCACCTAATAATGATAGACCACCAAATACTGTGATTA
TGAGCGATTTTTGAGCACCATATATAGATGCTTGTCGTTCGCGCCAGAATGAAATAAGTAAAAAACTAGAAAATGACGTTAGCTCCCAGA
ATAAATATAGAATAATAACATTATCTGAAAGTACGACACCTAACATTGCACCCATAAATAGTAATAAATAACAATAAAAATTCCCTAGTTG
TTCTGACTTACTTAAGTAGCCGATTGAATATAATACTACTAAACTGCCGATTCCTGAAATAAGCAAACTAAAGAGTAAACCTAAGCCATCA
AGATATAAATCAAAGTTCATACCAAAATGAGGCATCCAATTTAAGGTTTTCATTACAGTATTACCTGACATCGTCGTTTTAATTAATGTAA
GCATATAAATAAATATGACGATAGGGACAGGTAATACGAACCATCCTAAATGTATACGTTTAAAAAATCTATACAGGATAGGAATAATG
AGTGCGAATATTAACGGTAATATCACCGCAATATGTAACAAACTCAC

Gene: kapB (kinase associated protein B)

Contig: 06_NODE_54, position: 51946 to 52329, length: 384 nt, orientation: REVERSE

Perfect match to: (MW2-BA000033-[918173:918556:r], highly conserved allele)

Sequence:

TTATTTAAAATTTTCTTTGTATTGAAGTGAATAATCTTCTTTTAAGCGTGCTAAACTAGCTAAAGACATTTCAGCATGTTTTGTTTGCTGAGC
TTTAAGTTTAGTTTCTAAATCTGTAATTGCTTGTTGAAGTGAATCTTCATAGCGCAATACATCAACATTGAAGTCGCGTAATTGTGAACGTT
TCGTATAGCGTTTTTCAAAATGGCTTAATGCTTTGCGTTCATGGAAAAATACACCTTCAGTTTCAGTAGGGTTATGTAAATCACCTTGTTTC
GGGTGTTTGATAACTTGTTCAACTTTAACAAGGACATCGTCTCCATTTTCTTCAACAATCGTGACACCATAGCTACCTGTTTTGTGTGAAAA
TCGATATAGCTTCAT

Gene: prsA1 (extracellular chaperone)

Contig: 06_NODE_54, position: 52393 to 52986, length: 594 nt, orientation: FORWARD

Perfect match to: (ED133-CP001996-[921801:922394], highly conserved allele)

Sequence:

ATGGCTAACTATCCACAGTTAAACAAAGAAGTACAACAAGGTGAAATTAAAGTGGTTATGCACACAAATAAAGGTGACATGACATTCAA
ATTATTTCCAAATATTGCACCAAAAACAGTTGAAAATTTTGTGACACATGCAAAAAATGGTTATTATGATGGAATAACATTCCACCGTGTC
ATTAATGACTTCATGATTCAAGGTGGCGATCCAACAGCTACTGGTATGGGTGGCGAAAGTATTTATGGCGGTGCTTTTGAAGATGAATTT
TCATTAAATGCATTTAACTTATATGGCGCATTATCAATGGCTAACTCAGGACCTAATACTAATGGTTCACAATTTTTCATTGTTCAAATGAA
AGAAGTACCTCAAAATATGTTAAGTCAACTTGCAGATGGTGGCTGGCCTCAACCAATCGTTGATGCATATGGCGAAAAGGGTGGTACAC
CATGGTTAGATCAAAAACATACAGTATTCGGTCAAATCATTGATGGTGAAACTACTTTAGAAGATATTGCAAATACAAAAGTGGGACCAC
AAGACAAACCACTTCATGATGTTGTAATTGAATCTATTGATGTTGAAGAATAA

Gene: yugI (putative RNA degradation protein)

Contig: 06_NODE_54, position: 53401 to 53778, length: 378 nt, orientation: FORWARD

Perfect match to: (RF122-AJ938182-[887150:887527], highly conserved allele)

Sequence:

TTGAATAACTACAAAATTGGCCAACATATCAAGGTGCGTGTAACTGGTATTCAACCATACGGTGCGTTTGTTGAGACCCCTAATCATACTG
AAGGACTGATTCATATATCAGAAATTATGGATGACTACGTTCATAATTTGAAGAAATTTCTATCAGAAGGCCAAATTGTTAAAGCTAAAAT
TTTGTCTATAGATGATGAAGGAAAGCTTAATCTATCATTAAAGGATAATGATTACTTCAAAAATTATGAGCGTAAGAAGGAAAAACAATC
AGTATTAGATGAAATCAGAGAAACAGAAAAATATGGGTTTCAAACACTTAAAGAACGCTTACCAATCTGGATAAAACAGTCAAAGCGAG
CAATTCGAAACGACTAA

Gene: namA (NADH:flavin oxidoreductase)

Contig: 06_NODE_54, position: 54140 to 55267, length: 1128 nt, orientation: FORWARD

Perfect match to: (MW2-BA000033-[920367:921494], allele observed in CC1+CC22+CC59)

Sequence:

ATGAAAAGTAAATACGAACCATTGTTTGATAAAGTAGAATTACCAAATGGAGTAGAGTTGAGAAATCGATTTGTGTTAGCCCCTTTAACA
CATATTTCTTCAAATGATGATGGTACTATTTCAGATGTAGAACTTCCTTATATTGAAAAGCGTTCACAAGATGTTGGTATTACAATTAATGC
TGCGAGTAATGTGAGTGATGTCGGAAAAGCATTTCCAGGACAGCCATCAATCGCGCATGACAGTGATATTGAAGGACTAAAACGATTAG
CTACAGCAATGAAGAAAAACGGTGCCAAAGCACTCGTACAAATACATCATGGCGGTGCACAAGCATTGCCTGAATTAACACCTGGTGGA
GACGTCGTAGCACCAAGTCCAATTTCTTTAAAAAGTTTCGGTCAGAAACAAGAACATAGTGCTAGAGAAATGACGAATGAAGAGATTGA
ACAAGCAATCAAGGATTTTGGTGAAGCAACGCGACGTGCAATTGAAGCAGGATTTGATGGTGTTGAAATACATGGCGCGAATCATTACT
TAATTCATCAATTTGTATCACCATACTATAATAGAAGAAATGATGTTTGGGCAAATCAATATAAATTCCCGGTCGCTGTGATTGAAGAGGT
GCTTAAAGCGAAAGAAGCGTATGGCAATAAAGACTTTATAGTTGGATACAGATTATCTCCAGAGGAAGCGGAGTCTCCAGGAATCACAA
TGGAAATTACAGAGGAACTCGTTAATAAAATTAGCCATATGCCAATCGACTATATTCATGTTTCGATGATGGATACGCATGCAACGACAC
GTGAAGGTAAATACGCTGGACAAGAAAGACTGCCTTTAATTCACAAATGGATAAATGGTCGTATGCCACTTATCGGTATTGGTTCAATTT
TCACAGCTGACGAAGCTTTAGATGCAGTTGAAAATGTTGGTGTTGACTTAGTAGCCATTGGTAGAGAGCTACTACTGGATTATCAATTTG
TTGAAAAAATTAAAGATGGACGGGAAGATGAAATTATTAATTACTTTGATCCAGAGAGAGAAGATAATCATCACTTAACTCCTAATTTAT
GGCATCAATTTAATGAAGGATTCTATCCATTACCACGTAAAGATAAATAA

Gene: rocD2 (ornithine aminotransferase 2)

Contig: 06_NODE_54, position: 55575 to 56765, length: 1191 nt, orientation: FORWARD

Perfect match to: (MW2-BA000033-[921802:922992], highly conserved allele)

Sequence:

ATGACTAAATCTGAAAAAATTATTGAGTTAACAAATCATTACGGAGCACATAATTATTTACCATTGCCAATTGTCATTTCAGAAGCTGAAG
GGGTATGGGTTAAAGATCCTGAAGGCAATAAATATATGGATATGTTATCTGCATATTCCGCTGTTAACCAAGGTCATAGACATCCGAAAA
TTATTCAAGCATTAAAAGATCAAGCTGATAAAGTGACTCTAGTTTCACGTGCTTTTCATAGTGATAACTTAGGTGAATGGTACGAAAAAAT
TTGTAAACTGGCAGGTAAAGATAAAGCTTTACCAATGAATACAGGTGCTGAAGCAGTAGAAACAGCTTTGAAAGCAGCACGACGCTGG
GCATACGATGTTAAAGGAATTGAGCCAAATAAAGCAGAAATCATTGCATTTAATGGTAACTTCCATGGTCGAACAATGGCGCCAGTTTCA
TTATCTTCAGAAGCAGAATACCAACGTGGTTATGGTCCGTTATTAGATGGATTTAGAAAAGTTGATTTTGGAGATGTAGATGCATTGAAA

GCTGCAATTAATGAAAATACTGCAGCAGTTTTAGTAGAACCAATTCAAGGTGAAGCGGGTATAAATATACCGCCAGAAGGATATTTGAA
AGCAATTAGAGAATTATGTGATGAACATAATGTCTTATTTATTGCTGACGAAATCCAAGCAGGATTAGGTCGTTCGGGTAAATTATTTGCT
ACGGATTGGGATAATGTAAAACCTGATGTCTATATTTTAGGTAAAGCACTAGGTGGTGGTGTCTTCCCAATTTCTGTTGTATTAGCAGATA
AAGAAGTATTAGATGTCTTTACACCTGGCTCACATGGTTCAACATTTGGTGGTAATCCACTTGCTTGTGCTGCATCAATTGCTGCATTAGA
TGTTATCGTTGATGAGGATTTACCAGGGCGCTCTTTAGAATTAGGAGATTATTTTAAAGAACAATTAAAGCAAATTGATCATCCATCAATT
AAAGAAGTCCGTGGACGTGGTTTGTTTATAGGTGTGGAACTTAATGAAAGTGCTAGACCATATTGTGAAGCTTTGAAAGAAGAAGGCTT
ATTATGTAAAGAAACGCATGATACTGTCATTCGTTTTGCACCACCATTAATTATTACTAAAGAAGAATTGGACCTTGCACTTGAAAAAATA
AGACATGTATTTCAATAA

Gene: gluD (NAD-specific glutamate dehydrogenase)

Contig: 06_NODE_54, position: 56874 to 58118, length: 1245 nt, orientation: FORWARD

Perfect match to: (MW2-BA000033-[923101:924345], allele observed in CC1+CC5)

Sequence:

ATGACTGAGAACAATAATTTAGTAACTTCTACTCAAGGAATTATTAAAGAAGCATTGCATAAATTGGGATTTGACGAAGGAATGTACGAT
TTAATTAAAGAACCTTTAAGAATGTTACAAGTGCGTATCCCTGTACGAATGGATGATGGCACAGTTAAAACATTCACAGGTTACCGTGCG
CAACATAATGATGCTGTTGGACCAACAAAAGGGGGCGTGCGTTTCCACCCAGATGTTGATGAAGAAGAAGTAAAAGCATTATCAATGTG
GATGACTTTGAAATGTGGCATTGTAAACTTACCATACGGTGGTGGTAAGGGTGGTATCGTTTGTGATCCACGTCAAATGAGCATTCATGA
AGTTGAACGTTTATCACGCGGATATGTAAGAGCAATTTCACAATTCGTAGGTCCGAACAAAGATATTCCAGCACCAGATGTATTTACAAA
CTCACAAATTATGGCTTGGATGATGGATGAATATAGTGCATTAGATAAATTTAATTCACCAGGTTTCATCACAGGTAAACCAATTGTATTG
GGTGGTTCTCATGGACGCGACAGATCAACTGCACTAGGTGTAGTTATTGCAATTGAACAAGCTGCAAAACGTCGTAATATGCAAATTGAA
GGTGCCAAGGTTGTTATTCAAGGTTTCGGTAATGCCGGAAGTTTCTTAGCTAAATTCTTATATGATTTAGGTGCAAAAATTGTAGGTATCT
CTGATGCTTACGGTGCATTACACGATCCAAATGGCTTAGATATAGATTATTTATTAGACCGTCGTGATAGTTTTGGTACGGTAACAAATTT
ATTTGAAGAAACAATCTCAAATAAAGAATTGTTTGAATTAGATTGTGACATTTTAGTACCAGCGGCTATTTCAAACCAAATTACAGAAGAC
AATGCACATGATATTAAAGCTAGTATCGTTGTTGAAGCTGCTAATGGACCTACAACACCAGAAGCAACACGTATTTTAACTGAACGTGGT
ATATTATTAGTTCCAGACGTATTAGCAAGTGCTGGTGGTGTAACGGTTTCTTACTTCGAATGGGTACAAAATAATCAAGGTTATTATTGGT
CTGAAGAAGAAGTTAATGAAAAGCTACGTGAAAAATTAGAAGCGGCATTTGATACGATTTACGAATTGTCTCAAAACCGAAAAATAGAT
ATGAGACTTGCAGCATATATCATAGGTATTAAACGTACAGCAGAAGCAGCTAGATATCGTGGTTGGGCATAA

Gene: glpQ2 (glycerophosphodiester phosphodiesterase)

Contig: 06_NODE_54, position: 58508 to 59437, length: 930 nt, orientation: REVERSE

Perfect match to: (COL-CP000046-[964375:965304:r], highly conserved allele)

Sequence:

CTACTTAATGACTTCTTTATATTTATCAGCGAAATTTGTAAAGACACCATCAACGCCATATTTATTTAATCGTAACATATCAGCTTTTTCATT
CACTGTATAAGGATGTACTATAAATCCTAAGTCTTTTAAATGATGGGTATTTTGTTCAGTTAAATCTGTATAATCAGGACCTAATCCAATCG
CATAAGAGCGTATCTCTTTTAAGCGTTGGTCGTTAAATTGTTGTAGTTCACCTTTATCAACTAATTTTACTAATGGCACATGCTTATTTTGAC
GATGAATTTTCTTTAAACTTTCGTCAGAAAATGATTGAATCATTACATGTCCATTTTTTAATTTATTGTTATTTAAAAGGTGATGCTTTTTCA
ATGAAGCTAATAATTGTTCTTCCATTCCTGGGTATACATCAGGTGACTTTGTTTCAATATAATAGTTTGCATTCGGGCCATAACGTTCTAAA
ATTTCATCTAAAGTGGGTACTTTAGCATTTTTATAACTTGCTCTTGCGTATTTTGGATATTTTTTATTAAACCAACTTCCTGCATCTAACTGTT
TTAATTCATCAAGGGTATAATCCTCAACTTTACCGTGTCCATTTGTTGTACGGTTAACAGTTTCATCATGCATAGCAACTAAATGGCCATCT
TTGGTACGTTGTAAATCAATTTCGATATAAGATGCTTTTAACTCATTATGACTCTTATCATATGCTTGAAACGTATGCTCGGGTGCATAGCC
ACTTGCGCCACGATGTGCGATAGTAGTGAATCGCTCATTCGTTAAATTTGTATGCCATTGAATAGCCTGAGGTTTATTTGCAATTTGATTT
GTTTGTTCAGCGCCAGCAGTAGGTACTGATAAAAATCCCATAGTAAAAACAGCAGAAGCAGCCATAAATTTAGTGAAGCTTTTCGAAGA
GTTAGTCAT

Gene: argH (argininosuccinate lyase)

Contig: 06_NODE_54, position: 59679 to 61058, length: 1380 nt, orientation: REVERSE

Sequence:

TTATTGTGATAGTAATTGTTTAGCAACATCAAGTTGTTGTTTGACCGATGATTGACCTGTTGAACCGTAACTTTGACGTCGTTTTAAACAAT
TTTCAGGCTGCAAATAATCGTAAATATCGGCATCAATACTAGAATGATGTTGTTGATATGTTGCTAAAGGAACATCTAATAAATAATGACC
TTGTTGTATACATTCTAAGACGATTTTTCCTACAATTTCATGTGCAGTTCTAAATGGAATATTTTTAGTTACTAAATAATCTGCTAGTTCCGT
TGCATTTGAAAAATCTTCTTTAACAGTTTGATTGAGTCGTTCTTTATTAATTGTCATCGTTTGAATCATACCTTCGAAAATACGTAAAGAAC
CTTTAATTGTATGGACAGCATCGAATAAACCTTCTTTATCTTCCTGCATATCTTTGTTATATGCTAGAGGTAATCCTTTTAAAGTCATAAGC
ATGCTCATTAAATGACCAGTCGTTCGACCAACTTTACCTCTAATTAATTCTGCCATATCAGGATTTTTCTTTTGTGGCATAATAGATGAGCC
AGTTGAAAATGCATCTGATAATGTAATGAATTTAGCTTCGTCTGTGGACCAGAAAATAAATTCCTCTGCAAAGCGTGATAAGTGAACCAT
CGTTAAAGAAATATTATGCAATGTTTCAATAATATAGTCTCTGTCACTAACAGCATCTAGGCTATTCTCATAGAGACTGCCAAAGTTCAAC
AATGCTGTTGTCTCGTGTCTATCGATAGGGTATGTGGTACCACTTAAGGCTGCTGCACCTAAAGGATTAATATCGATTCGTTTTAAACTAT
CTTCAAATCGTTGTTGGTCTCGTTGTAACATCCAAAAATAAGTCATAATATGATGTGCAAATGAAATTGGCTGTGCACGCTGTAAATGAGT
ATAACCAGGCATAATTGTATCAACATTATTGGAAGCGATGTCTACAATTACACTTTGTAACGACTTAATTAATGCGATGATATCTTGCACT
TGTTTCTTAGTGTACAAGTGCATGTCTGTTGCAACTTGATCGTTTCTACTGCGTCCAGTATGCAACTTACCACCAGCATCACCAATACGTTT
AATTAATTCATGTTCAATATTTAAATGAATATCTTCTAATGATGCACTAAATTGAATTTGATCTTGATGATAATCATGTTGAATAGATTTTA
GTCCTTGTATAATTTGTTCGCTGTCTTGTTGACTAATAATGCCTTGATTCGCAAGCATAGTTGCATGTGCAATGCTGCCTTCGATATCTTGA
TTTATGAGCGTTTGATCAAAAGTAATGGATGCGTTAAAGTCGTCAACCCACTCTTCAGGTTGTACTTCAAATCTACCGCCCCAAGCTTTATT
GCTCAT

Gene: argG (argininosuccinate synthase)

Contig: 06_NODE_54, position: 61048 to 62253, length: 1206 nt, orientation: REVERSE

Perfect match to: (N315-BA000018-[927310:928515:r], allele observed in CC5+CC1+CC361)

Sequence:

TTATTGCTCATTGCTATAGCCTCCATGTAGCATCGAATTTACTTGAGTAGGTAAACCATAGATATCGATAAAGCCAACAGCAGCGTCTTGA
TTAAATGCATCTTCTTTTGTATAAGTTGCTAATTTTTCATCATATAATGTGTAAGGTGATTTTCTACCATTTACGATGGCATTACCTTTGAAT
AATTTAATTCTGACATCACCACTTACGTATTGCTGAGTACTATCAATAAATAATTTTAAGCTATCAGTTAAAGGTGAGAACCAAAGTCCATT
GTATAGTTGTTCAGCAAATTGCTTCTCGATGATTGGTTTAAAGTGTGCGACATCTTTCGTTAACGTAATCGTTTCTAATGCTTTATGCGCTT
TTAAAATAACTTCTGCAGCAGGTGCCTCATAAATTTCTCTTGATTTGATACCTACAAGTCTATTTTCTACATGGTCAATTCTTCCGATACCAT
GCTTACCAGCTAATGCATTCAACGTTAAAATTAAATCGTCTAATTCATATGTTTTGCCATCAATTTGAACTGGGATGCCTTTATCAAACGTT
AAAATGATTTCATCAGCAGCATCTGGTGTTTCTTCTAAAGCATTTGTTAGATCGAACGCATCCTCTGGTGGCGCAGCATAAGGATCTTCTA
AAATACCACATTCATTCGCTCTGCCCCATAGATTTTGATCGATAGAATAAGGTGAATCATGGTTGATTGATACAGGGATATTATGTTTAAT
TGCATAATCGATTTCTTCTTCACGACTCCATGCCCACTCACGTACAGGTGCGAATGCTTTCAATGATGGGTTTAATGCTTTAATGGCAACTT
CGAAACGTACTTGGTCATTCCCTTTACCAGTACAACCATGTGCAATACCTACTGAATTTGTTTTCTCAGCAATCTCTACTAATTTTTTAGCGA
TTAATGGTCTTGATAAAGCTGAAACTAATGGATATGCATTTTCATACATTAAATTTCCTTTGATTGCATAACTTACATACTCATCACTAAATT
CTTTTGTTGCATCAATAATATGACATTCAACTGCTCCCATATCTAAAGCTTTTTTATAAACGATGTCTAAATCTTTACCTTCACCAACATCTA
GGCAACAAGCTACAACGTCGTATCCTTTGTCGATAAGCCATTGAACGGCCACACTTGTATCTAGTCCTCCTGAATATGCTAAAACAATTTT
CTCTTTCAT

Gene: pgi (glucose-6-phosphate isomerase)

Contig: 06_NODE_54, position: 62604 to 63935, length: 1332 nt, orientation: FORWARD

Perfect match to: (MW2-BA000033-[928831:930162], allele observed in CC1+CC8+CC30-ST582+CC1290)

Sequence:

ATGACTCATATTCAATTAGATTTTAGTAAAACGTTAGAATTTTTCGGTGAACACGAATTAAAACAACAACAAGAAATTGTTAAATCAATTC
ACAAAACAATTCATGAAGGTACTGGTGCAGGTAGTGACTTCTTAGGCTGGGTTGATTTACCAGTTGATTACGACAAAGAAGAATTTTCAA
GAATTGTTGAAGCATCAAAACGCATTAAAGAAAATTCTGATGTTTTAGTAGTCATCGGTATTGGTGGTTCTTACTTAGGTGCACGTGCAG
CAATCGAAATGTTAACGTCATCATTTAGAAACAGCAATGAATACCCTGAAATTGTATTTGTTGGTAATCACTTATCATCAACATATACGAA
AGAGTTAGTTGATTATTTAGCAGACAAAGATTTCTCTGTAAACGTTATTTCTAAATCTGGTACAACTACAGAACCAGCAGTTGCATTTAGA
TTGTTCAAACAATTAGTTGAAGAAAGATACGGTAAAGAAGAAGCACAAAAACGTATATTTGCAACAACGGATAAAGAAAAAGGTGCTTT
AAAACAGTTGGCTACAAACGAAGGTTATGAAACGTTTATCGTACCTGATGATGTAGGTGGAAGATATTCTGTTTTAACAGCAGTAGGATT
ATTACCAATTGCAACTGCTGGAATTAACATCGAAGCTATGATGATTGGTGCTGCAAAAGCACGTGAAGAATTATCTTCAGATAAATTAGA
AGAAAACATTGCATACCAATATGCGACAATTCGAAACATTTTATATGCAAAAGGTTATACAACAGAAATGTTGATTAACTATGAACCATCT
ATGCAATACTTTAATGAATGGTGGAAACAATTATTTGGTGAATCAGAAGGTAAAGACTTCAAAGGTATCTATCCTTCAAGTGCCAACTAC
ACAACTGATTTACATTCTTTAGGTCAATATGTACAAGAAGGCCGTCGTTTCTTATTCGAAACAGTGGTAAAAGTAAATCATCCTAAATATG
ATATTACTATTGAAAAAGATAGTGATGATCTAGACGGATTAAATTATTTGGCTGGTAAAACAATCGACGAAGTTAACACAAAAGCATTCG

AAGGTACATTATTAGCGCATACTGATGGTGGTGTTCCTAACATGGTAGTGAACATTCCACAATTAGATGAAGAAACTTTCGGTTATGTCG
TATACTTCTTCGAACTTGCTTGTGCAATGAGTGGATACCAATTAGGTGTAAATCCATTTAACCAACCTGGTGTAGAAGCATATAAACAAAA
CATGTTCGCATTATTAGGTAAACCTGGTTTTGAAGACTTGAAAAAAGAATTAGAAGAGCGTTTATAA

Gene: yhjE (putative membrane protein)

Contig: 06_NODE_54, position: 64260 to 64835, length: 576 nt, orientation: FORWARD

Perfect match to: (N315-BA000018-[930522:931097], highly conserved allele)

Sequence:

TTGTCGTTTCATCAAGTAGAAGAATGGTTTGAGATATTTCGACAGTTTGGTTATTTACCTGGATTTATATTGTTATATATTAGAGCGATAAT
TCCAGTATTTCCTTTAGCACTCTATATTTTAATTAACATTCAAGCTTATGGACCTATTTTAGGTATATTGATTAGTTGGCTTGGATTAATTTC
TGGAACATTTACAGTCTATTTGATCTGTAAACGATTGGTGAACACTGAGAGGATGCAGCGAATTAAACAACGTACTGCTGTTCAACGCTT
GATTAGTTTTATTGATCGCCAAGGATTAATCCCATTGTTTATTTTACTTTGTTTTCCTTTTACGCCAAATACATTAATAAATTTTGTAGCGAG
TCTATCTCATATTAGACCTAAATATTATTTCATTGTTTTGGCATCATCAAAGTTAGTTTCAACAATTATTTTAGGTTATTTAGGTAAGGAAAT
TACTACAATTTTAACGCATCCTTTAAGAGGGATATTAATGTTAGTTGTGTTGGTTGTATTTTGGATTGTTGGAAAAAAGTTAGAACAGCAT
TTTATGGGATCGAAAAAGGAGTGA

Gene: spsA (signal peptidase I subunit A)

Contig: 06_NODE_54, position: 64840 to 65364, length: 525 nt, orientation: FORWARD

Perfect match to: (MW2-BA000033-[931067:931591], highly conserved allele)

Sequence:

GTGAAAAAAGTTGTAAAATATTTGATTTCATTGATACTTGCTATTATCATTGTACTGTTCGTACAAACTTTTGTAATAGTTGGTCATGTCAT
TCCGAATAATGATATGTCACCAACCCTTAACAAAGGGGATCGTGTTATTGTAAATAAAATTAAAGTTACATTTAATCAATTGAATAATGGT
GATATCATTACATATAGGCGTGGTAACGAGATATATACTAGTCGAATTATTGCCAAACCTGGTCAATCAATGGCGTTTCGTCAGGGACAA
TTATACCGTGATGACCGACCGGTTGACGCATCTTATGCCAAGAACAGAAAAATTAAAGATTTTAGTTTGCGCAATTTTAAAGAATTAGAT
GGAGATATTATACCGCCTAACAATTTTGTTGTGCTAAATGATCATGATAACAATCAGCATGATTCTAGACAATTTGGTTTAATTGATAAAA
AGGATATTATTGGTAATATAAGTTTGAGATATTATCCTTTTTCAAAATGGACGATTCAGTTCAAATCTTAA

Gene: spsB (signal peptidase I subunit B)

Contig: 06_NODE_54, position: 65380 to 65955, length: 576 nt, orientation: FORWARD

Perfect match to: (Strain_21343-AHKV01000006-[11030:11605:r], highly conserved allele)

Sequence:

TTGAAAAAAGAATTATTGGAATGGATTATTTCAATTGCAGTCGCTTTTGTCATTTTATTTATAGTAGGTAAATTTATTGTTACACCATATAC
AATTAAAGGTGAATCAATGGATCCAACTTTGAAAGATGGCGAGCGAGTAGCTGTAAACATTATTGGATATAAAACAGGTGGTTTGGAAA
AAGGTAATGTAGTTGTCTTCCATGCAAACAAAAATGATGACTATGTTAAACGTGTCATCGGTGTTCCTGGTGATAAAGTAGAATATAAAA
ATGATACATTATATGTCAATGGTAAAAAACAAGATGAACCATATTTAAACTATAATTTAAAACATAAACAAGGTGATTACATTACTGGGA
CTTTCCAAGTTAAAGATTTACCGAATGCGAATCCTAAATCAAATGTCATTCCAAAAGGTAAATATTTAGTTCTTGGAGATAATCGTGAAGT
AAGTAAAGATAGCCGTGCGTTTGGCCTCATTGATGAAGACCAAATTGTTGGTAAAGTTTCATTTAGATTCTGGCCATTTAGTGAATTTAAA
CATAATTTCAATCCTGAAAATACTAAAAATTAA

Gene: rexB (ATP-dependent nuclease subunit B)

Contig: 06_NODE_54, position: 66115 to 69591, length: 3477 nt, orientation: FORWARD

Perfect match to: (65-1322-ACJS01000027-[95107:98583], allele observed in CC30)

Sequence:

ATGACATTACATGCTTATTTAGGTAGAGCGGGAACAGGTAAGTCTACGAAAATGTTGACCGAAATAAAACAAAAAATGAAAGCAGATCC
GCTTGGAGATCCAATCATTTTAATTGCGCCAACTCAAAGTACATTTCAATTAGAACAAGCCTTTGTCAATGATCCGGAATTAAATGGTAGT
TTAAGAACAGAAGTGTTGCATTTTGAACGATTAAGTCATCGTATTTTCCAAGAAGTTGGTAGTTATAGCGAACAAAAGTTATCTAAAGCT
GCAACGGAAATGATGATTTATAACATTGTTCAAGAACAACAAAAGTATTTAAAACTTTATCAATCACAAGCAAAATATTATGGGTTTAGT
GAAAAATTAACAGAACAAATTCAAGATTTTAAAAAATATGCAGTAACGCCTGAACATTTAGAACACTTTATTGCTGATAAAAATATGCAA
ACTCGAACTAAAAATAAGTTAGAGGATATTGCTTTAATATACCGTGAGTTCGAACAACGCATTCAAAACGAGTTTATTACTGGTGAGGAT
TCATTACAATATTTTATTGATTGTATGCCGAAATCAGAGTGGCTAAAACGTGCTGATATATATATTGATGGTTTTCACAACTTTTCAACGAT
TGAGTATTTAATAATCAAAGGATTAATTAAATATGCGAAGAGTGTCACAATTATATTGACGACAGATGGTAACCACGATCAATTTAGTTTA
TTTAGAAAACCATCGGAAGTGTTACGACATATTGAAGAAATAGCAAATGAACTCAATATTTCTATTGAACGTCAATATTTCAACCAATTAT
ATCGCTTCAATAATCAAGATTTAAAGCATCTTGAACAAGAATTTGATGTACTTCAAATCAATCGAGTGGCATGTCAAGGTCATATCAATAT
TTTAGAATCTGCGACTATGAGAGAGGAAATAAATGAAATTGCGCGACGTATCATCGTTGATATTCGTGATAAGCAATTACGATATCAAGA
TATTGCTATTTTATATCGTGATGAATCTTATGCTTATTTATTTGATTCCATATTACCGCTTTATAATATTCCTTATAATATTGATACAAAGCGT
TCGATGACACATCATCCGGTCATGGAAATGATTCGTTCATTGATTGAAGTTATTCAATCTAATTGGCAAGTGAATCCAATGCTACGCTTAT
TGAAGACTGATGTGTTAACGGCATCATATCTAAAAAGTGCATACTTAGTTGATTTACTTGAAAATTTTGTACTTGAACGTGGTATATACGG
TAAACGTTGGTTAGATGATGAGCTATTTAATGTCGAACATTTTAGCAAAATGGGGCGTAAAGCGCATAAACTGACCGAAGATGAACGTA
ACACATTTGAACAAGTCGTTAAGTTAAAGAAAGATGTCATTGATAAAATTTTACATTTTGAAAAGCAAATGTCACAAGCGGAAACTGTAA
AAGATTTTGCAACTGCTTTTTATGAAAGTATGGAATATTTCGAACTGCCAAATCAATTGATGACAGAGCGAGATGAACTTGATTTAAATG
GTAATCATGAAAAGGCGGAGGAAATTGATCAAATATGGAATGGCTTAATTCAAATCCTTGATGACTTAGTTCTAGTATTTGGAGATGAAC
CAATGTCGATGGAACGTTTCTTAGAAGTATTTGATATTGGTTTAGAACAATTAGAATTTGTTATGATTCCGCAAACATTGGACCAAGTAAG
TATTGGTACGATGGATTTGGCTAAAGTCGATAATAAGCAACATGTTTTCTTAGTAGGTATGAATGATGGTACGATGCCACAACCAGTAAC
TGCGTCAAGCTTGATTACAGATGAAGAAAAGAAATACTTTGAACAGCAGGCTAATGTCGAGTTAAGTCCAACATCAGATATTTTACAGAT
GGATGAAGCATTTGTTTGCTATATTGCTATGACAAGAGCGCGACAAGATGTTACATTTTCTTACAGTCTAATGGGATCAAGTGGTGATGA
TAAGGAGATCAGCCCATTTTTAAATCAAATTCAATCATTGTTCAACCAATTGGAAATTACTAACATTCCTCAATACCATGAAGTTAACCCAT
TGTCACTAATGCAACATGCTAAGCAAACCAAAATTACATTATTTGAAGCATTGCGTGCTTGGTTATATGATGAAATTGTGGCTGATAGTTG
GTTAGATGCTTATCAAGTAATTAGAGATAGCGATCATTTAAATCAAGGTTTAGATTATTTAATGTCAGCATTAACGTTTGACAATGAAACT
GTAAAATTAGGTGAAACGTTGTCTAAAGATTTATATGGTAAGGAAATCAATGCCAGTGTATCCCGTTTTGAAGGTTATCAACAATGCCCA
TTTAAACACTATGCGTCACATGGTCTGAAACTAAATGAGCGAACGAAGTATGAACTTCAAAACTTTGATTTAGGTGATATTTTCCATTCTG
TTTTAAAATATATATCTGAACGTATTAATGGCGATTTTAAACAATTAGACCTGAAAAAAATAAGACAATTAACGAATGAAGCATTGGAAG
AAATTTTACCTAAAGTTCAGTTTAATTTATTAAATTCTTCAGCTTACTATCGTTATTTATCAAGACGCATTGGCGCTATTGTAGAAACAACA
CTAAGCGCATTAAAATATCAAGGCACGTATTCAAAGTTTATGCCAAAACATTTTGAGACAAGTTTTAGAAGGAAACCAAGAACAAATGAC
GAATTAATTGCACAAACATTAACGACAACTCAAGGTATTCCAATTAATATTAGAGGGCAAATTGACCGTATCGATACGTATACAAAGAAT
GATACAAGTTTTGTTAATATCATTGACTATAAATCCTCTGAAGGTAGTGCGACACTTGATTTAACGAAAGTATATTATGGTATGCAAATGC
AAATGATGACATACATGGATATCGTTTTACAAAATAAACAACGCCTTGGATTAACAGATATTGTGAAACCAGGTGGATTATTATACTTCCA
TGTACATGAACCTAGAATTAAATTTAAATCATGGTCTGATATTGATGAAGATAAACTAGAACAAGATTTAATTAAAAAGTTTAAGTTGAGT
GGTTTAGTTAATGCAGACCAAACTGTTATTGATGCATTGGATATTCGTTTAGAACCTAAATTCACTTCAGATATTGTACCAGTTGGTTTGA
ATAAAGATGGCTCTTTGAGTAAACGAGGCAGCCAAGTGGCAGATGAAGCAACGATTTATAAATTCATCCAACATAACAAAGAGAATTTT
ATAGAAACAGCTTCAAATATTATGGATGGACATACTGAAGTTGCACCATTAAAGTACAAACAAAAATTGCCATGTGCTTTTTGTAGTTATC
AATCGGTATGTCATGTAGATGGCATGATTGATAGTAAGCGATATCGAACTGTAGATGAAACAATAAATCCAATTGAAGCAATTCAAAATA
TTAACATTAATGATGAATTTGGGGGTGAGCAATAG

Gene: rexA (ATP-dependent nuclease subunit A)

Contig: 06_NODE_54, position: 69592 to 73245, length: 3654 nt, orientation: FORWARD

Perfect match to: (MW2-BA000033-[935819:939472], allele observed in CC1+CC25+CC239)

Sequence:

ATGACAATTCCAGAGAAACCACAAGGCGTGATTTGGACTGACGCGCAATGGCAAAGTATTTACGCAACTGGACAAGATGTACTTGTTGC
AGCCGCGGCAGGTTCAGGTAAAACAGCTGTACTAGTTGAGCGTATTATCCAAAAGATTTTACGTGATGGCATTGATGTCGATCGACTTTT
AGTCGTAACGTTTACAAACTTAAGCGCACGTGAAATGAAGCATCGTGTAGACCAACGTATTCAAGAGGCATCGATTGCTGATCCTGCAAA
TGCACACTTGAAAAACCAACGCATCAAAATTCATCAAGCACAAATATCTACACTCCATAGTTTTTGCTTGAAATTAATTCAACAGCATTATG
ATGTATTAAATATTGACCCGAACTTTAGAACAAGCAGTGAAGCTGAAAATATTTTATTATTAGAACAAACGATAGATGAGGTCATAGAAC
AACATTACGATATCCTTGATCCTGCTTTTATTGAATTAACAGAGCAATTGTCTTCAGATAGAAGTGATGATCAGTTTCGAATGATTATTAA
ACAATTGTATTTCTTTAGCGTTGCAAATCCAAATCCTACAAATTGGTTGGATCAATTGGTGACACCATACGAAGAAGAAGCACAACAAGC
GCAACTTATTCAACTACTAACAGACTTATCTAAAGTATTTATCACAGCTGCTTATGATGCTTTAAATAAGGCGTATGATTTGTTTAGTATGA
TGGATAGCGTCGATAAACATTTAGCTGTTATAGAAGATGAACGACGTTTAATGGGGCGTGTTTTAGAAGGTGGCTTTATTGATATACCTT
ATTTAACTGGTCACGAATTTGGCGCGCGTTTGCCTAATGTAACAGCGAAAATTAAAGAAGCAAATGAAATGATGGTCGATGCCTTAGAA
GATGCTAAACTTCAGTATAAAAAATATAAATCATTAATTGATAAAGTGAAGAGTGATTACTTTTCAAGAGAAGCTGATGATTTGAAAGCT
GATATGCAACAATTGGCGCCACGAGTAAAGTACCTTGCGCGTATTGTGAAAGATGTTATGTCAGAATTCAATCGAAAAAAGCGTAGCAA

AAATATTTTGGATTTTTCTGATTATGAACATTTTGCATTACAAATTTTAACAAATGAGGATGGTTCGCCTTCAGAAATTGCCGAATCATACC
GTCAACACTTCCAAGAAATATTGGTCGATGAGTATCAAGATACGAACCGAGTTCAAGAGAAAATACTATCTTGCATCAAAACGGGTGAT
GAACATAATGGTAATTTATTTATGGTTGGAGATGTTAAGCAATCCATTTATAAATTTAGACAAGCTGATCCAAGTTTATTTATTGAAAAGT
ATCAACGCTTTACTATAGATGGAGATGGCACTGGACGTCGAATTGATTTGTCGCAAAACTTCCGTTCTCGAAAAGAAGTACTGTCAACGA
CTAACTATATATTCAAACATATGATGGATGAACAAGTCGGTGAAGTAAAATATGATGAAGCGGCACAGTTGTATTATGGTGCACCATATG
ATGAATCGGACCATCCAGTAAACTTAAAAGTCCTTGTTGAAGCGGATCAAGAACATAGTGATTTAACTGGTAGTGAACAAGAAGCGCAT
TTTATAGTAGAACAAGTTAAAGATATCTTAGAACATCAAAAAGTTTATGATATGAAAACAGGAAGCTATAGAAGTGCGACATACAAGGA
TATCGTTATTCTAGAACGCAGCTTTGGACAAGCTCGCAATTTACAACAAGCCTTTAAAAATGAAGATATTCCATTCCATGTGAATAGTCGT
GAAGGTTACTTTGAACAAACAGAAGTCCGCTTAGTATTATCATTTTTAAGAGCGATAGATAATCCATTACAAGATATTTATTTAGTTGGGT
TAATGCGCTCCGTTATATATCAGTTCAAAGAAGACGAATTAGCTCAAATTAGAATATTGAGTCCAAATGATGACTACTTCTATCAATCGAT
TGTAAATTACATTAATGACGAAGCAGCAGATGCAATTTTAGTTGATAAATTAAAAATGTTTTTATCAGATATTCAAAGTTACCAACAATAT
AGTAAAGATCATCCGGTGTATCAGTTAATTGATAAATTTTATAATGATCATTATGTTATTCAATACTTTAGTGGACTTATTGGTGGACGTG
GACGACGTGCAAATCTTTATGGTTTATTTAATAAAGCTATCGAGTTTGAGAATTCAAGTTTTAGAGGTTTATATCAATTTATTCGTTTTATC
GATGAATTGATTGAAAGAGGCAAAGATTTTGGTGAGGAAAATGTAGTTGGTCCAAACGATAATGTCGTTAGAATGATGACAATTCATAG
TAGTAAAGGTCTAGAGTTTCCATTTGTCATTTATTCTGGATTGTCAAAAGATTTTAATAAACGTGATTTGAAACAACCAGTTATTTTAAATC
AGCAATTTGGTCTCGGAATGGATTATTTTGATGTGGATAAAGAAATGGCATTTCCATCTTTAGCTTCGGTTGCATATAGAGCTGTTGCCGA
AAAAGAACTTGTGTCAGAAGAAATGCGATTAGTCTATGTAGCATTAACAAGAGCGAAAGAACAACTTTATTTAATTGGTAGAGTGAAAA
ATGATAAATCATTACTAGAACTAGAGCAATTGTCTATTTCTGGTGAGCACATTGCTGTCAATGAACGATTAACTTCACCAAATCCGTTCCA
TCTTATTTATAGTATTTTATCTAAACATCAATCTGCGTCAATTCCAGATGATTTAAAATTTGAAAAAGATATAGCACAAGTTGAAGATAGTA
GTCGTCCGAATGTAAATATTTCAATTATATACTTTGAAGATGTGTCTACAGAAACCATTTTAGATAATAATGAATATCGTTCGGTTAATCA
ATTAGAAACTATGCAAAATGGTAATGAGGATGTTAAAGCACAAATTAAACACCAACTTGATTATCAATATCCATATGTAAATGATACTAA
AAAGCCATCAAAACAATCTGTTTCTGAATTGAAAAGGCAATATGAAACAGAAGAAAGTGGCACAAGTTACGAACGAGTAAGACAATATC
GTATCGGTTTTTCAACGTATGAACGACCTAAATTTCTAAGTGAACAAGGTAAACGAAAAGCGAATGAAATTGGTACGTTAATGCATACAG
TGATGCAACATTTACCATTCAAAAAAGAACGCATATCTGAAGTTGAGTTACATCAGTATATCGATGGATTAATCGATAAACATATTATCGA
AGCAGATGCGAAAAAAGATATCCGTATGGATGAAATAATGACATTTATCAATAGTGAGTTATATTCGATTATTGCTGAAGCAGAGCAAGT
TTATCGTGAATTACCGTTTGTAGTTAACCAAGCATTAGTTGACCAATTGCCACAAGGAGACGAAGACGTCTCAATTATTCAAGGTATGATT
GACTTAATCTTTGTTAAAGATGGTGTGCATTATTTTGTAGACTATAAAACCGATGCATTTAATCGTCGCCGTGGGATGACAGATGAAGAA
ATTGGTACACAATTAAAAAATAAATATAAGATACAGATGAAATATTATCAAAATACGCTTCAAACGATACTTAATAAAGAAGTTAAAGGT
TATTTATACTTCTTCAAATTTGGTACATTGCAACTGTAG

Gene: Q5HHB6 (putative fumarylacetoacetase)

Contig: 06_NODE_54, position: 73411 to 74313, length: 903 nt, orientation: FORWARD

Perfect match to: (MW2-BA000033-[939638:940540], highly conserved allele)

Sequence:

ATGAAATTCTTATCATTCAAGTATAATGACAAAACTTCATATGGCGTTAAAGTAAAACGCGAAGATGCTGTATGGGATTTAACACAAGTA
TTTGCTGACTTTGCAGAAGGAGATTTCCATCCTAAAACATTGTTAGCTGGTTTACAACAAAATCATACTTTAGATTTTCAAGAACAAGTAC
GTAAAGCAGTTGTAGCAGCAGAAGATAGCGGCAAAGCTGAAGACTATAAAATTTCATTTAATGACATTGAATTCTTACCACCAGTAACAC
CTCCGAATAATGTGATTGCTTTTGGTAGAAATTACAAAGATCATGCGAACGAATTAAATCATGAAGTAGAAAAATTATATGTATTTACAA
AAGCAGCGTCATCTTTAACAGGAGATAATGCAACAATTCCAAATCATAAAGATATTACTGATCAATTAGATTATGAAGGTGAATTAGGTA
TTGTTATTGGTAAGTCTGGTGAAAAGATTCCAAAAGCATTAGCTTTAGATTATGTTTACGGCTATACAATTATTAACGATATCACTGATCG
CAAAGCACAAAGTGAACAAGATCAAGCATTTTTATCAAAAAGTTTAACTGGCGGTTGCCCAATGGGTCCTTATATCGTTACTAAAGACGA
ACTACCATTACCTGAAAATGTAAATATTGTTACAAAAGTTAACAATGAAATTAGACAAGATGGTAACACTGGCGAAATGATTCTTAAAAT
TGATGAATTAATAGAAGAAATTTCAAAATATGTTGCACTACATCCGGGAGATATTATTGCAACTGGTACACCAGCAGGCGTTGGTGCAGG
TATGCAACCACCTAAATTTTTACAACCAGGTGATGAAGTTAAAGTGACTATTGATAATATTGGAACGCTGACAACTTATATCGCTAAATAA

Gene: UPF0344 (unknown protein fold UPF0344)

Contig: 06_NODE_54, position: 74642 to 75031, length: 390 nt, orientation: FORWARD

Perfect match to: (N315-BA000018-[940904:941293], highly conserved allele)

Sequence:

ATGTTACATTTACATATATTAAGTTGGGTATTAGCGATTATTTTATTTATCGCTACATACTTAAACATTTCAAAAAATCAAGGCGGATCACC
ATTTTTCAAACCGTTGCACATGATTTTACGCTTATTTATGCTGTTGACGTTAATTTCAGGATTTTGGATATTAATTCAGTCATTTATGAATGG
CGGGGCAAATCATATGTTGCTTACATTGAAAATGCTGTGTGGTGTTGCAGTAGTTGGATTGATGGAAGTGTCGATTGCTAAAAGAAAGA

GACATGAACAAAGTCACAAAATGTTTTGGATAACAATGGCATTAATTATCATCACAATGGTATTAGGTGTCATTCTACCGTTAGGGCCTAT
ATCAAAATTATTCGGTATTGGCTAA

Gene: cdr (coenzyme A disulfide reductase)

Contig: 06_NODE_54, position: 75203 to 76519, length: 1317 nt, orientation: REVERSE

Perfect match to: (N315-BA000018-[941465:942781:r], highly conserved allele)

Sequence:

TTATTTAGCTTTGTAACCAATCATATTGATTAAATCTTTAGGGTGGCTATATGGTGGTGCATAAGCCACTTCAAACTCAGTTAACTCATCTA
CAGTTAGCTGGTTCATCATTGCCATCGATAGTACATCAATACGTTTATCTGCACCTTCTTTTCCTACTGCAGCTGCTCTTAAAATCTGACGG
TTTGAAGTGTCATAATATACCCTTAAGTGTAAAGGGGAATTTCCTGGGTAATAATTCGCGTGTGCACCTTGAGTGACTTCCACCATTTTAT
AGTCAAATTGCTTTAGTTCATTTGGTTTAACGCCGACACTCGCAAATGTATAATCAAAGAACTTCACAATATTGTTGCCTAAGAAGCCTTT
GAATTCAATAGTGTCATTTCCAGCAATTTGTTCGGCAACAATACTTGCTGCACGGTGAGCGCCCCAAGCTAAAGGAACACTAGCCGGTAG
ATCGACATGTCGATAATGTGATGTTGCAATATCGCCTATTGCATAAATGTTTGGAACATTTGTTTCAAATTTATCGTTTACCGGTATGAAAC
CTTTTCGATCAAGTTTGATATTTGAACTTTCGATAAATTTTGAATTGGGGTGAGTACCGACACCTTCAATAATCATATCGTAATGTTCAACT
TTTCCTGATTTAAATGTAATTTCATTTCCATTGATAGCATCAATTTCCTCATTTAAACGGTATGGAATCTCCCGCTTATCTAATTCATCAAGT
ATAGGTTGATTCATGTCAGCATCCATTAATTTATTTATCTTATCAGATCGATGAATTAAAGTAGGGTGTAAACCACGTTCGTAAAGATTTTC
AAGAACTTCTAATGAAACATACCCTGCACCTACAACTAATACTTTATCAACTTGATTTGCTTTGATGAATTGATCGATAGCATCAGTGTCTT
CTAAATTTCTAAGTGTAAATGTAATATCACTTTCAAAGCCAAGGCTATTTGCACTTGCACCAGGGCTTAAAATGAGTTTATCGTAAGATTC
TTCAAATTGTTCGTTGGTCTTTCTATTTAATACAGTTACAGTTTGTCTTTCATCATTGATTGCAATAACTTCATGATAAGTTTTTACTGTAATT
TGCTTTCTATCATAAAATTTTTCAGGTGTATACGCTAAAGCATATTTTCTATCTTCAACAACTTCGCCAATGACATAAGGCAATGCACAATT
AGCAAAGCTCATATCACGATCTTTTTCAAAAATAATAATGTCACTTTCTTTATCTAAACGTCGAATTTGGCTGGCACATGTTGCACCGCCAG
CGACTGCTCCGACTACGACTATTTTGGGCAT

Gene: yitU (putative phosphatase)

Contig: 06_NODE_54, position: 76571 to 77395, length: 825 nt, orientation: REVERSE

Perfect match to: (RF122-AJ938182-[910810:911634:r], highly conserved allele)

Sequence:

TTAACAGTAATATCTAATATTTAAATTAAAGAAATCATTCAAATATCGACCAATGCCATCTTCATTATTGTTGAATGTAATATTGTTCGCTA
CATCTTTAAGTTCTTGCAAACCATTTTCCATAGCAACACCATGGCGGGCGTACTCAATCATTTCAATATCATTATCTTCATCACCGAATGCA
ATAATATTATTTCGGTCAATATTTAAAAATTGTCTAACTTGCTCAATGCCTCTTGCTTTATTAATACCAAGTTTTACAATTTCAATGACAGGG
AATGGTGCGCCCCAGCGTCGATGCTCAATATGATCGGCATAAAAATGAGTAAGCATATTTTTGATTTCAGGTATTTTACTTTCTTCGGCTT
CAATTAAAATTGAGGTAGGGGATTCTTTCAAGTGGACAAGTAAATTACCAGTTTGAATTCTTGGATTACCCATTGAAAAACCTTCAAATAA
TCTTGGATCATGATTGTTAATGAAAACATAATCTTTCACTTCTGCTATAATATTCGATACTTGATATTGTTGTAATCCTTGAATAATGTTTTG
TGCGATGCCTAAATCTAAAATTTCATGGCAAGTTTTGAAGTTTTTATCTTTAGGGTGATGTACGTAAGCGCCATTAAAATTAACAATTGGT
GTCGTTAAATTTAATTCATGATAATACATTTGACTTGCACGATAAGGTCTGCCAGTCGCAATCATAATTTGGTGTCCACGTTGTTGTAATTC
ATTTAATACTTGTTTAGTATATGATGAAATTTCTTTGTTATCGTTTAATAATGTTCCGTCTAAGTCTAGACATATTAAATGTGGTTGCAT

Gene: paaD-sufT (factor essential for maturation of holo-LipA)

Contig: 06_NODE_54, position: 77509 to 77817, length: 309 nt, orientation: FORWARD

Perfect match to: (MW2-BA000033-[943736:944044], allele observed in CC1+CC15+CC80)

Sequence:

ATGGAAGAGGCATTGAAAGATAGTATCTTAGGTGCATTAGAAATGGTAATTGACCCTGAATTAGGAATTGATATCGTTAATTTAGGTTTA
GTATACAAAGTGAATGTTGATGATGAAGGCGTATGTACAGTTGATATGACTTTAACATCAATGGGATGTCCAATGGGACCTCAAATTATT
GATCAAGTTAAAACAGTATTAGCAGAGATTCCTGAAATACAGGATACTGAAGTGAATATCGTATGGAGTCCACCTTGGACAAAAGATAT
GATGTCACGTTACGCTAAGATTGCACTTGGTGTGAGCTAA

Gene: Q5HHB1 (putative O-acetyltransferase)

Contig: 06_NODE_54, position: 78365 to 80179, length: 1815 nt, orientation: FORWARD

Perfect match to: (11819-97-CP003194-[976556:978370], highly conserved allele)

Sequence:

ATGAACAAAACAAAGGGTTTTACAAAGTATAAGAAAATGAGATATATGCCAGGGCTCGATGGTTTGAGGGCAATCGCTGTTCTAGGAAT
TATTATTTACCACTTAAACAAGCAATGGTTGACAGGTGGCTTTTTAGGTGTGGATACATTTTTTGTGATCTCTGGTTATTTAATTACAAGCT
TATTACTCAAAGAGTATGATGACACAGGTATCATTAAATTGAAAAGCTTTTGGATACGTCGTTTAAAACGTTTATTACCAGCAGTCATAGT
TTTATTAATGGTTGTAGGGACAGCAACCTTATTATTAAAATCAGATAATATCATTAGGGTTAAACATGATATTATTGCTGCGATATTTTATG
TATCAAACTGGTGGTATATAGCAAAAGATGTTAATTATTTTGAGCAATTTTCATTTATGCCATTAAAGCATTTATGGTCTTTAGCAATTGAA
GAACAGTTTTACATATTTTTCCCAGTTATTTTGGTTACATTATTGTTAACAATTAAAAAGCGATACAAAATAGGATTTATTTTTTGGGGAGT
ATCAATAATTTCTTTAGGGTTAATGATGTTTATCTACAGTATTAATGGGGATCATTCACGAGTGTATTTTGGTACAGATACTAGATTACAG
ACATTGTTACTGGGTGTTATTTTAGCTTTTTTATGGCCACCGTTTAAATTGAAAAATGATCCACCTAAAGTTGTAAAATATGTTATTGATAG
CATAGGTAGTTTATCATTTATAGTACTTATATTATTATTTTTCATTATTAATGATGAGACGAATTGGATATATGATGGTGGTTTCTATTTAAT
ATCCATATTAACGTTATTTATTATTGCCAGTGTCGTTCATCCATCTACATGGATAGCGAAGATATTTTCAAATCCAGTGTTAGTATTTATCG
GGAAAAGGTCTTATAGTTTATATTTATGGCATTTTGCAGTAATTAGTTTCGTACATAGTTACTATGTAGATGGACAGATACCTGTATATGT
GTACTTTATAGATATAAGTTTAACAATTATATTTGCAGAGCTATCATATCGCTTTATAGAAACTCCATTTAGAAAAGAAGGTATTAAAGCTT
TAAATTGGCGACCTTCTTATATACCACAATTTATAAGAATGGCAATTGTAGTAACCTTGTTAATTCCATTTATGTTGATTTTAGTAGGTGCA
TTCAATAAATATGGTAAAGACATTATTGGAGAAAAAGCGAATAGCTTTGATACCACTATTGAAGATAATTATTTAATGCGGATAGCACCA
ATTGATAACATTCACATTGATGGCTTAGTAAGTGAGAAGAAAAAGGAATCTTCCGACGTATATAATAATATTAAACCTCTTTTAATCGGTG
ATTCAGTAATGGTTGATATCGGTGAGTCATTTAAGTCATCAGTTCCTAAGTCTAGAATTGATGGAAAAGTAGGGCGTCAATTGTATCAAA
CCTTACCTTTAGTTAAAGCGAATTATTCACAATATAAAAAATCATCTGATCAAGTCGTATTAGAATTAGGTACAAATGGCGACTTTACTGT
CAAACAGCTCGACGATTTACTTAATCAATTTGGAAAAGCCAAGATTTATTTAGTTAATACACGTGTTCCAAGAATTTATGAGGCAAATGTA
AATCGATTATTAGCTGACGCGGCGAAACGAAAGTCCAATGTCACATTAATTGATTGGAATAAGCGATCACAAGGACATAGTGAATATTTT
GCACCAGACGGTGTACATTTAGAGTACAAAGGAGTCCTAGCTTTAAAAGATGAAATATTAAAAGCACTTAAAAAGAAATAA

Gene: clpB (chaperone-like protein B)

Contig: 06_NODE_54, position: 80382 to 82991, length: 2610 nt, orientation: FORWARD

Sequence:

ATGGATATAAATAAAATGACATATGCTGTTCAAAGTGCTTTACAACAAGCAGTTGAACTGAGTCAGCAACATAAATTACAAAATATAGAA
ATTGAGGCAATTTTAAGCGCTGCCTTAAATGAAAGTGAAAGCTTATATAAAAGTATTTTAGAACGAGCAAATATTGAGGTAGATCAATTA
AACAAAGCTTATGAAGACAAACTAAACACGTATGCATCTGTAGAAGGTGACAATATACAATATGGTCAATATATTAGCCAACAAGCAAAC
CAATTGATAACTAAGGCTGAATCATACATGAAAGAATATGAAGATGAATATATTTCAATGGAGCATATTTTACGTTCGGCAATGGACATT
GATCAAACAACAAAACATTATATAAATAATAAAGTAGAAGTTATCAAAGAAATTATTAAAAAAGTAAGAGGGGGAAATCACGTGACATC
ACAAAATCCAGAAGTTAATTACGAAGCATTAGCTAAATATGGCCGCGACTTAGTAGAAGAAGTTAGACAAGGTAAAATGGATCCTGTTA
TAGGAAGAGATGAAGAAATTCGAAATACGATTCGTATTTTAAGTCGTAAAACTAAAAACAACCCTGTGCTCATTGGTGAACCAGGTGTTG
GTAAAACTGCAATTGTTGAAGGATTAGCGCAACGTATAGTTAAGAAAGATGTGCCAGAATCATTATTAGATAAAACTGTTTTTGAGTTAG
ATTTAAGCGCATTAGTAGCGGGCGCTAAATATCGTGGTGAATTTGAAGAGAGATTAAAAGCAGTCCTAAAAGAAGTTAAAGAGTCTGAT
GGTAGAATTATATTATTTATTGATGAAATCCATATGCTTGTAGGTGCTGGTAAAACAGATGGTGCCATGGATGCAGGCAACATGCTAAAA
CCAATGTTAGCACGAGGAGAGTTACATTGTATTGGTGCAACAACTTTAAATGAATATCGAGAATATATTGAAAAAGATTCGGCATTAGAG
CGTCGTTTCCAAAAAGTAGCAGTTAGTGAGCCTGATGTTGAAGATACAATTTCAATTTTACGTGGTTTAAAAGAACGATATGAAGTGTAT
CATGGTGTGCGTATTCAAGATAGAGCCTTAGTTGCTGCCGCTGAATTGTCTGATCGTTACATCACTGATCGTTTTTTACCAGATAAAGCCA
TTGATTTAGTTGACCAAGCATGTGCAACAATTCGTACGGAAATGGGATCAAATCCAACTGAATTGGATCAAGTTAATAGACGTGTCATGC
AATTAGAAATTGAAGAAAGCGCACTTAAAAATGAATCTGACAATGCGAGCAAACAGAGATTACAAGAACTACAAGAAGAGCTTGCCAAT
GAAAAAGAGAAACAAGCAGCACTTCAATCTCGTGTAGAATCAGAAAAAGAAAAAATAGCAAATTTACAAGAAAAACGTGCGCAACTAG
ATGAAAGTAGACAAGCGTTGGAAGATGCACAAACAAATAACAATTTAGAAAAAGCTGCTGAACTACAATATGGAACAATTCCTCAATTG
GAAAAAGAACTTAGAGAATTAGAGGATAATTTCCAAGATGAGCAAGGTGAAGATACAGATCGAATGATTCGTGAAGTTGTAACAGACG
AAGAAATTGGCGATATTGTCAGCCAATGGACAGGCATACCAGTTTCAAAATTAGTTGAAACAGAACGTGAAAAATTACTTCACTTAAGTG
ACATCTTGCATAAACGTGTTGTAGGTCAAGATAAAGCGGTTGACCTGGTTTCAGATGCAGTAGTTAGAGCAAGAGCAGGTATTAAAGAT
CCAAACAGACCTATTGGTAGTTTCTTATTCCTAGGTCCAACTGGAGTAGGTAAAACTGAATTAGCTAAATCATTAGCTGCATCATTATTTG
ATTCTGAAAAACATATGATTCGTATTGATATGAGTGAATATATGGAAAAACATGCAGTATCAAGATTGATAGGGGCACCTCCAGGATATA
TTGGACATGATGAAGGGGGTCAATTAACTGAAGCGGTTCGTCGTAATCCATACTCAGTTATTTTATTAGATGAGGTTGAAAAAGCGCATA
CTGACGTCTTTAATGTATTATTGCAAATTTTAGATGAAGGCCGTTTAACTGATTCTAAAGGACGTAGCGTTGATTTTAAAAATACTATTATT
ATTATGACAAGTAATATTGGATCTCAAGTTTTATTAGAAAACGTAAAAGAGACTGGTGAAATTACAGAATCAACAGAAAAAGCTGTTATG
ACAAGTTTAAATGCATATTTCAAACCAGAAATTTTGAATCGTATGGATGATATCGTATTATTTAAACCATTATCTATTGATGACATGAGTAT

GATTGTAGATAAAATCTTAACGCAATTAAATATAAGATTATTAGAACAACGAATCTCAATTGAAGTTTCTGATGATGCTAAAGCTTGGCTA
GGTCAAGAAGCTTATGAACCTCAATACGGTGCAAGACCATTAAAACGTTTTGTACAACGCCAAATTGAAACACCATTAGCACGTATGATG
ATTAAAGAGGGATTCCCAGAAGGTACAACGATTAAAGTTAATTTAAATTCAGACAATAACTTAACGTTTAATGTTGAAAAAATTCATGAA
TAA

Gene: Q5HHA9 (transcriptional regulator, LysR family)

Contig: 06_NODE_54, position: 83050 to 83919, length: 870 nt, orientation: REVERSE

Perfect match to: (MW2-BA000033-[949277:950146:r], highly conserved allele)

Sequence:

TTATAATTGTTCATTGGCAATATACTTTTTAAATGATTTAATAAACACCAATATTTCTGGGCTTTCTTTTTTGCTGTATATATATGTAAATGA
AATAGGTGCTTGTAAAATTTTCGTGTTAATAACCGAAATATTGTAATCACTATTTGTTGTTATATAAAGAGGCAGGAATGATATACCTTGA
TTCATTTCGATTAATTTAATTGAAGTATGCACATCATTGATAGATAGAAATTGTGCTTTTTCATAAATATTTAAAATATTATTTTTAAGTGAT
GACCAATATTCTGGATGGTTATCACTTATTATTTTGTATTTTTCAAATAAAGATGCCTCAGTTAGAAGATGATTATTCTCTTTATTGGGAGC
AATCAATACAATTTTACCTTCGCATACTTTTTCAGAATGAACTTCTCTTAGTTTAGGTTGATTTCTGCTAATCCCGATGTCATACGTATGATT
ATTAATATCTTTTTCAATATTTTCATTTTTGACATGAAGAGAAACATCGATAAAAGGATGCTCGTTAAAGAAAGATTTTAAAAATTTGGGC
ATAATGAATGTCGCGATATATGAAGACACGACAACATTTAATTTCGATTGAAACATCGTTTTTTTAAGTTGGATATGTTTGATGCCACTTTC
ATATTGTTCAATAAAACTTTGCGCAATTGGAAGAAATGTATGACCATCTTCAGTCAAGATAATTTGATTTTTATAAGTTTCAAATAGTTTCA
CATTGAGATGCTGTTCTAAATTTTTAATTTGCTTATGTATAGAAGGTATAGTGAGATTAATTTCTTCACTAGCTAATCGATAGTTTAACGTC
TTCGCTAAAATGACAAATGTATAGTACCAATCTAAATTCAT

Gene: leuA2 (2-isopropylmalate synthase, locus 2)

Contig: 06_NODE_54, position: 84029 to 85173, length: 1145 nt, orientation: FORWARD

Perfect match to: (Strain_21201-AFTO01000020-[104908:106052:r], allele observed in CC5)

Sequence:

ATGATTCGAATTCAAGATAATACAATAAGGGATGGTATGCAACAAAGTAATGTTCGAAAAAGTCTAATTATAAAAAAAGAAGTATTGAA
ACAAATTAACAAGTTAAATATAAATTCTGTTGAAGTAGGCATGTGTACAACTATCGAGGATGAATTTAATATTCATCAATTCAGAGACATT
TTAAGTCCTGAAAAAGAATTAGTAGTATTGACTAGGCTTAATGAAAAAGAAATAAAAAAATAGTCAAATTGAAAATTCATAATTTAGTGG
TAAAAATACTATTGCCAATATCTGACTTGCATATAAAAGAAAAGCTTAATTTTTCAAATAAATATTATATTCAGAAAATCAAAGACTGCTT
GGATATATTAAAGAAAGATAAAAAAGGAGTAGATATTTGTTTTGAAGATGCAACAAGGACTTCTAGAGAAAAATTGAAAGAATACATGG
AAATTATTTCAAAATATCAAGTTAGAACAGTTACATTTGCGGACACTGTAGGATGTTCGACACCATTAGAATACGGAGATATTTTTAATTA
CTTTGTAAAAAAATATTCTAACATAATTTTTTCTGCTCATTGTCATAACGATCTAGGGTTGGCTACTGCAAATACATTAGCTGCAATTTTAA
ATGGTGCAAAGCAAATAGAAACTACATTTTTGGGAATTGGTGAGAGAGCGGGTAATGCTCCTATTGAGGAAATAATTACTATTTTGACAA
AAAAACAAATAGAAAGTACGGAATTCACTTTACCCGACGTATATAAAACTAGTATTAATATTTCTAAAATTCTCGATTTTCAAATATCAGA
AAACAAACCTATAATTGGTGAAAATATATTTAAACATGAATCAGGAATTCATCAAGATGGTACTAAAAAAAATATAAATATGTATCAATAT
TTAGTTCCTAGTGATTTAGGATTGAAAAATTCACAAGTTGTTCAGTTTCCAATAAGTAATATTTCTAGTAAGAAAATCTTGCACAATAAATT
TAAATCAATAGTTAACACTGAAGAAATTGATGAAAATATTTCTTTCTATAAACTTGTGAACAAGTTTTCACCTGAAGTAGCACCTGAAGAT
ACAGTGGATTTACTTCAGATAATAAAAAGGAGGAGTAAAGATGGAAATTTTAAATGA

Gene: Q5HHA7 (Sua5/YciO/YrdC/YwlC family protein)

Contig: 06_NODE_54, position: 85157 to 85798, length: 642 nt, orientation: FORWARD

Perfect match to: (MW2-BA000033-[951385:952026], highly conserved allele)

Sequence:

ATGGAAATTTTAAATGATTCTGTAAAAAGTTTTGAAAAACTTTATTTACAATTACAAGATGGTTTTCCAGTGATTGTTCCTACTGATACTAA
TTATAATTTATGTAGTTTACCTAACAACGATCTTTGTATCGATAAAATATTTGAATATAAAAAGCGATCGAAAGATAAGCCATTATCATTAT
TTATTGATAAGCCAGAGGATTGGAAATTGTATGGAGATAATCAAAATACGGAAATAGTTGATAAACTAGTTGAAATATTTTGGCCTGGAC
CATTAAATATTATTTTAAAAAATAAAACAAGCTATAATTATATGCTCAATAATTCAGATAGTATAGCTATAGGATGTGTACAAAATAAAAC
GATGAGAAGATTTATTTCATATATTAATTCACCTATAGCAATTACTTCAGCAAATATATCTGGAACTGCCGATGATATTTTAATAACTGAAA
ATGAAGCAATTAAGCACATGGGCGAAAATGTAAGATATATGCTAAGAAGTCAAAATAAAACTAACTATAAAACATCTAGTACGATTATTA

AAGTGACAGATAATAAAATTGAATTATTAAGAGAAGGAGATATAAAGTTTGAAGAAATAAAAGAAAGACTAGGTACAGGTATTATTTAT
GAATAA

Gene: Q5HHA6 (putative protein)

Contig: 06_NODE_54, position: 85791 to 86909, length: 1119 nt, orientation: FORWARD

Perfect match to: (MW2-BA000033-[952019:953137], highly conserved allele)

Sequence:

ATGAATAAGTTAATACTTGGGATTTATTTATACCGAATTTTTTCACGAGCATACTTTTATTTACCGTTTTTATTAATTTACTTTTTGATTCAAG
GTTATTCCATAATACAATTAGAAATATTAATGGCGTCTTATGGCATTGCAGCATTTTTATTCTCTCTATACAAAGAGAAGTGTTTTAAAATT
TGTAACTTAAAAGATTCTAATAAATTAGTTGTTAGTGAAATATTCAAAATCATCGGTTTATTGTTGTTATTATATCAAAATCAATATTTAATT
TTAGTAGTGGCACAAATATTATTAGGGTTAAGTTACTCAATGATGGCGGGTGTTGATACCGCAATAATTAAAAGAAATATAACAAATGAG
AAATACGTACAAAATAAGTCAAATAGCTATATGTTCCTATCATTATTAATTTCAGGGATTATAGGTAGTTATCTTTATGGAATAAATATTAA
ATGGCCTATAATAATGACTGGTATATTTTCAATTCTAACAATTATAATTATTCGATGCACATTAGTTGAAAATAGGGAATTAAATTTAATAG
GAGAAACAAAGGGAAAGATAAAGAAATTTCTACCAGAAGAGAAGTTTTGGATATTGCATTATTCTTTTTTAAGAGCGTTAATATTAGGAT
TTTTTATAGGATTTATTCCAATTAATATATATATAATGATTTAAAACTGAATAATTTACAATTTATTTCAGTATTAACTTGTTACACAGTTATGG
GTTTTGTATCTTCACGTTATTTAACTAAATACTTGAATTATAAGTTTGTGTCAGAAATTTGTTTAGTAATATTTTTAATAATATATACATATC
AAAGTTTCATAGCAGTTACTATTTCTATGATATTTTTAGGTATTTCTTCAGGGTTAACTCGTCCACAAACTATAAATAAACTTTCTAGCAGT
AGTAACTTAAGAGTGATGCTTAATTATGCAGAAACGTTATATTTTATTTTTAATATCGCATTTTTACTTATGGGTGGTTACTTATATACAAT
AGGAACTATTCAATACTTAATATTATTTATTTCGTTATTAATTTTTATATATTTAATAATAATATTTTATTTTACAAGGAGAGAGCAACATGA
AAATAAAAACTGA

Gene: Q5HHA5 (putative protein)

Contig: 06_NODE_54, position: 86893 to 87408, length: 516 nt, orientation: FORWARD

Perfect match to: (MW2-BA000033-[953121:953636], allele observed in CC1+CC8+CC239)

Sequence:

ATGAAAATAAAAACTGAATTTAAAGGGAACAATATACCATATGAATACGCAGCAGGTGCAGATGTGAGTGATTCTATTAACGGGAATCC
AATTAAGTCATTTCCATTTGAAGTAATTGAATTACCGGAAGGGACTAAATATCTTGCTTGGTCTTTAATTGACTATGATGCAATTCCTGTAT
GTGGCTTTGCTTGGATTCATTGGAGTGTAGCTAATGTAAGTGTTAGTGGCAATTCAATTTCTATAAAAGCAGATTTATCAAGAACAAAGG
GTGACTATGTACAAGGTAAAAATAGCTTTACTAGTGGGTTGTTGGCTGAAGATTTTTCAGAAATAGAAAATCACTATGTAGGACCTACAC
CACCTGATCAAGATCATCAATATGAATTAACAGTTTATGCGTTAGATCATTCTTTAAATTTGAAGAATGGGTTCTACTTGAATGAATTTTTA
AAAGAAGTAAATCAACATAAAATTGATCAAACAAGTATTAACCTTATAGGAAGAAAAATTTAA

Gene: eapH-2 (extracellular adherence protein homolog, locus 2)

Contig: 06_NODE_54, position: 87719 to 88153, length: 435 nt, orientation: FORWARD

Perfect match to: (MW2-BA000033-[953947:954381], allele observed in CC1)

Sequence:

ATGAAATTAAAATCATTTATAACTGTAACTTTGGCACTGGGCATGATCGCAACGACTGGCGCTACTGTGGCAGGTAATGAGGTATCTGCA
GCAGAAAAGGACAAACTACCGGCAACTCAAAAAGCTAAAGAAATGCAAAATGTTCCATATACAATTGCAGTAGATGGCATTATGGCTTT
TAATCAATCTTACTTAAATTTACCAAAAGATAGCCAATTATCATATTTAGATTTAGGAAATAAAGTTAAAGCTTTGTTATATGATGAACGC
GGTGTAACACCTGAGAAGATTCGAAATGCAAAATCTGCCGTTTACACGATTACTTGGAAAGATGGTAGTAAAAAAGAAGTGGATCTTAA
GAAAGATAGCTACACAGCAAACTTGTTTGATTCAAATTCAATTAAACAAATTGATATTAATGTAAAAACTAAATAA

Gene: A6U088 (putative protein)

Contig: 06_NODE_54, position: 88410 to 88595, length: 186 nt, orientation: REVERSE

Perfect match to: (N315-BA000018-[954670:954855:r], highly conserved allele)

Sequence:

TTATTGTGATGAATCTTTCGGCGGTTTAATTACTGCAGCAAAAATTGCTGTGAAAATCGTGAACAATACTGCCATGATAATTGGATTCACT
ACATTTAAGCTGTCTCCACCTACTAGGCTATTAAGTACAAAGTTAACCATTTGCATTAATAATAATGCCCAAAAGAATGTTACGAGGTGTT
TCAT

Gene: fabH (beta-ketoacyl-ACP synthase III)

Contig: 06_NODE_54, position: 88890 to 89831, length: 942 nt, orientation: FORWARD

Perfect match to: (MW2-BA000033-[955118:956059], allele observed in CC1+CC8+CC239)

Sequence:

ATGAACGTGGGTATTAAAGGTTTTGGTGCATATGCGCCAGAAAAGATTATTGACAATGCCTATTTTGAGCAATTTTTAGATACATCTGAT
GAATGGATTTCTAAGATGACTGGAATTAAAGAAAGACATTGGGCAGATGATGATCAAGATACTTCAGATTTAGCATATGAAGCAAGTTT
AAAAGCAATCGCTGACGCTGGTATTCAGCCCGAAGATATAGATATGATAATTGTTGCCACAGCAACTGGAGATATGCCATTTCCAACTGT
CGCAAATATGTTGCAAGAACGTTTAGGGACGGGCAAAGTTGCCTCTATGGATCAACTTGCAGCATGTTCTGGATTTATGTATTCAATGAT
TACAGCTAAACAATATGTTCAATCTGGAGATTATCATAACATTTTAGTTGTCGGTGCAGATAAATTATCTAAAATAACAGATTTAACTGAC
CGTTCTACTGCAGTTCTATTTGGAGATGGTGCAGGTGCGGTTATCATCGGTGAAGTTTCAGATGGCAGAGGTATTATAAGTTATGAAATG
GGTTCTGATGGCACAGGTGGTAAACATTTATATTTAGATAAAGATACTGGTAAACTGAAAATGAATGGTCGAGAAGTATTTAAATTTGCT
GTTAGAATTATGGGTGATGCATCAACACGTGTAGTTGAAAAAGCGAATTTAACATCAGATGATATAGATTTATTTATTCCTCATCAAGCTA
ATATTAGAATTATGGAATCAGCTAGAGAACGCTTAGGTATTTCAAAAGACAAAATGAGTGTTTCTGTAAATAAATATGGAAATACTTCAG
CTGCGTCAATACCTTTAAGTATCGATCAAGAATTAAAAAATGGTAAAATCAAAGATGATGATACAATTGTTCTTGTCGGATTCGGTGGCG
GCCTAACTTGGGGCGCAATGACAATAAAATGGGGAAAATAG

Gene: fabF (3-oxoacyl-[acyl-carrier-protein] synthase 2)

Contig: 06_NODE_54, position: 89843 to 91087, length: 1245 nt, orientation: FORWARD

Perfect match to: (Strain_21193-AFEG01000025-[101398:102642:r], allele observed in CC25+CC361+CC395+CC707)

Sequence:

ATGAGTCAAAATAAAAGAGTAGTTATTACAGGTATGGGAGCCCTTTCTCCAATCGGTAATGATGTCAAAACAACATGGGAGAATGCTCTA
AAAGGCGTAAATGGTATCGATAAAATTACACGTATCGATACTGAACCTTATAGCGTTCACTTAGCAGGAGAACTTAAAAACTTTAATATT
GAAGATCATATCGACAAAAAAGAAGCGCGTCGTATGGATAGATTTACTCAATATGCAATTGTAGCAGCTAGAGAGGCTGTTAAAGATGC
GCAATTAGATATCAATGAAAATACTGCAGATCGAATCGGTGTATGGATTGGTTCTGGTATCGGTGGTATGGAAACATTTGAAATTGCACA
TAAACAATTAATGGATAAAGGCCCAAGACGTGTGAGTCCATTTTTCGTACCAATGTTAATTCCTGATATGGCAACTGGGCAAGTATCAAT
TGACTTAGGTGCAAAAGGACCAAATGGTGCAACAGTTACAGCATGTGCAACAGGTACAAATTCAATCGGAGAAGCATTTAAAATTGTGC
AACGCGGTGATGCAGATGCAATGATTACTGGTGGTACAGAAGCACCAATTACTCATATGGCAATTGCTGGTTTCAGTGCAAGTCGAGCG
CTTTCTACAAATGATGACATTGAAACAGCATGTCGTCCATTCCAAGAAGGTAGAGATGGTTTTGTTATGGGTGAAGGTGCTGGTATTTTA
GTAATTGAATCTTTAGAATCAGCACAAGCTCGAGGTGCCAATATTTATGCTGAGATAGTTGGCTATGGTACTACAGGTGATGCTTATCAT
ATTACAGCGCCAGCTCCAGAAGGTGAAGGCGGTTCTAGAGCAATGCAAGCAGCTATGGATGATGCTGGTATTGAACCTAAAGATGTACA
ATACTTAAATGCCCATGGTACAAGTACTCCTGTTGGTGACTTAAATGAAGTTAAAGCTATTAAAAATACATTTGGTGAAGCAGCTAAACA
CTTAAAAGTTAGCTCAACAAAATCAATGACTGGTCACTTACTTGGTGCAACAGGTGGAATTGAAGCAATCTTCTCAGCGCTTTCAATTAAA
GACTCTAAAGTCGCACCGACAATTCATGCGGTAACACCAGATCCAGAATGTGATTTGGATATTGTTCCAAATGAAGCGCAAGACCTTGAT
ATTACTTATGCAATGAGTAATAGCTTAGGATTCGGTGGACATAACGCAGTATTAGTATTCAAGAAATTTGAAGCATAA

Gene: Q2YWV0 (putative protein)

Contig: 06_NODE_54, position: 91142 to 91513, length: 372 nt, orientation: REVERSE

Perfect match to: (Z172-CP006838-[1001244:1001615:r], allele observed in CC239)

Sequence:

TTATACTAAGATGAGCGACAGCACAATCGTCATAATAAAATATAAAATATTTATTAATAATAAAGGGATTATCCATGTAGAAACAAAGTA
ATGCTCTTTTTTTACCTCTTGTGGGTTGAAAAATGGATCATCAGAGATAGACTTCTTCTTTTTCGAAGATGACATTTGATACTTTAATCTTCT
AAAACCATAACTTGTCGCATCAAAAATGCCTTCTTGTACAAGTAAAATCAAAAATATGCTAATAAAAATAATTAATGAAACATAAAACAAT

ATATTTAAATATGTAATGATAGTATGGCTATTAAAAAGCCATATAATAAACGTTAATATTGGCGTTATTAGTGCCATTCCAAGCCATTTTTT
CAACAT

Gene: oppB (oligopeptide ABC transporter, transmembrane permease subunit)

Contig: 06_NODE_54, position: 91756 to 92682, length: 927 nt, orientation: FORWARD

Perfect match to: (MW2-BA000033-[957984:958910], allele observed in CC1)

Sequence:

ATGGGGAAATATATTTTCAAACGATTTATTTATATGCTTATTTCTTTATTTATTATTATTACAATTACATTTTTCTTAATGAAATTAATGCCAG
GTTCGCCATTTAACGATGCTAAATTAAATGCTGAACAAAAAGAAATTTTAAATGAAAAATATGGATTAAATGATCCTGTAGCTACGCAGT
ATTTACATTATTTAAAAAATGTTGTTACAGGCGATTTTGGTAATTCATTCCAGTATCATAATCAACCTGTGTGGGATTTGATTAAACCGAG
ACTACTACCTTCTTTTGAAATGGGTCTTACAGCAATGTTCATCGGTGTGATACTGGGACTTATTTTAGGTGTTGCAGCAGCTACTAAACAA
AATTCTTGGGTTGACTATACAACTACAGTTATTTCAGTTATTGCAGTATCTGTACCATCTTTTGTACTTGCTGTACTTTTACAATATGTATTT
GCAGTTAAATTAAGATGGTTCCCAGTAGCTGGATGGGAAGGTTTTTCGACCGCGGTATTACCGTCACTTGCATTATCTGCAGCTGTTTTAG
CAACTGTCGCCAGATACATAAGAGCAGAGATGATAGAGGTATTAAGTTCAGACTATATTTTATTAGCGAGAGCTAAAGGTAATTCGACA
ATGCGTGTACTTTTTGGACATGCACTTAGAAATGCTTTAATTCCAATTATTACAATTATCGTTCCCATGTTAGCAAGTATTTTAACAGGCAC
TTTAACAATTGAAAATATTTTTGGGGTTCCTGGATTAGGGGATCAATTCGTACGTTCAATTACAACAAATGATTTCTCAGTAATCATGGCA
ATCACACTATTATTTAGCACACTGTTTATCGTTTCTATTTTTATTGTAGATATTTTGTACGGTGTGATAGATCCACGAATTCGTGTTCAAGG
AGGTAAAAAAATAA

Gene: oppC (oligopeptide ABC transporter, transmembrane permease subunit)

Contig: 06_NODE_54, position: 92682 to 93752, length: 1071 nt, orientation: FORWARD

Perfect match to: (MW2-BA000033-[958910:959980], allele observed in CC1)

Sequence:

ATGGCTGAAAATAAAAACAATTTGTCGATTAACGACGATCATTCTAATGCAGCTATGACGCATACCTCTGACGCTATCTCATCATCTGATT
TTATTATTAGAGAATTAGATTTGAATCAGGAACCTGAAATGCAACGAGAAAGCAAAAACTTTTGGCAAGATGCTTGGGCTCAGTTAAAAC
GAAATAAGTTAGCTGTTGTCGGTATGATAGGTTTAATTATCATTGTAATATTTGCTTTTATCGGTCCAGTTATAAATAAACATGATTATGCT
GAACAAAATGTAGAACATAGAAATCTTCCGGCAAAAATACCTGTATTAGACAAAGTTCCATTTTTACCTTTTGATGGTAAAGATGCAGAT
GGCAAGGATGCTTATAAAGCAGCAAATGCTAAAGAAAATTATTGGTTTGGTACTGATCAGTTGGGTCGAGATTTATGGACAAGAACATG
GAAAGGTGCTCAAATTTCATTGTTTATCGGTGTTGTTGCAGCGATGTTAGATATTTTTATTGGCGTTGTATATGGTGCGATTTCTGGATTCT
TCGGTGGACGTGTCGATACGATTATGCAACGTATACTTGAAGTCATAGCATCTATTCCGAATTTAATTGTCGTAATTTTATTTGTATTAATT
TTTGAACCATCCATTTGGACAATTATATTGGCTATGTCTATCACAGGCTGGTTAGGCATGAGCAGAGTTGTACGTGGAGAATTTTTAAAAT
TAAAAAATCAAGAGTTTGTCATGGCTTCGAAAACATTGGGGGCTTCAAAATTCAAATTGATATTTAAGCATATTTTACCTAATACATTAGG
TGCTATCGTGGTTACATCAATGTTTACAGTACCTAGTGCTATTTTCTTCGAAGCATTTTTAAGTTTCATTGGTATAGGTGTACCCGCACCTC
AAACATCGTTAGGGTCATTAGTAAATGATGGGCGAGCAATGTTATTAATTTATCCACATGAATTATTTATACCAGCAATGATTTTAAGTTT
ATTAATTCTATTCTTTTACTTATTTAGTGATGGATTACGTGATGCATTTGATCCGAAAATGCGTAAATAA

Gene: oppD (oligopeptide ABC transporter, ATPase subunit)

Contig: 06_NODE_54, position: 93768 to 94850, length: 1083 nt, orientation: FORWARD

Perfect match to: (MW2-BA000033-[959996:961078], allele observed in CC1+CC5)

Sequence:

ATGACTGAAAGAATATTAGAAGTAAATGATTTGCATGTTTCCTTTGATATTACAGCAGGGGAAGTGCAGGCAGTGAGAGGTGTAGATTT
TTATTTAAACAAGGGGGAAACATTGGCAATTGTTGGTGAATCAGGTTCAGGTAAATCTGTAACAACAAAAGCAATTACAAAATTATTCCA
AGGGGACACAGGAAGAATTAAAAAGGGAGAAATTTTATTTTTAGGGGAAGATTTAGCAAAAAAACCTGAAAATGAGTTAATTAAATTAC
GTGGCAAAGATATTTCAATGATCTTTCAAGATCCAATGACATCTTTAAACCCAACTATGCAAATTGGTAAACAAGTCATGGAACCATTAAT
TAAGCACAAAAATTATAGTAAAGCACAAGCTAAAAAGCGCGCATTGGAAATACTAAATCTTGTAGGTTTACCAAATGCAGAAAAAAGAT
TTAAAGCATATCCACATCAATTTTCAGGTGGACAAAGGCAAAGAATTGTTATTGCAACCGCATTAGCTTGTGAACCTAAAGTGCTCATTGC
TGATGAACCAACGACTGCATTAGACGTAACGATGCAGGCACAAATTTTAGATTTAATGAAAGAACTACAACAAAAAATCGATACTGCAAT
TATTTTTATAACGCATGATTTAGGGGTTGTTGCGAATATTGCTGATAGAGTGGCAGTTATGTATGGTGGTCAAATGGTTGAAACAGGAGA

TGTTAACGAAATATTTTATGATCCAAAGCATCCATATACATGGGGATTATTATCGTCAATGCCTGATTTATCAACAACAAATGACACACCA
TTACTAGCGATTCCTGGAGCGCCACCTGATTTATTACACCCACCTAAAGGTGATGCATTTGCGAGACGTAGTCAATATGCATTAGATATTG
ATTTTAAAGTAGAACCACCGTGGTTTAAAGTTTCACCGACACATTTTGTGAAATCTTGGTTATTAGACGCACGTGCACCAAAAGTTGAACT
ACCCGAGCTGGTAAAACAACGTATGAAACCGATGCCTAATAATTATGAAAAACCACTCAAGGTAGAAAGGGTGTCGTTCAATGAAAAAT
GA

Gene: oppF (oligopeptide ABC transporter, ATPase subunit)

Contig: 06_NODE_54, position: 94840 to 95781, length: 942 nt, orientation: FORWARD

Perfect match to: (MW2-BA000033-[961068:962009], highly conserved allele)

Sequence:

ATGAAAAATGATGAAGTGCTATTATCCATTAAAAATTTAAAGCAATATTTTAACGCAGGAAAGAAAAACGAAGTGAGAGCGATTGAAAA
TATTTCGTTTGATATATACAAAGGGGAAACATTAGGTTTAGTAGGAGAATCGGGGTGTGGTAAATCTACAACTGGTAAATCAATTATTAA
ACTTAATGATATTACAAGTGGAGAAATTTTGTATGAGGGTATTGATATACAAAAGATTCGTAAACGTAAAGATTTGCTTAAATTTAATAAA
AAGATACAGATGATTTTTCAAGACCCATATGCGTCTTTAAATCCTAGGTTAAAAGTAATGGATATAGTAGCTGAAGGTATTGATATCCATC
ATTTAGCAACTGATAAACGTGACCGTAAAAAACGTGTCTATGATTTACTTGAAACTGTTGGATTAAGTAAAGAACATGCCAATCGCTATCC
TCATGAATTTTCAGGTGGACAACGCCAACGTATTGGAATTGCCCGTGCATTAGCCGTTGAACCAGAATTTATTATCGCGGACGAACCAAT
ATCGGCATTGGATGTTTCAATCCAAGCTCAAGTAGTTAATTTATTATTAAAATTACAACGTGAAAGAGGGATTACGTTCCTATTTATAGCT
CATGATCTATCAATGGTGAAGTATATTTCAGATCGTATTGCAGTGATGCATTTTGGGAAAATAGTTGAAATTGGACCGGCAGAAGAAATT
TATCAAAATCCATTACACGATTATACTAAGTCTTTATTATCAGCCATTCCACAACCTGATCCTGAATCAGAACGCAGTCGCAAACGATTTAG
TTATATTCATGATGAAGCAAATAATCATTTAAGACAATTACATGAAATAAGACCACAGCATTTTGTCTTTAGTACTGAAGAAGAAGCGGC
ACAACTACGAGAAAATAAATTGGTGACACAAAATTAA

Gene: oppA (oligopeptide ABC transporter, substrate-binding protein)

Contig: 06_NODE_54, position: 95800 to 97455, length: 1656 nt, orientation: FORWARD

Perfect match to: (M0239-AIWE01000002-[103488:105143], allele observed in CC188+CC1+CC22+CC188)

Sequence:

ATGACGAGAAAATTTAGAACACTTATTTTAATTTTGATTGCTACAATTGCATTAAGTGGTTGTGCTAATGATGATGGTATTTATTCAGATA
AAGGTCAAGTATTCAGAAAAATTTTGTCATCAGACTTAACATCCCTTGATACATCATTAATAACGGATGAAATATCTTCTGAAGTGACTGC
GCAAACATTCGAAGGTTTATACACATTAGGAAAAGGTGACAAACCGGTGTTAGGTGTTGCGAAAGCTTTTCCTGAAAAGAGTAAAGGTG
GTAAAACTTTAAAGGTTAAATTAAGAAGCGATGCTAAATGGAGCAATGGTGACAAAGTGACTGCGCAAGATTTTGTTTATGCTTGGAGA
AAAACAGTTGACCCTAAAACAGGTTCTGAATTTGCATACATTATGGGGGACATTAAAAATGCGAGTGATATTAGTACTGGTAAGAAACCT
GTAGAGCAATTAGGTATCAAAGCATTAGATGATGAAACATTACAAATTGATTTAGAAAAGCCGGTTCCATATATTAATCAATTATTAGCA
CTTAATACCTTTGCACCTCAAAATGAAAAAGTTGCCAAAAAATATGGTAAAAATTACGGTACGGCAGCTGATAGAGCGGTATACAATGGT
CCATTTAAAGTTGATGATTGGAAACAAGAAGATAAAACGTTACTATCTAAAAATCAGTATTATTGGGATAAAAAGAATGTAAAATTAGAT
AAAGTGAATTATAAAGTTATTAAAGACTTACAAGCCGGTGCATCATTGTATGATACTGAATCAGTAGATGACGCAGTTATTACTGCAGAT
CAAGTAAATAAATATAAAGATAACAAAGGATTAAACTTTGTGTTAACGACTGGGACATTTTTTGTAAAAATGAATGAAAAACAATATCCT
GATTTTAAAAACAAAAATTTAAGACTGGCTATCGCACAAGCAATAGATAAAAAAGGATACGTTGATTCAGTGAAAAACAATGGCTCAATT
CCTTCCGATACACTAACAGCCAAAGGAATTGCGAAAGCGCCTAATGGCAAAGATTATGCGAGTACCATGAATTCGCCTTTAAAATATAAT
CCTAAAGAAGCAAGAGCACACTGGGACAAAGCTAAAAAAGAGTTAGGTAAAAATGAAGTGACATTTTCAATGAACACAGAAGATACAC
CAGATGCAAAAATATCTGCTGAATATATCAAATCGCAAGTTGAGAAAAATTTACCAGGAGTTACTTTGAAAATTAAGCAATTACCGTTTA
AACAAAGAGTATCACTAGAACTGAGTAACAATTTTGAAGCATCACTTAGTGGTTGGTCTGCAGATTACCCTGATCCTATGGCTTATTTAGA
AACAATGACCACAGGTAGCGCACAAAATAATACAGACTGGGGTAATAAAGAATATGATCAATTACTTAAAGTAGCAAGAACCAAATTGG
CACTTCAACCGAACGAACGATATGAAAACTTGAAAAAAGCAGAAGAAATGTTCCTAGGAGATGCACCGGTAGCACCAATTTATCAAAAA
GGTGTTGCACATTTAACAAATCCTCAAGTAAAAGGATTAATTTACCATAAATTTGGTCCAAATAACTCACTTAAACATGTATATATTGATA
AATCGATAGATAAAGAAACAGGTAAGAAGAAAAAATAA

Gene: appA (oligopeptide ABC transporter, substrate-binding protein)

Contig: 06_NODE_54, position: 97667 to 99382, length: 1716 nt, orientation: FORWARD

Sequence:

ATGGGGAAGCTTATTAAATATATTTCAATACTTCTTATTGTCGTTTTAGTGTTGAGCGCTTGCGGAAAAAGCAGTAATAAAGATGAAGGA
GTAAAAGATGCTACTAAAACAGAAGCGTCAAAACATAAAGGTGGTACGCTAAATGTAGCGTTAACAGCACCGCCAAGTGGTGTTTATTC
TTCGTTATTAAATAGTACACATGCGGATGCTGTAGTTGAAGGATATTTTAATGAAAACTTATTAACAATTGATAAAAAAATACGTCCTAAA
TCATATATTGCTTCATGGAAGGACATCGAGCCGGCTAAGAAAATAGAATTTAAAATTAAAAAAGGTATTAAATGGCATGATGGTAATGA
ATTGAAAATTGATGATTGGATTTATTCAATTGAAGTCTTAGCTAACAAGGATTACGAAGGTGCTTATTATCCAAGTGTAGAAAATATCCAA
GGTGCAAAAGATTATCATGAAGGAAAAGCTGATCATATTAGCGGATTGAAGAAAATAGATGACTACACAATGCAGGTTACATTTGATAA
AAAGCAAGAAAATTACTTAACAGGATTTATTACTGGACCTTTATTAAGTAAAAAATATTTATCAGATGTACCAATTAAAGATTTAGCGAAA
TCAGATAAAATCCGAAAATATCCTATTGGTATTGGACCATATAAAGTTAAGAAAATCGTCCCAGGTGAGGCTGTTCAACTTGTTAAATTTG
ATGATTATTGGCAAGGTAAGCCTGCATTAGACAAAATCAATTTAAAAGTTATTGATCAAGCTCAAATTATTAAGGCAATGGAAAAAGGCG
ATATTGATGTTACGAATGATGCTACCGGTGCAATGGCAAAAGATGCTAAGTCATCTAATGCTGGTCTCAAGGTATTATCTGCGCCAAGCT
TAGACTACGGTTTAATAGGATTCGTATCTCATGATTACGATAAAAAAGCTAATAAAACTGGTAAAGTGAGACCGAAATATGAAGACAAA
GAATTACGTAAAGCAATGCTTTATGCAATTGATAGAGAAAAAATGGATCAAAGCATTTTTCAATGGTTACGCTAGTGAAATAAATAGTTTT
GTACCATCAATGCATTGGATAGCAGCTGATCCTAAGGAACTAAACGATTATAAATATGATCCTGAAAAAGCTAAAAAAAATCTTAGATAAG
TTAGGTTATAAAGATCGAGATGGTGACGGATTTAGAGAAGATCCTAAAGGTAATAAATTTGAGATTAACTTTAAACATTATTCAGGTTCA
AATCCTACTTTTGAACCAAGAACTGCTGCGATAAAAGATTTCTGGGAAAAAGTTGGCTTGAAAACAAATGTGAAGTTAGTAGAATTCGGT
AAATATAATGAAGACTTAGCAAATGCATCAAAAGATATGGAAGTGTACTTCAGATCATGGGCAGGTGGTACAGATCCAGACCCATCAGA
TTTATACCACACTGATAGACCTCAAAATGAAATGAGAACAGTTTTACCAAAATCAGATCAATATTTAGATGATGCGTTAGACTTCGATAAA
GTAGGCATTGATGAAAAGAAACGTAAAGATATTTATGTTAAATGGCAAAAATATATGAATGATGAGTTACCTGGATTACCAATGTTCCAA
GGTAAATCGATAACTATAGTTAATGATAAAGTACGAAACTTAGACATTGAAATTGGAACTGATCAAAGTTTATATAATTTAACTAAAGAA
GCTTAG

Gene: appD (oligopeptide ABC transporter, ATPase subunit)

Contig: 06_NODE_54, position: 99433 to 100419, length: 987 nt, orientation: FORWARD

Perfect match to: (O11-CP024649-[989450:990436], allele observed in CC130+CC8+CC30+CC239)

Sequence:

ATGAATAATGTATTGTTAGAGGTTAAAGATTTAGAAACATCATTAAAAATAAATAATGAATGGTTAGCAACTGTTGAAAATATTTCTTTTG
AATTATCTAAAGGAGAAGTTTTGGGTATAGTAGGGGAATCTGGTTGCGGTAAGTCCATATTAAGTAAGTCAATTATTAAATTATTACCAG
AAAAGATATCTAAACTAAGTAATGGAGAAGTTATATTTGATGGTAAACGAATCGATACGCTCAATGAGAAGCAATTGCTAGATATTCGA
GGAAATGATATTGCTATGATTTTTCAAGAACCTATGACTGCTTTAAATCCTGTATTTACCATAAAAAATCAACTTGTGGAATCTATAAAATC
ACATAAAAAAATTTCTAAAAAAGAAGCAAATAACTTAGCAAAAGATTTACTAAAAAAAGTTGGAATTGCTAGACAAGATGAAATATTAAA
TAGCTATCCTCATCAATTATCTGGTGGTATGAGACAAAGAGTAATGATTGCAATGGCCATTTCATGTTCTCCTAAATTATTAATTGCTGAT
GAACCTACAACAGCATTGGATGTCACGATTCAAGCGCAAATATTAGACTTATTAAAAGAATTGCAAAAGGAAACGCAAATGGCAATTAT
GATGATTACACATGATTTGAGTGTAGTTGCTGAGTTTTGCGATAAAGTCTTAGTTATGTATGCAGGTCAAATTGTAGAATTTGGAGGCAT
AAAAGAAATACTACACAATCCGAAACATCCTTATACCCAAAAATTATTATCAACAATTCCAAAACTTAAAGAAGAGCAGAAACGACTTGA
AACGATAGAAGGAATTGTGCCATCAATCCAAGCATTTCACGTTAATAAGTGCAGATTTGCAAATAGATGTAACAAAAAAATGGATATTTG
TAATAATCAATCTCCTAAAATGCATGTTTGTGAAGACGTCATTGTACGTTGTCATTTGTACAAAAATGAATATAAGGAGATATAA

Gene: appF (oligopeptide ABC transporter, ATPase subunit)

Contig: 06_NODE_54, position: 100422 to 101402, length: 981 nt, orientation: FORWARD

Perfect match to: (KLT6-APFH01000004-[410827:411807], highly conserved allele)

Sequence:

ATGGAAAATATTTTAGAAGTCAACCAAATAAAAAAATACTACAAAATTAAAACTGGATTATTACAAAAAACTCAGTACGTTAAAGCTGTT
GATGACGTATCGTTTTCAATAAAAAAAGGACAAACTTTTGGATTAGTAGGAGAATCGGGTTGTGGTAAGTCAACGTTAGGTAAAGTGAT
TATCAGGCTTGAAGATGCAACTTCAGGCTCAATAATTGTTAATGGTGAAGATATAACAAGATTACAAGGTAAAAAACTCAGAAAATCACG
ACAACAATATCAGATGATATTTCAAGATCCGTATGCATCATTGAATCCGATGCAAATGGTTGGAGATATCATTTCAGAACCTATTTTAAAT
TATAAAAAATTGCCAAAAGAAGAAATAAAAAAAGAAGTACTATATTTATTAAAATGTGTTGGCCTAAGTGAAGATGCATATTATAAATAT
GCACATGAATTTTCAGGTGGACAGAGACAAAGAGTGGGAATTGCAAGAGCATTGGCTTTGCGTCCGAGTTTAATTGTTGCTGATGAGCC
TGTAAGTGCATTAGATGTATCTGTTCAATCTCAAGTACTGAATTTATTAAAAGATTTACAAGAACAATTTAACTTAAGCTATTTATTTATCG
CACATGATTTAAGTGTAGTAAAACATATAAGTGATGTCATTGGAGTTATGTATTTAGGTCATATAGTTGAAATCGCATCTGATAAAGAAA
TTTATGAAAATCCCAAACATCCATATACAAAAGCGTTGATTTCATCAATACCACAAATTGATAAACATAATAACAATAGAATTATATTAAA
AGGAGAATTACCTTCGCCAAGTAATCCGCCGCAAGGTTGTCCTTTTCATACAAGATGTCCGATTGCAAAAGATAAATGTAAAGAAAATAT
ACCACAATTAAAGGACATTGGTGATGAACATCAAGTTGCTTGTTTTTATGTAAATAAAGTAGGTGATTTAAATGGTTAA

Gene: appB (oligopeptide ABC transporter, transmembrane permease subunit)

Contig: 06_NODE_54, position: 101395 to 102357, length: 963 nt, orientation: FORWARD

Perfect match to: (MW2-BA000033-[967623:968585], highly conserved allele)

Sequence:

ATGGTTAAATTAATATTAAAGAGATTAGGTTTAATGATTCCGTTACTAATTTTAATTTCTATTGTTGTATTTTCATTAGCTATCATTCAACCA
GGAGATCCATTTTCAGATTTACAAAACGGAAAAATAAAACAAGAAGCGATAAATGCACAAAGGGAAAAGTTAGGCCTCAACGACTCTAT
ATCACATCAATACATTAGATGGGTCAATCATGTTATACATGGTGATTTAGGGGAGTCAATCAAATATAAAAGGCCGGTAATTGATGTTAT
TGAGGAAAGAATTCCAAATACAATATTACTCGGTGCTATGTCATTAATTATTACTTATATTATCTCATTTGCTTTAGGAATAACGTCAGGTA
GATATTCTTACAGTTTGACTGATTATACTGTGCAAATATTTAATTATTTGATGTTAGCTATTCCATCTTTTATTGCGGGAGTATTTGCAATTT
TTATTTTTTCTTTTGAATTACAATGGTTTCCGTTTCAAGGTTCTGTTGATATTAACCTTAAAGAAGGTACTTTTGAATATTATATGAGTAAAA
TTTATCATACATTTTTGCCTGCATTAACTTTAGGATTATTATCTACTGCTGGTTATATTCAATATTTACGTAATGATATTATTGAAAATTCTA
AAAAAGATTATGTATTGACGGCAAGGTCAAAAGGATTATCTATGAATAAAATTTATAATAAACATATATTGAGAAATTCTTTAATACCTAT
TATTACATTTTTAGGTGCTGATATTGTAAGTATTTTAGGTGGAGCTGTGATTACTGAGACTATCTTTTCATATAACGGTATCGGTAAATTAT
TTTTAGAATCGGTAATAGGTCAAGACTATCCATTAATGATGGCATTAACGTTGTTTTTCTCATTTTTAGGTTTACTGGGTAATTTGATTTCT
GATATTACTTATGGATTTATAGATCCAAGAATTAGAAGTAACTAG

Gene: appC (oligopeptide ABC transporter, transmembrane permease subunit)

Contig: 06_NODE_54, position: 102369 to 103250, length: 882 nt, orientation: FORWARD

Perfect match to: (MW2-BA000033-[968597:969478], allele observed in CC1+CC12+CC188)

Sequence:

ATGCAAAATAAGTCAAAATCGCCTTTTAAAATTGCATTGTCTAGATTTATTCATAATAAAATTGCAATGTTATCGATTATTTTTTTATTAATC
ATAACTATTATATCAATTATAGCGCCATTAATAGCTCCTTTTCCAGTGAACCAACAAGATTTATTAAATATAAAAGGTGAAATGACAGCAC
AAAACATTCTTGGTACAGACTCTGGTGGTAGGGATAACTTTAGTCGTTTGTTATATGCAGGTCGTATTTCATTATCCATTGGAATTACATCT
ACAATAGGAATGCTTTTGATTGGAATTACAGTTGGAGTGATTTCTGGTTATTTTGGAGGTATTGTTGATACATTATTAATGAGAATAACCG
AATTTGTTATGTTATTTCCATTTTTAATATTTGCAATTGTATTAAATGCTGCACTTGGAGATAAAATTAAAAATCCTTATGGATCTGCCATAA
TTCTTGTTCTAGTTATTATCGTATTAAGTTGGGGAGGTATTGCAAGACTTGTTCGTGGTAAAGTACTTCAAGAAAAAGAAAATGAATACTT
TTTGGCAGCAAAATCAATTGGTACACCCACATATAAAATTATTTTGAAACATCTTTTGCCGAATATATTAAGTGTAGTTATCGTACAAGCA
ACATTGTTATTTGCCGGTATGATTGTAGTGGAATCAGGATTGAGCTTTTTAGGATTCGGAATTAGTAAAGCAATACCATCTTGGGGTAAT
ATGTTGAGTGATGCTCAAGAAGGGGATGTTATAAGTGGTAAACCGTGGATATGGATGCCACCTGCTATAATGATTACATTAACTATATTA
AGTATAAACTTTGTAGGGGAAGGGCTTAAAGATGCTTTTAATCCTAGAGGTAGACGTTAA

Gene: trpS (tryptophanyl-tRNA synthase)

Contig: 06_NODE_54, position: 103292 to 104281, length: 990 nt, orientation: REVERSE

Perfect match to: (MW2-BA000033-[969520:970509:r], highly conserved allele)

Sequence:

TTATCTCTTACGTCCTAAACCCATCGCTTTTTCCATTTTTTTGACAGTTTTAAATGAAACTTTGTGTGCTTTATCTCTACCTTGATCTAAAATA
TCATCAAGTTTATCTGAGTTATAGAAACTTTCGTATTTTTCTTGGAATTCTACTAAAAATGCTTTAACTATTTCAGCAAGGTCACCTTTAAAT
TTACCATAACCTTCGCCCTCATATTTTGCCTCAATATCTTTAATTGGCATGTCTGTTAATCCAGCGTATATTGAAATTAAATTTGTTATACCT
GGCTTGTTGTCACGATCAAATTTAATAATACCATCTGAATCAGTTACTGCGCTTTTAATTTTTTTAGCTGCAACATTCGGCTCGTCTAATAA
TGAAATGAAGTTTTTAGCATTATCATCACTCTTACTCATTTTTCTTGTTGGGTCTTGTAAACTCATGACACGTCCACCAACTTTAGGCATAC
GAATTTCAGGTTTCACAAGCACATCATTATAGCGACTATTAAATCTATCTACAAGGTTACGAGTCAATTCGATATGCTGCTTTTGGTCATCT
CCAACTGGAACGATATTAGTATTGTAAAGAACAATATCAGCTGCCATTAAAGGTGGATATGTTAATAGACCAGCAGGTATACCTTCAACT
GCTTTCTGAGCTTTATCTTTGTATTGCGTCATACGCTCTAATTCTCCAACAGAAGCAATCGTAGTTAACATCCATCCTGCTTGTACGTGTGC
AGGGACTTCAGATTGTATGAACAATGTTGCTTTGTCTGGATCTATACCAGAAGCTAAATAAATCGCTGCTAATTGTCTGGTCTGTTTACGT
AATTTTAAACGATCTTGTGGCATTGTAATTGCATGTTGATCTACGATACAGAAATAACAATCATAGTCATTTTGCACATCAACAAATTGTTT
TAGTGCGCCAATATAATTTCCAATAGTAGGAATTCCACTAGGTTGGATGCCTGAAAATAATGTCTCCAT

Gene: spxA (thiol/oxidative stress global regulator)

Contig: 06_NODE_54, position: 104576 to 104971, length: 396 nt, orientation: FORWARD

Perfect match to: (N315-BA000018-[970835:971230], highly conserved allele)

Sequence:

ATGGTAACATTATTTACTTCACCAAGTTGCACATCTTGCCGTAAAGCGAAAGCATGGTTACAAGAACATGACATTCCGTATACGGAGCGT
AATATTTTTTCTGAACATTTAACAATTGATGAAATTAAGCAAATATTAAAAATGACTGAAGACGGTACTGATGAAATCATTTCTACACGTT
CTAAAACATACCAAAAATTAAATGTTGATATTGATTCACTACCATTACAAGACTTATATTCAATCATTCAAGATAATCCTGGCTTATTACGT
CGTCCAATTATTTTAGATAATAAACGACTACAAGTTGGTTATAATGAGGACGAGATTCGACGTTTCTTACCTAGAAAAGTTCGTACGTTCC
AATTACAAGAAGCACAACGTATGGTTGACTAA

Gene: trfA (adapter protein)

Contig: 06_NODE_54, position: 105342 to 106061, length: 720 nt, orientation: FORWARD

Perfect match to: (MW2-BA000033-[971570:972289], highly conserved allele)

Sequence:

ATGAGAATAGAACGAGTAGATGATACAACTGTAAAATTGTTTATAACATATAGCGATATCGAGGCCCGTGGATTTAGTCGTGAAGATTTA
TGGACAAATCGCAAACGTGGCGAAGAATTCTTTTGGTCAATGATGGATGAAATTAACGAAGAAGAAGATTTTGTTGTAGAAGGTCCATT
ATGGATTCAAGTACATGCCTTTGAAAAAGGTGTCGAAGTCACAATTTCTAAATCTAAAAATGAAGATATGATGAATATGTCTGATGATGA
TGCAACTGATCAATTTGATGAACAAGTTCAAGAATTGTTAGCTCAAACATTAGAAGGTGAAGATCAATTAGAAGAATTATTCGAGCAACG
AACAAAAGAAAAAGAAGCTCAAGGTTCTAAACGTCAAAAGTCTTCAGCACGTAAAAATACAAGAACAATTATTGTGAAATTTAACGATTT
AGAAGATGTTATTAATTATGCATATCATAGCAATCCAATAACTACAGAGTTTGAAGATTTGTTATATATGGTTGATGGTACTTATTATTAT
GCTGTACATTTTGATAGTCATGTTGATCAAGAAGTCATTAATGATAGTTACAGTCAATTGCTTGAATTTGCTTATCCAACAGACAGAACAG
AAGTTTATTTAAATGACTATGCTAAAATAATTATGAGTCATAACGTAACAGCTCAAGTTCGACGTTATTTTCCAGAGACAACTGAATAA

Gene: trfB (putative protein)

Contig: 06_NODE_54, position: 106182 to 107168, length: 987 nt, orientation: FORWARD

Perfect match to: (MW2-BA000033-[972410:973396], allele observed in CC1)

Sequence:

ATGTTAGTAGCTTTAAATGAAGAAAAGGAACGTGTGTTAGCAACTACTGCATTGAGAAAGACACAATATTTTTGTCCGGTGTGTGGCAA
GCAAGTTATTTTAAAGCGTGGGCTCAAAGTAATTAGTCATTTTGCACATAAACATTTAGCGGAACAAAAATGTTTTAATAATGAAACGATT
AAACATTATAAAAGTAAATTGATTTTAGCACAGATGATACAGCAACAAGGATTTAAAGTAGAGATAGAGCCATTTTTAAAAGAAATAAAA
CAAATTCCGGATATTTTGATTAATAATAAATATGTTATTGAGCTACAGTATTCGCCAATTCCTTATAAACAGATTCTTCAACGAACGGAAG
GTTTAAAGAAAATGGGATATAAAGTAAGTTGGTTATTAAATGATGTTGATTATTGTCATAATAAAGTGAAGTTCAATCATTTTCAAAGTAT
GTTTATTAATCCATTCACTCGAAAACTTCATACGTTCAATTTAGAGAAAAAACAAATAATGATGTTTCAACAAATACAATATTTAGGCGGG
CACAAATATGTCGCTGAAAAAAGAAATGCCAAAATTAGTGAGTTGTTTAATGAGGCGCCTTGTGATTATCATGCTGTTTATAAATTATCAA
AGTTCGCAATTAATCAATATATCAAATATTGTCGCTGGCAAAATTCTGTTTTAGAACCCACTTTAAGTGCAATGTATCAATTACAGTTAACT
GATCAAGAAGTAGTGCACAATTATGGTTATATTTTTCCAGAGCAAATTTATATTAAAAATCATCCTATTGAGTGGCAATTACAAGTTGATT
TATGGTTAAAGAATGGAAAAAGCAAATTAGTAAATGACAATCTTAATTATTTTAAACTGAAAAAGTTTATTGTTGGTCTAGAAAGTAAAA
CAGCAATTATAGAAAAACTTATTAACAATTATTTAAATATTTGTTCAGATAGAGGTAATGACGTGCAAATTTTGTTCTAA

Gene: pepF1 (oligoendopeptidase F)

Contig: 06_NODE_54, position: 107216 to 109024, length: 1809 nt, orientation: FORWARD

Perfect match to: (MW2-BA000033-[973444:975252], allele observed in CC1+CC12)

Sequence:

ATGAGTCAACAATTATCTAGAGAAGAACAGGAACGTAAATATCCTGAATATACATGGGACTTAACAACAATTTTCAAAGATGATGAAGCT
TTTGAGGCTGCATTTAAAGAAGTTGAAAATGAGTTAGGCAAAGAAGAACAATTTAAAGGACACATTGGTGATAGTGCTGAGACATTATA

CAATGCATTAGAATTAGAAGATACATTAGGTACTAAATTAGAAAAAGTATATGTATACGCGCACCTAAAACAAGACCAAGATACAACGA
ACGACAAGTATACTGGTATGGAGTCAAGAGCACATCAATTAATTATTAAATTTAGCTCGGCATGGAGTTTCTTAGTGCCAGAGATTTTAC
AAATTGATGAAGATAAAATTCAATCATTTGTAAATTCATATGATAAATTACAAAAATTCGCATTTGATTTGAAGTTGATTAATGAAAAACG
TCCTCATATTTTAGATGCTGAAACTGAAAAGTTATTAACAGAAGCGCAGGACGCGTTATCAACGCCATCAAATGTATACGGTATGTTTAG
CAACGCTGATTTAGTATTTGAAGATGCGATTGATAAAGATGGAAATGCACACCCGTTAACACAAGGTACATTTATTAAGTATTTAGAATC
AGATGATCGCAAACTAAGAGAAAGTGCTTTTAGAAATGTATATAAAGCATATGGTGCTCATAATAATACGCTTGGCGCTACGCTAGCAG
GTGAAGTGAAGAAAAATGTATTTAATGCTCGTACACACAATTACAAAACTGCAAGAGAAAAAGCATTGAGTAATAATCATATTCCAGAAA
ATGTATATGACAATCTAGTAAAAACTGTACATAAATATTTACCATTGCTACATAGATATACTGAATTGCGCAAAGAATTGCTAGGTTTAGA
TGACTTGAAAATGTATGATTTATATACACCATTAATTAAAGATATTAAGTTTGAAATGCCTTATGAAGAAGCTAAAGAGTGGATGTTAAA
AGCTTTAGAACCAATGGGTGAAGAATATTTAAATGTAGTTAAAGAAGGCTTAAACAATCGTTGGGTCGATGTCTATGAGAATAAAGGTA
AACGTTCAGGTGGCTATTCATCAGGTGCACATTTAACTAATCCATTTATTCTACTTAACTGGTCTAATACTATTTCAGACTTATACACATTA
GTTCATGAATTTGGGCATTCAGCACATAGTTACTTCAGTAGAAAATTCCAACCGTCAAATTCTAGTGACTACACTATTTTTGTCGCTGAAG
TTGCATCAACTTGTAACGAAGCACTTTTAAGTGATTATATGGATAAACATCTTGATGATGAAAAACGCTTATTATTATTAAACCAAGAATT
AGAACGTTTCAGAGCTACATTATTCCGACAAACAATGTTCGCAGAATTTGAGCATAAAATTCATGCAATTGAAGAAGCAGGTGAACCTTT
AACACCAACTAGAATGAATGAAGAATATGCCAAATTAAATAAATTATACTTCGGTGATTCTGTAGAAACTGATGAAGATATTAGTAAGGA
ATGGTCACGTATTCCACACTTCTATATGAATTATTATGTATATCAATACGCAACTGGTTACAGTGCAGCTCAAAGCTTAAGTCATCAAATTT
TAACAGAAGGTAAGCCAGCAGTAGATAGATATATTAATGAATTCTTGAAAAAAGGTAGCTCAAATTATCCAATTGAGATATTAAAAAATG
CTGGTGTAGATATGACAACACCTGAACCAATTGAACAAGCTTGTGAAGTTTTTGAACAAAAATTGAACGCTTTTGAAAAATTAATGAAAG
CTTAG

Gene: yjbH (SpxA binding thioredoxin domain protein)

Contig: 06_NODE_54, position: 109484 to 110290, length: 807 nt, orientation: REVERSE

Sequence:

TTATTTTGATTTGATTTTAGTCATTTTAGATTTCCAGAAATCGCCATCTGGATATTTAAGTTTTTCAATTTTTTGTTGAATGGCTAACTTCTTT
AACTCTTTGTTTAAAAGTTTTTCTGGCCATTCATAAATAGTAAGTAATTCTTCCATCGTTACAAGTTGTTGTTGCTGTATATAAGTTTCTAAT
TTAGGAGGAAGATTCTTTTCGATAGGTTTACCCATCAATTCATTAATTATATAAGTATAGATGTGATATGGGTATAATCCTTCGACTTTTAA
ACCTTCTTCATGAACATCTTCACTAAAGAAAACGAGAGAAGGGGCTTGTTCGATTTCCATTTCTCTTGCAATATGCAAATCAATTTTCAAG
CTTTCGGTTAGTTTACTTTTTTGTAAGTCGTCTTTAAATACTTCTAAATCAATACCTGCATTTTGAATACAGTCACAAATCATTGATTCTGTA
ATAATATCTCTTTTAGGTATGATTTCATTTTGCATTAAATGTATAAATCGTTCGGCACGTACACGACCTTGTAACTCAGCTGCTTTATAAGC
TAGGGCGATGTTATCAAAGTTGGATGTACTTTGAGCTTGGCATTTCGTTAATACTTTTAACGAAGGATTTAATATATGTCTGATACGTATA
TATTGATTATATTCAATTCTTAATTTGGATAAGATTGCTGATAATTTGAAGCAATCGGAGCTAAATGGATCGAAAAATGAATAAATTTCGA
TTTTACTTACAGGTGATAGATTAATATCTTCACGACTCTTATTTTCCATTATTCGTAATTCTCCAGCCAT

Gene: yjbI (putative thiol management oxidoreductase component)

Contig: 06_NODE_54, position: 110313 to 110678, length: 366 nt, orientation: REVERSE

Perfect match to: (N315-BA000018-[976572:976937:r], highly conserved allele)

Sequence:

TTAGGAATTCACCATATGATTAGCAGTTAATCTTAAGCGCTCAAATAAATAATCTCCAACACCTTGTGGAAACGCAGCGCGATTAATTGCT
GTCTGCATATTTTCTAACCATGCATCTCTTTCAAATTCAGTGATTGTAAAATCCATATGTCTTTTTCTTAGCATAGGATGTCCGTGTTCTTCG
GTATAAATGTTTGGACCGCCCAAAAACTGTGTTAAAAATTGTTTTTGTTTACGACTTGTTTCTGCAAAATCTCCTGGAAACAGGTGATTAA
GTCGTTCATCTTTTTCTACAAGGGTGTAAAAATAATCAATCATATCGTATAACGCTTCTTTACCAATGATGTCATATGGTGTTGTTGTCAT

Gene: yjbK (putative RNA/thiamine triphosphatase)

Contig: 06_NODE_54, position: 110782 to 111375, length: 594 nt, orientation: REVERSE

Perfect match to: (MW2-BA000033-[977010:977603:r], allele observed in CC1+CC97+CC398)

Sequence:

TTAATTTATATTGTTTGAAAGTGTTTCTTTTTTCTTGAAAAAACGTTGAACTTTATTTAAAGGTTGATGATGTTCGAGGTTTAGTTCGTTTAA
TAAAGATTGGAACTTTTGTAAACCTTGATTATAGTCTTTAACTTCGAACTCTAACTCATAATCCGTAGTATCGAAATACTCACTTTTATCTAA

AACGAGTAAATCACCTTTATATTTAGTTTCTTGGCGATATGTCGTTAATGCACCAAGTATTGATAAAGTTGTATCCTTTACACCAAACTGTT
CAACTATAATTTGACGAATGTCATCTGGAAGATTGTCGTTTGAAATAATCAAGTTCATCTCTGGTTTAATGTCGACGATATAGTTGTATTCT
AATAGACCAACCTTTGCTGGTGTCTTTAAAGTCATTTCATATTGATTGTCTTTAACTCTTATGCGTAGTGCAGAGCGATGTTCCTTTAATTT
GAAATCGGGTGTATCAATATAGTAATTGATTTGCTTAAAAAGCACACTGTCTTTAAAATATTTCTCTTGCAATTTATTATAGATTGATGCAG
TTATCATTTGTTTAAATTCTATTTCATGATTTGTTGCCAT

Gene: yjbL (putative phosphatase)

Contig: 06_NODE_54, position: 111561 to 111908, length: 348 nt, orientation: FORWARD

Perfect match to: (MW2-BA000033-[977789:978136], highly conserved allele)

Sequence:

ATGCGTTTATATATTAATGAAATTAAAATTAAAGATGACATACTTTATTGTTATACAGAAGATTCTATTAAAGGATTATCTGAAGTAGGAC
AAATGCTCGTTGATAGTGATAATTATGCCTTTGCGTATACATTAGATGATGGTAAAGCGTATGCTTATCTCATTTTCGTACAAGAAACATG
GACGATGTTGCATGAAAACATGACTAAAAAAATTATTATCAATGATGAACTAGAATTGACTGAATTCCACCAAGAACTTACTTATATTTTA
GACAACATAAAAGGGAATAATAATTATGGTAAGGAATTTGTTGCAACCGTTGAAGAAACATTCGACATTGAATAA

Gene: relQ (ppGpp synthase III)

Contig: 06_NODE_54, position: 111925 to 112560, length: 636 nt, orientation: FORWARD

Perfect match to: (MW2-BA000033-[978153:978788], highly conserved allele)

Sequence:

ATGAATCAATGGGATCAGTTCTTAACACCTTATAAGCAAGCGGTTGATGAGTTGAAAGTGAAACTTAAAGGCATGCGCAAACAATATGA
AGTTGGTGAACAAGCGTCGCCAATAGAATTTGTTACTGGTCGTGTTAAACCAATCGCTAGTATTATAGATAAGGCAAACAAACGACAAAT
ACCATTTGATAGGTTAAGAGAAGAAATGTACGATATCGCTGGTTTAAGAATGATGTGCCAATTTGTTGAAGATATTGATGTTGTCGTCAA
TATTTTAAGACAAAGAAAAGATTTTAAAGTAATTGAAGAACGAGATTATATTCGTAACACTAAAGAAAGTGGTTACCGCTCGTATCATGT
CATTATTGAATATCCAATTGAAACATTACAAGGCCAAAAATTTATATTGGCTGAGATTCAGATTCGTACATTAGCAATGAATTTCTGGGCA
ACGATTGAACATACTTTACGATATAAATATGATGGTGCTTATCCGGATGAAATTCAACATCGTTTGGAAAGAGCGGCAGAAGCAGCGTAT
TTACTTGATGAAGAGATGTCTGAAATTAAAGATGAAATTCAGGAAGCTCAAAAATATTACACGCAAAAACGTTCTAAAAAACATGAAAAT
GATTAA

Gene: ppnK (putative inorganic polyphosphate/ATP-NAD kinase)

Contig: 06_NODE_54, position: 112577 to 113386, length: 810 nt, orientation: FORWARD

Perfect match to: (CN1-CP003979-[946126:946935], highly conserved allele)

Sequence:

ATGCGTTATACAATTTTAACTAAAGGTGACTCCAAGTCTAATGCCTTAAAGCATAAAATGATGAACTATATGAAAGATTTTCGCATGATTG
AGGATAGTGAAAATCCTGAAATTGTTATTTCAGTTGGTGGTGATGGTACATTACTACAAGCATTCCATCAGTATAGCCACATGTTATCAAA
AGTGGCATTTGTTGGAGTTCATACAGGTCATTTAGGATTTTATGCGGATTGGTTACCTCATGAAGTTGAAAAATTAATCATCGAAATTAAT
AATTCAGAGTTTCAGGTCATTGAATATCCATTGCTTGAAATTATTATGAGATACAACGACAACGGCTATGAAACAAGGTATTTAGCATTAA
ATGAAGCAACGATGAAAACTGAAAATGGCTCAACACTTGTTGTGGATGTTAACTTAAGAGGGAAACACTTTGAGCGATTTAGAGGCGAT
GGATTATGTGTATCAACACCTTCGGGTTCAACGGCTTATAACAAAGCGCTAGGTGGCGCACTGATACATCCTTCACTTGAAGCAATGCAA
ATTACAGAAATTGCCTCAATAAATAATCGTGTGTTTAGAACGGTAGGATCACCACTTGTATTACCAAAGCATCATACATGTTTAATATCAC
CAGTTAATCATGATACCATTAGAATGACGATAGATCATGTTAGTATCAAACATAAAAATGTTAATTCAATACAATACCGTGTAGCAAATGA
AAAAGTGAGGTTTGCACGTTTTAGACCATTCCCATTCTGGAAACGTGTACACGATTCTTTCATATCAAGTGATGAAGAACGATGA

Gene: rluE (ribosomal large subunit pseudouridine synthase)

Contig: 06_NODE_54, position: 113383 to 114237, length: 855 nt, orientation: FORWARD

Perfect match to: (MW2-BA000033-[979611:980465], allele observed in CC1)

Sequence:

ATGAAATTTAAGTATCATATATCACAACAGGAAACTGTTAAAACTTTTTTAGCACGACATGATTTTTCTAAGAAGACAGTGAGCGCCATTA
AAAATAATGGCGCTTTAATTGTTAATGATGAACCAGTCACAGTGCGTAAGCAATTAATGCCAAATGATATATTAGAAATTCATTTACCACG
AGAAATACCGAGTGTTAATTTAATACCTTATGCTCGTAAGCTAGAAGTATTGTATGAAGATGCTTTTATCATCATAGTTACTAAACCAAAC
AATCAAAATTGTACACCTTCGAGAGAACATCCTCATGAAAGTTTAATCGAACAAGTACTATATCATTGTCAGGAAAATGGTGAAAATATT
AACCCACATATTGTTACGCGTCTAGATCGTAATACAACTGGTATTGTGATATTCGCTAAATATGGATATATCCATCATTTATTTTCTAAAGT
AAACTTGAAAAAAATATATACTTGCCTTGTATATGGTAAAACCCATACATCTGGTATTATTGAAGCTAATATTAGACGGTCAAAGGATAGT
ATTATAACTAGAGAAGTTGCCTCGGATGGTAAATACGCTAAAACATCTTATGAAGTAATAAATCAGAATGATAAATACAGTTTATGCAAA
GTTCATTTGCATACGGGACGTACACATCAAATTCGTGTACATTTTCAACATATTGGGCATCCAATTGTGGGAGATTCTTTGTATGATGGTT
TTCATGACAAAATTCATGGTCAAGTACTGCAATGTACGCAAATATATTTTGTTCATCCAATCAATAAGAACAATATTTATATTACAATTGAT
TATAAGCAATTACTTAAATTATTCAATCAACTCTAA

Gene: mgtE (magnesium transporter E)

Contig: 06_NODE_54, position: 114258 to 115643, length: 1386 nt, orientation: FORWARD

Perfect match to: (CIG1605-AHKE01000007-[114363:115748], allele observed in CC30)

Sequence:

ATGTCAATGAACACAGATGAAAAAGAGCGTGTTCAAGAGGAATTATATGATGAGACATTATTAGATCAATACTTAGAAAATGATGATATT
GATCAATTTAGAGATGAATTTCTAGCATTACACACATATGAACAAAGTGAGTATTTTGAAGATACTACCGATGAAAATAGACAAAAGATT
TTTCAATATTTATCACCTGAAGAGGTTGCAAATTTCTTTGATCAATTAGATATTGATGACGATGAATATGAGTTGCTATTTGATAAAATGA
ATGCGACATACGCAAGTCACATATTAGAAGAAATGTCATACGATAATGCAGTAGATATTTTAAATGAGTTGACTAAACCAAAAGTTGCTA
GTCTTTTAACATTGATGAATAAAGATGACGCGAATGAAATCAAAGCATTACTTCACTATGATGAGGATACGGCCGGCGGTATTATGACGA
CGGAGTATTTATCACTTAAAGCGCATACGCCTGTTAAAGAAGCATTATTATTGGTCAAAGCGCAAGCACCAGACGCAGAAACAATATATG
TTATATTTGTCGTTGATGATGATGGTAAATTAGTAGGTGTTTTATCGCTAAGAGATTTAATTGTAGCTGAAAATGATGCTTATATTGAAGA
TATTATGAATGAACGTGTCATTAGTGTGAATGTAGCAGACGACCAAGAAGATGTTGCTCAAGTTATGAGAGACTATGATTTCATGGCTGT
ACCTGTTATAGATTACCAAGAACATTTGCTTGGTATCATCACGATTGATGATATTTTAGACGTTATGGATGAAGAGGCTAGTGAAGACTA
CTCTCGTTTAGCCGGGGTATCAGATATCGATTCGACTAATGATTCAATCATTAAAACAGCATTAAAACGTTTACCATGGTTGATTATTTTAA
CATTTTTAGGAATGATTACTGCGACAATTTTAGGGAGATTCGAAAAAACATTAGAAAATGTAGCGCTACTCGCAGCGTTTATTCCTATTAT
TAGTGGTATGTCAGGAAATTCAGGTACACAATCTTTAGCCGTTTCAGTTCGTAACATTACGACAGGGGAAATTAATGAGCAAAGTAAATT
TAGAATTGCATTAAGAGAAGCAGGAAGTGGTGTATTATCGGGTGTTGTATGTTCAACAATATTATTTACAATTATTGTTGCAATATATCAT
CAGCCACTTTTAGCATTAATCGTTGCAGGAAGTTTAACTTGTGCGATGACGGTGGGGACGTTTGTAGGTTCGATGATTCCATTATTGATG
AATAAATTAAATATCGATCCAGCAGTGGCTAGTGGACCATTTATTACAACAATTAATGATATTATTAGTATGTTGATTTATTTTGGTTTAGC
TACATCATTTATGGCTTACTTAATTTAA

Gene: cpaA (monovalent cation:H+ antiporter)

Contig: 06_NODE_54, position: 115653 to 117497, length: 1845 nt, orientation: FORWARD

Perfect match to: (MW2-BA000033-[981881:983725], allele observed in CC1)

Sequence:

ATGGAGTTTTTATCTTTAGTTATTGTTGTTTTAGCAGCGTTTTTAACTCCAATAATTGTCAATCGATTAAATATTAATTTCTTGCCAGTTGTT
GTTGCAGAAATTTTGATGGGGATTGTGATTGGAAATTCATTTCTAAATATAGTAGAAAGGGATTCAATTCTAAATATTTTATCAACATTAG
GCTTTATCTTTTTAATGTTTTTAAGTGGTTTAGAAATTGATTTTAAAGCTTTTAAAAAAGATAAACGCGCACGTCAAGGACAAAATAATGA
TGAATCCTCAATTCCAGGGCATCTTAATCTAGCGTTAACTGTATTTGCATTTATTATGATTATTTCGATTCTTTTAGCGTATGTATTTAAATG
GCTTGGATTAGTGGATGATGTGTTATTAATGGTCATTATCATTTCAACTATTTCCTTAGGCGTAGTTGTTCCAACTTTAAAAGAAATGAATA
TTATGAGAACAACTATAGGGCAATTTATCCTATTAGTAGCAGTACTTGCGGACTTAGTAACTATGATTTTATTAACGGTCTATGGTGCAAT
CAATGGTCAAGGCGGCAGTACAATATGGTTAATAGGTATATTAGTTGTTTTCACAGCAATTTCATATATTTTAGGTGTTCAATTTAAAAGA
ATGTCATTTTTACAAAAATTGATGGATGGTACGACGCAAATCGGTATTCGTGCGGTATTTGCATTAATAATATTATTAGTAGCCCTAGCAG
AGGGAGTTGGCGCAGAAAATATATTAGGTGCATTCTTAGCAGGTGTCGTTGTTTCATTATTAAATCCAGATGAAGAAATGGTTGAAAAGT
TAGACTCATTTGGTTATGGGTTCTTTATTCCTATTTTCTTTATAATGGTTGGTGTAGATTTAAACATACCTTCATTAATTAAAGAACCGAAAT
TACTAATTATCATACCGATTTTAATCGTTGCATTTATCGTTTCAAAATTAATTCCAGTCATGTTTATTCGACGTTGGTTTGATATGAAAACAA
CGATTGCATCAGCATTTTTATTAACATCAACATTATCGCTCGTGATAGCTGCAGCCAAAATTTCAGAAAGATTAAATGCTATTTCAGCTGA
AACGTCAGGTATATTAATTTTAAGCGCAGTCATTACATGTGTATTCGTTCCGATTATTTTCAAAAAACTGTTTCCAGTTCCAGATGAGTTTA
ACCGTAAAATTGAAGTTAGTTTAATTGGTAAAAATCAATTAACGATTCCTATAGCGCAAAATTTAACATCTCAGTTATATGACGTGACATT

ATATTATCGCAAAGACTTGAGTGATCGTCGTCAATTGTCGGATGATATCACGATGATAGAAATTGCTGATTATGAACAAGATGTTTTAGA
ACGACTAGGTCTGTTTGACCGAGACATAGTTGTTTGTGCCACGAATGACGATGATATTAACCGAAAAGTTGCTAAATTAGCCAAAGCACA
TCAAGTTGAGCGTGTCATTTGCAGACTTGAAAGCACAACGGACGATACAGAGTTAGTTGATTCAGGTATTGAAATTTTCAGTAGCTACTT
AAGTAATAAAATCTTATTAAAAGGTTTAATTGAAACACCTAACATGTTGAATTTATTAAGTAATGTTGAAACGTCACTATATGAAATTCAA
ATGTTAAATTATAAATATGAAAATATTCAATTACGTAATTTCCCATTCGGAGGAGACATCATCTTCGTGCGTATTATCCGTAATAATGAGT
CGATTGTTCCGCATGGAGATACACAATTGCGATATGGAGATCGCTTAATTGTTACCGGTGCTAAAGAATACGTTGATGAATTGAAGCAAG
AGTTAGAATTTTATTTTTAA

Gene: fabI (enoyl- (acyl-carrier-protein) reductase)

Contig: 06_NODE_54, position: 117775 to 118545, length: 771 nt, orientation: FORWARD

Perfect match to: (MW2-BA000033-[984003:984773], allele observed in CC1+CC12+CC188+CC692)

Sequence:

ATGTTAAATCTTGAAAACAAAACATATGTCATTATGGGAATCGCTAATAAGCGTAGTATTGCTTTTGGTGTCGCTAAAGTTTTAGATCAAT
TAGGTGCTAAATTAGTATTTACTTACCGTAAAGAACGTAGCCGTAAAGAGCTTGAAAAATTATTAGAACAATTAAATCAACCAGAAGCGC
ACTTATATCAAATTGATGTTCAAAGCGATGAAGAGGTTATTAATGGTTTTGAGCAAATTGGTAAAGATGTTGGCAATATTGATGGTGTAT
ATCATTCAATCGCATTTGCTAATATGGAAGACTTACGCGGACGCTTTTCTGAAACTTCACGTGAAGGTTTCTTGTTAGCTCAAGACATTAG
TTCTTACTCACTAACTATCGTTGCTCATGAAGCTAAAAAATTAATGCCAGAAGGTGGTAGCATTGTTGCAACAACATATTTAGGTGGCGA
ATTCGCAGTTCAAAACTATAATGTGATGGGTGTTGCTAAAGCGAGCTTAGAAGCAAATGTTAAATATTTAGCATTAGACTTAGGTCCAGA
TAATATTCGCGTTAATGCAATTTCAGCTGGTCCAATCCGTACATTAAGTGCAAAAGGTGTGGGTGGTTTCAATACAATTCTTAAAGAAATC
GAAGAGCGTGCACCTTTAAAACGTAATGTTGATCAAGTAGAAGTAGGTAAAACTGCGGCTTACTTATTAAGTGATTTATCAAGTGGCGTT
ACAGGTGAAAATATTCATGTAGATAGCGGATTCCACGCAATTAAATAA

Gene: UPF0118 (putative membrane protein)

Contig: 06_NODE_54, position: 118740 to 119825, length: 1086 nt, orientation: REVERSE

Perfect match to: (MRSA252-BX571856-[1023233:1024318:r], allele observed in CC30+CC25)

Sequence:

TTAGTCCTTAACATTACTGTTTGCTTTATCAATAATGCGTTGGCGGTATTTGAAAATATTACTAACAACCGTTTTAAGTACAGCATATAATG
GCACTGCAATCAGAACTAATGTAAAGCCACCTAAATCTCCTGCTGCTAAAATAACAACGATAATTGTTAGAGGATGGATACTTAAAGATT
TACCCATTACATTTGGCGTAATGATATTACCTTCAAGTTGGTGTGCAATTAATGTAATGATACAAACCCATATAAATGTAGTAGGACTATC
TATAATACCGAGTATTGCTGCAGGTGCAAATGATAACCATGAACCTAAGAAAGGAATTAAGTTTGCGACACCAGCAAATAGTACTAATAA
AGGAATATATGGTAAGTCAATAATTGAATAACCGATATATAAGAATATACCTAAAATAACACTGACAGTTACTTGACCTTGAATGTAAGA
TTTTAATGTAAAGTTTAAATCAGTTAATAAATCTACGAAAAATACTTTACGTTCACCTTTGAAAAATTTAGCAACAGCTGGGATAAATTTTT
CATGGTCTTTTAACATATAAATTAAGAAGAATGGAACCATAATCAATAAGAAGATGGTTGAAATTAATGATGTAATGTACTGTAATGAAT
TAGATAAAATATTAGTAACGCCATCACCCATTGATTTAACCATATTTGTAATTCTACTTGTTACATCTTCAGGTAATTTATCCATTTGAGCTA
GTGCGAATTTAATAATTTGCTCTGCTTCTTTTTGTAGGGCAGGTGTCTGACTAATTAAATTGTTGATATTTGAAATGATGATTGGTGCAAC
AAACGCAACAATTAAGCCGATAATAGCAAACAAAGCTAACATGATTGTTGTTATACTAGCCCATCTTGGAAAACCGACTTTTTCAAGTAA
GTTTTGAAAAGGTAGACAGATATAAAATAAAAAACCACTAATTAAAAATGGAAGAAATACAGAACCGATGATTGTAGCTATTGGAGTAA
ATACTTCGTGCACTTCCATAAATAGTTTGATGAGAATGAACAGCATAATCAGAGCGATGCCAGTTCGGAACCAAACCTTGTTTAACAT

Gene: yrbD (alanine or glycine carrier protein)

Contig: 06_NODE_54, position: 120167 to 121735, length: 1569 nt, orientation: FORWARD

Perfect match to: (MW2-BA000033-[986395:987963], allele observed in CC1)

Sequence:

ATGATTGAAAAAATTAGTAACCTTTTTAAATGAGGTTGTATGGAGTAAGCCATTAGTTTATGGTTTGCTAATTACTGGTGTGCTATTTACATT
GCGTATGCGATTTTTTCAAGTTAGACATTTTAAAGAAATGATTCGATTAATGTTTCAAGGAGAGAAGTCTCCTAATGGTATTTCAAGTTTT
CAAGCGATAGCCATGTCTTTAGCAGGCAGGGTTGGTACAGGTAATATTGTCGGTGTATCTACTGCAATATTTATAGGAGGACCTGGTGCA
GTATTTTGGATGTGGATTACTGCGTTTTTAGGTGCAAGTAGTGCTTTTATTGAATCTACACTTGGTCAAATATTCAAGAGAGTTGAAAATA
ATGAATACCGTGGTGGACCAGCGTATTATATTGAATATGGTATTGGTGGTAAATTTGGTAAAATTTACGGAATTATCTTTGCTATTGTTAC

GATTATCTCAGTAGGTCTATTGCTTCCTGGTGTGCAATCTAACGCTATAGCAAGTTCTATGCATAATGCGATTCATGTTCCACAATGGTTA
ATGGGTGCTATTGTTGTAGTTATTTTGGGATTAATTATTTTTGGTGGTGTACGTAGTATTGCCAATGTTGCAACAGCCGTTGTACCATTTAT
GGCAATTATTTACATACTGATGGCTGTCATTATCATTTGTATCAATATACAAGAAGTGCCAGCGTTATTTGCATTAATTTTCAAATCAGCAT
TTGGATTACAATCTGCTTTTGGTGGTATCGTTGGCGCAATGATAGAGATTGGTGTTAAACGTGGATTATACTCAAATGAGGCTGGTCAAG
GTACAGGTCCACACGCAGCAGCGGCAGCAGAAGTATCACATCCAAGTAAACAAGGTCTAGTACAAGCATTTTCAGTTTATATTGATACAT
TATTTGTATGTACTGCAACTGCTCTGATTATACTTATTTCTGGTACATATAATGTGACTGATGGTACGGTTAATGCGAATGGCACACCGCA
TTTAATTAAAGATGGCGGTATTTATGTTGAAAATGCAACAGGTAAAGATTATTCAGGTACTGCGATGTATGCACAAGCCGGCATTGATAA
AGCGTTCCATGGCAGTGGTTATCAATTTGATCCTACTTTCTCTGGCGTAGGTTCGTACTTTATTGCATTTGCTTTATTCTTCTTTGCATTTAC
TACAATTTTGTCGTACTACTACATTACAGAAACAAATGTTGCTTATTTAACGCGTAATCAAAATAATCAAGTTTCATCGATATTTATTAATA
TTGCTCGTGTGATTATTTTGTTCGCTACATTTTACGGTGCAGTTAAAACAGCTGATGTAGCATGGGCATTCGGTGATTTAGGTGTAGGTCT
AATGGCTTGGTTAAATATCATTGCGATTTGGATTTTACATAAGCCTGCCGTAAATGCTTTAAAAGATTATGAAATTCAAAAGAAACGTTTA
GGAAACGGTTATAATGCAGTTTATCAACCTGATCCGAATAAATTACCTAATGCTGTCTTTTGGTTGAAGACATATCCAGAACGTTTAAAAC
AAGCACGTGCCAAAAAGTAA

Gene: yjcH (putative esterase)

Contig: 06_NODE_54, position: 121880 to 122638, length: 759 nt, orientation: FORWARD

Perfect match to: (N315-BA000018-[988141:988899], highly conserved allele)

Sequence:

ATGTCAGAATTTAAAACTGGTAAGATTAATAAACATGTTTTATATAGTAATATTTTAAATAGAGATGTCACGATAAGTATTTATTTACCAG
AATCTTATAATCAACTTGTTAAATATAATGTCATTCTTTGCTTTGACGGATTAGATTTTTTACGTTTCGGGAGAATACAACGTACATATGAA
TCGTTAATCAAAGAAGCGCGTATTGATGATGCGATCATTGTTGGATTCCATTATGAAGACGTTGATAAGCGTAGAGAGGAATTTCATCCA
CAAGGAAGTCGTTCTCATTTAACTATTCAATCAGTCGGTAAAGAAATATTGCCATTTATTGACTCGACGTTTTCTACACTGAAAGTAGGTA
ATGCAAGGTTATTAGTAGGGGATAGTTTAGCGGGTAGTATTGCCTTATTAACGGCGTTGACCTATCCAACGATTTTTAGTCGTGTAGCAA
TGTTAAGTCCACATTCAGATGACAAAGTATTAGATAAGCTAAATCAATGTGCTAATAAAGAACAATTGACAATTTGGCATGTCATTGGTCT
AGATGAAAAAGATTTTACTTTACCAACAAATGGTAAGCGTGCCGATTTCTTAACACCGAATAGAGAATTAGCTGAACAAATTAAGAAATA
TAATATAACTTATTATTACGATGAATTTGAAGGTGGTCACCAATGGAAAGATTGGAAACCATTGCTGTCAGATATATTATTGTATTTTTTA
CGTAAAAACACAGATGATCAACATTATGAGTAA

Gene: UPF0477 (putative protein)

Contig: 06_NODE_54, position: 122832 to 123341, length: 510 nt, orientation: FORWARD

Perfect match to: (RF122-AJ938182-[955880:956389], highly conserved allele)

Sequence:

ATGATTTTAGGATTAGCATTAATTCCATCAAAGTCATTTCAAGAAGCGGTGGATTCTTACCGTAAAAGATATGATAAACAGTATTCACGAA
TTAAACCACATGTGACAATTAAAGCGCCATTTGAAATTGAAGATGGTGATTTAGATTCTGTCATTGAACAGGTTAGAGCTCGTATTAATG
GTATACCAGCAGTAGAAGTTCATGCTACAAAAGCTTCTAGCTTCAAACCAACGAACAATGTGATTTACTTTAAAGTTGCGAAGACGGACG
ACTTAGAAGAATTGTTTAATCGCTTTAATGGAGAAGATTTCTATGGAGAAGCTGAACATGTTTTTGTGCCACACTTTACAATAGCACAAG
GACTATCTAGCCAAGAATTCGAAGATATTTTTGGTCAAGTAGCATTAGCTGGGGTAGACCATAAAGAAATTATCGATGAATTAACTTTGT
TACGTTTTGACGATGACGAAGATAAATGGAAAGTTATTGAAACGTTTAAATTAGCTTAA

Gene: ltaA (glycolipid permease)

Contig: 06_NODE_54, position: 123454 to 124644, length: 1191 nt, orientation: REVERSE

Perfect match to: (GR1-AJLX01000008-[50880:52070:r], allele observed in CC361+CC5+CC25+CC361)

Sequence:

TTACTTAGCTTTTTCTCTATTTGCTATAAAGTAGCTTCCATAAAATACAGCTAAGACTAAAAAGATTAATGCCGAGAAATAAAATGTATTGT
TTAAATTGTTGGTAAATTGTGTAATTAATCCGCCAAATAATGGCCCTATCATTGAGCCGAATCCTTGGATACTATTAAAAACACCCCAAGT
TTCTTCTTGTTCATCTGATTTGATAAATCGTGCCATAAAGGTATTCCATGCTGGTAATAAGATGCCATACATTAGACCGATAGCTAAAGCG
ATAATCCACACGATGTGAATATTAACAATCATAGATAGAGTGAAAATTAATATCATGTATAAAATAAATCCGCTTAGAATAACACCATACA
TAAAGTTTCTGCTGCGGTTATCTATTAGTTTCGATAAAAATAGCATCGAAACTGCACAGCCGATACCACCAATAATGATTGCAACAGTATA

TTCAATTGTACTTACGTTAATAACCTTAGTAGCATATGTTGGTAATATAGGAACTAGGGCAGCAATTGCGGCACCTTGTAAAAGAATACC
AGGGAACAACAATAAATGGCGCTTTGTCACATCAACAATTTGTCTCAATTGAGCTTTAACTGGACGAGTATTATAATTTGTTAACTTTACA
TCGACAAAATAATATAATATCCATGCAATTAAAACGACTAAAGACATCATGAAGGCAAAGCGTGTTGGGTGCACTTTGATAAGTAGATTC
ATAAAAACCATACCTACCAATAGGCCTAACAACCATGAAAAATAAACATAGCCCATTTGTTTGCCACGTTTATCTTCTTCAACACTGGATA
ACATAATGACCCAAATAGGACTAACTGCAATACCGAGCATCATAGCACTAAATATGATTACAAAAGGTGATGCTGGAAACCAAATAACTA
AAAATAAACTTGTAAATGCTAAAATAAATCCAGTCGTTAAAACGATTTTTGTGCCGAATTTTTTCAGTAAAAATCCTATAACAAAGTTTGT
AGATGCATCAGCAATAAAATGTATTGAAAATGCTAGAGACGTTATTGCTACAGCAATGGATGTAACTGTTGGCAAGAAATTAATATAGCT
TAGGATATACATGCCTCTCGCAAATTCCATTAAAAATAAGATAATAAGCATTAAAATGAAATTTTTATGATTAGCGTAATTATTTAACGAA
GAATCTTGCAT

Gene: ugtP (processive diacylglycerol glucosyltransferase)

Contig: 06_NODE_54, position: 124622 to 125797, length: 1176 nt, orientation: REVERSE

Sequence:

TTATTTAACGAAGAATCTTGCATATAAAGGAACCTTTCCATAAATCTCTTGTGGTTGTGATGAATGACCGATTAAATCAAGTAAGTCTCGA
CATATTGTCTGTGTAGCATACTTAATTTTATCTTGTTCCATTGTACTAATCATATTAGTTAATTGCTCATTACCGTTCGTTAAACTTGCTACA
ATTTTTATTGCTTCTTCTGGAGTATCAGCGATTTTACCAAAACCTTTTTCTTCAAAGTAAAGGGCATTTTCAAGCTCTTGACCAGGTGCAGG
ATTTAGGAAAATCATTGGAATACAACGGGCGAAACCTTCAGTTATTGTGATACCACCAGGTTTCGTAATCATAAGTTGACTTGATGCCATC
CATTCATTCATGTGTTTGGTATAACCTAGAATCAATACATTCTCGTTAGATTTAAACTTAGCTGTTAAAGAACGCTTTAGCTCTTTGCTCTTA
CCACAAATCATAACTACTTGTGCATTTGCACTTTTCGCTAATATATCAGTGATCATCGTGTCAAAACCTTTAGATACACCAAATGCACCAGC
TGACATTAAAATAGTTTGCTTATCCGGATCTAAGTTGTTGTCTATTAACCACTGCTTTTGATTAATAGGCGTTTCAAATTTGTTATCAATAG
GAATACCTGTCACTTTAACTGTTGAAGGATCAATACCTACGTCTATGAAGTCTTGTTTCGTTTCTTTTGTTGCCACATAATATCTTGTTGAAT
ACGGCGTAATCCAGTTTTTATGTAAGCGATAGTCTGTCATCACTGTAGCAACTGGAATATTAATGTTAAATTGCTCAGTGAGTACCGACAT
AACTGGTGTAGGAAACGTTAATAATATTAAATCTGGCTTTTCTTTTATCAATAAATTGATTAACTTATTAAGTCCATAGTATTTGTAAAAAC
ATTTGTCTAGTTTATCTGGGCGGCTGTAATAAAACCCTTTGTACATATTTCTAAAATATTTAAAGCTATTGATATACCATTTTTTACAAATAG
AAGTCAAAATTGGATGAGCTTCCATAAATAAATCGTGCTCAATGACGCTTAAATGGTCTAGATTCATATCATTAAGTTGATTAACGATACT
CTGTGTAACTTGCATATGACCGTTACCGAATGAGCCAGTAATAATCAATATCTTTTTATTTTGAGTAACCAT

Gene: murE (UDP-N-acetylmuramoylalanyl-D-glutamate--2,6-diaminopimelate ligase)

Contig: 06_NODE_54, position: 126230 to 127714, length: 1485 nt, orientation: FORWARD

Perfect match to: (MW2-BA000033-[992458:993942], allele observed in CC1+CC45I)

Sequence:

TTGGATGCAAGTACGTTGTTTAAGAAAGTAAAAGTAAAGCGTGTATTGGGTTCTTTAGAACAACAAATAGATGATATCACTACTGATTCA
CGTACAGCGAGAGAAGGTAGCATTTTTGTCGCTTCAGTTGGATATACTGTAGACAGTCATAAGTTCTGTCAAAGTGTAGCTGATCAAGGG
TGTAAGTTGGTAGTGGTCAATAAAGAACAATCATTACCAGCTAACGTAACACAAGTGGTTGTGCCGGACACATTAAGAGTAGCTAGTATT
CTAGCACACACATTATATGATTATCCGAGTCATCAGTTAGTGACATTTGGTGTAACGGGTACAAATGGTAAAACTTCTATTGCGACGATG
ATTCATTTAATTCAAAGAAAGTTACAAAAAAATAGTGCATATTTAGGAACTAATGGTTTCCAAATTAATGAAACAAAGACAAAAGGTGCA
AATACGACACCAGAAACAGTTTCTTTAACTAAGAAAATTAAAGAAGCAGTTGATGCAGGCGCTGAATCCATGACATTAGAAGTATCAAG
CCATGGCTTAGTATTAGGACGACTGCGAGGCGTTGAATTTGACGTTGCAATATTTTCAAATTTAACACAAGACCATTTAGATTTTCATGGC
ACAATGGAAGCATACGGACACGCGAAGTCTTTATTGTTTAGTCAATTAGGTGAAGATTTGTCGAAAGAAAAGTATGTCGTGTTAAACAAT
GACGATTCATTTTCTGAGTATTTAAGAACAGTGACGCCTTATGAAGTATTTAGTTATGGAATTGATGAGGAAGCCCAATTTATGGCTAAA
AATATTCAAGAATCTTTACAAGGTGTCAGCTTTGATTTTGTAACGCCTTTTGGAACTTACTCAGTAAAATCGCCTTATGTTGGTAAGTTTAA
TATTTCTAATATTATGGCGGCAATGATTGCGGTGTGGAGTAAAGGTACATCTTTAGAAACGATTATTAAAGCTGTTGAAAATTTAGAACC
TGTTGAAGGGCGATTAGAAGTTTTAGATCCTTCGTTACCTATTGATTTAATTATCGATTATGCACATACAGCTGATGGTATGAACAAATTA
ATCGATGCAGTACAGCCTTTTGTAAAGCAAAAGTTGATATTTTTAGTTGGTATGGCAGGCGAACGTGATTTAACTAAAACGCCTGAAATG
GGGCGAGTTGCCTGTCGTGCAGATTATGTCATTTTCACACCGGATAATCCGGCAAATGATGACCCGAAAATGTTAACGGCAGAATTAGCC
AAAGGTGCAACACATCAAAACTATATTGAATTTGATGATCGTGCAGAAGGGATAAAACATGCAATTGACATAGCTGAGCCTGGGGATAC
TGTCGTTTTAGCATCAAAAGGAAGAGAACCATATCAAATCATGCCAGGGCATATTAAGGTGCCACATCGAGATGATTTAATTGGCCTTGA
AGCAGCTTACAAAAAGTTCGGTGGTGGCCCTGTTGGTCAATAA

Gene: yueH (putative protein)

Contig: 06_NODE_54, position: 127704 to 127955, length: 252 nt, orientation: FORWARD

Perfect match to: (MW2-BA000033-[993932:994183], allele observed in CC1)

Sequence:

TTGGTCAATAAAAGATTTATTGATGAAGGTAAAACTATTGATGTTTATTTATTCGAAGCATTAAATGACCAGATAATCATTGCTATACCAG
ATTGGTTTTGGTCATATCAGATGGCAATGACATTAGATGAAGAAACTTGTTTTGAAGCAATACTCATGCAATTGTTTGTTTTTAAAGAAGA
GGAAGAGGCAGAATCGATTGCATCACAACTAACAGATTGGATAGAAACATATAAAAAGGAGAAAGACTAA

Gene: prfC (peptide chain release factor 3)

Contig: 06_NODE_54, position: 127955 to 129517, length: 1563 nt, orientation: FORWARD

Perfect match to: (MW2-BA000033-[994183:995745], allele observed in CC1)

Sequence:

ATGAACTTAAAGCAAGAAGTTGAGTCTAGAAAGACTTTTGCGATTATTTCACATCCCGATGCAGGGAAAACAACGTTAACTGAAAAACTA
TTGTACTTCAGTGGTGCCATTCGTGAAGCGGGTACAGTTAAAGGGAAGAAGACTGGTAAATTTGCGACAAGTGACTGGATGAAAGTTGA
ACAAGAACGTGGTATTTCTGTAACAAGTTCAGTAATGCAATTTGATTACGATGATTATAAAATCAATATCTTAGATACACCAGGACATGAA
GACTTTTCAGAAGATACGTATAGAACATTAATGGCAGTTGACAGTGCTGTCATGGTCATAGACTGTGCAAAAGGTATTGAACCACAAACA
TTGAAGTTATTTAAAGTTTGTAAAATGCGTGGTATTCCAATCTTTACATTCATTAATAAATTAGACCGAGTAGGTAAAGAACCATTTGAAT
TATTAGATGAAATCGAAGAGACATTAAATATTGAAACATACCCTATGAATTGGCCAATTGGTATGGGACAAAGTTTCTTTGGCATCATTG
ATAGAAAGTCTAAAACAATTGAACCATTTAGAGATGAAGAAAATATATTACATTTGAATGATGATTTTGAGTTGGAAGAAGATCATGCAA
TTACAAATGATAGTGCTTTTGAACAAGCGATTGAAGAATTAATGTTGGTTGAAGAAGCGGGTGAAGCCTTTGATAATGACGCGCTGTTG
AGTGGAGACTTAACACCTGTATTTTTCGGTTCAGCTTTAGCTAACTTTGGTGTACAAAATTTCTTAAATGCATATGTTGATTTTGCGCCAAT
GCCAAATGCAAGACAAACAAAAGAAGAGGTTGAAGTAAGCCCGTTTGATGATTCATTTTCAGGATTTATCTTTAAAATTCAAGCCAACAT
GGACCCTAAACACCGTGATAGAATTGCATTTATGCGTGTCGTTAGTGGTGCATTTGAACGTGGTATGGATGTTACTTTGCAACGTACTAA
TAAAAAGCAAAAGATCACACGTTCAACGTCATTTATGGCAGACGATAAAGAAACTGTGAATCATGCTGTAGCAGGCGATATCATTGGAC
TATATGATACTGGTAATTATCAAATTGGAGATACTTTAGTTGGTGGAAAACAAACCTACAGTTTCCAAGATTTACCACAATTTACGCCAGA
AATTTTTATGAAAGTTTCTGCTAAAAACGTCATGAAACAGAAGCATTTCCATAAAGGTATTGAACAATTAGTACAAGAAGGTGCGATTCA
ATACTATAAAACATTACACACAAACCAAATTATTTTAGGTGCTGTTGGTCAGTTACAATTTGAAGTTTTCGAACATAGAATGAAAAATGAA
TATAATGTTGATGTTGTTATGGAGCCAGTAGGCCGTAAAATTGCACGTTGGATTGAAAATGAAGACCAAATTACAGATAAGATGAACAC
ATCAAGATCGATTTTAGTGAAAGATAGATATGACGATTTAGTATTCTTATTTGAAAATGAATTTGCAACAAGATGGTTTGAAGAGAAATT
CCCTGAAATTAAATTGTATAGTTTACTTTAA

Gene: terC (putative integral membrane protein, TerC family)

Contig: 06_NODE_54, position: 129819 to 130622, length: 804 nt, orientation: FORWARD

Perfect match to: (GR1-AJLX01000008-[57245:58048], allele observed in CC361+CC25-ST291+CC361)

Sequence:

ATGTTAATGGATCCAAGTTTGATCTTACCTTATTTATGGGTACTTGTCGTTTTAGTATTTTTAGAAGGCTTATTAGCAGCAGATAACGCGAT
TGTTATGGCTGTAATGGTTAAGCACTTACCACCCGAACAACGTAAAAAAGCTTTGTTTTACGGTTTGTTAGGTGCATTTGTATTTAGATTTT
TAGCATTATTCTTAATTAGTATTATCGCGAACTTTTGGTTTATTCAAGCTGCAGGAGCGGTTTACTTAATTTATATGTCAATCAAAAATCTG
TGGCAGTTCTTTAAACACCCAGAAATTGAAAGTCCTGAAGCTGGAGATAATCATCATTATGATGAATCTGGTGAAGAGATTAAAGCAAGT
AACAAATCATTCTGGGGAACTGTGTTGAAAATAGAATTTGCAGATATCGCATTTGCCATTGATTCTATGCTTGCTGCTTTAGCTATTGCTG
TAACACTTCCTAAAGTTGGTATTCACTTTGGTGGTATGGACTTAGGTCAGTTCGTAGTCATGTTCCTAGGTGGAATGATTGGTGTTATTCT
AATGCGTTATGCAGCAACATGGTTTGTAGAGCTATTAAACAAATATCCAGGACTTGAAGGTGCAGCCTTCGCGATCGTTGGTTGGGTAG
GTGTTAAATTAGTTGTCATGGTATTAGCGCACCCAGACATCGCTGTATTGCCTGAGCACTTCCCACATGGCGTATTATGGCAATCTATTTT
CTGGACAGTACTAATTGGATTAGTAATTATCGGTTGGTTAGGTTCAGTTGTTAAAAATAAAAAATCGCATAAATAA

Gene: htrA (serine protease)

Contig: 06_NODE_54, position: 130856 to 133165, length: 2310 nt, orientation: FORWARD

Perfect match to: (JH9-CP000703-[1121759:1124068], allele observed in CC5)

Sequence:

GTGGATATTGGTAAAAAACATGTAATTCCTAAAAGTCAGTACCGACGTAAGCGTCGTGAATTCTTCCACAACGAAGACAGAGAAGAAAA
TTTAAATCAACATCAAGATAAACAAAATATAGATAATACAACATCAAAAAAAGCAGATAAGCAAATACATAAAGATTCAATTGATAAGCA
CGAACGTTTTAAAAATAGTTTATCATCGCATTTAGAACAGAGAAACCGTGATGTTAATGAGAATAAAGCTGAAGAAAGTAAAAGTAATC
AGGATAGTAAGTCAGCATATAACAGAGATCATTATTTAACAGACGATGTATCTAAAAAACAAAATTCATTAGATTCAGTGGACCAAGATA
CAGAGAAATCAAAATATTATGAGCAAAATTCTGAAGCGACTTTATCAACTAAATCAACCGATAAAGTAGAATCAACTGACATGAGAAAGC
TAAGTTCAGATAAAAACAAAGTTGGTCATGAAGAGCAACATGTACTTTCTAAACCTTCAGAACATGATAAAGAGACTAGAATTGATTTTG
AGTCTTCAAGAACTGATTCAGACAGCTCGATGCAGACAGAGAAAATAAAAAAAGACAGTTCAGATGGAAATAAAAGTAGTAATCTGAAA
TCTGAAGTAATATCAGACAAATCAAATACAGTACCAAAATTGTCGGAATCTGATGATGAAGTAAATAATCAGAAGCCATTAACTTTGCCG
GAAGAACAGAAATTGAAAAGACAGCAAAGTCAAAATGAGCAAACAAAAACCTATACATATGGTGATAGCGAACAAAATGACAAGTCTA
ATTATGAAAATGATTTAAGTCATCATATGCCATCGATAAGTGATGATAAAGATAACGTCATGAGAGAAAATCATATTGTTGACGATAATC
CTGATAATGATATCAATACACCATCATTATCAAAAACAGATGACGATCGAAAACTTGATGAAAAAATTCATGTTGAAGATAAACATAAAC
AAAATGCAGACTCGTCTGAAACGGTGGGATATCAAAGTCAGTCAAGTGCATCTCATCGTATCACTGAAAAAAGAAATAATGCTATTAATG
ACCATGATAAATTAAACGGTCAAAAACCAAATGCAAAGACATCGGCAAATAATAATCAAAAAAAGGCTACATCAAAATTGAACAAAGGG
CGCGCTACAAATAATAATTATAGTGACATTTTGAAAAAGTTTTGGATGATGTATTGGCCTAAATTAGTTATTCTAATGGGTATTATTATTCT
AATTGTTATTTTGAATGCTATTTTTAATAATGTGAACAAAAATGATCGCATGAATGATAATAATGATGCAGATGCTCAAAAATATACGACA
ACGATGAAAAATGCCAATAACACAGTTAAATCGGTCGTTACAGTTGAAAATGAAACATCAAAAGATTCATCATTACCTAAAGATAAAGCA
TCTCAAGACGAAGTAGGATCAGGTGTTGTATATAAAAAATCTGGAGATACGTTATATATTGTTACGAATGCACACGTTGTCGGTGATAAA
GAAAATCAAAAAATAACTTTTTCGAATAATAAAAGTGTTGTTGGGAAAGTGCTTGGTAAAGATAAATGGTCAGATTTAGCTGTTGTTAAA
GCGACTTCTTCAGACAGTTCAGTGAAAGAGATAGCTATTGGAGATTCAAATAATTTAGTGTTAGGAGAGCCAATATTAGTCGTAGGTAAT
CCACTTGGTGTAGACTTTAAAGGCACTGTGACAGAAGGTATTATTTCAGGTCTGAACAGAAATGTTCCTATTGATTTCGATAAAGATAAT
AAATATGATATGTTGATGAAAGCTTTCCAAATTGATGCATCAGTAAATCCAGGTAACTCGGGTGGTGCTGTAGTCAATAGAGAAGGAAA
ATTAATTGGTGTAGTTGCAGCTAAAATTAGTATGCCAAACGTTGAAAATATGTCATTTGCAATACCTGTTAATGAAGTACAAAAGATTGTA
AAAGATTTAGAAACAAAAGGTAAAATTGACTATCCCGATGTAGGTGTTAAAATGAAGAATATTGCCAGTCTAAATAGTTTTGAAAGACAA
GCAGTTAAATTGCCAGGAAAAGTTAAGAACGGTGTTGTTGTAGATCAAGTTGACAACAATGGTTTAGCAGATCAATCTGGTCTGAAAAA
AGGTGATGTAATTACTGAATTAGATGGCAAACTTTTAGAAGATGATTTACGCTTTAGGCAGATTATATTTAGTCATAAAGATGACTTGAA
ATCAATTACAGCGAAGATTTATAGAGATGGTAAAGAGAAAGAAATTAATATTAAACTAAAATAA

Gene: ktrD (potassium uptake protein D)

Contig: 06_NODE_54, position: 133182 to 134540, length: 1359 nt, orientation: FORWARD

Perfect match to: (N315-BA000018-[999443:1000801], allele observed in CC5+CC1+CC361)

Sequence:

GTGTCAATTTTTAGCCAGTTTTTAAAAAGATCAAGCCCTCAACAAGGTATTGTATTGTACTATATCGTCGCAATTGTCATTGCATTTTTATT
ATTAAACTTACCGTATGTTCATAAACCAGGTGTAGAAGTAAATCCAATTGACACATTATTTGTTGCCGTATCCGGAATTAGTGTTACTGGA
TTGTCTCCGATAAGTATTGTCGATACCTATTCTACATTTGGACAATTAATTATCCTCGTGATATTAAATATTGGTGGAATTGGCGTCATGGC
AATTGGTACGATGTTATGGGTGGTACTAGGTAAACATATTGGAATTAGAGAACGTCAGTTAATTATGTTAGATAATAACAAAAACACAAT
GAGTGGTACCGTCAAATTGATTATTGATATTGTAAAATCAATATTTGTAATCGAACTCGTAGGAGCCATGTTATTAGCATTTTACTTTTATC
GAGATAATCCAGATTTAAAATATGCAATCATGCAAGGTGTTTTTGTTTCTATTTCTGCCACTACCAATGGTGGATTAGATATTACAGGTAA
GTCATTAATTCCTTATGCACATGATTATTTTGTACAAGCGATAGTTATATTTTTAATAATTTTAGGATCAATCGGCTTCCCAGTATTATTAGA
AGTTAAAGCTTATATTCAAAATAGGGTTACTAATTTTAGATTTTCATTATTTACTAAAATTACGACATCAACATATTTATTCCTATTTATTGT
TGGGGTATTAGCCATTCTATTATTTGAACATAACCATGCGTTCAAAGGATTAAGTTGGCATCAATCGTTATTCTATTCGCTGTTTCAATCAG
CGACTACAAGAAGTGCGGGTCTTCAAACAATTGATGTGACAACACTAAGTGACCCCACTAATATTATCATGGGTATTTTAATGTTTATAG
GATCTTCGCCAAGTTCGGTTGGTGGCGGTATTCGTACAACAACTTTCGCTATTTTAATTTTGTTTTTAATTAACTTTAGTAATAATGCCGAT
AAAACATCCATTAAAGTTTACAATAGAGAAGTACACATTATGGATATTCAACGTTCATTTGCAGTATTTACAATGGCGACAATTTTAACAT
TTTTAGGAATGCTAATTATATCAGCTACTGAAAATGGTAAGCTTACATTTTTACAAGTATTTTTTGAAGTCATGTCTGCATTTGGAACTTGT
GGACTATCGCTTGGTGTCACAAGTGATATTAGTGATATTTCTAAGGTCGTACTAATGATACTCATGTTTATAGGACGTGTTGGCTTAATAT
CATTTATCATTATGATAGCAGGACGTCGAGAACCAGATAAATTCCATTATCCAAAAGAACGTATTCAAATAGGATAA

Gene: Q1XY52 (putative 5'-nucleotidase)

Contig: 06_NODE_54, position: 134676 to 136190, length: 1515 nt, orientation: FORWARD

Perfect match to: (MW2-BA000033-[1000904:1002418], allele observed in CC1+CC239)

Sequence:

ATGATGGAAAAAAATGAAAACATTAATGTAGAGATTTTAACTACGTCAGATATGCATAGTCATTTCTTAAATGGTGATTATGGTTCAAATA
TTTATAGAGCTGGTACTTATGTTAACCAAGTAAGAGCACAAAATCATCGCGTCATTTTATTAGATAGTGGCGGAAGTTTAGCTGGCTCGTT
AGCGGCCTATTATTATGCTATTGTTGCACCTTATAAACGACATCCAATGATAAAGTTAATGAACAGAATGCATTATGATGCTAGCGGTGT
GAGTCCAAGTGAATTCAAGTTTGGTTTATCATTTTTAACTCGTTCAATTGCTTTGGCACGTTTTCCATGGTTATCAGCAAATATTGAATACA
ATGTTACTAAGGAGCCTTATTTTTCAACTCCATATTGTATTAAACATTTTGGTGACTTAAAAATTGCTATCGTAGGCGTTACAGCAGATGGT
TTAATGGAAAATGAGTATTCTGAAATGGAGCAAGATGTATCTATTGAAAAGACATTAGTGGCATCAAAACGATGGATTAGATATATCCAT
GAAGTTGAAGAGCCGGATTTTTTGATTGTAATTTATCATGGTGGATTGAATAAAATTAGTAATAGTACGAAAAATAAAAAGGCAAGTTCG
AATGAAGCTGAAAAATTAATGGAAGAACTCGGTGTTATAGATTTAATGATTACAGCTCATCAGCATCAAACAATAGTCGGTCAAGATCAT
GAAACGTATTATGTTCAGGCTGGTCAAGATGCAAAAGAGCTTGTACATCTTTCGATTAATTTTAAAAAGAGAACAACAACTTATGATGTT
GAAAGCATTGATTCTAAAGTGATTGACTTAAATGAGTATGAAGAGGACCAAGAATTATTAGATTTAACATTCTATGATAGAAAAGCAGTG
GCTTATTGGTCACAGGAAATCATAAGTGATAAAGGTTTGATGTTATCAGTAAATGGGTTACAAGATTTAGTCTGTCAAACACATCCATTTT
CACAATTATTACATGATGCAATTCACCTTGCATTTGATAATGATATAACATGTGTCCACGTGCCTATGAACGGAGAGAAGGGGTTGAGTG
GACAGATTCGAAATGAAGATTTGTATCATGCATACCCATATCCAGATAAGCCAATGGATATGACAATTAGTGGTCAAAATATCAAAGATA
TATTGGAGTATAGTTATTCACATTTAGATTTTGTTAACGAGCAATTAAGCTTAACAATTATTGATGAAACGTTATGTACAATGTGGCAAGG
ATTCAATTATGAGATTGATATGAATCAAGAACCTGGGCAACGAGTAATGTTAGATCAAATTGATTTGAGTAAGAGTTATAGAGTTACAAT
GACTGACTATTGTTATCGTAACTACAAGAATTATTTAAAAAATGCTATTATACATGAATCATACGATGAAACAATGAGTACATTAATTGCA
GAGAAGTTAAGAGATCCGAATTATCATATTTCATGTAGTGATAATTTTATAGTTAAAAACAGGTAA

Gene: comK (putative competence transcription factor)

Contig: 06_NODE_54, position: 136677 to 137246, length: 570 nt, orientation: REVERSE

Perfect match to: (N315-BA000018-[1002938:1003507:r], highly conserved allele)

Sequence:

TTATTTATCTTCAAATAAAGAATAGCGTGTCAATGCTTCAAACACATTCAATGTGGCTTTATTATAATCTATTGGTTGGTCGGGATTTTTTG
ATATCATGCGAGATTGTTTATCTACCATGTAATAGTAAAAAATGGAATTGTTATATTGATGCCATAAACTGTGGTATGAAACATGAAGAAT
GATTGATTCATTATTAATAAATGTCACTTTACATTTACGATTTTTTAATTCTTTAATACTTTCGATATAATGCATATTTAACCAAATATTTTCA
TTTTGTCTGTCAGAATGTGTTGGGAAAAAATAAGTTGGAAATAATGGTGTTAGTAAAATAGGTGGTTTACTAGAAATGCCAGTAATGCG
GTTTGTCTCTGCTTTCTTGCCAAGATAAGTATTACCATAAAATTTGCAAGAGCGTTCAATGATTTTCTGGACTTTAAAAGGATTTTGAATAC
GCGTTTTGTCAAACCGAATTATTTCACTACCGTTTCTTTGATCGTCATCATCAAATGCTGGTCGAATAACCATGTCTCCTTTGCGTATCACAT
AAATATTTTGAGAATACAT

Gene: Q6GI58 (putative protein)

Contig: 06_NODE_54, position: 137447 to 137674, length: 228 nt, orientation: FORWARD

Perfect match to: (RF122-AJ938182-[970507:970734], highly conserved allele)

Sequence:

GTGGTTTCTATGAAATACAATACTAATGTTAAACATACAACTTTAGAAGCGTTTGTCACAACCGTCAATGATTTGGGTATTGAATTAATTA
TCAATGAAGCACTTCGAGAGGTAAGAAAACGACAGCTCATAGAACTTATAGATGACGCACTCGTCAATAAAGATGAAGCAGCATTTAAT
CAATATACGGCAGAATACAAAAATTTGGAGGCATTTCTCGGTGAATAA

Gene: lplA2 (lipoate-protein ligase A, locus 2)

Contig: 06_NODE_54, position: 137755 to 138741, length: 987 nt, orientation: REVERSE

Perfect match to: (MW2-BA000033-[1003983:1004969:r], allele observed in CC1+CC30+CC121+CC130+CC133)

Sequence:

TTATGACATTAATCTAATTAATTCATGTCTATCTATGTCACCGAAATAGTGATATAAATCATATTCTGATAGTGCTTCTTCTATGTGCTCAAA
GTCGTGTAGACAGCCAACTAATGCATTTTCAAGATCAGTGACATCTCCGACACCAAAGAAATCACCGAAAATTTTTGCATGTTCGATTTTA
CCTCGTTTAACATCAAACTTAATTTGTACAAATCCTTTTTCAAATTTTTCCTCGCGTTCAAAGTTATATTTAGGGTTTCTACCATAATTCCATT
CCCAAGTTCTATATTTGTCGTTACTTAACTTTTCAATATTTTCCCAATCTTCATCCGTTAATTTATATTCTTCTACTTCAGTTTCTCCAAAGATA
GTTTTCAAGATGATTTTTTTAAATTCTTCAATTTCTAAAGGGTCATTTAAAAATTCTTGAATGTTTGCTACTCGTTTACGAACAGATTTAATA
CCTTTTGATTTAATCTTAGCTGGATTAACTTTTAGTGCATTCTGAACTTCGTCTAAATCACTATTTAACATCAACGTACCATGACTAAACATT

CTATTTTTAACTTTAACCATGGCATTCCCTGAGATTTTCGCTTGTCCAACTTGAATATCGTTACGACCAGTTAATTCAGCATTGACGCCTAA
AGATTGTAATGCTTGAACAATTGGTTCAGTGAATTTTTGGAAATTGTGGAAACTGTTACCATCATCATCTGTTATGAAACTAAAGTTTAAA
TTGCCAGTATCATGATAAACAGCGCCACCACCAGAAATTCTTCTTACTACATCGATGTTATGAGCATCTATATATGTCTGATTTACTTCCTC
TATCGTATTTTGATTCTTTCCAACAATGATAGATGGTCTATTTATGTAAAATAAAAAGTAACTTTCTTCTGCTGGTAAATTTTTTAAAACATA
TTCTTCCATTGCTAAGTTTAAAGTTGGATCTGTAATATTATTATTACTAATGAATTTCAT

Gene: Q6GI56 (putative protein)

Contig: 06_NODE_54, position: 138940 to 139116, length: 177 nt, orientation: FORWARD

Perfect match to: (RF122-AJ938182-[972000:972176], highly conserved allele)

Sequence:

ATGACTGTTGCAGAAGTGGGTAACATTGTTGAGTTTATGGATGGATTAAGAGGTCGTGTTGAAAAAATCAACGATAACTCTGTTATTGTT
GACTTAACAATTATGGAAAATTTTAATGACCTTGATTTACCGGAAAAAACTGTTATCAATCATAAACGATATAAGATTGTTGAATAA

Gene: Q5HH56 (putative protein)

Contig: 06_NODE_54, position: 139131 to 139733, length: 603 nt, orientation: FORWARD

Perfect match to: (MW2-BA000033-[1005359:1005961], allele observed in CC1+CC7)

Sequence:

ATGAATAAAATCTCGAAGGCTTTAACTTGGTTTATTATAAGTTTCATTATATTTCATCTCATATTATTTATTATGTGGGGCGAACACCAAGA
ATACTGGTATTTATATACAGGTATAATGCTAATTGCTGGTATCAGTTATGTATTTTTATCAAAGAGATATTGAATCTAAGCGGTTGCTTACAT
CAATTGGTGTTGGTATTATTACAGCAATTATTTTAATTATGCTTCAACTTTTATTCTCACTTATAACTTCTAATTTAAGTTATAGTTCATTAAT
TAAAGAATTAGCAAGAACAGGTGTCAATTGGAAGTGGCAAATGTTAGTAACTTTACTTTTTGTCATTCCATGTCACGAGTTATATATGAG
AACTGTTTTACAAAAAGAATTAACACATTTTTCAATACCGAAATGGGTAGCTATATTAATAACTGCAATTTGTTCTAGTTCATTATTTATTT
ATTTAGATAATTGGTGGATAGTAACATTTATATTTGTTGCACAAGTGATATTATCATTAAGTTATGAGTATACTCGACGTATAGCAACAAC
ATCCGTTGCACAAATTGTAGCTATTATATTGTTATTAATTTTTAATGCTTAA

Gene: rli28-L1 (Listeria sRNA rli28 homolog)

Contig: 06_NODE_54, position: 139812 to 140017, length: 206 nt

Sequence:

TTCATTATAGTGGTAATATTTTATAAAAATAGTATAATATTTTGTGGGCAAGTTGAATTGATGGTGGCTATCTGAGTAAAGGGGGGTGGT
GCCTATGGCATTACTTAACACTTTTGAAAGGAAAAGCCTATTGTGATATCTATTGCAAACGCATTACATTTAATGTTAAGTTTCGGTATGTT
TATCGTCACTTTCATTGGTATAGT

Gene: txpA-var1 (toxin involved in plasmid maintenance)

Contig: 06_NODE_54, position: 139944 to 140051, length: 108 nt, orientation: FORWARD

Perfect match to: (N315-BA000018-[1006264:1006371], highly conserved allele)

Sequence:

GTGATATCTATTGCAAACGCATTACATTTAATGTTAAGTTTCGGTATGTTTATCGTCACTTTCATTGGTATAGTAGTAGCAATAATAAATTT
AAGCAATAAAAAATAA

Gene: Q7A194 (putative exported bacteriocin)

Contig: 06_NODE_54, position: 140678 to 140973, length: 296 nt, orientation: TRNC-FRWD (no start codon)

Perfect match to: (N315-BA000018-[1006998:1007293], highly conserved allele)

Sequence:

AGGTGTTTTTATAGAAGAATAAAATTTTTGGCACTATATTATATTTAACTTTAGCACTTGGATTATCAACAGCAGCTTATGCATCTACAGAA
TACGCAGAAGGAGGCACTTGGAGTCACGGTGTCGGCAGTAAGTATGTTTGGTCTTATTATTATCATGGTCATAAAGGACATGGTGCAAC
AGCTATTGGAAAATATAGATCATTTAGTGGTTATACAAGAGCTGGTGTAAAAGCAAAAGCATCAGCTACTAAACATAATTGCTGGGTCAA
TAGAGCGTATTATAACATTTATTAA

Gene: yujE (putative bacteriocin-associated integral membrane protein)

Contig: 06_NODE_54, position: 141017 to 142980, length: 1964 nt, orientation: FORWARD

Perfect match to: (ECT-R2-FR714927-[965761:967725], allele observed in CC5)

Sequence:

ATGAAATGGTTCAAATTAATACTTGATGTTACAACCTTCATTCTGATTGCTATATTGTTATTTGTTTATACATATAAAGAAAACGAAGAAAT
ATTGCCTGATACTAAATACCCTATAGCGGTAACTGACTGGAATAAAAAATATAGTAAAAATGAGATTTATAAACGTATAAATCAATTCGCT
AAAAATGAGAACGTAGCAATCTATAAATCAACTTCAAATTATACAAACAAAAACGTAGATAAAGATATATATGTATTTAATAAATCAAAA
GCAACAACTATCACTCCTTTTAACGCTAAATATAACATTCATTATTTAAGTGACGACGAATTATTAAAAAAGATATCAAAGGAAGTTATTTT
GTAAAAGACAAAAATTTTGACGTGTCTAAATTCATAAATTTTTTAAAAGAATATGGTGTTACTGCTGAATCATACAAAATAGATCATATGA
TGATTGCCGTTGGTGTCATTAAACAAATGAATATAGAAGTTCCGTTATCCGCACTTTTAATCGTTTATTTTATTTATTATATATTTTCGAAAAG
AACATTAATTTCAAAGCGTATGCGATTAAGTATTTAAATGGTTTTACATTAAGAAAAATAATTTTTGAAAATTTTTCAAAAAAATGCACGT
ATTGGGTAACGTTAATCATAAGTCAAATTCTTTTAACTACAAGTGTACTTTGGATATTAAATTACACGGGTAATTTAGATTTATTCATATTA
AGATTAGTTCTGCTTTCATGTCTTTTCATTTTAACGATTAGTGTTATTAATTTATGGACTTTCTTAATGTTACTAAATTTAAATATTGCTAATA
TGATTAAAGGTAAGCAGCACTTTAAAACAATTCGTTTTATTAATACAGTTTGTAAAAGTATTCTCTTAGTACTAATAGCTAATGTAATGATA
GAAAATACTAGTGTTATTAAAGATTTGAATAAAATAAAAGAAACTGAGAAATATTGGAATGTATTAGATGATTATTACACGATTGAATTT
GCACCTTATCACGAAACAAAACAAAGTTTGATTGATAATATGGTGCGATCAGAACAATTAGTAAAGGCTAGTGAAGCAGAAAATAATGC
GATTTTATTCAAACCAAAGGGTGACTCCGTTGACAATGACAACTTTTCACCTGATGAGGGGAATGTAATATTAGTAAATAATCAGTTTTG
GTCGATTTATCACAAGCAGTTTCAACCTGATATTCCAATAAAAAATCAAAAAAATAATGTCGAAGTAATTATTCCACAAAAGTTTCATGCA
ATGCGTAATGAAATCAATCAAGCATATCATTCATGGTTTGAATTTGTACAAAATAAAAATAATAAAGAGAGTAAGTTATCTATACAGTTTA
TCAACAAAAATGATTATCGAATTTTTACATTTGATGCACGAGATAGTCGCCATTTGTCATTTATAGAGGCGCCAATCATTGTGAATGTTCA
GGCATCAGATTTATCGAATGATTTTTATTATGCCATGATCAGTCAAGGCGGGTATTTATTCAAAAATTATGACGCGCTAGTAAAAAATATT
GAAAAGTATCATCTTGATGGGGAAATCAGTGGAATAACCAATTATAAAGATAGCGTGATGGAAATGTATCATGAAAACAATTTGAAATT
AACAGTACTCAACTTTTCACAAATCATTATCGCAATCATTTTAATAATTATTATTTTATTTGATGTGAAATATTATTTTGAACAACATCGAAA
ATTACTCGTAATCAAAAAGCTATTTGGTTATTCAACATTAAGAGCCAATTACCAATACTTATTAATAAATAATATAGTTGTTGTTTTTATTG
GAATATTGACGAATGTAATTTTACATTCTCAGTATATAATGATGATATTTGCAACGATTATTGTTGTTCAAATATTATTGCAGATTTGCAGC
CTATACTATCATGGCCGACGTTTTAATGAAGTTATCAAGGAGTTTTAA

Gene: yxeA (putative protein)

Contig: 06_NODE_54, position: 142983 to 143303, length: 321 nt, orientation: FORWARD

Perfect match to: (MW2-BA000033-[1009211:1009531], allele observed in CC1)

Sequence:

ATGAAATATATCATTGCAATATTATTAGGATTAATCGTATCCATTACTATTGCTTTTACAATCATACATCATCCTATACTTGATCGTTTTAAT
CCTTTCTTAAAAACGGAGTATAGTTATGCCAAAGTGCCAAAAGGTACGCAACAATATGTTAATATTACGGCTTATAGTGAAAGAGGGGA
AAAGCTTGATTATAAATTAACATTTAATGGATTTTCACCTAGTAGAACGTATGTCGAAATAAAGCATAAAGGGCAATATGTCATATCGATC
ACATATGTTGAAAAAGAGGATACACCAAAAGAGGTAAGACAAGAATGA

Gene: yujD (ABC transporter, ATP-binding protein)

Contig: 06_NODE_54, position: 143300 to 143941, length: 642 nt, orientation: FORWARD

Perfect match to: (MW2-BA000033-[1009528:1010169], allele observed in CC1+CC12)

Sequence:

ATGATAGAATTAAAAGATTTAACCATACAAAAAGGTAATACACATATTTTGAAGAAACTTAATTTGAAATTTCAATGTGGAAAATCATATG
CACTTATTGGTAAAAGTGGATGCGGTAAATCTACATTATTAAATACTATTGCTGGACTTGAAAAAACGGGGAAACAATATGTCTATTTTAA
TGGTCAATTAGAACAATTTAAATCTAATTTTTACAGAGATAAATTAGGATATTTATTTCAAAATTATGGATTAATCGATAATTTGACAGTAA
ATGAAAATTTAGATATTGGATTAGCATATAAAAAAATAAGTAAGAAAGAAAAAGAACAATTAAAGATACGTTATATAGAACAGTTTGGT
CTGTCAAACAGTTTAAAAAGAAAAGTTCATACGCTAAGTGGAGGTGAACAACAACGTGTCGCTTTAATTAGAATGATGTTAAAAGATCCG
ATTGTTATGTTAGCTGATGAACCAACGGGTGCGTTAGATCCTAAAACAGGACAGATGATTATTCAATCATTATTTGATTTGGTCGATGAA
AATAAAGTGCTGATTTTAGCAACACATGATATGGCTATTGCAAATCAATGTGACGAAATAATAGATTTAGAACAGTATAGAAAAGTAGCA
TCTATGTGA

Gene: Q6GI49 (putative membrane protein)

Contig: 06_NODE_54, position: 144029 to 144319, length: 291 nt, orientation: REVERSE

Perfect match to: (Strain_10388-HE579059-[988975:989265:r], allele observed in CC5+CC772)

Sequence:

TTATGAAAGTAACAACGGCAAACTGAAAAAAAGCAATATAATAATATTTAATACGATTAGTAATAAATGTAATTGATTTTTCGTTTTAATA
TAGCAAAAAACAATGACTAAAGATAACAACAATATCATGATTGCACCTGGGAAGAACCATAATGCTGAAGCTGTGTACCAAATCATTGGT
AACGCCATAATAATAAAAGATATAAGACAAGAAAGGATACCAATAATATTATAAGTTCTAGTCAAAATTACACCTCCAATTTTCATTTTAA
AATGACATGCGTAAAACAA

Gene: Q6GI48 (putative protein)

Contig: 06_NODE_54, position: 144356 to 144463, length: 108 nt, orientation: TRNC-FRWD (no start codon)

Perfect match to: (MW2-BA000033-[1010584:1010691], allele observed in CC1)

Sequence:

GCGGTAGAAGAATTTGAAATTAAATTGAATTGGCTTATCTTTCGTAAACATAATTATGACGTAACAATCCTTATAAGTGGAACGAGTGTA
GTTATACCATCTGTATAG

Gene: Q6GI47 (putative protein)

Contig: 06_NODE_54, position: 144660 to 144947, length: 288 nt, orientation: FORWARD

Perfect match to: (MW2-BA000033-[1010888:1011175], allele observed in CC1)

Sequence:

ATGGAAAAGGGGATTTTCAATTACGATAATGCGAATGTATTAAAATTAGATACTAATCAGTTAAATGAAAACATAAAAGTCATTGACGAT
ATATTTAAAAATTATGAGCAGATAGATCCAACTATAGAAATCGAAAATGGAAATACAAAATTAAAATTAAATGGATATTTTATTGCGTCA
ATTATAAGCCCATTAAATTTAAATAAACTTAATAATTTATATGTTGAAGAAGAATTTTATCATACATATAACGAATTAATTGTTAAATATAC
TGAGGTAAAGGAATAA

Gene: tarM (alpha-GlcNAc appending glycosyltransferase)

Contig: 06_NODE_54, position: 144953 to 146434, length: 1482 nt, orientation: FORWARD

Perfect match to: (MW2-BA000033-[1011181:1012662], allele observed in CC1)

Sequence:

ATGAAAAAAATATTTATGATGGTACATGAGTTAGATGTCAATAAAGGTGGTATGACCTCTTCGATGTTCAATAGAAGTAAAGAGTTTTAT
GATGCGGACATACCTGCTGATATTGTTACTTTCGATTACAAAGGAAACTATGATGAAATTATTAAAGCTTTGAAAAAACAAGGTAAAATG
GATCGAAGAACGAAAATGTATAATGTATTTGAATATTTTAAACAAATTTCAAATAATAAACATTTTAAGTCTAATAAATTGTTATATAAAC
ATATTTCAGAAAGACTAAAAAATACGATTGAAATTGAAGAGAGTAAAGGTATTTCAAGATATTTTGATATAACGACTGGTACATATATTG
CCTACATTAGAAAAAGTAAATCTGAAAAAGTGATTGATTTCTTTAAAGATAATAAACGAATTGAACGGTTTAGTTTTATAGATAATAAAGT
GCATATGAAGGAAACATTTAATGTAGATAATAAAGTTTGTTATCAAGTATTTTATGATGAAAAGGGATACCCATATATTTCAAGGAATATT

AATGCTAATAATGGTGCTGTAGGTAAAACTTATGTGTTAGTTAATAAAAAAGAATTTAAAAACAATTTAGCACTGTGTGTTTACTATTTAG
AAAAACTAATAAAAGATTCTAAAGATAGTATTATGATTTGTGATGGACCAGGGAGTTTTCCAAAAATGTTTAATACAAATCATAAAAATG
CTCAGAAATATGGCGTTATTCATGTTAATCATCATGAAAATTTCGATGATACGGGTGCATTTAAAAAAAGTGAGAAATATATTATTGAGA
ATGCGAATAAAATTAACGGTGTAATTGTATTAACAGAGGCACAAAGATTAGATATTCTTAATCAATTTGATGTAGAAAATATTTTCACTAT
TAGCAATTTTGTTAAGATACATAATGCTCCAAAACATTTTCAAACTGAAAAAATCGTAGGTCATATTTCTAGAATGGTACCAACGAAGCGA
ATTGATTTGCTTATTGAAGTGGCTGAGTTAGTCGTAAAAAAAGATAATTCTGTTAAATTTCATATATATGGAGAAGGATCTGTCAAAGAT
AAAATAGCTAAAATGATTGAAGATAAAAATTTAGAAAGAAATGTTTTTCTTAAAGGATATACAACAACTCCACAAAAATGCTTGGAAGAT
TTTAAATTAGTCGTTTCTACATCTCAATATGAAGGTCAAGGGTTAAGTATGATAGAAGCAATGATTTCTAAAAGGCCTGTTGTTGCCTTTG
ACATCAAATACGGACCAAGTGATTTTATAGAAGATAATAAAAATGGTTATTTAATAGAAAACCATAATATTAAGGACATGGCTGATAAAA
TACTTCAGCTTGTTAATAATGATGTATTAGCAGCGGAGTTTGGTTCGAAAGCGAGAGAAAACATTATAGAAAAATATTCAACGGAATCAA
TATTAGAAAAATGGTTAAATCTTTTCAATAGCTAA

Gene: Q6GI46 (putative membrane protein)

Contig: 06_NODE_54, position: 146523 to 146879, length: 357 nt, orientation: REVERSE

Perfect match to: (N315-BA000018-[1011007:1011363:r], highly conserved allele)

Sequence:

TTAATAAAATTGTAGCCATTTCTTCCAGCTGTAACTACTAATACAAGCTAAGTTCATTACGATATGTATGATTTCAAATTCATAACCACTTA
CATATCCTCTTGTAAGTTTAAGTGTGAAGATTGAGCCTAACATAACACAAATATAAAAAACATTAACATAAGGCGTTAAAAAGCCGATAA
TAAGTGCTAAACCTCCAACAACTTCACCAATTGACATCAATACTGCGATGGTAGGTGGTAAACCTAAAGAGCCTAAGAAGTGAATTGTTC
CATCTAAATCAACAAATTTCAAAGTACCATGCACTAAGAATGATGTTGCTAACATCCATCTAATTAAAAGCATACCTAATTTCAT

Gene: Q5HH47 (ABC transporter, substrate-binding protein)

Contig: 06_NODE_54, position: 147367 to 148326, length: 960 nt, orientation: FORWARD

Perfect match to: (MW2-BA000033-[1013595:1014554], allele observed in CC1+CC130)

Sequence:

GTGAATAGGAATATCGTTAAACTAGTTGTGTTTATGCTAATCTTAGTTGTAGCAGTAGCGGGTTGTGGTCAAAAAGATACTGAAGAGAA
AACTGAAATGACGACAATAAAAGATGAATTAGGAACTGAAAAAATTAAGAAAAATCCTAAACGTGTTGTTGTATTAGAATATAGTTTTGC
TGATTATTTAGCAGCATTAGATATGAAACCTGTTGGTATTGCAGATGATGGCAGCAGTAAAAATATAACAAAGTCAGTAAGAGATAAGA
TTGGGGCATATGAATCGGTTGGATCTAGACCGCAACCGAATATGGAAGTGATAAGTAAATTAAAACCGGATTTGATCATTGCAGATGTT
AGCAGACATAAGAAAATCAAATCAGAATTGAGCAAAATTGCGCCGACAATTATGTTAGTCAGTGGTACGGGAGATTACAATGCAAATAT
TGAAGCATTTAAAACAGTCGCTAAAGCAGTTGGCAAAGAGAAAGAAGGCGAGAAGCGTCTGGAAAAGCATGATAAAAATATTAGCGGAG
ATTAGAAAGAAAATTGAACAGAGTACGTTAAAATCTGCATTTGCATTCGGTATCTCAAGAGCAGGTATGTTTATTAATAATGAAGATACA
TTTATGGGACAATTCTTACTTAAAATGGGTATTCAACCTGAAGTCACAAAAGACAAAACTACGCATGTTGGTGAACGCAAGGGTGGTCCT
TATATATATTTAAATAATGAAGAACTTGCCAATATCAATCCAAAAGTTATGATTTTAGCCACTGACGGGAAAACGGACAAAAATAGAACG
AAATTCATTGATCCTGCAGTTTGGAAATCATTAAAAGCTGTGAAAGATAACAAAGTTTATGACGTTGACCGAAATAAGTGGTTGAAATCA
AGGGGGATTATCGCAAGTGAAAGTATGGCAGAAGATTTAGAAAAAATTGCAGAAAAAGCAAAATAA

Gene: sbrA (sigma-B regulated small RNA A)

Contig: 06_NODE_54, position: 148321 to 148517, length: 197 nt

Sequence:

AAATAAAAATACAGCGCTACTCGTAAATCATATAAGAGTGGCGCTGTATTTTTAATTATGTTTATTATCTGTCGGATGTGATGATTTACCT
GAAAGTTTATTTCGAATAAATTTAATTACATAACCGACAAGGATTGTTTTAACAATTCTTTTAATGAATTGGCGCATCGTTACATACCTCAT
TTCTCTATATCTTA

Gene: shpA (small basic peptide)

Contig: 06_NODE_54, position: 148372 to 148488, length: 117 nt, orientation: REVERSE

Perfect match to: (RF122-AJ938182-[982280:982396:r], highly conserved allele)

Sequence:

TTAATTATGTTTATTATCTGTCGGATGTGATGATTTACCTGAAAGTTTATTTCGAATAAATTTAATTACATAACCGACAAGGATTGTTTTAA
CAATTCTTTTAATGAATTGGCGCAT

Gene: Q7A191 (short two-transmembrane helix protein)

Contig: 06_NODE_54, position: 148567 to 148782, length: 216 nt, orientation: REVERSE

Perfect match to: (MW2-BA000033-[1014795:1015010:r], highly conserved allele)

Sequence:

TTAATTTTTAGGGAATATAATATAACCATCTTTATCTGCTTTTTTAGTAAAAATGACAAAAATGGCATGTATTATTGAGATGATGGTAGGG
ATACCTGTCCAGAAAAATAATAAGTGAAAAAGACCTTGTCCAAATTTATCAGCATAAAATTTATGAATACCTAAACCTCCAAGAAATAATG
CAACAATAACATAAATGGCTTTATTGACTTTCAT

Gene: Q6GAH2 (GNAT family acetyltransferase)

Contig: 06_NODE_54, position: 148947 to 149498, length: 552 nt, orientation: FORWARD

Perfect match to: (MW2-BA000033-[1015175:1015726], allele observed in CC1)

Sequence:

GTGATACGTCAAGCACGTCCAGAGGACCGATTTGATATTGCGAAGTTAGTTTATATGGTGTGGGATGATATGGAATTAGAATTGGTAAA
GCATCTACCTAAAGACATGGTATTAGATGCAATTGAAAAAAGCTGTGTTGATGCAACATATCGAACTTTTTATCAGCATATTTTAGTTTAT
GAAGTAGAAAGTAAAGTAGCAGGTTGTATTATTAGCTATAGCGGTGAAAATGAATTGAAATATGAAAAAGCATGGGAACTACTTGACTT
GCCAGAAGAAATAAAACAATATGGCACGCCATTACCTGTAAAAGAAGCTAAAGACGATGAGTATTATATAGAAACAATTGCGACATTTG
AAGCATATAGAGGTAGAGGCATCGCGACAAAGTTATTAACGTCATTACTTGAATCAAGTACACATGTTAAATGGAGTTTGAATTGCGATA
TTAATAATGAAGCAGCATTAAAGTTATATAAAAAAGTAGGCTTTATATCTGATGGACAGATTGAATTATACAAGCACATGTATCATCATTT
AATTGTTAAATAA

Gene: menA (1,4-dihydroxy-2-naphthoate octaprenyltransferase)

Contig: 06_NODE_54, position: 149549 to 150487, length: 939 nt, orientation: REVERSE

Perfect match to: (N315-BA000018-[1014034:1014972:r], highly conserved allele)

Sequence:

TTAAATGCCTGCAAATAATGCACTAATATAAATACCTAATGCATATAATAAACCGAAAAATGTATTTGTTTTACCAGCAGCAGCCATTGCT
GGCATCATTGTAGGCGGTGTATCATTCTTCTTGAAACGTCTGATAACTTTAACAGGCATTGGGAATGATAACAACGCAAGTAAGTAAAAT
AATGAGCCACCAGGTTTAATAATGATCGTAAGTACAATAAAGGCATAAGCGATAAAGTACATGATTGCCATAAATGTTAAAGAAGCATTT
TTACCTAATAGAATGGGTAAAGTTTTGCGACCACTTGCTTTATCTTTGACACGGTCGCGAATATTGTTAGCCATATTAATTAAACCGATAG
TGATTACTATAGGTACACTTAACCAAATTACATAACTTTGAATATTGCCAGTTTGAATAAAGAATGCAATAACGATAATAAACATACCCAT
AAATACGCCTGAGAATAATTCACCGAAAGGCGTCCATGAAATAGGGAAAGGGCCACCTGTATATAGGTAACCAACAGCCATACATACTA
ATCCAACTGGTAATAACCAAAATGAAGAGTTAGCAGCTAAAAACAAACCTAATATTGCTGCTAAGATGTAAAATGCAATGGCTAATCGTA
GCACAAGCTCTGGGCTCATACCGTTGCGAACAATGGCACCACCAATGCCTACAGATTCATGATCATCAAGGCCTTTTTTATAATCATAGTA
TTCATTAAACATATTAGTTGCTGCTTGAATAAGTAAGCATGCTAGTAACATGGCAATGAATAGGCTGATTTTAATATGATCTTCGCTACCA
AGAAAATATATTTTAGATGCTGCTGTACCAACTAAAACGGGTACTACGGAAGCAGTTAATGTATGAGGACGCATTAAATGCCAATATTTC
TTAACTGTAGAATATTGCTGATATTGATTACTCAT

Gene: menF (putative menaquinone-specific isochorismate synthase)

Contig: 06_NODE_54, position: 150669 to 152030, length: 1362 nt, orientation: FORWARD

Perfect match to: (N315-BA000018-[1015154:1016515], allele observed in CC5+CC1+CC361)

Sequence:

ATGGCTACGGGCGTATTAGAGGACGATATTGTCAAAGAGATATATGGCAGCTCAAAGGAATGGGTTTCAGTTGAAGTGAAATTATCACA
GTCACTAGACCCGAGCACATTATTTCATCTCACTGACAATGAGGCAGGAGATCGCTTTTATATGCGTTTGAATGATAATCGAACGTCATAT
TTTGGCTACAAAGCAATTCAATTATTCAAAAATAATTCTAAAAATAAACAATCTATTTTTAAAGACTGGGAAAAATTAAAACATAACATCA
CATTTATACATCCGCAATCTGAGAAACATCATCTTCGAGTTGTTGGAGGATTCCAATTTTCGAGTCATAAATCAGATGATGAATGGCGAG
AGTTTGGACTAAATCATTTTGTATTACCTGAAGTTTTAATTTCAACTGATAATAATGGGACATTTTTAACTTATACAGTTAAAAGGGAAAG
TTTTACTGTTGAGGCATTGAACGATTTAATGGATTTGTTCAACAATATATCGGACATAGATGTGGACGAGCAAATTGGGGAAATTACTAG
AAATGAAGATATTTATAAAGATGACTGGCGTCAACTTGTAGTAGAAGCTATAGAATCTATTAATAATGAAGAAAAAAATTGTACTAGCACG
TAGACGGTTAATAAAGTTCGATAAAGATATCAGTATTCCATATATTCTAAAGCAAGCATATTCTAAAGAAAAAAACAGTTATATATTCTTG
TTAGAATCACAAGATTCTATATTCTTTTCACAAACACCTGAACAATTAATAAAGGTCAATAATAAAATACTATCGACTAAAGCTGTAGCAG
GTACAATTAAACGTTCACAAGATGAGGACGAAGATACAAAAAATGTTGAAGCATTTTTAAAAGATAATAAAAACTTAATCGAACATCGAT
TTGTTGTTGACAGTATTTTACATGATATTAAACCTTATATCACTGAATTACATTATGATAAGACGCCTAAAATTCTAAAAAATGATCATTTA
TATCACTTGTACACTGAAATAAAGGCGCCACTGAAGGATGATTCGTATATTAGTTTAATTGATAATTTACATCCAACACCTGCTTTAGGTG
GCTATCCAAAAGAATTTGCGATGGATTTTATTGAACAGAAAGAATTTGGTACACGAGGATTATATGGTGCGCCGGTTGGCTATATAGATA
TATATGATGATTGTGAATTTATTGTTGCAATTCGTTCGATGCTTATTAAGAAAGCACAAGCAACTTTATTTGCTGGGTGTGGCATTGTTAA
AGATTCTGATCCAGATAGTGAATTGGCAGAAACGAACCTTAAGTTCACACCTATGATGAATGCATTAGGAGTCGATATGAATGGGAAAT
CATAA

Gene: menD (2-succinyl-6-hydroxy-2, 4-cyclohexadiene-1-carboxylic acid synthase/2- oxoglutarate decarboxylase)

Contig: 06_NODE_54, position: 152017 to 153690, length: 1674 nt, orientation: FORWARD

Sequence:

ATGGGAAATCATAAAGCAGCTTTAACGAAGCAAGTTTTTACATTTGCATCTGAGTTATATGCGTACGGCGTAAGGGAAATAGTTATCAGT
CCGGGATCACGCTCAACGCCACTTGCACTTGCATTTGAAGCACATCCAAATATTAAAACATGGATACACCCCGATGAGCGAAGTGCAGCA
TTTTTTGCAGTTGGGTTAATTAAAGGTAGTGAAAGACCTGTCGCTATATTATGTACGTCAGGTACAGCAGCAGCGAATTATACGCCTGCA
ATTGCTGAAAGCCAAATTAGTAGAATTCCATTAATCGTTTTAACAAGTGACCGTCCGCATGAATTAAGAAGTGTAGGCGCACCACAAGCG
ATTAATCAAGTAAATATGTTTAATAATTATGTAAGTTATGAGTTCGATATGCCTATTGCGGATGATAGTAAAGAGACCATTAATGCAATTT
ATTATCAAATGCAAATTGCTAGTCAATATTTATATGGACCACATAAAGGGCCAATTCATTTTAACTTGCCATTTAGAGATCCGTTAACACCT
GATTTGAATGCAACAGAATTGTTAACTTCTGAGATGAAGATTTTACCGCACTATCAAAAAAGTATAGATGCATCGGCATTAAGACACATTT
TAAATAAGAAAAAAGGTTTAATTATTGTAGGGGATATGCAGCACCAAGAAGTTGATCAAATACTAACGTATTCAACGATATATGATTTGC
CTATTTTAGCTGATCCTTTAAGTCATTTAAGAAAATTTGATCATCCGAATGTTATCTGTACATATGATTTGCTGTTTAGAAGCGGCTTAGAC
TTAAATGTGGATTTCGTAATTCGTGTTGGGAAACCAGTGATTTCTAAAAAGTTGAATCAATGGTTAAAGAAAACTGATGCATTTCAAATAT
TAGTGCAAAACAATGATAAGATTGATGTCTTTCCGATAGCACCAGATATTTCATATGAGATTTCTGCGAATGATTTCTTTAGGTCATTAAT
GGAAGACACGACCATCAATCGCGTAAGTTGGTTAGAAAAATGGCAACGCTTAGAGAAAAAAGGGCGTAAAGAAATTAAATGTTATTTG
GAACAAGCTACAGATGAGAGTGCATTCGTTGGTGAATTGATTAAGAAAACATCTGAAAAAGATGCATTATTTATTAGTAATAGTATGCCT
ATCAGAGATGTAGATAACTTGTTATTGAATAAAAATATAGATGTCTATGCGAATCGTGGTGCGAATGGTATTGATGGTATCGTTTCAACT
GCACTGGGTATGGCTGTGCATAAACGAATAACATTATTGATAGGTGATTTATCATTTTATCATGATATGAATGGACTATTAATGTCAAAAT
TAAATAATATTCAGATGAATATTGTATTATTGAACAACGATGGTGGCGGTATTTTTTCATATTTACCACAAAAAGAAAGTGCAACTGACTA
TTTTGAACGGTTGTTTGGCACACCGACGGGATTGGATTTCGAGTATACAGCTAAGTTATATCAATTCGATTTTAAACGTTTTAACAGTGTT
TCAGAATTTAAAAATGCCACATTGTTATCTGAAACTTCGACGATTTATGAATTGATAACGAATCGCGAAGATAACTTTAAACAGCATCAAA
TTTTATATCAGAAATTGAGTGAAATGATTCATGACACATTATAA

Gene: menH (2-succinyl-6-hydroxy-2, 4-cyclohexadiene-1-carboxylate synthase)

Contig: 06_NODE_54, position: 153677 to 154480, length: 804 nt, orientation: FORWARD

Perfect match to: (CIGC93-AHVD01000012-[68834:69637], allele observed in CC15+CC1+CC15)

Sequence:

ATGACACATTATAATTACTATGAAGCAAACGTTGAGACCAATCAAGTTTTAGTATTACTGCATGGTTTTCTTAGCGACAGTCGTACTTATT
ATAATCACATCGATAAATATACTGATATCTGTCATGTCATCACTATAGACTTACCAGGACATGGCGAAGATCAGTCTTCAATGGATGAAAC
GTGGAATTTTGATTATATTACGACGTTGTTAGACCGAATTTTAGATAAATATAAAGATAAATCAATAACATTGTTTGGATATTCAATGGGT
GGACGTGTTGCATTATATTATGCAATTAATGGTCACATCCCTATATCTAATTTGATATTAGAAAGTACGTCACCAGGTATTAAAGAAGAAG
CAAATCAATTGGAACGCCGTCTTGTTGATGATGCACGTGCTAAAGTATTAGACATAGCAGGTATTGAATTATTTGTTAATGATTGGGAAA
AGTTGCCATTATTTCAATCGCAACTAGAATTACCAGTTGAAATACAACATCAAATAAGACAACAACGATTGTCCCAATCGCCACATAAAAT
GGCCAAAGCATTAAGAGATTATGGTACAGGTCAAATGCCAAACTTATGGCCGCGCCTGAAAGAAATTAAAGTACCAACATTAATATTAG

CTGGAGAATATGATGAAAAATTTGTACAGATTGCGAAAAAAATGGCAAATTTAATTCCTAATAGTAAATGTAAATTAATTTCTGCTACAG
GTCATACAATTCATGTGGAAGATAGTGATGAATTTGATACAATGATATTAGGATTTTTAAAGGAGGAGCAAAATGACTAA

Gene: menB (1,4-dihydroxy-2-naphthoyl-CoA synthase)

Contig: 06_NODE_54, position: 154473 to 155294, length: 822 nt, orientation: FORWARD

Perfect match to: (MW2-BA000033-[1020701:1021522], allele observed in CC1+CC1217)

Sequence:

ATGACTAACAGACAATGGGAAACACTTAGAGAATATGATGAAATCAAATATGAATTTTATGAAGGGATTGCTAAGGTAACAATAAATCG
CCCTGAAGTACGCAATGCGTTTACACCTAAAACAGTTTCTGAAATGATTGACGCATTTTCACGTGCACGTGATGATCAAAACGTTTCAGTT
ATCGTATTAACTGGTGAAGGTGATTTAGCATTCTGTTCTGGTGGTGACCAGAAGAAACGTGGACATGGTGGTTATGTAGGTGAAGACCA
AATCCCTCGCTTAAATGTATTAGATTTACAGCGTTTAATTCGTATTATTCCAAAACCGGTTATCGCGATGGTAAAAGGTTATGCTGTAGGT
GGCGGTAATGTACTAAATGTTGTTTGTGACTTAACGATTGCTGCTGATAATGCTATTTTTGGACAAACTGGTCCTAAAGTAGGTTCATTTG
ATGCGGGTTATGGTTCAGGATATTTAGCACGTATCGTTGGACATAAGAAAGCACGTGAAATTTGGTACTTATGTCGTCAATACAATGCAC
AAGAAGCTTTAGATATGGGTCTAGTAAATACAGTGGTACCTTTAGAGAAAGTTGAAGATGAAACTGTGCAATGGTGTAAAGAGATTATG
AAACACTCACCAACAGCGTTACGATTCCTTAAAGCAGCTATGAATGCTGACACAGATGGTTTAGCTGGTTTACAACAAATGGCTGGGGAT
GCAACACTGCTTTATTACACAACTGATGAAGCGAAAGAAGGCCGTGATGCGTTTAAAGAAAAACGTGATCCTGACTTCGATCAATTCCCT
AAATTCCCATAA

Gene: sspC (staphostatin B)

Contig: 06_NODE_54, position: 155532 to 155861, length: 330 nt, orientation: REVERSE

Perfect match to: (CN1-CP003979-[989812:990141:r], allele observed in CC72+CC8+CC22+CC72+CC239)

Sequence:

TTATACTAAGCGCTCATAAACGATTGGGCGCGAAGTGCCAATACCTTGTTTGGATGACGTTTGCATGAGGATATGCTGTGTATCATCATA
ATCAAGAATATAAATGATTTCTTCATTATCAATAGATGAAAATTTAATGCGTTGATGTGCCGTATCTATAAAAAGAATATGATATTGATTG
TGACTTGTATCATCGCCATGACGTATACAAATTGATTTTTGTAATTGATGATTACTCCAATTACCAATGAATAAATTGATATTGGTTTGTTC
TAGATGTGTGAGTTTGGTTGTGTCGTAAACTAAATTTATAAATTGTAGTTGATACAT

Gene: sspB (staphopain B)

Contig: 06_NODE_54, position: 155899 to 157080, length: 1182 nt, orientation: REVERSE

Perfect match to: (N315-BA000018-[1020384:1021565:r], allele observed in CC5+CC22+CC25+CC772)

Sequence:

TTAGTAACCTATCATTGAACCATACCAGTTATAATCACGATTGAATGATAAATGTAATAGGCTTGAATCTGCATCTTGGATTGATAATTCT
GTATCCCAAGGATTCCAGTAAATAAGTTTTTCTTGGTCATTAATTTTAGCATTACCAACAACTGCTAGGGCATGTCCTAAATGTGGATCATT
AGGGTTTTGAGATACACTTTGTGCAAGAATCATAATTCCTACATTATCTTTTGTAAGTTGATCAACTTGTTCATATGATGGTACGCCTTCTT
GATAATGAATATCTCTGCCTTGTGATTTACCGTATTCAATCATTTGATTAGGGAATGTTGAGCAATTAGGAAGGTCTTGCTCACTTACTTCA
GGGTATAATGTACGCATAATATCATGTGCATTATAAGTGTCTGTATTTTTAGTTGCATTTAATAATGCTGCCATACTAAATCCTGCACACCA
TGAGTTATCGAATTGTTGTTCTCTAATTTTGAAGTTTTTTAATGTATTTTCATATTGAACTTGATCTTCTTGTATCGCTTCGTTTTCTTCAACT
TTAGTAGGTGTTACTGTATTTTTTAACTCTTGTTTCAATTTTGATGAAACTGTTTTAGCACTTTCTTTTTCTTTTACATTACCAGGTAGTGGC
GTAGCTTTTACTAATCTAACTTTGCCATCTTCTTCAAAATAAAATCCTTTTTCATCAGTAAGAACAGTGATATTTGAATTTTTATCTTTAATTT
GGTCTAAATCTTTAGCGATGAAGTTTGAAATTTTAACACTGTAATTCATGTCTTCTTTTGATTTGTTTAAATCATCTTTATTTTTAGGGCTAA
GTGTTAAAGTATAAACAATTTTACCGTCTTTTATAACTGGATAATAGTAACTATTATCTTCTTCACCATTAAATTTATAAATTTTAAAAGCTT
CGCCAAGTTCATATTTACCAGTTTTTGCATTACTTTGTTTATCTAATGCTTTAGCATAACCAGCAAATTGTTGTTGTGCTAGATCTTTTACTT
TTTGAGGTACTTTGTCACTCTTAACATTAATTTCTAGCTGTTTAGAGTGTGAATCGGCTTTGGCTTTATTGTTATTAGCAAATGCGCCTAGT
GATAAAATAAGCATTGAAACCATTATGATGCTTATAATATTGAATACTCTAGATTTATATGAACTATTCAT

Gene: sspA (glutamyl endopeptidase)

Contig: 06_NODE_54, position: 157162 to 158136, length: 975 nt, orientation: REVERSE

Sequence:

TTATGCAGCGTCAGGGTTGTCTGAATTATTGTTATCGCCATTGTCTGGATTATCAGGGTTGTTAGGGTTATCAGGATTGTTAGGGTTATCA
GGATTATCTGGGTTATTAGGTTGGTCATCGTTGGCAAAATGGATATCTTCAATATTTTGTTTTAAGAAGTTGCGTACATTTTCATTAATAAA
TACCGCACCATTAAATTCATTTGGTACACCGCCCCAATGAATTCCGATCACTTCATTTTTTTCATTAAATACAGGTGAACCTGAGTTACCAC
CAGTTGTACTTAAATCATATTGCATAGCTTCGCCTTTGAGGTAAGTGATTTTTCCTTTACTTTCCCACATTGTTGCTACAGGTTTATCACCAG
GATATCCTGTTACAGTAATATTTTGGTTAACTTGTGTTTCAGCATTATTACTCATTGTTGCTGGTTTAACTACTTCACCAATATGTTTGTTTT
GCTCATTAGGGGAGAATTTAACTATTGCTAAATCACCTTCGCCTGAATATTTAGTGATTTGTTCAGCAGTGAAACCACCATTTGGATAATT
GTCTTGGTTAATTGCAGAAGGGAATGCTTTTAAAGCATGAGGATCACCGTGCGTAGCATCTACGACGTGTTTATTTGTTAAAAGAGTATC
TTTACCTACAACTACACCGGAAGCAATAAATGTACCAGTAGGTGCTTCAACTTGAATATAAGTTACGGGTGCATAATGACCATTCGTTGTA
TCTGTGATTTGGTGACGATCGTTATTTGGTAATATAACATTTGCGTGTTCACGTTGTTCTAATGGTTTAAGGTTACCGCCTTTTTGAATCTT
AGGTGTTTGCTGTTTGCTTGACTGTGATTGTTGCGGATGATTGTCCATAGCCTTTGAAGATAACGCGTTTGCTGCTGGAGAACTCACAAG
TGTCGCTGTTGTCAAAGTTGCAACGAATAAAGAACTAACTTTTAAAAATTTACCTTTCAT

Gene: aspC (putative aspartate transaminase)

Contig: 06_NODE_54, position: 158664 to 159818, length: 1155 nt, orientation: REVERSE

Sequence:

TTATTTAGTATTTAAATATTTAGCAAGTCTCTTCATACCTTCTTTTAAAACATCCATTTCATAAGCATAGGAAATCCTTACAAATCCTTTACC
GAATTCTGTAAAGGATGAGCCTGGAACGATTGCTAAATGTGTTGATTCAAGTAAATCGACACAAAATTCGAAATCGTCATCGGTGATATG
TTTAATACTTGGGAAAATATAAAACGCGCCTTCAGGTTGAGCGGTAATCTCAAAACCTAATTTAGTTAATTCAGATACTAAATAATTTCTTC
GTTCTACATAAGCTTCGTTCATATATTTAGGAGCTTCTAATCCTTCGTTAAGTGCTGTTATACATGCTATTTGAGCTGGAACATTGGCACAA
ATACAATTATAGGCGTGCATAAATGTTAATTTATCAATCAAATATTGAGGTCCTAATAGAAAACCAATTCTTATTCCGGTTGCTGAGTGTG
ATTTACTTAAACCACCAATTAATATTAATTGATCACGAATGTCTTCAAATTCAGCGAAGGATACATGTTTACCACTAAATGTATTTTCAGCA
TAAATCTCATCGCTAATAATAAATATCGGATATTTTTTTAATACATTTACGATATTTAAAACTTCATTTCTTTTTAAAACTACGCCAGTTGGA
TTAGTCGGATAATTTAACAAGACAGCTTTTGTCTTTGGAGAAATATGACTTTCTAATGCATCAGGTGTAATTTTAAATTGTGTTGCTGTTGT
ATCAATATAAATTGGTTTACCACCTAGTACTTCGATGAGTGGTATGTAGCCTGCATAAATAGGTCCCGGTATTATAATTTCATCTCCAGGC
TCTATGATAGAACGTAACGTTGTGTCTATTGCTTCACTTGCTCCATTTGTCACAATAATTTCTTCAGGATCATAGGAAAAATGATAACGATT
TTTGAAGTATTGACTAATTGCTTCGCGAGTTTCTAATAACCCTTTATTGTGAGAGTATGATGTCTTGTCATTGTTAATAGCATCAATATATG
CCTTTTTCACAACATCAGGCATCGGGAAGTCTGGTTGGCCAATAGTTAAATTAACACAATCATCCAAATTATTCATACGATTTGAAAATTG
GCGAATACTTGGTGCTCTTAAATATTTAGAATTAGAATTTAAAGAAAGTTTCAT

Gene: ykrP (putative membrane bound acyltransferase)

Contig: 06_NODE_54, position: 160014 to 161024, length: 1011 nt, orientation: REVERSE

Perfect match to: (M0239-AIWE01000002-[166017:167027:r], highly conserved allele)

Sequence:

TTATGAATCTTTAAACTGTGATGGTCTTTGTAAATTAATTATTGGATTTGTCCATTTACAAACAAAGTTTGTAGATAATACATATACGATGA
TTACAGATATACTTATTAAATAAAGATAAGTCATTAATGATATAGGATTATCGAATGGGTACCATTCAAATCCTCTAACAATGCCAATAAT
TAAACCATGTAATAAATATACGTATAGCGTACGACTACCAATATAAGTATATAATTTTTTCTTTGTTGACATTAAATTTAGAAACGCAGTCA
TTGCGATTAATATAATTCCATATAATATAAGTCGTTTAAAAGGACTGAATATACTCTGTCCTTCATTTTCAAGTGAAGTATATGGTGAACTT
CCCAATAACCAATCTGCATTGATAGGATGAATCACGTAAACGATAAAAAACAAAATAAAGGTAATGATAGATACTGGTATTAGTTTTTTA
TTTTTAAAAATAGCCGTATGTTTTTTGGTGAAAATGTAACCTAGATAAAATATTGGGAAAAATACGATTGTCCTTGAAATGCTTAAGTAGC
TATCGATGTTATCTGAAAAACCTGCTCCAATAGATATAATAATTGAAACTGATAGCACTTTATATGGATTAAATCTTCTAACTATTACTAAA
ATGACATGAAAGAAAAATAGCGTGATCAAAAACCATAACGCAAATACTGGGTTAAAAGGATCAAGTTGTAATTCGTCACTTTTACCTGTT
AAGAAATAATAAATTGAAAAGAATGCAAAAAATATCATATAAGGTACTATCAAACGTTTTGAAATTTTTTCTAAATAGTATGGTTTATCAA
TATTTTTCGCGAAATAACCAGATATAAACAAAAATGTTGGCATATGGAAACTATAAATAACTAGGTATAATGCTGATAAATATTTATCGCC
ACTAGTGTAGGGTTGTAACATATGTCCAAATACGACTAATAATATTAAAATTGCTCTTGCGTTGTCAAAAAAATAATCTCTCTCTTTTAATG
AAGTCAT

Gene: Q5HH32 (helix-turn-helix transcriptional regulator)

Contig: 06_NODE_54, position: 161177 to 161596, length: 420 nt, orientation: FORWARD

Perfect match to: (MW2-BA000033-[1027414:1027833], highly conserved allele)

Sequence:

ATGTATAAACAACTTGAAAAACTTATTACACTGACTAACAATGACTTAAACTTAGTGAATAGAAGATTTGGACAACGCACGGATATCACA
TCTGAACAGCTAGAACTTCTCCGTATTTTATTTAATTACGATCGCTTATCACAGTATGATTTGACGATGAAGATTAGCAGGGAACAATCTA
TAGTTTCAAGGTGGATTAAGAAATTAGTTTTGAAAGGGTACATCACAAGTCAACAATCTAGCGAAGATTTAAGATGTAAAGAATTAATTT
TGACTGATCAAGCACGTACATTAATTTCACAAATAAATAATGCACGTTGCGAATTGATTGAAGCAAGATGTCAATGTTTATCGGAAGTCG
AATTAGACAATTTAAATCAATTACTTGATAAGTTAAATCAACGACGCATATCGTTGTAA

Gene: atl (autolysin)

Contig: 06_NODE_54, position: 161803 to 165573, length: 3771 nt, orientation: REVERSE

Sequence:

TTATTTATATTGTGGGATGTCGAAGTATTTGCCGACTTCGCCAATTTTATCATAGTAGCCTTTGATGATTTTAGCATTGATGTTAGCCCAAT
CTATATCTGTAGCATATTGGTGTGTTCCTGGATGTGCAGGATTCCATCTCATTTTGTAAAGTGTATTTTGACCAGCTTTTACATATGAGTTG
CCGATGAATTTAGCACCACCAACGATTGCTTTTGATACTGTGTCCCAACCAGCTTGTTTAGCATATTTAATACCTTCACGTAAAGGATCGTT
ATCATATGCAGCAATACCAAATACGTTATGGTATTTCGTGTTTGAGTTAGTTACAACTTTGTTGTTCACTACATCTGCACCTTTCGCTAATT
GAGAAGTACCGTTACCTGTTTCTAATAGGGCATGTGAGATAAGATAAACTTCATTAATGCCATACATTTGAGCAGCTTTGTTAAATGCAG
CACCTTGGTTTTCTAATACACCTTTACCTTTTAAGAATTGATTGATTTTATCAATAGAAATATTTTGTGGTTGGTCTAAGCGTAAGAATTGA
TATTTTAATGCTGGATCTTGAGCTAAACGCTTCGTATCCATTGCATGCTTAACATCATTAAAGTTAGCATCTGTCCACTTACCTGGTACACG
TTGTACTTGTGGTTTATATTGTAAACCAGCTTGTATTTGAGCAACTTGGTTTAATGTCATACCTGTTTGATTATACTTAATTAATTCTTTAGC
TAAATCAGTTGATTTAATCCATGCTAATTTACCGTTAGATAATTTACCATAGTACCAAGTTTGTCCATTAATGACTTGTTCTTTAACAACTGC
GAATGGTTGTTCATTAAATGCTTTTAATGAGTATTTAGCTGTATCAGAATTTGGTGTTACATAGTAATAACCATTACCATTTTTAATTACAT
AAGTGTAGTTATAATCTTTGGCAGCTGATGTAGTTGGTTTAACAGCAGTTGGTGCAGTTAAATCTTTTGCATTTACCCAACCAGTGCGGTT
ATTAATAGTACCGTATAAATAAACATCTTTGCCTACAGATACTTGTTTCGTTGCATTAAATGTACCTTGAGCAATGTTATTGCCTGTTAAAA
TGACTTGGTTTTTAGTACCCCAAGGAACCATTGATAAGCCGTTATTTGATTTATTAACAGTATATTTTTGAGTCGTTTTAACTTCTTTGCCTA
AGTTTTGAACATTTAAGTCTTTTACATTGAACCAACCTAATGGGATGTTATGGCTTGTATTGTTTAATAATACATACGTTTCATTACCATGA
GCACGCTCTTTTGTTACATAGAACGTACGGTCTGCATATTTCGCACCGTTTTTCGCTGTTTTTTCATAAACAGAAGCACGAATACCAGTGTT
GTTTGGTTTAACTTGAGCAATCTTGCTAACTGTTTGAGTCGTTTGTGGTTTAGTAACAGTATAAGCTTTTACAGCTGTTTTTGGTTGTGCTA
CTGCTTTTTTAGGTGCAGCAGGTACAGCTAAATATGCTTTACTTACCCAACCAGATTTACCATTTACAGTTCCAAATAAATAGATAGATTTA
TCAATTTGTTGTTGCTTAGTCGCTTTAAAAGTTTGGTTACCTGTACCAGAAACTGCACCAGCTTCTTGTTTATAAGTGCCCCAAGGTACTGA
ATATAATTTAGTGCCTGGTTTTACTGTATATGTTTGCATTACATTTACAGGTGATTTTGCATTGTTATAAATAACGTCACCTTGTTTAACCCA
ACCAATTAAAGTTGGACTATTGTAATCTTTAACTAAGTAGAATTTGTTTCCACCTAAACTTGCTTCTTTTGTTACAGCAAATGTTTTTTGAAC
TTCTTTCGTTGGCTTACCAGTTTTGTCATAAACTGTAGTGAATAAGCCATTGTTTTTAGCATTAATTTGAGCAACACCGTTTAATGATGAAA
CTGTTAATTTATTATTTGTTGTAGGTGTTGATGGCTTAGGTGTTGGTGTAGGCGTAGGTTTAGCAGTATCAACTAAATATGCTTTACTTAC
CCAACCAGATTTACCATTCACAGAGCCATATAAATAAATTGATTTATCAATTTGTTGTTGCTTTGAAGCCTTAAATGTTTGGTTTCCAGAGC
CAGACACACTACCAGCAACTTGTTTAGATGTACCCCAAGGTACTGTATAAAGTTTCGTACCAGGTTTGATTGAATATGATTGATTTACATT
TACAGGTGATTTAGCTGTGTTGTAAACCACATCGCCTTCTTTAACCCAACCAAATTTATTACCAGAATTGTAATCTTGAACAAGATAGAATT
TTTGATTACCTAATGTAGCTGTTTTAGATACAGCAAATGTTTTTTGAACTTCATTAGTTGCTTTACCAGTTTTGTCGTATACAGTAGTATATA
AACCACTATTTGTTGGTTTGATTTGTGCGACACCATTGTTTGCAGCAACTGTTAATTTACCAGTTGATGGTTTCGACGGTGTTGTTGGTTTT
GATGGTGTAGTAGGGGTAGTTGTAGATTGCGTACCCCATGGCGCCACTTTACCCATTTTTATTAAATATTTTTCATTAATTAAGTCATATAA
TTGATCATAACTATAATTATGACTTCTTAAATATCCATGTGGATCGGCATGGTCAGTACCACCTAAATATTTACTTACAGCGTAGTGAGTCC
ATACTGTACCATTTCCATCATACTCAGCACTGTCTGGTTTTAAACCATAATATTGTAATTGTGTAGCTGCATAGTCAGCATAGTTATTCATT
GAACGTGCAAATGAAGCATAGTCGTGTGTGTGTACGATTTCAACATTGATGAATCTAGGGTTACCGACTGCACCGACACCCCAAGATAA
GTAATCCGTTGGTGCTGTTTCGATTATACGATCCCCATCAACAAATGCATGTACGAATGCGTTTTGATAGTTATTTTTCATATAACTAATTT
CACCATTTATCGTCGAACGATCATTAGCTGTATCATGAACTACGATACCTTCAGGACGACCTACGCCGTTACGGTATGCGTATTTAGGGAA
GTAAGATGTATAATCTTCTTCAATTTTAGGTGCTTTTAAGTTATTTTTACGAATGTAATCGTTAATTGAAGAGTTTACTTGTGGTTTATATTT
TGGCAAACTCGTTTTTGGTGTTGCAGCAACTGATCTTGGTTGTGCTGAAGCGCTAAAAGTAGTTACTTTAGGTGTCGCTTCAGTTTTAGCT
TTAGGTGCTGATGTAGTTGCAGCTTTAGGTGCTGCGGCTTTATATTGCGTTTCAAGAGCTGCAGGTTTAGCAGCTGATTTAATTAATTCTG
GATTAATTTGATTTTCTGAATTATCATCTTCATCATCAACTAAACTATAACCAGCATTTGTAACATTAGTGTTAGTTTTAGGTGCTGTAGTG
CTTGTTGACTTTGCAACAGGCTGCGTATTATTTGTAGTCGCTGATTGATTAGCACGAGTGTCACCATTTACTTGTGCAGTATCAACTTTTTG
ACTTACTTGAGCATTGCCTGTTTTGTTATTTGCTGTTTTTGGTTGGACAATAGCAGGGTCTTGATATACTTGAGTGCCAGAAATGTTTTGCG
TTGGATTTTTTACCTCAGCTTTTGCTTGTTCAGTAGTTGCTTTAACTTTATTACTATCTAAAACGTTTTTATTAGTAGTTTGATCTTGTGTCGT
CTCAGCTGCTTGAACTTGATGTGCAGTGACTGCTGAACCTACAAGCGTTAATGCAACCATTGATGGTAGTTTGTAATTGAATTTTTTCGCC
AT

Gene: UPF0039 (putative N-acteyltransferase)

Contig: 06_NODE_54, position: 165801 to 166235, length: 435 nt, orientation: REVERSE

Perfect match to: (N315-BA000018-[1030316:1030750:r], highly conserved allele)

Sequence:

TTAATTAAGCGAGGTTAACTTTTTTGTCATTTCAATATGCTCGATGCCTTCCTCAAGAAATATATTACCTCTCATTTTAAAGTTTAAACTTTC
ATAAAATGGGATAGCATGACATTGGGCATTCATAGTAGCTACGTAAAAACCTTCATCTTTAGCTAATGATTCTACAGCTTGCATAAGCATT
CTACCCATTCCTTGTCCACGATGTGATTTCATCACAGCTACTCGTTCTATTTTGACAGTTGTTTCATTAATAGGGCGTATTCGAGCAGTGGC
AACTGGCTGTCCATTATCATATCCAATGAGGTGAATAGATTCAGATTCATATTCATCAATTTCACTTTCCTCAGGGACGCCTTGTTCTTCTA
CAAACACTTTCTTTCTTATATAGAAGCAATCTTCTAACATCTTTTGATTGTTTACTTTTGAAAACAT

Gene: Q5HH29 (putative protein)

Contig: 06_NODE_54, position: 166390 to 166860, length: 471 nt, orientation: REVERSE

Perfect match to: (11819-97-CP003194-[1064617:1065087:r], highly conserved allele)

Sequence:

TTATAGTGCTTCTTTTCTAATAATTGCTGTATATTGCCAAAAGATTCGCATTTGAGCAATATTCCAATTATTCATTCCCATTGTAAATCCTGA
AGGCTTAAATATATTCACATCAGTTACTTCTAAAGCTGCTGCAACTAAATATTGTAATGGAATGCTAAGTGATTTCATTGTTTCGGTAATG
CCATTTTCATCATATACCCATGAAAATGGTAACTCTGATTCGAAAACATCAACACTATTGAAAATTTCAGGTAGGGCTAAAATATAACATG
CACCATCTACAATAGGATTGTCGATACTATTTTTGTAATAAATTAGGAGTGCTTCATAGTTTTCTCGATGCTCATGATTGACGTAAAAAAAT
TCACTTCTAAAATGAGCCATATCTTGGCTATGAATGATTTGTTGTTCTAACATATTAAACATGCCATTTATATTTGCAATCTTTTCGTATGTT
TTTCTACTCAT

Gene: tagV-lcpB (phosphotransferase)

Contig: 06_NODE_54, position: 166908 to 168125, length: 1218 nt, orientation: REVERSE

Perfect match to: (08-02119-CP015645-[1167658:1168875], allele observed in ST582+CC8+CC4803)

Sequence:

TTAATTTACAACACCATTTTGGTTATTTGAAGCTTGTGGCGCTTGTTGTGTGCCACCTTGATTTTGATTTAAGTTTTGATCTGTAGCAGGTT
GTTGTTGATTGCTGGAATCACTGTTATTAGTTGAATCATTGTTCTCGTTAGATGTCTTATCTTTATCTGTCGTATCATTTTGTTCTTTTCTCAA
TAAACTACTATCTAAAGGCGTTAATGGTATTAATGAACCATAATGATTAATGACACGTTGATCTAAGAAATCATTTTTATCATTAATAGGT
GATAATTCTAAGTCTTTACGAAGTAAGTTTGCATATTTTTGAATGCTTTCAACACTTGGATGATAATAGTAAATACCATTTAACATATCATC
TTTACCTTTTAATTGCGCAGTTTTAATTTCAACATCATTTGTTAAGTACATTTTTGCTAAAGCTTTAATTTCAGAGTTAGTTAAATTATGCTTT
GCATTTTTACCTACAATTTGAATCACGTTATCAAGTTTATCAATAGAATCAACTTCCTGTGCTTTTTGGAATAAAATCTTAATTAATTCCATT
TGACGTTGTCCACGTTTTAAGTCTGAATCATGATGTCTAGTTCTAGCAACTGCTAAAGCCTCATCACCATTTAATTTTTGGTACCCTTTTTTA
ATTTTAATCTTACCAGTATCATCTGTGTTAGGTTCATTTAAGTCGTATGGCACATCATAGTATATGCCACCAAGCTCATTTACAGCCTCGAC
AAATGCTTTCATATTGACTCTCACATAATAATCAACAGGTACATTCATGGTAGCTTCTACCGAATCCATTGCGGCAATTGGACCACCATAT
GCATGTGCATGGGTAATCTTATCGTAATAGCCAACTTTAGGAATGTAGCTGATAGTATCACGTGGAATACTAAGCATTCTAATTTGATGTT
TTGATTGATTAAAAGTAGTTAAAATCATAGCGTCTGATCTAGAGTGTTCAGCATCCTGTCCTTTTTTTTCTTCTTCCATCGTTATCATCGATAC
CTAAGAAAAGAATAGAGATAGGTTGTTCTTCGGGATTGACTTTATTATCTCTTAAGTTGGATTGACGATTAGCATTTTTGCTGTCTTGAGA
AGATTCGAATGCATCTTGGGACGTTTTAAAAAGTAACGTAGCGAAGACTATTGGAACAACAATGAGAACCAATGCTAGAAGGATCAAAA
AGTATTTTAAAAATTTATTCAT

Gene: fmtA (autolysis and meticillin resistant-related protein)

Contig: 06_NODE_54, position: 168576 to 169769, length: 1194 nt, orientation: FORWARD

Perfect match to: (COL-CP000046-[1074466:1075659], allele observed in CC8+CC5+CC772)

Sequence:

ATGAAATTTAATAAAGTAAAACTAGTTATACATGCGTGTGTACTATTATTTATCATTATTTCTATAGCGTTAATTTTTCATCGATTACAGAC
GAAGACACATTCTATAGACCCAATACATAAGGAAACAAAATTATCAGACAATGAAAAATATTTAGTGGATCGTAATAAGGAAAAGGTTG
CGCCGTCTAAACTAAAAGAGGTATATAATAGCAAGGATCCTAAATATAAGAAAATTGACAAGTATTTACAAAGTTCATTATTTAACGGTT
CAGTAGCTATATATGAAAATGGCAAATTGAAAATGAGTAAAGGTTATGGATATCAAGATTTTGAAAAAGGTATTAAAAACACACCGAAT
ACGATGTTTTTAATAGGTTCAGCTCAAAAATTTTCAACAGGGTTACTGTTAAAACAGTTAGAAGAAGAACATAAAATAAATATCAATGAT
CCAGTAAGTAAATACCTTCCATGGTTTAAAACATCTAAGCCTATCCCATTGAAAGATTTAATGTTGCATCAAAGTGGATTATATAAATATA
AATCCTCAAAAGATTATAAAAATTTAGATCAAGCAGTTAAAGCGATTCAAAAACGTGGTATTGATCCTAAGAAATACAAAAAGCATATGT
ATAACGATGGGAATTATTTAGTACTTGCGAAAGTAATTGAAGAAGTTACAGGTAAATCTTATGCTGAGAATTATTATACAAAAATAGGAG
ATCCTTTAAAACTTCAGCACACAGCATTTTATGATGAACAACCTTTTAAAAAATATCTAGCAAAAGGTTATGCTTATAATAGTACAGGACT
TTCATTCTTAAGACCTAATATTTTGGACCAATACTATGGTGCAGGTAATTTATATATGACACCAACAGATATGGGTAAATTAATTACTCAA
ATACAACAATATAAATTATTCAGTCCTAAAATAACCAATCCATTATTACATGAGTTTGGTACGAAACAGTATCCAGATGAATATCGATATG
GTTTCTATGCTAAGCCAACATTGAATAGACTTAACGGGGGATTCTTTGGACAAGTCTTTACTGTTTACTATAATGATAAGTATGTAGTTGT
ACTTGCATTAAATGTAAAAGGAAACAATGAAGTTCGAATCAAACATATTTATAATGATATTTTAAAACAAAATAAACCTTACAATACGAAG
GGTGTTATTGTTCAATAA

Gene: qoxD (cytochrome aa3 quinol oxidase subunit 4)

Contig: 06_NODE_54, position: 170306 to 170596, length: 291 nt, orientation: REVERSE

Perfect match to: (MW2-BA000033-[1036543:1036833:r], allele observed in CC1+CC12+CC15+CC80+CC772)

Sequence:

TTATAAGTGTGAAGAGTGACCGCCTTGCATAACCCAATATGTTCCGACAACGAAACAAAGTGTAATTACAAGAGCAAAGATAACTTTGAA
TGTTTGTAAACGTCCATCTTTACCTTCAGTTAAATGCATGAACATTAATAATTGAAGTCCTGCTTGGACGAATGCAAAGCCAAAGATAATT
GTCAACTTCGCGTGGAATGTTAATGACGTGTATAGTGTTACGTATACTGCTAAAAGCGTTAATACGATAGATGCGATAAATCCTACAGTA
TGTTTCATTATTGTACTCAT

Gene: qoxC (cytochrome aa3 quinol oxidase subunit 3)

Contig: 06_NODE_54, position: 170593 to 171198, length: 606 nt, orientation: REVERSE

Perfect match to: (RF122-AJ938182-[1004510:1005115:r], highly conserved allele)

Sequence:

TCATCCGCTATACACCATCCCTATCATATATACGGCAGTAAAGATGAAAACCCAAACAACATCTAAGAAGTGCCAGTATAAACTTACTATA
AATAATTTTGGCGCATTATATTTGTCTAATCCGCGTCGTTGGATTTGGATTAATAAACAAATGGCCCAAACGATACCTAGCGATACGTGAC
AACCATGCGTTCCTAATAGGATAAAGAAACTAGACCAGTAAGAACCAATTGTTGGGTTAACGCCTTCTGATGCATAGTGTGCGAATTCAT
AAATTTCGAATCCAACAAAGACTAAACCTAAAAGTAACGTAATGATCATCCAAAACATCATTAACTTTTGTTTTTCTTGGCGCATGTAGTA
AATAGCAATACCACATGTGTAAGAACTGAATAATAATGCAAACGTCATTATTAAAACAAGAGGCAATTCAAATAACTCAGTAGTCATTTT
ACCTGCATAATCGCCACCATGTTGCAAAGTTAATAGTGTCGCAAATAGGGTACCGAATAACGCAAATTCGGCTGTAATGAAAATCCAAAA
GCCAAGCTTATTTAATTCGCCTTCATGTGTGCGTGAATCAATAGTGTTTGTATCATGACTCAT

Gene: qoxB (cytochrome aa3 quinol oxidase subunit 1)

Contig: 06_NODE_54, position: 171188 to 173176, length: 1989 nt, orientation: REVERSE

Sequence:

TCATGACTCATGACTTACAGCCTCCCTTTCTTTAATACGCGCTTCTCTTAATCTAGCTTCAGTTTCAGCAACTTCAGCAGCAGGGATATGAT
ATCCGTGATCGATTTGGAAACTGCGATAAATCATAGTACCAAAAATACCGAATAAACAAATTAATGCTGGAATTACAGTTTCGAAAATTA
AGAAGAAACCGCCGATAGTCATAAAGATACCAATCCAGAATCCAACAGGAGTATTGTTTGGCATATGAATGTCTTTGTAATTATGGTTGT
CTAAGTAATGACGACCATGTTCTTTCATATCAACAAATGTGTCGTAGTCATTCCAATCTGGTGTAATGGCAAAGTTGTATTTAGGTGGAAT
TGCTGATGCAGTAGTCCACTCTAGAGTACGACCAAGGCCATCCCAGTTATCTCCAGTTGCTTCACGTGGAGATTTGAAGTGACTGTATAC
GATACTAACAACAAGGAATAAGAACCCGATTGCCATTAATAATGCACCGATAGTTGAAATTAAGTTTAATAAGAACCAACCATCTGATGG
CATATAAGTGTATAAACGACGTGGCATACCATCTAATCCAAGAATGAATTGTGGTAAGAAACAAACGTTAAATCCGATCATGAAGAACCA
GAAGCACCATTTGTTTAATGTTTCGTTTAATTTGTAACCCATCATCTTTGGATACCAGAAGATTAAACCAGCTAAGCAGGCAAATACAACA
CCAGTAACCAATGTATAGTGGAAGTGAGCTACTAAGAAGTACGTATTGTGATATTGATAGTCAGCTGATGCCATTGCTAACATTACACCC

GTAACACCACCTAAAAGGAAGTTAGGGATAAATGCTAATGAGAATAGCATTGGTGACTCAAATGTAATACGTCCTTTATATAATGTTAAT
AACCAGTTAAACAATTTCACACCAGTTGGAATACCAATCAGCATTGTTGAAATTGAGAAGAATGAGTTGATTAACGCACCATTACCCATT
GTGAAGAAATGGTGAACCCAAACTAAGAAACTAAGGAACGCGATACCGGCAGTTGCCCATACCATACTTTGATGTCCGAATAAACGCTT
ACGAGCGAATGTCGGGATAATTTCTGAGTAAATACCAAATGCTGGAAGGATAACGATATAAACTTCAGGGTGCCCCCATACCCAGAAGA
AGTTAGCCCAAAGCATTGGCATACCGCCATGTGCAACTGTGAAGAATGCTGTGTCAAATATTCTATCAGTTGTCATTAATGCTAACGCTAC
TGTTAAAGGAGGGAAAGCAAGAATAACAATTAATGTAGTAATAAATGTTGTTACTGTAAACATTGGCATTTGCATAAACTTCATAGTTGG
TGTTTTACATCTTAAAATTGTTACAAAGAAGTTGATACCTGTAGCTAAGGTACCAAGCCCTGAAATTTGTATAGCTATTAAGTAATAGTTA
ACACCCGGACCAGGACTGAATTCACCTGCTAGTGGCGCATAGTTTGTCCAACCAGCTGCTGGTGAACCACCAATAATAAATGACAGGTTG
AATAAAATCATACCTGCAAAGAATAGCCAGAAACTTACGTTGTTTAATACTGGGAATGCAACATCACGTGCTCCAATTTGTAATGGAACA
ACGATATTCCATAAACCAAAGATAAATGGCATTGCCATGAAGATAATCATGATTACACCATGTGTACTAAAAATTTCGTTATAGTGGTTAG
ATTCTAAAAATTTGTTATCAGGTACTGTTAATTGCGCACGAATAAGTAACGCATCAATACCACCACGGACGAACATTAATACGGCACAGA
TTAAATACATAATACCGATTTTCTTATGGTCTACAGATGTGAACCATTCTTTGTAAAGATATTTCCATAATTTAAAGTAAGTAATTACTGCG
ATTAAACCAATAACTAAGAATGGGGCACCAATTTGTGCCATTGTAATCATCCAGTTACCTTTAACTAGTAATTGATCCCATGGAAAATTCA
T

Gene: qoxA (cytochrome aa3 quinol oxidase subunit 2)

Contig: 06_NODE_54, position: 173176 to 174276, length: 1101 nt, orientation: REVERSE

Perfect match to: (MW2-BA000033-[1039413:1040513:r], highly conserved allele)

Sequence:

TTAATGTCCACCTCCATGATCATCATTGTCTTGATCTTGCGCATCTTTTGAAATTTTCTTCATTTCTTTCGCATTTTTCGATTCATCTTTCTTGA
ACTCATTGTTATATGGTTCGTCATTTCCAAGAATCATCAACTTCATACCATGTCGTTTATAGTTCGCATTTGTAATTTGAGCTTTACGAGCA
GGTATTAATGGTTTGTCTGATACATCTTTAAACATATTTTCTTCACTAGTGAAGTTTGGATCTTTCAATTCGAAATTGAAACGTTTATATGC
ATAGAAGATGTATTCTGGATCGGCTGCTGGATCAACAAACGCCATATGTGTACCATTAAATTCTAAAGCTTTATTAGGTGTGCTTGGTAAT
AATTGTTTATCAAATGTATCTTGATCTAACGTTTTCTTACCTTTAACTTCTTTCACCCATTTGTCGTAGTCTTTTTGACTAACGGCATTTACTT
TAAATGTTTGACGTGAGAATCCTTCACCATTGAAGTTAGAGTTACGACCTCTGAACGTACCAGTTTGAGATGCTTCTAACGTCCAATTCAT
TGTCATGCCAGTCATGGCATATTTTTGACCACCTAATTGTGGAATCCAGAAACTTGTCATTGTATCCATAGCTTGAAGCTTAAATACAACA
GGACGATCTTTAGGGATTGTTAATGTGTTAACAGTCTCTATATGTTCATCTGGATAAGCAAAGAACCATTTGTATCCTGCACTTACTGCAT
ATACAACCATTGGATCTTTCTCACTCTTCGGTGGTTTTTCGTAATCGTATAAAGTTTTAACTGTAGGAATAGCTAAAGCAGCAACGATTAT
GATAGGTATTACAAACCATATTGTTTCAATGATGGCATTATGGTGCATCTTACCAGATTCGGCATTCTTATTATAACTATACTTGTAAATAA
AAATGGCGAACATGCCAAGTACAACGAAACAAATAACAAGCATGAAGACGATTGAATAAAGAATCAAGAACTTCTGACTACTTGCTACT
GGCCCTTTTGCGTTGAAAATTTCTATATTTGAACAACCACTAAGTAAAATTAGTGTGCCAAATAATAGAAGCAAAGACTTAAATTTTGACA
C

Gene: iraE (chitinase B)

Contig: 06_NODE_54, position: 174848 to 175165, length: 318 nt, orientation: REVERSE

Perfect match to: (RF122-AJ938182-[1008766:1009083:r], highly conserved allele)

Sequence:

CTATTTAACCTCAATATACCTAGTGGAAATTTTAATTGCACCATCTGAATCAGTGACATGATATTTAATTTGATATTTACCAGATTTAGATG
TATCAATTTGGCCATCGACTTTAATTTTATCGGTTAAATCTCCATCTTCTTTATCAAATGCACTTATGCCGTTTAGAAGGTTATAATCTTGAC
CTTTCTTAATAACGATATCATTAGCGCCTTTAATTTGTGGTGTAGTATTCGTCGTTGCATCTGCATTTAAATTTGGTGTTACTAGTGTAGCA
GAAACACCGAGGGCTGATAATGACTGTAATAGTTTATTCAT

Gene: folD (bifunctional methylenetetrahydrofolate dehydrogenase and methenyltetrahydrofolate)

Contig: 06_NODE_54, position: 175890 to 176750, length: 861 nt, orientation: REVERSE

Perfect match to: (MW2-BA000033-[1042185:1043045:r], allele observed in CC1+CC5)

Sequence:

TTACGAATCAATACCTCGACGCATTTTTTCTGCAAGCAAAGTATTATTTAATACCATAGTAATTGTTAATGGACCAACGCCACCAGGAACT
GGTGTAATAGCTCCAGCAATTTCTTTAACCGCATCATAATCAACGTCACCTTTTAATTTGCCATTTTCATCTGGCGTATTGCCAACATCGAT

AATTACTGCTCCTTCTTTGACCACATCTTTTGTTACTAAACCAGGCTTACCAACTGCACTGACAATGACATCAGCATCTTTTAAATATGATG
CCATATCTTTTGAACGAGAATGTAAGATTGTTACTGATGCATTTTTTTGAAGTAGTAACTTAGAAACTGGTTGTCCGACAATATGACTTCG
TCCAATTACAACTGCATTTTTACCTTCTAAATCAATATCTGCATGTTTTAATATTTCCATGATGCCGAGCGGTGTGCAAGGTACAAAAGTTT
GTTCATCGATATATAATTTCCCTATATTTATTGGATGAAATCCGTCCACATCTTTTTCAGGATTGATTGCTTCTAATATTTTCTGTTCGCTAA
CTTGTTTTGGTAATGGTACTTGTACCAAAATACCACTTACAGAATCATCATTATTTAGTCTATTTAGTTCGTTTAATACTTCTTCTTCAGTAG
CTGTTTCTTCCAAATGTACGATTTCTGAAATCATACCAATTTTTTCAGCTGCTTTCTTTTTTGATCTAACATAACTTTGACTAGCGCCATCATT
ACCAACTAATATAACGGATAATTTAGGTGTAAAACCCTTTTCTTTTAGCGCTTCAACTTGATCTTGTAACCCCTGTCTGTAGTCTTTGGCAA
TTTGTTTACCATCTAAAATTTTAGCAACCAT

Gene: purE (phosphoribosylaminoimidazole carboxylase catalytic subunit)

Contig: 06_NODE_54, position: 176951 to 177433, length: 483 nt, orientation: FORWARD

Perfect match to: (MW2-BA000033-[1043246:1043728], allele observed in CC1+CC5)

Sequence:

GTGAAAGTAGCAGTCATTATGGGCAGTTCTTCCGATTGGAAAATTATGCAAGAGAGTTGTAACATGTTGGATTATTTTGAAATTCCGTAC
GAAAAACAAGTAGTATCCGCACATCGTACGCCAAAAATGATGGTTCAATTTGCTTCTGAAGCGAGAGAAAGAGGTATAAACATTATCATT
GCAGGCGCTGGCGGTGCGGCACATTTACCAGGTATGGTTGCATCATTAACGACGCTACCAGTTATTGGTGTGCCGATTGAAACAAAAAG
TTTAAAGGGTATAGATTCTTTATTATCAATTGTTCAAATGCCAGGAGGTATTCCGGTTGCAACGACTGCAATTGGTGCAGCAGGTGCTAA
AAACGCAGGTATACTTGCAGCAAGAATGTTAAGTATTCAAAATCCTTCTTTAGTTGAAAAACTAAATCAGTATGAATCTTCGTTAATTCAA
AAAGTGGAGGACATGCAAAATGAGCTTCAATAA

Gene: purK (phosphoribosylaminoimidazole carboxylase ATPase subunit)

Contig: 06_NODE_54, position: 177420 to 178544, length: 1125 nt, orientation: FORWARD

Perfect match to: (MW2-BA000033-[1043715:1044839], allele observed in CC1)

Sequence:

ATGAGCTTCAATAAATTAAAGTTTGGTGCGACTATTGGCATTATTGGTGGTGGTCAGCTTGGAAAGATGATGGCACAATCAGCTCAAAAA
ATGGGTTATAAAGTGGTTGTATTGGATCCTTCTGAAGATTGTCCATGTAGATACGTTGCACACGAATTTATACAAGCCAAGTATGACGAT
GAAAAGGCACTCAATCAATTAGGACAAAAATGTGATGTGATTACTTATGAATTTGAAAACATTTCAGCCCAACAATTAAAACTATTATGT
GAAAAGTACAATATTCCGCAAGGTTACCAAGCTATACAGTTATTACAAGACCGCTTAACTGAAAAAGAAACATTAAAAAGTGCTGGTACC
AAAGTTGTCCCGTTCATTTCAGTAAAAGAATCTACAGATATTGACAAAGCAATTGAAACATTAGGATATCCTTTTATTGTAAAAACTAGAT
TTGGTGGCTACGATGGCAAAGGTCAAGTTTTAATTAACAACGAAAAAGACTTACAAGAAGGTTTTAAATTAATTGAAACTAGTGAATGCG
TAGCTGAAAAATATTTGAATATCAAGAAAGAAGTATCTCTTACTGTTACAAGAGGAAACAACAATCAAATCACTTTTTTCCCATTACAAGA
AAATGAGCATAGAAATCAAATACTTTTCAAAACAATTGTTCCAGCGAGAATAGATAAAACAGCTGAGGCGAAAGAGCAAGTTAATAAAA
TTATCCAATCGATTCATTTCATTGGAACATTTACAGTTGAATTTTTTATAGATAGTAACAACCAATTGTATGTGAACGAGATAGCACCAAG
GCCTCACAATTCCGGACATTATTCAATTGAAGCATGTGATTATTCACAATTTGATACTCATATTTTAGCAGTTACCGGACAATCATTACCAA
ATTCAATTGAATTATTAAAGCCAGCAGTCATGATGAACTTACTAGGTAAAGATTTAGATTTATTGGAAAATGAATTTAATGAACATCCAGA
GTGGCACTTACATATTTATGGTAAGTCTGAGCGTAAAGATAGCAGAAAAATGGGGCATATGACTGTACTAACGAATGATGTAAACCAAA
CTGAACAAGATATGTACGCTAAATTTGAGGGGAGTAATTAA

Gene: purC (phosphoribosylaminoimidazole-succinocarboxamide synthase)

Contig: 06_NODE_54, position: 178548 to 179252, length: 705 nt, orientation: FORWARD

Perfect match to: (N315-BA000018-[1043201:1043905], highly conserved allele)

Sequence:

ATGACATTATTATATGAAGGAAAAGCGAAGCGCATTTTCTCAACAAATCAAGAAAATGAATTAAGAGTTGAATATAAAGATGAAGTTACT
GCTGGAAACGGGGCTAAGAAAGACACAATGGCAGGTAAGGGGCGATTAAATAATCAAATTACTTCTATTATATTTAAATATTTACAAGA
AAATGGAATAGAAAGTCACTTTATTAAACAATTATCTGAAACAGAACAATTAGTTAAGCCTGTGAAAATAATTCCATTAGAAGTAGTTGTT
CGTAATATTGCTAGTGGATCTATTACAAAGCGTTTAGGTTTTGAAAATGGTGAAGTTTTTAGAGAACCACTTGTAGAATTTTTCTATAAAA
ATGATGCGTTAAATGATCCGTTGATAACGGATGACCATGTTAAATTGCTCAATATAGCATCAGATGAAGATATTGAAATACTAAAATCCA
AAGCATTAAAGATTAATAATGTGTTGAAACAATTAATGGATGCTATGAATTTAAAATTAGTAGATTTTAAAATCGAATTTGGAAAGACTG

AGACTGGTCAAATTTTGTTAGCGGATGAAATATCTCCAGATACATGTCGAATTTGGGATAAAGCTACCAATGCAAACTTTGATAAAGATG
TATATAGAAATAACACTGGATCACTGATTGAAACATATCAAATATTTTTAAACAAATTGGAGGATTTAAAATAA

Gene: purS (phosphoribosylformylglycinamidine synthase)

Contig: 06_NODE_54, position: 179252 to 179515, length: 264 nt, orientation: FORWARD

Perfect match to: (N315-BA000018-[1043905:1044168], highly conserved allele)

Sequence:

ATGAAAACAATTGAACTACATATCACATTACAACCACAAGTATTAGATACGCAAGGACAAACGCTTACTCGAGCTGTACATGACTTAGGT
TATGCACAAGTGAATGATATTCGTGTAGGAAAAGTATTATATATGACAGTGGATGAGGTTAGTGATGAAAAGGTACACAACATTATTAC
AACTCTAAGTGAAAAATTGTTTGCAAATACAGTGATTGAAGAATATAGCTATAAAGTGTTAGATGATGAAAAGGAGAATGCATAA

Gene: purQ (phosphoribosylformylglycinamidine synthase 1)

Contig: 06_NODE_54, position: 179517 to 180188, length: 672 nt, orientation: FORWARD

Perfect match to: (MW2-BA000033-[1045812:1046483], allele observed in CC1+CC8+CC22+CC72+CC239)

Sequence:

ATGAAATTTGCGGTTCTTGTTTTTCCAGGTTCGAATTGTGATAGAGACATGTTTAATGCTGCTATTAAAAGTGGTGTTGAAGCGGAATATG
TAGATTATAGAGAAACATCACTAAGTGGATTTGATGGCGTACTTATTCCTGGTGGATTTTCATTCGGGGATTACTTAAGATCTGGGGCAA
TGGCTAGTGTAGCGCCGATTATTTCGGAAGTTAAACGTCTTGCAGCTGAAGGTAAGCCAGTATTAGGTGTTTGTAATGGGTTTCAAATTT
TAACTGAAATAGGCTTATTACCTGGTGCATTATTGCATAACGATTCACATTTATTTATTAGTAGAAATGAAGAGTTAGAAATAGTGAATAA
TCAAACGGCATTTACAAATCTTTATGAACAAGGTGAAAAAGTTATATATCCTGTAGCTCACGGTGAAGGTCATTATTATTGTACTGATGAA
ATATATCAACAATTAAAAGCTAACAATCAAATTATTCTGAAATATGTGAATAATCCGAACGGTTCATATGATGATATTGCAGGAATTGTTA
ACGAAAAAGGCAATGTATGTGGCATGATGCCACATCCTGAAAGAGCTTTAGAAACGTTGTTAGGTACTGATAGTGGTGTGAAATTATTT
GAAGCGATGGTAAAAAGTTGGAGGGAACAACATGTCTAA

Gene: purL (phosphoribosylformylglycinamidine synthase 2)

Contig: 06_NODE_54, position: 180181 to 182370, length: 2190 nt, orientation: FORWARD

Perfect match to: (MW2-BA000033-[1046476:1048665], highly conserved allele)

Sequence:

ATGTCTAAATTTATCGAACCAAGCGTTGAAGAAATTAAACTTGAAAAAGTATATCAAGATATGGGATTAAGTGATCAAGAATATGAAAAA
GTTTGCGATATTTTAGGCAGACAACCTAACTTTACAGAAACAGGTATCTTTTCTGTTATGTGGAGTGAACATTGCTCTTATAAACATTCTAA
ACCGTTTTTAAAGCAATTTCCTACGTCAGGTGACCATGTGCTTATGGGGCCTGGTGAAGGTGCAGGGGTAGTCGATATAGGTGATAATC
AAGCCGTAGTATTTAAAGTAGAGTCTCACAATCATCCATCAGCAATTGAACCATATCAAGGGGCTGCTACAGGCGTTGGTGGAATCATTC
GTGACATTGTCTCTATTGGGGCTAGACCTATTAATTTGTTAAACAGTCTTAGATTTGGAGAATTAGATAATAAACAAAACCAAAGATTACT
TAAAGGTGTTGTAAAGGGTATCGGAGGTTATGGTAACTGCATTGGTATTCCAACAACTGCTGGTGAAATCGAATTTGATGAACGTTATGA
TGGCAATCCACTTGTTAATGCAATGTGTGTTGGTGTTATCAATCACGACATGATTCAAAAAGGCACAGCAAAAGGTGTAGGTAATTCGGT
CATTTATGTTGGTTTGAAAACTGGTCGAGATGGTATTCATGGTGCTACTTTTGCATCTGAAGAATTGACGGAAGAAAGCGAAAGTAAACG
ACCTTCTGTACAAATCGGTGATCCATTTGTAGGTAAAAAATTAATGGAAGCAACACTTGAAGCAATTACATTTGATGAATTAGTTGGTATT
CAAGATATGGGTGCTGCTGGTTTAACATCTTCATCGTCTGAAATGGCGGCAAAAGGTGGTAGTGGGTTACATTTGAGATTAGAACAAGT
GCCAACACGTGAGCCAGGTATTTCTCCTTATGAAATGATGCTTTCAGAAACTCAAGAACGTATGTTACTAGTTGTTGAAAAAGGTACTGA
ACAAAAATTCTTAGATTTATTTGATAAGCACGAATTGGATAGTGCTGTTATAGGTGAAGTTACAGATACAAATCGTTTTGTTTTAACATAT
GATGACGAAGTTTATGCTGACATTCCAGTTGAACCACTAGCTGATGAAGCACCTGTATATATTTTAGAAGGAGAAGAAAAAGATTATAAT
ACTTCTAAAAATGATTATACACACATCGATGTTAAAGATACTTTCTTTAAATTACTTAAGCATCCGACTATAGCATCTAAACACTATTTATAT
GATCAATACGACCAACAAGTTGGTGCCAATACGATAATTAAGCCAGGACTTCAAGCATCGGTAGTACGTGTGGAAGGCACAAATAAGGC
AATTGCTTCAACAATTGATGGTGAAGCGCGTTATGTATATAACAATCCATATGAAGGTGGAAAGATGGTAGTAGCTGAAGCTTATCGAA
ATTTAATTGCCGTGGGTGCAACACCATTAGCAATGACAGATTGTTTAAATTATGGTTCTCCTGAAAAGAAAGAAATCTATCAACAGTTGAT
AGATTCAACGAAAGGTATGGCAGAAGCATGCGACATTCTTAAGACACCAGTAGTTTCTGGTAATGTATCTTTATATAACGAAACGAAAGG
TACTTCTATTTTCCCAACACCAGTTGTTGGAATGGTAGGTTTGATTGAAAATGTAAATTATTTAAATGATTTTGAACCTCAAGTTGGAGAT
AAATTATATTTAATCGGTGATACTAAGGACGACTTTGGTGGTAGTCAACTTGAAAAGTTAATTTATGGCAAAGTTAATCATGAATTTGAGT

CATTAGATTTGAGTTCAGAAGTTGAAAAAGGTGAATCAATCAAGACCGCTATTCGTGAAGGACTATTATCACATGTTCAAACAGTTGGTA
AAGGTGGCTTACTGATTACCTTAGCTAAACTAAGTGCGCATTACGGTTTAGGATTAAAATCTTCAATAGATATAACAAATGCACAATTGTT
TAGTGAGACGCAAGGCCGATATGTTGTTTCTGTTAAATCAGGTAAAACTTTAAATATTGATAATGCAATAGAAATTGGACTTTTAACAGA
TAGTGATAATTTCAAGGTAACAACACCATATACAGAGATTAGTGAAAATGTTTCAGATATTAAACAAATATGGGAAGGGGCAATTGCTCA
ATGTTTAACTACTCAGGATTAA

Gene: purF (phosphoribosylpyrophosphate amidotransferase)

Contig: 06_NODE_54, position: 182349 to 183833, length: 1485 nt, orientation: FORWARD

Perfect match to: (MW2-BA000033-[1048644:1050128], allele observed in CC1+CC8)

Sequence:

ATGTTTAACTACTCAGGATTAAACGAAGAATGTGGCGTGTTTGGTATTTGGAATCATCCTGAAGCAGCGCAACTAACATATATGGGACTT
CATAGTTTGCAACATCGTGGTCAAGAAGGTGCAGGTATAGTTGTTTCTGATCAAAATGAATTAAAAGGCGAGCGAGGATTAGGCTTACT
AACTGAAGCGATTAAAGATGATCAAATGGAACGATTAAAAGGATATCAACATGCAATTGGTCACGTCCGTTATGCTACTTCAGGTAATAA
AGGTATTGAAAATATTCAACCGTTTCTGTATCACTTTTATGATATGAGTGTAGGTATTTGTCATAATGGTAACCTCATTAATGCTAAATCAT
TGCGTCAGAATTTAGAAAAACAAGGTGCTATCTTCCATTCGTCTTCTGATACTGAAGTCATTATGCATTTGATACGTCGAAGTAAAGCTCC
TACTTTTGAGGAAGCGTTGAAAGAAAGTTTGCGAAAAGTTAAAGGCGGTTTTACATTTGCGATTTTAACTAAAGATGCGTTATATGGCGC
AGTAGATCCAAATGCTATCAGACCACTTGTTGTAGGTAAAATGAAAGATGGGACATACATCCTTGCAAGTGAAACATGTGCAATAGATGT
GTTAGGTGCAGAATTTGTTCAAGATATTCATGCAGGTGAATATGTCGTGATTAACGATAAAGGTATTACAGTTAAATCTTATACACATCAT
ACGACAACTGCAATTTCTGCGATGGAATATATTTATTTTGCTAGACCAGACTCAACAATAGCTGGTAAAAATGTCCATGCAGTACGTAAA
GCTTCTGGTAAAAAATTAGCCCAAGAAAGCCCTGTAAATGCTGATATGGTCATCGGTGTACCCAATTCATCGCTATCAGCTGCGAGTGGT
TATGCTGAAGAAATAGGTTTGCCATATGAAATGGGACTAGTTAAAAATCAATATGTTGCAAGAACATTTATTCAACCAACTCAAGAATTA
CGTGAGCAAGGTGTGAGAGTGAAGTTATCTGCGGTAAAAGATATAGTAGATGGGAAAAATATCATTCTTGTTGATGATTCCATTGTTCGC
GGTACGACAATTCGACGCATTGTGAAAATGTTAAAAGATTCTGGTGCAAATAAAGTGCATGTGCGTATAGCATCACCGGAATTTATGTTC
CCAAGTTTTTATGGAATCGATGTTTCAACTACGGCAGAATTAATTTCTGCAAGCAAATCACCTGAAGAAATTAAAGATTATATTGGCGCTG
ATTCATTAGCATATCTATCTGTAGATGGGTTAATTGAATCAATTGGTTTAGATTATGACGCGCCATATAGTGGCTTATGTGTAGAAAGTTT
CACTGGAGATTATCCTGCAGGGTTATATGATTATGAAGCAAATTATAAAGCACATTTAAGTCATCGACAAAAGCAATATATTTCTAAAAAC
AAACACTTTTTTGATAGCGAGGGAAATTTAAATGTCTAA

Gene: purM (phosphoribosylformylglycinamidine cyclo-ligase)

Contig: 06_NODE_54, position: 183826 to 184854, length: 1029 nt, orientation: FORWARD

Perfect match to: (MW2-BA000033-[1050121:1051149], allele observed in CC1)

Sequence:

ATGTCTAAAGCATATGAACAATCTGGTGTAAATATTCATGCTGGTTATGAAGCTGTAGAAAGAATGTCTAGTCATGTTAAACGTACGATG
CGTAAAGAAGTTATCGGTGGTTTAGGTGGATTCGGTGCTACATTTGATTTATCACAATTAAATATGACAGCGCCAGTTTTAGTTTCTGGAA
CAGACGGAGTAGGTACGAAATTAAAACTAGCTATCGACTATGGGAAACATGATTCGATAGGTATCGATGCAGTCGCAATGTGTGTTAAT
GATATTTTAACGACAGGTGCAGAACCATTGTATTTTTTAGATTACATCGCTACAAATAAAGTTGTTCCTGAAGTTATTGAACAAATTGTTA
AAGGTATTAGTGATGCATGCGTTGAAACGAATACTGCACTTATCGGTGGAGAGACTGCTGAAATGGGTGAAATGTATCACGAAGGTGAA
TATGATGTAGCCGGATTTGCAGTTGGAGCAGTTGAAAAGGATGACTATGTAGATAGTTCAGAAGTGAAAGAGGGACAAGTTGTTATAG
GGCTTGCGTCAAGTGGCATTCATTCAAATGGATATAGTTTAGTGCGCAAATTAATTAATGAATCAGGCATTGATTTGGCATCAAACTTCG
ATAATCGTCCATTTATAGATGTCTTTTTAGAACCAACTAAATTATATGTCAAACCTGTACTTGCTTTGAAAAAAGAAGTTTCTATTAAGGCA
ATGAATCATATTACTGGTGGAGGTTTTTATGAAAATATTCCACGTGCATTGCCAGCCGGATATGCTGCTAGAATTGATACTACATCATTTC
CAACGCCAAAAATATTTGATTGGTTACAACAACAAGGCAATATAGACACAAATGAAATGTATAACATTTTTAACATGGGTATTGGCTATA
CGGTTATCGTTGATGAAAAAGATGCATCACGCGCTTTGAAGATTTTAGCAGAACAAAATGTGGAAGCCTATCAAATTGGTCATATTATGA
AAAATGAGTCAACTGCAATTGAATTGTTGGGGGTATAA

Gene: purN (phosphoribosylglycinamide formyltransferase)

Contig: 06_NODE_54, position: 184857 to 185423, length: 567 nt, orientation: FORWARD

Perfect match to: (MW2-BA000033-[1051152:1051718], allele observed in CC1)

Sequence:

ATGGTTAAAATTGCGATTTTTGCATCAGGTTCAGGAAGTAACTTTGAAAATATAGTTGAGCATGTTGAATCAGGAAAACTTGAAAATATT
GAAGTTACGGCGCTATATACGGATCATCAAAATGCGTTTTGTATAGATAGAGCAAAAAAGCACGATATTCCTGTTTATATTAATGAACCA
AAACAATTTGATTCAAAAGCAGCGTATGAACAACATTTAGTATCACTATTAAATGAAGATAAGGTAGAGTGGATTATTTTAGCTGGCTAC
ATGCGTCTAATTGGTCCAGATTTATTAGCTTCATTTGAAGGTAAAATATTGAATATACATCCATCTCTATTGCCGAAATATAAGGGGATTG
ACGCAATAGGCCAAGCATATCATAGTGGCGATACTATTACTGGTTCGACAGTACATTATGTTGATTGTGGTATGGATACGGGAGAAATTA
TTGAACAGAGACAATGTGATATTAGACCGGACGATTCAAAAGAACAATTAGAAGAGAAAGTAAAAAAATTGGAATATGAGTTATATCCA
AGTGTTATTGCTAAAATTGTAAAATAA

Gene: purH (bifunctional phosphoribosylaminoimidazolecarboxamide formyltransferase/IMP cyclohydrolase)

Contig: 06_NODE_54, position: 185438 to 186916, length: 1479 nt, orientation: FORWARD

Perfect match to: (08-02119-CP015645-[1148841:1150319:r], allele observed in ST582+CC8)

Sequence:

ATGAAGAAAGCTATTTTGAGCGTATCAAATAAAACAGGTATTGTAGAGTTTGCTAAAGCGTTAACGCAATTAAATTATGAATTATATTCA
ACAGGTGGTACTAAACGTATATTAGATGAAGCAAATGTACCAGTTCGTTCTGTTTCAGACTTAACACATTTTCCAGAAATAATGGATGGC
CGTGTTAAAACATTACATCCGGCTGTTCATGGTGGCATTTTAGCTGATCGAAATAAACCGCAGCATTTAAATGAATTATCAGAACAACATA
TAGATTTAATTGATATGGTAGTAGTTAATTTATATCCATTCCAACAAACTGTTGCAAACCCTGATGTGACGATGGACGAAGCAATTGAAAA
TATTGATATTGGTGGTCCAACAATGTTACGTGCTGCAGCTAAAAACTATAAACATGTAACAACAATTGTACATCCGGCAGATTATCAAGA
AGTATTGACGCGATTAAGAAACGATTCGTTAGATGAGTCATATAGACAATCATTAATGATTAAAGTTTTTGAGCATACTGCAGAATATGA
TGAAGCGATTGTACGTTTCTTTAAAGGGGATAAAGAAACTTTAAGATATGGAGAAAATCCACAACAATCAGCGTATTTTGTGAGAACTTC
GAATGCTAAGCACACGATTGCAGGCGCTAAACAATTACATGGGAAACAATTAAGCTATAACAATATTAAAGATGCAGATGCTACACTAG
CTTTAGTTAAAAAGTTTGATACACCTGCTACAGTTGCGGTTAAACACATGAATCCATGTGGTGTTGGTATCGGTGACACGATAGAACAAG
CATTTCAACATGCGTATGAAGCGGATAGTCAATCAATATTTGGTGGAATTGTTGCATTAAACCGAGCTGTAACACCTGAGTTAGCAGAGC
AATTGCATAGTATCTTTTTGGAAGTCATTATTGCACCAAAATTTACAGATGAAGCATTAGATATTTTAAAACAAAAGAAGAATGTAAGATT
ATTAGAAATTGATATGACTATAGACAGTAACGAAGAAGAGTTTGTTTCAGTATCTGGCGGATATTTAGTTCAAGATAAAGACAATTATGT
CGTGCCAAAAGAAGAAATGAAAGTTGTTACAGAAGTAGCACCTACTGATGAACAATGGGAAGCAATGTTATTAGGATGGAAAGTTGTAC
CATCAGTAAAAAGTAATGCAATTATTTTAAGTAATAATAAACAAACTGTAGGTATAGGTGCTGGACAAATGAATCGTGTCGGTGCTGCTA
AAATTGCGTTAGAGAGAGCTATTGAAATCAATGATCATGTAGCGTTAGTATCTGATGGATTTTTCCCTATGGGAGATACAGTTGAACTTG
CAGCACAACATGGTATAAAGGCAATTATCCAACCGGGTGGTTCGATTAAAGATCAAGATTCAATTGATATGGCTAATAAACATGGTATTG
CAATGGTAGTCACAGGCACTCGACATTTTAAACACTAA

Gene: purD (phosphoribosylamine-glycine ligase)

Contig: 06_NODE_54, position: 186938 to 188185, length: 1248 nt, orientation: FORWARD

Sequence:

ATGAATGTATTAGTAATTGGTGCTGGTGGACGAGAACATGCACTTGCATATAAACTTAATCAATCGAATCTAGTTAAACAAGTGTTTGCC
ATTCCAGGTAATGAGGCAATGACACCTATAGCTGAAGTACACACTGAAATTTCAGAATCTGATCATCAAGCGATATTAGATTTTGCTAAA
CGGCAAAATGTTGATTGGGTAGTTATAGGTCCAGAACAGCCGCTAATTGATGGATTAGCAGACATTTTACGAGCGAATGGTTTCAAAGT
GTTTGGTCCAAATAAGCAAGCAGCTCAAATCGAAGGCTCAAAATTATTTGCTAAAAAGATAATGGAAAAATATAATATTCCAACTGCTGA
TTATAAAGAAGTTGAGCGAAAAAAGGATGCTTTAACATATATTGAAAACTGTGAATTGCCCGTTGTTGTCAAGAAAGATGGGTTAGCTGC
TGGGAAAGGCGTTATTATTGAAGATACTATTGAAGCAGCCAGAAGTGCTATTGAGATTATGTATGGTGATGAAGAAGAAGGTACTGTTG
TATTTGAAACGTTTTTAGAAGGTGAAGAGTTCTCGCTAATGACATTTGTTAATGGTGATTTAGCAGTACCTTTCGACTGTATTGCACAAGA
TCATAAACGCGCATTTGATCATGATGAAGGACCAAATACTGGTGGTATGGGGGCTTATTGTCCAGTACCACATATTAGTGACGATGTTTT
AAAACTTACAAATGAAACAATTGCACAACCCATTGCAAAGGCAATGCTTAATGAAGGTTATCAATTCTTCGGTGTATTATACATTGGTGCT
ATTTTAACTAAAAATGGTCCAAAAGTAATAGAATTTAATGCCCGTTTTGGTGATCCTGAAGCTCAAGTATTATTAAGTCGCATGGAAAGT
GATTTAATGCAGCATATTATTGATTTAGATGAAGGAAAACGTACTGAATTCAAATGGAAAAATGAATCTATTGTAGGGGTCATGTTGGCA
TCAAAAGGATATCCTGATGCATATGAAAAAGGGCATAAAGTAAGTGGCTTTGATTTAAATGAAAACTATTTTGTTAGTGGATTAAAGAAG
CAAGGTGATACCTTTGTTACTTCAGGTGGTAGAGTTATACTTGCCATCGGAAAAGGTGACAATGTACAAGATGCACAGCGAGACGCATA
CGAAAAAGTATCACAAATACAAAGTGACCATTTATTCTATCGTCATGACATTGCGAATAAAGCACTACAACTTAAATAA

Gene: ykoC (hydroxy-methyl-pyrimidine ABC transporter, transmembrane permease)

Contig: 06_NODE_54, position: 188450 to 189256, length: 807 nt, orientation: REVERSE

Perfect match to: (MW2-BA000033-[1054745:1055551:r], allele observed in CC1+CC5)

Sequence:

TTAACCTACACGACCAAGACGAACATCATCTATGCCCGTGATGGGTAAGGTGATTGAACAATAATATGCCAAAGTAATAATGGCAATTAA
AACTATAATAAAGATTATATCTTTATATGAGAAAGGTACGTTGTAATAGTAAGTACGAGGACCATCTCTAAATCCTTTGGACTCCATCGCA
ACTGATAATTGATGTGCCTTTCTAATATTTTGGCTTAATAGAGGTATAATTAAATGCTTAAATCGCTTTAACCCTCTATAATTTGCTGCGTCT
ATCATCTGATAGCGCATTTTTAAAGATCTGCGAAGCTGTATTAAAGAACTAATCATTAAAGGTATCATACGAATGGCAGCCATGAATGCA
TAAGCAACTTTTGATTTAACCTTTAAATGTTGCATTAAACTATAAAATATCATGACAACCTGAGATGTAAGTGCGATTAAGATACCGAAAA
ATGAAATAGCAATGGTTCTTAATGATACATGTAAACCACGAACTAAACTTTCTGTTGTAATATGGATAAATCCGAATTTCAAAATTGTATG
GCTACCATTCCCGTATAAAATCATGAACAGGGAAGAGAGTAATGCAAAGCCAATACTTATAGTTATAAAAATTGCTGTAATTTTAAACTG
AGTACCATTAAACATCAATAAGAAAACTAACATTAAGATAGTGATATAAAGCATAAAATCGAAATTATGCACAAATATAATAAAGAAAAA
TAGTATAATTCCAAGAAATAGTTTCGTTATAATGTTGACATCATCAACAAATGATTGCCGAACTTTCCATTGCTCATACAT

Gene: ykoD (hydroxy-methyl-pyrimidine ABC transporter, ATPase)

Contig: 06_NODE_54, position: 189249 to 190649, length: 1401 nt, orientation: REVERSE

Perfect match to: (MW2-BA000033-[1055544:1056944:r], allele observed in CC1+CC22+CC30)

Sequence:

TCATACATTCGTATCACCATCACAATCTAGTAACGCACCATCTGAAATTTTAAGTCTTCTTGATGGATAACGTTCAATTATTTCATCGTCAT
GTGTAACCATGACAATACTTTGTCCCAAATTAATTCGCTTTTGGAAAAGTTTGATCAACTGGAATGTATTATGGCTATCAAGTCCAAATGT
CGGTTCATCTAAAAAGATAATATCAGCTTTAGAACTTAGTGCGGTAGCTACGCTAAGGCGTCGTTTTTGACCAATAGACAACTCATAAGG
ATGTTGATCTTTTACATTTTGTAAATCTAAAAGTTTTAAAAGTTGTATCGTTTCATCATCACTTTGATCTTTAGAAAGGTGATTAAAATGAA
TGTTAATTTCATCATAAACCGAATTTGTTATAAATTGTAATTCTGGGTTTTGATAAACTAGGTACATGTGTTTTGCTGCATGTTTAATTTTTG
TTAAACGCTGATTTTCAAAATAAACATCACCTTGATATTTAATCAATTGCATAATTGATTCAAGCAAGGTTGTTTTACCACTACCATTTGCC
CCTGTAATTGTAATCCACTCACCTAGACCAATTTCTAAATCTGAGAATGAGAGCAATGTTGATTTACCGCGAATAATACGTCCATTTTTAA
ATTGTAATAAGTGTGAGTTTGTTGTTGGAAAGTCAACACGACTTGGTGCGAATTCCCATGCACGTGGATGCCACACACCATATTCACTGA
GTAAATGAACATACTTCTGTAATATGATTTCAGGACATTCATCGGCAATGATATTTCCGTTATAATCCATCAAAATGACGCGGTCGACATG
ATTCCAGATGTGTTTAACTTTATGTTCAACGATTACAACAGTTTGATCTTCCCAAAGTTCAATTAGTTTAGTCCATAAATCTTCTGTTGCTTG
AACATCTAACATTGCTGTCGGTTCATCTAAAAACAATGTTTTTGATTGTTGAAGAATGGTTTCAACAATTGCCAATTTCTGTTTCATCCCGC
CACTTAAATCTTTGATATACGTTTCAGGGGTAACATTTAAATTGACCATATTTAAAGCATTGATAATTAACGCATCCATGTCTTCACGTGGT
ACTTGTCTATTTTCTAAAACGAATGCAAGTTCTTCGTATACTTTTGGCATACAAAACTGGCTATCAGGGTCTTGGAAAATAACGCCACTTA
ATGGGTCAACGATTAGTTCATCATATTTCATAGGTAATTCAATTAAATTAGGAACAATACCACTTAATACATTCAGAAGTGTACTTTTACCG
CAACCAGAAGGACCGAGTAAAAGTACTTTTTCTTTGTCTTGAATAGTGATATTTAAATGATCGAAAATTTTACGCTGACCACTTGGATATT
TTAATCGTAAATCACTTACTTTTAACAC

Gene: ykoE (hydroxy-methyl-pyrimidine ABC transporter, substrate-binding protein)

Contig: 06_NODE_54, position: 190664 to 191239, length: 576 nt, orientation: REVERSE

Perfect match to: (MW2-BA000033-[1056959:1057534:r], highly conserved allele)

Sequence:

TTATAAGTTGTCGTAATCTTCTTTAGCAGCTGGTCTAAATAATTTTGTTACGCCTGTCTTATCTAAAGCTTTTACTAAAAGGTAAGATAGGA
CGCCGGCGACTACTGCACCACTAATTAATCTAAATACGATGAATAATGTTAAGTTCCAACCTGCAACTTCATTTAAATAACCATAGAAATA
ATCTATAGGGAAAGCCGCGATTGCTGTACAAAAACCTGCTAACATAGCTACCATAACTGAACGAGATTGATATTTAAAAATTGCAAAGAC
AAGTTCACACGCTAAACCTTGTATGAAAGCGTAAACGATTGTCGGAATATCGAAACGACCCATAATGATAGTTTCGCCGGCACCTGCAGC
AAATTCAGCCAGTAAAGCAATACCTGGTTTTGGAATAATTAGATAGCAGACAATCGCTGCCATGAACCAAACCCCGTTTGTTAATTGTTCG
AGGTGAAGGCCTGTAGCTTGCACACCATTGTAAACAAACCACCATAAATTGTAAATAACTGCGAATACTACTGAAATAAGTACGGTTACT
AGTATTTCAGATAGCTTTAAACCTTTTGACAT

Gene: graF (putative protein)

Contig: 06_NODE_54, position: 191760 to 191894, length: 135 nt, orientation: REVERSE

Perfect match to: (N315-BA000018-[1056731:1056865:r], highly conserved allele)

Sequence:

TTAATTTAGTGTATCTTGGATATCTTGTTTTGTTTGATTAATATCTTCTGTTTTTTCTTCTTTTTTATCTTTTAATTTTTCTTCAACTTCTTTAGC
TTTTTCTGCTGCTTTTTTATTTTGATTTTCATTAGACAT

Gene: Q5HH05 (polytopic membrane protein)

Contig: 06_NODE_54, position: 192047 to 193342, length: 1296 nt, orientation: FORWARD

Perfect match to: (GR1-AJLX01000008-[118099:119394], allele observed in CC361+CC239)

Sequence:

ATGTCTTTTCTTAGGAAACACGCCGAAATTATTTTTAGCTATTTAATCGGTATCGTTTCACTCTTCACTGGTCTCATTATTTTAATTAACTTG
CCATTAATTAAACAATTAAATGGTGGTAAAAAAGTTGATACACATGTTCATAATGTGTGGGAATTTCTGAATGCATTTTTCAGTGAAATTA
TTAAAGTAATGAGTCGATTTATAGGTAATTTCCCTATAGTTAGTGCAATTGTGATAATTATATTCGGTATTTTAGTTATGTTGATTGGTCAT
ACATTACTTAGAACTATTAAGTATGACTATGATATTTCTATCTTTTTCTTAGTTATCGGTATCATGTACTTTATTATTACTCTTATATTAATGA
CTCAAGTTTATGGATTCTTTGCAGTGATTTTCATTATTCCATTTACAATTCATATAGGATATATCGTCTATAAAGATGAATTGAATCAGGAA
AATGTAAAAAATCATTTCATGTGGATAATTGTGAGTTATGGTATAAGTTACTTAATTACACAAATTGCATTGTATGGCAGAATTGATGCTA
ATGAAATAGAGTCAATTGATATCTTAAGTGTCAATGCTTTCTTTATAATTATGTGGTTACTTGGTCAAATGGCTATTTGGAATTTCTTGTTC
TTGCGCCGAGCTTTACCTTTAACAAAGCAAGAATTAGGTGAAGAGGAGCCAGAATTATCAAGAACAAGTAAAGGGAATGTCACGAATCA
AACTAAAATTCACTTGAAACAACTCCAAGATAAGACTACAGAATATGCACGTAAGACAAGAAGAAGTGTCGATTTAGATAAAATTAGAG
CTAAAAGAGATAAATTCAAAAAGAAAGTTAATGATATTATCGATATTCAAGAAGACGATATTCCTGATTGGATGAGAAAACCGAAATGG
GTTAAACCAATGTATGTCGAACTATTTTGTGGTGTCGTCATCTTTTTATTCACATTTTTAGAATTTAATAATCGTAATGCATTATTTGTATCT
GGTGATTGGAAATTATCACAGACACAATATGTTATTGAATGGGTTACATTACTAATTCTGTTATTCATTATTATCGCATATATCGCTACAAC
GTTAACTTTCCACTTGAAAGGTAAGTTTTATTATTTACAACTATTTATGGGGAGCATTTTATTCTTTAAATTGTTAACGGAATTCATAAATAT
AATGATTCATGGACTACTACTTTCAGTGTTCATTACACCAACATTACTATTAATGTTATTGGCAATCATCATTTCTTATTCGTTACAATTACG
AGAGCGACCATAA

Gene: ywbD (S-adenosylmethionine-dependent methyltransferase)

Contig: 06_NODE_54, position: 193767 to 194939, length: 1173 nt, orientation: FORWARD

Perfect match to: (MW2-BA000033-[1060061:1061233], allele observed in CC1+CC5)

Sequence:

ATGAAAATAGCAACTCTGAACAAAGGCAAAGAAACAAAATATTTTAATGGATATCCTTTAATTGAAGAAGAGGATATCTATTCACAAGAT
CATTTAAAAGAAGGAGATATTTTTCAAATTGTGACTGATAAATCACAATATGTTGCAACGGCTTATGTTGGTCGCCAACATAAAGGATTA
GGTTGGGTTCTAACATACGATAAAGCTCAAGAAATCAACACAGCTTTCTTTGTGAAATTGTTTAATACTGCATTAGCAGAACGTGATTATT
ATTTTAATATAGATGGAACAAATGCTTTTAGATTATTTAATGCTGAAGGTGATGGTGTTGGGGGATTAACAATCGACAATTACGATGGTC
ATTTGTTGATTCAATGGTACTCAAAAGGTATTTATAAATTTAAATATGCCATTCTTGAAGCGGTTAGAAAAGTATTTGATTATAAATCTATT
TACGAAAAAGTAAGATTTAAAGACAGCGAATATAGTGGTGGTTTTGTTGAAGGAGATGCACCTGAGTTTCCAATTGTTATCGAAGAAAA
CTTCACATTTTATAATGTAGACCTTGAAGATGGTTTGATGACAGGTATCTTTTTAGATCAAAAAGAAGTGCGCAAGAAATTAAGGGATCA
ATATGCCAAAGAACGCCATGTTTTAAACTTATTTAGTTATACAGGTGCTTTTTCTGTAATAGCAGCAAGTGAGGCATCTTCAACAACAAGT
GTAGATTTGGCTAATCGTTCTCGTAGTTTAACTGAAGAAAATTTTGGATTAAATGCTATTGATCCTAAATCCCAATATATTTATGTCATGGA
CACTTTTGATTTCTATAAATATGCTGCACGACATGGACATAGTTATGACACGATCGTGATTGATCCACCTAGCTTTGCGCGTAACAAAAAA
CGTACATTTTCAGTGCAAAAAGATTATGACAAATTAATTAATGGCGCCTTAAATATCTTATCATCTGAAGGAACATTATTGTTATGTACAA
ACGCAAGTGTATATCCATTAAAGCAATTTAAAAATACTATTAAAAAGACGCTTGAAGAGAGTGGCGTTGATTATGAATTAACTGAAGTTA
TGGGATTACCAAAAGATTTTAAAACGCATCCACATTATAAGCCATCTAAATATTTAAAAGCTGTTTTTGTAAATATTAGACATTAA

Gene: Q5HH03 (putative protein)

Contig: 06_NODE_54, position: 194993 to 195535, length: 543 nt, orientation: FORWARD

Perfect match to: (MW2-BA000033-[1061287:1061829], allele observed in CC1+CC72+CC80+CC188+CC772)

Sequence:

ATGGGATTCAAAAACAATTTAACATCAAATTTAACAAATAAAATTGGTAATTCAGTCTTTAAAATAGAAAATGTTGACGGAAAAGGTGCA
ATGCCAACGACGATTCAAGAATTGAGAGAAAGACGACAACGTGCTGAAGCAATTGTAAAGAGAAAATCTTTAATGTCATCAACAATGAG
CGTTGTTCCAATTCCGGGTTTAGATTTTGGTGTTGATTTAAAATTAATGAAAGATATTATCGAAGATGTAAATAAAATATACGGATTAGAT
CATAAGCAAGTTAATAGCCTTGGGGATGATGTGAAAGAAAGAATTATGTCTGCAGCAGCAATTCAAGGTAGTCAATTTATTGGTAAAAG
AATTTCAAGTGCATTTTTAAAAATAGTTATTAGAGATGTAGCTAAACGTACTGCTGCAAAACAAACAAAATGGTTTCCTGTTGTAGGACAA
GCTGTGTCTGCATCTATTAGTTACTATTTTATGAATAAAATTGGAAAAGATCACATTCAAAAATGCGAAAATGTTATTAAAAATGTCATGT
AG

Gene: ptsH (phosphocarrier protein HPr)

Contig: 06_NODE_54, position: 195689 to 195955, length: 267 nt, orientation: FORWARD

Perfect match to: (N315-BA000018-[1060659:1060925], highly conserved allele)

Sequence:

ATGGAACAAAATTCATATGTAATCATCGACGAGACTGGTATTCACGCTAGACCAGCAACAATGTTAGTACAAACAGCTTCAAAATTCGAT
TCTGATATTCAATTAGAATATAACGGTAAGAAAGTAAACTTAAAATCAATCATGGGTGTTATGAGCCTTGGTGTTGGTAAAGATGCTGAA
ATTACAATTTATGCTGACGGTAGTGATGAATCTGACGCCATTCAAGCAATCAGTGACGTCTTATCAAAAGAAGGATTGACTAAATAA

Gene: ptsI (phosphoenolpyruvate-protein phosphotransferase)

Contig: 06_NODE_54, position: 195958 to 197676, length: 1719 nt, orientation: FORWARD

Perfect match to: (MW2-BA000033-[1062252:1063970], allele observed in CC1)

Sequence:

ATGTCTAAATTAATTAAAGGTATTGCCGCATCTGATGGTGTCGCAATTGCTAAAGCTTATTTATTAGTTGAGCCAGACTTAACATTCGACA
AAAATGAAAAAGTCACTGATGTTGAAGGAGAAGTTGCAAAGTTCAATAGCGCTATCGAAGCTTCTAAAGTTGAGTTAACTAAAATTAGA
AATAATGCAGAGGTTCAACTAGGTGCTGATAAAGCTGCTATCTTTGATGCACATTTATTAGTTTTAGATGACCCTGAATTAATTCAACCAA
TCCAAGATAAGATTAAAAATGAAAACGCTAATGCTGCTACAGCATTAACGGATGTAACAACACAATTTGTTACAATTTTTGAATCTATGGA
TAACGAATACATGAAAGAACGTGCGGCTGATATTCGCGACGTTTCTAAACGTGTGTTATCACATATTTTAGGTGTAGAATTACCGAATCC
GAGTATGATTGATGAAAGCGTTGTTATTGTAGGGAATGACTTAACGCCATCTGATACTGCTCAATTAAATAAAGAATTCGTACAAGGTTT
TGCTACAAACATTGGCGGAAGAACAAGTCACTCTGCAATTATGAGTCGTTCTTTAGAAATTCCAGCAATTGTTGGTACAAAATCAATTACT
CAAGAAGTTAAACAAGGCGACATGATTATCGTAGATGGATTAAATGGTGATGTAATCGTTAATCCAACTGAAGATGAGTTAATCGCTTAT
CAAGATAAACGTGAGCGTTATTTTGCTGACAAGAAAGAATTACAAAAACTACGTGATGCTGATACTGTTACAGTTGATGGTGTTCACGCA
GAGCTTGCTGCAAATATTGGTACACCTAATGATTTGCCAGGTGTTATTGAAAATGGTGCACAAGGTATCGGCTTATATAGAACTGAGTTT
TTATATATGGGTCGTGACCAAATGCCTACAGAAGAAGAACAATTTGAAGCTTATAAAGAAGTATTAGAAGCAATGGACGGTAAACGTGT
TGTTGTACGTACTTTAGATATAGGTGGAGATAAAGAATTATCATACTTAAACTTGCCTGAAGAAATGAATCCATTCTTAGGTTACCGTGCG
ATTCGTTTATGCCTTGCGCAACAAGATATTTTCAGACCACAGCTACGTGCATTATTACGTGCATCAGTTTATGGTAAGTTAAATATCATGTT
CCCAATGGTTGCAACAATTAACGAATTTAGAGAAGCTAAAGCTATATTATTAGAAGAAAAAGAAAACCTTAAAAATGAAGGTCATGACAT
TTCGGATGATATAGAATTAGGAATCATGGTAGAGATACCTGCAACAGCAGCATTAGCTGATGTCTTTGCTAAAGAAGTAGATTTCTTCAG
TATCGGTACAAATGATTTAATTCAATACACATTAGCTGCTGACCGTATGTCAGAGCGTGTATCATATCTATACCAACCATATAACCCTTCAA
TCTTACGTTTAGTTAAACAAGTTATTGAAGCGTCACATAAAGAAGGTAAATGGACAGGTATGTGTGGTGAAATGGCTGGAGATGAAACA
GCTATTCCATTATTGCTTGGTTTAGGTTTAGATGAGTTCTCTATGAGTGCAACGTCTATTCTGAAAGCAAGAAGACAAATTAATGGTTTAA
GTAAAAATGAAATGACTGAACTTGCTAACCGTGCAGTCGACTGTGCAACGCAAGAAGAAGTTATTGAATTAGTTAACAACTACGTAAAAT
AA

Gene: nrdH (glutaredoxin)

Contig: 06_NODE_54, position: 197913 to 198146, length: 234 nt, orientation: REVERSE

Perfect match to: (MW2-BA000033-[1064207:1064440:r], allele observed in CC1+CC7)

Sequence:

TTAATCTTGGATATTTAATACTTTGTTGATTTCATCAAGATCAACATGGTACATTGGATTACCATTCAACAAAATAAACGGAGTTGAAAAA
GCATCAAAATCTATCATTTCGTTTCGATATTGTTGATTGTTGATATTTCTCTCTTCAAAATCAATTTGATGCTCATTAAGATAATTTTTTACA
AATGTACAAGGTGGGCAATCATTCTGCGTATAAACGATTATTTCTGACAT

Gene: cydA (cytochrome bd quinol oxidase subunit 1)

Contig: 06_NODE_54, position: 198344 to 199705, length: 1362 nt, orientation: FORWARD

Perfect match to: (MW2-BA000033-[1064638:1065999], allele observed in CC1)

Sequence:

ATGGATACAGTTGAAATCAGTCGGTTTTTGACAGCTATGACTTTAGCAGTTCATATCATTTTTGCAACGATTGGTGTTGGTATGCCTTTAAT
GTTCGCAATTGCAGAATTTTTAGGTATTCGCAAAAATGATCTTCAATATATAGCTATGGCCAAAAGATGGGCTAAAGCTTATACAATTACT
GTAGCAGTGGGAGTTGTTACAGGTACAATTATAGGACTTCAATTATCATTGATTTGGCCTACATTTATGGAAATGGGTGGACACGTTATT
GCACTTCCATTATTTATGGAAACATTTGCGTTCTTCTTTGAAGCTATTTTCTTAAGTATATATTTATATACTTGGGATCGTTTTAAAAATAAA
TGGACACATTTCTTAATTAGTATACCAGTAATTATTGGTGGCTCTTTCTCAGCATTCTTCATTACTTCAGTGAATTCATTTATGAATACGCCT
GCAGGTTTTGAGTTGAAGAATGGAAAGATGGTCAATGTTCAACCTATAGAAGCGATGTTTAACCCATCGTTTATAGTTCGATCATTTCAC
GTAATTACTACAGCCGGTATGACGATGGCATTTGTTATTGCATCAATAGCAGCTTTTAAATTATTGCGTAATCGTCAACCTAAAGATACTG
TCTACCATAAGAAAGCTTTGAAAATGTCTATGATAGTTGGATTCTTTTCAACATTACTTTCTATGTTGGCAGGGGATTTATCTGCAAAATTT
TTGCATAAATTCCAACCTGAAAAATTAGCAGCTTATGAATGGCATTTCGATACATCTTCCCATGCTAAATTATTATTATTTGGTGTGTTAGA
TGAAAAGACTCAGCAAGTTAAAGGTGCGATTGAATTACCTGGACTATTGAGTTTCTTAGCAGATAATAGTGTCAAAACTAAAGTGCAAG
GGTTAAATGATTTTCCAAAAAGTTTACATCCGCCTATGATTGTCCATTATTTCTTTGATTTAATGGTAACGATGGGAATTTTATGTTTTGTC
ATTTCAGGTGTTTATGTTTTAACTTTAATGTTTAAAAAGCTTAGAAAGTTTTCTACTCATAAATGGATGCTTTACGGAATATTATTAACAGG
ACCAGCTTCAATGCTAGCTATAGAATTTGGATGGTTCTTAACAGAGATGGGTAGACAGCCTTGGATTGTTCGTGGTTATATGCGCGTGGC
AGAAGCAGCAACACAAGCAGGCGGAATAACCTTCGTTACAATTTTATTTGGCATATTGTACATCATTTTAATGTATACATGTGCATACGTA
TTAATTCGTATGTTTAAAAATAAACCGGCGTATGAAGATGTAAATCGTTTAGCCAAGAAGCAAGGAGGAGAAATAGAAAAATGA

Gene: cydB (cytochrome bd quinol oxidase subunit 2)

Contig: 06_NODE_54, position: 199702 to 200721, length: 1020 nt, orientation: FORWARD

Perfect match to: (MW2-BA000033-[1065996:1067015], allele observed in CC1+CC8)

Sequence:

ATGATTTATGCATTTATAGGTATAACAGTGTTATGGTTGTTTTTATTTTGCTATATCATTATTGCTTCTATAGATTTTGGGGCAGGTTTCTTC
GCATTGCATTCAAAGTTAACTGGTGATGAAAAGAAAATTAATCACTTAATTTCACGTTATTTAAACCCAGTTTGGGAAGTTACGAATGTAT
TCTTTGTTTTCTTCTTCGTAGGATTCGTAGGTTTCTTTCCAGAGTCAATCAAATATTTAGGCACGGTATTGTTAATACCAGGTTCAATAGCA
CTGATTATGATATCGTTGAGAAACAGTTTTTATGCATTTGAAAATTATGGTCAAGATACAAAATTAGCATGGATGATCATGTATGGGGTA
AGTGGATTATTAATTCCAGCTTCATTATCTACTGCTTTAACTATTACAGAAGGTGGCTATATTAATGTTCGAAACAATGTTATCGATCTAGA
TTGGGTGCAGTTACTATTAAGCCCATTTGCTTGGTCTGTAGTATTCTTGGCAATTATTTCAGTTTTATATATTTCATCAGGATTTTTGACATA
TTATGCTAAAAAAGCAAATGACGAACCAGCATATAATTTAACAAGACAATGGCACATATTTTTAGGGCCGCCGATGATTATCATTTGTTTA
TTCGTATTTCTATCATTACGCATTCAAAATTCTGAACATTTTTATTCAGCTGTTTTTGACTATTGGTGGATGTTTGCTATAAGTTTCTTATTCT
TTGCATTAGCTTCATTGTTAACATTCTTTAAGAAAAAACATGGTTTGGCTTTTGTATTTGTTATTTTACAAATGATGTTCGCGTTCTTTGGCT
ATGGAATTAGTAAATTGCCATACCTTTTATATCCGTTTGTAAAAATTACAGATGCATATGTTAATCCAGAAATGGGCTGGACATTAGTGAT
TGTCTTTATTTTAGGTTTACTTTTATTACTTCCATCGTTAATATTATTATTAAGATTATTTGTTTTCGACAAAGAATATGTTGAAGGAAAGAA
ATCATAA

Gene: ktrA (potassium uptake protein A)

Contig: 06_NODE_54, position: 200854 to 201516, length: 663 nt, orientation: FORWARD

Perfect match to: (N315-BA000018-[1065824:1066486], highly conserved allele)

Sequence:

ATGGGTAAAGAATATGTAGTCATCGGTCTAGGTCGTTTTGGAGGTAGTATCGTTCGTGAGTTGAATGCATTAGACATGGATGTAATGGC
CATCGACCATGATGAAAATAGAGTGAATGAATATAGTGATATCGCTACTCATGCGGTTGTTGCAGACACAACAGATGAAGCAGTTATGA
AAAGTTTAGGTATCCGTAACTTTGATCATGTCATTGTGGCAATTGGTGAAAATATTCAATCAAGTACGTTGACGACTTTAATTTTAAAAGA
GTTAGGTGTAAAAAAAGTAACTGCTAAAGCACAAAATGATTATCATGCAAAGATTTTAAATAAAATTGGAGCAGATACGGTTGTGCACCC
TGAGCGTGATATGGGTAGACGTATTGCGCATAATGTTGCGAGTGCAAGTGTACTTGATTATCTTGAGTTGGCAGACGAGCATTCTATTGT
AGAATTGAAAGCAACTGAAAAGATGGCGGGGCAGTCTATCATTGATTTAGATATAAGAGCACAATATGGAATTAACATTATTGCAATTA

AACGAGGCAAAGAGTTCATCATTTCACCAAATCCAAATATTAATTTAGAAATAGGTGATATTTTAATCATGATTGGACATGATAATGATTT
AAATCGCTTTGAAAAAAATATTGCGACGAGATAA

Gene: tx_universal2 (rho-independent terminator)

Contig: 06_NODE_54, position: 201687 to 201725, length: 39 nt

Perfect match to: (Strain_21331-AGTV01000040-[104403:104441], allele observed in CC398)

Sequence:

TAGAATTGAAAAAAGCTTGTTACAAGCGCATTTTCGTTC

Gene: rnjA (ribonuclease J1)

Contig: 06_NODE_54, position: 201865 to 203562, length: 1698 nt, orientation: REVERSE

Perfect match to: (MW2-BA000033-[1068159:1069856:r], allele observed in CC1+CC7+CC12+CC25+CC88)

Sequence:

TTATTTATTGTTTGATTCTTTTTGTTCGTTTACCTTCATAATGACTGGTAAAATCATTGGTTTTCTAGCTGTTTTTCAAATAAATAAGGTTGT
AATGTTTCAATGATAGAAGATTTAATCTGATGCCATTGAATATCTTTATTTTGATTTAACTTACTAATAACATCAGTTTTGATTTTGCGTTGT
GCATCATAAATTAATTGACCTGATTCCCTCATATATACAAATCCTCGAGAAATAATGTCTGGACCAGAAAGTAATTTATTTGTATTAAAATC
AATACTAACAACAACGATAACTAAACCTTCTTCAGATAATAGCTTACGGTCTCTTATTACAACATTACCGATATCACCGATACCACTACCAT
CAACAAGTACATTACCAGATGGAATGCGACCAGCTTTACGTGCTGAATCGTGTGTTAAAGCTAAGACATCTCCAATATCAAAGATGAAGA
CATTATCTTCTTCAACGCCGCATTCAACACCAGTCTCACCATGTGCTTTTAACATACGGTATTCACCATGAATAGGTAAGAAATATTTCGGC
TTGATTAATCGAAGCATTAATTGTTGATCACCTTGAGAACCATGCCCTGAAGTATGGATGTTAGAAATCTTGCTATGGATAACATCTGCAC
CAGCTTTATACAAGGAATTAATAGTTCTGTTAATACTTTTTGTATTACCTGGGATAGGTGATGAACTAAATACAACGGTATCTTCAGGTAT
AATTTTAATTTGCTTATGAGTACCATTAGCAATTCTAGATAATGCTGCCATTGGTTCACCTTGTGAACCAGTACATAGTATCAATAACTCAT
GCTTCGGTACGGTATTAATTTTATTAGGTTCAATAAATGTTTCAGGTGGTGCTTTAATATAACCAAGTTCCATACCGATTTTAATATTGTTT
TCCATCGAACGACCGAACGTAACAATTTTACGGTTATTTTTGATAGCAGCTTCAACTGCTTGTTGAACTCGGTAAATATTAGAAGCGAAG
GTAGCAAATATAATACGACCTTTACAATTACGGAAGATCTTATCTACGTTTTGACCAACTTCACGTTCGCTTAAAGTAAAATCAGGCACAA
GTGAATTTGTTGAGTCTGAAAGTAAACATAGAACGCCTTCTTCGCCTAATTGAGCCATTTTAGCAATGTTTGCTGGTTTGCCTACAGGTGT
AAAATCAAATTTAAAGTCACCGGTATGAACTACTTTTCCTTCAGGTGTATCTACGATGACGCCATAAGTTTCAGGAATACTATGCGTAGTT
AAGTAGAAAGAAATCGTAAAGTGCTTAGATTTAATCACACTGTCCTCATTGATTTCATTTAGTTTAGCAGTACGTAATAAATGATGTTCTT
CAAGTTTATTACGGATTAAACCTAATGCTAAAGGACCACCATAAATAGGTATATTAAGTTGTTTTAATAGGAAGGGCACACCGCCTATAT
GGTCTTCGTGACCATGTGTTATAAATAGGCCAACAATTTTATCTTGGTTTTGAACTAGATATGTGTAGTCAGGTATAACATAATCAATCCC
TAATAAGTTATCATCTGGGAATTTGATACCTGCATCGATAATGACAATTTCGTCTTTATACTCAACTGCATAAGTATTTTTACCGATTTCAC
CTAGACCTCCAAGTGCATATACACCTACTTCATTTGGATGTAATTGTTTCAT

Gene: rnpZA (DNA-directed RNA polymerase subunit omega 1)

Contig: 06_NODE_54, position: 203562 to 203780, length: 219 nt, orientation: REVERSE

Perfect match to: (N315-BA000018-[1068429:1068647:r], highly conserved allele)

Sequence:

TTATTTAGCAATCTCCACATTAAAGTGTTCTGAGTTTTCTTTTTCGTAATCTAAATGTGCGCCCTCTAATTTAGTGATAAATTCGATATTAAA
ATTACGATCTTTCAAGTAACGACGTACTTGTTCTTCTGTTTGAGCTTCAACATAAAGTGATTGTGTATTTTCACGCACAATTACCTCGTCTCT
GTTATGTTGATAAAAAACTTTAAATACTGCCAT

Gene: def1-defB (N-formylcysteine deformylase)

Contig: 06_NODE_54, position: 204262 to 204813, length: 552 nt, orientation: REVERSE

Perfect match to: (TW20-FN433596-[1157645:1158196:r], highly conserved allele)

Sequence:

TTAAACTTCTACAGCATCTGTATGTGGTTGTAATGGGTGATCTTTGTCAATGTGATCATAGAACATTACACCATTTAAATGGTCAATTTCAT
GTTGGAAAACAATTGCTGGATATCCTTTTAGACGTAATTGTATATCATTACCTTCGATGTCTTTGGCTTTAATTGTAATTCTATTATGACGG
TGAACTAGACCAGCAACATTATCATCGACACTAAGGCAACCTTCACCAGTTGGTAAATAAGCTTCTTGAACGCTATGACTTACAATTTTTG
GGTTCACAAGCATATAGTCATAAGATTTGCCACTGCCATCATCTGGTATTAAAACAGCAATCATACGTTTAGAAATATTAATTTGAGGTGC
AGCCAAACCAACGCCTGAACGTAAACCATATCGTTTCGCGATTTCCTCATCTTGACTATTTACTAAAAACTCTCTCATGGCGATTAATGTTT
CTTTTTCTTCTTTAGTTAATGGTAATTCTAACTCAGCTGCTTTTTGACGCAAAGTTGGATGACCATCTCTAATGATGTCTTTCATTGTTAACA
T

Gene: ykyA (putative cell-wall binding lipoprotein)

Contig: 06_NODE_54, position: 205178 to 205804, length: 627 nt, orientation: FORWARD

Perfect match to: (MW2-BA000033-[1071472:1072098], allele observed in CC1+CC80)

Sequence:

ATGAAATTTGGAAAAACAATCGCAGTAGTATTAGCATCTAGTGTCTTGCTTGCAGGATGTACTACGGATAAAAAAGAAATTAAGGCATAT
TTAAAGCAAGTGGATAAAATTAAAGACGATGAAGAACCAATTAAAACTGTTGGTAAGAAAATTGCTGAATTAGATGAGAAAAAGAAAA
AATTAACTGAAGATGTCAATAGTAAAGATACAGCAGTTCGCGGTAAAGCAGTAAAGGATTTAATTAAAAATGCCGATGATCGTCTAAAG
GAATTTGAAAAAGAAGAAGACGCAATTAAGAAGTCTGAACAAGACTTTAAGAAAGCAAAAAGTCACGTTGATAACATTGATAATGATGT
TAAACGTAAAGAAGTAAAACAATTAGATGATGTATTAAAAGAAAAATATAAGTTACACAGTGATTACGCGAAAGCATATAAAAAGGCTG
TAAACTCAGAGAAAACATTATTTAAATATTTAAATCAAAATGACGCGACACAACAAGGTGTTAACGAAAAATCAAAAGCAATAGAACAG
AACTATAAAAAGTTAAAAGAAGTATCAGATAAGTATACAAAAGTACTAAATAAGGTTGGTAAAGAAAAGCAAGACGTTGATCAATTTAA
ATAA

Gene: pdhA (pyruvate dehydrogenase E1 component, alpha subunit)

Contig: 06_NODE_54, position: 205975 to 207087, length: 1113 nt, orientation: FORWARD

Perfect match to: (MW2-BA000033-[1072269:1073381], allele observed in CC1+CC8+CC239)

Sequence:

ATGGCTCCTAAGTTACAAGCCCAATTCGATGCAGTAAAAGTTTTAAATGATACTCAATCGAAATTTGAAATGGTTCAAATTTTGGATGAGA
ATGGTAACGTCGTAAATGAAGACTTAGTACCTGATCTTACGGATGAACAATTAGTGGAATTAATGGAAAGAATGGTATGGACTCGTATCC
TTGATCAACGTTCTATCTCATTAAACAGACAAGGACGTTTAGGTTTCTATGCACCAACTGCTGGTCAAGAAGCATCACAATTAGCGTCACA
ATACGCTTTAGAAAAAGAAGATTACATTTTACCGGGATACAGAGATGTTCCTCAAATTATTTGGCATGGTTTACCATTAACTGAAGCTTTC
TTATTCTCAAGAGGTCACTTCAAAGGAAATCAATTCCCTGAAGGCGTTAATGCATTAAGCCCACAAATTATTATCGGTGCACAATACATTC
AAGCTGCTGGTGTTGCATTTGCACTTAAAAAACGTGGTAAAAATGCAGTTGCAATCACTTACACTGGTGACGGTGGTTCTTCACAAGGTG
ATTTCTACGAAGGTATTAACTTTGCAGCAGCTTATAAAGCACCTGCAATTTTCGTTATTCAAAACAATAACTATGCAATTTCAACACCAAGA
AGCAAGCAAACTGCTGCTGAAACATTAGCTCAAAAAGCAATTGCTGTAGGTATTCCTGGTATCCAAGTTGATGGTATGGATGCGTTAGCT
GTATATCAAGCAACTAAAGAAGCACGTGACCGCGCAGTTGCAGGTGAAGGTCCAACATTAATTGAAACTATGACATATCGTTATGGTCCT
CATACAATGGCTGGTGACGATCCAACTCGTTACAGAACTTCAGACGAAGATGCTGAATGGGAGAAAAAAGACCCATTAGTACGTTTCCG
TAAATTCCTTGAAAACAAAGGTTTATGGAATGAAGACAAAGAAAATGAAGTTATTGAACGTGCAAAAGCTGATATTAAAGCAGCAATTA
AAGAGGCTGATAACACTGAAAAACAAACTGTTACTTCTCTAATGGAAATTATGTATGAAGATATGCCTCAAAACTTAGCAGAACAATATG
AAATTTACAAAGAGAAGGAGTCGAAGTAA

Gene: pdhB (pyruvate dehydrogenase)

Contig: 06_NODE_54, position: 207091 to 208068, length: 978 nt, orientation: FORWARD

Perfect match to: (N315-BA000018-[1071958:1072935], highly conserved allele)

Sequence:

ATGGCACAAATGACAATGGTTCAAGCGATTAATGATGCGCTTAAAACTGAACTTAAAAATGACCAAGATGTTTTAATTTTTGGTGAAGAC
GTTGGTGTTAACGGCGGTGTTTTCCGTGTTACTGAAGGACTACAAAAAGAATTTGGTGAAGATAGAGTATTCGATACACCTTTAGCTGAA
TCAGGTATTGGTGGTTTAGCGATGGGTCTTGCAGTTGAAGGATTCCGTCCGGTTATGGAAGTACAATTCTTAGGTTTCGTATTCGAAGTA

TTTGATGCGATTGCTGGACAAATTGCACGTACTCGTTTCCGTTCAGGCGGTACTAAAACTGCACCTGTAACAATTCGTAGCCCATTTGGTG
GTGGCGTACACACACCAGAATTACACGCAGATAACTTAGAAGGTATTTTAGCTCAATCTCCAGGTCTAAAGGTTGTTATTCCTTCAGGCCC
ATACGATGCGAAAGGTTTATTAATTTCTTCTATTAGAAGTAATGACCCAGTCGTATACTTAGAGCATATGAAATTGTATCGTTCATTCCGT
GAAGAAGTACCTGAAGAAGAATATACAATTGACATTGGTAAGGCTAATGTGAAAAAAGAAGGTAATGACATTTCAATCATCACATACGG
TGCAATGGTTCAAGAATCAATGAAAGCTGCAGAAGAACTTGAAAAAGATGGTTATTCTGTTGAAGTAATTGACTTACGTACTGTTCAACC
AATCGATGTTGACACAATTGTAGCTTCAGTTGAAAAAACTGGTCGTGCAGTTGTAGTTCAAGAAGCACAACGTCAAGCTGGTGTTGGTGC
AGCAGTTGTAGCTGAATTAAGTGAACGTGCAATCCTTTCATTAGAAGCACCTATTGGAAGAGTTGCAGCAGCAGATACAATTTATCCATT
CACTCAAGCTGAAAATGTTTGGTTACCAAACAAAAATGACATCATCGAAAAAGCAAAAGAAACTTTAGAATTTTAA

Gene: pdhC (dihydrolipoyllysine-residue acetyltransferase component of pyruvate dehydrogenase complex)

Contig: 06_NODE_54, position: 208159 to 209451, length: 1293 nt, orientation: FORWARD

Perfect match to: (N315-BA000018-[1073026:1074318], allele observed in CC5+CC1+CC8)

Sequence:

GTGGCATTTGAATTTAGATTACCCGATATCGGGGAAGGTATCCACGAAGGTGAAATTGTAAAATGGTTTGTTAAAGCTGGAGATACTATT
GAAGAAGACGATGTTTTAGCTGAGGTACAAAACGATAAATCAGTAGTAGAAATCCCATCACCAGTATCTGGTACTGTAGAAGAAGTTAT
GGTAGAAGAAGGTACAGTAGCTGTAGTTGGTGACGTTATTGTTAAAATCGATGCACCTGATGCAGAAGATATGCAATTTAAAGGTCATG
ATGATGATTCATCATCTAAAGAAGAACCTGCGAAAGAGGAAGCGCCAGCAGAGCAAGCACCTGTAGCTACTCAAACTGAAGAAGTAGA
TGAAAACAGAACTGTTAAAGCAATGCCTTCAGTACGTAAATACGCACGTGAAAAAGGTGTTAACATTAAAGCAGTTTCTGGATCTGGTAA
AAATGGTCGTATTACAAAAGAAGATGTAGATGCATACTTAAATGGTGGTGCACCAACAGCTTCAAATGAATCAGCTGCTTCAGCTACAAG
TGAAGAAGTTGCTGAAACTCCTGCAGCACCTGCAGCAGTAACATTAGAAGGCGACTTCCCAGAAACAACTGAAAAAATCCCTGCTATGC
GTAGAGCAATTGCGAAAGCAATGGTTAACTCTAAGCATACTGCACCTCATGTAACATTAATGGATGAAATTGATGTTCAAGCATTATGGG
ATCACCGTAAGAAATTTAAAGAAATCGCAGCTGAACAAGGTACTAAGTTAACATTCTTACCTTATGTTGTTAAAGCACTTGTTTCTGCATT
GAAAAAATACCCAGCACTTAACACTTCATTCAATGAAGAAGCTGGTGAAATCGTTCATAAACATTACTGGAATATCGGTATTGCAGCAGA
CACTGATAGAGGATTATTAGTACCTGTTGTTAAACATGCTGATCGTAAGTCTATTTTCCAAATTTCAGATGAAATTAATGAATTAGCTGTT
AAAGCACGTGATGGTAAATTAACAGCCGATGAAATGAAAGGTGCTACATGCACAATCAGTAATATCGGTTCAGCTGGTGGACAATGGTT
CACTCCAGTTATCAATCACCCAGAAGTAGCAATCTTAGGAATTGGCCGTATTGCTCAAAAACCTATCGTTAAAGATGGAGAAATTGTTGC
AGCACCAGTATTAGCATTATCATTAAGCTTTGACCACAGACAAATTGATGGTGCAACTGGCCAAAATGCAATGAATCACATTAAACGTTT
ATTAAATAATCCAGAATTATTATTAATGGAGGGGTAA

Gene: pdhD (dihydrolipoamide dehydrogenase)

Contig: 06_NODE_54, position: 209455 to 210861, length: 1407 nt, orientation: FORWARD

Perfect match to: (RF122-AJ938182-[1043190:1044596], highly conserved allele)

Sequence:

ATGGTAGTTGGAGATTTCCCAATTGAAACAGATACTATAGTAATCGGAGCAGGTCCTGGTGGATACGTTGCAGCAATTCGTGCAGCTCA
ATTAGGACAAAAAGTAACAATCGTTGAGAAAGGTAATCTTGGTGGTGTTTGCTTAAACGTAGGATGTATTCCTTCAAAAGCATTACTACA
TGCTTCTCACCGTTTTGTTGAAGCACAACATTCTGAAAACTTAGGTGTTATTGCTGAAAGTGTTTCTTTAAACTTCCAAAAAGTTCAAGAAT
TCAAATCATCAGTTGTTAATAAATTAACTGGTGGTGTTGAAGGCTTACTTAAAGGTAACAAAGTTAACATCGTTAAAGGTGAAGCATATT
TCGTAGATAACAATAGCTTACGTGTTATGGACGAAAAGAGCGCACAAACATACAACTTTAAAAATGCAATCATTGCAACAGGTTCAAGAC
CAATTGAAATTCCTAATTTCAAATTCGGTAAACGTGTTATCGACTCAACAGGTGCTTTAAACTTACAAGAAGTACCAGGTAAATTAGTTGT
AGTTGGTGGAGGATACATTGGATCAGAATTAGGTACAGCATTTGCTAACTTTGGTTCAGAAGTAACCATCCTTGAAGGTGCTAAAGATAT
CTTAGGTGGCTTCGAAAAACAAATGACACAACCTGTTAAAAAAGGTATGAAAGAAAAAGGTGTTGAAATCGTTACTGAAGCTATGGCTA
AATCAGCTGAAGAAACAGATAACGGAGTTAAAGTTACTTATGAAGCTAAAGGCGAAGAGAAAACAATCGAAGCTGATTATGTATTAGTA
ACTGTAGGTCGTCGTCCAAACACAGACGAATTAGGCCTAGAAGAATTAGGTGTTAAATTCGCTGACCGTGGATTATTAGAAGTTGATAAA
CAAAGCCGTACGTCTATCAGCAATATCTATGCAATTGGTGATATCGTTCCAGGTTTACCACTTGCTCACAAAGCTAGCTATGAAGCTAAAG
TTGCTGCTGAAGCAATTGATGGTCAAGCTGCTGAAGTTGATTACATTGGTATGCCAGCAGTATGCTTTACTGAACCAGAATTAGCTACAG
TTGGTTATTCAGAAGCGCAAGCTAAAGAAGAAGGTTTAGCAATTAAAGCTTCTAAATTCCCATATGCAGCAAATGGTCGTGCATTATCAT
TAGATGATACTAACGGATTTGTTAAACTTATTACACTTAAAGAAGATGATACTTTAATCGGTGCTCAAGTAGTTGGTACTGGTGCATCAGA
TATTATCTCTGAATTAGGTTTAGCAATTGAAGCTGGTATGAATGCTGAAGATATCGCATTAACAATCCATGCACATCCAACATTAGGTGAG
ATGACTATGGAAGCAGCAGAAAAAGCTATCGGATACCCAATCCATACAATGTAA

Gene: UPF0223 (putative protein)

Contig: 06_NODE_54, position: 211029 to 211304, length: 276 nt, orientation: FORWARD

Perfect match to: (N315-BA000018-[1075896:1076171], highly conserved allele)

Sequence:

ATGGAATATGAGTATCCAATTGATTTAGACTGGAGTAATGAAGAGATGATTTCAGTGATAAATTTCTTTAATCATGTAGAGAAGTATTAT
GAATCCGGCGTGACGGCAGGCGACTTTATGGGTGCATATAAAAGATTTAAAGAAATTGTGCCTGCTAAAGCAGAGGAAAAACAAATTTT
TAATACTTTCGAAAAAAGTAGTGGCTATAATAGTTACAAAGCAGTTCAAGATGTAAAAACTCACTCTGAAGAACAAAGAGTAACAGCTAA
AAAATAA

Gene: puuR (transcriptional regulator)

Contig: 06_NODE_54, position: 211448 to 211987, length: 540 nt, orientation: FORWARD

Sequence:

ATGAACATAGGTAATAAAATTAAAAATCTTAGAAGAATTAAAAATTTAACGCAAGAAGAACTTGCTGAACGTACAGACTTATCGAAAGG
CTACATTTCACAAATAGAAAGTGAACATGCCTCACCAAGTATGGAAACTTTCTTAAATATTATAGAGGTGTTAGGAACGACGCCAAGTGA
ATTTTTTAAAGACAATGAAAATGAAAAAGTATTATACAAGAAGGAAGAACAAGTTATTTATGATGAGTATGATGAAGGTTATATATTAAA
TTGGTTAGTTTCAAAGTCAAATGAATATGATATGGAGCCATTAATATTAACTTTAAAGCCTGGAGCATCATATAAAAATTTTAATCCATCA
GAGTCTGATACGTTTATTTATTGTATGTCAGGTCAGATAACACTTAATTTAGGCAAAGAGATATATCAAGCACAAGAAGAAGACGTTTTG
TATTTTAAAGCACGAGATAATCATCGTTTGTCAAACGAATCAAACAATGAAACACGAATACTTATTGTAGCGACAGCTTCATATTTATAG

Gene: potA (spermidine/putrescine ABC transporter, ATP-binding protein)

Contig: 06_NODE_54, position: 212000 to 213094, length: 1095 nt, orientation: FORWARD

Perfect match to: (MW2-BA000033-[1078294:1079388], highly conserved allele)

Sequence:

TTGGAACCGTTATTATCATTAAAATCAGTTAGTAAAAGCTATGATGATCTTAATATCTTAGATGACATAGATATTGATATTGAATCAGGAT
ACTTTTATACATTATTAGGTCCTTCAGGTTGTGGTAAAACAACAATTTTAAAATTAATTGCAGGGTTTGAATATCCTGACAGTGGTGAAGT
GATTTATCAAAACAAACCAATTGGTAATTTACCACCAAATAAACGTAAAGTGAATACAGTCTTTCAAGATTATGCATTATTTCCACACTTAA
ACGTCTATGATAATATCGCTTTTGGTTTGAAATTAAAAAAATTATCAAAAACCGAAATTGATCAAAAAGTAACTGAGGCATTAAAATTAGT
AAAACTTTCAGGTTATGAAAAAAGAAATATTAATGAAATGAGTGGCGGACAAAAGCAACGTGTTGCAATTGCACGTGCTATCGTAAATG
AACCAGAAATATTATTGTTAGATGAATCTTTATCCGCATTAGATTTGAAATTGCGTACTGAAATGCAATATGAATTACGAGAATTGCAATC
TAGATTAGGTATTACATTTATATTTGTAACACATGATCAAGAAGAAGCGTTAGCATTAAGTGACTTTCTTTTTGTATTAAAAGATGGGAAA
ATTCAACAATTTGGCACACCAACAGATATATATGACGAACCAGTGAATCGATTTGTAGCTGATTTTATTGGAGAATCTAATATTGTTGAAG
GGCGCATGGTTAGAGATTATGTCGTGAATATTTATGGGCAAGATTTCGAATGTGTCGATATGGGTATTCCTGAAAATAAAAAAGTAGAA
GTCGTTATTCGACCAGAAGATATATCATTAATCAAAGCTGAAGAAGGATTATTTAAAGCAACTGTTGATTCTATGTTATTTAGAGGGGTCC
ACTATGAAATATGTTGTATAGACAATAAAGGTTATGAATGGGTAATACAAACGACTAAAAAAGCTGAAGTAGGCAGTGAAGTTGGTCTT
TATTTTGATCCTGAAGCCATTCATATTATGGTTCCTGGAGAAACAGAAGAAGAATTTGATAAACGTATTGAAAGCTATGAGGAAGTAGAC
AATGCGTAA

Gene: potB (spermidine/putrescine ABC transporter, transmembrane permease)

Contig: 06_NODE_54, position: 213087 to 213884, length: 798 nt, orientation: FORWARD

Perfect match to: (MW2-BA000033-[1079381:1080178], allele observed in CC1)

Sequence:

ATGCGTAATACTAATAAATTTCTCTTAATCCCGTATTTACTATGGATGGTTATATTTATTATTGTACCAGTTGTATTACTCATTTATTTTTCAT
TTTTAGATATCAATGGACATTTTAGTTTCACGAATTATCAACAAATTTTTACTACAAAATATTTGAAAATGTTTGCATATTCAATTTTATATG
CCGCTTTAATAACAATTATTACCTTGACTATCAGTTATCCAGCTGCCTATTATATTACTCGTTCGAAATTTCAAAATATCTTATTAATGATAA
TGATTATTCCAACATGGATAAATTTATTGTTAAAGACATATGCTTTTATAGGTTTATTAAGTCATGATGGCGTGATTAATCAATTTTTCCAC
TTATTTAATTTACCATCATTCAATTTGTTATTTACAACTGGTGCGTTTTTAGTAGTGGCAAGTTACATTTATATACCATTTATGATTTTACCTA

TATTTAATAGCATGAAAGCAATTCCTAATAATTTATTGCAGGCCTCAAGTGATTTAGGTGCCAGTCCTTTCTATACTTTTAGAAAAGTAATC
ATGCCGTTAACAAAAGAAGGTGTTATGACTGGGATTCAAGTAACCTTTATTCCATCACTTTCATTATTTATGATTACAAGATTAATTGCAG
GTAATAAAGTCATAAATATAGGTACGGCAATAGAGGAACAATTTTTAACAATTCAAAATTATGGTATGGGATCAACTATAGCTATATTCTT
AATTGTATTTATGGCATTCATTTTAATCATTACAAAATCATCTAATGGGAGAGGGTGA

Gene: potC (spermidine/putrescine ABC transporter, transmembrane permease)

Contig: 06_NODE_54, position: 213890 to 214699, length: 810 nt, orientation: FORWARD

Perfect match to: (MW2-BA000033-[1080184:1080993], allele observed in CC1+CC5)

Sequence:

ATGAAATGGTATGGAAAGCTGTATATCGGGATACTTTTAGCGATTTTATACATCCCAATATTCTTTTTAATGTTCTATTCATTCAATTCGGC
TGGTAATATGATTCACTTTGAACATTTTACATTAGAGCATTATCAATCATTATTTCAAAATGATCATTTAATGTCGGTCATTTTTAATACGAT
AGCTGTAGCACTTTTAGCAGCCTCAATTTCTACAGTTATTGGTACATTTGGTGCCATTGCTATTTATTATTTAAGAAATAAAAAGTTTAAAG
TAACTTTACTAACATTGAATAATGTCTTGATGGTATCATCCGACGTTGTCATAGGTGCATCATTCTTAATTATGTTTACAACGATTGGCCAT
TTTACTGGTCTTGGTTTAGGATTTTGGACAGTTCTAATATCTCATATTGCATTTTGCATACCTATAGTTGTGATTATCGTCTTACCACAACTG
TATGAAATGAATAATAATATGTTAAATGCTGCAAGAGATTTAGGAGCGACTGAACCACAATTATTAAGCAACATTATTATTCCTAATATTT
TACCTTCTATTATAGGAGGATTCTTTATGGCTTTAACTTATTCACTAGACGATTTCACAGTAAGTTTCTTCGTTACTGGTAATGGCTTTAGT
GTGTTATCAGTTGAAGTTTATGCTATGGCGAGAAAAGGAATTAGTATGGAAATTAATGCGATTTCAACATTACTGTTTGCTGTTATTGTAT
TAGGAATACTAGGATATTATTTGATTCAATACGTGATAAATAAGAAAAAACTAATCAAGCGAGGTGTAAAATAA

Gene: potD (spermidine/putrescine ABC transporter, substrate-binding protein)

Contig: 06_NODE_54, position: 214699 to 215772, length: 1074 nt, orientation: FORWARD

Perfect match to: (ED133-CP001996-[1082821:1083894], highly conserved allele)

Sequence:

ATGAAACGTTTTTTACAACTCATTATAGGTGCATTAGTTGTGGGTATGCTTTGTCTTACTTTAAGTCATTGGTTTAAATCTAAAGAACAAGT
GCATACAAATCAAAAAATTTACGTATACAATTGGGGCGAATATATTGATCCAGAGTTAATTAAGAAATTTGAAAAAGAAACTGGCATTCA
AGTCGTTTATGAAACTTTCGATTCAAATGAAGCGATGGAAGCCAAAATTCGCAATGGCGGTACACATTATGATGTTGCTTTTCCTAGTGA
ATATACAGTTCAAAAATTGAAAAGAGATCATTTATTGTTACCAATAGATCATAATAAGGTACCTAATATTAAAAATTTAGATTCAGATTAT
ATGAATATGTCATTTGATAGAGGCAATAAATATTCTTTACCTTATTTCTTTGGAACTGTAGGTATTTTATATAATAAAGAAAAGTATCCAAA
TGAATCATTTGATAGTTGGAAGTCATTGTATAATCCTAAATTTAAAAACCAAATTTTACTAGTTGACGGTGCTAGAGAGATTATAGGCATG
AGTTTGAATAAACTTGGGTATAATCTTAATGACCGTAATTCGCACCATTTAAAAGAAGCAGAGCGAGATTTAACCAAACTAGCACCACAA
GTAAGAGGTGTCGTAGGTGATGAAATTACCATGATGCTTCAACAAAATGAAGGTAACATAGCGGTTGTTTGGAGTGGTGTTGCAGCACC
TCTAGTGCAAGAAGGGGATAAATATAATTATGTTATTCCTAAAGAAGGATCGAATTTATGGTTCGACAATATGGTAATTCCAAAAACGGC
ACAAAATAAAGAGGGTGCATATAAATTTATGAATTTTTTATTAGATGCTAAAAATAACAAGCAAAATACAGAATTCGTAGGCTATGCAAC
GCCAAACAAGGCTGCTCGACAATTGTTGCCTAAAGAGATTAAAGACGACCATCGTTTTTATCCGACTAAGAAAGAGCAAGAACGCCTTG
AAGTTTATAAAGATTTAGGACCTGAAGTTTTAAGTGAATACAATGAAAACTTTTTGAATTTCAAAATGTCATTAAAATAA

Gene: Q5HGY1 (polytopic membrane protein)

Contig: 06_NODE_54, position: 215846 to 216865, length: 1020 nt, orientation: FORWARD

Perfect match to: (MW2-BA000033-[1082140:1083159], highly conserved allele)

Sequence:

ATGACTGGAGAACAATTTACTCAAATTAAACGTCCAGTAAGTAGATTAACTGAAAAAGTTCTAGGTTGGTTATGTTGGGTGATGTTATTA
GTGCTTACTGTCATTACGATGTTTATAGCACTTGTTTCATTCAGTAATAACACATCGATTGCTAATCTTGAAAATACATTAAACAATAATGC
ATTTATCCAGCAATTATTAGCTGGAAATGGCTATAATACAACACAATTTGTAATATGGTTACAAAATGGTATATGGGCTATTATCGTTTAC
TTTATTGTTTGTTTGTTGATTTCATTTTTAGCTCTCATATCTATGAATATAAGAATCTTGTCAGGTTTCTTATTCTTAATATCAGCAATTGTAA
CGATTCCTTTAGTTTTACTTATCGTTACTTTAATCATTCCGATATTATTCTTTATTATTGCGATGATGCTATTTATAAGAAAAGATAAAGTTG
AAATGGTTGCGCCACAATATTATGAAGAGTATAACGGACCGATTTATGATTATCGTGAACCTGTGTATGAGCGCCCCCAACCGAAAGATG
ATTATTATGATGTGCCTAAATATGAAAAAGAATTGGATAAATCAAATACTGTATATGATCAAGAACAGGAAAGAGATAAATATGATCAAT
TTCCTAAACGTGCAGTTGAAAGTGAATATAATCATGATGAGCGCACTGAGGAAGAACCATCAGTATTATCCAGACAGGCTAAATACAAA

CAAAAAAGTACTGAAGAACTAGGTATTGAAGATGATGGTTATTATGCAGAACCTGAAGTTGATCCAAAAGAATTGAAAGCACAACAAAA
GCGAGAAAAAGCTGAAATTAAAGCTAAGAAAAAAGAAAAACGTAAAGCTTATAACCAACGAATGAAAGAACGTAGGAAAAATCAACCT
AGCGCAGTTAGTCAACGTCGAATGAATTTTGAAGAGCGACGTCAAATTTACAACAATGATATTTCTGAAGAACGCAATTCAAGTGAAGTT
AAGGACAAAAAAGAGCAAGAATAA

Gene: DUF4064 (protein of unknown function)

Contig: 06_NODE_54, position: 217148 to 217567, length: 420 nt, orientation: FORWARD

Sequence:

ATGAATAGAAAACCTGAATTAATCATGGCTTGGATTGCAAATAGTATTAGTATTATTTATTTATTAGTTATGGGGCTATCCTATTTTTCTTT
AAAAAGTGGTAATGCATCACAACGTGAAGAATTAGCGAAGCAATTATCTCAGAACGGTGGCAAGGTTTCTTTAGATATGCTTCAGACAA
CAATGGGTGCATTAGCAATTATTTTATTAATTTCAACACTTTATGGTATATTTGCGACAATTTGTATTAAAGGACGTAGAAAATTATCGATT
ATACTTTTTGTTATCGCGATAATTGTAAGTTTGATGGCTCTTAATTTAATTGCAATTGTCTTATGGGTTATCGTGATGATTATGTTGATTTCT
AAAAAAGAATCAAAAGAAACTACACATAAGGACGATGAGTATATTTATCATTAA

Gene: mntH (divalent metal cation transporter)

Contig: 06_NODE_54, position: 217649 to 219001, length: 1353 nt, orientation: REVERSE

Perfect match to: (MW2-BA000033-[1083943:1085295:r], allele observed in CC1+CC22+CC30)

Sequence:

TTAACTTTGTAGTTCTTGGAATGTTTGAACGATTAAATAGACATTTAAAATACTTAGAATAATAATAAGTGTCCATGAAATGATATTAACC
CATGTTTTATTATAGAAAGGTCCCATCAAGTCTTTATTACTAGTAGCCAATTGTAATGGTATTAAACAGAATGGCAGAGCAATACTTAAAA
ATACTTGAGAGAATACAAGTAGTTGCTCAATTTTAGCTGCATTACCTTTAAAAATGATTAAGCATACGATAACAGGAATGACAGCAAGGG
AACGTGTAATTAAACGTCTTAACCAATTTGGTATGTGTAATCTTAAAAATCCTTCCATTACAATTTGTCCTGCTAAAGTACCAGTAATCGTT
GAATTTTGACCTGACGCTAATAATGCAACTGCAAATAATGTACTCATGATTGCACCCATTGTTGCACCTAGTACAGGTTCAGTTTTTAAGG
CGTGATATAAATCATAGAAACCACCTAAATCGTCAGCGTTTGAGTTGAAAAATAGTGATGCACCTAACACTAATAATAAGCAATTGACTA
CAAATGCGATTGATAACTGAATGTTCGAATCTATCGTAGCAAATTTAATCGCTTGCGCCTTTTCTTCATTGTTATGTCTTGAGTATGTTCTA
GATTGTACAATTGATGAATGTAAGTACAAATTATGAGGCATAATTGTAGCGCCAATAATACCTAATGCAATATAGAGAATGCCGTTATTT
GTAATGATTTCACTATGTGGTATAAATCCATTTAACACAGCATTCAACTGTGGTGATGAAATATAGACTTCAAATATAAAGATGAATAACA
CTGTGAAAATTAATGTACCAACAATAGCTTCAATTTTTCTAAAACCATATTTCATTATAAATAGTAGTAAAAATACATCAAGTACAGTTATT
AGTGCACCGACGATTAAAGGTATGTTAAATAGGAGATTAAGAGCAATAGCACTACCAATAACCTCAGCAATATCTGTAGCGATAATTGCT
AGTTCTGCAATGATCCAAAAGATTATAGCAATAGGTCTTGATAAATAATGTCGTGTCATTTGAGCTAAGTCCATACCTGTTGCTATTCCTA
ATCTCACTGTCATGCTTTGAAGTAACATTGCTGATAAACTTGAAATAAGAATTACGAATAGCAAAGTATAGCCATATTGCGCGCCACCTTG
CATTGATGTTATCCAGTTTCCGGGATCCATGTAACCAACAGCGACTAATAACCCAGGCCCAAGAAATGATAAAAATTTCTGTTTATTTGAA
CTGCGATGATCGAATTTAATTGTATTGTTTATTTCGTCTAAACTTAATTGTTCATTTGTTGAATGTCGTTTATTATTCAT

Gene: yktB (conserved hypothetical protein)

Contig: 06_NODE_54, position: 219185 to 219799, length: 615 nt, orientation: REVERSE

Perfect match to: (MW2-BA000033-[1085479:1086093:r], allele observed in CC1+CC9+CC15+CC188)

Sequence:

TTATGCAGAATAAAATGGTAAGAACTGATCGAAGGTTTCTTCTAAAAATGCAATAAATGCTTTGTCACTTTTTAATCTTTTATCTTGTGGTG
TGATTGCACGCGCTATAAAGAATTCACCTTTTTTCACATTGATGGCTCTTTGTATCGCTTCTTTTAAATCATCATCCGTTAAATCTTTAATAA
ATGGTTTATCAGGTTTCATATGATCTAAGCAAACACGATAATCATCAGGTAATTGTTGAATAGCTTTAAAGTTTCTTTCAAAAACTTTTGCA
CGTGTTGCTTTATCTTTTGCTTCATGCATGATTCCAAACATAACAAACAGTTGATCTTCAAACATACCAATTTGGAAATGAGGTAACATTTT
ATAGCCTCTTTTGTTTGTTGCAAAAGCAACCCATGTATCTTTAGGAGGATTCACACTTCTTCTAGCATGCTTTGCTACGTGAGGATAAAAT
GTTTCACCAGTTTGACTTGTAAAGAAGTCACTAAAATATTCTCCTAATTCATGGAGTTGTGGTCGTATGTATTCGTTTAAAGCTTCCATTCG
TGCGTCTAAGCCTTCTACGTTGAACGCTTTGAAATCTTTAGGTTTAAATGTATATTTTGTCAT

Gene: suhB1 (inositol monophosphatase)

Contig: 06_NODE_54, position: 219956 to 220783, length: 828 nt, orientation: FORWARD

Perfect match to: (MW2-BA000033-[1086250:1087077], allele observed in CC1+CC22+CC30+CC772)

Sequence:

ATGGCACTTTATGGATTTGCCCAAGGACTTATTCAAGAAGCAGGAATTAGAATTAAACAATTGATGGAGCAAAATTTAACAATTGAAACA
AAGTCAAATCCGAATGACCTTGTTACAAATGTAGATAAAGCAACAGAAGATTTTATTTTTGATACAATTTTAGAAACATATCCCAATCATC
AAGTATTAGGTGAAGAAGGGCATGGTCATGACATCGATACTTCCAAAGGTACGGTATGGGTTGTTGACCCAATAGACGGTACATTGAAT
TTTGTTCATCAACAAGAAAATTTCGCAATTTCAATTGGTATTTATATCGATGGTAAACCTTATGCAGGTTTTGTATATGATGTTATGGCTGA
TGTCTTATATCATGCTAAAGTAGGGGAAGGTGCATATCGTGGTAGCCAACCCTTGAAACCATTGAATGATTCTAATCTAAGACAAAGCAT
TATTGGGATCAATCCGAACTGGTTAACTAAACCAATTTTAGGAGAAATCTTTAAAGAAATTGTTAATGATTCTAGAAGTGCAAGGGCATA
TGGTAGTGCAGCGCTTGAAATCGTTTCAGTTGCTACAGGTAATTTAGAAGCATATATGACGCCAAGACTTCAACCATGGGATTTTGCTGG
CGGATTGGTTATTTTATATGAAGTAAATGGACAAGCTTCCAATTTACTAGGAGAACCATTAACAATTAGTGGTCCAAATTCAATCTTAGTT
GGAAATCGTGGTCTCCATCAAGAAATTAGCAATGATTATTTAGAGCCCCACCATGATGCGTTAATACAATTACATGAACAACGATTTAAA
AGAAAATCAAAATAA

Gene: ylaF (putative membrane protein)

Contig: 06_NODE_54, position: 220937 to 221128, length: 192 nt, orientation: REVERSE

Perfect match to: (N315-BA000018-[1085801:1085992:r], highly conserved allele)

Sequence:

TTATAGCCAATCGTTTTCTCGATATTTTTTCTTTGTAGTAAATCCAATTCCAAAGGTTGCAACGAGTAATATAAATGTTAAAATCATCATTG
GTACATTTGATGCACCAACAGCAAAACTAAATAATACTAAGAAAACAACAGCTAATATAGAAAATACCCAAAAGATATTTTTAGATTTTTT
TTGTTTCAT

Gene: typA (GTP-binding protein)

Contig: 06_NODE_54, position: 221230 to 223077, length: 1848 nt, orientation: FORWARD

Perfect match to: (MW2-BA000033-[1087524:1089371], highly conserved allele)

Sequence:

ATGACTAATAAAAGAGAAGATGTCCGCAATATAGCAATTATTGCTCACGTTGACCATGGTAAAACAACTTTAGTAGATGAGTTGTTAAAA
CAATCTGGTATATTCAGAGAAAATGAACATGTCGATGAACGTGCAATGGACTCTAACGATATCGAAAGAGAGCGTGGAATTACGATTCT
AGCCAAAAATACGGCTGTTGATTATAAAGGTACACGTATTAATATTTTGGATACACCAGGACATGCAGACTTTGGTGGAGAAGTAGAAC
GTATTATGAAAATGGTTGATGGGGTTGTCTTAGTAGTAGATGCGTATGAAGGTACAATGCCTCAAACACGTTTTGTACTTAAAAAAGCGC
TAGAACAAAACCTGAAACCTGTTGTTGTTGTTAATAAAATTGATAAACCATCAGCACGTCCAGAGGGTGTTGTAGATGAAGTTTTAGATT
TATTTATTGAATTAGAAGCAAACGATGAACAATTAGAATTCCCTGTTGTTTATGCTTCAGCAGTAAATGGAACAGCTAGCTTAGATCCTGA
AAAACAAGATGATAATTTACAATCATTATATGAAACAATTATTGATTATGTACCAGCTCCAATTGATAACAGTGATGAGCCATTACAATTC
CAAGTAGCATTGTTGGACTACAATGATTATGTTGGACGTATTGGTATTGGTCGTGTATTCAGAGGTAAAATGCGTGTCGGAGATAATGTA
TCACTAATTAAATTAGACGGTACAGTGAAAAACTTCCGTGTAACTAAAATCTTTGGTTACTTTGGATTAAAACGTTTAGAAATTGAAGAAG
CACAAGCTGGAGATTTAATTGCTGTTTCAGGTATGGAAGACATTAATGTTGGTGAAACTGTAACACCACATGACCATCAAGAAGCATTGC
CAGTTCTACGTATTGATGAGCCTACTCTTGAAATGACATTTAAAGTTAACAATTCTCCATTTGCTGGCCGTGAAGGTGACTTTGTAACAGC
GCGTCAAATTCAAGAACGTTTAAATCAACAATTAGAAACAGATGTATCTTTGAAAGTTTCTAACACAGATTCTCCAGATACATGGGTAGTT
GCTGGTCGCGGTGAATTGCATTTATCAATCCTTATTGAAAATATGCGTCGTGAAGGTTATGAATTACAAGTTTCAAAACCACAAGTAATTA
TTAAAGAAATAGATGGTGTAATGTGTGAACCATTTGAACGTGTGCAATGTGAAGTGCCACAAGAAAATGCAGGTGCTGTTATTGAATCA
TTAGGTGCACGTAAAGGTGAAATGGTTGATATGACTACAACTGATAATGGACTTACACGTTTAATCTTTAATGTACCGGCTCGTGGTATG
ATTGGTTATACGACTGAATTTATGTCAATGACAAGAGGTTACGGTATTATTAACCATACATTTGAAGAATTTAGACCACGTATTAAAGCAC
AAATTGGCGGTCGTCGTAATGGTGCATTAATTTCAATGGATCAAGGTTCTGCAAGTACTTATGCCATTTTGGGACTTGAAGATAGAGGTG
TAAACTTCATGGAACCTGGTACTGAAGTTTATGAAGGTATGATTGTTGGTGAACATAATCGTGAAAATGATTTAACTGTTAACATCACTAA
AACAAAACATCAAACTAACGTACGTTCTGCAACGAAAGACCAAACACAAACAATGAATAGACCGCGTATTCTAACATTGGAAGAAGCGT
TACAATTCATTAATGATGATGAACTTGTTGAGGTTACACCAGAAAGCATACGTTTAAGAAAGAAAATTTTAAACAAAAATGTTCGTGAAA
AAGAAGCAAAGCGTATCAAACAAATGATGCAAGAAAACGAATAA

Gene: ylaL (protein of unknown function)

Contig: 06_NODE_54, position: 223386 to 223868, length: 483 nt, orientation: REVERSE

Perfect match to: (N315-BA000018-[1088250:1088732:r], allele observed in CC5+CC361)

Sequence:

TTAAATCACCTCAATATGAGTTACATGGTTGATTCGATATGAATAACCATCTTCTTGATTGTAAATAAAACTATCTACACCATTATCGCTGT
AAAGTCGTTTACCGTCTTTTGCAAATTGGAAAAATAAATAGGGTAATAGATCTATCGGTATATCTAAATGATCATGCTCATTTGATAATCG
AATAGTAGTTGCAGAGTCATGTGGTTCGGAATGTTTGAAAAAAGGTGTCATATTAATGACAAATGAACCTTCTAACATGGCACGTTTTTT
ATATTTTATTTCTGAATTTAAAGTAGGCGGATTAGTTTGTCCTTCTAGGATAGCACGATTCCATTCATGATTATCTTCAAAGTCGATTGGTT
TTGAACCATCAAATACACCTTTTTCTAAATCTTCGATGCTAACTTTTCTATCATCGAAAATCCAAGTCGTACTATCTAATGTTATAGGAAACT
TTACGGCTCCTTTAATTTGTATCAT

Gene: ylaN (putative conserved protein)

Contig: 06_NODE_54, position: 224009 to 224284, length: 276 nt, orientation: FORWARD

Perfect match to: (N315-BA000018-[1088873:1089148], highly conserved allele)

Sequence:

ATGGCGAAACAAGCAACAATGAAAAATGCAGCTTTGAAACAATTGACTAAAGATGCTGATGAAATCTTGCATCTGATTAAAGTTCAACTA
GATAATTTAACATTACCTTCATGCCCATTATATGAAGAAGTACTAGATACACAAATGTTTGGACTTCAAAAAGAAGTTGATTTTGCTGTTA
AATTAGGTTTAGTTGACCGCGAAGATGGCAAACAAATTATGTTACGTCTTGAGAAAGAACTTTCAAAATTACATGAAGCTTTTACACTTGT
TTAA

Gene: ftsW2 (cell division protein W2)

Contig: 06_NODE_54, position: 224598 to 225824, length: 1227 nt, orientation: FORWARD

Perfect match to: (MW2-BA000033-[1090892:1092118], allele observed in CC1+CC5)

Sequence:

ATGAAGAATTTTAGAAGTATTTTACGGTATATTGGTAAAACCTCAAAGTTTATTGATTATCCGTTATTAGTTACATATATTGTATTGAGTTT
AATTGGATTAGTGATGGTATATAGTGCAAGTATGGTTCCAGCAACTAAAGGCACATTGACTGGTGGTATCGATGTTCCAGGAACGTATTT
TTACAACCGACAATTAGCATATGTCATAATGAGTTTTATAATTGTATTTTTTATTGCATTTTTAATGAATGTTAAATTACTGAGTAATATTAA
AGTGCAAAAAGGTATGATTATAACTATCGTCTCACTATTATTACTGACGTTAGTAATAGGTAAAGATATTAATGGTTCTAAAAGTTGGATA
AACTTAGGATTTATGAACTTACAGGCATCTGAGTTATTAAAAATTGCAATCATATTATATATCCCATTTATGATCAGTAAAAAAATGCCTA
GAGTATTAAGTAAACCAAAATTAATTTTAAGTCCTATTGTATTAGCATTAGGTTGTACGTTTTTAGTTTTCCTACAAAAAGACGTAGGGCA
AACATTACTAATATTAATTATTTTAGTTGCGATCATTTTTTATTCAGGAATTGGGGTAAACAAAGTCCTAAGATTTGGTATACCAGCAGTAC
TAGGATTCTTAGTAGTATTTGTCATTGCATTAATGGCTGGTTGGTTACCAAGTTATTTAACTGCCAGATTTAGTACGCTAACAGATCCATTC
CAATTCGAATCAGGAACTGGATACCATATTTCCAATTCATTGCTTGCGATAGGTAACGGTGGCGTATTTGGAAAAGGATTAGGAAATAGT
GCAATGAAATTGGGCTATTTACCAGAACCACATACAGATTTTATTTTTGCAATTATTTGCGAAGAATTAGGTTTAATCGGAGGATTGCTAG
TTATTACTTTAGAGTTCTTTATTGTATATCGTGCCTTCCAGTTTGCAAATAAAACATCATCATATTTTTATAAACTTGTGTGTGTTGGGATTG
CCACATACTTTGGAAGTCAAACGTTTGTAAACATTGGCGGTATTTCGGCAACAATTCCATTAACTGGTGTGCCATTGCCATTTATCAGCTTT
GGTGGATCATCAATGATTAGTTTAAGTATTGCTATGGGATTACTTCTGATTGTAGGTAAACAAATCAAAGTAGACCAGCAACGAAAGAAA
CAACAACAAAAAGTTGATATAAGAAGACAATTTAATTAA

Gene: pycA (pyruvate carboxylase)

Contig: 06_NODE_54, position: 226378 to 229830, length: 3453 nt, orientation: FORWARD

Perfect match to: (N315-BA000018-[1091242:1094694], allele observed in CC5+CC1+CC361)

Sequence:

TTGAAACAAATAAAAAAGTTACTTGTTGCTAACCGTGGAGAAATTGCAATTCGTATATTCAGAGCGGCGGCAGAATTAGACATCAGCACA
GTTGCAATTTATTCGAATGAAGACAAAAGTTCATTACATAGATATAAAGCAGATGAATCCTATTTAGTTGGAAGTGATTTAGGTCCTGCT

GAAAGTTATTTAAATATTGAGCGTATCATTGATGTAGCAAAACAAGCGAATGTGGATGCGATTCATCCTGGCTATGGATTTTTAAGTGAA
AATGAACAATTTGCGCGTCGTTGTGCAGAAGAAGGAATTAAATTTATTGGTCCTCATTTAGAACATTTAGATATGTTTGGAGATAAAGTT
AAAGCTCGTACAACGGCTATCAAGGCAGATTTACCAGTTATTCCTGGTACAGACGGTCCAATTAAATCATATGAATTAGCAAAAGAATTT
GCAGAAGAAGCTGGTTTCCCGCTAATGATTAAGGCCACAAGTGGTGGCGGCGGTAAAGGTATGAGAATCGTTCGTGAAGAAAGTGAAT
TAGAAGATGCTTTCCATAGAGCAAAATCAGAAGCTGAAAAATCATTTGGTAATAGTGAAGTTTACATAGAGAGATACATTGATAATCCAA
AGCATATTGAAGTACAAGTTATAGGTGACGAATATGGAAATATCGTACACTTATTTGAACGTGATTGTTCAGTACAACGTCGTCATCAAA
AAGTTGTAGAAGTTGCACCATCAGTTGGATTATCACCAACATTACGTCAACGTATTTGTGATGCTGCAATTCAATTGATGGAAAATATTAA
ATATGTCAATGCAGGTACTGTTGAATTTCTAGTATCTGGTGACGAATTCTTCTTTATAGAAGTTAACCCTCGTGTACAAGTTGAGCATACA
ATTACAGAGATGGTAACAGGAATTGATATTGTTAAGACACAAATTTTAGTTGCAGCAGGTGCCGATTTATTTGGTGAAGAAATTAATATG
CCGCAACAAAAAGATATTACAACATTGGGCTATGCCATCCAATGTCGTATTACAACAGAAGATCCGTTAAATGATTTCATGCCGGATACT
GGAACAATCATTGCTTATCGTTCAAGCGGTGGCTTTGGTGTACGTCTCGATGCTGGAGATGGTTTCCAAGGTGCTGAGATATCACCTTAT
TATGATTCATTACTCGTAAAATTATCTACACACGCGATATCATTTAAACAAGCAGAAGAAAAAATGGTACGTTCATTACGAGAAATGCGT
ATTCGTGGTGTTAAAACTAATATTCCATTCTTAATTAATGTAATGAAGAATAAAAAGTTCACAAGTGGTGATTACACAACTAAATTTATTG
AAGAAACACCAGAACTTTTCGACATTCAGCCGTCTCTAGATAGAGGTACTAAAACATTAGAATATATAGGTAATGTAACAATTAATGGTT
TCCCAAATGTTGAGAAACGTCCGAAACCAGACTATGAATTAGCATCAATTCCAACTGTATCTTCAAGTAAAATCGCTTCATTTAGTGGTAC
GAAACAATTGCTTGATGAAGTAGGTCCAAAAGGTGTAGCTGAATGGGTTAAAAAGCAGGATGATGTTTTACTAACAGATACAACCTTTA
GAGATGCACACCAATCATTATTAGCTACACGAGTTAGAACTAAGGATATGATTAATATCGCATCCAAAACAGCGGACGTATTTAAAGATG
GTTTCTCACTTGAAATGTGGGGCGGTGCTACATTTGATGTGGCATATAATTTCTTGAAGGAAAACCCATGGGAACGACTTGAACGTCTAC
GTAAAGCTATTCCAAATGTATTATTCCAAATGTTGTTACGTGCTTCAAACGCAGTTGGTTATAAAAACTATCCTGATAATGTTATTCATAAA
TTCGTACAAGAAAGTGCTAAAGCAGGCATAGATGTCTTTAGAATTTTCGATTCTTTAAACTGGGTAGATCAAATGAAAGTTGCCAATGAA
GCAGTACAAGAAGCGGGCAAAATCTCTGAAGGTACTATTTGTTATACAGGTGACATTTTAAATCCTGAGCGATCAAACATTTATACTTTA
GAGTATTATGTCAAACTAGCTAAAGAGTTAGAACGTGAAGGTTTCCATATTTTAGCGATTAAAGATATGGCAGGCTTATTAAAACCTAAA
GCCGCTTACGAATTGATTGGTGAGTTAAAATCAGCTGTAGATTTACCAATTCATCTTCACACTCATGATACAAGTGGTAATGGTTTATTAA
CATACAAACAAGCAATAGATGCTGGTGTCGATATCATTGATACTGCTGTTGCTTCAATGAGTGGTTTAACAAGTCAGCCAAGCGCCAATT
CGTTATATTATGCATTAAATGGCTTCCCACGCCACCTTAGAACTGATATTGAAGGTATGGAGTCACTTAGTCATTATTGGTCAACTGTACG
TACTTATTATTCAGACTTTGAAAGTGATATCAAATCACCGAATACTGAAATTTATCAACATGAAATGCCTGGTGGACAGTATTCGAATTTA
AGTCAACAAGCTAAAAGTTTAGGTTTAGGCGAAAGATTTGATGAAGTCAAAGATATGTATCGCAGAGTGAATTTCTTATTTGGTGATATC
GTAAAAGTAACTCCATCGTCTAAAGTAGTTGGTGATATGGCACTTTATATGGTACAAAATGATCTTGATGAACAATCCGTGATTACAGAT
GGCTATAAATTAGATTTCCCAGAATCAGTAGTGTCGTTCTTCAAAGGCGAAATAGGACAACCTGTAAATGGTTTTAATAAAGATTTACAA
GCGGTTATTTTAAAAGGCCAAGAAGCACTAACAGCTCGTCCAGGTGAATATCTAGAGCCAGTTGATTTTGAAAAAGTCCGTGAGTTGCTT
GAAGAAGAGCAACAAGGTCCTGTTACGGAGCAAGATATTATTAGTTATGTATTATATCCAAAAGTATATGAACAATATATTCAAACTAGA
AATCAATACGGAAACTTATCGTTACTTGATACGCCTACATTCTTCTTTGGAATGCGTAATGGTGAAACAGTAGAAATCGAAATCGATAAG
GGTAAACGATTAATTATTAAACTAGAAACGATTAGTGAACCAGATGAAAATGGTAATAGAACGATTTACTATGCGATGAATGGTCAAGC
GAGACGTATTTACATTAAAGATGAGAATGTGCATACAAATGCGAACGTTAAGCCAAAAGCAGATAAGAGTAATCCAAGTCATATCGGTG
CGCAAATGCCAGGTTCAGTAACTGAAGTCAAGGTTAGTGTAGGTGAATCTGTGAAAGCTAACCAGCCGTTGCTAATTACTGAAGCTATG
AAAATGGAAACAACAATTCAAGCACCATTTGACGGTGTGATTAAACAAGTAACTGTAAATAATGGTGACACAATAGCGACAGGCGATTT
ATTAATCGAAATTGAAAAAGCAACTGACTAA

Gene: ctaA (heme A synthase)

Contig: 06_NODE_54, position: 229970 to 230881, length: 912 nt, orientation: REVERSE

Perfect match to: (N315-BA000018-[1094834:1095745:r], highly conserved allele)

Sequence:

TTATTGCTTGTCACTTCTTACTGATCGTAACATTAGCATGATAAAGTATGTTGTCATACCAAATAAATAAGTGATAAATAATGCATGGAAA
AGTGCGATTATCAGGTTAACATTTGTCATAATAGATAATGCACCTGTGATAACTTGTAATATAACAAGTATAAATGCAGCTGTATAACCAT
AATGAACAGTACGGTTATTTGGATAATTTTTAACAGCGTGAATATAAGTAATCATAATAATCGTAAACACTATAAAAGCCATGATACGAT
GCGTGAGTTGAACCCAATCTTGTTCTGAATGTGGTACAAGATCGTGGAATGGCAATGGCCAACCACCATATGCTAAACTTGCATCCGCAT
GTCTCACTAGTGCACCAGTATAAACACCACAATAAATGATGATTGCCATTAACCATGTTAAACGTCTTAATGGCTTTTTGATATATAATTCG
TCAGCTTCATATTTTTGATCTATAGAGAAAATAATCAATGTTATTAAAAATACAGATGAGAAACTGATTAATGATATACCAAAGTGCAATG
CTAAAACGTAATCGTTTTGTTGCCAAATAACAGCAGCAGCTCCGATTAATGCTTGCAATAATAAGAATCCAACACTAATGATTGATAAAG
GTTTAATTTCTTTAATATAGCCTATATGTTTCCATGCAGTGATAACTAACCATAAGACCATTAATAAAGACAAAGCTGAAACGGCTCTATG
ACTTAACTCAATAATCGTATCAATAGGAAAGAATTCTGGAATCAACGCACCATGACATAGTGGCCAAGAAGAACCACAACCATCAGCTGA
TCCGGTTTTGGTAACTAAGGCTCCACCAAGTTGTACAAATGTCATCATTAACGTTGCTACGACACCTAACCATTTTAAATTCTTTTTGCCAA
ACAA

Gene: ctaB (protoheme IX farnesyltransferase)

Contig: 06_NODE_54, position: 231321 to 232232, length: 912 nt, orientation: FORWARD

Perfect match to: (RF122-AJ938182-[1065050:1065961], highly conserved allele)

Sequence:

ATGAGCAAAGAGCATACTTTGTCACAAAATATTAGCAGAGTTAACTTCAAAGAATTGCAACAGATAATTAAAATGGGACTTGTTCAAGGT
AACTTAATTCCGGCTTTTGCGGGCGCATGGCTAGCAGTTGTAATGACAAATCATTCCTTCTTATCATCAATACCTCAAATTTTATTAATGCT
ATTAGGATCTACTTTAATTATGGGTGGCGCATGTGCGTTAAATAATTATTACGACCAAGATATTGATCGTATTATGCCTAGTAAACAAAAT
AGACCAACTGTAAATAATAGAATTACAGATCAAAATTTATTATTATTAAGTTTTGGTATGATGTTAGTTGGAGAAATTTGTTTGTTTTTATT
GAATATACCATCAGGCGTACTTGGTCTTATGGGGATTGTAGGTTATGTGTCTTATTACTCAATATGGTCTAAAAGACATACAACATGGAA
CACAGTGATTGGGAGTTTTCCTGGAGCAGTACCACCACTAATTGGATGGGTTGCAATTGAAGGACAAATTAGTTTAACAGCGATTGCGCT
GTTTTTAGTTGTATTTTGTTGGCAACCAATTCATTTTTATGCCTTAGCTATTAAACGTAAAGATGAATATGCACTTGCAAATATTCCAATGTT
ACCATCAGTTAAAGGCTTTAAACGTACACGTGTCAGTATGTTTATCTGGTTGATTATTTTATTGCCAGTACCTTTATTACTAATAAATTTAG
GTGTCGTATTCGTAGTGTTAGCTACATTATTAAATTTAGGATGGATTGCATTAGGTTTAACAACATTTAAGAAAAATTCAGATCAAACAAA
ATGGGCAACACAAATGTTTATATATTCACTAAATTATTTAGTGATCTTTTTCGTGTTAGCTGTGATTGTTTCATTACTTACTTTGATCTAG

Gene: yozB-ctaM (factor required for functioning of QoxABCD)

Contig: 06_NODE_54, position: 232257 to 232718, length: 462 nt, orientation: FORWARD

Perfect match to: (MW2-BA000033-[1098551:1099012], allele observed in CC1)

Sequence:

ATGGGCGTTCCAATTTTACCAACGATTAGTACGACATGTATTGTCATTAGTGCAATTTTAATTGCCATTGGTTGGAGACTTATTTGGAAAA
GGGAAATAAATAAACACAAAAATGTTATGTTAGCTGCAGCTGTTTTTGCTTTAACTTTTTTCTTAATCTATGCAAGTAGAACGATTTTCATC
GGTAATACAGCATTTGGCGGACCAGCATCAATTAAGAAATATTATACGATTTTCTTATTTTTCCACATTAATTTAGCAACAATTGGTGGTAT
TCTAGGCCTAGTTCAAATTATTACTGCATTTAAAGATAAATATAATGTACACCGCAAATTCGGGCCATTCGCTTCAGTTATATGGTTCTGTA
CTGCAATTACAGGTGTAGCAGTTTACTTATTATTATATGTATTATATCCAGGTGGAGAAACGACATCACTGATTAAAGCAACATTTGGTCA
TTAA

Gene: ylbC2 (putative protein)

Contig: 06_NODE_54, position: 233183 to 234082, length: 900 nt, orientation: FORWARD

Perfect match to: (MW2-BA000033-[1099477:1100376], highly conserved allele)

Sequence:

GTGAATAAATCAAATAACCATGCTGAAAATCCAAAGCCTAAAGAAGGTGTTGGTACATGGGTAGGTAAAGATATTAAAGTGCTTACTTCT
AAATTTGGACAAGCAGATCGTGTCTACCCTTTTAGAGATGGTTACAAAAATTATGTGTTTAAAGACAAAAACAGTTATTACATTGTTTCAA
CTAAACGTGAAGAAATCGTTTCAGTGTATGCTACAGGTGAGAAAGTCAATGTTAGTCCGTTAAAAATAGGACAACATTCTGCAGAAATTT
TTAATCATACAAGTATTAATCCAGAACCGTCCTTTAAAGTTGATGGTAAAAAATATGAATTTGAACTTTCAGATGAAGATTTAAAAACACA
AACACTGATTAAATATGGCGACATATATGCACAAGTGTATTCTGATCAACAATCGAAAAAGGTGCTAAGTGTACGATTTTTAACAAAAGA
AATGTTAGCAGATATTGAACCTTATCAATTAAATTCTAATTCTACGTCAGAAGAGCATAATAAGCGTCCAGTTGAGCAAAATCCAAATCAA
TTAATTTCTCTTTATGAAGTAACGAATGAAATGAGAAAATTAAAAGGATTAAAACCATTGAAAATCAATAGCGATTTAGCACATATTGCAT
CTAATAACTTATATGAAGCGACCTCTAATGGTTCTGATAGTGTTGAATTTACAGAGGACGCATTAAGAGGGCAATTAGATAAAAATCACG
TTACTTATAAAACAACTGCTCAAAATGTTGGTTATGCGTTTAATGATGTACCAACATTAATCCATAGTTGGATGAATTCAGATATACATCG
ATCTCGTCTATTAAATTCAAAATACGATGAGATGGGTGGAGATGTAATGAGAGATTATTATTCACTAATTTTCTTAGAAAAATAA

Gene: ylbF (putative regulator protein)

Contig: 06_NODE_54, position: 234098 to 234532, length: 435 nt, orientation: FORWARD

Perfect match to: (N315-BA000018-[1098962:1099396], highly conserved allele)

Sequence:

ATGATTAATGAAGCTTCACTAGCGATATTAGATGATATTGATGAACTAGCTGATATGATAGTTGCATCAGATATTTATGCATCATTTGAGC
AAGCTAAACAGGCGCTCGAAAACAATGACGAAGCACATTTACTTTATCAATCATTTTTAAAATCAAAAGAGAAATATGATGAAGTAATGC
GTTTCGGGAAATATCATCCTGATTATAAGAAAGTCATGTTAGAGACAAGACAACGCAAAAGAGCATATGAGATGCTTGACGTTGTGATG
CATTACAAAGCTAGGGAAATGGCACTTCAACATTTAATTGATGAAGTTGTTACGAAAATTGCGTACGCTGTTTCAGAGCATGTCAAAATA
GAAACAGGTAATCCATTCTTTCAAACATCACATAGTGGTTGTGCGACGGGCGGATCCTGTAATTGTTCATTATAA

Gene: yhdW (putative glycerophosphodiester phosphodiesterase)

Contig: 06_NODE_54, position: 234574 to 235521, length: 948 nt, orientation: REVERSE

Sequence:

CTAGCATCTGACTCGATGTTTTTATTTATTCGGGATTGTTTGTTTGAATTGTTGTGCTAAATCTGGTCGATCTGTCACAATCGTGTGTGCAC
CTTTTTGGTATAAATCATTCATCAGATCTATACTATTTACGCCATAATAGCCTGGAATGATATTCATATCATTTAACCATTTGATAAAACGA
GATGAAGTCAAATCAATGCCTTTAAAATGAGTAGGCATTTGGAACGTTTGTGCTAATGGTTGGTAGTATCTACCACCTAATAAATGATATT
TTAAAAATGCTTCTGTAACTTCCTGTTGGCTAGCACCAATTGCGACGGATCCTTGTGCAATTTTATTAAAACGAACGATTTGTTCTTTATAA
AAACTTGTCACAAGAACGCGGTCAAATGCTTGATTTTCTGCAATTGTATCAAACATAATTTGTGGTGCGATTGAGCCTTCATAGGATTCAG
GAGCATCTTTTAAGTCTACGTTTATATACATATCAGGATATTGCTTCAGCAACTCATCGAAGGTTAGTATAGCTGTGTGTGCATGACCACG
ATATGGTGTTAGTCCATTGATATCTTTGAAGTGATAAGCTGCGTCTAATTTCTTTAATTCTGCTAATGTATGGGCACTAACTTTTCCAGAGC
CGTTCGTCGTTCTATCAACAGTTGCGTCATGAAAAACGATAAGCTGTTGATCTTTTGTGAGTCTCACATCCGTTTCAAAGCCATCAACGCC
TAATTGTTTAGCATAGTCAAATGCAAGTTGCGTTTGCTCTGGTCTTAAAGCCATACCACCGCGATGCGCAAATATATATGGTGCATTGCCT
TTGAAAAAAGCAGGGATGGTTTGCTTTTTAGTAATCACTTTATTTTTATTGATCATTAATAGACTACTTACAAATCCAGCACCGACTAGTAC
CGCATTTAAAATGTTTCTGTTTACTTTTTTCAT

Gene: ylbG (putative conserved protein)

Contig: 06_NODE_54, position: 235760 to 236014, length: 255 nt, orientation: FORWARD

Perfect match to: (MW2-BA000033-[1102054:1102308], highly conserved allele)

Sequence:

ATGAATTTAATCCCAAGAACTAGTATTGTAGTTTATTTAAAACATATGAAACATGAACGACAAATCCGAAAATATGGACATATCGTTCATT
CAAATAGAGATCGTAAATTTGTAATTATGTATGTGAATGAGCAAGATGTTGATCAAATTGTACATAAACTAATGCAACTTAAATACGTTA
GACACATTGATGGCTCACCATATAAATACTTAAAGAAAACTTACGAAAAAGAGAAACACGAAATATATAATTAA

Gene: Q5HGW1 (putative protein)

Contig: 06_NODE_54, position: 236017 to 236406, length: 390 nt, orientation: REVERSE

Sequence:

TTATTGAAGTTCTGGTATCCATTTTTGGAGCCTTAACACACTAATTAAATAATTATAATATAACGATAAATCATGAAATGCTTGAGGGGGC
TGTACATCGATACCTAAAATTGGTATGCCGAATTTTTCAGCACAATCATTAATCATTTGAAACTTTTTATTTGAATCTGGATATGGATATTT
AATAGCGTTAATCATGATATAAAATGCTTGGAAATGATTGCCAGTTGTAGGGTTCATAATGATTGCTACAGATGACTTTTTATTTTCAGCT
TGTGGTGAATGAAAATAGATAATTTTATCAATACGTTGATGGTTATATTCAATCATCGTCATGAATTCGAACAAATCTGTTAGACCTTCAC
CAAGTGTAATAAATGTTTGTTTCAT

Gene: rsmD (RNA methyltransferase)

Contig: 06_NODE_54, position: 236476 to 237018, length: 543 nt, orientation: FORWARD

Perfect match to: (MW2-BA000033-[1102770:1103312], allele observed in CC1)

Sequence:

ATGCGCGTCATTGCAGGTAAACATAAAAGTAAAGCTTTAGAAAGTATGGAAGGCCGTAATACGAGACCAACTATGGATAAAGTTAAAGA
AGGTATCTTTAATAGTTTATATGATGTGTCAGGTATAGGTTTAGATTTATTTGCAGGAAGTGGGGCGCTTGGAATAGAAGCACTCTCTCG
AGGTATGGATAAGGTAATCTTTGTTGATCAAAATTTTAAAGCTGTAAAAGTTATTAAATCAAATCTTGCGAATTTGGATTTAGAGGCACA

ATCTGAAGTTTATAAAAATAATGCAGATAGAGCTTTAAAAGCATTGTCAAAACGGGATATTCAATTTGATGTCATTTTCTTAGATCCACCT
TATAATAAAGGTCTCATTGATAAAGCTTTAAAACTAATTTCAGAGTTTAATTTATTGAAAGAAAATGGTATCATCGTTTGTGAATTTAGCA
ATCATGAAGAAATAGACTATCAACCGTTTAATATGATTAAACGTTACCATTATGGGTTGACAGACACATTGTTATTAGAAAAGGGAGAAT
AG

Gene: coaD (phosphopantetheine adenylyltransferase)

Contig: 06_NODE_54, position: 237020 to 237502, length: 483 nt, orientation: FORWARD

Perfect match to: (RF122-AJ938182-[1070749:1071231], highly conserved allele)

Sequence:

ATGGAACATACAATAGCGGTCATTCCGGGTAGTTTTGACCCCATTACTTATGGTCATTTAGACATTATTGAGAGAAGTACAGATAGATTT
GATGAAATTCATGTCTGTGTTCTTAAAAATAGTAAAAAAGAAGGTACGTTTAGTTTAGAAGAGCGTATGGATTTAATTGAACAATCTGTT
AAACATTTACCTAATGTCAAGGTTCATCAATTTAGTGGTTTACTAGTCGATTATTGTGAACAAGTAGGAGCTAAAACAATCATACGTGGTT
TAAGAGCAGTCAGTGATTTTGAATATGAATTACGCTTAACTTCAATGAATAAAAAGTTGAACAATGAAATTGAAACGTTATATATGATGT
CTAGTACTAATTATTCATTTATAAGTTCAAGTATTGTTAAAGAAGTTGCAGCTTATCGAGCAGATATTTCTGAATTCGTTCCACCTTATGTT
GAAAAGGCATTGAAGAAGAAATTTAAGTAA

Gene: ylbM (HIGH nucleotidyl transferase)

Contig: 06_NODE_54, position: 237564 to 238703, length: 1140 nt, orientation: REVERSE

Perfect match to: (MW2-BA000033-[1103858:1104997:r], allele observed in CC1)

Sequence:

TTAACGATATTGCTGTATGACAGGCGTGTTGAAATCTGTTTGTTGTTGCCCGCTTATTGCATTGTATATGTGTGTTGCTTTGATTTCATTTG
TGAAGTAATGTGCATTGCTTTTGTTAATATTGGTTATATATTGTCTTTCTGGGAACGCTGTTTTTAAATGCTTTAAATATTGTCTGCCACGG
TCGTTCATCGCTAATACTTTAACTGCGTGAATGTTACTCGTAACATCTGTAGGTTTAATGTTTAATAATACATTCATTAACAGTCTTTGGAT
ATGCGTATATGTATAACGCTTTGTTTTTAGTAATTTTACAAAATGATGAAAATCAGTTGCTTCATAAATGTTAGATTTCAAACGATTTTCAA
AACCTTCAGTAACAGTATAAATATTTTTTAATGAATCTGTAGTCATAGCTATGATTTGATATTTCAAATATGGAAATATTTGATTTAATGTT
ATATGAGGTGTTACGTACAAGTGTTGAATATCTTTAGGTACCACATGATGCCAATGATCATCTTGACTAATGATTGATGTTCTAATAGATG
TACCACTTGCAAACTGATGGTGTTGAATTAATGAATCATGATGTTGAGCATTTTCTCGTTTGATAGAAATTGCATTGATGTTTTTAGCATTT
TTAGCAATTGCTTTCAGGTAACTAATACCAAGTATGTTGTTAGGACTTGCTAGTGCTTCATGATGCTCTAATAATTCGCTAATGATACGAG
GGTAGCTTTTACCTTCTTTTACTTTTTGTGAAAAGGATTCAGATTGTTCAATTTCATTAATGCTGTGTGCTAATTGCTTTAATGTTTTGATAT
CATTATTTTCACTACCAAATGCAATTGTATCGACACTCATATAATCTGCGACTTTAACTGCTAGTTCGGCAAAATGATCGCCAGATGATAA
ACTGGCAGTTGCTGGTAGTTCGATAACTAAATCAGCTGTTGATAACGCCATTTTTGCACGAGTAAACTTATTATAGATTGCTGGTTCGCCA
CGCATGACAAAGTTACCACTCATTATTGCAATAGTAACGTCAGCATTTGTAAGTTTTTTAGATTGATTAATATGATATTGATGCCCATTATG
AAAGGGATTATATTCTGTGATTAAGCCAACGCTTTTCAT

Gene: Q4L5E0-ylbN (putative DNA binding protein)

Contig: 06_NODE_54, position: 238830 to 239387, length: 558 nt, orientation: FORWARD

Perfect match to: (N315-BA000018-[1103694:1104251], highly conserved allele)

Sequence:

ATGAAATGGTCAATTACGCAATTAAGGAAATATCAAGGTAAGCCATTTGAATTTGATCAAACGGTGAGTTTTGACAATTTAAAAGAATCA
TTAGATTTAATTGATTTATCTCCAATTACAATCCAAGGTCAGTTAACCATTAAGTCAACAGAAGTCGTTGCGGATATTCACATTACTGGAA
CGTATACAATGCCTTGTGCACGTACTCTTGTACCAGTAAAAGTCCCACTAGATGTAACTACTACAGAAGTATTTGATTTAGAAGGGTACAA
TCAGTATAACGATGATCAAGATGATGTAGATGAACACTATCACATTATTAAAGATGGTATGGTTAATCTTCAGGATATTGTCGAGGATAT
AGTTATTATTGAGAAACCAATGAGAGCTTATTCAGAGCAAAGTGACCAAATGTTGACAGTAGGTAATGGTTGGGAAGTAATCGATGAGG
ATCAATTAGATGAGCTTGCTAAACAGCAAGAACAAGATGATTCAGAATCACGACAAGTTGATCCAAGGCTTCAAAAATTACAACAATTAT
ATGATAAAGAGCAATAA

Gene: rpmF (50S ribosomal protein L32)

Contig: 06_NODE_54, position: 239467 to 239640, length: 174 nt, orientation: FORWARD

Perfect match to: (RF122-AJ938182-[1073196:1073369], highly conserved allele)

Sequence:

ATGGCAGTACCAAAAAGAAGAACTTCTAAAACTAGAAAAAACAAACGTCGTACGCATTTCAAAATTTCAGTACCAGGTATGACTGAATGC
CCAAACTGTGGCGAATACAAATTATCACACCGTGTATGTAAAAACTGTGGTTCTTACAATGGCGAAGAAGTAGCAGCTAAATAA

Gene: tx_rpmF_isdB_2 (rho-independent terminator of rpmF and isdB, locus 2)

Contig: 06_NODE_54, position: 239698 to 239734, length: 37 nt

Perfect match to: (Newman-AP009351-[1098839:1098875:r], allele observed in CC8)

Sequence:

TTATATTGCGAAAAATAATTGGCGAACGAGGTAACTG

Gene: isdB (extracellular hemoglobin-binding protein)

Contig: 06_NODE_54, position: 239794 to 241731, length: 1938 nt, orientation: REVERSE

Sequence:

TTAGTTTTTACGTTTTCTAGGTAATACGAATGCAACGATGCTACTTAAAGCTAATAATGCCATTAATGGTAATGTCATATCTTTATTTGATT
CTTCACCAGTTTGTGGTAATGATTTTGCTTTATTTTCTTGTGTATTTTTATTGTTTTGGCTTTGAGTGTGTCCATCATTTGTGTTTTTAATGTT
TGCTTTTTGTAATGGAGCACTATCTTTTGCTTCGCTAGAACCTGCTGAAGTTTGAACAACATCTTTTGTTGTTTTTGATGAAGCAGTTGTTG
GTTTTGCAACATTTTGAGTCGTAGATACTACCTTAGTTGGAGTTGTACTACTTGATTCTACTTCACCTTTAGTTGGTTTTGTAGCAGGCGTT
TTGTCTTTACCTGACTCACTAGATGCGTCATTTTCTTTTTCAACACTTGGTAATTGTTTATTGTCATCTTTTTGGCTGTCTTGTTTTTGTGATT
CTTTTTCAACAGGTGATGGTGTTGGTTTGCTAGGCGTAGCTGGAGTAGCTTCCTTCTTAGCTGAGTTATCTTGTTGTTCTTTTTTGTTAGTT
TTATCGGTATTGGCTTTTGTAAATGCTTCTTTATCAACGATTCTGACATGGTATTGTCCATCATAATCAATCGTTTTTACGTGAACTTTAACG
ATAGCATCATATAGAGTTTTACCTTCAACATATGGGAAAATAATTGTTCTAGTATTATTTTTAGCATCTTTGCTTATAGTTCTAACACGTTG
ACCTTCAACCATGAAATCTTTCCAGTAATCGTCATTAGTAGTTTCCATGACCATATATTTTTTGCCGTTAAGCATACCTGTTTTAATAGGGT
GTTTAACAAAAGTATCCATCATAGATTCGTTATTCTCAACACTTTCATAAACAACATATTTTGTATCTTGTAAATCAGTCATTTTTTCATTTG
TTGGTTGTACATTTTGGAATTCAGTAATAGCTGATTTCACTTGCTCATCTAAAGCTTTCTTTGTATCCTCTAATTTCTTCTTGTACTCAGCCTT
TAATTTTTCAGGAAGTTTATCTTGAATTTTATTTAATTCATAAACTTGTCTTTCTAGTGTTTTCGCTTTTTTATATGGCGCTAATAATTTTTCA
GCTTTATAATCTTCTTCAGTTTTGAATTTATCTGCACTGTTATAAATTGGTTGTGCGAATTCCATTAATGTGTAATCGTATTTTTCTTCTTTGT
TATTGAAGTGTGTTGAACTAACAATTTTAACAGCTTTTGTTCCGTTTGATACAGAGAAGCGAATGTAAGCATAATCTTTAACAGTATCGTA
TGATACTAATTTAATTGGCAACTTTTTGTCACCTTCATAAACTTCAAATTTTCTCCAAAATTGACCTGATTGTAATCCTAATTCAATTTCTGG
TTTTGAATCAGTGAAAATAACTCTAGCAGGTTTAACAGAACTTGCATAATGATAAAACTGTTGAGTTCCATCTTTCTTTTTCATTTCAAAAT
CAATTGGACGAGAGTTTGGTGCGCTATGATCTTTGTCTTTTATTGCAGGGTTTTTAATCGCTTCTCTAAGTTCCTGATTCAAAATAGGATAT
GTATTGTTAGTGGCTTTTGCTGCTGGTTTAACTTCTTTTGTTTCCTTAGGGGCTTTAACTTCTTTAACTTCTTTAGCTTCTTTTGTTTCAGAAG
TAGGAGCCTCAACTTCTTTATTAGATACTGAGACAGCATTAGCTACTGGTTTAGTTTCTGGAGCTTTTTCAGATGTTGTTGTTGGACTTGCA
ACTGCTTCAGTTTTTGGTTGTGCTTCTGTATTTGTACCACCTGTTTCTTCAGCTGCTGCTTGTGCTTCGCCATTTGACATTAATAATAAAAGT
GTACTAATTGCTACAGATGCAACGCCTAGTGATGACTTTCTAATTGAATAAAATGATTTAAATTCTTTTTGCTGTTTGTTCAT

Gene: isdA (extracellular transferrin-binding protein)

Contig: 06_NODE_54, position: 241934 to 242986, length: 1053 nt, orientation: REVERSE

Perfect match to: (11819-97-CP003194-[1140555:1141607:r], highly conserved allele)

Sequence:

TTATTTAGATTCTTTTCTTTTGAAAAGTAATAAAGATAATGAACCTAAAAGGGCAAGTGTTGCGAAGGCAACTGTGCTAATAAAGTTATCA
ACTGAAGTTAAACCAGTTTTTGGTAATTCTTTAGCTTTAGATGCTTGTTTAGGCGTTTCGTTATGTTTTGTAACTTTGTTAGTTTGTTGTGAT
TTATTATCACTTACAGCTTGATTGTTGCTTTCAGATTTCGCTGTTGCAACATCTTTAACAGGTGTTTGAACTTTATTTTGTTCTTGAGCAGTT
TGTGCTGTTTTAACTGTATGAGCAGTTTGTGTTTTAGTTTGATCTTTTGTTGTGTCAGTACTTACAACTTTAGTAGAGTGATTGTCTTCAACT

TTGCTTGTTGTAGTTACAGTAGGTTTAACTTTTTCAACTTTAGGTTGAACTGGTTTAGTTTGCTCAGTAGGTGTTTTAGGTTGAGCTGGTTT
TGGTTGAACCGGTTTAACATTGTTTGGTTTTGCTGCGTCAGCTAATGTAGGAATTGCTTTTTCAAATTCCAAATGCGTAGTATATCTATGAT
TGTAATTAATTTGTGGCACGACAATATGTACTTTAGTAGTTAAGCTCTTATATCCAGGTTCAACTGCAACATTGATTGTTCTAGTATCCGCT
TTTTTATTATCGTTAACAACAGTTGTTGCTAATTCTTGATTGTTTGCATTGTAAAATTTGTATTCTTTCCAGAATGATGCATTGTTTAACACG
GTTTGGAAATAATATTTATTATTTTGTTTAATTACTTTACCAGGGTGTTGCATATAGTCATCCATGTGTGACTTCTCTGAAGAGCCATCTTTT
TGCACTTGGAAATTAATTGGTTGTGATGTTGCTTGAGAAACTTGTGTGCTTTGATTATTAGTTGCGTTCGTAGCTTCTGTTGCCGCATTGA
CTTGTTGGCTGTCTGCGCCTATGTATACAAGGGAACCTAAAATGATAGATGCTGTACCCATTGTAATCTTTTTCATAGCTGATGAACGTTG
TTCTGATTGATACTTACTGTTTAAATAATGTTTTGTCAT

Gene: isdC (extracellular heme-binding protein)

Contig: 06_NODE_54, position: 243195 to 243878, length: 684 nt, orientation: FORWARD

Perfect match to: (N315-BA000018-[1108059:1108742], highly conserved allele)

Sequence:

TTGAAAAATATTTTAAAAGTTTTTAATACAACGATTTTAGCGTTAATTATCATCATCGCGACATTCAGTAATTCTGCAAATGCCGCAGATAG
CGGTACTTTGAATTATGAGGTTTACAAATACAATACCAATGACACGTCAATTGCTAATGACTATTTTAATAAACCGGCAAAGTACATTAAG
AAAAATGGTAAATTGTATGTTCAAATAACTGTCAACCACAGTCATTGGATTACTGGAATGAGTATCGAAGGACATAAAGAAAATATTATT
AGTAAAAACACTGCCAAAGATGAACGCACTTCTGAATTTGAAGTAAGTAAGTTGAACGGTAAAATAGATGGAAAAATTGACGTTTATAT
CGATGAAAAAGTAAATGGAAAGCCATTCAAATATGACCATCATTACAACATTACATATAAATTTAATGGACCAACTGATGTAGCAGGTGC
TAATGCACCAGGTAAAGATGATAAAAATTCTGCTTCAGGTAGTGACAAAGGATCTGATGGAACGACTACTGGTCAAAGTGAATCTAATA
GTTCGAATAAAGACAAAGTAGAAAATCCACAAACAAATGCTGGTACACCTGCATATATATATGCAATACCAGTTGCATCCTTAGCATTATT
AATCGCAATCACATTGTTTGTTAGAAAAAAATCTAAAGGCAATGTGGAATAA

Gene: isdD (heme ABC transporter, membrane component)

Contig: 06_NODE_54, position: 243878 to 244954, length: 1077 nt, orientation: FORWARD

Perfect match to: (08-02119-CP015645-[1062788:1063864:r], allele observed in ST582+CC8)

Sequence:

ATGAGAAATGTTAAACAAATTGCTACAAAATCTATTATAGCTATTATTAGCTTAGGTATACTTACATATACAACAATGATTGGTAGCGTGT
TGGCTGATGAGATAAAATATCCATCAGCCAAATTTAATCAACCTGAAGCAAAAGATAAAACAGAATTAACTACATCAATTTTTGATGAAA
AGATAAAAGAGAATAAAGCGTTAGAGTTACTAATTTTTAATCAAGAAAATAAAAATGTAACTGAGGAACAACAACTAGTTGACGAAAAG
GCGCAATTGATTTCAGATATGACTGGTAAAATTTACTTGCAAGTAAAGCTAAAAGGTCAAATAGATAAAGAACAACTTGTTTTTCAAAAT
GACAAAAATGAAGAATTTCCTTTTGTTATAAAAGATGAAAAGGATGACACAATAGTAAGAATTTTAATTGAACAGCATATGGATAAAATC
AATATGCATGTTAAAACGTTGGCTGAAAAGAAAAATCTAGATAACAAAGAAATGGTGTATTCTATTCATTTTAAAGAGAAAAAAGTACAA
CATGATGATGCAAAAGAAGTGCCTTCAAAACATCAAAATCAAGAAAATAATCAAGATCAGCTTAAAAAAGATATTGATGACAAAAAAGA
TAGTCAAAAATCAGATACTAAGGAAAGACGTACTAGCCTTTTTACTGAAAAAGGATTAAATGATATTCCTGTACAAAAAGATAAAGTGCA
ACAAGACAGTAATAAAAAGATTGAAAATGAGCGACCTAAAGCATCAGGTACATTAAAAGTTGAAAATAGCCCTCCAACAGTAAAAAAGG
TTGAAAATAATCACAAAGAGCAACCGAAACATAAAGATGAAAAATCAAAAAAGGAAAAGAAAAAAGTAGTTGAAAAAGAAAAAGCGTT
ACCAGCTTTTAATAGAGATGATGATAGCAAGAATAGTAGTCAATTATCTAGTGATATTAAAGAACTTGATGAACCAAATCATAAAAAGCA
ATATATTTTATTTGCAGCTGGCATTGTGTTAGCAACTATTTTACTTATTTCGGCACATTTATACAGCAGAAAGAGAGGTAACCAAGTTTGA

Gene: isdE (heme ABC transporter, substrate-binding protein)

Contig: 06_NODE_54, position: 244951 to 245829, length: 879 nt, orientation: FORWARD

Perfect match to: (K12S0375-JYGF01000009-[53026:53904], allele observed in CC692+CC30)

Sequence:

TTGAGAATCATAAAGTATTTAACCATTTTAGTGATAAGCGTCGTTATCTTAACCAGCTGTCAATCTTCCAGTTCTCAAGAATCAACTAAATC
CGGCGAATTCAGAATCGTACCAACAACTGTTGCATTGACAATGACATTGGACAAATTGGATTTACCAATTGTCGGCAAACCCACGTCATA
TAAGACATTGCCTAATCGTTATAAAGATGTACCGGAAATTGGTCAACCAATGGAGCCGAATGTTGAAGCTGTTAAAAAATTAAAACCAAC
ACATGTTTTGAGTGTGTCAACGATTAAAGATGAAATGCAACCATTTTACAAACAATTAAATATGAAAGGCTACTTTTATGATTTTGATAGT
TTAAAAGGGATGCAAAAGTCGATTACACAATTAGGTGATCAATTTAATCGTAAAGCACAAGCAAAAGAATTAAATGACCATTTAAATTCT

GTAAAGCAAAAAATTGAAAATAAAGCAGCTAAACAAAAGAAACATCCCAAAGTATTAATATTAATGGGTGTACCGGGTAGCTATTTAGT
AGCAACTGATAAATCATATATTGGTGATTTAGTTAAAATAGCAGGTGGAGAAAATGTTATTAAAGTGAAAGATCGTCAATATATTTCGTC
TAATACTGAAAATTTGTTGAATATCAATCCAGATATTATTTTAAGATTACCACACGGAATGCCTGAAGAAGTTAAGAAAATGTTTCAAAAA
GAATTTAAACAGAATGATATTTGGAAACATTTTAAAGCTGTGAAAAATAATCATGTTTATGACTTAGAGGAAGTGCCATTCGGTATTACA
GCAAATGTTGATGCTGATAAGGCAATGACTCAATTATATGATTTATTTTATAAGGATAAAAAATAG

Gene: isdF (heme ABC transporter, transmembrane permease)

Contig: 06_NODE_54, position: 245842 to 246807, length: 966 nt, orientation: FORWARD

Perfect match to: (MW2-BA000033-[1112136:1113101], allele observed in CC1)

Sequence:

ATGATAAAAAATAAAAAGAAACTACTATTTTTATGTTTGTTAGTCATTTTAATCGCAACTGCTTATATTTCGTTTGTAACCGGTACAATTAA
ATTGTCATTTAATGACCTAATTACAAAATTTACAACTGGTAACAATGAAGCAGTGGATTCAATCATTGATTTGCGATTGCCACGTATATTA
ATTGCATTGATGGTTGGCGCAATGTTAGCAGTTTCTGGAGCATTATTACAAGCAGCACTACAAAATCCTTTGGCAGAGGCGAATATCATT
GGCGTTTCCTCAGGTGCACTTATAATGAGAGCACTTTGTATGTTGTTTATTCCACAATTGTACTTTTACTTACCATTATTAAGTTTTATTGGA
GGTTTAATACCATTTTTAATAATTATATTGTTGCATTCTAAATTTAGATTCAATGCTGTAAGTATGATATTAGTAGGTGTTGCGTTATTCGT
ATTATTAAATGGTGTTTTAGAAATTTTAACTCAAAACCCTTTAATGAAAATTCCTCAAGGCTTAACAATGAAAATATGGAGCGACGTATAC
ATATTAGCAGTATCAGCATTATTGGGATTAATATTAACATTACTATTGTCCCCTAAATTGAATTTACTAAATTTAGACGACATACAAGCGCG
AAGTATCGGTTTTAATATTGATCGTTACAGATGGTTAACAGGTTTATTAGCAGTATTTTTAGCAAGTGCAACTGTTGCGATTGTTGGACAA
CTAGCCTTTTTAGGTATTATTGTGCCACATGTGGTTAGAAAGCTAGTTGGGGGCAATTACAGAGTACTTATTCCGTTTTCTACAGTTATTG
GTGCATGGCTATTGTTAGTGGCTGATTTATTAGGACGAGTGATACAGCCTCCTCTAGAAATTCCAGCCAATGCTATTTTAATGATTGTCGG
TGGTCCAATGCTAATTTACTTAATTTGTCAAAGTCAACGAAATCGAATCTAA

Gene: srtB (sortase B, NPQTN specific)

Contig: 06_NODE_54, position: 246869 to 247603, length: 735 nt, orientation: FORWARD

Perfect match to: (MW2-BA000033-[1113163:1113897], allele observed in CC1+CC12+CC188)

Sequence:

ATGAGAATGAAGCGATTTTTAACTATTGTACAAATTTTATTGGTTGTAATTATTATCATTTTTGGTTACAAAATTGTTCAAACATATATTGA
AGACAAGCAAGAACGCGCAAATTATGAGAAATTACAACAAAAATTTCAAATGCTGATGAGCAAACATCAAGCACATGTGAGACCACAAT
TTGAATCACTTGAAAAAATAAATAAAGACATTGTTGGATGGATAAAATTATCAGGAACATCATTAAATTATCCAGTACTACAAGGTAAGA
CAAATCACGATTATTTAAATTTAGATTTTGAGCGAGAACATCGACGTAAAGGTAGTATTTTTATGGATTTTAGAAATGAATTGAAGAATTT
AAATCATAATACTATTTTATACGGGCACCATGTCGGTGATAATACGATGTTTGATGTGTTAGAAGATTATTTAAAGCAATCGTTTTATGAA
AAACACAAGATAATTGAATTTGACAATAAATATGGTAAATATCAATTGCAAGTATTTAGTGCATATAAAACTACTACTAAAGATAATTACA
TACGTACAGATTTTGAAAATGATCAAGATTATCAACAATTTTTAGATGAAACAAAACGTAAATCTGTAATTAATTCAGATGTTAATGTAAC
GGTAAAAGATAGAATAATGACTTTATCAACGTGCGAAGATGCATATAGTGAAACAACGAAAAGAATTGTTGTTGTCGCAAAAATAATTA
AGGTAAGTTAA

Gene: isdG (heme oxygenase)

Contig: 06_NODE_54, position: 247622 to 247945, length: 324 nt, orientation: FORWARD

Perfect match to: (MW2-BA000033-[1113916:1114239], allele observed in CC1+CC692)

Sequence:

ATGAAATTTATGGCAGAAAATAGGCTGACGTTAACAAAAGGAACAGCAAAAGATATTATAGAACGATTTTACACGAGACATGGGATTGA
AACATTAGAAGGCTTTGATGGCATGTTTGTTACACAAACTTTAGAACAGGAAGATTTTGATGAAGTGAAAATTTTAACAGTTTGGAAATC
AAAGCAAGCTTTTACGGATTGGTTAAAATCTGATGTCTTTAAAGCAGCACATAAACATGTTAGAAGTAAAAATGAAGATGAAAGTAGCCC
GATTATCAATAACAAAGTAATTACATATGATATAGGCTATAGTTACATGAAATAA

Gene: spoU (putative tRNA/rRNA methyltransferase)

Contig: 06_NODE_54, position: 248329 to 249069, length: 741 nt, orientation: FORWARD

Perfect match to: (08-02119-CP015645-[1058673:1059413:r], allele observed in ST582+CC5+CC8+CC1290)

Sequence:

ATGGAACAAATTACTTCTGCACAAAATAATAGAATTAAACAAGCGAACAAGCTAAAAAAGAAACGTGAGAGGGATAAAACTGGATTAGC
TTTAATTGAAGGTGTGCATTTAATTGAAGAAGCTTATCAAAGTGGAATTGTAATTACACAATTATTTGCAATTGAACCGGCAAGATTAGAT
CAGCAAATTATCGCATACGCGCAAGAAGTTTTTGAAATAAACATGAAAGTTGCTGAATCTTTATCAGGTACAGTGACACCACAAGGGTTT
TTCGCAATCATTGAGAAGCCGCACTATGATATTTCTAAAGCACAACAAGTATTGCTCATCGATCGTGTTCAAGATCCTGGAAATTTAGGCA
CATTAATTAGAACTGCGGATGCTGCTGGAATGGATGCTGTAATAATGGAGAAGGGTACGACAGATCCTTATCAAGATAAAGTGTTGCGA
GCGAGTCAAGGTAGTGTTTTCCATTTGCCAGTTATGACACAAGATCTCGATACGTTTATTACTCAATTTAATGGTCCTGTTTATGGTACAG
CACTTGAAAACGCAGTGGCATACAAAGAAGTTACTTCAAGTGATTCTTTTGCATTACTATTAGGTAATGAGGGAGAAGGTGTTAATCCTG
AATTATTAGCACATACTACACAAAATTTAATCATACCTATTTATGGTAAAGCTGAAAGTTTAAATGTAGCGATTGCAGGTAGTATTTTACTT
TATCATTTGAAAGGTTGA

Gene: tbox04 (T-box leader element)

Contig: 06_NODE_54, position: 249150 to 249361, length: 212 nt

Sequence:

TAAACCAATAAAAAGGCATGGACATTTATATAAATAATTGTTTTAGGGAGAATAATCGTGACTGCAAGTTATTCCAATTATTTAAAGTCTT
TTCACCTTTTTGGTTACTTAAAGAGATTTAAGTCGGAAAGACAATCCGTTATCAATATTAAACAAGTGTATGCTTAGGCATAAATTTGGGT
GGTACCACGGAAATGACTTTCGTCCCTTAT

Gene: pheS (phenylalanyl-tRNA synthase alpha chain)

Contig: 06_NODE_54, position: 249450 to 250508, length: 1059 nt, orientation: FORWARD

Perfect match to: (MW2-BA000033-[1115744:1116802], allele observed in CC1+CC22+CC445+CC692)

Sequence:

ATGTCTGAACAACAAACAATGTCAGAGTTAAAACAACAAGCGCTTGTAGATATTAATGAAGCAAATGATGAACGTGCACTGCAAGAAGT
TAAAGTGAAATACTTAGGTAAAAAAGGGTCAGTTAGCGGACTAATGAAATTGATGAAGGATTTGCCGAATGAAGAGAAACCTGCATTTG
GTCAAAAAGTGAATGAATTGCGTCAAACAATTCAAAATGAATTAGATGAAAGACAACAGATGTTAGTTAAAGAAAAATTAAATAAGCAA
TTGGCTGAAGAAACAATTGATGTATCATTACCAGGTCGTCATATTGAAATCGGTTCAAAGCATCCATTAACACGTACAATAGAAGAAATT
GAAGACTTATTCTTAGGTTTAGGTTATGAAATTGTGAATGGATATGAAGTTGAACAAGATCATTATAACTTCGAAATGCTGAATTTACCTA
AATCACACCCTGCACGTGATATGCAAGATAGTTTCTATATTACGGATGAAATTTTATTACGTACGCATACATCACCAGTGCAGGCACGTAC
GATGGAATCACGTCATGGTCAAGGTCCAGTTAAAATTATTTGCCCTGGTAAAGTGTATCGTCGTGACTCTGATGATGCGACACATAGTCA
TCAATTTACACAAATCGAAGGATTAGTTGTTGATAAAAACGTTAAAATGAGTGATTTGAAAGGTACTTTAGAATTGTTAGCTAAGAAATT
ATTTGGTGCTGATCGTGAAATTCGTTTACGTCCAAGTTACTTCCCATTCACTGAACCTTCTGTAGAAGTTGATGTGTCATGTTTTAAATGTA
AAGGAAAAGGTTGTAATGTGTGTAAACACACAGGATGGATTGAAATTTTAGGTGCTGGAATGGTACATCCTAATGTATTAGAAATGGCT
GGTTTTGATTCTTCAGAGTACTCTGGATTTGCATTTGGTATGGGACCAGACCGTATTGCAATGTTGAAATATGGTATAGAAGATATTCGTC
ATTTCTATACTAATGATGTGAGATTTTTAGATCAATTTAAAGCGGTAGAAGATAGAGGTGACATGTAA

Gene: pheT (phenylalanyl-tRNA synthase beta chain)

Contig: 06_NODE_54, position: 250508 to 252910, length: 2403 nt, orientation: FORWARD

Perfect match to: (ATCC51811-ADVP01000010-[55786:58188:r], allele observed in CC1)

Sequence:

ATGTTGATATCAAATGAATGGTTGAAAGAATATGTAACAATCGATGATTCTGTAAGTAATTTGGCAGAACGTATTACGCGCACAGGTATT
GAAGTGGATGATTTAATTGACTACACAAAAGATATCAAAAATTTAGTTGTCGGCTTCGTTAAGTCAAAAGAGAAACATCCTGATGCAGAT
AAATTAAATGTTTGCCAAGTTGATATCGGAGAAGACGAACCTGTACAAATAGTATGTGGTGCACCGAACGTTGATGCAGGACAATATGT
CATTGTTGCTAAAGTAGGTGGAAGATTGCCTGGTGGTATTAAAATTAAGCGTGCCAAATTACGCGGTGAACGTTCAGAAGGTATGATTT
GTTCGTTACAAGAAATTGGTATTTCAAGTAATTATATACCGAAAAGTTTTGAATCAGGCATTTATGTATTTAGTGAATCCCAAGTTCCAGG

AACAGATGCCTTACAAGCTTTATATTTAGATGATCAAGTAATGGAATTTGATTTAACGCCGAATCGTGCAGATGCTTTAAGTATGATAGGT
ACTGCTTATGAAGTTGCAGCATTGTATAATACAAAAATGACTAAGCCAGACACAACATCAAATGAGCTTGAGTTATCTGCAAATGATGAA
CTGACTGTGACAATAGAAAATGAAGATAAAGTACCATATTATAGTGCACGTGTTGTTCACGACGTGACAATTGAACCCTCGCCAATTTGG
ATGCAAGCACGCTTAATAAAAGCGGGTATACGTCCTATTAATAATGTTGTTGACATTTCAAATTATGTGTTATTAGAATACGGTCAACCAT
TGCACATGTTTGATCAAGATGCGATTGGTTCACAACAAATTGTTGTTCGTCAAGCTAATGAAGGCGAAAAAATGACAACATTAGATGATA
CAGAACGTGAATTATTAACGAGCGATATTGTCATTACTAATGGACAAACTCCAATTGCATTAGCTGGTGTTATGGGTGGCGATTTTTCAG
AAGTTAAAGAACAAACATCAAATATAGTGATTGAAGGTGCTATTTTTGATCCAGTTTCAATTCGTCATACATCAAGACGTTTAAATTTACG
CAGTGAATCATCTAGTCGTTTTGAAAAAGGAATAGCTACTGAATTTGTAGATGAAGCAGTCGACCGTGCATGTTATTTATTACAAACTTAT
GCAAACGGAAAAGTGCTAAAAGATAGAGTGTCTTCAGGAGAACTTGGTGCATTTATTACACCAATCGACATCACTGCTGATAAAATTAAT
CGCACTATTGGATTTGATTTGTCACAAAATGATATTGTTACTATTTTTAATCAACTAGGGTTTGATACAGAAATAAATGATGATGTTATTAC
AGTGCTAGTACCATCACGTCGTAAAGATATTACAATTAAAGAAGATTTAATTGAAGAAGTTGCACGTATATATGGATACGACGATATTCC
ATCAACGTTACCTGTCTTCGATAAAGTTACTAGTGGTCAGCTAACTGATCGCCAATATAAAACGAGAATGGTTAAAGAAGTGTTAGAAGG
TGCTGGATTAGATCAAGCTATTACGTATTCGTTAGTTTCTAAAGAGGATGCTACTGCATTTTCGATGCAACAGCGTCAAACAATTGATTTA
TTGATGCCAATGAGTGAAGCGCATGCGTCATTACGTCAAAGTTTATTACCACATTTAATCGAAGCAGCATCATATAATGTGGCACGCAAA
AATAAAGATGTAAAATTATTTGAAATCGGCAATGTCTTCTTTGCTAATGGAGAAGGTGAACTACCAGATCAAGTTGAATATTTAAGTGGT
ATTTTAACTGGAGATTATGTAGTCAATCAATGGCAAGGTAAGAAAGAAACGGTTGATTTCTATTTAGCAAAAGGTGTCGTGGATCGAGT
ATCTGAAAAGTTAAACCTTGAATTTAGTTATCGTCGTGCCGATATTGATGGATTACATCCAGGTCGTACAGCTGAAATCTTATTAGAAAAT
AAAGTTGTTGGTTTTATTGGTGAATTACATCCAACATTAGCAGCTGATAATGATTTAAAACGTACGTATGTTTTTGAGTTGAATTTTGATG
CATTAATGTCTGTGTCGGTAGGTTACATTAATTACCAGCCAATTCCGAGATTCCCAGGCATGTCTCGTGACATTGCATTAGAAGTAGATCA
AAATATTCCAGCAGCTGATTTATTATCAACGATTCATGCACACGGTGGCAATATATTAAAAGATACACTTGTCTTTGATGTATATCAGGGC
GAACATTTAGAAAAAGGTAAAAAATCAATTGCAATACGTTTAAATTATTTAGACACAGAAGAAACATTGACAGATGAGCGCGTTTCAAAA
GTACAAGCGGAAATTGAAGCAGCATTAATTGAACAAGGTGCTGTTATTAGATAA

Gene: rnhC (ribonuclease HIII)

Contig: 06_NODE_54, position: 253145 to 254083, length: 939 nt, orientation: REVERSE

Perfect match to: (MW2-BA000033-[1119439:1120377:r], allele observed in CC1+CC15+CC361)

Sequence:

TCAAAGAGGCTTTAAAATTTTTTGGGCTTTTTCACGATTTTTAAAATGCTTTTTTGAAATGGTATCTAAATGTGAAAGACCGTATTTTTTTAT
AATTTTGGCGGCGATTACATCGACTTTAGCACCGGCACCTTTAGGAATCGTCATATTAATATTTTTTGATATTTGATCCATATATGTAACAA
ATGCGTATCGAGAGATTATGCTTGCCACTGCAATGGCTAATGACTTCGATTCTCCTTTTGTTTCAAATTTTGTTTTCTTTGGAAGTGGTATA
TCGGATAATGCGTAATGGTTATACACTTCGCGTTTTGCGAACTGATCAATGACGATATAGTCTAATTGAGACGAATCAATTTTTTCAAGTA
CATTTTTGATGGCTTCATTATGAAGAACAGCTTTCATTTTTACTTGAGTCCAGCCTTTTGCTTGCTGAATATTATATTTTTCATTGTGTAGTG
TTAATAATGAATGTGGGATGAAAGTAACCAATTGCTCAGCAAGTTCTACAATTTTGGTATCGGTTAATTTTTTTGAATCATCTACACCCAA
AGTTTTTAAAATAGGGACATGCTCTTTGGTAACGAAAGCAGCACATACAGTCAATGGACCAAAGTAATCGCCACTTCCAGCTTCATCACT
ACCAATACAGTTAAATTGATCATACATTAAAGTTTGTTCAAGAAAAGAATTAGCCATATTTTTCTTTTTAGTTTTATTAGTATTCAATTGAG
AATGCTGCGGTAGAAGTTCTTTAGACACAGCTTCTGCATGATTACCTTGAAACATGACTTTACCTGATTGGTAAATATTTACAGTTGTATTT
TGATACTTTGCACGTGCTTTCATACCTTGAGGTAAATTCTCAGTATCAAAAGTAATGCGTGACATTAATGTCGTTATGTCTTTATCCGACAA
TTTAAAAACGATATTCGCCAT

Gene: zapA (regulator of Z-ring formation)

Contig: 06_NODE_54, position: 254453 to 254719, length: 267 nt, orientation: FORWARD

Perfect match to: (N315-BA000018-[1119513:1119779], highly conserved allele)

Sequence:

ATGGCACAGTTTAAAAACAAGGTAAATGTATCAATTAATGATCAGCTTTTTACAATTGTTGGGGAAGATAACCCAGAGCACATACGATAT
GTAGCACATTTAGTTGATGATAAAATAAAAGAATTAGGGTATAAAGCAGCAGGTTTAGATACTTCAAGAAAAGCAATACTAACTGCTGT
GAATATTATGCATGAAAAAGTACTACTAGAAGAAGAAAATCGACGTTTGAAACAACAAATTCACAAATTGCAGCAGCGTGAGCAATAA

Gene: yshB (uncharacterized transmembrane protein)

Contig: 06_NODE_54, position: 254720 to 255241, length: 522 nt, orientation: FORWARD

Perfect match to: (MW2-BA000033-[1121014:1121535], allele observed in CC1+CC15)

Sequence:

ATGGTCATTGATTTTATCATAATCATTTTCTTTGTGTATTTTGTCATGGTTGGATTCAGACGAGGTTTTTGGTTATCTATGATACATTTGAGT
GCAACGATTGTATCATTGTGGATTGCCAGTCAATTTTACAAATCTATTGTAGAAAGATTAATTGTATTTATTCCATATCCTAAAACAACAGC
ATTTAATACAACTTTTGCGTTTCATTTTAATCATCTACAAAATCGATTTGAAGCGATTGTAGCTTTTTTAATGATTACATTGTTTTGTAAGTT
CATTTTATATCTAATTATCGTAACTTTTGATAAAATAATAGCGTATCAAAACATTCATATTTTCAGTCGTGCAATGGGAATGATAGTTGGTG
TGTTTATGACGATAATTGTCTTACACTTTACGTTATATCTATTGGCATTATATCCTAACGAAGCATTACAACATCAGCTTAAAATATCTATTG
TGAGTCATTCATTGATTTTTCACATCCCATATTTATCGGCTTTCACCATTAATTTATAA

Gene: polX (DNA-directed DNA polymerase IV beta subunit)

Contig: 06_NODE_54, position: 255314 to 257026, length: 1713 nt, orientation: FORWARD

Perfect match to: (COL-CP000046-[1161345:1163057], highly conserved allele)

Sequence:

ATGACAAAAAAAGATGTTATCAAACTATTAGAACAAATTGCTACTTATATGGAATTAAAAGGGGAAAATACTTTTAAAATATCAGCGTAT
CGAAAAGCAGCTCAAAGTCTTGAATTAGATGAACGACCATTAGATGAAATATCTGATGTAACGGAGTTAAAAGGCATTGGTAAAGGTGT
TGCAGAAGTAATCAATGATTACCGTGAGACCGGTGAATCTCAGTATTTACAGCAATTACAGGAAGAAGTTCCGGAAGGTCTTATTCCACT
TTTGAAAATTCAAGGACTTGGAAGCAAGAAAATTGCTAAGCTATATAAAGAGTTGAATATTGTTGATAAAGCGTCACTTCAAGTTGCTTG
TGAAAATGGAAAAGTTAGTGAATTAAGCGGATTTGCTAAGAAAACGGAACAAAACATATTAGAAGCTGTGAAACAACTTGGTGCTAAGA
AAGATAGATATCCAATTGATCAAATGAGAAGACTTAATCAAGAAATCATTGATTATATAGATACATTAAATTATATCGATCAATATTCATC
TGCAGGAAGCTTCCGTCGTTTTAAAGAAATGAGCAAAGATTTAGATTTCATAATAAGTACCGATAACCCAAAAGCAGTGCAGCAGCAATT
ATTAAATATTCCCAATAAAGTAAAAGAAGTTGCAGTGGGGAACACAAAAGTTTCATTAGAATTAGCGTATGATGATGAAACGATTGGTG
TCGATTTTCGATTAATTGAACCAAGTGCTTTTTATCATACATTGCAGCATTTTACTGGGTCAAAAGAACATAATATAAGAATTCGACAACTT
GCTAAAGCACGTGATGAAAAAGTAAGTGAATATGGAATTGAACAAGCTGATGGTACATTAATTCAATATGATAGTGAAGCCAAGATATA
TGAACATTTTAATGTGAATTTTATACCACCTGCTATGCGAGAAGATGGTAGCGAATTTGATAAAGATCTAAGTAATATCATTACAATAGAT
GATATTAATGGTGATATTCATATGCATACAACGTATAGTGATGGTGCGTTTTCTATTCGAGACATGGTAGAAGCAAATATCGCAAAAGGT
TATAAATTCATGGTAATTACTGATCATTCACAAAGTTTACGTGTTGCTAATGGCTTACAAGTAGAAAGACTTTTAAGACAAAACGAAGAA
ATTAAGGCTTTAGATAAAGAATATAGTGAAATTGATATTTATTCAGGTACAGAAATGGATATATTACCTGATGGCTCGCTGGATTATGAT
GATGAAATTTTAGCACAACTTGATTATGTAATTGGAGCTATTCATCAAAGCTTTAACCAATCAGAAGAACAAATTATGGAACGATTAGCTA
ATGCATGTCGCAATCCATACGTGCGACATATAGCGCATCCAACAGGGCGTATTATAGGTAGAAGAGATGGTTATAAACCGAATATTGAA
CAATTAATGGCATTAGCTGAAGAAACGAATACAGTATTAGAAATTAATGCCAATCCACATCGACTGGATCTGAGCGCTGATATCGTTCGT
AAATATCCAAATGTGAAATTAACTATTAACACTGATGCGCATCATACAAATCATTTAGATTTTATGAATTATGGCGTAGCAACTGCGCAAA
AAGGATTTGTAACAAAAGATAGAGTGATTAACGCATTATCGCGTGAAGCTTTTAAAGACTTTATTGAAAATAATACAAAACTTAAGAAAT
AG

Gene: mutS2 (DNA mismatch repair ATPase)

Contig: 06_NODE_54, position: 257036 to 259384, length: 2349 nt, orientation: FORWARD

Perfect match to: (MW2-BA000033-[1123330:1125678], allele observed in CC1+CC12)

Sequence:

ATGAGACAAAAAAACATTAGACGTCTTAGAATTTGAAAAAATAAAATCACTCGTTGCCAATGAAACTATTAGTGACTTAGGCTTGGAAAAG
GTCAATCAAATGATGCCAGCTACTAATTTTGAAACGGTTGTTTTTCAAATGGAAGAAACGGATGAGATTGCTCAAATTTATAATAAGCATC
GTTTACCAAGCTTGAGTGGCTTATCTAAAGTATCAGCATTCATTCATCGCGCTGATATTGGCGGCGTTTTAAATGTATCAGAGCTTAACTT
GATAAAAAGATTAATTCAAGTACAAAATCAATTCAAGACATTTTATAATCAATTGGTTGAAGAAGATGAAGGTGTTAAATACCCAATATT
AGATGACAAGATGAATCAATTACCTGTGTTAACTGATCTTTTTCATCAAATAAATGAAACATGCGATACGTATGATTTATATGATAATGCG
AGTTATGAATTGCAAGGGATTAGAAGTAAAATTTCTAGCACGAATCAACGTATTAGACAAAATTTGGACCGTATTGTTAAAAGCCAAGCA
AATCAGAAAAAATTATCAGATGCTATTGTAACAGTTAGGAATGAAAGAAACGTTATACCTGTCAAAGCTGAATATCGACAAGATTTTAAT
GGGATTGTACATGATCAATCTGCTTCAGGACAAACATTGTATATTGAGCCATCATCAGTTGTTGAAATGAATAATCAAATTAGTCGATTAC
GTCATGACGAAGCAATTGAAAAAGAACGCATTTTAACGCAACTAACTGGTTATGTGGCTGCGGACAAAGATGCACTACTTGTGGCAGAA
CAAGTCATGGGTCAGTTAGATTTTTTAATCGCAAAAGCGAGATATAGTAGAAGTGTTAAAGGAACAAAGCCGATATTTAAAGAGGACCG
TACTGTATATTTACCTAAAGCATACCATCCATTATTAAATCGTGAGACTGTTGTAGCTAATACCATCGAATTTATGGAAGATATTGAAACG
GTAATTATTACAGGACCGAATACAGGTGGTAAAACTGTAACATTAAAAACATTAGGTTTAATTATTGTTATGGCTCAATCAGGATTGTTG

ATTCCCACACTTGATGGTAGTCAGTTAAGTGTATTTAAAAATGTATATTGCGATATCGGAGATGAACAATCAATAGAACAATCATTATCAA
CTTTTTCATCTCATATGACGAATATAGTTGAAATTTTAAAGCATGCAGACAAACATAGTTTAGTTTTATTTGATGAATTAGGTGCAGGTAC
AGATCCGAGTGAAGGTGCTGCATTAGCAATGAGCATTTTAGATCATGTTAGAAAAATTGGTTCTCTAGTAATGGCAACGACGCACTATCC
TGAACTTAAAGCATATAGTTATAATCGAGAAGGCGTTATGAATGCGAGTGTAGAATTTGATGTAGATACTTTGAGTCCAACGTATAAGTT
ATTAATGGGTGTGCCGGGTCGTTCAAATGCTTTTGACATTTCTAAAAAGTTAGGTCTTAGTTTGAATATTATTAATAAGGCTAAGACGATG
ATTGGTACTGATGAAAAAGAAATAAATGAAATGATTGAATCATTAGAGCGTAATTACAAACGTGTAGAGACACAGAGGTTAGAACTGGA
CCGTCTTGTAAAAGAAGCGGAGCAAGTGCATGATGATTTATCTAAGCAGTATCAACAATTCCAAAATTATGAAAAGTCTCTAATAGAGGA
AGCGAAAGAAAAAGCAAATCAGAAGATTAAGGCTGCAACAAAAGAAGCTGACGATATTATTAAAGACTTAAGACAATTGCGTGAACAA
AAAGGTGCAGATGTTAAAGAACATGAATTGATTGATAAGAAGAAACGATTAGATGATCATTATGAAGCGAAATCTATAAAGCAAAATGT
ACAAAAGCAAAAATACGATAAAATTGTTGCTGGTGATGAAGTAAAAGTATTATCTTACGGTCAAAAGGGTGAAGTTTTAGAAATTGTCA
ATGATGAAGAAGCAATTGTTCAAATGGGAATTATTAAAATGAAGTTACCTATTGAAGATTTAGAGAAAAAACAAAAAGAAAAAGTTAAG
CCAACGAAAATGGTTACACGTCAAAATCGTCAAACAATTAAAACTGAACTTGACTTACGAGGCTATCGTTATGAGGATGCTTTAATTGAA
CTAGATCAATATTTAGATCAAGCCGTTTTAAGTAATTACGAACAAGTTTATATCATTCATGGTAAAGGTACAGGTGCACTTCAAAAAGGTG
TACAACAACATTTGAAAAAGCATAAAAGTGTTAGTGACTTTAGAGGTGGTATGCCAAGCGAAGGTGGATTTGGCGTTACCGTTGCAACA
CTAAAATAA

Gene: trxA1 (thioredoxin)

Contig: 06_NODE_54, position: 259557 to 259871, length: 315 nt, orientation: FORWARD

Perfect match to: (N315-BA000018-[1124617:1124931], highly conserved allele)

Sequence:

ATGGCAATCGTAAAAGTAACAGATGCAGATTTTGATTCAAAAGTAGAATCTGGTGTACAACTAGTAGATTTTTGGGCAACATGGTGTGGT
CCATGTAAAATGATCGCTCCGGTATTAGAAGAATTAGCAGCTGACTATGAAGGTAAAGCTGACATTTTAAAATTAGATGTTGATGAAAAT
CCATCAACTGCAGCTAAATATGAAGTGATGAGTATTCCAACATTAATCGTCTTTAAAGACGGTCAACCAGTTGATAAAGTTGTTGGTTTCC
AACCAAAAGAAAACTTAGCTGAAGTTTTAGATAAACATTTATAA

Gene: uvrC (ultraviolet response system subunit C)

Contig: 06_NODE_54, position: 260195 to 261976, length: 1782 nt, orientation: FORWARD

Perfect match to: (MW2-BA000033-[1126489:1128270], allele observed in CC1)

Sequence:

TTGGAAGACTATAAGCAACGAATTAAAAATAAATTAAATGTCGTACCTATGGAACCAGGCTGCTATTTAATGAAAGATCGTAATGATCAA
GTGATATATGTTGGCAAAGCTAAAAAGCTAAGAAATCGATTGAGATCATATTTTACGGGTGCTCATGATGCTAAAACAACGAGACTGGTT
GGTGAAATACGTCGCTTTGAGTTTATTGTCACGTCTAGTGAAACAGAGTCACTTTTACTTGAATTGAATCTGATTAAACAATATCAACCAA
GATATAATATATTATTAAAGGATGATAAAAGTTATCCATTTATTAAAATTACGAAGGAGAAATATCCTAGACTACTAGTGACGAGAACTG
TAAAACAAGGTACTGGCAAATATTTCGGACCGTATCCGAATGCATATTCTGCTCAAGAAACTAAAAAGTTATTAGACAGAATATATCCAT
ATCGCAAATGTGATAAGATGCCAGATAAATTATGTCTTTATTACCATATTGGACAATGTTTAGGACCATGTGTATATGACGTTGATTTGAG
TAAATACGCACAAATGACGAAGGAAATTACTGATTTTCTGAATGGGGAAGACAAAACAATTTTAAAAAGTTTAGAAGAGCGAATGTTAA
CTGCAAGTGAATCACTTGATTTTGAACGGGCTAAAGAATATAGAGATTTAATTCAACATATTCAAAATCTGACAAACAAACAAAAAATTA
TGTCATCAGATAAAACGATTCGTGATGTCTTTGGTTATAGTGTTGATAAAGGATGGATGTGTATCCAAGTTTTCTTTATACGACAAGGTAA
TATGATAAAGCGAGATACAACGATGATTCCATTACAGCAAACAGAAGAAGAAGAATTTTATACATTTATTGGACAATTTTATAGCTTAAA
CCAACATATTTTACCTAAGGAAGTTCATGTACCACGTAATTTGGATAAAGAAATGATTCAATCTGTTGTGGACACTAAAATCGTTCAACCC
GCGCGAGGTCCCAAAAAAGATATGGTTGACCTAGCTGCACATAACGCTAAAGTATCCTTAAATAATAAATTTGAATTAATATCACGTGAT
GAGTCAAGAACGATTAAAGCTATTGAAGAACTTGGAACACAAATGGGAATTCAAACACCAATTAGAATTGAAGCATTCGATAATTCTAAT
ATTCAAGGTGTGGATCCAGTGTCAGCAATGGTTACATTTATCGACGGTAAACCAGATAAGAAAAATTATAGAAAGTATAAAATCAAAAC
GGTTAAAGGTCCAGATGATTACAAATCAATGAGAGAAGTAGTAAGACGACGATATTCTCGCGTTTTAAACGAAGGATTACCATTACCTGA
TTTAATAATAGTAGATGGTGGTAAAGGACATATGAACGGGGTTATTGATGTGCTACAAAACGAATTAGGTCTTGATATCCCTGTTGCAGG
TTTGCAGAAAAATGATAAACACCAAACATCTGAATTATTATATGGCGCTAGTGCAGAAATTGTACCACTGAAGAAAAATAGCCAGGCATT
TTATTTGTTGCACCGTATCCAAGATGAGGTTCACAGATTCGCAATCACATTTCATAGACAAACACGTCAAAAGACAGGCTTGAAATCAATA
CTTGATGATATAGATGGTATCGGTAACAAACGTAAAACATTATTATTGCGTTCATTCGGTTCAATCAAGAAAATGAAGGAAGCTACACTT
GAAGATTTTAAAAATATAGGTATTCCTGAAAACGTCGCAAAGAACCTACATGAACAATTGCATAAATAA

Gene: sdhC (succinate dehydrogenase cytochrome b-558 subunit)

Contig: 06_NODE_54, position: 262300 to 262914, length: 615 nt, orientation: FORWARD

Perfect match to: (MW2-BA000033-[1128594:1129208], highly conserved allele)

Sequence:

TTGGCTCAATCAAAAAATGAATTTTATCTAAGACGTATTCACTCGTTATTAGGTATTATCCCAATAGGTGCATTTTTGGTCGTTCATTTATT
AGTGAATCACCAAGCAACACAAGGTGCTGAAGCGTTTAATAAGGCATCTAACTTTATGGAATCATTACCATTTCTAATTATTGTAGAATTT
TTATTTATATACATTCCGTTGTTATATCACGGTTTGTTTGGTATACACATTGCATTTACAGCAAAAGAAAATGTTGGACATTACTCGATTTTT
AGAAACTGGATGTTCTTCTTCCAAAGAGTGAGTGGTATCTTAACATTTATCTTTATTGGTATCCATTTATGGCAAACACGTTTACAAAAAG
CATTTTACGGCAAAGAAGTGAATTACGATTTAATGCACGAAACATTGCAACATCCTGGATGGGCAATATTTTATATTATTTGTATTATTGC
TGTTGTGTTCCACTTTGCAAATGGCTTATGGTCATTCTTAGTTACTTGGGGTGGACTTCAATCTCCAAAATCACAACGAGTATTTACATGG
GTTTCATTAATCGTATTTTTAGTTATTTCGTATATTGGTGTTACTGCAATTATTGCCTTTATGTAA

Gene: sdhA (succinate dehydrogenase flavoprotein subunit)

Contig: 06_NODE_54, position: 262966 to 264732, length: 1767 nt, orientation: FORWARD

Perfect match to: (65-1322-ACJS01000030-[162293:164059], allele observed in CC30+CC80)

Sequence:

ATGGCAGAGAAACATCTTATTGTTGTCGGAGGTGGCCTAGCGGGCTTAATGTCAACAATTAAAGCGGCAGAAAAAGGTGCACATGTAGA
TTTGTTCTCAGTTGTACCAGTAAAGCGTTCGCACTCTGTTTGTGCCCAAGGTGGCATTAATGGTGCGGTCAATACTAAAGGGGAAGGCGA
TTCTCCTTGGATTCACTTTGATGATACAGTGTATGGTGGCGACTTCCTTGCAAACCAACCACCTGTTAAAGCGATGACAGAGGCAGCACCT
AAAATTATTCATTTATTAGACCGTATGGGCGTAATGTTCAATAGAACAAATGAAGGTCTATTAGATTTTAGACGTTTCGGTGGTACATTAC
ATCACAGAACAGCATATGCAGGGGCAACAACTGGACAACAATTATTATATGCATTGGATGAACAAGTTCGTGCATATGAAGTAGATGGA
TTAGTTACGAAGTATGAAGGATGGGAATTCCTTGGCATAGTTAAAGGTGACGATGATAGTGCAAGAGGTATCGTTGCACAAAATATGAC
AACTGCTGAGATTGAAACATTTGGTTCAGATGCAGTTATTATGGCAACGGGTGGCCCTGGTATTATTTTCGGTAAAACAACAAACTCAAT
GATTAATACAGGATCAGCGGCTTCCATTGTTTACCAACAAGGCGCTATTTATGCTAATGGTGAGTTCATTCAAATTCATCCTACTGCAATC
CCTGGTGATGATAAACTGCGACTAATGAGTGAATCAGCACGTGGTGAAGGTGGACGAATTTGGACATATAAAGATGGTAAGCCTTGGTA
CTTCTTAGAAGAGAAATATCCTGATTATGGTAACTTAGTACCTCGTGATATCGCAACGCGTGAAATTTTCGATGTATGTATTAACCAAAAA
TTAGGTATAAATGGCGAAAACATGGTATATCTTGATTTGTCACATAAAGATCCACATGAGTTAGATGTAAAACTAGGTGGTATCATTGAG
ATTTATGAAAAATTCACTGGTGATGACCCACGCAAAGTACCAATGAAGATTTTCCCAGCTGTTCACTATTCAATGGGTGGTCTATATGTAG
ATTATGATCAAATGACAAATATTAAAGGGTTATTTGCAGCTGGAGAATGTGACTTCTCTCAACATGGTGGTAACCGCTTAGGTGCCAATT
CATTGTTATCAGCGATTTATGGTGGTACAGTAGCAGGTCCAAACGCGATTGATTATATTTCAAATATTGATCGATCATATACTGATATGGA
CGAAAGTATTTTTGAAAAACGTAAAGCTGAAGAGCAAGAACGTTTTGATAAATTATTAGCTATGCGCGGTACAGAAAATGCCTATAAATT
ACACCGTGAACTTGGTGAAATTATGACAGCAAATGTAACTGTTGTTCGTGAAAATGAAAAACTGTTAGAAACAGATAAAAAGATTGTTG
AATTGATGAAACGTTATGAAGATATTGATATGGAAGATACTCAAACTTGGAGTAACCAAGCGGTATTCTTTACCCGTCAACTATGGAACA
TGTTAGTACTTGCACGTGTTATTACGATTGGTGCATATAACCGTAACGAATCACGCGGTGCCCATTATAAACCAGAATTCCCAGAGCGTA
ATGATGAAGAGTGGTTAAAAACGACAATGGCCTCATTCCAAGGCGCATTTGAAAAACCACAGTTTACTTATGATGACGTCGATGTGAGTT
TAATACCACCTCGTAAACGTGATTACACAAGTAAGTCTAAAGGGGGTAAAAAATAA

Gene: sdhB (succinate dehydrogenase iron-sulphur protein subunit)

Contig: 06_NODE_54, position: 264732 to 265547, length: 816 nt, orientation: FORWARD

Perfect match to: (MW2-BA000033-[1131026:1131841], allele observed in CC1+CC5+CC8)

Sequence:

ATGACTGAACAATCAGTGAAAAACACTCCACAACATGAAACACAATCTAAACCGAAACAAAAAACAGTAAAATTAATTATTAAACGACAA
GATACAAGTGATTCTAAGCCTTATGAAGAAACATTTGAAATTCCATATCGTGAAAATTTAAACGTCATTGCTTGTTTAATGGAAATTAGAC
GTAACCCAGTTAATATTAAAGGTGAAAAAACAACACCTGTTGTCTGGGATATGAACTGCTTAGAAGAAGTATGTGGAGCATGTTCTATGG
TTATCAATGGTCGTGCAAGACAATCTTGTTCTGCGATTGTTGATCAATTAGAACAACCTATTCGTTTAGAGCCAATGAATACTTTCCCAGTT
ATCCGTGACTTACAAGTTGATCGTTCTAGAATGTTCGATAACTTAAAACGTATGAAAGCATGGATCCCAATTGATGGAACGTATGATTTA
GGTCCGGGACCACGTATGCCAGAGAAAAAACGTCAAACAGCTTATGAATTATCTAAATGTATGACATGTGGTGTATGTTTAGAGGTTTGT
CCTAATGTTACTGAAAATAATAAATTCGTTGGTGCGCAAGCAATCTCGCAAGTTCGTTTGTTTAATTTGCACCCAACAGGATCTATGACTA
AAGATGAACGTTTAAATGCATTAATGGGTACTGGTGGCTTACAGCAGTGTGGTAATTCACAAAACTGTGTTAATGCTTGCCCTAAAGGTA

TTCCATTAACAACATCCATTGCAGCAATGAACAGAGAAACAACATTCCACATGTTTAAATCATTCTTTGGTTCAGACCATGAAGTAGAATA
A

Gene: murI (glutamate racemase)

Contig: 06_NODE_54, position: 265783 to 266583, length: 801 nt, orientation: FORWARD

Perfect match to: (N315-BA000018-[1130843:1131643], highly conserved allele)

Sequence:

ATGAATAAACCAATAGGTGTAATAGACTCTGGTGTCGGAGGTTTGACAGTAGCTAAAGAAATTATGCGTCAGTTGCCAAATGAGACGAT
TTATTACTTAGGTGATATTGGACGATGTCCATATGGGCCAAGACCAGGAGAACAAGTAAAACAATATACAGTTGAAATCGCTCGTAAATT
AATGGAATTTGATATAAAAATGCTCGTGATTGCTTGTAATACAGCAACTGCTGTAGCTTTAGAATATTTACAAAAGACCTTATCAATCCCA
GTGATTGGCGTAATTGAACCAGGTGCTAGAACAGCAATAATGACTACTAGAAATCAAAATGTATTAGTACTAGGAACGGAAGGCACAAT
TAAATCTGAAGCATATCGTACGCATATTAAACGTATCAATCCACATGTAGAGGTACATGGCGTTGCCTGTCCAGGTTTTGTGCCACTTGTA
GAACAAATGAGATATAGTGATCCAACAATTACAAGCATTGTCATTCATCAAACACTGAAACGTTGGCGTAATAGTGAGTCTGATACTGTC
ATTTTAGGATGTACCCACTATCCATTGCTCTATAAACCTATCTATGATTATTTTGGTGGTAAAAAGACAGTGATTTCGTCTGGATTAGAAAC
GGCTCGTGAAGTTAGTGCATTGCTAACATTTAGTAATGAACATGCAAGTTATACTGAACATCCAGATCATCGATTTTTTGCAACAGGTGAT
CCTACTCACATTACTAACATTATCAAAGAGTGGTTAAATTTATCTGTCAATGTGGAACGTATATCAGTGAATGACTAG

Gene: ntpA (nucleoside-triphosphatase)

Contig: 06_NODE_54, position: 266595 to 267182, length: 588 nt, orientation: FORWARD

Perfect match to: (MW2-BA000033-[1132889:1133476], allele observed in CC1)

Sequence:

ATGAAAGAGATTGTTATTGCATCGAATAATCAAGGGAAAATAAATGACTTTAAAGTAATATTTCCAGATTACCACGTAATAGGTATTTCA
GAACTAATACCAGATTTTGATGTGGAAGAAACAGGATCAACATTTGAAGAAAATGCTATATTAAAATCAGAAGCTGCTGCAAAAGCATT
GAATAAAACAGTCATAGCTGATGACAGTGGACTAGAAGTTTTTGCATTAAATGGTGAGCCAGGTATATACTCTGCACGTTATGCTGGTGA
AAATAAAAGCGATGAAGCAAATATTGAAAAATTATTAAATAAGCTTGGTAATACAACTGATCGTCGTGCGCAATTTGTTTGTGTCATAAG
TATGAGTGGCCCTGATATGGAAACAAAAGTATTTAAAGGTACTGTTTCAGGTGAAATTGCAGATGGAAAATATGGCGAAAATGGTTTCG
GATATGATCCGATATTTTATGTACCGAAATTAGATAAAACCATGGCTCAACTTTCAAAAGAACAAAAAGGGCAAATTAGTCATAGACGAA
ATGCGATTAATTTACTTCAAGCTTTTCTTGAAGGTGATAAAAATGTCTAA

Gene: ysnB (metallophosphatase)

Contig: 06_NODE_54, position: 267175 to 267678, length: 504 nt, orientation: FORWARD

Perfect match to: (ATCC51811-ADVP01000010-[41018:41521:r], allele observed in CC1)

Sequence:

ATGTCTAAATGGATTATTGTGAGTGATAACCATACTGAATCAGGCGTTTTATATCAAATTTATGAAATGCACCCAGATGCAGATGTATATT
TACATTTAGGAGATTCAGAATTCGCGTATGATGATACGGAACTTAGCTTATTTAATAGAGTAAAGGGCAATTGTGATTTTTACCCAGAATT
TGAAAATGAAGCGGTCGCAAAATATAATGACGTGAAAGCATTTTATACTCATGGACATTTATATCAAGTCAATCGAACAAGAGATTTATT
AGCTGAAAAAGGACTTGAATTAGGTTGTTTGTTTGCATTTTATGGACATACACATGTGGCAAAATATGAGTATATTAATGGTGTTCATGTT
ATTAATCCTGGAAGTATATCTCAATCTAGAAGTTCAATGGAAGAAACATATGCTGAAGTTATTATTGATGATCAAACTTTACATGGCACCA
TCAATTTCAAAAATCGACATCACGAAACAATCAGTCATACTACTTTTTAA

Gene: Q6GA61 (putative protein)

Contig: 06_NODE_54, position: 267806 to 267973, length: 168 nt, orientation: FORWARD

Perfect match to: (MW2-BA000033-[1134100:1134267], allele observed in CC1)

Sequence:

ATGAAAAGAATTGTAATTTTGGAGAATGTGATTGTTGATGACCCTATAAGGTTGAATCATGTTGATTATTTGGTTAAAAGTGTATTATTAA
AAAGTATAAAATTAAGATTCCAAGAAGTCAGGAAATTTGAAATTTTAGTTAAAATCATGAATCGTATACATATTTAA

Gene: ecb (extracellular complement-binding protein)

Contig: 06_NODE_54, position: 268167 to 268484, length: 318 nt, orientation: FORWARD

Perfect match to: (MW2-BA000033-[1134461:1134778], allele observed in CC1)

Sequence:

ATGAAAAAGAATTTTATTGGGAAATCAATTTTAAGCATAGCTGCTATTAGTTTAACGGTATCAACGTTTGCCGGTGAATCTCATGCACAAA
CTAAGGTTGAAAAATATAACGAGTATCAAACAAACTTTAAAAAACAAGTAAATAAAAAAGTTGTGGACGCACAAAAAGCTGTAAACTTG
TTCAAACGTACAAGAACTGTTGCAACACACCGTAAAGCACAAAGAGCTGTTAACTTAATTCATTTCCAACACAGCTATGAAAAGAAAAAA
TTACAAAGACAAATCGATCTAGTTTTAAAATATAATACTTTAAAATAA

Gene: flr (formyl peptide receptor inhibitory protein)

Contig: 06_NODE_54, position: 268854 to 269252, length: 399 nt, orientation: REVERSE

Perfect match to: (MW2-BA000033-[1135148:1135546:r], allele observed in CC1+CC12)

Sequence:

TTAATACCAAGTAATCGAATCGATTTCGCCGTTTTTAACCACTGGAATATTTTTTCTATGTTCTTCTAATGGGCGAGTGATATCGAAGGTAT
AATAACCATTTTTACCACCGTCTTTGATTTTTACTACAGCTATTGTATTGCCTTTACCCATCTTTTTAGCTATTACATATAAGTCTTCAACAGT
TTTACCTTTATAGTGTTCGTTTTTTTCATCGGCTTCTTTTATTAATTTATCAGCACGTTCATCGTCCTTTTTAGCTTGGTCTGCTAAATTTTTA
GCGATTTCTAAACCTTTCCATTCATAACTAAAGAACGCCTTAGCATCATTAGTTTGAGTTAGAAGACCTGTAGCGATGACTGTAGATGCAA
TAATTACTTTTGTGATATTTTTTTTCAT

Gene: Q2YXB9 (putative membrane protein)

Contig: 06_NODE_54, position: 269923 to 270429, length: 507 nt, orientation: FORWARD

Perfect match to: (MW2-BA000033-[1136217:1136723], allele observed in CC1+CC72+CC80+CC188)

Sequence:

ATGATAATTAAAATTTTAACAATTCTATTACTACTTTGTATATTGAGCTATTTGGTTACAAATAGAAAGAAGCCTTTTCTGTTCTTAAAAAC
ACTCTTTATGGGTGTGGTATTTATCTTTATAGGATATATTTCACTGGCAATATCTGCCGTAATTATTTATGGCATTATTCAATTTATCACAAT
TGATTTTGGTAGTTTTTTCTTAATGGGTATTATATTAATCTTGATTTCAAGTATATTCCAATTATTTATAGTTAGATTACTTTTTAGAAAAAA
GAATGTCGATTTGACAGAGGTTGTCGTTTTAGAACATTTAATTCAATGGTTCTTAGTTTACTTTGCGATCTATCAAGCAGTAAATGAAAAA
ATGGACATTAATGATATTAATATCGACAATTTTCAATCTGTCTTTTTTGACGTGTCTAATTTGAATTTAGTAATTCTACCAACGTTAATCATT
AGCTGGGTCACAATATTTAACTATAGAATGAGAAGTTACAAATAA

Gene: efb (extracellular fibrinogen-binding protein)

Contig: 06_NODE_54, position: 270688 to 271185, length: 498 nt, orientation: FORWARD

Sequence:

ATGAAAAAATAAATTGATAGCAAAATCTTTATTAACAATAGCGGCAATAGGTATTACTACAACTACAATTGCGTCAACAGCAGATGCGAGC
GAAGGATACGGTCCAAGAGAAAAGAAACCAGTGAGTATTAATCACAATATCGTAGAGTACAATGATACTACTTTTAAATATCAATCTAGA
CCAAAATTTAACTCAACACCTAAATATATTAAATTCAAACATGACTATAATATTTTAGAATTTAACGATGGTACATTCGAATATGGTGCAC
GTCCACAATTTAATAAACCAGCAGCAAAAACTGATGCAACTATTAAAAAAGAACAAAAATTGATTCAAGCTCAAAATCTTGTGAGAGAAT
TTGAAAAAACACATACTGTCAGTGCACACAGAAAAGCACAAAAGGCAGTCAACTTAGTTTCGTTTGAATACAAAGTTAAGAAAATGGTCT
TACAAGAGCGAATTGATAATGTATTAAAACAAGGATTAGTTAAATAA

Gene: scc (similar to complement inhibitor)

Contig: 06_NODE_54, position: 271339 to 271689, length: 351 nt, orientation: FORWARD

Perfect match to: (N315-BA000018-[1136409:1136759], highly conserved allele)

Sequence:

ATGAAATTTAAAAAATATATATTAACAGGAACATTAGCATTACTTTTATCATCAACTGGGATAGCAACTATAGAAGGGAATAAAGCAGAT
GCAAGTAGTCTGGACAAATATTTAACTGAAAGTCAGTTTCATGATAAACGCATAGCAGAAGAATTAAGAACTTTACTTAACAAATCGAAT
GTATATGCATTAGCTGCAGGAAGCTTAAATCCATATTATAAACGTACGATTATGATGAATGAATATAGAGCTAAAGCGGCACTTAAGAAA
AATGATTTCGTATCAATGGCTGATGCTAAAGTTGCATTAGAAAAAATATACAAAGAAATTGATGAAATTATAAATAGATAA

Gene: SIRU01 (staphylococcal interspersed repeat unit 1)

Contig: 06_NODE_54, position: 272010 to 272175, length: 166 nt

Sequence:

GGCCACAACAGAGAGAAATAGGATCACCAATTCCAACAGACAATGCAAGTTGGCGGGGCCCCAACATAGAGAAATTGGATCACCAATTT
CAACAGACAATGCAAGTTGGGGTGGGGCCCAACACAGAAGCTGGCGAAAAGTCAGCATACAAAAATGTGCAAGTTGG

Gene: repeat_nySAgamma (repeat element)

Contig: 06_NODE_54, position: 272061 to 272242, length: 182 nt

Sequence:

GGCGGGGCCCCAACATAGAGAAATTGGATCACCAATTTCAACAGACAATGCAAGTTGGGGTGGGGCCCAACACAGAAGCTGGCGAAAA
GTCAGCATACAAAAATGTGCAAGTTGGCGGGGCCACAACAGAGAGAAATAGGATCACCAATTCCAACAAACAATGCAAGTTGGCGGGG
CCCCAA

Gene: STAR (Staphylococcus aureus repeat element)

Contig: 06_NODE_54, position: 272177 to 272451, length: 275 nt

Sequence:

GGGGCCACAACAGAGAGAAATAGGATCACCAATTCCAACAAACAATGCAAGTTGGCGGGGCCCCAACATAGAGAAATTGGGTCACCAA
TTTCAACAGACAATGCAAGTTGGGGTAGGACATCGATAAAAAAATACTTTTTCTTTAGAAATTAGTATTTCTTATGCATGAGTTTTACTCAT
GTATTCATTTTTTAAGTAGACATTAGCTACAGCTAATGATAAAGAACCACTACATAATAAATCATTAGTGGTTCTTTATCATTTCTATCTCA
CTC

Gene: A5IS45 (putative protein)

Contig: 06_NODE_54, position: 272454 to 272639, length: 186 nt, orientation: REVERSE

Perfect match to: (N315-BA000018-[1137525:1137710:r], highly conserved allele)

Sequence:

TTACTGGAAGAAAAAGTTTACGTTTGTAGAACATGCCACAATACCAAAAATAATTAAGAAAAATAAGACGATAAGCATGATGACACTTTT
CAAACAACCTCTATCAGTTTCTTCCGATTTTCTTTGTTGAACCTTTTTATAATCTTCAAGTAGTTTTGTCGCTTTTTTATTTATATGTTTATTCA
T

Gene: Q6GHS5 (putative uncharacterized protein)

Contig: 06_NODE_54, position: 272846 to 273094, length: 249 nt, orientation: REVERSE

Perfect match to: (N315-BA000018-[1137920:1138168:r], highly conserved allele)

Sequence:

CTAGAATACATAAATGAATAGTAATGGTGCACAGTATAGAATAATTAAGGCTATATTAAGTATAAATATCGTTAACTGTAAGCTATCTTTA
GTTTTAATATAAACTATTAGGATAATCGACGTAAGAAGAATCATATATATTAATGATGAAGAAGTCCATACAAAATCCGCATCATTTGTTG
TTAATAATGGGACTATAATTAATCCGAAATTAATCATGCATGCTATATATACTATAATGTTATACAC

Gene: A5IS47 (putative protein)

Contig: 06_NODE_54, position: 273258 to 273491, length: 234 nt, orientation: REVERSE

Perfect match to: (MW2-BA000033-[1139552:1139785:r], allele observed in CC1+CC97)

Sequence:

TTAATGCTCATCAACATTTGGATTTTGAATATTCAATTCAAAAACTTTATTAGCTACGTCAATTGTAAAATCAGAACCATAGTTGACATGAG
CTACTTTTAATTTTCCATCTAAATAATAGATTGCGATTGCAACATCGTAAAATTCGTCAATGACAAATAAACTCTTTTCATTTGTTACAACCT
CATGCTCTCCTGAGTATACAACGTTAATTTCCCAATCATTAAAAACCAT

Gene: hla (haemolysin alpha)

Contig: 06_NODE_54, position: 273968 to 274926, length: 959 nt, orientation: REVERSE

Perfect match to: (ATCC51811-ADVP01000010-[33770:34728], allele observed in CC1)

Sequence:

TTAATTTGTCATTTCTTCTTTTTCCCAATCGATTTTATATCTTTCTGAAGAACGATCTGTCCATTTATCTTTAGTATTGGTACCTTTCCAATTTG
TTGAAGTCCAATGCAATTGGTAATCATCACGAACTCGTTCGTATATTACATCTATATTTGTTTGTTGTTTGGATGCTTTTCTATCCATAGTAA
TAACTGTAGCGAAGTCTGGTGAAAACCCTGAAGATAATAGAGAACTTGCTTTGTTAGGATCAAGGAAGTTATCTGCTGCTTTCATAGAAC
CATTTCTAGTTTTCATGAAAAGTTGATTGCCATATACCGGGTTCCAAGAATCTCTATCATATGGTCCCCAATTTTGATTCACCATATTGTTA
AATATCACTTTCCAGCCTACTTTTTTATCAGTTGGGCTCTCTAAAATTGTTTTGAAATCAGGTTGAACATATTTCAGTGTATGACCAATCGA
AACATTTGCACCAATAAGGCCGCCAATTTTTCCTGTATCATCACCAGTAACATTACCGTTGAATCCATAAGTTAAAGTACTCATATACTCTT
TTGTATCAATCGAATTTCTTGGATAGTAATCAGATATTTGAGCTACTTCATTATCAGGTAGTTGCAACTGTACCTTAAAGGCTGAAGGCCA
GGCTAAACCACTTTTGTTAGCACCTTCTTCGCTATAAACTCTATATTGACCAGCAATGGTACCTTTCGTTCTAATAACTAGCAGTTTTTTATT
GTGATTTTTATCATCGATAAAACTATAAAATACTTTTTTGTGCATGCCATTTCTTTATCATAAGTGACTAAATCACCTGTTTTTACTGTAGTA
TTGCTTCCAATATCTGTAGTACCGGTTTTAATATTAATATCAGAATCTGCGGCATTAGCGACAGGATTCATTAATATGGAACCTAGCAATA
GTGTTGTTGTTACTGAGCTGACTATACGTGTTTTCAT

Gene: A6U0Y3 (putative protein)

Contig: 06_NODE_54, position: 275599 to 275745, length: 147 nt, orientation: FORWARD

Perfect match to: (MW2-BA000033-[1141894:1142040], allele observed in CC1+CC15+CC72+CC88)

Sequence:

ATGAAACTATTTTATATCGTATTTCTTATTATTATATGGCTGAATATATTTTTAGGAAATGAAATCATCCATACACTGACTGTTTTAATAACA
ACATTATATATTGTTAATTCAAGAAAGGGGATTAAAAATGACAGAGTTGAATAA

Gene: Q2FZB4 (putative protein)

Contig: 06_NODE_54, position: 275729 to 275926, length: 198 nt, orientation: FORWARD

Perfect match to: (RF122-AJ938182-[1109354:1109551], highly conserved allele)

Sequence:

ATGACAGAGTTGAATAATATTATAAACTCTCTTCAATCTTTGTTTGAGTCTGAATCAGGCTATAAAATTTCGAAAAATTCAGGAGTTCCAT
ATCAAACAGTACAAGATTTAAGAAATGGGAAAACCAAACTAGAAGATGCTAGATTTAGAACGATTATTAAACTTTATAGTTACTATGTCT
CATTAAAAGAACACTAA

Gene: SIRU01 (staphylococcal interspersed repeat unit 1)

Contig: 06_NODE_54, position: 276087 to 276252, length: 166 nt

Sequence:

GGCCCCAACACAGAAGCTGACGAAAAGTCAGCTTACAATAATGTGCAAGTTGGGGATGGGCCCCAACACAGAGAATTTCAAAAAGAAA
TTCTACAGACAATGCAAGTTGGCGGGGCCCCAACATAGAAGCTGGCGGAAAGTCAGCTTACAATAATGTGCAAGTTGG

Gene: setB3 (staphylococcal enterotoxin-like toxin B locus 3)

Contig: 06_NODE_54, position: 276483 to 277199, length: 717 nt, orientation: REVERSE

Perfect match to: (GR1-AJLX01000008-[202482:203198:r], allele observed in CC361+CC5+CC239+CC361)

Sequence:

CTAATCAAAGAGGACGTCAACAGTTATTTTATTAGGATTTTTAACATAAACATTTGCTAGATCTGAATGTAATCTTTTGCTTAAATCAATAG
TGTAGTTATTACCGCCACCGGTGATCTTAAGCTTACCTTTATTACGATTTTCGTTATATAATATTTTATTTTTTATTAACGCTTCTCGTGCAC
GGAAATCGATTTCTTTCAATGTTAATACTGGTTTATTGCCTTGGTATATTTTATGCGCACCAATAATCGTTTGTAGTTTATCTTTGTATTGCA
CAAAAAGATTATAAGTTTTATCAGAAGGTTTTGCGGCTGGTGTAACGCCACCTGTAAATGTCTCTCTATAAGACCACCATAACTGATCAGT
ATCTTTGTCTTTTAGTCCAAACACATCTACGTAACGACCTTTTAACTGATTAATATTTCCCCAACTTTCAGCGCCCCATAAAGATATATGTGC
TGACCATGAATATCTCTTAAGTTCAACATAAATGTTTCCATTATCATATTGATATAGCCATTTATTTGAAAATGAAAAATGAGGCTGTGTGT
AATATTTAATTAATTCATTGATGTTAGTTTCATCTTGACCAATGCTATAAGCTTTAGCTTCAGAGTAAAAACTAAATACTGGTTTTTGATTTT
GAGGTAATACAGTACCTAATAGTAATAATGTTGTCGTTAAAATTATATTTTTCGTGATGTTCTTACTCAT

Gene: setB2 (staphylococcal enterotoxin-like toxin B locus 2)

Contig: 06_NODE_54, position: 277307 to 278032, length: 726 nt, orientation: REVERSE

Perfect match to: (MW2-BA000033-[1143602:1144327:r], allele observed in CC1+CC8+CC239)

Sequence:

TTAGTTTGATTTTTCGAGGATAACTTCAATTTTTGCATTTTGAGGTTTTTTAACATATCTATTTGCATCAGTTGATGGCAACCTTTTACTTAA
ATCTATTGTGTAGTTATTGTCTGCACCTGTTATTTTAATTTGTCCTTTATTATAAGAATTATTATATAATTTTTTACTTTTAATTAATGTTTGA
CGAATACGAAAATCTAATTCTTTTAAAGTTAAAACAGGCTTATTGCCTTCATAAACTGGAAATCCGCCAGTAAACGTTTCTGCTTTATCTTT
ATATGTCACATTCAGTTTATAGTGTTTATCGTTAGATGTTGCTGCAGGAGTAACACCACCAGTAAACGTTTCTTGAGATAATGCAAAAGAA
TCAATGGTTTCTTGGTCTTTTATGCCAAAAATATCAACGCTTTTATTTCTTAATTGGTTGATATTGCCCCAACTTTCAGGTCCATAAACTTGA
ATATGACTATACCAAGAAAACTGTAACAACGTTGCATGAATCGTACCGTTATCTTTTTGCCATAACGTACTGTTAGAGAAGGTTAAATATT
TTTGCGAGTAATATTTAGTTAACTCATTAACGTTAGTTTCGTTTTGATTTATATAATAAGCTTTCGCTTCAGATGAAGAATTGATAGGTGTA
TTAGGAAATTGTGTAGATGCTGTACCTAATAGTAACAATGTTGTTGATAAAATAATTTTTTTCGTGATGTTATTGTTCAT

Gene: setB1 (staphylococcal enterotoxin-like toxin B locus 1)

Contig: 06_NODE_54, position: 278127 to 278852, length: 726 nt, orientation: REVERSE

Sequence:

TTAGTTAGATTTTTCGAGTATGACTTCAATTTGTGCATTTTTAGGATTTTTAACATAACGGTTTGTGTCAGTTAATTTTAACTTTTTACTTAA
ATCAATCGTGTAATTATTTCCATCAGCAGTTATCTTAATTTGACCTTTATTAAATTCTCCGTTATATAACTTTTTATTTTTTATTAATGTTTGA
CGAATACGGAAATCTAATTCTTTTAAAGTTAATACTGGTTTATTTCCTTTGTAAAATTCATGTCCACCGATGATAGTTTGTTGTTTATCACTA
TATTTTAAAAATAGTCTATAAGGCTTATCAGATGAAGTAGCTGCTGGCGTAACACCACCAGTAAATGTTTCATCATAAGTCCAGTAACCTT
CAACTGTGTCCTCATCTTTAGTTCCAAATATATCAACGTATTTATTTCTTAACTGATTAATGTTTCCCCAACTCTCGGATCCAAACACTTGAA
TATGACTATACCAAACCCACGTTTGCAATGTTGCATGAATGCTACCATTGGGCTTTTGCCATAACCATTTATTTGATAGTGATAAATGAGG

CTGTGTATAATACTTTATTAACTCATTGATATTAGTCTCGTTTTCACTGATATTATAGGCTTTTGCTTCAGATGAAAAACTGATTGGTGTTTT
AGGAAGTTGTGTTGATGTAGTTCCTAAAAGTAACAATGCTGTTGATAAAACTAGTTTATTCATGATGTTCTTTTTCAT

Gene: arcB-L1 (ornithine carbamoyltransferase locus 1)

Contig: 06_NODE_54, position: 279290 to 280291, length: 1002 nt, orientation: FORWARD

Perfect match to: (MW2-BA000033-[1145585:1146586], allele observed in CC1)

Sequence:

ATGAAAAAATTTACGAAACAGAAGTTTTTTAACTTTATTAGACTTTTCACGACAAGAGGTAGAATTCTTATTAACACTCTCCGAAGATTTAA
AACGTGCTAAATATATTGGCACTGAAAAGCCTATGTTAAAAAATAAAAATATTGCACTGTTATTTGAAAAAGATTCTACAAGAACGCGAT
GTGCATTTGAAGTTGCAGCGCATGATCAAGGTGCAAATGTAACTTATTTAGGCCCAACTGGATCACAAATGGGTAAAAAAGAAACAACT
AAAGATACTGCACGTGTGCTTGGTGGAATGTATGATGGCATTGAATACCGTGGTTTTTCACAAAGAACAGTAGAAACTTTAGCTGAGTAT
TCAGGCGTACCAGTGTGGAATGGTTTAACTGATGAAGATCATCCTACTCAAGTTCTTGCTGATTTCTTAACAGCAAAAGAAGTCTTAAAA
AAAGATTATGCAGATATTAACTTTACATATGTTGGAGATGGTCGTAATAACGTTGCAAATGCATTAATGCAAGGTGCTGCCATTATGGGT
ATGAACTTCCATTTAGTTTGTCCAAAAGAATTAAATCCAACAGATGAATTATTAAATCGCTGTAAAAATATTGCCGCTGAAAATGGTGGCA
ACATATTAATCACAGATAATATTGACCAAGGTGTAAAAGGTTCGGATGTAATTTACACTGACGTTTGGGTATCAATGGGTGAACCTGATG
AAGTATGGAAAGAACGACTTGAATTATTGAAACCATATCAAGTAAATAAAGAAATGATGGATAAAACTGGTAATCCAAATGTTATTTTTG
AGCATTGCTTACCATCTTTCCATAATGCTGATACGAAAATTGGTCAACAAATTTTTGAAAAATATGGAATTCGAGAAATGGAAGTTACAG
ATGAAGTATTCGAAAGTAAAGCTTCAGTTGTATTCCAAGAAGCTGAGAACAGAATGCATACAATCAAAGCAGTGATGGTTGCTACATTG
GGTGAATTTTAA

Gene: arcC-L1 (carbamate kinase, locus 1)

Contig: 06_NODE_54, position: 280314 to 281246, length: 933 nt, orientation: FORWARD

Perfect match to: (Tager_104-CP012409-[405894:406826:r], allele observed in CC49+CC5+CC8+CC4803)

Sequence:

ATGGCGAAAATAGTAGTAGCATTAGGTGGTAATGCTTTAGGAAAATCACCTCAAGAACAACTCGAGCTTGTTAAAAATACTGCGAAATC
ATTAGTAGGATTAATAACAAAAGGACATGAGATTGTTATTAGTCATGGTAATGGACCACAGGTTGGAAGCATTAATTTGGGACTTAACTA
TGCTGCAGAACATAACCAAGGTCCGGCATTTCCATTTGCTGAATGTGGCGCAATGAGTCAAGCTTACATCGGCTATCAATTACAAGAAAG
CTTACAAAATGAATTGCATTCTATTGGAATGGATAAACAAGTGGTAACACTAGTGACACAAGTTGAAGTTGATGAAAATGATCCGGCATT
TAACAATCCTTCAAAACCAATTGGGTTATTTTACAACAAAGAAGAAGCTGAACAAATTCAAAAAGAAAAAGGATTTATATTTGTTGAAGA
TGCTGGAAGAGGATATAGACGCGTTGTTCCTTCACCACAACCCATCTCTATTATTGAATTAGAGAGTATTAAAACACTTATTAAAAATGAT
ACACTCGTTATTGCTGCTGGCGGTGGAGGTATACCAGTAATTAGAGAGCAACATGATGGTTTTAAAGGTATTGATGCAGTTATAGACAA
AGATAAAACAAGTGCATTGTTGGGTGCTAATATTCAATGCGATCAATTGATTATTTTAACAGCAATTGATTATGTATATATTAATTTTAACA
CTGAAAACCAACAGCCTTTGAAAACAACAAATGTTGATGAATTAAAACGATATATAGACGAAAATCAATTTGCAAAAGGAAGTATGTTAC
CAAAAATTGAAGCAGCCATATCATTTATTGAAAACAATCCAAAAGGAAGCGTGCTTATAACATCATTAAATGAATTAGATGCTGCCTTAG
AGGGTAAAGTAGGTACTGTGATTAAAAAGTAA

Gene: arcD1 (C4 transporter, TRAP family)

Contig: 06_NODE_54, position: 281418 to 282974, length: 1557 nt, orientation: FORWARD

Sequence:

GTGGAAAATACAATTAATGAAAGTGAAAAGAAAAAACGATTTAAATTAAAAATGCCAGGTGCATTTATGATTTTATTCATTTTAACGGTT
GTTGCAGTTATAGCAACATGGGTTATTCCTGCTGGTGCATATTCTAAACTTTCTTACGAACCTTCATCCCAAGAACTAAAGATAGTTAACC
CTCATAACAAAGTGAAAAAGGTTCCGGGTACGCAACAGGAACTAGACAAAATGGGAGTTAAAATTAAGATTGAACAATTTAAATCAGGT
GCAATTAATAAGCCGGTATCAATTCCGAATACTTATGAAAGATTAAAGCAACATCCAGCTGGACCAGAACAAATAACAAGTAGCATGGTT
GAAGGTACGATAGAAGCGGTCGATATCATGGTATTCATTCTTGTACTAGGGGGACTTATTGGCGTAGTTCAAGCCAGTGGTTCTTTTGAA
TCGGGATTGTTAGCTTTAACGAAGAAAACAAAAGGGCATGAATTTATGCTAATTGTGTTTGTATCAATACTAATGATTATCGGCGGGACG
TTATGTGGTATTGAAGAAGAAGCTGTAGCATTCTATCCGATTTTAGTCCCTATATTTATAGCGTTAGGATACGATTCTATCGTTTCAGTTGG
TGCCATATTCCTTGCCAGCTCTGTCGGTAGTACATTTTCAACTATTAACCCGTTCTCGGTTGTAATTGCCTCTAATGCCGCTGGTACAACTT
TTACGGATGGCTTGTATTGGAGAATAGGTGCTTGTATTGTCGGTGCGATTTTTGTTATTAGTTATTTATATTGGTACTGTAAAAAAATTAA

AAACGATCCTAAAGCGTCATATTCTTATGAAGACAAAGATGCTTTTGAACAGCAATGGTCTGTATTAAAAGATGACGATAGTGCCCATTTT
ACTTTGCGTAAGAAGATAATCCTTACATTATTTGTACTACCATTTCCAATTATGGTATGGGGAGTTATGACGCAAGGTTGGTGGTTCCCAG
TTATGGCTTCAGCATTTTTAATATTTACAATTATAATAATGTTTATTGCTGGGACAGGTAAATCTGGATTGGGAGAAAAAGGAACTGTAG
ATGCATTTGTCAATGGCGCATCAAGTTTAGTAGGTGTATCTTTAATTATTGGTTTAGCTCGAGGTATTAATTTAGTGTTGAATGAAGGTAT
GATTTCAGATACAATCTTACACTTTTCATCATCTTTAGTTCAACATATGAGTGGACCATTATTTATCATCGTATTACTATTTATTTTCTTCTGT
TTAGGTTTTATCGTGCCATCATCTTCTGGATTAGCGGTATTATCAATGCCTATCTTTGCACCACTAGCTGATACAGTAGGTATACCAAGATT
CGTCATCGTTACGACATATCAATTTGGTCAATATGCGATGTTATTCTTAGCGCCGACTGGACTTGTTATGGCCACACTACAAATGTTAAAC
ATGCGATATTCACATTGGTTCCGATTTGTATGGCCGGTAGTTGCTTTTGTATTGATTTTCGGTGGCGGACTACTAATTACGCAAGTACTAA
TTTATTCATAA

Gene: A5IS57 (putative membrane protein)

Contig: 06_NODE_54, position: 283281 to 283508, length: 228 nt, orientation: FORWARD

Perfect match to: (MW2-BA000033-[1149576:1149803], allele observed in CC1+CC25+CC72)

Sequence:

ATGACACATTTGACAAAGGTTTTAGATACACTAACTGGAATATGCGTAGTATTATTATTTAGTAAATATTTTGTGGCGTATGCAAATATGG
TGTTTGATTGGAATTTAAGATGGTATTTGCTAGAAAACATACCACATTTGCCAATTATATTATTTATTCTGATGTTTATTTTCGGAGTACCTT
CTGAAATGATAAAAGATAGGCAAAGGAAAAATAACGGCGTTTAA

Gene: per (putative permease)

Contig: 06_NODE_54, position: 283814 to 284761, length: 948 nt, orientation: REVERSE

Perfect match to: (N315-BA000018-[1150342:1151289:r], allele observed in CC5)

Sequence:

TTACTGAGACGCATTACGAAGAAATTTATCTTGTTTTTCCTTTTTTAAAAAGAAGTGAAGGTATCCTATAAAGACTCTAAGTACTATTACAG
TGGCTAATAACACTTCGATAAACAAAAGACCTTTCCAAATGTCTGGAAACATAAGTACAGGCAAACTGTTCTTTAAAGCAGTTGCTGAGA
TTACTAAAGGGAATGTGAAAGCTGAAAATACGGGCGAAAAAGGTTCTTTTAGTAATTTAGGCAATTGTATAATGATATAAAAATAAAATA
TTTGAGCTAATATTAAAAATATAATTACGATAAAAGCATTCGCCTTAGGAAAGGCTATAACATATGCTGCAGCGACTAAAGAAAATGGTG
CACAAATTGTCGATGTGTTCGGTTTGATTGACGTCTGCAATGGAAATGCTTTTAATCGCTTGAAAACTATAGGTAAGACAATACAAGTAG
CTACAAAGCCATATATTACTGTTAATTGACCTATGAAAAAATATCCGCTAACGGGTGCCGTCAATCCTGCGATAGCAATACCAATAAAAA
GTACAGTCCACGAAGGATAAACATTTTCAAGTGAAAAATCTTTTAAATATTTTATTGAAAAAATAATCATATGCGTCATAATTCCCACAAG
GCATAAAATCCAAATAGGCGTTATTAAGCTATTGATAAAAGTTATGTTACTAAAAAATGTATTTAAATAAGTAGTACCTAAAAAGCCAGA
CATGAAAAATGTTGTGAACACTGATGAAACTAGAGGACTGTTTAATTGTTCTTTCACATTATTAAAATATTTGATCATAGTACATAAAAGG
TGAATCCAAATCAAGAAAGCAAAGATTCCGCAAACAGCGTTTAAAGTAAGAGATAAGTCTTTTAATAGATTACCCAGGCCTAATAATCCT
AAGACTAGTCCTGACGTTACTAGAGGTGCTTTTTGAAGTCTCAT

Gene: A6QG73 (putative DNA-binding protein)

Contig: 06_NODE_54, position: 285010 to 285198, length: 189 nt, orientation: FORWARD

Perfect match to: (MW2-BA000033-[1151305:1151493], highly conserved allele)

Sequence:

ATGAGAAATCAAATTCAAAAACTATTAGACAGTGATTTGAGCAGTTTACATATATCGAAACAAACAGGAGTTCCACAAAGCACAATACAC
AGAATGAGAAAAAATGAAAGATCATTAGACAATATGTCATTGAAAAACGCTGAACTACTTTATAAATTTGCCAATAGTATATTTAGCAAT
GAAAATTAA

Gene: psmB1 (phenol soluble modulin beta 1)

Contig: 06_NODE_54, position: 286110 to 286244, length: 135 nt, orientation: FORWARD

Perfect match to: (N315-BA000018-[1152631:1152765], highly conserved allele)

Sequence:

ATGGAAGGTTTATTTAACGCAATTAAAGATACCGTAACTGCAGCAATTAATAATGATGGCGCAAAATTAGGCACAAGCATTGTGAGCATC
GTTGAAAATGGCGTAGGTTTATTAGGTAAATTATTCGGATTCTAA

Gene: psmB2 (phenol soluble modulin beta 2)

Contig: 06_NODE_54, position: 286301 to 286435, length: 135 nt, orientation: FORWARD

Perfect match to: (N315-BA000018-[1152822:1152956], highly conserved allele)

Sequence:

ATGACTGGACTAGCAGAAGCAATCGCAAATACTGTGCAAGCTGCACAACAACATGATAGTGTGAAATTAGGCACAAGTATCGTAGACAT
CGTTGCTAACGGTGTGGGTTTACTAGGTAAATTATTTGGATTCTAA

Gene: Q1Y2B3 (putative haloacid dehalogenase-like hydrolase)

Contig: 06_NODE_54, position: 286559 to 287254, length: 696 nt, orientation: FORWARD

Perfect match to: (N315-BA000018-[1153080:1153775], highly conserved allele)

Sequence:

ATGGGGAAATTGGGATATAAAAATATTTTGATAGACTTTGATGATACAATTGTTGATTTTTATGATGCAGAAGAATGGGCGTTTCACTAT
ATGGCGAATGTTTTTAATCATAAAGCAACAAAGGATGATTTTTTAACATTTAAAAAAATCAATCACCAACATTGGGAAGCTTTTCAACAAA
ATAAATTAACGAAGTCTGAAGTATTATCAGAACGATTTGTGAATTACTTCAAACATCATCAAATGGAAGTTGATGGGCATCGTGCAGATG
TGTTATTTAGAAATGGATTAGCAGAAGCTAAAGTTAAATACTTTGATCAAACATTAGAAACAATTGTCGAATTATCGAAAAGACATGATTT
ATATATTGTTACTAATGGTGTAACCGAAACGCAAAAGAGAAGGTTAAATCAGACGCCGTTGCATAAATATATTAAAAAGATATTTATATC
TGAAGAAACAGGATATCAAAAACCTAATCCGGAATTTTTTAATTATGTTTTTAATGATATTGGTGAGGATGAAAGACAGCACTCGATTAT
AGTTGGAGATTCTTTAACATCTGACATTCTAGGTGGAATCAATGCGGGTATAGCTACTTGCTGGTTTAATTTTAGAGGATTTGATCATAAT
CCAGGAATTATACCTGATTATGAAATTAATTCATGGAAACAACTAAATGATATTGTACGTTAA

Gene: Q931T2 (N-acetyltransferase, GNAT family)

Contig: 06_NODE_54, position: 287363 to 287803, length: 441 nt, orientation: REVERSE

Perfect match to: (RF122-AJ938182-[1120882:1121322:r], highly conserved allele)

Sequence:

TTATGGTTTCCACAATAAGACATCATGTCCCTCTATATTTTTAGCTTCCACATCTGTAAATCCATGATGTAAAAAGAAATCCTTAGAATCAT
TTCTGCCAATGGCTTTAATTGGCATATTGAAACTCTTTGCAAAATCAATCAATTCTGAAGCGTACCCTCTGTTTTGATATTTTGGTAATACTT
CTAACTTCCATAATAATATATAATCTTCAAAATCTGGGAAGTAGATTTCTTCGACATCACCTTTTTTTAGTAATGCCATTCTAGCTCCTAATT
GATCTCCGACAAATATGCCATAAAATGGTGAATCTGAACTTGCATCAATCATTTGACCGTTTAACTCATTGACCATGTATAAGTCTTTGTTG
CCAAACGCTCTAAAGTTTTCGAATAATTCGTCAGTTTTGTAATTAATTTCAAGACGTTTGATTTCACTCAT

Gene: bshC (bacillithiol cysteine-adding enzyme)

Contig: 06_NODE_54, position: 287998 to 289611, length: 1614 nt, orientation: FORWARD

Perfect match to: (MW2-BA000033-[1154293:1155906], highly conserved allele)

Sequence:

ATGGACTGTAAAGTAGTTAGTTTAAATGAAAAAGATCAGTTTATACCAAAAATAAAGAGCAGTGACCCTGTAATAACAGGATTATTTCAA
TATGATGCAGCTCAACAAACTAGTTTTGAAAAAAGGATGTCTAAAGAAAATAATGGAAGAGAAGCGGCATTAGCGAATGTTATTCGTGA
ATATATGAGTGATTTAAAGCTTTCAAGTGAACAAGAATTAAACATACAACATTTAGCTAATGGTTCAAAAGTTGTGATTGGTGGACAACA
AGCAGGGCTTTTCGGGGGACCATTGTATACATTCCATAAAATATTTTCAATCATTACTTTATCTAAGGAATTAACGGATACACATAAGCAA
CAAGTAGTACCAGTTTTTTGGATTGCAGGAGAAGATCATGATTTCGATGAAGTGAATCATACATTTGTTTATAACGAAAATCATGGGTCG

CTGCATAAGGTTAAATATCATACAATGGAGATGCCAGAGACGACTGTCTCTAGATATTATCCTGATAAGGCTGAGTTGAAACAAACTTTA
AAAACGATGTTCATTCATATGAAAGAAACTGTTCATACACAAGGTCTACTGGAGATTTGTGACAGAATTATTGACCAATATGACTCGTGG
ACTGATATGTTTAAAGCACTACTGCATGAAACATTTAAAGCATATGGCGTTCTATTTATAGATGCGCAGTTTGAGCCGTTAAGAAAAATG
GAAGCGCCTATGTTTAAAAAGATTTTGAAAAAACATCAGTTGCTTGATGATGCTTTTAGAGCAACACAACAACGTACTCAAAATCAAGGC
TTGAATGCGATGATACAAACAGATACAAATGTTCATTTATTCTTACATGATGAAAATATGCGTCAATTAGTTTCGTATGATGGTAAGCATT
TTAAATTAAATAAAACAGATAAGACATATATAAAGGAAGAAATTATAAATATTGCGGAAAATCAACCTGAATTATTTTCTAATAATGTAGT
GACAAGACCATTAATGGAAGAATGGTTATTTAACACGGTGGCATTTGTTGGAGGACCGAGTGAAATTAAGTACTGGGCTGAACTAAAAG
ATGTATTTGAACTATTTGATGTTGAAATGCCTATCGTGATGCCAAGGCTTAGAATTACTTATTTAAATGACCGTATAGAAAAATTACTTTC
GAAATACAATATTCCATTAGAAAAAGTGTTAGTCGATGGTGTTGAAGGAGAAAGAAGTAAGTTTATTAGAGAACAAGCATCACATCAAT
TTATTGAAAAGGTAGAAGGTATGATTGAACAACAGCGTCGTCTAAACAAAGACTTATTAGATGAAGTGGCGGGGAATCAAAATAATATT
AACCTTGTGAATAAAAATAATGAAATTCATATACAACAGTATGATTATTTGTTAAAACGTTATCTTTTAAACATTGAAAGAGAAAACGACA
TCAGTATGAAGCAATTTAGAGAAATTCAAGAAACACTCCATCCAATGGGAGGATTACAAGAAAGAATATGGAATCCACTTCAAATTTTGA
ATGATTTTGGGACAGATGTGTTCAAGCCCTCCACCTATCCACCACTTTCTTACACTTTTGATCATATTATTATAAAACCTTAA

Gene: mraZ (putative transcriptional regulator)

Contig: 06_NODE_54, position: 289755 to 290186, length: 432 nt, orientation: FORWARD

Perfect match to: (RF122-AJ938182-[1123275:1123706], highly conserved allele)

Sequence:

ATGTTCATGGGAGAATACGATCATCAATTAGATACAAAAGGACGTATGATTATACCGTCCAAGTTTCGTTATGACTTAAATGAGCGTTTTA
TTATCACAAGAGGCCTTGATAAATGTTTATTCGGTTACACTCTAGACGAATGGCAACAGATTGAAGAGAAAATGAAAACCTTACCTATGA
CAAAAAAAGACGCACGTAAGTTTATGCGTATGTTCTTCTCTGGTGCTGTTGAAGTAGAACTTGATAAGCAAGGGCGTATTAACATCCCTC
AAAACTTGAGGAAATACGCTAATTTAACTAAAGAATGTACAGTAATCGGTGTTTCAAATCGTATTGAGATTTGGGATAGAGAAACTTGGA
ATGATTTCTATGAAGAATCTGAAGAAAGTTTCGAAGATATTGCTGAAGATTTAATAGATTTTGATTTTTAA

Gene: rsmH-mraW (16S rRNA methyltransferase H)

Contig: 06_NODE_54, position: 290223 to 291137, length: 915 nt, orientation: FORWARD

Perfect match to: (MRSA252-BX571856-[1198276:1199190], highly conserved allele)

Sequence:

ATGTTAAACGAAACCATTGATTATTTAAATGTAAAAGAAAATGGTGTGTACATTGACTGTACGCTAGGTGGAGCGGGACATGCCCTTTAT
TTACTAAATCAATTAAATGACGACGGAAGATTAATAGCAATCGATCAAGACCAAACTGCAATTGATAATGCTAAAGAGGTATTAAAGGAT
CATTTGCATAAGGTGACTTTTGTTCATAGCAACTTCCGTGAATTAACTCAAATATTAAAAGACTTAAACATTGAAAAAGTAGATGGAATTT
ATTACGACTTGGGTGTTTCAAGCCCACAACTCGACATTCCAGAACGAGGATTCAGTTATCACCATGACGCAACATTAGACATGCGTATGG
ACCAAACACAAGAACTAACAGCATATGAAATTGTTAACAATTGGTCATATGAAGCGTTAGTGAAGATTTTTTATCGCTATGGCGAGGAGA
AATTTTCAAAACAGATAGCTCGAAGAATCGAAGCACATCGCGAACAACAACCAATAACAACAACATTAGAATTAGTTGACATTATAAAAG
AAGGTATTCCTGCAAAAGCAAGAAGAAAAGGCGGACATCCTGCAAAACGAGTATTTCAAGCACTACGAATTGCAGTAAACGATGAATTG
TCAGCTTTTGAAGATTCAATAGAACAAGCGATTGAATTAGTGAAAGTAGATGGCAGGATTTCGGTAATCACTTTCCATTCTTTAGAAGAT
CGTTTATGTAAACAGGTGTTCCAAGAATATGAAAAAGGTCCAGAGGTACCAAGAGGATTACCAGTTATACCAGAAGCATATACACCTAA
GTTAAAGCGTGTTAATCGTAAACCGATTACCGCTACAGAAGAAGATTTAGATGACAATAACAGAGCACGAAGCGCGAAATTACGTGTAG
CTGAAATACTTAAATAA

Gene: ftsL (initiator of cell division)

Contig: 06_NODE_54, position: 291151 to 291552, length: 402 nt, orientation: FORWARD

Perfect match to: (N315-BA000018-[1157672:1158073], highly conserved allele)

Sequence:

ATGGCTGTAGAAAAAGTGTACCAACCATATGACGAACAAGTTTATAATAGTATACCGAAGCAACAACCACAAACTAAGCCCGAAAAGAA
GACTGTTTCGAGAAAAGTGGTTGTACAATTAACTAAATTTGAAAAAGTTTTATACATAACTTTGATTACTGTAATTGCTATGTTAAGTATTT
ATATGCTATCTTTAAAAATGGATGCGTATGATACGCGAGGAAAGATTGCAGATTTAGATTATAAAATAGATAAACAATCAAGTGAAAACA

GTGCTTTACAATCTGAAATCAAAAAGAATTCTTCTTATGAACGCATATACGAAAAGGCTAAGAAACAGGGGATGAGCCTTGAGAACGAT
AATGTAAAGGTAGTGCGTAGTAATGGCGAAGCAAAAAATTAA

Gene: pbpA (penicillin binding protein A)

Contig: 06_NODE_54, position: 291533 to 293767, length: 2235 nt, orientation: FORWARD

Perfect match to: (Tager_104-CP012409-[393367:395601:r], allele observed in CC49+CC5+CC49)

Sequence:

ATGGCGAAGCAAAAAATTAAAATTAAAAAAAATAAAATAGGGGCAGTCCTACTTGTTGGTTTATTCGGACTGCTCTTTTTTATATTGGTTT
TAAGAATTTCATATATCATGATTACTGGACATTCTAATGGTCAAGATTTAGTCATGAAGGCAAATGAAAAGTATTTAGTTAAGAATGCACA
ACAACCAGAACGAGGAAAGATATATGATCGTAATGGTAAAGTGCTAGCAGAAGATGTAGAAAGATATAAACTTGTTGCAGTAATAGATA
AAAAGGCGAGTGCCAATTCTAAAAAACCTAGGCATGTAGTTGATAAAAAAGAGACTGCAAAGAAATTATCTACAGTCATTAATATGAAG
CCAGAGGAAATTGAAAAGAGACTTAGTCAAAAGAAAGCTTTCCAAATTGAATTTGGACGCAAAGGAACAAATTTAACGTATCAGGACAA
ATTGAAAATAGAGAAAATGAATTTGCCTGGTATTTCTTTATTGCCTGAAACAGAACGCTTTTATCCAAATGGCAATTTTGCATCACACTTA
ATTGGTAGAGCTCAGAAAAATCCGGATACTGGTGAACTTAAAGGTGCACTTGGAGTTGAAAAGATTTTTGATAGTTATTTAAGTGGATCT
AAAGGATCATTGAGATATATTCATGATATTTGGGGATATATCGCACCAAATACTAAAAAAGAGAAGCAGCCTAAACGTGGTGATGATGT
CCATTTAACAATCGATTCAAATATTCAAGTATTTGTTGAAGAAGCTTTAGATGGCATGGTTGAAAGATACCAGCCGAAAGATTTATTTGCG
GTTGTCATGGATGCCAAAACTGGAGAAATTTTAGCATACAGTCAGCGACCAACATTTAATCCTGAAACTGGTAAAGACTTTGGTAAAAAG
TGGGCAAATGACCTTTATCAAAACACATACGAGCCTGGATCAACATTTAAATCATATGGGTTAGCAGCTGCTATTCAAGAAGGTGCTTTT
GATCCTGATAAGAAATATAAATCTGGACATAGAGATATTATGGGTTCACGTATTTCAGACTGGAATAGAGTCGGTTGGGGTGAAATCCC
AATGTCACTCGGATTTACTTATTCATCTAATACATTGATGATGCATTTACAAGATTTAGTTGGTGCAGACAAAATGAAATCTTGGTATGAA
CGATTTGGATTTGGAAAATCAACTAAAGGTATGTTTGATGGAGAAGCACCTGGTCAAATTGGATGGAGTAATGAGTTGCAACAAAAAAC
GTCATCATTTGGTCAATCGACAACAGTAACACCTGTTCAAATGTTACAAGCGCAATCAGCGTTCTTTAATGATGGTAATATGTTAAAACCA
TGGTTTGTGAATAGCGTTGAAAATCCTGTTAGTAAAAGACAATTTTATAAAGGGCAAAAACAAATCGCAGGCAAACCAATAACAAAAGA
TACTGCTGAAAAAGTTGAAAAGCAATTGGATTTAGTTGTGAATAGTAAGAAGAGTCACGCTGCAAACTATCGTATTGATGGTTATGAGG
TCGAAGGTAAGACTGGTACAGCACAAGTCGCTGCACCTAATGGTGGTGGATACGTTAAAGGTCCAAACCCATATTTTGTAAGTTTTATGG
GTGACGCGCCGAAGAAAAATCCTAAAGTTATTGTATACGCTGGTATGAGCTTGGCACAAAAAAATGACCAAGAAGCTTATGAATTAGGT
GTTAGTAAAGCGTTTAAACCAATAATGGAAAATACTTTGAAATATTTAAATGTAGGTAAATCAAAAGATGACACATCTAATGCAGAGTAT
AGTAAAGTGCCAGATGTTGAAGGTCAAGACAAACAAAAAGCTATTGATAATATGAGTGCAAAATCATTAGAACCAGTTACTATCGGTTCT
GGCACACAAATAAAAGCACAATCTATAAAAGCAGGGAATAAAGTCTTACCTCATAGTAAAGTACTGTTATTAACAGACGGAGACTTAACT
ATGCCTGACATGTCAGGATGGACGAAAGAAGATGTCATTGCTTTTGAAAACCTAACAAATATTAAAGTAAATTTAAAAGGTAGCGGTTTT
GTGTCCCACCAATCAATTAGTAAGGGACAAAAACTTACTGAAAAAGATAAAAATAGACGTAGAATTTTCATCAGAGAATGTAGACAGCAA
TTCGACGAATAATTCTGATTCAAATTCAGATGATAAGAAGAAATCTGACAGTAAAACTGACAAGGATAAGTCGGACTAA

Gene: mraY (phospho-N-acetylmuramoyl-pentapeptide-transferase)

Contig: 06_NODE_54, position: 294059 to 295024, length: 966 nt, orientation: FORWARD

Perfect match to: (MW2-BA000033-[1160354:1161319], allele observed in CC1)

Sequence:

ATGATTTTTGTATATGCGTTATTAGCGCTAGTGATTACATTTGTTTTGGTACCTGTTTTAATACCTACATTAAAAAGGATGAAATTTGGTCA
AAGTATTCGAGAAGAAGGTCCACAAAGCCATATGAAGAAGACTGGTACACCAACGATGGGTGGACTAACATTTCTATTAAGTATTGTGA
TAACGTCTTTGGTGGTTATTATATTTGTGGATCAAGCTAATCCAATCATACTGTTATTATTTGTGACGATTGGTTTTGGGTTAATTGGTTTT
ATAGATGATTATATTATTGTTGTTAAAAAGAATAACCAAGGTTTAACAAGTAAACAGAAGTTTTTGGCGCAAATTGGTATTGCGATTATTT
TCTTTGTTTTAAGTAATGTGTTTCATTTGGTGAATTTTTCTACGAGCATACATATTCCATTTACGAATGTAGCAATCCCACTATCATTTGCAT
ATGTTATTTTCATTGTTTTTTGGCAAGTAGGTTTTTCTAATGCGGTAAATTTAACAGATGGTTTAGATGGATTAGCAACTGGACTGTCAATT
ATCGGATTTACAATGTATGCCATCATGAGCTTTGTGTTAGGAGAAACGGCAATTGGTATTTTCTGTATCATTATGTTGTTTGCACTTTTAG
GATTTTTACCATATAACATTAACCCTGCTAAAGTGTTTATGGGAGATACAGGTAGCTTAGCTTTAGGTGGTATATTTGCTACGATTTCAAT
CATGCTTAATCAGGAATTATCATTAATTTTTATAGGTTTAGTATTCGTAATTGAAACCTTATCTGTTATGTTACAAGTCGCTAGCTTTAAATT
GACTGGAAAGCGTATATTTAAAATGAGTCCGATTCATCATCATTTTGAATTGATAGGATGGAGCGAATGGAAAGTAGTTACAGTATTTTG
GGCTGTTGGTCTGATTTCAGGTTTAATCGGTTTATGGATTGGAGTGCATTAA

Gene: murD (UDP-N-acetylmuramoylalanine-D-glutamate ligase)

Contig: 06_NODE_54, position: 295026 to 296375, length: 1350 nt, orientation: FORWARD

Perfect match to: (Strain_21334-AGTW01000008-[273414:274763], allele observed in CC9+CC239)

Sequence:

ATGCTTAATTATACAGGGTTAGAAAATAAAAATGTATTAGTTGTCGGTTTGGCAAAAAGTGGTTATGAAGCAGCTAAATTATTAAGTAAA
TTAGGTGCGAATGTAACTGTCAATGATGGAAAAGACTTATCACAAGATGCTCATGCAAAAGATTTAGAATCTATGGGCATTTCTGTTGTA
AGTGGAAGTCATCCATTAACGTTGCTTGATAATAATCCAATAATTGTTAAAAATCCTGGAATACCTTATACAGTATCTATTATTGATGAAG
CAGTGAAACGAGGTTTGAAAATTTTAACAGAAGTTGAGTTAAGTTATCTAATCTCTGAAGCACCAATCATAGCTGTAACGGGTACAAATG
GTAAAACGACAGTTACTTCTCTAATTGGAGATATGTTTAAAAAAAGTCGCTTAACTGGAAGATTATCCGGCAATATTGGTTATGTTGCATC
TAAAGTAGCACAAGAAGTAAAGCCTACAGATTATTTAGTTACAGAGTTGTCGTCATTCCAGTTACTTGGAATCGAAAAGTATAAACCACA
CATTGCTATAATTACTAACATTTATTCGGCGCATCTAGATTACCATGAAAATTTAGAAAACTATCAAAATGCTAAAAAGCAAATATATAAA
AATCAAACGGAAGAAGATTATTTGATTTGTAATTATCATCAAAGACAAGTGATAGAGTCGGAAGAATTAAAAGCTAAGACATTGTATTTC
TCAACTCAACAAGAAGTTGATGGTATTTATATTAAAGATGGTTTTATCATTTATAAAGGTGTTCGTATTATTAACACTGAAGATCTAGTATT
GCCTGGTGAACATAATTTAGAAAATATATTAGCAGCTGTGCTTGCTTGTATTTTAGCTGGTGTACCTATTAAAGCAATTATTGATAGTTTA
ACTACATTTTCAGGAATAGAGCATAGATTGCAATATGTTGGTACTAATAGAACTAATAAATATTATAATGATTCCAAAGCAACAAACACG
CTAGCAACACAGTTTGCCTTAAATTCATTTAATCAACCAATCATTTGGTTATGTGGTGGTTTGGATCGAGGGAATGAATTTGACGAACTCA
TTCCTTATATGGAAAATGTTCGCGCGATGGTTGTATTCGGACAAACGAAAGCTAAGTTTGCTAAACTAGGTAATAGTCAAGGGAAATCG
GTCATTGAAGCGAACAATGTCGAAGACGCTGTTGATAAAGTACAAGATATTATAGAACCAAATGATGTTGTATTATTGTCACCTGCTTGT
GCGAGTTGGGATCAATATAGTACTTTTGAAGAGCGTGGAGAGAAATTTATTGAAAGATTCCGTGCCCATTTACCATCTTATTAA

Gene: ftsQ (cell division protein Q)

Contig: 06_NODE_54, position: 296391 to 297710, length: 1320 nt, orientation: FORWARD

Perfect match to: (MW2-BA000033-[1162686:1164005], allele observed in CC1)

Sequence:

ATGGATGATAAAACGAAGAACGATCAACAAGAATCAAATGAAGATAAAGATGAATTAGAATTATTTACGAGGAATACATCTAAGAAAAG
ACGGCAAAGAAAAAGATCAAAGGCTACACATTTTTCTAATCAAAATAAAGATGATACATCTCAACAAGCTGATTTTGATGAAGAAATTTA
CTTGATAAATAAAGACTTCAAAAAAGAACAAAGCAATGATGAAAATAATGATTCTGCTTCTAGTCGTGCAAATAATAATAATATCGATGA
TTCTACAGACTCTAATATTGAAAATGAGGATTATAGATATAATCAAGAAATTGACGACCAAAATGAATCGAATGGAATTGCATTCGACAA
CGAACAACCTCAATCAGCTCCTAAAGAACAAAATGGCGACTCGAATGATGAGGAAACAGTAACGAAAAAAGAACGAAAAAGTAAAGTA
ACACAATTAAAGCCATTAACACTTGAAGAAAAGCGGAAGTTAAGACGTAAGCGACAAAAACGAATCCAATACAGTGTTATTACAATATT
GGTATTGTTGATTGCTGTTATATTAATTTACATGTTTTCACCACTTAGTAAAATTGCGCATGTAAATATAAATGGAAATAATCACGTTAGTA
CTTCAAAGATAAACAAAGTTTTAGGTGTTAAAAATGATTCAAGGATGTATACGTTTAGTAAAAAAAATGCTATTAATGATCTCGAAGAGA
ATCCATTAATCAAAAGTGTTGAGATACACAAGCAATTACCAAACACATTAAACGTAGATATCACAGAAAATGAAATTATTGCTTTAGTGA
AATATAAAGGTAAATATTTACCTTTATTAGAAAATGGTAAATTGCTTAAAGGTTCAAATGATGTCAAAATTAATGATGCACCTGTCATGGA
TGGTTTCAAAGGTACAAAAGAAGATGATATGATTAAGGCGTTATCTGAAATGACACCTGAAGTTAGACGATATATTGCCGAAGTGACAT
ACGCCCCAAGTAAAAACAAACAAAGCAGAATTGAATTGTTTACGACAGATGGACTTCAAGTAATCGGTGATATTTCGACGATATCTAAGA
AAATGAAATATTATCCGCAGATGTCACAATCATTATCAAGGGATAGTTCGGGTAAACTAAAAACACGAGGCTATATTGATTTATCAGTCG
GTGCTTCATTTATCCCATACCGTGGAAACACGTCTAGTCAATCAGAAAGCGATAAAAATGTGACTAAATCATCTCAAGAGGAAAATCAAG
CAAAAGAAGAATTACAAAGCGTTTTAAACAAAATTAACAAACAATCAAGTAAGAATAATTAA

Gene: ftsA (cell division protein A)

Contig: 06_NODE_54, position: 297816 to 299228, length: 1413 nt, orientation: FORWARD

Perfect match to: (Strain_21193-AFEG01000027-[49708:51120:r], allele observed in CC25+CC8+CC25+CC239)

Sequence:

ATGGAAGAACATTACTACGTAAGTATTGATATTGGATCATCAAGCGTAAAAACAATAGTAGGCGAGAAATTTCACAATGGTATAAATGT
GATAGGTACAGGACAAACCTACACGAGCGGTATAAAAAATGGTTTAATTGATGATTTTGATATTGCGCGACAAGCAATCAAAGACACAA
TTAAAAAGGCATCAATCGCTTCGGGTGTTGATATTAAAGAAGTTTTCCTGAAATTACCTATCATTGGAACGGAAGTTTATGATGAATCAA
ATGAAATCGACTTTTATGAGGATACAGAAATCAACGGTTCACATATCGAAAAAGTATTAGAAGGTATTAGAGAAAAAAATGATGTGCAA
GAAACAGAAGTAATTAATGTGTTCCCGATTCGTTTTATAGTCGATAAAGAAAATGAGGTTTCAGACCCTAAAGAATTAATTGCCAGACAT
TCATTAAAGGTTGAAGCAGGCGTAATTGCTATTCAAAAATCGATTTTAATTAATATGATTAAATGCGTAGAAGCATGTGGTGTTGATGTA
TTAGATGTTTACTCTGATGCATATAACTATGGTTCAATCCTAACAGCTACTGAAAAAGAGTTAGGTGCATGTGTCATTGATATTGGTGAAG

ACGTTACGCAAGTTGCTTTTTATGAACGCGGTGAATTAGTAGATGCTGATTCTATCGAAATGGCAGGGCGTGATATTACAGACGATATTG
CACAAGGATTAAACACTTCTTATGAAACTGCTGAAAAAGTTAAACACCAATATGGTCATGCATTCTATGATTCTGCTTCAGATCAAGATAT
CTTCACTGTTGAACAGGTTGATAGTGATGAAACAGTACAGTATACTCAAAAAGATTTGAGTGACTTTATTGAAGCGCGTGTAGAAGAAAT
ATTCTTCGAAGTATTTGATGTTTTACAAGATTTAGGATTAACAAAAGTAAATGGTGGGTTTATTGTAACTGGTGGATCTGCAAACTTACTT
GGCGTAAAAGAATTATTATCAGATATGGTAAGTGAAAAAGTTAGAATTCACACGCCATCACAAATGGGAATTAGAAAACCTGAATTTTCT
TCAGCAATTTCTACAATTTCTAGTAGTATCGCTTTTGATGAGTTATTAGATTATGTTACAATTAATTATCATGATAGTGAAGAAACTGAAGA
AGATGTTATTGATGTGAAAGACAAAGATAACGAATCTAAATTAGGCGGATTTGATTGGTTTAAACGTAAAACAAACAAAAAAGATACTC
ATGAAAATGAAGTAGAGTCAACAGATGAAGAAATTTATCAATCAGAAGATAATCATCAGGAACATAAACAGAATCATGAACATGTTCAA
GACAAAGATAAAGATAAAGAAGAAAGTAAATTCAAAAAACTAATGAAATCTCTATTTGAATGA

Gene: ftsZ (cell division protein Z)

Contig: 06_NODE_54, position: 299261 to 300433, length: 1173 nt, orientation: FORWARD

Perfect match to: (08-02119-CP015645-[1007221:1008393:r], highly conserved allele)

Sequence:

ATGTTAGAATTTGAACAAGGATTTAATCATTTAGCGACTTTAAAGGTCATTGGTGTAGGTGGTGGCGGTAACAACGCCGTAAACCGAAT
GATTGACCACGGAATGAATAATGTTGAATTTATCGCTATCAACACAGACGGTCAAGCTTTAAACTTATCTAAAGCTGAATCTAAAATCCAA
ATCGGTGAAAAATTAACACGTGGTTTAGGAGCAGGAGCTAATCCTGAAATCGGTAAAAAAGCTGCAGAGGAATCTCGTGAACAAATTGA
AGATGCAATCCAAGGTGCAGACATGGTATTTGTTACTTCTGGTATGGGTGGCGGAACTGGTACTGGTGCAGCACCAGTCGTTGCTAAAA
TTGCAAAAGAAATGGGCGCATTAACTGTTGGTGTTGTAACTCGTCCATTTAGTTTTGAAGGACGTAAACGTCAAACTCAAGCTGCTGCTG
GAGTAGAAGCTATGAAAGCTGCAGTAGATACATTAATCGTTATACCAAATGACCGTTTATTAGATATCGTTGACAAATCTACGCCAATGA
TGGAAGCATTTAAAGAAGCTGATAACGTGTTACGCCAAGGTGTACAAGGTATCTCAGACTTAATCGCTGTTTCTGGTGAAGTAAACTTAG
ACTTTGCAGACGTTAAGACAATTATGTCTAACCAAGGTTCTGCATTAATGGGTATTGGTGTTTCTTCTGGTGAAAATAGAGCGGTAGAAG
CTGCTAAAAAAGCAATCTCTTCTCCATTACTTGAAACATCTATCGTTGGTGCACAAGGTGTGCTTATGAATATTACTGGTGGCGAGTCATT
GTCATTATTTGAAGCACAAGAGGCTGCTGATATTGTCCAAGATGCTGCAGATGAAGACGTTAATATGATTTTCGGTACAGTTATTAATCCT
GAATTACAAGATGAGATTGTTGTAACAGTTATTGCAACTGGTTTTGATGACAAACCAACATCACATGGTCGTAAATCTGGTAGCACTGGA
TTCGGAACAAGCGTAAATACTTCTAGCAATGCAACTTCTAAAGATGAATCATTCACTTCAAATTCATCAAATGCACAAGCAACTGATAGTG
TAAGTGAAAGAACACATACAACTAAAGAAGATGATATTCCTAGCTTCATTAGAAATAGAGAAGAAAGACGTTCAAGAAGAACAAGACGT
TAA

Gene: ylmD (laccase domain protein)

Contig: 06_NODE_54, position: 300693 to 301484, length: 792 nt, orientation: FORWARD

Perfect match to: (MW2-BA000033-[1166988:1167779], highly conserved allele)

Sequence:

GTGAATGATAATTTTAAAAAGCAACCGCATCATTTAATATATGAAGAGTTATTACAACAAGGTATTACTCTAGGTATTACAACTAGAGGC
GATGGTTTAAGTGACTATCCTAAAAATGCTTTTAATATGGCGAGATATATTGATGATCGCCCATATAATATTACTCAACATCAATTGCAAT
TAGCTGAAGAAATTGCGTTTGATAGAAAAAATTGGGTGTTTCCCATTCAAACACATGAAAATAAAGTCGCTTGTATTACAAAGGATGATA
TAGGCACAAATATAGACACTTTAACTGATGCGCTTCATGGTATTGATGCGATGTACACATATGATAGTAATGTCTTATTAACGATGTGTTA
TGCAGACTGTGTACCAGTATATTTTTATAGTACAAAACATCATTTTATTGCATTGGCGCATGCAGGTTGGCGTGGTACCTATACTGAAATT
GTAAAAGAAGTGCTAAAACATGTGAACTTTGATTTGAAAGACTTACATGTCGTTATTGGACCATCTACATCATCAAGTTATGAAATTAATG
ATGATATTAAAAATAAATTTGAAACATTGCCAATTGATAGTGCCAACTATATTGAAACTAGAGGACGAGATCGTCATGGTATTGATTTGA
AAAAAGCCAATGCTGCATTATTAAATTATTATGGTGTTCCTAAAGAAAATATTTATACGACAGCGTATGCTACATCTGAACATTTAGAATT
ATTTTTCTCTTATCGATTAGAAAAAGGTCAAACAGGACGCATGTTAGCATTCATTGGTCAACAGTAA

Gene: ylmE (putative protein similar to Bacillus subtilis YlmE)

Contig: 06_NODE_54, position: 301502 to 302176, length: 675 nt, orientation: FORWARD

Perfect match to: (MW2-BA000033-[1167797:1168471], allele observed in CC1+CC25)

Sequence:

TTGCGTGTGAAAGATAATTTACAACAAATCTCAACACAAATTAATGACAAAAGTGAAAAAAATAATTTTTCAACAAAACCAAACGTGATT
GCAGTTACAAAATATGTTACAATAGAGCGAGCTAAAGAAGCGTATGAGGCTGGAATAAGACATTTTGGTGAGAATAGATTGGAAGGCTT
TTTACAAAAGAAAGAAGCATTACCATCAGATGCGGTGATCCATTTTATAGGATCATTACAATCTCGAAAAGTTAAGGGCGTTATAAACGA
CGTAGATTATTTCCATGCTTTAGATCGATTGAGCTTAGCCAAAGAAATTAACAAACGTGCAGAACATAAAATTAAATGTTTCTTGCAAGTG
AACGTTTCGGGAGAAGCTTCTAAACATGGTATTGCTTTAGAAGATGTTGATCAGTTTATAGATGATCTTAAAAAATATGACAAAATCGAA
ATTGTAGGTTTAATGACGATGGCACCATTGACAGATGATGAAGCATATATTAGATCGTTATTTAAACAGTTACGTTTGAAAAAAGAAGAA
ATACAACGACTCAATTTAGAATATGCGCCTTGTGATGAATTATCAATGGGAATGAGTAATGACTATCTTATTGCAGTTGAAGAAGGTGCG
ACGTTTGTTAGAATTGGGACTAAACTTGTAGGAGAAGAGGAGTGA

Gene: sepF (cell division protein)

Contig: 06_NODE_54, position: 302173 to 302736, length: 564 nt, orientation: FORWARD

Perfect match to: (N315-BA000018-[1168694:1169257], highly conserved allele)

Sequence:

GTGAGCCACTTGGCTTTAAAAGATTTATTTAGTGGATTTTTTGTAATAGATGATGAAGAGGAAGTAGAAGTACCTGACAAACAACAACAG
GTCAATGAAGCGCCAGCAAAAGAGCAGTCACAACAAACAACAAAACAAAACGCAATCAAATCAGTCCCTCAAAAATCTGCATCAAGATA
TACAACAACGTCAGAAGAAAGGAATAACCGTATGTCTAATTATTCAAAAAATAATTCACGTAATGTTGTAACTATGAACAATGCTACACC
AAACAATGCATCACAAGAAAGTTCAAAAATGTGTTTATTCGAACCACGTGTTTTTTCAGATACACAAGATATTGCTGATGAGCTTAAAAAC
CGCCGTGCGACACTTGTCAATTTACAACGTATTGATAAAGTATCAGCGAAAAGAATTATTGATTTTTTAAGCGGTACTGTTTATGCAATCG
GTGGAGATATCCAACGTGTAGGTACTGATATTTTCTTATGTACGCCTGATAATGTGGAAGTAGCTGGAAGCATTACAGACCATATTGAAA
ATATGGAACATTCATTCGACTAA

Gene: ylmG (putative protein)

Contig: 06_NODE_54, position: 302748 to 303038, length: 291 nt, orientation: FORWARD

Perfect match to: (N315-BA000018-[1169269:1169559], highly conserved allele)

Sequence:

ATGGATATAAATGTGCTAGCTACAATATTTAAATTTATCCTTTTTGTTGTTGAAATTTATTATTTCGGCATGATTATATATTTCTTTACATCTT
GGGTACCAAGTATTAGAGAAACTAAGGTAGGTTATTTTTTAGCGAAAATATATGAACCTTTCTTACAACCATTTAGAAAAGTAATTCCACC
TATTGGAATTATCGACATATCATCAATCGCTGCAATTATCGTTTTAGTATTATTCCAAAAAGGGTTACTCCAAATCTTTAATTGGATTTTAA
TTCAATTACAATAA

Gene: ylmH (putative protein similar to Bacillus subtilis YlmH)

Contig: 06_NODE_54, position: 303121 to 303927, length: 807 nt, orientation: FORWARD

Perfect match to: (MW2-BA000033-[1169416:1170222], highly conserved allele)

Sequence:

ATGGTATTTTATAATTTTTTTAAGGTAGTGATTAACATAGATATTTATCAACACTTTAGACAGGAAGAATACGAATTAATTGATCAGCTAA
CGGATAAATGTGATCAAGCGGAACAGCATTATGCACCAGTATTAACGCATTTTTTAGATCCAAGAGGGCAATATATATTGGAAGTGATTT
GTGGCAGTTATGAAGATTTAAACGTATCTTTTTATGGTGGACCTAATGCTGAAAGAAAAAGAGCAATCATTTCGCCGAACTATTATGAAC
CTAAAGAAAGCGACTTTGAATTAACTTTAATGGAAATAGATTATCCTGAAAAAATTCGTCACTTTAAAACATCAACATATTTTAGGGACATT
AATGTCTTTAGGTATCGAACGCGAACAAGTTGGAGATATAATTGTGAATGAACGAATTCAATTTGTTTTGACAAGTAGATTGGAATCATT
TATTATGTTAGAATTACAACGTATTAAAGGCGCATCAGTTAAACTTTATACTATTCCAGTAACAGATATGATACAATCTAATGAGAATTGG
AAAAATGAAAGTGCAACAGTTAGTTCTTTAAGGTTAGATGTTGTTATTAAAGAAATGATACGTAAATCACGTACGATTGCGAAACAACTA
ATCGAAAAAAAAACGTGTTAAAGTGAATCACACTATTGTTGATTCAGCAGATTTTCAATTACAAGCAAATGATTTAATATCCATCCAAGGTT
TTGGTAGAGCACACATTACTGACTTAGGTGGTAAAACTAAAAAAGATAAAACGCACATTACCTATAGAACATTATTCAAATAG

Gene: divIVA (cell-division initiation protein)

Contig: 06_NODE_54, position: 303951 to 304568, length: 618 nt, orientation: FORWARD

Perfect match to: (JKD6159-CP002114-[1150361:1150990], highly conserved allele)

Sequence:

ATGCCTTTTACACCAAATGAAATTAAGAATAAAGAGTTTTCACGTGTAAAGAATGGTTTAGAACCTACTGAAGTTGCTAATTTTTTGGAGC
AACTAAGCACTGAAATTGAACGTCTTAAAGAAGATAAAAAACAACTTGAAAAAGTAATCGAAGAGAGAGATACTAATATTAAGTCTTATC
AAGACGTGCATCAATCTGTAAGTGATGCTTTGATACAAGCTCAAAAAGCTGGTGAAGAAACTAAGCAAGCTGCAGAAAAACAAGCTGAA
GCGATTATAGCTAAGGCAGAAGCGCAAGCTAATCAAATGGTTGGTGACGCGGTAGAAAAAGCACGCCGTTTAGCATTCCAGACTGAAG
ATATGAAACGTCAATCAAAAGTATTTAGATCGCGTTTCCGTATGTTAGTTGAAGCGCAATTAGACTTATTAAAAAACGAAGATTGGGATT
ACTTGTTGAATTATGATTTAGACGCTGAACAAGTGACGCTTGAAAATATTCATCATTTGCATGAAAATGATTTAAAGCCAGATGAAGTTG
CAGCAAATGCACAAAATAATGCATCAAATACATCAGACAATAATCAACAATCCAATGATTCAGAAACAACTAAGAAGTAA

Gene: tbox11 (T-box leader element)

Contig: 06_NODE_54, position: 304574 to 304746, length: 173 nt

Sequence:

AAATAAAGACAGACGCGTAATATACATTTAACTTTTCACAGCGAATTAGGTAATGGTGAGAGCCTAGTAAAAGCATGTATGTTATATCAC
TGGCTTTTTAATATTTAAATAATGTAATGAGAGAACTCTAAGTTGAGTTAATAAGGGTGGTACCGCGAGCAATCGTCCCTTTT

Gene: ileS (isoleucyl-tRNA synthetase)

Contig: 06_NODE_54, position: 304789 to 307542, length: 2754 nt, orientation: FORWARD

Sequence:

ATGGATTACAAAGAAACGTTATTAATGCCTAAAACAGATTTCCCAATGCGAGGTGGTTTACCAAACAAGGAACCGCAAATTCAAGAAAA
ATGGGATGCAGAAGATCAATACCATAAAGCGTTAGAAAAAAATAAAGGTAACGAAACATTCATTTTACATGATGGCCCACCATACGCGA
ATGGTAAATTACATATGGGACATGCCTTGAACAAAATTTTAAAAGACTTTATTGTACGTTATAAAACTATGCAAGGGTTCTATGCACCATA
CGTACCAGGTTGGGATACACATGGTTTACCAATTGAACAAGCATTAACGAAAAAAGGTGTTGACCGAAAGAAAATGTCAACAGCTGAAT
TCCGTGAGAAATGTAAAGAATTTGCTTTAGAACAAATTGAATTACAGAAAAAAGATTTTAGACGTTTAGGTGTTCGTGGTGACTTTAATG
ATCCATATATTACATTAAAACCTGAATACGAAGCTGCACAAATTCGTATTTTTGGAGAAATGGCAGATAAAGGTTTAATTTATAAAGGTAA
AAAGCCAGTTTATTGGTCTCCTTCAAGTGAGTCTTCATTAGCAGAAGCAGAAATTGAATATCACGATAAACGTTCAGCATCAATTTACGTT
GCATTTGACGTTAAAGATGACAAAGGTGTCGTAGATGCAGATGCTAAATTTATTATCTGGACAACAACGCCATGGACAATTCCATCAAAT
GTTGCGATTACCGTTCATCCTGAATTAAAATATGGTCAATACAATGTAGATGGCGAAAAATATATTATTGCAGAAGCCTTATCTGACGCTG
TAGCAGAAGCACTGGATTGGGATAAAGCATCAATCAAATTAGAAAAAGAATACACAGGTAAAGAATTAGAGTATGTTGTAGCACAACAT
CCATTCTTAGACAGAGAATCGTTAGTGATTAATGGTGATCATGTTACTACAGATGCTGGTACAGGTTGTGTACATACAGCACCAGGTCAC
GGGGAAGATGACTATATTGTTGGTCAAAAATATGAATTGCCAGTAATTAGTCCAATCGATGATAAAGGTGTATTTACTGAAGAAGGCGG
CCAATTTGAAGGAATGTTCTATGATAAAGCTAATAAAGCCGTTACTGATTTATTAACAGAAAAAGGTGCACTATTAAAATTAGACTTTATT
ACACATAGCTATCCACACGACTGGAGAACAAAAAAACCTGTAATCTTCCGTGCTACACCACAATGGTTTGCCTCAATCAGTAAAGTAAGA
CAAGATATTTTAGATGCAATCGAAAATACAAACTTCAAAGTAAATTGGGGTAAAACACGTATTTACAATATGGTTCGTGACCGTGGCGAA
TGGGTTATTTCTCGTCAACGTGTGTGGGGTGTACCGTTACCAGTATTTTATGCTGAAAATGGCGAAATTATCATGACGAAAGAAACAGTG
AATCATGTTGCTGATTTATTTGCAGAACACGGTTCAAATATTTGGTTTGAAAGAGAAGCGAAAGACTTACTACCAGAAGGATTTACACAT
CCAGGCAGCCCTAACGGTACATTTACTAAAGAAACAGACATTATGGACGTTTGGTTTGATTCTGGTTCATCACACCGTGGCGTGTTGGAA
ACAAGACCGGAATTAAGTTTCCCAGCGGATATGTATTTAGAAGGTAGTGACCAATATCGTGGTTGGTTCAACTCTTCTATCACAACTTCAG
TTGCTACAAGAGGAGTATCACCTTATAAATTCTTACTTTCTCATGGTTTTGTTATGGACGGTGAAGGTAAGAAAATGAGTAAATCTTTAGG
TAATGTGATTGTACCTGACCAAGTGGTTAAACAAAAAGGTGCTGATATTGCGAGACTTTGGGTAAGTAGTACGGACTATTTAGCTGATGT
TAGAATTTCTGATGAAATTTTAAAACAAACATCTGATGTTTATCGTAAAATCAGAAATACATTAAGATTTATGTTAGGTAACATTAACGAT
TTCAATCCTGACACAGATAGCATTACTGAATCAGAGTTATTAGAAGTGGATCGTTACTTGCTAAATCGTTTACGTGAATTTACTGCAAGTA
CGATTAACAACTATGAAAACTTTGACTACTTAAATATTTATCAAGAAGTTCAAAACTTTATCAATGTTGAGTTAAGTAATTTCTATTTGGAT
TACGGTAAAGATATTTTATATATTGAACAACGAGATTCTCATATCCGTCGTAGTATGCAAACAGTGTTATATCAAATTTTAGTTGATATGA
CGAAGTTGTTAGCACCAATCTTAGTGCATACAGCTGAAGAAGTTTGGTCTCATACACCACATGTTAAAGAAGAAAGTGTTCACTTAGCAG
ACATGCCTAAAGTTGTAGAAGTAGATCAAGCTTTATTGGATAAATGGCGTACATTTATGAATTTACGTGATGATGTGAACCGTGCATTAG
AAACTGCTCGTAATGAAAAAGTTATTGGTAAATCATTAGAAGCTAAAGTTACGATTGCTAGTAACGATAAATTTAATGCATCTGAATTCTT
AACTTCATTTGATGCATTACATCAATTATTTATCGTGTCACAAGTTAAAGTTGTAGATAAGTTAGATGATCAGGCAACAGCTTATGAACAT
GGTGATATTGTCATCGAACATGCAGATGGTGAAAAATGTGAAAGATGTTGGAACTATTCAGAGGATCTTGGTGCTGTTGATGAATTGAC
GCATTTATGCCCACGATGCCAACAAGTTGTAAAATCACTTGTATAA

Gene: PF00903 (glyoxalase family protein)

Contig: 06_NODE_54, position: 307832 to 308629, length: 798 nt, orientation: FORWARD

Perfect match to: (MW2-BA000033-[1174127:1174924], allele observed in CC1+CC239)

Sequence:

ATGTATCATAACAGTAACGCAAACTTTGTCAATGGTATCACTTTAAATGTGAGAGATAAGAATGAATTAAAGCCATTTTATGAGGACATA
TTAGGATTAAATATTATAAATGAGACATTAACATCGATACAATATGAAGTAGGTCAAAATAATCATGTCATTACACTTGTTGAATTACAAA
ATGGACGTGAACCTTTAATGTCCGAAGCGGGACTGTTTCATATCGCAATTAAACTACCTCAAATTAGTGATTTAGCTAATTTACTAATTCA
TTTAAGCGAATATGATATTCCAGTTAACGGAGGTATACAGCCTGCTTCGTTATCATTATTTTTTGAAGACCCGGAAGGAAACGGTTTTAAA
TTTTATGTTGATAAAGACGAAGCGCAATGGACGAGGCAAAATGATTTAGTAAAAATTGATATTAGACCATTAAATGTACCGAGATTAGTG
AGTCATGCAACAAAATTGTTATGGTTAGGTATTCCAGATGACGCTATTATAGGTGCATTGCATATTAAGACAATTCATTTATCAGAGGTAA
AAGAGTACTACCTCGATTATTTTGGATTAGAGCAATCGGCATATATGGATGATTATTCAATATTTTTAGCATCGAATGGCTATTATCAACA
TTTGGCCATGAATGATTGGGTATCAGCAACGAAACGTGTAGAAAATTTTGATACGTATGGATTAGCAATTGTTGACTTTCATTATCCTGAA
ACAACACATTTAAATTTACAAGGTCCGGATGGTATCTATTATCGCTTTAATCATATCGAAGTTGAAGATTAG

Gene: lspA (lipoprotein signal peptidase)

Contig: 06_NODE_54, position: 309482 to 309973, length: 492 nt, orientation: FORWARD

Perfect match to: (MRSA252-BX571856-[1217944:1218435], highly conserved allele)

Sequence:

ATGCACAAAAAATATTTTATTGGCACTTCCATTTTAATAGCAGTATTTGTCGTTATATTTGACCAAGTTACTAAATATATTATAGCTACTAC
AATGAAAATTGGAGATTCATTTGAAGTGATACCGCACTTTTTAAACATAACATCACATCGAAATAATGGTGCTGCATGGGGAATATTGAG
TGGAAAAATGACATTTTTCTTTATTATTACCATTATTATATTAATAGCCTTAGTATATTTCTTTATTAAAGATGCTCAATATAATTTGTTTATG
CAAGTTGCTATTAGTTTACTTTTTGCAGGTGCACTTGGAAACTTTATTGATAGAATTTTAACAGGAGAAGTTGTTGACTTTATTGATACAA
ATATTTTTGGTTATGATTTTCCAATATTTAATATCGCAGATTCAAGTTTAACAATTGGTGTAATATTAATTATTATTGCCTTATTAAAGGATA
CTTCCAATAAAAAGGAGAAGGAGGTTAAGTAA

Gene: ylyB (ribosomal large subunit pseudouridine synthase)

Contig: 06_NODE_54, position: 309973 to 310890, length: 918 nt, orientation: FORWARD

Perfect match to: (O11-CP024649-[1199118:1200035], highly conserved allele)

Sequence:

ATGGAGACTTATGAATTTAACATTACAGATAAAGAACAAACAGGTATGCGTGTAGATAAGTTGCTGCCTGAATTAAATAGTGATTGGTCT
CGTAACCAGATACAAGATTGGATTAAAGCAGGTTTAGTCGTTGCAAACGATAAAGTTGTTAAATCTAATTATAAAGTGAAACTTAATGAT
CATATAGTTGTCACTGAAAAAGAAGTGGTTGAAGCTGATATTCTACCTGAAAATTTAAATTTAGATATTTATTATGAAGATGACGATGTTG
CAGTTGTATATAAACCGAAAGGCATGGTAGTTCATCCATCACCAGGGCATTATACCAATACATTAGTTAATGGTTTAATGTATCAAATTAA
AAATTTGTCAGGTATTAATGGAGAAATTCGTCCAGGTATTGTTCACCGTATAGATATGGATACTTCTGGTTTATTAATGGTTGCTAAAAAT
GATATTGCTCATCGTGGGCTTGTAGAACAATTAATGGATAAATCTGTTAAAAGAAAATATATCGCTTTAGTTCACGGGAATATTCCTCATG
ATTACGGTACAATCGATGCGCCAATTGGTAGAAACAAAAATGATCGTCAATCTATGGCTGTTGTTGATGATGGTAAGGAAGCAGTGACA
CATTTTAACGTACTAGAACATTTTAAAGATTATACGCTTGTTGAATGTCAACTTGAAACAGGACGTACGCATCAAATCCGTGTGCACATGA
AATATATTGGCTTCCCATTAGTTGGTGATCCAAAGTATGGACCGAAAAAGACATTGGATATTGGTGGTCAAGCTCTACATGCTGGACTTA
TTGGATTCGAACATCCAGTAACAGGTGAATATATTGAAAGACATGCTGAATTACCACAAGACTTTGAAGATTTATTAGATACAATTCGAA
AAAGAGATGCATAA

Gene: pyrR (pyrimidine operon regulator and uracil phosphoribosyltransferase)

Contig: 06_NODE_54, position: 311290 to 311817, length: 528 nt, orientation: FORWARD

Perfect match to: (MW2-BA000033-[1177585:1178112], highly conserved allele)

Sequence:

ATGTCTGAACGTATCATAATGGATGATGCCGCAATACAACGTACAGTGACGAGAATCGCTCACGAAATTTTGGAGTATAACAAAGGTACT
GATAATTTAATTCTTTTAGGTATCAAAACAAGAGGTGAATATTTAGCGAATCGTATACAAGATAAAATTCATCAAATTGAGCAACAACGT
ATACCTACTGGAACAATTGATATTACATACTTTAGAGATGATATAGAGCACATGTCATCACTTACGACAAAAGACGCAATAGACATCGAC
ACAGATATTACAGATAAAGTAGTCATCATTATTGATGATGTGCTGTATACTGGTCGAACGGTTCGTGCTTCACTTGATGCTATTTTGCTAA
ATGCTAGACCTATTAAAATTGGTTTAGCTGCTTTGGTTGATCGAGGACATCGTGAGTTACCAATTCGAGCAGATTTTGTTGGTAAAAATAT
ACCTACTTCTAAAGAGGAAACGGTAAGTGTCTATTTAGAAGAAATGGATCAAAGAAATGCAGTTATAATTAAATAA

Gene: pyrP (uracil:cation symporter)

Contig: 06_NODE_54, position: 312035 to 313342, length: 1308 nt, orientation: FORWARD

Perfect match to: (MW2-BA000033-[1178330:1179637], highly conserved allele)

Sequence:

ATGCAAAATGATGAAATGTTTGAACGAACAGTAAAACCTGTACTAGATGTAAATGAAAAACCACAACCAGCGCAATGGGCATTTTTAAG
CTTACAACATTTATTTGCGATGTTTGGCGCAACAGTACTAGTACCATTCTTAACAGGATTACCAATATCCGCAGCGTTACTAGCTTCGGGA
ATCGGTACATTACTTTATATCTTAATAACGAAGGCGCAAATACCAGCATACTTGGGATCTAGCTTTGCATTTATCACGCCAATTATCACGG
GATTAAGTACGCATAGCTTAGGAGACATGCTTGTAGCATTATTCATGAGTGGTGTTATGTACGTCATCATCGGGATTCTAATCAAATTAA
GTGGGACAGCATGGTTAATGAAATTATTACCACCAGTTGTTGTTGGACCAGTCATCATGGTAATTGGTTTAAGCTTAGCGCCTACTGCAG
TCAATATGGCAATGTATGAAAATCCTGGAGATATGAAAGGTTACAATATCAGTTTCTTAATTGTTGCAATGATAACCTTGCTTGTAACAAT
AGTCGTTCAAGGATTTTTTAAAGGATTCCTATCTTTAATTCCAGTACTTGTAGGTATTATCGTAGGTTATGTGGTAGCGATTTTTATGGGG
ATTGTGAAATTTGATGCAATTATGTCAGCAAAATGGATAGATTTCCCTCATATTTATCTGCCATTTAAAGATTATGTACCTTCATTTCACTTA
GGACTTGTACTGGTAATGATTCCGATTGTGTTTGTAACAGTAAGTGAACATATTGGGCACCAAATGGTATTGAATAAAATCGTAGGTAGA
AACTTCTTTGAAAAGCCAGGACTTGATAAATCAATCATTGGTGATGGTGTTTCTACAATGTTTGCCAGTATTATTGGTGGACCACCAAGTA
CAACATACGGTGAAAATATCGGTGTATTAGCGATTACCAGAATATACAGTATTTACGTCATTGGTGGTGCAGCAGTTATAGCAATTGTTTT
AGCATTCATTGGTAAGTTCACTGCATTAATTTCTTCTATACCTACACCAGTTATGGGAGGAGTATCTATATTACTTTTCGGTATTATTGCAG
CAAGTGGCTTAAGAATGCTAGTTGAAAGCAAAGTAGATTTTGCGAACAATCGAAATTTAGTTATAGCTTCAGTAATTTTAGTTGTAGGTA
TCGGTAATTTAGTATTTAACTTAAAAGAAATTGGTATCAACCTTCAAATTGAGGGGATGGCATTAGCTGCACTTTCAGGAATTATTTTGAA
CTTAATCTTACCTAAAGAGAAAAAACAAAACAATTAA

Gene: pyrB (aspartate carbamoyltransferase)

Contig: 06_NODE_54, position: 313370 to 314251, length: 882 nt, orientation: FORWARD

Perfect match to: (MW2-BA000033-[1179665:1180546], highly conserved allele)

Sequence:

ATGAATCATTTATTATCAATGGAACATTTATCTACAGATCAAATATACAAACTTATCCAAAAGGCAAGTCAATTTAAATCTGGTGAACGTC
AACTACCAAACTTTGAAGGGAAATATGTCGCAAATTTATTCTTTGAAAATTCTACTCGAACAAAATGTAGTTTTGAAATGGCAGAACTTAA
GCTAGGGTTAAAAACGATTAGCTTTGAAACATCAACATCATCTGTTTCAAAAGGTGAATCTTTATATGACACATGTAAAACTTTAGAAAGT
ATTGGCTGTGATTTATTAGTCATTAGACATCCGTTTAATAACTACTATGAAAAATTAGCGAATATTAACATCCCAATTGCGAATGCTGGTG
ATGGTAGTGGACAACATCCAACACAAAGTTTACTTGATTTAATGACGATATATGAAGAATATGGATATTTTGAAGGCTTGAATGTATTGA
TTTGTGGAGACATTAAAAATTCACGTGTCGCACGTAGTAATTACCATAGTTTAAAAGCATTAGGTGCAAACGTAATGTTTAATAGCCCAA
ATGCTTGGATTGATGATTCTTTAGAAGCACCTTATGTAAATATAGATGATGTTATAGAAACAGTAGATATAGTTATGTTATTAAGAATTCA
ACATGAAAGACATGGGCTTGCAGAAGAAACTAGATTTGCAGCAGATGATTATCATCAAAAGCATGGCTTAAATGAAGTGCGCTATAACA
AATTACAAGAACATGCTATTGTTATGCATCCGGCACCTGTGAATAGAGGAGTAGAAATACAAAGCGATTTAGTAGAAGCTTCAAAATCAA
GAATTTTTAAGCAAATGGAAAATGGCGTTTACTTAAGAATGGCAGTCATTGATGAATTATTAAAATAG

Gene: pyrC (dihydroorotase)

Contig: 06_NODE_54, position: 314269 to 315543, length: 1275 nt, orientation: FORWARD

Perfect match to: (MW2-BA000033-[1180564:1181838], allele observed in CC1+CC239)

Sequence:

ATGAAATTAATTAAAAACGGTAAAGTATTACAAAATGGCGAATTACAACAAGCAGATATTTTAATTGATGGTAAGGTAATTAAACAAATT
GCACCTGCAATTGAACCAAGCAATGGTGTTGACATCATAGATGCGAAAGGTCACTTTGTGTCACCTGGATTTGTCGATGTTCATGTTCATT

TACGTGAACCTGGTGGTGAATATAAAGAGACAATTGAAACTGGTACTAAAGCTGCTGCTAGAGGCGGATTTACAACTGTATGTCCAATG
CCTAACACAAGACCGGTACCAGATTCTGTAGAACATTTTGAAGCTTTACAAAAATTAATCGATGACAATGCTCAAGTACGTGTATTACCTT
ATGCTTCAATTACAACACGTCAATTAGGTAAAGAATTGGTTGATTTCCCAGCACTAGTAAAAGAAGGTGCCTTTGCGTTTACAGATGACG
GTGTAGGAGTACAAACTGCAAGCATGATGTATGAAGGTATGATTGAAGCTGCAAAAGTAAACAAAGCCATCGTAGCACACTGTGAAGAT
AATTCATTAATCTATGGTGGTGCAATGCATGAAGGGAAACGCAGTAAAGAGTTAGGTATACCAGGTATTCCAAACATTTGTGAATCTGTT
CAAATCGCAAGAGATGTACTATTAGCTGAAGCAGCAGGTTGTCATTATCATGTATGTCATGTTTCTACTAAAGAAAGTGTTAGAGTCATTC
GTGATGCTAAACGCGCAGGCATTCATGTTACAGCTGAAGTTACACCACATCATTTATTGTTAACAGAAGATGATATTCCTGGTAATAATGC
CATTTATAAAATGAATCCACCATTGAGAAGTACTGAAGATAGAGAGGCTTTGTTAGAAGGGTTACTAGACGGTACAATTGATTGTATCGC
AACAGACCATGCACCACATGCACGTGATGAAAAAGCGCAACCAATGGAAAAAGCACCATTCGGAATTGTTGGTAGTGAAACAGCATTCC
CATTATTATATACGCATTTTGTAAAAAATGGTGATTGGACATTACAACAATTAGTAGATTACTTAACAATTAAACCATGTGAGACATTTAA
TTTAGAATACGGCACATTAAAAGAAAATGGTTATGCAGATTTAACAATCATTGATTTAGATAGTGAACAAGAAATTAAAGGAGAAGATTT
CTTATCAAAAGCAGATAATACACCATTTATCGGCTATAAAGTTTATGGAAATCCGATCTTAACAATGGTTGAAGGCGAAGTTAAATTTGA
GGGGGATAAATAA

Gene: pyrAA (carbamoyl-phosphate synthase short chain)

Contig: 06_NODE_54, position: 315545 to 316645, length: 1101 nt, orientation: FORWARD

Perfect match to: (MW2-BA000033-[1181840:1182940], allele observed in CC1+CC8+CC30)

Sequence:

ATGCAAAGCAAACGTTATCTAGTGTTAGAAGACGGTTCTTTTTACGAAGGCTACCGTTTAGGATCTGATAACTTAACTGTAGGAGAAATT
GTATTTAATACAGCGATGACAGGTTATCAAGAAACTATTTCAGATCCATCATATACAGGTCAGATCATTACTTTTACGTATCCATTAATCG
GTAATTATGGTATCAATAGAGACGATTTTGAATCATTAGTACCTACATTAAACGGTATTGTAGTGAAAGAAGCGAGTGCGCATCCAAGTA
ATTTTAGACAACAAAAGACACTTCATGACGTTTTAGAATTGCATCAAATTCCAGGGATTGCAGGTGTTGATACAAGAAGTATTACGCGTA
AAATTCGACAACACGGTGTGTTAAAAGCTGGTTTTACTGATCGAAAAGAAGATATTGATCAACTTGTCAAACATTTACAACAAGTAGAAT
TACCTAAAAACGAAGTAGAAATCGTTTCGACTAAAACACCGTATGTTTCGACAGGTAAGGATCTAAGTGTCGTACTTGTAGACTTTGGTA
AGAAGCAAAATATTGTTCGAGAATTAAACGTCAGAGGTTGTAACGTCACAGTTGTACCATATACAACTACTGCCGAAGAAATTTTAGCAA
TGGCTCCAGATGGCGTTATGCTATCAAACGGACCAGGTAATCCTGAAGTTGTAGAATGTGCGATTCCAATGATTCAAGGAATTTTAGGGA
AAATTCCGTTCTTTGGTATCTGTCTAGGACATCAACTTTTTGCATTATCTCAAGGAGCAAGCTCATTTAAAATGAAGTTTGGTCATCGTGGT
GCGAACCATCCAGTTAAAAATTTAGAGACTGGAAAAGTTGATATTACGAGTCAAAACCATGGATATGCAATAGATATAGATTCGTTAAAA
AGTACTGATTTAGAAGTTACTCATCTTGCATTAAATGATGGTACTGTAGAAGGTTTAAAACATAAAACATTACCAGCATTTTCTGTTCAAT
ACCATCCTGAAGCAAATCCAGGACCGTCAGATTCAAACTATCTATTTGATGATTTTGTAGCAATGATGACTAATTTTAAGGAAAAGGAGC
GTCATATCAATGCCTAA

Gene: pyrAB (carbamoyl-phosphate synthase large chain)

Contig: 06_NODE_54, position: 316638 to 319811, length: 3174 nt, orientation: FORWARD

Perfect match to: (N315-BA000018-[1183131:1186304], allele observed in CC5+CC1)

Sequence:

ATGCCTAAACGTAATGATATCAAAACAATTTTAGTAATAGGGTCTGGGCCAATTATCATAGGTCAAGCAGCTGAATTTGATTATGCTGGA
ACACAAGCATGTCTAGCTTTAAAAGAAGAGGGATATCGAGTTATTCTTGTAAATTCAAATCCAGCGACAATCATGACTGATAAGGAAATT
GCGGATAAAGTATATATCGAACCGTTAACTCATGATTTTATAGCGCGAATTATACGTAAAGAGCAACCTGACGCTTTACTTCCAACTTTAG
GTGGTCAAACAGGTTTAAACATGGCGATTCAACTACACGAAAGTGGTGTGCTTCAAGATAATAACGTCCAATTATTAGGAACTGAGCTAA
CATCAATTCAACAAGCAGAAGACCGTGAAATGTTTAGAACATTAATGAATGATTTAAACGTTCCTGTACCAGAGAGTGACATTGTAAATA
CAGTAGAGCAAGCCTTTAAATTCAAAGAGCAAGTGGGATACCCGCTAATTGTTAGACCGGCATTTACGATGGGTGGTACCGGAGGCGGT
ATTTGTCATAATGATGAAGAATTACATGAAATCGTCTCAAATGGTCTTCATTATAGTCCAGCAACGCAATGTTTATTAGAAAAATCTATCG
CAGGTTTTAAAGAAATCGAATACGAAGTAATGCGTGATAAAAACGATAATGCCATCGTTGTATGTAACATGGAAAATATTGATCCAGTTG
GTATTCATACTGGCGATTCAATTGTTGTGGCTCCTAGTCAAACATTATCAGATGTTGAGTATCAAATGTTACGTGATGTTTCATTAAAAGTT
ATTCGAGCTTTAGGTATCGAAGGTGGTTGTAATGTTCAATTAGCATTAGACCCCCATTCATTCGATTATTATATTATAGAAGTAAATCCGC
GTGTATCACGTTCATCAGCGTTAGCTTCAAAAGCAACAGGATATCCTATTGCAAAATTAGCTGCTAAAATCGCGGTTGGTCTAACATTAG
ATGAAATGTTAAATCCAATTACAGGAACATCTTATGCAGCGTTTGAACCAACTTTAGACTATGTGATTTCAAAAATACCAAGATTTCCTTTT
GATAAATTTGAAAAAGGAGAACGAGAGCTTGGCACACAAATGAAAGCAACAGGTGAAGTTATGGCCATTGGTCGAACTTACGAAGAAT
CATTGTTAAAAGCAATTCGATCACTTGAGTATGGTGTGCATCACTTAGGATTACCAAATGGTGAAAGCTTCGATCTTGATTATATTAAAGA
ACGTATTTCACACCAAGATGATGAACGATTATTTTTCATCGGCGAAGCAATTAGAAGAGGCACAACATTAGAAGAAATTCATAATATGAC
TCAGATTGATTACTTCTTCTTACACAAGTTCCAAAACATTATTGATATTGAGCATCAACTAAAAGAGCATCAAGGTGATTTAGAATATCTTA

AATATGCAAAAGATTATGGATTTAGTGATAAAACAATAGCGCATCGCTTTAATATGACGGAAGAAGAAGTATATCAATTGCGTATGGAA
AATGATATTAAACCTGTTTACAAGATGGTTGATACTTGCGCAGCTGAATTTGAATCTTCAACACCATATTATTATGGTACATACGAAACTG
AAAATGAATCCATAGTTACTGACAAAGAAAAAATCTTAGTATTAGGCTCTGGACCAATTCGAATCGGCCAAGGTGTAGAATTTGACTATG
CGACAGTTCACGCCGTTTGGGCAATTCAAAAAGCAGGGTACGAAGCGATAATTGTGAATAACAATCCAGAAACAGTTTCAACAGACTTCT
CAATTTCTGACAAATTATACTTTGAACCTTTAACTGAAGAAGATGTGATGAATATCATTAATTTAGAAAAACCTAAAGGTGTCGTTGTACA
ATTTGGAGGACAAACAGCGATTAATTTAGCAGACAAATTGGCTAAACATGGTGTTAAAATACTTGGTACTTCACTAGAAAATCTAAATCG
TGCTGAAGATAGAAAAGAATTTGAAGCACTATTAAGAAAAATTAACGTGCCACAGCCACAAGGGAAAACAGCTACATCACCTGAGGAAG
CATTAGCGAATGCTGCAGAAATCGGATATCCGGTTGTAGTAAGACCTTCTTATGTATTAGGTGGTCGCGCAATGGAAATTGTAGACAATG
ACAAAGAGTTAGAAAACTATATGACCCAGGCTGTAAAAGCGAGTCCGGAACATCCGGTACTAGTCGATAGATATTTAACTGGTAAAGAA
ATTGAAGTTGATGCGATTTGTGATGGAGAAACGGTCATTATTCCAGGAATAATGGAACATATTGAACGTGCTGGTGTGCATAGTGGTGA
CTCAATCGCTGTATATCCACCACAAACTTTGACAGAAGACGAGTTAGCAACACTTGAGGACTATACTATAAAATTAGCTAAAGGTTTAAA
CATCATTGGCTTAATCAACATTCAATTCGTTATAGCTCACGATGGTGTGTATGTTTTAGAAGTAAATCCACGTTCTAGTAGAACGGTACCA
TTCTTAAGTAAAATTACTGATATTCCAATGGCACAATTAGCTATGCGAGCAATCATTGGGGAAAAACTAACAGATATGGGTTATCAAGAA
GGGGTTCAACCATATGCTGAGGGTGTCTTTGTGAAAGCACCGGTATTTAGTTTCAATAAATTGAAAAATGTTGATATTACTTTAGGACCT
GAAATGAAGTCAACAGGTGAAGTGATGGGGAAAGATACTACATTAGAAAAGGCGTTATTCAAAGGGTTAACAGGTAGTGGTGTTGAAG
TTAAAGATCACGGTACAGTATTAATGACCGTCAGTGACAAAGATAAAGAGGAAGTTGTTAAATTGGCACAACGCTTAAATGAAGTTGGC
TATAAAATTTTAGCAACGTCTGGAACAGCTAATAAATTAGCTGAGTATGACATACCTGCAGAAGTAGTAGGCAAAATTGGTGGCGAAAA
TGATTTATTAACACGTATTCAAAATGGTGATGTTCAAATCGTTATAAATACAATGACTAAAGGTAAAGAAGTAGAAAGAGATGGCTTCCA
AATTAGACGTACTACAGTTGAAAATGGTATTCCATGTTTGACATCTTTAGATACAGCTAATGCCTTAACGAATGTAATTGAAAGTATGACA
TTTACAATGCGTCAAATGTAA

Gene: pyrF (orotidine 5'-phosphate decarboxylase)

Contig: 06_NODE_54, position: 319921 to 320613, length: 693 nt, orientation: FORWARD

Perfect match to: (MW2-BA000033-[1186216:1186908], highly conserved allele)

Sequence:

ATGAAAGATTTACCAATTATTGCATTAGATTTTGAATCAAAAGAAAAAGTAAATCAATTTTTAGATTTATTTGATGAATCATTATTCGTAAA
AGTAGGTATGGAACTTTTTTATCAAGAAGGTCCTCAATTAATTAATGAGATAAAAGAAAGAGGCCATGATGTATTTTTAGATTTAAAATT
GCATGATATTCCTAATACAGTAGGTAAGGCGATGGAAGGACTAGCTAAATTGAATGTTGATCTGGTAAATGTTCATGCTGCTGGTGGCG
TAAAAATGATGTCTGAGGCCATTAAAGGATTAAGAAAACATAATCAACATACAAAAATTATTGCAGTAACACAGCTTACGTCAACAACAG
AAGACATGTTACGACACGAACAAAATATACAAACATCGATTGAAGAGGCCGTTTTAAATTATGCCAAGTTAGCAAATGCAGCTGGTTTAG
ATGGCGTTGTTTGTTCACCTCTTGAAAGTCGTATGTTGACTGAAAAGTTAGGTACATCATTTTTAAAAGTAACACCAGGTATTAGACCTAA
AGGTGCATCTCAAGATGACCAACACCGTATTACGACACCGGAAGAAGCAAGACAGCTTGGTTCGACGCATATTGTAGTCGGTAGACCGA
TTACACAAAGTGACAATCCAGTCGAAAGTTATCATAAAATTAAAGAAAGTTGGTTAGTATAA

Gene: pyrE (orotate phosphoribosyltransferase)

Contig: 06_NODE_54, position: 320613 to 321224, length: 612 nt, orientation: FORWARD

Perfect match to: (Strain_21193-AFEG01000027-[27800:28411:r], highly conserved allele)

Sequence:

ATGGCTAAAGAAATTGCAAAATCATTATTAGATATTGAAGCTGTAACATTATCACCAAATGATTTATATACATGGAGTTCAGGTATTAAAT
CACCGATTTACTGTGATAACCGTGTTACGTTAGGTTATCCTTTAGTTCGAGGCGCAATCCGCGATGGTTTAATTAACTTAATTAAAGAACA
CTTTCCTGAAGTAGAAGTTATTTCTGGTACTGCAACAGCTGGTATTCCACATGCAGCTTTTATTGCTGAAAAATTAAAATTACCAATGAATT
ATGTTCGTTCATCAAATAAGAGTCATGGTAAGCAAAATCAAATCGAAGGTGCTAAAAGTGAAGGTAAAAAAGTAGTTGTGATAGAAGAT
TTAATTTCGACAGGGGGATCTTCAGTCACAGCAGTTGAAGCCTTAAAACTAGCAGGTGCAGAAGTATTAGGTGTTGTAGCTATCTTTACT
TACGGTTTGAAAAAAGCAGATGATACATTTAGCAATATTCAACTACCTTTTTACACTTTAAGTGATTACAATGAATTAATTGAAGTAGCTG
AAAATGAAGGTAAAATTTCTAGTGAAGATATCCAAACATTAGTTGAATGGAGAGACAACTTAGCATAA

Gene: Q5HGM6 (putative protein)

Contig: 06_NODE_54, position: 321254 to 321466, length: 213 nt, orientation: FORWARD

Perfect match to: (MW2-BA000033-[1187549:1187761], allele observed in CC1)

Sequence:

ATGAATGACAAAACATCTAATGATTTATATGGGAAGATAAAACATTGTAACGAATTTATCAATCATTCAAATGATTCCAATCTATCTAGTA
GTCACGATGTCGACGAAAGTTCAACGAAGCAAAAACATATAAAAAATAAAACAACTATAGACCATAATGATGATTTATTTAAACATGTAA
AGGATATATTACGTAAACAAGGACAAATTTAA

Gene: Q5HGM5 (putative protein)

Contig: 06_NODE_54, position: 321903 to 322304, length: 402 nt, orientation: FORWARD

Perfect match to: (MW2-BA000033-[1188198:1188599], highly conserved allele)

Sequence:

ATGGATATTCCAAAAATCACGACATTTTTAATGTTTAATAACCAAGCTGAAGAAGCTGTTAAACTATACACAAGCTTATTTGAAGATAGTG
AGATTATAACAATGGCTAAGTATGGTGAAAATGGACCTGGTGATCCCGGGACTGTACAACACTCAATATTTACATTAAATGGACAAGTAT
TCATGGCGATTGATGCTAATAGTGGCACAGAATTACCAATGAATCCTGCGATTTCATTATTTGTTACAGTAAAAGATACTATTGAAATGGA
ACGACTATTTAATGGATTAAAAGATGAAGGTGCCATTTTAATGCCAAAAACGAATATGCCACCATACAGAGAGTTTGCTTGGGTTCAAGA
TAAGTTTGGAGTAAGTTTTCAATTAGCATTACCTGAGTAA

Gene: fbpA (putative fibronectin/fibrinogen-binding protein)

Contig: 06_NODE_54, position: 322567 to 324264, length: 1698 nt, orientation: REVERSE

Perfect match to: (MUF256-AZSE01000020-[46428:48127:r], allele observed in CC1)

Sequence:

TTATGATTGTTTCATTTTTTGAATAAGTTCATAATCAGGTGTAGCATACAAAGTTTTTTGATTGTCATATGTTACAAACCCAGGCTTTGCAC
CTGATGGTTTGTGCACATTTTTAATTAATGTGTAATCAACAGGTATTTGTCCAGAATTACCAGCTTTTGAAAAGTATCCTGCTAACATAGCC
GCTTCCTTGATTGTCGTATCACTTGGTGCATCATTAAATATAACGACATGTGAACCAGGAATATCTTTTGTGTGTAACCATGTGTGAGTTTT
TTTAGCTTTTTTATTTGTTAAATAATCATTTTGCTTGTTATTCTTGCCAACATATATATCGTCGCCATCAGTTGATACATAATGTTGTAATTG
AATCTGCGCTTTCTTTTTCTTAGTTTGATTTTTACGCTGTTTCATAAAGCCTTGTTCTGCTAATTCATCTCTAATTTCATCAATGTCATGGACA
GAAATATGATGTAATTGTTGTTCGATTGTTGAAAAATAATCTATATTGTCTTTCGTCAATTGAATTTGATGTTGTAATTCACGTTCTCTCGTT
TTCATACGATTATATTGTTTATAATAATATTGAGCATTTGCTGATGGGGATTTTGTAGGATTTAAAGGAATGACAACTTCTTCATTCGTATA
ATAATTCAATGCCGTCACTTCTTTATCGCCTTGCTTAATTCGATATATATTAGCAGTGATCAATTCACCATATAACTGTTCAGTATCTTTATT
TTTAGACTGCTCATATTCTTCAATCAACTTCGCTAATTTATTTTGATATTTGTGCAACTGCTGTTGAACAAATCGAACTAAATCATTCGCACG
TTGTTTAACGCGTTCACGTTCGCCACGCGCATCATAAAAACGATCAAGTAAATCATTTAATGAATCGTATGTAACTGTATCATCATTAAATT
GATTTAACTTTATAAAATAGAAATCCTCTTTACCTGTTTCATGATTTTTATGAAAAATAGGAGTAGGTGGTAACTTGGTTTCTGCCATTACT
TCGTCAAATGCTTCTGGTAATGTTGATGAAGTCATAAATTGACGACGACTAACAATTTCATTCGTAATTAAAGGGCTAAATCCTTCAAACT
GATTCAATAATTGTTTAGCAATATTACCTGCGTTAAAATCGATATATTTCAACACCTCTGCACCTGTAATATCATACGGATTTATTTTGTGCT
GAGTAGGTGGTGCTTCATAATTAAATCCTGGCATTACTGTACGATAGTGATTCGTATTTGGTGTTAAGTGTTTAAATCCTTCAATTATTTTG
CGATTTTCATCTACTAAAATTAAGTTACTATGTTTACCCATAATCTCAAGGATGACAGTGCGGTAAATAGTATCGCCAATTTCATCTTTACT
CTTTATTTCGATTTCAATGCGACGATCATTACCAATTTGCTTAATCGATTTGATAATACCACCTTCTAAGTGTTTTCTAAAAACACGCGCAA
ACATGGGTGGATTAAATGGATTATCATATTTTTTAGTAGTTAATTGTAATCTTGAAAAGTTTGGATGGATTGACAATAACAATTGATGGTT
TTGTCTATTTTGACGTACAACCATTAGTATCGTGTCATTATCAGGTTGATTGATTTTGTGAACGCGTCCTGTTGTTAAAAATTGTAGAGACT
CAACCATTTTCTTTGTAAATAAGCCATCATAAGCCAT

Gene: gmk (guanylate kinase)

Contig: 06_NODE_54, position: 324539 to 325162, length: 624 nt, orientation: FORWARD

Perfect match to: (MW2-BA000033-[1190834:1191457], highly conserved allele)

Sequence:

ATGGATAATGAAAAAGGATTGTTAATCGTTTTATCAGGACCATCTGGAGTAGGTAAAGGTACTGTTAGAAAACGAATATTTGAAGATCCA
AGTACATCATATAAGTATTCTATTTCAATGACAACACGTCAAATGCGTGAAGGTGAAGTTGATGGCGTAGATTACTTTTTTAAAACTAGG
GATGCGTTTGAAGCTTTAATCAAAGATGACCAATTTATAGAATATGCTGAATATGTAGGCAACTATTATGGTACACCAGTTCAATATGTTA
AAGATACAATGGACGAAGGTCATGATGTATTTTTAGAAATTGAAGTAGAAGGTGCAAAGCAAGTTAGAAAGAAATTTCCAGATGCGCTA

TTTATTTTCTTAGCACCTCCAAGTTTAGAACACTTGAGAGAGCGATTAGTAGGTAGAGGAACAGAATCTGATGAGAAAATACAAAGTCGT
ATTAACGAAGCGCGTAAAGAAGTTGAAATGATGAATTTATACGATTACGTTGTAGTTAATGATGAAGTAGAACTTGCGAAGAATAGAAT
TCAATGTATTGTAGAAGCTGAGCACTTAAAAAGAGAGCGCGTAGAAGCTAAGTATAGAAAAATGATTTTGGAGGCTAAAAAATAA

Gene: rpoZ (DNA-directed RNA polymerase subunit omega)

Contig: 06_NODE_54, position: 325162 to 325380, length: 219 nt, orientation: FORWARD

Perfect match to: (N315-BA000018-[1191655:1191873], highly conserved allele)

Sequence:

ATGTTAAATCCACCTTTAAACCAATTAACGTCACAAATTAAATCAAAGTATTTAATTGCAACAACTGCAGCGAAAAGAGCGCGTGAAATT
GATGAACAACCTGAAACTGAATTATTAAGTGAATATCATTCATTTAAACCAGTTGGTAGAGCGTTAGAAGAAATTGCTGACGGTAAAATT
CGCCCTGTTATTTCAAGTGATTATTATGGTAAAGAATAG

Gene: coaBC (bifunctional phosphopantothenoylcysteine decarboxylase/phosphopantothenate--cysteine ligase)

Contig: 06_NODE_54, position: 325596 to 326795, length: 1200 nt, orientation: FORWARD

Perfect match to: (MW2-BA000033-[1191891:1193090], allele observed in CC1+CC5)

Sequence:

ATGAAGAAAATATTATTAGCCGTTACAGGTGGCATTGCGGCATATAAAGCAATTGATTTGACAAGTAAGTTAACACAATCTGGGTATGAA
GTTCGCGTTATGTTAACGAATCACGCACAAAAATTTGTGACACCATTAGCATTTCAAGCAATAAGTCGAAATGCTGTTTATACAGATACTT
TTATAGAAGAAAATCCTTCAGAAATACAGCATATTGCATTAGGTGATTGGGCAGATGCAATCATTGTTGCACCTGCAACGGCAAATACAA
TTGCAAAATTGAGTGTAGGTATTGCTGATGATTTGGTGACATCAACGTTGCTAGCAACAGAGACACCGAAATTTATTGCGCCTGCTATGA
ATGTGCATATGTATGAAAATAAACGTACGCAGCAAAATATTAATATTTTAAAAGAAGATGGGTATCATTTTATCGAACCAGGAAGCGGAT
TTCTAGCATGTGGTTATGTTGCTAAAGGACGTATGGAAGAACCGCTTCAAATCGTTTCTGTAATTGATGCTCATTTTCAAAATAGTAATCG
TTTAGCTAATAGTTCATTTCAAGATAAACGCGCATTGGTTACAGCAGGACCAACTATTGAAGTTATCGATCCAGTCAGATTTGTATCCAAT
CGTTCTTCTGGAAAAATGGGTTATGCAATAGCTGAAGCATTGCGAAATCGAGGAGCTATCGTGACGTTAGTTGCTGGTCCTACAACACTA
GAGGATCCAAAAGATATTGAAGTTATTCATGTTCAAAGTGCTGAAGAAATGTTTGAACAAGTGACAAGCCGATTTGACGAACAAGATAT
TGTTGTAAAAGCAGCAGCCGTATCTGACTATACACCGGTTGATGTATTAGAACATAAGATGAAAAAGCAGGATGGTGATTTGTCAGTATC
TTTTAAACGTACTAAAGACATTCTTAAATATTTAGGTGAACATAAAACATCACAGTATTTAATAGGCTTTGCAGCAGAGACTGAAGATATT
GAAAATTATGCACAACAAAAATTACGCAAGAAAAATGCAGATGTGATTATTTCAAATAATGTTGGGGATATGTCTATCGGATTTAGTTCT
GATGATAATGAATTGACAATGCATTTTAAAAATAATGAAAAGGTAAATATCAAGAAAGGAAAAAAAGTAGTATTAGCTGCACAAATTTTA
GATGAACTAGAAACTAGGTGGCAATAA

Gene: priA (primosomal protein N')

Contig: 06_NODE_54, position: 326795 to 329203, length: 2409 nt, orientation: FORWARD

Perfect match to: (Strain_21202-AGRO01000049-[114187:116595:r], allele observed in CC395+CC239)

Sequence:

ATGATAGCGAAAGTCATAGTCGATGTCGCGTCGAAGAGCGTTGACTATAAATTTGATTATATAATTCCCGAACAACTCGAATCTGTCATC
CAACCTGGTGTGCGTGTAATTGTACCTTTTGGACCAAGAACGATTCAAGGTTATGTAATGGAAGTAACAGCAGAACCTGATGCACAACTT
GACGTTTCGAAGTTAAAAAAAATCATAGAAGTGAAAGATATACAACCAGAATTAACATCAGAATTAATAGCTTTAAGTGAGTGGATGGG
TTCAACTCATGTCATTAAACGTATTTCTATGCTAGAAGTGATGCTTCCGAGTGCTATTAAAGCGAAGTATAAAAAAGCATTTAAGATGAAA
GATGACATAGAGCTACCTTCAGCTTTATTACAAAAATTTGATAAGCATGGTTACTATTATTATAAAGATGCGCAAAAAAATAATGATATTC
AATTGCTTATGAAGTTGTTAAAAGATGATATCGTTGAAGAAAAAACGATTCTCACACAAAATATAACTAAAAAAACCAAGCGTGCTGTTC
GTGTCATTGAAGGGTATCATCCTGATGAAGTATTAGCTAAGTTGGAGAAAGTTATTAAACAATACGATTTGTATGCTTACTTGTCTGAAG
AACAACATAAAACAATATTTTTAACTGATATTGAGGATATGGGCTTTTCAAAATCCAGTTTAGATGGACTTATCAAAAAAGGTTATGTTGA
AAAATATGACGCGGTTGTTGAAAGAGACCCATTTAAAGATCGTGTTTTCGAACAAGAATCAAAACAGCAATTAACAGAAGACCAATATA
AAGCATATGAAGCGATTAAAGCTAAAATTGTAAGCCAAGAGCAAGAAACATTTTTACTTCATGGTGTGACGGGATCAGGTAAAACAGAA
GTATATTTACAAACGATAGAAGATGTTTTAAGCCAAGGAAAACAGGCGATGATGTTAGTTCCTGAAATCGCTCTAACACCGCAAATGGTT
TTACGCTTCAAACGTCGATTTGGTGATGACGTTGCTGTATTACATTCTGGCTTATCTAATGGGGAACGTTATGATGAGTGGCAAAAAATTA
GGGATGGTCGTGCGAGAGTAAGTGTTGGTGCAAGGTCAAGTGTGTTCGCACCTTTCAAAAATTTAGGGTTAATCATCATTGATGAAGAA

CATGAATCTACATATAAACAAGAAGATTATCCGAGATATCACGCTAGAGAAATTGCCCAATGGCGAAGTGAATATCATCACTGTCCAGTC
ATTTTAGGAAGTGCAACACCATGTCTTGAAAGTTATGCACGAGCTGAAAAAGGCGTTTATCATTTGCTATCATTACCAAACAGAGTGAAC
CAACAAGCTTTACCTGAAATTGATATAGTAGACATGCGTGAAGAATTGAGTGAAGGTAATCGGTCAATGTTTTCAAAAGATTTACGTGAA
GCCATACAATTAAGATTAGATCGACAGGAACAAGTTGTTTTATTTTTAAATCGACGTGGTTATGCATCGTTTATGTTATGTCGGGATTGTG
GATATGTACCGCAATGTCCAAACTGTGATATTTCATTAACGTATCATAAAACGACAGACTTATTAAAATGTCACTATTGTGGTTACCAAGA
GACGCCACCGAATCAATGTCCAAATTGTGAGAGTGAACACATTCGACAAGTAGGTACTGGTACTCAGAAAGTTGAAGAACTATTGCAAC
AAGAATTTGAAGATGCGCGCATAATTAGGATGGATGTAGATACAACCTCAAAGAAAGGTGCACATGAAAAGTTATTGACTGAATTCGAA
AAAGGTAACGGTGACATTTTACTAGGTACTCAGATGATTGCGAAAGGATTAGATTATCCAAATATTACTTTAGTTGGTGTGCTGAATGCA
GATACAATGTTAAATTTACCTGATTTTCGGGCGAGCGAACGTACTTATCAACTATTAACGCAAGTGGCTGGTAGAGCTGGTCGTCATGAA
AAGGCAGGTCAAGTCATCATTCAAACGTATAATCCAGATCATTATTCAATATTGGATGTTCAAAAAAATGATTATTTAACATTTTATCGTCA
GGAAATGGAATATCGTAAATTAGGAAAGTATCCACCGTATTATTATTTGATTAATTTCACAATCTCACATAAAGAAATGAAGAAGGTTAT
GGAAGCATCGCAGCATGTTCATAAAATTTTATTACAGCATTTAACAGAAAAAGCGCTTGTACTAGGTCCATCTCCGGCAGCACTTGCGAG
AATCAACAATGAATTTAGATTCCAAATTTTAGTGAAATATAAAAGTGAACCTGGATTATTACAAGCCATTCAGTTTTTAGATGACTATTAC
CATGAAAAATTTATAAAAGAAAAATTAGCATTGAAGATTGATATTGATCCACAGATGATGATGTAA

Gene: Q5HGL9 (putative lipoprotein)

Contig: 06_NODE_54, position: 329706 to 330659, length: 954 nt, orientation: FORWARD

Perfect match to: (N315-BA000018-[1196202:1197155], highly conserved allele)

Sequence:

ATGAAAAAGACACTGGGATGTTTACTTTTAATTATGCTTTTAGTCGTAGCAGGTTGTTCTTTTGGTGGGAATCATAAATTATCATCAAAGA
AATCAGAAGAATCAAAACAAGAAACTGTAAAAAAAGAATCGGAAGAAGAGAAAGATCCAGATTTAGAGAAATATGAAGAAATAGAGA
AGAAAATGAAAGGAATTAAAGATGCGCCATCTCTTGATAAGTTGGATCCATTAATGACAGAAAAGTCGTTTACGAATAGTAAAGGGATT
CAAGGATGGAAAGATTACAAAGAATTAATGGGTAAAGTGGAACTTGCAGATTATAGATTTACTAAAGATTCAAAAGGATCTTCAATAAA
AGATGTTGATGCATTCTTTAAAGGTAAGAAAGGTATAAAAAGGAAAGTGATTGAAACACACGATGATGTAAAACAAGTTGATTATTGGT
ATGTAGATCCAGATGGAAAGAAAATTGGCAATTCAAACACACCTGTTTTTTACGCAGAAATTATGACAAAATATAAAGATGGAAAGTTAG
TTTATGCATCAGTCGAACCAGGATCTTACGTAATACATAAAGATGATGCAATTAAATATGACGATTATTCTAAGTTAAAAAAATTAAGTCA
GCTAACTAAACTTGATCATCCAAAACCAGTTCCATATAGCGTAGCTCAAATCAAATCTTTCGGAGTACCTTTAACAAGCGTTTCATTTATGA
CACATGGATCAAAGGATACTAAAGATGAAGTGTTGCCGGCATTGGCCTATTTCACTTTTTCACCAAAAAATTATGAAGACAAGTCTAATCC
AGATCCAAAAGTTTTAAATTTAGTACATATGGATTTCTTAAATGCATCTAGTGATTTTGGTAACGCACATTTTGTTGTTTTAAGTAAATATA
TTAAAGAGTATGAATCAAACTATGAAACAGCGTCAGATGATTCTTTAAAATAG

Gene: Q5HGL8 (short two-transmembrane helix protein)

Contig: 06_NODE_54, position: 330822 to 331100, length: 279 nt, orientation: REVERSE

Perfect match to: (MW2-BA000033-[1197117:1197395:r], highly conserved allele)

Sequence:

TTATAAAGTGACATTACCTTGTTCATCAGCAGGTTTGAAAACAGTAATCACTGCACTAATAATTGCTAAAATGTGTGGGATACCTGTCCAA
CAGAATATTAAGTGTAGAATACCTTGCATATTCTTGCCGGCATAAAATTTATGAATACCAAAACTACCTAAGAACAATGCTAATAAAATAT
AAATAACTTTGTTTACTTGCATTTCTTTCCCTCCAGTTGAATTGCTTATAATGACATTAGCTTCTCTTTTTATTATACCCACTTTTAGTTCAAA
CAT

Gene: def2-defA (formylmethionine deformylase)

Contig: 06_NODE_54, position: 331324 to 331812, length: 489 nt, orientation: FORWARD

Perfect match to: (MW2-BA000033-[1197619:1198107], allele observed in CC1+CC22+CC80+CC188+CC772)

Sequence:

ATGGCGATTAAAAAGTTAGTACCAGCATCGCATCCTATTTTAACGAAAAAAGCGCAAGCAGTTATAAAATTTGATGATTCGTTAAAAAGA
TTATTACAAGATTTAGAAGATACAATGTATGCACAAGAAGCAGCTGGCTTATGTGCACCTCAAATTAATCAGTCATTGCAAGTGGCAATC
ATTGATATGGAAATGGAAGGATTATTACAACTTGTTAATCCGAAAATTATTAGTCAATCAAATGAAACGATAACAGACTTAGAAGGTTCA
ATTACATTGCCAGATGTTTACGGCGAAGTGACAAGAAGTAAAATGATAGTTGTCGAAAGTTATGACGTCAATGGGAACAAAGTTGAACT

AACTGCACATGAAGATGTAGCAAGAATGATTTTGCATATTATAGATCAAATGAACGGTATCCCTTTTACAGAACGTGCGGACCGTATTTT
AACAGATAAAGAAGTGGAGGCATATTTTATAAATGACTAA

Gene: fmt (methionyl-tRNA formyltransferase)

Contig: 06_NODE_54, position: 331805 to 332740, length: 936 nt, orientation: FORWARD

Perfect match to: (RF122-AJ938182-[1165897:1166832], highly conserved allele)

Sequence:

ATGACTAAAATAATATTTATGGGTACACCAGACTTTTCAACAACTGTTTTAGAAATGCTTATTGCAGAACATGATGTCATTGCAGTCGTAA
CGCAACCAGATCGACCTGTTGGACGTAAACGTGTTATGACACCACCACCAGTTAAAAAAGTTGCAATGAAATATGATTTACCTGTATATC
AGCCTGAAAAATTAAGTGGATCAGAAGAATTAGAACAATTGCTTCAATTAGATGTAGATTTAATTGTAACTGCTGCTTTTGGACAATTATT
ACCTGAATCATTGTTGGCATTACCAAAACTTGGGGCAATTAATGTACATGCATCATTGTTACCGAAGTATAGAGGTGGTGCACCAATTCA
TCAGGCAATTATCGATGGTGAACAAGAAACCGGCATAACAATTATGTATATGGTTAAAAAATTAGATGCGGGTAATATTATTTCGCAACA
AGCAATTAAAATAGAAGAAAATGATAATGTCGGTACGATGCATGATAAATTAAGTGTATTAGGGGCAGATTTATTAAAAGAAACTTTACC
ATCTATTATAGAGGGCACAAATGAAAGTGTACCTCAAGATGATACGCAAGCAACATTTGCTTCCAATATTCGACGCGAAGATGAGCGAAT
TAACTGGAATAAACCAGGAAGACAAGTGTTTAATCAAATTCGTGGATTATCACCATGGCCAGTTGCTTATACAACTATGGATGACACTAA
CTTGAAAATATACGATGCTGAACTCGTTGAGACTAATAAGATAAACGAGCCTGGAACCATTATAGAAACGACTAAAAAAGCCATTATTGT
TGCTACAAATGATAATGAAGCTGTTGCAATTAAAGATATGCAATTAGCTGGGAAAAAGAGAATGTTAGCTGCCAATTATTTAAGTGGTGC
GCAAAACACACTAGTAGGGAAGAAACTTATATGA

Gene: sun-rsmB (ribosomal RNA small subunit methyltransferase B)

Contig: 06_NODE_54, position: 332737 to 334044, length: 1308 nt, orientation: FORWARD

Perfect match to: (MW2-BA000033-[1199032:1200339], allele observed in CC1)

Sequence:

ATGATAGAAAACGTGAGAAGTCTTGCTTTTGACACGATTCAAGATATATTAAATGAAGGTGCGTATAGTAACTTGCGTATCAATGAAGTG
TTGTCAGAAAATGAATTAAATGCAATGGATAAGGCTTTATTTACAGAAATTGTCTACGGAACCGTTAAAAGAAAATTTACGTTAGATTTTT
ATTTAAAGCCTTTTGTGAAAACAAAAATTAAGGCATGGGTTAGGCAATTATTATGGATGAGTATTTATCAATATGTTTATTTAGATAAAGT
TCCAAATCACGCCATCATTAATGAAGCAGTTGAAATAGCAAAAGAACGCGGTGGCTATCATAATGGTAATGTCGTAAATGGTATTTTACG
TACAATGATGCGTAGTGACTTACCTGATTTTAATGAAATTGCAGATCCTAAAAAAAGAATGGCAATCGAGTATAGTATGCCGAAGTGGAT
TATAGATCATTGGGCAACACATTATGGTCTCGAAAAAACTGAAACAATTTTACAGTCATTTTTAGAAACGACATCAACAACTGTGCGTGCC
AACCTGACGCGAGCATCATTAGATGATATTATTGAAAAGTTGCAAGACGAAGGTTATGACGTTGAAAAAGATCATGACTTACCTTATTGT
CTCCATATAGGAGGACAACCAATTATTCATTCTCGTTCATTTAAAGATGGATTCGTTTCAATTCAAGATAAAAGCTCAATGTTTGTTGCACA
CATTATGAATGTAGACCGACATGATCACGTATTAGATGCATGTAGTGCACCTGGCGGTAAAGCTTGTCACATTGCTGAAGTTTTAATGCC
AGAAGGGCAAGTTGACGCTTCAGATATACATGATCACAAAATAGACTTAATTAATTTTAATATAAAAAAATTACGATTAACAAATATTAA
AGCTTTTCAACATGATGCGACAAAACCTTATGATAAAACATACGATAAGATACTTGTTGATGCACCATGTAGCGGATTAGGTGTAATGAG
ACATAAGCCGGAGATTAAGTATACTCAAAGCAAACAACATATTGAGTCACTAGTTGAATTACAGCTTGAAATATTGGAAAATGTAAAAAA
CAATGTAAAAATAGGTGGCGAAATCATCTATTCAACATGTACAATTGAGCAACTAGAAAATGAAAACGTGATTTATACGTTTTTGAAAAA
TAATAAAAACTTCGAATTTGAACCGTTTCAACATCCGATAACTGGAGAGTTGGTCAAAACGTTACAAATCATGCCGCAAGACTTTAATTCA
GATGGATTCTTTATCACTAAGATAAAAAGAAAGGACAATTAG

Gene: rlmN (ribosomal RNA large subunit methyltransferase)

Contig: 06_NODE_54, position: 334047 to 335141, length: 1095 nt, orientation: FORWARD

Perfect match to: (CIG1769-AHVG01000014-[62429:63523], allele observed in CC5)

Sequence:

ATGATAACTGCTGAAAAGAAAAAGAAGAATAAATTTCTTCCAAATTTTGACAAGCAATCAATATATTCATTGCGATTTGACGAAATGCAA
AACTGGCTCGTTGAACAAGGTCAACAAAAATTTCGAGCGAAACAGATTTTTGAATGGTTATATCAAAAAAGAGTAGATTCGATTGATGAA
ATGACGAACTTATCGAAAGACTTACGACAGCTTTTAAAAGATAACTTTACTGTTACAACTTTAACAACTGTAGTAAAACAAGAAAGTAAA
GACGGTACAATTAAATTCTTATTTGAATTACAAGATGGCTATACAATTGAAACTGTTTTAATGAGACATGATTATGGAAATTCAGTATGTG
TAACGACACAAGTAGGTTGTCGTATCGGATGTACGTTTTGTGCTTCTACACTTGGCGGCTTAAAAAGAAACCTTGAAGCTGGCGAAATTG

TTTCACAAGTTTTAACAGTTCAAAAAGCCCTTGATGCTACAGAAGAGCGCGTATCTCAAATTGTCATAATGGGTATCGGTGAACCATTTGA
AAATTATGATGAAATGATGGACTTTTTAAGAATCGTCAATGATGATAATAGTTTAAATATTGGTGCACGTCACATTACAGTATCAACATCA
GGTATCATTCCTAGAATATACGACTTTGCGGATGAAGATATCCAAATTAATTTTGCTGTAAGCTTACACGCCGCAAAAGATGAAGTGCGA
TCACGCTTGATGCCAATTAACCGTGCATATAATGTTGAGAAGTTAATCGAAGCAATTCAATATTATCAAGAAAAAACAAATCGTCGTGTTA
CTTTTGAATATGGTCTGTTTGGTGGTGTGAATGACCAACTAGAACATGCAAGAGAATTAGCACATTTAATAAAAGGCTTAAACTGCCATG
TTAACTTAATTCCTGTCAACCATGTTCCAGAAAGAAATTATGTGAAAACGGCTAAAAATGATATCTTTAAATTTGAAAAAGAATTAAAGAG
ACTAGGAATTAATGCCACAATACGTCGTGAACAAGGTTCGGATATTGACGCAGCTTGTGGTCAATTAAGAGCAAAGGAACGACAAGTAG
AAACGAGGTAA

Gene: stp1-prpC (serine/threonine protein phosphatase)

Contig: 06_NODE_54, position: 335148 to 335891, length: 744 nt, orientation: FORWARD

Perfect match to: (MW2-BA000033-[1201443:1202186], allele observed in CC1+CC15+CC772)

Sequence:

ATGCTAGAGGCACAATTTTTTACTGATACTGGACAACATAGAGATAAGAATGAAGATGCGGGTGGTATTTTTTATAATCGAACTAATCAA
CAACTTTTAGTTCTGTGTGATGGTATGGGCGGCCATAAAGCAGGAGAAGTTGCAAGTAAATTTGTTACAGATGAGTTGAAATCCCGTTTT
GAAGCGGAAAATCTTATAGAAGAACATCAAGCTGAAAATTGGTTGCGTAATAATATAAAAGATATAAATTTTCAGTTATATCACTATGCA
CAAGAAAATGCAGAATATAAAGGTATGGGTACAACATGTGTTTGTGCACTTGTTTTTGAAAAATCAGTTGTGATAGCAAATGTCGGTGAT
TCTAGAGCCTATGTTATTAATAGTAGACAAATTGAACAAATTACTAGTGATCACTCATTTGTTAATCATCTTGTTTTAACGGGTCAAATTAC
GCCGGAAGAAGCATTTACACATCCACAACGTAATATTATTACGAAGGTGATGGGCACAGATAAACGTGTGAGTCCAGATTTGTTTATTAA
GCGATTAAATTTTTATGATTATTTATTATTAAATTCAGATGGATTAACTGATTATGTTAAAGACAATGAAATTAAGCGTTTGTTAGTAAAA
GAAGGTACAATAGAAGATCATGGTGATCAATTAATGCAATTGGCATTAGATAACCATTCGAAAGATAACGTTACTTTCATACTCGCGGCT
ATTGAAGGTGATAAAGTATGA

Gene: pknB-prkC (muropeptide sensing serine/threonine protein kinase)

Contig: 06_NODE_54, position: 335888 to 337882, length: 1995 nt, orientation: FORWARD

Perfect match to: (XN108-CP007447-[1258945:1260939], highly conserved allele)

Sequence:

ATGATAGGTAAAATAATAAATGAACGATATAAAATTGTAGATAAGCTTGGCGGCGGTGGCATGAGTACCGTTTATCTTGCTGAAGATAC
GATACTTAACATTAAAGTTGCAATTAAGGCGATTTTTATACCACCTAGAGAAAAAGAAGAAACATTAAAACGTTTTGAACGAGAAGTACA
TAACTCATCACAGCTATCACATCAAAATATAGTAAGTATGATCGATGTTGATGAAGAAGATGACTGTTACTACTTAGTAATGGAATATATT
GAAGGTCCGACTTTGTCTGAGTATATTGAAAGTCATGGGCCATTAAGTGTTGACACGGCGATTAATTTTACGAATCAAATATTGGATGGT
ATTAAACATGCGCATGATATGCGTATTGTACATAGAGATATTAAGCCACAAAATATATTAATTGACAGCAATAAAACGTTGAAAATATTT
GATTTTGGAATTGCTAAAGCTTTAAGTGAGACGTCTTTAACTCAGACTAATCATGTGTTAGGTACTGTGCAGTACTTTTCGCCAGAACAAG
CAAAAGGTGAGGCAACGGATGAATGTACAGATATTTATTCTATAGGTATTGTGTTATATGAAATGCTTGTTGGTGAACCACCCTTTAATG
GAGAAACTGCAGTTAGCATTGCGATTAAACATATTCAGGATTCTGTGCCAAATGTGACAACAGATGTACGTAAGGATATTCCGCAATCTT
TAAGTAATGTCATTTTACGCGCTACAGAAAAAGACAAAGCGAATCGTTACAAAACAATTCAAGAAATGAAAGATGATTTGAGTAGTGTTT
TACATGAAAATCGAGCGAATGAAGATGTCTATGAACTCGATAAAATGAAAACGATAGCGGTACCTTTGAAAAAAGAAGATCTAGCAAAG
CATATTAGTGAACATAAGTCGAATCAACCTAAACGTGAAACGACGCAAGTACCTATTGTAAATGGGCCTGCTCATCATCAGCAATTCCAA
AAGCCAGAAGGTACGGTTTACGAACCAAAACCTAAAAAGAAATCAACACGAAAGATTGTGCTCTTATCACTAATCTTTTCGTTGTTAATG
ATTGCACTTGTTTCTTTTGTGGCAATGGCAATGTTTGGTAATAAATACGAAGAGACACCTGATGTAATCGGGAAATCTGTAAAAGAAGCA
GAGCAAATATTCAATAAAAACAACCTGAAATTGGGTAAAATTTCTAGAAGTTATAGTGATAAATATCCTGAAAATGAAATTATTAAGACA
ACTCCTAATACAGGTGAACGTGTTGAACGTGGTGACAGTGTTGATGTTGTTATATCAAAAGGCCCTGAAAAGGTTAAAATGCCAAATGTC
ATTGGTTTACCTAAGGAGGAAGCCTTGCAGAAATTAAAATCGTTAGGTCTTAAAGATGTTACGATTGAAAAAGTATATAATAATCAAGCG
CCAAAAGGATACATTGCAAATCAAAGTGTAACCGCAAATACTGAAATCGCTATTCATGATTCTAATATTAAACTATATGAATCTTTAGGCA
TTAAGCAAGTTTATGTAGAAGACTTTGAGCATAAATCCTTTAGCAAAGCTAAAAAAGCCTTAGAAGAAAAAGGGTTTAAAGTTGAAAGT
AAGGAAGAGTATAGTGACGATATTGATGAGGGTGATGTGATTTCTCAATCTCCTAAAGGAAAATCAGTAGATGAGGGGTCAACGATTTC
ATTTGTTGTTTCTAAAGGTAAAAAAAGTGACTCATCAGATGTCAAAACGACAACTGAATCGGTAGATGTACCATACACTGGTAAAAATGA
TAAGTCACAAAAAGTTAAAGTTTATATTAAAGATAAAGATAATGACGGTTCAACTGAAAAAGGTAGTTTCGACATTACTAGTGATCAACG
TATAGACATTCCTTTAAGAATTGAAAAAGGAAAAACAGCAAGTTATATTGTTAAAGTTGACGGTAAAACTGTAGCTGAAAAAGAAGTCA
GCTATGATGATGTATAA

Gene: cpgA-engC (ribosome small subunit-dependent GTPase A)

Contig: 06_NODE_54, position: 338110 to 338985, length: 876 nt, orientation: FORWARD

Perfect match to: (N315-BA000018-[1204606:1205481], highly conserved allele)

Sequence:

TTGAAGACAGGTCGAATAGTGAAATCAATTAGTGGGGTATATCAAGTAGACGTTAATGGCGAACGTTTCAATACAAAACCACGAGGATT
ATTTAGAAAGAAAAAATTTTCACCGGTAGTTGGTGATATAGTGGAATTTGAAGTACAAAACATTAACGAAGGCTATATTCATCAAGTGTT
TGAGCGGAAAAATGAGTTGAAAAGACCACCTGTAAGTAATATAGATACACTAGTAATTGTAATGAGTGCTGTCGAGCCAAATTTTTCAAC
GCAATTATTAGATCGATTTTTAGTTATTGCACATTCGTATCAGTTAAATGCGAGAGTTTTGGTGACTAAAAAAGATAAAACACCAATTGAA
AAGCAGTTCGAAATTAATGAGTTGTTGAAAATATATGAAAATATTGGCTATGAGACTGAATTTATTGGAAATGATGATGATCGAAAAAAA
ATTGTAGAAGCTTGGCCAGCTGGACTTATAGTACTTAGTGGTCAATCAGGTGTCGGTAAGTCCACTTTCTTAAATCATTATCGTCCAGAAC
TTAATCTTGAGACAAATGATATATCAAAATCATTAAATCGAGGAAAGCATACTACAAGACATGTCGAACTATTCGAACGTCAAAACGGTT
ATATTGCAGACACACCTGGATTCAGTGCTTTAGATTTTGATCATATAGATAAAGATGAAATAAAAGATTATTTTCTTGAATTAAATCGATA
TGGTGAAACATGTAAGTTTAGGAATTGTAATCATATCAAAGAACCTAATTGTAATGTTAAGCATCAATTAGAGATAGGGAATATTGCGCA
ATTTAGATACGACCATTATTTACAACTATTTAATGAAATTTCAAATAGAAAGGTTAGATATTAA

Gene: rpe (ribulose-5-phosphate 3-epimerase)

Contig: 06_NODE_54, position: 338986 to 339630, length: 645 nt, orientation: FORWARD

Perfect match to: (MW2-BA000033-[1205281:1205925], highly conserved allele)

Sequence:

ATGACAAAACTATATCCATCATTATTATCTGTTGATTTTTTGGATTTACAACATGAATTAAAACGACTTGAAGAAGCAGGTGTCGACGGAG
TTCATTTTGATGTTATGGATGGTCAATTTGTGCCTAATATATCTATTGGTTTACCAATATTAGATGCAGTAAGAAAAGGCACAACATTACCT
ATAGACGTACATTTGATGATTGAAAATCCAGAAAAGTATATTGCATCATTTGCAGAACATGGTGCCGATATGATTTCAATTCATGTCGAAT
CAACGCCTCATATTCATCGTGCTATTCAAATGATTAAACATTTAGATAAAAAAGCTGGTGTAGTAATTAATCCTGGTACACCAATATCACA
AATTGAACCTATTTTAGACATTGTTGATTATGTACTAGTGATGACAGTTAACCCAGGGTTTGGTGGTCAATCATTTATTGATCAATGCGTA
GAAAAAATAGCGGGTCTTAATGCTATTAAAATGGAACGTCAATTAAACTTTGATATTGAAGTTGATGGAGGCGTAAATACCGATACAGC
GAAAGTTTGTGTTGAAAATGGTGCTACAATGCTAGTAACAGGTTCATTTTTCTTTAAACAAGAGGATTATAAAAAAGTCACACAAGCATT
GAAAGGTTGA

Gene: thiN (thiamine pyrophosphokinase)

Contig: 06_NODE_54, position: 339637 to 340278, length: 642 nt, orientation: FORWARD

Perfect match to: (MW2-BA000033-[1205932:1206573], highly conserved allele)

Sequence:

ATGCATATAAATTTATTATGTTCTGATCGACACTTGCCGCAAGATATTTGGGCCAAAAGTAATGAAGGTAAATGGGGCGGCGTTGATAGA
GGTGCTTTGATTTTATTGAAGCATCAAATTATCCCTTTTTTCTCAGTGGGAGACTTTGATTCAGTCAGTAAAGAAGAACGCCAACTTCTAA
CAGAACAGTTACAAATCAAACCAGTTCAAGCTGAAAAAGCTGATACGGATTTAGCTTTAGCGGTTGATAAAGCTGTTGCACTTGGATTTG
ATAGTATTACAATTTATGGTGCAACAGGCGGACGATTAGATCACTTTTTTGGGGCAATTCAGTTATTATTGAAAAAAGCATATTATAAACA
TGATGTTCATATAGAAGTTATCGATCAACAAAATAAAATTGAATTATTGCCTAAAGGTCAACATACAGTTGAAAAAGATAAGAGTTATCC
GTACATTTCATTTATACCGATGACTGATGATGTAGAACTTTCTCTAGCAGGTTTTAAATATAATTTAGCTAGACAAATGCTTAATATAGGTT
CTACTTTAACTATTTCAAATGAAATTGAGTCTTTGCAAGCGAAAGTAACTGTACATGATGGGTTGATTTTGCAAATTAGAAGTACAGATTT
AAATTAA

Gene: rpmB (50S ribosomal protein L28)

Contig: 06_NODE_54, position: 340659 to 340847, length: 189 nt, orientation: REVERSE

Perfect match to: (RF122-AJ938182-[1174752:1174940:r], highly conserved allele)

Sequence:

TTAAACTCTAGTTACTTTACCAGATTTTAAAGCACGTGCAGAAACCCAAACTTTTTTAGGTTTACCGTCAACTAGGATTCTAACTTTTTGAA
GGTTAGCGTTCCATCTACGTTTAGTAGAGTTTAAAGCGTGTGAACGTCTGTTACCAGTCGAAGCTTTACGACCTGTTACGAAACATTGTTT
ACCCAT

Gene: yloU (similar to alkaline-shock protein)

Contig: 06_NODE_54, position: 341290 to 341664, length: 375 nt, orientation: FORWARD

Perfect match to: (RF122-AJ938182-[1175382:1175756], highly conserved allele)

Sequence:

ATGACATTAGAGATTTCAAATGATTACGGTAAAATTGATATTTCAAACGAAGTGATTGCTTCGGTTGTAGGTGGAAAGGCCGTTGAATGT
TATGGTATTGTAGGTATGGCATCTAGACAACAAGTTAGAGATGGTATTGCGGAAATACTAGGACATGAAAATTATGCTAAAGGCATCAA
AGTAACTGAAAATAATGGCGTAGTGGATATAGATATGTACATTATTGTAAGTTACGGTGTGAAAATATCTGAAGTTGCCAATAATGTACA
ATCAACAGTGAAATATACTTTGGAAAAATCACTTAATGTATCAGTAAATTCAATCAATATATATGTACAAGGTGTACGTGTGAATAATACA
GGCAAGAAAGCTTAG

Gene: yloV (putative glycerone kinase)

Contig: 06_NODE_54, position: 341679 to 343325, length: 1647 nt, orientation: FORWARD

Perfect match to: (ED133-CP001996-[1251869:1253515], highly conserved allele)

Sequence:

ATGATTAGCAAAATTAATGGTAAATTATTTGCCGATATGATTATACAAGGGGCACAAAATTTATCTAACAATGCAGATTTGGTAGATTCTT
TGAATGTGTATCCAGTGCCAGATGGTGATACAGGAACAAATATGAATCTTACTATGACTTCAGGTCGCGAAGAAGTAGAGAATAATTTGT
CGAAAAATATCGGCGAATTAGGTAAAACATTCTCGAAAGGTTTACTAATGGGTGCAAGAGGTAACTCTGGTGTCATCTTGTCACAATTAT
TCAGAGGATTTTGTAAAAATATTGAAAGTGAATCTGAAATTAATTCAAAATTGTTAGCTGAAAGTTTTCAAGCTGGTGTTGAAACGGCAT
ATAAAGCTGTTATGAAACCAGTTGAAGGTACAATACTTACAGTTGCAAAAGATGCTGCGCAAGCTGCAATAGAAAAAGCAAATAATACT
GAAGATTGTATAGAATTAATGGAGTACATTATTGTAAAAGCCAATGAATCACTTGAAAACACACCAAACTTATTAGCTGTACTTAAAGAA
GTTGGTGTTGTTGATAGTGGCGGTAAAGGTTTGTTATGCGTTTACGAAGGATTCTTAAAAGCGCTTAAAGGTGAAAAAGTTGAAGCCAA
AGTTGCAAAGATAGATAAAGATGAATTTGTACATGATGAACATGATTTCCATGGTGTAATTAATACTGAAGATATTATTTATGGCTATTGT
ACTGAAATGATGGTTCGTTTTGGAAAGAATAAAAAAGCCTTTGATGAACAAGAATTCAGGCAAGATATGAGTCAATTTGGTGATTCTTTA
TTAGTCATTAATGATGAAGAAATTGTGAAAGTTCACGTGCATACCGAATACCCAGGTAAAGTGTTTAATTATGGTCAACAATATGGTGAA
TTAATTAAACTTAAGGTTGAAAATATGAGAGAACAGCACCGTGAAGTGATTCGAAAAGAACAGCACACAGCTAAACCGAAAATGGAAAC
GGTTGAAACAGCAATTATTACTATTTCTATGGGTGAAGGTATTTCAGAGATATTCAAATCAATGGGTGCCACACATATCATTAGTGGTGG
ACAAACGATGAATCCTTCTACAGAAGATATCGTTAAAGTCATTGAACAATCAAAATGTAAACGTGCAATTATTTTACCGAATAATAAAAAT
ATTTTAATGGCAAGTGAACAAGCAGCGAGTATTGTTGATGCAGAAGCAGTTGTTATTCCAACGAAATCTATTCCTCAAGGTATAAGCGCA
CTATTCCAATATGATGTGGACGCAACACTTGAAGAAAATAAAGCGCAAATGGCTGATTCAGTAAATAACGTTAAATCTGGTTCATTAACG
TACGCTGTTCGTGATACGAAAATTGATGGCGTTGAGATTAAAAAAGACGCGTTTATGGGCTTGATTGAAGATAAGATTGTAAGCAGCCA
AAGTGATCAATTAACAACGGTTACTGAGTTGTTAAATGAGATGTTAGCAGAAGATAGTGAAATATTGACTGTGATTATTGGTCAAGATGC
AGAGCAAGCAGTTACAGATAACATGATAAACTGGATCGAAGAGCAATATCCAGATGTAGAAGTGGAAGTTCATGAAGGTGGACAACCA
ATTTATCAATATTTCTTTTCAGTAGAATAA

Gene: recG (ATP-dependent DNA helicase)

Contig: 06_NODE_54, position: 343515 to 345575, length: 2061 nt, orientation: FORWARD

Sequence:

TTGGCTAAAGTAAACTTAATAGAAAGTCCATATTCTCTTTTACAATTAAAAGGTATAGGTTCTAAGAAAATAGAAGTATTGCAACAACTAA
ATATTCATACAGTGGAAGATCTTGTTCTTTATTTGCCAACTAGATATGAAGATAATACAGTGATTGATTTGAATCAAGCAGAAGATCAATC
TAACGTTACGATAGAAGGACAAGTATATACAGCTCCAGTAGTTGCATTTTTTGGAAGAAATAAATCAAAATTAACCGTTCATTTAATGGTA
AATAATATTGCTGTCAAATGTATTTTTTTCAATCAACCGTATTTAAAAAAGAAAATCGAATTAAATCAAACTATAACTGTTAAAGGTAAGT
GGAATAGAGTTAAACAGGAAATTACTGGTAATAGGGTTTTCTTTAATTCACAAGGGACACAAACTCAAGAAAACGCAGATGTTCAATTA
GAACCAGTCTATCGTATTAAGGAAGGTATTAAACAAAAGCAAATACGAGACCAAATTAGACAAGCGTTAAATGATGTGACAATTCATGA
ATGGTTAACTGATGAACTAAGAGAAAAATATAAATTAGAGACCTTGGACTTTACTTTGAACACATTACATCATCCTAAAAGTAAAGAGGA

TTTATTACGTGCTCGTAGAACCTATGCATTTACTGAACTGTTTTTATTCGAATTACGTATGCAATGGCTAAATAGATTAGAAAAGTCATCTG
ACGAAGCAATTGAAATTGATTATGGCTTAGACCAAGTTAAATCATTTATTGATCGTTTACCTTTTGAACTAACTGAAGCACAGAAATCCAG
TGTTAATGAAATTTTTAGAGATTTAAAAGCACCAATACGTATGCATCGATTACTTCAAGGTGATGTAGGTTCAGGAAAAACAGTAGTTGC
TGCAATTTGTATGTATGCGTTAAAAACGGCTGGTTATCAATCAGCATTGATGGTACCAACTGAAATTTTAGCAGAGCAACATGCTGAAAG
TTTAATGGCTTTATTTGGAGATTCTATGAACGTTGCATTGTTAACTGGGTCAGTAAAAGGTAAGAAACGAAAGATACTTTTAGAACAACTT
GAAAATGGTACGATTGATTGTTTAATTGGAACCCATGCTTTGATTCAAGATGATGTGATTTTCCATAATGTTGGTTTAGTAATTACAGATG
AACAACATCGATTTGGTGTGAATCAACGCCAGCTTTTAAGAGAAAAAGGTGCAATGACGAATGTGTTATTTATGACAGCAACCCCGATAC
CAAGAACACTAGCAATATCAGTTTTTGGTGAGATGGATGTGTCTTCAATTAAACAATTACCAAAAGGTCGTAAACCTATCATTACTACTTG
GGCAAAGCATGAGCAATACGATAAAGTTTTGATGCAAATGACCTCAGAGTTGAAAAAAGGTCGTCAAGCATATGTCATTTGCCCGCTAAT
AGAAAGTTCTGAGCATCTCGAAGATGTTCAAAATGTTGTCGCATTGTACGAGTCTTTACAACAGTATTATGGTGTTTCCCGTGTAGGGTTA
TTGCATGGTAAGTTATCTGCCGATGAAAAAGATGAGGTCATGCAAAAGTTTAGTAATCATGAGATAGATGTTTTAGTTTCTACTACTGTTG
TTGAAGTAGGTGTTAATGTACCGAATGCAACTTTTATGATGATTTATGATGCGGATCGCTTTGGATTATCAACTTTACATCAGTTACGCGG
TCGTGTAGGTAGAAGTGACCAGCAAAGTTACTGTGTTTTAATTGCATCCCCTAAAACAGAAACAGGAATTGAAAGAATGACAATTATGAC
ACAAACAACGGATGGATTTGAATTGAGTGAACGAGACTTAGAAATGCGTGGTCCTGGAGATTTCTTTGGTGTTAAACAAAGTGGGTTGC
CAGATTTCTTAGTTGCCAATTTAGTTGAAGATTATCGTATGTTAGAAGTTGCTCGTGATGAAGCAGCTGAACTTATTCAATCTGGCGTATT
CTTTGAAAATACGTATCAACATTTACGTCATTTTGTTGAAGAAAATTTATTACATCGCAGTTTTGACTAA

Gene: fapR (fatty acid and phospholipid biosynthesis transcriptional regulator)

Contig: 06_NODE_54, position: 345793 to 346350, length: 558 nt, orientation: FORWARD

Perfect match to: (RF122-AJ938182-[1179874:1180431], highly conserved allele)

Sequence:

TTGAAACTAAAGAAAGATAAACGTAGAGAAGCAATCAGACAACAAATTGATAGCAATCCCTTCATCACAGACCATGAACTAAGCGACTT
ATTTCAAGTGAGTATACAAACAATTCGTTTAGATCGCACTTATTTAAACATACCAGAATTAAGGAAGCGTATTAAATTAGTTGCTGAAAAG
AATTATGACCAAATAAGTTCTATTGAAGAACAAGAATTTATTGGTGATTTGATTCAAGTCAATCCAAATGTTAAAGCGCAATCAATTTTAG
ATATTACATCGGATTCTGTTTTTCATAAAACTGGAATTGCGCGTGGTCATGTGCTGTTTGCTCAGGCAAATTCGTTATGTGTTGCGCTAATT
AAGCAACCAACAGTTTTAACTCATGAGAGTAGCATTCAATTTATTGAAAAAGTAAAATTAAATGATACGGTAAGAGCAGAAGCACGAGT
TGTAAATCAAACTGCAAAACATTATTACGTCGAAGTAAAGTCATATGTTAAACATACATTAGTTTTCAAAGGAAATTTTAAAATGTTTTAT
GATAAGCGAGGATAA

Gene: plsX (glycerol-3-phosphate acyltransferase)

Contig: 06_NODE_54, position: 346355 to 347341, length: 987 nt, orientation: FORWARD

Perfect match to: (MW2-BA000033-[1212650:1213636], allele observed in CC1+CC239)

Sequence:

ATGGTTAAATTAGCAATTGATATGATGGGTGGCGACAATGCGCCTGATATCGTATTAGAAGCCGTACAAAAGGCTGTTGAAGACTTTAA
AGATCTAGAAATTATACTTTTCGGTGACGAAAAAAAGTATAATCTGAACCATGAACGAATCGAATTTAGACATTGTTCTGAAAAGATTGA
AATGGAAGATGAGCCTGTTAGAGCGATTAAACGTAAAAAAGATAGCTCAATGGTAAAAATGGCTGAAGCTGTGAAATCTGGTGAAGCA
GATGGATGTGTGTCAGCAGGTAATACTGGTGCTTTAATGTCAGCTGGTTTATTCATTGTTGGACGTATTAAAGGTGTAGCTAGACCGGCT
TTAGTAGTAACATTGCCAACGATTGATGGAAAAGGTTTTGTATTTTTAGACGTTGGTGCAAATGCTGATGCTAAACCTGAACACTTATTAC
AGTATGCGCAACTAGGGGATATTTATGCTCAAAAAATCAGAGGTATTGATAATCCGAAAATCTCATTATTAAATATAGGTACCGAGCCAG
CTAAAGGTAATAGTTTAACGAAAAAATCATATGAGTTATTAAATCAAGATCATTCATTGAATTTTGTTGGGAATATTGAAGCGAAGACATT
AATGGATGGCGATACAGATGTTGTAGTTACCGATGGCTATACTGGGAACATGGTCCTTAAAAATTTAGAAGGTACTGCAAAATCAATCG
GTAAAATGTTAAAAGATACGATTATGAGTAGTACTAAAAATAAATTAGCAGGTGCAATATTGAAGAAAGATTTAGCAGAATTCGCTAAA
AAGATGGATTACTCAGAATACGGTGGTTCAGTATTATTAGGATTGGAAGGTACTGTAGTTAAAGCACACGGTAGTTCAAATGCTAAAGC
TTTTTATTCTGCAATTAGACAAGCGAAAATCGCAGGAGAACAAAATATTGTACAAACAATGAAAGAGACTGTAGGTGAATCAAATGAGT
AA

Gene: fabD (malonyl CoA-acyl carrier protein transacylase)

Contig: 06_NODE_54, position: 347334 to 348260, length: 927 nt, orientation: FORWARD

Perfect match to: (04-02981-CP001844-[1248419:1249345], allele observed in CC5)

Sequence:

ATGAGTAAAACAGCAATTATTTTTCCGGGACAAGGTGCCCAAAAAGTTGGTATGGCACAAGATTTGTTTAATAACAATGATCAAGCAACT
GAAATTTTAACTTCAGCAGCAAAGACGTTAGACTTTGATATTTTAGAGACAATGTTTACTGATGAAGAAGGTAAATTGGGTGAAACTGAA
AACACGCAACCAGCTTTATTGACGCATAGTTCGGCATTATTAGCAGCGCTAAAAAATTTGAATCCTGATTTTACTATGGGGCATAGTTTAG
GTGAATATTCAAGTTTAGTTGCAGCTGACGTATTATCATTTGAAGATGCAGTTAAAATTGTTAGAAAACGTGGTCAATTAATGGCGCAAG
CATTTCCTACTGGTGTAGGAAGTATGGCTGCAGTATTGGGCTTAGATTTTGATAAAGTCGATGAAATTTGTAAGTCATTATCATCTGATGA
CAAAATAATTGAACCAGCAAACATTAATTGCCCAGGTCAAATTGTTGTTTCAGGTCACAAAGCTTTAATTGATGAGCTAGTAGAAAAAGG
TAAATCATTAGGTGCAAAACGTGTCATGCCTTTAGCAGTATCTGGTCCATTCCATTCATCGCTAATGAAAGTGATTGAAGAAGATTTTTCA
AGTTATATTAATCAATTTGAATGGCGTGATGCTAAGTTTCCTGTAGTTCAAAATGTAAATGCGCAAGGTGAAACTGACAAAGAAGTAATT
AAATCTAATATGGTCAAGCAATTATATTCACCAGTACAATTCATTAACTCAACAGAATGGCTAATAGACCAAGGTGTTGATCATTTTATTG
AAATTGGTCCTGGAAAAGTTTTATCTGGCTTAATTAAAAAAATAAATAGAGATGTTAAGTTAACATCAATTCAAACTTTAGAAGATGTGA
AAGGATGGAATGAAAATGACTAA

Gene: fabG1 (beta-ketoacyl-[acyl-carrier-protein] reductase)

Contig: 06_NODE_54, position: 348253 to 348987, length: 735 nt, orientation: FORWARD

Perfect match to: (RF122-AJ938182-[1182334:1183068], highly conserved allele)

Sequence:

ATGACTAAGAGTGCTTTAGTAACAGGTGCATCAAGAGGAATTGGACGTAGTATTGCGTTACAATTAGCAGAAGAAGGATATAATGTAGC
AGTAAACTATGCAGGCAGCAAAGAGAAAGCTGAAGCAGTAGTCGAAGAAATCAAAGCTAAAGGTGTTGACAGTTTTGCGATTCAAGCA
AATGTTGCCGATGCTGATGAAGTTAAAGCAATGATTAAAGAAGTAGTTAGCCAATTTGGTTCTTTAGATGTTTTAGTAAATAATGCAGGT
ATTACTCGCGATAATTTATTAATGCGTATGAAAGAACAAGAGTGGGATGATGTTATTGACACAAACTTAAAAGGTGTATTTAACTGTATC
CAAAAAGCAACACCACAAATGTTAAGACAACGTAGTGGTGCTATCATCAATTTATCAAGTGTTGTTGGAGCAGTAGGTAATCCGGGACA
AGCAAACTATGTTGCAACAAAAGCAGGTGTTATTGGTTTAACTAAATCTGCGGCGCGTGAATTAGCATCTCGTGGTATCACTGTAAATGC
AGTTGCACCTGGTTTTATTGTTTCTGATATGACAGATGCTTTAAGTGATGAGCTTAAAGAACAAATGTTGACTCAAATTCCGTTAGCACGT
TTTGGTCAAGACACAGATATTGCTAATACAGTAGCGTTCTTAGCATCAGACAAAGCAAAATATATTACAGGTCAAACAATCCATGTAAAT
GGTGGAATGTACATGTAA

Gene: SIRU13 (staphylococcal interspersed repeat unit 13)

Contig: 06_NODE_54, position: 349174 to 349202, length: 29 nt

Perfect match to: (MW2-BA000033-[1215597:1215625], allele observed in CC1)

Sequence:

TAAGAAACACTAATCAATAAATTGATAAG

Gene: SIRU13 (staphylococcal interspersed repeat unit 13)

Contig: 06_NODE_54, position: 349238 to 349266, length: 29 nt

Perfect match to: (MW2-BA000033-[1215597:1215625], allele observed in CC1)

Sequence:

TAAGAAACACTAATCAATAAATTGATAAG

Gene: acpP (acyl carrier protein)

Contig: 06_NODE_54, position: 349357 to 349590, length: 234 nt, orientation: FORWARD

Perfect match to: (RF122-AJ938182-[1183276:1183509], highly conserved allele)

Sequence:

GTGGAAAATTTCGATAAAGTAAAAGATATCATCGTTGACCGTTTAGGTGTAGACGCTGATAAAGTAACTGAAGATGCATCTTTCAAAGAT
GATTTAGGCGCTGACTCACTTGATATCGCTGAATTAGTAATGGAATTAGAAGACGAGTTTGGTACTGAAATTCCTGATGAAGAAGCTGAA
AAAATCAACACTGTTGGTGATGCTGTTAAATTTATTAACAGTCTTGAAAAATAA

Gene: rnc (ribonuclease 3)

Contig: 06_NODE_54, position: 349706 to 350437, length: 732 nt, orientation: FORWARD

Perfect match to: (MW2-BA000033-[1216065:1216796], highly conserved allele)

Sequence:

ATGTCTAAACAAAAGAAAAGTGAGATAGTTAATCGTTTTAGAAAGCGCTTTGATACTAAAATGACAGAGTTAGGCTTTACTTATCAAAAT
ATTGATTTATACCAACAAGCATTTTCGCATTCGAGTTTTATTAATGATTTTAATATGAATCGTTTAGACCATAATGAGCGTTTAGAGTTTTT
GGGTGATGCGGTATTAGAATTGACGGTTTCACGATATTTATTTGATAAACATCCCAACTTGCCAGAAGGGAATTTAACAAAAATGCGTGC
CACTATTGTATGTGAGCCCTCACTTGTAATATTTGCGAATAAAATTGGATTGAACGAAATGATTTTACTTGGTAAAGGTGAAGAGAAAAC
AGGGGGACGTACAAGACCATCATTAATATCAGATGCATTCGAAGCATTTATTGGGGCATTGTATTTGGATCAAGGACTAGATATAGTTTG
GAAATTTGCTGAGAAAGTCATTTTCCCACATGTAGAACAAAATGAGTTATTAGGCGTGGTAGATTTTAAAACACAATTCCAAGAATATGT
GCACCAGCAAAATAAAGGTGATGTAACCTATAATTTAATAAAAGAAGAGGGACCGGCACATCATCGTCTATTCACTTCAGAAGTTATTCT
GCAAGGGGAAGCAATAGCTGAAGGTAAAGGGAAAACGAAAAAAGAATCAGAACAACGTGCTGCTGAAAGTGCCTATAAGCAATTAAA
ACAAATTAAATAG

Gene: smc (chromosome segregation protein SMC)

Contig: 06_NODE_54, position: 350584 to 354150, length: 3567 nt, orientation: FORWARD

Perfect match to: (08-02119-CP015645-[953309:956875:r], allele observed in ST582+CC8)

Sequence:

ATGGTTTATTTAAAATCAATAGATGCCATTGGATTTAAGTCTTTTGCAGATCAAACCAATGTTCAATTCGATAAAGGTGTAACTGCAATTG
TTGGTCCAAATGGAAGCGGTAAAAGTAATATTACAGATGCTATTAAATGGGTGTTGGGTGAACAATCGGCTAAATCATTACGTGGCTCA
AAAATGGAAGATATTATCTTCTCAGGTGCAGAACATCGCAAAGCTCAAAATTATGCTGAAGTACAGTTAAGATTAGATAATCATTCTAAA
AAGCTCAGTGTTGATGAAAACGAAGTTATTGTAACAAGAAGATTGTATCGAAGTGGTGAAAGTGAGTACTACATAAATAATGACCGTGC
AAGATTAAAAGATATTGCCGATTTATTTTTAGATTCTGGATTGGGAAAAGAAGCGTATAGCATTATCTCGCAAGGTAGAGTTGATGAAAT
ACTAAATGCTAAACCAATTGATAGACGTCAAATTATTGAAGAATCGGCTGGTGTACTTAAATATAAAAAACGTAAAGCTGAATCATTAAA
TAAACTTGACCAAACAGAAGATAATTTAACGAGAGTAGAAGACATTTTATATGATTTGGAAGGTCGTGTAGAACCTCTAAAAGAGGAGG
CAGCTATAGCTAAAGAATATAAGACACTTTCACATCAAATGAAACATAGTGACATTGTAGTTACAGTGCACGATATTGATCAATATACAA
ATGACAATAGACAATTAGATCAACGTTTAAATGATTTACAAGGCCAACAAGCAAATAAAGAAGCTGACAAACAACGTTTAAGCCAACAA
ATTCAACAATATAAAGGTAAACGTCATCAACTTGATAATGATGTTGAATCGCTTAATTATCAATTAGTAAAAGCTACGGAAGCCTTTGAAA
AATATACGGGACAATTAAATGTTTTAGAAGAACGTAAGAAAAATCAATCTGAAACAAATGCACGATATGAAGAAGAACAAGAAAATTTA
ATGGAGCTTTTAGAAAATATATCAAATGAGATTTCTGGAGCTCAAGATACTTATAAGTCTCTGAAAAGTAAACAAAAAGAACTCAATGCT
GTCATTCGTGAACTTGAAGAACAACTTTATGTTTCAGACGAAGCACATGATGAAAAAATTGGAAGAAATTAAAAACGAATACTATACATTA
ATGTCAGAGCAATCAGATGTTAACAATGATATTCGTTTTTTAAAGCATACTATAGAAGAGAATGAGGCTAAAAAATCAAGACTAGATTCT
CGATTAGTTGAAGTTTTTGAGCAATTGAAAGATATTCAGGGTCAAATAAAAACGACAAAAAAAGAATATCAACAGACCAACAAAGAACT
TTCTGCTGTAGATAAAGAAATTAAAAATATAGAAAAAGATCTCACTGATACAAAAAAAGCACAAAATGAATACGAAGAGAAATTGTATC
AAGCATATCGATATACCGAAAAAATGAAAACACGTATTGATAGTTTGGCAACGCAAGAGGAAGAATATACTTATTTTTTCAATGGCGTCA
AACATATTTTGAAAGCTAAAAATAAAGAATTAAAGGGTATTCATGGTGCAGTTGCGGAAATTATTGATGTGCCATCTAAATTAACTCAGG
CAATTGAAACAGCATTAGGTGCTTCATTACAACATGTCATTGTAGATTCAGAAAAAGATGGACGCCAGGCTATTCAATTTTTAAAAGAAC
GTAATTTAGGTCGTGCGACGTTTTTACCATTAAATGTTATACAGAGTAGAGTGGTAGCGACTGATATTAAATCTATTGCTAAAGAGGCAA
ACGGATTTATTAGTATTGCTTCGGAAGCAGTTAAAGTAGCACCAGAATATCAAAATATTATCGGGAATTTATTAGGTAATACGATTATCGT
TGATCATTTAAAGCATGCAAATGAATTGGCACGTGCGATTAAATATCGAACTCGTATTGTTACTTTGGAAGGTGATATTGTAAACCCTGGT
GGTTCTATGACTGGTGGTGGCGCTCGTAAGTCCAAAAGTATTCTGTCTCAAAAAGACGAGTTGACAACAATGAGACACCAATTAGAAGA
TTACTTGCGTCAAACAGAATCATTTGAACAACAATTTAAAGAGTTGAAGATAAAAAGTGATCAATTAAGTGAACTGTATTTTGAAAAAAG
TCAAAAGCATAATACACTTAAAGAGCAAGTGCATCATTTTGAAATGGAGCTCGATAGATTAACTACACAAGAAACACAAATAAAAAATGA
TCATGAAGAATTCGAATTTGAAAAAAATGATGGTTATACGAGTGACAAAAGTCGACAAACTTTGAGTGAAAAAGAAACTCATTTAGAAA
GTATTAAAGCATCTTTAAAACGACTAGAAGATGAAATTGAACGCTACACAAAACTTTCTAAAGAAGGTAAGGAAAGCGTAACTAAAACA
CAACAAACGTTACATCAGAAACAATCTGATCTTGCTGTGGTTAAAGAGCGTATTAAAACACAACAACAGACAATAGATCGATTAAATAAT
CAAAATCAACAAACTAAACATCAATTAAAAGATGTTAAAGAAAAAATTGCATTGTTTAATTCGGATGAAGTGATGGGCGAACAAGCTTTT
CAAAATATTAAAGATCAAATTAATGGTCAACAAGAAACGAGAACACGCTTATCTGATGAATTAGATAAATTGAAACAACAACGTATTGAG

TTGAATGAACAAATCGATGCGCAAGAAGCTAAACTACAAGTTTGTCACCAAGATATTTTAGCTATCGAAAATCACTACCAAGATATTAAA
GCTGAACAATCAAAGCTAGATGTATTAATTCATCATGCGATAGATCATTTAAATGATGAATATCAATTGACTGTTGAACGTGCGAAATCTG
AATATACGAGTGATGAATCGATTGACGCATTACGTAAAAAAGTTAAGTTAATGAAGATGTCGATTGATGAACTAGGTCCTGTAAACTTAA
ATGCAATTGAACAATTTGAAGAGTTAAATGAACGTTATACATTTTTAAGTGAACAACGTACAGATCTTCGTAAAGCTAAAGAAACATTAG
AGCAAATTATAAGTGAAATGGATCAAGAGGTTACTGAAAGATTTAAAGAAACTTTCCATGCTATTCAAGGACATTTTACAGCTGTGTTCA
AACAATTGTTTGGTGGAGGCGATGCAGAATTGCAATTAACTGAAGCCGATTATTTAACAGCTGGTATTGATATTGTGGTACAACCACCGG
GTAAAAAGTTGCAACATTTATCGTTACTGAGTGGTGGTGAGCGTGCATTAACTGCTATTGCTTTACTATTTGCAATTTTAAAAGTAAGATC
TGCACCTTTTGTTATATTAGATGAGGTTGAAGCTGCACTAGATGAAGCAAATGTTATTAGATACGCAAAATATTTAAATGAGTTATCAGAC
GAAACACAATTCATTGTTATTACACACCGTAAAGGAACAATGGAATTTGCGGATAGGTTATACGGTGTAACAATGCAAGAATCAGGTGTT
ACTAAACTTGTGAGTGTGAATTTAAATACAATAGATGATGTGTTGAAGGAGGAGCAATAA

Gene: ylxM (modulator of signal recognition partical GTPase activity)

Contig: 06_NODE_54, position: 355387 to 355719, length: 333 nt, orientation: FORWARD

Sequence:

ATGGGCCAAAATGATTTAGTTAAAACGTTACGAATGAATTATTTGTTTGATTTTTATCAATCCTTATTGACGAATAAACAACGTAATTATTT
GGAATTATTTTATCTTGAAGATTATTCTTTAAGTGAAATCGCAGATACTTTTAATGTGAGTAGACAAGCAGTTTATGATAATATAAGAAGA
ACTGGCGATTTAGTTGAAGATTATGAAAAGAAATTGGAATTATACCAGAAATTTGAGCAACGCCGAGAAATATATGATGAAATGAAACA
ACATTTAAGTAATCCAGAACAAATACAACGTTATATTCAACAATTAGAAGACTTAGAATAG

Gene: ffh (signal recognition particle protein subunit)

Contig: 06_NODE_54, position: 355745 to 357112, length: 1368 nt, orientation: FORWARD

Perfect match to: (DAR4145-CP010526-[1274410:1275777], allele observed in CC772+CC1+CC239+CC772)

Sequence:

ATGGCATTTGAAGGGTTATCAGAACGCTTGCAAGCGACGATGCAAAAAATGCGTGGTAAGGGTAAACTTACTGAAGCTGATATAAAGAT
AATGATGCGTGAAGTAAGATTAGCGTTACTTGAGGCTGACGTAAACTTTAAAGTGGTAAAAGAATTTATTAAAACAGTATCAGAACGCG
CATTAGGTTCCGATGTAATGCAATCATTAACACCAGGGCAACAAGTTATTAAAATAGTTCAAGATGAATTAACGCAGTTGATGGGTGGAG
AAAATACGTCGATTAATATGTCAAATAAACCACCTACTGTTGTTATGATGGTTGGTTTACAAGGTGCTGGTAAAACAACAACTGCAGGTA
AATTAGCATTATTGATGCGTAAAAAATACAACAAAAAACCTATGTTAGTTGCAGCAGATATTTATCGTCCAGCAGCGATAAATCAATTACA
AACAGTAGGGAAACAAATTGATATTCCTGTATACAGTGAAGGAGATCAAGTAAAGCCACAACAAATTGTAACTAATGCATTAAAACATG
CTAAAGAAGAACATTTAGACTTTGTAATCATTGATACAGCAGGTCGATTACACATCGATGAAGCATTGATGAACGAATTAAAAGAAGTAA
AAGACATTGCTAAACCAAACGAAATTATGTTAGTTGTCGATTCAATGACGGGTCAAGATGCTGTCAATGTTGCAGAATCTTTTGACGATC
AACTTGATGTCACAGGTGTTACCTTAACTAAATTAGATGGTGATACACGTGGTGGTGCAGCTTTATCTATTCGTTCGGTGACACAAAAACC
AATTAAATTTGTTGGTATGAGTGAAAAGTTAGATGGTTTAGAGCTATTCCATCCTGAACGTATGGCATCACGTATTTTAGGTATGGGTGA
TGTGTTAAGTTTAATTGAAAAAGCGCAACAAGATGTGGATCAAGAAAAAGCAAAAGATTTAGAGAAAAAGATGCGTGAGTCATCGTTTA
CTTTAGATGATTTTTTAGAACAACTTGATCAGGTGAAAAATCTAGGACCACTGGATGATATTATGAAAATGATTCCAGGTATGAATAAAA
TGAAAGGGCTAGATAAGCTTAATATGAGTGAAAAGCAAATTGATCATATTAAAGCGATTATCCAGTCAATGACGCCGGCTGAAAGAAAC
AATCCAGACACATTGAATGTATCACGTAAAAAGCGTATTGCTAAAGGGTCTGGTCGTTCATTACAAGAAGTCAATCGTTTGATGAAACAA
TTTAACGATATGAAGAAAATGATGAAACAATTCACTGGTGGCGGTAAAGGTAAAAAAGGTAAACGCAATCAAATGCAAAATATGTTAAA
AGGTATGAATTTACCGTTTTAA

Gene: rpsP (30S ribosomal protein S16)

Contig: 06_NODE_54, position: 357547 to 357822, length: 276 nt, orientation: FORWARD

Perfect match to: (N315-BA000018-[1224108:1224383], highly conserved allele)

Sequence:

ATGGCAGTTAAAATTCGTTTAACACGTTTAGGTTCAAAAAGAAATCCATTCTATCGTATCGTAGTAGCAGATGCTCGTTCTCCACGTGACG
GACGTATCATCGAACAAATCGGTACTTATAACCCAACGAGCGCTAATGCTCCAGAAATTAAAGTTGACGAAGCGTTAGCTTTAAAATGGT
TAAATGATGGTGCGAAACCAACTGATACAGTTCACAATATCTTATCAAAAGAAGGTATTATGAAAAAATTTGACGAACAAAAGAAAGCTA
AGTAA

Gene: rimM (16S rRNA processing protein)

Contig: 06_NODE_54, position: 358010 to 358513, length: 504 nt, orientation: FORWARD

Perfect match to: (N315-BA000018-[1224571:1225074], highly conserved allele)

Sequence:

ATGAGAGTTGAAGTTGGTCAAATTGTTAACACACATGGTATTAAAGGTGAAATTAAAGTAAAATCCAATTCAGACTTTACAGACGTTCGT
TTTCAACCCGGTCAAGTGCTGACAGTTGTGCATAACAATAACGACCTTGAATATACTGTTAAGTCACATAGAGTGCATAAAGGGCTTCAT
ATGCTTACATTTGAAGGTATTAATAATATTAATGATATTGAGCATTTAAAAGGGAGTTCTATTTATCAAGAGCGTGATCATGAAGATATCG
TACTTGAGGAAAATGAATTTTATTATTCAGATATTATAGGATGTACAGTTTTTGATGATCAAGAAACACCAATAGGTCGTGTAATTAATAT
ATTTGAAACAGGTGCGAATGATGTGTGGGTGATTAAAGGATCTAAAGAATATTTGATTCCTTATATTGCTGATGTTGTAAAAGAAGTGGA
TGTTGAAAATAAAAAAATTATCATCACGCCAATGGAAGGATTGTTGGATTAA

Gene: trmD (tRNA  (guanine-N (1)-)-methyltransferase)

Contig: 06_NODE_54, position: 358513 to 359250, length: 738 nt, orientation: FORWARD

Perfect match to: (RF122-AJ938182-[1192339:1193076], highly conserved allele)

Sequence:

ATGAAAATTGATTATTTAACTTTATTTCCTGAAATGTTTGATGGTGTTTTAAATCATTCAATTATGAAACGTGCCCAAGAAAACAATAAATT
ACAAATCAATACGGTTAATTTTAGAGATTATGCAATTAACAAGCACAACCAAGTAGATGATTATCCGTATGGTGGCGGACAAGGTATGGT
GTTAAAGCCTGAACCTGTTTTTAATGCGATGGAAGACTTAGATGTCACAGAACAAACACGCGTTATTTTAATGTGTCCACAAGGCGAGCC
ATTTTCACATCAGAAAGCTGTTGAATTAAGCAAGGCCGACCACATCGTTTTCATATGCGGACATTATGAAGGTTACGATGAACGTATCCG
AACACATCTTGTCACAGATGAAATATCAATGGGTGACTATGTTTTAACTGGTGGAGAATTGCCAGCGATGACCATGACTGATGCTATTGT
TAGACTGATTCCAGGTGTTTTAGGTAATGAACAGTCACATCAAGACGATTCATTTTCAGATGGGTTATTAGAGTTTCCGCAATATACACGT
CCGCGTGAATTTAAGGGTCTAACAGTTCCAGATGTTTTATTGTCTGGAAATCATGCCAATATTGATGCATGGAGACATGAGCAAAAGTTG
ATCCGCACATATAATAAAAGACCTGACTTAATTGAAAAATATCCATTAACTAATGCAGATAAGCAAATATTAGAAAGATATAAAATAGGA
TTGAAAAAAGGTTAG

Gene: rplS (50S ribosomal protein L19)

Contig: 06_NODE_54, position: 359353 to 359698, length: 346 nt, orientation: TRNC-FRWD (no stop codon)

Sequence:

ATGACAAATCACAAATTAATCGAAGCAGTAACTAAATCACAATTACGTACAGACTTACCTAGTTTCCGTCCTGGTGATACTTTACGTGTAC
ACGTACGTATCATTGAGGGTACTCGTGAGCGTATCCAAGTATTCGAAGGCGTTGTAATTAAACGTCGTGGCGGTGGCGTTTCTGAAACGT
TTACAGTTCGTAAAATTTCATCAGGTGTTGGCGTGGAACGTACATTCCCATTACACACACCAAAAATTGAAAAAATCGAAGTTAAACGTC
GTGGTAAAGTACGTCGTGCTAAATTATATTACTTACGTAGTTTACGTGGTAAAGCTGCTAGAATCCAAGAAATTC

Gene: SIRU01 (staphylococcal interspersed repeat unit 1)

Contig: 06_NODE_54, position: 359990 to 360157, length: 168 nt

Sequence:

GGCCCCAACACAGAAGCTGACGAAAAGTCAGCTTACAATAATGTGCAAGTTGGGGTGTGGGCCCCAACAAAGAGAATTTCGAAAAGAA
ATTCTACAGGCAATGCAAGTTGGGGATGGGCCCCAACAAAGAGAATTTCGAAAAGAAATTCTACAGACAATGCAAGTTGG

Gene: STAR (Staphylococcus aureus repeat element)

Contig: 06_NODE_54, position: 360044 to 360264, length: 221 nt

Sequence:

GTGTGGGCCCCAACAAAGAGAATTTCGAAAAGAAATTCTACAGGCAATGCAAGTTGGGGATGGGCCCCAACAAAGAGAATTTCGAAAA
GAAATTCTACAGACAATGCAAGTTGGCGGGGCCCCAACATAGAAGCTGGCGGAAAGTCAGCTTACAATAATGTGCAAGTTGGGGTGGG
ACGACGAAATAAATTTTGCGAAAATATCATTTCTGTCCCACTCCC

Gene: SIRU01 (staphylococcal interspersed repeat unit 1)

Contig: 06_NODE_54, position: 360049 to 360213, length: 165 nt

Sequence:

GGCCCCAACAAAGAGAATTTCGAAAAGAAATTCTACAGGCAATGCAAGTTGGGGATGGGCCCCAACAAAGAGAATTTCGAAAAGAAAT
TCTACAGACAATGCAAGTTGGCGGGGCCCCAACATAGAAGCTGGCGGAAAGTCAGCTTACAATAATGTGCAAGTTGG

Gene: yfhO (putative membrane protein)

Contig: 06_NODE_54, position: 360287 to 362893, length: 2607 nt, orientation: REVERSE

Perfect match to: (08-02119-CP015645-[944564:947170], allele observed in ST582+CC12+CC20+CC1156)

Sequence:

TTATTTTTGTCTTGCCCATCTCGTGAAAATAATACTACATATAATGCCAAATATAGTAATTGTTATAAGTAAATAATAGTATGGTGGGGCA
TAGCTCAATTGAATCTTTGTTATATTTTTAGGTGCTTTAATGCCGGTCATAACACCATTTACTTGTTCAACTTTAAGACTTTGATCACCTGAT
GTCGCTTTCATACCTTGATTATATGCTGTTGGCAAAACAATATACCCAGATGAATTTTTATTTTTAGTAATAGTATAACCTTGCTTTGTCTTA
CTAACTTTGACAGCCTCTAATGAATTTGAAGCGTCTTTAAGCGTGGTATAATCTTCGCCGTATATCCCTTTTAAATTTACACGATACTTACC
TTTAGGCAATGATAATCTAATCCTATCTGAAGCTTTAATGCGTATTGTTACGGGTGTTACAAAGCGTCGATATTTATAAGTGAGTTTATTTC
TTTCTTGTGTATATTCATTCACTTTAACATCATGAGCTTTATCCGGCGAAAGTAATTCTAAATCCATTTCAAAATACAAATCTTTAAATTGAT
TAGAAACTGATTTTGGCAACTGTACAGTTAGACCACCATTATTTTGTTTAACTTGTAATAAATGTTTTGTAGGAGATTGCCAGGCTGCACT
ATTTAATTTAATTGTTGAATCTGATAGTAAATTTTTATTGGCTTTAAAATGTGTATTAACATCTTTAGTCTTGTTAGAAACAATCCCTTGCAA
CATTGCTTGTTCTTTATCTAATGGAGATTTTAATTCTTTATTGGAAAAGACCTTATTTGTAATATGTGCACTTGGATAATGGATGGTATTTTT
AGAATGAATCCAACGAACTTTATTGTCTTTGTGTTCAGACTTAATTTTAAATCCATATGGTAAGTTGTCATCATGATTCACTCTAATTCGAT
CATTAACATTCCAAAGTGATAGTAAATTTTGACGATTGCCAAGTAATCTATAAGTGCTGTTTTTATCGATTGGCATATTAATTTGGAGTGTC
TTGTCATAATATTTTAATATGTCTCCATTAAAAATACTAGAATATAATGAAATGCCATTATAATGATATATAAATGGTGAATTTAATGCATA
GTCTGACATATAATCAATGCGATTAAATGGACCAGTTGCATTTTGATTTATCTTTTTTATAAGCTGGTTTACATAGTTACTATGGTAATCAT
GTTGTTTCAACGTTGATAATGATTGTTGATAAGGTTTGATTGCCATATTTTTATTGTTATCTAAAATGACGATTTGTTGAATCATAACGATT
AATACTAATATTGCAACGGTTAATTTTTTATAACGCCATAAACTAAATTTTAAAATAACGGCAAGCACCATTAGCAGGATAATACCTACTA
TAAGTGCAAGTGGGTGTGTCGGTGATAGTAATACATAAAGTAATGCGATGATGCTTACTGGTATTGTTCTGATTAAATAATATTTCATATT
TAATGTTGATAAATGTTGAATAAACAATCCGCAAAGAGCGCTTGATGATAGTGCTAAGATATACACCCAACGCCTTTCTGGAAATGAAAA
ACCATTAAAAGCACTGTCGAAATACTGTGATAATGAACCAATAAATAATAACCATGTTACTATTGCGAAAAGTCTATAAAAGTAAAAACG
ATACAGTTTGAATGACAATAATGCAACGATAGTAAGAATTGAAATCGTAATATAAAATCCATCGCTAAAGAAAAAATAATGGTAATCAAG
TGGTGTCAAAAACGGTATATCAACATTGGGATTTTGCTTTCTGTCATTTTCCAAAAACGCAGAAATGCCAGTGAATAAACCAAATACACTT
GATAACACACTCAAAACTGTAGCAGATATGACGCAAATTAATTTTTGTGTTCTAGAGACAATGTCATATTTGTAAGTGAAAATGAGTCGA
TATAAATAGTAGCAACCTATAATAATAGCTTGATAATAACTGAAATAAAAATTGCTAAATAGTGTTAAGGCTATCGCAACAATGAAAATA
CCGATTTTGCGTTGTTGAAAATATCTTTCCAAACCAAGAATCGATAATGGTAATAAATATAATAAACTTCCATAAAATGACCAAGTAAAAT
TAAAGTATATAACGACAGTTGACATGCCGTATAAAATCGTAGCGATCATATTTGTTGAGCGTTTAAAGTGTAATATTTTAAATAAGTAGAA
GGTCACGACAAATGTTATGATAGCTCTTATCATGGCCATAATAAGTTGGTTTGTCGGCCAAAAATGTATTGTTGTCGGATTAAATATACCA
ACCGTTTCTCCTATTTTAATGAATAGAAAATTTAGCCACATTAAAGGTGACAGCGAATAATAATATGATAGTCCTTTCATATAATCGCCACC
TAATCCAAACGATGCATCATATAAACTAGAAAAACTACTTAAATGTTCATACAAATACATTTGAAATGGCATCATTTGACGGAATCCATCT
CCAGCCCCACTAAAAACAGTACCATTCACAATATAATCATAGATATGAGTAGAAAATAAAATAAGCGTTAATATTACACTAATGAAAGTT
ATAACAAAGAATTGTTTGACGTTTGAATTTAGCCACTTTTTTAACAC

Gene: rbgA (putative GTP-binding protein)

Contig: 06_NODE_54, position: 363294 to 364178, length: 885 nt, orientation: FORWARD

Perfect match to: (MW2-BA000033-[1229652:1230536], highly conserved allele)

Sequence:

ATGGTTATTCAATGGTATCCAGGACATATGGCGAAAGCCAAAAGAGAAGTAAGTGAACAATTAAAAAAAGTAGATGTAGTGTTTGAACT
AGTAGATGCAAGAATTCCATATAGTTCAAGAAACCCTATGATAGATGAAGTTATTAACCAAAAACCACGTGTTGTTATATTAAATAAAAA
AGATATGTCTAATTTAAATGAGATGTCAAAATGGGAACAATTTTTTATTGATAAAGGATACTATCCTGTATCGGTGGATGCTAAGCACGG
TAAAAATTTAAAGAAAGTGGAAGCTGCAGCAATTAAAGCGACTGCTGAAAAATTTGAACGTGAAAAAGCAAAGGGACTTAAGCCAAGA
GCGATTAGAGCGATGATTGTTGGAATTCCAAATGTTGGTAAATCCACATTAATAAATAAACTGGCAAAGCGTAGTATTGCGCAGACTGGT
AATAAACCAGGTGTGACCAAACAACAACAATGGATTAAAGTTGGTAATGCATTACAACTATTAGACACACCAGGGATACTTTGGCCTAAA
TTTGAAGATGAAGAAGTCGGTAAGAAGTTGAGTTTAACAGGCGCGATTAAAGATAGCATTGTGCACTTAGATGAAGTAGCTATATATGG
ATTGAACTTTTTAATTCAACATGATTTAGCGCGATTAAAGTCACATTATAATATTGAAGTTCCTGAAGATGCAGAAATCATAGCGTGGTTT
GATGCGATAGGGAAAAAACGTGGCTTAATTCGACGTGGTAATGAAATTGATTACGAAGCAGTCATTGAACTGATTATTTATGATATTCGA
AATGCTAAAATAGGAAATTATTGTTTTGATATTTTTAAAGATATGACTGAGGAATTAGCAAATGACGCTAACAATTAA

Gene: rnhB (ribonuclease HII)

Contig: 06_NODE_54, position: 364162 to 364929, length: 768 nt, orientation: FORWARD

Perfect match to: (MW2-BA000033-[1230520:1231287], allele observed in CC1+CC15+CC188+CC772+CC1156)

Sequence:

ATGACGCTAACAATTAAAGAAGTTAAGCAGTTGATTAATGCGGTTAATACAATAGAAGAATTAGAAAATCATGAATGCTTTTTAGATGAG
CGAAAAGGTGTTCAAAATGCCATAGCTAGGCGCAGAAAAGCGTTAGAAAAAGAACAAGCTTTAAAAGAAAAGTATGTTGAAATGACTTA
CTTTGAAAATGAAATATTAAAAGAGCATCCTAATGCAATTATTTGTGGTATTGATGAAGTTGGAAGAGGACCTTTAGCAGGTCCAGTCGT
TGCATGCGCAACAATTTTAAATTCAAATCACAATTATTTGGGCCTTGATGACTCGAAAAAAGTACCTGTTACGAAACGTCTAGAATTAAAT
GAAGCACTAAAAAATGAAGTTACTGCTTTTGCATATGGTATCGCGACAGCTGAAGAAATAGATGAATTTAATATTTATAAAGCTACTCAA
ATCGCCATGCAGCGAGCTATTGATGGATTATCAGTACAACCAACGCATTTATTGATAGACGCGATGACGCTTGATAATGCACTGCCTCAA
GTATCTTTAATCAAGGGTGATGCAAGAAGTGTATCTATTGCAGCGGCAAGTATCATGGCAAAGGTTTTTCGTGATGATTATATGACACAG
TTATCTAAAGATTATCCTGAATATGGTTTTGAAAAAAACGCGGGTTACGGTACCAAACAACATTTACTAGCAATCGATGATATTGGCATTA
TGAAAGAGCATAGAAAAAGCTTTGAACCTATAAAATCGTTACTGTAA

Gene: sucC (succinyl-CoA synthase subunit beta)

Contig: 06_NODE_54, position: 365038 to 366204, length: 1167 nt, orientation: FORWARD

Perfect match to: (JKD6008-CP002120-[1273237:1274403], allele observed in CC239)

Sequence:

ATGAATATCCACGAGTATCAAGGTAAAGAAATATTTCGTTCAATGGGCGTTGCAGTTCCAGAAGGACGAGTAGCATTTACTGCTGAAGA
AGCGGTGGAGAAAGCAAAAGAATTAAATTCTGATGTTTATGTTGTAAAAGCACAAATTCATGCTGGAGGTAGAGGTAAAGCAGGCGGA
GTAAAAATTGCTAAATCTTTATCTGAGGTAGAAACATATGCAAAAGAATTATTAGGGAAAACTTTGGTGACACATCAAACTGGTCCAGAA
GGTAAAGAAATTAAGCGTTTATATATCGAAGAAGGTTGTGCTATTCAAAAAGAATATTACGTTGGATTCGTTATTGATCGTGCGACTGAC
CAAGTAACATTGATGGCGTCTGAAGAAGGGGGCACTGAGATTGAAGAAGTTGCTGCGAAGACTCCTGAAAAGATCTTCAAAGAAACTAT
CGATCCAGTAATCGGACTTTCACCATTCCAAGCAAGACGAATTGCGTTTAATATTAATATTCCTAAAGAATCTGTTAACAAAGCAGCTAAA
TTCTTATTAGCACTTTATAATGTATTCATTGAAAAAGATTGTTCAATCGTAGAAATCAACCCATTAGTTACAACAGCTGATGGTGATGTATT
GGCATTAGATGCTAAAATTAATTTTGATGATAATGCATTATTCAGACATAAAGATGTTGTAGAATTACGTGATTTAGAAGAAGAAGATCC
GAAAGAGATTGAAGCGTCTAAACATGATTTATCATACATTGCATTAGATGGTGACATCGGATGTATGGTTAATGGTGCAGGTTTAGCCAT
GGCAACAATGGATACGATTAATCATTTCGGTGGAAACCCAGCCAATTTCTTAGATGCAGGCGGAAGCGCTACTAGAGAAAAAGTAACTG
AAGCATTTAAAATCATTTTAGGTGATGAAAATGTTAAAGGTATTTTTGTAAACATTTTCGGTGGCATTATGAAATGTGATGTTATCGCAGA
AGGTATCGTTGAAGCTGTAAAAGAAGTAGATTTAACTTTACCACTAGTTGTACGCTTAGAAGGTACAAATGTTGAGTTAGGTAAAAAAAT
CTTAAAAGACTCAGGATTAGCAATTGAACCAGCAGCAACAATGGCTGAAGGTGCACAAAAAATTGTTAAACTAGTCAAAGAAGCATAA

Gene: sucD (succinyl-CoA synthase subunit alpha)

Contig: 06_NODE_54, position: 366226 to 367134, length: 909 nt, orientation: FORWARD

Perfect match to: (11819-97-CP003194-[1264776:1265684], highly conserved allele)

Sequence:

ATGAGTGTATTTATAGATAAGAATACTAAAGTAATGGTACAAGGTATTACAGGGTCTACTGCCCTTTTCCATACAAAACAAATGCTTGATT
ATGGTACGAAAATAGTAGCAGGTGTGACGCCTGGTAAAGGTGGTCAAGTTGTTGAAGGCGTTCCTGTTTTCAACACTGTTGAAGAAGCT
AAAAATGAAACCGGGGCAACGGTTTCAGTCATTTACGTTCCAGCACCATTTGCTGCAGACTCAATTTTAGAAGCAGCTGATGCAGACTTA
GATATGGTTATTTGTATCACTGAACATATTCCTGTATTAGACATGGTTAAAGTTAAACGCTACTTACAAGGTAGAAAAACACGTTTAGTTG
GTCCAAACTGTCCAGGTGTGATTACAGCAGATGAATGTAAAATTGGTATTATGCCTGGCTATATTCACAAAAAAGGTCATGTTGGTGTAG
TATCTCGTTCAGGTACATTAACATATGAAGCAGTGCACCAATTGACTGAAGAAGGTATTGGTCAAACTACAGCTGTTGGTATTGGTGGAG
ACCCAGTCAACGGAACAAACTTTATTGATGTTTTAAAAGCATTCAATGAAGATGACGAAACGAAAGCAGTTGTTATGATTGGTGAAATCG
GTGGTACGGCTGAAGAAGAAGCAGCTGAATGGATTAAAGCGAATATGACAAAACCAGTTGTAGGCTTTATCGGTGGACAAACAGCACC
TCCTGGAAAACGTATGGGACATGCTGGTGCAATCATTTCAGGTGGTAAAGGTACTGCTGAAGAGAAAATTAAAACATTAAATAGTTGTG
GTGTGAAAACAGCGGCAACACCTTCAGAAATTGGTTCAACATTAATTGAAGCTGCTAAAGAAGCAGGTATTTATGAAGCATTATTAACTG
TTAATAAATAA

Gene: lytN (cell-wall hydrolase)

Contig: 06_NODE_54, position: 367328 to 368479, length: 1152 nt, orientation: FORWARD

Sequence:

ATGTTTGTATATTATTGTAAGGAGTGTTTCATCATGAATAAACAACAAAGTAAAGTACGCTATTCAATTAGAAAAGTTAGTATTGGAATTT
TGTCAATTTCAATAGGTATGTTTTTGGCATTGGGTATGTCGAACAAAGCATATGCAGATGAAATTGATAAATCTAAAGATTTTACAAGAG
GGTATGAGCAAAATGTATTCGCGAAATCAGAGTTAAATGCTAATAAAAATACGACAAAAGACAAAATAAAAAATGAAGGTGCTGTTAAA
ACATCGGACACAAGTTTAAAGTTAGACAACAAATCAGCAATTTCAAACGGAAATGAAATTAATCAAGATATAAAGATTTCAAATACTCCG
AAAAACTCAAGCCAAGGTAACAATCTAGTTATTAATAACAATAAACCTACTAAAGAAATTAAAATTGCAAACTTGGAAGCTCAAAATTCT
AATCAGAAGAAAACGAATAAAGTTACTAATAATTACTTTGGTTACTACAGTTTTAGAGAAGCTCCAAAAACACAAATCTATACTGTAAAA
AAAGGAGACACACTTAGTGCTATAGCATTAAAATACAAAACTACAGTTTCAAATATTCAAAATACAAATAATATAGCAAATCCTAATTTAA
TATTTATTGGTCAAAAATTAAAAGTGCCAATGACACCATTAGTAGAACCAAAACCAAAAACAGTGTCTTCAAATAATAAAAGTAATAGTA
ATAGCAGTACATTAAATTATTTGAAAACATTAGAGAATAGAGGATGGGATTTCGACGGTAGTTATGGATGGCAATGTTTCGATTTAGTTA
ATGTATATTGGAATCATCTTTATGGTCATGGATTAAAAGGATATGGAGCTAAAGATATACCATATGCAAATAATTTTAATAGTGAAGCTA
AAATTTATCACAACACACCAACTTTCAAAGCTGAACCTGGGGACTTAGTGGTTTTTAGTGGAAGATTTGGTGGAGGATATGGTCATACAG
CTATTGTCTTAAATGGTGATTATGATGGAAAATTAATGAAGTTCCAAAGTTTAGATCAAAACTGGAATAATGGTGGATGGCGTAAAGCAG
AGGTTGCACATAAAGTTGTTCATAATTATGAAAATGATATGATTTTTATTAGACCATTTAAAAAAGCATAA

Gene: fmhC (endopeptidase resistance gene)

Contig: 06_NODE_54, position: 368507 to 369750, length: 1244 nt, orientation: FORWARD

Sequence:

ATGAAATTTTCAACTTTAAGTGAAGAAGAATTTACCAACTACACCAAAAAGCACTTCAAACATTATACACAGTCTATAGAATTATATAATT
ATAGAAATAAAATAAATCATGAAGCACATATTGTGGGAGTGAAGAATGATAAAAATGAAGTTATAGCTGCATGTTTATTAACAGAGGCA
CGAATTTTTAAATTCTACAAATATTTCTACTCTCATAGAGGTCCTTTACTTGATTATTTCGATGCTAAATTAGTTTGTTACTTTTTAAAGAAT
TATCTAAATTCATTTATAAAAATAGAGGAGTATTTATTCTTGTTGATCCATATTTAATAGAGAATTTAAGAGATGCAAATGGTAGGATAAT
AAAGAATTATAATAATTCAGTGATAGTAAAGATGCTAGGGAAAATTGGGTATCTCCATCAAGGTTATACAACAGGATATTCAAATAAAAG
TCAAATTAGGTGGATTTCTGTATTGGATTTAAAAGATAAAGATGAGAATCAACTTTTAAAAGAAATGGAATACCAAACTAGAAGAAATAT
AAAAAAGACTATTGAGATTGGTGTTAAGGTTGAAGATTTATCTATTGAAGAAACAAATCGATTTTATAAATTGTTTCAAATGGCTGAAGA
AAAACATGGTTTTCATTTCATGAATGAAGATTATTTTAAACGAATGCAAGAAATATATATAGATAAGGCAATGTTAAAGATAGCTTGTATA
AATCTTAATGAATATCAAGATAAATTAAAAATACAATTATTGAAAATCGAAAATGAAATGATGACTGTGAACAGAGCATTAAATGAAAAT
CCAAATTCTAAAAAAATAAATCAAAATTAAATCAGTTAAATATGCAATTATCTAGTATTAATAATAGAATTAGTAAAACCGAAGAACTAAT
ATTTGAAGATGGACCTGTTTTGGATTTAGCTGCTGCTTTATTTATATGTACTGATGATGAAGTTTATTATCTATCAAGTGGATCAAATCCGA
AATATAATCAGTATATGGGTGCATATCATCTACAATGGCATATGATAAAATATGCAAAATCACATAATATTAATAGGTATAATTTTTATGG
AATAACAGGCGTCTTTAGTAATGAGGCGGATGATTTTGGTGTTCAACAATTTAAAAAGGGTTTTAATGCACATGTTGAAGAATTAATTGG
TGATTTCATCAAACCAGTAAGACCAATTCTATATAAATTTGCAAAACTTATTTATAAGGTTTAA

Gene: dprA (DNA processing protein)

Contig: 06_NODE_54, position: 369923 to 370795, length: 873 nt, orientation: FORWARD

Perfect match to: (MW2-BA000033-[1236282:1237154], highly conserved allele)

Sequence:

TTGATTAAACTATTTTTGCTTAAGTTATACTGGGCACACTTTTCGACTAAACAAATTCATCAATTTTTAATGGCATATCCTAATGTAATTAAA
GAGGAGGGAAGAAAAAAAGATAGTTATTTATGTGAATGGGTGAATAGGGAAGAAAATGTTCATTTATTACGTAAATACTATGCTTTTAT
AAAACTTGATCATAACGATATTATTAAAGAACTGCAGAAATTAAAAGTAAGTTACATTACATATATGGATTCTGAATACCCAGTGCTATTA
AAAGAAATATATCAATTTCCATTACTTCTTTTCTATAAAGGGAACATCAAATTAATAAATAATATGCATCATTTGGCAGTAGTAGGTGCAA
GAGATTCTACAAGTTATACCCAACAGTCTTTAGAATTTTTATTATCAAATGATAAAAGCAAATATTTAACAATTGTTTCCGGCCTTGCTCAA
GGAGCTGATGCAATGGCACATCAAATAGCTTTAAAATACAATCTCCCTACAATTGCAGTTTTAGCCTTTGGCCATCAAACACATTATCCCA
AAAGTACATTAGCATTAAGAAATAAAATAGAAGAAAAAGGTTTAGTTATATCCGAATATCCACCACATACACCAATTGCTAAATATAGAT
TTCCTGAGCGCAATAGAATTATCAGCGGTTTGTCAAAAGGGGTTTTAATTACTGAGGCTAAGGAACAAAGTGGCAGTCACATCACGATA
GATTTTGCATTAGAGCAAAATAGAAATGTTTATGTTTTACCTGGATCTATGTTTAATCCTATGACAAAAGGTAATTTATTACGTATCCAAG
AAGGTGCTAAGGTAGTATTAAACGCTAATGATATATTTGAAGACTACTATATTTAA

Gene: topA (DNA topoisomerase I (type IA))

Contig: 06_NODE_54, position: 370975 to 373044, length: 2070 nt, orientation: FORWARD

Perfect match to: (MW2-BA000033-[1237334:1239403], highly conserved allele)

Sequence:

TTGGCAGATAATTTAGTCATTGTTGAATCGCCTGCAAAAGCAAAAACCATTGAAAAGTATTTAGGTAAGAAATATAAAGTTATAGCTTCA
ATGGGACACGTCAGAGACTTACCAAGAAGTCAAATGGGTGTCGACACTGAAGATAATTACGAACCAAAATATATAACAATACGCGGAAA
AGGTCCTGTTGTAAAAGAATTGAAAAAACATGCAAAAAAAGCGAAAAACGTCTTTCTCGCAAGTGACCCCGACCGTGAAGGTGAAGCAA
TTGCTTGGCATTTATCAAAAATTTTAGAGCTTGAAGATTCTAAAGAAAATCGCGTTGTTTTCAACGAAATAACTAAAGACGCTGTTAAAGA
AAGTTTTAAAAATCCTAGAGAAATTGAAATGAACTTAGTCGATGCACAACAAGCGCGTCGAATATTAGATAGATTGGTTGGCTATAACAT
CTCGCCAGTTCTATGGAAAAAAGTAAAAAAAGGTTTGTCAGCGGGTCGAGTTCAATCTGTTGCACTTCGTTTAGTCATTGACCGTGAAAA
TGAAATTCGAAACTTTAAACCAGAAGAATATTGGACTATTGAAGGAGAATTTAGATACAAAAAATCAAAATTCAATGCTAAATTCCTTCAT
TATAAAAATAAACCTTTTAAATTAAAAACGAAAAAAGATGTTGAGAAAATTACAGCTGCACTAGATGGAGATCAATTCGAAATTACAAAC
GTGACTAAAAAAGAAAAAACGCGTAATCCAGCAAACCCATTTACAACTTCTACATTACAACAAGAGGCGGCACGTAAATTAAACTTTAAA
GCAAGAAAAACAATGATGGTCGCACAACAATTATATGAAGGTATAGATTTGAAAAAACAAGGTACGATTGGTTTAATAACATATATGAG
AACCGATTCTACACGTATTTCAGATACTGCCAAAGCTGAAGCAAAACAGTATATAACTGATAAATACGGTGAATCTTACACTTCTAAACGT
AAAGCATCAGGGAAACAAGGTGACCAAGATGCCCATGAGGCTATTAGACCTTCAAGTACTATGCGTACGCCAGATGATATGAAGTCATT
TTTGACGAAAGACCAATACCGATTATACAAATTAATTTGGGAACGATTTGTTGCTAGTCAAATGGCTCCAGCAATACTTGATACAGTCTCA
TTAGACATAACACAAGGTGACATTAAATTTAGAGCGAATGGTCAAACAATCAAGTTTAAAGGATTTATGACACTTTATGTAGAAACTAAA
GATGATAGTGATAGCGAAAAGGAAAATAAACTGCCTAAATTAGAGCAAGGTGATAAAGTCACAGCAACTCAAATTGAACCAGCTCAACA
CTATACACAACCACCTCCAAGATATACTGAGGCGAGATTAGTAAAAACACTAGAAGAATTGAAAATTGGGCGACCATCAACTTATGCACC
GACAATAGATACGATTCAAAAGCGTAACTATGTCAAATTAGAAAGTAAGCGTTTTGTTCCTACTGAGTTGGGAGAAATAGTTCATGAACA
AGTGAAAGAATACTTCCCAGAGATTATTGATGTGGAATTCACAGTGAATATGGAAACGTTACTTGATAAGATTGCAGAAGGCGACATTA
CATGGAGAAAAGTAATCGACGGTTTCTTTAGTAGCTTTAAACAAGATGTTGAACGTGCTGAAGAAGAGATGGAAAAGATTGAAATCAAA
GATGAGCCAGCCGGTGAAGACTGTGAAGTTTGTGGTTCTCCTATGGTTATAAAAATGGGACGCTATGGTAAGTTCATGGCTTGCTCAAAC
TTCCCGGATTGTCGTAATACAAAAGCGATAGTTAAGTCTATTGGTGTTAAATGTCCAAAATGTAATGATGGTGACGTCGTAGAAAGAAAA
TCTAAAAAGAATCGTGTCTTTTATGGATGTTCGAAATATCCTGAATGCGACTTTATCTCTTGGGATAAGCCGATTGGAAGAGATTGTCCAA
AATGTAACCAATATCTTGTTGAAAATAAAAAAGGCAAGACAACACAAGTAATATGTTCAAATTGCGATTATAAAGAGGCAGCGCAGAAA
TAA

Gene: gid-trmFO (methylenetetrahydrofolate--tRNA- (uracil-5-)-methyltransferase)

Contig: 06_NODE_54, position: 373200 to 374507, length: 1308 nt, orientation: FORWARD

Perfect match to: (MW2-BA000033-[1239559:1240866], highly conserved allele)

Sequence:

ATGACTCAAACTGTAAATGTAATAGGTGCTGGTCTTGCCGGTTCAGAAGCGGCATATCAATTAGCTGAAAGAGGAATTAAAGTTAATCTA
ATAGAGATGAGACCTGTTAAACAAACACCAGCGCACCATACTGATAAATTTGCGGAACTTGTATGTTCCAATTCATTACGAGGAAATGCT
TTAACTAATGGTGTGGGTGTTTTAAAAGAAGAAATGAGAAGATTGAATTCTATAATTATTGAAGCGGCTGATAAGGCACGAGTTCCAGC
TGGTGGTGCATTAGCAGTTGATAGACACGATTTTTCAGGTTATATTACTGAAACACTTAAAAATCATGAAAATATCACAGTTATTAATGAA
GAAATTAATGCCATTCCAGATGGATACACAATTATCGCAACAGGACCACTTACTACAGAAACCCTTGCGCAAGAAATAGTGGACATTACT
GGTAAAGATCAACTTTATTTCTATGATGCGGCTGCTCCAATTATTGAAAAAGAATCTATTGATATGGATAAAGTTTACTTAAAGTCCCGTT

ATGATAAAGGTGAAGCTGCATATTTAAACTGTCCTATGACTGAGGATGAATTTAATCGCTTTTATGATGCAGTATTAGAAGCTGAAGTTG
CGCCTGTAAATTCATTTGAAAAAGAAAAATATTTCGAGGGTTGTATGCCTTTTGAAGTAATGGCAGAACGCGGACGCAAGACATTACTAT
TTGGACCAATGAAACCAGTAGGATTAGAAGATCCAAAGACTGGGAAACGTCCTTATGCAGTGGTTCAATTAAGACAAGATGATGCTGCT
GGCACACTCTACAATATTGTCGGCTTCCAAACGCATTTAAAATGGGGAGCTCAAAAAGAAGTTATTAAATTAATTCCAGGTTTAGAAAAT
GTTGATATTGTTAGATATGGTGTGATGCATAGAAATACCTTCATTAACTCACCGGACGTATTAAACGAGAAATATGAATTGATTTCACAAC
CAAACATACAGTTTGCGGGACAAATGACTGGTGTTGAAGGTTATGTAGAAAGCGCAGCTAGCGGCTTAGTTGCAGGTATCAATCTTGCG
CATAAAATATTAGGCAAAGGTGAGGTAGTATTTCCTAGAGAAACGATGATTGGAAGTATGGCTTACTATATTTCTCATGCTAAAAATAAT
AAGAATTTCCAACCTATGAATGCTAACTTCGGGTTATTACCATCTTTAGAAACTAGAATTAAAGATAAAAAAGAACGCTATGAAGCACAA
GCTAATAGAGCTTTGGATTACTTAGAAAATTTCAAAAAAACTTTATAA

Gene: xerC (site-specific recombinase C)

Contig: 06_NODE_54, position: 374924 to 375820, length: 897 nt, orientation: FORWARD

Perfect match to: (N315-BA000018-[1241147:1242043], allele observed in CC5+CC1+CC15)

Sequence:

TTGAATCATATTCAAGAAGCGTTTTTAAATACATTGAAAGTTGAACGGAATTTTTCGGAACATACATTGAAATCATATCAAGATGACTTAA
TTCAGTTTAATCAATTTTTAGAACAAGAACATTTACAGTTGAAAACTTTTGAATACAGAGATGCTAGAAATTATTTGAGCTATTTATATTCA
AATCATTTGAAAAGAACATCTGTTTCTCGTAAAATCTCAACGTTAAGAACTTTCTATGAATATTGGATGACGCTTGATGAGAACATTATTA
ATCCATTTGTTCAATTAGTACATCCGAAAAAAGAAAAATATCTTCCGCAATTCTTTTACGAAGAAGAAATGGAAGCGTTATTTAAAACTGT
AGAAGAGGACACTTCAAAAAGTTTACGGGATCGAGTTATTCTTGAATTGTTATATGCTACAGGTATCCGTGTTTCGGAATTAGTTAATATT
AAAAAACAAGATATAGATTTTTACGCGAATGGTGTTACCGTATTAGGAAAAGGGAGCAAAGAGCGCTTTGTACCGTTTGGTGCTTATTGT
AGACAAAGCATCGAAAATTATTTAGAACATTTCAAACCAATTCAGTCATGCAATCATGATTTTCTTATTGTAAATATGAAGGGTGAAGCAA
TCACTGAACGCGGTGTACGATATGTTTTAAATGATATTGTTAAACGAACAGCAGGCGTAAGTGAGATTCATCCCCACAAGCTCAGACATA
CATTTGCAACGCATTTATTGAATCAAGGTGCAGACCTAAGAACAGTACAATCGTTATTAGGTCATGTTAATTTGTCAACAACTGGTAAATA
TACACACGTATCTAACCAACAATTAAGAAAAGTGTATCTAAATGCACATCCTCGAGCGAAAAAGGAGAATGAAACATGA

Gene: hslV (heat shock protein, ATP-dependent protease)

Contig: 06_NODE_54, position: 375817 to 376362, length: 546 nt, orientation: FORWARD

Perfect match to: (MW2-BA000033-[1242176:1242721], highly conserved allele)

Sequence:

ATGAGTAATACAACATTACATGCAACAACAATTTATGCTGTAAGACATAATGGGAAAGCAGCTATGGCTGGAGATGGGCAAGTAACGCT
TGGTCAACAAGTCATCATGAAACAAACGGCAAGAAAAGTGCGACGTTTATATGAAGGTAAAGTGTTAGCTGGTTTCGCAGGTAGTGTAG
CAGATGCGTTTACGTTATTTGAAAAATTCGAAACAAAATTACAACAGTTTAGTGGTAACTTAGAAAGAGCTGCTGTTGAATTGGCACAAG
AATGGCGAGGCGATAAACAATTACGTCAATTAGAAGCTATGCTAATTGTAATGGATAAAGATGCTATTTTAGTTGTCAGTGGAACTGGC
GAAGTTATTGCCCCAGATGATGACCTTATCGCTATTGGATCAGGAGGCAACTACGCATTAAGCGCAGGACGTGCATTGAAACGCCATGC
ATCGCATTTGTCTGCTGAAGAAATGGCATATGAGAGCTTGAAAGTAGCGGCTGATATTTGTGTCTTTACCAACGATAATATTGTTGTTGA
AACACTATAA

Gene: hslU (heat shock protein, ATPase subunit)

Contig: 06_NODE_54, position: 376428 to 377831, length: 1404 nt, orientation: FORWARD

Perfect match to: (MW2-BA000033-[1242787:1244190], allele observed in CC1)

Sequence:

ATGGATACAGCTGGAATAAGATTAACTCCAAAAGAAATCGTATCTAAATTAAATGAATACATCGTTGGACAAAATGATGCTAAACGTAAA
GTGGCAATTGCCTTACGTAATCGATACAGAAGAAGTTTATTAGATGAGGAATCAAAGCAAGAAATTTCACCTAAAAATATTTTGATGATT
GGACCAACTGGCGTTGGTAAAACTGAAATTGCAAGAAGAATGGCCAAAGTTGTCGGCGCGCCATTTATAAAAGTAGAAGCTACTAAATT
TACTGAGGTAGGTTATGTAGGACGAGATGTTGAAAGTATGGTTAGAGATCTTGTTGATGTTTCAGTAAGATTAGTCAAGGCACAGAAAA
AATCATTGGTACAAGATGAAGCAACAGCTAAGGCCAATGAAAAACTTGTTAAGTTATTAGTTCCAAGTATGAAAAAGAAAGCGTCTCAA
ACGAATAATCCTTTAGAGTCACTTTTCGGAGGTGCAATTCCAAATTTCGGACAAAATAACGAAGATGAAGAAGAACCACCTACTGAGGA
AATTAAAACAAAACGTTCTGAAATTAAGAGACAGCTAGAAGAAGGCAAACTTGAAAAAGAAAAGGTAAGAATTAAAGTCGAACAAGAT

CCTGGTGCTTTAGGTATGCTAGGTACAAATCAAAATCAGCAAATGCAAGAGATGATGAATCAATTAATGCCTAAAAAGAAAGTTGAGCG
AGAAGTTGCTGTTGAGACGGCAAGGAAAATCTTAGCTGATAGTTATGCGGATGAACTAATTGATCAAGAAAGCGCTAACCAAGAAGCGC
TTGAATTAGCAGAACAAATGGGTATCATCTTTATAGATGAAATCGACAAAGTTGCGACGAATAATCATAATAGTGGTCAAGATGTCTCAA
GACAAGGTGTTCAAAGAGATATTTTACCTATACTTGAAGGTAGCGTTATTCAAACCAAATATGGTACTGTGAATACTGAACATATGCTGTT
TATAGGTGCTGGAGCTTTCCATGTATCTAAGCCGAGTGACTTGATACCAGAATTGCAAGGTCGTTTTCCGATTAGAGTTGAACTTGATAG
TTTATCGGTAGAAGATTTTGTAAGAATTTTGACAGAACCAAAATTGTCATTAATTAAACAATATGAAGCATTGCTTCAAACAGAAGAAGTT
ACTGTAAACTTTACCGATGAAGCAATTACTCGTTTAGCTGAGATTGCTTATCAAGTAAATCAAGATACAGACAACATTGGTGCACGTCGA
CTTCATACAATTTTAGAAAAGATGTTAGAAGATTTATCATTCGAAGCACCAAGTATGCCGAATGCAGTTGTAGATATTACCCCACAATATG
TTGATGATAAATTAAAATCAATTTCAACAAATAAAGATTTAAGTGCATTTATTCTATAA

Gene: codY (GTP-sensing transcriptional pleiotropic repressor)

Contig: 06_NODE_54, position: 377856 to 378629, length: 774 nt, orientation: FORWARD

Perfect match to: (MW2-BA000033-[1244215:1244988], highly conserved allele)

Sequence:

ATGAGCTTATTATCTAAAACGAGAGAGTTAAACACGTTACTTCAAAAACACAAAGGTATTGCGGTTGATTTTAAAGATGTAGCACAAACG
ATTAGTAGCGTAACTGTAACAAATGTATTTATTGTATCGCGTCGAGGTAAAATTTTAGGGTCGAGTCTAAATGAATTATTAAAAAGTCAA
AGAATTATTCAAATGTTGGAAGAAAGACATATCCCAAGTGAATATACAGAACGATTAATGGAAGTTAAACAAACAGAATCAAATATTGAT
ATCGACAATGTATTAACAGTTTTTCCACCTGAAAACAGAGAATTATTCATAGATAGTCGTACAACTATCTTCCCAATTTTAGGTGGAGGAG
AAAGATTAGGTACATTAGTACTTGGTCGAGTACATGATGATTTTAATGAAAATGATTTGGTACTAGGTGAATATGCTGCTACAGTTATTG
GTATGGAAATCTTACGTGAGAAGCATAGTGAAGTAGAAAAAGAAGCGCGCGATAAAGCTGCTATTACAATGGCAATTAATTCATTATCTT
ATTCTGAAAAAGAAGCGATTGAACATATCTTTGAAGAACTTGGCGGTACGGAAGGCCTATTAATCGCATCAAAAGTTGCAGATAGAGTT
GGTATTACTAGATCTGTAATTGTAAATGCACTACGTAAATTAGAAAGTGCTGGTGTAATTGAATCACGTTCTTTAGGAATGAAAGGTACT
TTCATTAAAGTTAAAAAAGAAAAATTCTTAGATGAATTAGAAAAAAGTAAATAA

Gene: rpsB (30S ribosomal protein S2)

Contig: 06_NODE_54, position: 378971 to 379738, length: 768 nt, orientation: FORWARD

Perfect match to: (N315-BA000018-[1245199:1245966], highly conserved allele)

Sequence:

ATGGCAGTAATTTCAATGAAACAATTACTAGAAGCGGGTGTTCACTTCGGTCACCAAACACGTCGTTGGAACCCAAAAATGAAAAAATAT
ATCTTCACTGAGAGAAATGGTATTTATATCATCGACTTACAAAAAACAGTGAAAAAAGTAGACGAGGCATACAACTTCTTGAAACAAGTT
TCAGAAGATGGTGGACAAGTCTTATTCGTAGGAACTAAAAAACAAGCACAAGAATCAGTTAAATCTGAAGCAGAACGTGCTGGTCAATT
CTACATTAACCAAAGATGGTTAGGTGGATTATTAACTAACTATAAAACGATCTCAAAACGAATCAAACGTATTTCTGAAATTGAAAAAAT
GGAAGAAGATGGTTTATTCGAAGTATTACCTAAAAAAGAAGTAGTAGAACTTAAAAAAGAATACGACCGTTTAATCAAATTCTTAGGCG
GAATTCGTGATATGAAATCAATGCCTCAAGCATTATTCGTAGTTGACCCACGTAAAGAGCGTAATGCAATTGCTGAAGCTCGTAAATTAA
ATATTCCTATCGTAGGTATCGTTGACACTAACTGTGATCCTGACGAAATTGACTACGTTATCCCAGCAAACGACGATGCTATCCGTGCGGT
TAAATTATTAACTGCTAAAATGGCAGATGCAATCTTAGAAGGTCAACAAGGCGTTTCTAATGAAGAAGTAGCTGCAGAACAAAACATCG
ATTTAGATGAAAAAGAAAAATCAGAAGAAACAGAAGCAACTGAAGAATAA

Gene: tsf (translation elongation factor Ts)

Contig: 06_NODE_54, position: 379920 to 380801, length: 882 nt, orientation: FORWARD

Perfect match to: (MW2-BA000033-[1246279:1247160], highly conserved allele)

Sequence:

ATGGCAACTATTTCAGCAAAACTTGTTAAAGAATTACGTGAAAAAACTGGCGCGGGTATGATGGATTGTAAAAAAGCGCTAACTGAAAC
TGATGGTGACATCGATAAAGCGATTGATTACCTACGTGAAAAAGGTATTGCTAAAGCAGCTAAAAAAGCAGACCGTATTGCGGCTGAAG
GTTTAGTACATGTAGAAACTAAAGGTAACGACGCAGTTATCGTTGAAATCAACTCTGAAACAGACTTTGTTGCTCGTAACGAAGGATTCC
AAGAGTTAGTTAAAGAAATCGCTAATCAAGTATTAGATACAAAAGCTGAAACTGTTGAAGCTTTAATGGAAACAACTTTACCAAATGGTA
AATCAGTTGATGAAAGAATTAAAGAAGCAATTTCAACAATCGGTGAAAAATTAAGTGTTCGTCGTTTTGCTATCAGAACTAAAACTGATA
ACGATGCTTTCGGCGCTTACTTACACATGGGTGGACGCATTGGTGTATTAACAGTTGTTGAAGGTTCAACTGACGAAGAAGCAGCAAGA

GACGTTGCTATGCATATCGCTGCAATCAACCCTAAATATGTTTCTTCTGAACAAGTTAGCGAAGAAGAAATCAACCACGAAAGAGAAGTT
TTAAAACAACAAGCATTAAATGAAGGTAAACCAGAAAACATCGTTGAAAAAATGGTGGAAGGACGTTTACGTAAATACTTACAAGAAAT
TTGTGCTGTAGATCAAGACTTCGTTAAAAACCCTGATGTAACAGTTGAAGCTTTCTTAAAAACAAAAGGTGGAAAACTTGTTGACTTCGT
ACGCTATGAAGTAGGCGAAGGTATGGAAAAACGCGAAGAAAACTTTGCGGATGAAGTTAAAGGACAAATGAAATAA

Gene: pyrH (uridylate kinase)

Contig: 06_NODE_54, position: 380938 to 381660, length: 723 nt, orientation: FORWARD

Perfect match to: (RF122-AJ938182-[1212184:1212906], highly conserved allele)

Sequence:

ATGGCTCAAATTTCTAAATATAAACGTGTAGTTTTGAAACTAAGTGGTGAAGCGTTAGCTGGAGAAAAAGGATTTGGCATAAATCCAGTA
ATTATTAAAAGTGTTGCTGAGCAAGTGGCTGAAGTTGCTAAAATGGACTGTGAAATCGCAGTAATCGTTGGTGGCGGAAACATTTGGAG
AGGTAAAACAGGTAGTGACTTAGGTATGGACCGTGGAACTGCTGATTACATGGGTATGCTTGCAACTGTAATGAATGCCTTAGCATTAC
AAGATAGTTTAGAACAATTGGATTGTGATACACGAGTATTAACATCTATTGAAATGAAGCAAGTGGCTGAACCTTATATTCGTCGTCGTG
CAATTAGACACTTAGAAAAGAAACGCGTAGTTATTTTTGCTGCAGGTATTGGAAACCCATACTTCTCTACAGATACTACAGCGGCATTACG
TGCTGCAGAAGTTGAAGCAGATGTTATTTTAATGGGCAAAAATAATGTAGATGGTGTATATTCTGCAGATCCTAAAGTAAACAAAGATGC
GGTAAAATATGAACATTTAACGCATATTCAAATGCTTCAAGAAGGTTTACAAGTAATGGATTCAACAGCATCCTCATTCTGTATGGATAAT
AACATTCCGTTAACTGTTTTCTCTATTATGGAAGAAGGAAATATTAAACGTGCTGTTATGGGTGAAAAGATAGGTACGTTAATTACAAAA
TAA

Gene: frr (ribosome recycling factor)

Contig: 06_NODE_54, position: 381679 to 382233, length: 555 nt, orientation: FORWARD

Perfect match to: (N315-BA000018-[1247907:1248461], highly conserved allele)

Sequence:

ATGAGTGACATTATTAATGAAACTAAATCAAGAATGCAAAAATCAATCGAAAGCTTATCACGTGAATTAGCTAATATCAGTGCAGGAAGA
GCTAATTCAAATTTATTAAACGGCGTAACAGTTGATTACTATGGTGCACCAACACCTGTACAACAATTAGCAAGCATCAATGTTCCAGAAG
CACGTTTACTTGTTATTTCTCCATACGACAAAACTTCTGTAGCTGACATCGAAAAAGCGATAATAGCGGCTAACTTAGGTGTCAACCCAAC
AAGTGATGGTGAAGTGATACGTATTGCAGTACCTGCCTTAACAGAAGAGCGTAGAAAAGAGCGCGTTAAAGATGTTAAGAAAATTGGT
GAAGAAGCTAAAGTATCTGTTCGAAATATTCGTCGTGATATGAATGATCAGTTGAAAAAAGATGAAAAAAATGGCGACATTACTGAAGA
TGAGTTGAGAAGTGGTACTGAAGATGTTCAGAAAGCAACAGACAATTCAATAAAAGAAATTGATCAAATGATTGCTGATAAAGAAAAAG
ATATTATGTCAGTATAA

Gene: uppS (undecaprenyl pyrophosphate synthase)

Contig: 06_NODE_54, position: 382606 to 383376, length: 771 nt, orientation: FORWARD

Perfect match to: (MW2-BA000033-[1248965:1249735], highly conserved allele)

Sequence:

ATGTTTAAAAAGCTAATAAATAAAAAGAACACTATAAATAATTATAATGAAGAATTAGACTCGTCTAATATACCTGAACATATCGCTATTA
TTATGGATGGTAATGGGCGATGGGCTAAGAAGCGAAAAATGCCTAGAATTAAAGGTCATTACGAAGGTATGCAAACAATAAAAAAAAT
TACTAGGGTAGCTAGTGATATTGGTGTTAAGTACTTAACTTTATACGCCTTTTCCACTGAAAATTGGTCAAGACCTGAAAGTGAAGTAAAT
TATATTATGAATTTGCCTGTCAATTTCTTAAAGACATTCTTACCGGAACTAATTGAAAAAAATGTCAAAGTTGAAACAATTGGATTTACTG
ATAAGTTGCCAAAATCAACGATAGAAGCAATTAATAATGCTAAAGAAAAGACAGCTAATAATACCGGCTTAAAATTAATATTTGCAATTA
ATTATGGTGGCAGAGCAGAACTTGTTCATAGTATTAAAAATATGTTTGACGAGCTTCATCAACAAGGTTTAAATAGTGATATCATAGATG
AAACATATATAAACAATCATTTAATGACAAAAGACTATCCTGATCCAGAGTTGTTAATTCGTACTTCAGGAGAACAAAGAATAAGTAATTT
CTTGATTTGGCAAGTTTCGTATAGTGAATTTATCTTTAATCAAAAATTATGGCCTGACTTTGACGAAGATGAATTAATTAAATGTATAAAA
ATTTATCAGTCACGTCAAAGACGCTTTGGCGGATTGAGTGAGGAGTAG

Gene: cdsA (phosphatidate cytidylyltransferase)

Contig: 06_NODE_54, position: 383383 to 384165, length: 783 nt, orientation: FORWARD

Perfect match to: (TW20-FN433596-[1337555:1338337], allele observed in CC239)

Sequence:

ATGAAAGTTAGAACGCTGACAGCTATTATTGCCTTAATCGTATTCTTGCCTATCTTGTTAAAAGGCGGCCTTGTGTTAATGATATTTGCTAA
TATATTAGCATTGATTGCATTAAAAGAATTGTTGAATATGAATATGATTAAATTTGTTTCAGTTCCTGGTTTAATTAGTGCAGTTGGTCTTA
TCATCATTATGTTGCCACAACATGCAGGGCCATGGGTACAAGTAATTCAATTAAAAAGTTTAATTGCAATGAGCTTTATTGTATTAAGTTA
TACTGTCTTATCTAAAAACAGATTTAGTTTTATGGATGCTGCATTTTGCTTAATGTCTGTGGCTTATGTAGGCATTGGTTTTATGTTCTTTTA
TGAAACGAGATCAGAAGGATTACATTACATATTATATGCCTTTTTAATTGTTTGGCTTACAGATACAGGGGCTTACTTGTTTGGTAAAATG
ATGGGTAAACATAAGCTTTGGCCAGTAATAAGTCCGAATAAAACAATCGAAGGATTCATAGGTGGCTTGTTCTGTAGTTTGATAGTACCA
CTTGCAATGTTATATTTTGTAGATTTCAATATGAATGTATGGATATTACTTGGAGTGACATTGATTTTAAGTTTATTTGGTCAATTAGGTGA
TTTAGTGGAATCAGGATTTAAGCGTCATTTCGGCGTTAAAGACTCAGGTCGTATACTACCTGGACACGGTGGTATTTTAGACCGATTTGA
CAGCTTTATGTTTGTGTTACCATTATTAAATATTTTATTAATACAATCTTAA

Gene: rseP (membrane-associated zinc metalloprotease)

Contig: 06_NODE_54, position: 384377 to 385663, length: 1287 nt, orientation: FORWARD

Perfect match to: (MW2-BA000033-[1250736:1252022], allele observed in CC1)

Sequence:

GTGAGCTATTTAGTTACAATAATTGCATTTATTATTGTTTTTGGTGTACTAGTAACTGTTCATGAATATGGCCATATGTTTTTTGCGAAAAG
AGCAGGCATTATGTGTCCAGAATTTGCGATCGGTATGGGACCGAAAATTTTTAGTTTTAGAAAAAACGAAACACTTTACACTATTAGGTT
ATTGCCTGTTGGTGGATATGTTCGTATGGCAGGAGATGGCTTAGAAGAGCCACCAGTCGAGCCCGGTATGAACGTTAAAATTAAACTGA
ATGAAGAAAATGAAATAACACATATCATATTAGATGATCATCATAAGTTTCAACAAATTGAAGCAATCGAAGTTAAAAAATGTGATTTTA
AGGATGACTTATTCATAGAAGGTATCACTGCTTATGATAATGAAAGACATCATTTTAAAATTGCTAGAAAGTCTTTCTTTGTTGAAAATGG
TAGCTTAGTTCAAATTGCTCCGAGAGACAGACAATTTGCACATAAAAAGCCATGGCCGAAATTTTTAACATTATTTGCGGGACCGTTATTT
AACTTTATATTAGCTTTAGTCCTATTTATTGGTCTTGCATATTATCAAGGCACGCCTACGTCTACTGTAGAACAAGTCGCAGATAAGTATCC
AGCTCAACAAGCAGGATTACAAAAAGGTGATAAGATCGTCCAAATTGGCAAATATAAAATATCTGAATTTGATGATGTTGATAAGGCGTT
AGATAAAGTTAAAGATAATAAGACGACTGTTAAATTTGAACGTGATGGTAAAACAAAGTCAGTTGAATTAACACCTAAAAAGACTGAAA
GAAAACTGACTAAAGTAAGTTCAGAGACGAAGTATGTTCTCGGATTCCAACCAGCGAGTGAACGTACGCTCTTCAAACCAATTGTATATG
GATTTGAAAGCTTTTTGAAAGGTAGTACACTAATTTTTACAGCTGTAGTCGGTATGTTGGCTAGTATATTTACGGGCGGATTCTCATTTGA
TATGTTAAATGGTCCGGTTGGTATTTATCATAACGTCGACTCAGTTGTTAAAGCGGGTATCATTAGCTTAATTGGTTACACTGCGTTATTA
AGTGTAAACTTAGGTATTATGAATTTAATTCCTATTCCTGCACTAGACGGTGGTCGTATTTTATTTGTTATATATGAAGCGATTTTCAGAAA
ACCAGTTAATAAAAAAGCGGAAACAACGATTATTGCTATTGGTGCCATTTTCATGGTCGTTATTATGATATTAGTAACGTGGAATGATATT
CGACGATATTTCTTATAA

Gene: proS (prolyl-tRNA synthase)

Contig: 06_NODE_54, position: 385683 to 387386, length: 1704 nt, orientation: FORWARD

Perfect match to: (Strain_16035-HE579065-[1230404:1232107], allele observed in CC5+CC361+CC445)

Sequence:

ATGAAGCAATCCAAAGTTTTTATACCAACGATGCGTGATGTGCCATCAGAAGCAGAAGCACAAAGTCATCGTTTATTATTGAAATCGGGT
TTGATAAAACAAAGTACAAGTGGGATTTATAGTTATTTACCGCTAGCAACACGTGTGTTAAATAATATTACTGCAATTGTGCGACAAGAA
ATGGAACGTATCGATTCTGTTGAAATTTTAATGCCAGCGTTACAACAAGCTGAATTATGGGAAGAATCAGGACGTTGGGGTGCATATGG
CCCAGAATTAATGCGTTTACAAGATAGACATGGAAGACAATTTGCATTAGGTCCAACACATGAAGAATTAGTTACATCAATAGTAAGAAA
TGAATTGAAATCATACAAACAATTACCGATGACATTATTCCAAATTCAATCTAAATTCCGTGATGAAAAGAGACCACGTTTTGGTTTACTT
CGTGGGCGTGAATTTATTATGAAAGATGCGTATTCATTCCATGCTGACGAGGCATCATTAGATCAAACGTATCAAGATATGTATCAAGCG
TATAGCCGTATTTTTGAGAGAGTTGGCATTAACGCAAGACCAGTAGTTGCAGATTCAGGTGCTATAGGCGGTAGCCATACACATGAATTT
ATGGCATTAAGTGCTATCGGTGAGGATACAATCGTTTACAGTAAAGAAAGTGACTATGCTGCTAATATCGAAAAAGCAGAAGTCGTTTAC
GAACCAAATCATAAGCATTCTACTGTGCAACCTTTAGAAAAAATTGAAACACCAAATGTTAAGACTGCACAAGAATTGGCAGACTTCTTA
GGTAGACCAGTAGATGAAATCGTTAAAACGATGATTTTCAAAGTTGATGGCGAATATATTATGGTTTTAGTGCGTGGCCATCATGAAATT
AATGACATTAAATTAAAATCTTATTTCGGCACAGATAATATTGAATTAGCAACACAAGACGAAATTGTTAATTTAGTTGGTGCAAATCCGG
GTTCACTAGGTCCTGTTATTGATAAAGAAATCAAAATTTATGCAGATAATTTTGTGCAAGATTTAAATAATTTAGTTGTCGGTGCTAACGA
AGATGGCTATCACTTAATTAATGTAAATGTAGGTAGAGACTTCAACGTTGATGAATATGGCGATTTCCGTTTTATTTTAGAAGGCGAAAA

GTTAAGTGATGGTTCAGGCGTTGCACATTTTGCTGAAGGTATTGAAGTTGGTCAAGTATTCAAATTGGGTACTAAGTATTCAGAATCAAT
GAATGCTACATTCTTAGATAACCAAGGAAAAGCCCAACCTTTAATTATGGGTTGTTACGGTATTGGAATTTCTAGAACGCTGAGTGCGAT
TGTTGAACAAAATCACGATGATAATGGAATTGTTTGGCCTAAATCAGTTACTCCATTTGATTTACATTTAATTTCTATTAATCCTAAGAAAG
ATGATCAACGAGAACTAGCGGATGCACTATATGCTGAATTTAATACTAAATTTGATGTGTTGTACGATGATCGTCAGGAACGTGCGGGTG
TCAAATTTAATGATGCCGATTTAATTGGTTTACCACTGCGAATTGTTGTTGGTAAACGTGCATCGGAAGGTATTGTAGAAGTTAAAGAAC
GTTTAACAGGTGATAGCGAAGAAGTTCACATTGATGACTTAATGACTGTCATTACAAATAAATATGATAACTTAAAATAA

Gene: polC (DNA-directed DNA polymerase III alpha chain)

Contig: 06_NODE_54, position: 387650 to 391960, length: 4311 nt, orientation: FORWARD

Perfect match to: (Strain_21331-AGTV01000007-[29659:33969:r], allele observed in CC398)

Sequence:

ATGACAGAGCAACAAAAATTTAAAGTGCTTGCTGATCAAATTAAAATTTCAAATCAATTAGATGCTGAAATTTTAAATTCAGGTGAACTG
ACACGTATAGATGTTTCTAACAAAAACAGAACATGGGAATTTCATATTACATTACCACAATTCTTAGCTCATGAAGATTATTTATTATTTAT
AAATGCAATAGAGCAAGAGTTTAAAGATATCGCCAACGTTACATGTCGTTTTACGGTAACAAATGGCACGAATCAAGATGAACATGCAA
TTAAATACTTTGGACACTGTATTGACCAAACAGCTTTATCTCCAAAAGTTAAAGGTCAATTGAAACAGAAAAAGCTTATTATGTCTGGAAA
AGTATTAAAAGTAATGGTATCAAATGACATTGAACGTAATCATTTTGATAAGGCATGCAATGGAAGTCTTATCAAAGCGTTTAGAAATTG
TGGTTTTGATATCGATAAAATCATCTTCGAAACAAATGATAATGATCAAGAACAAAACTTAGCTTCTTTAGAAGCACATATTCAAGAAGAA
GACGAACAAAGTGCACGATTGGCAACAGAGAAACTTGAAAAAATGAAAGCTGAAAAAGCGAAACAACAAGATAACAACGAAAGTGCT
GTTGATAAGTGTCAAATTGGTAAGCCGATTCAAATTGAAAATATTAAACCAATTGAATCTATTATTGAGGAAGAGTTTAAAGTTGCAATA
GAGGGTGTCATTTTTGATATAAACTTAAAAGAACTTAAAAGTGGTCGCCATATCGTAGAAATTAAAGTGACTGACTATACGGACTCTTTA
GTTTTAAAAATGTTTACTCGTAAAAACAAAGATGATTTAGAACATTTTAAAGCGCTAAGTGTTGGTAAATGGGTTAGGGCTCAAGGTCGT
ATTGAAGAAGATACATTTATTAGAGATTTAGTTATGATGATGTCTGATATTGAAGAGATTAAAAAAGCGACAAAAAAAGATAAGGCTGA
AGAAAAGCGTGTAGAATTCCACTTGCATACTGCAATGAGCCAAATGGATGGTATACCCAATATTGGTGCGTATGTTAAACAGGCAGCAG
ACTGGGGACATCCAGCCATTGCGGTTACAGACCATAATGTTGTGCAAGCATTTCCAGATGCTCACGCAGCAGCGGAAAAACATGGCATT
AAAATGATATACGGTATGGAAGGTATGTTAGTTGATGATGGTGTTCCGATTGCATACAAACCACAAGATGTCGTATTAAAAGATGCTACT
TATGTTGTGTTCGACGTTGAGACAACTGGTTTATCAAATCAGTATGATAAAATCATCGAGCTTGCAGCTGTGAAAGTTCATAACGGTGAA
ATCATCGATAAGTTTGAAAGGTTTAGTAATCCGCATGAACGATTATCGGAAACGATTATCAATTTGACGCATATTACTGATGATATGTTAG
TAGATGCCCCTGAGATTGAAGAAGTACTTACAGAGTTTAAAGAATGGGTTGGCGATGCGATATTCGTAGCGCATAATGCTTCGTTTGATA
TGGGCTTTATCGATACGGGATATGAACGTCTTGGGTTTGGACCATCAACGAATGGTGTTATCGATACTTTAGAATTATCTCGTACGATTAA
TACTGAATATGGTAAACATGGTTTGAATTTCTTAGCTAAAAAATATGGCGTAGAATTAACGCAACATCACCGTGCCATTTATGATACAGAA
GCAACAGCTTACATTTTCATAAAAATGGTTCAACAAATGAAAGAATTAGGCGTATTAAATCATAACGAAATCAACAAAAAACTCAGTAAT
GAAGATGCATATAAACGTGCAAGACCTAGTCATGTCACATTAATTGTACAAAACCAACAAGGTCTTAAAAATCTATTTAAAATTGTAAGT
GCATCATTGGTGAAGTATTTCTACCGTACACCTCGAATTCCACGTTCATTGTTAGATGAATATCGTGAGGGATTATTGGTAGGTACAGCGT
GTGATGAAGGTGAATTATTTACGGCAGTTATGCAGAAGGACCAGAGCCAAGTTGAAAAAATTGCCAAATATTATGATTTTATTGAAATTC
AACCACCGGCACTTTATCAAGATTTAATTGATAGAGAGCTTATTAGAGATACTGAAACATTACATGAAATTTATCAACGTTTAATACATGC
AGGTGACACAGCGGGTATACCTGTTATTGCGACAGGAAATGCACACTATTTGTTTGAACATGATGGTATCGCACGTAAAATTTTAATAGC
ATCACAACCCGGCAATCCACTTAATCGCTCAACTTTACCGGAAGCACATTTTAGAACTACAGATGAAATGTTAAACGAGTTTCATTTTTTA
GGTGAAGAAAAAGCGCATGAAATTGTTGTGAAAAATACAAACGAATTAGCAGATCGAATTGAACGTGTTGTTCCTATTAAAGATGAATT
ATACACACCGCGTATGGAAGGTGCTAACGAAGAAATTAGAGAACTAAGTTATGCAAATGCGCGTAAACTGTATGGTGAAGACCTGCCTC
AAATCGTAATTGATCGATTAGAAAAAGAATTAAAAAGTATTATCGGTAATGGATTTGCGGTAATTTACTTAATTTCGCAACGTTTAGTTAA
AAAATCATTAGATGATGGATACTTAGTTGGTTCCCGTGGTTCAGTAGGTTCTAGTTTTGTAGCGACAATGACTGAGATTACTGAAGTAAA
CCCGTTACCGCCACACTATATTTGTCCGAACTGTAAAACGAGTGAATTTTTCAATGATGGTTCAGTAGGATCAGGATTTGATTTACCTGAT
AAGACGTGTGAAACTTGTGGAGCGCCACTTATTAAAGAAGGACAAGATATTCCGTTTGAAACATTTTTAGGATTTAAGGGAGATAAAGTT
CCTGATATCGACTTAAACTTTAGTGGTGAATATCAACCGAATGCCCATAACTACACAAAAGTATTATTTGGTGAGGATAAAGTATTCCGTG
CAGGTACAATTGGTACTGTTGCTGAAAAGACTGCTTTTGGTTATGTTAAAGGTTATTTGAATGATCAAGGCATCCACAAAAGAGGTGCTG
AAATAGATCGACTCGTTAAAGGATGTACAGGTGTTAAACGTACAACTGGACAGCATCCAGGGGGTATTATTGTAGTACCTGATTACATG
GATATTTATGATTTTACGCCGATACAATATCCTGCCGATGATCAAAATTCAGCATGGATGACGACACATTTTGATTTCCATTCTATTCATGA
TAATGTATTAAAACTTGATATACTTGGACACGATGATCCAACAATGATTCGTATGCTTCAAGATTTATCGGGCATTGATCCAAAAACGATA
CCTGTAGATGACAAAGAAGTCATGCAAATATTTAGTACACCTGAAAGTTTAGGTGTTACTGAAGATGAAATTTTATGTAAAACAGGTACG
TTTGGGGTTCCAGAATTCGGTACAGGGTTCGTGCGTCAAATGTTAGAAGATACAAAGCCAACAACATTTTCTGAATTAGTTCAAATCTCA
GGATTATCTCATGGTACAGATGTGTGGTTAGGCAATGCTCAAGAATTAATTAAAACCGGTATATGTGATTTATCAAGTGTAATTGGTTGT
CGTGATGATATCATGGTTTATTTAATGTATGCTGGTTTAGAACCATCAATGGCTTTTAAAATAATGGAGTCAGTACGTAAAGGTAAAGGT
TTAACTGAAGAAATGATTGAAACGATGAAAGAAAATGAAGTGCCGGATTGGTATTTAGATTCATGTCTTAAAATTAAGTACATGTTCCCT
AAAGCCCATGCAGCAGCATACGTTTTAATGGCAGTACGTATCGCATATTTCAAAGTACATCATCCACTTTATTACTATGCATCTTACTTTAC
AATTCGTGCGTCAGACTTTGATTTAATCACGATGATTAAAGATAAAACAAGCATTCGAAATACTGTAAAAGACATGTATTCTCGCTATATG
GATCTAGGTAAAAAAGAAAAAGACGTATTAACAGTCTTGGAAATTATGAATGAAATGGCGCATCGAGGTTATCGCATGCAACCGATTAG

TTTAGAAAAGAGTCAGGCGTTCGAATTTATCATTGAAGGCGATACACTTATTCCGCCGTTCATATCAGTGCCTGGGCTTGGCGAAAACGT
TGCGAAACGAATTGTTGAAGCTCGTGACGATGGCCCATTTTTATCAAAAGAAGATTTAAACAAAAAAGCTGGATTATCTCAGAAAATTAT
TGAGTATTTAGATGAGTTAGGCTCATTACCGAATTTACCAGATAAAGCTCAACTTTCGATATTTGATATGTAA

Gene: rimP (ribosome maturation factor)

Contig: 06_NODE_54, position: 392250 to 392717, length: 468 nt, orientation: FORWARD

Perfect match to: (MW2-BA000033-[1258609:1259076], highly conserved allele)

Sequence:

ATGAGTAAAATTACAGAACAAGTAGAAGTGATTGTTAAACCAATTATGGAAGACTTGAATTTTGAACTTGTAGACGTTGAATATGTCAAA
GAGGGTAGAGATCATTTTCTTAGAATCTCTATTGATAAAGAAGGTGGCGTAGATTTAAATGATTGTACGCTAGCTTCTGAAAAAATAAGT
GAAGCTATGGATGCAAATGATCCTATTCCTGAAATGTATTATTTAGACGTAGCGTCACCTGGTGCAGAACGTCCAATTAAAAAAGAACAA
GATTTCCAAAATGCAATAACTAAACCTGTATTTGTTTCTTTATATGTACCAATTGAAGGTGAAAAGGAATGGTTAGGCATTTTACAAGAAG
TCAATAATGAAACAATTGTAGTACAAGTTAAAATCAAAGCAAGAACGAAAGATATAGAGATACCGAGAGACAAAATAGCAAAAGCACG
TCACGCAGTTATGATTTAA

Gene: nusA (transcription antitermination protein)

Contig: 06_NODE_54, position: 392738 to 393913, length: 1176 nt, orientation: FORWARD

Perfect match to: (TW20-FN433596-[1346910:1348085], allele observed in CC239+CC5+CC25+CC239)

Sequence:

GTGTCAAGTAATGAATTATTATTAGCTACTGAGTATTTAGAAAAAGAAAAGAAGATTCCTAGAGCAGTATTAATTGATGCTATTGAAGCA
GCTTTAATTACTGCATACAAAAAGAATTATGATAGTGCAAGAAATGTCCGTGTGGAATTAAATATGGATCAAGGTACTTTCAAAGTTATC
GCTCGTAAAGATGTTGTTGAAGAAGTATTTGACGACAGAGATGAAGTGGATTTAAGTACAGCGCTTGTTAAAAACCCTGCATATGAAATT
GGTGATATATACGAAGAAGATGTAACACCTAAAGATTTTGGTCGTGTAGGTGCTCAAGCAGCGAAACAAGCAGTAATGCAACGTCTTCG
TGATGCTGAACGTGAAATTTTATTTGAAGAATTTATAGACAAAGAAGAAGACATACTTACTGGAATTATTGACCGTGTTGACCATCGTTAT
GTATATGTGAACTTAGGTCGTATCGAAGCTGTTTTATCTGAAGCAGAAAGAAGTCCTAACGAAAAATATATTCCTAACGAACGTATCAAA
GTATATGTTAACAAAGTGGAACAAACGACAAAAGGTCCTCAAATCTATGTTTCTCGTAGCCATCCAGGTTTATTAAAACGTTTATTTGAAC
AAGAAGTTCCAGAAATTTACGATGGTACTGTAATTGTTAAATCAGTAGCACGTGAAGCTGGCGATCGCTCTAAAATTAGTGTCTTCTCTG
AAAACAATGATATAGATGCTGTTGGTGCATGTGTTGGTGCCAAAGGCGCACGTGTTGAAGCTGTTGTTGAAGAGCTAGGTGGCGAAAA
AATCGACATCGTTCAATGGAATGAAGATCCAAAAGTATTTGTAAAAAATGCTTTAAGCCCTTCTCAAGTTTTAGAAGTTATTGTTGATGAA
ACAAATCAATCTACAATAGTTGTTGTTCCTGATTATCAATTGTCATTAGCGATTGGTAAAAGAGGACAAAACGCACGTCTAGCTGCTAAAT
TAACCGGCTGGAAAATTGATATTAAATCAGAAACAGATGCGCGCGAAGCGGGTATCTATCCAGTAGTTGAAGCTGAAAAAGTAACTGAA
GAAGATGTTGCTTTAGAAGATGCTGACACAACAGAATCAACCGAAGAGGTAAATGATGTTTCAGTTGAAACAAATGTAGAGAAAGAATC
TGAATAA

Gene: ylxR (putative protein)

Contig: 06_NODE_54, position: 393934 to 394218, length: 285 nt, orientation: FORWARD

Perfect match to: (MW2-BA000033-[1260293:1260577], allele observed in CC1+CC7+CC188)

Sequence:

ATGAAAAAGAAAAAAAATTCCGATGCGAAAATGTATTCTTTCAAATGAAATGCATCCCAAAAAAGATATGATTCGTGTTGTTGTTAATAAA
GAAGGCGAAATCTTTGCGGATGTTACTGGAAAGAAACAAGGCCGTGGCGCATATGTTTCTAAAGATGTTGCTATGGTTGAAAAAGCACA
ACAAAAAGAAATTTTAGAAAAATATTTTAAAGCATCTAAAGAGCAATTGGATCCTGTTTATAAAGAAATTATTAGATTAATTTATAGAGAA
GAGATCCCAAAATGA

Gene: ylxQ (RNA binding protein)

Contig: 06_NODE_54, position: 394215 to 394532, length: 318 nt, orientation: FORWARD

Perfect match to: (MW2-BA000033-[1260574:1260891], allele observed in CC1+CC7+CC188)

Sequence:

ATGAGTATAGATCAAATATTAAACTTTTTAGGATTAGCAATGAGAGCTGGTAAAGTAAAAACAGGTGAATCAGTCATTGTTAATGAGATA
AAAAAAGGAAATTTAAAGCTCGTTATTGTTGCAAATGATGCGTCTGATAATACAGCTAAATTAATTACAGATAAATGTAAGAGTTACAAA
GTTCCATTCAGAAAGTTTGGAAATCGAAATGAATTGGGAATAGCACTTGGAAAAGGTGAGCGTGTTAATGTAGGGATTACTGACCCAGG
CTTTGCTAAAAAGTTGCTATCAATGATAGATGAATATCATAAGGAGTGA

Gene: infB (translation initiation factor 2)

Contig: 06_NODE_54, position: 394537 to 396654, length: 2118 nt, orientation: FORWARD

Sequence:

ATGAGTAAACAAAGAATTTACGAATATGCGAAAGAATTAAATCTAAAGAGTAAAGAGATTATAGATGAGTTAAAAAGCATGAATATTGA
GGTTTCAAATCATATGCAAGCTTTGGAAGATGACCAAATTAAAGCATTAGATAAAAAGTTCAAAAAAGAACAAAAGAACGACAATAAAC
AAAGCACTCAAAATAATCACCAAAAATCAAACAATCAAAACCAAAATAAAGGGCAACAAAAAGATAACAAAAAGAATCAACAACAAAAT
AATAAAGGCAACAAAGGCAATAAAAAGAATAATAGAAATAATAAGAAAAATAACAAGAATAATAAACCACAAAATCAACCAGCTGCTCC
AAAAGAAATACCATCAAAAGTGACATATCAAGAAGGTATTACAGTAGGCGAATTTGCGGATAAATTAAATGTTGAATCATCAGAAATTAT
CAAAAAATTATTCTTACTTGGTATTGTTGCTAATATCAATCAATCATTAAATCAAGAAACAATCGAATTAATTGCCGATGATTATGGCGTTG
AGGTTGAAGAGGAAGTTGTGATTAATGAAGAAGATTTATCAATCTACTTCGAAGACGAAAAAGATGATCCAGAAGCAATTGAGAGACCA
GCAGTTGTAACAATTATGGGACATGTTGACCATGGTAAAACTACTTTATTAGATTCAATTCGTCATACAAAAGTTACAGCAGGTGAAGCA
GGTGGAATCACTCAACATATTGGTGCATATCAAATTGAAAACGATGGCAAAAAAATCACTTTCTTAGATACACCGGGACATGCTGCATTT
ACAACGATGCGTGCGCGTGGTGCACAAGTAACAGATATTACAATTTTAGTAGTAGCAGCTGACGATGGTGTTATGCCACAAACAATTGA
AGCAATTAACCATGCTAAAGAAGCAGAAGTACCAACTATTGTTGCAGTAAATAAAATTGATAAACCAACTTCAAATCCTGATCGAGTTAT
GCAAGAATTAACTGAATATGGTTTAATTCCTGAAGATTGGGGTGGCGAAACAATTTTCGTTCCACTTTCTGCATTAAGTGGTGATGGTATC
GACGATTTATTAGAAATGATAGGATTAGTTGCAGAAGTTCAAGAACTTAAAGCAAATCCTAAAAACCGTGCTGTTGGTACAGTTATCGAA
GCTGAATTAGATAAATCACGTGGTCCTTCTGCATCATTATTAGTACAAAACGGTACATTAAATGTTGGGGATGCGATTGTAGTTGGTAAT
ACTTACGGCCGTATTCGTGCAATGGTTAATGACTTAGGTCAAAGAATCAAAATGGCTGGTCCATCAACGCCTGTTGAAATTACAGGTATT
AATGATGTGCCACAAGCTGGGGATCGCTTTGTTGTATTTAGTGATGAAAAACAAGCTCGTCGTATTGGTGAATCAAGACACGAAGCTAG
CATTGTTCAACAACGTCAAGAAAGTAAAAATGTTTCATTAGATAACCTGTTTGAACAAATGAAACAAGGTGAAATGAAAGATTTAAACGT
TATTATTAAAGGTGATGTTCAAGGTTCTGTTGAAGCTTTAGCTGCATCATTAATGAAAATTGATGTTGAAGGCGTAAATGTTCGTATCATT
CATACAGCGGTTGGTGCAATTAATGAGTCAGACGTGACACTTGCTAATGCCTCAAATGGTATTATCATTGGTTTCAATGTTCGTCCAGACA
GTGGTGCAAAACGTGCTGCAGAAGCTGAAAATGTTGATATGCGTTTACACAGAGTTATTTATAATGTTATCGAAGAAATTGAATCAGCGA
TGAAAGGTTTACTTGATCCAGAATTTGAAGAACAAGTTATCGGACAAGCTGAAGTTCGTCAAACATTCAAAGTTTCTAAAGTTGGTACTA
TTGCTGGATGTTATGTTACTGAAGGTAAAATTACGCGAAATGCTGGTGTACGTATTATTCGTGATGGTATTGTTCAATATGAAGGCGAAT
TAGATACACTTAAACGTTTCAAAGATGATGCTAAGGAAGTTGCAAAAGGTTATGAATGTGGTATTACAATTGAAAACTACAATGACCTTA
AAGAAGGCGATGTTATCGAAGCATTTGAAATGGTTGAAATTAAGCGTTAA

Gene: rbfA (ribosome-binding factor A)

Contig: 06_NODE_54, position: 397040 to 397390, length: 351 nt, orientation: FORWARD

Perfect match to: (N315-BA000018-[1263268:1263618], highly conserved allele)

Sequence:

ATGAGCAGTATGAGAGCAGAGCGTGTTGGTGAACAAATGAAGAAGGAATTAATGGATATCATCAACAATAAAGTCAAAGATCCTCGAG
TTGGTTTTATTACAATTACAGATGTTGTTTTAACAAATGATTTATCGCAGGCTAAAGTATTTTTAACTGTATTAGGTAACGATAAAGAAGT
AGAAAATACATTTAAAGCACTTGATAAAGCAAAAGGCTTCATTAAGTCTGAATTAGGTTCTAGAATGCGATTACGTATTATGCCGGAATT
AATGTATGAATATGATCAATCAATCGAATATGGTAATAAAATTGAACGAATGATTCAAGATTTACACAAACAAGATAGATAA

Gene: truB (tRNA pseudouridine synthase B)

Contig: 06_NODE_54, position: 397560 to 398477, length: 918 nt, orientation: FORWARD

Perfect match to: (N315-BA000018-[1263787:1264704], highly conserved allele)

Sequence:

ATGTATAATGGGATATTACCAGTATATAAAGAGCGCGGTTTAACAAGTCATGACGTTGTATTCAAATTGCGTAAAATATTAAAAACTAAA
AAAATAGGTCACACGGGTACGCTTGATCCCGAAGTTGCAGGCGTGTTACCGGTATGTATAGGTAATGCAACGAGAGTTAGTGATTATGT
TATGGATATGGGCAAAGCTTATGAAGCAACTGTATCGATAGGAAGAAGTACAACGACTGAAGATCAAACGGGTGATACATTGGAAACA
AAAGGTGTACACTCAGCAGATTTTAATAAGGACGATATTGACCGATTGTTAGAAAGTTTTAAAGGTATCATTGAACAAATTCCGCCGATG
TACTCATCCGTCAAAGTAAATGGTAAAAAATTATATGAATATGCGCGTAATAATGAAACAGTTGAAAGACCAAAGCGTAAAGTTAATATT
AAAGACATTGGGCGTATATCTGAATTAGATTTTAAAGAAAATGAGTGTCATTTTAAAATACGCGTCATCTGTGGTAAAGGTACATATATT
AGAACGCTAGCAACTGATATTGGTGTGAAATTAGGCTTTCCGGCACATATGTCGAAATTAACACGAATCGAGTCTGGTGGATTTGTGTTG
AAAGATAGCCTTACATTAGAACAAATAAAAGAACTTCATGAGCAGGATTCATTGCAAAATAAATTGTTTCCTTTAGAATATGGATTAAAG
GGTTTGCCAAGCATTAAAATTAAAGATTCGCACATAAAAAAACGTATTTTAAATGGGCAGAAATTTAATAAAAATGAATTTGATAACAAA
ATTAAAGACCAAATTGTATTTATTGATGATGATTCAGAAAAAGTATTAGCAATTTATATGGTACACCCTACAAAAGAATCAGAAATTAAAC
CTAAAAAAGTCTTTAATTAA

Gene: ribF (riboflavin biosynthesis protein)

Contig: 06_NODE_54, position: 398492 to 399463, length: 972 nt, orientation: FORWARD

Perfect match to: (MW2-BA000033-[1264851:1265822], allele observed in CC1+CC15-ST582)

Sequence:

ATGAAAGTCATAGAAGTGACACATCCTATACAATCAAAACAGTATATTACAGAGGATGTTGCAATGGCATTCGGATTTTTCGATGGCATG
CATAAAGGTCATGACAAAGTCTTTGATATATTAAACGAAATAGCTGAGGCACGCAGTTTAAAAAAAGCGGTGATGACATTTGATCCGCAT
CCGTCTGTCGTGTTGAATCCTAAAAGAAAACGAACAACGTATTTAACGCCACTTTCAGATAAAATCGAAAAAATTAGCCAACATGATATT
GATTATTGTATAGTGGTTAATTTTTCATCTAGGTTTGCTAATGTGAGCGTAGAAGATTTTGTTGAAAATTATATAATTAAAAATAATGTAA
AAGAAGTCATTGCTGGTTTTGATTTTACTTTTGGTAAATTTGGAAAAGGTAATATGACTGTACTTCAAGAATATGATGCGTTTAATACGAC
AATTGTGAGTAAACAAGAAATTGAAAATGAAAAAATTTCTACAACTTCTATTCGTCAAGATTTAATCAATGGTGAGTTGCAAAAAGCGAA
TGATGCTTTAGGCTATATATATTCTATTAAAGGCACTGTAGTGCAAGGTGAAAAAAGGGGAAGAACTATTGGCTTCCCAACAGCTAACAT
TCAACCTAGTGATGATTATTTGTTACCTCGTAAAGGTGTTTATGCTGTTAGTATTGAAATCGGCACTGAAAATAAATTATATCGAGGGGTA
GCTAACATAGGTGTAAAGCCAACATTTCATGATCCTAACAAAGCAGAAGTTGTCATCGAAGTGAATATCTTTGACTTTGAGGATAATATTT
ATGGTGAACGAGTGACCGTGAATTGGCATCATTTCTTACGTCCTGAGATTAAATTTGATGGTATCGACCCATTAGTTAAACAAATGAACG
ATGATAAATCGCGTGCTAAATATTTATTAGCAGTTGATTTTGGTGATGAAGTAGCTTATAATATCTAG

Gene: rpsO (30S ribosomal protein S15)

Contig: 06_NODE_54, position: 399578 to 399847, length: 270 nt, orientation: FORWARD

Perfect match to: (RF122-AJ938182-[1230825:1231094], highly conserved allele)

Sequence:

ATGGCAATTTCACAAGAACGTAAAAACGAAATCATTAAAGAATACCGTGTACACGAAACTGATACTGGTTCACCAGAAGTACAAATCGCT
GTACTTACTGCAGAAATCAACGCAGTAAACGAACACTTACGTACACACAAAAAAGACCACCATTCACGTCGTGGATTATTAAAAATGGTA
GGTCGTCGTAGACATTTATTAAACTACTTACGTAGTAAAGATATTCAACGTTACCGTGAATTAATTAAATCACTTGGTATCCGTCGTTAA

Gene: pnpA (polyribonucleotide nucleotidyltransferase)

Contig: 06_NODE_54, position: 400215 to 402311, length: 2097 nt, orientation: FORWARD

Perfect match to: (SA40-CP003604-[1255395:1257491], highly conserved allele)

Sequence:

ATGTCTCAAGAAAAGAAAGTTTTTAAAACTGAATGGGCAGGAAGATCTTTAACGATTGAAACAGGGCAATTAGCTAAACAAGCAAATGG
CGCTGTATTGGTTCGTTATGGAGATACAGTCGTGTTATCGACGGCAACTGCATCAAAAGAACCTCGTGATGGAGATTTCTTCCCATTAAC
AGTGAACTATGAAGAAAAAATGTACGCTGCGGGTAAAATTCCTGGTGGATTTAAAAAGAGAGAAGGACGTCCTGGTGACGATGCAACA
TTAACTGCGCGATTAATTGATAGACCAATTAGACCTTTATTCCCTAAAGGATATAAGCATGATGTTCAAATTATGAACATGGTATTAAGTG
CAGATCCTGATTGTTCACCACAAATGGCTGCAATGATTGGTTCATCTATGGCGCTTAGTGTGTCGGATATTCCATTCCAAGGGCCAATCGC
CGGTGTAAATGTGGGTTATATTGACGGTAAATATATCATTAACCCAACAGTAGAAGAAAAAGAAGTTTCTCGCTTAGACCTTGAAGTAGC
TGGTCATAAAGATGCAGTAAACATGGTAGAGGCAGGCGCTAGTGAGATTACTGAACAAGAAATGTTAGAGGCGATTTTCTTTGGTCATG
AAGAGATTCAACGTTTAGTTGATTTCCAACAACAAATCGTCGACCACATTCAACCTGTTAAACAAGAATTTATTCCAGTAGAGCGTGATGA

AGCGCTAGTTGAACGTGTAAAATCTTTAACCGAAGAAAAGGGACTTAAAGAAACAGTTTTAACATTTGATAAACAACAACGAGATGAAA
ATCTTGATAACTTAAAAGAAGAAATCGTCAATGAATTTATCGATGAAGAAGATCCAGAAAATGAATTACTTATTAAAGAAGTTTATGCAA
TTTTAAATGAATTAGTGAAAGAAGAAGTTCGACGTTTAATTGCAGATGAAAAAATTAGACCAGACGGCCGTAAACCTGATGAAATCCGTC
CATTAGATTCTGAAGTTGGTATTTTACCTAGAACGCATGGTTCAGGTCTATTTACACGTGGTCAGACTCAAGCACTTTCAGTTTTAACATTA
GGTGCTTTAGGCGATTATCAATTAATTGATGGTTTAGGACCTGAAGAAGAAAAAAGATTCATGCATCATTACAACTTCCCGAATTTTTCAG
TAGGTGAAACTGGTCCAGTACGTGCGCCAGGTCGTCGTGAAATTGGACATGGTGCGTTAGGTGAAAGAGCATTAAAATATATTATTCCT
GATACTGCTGATTTCCCATATACAATTCGTATTGTAAGTGAGGTACTTGAATCAAATGGTTCATCATCTCAAGCGTCAATTTGTGGATCAA
CATTAGCATTAATGGATGCGGGCGTACCGATTAAAGCACCAGTTGCTGGTATTGCTATGGGCCTTGTTACACGTGAAGATAGCTATACGA
TTTTAACTGATATCCAAGGTATGGAAGATGCATTAGGTGATATGGACTTTAAAGTCGCTGGTACTAAAGAAGGTATTACAGCAATCCAAA
TGGATATTAAAATTGACGGTTTAACGCGTGAAATTATCGAAGAGGCTCTAGAACAAGCGAGACGTGGTCGTTTAGAAATAATGAATCAT
ATGTTACAAACAATTGATCAACCACGTACTGAATTAAGTGCTTACGCGCCAAAAGTTGTAACTATGACAATTAAACCAGATAAGATTAGA
GATGTTATCGGACCTGGTGGTAAAAAAATTAACGAAATTATTGATGAAACAGGCGTTAAATTAGATATTGAACAAGATGGTACTATCTTT
ATTGGTGCCGTTGATCAAGCTATGATAAATCGCGCTCGTGAAATCATTGAGGAAATTACACGTGAAGCGGAAGTAGGTCAAACTTATCA
AGCCACTGTTAAACGTATTGAAAAATACGGTGCGTTTGTAGGCCTATTCCCGGGTAAAGATGCGTTGCTTCACATTTCACAAATTTCAAAA
AATAGAATTGAAAAAGTGGAAGATGTATTAAAAATCGGTGACACAATTGAAGTTAAGATTACTGAAATTGATAAACAAGGTCGAGTAAA
TGCTTCACACAGAGCATTAGAAGAATAA

Gene: rnjB (ribonuclease J2)

Contig: 06_NODE_54, position: 402547 to 404220, length: 1674 nt, orientation: FORWARD

Perfect match to: (08-02119-CP015645-[903234:904907:r], allele observed in ST582+CC8+CC22+CC239)

Sequence:

TTGAGTTTAATAAAGAAAAAGAATAAAGATATTCGCATTATACCATTAGGCGGTGTTGGCGAAATTGCTAAAAATATGTATATCGTTGAA
GTAGACGATGAAATGTTTATGTTAGATGCTGGACTTATGTTTCCAGAAGACGAAATGCTAGGTATTGATATTGTTATACCAGACATTTCAT
ACGTACTTGAAAATAAAGATAAATTGAAGGGTATATTCCTTACACACGGACATGAGCACGCGATTGGTGCAGTGAGTTATGTTTTAGAAC
AATTAGATGCACCAGTATATGGATCTAAATTGACAATAGCGTTAATTAAAGAAAATATGAAAGCCCGTAATATTGATAAAAAAGTTCGCT
ACTACACAGTTAATAATGATTCAATTATGAGATTCAAAAACGTGAATATTAGTTTCTTTAATACGACACACAGTATTCCTGATAGTCTAGG
TGTTTGTATTCACACTTCATATGGTGCCATTGTGTATACAGGTGAATTTAAGTTTGACCAAAGTTTACATGGACATTATGCACCAGATATTA
AACGTATGGCAGAGATTGGTGAAGAAGGTGTATTTGTCTTAATCAGTGATTCTACTGAGGCAGAGAAACCTGGATATAATACTCCGGAA
AATGTGATTGAACATCATATGTATGACGCTTTTGCAAAAGTGCGAGGTCGCTTGATTGTTTCATGTTATGCTTCGAACTTTATACGTATAC
AGCAAGTTTTAAATATTGCTAGCAAGCTAAATCGTAAAGTGTCATTTTTAGGAAGATCACTTGAAAGTTCATTTAATATTGCTCGTAAAAT
GGGGTATTTCGACATTCCTAAAGATTTGCTAATTCCTATAACAGAAGTTGATAATTATCCTAAAAATGAAGTGATAATTATAGCTACTGGT
ATGCAAGGAGAACCTGTAGAAGCCTTAAGTCAAATGGCGCAACATAAGCATAAAATTATGAATATCGAAGAAGGCGATTCTGTATTTTTA
GCAATTACGGCTTCTGCAAATATGGAAGTTATCATTGCGAATACATTAAATGAGCTTGTACGTGCTGGCGCACATATTATTCCAAATAACA
AGAAGATTCATGCTTCAAGTCATGGTTGCATGGAAGAATTAAAAATGATGATTAATATTATGAAACCTGAATACTTTATTCCTGTACAAGG
TGAATTTAAAATGCAGATAGCACATGCGAAGCTAGCAGCTGAAGCAGGTGTTGCACCAGAAAAGATTTTCCTTGTGGAAAAAGGAGATG
TCATTAATTACAACGGTAAAGATATGATATTAAATGAAAAGGTAAATTCAGGAAATATTTTAATAGATGGTATTGGTATTGGGGATGTAG
GAAATATCGTGTTGAGAGACCGTCATCTTTTAGCAGAAGATGGTATCTTTATTGCTGTAGTAACGTTAGATCCTAAAAATAGACGTATAG
CTGCGGGACCTGAAATTCAATCTCGTGGGTTTGTATATGTACGTGAAAGTGAAGACTTATTACGTGAAGCAGAAGAGAAAGTACGTGAA
ATAGTAGAGGCTGGTTTACAAGAAAAACGCATAGAATGGTCTGAAATTAAGCAAAATATGCGTGATCAAATTAGTAAACTATTATTCGAA
AGTACAAAACGTCGTCCTATGATTATTCCAGTAATTTCTGAAATTTAA

Gene: ftsK (cell division protein K)

Contig: 06_NODE_54, position: 404603 to 406846, length: 2244 nt, orientation: FORWARD

Perfect match to: (MW2-BA000033-[1270962:1273205], allele observed in CC1+CC93+CC239)

Sequence:

ATGGTGTTGGGTGTTTTCCAATTAGGAATAATAGGTCGTCTAATTGACAGCTTCTTTAATTATTTATTTGGGTACAGTAGATATTTAACATA
TATTTTAGTACTCTTAGCAACTGGTTTTATTACATACTCTAAACGTATTCCTAAAACTAGACGAACGGCTGGTTCGATTGTATTGCAAATTG
CATTGCTATTTGTATCACAGTTAGTTTTTCATTTTAATAGTGGTATCAAAGCTGAAAGAGAACCTGTACTTTCTTATGTGTATCAGTCATAC
CAACACAGTCATTTCCCAAATTTTGGTGGCGGTGTATTAGGCTTTTATTTATTAGAGTTAAGCGTACCTTTAATTTCATTATTTGGTGTATG
TATTATTACTATTTTATTATTATGCTCAAGTGTTATTTTATTAACAAACCATCAACATCGTGAAGTCGCAAAAGTTGTACTGGAAAATATAA
AAGCTTGGTTTGGTTCATTTAATGAAAAAATGTCGGAAAGAAACCAAGAAAAACAATTGAAGCGTGAAGAAAAAGCGAGACTTAAAGA
AGAACAAAAGGCACGTCAAAATGAACAGCCACAAATAAAAGATGTGAGTGATTTTACGGAAGTGCCTCAAGAAAGAGATATTCCAATTT

ATGGGCATACTGAAAATGAAAGTAAAAGTCAGAGTCAACCAAGTCGGAAAAAGCGAGTGTTTGATGCAGAGAATAGTTCGAATAACAT
CGTAAATCATCATCAAGCAGATCAGCAAGAACAATTAACAGAACAAACTCATAACAGTGTTGAAAGTGAAAACACTATTGAAGAAGCTG
GTGAAGTTACGAATGTATCGTATGTTGTTCCACCGTTAACTTTACTTAATCAACCTGCAAAACAAAAAGCAACATCTAAAGCTGAAGTGCA
ACGTAAAGGACAAGTACTAGAGAATACATTAAAAGATTTTGGGGTAAATGCAAAAGTGACACAAATTAAAATTGGTCCTGCAGTAACTC
AATATGAAATTCAACCAGCTCAAGGGGTTAAAGTGAGTAAAATTGTAAACTTGCATAATGATATTGCATTAGCTTTAGCAGCAAAAGATG
TTAGAATCGAAGCGCCAATACCTGGTCGCTCTGCAGTAGGTATTGAAGTGCCAAATGAGAAAATTTCATTAGTTTCACTAAAAGAAGTTT
TAGATGAAAAATTCCCGTCTAATAATAAACTAGAAGTTGGATTAGGAAGAGATATATCAGGTGATCCAATTACTGTTCCACTAAATGAAA
TGCCACACTTATTGGTGGCAGGATCGACGGGTAGTGGTAAATCTGTTTGTATAAATGGTATTATTACAAGTATTTTATTAAATGCTAAGCC
GCATGAAGTTAAACTTATGTTAATCGATCCGAAAATGGTTGAACTAAATGTTTATAACGGAATTCCACATTTATTAATTCCGGTTGTTACA
AATCCTCATAAAGCTGCTCAAGCTTTAGAAAAAATTGTAGCTGAGATGGAAAGACGTTATGATTTATTCCAACATTCATCAACTAGAAATA
TTAAAGGTTATAACGAATTAATCCGTAAGCAAAATCAAGAATTAGATGAGAAGCAACCAGAATTACCTTATATCGTTGTTATTGTAGATG
AGCTTGCAGATTTAATGATGGTAGCTGGTAAAGAAGTTGAAAATGCGATTCAACGTATTACACAAATGGCACGTGCAGCAGGTATACAT
TTAATTGTAGCGACACAAAGACCTTCTGTGGATGTAATTACAGGTATCATTAAAAATAATATTCCATCTAGAATTGCTTTTGCTGTAAGTTC
TCAAACAGATTCAAGAACTATTATTGGTACTGGCGGCGCAGAAAAGTTACTTGGTAAAGGTGACATGTTATACGTTGGAAATGGTGATTC
ATCACAAACACGTATTCAAGGGGCGTTTTTAAGTGACCAAGAGGTGCAAGATGTTGTAAATTATGTAGTAGAACAACAACAGGCAAATT
ATGTAAAAGAAATGGAACCAGATGCACCAGTGGATAAATCGGAAATGAAAAGTGAAGATGCTTTATATGATGAAGCGTATTTGTTTGTT
GTTGAACAACAAAAGGCAAGTACATCATTGTTACAACGCCAATTTAGAATTGGTTATAATAGAGCATCTAGGTTGATGGATGATTTAGAA
CGCAATCAGGTAATCGGTCCACAAAAAGGAAGCAAGCCTAGACAAGTTTTAATAGATCTTAATAATGACGAGGTGTAA

Gene: ymfC (putative transcriptional regulator, GntR family)

Contig: 06_NODE_54, position: 406851 to 407564, length: 714 nt, orientation: FORWARD

Perfect match to: (MRSA252-BX571856-[1314914:1315627], allele observed in CC30+CC1+CC25+CC30+CC239)

Sequence:

ATGTCAGAAATGAATGCGGTATATAACGTTAAACAATACATTTTAAATTTGATTAAGCAAAATAAATTGGAATATGGTGACCAACTTCCA
AGTAATTTATCAATTGCCAGAGAATTAAATGTAAAAACCGACGATGTTTATGAAGCAATTCAGGCATTGATTACTGAACAAGTCATTAAA
GATAATTTTGAAGAGGGCACAAGTGTTAAGTCACTGCCCCCTTTCTTTTATCCATTGAATGAACTTATAAGTATTGGGCAAATGATTAAAA
ATGCAGGATTTGAATGCGGAACTGAATACTTAAATTTTGATGAGCAACCAGCAACTATGTTAGATGCAAATTTGTTGAGCGTTGAAGAAG
GATATCCAGTAACCATTATAGAACGATTACGAACTGCCGATGGAGAACCGGTCGTCTATTGTTTAGATAAAATTGCTAAAAAAGAATTAA
CATGTACAGAGTATCAAATGAGCAATGGATCGATACTAAGTGCGATAAAAGAACAAAGTAATCATAATATTTGTTACGCTGATACAGAA
ATTGAAGCGGTAAATTATGAACCTCGAATATCCGAAGTACTGAATGCTTCGCCACACGAAGGTTTGATTTTATTAAAAATTACGCACTACA
ATGAATTAGATGAACCTATTTTGTATTCATTAAATTATATGAAAAATAGCTTAGTTCAATTTAAAATCACTAGAAAAATATAG

Gene: ymfF (putative metalloprotease)

Contig: 06_NODE_54, position: 407595 to 408860, length: 1266 nt, orientation: FORWARD

Sequence:

TTGAGTAGACAATCTCAACCAAATATACATATCAAAGTTTCACCAACAACTAAATTTAAAACAACTACTATAGTTTTTAAATTTATGGCACC
TTTAGAATATGACACAACTACAGCTAGATCATTATTAAGCAAATTATTAGTTCGAGCAACTAAGAAATGGCCAACCGATAAGTCGTTTAAT
AATCACTTAGCCGATTTATATGGTGCATATGTGAATAGTACAATTTCAAAATTCAAAGGTCAGCATGTCATTACATTTTCATTAGAAATTGT
AAATGAACGTTATTTAAGAAACGGTGAATCATTATTTAATCAAGGATTAGATTTATTACAAGAAATCATTTGGAATCCATTAATTGAAAAT
AAAGCATTCAATGATAATTTTGTTAACCAAGAGAAAACATTATTAGCCAAAAAAATAGAAGCAATGGTAGATAACAAAGCACAATATTCG
TTTTTAAAATTACTCGACCATATGTTTGAAAATGAAGCATACAAATACTTATCTACAGGACAACTAGAACAAATCCCACATATTACTGCTG
AAACACTATATCATACATATCAATCAATGATTAATAATGATCAATGTTCTGTTTATGTTGTCGGCAATGTAGAACCTGAAAGTGTTGAGAA
ACAAATACGTGAAAAATTTGCACTTAAACCATTCGATAAACATCAATTCCAACATTCTACACATCATTTACACGATGAAGAAGTTGATTAT
ATTGTTGAATATGATGACGTGGATCAAGCTAAATTAAATATGGGATATCGTTTTCCAACACAATATGGACAAAGTGGATATGCTGCTTTT
GTTGTATTTAACATGATGTTTGGAGGAGATCCTTCATCTGTTTTATTTAATGAAGTGCGAGAAAAGCAAAGTTTAGCGTACTCTATACATT
CACAAATTGATGGCAAAAATGGCTATTTATTTGTTTTGAGTGGGGTTTCAAGTGATAAGTACGAAACTGCAAAAGACACTATTATAAGTG
AATTTGAAAAAATAAAAGCAGGAGATTTCACTGAAGAAAAATTAGAGTTAGCTAAAAAAGTAATCATTTCTCATCGATATGAATCTGAAG
ATCGTCCGAAAAGTATTATAGAGATTATGCATAACCAAATATTATTAGAGCAACCACAAAGCAAAGAAACATTTATAAATGATATACAGA
AGGTAAGTCGCGAAGATATTGTTTCTGTTGCTGAAAAAGCATTTTTAGATACAATCTATGTGTTGACAAAAGGAGGGGATAAATAA

Gene: fabG2 (3-oxoacyl-[acyl-carrier-protein] reductase)

Contig: 06_NODE_54, position: 410146 to 410850, length: 705 nt, orientation: FORWARD

Perfect match to: (MW2-BA000033-[1276505:1277209], allele observed in CC1+CC5+CC8+CC80+CC239)

Sequence:

ATGAAAGCATTAGTATTAGGTGGTTCTGGTTCAATTGGTTCTGAGATAGTCAAACAATTATTAACTGATGGATTTGAAGTTTATGTGCAAT
ATTATCGTACTGATATAAATGAATTAACTAGCAAATTTAATGATGATAAAGTTCGTTTTATACAAGCGGATTTATCTCAAACAATTGATATT
GACAAAACATTTGGTGACATTAAATCATTAGACTGTTTAATATATGCAAGTGGTCAGTCTTTATATGGTGTTTTACAAGATATGAAAGACC
ATGATATTGATGCATGTTATCAGTTAAATGTCTTGCAATTAATTCGATTATGTAGATATTTCGTTGATGTTTTACGTCAAAGTGACAATGGA
AGAATTATTGTAATTTCATCAATTTGGGGTGAGACAGGAGCTAGTATGGAAACTATTTATTCGACGATGAAAAGTGCACAATTAGGTTTC
GTTAAGGCGCTTAGTCAAGAGCTTGCACTAACATCAGTGACAGTAAATGCTATCGCACCTGGATTTGTAGCCGGTAATATGGCAAGTGA
GTGGCAAGAAGATGAACTTCAAGCAATGATAACTGAATTACCACAACAGCGATTGATTTTACCGAGTGAGGTTGCTCATACATGCGCCTA
TTTATATCACCCAAATGCTAGAAGTGTTACTGGAACTATACAGAAAGTTAATGGTGCTTGGTATATTTAA

Gene: ymfK (ACT domain regulatory protein)

Contig: 06_NODE_54, position: 410955 to 411782, length: 828 nt, orientation: FORWARD

Perfect match to: (MW2-BA000033-[1277314:1278141], highly conserved allele)

Sequence:

ATGACAGTTGCAGAGAAAAAAGAATGGTACCTAGAATACGAAATTGAAATTAATAGACCGGGTCTTTTAGGTGATGTATCTAGTTTATTA
GGTATGTTAGGTATAAGTATTGTTACAATTAATGGTGTTGATCAAGGTAAACGAGGCCTTTTAATTAAAACAGACAATCTTGAAAAAGTT
GAACGTTTTGAGCAAATAGCTCGTGGTATAAATGAAATTGAAATAACAAAGCTTAAAAAACCAGAATTAAGAGACCGTCTTGCAGTAAG
ACATGGTAGATATATTGAGCAAGATGCAAAAGATAAGAAAACCTTTCGATTTGAGCGTGAAGATTTAGGCTTGTTAGTAGACTTTTTAGC
TGAATTGTTCAAAGAAGAAGGTCATAAGTTGATTGGCATTAGAGGTATGCCACGAGTTGGTAAAACTGAATCAATTGTTGCGGGAAGTG
TTTGTGCACATAAGAGATGGTTATTTATTAGTTCTACTTTAATAAAACAAACTGTACGTAGCTCTCTAATTAAAGGGGAATATGATGCCAA
TCATGTATACATTATTGATGGTGCAGTTACTGCCAGAGAATCTAATCCAAAACATCAAGAGCTTGTTAACGAAGTTATGACGTTACCATCA
ATCAAAGTCGTTGAACATCCAGATTTATTTGTTGAAACAAGTACTTGTACAATGGAAGATTTTGACTATATTATCGAATTGAGAGAAAATG
AAAATCAAGAAATACATTACGAAGAAATGAAGAAACAGACAGTCCAAAGTAAGAATAATTTAGATTTTGGAGATCCGTTTGGTGGTGGT
TTTGGTTTCTTCGAGTAA

Gene: ymfM (transcriptional regulator)

Contig: 06_NODE_54, position: 411801 to 412193, length: 393 nt, orientation: FORWARD

Perfect match to: (N315-BA000018-[1278026:1278418], highly conserved allele)

Sequence:

TTGAAAACGGTCGGTGAAGCGCTAAAAGGTAGACGTGAAAGGTTAGGAATGACTTTAACAGAATTAGAGCAACGTACTGGAATTAAAC
GTGAAATGCTAGTGCATATTGAAAATAATGAATTCGATCAACTACCGAATAAAAATTACAGCGAAGGATTTATTAGAAAATATGCAAGCG
TAGTAAATATTGAACCTAACCAATTAATTCAAGCTCATCAAGATGAAATTCCATCGAACCAAGCCGAATGGGACGAAGTAATTACAGTTT
TCAATAATAATAAAGACTTAGATTATAAGAGTAAATCAAAAGAGCCAATACAATTATTAGTAATCATGGGTATTACAGTTTTAATAACTTT
ATTGTTATGGATCATGTTAGTTTTAATATTTTAA

Gene: pgsA (CDP-diacylglycerol--glycerol-3-phosphate 3-phosphatidyltransferase)

Contig: 06_NODE_54, position: 412227 to 412805, length: 579 nt, orientation: FORWARD

Perfect match to: (N315-BA000018-[1278452:1279030], highly conserved allele)

Sequence:

ATGAATATTCCGAACCAGATTACGGTTTTTAGAGTAGTGTTAATACCAGTTTTTATATTGTTTGCGTTAGTTGATTTTGGATTTGGCAATGT
GTCATTTCTAGGAGGATATGAAATAAGAATTGAGTTATTAATCAGTGGTTTTATTTTTATATTGGCTTCCCTTAGCGATTTTGTTGATGGTT
ATTTAGCTAGAAAATGGAATTTAGTTACAAATATGGGGAAATTTTTGGATCCATTAGCGGATAAATTATTAGTTGCAAGTGCTTTAATTGT
ACTTGTGCAACTAGGACTAACAAATTCTGTAGTAGCAATCATTATTATTGCCAGAGAATTTGCCGTAACTGGTTTACGTTTACTACAAATT
GAACAAGGATTCGTAAGTGCAGCTGGTCAATTAGGTAAAATTAAAACAGCAGTTACTATGGTAGCAATTACTTGGTTGTTATTAGGTGAT

CCATTGGCAACATTGATTGGTTTGTCATTAGGACAAATTTTATTATACATTGGCGTTATTTTTACTATCTTATCTGGTATTGAATACTTTTAT
AAAGGTAGAGATGTTTTTAAACAAAAATAA

Gene: cinA (competence-damage inducible protein)

Contig: 06_NODE_54, position: 413032 to 414183, length: 1152 nt, orientation: FORWARD

Perfect match to: (08-02119-CP015645-[893272:894423:r], highly conserved allele)

Sequence:

ATGTCAATTGCCATTATTGCTGTAGGCTCAGAACTATTGCTAGGTCAAATCGCTAATACCAACGGACAATTTCTATCTAAAGTATTTAATG
AAATTGGACAAAATGTATTAGAACATAAAGTTATTGGAGATAATAAAAAACGTTTAGAATCAAGTGTACGTCATGCGCTAGAAAAATAT
GATACTGTTATTTTAACAGGTGGCTTAGGTCCTACGAAAGATGACTTAACGAAGCATACAGTGGCCCAGATTGTTGGTAAAGATTTAGTT
ATTGATGAGCCTTCTTTAAAATATATTGAAAGCTATTTTGAGGAACAAGGACAAGAAATGACACCTAATAATAAACAACAGGCTTTAGTA
ATTGAAGGTTCAACTGTATTAACAAATCATCATGGCATGGCTCCAGGAATGATGGTGAATTTTGAAAACAAACAAATTATTTTATTACCAG
GTCCACCGAAAGAAATGCAACCAATGGTGAAAAATGAATTGTTGTCACATTTTATAAACCATAATCGAATTATACATTCTGAACTATTAAG
ATTTGCGGGAATAGGTGAATCTAAAGTAGAAACAATATTAATAGATCTTATCGATAAACAGACTAATCCTACGATTGCGCCTTTGGCGGG
AAGTCATGAAGTATATATTAGATTGACTGCAAATGCCGACTCAAAAGAACAAGCACAATCATTGATTCAACCTGTTAAACAAGAAATTCT
TGATCGTATTGGAGAATATTATTATGGTTCAGATGACACATTAATTGAGCAAGCTGTAATAAAGAAAATTCATGAACCTTTTGTAATATAT
GATGGTATTACTAATGGTGCTTTATATCATCGATTGAAAGAAGTGGATTTAAACGATGTTCTAAAGGGTATGATTAATCACAATGAAAAC
TTTGTTGATATTAATAAACCTATTGAGCAGCAATTAAAAGATGCAGTGCAATTTGTTAATAAATTGTTTAATGTGTCATCAGCAATTATTCT
ATTAGAGTATGATGGTGTAGTCCATATAGGCTATGATAATAACTTTGAATTTAAAACTGAGCAATTTAAAATGTCTAAATCTAGAAATTTA
TTAAAGAACAGAAGTCAAAATTATGCGCTCATAAGATTATTAAATTGGCTTAGAACAACAAATTAA

Gene: recA (recombinase A)

Contig: 06_NODE_54, position: 414348 to 415391, length: 1044 nt, orientation: FORWARD

Perfect match to: (08-02119-CP015645-[892064:893107:r], allele observed in ST582+CC8+CC30+CC4803)

Sequence:

TTGGATAACGATCGTCAAAAAGCTTTAGATACAGTAATTAAAAATATGGAGAAATCTTTCGGTAAAGGTGCCGTAATGAAGTTGGGTGA
CAATATAGGTCGCCGAGTTTCAACTACATCAACTGGTTCAGTTACATTAGATAATGCGCTAGGTGTAGGTGGCTATCCTAAAGGACGAAT
TATTGAAATTTATGGTCCTGAAAGTTCTGGTAAGACAACAGTAGCGCTTCACGCTATTGCTGAAGTACAAAGTAATGGCGGGGTGGCAG
CATTTATCGATGCTGAACATGCTTTAGATCCAGAATATGCTCAAGCATTAGGCGTAGATATCGATAATTTATATTTATCGCAACCGGATCA
TGGTGAACAAGGTCTTGAAATCGCCGAAGCATTTGTTAGAAGTGGTGCAGTTGATATTGTAGTTGTAGACTCAGTTGCTGCTTTAACACC
TAAAGCTGAAATTGAAGGAGAAATGGGAGACACTCACGTTGGTTTACAAGCTCGTTTAATGTCACAAGCGTTACGTAAACTTTCAGGTGC
TATTTCTAAATCAAATACAACTGCTATTTTCATCAACCAAATTCGTGAAAAAGTTGGTGTTATGTTCGGTAATCCAGAGACTACACCAGGT
GGACGTGCATTAAAATTCTATAGTTCAGTAAGACTAGAAGTACGTCGTGCAGAACAGCTTAAACAAGGACAAGAAATTGTAGGTAATAG
AACTAAAATTAAAGTCGTTAAAAATAAAGTGGCACCACCATTTAGAGTAGCTGAAGTTGACATTATGTATGGACAAGGTATTTCTAAAGA
GGGTGAACTTATTGATTTAGGTGTTGAAAACGACATCGTTGATAAATCAGGAGCATGGTATTCTTACAATGGCGAACGAATGGGTCAAG
GTAAGGAAAATGTTAAAATGTACTTGAAAGAAAATCCACAAATTAAAGAAGAAATTGATCGTAAATTGAGAGAAAAATTAGGTATATCT
GATGGTGATGTTGAAGAAACAGAAGATGCACCAAAGTCATTATTTGACGAAGAATAG

Gene: rny (ribonuclease Y)

Contig: 06_NODE_54, position: 415745 to 417304, length: 1560 nt, orientation: FORWARD

Perfect match to: (MW2-BA000033-[1282104:1283663], allele observed in CC1+CC101)

Sequence:

GTGAATTTATTAAGCCTCCTACTCATTTTGCTGGGGATCATTCTAGGAGTTGTTGGAGGGTATGTTGTTGCCCGAAATTTGTTGCTTCAAA
AGCAATCACAAGCTAGACAAACTGCCGAAGATATTGTAAATCAAGCACATAAAGAAGCTGACAATATCAAAAAAGAGAAATTACTTGAG
GCAAAAGAAGAAAACCAAATCCTAAGAGAACAAACTGAAGCAGAACTACGTGAAAGACGTAGTGAACTTCAAAGACAAGAAACCCGAC
TTCTTCAAAAAGAAGAAAACTTAGAGCGTAAATCTGATCTATTAGATAAAAAAGATGAGATTTTAGAGCAAAAAGAATCAAAAATTGAA
GAAAAACAACAACAAGTAGATGCAAAAGAGAGTAGTGTTCAAACGTTAATAATGAAGCATGAACAAGAATTAGAACGCATCTCCGGTCT
CACTCAAGAAGAAGCTATTAATGAGCAACTTCAAAGAGTTGAGGAAGAACTGTCACAAGATATTGCAGTACTTGTTAAAGAAAAAGAAA

AAGAAGCTAAAGAAAAAGTTGATAAAACAGCAAAAGAATTATTAGCTACAGCAGTACAAAGATTAGCAGCAGATCACACAAGTGAATCA
ACGGTATCAGTAGTTAACTTACCTAATGATGAGATGAAAGGTCGAATCATTGGACGAGAAGGACGAAACATCCGCACACTTGAAACTTT
AACTGGCATTGATTTAATTATTGATGACACACCAGAAGCGGTTATATTATCTGGTTTTGATCCAATAAGAAGAGAAATTGCTAGAACAGC
ACTTGTTAACTTAGTATCTGATGGACGTATTCATCCAGGCAGAATTGAAGATATGGTCGAAAAAGCTAGAAAAGAAGTAGACGATATTAT
TAGAGAAGCAGGTGAACAAGCTACATTTGAAGTGAACGCACATAATATGCATCCTGACTTAGTAAAAATTGTAGGGCGTTTAAACTATCG
TACGAGTTACGGTCAAAATGTACTTAAACATTCAATTGAAGTTGCGCATCTTGCTAGTATGTTAGCTGCTGAGCTAGGCGAAGATGAGAC
ATTAGCGAAACGAGCTGGACTTTTACATGATGTTGGTAAAGCAATTGATCATGAAGTAGAAGGTAGTCATGTTGAAATCGGTGTAGAAT
TAGCGAAAAAATATGGTGAAAATGAAACAGTTATTAATGCAATCCATTCTCATCATGGTGATGTTGAACCTACATCTATTATATCTATCCTT
GTTGCTGCTGCAGATGCATTGTCTGCGGCTCGTCCAGGTGCAAGAAAAGAAACATTAGAGAATTATATTCGTCGATTAGAACGTTTAGAA
ACGTTATCAGAAAGTTATGATGGTGTAGAAAAAGCATTTGCGATTCAGGCAGGTAGAGAAATCCGAGTGATTGTATCTCCTGAAGAAAT
TGATGATTTAAAATCTTATCGATTGGCTAGAGATATTAAAAATCAGATTGAAGATGAATTACAATATCCTGGTCATATCAAGGTGACAGTT
GTTCGAGAGACTAGAGCAGTAGAATATGCGAAATAA

Gene: Q5HGE4 (putative protein)

Contig: 06_NODE_54, position: 417601 to 417816, length: 216 nt, orientation: REVERSE

Perfect match to: (N315-BA000018-[1283823:1284038:r], highly conserved allele)

Sequence:

TTATTTTAATTGTAAATCTGTTTTCTTTAATTCTTTTATAACTTCTGCAGTATCATAACAATTTGTTGCAATTGTTGAATATCTCTCTGCTAAA
CGATATGCATTAATGTAAAGCTTTAAACTTTCTTTAGCTATATCCTCTGCATCTTCGAATTTTGATGGGTTAGACATAACCACTAATTCTGC
AAATTTTTCTGGATCAATATTAATAGACAT

Gene: ymdB (putative hydrolase)

Contig: 06_NODE_54, position: 417990 to 418787, length: 798 nt, orientation: FORWARD

Perfect match to: (MW2-BA000033-[1284349:1285146], allele observed in CC1+CC15+CC72)

Sequence:

ATGAGAATAATGTTTATAGGGGATATCGTAGGTAAAATTGGACGAGACGCAATTGAAACGTACATACCTCAACTGAAGCAAAAGTATAA
ACCAACAGTTACAATTGTAAATGCTGAAAATGCAGCACATGGTAAAGGTTTGACTGAAAAAATATATAAACAATTACTAAGAAATGGTGT
AGATTTCATGACTATGGGTAATCACACATATGGTCAACGTGAAATTTATGATTTTATAGATGAAGCAAAACGTCTAGTAAGACCAGCGAA
TTTTCCGGATGAAGCGCCGGGAATTGGTATGAGATTTATACAAATTAATGATATTAAACTTGCAGTTATTAATCTGCAAGGAAGAGCGTT
TATGCCAGATATTGATGATCCTTTTAAAAAGGCAGATCAATTAGTCAAGGAAGCACAAGAACAAACTCCGTTTATATTTGTTGATTTTCAT
GCAGAAACAACTTCTGAAAAGTATGCAATGGGATGGCATTTAGATGGTAGAGCTAGCGCTGTTGTTGGAACGCATACACACATTCAAAC
AGCAGATGAACGTATTTTACCAAAGGGGACAGGGTATATAACGGATGTTGGTATGACAGGTTTTTATGACGGCATTTTAGGAATAAATA
AAACAGAGGTAATTGAGCGTTTTATCACTAGTTTGCCACAAAGACATGTTGTTCCAAATGAAGGTAGAAGTGTATTATCTGGTGTTGTTA
TTGATTTAGACAAAGAAGGTAAAACAAAGCACATCGAACGTATATTGATAAATGATGACCATCCATTTTCAACATTTTAA

Gene: porA (pyruvate ferredoxin oxidoreductase alpha chain)

Contig: 06_NODE_54, position: 418927 to 420687, length: 1761 nt, orientation: FORWARD

Perfect match to: (MW2-BA000033-[1285286:1287046], highly conserved allele)

Sequence:

ATGAAACCACAATTATCGTGGAAAGTTGGCGGTCAACAAGGCGAAGGTATTGAATCAACTGGGGAAATCTTCGCTACGGCTATGAATAG
AAAAGGATATTATTTATATGGATATAGACATTTTTCAAGTCGTATCAAAGGTGGACATACGAATAATAAAATTAGAGTTTCTACGACGCCT
GTTCATGCAATTAGTGATGATTTAGATATTTTGATTGCATTTGACCAAGAAACAATTGATGTTAACCATCATGAAATGAGAGAAGACAGT
ATTATTTTAGCTGATGCCAAGGCTAAACCTGTGAAACCAGAAGGATGTCATGCACAGCTTATTGAATTACCTTTTACAGCAACCGCTAAAG
AATTAGGTACAGCATTAATGAAAAACATGGTTGCAATAGGTGCTACTAGCGCATTGATGAATTTGAATACAAATACATTTGAAGAACTTA
TTACTAATATGTTTTCTAAAAAAGGTGACAAGGTAGTTGAAGTCAATATCCAAGCATTAAACGAAGGTTATCAATTAATGCAATCCCGCTT
ACCTGAAATCGACGGGGACTTTGAATTAGAGTCAACAGATGCACTACCACATCTATATATGATTGGTAACGATGCCATTGGATTAGGTGC
AATTGCTGCAGGTTCACAATTTATGGCGGCATATCCTATTACACCTGCGTCTGAAGTTATGGAATATATGATTGCCAATATATCTAAAGTA
AACGGAGCGGTTATTCAAACAGAAGATGAAATTGCTGCTGTAACTATGGCTATTGGTGCAAATTATGGTGGAGTCAGAGCGTTTACGGC

TAGTGCTGGTCCAGGTTTATCTTTAATGATGGAAGCAATTGGATTATCTGGTATGACTGAAACGCCATTAGTCATTATTAATACTCAACGA
GGTGGACCTTCTACTGGATTACCTACGAAACAAGAACAGTCAGATTTAATGCAAATGATTTATGGTACACATGGTGATATTCCAAAAATA
GTTGTAGCACCAACTGATGCAGAAGATGCATTTTATTTAACTATGGAAGCCTTTAATTTAGCAGAACAATATCAATGCCCTGTTATAGTTT
TAAGTGATTTGCAATTATCTTTAGGTAAACAAACTGTTGAAAAATTAGATTATAATCGCATTGAAATTAAACGTGGTGAAATCATTCAATC
TGATATTGAACGTGAAGAAGATGATAAAGGTTATTTCAAGCGTTATGCGTTAACATCCAATGGTGTTTCTCCTAGACCTATCCCCGGTATT
AAAGGAGGTATTCATCATATAACTGGTGTGGAACACAATGAAGAAGGTAAACCTAGTGAATCTGCATCAAATAGACAACAACAAATGGA
AAAACGAATGCGTAAAATTGAGCAGTTACTAATTGAATCGCCAGTAGAAGCTAACTTACAACATGAAGATGCAGATATTCTTTATATCGG
TTTTATTTCTACAAAAGGTGCAATTCAAGAAGGTAGTAACCGTTTGAATCAACAAGGCATAAAAGTTAACACTATACAAATTAGACAATT
GCATCCATTCCCAACAAGCGTTATTCAAGATGCAGTTAATAAAGCGAAGAAAGTCGTTGTAGTGGAGCACAATTATCAAGGACAATTGG
CTAGTATTATAAAAATGAATGTCAATATTCATGATAAGATTGAAAATTATACAAAGTATGATGGGACACCTTTCCTACCACATGAAATCGA
AGTAAAAGGCAAAATAATTGCTACTGAAATAAAGGAGATGGTATAG

Gene: porB (2-oxoglutarate ferredoxin oxidoreductase subunit beta)

Contig: 06_NODE_54, position: 420688 to 421554, length: 867 nt, orientation: FORWARD

Perfect match to: (ED133-CP001996-[1329773:1330639], highly conserved allele)

Sequence:

ATGGCGACATTTAAAGATTTTAGAAATAATGTTAAGCCTAACTGGTGCCCTGGATGTGGCGATTTCTCAGTACAAGCTGCAATTCAAAAA
GCAGCCGCAAATATAGGGTTAGAACCTGAAGAAGTAGCTATCATCACCGGTATAGGATGTTCTGGCCGTCTTTCAGGATATATTAATTCT
TATGGCGTTCATTCTATTCACGGACGTGCATTACCTTTAGCTCAAGGTGTAAAAATGGCGAATAAAGATTTAACTGTTATTGCATCTGGAG
GAGATGGTGATGGTTATGCTATAGGTATGGGGCATACAATCCATGCTTTAAGAAGAAATATGAACATGACGTATATAGTCATGGATAAT
CAAATTTATGGTTTGACAAAGGGACAAACATCGCCGTCATCAGCAGTAGGATTTGTTACTAAAACAACGCCAAAAGGTAATATAGAAAA
AAATGTTGCGCCTTTAGAATTAGCATTATCATCTGGTGCCACATTTGTAGCCCAAGGTTTTTCAAGCGATATTAAAGGATTAACAAAACTA
ATTGAAGATGCAATTAATCATGATGGATTTTCATTCGTTAATGTCTTTTCACCATGTGTGACTTATAATAAAATTAACACATACGATTGGTT
TAAAGAACATTTAACAAGTGTTGATGACATTGAAAATTATGATTCTACAGATAAACAATTAGCGACTAAAACTGTTATTGAACATGAATCT
TTAGTAACTGGTATTGTTTATCAAGATAAAGAAACACCATCATATGAATCTCAAATTAAAGAGTTAGATGATACACCACTTGCTAAAAGA
GATATCAAAATTACTGAAGACACCTTCAATGCATTAACTGAACAATTTATTTAA

Gene: DUF77 (putative protein)

Contig: 06_NODE_54, position: 421648 to 421941, length: 294 nt, orientation: FORWARD

Perfect match to: (MW2-BA000033-[1288007:1288300], allele observed in CC1+CC72)

Sequence:

ATGAAAGACACATTAATGAGTATACAAATAATTCCTAAAACACCAAACAATGACAATGTTATACCTTACGTAGACGAGGCGATTAAAATA
ATTGACGAATCTGGTATGCATTTTAGAGTAGGTCCGTTAGAAACGACAGTACAAGGAAATATGAATGAATGTTTAATTTTAATACAATCA
TTAAATGAACGAATGGTGGAACTTGAATGTCCAAGTATTATTAGCCAAGTTAAGTTTTATCATGTGCCAGATGGCATCACTATTGAAACTT
TAACTGAAAAATATGATGAATAA

Gene: miaB ( (dimethylallyl)adenosine tRNA methylthiotransferase)

Contig: 06_NODE_54, position: 422075 to 423619, length: 1545 nt, orientation: FORWARD

Perfect match to: (ATCC51811-ADVP01000009-[107244:108788:r], allele observed in CC1)

Sequence:

GTGAACGAAGAACAAAGAAAAGCAAGTTCTGTAGATGTTTTAGCTGAGAGAGATAAGAAAGCAGAAAAAGATTATAGTAAATATTTTG
AACATGTTTATCAGCCGCCTAATTTAAAAGAAGCAAAAAAAAAGAGGTAAACAAGAAGTTCGTTATAATAGAAATTTTCAAATTGATGAAA
AATATCGCGGTATGGGGAACGAGCGTACATTTTTAATTAAAACATATGGATGTCAAATGAATGCACATGACACTGAGGTCATTGCTGGTA
TACTTGAAGCATTAGGCTATCAAGCAACGACTGATATTAACACTGCAGATGTTATTTTAATTAATACATGTGCGATTAGAGAAAATGCCG
AGAACAAAGTGTTTAGTGAAATAGGTAATTTGAAGCATTTGAAAAAAGAACGACCTGATATTTTAATCGGTGTTTGTGGTTGTATGTCAC
AAGAAGAGTCAGTAGTGAATAAAATTTTAAAATCGTATCAAAATGTAGATATGATATTTGGTACACATAATATTCATCATTTACCAGAAAT
TTTAGAAGAAGCATACTTATCTAAAGCAATGGTTGTTGAAGTATGGTCTAAAGAAGGAGACGTTATTGAAAATCTTCCAAAAGTCCGTGA
AGGCAACATTAAAGCATGGGTCAATATTATGTATGGTTGTGATAAGTTTTGTACATATTGCATTGTTCCATTTACAAGAGGTAAAGAACG

AAGCCGCAGACCTGAAGACATTATAGATGAAGTACGTGAACTTGCTCGTGAAGGTTACAAAGAAATAACGCTTTTAGGTCAAAATGTAA
ATTCTTATGGTAAAGATTTACAGGATATAGAATATGACTTAGGAGATCTTTTACAAGCAATTTCTAAAATAGCGATTCCAAGAGTTCGTTT
CACAACAAGTCATCCTTGGGACTTTACAGATCACATGATTGATGTTATTTCAGAGGGTGGTAATATCGTTCCTCATATCCACTTGCCAGTT
CAATCTGGAAATAATGCAGTATTAAAAATAATGGGTAGAAAATATACACGAGAAAGTTATTTGGATTTAGTAAAACGAATCAAAGATAG
AATTCCTAATGTAGCATTAACTACAGATATTATTGTAGGGTATCCAAATGAATCAGAGGAACAATTTGAAGAAACTTTAACTCTGTATGAT
GAAGTTGGTTTTGAACATGCATATACGTATTTGTATTCACAACGTGATGGTACGCCTGCTGCTAAAATGAAAGATAATGTACCTTTAAATG
TCAAAAAGGAACGATTGCAACGTTTGAATAAAAAAGTTGGTCATTATTCACAAATAGCTATGAGTAAGTACGAAGGACAAACTGTAACA
GTACTTTGTGAAGGTAGTAGTAAAAAAGATGATCAGGTTCTTGCTGGCTACACTGATAAAAATAAGCTAGTTAATTTCAAAGCGCCTAAA
GAAATGATTGGTAAACTAGTGGAAGTACGAATAGATGAAGCTAAACAGTATTCATTAAATGGCAGTTTTGTAAAGGAAGTAGAGCCGGA
AATGGTGATTCAATAA

Gene: ymcA (putative membrane protein)

Contig: 06_NODE_54, position: 423620 to 423985, length: 366 nt, orientation: FORWARD

Perfect match to: (MW2-BA000033-[1289979:1290344], highly conserved allele)

Sequence:

ATGTATAATAAAGATGACGTGTTGAAACAAGCGGATAATATTGCAAATAAAATTAAAAATTTGGATACTATCAAAACATATCAACAAATT
GAAGCACAGATTCATCAGAACCAAACGATAAAGACTAAAATGGATATGTTAAAAAAGCATCAAAAACAAGCAGTAAACTTTCAAAATTA
CGGGAAACAAAATGCGCTAGAACAGTCGGAACATACCATTCAAAGTATAGAAGCAGAAATAAATACATTGCCCATAGTTGAACAGTTTC
AAACTTCACAATATGAAGCGAATCAATTATTGAAAATGTTTGTATCAACAATGGAAACACGTTTAAATGACCATAATAAAGCCAAGCATA
GTGATTAA

Gene: thiW (ThiW family protein)

Contig: 06_NODE_54, position: 424012 to 424503, length: 492 nt, orientation: FORWARD

Perfect match to: (N315-BA000018-[1290234:1290725], allele observed in CC5+CC1+CC15+CC188-ST582)

Sequence:

ATGAAATCAAGAAAACTGGCTATAACTGCACTTTTAATTGCAATAAATGTTGTATTAAGCAGTATTATCATCATTCCTCTAGGACCAGTTA
AGGCAGCACCAGTACAGCATTTTGTAAATGTATTAAGTGCGGTCATAGTAGGTCCTTGGTATGGATTAGCTCAAGCGCTTATATCATCAA
TTTTAAGAGTTCTTTTTGGTACTGGTACAGCTTTTGCATTTCCAGGTAGTATGATTGGAGTTTTATTGGCTAGTATGTTTTACATATATCGT
AAACATATATTCATGGCCGCGGTCGGTGAAGTACTTGGAACTGGTGTCATCGGAAGTTTAATTTGTATACCATTAGCATATTTCCTTGGGC
TTCAAGACTTCTTCATTAAACCGTTAATGATTACGTTCATAGTCTCAAGTGCTATCGGATCTATTATAAGTTATTTCTTATTAATTACTCTAA
AAAAACGTGGTATTCTTCAAAGGTTTATAAAATAA

Gene: mutS (DNA mismatch repair protein)

Contig: 06_NODE_54, position: 424806 to 427424, length: 2619 nt, orientation: FORWARD

Sequence:

ATGTCTAATGTTACACCAATGATGCAGCAATATTTAAAAATAAAATCAGAATACCAAGATTGCTTATTATTTTTTAGACTAGGTGATTTCTA
TGAAATGTTTTATGAAGATGCCAAGGAGGCATCACGTGTACTTGAAATTACTTTAACTAAAAGAGATGCTAAAAAAGAAAATCCAATTCC
GATGTGTGGTGTTCCGTATCATTCTGCAGATAGTTATATAGATACACTTGTTAATAATGGATATAAAGTAGCTATTTGTGAACAGATGGA
AGATCCGAAACAAACGAAAGGTATGGTTAGACGTGAGGTAGTAAGAATTGTGACTCCAGGAACTGTGATGGAGCAAGGTGGTGTAGAT
GATAAACAAAATAACTATATTTTAAGTTTTGTTATGAATCAACCTGAAATTGCGCTTAGTTACTGTGATGTTTCTACTGGCGAATTAAAGG
TTACACATTTTAATGATGAAGCGACTTTATTAAATGAAATTACGACGATAAACCCTAACGAAGTTGTTATCAATGACAATATTTCCGATAA
TTTAAAAAGACAAATTAATATGGTGACAGAAACAATAACAGTCAGGGAAACGTTATCATCAGAAATCTATAGTGTGAATCAAACTGAACA
TAAATTAATGTATCAAGCGACACAATTATTGCTAGATTATATTCATCATACACAAAAACGTGATTTATCGCATATCGAGGATGTTGTTCAA
TATGCAGCTATAGATTATATGAAAATGGATTTTTATGCTAAGAGAAACCTTGAGTTAACGGAAAGCATTCGATTAAAATCAAAAAAAGGA
ACGCTACTTTGGCTAATGGACGAAACGAAAACACCAATGGGAGCACGCCGCTTAAAACAATGGATAGATAGACCACTAATAAGTAAAGA
ACAAATTGAAGCACGATTAGATATCGTTGATGAATTTAGTGCTCATTTCATAGAAAGAGACACCTTAAGAACATATCTTAATCAAGTGTAT
GATATTGAACGTCTTGTTGGGCGTGTTAGTTACGGAAATGTTAATGCGAGAGATTTAATTCAACTTAAACATTCCATTTCTGAAATACCGA
ATATTAAAGCATTACTAAATTCTATGAATCAGAATACGCTTGTACAAGTTAATCAACTAGTACCCCTTGATGATTTACTTGATATATTAGAA

CAGAGTTTAGTAGAAGAACCACCAATTTCAGTTAAAGATGGCGGACTATTCAAAGTTGGTTTTAATACGCAATTAGATGAATATCTTGAA
GCTTCAAAAAACGGAAAAACATGGTTAGCAGAATTACAAGCCAAAGAAAGACAACGTACAGGAATAAAATCATTGAAAATAAGCTTTAA
TAAAGTGTTTGGTTATTTTATAGAAATAACACGTGCCAACTTGCAAAATTTTGAACCAAGTGAATTTGGTTATATGAGGAAGCAAACGTT
ATCGAATGCTGAACGTTTTATAACTGATGAACTTAAAGAAAAAGAAGATATCATTTTAGGTGCGGAAGACAAAGCCATCGAATTAGAAT
ATCAATTATTTGTTCAGCTACGTGAAGAAGTTAAAAAATATACTGAACGTTTACAACAACAAGCTAAAATTATTTCAGAGCTAGATTGTTT
ACAGAGCTTTGCAGAAATTGCTCAAAAATATAATTACACTAGGCCTTCATTTAGTGAAAATAAAACATTAGAATTAGTGGAATCTAGGCA
CCCAGTAGTGGAAAGAGTAATGGATTATAATGACTATGTGCCTAATAATTGTCGATTAGATAATGAAACATTTATATATTTAATTACAGGT
CCGAATATGTCTGGTAAATCGACATATATGAGACAAGTTGCCATAATTAGTATAATGGCCCAAATGGGAGCTTATGTCCCTTGTAAAGAG
GCAGTGTTACCTATATTTGATCAAATATTCACTAGAATAGGTGCGGCAGATGATTTGGTTTCAGGTAAGAGTACGTTTATGGTAGAAATG
CTAGAAGCACAAAAGGCATTAACTTATGCAACAGAGGATAGTTTGATTATTTTCGATGAAATTGGACGTGGTACTTCAACGTATGACGGT
TTAGCTTTAGCGCAGGCAATGATAGAGTATGTAGCTGAAACATCACATGCTAAAACGTTATTTTCAACACATTATCATGAATTGACAACAT
TAGATCAAGCATTACCAAGTCTAAAAAATGTTCACGTCGCTGCTAATGAATATAAAGGTGAACTTATATTCTTGCATAAAGTCAAAGATG
GTGCAGTTGACGATAGTTATGGTATTCAAGTTGCGAAATTAGCTGATTTACCTGAAAAAGTTATTAGCAGAGCACAAGTGATTCTAAGCG
AGTTTGAAGCGTCTGCTGGTAAAAAATCATCGATATCAAATTTAAAAATGGTCGAAAATGAACCTGAAATTAATCAAGAAAATTTAAACT
TAAGTGTTGAAGAAACAACTGATACTTTATCTCAAAAAGACTTTGAACAAGCATCATTTGATTTGTTTGAAAATGATCAAGAAAGCGAGA
TTGAACTACAAATTAAAAATTTGAATTTATCTAATATGACACCAATTGAGGCATTGGTGAAGTTAAGTGAATTACAAAATCAATTAAAATA
G

Gene: mutL (DNA mismatch repair protein)

Contig: 06_NODE_54, position: 427437 to 429446, length: 2010 nt, orientation: FORWARD

Perfect match to: (Strain_21193-AFEG01000003-[92112:94121:r], highly conserved allele)

Sequence:

ATGGGGAAAATTAAAGAACTCCAAACCTCATTAGCAAATAAAATCGCAGCAGGTGAAGTAGTTGAAAGACCGAGTTCTGTTGTGAAAGA
ACTGTTGGAAAATGCTATAGATGCAGGCGCTACAGAAATAAGCATTGAAGTAGAGGAATCTGGCGTCCAATCTATTCGCGTAGTCGATA
ATGGAAGCGGAATTGAAGCGGAAGACTTAGGATTAGTATTTCATAGACATGCGACTAGTAAATTAGATCAAGATGAAGATTTATTTCATA
TTAGGACATTAGGATTCCGTGGTGAAGCACTAGCCAGTATTTCATCAGTTGCTAAAGTAACATTGAAGACTTGCACGGATAATGCTAATG
GAAATGAAATATATGTAGAAAATGGTGAAATATTAAATCATAAGCCTGCAAAAGCGAAAAAAGGAACAGATATACTTGTAGAATCATTA
TTTTATAATACACCAGCACGTTTAAAATATATTAAAAGTTTATACACTGAACTAGGTAAAATAACAGATATTGTCAACAGAATGGCAATGA
GCCATCCGGACATTCGAATAGCACTCATTTCAGATGGCAAAACAATGTTAAGTACAAATGGTTCAGGACGAACTAATGAAGTGATGGCA
GAGATTTATGGGATGAAAGTTGCACGAGATTTAGTACATATATCTGGAGATACAAGTGATTATCACATTGAAGGTTTTGTTGCAAAGCCT
GAACATTCTAGAAGTAATAAGCACTATATTTCTATTTTTATTAATGGACGATACATTAAAAACTTTATGCTAAATAAAGCGATTTTAGAAG
GCTATCATACACTCTTAACAATAGGTAGGTTCCCGATTTGTTATATTAATATTGAAATGGATCCAATCTTAGTAGACGTAAATGTTCATCCA
ACAAAACTAGAAGTGCGTTTATCAAAAGAAGAGCAACTATATCAATTGATAGTGAGCAAAATACAAGAAGCATTTAAAGACCGTATATTA
ATTCCTAAAAATAACTTGGATTATGTGCCGAAAAAAAAATAAAGTGTTACATTCATTCGAACAACAAAAAAATCGAATTTGAACAAAGACAA
AACACAGAGAATAATCAAGAGAAGACGTTTTCATCTGAAGAAAGTAACAGTAAGCCATTTATGGTAGAAAATCAAAACGATGAAATAGT
TATAAGAGAAGATTCATATAATCCATTCGTAACGAAAACGTCTGAAAGTTTAATAGCTGATGATGAATCTTCCGGTTATAATAATACACGT
GAAAAAGATGAAGACTACTTCAAAAAGCAACAAGAAATTCTACAAGAAATGGATCAAACATTTGATTCGAATGACGATACATCTGTGCA
AAATTATGAGAATAAAGCGTCTGATGATTATTATGATGTAAACGATATTAAAGGAACAAAAAGTAAAGACCCTAAACGAAGAATTCCATA
TATGGAAATTGTTGGCCAAGTACATGGAACGTATATTATTGCTCAAAATGAATTTGGCATGTACATGATTGACCAGCATGCAGCTCAAGA
AAGAATAAAATATGAATATTTTCGAGATAAAATAGGTGAAGTTACCAATGAAGTACAAGATTTATTAATCCCGTTAACATTTCATTTTTCA
AAAGATGAACAATTAGTCATTGATCAATATAAAAATGAGCTTCAACAAGTAGGTATCATGTTAGAACATTTTGGTGGTCATGATTATATTG
TAAGTAGCTATCCAGTTTGGTTCCCTAAAGATGAAGTAGAAGAAATTATTAAAGATATGATTGAGCTAATTTTGGAAGAGAAAAAAGTA
GATATCAAAAAATTACGTGAAGATGTAGCAATCATGATGTCATGTAAAAAATCTATTAAAGCGAATCATTATTTACAAAAACATGAAATG
TCTGATTTAATTGATCAATTAAGAGAAGCGGAAGATCCATTTACATGTCCACATGGTCGTCCAATTATCATTAATTTTTCAAAATACGAATT
AGAAAAATTATTTAAGCGTGTGATGTAG

Gene: glpP (glycerol-3-phosphate responsive antiterminator)

Contig: 06_NODE_54, position: 429461 to 429994, length: 534 nt, orientation: FORWARD

Perfect match to: (MW2-BA000033-[1295820:1296353], highly conserved allele)

Sequence:

GTGAATAACAACATATTGCCTGCCATAAGAAACATTAAAGATTTAGAGAAACTGATTAAAACAGATTATAAAATGTGTGTGCTTCTAGAT
ATGCATATAGGACATATAAAAAGTATTATGGAATTGCTGAAGCAAAATCATATAGAGTGTTTTATTCATATAGATTTGATAAAAGGTTTAA

GCCACGATGAATTTGCAAGTGAATTTATTATTCAGCAATACAAGCCAAAAGGTATCGTATCGACTAAATCTAAAGTAATAAAAAAAGCTA
AATCATTAAATACTTTAACGATTTTTAGAGTATTTATTATTGATAGTCAAGCATTGAAACGCAGTATAGATTTGATAAAAAAAGTTGAACC
TGATTTTGTTGAAGTACTTCCAGGTGTTGCGAGTAAAGCGATTCATCATATTCAGAAAGAAACAAACACACAAGTCATTGCAGGTGGCCT
AATTAATACAATAGATGAAGTCAATGAAGCTGTTAAAAATGGAGCGAAATATGTAACAACTAGTTATGATAAACTTTGGTAA

Gene: glpF (glycerol uptake facilitator)

Contig: 06_NODE_54, position: 430469 to 431287, length: 819 nt, orientation: FORWARD

Perfect match to: (N315-BA000018-[1296691:1297509], highly conserved allele)

Sequence:

ATGAATGTATATTTAGCAGAATTCCTAGGAACTGCAATCTTAATCCTTTTTGGTGGTGGCGTTTGTGCCAATGTCAATTTAAAGAGAAGTG
CTGCGAATGGTGCTGATTGGATTGTCATCACAGCTGGATGGGGATTAGCGGTTACAATGGGTGTGTTTGCTGTCGGTCAATTCTCAGGT
GCACATTTAAACCCAGCGGTGTCTTTAGCTCTTGCATTAGACGGAAGTTTTGATTGGTCATTAGTTCCTGGTTATATTGTTGCTCAAATGTT
AGGTGCAATTGTCGGAGCAACAATTGTATGGTTAATGTACTTGCCACATTGGAAAGCGACAGAAGAAGCTGGCGCGAAATTAGGTGTTT
TCTCTACAGCACCGGCTATTAAGAATTACTTTGCCAACTTTTTAAGTGAGATTATCGGAACAATGGCATTAACTTTAGGTATTTTATTTATC
GGTGTAAACAAAATTGCCGATGGTTTAAATCCTTTAATTGTCGGAGCATTAATTGTTGCAATCGGATTAAGTTTAGGCGGTGCTACTGGTT
ATGCAATCAACCCAGCACGTGATTTAGGTCCGAGAATTGCACATGCGATTTTACCAATAGCTGGTAAAGGTGGTTCAAATTGGTCATATG
CAATCGTTCCTATCTTAGGACCAATTGCCGGTGGTTTATTAGGTGCAGTGGTATACGCTGTATTTTATAAACATACATTTAATATTGGTTGT
GCAATTGCAATTGTTGTAGTTATTATTACTTTGATTTTAGGTTACATTTTAAATAAATCATCAAAAAAAGGTGATATCGAATCAATTTACTA
A

Gene: glpK (glycerol kinase)

Contig: 06_NODE_54, position: 431416 to 432912, length: 1497 nt, orientation: FORWARD

Perfect match to: (Strain_6850-CP006706-[1276671:1278167], allele observed in CC50+CC1)

Sequence:

ATGGAAAAATATATTTTATCTATAGACCAAGGAACAACAAGCTCAAGAGCGATTTTATTCAATCAAAAAGGGGAAATTGCAGGGGTAGC
ACAACGTGAGTTTAAGCAATATTTTCCACAATCAGGTTGGGTTGAACATGATGCAAATGAAATTTGGACATCTGTGTTAGCTGTAATGAC
GGAAGTAATTAATGAAAATGATGTTAGAGCTGATCAAATTGCAGGTATCGGTATTACAAACCAACGCGAAACAACGGTGGTTTGGGACA
AACATACTGGCCGCCCAATTTATCACGCAATTGTTTGGCAATCACGTCAAACACAATCAATTTGTTCAGAATTAAAACAACAAGGATATGA
ACAAACATTTAGAGATAAGACAGGATTACTTTTAGATCCGTATTTTGCAGGTACAAAAGTTAAATGGATTCTAGACAATGTTGAAGGTGC
ACGAGAAAAAGCAGAAAATGGCGATCTATTATTTGGAACGATTGATACTTGGTTAGTATGGAAATTATCAGGAAAAGCTGCGCATATTA
CTGATTATTCAAATGCGAGTCGTACATTAATGTTTAATATCCATGATTTAGAATGGGACGATGAGTTATTAGAACTACTTACAGTACCTAA
AAATATGTTGCCAGAAGTTAAACCTTCAAGTGAAGTATATGGTAAGACAATTGATTACCACTTCTATGGTCAAGAAGTACCAATCGCTGG
TGTAGCTGGTGATCAACAAGCAGCATTATTTGGACAAGCTTGCTTCGAACGTGGTGACGTGAAAAACACATATGGAACTGGTGGCTTCA
TGTTAATGAATACAGGTGACAAAGCGGTTAAATCTGAAAGTGGTTTATTAACAACAATTGCTTATGGTATTGATGGAAAAGTAAATTATG
CGCTTGAAGGTTCCATCTTTGTTTCGGGTTCAGCAATCCAATGGTTACGTGATGGATTAAGAATGATTAATTCAGCACCACAATCAGAAA
GTTATGCGACACGAGTTGACTCTACTGAGGGTGTTTATGTTGTTCCAGCTTTTGTAGGTTTAGGAACACCATATTGGGATTCTGAAGCAC
GTGGTGCGATTTTCGGTTTATCACGTGGAACTGAAAAAGAGCACTTTATCCGTGCAACTTTAGAATCACTATGTTACCAAACTCGTGACGT
TATGGAAGCAATGTCAAAAGACTCTGGCATTGATGTCCAAAGTTTACGTGTCGATGGTGGTGCAGTTAAAAACAACTTTATTATGCAGTT
CCAAGCAGACATTGTTAATACTTCTGTTGAAAGACCTGAAATTCAAGAAACTACAGCTTTAGGTGCTGCATTTTTAGCAGGATTAGCAGTT
GGATTCTGGGAGAGTAAAGATGATATCGCTAAAAACTGGAAATTAGAAGAAAAATTCGATCCGAAAATGGATGAAGGCGAAAGAGAAA
AATTATATAGAGGTTGGAAAAAAGCTGTTGAAGCAACACAAGTTTTTAAAACAGAATAA

Gene: glpD (aerobic glycerol-3-phosphate dehydrogenase)

Contig: 06_NODE_54, position: 433022 to 434743, length: 1722 nt, orientation: FORWARD

Perfect match to: (Strain_21235-AFTQ01000042-[8906:10627:r], highly conserved allele)

Sequence:

ATGTGGGACAAGTCTCTCGTTTTTACATTTTTTAGGAGGCGTTTTGGAATGGCATTGTCTACTTTTAAGAGAGAACATATTAAAAAGAATT
TAAGAAATGATGAATATGATTTAGTAATTATTGGTGGCGGTATTACAGGTGCAGGTATTGCACTAGACGCGAGTGAAAGAGGAATGAAA

GTTGCATTAGTTGAAATGCAAGACTTTGCACAAGGAACAAGCTCAAGATCTACAAAATTAGTCCATGGTGGTTTACGTTACTTAAAACAA
TTCCAAATTGGAGTAGTTGCCGAAACTGGTAAAGAACGTGCGATTGTTTATGAAAATGGACCTCATGTTACGACTCCAGAGTGGATGCTT
TTACCAATGCATAAAGGTGGAACATTTGGTAAATTCTCAACATCAATTGGTTTAGGAATGTATGATCGTTTAGCAGGTGTTAAGAAGTCT
GAACGTAAAAAAATGTTATCTAAAAAAGAAACTTTAGCTAAAGAACCATTAGTTAAAAAAGAAGGTCTAAAAGGCGGCGGTTACTATGT
TGAATATCGTACTGACGATGCGCGTTTAACTATTGAAGTTATGAAGCGTGCTGCTGAAAAAGGCGCAGAAATTATCAACTATACTAAATC
TGAACACTTCACTTATGATAAAAATCAACAAGTAAATGGTGTTAAAGTTATAGATAAATTAACTAATGAAAATTATACAATTAAGGCTAAA
AAAGTGGTTAATGCAGCAGGTCCATGGGTTGATGATGTTAGAAGTGGTGATTATGCACGCAATAATAAAAAATTACGTTTAACTAAAGG
TGTACATGTTGTTATTGATCAATCAAAATTCCCATTAGGTCAAGCAGTATACTTTGATACTGAAAAAGATGGAAGAATGATTTTTGCAATT
CCACGTGAAGGAAAAGCGTATGTAGGTACTACAGATACATTCTATGACAATATCAAATCTTCACCATTAACTACACAAGAAGACAGAGAC
TATTTAATCGATGCGATTAATTACATGTTCCCTAGTGTTAATGTTACAGATGAAGATATTGAATCAACATGGGCAGGAATTAGACCATTAA
TTTACGAAGAAGGCAAAGACCCTTCTGAAATCTCTCGTAAGGATGAAATTTGGGAAGGTAAATCAGGTTTATTAACTATTGCAGGTGGTA
AATTAACAGGCTATCGTCACATGGCTCAAGACATTGTTGATTTAGTATCTAAACGCTTGAAAAAAGACTACGGTTTAACATTTAGTCCATG
TAATACAAAAGGTCTGGCAATTTCAGGTGGCGATGTAGGTGGTAGCAAGAACTTTGATGCGTTTGTAGAGCAAAAAGTAGATGTAGCTA
AAGGATTCGGCATTGATGAAGATGTTGCAAGACGTTTAGCATCTAAATATGGTTCAAATGTTGATGAATTGTTCAACATTGCGCAAACAT
CTCAATACCATGATAGCAAGTTACCATTAGAAATTTATGTAGAACTTGTTTATAGTATTCAACAAGAAATGGTATACAAACCTAACGATTT
CTTAGTTCGTCGTTCTGGTAAAATGTATTTCAATATTAAAGATGTATTAGATTATAAAGATGCTGTCATCGATATTATGGCAGATATGCTT
GATTACTCTCCAGCTCAAATTGAAGCATATACTGAAGAAGTTGAGCAAGCAATTAAAGAAGCGCAACATGGAAATAATCAACCAGCAGT
TAAAGAATAA

Gene: pldB2 (putative lysophospholipase, locus 2)

Contig: 06_NODE_54, position: 434893 to 435807, length: 915 nt, orientation: FORWARD

Perfect match to: (N315-BA000018-[1301115:1302029], highly conserved allele)

Sequence:

ATGACTGAAAAACAATTTAAATTAACTGTACAAGATAATACGAATATTGAAGTTAAAGTGAATTTTACAGATGTAGATTCAAAAGGAATT
ATTCATATATTTCATGGTATGGCTGAACATATGGAACGTTACGATAAATTAGCACATGCACTTTCAAAGCATGGCTTCGATGTGATACGTC
ATAATCATCGAGGACATGGTATTAATATTGATGAATCAACAAGAGGGCATTACGATGATATGAAACGAGTTATCGGTGATGCCTTTGAA
GTAGCGCAAACAGTGAGAGGCAATGTTGATAAACCATACATTATAATCGGACATTCAATGGGATCCGTTATAGCTAGATTGTTTGTAGAA
ACATATCCGCAATATGTTGATGGTCTAATTTTAAGTGGTACTGGTATGTATTCATTATGGAAAGGTTTACCAACCGTTAAAGTGTTACAAC
TGATTACAAAAATTTATGGTGCTGAGAAACGAGTTGAATGGGTTAACCAGTTAGTATCAAATAGTTTTAATAAAAAAATACGTCCATTAC
GTACACAAAGTGATTGGATTTCTAGTAATCCAATTGAAGTAGATAACTTTATTAAAGATCCATATAGTGGATTTAATGTGTCAAATCAATT
ATTATATCAAACAGCCTATTATATGCTACATACATCACAATTAAAAAATATGAAAATGTTAAATCATGCCATGCCTATATTATTAGTTTCAG
GATATGACGATCCTTTAGGTGATTATGGTAAAGGGATTTTAAAATTGGCGAATATATATAGAAAAGCTGGCATTAAAAATGTTAAAGTGA
ATCTTTATCATCATAAACGTCATGAAGTGTTATTTGAAAAAGATCATGACAAAATTTGGGAAGACTTGTTTAAATGGTTGAATCAATTTTA
TAAAAAATAA

Gene: miaA (tRNA delta (2)-isopentenylpyrophosphate transferase)

Contig: 06_NODE_54, position: 435825 to 436760, length: 936 nt, orientation: FORWARD

Perfect match to: (N315-BA000018-[1302047:1302982], highly conserved allele)

Sequence:

ATGAATAAAAATAAGCCTTTTATTGTAGTAATTGTGGGGCCAACTGCTTCAGGTAAAACAGAGCTTAGCATAGAACTCGCGAAGCGTATC
AATGGTGAAATCATAAGCGGTGATTCTATGCAAGTTTACAAACATATGAATATTGGAACTGCAAAAGTAACACCTGAAGAAATGGATGG
TATTCCACATCATTTAATTGATATCTTGAATCCTGATGATACATTTTCAGCATATGAATTCAAGCGATTAGCAGAAGATTTAATTACTGATA
TAACGAATAGAGGTAAAGTTCCAATCATAGCAGGTGGAACAGGCTTATATATTCAATCATTAATATATAATTATGAATTAGAAGATGAAA
CAGTTACACCTGCACAATTATCCATAGTTAAACAAAAGTTATCTGCATTAGAACATTTAGATAATCAGCAACTACACGATTATTTAGCTCA
ATTTGATGCGGTTTCTGCAGAAAATATTCACCCTAACAACCGCCAAAGAGTGTTGCGCGCTATTGAATATTATTTAAAAACAAAAAAACTT
TTGAGTAATCGCAAGAAAGTGCAACAATTTACTGAAAATTATGATACATTATTATTAGGGATTGAAATGTCGCGTAAAACATTATATTCAA
GAATAAATAAACGTGTTGATATTATGTTGGATCACGGATTATTTAGAGAAGTGCAACAACTTGTTGAACAAGGCTATGAATCTTGCCAAA
GTATGCAAGCTATTGGATATAAAGAATTAATACCTGTGATTAACGGACAAATGATTTATGAAGATGCTGTCAATGATTTAAAGCAACATT
CACGCCAATATGCAAAACGACAAATGACATGGTTCAAGAATAAAATGAGTGTTCATTGGTTAGATAAAGAAAATATGTCACTTCAAATGA
TGTTAGATGAGATTACAACCCAGATTAAGTAA

Gene: hfq (RNA chaperone)

Contig: 06_NODE_54, position: 436775 to 437008, length: 234 nt, orientation: FORWARD

Perfect match to: (N315-BA000018-[1302997:1303230], highly conserved allele)

Sequence:

ATGATTGCAAACGAAAACATCCAAGACAAAGCACTAGAGAATTTTAAAGCAAACCAAACTGAAGTAACTGTATTCTTTCTAAACGGTTTC
CAAATGAAAGGTGTTATTGAAGAATACGACAAGTATGTCGTAAGCTTAAATTCTCAAGGCAAACAACACTTGATTTACAAACATGCGATC
AGCACTTATACAGTAGAAACTGAAGGTCAAGCATCTACTGAAAGTGAAGAATAA

Gene: gpxA-L1 (glutathione peroxidase locus 1)

Contig: 06_NODE_54, position: 437230 to 437706, length: 477 nt, orientation: REVERSE

Perfect match to: (MW2-BA000033-[1303589:1304065:r], allele observed in CC1+CC22)

Sequence:

TTATAATAATTTTTCTATTTCTCTTTCGATTTGAACAGGTTTTTTTTGAGGTGCAAATCGTTTAACAACGTTACCTTCGCGATCCACTAAAAA
CTTAGTGAAATTCCATTTGATTTTCTCATTAAAGAATCCGTGTTGTGCCGCAGTCAAATATCTAAATAAAGGTAATTGATGTTCCCCTTTTA
CGTCTATTTTTTGATGCATAGGGAAGGTAACACCATAGTTTAATTTACAGTTTTGAGCTGCTTCTTCGCCTGATCCAGGTTCTTGGCCACCA
AATTGATTACAAGGGAAACCTAGAATTACAAACCCTTGATCTTTGTATTTCTCGTATAATGATTGCAAACCTTCAAATTGTGAAGTAAAGC
CACATTCGCTAGCTGTATTAACAATTAGCATAACGTCACCCTTATATGCATCTAATTTGTAAGTAACACCTTTATTTGTTTCTACTACAAAAT
CATAAATTGTCTCCAT

Gene: ynbA (GTP-binding proteinase modulator)

Contig: 06_NODE_54, position: 437818 to 439056, length: 1239 nt, orientation: FORWARD

Perfect match to: (MW2-BA000033-[1304177:1305415], allele observed in CC1)

Sequence:

ATGGCTCAGCAACAAATTCATGATACTAAAAATAAACTAGAAAAAGCTGTCTTAGTCGGTGTACATGCTCAAGATGATAAGCAATTTAAT
TTTGAGTCTACAATGGAGGAATTATCATCTTTATCAGAGACTTGCCAACTTGAAGTGTTGGGTCAAATTACTCAAAACAGAGATCGTGTA
GATCGCAAATATTATGTTGGTAAAGGTAAAATGGAAGAAATTCAAGCATTTATTGAGTTCAAAGATATTGATGTAGTCATCACAAATGAT
GAATTAACGACTGCACAATCCAAATCACTAAATGAAGCTTTAGGTGTAAAAATTATTGATAGAACTCAGTTGATTCTTGAAATATTTGCAT
TAAGAGCAAGAAGTAAAGAAGGTAAATTGCAAGTAGAGCTAGCACAACTTGATTATTTATTACCTAGATTGCAAGGCCATGGTAAAAGT
CTTTCTCGTTTAGGTGGCGGTATTGGAACTAGAGGCCCTGGTGAAACGAAGTTAGAGATGGATCGCAGACATATTCGAACTCGTATGAA
TGAAATTAAACATCAATTGCGGACGGTAGAAGAACATCGCGAAAGATATCGAAATAAAAGAAATCAAAATCAGGTGTTTCAAGTAGCTT
TAGTTGGTTATACAAATGCTGGTAAATCATCATGGTTTAATGTTTTAGCAAATGAAGAGACATATGAAAAAGATCAATTATTTGCAACGTT
AGATCCTAAAACACGACAAATTCAAATAAATGATGGATTTAATTTAATTATTTCAGATACTGTTGGTTTTATACAGAAACTACCTACGACG
TTAATTGCAGCTTTTAAATCAACTTTAGAAGAGGCTAAAGGTGCAGATTTATTAGTACATGTCGTAGATAGTAGCCATCCTGAATACCGTA
CGCAGTATGACACAGTTAATGATTTAATCAAACAATTAGATATGAGTCATATTTCTCAAATAGTTATTTTTAATAAAAAGGACTTATGTGA
TCATGCATCAAATTGTCCAGCAAGTGATTTGCCTAATGTTTTTGTTTCTTCTAAAAATGATGGTGATAAATTACTTGTTAAGACGTTATTTA
TTGATGAAATCAAAAGGCAATTAACTTATTATGATGAGACAATTGCGACGAATAATGCAGATCGATTATATTTTCTAAAACAACATACATT
AGTGACTGAACTTAAATATGATGAAATTGAAAATGTTTATCGTATAAAAGGATTTAAAAAATAA

Gene: ynbB (putative C-S lyase)

Contig: 06_NODE_54, position: 439075 to 440313, length: 1239 nt, orientation: FORWARD

Perfect match to: (MW2-BA000033-[1305434:1306672], allele observed in CC1)

Sequence:

ATGAAAGATATAAGTAAGATAGTAGCTGACGTCGAATCAACGTTAGCACCATATTTTAAAGAAATTGAAGAAACAGCATATATTAATCAA
GAAAAAGTATTAAATGCATTTCATCATGTCAAAGCAACCGAAAGTGATCTACAAGGATCAACAGGATACGGATATGATGATTTTGGACGT
GATCATTTAGAAGAAATATATGCGCAGGCATTTAAAGCAGAAGATGCAATTGTTCGTCCGCAAATTATTTCAGGTACGCATGCGATTACT

ATTGCATTACAAAGTTTATTAAAACATGGTGATGAATTAATTTATATAACGGGAAGTCCATATGACACTTTACTTGAAGTCATTGGTGTAA
ACGGAAATGGTATTGAAAGTTTAATGGAGCACGGTGTATCGTATAAAGATATTGCACTTAAAGAAGGTAAGATCGATATTGAAAGTGTG
TTAGATGGGGTTTCTGAGCGTACCAAAGTAATAGCGATTCAACGTTCGAAAGGCTATGATCAAAGACCTTCAATTCCTCTAGATGAAATT
GAACAGGTGATTACTAGGTTGAAAGAAGTGCATCCTAATATTTTAATATTTGTGGATAACTGTTATGGGGAATTTGTTGAAAGACGTGAA
CCTATAGAATGTGGTGCCGATTTAATAGCAGGATCATTAATTAAAAACCCTGGCGGTGGTTTAGCTAAGATTGGTGGATACATTGCTGGT
AGAAAAGATTTAATTGAACGATGTGGTTATAGATTGACAGCACCTGGTATTGGTAAAGAAGCAGGTGCATCATTAAATGCATTGCTTGAA
ATGTATCAAGGTTTCTTTTTAGCACCACATGTTGTCAGTCAGAGTCTTAAAGGGGCATTGTTTACTAGTTTATTTTTAGAAAAAATGAATAT
GAACACAACGCCGAAGTACTACGAAAAACGAACTGATTTAATTCAAACAGTTAAATTTAAAACGAAAGAACAAATGATTTCATTTTGTCA
AAGTATTCAACACGCATCCCCAATTAATGCACATTTTAGTCCAGAACCGAGTTATATGCCTGGTTACGAAGATGATGTTATTATGGCTGCT
GGTACGTTTATTCAAGGTTCATCGATTGAATTATCTGCAGATGGACCTATTCGTCCTCCTTATGAAGCATATGTTCAAGGAGGATTAACAT
ATGAACACGTTAAAATTGCTGTGACAAGAGCTGTTAATCAGTTGAAAGAACAAGGACTTATATAA

Gene: glnR (glutamine synthase repressor)

Contig: 06_NODE_54, position: 440557 to 440925, length: 369 nt, orientation: FORWARD

Perfect match to: (RF122-AJ938182-[1273563:1273931], highly conserved allele)

Sequence:

ATGATATCGAATGATGCAATCAGACGAAATATGGCTGTCTTCTCTATGAGTGTAGTAAGTAAGTTAACGGATTTAACGCCAAGGCAAATA
CGTTACTATGAAACACATGAACTCATCAAACCTGAAAGAACAGAAGGTCAAAAACGTCTGTTCTCACTCAATGATTTGGAAAGATTACTA
GAAATTAAATCATTATTAGAAAAAGGATTTAATATCAAAGGGATTAAACAAATCATTTATGACTCACAAGAGCATTTAACAACAGATGAA
CAAGAGATAAGAAAAAAGATGATTGTAGATGCCACGCAAAAGCCTATTGGAGAAACTTTGCCAATAAATCGTGGTGATTTATCCCGATTT
ATTAAATAA

Gene: glnA (glutamine synthase, type I)

Contig: 06_NODE_54, position: 440944 to 442284, length: 1341 nt, orientation: FORWARD

Perfect match to: (XN108-CP007447-[1363512:1364852], allele observed in CC239)

Sequence:

ATGCCAAAACGTACTTTCACTAAAGACGACATTCGTAAATTTGCAGAAGAGGAAAATGTAAGATATTTAAGATTACAATTCACTGATATTT
TAGGAACAATTAAAAATGTTGAAGTGCCTGTAAGCCAATTAGAAAAAGTACTTGATAACGAAATGATGTTTGACGGTTCTTCTATCGAAG
GTTTCGTACGTATCGAAGAATCAGATATGTACTTACATCCAGATTTAGATACTTGGGTAATCTTCCCATGGACTGCTGGACAAGGTAAAG
TTGCACGTTTAATTTGTGATGTATATAAAACAGATGGAACACCATTTGAAGGGGATCCTCGTGCAAACTTAAAACGTGTATTAAAAGAAA
TGGAAGATTTAGGCTTCACAGACTTTAACCTAGGGCCTGAACCAGAATTCTTCTTGTTTAAGTTGGATGAAAAAGGGGAACCAACTTTAG
AACTTAATGATGATGGTGGATATTTCGATTTAGCACCTACAGATTTAGGTGAAAACTGTCGTCGTGATATTGTTTTAGAATTAGAGGATAT
GGGCTTCGATATTGAAGCTAGTCACCATGAAGTTGCCCCTGGTCAACATGAAATTGACTTTAAATATGCAGATGCTGTTACAGCATGTGA
TAATATCCAAACATTTAAATTGGTTGTTAAAACAATCGCACGTAAACATAATTTACACGCAACATTTATGCCTAAACCATTATTTGGTGTGA
ATGGTAGCGGTATGCACTTTAACGTTTCATTATTCAAAGGTAAAGAAAATGCATTCTTTGATCCGAATACTGAAATGGGCTTAACAGAAA
CAGCTTACCAATTCACAGCTGGTGTACTTAAAAACGCACGTGGATTTACAGCGGTATGTAACCCATTAGTAAACTCATACAAACGTTTAGT
TCCTGGTTATGAAGCACCATGTTATATTGCATGGAGCGGTAAAAACCGTTCACCGTTAATCCGTGTACCATCTTCAAGAGGATTATCAACT
CGTATTGAAGTACGTTCAGTTGACCCAGCTGCGAACCCATATATGGCGTTAGCTGCAATTTTAGAAGCTGGTTTAGACGGTATTAAAAAT
AAATTAAAAGTTCCAGAACCTGTTAACCAAAATATTTACGAAATGAATCGTGAAGAACGTGAAGCAGTAGGCATTCAAGACTTACCTTCA
ACACTTTACACAGCATTAAAAGCAATGCGTGAAAATGAAGTTATAAAAAAAGCTTTAGGAAATCATATCTATAATCAATTTATTAATTCAA
AATCAATTGAATGGGATTACTACAGAACTCAAGTATCTGAATGGGAAAGAGATCAGTACATGAAGCAATATTAA

Gene: A6U1C8 (putative protein)

Contig: 06_NODE_54, position: 442787 to 442984, length: 198 nt, orientation: FORWARD

Perfect match to: (N315-BA000018-[1308998:1309195], allele observed in CC5+CC1+CC8+CC96)

Sequence:

ATGAATTGCTATGATGAAATATTCAATACAATCAAAAAATTGATAGAAAACAAAGAGATATCAAGTTATCAAATTAATAAAGATACTGGG ATAAGTTACGGTAATATTAATGCTATGCGCCGTGGGGAAAGAAGAATAGAAAATTTAAGCTTAAAGAATGCAAAGATCTTATATGAATA TGCGAAAAAGGTATTGTAA

Gene: Q2FYY4 (putative protein)

Contig: 06_NODE_54, position: 443672 to 443896, length: 225 nt, orientation: FORWARD

Perfect match to: (MW2-BA000033-[1310031:1310255], allele observed in CC1+CC15+CC97)

Sequence:

TTGACAAAAAATTATGAACCAGTTTAAAGAAATTTATAATACAATAGAAAAATTACTAAATGATAAATCAATATCTAATTATAGAATTAATC AAGACACTGGTGTTTCTTATGGTGGTATAAGTGAATTAAGAAGCGGGAAAAGAAAAGTGAATAATTTAACTTTAGAAACAGCGGAAAAA CTCTATAATTACCAAAAACAATTAGAAATAATGATTTAAGATTAA

Gene: A5ISJ3 (putative protein)

Contig: 06_NODE_54, position: 444187 to 444393, length: 207 nt, orientation: FORWARD

Perfect match to: (MW2-BA000033-[1310546:1310752], highly conserved allele)

Sequence:

ATGAATGAGATTGAAACTATTATAAGTGAAATAGAAAAGTTATTAACTAACAATACACCATATAGTATTTCAAAAAACTCAGGTGTACCA CGTCAAACAGTTACTGATTTAAAGGTAGGTAAAACTAAAATAAAAGAAGCTAAATTTAAAACGATAATCAAGTTATATGAATATCAAAGA ACATTAGAAAATAAAACAGAATGTTAA

Gene: Q2YXQ4 (putative protein)

Contig: 06_NODE_54, position: 445632 to 445816, length: 185 nt, orientation: FORWARD

Perfect match to: (MW2-BA000033-[1311991:1312175], allele observed in CC1+CC97)

Sequence:

ATGTCAGAATACAAGAAAAAAATAATTGAATTAATTGAAAGTAATTTAACAGGATATGAAATTTCTAAAAAACTGGAGTTTCTCAATACG TACTTTCACAATTAAGACAGGGCAAACGCGAAGTAGATAATCTAACCCTGAATACAACAGAAAAATTATATGAATATGCCAATAAAGTTT TGTAA

Gene: Q2FYX8 (putative protein)

Contig: 06_NODE_54, position: 446560 to 446811, length: 252 nt, orientation: FORWARD

Perfect match to: (N315-BA000018-[1312768:1313019], highly conserved allele)

Sequence:

ATGCTTACATTAATAAAATTGAAAGAAGATGAACAGGTTATAATATATGAATATATACCTGAAGATGATATAAGTAACGGTAAAGGTTCA GTAACTTTTAATAAAAAAGATGCAGAGGTTATAGATTTCTCATTATCTGAAATAGAAAATGAAGAATATTTTATGTTATATCGTAATAAGT CTTTTTCTGTAGTAAGAGACTTTATCGAGAAGCAAGAATTTCCCGAAAATTATAAAATAGCATGGTATTAA

Gene: Q8NWX1 (putative protein)

Contig: 06_NODE_54, position: 447286 to 447720, length: 435 nt, orientation: FORWARD

Perfect match to: (11819-97-CP003194-[1345824:1346258], highly conserved allele)

Sequence:

TTGCTTAAAGATGCCAAAGAAAACAATGATAGCAATGAAGTAGCTTATTTATTAAAAGATGGTAAAGTTACAAAAGTATATGGTGATCAA
GATAGTGTATCTTTTGCACCAGGGGAAAAAGCAACAGAATTGTTATTTAACAGTAAACCGAATTCAATTGTTATGTTACATAACCATCCTG
GGCAGTCTAGTTTTTCTCTTACAGACTTGTATTTATTTATATTTAATAATTCTATTAAAACACTGACAATTGTTACAAATAAAGGTCAAACA
AAGTACTTAACTAAGACAAAAGAATATTGCAAATCAACTTGTATTGATTGTATTAAAAAATATAATAAAAATAAGAATATAAAAAAATTCA
ATCATAAGGATATTGATATGATTCTAAAGAGATTATATAATAGTGGTAACATAATATATAAAGTTAGGTGA

Gene: A0A0H3JW92 (bacteriophage capsid protein)

Contig: 06_NODE_54, position: 448008 to 448220, length: 213 nt, orientation: FORWARD

Perfect match to: (MW2-BA000033-[1314367:1314579])

Sequence:

ATGGAGAAAAATGAAAGTAATATTACTGACGTAACTCAGAACGAAGAGCAACTAGACAACAGTGATGAACAATCACAACAGAATGAGA
AAACATTTTCTCAAGAAGAAGTATCACAATTGATTAAAGAGCGTATAGCTAGAGAACGCAAAAAATCAGATGAACGTATTAAAGATGCG
GTTCAAGAAGCTGAGAAGTTAGCTAAAATGAAGTAG

Gene: Q8NWW7 (putative protein)

Contig: 06_NODE_54, position: 449001 to 449462, length: 462 nt, orientation: FORWARD

Perfect match to: (MW2-BA000033-[1315360:1315821], allele observed in CC1+CC72+CC80+CC97)

Sequence:

GTGCCACATGTTGGCAGTTGGCGAGACAAGTTCTTTAAAGAGCGTGAAGGTAAATATCAGGTAGAAGTAAAAGAAGCTAAATTACAAG
AAAAAGCTAAAAACCAGATGAAAGAAATGATTGAAAGTTGTAAAATGAAAATAGAAATAAATTGTGAAAAACAAAATGGACATATGTTA
GGTCATCATCTATATAATGAAAATAAAAAAAGAGCCATTTTAAATAATAAGAAATTGCCTAGCTATACAATACTTTCTATAGATTTATTGA
ATGAATTGTTAAGAGAAAAAAATGTCAACAGGCAATCTAATATTAAGTGATGAGCTATTTGATATGAAAGAGATTATTAATTTTAATCAAA
TTATTGGAAAAGTACATATCGATAATGTGTATATTGAAACCAGAAAAGGAAAAGTGCATTATTCGAAGACAGGTGCTCATATAGTACCTT
ATATTGATAAGTAG

Gene: A6QGL8 (aromatic amino acid beta-eliminating lyase/threonine aldolase)

Contig: 06_NODE_54, position: 450228 to 451253, length: 1026 nt, orientation: REVERSE

Perfect match to: (MW2-BA000033-[1316587:1317612:r], highly conserved allele)

Sequence:

TTAGATTAATTCTAATAGTTTATTTAAATTTTCTTCGGTTGTCGCCCAGCTGGTTGCGAATCTAACAACACGATGTTGATCATCGTATTTTTC
CCAAACAGCAAATTTAACTTTTTGTTCTAACTCTGCTATTTTCTCGTTACTTAAAATAAAAAATTGTTGATTGGTTGGAGAATCAAAGTAAA
GACGATAGCCTTTATTTATAAACCCGTCTTTCATCTTATTTGCCATTTCGATAGCATGTCTGCTTATATTAAAATATAAATTATCCGTAAATA
ATTCTAAAAATTGTATGCCTGTTAACCGTCCTTTTGCTAAAAGTGCACCGTGATGTTTGATTCGAGTGGTAAATTGTTTCGGTTCATTATTC
TTCGTAAAAACAATTGCTTCTCCGCATAATGCACCTATCTTCGTACCACCTATATAAAATACATCACAATATTTAGCGATGTCTTTAATAGT
CATATCTGATTGATCACTCATCAATCCATACCCTAATCGTGCACCATCCATAAATAATGGAAGCTGATATTGCTTACATACTTTGCATAACT
CTTCCAATTCTGATTTAGAGTATAATGTGCCATATTCTGTAGGATGAGAAATATATACCATTCCTGGGAATACCATATGGTCCTTTTTAAAA
TCACTTTTAAATGTCTCCATGTAAGTTTCAACATCTGAAGCACTAACTTTTCCTTCCTTAGAGGGTATAGTAATTACTTTATGTCCACTATAT
TCAATTGCACCGCCCTCATGCACAGCAACATGACCAGTGTCTGCTGAAATAACCCCTTCGTAACTTTCTAACATTGAATTAATAACAACCT
GATTGGTTTGCGTTCCACCTACTAAAAAACGGATAGTAGCATTTGGACAATCAATTGTATCTTTAATCTTTTCAATTGCCTGAGCTGTGAAT
TGATCAAAGCCATATCCCGAAGCTTGTACAAGATTTGTATCTACTAATCGTTTTAATACTTTTTCATGAGCACCTTCTAAATAATCATTTTCA
AATGAGATCAC

Gene: A5ISJ9 (putative protein)

Contig: 06_NODE_54, position: 451523 to 451720, length: 198 nt, orientation: FORWARD

Perfect match to: (N315-BA000018-[1315775:1315972], highly conserved allele)

Sequence:

ATGGAACAAATTAAACTTAAAACTTTTACAGCTGAGACTTTAGAGTTATTAGAAAAAAACATTAATGCTTTTTTAAGTTCTGAAGAAGCTA
CAAATTTAAAATTAGTAAATATTACTATTAAAGAAATAGAAGAAAGAACATTCCCAAATAATGAAGAAGAATTCAATGCAATTTTAACTTT
ATCTGTGAATAAATAA

Gene: cls1 (cardiolipin synthases 1)

Contig: 06_NODE_54, position: 451777 to 453258, length: 1482 nt, orientation: FORWARD

Sequence:

ATGCGATATACATTTTCAAATGATTTAGGACCACTTTTTACCATTATTTTAGCCATTGGATTCATCATTAATTTAGTATTGGCTTTTATTATT
ATCTTTTTAGAAAGAAATAGACGTACAGCGAGTTCAACTTGGGCATGGCTATTTGTACTTTTTGTCTTACCATTGATTGGTTTTATTCTTTA
CTTGTTTTTTGGTAGAACCGTTTCGGCACGTAAATTGAATAAAAACAATGGTAAGGTGTTAACGGATTTCGATGGACTTTTAAAACAACA
AATAGAAAGCTTTGATAAAGGTAATTATGGTACTGATAACAAACAAGTTCAAAAACATCATGATTTAGTACGTATGCTTTTGATGGATCA
AGATGGTTTTTTAACTGAAAATAATAAAGTTGATCATTTCATTGATGGAAATGATTTATATGATCAAGTTTTACAAGATATTAAAAATGCT
AAAGAATACATCCATTTAGAGTATTATACATTCGCTCTAGATGGCTTGGGCCAAAGAATTTTAAAAGCGTTAGAAGAGAAATTGAAACAA
GGTCTAGAAGTGAAAATTTTATATGATGACGTTGGTTCAAAGAAAGTTAAGATGGCTAATTTTGATCATTTTAAATCGTTAGGTGGAGAA
GTTGAAGCATTTTTTGCTTCTAAATTACCGTTATTGAATTTCAGAATGAATAACAGAAATCATAGAAAAATCATCGTAATCGATGGTCAAC
TAGGTTATGTCGGAGGATTTAACATTGGTGATGAATATCTAGGATTAGGTAAACTAGGGTATTGGAGAGATACGCATTTACGTATACAA
GGGGATGCGGTTGATGCACTGCAGTTGCGATTTATTTTAGACTGGAATTCGCAAGCGCACCGTCCACAGTTTGAATATGATGTTAAGTAT
TTCCCTAAAAAGAACGGACCATTGGGCAATTCACCAATTCAAATAGCTGCAAGTGGTCCGGCTAGTGACTGGCATCAAATTGAATATGGT
TATACAAAAATGATTATGAGCGCTAAGAAGTCGGTATATTTGCAATCGCCTTATTTCATTCCGGATAACTCATATATAAATGCCATTAAAA
TTGCAGCTAAATCAGGGGTAGATGTTCATCTAATGATTCCATGTAAGCCAGATCATCCTTTAGTTTATTGGGCGACATATTCAAATGCTTC
AGATTTACTATCTAGTGGCGTAAAAATTTACACGTATGAAAATGGATTTATACATTCTAAAATGTGTTTAATAGATGATGAAATGGTTTCA
GTCGGAACTGCTAATATGGACTTTAGAAGTTTTGAATTGAATTTCGAAGTCAATGCTTTTGTATATGATGAAAAGCTTGCTAAAGATTTAA
GGGTGGCTTATGAACATGATATTACAAAATCAAAACAACTAACCAAAGAATCATATGCCAATAGACCGCTGTCTGTTAAGTTTAAAGAAT
CGTTAGCTAAATTAGTTTCGCCAATTTTATAA

Gene: A6QGM1 (ABC transporter, ATP-binding protein)

Contig: 06_NODE_54, position: 453442 to 454341, length: 900 nt, orientation: FORWARD

Perfect match to: (N315-BA000018-[1317694:1318593], highly conserved allele)

Sequence:

TTGATTCAAATATCTAATATCAACAAGTCATTTAATAAAAGATGTGTTCTAAAAAATATTTCGTTCGATATTGAACAAGGTAAATGTATCG
CTTTAATTGGAAAAAATGGTGCTGGAAAGTCAACGTTAATTGATATATTAATTGGTAATGTTAATGCTAATTCTGGTGAGATATTTGATAA
AGACAAGTTATTACAAAGTGAAAATCGCAGTATAATGTTCCAAAAAACGATGTTTCCAGATCAATTAAAAGTTATTGAGATTATCAACTTA
TATCAATCATTTTACGAAAATCCATTACCATTGGAAGAAATAATAGAACTGACGAAATTTGATTCTAGTCAACTGAACCAATTTGTAAATA
AACTTTCTGGTGGTCAACAACGATTACTCGATTTTGTATTATCTTTAATCGGACAACCACAATTGATCTTATTAGATGAACCAACATCGACT
ATGGATATAGAAATTAGAGAATATTTTTGGTCAATTATTGAAAATTTAAAAGAAGATAATCGAACGATACTCTATACATCGCACTATATTG
AAGAAGTCGAACGTATGTCAGACAAAATTATTCTCATTGAAAATGGAGAAATAATACTTAATGATTCAACGTCACATATTAGAACCAATC
AGCAATCTCAGATTACGTTATCCGATGAATATATAAGAAAGTTAAAACTAGATAAAGATGATTTAGTTATTCAAAAAAATCATAATGGCA
CTATCAAAATTATTACTTCAAATGTAAATGATACGATTTTATATCTTCAACAACTTCATATTAATTTGGATGATATTGAAATACAAAAAGTC
TCAATTGTTGATTCATACTTCAACAATAAAAAGCAAAGGGGATCTAATTATGATACTAAGTTACTTGAAAATCGAATTTAA

Gene: Q5HGA1 (ABC transporter, transmembrane permease)

Contig: 06_NODE_54, position: 454310 to 455041, length: 732 nt, orientation: FORWARD

Perfect match to: (MW2-BA000033-[1320669:1321400], highly conserved allele)

Sequence:

ATGATACTAAGTTACTTGAAAATCGAATTTAAAGTTATAATGCGTAAAAAAACAACATTAATATTATCTATTTTATTTCCTGTTATATTCTAT
ATATTATTTACTTCGATATTGGAATTGCCGGAAGATGTTAAACCTAAATTTTATAAAGAGTATATGTATAGTATGACGGTTTATAGTTTGTT
AAGTTTTAGTTTACTAACTTTTCCATTAGATATTATTAATGAAAAACAAAATGAATGGCGCCAAAGATTAATGGTAACACCATTTACTTTTA

CTAGTTATTATATTTCAAAAGTAGTGAAAACTATGCTGCAATTTGCAATAGCGATATTAGTTATTTTTATGGTTGGACATTTTTATAAAGGT
GTTGCAATGAGTGCAGTTCAATGGTTAGAGTCAGGAATATTTTTATGGTTAGGTGCGTCTCTATTAATAACTTTTGGCATATTATTTTCTTT
GTTAAATGATATTCAAAAAACAAGTGCTTTAGCTAATATCGTAACAATTGGTTTAGCAGTATTAGGTGGATTGTGGTTTCCGATAAACACA
TTTCCAAATTGGCTTCAACATGTTGCTCATGTTTTACCGAGCTATCATTTGCGTAAACTAGGTGTAGATATTGCTTCAAATCATCATATCAA
TTTAATATCATTTGCTATAATACTCTTGTATGCTTTAGGGAGTATATTAGCAGTATATTGTATTAGTCATTTTAAAAGGGCGGAATAA

Gene: desK (two component sensor/regulator, sensor histidine kinase)

Contig: 06_NODE_54, position: 455045 to 456136, length: 1092 nt, orientation: FORWARD

Perfect match to: (COL-CP000046-[1361000:1362091], highly conserved allele)

Sequence:

ATGAAATTTTTAAAAGATACTTCAATTGCTGAAATATCGTCTATACTTTATCTGATTTTTCCTATTGCCGGTATATTTTTTAATGAAGTATAT
GGTCCCAAATGGTTGTATATTATATCAGTCATTGTCTTTTCGTTGTCGTATCTTATATTAGTTATAGTAAATAATAGACTTAATACATTAATG
TTTTACATTTTGTTGATTATTCATTATTTTATTATTTGTTATTTTGTTTTCAGTGTACATCCAATGCTAAGTTTGTTTTTCTTTTATAGTGCTTT
TGCCGTTCCATTTACTTTTAAAAATAATGTTAAAAAAACGGCAACTAATCTTTTCATACTAACAATGATTATATGTACAATAATAACGTACT
TATTGTATAACAACTATTTTGTTGCAATGATGGTTTATTATGTCGTTATATCGTTAATAATGCTAGATAATTTTAAAAAAATGAAAAACCGT
GAATATCAAAAAGAAATAGCAGAAAAAAATAGACATATTAATACATTAATTGCTGAACAAGAGCGACATAGAATTGGTCAAGACTTACA
TGATACGTTAGGGCATGTGTTTGCAAGTTTATCATTAAAATCAGAATTAGCTTATAAACTAATAGATGCTGATGTAGAAAAAGTAAAAGC
TGAATTATTAGCAATTAATAAATTATCTCGTGAATCATTGAACAAAGTTCGAGAAATTATTGATGATGTAAAATTACCATCATTTATTGAA
GAGATTGATAGTATACGTAAAGTTTTAAAAGATGCTGATATTGATTTTACATTTGAAAATAAAGAATTAGCGCAAGTATTAAGTCCTACTA
AACAATCTATGTTAGTTATGATTACGCGTGAAGCGATAAATAATGTTATTAAACATGCAAATGCTTCAAAAGTTCATGGTAAATTAAAAAC
TGTAAACAATCATAAATTACTGCTTATGATTGAAGATGATGGCAAAGGTATCGATAGTGATTGTGAGGTGAAAAGTATTTCACAGCGTGT
ACAACATTTAAATGGAACTTTAGCAGTCGACTCAACAAATGGAACTAAAATAATCATTGAAATCTCAACAGGAGGAATAGCATGA

Gene: desR (two component sensor/regulator, transcriptional regulator)

Contig: 06_NODE_54, position: 456133 to 456735, length: 603 nt, orientation: FORWARD

Sequence:

ATGACATCTTTAATTATTGCAGAAGATCAAAATATGTTACGACAGGCAATGGTTCAATTAATTAAACTACATGGTGATTTTGAAATTTTAG
CAGATACTGATAATGGTCTCGATGCAATGAAACTTATTGAAGAATATAATCCTAACGTTGTTATTTTAGATATAGAAATGCCAGGCATGAC
TGGACTTGAAGTTTTAGCGGAAATTAGAAAAAAGCATTTGAATATTAAAGTGATTATTGTAACAACTTTTAAAAGACCGGGATACTTTGA
AAAAGCAGTTGTGAATGATGTGGATGCATATGTTTTAAAAGAACGTTCTATAAAAGAATTGGTGGAAACCATTAATAAAGTAAATAACG
GAGAGAAAGAATATAGCGCCACATTGATGACTTCATTTTTTGTAGATAAAAACCCATTAACGCCCAAAGAACAAATTGTATTAAGGGAAA
TTGGCAATGGTTTAAGTAGTAAAGAAATAAGTGAAAAATTATTTTTGACAGATGGAACAGTTAGAAATTATACATCTGTTATAATTGATA
AATTATTTGCAGATAATCGTTTTGATGCTTGGAAAAAGGCAAATGAAAAAGGCTGGATCTAA

Gene: Q99UF3 (putative protein)

Contig: 06_NODE_54, position: 456848 to 457036, length: 189 nt, orientation: REVERSE

Perfect match to: (N315-BA000018-[1321100:1321288:r], highly conserved allele)

Sequence:

CTAATTTTTTCGCTTATTATAAAACGAAACAATCAAGTAAACGATAAAGCCTACAAAGATACCCAATAAAATAGATAGTACTGCCGTCACT
ATTAATGGTAATTTGAAAAATATTTGTAGGAAAATACCAATGATAATTGCGATAATTACTGCAATTAATGTGACTGTATTTTCATTTGAAA
TGTTCAT

Gene: nuc2 (thermostable extracellular nuclease locus 2)

Contig: 06_NODE_54, position: 457175 to 457708, length: 534 nt, orientation: FORWARD

Perfect match to: (N315-BA000018-[1321427:1321960], highly conserved allele)

Sequence:

ATGAAGTCAAATAAATCGCTTGCTATGATTGTGGTAGCCATCATTATTGTAGGTGTATTAGCATTTCAATTTATGAATCATACGGGTCCTTT
CAAAAAGGGGACGAATCATGAAACTGTACAAGATTTAAATGGTAAAGATAAAGTACATGTTCAAAGAGTTGTGGATGGTGATACATTTA
TTGCAAATCAAAATGGTAAAGAAATTAAAGTTAGGCTTATAGGGGTTGATACGCCAGAAACGGTGAAACCGAATACGCCTGTACAACCA
TTTGGCAAAGAAGCATCAAATTATAGTAAGAAGACATTAACAAATCAAGATGTTTATTTAGAATATGATAAAGAAAAACAAGATCGCTAT
GGTAGAACATTGGCGTATGTATGGATAAGTAAAGATCGTATGTACAATAAGGAATTAGTGGAAAAGGGACTTGCTAGAGAGAAGTATTT
TTCACCAAATGGCAAATATAGAAATGTATTTATAGAAGCACAAAATAAAGCTAAACAACAGAAATTAAATATTTGGAGTAAATAA

Gene: Q5HG96 (putative protein)

Contig: 06_NODE_54, position: 457851 to 458705, length: 855 nt, orientation: REVERSE

Perfect match to: (COL-CP000046-[1363806:1364660:r], highly conserved allele)

Sequence:

TTACTTGAATATAGCTGTGATTTGATCAACCTGTTCTTTATTTAAAGGGTGTTTAGATAGCTCTATGGCTTCGCTAATAATACGTCGTAACG
AAGATTTTGTTGAAACTGTATCAACATATTTATCTCTTTCTTCAGAACCTTGAATGATATTAACTGTACCATCACTAAAGTAAATAACACCG
GTACTTTGGACTTTAAAGTTAAAGTTTTGTTCAATATGATCCTTTAATGCTTTCGCATTATTTGCTGCTAATTGATACGGATCATAATCATAA
AAATCATAAATAACACGATTTGGTTGAACTGTTTCTGTAAAAGTATAGATAGACTTTCTTGATGAATTAAACTGATCATGATATTGCTGGC
TAATGTAATGACCGACAACTTTTTCAATATTACTATTGCTTATTTCTGTATCATGCTCATCAGGTACATCAAAGTGATAAAATGTTTTTTCAC
CCCAGCTTTTTACATCAACGTTTATTAAACCGATATCTGAAACAATGATAAAATCAAATGAACGTGCATACTCGAAAAACGGATGTTTAGT
AGCTAAATTGCTTGTAACGATAATATCGTAATATTTTAATTTACCATTTTCGAGATAGCTATCTAAAATCTTGCGTAATTCTCTTTTAGCATT
TTTGTATGCATGGTGGCCAACATCATTATTAGAACTCAGCATTTGACTTCTTAATTCAGCATTTTCTGCACTTAATGCTTTGTTCTTTTTAAT
AAGTTGCTTTCTTGCATAAACTTCGGTATCTATTTTACTATTACTATACCTTTGATTTAAAACTAATATACCAATTAATGCTACAATGATAAT
GATAAGTACAACATAAAAAGACAT

Gene: Q5HG95 (putative protein)

Contig: 06_NODE_54, position: 459021 to 459611, length: 591 nt, orientation: FORWARD

Perfect match to: (MW2-BA000033-[1325380:1325970], allele observed in CC1+CC8+CC30)

Sequence:

TTGAGAGTGAATGAAATGAATGCTAAAGAACAATTAGTGGACAATTTAATGAAAACATCATCGCAATTATTTAAATTTCACGGTGAAGTT
GCCATGCAGCTTTTCTTAAATGATGAATTAAAATTACCTTCTATTGTTGAAATATGCGTGGAACGTAAGCGTTTAAGTGATATTGTGAAAG
TTATTCCGCAATCATATGCGTTACTATACATAGATAAGCAAGATCAAGCAATAGCTAAAGAAGATTTATCACTTTCAAAAATTGCAAAAGT
TTATGTGCAATATGATGATACAACAATAATGAGTATTTTCGTTTATGATGTAGTAAACGATGAATGGATTTTTAGATTGGATCCGAATATA
CGTATACCTAAGAGTAACATATACTTCCATAGTTTAAATTGGGATGTGGATTATATTAAACCGGAGATTGTTCTAATGTATGATCTAATGC
AACACCATCAGTATCATCATTATTCCAATTATAAACGAGTCATAGATGCATTAAGCTACTATCAATTTTTTATTTTAAAATTTGTAGTAGGT
GAGCAACGTATTAAGGATGCAATCCAGAGAACAATAAATAATTAA

Gene: thrA (aspartokinase)

Contig: 06_NODE_54, position: 459665 to 461047, length: 1383 nt, orientation: REVERSE

Perfect match to: (08-02119-CP015645-[846420:847802], allele observed in ST582+CC5+CC8+CC97+CC398)

Sequence:

TTAGTTTTTATCACACAAGTTTTTTAATGCAACCCCGTGATAGCAAAACTCATATGTAGATAATACAGCTTTTTCAGCATCATCTACATGAA
TTCCAAACATCATCGAGATTTCCGAAGCACCTTGGTTAATCATTTTTAAGTTAATTTTTGATTCAGCTAAGGCATGTGTAATTTTATTTGCA
GTACCGATGACTTTATTCATACCTTCGCCGACAATCATTAATATTGCTAAATCATGCTCAATACTTAGCTCATCAACATCACATTTTTGACG
AATTTCATTTAATACTTTTGTTTCTTTATTTTGAATTTGTTTTGAACGCATAACGATACTGATAGTATCAATACCTGAAGGCATATGATCAAA
TGAAATATTATTATCCTCTAAGACACCTAATATCTTTCTAGTAAACCCGACTTGTCTATTCATTAAATACTTTTTGATATTAATAACGGTAAA
ATCTTTATCACAACTTATACCGCTAATCACATTTTTCGCATTTATTTCTCTATCATGCACTATAAATGTACCTTTATCTTGAGGGCGGTTCGT
ATTTTTAATCACCACAGGGATGCGATCTTTATAAAGTGGTTGTAAGGCTTCATCATGGAAAACACTAAAACCAGCATAAGATAATTCACG
CATTTCTCGATAAGTGATTTCTTCGATTAATTCAGGATCTTTGATGATATTTGGATTAGCTTTATAAATACCAGACACATCGGTGAAATTTT

CATAAATTGTAGCTCTAACACCACTTGATATGATGGCGCCAGTTATATCTGATCCGCCACGTGGAAATGTAACTATATATCCTTCATGAGA
TACGCCGAAAAATCCTGGGATAATTAGTTTCTCATCATAATCTCTTAATTTTTTAATTTCAGAGTAAGCACTATCTAATATTTGTGCTTCTTG
TGGGACGTCAGTAACAAAAATACCCGCTTCCTTCGGTGATATATATTTTGTTGGTATACCTTGACTATTATTATATAAAGCTATCAATTGCG
CATTAAAATCTTCACCACAAGAAAGTAATGCATCTAATAGTCTCTTTGGTTCATTTTTTAATTGATTAATATAATGTTCCAAAGTCACATCTA
TCGTCCGTAAAATACTTTCATCCATTTGCAATTCTTTTACAATATCATCATAACGCTGAATAATTTCTCTTTTTTTATCATGATAATCAAGAT
GATTAATGACCTTTTCATATAATCTGATTAACAAATCAGTTGTTTTAATATCATTATCATGTCTTTTACCTGGAGCAGAAACGATAACAATC
TTTCGCTCTGGATCAGAATTAACAATATTTAAAACCTTTTTAATTTGAGTAGCATTGGAGACGGAGCTACCACCGAATTTGGAAACTTTCA
T

Gene: hom (homoserine dehydrogenase)

Contig: 06_NODE_54, position: 461238 to 462518, length: 1281 nt, orientation: FORWARD

Perfect match to: (CA347-CP006044-[1334261:1335541], highly conserved allele)

Sequence:

ATGAAAAAAATTAAATATAGCATTATTAGGATTAGGTACTGTCGGATCTGGTGTTGTTAAAATCATCGAAGAGAACCGACAGCAAATTCAA
GATACATTAAATAAAGATATTGTCATAAAGCATATTCTTGTTCGAGATAAATCTAAAAAGAGACCGCTAAATATTAGCCAATATCATTTAA
CTGAAGATGTTAATGAAATTTTAAATGATGATTCATTAGATATTATCGTTGAAGTCATGGGAGGAATTGAACCAACTGTAGATTGGTTAA
GAACAGCACTTAAAAATAAAAAACATGTTATTACCGCAAATAAAGATTTATTAGCAGTACATCTTAAACTTTTAGAAGATTTAGCAGAAG
AAAATGGTGTAGCTTTAAAGTTTGAAGCGAGTGTAGCAGGTGGTATTCCGATCGTAAATGCCATAAATAATGGTTTGAATGCGAATAAT
ATTTCAAAATTTATGGGAATTTTAAATGGTACCTCTAATTTTATTTTATCTAAAATGACTAAAGAGCAAACGACATTTGAGGAAGCACTTG
ATGAAGCGAAAAGACTTGGTTTTGCTGAAGCGGATCCAACTGATGATGTAGAAGGGGTAGATGCAGCGCGTAAAGTTGTCATTACATCA
TATTTATCATTTAACCAAGTCATTAAATTAAACGACGTTAAACGAAGAGGAATTAGTGGCGTAACTTTAACTGATATTAATGTAGCCGATC
AACTGGGGTATAAAATTAAATTGATTGGTAAGGGAATATATGAAAATGGCAAAGTTAATGCATCGGTAGAACCAACGTTAATTGATAAA
AAGCATCAATTAGCAGCTGTAGAGGATGAATATAACGCGATTTATGTCATTGGTGATGCCGTTGGTGACACGATGTTTTATGGAAAAGG
AGCAGGCAGTTTAGCAACAGGTAGTGCCGTTGTCAGTGATTTATTGAATGTAGCATTATTCTTTGAATCAGATTTACACACATTGCCACCA
CATTTTGAAATAAAGACAGATAAAACACGGGAAATGATGGATTCAGATGCAGAAATTAATATTAAAGAAAAATCCAATTTCTTTGTAGTA
GTGAATCATGTCAAAGGTTCAATTGAAAATTTTGAAAATGAGTTAAAGGCAATATTACCATTTCACCGATCATTAAGAGTTGCAAATTACG
ATAATCAATCATATGCCGCTGTTATAGTTGGATTGGAATCATCACCGGAAGAATTAATCACTAAGCATGGATACGAAGTTGACAAAGTAT
ACCCAGTAGAAGGAGTTTAA

Gene: thrC (threonine synthase)

Contig: 06_NODE_54, position: 462524 to 463585, length: 1062 nt, orientation: FORWARD

Sequence:

ATGAGAAGATGGCAAGGATTAGTAGAAGAGTTTAAAGCACATTTACCAGTAAATGAAAATACACCAAAATTAACATTGAATGAGGGAAA
TACACCACTCATTCATTGTGAAAATATGTCTAAAATACTAGGTATAGATTTATATGTGAAGTATGAAGGTGCCAATCCGACAGGTTCATTT
AAAGATCGCGGTATGGTAATGGCTGTGACAAAAGCAAAAGAGCAAGGTAAGAAAATTGTAATATGCGCTTCGACTGGAAATACATCAG
CGTCTGCAGCAGCATATGCAGCGAGAGCAGGTTTAAAAGCTATCGTCGTAATACCAGAAGGTAAAATTGCATTAGGTAAATTGTCGCAA
GCAGTAATGTATGGTGCAGAAATCGTTTCTATTGAAGGAAACTTTGATGAAGCTTTAGAAATTGTAAAAGAAATTGCAAAAAGTGGCGA
AATAGAGCTTGTAAACTCTGTCAATCCATTTAGAATCGAAGGGCAAAAGACAGGCTCATTTGAAATTGTACAACAATTAGACGGTGAAGC
ACCTGATATTTTAGCGATTCCTGTAGGTAATGCAGGTAATATTACTGCATATTGGAAAGGCTTTAAAGAATATCATGAAGCTAAAGGTTC
ACAATTGCCGAAAATGTTTGGCTTCCAAGCTGAAGGCGCATCACCAATTGTTCAAAATAAAGTCATTAAAAATCCTGAAACGATTGCAAC
TGCTATTCGAATTGGTAATCCTGCTAGTTGGGATAAGGCGACTAATGCTCTTAAAGAATCAAATGGATTAATAGATAGTGTTACTGATGA
TGAAATTCTAGAAGCATATCAGTTAATGACAACTAAAGAAGGTGTCTTTAGTGAACCAGCGAGTAATGCTTCTATTGCAGGTTTAATTAA
ATTGCATAGACAAGGTAAATTACCTCAAGGTAAAAAAGTAGTTGCTATTTTAACTGGTAATGGATTAAAAGATCCTGATACTGCTATTTCA
CTACTAGATAATCCGATAAAGCCATTGCCAAATGATAAAGATAGCATTATCGATTATATTAAAGGAGCTTTATAA

Gene: thrB (homoserine kinase)

Contig: 06_NODE_54, position: 463587 to 464501, length: 915 nt, orientation: FORWARD

Perfect match to: (NN50-BAEA01000026-[82209:83123:r], allele observed in CC4803+CC8+CC72+CC239+CC772)

Sequence:

ATGTCGAATGTTTTGGAGTTAACAATTCCTGCATCAACAGCCAACCTTGGAGTTGGCTTTGATTCTATAGGTATGGCTTTAGATAAATTTTT
GCATCTGTCTGTAAAGGAAACATCAGGGACAAAATGGGAATATATTTTCCATGATGATGCATCTAAGCAATTGCCTACTGACGAAACAAA
CTTTATTTATCATGTAGCACAACAAGTTGCTTCTAAATATAGTGTTGACTTGCCTAATTTATGTATCGAAATGAGAAGTGATATTCCATTGG
CAAGAGGGTTAGGTTCGTCAGCTTCTGCTTTAGTAGGAGCTATATATATCGCAAATTATTTTGGTGATATCCAACTGTCTAAACATGAGGT
ATTACAATTAGCGACTGAAATCGAAGGACATCCTGATAATGTTGCGCCGACCATTTATGGTGGTTTAATCGCTGGATATTATAATGATGTC
TCGAAAGAAACGTCAGTTGCACATATCGACATACCAGACGTGGATGTGATTGTAACGATACCAACTTATGAACTAAAAACAGAAGCATC
AAGACGTGCTTTACCACAAAAATTAACACATAGTGAAGCGGTTAAAAGTAGTGCAATTAGTAATACAATGATTTGTGCATTAGCACAGCA
CAATTATGAATTAGCAGGTAAACTCATGCAACAAGATGGCTTTCATGAACCGTATCGTCAGCATTTAATTGCTGAATTTGATGAAGTGAA
AACAATTGCTATTCAACATAATGCCTATGCAACTGTAATTAGTGGTGCTGGACCAACTATTTTAATATTTAGTCGTAAAGAAAATAGTGGG
GAATTGGTTCGCTCTTTAAATAGTCAGGTAGTATCATGCCATTCTGAATTGGTCGATATTAATATCAGTGGTGTTAAAGAACGAATTGTAT
ACCAATAG

Gene: yxeH (putative hydrolase)

Contig: 06_NODE_54, position: 464559 to 465362, length: 804 nt, orientation: FORWARD

Perfect match to: (MW2-BA000033-[1330918:1331721], highly conserved allele)

Sequence:

ATGACAAATTATAAAGTTGTCGTTTTAGATATGGATGACACATTGCTAAATTCAGATAATGTGATATCAGAAGAAACTGCAAATTATTTAA
CAGCAATTCAAGATGAAGGTTATTATGTTGTTTTAGCATCTGGTAGACCTACTGAAGGTATGATTCCAACTGCTAGAGATTTAAAATTACC
TGAACATCATAGCTATATTATTAGTTATAACGGTAGTAAAACGATTAACATGACTAATGAAGAAGTAGAAGTAAGTAAATCGATTGGTAA
GCAAGATTTCGATGAAATTGTAGATTATTGTCGAGATAGAGGCTTTTTCGTTCTTACATATCATGATGGTCAAATTATTTACGACAGCGAA
CATGAGTATATGAATATTGAAGCAGAATTAACAGGTTTACCGATGAAACGTGTTGATGATATCAAAGCGTATATTCAAGGCGATGTACCC
AAGGTCATGGGTGTAGATTATGTAGCGAATATTACAGAAGCTAGAATTGATTTGAATGGTGTGTTCAATGATAATGTAGATGCTACGAC
AAGTAAGCCATTCTTCTTAGAATTTATGGCCAAAGACGTTTCAAAAGGTAATGCAATTAAAGCGTTATGTCACAAATTGGGATATTCGGT
GGATCAAGTCATTGCTTTTGGTGATAGTATGAATGATAAATCAATGTTTGAAGTCGCAGGTCTAGCTATTGCTATGGGGAATGCATCAGA
TGAACTTAAGCAATATGCAAATGAAGTTACGTTGGATCATAATGAAAATGGTATTCCACATGCGCTCAAAAAATTGTTATAA

Gene: Q5HG88 (putative protein)

Contig: 06_NODE_54, position: 465655 to 465969, length: 315 nt, orientation: REVERSE

Perfect match to: (MW2-BA000033-[1332014:1332328:r], highly conserved allele)

Sequence:

TTATTTAGCTGTTTGTGTCCAGTCATGCTTACCTCTGAAACGTTTTACATGAGCATATATTTGGTCAGCAGTATAATCTAAATTTGTATAAA
CTGTAATACTGAACGCTGATTGGTTTTCAGCAAATGATTCTTCGGAAAATAATTCCTTTAAGTGCATAAATAAATCATTCATTTGTTTATGG
TTTAAACCGGGATATTCCCTTAAAGTTCTCTTTAATAATTGTCCGTTCTTTTCTTCTAATGCTTGAACTACAATAACAAATCTTTCATCTTGCT
TTAGTACATCATCAAATAAATTTGTTTGTCCAATCAT

Gene: Q5HG87-lysP2 (putative amino acid permease)

Contig: 06_NODE_54, position: 466187 to 467641, length: 1455 nt, orientation: REVERSE

Perfect match to: (MW2-BA000033-[1332546:1334000:r], highly conserved allele)

Sequence:

TTATTTTTGATTGTCATAATCGTGTGGTTTTAAATTAATTGTTTCTAGCTTTACAAATTTTGTTTTGTGAATGATTTTATGAATAAAGTAAAT
CAACGCTAGAATGATTAAAGGTAAAAAGTTTTTAAAAGCATTTAACCATTGATCTTTTAAAATATATTCAACTGAGCCACCAAATAGCAAG
AATAATAGTGTAGTGATGACAATGATTGGTCCTAATGGATAAAAAGGTGCTTTATATGGTAGGACCTTATTAGGGTCTTGACCTTGTTTTT
TAATAGCTTGTCGCAATCGTATTTGTGACCAAATGCTTGATCCCCAAACAACTATAATCATTGAACCAATAATTTCAAGTAAATTAAAAAC
GGCATTTGAATTAAAGTTTGCATAAATAATAACAATAACAACGACTGCATAAGTAGTTAATAATGCTCTTAAAGGTAACTTAGTTGTCTTG
TTTAATTTACTTAAAAATTGGGGTGCTTTTTTGTCTGAACTTAAGGAATACAACATTCTGCCTGTTGTATAAACACCTGAATTTGCAGCGGA
TAATAATGAAGTTAAAATAACCGCGTTGATTACTGATGCTGCAAAGGCTATGCCTACTCTATCGAATACAATTGTAAATGGGCTTTGACTT
ATTGAACTACTTGCTCTTAATAATGATGGATCTGTGTACGGAATAATTGCACCAATTACTGCAATTGATAAGACATAGAATAAAAGAATAC
GCCAAAATACTTGTTTAATTGCCTTAGGCATAGACTTTTTAGGGTCATCTGATTCACCAGCAGTTACTGCTACTACTTCTGTACCACCAACC

GAAAATCCGGCGACTAATAATACGCCTAAGAAACCAGAGATACCACCAACAAACGGTGCTTGGCCTTTTGTATAGTTTTCAAATCCATAT
GTATGACCACCTAAGATACCGAAAATCATTAAAAAGCCAAAAATAACGAATACGATAATTGTTAACACTTTAATCAATGATAACCAAAAC
TCAGTTTCTCCAAATGATTTTACAGAAAAAATGTTTAATAATAGTAAAATTGTAATAAAGATTAAGCTCCAAGTAATGGGGTGGAAAAAT
TTAAATGTGTCCCAGAAATAAAGCACATTTGACGCTACTATGACATCAACACTTGTAACTAATGACCACAATGCCCAATACAACCATCCCA
TGGTAAAGCCAAGAGATGAGTCAATAAAGCGTGTTGAATAAGAGCTGAATGAACCTGATACTGGATAAAATGTTGCCAACTCTCCAATT
GATGACATTAAGAAATATAGCATGACACCAATAACAAGATAAGCGAGTATAGCGCCTCCAGGACCAGCTTGAGAAATGATATTACCAGT
AGCTACAAATAGACCAGTCCCAATTGCACCACCTATAGCAATCATGGAAATGTGTCTTGAGTTAAGACTACGGTTCATTTTATTATCTTCC
AT

Gene: katA (catalase A)

Contig: 06_NODE_54, position: 467845 to 469362, length: 1518 nt, orientation: FORWARD

Perfect match to: (08-02119-CP015645-[838105:839622:r], highly conserved allele)

Sequence:

ATGTCACAACAAGACAAAAAGTTAACTGGTGTTTTTGGGCATCCAGTATCAGACCGAGAAAATAGTATGACAGCAGGGCCTAGGGGACC
TCTTTTAATGCAAGATATTTACTTTTTAGAGCAAATGTCTCAATTTGATAGAGAAGTAATACCAGAACGTCGAATGCATGCCAAAGGTTCT
GGTGCATTTGGGACATTTACTGTAACTAAAGATATAACAAAATATACGAATGCTAAAATATTCTCTGAAATAGGTAAGCAAACCGAAATG
TTTGCCCGTTTCTCTACTGTAGCAGGAGAACGTGGTGCTGCTGATGCGGAGCGTGACATTCGAGGATTTGCGTTAAAGTTCTACACTGAA
GAAGGGAACTGGGATTTAGTAGGGAATAACACACCAGTATTCTTCTTTAGAGATCCAAAGTTATTTGTTAGTTTAAATCGTGCGGTGAAA
CGAGATCCTAGAACAAATATGAGAGATGCACAAAATAACTGGGATTTCTGGACGGGTCTTCCAGAAGCATTGCACCAAGTAACGATCTT
AATGTCAGATAGAGGGATTCCTAAAGATTTACGTCATATGCATGGGTTCGGTTCTCACACATACTCTATGTATAATGATTCTGGTGAACGT
GTTTGGGTTAAATTCCATTTTAGAACGCAACAAGGTATTGAAAACTTAACTGATGAAGAAGCTGCTGAAATTATAGCTACAGATCGTGAT
TCATCTCAACGCGATTTATTCGAAGCCATTGAAAAAGGTGATTATCCAAAATGGACAATGTATATTCAAGTAATGACTGAGGAACAAGCT
AAAAACCATAAAGATAATCCATTTGATTTAACAAAAGTATGGTATCACGATGAGTATCCTCTAATTGAAGTTGGAGAGTTTGAATTAAAT
AGAAATCCAGATAATTACTTTATGGATGTTGAACAAGCTGCGTTTGCACCAACTAATATTATTCCAGGATTAGATTTTTCTCCAGACAAAA
TGCTGCAAGGGCGTTTATTCTCATATGGCGATGCGCAAAGATATCGATTAGGAGTTAATCATTGGCAGATTCCTGTAAACCAACCTAAAG
GTGTTGGTATTGAAAATATTTGTCCTTTTAGTAGAGATGGTCAAATGCGCGTAGTTGACAATAATCAAGGTGGAGGAACACATTATTATC
CAAATAACCATGGTAAATTTGATTCTCAACCTGAATATAAAAAGCCACCATTCCCAACTGATGGATATGGCTATGAATATAATCAACGTCA
AGATGATGATAATTATTTTGAACAACCAGGTAAATTGTTTAGATTACAATCAGAGGACGCTAAAGAAAGAATTTTTACAAATACAGCAAA
TGCAATGGAAGGCGTAACGGATGATGTTAAACGACGTCATATTCGTCATTGTTACAAAGCTGACCCAGAATATGGTAAAGGTGTTGCAA
AAGCATTAGGTATTGATATAAATTCTATTGATCTTGAAACTGAAAATGATGAAACATACGAAAACTTTGAAAAATAA

Gene: rpmG2 (50S ribosomal protein L33, locus 2)

Contig: 06_NODE_54, position: 469453 to 469602, length: 150 nt, orientation: FORWARD

Perfect match to: (RF122-AJ938182-[1299702:1299851], highly conserved allele)

Sequence:

GTGCGCGTAAACGTAACATTAGCATGCACAGAATGTGGCGATCGTAACTATATCACTACTAAAAATAAACGTAATAATCCTGAGCGTATT
GAAATGAAAAAATATTGCCCAAGATTAAACAAATATACGTTACATCGTGAAACTAAGTAA

Gene: rpsN (30S ribosomal protein S14)

Contig: 06_NODE_54, position: 470056 to 470325, length: 270 nt, orientation: FORWARD

Perfect match to: (RF122-AJ938182-[1300305:1300574], highly conserved allele)

Sequence:

ATGGCTAAGAAATCTAAAATAGCAAAAGAGAGAAAAAGAGAAGAGTTAGTAAATAAATATTACGAATTACGTAAAGAGTTAAAAGCAA
AAGGTGATTACGAAGCGTTAAGAAAATTACCAAGAGATTCATCACCTACACGTTTAACTAGAAGATGTAAAGTAACTGGAAGACCTAGA
GGTGTATTACGTAAATTTGAAATGTCTCGTATTGCGTTTAGAGAACATGCGCACAAAGGACAAATTCCAGGTGTTAAAAAATCAAGTTGG
TAA

Gene: guaC (GMP reductase)

Contig: 06_NODE_54, position: 470482 to 471459, length: 978 nt, orientation: FORWARD

Perfect match to: (M1015-ACST01000016-[28505:29482], allele observed in CC30)

Sequence:

GTGAAAATATTTGATTACGAAGATATTCAATTAATACCTAATAAATGCATAGTTGAAAGTAGGTCTGAATGTGATACAACTATCCAATTTG
GTCCGAAAAAATTCAAGCTACCTGTAGTTCCTGCAAATATGCAAACAGTTATGAATGAGAAATTAGCGAAATGGTTTGCTGAAAATGATT
ACTTTTATATCATGCATCGTTTTGATGAAGAAGCAAGAATACCTTTTATAAAACATATGCAAAATTCAGGCTTATTTGCATCTATTTCAGTT
GGTGTAAAGAAAGCGGAATTTGATTTTATTGAAAAGTTAGCTCAAGAAAAATTAATCCCCGAATATATTACAATAGATATTGCGCATGGT
CACTCAGATTCAGTGATAAACATGATTAAACATATAAAAAACCATATACCTGATAGTTTTGTTATTGCTGGTAATGTTGGCACGCCAGAAG
GTGTTAGAGAATTAGAAAATGCTGGTGCTGATGCTACCAAAGTCGGTATAGGTCCTGGTAGAGTTTGTATTACAAAGATTAAAACAGGT
TTTGGTACTGGTGGTTGGCAGTTAGCGGCATTAAACATATGTAGTAAAGCAGCTCGTAAACCTTTGATTGCCGATGGTGGTATAAGAACG
CATGGCGACATTGCTAAATCAATTAGATTTGGTGCATCAATGGTCATGATTGGTTCATTATTTGCTGCACACGAAGAATCACCTGGTGAA
ACTGTAGAACTTGATGGTAAACAGTATAAAGAATATTTTGGTAGTGCATCTGAATTTCAAAAAGGCGAACATAAAAATGTAGAAGGTAA
AAAAATGTTTGTAGAACATAAGGGTTCATTAATGGATACCTTAAAAGAAATGCAACAAGATTTACAAAGCTCAATTTCATATGCCGGTGG
AAAAGACTTGAAATCATTACGTACTGTAGATTATGTTATTGTTAGAAACTCTATTTTCAACGGTGATAGAGATTAA

Gene: ylbC1 (putative protein)

Contig: 06_NODE_54, position: 471634 to 472497, length: 864 nt, orientation: FORWARD

Perfect match to: (MW2-BA000033-[1337993:1338856], allele observed in CC1+CC5+CC80+CC239)

Sequence:

TTGGATACACCGAAGAAACAACAATTTGCCTTTAATAATATACAAATGAACATGTCGAAATCAGATGTTGAGAAAACATTAAATAAACCA
AAAAGAGTGACATTTAATGAATATGGTACGAAGTGGTATACGTATTATGATGACGATTACAATAATTTTATAATGATAAGTTACATGAAA
GATAAAGTTAATGCGTTATATACAAATCAAAATATAATCACTTCAAAATCAAAAATTAAATACAATACACCTAAATCGGTTGTAAGGCAAA
GATTAGGCGAACCAGAAACAGAGATTGTTAAAGGTAGAGTGCGTTACGAACAAAATAATAAAGAATATGATGTTTTCCATAAAAATCAC
ATTTATACGACGGTATTTTATGATAAGCATCGACGTAATAATGTAACAGCTGTTTTACAAGTAAGTGATGCTATGGAAAATAGATTAAAA
GAACAATATGGAGCACCTTCGAAATCGCTTGCAGATAGTTTTGAACTACAAAATTTTGATTTAGTTAATGCTGAAAGAAAACAACATCAA
TTATTTACATTGAAGTATTCTAAACAGAATTCTGAAACTGCACGTAAGCATAGTAAAGATATGGCCAACAATCATTATTTTGATCATACAA
ATTTAAAAGGTCAATCACCATTTGATCGATTGAAAAAAGATGGTATTACATTTAACTCAGCCGGAGAGAATTTAGCATATGGTCAAGTTA
GTAGTATCTATGCACATCAAGGATTAATGAATTCTATTGGTCACAGAAAAAATATTTTAAATGATACGTTTAAAATATTAGGTGTTGGTGT
TGATTTTAATGATGAAAAACAACCTTTTTGGACAGAAAATTATACTGGTTAA

Gene: lexA (DNA damage-inducible LexA repressor)

Contig: 06_NODE_54, position: 472877 to 473500, length: 624 nt, orientation: REVERSE

Perfect match to: (MW2-BA000033-[1339236:1339859:r], highly conserved allele)

Sequence:

TTACATTTCGCGGTACAAACCAATTACTTTCCCAATTACAGCAACATTGTCTAGGTAAATTGGCTCCATTGTACTATTTTCAGGTTGTAATC
GATAACGATTTTTTTCTTTATAGAAGCGTTTGACAGTTGCTTCATCTTCCTCAGTCATAGCAACAATAATGTCTCCATTTTCTGCTATGGTTT
GACTGCGAACAATTACTTTGTCTCCGTCTAATATACCAGCCTCAATCATACTGTCGCCTACGACGTTTAATATGAATATGTCGCTATTGTGT
GTCGATGTTAAGTGTTCAGGTAATGGAAAATATTCTTCAATATTTTCTACTGCGGTAATAGGAACACCTGCTGTGACTTTACCAATAACTG
GCACATGAATCGTTTCTTCCATATTAATATTATCATTTGTTTGATCACTTACAATTTCTATAGCACGTGGTTTCGTTGGATCTCTTCTTATAT
AGCCTTTTTCTTCAAGACGTGAAAGGTGACCATGAACAGTTGAACTGGATGCTAAGCCAACTGCTTCACCAATTTCGCGAACACTAGGCG
GATAACCTTTCGTTTGAACAACTTGTTTAATATAGTTATATATTTCGCTTTGTCGTTTTGTTAATTCTCTCAT

Gene: Q5HG79 (putative membrane protein)

Contig: 06_NODE_54, position: 473643 to 473876, length: 234 nt, orientation: FORWARD

Perfect match to: (MW2-BA000033-[1340002:1340235], highly conserved allele)

Sequence:

ATGTTTTACAATAAATATAAAAACGTATCAACATATATCATCATATTTTTAGTTTCAAGTGCAGCCTTTGCAATATTCTTGTTAAGTGCGAA
CATTAGTGCTCACTCGGAACAAGTGTACGAAATGACTGACCATCAAATTAAGAACAATACGATAAATAAAGCATACGAACATAAAGACCC
TACAAACAATAGCGAACAAAGAGATGGGAAAGTGTTCGCTTTAATAAATTGA

Gene: DUF896 (protein of unknown function)

Contig: 06_NODE_54, position: 474012 to 474251, length: 240 nt, orientation: FORWARD

Sequence:

TTGAGTAATTCTGATTTGAATATCGAAAGAATTAACGAGTTAGCTAAAAAGAAAAAAGAAGTAGGATTAACTCAAGAAGAAGCAAAGGA
GCAAACAGCCTTAAGAAAAGCTTATCTTGAGAGTTTTAAAAAAGGGTTTAAACAACAAATTGAAAATACTAAAGTAATTGATCCAGAAG
GTAATGATGTAACACCTGAAAAAATTAAAGAGATACAACAAAAAAGAGATAATAAAAATTAA

Gene: tkt (transketolase)

Contig: 06_NODE_54, position: 474372 to 476360, length: 1989 nt, orientation: FORWARD

Perfect match to: (MW2-BA000033-[1340731:1342719], allele observed in CC1+CC239+CC4803)

Sequence:

ATGTTTAATGAAAAAGATCAATTAGCTGTTGATACGCTACGTGCACTAAGTATCGACACAATCGAAAAAGCGAATTCTGGTCATCCAGGA
TTACCTATGGGAGCTGCCCCAATGGCTTACACTTTGTGGACACGTCATCTGAATTTTAATCCACAATCTAAAGATTACTTCAATAGAGACC
GTTTCGTATTATCTGCAGGGCATGGTTCAGCATTATTGTATAGCTTGTTACATGTTTCTGGTAGTTTAGAATTAGAAGAATTAAAGCAATT
TAGACAATGGGGTTCTAAAACACCAGGTCATCCTGAATACAGACATACAGATGGTGTAGAAGTTACTACCGGACCACTTGGACAAGGTT
TTGCTATGTCAGTAGGATTAGCTTTAGCAGAAGATCACCTAGCAGGGAAATTTAATAAAGAAGGATATAATGTTGTAGATCATTACACAT
ATGTATTAGCTTCTGACGGTGATTTAATGGAAGGTATATCGCATGAAGCAGCTTCATTTGCTGGACATAATAAATTAAGTAAATTAGTTGT
TTTATACGATTCAAATGATATTTCATTAGATGGCGAATTAAACAAAGCTTTTTCTGAAAACACAAAAGCTCGTTTTGAAGCATATGGTTGG
AATTACTTACTAGTTAAAGATGGTAATGATTTAGAAGAAATTGATAAAGCGATTACTACAGCTAAATCTCAAGAAGGACCAACGATTATT
GAAGTTAAAACAACAATCGGATTTGGTTCACCGAATAAAGCAGGAACTAATGGTGTTCATGGGGCACCTTTAGGTGAAGTTGAAAGAAA
ATTAACATTCGAAAATTACGGTTTAGATCCTGAAAAACGTTTTAATGTTTCAGAAGAGGTATACGAAATTTTCCAAAATACTATGTTAAAA
CGTGCTAATGAAGATGAATCTCAATGGAATTCATTATTAGAAAAATATGCAGAAACATATCCTGAATTAGCAGAAGAATTTAAATTAGCG
ATTAGTGGTAAATTGCCTAAAAATTATAAGGATGAATTACCACGTTTTGAACTGGGTCATAATGGTGCATCTCGTGCTGATTCTGGTACTG
TTATTCAAGCAATCAGTAAAACTGTCCCTTCATTCTTTGGTGGATCAGCAGACCTTGCTGGTTCAAACAAATCCAATGTAAATGATGCAAC
AGATTATAGTTCTGAAACACCTGAAGGTAAAAATGTGTGGTTTGGTGTACGTGAATTTGCTATGGGTGCTGCTGTAAATGGTATGGCTGC
ACATGGAGGTTTACATCCATATGGTGCAACATTCTTCGTATTTAGTGATTATTTAAAACCAGCGTTACGTTTATCATCAATTATGGGATTAA
ATGCAACGTTCATCTTCACACATGATTCAATTGCAGTAGGTGAAGATGGTCCTACTCATGAACCAATTGAACAATTAGCTGGATTAAGAG
CTATTCCAAATATGAATGTTATCCGTCCTGCTGATGGTAATGAAACAAGAGTAGCATGGGAAGTTGCCTTAGAATCTGAATCTACACCTA
CTTCATTAGTATTGACACGTCAAAACTTACCGGTTTTAGATGTACCAGAAGATGTAGTTGAAGAAGGCGTTCGAAAAGGTGCCTATACAG
TTTATGGCTCTGAAGAGACACCAGAATTCCTATTATTAGCTTCAGGTTCAGAAGTTAGTCTTGCAGTTGAAGCTGCTAAAGATCTTGAAAA
ACAAGGTAAATCAGTGCGTGTTGTTTCAATGCCTAACTGGAATGCATTTGAACAACAATCTGAAGAATATAAAGAATCAGTTATTCCATC
AAGCGTAACAAAACGTGTTGCGATTGAAATGGCTTCACCGCTTGGATGGCATAAATATGTAGGTACTGCAGGTAAAGTTATTGCTATTGA
CGGCTTTGGCGCAAGTGCACCTGGCGATTTAGTAGTTGAAAAATATGGATTTACTAAAGAAAATATCTTAAACCAAGTTATGAGCTTATA
A

Gene: UPF0154 (protein of unknown function)

Contig: 06_NODE_54, position: 476638 to 476880, length: 243 nt, orientation: FORWARD

Perfect match to: (RF122-AJ938182-[1306887:1307129], highly conserved allele)

Sequence:

ATGGCAACTTGGTTAGCAATTATTTTTATAGTAGCTGCATTAATTTTAGGTTTAATTGGAGGTTTCCTTTTAGCTAGAAAATATATGATGGA
CTACTTGAAGAAAAACCCACCAATCAACGAAGAAATGCTTCGTATGATGATGATGCAAATGGGTCAAAAACCTTCTCAGAAGAAAATTAA
TCAAATGATGACGATGATGAATAAAAATATGGATCAAAATATGAAGAGTGCGAAAAAGTAA

Gene: ccdC (membrane protein involved in cytochrome C biogenesis)

Contig: 06_NODE_54, position: 477059 to 477526, length: 468 nt, orientation: FORWARD

Perfect match to: (N315-BA000018-[1341314:1341781], highly conserved allele)

Sequence:

GTGCTCTATTTAATATTTTCAATCATTGTAGCTTTATTTATGGGAACTATAGTTATAGTTATTCGTATGAAAGCTCAAAATTATCCGGTAAA
TGAGAAAAAAATAGTTTTGCCACCGTTTTTTATGGCGACCGGTGCATTGATGTACGTCGTTCCATATTTTAGGCTAACAGGATCGGAAAT
GCTAGAAGCCTTTATAATTGGTTTGCTTTTTTCTACAGTTCTAATTTGGACTTCTCGATTTGAAGTCAAAGGTACAGAAATTTATATGAAAC
GATCTAAAGCATTTCCAGTTATTTTGATTTCATTACTTATCATTCGTACTGTGATGAAAATATTCATTAGTAATGAAATAGATCCTGGAGAA
TTAGGCGGCATGTTCTTTTTATTAGCATTCTGTATGATTGTTCCTTGGAGAGCAGCAATGCTATATAAATACAAAAAACTAAAGAAAACAT
TAATCAATTAA

Gene: sbcD (nuclease SbcCD subunit D)

Contig: 06_NODE_54, position: 477650 to 478771, length: 1122 nt, orientation: FORWARD

Perfect match to: (MW2-BA000033-[1344009:1345130], highly conserved allele)

Sequence:

ATGAAAATTATACATACAGCAGACTGGCACTTAGGGAAAATATTAAATGGCAAACAGCTTTTAGAAGATCAAGCGTATATTTTAGATATG
TTCGTAGAAAAAATGAAAGAAGAAGAACCTGATATCATTGTGATAGCTGGAGATTTATATGACACAACATATCCAAGTAAAGATGCAAT
CATGTTATTAGAACAAGCGATTGGAAAGTTAAATTTAGAACTGCGTATACCAATAATTATTATTAGTGGAAATCACGATGGTAAAGAGAG
GTTAAACTATGGGGCGAGTTGGTTTGAACATAATCAGTTATTTATAAGAACAGATTTTACATCGATTAATTCACCAATAGAGATAAATGG
GGTTAATTTTTATACACTCCCTTATGCTACTGTGAGCGAAATGAAACACTACTTTGAAGATGACACCATTGAAACACATCAACAGGGAATT
ACGCGCTGTATTGAAACAATAGCACCGGAAATTGATGAAGATGCCGTCAATATTTTAATTAGTCATCTGACTGTTCAAGGTGGAAAGACA
TCTGATTCTGAAAGACCATTAACTATTGGAACGGTTGAATCAGTTCAGAAAGGTGTTTTTGATATATTTGATTATGTCATGCTAGGTCACT
TGCATCATCCATTTAGTATAGAAGACGACAAAATTAAATATAGTGGCTCCTTATTGCAGTATTCATTTTCGGAAGCGGGTCAAGCTAAAG
GGTATAGACGTGTAACAATTAATGATGGCATTATTAACGATGTATTTATTCCTCTTAAGCCACTTAGACAATTGGAAATTATCTCAGGCGA
ATATAATGATGTTATTAATGAAAAAGTTCATGTGAAAAATAAAGATAATTATTTACATTTTAAACTTAAAAATATGTCTCATATTACTGATC
CAATGATGAGTTTAAAACAAATTTATCCTAATACTTTAGCGCTGACGAATGAAACTTTTAATTACAATGAAGAAAATAATGCTATAGAAAT
AAGTGAAAAAGATGACATGTCAATTATCGAAATGTTTTATAAACATATAACTGATAAAGAATTATCGGATATCCAATCTAAAAAGATAAA
AAATATTTTAGAAAACGAATTGAGAAAGGAGGATTAA

Gene: sbcC (nuclease SbcCD subunit C)

Contig: 06_NODE_54, position: 478775 to 481804, length: 3030 nt, orientation: FORWARD

Perfect match to: (Strain_21305-AFNO01000034-[50282:53311], allele observed in CC25+CC239)

Sequence:

ATGAAACCATTACATTTAAAGTTGAATAATTTCGGCCCCTTTTTAAAAGAAGAAATTGATTTTTCTAAAATTGATAATAATGAATTGTTTTT
AATAAGTGGTAAGACTGGATCGGGTAAAACAATGATTTTTGATGCAATGACTTATGCCTTGTTTGGTAAAGCATCAACTGAACAAAGAGA
AGAAAATGATTTGAGAAGTCATTTCGCTGATGGTAAACAGCCGATGTCAGTAACATTTGAATTTCAATTAAATCATCGAATTTATAAAGTG
CATAGACAAGGCCCTTATATCAAAGAAGGTAATACAACAAAAACGAACGCTAAATTTGATGTATTTGAGATGGTGGATGGCAAGTATGA
AATTAGAGAAAGTAAAGTAATTTCAGGTACCCAATTCATTATTGAATTATTAGGAGTAAATGCAGATCAATTCCGACAATTGTTTATTTTG
CCTCAAGGTGAATTCAAACGCTTTTTAATATCAAACAGTCGTGAAAAGCAAGGGATATTAAGAACACTGTTTGACAGTGAAAAATTTGAA
GCTATACGAGAAATATTAAAAGAAGAAGTAAAAAAAGAAAAAGCTCAAATCGAGAATAGATATCAACAAATTGACCTTTTATGGCAAGA
AATTGAATCATTTGATGATGACAATATAAAAGGCTTATTAGAGGTTGCCACTCAACAGATAGACAAATTGATTGAAAATATACCACTTTTA
CAAGCTAGGTCGAAAGAAATACTAGCATCTGTAAATGAAAGTAAAGAAACTGCTATTAAAGAATTTGAAATAATAGAAAAGAAAACATT
AGAAAATAATATATTAAAAGATAATATTAATCAACTCAACAAAAATAAAATTGATTTCGTTCAATTGAAAGAACAACAACCTGAAATAGA
GGGAATTGAAGCTAAGTTAAAGTTGTTACAAGATATTACAAACCTATTGAATTATATTGAAAATAGAGAAAAAATTGAAACTAAAATTGC
TAATAGCAAAAAAGATATTTCTAAAACCAATAATAAAATATTGAATCTTGATTGTGATAAGCGAAACATAGACAAAGAGAAAAAAATGTT
AGAAGAAAATGGAGATTTAATTGAAAGTAAAATCTCTTTTATTGATAAAACTAGAGTATTATTTAACGATATTAATAAGTATCAACAAAGT
TATCTCAATATTGAACGCTTGAGAACTGAGGGTGAACAATTAGGTGATGAATTAAATGATCTAATTAAAGGTTTAGAAACGGTCGAAGAT

TCAATAGGTAATAACGAAAGTGATTACGAGAAAATTATCGAACTAAATAATACGATAACGAACATAAATAATGAAATTAATATAATTAAA
GAAAATGAAAAAGCTAAAGCTGAATTAGATAAACTATTAGGTAGTAAGCAAGAGTTAGAGAATCAAATTAATGAAGAAACATCTATATT
GAAGAATCTCGAAATAAAATTAGATCGCTACGATAAAACAAAATTGGACTTAAATGATAAAGAAAGCTTTATAAGTGAAATTAAATCTGC
TGTAAATATTGGAGATCAATGTCCGATATGTGGTAATGAAATTCAGGATTTAGGGCATCATATTGATTTTGACAGTATTGCTAAACGTCA
AAATGAAATTAAAGAAATTGAAGCAAATATCCACGCTATAAAATCGAATATTGCTGTGCATAATTCTGAAATTAAATTTGTTAATGAAAA
AATATCGAATATTAATATTAAAACGCAAAGTGATTTTTCACTTGAAGTATTGAATAAGCGTCTGCTAGAAAATGAAAATGCATTGAATAAT
CAAAGAGATCTTAATAAATTTATAGAACAAATGAAAGAAGAAAAAGATAATCTAACGTTGCAAATTCATAATAAACAATTGCGTCTAAAT
AAAAATGAATCTGAGTTGAAATTATGTCGAGATCTCATCACTGAATTTGAAACACTCTCGAAATATAATAATATCACTAATTTTGAGGTGG
ATTATAAGAAGTATGTTCAAGATGTGAATCAGCATCAAGAACTCTCAAAGGAGATTGAAGATAAGTTAATGCAATTGTCTCAAAGAAAGT
TAATTGAGCAAAATAATCTAAATCATTATGAAAATCAACTAGAAACTTACAATAATGACTTAGAATTGAATGAACAGTCTATTGAAATGG
AAATGTCGAGGCTGAATTTAACTGATGACAATGATATAGATGAAATAATAGCCTGGAGGGGCGAGCAAGAGGAATTAGAGCAGAAAAG
GGATACTTATAAAAAACGTTATCATGAATTTGAAATGGAAATAGCTAGGTTAGAATCATTAACTAAGGATAAAGAGTTATTGGACTCTGA
TAAATTAAAAGATGAATATGAGCTAAAAAAAGGAAAGATGAATACACTGATAGATGAATACTCTGCTGTTCATTATCAATGTCAAAATAA
TATTAATAAAACACAATCTATAGTTTCGCATATTAATTACTTAAATCAAGAATTAAAGGATCAACAAGAAATATTTCAATTGGCTGAAATT
GTCAGTGGTAAGAATAACAAAAATCTTACATTGGAAAACTTTGTCTTAATTTACTATTTAGATCAAATTATTGCTCAAGCAAATCTGAGAT
TAGCAACAATGTCAGATAATCGATACCAACTAATTAGGCGAGAAGCGGTTTCTCATGGTCTTAGTGGCCTAGAAATTGATGTATTTGATT
TGCATTCAAATAAGTCTAGACATATTAGCTCGTTATCAGGTGGAGAAACTTTCCAATCGTCGCTTGCATTAGCTTTAGGGTTAAGCGAAAT
TGTACAGCAGCAATCAGGAGGTATTTCACTAGAATCAATATTTATTGATGAAGGATTCGGTACATTAGATCAAGAAACGCTTGAAACAGC
GTTAGACACTTTATTAAATCTTAAATCAACTGGTAGAATGGTTGGGATTATTTCACATGTGAGCGAATTGAAAAATAGAATACCTTTAGTT
TTAGAAGTGAAATCAGATCAATATCAGAGTTCAACAAGATTCAAAAGAAATTAA

Gene: mscL (large-conductance mechanosensitive ion channel)

Contig: 06_NODE_54, position: 481875 to 482237, length: 363 nt, orientation: REVERSE

Perfect match to: (MW2-BA000033-[1348234:1348596:r], highly conserved allele)

Sequence:

TTATTTTTTCTCACGTAATAAATCTCTGATTTCAGTTAATAACACAACATTTTCTTCCACAACTGCTTCTTCTTCGGCTTCTTCTTTCTTCATTA
ATGTATTTGCAATCTTAACAAAGATGAATAAAGCAAACGCGATGATAATAAAGTCGATAACAGATTGGATAAATAAACCGTATTTAATAC
CCCAGAATGACCATTCTTTAGCAAAATCAACTGATCCGAAAATTTTACCAATTAATGGCATAATGATATTTTCTACTAATGAAGATATAATC
TTGTTGAAAGCTGCACCCATCACAACAGCAATTGCTAAATCTAAGACGTTACCTTTTAAGGCGAACTCTTTGAATTCTTTTAACAT

Gene: opuD1 (glycine betaine transporter 1)

Contig: 06_NODE_54, position: 482441 to 484087, length: 1647 nt, orientation: FORWARD

Perfect match to: (JKD6159-CP002114-[1333849:1335495], highly conserved allele)

Sequence:

ATGAATTCTTCTTCACCAGAGAATCCAAATGGAAAGAAGTATTCACCAGTCTTCATCTATAGTGCAATTGTTGTTGCTATAGTCGTATTACT
TGGTGCATTTTTACCTGAACAATTCAACTATGTTACCAATAATATTAAAATGTGGATTACAGAAAAGTTAGGTTGGTATTATCTTATTCTTA
CTACGATTATCGTGTTCTTCTGTATATTCCTTATTTTTAGTCCTATTGGAAAACTTAAACTAGGTAAACCAAATGACAAACCTGAGTTTAAT
ACAATTTCATGGTTTGCTATGTTGTTTAGTGCTGGTATGGGGATAGGTTTGGTGTTTTATGGTGCAGCTGAACCGATGGCGCACTTTGCTA
CGCCACCTACAGCAGATCCCAAAACTACTGAAGCTTATACTGAAGCTCTACGTTCAACATTTTTCCATTGGGGATTCCATGCTTGGGCTGT
TTATGGTGTTGTTGCGTTAGCGTTGGCATATTCGCAATTCCGTAAAGGTGAACCAGGTTTATTATCTAGAACTTTACGTCCTCTTTTAGGT
GATAAAGTAGAAGGTCCTATTGGGATTTTTATTGACGTTTTATCTGTATTTGCGACAATCGTTGGGGTAGCCGTTTCGTTAGGTATGGGT
GCTCTACAAATTAATGGTGGTTTACATTACTTGTTCAATGTTCCAAACAATACGTTTGTACAAGCGATTATCATCATTGTTGTTACTATCTTA
TTTATAGCAAGTGCATGGTCTGGATTAAGTAAAGGTATTCAATACTTAAGTAACTTGAACATTGGTTTAGGTACTATTTTAATGGTAGCTG
CTTTAATTGTTGGACCAACTGTTCTTATTTTAAATATGTTAACTAGCTCTACGGGTAGTTTACTAAACACATTCTTGTTTAATAGTTTTGATA
CAGCAGCTTTAAATCCTCAAAAACGTGAATGGATGTCTTCATGGACACTTTATTACTGGGGTTGGTGGTTAAGTTGGAGTCCATTCGTTG
GAGTGTTTATTGCACGAGTTTCAAAAGGACGTTCAATTAGAGAGTTCATTTCTGGTGTCTTGCTAGTTCCAGCAATTGTTAGTTTTGTTTG
GTTTAGTGTCTTTGGTGTATTAGGCATCGAGACAGGTAAGAAACACAAAGAAATTTTTGATATGACTCCTGAAACACAGCTATTTGGAGT
GTTTAATCATGTGCCATTTGGCATTGTTTTATCGTTGATTGCATTATTATTAATTGCATCATTCTTTATTACATCTGCTGACTCAGCAACATT
TGTATTAGGAATGCAAACAACATTTGGTTCATTAAATCCATCTAGTATGGTAAAAGTTGTTTGGGGAATTTCACAGGCCTTAATAGCATTT
GTACTTTTATTAGCTGGTGGCGGTAACGGCGCTGAAGCTTTAAATGCGATTCAAAGTGCTGCAATTATAAGTGCATTCCCATTCTCCTTTG
TCGTCATACTCATGATGGTAAGTTTCTACAAGGATGCGAACCAGGAACGTAAATTCCTAGGTTTAACATTGACTCCGAATAAACATCGCTT

ACAAGAATATATCAAGAGTCAACAAGAAGATTATGAATCTGACATTCTTGAAAAGCGTCAGTCACGTAGAAATATAGAGAAAAAAGATA
ACTAA

Gene: tx_universal2 (rho-independent terminator)

Contig: 06_NODE_54, position: 484413 to 484451, length: 39 nt

Perfect match to: (Strain_21331-AGTV01000040-[104403:104441], allele observed in CC398)

Sequence:

GAACGAAAATGCGCTTGTAACAAGCTTTTTTCAATTCTA

Gene: STAR (Staphylococcus aureus repeat element)

Contig: 06_NODE_54, position: 484456 to 484677, length: 222 nt

Sequence:

GGGGCCCCAACACAGAGAATTTCGAAAAGAAATTCTACAGGCAATGCGAGTTGGGGTGAGGGCCCCAACACAGAAGCTGACGAAAAGT
CAGCTTACAATAATGTGCAAGTTGGGGATGGGCCCCAACAAAGAGAAATTGGATTCCCAATTTCTACAGACAATGCAAGTTGGGGTGGG
ACGACGAAATAAATTTTGCGAAAATATCATTTCTGTCCCACTCCC

Gene: SIRU01 (staphylococcal interspersed repeat unit 1)

Contig: 06_NODE_54, position: 484458 to 484626, length: 169 nt

Sequence:

GGCCCCAACACAGAGAATTTCGAAAAGAAATTCTACAGGCAATGCGAGTTGGGGTGAGGGCCCCAACACAGAAGCTGACGAAAAGTCA
GCTTACAATAATGTGCAAGTTGGGGATGGGCCCCAACAAAGAGAAATTGGATTCCCAATTTCTACAGACAATGCAAGTTGG

Gene: acnA-citB (aconitate hydratase)

Contig: 06_NODE_54, position: 484979 to 487684, length: 2706 nt, orientation: FORWARD

Perfect match to: (N315-BA000018-[1349289:1351994], highly conserved allele)

Sequence:

ATGGCTGCAAATTTTAAAGAGCAATCAAAAAAACATTTTGACTTGAATGGCCAAAGTTATACTTACTATGATTTAAAAGCTGTAGAAGAG
CAAGGTATTACTAAAGTTTCCAATTTACCTTATTCAATTCGTGTTTTGTTAGAATCTTTACTTCGTCAAGAAGATGATTTTGTAATTACAGAC
GATCATATTAAAGCTTTAAGTCAGTTTGGAAAAGATGGAAATGAAGGCGAGGTACCATTTAAACCTTCTCGTGTTATTTTACAAGATTTCA
CAGGTGTACCAGCCGTAGTTGATTTAGCTTCTTTACGTAAAGCAATGGATGACGTTGGGGGAGATATTACTAAAATTAATCCAGAAGTAC
CGGTGGATTTAGTTATTGACCACTCAGTTCAAGTGGATAGCTATGCAAATCCAGAAGCTCTTGAACGTAATATGAAATTAGAATTTGAAC
GTAACTATGAACGTTATCAGTTTTTAAATTGGGCAACGAAAGCATTTGATAATTACAATGCAGTTCCTCCTGCAACTGGAATAGTTCACCA
AGTTAACTTAGAATATTTAGCAAGTGTTGTACATGTTCGTGATGTAGATGGTGAAAAAACTGCATTTCCAGATACATTAGTTGGTACTGAT
TCACATACAACAATGATAAATGGTATTGGCGTACTAGGATGGGGTGTTGGTGGTATTGAAGCTGAAGCTGGAATGCTTGGACAACCTTC
TTATTTCCCAATTCCAGAGGTTATTGGTGTACGACTAGTAAATTCATTACCACAAGGCGCAACAGCAACTGATTTAGCGTTAAGAGTAACT
CAAGAGCTACGTAAAAAAGGTGTTGTTGGTAAATTTGTGGAGTTCTTTGGTCCAGGTGTACAACATTTACCACTAGCAGACCGTGCTACA
ATTGCAAACATGGCACCAGAGTATGGAGCAACTTGCGGATTCTTCCCAGTTGATGATGAATCTCTTAAATATATGAAGTTAACTGGTAGA
TCAGACGAACATATCGCGCTAGTAAAAGAATATTTGAAACAAAACCATATGTTCTTTGATGTTGAGAAAGAAGATCCTAATTATACAGAT
GTTATCGAATTGGATTTATCAACAGTTGAAGCATCGCTTTCAGGACCAAAACGTCCTCAAGATTTAATTTTCTTAAGTGATATGAAATCAT
CATTTGAAAATTCTGTAACAGCTCCAGCAGGCAACCAAGGACACGGTTTAGATAAAAGTGAATTTGATAAGAAAGCTGAAATTAACTTTA
AAGATGGATCAAAAGCTACAATGAAAACAGGTGATATTGCAATAGCAGCAATTACATCATGTACAAATACATCTAACCCTTATGTAATGT
TAGGTGCAGGTTTAGTTGCTAAAAAAGCAGTTGAAAAAGGCTTGAAAGTTCCTGAATACGTTAAAACTTCTCTAGCACCAGGATCAAAAG
TTGTTACCGGATATTTAAGAGATGCTGGCTTACAACCTTATTTAGATGATTTAGGCTTCAACTTGGTTGGTTATGGATGTACAACTTGTATC
GGTAATTCAGGTCCTTTATTACCAGAAATTGAAAAAGCGATTGCTGATGAGGACCTATTAGTGACATCTGTATTATCTGGTAACCGTAACT
TTGAAGGTCGTATCCATCCTCTTGTTAAAGCCAATTACCTAGCTTCACCACAGTTAGTTGTTGCTTATGCATTAGCTGGAACGGTTGATATT

GATTTACAGAATGAACCTATTGGTAAAGGTAATGACGGTGAAGATGTATATTTGAAAGATATTTGGCCATCAATTAAAGAAGTTTCAGAT
ACCGTTGATAGTGTTGTAACACCTGAATTATTTATTGAAGAATATAATAACGTATACAATAACAACGAATTATGGAATGAGATTGATGTA
ACTGATCAACCTCTATATGACTTTGATCCTAATTCAACATACATTCAAAATCCATCATTCTTCCAAGGATTATCTAAAGAACCGGGTACGAT
TGTTCCATTAAATGGTTTACGTGTTATGGGTAAATTCGGTGATTCTGTGACAACTGACCACATCTCTCCAGCAGGTGCAATTGGTAAAGAT
ACGCCAGCTGGTAAATATTTACAAGATCATCAAGTGCCTATTCGTGAATTTAATTCATATGGTTCAAGACGTGGTAATCACGAAGTAATG
GTTCGAGGTACGTTTGCTAATATACGTATTAAAAACCAATTAGCGCCAGGTACTGAAGGTGGTTTTACAACTTATTGGCCAACAAATGAA
GTAATGCCTATCTTTGATGCTGCAATGAAATATAAAGAAGATGGTACAGGTTTAGTTGTATTAGCTGGTAACGATTATGGTATGGGTTCA
TCTCGTGACTGGGCAGCAAAAGGTACAAACTTATTAGGTGTTAAAACAGTTATTGCACAAAGTTATGAACGTATCCATCGTTCAAATTTA
GTTATGATGGGTGTATTACCATTAGAGTTTAAAAAAGGTGAATCAGCTGATTCTCTTGGTCTAGATGGTACAGAAGAAATTTCTGTTAAT
ATTGATGAAAATGTTCAACCACATGACTACGTCAAAGTTACTGCTAAGAAGCAAGATGGTGATTTGGTAGAATTTGACGCTATGGTTCGT
TTTGACTCACTTGTTGAAATGGATTACTATCGTCACGGTGGAATTTTACAAATGGTTTTAAGAAATAAATTAGCGCAATAA

Gene: yneP (putative thioesterase)

Contig: 06_NODE_54, position: 487864 to 488331, length: 468 nt, orientation: FORWARD

Perfect match to: (MW2-BA000033-[1354165:1354632], allele observed in CC1+CC8+CC30)

Sequence:

ATGATATATAGTATTACAGAAATAGAAGCGCGTTATGCTGAAACTGATAAGATGGGTGTAATTTATCACGGGAATTATGCAACTTGGTTT
GAAGTTGCGCGGTTGGATTATATATCGAAGTTAGGTTTTAGTTATGCTGATATGGAAAAACAAGGAATCATTTCACCTGTGACTGACCTC
AATGTCAATTATAAAAAGTCTATTTTTTATCCAGAAAAAGTTAAAGTTAAAACTTGGGTTGAAAAATATTCGAGATTACGTTCAGTGTATA
AATATGAGATTTTTAATGAAAAAGGTGAACTTGCAACTACAGGTTCCACAGAATTGATTTGCATTAAAGAAGATACTTTTAAGCCTATACG
GTTGGATCGTTATTTCCCAGATTGGCATGAAGCTTATAGTAAAGTGCAAACGCTCAATAATGAAGGGAAAACAGTAGAGATAATGGATG
ATATTGATTCTTTATAA

Gene: yneR (putative iron-sulphur cluster biogenesis protein)

Contig: 06_NODE_54, position: 488538 to 488834, length: 297 nt, orientation: REVERSE

Perfect match to: (N315-BA000018-[1352848:1353144:r], highly conserved allele)

Sequence:

TTATTTTGTGGAATAAGAAATTTCATCTTCGTGATCAACTACATTTACAATAATGTGGTCATCTTCAAAGTACCACAAATCTTTTTCCGCTAC
GACAACATTTAAATCGTCATATTGTTGTTCATAGCCAATATCAACATCTTCCTTTGGTTCAACTGTAAAAGCAGGACTAAATCCTTGCTTGA
GTTGGAATTCGCCACCATATCTTACAAAAAACACGAGCACTTTATTATTTTCAGGCAACTCAAGTTCATTTTTAAACCAAGTTACTGCTGCA
TCAGTAAGTTCTATTTGCAT

Gene: plsY (glycerol-3-phosphate acyltransferase)

Contig: 06_NODE_54, position: 489209 to 489817, length: 609 nt, orientation: REVERSE

Perfect match to: (RF122-AJ938182-[1319158:1319766:r], highly conserved allele)

Sequence:

TTACATCCATTTTATTTTAGGTTCTTCGCCTCTAAAAATCCTTGCGATATTAGAGCGATGTCTAATTATCAATATGATTGAAACTAAGAAAC
TAACGACTAATAAAATATAGTCTTGAATGATAAGCGAGCCAATCACACAGCAAATTGCTGCAACGATACTTGCTAAAGAAACATATTTAA
AAATCTTCAATACAATAAAGAAGATAATTGCAAGTATTAGTAAAAGTATCGGATTGACTCCCAAGACGACACCTGCACTAGTTGCAACAG
CTTTGCCACCTTGGAATTTTAAATAAACAGGATAAACGTGTCCAAGTATAGCGAATAAGCCAACAATTAAACCATTTGTAAAAAAAGTAC
TAATAGGGCCATCTGCGTGAACTGGTAACCATAAAGGGAAGAAAACAGTTATGAACCCCTTTGAAAATATCTAGAAATGTTACCAAGAAT
CCTGCAGGACGACCTAATACTCTAAAGCTATTAGTAGCGCCAGTATTACCACTACCAAATTGTCTAATATCTTTTTTGAAAAATAATTTTCC
AATTACGAATCCACTTGGGAAAGCGCCGATAAGATAACTTAGTAGTAACATGACGATTATCATCAT

Gene: grlB (DNA topoisomerase IV, subunit B)

Contig: 06_NODE_54, position: 490023 to 492014, length: 1992 nt, orientation: FORWARD

Perfect match to: (MW2-BA000033-[1356324:1358315], allele observed in CC1+CC5+CC8+CC30-ST582)

Sequence:

ATGAATAAACAAAATAATTATTCAGATGATTCAATACAGGTTTTAGAGGGGTTAGAAGCAGTTCGTAAAAGACCTGGTATGTATATTGGA
TCAACTGATAAACGGGGATTACATCATCTAGTATATGAAATTGTCGATAACTCCGTCGATGAAGTATTGAATGGTTACGGTAACGAAATA
GATGTAACAATTAATAAAGATGGTAGTATTTCTATAGAAGATAATGGACGTGGTATGCCAACAGGTATACATAAATCAGGTAAACCGAC
AGTCGAAGTTATCTTTACTGTTTTACATGCAGGAGGTAAATTTGGACAAGGCGGCTATAAAACTTCAGGTGGTCTTCACGGTGTTGGTGC
TTCAGTTGTAAATGCATTGAGTGAATGGCTTGAAGTTGAAATCCATCGAGATGGTAATATATATCATCAAAGTTTTAAAAACGGTGGTTC
GCCATCTTCTGGTTTAGTGAAAAAAGGTAAAACTAAGAAAACAGGTACCAAAGTAACATTTAAACCTGATGACACAATTTTTAAAGCATC
TACATCATTTAATTTTGATGTTTTAAGCGAACGACTACAAGAGTCTGCGTTCTTATTGAAAAATTTAAAAATAACGCTTAATGATTTACGCA
GTGGTAAAGAGCGTCAAGAGCATTACCATTATGAAGAAGGAATCAAAGAGTTTGTTAGTTATGTCAATGAAGGAAAAGAAGTTTTGCAT
GACGTGGCTACATTTTCAGGTGAAGCAAATGGTATAGAGGTAGACGTAGCTTTCCAATATAATGATCAATATTCAGAAAGTATTTTAAGT
TTTGTAAATAATGTACGTACTAAAGATGGTGGTACACATGAAGTTGGTTTTAAAACAGCAATGACACGTGTATTTAATGATTATGCACGT
CGTATTAATGAACTTAAAACAAAAGATAAAAACTTAGATGGTAATGATATTCGTGAAGGTTTAACAGCTGTTGTGTCTGTACGTATTCCA
GAAGAATTATTACAATTTGAAGGACAAACGAAATCTAAATTGGGTACTTCTGAAGCTAGAAGTGCTGTTGATTCAGTTGTTGCAGACAAA
TTGCCATTCTATTTAGAAGAAAAAGGACAATTGTCTAAATCACTTGTGAAAAAAGCGATTAAAGCACAACAAGCAAGGGAAGCTGCACG
TAAAGCTCGTGAAGATGCTCGTTCAGGTAAGAAAAACAAGCGTAAAGACACTTTGCTATCTGGTAAATTAACACCTGCACAAAGTAAAA
ACACTGAAAAAAATGAATTGTATTTAGTCGAAGGTGATTCTGCGGGAGGTTCAGCAAAACTTGGACGAGACCGCAAATTCCAAGCGATA
TTACCATTACGTGGTAAGGTAATTAATACAGAGAAAGCACGTCTAGAAGATATTTTTAAAAATGAAGAAATTAATACAATTATCCACACA
ATCGGGGCAGGCGTTGGTACTGACTTTAAAATTGAAGATAGTAATTATAATCGTGTAATTATTATGACTGATGCTGATACTGATGGTGCG
CATATTCAAGTGCTATTGTTAACATTCTTCTTCAAATATATGAAACCGCTTGTTCAAGCAGGTCGTGTATTTATTGCTTTACCTCCACTTTAT
AAATTGGAAAAAGGTAAAGGCAAAACAAAGCGAGTTGAATACGCTTGGACAGACGAAGAGCTTAATAAATTACAAAAAGAACTTGGTA
AAGGCTTCACGTTACAACGTTACAAAGGTTTGGGTGAGATGAACCCTGAACAATTATGGGAAACGACGATGAACCCAGAAACACGAACT
TTAATTCGTGTACAAGTTGAAGATGAAGTGCGTTCATCTAAACGTGTAACAACATTAATGGGTGACAAAGTACAACCTAGACGTGAATGG
ATTGAAAAGCATGTTGAGTTTGGTATGCAAGAGGACCAAAGTATTTTAGATAATTCTGAAGTACAAGTGCTTGAAAATGATCAATTTGAT
GAGGAGGAAATCTAG

Gene: grlA (DNA topoisomerase IV, subunit A)

Contig: 06_NODE_54, position: 492014 to 494416, length: 2403 nt, orientation: FORWARD

Perfect match to: (ATCC51811-ADVP01000009-[36505:38907:r], allele observed in CC1)

Sequence:

GTGAGTGAAATAATTCAAGATTTATCACTTGAAGATGTTTTAGGTGATCGCTTTGGAAGATATAGTAAATATATTATTCAAGAGCGTGCA
TTGCCAGATGTTCGTGATGGTTTAAAACCAGTACAACGTCGTATTTTATATGCAATGTATTCAAGTGGTAATACACACGATAAAAATTTCC
GTAAAAGTGCGAAAACAGTCGGTGATGTTATTGGTCAATATCATCCACATGGAGACTCCTCAGTGTACGAAGCAATGGTCCGTTTAAGTC
AAGACTGGAAGTTACGACATGTCTTAATAGAAATGCATGGTAATAATGGTAGTATCGATAATGATCCGCCAGCGGCAATGCGTTACACT
GAAGCTAAGTTAAGCTTACTAGCTGAAGAGTTATTACGTGATATTAATAAAGAGACAGTTTCTTTCATTCCAAACTATGATGATACGACAC
TCGAACCAATGGTATTGCCATCAAGATTTCCTAACTTACTAGTGAATGGTTCTACAGGTATATCTGCAGGTTACGCGACAGATATACCACC
ACATAATTTAGCTGAAGTGATTCAAGCAACACTTAAATATATTGATAATCCGGATATTACAGTCAATCAATTAATGAAATATATTAAAGGT
CCTGATTTTCCAACTGGTGGTATTATTCAAGGTATTGATGGTATTAAAAAAGCTTATGAATCAGGTAAAGGTAGAATTATAGTTCGTTCTA
AAGTTGAAGAAGAAACTTTACGCAATGGACGTAAACAGTTAATTATTACTGAAATTCCATATGAAGTGAACAAAAGTAGCTTAGTAAAAC
GTATCGATGAATTACGTGCTGACAAAAAAGTCGATGGTATCGTTGAAGTACGTGATGAAACTGATAGAACTGGTTTACGAATAGCAATT
GAATTGAAAAAAGATGTGAACAGTGAATCAATCAAAAATTATCTTTATAAAAACTCTGATTTACAGATTTCATATAATTTCAACATGGTCG
CTATTAGTGATGGTCGTCCAAAATTGATGGGTATTCGTCAAATTATAGATAGTTATTTGAATCACCAAATTGAGGTTGTTGCAAATAGAAC
GAAGTTTGAATTAGATAATGCAGAAAAACGTATGCATATCGTTGAAGGTTTGATTAAAGCGTTGTCAATTTTAGATAAAGTAATCGAATT
GATTCGTAGCTCTAAAAACAAGCGTGACGCTAAAGAAAACCTTATCGAAGTATACGAGTTCACAGAAGAACAGGCTGAAGCAATTGTAA
TGTTACAGTTATATCGTTTAACAAACACTGATATAGTTGCGCTTGAAGGTGAACATAAAGAACTTGAAGCATTAATCAAACAATTACGTCA
TATTCTTGATAACCATGATGCATTATTGAATGTCATAAAAGAAGAATTGAATGAAATTAAAAAGAAATTCAAATCTGAACGACTGTCTTTA
ATTGAAGCAGAAATTGAAGAAATTAAAATTGACAAAGAAGTTATGGTGCCTAGTGAAGAAGTTATTTTAAGTATGACACGTCATGGATA
TATTAAACGTACTTCTATTCGTAGCTTTAATGCTAGCGGTGTTGAAGATATTGGTTTAAAAGATGGTGACAGTTTACTTAAACATCAAGAA
GTAAATACGCAAGATACCGTACTAGTATTTACAAATAAAGGTCGTTATCTATTTATACCGGTTCATAAATTAGCAGATATTCGTTGGAAAG
AATTGGGACAACATGTATCACAAATAGTTCCTATCGAAGAAGATGAAGTGGTTATTAATGTCTTTAATGAAAAGGACTTTAATACAGATG
CATTTTATGTTTTTGCGACTCAAAATGGCATGATTAAGAAAAGTACAGTGCCTCTATTTAAAACAACGCGTTTTAATAAACCTTTAATTGCT
ACTAAAGTTAAAGAAAATGATGATTTGATTAGTGTTATGCGCTTTGAAAAAGATCAATTAATTACCGTCATTACTAATAAAGGTATGTCAT
TAACGTATAATACAAGTGAACTATCAGATACCGGATTAAGGGCAGCTGGTGTTAAATCAATAAATCTTAAAGCTGAAGATTTCGTTGTTA
TGACAGAAGGTGTTTCTGAAAATGATACTATATTGATGGCCACACAACGCGGCTCGTTAAAACGTATTAGTTTTAAAATCTTACAAGTTGC

TAAAAGAGCACAACGTGGAATAACTTTATTAAAAGAATTAAAGAAAAATCCACATCGTATTGTAGCTGCACATGTAGTGACAGGTGAAC
ATAGTCAATATACATTATATTCAAAATCAAATGAAGAACATGGTTTAATTAATGATATTCATAAATCTGAACAATATACAAATGGCTCATT
CATTGTAGATACAGATGATTTTGGTGAAGTAATAGACATGTATATTAGCTAA

Gene: alsT (sodium:alanine symporter family protein)

Contig: 06_NODE_54, position: 494666 to 496126, length: 1461 nt, orientation: FORWARD

Perfect match to: (MW2-BA000033-[1360967:1362427], highly conserved allele)

Sequence:

TTGAAAGATTTCGATAGTTTAATTCCTGGATGGTTTAAAGAATTTGTCCATGTTGGTACCGATTTAATATGGTCTCAATATTTAATTGGTCT
ATTATTGACAGCTGGATTCTTCTTTACAATTAGTTCTAAATTCGTCCAATTACGAATGTTACCTGAAATGTTTAGAGCTTTAGTAGAACGTC
CAGAAACTTTAGAAGATGGTAAGAAGGGTATTTCGCCATTCCAAGCATTTGCGATTAGTGCTGGTTCGAGAGTTGGTACTGGTAATATTG
CTGGTGTTGCGACTGCGATTGTTTTAGGCGGTCCAGGTGCAGTGTTTTGGATGTGGGTTATTGCATTTATAGGTGCAGCGAGTGCATTTA
TAGAAGCGACTTTGGCTCAGGTTTATAAAGTACATGATAAAGATGGTGGATTCCGTGGTGGTCCAGCTTACTATATTACTAAAGGTTTAA
ATCAAAAATGGCTAGGTATCGTATTTGCGATTTTAATTACAATTACATTTGCATTTGTATTTAACACAGTGCAATCTAATACAATTGCGGA
GTCGTTAAATACGCAATATAATATTAGTCCAGTAATCACAGGTATTATTTTAGCAATCGTAACAGCTATTATTATATTTGGTGGTGTACGT
AGTATTGCTACGTTATCTTCGTTAATTGTACCGATTATGGCTATCATTTACATTGGTATGGTTTTAGTAATATTGCTATTTAATTTAGATCAA
ATTGTTCCTATGATAGGTACGATTATTAAAAGTGCATTTGGTATCGAACAAGTAACTGGTGGCGCTGTAGGTGCTGCGGTTCTTCAAGGT
ATCAAACGTGGTTTATTCTCTAACGAAGCTGGTATGGGTTCTGCGCCGAATGCAGCGGCAACTGCTGCCGTACCACACCCTGTTAAGCAA
GGTTTAATCCAATCATTAGGTGTGTTCTTTGATACAATGTTGGTTTGTACAGCAACTGCAATCATGATTTTACTATATTCAGGACTGAAATT
TGGTGATAACGCACCTCAAGGTGTTGCAGTTACTCAATCAGCACTTAATGAGCATTTAGGTTCTGCTGGAGGTATTTTCTTAACAATAGCA
GTTACACTGTTTGCATTTTCATCTGTGGTAGGTAATTACTATTACGGTCAATCTAATATTGAATTTTTATCAACAAACCGTGTAATATTATTT
ATCTTTAGATGTCTTGTTGTAGTACTTGTCTTTGTCGGTGCAGTTGTAAAAACAGAAACAGTATGGAATACGGCAGACTTATTTATGGGCT
TAATGGCAATTGTAAACATTATTTCCATTATAGGACTGTCCAATGTAGCTTTTGCATTGATGAAAGATTATCAAAAGCAGAAAAAAGAAG
GCAAGAACCCTGTCTTTAAACCTGAAAACTTAGAAATTAACTTATTTGGAATTAGTGCTTGGGGCGCTAACAAATATAAGAACTCTGATA
AATAA

Gene: glcT (transcriptional antiterminator)

Contig: 06_NODE_54, position: 496626 to 497477, length: 852 nt, orientation: FORWARD

Perfect match to: (MW2-BA000033-[1362927:1363778], highly conserved allele)

Sequence:

ATGGGAGAATATATTGTTACTAAAACATTGAACAACAATGTCGTAGTATGTACTAATAATGATCAAGAAGTTATTTTAATCGGTAAAGGT
ATTGGTTTTAACAAAAAAGAGGGAATGGCGTTAAACGACCAAACTATTACAATAGAGAAAATTTATAAATTAGAGAGTGAGCAACAAAA
AGCACATTATAAAAGTTTAGTTGAAATCGCTGATGATAATGTATTACAAGTAATTATTGATTCGTTGAATTTTATTTCTAATACTGCGATGA
ATGTTGATTCAAAACAACTTGTAGTTTCATTAACGGATCATATTATATTTGCTTATAAACGCTTAAAACAAAATCAAGTTATTAGCAATCCA
TTTGTTATGGAAACTATGCAGTTATATAGTGATGCATATCATATTGCTAAACAGGTGATTGATCAGTTAAATGCAGCATTAGATGTACATT
TTCCTGAAGATGAGATAGGATTTATTGCATTACATATTGCATCTAATACAGAAGATTTATCTATGCATGAGATGACCTTGATCAATAATGT
TATTAAAAAAAGGTATAGATATCATTGAATCAGACCTTGTGACAACTGTTGATAAGGAATCATTACAATACCAACGTTTTATAAGGCACGTA
CAATTTTTAATTCGCCGATTAAGAAGAAAAGAATATATACATGCACAAGATGATTTTGTGTCTATGATTAAAAATCACTATCCGATTTGCT
ATAACACAGCATATAAAATTTTAACTATGATACAAAAACAATTTGATGTTAATATCAGTGAGTCTGAAATTATATATTTAACATTACACATT
CATCATTTTGAAGAAAGGATTAATCAATCCTAA

Gene: yubA (putative membrane protein)

Contig: 06_NODE_54, position: 497612 to 498820, length: 1209 nt, orientation: FORWARD

Perfect match to: (N315-BA000018-[1361923:1363131], highly conserved allele)

Sequence:

TTGAATGAAAATGAAAAGAATATAAGAAAGAATTTTTTAAAATTTACCGAATCACGGTATATGAAGTTTGTTGGTGGGAATGATTTAGTC
TTCTCATTAATAGCGCTAGTATTGTTGGGTATTGTTATTTTTATTTTCGAAAAAGTATCATATGTTTTTGATCCTTTTATCATCGTTTTTAAGA
CGATAGCAGCACCTATCATCGTCTCTTTAATTCTATTCTATCTATTTAACCCAATCGTAAATATGATGGAACGTTATAGAATACCAAGAGTT

GCAGGTATTTCTATTATTTATCTAGCTGTAGTAGGTGTTATTACGTTAATTGTTAATTTATTGATACCTATTATTGGTTCGCAAGTAGATAG
TTTAGTTAAAAATTCACCGCAATATCTAGAAAAATTAATTAATTCTATTGATAAAATAGCAAATAATACGTTTTTCTCTTCGTATTATAGTCA
AATTAATGATTGGTTAAATTCTTTACCTAAGAAAATACCATCTATGTTAAGTGAATTTACAGATGGCTTTGGGTCTAAAATTGCAACGTTT
GCAGAAACGATTGCTAATATTGGCGTTGTGATTGTCACAACACCATTTGTACTATTCTTTATGCTTAAAGATGGACATCACTTCAAAGAAT
TTTCAACGAATATTATGCCACCGAAATTCCGAAAAGATTTTCATGATCTACTTGAAAAAATGAGTGTTCAAGTTGGTTCATACATTCAAGG
ACAAATTATCGTTTCATTCTGTATCGGTATACTGTTGTTTATCGGTTATTCGGTTATCGGGTTGAAATATAGCTTAGTATTAGCTAGTATTG
CGGCAGTTACAAGTGTTGTACCATATTTAGGGCCTACTATAGCGATTTCTCCAGCTATTGTAATAGCTGCTATAACATCGCCGTGGATGCT
CTTAAAATTAGCAGTAGTATGGACTTTAGTACAATTTGTTGAAGGGCACTTCATTTCACCAAATATCATGGGTAAAACACTTAAGATTCAT
CCACTTACAATCATTTTCATTTTACTGTGTGCAGGCAAATTGCTTGGTATTGTAGGCGTTATTTTAGGTATTCCGGGATATGCTATTTTAAA
AGTATTAGTTACTCATTTATTCCAATTATTTAAACGTCGATACAATCGTTTCTATGGTAATGATGTAGGTGAATATGATATTAAAGAAAGT
AATAAAATAGTTGAATAA

Gene: mprF (lysylphosphatidylglycerol synthetase)

Contig: 06_NODE_54, position: 499301 to 501823, length: 2523 nt, orientation: FORWARD

Sequence:

ATGAATCAGGAAGTTAAAAACAAAATATTTTCAATCTTAAAAATTACGTTTGCTACAGCTTTATTTATTTTTGTAGTAATCACATTGTATCG
GGAGTTATCTGGTATTAACTTTAAAGATACGTTGGTTGAATTTAGTAAGATTAACCGTATGTCCTTAGTTTTACTATTTATTGGTGGTGGG
GCATCGCTTGTTATTCTATCAATGTATGATGTGATTTTATCTAGAGCTTTAAAAATGGATATATCCTTAGGCAAAGTTTTAAGAGTAAGTTA
TATCATCAATGCATTGAATGCGATTGTAGGTTTCGGTGGCTTTATTGGTGCAGGCGTTAGAGCTATGGTTTATAAAAACTATACGCATGAT
AAAAAGAAATTAGTTCACTTTATATCCTTAATACTTATTTCAATGTTGACAGGTTTAAGCTTATTATCATTGTTAATTGTATTCCATGTTTTC
GATGCATCTTTAATTTTAGATAAGATTACATGGGTAAGATGGGTATTATATGTAGTGTCATTTTTCTTACCATTATTCATTATTTATTCAATG
GTTAGACCACCTGATAAAAACAATCGTTTTGTAGGATTGTACTGCACTTTAGTGTCGTGTGTTGAATGGTTAGCAGCTGCAGTTGTATTAT
ATTTCTGTGGTGTAATTGTTGACGCTCATGTATCATTCATGTCCTTTATTGCAATATTTATCATTGCTGCATTATCAGGTTTAGTCAGCTTTA
TTCCTGGTGGTTTCGGCGCTTTCGATTTAGTTGTATTACTAGGATTTAAAACTTTAGGTGTCCCTGAGGAAAAAGTATTATTAATGCTACTT
CTATATCGTTTTGCGTACTATTTTGTACCGGTAATTATTGCATTAATTTTATCATCATTTGAATTTGGTACATCAGCTAAGAAGTACATTGA
GGGATCTAAATACTTTATTCCTGCTAAAGATGTTACGTCATTTTTAATGTCTTATCAAAAGGATATTATTGCTAAAATTCCATCATTATCATT
AGCAATTTTAGTATTCTTTACAAGTATGATCTTTTTTGTAAATAATTTAACGATTGTTTACGATGCTTTATATGATGGAAATCACTTAACGTA
TTATATTCTATTGGCAATTCATACTAGTGCTTGTTTATTACTTTTACTGAATGTAGTTGGTATTTATAAGCAAAGTAGACGTGCCATCATCTT
TGCTATGATTTCAATTTTATTAATCACAGTGGCGACATTCTTCACTTACGCTTCATATATTTTAATAACATGGTTAGCTATTATTTTTGTTCT
GCTTATTGTAGCTTTCCGTAGAGCACGTAGGTTGAAACGCCCAGTAAGAATGAGAAATATAGTTGCAATGCTTTTATTCAGTTTATTTATT
TTATATGTTAACCATATATTTATTGCTGGAACGTTATATGCATTAGATATTTATACGATTGAAATGCATACATCTGTATTGCGCTATTACTTC
TGGCTTACGATTTTAATCATCGCTATCATCATAGGTATGATTGCATGGTTGTTTGATTATCAATTTAGCAAAGTACGCATTTCTTCTAAAAT
TGAAGATTGCGAGGAGATTATTAATCAGTATGGCGGTAATTATTTGAGTCACTTGATATATAGTGGTGACAAGCAGTTTTTCACTAATGA
AAATAAAACAGCATTTTTAATGTATCGTTATAAAGCAAGTTCATTAGTGGTTCTTGGAGATCCGTTAGGTGATGAAAATGCCTTTGATGAA
TTGTTAGAAGCATTCTATAATTACGCTGAGTATTTAGGCTATGATGTTATATTCTATCAAGTTACAGATCAACACATGCCTTTATATCATAA
TTTCGGTAACCAATTTTTCAAATTAGGTGAAGAAGCAATTATTGATTTAACGCAATTTTCAACTTCAGGTAAAAAACGCCGTGGATTTAGA
GCGACTTTAAACAAATTCGATGAACTTAATATTTCATTCGAAATTATTGAACCACCGTTTTCAACTGAATTTATAAATGAACTTCAACATGT
AAGTGATTTATGGCTAGATAATCGTCAGGAAATGCATTTCTCTGTGGGTCAATTTAATGAAGAATACTTATCTAAAGCGCCAATTGGTGT
AATGCGAAATGAAGAAAATGAAGTAATTGCATTTTGTAGTTTAATGCCAACATACTTTAATGATGCCATTTCAGTCGATTTAATTAGATGG
TTGCCAGAGTTAGATTTACCATTAATGGATGGTCTATACTTGCATATGTTACTTTGGAGTAAAGAACAAGGTTATACAAAATTTAATATGG
GTATGGCAACGTTATCGAACGTTGGTCAATTGCATTATTCATATTTAAGAGAACGACTTGCAGGCCGTGTCTTTGAACATTTCAACGGTCT
ATATCGTTTCCAAGGATTACGTCGTTATAAATCTAAATATAATCCGAATTGGGAACCACGCTTTTTAGTTTATCGTAAAGATAATTCGCTTT
GGGAATCACTTTCTAAAGTAATGCGTGTAATACGTCACAAATAA

Gene: msrA1 (peptide methionine sulfoxide reductase A1)

Contig: 06_NODE_54, position: 502026 to 502535, length: 510 nt, orientation: REVERSE

Perfect match to: (MW2-BA000033-[1368327:1368836:r], allele observed in CC1+CC445+CC772)

Sequence:

TTATTGCTTATTTTTGTATTCTTGGCGTATTTTTTGTTCTTCTGCATAGCGCTCTGGATTTTTCTTATAAAAATCTTGGTGATAGTCTTCGGCT
TTGTAAAATTGTGACGCGGGTAATATTTTTGTTGCAATTGCCTTATCAGCATTAATCGTATTTTTAAGCTGCTCGATATAAGTCTCAGCGAG
TTCTTTTTGATGATCATTAGTGTAGAAAATAGCTGTTTGATATTGAGGACCACGGTCTTGATATTGACCCCCTGTATCTAATGGGTCAATG
ACTGAGAAAAATATTTCTAATAACTTATTGTATGAGAATAATGCAACATCATATTGAATTTCAACAGTTTCTAAATGACCACTCGTACCTGA

TTTTACTTGTTCGTAAGTAGGATTTTCAATATGTCCGCCCATATATCCAGAAGTTACTTTTTCTATGCCGTCAAAGGTGTCAAATGGTTTCG
TCATACACCAAAAGCAACCTCCGGCAAAATAAGCTGTATTAATATTCAT

Gene: tagT-lcpA (phosphotransferase)

Contig: 06_NODE_54, position: 502672 to 503655, length: 984 nt, orientation: FORWARD

Sequence:

ATGGATAAAGAAACTAATGACAACGAATATAGACGTCAAAGTGAACATCGCACTTCGGCGCCTAAGCGAAAAAAGAAGAAGAAAATTA
GGAAATTACCTATCATTCTTCTGATTGTTGTAATTTTACTTATCGCATTAGTTGTATATATTGTGCATAGTTACAATAGCGGTGTAGAATAT
GCCAAGAAACATGCGAAAGATGTTAAAGTACATCAATTTAATGGACCAGTAAAAAATGATGGTGAAATTTCTATTCTTGTACTCGGTGCA
GATAAAGCACAAGGTGGACAATCAAGAACAGATTCTATCATGGTTGTTCAATATGACTTTATCAATAAAAAGATGAAAATGATGTCTGTC
ATGCGTGATATTTATGCAGATATTCCAGGATATGGAAAACACAAAATTAATTCAGCATACGCTTTAGGTGGTCCAGAGCTACTTAGAAAA
ACACTTGATAAAAATTTAGGAATTAATCCTGAATATTATGCAGTAGTTGATTTTACTGGATTTGAGAAAATGATTGATGAATTAATGCCAG
AAGGTGTACCAATTAATGTCGAAAAGGATATGTCGAAAAATATTGGTGTATCTTTGAAAAAGGGTAACCATAGGTTGAATGGTAAAGAA
TTACTTGGTTATGCAAGATTCCGTCACGACCCTGAAGGTGACTTCGGACGCGTGCGACGTCAGCAACAAGTGATGCAAACATTGAAAAA
AGAAATGGTTAATTTTAGAACAGTTGTTAAATTACCAAAAGTTGCAGGTATTTTAAGAGGCTATGTGAATACAAACATTCCTGATTCAGG
GATTTTCCAAACAGGTTTGAGTTTTGGTATCCGAGGTGAAAAAGATGTTAAGTCATTGACTGTGCCAATCAAGAACTCATACGAAGATGT
CAATACAAATACTGATGGTAGTGCATTACAGATTAATAAAAACACAAATAAACAAGCTATTAAAGACTTTTTAGATGAAGATTAA

Gene: txbi_lcpA (bidirectional rho-independent terminator of lcpA)

Contig: 06_NODE_54, position: 503648 to 503666, length: 19 nt

Perfect match to: (MRSA252-BX571856-[1431394:1431412], allele observed in CC30)

Sequence:

AAGATTAAAAATAAACAAG

Gene: dmpI (4-oxalocrotonate tautomerase)

Contig: 06_NODE_54, position: 503743 to 503928, length: 186 nt, orientation: REVERSE

Perfect match to: (N315-BA000018-[1368054:1368239:r], highly conserved allele)

Sequence:

TTATTGATCTGACTTTCTTACGCCAGCCACACCATAATGGTTTGGTTTCATTTCTTCTATAACAACGTGAATTGCTTGTCTATTTGCCCCCGT
TGTTTTTTCTACGGCGTCAGTTACTTCGCTAACTAAATTTTTTAATTGTTCATCCGAACGACCTTCTAATAATTTTACATTGACGATTGGCAT

Gene: uvrX (DNA-damage repair protein)

Contig: 06_NODE_54, position: 504076 to 505338, length: 1263 nt, orientation: FORWARD

Perfect match to: (MW2-BA000033-[1370377:1371639], highly conserved allele)

Sequence:

GTGTATAATTATCATTTATTAGAAGATAGGGATGTTCTATGTATTGACCAAAAAAGTTTTTTTGCGAGTGTTTCTTGTATTGAAAAGGGGC
TAGATCCATTAGAAACAAAGCTAGCTGTTGTTGCAGATACTAAGCGTCAGGGTTCTGTAATATTGGCTGCGACACCTAAATTAAAAGAAT
TAGGCATCAAGACAGGGTCGCGATTGTTTGAAATACCACATAGAAATGATATTTACATTATCAATCCAAGTATGCGTAAATATCTTAATGT
TTCAGTTGCTATTTCTAAGATTGCATTGCGTTATATTCCACCTGAAGATTTACACCAATATAGTATTGACGAATTTTTTATGGATGTTACTG
ATAGCTATCATAGATTTAGTTCTACAGTACATGCATTTTGCGAAAGACTTAAACGTGAAATTTATGAAGAAACAGGCATTTATTGTACTGT
GGGCATTGGTTCTAATATGTTATTAAGTAAAATTGCTATGGATGTTGAAGCGAAGCATAGTCAAAATGGTATAGCTGAATGGCGATATCA
AGATGTACCAACGAAATTATGGCCAATTCAGCCCTTGCGAGATTTTTGGGGTATTAATCGTCGAACAGAAGCCAAATTGAATAAAAGAG
GAATTTTTACTATAGGAGATTTAGCGAAATATCCATATAAATTTTTAAAAAAAGAGTTCGGTATTTTAGGTGTTGATATGCATCTACATGC
GAATGGGATAGATCAGAGTAAAGTACGTGAAAAGCACAAGATCAGCAATCCATCGATATGCAAAAGTCAAATATTAATGAGAGATTATC

ATTTTGATGAAGCAAAAGTAGTAATGCAAGAGTTAATTGAAGATGTTGCTAGCAGAGTTCGAGCAAGAAAAAAAGTGGCAAGAACGAT
ACATTTTGCCTTTGGCTATAGTGATGAAGGCGGTGTACATAAGCAATATACTTTGAAAGATCCAACAAACTTAGAAAAAGATATTTATAA
AGTAGTAATGCATTTCGCAGATAAATTATGTAATAAACAAGCACTATATCGTACGCTAAGTATATCTTTGAGTCAATTTATTAATGAGGAT
GAGCGACAGTTAAGTCTGTTTGAAGATGAATACCAACGCAAACGTGACGAATGTCTAGCTAAAACGATAGACCAATTACATTTGAAATAC
GGCAAAGGTATTGTGTCCAAAGCAGTATCGTTTACAGAAGCAGGTACAAAACACGGCAGATTAGGTTTAATGGCTGGACATAAAATGTA
A

Gene: tyrA (prephenate dehydrogenase)

Contig: 06_NODE_54, position: 505474 to 506565, length: 1092 nt, orientation: REVERSE

Perfect match to: (MW2-BA000033-[1371775:1372866:r], highly conserved allele)

Sequence:

TTATTGGATATAACAATCAAAATCACTCAATGCTTGCATACCGCGTTCTCGGTCAGTAGGGTTTTTGAAACTAATTTTTAAAGCACCGTAT
ATATCTTCGCGTACTTCTAAGATTCTTAAGTTGCTTATAGATATGTTATGTAAACTCAGGATATAAGTCACTTTACTTATCATACCTGATTCA
TCCGGAATGTCTACATATAGATCATACGCAGTATTTAGTCCACCTAGTTGTTTAGCGGGTAGTGCGTCGCGATACGATTTAGCTTGGGCA
AAAAATGATAACAATTTTTCAGAATCATTGCTTTCAATTAGTCTTTCTAAATCTTGAAACTGACTTTTTAGCTGTCGAATCATTTCTAAAATA
TACGTTTTATTACTCAAGGTGATATCTTTCCACATTTGTGCATTACTACTAGCTATACGAGTGATATCACGAAAACCACCAGCTGCAAGTTT
ATTAACTAAATGATGTTCTTGACCGTTCTTTTGACTAACATGAACTAAACTAGATGCAACGATATGAGGTAAATGACTTACGACGCTTGTT
ACGTAGTCGTGTTCTTCAGCAGTAGTTACAATAAATTTAGCAAGAGTAGGTGATAACAGTTCTTTTAACGTGTTTGCTGCTTGCTCATTTCT
TGGCTCATTGTAGACTAAAATATAATAAGCGTTTTCAAATAAGTGCTTTTTAGCATTTAGTACACCAGATTTATGACTACCAGCCATTGGA
TGACCACTGACTAAATGAATATTATGCTTTAATAAATTGCATTCGTGTTGCTGTATCATTGCTTTAGTACTACCAGTATCAGAAACAATAAC
ACCAGGTTTAGTTGGCATATCTATAAGCTCGCTAAGATATTTATTTGTGATAGCAACAGGTGTTGCATAAATAATTACATCGGCTTTTTTA
ATAGCTTCACTATAATTTAAACATTTTTCATTAATAATGCCGATTGATTTAGCTTTATCTAACTGAGAAGTATCTGCATCGTATGCAATAAT
ATTAGTATTAGGGTTATGGTATTTTATATTGCTAGCAAGACTTCCACCAATTAATCCAAGCCCAACAAATAAAACTGTTGTCAT

Gene: yhfE (putative endoglucanase)

Contig: 06_NODE_54, position: 506730 to 507761, length: 1032 nt, orientation: FORWARD

Perfect match to: (N315-BA000018-[1371042:1372073], allele observed in CC5+CC1)

Sequence:

ATGGAACCAATATTAGAAATGATTAAAACATTAACAGGTATTAATAGTCCTTCAGGAGACACAGAAGAAGCAATTCAATTTGTCGAAAAA
TATGCAAAAGACTTGGGTTATCAAACAACACTAACAAATAAAGGTGCGTTATTAATAACAGTGCCAGGCAAAAATGATGAAGTACAACG
CTGTATTACTGCTCATGTTGATACTTTAGGTGCAATGGTTAAAGAAATTAAAGAAGATGGTCGCTTAGCAATAGAATTAATTGGAGGATT
CACGTATAACGCGATTGAGGGTGAATATTGCCAAATTAAAACTGATGCTGGTCAAATATATACAGGAACAATTTGTCTGCATGAAACAAG
TGTTCATGTATATAGAAATAATCATGAAATACCTAGAGATCAAAAGCATATGGAAATAAGAATTGATGAAGTAACTACATCAGAAGAAG
ATACAAAGAGTTTAGGTATTTCAGTAGGTGATTTTGTTAGCTTTGATCCACGTACAGTTATCACGTCATCAGGTTTTATTAAATCTCGTCAT
TTAGATGATAAAGCTAGCGTAGCGATTATACTACAATTACTAAAGAAATTAAAAGAAGAGCAAATAATATTACCACATACAACGCAATTT
TATATTTCTAATAACGAAGAAATAGGTTACGGTGCAAATGCATCAATTGATTCGAAAATCAAAGAATATATTGCATTAGATATGGGCGCG
TTGGGAGACGGTCAAGCATCGGATGAATATACAGTTTCTATTTGTGCCAAAGATGCTTCAGGTCCATATCATAAGCAATTGAAATCGCAC
CTAGTTAATCTTTGCAAAATAAATAACATTCCATATAAAGTCGACATATATCCATATTATGGTTCAGATGCTTCAGCAGCTTTACATGCTGG
TGCGGATATCAGACATGGTTTATTTGGCGCTGGCATTGAATCATCTCATGCAATGGAACGAACACATATTGATTCTATTAAAGCGACAGA
GAAATTACTATATGCATATTGCTTATCACCAATTGAGTAA

Gene: tbox05 (T-box leader element)

Contig: 06_NODE_54, position: 507823 to 507996, length: 174 nt

Sequence:

AATTTTCTAAAGAAATAGTAGCAGATATGAAACGTAGCAAATAGAAAGCTAATGGGTGATGGGAATTAGCACGCCATATCTTGTGAATT
GGACTTTGGAAAACAATTGAATGAGTTTTGAAAGTGAACATGAATTATGTTAACTAAGGTGGCACCACGGTAACGCGTCCTTACA

Gene: tbox06 (T-box leader element)

Contig: 06_NODE_54, position: 508024 to 508210, length: 187 nt

Sequence:

ATTTAGACAAAATGTAGTAGTTAATTAAAGGTAGCAACAGAAAGTTAGTGGATGATGTGAACTAACACCGAGATTAATGAAATTGGGTT
TTGTCTGCAACAGAAAAATTATATATAGTAAAGAGTGAACTATGAATATTTCGAATATTCGGTTAATTTAGGTGGTACCACGCGTCAGCG
TCCTTTAT

Gene: trpE (anthranilate synthase component I)

Contig: 06_NODE_54, position: 508253 to 509659, length: 1407 nt, orientation: FORWARD

Sequence:

ATGGATATATTTTATAAAAAAATAAAAGCAAATGTAACGCCAGAAGTTTTAGCACAACTTCATTCCAAGAAGATCATTTTGGAAAGTACA
AATCAACAACAAACTAAAGGTCGCTATTCAGTTGTTATTTTTGATATTTATGGCACTTTAACTTTAGATAATGATGTATTATCAGTAAGCAC
TTTAAAAGAATCGTATCAAATCAGTGAAAGACCGTACCATTATTTAACGACTAAAATAAATGAAGACTACCATAATATTCAAGATGAGCA
ACTTAAGTCATTACCGTTTATATCTGGATATGTTGGGACGTGTAGCTTTGATTTAGTAAGACATGAATTTCCTAAATTGCAATCAATACAAT
TAGAAGATCACAAGCAGCACGATGTAAGGTTATATATGGTTGAACAAGTTTATGTATTTGACCATTACAAAGATGAGTTATATATCATCG
CGACGAATCAATTTTCAAATTCAACAAAATCAGATCTTGAGAATCGAGTTAATAAGTCTATCGAAGACTTAACTAAAATCCAACCATTCAT
GCCTACACAAGATTTTGATTTTAAAACTAAAGAAATTCAATCAAACATTTCTGAAGAAAGATTTATCGAAATGATTCAGTATTTCAAAGAG
AAAATAACAGAAGGGGATATGTTTCAAGTTGTGCCATCAAGAATTTACAAATATGCGCATCATGCTAGTCAGCATTTAAATCAACTTTCGT
TTCAACTGTATCAAAATTTAAAACGACAAAACCCAAGTCCATATATGTATTATCTTAATATCGATCAACCATATATTGTCGGTAGTTCTCCC
GAAAGTTTCGTAAGTGTCAAAGATCAAATTGTAACAACTAATCCTATTGCAGGTACGATTCAACGTGGTGAGACGACACAAATAGATAAT
GAGAATATGAAACAACTACTTAATGATCCAAAAGAATGCAGCGAACATCGTATGCTAGTTGATTTAGGGCGTAATGATATTCATAGAGTA
AGTAAAATCGGTACCTCAAAAATTACTAAATTAATAGTTATTGAAAAATATGAACATGTTATGCATATCGTAAGTGAAGTCACAGGTAAA
ATAAATCAAAATTTATCGCCAATGACAGTTATTGCGAATTTATTACCAACAGGTACCGTTTCAGGTGCACCAAAATTACGTGCAATTGAAA
GAATATATGAACAATATCCACATAAACGGGGCGTTTATAGTGGTGGTGTTGGATACATAAATTGTAATCATAACTTAGATTTTGCATTAG
CAATTCGAACGATGATGATAGATGAGCAGTATATCAACGTAGAAGCTGGTTGTGGCGTTGTATATGATTCTATTCCTGAAAAAGAACTGA
ATGAAACGAAATTGAAAGCTAAAAGCTTATTGGAGGTGAGCCCATGA

Gene: trpG (anthranilate synthase component II)

Contig: 06_NODE_54, position: 509656 to 510222, length: 567 nt, orientation: FORWARD

Perfect match to: (MW2-BA000033-[1375957:1376523], allele observed in CC1+CC22+CC361)

Sequence:

ATGATCTTAGTTGTAGATAATTATGATTCCTTTACATATAACCTAGTGGATATTGTTGCTCAACATACTGACGTCATTGTTCAATACCCTGA
TGATGATAATGTGCTGAATCAATCGGTGGACGCTGTTATTATATCTCCTGGTCCAGGGCATCCATTAGACGATCAACAGTTGATGAAAAT
CATATCAACCTATCAACACAAACCCATTTTAGGTATATGTTTAGGGGCTCAGGCACTGACTTGTTACTACGGTGGAGAAGTCATTAAAGG
CGACAAGGTTATGCACGGCAAAGTTGATACACTAAAGGTTATATCGCATCATCAACATCTGTTATATCAAGATATACCAGAACAGTTTTCA
ATTATGAGATATCATTCATTAATAAGTAACCCTGACAATTTTCCAGAAGAATTGAAAATTACTGGACGTACCAAAGATTGTATACAGTCAT
TCGAGCATAAAGAAAGACCGCATTATGGTATTCAGTACCATCCTGAATCATTTGCTACAGACTATGGTGTCAAAATAATTACAAATTTCAT
TAATCTAGTGAAGGAAGGATGA

Gene: trpD (anthranilate phosphoribosyltransferase)

Contig: 06_NODE_54, position: 510228 to 511226, length: 999 nt, orientation: FORWARD

Perfect match to: (08-02119-CP015645-[796237:797235:r], highly conserved allele)

Sequence:

ATGACATTACTAACAAGAATAAAAACTGAAACTATATTACTTGAAAGCGACATTAAAGAGCTAATCGATATACTTATTTCTCCTAGTATTG
GAACTGATATTAAATATGAATTACTTAGTTCCTATTCGGAGCGAGAAATCCAACAACAAGAATTAACATATATTGTACGTAGCTTAATTAA
TACAATGTATCCACATCAACCATGTTATGAAGGGGCTATGTGTGTGTGCGGCACAGGTGGTGACAAGTCAAATAGTTTCAACATTTCAAC

GACTGTTGCTTTTGTTGTAGCAAGTGCTGGCGTAAAAGTTATAAAACATGGTAATAAAAGTATTACCTCAAATTCAGGTAGTACGGATTT
GTTAAATCAAATGAACATACAAACAACAACTGTTGATGATACACCTAACCAATTAAATGAAAAAGACCTTGTATTCATTGGTGCAACTGA
ATCATATCCAATCATGAAGTATATGCAACCAGTTAGAAAAAATGATTGGAAAGCCTACAATATTAAACCTTGTGGGTCCATTAATTAATCCA
TATCACTTAACGTATCAAATGGTAGGCGTCTTTGATCCTACAAAGTTAAAGTTAGTTGCTAAAACGATTAAAGATTTAGGTAGAAAACGT
GCAATCGTTTTACATGGTGCAAATGGTATGGATGAAGCAACACTATCTGGTGATAATTTGATATATGAATTGACTGAAGATGGAGAAATC
AAAAATTACACATTAAATGCGACTGATTATGGTTTGAAACATGCGCCGAATAGTGATTTTAAAGGCGGTTCACCTGAAGAAAATTTAGCA
ATCTCCCTTAATATCTTGAATGGTAAAGATCAGTCAAGTCGACGTGATGTTGTCTTACTAAATGCGGGTTTAAGCCTTTATGTTGCAGAGA
AAGTGGATACCATCGCAGAAGGCATAGAACTTGCAACTACATTGATTGATAATGGTGAAGCATTGAAAAAATACCATCAAATGAGAGGT
GAATAA

Gene: trpC (indole-3-glycerol phosphate synthase)

Contig: 06_NODE_54, position: 511228 to 512010, length: 783 nt, orientation: FORWARD

Perfect match to: (COL-CP000046-[1417185:1417967], highly conserved allele)

Sequence:

ATGACGATTTTATCAGAAATTGTTAAATATAAACAGTCACTTTTACAAAATGGCTATTATCAAGACAAACTTAATACCTTGAAAAGTGTGA
AGATTCAGAATAAAAAATCTTTTATAAACGCAATTGAGAAAGAACCAAAGCTAGCAATTATTGCAGAAATTAAATCGAAGAGTCCTACAG
TTAATGACTTACCTGAACGAGATTTATCGCAACAAATCTCAGATTATGACCAATATGGTGCAAATGCCGTGTCCATTTTAACTGATGAAAA
GTACTTTGGTGGTAGTTTTGAAAGATTACAAGCATTGACGACAAAAACAACATTACCCGTATTATGCAAAGACTTTATTATAGACCCGCTT
CAAATTGATGTTGCTAAACAAGCTGGTGCATCTATGATTTTATTGATCGTTAACATCTTATCTGATAAACAATTGAAAGATTTATATAACTA
CGCTATATCGCAAAATCTAGAAGTGTTAGTTGAAGTACATGATCGCCATGAATTAGAACGTGCCTATAAGGTTAATGCTAAATTGATTGG
TGTAAATAACAGGGACTTAAAACGATTTGTTACAAATGTGGAACATACAAATACTATTTTAGAAAATAAAAAAACAAATCATTATTATATT
TCTGAAAGTGGTATTCACGATGCATCTGATGTAAGAAAAATCTTGCATAGTGGTATCGATGGCTTACTAATAGGTGAGGCGCTTATGCGT
TGTGACAATCTATCTGAATTTTTACCACAACTGAAAATGCAAAAGGTGAAGTCATGA

Gene: trpF (N- (5'-phosphoribosyl)anthranilate isomerase)

Contig: 06_NODE_54, position: 512010 to 512642, length: 633 nt, orientation: FORWARD

Perfect match to: (N315-BA000018-[1376322:1376954], highly conserved allele)

Sequence:

ATGAAATTGAAATTTTGTGGCTTTACATCAATAAAGGATGTTACAGCGGCCAGTCAATTACCTATTGATGCGATAGGTTTCATCCATTATG
AAAAAAGTAAAAGGCATCAAACAATTACCCAAATAAAAAAGTTAGCGTCTGCTGTTCCAAATCATATCGATAAAGTATGTGTCATGGTAA
ATCCTGATTTAACAACAATTGAACACGTATTAAGCAATACGTCAATTAACACAATACAGTTACACGGCACAGAATCTATTGATTTTATACA
GGAAATTAAAAAGAAATATTCAAGCATTAAAATCACTAAAGCTTTAGCTGCAGATGAAAACATAATCCAAAACATAAATAAATATAAAGG
GTTCGTAGATTTATTTATTATCGACACACCCTCAGTGTCGTATGGTGGTACCGGTCAAACATATGACTGGACTATTTTGAAGCACATAAAA
GACATACCTTATTTGATAGCAGGAGGCATTAACTCTGAAAATATTCAAACAGTTAATCAACTTAAATTATCACATCAAGGTTATGATCTTG
CATCAGGTATAGAAGTAAATGGGCGAAAAGATATAGAAAAAATGACAGCAATTGTAAATATTGTGAAAGGAGATAGAGACAATGAATA
A

Gene: trpB (tryptophan synthase beta chain)

Contig: 06_NODE_54, position: 512635 to 513849, length: 1215 nt, orientation: FORWARD

Perfect match to: (TW20-FN433596-[1468447:1469661], highly conserved allele)

Sequence:

ATGAATAAACAAATACAAACAGAAGCAGATGAATTAGGTTTCTTTGGTGAATATGGAGGGCAATATGTTCCAGAAACATTAATGCCAGC
AATTATTGAGTTGAAAAAAGCTTATAAAGAGGCAAAAGCAGACCCAGAGTTTCAAAGAGAACTGGAATACTATTTATCAGAGTATGTAG
GACGCGCGACACCACTTACATATGCTGCATCATATACTGAAAGCCTAGGTGGCGCTAAAATATATTTGAAACGAGAGGATCTAAATCATA
CAGGCGCCCATAAAATTAATAATGCGTTAGGTCAAGCGTTGCTTGCTAAAAGAATGGGCAAGAAGAAGCTTGTTGCTGAAACTGGTGCG
GGTCAACATGGTGTAGCTAGTGCTACGGTTGCTGCATTATTTGATATGGAACTTGTTGTCTTTATGGGAAGTGAAGATATTAAAAGACAA
CAACTTAATGTATTTAGAATGGAATTACTTGGTGCAAAGGTTGTGGCAGTTGAAGATGGTCAAGGGACTTTATCGGATGCAGTTAATAAA
GCATTGCAATATTGGGTAAGTCATGTAGATGATACACATTATTTATTAGGTTCTGCATTAGGTCCAGACCCGTTCCCAACGATTGTTAGAG

ATTTTCAGAGTGTGATTGGTAAAGAAATAAAATCACAGATATTGAAGAAAGAAGGTCGACTTCCGGATGCAATTGTAGCATGTATCGGT
GGTGGCTCAAATGCAATCGGTACATTTTATCCATTTATTAAAGATGATGTTGCATTATACGGTGTTGAAGCCGCAGGTCAAGGCGATGAT
ACTGATAAACATGCACTTGCAATTGGCAAAGGATCACCTGGCGTATTACATGGTACTAAAATGTATTTAATTCAAGATGAAGATGGGCAA
GTGCAACTAGCACATTCTATTTCAGCAGGACTTGATTATCCTGGTATTGGACCAGAACATTCTTATTACCACGACATTGGTAGAGTAACTT
TTGAAAATGCTAGTGATACACAAGCAATGAATGCTTTAATCAACTTTACAAAACATGAAGGTATTATACCTGCAATTGAAAGTGCACATG
CACTGAGTTATGTTGAAAGACTAGCGCCTACGATGTCGAAAGAAGATATTATTGTAGTAACTATTTCTGGACGTGGCGATAAAGATATGG
AAACAATTAGACAATATATGGTAGAGCGAGGTCTTGCAAATGACTAA

Gene: trpA (tryptophan synthase alpha chain)

Contig: 06_NODE_54, position: 513842 to 514570, length: 729 nt, orientation: FORWARD

Sequence:

ATGACTAAATTATTTATACCTTATATTATGGGCAATAAAGATTTGATTGAAAATGCAACATTGTTGAGTGAAAATGGTGCAGATATAATTG
AAATTGGAGTACCTTTCTCTGATCCGGTTGCTGATGGTCCAGTTATCATGGAAGCAGGTCAACAAGCGATTAAACAAGGCATCACGATAG
ATTATATTTTCAATCAATTAGAAAAACATGGTGATCAAATTAAGTGTAACTATGTATTAATGACGTATTATAATATTATTTGTCATTATGGA
GAACAAGCGTTTTTTGAAAAATGTCGAGATACTGGTGTCTACGGCTTAATTATTCCTGATTTACCATATGAATTATCGCAGCGTTTAAAAC
AACAATTTAGTCACTATGGCGTCAAAATCATATCGTTAGTTGCGATGATTACTGATGACAAACGTATAAAAGATATCGTATCCCATGCGG
AAGGCTTTATTTATACTGTGACGATGAATGCGACAACAGGGCAAAACGGTGCGTTTCATCCAGAATTAAAACGAAAAATTGAGTCAATTA
AAGCGATAGCCAATGTGCCAGTTGTCGCAGGATTTGGTATAAGAACACCACAACATGTTGCAGATATAAAAGAGGTTGCAGATGGCATT
GTCATTGGTAGCGAAATCGTTAAGCGATTTAAATCTAACACGCGTGAGGAAATCATTAAATATTTACAATCTATCCAACAAACATTGAATA
ATTAA

Gene: femA (aminoacyltransferase)

Contig: 06_NODE_54, position: 514892 to 516154, length: 1263 nt, orientation: FORWARD

Perfect match to: (MW2-BA000033-[1381193:1382455], allele observed in CC1)

Sequence:

ATGAAGTTTACAAATTTAACAGCTAAAGAGTTTGGTGCATTTACAGATAGCATGCCATACAGTCATTTCACGCAAACTGTTGGCCACTATG
AGTTAAAGCTTGCTGAAGGTTATGAAACACATTTAGTGGGAATAAAAAACAATAATAACGAGGTCATTGCAGCTTGCTTACTTACTGCTG
TACCTGTTATGAAAGTGTTCAAGTATTTTTATTCAAATCGCGGTCCAGTGATTGATTATGAAAATCAAGAACTCGTACACTTTTTCTTTAAT
GAATTATCAAAATATGTTAAAAAACATCGTTGTCTATACCTACATATCGATCCATATTTACCATATCAATACTTGAATCATGATGGCGAGAT
TACAGGTAATGCTGGTAATGATTGGTTCTTTGATAAAATGAGTAACTTAGGATTTGAACATACTGGATTCCATAAAGGATTTGATCCTGTG
CTACAAATTCGTTATCACTCAGTGTTAGATTTAAAAGATAAAACAGCAGATGACATCATTAAAAATATGGATGGACTTAGAAAAAGAAAC
ACGAAAAAAGTTAAAAAGAATGGTGTTAAAGTAAGATTTTTATCTGAAGAAGAACTACCAATTTTTAGATCATTTATGGAAGATACGTCA
GAATCAAAAGCTTTTGCTGATCGTGATGACAAATTTTACTACAATCGCTTAAAATATTACAAAGACCGTGTGTTAGTACCTTTAGCGTATA
TCAACTTTGATGAATATATTAAAGAACTAAACGAAGAGCGTGATATTTTAAATAAAGATTTAAATAAAGCGTTAAAGGATATTGAAAAAC
GTCCTGAAAATAAAAAAGCACACAACAAGCGAGATAACTTACAACAACAACTTGATGCTAATGAGCAAAAGATTGAAGAAGGTAAACGT
CTACAAGAAGAACATGGTAATGAATTACCTATCTCTGCTGGTTTCTTCTTTATCAACCCATTTGAAGTTGTTTATTATGCTGGTGGTACATC
AAATGCTTTCCGTCATTTTGCCGGAAGTTATGCAGTGCAATGGGAAATGATTAATTATGCATTAAATCATGGCATTGACCGTTATAATTTC
TATGGTGTTAGTGGTAAATTTACAGAAGATGCTGAAGATGCTGGTGTAGTTAAATTCAAAAAAGGTTACAATGCTGAAATTATTGAATAT
GTTGGTGACTTTATTAAACCAATTAATAAACCTGTTTACGCAGCATATACCGCACTTAAAAAAGTTAAAGACAGAATTTTTTAG

Gene: femB (aminoacyltransferase)

Contig: 06_NODE_54, position: 516173 to 517432, length: 1260 nt, orientation: FORWARD

Perfect match to: (Strain_21310-AFNP01000066-[56326:57585:r], allele observed in CC22+CC8+CC22)

Sequence:

ATGAAATTTACAGAGTTAACTGTTACCGAATTTGACAACTTTGTACAAAATCCATCATTGGAAAGTCATTATTTCCAAGTAAAAGAAAATA
TAGTTACCCGTGAGAATGATGGCTTTGAAGTAGTTTTATTAGGTATTAAAGACGACAATAACAAAGTAATTGCAGCAAGCCTTTTCTCTAA
AATTCCTACTATGGGAAGTTATGTTTACTATTCGAATCGTGGTCCAGTAATGGATTTTTCAGATTTAGGATTAGTTGATTATTATTTAAAAG
AGTTAGATAAATATTTACAGCAACATCAATGTTTATATGTTAAATTAGATCCGTATTGGTTATATCATCTATATGATAAAGATATCGTGCCA

TTTGAAGGTCGCGAGAAAAATGATGCCCTAGTAAACTTGTTTAAATCACATGGTTACGAGCATCATGGCTTTACAACTGAGTATGATACA
TCGAGCCAAGTACGATGGATGGGCGTATTAAACCTTGAAGGTAAAACACCCGAAACATTGAAAAAGACATTTGATAGTCAACGTAAACG
TAATATTAATAAAGCGATAAACTATGGTGTTAAAGTCAGATTCCTTGAACGTGATGAGTTCAATCTTTTCCTAGATTTATATCGTGAAACT
GAAGAGCGTGCTGGATTTGTATCAAAAACAGATGATTATTTTTATAACTTTATTGACACATATGGAGATAAAGTATTAGTACCATTAGCAT
ATATTGACCTTGATGAATATGTGTTAAAGCTGCAACAGGAATTGAATGACAAAGAAAATCGTCGTGATCAAATGATGGCGAAAGAAAAC
AAATCAGATAAACAAATGAAGAAAATTGCAGAATTAGATAAGCAAATTGATCATGATCAGCATGAATTATTGAATGCAAGTGAATTGAG
CAAAACGGACGGCCCAATTCTAAACCTTGCTTCTGGTGTTTATTTTGCAAATGCATATGAAGTGAATTATTTCTCTGGTGGTTCATCAGAA
AAATATAATCAATTTATGGGACCATACATGATGCATTGGTTTATGATTAACTATTGCTTCGATAATGGTTATGATCGTTATAATTTCTATGG
TTTATCAGGTGATTTTACGGAAAACAGTGAAGATTATGGCGTATACCGCTTTAAACGTGGATTTAATGTACAAATCGAAGAATTAATAGG
GGATTTCTATAAACCAATTCATAAAGTGAAATATTGGTTGTTCACAACATTGGATAAACTACGTAAAAAATTAAAGAAATAG

Gene: Q99UA6 (putative membrane protein)

Contig: 06_NODE_54, position: 517721 to 518466, length: 746 nt, orientation: FORWARD

Perfect match to: (04-02981-CP001844-[1416624:1417370], allele observed in CC5+CC8)

Sequence:

ATGAATCGAATTGAAAAACATGCCAAAAACACTTTTATTATTCTGATGCTCATTATGTTATTTTGGATTTTTATGAGTTTTATTTTCCAGAAA
TTATTATTTCCACCTTCGAAAAATAATTTAACTACATATGAAGCATTAAAATATTATACACATTTAAAGGGATATTACGGTTTGGATCATAT
ATCAAAAGGAATAGCATACATTGCCTGTGTGTTAATTCCATTCAATTTCTTTTTTAGATTCAATGACATCAAAAAAGATAATAACTATAATA
ATATTATAAGTACGTTATTCCTATTATTATACTTTTTAGTTAATGGAATTTCGTTGATTATTCAAGGATTCACTGCAGAATTTACAATTAGTT
TAATTAGTGAGTCAAATATTCATAATAATCATGAATTTGCTGTGAACTTATTTAGATATGTCATACAAGAAGGTGGTATTTCTTTTAGTACA
TATCTAGTGTGTAACTTTTCTATAATAATGTGGTTATTCTTTTCGTGTTCTTTATTAAAAGAACGAAAACCAGTTGTCAGATGTTTACCATTA
ATTATATCTTGTTTAAAATTAATATTAATCCTACTTTTTTACTTTCAATACTTTTGGTCATATATCAAACTCAATCTGCACAAATCTTATTTATT
TTTATAGATTTTCTAAATTTTGTCGCGTTAATTTTAGTTTATTTATGTACTAATCCTAATAATAGAGGTATTGATAAAATAGCATGTGTTAAA
TAA

Gene: cof (Cof-like hydrolase)

Contig: 06_NODE_54, position: 518698 to 519415, length: 718 nt, orientation: FORWARD

Perfect match to: (MW2-BA000033-[1384999:1385716], allele observed in CC1)

Sequence:

ATGAGATTTGTCTTTGATATTGATGGTACGCTTTGTTTTGACGGCCGATTAATTGACCAGACTATTATTGATACATTGTTACAATTACAACA
TGATGGTCATGAACTTATATTTGCATCAGCACACATTAATTGGCGCAAATGGTGCTATGATTTCACAGCAATCAAAGATTTCTGTTATCAA
ACCAATTCATACAGATACATATCACCATATCTTAAAAATAATTCAAAAGTATGAGTTAGATTATATTATTGATGATGATTGGAATTATGCT
GCACAACTTGACGCTGAGAACGCGATTTTTGAGCGTTTAGATCCACATAAGCTGGCCAGTTGTATTGATGTTGCAAATATCGACACGCCA
ATCAAAGTTATTTTATTAAATATAGACCCGGCACAAATTACAACTATATTAGACGAGCTAAATAAATACCATCAAGAATTGGAAATGATTC
ACCATTCAAATGAGTATAACATTGATATAACAGCGCAAAATATTAATAAATATACTGCATTACAATATATATTTGATGCAGATGTTAAATA
TATAGCATTTGGTAATGACCACAATGATATTGTCATGTTACAACATGCTAGTAGTGGCTATATTATAGGACCATCAGAAGCATACACACAC
GCAATATTGAAACTTGATAAAATCAAACACATCAATAATAATGCACAAGCTATTTGCAAAGTCTTAAAATCATATAAATAA

Gene: Q5HG42 (putative protein)

Contig: 06_NODE_54, position: 519464 to 519937, length: 474 nt, orientation: REVERSE

Perfect match to: (MW2-BA000033-[1385765:1386238:r], highly conserved allele)

Sequence:

TTAATAAAATTCGTCCTCGAACATTTCTTCCTCTTCATCTAATCCAAATAATTCTGCCATTTCTCCATGTTCAATTAACATGTTTAAATATGCA
TCGCGGAGTTCTTCTTCACTCATATCATTAATCATTTCTTTAAGACTATCAATCCACATATTTCTGCGTAATTGATAGTCTTCTTCAACTTCGT
TTAACATCATTATATGTTTATTTGCTGCTTCTGGACTAGCTGTAAAGAGTAATGCAATCATATGTTTACATATCACTCGTCTTCCCATCAGCAT
GAGGACAATTACATTTGGATTTTCTAGGATGTTCCATATCAATATAACAACGATATACTTTGTTGCCACTGCCCTTTACTTCAGCCTCATGC
TGCGTTTCTGAAAATGATTTTAAGTTAATGACGCATTCACTTTGATAATAATTAAAGCCTCTTTCTATAGAACGAATACTTGCAATATCAAG
TAATCCCAT

Gene: nikF (oligopeptide ABC transport, ATP-binding protein)

Contig: 06_NODE_54, position: 520061 to 520762, length: 702 nt, orientation: REVERSE

Perfect match to: (MW2-BA000033-[1386362:1387063:r], allele observed in CC1+CC7)

Sequence:

CTAATATGAAAATGCTTGCACTAATTCTTTTGTATAAGGGTGTCTATCAACATTAAATAATTCCTCTATTGCAAAATCATCGACTATCATGC
CATCCTTAAGAACGATAATTCTATTAACTAAGCGTTGTAACACGGATAAATCATGAGAAATAACGATAAAATGATTTAAGTTCGTAATCGT
TTGCGCTTTTAATATATTTATTACATTTTGTTCAGCTATAACATCTAAATTTGAAGTTATCTCATCACATATTAAAACGCGAGGCTGTGCTAA
TAACGAACGCATGACATTAAATCTTTGTAATTGTCCGCCACTCACTTCGCTTGGTAATTTAGTCAATAATTGCGCGTTTAACTCAAAAGTA
GATAAATGTTGTAATAATAATTGATCCTGAGCAGTATTATCAGTTAGGCCTCTGTAATAATATAACGCTTCTTTTAATGAGGTCTCAATCGT
CCAATCAGGGTTAAAGCTAGTTAAAGGGTGTTGGAAAATCGGTAACACAGCATTGTCACTTAAGTAAATTTCTCCTTTAGCAGGTTTAAA
CAAGCCAAGAACCAATGAAGCGAGCGTACTTTTACCACAGCCACTTTCGCCTAAAATACCAACATTTTCTCCATCAGGTATAGTAATATTG
ATATCTTGTAGCACCATCTGCTTTTTATTATAACCAAAAGTCACATGTTTTAACTCAATCAT

Gene: nikD (oligopeptide ABC transport, ATP-binding protein)

Contig: 06_NODE_54, position: 520755 to 521528, length: 774 nt, orientation: REVERSE

Perfect match to: (MW2-BA000033-[1387056:1387829:r], highly conserved allele)

Sequence:

TCAATCATAGTAATCCCTCTTTAATTGTGTTCTATATTTAATTAGACGTTCAGTATACGGATGCAAATGCTCATACTTGAAATGATTAATAT
TACCTCGTTCAATGATTTGACCTTCTTTTAAAACATAAATGTACTGACAATATTTCAATACATGACTTAAGTTATGTGTGATAATAAATAAT
GTTTGACCATGTTCTAATACAATATGCTGTAATAAATCCATCACTTGATTACCGTTCAAAGCATCCAATGATGCAACTGGTTCGTCTGCAAT
GATTAATTTAGGCTCCAACATGAGAACGCTTGCTATGTATACGCGTTCAAGTTGGCCCCCAGAAAGTTGGAAACTATATTTATTTAATATA
TCTTTGCTTTGTAAATTAACCCACGACAAAGCCTTATCAACTTTGGACAAAGCCTCTTCTTTACTACCTTTATAATGCTTACGATAAATCGCA
GTTAACTGTTTACCTAATTTAGTATGGTCGTTAAAACTTTCTGCATAATTTTGAGAAATATAGCCAATTGTATGACCATAATATTGACTCAA
TCTACTAACATTTTCCCCATCAAATTGGTACGAATCATACGTGCAGCTTAAATCAAATGGTAAATATTCAAGTAAAGCTTTAGCAATCAAA
CTTTTTCCAGCGCCGCTCTCTCCAATCAAGGCATTAATCTGTTGACTAAAAATTTTCAAATCAATCCCTTTAATAAGAGATTTCTCACTAGTA
TTCTTTATTGTTAAATTTTGTATATCAATGAGACTCAT

Gene: nikC (oligopeptide ABC transport, transmembrane permease subunit)

Contig: 06_NODE_54, position: 521515 to 522345, length: 831 nt, orientation: REVERSE

Perfect match to: (N315-BA000018-[1385875:1386705:r], allele observed in CC5+CC49)

Sequence:

TCAATGAGACTCATCATATTCACCCCGTTGTTTCAGCAATCTATCTCTTAGTGCATCACCGGTTAAATTAAAAATTAAAATAGTTATAGCAA
TGACTGAAGCAGGTGCAATCAACATAATTGGATGAGACGAAATAAAATCACGACCTTGTTGCAACATAGCGCCCCACTCTGGTGTTGGC
GGTTGTGCACCTAACCCAATAAATGATAGTGAACTTATATATAGAATGATTTTACCGAAATCAACGACCATCAAAACGATAATAGCCGGT
ATAATTTTAGGTGTTAAATGACGTATTAATATTGTTCTTGTTGGTACATGAAATAATTGTGCCATTTTTATATAAGGCTTATTCATTTCGCTA
TTAACTATACTTCTAGTCAACCTTGTGTAATTCATCCATTTTATTAATGTAATTGAGATAACTAAATTCCATAAAGATGGTTGAAAAAAACT
TGCTAAAGCAATCATGATGATAAATTCTGGAATACTCAGACCAACATCAATAAACCTTAACACTAATCGTTCAATCCACCCTTTTTTATATC
CGGCAAATAGACCTAGTGTAACACCTATGACAACGATAGCTATTAATGTTAAAACAGTAACAAACAATGTTGAACGTGCACCGATAATAA
TTCGGGTAAATAAATCTCTCCCATAATCATCAGTTCCTAATAAATGCAACCAACTAATAGGTTCAAAAGTTTGTGATAAATTGACTTTGGTT
GCATTTTCACTACTGACAAAGAATTGCAGTACAATTACCACAAAAATAAATGCAACGAATACAAAAAATATCAGGTTATTCTTTGAAAATA
TTTTATGCAT

Gene: nikB (oligopeptide ABC transport, transmembrane permease subunit)

Contig: 06_NODE_54, position: 522338 to 523324, length: 987 nt, orientation: REVERSE

Perfect match to: (MW2-BA000033-[1388639:1389625:r], highly conserved allele)

Sequence:

TTATGCATGACGGTCACTACTTTCTGATATCAATGGTGTATTGGTTTTGATTTTTGGATTTCCTAATTGTAAACGCTGCTTCGGATCAAGTA
ATAACGTTAATAAATCAGCAATCGTATTGATAATAACAACGAAGAAGCCAATAAATAACACGCATCCTTGAATAACAGGATAATCTCGAG
ATTTAATACTATCCATTAATAGATAACCAATACCAGGTATATCAAATAAATTTTCAATCACTACAGTACCACCTATTAGACTGCCAAGTGAA
ATCCCTAGTAATGGGATAATCGGCAAAATTGTTGGTTTTAGTAAATCATGAATTAAAATATAACGTTCATTCATACCGCGTAATCTTGATG
CTTGTACGATATTACTTTGCAATAACATCAATAAATTAGAACGCACTAAACGAATGATGTATGCACACATACCTAAAGATAGCGTGATTAC
AGGTAATATAAACTGACTTAGTATAACGCTATCTATATTCATTAAATTTGTGACAATAAATAATAAAATAATACCGATAAAGAACGCTGGT
AAACTAATCGATAGTGTTGAGATCACTCTAATCACTTTATCCGTCCACTTATGAAATCGTTTGGCTGCTATAATGCCGAGCGGTATAGATA
TGCATAACGACACTACTAATGTTGAAAATGATATGAGTAATGTTATGGGTGCATAGTTGAATAATATCTGTGTTACCGGTTCTTTTGATTC
AAAACTTTTTCCTAAATTAAAATGTAATAAATGATTCATCCAATGCCACCACTGTACCAATAAAGAATCATTTAATCCCAATTTATCTTTGG
TTGCATTTATTTGTTCCGTCGACACTTGTGCTACATCAAGATGTAATATTTTATCAACAGGATTGCCTGGTGATAATTTCATTAAAATGAAT
GTAAGTGTAGAAATAACAAATAAAACAACTATCATTTGCATCAGTCTATACAACATAGACTTTATTATGAACAT

Gene: Q5HG37 (putative protein)

Contig: 06_NODE_54, position: 523628 to 523972, length: 345 nt, orientation: REVERSE

Perfect match to: (N315-BA000018-[1387988:1388332:r], highly conserved allele)

Sequence:

TTATTTTTTATCTTTACGGCGAAGTTCAGCGCCCTCATAGCCGTATTTTTCAATTTGCTTTTCTAATTTACGCGCTTTTCTTTCTTTACGCCAA
TTTCTAGTAAAATACCATAATAGAAAACTAATTAATAAACTCATAATCGCTAAAAATGCAGCGTATCCTAATAATGGTTGATATTTTATATC
TTGAAAATTTGGAATAAAAAATGCAAGCACACCTAATATAACAAATGTAATTACTGCAGATACAAACCATTTATTTAAAACTAAGCAACA
GAATATTGTTAATAAAATCATTATTAATGTTGTGATCCATAAATAATTAGGCATATCGAATAATGTCAT

Gene: pepF2 (oligoendopeptidase F, M3 family)

Contig: 06_NODE_54, position: 524177 to 525991, length: 1815 nt, orientation: FORWARD

Perfect match to: (Strain_21193-AFEG01000026-[681:2495], allele observed in CC25+CC239+CC361+CC772)

Sequence:

ATGTCTCAAGGTTTACCTTTAAGAGAAGATGTTCCTGTTTCAGAAACATGGGATTTAGTAGACTTATTTAAAGATGATCAACAATATTATG
AAAGTATTGACGCTCTAGTACAACAAGCAAATCAATTTCATCATACATATGCAACAACATTAAATTCAATCGAACAAATTAATACTGCTTT
AGCTGAATTAGAAAATATTTTAATTGCCTTAGATCGCTTAAGTAATTATGCAGAACTACGTTTAAGTGTAGATACTAGTAATATCGAGGCA
CAAGTATTGAGCGCTAAATTATCTACTACATACGGTAAAATTGTTAGCCAATTATCATTTGTAGAGTCAGAAATACTTGAATTACCAGAAG
AAATACTTCAACAATTAGAAGAATCATGTCCATATCAACACTATATTAAACAGTTAATAAAACAAAAGCCATTCCAATTATCTGCGTCGGT
AGAACAAGTATTAGCAACTTTATCACCTACGCTAAACAGTCCTTACGATTTATACGGCACGACAAAAATGCTAGATATTACATTCGATTCA
TTTGAACATGATGGTACAACGTACCCTGTCGACTATGCTACGTTTGAAAATGATTATGAAGATAATAAAGAGCCTGAGTTTAGACGTAAA
AGTTTCAAATCGTTTAGCGATGGGATTCGAAAATATCAGCATACTACCGCGGCTACATATAATATGCAAGTACAACAAGAAAAAATTGAA
GCTGATTTACGTGGATTTGAATCAGTCATCGATTATTTATTACATAGTCAAGAAGTAACGCGTGATATGTTTGACCGTCAAATCGATATGA
TTATGCGTGACTTGGCACCAGTTATGCAGAAATATGCTAAACTTTTACAACGTATTCACGGATTAGATAACATGCGTTTTGAAGACTTGAA
GATTTCTGTAGACCCTGATTATGAACCAGAGATTTCAATTGAAGACTCAAAAAATTATATTTTCGGTGCGTTAAGTGTTTTAGGTGATGAC
TATACAAACATGTTACGTGAAGCATACGATCAGCGATGGATTGATTTTGCACAAAATAAAGGTAAAGATACAGGCGCATTTTGTGCAAGT
CCATACTTTACACATTCATATGTGTTTATTTCTTGGACTGGTAAAATGGCTGAAGCATTTGTCTTAGCACATGAATTAGGTCATGCAGGTC
ATTTTACATTAGCTCAAAAACATCAACCATATCTTGAATCAGAAGCATCAATGTACTTTGTTGAAGCCCCTTCTACAATGAATGAAATGTTG
ATGGCCAATTATTTATTTAACACAAGTGATAATCCAAGATTTAAGCGTTGGGTTATTGGCTCAATTTTATCTAGAACATATTATCATAATAT
GGTTACCCATTTATTAGAAGCTGCTTATCAACGTGAAGTGTATCACAAAGTAGATCAAGGTGAATCTTTAAATGCGCCGACATTAAATGA
AATAATGCTAAATGTTTATAAACAATTTTTTGGAGATGCAGTAGACATGACTGAGGGTGCTGAATTAACATGGATGCGTCAACCTCATTA
CTATATGGGATTATATTCGTATACGTATTCTGCTGGCTTAACAATCGGAACTGTCGTTTCTCAAAAGATTAAAAATGAAGGCCAACCAGCT
GTTGATGCTTGGTTAGAAACATTGAAAAAAGGTGGTAGTGTATCACCTGTCGAACTTGCAAACATTGCAGGTGTAGACATTACTACAGAA
CAGCCACTTAAATCTACAATTCAATATATTTCTGATTTAGTCGATGAAGTTGAAAAATTAACAGATGAAATTGAGCAAGCAAATAACTAA

Gene: phoU (phosphate ABC transporter, regulatory protein)

Contig: 06_NODE_54, position: 526130 to 526771, length: 642 nt, orientation: REVERSE

Perfect match to: (N315-BA000018-[1390491:1391132:r], highly conserved allele)

Sequence:

TTATTGTTCGTAATGTGTACCTGTTAAATAAAAATAAACACTTTCAGCGATGTTAATAATATGATCACCAATACGTTCTAAATGTCTTGCTG
CTAAATGAGCTTGTGCAGCGACAAATGGATCGTTATCAATAAGATACGTTGCGTTAATAATATGACTATATAAGTCATCGATATCTTCATC
ACGCTCAATTATTTCTCTTATTAATACGGTATCTTTCTTTTTAAATGCTTGATCTAAGTCCTTTAACATTAACATAGCTAATTTACCCATTGTC
TTTAAACGGGTTAACACATAATCATCTGTAATCTTTGTACGCAATCGAATATTGGCAATACTCGATGCATTATCTCCTATTCTTTCTAAATC
GGAGGCGATTTTTAATGAAGAAATCATCATACGCAAATCACTCGCAATGGGCTGTTGCTTTGTAATTAACATGATAACTCGCTCATTAATA
TCATAATTTAATTGATTGATATGTTTATCGTTTTTAACTGTTTGTCGTGCAAAGCCTCTATCGTCAATACTTAATGATTTTATACCATTTTCA
ATACTCACATAGACATTTGCACCTAACCGACGTAATTCCTTTATTAAATCATCAAGTTGCTCCTGATATCGCTGTCTAATTATTGCCAT

Gene: pstB (phosphate ABC transporter, ATP-binding protein)

Contig: 06_NODE_54, position: 526778 to 527629, length: 852 nt, orientation: REVERSE

Perfect match to: (N315-BA000018-[1391139:1391990:r], highly conserved allele)

Sequence:

TCAACCAAACCTTCCTGAAATATAATCTTCTGTTTTCTTGTTTGATGGGTTAGAGAAAATTTTATCAGTATCATCATATTCATTGACATAACC
ATTTAAGAAAAATGCAGTTTTATCTGATACACGAGCTGCTTGTTGCATATTATGTGTAACCATAATAATTGTATACTTTTCTTTTAGTTCTTG
AACCAACTCTTCTACTCTTAATGTTGAGATTGGATCTAATGCTGATGTCGGTTCATCCATTAAAATGACTTCAGGTTCAATTGCTAAACAAC
GCGCGATACAAACACGTTGTTGTTGCCCACCGGATAAACTATATGCATTTGTGTGCAACCTATCCTTTAATTCATCCCAAATTGCAGCGCC
ACGTAATGATTTCTCAACGATTTCATCAAGAACTTTTTTATTTTTAATACCGTGAATCTTTGGACCGTAAGTAATATTATCGTATATTGATTT
TGGAAATGGATTAGGTTGTTGAAAGACCATGCCCACATTTGTACGTAATTGTTCTTTAGAATATTTTTGATCAAAAATGTCTTGATCTCGA
TATAATATTTTACCAGCTGTTTTCACAGAAGGTACTAACTCAACCATTCGATTCAAAGTTTTAATATATGTTGATTTGCCACAACCAGATGG
ACCTATAATGGCAGTAATTTGGTTTTCATAAATATCTAAATTAATATTTTGTAATGCATGATTTTCGCCATACCATAAGTCTAAATTTTGTGT
TGAATATATAACAGAATGTGAGTTTGTATCATCTGGTGTTTTATGATGACTTTGTGAGACATCAAACGTATGACTTTGAGATATTTGTTTA
GTTTGTGCAAGTGTTTGCGCCAT

Gene: pstA (phosphate ABC transporter, transmembrane permease subunit)

Contig: 06_NODE_54, position: 527676 to 528593, length: 918 nt, orientation: REVERSE

Perfect match to: (08-02119-CP015645-[779401:780318], allele observed in ST582+CC20+CC239)

Sequence:

TTAGAATTTTTTACTAAATTTGTTACGTAAAATAATCGCAACGCCATTCATTAAGATTAAGATAACTAGTAAAACGATAATGCCTGCCGAT
GCAACATTCTGGAATTCTTCTTGAGGCATTTTCGCCCAAGTAAATATTTGGATTGGTAATGCTGAAAATTGATCCAATATACTTCTAGGTG
TTGCCAATAATATAGTCGGTATACCGATTAGCACAAGTGGCGCTGTTTCTCCCAGTGCTCTTGAAAGAGACAAAATGAATCCAGTTAAAA
TACCAGGTAACGCTGCTGGTAAGACAACACGTCTTATCGTTTGCCATTTATTAGCACCTAAACCATAAGAAGCTTCGCGTACTGAGTTAG
GTACAGCTCTAATTGCTTCCTGACTTGAAACAATAATAATTGGTAATATCAGTAAGGTCATTGTTAGCGCTGCTGCCAATATACTGTTACC
CATTTTCAAGGCTTCAATCCCCGCACCACCAACGAACAAAGTGTAACCTAATATCCCAAATACAACTGATGGTACACCAGCTAAATTGGAA
ATACTGATTTTAACAAACTGAGTAAATTTGTTGTTTTTCGCATATTCTTCTAAGTATATAGCTGTACCTATTCCTAGGATGATTGATAATGG
AATGATACTTAACATTAACCAAAGTGAACCGATTAACGCGCCTTTAACGCCAGCCATAGATGGTGTTGAAGAAGAAAAATTAGTGAAAA
ACTGTAAATTTAAATGACTTACCCCTTTAATCAATGTTTGAGTTAACAACGCAATAAGTACGACAAGTCCTAATAATGTACATGCTAAAAA
TATGAGTTTGAACACTTTATTTTTAACCGTTCTGGATGATAAATGTTTTTGGACAAGTTGTTGATCGACGAGTGATTGTCTATTATTATCTG
TCGTTTCCAT

Gene: pstC (phosphate ABC transporter, transmembrane permease subunit)

Contig: 06_NODE_54, position: 528595 to 529521, length: 927 nt, orientation: REVERSE

Perfect match to: (11819-97-CP003194-[1423916:1424842:r], highly conserved allele)

Sequence:

TTAATACTCCTCCCTAAAACGCTTAGAAATCCACTGAGAAAGTAAATTCATGATTAAGGTAAAGATAAATAGTGTGAACCCTACAGCATA
AATACTGTAATAAATATTTGATCCAAATGTTGCATCACCTGTCGCTATCTCAACAATATATCCAGTCATTGTTTGAATCGAACTTGTTAAAC
TTAATGAAGCTGTTGGCGAACTACCTGCCGCTAATGATACAATCATCGTTTCTCCAATTGCTCTTGAAATCGCGAGAACGATTGAAGCTAC
AATACCTGATGTTGCTGCGGGAAGTACGACTTTAGTTGCTACTTCTAATTTAGTTGCTCCAAGTCCATAGGCACCTTCTCGAATTTTATTTG
GTACAGATGCCATTGCATCCTCACTCAAACTTGTGATGAGAGGGACAATCATAATACCGACAACTAAGCCGGGACTTATAGCATTAAACT
CTCCAAGACCTGGTATGAAAGATCTTAATACTGGTGTAACAAAGGTTAATGCAAAGAAACCAAACACAATTGTTGGTATTCCTGCTAAAA
TTTCTAATATCGGTTTAATTATGCGTCGTGCACGGTCACTTGCATATTCACTTAAATAAATTGCTGCACCAAGCCCGACTGGAACTGCAAA
TATAGTCGCAATAACTGTGATTTTTAAAGTCCCTATTATCAATGCCCAGATACCAAACTTAGGGTCTGAACCGGTAGGATTCCAAGTAGTA
GAAAATAGAAATTCAGTTATTGGAATTCTGGTGAAAAAAGTGATGGTTTCTAAAAGCAATGTGATTAATATACCTAGTGTTGTTAAAATG
GAAATCGCTGAAATTGCGGCTAAAATAACTGGTATAATTTTGTCATTATGCTTCCCTTTTTTATTATTATTTTTTTCGATTAAAGCTTTAACA
TTAGTAGATGAAGTCAT

Gene: pstS (phosphate ABC transporter, substrate-binding protein)

Contig: 06_NODE_54, position: 529712 to 530695, length: 984 nt, orientation: REVERSE

Perfect match to: (08-02119-CP015645-[777299:778282], allele observed in ST582+CC1+CC5+CC22+CC239)

Sequence:

TTATTTTTTGTCTTCAGACTTTTTATCATCAGATTTCTTGTCGTCTGATTTTTGATTTTTATCAATAAATGCTTTTAAATCATCTAATTGTGATT
TGTATGTTTTCTCTGGTGCTGCTACATATCCAGCTTCTTCAGCTGCTTTACCTTTATCTTCTAAGACGAATTTGATAAATTCTGACATTACTT
TATTATCTTTCAATGCTTTTTCATTTACATAAATGAATAATGGTCTACTTAATGCATAAGAGTTATCTTGAATTGTTTTTTTCGTAGGCTCTG
TTGCTTTACCATTTTCATCTTTGATTTTAACTTCTTTTAATTTATCTTTATTTTGTACGTAGAAGTTATATCCAAAGTATCCGATTCCCTCTTTG
TTTTTCGTTACAGAAGAAACGATAGCATTTGTATCAGCATTTTTTTCTGCTTTAATATCTTCTTTATTCATTACTTCATTTTCAAAGAAGTCAT
AAGTACCATGACTTGAGTTTGGTGATACAGCATTTATTTTTTTATCTGGCCATTTACTATTAACATCTTTCCATGTTTTAGCTTTTCCAGAAT
AAATTGCTTTTAATTGCTGTTTGTCTAATTCATCTACAAAATCATTTTCTTTATTTACAGCAACCGTTACACCATCTTGCGCAATTTTAAATTC
TTTGTATTTGATATTCTTATCTTGTAATTTTTGCTTCTCTTCATCTTTAATTGGTCTAGAAGCATCAGCGAAGTCGATATCTCCTGCAATGAA
TTTTTGGAAACCAGCACCTGTACCAGCTTGTCCTGCTGAGATTTTAGCATCCGAGTGATCTTGAGCCCATTTTTCATTTAATTTCTCCACAA
TTGGTGCTACTGTTGATGAGCCATCACCTTTAGCTTCCCCTTTTAAATCACTATTACCACTGCCACCATTACCGCCACCACAAGCACCTAAT
AATAGTGTTGCACCTAAAGCTGTAGTACCAACAAATTGCCATTTTTTCAT

Gene: cvfB (conserved virulence factor B)

Contig: 06_NODE_54, position: 531671 to 532573, length: 903 nt, orientation: REVERSE

Perfect match to: (CN1-CP003979-[1366128:1367030:r], allele observed in CC72+CC1)

Sequence:

TTATTCTTTTGAGTCCATTCGACTCCAACCTTTTTTAGTTAAAGTGATTTTACCTGTTTCTATATTAATAATCTTCTGTTTATATAAGTGACCG
ATTGCACGTTTGAATGAACCTTTACTCATATTGAATACTTCTTTAATCGCTTCAGGGCTTGATTTGTCCCAGAATGGTAATTCACCATCATA
TTCAACTAGTAAATCAAAGATGACTTGGCCGTCATCGTCTAAACGTTCATGTGCAAGTGGTAAAAATGAACCATTTAACTCACCTTTATCA
TTATGCCCGATAATTCTAACTTGAACAGATTCACCTAATCTTGGTTCAGCTTTACGTTCTGATTCATGTACGAAAATTTTGTAACCTGATTC
GCTTAATAAAAAGCTACCAATTCGTAATACGCGGTAAGGTTTGGCTTCAATGACTTCGTTTTTTAAATTATCGTCGTGTACAGGTGTAAAC
ATATTTTCTACAACAGATTCACTCGCTAATCTTCCATACATATGATTCTCACGGTCAATTCGTAATGTGACTAGCAAATGATCACCAGGTTG
TGGCCATAGTGATTTCACTTTTGGTAAATCTTCCCATGGTACTAACACTTCACGGGGTAATCCAACATCTATACGTGCCCCATCGCGATCC
GTTTTAAGTACTTTAGCAAAGTCATATTTATCTTTCGTAATATCAGGCATATTTTGAGTTGCAAATAATTCACCTGAACGGTTTGGATAAAT
GAAGAAACTATATTCTTCACCTACTTCTAATTCATCATCATCGTTCATTTCTGATTGGTTTAACTTTACGTTTTCACCGTTTGGTCCTTTTAAA
AGGTAAGTTGAGCCTTGTAACCCTACTACTTCAAGGAATTCTATAGAACCTACTATATCTTTGTCTAATGCCAT

Gene: ykpA (ABC transporter, ATP-binding protein)

Contig: 06_NODE_54, position: 532720 to 534321, length: 1602 nt, orientation: FORWARD

Perfect match to: (08-02119-CP015645-[773677:775278:r], allele observed in ST582+CC1+CC5+CC15)

Sequence:

ATGTTACAAGTAACTGATGTGAGTTTACGTTTTGGAGATCGTAAACTATTTGAAGATGTAAATATTAAATTTACAGAAGGTAATTGTTATG
GATTAATTGGTGCGAATGGTGCAGGTAAATCAACATTCTTAAAAATATTATCTGGTGAATTAGATTCTCAAACAGGACATGTTTCATTAG
GTAAAAATGAACGTCTAGCTGTTTTAAAACAGGACCACTATGCTTATGAAGATGAACGCGTGCTTGATGTTGTAATTAAAGGTCACGAAC
GTCTTTATGAGGTTATGAAAGAAAAAGATGAAATCTATATGAAGCCAGATTTCAGTGATGAAGATGGTATCCGTGCTGCTGAACTTGAA
GGTGAATTTGCAGAAATGAATGGTTGGAATGCTGAAGCTGATGCTGCTAACCTTTTATCTGGTTTAGGTATCGATCCAACTTTACACGAT
AAAAAAATGGCTGAATTAGAAAACAACCAAAAAATTAAAGTATTATTAGCGCAAAGTTTATTCGGTGAACCAGACGTACTATTACTGGAT
GAGCCTACTAACGGTCTCGATATTCCAGCAATCAGTTGGTTAGAAGATTTCTTAATTAACTTTGATAATACTGTTATCGTAGTATCGCATG
ACCGTCATTTCTTAAATAACGTATGTACACATATCGCTGATTTAGACTTTGGTAAAATTAAAGTTTATGTTGGTAACTATGATTTTTGGTAT
CAATCTAGTCAGTTAGCTCAAAAGATGGCTCAAGAACAAAACAAGAAAAAAGAAGAAAAAATGAAAGAGTTACAGGACTTTATTGCTCG
TTTCTCAGCTAACGCTTCTAAATCTAAACAAGCAACAAGTCGTAAAAAACAACTTGAGAAAATTGAATTAGATGATATTCAACCATCATCA
AGAAGATATCCTTTCGTTAAATTCACGCCTGAGCGTGAGATTGGTAATGACTTATTAATCGTTCAAAATCTTTCTAAAACGATTGACGGTG
AAAAAGTATTAGATAACATTTCATTCACAATGAATCCAAATGATAAAGCAATTTTAATTGGAGATAGTGAAATCGCGAAAACAACATTAC
TTAAAATATTAGCTGGTGAAATGGAACCAGACGAAGGTTCTTATAAATGGGGTGTAACAACATCATTAAGTTACTTCCCTAAAGATAACT
CAGAATTCTTTGAGGGCGTTAATATGAACCTTGTAGATTGGTTAAGACAATATGCACCGGAAGATGAACAAACAGAAACATTTTTACGTG
GTTTCTTAGGTCGTATGTTATTTAGTGGTGAAGAAGTTAAGAAAAAAGCTAGTGTGCTTTCAGGTGGAGAAAAAGTACGTTGTATGCTAA
GTAAAATGATGTTATCAAGTGCGAATGTACTTTTACTTGACGAACCTACTAACCACTTAGACTTAGAAAGTATTACTGCTGTCAATGATGG
TCTTAAATCATTTAAAGGTTCTATCATCTTTACTTCTTATGACTTCGAATTTATCAACACGATTGCAAACCGTGTTATCGATTTAAATAAACA
AGGCGGCGTTTCAAAAGAAATTCCATATGAAGAATACTTGCAAGAAATCGGCGTTTTAAAATAA

Gene: RF00168 (lysine riboswitch)

Contig: 06_NODE_54, position: 534931 to 535106, length: 176 nt

Sequence:

ATATTTTGATGAGGCGCATCAATCATGAGTAAAGTTTAGATTACTGTCTGCTAACAGCTAAATTTGAAAGGGTGCGATGCCGAAGCGATT
ATAATAGCAGTTATAATTTGTTGGACTTTTTGGTTAAGAGCTGAGAGTTTGTCATTATTTAAAAATAATGGAGTGCATCACTTGTA

Gene: lysC (aspartate kinase)

Contig: 06_NODE_54, position: 535188 to 536393, length: 1206 nt, orientation: FORWARD

Perfect match to: (TCH959-AASB02000236-[2100:3305], allele observed in CC7+CC1)

Sequence:

ATGGTAACAAGAAGTGTGTTGAAATTTGGCGGATCATCCGTCAGTGATTTTACAAAAATAAAAAGGATCGCTGAAATGTTAAAGGAGCG
GGTCAATCAAGATGAACAATTAATTGTCGTTGTAAGTGCTATGGGTAACACAACAGATCAATTAATGACGAATGTATCGACCTTGACTAA
AGCACCAAAACAACAAGAACTGGCATTATTATTGACAACCGGAGAGCAACAAACTGTATCTTATTTATCAATGGTATTAAATGATATCGG
TGTGAATGCCAAAGCAATGACTGGCTATCAAGCGGGTATTAAAACCATTGGCCATCATTTAAAAAGTAAAATTGCTCAAATTAATCCTCA
AACATTTGAACAAGCCTTTCAAGAAAACGATATTTTAGTAGTTGCTGGATTTCAAGGCATCAATGAACATCAGGAATTAACAACTTTAGG
CAGAGGCGGTTCTGATACGACCGCTGTGGCACTTGCTGTTAGTAATCAAATACCTTGTGAAATTTATACCGACGTTGATGGTGTGTATGC
CACTGACCCAAGACTTTTACCAAAAGCTAAACGACTAGACATCGTCTCATATGAAGAAATGATGGAAATGAGCGCTTTAGGTGCTGGTGT
ACTTGAAACAAGAAGTGTTGAATTAGCTAAAAACTATAATATCCCTTTATATTTAGGAAAAACTTTATCGAACGTGAAAGGAACATGGAT
TATGTCAAATGAAGAAATATTAGAGAAAAAAGCAGTTGCTGGTGTGGCTTTGGATAAACATATGATGCATGTAACAATTAGTTATCCCCT
ACCTGACAATCAGCTACTTACCCAACTATTTACGGAACTTGAAGAAGGTGCTGTAAATGTTGATATGATTTCACAAATCGTCAACTTGGAT
GGACTACAACTATCCTTCACGATTAAAGATAGTGATTTTCATCAAATTTCTATGATTCTTGAAACATTAAAGAATCAATATGAAGCATTAG
CTTATAAAATCAATGAGCATTATGTCAAAATTTCATTAATTGGCTCAGGCATGCGTGATATGTCAGGTGTGGCATCAAAAGCATTTTTGAC
ATTAATTGAAAATAATATACCTTTCTACCAAACAACAACTTCTGAAATAAGTATTTCATACGTCATTGATGATTTTAATGGGCAACAAGCG
GTGGAAAAACTATATGACGCATTTAACATTTAA

Gene: asd (aspartate semialdehyde dehydrogenase)

Contig: 06_NODE_54, position: 536457 to 537446, length: 990 nt, orientation: FORWARD

Perfect match to: (MW2-BA000033-[1402758:1403747], allele observed in CC1+CC7+CC80+CC96)

Sequence:

ATGACAAAATTAGCAGTTGTGGGTTCAACAGGATTAGTAGGTACAAAAATGTTGGAGACATTAAATCGTAAAAATATTCCTTTCGATGAA
TTAGTATTATTTTCATCAGCACGTTCTGCAGGGAAAGAAGTTGAATTTCAAGGAAAAACATATACAGTTCAAGAATTAACTGATGCTCGT
GCAAGTGAACATTTCGATTATGTATTAATGAGTGCTGGTGGCGGTACAAGCGAACACTTTGCCCCACTCTTTGAAAAAGCTGGTGCAATC
GTTATAGACAATTCAAGTCAATGGCGTATGGCAGAAGATATTGATTTAATCGTTCCGGAAGTCAATGAACCTACTTTTACAAGAGGTATC
ATTGCCAATCCAAACTGCTCTACGATTCAGTCTGTTGTGCCATTAAAAGTATTGCAAGATGCTTATGGTTTAAAACGAGTGGCATATACAA
CATATCAAGCTGTATCAGGTTCAGGGATGAAAGGTAAGAAAGATTTAGCTGAAGGTGTAAATGGTAAAGCACCAGAAGCATATCCACAT
CCAATTTATAATAATGTGTTACCGCATATTGATGTGTTTTTAGAAAACGGATATACAAAAGAAGAACAAAAAATGATTGATGAGACGAGA
AAAATTTTAAATGCGCCAGACTTAAAAGTAACAGCAACATGTGCACGTGTGCCTGTTCAAGATAGTCATAGTGTTGAAATTGATGTAACG
CTTGAAAAAGAAGCGACTGCAGAAGATATTAAAGCATTATTTGATCAAGATGACCGCGTTGTTTTAGTAGACAATCCAGAGAACAATGA
ATATCCAATGGCAATCAATTCTACTAATAAAGATGAAGTGTTTGTTGGCCGTATACGTAGAGATGATTCATTAGAAAATACTTTCCATGTA
TGGTGTACATCAGACAATTTATTAAAAGGTGCTGCATTAAATGCTGTACAAGTATTGGAACAAGTTATGCGTTTAAAAGGAGCGAATTAA

Gene: dapB (dihydrodipicolinate reductase)

Contig: 06_NODE_54, position: 538332 to 539054, length: 723 nt, orientation: FORWARD

Perfect match to: (MW2-BA000033-[1404633:1405355], allele observed in CC1+CC15+CC72+CC361+CC772)

Sequence:

GTGAAAATATTACTAATTGGCTATGGTGCAATGAATCAACGCGTTGCTAGATTAGCAGAAGAAAAAGGACATGAAATCGTTGGGGTCAT
TGAAAATACACCGAAAGCAACAACGCCATATCAACAATATCAACATATTGCAGATGTTAAAGATGCCGATGTTGCAATAGATTTTTCAAA
TCCAAATCTGCTTTTCCCTTTATTAGATGAAGAGTTTCATTTGCCATTAGTTGTGGCAACAACTGGCGAGAAAGAAAAACTACTTAATAAG
TTAGATGAATTGAGTCAAAATATACCTGTATTTTTCAGCGCGAACATGAGTTATGGCGTTCATGCACTGACTAAAATTTTAGCAGCTGCTG
TTCCCCTACTTGATGAATTCGACATCGAATTAACTGAGGCACATCATAATAAAAAAGTAGATGCACCAAGTGGTACGTTAGAAAAATTGT
ATGATGTGATCGTATCTTTGAAAGAAAATGTAACACCTGTGTATGATAGACATGAATTAAATGAAAAACGCCAGCCACAAGATATTGGTA
TACATTCTATTCGTGGAGGTACGATTGTCGGTGAACATGAAGTTCTATTTGCTGGCACTGATGAAACGATTCAAATCACGCATCGTGCAC
AATCAAAAGATATTTTTGCGAATGGTGCAATACAAGCAGCAGAACGCTTAGTTAATAAACCAAACGGCTTTTATACGTTTGACAACTTATA
A

Gene: dapD (2,3,4,5-tetrahydropyridine-2,6-dicarboxylate N-acetyltransferase)

Contig: 06_NODE_54, position: 539081 to 539800, length: 720 nt, orientation: FORWARD

Perfect match to: (MW2-BA000033-[1405382:1406101], highly conserved allele)

Sequence:

ATGGTACAACATTTAACAGCTGAAGAAATTATTCAATATATAAGTGATGCTAAAAAGTCTACACCAATAAAAGTATATTTAAATGGTAATT
TTGAAGGCATCACATATCCAGAAAGTTTTAAAGTATTTGGTTCAGAACAATCTAAAGTAATCTTTTGTGAAGCGGATGATTGGAAACCTTT
TTACGAAGCATATGGTAGTCAATTCGAAGATATAGAAATTGAAATGGATCGTCGCAATTCTGCCATTCCATTAAAAGACTTAACAAATAC
GAATGCACGAATTGAACCAGGTGCGTTTATTAGAGAACAAGCCATTATTGAAGATGGTGCTGTCGTTATGATGGGCGCAACAATTAATA
TTGGCGCAGTCGTTGGCGAAGGTACAATGATTGATATGAATGCTACTCTCGGTGGTCGTGCTACAACTGGTAAAAATGTACATGTAGGG
GCTGGCGCAGTATTAGCAGGTGTGATTGAACCCCCTAGTGCATCACCGGTTATAATCGAGGATGATGTATTAATCGGTGCAAATGCAGTT
ATTTTAGAAGGTGTACGTGTTGGTAAAGGTGCTATTGTTGCAGCTGGCGCGATTGTGACACAAGATGTACCAGCTGGTGCAGTTGTTGCT
GGTACACCTGCAAAAGTGATTAAGCAAGCTTCTGAAGTACAAGATACTAAAAAAGAGATTGTAGCAGCATTAAGAAAACTGAATGACTA
G

Gene: hipO (hippurate hydrolase)

Contig: 06_NODE_54, position: 539943 to 541094, length: 1152 nt, orientation: FORWARD

Perfect match to: (MW2-BA000033-[1406244:1407395], allele observed in CC1)

Sequence:

ATGAATGAATTAGAATTTGTTACGAAACATCGCCGTCATTTACATCAACATCCTGAATTAAGCTTACATGAATTTGAAACAACTGCTTATAT
TAAAGCGTTTTTAGATAGTTTAAATATTAAATACGATTGCCCATTGGAAACTGGCGTCATTGCATACTTAGAAGGTAATGGCTCACATACG
ATAGCGTATAGAGCTGATATTGATGCCTTACCTATTTTAGAGGAAAATGATGTGCCTTATCGCAGTCAATCTGATCATGTGATGCATGCTT
GTGGACATGATGGTCATACAACTGCATTAATGCTTTTTGTACAACGTTGCAAAGACATGCAAGACGCAGGTCAATTACCGCAAAATGTCG

TTTTCATTTTCCAACCTGCAGAAGAAACTGGTGGCGGTGCAAATCGATTAATAAAAGCCGGTGCCTTTGATAAGTATCCAATTGAAGCGG
TATTTGGTATTCATGTTAACCCATTTGCTGATGAAGGCATTGCAGTGATAAGAGATGAAGAAATTACGGCCAGCGCAACAGAGTATCGCT
TTTTCTTAACAGGCCTGTCAAGTCATGTTGCTGATAAAGAACAAGGTCATTCTTGTGGTGAAGCATTACAACATGTATTAACTCAAATATC
ACAAATTCAACAATTTCACCTTAACGGTTTGAAACGAAATATTGTTCATATTGGTCATTTTAAAGCTGGTGAAGCGATTAACACTGTACCA
AGTAATGGTTATTTAGAAGGTACAATTCGTACATATGATATTGATGATTTAACAATCGTTAAAAATCAAATGCACAAGATAGCAGAAAGT
GTCAAGCTTCTGTTTAATGTAGATTGTGAAGTTAAATTTGCAGAAGGTTATCCCCCTACAATCAATAGTCCGAAATTACGTACTCAAATAG
AGGACGCCTTAATAAAAGCTGATTTAAATGTCTATGACAAACCAACGCCATTCTTATTTGGGGAAGATTTTAGTTTTTATGGTCAACAACT
AGCTCCAGCTTACTTTGTTTTTATAGGAACACGAAATGAAGATAAAGGTTTTGTAACTGGTTTGCACACATCACATTTAAATTTTGATGAA
AAAGTGTTAATAAACGTGGTTAATTTTTACGAAAATTTATTAAATAATTACAAAGAGGTGTAA

Gene: alr2 (alanine racemase, locus 2)

Contig: 06_NODE_54, position: 541099 to 542184, length: 1086 nt, orientation: FORWARD

Perfect match to: (MW2-BA000033-[1407400:1408485], allele observed in CC1)

Sequence:

TTGACAGCAACATGGTCTGTAAATAAGAAAATATTTTTACAAAATGCAATCACAGTCAAAAACAATCAGCCATTAATGGCAGTTGTTAAA
AATAATGCATATCACTATGACCTAGAATTTGCTGTAACTCAGTTTATCCATGCAGGTATAGATACATTTAGCACAACATCACTACGAGAAG
CAATTCAAATTAGACAACTTGTTCCAGATGCAACAATCTTTTTAATGAATGCAGTTTACGAGTTTGATTTAGTCCGTGAACATCAAATACA
CATGACTTTGCCGTCGTTGACATATTACTATAACCATAAAAATGATTTAGCAGGTATTCATGTTCACTTAGAATTTGAAAATTTATTACATC
GGTCTGGATTTAAAGATTTAAACGAAATTAAAGAAGTATTGAAAGATCACCATCATAATCAAAATGCAAAAATGATTATTAGTGGTTTAT
GGACCCATTTTGGATATGCTGATGAATTCGATGTGTCAGATTATAATGTTGAACGTTCACAATGGATGGAAATTGTTGAAGCACTTTTATC
TGAAGGTTATCAGTTCGACCTAATCCATGCTCAAAATAGTGCGAGTTTTTATCGGGAAGGACAAATATTACTACCCCACCATACACATGCA
CGTGTAGGTATTGCGTTATACGGTTCAAGACCATATAGTTCACTGAATCAACATGATATAGTTCAGTCATTAACTGTAAAAGCACATGTTA
TTCAAGTGCGCGAAGTACAAGCTGGTGATTATTGCGGTTATAGCTTTGCCTTTGAAGTGACTAAAAACAATACAAAATTAGCTGTAGTTG
ATATCGGTTATGGCGATGGAATTTTAAGAACACGTGCTAAACATGAAGCACTTATCAATGGTAAACGCTACCCGATACGTGCATTAATGA
TGAGCCATATGTTTGTTGAAGTAGATGGCAATGTACATGCACAAGATGAAGTTATTCTTTATAATAATGATATCCGCATCGATGAATATAC
CTTTAAAGGTGTTGGTGCAAATTCTGAACAATTAAGTGCTATGAATCATGATTCTTTAAAAAAGGAGTACATTTCAAATGACTGTTAA

Gene: lysA (diaminopimelate decarboxylase)

Contig: 06_NODE_54, position: 542174 to 543439, length: 1266 nt, orientation: FORWARD

Perfect match to: (Strain_21193-AFEG01000004-[10700:11965], allele observed in CC25+CC1+CC25+CC479+CC707)

Sequence:

ATGACTGTTAAATATAATCAAAATGGCGAATTAACAATGGATGGTATTAGTTTAAAAACGATTGCGCAAAGCTTTGGTACACCTACCATT
GTTTATGATGAACTACAAATTAGAGAACAGATGCGCCGTTACCATCGCGCATTTAAAGATAGTGGATTAAAATACAATATTTCATACGCCT
CAAAGGCATTTACTTGCATTCAAATGGTCAAACTTGTAGCTGAGGAAGATTTACAGTTAGATGTTGTTTCTGAAGGTGAATTATATACAG
CTTTAGAAGCAGGTTTTGAACCGAGTCGCATCCATTTCCATGGTAACAATAAAACGAAACATGAAATTAGGTATGCTTTAGAAAATAATA
TCGGTTATTTTGTTATAGATTCATTAGAAGAAATTGAATTAATAGACCGCTATGCTAATGATACGGTGCAAGTTGTATTACGAGTTAATCC
AGGTGTTGAAGCACATACACACGAATTTATTCAAACTGGGCAAGAAGATAGTAAGTTTGGATTATCAATTCAATATGGCTTAGCTAAAAA
AGCAATTGACAAAGTCCAACAATCTAAACACTTAAAATTAAAAGGTGTACATTGTCATATTGGTTCACAGATTGAAGGTACAGAAGCATT
TATTGAAACTGCTAAAATTGTTTTACGTTGGCTTAAAGAGCAAGGCATTCAAGTTGAATTATTAAACCTTGGTGGTGGCTTTGGTATTAAA
TATGTTGAAGGTGACGAAAGTTTCCCTATCGAAAGTGGTATTAAAGATATTACAGACGCAATAAAATCCGAAATTAAAGTTCTAGGTATA
GATGCACCAGAAATAGGTATTGAACCGGGACGATCAATTGTAGGTGAAGCTGGCGTTACTTTATATGAAGTTGGAACCATTAAAGAAAT
TCCAGAGATTAATAAATATGTTTCAATCGATGGCGGTATGAGTGATCATATCAGAACTGCACTTTATGACGCAAAGTATCAAGCATTGCTT
GTTAATAGAAATGAAGAAGCAGATGACAGTGTAACTATAGCTGGAAAATTATGTGAGTCTGGTGATATCATTATTAAAGACGCTAAATTA
CCTTCATCAGTCAAACGTGGAGACTATCTTGCTATATTATCAACTGGTGCATATCATTACTCTATGGCATCCAATTACAATCAAATGCAAAA
GCCTTCTGTGTTTTTCTTAAAAGATGGCAAAGCACGTGAAGTTATAAAGCGACAATCGTTAAGACAACTCATTATTAATGATACAAAATAA

Gene: msaC (RNA modulating SarA production)

Contig: 06_NODE_54, position: 543679 to 544080, length: 402 nt

Sequence:

TTAGATTTTATGCTTTACTTGCTTTTTCAAATAATATAAAACGATAAGAATTAACAACAAATATAATGTATAAAACAGAATTTCATCAAATC
CAAAATCTTTACTTGTAATTAATACATCAATATCTTCTAATTCCATCATCTCTGCTTTAATATAATTATTAAACTTTGGCAAAGTCATTAAAT
GTACATTGTAAATATAGTAATAAAAGTCGATAAATATGATGTTAAAAAATATGTATGTTTTATCAATATTAGTTTTGAAAAATAATATCCC
AGAAATCATTATCGGAATCACTAATACAATCATCATTGCTGCTAAAATATTAATATTTAATCCAATTGCAGATAATACACCGAAAACTAAT
AAATTAGCAACTAAACTAAGGATAAGATATTTCAT

Gene: msaB-cspC-L2 (RNA chaperone locus2)

Contig: 06_NODE_54, position: 544277 to 544477, length: 201 nt, orientation: REVERSE

Perfect match to: (RF122-AJ938182-[1373964:1374164:r], highly conserved allele)

Sequence:

TTATAGTTTAACAACGTTTGCAGCTTGTGGACCGCGGTCGCCTTCAACTACTTCAAACTCAACAGCTTGACCTTCTTCTAATGATTTGTAAC
CATCTTGGTTAATTGCTGAAAAATGTACGAATACGTCATTTTCTCCTTCAACTTCGATAAAGCCGAATCCTTTTTCAGCGTTAAACCATTTA
ACTGTACCTTGTTTCAT

Gene: msaA (domain of unknown function 1033-containing protein)

Contig: 06_NODE_54, position: 544648 to 544956, length: 309 nt, orientation: REVERSE

Perfect match to: (N315-BA000018-[1409203:1409511:r], highly conserved allele)

Sequence:

TTAATCAATTATTGGTAGTTTATAATATACTTCATTATTTTGTAAAACAATGAAACTATAAAATATTTGTAAATCCTCTTCACAATCTTCGCA
ATAGTTCATGTCACAATTATTATAAAATGCATAAATATTATATTTGCCTGTGACATAATTATCGAACTGAACTTTACATTGATTAATCAATT
GTTGATAGTGCTTAAACATGTCATCAAAATCTTGATATTGATATTTTTCAACAATGTTTTCTGGCCAGTCACTGAATAACCACCATCCCTCA
TAATCGGCTCTAATTTTGGTAACTGTCCACAT

Gene: acyP (acylphosphate phosphohydrolase)

Contig: 06_NODE_54, position: 545119 to 545388, length: 270 nt, orientation: FORWARD

Perfect match to: (N315-BA000018-[1409674:1409943], highly conserved allele)

Sequence:

ATGAGACATATACATTTACAAGTATTCGGACGCGTTCAAGGCGTCGGATTTAGATATTTTACACAACGCATTGCAATGAACTATAACATTG
TCGGTACTGTTCAAAATGTAGATGACTATGTAGAGATATATGCACAAGGGGATGACGCAGATATAGAGAGATTTATTCAAGGTGTAATT
GAAGGTGCCTCACCAGCATCAAATGTAACAAGCCATCAACTTGAAGAGTTAGAACTAAATCAAAAATTATCGGATTTTCGATCAATATAA

Gene: xpaC (5-bromo-4-chloroindolyl phosphate hydrolysis protein)

Contig: 06_NODE_54, position: 545407 to 546036, length: 630 nt, orientation: FORWARD

Perfect match to: (RF122-AJ938182-[1375090:1375719], highly conserved allele)

Sequence:

ATGACAGTGAGATATAATATTTCTCATATATTTGGGGTGTTAGTGGGAATTCCTGTAGCGTTTTTAACAAGCATATTTGGGATGATTGCAC
TTGATGTATCTTTTTTAATTGATATGTCTATTGGTATTGTTGGCTTTTTAATGACATACCTACCGATACAAAAACTCACTTCACGCAAATATT
TAAACGAAATTGGTTTGACTAGAAAAGACTATCGCTATATTCGAAATCAGTTAAATCATACACACCAAAAACTTAGAGGTATTTTAAAAAC
GTATGTCAATATAAGATCAATTAAAGATTTTAGGCAGATTAATGATATATACCAAATTTCACGTTCTATTTATACGACAGTTAGACAGAGA
CCTGCATCATTTTATAAAGTTGAAGGCTTTTTTTATTCTCATATTGATAATGCTTTAAATTTGGTTGATGCATATACACGTCTAGCAAAAAT
GCCCAAAAAATCAATTAATGAACAGCAAAAGTTAGAACAAACACGAATTACTTTGGATGAGGTCAAACGAACATTAATCGCTGATTTAAA
GCGTCTCAACGAAGATGATTATGAACGTTTAGATATTGAAATGGAATTAAATAAGTTACATCAAAAACATCATCAAGATTGA

Gene: terA (putative tellurite resistance protein)

Contig: 06_NODE_54, position: 546068 to 547204, length: 1137 nt, orientation: FORWARD

Perfect match to: (N315-BA000018-[1410623:1411759], allele observed in CC5+CC361)

Sequence:

ATGACTGAAAATAAAAGTTTCAAAGAAAGCCATCCACTAGATGATTTTATAAGCGATAAAGAATTATCGAATACTACTATTCAAAAAGAA
AAGTTAACAATTGAACAACAAAAACAGGTAGACACAATCAGTAAACAAATTAACCCTTTAGACAATGAAGGTTTATTAGCGTTTGGTTCT
GATTTACAGAAACAAATGTCTCAATTTTCACATCAAATGTTGGATGAAGTACAAAGTAAAGATGTTGGTCCTATTGGAGATACTTTGTCA
GATCTAATATCAAAACTAAAGTCAGTTAATCCAAATGAGTTAAATACTGATAAACCATCTATGTTAAAAAGAATTTTTAGCAGAGCAAAGT
CGTCTATCAATGAAATCTTTTCAAGAATGCAATCAGTTAGTGCTCAAGTCGATCGCATAACGATTCAACTGCAGAAACATCAAACACATTT
AACAAGAGATATTGAATTATTAGATACGCTATATGATAAAAACAAACAATACTTTGATGACTTATCATTGCATATCATTGCTGCACAGCAA
AAAAAGTTGCAATTAGAAAATGAAAAGCTACCACAATTGCAACAGCAAGCGCAGCAATCCACTAATCAAATGGATATTCAACATGTTGCA
GATATGCAGCAATTTATAGATAGACTAGATAAACGCATATATGACTTACAGCTTTCAAGACAAATAGCTTTGCAAACTGCGCCACAAATTC
GTATGATTCAAAATGTTAATCAAGCACTTGCCGAGAAGATACAAAGTTCAATTTTGACAAGTATTCCACTATGGAAAAATCAAATGGCCA
TTGCGCTTACATTAATGAGACAGCGTAATGCAGTTGCTGCACAACGAGCTGTCACTGATACAACTAATGATTTATTAACAGCAAATGCTG
AAATGTTGAAACAAAATGCGATTGAAACTGCAACAGAAAATGAGCGTGGCATTGTTGACCTTGATACATTGAAACGTACACAGCGTAAC
ATTATTGAGACAATTGAAGAAACATTAATTATTCAACAACACGGTCGCGAAGAACGACAATTAGCTGAAAAAGAATTACAACAATTAGAA
CAAGATTTAAAGTCACATTTAGTGAACATCAAAGGAACGAATAAACAATCATAA

Gene: brnQ3 (branched-chain amino acid transport system II carrier protein)

Contig: 06_NODE_54, position: 548217 to 549560, length: 1344 nt, orientation: REVERSE

Perfect match to: (MW2-BA000033-[1414518:1415861:r], allele observed in CC1+CC22+CC30+CC772)

Sequence:

TTATTCCTGTTGATATTTAATTGGATCTTGTTTTACAAATATGCCGACTAGATAGCCTAATATCGTTGCAATAATTGCTACTGGGAACCACT
CTAAAGAATACGCTCTTAGAGGCAATGATTCTATAAAGTTAATTTTCAGCCAACCTAACTTACTAATAACACTGAAAATCGACAATATAAA
TACGATAATAACTGGAATTTGTTGTGAAATGCGTTTTGTCGGTATGAATTTGGCAATTAAAATTAATAATACAACAGTTATTGCTACTGGG
TATACAATGCTTAATACCGGAATTGACATTGAGATAACAGCATTTAAACCTTGGTTAGCAATAATAAAACTCATTAAAATGAAAACTAATA
CAAATGCTTTGTATGATACTTTAGGTACGATTCTATGGAAATATTCAGAAACTGCAACAATAAGCCCGCATGCTGTAGTTAGACATGCCA
GAGCCACAATGATGCCCAATAAATATTTTCCGAATGAACCAAATCCTGTTGAAGCCATTGTCGTTAATAAATATGTCCCAATGTTTCGATC
TTTGGATTTCAATTGATCTAACGTCATGTCACTTACTGGCATATGATTACCAATATAACCTAATGAAATATATATGAAAATTAAAGCTACG
GCTGCAATTAAACCAGCAGTCAAAGTTTGTTTGAATATTTGATTTGTTTTAGTAATGCCTGTTAGTTTTACTGCATTAACAACAATCATTGA
AAAAGCAATTGCTGCAATGGCATCCATTGTTAAATAGCCTTGTGTAAAGCCTTCAGCAAAACTTGAAAAATTAGAATGATATAGTGCTTC
ATTGCCCTTTCCAGCACTATTACCGCTAAAGTCTAAGTATCCTTTAATAATCATCGCTAAAATAGTAATCAATAATAATGGTGTTAATAATG
AACCAATACGATCGATTAACTTAGATGGATTTAAACAAATATACAAAACGACTATGAAGTAGATAATCGTAAATATAAATAAAGCGATAC
TACTATTGCTATGTATAATTGGTGTAATTGTCATTTCAAAAGATGTAGATGCAGTTCTAGGTATTGCGAAAAGTGGTCCTATAGTCAAATA
AATGATGATTAAGAACAATATTGAAAATTTAGGTGAAATTTTATTTAATGCGCCAATATATCCTTCTTTATCAAGTGCACCTACAATCACAC
CTAATAATGGTAAACCAATCCCAGTTAGAACAAACGCTAAAATGGCGGGCCAGAAAAATTGACCACTATCCAATCCGAGATTAGGAGGA
AAAATTAGATTGCCTGCGCCAAAAAACATAGCGAAGAGCGTAAACCCAATGACCCATGTATTTTTATTCAT

Gene: cobT (putative cobalamin biosynthesis protein)

Contig: 06_NODE_54, position: 549785 to 551671, length: 1887 nt, orientation: REVERSE

Perfect match to: (MW2-BA000033-[1416086:1417972:r], allele observed in CC1+CC239)

Sequence:

TTATAAAGATTTAAGTAGTAATTTTTTTAATAATGGAGAGAGATGTCCAGGTAAATGCGCAACACCTTCAACGAAAATAGCATATTGACC
ATAAATATTATGAATTGTTTGTTCAACATCTTCAGTTATTGGATCTTGACTCAAAAATACATTAAATACTTCAATACCAAATTTACGTGACA
TTTCTACAGCTTCATACGTATCAATAATACCATCTTGACTATAATTAAATGCAGACGGTTCGCCGTCTGAAAATACGATTAAAAATCGTTG
ATGTTGATTTCGACGCATTAATCGTTCACTTGCAACTCTAATAGCAACACCATCACGATTATCATCTTGAGGTTCAAGTGCCATAATACGT
GGGCCATCTTTTTCAAAGGTTGAGTAATCATAGTTAATAATTTCATTAATGATATTTGGTTGTGCATGATCGTCTGAATCAAAGGCATCCT
CACTGAATGATAAAATTTCATGTTTAATGTTCAATGCTTTTAACGTCTCGTGGAATAACACTACACCTTTCTTCGTTTCAGCCATTTTATCAT
GCATACTTGCTGACGCATCAATTAACAATGTAAACGTCGCATCAAATGATTTACTTAAATCTTGTTTTTTGTAAAACAATTTATATTGATCG

TCGATAAACCAATTAATTAAATCCTTTTGTAATCGTCCTTTTGTTAAATTAAAACGTGCATCTCGTTGTTCTCGTTCAATCGTTTTCTTTATAA
TTTGAATTAAGTCTTTGATTTCATATTGTACGTCTTGTTTCGATTCTTGATATTCTAAAACATATTGTGGCTCAATTTCAGGAATTTGCCATT
TTATTTCTACATTTTTGTTCACACCATCAAGTTGAAATGCTTGGCTTTGACCAACAGCATCGCCTTCTTCACGATTTAGCGTATCGTTTGAAC
CTTTGCCCTTTTTGGTCATCATATCAGTCATATCATCTGTAGCATCGCCTTCACGTGCTTCATCATTACCCAATGTTTCACTATTTTGTCCTTC
GTGAAGTTCCATTTCTAAATAGGCGCCACCTTTTGACTCACTATCTGCAGATTTAGAGTCAGCTTTTTCACTTTCGTTATCATCATCTTCTGA
AGTATCATCTTGCCCATCGACTTGACTTGCATCAGTTCTCTTTAAATCATCAAACTCTGGACTAGCTAATGTATTATATATAGTGTTTTAGGTA
AGTAATAATATTCGTTTAGCATATCTTCTTTTAAAATATCATCTACTTGATACATAATTCTTTGAGCCAGATACATATTATCTTCAGAATTTT
GATTTTGGAAAAAATTAGGTAAATATAAGAACATATTAACTACAATATCATCTAAATCTGAATGTATAGATGGTATATCAAAGAAATCTTG
GCTTAAAAATGCATGTTCTAAATACAAAAATAATAAATCTGTATATTGTGTTTTAGTACGATACACTTTAATTTGAGATTCCGTATATGATA
TACGTGTATCTAAGCGAAGATCAATTAATTTAGCAGTACTTGGGCGCTCAACTTTAATAGAATTTAATACGCGCATATCTTCTAATAATTTA
AAAAGTTGTTGATAAAATTTAGGGTGTTTAAAAGTTTTATCTTGTACTACTTCATTTACAATTTGTACATCCATCATATGATAACCGTAAGC
AGCTAACATAACATCTGTTTTTAAACCAGCCATTTCGATATGGCTTGGTCGATGTGACCAAAACCAACTCGTAATCAAAACATTTTGAACA
GGATTATAGTAAGGGAATTTTTGGATTTTAACTTGTGTTTGTTCATTTTTTAATAAGAGTCGAGCTAAGTCTTGCAACATCATTACTTGCTT
GGCATCTAACTGTTCGTCATTAAATTTTATGAAACGATCACTCAT

Gene: norQ (putative nitric-oxide reductase activation protein)

Contig: 06_NODE_54, position: 551685 to 552476, length: 792 nt, orientation: REVERSE

Perfect match to: (N315-BA000018-[1415301:1416092:r], highly conserved allele)

Sequence:

TTAAAAGTTTAGTTCTACAGCATTATATATTGCTTGTTGTTCACGTTCATCTTCCAATTTATCAATAATTGTACGTTTAATTGCACGTTCAAC
TGGCATTACAGTGATTAAATCACACAAGTCTAATAATGCACGGATACTAGCGGCTTCTTCAGAAATTTGTCCCTGCTTAGACATAGTACGT
AAATCTTCATTAAACTTAATAATTTGTTCGATTTGTTTATCATCTTGTAATAAACTTTGCTCTTTAATCACATTTTTTAAAATGTCCCCATCAA
TATAATCAACGTGAATAACAACAAAGCGATTTTTTAGTGCTTCATTCATTGGCAAAGTACCAACATAACCTTCATTTATCGCTGCTATAACG
TTAAATCCTGGTACAGCTTTGATTACTTCACCAGTGTATGGATTCGTAATTTGACGACGATAATCTAAGACCCCATTTAATACAGGCAATG
TTTCAGGTTTAGCCATATTTATTTCATCAATATATAAAATATGCCCCTCTTTCATAGCTTTAATAACTGGACCATCTACAAAGACAATTTCTT
GTTGACCTTCCGCATTTGTTTTAATTGTTTTAAAGCCTAATAAGCTTTCTGTATCTAAATCAACAGAACAATTGACTTGATGCATGGGTGTA
TCAACAACTTCACTTAATGTTTCTGCCAACTTTGTTTTCCCTGAACCTGTTGGACCTTTAAGTAAAATATTTTTATTTAAATCAAATAATGCC
TTCGCATCATTGAAAACTGTTGAATCTGAATTCTTATAATGTTTTAGTGCCAT

Gene: yozC (protein of unknown function)

Contig: 06_NODE_54, position: 552657 to 552860, length: 204 nt, orientation: REVERSE

Perfect match to: (N315-BA000018-[1416273:1416476:r], highly conserved allele)

Sequence:

TTAAACTTCTTTGAAATATGTTTTATAGTAACCGCCAACTAATCCAGAATTATCTATTATTTGATAATATTCTTCAGTTTCGTTGATAACATC
ATATTGTTTGCCAACCGTTAACATGTCAGAAACTGTAAATTTCTTTGCATCTGTATTAATGACTTCTACTTTCTTGATTGGTGTACGTTCTTT
CCAAGTTTCATGTAACAT

Gene: Q5HG08 (putative metalloenzyme)

Contig: 06_NODE_54, position: 552889 to 553698, length: 810 nt, orientation: REVERSE

Perfect match to: (Strain_21193-AFEG01000004-[20475:21284:r], highly conserved allele)

Sequence:

TTATTTTTGTAAAGTGAATATAAACGATAATCCACTGTTTTCAGTAATTCTAATTGATTCAATATCATCTTGCTCAAGCGTTTGATATGGTAT
TTCCACTTCATCTAAGCGCGATTTCGCTTGATTAAAGAAATCTGCATCTTTCGTTTCAAACTCAACTTGATCGACTGCGCCATATTCCGGCA
TTGTCATTTCGGTTTCTACAGGCATTAAATGTATTTCGCCACCTAGACCACCCGTTCCAACTTTGAATACTTGGACATGATAGTCAGCATTG
TCGAAGGGTTGGTATTCTGCAAATACTTCAAGTCCGAATATATTTGTTAAAATTTGACCTGTAATATCTACATGATTCACTTTAAGAATCAC
TGGTCCTAAACCTTGCACTTGATGTAACGGATTGACCGCACTCTCAAAAGAAGGCATACCTAAACCAACGCCATAATTATTCTCGTTTGAA
TATATTGAGAAAATATGACCATTGTTATCTTCGAGACTGAAATATTTATTGCCATTTAATTCTTTAACTGTTGTAAATGGAATATCCTTATTC
GATAATATTTCCGCATACTCCTCTAAACCTGAGTCAGTTGGTGTACGTAATCCAATACCCACAAAGTGAGATTCTTCTAATTGCTCACTTGG

AACTTGTATAAATTGAAGTCTTGTTCCTGGGCTTATATCTGCATCGCCGAAACGAATTGAGTTTTCAAGAAGTTCTTCATAATTTAATCCTA
ATATGTCAACCATGAAATGTTTTGTCTGTTCTATATTTGTTGTACCTAATGTTATACTTCTAAGTCCACACAT

Gene: odhB (2-oxoglutarate dehydrogenase complex subunit E2)

Contig: 06_NODE_54, position: 554278 to 555546, length: 1269 nt, orientation: REVERSE

Perfect match to: (N315-BA000018-[1417894:1419162:r], highly conserved allele)

Sequence:

TTAAGATTCTAATAATAAGTCTTCTGGGTTTTCAATTAATTCTTTAATTGTTTTTAAGAATCCAACTGCTTCTTTACCGTCAATAATTCTATG
ATCATAGCTTAATGCAATATACATCATTGGACGATTTTCGATTGTATCTTGATCAATCGCAATTGGTCTTGTAATAATTGAATGCATGCCTA
AGATTGCAGCTTGATTACCATTGATAATTGGCGTACTCATCATTGATCCAAAAATACCGCCATTTGTAATCGTAAATGAACCATTAACCAT
ATCATCTAAGCCAAGTTTTTTCTCTCGTGCTTTAACTGCTAAATTAGCAATTTCTGCTTCGATTTCTGCAAAATTCTTTTTATCACAATCTCTT
ACAAATGGTACTAATAATCCATCATCTGTAGAAACAGCTACACCAATATCATAATATTGTTTCGTAATCATGTCGTCGCCGTCGATTTCTGC
ATTAACTTCTGGATACTTTTTCAAAGCTGCTACAGAAGCTTTAGTAAAGAATGACATAAATCCTAATTTAGTACCATCATGATCTTTCATAA
ATTGTTCTTTCTTACGTTTACGCAATTCCATAACATTTGTCATGTCAACTTCGTTAAATGTTGTTAACATAGCTGTATTATTAGATACCTCTA
ATAATTTTTTGGCAGCTGTTTTCTTTCTACGTGACATTTTTTCACGAATCACTGGTTTTGTAGGATATTGATTGTATTTTTTCTCTTCTTTTGC
AGATGCTTGTTGTGTTGTTTGTGTTGATGCCGGTGCCTGTTGTTTCTTATCAATATCTTCTTTACGAACCACATCATTTGTTTTCGGACTTAC
TTCAGCAAGATTCACACCATTTTCACGAGCATATCGACGCGCAGAAGGCGTAGCATTAATACGTTGCTGATTGTCATCATTTGCTTGATTT
ACTTCAGCTTTATCTACCGAATTATTTGTTGTTTCTTCTTTTTTATTATTTGTTTCTTCATTTTGTTGTGGAGTATTGTCGTTACTATTTTCTTT
AGAAGCATTGCCACTACCTTCGCCGATGATAGCAATTGCTTGTCCAACTTCTACAGTGTCGCCTTCACTTGCAAGTTGTTCAGATAATACA
CCTGCTTCTTCAGATACAACTTCGACATTAACTTTATCAGTTTCTAATTCAAGAATAGCTTCACCTTTTTCTACGCTATCCCCTACGTTTTTCA
ACCATTCTGCAATGGTACCTTCTGTAATAGATTCTGCTAATTCTGGAACTTTAACCTCTGGCAT

Gene: odhA (2-oxoglutarate dehydrogenase complex subunit E1)

Contig: 06_NODE_54, position: 555560 to 558358, length: 2799 nt, orientation: REVERSE

Perfect match to: (IS-125-AHVC01000084-[8283:11081:r], allele observed in CC239+CC1+CC239)

Sequence:

CTAGTTATTTTTTAATGCATTTTCTATAATTTTATTTTGAACAAGTTTATGAATTTCTCCATCGCCTTCAGCTGGAGCAGCCCTTTGAATTCT
GCCATGATAACTTAAATCATATTTATCTGCAACTAGCACTTTAACATATGGATAGACATATAACCAGGCACCTTGATTTTTAGGTTCTTCTT
GTACCCATGACACTTCTTCAAGGTTTGGCAATTGTGCTAGTAATGCTTCAATCTCTTCCTCTGGGAATGGATACAATCTTTCAATCGCAACG
AGTAATACTGATTCGTCTGGATTTTTAGCTAATGCTTCTTTTAAATCAATGAACATTTTACCAGTTGCCAAAATAACTTTTGTAACCTTATCC
GCTTGATATGATTCTGTCAAAATTGGCTCAAATCCACCAGAAGTAAATTCATCAATTGGTTTTGCAACTGTTTTATTTCTCAGTAAGCTTTT
TGGTGACATAACAACCAATGGTCGCATTTGTTCAGAATCTAAACTAGCCGCTTGTGCACGCAATAAGTGGAAATAATTACTTGAACTAGA
TAAGTTGACAACTGTGCAATTATTTTCAGCAGCTAATTGTAAAAATCGCTCTAATCTTGCTGATGAATGTTCAGGCCCTTGACCCTCATATG
CATGAGGTAAGAATAATGTTAATCCTGAACGTTCTCCCCATTTTGAGCGAGAACTGAATAAGAAGTTGTCAAAAATCATTTGTGACATATT
TGCAAAATCACCATATTGTGCTTCCCAAATATTGAAGCTTTTTTTGTTTTCCACATTATAGCCGTATTCAAAACCAACTACTGCTGCTTCTGA
AAGCGGAGAATTGTGTATATCAAATGTCGCTTTTTGATCAGGAACATGATGTAAAGGTGTATATGTTTCACCTGTTTGCTCATCATGTAAC
ACGGCATGCCTATGACTGAATGTACCACGTTCACTATCTTGACCAGTTAAGCGAATCGGTGTACCATCTTGTAAAATTGTCGCAAATGCAA
GTTGTTCTGCTTGTGCCCAATCAACTAAACCATCTTCTTTATTAAACGGCTCATGACGCTTCTCAAGAACTTTGTTTAACTTTTTCAAAATGT
TAAAGCCATCCGGATATGTTAACAATGCATCATTTATTTCTTTCAAGTGATCAAAAGTAAATGATTGTTCGTCTGCTTGTAACGGTAATGC
AAGATCTGCAGGCTTTTCCATATCTGGATTATCCATTTTATCAGCTTTATTAATTTTATCATGAGCTTGTCTTAGTTCCTTTTGGACTTGTTCT
ATAAATGAATGCATTTCATCTTCTGAAATGACACCTTCATTAACAAGCTTTTTACCAAACACATATTCAACAGAGTCATGTTTGCGAATATT
CTGATAAGGAACTGGATTAGTAATTGATGGTTCATCCATTTCGTTATGTCCGAAACGACGATAACCTACTAAATCAATAACGACGTCTTTA
TGAAACTCTTTTCTAAATTCCATTGCAATATCAATTGCTTCAATAGTAGCTTCAACGTCATCTGCATTGACATGGAATATTGGCACATCATA
ACCTTTGGCCACATCTGTAGAATAAGTTGTTGAACGTGCATCAATTGGTTCTGTAGTAAATCCAATTCTATTGTTAGTAATAATATGCAAT
GAACCACCCGTAGAATAGCCTTTCAAGTTTCCTAAGTTCATTGTTTCGAAGTTAATTCCTTGACCAGGATAAGCAGCATCGCCATGTATAA
TAATTGGCATTGCTTTATGATGATCAGTCGTCGGAGCCCCAGCTCGTTGTGTATCATCTTGTGCTGCTCTCGTACGCCCCTCAACAACAGG
TGCAACAATTTCCAAGTGACTTGGATTGTTAGCCAGTGCAATACGCTGCATTGTACCGTATGAATCAGTAGTTTTAATGCCACCAAGGTG
ATATTTCACATCACCAGTCCATCCAGCAGTTAACTGCAAGCTACCATCTTCAGGTAAGAATTTCATTGGATCTGTATGCATAAATTCTGAA
ATCATCATTTCGTACGGTTTTTCTAAGACATGCGTTAAAACGTTTAAACGTCCACGGTGAGCCATGCCTATTTGTATATTTTTAATACCTTCT
TTCGCAGCAATCGTAATAGTACGTTGTAACATCGGTACAAGTGCGTCTACCCCTTCAATTGAAAAACGCTTTGCACCAACGAAGTTTTTAT
GAAGATATTTTTCAAACCCTTCAACATACGCTAATTGTTTGAATAGTGCCCTTTTTTCGTTATTATTTAACGTTACTTTATATGGCGTTTCAA
TTCTTCTTTTTAACCAACCACGTTCGGTATTGTTATTAATATGTGTATACTCAAATGCAATTGGTCCTTTGTAACGTTTTTCCATTCTTAAAAT

TGCTTCATAAGCATTATCATAAATGTCGGCAAAGTGATCTGAAACAATTCCTGCTGATATACCTTCCAAAGTCTGTTGATCTAAATCAAAG
TCTTCAATCTCTAATTTAGGTACATGTTTCCTTTTTGGAGGATTTACAGGATAAATATCGGCTTTAAGATGCCCGTATTGGCGAATATTATC
AATTAAACGCATGACACGCTTAATTGTGCCGTCGCTATTTTGACTACTTGTACTTTTTAAAGCTGGTACAATTGAGTCATCATTCTTAATTG
TGCTGAATAAGACTTGTAAATCTTCTGGTACAGATGATGGATCTTGTAAAAAGTCATCATATAGATCTAACATTAGACCTAAATTCGCACC
GAAGTTTACAGGAGCCTCTGAAACTTCTTTTCTTTCGTTAGTCAT

Gene: arlS (two component sensor/regulator, sensor histidine kinase)

Contig: 06_NODE_54, position: 558642 to 559997, length: 1356 nt, orientation: REVERSE

Perfect match to: (N315-BA000018-[1422192:1423547:r], highly conserved allele)

Sequence:

TTAAAATATGATTTTAAACGTTGTTCCTTTGTTAATTTCACTTTTAATTTTAATCGATCCTCCGTTTAATTGAATGATTTTTTGAGCAATAGAT
AATCCGAGTCCATTACCGCCTTGACTTCTTGAACGAGATTTATCCACTCGATAAAAGCGATCAAAAATGAAATCTTGATCTTCCTCTGGAA
TACCAATTCCATGATCTGTAATTTCAATTATTTTTTGCTTATTTTTTAACCTTGTCTTAACTTTAATTTTCTTATTCTTCACATCATATTTGATT
GCATTATCAATAAAGATTAAAAATAATTGTTCGAATTGATGAGGTTTCATTTTAATTTCTAGATTTTTAGATGTCAGATCCGTATCAAATTG
ATAATCAGGATGCAATTGTTTTAATGAGTGTATTCGCGAGCGAATTTCATCATTAATATGCACGGTCTGTGCTTCAGAAGAAATGTCATTT
ACATCTCCTTTAGTCAATTCAAGTAATTCTTCGACTAATTTTATGATACGATTCATTTCTTCAATAGAAATATTTAACGATTCTTCTAATACT
GCTGGGTCTTTTTTTCCCCATCGCTGAATCAAATTTAAATGACCTTGAATAATTTGTAATGGTGTTCGTAATTCATGTGACGCATCTTCAAC
AAATTGTCTTTGTTGATTAAATGATTCTTCAATTTGGCTCATCATCTCATTAAACGTATTTGCTAAATTATCTATTTCTTCATAATTTGTATTT
AATTGCAATTTATTTTGAAAACCATCTCGTCGAATCTCAATCATTTTATTTGATAAACTGACAAGCGGTTTAGTAATTTGTGTTGAAAATAC
ATAACTGATTGTGGCAGTTATAATTGTTGCAATCACTCCAAATGCCAGCGCAATGATATACAATGATTTTACGATGTTATCATAATTTTCTA
GTGAATGAATTAACAAGCTATACCCTTTGAAATCTTGCGTTGTAATTGGTTCTTTAATAATTAAATATTCAATGCCTTTATAGCGTTTTTTA
TTACGCGGTCAAAATAACGGTGTTCATAACCTGGTTCAACTCTCACTGTGTTATCATTCGATGTCTCAAATAATTTATTATTATGCTCATCA
TAAATAATTATCTCTTGAAAATTACCTAAAGATGCATTCAAGTCTAATGCAGATATATCTTTAACAGGCTTAGAATGAAATAAATTATTAAT
ATCGCTTGAGCTTCGTTCTGCATCATCAAGCTCACTATTATGCAGTGTATCTTTCAAGAAAAAAATAATAATTAAACAAAACAAAAATATC
GTGACAAACGTAATCATCGTGGTAACAATAATCCAGTTATTGCGCAATTTACGTTTTGTCAT

Gene: arlR (two component sensor/regulator, transcriptional regulator)

Contig: 06_NODE_54, position: 559994 to 560653, length: 660 nt, orientation: REVERSE

Perfect match to: (CIGC93-AHVD01000017-[74116:74775:r], allele observed in CC15+CC30+CC34+CC772)

Sequence:

TCATCGTATCACATACCCAACGCCACGAACTGTTTCAATCATTTTGTCACGATCGTATGGTTTTAATTTGTTTCGTAAATATCTTATATAAAC
ATCTACGACATTTGTTTCTACTTCACTATTATAACCCCATACATGATTTAAAATTTGTTCCCGTTGCATAACATGGTTTTTATTTTCAGCAAG
AAGATATAGTAAATCATACTCTGTTTTTGTTAATTCAATTTCTGCGCCATTTACCGTCACTTTAAAAGCATTCTTATCAATTGTAATACCATT
GACATCGATAATATCTTTTTGTGGCTGACGACGTAAAATCGCACGTATTCTTGCTAAAAGTTCTTCAATATCAAATGGCTTAACTATATAAT
CGTCTGCACCGTAATCAAGCCCAGCAACTTTGTCATACGTATCACTTTTCGCTGTAATTATAATGATAGGTGTAGATTGTTGTTGTCTAATT
TTGCGACAAATTTCTAAGCCATTAATTGACGGCAACATTAAATCTAATATGATTAAATCATAGTAATGGCTAAGCGCTTTATCTAAACCGT
CTTGTCCATCATACTCTGTGTCCACATTGTAATTTTCATGTGTGAGTTCCAATTCAAGAAATCTTGCTAAGTTTTGTTCATCTTCTACTATTA
AAATTTGCGTCAT

Gene: pgpB (putative phosphoesterase)

Contig: 06_NODE_54, position: 561440 to 562054, length: 615 nt, orientation: REVERSE

Perfect match to: (MW2-BA000033-[1427741:1428355:r], allele observed in CC1)

Sequence:

TTAATTTATTAATTTATTTCTAAGTAATAACGTTGATAAAATAATGCAAAATACGCCACCAATAATGCCGGCAATAATATCTGTTGGATAAT
GTACACCTAGATATACACGTGATATGGAAATCAATAAAATCATAGCTGCACATAACCCTATAAGAATACCTTTTGAATTACCTTGATTTAA
TCGATTTAATAGATAGATACCACTTCCAAAATATGCAGTTGATCCCATAGCATGACCGCTAGGAAAACTAAATCCTGTTATATCAATTAAA
CGCAGCAATGTAGGTCTTTCTCTATCGAATATATTTTTTAATGCTGGATTCAAAATTCCAGATAATGCCATTGTTAATGCAAAAAATAATGC
TTCAATTTTGTGGCGCTTTAACATGAGATATGCCACAAGAAGTAATGAAATACATAACATTGCCCAGACTTCACCTACTTTAGTAGCCCCA

AGCATGATAGATGTCGTTATGAAGCTCTCTGATGAATATATAAATTCATAAACTTCATTATCAATCCATTTCCCTAGTCTTGATTCGTGGAA
AAACGCGATAATTCCAAAAACCAATGTAAAAACGATGAGCAAAGAGATACGTTTCCATTGACTCAT

Gene: murG (undecaprenyldiphospho-muramoylpentapeptide beta-N-acetylglucosaminyltransferase)

Contig: 06_NODE_54, position: 562071 to 563141, length: 1071 nt, orientation: REVERSE

Perfect match to: (MW2-BA000033-[1428372:1429442:r], allele observed in CC1+CC5+CC45I+CC80)

Sequence:

TTAATTCAATGCATCTTTAATCATCTTATCAAATAAAGCTTCTTTCGTATAACTTTGTTCATACGATTTCATATTATTGATAATTCGAGTTCTT
TCCTGTTCCATTTCATTTAGTTCTTGTAATAAAATTTGTGCTGTTAATTGTTCTTCATCAATCGCTTTAGCATAACCTTTATCAGCAAAATGA
TTTGCATTGTCAATTTGGTCGCCTCGGGATTGATCTAAACCTAATGGTACTAATAACATTGGTATACGTAATGTTAAGAACTCATAAATCG
CATTTGATCCTGCTCTACTTATTACTGTGTCCGTAATTGCTAATAAATCTGTTAAATCCTCTTTAACAAATTCATATTGTATATATCCTGATTT
TTTAACTTGAGCATCTTTTAATCCTTTACCAGTTAAATGTATCACTTGATATTGTTGTAATAATGCATCTAAGTTTTCGCGAATAATGCTATT
TAATTTTTTACTTCCTAAGCTTCCACCCATAACGAGTAAAACTTTTTTATTTTCATTAAAGCCTGTTAATTGATAACCATTATGTGCATTACC
ATTTTTTAAATCTTCTCGAATTGTTGCTCCAATAAAATCAGCTTTCTCTTTAGGTAAGTAGTTTAGCGTTTCTTCAAATGTTGTATATATTTTC
TTGGCAAATTTAAGTGCTATCTTATTCGCTAATCCTGGTGTTAAGTCAGATTCATGAATAATAGTTGGTATATTTAATGATTTGGCTGCAAT
AACAACAGGCACAGATACAAATCCACCTTTTGAAAATAATAGATCAGGTTTTTCTTTTTTCAAAACTTTACGAGCATCAAGAATACCTTTCA
ATACTTTAAATACGTCTTTGGCATTTTCTAAAGAAATATATCTTCTTAATTTACCACTCGAAATAGGATAATACTTAATTTCTGGTAGTTGTG
ATTCAATCATTTCTCTTTCAATACCATTTTTAGAACCAATATAAAGCGCTTCATAACCTTGTGATAATGCAGTTGGAATTAAACTTAAATTTA
CTGATACGTGTCCAACTGTTCCCCCTCCGGTAAATGCGATTTTCGTCAT

Gene: Q7A0W4 (putative acetyltransferase)

Contig: 06_NODE_54, position: 563153 to 563662, length: 510 nt, orientation: REVERSE

Perfect match to: (MW2-BA000033-[1429454:1429963:r], highly conserved allele)

Sequence:

CTATTCTTTTAAATTTTTATAATATGCATAAAATGGTTCACCTTTATCATATGGCGGATATTCCATTAATTGTTCACCGACCTTATGAAATCC
AAATTTCGCAAATAAACCTTGTGCAGGTTTGTTTAACGCAAAGGTGTCCGTTAAAATAACTTCTGCACCACGTGCTTTAACTACATCAATA
ACATAATTGAATAATTCTGTAGCAGCTCCTTTATATTCTTTCGAACCAGTTAATCGATGAATAACAAAGGCGCCTTCTCTATTTACTGGCCA
GTCAATGTCATCATACCATTCTGCTTGGTCTTGGTCGACAACAATAAAGCCATAAATTTTGTCATTTTCCTCTAATACGTACAAATAATCTTT
AGCAATATCTTCTTCAAAATGTTCTAAAAGAGGGTACTGATCGTCCCATTGCTCGTTGTCGTGTTCTTTCATTAATTCTTTTGCTTCTTCTAC
TAGATTTAAGATTTGATCTAAATCTGACATTTTACCTAGACGGATCAT

Gene: ctpA (carboxy-terminal processing protease)

Contig: 06_NODE_54, position: 564849 to 566339, length: 1491 nt, orientation: REVERSE

Sequence:

TTATTTTAAAATATTAATCAATTTATCGAGAACATCATCATGTTTATTAGCTTTTTCAACTAATAACTCAGTAAATTTATTATTCGTTTCTTTA
TTAAATTCACCAGTTACCTCGAGTTTATTCGCTTGTTGGAAAGCTTTAACTTGATTTTCTAAAGCTTGATCAAATTGCGTTGTTTCATTATCA
ACTTTATAACCTAAAGCTGACAAACCAATTTTAATAGTTTTAATATTTTTATCATCGTCTCCAACTTTAAATGTTTTCGTATTAGGAATGACA
TTTAAAGATTGATATTTAGGTGTGTCAATAGTAACGTCTGGTTTAATGCCTTTACCGTGAATATAATGACCATCTGGCGTTAACCATTTCAT
TTCAGTATATTTTAACAATGAACCATCCTTAAACTCTCTTGTAGTTTGTACGACACCTTTGCCGAATGTTTTTGACCCATAAACTTTAGCTTT
ATTATAGTCTTTTAGCGCACCAGTAAACACTTCAGAAGCGCTAGCTGAACCTTCATTCACTAAGATGGATATATCCATGTCTTTCGCTTCTT
TTAACGCATCATTAGAAGTTTGAATTGCTTCAGTATCTTTACCTTTTTCTAGTTTAACAACAGTTTTTCCTTTATCGATAAAAATATTTGCCA
TTTTAACAGCTTCATCTAGTAGTCCACCTGGATTATTTCTTAAATCTAAAACAATCTTTTTCAAACCATCTTTGTGAGCTTTTAGAACTGCAT
CTTTCAATTCACCTGATGTATCATTCTGGAATTTATTAATAGTAATAACTCCAACTTTACCTTTTTTCTTATACTCAACACTTTTAACATGAAT
TTTTTCACGTTTAATCTTAACGTCTTTTTCTTCACTACCTCGTTGAACAGTTAAAGTGACTTCAGTGTTTTCTTTACCACGAACATCTTTGACA
ACTTCATCTAATGCTTTTCCTTTAATTGATTTTCCATTTACTTTAGTAATGACATCTTTAGGACGAATGCCAGCACGTTCTGCTGGAGATCCC
TTCATAGGACTAGTAACCATAATTTGATCATTTTTCTTTTGCATTTCTGCACCAATACCTACAAAATTACCTGAAACACCTTCATTAAAGGAT
TTCGTTTGTTCTTTTGTTAAATATTCAGAATAAGGATCTTTTAATTCTTTGACCATGCCATCAATTGCAGCTTTACTTAACTTGTCAGAGTCC
TGTTTTTTGTAATAATCACTATTTAAGATTTTATACACATTTTCAATTTTATTTAAGTTTGCTTGATCAGTTTTGTTTAAACCACTTATTTTTTG

ATTTATAAAAATATATGCAACAACTGTGATGACAGCTGTTATTAGGATTGTACCAATTAATATTGATATGAATTGCCAACGTTTTAAGTGA
ACGCGTTTCGATGAATTAGTTTCTTGGTCTTGATTGCTTGTTGCAATTTCAGCGCGTTCATCATCGGATGAAGATGTGTGTTGCTTATCATC
CAT

Gene: yozE (putative RNA binding protein)

Contig: 06_NODE_54, position: 566531 to 566752, length: 222 nt, orientation: REVERSE

Perfect match to: (N315-BA000018-[1429279:1429500:r], highly conserved allele)

Sequence:

TTAAAATTTTAGCCATTCCGTATATTCTTCATATAAATCATCAAATACAGACATTGGCAATGTGAAATCACCATGTGTCTCAATATAATCAG
ACAGTATGTTAAAATCATCATCGTGTTTTGGGAAAGCAAGATCGTCAAATATCTCTTCTGCTAGACGACCTTTATCGTCGTGTCGACCACG
AACTGTCATGACAAATTGATAAAACGAATAGTTTTTCAT

Gene: crr (glucose-specific phosphotransferase system IIA component)

Contig: 06_NODE_54, position: 566752 to 567252, length: 501 nt, orientation: REVERSE

Perfect match to: (MW2-BA000033-[1433054:1433554:r], allele observed in CC1)

Sequence:

TTAGTTCATTGTCACATCAATCACTTTTGTTTCACCTTTAATCACAGCATTTTCATCATAAATATTAATTGAAGCTGCTTGATCAGTGTTAGT
AATTATAATTGGTGAAATTACAGATTTAGCGTTATTATTAATATATTCAAGGTTGAATCTTACTAATGGATCTCCGACGTTAACTTCGTCAC
CACTAGACACTAACACTTCAAATCCTTCACCGTCTAATTGAACTGTGTCTAAACCGATATGAACTAATAATTCTAATCCGTTATCTGCTTTT
AACCCAATCGCATGCTTAGTTGGAAAGACATTGTCAACACGTCCTGCAATTGGAGACACAACTTCTCCTTCAGTTGGATTAATACCAAAAC
CTTCGCCCATCATTTTTTGTGCGAATACAGGATCTGGAATATCTTCAATTTTCACGAATTCTCCAGTTAATGGTGTATAAATTGCGATATCT
TTCTGAACTTCTTTGCCTTTTCCGAATAATTTTTTAAACAT

Gene: msrB (peptide methionine sulfoxide reductase B)

Contig: 06_NODE_54, position: 567264 to 567692, length: 429 nt, orientation: REVERSE

Perfect match to: (MW2-BA000033-[1433566:1433994:r], highly conserved allele)

Sequence:

CTACTTATCAAAATGTGATATTAAATCGCCATAACCCAATTCTTCTAACTTTTCATATGGAATAAATTGAATTGCAGCGGAATTGATACAGT
ATCTTAAGCCGCCACTTTCTTTAGGTCCATCATTAAAGACATGTCCTAAATGACTATTTGATTCTTCTGAACGCACTTCAGTTCTCAACATAC
CAAATGATTTGTCGACTAATTCTATAATTTCATCGTCATCAAGCGCTTTGGAAAAGCTAGGCCATCCACATTCAGAATGAAACTTTTCTTCA
GATGTAAATAAAGGTTTACCAGAAATTTTATCTACATAAATTCCTTTAGCAAAATGATTCCAATATTCATTCATAAATGGTGGTTCAGTGCC
GTTTTCTTGTGTAACAATATATTCTATATCTGTTAGTTCACTTTTATCTTTTTTAAGCAT

Gene: msrA2 (peptide methionine sulfoxide reductase A2)

Contig: 06_NODE_54, position: 567685 to 568218, length: 534 nt, orientation: REVERSE

Perfect match to: (MW2-BA000033-[1433987:1434520:r], highly conserved allele)

Sequence:

TTAAGCATTTTGATTCCCCCAATGTGATTCTATAAACGCTTTTCTACCTGAACCACGTTGATATTGGTAATAATGTACCGGGTTCTTTTTGTA
ATAATCTTGATGGTAGTCTTCAGCTGGATAGAAATTTTTATATGGTTTAATAGGTGTAATCACTGGTTTCTTGAAAATACCTTGTTCATTTA
ATTGTTGCTTTTTAAACTCAGCAGCCTTTTTCTGATGTTCATCATGATAGAAAATGACTGGTTGATAGCTTTCGCCTCTATCGAAAAATTGC
CCTTGATCATCAGTTGGGTCAAATGTTTTGAAATATATGTCTAATATATTTTCAAAGGAAGTAACCTCTGGATCAAACGTAATTTGTACTG
CTTCGACATGGCCGGTTTGATTCGTACATACCTGTTCATAAGTTGGGTTGTCAACATGACCGCCACTATAACCAGATACGACTGACTTGAT
GCCTGGATATGATGTAAATGGTTTAACCATGCACCAGAAACATCCTCCTGCTAATGTTGCATATTCTTTTGTCAT

Gene: degV2 (DegV domain-containing fatty-acid-binding protein)

Contig: 06_NODE_54, position: 568304 to 569143, length: 840 nt, orientation: REVERSE

Perfect match to: (N315-BA000018-[1431052:1431891:r], highly conserved allele)

Sequence:

TTACTTCTTAAGGACTACGAGGCCAATCGCACCTTGACCAGTATGTGCAGAAATAACTGGTGTAGTTACATTTATATCGTAATTATTCACA
TGAAAAGCTTCATTAAAAACTTTCTTCAATTTATCAACATATTCAATGACGTTAGCATGTGCGACACCAATGGATTTGATTTCATGATCTCC
TATAAATTCAGCAATTTCCTTTTTCAAGTATTGGATACTAGAATTTTGAGTTCTCGCATTGTGCACAAGCTCTAAGCGACCATCATCTAGTG
TACCAATTGGTTTAATTTTCATAAGATTACCAATCAAACCTTTTGTTTTACTAATTCTGCCACCTTTAATTAATTGATTCAATTGCCCTATAAC
TACAAATAATTTAATGTTTTCTCTTAAATGATTTAACTTTTTAACTATTTCAGAAGTTGAGACACCTTCTTTTACAAGCTCTACTAGGTGTTG
TATTTGATACCCTAAACCAAAAGAAATAGATTTTGAATCAATAACAGTTACATTAGCATCTACCATTTGACTTGCTTGGTAAGCAGTGTTAT
ATGTACCACTTAATCCTGAAGAAAGATGAATACTTATGATTTCAGAGCCATCTTTTCCTAGTTCTTCATAAGCAGATATAAATTCACCTATG
GCTGGCTGACTTGTCTTTACATCTTCATCATTTTCAATATGATTAATAAATTCTTCTGATGTAATATCTACTTGGTCAACGTATGAAGCTCCT
TCAATAGTTAAACTTAAAGGAATTACATGAATGTTGTTTGCTTCTAAGTATTCTTTAGATAAATCGGATGTTGAGTCTGTTACTATAATCTG
TTTTGTCAT

Gene: folA (chromosomal dihydrofolate reductase)

Contig: 06_NODE_54, position: 569158 to 569637, length: 480 nt, orientation: REVERSE

Perfect match to: (MW2-BA000033-[1435460:1435939:r], allele observed in CC1)

Sequence:

TTATTTTTTACGAATTAAATGTAGAAAGGTATGTGGAATTGTATTTTTCTCATCTAGTTTACCTTCAACTGAAGAGGCAACTTCCCAGTCTT
CAAATGTATAAGGTGGAAAGAACGTATCACCACGGAATTTACCTTCAATAACAGTAATATACATGTCGTCCACTTTATCAATCATTTCTTC
AAATAATATTTGCCCTCCAAATATGAAAACATGGCCCGGTAGTTGGTAAATATCTTCAATAGAGTGAATTACATCAACGCCCACTACGTTG
AAACTTGTATCTGAAGTAAGTACAACATTTCGACGATTCGGTAGTGGTTTACCAATCGATTCAAATGTCTTACGACCCATTACTAAAGTAT
GACCTGTTGATAATTTTTTAACATGTTTCAAATCATTTGGTAGGTGCCAAGGTAATTGATTTTCAAAACCAATTACTCGTTGCAAGTCATGT
GCAACTAGAATGGATAAAGTCAT

Gene: thyA-chr (thymidylate synthase)

Contig: 06_NODE_54, position: 569837 to 570793, length: 957 nt, orientation: REVERSE

Perfect match to: (MW2-BA000033-[1436139:1437095:r], allele observed in CC1)

Sequence:

CTACACTGCTATTGGAGCTTTTATTGCTGGATGTGATTCATAGTCAACAATTTCCAAATCTTCATAATTTATGTCGAAAATAGACTTGTCAC
TGTTAATTTTTAATGTTGGAGGATTGAAGCTTTCACGTGCTAATTGTGTTTGAATCGCATCAATATGATTTGAATAAATATGTGCATCTCCA
AATGTATGCACAAATTCTCCTACTTCAAGTCCACATTCTTTGGCAATAAGGTGTGTCAATAAAGCGTAGCTTGCGATATTAAATGGCACAC
CTAAAAAGATATCTGCGCTACGTTGGTATAACTGGCAACTTAACTTACCATCTTGGACATAAAACTGGAACATGGTATGACAAGGCGGAA
GTGCCATTGTATCAATTTCTGTTGGATTCCATGCAGATACGATGTGTCGTCTTGAATCTGGATTATGCTTAATTTGTTCAATTACTGTTTTA
AGTTGATCAAAATGATTACCATCTTTATCAACCCAATCTCGCCATTGTTTACCATAAACATTTCCTAAATCACCGAATTGCTTCGCAAAAGT
ATCATCTTCAAGAATACGTTGCTTAAATTGTTTCATTTGTTCTTTATATTGTTCGTTAAATTCAGGATCACTCAATGCACGATGCCCGAAATC
TGTCATATCTGGACCGTTATACTCGTCTGATTTTATATAATTTTCAAAAGCCCATTCGTTCCAAATATTATTATTATATTTTAATAAGTATTG
GATGTTTGTATCTCCTTTAATGAACCATAATAATTCGGTTGCTACTAATTTAAAAGAAACTTTCTTTGTCGTTAATAGTGGAAATCCTTTAG
ATAAGTCAAAGCGAAGTTGATGACCAAATTTCGAAATCGTACCTGTATTTGTGCGATCATTTCGTGTATTTCCTATTTCTAAAACTTCTTCA
CAAAGACTGTGATATGCTGCATCAAATGAATTCAACAT

Gene: cvfC4 (conserved virulence factor C operon, protein 4)

Contig: 06_NODE_54, position: 571217 to 571654, length: 438 nt, orientation: REVERSE

Perfect match to: (N315-BA000018-[1433965:1434402:r], highly conserved allele)

Sequence:

CTATTTACAATTTTCGTCAAAGGCATCCTTTAAGTCCATTGCAATGTCATTAATATCTCTACCTTCGATAAATTCTCTAGGCATAAAATAAAC
TAAATCTTGACCTTTGAATAAAGCATACGAAGGACTAGATGGTGCTTGCTGAATGAATTCTCGCATTGTAGCAGTTGCTTCTTTATCTTGC
CCAGCAAAAACTGTAACTGTATTTGTAGGTCTATGTTCATTTTGTGTTGCAACTGCTACTGCAGCTGGTCTTGCTAATCCAGCTGCACAGC
CGCATGTAGAGTTAATAACTACAAAAGTAGTGTCATCAGCATTTACTTGGTTCATATACTCCGATACTGCTTCGCTCGTTTCTAAACTTGTA
AAACCATTTTGAGTTAATTCGCCACGCATTTGTTGCGCAATTTCTTTCATATAAGCATCATATGCATTCAT

Gene: cvfC3 (conserved virulence factor C operon, protein 3)

Contig: 06_NODE_54, position: 571670 to 572794, length: 1125 nt, orientation: REVERSE

Perfect match to: (N315-BA000018-[1434418:1435542:r], highly conserved allele)

Sequence:

TTAAATTGTTCTGTTTGCCATTTGTTTCCATACTGAACCAAGTGCCTCATCTCCGTTTTCAATACGAGATATGGCCATTTCAATTTGTAATTT
AACTTCAAACGCATTGTCATTAATATGGGCTTTTAGTGCGGGAAGCTGCTCTGCATTACCTTCATCAAAGATAAACATAGCAGCACGCCAC
CTAACGATTTTCTGTGGATCATCTAATAGTAGCACCATTTCTGGTAGTGCCTCTGGATACCCTAAATCGCTTATGCAATCCCCTGCTGTTCT
TCTTACAGCAGGACTTTTATCACGAAGCCCCTTATATAAATACGGTAAAATTTCTTTACTTTCAATCATACCTAATAATACAATCGCTTGAC
GTCTAACCGGTACTTTTTCATCAGATAAAGCTAAATCAAGTAGCGGTATATCTTCAAAAGTCGGCTTTGGAAAATGGTTTAACATTCGTAA
TCGAGTCTTCCAATTATCAGTTGCATGATATTCATCCAAAGAGACATGTCGATAGAAATGATAATTATTAGTTGCGTGATTTTCTTCTAAA
GCATGTTTTACCAATACGGGTAACTGTGATTCTGGATAGGTAGCTAGCACTTCTTCTAGGACACCATCCATTACTTCTTCAATATTTCCATA
GCGATTTCCTAAATCTAGCCATTTACGCATAAAAACAATATTGTCATGTGCTGTTTGCGCTTGAGTCATATGATCAACATATGTTTGTGGTA
ATTGTTCTCTTAATTCTTGGTCAGCAGAAGTTAGCTTAATTTGATACGGTATACCCTTAAAAGTTAATAATTCAGCTTTAATTTCACCAAAA
TGATTGTCAATTTGAGGTTCATTTACAGATTCTAAAACCTTATTCGCATCAGAAAAAGCAGCTTTAATATCAGGTAATATGACTTCCCAATC
AGCTTTTGGTGCCTTATCAACAGCTAAGAAGTTCATGACATGAAAAATGGAAGTGATACCATCTATAGATAACAACTGATTTATAAATCTT
GGTTGTGTTTCTTCTACTTTTTTATAAGTATTAGATAACTTGTCTTCTCTTGTATATGACAAAACAACTTTCATTGTATTTGGACTTGGTGTT
GGCTCTATACGTAAAATTTCCAT

Gene: cvfC2 (conserved virulence factor C operon, protein 2)

Contig: 06_NODE_54, position: 572832 to 573083, length: 252 nt, orientation: REVERSE

Perfect match to: (N315-BA000018-[1435580:1435831:r], highly conserved allele)

Sequence:

TTATTCGAATACAGCCTCTACTTTTGGCAATACTGTTTCCCAATTTGCGTCATTTTCTTTATCTACTGAAATAAAGTCCATAACATGGAAAAT
TGATTTAACGCCTTCAACCTTTAAGATGTCATTAATAAATGCTGGCTGTGAATCATCAACTTTAGTATACGTATCTGATGTCATACCTTCTC
TGCTTTCACTAAGTGTAATCTTCATTGTGTTGTGGTTCGGTGTTTCTGATATAGATATAATTTTCAT

Gene: cvfC1 (conserved virulence factor C operon, protein 1)

Contig: 06_NODE_54, position: 573095 to 573289, length: 195 nt, orientation: REVERSE

Perfect match to: (RF122-AJ938182-[1401344:1401538:r], highly conserved allele)

Sequence:

CTATTGAAGTTCATTTCCTAATTGTTTTATTTCTTTCCCTATTGAACACTCATTGATACAAAAATGATGCGCCTTCGTTTTCCCCTCTAATTTA
CGCAGACGAGTTTTGATTGGACATTGATTGCAATAAGTATTCATTAAATCATCTATTTTCGCGAGTGCATTTTGCTCTGAAAAAGTTAAAA
TACGCGACAC

Gene: Q5HFZ1 (putative membrane protein)

Contig: 06_NODE_54, position: 573534 to 574238, length: 705 nt, orientation: FORWARD

Perfect match to: (N315-BA000018-[1436282:1436986], allele observed in CC5+CC1)

Sequence:

ATGTATAATGAAATTTTAGGACTAGTTACGTTTATTGCAACGTTCGTACTTATGGTACTTATGTATCGCTTTTTTGGTAAACAAGGTTTAAT
TGCATGGGTTGCAATTGGCACAATCATTGCCAACATACAAGTGATTAAAACTGTAGAAATCTTTGGTATTTCAGCAACTTTAGGTAATGTC
ATGTTTGCTTCTATTTATTTAGCAACAGATATTTTAAATGATATTTATGGGCGTAGAGTTGCAAAAAGAGCAGTTTGGTTAGGCTTTTCATC
AACATTAATTATGATTATTGTTATGCAATTGTCATTACATTTTATTCCTGCACCAGAAGATATGGCACAAAAAGCATTACACGCAATCTTTG
ATGTTGTGCCACGTATTGCTTTAGGCTCAATTGTCGCATATATTATTGGTCAACATATTGATGTATTTATCTTTTCACTAATTAAAAAAGTAT
TTAGTTCTGATAAAACGTTTTTCATCCGTGCATATGGTAGTACATTTTTAAGCTCAATAATTGATACAGCTTTATTTGTAGCTATCGCATTTA
TCGGAAGTTTACCTGGTACAGTTGTATTTGAAATATTTATTACAACGTACGTATTAAAATTAGCTTCAACAGTTTTCAATGTACCATTTGGA
TATATTGCTAAATCATTTTATCGTAAAGGTAAGATTGATAAGTTAGATCAAGGCTATTAA

Gene: SIRU01 (staphylococcal interspersed repeat unit 1)

Contig: 06_NODE_54, position: 574321 to 574489, length: 169 nt

Sequence:

CCAACTTGCATTGTCTGTAGAAATTGGGAATCCAATTTCTCTTTGTTGGGGCCCATCCCCAACTTGCACATTATTGTAAGCTGACTTTTCGT
CATCTTCTGTGTTGGGGCCCACACCCCAACTCGCATTGCCTGTAGAATTTCTTTTCGAAATTCTCTGTGTTGGGGCC

Gene: repeat_nySAgamma (repeat element)

Contig: 06_NODE_54, position: 574379 to 574549, length: 171 nt

Sequence:

CCAACTTGCACATTATTGTAAGCTGACTTTTCGTCATCTTCTGTGTTGGGGCCCACACCCCAACTCGCATTGCCTGTAGAATTTCTTTTCGA
AATTCTCTGTGTTGGGGCCCACACCCCAACTCGCATTGCCTGTAGAATTTCTTTTCGAAATTCTCTGTGTTGGGGCCCC

Gene: rnhA (ribonuclease HI)

Contig: 06_NODE_54, position: 574836 to 575237, length: 402 nt, orientation: FORWARD

Perfect match to: (COL-CP000046-[1479407:1479808], highly conserved allele)

Sequence:

ATGGCGAAAATAAATTTTGATGCTGCGACGAAAGGAAATCCAGGCATAAGTACATGTGCCATTGTAATCAAAGAAGATGAGCAGCATTA
TACATATACACATGAGTTAGGCGAAATGGATAACCACACTGCAGAATGGGCTGCATGTATTTATGCACTAGAACATGCACGTGAATTAAA
TGTTCAAAACGCACTATTATATACAGACTCAAAGCTAATTGCAGATAGCATTGAAGCTGGTTATGTGAAAAACGCAAATTTCAAACCTTAT
TTTGATCAAATAGAAATATTTGAAAAAGATTTTGATTTATTATTTGTTAAATGGATACCGAGAGAACAAAACAAAGAAGCGAATCAACAC
GCTCAACAGGCATTGTATAAATTAATTAAAAAGAATAAATAA

Gene: ebh (cell wall associated fibronectin-binding protein)

Contig: 06_NODE_54, position: 575296 to 606918, length: 31623 nt, orientation: REVERSE

Sequence:

TTATTTTTTATTTTTCTTAGATTTCTTTTTCTTCTTTTTGGCTGCTACCTTTTTAGAAGTATTTTTTGATGCTGACTTTTTGTCTTTGGATTGGTT
ATCTTTTTGATTTTTCTTTTTAGCCAATAATAAAGGAACGTCCTCATCTTTAGATTCAATACTAGTTGTTGTTTCAACATCTTCCTCTTTATCT
TTGCGTCGTTTTGCGAATAAGAGTGGTGAATGTTCGTTATCTTTCAACACTTTTTCATCTGTGTTTTCATTTGTAACTTCCACATCTTCTTCA
TCTTCTTTACGACGACGTTTTGGTAAGAAGAAAGGCGTATGTTTAACTTTATCGAGTGACTCGCCATTATTTAGCGAATCTTTTTCTTCAAC
AGTAACATCTTCTTCATCTTCTTTTCTGCGACGTTTCGCAAATAAAAGTGGTAAATGTTTTGTATCGTCTAAAGTCTCTTTTATTGAATCTTT
ATTATTATCTCTTATTTCTAATTCTTCCTCTTCATCTTCTTTACGACGACGTTTGGCAATGAAGAACCAGAAACTAGCTAGTAAGCCAGAGA
TACCCACCACACCAATAGCGTTTTTAATAACATTACCGAAATTATTACTAAAGTGACGCATATGAAGTGGATCAATGTCATCATCTTCATCA
ATTTTCTTTTTATGTCCAATAGTCGAACTGTTAAATGGATGATTTGCAGTTCCATAACCAATTGTTAAATGGCTATTAGATTCATTTCCAGT
ACTTGAAGATTGTTTAGCACGATCAGATATTACAATTTGTACTGCTGAAATTCGAGCAATACCATTGTTCAATGCAGCCTCAACTTGCTGT
AATGTATGCGCATTATTAATATCTCTAATTGTTTCAAGCACAATTTCGTTAATTTGATTCATTGCTGCTTGTTTTTCATCAGCTGTGCCAATT

TCACTATTTCGAATACTATTCATTTTTTCATTTGAAAAGTCTTTAATTCTACTAATAGCTTCTTGCTTGCGTTTAGCTAGTTCTTTTGCTGTTG
GATTAGCTGCTTTCATTTGAGCTTTAAATTGTTCCAATTGCTCAGTTATTTCATCGATGCTTTGCGCACGTTGAATCGCTTGAATTGCTTGTT
CTTTTAATTGATTTAACTTAGCAATAGCTTCTTGCTTCTCTTTATCTGTTAGATCAGTACGAGCTTTGATTTCATCAATCATATGTTGAATTG
CATCTTCAATCGATTTAATTGCATTTGATTTTGCTTGTTCAATCGTAAATTGTTCTGGATTAAATTGTTCAATATGCGCTTGTTCTTGTTGTT
GAATTTCTTCAACTGAATGAACATCAGGTGCATTGTTAATATGATCAATTGCTTGTTGTTTAAGCTTATTAACTTCTGCAATTGCAGCTTCT
TTTTGTTCCAGTGTTAATGTCACACTATTATTGATTTCATTAATCTTTTGTTGTGCCGCATTTTCGATTGATTCAATTGCCTGTTGTTTGACTA
CTGACAATTCTTTTTCTACAACTTTTACAGGAACATTAACAATCACTTTTGATCCATCAAGCAATGTAACTTCAACTTTTGCTGTTAAGCTAT
CAGAAATCGTTGCAGTTGATGGTGTATCAATAACTTCTGCTGAAAGCTGATCAATTAAGTTTATGTGTGCAAGAATATCTTGTTCTGTAAT
GATGTCATCACGATGTACAATGAGTTCACCATTAACTAGGATTTGCTCAGGTGTTGCTTCAAAAATTTCATTTACAGCAGGTTGTTCATCA
ACCTCCCATACATGTGTATTTCTAATGTCATCTAAACCTAAGTTTAATCCTCGATTTAATTGATCTATAGTTTGAGCATTCTCAACGTTTTGT
AGTGCTCGTTTTTCAGCTTCGTCAATTTCTTTGAGCGCTTTTGCTTTTTGCTCAGGTGTTAAATCTGGATTTGAATTGATTTGATCACGCTTC
GCATTAGCTAAGGCTTTTATTGCATTTTTCGCATCTTCTTTAGCTTTCACTAAATCTTTGATGTCTTGTAATGCTTGTGCAAGTTGTGCTTTG
GCTTGTTCAATTTCTTCTTTAGTCATCGCATTGTTAATGTCGTTATGACCTTGTTGAAGTATTTGGTTAATTCGATCTTTAAGTGCTTGTTTTT
CCTTATCTGTTAGATTTGGATTTTGATCGATTTCGTCAATTAAAGCTTGAACTTGCTTGTCAACATCTTGTTTCGCATTTTCTTTAGCTTTCAC
TAAATCTTTAATTTCTTGTAATGCTTGCGCAAGTTGTGCTTTGGCTTGTTCAATTTCTTCTTTAGTCAGCGCATTGTTAATGTCGTTATGACC
TTGTTGAAGTATTTGATTAATACGATCTTTAAGTGCTTGTTTTTCCTTATCTGTTAGATTTGGATTTCGATCAATTTCGTCAATTAATGCTTG
AACTTGCTTGTCAACATCTTTATTCGCATCAATTTTAGCTTTCGGTATTTCATTGGCATGCACTTGTTCAATCGCATGGTTGCCAGCTGTTTG
AACTTGAGATACATCCTGATTACTTGTTGCTTTATTAATGTTGTTGATGATGCTGTTTGCCAATTCTTCTGCTTTATTTTTCGCAATAAGCTT
GTCTTGATCCGTCGCATTTGAAGCTTCGATTTCTTTTAGCTTATTAGCTAAAGCTTGATTAATAGATTGAATTGCCTTGTCTTTAGCATCTTG
TAGTCGTTGATCACCATTAAGATTATGGATTGCATCATTGACTGCTTGGATTGCGCCATTGATATCATTCACATTTGTGTTATCACTATTTA
GCAATGCATTTGCTAGACGTTTGGCATCATCGAAGTTTGTTTTAGCATTATCGTCAGCGTTTTGGTAATTGACAGTTTGCTCTGCATTTGG
AATTTCATTGTCAACTAAATGTTTCAATGTTTCCATTGCATCATTTAAGTCAATTTGATTATTAACAATATCTGTTACATCTGATACAGTATC
GGCATTGTTAATTGCTTTATGTGCAAGATCTTGTTGCTGTTGATTTAATCCATTTAACGAATTAACAAACGCATTTGCTTTATCTTTGGCATT
TGCAAGGTTTTGGTCTCCATTTAATGCATTTTGAGCATCGATAATATTTTGTTTCAATTGCTCTGCTTCAGCTTTTGCAATTGCATTACCTTG
CACTTTATCTAACTCATGTGCTGCATTCGCAATTGCATTATCATAATTTGCTTTCAAATTGTCATCTGCATTGATGTAATTTGTGCTGCTGAT
AACACCAAGGTGCTGTTCGTCAATATATTGTTGTACTGCACCCATTGCATTATCTAATTTAGTTGCTCTATTAATTGCTCGAGATGCTTTATT
TAGCGTTTCAGCATTATTAATTTGTTGGATTGCTAATTGTCTTTGAGCATTGTTTAGATGTGTTAATTGATCCAATCTTTGTAATGCTTCAG
ACTTACGATTATTAAGTCTTTCTTCACCATTTAATGCCTGTTTCGCTGCCGTTACTGCATCATTTAATTTGATAACTTGTTCTGCAGTTAAGT
TTTGACCAGTCGATTTATCAAGTGCTTGTTTCGCTTTATCAACCGCTTCATCATATACTTGTTTGACTTGTGTATCTGCATTGACATAGTTCG
TGCTACCTTTCGTGCGTCCTTCATTGCCAGTGATTTCTTCTGACAATTGATCCATTGCACGATTAAGTGCCTTAGCCTCATCTACAATTTGTT
GGATTTGATTTAAATCGTTTGATTGATCGATGCGACCTTTAAATCCATCTTGTTGAGCATTGTTTAATGCATTCAATTGGTCGATGTCATGT
TTACCATTTGTTTTAGCAAGTGCTACACGTTCATCACCATTTAATGCTTGTTTTGCATTTAAAATATTTTGAAGTGCTTGATCCACTTGTTGC
TTATTCGCATTTTGTTTCAATACATTTTCAGCATCAGCAATCGCTTGTTTATAAGCCTTTTGCTTATCTGAATCTGCTTGCGTGTAATCAACT
GTTTGCTCAACATTTGTATGATCATTAACAGCTTGTTGTAATTGATCCATTGATTGGTTCAATTGCGTTGCTTGATCTACTAATTCAGCGAT
TGGTTGAAGTTGCGTCGCTTGATCAATGTTTTGTTTAGCAGTTGCGATTTGATCAGCATTTAAATGTGTTAATTGGTCAATATTTTGTTTCG
CTTGTGTTTTAGCTTCAGCGACTCTCTCATCACCATTTAACTCATTTACTTTTTCTTGAAGCTTAGTTAATGCTTGTTCTACAGCGTCTTTATT
CGCATTTGAACCATTAGTTGGATCTGTAATGCTTTCTGCAGCTTGTAACGCTTGGTCTACTGCTTCTTTTTTATCAGTTGACGCTTCAGTGT
AATTTGGTTGAGCCTTATCTGTATTTACTTGATCAACTTTATTTTTCAATGTTTCCATCGCGTGATCAAGTTCAGTAGCAGTTTGAACATGTT
GTGCAACCTCTGTTCTTGTAGGCGCTGCATTTATAGCATCAGTTAATGCTTGTTGTTGTGCATGATTTATGTTTGGCAATGCATTTACAGTT
GTTACTGCTTGTTGTTGATCACGAGCAAGTTTTTGATCACCATGTAGGTTATCTTTTGCAGTTGTTACTGCTTGTGTCAATTGTTCAACTTG
TGCTTTATCAAGCGTTGGATTACCTGTTTGGTTAATTAAATCTTTTGCATTTTGAACTGCTGCTTGGTAAGCATCTTTTTGCGGTTTATCTTC
ATTGATAAACTTGCTACCAGATTCTGTTTGTTGTTGATCTTGAATACTATTACGTAATGCTTGCATTGCTTGATCAAGCGCTTTTGCTTCAG
CTAATTTTTGCGCTACTTCATCACGAGTTGCTGCGTTGTTTATTTGGCTTTCAAGTGCTTGACGTTGCGGATTATTCAAACCATTTAATTGA
TTTAAATCAGTAACCGCATGTTGTTTATCGTCTGCAAGTTTTTGATCACCGTGTAGGTTATCTTTAGCTTGGTTAACAGCTTGTGTCAATTG
TTCAACTTGTGCTTTATCAAGCGTTGGATTGTTAGTTTGATTAATTAAATCTTTTGCATTTTGAACTGCTGCTTGGTAAGCATCTTTTTGTGG
TTTATCTTCATTGATAAACTTGCTACCCGCTTCCGTTTGCTGTTGATCTTGAATGCTATTACGTAAAGCTTCCATTGCTTGGTTAAGTGCTTG
TGCTTCAGTTAATTTTTGTGCTACTTCGACACGAGTTGCTGCATTATTGATTTGATTTTCAAGTGCTTGACGTTGTGGTGTATTCAAGTTAG
ACAAGTTATTTAACGTTTCTGTTGCACGTTGCTTATCTTGAGCTAGTTTTTGATCACCATGTAAATTGTTTTTAGCATCAGTCACTGCCTGTG
TCGCTTGATCAATGATTGATTTGGCTAATGTAGGATCAGTTGTTTTGTTAATCAAATCTTTCGCGTGTTGTACTGCTTGCGTATAAGCATCT
TTTTGCGCTTGATCCTCGTTAATAAATTTACTACTTGCTTCAGTTTGTGGTTGATCCTTAATACTTTCTTTTAATGCTTTTATCGCTTCATTTA
ATGCTTGCGCTTGTGCAATGATTTCAGCCACTTTATCACGAGTTGTTGCATTATTAATTTGATTTTCTAGCGCTTGTTGTTGAGCTGGTGTT
AATTGATCTAAATGATTTAGAGAATTTCCAGAAGTTTGCTTATCTTGAGCTAATCGTTCAACACCATCTAATGCATTTTTAGATGATGTTAC
TGCTTGAGTCGCACTTGATACATTACCTTTATTGATAGTTGGATTATTTAATCCTGCAATGATAGACTCAGCATTTTGAACTGCTTCATCAT
AGGCTTGTTTTTTATTCGGTTCTGCATTGACATATGCACTGCTCGCACGTGTTGCATCTTTGTCAGCAATACTTTGTTGTAATGCATCCATA
AGTTGATCTAATGCTTGTGCTTCAGTCAAATCTTGCTTAACTGCTGTTCTAGTTGTTTCGCTATCAATTAACGTATCTTCCATATGTTTTTGT
GCATTGTTTAGATGTGCTAATTGACTAACCGTTTGTTTAGCATGATCTTTATCATTTTGTAATTTCACATCACCATGTAATGCTGCTTTCGTT
GTATTCACAGTTGCCGCTGCTTGATTAATCGCATTATTATCTAATGTTGCAGTTTGTTCATTGATAATATTATTTGCGGCTTGAACAGCTTG
ATCATAGTTTTGTTTCTCTGGTTGATCTTCGTTGATATATTTGCTATTTGCTTTAGTTGCTGCGTTATCTTGAATGCTCTGTCTTAAACCATG
CATTGCTTGGTTAAGCGTATTCGCATTTTGTTCAATTTGATGAACTGCAGTTACTAAAGTTGCAGCTGTAACTTGGTCTTTTAATGCTGTTT
TTTGAGGATCATTTAAGCTTGTTAAAGCATCAATTGCTTGCGTCGCATTTGTTTTAGCAACTTGTAAATTATGATCTCCATTTAACGCTTGA

AGTGTTGTTTGAACTTTAGTAATAGCTTGTTCTACTTCTGTTTGGTTCGCATTTTGACCAGTTTGTTTGTTAATCATTGCTGCCGCTTCATTT
ACCGCTGTATTATAAGCAGTTTGTTTATCAGTATCAGCATTGATGAAGTTACCACGTTGTTCAACGGCTTGATGATCTGCAATCGCATTAA
TTAAGTTACCCATTGCTGTATTTAAAGTTTCTGCTGTTTGCTTGTGGCCATTAACTGCAGCAATATCAGATGATTGCGTAATTTGTGATTGT
AACTGTTGCTTTTGCGCATTATTTAAATGATCTAATTGATCAAGTCTATTGTTAGCTTGTTGCTTAGCTGCTGCAAGGTTTTCATCACCATCT
AATGCAACTTTCGTATTATTCACTTGTGTCGTTGCACCATTAACATCATCTGGATTCATCGTAGGCGATGTAGTTTGATTAATGATATTATT
AGCAGCTGTTACAGCTTGGTTATAGTCATTTTGTCTTTGCGCAGTTGCATCATGATAATCTTCAGATGCTAATGTCGTTTGATTATCCGCAA
CACTATTTCTTAATAATTCCATCGCATGATCTAACGTTTGTGCTTTTTGATTAATCGTAGTGACATCTGTAACAAGTGGCGCTTGTGTAATT
TGACTTTCAAATGATTGTTTTTGTGCATCATTTAAGTGTGTTAAGTTCGCCAATTGTTGTTTTGCTTGTTGTTTTGCATTTGCCAAATTGTCA
TTACCATTTAATGCTCGTTTCGCTGCGTTCACTTCATTAGTGATGCTTTCAACTTGTGCTTGTACTTCATTAGTACCACTTTGTTTATTCAATA
ATGCTTCAGCTTTTGCTACTGCCTGTTTATACGCATTTTGTTTGTCAGGATCTGCATTTAAGTAATTACCGTCCGCTAATGTTTGAGCTTTAT
CATTAATAGCTTGTGATAATTGATCCATTGCATGATCTAGGCTCGTTGCTGTTTGTTTAACTTGGTTCACTGTAGCAATTTCAGGCGATTGA
TCGATTTGACCTTTTAATGCTTGTTTTTGAGCATCGTTTAAATGCGTCATTGCATTAACGGCATCTTTAGCATTTTGCTTTTCAGTGGCTAAT
TTAGCTTCACCGTTTAAGCTATTTTTAGCATCAGTCACTTGAGTTAATGCACGAGTAATGTCATCTGGGTTAAGCGTTGGATTTGTCGTTTG
ATTGATAATACCTTCCGCTTGAGACACTGCATTTGTATATGCTGTTTGCTTATCGACATCAGCATCGTGGTAGTTCTCACTTGCTTTCACAG
TATCTTTATTTGCAATACCTTGTTTCAAGTTACCCATTGCTGTATTCACATTTTGTGCATTTTGTTTAGTTTGTTCAACTGTAGCTAAAGCTTG
TGCTTGATTGATTTGGTTTCGTAATGCATCACGTTGTGGTTGATTTAAGTCACGTAACGTATCAAGATTTGCTAAAGCATCTTGTTTCGCTT
GCGCTAATTTTTCATCACCGTTTAATGCATCTTTCGCTTGATTCATAGTTGTAACTGCATGTGTTACTGTATCAGGCGCCAATACAGGTGTT
GGTGTGCCATTTGCGATTTGTTGCGCTTGGTTAGCTGCATTGTTATAAGCTTGTTGTTTGTCTTGATCTGCTTGTGTAAAGTCAACTGACTG
TGGTACTTGACTGTTCGCTTGAATTTGTTGTTTCAATGTACCCATTGCATTATTTAACGCATCAGCATTTTGCTTAATAGCATTAACACCTGT
AACAAGTTCTGCATGCGACACTTGGTCTTTTAATGCTGTTTTTTGTGGTTGATTTAAGTTTGGTAACTGATCAATGGCTGTATTTGCATTGT
CTTTAGCAACTTGTAAGTTATGGTTACCGTTTAAATCACCCTTAGCTTGAGCCACTTGTTGTGGATCCACGTTTGCATTTGGTGTACCACTA
ATGATTTGTTCAGCATGTGCTACCGCATTGTTATATGCATTTTGCTTATCAGTATCAGCATTGACAAAGTTACCATTTGCTTTAGTTTGGTC
TTTATCTGCAATACCTTGTTTCAATTGTGTCATTGCTTGATTTAAATCTTGTGCTGTTTGCTTAATCGTGTTTACATTTGCAACAGTTTGCGC
AGCATCGACTTGTTGTTTTAATGCATCTTTTTGCGCTTGATTTAAATCATTTGCATTTGTAATCACTTGTTTCGCTGCATCTTTTGCACGTTG
TACATTGTCATTACCATTCAATGCTTGTTTCGCTTCGTTCACACGTTGCATCGCTTGTTCCACTTCAGTTTGACTTGCATTGCTGCCATTTGC
TTTAGATAAAATACCTTCTGCATGTGAAACCGCATTAGTATAAGCATCTTTCTTAGCTTGATCTGCATCAGTAAAGTTAATGCTATTTAATG
TCTCAGTTTTATCTTGTAATGCATTGTTTAAGTTAGTCATAGCATTATTCAGATTTTGAGCATTTTGCTTAACATTATTAACTGTTGCAATAT
CTGGTGCTTGTTCAACTTGAGTTGTTAACGCTTGTTTTTGCGCATCATTTAAATGATCTAATGTACCAAGATTTGTCTTAGCAGCTTGTTTG
GCATCAGCTAATTTTTGATTACCGTTAAGTGCTTGATTTGTATCATTCACTTTAGTTAATGCTTGTTGAATTGTATTTGGATCCATTGTTGGA
TTTGTTGTTTGTTTCAACAATTCTTCTGCTTTTGTTACTGCATTATCATATGCTGTTTGCTTGTCAGTATCAGCATCGTGATAGTTCTCGCTAC
CTTTAATTTGTGCTTTATTCGCAATACCTTGTTTCAATTGTGTCATCGCGTCATTAAGCGTTGTTGCCGCTTGTTGAATAGCATTGACGTTTG
GTACATGTGTTGCTTGCGTAATTTGTTTGTTGTATTCATCACGTTGCGCTTGATTCAAGTTTGGCAATTGATCAATAGCATGTTGAACATTT
TGTTTCGCCGTTGCTAAGTTCGTATTACCATTTAAATCATTTTTAGCTTGCGTAACTTTATTTAAAGCTGCAGTAATTTCGCTAGGTGTAAC
GACTACATCAGGCGTACCACTAATTAATGCTTCAGCTTTTGCTACTGCTTGATTATATGCATTTTGCTTGTCAGGATCTGCATTGACAAAGT
TACCATCAGCTTTTGTTTGTTCTTTATCTGCAATGCCTTGTTTTAATTGTGTCATCGCATTGTTTAACTCTTGTGCTGTTTGTTTAACATTGTT
TACACCAGCTACAGTAGTTGCATTTTGAACTTGTTGTTTTAATGCATTTTTCTGTGCTTGGTTAAGGTCATTAGAGCTATTAATTAATGCTG
TTGCTTCGTCTTTTGCATGTTGAACGTTGGCATTACCATTTAATGCTTGTTTTGCTGCATTTACTTGTTGGATTGCTTGTTCAACTTCAGTTT
GTGTTGCATTGCCACCATTAGCTTTTGAAATAATGTTTTCAGCATTTGTAACAGCTGTGTTATATGCTTGTTGTTTTGCTTGATCTGCATCA
GTGAAGTTAACTGTTGCTTTCGTAGCATCTTTATCGGCTATCGCTTGTTTCAAGTTACCCATCGCAGTATTTAAGTTTTGCGCATTTTGCTTA
ATTTGATTTGCTTCATCAACAGTATGCGCACCATTAATTTGATCAGTAACAGCTTGTTTCTGCGCATTATTTAAGTTGTCTAATGAACCTAA
AGATTGCGTTGCTTGTTGCTTCGCTGCCTCTAAGTTTTCATTACCATTTAATGCATTTTTAGTTGTGCTTACTTGTCCAGCAGCTTGATTGAT
AACAGTCGGATCTAAAGTTGGACTTGTAGTTTGATCAATAATACCTTGTGCAGTTGTGACAGCATTATTGTACGCATCCTTTTTATTCGGA
CTTGCATCAGTATAATTTTGGTTTTGTTTTGTTGTCGCATTATCTGCAATACTTTGACGTAATTTGTCCATTGCTGCATCAACATTGTTTGCT
TTTTGTTCATTACCTTGTGCTTCTGCAACAGTAGTCGATTGTTGTACCAATTGTTTTAATGCCTCTTTTTGTGCATTTGTTAAATGGCTTAAA
CCGTCAATTGCTGTATTTGCGTGTTGTTTAGCTTTTTCAAGGTTTTGAGTACCATTAAGCGCTGCTTTTGTAGTATTCACTTGATTCATAGCT
GCTTCAACTTGATCTTTAGGCACGTTTGTACCTGTAGATTTATTTAAAATATTTTCAGCATTACGAACCGCTTCATTGTATGCATTTTTCTTC
TCTGGATCTGCATCTGCAAAGTTTTGACTGCCAAGTGTAGTGTCTTTATCATTCAAGCTATTTTTCAAGTTACCCATCGCTGTATTCAGTTC
CTGAGCGGTTTGAATGGCTTGCGTTACTTCAGCTACAGTGTGCGCTTGATTGATGTTTCCAGTAACAGCACCTTTTTGTGCATTGTTTAAG
TTATCTAACGTAGATAATGTTTGGTTTGCATTGTTTTTAGCATCTGTTACTTTTCTATCACCATTCAACGCTTGTTTAGTAGTTGTTACAGTT
GAAAGTGCTGCTTCAACTTGTGATTGGTTCGCATTTGTACCATTTGCTTGATTAATAATATTTTCAGCAGCAGTTACCGCATTGTTGTATGC
TGTTTGTTTTTCTGGATCTGCATCTGTAAAGTTAACACTTTGTTTCACTTGATCTTTGTCTTGGATTCCGTTGATTAAACGTTCCATTGCTTG
ATCTAATTGTTCAGCTTTAGTTTTCACTTGATTTACTGTGCTAATATTTGGTGCATTTGTCACTTGTGTATTTAAATCTTGCTTTTGAGCATTA
TTTAAATGACTTAATTGGTTGATGTTAGCAGTTGTTTCTTGCTTTTTATTTGCTAAGTTTTGCGCACCATTAAGCGCAGTTTTGTTAGTGTTC
ACTTGATTAGCTGCTTGTGTTACTGCAGATGGATCAAGTGTTGGCTGACTCGTTGCATTAATGATATTTTCAGCACTTGTAACTGCTGTATT
GTATGCATTTTGTTTATCTGTATCTGCATCAACATATGGTTGACTCGCTTTTACTTCATCTTTATTCGCAATACTATTTCGAAGTTGATTCATT
GCATTGTCAAGATTTGTTGCATTGTCTTTCACACTTTGTACACCAGTTACTTGTGTCGCGCTATCAATTTGACCAGTAATACTTTGTTTTTGT
GCATCAGATAAATGTGTCATTTGACGTAATGCATCTTTAGCTTGTTGCTTCGCAGTTGCTAAGTTTGAATCACCATTTAAACTTGTTTCAGC
ATTATTGACACGTTGTGTTGCCGCAGTAACATCTGCTGCATTTGTAATCACATTAGGTGAACCATTTACAATGTTTTTCGCAGCATTATATG
CATCAGTATAAGCACCTTGCTTATCAGGACTTGCGTTAGTGTAATTACCACCAGCAACAATTGTGTCATGATCAGCAATTCCTTGTTTTAAT
TGATCCATCGCATTATCAAGTGATGTAGCACTATTTTTCAGATCAGTTACACCTGATACACGTTGCGCAGCGTTCACTTGTTGTTTTAATGC

TGTTTTTGTGGTGTATTCAAATGTGTCAAGTTATCGATAGCTTGTGTTGCTTCAGTTTTAGCGCGTTGTAAGTTCGCGTCACCGTTCAAAG
CATTTTCAGTAGACGTTACACTTTGAATTGCTTGTTCAACAGCTTGCTTATCTTTATTCGTACCATGATCTTTATTTAAGTAAGATTCAGCTT
GTGTGATAGCATTATCAAATGCTGTTTTCTTAGTTGGATCTGCGTCGATGTAGTTTTCGCTCGCTTTAGTTTGATCTTTATTTGCGATTGCT
GACTCTAATCGTTGCATTGCACCATCTAAGTCTTGTGCTTTCGTTTTAACACTATTTACACCGTTAACTGTTGTTGCACCTTCGATTTGTGAT
GTTAAATCTTGTTTTTGTGCATTATTTAAGTGTGTTAATGTACCTAAATTTGCTTTCGCATCTGATTTTGCTTGTCTTACATTTGCATCACCGT
TTAAAGCATTCTTAGCAGTCGTTACTTGATTTAATGCAGCTTCAACTTGTGCTTTTGTCATATTTTGACCGTTTGCTTTATCTAAAATACCTT
GAGCATTCGTAACAGCATTTGTGTAAGCATCTTTCTTACCTTGGTCAGCATCTGTAAAGTTCACTTGTTGTTTAACAGTGTTTTGGTCATTA
ATACCATTTTGAAGTTGACCCATTGCTGTATTTAATTCATTAGCAGTATTTTGTGCAGCAGTTACACCTGCTACTGTCGTTGCATTGTCAAT
TTGACTAGTTGCTGCAGTTGATTGCGCATTATTCAAGTTACTATATGAAGCTAATGCACGTTTCGCTGACTCTTTTGCTTGAGCAACTTTAT
TATCACCATTCAAGTCATTTTTAGCTTGATTCAATTGGTTTAGTGCTGCTTGAACTGTTGTTTCATTCGCGTTGCTGCCATTAGCATTAGTAA
TTGCTTCTGCTTGTGTAACCGCAGTATCATACGCTTGTTGTTTTGGTTGATCCGCATCTGTGTAATTGACACTACGTTTCGTATTATCCTTTT
CAGCAAGTGCATGTTTCAAATTACCCATCGCTGTGTTTAACGATGTTGCTTTATTTTTAGCCGCTGTGACAGCTGCAAGATTTGGTGCTTG
ATCCACTTCAGCATTTGCCGCAGTTCGTTGTGCATTGTTCAAGTCTGTCAAACGACCGATATCTGATTTCGCAGTTTGTTTTGCTGCTGCTA
ATTTTTCATCACCGTTCAATGCAGACTTCGCACTGTTCACTTGGCTCGCTTTTTGGTTAATTGTATCAGGATTCATTTCAGGGTTATTCGTTG
CACTAATAATACCTTCAGCATTAGTTACCGCATCATTGTATGCATTTTGTAAATCTGCATTCGCGTCTTGATAATCTTCGCTTGATTTAGTCG
CATCTTTAGAAGCAATACTTTGTCTTAATTGATTCATTGCTTGATCTAAAGTACCGGCATTAGCTTGAATACCTTGAACACCTGCAACAGTC
GTACCACTTTCGATTTGCGATGTTAAGTTTGCTTTTTGAGCATCAGTTAAGTGTGACATTGTCGCTACTTGTTGTTTAGCTGTGTTCTTCGC
TTCATTTAAGCGCGCATCACCATTTAATGCAGTTTTAGCAGTATTCACACGTTGTAATGCTTGTTCAACGGCTGCTTTATTTTCATTTGTGCC
AGCTGTTTTATCTAATAACGTTTTAGCTGCCGTTACAGCATCATTGTATGCAGTTTGTTTATCTCTATCTGCATCAGTATATTTCTGAGCTGC
TTTTGTAGCTGCTTCATCGTTAATACCACGTTGTAAGTTGCTCATTGCAGTATTTAATTCAGATGCTGTTGTAGACACTTGATTTACACCTG
CAACTGTTGTAGCACCTTCAATTTGTGATTTCAACGCTTCTTTTTGTGCGTTGTTGATTGATGTTAAGTTATTCAATGCATTTTTCGCAGTTG
TCTTAGCATTCGCAACATTTTGATTACCGTTCAATTCGTTTTTAGCACGTTGTACATTTTGTAACGCAGATTCAACATTGTCTTTTGCAGTAT
TTGGACCTTGTGCTTTATTTAAAATTTGTTCAGCTTGCGTCACTGCATTTGTATATGCATTACGTTTCGCTTCATCTGCATCAGTGAAGTTAA
CACCTTGCTTAATCGTATTCTGATCTTGAATACCATTTTTCAAGTTCGTCATAGCTGTATTTAATGCATCCGCATTATTTTGTGCTTGTGTTA
CTTCATTAACATGCGTTGCACCCTCGATTTGACGTTTCGCCGCTTCTTTTTGAGCATTAGTTAAGTCACTTAAGCCATTCAAATGTTGCTTC
GCATTTGTTTGCGCAGTTCTTAAGTTTTCTTGACCGTTTAACGCTTGTTGTTTAGCTGTCACTTGGTCTTTCGCTTGATTAATTTCTTGCGCA
TTCATTGATGGACTAGTTGTTTGACCAATGATTGCTTTTGCTGCAGTGACTGCACTATCGTATTCACTACGATTATTAGGACTTGCATCTGT
GTAGTTTTGACCTGCTTTAATCGTTGCTTCATTCGCAATGCTATCTCGTAGACCTTTCATTGCAGTGTTTAATGTTTGTGCATTATCACGAAC
AGTTTGAACATTTGGCAACGTCGTCGCTTGACCCACTTGTTCTTTAAGTTTTGCTTTTTGCGGACCATTTAAAGAAGTTAAACCATCAATTG
CTGTGTTAGCATTTTGTTTCGCTACTTCTAAATTATGATTACCGTTTAACTGCGTCTTCGCATTCGTTACTTGCGTTGCTGCATTTGTAACAT
CTGATGGTGTTAACGTTGGTGTTGGTGTACCATTAACGATACTTTCGGCAGCTGTCACTTTTTCATCATATGCTTGACGTTTATTCGCATCA
GCATTGACATAGTTACCACTAGTTTTTGTGTCAGCTTTATCAGCAATAGCACGTTTTAAAGCTGTCATCGCAGTATTTATTTCAGTCGCAGT
ATGTTCAACACCATTTGCTGCAGATACGCGTCCTGCACTTGTTACTTGTGCTTTTAATGCTTCTTTTTGTTTTGTATTTAAGTCCGAAGCGTT
TGTAATCGCTGTGTTAGCAGCCTGTTTCGCACGTTCTAAGTTTTGAATACCGTTTAATGCAGTATTTGCTTGTGCAACAGCTTGCATTGCTC
TTTCAACATCTGCTTTAGGTGTATTACCGCCAGCTGTTTTATTTAAAATAGTTGCTGCTGCATTTACTGCTTGAGAATAAGCCGTTCGTTTA
GCATCATCAGCATCTTGATAATTTTGACTTTGTAACGTCGTGTCTTTATCACGAATTGATGTTTCTAATTGACCCATAGCACCATCTAATTGT
TGCGCTTTGGCTTTAACTGTATTAACACCTTCAACATTTGTTGCTTGTGTAATTTCATTATCTAACGCATTACGTTGTGCATTATTAATGTGT
GTTAATGTACCTAACGTTTGTTTCGCAGCAGCTTTAGCTTCATTTAATTTCGCATCACCGTTCAACGCCGTCTTCGTACTGTTCACATTTTGT
AATGCTTGTTCAACTGCTGCTTTGTCTACATTTTGACCACTAGCTTTTGTTAAAATTGCTTTCGCTGCATTTACAGCTTGATCATAAGCTGAT
TTCTTATTTGGCTCTGCATCTAGGTATTTCTGAGTTTGTTTTGTTTGTGTCTCATCATTGATACCATTTTGTAAACTATGCATTGCGTTATTTA
ATTCTGTTGCTTTGGCAGTTTCTTGATTTACACCAGCTACTGTAGTTGCACCATCAATGCTACGCGTTAACGCATCTTTTTGTGCATTGTTA
ATTGATGTTAAGTTATTTAAGTTGTTTTTCGCAGTTGTCTTAGCTTGAGCTAAGTTTTGCGCACCATTTAATGCATGTTCTTTAGTTGTCACT
TGTGATGTTGCTTGCGTAATAGTATTTGGTTCCATCGTTGGGTTCGATGTTTGATTAATGATTGCTTTTGCTGCAGTAACTGCGCTGTCGTA
CTCGTTACGATTAGTTGGACTTGCGTCAGTGTAGTTTTGACCTGCTTTAATTGTTGCTTCATTCGCAATACTATCTCTTAAGCCTTTCATCGC
TGTATTCAACGTTTGAGAACTATTTTTAACAGTTTGAACACCTTCTAATGTAGTTGCACTTTGAACTTGTTCTTTTAATTTTGCTTTTTGTGC
ATTATTCAATTGTGCTAAGCCATCAATTGTATTATTAGCATTTTCTTTAGCAACACGTAAGTTGTTATCGCCATTTAATGCTGTTTTAGCCGC
ATTGACTTGTGAAGTCGCACTATTTACATCATTGACTGTTAATGTCGCATTCGGTGTACCATTAATGATATTTTCAGCGTTAGTCACTTTTG
AATCATACGCTTGACGTTTTTCTTGATCAGCATTGACATAATTACCGCTTGCTTTTGTTCTTTCTTTATCAGCAATAGCTGCTTTAAGTGCTG
TCATCGCACCGTTCAATTCAGTAGCTGTATGTTGAACGTTATTTGCATCAGATACACGTTGTGCATTGTTAACTTGTGTTTTTAATGCATCT
TTTTGTTTTTGGTTTAAGTCATGTGCGTTGTTAATTGTGTTCGTCGCATTTGTTTTAGCTTGAGCTAAGTTTTGTACACCATTTAACGCTGCT
TTTGTTGTATTGACTTGATTTGCCATGCCATTAATCGCATTAGCATCCATATTTGGATTGTTCGTTGCATTAATAACACCATTTGCATTATTT
ACAGCAGTATTGTAATTATTTTTGTTGCTATCAGATGCATCAAGATAATTTTGACTTGTTTTCGTCGTATTATCATTTTGGATACTTGTACGT
AATGCACCCATGGCAGTATTTAAGGTATTACCTTTATCTTTAACACCATTTACACCTGCTACATTTTGCGCTTGTTCAACTTGATGCTTCAAG
TTGTCTTTTTGTAATTGTGTTAAGTTAGGTAAACCATTAATCGTATTTGTTGCTGAAGTTTTCGCATTTCTTAAGTTTTCAGCACCATTTAAA
GCCGCTTTCGCTCTTGTAACTGCATTTAATGCATTATCAACGTCTGCTTTAGATGTGTTACCACCAGTTTGTTTATTTAAAATGCCTTCAGCC
GCTGTGACAGCTTGCGTATATGCATTTCGTTTTGATTCATCTGCATCAAGATAATTTTGATTTCTTAATGTAGCATCTTTATCATTGATTGCA
CCTTGTAAGCTATTCATTGCGTCGTCTAATGTATTGGCATTTGTTTTTACAGTATTAACGCCATCAACAGTCGTCGCTTGATTGATTTGACC
TTCTAACGCAGTACGTTGTGCATTCGTAATATGGTTTAAAGTGCCTAAGTTTTGTTTAGCTGCCGCTTTCGCTTCTGCCAGTTTTGCATCAC
CATTCAATGCATCTTTCGTACTTGTTACTTGTTGTAATGCACGGTCAACTGCTGCTTTATCTGAATTTGAACCTGTTTGTTTATTTAAAATTG
CTTTCGCAGCAGCTACAGCTTGATCATAAGCAGTTTTCTTACTTTGTTCAGCGTCACGATATTTTTGAGTTTGTTTTGTTGCATTTTCATCAT

CAATACCATGTTGTAATTGACCCATAGCTGTATTAAGTTCATTTGCAGTTTGTTGGATACTTGTTACATTTGCAACACGTTGCGCACTTGTC
ACTTGTGCTTTCAACGCATCTTTTTGCGCTTTATTTAAGTTAGTAGCACCATCGATGGCATTTGTTGCTGTTTGTTTCGCTTGCGTTAAATTA
TGTGTACCATCTAATGCATTTTTCGTTGATGTCACTTGTGTAGCGATTTGGTTAATTGCATTAGCATCCATATTTGGATTGCTTGTTGCATT
AATGACACCATTAGCACTATCAACAGCATTGTTATAGTTTGTTTTGTTACTTCCTGTAGCATCAAGATAGTTTTGGCCATTTTTCGTAGCTG
TGTTATCTTGAATGCTATTTCTTAACGTACCCATAGCACCATTTAATGTGTTGGCACTTGATTTAACAGTATCTACACCTGCAACATTTTGTG
CTTGTTGCACTTGTTGTTTCAATTTATCTTTTTGAGCAGTTGTTAAATGTTGAAGTCCGTTTATAGTAGTGTTTGCATTTGATTTAGCTTGCT
CTAAATTATGGTTACCATTTAGGGCACCTTTCGCATTAGCAACTTGTGACAACGCGTTTTCAACGGCAGCTTTATCTAAATTTGATCCACTA
TTTTTATTTAAAATATTTTCTGCGTTCGCAACGGCTTGAGTATAAGCAGTTTTCTTATCTGAATCAGCATCATGATAGTTTTCACTTGCTAGT
GTATTTGCTTTATCATTAATACCATTTTGCAAGTTAGCCATCGCTGTATTCAATTGTGTACCTGTATTAGATACAGCTTCAACACCAGCTAC
AGTTGTTGCTTGATCAATTTTAGTTGTTAAGTCACGTTTTTGTGCATTATTAATTGACGTTAAGTTATTTAATGCATTTTTAGCTGCATTCTT
CGCATTTGTAACGTTTTGATCACCATTCAATGCATTTTTAGCACTTGAAACATTTTGAATAGCCGCTTCAACATCTTGTTTAGACGTATTTGC
ACCTTGCGTTTTATTCAGAATTGCTTCAGCTCTGCTTACCGCATTTGTATACGCATCACGTTTAGCTTTATCTGCATCAGTGAAGTTAACAC
CTTGTTTAACTGTGTTTTGATCATGGATAGCTTGTTCCAAGTTGCCCATTTGCGTGTTTAACTCAGTTGCTGCATTTTTAGCAGCTGTTACCT
CACTCACATGACCTGCACGGTCAATTTGTGATGAGATGGCAGATTTTTGGTTATTTGTTAAGTGTGATAACGTATTTAAGTTTTGCTTAGC
AGTGTTTTGTGCATTTCTTAAGTTTTCAGCACCGTTTAAACCATTTTTAGCATTATTCACTTGTGTTGCAGCTTGGGTAATCGCACTAGCAT
CCATAGTCGGATTGTTAGTTTGATTAATGATACCTTTCGCATTTGACACAGCGCTATTATATGTTGATTGGTTATTCGGACTTGCGTCTGTA
TAGTTTTGACTTGCTTTGACTGTTGTTTCGTTAGCAATACTATCTCTTAAGCCTTTCATTGCATTGTTCAATGCTTGTCCATTTGTTTGAACA
GATTGTACGTCTTCTAATCTATTGGCTTGTCCCACTTGTTCTTTTAATTTAGCTTTTTGAGGAGTATTTAATTGCGTTAATGCATCAATAGCA
GTATTGGCGTTTTGTTTTGCTACACGTAATCTTTCGTCACCATTTAATTCTTGTTTCGCGCTGTTTACTTGATTAGCTGCAGCTGTTACTTCT
GAAGGTGTCGTAACAACCGTTGGCGTACCGCTAATAATATGTTCAGCATTGGTAACTTTAGTTGTGTAAGCATTTTGTTTAGTGCTATCGG
CGTTGACATATTTACTGCTTGCTAACGTATTCGTCTTATCTGCGATGGCATGTTTTAATGTGCCCATCGCCGTGTTCAGTTCAGTCGCATTA
CGTTGTACATCTTGTGCATTAGATACGCGTTGAGCACCATTAGCTTGTGCTTTTAATGCATCTTTTTGATGTTGATTTAAATCAGATGCGCC
ATTGATTGCTGTAATCGCTGCTTGTTTCGCATTGTTTAAGTTTTGCGTACCATTTAATGCATGTTTCGCATTATTAACATTATTAAGTGCTTG
TTCGACTGCTGTTTTCGCTGTATTCGGTCCAGTTTGTTTATTTAAAATGGTTTCGGCTGCTGATACAGCTTGATTGTATGCATTACGTTTTTG
CTCATCAGCATCCAAGAAGTTTTGGCTCGCTAATGTTCCTGACTTATCGTTGATAGCCGTTTGTAAGTTACCCATAGCACCATCTAAACTAT
TCGCATTTTGTTTAACAGATTCAACACCAGCTAAGTTTGTAGCTTGTGAAATTTGATTTGTTAAATCATTACGTTGAGCTGTAGTGATATGC
GTTAAAGTACCTAAATGTTGTTTCGCTGCGTTTTGAGCTGCAATTAATTTTGCATCGCCATTCAATGCATCTTTCGCAGTATTCACACGTTG
CAATGCTGCTTCAACTGCATTTTGCGCAGTGTTTGGACCATGTTGTTTATTTAAAATCGCTTTCGCTGCAGTAATAGCATTATCATACTCTT
GTTGTTTATTTGTATCAGCATCACGATATTTCTCAGATGATTTAACTTGAGATTCGTTGTTAATACCACTTTGCAAGCTAGCCATAGCTTGA
TCTAGATGTTGTGCATTTTGTTTTACAGTATCAACACCACTAACTCTTGTCGCACTACTAATTTGACTAATCAAATTGTTCTTTTGAGCATCT
GTAATACTCGTTAATGTGTTTAAGTACGTTTTCGCATTTTGTTTTGCCGTAGCTAAGTTTTCATCACCGTTAAGTGCCGTTTTAGAACTATTC
ACTTGCTCTGCTTTTTGTGTAATCGTACTTGGATTCATTTCTGGATTACTATTTGCATTAATAATCGTTTCAGCAGCAGCTACTGCGTTGTTA
TATGCTGTTTGCTTATCATTATTAGCATCTACGTAATCTTCACTTGCTTTAGTCGCATCTTTGTTGGCAATACTTTGTCTTAACGTATTCATG
GCTTGATCTAATGTATTGGCATTTGATTGAACCGTATGAACACCAGCGACAGTAGTACCACTATTAATTTGGTTTGTTAAATTCGTTTTTTG
TGCAGTTGTTAAATGCGTCATATTATTTAACTGCTGTTTCGCTGTTTGCTTCGCTTGATCTAATAAACGCGTACCATCTAAGTTATTTTTAGC
CGAATTCAATTGATTCATCGCTTCAGTAACTTGATCTTTCGTTTTATTTTGACCATTTGATTTATTTAAAATATCTTTCGCAGCTTGTACCGC
ATTTGTGTATGCTGTTTTCTTACTAGGTGTCGCATCTTGATAGTTTTGACTATTAAGCGTCGTTTGTTCATCATTGATTGCACCTTGCAAGTT
ACCCATCGCTGTATTTAAATCTGTACCTTGTTGTTTAACAGTATTTACGCCAGCAATATTTGATGCAGCATTAATTTTAGATTTAACATCTGC
TTTTTGTGCATTATTTAAATGTGGTAATGCATCAATTGCTCTTGCAGCATCTGTTTTAGATTGTGCTAATTTTTCATCACCATTTAATGCATT
TTTATTAGAAGTAACAGCTGAAGTTGCACGATTAACATCATCAACAGACATCGTTGGATTTGTTGTTTGATTAATGATTGTTTCGGCACTT
GAAACTGCACTGTTATATGCATTTTGTTTAGCTGTATCTGCATCCGCATAATCTTCTGTACGTTTCACTTGATCTTTATCTGCAACAGCTTGT
CTTAAGTTACCCATTGCACTATTTAAGTTTGTTGCATTTTGCTTAATCGTATTAACTGTCTCGATTTGATGTGCACTATTAATTTGCGATTGT
AAGTTTTGACGTTGCGCATTATTCAAGTTATTTAATTGACCTAATGCAGTGTTCGCTTCTTGTTTCGCAGCATTTAACTTAGCTTCACCATTC
AATGCATGTTCTTTACTAGTGACATTTGATAAAGCATTGTTAACATCACTTGGTGTTATAACTGGATGTTGTGCATTACCATTAATAATGTC
ATTCGCATGGTTGTATGCATTGTTGTAATCATTTTTCTTATTAGTATCTGCGTTGACATAATTATCACTTTGTACGACTTGATTATGATTAGC
AACGCCACGTTTTAAACCTGTCATAGCAGTATTTAAGCTTTGAGTCGTTTGTTTAATATCATTTACTGCATGTACATTTTGTGCACTATTCAC
TTGTTGTGTTAATGCATTCTTTTGTGCTTGGTTTAAATCACTTGCATGCGTAATCGCATTTGTTGCTTCTGTTTTCGCACGTTGTAAGTTTTG
TTGACCATCTAAAGCATCTTTCGTCGATTTAACAGATGCTGCTTTTTGGTTCACTGTGTTAACATCCATCGTTGGATTAGTCGTTTCTCCAAT
GACACCTTTAGCCGCATTAACTGCATTATCATACGCTTGTTTATTAGCTGGTGTTGCGTCAGTGTAATTTTGACCTGATTTAATTGTATTAT
TATCCGCAACACTGTCTTTTAATTTCGTCATCGCAGTATTTAAAGCCGTAATGTTAGACTTAATTGTTTCAAGAGCAGCATGATTTTGAGCA
GCATTAATTTGTTGCGTGAAATTATTTTGTTGTGCTTGGTTTAAGTTAGATGCACCATGTAATGTTGTTAACGCTGGCTGTCTATCTAATGT
TAAACCTTGACGTGCTGTATTTAATTGATCCTTAGCTTGGTTTGCCTCTGTCACTTTATCATTGATATTTTGGACAGTTGGGTTGCCATTCA
ACACTTGATTAATTTCAGTTAACTTTTGACGCGCTTCTTGTAATTTTTGGTTGTACGCATTTAACGAAGCGGTCGTCATACCTGTTGTATCC
GTTGGTTGATTAATGCTTTGTTCTAATTGCGTTTTCGCAGTTTGAAGCGGTGCTTTATCTGGTGTTAACGCTTGACGTGCATGATCTAAATC
AGATTTAGCTTGATTTGCTGCAGACGTATTTGTATTAATTTGTTCTACAGTCGGTGAACCTGCTAATACTTGATTGATTTGTTGAATCTTAT
TACGTGCAGCTGTTAACTTCGCATTGTATGCATTTATAGAGTCTTGTGTCATACCAGTTGTACTTGTTTGCGTGTCAATACTATGTTGTAGT
TGATTTTTCGCATTTTCTAAAGGCGCTTTATCAACTGTTAAGCCATTGCGTGCTTGATCAAGTGCTGTTTTAGCAGCATTCGCTGCTGTCAC
GTTTTGACGAATCGTTGCAACATCTGGATGAGATGCCAGTACGCGATCAATTTCTTGAATTTTAGTTCTAGCTGCTGAAAGTTTTTCATTA
AATGCTGCAATAGATGTGCTTGTCATACCAGTCGTACTCGTTGGCTGATCAATATCATTTTGCAACTGAGTTTTTGCAGTTTGTAATGGTG
CCAAGTCTGGTGTTAATCCAGCAATTGCTTGTTTTAAGCTATTATATTTTTCTTCTACTTTTGTTTTTCTTCGGCAATTTGTTGGTCTGTTGC

GTCACCATTGTTAATAACATTTTGTGCATTTGTTGATTCTGTTTGACCCGCACGTTTAGCATTTTCATATGCTTGGATTGATGATTGTGTCAT
ACCATCAGTAGTTACTGATTTATTGATTTCTTCATCAAGTTTCGTCTTAGCAGTTCTTAAAGCACTATTATCAGCTAAAGGTACTAATTGATT
AATTGCTTGCGTTAATCGCTCATTAACACGATTTACATTTGTTAATGCTGTTTGTACCTCTTGCACTGATCTTATTGGCTTCTGAATGATAGC
ATTAGCACTATTTTTAGCACTTGTAAGTTCTGCTTGAAGCGCATGCATTGAATTATTATACGCAGTAATACTTGCTGGTTTCTTACCAGTCG
TTGTACCTGTGCGATTCAATTGTTGCACTGCTTGCTCTAAGGTATGTGTATCTGCAGTTAAATTTTGTTTCGCTTGGTTTAATGCTGTTAAT
GCGTTATCGACACGATGTTTTTCCTCTGAAATTTGCTGCGGTGTTGCATCTCCATTGTCTATAACACGTTGTGCTGCAGTTATTTCAGTTTC
TGCTTCACGCTTCTTCGCATTATAGTTATCAATACTTTGTTGCGTCATACCAGCAGTTGATGGTACTTGGTTCACAGAACTTTGTAAGTTAT
TTTTAGATGTTACTAATTGGCTATTATCTTCTTTATTTTGAAGTAATGCTTTAGCTTGATCAATCTTAGTTTGTGCTGCACGAACTTTAGTTA
ATGCGTCAGAAACTTGTTGTGGTGTTGCACGATCATTATTAATCACTTGTTGCGCTTCTGTTTTTGCAGTATTGATTTGTTGTTGCGCATTA
TGAATTGCATTATTGTACTGCGTAATTGTACCTGGCTTTTTACCTTCAGTGCTTACTGGATCATCTAAATGATTTTTAGCTGTGATTAACTCA
CGTTTATCCGCTTTTGTGAAAATGGACTCTTGTACTTCTTCAGTACTACCATCATTGTAAGTTACTGTCACAGGAATCGTCGTTGTGCTACC
ACCAGCTAAATTAGTAGGCATTGCTGTGCCATTTTTAATCGTTGCAGTACGTTTATTAGCAACTTGAACTGCATTGTTAATTTCAGCTGCTG
TTACATTTGAACCATAATCTTTCACAATTTCAGTTGTGTTGACAGTATGAGCTGCCGGTGCAGTTAATGTACTTGGATTACTACTTACTGAG
TGACCTGTACCTGCTTTCGGAGTAATTGTGATTGATGAATTTGGTTTTATAGTATTGGCATTGAACGTCACTTTACCAGTTTGTGCATCTAA
CGTTACATAGTCAGGCTTATTCGCAATTGTCCATTGATTATTTTGACCACGAACAACATTAATTGTCTTACTATGTTCTGCACCATTACCCAC
TTTTTCAGTGTAAGCAATATCCATTGCTTGAGTTGGATTAATTAAATGTCCTGATGGATTATTAGGCGTGATATCAATATGACCATTTTGCC
AAATCTTAGTAGTCGCTTGGTTCGGTTGTGGTGCGACAACTGTGAAATCATCACTACGTTGCTCATCACTTACTGTCTCTCCGCTTCCTTGC
GTTGCAACAACTTGAATTGTATCAGCAGGGTTGAAAGTACCTGCTGCAACAGTAATACCATTATTAGTTCCAGCAATACCTGCTACAGTTG
CTGCAGATGCTTCTTTCACCCATGGACTTGTATTATTGCGACGTGTAAATGTCGTCACAACGTTACCATTACGTTTAATAACTAATTTATCA
GCGTATGTCGTTACGTTACCTGCATGTGTATTCACTGTTTGGTTTGCTCCAGGTGCAATTGTAATCGCTCCTGCCGCTGTTTCAGTCACAGT
TGGTTTCGCTGGTTGCACATCTTTTACTACAAATTTCGCTGGTAAAGATGTTGCAAAAGTATGTCCGTTATAGATGACGTCATATTTTGCG
TTAACGACTTTAGCCACATTCGGTTTATTCATAGCTGACCAGTTTGCGTCATTTGTACCTGTAGTATCACGATTCCATTTATACGTAAATCC
ATCTGTTGGTAAACCAGTAGCATTTTGCATATGTGCATATCCTGATGCTTGCGTACCACTTGCTAAAGTGCCTCCAACCGTTGTCGTATAA
GTAGTTTGAGGGAATTCAAATTGATATACATTAACAGTAACAGGAACTCGTTTAGCAGCTGAAATACCTGGATATGTGACATCGACATTT
AAATGTTGCACGCCTGCTTGTTGGTTATTTGGTTGTTGTCTATTTGCCCATGCTGCAGTGATACCATTTGTGTTTGTATTTGGATCAAATGT
AATGTAGTTCATCGCATCCGTTCCATTAGTCAAATTTTGACCTTTCACATCACGTGATGGCGCCTTTGCATTAGCAACTGGATAAACTTTGA
CTGGAACTTCAACATTACGCGTACCTTGACCATTAGGTAATGTTACAACCGCAGTTTTATGAGTGTTACCGACTGTATTCTTCCATGTATCT
GGACTATCATGCCATGCAACCGTTGCCCCATGTGGCGGGTTTTGAATAAATCTTTCTACGTGTCCGAAATCAAAACCGTCGCCACCTTTAA
TAAATACAGCACCTTCAGTAGTTGCTTGTAATTGTGGTGTCACTGTTACTGTTGCACTGTCATTTGAATCAACAGATTCATTTCTTGTTACA
TCTATCGCTCGACCATGTTCATCTTGAGTAGTGTACGTCACATTATTCATTGTGATAGAAGACCTTGCCTTAATCACACCATTTGGTAACGC
ATCACTTACCGTTACAACTGCAGTATTACCACTACCATAAGTCGTGTTTGTAATCGTTAATGGCGTATTATCTGCTTTAAATAATTTAATAG
ATGAACTACTTAACACATTATTAATTTTAATTTGTTGGTTAGTAAGACCTGCTTTATAAGTCACAGAGTTGCCATCAATTCGCGGTGGATCT
GGTTTAACTTTTGCTAAAATCTTGATAATTTGCGTTTGGTGATTATCATAAGTAACTCGAATATCCCTTGTTGAAATACCAACCACATCTTT
ACTTGGTCCATATGAATTTGTAAATTGGAAATTCATACGTTGCGCATTTTGTGTCCAAGAATCATTAACTGGTTTAACATATTGTTTCGCAT
CATACACATCAGATACACCTGGGAACACAGCACCTCTATTCGTCTCAAAGACATGCTTCGGTACTGTACTTACTACTTTATATGTTGCTGTT
TTCGTAATCGGCGTTGTTTCACCATCAATTAAGATATGTGCAGTTACTGTTATATCTTCACCAATACGTGTATTATCTTTATTTGGCGCTTGT
CCACTTACCCATGTAATAGTTGCATCTGGAATGGCACTACCATTAGATAACTTAAAGTAATCTGATGCACTAGAACCATTACCTGCTGGGA
AGTCTTGGCCTTGTACAGTGTAATGCGAATGTGCAACGATTTCTGGAATGACATGCTTTACAGGTACAGTCACTGTTGATGTTGTTCCATC
TTGATAAGTAGCAGTTACTGTAACATTGGCATTATTTCCAGTACGACTGACATTACTAACTGTTTTACTAGTGATTTCATTCGCACTTGCTC
TTGCATAACTTCTATTTGGTACTGTTTCAGTAAACGTTAATGAATTAATAATTGCTGTTTGATCAGCTTGTGATACTGTCGCATTATTCGAA
ATATTGGCAATTCTCACAGGATTAGCAGCCGTTGATGAAGTACCAACTCGATATTTATCACGCAAAGGTTTCACTGTTACATTGAACGAA
GTTGTAGCTGTATTGCCGCTTGTATCAGTTGCTAATAAATTGATTGTCTTATTAGTTGCTGATGTCACATTTGGTGCCGTTGCAGAAACAT
GTTGATGGTTATTATCAACAGTGCCTGTAATTTGTGATGTATTTGGTACAGTTACAGATTGCACACCAAAGTTATCATTCGCTGTAATAGT
ATTTGTAAATGTTTCACCTGAGAACACTTGATGATTAGTGTAATTACCTACTGAAATTGTTGGATTCACTAAATCACTTGGTACAAAATAA
ATGTTAATAGCGTCAGTAGTATTTCCTGTATTTTGATTTAAATGTTCAACATATTGTTTTGGACCATATGGCGTTAAGTATAACTGTGCTTT
ATAAACTGCATCACTTGCATTATGTGTAGAATTACTTTTTACAACGTGGTCAATTGTAAATGCGCTAGCACCATTAGCTGCCGGTTCGTTTA
CGTTAACAACAGTTGAGTTACTATGGTTTGCACGAGTATTTGAATTTGTAACAGGTTGCCCACCATAACCGTTTGAAGGGTTGATGATGTC
TAACACTTGAATCACTTGACCATTCAACGTAAATTGACGTTGACCATCAGTTGTCGCATTTGATTGTGAATAACCAGTCGTTCTAAAGTTA
GGTCTTCCGCCAGCTTCTGCTTGTGCTTTTTCATTACCAGTAATATTATTACGCAATCCAGGCACTGTAGTAGTCGCTTTTAGCATTGTTAA
AATATCATTAGTTGTAATTTGTGTGCCTAATGTAGCATCATAACGATAAATTAAAGATTTATGGTCATCAGACCATGATAAACCACCATCT
GTTGGTCTGTTTGCAATTTTTGCATTCGTCCATGTCACTGTATAATCTTGCGGGAAATTAACCCAACGCAAGAAATTCATATTAGCTAGGTT
TGACGCGAATGATTTCGTTAATCGACCTTTATTAATATTTACTGTAATAGTACTTACATTAGCACCATTAGGTGTATTTGTAACTGAAATAT
TACCCTCGGCAAGCGTAATTACATCTCTATTTGCATTAATAAAAGCTTGTTTTACTTTTGAGATTTCATCAGTTGTTAAATCATATGTTGAC
GTACGATAGACACGTTGATCGCCTTCAGGTTGTTTCACATCAACGATTTTTAAACTAATATAGAAATCTTCAGTGTCTTTATGATATGCATT
TGAAGCAACTATATGATATAACTGCGTGCCTGCATTCATTTGACTAACTTCTTGTGCAGTTGGAATAGTTCTATCAGATGTAATATTTGTAA
ATGTGCCACTTGGAATAGCTTGTCCATTACTTAAAGTAAAATATTGTTTAATATCACCAATATTAAATGATTGTCCTTTAGCAATCGTTACC
GTTGCTGTTTTAGCTGCATTGGCAGTTTGATATTCAGATTTCACAACTGGTTCGTATGTCATCACATTTGTAGCATCAAGCGTTGTTGATGA
GCCATCACGGTAATGTAATGTGACATTACCATTATTATCGACAGTTACTGGATCAGTTGATGCTAAATATCCTCTTATATTTTGGTTTTTAT
TCATAAAGGCAGTAATTATGCTTTGCTTTTCATCATTAGATACAGCAGTCGGATTGACTACTACAACTTTCTCAGTATTTCCTAATACAATC
GGATGAGCATCTTCACTAATTTTACCTACATGAATTGACACATGTTTAGATTGACTATCATTTGTCGTATTATTGACATTATCTGTCGCTGA

TACTTTAAATGTAATATCACTGTTAAACGCCTGATTCATACTCACTCTACCTGTAATAGCTAATGAGCCGTTTTTGTTGTCGGATTTAGTTA
AATTTGATGTCCAACCATTCGGCAAAGTAGTAATAGTTGTATTTTTTATACCAGAATGTCTATCAGAGACTCCCATTGTAAAGTTGACTTC
GTCGCCTCGATAGTATTTGGCATTTATTCCAACAGGATTATTGATTGTTGGTGGAATGTCATCTGGCAAAATAGTAACAGCATTACTCATA
TTAGATGCAACTACTTTATTATAATAAACTACTGCTCTAATTTGTTTACCTACAGGTGGTATGAATACAACATAATCTTGCGGTCGTATAGT
AATTGTATTATGCACCCCATCAGGTGTACCAGTCGCAAGTATCGTATAACCAGATGGTATTTGATCTGCCTCACTACCACCATTCGTAGTTT
GATTTGTTGGTGCAACTAAATAAACCAATGCCGATGGATCCAACGGTATTCCCGAAATATTAACAGGCACTTTTTGTAATGCTGTACCTCT
TAATTGCTCAGCTGTCGTTGTAATTGTTGGTGTATGTGGTTTAACACGAATTTCGAATAATTGTTCTTTACGTCCAAAACGTATCGTTCCAA
AGTAATCCCCTGGTAATAAATTACCAACAAGTTCAATGTTACGTTCTCTAGCATTTTTAGTTCTATTTGTCGAGTTTTCAATGTGTAATGTA
CGCGGAATACCTGTAATTTGCCAATCTTTATCTGTAAAATTCTCATTACTTGTTAAATTAATACGTAATGGTTGAATAGATGTTACGCTACC
ACCTAAATTCACGGAAGCATTCCCTTGGTTACCTGCATATACATAGAAAACTGGATGCCCAGCTTGTTTTGAAAATGGTTTTTCAGCAATG
TTTTGAGTATCGTCATTTTTAAAGAATTTAATATTATGTTGGATTTCAGGTAATACCGTTAAGTTAACAGGAACTAAAGTTTCACTATTACC
ATAACCATCAGGATAGTCGAAACCAATCATTAATCTTATTGGGTTTCTAGTTCTTGCTGCTTCTTGTAATTTTTGATCAGTAAATAATTGAC
TTAATTGATATTCATTTAATTCCTGAACGCCCCCCGGTCTAAAAGCGTTAGTAGTTGCATTAGTTGGGTAAAAACCTAACACTTTATTACTC
GGCGTCTTAACATAACTTAATGGATCATCAACAAAATCATCTATATTTAGGTTTGTACTTTCTAATACCTTTTTCGTTTGTACATTTCCTTGA
CTCACTTCTACACGTTTAGTTGTACCTATGCGATATGTACGATTAACCACTTGTCTTAAACCATTAAGACGTTGTTGATGGTCTTGCGTTGA
TTCGACGTATAGTTGACTGTACATAAATAATTGATTGTACTCTAAAGCACGCCCACCTGCTGCATAGTACAATTTATTAACAGTTGGACCT
TGTGTTTTAAATGAAATTGTATAACTTGCATCACCTTGACCGATGAAAGTATAAACTTTAGGAACACCCGGTGATTCAGCAGGTTTTTGAC
CATTAATATATTCAAAATTTTGTCTACCAAATGCTTTAGTAGCAGCTGTTGCTCCAGCTTCACTAAAATAATCACTCGCTCTAGATAAATCT
TCTAAAGAATAAACATGGACAGTTGGCCAGCTATATATTACTTGGCCACTTCTATTTCTTATTTTAAAGTCATGTGAACGATCAGGATCATT
TACTCCATATTCCCACATTTGTTGAAGTGGTTTATTTGCACCTGCTCCTGCTCCATGACTCCATTGTACATTTGTTCCATCTGAATTAACTGT
TACAAAGTCAGTTCTTCCTACTGGCACTTGGTCTGCTGGTAATGCAAACCAGAAAATCATATTTTGATGTGGAGCATGTCCTTTATTAAAC
TTAATAACCCATTGGTAGCCATCTCCAGTTTGTTTCGCTGTCATAAGAGCAATTTCCTTTGAGTCAGAGGCCTTCAATGTATTCCATGGTGT
AGTTGGTAAATTATATGGCGCCCCTTTACTAGAAGCATTGATATATGTACCTACAGGATATCCTGAACCATCATTAGCATTTTCAACATAA
TATGATGAATTTGCATTTTGATAGTCTGATACTGATCTAGGCTCTGCAGCTCTAGACTCAACAGAACGCGTTTGAATTCTGTTTGATCGAC
GGCTAGTTTGTTGACGTTGAGGCATTGTACGAATTAAAGCAACAGCTTGTTCCAATTCTTTGTATGCAAGTTGCAAGTCTTTATTGTTTATT
GGATCATTACTGCCTAATAACGTATTAATCTTTTCAAAAATGCCTTTTACATAGTTAAATGTGCCAGAATCTGCATTTTCACGGTCAATAAA
GCGATAATCATGATAATTTGCATCAAAAAATGCTTGTAATTCATTTCGGTCATGTTGTACGTTACCATTATCAGATGAATCTGAAGCTGTT
ACTACATTTGCTTCATGATTTTCTAAATGAGCCGCTCTATTGTCGTTTCCATTTTTATTAGCAGATTGACTTTCGTTTTGTTTATGCTTACCTT
CTTCTTTATCTGGTTGTGTTGTCGCAGCCGTTGCCGAATCTTTCTTTGTATTTACATTTTGAGATGCTGGTTGTTCATCATTAGTAGTAGATG
ATTGCGCTACTTTTTGATCTACTAAATTAGCTTGACTAATATTTGGTTGCTCATTTTCATGAGTAGCAGATAATGATTGACTATTTTGTGAA
TTTTGAGAATTTTGTACTTGAGATTCTCGATTCTCAGTCTGTTCATTATTACTTTGTTGTTTCTGTTTAACCACGCTTGCTGGTTGATTTGTTT
CAGCAGCATGTGCTTGTGATGTATTGAATCCTAAAAATACCAATGTCGCAATGACAGTTGAAAATGTACCAACTGTATATTTACGAATACT
AAACTTTTGAATTTTATCACGATAATTCAC

Gene: norB (multidrug efflux pump)

Contig: 06_NODE_54, position: 607302 to 608707, length: 1406 nt, orientation: REVERSE

Sequence:

TTAATTCTCAATTATCATAATTGAGTGTCGTTTTGTTTAGGCACAAGTAACAAAATGATAACGAATGATAATATTCCCATACCTGCATTTAA
CCATAATGCAATCATTGCACCTGTATAAATGTTTGTCATATTTGATACGATTGCATATACTGCACCACTCAATGCGACGCCAAATGCTCCAC
CTAATGCAGAAGCCATTTTATAGATACCTGCAGCAACGCCTACTTTTTCTAACGGTGCATTTGCAATTGCTGTATCTGTTGATGGTGTAGC
ATATATCCCTAGTCCTAAACCAAAGAATAAATAACCTATAATACAACAAATGACATACAATATTTCTGGCAAGAAAGTTAATGAAATGAG
ACATTCTCCGACAATAAGAACTCCTGTTCCAATTAACATTGGTTTCTTGCATCCGAGTGTTTGAAGTAACTTTTCACCAACACGAATCATAA
TTAGTACCATTACTAAATAAGTGATTGATAAACTTCCTGCTTGCAATGAAGAATATCCTAAACCTCTTTGAACAAATGTGTTGGCTACTATT
AATGTTCCTGCAACACCATTTAACAAAAAGTTTGAAGCTGTTGCACCTGTGTAAGCTTTATTTTTAAATAATTTAAAATCGATTAAAGGATT
TGTAGCACGCTTTTCAAGAACTATAAATAAACTAAAAGATCCAATTGCAATTGCTAATAAAGTGATAAAAAGAAGTGAGCTAACACCTAA
TTCTGATCCTTTAGTAATTAAAATATTTAAACTGAGAAGCATAATGACTAAAAGAACCAGACCTTTAATGTCAAATTTATTTAGAGAAATC
GATTTAGATTTAGTTTCAGGTGTGCCTTTAATAAGAAAAAGCGCAATTAATGAAATTATAATTGATAGGATGAAAATCCAACGCCAACCT
AAAAGCGTTGCAACTGCACCTCCAAAAAATGAACAAACACCAGAGCCGCCCCATGAGCCAATTGACCAATAACTTAAAGCGCGTTGTCTA
TCTTTCCCAATGTAATATGACTTAATAATAGACAAAGTTGCAGGCATAATACATGCTGCTGAAAGTCCTTGAATTAATCTTCCTATAATAA
GTAATAAAGGAATATTTGAAATAATGATTAATAATGAACCCAATATATTTAAGATAATACCAATGTTCGTGAGTTTAATTCTGCCATATTT
ATCAGCAAGACCACCTGCTCCTACTACAAACATTCCTGAAAATAAAGCAGTTATACTAACGGCGATATTAACCGTTCCAATATCTGTATTG
AAACTATCTTCAAGTATTGGTACAACATTAACCAATGATTGTGCAAATAGCCAAAACGTTATTACACTTAAAACAATTCCTATTAACAACTT
ATTATTGCCTTCAAATGCCTCTCTTGACGGCTTTTCCAT

Gene: steT (serine/ threonine exchanger transporter)

Contig: 06_NODE_54, position: 608864 to 610186, length: 1323 nt, orientation: REVERSE

Perfect match to: (N315-BA000018-[1471747:1473069:r], allele observed in CC5)

Sequence:

TTATGCTGCTTTTTGTTTCTTCTTATAATAATAAACTGGTATACCAAGTGCTGTTATTAGAATTCCAATGATTGCTAGTATAAATTGTGTAAA
CAGTGTATTAATTAATACAAATGACCCTGCCAAAATTGCAATTAAAGGTATGATTGGATATAACGGTACTTTATATGGTCGCTCCATATTT
GGTTCGCGTTTCCTTAAAATTATTACCGCAACAAATGACATACAATAGAACAACCAAATAACAAAGATTAGCATGTTTGTAATTGTATCAA
ATGCTCCCATTGACATCATGATGATAGCGATTATAAGTTGTATAATTGCGCCAAACCATGGTGCGCCAGATTTTGTTAATTTTGCGAATAA
GTGGCTAAATGGCAATAATTTTCTTTCAGCCATTGCATATGGTACGCGCATACCAGTCATAGTATAGCCATTGATCGTACCATAAACAGAA
ATTAATATACCGATTGTAATAATCTTACCGCCATTTTCACCAAATAATATTTTTGATGTATCTGAAGCCGCATTTAAATTACCAGCAAGTAG
TTCTATTGGCAACGTTAATAAGAATGTAGCGTTAATTAATAAATACACAGCCATAATACAACCGATACCAACTGAAATCGCTAAAGGTAA
ATCGCGTTTAGGATTTTTAAGTTCCCCCGCAACATTTCCTACATGAATCCAACCATCATATGCAAACATAGTTGCTAATAAACCACTACCAA
TTGCTGTAAAGAAACCATTTCCTGAATTACCTGTAGTTGGAATTAATGAAAAAGTGATATCTCCAGATTGAAAAATACCAAAAATTACAAT
AACGATGATTGGAATCAGTTTAATTACTAAAGTAACTGATTGTAAAATTCCGCCTGCTTTTGAACCTAGGAAATTTATCAACACAATAGAT
AACGCAGATGCGATTGCTATTGGTATTAACGAACCTATAGATAAATGGAATAAATTAATTAGCTGTGTCGCAAATACGATAGACAATGCT
GCTACGTTAGCTGGAAAATAAATAAATGATTGCGCCCAACCTGATAGGAAGCCCCAGAAATCACCGTATGTATATTCTATATACTTCGTTA
AGCCACCTGTTTCAGGGATTGCAGCAGCAAGTTCTGCTGCTGTTAACCCCGCACAAATGGTAATGATGCCGCCTAGGAACCATACAAACA
AGGCCATTCCTGCTGTTCCTGTTACTTCTGTTACGTTTGATATTTTAAAGAATACTCCTGAACCAATAACTGTCCCCATAACAATAGCAAAC
GCTGAGAAGAAACCTATATTTTTTTGTAATTCTTTACCATTTGACAT

Gene: ilvA1 (threonine dehydratase locus 1)

Contig: 06_NODE_54, position: 610217 to 611257, length: 1041 nt, orientation: REVERSE

Perfect match to: (MW2-BA000033-[1474553:1475593:r], allele observed in CC1+CC8)

Sequence:

TTAACCTACCACACCCTTGCTTGTATCTGCAATATTCAGTCCATGTTCAATGACACCTGAAACTCTAGTTAAGTCAACATTCCCGCCTGAAA
CTAAAGCAACAACATTTTTATCTTCAAGCCATTTATTGTTTATTTTTCCACTTAAAATTGCAGCTGTTGGTAATGCGCCTGCACCTTCAGTAA
TAATTTTGGCACGCTGCATTAAATCTTTCATAGCATGTTCAATTTCTTCTTCAGTAACAAGAATAAATTCATCTACTAAATGTTTAACTACTT
CATATGTTTGTTCACCAGGAACTTTTACATCACAACCATCTGCTATCGTGCTATCCACTCGATGTTCAGTTAAATCTCTCTTATAGAAAGAC
TCAGCCATACCATGAACATTCTCAGATTGAACACCGATAATATGAATTGAAGGGTTAAATGATTTTAATGCGGTGGCAATACCTGCAATT
AATCCTCCACCGCCAACTGGTACGATGACTGTATTCACATTCCAAATATCATCTAAAATTTCTAAACCAATTGTTCCTTGGCCTGCCATTAC
AAACTTATCGTCATATGGATGAACGATTGTCATGCCATTTTCTTTCGCTAATTCTTCCATATAAAGTCTAGTTTCGTTAAAGTTTTTACCTTT
TAAAATAACCTTTGCCCCATAGCCTTTTGTTGCTTGTTGTTTCGCTTGTGGTGCTGTTTCAGGCATTACGATAGTTGCATCAATGCCTAATA
ATTTAGCTGTTAAAGCAACACCTTGTGCATGGTTCCCAGCAGATGCTGCGATAATGCCTTTTTCTTTTTGTTCATCTGTTAAGTGATTAATT
TTATTGCTAGCGCCTCTAAATTTAAAAGATCCTGTGAATTGCATATTTTCTAATTTTAGAAATACATTCCCTTTAGTTATACTTTGGCTTAAA
TACATTGATTTAATTAGAGGTGTTCGACGAATAAATGGTTTAATGCTTGCTTTAGCTTCTTCGATATCTCCTAAACTTACAATATGTGCTGT
TTGTAATGTAACTGTGTTGGTTGTCAT

Gene: ald1 (alanine dehydrogenase 1)

Contig: 06_NODE_54, position: 611352 to 612470, length: 1119 nt, orientation: REVERSE

Perfect match to: (MW2-BA000033-[1475688:1476806:r], allele observed in CC1)

Sequence:

CTATTCGATAACATTTAATATTTCTTTATAATCTAGGTCATGTGATGAAGCTAATCCTTGATTTGTCACTTGTCCTTGGTAAATGTTTACACC
AGTACTTAAGGCTTCATTATCTTTAATTGCTTGTTCTAAGCCTTTGTCACAAATTTCTAATATATAATCAATATTTCCTTGTGCTAATGCCATT
GTTGAAGTTCTTGGGACTGCTCCTGGTTGGTTTGGTACACCATAATGAATCACACCTTCTTCTTCATACACTGGATCAGAAATTGTAGTTG
GTCTAATTGTTTCAATAGTTCCACCTTGGTCAATAGCTATATCGATTAATACTGAACCTTTTTTCATTGATTTAACCATCTCACGAGTAACCA
ATTTTGGCGGTTTCGCACCAGGAATTAAAATTGTAGAAATAAATACATCTGCTTTTTTTATTTGTTCTGCTAAATTTTCTGGTGTTGATTTG
ACTACTGTGACATCTTTTTCTGCATACATATCTTGTAAATATTTAATGCGATCATCGTTTAACTCGATAATGATTACTTTAGCATTTAGTCCC
AAGGCAACATTTGCTGCATTTGTTGCTGCTACTCCACCACCGAAAATCACATATGTACTACCAGGTATATCCACATTTTCATGTACACCAGT
CACTAAAGTACCTTGACCACCATGTTGTGCTTCAGAGTAGTAAGCTCCCATAATTGCTGAGCGTTGACCTGCTATAGCACTCATTGGCGCT
AATAATTCTGCTTTTCCATTTTTTATAATGGTTTCACCACTAATCGCAGTTACACCAACTTCTTGCATTTTTTCTACTATTTCTTTTGAAGATG
CTAAATGTAAAAAATCCCCAGATAATTTGATTCTTTTTGAAATATTGATATTCGCTTTCATGAGGTTCTTTTACTTTGATAACAAGATCAGCTT

CCCATGCTTGTTCATGAGTTACGATCTTAGCGCCTTCTTTTTCATACATATCGTTAGAAAATCCTGAACCAATGCCAGCATTTTTTTCAACAA
TTACTTTATGTCCTGCATCCGTTAACTTACGCACATTTTCGGGTGTGCAAGCTACACGTCCTTCACCTTGTTTTAATTCTTTGACTACTGCAA
CTAACAT

Gene: exo53 (putative 5'-3' exonuclease)

Contig: 06_NODE_54, position: 612946 to 613824, length: 879 nt, orientation: REVERSE

Sequence:

TTAAAAATGGGATGAAATATATTTTCCTGAAACATGTAATTCATGTTCGTTACAAATTGATAAAATATGATTAAGTGTTGTTGCAAATGAC
ATTTTCTCAAAAAGCGATTCACTATCAATTGGTACTTGTGTGTGAATTTCAGCAAGTCGCTTCGATAAATATAGTTCATCTAAATTGTCATT
AATCTTATTACGTTGCCCAGCTGATAATGCATCAATGTTTTCAACCACATTTTCAACGCTTTGATATTGCTGAATTAACTTAATAGCCGTTTT
CTCACCAATTCCTTTAACACCTGCATATCCATCTGCTGTATCACCCATAAACGCTTTAATATCGATTAGTTGTTGTGGTTCAAGGGCATATT
CTTCGTTAAAACGATGTAATGTATATCTATTATAAATGTTAAAACCTTTTTTAATTAGCCAGACTTCAACATTGTCATTAATACATTGCAGT
AAATCTTTGTCGCCCGTAATAATATAGACATCGTTATCAGTTGAATATTGTTGTGCTAATGTACCTATAACATCATCCGCTTCATAGTTTTT
AACGCCAATATTTACAAAGCCAAATTGCTCTGAAATTTCTTTTACATAATCAAATTGTGGTATCAATTCTTCTGGTGGTGCAGAACGATTTT
GCTTATAACCATCAAACATATCATTTCTAAAAGTTGATTGTCCCATATCCCAACATACAGCTACATGTGTAGGGCGTATTTCATGTATTGCC
GAAAAGATATGACGCACAAACCCTTGTATTCCATTTGTAGGTACACCTTGTGAATTGTACATAAATTGTTTATGAAGACTTGTAGCGTAGA
AATGTCTAAATAATAGTGCCATACCATCTACAAGTAATATTTTATTAGGCAT

Gene: Q2FGZ8 (putative protein)

Contig: 06_NODE_54, position: 613844 to 617284, length: 3441 nt, orientation: REVERSE

Sequence:

TTAATATAAAATAGTTTTCAGTTGTTGATGTTTATCTTTCAATTGATTGATAAGTGTTGTATCTAAATCAAATGCTAATAAATCGTTTATTTG
CGTTTGAATATTAGCTTCAAAACTTTCAAATTGCGATTCAGCTTCTACAGCCATTTGCTTAACATATAATTCAAGTTGTTGCCTCAATAATCC
CATTTGTGGTTGTAATAATCCTAACGTACTTTGACAAATTGATTCATGTATATCTCTTTGCCCATTTGGATTCAAAATTTTACGTTTTGTTAA
TTGTTTAGGCAATGCATTTAGCATATCATTGAAATCGATGTGCAATAATGGTTGCTCTATATTAGCTGATTCAAAGTTAAACTGAGGATTA
ACAATGACATGTAAATCTGCTAATTGTTGAACGATTGGTGCAATTTGCTCAGTGAGTTGCTTATTAAAGTATTTTTTTATACGTTCTGTAAT
TAAAGATTGCTCTAAAAACAATCGTTGATGAATTTGATCTAAGTATACTTTCGTAGACACTTTCTTTTCTTCATTAAAATCACTATTTTGCGT
CATTTGAGAATTAAACACTGATTTAACATCGTCAAGTAGTTGTAATTTTAAACGTGCATTTAAATGATAAACTTGTTCTTCAACTTCGTTGT
CTGTATGTTGTAACGTCGCATCAATTAATTGATGTTGTAAACGCTGTTTATCTTTATAGACTGTTAACCTTTGTTGGCGACGTGAAATATCA
GCTTTATTTGTTTCAAATTCTGTAATCATCTCTACATAAGAACGATCCATTTGTTTAAGCTGATGAATCATTTGTTGTTCTAAAATTGATTTA
GATTCAACATCAACAAATTGTTGTATGCTTTGTTTTAATTGATCAATGCCCTTATCTTCAGCTTGTAATGCATTTCGACTTGATACAGCAAA
AATGTCTGATTGTAAGTGTACTTGTCTTAATGCATCTGATACATATGTTTCAACCGCTTCAAGATCATCTTGACTTTCTGCTAAATCAGCAG
CATTAATTACCATTTTAAATGCTTGGTTTTCATTCAACTGGTTCATATCTTTCATGTGTTCTATAAACGCCTTGTCATTATCAGTAAATGAAT
GATTAAAATAACTTACATACAATATTAAGTCTGAAGAAGTTAAAATTTGCTCGGTTTCATTTGTATGCCTTTGGTTATTTGAGTGTAGCCCT
AATGAATCAACAATTATTTTACCTTTTAACCAATCATGCATTAATGCAATGTGTACTGTCTTAACAAATGTTGCATATTCATCTTCTGCACTC
CACTTTTTCAATTCCTGTTGATTAATGGCATGTTTTTCTCCATTTTCCAACATATTGACATACAATTTATAATGTTTTTCAACTGCCTGAATAA
AAGCGAGTTGATTTTTATTTAAATGTGATTTTAACTTTTCTAAATCTGAATTAATAAAGTCTTCTATAGTCGAATAAGACATATCTTGGTAT
TCAACTACTGCATTAATTTCATCTAATAATTGCGATTGTGATTTTAGCGTTATATAACTCTCGTCTCCATAAGATATTTCTGTAGTAGCTGCT
GTAGTAGGATTTGGAGAACTGACTAAAATATGCTCGCCTAATAATGCATTTATCAAACTACTTTTACCAGCACTAAATGTTCCAAATACAC
CTATTTTTATTAATTTATTATCTATACGTGTTAATGTTTCGTGGATATCTTGCTTTGTACGCTTGAACAAAGGCACATCCGAAATTATATCAA
GCGCTTTTTGAATATCTATAGACATATTTGTTGTAGCTGTATTTTGATTTAGCTGCTGATTATCACGATTATCCTGAGTATTATCAGTAGCC
ACTTGATATGTTGTCTCTTGTCGACCTATTAATTTATCTAGAGATTCATCTAAATGAATATAGTAGTGACGATAATTCTTAGTCGTCAATGA
CTGACGCAGCTCATTTAATTCTGTATAACGTTGATATTCTTTTAAATCATCACTTTCTTCTGTTGGTAATTCATCTGCCTGTACATTTTCTATT
ATTTCTTTAAAAATTGGTGTTGATTGTTGTTCAACATATTTCTTAATGGCTTTAACCACTTCGTCTGAAAATGTAAGTACATAAGTATTGCT
GATTGATGTTTGTGGTTGATATAAATCTTCAATCATTTCCGGCTTAACGTCATAATGCTGATTTAATACTTTATCTGAAGCTTCTTTTTTATT
GATAAAACGCGTTACAAATGACATATCTTCTCGCATTGGTTGACGAATTTGTTGATTAACATGTTCTTGTAACGCATCTGTCGCTGTTAATA
ATCGCTGTTGTTGAATTTGTAGCTTTTTCTTCTTTTTATTAAAAAACCCACCAACATTAAAGTCTTGAGACATACTTTCCAAATAACTTCGTA
ACATTTCTCTCATATTATGCGGCATAATGTATGCATTTTCTAAAATATTTTTACGCTTATTCTTTAAAAATGCCATTAATTCATCTGGATTATT
TAAAAGTTGTGCCTCTTCACTAATTGCTTGATGTTGTTGACTATTTAAAAATGCTTGTTCAAACTCCGCTTCTTCGATACCTAAATCTTCTAG
TACTTCCTGAATTTCAGACTGAATGTAATCTAGCTGAGCTTCGGTAATGTATTCAACCGTTCTTGATGTATAATCCTCTATTGTCTCTCTATG
TTGATCTAATGAAATTAGATAACTTGATAATACTTCAAGTTCATTTTCAGGGTGATCAAATTTAGATACATAAAAGGTGCGTTCTAATTTA
ATACCCCAATCTGCAATTGATTTTTCAACTCGAGATTTAAACGTAGAGAATGACAATTCATCGTCTTGATGCTTGTCAATTTGATTAATGAT
AAACACAACAGGTATTCCAACATCATTTATATGCTTCATAAACTTAAAGTTAAGTTCAGATTGAACGTGGTTATAGTCAACCGTATAAAAT

ATCATATTACTTGTATACATATATTGTTCTGTTATTGACTGATGTGATGCAACATTTGAATCAACACCTGGTGTATCTTGCAACGTAAACCC ATTTTCAAATTTAGCTGATTGAAAATTAATTTCTACAGATTCAACGTCGACATTTTGGCGATTCATTTCCCTTACTTCATCATAATTAGATAA TTTGGCATACGTTTGATTCGGCAAATTAGCAATAATATCGTGATTGTCTGAAACTGACACAATAGCAGTATTACTTGTCGTTGGTACAGGA GAACTTGGTAAGATATCTTGTTCAATTAATAAATTTATCATTGTCGATTTACCTGCAGAAAAATGTCCAACGAACGAACATGTATATTGCT GCAAATATACTTTCTTAATTACTTGGTTAATTGTATGTAAAAGTGCTTCATTTCGCGACTTTTCAACTTCTTTTTTTAATTTATATAAAAGATC TAATTGTTCTTTATTAATCAT

Gene: piuB (iron-regulated membrane protein)

Contig: 06_NODE_54, position: 617588 to 618928, length: 1341 nt, orientation: FORWARD

Perfect match to: (TW20-FN433596-[1572392:1573732], highly conserved allele)

Sequence:

ATGAAAAAATACATTTAATCCATTACAAAGATTACATTTCTATGCAGCAATATTTATTGCTCCACTGTTAATCACTTTAACCATTTCGGGCATT GGTTACTTGTTCTTTCCAGAAGTTGAAAATAATATTTATAAGAATGAGTTTTTTGGTGACAGTGATGTAAAAACGCATCAAACATTAAATG ATGCAGTACATCAAGTTGAACAACAATATGAAGGATTCTTTGTAAGTAAAGTTAGCATACTTGATGAACCATATAACAAACGAATTACAC TGAGTGATATGGCAGGAAATCAACGTTACGTCTTTCTAGATCATAACAATCAATTTGTTGCAGATCAAAATGCGAAACATACGTATTCTAA TGTGATGCGAAGTATACATAGTTCTTTGTTTACTGAAAATACTATTATTAATTATTTAGTAGAGTTAACCGCATGTTGGATGATATTCATGA TTTTATCTGGTACTTATTTACTCATTAAGAAGCATTTAATTTCTAACAAAAGTAAGGCACTTCGTTGGCAAAAGTGGCACGCAATGATTGG AGTTATCATTGCAATTCCAGTATTTGTATTAGTCTTAACTGGATTGCCATGGTCTGGTTTTATGGGCAGTAAAATTGCCGGTATGATGGAC ACAAACGGTGACCTTGGTCAAGGTGAATTAGCGATTAATCCACCTAAATCAGATTTGAACGAATTACCTTGGGCTACACGTAAAAATAAA CAGCCAGCTTCATCCGAAAAAGGTTCAAGTGGTCATCATGGTAATGCAGCAATGCCTCAAACCAAATTAGATTATCAAATATCTATTGATA AGGTCGTTGAACAGGCGCAAAAAGCTGGTATTAAAAAGCCGTTTTCAATCGTATATCCAAGTGATAAAAATGGTACCTTTATTGTATCTA ATACTAGTAATTCAGGTGTTACTGGGCTAGATGTATCACCATACAAGGAACAAACACTTTATTTCGATCAATATAGCGGTAAAAAGCTAG GTACGATTAAATATGATGACTACGGTATTATTGCTAAATGGTTTACATGGGGCATTCCGCTTCACGAAGGTCATTTATTCGGCATTTTAAA TAAAATCATTAATTTATTTGTATGTATCGCTTTATTAGTAGCCATTGGCATGGGGTTTGTCTCTTGGATAAAGCGTACAAAAAATACTGCA GTAAAAGTACCACATCGCGTAAAAAAACCAGCATCTATATCACTCATAATATGTTTAATTGTATTAGGATTATTAATGCCATTATTTGGATT ATCACTTATCCTTGTATTTATAATTGAATTAATATTATATATTAAAGATCGTCGTGCTAAACAATAA

Gene: tx_universal2 (rho-independent terminator)

Contig: 06_NODE_54, position: 619080 to 619118, length: 39 nt

Perfect match to: (Strain_71193-CP003045-[770837:770875:r], allele observed in CC398)

Sequence:

GAATGAAAATGCGCTTGCAACAAGCTTTTTTCAACTCTA

Gene: STAR (Staphylococcus aureus repeat element)

Contig: 06_NODE_54, position: 619182 to 619459, length: 278 nt

Sequence:

GGGCCCCAACACAGAGAATTTCGAAAAGAAATTCTACAGGCAATGCGAGTTGGGGTGTGGGCCCCAACACAGAGAATTTCGAAAAGAA ATTCTACAGACAATGCAAGTTGGGGTGGGACAACGATAAAGAAATACTTTTTCTATAGAAATTAGTATTTCTTATGCATGAGTTTTACTCA TGTATTCCTATTTTTAAGTACACATTAGCTGTGGCTAATGTGTAAGAACCACTACATAATAAATCATTAGTGGCTCTTTATCATTTCTGTCCC ACTCCC

Gene: Q5HFY0 (putative protein)

Contig: 06_NODE_54, position: 619557 to 619889, length: 333 nt, orientation: REVERSE

Perfect match to: (ATCC51811-ST1-ADVP01000007-[46795:47127], allele observed in CC1)

Sequence:

TTAATTCATATGCATGTCGTGAATTTTCATCATTAAACCGTGTGGGACGTCATCATGTTTAACAATCTCTTGTTTATAAAATGTCTTCCCATC
ATCTTTAGAAACTAATTTTACAAAGTCTCCTTTTTTCGGCTTGAAATCATTAGATGGTTCTACTTTGATATTATGTTTGACTAATCCATCTTTT
TCACTTACAACTTTTTCTGCATTTGTATTACTATTCATATAACCAAAATAAGTCGTTTTTTGACTAGAAAAAATCATTAAACATACAACGGC
AATACCAATAACAATCAATGCTATAGATAAACCTAGACCCATTTTTTTACCCAT

Gene: ypsC (site-specific DNA-methyltransferase)

Contig: 06_NODE_54, position: 619973 to 621118, length: 1146 nt, orientation: REVERSE

Perfect match to: (Mu3-AP009324-[1560245:1561390:r], allele observed in CC5)

Sequence:

TTAGTTTTCGATAGTTTTACGTTCTGTCTTTTTACCCCAGTATTGATAATAAGTACATTCTATATATCCATTAAATAACTTACGTCGCTTCGTT
GCTTTACGATCTACTAAATATTCAAATTCTTTATTACTTGTTAAAATGTATGTAGATAAAAATGGATGTTGTTTCATTAGTTTACCAATATAA
CGGTACATTTCTTCAACTTCTTCACGATCACCAATACGTTCACCATATGGAGGATTTCCAATTAACGCCACCGGTTCTTCTGTATCAATTGT
TAATGTATTGACATCTTTTACACTAAATTTAATAATATCAGACAACCCAACTTCTTCAGCGTTACGCTTAGCAATCTCTACCATTTCTGGATC
AATATCAGAAGCATATACTTCGATTTCTTTATCATAATCAGCCATCTTATCCGCTTCATCACGGTAATCATCATAAATATTTGCTGGCATGA
TGTTCCATTGCTCTGATACGAACTCGCGATTAAAACCAGGTGCGATATTTTGAGCAATTAAACAAGCTTCTATAGCTATTGTACCCGAACC
GCAAAATGGATCAATTAAAGGTGTATCACCTTTCCAGTTTGCAAGACGGATTAAACTTGCTGCCAACGTTTCTTTAATTGGTGCTTCACCT
TGTGCTAATCTATATCCACGTCTGTTCAAACCAGAACCTGATGTGTCTATAGTCAATAATACATTATCTTTTAAAATGGCAACTTCAACAGG
GTATTTGGCACCTGATTCATTTAACCAACCTTTTTCGTTATATGCGCGACGTAATCGTTCAACAATAGCTTTCTTAGTTATCGCCTGACAAT
CTGGCACACTATGTAGTGTTGATTTAACGCTTCTACCTTGAACTGGGAAGTTACCTTCTTTATCAATTATAGATTCCCAAGGGAGCGCTTT
GGTTTGTTCGAATAATTCGTCAAACGTTGTTGCGTTAAAACGTCCAACAACAATTTTGATTCGGTCTGCTGTGCGCAACCATAAATTTGCC
TTTACAATTGCACTTGCGTCTCCTTCAAAAAATATACGACCATTTTCAACATTTGTTTCATAGCCTAATTCTTGAATTTCCCTAGCAACAACA
GCTTCTAATCCCATCGGACAAACTGCAAGTAATTGAAACAT

Gene: rnpB (ribonuclease P RNA component)

Contig: 06_NODE_54, position: 621313 to 621709, length: 397 nt

Sequence:

ACTAGTAGTGATATTTCTATAAGCCATGTTCTGTTCCATCGTACTCATCACGTGCACTAGTCACACTGGTACTCAGGTGATAACCATCTGTC
TACACCACTTCATTTCGCGAAGTGTGTCTCGTTTATACGTTGAATTCCGTTAAACAAGTGCTCCTACCAAATTTGGATTGCTCACTCGAGG
GGTTTACCGCGTTCCACCTTTTATATTTCTATAAAAGCTACGTCACTGTGGCACTTTCAAATTACTCTATCCATATCGAAAGACTTAGGATA
TTTCATTGCCGTCAAATTAATGCCTTGATTTATTGTTTCATCAAGCACGAACACTACAATCATCTCAGACTGTGTGAGCATGGACTTTCCTC
TATATAATATAGCGATTACCCAAAATATCA

Gene: gpsB (cell cycle protein)

Contig: 06_NODE_54, position: 621769 to 622113, length: 345 nt, orientation: REVERSE

Perfect match to: (N315-BA000018-[1484229:1484573:r], highly conserved allele)

Sequence:

TTATTTACCAAATACAGCTTTTTCTAAGTTTGAAATACGTTTTAAAATATCTACATTATTTGAAGATGTATTTGTTGTTGTATTATTCGAAGA
AAAACTTTTATTGTCCTGAGGTCTTGATGTTGCTACACGTAGTCTTAATTCTTCTAATTCTTTTTTAAGTTTATGATTCTCTTCTGATAATTTT
ACAACTTCATTATTCATATCGGCCATTTTTTGATAATCAGCAATAATGTCATCTAAAAATGCATCTACTTCTTCTCTTCTATAGCCACGAGCC
ATCGTTTTTTCAAAATCTTTTTCATAAATATCTTTTGCTGATAATTTCAATGAAACATCTGACAT

Gene: ypsA (conserved hypothetical protein)

Contig: 06_NODE_54, position: 622127 to 622690, length: 564 nt, orientation: REVERSE

Perfect match to: (MW2-BA000033-[1486463:1487026:r], highly conserved allele)

Sequence:

TTAGAAACTTTGATCTTCAGACCACTGTAAGTCATTGATGAATGCTGTTAATTCATCGAACGTCACAATATCACAAGTATAGTTTGTTTTAT
CCATAAAATCAACTAACATCTGCTTGAAGAACTTAGGACTTGCCTCTTGTTCCTCATCATAAATGAGCAATGTTTGATCCGAATGTTCAAG
CATAAATTGATCTGCTTGTTTAAATTGAAAAGGACCCTGATACGACGTATGAAAAATACTATCAACATAATCTGCATGCTTAATTATGTTG
GCATACTTACTTTGATTATGTTCATTCCACTTTTCTGTATGTCCTTGGAACGGTGTAATTACAGCAAACTTTAACGAATCATATGTTCGTTGT
AATTCAATAACAACTTCTGCAGTCCATAATTCTATACCCATTTGCCCTTGTATTAACACCCATTCTAATCCTTCATCCAACAGTTGTTCAATT
TTATGTTTTATAAATTGTTTTAAATAATGTACTTCAGGTGCGTCATCTTTAAAAATGTTTAATTCGAATGATTTGTAACCTGTTACATAAACT
GTTTTAACCAT

Gene: yppE (conserved hypothetical protein)

Contig: 06_NODE_54, position: 622683 to 623033, length: 351 nt, orientation: REVERSE

Perfect match to: (MW2-BA000033-[1487019:1487369:r], allele observed in CC1+CC7+CC15)

Sequence:

TTAACCATCAGTACCCTCTTTTGTTACGCCATCTAATATATTTTGTAAATCATATTGAACACTTTTAAGCTTTTCTATAAATAACTTTCGACTT
GTTCGCTTAAAATGACATTCGACAGAAAGTTGTTCAATGTTAGCAATCAGTAGCTCAAATTTCCTTGAATTCATATAAGGTACTTCTATAAT
AAATTCACGATGTAATTTGATCTCATTGAGCATGCTATCAATATGTTCAGTATATGGCTTTACAGTTTGGTAAAAATCATGATCTTGTTGTT
GTGATTTCACATTTTCAAAATTTTGTTGCATGTTGTTAACTTCATAAATTAGTGATTCGACTAAATCATTCAT

Gene: recU (Holliday junction resolvase)

Contig: 06_NODE_54, position: 623573 to 624199, length: 627 nt, orientation: FORWARD

Perfect match to: (N315-BA000018-[1486033:1486659], highly conserved allele)

Sequence:

ATGAATTATCCAAATGGTAAACCATATCGTAAAAATAGTGCTATAGACGGAGGGAAAAAGACCGCTGCCTTTAGTAATATTGAGTATGGT
GGACGTGGTATGTCACTTGAAAAAGATATCGAACATTCAAATACGTTTTATCTTAAAAGCGACATTGCAGTTATTCACAAAAAGCCTACG
CCAGTACAAATAGTTAATGTCAACTATCCTAAGCGGAGTAAAGCTGTGATTAACGAAGCTTATTTTCGTACACCTTCAACAACTGATTACA
ACGGCGTTTATCAAGGTTATTATATTGATTTTGAAGCAAAGGAAACTAAAAACAAGACGTCCTTTCCTTTAAATAATATTCATGACCATCA
AGTCGAACATATGAAAAATGCATATCAACAAAAAGGTATTGTGTTTTTAATGATTCGTTTTAAAACGCTAGATGAAGTTTATCTTTTACCC
TATTCAAAATTCGAAGTATTTTGGAAGAGATATAAAGATAATATTAAAAAGTCTATAACAGTTGATGAAATACGAAAAAATGGTTACCAT
ATTCCTTATCAGTATCAACCAAGATTAGACTATCTAAAAGCAGTTGATAAGTTGATATTAGATGAAAGTGAGGACCGCGTATGA

Gene: pbpB (penicillin binding protein B)

Contig: 06_NODE_54, position: 624196 to 626379, length: 2184 nt, orientation: FORWARD

Perfect match to: (MW2-BA000033-[1488531:1490714], allele observed in CC1+CC22+CC395)

Sequence:

ATGACGGAAAACAAAGGATCTTCTCAGCCTAAGAAAAATGGTAATAATGGTGGGAAATCCAACTCAAAAAAGAATAGAAATGTGAAGA
GAACGATTATTAAGATTATTGGCTTCATGATTATTGCATTTTTCGTTGTTCTTTTACTAGGTATCTTATTGTTTGCTTATTATGCTTGGAAAG
CACCTGCTTTTACCGAAGCTAAATTACAAGATCCGATTCCTGCAAAGATATATGACAAGAACGGAGAACTTGTTAAAACATTAGATAATG
GCCAAAGACATGAGCATGTAAATTTAAAAGACGTGCCGAAATCAATGAAAGACGCAGTACTTGCAACTGAAGACAATCGTTTCTACGAA
CATGGCGCACTTGATTATAAACGTTTATTCGGTGCAATTGGTAAGAACTTGACTGGTGGATTTGGTTCTGAAGGTGCCTCAACATTAACA
CAACAAGTTGTTAAAGATGCATTTTTATCACAACATAAATCTATTGGACGTAAAGCTCAAGAAGCATACTTATCATATCGTTTAGAACAAG
AGTATAGTAAAGATGATATCTTCCAAGTATATCTAAATAAAATTTACTATTCTGATGGCGTAACAGGTATTAAAGCTGCTGCTAAGTATTA
CTTTAATAAAGATTTAAAAGATTTAAACTTAGCGGAAGAAGCTTATTTAGCCGGTTTACCTCAGGTTCCAAACAACTATAATATTTATGAT
CATCCAAAAGCTGCTGAAGATCGTAAAAACACTGTTTTATACTTAATGCATTATCATAAACGCATTACAGATAAACAGTGGGAAGATGCT
AAGAAAATCGATTTAAAAGCGAACTTAGTAAATCGTACTGCTGAAGAACGTCAAAACATTGATACAAATCAAGATTCTGAGTATAATTCA
TACGTTAACTTTGTAAAATCTGAATTAATGAATAATAAAGCATTCAAAGATGAAAATTTAGGTAATGTATTACAAAGTGGTATTAAAATTT
ATACAAACATGGATAAAGATGTTCAAAAAACATTACAAAATGATGTTGATAATGGTAGCTTCTACAAGAATAAAGACCAACAAGTTGGT
GCAACGATTCTTGATAGTAAAACTGGTGGTTTAGTTGCTATATCTGGTGGACGTGATTTCAAAGACGTCGTTAACAGAAACCAAGCAACA
GATCCTCACCCTACTGGTTCATCTTTAAAACCTTTCTTAGCGTATGGACCTGCCATTGAAAATATGAAATGGGCAACAAACCATGCGATTC
AAGATGAATCTTCATATCAAGTTGATGGTTCTACATTTAGAAACTATGATACGAAGAGTCACGGTACTGTATCTATTTATGATGCTTTACG

ACAAAGTTTCAATATACCAGCTTTAAAAGCTTGGCAATCAGTTAAGCAAAATGCTGGTAATGATGCACCTAAGAAATTCGCTGCCAAACT
TGGCTTAAACTACGAAGGCGATATTGGTCCATCTGAAGTACTTGGTGGTTCTGCTTCAGAATTCTCACCAACACAATTAGCATCAGCATTT
GCTGCAATCGCTAACGGTGGTACTTATAACAACGCGCATTCAATTCAAAAAGTAGTTACTCGTGATGGTGAAACAATCGAATACGATCAT
ACTAGCCATAAAGCGATGAGTGATTACACTGCATACATGTTAGCTGAGATGCTAAAAGGTACATTTAAACCATATGGTTCTGCATATGGC
CATGGTGTATCTGGAGTAAATATGGGTGCTAAGACAGGTACTGGTACTTACGGTGCTGAAACTTATTCACAATATAATTTACCTGATAAT
GCAGCGAAAGACGTGTGGATTAACGGCTTTACACCTCAATACACTATGTCAGTGTGGATGGGCTTCAGTAAAGTTAAACAATATGGTGA
AAACTCATTTGTGGGACATAGCCAACAAGAATATCCACAGTTCTTATATGAAAATGTGATGTCAAAAATTTCATCTAGAGATGGCGAAGA
CTTTAAACGTCCTAGCTCAGTAAGTGGTAGTATCCCATCAATCAATGTTTCTGGTAGTCAAGATAACAACACTACAAATCGTAGTACACAC
GGTGGTAGTGACACATCAGCAAACAGCAGTGGTACTGCACAATCAAATAACAATACTAGATCTCAACAATCTAGAAACAGCGGTGGATT
AACAGGTATATTCAACTAA

Gene: Q1YBU7 (putative protein)

Contig: 06_NODE_54, position: 626943 to 627284, length: 342 nt, orientation: REVERSE

Perfect match to: (MW2-BA000033-[1491278:1491619:r], highly conserved allele)

Sequence:

CTATACCTGCTTATTCTTTAACCTTGTTTGATAACTAGCATTCATTTTAATCAATTCAGTTTTAAAGGTCTTCATTTGTCGATAGCCCATAAA
ATGATATTTACGTTCAATCATATAATCATATCTTTCTTTAAAATTCATTGGCACAACAGGATATTGATTTAATTTATCAAAATCAATATTGCC
AGTTATGTCATTAACCTGCTTAAAATAATCAATAATTTTACTATGGTATTCATCCAAGTAGGGCTTTGCTTCACTAGAACCTAATGTCTTAT
TTTTGGCAAATACATCAATCTCTTGTTCTAAATTATCAAATACCTCTTTTGTAATATTTGTCAT

Gene: nth1 (putative endonuclease III, locus 1)

Contig: 06_NODE_54, position: 627289 to 627948, length: 660 nt, orientation: REVERSE

Perfect match to: (COL-CP000046-[1531312:1531971:r], highly conserved allele)

Sequence:

TCACGCTTCTTTCAAACTAGCTTTATAACGTTTTTGTCCTTCTCTACAATCTTCTAATAGTGGACAAATATCGCATTTAGGTTTTCGGGCTAA
ACAGTGGTATCTTCCAAAGAAAATGAGTTGATGATGGCTCCTATTCCATCTATCTCTAGGTATGACAGAACATAAACGGTCTTCTACCTGT
CTCACATTATCTTTCCAACGATTAATACCTAAGCGTTTAGAAACACGTTCTACATGCGTATCAACAGCTAATGAAGGTTCATCAAATGCTAC
ACTCATGACTACATTAGCAGTTTTACGTCCTACACCTGCTAAACTTTCTAATTCCTTATGTGTTTGTGGTATTTCTCCATTAAATTGATCAAT
CAAAGATTGACAAAGTTTCTTAATATTCTTAGCTTTGTTACGATACAGACCGATAGAACGAATATCATTCATAAGTTCTTCATCACTGACTG
CCAAATAATCTTCAGGCGTTTTGTATTTTTTAAACAGCTCAGTTGTTACTCTATTTACTAGAACGTCTGTACATTGCGCTGACAATAATACA
GCAATAGTTAATTCGAACGGATTATCATGTTTTAATTCACATTCTGCATCCGGAAACATATTTGCTATAACATCAATCATTTCTAATGCTTTT
TTCTTACTTACCAT

Gene: dnaD (primosome, DnaD subunit)

Contig: 06_NODE_54, position: 627938 to 628624, length: 687 nt, orientation: REVERSE

Perfect match to: (RF122-AJ938182-[1443496:1444182:r], highly conserved allele)

Sequence:

TTACTTACCATCAAGGTTCTCCCCGTTTAACCAATCAAATTTAGGTACCGTTTTAACTGTGTGCGTCATTTTCGGTTTATTAAATTTTTCTCTT
ATTTTTCTAGAATCGTCAATTGTTTTGACATTGTTTTTCTTCCAATTAAGTAAAATACGATCCATATATTTAAAGCTAAGTTTATTCAAACTA
TTCGCCTCGTCTAATGCCGCTTGTATAATTGCACTATCGTGTTTATCAACATCAATCCATTGATTTAACGTTTCTATTTCATATGGAGATAAC
GGCCTTGCAAATGTATCCTCTAAAACTCTAAATAATTGTTTAAATTTTTCTTTACTATTTTGCTCTTTCGTTTCCATACTTTGTTGCTTCAATA
TATGACTTAATTTTTCGAAAAAAGGATCTAGATTCATATATTCGGTAAATCTACCTTCTTCATCTTTTTGAACTTGTAATTCTAGCAATTCAC
GTTGTATCAAATTTTGAATAACCATTGTAATATCGCGTGGTTGCATAGTTGAGCCCTTCTGAAGTAATTCAATTGAAGGCTGTTTATTTGAT
GTTTCGGAAGCATAAATCAATTTAAGCAAAATGACTAAATCTTGCTCATCTAAACCTAAGTCACTGTAATGGTCTAATAATTCTCTTCGTAT
CACTACAGGTCTTGCTTTTAATTGATATTTATCCAT

Gene: asnS (asparaginyl-tRNA synthase)

Contig: 06_NODE_54, position: 628952 to 630244, length: 1293 nt, orientation: REVERSE

Sequence:

TTATGGATATAAACGGTTTAATAATCTTGGGAATGGCGCTGTTTCACGAACGTGTTCAACACCAGAAATCCATGCTACTGTACGTTCTAAA
CCTAAACCAAATCCACAGTGTGGCACACTACCATAACGACGTAAGTCTAAGTAGTAACTATATGCTTCTTCGTCTAATCCATGTTCTTTAAC
GCGTTGTTCTAACAATTCTAAGTCATCCACACGTTCAGATCCACCAATAATTTCACCGTATCCTTCAGGTGCAATTAAGTCTGCACATAATA
CAGTTTCTTCATTTTCAGGATTTGGTTGCATATAGAAAGGCTTAATTTTAGTTGGATAATTAGTAATAAACACCGGTAAATCATAATGATT
AGCAATGGCTGTTTCATGTGGCGCACCAAAATCTTCGCCCCATTCAATATCATCAAAGCCTTCTGATTTTAAGAATTCAATTGCATCATCAT
ATGAAATTCTAGGGAATGGTGTCGCAACTTTTTCAAGTTTTGATGTATCACGCTCTAAAATTTTCAACTCTAGTTTACAATTTTCTAAAACT
GATTTTACAACATGTGTTACATATTGTTCTTGAATTTCTAAACTTTCAGCATGATTTGTGAAAGCCATTTCCCCTTCAATCATCCAGAACTCG
ATCAAGTGTCTACGTGTTTTTGATTTTTCAGCTCTGAAAGTTGGACCAAATGAAAATACTTTTCCGTGTGCCATTGCTGCAGCTTCTAAGTA
TAACTGACCACTTTGAGATAAAAACGCATCTTGATCAAAGTATTTAGTATGGAATAATTCACTTGTACCTTCTGGCGCACTTGCTGTCAAA
ATTGGTGGATCAACCTTTGTAAATCCATCTTTGTTGAAAAATTCATACGTTGCACGAATAACTTCATTTCTAATTTTCATTACAGCATGTTGT
TTTTTAGAACGTAACCATAAATGACGGTGATCCATTAAGAATTCTGTACCATGATTTTTAGGTGTAATCGGATAGTCATGCGCTTCTGAAA
TAACTTCAATTGATTTCACTTGCATTTCGTATCCTAAGTCAGAACGATTATCTTCTGTAATTGTGCCTGTAACGTATAGAGATGATTCTTGA
GTAATTTCTTTCGCAAGTTTGAATACCTCTTCATCAACTTCTGATTTAACTACTACGCCTTGCATAAAGACTGTTCCATCACGTAATTGTAAA
AAGGCGATTTTACCACTTGAACGTTTATTTGTTAGCCAAGCACCAATTGTAACGTCTTGGTTTAAATGATCTTTCGCTTGTTTAATCGTTGT
TTTCAT

Gene: dinG1 (damage inducible ATP-dependent 3'->5' nuclease, locus 1)

Contig: 06_NODE_54, position: 630566 to 633259, length: 2694 nt, orientation: REVERSE

Perfect match to: (N315-BA000018-[1493028:1495721:r], allele observed in CC5+CC1)

Sequence:

TCACTTTTTCTTTTTTTGAATTTGTCTTAATAATTTTCCAAACTGTTGAATGTCGCCTTTTTTCTGACGATAATTTTCAAGTGTTTGTTCAAAA
AAGTTTTTATAATTACTGTTTATAAGTCGATCATCAAATGAAACTATTATGCCGCGATCATTTTCACTTCTAATTAATCTTCCAAGTCCTTGT
CTAAAACGTGTAACTGCATCAGGTAATACATATTCCTTGAAAGTTGAAGTGAATTCAGAATCCATAAGCCAATATTTTGCATTATGCTTGT
TCATAAACGGTAACTTCGCTATCATCACACATTTAATACCATTTGCTTGAAAATCAAAACCTTCAAAAAATGTTGACGTACCAAGCAGTAT
GGCCTTATCAAAATTATTAAACTGTTGTACTATTTTATAATTTTGGTTCTGCTGTTGTGTTAATACAACATAATCTTCAAATTCTGGCAATTC
ATTTAGCATATCTTGTACCATATGCATCATTTTATAACTCGTAAATAAGACTAAACATTTTGATGACGTTATAGTCGTATATTCAATAATAT
AGCTTACAATCGATGCTACATACTCATCTATATTTTTATATTGATAAGATGCTACATCACTCGGTATAAATACACTTGTATTTTTTGCACTTT
GTAACGACGTGTTAACTTCAAATGTATTAAAGTGAACATCTTTGTTGAATAACTGTTTAAAAGCTTCAAACGAATGATTAAATTTTAAAGT
ACCAGATATAAAAATGAGTGATTTAAATTTTTCCAATACTTGTTTCGTTAATACATCTTTTACAGCGTAATCTTTCACATATAAACGTATTGT
TGATTTTTGAGATAAATTTTTAATCGAAATGAAACTAGTATGTCCAGCTTTTAAACTTTGTTCGATATTTTTAAATTTATCTTTTAAATACAA
CAACTGTTTCCGTAATGATTTAACTGTTTTATGACTAATGCCATTGAATATTTCTAGCGTTTTATTTAACTTATCGATAATCGCATGTAAATC
CTTCAAAATGTCTTTTGTTTCAAAAGTAAATACATTATGGAAGCGATGAATATCATCATCATAAACATCAGAATCATTGATAATCGTAAAT
ATCGTTGAGAACAATTGCTCATTTAACTCATGAATCTCATTCATACTAGCCTTCAAGCCAAAAATATCAATTGGTGCAATATCTAATTTTTC
CAAAATTCGCTGCTTTTCCAGTTGATCAATTGCCTTTAACAATTTTTCATTTTCGTTTTTACCAATCAAACCAAGCTGATATTTAATATCAGC
ATAACTCAACTCATTTGTCACTTGATTTAAGGCATAGTCTGGTAAGCGATGTGCTTCATCCACTATACAATCATCAAACAATTGATATATTG
AATTTTCAACATCAGAATGAATTAAATGTGCATGATTTGTAATACCAATTTGAATGTTCTGTGCATTTCGCTTAATAAAATTATAATAATGA
ACATCGTGACGTGCCGGTACATATGTTTCAATTTTCTGGTCAAAATACATCTTTTGACCACCTTTTAAATTTAATTCCTGTATATCTCCGGAC
GGCGTCTCTGTAATCCAAATCAGCAATTGCATTTTCAAGATATTCACTTCGTAATTACTTGTGTCATCTTTTAAAATTTGACTAATAAGCCC
CAATGAAATGTAATCACTTTTACTTTTAATCAATAGTGCATTAATTTTAAAATTCAACGCTTCATTCATTGCTGGAATATCTTTTTCTAACAA
TTGACTTTGCAGTAATTTAGTATTGGTAGAAATCATGACATGCTTCCCAGTTTCAATATTATACATCAAGGCCGCAAGTAAATATGCTAAT
GATTTACCACTGCCTAGTGATGCTTCAATCATTGCTTTTTCACTATGCATGAGCTGATCTAATATAGTTTCCGCTAAATATAATTGTTGCGG
TCGATATGTTAAGCCAAGTTGATCTACAGCTTTGCTATATAAAGACTTCAAGCTGCCATTATAATTTGTTGTCGGCTTTTTAAAATCAACTT
GCTTACGATAGATAATCTGTTCGAACTTTTCGTACGATTTATCCAATGGCTTTGCATCATATTGCCTAACCATCTCAAAGAAAATATCATAC
AAATCGTATTTCAACTGTTTACTTAAATAATATAATTGCTTCAAAGTATCTAACGGTAACTTTTCAAATTTTTCAAAAGCTAATATCATCAAT
TTAGCAGTAGTAGCGGCATCTTCGTCAGCTCGATGGGCATTTGCTAAGGTAATACCATGTGCCTCTGCTAATTCACTTAATTGATAGCTTT
TATCTGTAGGAAAAGCTATTTTAAAGATTTCTAGTGTATCTATAACTTTTTTGGGACGATATTGAATATTACAATCTTTAAATGCCTTTTTAA
TAAAATTCAAATCAAAATCTACATTATGAGCTACAAAAATGCAATCTTTTATCTTATCGTAGATTTCTTGTGCAACTTGATTAAAATATGGC
GCTTGTTGTAGCATATTTTCTTCAATGGATGTTAACGCTTGAATGAACGGCGGAATCTCTAAATTTGTTCTAATCATAGAATGATATGTATC
AATAATTTGGTTATTGCGCACAAACGTTATACCAATTTGAATGATATCGTCAAAATCTAATTGGTTGCCTGTTGTTTCCAAATCCACAACG
GCATAGGTTGCCATACCCAT

Gene: birA (bifunctional protein BirA)

Contig: 06_NODE_54, position: 633283 to 634254, length: 972 nt, orientation: REVERSE

Perfect match to: (MW2-BA000033-[1497618:1498589:r], allele observed in CC1)

Sequence:

TTAAAAATCTATATCTGCACTAATTAAACGGTGTGATTCACCCGCTTCATCTCTAACAATTAGATAGCCATCGTAATCTAAATCAATTGCTT
GTCCTTTAAACTGTTTATCATTTTCTGTAAATAGCAACGTTCTATTCCAAATATTAGAAGCTGCAATATATTCTTCACGAATTTCAGAAAAA
GGTAACGTTAAAAATTGATTATATCTTTTTTCAATTTCTTGAAGTAATCTCTCTAAAAATTGATATCTATCTAATTTATTTTTATCATGTAATT
GTACACTTGTTGCTCTATGTCTAATACTTTCATCAAAGTTTTCTAGTTGTTGCGTCAAATTAATACCTATACCACATATTATTGCTTCTATAC
CATCATTATTAGCAACCATTTCAGTTAAGAAACCACACACTTTACCATTATCAATATATATATCATTCGGCCATTTCACTTTGACTTCATCTT
GACTAAAATGTTGAATCGCATCTCTTATCCCTAATGCAATAAATAAATTAAATTTAGATATCATTGAGAATGCAACGTTAGGTCTTAACAC
GACAGACATCCAAAGTCCTTGCCCTTTTGAAGAACTCCAATGTCTATTAAATCGCCCACGACCTTTCGTTTGTTCATCACTCAAGATAAAAA
ATGAAGATTGATTTCCAACAAGTGACTTTTTCGCAGCAAGTTGTGTAGAATCTATTGAATCGTATACTTCACTAAAATCAAACAAAGCAGA
ACTTTTTGTATATTGGTCTATTATACCTTGATACCAAATATCTGGGAGCTGTTGTAATAAATGCCCTTTATGATTTACTGAATCTATTTTACA
TCCCTCTAACTTTAATTGGTCAATCACTTTTTTTACTGCAGTGCGCGATATATTAAGTGATTCCGCAATGCTTTGTCCAGATATATAATTCG
GTTTATTTTTATAGAGTAATTGAAGTACATCTTGACTATATTTTGACAT

Gene: cca-papS (tRNA CCA-pyrophosphorylase)

Contig: 06_NODE_54, position: 634241 to 635443, length: 1203 nt, orientation: REVERSE

Perfect match to: (Strain_21178-AGRN01000008-[28008:29210], allele observed in CC239)

Sequence:

CTATATTTTGACATGATTATCCACCCATTTCAAAATTTCAGTTTCTTCGTTGCTTACTTTACCTGTTACAATCGCTATCTCAATTTGTCTTAGC
ACATCTTTTAACCACGGACCACTTTTGGCATTTAAATGTGCCATAAGTACACCGCCATTAACCATCATGTCTTTTCTATTATGCATAGGTAA
ACGATGTAATGTTTCATCAATCGTTTGAAGGTTAACGATTAATGGTTCATGTCCTTGAATATCATTTGCCTTTATCACGTCTGCTGCAACCA
TTACATTTTTAATGAGATGCGTATCATAATCATAAACAAACATTTTTAATTGTTCTTTTGTAATAATACTTGGTAATGCATTCATAATTTGAA
TATATTGATTGATATCTTTTACTTGTCGGTTACTTAGCTTTAAAGGCTTCAATGAGTAATTAATATCAAATTTAACTGATACTATAGCAATC
AACAATTCTAAATCAATTGCTTCAGTTACATTAATTTGATTCATATCAAGATGTTCGAAATACGGCATATAATTAAATGCTTTCAGCGATTT
TAAATGATTAAAACTCTTTTCAACATTAATACCTCGCATTAATTTAGTTAGTTCAATCACTATACGCTCAATTGATAAAAATTTAATATCTGC
CATTTGTATACGCATCGCTTCGAATGTTTCCGTTGCAATATCAAATGATAATTGTGACTGGAACCTTAAACATCGAATCATACGTAAAGCA
TCTTCTTGGAATCGTTCCTCAGCTATACCTACAGTTCTTATTATTCGATTATTAATATCTTGTTGACCATCAAAATAATCATACAATTTGTAT
GCTGTATCCATTGCTATCGCATTCATCGTGAAATCTCGTCGTTGCAAATCTTCGTATAAATCACGAACAAATGTAACACCACTTGGTCTAC
GGTGATCGACATAATCTTCTTCAGCCCGGAATGTTGTCACTTCATAATTTTCATCATTAAAAACTACATTTATCGTGCCATGTTCTTTACCTA
CAGGTATCGTATGACTAAAGATAGATTCTATTTCATCCGGCGTTGCACTTGTTGTGATATCTATATCATGAATATTTCTTCCCATGACATAA
TCTCTTACAGAGCCACCTACATAATATGCTTCAAAACCATTGTCTTGAATTTGTTCTAATATAGGCCTTGCCTGTTCAAATAATGATTTATCC
AT

Gene: bshA (N-acetyl-alpha-D-glucosaminyl L-malate synthase)

Contig: 06_NODE_54, position: 635448 to 636590, length: 1143 nt, orientation: REVERSE

Perfect match to: (MW2-BA000033-[1499783:1500925:r], allele observed in CC1)

Sequence:

TTACTCGCCTTTACTTTTGTTATGCTCATTTAGCATTTTTTGATAATAATACTCATATTGATCTGTAATAAGTTCTGATCCAAAACGTTCAGC
AATATCTGCTAGCATGTTTTTCTGAAGTTTGTTGTATAACACCTTATCTTCAAGTAATCGGATAGCATAGTCACTCGCTGAATCACAATCAC
CCACATCTACGACAAATCCAGTTTCACCATGTTTAATAACCTCTTTAATTCCACCGGCATTTGAACCAATTGGAACGACGCCTGTTTTCATA
GCCTCAAGTAAAGTTAGTCCAAAGCTTTCTTTTTCACTTAATAATAATACTAAGTCAGATAATTGGTAAAATTCACTTACGCAATCTTGTTT
CCCTAAAAATAAAACATCCTCTTCTACGTTTAACTCTTTCGTCAATTGACGCATTGGCACTAATTCAGGACCATCTCCAAGTAAAATTAATT
TACTAGGTATCTTTTCACGTACTTTTGCAAATGTTTCTATAATAGTATCTATGCGTTTTACTTGTCTAAAATTCGATACATGTATTAACACTT
TTTCATCTGGTGCTATACCAAATTGTGATTTTAATGCTGTGTTATGTTTAGTTGGAAACTCATTTTCACGTACAAAATTATAAATCGGTATA
ATTTCTTTGTTAGTTTCAATAATTTCATGTGTTTCTTGTGCTAAAGATTTACTCACACTTGTCACAATATCACTTTTTTCAATGCCAAATTTAA

TTGCACCTTGGAGTGAATGATCATAGCCCAAAACAGTAATATCAGTACCGTGTAGCGTTGTCATAATTTTTATATCTTTACCTGACATCTCA
CGAGCTAAAATCCCACAAATTGCATGAGGTACAGCATAGTGCATATGCAACAAATCAAGACCATATTCTTTAATAACTTCAGCGATTTTAG
TACTTAACGTAATATCATACGGTGGATACTGAAATACTGCATATTGATTCACTTCAACTTGATGAAAAATCATATTCGGTAATGGTTTTCTT
ATTCTAAACGGGATATTTGAAGTGATAAAATGTACTTCGTGACCTCGCTCTGCTAATTTAATTCCTAATTCTGTGGCAATAATTCCAGAACC
ACCCATGGACGGGTAACATGTTATACCTATCTTCAT

Gene: ypjD (MazG nucleotide pyrophosphohydrolase)

Contig: 06_NODE_54, position: 636832 to 637149, length: 318 nt, orientation: REVERSE

Perfect match to: (MW2-BA000033-[1501167:1501484:r], allele observed in CC1+CC8+CC30+CC239)

Sequence:

TCACTTTCTTTCGAATCGATTTTTATCTCTTGTATTAAACTTTTCCATAGTTTCATTAAAGCTCTCTGTCATATCTATTCCCATTGAATTCGCT
AAACATAACAACACAAATAAATTATCACCTAATTCTGCTTTAATCGTATTTGCTTCCTCTGAATCTTTCTTCTTTTTTTCACCATAGGTATGAT
TTATTTCACGTGCAAGTTCGCCCACTTCTTCAGTCAATCTAGCTAAGTTAGCTAATGGTGAAAAATATCCTGTTTTAAATTGTCCAATATAT
TCATCAACTTCACGTTGCATTTCTACCATTGATTTCAT

Gene: yugP (putative metal-dependent protease/peptidase)

Contig: 06_NODE_54, position: 637485 to 638192, length: 708 nt, orientation: REVERSE

Perfect match to: (N315-BA000018-[1499949:1500656:r], highly conserved allele)

Sequence:

TTAATCACTTGAACGCGCAAGCAAAATGATACGTACAAGCTCTGCTACAGCGACAGCAGTTGCTGCAACATAAGTCATTGCTGCTGCAGA
TAATACTTTACGCGCATGCTTGTATTCTTTTTCATTTACAATGTTCAATGCCGTAATTTGTTTCATCGCTCTTGAACTCGCATCAAACTCAAC
TGGTAACGTAACAATTGAGAATAATACCGCTAATGACATTAAACCAGCACCAATCCATAAAGCAGTTGAACCAAATGCACTACCTATCGC
TGTTAAGATAATACCTAACATGATGATCATATAACTTAATGAACTCCCTAGGTTTGCAACAGGTACTAATGCTGCTCTGAATCTTAAGAAC
CAATATCCTTGGTGATCTTGAATGGCATGACCAACTTCGTGGGCTGCAATTGCAGTTCCAGCAACTGATGGTCTGTCATAGTTTGCAGGA
GATAGTGAAACAACTTTCTTTTTAGGATCGTAATGATCTGTTAAGAATCCTTCACCTTTAACAACTTCGACATCATAAATACCGTTTGCATG
TAAAATTTCTAATGCAACTTCACGACCCGTTTTACCACTAGTTGATCTAACTTGTGAATATTTCTCATAGTTAGATTTAACTTTGTGTTGTGC
CCATAAAGGAAGCACCATTAATATTACGAAATAAATTATCATAGTAAAAATTGAAGACAATAAACTCAC

Gene: ypjA (putative integral inner membrane protein)

Contig: 06_NODE_54, position: 638237 to 638824, length: 588 nt, orientation: REVERSE

Sequence:

TCATTTACACTTTAATAATTTGTTTAATTCAATATAAAGCAAAAGTCCAAAAACACTCAGACAACATGATAATACACCAATTTGCCACACAT
GTGTAGTTATAAAATCATAATATGGAAATTGAAGGTGAAAATAGTCAATATAATCATTCAAAAATACCCAAGTCATCGCTACACTGATTCC
AATCATAGAACGTTTAAACCTAGGATAGAAGTAAATTGCCTGAACGGCCATTATACTGTGGGAAAACATTAATATCAGGCCATTTACTGT
AATATCACCTTGTTCAATAATAAATAATATATTCATAATAACTGCCCAAATCCCATATTTGAATAATATTACAAATGCCAGTGCATCGATAA
TACTATTTTGTTTTTGGATTAATATCATTGAGATAGAAATAACTAAGAATAATATTGCAGTTGGGCTATCTGGAACAAAAATCTTAAAATG
CCACGGCGTATGACTTAATTGTTCACCATACCATATATAACCATAAATCATCCCTAATATATTACAAATGAGTAGCATCATTAACCAAGAA
CGTTGATAAAGTGTATATTGCCAAAATGTATTAATCGTCAT

Gene: ypiB (conserved hypothetical protein)

Contig: 06_NODE_54, position: 638814 to 639389, length: 576 nt, orientation: REVERSE

Perfect match to: (MW2-BA000033-[1503149:1503724:r], highly conserved allele)

Sequence:

TTAATCGTCATCTGCTAAGTCCTCAAATTGATTATGTTTATTTACTAGCTTGAGTGTATTTAAAATTTGCGTTAGTTGATAAAAACGTTGCTT
TTCATTCATCTGTAAACTTAAATCAATATTGTGTAACAAGTAATCTATTAATAACGCATGTTTATGCCGATCTATAGCCATACTATTTAAGT
CATGAAGATAAGTTTGATAACTGGGCGATTCCGTTAGTTGAGCAACTATTAAATCATCTAGCCTTTGCTCACGCTTTGACACGTTTGCGAA
GTGAATTTGAATATCAAAAGCACAGTTATGATTAGCGATATAATCAAATATTTCATTTGTATTCATTAACTTTATATTACGCTTAGTAAATT
GAATTGCAGAAGCGTGACTTCCCACTTCTGCAATTTCTAATGTTTCATGATGATTAATTTTTGTATCTACAAAATGAATGTTTGCCAATTTC
GCCTCATTCACTTTTATATAGTTAAGCACCCAAACTGCAATACGCGACTTAAATCGATATTGAAAAAGTAAATATTCAATAAAACTTTCTTT
AATTTGATTGAGTGTCTCTGACAT

Gene: ypiA (putative protein)

Contig: 06_NODE_54, position: 639403 to 640647, length: 1245 nt, orientation: REVERSE

Perfect match to: (MW2-BA000033-[1503738:1504982:r], allele observed in CC1)

Sequence:

TTAAGATTGCAATCTTGATAATTCGTCATGCCAATTTTCGTTACTTGGCTCTAGTTCCAACAATTGATTTAAAATAGTAATTGCTTGTTCCTT
TTGACCAATTTCAGTTAAATAGAAATAATAATCACTCATAAAATCAATATTTGTTTTCATCGTTGGATATGCTAATTCAAAGAAATGTTGAG
CTTCTTTATCTCGCTCTTCTTGACCATAGGCGAACGCTAAATGCCACATGAATGTAGGATCCAAATCTTCTTCATCTACATATGTTAATAAT
TCAATGATTGCTTCATAATCTTCTTCATTACGATATAAATCGCTTAAAATCAATAAAGGTTCTTGGTAAGCATTATCAACCTCTAATGCTTG
TCTTAACAATAATACACCTTCATTAGCATCGCCGTGTTCTATTTCCAAACATCCAGTTGTATACATTAACTCTTTATAAAATTGACTTAGTCG
TAATCCTTCTTTACCCGTCTCAATGGCATCTGGATAATTTTTTTCATTTTCATATAATGATTGTAAATACAAGTAGCCTTGAATATAATCAGG
ATCTTTAGAAAGTAATGTAGTCATTATTTTAATTGCTTCTTGAGTGATGTCATTTTTATCGTAAGAAATGGCTTTTTTGAGATAATCTTCTG
AAGTCATTTCATCTTCATTAATTTCATCGTATAAGCGAATCGCATCACTATAGTTACCACTTTGTAAACTACAATCTGCCATACGAGAGAAT
AAGTTTACACCATTAACTTGATATTCACCAGTTTCTAAAACGGTTTCGTATTCAGAGGTAGCACGTAAATATTGACCATCATAATATAACAT
TTCAGCCAATGCAAAATGGATTATTGGATCATTTGGCTCTAGTTCAAGTGCTTCTTGTAATTTAGCAATAGCAACTTCCATCATATTAATTT
GTTGATATAAATCTGCTTCTAACATCAACTTTTCAGGTGATGGTTCAACATAACTTAAATATTCTAACGCTTCGTCAGTTTGATTTTCAGAC
ATTAAACCTTCAATAAAATAAATCAGCAATTCACTTTCGTCTGGATATTTGTGATATAACACGCGAAATACTTCCAAACCTTGTGGCATTAA
TCCAAAATTGTAAAGTGTCTCTCCTAGAATAAATAATGCGTCATCGTTGTCAGTAGTTATTGCTTCATTAACACGAGAGTCTAAATTTTCTA
GTTTTTGTAGATTGATATCGTCTATTAATTTATAGATATCTTCCAT

Gene: aroA (5-enolpyruvylshikimate-3-phosphate synthase)

Contig: 06_NODE_54, position: 640654 to 641952, length: 1299 nt, orientation: REVERSE

Perfect match to: (11819-97-CP003194-[1534624:1535922:r], highly conserved allele)

Sequence:

TTATCCCTCATTTTCTAAAAGCTTTAGTTTTGGTAAAAATCCTGGAAATGATACATTTACAGCATCAAATTGTTTGATTTTGACAGGCTCGC
TTGAAAGTAGAGAAGCAACTGCAAGCATCATTCCTATTCGATGATCAGTTAAACTATCAACTGTTGCATTTGTTTTAAATTCTGACGGATG
AATAATCAATCCATCATTAGTTGGTTGTAATTCAAACCCTAACAAGTTTAACATATCAGCCGTTGTATCAATTCTATTTGTTTCTTTTACTTT
TAATTCCTCGGCATCTTTAATTGTACTCGTGCCAACTGCTTGTGTACAAAGTAATGCTATTACAGGCAGTTCATCAATTGCTTTTGGAACTA
ATTCTCCTTCGATTGTTATTGGTTGAAGCATTGGTGTGTATTGAATACGAATAGAAGCAGTAGGTTCAGCACCAGTTGTTTGATTGAAAA
GTTGGATATTACCGCCCATTTTTTCAACAATATCAATAATACCTGAACGTGTTGGATTGATTCCAACATTATGAATTGTTACATCACTTCCT
GGTGTGATAAGTGCTGCAACAATAAAGAACGCTGCAGATGAAATATCGCCAGGAACATGAAAATCTGCAGGTTTAATGTATCGAATTGC
TTCAGGGGTTGTATTAATTGATAACCCTTCTGCTTCAATTGGAATATTAAAATGTTTGAACATCGTCTCAGTATGATTTCGACTTACATCTA
ATTCTTTAATGATGGTCGGTTCCTTAGAAAACAAACTTGCAAATAAAATGGCACTTTTTACTTGTGCACTTGCAACTTCCATTTGATAATTT
ATACCTTTTATGACAGATGGCTTAATAATTAATGGTGTATAATTATCTTCAATACCTTCAATATTCGCATCCATAAGTTTCAATGGTCTCAA
GACACGATCCATTGGCCTTTTACCAATTGAAACATCGCCAGACAAAACACTTTCAATACCTAAACCACTTAACAAACCTGCCAATAATCGT
GTTGTCGTACCAGAATTACCTGTATACAATACTTGATGTGGCGTGTTAAAAGATTGATATCCTGGGGAAGTCACAACTAATTTTTCATCAT
CTTCTTTGATTTCTACACCTAACAGTCGGAAAATGTCCATCGTACGACGACAATCTTCGCCAAGTAGTGGCTTATATATAGTAGATACACC
TTCAGCTAGCGACGCCAACATGATTGCACGGTGTGTCATTGACTTATCGCCCGGCACTTCTATTTCGCCCTTTAACGGACCTGAAATATCA
ATGATTTGTTCATTTACCAT

Gene: aroB (3-dehydroquinate synthase)

Contig: 06_NODE_54, position: 641962 to 643026, length: 1065 nt, orientation: REVERSE

Perfect match to: (MW2-BA000033-[1506297:1507361:r], allele observed in CC1)

Sequence:

CTACTTAAAATATGTTTTTAATTGTTCACATGCATGTTGTAATGTTAGTTGATCAACATGTTGTACAACGATATCTCCAAATTGTCTAATCA
AGACCATTTGTACACCTTGCTTATCATTCTTTTTATCACTTAGCATATATTCGTATAACGTTTCAAAATCCAAGTCAGTTATCATGTCTAAAG
GATAACCGAGTTGTATTAAATATTGAATATAATGATTAATTTCATGCTTAGAATCAAACAAAGCATTCGCAACTATAAATTGATAGATAAT
GCCAACCATCACTGCATGACCATGAGGTATTTTATGATAGTATTCAACAGCATGCCCAAATGTATGACCTAAATTTAAAAATTTACGTACA
CCTTGTTCTTTTTCATCTGCAACAACAATATCCAGCTTCGTTTCAATACCTTTAGCAATATATTTATCCATACCATTTAATGACTGTAATATCT
CTCTATCTTTAAAGTGCTGTTCGATTTCTTGCGTCGTTGATTCACCATTCAATAACGCATGCTTATAAACTTCTGCATAGCCACTTAATATTT
GCTCAAATGGTAACGTCTTTAAAAAGTCTAAATCATAAATCACAGCAGTTGGACGATAAAATGCACCGATAAGGTTTTTACCTTGTTTTGA
GTTAATACCCACTTTACCGCCAACACTAGAATCATGCGCCAAAATCGTTGTAGGAACTTGTATAAAATGGACACCTCTTAATAGTGTTGCT
GCTACAAATCCTGCAAAATCACCAGTTGCACCACCACCAACAGCAATAATTGCTGTATTACGAGTCACATGATGGGATAAAATATACTCTA
ATGTTTCTTGATATTGATGAAATGTTTTTGTCTTTTCACCTGCTGGAATAATGACTTTATGCACATTTTCATATGATAAAATATCATCAAATT
TATTCGCAAAATATTGATTTACATATTCATCAATTAATATAAAACTTTGATCAAACTGATCAATATACGTGCTAATATGGTCAATTGCACCG
CGTTCAACATATATTGGATAATTATTTGAAGGGTATGTTGTTTGTAATTTCAT

Gene: aroC (chorismate synthase)

Contig: 06_NODE_54, position: 643052 to 644218, length: 1167 nt, orientation: REVERSE

Perfect match to: (MW2-BA000033-[1507387:1508553:r], allele observed in CC1)

Sequence:

TTAAAACTCAATATTTAATTGTCTGCGCTCAATAATTTGTTGTTTAAGTTGCTCAATATGATTTGATTGGAATTCTTCCAATAATGCTTTCGC
AATTTCAAATGCTACGACATGTTCGCAGACGATACTTGCTGCAGGAACAGCACAACTATCAGAACGTTCAATTGTTGCTTTAAAGTCTTCT
TTAGTATTAATGTCTACTGAATTTAATGGTTTATATAACGTTGGAATTGGTTTCATTACACCATTAACGATAATTGGCATTCCATTTGACAT
ACCGCCTTCTAAACCACCTAAGTGATTTGACCCACGATAATAGCCCAATTCAGTATTGTAGAGAATTTCGTCTTGAATTTCGCTACCAGGC
TTTTCAGCTGCTTTAAATCCTTCTCCAAAACTTACACCTTTAAATGCATTAATACTAACGACACCCTGTGCTATTCTTCCATCTAATTTACGA
TCATAATGTACATAACTACCTACACCAACAGGCATATTTTCAACTACAACTTGAACTACTCCCCCTATTGAATCACCATCGTTTTTAGCTTCG
TCAATTTTATCTCGCATTGCTTGTGCGATACTGTCATCAATTACACGAACATCATTACGATCAAGATTTGCTTTAAATGTTTTTGAATCATAA
AAATCTTTATCTTTAATTCCACCTATTTCAACAACACGACTGTATATATCGATATCTAACTGTTGTAATAACACTTTACATAAGGCACCGACT
GCAACTCGAGCTGCTGTTTCTCTAGCAGATGATCGCTCTAGCACATTTCGTAAATCACGATGATTATATTTCATACCTCCAACCAAATCTGC
ATGACCAGGTCTTGGTTTTGTAATAGTACGTTTCATATTTTCACGTTCTTCTTCACTTATTGGAGCTGCTCCCATAATTTTTCTCCAATGCGT
AAAGTCATCATTGGTTACAACCATAGTAATTGGACTACCTAATGTATAACCATTTCTAACGCCTGATACTATTTCTACTGTATCTTTCTCAAT
TTGCATGCGTCGGCCACGACCATAACCACCTTGACGCTTGAACATTTCTTTATTAATATCTTCAACTTTAATTTCTAAATTTGCTGGTATACC
TTCGACAATAACTGTTAATTGAGGTCCATGTGATTCTCCAGAAGTTAGATATCTCAT

Gene: Q2FYG8 (putative membrane protein)

Contig: 06_NODE_54, position: 644605 to 644805, length: 201 nt, orientation: REVERSE

Perfect match to: (N315-BA000018-[1507068:1507268:r], highly conserved allele)

Sequence:

TTAAGCGCTTTTGTTATACTTTGACTTCAAAAATATAATCAATTCTTTTACTAATACATAAAAAATATAAATGAGAGTCAGTATAAAAATAA
TTTTTGTTATAGGATTAAGTGGTTTATCTAAAAATATAGCACCATATAGTAATTCCATTAATTTTAAGAACATTAAAATCCCCAAAATAATC
GAAATAATATTCTTCAT

Gene: ndk (nucleoside diphosphate kinase)

Contig: 07_NODE_2, position: 308 to 757, length: 450 nt, orientation: REVERSE

Perfect match to: (08-02119-CP015645-[664824:665273], allele observed in ST582+CC15+CC20+CC22)

Sequence:

TTATTCATATAACCATGCATCACGTGGTGAAGCATAGCTAGTAATTTCATTTTCATTAAACCATAGATTAATTTCACGTTCAGCAGAATCTA
ATGAATCTGAACCGTGAATGATATTTCTACCAACAGTTAAACCTAAATCACCTCTAATTGATCCTGGTGAAGCTTCTGAAGGATTGGTGCT
GCCAATAATATGTCTAGATACATTAACTGCATCTTCACCTTCAACTACCATTGCGAACACTGGTGCTGATGTAATAAATGAAATTAAATCA
TTATAAAATGGTTTACCTTGGTGTTCACCATAATGTGTTTCAGCAAGTTCCATTGGTACTTGCATTAATTTACCACCGACAAGTTTTAGTCC
TTTTCTTTCAATTCTTGAAATTACTTCACCAATTAGATTTCTTTGTACTGCATCTGGTTTAATCATTAAAAATGTACGTTCCAC

Gene: gerCC (heptaprenyl diphosphate syntase component II)

Contig: 07_NODE_2, position: 849 to 1808, length: 960 nt, orientation: REVERSE

Perfect match to: (MW2-BA000033-[1509891:1510850:r], allele observed in CC1+CC15+CC30+CC133+CC1464)

Sequence:

CTACGTGTTTCTTGAACCCATTTTTTTCGTCAAACTTAAAAGTAGTGATTTCGGATGTCCATCTGGTAACTCAGAAATCAAATCCAAAGCTT
TACTTAAATACTTCGAACTTACTGCTTTAGATTCATTAATACTGTCAGACTTTCTAATAATTTGAATACATTCTTCAAATTCTTTTCGATCGCT
GTCACGTCGTAATTGCTCAATTTTCAATTTGAAGTCTGGGTTTTTACGCATTTCTAATAAAATCGGTAACGTAATATGACCATTAAGCAAAT
CACTTCCGACCGGCTTACCTAATTTCTTTTCGGTACTTGTGAAGTCTAATACATCATCAATGATTTGGAAGCTCATACCTATATAATGACCA
ATCATTTTCAATTTTCGTACAGTCTCTTTATCAGATTGAGATGTAATTGCACCAACTTCAGTTGATATTTGAATTAACAGTGCTGTTTTGCG
ATTGATACGTCGTAAATAATTGATAATTGTCTGTTGACTGTTAAATTGGTCTTGAAATTGGAAAAGTTCCCCTCTACAAACATCAACGATA
GATTCAGATATCAATTGATGTACACGATTATCTTTAACGGCCATTAAGTGTTCAAGTCCTAATGCCAATAAAAAATTCCCAGTTAAAATAG
CAGTTGTCTGATCCCATTTCTTTGATATGGTTAACTTGCCTCGACGCTTGTCGCTTTTATCAATAACGTCATCATGAACAAGTGTTGCCATA
TGAATTAACTCTAATGCGACTGCAACTTGATACGTTTGTTCAGACGTTTGTTCATCTTTGCCAAATTGGCTACTCAGAATTACAAATGCTGG
GCGTACTCTTTTACCACCAGAAGACAATAAGTGTAATGATGCCTGTTCTAATACAGAATCTTTACTTTTTATTGCCTTTTCAAGTCGTTGTTC
CACTTTCTTAATTTCATTGTTCATGTTTAACTTTGCCAC

Gene: ubiE (menaquinone biosynthesis methyltransferase)

Contig: 07_NODE_2, position: 1810 to 2535, length: 726 nt, orientation: REVERSE

Perfect match to: (08-02119-CP015645-[663046:663771], allele observed in ST582+CC1+CC101+CC1156)

Sequence:

TTAATCACCTTTGGTATTATCTTTTTCTTTATAGCCAAGGTGCATTGCAGCAACGCCCCCTGTAAAACTACGTACTCTTACATTTATGAAACC
CGCTTCTTCAAACATGCGCTTCAACTCTTCTTTTCCAGGAAAATTAAACGTAGATTGCTGTAACCATTCATATTCTTCTTTTGATTTTGCAAA
TAATTTCCCAAAAATAGGCATAACAAATTTAAAGTATAATGCATACATTTGTTTAAAGACTGGCAAAGTTGGTTGGCTCGTTTCAAGACAT
ACCACCATACCACCTGGTTTAAGTACTCTATTCATTTCTTTTAACGCGACTAAATAGTCTGGCACATTCCTTAATCCAAACCCAATTGTTACA
TAATCAAAAGAATTGTCTTCAAACGGCAATTCCATTGCATCACCATGAACAAGTTTAACATTTTCCATTGAAGCAGTTTTTTCTTTTCCTACT
TCTAACATATTCTCACTAAAGTCAATACCAGTAACTTCACCTGTTGGTCCTACAGCTTTGCTTAATGCGATTGTCCAATCACCAGTACCACA
ACAAACATCTAATGCTTTCGTCCCTTTTCTAACGCCCATGTCTTTCATGACGCGTTTTCTCCATACTTTATGCTGCTCAAAACTAATAATATT
ATTTAATCTATCATATTTTTTTGAAATATTTTGAAAAACGCGATGTACTTGCTCTTTATTTGCTTTATTGTCAGCCAT

Gene: gerCA (putative protein)

Contig: 07_NODE_2, position: 2538 to 3110, length: 573 nt, orientation: REVERSE

Sequence:

TTAATTACCTCTACTTTTTAAATAACTTTTTTGGATATCGTGTAAGTAATGCTTTACTTCACTTTGATTATATTTCTTGAAGTATGATGGATA
GTAATCAGACATATCTTCAAATAAATAATTATATATTTCCGACTCATCAATATTGATACCGAAATGAGATAACGTAATATACGGGAAAAGT
GTTTCAATTTTTACTATTGCTTGAGAAATTTCATAATCATTTAAAGCTTGATGATGTAATGAAGATTTCAATTCATTAATTTCAACAATTGCT
TTACTAATTTCATTTTGAAATGATAAATCATTGATTTCTGCTAGTAGCGTATAAAAATGTGCACTAATTAAATCTCCAATCAAAATGGAATG
TTTAGACAAATGATTATATGTAATGTCATCAAGGTGTCTCATTGATGTGTCAATTGTGAGGCATGCTACTTTGGCAACATCTGGAATATCA
TATGAATCAAGTAACTTACCTAACCGATGATTAATATTAATGGATTCATATTCTGATACACCCTTTAATCTTTCTTCTATTTGTCTTTCCAATT
TGCTAACAGTTGTTTCCAT

Gene: hup (DNA-binding protein HU)

Contig: 07_NODE_2, position: 3541 to 3813, length: 273 nt, orientation: REVERSE

Perfect match to: (RF122-AJ938182-[1463493:1463765:r], highly conserved allele)

Sequence:

TTATTTTACAGCATCTTTTAATGCTTTACCAGCTTTGAATGCTGGAACTTTACTTGCTGGGATATCAATTTCTTTACCAGTTTGAGGGTTAC
GACCTTTACGTGCAGCACGTTCACGTACCTCAAAGTTACCGAAACCAATTAATTGTACTTTTTCACCTTTAGCAAGTGAGTTTTGGATTGAT
TCGAATACAGCATCTACTGCTGAACCAGCTTCTTTTTTAGTTAAATCAGCTTGCTCTGCAACTGCATTGATTAAATCTGTTTTGTTCAT

Gene: gpdA (glycerol-3-phosphate dehydrogenase)

Contig: 07_NODE_2, position: 3984 to 4982, length: 999 nt, orientation: REVERSE

Perfect match to: (MW2-BA000033-[1513026:1514024:r], allele observed in CC1+CC5)

Sequence:

TTATTCAGATTTTTTATCGCGCTCCATTAAATCTTTTACGCATTCTTTTACTGAGATATTTTCAAATAATACTCTATATAATGCATTTGTAATT
GGCATATCCACATTTTTTTCTTTAGCTAAATGATAAACTGATTTAGTTGTATAAATACCTTCAACGACCATATTCATTTCAGATAATGCTTGA
TCCATTGATTCACCTTGTCCAAGTTTATATCCTAATGTGAAATTTCGAGAATGTGTTGATGTGCAAGTAACGATTAAGTCACCGATACCAC
CTAAACCTAGAAATGTCATAGGATCGGCACCTAACTTTTCACCTAATCTACTAATTTCCGCTAAACCACGAGTCATTAATGCAGCTTTTGCA
TTATCACCGTAGCCAATTCCAGCTACGATACCACTTGCTACTGCGATGATATTTTTCAATGCACCACCAAGTTCAACACCAATCAAGTCATC
ATTCGTGTACACACGCAAATAATCATTCATAAATAAATCTTGCGTTAATTTACTTACACTTTTATCTTTTGATGAAGCAGCAACTGTAGTTG
GTTGCTTGACTACAACTTCTTCCGCATGACTTGGCCCTGACAACACGCCAATACCTGCATTATATTCAGGTGAAATAGAATCTTCAATCATT
TCTGACACACGTTTAAACGTCCCATTTTCAATACCTTTAGCAACATGTATAAAAGTCTTTTTAGAGGTCAGCTTATCATTAATTTGAGAAGC
AACTTCTCGCATTGCTTTAGTAGGTAAAGCCATTAAGTAAATATCTGCAAATTGAATTGCTTTGGTCATATCTGAAGTAGCGATGATGTTA
ACATCTAATTTCGCGTATTTTAAATACTTTTTATTTGTATGACATGTATTTAATTCATCAACAGCATCTTGATTTTTACCCCACATCAAAACAT
CATGTCCATTTTCTGCAAGAACATTGGCAAGGGCTGTCCCAAAACTTCCCATACCAAAAACGGTAATTTTAGTCAT

Gene: engA (GTP-binding protein)

Contig: 07_NODE_2, position: 4999 to 6309, length: 1311 nt, orientation: REVERSE

Perfect match to: (MW2-BA000033-[1514041:1515351:r], highly conserved allele)

Sequence:

TTAATTTCTCTTTCGAGCTATAATATGAATTGGTGTACCTTCAAAACCAAAAGCGGCACGGATTTGATTCTCTAAATAGCGTTTATAAGAA
AAATGCATTAATTCTACATCATTAACAAATACAACAAATGTCGGTGGTTCTATAGCAACTTGTGTTGCATAAAAGACATTCAAACGTCTAC
CTTTGTCTGTTGGTGTAGGGTTCATGGAAATTGCATCAGTAACAACTTCATTTAAAGTTGAACTTTGAACACGTTTTTTATGGTTTTCACTT
GCTTCATTGATGTAAGGGAATAATGTACGTAATCTTGTGCGTTCTTTAGCAGACACAAAAGCAATTTGTGCATAATCTAAAAATTGGAATT
CTTTACGTACTTCATCTTCAAATTTCTTCATCGTTTTACTATCTTTTTCCACAGTATCCCATTTATTTACGACAATCACGACTGCTTTACCTTGT
TCATGTGCATATCCTGCAACACGTTTATCTTGTTCAATGATGCCTTGTTCTGCATCTATGACCACTAAAACAACATTTGAACGTTCAATCGC
TTTTAAAGCTCTTAATACTGAATATTTCTCAGTTGATTCATATACTTTTCCTTTTTTACGCATACCAGCAGTATCGATTAAAACATAATCTTG
TCCATCATAACTATACTCTGTATCAATAGCGTCTCTCGTTGTCCCTGCAACATTAGAAACGATAACGCGATCTTCACCTAAAATAGCATTTA
CTAAACTTGATTTACCTACGTTTGGTCGTCCAATAATGGATAGTCGAATTGTATCTTCATCATAAGGATCTTCTTCCTCTTCACCAAAATGA
GAAACAACTGCATCTAACAAGTCACCAAGACCTAAACCATGTGACCCTGATATCGGATACGGTTCACCAAATCCTAATGAATAGAAATCA
TACACGTCTGTACGCATTTCCATATTATCTACTTTGTTAACCGCTAATACGACCGGTTTTTTAGATTTGTATAAAATTTGAGCGACCATTTCA
TCGCTTTGTGTCAATCCTTCACGCACGTTAACCATAAAAATAATAACATCCGCTTCATCTATGGCGATTTCTGCCTGCGCTCTAATTTGTGT
TTGGAATGGTGCATCACCAATTTCAATACCACCTGTATCAATAATATTGAAATCATGTGTTAACCATTCACCTGAAGAATAAATACGATCT
CGTGTTACACCTGGCGTGTCTTCCACAATCGAAACACGTTCTCCAACTATTCTATTAAAAATTGTAGATTTACCTACATTAGGCCTACCTAC
AATAGCTACTATAGGTTTAGTCAT

Gene: rpsA (30S ribosomal protein S1)

Contig: 07_NODE_2, position: 6531 to 7706, length: 1176 nt, orientation: REVERSE

Perfect match to: (MW2-BA000033-[1515573:1516748:r], highly conserved allele)

Sequence:

TTATAGTTTAAGATTTTTAAGTTTATCACCAATCATATCGCCAATTGTTGGATTATCTTCTTCTTCGTTTTCTAAGTACGCCTTAGTCGTAGA
AGGATCACTTTCAACAACATCTTCGTTTGGTAATGTTGCTTTAATAGATAGTGATACTCTTTCATTCTCTTCATCAATACCTAATATTTTAAC
ATTTACTTGTTGACCAGGTTCTAACACTTCACCTGGCGTACCAATGTGTTTGTGTGCAATTTCAGAAATATGTACAAGTCCTTGTACACCTG
GTGCAATTTCAACAAATGCACCAAAGTTTGCCAATCTTACTACGACACCTTCAATGACATCATTTTCGTGGAATTGACCTTTAATATTTTCG
AAAGGTGTTGGTAACGTATCTTTGATTGATAATGAAATACGTTCTGTATCTCTATCAATAGATTTAATTTTAACTTTAACATCTTGACCAAT
TGAAACTACTTCTTCTGGTGTTTGAACATGTTCGTGAGAAAGTTCAGATACATGCACTAAACCATCAACACCGCCAATGTCTATAAATGCA
CCAAATTGAGTTAAACGCGCTACTTTACCATCAATAACATCGCCTTCATTTAAAGATTGTAATAATTGATCTTTTTTAGCATCGTTTTCTTCT
TGTTCAACTGCTTTACGGCTTAAAATGACTCTATTATTTTCAGGATCCAATTCTTCAACTTTAATACGAATTGTTTGTCCATCAAACACAGA
GAAATCCTCAATGAAGTCTGTTGAAATTAGTGAAGCCGGAACAAAACCTCTTTGTCCTACATCAACAACCAAACCACCTTTAACTACTTCT
GTTACTTTCGCTTCGATGATTTCATTATTATCTAATTTTTCTTGTAAATAACTATAAGACTTCTCAGTTTCAAGTTGTCTTCTAGATAAGATG
TAAGCTCCAGTTTCATTTTCTTCATCAAACTCAACTTTAGTGACATATGCTTCAACTTCGTCGCCCTCTTTTACAACTTCACTTGGGCTATCA
ATATGATGCGTAGATAGTTGACTAATAGGAATAATCCCATTAAATTTACCACCGTTGATATGAACAACAACTTGCTTGTCTTCAACTTGTT
GTACCTCGCCAGTGACTTTGTCACCTTCTTTAATATCGTTAATCATTGATTCATTGAATTCTTCAGTCAT

Gene: cmk (cytidine monophosphate kinase)

Contig: 07_NODE_2, position: 8404 to 9063, length: 660 nt, orientation: REVERSE

Perfect match to: (RF122-AJ938182-[1468371:1469030:r], highly conserved allele)

Sequence:

TTATTTAATTTGACTCACCATCGCTAAAATTTCGTCAGTAACTTCTTCAATCGACTTGCCTGTCGTATCTAATGTCACTGCATCATCTGCTTT
TCTTAATGGTGATATTTCACGGTTCATGTCATATTGATCACGAGCTTCAATATCACGTTTTAAATCTTCAAAATTTGATTCGATACCTCTTAA
TTGATTATCTTTATATCTTCTTTCTGCTCGCTCTTCAACTGATGCAATCATATATACTTTTAAATCTGCATCTGGTAGCACTACAGTTCCGAT
ATCGCGACCATCCATTACGATACCTTTTTCTGCAGCTAACTCTTTTTGTTTTTTAACGGCGAATGAACGTACTGGCTCTTTAGATGCAACGT
ATGAAACATGTTGCGTCACATCATTATTTCTTAAAAAGTCTGTTACATCTTCGTTATCTAAAATGACACATTGACCTTTATCTGCTTTATAAG
TTAAATCTAATGTTGTTTGGTCAACTAGTTTTGCAAAGTCCTCAGTTTTGTTTAATTTTAAATATTTGTATGTTAATGCACGATACATTGCTC
CTGTATCGACATAAATCATTGATAGTTCGCTGGCTACACGTTTCGCAATTGTACTTTTTCCGGCAGCAGCTGGACCATCTAATGCAATATT
AATGGCTTTCAT

Gene: ansA (L-asparaginase)

Contig: 07_NODE_2, position: 9140 to 10108, length: 969 nt, orientation: FORWARD

Perfect match to: (MW2-BA000033-[1518182:1519150], allele observed in CC1+CC15-ST582+CC1217)

Sequence:

ATGAAACATCTACTTGTTATTCATACTGGTGGCACCATTAGTATGTCACAAGACCAATCTAATAAAGTAGTAACAAATGATACCAACCCTA
TTTCAATGCATCAAGATGTCATAAATCAATATGCACAAATAGATGAATTAAATCCTTTTAATGTACCATCACCTCATATGACAATCCAACAT
GTTAAACAATTAAAGGATATTATTTTAGAAGCAGTAACAAATAAATATTATGATGGTTTCGTTATCACGCATGGTACCGATACGTTAGAA
GAAACTGCCTTTTTACTTGATTTAATATTAGGTATCGAGCAACCTGTTGTTATTACTGGCGCAATGCGCTCGTCTAATGAAATTGGTTCTGA
CGGATTATATAATTATATTTCCGCTATTCGCGTTGCCTCTGATGAAAAGGCCCGTCATAAAGGCGTGATGGTTGTATTTAATGATGAAATT
CATACGGCGCGTAATGTTACCAAAACACATACGTCTAATACAAACACATTTCAAAGTCCAAATCATGGTCCGCTAGGTGTATTGACAAAG
GATCGTGTGCAATTCCATCATATGCCATATCGCCAACAAGCATTGGAAAATGTCAATGACAAACTAAATGTACCATTAGTAAAAGCATAT
ATGGGTATGCCAGGTGACATTTTTAGTTTTTATAGTCGAGAAGGTATCGATGGTATGGTTATTGAAGCGTTAGGACAAGGCAACATACCT
CCAAGCGCATTAGAAGGCATTCAACAATTAGTATCTTTAAATATACCTATTGTGCTAGTTTCACGTTCCTTTAATGGTATTGTGAGTCCAAC
TTACGCATACGATGGTGGTGGTTACCAACTCGCACAACAAGGTTTTATTTTTTCTAACGGTTTGAATGGTCCAAAAGCAAGATTAAAATTA
TTAGTTGCGTTAAGCAACAATTTAGATAAAGCTGAAATCAAATCATATTTTGAATTATAA

Gene: ypdA (FAD-dependent pyridine nucleotide-disulphide oxidoreductase)

Contig: 07_NODE_2, position: 10223 to 11209, length: 987 nt, orientation: REVERSE

Perfect match to: (MW2-BA000033-[1519265:1520251:r], allele observed in CC1+CC772)

Sequence:

TTATGATTCTAACGGCGTTTGTTTCTTAGCTAGCATGCTTTGAGCAATAATGCCACCGTGGAATTTACCATTTTCAATAAAAATGGTATTCG
CATCATTCCCTGCAGCAATCACACCTGCAATGTAGCAATTTTCGATATTTGTTTCGTATGTCTCTTTATTATACATAGGCGCTGTTCCAAATT
CATTTGTATTAATTTGAATACCTACAGATTTTAAAAATTCATAATCGGGATGATAACCAATCATCGCAAAAACATAATCATTGTGTATCGTT
TTACTTTCACCATTTACTTCATAAGTCACAGTATCTTCAGTTATTTGGGTAACATTAGCATTAAATTCCATGTCAATTTTTTCATGATTTACTA
ATGCTGTGAAATTTGGAAGTATCCACGGTTTAATTGAAGGCGAATAATCTCCACCACGATATAGAACCGTCACGTTAGCACCAGCTTTTTC
CAACTCCAAAGCAGCATCGATAGCCGAATTCTTACCACCAATAATAACAACATCTTGATCAAAATACGGATGTGCCTCTTTAAAATAATGG
AACACTTTAGGTAAATCCGCACCTTCAACCTCTAATGTATTATGCTGACCATAATAGCCTGTCGCGATTGTTAAAAATCGACATTCATAAAC
ATCTTTCGTCGTAGTAATTGTAAATTTATTATTCATTTTTTTAACAGTTAATACTTCTTCAAATGCATTTACTTTTAATTGATGATGTTTTACA
ACTTCTCGGTAATAAACTAGCGCTTGATTACGTCTTGGTTTACTTTCTTCAACGATAAACGGTACGTCCCCAATACTTAATTTATCACTTGA
TGAGAAAAATGTTTGGTGAGTAGGATAATTATATATTGATTCAACGACATTACCCTTTTCAATAATTAAGGTATCAATACCTTTTCTTTTTT
GTTCAATAGCCGCACTTAATCCGCATGGCCCTCCACCAATTATGATACTTTCAACTTTTTGCAT

Gene: ebpS (cell surface elastin binding protein)

Contig: 07_NODE_2, position: 11525 to 12985, length: 1461 nt, orientation: REVERSE

Sequence:

TTATGGAATAACGATTTGTTGACCGTTTCTAATATTGTTACCACTTAAACCATTGGCACGTCTAATTTTTTCAACATTTTCCGGTGAACCTG
AACCGTAGTATTGAATTGCGATACGGTATAAGTTTTCTTGACCATTCACTGTATGTCTTTGGCCACCACCTTGACGTTGTTGTTGCTGTTGT
TGATTTTGATTAGCTTGTTGTTGATTTTGATTATTTTGTGCTTGATTGTTCGCTTGATTAGCGTTGTTTTGATCATTATCAGATTCATCTTTA
GTCGCTTTGTCTTGATCCTCTTTTGATTTATCACTGTCTGTAGATTTTGATTTATCTTTAGAAGCGTCTTTAGATGTGTCTTTGTCTTTACTTT
CATCAGCATTATTTTTATTTGTATTCGCGATTTTATTTTCTTTTGTACCATTATTATGATTGTTTAATGCCATGCCTCCAAATATCGCTAATGC
ACCGATAATTAGTACAGCTGCAATTAATGGTAACAATACTTTGGCCATGCCACCTTTTTTACGTTCTTTATCTCTGTCATGATTGTCATGTTC
ATCATGGTTTTGGCTTGCATTATTAGAGGCATGTGGTTTTGAAGCGGCAGAAGCACTTTTACTTGCTGCGCCTCCAGCCAAACCTGCTGTT
CCAGCACCGATCGCTGCACCTTTTTTGCCATTATGATGATCTTTAGACTTATCTTGAGACACTTTATCCTCAGTCGAGTTATTCGACTTGTC
AGAATTACTTTTGTTTTGAGCGTCATTTGAATGTTTCTTAGCTTTAGAAACACCCATTGCACCAGCTGCACCTGCAACACCCGCTGTTCCAG
CACCAATTGCTGCGCCTTTTTTACCACTATGATGATCTTTAGATTCATCTTTGTCTTGCTTAACAGTTACATCATCATGTTTATCTTTTGATGT
ATTTGCTTGGTTAGCACCTGTTGCAAAATATGGTTTAGGTTGCTGAGATTGTTCAGCTTCACTCTTATCAGAAACTGTTGAATGCTCAGTG
TTATTTTCTGCTTCTTTAATAGTCTCGTGTTTATCATTGTCTTCGATTGGTTCTGGATGTGATTTATCCATTGCAAAAGCATTCTTATTATAAT
ATTCCTCTTCATGTTGAGGTGTACTGTCTTGATGGCTAGGTTCTTGACTTTCAGTACTGTGTGATGATTCGACTTGACGATCATCTATTGTG
CCAGCCTCATTTTGAACATTGTCTTCAGATGTTTGTGATTCATTGTGAACTTGTTTATTATGATTCGTTGCTAAATCACGGCGTCTTTTTCTT
CTTTGGGCATTTCTTGGCGGAAACTGTTGCTCCGTATTCTTTATTGTATCCTGATGTTCTAATTCTGATTGGTCTTTTTCAACATCTTCCGTA
TGGTCTTGATGTGAATTTGTGTCTATCGATTGACGATTTTTTTCAAAGTCATCTTTAAAATTATTAGACAT

Gene: recQ2 (ATP-dependent DNA helicase)

Contig: 07_NODE_2, position: 13138 to 14517, length: 1380 nt, orientation: REVERSE

Perfect match to: (MW2-BA000033-[1522266:1523645:r], allele observed in CC1+CC239)

Sequence:

CTATCTGAGAAATAAATTTTGCAACTTTTCATCAAATCCAATACTTCTAATTACCTTCTTCTTATTTAAAATTGCGATATCAGTTATATTAGA
ATCATTGTCACAACATCGATCTTGTGCCGGTGGATATTCACCGAAAAATTCTAATAAATACTTCCGTCTACATTGATCCAATTTGCAATAGC
CAATCATGCGAAAGAATCCTAATTGCTTTCGTTTAAATGATTGCTTAAATATCTGTTTCAAGGCGCCGATACTATAGAATGAATGCAACGT
TGTCAAAACGTCTTGTTTATCGGGAGCTAAAAATTCTCCTATTTCGAAATTTTGTACATCTTCTTCTGTTATCATATCTGCAAATAATAACGT
TTCTAAAATATATTTATCGTCCGGTTGGAATAAACTAATTGCCTGACTTAGTTCACCATCGCGACCCGCACGGCCAATTTCTTGAATGTAGT
TAGAAGGACTTGTTGAAAGATGAAAGTGAATGATTGTGCGAATATCTTTTTTATTAATTCCCATACCAAAAGCACTCGTTGCGACTATAAT
CGGAATATCATTATTTAAAAATTGTTGTTGAACTGTGTGTCGCTCTTGATAATTCATATCACCATGATAAATACCTGTAAGAAAACCTGAA
TCATAAATAAGTTGCGCTAAATTCAGACACATCTTTTTCGATGAGACATAAATAATCGTTGGTCCCGACTGTTGTAGAAACGGCAGCAACC
ACTCAATTTTATCTTCGTCATCATGAAAATTAAGATGCTTAAAGCTTATGTTTGGGCGATTCATTGTAGTTTTAATAACGTTAAATTGAATC
GCTAACATTTCCGTCAAATCATCTTGTAAATGCGGTGGTGCAGTTGCTGTCAATGCTAAGACAACCGCTTCTTTAAAATGCTTTGTTACTTT
TCCTATTAGAGCATAATGTGGTCTGAAATCATATCCCCATTCAGATAGGCAATGTGCTTCATCTAGAACAATCATGCCAAAGTCTATCATA
GATATTAATTTAAAATTTGACGGTTGCAAGAGAAATTCTGGACTTAGAAAGATGAAGCGGCTATGTCGTAAACATTTAATATTATGCTTTT
TCTCAATTTCATCCATACCAGAGTGAATACATGTTACACGTTTTTCTCCATTTATTTTCAACTGCATAACTTGGTCATCCATTAAAGATATTA
ACGGTGAGATAATTAATGTCGGCTTACCTGATAAATACGTAGGTATTTGATAACACAAACTCTTTCCACTTCCAGTTGGAAGTATACCTAG
AGTGTGTTGTTGAGACATTATACTTTCTATAATTTCCTGTTGTCCCGGTTTAAAACTCTCGAATCCAAATTTGTTTCGTAAAATATCATGCA
ACAT

Gene: A5IT10 (putative protein)

Contig: 07_NODE_2, position: 14507 to 15460, length: 954 nt, orientation: REVERSE

Perfect match to: (N315-BA000018-[1521776:1522729:r], allele observed in CC5+CC1+CC8+CC239)

Sequence:

TCATGCAACATTCAGATCACCTCTTTCAAATCCAACGATTAATACTTTTAATTGAAAATATGATAACGTGTCAAATTGTTCTTTGTAAAATTT
TAATCGTTCGCCACGATGCTGTTGATAAAAATTCAAAAACTGGAGTTGATCTTCTAGTTCAACATAATCATCGTAATTAGACATGTAACCT
TTGATTAAGATTTCAAGTACATGATCTTCGATAGTATTGATTTTAACTTGTTGTTGAGCCGCTATATCTTCCATCGTGAATTGTTCAAGCAA
TTTTGTATAAGTAATATAAGTTTGATTTAATAATGTAGGTTTCATTATTATTTTTGATAAAATAGTATATTCTCCACTTTCTAATTCAAACAT
CATTGTCACTAAATTATTCATTTCGTATTCAAACAATTGCTGTTGAGATAGCTTTTCTATTAAACTAACTTGCTGTCTCGTATACATTGGTTC
ATCATAGCCTTGCAAATAATAATGTAAGTAACATGGCCCTTTTAAAGTTATTGCTTTAAACAAGTTATGTAGTTCATTTTCAAAATCAATTT
GTAATTGGTTTTCTTTAATGTAGTTATAGACAATCTTTACAGTTTCTTGTATCTTATTATTTTGAGAGATTGGTACAAAATGAAAAACATGT
TGTTTGGTATTAGACATGGTTTGCACTAATAGTTGAATTGCTTGAAATGTTTGCCCCATGCTTTCAAATGTATATCTAGGATGCAACATGA
TTTCCATTTCAGCATTAAATTCATTGATTTTTTCTAAAAATAGCTCAAAAGACGGATATTTTAATAGTGGTAAACTGTGATATAATGACAAC
TGTTGTTGACTACAAGCGTCAAAAAAGGTTTGGTGAGATTTCTTACCAACTAAGATATTGTAAATACTTTTATTTGTTTTATAGTTAAATGT
TTGTTGTAATGCTGTTTTTATAATGTGTTGCAA

Gene: fer (ferredoxin)

Contig: 07_NODE_2, position: 15568 to 15816, length: 249 nt, orientation: FORWARD

Perfect match to: (RF122-AJ938182-[1475595:1475843], highly conserved allele)

Sequence:

TTGGCAAAATATACAATCGTTGATATGGATACTTGTATTGCATGTGGTGCATGCGGTGCAGCAGCGCCAGATATATATGATTACGACGAC
GAAGGTATTGCTTTCGTAATCCTTGACGATAACCAAGGTACTGCAGAAGTACCTGAGGAATTATATGAAGATATGGAAGATGCAATTGAT
GGATGCCCTACAGATTCTATTAAAATTGCAGACGAATCATTTGATGGGGACGCTTTAAAATTTGAATAA

Gene: ribU (riboflavin ECF transporter, substrate-specific component)

Contig: 07_NODE_2, position: 15922 to 16467, length: 546 nt, orientation: REVERSE

Perfect match to: (MW2-BA000033-[1525050:1525595:r], highly conserved allele)

Sequence:

TTAAATTCTTTTCAAGAAATTCGCAAGCCTTCTATATAGTAAAATAAATACAATAGAAATAACGATACCTTTAATAATATTGAATGGTATAA
TTCCTGAAACAATGATTACTTTAAGATTATTTGCGATATCAGCTAAGTTAAATATCATACCGTACAAAGGTAATAGAACGAAATAGTTCAA
AATACTCAACACGATAGTCATAACGATTGTTGCAATGATTAATCCAGTAATCAAAGATTTTGTTGAACGTTTATTTTTATAGATGGCGTAA
GCAGTTAATAAGAAACTTGCGCCTGCTAAAAAGTTAGCAAATGGTCCAACTGGATCGCCCATACTAAATAAGTAGTTCAATAAATTTTTA
ACCAGTGCAACTACGATACCGGCAACTGGTCCAAACGTAAATGTAGCTAGTAGTGACGGTACATCACTAAAATCTAAAGTTAAGTATGGT
GGCAAAAATGGTATAGGAAACTTGATAAAAGTTAACACAAACGCAATCGCGCTCAACATACTTATTGTGATAAGACGTTTATTTTGTTGC
AT

Gene: graD04 (putative lipoprotein)

Contig: 07_NODE_2, position: 16877 to 17791, length: 915 nt, orientation: REVERSE

Perfect match to: (DAR4145-CP010526-[1596900:1597814:r], allele observed in CC772+CC1+CC72+CC772)

Sequence:

TTATTCATGATTAAATACACCTACTTCTATTACATCCTTCTTACTATAATTAGGTCTTGTTGTATTTATATATTTATTAGAAATTTGAAGAGA
AACATTCAAATCATGAGGAATATTCTTTTTCTTTTTTAGCACTTCAGAAAAATCAATCATCTCGTCTATTGTATTATCTTTAGTAAAATTATC
TTTAGTACTAAATAAAGTTGTAACAACATATGGACGTGATTTATCTTTAAGTTGTTTTTTTGAGTTTCTCATACCGTCCCTAAAAGACTTATC

ATCCTCTTTGATTAAGGGTTGAAAATACTTTTTATATTCATCTAAAGTATATGGAATTGCAGTCAAATAATAATACTCATTTCTAAAACCAC
TATTCTGCGTTTTCATAATTGCATTTTCAGTATAACCAGTATATTTATATTTACTTTTATATTCTTTTAAAACTTCTGTTAAATTATCCAACTCT
TCCTTATGTGCTCGATATTCAAAGCCACTCAACACTGTACCAACTAAAGTACTCATATCATCGCCTTTATCCTCACTTCTTAATGAGCTATCA
CTGTCAATAATTGATTTGTCAAATGGAATACTCGCATTAAATACGATATCGTGGTCATCACAATGCACGTATACTTCTACGCCGTCGCCAC
TACCTACAACATTCGTAGCTTTAACTTTTAGACCGAAGTTATCCATAAAAAATTGTTCGCCACGTTTAGCAATTTTATCTTTATGCTTCTTCG
CAAATTCAATCGCATCTTTTTCTGCAGGTGGTTGGAAGCCTTGACCTACATATTTTGAAGCTTCCATTTCTTCTGGTACTGATTTTGTTTCTT
TATTAGATTCGTTATTGGTAGTTGAACATCCTGATAGTATTATCGTTGCTATTAAGATTAGTTTAATTCTTTTAAACAT

Gene: graD03 (putative lipoprotein)

Contig: 07_NODE_2, position: 17848 to 18765, length: 918 nt, orientation: REVERSE

Perfect match to: (MW2-BA000033-[1526976:1527893:r], allele observed in CC1+CC72)

Sequence:

TTATTCACCTTTACTGTGCACCCCATATTCTATTGGACGATTATCATCATAAAAGGCTTTTTTAGTATTAATAGTAGGCTTACCTATTTGAAT
TGTAATTGTAGATTTATCTGGTTTATTTTTTAAATTATATAATTTATCACTTAGTTCAATTACATCATCTTCTGTATTGTCATTAGTAAAATTT
TTCTTTGTACTAAACAGTGTCGCTACTGCATCTGTATCAGCGGCATAATTCACTTCTTTTCTTGCTCGTTCCATTCCTTCTTTGAATTCCTTAT
CATTTTTTTGAATCAAAGGTTCATAATACTTTCGATATTCTTTTAAACTTCTAGAAGAGTATGTAATATAAAAATATTCATTTTTATATCCAA
CGTTTTGTGTCTTATTAATTGCTTCTTTAGTAAAACCTGTATATTGATATTTCTTTTCATTTTCTTTGAAAAATTTATATAAGTTATCATACTTT
TCTTTTTGCGCTCGATATTCAAAGCCACTCAGCACTGTACCCACCATCATACTCATATCATCACCGTTGTCATTACTACGCATTGATCCTTTT
TGATGGATGGCATCTTTGTACAAGGGCAAACTTGCATTAAATACAATGCCATGATCATCGCAATGCACATAAACTTCTATACCATCATCTT
TACCTACAACATTGGTAGCCTTTACCTTCAGTCCAAAGTTGTCTTTAAAGAATTGTTCACCTACTTTTTCAAATTCTTTACGATGCTTCTTCG
CAAATTCAATCGCATCTTTTTCTGCAGGTGGTTGGAAGCCTTGTCCAACATATTTTGAAGCTTCCATTTCTTCTGGTACTGATTTTGTTTCAT
TGTTGGATTCGTTATTGGTAGTTGAACATCCTGATAGCAGTAATGTTGCTATTAAGATTAGTTTAGTTCTTTTAAACAT

Gene: graD02 (putative lipoprotein)

Contig: 07_NODE_2, position: 18823 to 19728, length: 906 nt, orientation: REVERSE

Perfect match to: (MW2-BA000033-[1527951:1528856:r], allele observed in CC1)

Sequence:

TCATTCGACCTCAATCCTTATAGACTCATTATCACTGTAATTAACTCGATTAGTACTAATAGTAGATTTTGCTAGTTGTAAAAATATTTTCGT
ATTTTCAAAATTAAGGTGTAACTTTTTCGTACTTTCAGACAAATCTATAACATCATCTAATTTTTTGTCCTTTGAAAAGTTACTACTTCTCGA
AAACAATGTTGTATGTACTTCTATTGCAGCTTTATAGCCTACTCCTTTCCTTGCTTGTTTCATACCTTTTTTAAAATTCAGATTATTTTTCTTT
ATTAGGGGTTCGTAATATTTCCTATATTCTTGGAGCGTCGGTATATTAGCAACTATATAAAAATATTCATTTTCATATCCACTATTTTGTGTT
TTGTTAATTGCCTCTTTAGTAAATCCTGTATATTGATATTTCTTTTCATTTTCTTTGAAAAATTTATATAAATTATCATACTTTTCTTTTTGCGC
TCGATATTCAAAGCCACTCAACACTGTACCAACTAAAGTACTCATATCATCGCCTTTATCCTCACTTCTTAATGAGCTATCACTGTCAATAA
TTGATTTGTCAAATGGAATACTTGCATTAAATACGATGTCGTGGTCATCACAATGCACGAATACTTCTACACCGTCGCCACTACCTACAAC
ATTTGTAGCTTTAACTTTTAGTCCAAAGTTATCCATAAAAAATTGTTCGCCACGTTTAGCAATTTTATCTTTATGCTTTTTCGCAAATTCAAC
CGCATCTTTTTCTGCAGGTGGTTGGAAGCCTTGACCTACATATTTTGAAGCTTCCATTTCTTCTGGCACAGATTTTGTTTCATTGTTGGATTC
GTTATTGGTAGTTGAACATCCTGATAGTAGAATCGTTGCTATTAAGATTAGTTTAGTTCTTTTAAACAT

Gene: A5IT17 (putative protein containing attachment site of PVL-phage)

Contig: 07_NODE_2, position: 19819 to 20023, length: 205 nt, orientation: TRNC-RVRS (no start codon)

Perfect match to: (CGS01-ABWT01000007-[41438:41642], allele observed in CC8)

Sequence:

TTAGTATACAGCTAGTTTTTCTAATTGTTCTTTAACTTGAATTAAGTTTGACCGTATTAGAGAGGCAGATTGATCCATCGTTTGAATTGCTT
GTCCTTCATTTTCGTTCAAGCCATTACAAACAACTTCAAACTGTTGTGCCATTTGATCAAGACGCGCATGAGCTTGTGTGTTTAAAATAAAC
ATATCGTCATAATGTGATGGT

Gene: lukF-PV (Pantone-Valentine leukocidin subunit F)

Contig: 07_NODE_2, position: 20253 to 21230, length: 978 nt, orientation: REVERSE

Perfect match to: (MW2-BA000033-[1529381:1530358:r])

Sequence:

TTAGCTCATAGGATTTTTTTCCTTAGATTGAGTATCTATTAATTTAACTGTATGATTTTCCCAATCAACTTCATAAATTGATGTATGAGTTGC
TCTATTTTCATCTTTATAATTATTACCTATCCAGTGAAGTTGATTCCAAAAGTTTGTATATCTATCCATTTCTCTTTGATAAGTAACAGTAATT
TTCGATTTTTTTGCAGCGTTTTGTTTTCGAGATAGGACACCAATAAATTCTGGATTGAAGTTACCTCTGGATAACACTGGCATTTTGTGATA
TTCCAAGAAGTTTTGTCCAGCATTTAAGTTGCTTTGTCTTGAGCCTAAAAACATTTCATTACCATAAGTTGAATGATAACTATCTCTGCCAT
ATGGTCCCCAACCATTATTCATAATTTTATGTGCTTCAACATCCCAACCAATTTTTTTGAAATTAGTTCTTTTATCTAAGCTAGTTCTATAGCT
TTCTTGTTTATAGTTAATTGTCTCTGAAAAAGATTTTGAACCATTACCTCCACCTGATAAGCCGTTAGAGATATTAATATCTCCACCATAAG
AATAACCTACCGTTTGTTGTACTTGAAATTCTTCATTTTGATTTTTAGGTGCATAATCTACAACGTTTACTGAGTCATTAGAATCTGAATTAA
TTGAAATGTTGTACTTAGAACCCCAATAAAATTGAGAACTAATAGTGTCTTTTGGATTTGGCTTTGTATAGCCAGAATAAATGTTTCCAGC
AGCTTTGAGTATTAATGTATCTTTATCATAACTTTTATCTTTAATAAAATTAAAAGTTAAAATCTGAGAAATTTTTAACTTATCGGAATCTGA
TGTTGCAGTTGTTTTGTACAAAGTAATTTTATCATCAACCTTTTTCTCACTTACAGGTGTGATATGTTGAGCTGCATCAACTGTATTGGATA
GCAAAAGCAATGCAATTGATGTAACAACTGATGATTTGACTATTTTTTTCAT

Gene: lukS-PV (Pantone-Valentine leukocidin subunit S)

Contig: 07_NODE_2, position: 21232 to 22170, length: 939 nt, orientation: REVERSE

Perfect match to: (MW2-BA000033-[1530360:1531298:r])

Sequence:

TCAATTATGTCCTTTCACTTTAATTTCATGAGTTTTCCAGTTCACTTCATATTTAACTGTGTAATTTCTGTTTACAAATGCGTTGTGTATTCTA
GATCCTTCTAAATAACTATTGCCATAGTGTGTTGTTCTTCTAGTAGCATGAGTAACATCCATATTTCTGCCATACGTTATTTCAAATTCACTT
GTATCTCCTGAGCCTTTTTCATGAGAAACAGTTGCAATAAATGAAGGATTGAAACCACTGTGTACTAATGGGGGTAATTCATTGTCTGGA
ACAAAATAGTCTCTCGGATTTTGACTATATGGTTTATATCCAACAAATAAATTTGGATCATGTCCAGACATTTTACCTAATGATGTGATAAA
TGAATTAGCTTTTATTCCCCATTGAACACTTTTTGAATTTTGACGTTCTACTTCACTGATATAGTTTTGTTGATTATAACTAATTGTTTTTGAA
TAATTAAATGAACCATTACCTCCTGTTGATGGACCACTATTAAAATTACCACCTATGTTATAACCTAATGTTTGACTAACATTTACTGAATC
TATTTTATTTTTAGGTAGATAATTTATTAAATCTACATTGGGGTCATTTGTTTTGAGACCAATATTGTATTGGAAAGGCCACCTCATTGCTT
TTATATGATCTGTGTTTTTGTAATTGTAATAAGTAGTCTTTGAATTGATAAAACCTTGCATTTTTAAAATCAAAGCGTCTTTGTTATACTTTT
TATCTTTAACAAAATCAAACTGAATATTTTGTGTGACCCCCCACTTATCGCTACTTGTATCTTCTGTTCTTTTGACTACCTCAGCGCCATCAC
CAATATTCTCAATATTGTTATCAGCTTTAGATTCATGAAACGAAGTAGCAATAGGAGTGATTATTCCTAACGACAATGTTGCAGCTAATAG
TCTTTTTTTGACCAT

Gene: O80065 (putative protein)

Contig: 07_NODE_2, position: 22278 to 22410, length: 133 nt, orientation: REVERSE

Perfect match to: (KT/314250-AOCP01000026-[47636:47768:r])

Sequence:

CTACAAATGTGTTTTTTTACTATCATTTCGAAAAAAACCCGTTCATTACCAAACAAATTATAAATAATTTATATTTTGATTGATAATAAACAT
AAAACCACGATATAGTTGACCATAACAACTTTTTAATCAT

Gene: amidase-1

Contig: 07_NODE_2, position: 22560 to 24014, length: 1455 nt, orientation: REVERSE

Perfect match to: (JKD6159-CP002114-[1515820:1517274:r])

Sequence:

TCAAACTGCACTAAACTTACCAAAACTGCTTATTCGGTTGCCTGCTATGTCTACCTCTCCGGTCGCAATATAGCGACGTTGTCCACTATTAG
CAATATAAGTAATCCATCTATAACCATTAATACAATATGCACCGTCATACGTAATTGTTGTGTTGTTGGGTAATACCCCTGTAATTCTTGAA

TTAGTTGAATAACCGTCTCTTACATTATTACCTTTAACATTAGCTACTGTGTAATTGCCTTGTTCTTTTTTATAAGGGACATTATTCTTATCG
AGTGTATAACCTGCTGGCACTGGCGGATTCTTTTCGTTTTTAACTGGCGCTTTGACCTCACTAGCTACCACTCCACCAATTGGCTTACCATG
AATCGCACCAGCTATTAGTTTAGAGTACAAGTCGTAATTTTTCTTAATCCAATCCATATCATTTTTATTAGTAATAAAACCTAATTCAGATA
AGCGATAATTGATATTTATTTCTGCTGATACATTAACATTTAGTAAATCGTTACGAGGTGTTACACCTCTTATTTGTCCTAAATTATTTTTAA
TAACATCTTGTATACTTTTATCAATAGTATCTGCATTGAATTGACTTGAGATAATAACATGCCCACCACTTGCGCTTTCTCCTGCTGCGTCTA
AATGTATTTCTAGAACAATGTCATACCCCTGTGATTTAACCCAATATAAGCCATAATCTTTTTTATTGCCTACATTAACACCGTATGCAGTA
TCTTGATACATATCTTGTGATTGACTTGAGCCACCGTATAATGCAACTTCATGTCCTGCATGTCTTAAATACTTAGCGATATTAGGCGTTAT
ATATTTACGTATAAAATCGCGTTCGTTTGTTCCGTTTCCTACTGCTCCAGGATCGTTATAACCATGACCGGCTACAAGCATAATTTTTTTAG
GTTTAATTACTGCCTCTTTTTTTGTAGTCGCTTGCTTAATAATACCTTTAGCTTTATTGCCAACGCTTAAGTTGTTAGGGAAGTTTAACCTAA
TAAAATACATTGGATTGTCATAATAATGAACATGTCTTGTCACAGTTTCAGGACCCCAACCAGGTTGCGCAACGCCATTAGTCCAACCTTT
ACCGTTCCAGTTTTGACCAAATGATGTGAAAGTGTTTAAATTTGCGCTCTCAACAATTTCAACGTGTCCAGCTCCGCCACCATACTTTGAC
GGGAAAACGACAATATCCAACTTTTGCGGTAAAAAGCTGTCATAGTTTTTAATTATTTGACCATATTTTTCAATCTTTGCTTTATTATCAAA
CGGGATATTATAAGCATATAAACCTTGCAGCCTTTCGCCTGTCGCTAACATAAAGAACATATTGGCGTAATCATAACACTGAAATCCATAA
AACAAATCAGGATTGAACTGCTTCCCTAATGAATTATCAAACCATTTTTCTGCTTGGTTTTTTGTTATCAACAT

Gene: holA-1 (holin)

Contig: 07_NODE_2, position: 24025 to 24327, length: 303 nt, orientation: REVERSE

Perfect match to: (JKD6159-CP002114-[1517285:1517587:r])

Sequence:

CTACCCTAAATCATTTGTGTCGTTCATATTCGTAGGTGTCATTACTTCTTTAATTGGCGCTTGCCCTGTTGCTTTTCTATATTTACTTTCAGCT
TTATATTTCTTTAATTTTTGATTCGCCCATTTCCCTTCTTGAGATGTTGGATTATCTTTATATGTAGTATATAAAGCAACAACTGTTAAGATA
ATCGATGAAACACTTTCTTCATCTACTGGTATCGGACTTATACCTTTATTCGCTAAGAATTGATTTACTAATGCTAAGATCAATACGATGTA
TCTTGTTATTACTTTTGCATCCAT

Gene: Q9MBN6 (putative bacteriophagal protein)

Contig: 07_NODE_2, position: 24463 to 24762, length: 300 nt, orientation: REVERSE

Perfect match to: (MW2-BA000033-[1533591:1533890:r])

Sequence:

TTAAATGCCAAAAATAGTTTTTAACAAGGCTATAACAAATGTACTTAGAATCGTCCCTATTAATCCTAGAATCCACATCTTGATGTCTCTAA
TATTTTTAGCATTTTTCTCTTTATTTTTTTCATCTTCTTCTTTGTCACGCCTTAGTTCTTCGAAATTTCTATCTAACTTGTCATAAATTTTTTCTT
GCGTTCTCAGACTGTCTTCTATTCTGTCGAATTTTTCAAACATAGTCTTATCATTTTCTTCTAATCGCGTTAAACGCCAATCTTGTTCGTGTC
GTTTGGTAAATCCAAACAT

Gene: hypothet. phage protein

Contig: 07_NODE_2, position: 24808 to 24972, length: 165 nt, orientation: REVERSE

Perfect match to: (Phage-3A-ORF089-[AY954956.1-[[21287:21451]])

Sequence:

CTATGACTCTAGATTTTCTGGATACTTTTCTCCTGTAATAATTGCATATTCCTCTTTATCTATAACTTCCATATCTACATACCACGCTATATCT
TCTTTACTATATTCTTTCAATTGATACCATGTTTTAATATCTTCGAATGTTGGTGAAATTAATTTAAGCAT

Gene: DUF2479 (putative bacteriophagal protein)

Contig: 07_NODE_2, position: 25354 to 26820, length: 1467 nt, orientation: REVERSE

Perfect match to: (CP007659-[1540263:1541729:r])

Sequence:

TTATTTTCTCACTCCTATAATTTTGTTAATTGTCCCTCTATTTGCGTTCGCACCAGAGCCTCTTTGACTTCCTAAGTCGAAATAGACATCGTT
TGATATAGTTAAAGATGTACGACTAGATTTAGTTAATCCAAACTCATAAACACCTCCACCATTTCCATCACCATCTGGAAGATTTGAGGGA
TTCAATGAAATCTTTCCTCCTCCAAAAGGACTGCCAAACTCTGTAAAGTCACCACCTGGAAAAGTCCCATAAAAAATTAATAAAATAAATT
GGTCTAAACTCTCATTTAAGTACAATGTAGAGCCCACACCATTTGCTGTTCCATCAAAAATAACCGAATACCTTTTATTAAACTTGTCATCT
GCGTATAATTTAGCGTTACTTTCGGCCATATTAGCTTTTGATTGGGCACTTTGAACAGTTTCAAAAGGTGTATTGTAATCATTAATAGCTAA
TTCTGACCACTCAGACCATGAACCCGCTTCTTTTCTTTTAACAAACACTTTATTTGTACCGTTCGGTCGATAAGTCATACGCTTGTAATCTGA
AGTTACTACTAAATATTCGACAGTACCGTTAGTACTAACATCTCTTGGATAATTTATAGCTTGCGAAACATAAATAAATTGGGTTGAATCA
CCTATTCTTTGTTCTGGATTATTAAAATCAAATCCAGTAATCTGCATTATCTTACCATCATCTTTAGTAATCTTAGCTTTTTGCCAATTTGAA
GTAGAACCACTTGTGACTAAACCACCACTATTCACTGACTGCTTGAAGGCTTCATGTTTCTCATCCATATATCGCTTTTGCTCATCGAATGT
TCTTGAATATGCTTGCGCTTTATTTTCCAAATCAGATATACGGCTATTAGCAAGTTGCTTTAATTCATCAATACTTGAAGATTTTGCTATTTG
AATATCTGATAGACCTTTTTCTTTAGCTTTTTCAATCAGACTCGCATAATCCTCACCATTTTTTATAGCCTCGTCCATTGCTTTCGCGCGATC
CATAATAGTTTTTTCTAATTCTTGAAACTCAACAATATAGTGTAGTTTTGTTTCAGAGGGAATCTTGCTAAACAAACTTTTTTCAACGTTAA
ATGTGATAGTTCTCTCTACAACTACCACGTCTGAATTACCTAATTCTGCAACCGAAACTTGAGCTTGATAACTTCCATCTCTTTTAATTACAT
CATTAGGTAATTGAAATTTTAAAATACCTTTAAATGGATCTAATATTTCTAGTGGAGCAACTACCATTACTCCTTTACCTCGAATCGCTATT
CGTGCTTTGATATTTTCTTCACTCAGTAATAACGGTTGATTATTTTTAATGATATTAAAAAGAAGAACAGAAGAATCACTCTCTCCTGTTCT
AAAAGTTATATCTAGATTTGAAATATTTTCATAATGCGCTGTGTTTTCTAAATTTATAGCTACAGATTTCTCTAAATTACTCAT

Gene: ORF636 (WTA-alpha-GlcNAc binding protein)

Contig: 07_NODE_2, position: 26820 to 28730, length: 1911 nt, orientation: REVERSE

Perfect match to: (JKD6159-CP002114-[1520080:1521990:r])

Sequence:

TTAACTTATAATTCTCCCTTCGTGTAAAGTCCATGGCCCTGAACTTGTTTTACTATCATAATTTTTCAATAGTATCTCAGCAGATGCTGTAAC
ACTATTACGAACTAGCCTATGAACAAAGCCACCTGTGTTTGAAGCTTCTACATATAAGTTCCAACCAGCTACCCCTTTACGTTCAGTTGGA
AAATCTGTAAAACGTTTTGTATCATCCGTAGTTAAATAAAACGACATGCCTACTATGTTAATATCTGACATTTTTGTGATGAATGAAGGTA
CTCTCTCCCATTTACCACTATTTTTAGGCACATAATTCCAGTCCGAAATGTCTCCAGTTCTTCCAGAAAGCACCCTTTCAAAAGTCATCATAT
TCCTTGCATAACTATTACGCGTCAATATCTGAATTACATCACCGCCAGTTTGTGGTGGCTTAACTTCCAAGAACCAACCTGCATCACGCCAT
TCTCTTGGTAATGGGAAATCATCGATTTGAACTGTATGATCAGTGTATAAATAGTAAAGACCTGGCTCTGTTAACATCCCAAGATTCTTAA
GTTTATCAGGCCTCATTGGTAAAGGTTTAACTCTACCACCTGTGTCACTCATGATAAAAGGAACGCCTCTTGAGTGAAGTATTTCTAAAAT
ACCTCTTTGCCCAATCATGAAAATACGATGTGTTCTATTTCCATCACCACCGACAGTAACACCTAGCATCAAAGCTTTTTTACCACTATCTTT
GTCATAGTATATTTGCAAACCTTCTGCTTCCGCAAATTCGCCAGGAAATGAATCTAGTGTTCCACCATAGTCAGCATTAACCTGATACGCT
TCTTCTCCTGTTTCTAAATCGAAAGCCGTTAAATAGTTTCTATTATTTGGATTACTGTCTCCTGTATACCAATACAAGTATTTTTCATCAAAA
GTCACACCCTGCATTGGTTGGGTTTCGTTTGTTAGTCTCATAGGGATACTGATTTTATGCAAAACTTTATCAATATTTTTATCAACATCGTC
TAAACTTCTTATCTCTATATAATTCATTGAGTTTTCAAGTTCCCACTGACTTCTAGGTCTCTCAATTCTGTATAGAATTTTATTTTCTTTTTCAT
TTATGACAGGGGTGATGTAGGGTTTTTCTGGGTGTCCTGTAAATACATCTTGCATACCATACTTGCCATAGCTAATTTCCACATTAGGCGT
ATACTTGAAACGAACTAATGTATTCTCATTATTACCATTTAAGATAAAACTATAAATCCATAACTCATCATCAATATATCTATAACCGTTAT
GTGTACCATGACCCCCACCTACAATCAATGAGCTGTCTATAAATTGACCATTAGGTCTTAAACGACTTAGCATATAGCCATTATTTCTAGCT
TGTGTCATGTATACTATGCCTGTTCTATTATCAAACCAGAAGGATTGCATTACTGCATTTGTAAGAGGTGCAAGTTCTGTCACAAATAAAA
ACTCTTGCTTATCAGGTTCAAAACGATACTCGATATCAAGAATTTCTTGTTTGGTCTTATTTAATTCTCTTATAGTTTCCTCTTTATTAATTTG
AGTTTTGGTTTCCCAATCGTCTAAATGTTCTTTTAATGTGTCAAAGGTTTCGCCGTTTACATTAACTCGAGCTTGAACAATCTCATTAGCAC
TGTTATTACGTGGTGCCACAACAAGTGCGTTAATTTGACTTTGTAAAGATTTGTTTACTGCTGCTTGCGATCTACCATTATAATAAATTTGC
TCAGCGAAGTGTTGCATTGTTTTAGCTTTCTGATGCAACTTAAACTCTGTTGTCAAGCCAAGCGCAAATTGCTCTATTCTTTGCAGGTTTTG
AATTTCTTTAGCTCTATAATCTCGACCTGCTAAAGCTCCCAAATCCTTTATTAAATACAAATTTTCCAT

Gene: minor-phi (bacteriophage minor structural protein)

Contig: 07_NODE_2, position: 26820 to 28730, length: 1911 nt, orientation: REVERSE

Sequence:

TTAACTTATAATTCTCCCTTCGTGTAAAGTCCATGGCCCTGAACTTGTTTTACTATCATAATTTTTCAATAGTATCTCAGCAGATGCTGTAAC
ACTATTACGAACTAGCCTATGAACAAAGCCACCTGTGTTTGAAGCTTCTACATATAAGTTCCAACCAGCTACCCCTTTACGTTCAGTTGGA
AAATCTGTAAAACGTTTTGTATCATCCGTAGTTAAATAAAACGACATGCCTACTATGTTAATATCTGACATTTTTGTGATGAATGAAGGTA
CTCTCTCCCATTTACCACTATTTTTAGGCACATAATTCCAGTCCGAAATGTCTCCAGTTCTTCCAGAAAGCACCCTTTCAAAAGTCATCATAT
TCCTTGCATAACTATTACGCGTCAATATCTGAATTACATCACCGCCAGTTTGTGGTGGCTTAACTTCCAAGAACCAACCTGCATCACGCCAT
TCTCTTGGTAATGGGAAATCATCGATTTGAACTGTATGATCAGTGTATAAATAGTAAAGACCTGGCTCTGTTAACATCCCAAGATTCTTAA

GTTTATCAGGCCTCATTGGTAAAGGTTTAACTCTACCACCTGTGTCACTCATGATAAAAGGAACGCCTCTTGAGTGAAGTATTTCTAAAAT
ACCTCTTTGCCCAATCATGAAAATACGATGTGTTCTATTTCCATCACCACCGACAGTAACACCTAGCATCAAAGCTTTTTTACCACTATCTTT
GTCATAGTATATTTGCAAACCTTCTGCTTCCGCAAATTCGCCAGGAAATGAATCTAGTGTTCCACCATAGTCAGCATTAACCTGATACGCT
TCTTCTCCTGTTTCTAAATCGAAAGCCGTTAAATAGTTTCTATTATTTGGATTACTGTCTCCTGTATACCAATACAAGTATTTTTCATCAAAA
GTCACACCCTGCATTGGTTGGGTTTCGTTTGTTAGTCTCATAGGGATACTGATTTTATGCAAAACTTTATCAATATTTTTATCAACATCGTC
TAAACTTCTTATCTCTATATAATTCATTGAGTTTTCAAGTTCCCACTGACTTCTAGGTCTCTCAATTCTGTATAGAATTTTATTTTCTTTTTCAT
TTATGACAGGGGTGATGTAGGGTTTTTCTGGGTGTCCTGTAAATACATCTTGCATACCATACTTGCCATAGCTAATTTCCACATTAGGCGT
ATACTTGAAACGAACTAATGTATTCTCATTATTACCATTTAAGATAAAACTATAAATCCATAACTCATCATCAATATATCTATAACCGTTAT
GTGTACCATGACCCCCACCTACAATCAATGAGCTGTCTATAAATTGACCATTAGGTCTTAAACGACTTAGCATATAGCCATTATTTCTAGCT
TGTGTCATGTATACTATGCCTGTTCTATTATCAAACCAGAAGGATTGCATTACTGCATTTGTAAGAGGTGCAAGTTCTGTCACAAATAAAA
ACTCTTGCTTATCAGGTTCAAAACGATACTCGATATCAAGAATTTCTTGTTTGGTCTTATTTAATTCTCTTATAGTTTCCTCTTTATTAATTTG
AGTTTTGGTTTCCCAATCGTCTAAATGTTCTTTTAATGTGTCAAAGGTTTCGCCGTTTACATTAACTCGAGCTTGAACAATCTCATTAGCAC
TGTTATTACGTGGTGCCACAACAAGTGCGTTAATTTGACTTTGTAAAGATTTGTTTACTGCTGCTTGCGATCTACCATTATAATAAATTTGC
TCAGCGAAGTGTTGCATTGTTTTAGCTTTCTGATGCAACTTAAACTCTGTTGTCAAGCCAAGCGCAAATTGCTCTATTCTTTGCAGGTTTTG
AATTTCTTTAGCTCTATAATCTCGACCTGCTAAAGCTCCCAAATCCTTTATTAAATACAAATTTTCCAT

Gene: hypothet. phage protein

Contig: 07_NODE_2, position: 28746 to 29036, length: 291 nt, orientation: REVERSE

Sequence:

CTAATAAAATAGCACTGTACCAAGTTTCCCACTATCGTCAACTGTTATTTTCCACAATTTACCGTTTGGGGATTTCTGTACAATGCTATTTTG
AATAATTCCTGCTTCGCCTATTTTTAATTTATCTAATTTATTTTTATCATCTACCGAAATGATACCGTCTTGAGGCAATCCATCAATATCACT
ACTGCCTGCATAAGGTATCCCATTTATAGCTTTCCAGTGTGTAGCTGGAAAGTACTGTTTATCGTTTTCAAGTAGCGCTTTGATTTTAACTT
CTTCTGTTGCCAT

Gene: Q8SDP1 (phiSLT orf 527-like protein)

Contig: 07_NODE_2, position: 29036 to 30619, length: 1584 nt, orientation: REVERSE

Perfect match to: (MW2-BA000033-[1538164:1539747:r])

Sequence:

TTATATTAATACACTCCCTATATCCATTGTCTCGAAAGGAGAATTCAAAGTACTAGTGTATAAATGATTTATACGATTTGCTTGATAGTTAT
ATCTATTATCTTGTGCAATAACTCGTCTGTTAAGTGCTTGTTGAATTTGTACCATATCTTTTATTTCATTGCTGAAAGACACTTCATCTATTG
CGTTTACAAATGGATGTGACCTATCAAGTTTAACAACCTTTAATTCAGTGTTATATCCCATTAATTCATGAACAAAAAAATACGCTATCTCTT
GGCTCTATTTTTTCATAACCTATATAATTAACATCTAATTCAGTCTTAGGAGTATCATTTATTTGCTTTTTTGCAAATTCTAACAGCTTATCCT
GTGTTTCGATATCTTCATTTGTTTGCGTATTAGCATATCGAATCCCAAACTGCTTTGCACTATCTGCGACGTAGTCGACAATTGCTTTGTAT
TGATTGCGACCTGAATTATCAGCAATTAAATTTAAGACTGTTGATTTTTCAGTTCCAACATACATACAAGGCTTAGCTTTTTTATTTGAAGA
TATATCAATTCTATTTTTGGGGTCTTCTCCTAAAAAATATCATTTCTAAAACGTGCTTGCCTTTATCAATATTTTTTATTAAATCTATTGTTTCA
GACTGAACCGACTTAGCAAAACAAGAAATTTGCTTAATTTGCTTGCCGTCTAAAATCAACTTATATATTCCACCTTGAGAGCCCTTTTTTAT
TGTAAATCTAACTGTTTCATTACCATACTTGCAATCAAAGTTAATAGTAGCTTTAGACCCAATTGTTTCGGTACGATAAGTACCTTCTTTTAT
AAAACCATTTGAATATTTAATGTCAGTTGTTCTAATAGGATTATAATTTTTCTTTTCCTCAGCTGTATACTTTTTTCCAAAAACTTTTATAGCT
GTTCTTAATTCCAATGTACTGACAGTTGCAGATACAGTATCAGTATTATATTGATATCGAATCACTTTCTCGCTTCTTTGATAAAATGTTTCA
GGAGAATAAAAACCAATCTCTGTATCATTTGGGTAAATTATACAGCCAAACAGGTCTACCGCTTCTTTACAGTATTCTAAGCCGTTTTTGTT
ACCTAATTCGTCAATCGGTACTTTTCGCTTAAAATCTCCAATTATTTTATAGGTCATTTTGACCGAAGTTTTTTGATTTGCAAATCCATATCT
TAAGTACTCATCTAAAGAGTATTCTGGCGTTTTACCAGTTTCGCTACTGTCGTCATCAAGCTTATTTGATTCCACTGAGTGATTTTGAAATT
CATACATTATGTGATATGCCGTAACTTCAATAAAAACTTTATCACCTTCAACCTTTGGCGCTGTCTGCTTAATTGTATATTTTTCACCATGAT
AAATTATGAAGTTTTCACAAATCAATAAATCAAAAACAAAACTATTATGAGTAGTTCTATAAACTGTAAAGGTGATGTACCTAGCTTCATT
CAGTTCATAATATTCTTTAAAAGAACCATAATCTACATCTAGTAAATTTTCACAAATCAATTCATTAAAATCCATTACTGATAAATGATCAT
GATAATCCAT

Gene: Q4ZCS6putative bacteriophagal protein

Contig: 07_NODE_2, position: 30628 to 31452, length: 825 nt, orientation: REVERSE

Perfect match to: (JKD6159-CP002114-[1523888:1524712:r])

Sequence:

CTACCTATAAATAAAAGGAAACTTAAATGTAGTTTTAATATCACTGACGTCTCCTTTAATCTTAAATTCATTTTTACCTGGCGCTAATGTTAT
AATGCCTCTATTTGTATCAATTCCCACTCTATTTATATCTCGATATGCATACACACCATCTAAAACAAAATCAGTGTTTTTATCTATACTTTT
GTTGTACTTAAAAATATCACCTGTTGTATAGTTAACCAGTTCAAATCCTCCACTCGCATTTAAATTAATTAATATTTTCAAATCGTGCTTGAA
TCGTGGATTTATCGTATCAGTAGAACCGTTCCAAATAGTAAATTGATTTGATGTATGAGTATATTTAGGTGTGAAATCAAGAGGAATTCC
ATTTTCAAACATCCAATTAGAGTCGAATAAGAACTCGCTATCGGTCCAATTAACTGATTCAGAATACCCTTTATAAACATTTAAACTTACTT
CAATTTCAGTTGAAGAACCATCTTTTAAATTAGATGTAACATTAGCTGTATTCACTGCATATTTAACACCAGGCATTTGAGAAGTAATAAC
ATAATAAGGATGTCTGCGATTAAACACAGATCTAAACCAATGCTCAAATAAATTTAAATCTATAACATCTATACCATCATAGCCAAACCTT
AATACTAATGAAAAAGGCGCAAAACTAATTGCGCCCGGTAAAATACCATCTACTCCGTTAATAGTTACACTGTTATCATTGGTGTTTGGAC
TTTCAGCCCTTGCATCTAAAAATATAAGCTGATTAAAATCTGTTATTACTTCTTCCTTGTAACCATCTATGATTTTTACAAAAGATTGCAT

Gene: tailfiber-6200-phiSLT-1 (bacteriophage tail fiber protein)

Contig: 07_NODE_2, position: 31452 to 37652, length: 6201 nt, orientation: REVERSE

Perfect match to: (EU861005-[28489:34689])

Sequence:

TTAATTAGTCAAACCTCCCATATAATTATTTGCATTTGCTCTATGCCCACTTTGTTTTGACAATATTTTTTCTAAACCTCTAATTGCATCATTA
GAACCTAAGTTATTATCCTGAGAAGAAACAGTTTGAATCAATGCATCTGTTAATTTATTTCCTTTATCACTTAACATAACAATTTGTTTCAA
CAATTTTTCAACTGTTGAAGTATCATTATTTACAGTGATGTTATTTGGCTTGCCATCCATACCGATGATGCGCATAACCTGTTCAGTTAATT
GAATTGCTCGTTTACGTCTAGTTAAAGGGATAACCATCTCCTGTTTATCTCCTTCACCCACTTCAGCAAGTTGATGCTTTGTAATCAAACCA
CCATTCGCATATCTTCTTGGACCACTTGGAGACCAACCACCTCTTGGGTTAAACTGTGAGCGCCAATATCTGTTGTTAAAGAACGCTAATA
ACTGATCGTAACCACTATATATATTGTTGTGACCTCTAACAGCATAATGTCTAAATGTTTGTGGGATATATTGAAGCAATCCTTTTGCTGG
ATTGCCCTGTAAAACGTTGATGTCTCTAAGCGCACTAGATTGAGTTATACCTGCATTTCCTCCTGATTCGTGTTGAATCAAGCTAATAATAT
TTCCTACATCACCCGAAGTAACATTAACACCCATTCGTTTTGCTGCACGACGTATATCGCCTGCCCAAGCAGATGCAGCCTTATTAACACCT
GAACCACTTCGAACGCCACTACCTTTAAGTGACTTCAACCATTTTTCTGGATCTTTAGCTGTATCATTCCCTGGATGTGACCCTTGCATCAA
TTGGAAATGTAAGTGTGCTCCTCTAACGAAATTACCTGTAGCACCTGATTTCCCTATCAGTTGACCAGCTTTAATACGTTGGCCTTGTCTTG
CTAATTGCTTAGATAAATGCATATACCAGTTCCATTCATTAGCACCAGTCTTAATTTGTATAGAATTACCGCCACCGTAATCAGTCCATACC
TTATCTGCTATACCACCTTTAACGGCATAAACGTTTGTTCCAGAAGGCATACCAAAGTCTATACCATAGTGACGACCGCCATTAAAGTTAA
GTCCACCTGTGTAGCGTCCAAATCTTTGCCAGATTGGATATTCAAATAGATAGCTTCCATCGCCTCCACCACCGAAATCTTCAAACCACGA
TTTTACTTTGTCTACTAATTTCTTTTTGAGCAATGAGTATGCGCCTTTAGCTATTTTTACTGTAGCGTTAGCTCCGCCTCCAAAATTAATATT
TAAACCTGACATTACTTTATTTACTAGTTTCCCTGGATGTTGTACGTAATCCCACACATCGCCGATTTTATCGCCTAACCAAGATGCACCAT
CTTTGATTTTATCGCCTGCTGCTTCAACCATTTCTTCTGCACCTTTTTTGATATTATGCGCTGTGTTTTTAGCTGTAGCTCCAAATTCTCCTGC
TTTTTTACCTATATTACCTTTAAGTTGGTCTAGCCAATCTTTCTTTTTCGTACCTCCATGAAACTTTGGTAAAACACCCATACGCTGTAACTTC
AGAGTGTCATTAGCATTTATTACGCTATCCCCAACTCCTAGTGGAACAACCACATCTCGTCCTTGGGGTGCATGGAATGTTCCGTCAGCCC
TGTGAATTACTTCTTGAACTCCACCACCTGGGGCGTTTCCAGAACCTCTATCATTTAATACAGCAAATGTCGGTTGCGTTAATGCTCCCGA
ATTATCGGTAGCTACACCCTTTCCTGCTAAAGTACCAGTAGACAATGTAGGTATTGGCTTGATGAGATTTTTATCAGTAATGGCTTTAGAT
ATTTTATTAATACCGCCAATCATGCTATTCAAACCGCCAATAGCTTTATTAGCAACATTTTTACCTAAATCAGCCGCAGCTCTTCCCATGTCT
TTACCAATATCTCTAATCCAATCATATGTTTTGGATAGCCATTTTCTAAAACCATTAAATACTGATTTAGCGTTAGACCATGCCGAACTTGA
AATTGCATCAAAACGATCGTGGGCTCTTGAATACATATCCCCAGTCCAATCTTTTAAAGATTTGTATGAGTTACTAAACCATTTCGATGTTC
CTTTCCAAACGGATTTTGCATTCGACCAAGCTGTACTAGAAATATTATCCCATTTCGCGCGAGATTTATTAGCCATATCCGTTAGCCAGCCC
TTTGCACTTTTATATGCATTGCTAAACCATTTTGATGTGCCTCTCCAAATAGATTTTGAATGCGCCCAAGCTTTATCTGAAGCATCTGAATA
CTTTTGCTTAGTTTGATTGTAAATACTTCCTGTTGTCGATTTAACAGATTGCCAAGCTTTTCCAAACCATTTACCAGTACTATTAGCTATAGC
CTTAGTGTGGTATCCTACAGAACTTTTGGCTGAGCTCCAACCTGAACTTAATTTGCTTGGAATCCCTTTGATTCCGCTCCACATTTTTTTCAT
TTCGCCGCCAAAATGATTAGCATTTCTGCCCATTTTACTAAAGGCTTCGCCAGTTTTACTTTTTACGCCGTCCCAAGCATTTCCAAACCATTT
CTTTATATTTTCTCTGTTTCTACGAGCTGTTTCTTCTTGTTCTTTAGCGTATTTATCGCTTTTTTTCTTTTGGTCTTCTCTAAAGTTAGACCACC
AACTTTTAAGGCCATTCCACCACTTTTCAGTATTTTTATATACACGTCCACTGGATAAATCCATTTCTTTATCAATATCTTTATTTTGCTTTTT
AACAACATCTACTACAGCATCTTTTTTAGATTTTGCTTTTCTTACTTCATCTTTATGTCTTTGATCAGCAATAGCTAACAATTTATCTTTTTCA
GACTTAGAAAGGTTGACGTTATTTTTTATAGCAATGACATCATCTTCATATTGCTTGTCCACTTCTTTTTTTCTTGCTTTTCTTGCTTTTTCTG
CTTCTTTAATTGCTTTGCTCGCTTCGTCTATTGAATAAGCATTTCTGTTTCTTTGCATTCTTACTAAAATACGCTCTTGCTCTTTTTCAGTCTTA
CTCAATTCTTTAACAGTGATATCACGTCTTTGATTTTCAAGCTTTTCAATTTCTTTTCTTTCATTTTCTGAAATCTGACCATCACTCAAAGCTT
TTTCTTTCAATTCTTTGATTTTCTGATTGAGTTCTTGCTCTTTTTTAATTCGCAAGTCATTTTTTTCTTTAGTTCGAGTTAAAATGTTTTGCTTT
TCTTGTTCATCGAATGCACTATACTTATCAATAAGTTCTTGAGTTTTTTCGAGTTCCTTTTTATTTCTTTTTTCTATTTCAGCTATAAGGTTATT
AGATAAATCCGTTTCAATTTTCAAAAGTTTTTTTGCTTTGTCTTCTGATATCTGACCCGAGTTTAAACGTACTTTTTCCATGATTCTGCTATTT
TCTTCAGAATAATGCACATATTTTTCTAAAGCTTTTTCTGTTTCTTTTGAAACACCTTTCCCTAACACTTTTACAGTATCAGACGCTTTTTTAG
AAGCTGTGCCCATGGTTTGCATAAATCCTTTAAACTTGTTGACTCCTACTTTGAGAAGGTCATCGTCGCTTAATGATTTATAACCATCTTTC
ATATCTTTTGAAAACTTTTCTTTGAAGCTTTTGCCGATACTTCCAAGATAGTTTTTAAACTCTCCTAGCTTTCTAACAGCGCCGCCAATAATT

TTACCACCAAAAAACTTTATAGTTTCTCCTAAACCGTTAATACCGTTTCTGAACCATTCCACACGATCATATGCGGTTTTAAAAACTTTATAC
GCAATTGTAATAGCAGTTATTGTAGCACCTATAGGTCCTGTTAAAAACCTTAAGGCTACACCCGCAAATCTTGCGCCTCCACTTACTGCAA
ATAAAGATTTTGCAGCTAATCCTAAACCGTTTTTCAAAAGTTTGAACGGTAAAATTGCTAGCTTTGCAGAATTTTTCAAAACATTTATAGGT
TTTAAATTAAACATCATAGCTCCGGCTAACCCTTTAAAGCCTTTTGACGTTTTTCCTGTTGTAGAACCAAGAAATAATGTTTGAAGACCTAA
AGATTTCATTGCTTTTGAATTAGTATTTGAAAGGATTGTATTTTCAGCAATACGTCTATTTAATGACGCATATCCTTTAGCAGCACTTCCAA
CTGTACGTATTAATAACCCTCCAGCAAGAACTGCAGGTCCAATTGCTGCACCAAAAAGTGCTAATCCTACTGAAGCTTTTCTAACCCAACC
AGGGAGATGTGTAAATCCATCAACTAATTTTGTTAAACCTTCCGCTCCTGCTCTAATCATAGGCGTTAAATCTTTACCGACTTCGATTGCTA
ATGATTCAAAAGCGCCACCTAATTGTTCCAGAGCGCCTTTGAGATTATCTTTCATCAAATCTGCTGCTTTTTTACTTTCGCCATTGGAATTCT
TTAAGGATTTACTATAGCTATTAATTTTATCTGGTCCCGCTTCAATCAAGGCTAAAAATCCACTTGCTGCTTCAGTACCAACTATTGTAGCC
ACTGTAGCTAGTTTTTGTTCTCTCGTCATGCCTTTCATATTATCTTGGAACTGTCTAATCAATTCACCCATGCCAACAAATTGACCTTTAGCA
TCAGACAAATGAATACCTAATTTTTTCATTTCCTTAGCTGTATTTTTACTTGGATTAGCTAGCCTGATAAATGAAGCTCTTAGGGCAGTACC
TGCTTGAGAACCCTCTAAACCTGAGTTAGATAAAACTTCAATTGCTGCGGAAGTGTCCTCTATTGAAACTCCTAATGCTTTTGCAGGAGTA
CCAGCATACTTCAATGCATCTCCCATGTACTGAATATCTGCAGCACTATCATTTGCTGATCTCGCAAGTAAATCAGCAACATGATTTGCATC
AGATGCTTTTAAACCGAAAGAGTTAATCGCTGAAGCCATTACAGTTGCAGTTGTAGCCATTTCTGCACCACTTGCTTCTGCTGCACTGATA
ACACCTGGCATAGCCTCCATTGTTTGTTTGGCATTAAAGCCTAAAGCTGCCAATTCTTCCATACCTTTAGCAACTTCGTTAGCACTTTTACT
GGTTTTAGCTCCTAAGTCAACTGCTTGATTAGACATGCTTTTCAAGTCTTTACTGCTTGCTTGCGCAATCGCTCCAACTCGAGACATTTGGC
CTTCAAAGTCTGCACTTGTTTTTAATGCTGCACCTAACCCTAAAGTAATTGGTGTAGATACGCCCATCGTCATTGTACGTCCCAGGGAAGT
CATTTTGTCTCCAATAGAACTAAATTTCTTTGACATGACATCCGCTTGACTTGCAAGTTTACCGAAATGACTTTGAGCTATCATTTGTTCTTT
GTTAAAAGTCTTCATTTCGGATGAAGCTTTATCTATTGAACGCTCCAAATTATTTAAAGCAGCTTTTTCTTTATTAACAGCTGTTTCAGCTTT
TGCGACATTAGCGCTATGATTCTTAATAGTATTGTTTAAATCATTAAATTCTTTTTCTGTTTGCTTTAATTTAGTATTAGTTTTAGCGTAAGA
ACTTTCAATTTTATCATTTGATTTTGAAAGATTGTCATTTTGCACTTTTAGTTTTTGAACTTGATTGCCTTCTTGTTTATATTGTTCAACAAGT
GCTTTATGCTTAGCGGACTGCTTCTGTACTGCGTCACTTGCTCTTTTTAGTTGTGCAGTAGTAGCTTGGTTACTATTCTTAAGCTTTTGTTCT
GCATCTCTCAACTGTTTAAGTTTTTGATACGCATCTTGTTTACGTTGATTTGTACGTTTATATTGATTTTCAGCTTTTTTAAGTTCTGTATTCG
ATGATTTTAAGGCTTCTTTAGATTTATCAAGAGCTAATTTTTCTTTTTTATTGGCTTCTACTAACTTTAAATATGCTTTCTCAACATCTTTTAC
ACTGGATTTAGCTTTTTGGTAATTAGCGTTAACTTGTTTAAGCTCATCTTCTACTTGAGAATACATCTTTTTTTGAACTTTAAGCCTATCATT
TAACCCCTTAATTCTCGCCTGATATTTTTCCATTGATTTTTCAGACTTATCAAATGCTGACAGATTAGCTTTCATTTCACTATTAACAACACCT
AATTGTCGCTTTAAACCTTTCATGCCTTCTTGGACACCTAAATGGTCTAATTTCAGCTCCAAGGTCATGCCTTCTACTTTTTCATTCAT

Gene: hypothet. phage protein

Contig: 07_NODE_2, position: 33262 to 33768, length: 507 nt, orientation: FORWARD

Sequence:

ATGAGATTTTTATCAGTAATGGCTTTAGATATTTTATTAATACCGCCAATCATGCTATTCAAACCGCCAATAGCTTTATTAGCAACATTTTTA
CCTAAATCAGCCGCAGCTCTTCCCATGTCTTTACCAATATCTCTAATCCAATCATATGTTTTGGATAGCCATTTTCTAAAACCATTAAATACT
GATTTAGCGTTAGACCATGCCGAACTTGAAATTGCATCAAAACGATCGTGGGCTCTTGAATACATATCCCCAGTCCAATCTTTTAAAGATT
TGTATGAGTTACTAAACCATTTCGATGTTCCTTTCCAAACGGATTTTGCATTCGACCAAGCTGTACTAGAAATATTATCCCATTTCGCGCGA
GATTTATTAGCCATATCCGTTAGCCAGCCCTTTGCACTTTTATATGCATTGCTAAACCATTTTGATGTGCCTCTCCAAATAGATTTTGAATGC
GCCCAAGCTTTATCTGAAGCATCTGAATACTTTTGCTTAGTTTGA

Gene: Major-tail (major tail protein)

Contig: 07_NODE_2, position: 38274 to 38729, length: 456 nt, orientation: REVERSE

Sequence:

CTATCCGTTTGTTACTGTCACTGAAATTTGTCCTGACTTATCGCTTCCATCAGTAGACATAGCAGTGATTACTGAAGTACCTTCAGCTACAC
CGTGAATTGCTCCTGTATTTTCATCTACAGTAACAAATTCTGGATGTTCACTTGTATATTTCAATATTTTATTCGTTGCTGTGCTTGGTGCAA
TGTTTGGCTCAACATTGTCATCGGTATTTACCATAATTGATTTAGTTTCTGGTGTAAATGATACGCCTGAGACTAGAATTGGATTGGTTTT
GAATTGAGGTACATCAACTTTACTAGATTCTTTACCATTTTCTTCCCATGCCACTTGGTAAGTACCTTTTGGATAAGTTGTATCCGCTTCTAA
ATTAGATAAAGTTACTGACACTTTGCCTTCACCTTGTTCAGAAGCTACGACGTCGTCTCCTTTATAAACCTTTAAAGTTTTAGTCAT

Gene: hypothet. phage protein

Contig: 07_NODE_2, position: 38763 to 38963, length: 201 nt, orientation: REVERSE

Perfect match to: (Phage-42e-ORF083-[AY954955.1-[[7227:7427]])

Sequence:

CTATTCTGCTGAAACTGTTGCAGATTTTGAATTAACTGCTACTTCAACATTTTGGGGATTAGCTGGGTAACGAACCTGCAGAATCCTCTGA
ATGATCTTCACTGTCCGTGTATCCAACGAATACTTTTTTGAAGAATTCTGCTTCTCCTTCTTTACCTTCATGATAACCGTATACAATACCTTG
TGACGTTCCATCAACAT

Gene: Major-tail (major tail protein)

Contig: 07_NODE_2, position: 38821 to 39462, length: 642 nt, orientation: REVERSE

Perfect match to: (Phage-42e-ORF021-[AY954955.1-[[6728:7369]])

Sequence:

TTAGCTGGGTAACGAACCTGCAGAATCCTCTGAATGATCTTCACTGTCCGTGTATCCAACGAATACTTTTTTGAAGAATTCTGCTTCTCCTT
CTTTACCTTCATGATAACCGTATACAATACCTTGTGACGTTCCATCAACATCAACTTTTCTATTCATCCAGTCACCTGTTAATTTTGTAGGTT
CTGGGGCTTCTGCTTTTTCACCTCGTGTTTTAAATTCAATTGAATCTAAACTAAAAGTACCTTTAAGTAAGGCTACATATACCGGCTGACCT
GTTAAACCATCTTCCGATTCGCCAATTACTGTTACATACGGTGCTCTTGTATTCTCTCCTACCCAAGATGTACCATTTTTATCTTTAGTACGT
CCAATAACTGTGTTTAAATCATCACTTGGAATATTGAAAATACTCATGTCAGACTTAACTTCATTAGTACCTTGTTTTTTCATCCATACACGT
TTGTTAGATGCAAACATATCTACTAAATCTGGTGCTAAACCTGTGATATTTAGGTCAACTGTACCACCTTTTTCATCTTCCCATGTCATGCG
TTTAACTACTTTTGTTGCTTCTGGGTTAAAAACTCCAACGTATAATCTTTTAAAACCTACTTTATAAGAACCTTGTCCTTCTGCCAT

Gene: hypothet. phage protein

Contig: 07_NODE_2, position: 39497 to 39892, length: 396 nt, orientation: REVERSE

Perfect match to: (Phage-42e-ORF035-[AY954955.1-[[6298:6693]])

Sequence:

CTATTCGATGCGCTGATTTTTATAATATATATTTTTGGGTATGCCTTGATATCGTCTCGACATCACATAACGTTTAGTTTCTTCAAAATAAGC
ATCTAACTGACTAGATGCTTGAATTAAATTTTGTTGATATAACAGGTATCTTATTCGTTTTGTTATATCAATTGTTTTCTGATTATTTGAAGA
TTCTACATCTATTTGAATTAAGTATTCTTCACTGAGATATTTATCAGACATAAAGTCTGAAGGCAAATCATAAACAGGTGTAATAACAACA
AAGGGTTTGGAAGTTTCAGCGTTTTCAGTGACTTTGTAATAGTATATTCTAGAATTTATATGTGTTTTGAGCTCTGCATCAGATAATAAAA
TTCCTTTTATGGTGTTTAATATATTCAT

Gene: hypothet. phage protein

Contig: 07_NODE_2, position: 39893 to 40294, length: 402 nt, orientation: REVERSE

Perfect match to: (Phage-47-ORF028-[AY954957.1-[[5897:6298]])

Sequence:

TTATCTGGCCAACTCCTTTTTTATAATTTCTCTATACTTACGTTCGCTAGCAGCTAATGTTTTTGCAATAACTCCAAAACCTCTTGGTGTATA
TTTTTTTCCATCTCTTGTATAACCATGTTCATTCAAGTGAATAATGTTTTTGCGATTCATAGGGCCTACCCATTCAATTAAAACAGCCCTTTC
TTGACTGCCAACTTTTGTATAAGGCTTAGATTTAGTCATTTCTTCTATACTGGCACCCGTATCTTTAAAGCTCTCGAACTCTTTCTTTAAAGC
CTTTATAAAAAAATTCAGATGCTTCATTTAAAGCTTTATCACTCTTAGCCTGCATTGCTTGTTTACCGTATACCGATTCTAATTTATTCAACAC
TTCAGGTATCCCTTTAATTTCTACACTCAT

Gene: DNA-packaging

Contig: 07_NODE_2, position: 40635 to 40913, length: 279 nt, orientation: REVERSE

Perfect match to: (Phage-42e-ORF055-[AY954955.1-[[5277:5555]])

Sequence:

CTACCACTTTTTTAATTTCAAAATCATTTTTTGCAATCCTTTTTCATTAAACACCTTGCTTCTAGATTGGTCATTTGAGTATCCACGACTTTCA
TAATCTCTTGCAATGATATATTTAATCGCTGTACAAAAAAGCGGGTATTCCAAGTCATCTTTGTCATAATCTGGAACCCCACTTAATAGTAA
TTCAGACTTAGCCGATTGAATGAGACCTTCAATTAAATCATTTTCGAAATTATAGTCAATTCTCAACCACAATTTAATTTCTTCTAAACTCAT

Gene: hypothet. phage protein

Contig: 07_NODE_2, position: 40658 to 40795, length: 138 nt, orientation: REVERSE

Perfect match to: (Phage-42e-ORF110-[AY954955.1-[[5395:5532]])

Sequence:

TCATTTTTTGCAATCCTTTTTCATTAAACACCTTGCTTCTAGATTGGTCATTTGAGTATCCACGACTTTCATAATCTCTTGCAATGATATATTT
AATCGCTGTACAAAAAAGCGGGTATTCCAAGTCATCTTTGTCAT

Gene: capsid-L (phage capsid protein)

Contig: 07_NODE_2, position: 40982 to 42145, length: 1164 nt, orientation: REVERSE

Perfect match to: (MRSA252-BX571856-[1610781:1611944:r])

Sequence:

TTAGCTGGGTAATGGACCTGTATTTTCTTTTGCTTTTGCAATTCTGAATGCACTGTCTAATGTACGTTGCTGATCATACCATGCTGTTAATA
CAAACAAATATTCGCCTTTTTTAACATCTTTATCAGTGTCATAAGTTGTTCCATCATAGTTAATTCCAAAATAATTGAAATCTCCCACAATAG
GTTTAACTGCTGCATCTGTAAATACTACTGGTTTGCCAAATACTTTTTCTGCTGGTGTGTCAAAGAAATTTGTTGTTCCATTTGAAAGAACA
CTAATAATTTTGACATAATCTGCATATCGCATATAAATTGTTGCGTTATCACGATAATCTTCATGTAAATCTGCTAAAGCGTTAATAATAGC
ATCATACATGTCTGCTCCCTCAACTTCTTTAACAGATCCATTATAAAATGACATGTGTTCTAATCCAGATTTAGGACTTACTGCTAAGGCAT
CTTTACGCTCTTTAGCTGCTAATCCTGATTGTAGTGCGTTTTCAACCCAGTTTACTAAATCTACATCTGATCCATGAATTACAGTATCTGAA
ATTGCAGCAAATACTTTGAATTTATTAGTAGTGAACTTGACTGTATCACCTTTTGCTTTTAATTCTTTTGCTGTTTCTACGTCTGTAATGAAA
TCATCATCGTCTAAAGTGTATGAAACTCTTGGAATCTCTAAACCTTTAATGTTAGTTAGACGAGCTTTTTCACGTAATTGGTTTTTAGCAAA
TGGTTCTGAAACAATTTCTTTAGAAAGTGTTTTTGGTAAGAGCTTATCTCCACCTGAATCATTTCCTGTTGGTAAAGCGTGTAATAAACGTT
GTGCCTCCATTGAAGGTTTTTCAAATTCATTTGGTAAAATCGCGTGACGATAAAACTCTGCCTTAGCTTTAACCATCTTCTCATTATCACTT
AAAGATTGATAAGCTTCTCCTTTATCTTTAACTTTCGCTTTTTCTTTCTCTTCAATGTCTTGCACTTGTCTTTCAACAATGTTAAATCTTTGTT
GTAAACCTGCTTTTTCTGTTTCTAGTTGTTTGATGTCTTCCATATCAATATTTGGATCTGTTGCTTTCTGACTCAATTCATCATTTTTATTTTTT
AATTGTTGTCCAATCATACCTAAGGATTGTTTTAATTCATATAATGTCGGCAT

Gene: Q5HIZ6 (bacteriophage prohead protease/Clp-protease)

Contig: 07_NODE_2, position: 42157 to 42930, length: 774 nt, orientation: REVERSE

Perfect match to: (FPR3757-CP000255-[1568074:1568847:r])

Sequence:

CTAATAATTCATTGTCATTTTTAAAATTTCGCATTCGCGTTTAATTTTTTCTCTTTTTTCTTTTTCTTCTAGTGACATACTTTCTTTAGGTGTTT
CAACCAATTCAGATGTATCTACATCATCAATTTTAGTGATTTTGTCTACATCTTTCTTTAAATCTTCTGGGACGTTCTCGAAACGCTTATATT
GCTCTTTAGAGATACTAGCAGCTATTTCATTAGCTCCTAAAATTTCATCTATCAAGCCGAAAGACAAGGCTTCTTCTGCAGTAAGCCAAGT
TTCTGCATCTAACATCTGTTTTAAGTGTTCTTGATCTAAATCTTTTGCTTTATCTAAATAAGCTGAATTACTAACAGCATCTGTTTTTTCAAG
TAAATCCGCTGTCTTTCTTAATTCTTCTGCATTACCTACAGTCATAACCCATGAATTATGAATCATTAAAAAACTATTTTTGTGCATAAAAAT
AGTGTCACCACTCATAGCGATAACACTAGCAATTGATGCCGCTAAGGCATCGACATAGATATTAATTTTTGCAGGATGCATTTTTAGCATA
TTGTATATTGCATGCCCTTCAAATACACTGCCTCCAGATGAATTTATATGAACATCTATTTCACTGATGTCTCCTAGTTCATCTAGTTTATTT
TTGAAATCTGTAGCAGTTACATCACTTTCAAACCATTTATCACTTACAATATCACCATAAATAAATATTTCACCTTTACTTTTTGATTTTCTTT
TCATTTGAAAATACTTAGCTTTCATTGACAT

Gene: portal2-phi2958PVL (bacteriophage portal protein type 2)

Contig: 07_NODE_2, position: 42914 to 44152, length: 1239 nt, orientation: REVERSE

Perfect match to: (TCH1516-CP000730-[1582778:1584016:r])

Sequence:

TTAGCTTTCATTGACATTTTTATCACCACCTTTCAAAGATTTTCTTAATTCAAGTGGCGTGTCAATTGGGTATAAATCACCGCTTATTAGCG
GCTTATCTCCACCTTCAACTGGTGGTAAATCTTCCCACTCTCTAATGTCATTTATAGTGTAGTAACCACTACGAACTGCTTTAAAGTACACT
TCTGCTTGTGTTGCACTATCAGCCCTTAAATAAGATTTAACGTTAAATTTAAAATACCTATTTTTTTCTCTGTCTGTTTTAGTAAGTAGTTTC
CGATTAAATTCTTCTTCATACTGTTTGACGATTGGCAATAAGGTATGCTGCAAGTAAAATCTGTTTAACTCTTCATTTTTCGCGAAATTTGT
ATTTGATCTTGCATTTAAGAATACTGAGGGCAATTGAAAAACGTTAGCTACTCTTTCTCTTGTTAAATTCTCGCTTGCCACTATATCTTCAG
AGACATATTTTTTAGGTAACGGTTCGATTTCAACACCAGGCTCTTGGAATAATATTCCACCGTTTTCTTCATAGTACTGTTTGAAATCTTCT
AACACTTGCTGCCTTTTTTCTTTACCTACATTGGAACCATATTTAAGCATGAAAGAATCAGGTTTTTGCATTTCTGTAAGATTAAAGGTTCT
TACTGCATTATCAAAATCAGTTGTATTCTTCAACACATCAATCGGACTAATGCCTTGCACCATATTAGATGCCACGATGTGTTTAAAATGCA
ACATGTCCATATTATGAACAATCAATTTATTTCCAGTTGCAGCATGAATGGAATAATAAAGTTCACGTGATTGGTTTTCAATTAACATTTCA
ACAACATCTGGATTTAATAAGAAAAGCTTTGATGGTTGATGATAGATGTCTCGTTCAATTAGCACATATGCATTACCTTTTTCATTTCTGAT
TGTTTCAATTTGATTAATAAAATCAAAACTGCTCAGAGAATTATTCGGTGACACTGTAAGTAAATCAGATACTTCTGTATTAACTACTTTAT
AATCTTCATACATTTTCAAGGGCAAACTAGCCATCGAATTAGATAACTTTGTAATAGCTGAAAATATCGTTTCATTAGTTTCAAGCGTATTA
TTGATTACACCCCAAAAAGATTTATTTTTCCATGGGCTAAAGTCATAAAGCTTAGAAGCTGACTGATCAATCCAATTGTCTATCAATTTTTT
CTTTATGCGTGTGACAATATTCTCTTTTGCGATAACATTCAC

Gene: terminaseL2 (bacteriophage terminase large subunit)

Contig: 07_NODE_2, position: 44157 to 45848, length: 1692 nt, orientation: REVERSE

Perfect match to: (JKD6159-CP002114-[1537416:1539107:r])

Sequence:

TTAACGCATTATATCTTTAATACTAATAAATTCTATGTTTCCTTCACCCTTGTCAGAAACAACTTTATTCATAATATCTGTATATGTGTTTAAA
AATGCTGCAAAGCCATCTATTTTACGATATCTGCTTTGCTTAGACGGCAACCAGTTTCCGTTTCTGTCTAGTTTCAACTGAACATTATTGAT
ATACCATTTCATTAAAGGATTATTATTAAATATTATTTTCCCATCTAAAAACATTTCTTTTAAATCCTTCAATGCAGGGCTCAAGGTCAAAG
CTCCTTGTCTTGTTTCTTCCGTTTCAAACCCGTAATTTTTTAACTCTTGATTTAGTTTGAATGCGTTCGCTCTATCATAAGTAATTTTTTCTAC
TACATAATGCTCATTCATCTTAATTATCCAATTTAAAACATCTTGGTAGTCAATATAAGGCTTATCTTGCACTGTTAATAAGCCATCTTCTTC
CCATTCTCTATAGGGTATTTTTTCGTTAGAATATTCAACTTTGTGCTTAGGAATCCATGAATGCGATAAAACTGCAACTTTACCATTATCTA
ACGCAAAAGTAGCACACGCGGCTGTAAAGTCCTCTGTTTCTGATAAATCATAACCAATCGTGCATGGTCTGCCTTCCAGCTCTTCTAAAGA
AACAATTTCATTATTTTTTTGGAGTGTTGGGTAATCAATAAAACTCATCTCGTCATTATTAGCAAAGATATTAAACCTTTTGGTTATAAAAT
CTCCACGTTCAGCTGGTGTTCTCTTAGCTTTTTCCCACTCTTCTTTCATCTCATCTAAATTTATAGAGACACCTAAGTTGGGATTTGCTTTTAT
CCAGTTCGACGAATCATTAATATCATCGTCATCATCCAAAGATGCTAAATAATAAAAAGTTCTTTCGTCTTCTATGATTTGATCTAAGGTGT
CTCTTCCCGCTTCTACCATATCAACAAGTGGACCATCTAATTGATACCCTGCTGTCGTAATGTAGATGAGAAGAGGTTGTAACCTTGCAGC
TCTTGAGTTTTTTATAACTGAAATCAATTTATAGTCTTTAAATTCATGAATTTCATCAAAAATCCCCATGTGTGTATTCAATCCATCTAACTT
ATCGCTATCTGATGCTTGGGGCATAATTTTTGATATCGTTGCGTCATAATGGATTTCATCTCTTAATGTTCTGAAATTTTTATCAAGCTTTG
GGCTAGCTTTTATCATCGCCTTAGATTCATCGAATAATATTCTAGCTTGTTTCATTACGTTTGCTAAAAGATGGATTTCAGCGCCGTTTTCT
CCATCTTGAGAAACAGCATAGTTAGCAACACCAGATATAGTAGTTGTTTTACCATTTTTTCGCCCCATAAATATCAAAGCTTCTTTAAACCT
GCGCAGTTTTGTTTCTTTATGAACCCAACCAAACAAACTGCCAATAATAAAATGTTGCCATGGTTGTAATACAAGTTGACGTTTAGATCCT
TTGGAAGGTTTACAAAACTTTTCTATGAATCGAATAGGACGGTGCGCTAATTCTTCATCAAATACCCATTTACCTCCATTTTCTAGATATCT
AAGGTGTCTCTCACATTCTTTTTTAACATATTTGCTTGTTTTTATTTTCCCTTGAGTGACTTGCTCTGCATACCATGTTGTTAATAGTTTTGGT
GAAGGTTCATTTAAAACTTTAATAGTCAC

Gene: terminase-S

Contig: 07_NODE_2, position: 45838 to 46143, length: 306 nt, orientation: REVERSE

Perfect match to: (Phage-42e-ORF043-[AY954955.1-[[47:352]])

Sequence:

TTAATAGTCACCGAATCCACCTTCTTCTTGAACTATCTTTTTTCTTTGTGCTGCAGTTAAACCCATAGACTTGAGTAAGTTATTTAGTGTTTG
AACTGTTTTTGTCAGTTCTATGCTTAATGGATTCTTAACAATATTGCTCGCACCAGCCTTGTTTGTATGCTCTATCATCAAATCACTATTTTT
AAGTTCATCTCTTAACCGACAATAAAATTCATACGTTTCTATATACAAATTAATTAATATGTCATCAGATTTTTTATAATCCTCTATATATTCT
TTCAGCTGTTTTTTTGTTAATTTCAT

Gene: nuclease (HNH-endonuclease)

Contig: 07_NODE_2, position: 46270 to 46584, length: 315 nt, orientation: REVERSE

Perfect match to: (JKD6159-CP002114-[1539529:1539843:r])

Sequence:

TTAAATTTTTAGAACTCTAATTTTCTTAAGATTACTTTTGTCATTACCATTTGCATGAATTTTGTTATGACAGCCATAACAAACTGACATTAG
ATTATCTAATTCTAAAGCTTTGTTAAAATCTTCATCAACATAAATAATGTGATGCACTATGTTTGCATCTGTTACAATATCTTCGCGTAAAC
ACATTTGACAAAGATGATTATCTCTATCTAATGCTATCTCCCTTAACTTCTTCCATGCTTTTGAATGATAGAACCAATCGTATTGATATGACT
TACGACCATGCTTATAAATGTTATTGTGCTTGGTCAT

Gene: Q4ZCF5 (phage transcriptional regulator/activator)

Contig: 07_NODE_2, position: 46741 to 47178, length: 438 nt, orientation: REVERSE

Perfect match to: (MW2-BA000033-[1555870:1556307:r])

Sequence:

TTATTTGATACCTGCATGATACGCTACCGCTTTAACAAAGTTCTTTCGTATAGTAGTAACAGTATTACGATGCATATGGCATTCATGTCCTA
TCTGTTCCATCTTTAACTTCTTTTCTTTATTCCAATACTTGAGCCTTATTACTTTCTTATGATCTTCAGGCAACTTTAAGTATTCACTCTCAACT
GCTTCGACCATTTCTTCAAGGTTTCGTAACATCTTATTAGTTAATAATCTAGTTGCCATTAGTTCAGTTGTTCTAACTGGTTCGCCTTTTTGT
AATGGTCCATATACAATATTGGAATCTTGTTCCTTCGTTGGATTAAGTATTTCCAACCTCAATCTTTTTATTTCTTTCTTGTTCTCATTTAAAT
TATATATTTCTGATTCAATATATTTAAATGTTCCTGGCTTGATATCATATATTGTGTTCCCCAT

Gene: helc-1350 (helicase 1  (ca1350nt))

Contig: 07_NODE_2, position: 47191 to 48549, length: 1359 nt, orientation: REVERSE

Perfect match to: (93b_S9-CP010952-[336391:337749])

Sequence:

TTACTTATGCTTAGCTATTCTTGCTTTAATAGCTTTCATTAATTCTTCTTGCGTTAGTTCTTTATTTTGTAAAGCTTTATATACTCTTTGATCTA
TTGTGTTATCGGTCATGATATGATGAATAATAGTCGTATGATTTTGTCCTTGTCTGTATAATCTAGCATTTGCTTGTTGGTATAATTCCAAG
GACCATGTAAGTCCAAACCAAACAATAATGTGCCCACCTTGTTGTAAGTTTAATCCATGTCCTGCACTTGCTGGATGTGCTATAAGCAACT
TAATGTCTCCACTGTTCCAACGTTCTTTATAGTTTGAATCCTCTAATGTGGTTGCTTCCTTAAACCTTTGAAGTATTCTTTCTTTATCGTGTTT
GAAGTTATAAAACAATAGTATTGGTTGGCCTTGAGACTCCTCTATAATTTCCTCTAACTTATCTAACTTCTTATCATGTATAAGTCTTACATC
TTCCTCATCTGTATAAACTGCGCCGTTAGATAGTTGAAGTAGTTTCTGACTTAATGATGCCCCATTTTGAGCTACAACTGTTCCTTCTTCTTC
CGATTCTAAAATATAGTTTTTTTCTAATTCTTCATATACTTTTCTTTCTTTTTCTGATAAGACTACTGTTTGTTTAGTATCAACTCTGTCAGGC
ATATCCAGATAATCTTTCGCTTTCATGCTTAAACATATATCTTCTATTTGTTTATATATCTTTTCTTCAGATCCGTCTCTTAGCTCCCACTTAA
AAATATGTTCGCTAACTTGATGAGTTGGTTTAAAGTACCTTTCTCGATAACGACTGAATGAAGACTCAAGTCTTTCACCTCTGTCTATCAAA
TAAACTTGAGCCCATAAATCCTGTAAACTATTTGGACTAGGTGTTCCTGTTAATCCTATAAATCTATTAATGAGTGGTAATTTCTTTTTAAT
AGATTTAAACCTTTGACTCTTAGGACTTTTAAATGTAGACAGTTCATCAATCACAACCATGTCAAATGGCCATTCTTTTTTATATTGATCAC
ATAACCATTTAGTATTTTCTTTATTGGTTACATAGATATCAGCCTCTGTGTTTAATGCATCATTTCTTTCTTTAGGTGTTCCTAAAACTAAAG
ACACTTTCAGATGATTTAAATGGTTCCACTTATCAACTTCATCAACCCATGTATCTTTAGCAACTTGTTTAGGTGCTATAACTAACATTTTTT
TAGTGTCTAACAACTGCAATTCACTAAATGCTGTAAGTGTTGATACTGTTTTCCCTAGACCCATATCTAAAAACAGACCGTATTTCTCATTA
TCTATCACTTTATCTATTGCATACTTTTGATAGCTATGTGGTTTGAAGTCAATCGCCAA

Gene: Q4ZCF7 (putative bacteriophagal protein)

Contig: 07_NODE_2, position: 48539 to 48829, length: 291 nt, orientation: REVERSE

Perfect match to: (MW2-BA000033-[1557668:1557958:r])

Sequence:

TCAATCGCCAAATGTTCCACCTACCATTCTGATAAAAGTATTTACTTGTTCTTTATTCCATAACACATATACTTTATGATCTCTATTTTCAAAT
TGTCTATGCACATATTTTTGTAAAGGATGCAACTTTCCTTTTTCTTGCTTCATTTCTACAAAATATGTTTTTCCTTCTGGCATAATAATAATTC

TATCTGGCACACCTCTTGTTCCAGGTGCAACCCATTTTAAACATAAACCGTTTAGCTTTGATATCTCTTTCACTAAATATTTTTCTAATGTCG
ATTCTTTCAT

Gene: virE (bacteriophage virulence-associated protein E)

Contig: 07_NODE_2, position: 49170 to 51617, length: 2448 nt, orientation: REVERSE

Perfect match to: (93b_S9-CP010952-[333323:335770])

Sequence:

TTATATTAAATCTTCTAAACTTTCATCTCTTACATAAGCTATCTGTACACCATAATCTTTTCCAAATCGAATTTTCCCACTTTTATTACCATCA
TATACAGACCAATTGTCTAATTGTCTTAAGATGTTTGAAATCTTTCTAATTTCCATAGATCCTCTACTATCTCCCTTATCTTTACCAAAACATT
CAACAAACACTTCAAGCGCACAGACCTTATTTCTTTCAACGTAATCTACATTTCCTGTTGGTAACATATCAACATCACCTTGATAAAATCGT
CTTCGTTCAAAGATAGTTAAGTCATCCCAATTGCTAGGAATTGGTGTGTTAAGATATTCATCAATAATGCCTGTATATGGAGATTCCTCAG
TATGTTTGCTTTGTATTGAACGCATTTCTTCTTCTAGTTCAGGGTTGAGGAATAACTCTTCTCCTTGTTCATAATAATATTTAGCTTCTGCCC
AAATTTGGTCGATCTCTTCTTTGGTTAGTTTAGACCAGTTCACTTCAACTCTCTCTGGATTTACAGTCATTGGCCAAAAACGTCTTCCACCA
GTTTCATCTCTTAAGAAATCAACTTTATTAGTTGTACCAATGAAAATACATTGCCTTGGAAAATCTTCAATATAATGTCCATAAGCAACACG
AAACCGGTCAACTTGTTTAGATATGAAATGCTTAATAGCTTCAACTTCAGCTTTTCTTGTAGCTGCAAGTTCTGCCATTTCCATTAGCCAAA
CGCCTTGTAAGGCCTCATAGGCTTCTTTACCTGTAACAGAAACTAAACTGTCAGAAAACCATGCACCACCTAATTTTTTTAGCAAAGCAGA
TTTACCTACACCTTGAGGACCATAAAGTGTAAGCATATAGTCAAATTTACATCCAGGCTCCATTACTCGAGCAATTCCAGCAGTCAATGCT
TTTTTGGTAGTTGTTCTATTCACTTCAGTATCTTCAACACCTAAGTATTTGATAAATAACTTTTCAAGACGTTTATGTCCATCCCACGATATT
TTATTTAGATAATCCCTTACTGGATGATAAGCATTTTGCATTGCTACGCTTATAATGGCATCTTTTGTTTTACCTGAATGGTGTATGTCATA
AATCTTTTCGATATAACTTCTTAAACTGCTATCATCACCGTCTTGCCATTGACGTGTCTTAAAATTAGTATTCCATGGCACTTTCCCTAAGCA
TTCAATTTGTTTTGTAAATTCATTAAATGCTATTTTTCCTTTTAAATTTGGATCATTACGCAATATAATTTCTATATTTGGGATACTAGCTTTG
AAAGTACCTTTCGAAGTAATTTCTAACGTCTCAGACCATGCATCATCGCTATTTACTATTTCATCGAAATCCTGCATTGCATCAGACATTTT
GTCGTTAATTAATTGCTTTTTAACAACTTCATCATTTTGCGCTCTTTGCTGCATTGCTTTATAACTAGGTAGTCGATTAACCGGAGTATCTGT
TTTAGCGTCTTCATCTTGAGCACCATATAAGTGTATGCGTACTAAATCAAAACTGTTCACAAGCATACCGCTAACGGGATCCGTATTATGA
TGAGAATAGGCAAACTTGTTATTTTCGTATAACACCAATCCACCTGCAGTTGAACCTTCATGATAGGTATAACGGTTAGTAGAATGTTTTT
CGTATAAGTCAGGAATAAAAGTTGATATAGCTTCTTCTATCGTATAGGCTCTACAAAATGCGCCAACAATTCCCGGCTTTTCTTCTGGGTC
ACCTTGCTTATCTGCTAATCTTTTAGTCTTACTCTCTTCCTTTGAAGACGTTGGCCATTCTAATGTGTCAGTCCAATCAACATATTCATTTAA
TATTTTATCTGGATCTAACAAAGGTAAATCTTCATAGGTAAAGAAAAATTCTGCATCATTGCTAGTTGAAGGCCAATACATTAACCTATGT
GGTTGATAAGTTGTATCATCGAAGTAATCCATGCCAACGATATCTGCGACTTTACGCCCAATAGCTTCATACTCATCTGCATTTACATTTCG
TTTTAAAGGAATCACTAAACGCAGTCTTGGACTTATCTCTCTATGCTTATGTGTTGAATATAAACAATATGCAAAATCATAAAACATAGAT
AATATGTCAGTCATATCTTGAGCAGCATAATCGATATCAAGTGTTAGCATTGAACGATTCATGACTTGACCAGCACGTCGTTTGCCTTCTT
TTAAATATCCACCGACAAATCCGCCAACATCTTTTATATCTGCTTGTTCAGACTTAGACATTTTATTGTACTCAGTTAAATCTTCTTTAGTTC
TAACTGTTTGTGCTAGCTTCTGCATAAAGTCAGACCAAGCCATATTGTGATTAGTCCAATATGTGGATAAACGACTAGCAGCATAAGAAT
ATGAGACATCACGATCATATTTAATTGTTTCTATTTGAGTGACTTTGTCTAACAT

Gene: Q4ZCN3 (putative bacteriophagal protein)

Contig: 07_NODE_2, position: 51905 to 52105, length: 201 nt, orientation: REVERSE

Perfect match to: (MW2-BA000033-[1561034:1561234:r])

Sequence:

TTAGCTTTTGAATCTTTTTCTAATTCGTTCAACTTCATTTTCATAATCTTCTAAACCTTCAACACCATTATTTTTTACTAACTGCTTGAAAAGA
TAAGAATTCATATACTCCAATGCTTCTATGGTTTTCATCTTATGAGAAATGCTACTTAACAAGATCAATAAAAATATAGCTAAAACGATTG
AAATGACAATCCACAT

Gene: rinB (Transcriptional-activator)

Contig: 07_NODE_2, position: 52173 to 52325, length: 153 nt, orientation: REVERSE

Perfect match to: (JKD6159-CP002114-[1545432:1545584:r])

Sequence:

TTACCATGAAACTTCAGCTCTGATTTTTTCGTAATCACTCGGCGCCTCTACATCATCATTAGCCGTCATCATAATATACACTTGCTCAGTTAC
ATACTTACCTAACTCATACATTGCTAGTAAGAATAATAGTCTTAGTATTTGTTTAATCAT

Gene: hypothet. phage protein

Contig: 07_NODE_2, position: 52322 to 52711, length: 390 nt, orientation: REVERSE

Sequence:

TCATTGTTTATCTACCTTCTTTACTTCGTATAAGACCGGATATAAATTTAAAAAGTGTATTCTATAACCGATTGTTTTAACTTCTACCTTATC
GCCTACTTTTAACCTAGCTTGTATATCTGCGCTATCAAATTTCTTTTTGAATAATAAGTCGGAATTTTCAATGACTTGTTTGTTGTCTAATAC
AATATAGAACTTGTCTTCTTTATCTTGTCTCTTGTTATATTTATCTGTAATAGTTCCTTGATGTAATTCTTTGTGTTGGTAACTAGCCACTGTA
TAGATAGGCAATGTGATAACAAGTAGCAATGCGAATATACCGAATAATGACAGTATTCCAACAATAAAGATGTCGAACCCATCCATATTT
TTAAGTTTTTTAATCATCAT

Gene: hypothet. phage protein

Contig: 07_NODE_2, position: 53373 to 53909, length: 537 nt, orientation: REVERSE

Sequence:

TTACACGTATCCTTTTCCTGCGTCTGCTGTTCCATCTTGTCTTTCATGATTCCTTTTCATTTTCTTTTTGTATGCGTCAATGAGCTGGTCGATA
GAATATAAGTTGAAAGCTATGTCTATCGCTATTACAATTGCCAATTGGTCAGGATAAAATTCTTTGAATATTATCTGTGGTGTGCTAACAA
CTGCGTCTTGAGCAAATTCTTTATCTTTAAAATTAAACATGTTGTGAAATTCACTATTTTTAAAACTTGATTCAATCGCTTCTTTTATCTCTTC
TGATGACACTCCTACTTGATTCGCAATACTCAATCCAAACGCCAACATGTCCGCTAATTCATCTAACTGAACATCTAACGGCTTACCTGGTT
TCTTCTTCCAGTTCTTAAACGTTTCCAATGTATTAAACCATTCAAAGAATTCAACTACATATGCTATTTTGCTATCTCGTAAGTTCAGCGTTG
GTATTCTATCGTCGAACTCCTTTTGTATTTGTAATAACTCTTGTAACTGATCAATTGTTAATGTATTAGTCAT

Gene: DNA-binding_protein

Contig: 07_NODE_2, position: 54882 to 55283, length: 402 nt, orientation: REVERSE

Sequence:

TTATGCTTCACTCCATTTCTTGAACATTTGGTTATAAGTGACATCGAACCAGTACGGATCACGTGAATGTTTTTGAGGCGTTCCATCATAAA
GCCATGGTCTCAATCTTCTCTTTCTTTCTTCTTCATATTCCGCTCTCACATTTCGTTGGTATAGGTTCAAAATCGCTTTTTTTCTGATTTTTTCT
CTCTCTTTTTCTTCATCTTTTATTTGACTCTTCATATATTCAACTTCATCTTTAGATTTTGAGTCTTTTCTTCCACACAATAATTCATCGCCGCG
CATTTTATGTTTGTATCTGTATCTAAGAAGTTCTGGAGATATATGATATTTTTCTGAAACTTCTCTCAATGTCATTAGTTTTCCTTTGATACG
CACTCTTATAACTTTTCTTCTAGCCAT

Gene: hypothet. phage protein

Contig: 07_NODE_2, position: 55283 to 55468, length: 186 nt, orientation: REVERSE

Sequence:

TCATTCCACCTCTAAATCTAAAACCTTGATATTTATAACGTTATATTTTAATAGTTCACCTGGATTATTAAATAAATAGTCCGCCAAATTCTC
TTTTTCTTTATCAATCTGATTGTAATTAACACTTTCGACTTCTGTAGGAATTCTAATGTCAACAGAAGCATTGATATAAGCTTGATGTTGCA
T

Gene: pol-phi12 (bacteriophage DNA polymerase)

Contig: 07_NODE_2, position: 55481 to 57442, length: 1962 nt, orientation: REVERSE

Perfect match to: (MW2-BA000033-[1564836:1566797:r])

Sequence:

CTAATCCTTCATATAAAACGGAGAAGTAAATCCGTCACTATTCAAATTCAATCCTTTTGCCCAATCGACAGGCTTATTCATGATAGTTTCGA
TTTCCTTAAGTCCATTTGAACCTCTAGGTATTTCTACAATTACTTCATCATGGACATGTCCAACTATTTTAAAACCTGATGCTTCAAGCCTAG
CTATAGAAATCGCAAGTAAATCCCTTGCAGTTGCTTGAACAATATTCTCGACTAACTTCCCACCATACGTTTTTAACTTTGACCATTTACGG
TTAAGATCTAAGCCCATAAATTCAACAACTTGACTACCCCAACTATTTTCACCAACTGAAGCTTTTGGATAAGCTAAAGCTCTTCCACTAGG
CAGTTCAATCATTAGAAAACCTTTTTTCATATAAAATCTAACTCCATGCGTATGATGCGTCTTTCGGGATTTCACAGTATTAATTGCAGCCT
CTTGGCAAGCCTTCCAAAAATTAACTATGTTAGGATTTGCGTTACGCCAACTATCAACTAAACCTTGTAATTCATTTTCTTCAATGCCCATTT
CCAATGCGCCCATCGCTTTTAAAGCTCCAGCGCCACCTTGATAACCTAAAGCTAATTCGGACACTTTTCCCTTTTGTCTGAGAGGGTCGCC
TTTAGTTATGCTTTCTACCGGGACATTAAACATTTGAGAAGCCGATGCTTCATATATCTTTCCGTGTGTGTTGAACACATCTAAACGCCATT
GTTCTTTTGCATACCATGCTATGACTCTTGCCTCTATTGCAGAAAAATCACTTACTGCTAGTTCATTACCTTCTTCAGCAGTAAATGTCGTCC
TAACTAATTGACTTAATAAGTCTTGAGGATGAACATTGAGTAATAAATCTAAATCGTCAAAACGTTGTTCTTTAATAAGATCTCTTGCTATT
TCTAATTCAGTATCTGAAATATAATGCTTTGTTAAATTCTGAAGTTGTACACCTCTACCTGCCCATCTTCCAGTACCGGCACCGTAAAATTG
AAACAGACCTCTTACCCGTTCATCACTGCACATCATGTCATGCATTTTGTTGTATTTTTTCACACTGGTTTTAGACATTTGCAATCTAATTTC
TAGCATTTTTTTAGCTTTTCCTGTTGCTTCTTTTAAGTACTCCTGAACCGTTTTCTTTTGTAAATTAGGTATATCTAATCCTTGGTCATCCTTT
AACCAAGCCAATAATTGTGTAGGACTATTAGGATTTTCTAAACCTGTTATATGTTTAGCTTGATTAAGCAATTCTTCTTTACTCTGCTTATC
GAGCACATTAGCTCCTAACATCAATGATTTAGAAAGCTTAATACCTCTGTCGTTTATATGTTGGTCAAAAACCCAATATGCTTGTTCAATTG
CAGTTACTGGAAAGTCTTTAATTTTATGAGCAATCGTCATTTCTACTTCTACATCTCGAATACAGTAATCTATAAATTGTTGCCATTTTTCAA
GATCATGTTCAGGCAAGTTTCTTGTTCTTCCTCCATTAACTTTTGTTGGTTTACAAGGTATAGAGAAATAACGAATTAAATTTTTACCTGCT
TTATCTTTTTGGTTTTGTAGTCTTAAAACTTCTCCAACTTTATCAAGCGAAGCAGGTAAGCCAATACGCATTGAATTAACCATTGTGCAAAT
CCATTCTTCAGGTGGCATCTGTTTATTAAAATGTTTAGCAAGACAAGTTCTTTCGAAATTAGCATTGAATGCATACTTTTTTACAGCAGGAT
CAAAAAGAGCAATTTTAAACGTCTCAAAATCAGCGTGGAAAGGCTCATTATCTACTTTAGTCATGTCAATCGCACTAATCGCTCCACCATC
TATTGAATAAGCTATAATTAAAATTTCGAAATCTTCAGCTTCTGTGTATTTATAGGCACCACATTTCGAAATATCATTACTGCTATATGTTTC
AATATCTATATTCATAAATCTCAA

Gene: hypothet. phage protein

Contig: 07_NODE_2, position: 57501 to 58058, length: 558 nt, orientation: REVERSE

Sequence:

TTATAAGAAATCCTCATCATCAGTGTCTAATTCATCGAAATCATCTTCTGCTGCACTTGCACCGCCAAGAGGTTCGCCTTTTTCTACAAGTT
GAATGTTGTTCAATCCAACTGCGATACCCTTATTACCATTTGTGTTGAAAGGAAATAGATTAATTGAAGCTCTAATATAATCACCACTTAC
AACAGTTCCAGAATCCGTTAATCTAATTTTGTTTTGGTCAATAATACCAGGTGCTTGTTTGCTTGATGCGTTAATAAAATAAGCGTCTTGAT
AATTGACATCATCTTCTCTTTCAGTATCTCCATCACGTAATGGAAGTTTCAGATTTGCAGGAACTTTGCCTCCAAACTTACTAACTTTTCCTT
CTTCTTTAGCAGCTTCTATAGCTTGTTCAATGGCTTTTATCGTACTTGTATCTGATTTAGGAATGATTAAACTGATTGAATACTTTGATTCTT
GCCCTTCTTGCATACTGTGAGGTTCAAAAATATGTGCATATGATGCTCTTACTTTTCCTGTAATCACTTTAGTTTTATTTAATACTTTTGCTTT
CAT

Gene: Q4ZCH8-phi12 (bacteriophagal protein)

Contig: 07_NODE_2, position: 58084 to 59250, length: 1167 nt, orientation: REVERSE

Perfect match to: (MW2-BA000033-[1567439:1568605:r])

Sequence:

TTATAGTTTGTCAAAATCATCTTCAGCAGATTGCTTTATAGCTGGTCGTTTATCCGACTCGGTAGCAAGTGTTAATTTACCTTGCGGCTTTT
CTATAAAACCCTCTGTAATTTTAGAAAATGCTTTTTTACCAATTAATTTTTCTAAATTCGTAATGCTAAGTAACTTGGTTTCTGTAATATCTT
CAGGTTTATAACCCGCTTCAACTAACTTTTCAAGCATTGCTTTTGTATCAGTTATCATTCTTCGTGAACGACCTTCTACAAGCTTCCAACCAG
GATAGTTTTTATCATTTTCTTTCGCTTGATTTAGTGCATATTGTTCTACTTCATCAGCCCATTTTTTGATATCAGGCAGTTTATATAAAAGTTC
TGCAATCTCTTCATCACTTAACAAATGTGGTGGCTTTTGAGGCACATTTTGCATGTATTCTGCACGTGTTCTACATGAATGATTTATCTTAC
AGAATCTACAATGACTACCTGCTTTAAACTCTCCTCCACCGTTATAAGCAAGTCTGGCTAATGGTTTAACAAAATCGGCTCCCCATTGAAG
TAATCTTGATATTGGTAACTCTTCAGTAGAAAAGTTATCTATTCGAGGTTGTATGATAGTCATGCGAATTGTATGAATGTCATACATTAAA
CTAAGCAGTTCATATGCGCCCAAGCCATATAATCTAAGTTGAGGATTATCTATAGCTGAAACTTCAATGCCTTTACCATATTTAAGGTCAA
TAATTTCAAGTACACCACCTGAAAATATAATGACATCACCAGTTCCAAAAGATTCAGGGACGTATTTACCTAAATCTAATTTTGTTTCAAAT
AAAGCTATTACATCATTATCCCTACTCAAAGCTTCGTTATATTTTTCTTCTACATTAGCTACATACTCTTCCACATATTCACGCAATTCTTCGC
TGTAATATTGATTTCTCTTATAATTTTGAAAAGCCTTATTAAACTCAAACTGTGTTAGGCCTTCATATTTAAGGCTGAAATATAACTCACTTA
ACTCATGGGCGAATGTACCTTCTTCGGCAAAAACTGTACTTTTATCTGCAATACCTTCACTTGCCTTAATACTCGGTGGGCAGTTTAGCCAT
TGTTTTGCTCCACTTGCACTAAGCTTTGCATGAGCTCTATTTGAGTGATCTAGCTTCAT

Gene: hypothet. phage protein

Contig: 07_NODE_2, position: 60593 to 60754, length: 162 nt, orientation: REVERSE

Sequence:

TTATTCTTCATAAAAGTATTCTTTGTAGTATATGAATGTTGCGATACTTGCGAATCCCGCAATTGACCATGCTGTAGTGAAGTACAGCAAT
GGCATAAGCACAATCGCTAAGACTGTGAAGCACAGTACTGCTATTAAGTAGCTTTTATATGTGTCGCTCAT

Gene: DNA-binding_protein

Contig: 07_NODE_2, position: 60766 to 61029, length: 264 nt, orientation: REVERSE

Perfect match to: (Phage-ROSA-ORF058-[AY954961.1-[[31643:31906]])

Sequence:

CTAATACCATTTTTTATGCTTTCTGATCAAATACTCTTCCAATTTAGAAATATTAATCAGAGTGCCTGTTGGTGAATAATCAATGTATAAATT
TTCTACACCTAAATTATCTTTGCGGTAATATTTCAACCAGTTGTATACTGTACTTCTACATACTCCAAACAATTGATGGATTTGTGTAGGTG
TTGCGTATAACTTTTTCACAAATTTTTCTTCGCCTCTATATGTGTTTTCTGGTGTTGGTGGTACTATGATTTTTGGCAT

Gene: DUF955 (putative bacteriophagal protein)

Contig: 07_NODE_2, position: 62506 to 62967, length: 462 nt, orientation: FORWARD

Perfect match to: (JKD6159-CP002114-[1555765:1556226])

Sequence:

TTGGGATTATACGAAGAACTTTGCATAAATAACGAAAAAATAAAGATAGAAGAAACTGACCAGCTTCCAAATTTCCAACCTGGATGCTAT
ATGAACGGAAAAAATTTATATAAGGCGTAATTTATCAGAAGTACGTAAAGCAGAAGTGTTATATGAGGAACTTGCCCCACCACAAGTTGAC
GTATGGCAACATTTTAGACCAAACAAAATGGATTAATAGAAAATTTGAAAATTATGCACGTAGACATGGTTTTACTTCAGCTGTACCGCTA
CATGAAATTGTAGAAGCTCACAACTACGGTGTTCGTAATTTGTACGAGTTGTCAGAGTATCTGCAGTTGAGTGAATCATACATACTAGAA
GCTATAGAACAATATAAAAAGATATATGGTATTGGAACTCACTATGGCGAGTATTCTATTACGTTTGAGCCGTTGAGAGTTTTTAAATATA
AGGAAATATAA

Gene: Na-K-ATPase

Contig: 07_NODE_2, position: 64076 to 64690, length: 615 nt, orientation: REVERSE

Sequence:

TTAGTATTCATTCATTAAATATAAATCCAATTTAATTTGTTGTTTAAGGTCTACAAGCGTATGTTTAATATACAATTCATCGTTTGACGGTAA
ATCAGATACTTTGAAATCTTGTCGCTCAACTTCTAGTAAATCGAAATCGCTACCAGCTGAATTATAGGTTTTAAGTTCACCCTCTTCAATGA
TTCTGTTTTCAAAGTCTTTTATAACTATAAATACTGGTTTACCGTTGTTATTAAACAACTTGTCTCTTTTGTCTAATAAGCTTATACAATCCAA
TTTCATAAACTTTCTGGTTATATTAATTAACCAGATAATAAATTTAACAATTAAAGGATTAAATACAAACACTGTTAAAACAAAAATAAATA
GAAACAAAATATTTGCTTTTAGACCTGTAAGCAACTGAATTAAATTCAAATTTTTTAAATCAACATTATTAAAAATTATAAAACTATAAAAC
CATATCAAACATGTTTCAATAGAAAAAATCAATAATACAGGAGTATTGATAACCTTGTTTTTTTCACTAACTAAACCTATCATTGTTAGATA
TTTATATGGTATGTAACCTAAAACTCCTGTAAGAAGAAGCGCCCCTAGAAATTGAGTCAT

Gene: int-1 (integrase)

Contig: 07_NODE_2, position: 64816 to 66021, length: 1206 nt, orientation: FORWARD

Perfect match to: (JKD6159-CP002114-[1558075:1559280])

Sequence:

ATGTGGATTGAAAAATTTAAAAACAAAAATAACGAAACTAAATACAGATACTACGAGAAGTATAAAGATCCATACACAGATAAATGGAA
GCGCGTAAGTGTTGTGTTGAACAAGAATACAAAACAATCTCAAAAAGAAGCAATGTTTCGTTTAGAAGAAAAAATAAAAGAAAAACTGA

ACAACAAGTCGTCAAGCGAATTAAAAACTTTGACTTTTCACGCGCTATTAGATGAATGGCTTGAATATCATATAAAAACATCAGGTTCAAA
GTTGACTACTCTTAATAATATAAAAATAAGAATTAGAAACATTAAACGATACAGCTCTGAGAACTTGCTTTTAAACAAACTAGATACAAAA
TATATGCAGATATTTATTAATAAATTATCAGATATCTATTCTCAAAATCAAGTAACCCGTCAACTCGGAGATATGAAAGGAGCTATTAAAT
ATGCAGTTAAATTTTACAATTATCCAAATGAATATTTGTTAACTAATGTCAAAATTCCTAAAAGAAGAAAAACAATAGAGGATATCGAAA
AAGATGAATCTAAAATGTACAACTATTTAGAAATGAACCAAGTCCTACAGATACGTGATCATATACTAAATGATAATAAGTTACACAAGC
GAAATCGCATTTTAATTGCCAGCATCTTAGAAGTACAGGCTTTAACTGGTATGCGCATAGGAGAACTACAAGCACTGCAGGAAAAAGAT
ATAGATTTATTAAACAAAACTATCAATATAACAGGTACAATTCACCGCATTAAATACGAGGAAGGATTCGGATACAAAGACACTACAAAG
ACTATAAGTTCAAAAAGAAGTATCAGCATCAATTCTAGAACCGTAGAAATTTTTAAAAAGATAATACTGGAAAACAAAATGTTGAAAAGA
TGGAATTCGAGCTATGTTGACAGAGGGTTCATATTCACAACAAAAAAAGGGAATCCTTTATGTAATAATCAAATCGCCGGTGTGCTTAAG
AAAACTACAAAAGCTTTAAATATGAATAAGAAAGTTACCACGCACACATTTAGACATACACACATAACTTTATTAGTAGAAATGAATGTTT
CTTTAAAAGCAATTATGAAAAGGGTAGGACATGTAGATGAAAAAACAACCATTCGCATATATACTCATGTAACTGAAAAAATGGATAGA
GAACTAACTCAAAAACTCGAAAACATTCCAAGTTAG

Gene: A5IT17 (putative protein containing attachment site of PVL-phage)

Contig: 07_NODE_2, position: 66105 to 68091, length: 1987 nt, orientation: TRNC-RVRS (no stop codon)

Sequence:

AAATAAACATATCATCATAATGTGATGGTTCAAATATCATTTGTACAATCAAAGGCTTCATGTTCTTAACAATATCATCTAAATGGTTATCT
AAAATTGGTGACACTGCTTTTAAATCATTAAGAAAAGGCTCCCATTTGCCTAAAGTATTATCTAATTCTTCTAATTTAGTTTTAATATAATTA
CAAGTTACATTAGGAATCAGGGACAAAAATTCTTTCTTTTTTACATTTAACATTTCAATTGCATGTCTTAAATTCTTACGTATTTTGGGAATT
GTATTAATCAAATATTTTATTACATCGACAATTTTCGATGCATATTCATCATATATACCTTGAACATAGTCTGCTATTTTTTTAATACCATCAT
CGATATGGTCTTTTAATATTTTCATTTTTCTTCCTAAATAATTAGAAGGTAAAGCTAGACCCTGCACCATATTTTCACCGCTATAATTAATTT
GATAATTTCCATCTAAAATTGTTGCATCTTGTTGCTTCATAATACTTCTAATATCTGCAATTTGCCTACCATAAATATCATTTTGATTTTTTAT
TTGCTCTATATTCTGTTTCACTACTTTCAAATGTTTCATCATTTCTTCAGATACTCCATCTCTGAAGTCGTGATCTATATTTTTGAAAATTTCT
AAAATTTCATTATCTATACTATCATACACTTTTTCTATAAAAGATTTTATACCTTTAAACAACTCATTAATTCTTTCTTTTAATGCATCCAATG
CAAAATCAGGTAATAAGTGTTTAACAGCACTAATACTTTCTATTGTTTCATCTGCAACTTCTTCAAGTGCGTTTATTTTACTAATTAAAGTTC
TTTCCATTTCTTCTAATTGAAATAAGTTAATCTTATCCTTAAATCCTTCTGATAATTGTTTCTTTCTATCTGCAAAATTTTTATTTTCATTTTCT
GAGATGTTAAAACTTTCATTTAAAAAGATTACGCATTCTGCTAACATACCACTTGTTTCACCAGTAATCAGTTTACTCAACGCATCAAGATT
TTCTAAATTAAGTTTAATTAAAGTTCCTTTTCCGGAACGTGCAATCGAATCTCCTGTCCAAACATTTATTGGAATTCGCCCATCCATATCTA
ATGTTATGTTAATAGTCTTTTTTACTTTTTTTCCATTTTTAATTTCTGTATCTTTTACCGACTTAATTTTGATTAGTGGTACAGTATCGTATGT
GTTATCTTTTCTATTTAACTTCCTTTTATAACCTACATGGCTGTCTATTAAAGCATCTAACCTGGGCACACCATCACTAATGTTAACGCGTTT
TCCTGGCATATCTTTGATGAATGGATCTTGTAACCATGTTAATAAATCGTTGATACTATTAAAACTAATCATATTATCAAAGCGTGGTCTAG
CAAATTTCTGCCAAGCAGCATAAGGAATCATTGCTGAGTCAGTAGCAACAACTTTTTCATTCGGATGTTTCGCTCCTTGATATTTTGCTCCT
GCACCACCTTCCGAATTACCACCATCCGCCACAATTGTTTTGTTTTTGTAATTATAAGGTTTAGTTTTGTATTTTCTTCTAAATTCATTTTCAT
CAAGAACAGAAGCATCTTTAATTTTTTGTCGATAACTATTTGCGAATTCTTCTACTTGCTTTAAATAATCTGTGCTTTCATTATCATTATTCA
TTAATTTAGCATTTTGGAGCCAATCATCTCCAAAACCCGATGATTTTAATGGATTATTTGGGTTAATTGCCTCATTAGATGTTCCTTGATAA
ATTATTGTCTGTTGACCAGTTGGATTCCCCTGATCATTAAGTAACTCGTATGTTTTAATATCAGCAGCACCATTCATATTATCATTACTATTA
TCATTATAATTATCTATTTGTTTGAACCGTTTTCCATTTACTTTAAAATCTTTTTTTTCATTTATATCTTGATAAACCCAATAACTGCTCAATTC
TGCTATGTCCCTATCATTTATTTTATTCAC

Gene: graD01 (putative lipoprotein)

Contig: 07_NODE_2, position: 68103 to 69011, length: 909 nt, orientation: REVERSE

Perfect match to: (MW2-BA000033-[1577011:1577919:r], allele observed in CC1)

Sequence:

TTACCCTTCAATTGGATTTATATCGTCATAAAATGGTTTTTTAGTATTTATACTAGATTTTCCTAATTGAATAGTAACTTGAGATTTTTGAGG
CATATTGGGTTTTTCTTTTAATTTATTACTCAATTCAATCACATCATCTACTGTATTGTCTTTAGTAAAGTTTTTCTTAGTTGAAAAAAGTGT
AGAAACTGCATCAGTATTGGCAGTATAATCTAACTCTTTTCTAGCTTGTTGCATACCTTCTTTAAATTCTTTATCATTTTTATGAATCAACGG
TTCGTAATATTTACGATATTCTTTTAAGTTTCTTGATAAATATGTGATATAAAAGTATTCATTTTGATATCCAACGTTTTGTGTCTTGTTAAT
TGCCTCTTTTGTAAAGCCTGTATATTGATATTTCTTTTCATTTTCTTTGAAGAATTTATATAAGTTATCATACTTTTCTTTTTGCGCTCGATAT
TCAAAGCCACTCAGCACTGTACCCACCATCATACTCATATCATCACCATTGTCATTACTGCGCATTGATCCTTTTTGATGGATGGCATCTTT
GTACAAAGGTAGACTTGCATTAAATACAATGCCATGATCTTCACAATGCACATAAACTTCTACACCATCATCTTTACCTACAACATTTGTAG
CTTTAACTTTTAGTCCAAAGTTATCTTTAAAGAATTGTTCACCTACTTTTTCAAATTCTTTACGATGCTTCTTCGCAAATTCAATCGCATCTTT

TTCTGCAGGCGGTTGGAAGCCTTGGCCTACATATTTTGAAGCTTCCATTTCTTCTGGTACAGATTTTGTTTCTGTGTTTGTGTCTTTATTTGA
TTCATTTTCCATCGCGGAACATCCCCCTAAAATTAATGTCGTGGTTAAAACTGATCCAATGAACTTTTTCAT

Gene: srrB (two component sensor/regulator of respiratory response, sensor histidine kinase)

Contig: 07_NODE_2, position: 69256 to 71007, length: 1752 nt, orientation: REVERSE

Perfect match to: (MW2-BA000033-[1578164:1579915:r], allele observed in CC1-ST582)

Sequence:

TTATTCTGGTTTTGGTAGTTTAATAATAAATGTTGTGCCTTTCCCTAATTCGCTTTTAACATCTATGGAACCACCATGCTCTTCGATAATCAT
TTTACAAATGAACAAACCTAAACCGGTACCTTGTTTACCTCGCGTTCTCGCTGCATCAACTTTATAAAAACGATCAAATACTTGTTGTAAAT
GTTCTGGTGCAATGCCTGTACCTGTATCTTTAATGTATAAAATATCTTCGCTTTCATTTTCATCACAAGTAATTGCAATTTCATCTCCAGGTT
TCGTATAACGTGATGCATTATCAATTAAGTTCGTTAGTACTTGGTCCATGCGATCCATATCATAACTCCAAACACGCTTCTTACAATAATTA
AAAGTCATATTTAGACCTAAATCATCAGCTTGTTGGCGATACTTAATTTTCATCTTATCTAGTAACGCTGCAATAGGCTGAACTTCTTTATT
TACGGATAACCCTTCAGCATCCATGCGTGCGACATTTAACAATTCATTAACTAAACGATTTAAACGTTTCGATTCATCAAGGACAATGGCA
AGCGATTCTTTTATTTCATCCGGTTCTGTAACAATACCATCTACAATTGATTCAGTATAACCTTGAAGTAATGATATCGGTGTACGTAATTC
ATGTGATACATTAGCAATGAAATCTTTCTTCATTTGATCTAGATTGTGCTCATTAGTCATATCACGAACTGTCACAACAACACCACTTTTAC
CTCCCTGTTCAATCTTGTCGATATAGCTTGTGGTCACAACAAAGAATCGTGCATTCATTTCTAAATCACGCATTTCCGTTTGTTTTGATTTAA
AAGTATCTTCAATTTGTCTTAATAAGAAAGCTTTAGCATCTTCATCAATATTGTCCATAATATCATTCGCCATCTTATTAGATAAGATAATTT
GTCGACTCTCATTAATACCTAGGACACCTTCTACCATAGAGTTAATTAAGCTGTCTCTAATATTTTTAGATGTGGATAATGCGTCGACATGC
TCTTCGATTTCTGTACTCATCTGATTAAATGCCTGCGATAATTGACCAATTTCATCTTTCGTTGTGACAGAAGGTTTATAAGAGTAATCCCC
TTCAGATACACGTGTAGCTTGGTCTCTTAAACGTCTTAAAGGTTTTGTAATTCTTGATGATAAGAAAAACGCAAAGACTGTTGTAATTGTT
AAGAAAATAACAGCCGTAATTATAGTGATAATCGTAATAGCATTATTTGTATCTTCGATTGATTTCAAGTCTTTATATATAAAGACTCCACT
ATATTTGCTATGACTATTCTTCTGTGCTTTTGTTGGATAGCCTAACAAAATATATGTTTGAGATGAGCCCTTTTCTTTAATCGTTACATTTCG
AGTAACAGATTTACCTTTATCAAACACATCGTCAAAATGGTCGTTGTTGACTACTTCATTCAACATTTGCTTTTTAATATTAGAAAGTGAAG
CCGTTGATTGACGATGTTTATTATTTATAATCATCAACCCACCAGGATTTTCAATTAATGTTTGACTATATTTTATTGCTTCTTCTTTATTATG
TGATTGTTCGACCAGTGAACTTATACGTCTAGCATCTTCTCTTATGGCATTTTCGGTTTCTTGTGTGAAATAGTATTGCATAAAGGTAATTA
AAGCAATACTTAATAAAATTAAAACTGTCGTCACTATTAAAATAATAGTTAACCACAGTTTAATTACGACACTATTTAGCCGGCTCATCAT

Gene: srrA (two component sensor/regulator of respiratory response, transcriptional regulator)

Contig: 07_NODE_2, position: 70988 to 71713, length: 726 nt, orientation: REVERSE

Perfect match to: (TW20-ST239-FN433596-[1624445:1625170:r], highly conserved allele)

Sequence:

CTATTTAGCCGGCTCATCATTAGATTTAACCTCAAATTTATACCCAACGCCCCAGACTGTTTGAATCATATGCGCAGCTTCACTAGACACAC
GATTTAACTTTTCTCTAAGTCGTTTAACATGAGTATCAACTGTTCTTAAATCACCATAGAATTCATAATGCCAAACTTCTTTTAATAATTGTT
CACGGTCAAATACTTTATTTGGTGTTTTAGCTAAATATATTAATAATTCGTACTCTTTAGGAGTCAAATTAACTTCTTGATTATCAGCAAGT
ACGCGATGTGCATCATTATCTATTTCTAAATGTTTAAATTCAATCACATCACGTGCGTGAGGTTCGCTTTGTTCTACAGTTGTAGATTGCGT
TCTTCTTAGAAGTGCTTTAACTCTTAAGACTACTTCTCTTGGTGAAAATGGTTTGACGATATAATCATCTGCACCAGATTCAAAACCTTCAA
CACGGTTTGTTTCTTCACCTTTAGCAGTCAACATAATAATCGGTGTTTGTTTATGTTCACGCAATTTAGTTGCCACCTGGATACCATCCATTT
CAGGCAACATTAAATCTAGTAGTATGCAAGCATAATTATTCTCCATTGCAAGTTCATAAGCCTCTTGGCCATTACTTGCTTCATGGATTTCA
AAAGATTCTCTTTCTAAATACATTTTAAGTAATCTTCTGATTCTATCCTCATCATCTACGATAAGTATTTCGTTCGACAT

Gene: rluB (23S rRNA pseudouridine synthase)

Contig: 07_NODE_2, position: 71846 to 72583, length: 738 nt, orientation: REVERSE

Perfect match to: (MW2-BA000033-[1580754:1581491:r], allele observed in CC1+CC22+CC93)

Sequence:

TTATTTCCCATGTTCTGCTAAATGACGCATCACTTTTACTTCATGAGGCGTCAATACACGTCCTTCACCAGCATTCAAACCGACAACATTTA
AAGGCCCATATTCAATACGAGACAGTTTCGTCACTTGATGACCAAAATGTTCGAACATTCTTCTGACTTGGCGATTACGACCTTCTGTAAT
TGTAATTTCAACCAATGTTGTGTTTTTATCTTTATCTTGTTTCTTAACTTTCACTTCAGCCGGTTGCGTCATACCATCTTCTAATTCAATACCT
TTTTCTAGGGCTTTCACTTCTTCTCTCATTAAGTAACCTTTTAATTTTGCTACATATTTCTTCTTAATTTGATATCTTGGATGTGTCATTAAATT

AGTAAATTCACCATCATTTGTAAGTAATAACAATCCAGAAGTATCATAGTCTAAACGACCAACAGGATAAATACGTGCTTCTATATCTTTA
AAGTAATCCGTAACCACTGTACGTCCTCTATCATCTGATACACTTGTTATCACTTGAGTTGGTTTATGGAATAAAATGTAAATTTTGTCTTC
TAGTTCTATTTTAATACCTTCAACTTCAATCGTATCTGATGGCTTTACTTTTGTTCCTAATTCAGTGACAGTCGTACCATTCACTTTCACTTTT
CCTTCAGAAATTAAAGTTTCTGCCTTACGTCTTGAAGTATAACCGCTATTAGCTATACGTTTTTGTAATCGTTCTAATTCTTTAGTCAT

Gene: scpB (chromosome segregation and condensation protein B)

Contig: 07_NODE_2, position: 72576 to 73118, length: 543 nt, orientation: REVERSE

Perfect match to: (MW2-BA000033-[1581484:1582026:r], allele observed in CC1)

Sequence:

TTAGTCATTATTTTCTCCTTTTTGATTGACTAGATTACTGAAGAAAGCATCCATTTCTTCATCGTCTTCTTCAGTTGTCGGCAAATCTTCTAT
ATTGGAAATACCAAATACATTTAAAAATAAATCAGTAGTAATTAACTGTTGGCTACGCTGTTCATTGACCACTTTAGCCTCAACTAGTCCTT
TGGCAATCAATGTCTTGACCGCACCATCTGAATTGATACTACGAATTAATTCAATATCACTTCTTGATAATGGCTGGTTATAAGCAATAATT
GATAGAACTTCCATTGCTGCTTGTGATAATTTCATTTGTGACTTTTGTTCAATTAATTGTTCAATATACGTTGCCGCTTCTTTTTTAGTCGTT
AAAACATACGTCGTTCCAAATCGTTGTATCATTAATCCATGTGATGAATAATTTTCAATTAATTCAACGAGTTGATTTTTCGACATATCTAA
TATTTCTAATAGTTGTTTTTCATCTAAACCTTCATCGCCAGCTGTAAATAATAGCGACTCTAATATACCATGATTATCCAA

Gene: scpA (chromosome segregation and condensation protein A)

Contig: 07_NODE_2, position: 73111 to 73842, length: 732 nt, orientation: REVERSE

Perfect match to: (MW2-BA000033-[1582019:1582750:r], allele observed in CC1+CC130+CC133+CC395)

Sequence:

TTATCCAAAATGGTAGTTCACTCCTCTAATAATGTTAATATCTTCAAAATTACGTTGTTGCTCAATATTAATTATTCCTGCTTTTGACATCTCT
AAAATAGCTAAAAAGTGAGTGACTACTTGTTCAATTGGCTCAGAAAACGTAAACAGACTAAAGAAGTTAAAATGATCTTTATCTTTCAAT
CTCGATGTCACTTGTTCTGTAGCTTGTTGAATGGTAAATGTCTCTTTTCGGATTTCAACAGATTTAGGTGTATTTAACTCAACTCTATTTTTA
ACTCTTTGATAAGCTACAATTAATTCAGTTAAATCAATCGTATGATTTGGATCCCAAGATTCATCCGTTTCCAAATGAGATAAATCTGTCGG
TCTTTTAGTAAAATAAAAATCTCTTTCTTCTTTCATGTCATTTAAAATAGCAGTATATTCTTTATAATTTTGATATTCTATTAAACGCCCAACT
AAATCTTCCCGTGGGTCATCATCAACATCCATATCTGATGTTGATTGTGGTAATAGCATCTTACTTTTAATCATTAAGAGTTCTGACGCTAA
TACTAGGTATTCACTTGCAATATTAATTTCAAGCTGTTTCATTGCATGAACGTACTGCATATACTGCTCTGTTAATGCTTGCATAGGAATAT
CATAAATATCTATTTCAAATTTTTGGATAAGATGCAGTAATAAATCTAATGGTCCATTGAAAGCATCTAATTTAACTTCATACAT

Gene: Q2FY75 (putative protein)

Contig: 07_NODE_2, position: 73934 to 74440, length: 507 nt, orientation: FORWARD

Perfect match to: (MW2-BA000033-[1582842:1583348], allele observed in CC1+CC30)

Sequence:

ATGCAACAGGCACTGATAAATTTCTATTATCAATTTCATACTAAGCAACATTATTTCCTATGTCATGACATTTTAGAAGATGCATGGAAAG
CTGAAAATAACTACAGTAAGCAAGATGCAGTTGTTAGTTTAATCTTGTTTGCAACTGCTTGTTACCATTATCGCCGCAATAATTTAAAAGG
CGCCTATAAGTCTTTTAATAAATCTAAAGAAATAATACAAAATGCTAAAGATAGAGATGCGTTATACCTTAATTTAAATGATTATCAATTG
TTAATTGAGCAACAGATTGCAAAATTGAATGCAGCAAAGCCTTTTTCATCTGTTATATTACCAATAACTCCAGACTTTGAACGCATCATCA
AGGCCAACTATCCAGACTATGATTATAATCAGGAAACATCAACAGATCCTTTTATTGTAGACCATCACATGCGGCGTGATCGTTCAGAAG
TGATTTCAGCAAAAGAAGAAGCGATTCAATTAAGAAAACATAGACGTAATTAA

Gene: xerD (chromosomal tyrosine recombinase D)

Contig: 07_NODE_2, position: 74518 to 75405, length: 888 nt, orientation: REVERSE

Perfect match to: (GR1-AJLX01000009-[241278:242165], allele observed in CC361+CC239)

Sequence:

TTATGCTCTAGGATGAAATTGGTTATACATTTTTCTAATTTGAGATTTTGAAACATGTGTATAGAGTTGCGTAGTAGATATGTCAGAGTGA
CCTAACATCTCTTGCACTGCTCTTAAATCTGCGCCATTTTCCAATAAATGTGTCGCAAAAGAGTGGCGTAACGTATGTGGCGTTAACGTCT
TTTTAATGTTTGCCTTTACACCATTTTGTTTAATCATTTTCCATATTGCTTGTCGTGATAAAGGTTTACCATGCATATTTAAAAATAAGACTT
CGGTAACAGTCTTTTTTAAAAGTTGCGGTCTAATCGTTTCAATATAAGTAGTTAAGTACTCAATGACTGCGTCGCCTAATGGTACAATTCTT
TCTTTATCGCCTTTACCAAATACGCGTACAAATCCCATTATTAAGTTCACGTTTTCTAACTCTAAATGTATCAATTCAGATACACGCATTCCC
GTTGCGTACAGAAGTTCTAACATCGTACGATCACGATAACCATTAATTTTATTTAGATCCGGCGTTTCTAATAAAGCCAACACTTCGTCAA
CATTTAAAACGTCAGGCAATTTTTTGTCATATTTTGGTGAATCTAATAATACCGTTGGATCTTTCGCCGCATATTTTTCTCTTATAGCAAATT
GATGAAAACTACGGATTGTTGAAATAAATCGCGCAATAGATTTAGCAGATTGCCCTTGGTCTATTAAATGCCCCAAACACTCTTGAATTAA
TTGTCGATCTATAAAATCAATATGCGAGATATGATGTTCAGTCATATAATCTTGATACTTTTTCAAATCACGTCTATAAGCACCAATTGTGT
TAGAACTTAGTCCTTTTTCAATTTGTATAAAACGCAAATATTCTTCAATAATTGTTTCCAT

Gene: fur (ferric uptake regulator)

Contig: 07_NODE_2, position: 75454 to 75903, length: 450 nt, orientation: REVERSE

Perfect match to: (MW2-BA000033-[1584362:1584811:r], highly conserved allele)

Sequence:

CTATCCTTTACCTTTAGCTTGGCACGTTTCACACACACCATGGAAAGTTAAACGATGATCTAAAATTTTAAAATTGAACTCATTTTCAACTC
GATTTTCAACTTCTGGTAACAAATCTTCATCGATTTCATCTACACGACCACATTCCATACATACTAAATGATGGTGGAAATGTTTTGCGCCT
TCTTTTCTTAAATCAAAACGAGCGACGCCATCACCAAAGTTAATTTTGTCGACAACTTTTAGTTCAGCTAACAACTCTAACGTTCTGTATAC
TGTCGCCAAGCCAATTTCAGGCGCTTTATCTTTTACTTTCAGATATACGTCTTCAGCACTTAGATGATCTTTTTCATTTTCAATTAGAACTCT
AACAGTAGCTTCGCGTTGTGGCGTTAGCTTATATGATGATTGTTGTAATTGTTGCTTAACGCGATTTAATCGTTCTTCCAA

Gene: nudF (nucleoside diphosphate phosphohydrolase F)

Contig: 07_NODE_2, position: 76008 to 76550, length: 543 nt, orientation: REVERSE

Perfect match to: (MW2-BA000033-[1584916:1585458:r], allele observed in CC1)

Sequence:

TTATTTAGAATGATTATAATTTAATAATAGGTGTTGCAATGCAATGATAGTCTTCGCATCTTCTATTTCTTTATTCATTAGCATCGATTTAAC
ATTCTCAATCGGAACTTTAATGACTTCGACAAATTCATCTTCATCTAAGTGAACTGTACCTTCTTCTAAATTATCCGTAAAATATATTGATAA
TTGTTCATCGCAAAAACCTGGTGAACCATACATATCTACAACATGTGTTAACTCTTTGGCAATATATCCTGTTTCTTCTTCTAATTCACGCTT
TGCCGCTTCGACTCTATCTTCATCATCTTCTAATTTACCAGCTGGAATTTCTAGCAATGGTTTTTCTACTGGTTTACGATACTGTTTCACTAA
TACGACTTCTTTTTTAGGTGTCACTGCACAAACAGCAACTGCACCATTATGATAAACTAATTCTCTTGTTGATGTTTCACCGTTTGGTAATG
TCACTGTATGAATTTCTACATCTACAATTTTGCCATTATAAATAACTGTTCGATCAATTGTTTTTTCATTTAAATCCAT

Gene: yhdN (putative oxidoreductase)

Contig: 07_NODE_2, position: 76632 to 77540, length: 909 nt, orientation: FORWARD

Perfect match to: (MW2-BA000033-[1585540:1586448], allele observed in CC1+CC12+CC22+CC80+CC188)

Sequence:

ATGCAAAAAAATATATTAAAAAGTGGTATTTCATTATCTGAATTAGGTCTAGGTTGCATGAGTTTAGGTACAGATTTAAAAAAAGCCGAA
CAAATTATAGATTGTGCTGTTGAAAATGGTATCACTTATTTTGATACAGCAGATATGTACGATAAAGGTATAAATGAATCAGTTGTTGGTA
AGGCACTTCTTAAATATCAACAACGCGATGATATTGTCATTGGTACAAAAGTAGGCAATCGTTTAACAAAAGATGGCAGTACAAAATGG
GATCCGAGTAAATCCTATATTAAAGAGGCAGTTAAAGGTTCACTAAAGCGTTTAGGTATCGATCATATCGATTTATATCAACTTCATGGCG
GAACCATTGATGACCCATTAGACGAAACAATAAGCGCATTTGATGAATTGAAACAAGAAGGAATTATACGTGCTTACGGTATTTCTTCTA
TTCGCCCAAATGTAATTGATTATTATTTAAAACATAGTCAAATCGAAACGATAATGTCTCAATTCAATTTGATTGATAATCGTCCAGAATCA
TTATTAGATGCAATTCACAACAATGATGTTAAAGTATTGGCAAGAGGACCTGTGTCTAAAGGATTATTAACTTCAAACAGTGTTAATGTG
CTCGACAATAAATTTAAAGATGGTATTTTTGATTATTCTCATGATGAATTGGGTGAAACAATAGCCTCTATTAAAGAAATTGAAAGTAATT
TATCTGCATTGACATTTAGTTATTTAACATCACATGACGTGCTTGGTTCCATCATTGTAGGTGCAAGTAGCGTCGACCAATTAAAAGAAAA
TATTGAAAACTATCATACTAAAGTTAGTTTAGATCAGATTAAAACAGCAAGAGCTCGTGTAAAGGATTTGGAATATACCAATCATTTAGT
GTAG

Gene: Q5HFS1 (putative protein)

Contig: 07_NODE_2, position: 77756 to 78004, length: 249 nt, orientation: REVERSE

Perfect match to: (MW2-BA000033-[1586664:1586912:r], allele observed in CC1+CC188)

Sequence:

TTAAAACATCTTTCTATATTTCACTTCGCATGTTGATTCATCATTATTAGTTATTATTTGTACACCCAGCACATTTCCTTGCAACACAAGTAG
TTTGAATTTTTCACAAGTATAATATAATGTACTGTCTGAAATTTGGTCTACAGAAATATCGCCTAAAATATCCAGCACTGTAAATTCTTCAA
ACACTGATAGTTGTTCCGCATATCGTACACAAAATCTTACCACACTCTCCGATTGACAGTTCAT

Gene: yqjQ (oxidoreductase)

Contig: 07_NODE_2, position: 78020 to 78775, length: 756 nt, orientation: REVERSE

Perfect match to: (MW2-BA000033-[1586928:1587683:r], allele observed in CC1+CC8+CC88+CC239)

Sequence:

TTATGCTTTATTTTTAAATAATTTAGGGAAACATCGTTCAAAAAATCTAGGCGCAATTTGATACATTTTCAACGCATGATGCATCCATTTAG
GCCGATTAATTTCCAATTGTTTTGTTTTAATGCCATAAATGATATCTTCTGCAAGCTGATTAGCATCAAGCATAATTTTCCCCATCCTTTTAG
CATACTTCATTGATGGGTCGGCTTTTTGATGAAAAGGTGTATCAATCGGGCCAACATTAACTGTCATGATATGTAAGTTTGGTGACTCTAG
TCTTAAAGCATTCATTAATGCATAAAACCCTGCTTTCGATGCCCCATAATGTGCAGCATTTGCTTGTGTGGAAAATGCAGCTTGACTTGAA
ATACCTACAATATGTGCGTTAGATGTTAAATATGGTCTCAACACAGTATATAAAACATTAAAACTAATTAAATTAAGCTGATACGTTTCAA
TCATTTCTGAAAAACTATGGTCTGAAATAGATTTGAAATAACCTAAACCTGCACTATAAATGAATCCATCGAATGATGTATTGTCTTCAAA
TTGCAGTGCCTGTATCGACTTCAAATCATTTAAGTCACAAGGAATAACATTTATAGTTTTCCCCAATTCCTGTTCAAAGATTCTAGTTGCTT
TATCAACATCACGCACCAACAACGTTACATGCACTTTATTTTCTAGTAACTTTCGGACAATCGATAAACCTAAACCACTCGTACCACCAGTC
ACTATAAAATGTTGTCCTTTCAT

Gene: proC (pyrroline-5-carboxylate reductase)

Contig: 07_NODE_2, position: 78917 to 79732, length: 816 nt, orientation: FORWARD

Perfect match to: (MW2-BA000033-[1587825:1588640], allele observed in CC1)

Sequence:

ATGAAACTCGTATTTTATGGAGCTGGTAATATGGCACAAGCTATATTTACAGGAATTATTAACTCAAGCAACTTAGATGCCAATGATATAT
ATTTAACAAATAAATCTAATGAACAAGCTTTAAAAGCATTCGCTGAAAAACTAGGTGTTAACTATAGTTATGATGATGCGACATTATTAAA
AGATGCAGATTATGTTTTTTTAGGTACCAAACCACATGACTTTGATGCTCTAGCAACACGCATCAAACCACATATCACAAAAGACAATTGC
TTCATTTCAATTATGGCAGGTATTCCGATTGATTATATTAAACAACAATTAGAATGCCAAAATCCAGTTGCTAGAATTATGCCAAACACAA
ATGCGCAAGTCGGACACTCTGTTACTGGCATTAGTTTTTCAAACAACTTTGACCCTAAATCTAAAGATGAAATTAACGATTTAGTTAAAGC
ATTTGGTTCTGTAATTGAAGTATCAGAAGATCATTTACATCAAGTAACAGCTATCACCGGAAGCGGCCCAGCATTTTTATATCATGTATTC
GAGCAATACGTTAAAGCTGGTACGAAACTTGGTCTAGAAAAAGAACAAGTTGAAGAATCTATACGCAACCTTATTATAGGTACAAGTAA
GATGATTGAACGTTCAGATTTGAGCATGGCTCAATTAAGAAAAAATATTACCTCTAAAGGTGGTACGACACAAGCTGGCCTTGATACATT
GTCACAATATGATTTAGTATCTATTTTCGAAGATTGTCTAAATGCTGCCGTCGACCGTAGTATTGAACTTTCTAATGTAGAAGACCAATAA

Gene: rnz (ribonuclease Z)

Contig: 07_NODE_2, position: 79835 to 80755, length: 921 nt, orientation: REVERSE

Sequence:

TTAGACTTTAAAACTATCAAAATCTTTTACAAAATTAAAATTAGGTGTATCTTCATTTTGTATCAATGTTTGATAAATTTCATTTATATCTTCT
GTATTATAGCGATTGCTCAAATGTGTAATCAACGTACGTTTAACATTGGCTTCTTTTATCAATGCAAATACGTCTTCAATATGGCTATGATG
ATAATTGTTGGCTAAATGCTTTTCACCATCTATATAGGTCGCTTCATGTACCATCACATCAGCATCTCTAGAAATCACACGTTCATTAGAAC
ATGGTTTTGTATCACCAAAAATTGCTACAACTGGACCCTGTTTGGACTCACCTCTAAAATCTTTTGATTGATAAACTTGACCATTATGTTCA
AATGTATCATGAGATTTTACTTCTTGATATTTAGGACCTGGTTCAAGACCAATGTTTTTTAACGCTTCAACATTGATTGTACCTGTAGTTTC

AGGTGCCATTACTCTATATCCATATGATGGAACACCATGATTAAGTAAATGCGCCTCTACAGTAAAACCATCATGATGATATGTCAGATGA
TCATCGATTTCAATATATGTAATTGGATAGTTTAAATGTGACTCTGATAAATTCATAGACATTTCCACATATGCTTTAATTCCTTTTGGTCCA
ACCAATGTAAGCGGCTTCTCTTCACCGCCCTGAAAAGAACGACTAGAAAGTAATCCTGGCAAACCAAAAATATGATCGCCATGCATATGA
GTAATAAATATATGTGTCACTTTTCCTAATTTAATTGCATGATGTAAAATTTGGTGCTGTGTACCTTCACCAACGTCGAAAAGCCATATGG
AATTGGAATATGGTTCTAAATTTAAGGCGATTGCTTGTGTATTTCTCTCTTTTGTAGGCAAACCTGCACTCGTTCCAAAAAATGTAACTTCC
AT

Gene: zwf (glucose-6-phosphate 1-dehydrogenase)

Contig: 07_NODE_2, position: 81068 to 82552, length: 1485 nt, orientation: FORWARD

Perfect match to: (MW2-BA000033-[1589976:1591460], allele observed in CC1+CC30)

Sequence:

TTGAGTACTAAAAACAAACACATCCCTTGTTTAATCACAATCTTTGGTGCAACTGGTGACTTAAGCCATCGTAAGTTGTTTCCATCAATATT
CCATCTCTACCAACAAGACAATTTAGATGAACATATTGCCATCATCGGTATTGGACGTCGTGACATTACTAATGATGATTTCCGTAATCAA
GTAAAATCATCAATTCAAAAGCACGTAAAAGATACAAACAAAATTGACGCGTTTATGGAACATGTCTTCTATCATAGACATGATGTTAGT
AATGAAGAAAGCTATCAAGAATTACTAGATTTTAGTAATGAATTAGATAGCCAATTTGAATTAAAAGGTAATCGACTATTCTATTTAGCAA
TGGCACCACAATTCTTTGGCGTTATTTCTGATTATCTAAAATCTTCTGGTCTTACTGATACAAAAGGATTTAAACGCCTTGTTATCGAAAAA
CCATTCGGTAGTGATTTAAAATCAGCCGAAGCATTAAACAATCAAATTCGTAAATCATTTAAAGAAGAAGAAATTTATCGTATTGACCACT
ATTTAGGAAAAGACATGGTTCAAAATATCGAGGTATTACGTTTTGCGAATGCGATGTTTGAACCATTATGGAATAACAAATATATTTCAA
ACATCCAAGTTACATCATCTGAAATACTAGGTGTTGAAGATCGTGGTGGTTATTATGAATCAAGTGGCGCGCTAAAAGATATGGTGCAAA
ACCACATGTTACAAATGGTTGCATTATTAGCTATGGAAGCACCTATTAGTTTAAATAGTGAAGATATCCGTGCTGAGAAAGTAAAAGTAC
TTAAATCACTGCGTCATTTCCAATCTGAAGATGTTAAAAAGAACTTTGTTCGTGGTCAATATGGCGAAGGCTATATCGATGGTAAACAAG
TTAAAGCATACCGTGATGAAGATCGCGTTGCAGATGACTCTAACACACCTACCTTTGTTTCAGGTAAATTAACAATTGATAACTTTAGATG
GGCTGGTGTACCATTCTATATTCGTACTGGTAAACGTATGAAATCTAAAACAATTCAAGTTGTCGTTGAATTTAAAGAAGTACCAATGAAC
TTATACTATGAAACTGATAAACTATTAGATTCAAACCTATTAGTAATCAATATCCAACCTAATGAAGGTGTATCTTTACATCTAAATGCTAA
GAAAAATACACAAGGTATCGAAACTGAACCTGTTCAATTGTCTTATTCAATGAGCGCTCAAGATAAAATGAATACTGTAGATGCATATGA
AAATCTATTATTCGATTGTCTTAAAGGTGATGCCACTAACTTCACGCACTGGGAAGAATTAAAATCAACATGGAAATTTGTTGATGCAATT
CAAGATGAATGGAATATGGTTGATCCAGAATTCCCTAACTATGAATCAGGTACTAATGGTCCATTAGAAAGTGATTTACTACTTGCTCGT
GATGGTAACCATTGGTGGGACGATATTCAATAA

Gene: graE (putative transcription regulator, AraC/XylS family)

Contig: 07_NODE_2, position: 82782 to 83648, length: 867 nt, orientation: FORWARD

Perfect match to: (MW2-BA000033-[1591690:1592556], allele observed in CC1+CC30)

Sequence:

TTGGACGTTATCAAGCAAATACAACAGGCAATTGTTTATATTGAAGATCGTTTATTAGAGCCTTTCAATTTGCAAGAATTAAGTGATTACG
TTGGTCTTTCGCCATACCATCTTGATCAATCATTTAAAATGATTGTCGGCTTATCTCCAGAAGCTTATGCACGCGCGCGTAAAATGACACTC
GCTGCAAATGATGTGATTAATGGTGCTACACGACTTGTAGATATCGCTAAAAAATATCACTATGCAAATTCAAATGATTTTGCAAATGATT
TTAGTGATTTTCACGGCGTATCACCTATTCAAGCTTCTACTAAAAAAGATGAATTACAAATTCAAGAGCGATTATATATCAAATTATCAACT
ACTGAGAGAACACCCTATCCATACAGATTAGAAGAGACAGATGATATTTCATTGGTTGGATATGCACGATTTATAGACACTAAGTATTTG
TCACATCCTTTTAATGTTCCGGATTTTTTAGAAGACTTGCTCATTGATGGTAAAATTAAAGAGTTACGACGATATAATGACGTTAGTCCATT
TGAACTATTTGTTATTAGTTGTCCTCTTGAAAATGGTTTAGAAATATTTGTAGGTGTACCAAGTGAACGTTATCCTGCACACTTAGAAAGC
CGATTTTTTACCTGGCAAACATTGTGCGAAATTCAATTTACAAGGTGAAATTGATTATGCAACTAATGAAGCTTGGTACTATATTGAATCAA
GTTTGCAGTTAACATTGCCATATGAACGAAATGATTTATATGTTGAAGTGTACCCTCTCGATATTTCATTTAATGACCCATTCACTAAAATT
CAGCTTTGGATTCCTGTTAAACAGAGTCCTTATGACGAAGATTAA

Gene: malA-yugT (alpha-D-1,4-glucosidase)

Contig: 07_NODE_2, position: 83730 to 85379, length: 1650 nt, orientation: REVERSE

Perfect match to: (ED98-CP001781-[1559561:1561210:r], allele observed in CC5)

Sequence:

TTAATTTAGTTCGATAACACATGCTTCATATGGACGTAACTGTTTTAAATTAACTTTGGCATCATAATTAAATAGCTTTACTTCTCCATGGCT
TAAATCAAATGGTACAGTTAATTCTGCTTCGTGGTTAGTAAGATTACCTACAATAAGAACTTGCTTTTCATTTAATGTTCTCGTGTACGCAA
AAACTTGTGAATTTTCAGCATCTACTAAATCAAATTGACCATATACGTATACATCATTAGACTTTCTTAATTGAATTAAATCTTTATAAAATT
GTAATACTGAATGCTCATCTTCTAATTGTTGTGCAACATTGATAGTTTTATAATTCGGATTCACTGGGAACCACGGTTCACCATTTGTAAAT
CCTCCATTTAACGTATCATCCCATTGCATTGGTGTGCGAGAATTATCTCGGTTCTCATCTTTATATTTCGCAAGTAAAGCGTCTACATCTCC
ACCTTGAGCTTTCACTATTTGATAGTCATTTTTAACAGCAACATCGTTAAACGTTTCAATACTTTCAAATGGATAATTCGTCATACCAATTTC
TTGACCTTGATAAATGAATGGCGTACCTTGTTGCAAGAAATAAACAGCTGCATGACTTGTTGCTGATTCATACCAATACTTGTCATCGTCA
CCCCACGTCGATACACGTCGTGGTTGGTCGTGATTTTCAATAAACAACGCATTCCAACCTTTATTTTCAAGTTGTTTTTGCCATCTATTTAAT
ACAGATTTATACGAATTTACATCAAAGTGAGAATCACCACTATTCCACAGTCCCAAATGTTCAAATTGGAATATCATATTAAATTTACCATT
TTCTTCCCCGACCCAGTCATCAGCATCATCAGGGCTTACACCATTCGCTTCACCAACAGTCATAATGTCATACTTACTTAATGAGCGATCTT
TCATCTCTTGTAACCAAGTTTGTATACCTGGCTGATTCATATCTACATCAAATGCTGGGGCATATGTTTTACCCTCAGGTACAGGTAAGTCA
CCCGCTTCAAACGTCTTCTTAATATGCGTAATTGCATCTACTCTAAATCCATCAATGCCTTTATCAAACCACCAGTTCATCATTTCAAATACA
GCATCTCTAACTTCCGGATTACCCCAATTCAAATCAGGTTGTTTTTTACTGAATAAATGGAAATAATATTGCTCAGTATTAGCATCATATTC
CCATGTAGATCCATTAAATATACTTTCCCAGTTGTTAGGTTCAGAGCCATCTGGCTTTGGATCTTGCCAAATGTACCAATCACGTTTGGGA
TTGTCTTTACTAGATTTGGATTCTATAAACCAAGGATGTTCATCAGATGTATGATTTACAACTAAATCTAAAATAAGCTTCATGCCTCTATC
ATGAACACCTTTTAATAAACAATCAAAGTCTTCCATCGTTCCAAATTCATCCATAATTTCTTGATAGTCACTAATATCATAACCATTGTCATC
ATTAGGTGATTTAAACATTGGACTGAGCCAAATGACATCGATACCTAAATCTTTTAAGTAGTCCAATTTATCAATCATTCCAGGTAAATCC
CCAATACCATCGTGATTACTATCATTAAAACTTCTTGGATATACTTGATATGCTACTGCTTCTTTCCACCATTGCTTATTCAT

Gene: malR-kdgR (transcriptional regulator of malA)

Contig: 07_NODE_2, position: 85395 to 86414, length: 1020 nt, orientation: REVERSE

Perfect match to: (NN50-BAEA01000025-[135697:136716:r], allele observed in CC4803+CC1+CC8+CC239)

Sequence:

CTATCGCTGTGTTGATTTTCTTATTTTTAATTCTGTATCTATAATGACGAGTTCAATAACATCCTGTGCTTTGTTTTTCAATATATTTAAAATT
GCTGAACCAGCCTGTTGACCTAACATTCGAGGCTTGATGTCAATACTGGTTTGAGGTGGTGACGCAATTTCGGTTAAATAAGAATCATTG
AATGTTGCTGTCATTACATCTTTTGGAATTTCAATATTAAGTTCATATAGCACACTTAAAATCGCTAAATGTAACATAGCATCTAACGAAAT
GATTGCCTGTTTAATATTTGGGTCCTTCAAGCGCGTATGTAGATTTTGCATGTAATTAAAAATAACTTTTCTTTCATTACTAGTCTCAATAAT
TTGATAATCCAGATTAAATTGTGATGCAACCGTTTCAAATCCTTGAATTCTATCTTTTGAAACTTCAAAATTTCCTTTTTCTGTAATAAATAT
TAATTCATCTACACCTTGTTCAATAACATGTCGTGTCAAATTTTCAGAAGCTAATATATTATCATTATCTATGTGTGTAAATTGATGATCTAT
ATCCGATGTAGGCTTACCAATCACAATAAATGGCATGCTTTCATCAATTAACATTTGTTTAATTGGATCATTTTCTTTTGAATAAAGCAGTA
TAAACGCATCAACCATTCGTTGTTTAATCATTTTATAAACTTCATCCATTAAATCATTCATATTATTTGAGACTGTCGTTTGTGTACCATAGC
CATGCTGGTTACACGTTTCAGAAATTCCTAGCAATACATTGATGTAGAATGGATTCAGTCGAATAGGCTCCTCAGACCCTTTTAACACTAA
ACCAATTTTATATGTTTGGTTTGTAATTAAAGTTCTAGCAGCGGTATTAGGAAAATAATTCAATTCTTCCATAACTTTCTTCACTTTTGAAAT
TGTCGCTTCGCTAATACGTTGATTTCCTTTTATAACTCTTGAAACTGTCGAAGGAGAAACACCGGCTTTTAGTGCAACATCTTTAATCGTAA
CCAT

Gene: Q5HFR3 (glyoxalase family protein)

Contig: 07_NODE_2, position: 86608 to 86979, length: 372 nt, orientation: REVERSE

Perfect match to: (MW2-BA000033-[1595516:1595887:r], highly conserved allele)

Sequence:

TTAGTCACTAACTGCAAAATAGTTACCTTGGCCATCTTGAAAATTAAATACACGTTGACCATTCATTTCTACTATATCATGCCCAGTTAAAC
CTAAATCATTTAATTTTGAGTATAATGCATCAAAGTTTTTCTCTTTAAACATTAAAGATGGTGTTCCTAGGTTCACTTCCGGGCTATACTTTT
CAATAAATTCTTTTGCCATAATCGTCAATGACGTTTCAGCATCTTTCGTAGGTGATACTTCAACTGCAACATAGTCCTCAGCTAACGGTGTT
TCACTTACAACAACAAATTCTAAAGTTTCTGTCCAAAATGCTTTCGCTTTGTCAACATCATCAACATATAACATAACTTGATTTAACTTTTCC
AT

Gene: gnd (6-phosphogluconate dehydrogenase, decarboxylating)

Contig: 07_NODE_2, position: 87198 to 88604, length: 1407 nt, orientation: REVERSE

Perfect match to: (MW2-BA000033-[1596106:1597512:r], highly conserved allele)

Sequence:

TTATTCTTCAATCCATTGTGTATGGAATACGCCTTCTTTATCTTTTCTTTCGTACGTATGAGCACCGAAGTAGTCACGTTGTGCTTGAATTAA
GTTTGCAGGTAAATCAGCTGCACGGTAACTATCATAGTAATTAATACTTGATGAGAAACCAGGTGTTGGTACACCATTTTGAACACCAGT
TGCGACAACATCACGTAACGCATCTTGATATTCAGTAACGATGTTTTTAAAGTAAGGATCTAGCAATAAGTTTTGTAATCCTGGATTATTA
TCGTAAGCATCTTTGATCTTTTGTAAGAATTGTGCACGGATAATGCAACCTTCTCTCCAAATCATAGCTAAATCACCAAGTTTTAAATTCCA
TTCATTATCTTCACTTGCTTTACGCATTTGCGCGAAACCTTGTGCATAAGAACAAATTTTACTCATATATAATGCTTTACGAATTTTTTCTAA
AAAGTCTTTCTTGTCACCATCAAATGATGCTTTTGGACCATTTAATTCTTTAGAAGCATTTACGCGCTCTTCTTTGATTGAAGAGATAAAAC
GTGCAAATACAGATTCAGTAATGATTGTTAATGGAATACCTAATTCTAATGCGTTAATTGAAGTCCATTTTCCTGTACCTTTTTGACCTGCA
GTATCAAGAATTTTTTCAACTAATGCTTCTTTATTTTCATCTAATTTCATGAAAATATCACCAGTGATTTCAATTAAATAACTTTCTAATTCAC
CAGCATTCCAGTCTTTGAACGTTTGAGCAATGTCTTCATGAGACATGCCTAATAATTCTTTCATCATAGCATAACTTTCTGCAATTAATTGC
ATGTCAGCATATTCAATACCATTATGTACCATTTTCACATAGTGTCCAGCACCATTTGGTCCAATATAAGTAACACATGAAGCACCGTCTTT
TGCCTTTGCAGCAATTGCATCAAGAATATCTGCAACTTTGTTATAAGCTTCTTCTTGTCCACCCGGCATTAATGACGGACCAGTTAACGCTC
CAATTTCACCACCAGAAACGCCCATACCAATAAAGTTGATTGCACTTTGTGCTAATGCTTTATTACGTCTGATAGTATCTTGATAGTTTGTA
TTACCACCATCAATTAAAATATCTCCATCATCTAATAAAGGTAACAAACTATCAATCGTTGCATCCGTAGCTTTACCTGCTTGAACCATTAA
TAAAATTTTACGTGGTTTTTCTAAAGAATTAACAAATTCTTCCAATGAGTACGTTGGATGAATATTTTTCCCTTTTGATTCTTCAACCATTAA
ATCAGTTTTTTCACTTGAGCGGTTAAATACAGATACACTATATCCGCGTGATTCAATATTCCAAGCTAGGTTTTTACCCATAACGGCTAAAC
CAATAACTCCAATTTGTTGTGTCAT

Gene: Q5HFR1 (peptidase T-like protein)

Contig: 07_NODE_2, position: 88672 to 89805, length: 1134 nt, orientation: REVERSE

Perfect match to: (MW2-BA000033-[1597580:1598713:r], allele observed in CC1+CC239)

Sequence:

TCATTTCGATTGTCGCGCAACTATTTTGATTATTTCTAACACTTGACTTGCAAGCAAGTTCAATGATTTAATCGGCATTCTCTCATTTGTTGT
ATGGATTTTTTCATAACCCACTCCTAAAATGACTGAAGGAATACCAAATGTATTAATAATACTGCCGTCTGAACCGCCACCAGAAATAATT
GTATTTGCAGATAATCCTAAATTACGAGCACTTTCTTGTGCAATTTTAACTACCGCTTCATTATCATTAATTTTAAATCCTGGATAACTTTGC
TCCACTGTAACTTCTGCTTTCCCACCTAATTCTGATGCAGTAGTTTCAAACACATCAGTCATATGTTTGACTTGTGTTTTTATTCTTTCTGGA
TCGTGAGAACGTGCCTCTGCTTCTAAAATGACTTCATCTGCAACAATATTCGTAGCTGAACCGCCATGAAACTTACCAATATTGGCAGTAG
TTATTTCATCAACTTGTCCTAATTTCATTCGACTAATTGCTTTCGCCGCAATATTAATAGCACTAACACCCTCTTTTGGCGTACTTGCATGAG
CCGTTTTGCCAAAAAATTTTAGCTGAAATTAACATTTGCGTCGGTGCACCTACAACCGTAGTGCCGACATCAGCACTTGCATCAATAGCATA
ACCAAAGTCCGCGTCCAACAACTCTGAATTTAATTCTTTAGCACCAATTAAACCTGATTCTTCCCCAACAGTAATCACAAATTGAATTTGTC
CATGTGGGATTTGTTGTTCCTTTATCACTTGCAAAACTTCAAGCATCGCTGCTAATCCTGCTTTATCATCTGCACCTAGTATAGTCGTACCA
TCAGAGTATATGTAGCCGTCATCTTTTACAATTGGCTTTACATTAATTGCGGGTACAACAGTATCCATATGGCTCGTCAAATATAATTTAG
GTACTTCGCCTTCTTCGATAGTACTATTCATTGTACACACTAGATTATTGGCACCTAATTTAGGATGTTTAGCCGCTTCATCTTCTTTAACAT
CTAACCCTAATGCTATGAATTTTTCTTTTAAAATAGGTTGGATTGTTGATTCATTCCCTGTCTCAGAATCGATTTGTACAAGTTCAAAAAAC
GTATTAAGTAATCTTTGCTCATTAATCAT

Gene: yqjA (YqjA like protein)

Contig: 07_NODE_2, position: 90374 to 91354, length: 981 nt, orientation: REVERSE

Perfect match to: (MW2-BA000033-[1599282:1600262:r], allele observed in CC1+CC45I+CC398+CC425)

Sequence:

TTAAGCTACTTCACTATGCATTTTCAATGAACCAAATTGCGATTTGATTTGTAAATATTCTTCTAATTCATTTAATATTTGAATAATACTTGC
TCTCGAGTTAAGCGCTTTGTGTGTTGTTGGCAATGGAAGTTCATCCAATTTCAAACGCGTCTCATACAAATTGTGTAAACGCATTGCTGTA
TAGTCATTACTATTCACATTTAGACCAATTTCTTTCAGCAGTGACGCAACATCATTTAAAAGTGGATCTTTATGACAGATACTTTCGATGAG
CGGTTTCATTCTCATTAACAATTCCACTTGCTCTTCTCGCATATCAAAATAATGATAGTATGAATTTTCGTTTCTAACAAAATGATTTTTAAC
ATCTCGGAACGCGATAGACTTCGCCTTTTTAATATTTAAAAGTAACACTTCAAATTCAATCGCAATGGTATCTTCATACTTTTCACAAATAT
AACTATATTTACTAAAAATATCAGCAATTTGTTGCTCAATTTTACATTTGTATTCGTCTAGTTGTTTGTCTAAACTTGGCATCATTAAATTCA
TTGTAAATGCAATGCTCAGTCCAATTAACAGTAATAATGTTTCATTAACAATTAAATGTGCATCAATTGATTTTGCATTAAAAACATGAAGT
AATATAACGCAACTCGTAATGACACCTTCTTGTACTTTTAATACGACAGTTAATGGTATAAATAACAATACGATAATACCGAGTACAATTG
GACTCTGACCTAATAAACTAAATATTGCTGAACCTAAAAACAATACTAAAAAACATGATACTAATCTTGAAATAATCGCTTGTAGCGAATG
TACTTTTGTATGTTTAATACATAATACGACTAATATGGCGCTTGAAGCATAATTATCTAAACCTAACAGCTTACTAATAATTACACCTAAAG
TCATACCCACTGCTGTTTTTATTGTTCTAAATCCAATCTTGTAAGGATTTAACTTTAACAT

Gene: yqiW (putative protein)

Contig: 07_NODE_2, position: 91368 to 91805, length: 438 nt, orientation: REVERSE

Perfect match to: (RF122-AJ938182-[1509914:1510351:r], highly conserved allele)

Sequence:

TTATCTTTCTTCACAATATTTATTGAATAATGTTTGTAATTGATTAATTACGTTCATCACATCATGACCTTCGATTTGATGTCTTTCAATCATT
TCTGTAATCTTTCCATCTTTTACTAATGCAAATGACGGACTTGAAGGCGCATAACCTTCGAAGTATTCACGCGCTCTTTGTGTCGCTTCTTT
ATCTTGTCCAGCAAATACTGTCACTAGACGATCAGGTAATACGTCATAATGTAAAGCATGTGATGCTGCTGGTCTTGCGATACCACCTGC
ACAACCACATACAGAATTGATCATAACTAGTGTTGTACCATCTTGTTTAAGAACTTTGTCAACATCTTCTGCAGTAGTTAATTGCTCATATC
CCGCAGATTCAATTTCATTCCTTGCTTGTTCTACAACACCGTTCATGTATAAATCGAAATTCATGTCCAT

Gene: bfmBB (branched-chain alpha-keto acid dehydrogenase E2)

Contig: 07_NODE_2, position: 92156 to 93430, length: 1275 nt, orientation: REVERSE

Perfect match to: (N315-BA000018-[1555520:1556794:r], allele observed in CC5+CC1+CC25)

Sequence:

CTAATATATATTTGTATTTTCTAAAGTATACTGTTCGATACGCTGTTTAATATGATTCATAAATTTACCTGTTTGTAAACCATCTAAAATACG
ATGATCAATTGAAATACATAAATTAACCATGTTACGAATTGCAATCATATCATTAATTACTACTGGCTTTTTAACGATTGATTCTACTTGTA
AAATCGCTGCTTGTGGATGATTTATAATACCCATTGATGATACTGAACCAAATGTACCAGTATTATTTACCGTAAATGTACCGCCCTGCAT
ATCTTCAGCTGTCAATTGCTTATTACGCGCTTTCGTTGCTAAAGTATTAATTTCTCTAGCTATACCTTTGATTGACTTTTCGTCTGCATGCTT
AATCACAGGTACGTATAATTTATTTTCATCAGCAACAGCAATTGAAATATTAATGTCTTTATGTAAGACAATTTCATTTCCTTGCCAGCTAC
TATTTAATAAAGGATATGCTTTTAAAGCATCTGCTACAGCTTTTACAAAGAAAGCAAAGAACGTTAGATTATATCCTTCTTTATTTTTAAAG
CTGTTTTTATAATGATTTCTCGTATTCACAAGATTTGTAGCATCTACTTCAATCATCATCCATGCATGTGGAATCTCTGTTACACTATTAACC
ATATTTTGCGCAATTGCTTTACGCACACCATTTACTGGTATTGTGCTGTTTTCACTATTGTCTTCAGATGATTGGTTACTTGATGTATCTACT
GATGTTGATTTTGTTTGAACTTGTTTGTCAGATTGAGCTGTGGTACCACCATTTTCAATAACTGACATTATATCCTTCTTAGTTACACGACC
TTCAAATCCACTACCTACAACTTGTGATAAATCAATGTCATGCTCTGAAGCGAGTTTAAATACAACAGGTGAAAAGCGACCATTATTACGA
GGTTGATTTTGTTTAGCAGTAAATGTCTGTTCCACTGTTGCACTAGCTTTTTTAGTAGATTTCTGAGTATGCTCATCCACTTTTGCTTGTATC
TCTTCAGTTGTTTCATTTGTCTTTTCATCAGCAGTTTCAATTTTACAGATAATTGTATCAATAGCTACTGTCTGCCCCGCTTCAACTAAAATT
TCTGTAATTGTTCCTGATATCGTGGAAGGGACTTCAGCTGTCACTTTATCTGTAATAACTTCACATAATGGTTCATATTCATCAATATGATC
ACCAACAGAAACTAACCATTGTTCAATGGTACCTTCATGAACACTCTCACCTAACTTAGGCATTGTTATTTCCAT

Gene: bfmBAB (2-oxoisovalerate dehydrogenase beta subunit)

Contig: 07_NODE_2, position: 93443 to 94426, length: 984 nt, orientation: REVERSE

Perfect match to: (N315-BA000018-[1556807:1557790:r], highly conserved allele)

Sequence:

CTAGAATTCTGCTAATTCACGCATTTTATTTAAGATTTTTTCTGGATTCATCATAATTTCATTTTCTAATACAGGAGAAAATGGCATAGATG
GTACATCTGGAGCAGCTAAACGCATGATTGGTGCATCTAAATCGAACAAGCAATGCTCTGCAATAATCGCTGACACTTCTGACATAATAC
TACCTTCTAAATTATCTTCAGTTACAAGTAAAACTTTACCTGTATTTTTAGCACGATCAATAATTGTTTCTTTATCTAATGGATAAACAGTTC
GTAAATCAACGACTTCAACGTTGATACCGTCTGCAGCTAAAATATCCGCTGCTTGTAAACAATAATTGACCATTAATCCATAACAAAATAC
TGTTAAATCTTCACCTTCACGTTTAACATCTGCTTTTCCTAAAGGTACAGTGTAATATTCTTCTGGCACTTCTTCCTTTAAGAAACGATAAGC
TTTTTTATGCTCAAAGTACAATACTGGATCATTTGATTCGATAGATGATAATAAAAGCCCTTTAGCATCATACGGTGTGGAAGGAATAACA
ATTGTTAAACCTGGTGATGAAGCAAATATACTTTCAATACTTTGTGAATGATATAGTCCTCCGTGAACACCGCCACCAAATGGTGCACGAA
TCGTTAATGGGCATTGCCAATCATTATTTGAACGATAACGCATTTTCGCAGCTTCACTAATAATTTGATTTGTCGCAGGTAAAATAAAATCT
GCAAATTGAATTTCTGCAATTGGTCTTTTACCTACCATAGCTGCACCAATGGCAGTTCCAACAATATTTGACTCAGCTAATGGCGTATCGA
TAACTCTGTCTTCACCATATTGTTGTTGTAGTCCTTGAGTAGTACCAAATACGCCACCTTTTTTACCAACATCTTCACCAAGAATAAACACA
TCTTTATTTTGTTGTAATGCTAAGTCTTGTGCCTGGCGTATCGCCTCTAAATAAGATAATTTAGCCAT

Gene: bfmBAA (branched-chain alpha-keto acid dehydrogenase E1)

Contig: 07_NODE_2, position: 94426 to 95418, length: 993 nt, orientation: REVERSE

Perfect match to: (TW20-FN433596-[1647886:1648878:r], highly conserved allele)

Sequence:

TTAGTTAAGACTCCCTTCTTCGTACACAAATGCATAGGCTTCTTCGACACTTGGATATGGCGCGTCTTCAGCAGCCTTTGTCGCTTTATTGA
TGATGTCTTTATGCTCCGCTTCTATTTCTGCCAACCAAGCATCATCGATAATGCCAGCTGAAAGCAACTCTTTTTTGAACTTTTCATTGCAGT
CAGCTTTTTTAAGCGCTTCACGCTCTTCTTTCGTACGATATTGGTCGTCATCATCTGATGAATGAGCTGTCATACGACTTGTTACTGCTTCA
ATCAAAGTTGAACCTTGACCAGAAATAGCTCGATCTCTTGCTTCTTTCATCGCTTTATACATTGCTAATGGATCATTACCATCTACTTGTTCA
CCATGTATACCGTAACCAAGTGCTCTATCCGATAATTTTTCAGCTGCGTATTGTAATGAATCAGGTACTGAAATTGCATATTTATTATTTAT
AATGACACATACAAAAGGAAGTTTGTGTACACCCGCGAAGTTTAAACCTTCATGGAAGTCACCTTGGTTTGAGCTACCTTCACCAACAGTT
GCTGTTGCAATTTTCTTCTTACCATCCATTTTTAAAGCTAAAGCAGCACCAACAGCATGGGGTATTTGAGTTGCTACCGGTGAACTTTGAG
ACAAAATATTCTTAGCTCTACTACTAAAGTGTGATGGCATTTGTTTTCCACCAGAGTTAACATCGTCTTTCTTTCCAAACGCTGATAAAAAC
GTATCATACGCTGAGATACCCATATAAGTAACGAAAGCTAGATCTCTATAATAAGGCGCTGTAATATCACCTTCTTCTAATGCGTATGCCA
TCCCAATCTGAGTTGCTTCTTGTCCTTGACCACTTACAACAAATGGAATTTTACCTGCACGGTTCAATAACCACAGTCTTTCATCTATTTTTC
TACCTAAATCCATCCATTTATATATTACTTTTAGGTCTTCTTCGCTAAGGCCTAATGATTTATAATCAATCAT

Gene: lpdA (dihydrolipoyl dehydrogenase)

Contig: 07_NODE_2, position: 95434 to 96855, length: 1422 nt, orientation: REVERSE

Perfect match to: (08-02119-CP015645-[567377:568798], allele observed in ST582+CC12+CC101+CC1217+CC1290)

Sequence:

TTATACGTGAATAGCTCTACTTTCTGCTTTCAATCCTAATTCCATCAACACTTCAGAGATGGAAGGATGTGCGTGTGTTGTTAGTCCTAATT
CTAATGCCGAGCCATTCATGAACTGCAACAGTGATGCCTCATTAATCAATTCTGTTACATGTGGACCAATCATATTAATACCCACAATTTCT
TCAGTTGATTGATCAATCACCATTTCGCTATACCCTTCGTTTGTGTCATGGCTATCAATCACTGCTTTACCAATTGCTTTAAATGGTACTTTA
AAACTTTTAACTTTCATTCCCTCTGCCTTTGCTTGTTCAATGTTTAAACCGATAGAAGCAATTTCAGGTTGTGAATAAATACACTTAGGCAT
CATGTTATAGTTTACTGGGATTGGGTTCCCCTCAAACATATGATCAACAGCCACAACACCTTCTTTTGATCCAACATGTGCCAATTGTAATT
TTCCAATACAATCACCAGCTGCATAAATATGTTTATCTTCAGTTTGTTGAAATTCGTTCGTTAAAATATGTCCTGATGTTGAAAGTTTTATTT
TAGTGTTGTTTAAACCTATATCTGATGTGTTAGGTTTTCTACCAATCGATAGCAACACTTTATCTACTTTAATTATGTCTGAGGAAATTTCA
AACGTAACACCATCTTCGTTAACATTTATATCATTTTCAGAAAGTTTTATTCCCTCATAGAATTTAACACCACGTGCTGACAATGATTTTTTT
AATAGTTGTGAAGCTTGTTTACTTTCAGTTGGTAAAATTCTTTCACCTGCTTCTATAACTGTTACGTCAACACCTAAATCTATCATCAATGAT
GCAAATTCCATTCCGATAACACCACCACCAATAATACCAATACTTGATGGTAACGTCTTTAATGATAATATATCATCGCTAGATAAAATTTT
ATCATGATCAAATGATAAGAATGGCAACTCTGCAGGCGAAGAACCAGTTGCAATTAATACAAATTGGTTTGGTAATAAGTCTGATTCACC
ATCTTCATATTCGACAGAAATTGTGCCACTTTGAGGTGAAAATATAGATGTACCTAGAATACGTCCCGTGCCATTATAAATGTCAATGTGA
TTGTGTTGCATTAAATGCTTTACACCTTGATACATTTGATTAATAATGTCTTCTTTTCGTGCCAACATATTTTCAAAATTAACATTAGCATCT
TTGACATCAACGCCAAACATTGCTGCCTGTTTTACTGTTTGAAATACTTCAGCAGATTTAAGCAGCGATTTAGTAGGAATACAACCTTTAT
GGAGACAAGTACCTCCTAATAGTTGTCGTTCTACTATTGCCACTTTTTTACCTAATTGAGACGCACGTATCGCAGCAACATATCCTGCAGT
ACCTCCACCGAGAACGACTAAATCATATTGTTTCTCTGACAT

Gene: recN (DNA repair protein N)

Contig: 07_NODE_2, position: 97006 to 98685, length: 1680 nt, orientation: REVERSE

Perfect match to: (MW2-BA000033-[1605914:1607593:r], highly conserved allele)

Sequence:

TTATCTACGTCTTTGATTGTGTTGTATCATTTCTCTTGCATTTTCTCGAGTTAAATCAGTAACACTTGCACCTGAAATCATTCGTGCAATTTC
ATCTACTTTATCATCGCTAATTAACTCTTGAACTTGTGTTGTTGTACGATCATCTTTTGATGATTTCGAAATTAATAAATGATGGTCGCTCAT
CGATGCAACTTGTGGTAAGTGAGAGATACAAATAACTTGTATATATTCAGCTATATCTCGCATTTTCTCTGCCATTTTTTGTGCAGCTTGTC
CAGATACACCTGAGTCAACCTCATCGAATAAAATTGCAGTTTGACCTCTCGATTTAACAAAAATACTTTTTAACGCTAACATAATTCTAGAA
AGTTCTCCACCTGACGCAATTTTATTTAAACTTTTTAATGGTTCCCCTTTATTTGGACTGATTAAAAATTCTACAAATTCGATTCCATCAATA
TTCGGTTCTTCTAATTTTTTAAATGAAATCTCAAGATTTGCGTCTTTCATTTGTAAGTTTTGAATTTCCGATACAATGTGGTCTCTTAACTCC
CTAGCGACTATACGACGCTGCTTTGATAACGCTTGTCCAACCTCTATAACTTGATTATACAATGCATTTATTTCTTCTCGTAACTGCGATGT
ACTTTGTTCATAGTTTTCAATTTTATTGATTTCATTATTAAGCTTTTCTTGATATGCGATTAATTCTGAAATATCTTTTCCATATTTACGTTTTA
AATTATTCAGCAAATTCATACGAGATTCATACTCGTTTAACACTTGTTCGTCGAATTCTGTATTAGCCATTTCATCATATAACTCATGTTTTG
CATCTTCTAAAATGTAGTAAAATTGATCAATATCTTCTTTTAATTTGTCATATTTGTTTGGAACTATATCGTTTATTGTTAACAAATGGTTGC

TTAGTTCATACAAACGATCAGTGATAGCATTTTCATCCGTTAATGTCATATGTGCGTTATTAAGCGCTAAGCTTAATTTTTCAGAGTTTTGA
ATGCGTTTAATATCTATTTCAAGTTGCTCTATTTCGCCTTCTTTTAGATGTGCTTCAGACAATTCTTCTAATTGGAATTTCATTAAATCTAAA
CGCTGCAGCAATGCTTGGTCTGCTGATTCTAAATCTTCTAACTCTTGCTTTTTGGCTTTATAATTTTGAAAAGTTTGATGATATTTATCCAAC
AAATCTTGATAACGTGATTCTGCGTAATTATCCAATAATGTTAAATGGTATTTTTGTTTCAACAAAGACTGCGTTTCATGTTGGCCATGAAT
ATCTAATAATTCTTGCATAACTTTTCGTAAATCTTGTAAAGTAACTGTTTGATTATTAATTTTACAAAGACTTTTACCAGAGCTGAAAATTTC
CCGTTTAACTAATAAAAAATCTTCATCTACATCAATATCCATATTTTTCAATATATGTATAGCATCTTTACTCTCGTCAATATCAAATATACC
TTCGATGACAGCCTTTTTTTCACCATGTCTTACAAAATCAGATGAAGCTCTCATTCCAATTAATTGTCCAATTGCATCTATAATAATTGACTT
ACCTGAACCCGTTTCACCACTTAAAACAGTTAAACCATCAGAAAATTGAATTTCTAACTCTTCAATAATAGCAAATTGCTTGATTGATAAG
GTTTGTAACAT

Gene: ahrC (arginine repressor)

Contig: 07_NODE_2, position: 98701 to 99153, length: 453 nt, orientation: REVERSE

Perfect match to: (JKD6008-CP002120-[1609982:1610434:r], allele observed in CC239)

Sequence:

TTATAACAAATTGAAAATTCTTGACTTGATTTCATCACTTGCCTCTTTGCTTCGACAAATAATTAAACAAGTATCATCACCACAAATTGTGC
CTAGTACTTCTTCCCAATTGATTTGGTCTAATATAGCTCCAATAGATTGTGCATTACCAGGTAACGTTTTTAGAACAAGTAAATTATCAGTA
CCATCTATATTAACAAAGGAATCCATTAAATAACGTCCCAATTTTTCTAAAGGATGGAATTTTCTATCATTTGGTAAACTATAAACATATTG
ACCTGAAGGTATAGGTACTTTAATAAGTTGTAGTTCTTTAATATCACGAGAAACAGTTGCTTGAGTGACATTTAAATCATAATCGTTTAAT
CGTTTAACTAATTCATCTTGTGTCTCTATCTGTTCATTTGAAATAATTTCTCTAATTTTTATATGCCTAACCGATTTTTTGGGCAC

Gene: ispA (geranyltranstransferase)

Contig: 07_NODE_2, position: 99585 to 100466, length: 882 nt, orientation: REVERSE

Perfect match to: (RF122-AJ938182-[1518129:1519010:r], highly conserved allele)

Sequence:

TTAGTGATCCCTGCTATAAAATAAATCAACGATTTCTAATAAGTGTTTTGTATTGAATTGTTCATCAATTTGCGTTAGTTCATCCACTGCTG
CGTCTCTATGATAAGTCAATTTATCTTCTGCGCCATCTTTCCCTAATAAACTCACGTACGTACTTTTATTATTTTCAAGATCGCTGCCCACTTT
TTTACCTAACTTTGCTTCATCACCATAGCAGTCTAATAAATCATCTTTAATCTGGAACATCATACCTAAATGATAACTATAACTTTCTAAATG
TTCTTTAGTTGCATCATCGACATTAGCGATATCTGCTGCACTCATAACCGCAAAAGTTAATAATGCTCCTGTTTTTGTTTTGTGTATCATTTC
CAAAGTTTCAAGATCAATTGGTTGGCCTTCGCTTTGCATATCTAACATTTGACCGCCGACCATTCCAACATGACCACTTGCTATTGACAGCC
GTTGTAGAACTTTTATTTTTACTTCATCAGTTAATCTATCATCACTTGAAATAAGTTCAAATGCTTTAGTTAATAAAGCATCACCTGCTAATA
TCGCAGTCCACTCACCATATACTTTATGATTTGTTAATTTTCCTCGTCGATAATCATCATTATCCATCGCTGGTAGGTCATCATGAATAAGT
GAATATGTATGAATCATTTCTAGTGCAATTGCGCTCTTCATACCTAACTCATACTCGGTATTTAGTGAATCTAAAGTGAGTAATAACAGAA
CTGGTCGGATGCGTTTACCTCCAGCATTTAATGAATACAACATACTTTCTTCTAGCTGAGTATCCATTACTGATTTATTTATCGCAACCGAT
AATTCATTATTGACTTCATCTATTAATTTATTCATCGGTAGATTCGTCAT

Gene: xseB (exodeoxyribonuclease 7 small subunit)

Contig: 07_NODE_2, position: 100444 to 100674, length: 231 nt, orientation: REVERSE

Perfect match to: (N315-BA000018-[1563808:1564038:r], highly conserved allele)

Sequence:

TTATTCATCGGTAGATTCGTCATTTTTTACATCCTCAGCTTCTTCTTTTATTAAGTCATTCACCTTTTTTTCGGCATTTTTTAAAGTTGTGTCAC
AAGCTGCTGATAGTTTCATACCACGTTGATATAAATCTAATGATTCCTCTAAAGATACTGTTTCATTATCTAATTTTTGAACAATTTGCTCTA
ATTCTTGCATCATTTCTTCAAAACTTTGCGTTTCTTTAGTCAT

Gene: xseA (exodeoxyribonuclease 7 large subunit)

Contig: 07_NODE_2, position: 100667 to 102004, length: 1338 nt, orientation: REVERSE

Sequence:

TTAGTCATTATTACACCTTACTTTCGTAACTTTTGCATCTACTAAGCCATCTTTCATTGTTAACGTCAATTGATCATTTTCTGTTAAATCTTTA
GTACTCGTAATGACTTCGTCTTTTTTATTAACAATTGCATATCCACGCAACATTGTATTAGTTGGACTTAAATTGTTTAAGTTTTCTACTTTA
TTTTTCAAATCATTTTTATAACTTAATATCTTAGAATTCAATAATTTAACAAGTTGGTTTGTCAATTGAAGATTATTTTGTTGTTCTTGATTAA
CACTACTTAGTAATGCTTTTAAATTATAACGTTGTTGCAACAGCATTAATCGATGTCTCTGTTGTTCAAAAGTTGCCTGAATTTGTTGTTTC
AGTCTCTTTTCTAAATCATCTCGACGTTGTATCTGTTGATCATACAATAAAGTTGGTTGTTTAAACTTGTAATACGATGACAAATGTTCAAC
ATGTTTACGTTGTTGTTCTAAATGTTTCTTGATGAAACGAGTCAATGTAAACTGATATTGCTGTATTTGTTGCAGCAATTCATATTGGTCTG
GTGTTGCAATAACAGCAGCTTGAGTTGGAGTCGCAGCTCTGATGTCTGCAGCAAAATCACTTAATGTAAAGTCTGTTTCATGACCTACTG
CTGATATAATCGGTGTCTTACAATTATATATTGCACGGACGACAGCTTCTTCGTTAAAATTCCATAGATCTTCTATGGATCCACCGCCTCGA
CCTACAATAATGGTATCTACACCTAAACTATCTGCATATTCAATTTTTTCAATAATGTCGTCCTTTGCTTTTTCACCTTGAACCAAAGTACTA
ATTTGTATTTGTTCAGCTAATGGAAAACGACTATTTATCGTTGAATGGATATCTCGAATTGCGGCACCTGTACTCGCTGTTAAAACTGCAA
TTTTTTTAGGAAACTTAGGTATTGATTTCTTATTCGCTTTATCAAAACAACCTTCTTCAGTTAATTTTTTCTTTAATGCTTCTAATTTTTGATAT
AAGTTCCCTATACCATCTAATTGCATTTTATTTACATAAATTTGATAGTTTCCACGACGTTCAAAAACAGAAACACGTGCTTCTAATAAGAC
TTCATCTCCTTCTTTAGGTTCGAAGTTTAATTTAGAAGCACTACCTTTGAACATCATGGCACTTATAACGCTTTCTTTATCTTTCACATTAAA
GTATAAATGACCACTTGAATGCTTTTTGAAATTTGAAAGCTCACCTTTAATCAATACAGATTGGAGATGTGGATCTTGATCAAATTTATATT
TAATATATTTCGTTAAAGCTGAAACACTTAAATAATCTGACAT

Gene: nusB (transcription antitermination protein)

Contig: 07_NODE_2, position: 102021 to 102410, length: 390 nt, orientation: REVERSE

Sequence:

TTATTTTTTTATATTACTCAATACACCATTTATAAATTTATAATGATCATCATCACTAAATTGTTTTGTTAATTCAACTGCTTCATTCATTACG
ACTTTAGCAGGTGTATCACTGTGTAATATTTCATATGTTGCCATTCTTAAAATAATACGATCCGTTTTTAATAAACGTGCAATAGTCCAATC
TTTTAAATAAGGACTAATTGTCTCGTCTAATACAGGTTCGTGATCTTTAACGCCAGAAACTAGCCAATGAATAAATTCGAAGTCTAAATCT
GGATTATCGTCTTTAATAAAGCTTATCGCTTCATTTATCGTTAAATCACTGTCCTCCATTTCTAATTGAAATAAAGTTTGAAAAGCTTGCACT
CGGGATTCTTTACGACTCAT

Gene: yqhY (putative protein)

Contig: 07_NODE_2, position: 102470 to 102832, length: 363 nt, orientation: REVERSE

Perfect match to: (RF122-AJ938182-[1521014:1521376:r], highly conserved allele)

Sequence:

TTACTTTTCAATAACGATTTGTGTAATGTGAATATTAATTTGCTTAGGTTCTATCGCTGTCATATTAGAAATTGAATTAAAAATTGACGTTT
GAATTTTGTTTGCAGTTTTTGAAATATTAACACCATGTTTTAATGCACAATATACATCTATATATATGCCATCTTCTTTACTCTCGATTTTTAA
ATCACGGCTTAAATTTTTACGACTAACTTTTTCTAAATTTGTTTCTTTTAATTCAGCAAAATGGCCAGTGATGCCTTCGACTTCCGAAGTAG
CTATACTTGCAATAACAGATAGCACTTCTGGCGCTATTTCTACTTTACCTAATTTTGAATTTGAATAATCAGTTACTTTGACCAT

Gene: accC (acetyl-CoA carboxylase, biotin carboxylase)

Contig: 07_NODE_2, position: 102847 to 104202, length: 1356 nt, orientation: REVERSE

Perfect match to: (MW2-BA000033-[1611755:1613110:r], allele observed in CC1+CC30+CC80)

Sequence:

TTAACCTTCATCATTCATAATGCTATTTTGCTCTAAAAAGTTTGTATTAAATTTACCGCTTCTAAATATATCGTTATTCAATAATTTAATATG
GAATGGAATAGTTGTATCAATACCAAGAACCACAAATTCACTTAATGCACGAATGCCAGCCATAATCGCTTCATCTCGTGTCGGTTCATGT
ATGATTAATTTCGCTACCATCGAATCATAATATGGCGGTATCGTATAATTAGTATAACATGCTGACTCTATTCGAACACCATATCCACCTG
GTGCAAGATATTGCTCAATTTTACCTGGTGATGGCATAAAGTTCTTGTAAGGATTTTCAGCATTAATTCTAAATTCAATTGCGTGTCCTGTT
AATTTAATATCTTCTTGTTTATACGGTAACACGTCACCCATAGCAACCTGTAATTGTAATTTAACTAAATCAATTCCTGTTACCATTTCAGTT
ACAGGATGTTCTACTTGAATACGTGTATTCATTTCCATAAAATAAAATTTATTATCATTTAAATCATATATAAACTCAATTGTTCCCGCATTT
TCATAATTTACAGCTTTCGCTGCACGCACTGCGGCATTTCCCATTTCACGACGTGTTTCATCATCTAAAATTGGGGAAGGTGCTTCTTCCAC
TAATTTCTGCATACGTCTTTGAATTGTACAATCACGTTCTCCTAAATGAATTACATTACCATAGCTGTCCCCAACAATTTGGATTTCAATATG
GCGGAAGTTTTCGATGAATTTCTCCATATAAAGTCCACCATTACCAAATGCAGTTTGAGCTTCTTGTTCTGTCATTCGGAAGCCAGTTTCAA

GTTCTTTTTCATCACGAGCAACACGAATACCTTTTCCGCCACCACCAGCAGTAGCTTTAATGATGACCGGATAGCCAATTTTTTTAGCGATT
TTCTTAGCTTCTGAGACGTCTTTCATTAAACCATCACTACCAGGAACAACTGGAACATTGGCTTTGATCATTTCTGCCTTAGCAACATCTTT
GATACCCATTTTTTGGATAGATTGATAACTTGGTCCAATGAACTTCAATTGGCATGCTTCGCATAATTCTGCAAAGTCAGCATTTTCAGCTA
AAAAGCCATAACCCGGATGAACGCCATCACAACCTGTAGAAGTTGCAATAGATAAGATGTTCGGAATATTTAAATATGAATCTTTAGACA
AAGTGGGACCTACGCAATATGCTTCATCAGCAATTTGAGTATGTAGCGCATCTTTATCCCCTTCAGAATAGATTGCAACAGTTTGGATGCC
TAAATCACGACAAGCGCGAATAATCCTAACTGCGATTTCACCGCGGTTTGCAATTAAAACCTTTTTCAT

Gene: accB (acetyl-CoA carboxylase, biotin carboxyl carrier protein)

Contig: 07_NODE_2, position: 104202 to 104666, length: 465 nt, orientation: REVERSE

Perfect match to: (MW2-BA000033-[1613110:1613574:r], highly conserved allele)

Sequence:

TTATTTCACCTTAAATAACGGTTGGCCATACTCTACCATTTGTCCGTCTTCTACTAAGATTTCAACAATTTCACCTGAAATTTCTGCTTGAAT
TTCATTAAATAGTTTCATTGCCTCTAAAATACACACTGTTGTTTCATTTGAAACAGTGTCCCCAACTTGCACATATGCTTCTTCGTCTGGAG
ATGGCGATTTGTAAAATGTACCTACCATAGGTGCATTAATTGTTTTGTGATTATCTGAAGTTGGCTTTGGAGCTTCAGTTTTATTGCTATCA
GTTGATTGTACTTGAGGCATAGGCATTGCCGCAGCTTCAACTGGCATTTGTGAGATTTGTGGCGTGATAATCTCAGTTTCTTTTTCTTTCTT
AAGCGTCACTTTGCCTTTAGTATCTTCAATATTGATTTCCGTTAAAGTTGATTTATCCAGAATTTCAATTAATTCTTTGATTTCTTTAAAGTTC
AT

Gene: efp (translation elongation factor P)

Contig: 07_NODE_2, position: 105140 to 105697, length: 558 nt, orientation: REVERSE

Perfect match to: (MRSA252-BX571856-[1679252:1679809:r], highly conserved allele)

Sequence:

TTATCCTCTTGAAATGTAGCTTCCATCACCAGTGTTGATAATTAAAACGTCACCTTCGTTTACAAATAAAGGTACATTTAATGTATAACCAG
TTTCAACAGTTGCCGATTTAGTAGCACCAGTTGCAGTATCACCTTTAATACCAGGTTCTGTTTCAGTTACTGTTAATTCAACAGTTTTAGGT
AATTCAACACCGATAGTTTCACCTTCGTATGTTTGAATTTGTACTTCCATACCTTCTTTTAAGTAATTCAATTCTTCTTTTAAGTAATCACTTG
AAAGTTCTGTTTGTTCAAAGCTTTCATTATCCATAAATACATGATTATCTCCGTCAGCATATAAATATTGCATGCGACGATTTTCAATCATT
GCTGGTTCAACTTTTTCACCAGCTCTAAACGTTTTCTCTTGAATTGCACCAGTTCTTAAATTACGTAATTTTGAACGAACGAATGCTGAACC
TTTACCAGGCTTTACATGTTGGAAGTCTATAACTTTCCAAATAGCGTTATCAACAGAAATTGTTAAACCTGTTTTAAAATCATTAACCGAAA
TCAT

Gene: yqhT (putative aminopeptidase)

Contig: 07_NODE_2, position: 105723 to 106784, length: 1062 nt, orientation: REVERSE

Perfect match to: (Strain_21334-AGTW01000023-[38585:39646:r], allele observed in CC9+CC1+CC188+CC361)

Sequence:

TTATGTTAAAACTATAAGGTCTTTTGTGCATTTAGTAAAGACTTGACAACCATTTTCTGTAATTAAAATATCATCTTCTATTCTTATACCGCC
CAAACCTTCTATATAAACACCAGGTTCTACTGTAACACAGTTGTTAACTTGAAGTTTATCTTGTATCGTACGAGCCAGCATTGGCCCTTCAT
GGATTTCTAAACCAATACCATGTCCTAGTGAATGTCCAAATTCTTTTCCATACCCTTTTGACTCTAAATAGTTTCTTGAAATGGCATCAGCTT
CTGCACCAGTCATGCCAGGTCTAATCTCATTAATTGCTTTCATTTGAGATTCAAGTACTATTTGATATATTTCTTTCAGTTTAGGATCTGGTT
CTCCAATAGCAAATGTTCTAGTAATATCTGAACAATAGCCGTTATAATACGCGCCAAAATCTAATGTAATCATGTCGCCTTTTTCAATAATT
TTGTCACTTGCAACACCATGTGGTAATGCACCTCTATGACCAGATGCTACAATCGTATCGAATGATGGTCCATCTGCTCCTAATTCTAGCA
TTTTACTTTCTAATATTGCCTTTAATTCTTTTTCAGTCATACCTGCTTTTACAACAGTTAAAATATATTCATATGTTTCATCAACAATATTAGC
TGCTTTTTGAATTAAAGCAATTTCCTCAGCATCTTTGACGTCTCTAATTTTATCTACAGTATTAGAAATGCTTATTAATGATATACGGCTTTT
ATTTAATTCAAGGTATGTATCATAACTTACATGGTGCCCCTCAAAACCTACATTTTCAAAATTTTCTTGGTGTAGCAATTCTTTAATCTCACC
AATAATAGTAGATTTACGATTAATAATTTCATAATTTGGCGCTTGCTTAGTTGCTTGATCAATATATCTAAAGTCTGTTATCAAATATTGTTT
ATCTTTAGATATGATAAGTGCTCCACTGGTACCAGTAAAACCTGATAAATATCTTCTATTGTAATCCGAAAGAATGATAATCGCATCTAAA
TGTTTTTGTTCTAAAATACGATGCACTTGTGTTATTCTGCTCAT

Gene: Q5HFM8 (putative lipoprotein)

Contig: 07_NODE_2, position: 106889 to 107470, length: 582 nt, orientation: FORWARD

Perfect match to: (RF122-AJ938182-[1525433:1526014], highly conserved allele)

Sequence:

ATGAAAAAATTGGTTTCAATTGTTGGCGCAACATTATTGTTAGCTGGATGTGGATCACAAAATTTAGCACCATTAGAAGAAAAAACAACA
GATTTAAGAGAAGATAATCATCAACTCAAACTAGATATTCAAGAACTTAATCAACAAATTAGTGATTCTAAATCTAAAATTAAAGGGCTTG
AAAAGGATAAAGAAAACAGTAAAAAAACTGCATCTAATAATACGAAAATTAAATTGATGAATGTTACATCAACATACTACGACAAAGTTG
CTAAAGCTTTGAAATCCTATAACGATATTGAGAAAGATGTAAGTAAAAACAAAGGCGATAAGAATGTTCAATCGAAATTAAATCAAATTT
CTAATGATATTCAAAGTGCTCACACTTCATACAAAGATGCTATCGATGGTTTATCACTTAGTGATGATGATAAAAAAACGTCTAAAAATAT
CGATAAATTAAACTCTGATTTGAATCATGCATTTGATGATATTAAAAATGGCTATCAAAATAAAGATAAAAAACAACTTACAAAAGGACA
ACAAGCGTTGTCAAAATTAAACTTAAATGCAAAATCATGA

Gene: Q5HFM7 (putative protein)

Contig: 07_NODE_2, position: 107484 to 107702, length: 219 nt, orientation: FORWARD

Sequence:

ATGCGTAATATAATATTTTATCTTGTACTTATTATTGCTGCGATTGGATTAGTAATGAATCTAGATGCCTTTATTTTTTCAATCGTCAGAATG
TTAGTCAGCTTTGCTGTAATAGCTGGTATTATTTATCTGATTTATTATTTCTTCATCTTAACTGAAGACCAACACAAATATCGCAAAGCGAT
GCGTAAGTATAAAAGAAATCAAAGAAGAAAATAG

Gene: lipM (octanoyltransferase)

Contig: 07_NODE_2, position: 107766 to 108596, length: 831 nt, orientation: REVERSE

Perfect match to: (MW2-BA000033-[1616677:1617507:r], highly conserved allele)

Sequence:

CTACTTTCTAAACATCCATTCATCTGAACGATATTTTTCAGTTAATTCTTCCACTTCTGCCAATTGAGCTTCTGTTAATTCAAGTGGCTTTAAT
TCTATATTTAAACCTTTCTTAAAACCTTTCTCGAAAGCTTCTTCCATTTGACTAATAGTAATGTGTTTATCTGAAATATCATTGATGGCAACT
GCTTTTTCAACGAATGCCTCTTTCATTTTTAATTTTAATCTTTCATTTTTATAAATAAACATATCAAACAGTTCATCAATATCAATATCTTGTA
AAATCGAACCGTGTTGGAGGATTACGCCCTTTTGTCTCGTTTGAGCACTCCCAGCAATCTTACGGCCTTCAACAACTAGCTCATACCAACT
TGGTGCATCAAAACACACTGAACTTCGAGGTTGTTTTAATTTTTGACGCTCTTCAGGCGTTTTAGGTACCGCAAAATAAGTATTAAATCCT
AAGTTTTTAAATCCTTCTAATAATCCTTGTGAAATCACTCTGTACGCTTCTGTAACTGTAGAAGGCATATTCGGATGCGATTCAGGCACAA
TCACACTGTAAGTTAACTCTTTATCATGTAGCACCCCACGGCCACCAGTTTGACGCCTTACGAGACCAAAACCTTTCTCTTTAACCTTATCA
ATATCAATTTCTTTTTGTAGCCTTTGGAAATACCCTATTGATAATGTTGCAGGATTCCATGTGTAAAAACGTATAACTGGATCAATTTCACC
TCTAGAGACAAAATTTAATAACGCTTCATCCATTGCCATATTATAATATGGGTCTTTACTTCCTGTATTAATAAAATTCCAAGTTTCAGTCAT

Gene: yqhL (putative sulfur transferase)

Contig: 07_NODE_2, position: 108754 to 109140, length: 387 nt, orientation: FORWARD

Perfect match to: (RF122-AJ938182-[1527298:1527684], highly conserved allele)

Sequence:

ATGAGTGCTAGTTTGTACATCGCAATAATTTTAGTTATAGCAATTATTGCTTATATGATTGTTCAACAAATTCTTAACAAGCGAGCTGTTAA
AGAATTAGATCAAAATGAATTCCATAATGGGATTAGAAAAGCTCAAGTCATCGATGTTAGAGAGAAAGTTGACTATGACTACGGTCACA
TTAATGGGTCTCGCAATATTCCTATGACAATGTTCAGGCAACGATTCCAAGGATTAAGAAAAGACCAACCGGTATACTTATGTGATGCCA
ATGGGATTGCTAGCTATAGAGCCGCTCGTATTTTGAAAAAGAATGGATATACAGATATCTATATGTTAAAAGGCGGCTATAAAAAATGG
ACTGGAAAAATAAAGTCTAAAAAATAG

Gene: gcvPB (glycine cleavage system P protein subunit B)

Contig: 07_NODE_2, position: 109505 to 110977, length: 1473 nt, orientation: REVERSE

Perfect match to: (MW2-BA000033-[1618416:1619888:r], allele observed in CC1)

Sequence:

TTATTTTTCCTGTTTAAGATTTTCAAACTTTAATATTGGTTTACGAGCAGCTGTAGCTTCGTCTAATCGATCAATCACAGTTGTATGTGGTG
CTTCTAGCACTTTATCAGGATCATTTTTAGCTTCTTCAGCAATACTAATTAATGTATCGATAAAATAATCAAGTGTTTCTTTAGACTCTGTCT
CAGTCGGTTCAATCATCATACCTTCTTCAACATTTAATGGGAAGTATATTGTTGGTGGATGTACACCGAAATCTAATAATCGCTTAGCCAT
GTCTAAAGTACGTACACCAAATTCTTTTTGACGCACACCACTTAACACAAACTCGTGTTTACAATATTGTTTATAAGGTATTTCAAAGTGTT
TAGATAAACGTGCTTTAATATAATTCGCATTAAGAACCGCTGCTTCAGAAACCTCTTTAAGTCCAGTTGCTCCCATAGTTCGAATATACGT
ATAAGCTCTTAAGTAAATACCAAAGTTACCATAAAATGGTTTTACACGTCCGATAGAATTTTTAATGTCATTATCATATTTAAATTTGTCGC
CATCTTTAATAACCATTGGCTTTGGTAAGTAACTTGCTAGTTCTTTTACTACACCGACTGGACCTGAACCAGGACCGCCACCACCATGTGG
ACCAGTAAATGTTTTATGCAAGTTTAAATGAACAGCATCAAATCCCATATCTCCTGGGCGAACTTTGTCCATAATAGCGTTTAAATTCGCA
CCATCATAATATAATAGACCCCCAGCATTATGGACGATTTCACGGATTTCCATAATATTTTTTTCGAAAATACCTAAAGTGTTTGGATTAGT
TAACATAATAGCTGCTGTATTTTCATTTACAACACGTTTCAAGTCATCAATATCAACTTCGCCACGTTCGTTTGATTTTACAGTAACTGATTT
AAATCCTGCAAATGAAGCTGAGGCTGGATTCGTACCATGCGCAGAATCTGGCACAATGACTTCATCACGATGACCTTCACCATTATTCTCA
TGATAAGCTTTAAATATCATCAATGCAGTCCATTCACCATGTGCGCCAGCAGCTGGTTGTAATGTCACCTCATCCATACCAGTAATTTCTTT
TAATTCTTCTTGCAAACTATAAATAATTTCTAATGAACCTTGAACTTGGTCTTCATCTTGTAATGGATGTGATTCACTAAATCCTGGTATTCT
AGCAACCTTTTCATTAATTTTAGGGTTATACTTCATCGTACATGAACCTAATGGATAAAATCCGTTGTCTACACCGAAATTTTTATTTGAAA
GTTCAGTATAATGACGTACTAAGTCTAGTTCAGCAACTTCAGGAAACTCCGCTTTGTTTTTACGAATAAATTTATCATCTAACAATGACTCA
ACAGAATTTGTTTTAATATCACTTTTTGGTAATGAATATGCATATCTGCCTTCACGAGATCTTTCAAAAATTAATGGACTTGATTTACTAGT
CAT

Gene: gcvPA (glycine cleavage system P protein subunit A)

Contig: 07_NODE_2, position: 110970 to 112316, length: 1347 nt, orientation: REVERSE

Sequence:

CTAGTCATTTAACTCACCAGCCTTTTCTACAAATGTATCGATTTCATCTTTTGTTCTTAATTCAGTTACAGCTATTAACATGTGATTTTTAAA
GTCGTCTGAAACAACACCTAAATCAAAACCACCGATAATATTGTACTTCACTAATTCCTCGTTAACTTGTTGAATTGGTTTGTCAAATTTGA
CTACAAACTCATTGAAAGATGTACCATCTAATACTTCAAAACCTTTTTTAATAAATTGCTGTTTAGCATAGTTAGCATGTTCTATATTTTGAA
CTGCAATATCATAGATACCTTGTTTACCAAGTGCTGACATTGCAATTGATGACGCTAGAGCATTTAATGCTTGGTTAGAACAAATATTAGA
TGTCGCTTTATCACGTCGAATATGTTGTTCACGTGCTTGTAATGTTAATACAAAGCCACGATTACCTTCATCATCTTGTGTTTGACCGACTA
ATCTACCTGGCACTTTACGCATTAACTTTTTCGTCGTTGCAAAATATCCACAATGTGGCCCACCGAATTGAGCAGGAATTCCGAATGGCTG
AGTATCACCTACAACAATATCTGCACCAAATGAACCTGGAGGTGTAAGTAATCCCAATGCTAGTGGATTTGCATATACGATAAATAATGC
TTTTTTATCTTCAATAAAGCTATGAATCTTTTCAAGATCTTCAATTGAACCGTAGAAGTTTGGATATTGTACTGCAACAGCTGCTGTTTCAT
CATCCACTGCTGCTTCTAATTTTTTCAAATCTGTAACAGTGCCATCTAAATCGATTTCCACTACTTCGAATTCCTTACGCGTCTTAGCATAAG
TATGAAGTACTTGTAATGCTTGATAATGTAAACCTTTTGAGACTACAATTTTATTTTTCTTTGTTTGACTAAATGCTAAGATACATGCTTCA
GCAAAGCTAGTCATCCCATCATACATAGAAGAATTTGCTACATCCATATCTGTTAATTCACAAATTAAAGTTTGGAACTCAAAAATGGCTT
GTAATTCACCTTGAGAAATTTCCGGTTGATATGGCGTATATGCTGTGTAAAATTCTGATCTTGAAATCATAGCATCCACAACTGATGGCGC
GTAATGATCATAAACACCAGCACCCAAAAATGATGTATGCGTTTCTTTAGTGATATTCTTGCTTGCAATGCGATTTAATCTTCTAAGTAAC
GTTGTTTCCGCTTCGCCTTCAGCAATATTTAAATCTCTATTTAATAAAATGTCACTTGGTACATCACCGAATAATTCTCCTATAGATTTTGCA
CCAATTGTTTGTAACATTTCTTGCTTGTCTTTTTCAGTTAAAGGTATATAACGATGACTCAC

Gene: gcvT (glycine cleavage system T protein)

Contig: 07_NODE_2, position: 112336 to 113427, length: 1092 nt, orientation: REVERSE

Perfect match to: (MW2-BA000033-[1621247:1622338:r], highly conserved allele)

Sequence:

TTATTTATCAATTTGATTTTTCTTAACAATTTTCGCTTTTAATTGACGCTTACGAACTTGAACAAGCAACTCTCTACCCATTTCAAACTCATCT
CTTTTTATCATTGCAAGTGCAATTGATTTTCCTGATGATGGAGACTGTGTTCCTGAAGTTACTTCTCCAATAATATTTCCATCTAAATCCATA
ACTTCATAACCAGTTCTTGCAATTCCTTTTTCAAGTAATTCTAATCCCACTGTTCTTCTTGGTGCACCATTTTCTTTTTGATCTTTTAATACAG
ATTTACCAATAAAATCAGCATCAATTAATGGTTTACTTGCAAAAGCGATACCACCTTCATATGGTGTAATTGATTCAGTTAAATCTTGTCCA
TGTAATGGCAATCCAGCCTCTAATCTTAATGTATCACGAGCGCCTAACCCACATGGCATAACATTATACTCTAATAAACCATCCCAAATTTT
TTCAGTATCATCGATATTACAATAAATTTCAAACCCATCTTCACCTGTGTAACCTGACTGAGATAAAATGACGTTTGCTCCAAATAATTTGA

CACCCTGTTTAAATTCAAACATTTTCATTTCAGTTACATCTTCATCAACTAATTGATTAATTAAATCTCTAGCTTTTGGTCCTTGTATTGCTAA
TTGACCATATTGGTTTGATACATTTTGTACTTCAACATCAAATTTCTCTTTGTGTTTTAAAATCCAATTAAAATCTTTTTCAGTATTAGCAGC
ATTAACAACTAATAAATAATTGTCGTCAGCTAATTTATATATTACTAAATCATCAATAATACCGCCTTCTTCATTACATAAAGCAGTATATA
ATGCTTTTGAAGTAGTTAAATTATCAGTATCATTTGATAATAAATATTGCACAAACTGACTAGCATCTTTACCTGTTACTTCAATTTCACCCA
TATGACTAACATCAAACAGGCCAATTTCGTATCGAACAGCATTATGCTCCTCTTTAATACTTGAAAATTGAACAGGCATCGCCCATCCTCC
GAATTCCACAATTTTTGCACCTCTATCAACATAATTTTGATATAAAGGTGTTTGTTTTAAATCACTTGACAT

Gene: aroK (shikimate kinase)

Contig: 07_NODE_2, position: 113586 to 114110, length: 525 nt, orientation: REVERSE

Perfect match to: (CN1-CP003979-[1543752:1544276:r], highly conserved allele)

Sequence:

CTAATACTGATCACTCGCTTTTATTAAATTTAGCAATTCATAATATATTTCTGAAATTGATAGCAAATGACTATCAAATTTCTTGAATGCGA
TTTCATTATATCTTAAATTCCGCGAGCAATACAAGTCATTTAACTGCTTGATTGTCTTATTATTTGCATTAGGTCGATGTGGGTCATCATTG
ATTCGACTATATATAATATCAATATTACAATCTAACCAAATAATGTTTTTTTGATTTTTCAAAAAATTAAATGCCTCTTCACTCTCAATAATA
CCACCACCAGTAGCAATTATATCTGCAGTGTTAATACATTCTTGCAAACATGTGAACTCTAAATTCCTGAAATATTGTTCACCATGTTTACT
AAATATTTCTGGTATTGTTAACTTATACTTCTCTTCGATATATGAATCTATATCAATAAATGATAAATTTTGCTCATCTGCAACGTATTTACC
AATCGTAGATTTACCGGTACCCATGAAACCAATTAAAATTATTGGTGATTTATCATGATTCAT

Gene: comGF (late competence protein F)

Contig: 07_NODE_2, position: 114343 to 114840, length: 498 nt, orientation: REVERSE

Perfect match to: (MW2-BA000033-[1623254:1623751:r], allele observed in CC1+CC8+CC4803)

Sequence:

TTATACATAAATAGTTTTAGTCTGCACATTTGTACCGACTTTAACTGTTATCGTTATTTTAATAATGGATTTATAGTAGATATTTGCAGTAAA
TGCTGTAACATTATTAATCATTGTTATATTTCCTCTGTCATTTACAACTTTAATAATTTTATTATTAATTAATTTGTATTCGATCATTTCTTCAC
CTTTATGTAAAATGATACGGTGTTGCCTAATTTCAATATCGTTTCTATCTACTCCTTTAAAATCATCTAGAATATCTCTTGAGAAAAATTCGA
AATCTACAGTTGTTAAATCCCTACTTTCAATTAGAAAAGTTTTACTAAGTCTAATTAAGTCTGGAACAATTAGTAAAGTTATACTTATAACC
ATCATCGCTACTAACATTTCAATGAGCGAAAAAGCTTTGACATTAATACTGTATACATGTCTTTTGATAAGAAGTGGTATTTTTTGAAATA
GCACAAATTTGTTGGTCACTTTGCTTAATATCAT

Gene: comGE (late competence protein E)

Contig: 07_NODE_2, position: 114758 to 115057, length: 300 nt, orientation: REVERSE

Perfect match to: (COL-CP000046-[1631200:1631499:r], allele observed in CC8+CC1+CC8+CC239+CC4803)

Sequence:

TTAATACTGTATACATGTCTTTTGATAAGAAGTGGTATTTTTTGAAATAGCACAAATTTGTTGGTCACTTTGCTTAATATCATACTTCCCTAT
AGTTACCCCCTTCTTAAGTTCTTCTTTATTAATTTTAGATACAGTCGTCAAAATTACTTTAGAAGCATCAATTGTTTGTAGTTTATGGTTTAT
ACTCGCTTGCATTTGATTCATCATTGGTATCAATAGTAATGTAATCAATCCAATTAGCAAAAATCCAGCCATACTATCTATTAAGAATGAAC
CTTTACACTTATAGCTTTTCAT

Gene: comGD (late competence protein D)

Contig: 07_NODE_2, position: 115044 to 115490, length: 447 nt, orientation: REVERSE

Perfect match to: (MW2-BA000033-[1624000:1624446:r], highly conserved allele)

Sequence:

TTATAGCTTTTCATAACGAATTCTTCCTTTTTCAATATGGAATATTATTCTATAAATTGAATTGTTATTGTCAATTGTTATGCTACCAAATTTA
TTGATATTCCCTTTTTTATCAAAGGCAATAATATCAACTTTTGCAACATTAATTATTTTGCCTACTTTTAATTTTAGAAATCGTATTTTATTAT

TCTCTATTACTTTAATAGTGTCACTGTTTTCATAAAATCTAACATTGATATATCCTTGATTTGCTATAGCTTGCGACTTAATATAATTCAATTC
AGTAATAAAAGAAATGATATTTGCCTCATCATCTATTACTCTAAGATTGCTTAATCCTTTAGATGTCATTGTCAAAAGTAGAAATATACTGA
TTAACATCATTACCACAAGCATCTCAATCATAGTAAATGCTGACTGCTTTCTAATTTGCAACTGCTTCTCCAT

Gene: comGC (late competence protein C)

Contig: 07_NODE_2, position: 115468 to 115779, length: 312 nt, orientation: REVERSE

Perfect match to: (N315-BA000018-[1578832:1579143:r], highly conserved allele)

Sequence:

CTAATTTGCAACTGCTTCTCCATTACTAATTGTGATTGTCTCTCCTGATTTACATGTCTTTTGTGCTTCTTTTATAAAACCATCTGCAATTAAG
TCTTCAATAGACGATGGATTTCTATTATGTTTCAATGCATACGCTTCAATTTGACTATTAACCATTTTTACCTGTGCATTACAACCTGTTGAT
TGTATGTGAGCAGTTTGTTTAGCAATATTTGGAATGATTAAAATTAATAATAAACTGATGATTAATAACACTAATAGCATCTCTATCAATG
TAAACGCTTGAGTTTTCTTAAGAAATTTAAACAT

Gene: comGB (late competence protein B)

Contig: 07_NODE_2, position: 115793 to 116863, length: 1071 nt, orientation: REVERSE

Perfect match to: (N315-BA000018-[1579157:1580227:r], allele observed in CC5+CC1)

Sequence:

TTATTTTATACTTTGCATCATTTGAAACATTGGTAACATAATTACTAAATAAATTGCGACAATAAATAAACCTAAAATCAAAAATAAAATAG
GCTGTAAAAACTGAGTCTGTTTTATCGCTTTATCTTCTATTTGTTTTACTAATATTTGTGAATATAACTTTAGTTCTACTTCTAGCTTCCCTCT
CTTTTCACCTTGTAGCACAAACTTAATTAATTGAGGCTTAAAGCATTTTAGTTTTTCTAAAATTTGAGGCAAACCATATCCCATTTCTGAAT
AAGTTAATAAGTATTTACCTAGAAACTGTCTAAATGGATCACTACTATGGTTAATATAAACGTCTACTATTGATTGAAGTGTAATACCATTT
TTATAAAACAACACTAATTCATTAGTTACAAAATAAGTTTTAAATAATTGGAAATAGCCTGATATTAGCGGTAGTTTCATCACAAAGTTTAT
CTTATTGAGCATATTTAAATTGTTATAAATTAATTTCATAATAATAGCCAACATAGATACTATTATGAGCATTACTACAATTATAGTAGGTA
AGCTGGTAATGAAAAAAGACAATGTTTTTTGAAAAGAAGATAGTTGAATATTCATAGAAGTATATAATTGTTGAAACTGTGGAATTACTG
TGAGGTTTAATATAATAATCATAGCAATAAAGATAGAAACTAGTATTAAGGGGTATTGCAGTGTCTTTAACAATCGTTGTTCTGACTTTCT
ATTCACTTTCATATAATTTACGGTTTCTTCTAGAACGTCTATAATATTGCCAAATCTTTCTGCCAAATATACTTGCATGACGATAGTATCGCT
ATAACCTATCAGTGATAATATTTGATTGCATGGTGCACCATTTGAAATTTCACTTAGAATGGTGGTACCTAATTGCTTATTTTTATATGTCA
TTTGAAGATTTAAAAATTGAAAACTTTGATACAGAGTGAAACCATATTTCAACAAATTACATAAATTTGAAAGTAAATCGATTTGTTGGGC
CTTACTTAATTGTCGCTTCTTAGAATGTAGTTTAAATGTATTTATCCATTGTAGTTTCAC

Gene: comGA (late competence protein A)

Contig: 07_NODE_2, position: 116835 to 117809, length: 975 nt, orientation: REVERSE

Perfect match to: (MW2-BA000033-[1625791:1626765:r], allele observed in CC1+CC30)

Sequence:

TTAAATGTATTTATCCATTGTAGTTTCACAAATGACACCTGCTTTTGTCATATCATCAAGTTTATCTTCTAAGTTCTTAAATGATGATGGTAA
TGAATGATTATGGGAAAAGAAATATCGGAGTTGTTGCTGAGATAGAATTTCGCATACTAATTGTCGCTGTTGCTTAATAGTAGTTACAAG
TCGTTGGTTTATAATTAAGTTAGTTGCCTGTATCAATTCTTGTACAGAAATGCCCATTTCTAATAGCCTTAAAATAGCACCTTTACAATCAG
TTGCATGCAATGTAGTCAGAACAAGGTGACCACTTAAACTAGCCTGTATAACACACTTGGCAACATCTTTATCTCTGATTTCACCTATTAA
AATAACATCAGGATCACATCTTAAAATAGCTTTAAACGAATTTACATAGTTAATGCCAGCTTTATCATTCACATTAATTTGGACGATACCAG
GAATTTGCATCTCTACAGGATCCTCTATAGAAATTACATTTAAATTCAAGGCTTTATTCGCGTATGAGACCATTTGATACATTAATGTACTC
TTTCCTGAACCAGTTGGCCCACTAAACAATAGTAATCCTTGTTTCTTATTCATGAGGTGTTTAAAATCATTGAATTTATAAGTTGATTTTTGT
TGTTGAAAAAATTGAGGTACAATTCTGATAACACAACTTTCTTGGCCAAGTGACAATGGTAAAGTTGATATTCTCAAAAAATATATTTTAT
TGAAATGGTAACTATATCGACCGCTCTGTGCGACTTGCTGTGTAGAAACATCAAGCCCAGCTTGAAACTTCATATAAACTAATAACTTTTG
ATAAATGCTATTCCCAATTTGTTCATACTGCTCCAAGTTATCATTAATTCTAAATTTAATACTTACTTCATTTTTAACTGGAATAAAATGTAC
ATCACTCGCTTTCATTTCTATCGCTTTATTAATTATTTCTTGAAATAGAATCTTCAA

Gene: yqgX (putative metal-binding hydrolase)

Contig: 07_NODE_2, position: 117861 to 118484, length: 624 nt, orientation: REVERSE

Perfect match to: (MW2-BA000033-[1626817:1627440:r], allele observed in CC1)

Sequence:

TTAACCGTGTAAAAATGGATTTAATTGTTCATCATCAACCGTCGTATATGGACCATGTCCAGGGAATAAAGGTAAATCGCCTTCTAATTCA
AATATTTTATCTTGAATAGAATCAACTAGCGTTTCATAATCACCTTTATATAAATCTGTACGTCCGATTCCATTATTAAATAATGTATCTCCA
ACAACTGCGAATTCATCGAACACATATGTTAAACTTCCTGGTGAATGTCCAGGTGTGTGTAACACATTAAACTTAAATCCTTCTATTTCTGT
GCTACCTTCGTTTAACTTTTCAGGAGTTACCTTACTTGTAATAATTGGTAATCCATATTGCTTAAATTTATCTGCCCCATTTTTAACGGGATC
TTTTAGAAAATCAAACTCTGCTTCATGCATATAAACCGGGACATCGAATCGATCAACTATATCATCGACTGCTCCGATATGATCAAAGTGT
GCATGTGTTAATAAAATAGCTTTTAACGGTTTATTTATTTGGTTTAATTTTTTAATAATTTTTCCACTTTCACCTGAAGGGTCAATCAGAATA
ACAGCTTTGTCATTTTCGATGAAATACGTATTAGTATCAACTAAGCCTAAAGTTAAGCTTGAAATCCTCAT

Gene: yqgV (putative protein)

Contig: 07_NODE_2, position: 118481 to 118810, length: 330 nt, orientation: REVERSE

Perfect match to: (N315-BA000018-[1581845:1582174:r], highly conserved allele)

Sequence:

TCATAGGTTTTCACCACTATTTTCTAAATGTTTTTGTACTGATGTTAGTTTATCATTCATTTTTCTAGATTTGTCTCGTCGGTCATCAATACGG
ATATTTGTACAAACTCTACTTAAACCTTTATCAAAAGGTAATTCATGTATCACTTGCACAACTTCTAATACATCGCTTAATTCACCTTCAATT
AGAGTATTCATTGGTGTTAATTGAAAATCAATTTTACCCATTGCTTTATATTCTTGAAGTTTTTTCTGAATATCTGCAATATATTTACTAACA
CTCGGACCTTCCGTTCCAACTGGAATAACAACCACATCAACAATAGCCAT

Gene: glk (glucokinase)

Contig: 07_NODE_2, position: 118810 to 119796, length: 987 nt, orientation: REVERSE

Perfect match to: (N315-BA000018-[1582174:1583160:r], highly conserved allele)

Sequence:

TTATTTTACCCCCTCTTTATCTAATACATAGGTCTTGATTAATCCTGCTGCTCCTGTAATACCTGCATCATTACCTAATTTCGCTTGTACAATT
TCAGTTTCAAATTGAGCAGGTGCAAATGTTAAATTATGATATTCTGTTTTAATATTTTCAATTAAAATAGGTCCTGCAGTAGACATTCCTCC
ACCTAGAACGATATATTTCGGATTACTTGTAACACTAATAATACTACATAAATATCCAATATAGTTTGCAACCTTTTCAGTAATGAAAATAC
AGAATTGGTCACCAGCTTTTGCCGCATCAAAAACAGCTTTTGCTGTAACCTTATTTTCTTTAATCAATTCTAATATAGAAGATCTAAACGTC
AACTTCGGATAGTAGAAGTTAACTAAGTTAACAACGCCTGTCGCTGAAGCAACTGTTTCAATACATCCAGAACGACCACAATTACATTTAA
ATCGTTGATCGAAGTCTGCTCTAAAATGACCTATTTCTGCGCCAGAGCCATTATGACCATGTACGATTTCACCATTTGAAATAATTCCTCCA
CCTAGACCTGTACCAAGTGTGATGGCAACAACATCATCGGCACCTTCACCAGCACCTTTGTGTTTCTCCCCTAAAGCAGCTATGTTAGCAT
CATTATCTACATACACTGGACAATCAACGAATTGTTCAAAAATCTCACGTACATTAACTTTTTCTGGCCAATATAAGTTTACTGCTCCATTT
ACTGTACCTTTTTCAAAGTCAACAGGACCTGGTACACCAATACCTACGCCAAGTACATTTGAAAAATTATAATTATTTTCATTTACTTTTTCA
ACAAACGAATCATAAATTCCTTTCAAAAGTGTATATCCTGTACTATCAGATGTATCAGTGTGAATAGACCATTTATGTAATTGTTCTAATTC
AGGTGTGAAAATACCTAATTTACAAGTCGTCCCGCCTACATCAGCTGCTAAAATAATTTTTGTCAT

Gene: yqgQ (putative single-stranded nucleic acid binding protein)

Contig: 07_NODE_2, position: 119793 to 119996, length: 204 nt, orientation: REVERSE

Perfect match to: (RF122-AJ938182-[1538336:1538539:r], highly conserved allele)

Sequence:

TCATTTCTGTTCATTCCTTCTCTGATTAATTATCAACGTACATTTCAAATATTCTTCCTTAGATAACAGTTCATATTGATACAATGATGAAAT
CTCCTGTTGAATCATTTCGTACATATCTTCTGGATTTTTAAAATATATTAGAAATCCGTAACTTTTCAATAACTGTTGTACATCATAAAAGTT
ATTTATTTTTCGACTCAT

Gene: gluP (transmembrane rhomboid domain peptidase)

Contig: 07_NODE_2, position: 119977 to 121440, length: 1464 nt, orientation: REVERSE

Perfect match to: (MW2-BA000033-[1628933:1630396:r], allele observed in CC1+CC25+CC30+CC772)

Sequence:

TTATTTATTTTTCGACTCATTTGATTTAGTCAACTCTTTTTCTAAGTTAATATAATCTGCATTTTTAGGGTCTGCATTTAATGCTTTACGCACA
TATTTTAATGCTTTTTCATCATCATTTAATGAACGATTTGCTATCGCTAACTCAAAATTTAATAAACCTGATTTAGGAAACATTCTAAGTCCT
CGCTCCCATTCTGTCATACCTTCGGACTTAGAATTGATAGTAGCCATAATCATACCACTTAAATAATATGTTTGATCATCGGCATAATTTTT
ATTTATTGTCTGCTTCACAATATTTTGAGCATTATCATAATTACCACTAGTCATATCATCTTTGATCAATTTATTATAAATATTATCTTCTTTA
ATTGTAAAAATTCTAATTTGAAGTGCAATAAATATAACAAGCATACCAATTAGTAAAATCCAAAAAATATTACGATTCACTTTATAGTAAT
AGCCAATTAAAGTTATTAATAAACCACCAATGAATCCTCCAATATGCGCCACAATATTTATATTTGACATAAACAGAGAAACACCAACTAA
TATCACTAATGCAATTAATAACTGTCCTAACATTTTTTTGTTAAATGTTTTTGAAACATACATCATCGCAAAAATTGATCCAATCAGACCAA
ATATAGCACCACTAGCCCCAACTGAAATTGTAGTCGTATTAAATGATAGTGATACAAAGTTTCCAAACAACCCTGCAATAAAGTATACAGT
TAACATCCGCCATGAACCAATAATTGCTTCGACTATTTTACCAAAAATAAATAATGAAAGCATATTCATAAGTATATGTTCAAAACTAAAA
TGTAAAAACATCGATGTAACAATTCGATACCATTCACCATGTACGACATTAAAATGCACTAACCCGCCAACATCTAATAATTTTACATCCG
AAAAATTATTTAAATATAAAATCATACATAACCATATTAAGACATTTACAAATATTATTGTGTATGTTGCCGGTGAAAATTTCTGCATATAT
TTATCTAAAAAGTTATCGGTTAATACTTTGCGTTTATAGAACATATATGTCTTTTTATCATTATCTTCTGAAATAAGTCTAGCCAAAAATATA
TTCGGCATATGCTTTATCAAATCTTTTGTGTGTCTTATAACATTGAATTTAATTCTAATTGGCGAAACTTCATTTAATTGTTCTTCTGAAAAT
TCCGATTCTGTAAAATAATAAAATTCAAAACTTTGTGGTTCGAAAGAAATAAAATTTGCTATTTCATCTTTGTGTTCAAGCACTTTCGCTTT
GTCAAAACGTATTTCTTGGGTAGACTTGATATGTTGTTTAAAAATGACTACTTGTTTTTTTCTTTTATGGGCTAACCAAATTTCTTGGTCATC
TTTTTCCCTACTGACAATATCAAAATTCAAGTACCTAATCCAATAATATATTGTTTTCCAAAATTGTTTGTCTATGTTCAT

Gene: yqgN (putative 5-formyltetrahydrofolate cyclo-ligase)

Contig: 07_NODE_2, position: 121452 to 121991, length: 540 nt, orientation: REVERSE

Perfect match to: (MW2-BA000033-[1630408:1630947:r], highly conserved allele)

Sequence:

CTATGCTGATTGATATATAATCAATTTATCGACTGGTTGATCGAATGATTCCGGTTCAAATGATGTTATTTGAAAATCGTATAATAAGCTTA
TTGTCTTTGTCTGATAATTAGCTAAAAACCTGTCGTAATAGCCACCACCATACCCAATTCTATATCCATCGTCTTGAAATCCAACACCAGGA
ACAACAATTAAATCTAGGTTATTCGTTGTTTCACCTTTTGAAGTTGGATAGTAAATCCCCTTATTATCGACATCAATATCTTTGAGATTAAA
TATTTCTTTAAAAGTCATTTGATGATTTAAATAATCCATTTTCGGTACAAAAATACGTTTATGATCCATTAAGGCTTGTTCAATAATAGAAA
AAGTATCTACTTCATGATTAAAAGAAAGAACTAGCGCAATTGCGTTTGCTTCTTTGTATTCTTCAGTTGCAAAAAATTGATTTCTTAACCAT
GTGTCTGCTTTTCGCTTTTCAGCTTTATTAAAATTCTTCATTTTATGTAAAATGTATTTTCTAATCTCATTTTTAGTCAC

Gene: rpmG1 (50S ribosomal protein L33, locus 1)

Contig: 07_NODE_2, position: 122200 to 122349, length: 150 nt, orientation: REVERSE

Perfect match to: (RF122-AJ938182-[1540743:1540892:r], highly conserved allele)

Sequence:

TTATTTTGTTTCACGGTGTAAAGTTTGTTTGTTTTCACGTGAACAGAATTTCTTCATTTCAACACGTTCTGGATTATTTCTTTTGTTCTTAGTT
GTAATGTAGTTTCTGTCACCACATTCCGTACAAGCTAAAGTTACGTTTACGCGCAT

Gene: pbpC (penicillin binding protein C)

Contig: 07_NODE_2, position: 122462 to 124537, length: 2076 nt, orientation: REVERSE

Sequence:

TTATTTGTCTTTGTCTTTATTTTTATCATCTTTACCTAACTGCTTAAAGTAGTAGTTAATTACATCTCTACCTAAGTCTCCACCTGTTAACCAT
GGTGGTGGTACAGGCTGATTTGTATATACAATTGAAAACGCTAATTTTGGATCATCAATTGGCGCGTATCCTATATAAGTAGAGTTAACT

CTTGGCTCTCCGTTTTGGAACACTTCAGCGGTACCCGTTTTACCAGCAGTAGGTACTACTGTATCTTTAAAACTAACATATCCAGTACCATC
TTTATCATTAAATGCCATTTTGAATCCTTCTTGAATTTGTTTGATTTCCTTTTCAGTATTATTAACCTTGTTCAAGACAGTGCCATTAATTTTC
TTCTTGAGTGGACCAACCTCATCTTTATTAGTTGATTCATGAATCGTTAATCCAATGTGTGGCTGTATTCTATAACCATCATTCGCTATAGTT
GAAACATATTGTGATAATTGTAATGGTGTATAGGTATCATATTGACCAATTGATAAATCTAGATAATTACCTGGATTATTTGTTAATGGTT
CGATTTGACCTCTTGTTTCATTTGGTAAATCTATCCCTGTTTTCACACCTAAGCCTACTTGATTTAATCCTCTTCTTAGCTTTTGGGCAGGTG
AACTTATGTCTGAAGGTAAAGCCATACCAGAATAATAAGGGTCTCCCGCTAATTTTAATGCTGTTTTAAACATATATACGTTTGATGAATG
CATCAAAGCTTGCTTATCATTAATAGATACATGCCCGTTTTTATTGAAGTATGATCGTTTTGTCAAACCACCTTGGAAATGTAATGGTTCAT
CGACCATTGTTTCTCCAACTTTGATAGCTTTATTCTGATAACCAGCTAATAATGTTCCACCTTTTACAGAAGATCCAACCGCAAATTGAGAA
GTAAACGTACCAATGTCATAATCAGTCATTTTACCACTCTTATTAATCTGCTTTCCGGCAAGCGCAAGAATGTCTCCATTTTTAGGATTTTG
TACAACCATCATTGCATTATCCATATCTTTGGCACCTTGACTGCGAAGCTTCTTAATTTGTTTATCTAATAATGCTTCTACTTCTTTTTGAAG
ATCTATATCGATCGTTAATTTCAAATCTTGACCGCGAGCGCCAGGATTTAACACTTCTGAAGATGTAACTTTACCAGATTTGTCCGTTGTGT
ATTTCATTTCTTTCTTCTTACCACGCAATACATCTTCATATTGATATTCTAGGTAAGATTTTCCAACACGATCATTGCGTGAATATCCTTTGG
ATAAGTAATGTTCTGTCAATTCTTTTGGAATACCTTCAGCAGGTGTCGATACATCTCCGAATATACCTCTTAAAGTATCGCCATATGGATAT
TTTCTATCCCAATCCATAGACGTGTTAACACCTGGTAATTTGGAAAGTTGCTGAGAAACTGCTGCATACTCTTTTTCACTGACATCTTCATT
TTTTATCATTTGTGGATCTAAAACTGTTCCTGCATTCATCTCTCGAAAAATAGCTAAAACTTGTAAATCTTTAGAAGACAATTCATCTAATT
GTGATTTTCTGATTTTCGATAACAGTTGTTTATCATATTGATCTTGTTTAATACTTCCATCTGCTAACATACCTTGTTCTTTTGTCATCATTGC
TTTTGCTTTTTTAGGATGCAACTGAATCCAGAAATCTTTCTTATCACGTTCTGTAATTTTCTTAGTATCCATCTTGATTAGCTTTGATAACTTT
TCAGCCGTATCCAACATTTCCGATTGTGTTGTTTTTCGACCCCTAGTATATGTAATAGCCATTTTAGAAGCATTATCAACTAAAACTTTCCC
ATTTCTGTCTAAAATACGACCTCTTGGCACAGACTCATTCACTGTAATGTTTTCATCATTTTTTATAATTTGTTTATAATGTGAGCCTTGTGC
GATTTGTAAATAACCTAAACGTAGTACTAGTACTGCAAAAATAAATACAATCACACCAAATATAAAGTTAATTCTCTTGTTAATTGTATTTT
GAACGATTTCATCATTTGATTTTTCTTTTAGTCTTTTTAACAA

Gene: sodA-L2 (superoxide dismutase, locus 2)

Contig: 07_NODE_2, position: 124658 to 125257, length: 600 nt, orientation: REVERSE

Perfect match to: (MW2-BA000033-[1633614:1634213:r], allele observed in CC1+CC7+CC9)

Sequence:

TTATTTTGTTGCATTATATAATTCGTCAACTTTTTCCCAGTTAACTACATTCCAAAATGCGCCAATGTAGTCAGGGCGTTTGTTTTGATATTT
TAGGTAATAAGCGTGTTCCCATACGTCTAAACCTAAAATAGGTGTTTTACCCTCAGTTAATGGATTATCTTGGTTTGGTGTAGTCACAATTT
CTAACTGGCCATTGTTTACGACTAACCAAGCCCAACCTGAACCAAAGCGTGCAGCTGCTTTGTCAGCAAATTCTTTTTTAAATTCTTCTAAA
GAACCCCATTGTTCTTTAATTTTTTCTACTACAGTACCTTTTTCTTCTGAGTTTGGTGAAAGTAACTCCCAGAATAATGAATGGTTTAAATGT
CCACCGCCATTATTACGTACAGCAGTTTGGATGTTAGCTGGTACACTGTCTAAATTAGCAACAATTTCTTCAATAGATTTAGATTCTAAATC
TGTACCTTCTACTGCAGCATTTAATTTCGTAACATAAGTGTTATGATGTCTGTCATGATGAATTTCCATAGTTTCTTTGTCAAAATGTGGTT
CTAATGCATCAAATGCGTATGGTAATTTTGGTAATTCAAAAGCCAT

Gene: zur (zinc uptake regulator)

Contig: 07_NODE_2, position: 125533 to 125943, length: 411 nt, orientation: REVERSE

Perfect match to: (MW2-BA000033-[1634489:1634899:r], allele observed in CC1+CC15+CC97)

Sequence:

TTAATCTTGGCAAGACTCACATACACCATAAACTTCAAGTTTGTGTTTGTGAATATTAACACCAGGTAGTGATAACTTTATCTGATCTATTG
GACAATAATCTATTACCTTTGTATCTCCACACTTTTCACAGATAAAATGATGATGATGATGGTTTGTACAAGCGATTCTAAACTTCATTTCA
CCATCAAGTTCTGTATTTTCAATAATCCCTAAATCTTTAAATAAGTGCAGGTTTCTATATATTGTGTCGAATGAAATTCCAGGATAATTTTC
ATCCATAACTTGCTGTATATACTTTGCGTTTATATACTTATCTTCTTCGACAAAAATATCTAACATATCTTTACGTTTATCTGTATATTTTAAA
CCGTTCTCTTTTAAAATTTTAATAGCATCATTTGTATTCAT

Gene: znuB (zinc ABC transporter, transmembrane permease)

Contig: 07_NODE_2, position: 125930 to 126763, length: 834 nt, orientation: REVERSE

Perfect match to: (MW2-BA000033-[1634886:1635719:r], highly conserved allele)

Sequence:

TCATTTGTATTCATTGATATTAGCTCCCTTTTTAAACTTCATTCGCATTTTCTGATAAGCCATTGTAATCATAAGTAAAATAACAAGTAGAAC
TACAATTACACCACCCGGAGAAATGTCCATATAGAAAGCTAGGACTAAGCCTAATATTACTGATAATTCACCTAAAAATACACTTAGTAAT
ATCAATTGCTTAAAACTTTTTGTTATTCGCATACTTATTGCAATTGGTAACGTGATTAACGCACTTACTAACAGTATCCCTACAACACGCAT
TGAGGCAGAAATAACCATCGCTACAATAACAATAAATAAAAATTGAATCCATTTAGGAATGCCAATGACTTTACTATATTCCTCATCAAAT
GACAATATAAATAATTCTTTATAAAACAATGTAATAAACAGAACAACTATGATGGCAATGACAATAATCGTTGTTAAATCACTTATATTCA
CTGCGCTTATTGAGCCAAATAGCAATCCAACAATTTCTTGATTGAACCCATCAGCTAATGAAATGAAGATTGCACTCAAGGCGATACCAG
CACTCATTATAATTGGAATAGCAATTTCTTGGTAAGCAGTGTATGACGTTCTTAATTTTTCAATTAGAAGCGCACCTACTATTGCGAATAA
GATTCCAAACCACATTGGATTAATAAATACTAGTGTTGGCATAATAGTAAGTAAAAACATACCGAAAGATATACCACCTAAAGTTACATG
ACTTAGAGCATCAGCTATAAGTGATAGTCGTCTAACAACGATAAAAGCACCGATTAGAGGCGCAATAAAACCTATCAAGATACCACTAAT
TAAAGAGTACCTCAT

Gene: znuC (zinc ABC transporter, ATP binding protein)

Contig: 07_NODE_2, position: 126835 to 127620, length: 786 nt, orientation: REVERSE

Perfect match to: (MW2-BA000033-[1635791:1636576:r], highly conserved allele)

Sequence:

TTAATTACAACATTCTCGATTATGCTGATGATCGACAAAACGTACAGGATGTCCATAAATTTTTGAAATTTCAACTTCATCAAGTGATTTAA
ACTCATCAGTTGTACCATGGAAATGCAAATGCTTATTTAAACATGCTACTTCAGTAGCAGTATCTGCTACAACACCGATATCATGAGTAAC
TAAGATAATGGTGATACCTTCTTGTTTTAATTGATCTAAAGTATTATAAAATTCACTTACATGTTTTGCATCAATACCATTCGTTGGTTCATC
AAGTACTAATACTGCAGGTTCTGAAATCAATGCTCGAGCAATCATTACACGTTGTTGTTGACCACCTGATAATTCTGCTATATTTTTATGAA
TTAAATCACTTATATTCAGTCTTTCTAGTACTTTAATCACTTTTTCATTATCTTTGCTATTAAATGTTTGGAAAAGACGTTTTGTCTTTGTTAA
TCCGCTTAAAACAACTTCTTTAACACTTGCTGGGAAACCTGAATTAAAGGCATTTGCTTTTTGTGATACATAGCTTAATTTAATTGATGTTT
TCTTATTTTTAAAATCAATACCTTCAACAAAAATCTCACCACTTTGTAAAGGTAATAACCCTAGAATCAACTTCAATAATGTTGATTTACCA
GCACCATTTGGTCCAACAATTGCTAAAAATTCACCTTTATTTATTTTAATGTTTATATTTCTAACACTTTTTTATGATCATAGTAGTAATTGA
CATTTTTCAATTCAAAGACTGGTGTCATCGTATTCTCACCTCGCAT

Gene: nfo (endonuclease IV)

Contig: 07_NODE_2, position: 127746 to 128636, length: 891 nt, orientation: REVERSE

Perfect match to: (N315-BA000018-[1591110:1592000:r], highly conserved allele)

Sequence:

TTATTGTTGCATAACCTTATTTTTTAATTCTGGGTCAAATTGCTGTTGTTTTAACATTTCAATTTCAAGTTTATATGGCGGTTTTTTATTTTTC
TTATCTTCACCAACATAAGGTGTTTCTAAGATTTTCGGAATATCTTTAAAACTATCATGATGCACAATGTAATTTAATGCATCAAAACCAAT
GTAACCGAAGCCAATATTTTCATGTCGGTCTTTTTGAGCGCCACGGTCATTTTTAGAATCATTGACATGAACAACTTTGATTCTGTCGACTC
CAATAATTTTATCAAATTCATTTAATACGCCATCAAAGTCCTCTTTCACATTATATCCAGCATCATGCGTATGACATGTATCAAAACATACT
GATAAACGTTCGTTATTATGAACTCCATCAATAATACGTGCTAACTCTTCAAACGAGCGACCAATCTCTGTACCTTTACCTGCCATCGTTTC
AAGCGCAATACGTACATTATTGTCATTCGTTAAAACTTCATTTAATCCTTCAATAATCTTATTAATTCCGGCATCAACACCAGCTCCAACAT
GCGCACCTGGATGTAATACAATATCTTTAGCGCCTATAGCTTGCGTTCTTTCAATTTCTTGTTGCAAGAAATCTACACCAAGATTAAACGTT
TCTGGTTTGGTTGTATTTGCAATATTAATGATGTATGGTGCATGAACAACAATATTAGATAAGCCATATTTTTCCATCACTTCATGACCTTT
AGTTATATTTAAATCTTCAATACTTTTACGGCGCGTGTTTTGAGGTGCACCAGTATAAATCATAAATGTTGTTTCACCATATTCATACGCTT
CTATAGCAGAACCTTCTAACATCTTTTTACCACTCATTGAAACATGTGATCCTAATAACAT

Gene: cshB (DEAD-box ATP dependent RNA helicase)

Contig: 07_NODE_2, position: 128646 to 129992, length: 1347 nt, orientation: REVERSE

Perfect match to: (Strain_21193-AFEG01000006-[406:1752:r], highly conserved allele)

Sequence:

CTAACCTTTTTTGTTTTGCTTACGTTTTTGTCTATTTTGCTGCTTACTAAATTGTTTACGCTCTTGACGTTTCATTTTTTCAACTTCTTGTTTAA
ATTTCTTCTTATAACCTGGTTTAACTTTGTTTTTAATTTTACTTCGAACTTTGTTCTTCACTTGATTAGTTAAATGGTCATCTTTGCGCATTCTT
GCTTGACGCTGATTGTGCGCTTTAACTTCTTTTAACTCACCATCTTTAATATCAACAGTATTGAATACAAAACCGCGATCTTCTATTAATGA
AATATTGTGTTCTTCATCAGGACTATAAAGCGTAATTGCTACACCTTTATAATTCCCACGACCAGTTCGTCCAACTCTATGCGTAAAGAAGT

CAATATCATTTGGCACATCAAAATTGATGACATGACTAACACCTTCAATATCAATACCACGAGATGCTAAATCGCTGGCAATAACGTATTG
GAATTCTAAATTACGTATACGTTTCATTTGTTGTTTACGTTCACGTGGCGTTAAACCACCATGAATCATACCAACTTTAATACCAGCTTCAT
TTAGTGAACGTGCTAAATCATTTGCATTATCTCTACTATTACAGAAAATAATACATAAGTATGGATTTAGTATATCAATTAAATTTAAAGTT
TTTTCAACTTTAGCTGCACCTTTAGTAGGTATTAAAAAGAATTCGATGTTCTTTTTATTTTGTTTTTTACTGTCGACAGCTACATATTCTGGA
TGACTTAAATATTTATTTAAAAATGGTTGTAACTGTTGTGGAATTGTAGCACTAAACACCGCAATATTTGCATTATCTTCCAATCTTGCAGC
AATGTAATCTACATCTTCAATTAATCCTAAGTCAATCATAAGATCCGCTTCATCAATAACTAAATATGATGCTAAGTGCACATGTAAATGTC
CCGTTTTAGCTAAGTCATTAATTCTAGTAGGGGTGCCTATAATCAATTGTGGTTGTGCATTACAACGTTGTCTATCTTTCTCTATATCTGTA
CCACCAATAAAAACTTTAACTGAAACACCAGCTTTAAATTGGCTTAAATGGTTCGCTGCATCGTATAGTTGTTGTGCAAGTTCTCTTGTTG
GTGCAACTACGATTGCTTGTGGTTCTTTTATTTCACTATCAATTAACTGCATTAATGGTAATAAAAATGCATGAGATTTCCCTGTACCCGTT
TGAGATTGACCAATTAAATTTGTTCTCTTTAGTATTCTTGGAATAATTCGATTCTGAATTTCAGTTGGTTTTTCAAAATTAAGGTCTTTCACA
GCGTCAATTAAACTAGATTCTAGATTAAATTGTTCGAATGGATGTTTTGCCAT

Gene: ybgI (protein of unknown function)

Contig: 07_NODE_2, position: 130106 to 131206, length: 1101 nt, orientation: REVERSE

Perfect match to: (GR1-AJLX01000009-[185476:186576], allele observed in CC361+CC8+CC25+CC80+CC239)

Sequence:

TTAAATATATTGAAATGGATCTGTATTAATTGTAGATGCTTCAACATCTATATTTATTTTTTCTGTATTGAACCAATTCATTAGTAACGTTTT
TAAACCTTCTTTCATCACATATTCGCTGTAATGATTAATATCAATTAAATTCACACCATGAATTTTAGCATCTAAGGCATCATGATGTTTAAT
ATCACCTGTAACAAAGACATCTGCGCCTTGTTGGACAGCTTGATATTCATATCCAATACCTGAACCACCAATAATTGCAATACGTTTAATTT
TCTGATTAGACTCACCAACAAAACGGACACTTGGGATATTTAATTTAGATTTAATATCAGCTGCGAAATCTTCCAATGTCATTTGATTATCC
ACTTCTGCCATAACGCCAAGTCCATAAAGGGATGTTTGTTTTATCTCAATAAAATCAAATACCGGTGTTTCATATGGATGGTATTGTTTAAT
TAATTGCTCAGCCCTTGACTTTTGATATGCATCTATCATAAATTCAATTTTAACTTCATCTACATATTCAATTTTATCAATTTGTCCTATTGTT
GGATTAGCTTCACCAACTGGTTTGAATTGCCCTCTTCCTTCACTTTCAAAGAAACAATATTCATAATTACCTTCTTGCGCTAATCCATTTTCA
CTAAGCTTATCTTTAAATGGTCCAACATTATCCTTAGGTATATATGTTTGAACTTTATAGTATACATCTTGTTGATTATTTATTATTGAAATG
TTCTTCAAACCCATCGCCTTCGCCAACATCATATTGACACCATACGGATTTACATCTAAATTTGTATGCATCGCTATTAAATTAATGTCATGT
TGAATTAGTTTTCTAATGATCAAACCATAACCATTAGCTTTTAATGATGTTACGCCTTTAAAGATTAGAGGATGATGACTAATAATAGTATT
ATAACCTTTTTCGATTGCTTCATTTACTACTTCCAACGTACAGTCTAATGCTGTTAAAACACCAGTAACTTCAACATCTTCATCACCTATTAA
CAATCCTACATTATCCCAAGATTCAGCAGTACTAAATGGCACATGATGATCTAACAATGTCATTAAATCAGCTATTTTCAT

Gene: trmK (tRNA  (adenine (22)-N (1))-methyltransferase)

Contig: 07_NODE_2, position: 131209 to 131886, length: 678 nt, orientation: REVERSE

Perfect match to: (MW2-BA000033-[1640165:1640842:r], allele observed in CC1)

Sequence:

CTATAACACCCTTTCAATTACAGCAATTTCGTCATTAATTTGAGCTAAACGTTGATGATGTTGTTCAGTATTGAGTTTCGATTTAATATGAT
AAAGTGCTTCTAACTCTCTTTGCCATTTTTTTATAAAATATTCATTTTTGTTGTTCAGCAATTTTGGTCCGAATTTCAATCCATCAGATGATA
GCTCTATTAATTGTGTAGAATATTCTGCTACAACAATTTCATAAATATGGCCTTTTTCTTCCATTATTATTTCATCAATTATTTCATAATTCAA
TTGTTGTAATGTTTGTCTTAAATTTTCAGTTTGGATATTACTTTGTAAAATCAACCTTGGATGTTGACTTAACTTATCTTGCCCATCTTTTAA
AATTTTAGCAATAAGTGGTCCGCCCATACCACATATTGTGATATTATCGATTACGTCCTCAGGTTGAATAACACTTAAGCCATCCCCTAAA
CGTACATCAATTCTATCTACTAATTGGTTTGCAGCTACATTTTTTACAGCAGCTTGAAAAGGGCCTTGAATAACTTCTCCAGCAATACCGCA
TTCGCATAAATGGTTTTGAATTGCATAGATTGGCAAATAAGCATGATCTGAGCCAATATCCGCGATTGTACCTTGTTTTAAAAATCGACTT
ACCGTCGTTAATCGGTTATTTAACGAAATCAT

Gene: sigA (sigma factor A of RNA polymerase)

Contig: 07_NODE_2, position: 132017 to 133123, length: 1107 nt, orientation: REVERSE

Perfect match to: (MW2-BA000033-[1640973:1642079:r], allele observed in CC1+CC15)

Sequence:

TTAATCCATAAAGTCTTTCAAACGTTTACTACGACTTGGATGTCTTAATTTTCTAAGTGCTTTTGCTTCAATTTGTCGAATACGTTCACGTGT
AACACCGAAAACTTTACCAACTTCTTCAAGTGTTCTTGTTCTGCCGTCATCAAGACCAAATCTTAATCGTAATACATTTTCTTCTCTATCAGT

TAATGTATCAAGCACATCTTCTAATTGCTCTTTTAATAATTCATAAGCAGCATGATCTGAAGGACTTTGTGCTTCCTGATCCTCAATAAAGT
CTCCTAAATGACTATCATCTTCTTCACCAATTGGCGTTTCTAATGAAACAGGTTCTTGCGCAATTTTTAAAATTTCACGAACTTTTTCTGCTG
GTAAATCCATTTCTTCACCAATTTCTTCTGGTGCTGGATCTCGACCTAAGTCCTGTAATAATTGACGTTGAACACGAATTAATTTATTAATT
GTTTCTACCATATGCACAGGGATACGAATCGTACGTGCTTGGTCAGCAATTGCACGAGTGATTGCTTGTCTAATCCACCATGTTGCATATG
TTGAAAACTTAAATCCTTTGTTAAAGTCAAATTTTTCAACAGCTTTAATAAGACCCATATTACCTTCTTGGATTAAATCAAGGAATAACATA
CCACGACCTACGTATCTTTTAGCAATACTTACAACTAAACGTAAGTTCGCTTCTGCTAGTCTTGATTTTGCTACTTCATCACCTTGTTCAATA
CGTTTGGCTAATTCGATTTCTTCTTGTGCACTTAATAAGTTAACACGCCCAATTTCTTTAAGGTACATACGAACTGGGTCATTTATTTTAAC
ACCTGGAGGGGCACTAAGATCACTTGGATTCAGTTTCTCGTCAGTATCTGAACTATCTTTTTCATTAACTAGTGAAATATCATTATCATTTA
ATTGATCAAAGAAATCATCCATTTGATCAGAGTCGATATCAAAATTCTGAAGTTTTTCAGCAATTTCTTCATGACTTAAATGACCCTCTTTT
TTACCCTTTTCAATTAATTGCTTCTTAACATCTTCTAATGTTAATGTCGGATCAATTGTTTGTTTTTTAATTTTAACTGTGTTATCAGACAT

Gene: dnaG (chromosomal DNA primase, variant 1)

Contig: 07_NODE_2, position: 133347 to 135164, length: 1818 nt, orientation: REVERSE

Perfect match to: (Mu50-BA000017-[1673123:1674940:r], allele observed in CC5)

Sequence:

CTACATGCGTTCTTTATTCTTAGCAACAATTTGCTGTAAATAGTATTTTTGTAATTCTACATCGCCAATCCTTGTAGCTTCCCTTAATTTATG
ATTCAATGACTCAATTGTTTCTTGTCCTTTTTCATTAATAACATTGACATAATCATCAATTTCATTTTCATATGGTTCGTCATTCAAATTATAT
TGTTCTAAGCTAATTAGTGTTTCTCTCAACTCATTTGAATTAACATACTGCACAGCATCACTGATATTATATTGATCATTTTCCGCATAAAAA
TCATGTAAGACTTCGAATACATATTTAAAATGCTGATTTGTGAAGTTATCCTTATCAACACTTTCATAATAATTTAAAAATGTATCTTTATCT
CTCATTAAATGTTTTAAAAATGCTCGCTCCGCTTTTTCTTGACGGCTCAAATTGTCAAATTGTGCCATACCAATTGGCTCAGGTTCAATATA
ACCGCCATACTCATCATAACCGCCATACTCATCATCTGGATAATAATTGGCTGGTGCTTGATTGAATTGTATTTCGTTAGCTAATTGCTCAG
GACTAACATTGAAAAATGGTGCAACATCATTTAAAGCCTTTTGTTGCAAAATCGATGATTTCATAAGCGAAATATCATGACTTAGTTCTTT
CAAATAACGTTCATATGAAAGGTCATTATGTGCAATTTCATCTTTTAATATACTCACTTTATAATGTGCAAATGACTTTTTGTCATTTTTTAC
AAAAGCAGTAAATGCGTCGTTGCCATACTTACCAATGTATTCATCCGGATCCATGCCTGATGGCAATTGTATAACAAATACATTTAGCCCT
TGCTGTAATAAATGTTGACCTGTTTTAAGTGTTGCTTCACTACCCGCAAAATCCCCATCAAACATTAATGTTATATTTGATGTTAACTTTCGT
ATAAAAGTAATATGTTCATCTGACAACTGTGTCCCCATTGTTGCAACAACGTTTTTCAAGCCAGCAGTATCAGATTTTATAACATCCATAAA
ACCTTCTAGTAATACGATTTCATCTAATTTTCTAATTGATTTACGCGCTTTATCTAAGTTGTATAACAACTTTCTTTTTTGAAAGATAGGTGT
TTCAGGACTATTTAGGTATTTTGGTTCTTGACCGGTATATGTTCGACCTGAATATCCAACAATTCTTCCTTGCGCATTTTTCAAAGGAAACA
TAATACGATTTCGAAATCTATCGTAATAACTGAAATTTTCTTCGTTACGTGATAATAATCCGGCTTCATATGCTAATTCAATATCGTAACCC
TTTTTTTGAAGAAAATCATGACAAAAATGTGAGCTATCGGGTGCAAAGCCAATGCCTCGCTCTTTAATAAGCGCATCTGTAAAACCACGTT
CTTGTAAGTATGTTAATGCTTGTTCGCCTTCGACTGTCTTTGTTAAAGCGTAATAATAAAATTCTTGTATTAACTCATGCATTTCAATCATTT
GTAAATCATCAGAAGCAATTTGAACATTTGAGTTAAATTGTGTTGCCTCAATATCTACAGCAACATTAACTCTATCACCTAATTCTTTAACC
GCTTCAACAAATGATATGTCTTTAATTTCTTGAGTAAATTGAAAAACATTGCCACCTTTTTTACAACCAAAACAATGACAGATTTGTTTATC
TTCAGAAACTGTAAATGAAGGTGTCTTTTCATCATGAAAAGGACACAAACCTATATAATTGCGTCCTCTCTTTTCTAGTTTTACATATTCAC
TTACCAAGTCTAAAATGTCGGTTTTATCTTTTATTTCATTAATGATCGATTGATCTATTCGCAA

Gene: yqfL (positive regulator of gluconeogenesis)

Contig: 07_NODE_2, position: 135225 to 136043, length: 819 nt, orientation: REVERSE

Perfect match to: (MW2-BA000033-[1644181:1644999:r], allele observed in CC1+CC239)

Sequence:

TCATTTCGATTTATTTTGTTCAATATAATGGATTATATCGTTTGCTGTTTCTTCGATTGCTTTTTGAGAAACATCAATGACAGGACATCCAAT
TTCACTTACGATTTCTTCAAAGTAATTCAATTCTTCTTGGATTCGAGCTTCTGTTGCATAACGAGCTGTGTCACCTAGTCCCAATTGTTTTAA
TCGCTCTTTTCTAATGCGATTTAATTTTTCTTCACTTATTTTAAGTGCGATACATTTCTTTGGATCAATATCATATAAGCCATCTGGCGGTGT
CACTTCTGGTACAATCGGTACATTCATAACTTTGTAACTTTTATGCGCTAAATACTGGGATAATGGTGTCTTTGAAGTTCTCGAAATACCAA
GTAAAACAATATCAGCTTTAGGTAATCCTTTAGGATCTTTACCATCATCGTATTTAACTGCAAACTCTATCGCATCAATTTTCTTGAAATAT
GCATCATCTAATCTATGAACGATACCTGGCTCATTATAAGGTTTTTCTTCAACCGAAGCTGATAATAAATCCATTAATGGCCCCATGATATC
GACAGACTTCAATTGGAATTCTGCTACTTTCTCACTCATATATTGCTTCATTTCAGGTTTAATAAGTGTATAAACAATGATAGCATTTGTAT
CTTTTGCAACTTGAATCACTTCATCAACATCTTCAAAAGATTCAATATATGGATATCTTAATAATTCATTTTTACATTGCTTAGGATTGAATT
GTGAAATACCTGCCCTAGCAACTAACTCTGCCGTTTCACCTATAGAATCTGAAGCTACGATAATTTTAATTTTTTCCAT

Gene: ccpN (catabolite control protein N)

Contig: 07_NODE_2, position: 136054 to 136677, length: 624 nt, orientation: TRNC-RVRS (no start codon)

Perfect match to: (11819-97-CP003194-[1674432:1675055:r], highly conserved allele)

Sequence:

CTATTCTTTATATAATGCTACTAATAACTTAGCTATTGTTGTTTTGGAAATTCTTCCAATTACTTCATACTTTTGATTATCTTTTTTTCTTACAA
TTGGAATCGAATCAATTTCTTTTTCAATCATTCTATCTGCTGCGTATATGACTAATTCGCTTTCCTCTAAATAAGTGACATTAGGCATACGT
GTCATATTTACACTGATAGGTACTGTATGAATATCTGCTCCAATCATTGAAGCTCTTAATAAATCTTTTCTTGAACACACACCAACAAAATC
GTTATCTTCATTAATAATAAATAATGTACTTACATCTTCTAAAAAAATTGTACAAATAGCGTCATAAACTGTTGTATTCTCTCTTAGCACAAC
AGGTTGAGACATATAGTCCTTAACCTCAAATTGTCGAAGTTTTTCATTAAAAAATTTACCTTTTGATTTACCTGAATAATAATATCCAACTC
GGGGACGCGCTTCTAAAAAACCTGACATTGTTAATATCGCTAAATCTGGTCTAAGCGTTGCTCTTGTTAAATTCAACTTATCTGCTATTTGT
TCACCAGTAATGGGTCCTTTAGTTTTAACAATTTCGATGATTCGTTCTTGTCTTTGACTGAGTTCTAT

Gene: glyS (glycyl-tRNA synthetase)

Contig: 07_NODE_2, position: 137012 to 138403, length: 1392 nt, orientation: FORWARD

Perfect match to: (MW2-BA000033-[1645968:1647359], highly conserved allele)

Sequence:

ATGGCAAAAGATATGGATACAATTGTTTCATTAGCAAAACACAGAGGTTTTGTGTTCCCTGGTAGTGATATTTACGGTGGTTTATCAAAC
ACATGGGATTATGGTCCTTTAGGTGTTGAATTAAAGAATAATGTTAAAAAAGCTTGGTGGCAAAAATTCATTACACAATCACCGTTTAAC
GTTGGTATCGATGCTGCAATCTTAATGAATCCAAAAGTATGGGAAGCTTCAGGACACTTAAACAACTTCAACGACCCAATGATTGATAAT
AAAGATAGTAAAATTCGATATCGCGCTGATAAATTAATTGAAGATTATATGCAAGATGTTAAAGGTGATGAAAACTTCATTGCCGATGGT
TTAAGTTTTGAACAAATGAAAAAAATTATTGACGATGAAGGTATTGTTTGTCCTGTAAGTAAAACTGCTAACTGGACTGAAATTCGCCAAT
TCAATTTAATGTTTAAAACATTCCAAGGTGTAACTGAAGATTCTACAAATGAAATTTTCTTACGTCCTGAAACAGCACAAGGTATTTTTGTA
AACTATAAAAACGTGCAACGTTCAATGCGTAAAAAATTACCATTTGGTATCGGTCAAATTGGTAAATCATTCCGTAATGAAATCACTCCAG
GTAACTTCATTTTCAGAACAAGAGAATTTGAACAAATGGAACTTGAATTCTTCTGTAAACCTGGAGAAGAAATCGAATGGCAAAATTATT
GGAAAACTTTTGCAAGTGACTGGTTAACAAGCTTAAATATGAGCAGTGAAAATATGCGTTTACGTGATCATGATGAAGATGAATTATCTC
ATTACTCAAATGCAACAACTGATATTGAATATAAATTCCCATTTGGTTGGGGTGAGTTATGGGGTATCGCAAGTCGTACAGACTTCGACTT
ACGTAAACATGCTGAACACTCTGGTGAAGATTTCAGATACCATGATCCAGAAACGAACGAAAAATATATTCCATATTGTATCGAGCCATC
ACTTGGTGCAGATCGTGTAACATTAGCTTTCTTATGTGATGCATATGATGAAGAAGGCGTTGAAGGTAGTAAAGATGCACGTACAGTTTT
ACACTTCCATCCTGCATTAGCACCATATAAAGCAGCGATTTTACCTTTAAGTAAGAAATTATCTGGCGAAGCGATTAAGATTTTTGAGCAA
TTAAGTTCTAAATTCTCAATCGATTTCGATGAATCACAATCTATCGGTAAAAGATACCGTCGTCAAGATGAAATCGGTACACCTTATTGTG
TAACATTCGACTTTGATTCATTAGAAGATAATCAAGTTACAGTACGTGACAGAGATTCAATGGAACAAGTTCGTATGCCAATCTCAGAGTT
AGAAGCTTTCTTAACTGAAAAAACAAAATTCTAA

Gene: recO (DNA repair protein O)

Contig: 07_NODE_2, position: 138554 to 139306, length: 753 nt, orientation: REVERSE

Perfect match to: (MW2-BA000033-[1647510:1648262:r], allele observed in CC1+CC188)

Sequence:

TTATTGTTCCAATCTTTTTAATTGGTTGATTAGTTTCTGACTTTTAAAAAACATACCTGCATATTCACGATATAACATTAAAATGATATCTGA
CATTTCATCAATAATTTCTTGATGGATATTCAATGAATTCATTTTATCTATCGGTAATTTTTGTAATACATCTAATAAATATAGTGTTTTATTC
GATAATATAACTGCATGTACATCTTTAGAAGCCTCTTGCCTTGAAATCGCACCGTCAAACTTAAAACTATAACCTATTAAATCTGCTTGTGT
GTCATTACCACTCACAGCACAGCGATTAAATGATGCAGTAAAACCAAATCGTTTCATACACTTTAACATAACTACGACTGACATTAACTGT
GCAGATGTACCTGATTCTATTTTTTCAAGAACAAATTGTAATAATTGATAGTTATATGGTGCAATGTCACCTTCATCCATTGAGCGCTCAAT
AGTTTCAGCTGCCAGAGACGCATAACTGCTTACGAAAAGGTCCATTTGTAATTTATAATGTTGACTAATAACATCTACAGAATTTAACGTT
CCCATACCTCGCCACTGATTGTAAATAAACAAACCATAAACAAACAATTGCGTTTGCGCTTGTAAACCCGTCTTAACTTTTTTAGCACGCCT
AGCCATAAGTGGTACTTTTGCACCATGCTCATTTAAAATCGTGATAATTTTATCAGATTCACCATAATCAACTGCTTTGATGATAATCCCTT
TTTGGCGCATTAACAA

Gene: era (GTP-binding protein)

Contig: 07_NODE_2, position: 139328 to 140227, length: 900 nt, orientation: REVERSE

Perfect match to: (MW2-BA000033-[1648284:1649183:r], allele observed in CC1+CC188)

Sequence:

TTAATCTTGGTCTTCAACATAACCAATTTGGCGAATAAAGTTAACTTTGTTTCGCCAGTCTCTTTGAACTTTGACCCATAATTCTAAGTAAA
CTTTAGAGCCTAGAAGCATTTCTATATCACGTCTCGCACGTTTCCCTACTTCTTTTAACTTTTTACCGCCTTTTCCAATGACAATTCCTTTTTG
CGAATCTCTTTCAACATATATAGTTGCTTCGATATGAACACGATCTTCGCTTTCTTTAACCATACGGTCCACATTAACACCAATCGCATGAG
GGATTTCTTCACTTGTAAGATGAAGGATTTTTTCACGAATGATTTCACCCACTACAAATTGTTCAGGATGGTCTGAAATTTGATCATCTGG
ATAATATTTAGGTCCTTCGGGTAAATACGTCTTTAAAACATCAATAAAATGATCTACATTTAGCCCTTCTAATGCTGAAATAGGTACAATCT
CTGTAAAGTCCATATAACTTTGATATTCTTCAATCTTTGGCATTAATTCATCTGGATGCACTAAATCTATTTTATTTAATACTAAAAATACTG
GTGTCTTAACATTTTTCAACATTTCTATAATATATTCATCACCTCGTCCAATTTCCTCATTGGCATTAACCATAAACATGATTGCATCTATCTC
AGATAATGTATTTTTAGCGACTTTCATCATATAGTCACCTAATTTGTGTTTAGGTTTATGAATACCTGGCGTATCAATGAATATAATTTGCG
CGTCATCTCTTGTCATAACACCTTGAATTTTATTTCTAGTTGTTTGAGCTTTATCGGACATGATTGCTATTTTATGGCCGATCACTCTATTAA
CAAATGTTGACTTCCCTACATTTGGTCTACCTATAATTGAAACAAATCCTGATTTATGTTCTGTCAT

Gene: cdd (cytidine deaminase)

Contig: 07_NODE_2, position: 140228 to 140632, length: 405 nt, orientation: REVERSE

Perfect match to: (MW2-BA000033-[1649184:1649588:r], allele observed in CC1)

Sequence:

TTATTCTAAATCCTTTCCTGAAAATCCAAATGGTAGTAACTCTGCGACTGTCATCATAACCATATCTCCTTTATGATTTGTCATATACACAG
GCATATCATCATCACATAATTCCTTTAAAACTTGACGACATGCACCACAAGGTGATGACGGTTTATCTGCATCTACGGTTACAGTTATTGA
TTCAAAATCACCTGGTCTGTATCCTTGAGAAATTGCCGATACCAAACTAGCTCGTTCAGCACATATCGATAATGGATAAGAAGCATTTTCT
ACATTGGTACCATAAAAAGTTCTACCGTCTTTCGTTTTTAAATAAGCTCCTACTTTAAATTGACTGTATGGCGAATATGATTCTTGTTGTGC
TTTTCTAACTTCTTGAAAATAATGAGGTTGATAACTCAT

Gene: dgkA (diacylglycerol kinase)

Contig: 07_NODE_2, position: 140643 to 140987, length: 345 nt, orientation: REVERSE

Perfect match to: (N315-BA000018-[1604006:1604350:r], highly conserved allele)

Sequence:

CTAAAATAACGCTATAAAATGTGGTAAAAATACTATTAAACCTATAATAAATGCTAATATTGAAACTATAAGTACACTAAAAGCCGCTATA
TCTTTTGCGTATTTAGCTAAATCATGATATTCAACGGTCACTAAATCGACAACATATTCAATAGCAGTGTTTAAAGCTTCAACAGTGAGAA
CTAATGCAATAGCAATGAGTATAAATATCCACTCAATACGATTAATATTTAGTACGAGACCAAAGACAATAGCAACAATCATTGCAAACA
CATGTAAAAGAAATTTATAGTCTTTTTGAATTAAGATTTTCAGCCCATCAAGTGCATATTTAAACCTTTTCAT

Gene: ybeY (ribosomal RNA endonuclease)

Contig: 07_NODE_2, position: 140990 to 141457, length: 468 nt, orientation: REVERSE

Perfect match to: (Strain_15532-HE579063-[1583190:1583657:r], allele observed in CC5+CC1)

Sequence:

TTAGCCTCGTGTTAATCCATATGCGTTTAATATTGTATCTTGTCGACCAAACATTTCCTTTTCATCCGCTTCAGTCATATGATCATAACCTAA
TAGATGCAAAAATCCATGTAATGCTAAAAATCCTAATTCTCGTTCAAAAGAATGTCCGTAATTGTTTGCTTGTTCTTGCGCTACATCCGTAC
AGATAATTATATCCCCTAAAACACGTGGTATATCAAGACCACTAAAATCAATCTCTGGCTCATCTTCTTCTAAAGCAAATGAGATTACATCT
GTAACTTTATCTTTATCTCTATATGTTCGATTAATTTCTTGTATTTCTTGTTTATCTACAAATGTAACAGAAAGCTCAGCATCGTCTTCTATAT
GCTCTTCTTTTTTAGCAAATTCTAATAAATCTTCAATTTGTTTATACCACGCATCTTTAACTAAGCCTGTGTGATCGCTAAAATCTATCGTAA
ACAT

Gene: phoH (phosphate starvation-induced protein)

Contig: 07_NODE_2, position: 141458 to 142378, length: 921 nt, orientation: REVERSE

Perfect match to: (MW2-BA000033-[1650414:1651334:r], allele observed in CC1+CC188)

Sequence:

TTAATTCTCTCCTTCATAATGTTCAATGATCTTACTTACCAATGGATGTCTTACTACATCGCTCTGATCTAATTTCAATATACTTATACCTTTA
ACGTTGTGTAACCTACTGACCGCTTCCTTAAGTCCACTTTTAACACCTTTAGGTAAATCGATTTGAGTTTGGTCACCAGTAACTACCATTTT
TGAGCCAAAACCTAGTCTTGTTAAAAACATTTTCATTTGCGCATGTGTCGTATTCTGCGCCTCATCAAGAATTACAAATGCATCTTCTAATG
TTCGTCCGCGCATATATGCAAGTGGCGCTATTTCGATAATGCCTCTTTCAATAAATCGCTCTGTTTGTTCACGCCCAAGAACAGTATATAG
ACCATCATATAAAGGTCTTAAATATGGATCTACCTTTTCTTTCAAATCTCCTGGTAAAAATCCAAGTGACTCTCCTGCTTCAACAGCAGGTC
TTGTTAATACAATACGTTTAACAGCACCTTTACGGAGTTGCTTTGCTGCATAAACTACAGCTAAGAATGTCTTACCTGTACCAGCAGGACC
TATACCAAATACTAAATCATTATTTTTCATGGCATTAACATATATACGTTGCCCCATCGTTTTCGCACGAATCGTCTTACCAAATGCATCTTT
AGTTATCTCTTCATCATATAAATCTAACAGATGTTGAATTGTGTTATTATGCGCCATTTTAATAGCTGCTTCAACATCTTTAATTGTAATATT
ATTACCTAAATCAATAACCTTCAGCAAATTGATTAATACTGATTCCGCTTTTTCTACGTTTTCTATTTTTGTACCTTTAACGGCAACTTCTTGT
CCTCTTGCATGGATGACAACATCGAAACTCTCTTCAATTGCTTTTAAATGTTCATCATTATTTCCAATTAAAGCTTGAGATTGATTCAT

Gene: Q5HFI8 (putative protein)

Contig: 07_NODE_2, position: 142709 to 143407, length: 699 nt, orientation: REVERSE

Perfect match to: (RF122-AJ938182-[1561230:1561928:r], highly conserved allele)

Sequence:

TTATAATTGTTTTGGTTTAGCTAAAATTTCTGACCATATCATACCATTGATTACTTCATCTTTATCAAATTGGAACGCTGATTTAGTCAAATC
TTTTTCGACATTTTGAGAATTAAGCAACTGCTTTAATTTCAAACGTTTTGTACGTTCAGATAAGTATTTATCCTCAATAATATCTCTAGCACG
TTTTTCCATTTTAGCAATTTGCTTTTCTTTTTCTCTATCAATGTCAGTACGAATCGTTTTAATATCATCTCTAAGTGATTTTTCTAATTGACGT
CTAATTTCGTCAGAATTATCTTGTCTAGAAGGTCTATGTTTCTGCTCCACCGGTTCTGCTTTAGGCACTGTAATTGGTTTCTCTGTCATCGG
TTTTGTTTCTGGTTGTTTTTTAGGTGCCATAGGTTCTACAACAGGTTTCGATTTAGGCTCTTCCTTTGGAAGTTCATCGAATAAAGGTGGTA
ACGTATCATCATATTTTCGTTTCGATGATTTCTTTTCTTCTTCATTTAATTCTTCACTTATTTCTTTAAACGTTCGCTCAATTTCTTCAAAAAAG
CCACCTTTTTTTGGTTCATTATCGGTAGATGTTTTTTGAGGTGGCTTTTGATTTTGTCTATCTTTATGACTATTTTCGCGCATAGTAGTAATG
ATAGAAATGATCACTGATATGACAAAAATTAGAATACCGACACTCAT

Gene: floA (flotillin A)

Contig: 07_NODE_2, position: 143424 to 144413, length: 990 nt, orientation: REVERSE

Perfect match to: (MW2-BA000033-[1652380:1653369:r], allele observed in CC1+CC15+CC80+CC188+CC772)

Sequence:

TTAATGTTCAGGTGACTCATCATCACTTTGATCAGTTCGTTTATTAATTGCATTTCTCATGCCTGTATCAGCTTCGATATTTTTCAAATTATA
ATAATCTTTAACACTGATATTACCTGAACGTAATGCTTCAGCCATAGCTAATGGTACTTCAGATTCGGCTTCAACTACTTTAGCATGCATTT
CTTGTACACGCGCTTTCATTTCTTGCTCAGTTGCTACAGCCATAGCTCTACGTTCTTCAGCTTTTGCTTGTGCAATATTTTTGTCTGCTAATG
CTTGTTCAGTTTGTAAGTCTGCACCAATATTTTTACTAATATCAACGTCAGCAATATCAATTGATAAAATTTCAAATGCAGTACCTGAATCT
AAACCTTTGCTTAAAACTGTTTTAGAAATATTATCTGGGTTTTCAAGTACTTCTGTATGATGCTTACTAGAACCAATTGTTGAAACGATACC
TTCACCAACACGTGCGATGATTGTTTCTTCACCAGCACCACCAACAAGTCGAGCAATATTAGCTCTAACTGTGATACGAGCTTTGGCTTTC
ACTTCAATACCGTTCATTGCTACACCTGCGATAAATGGTGTTTCAATGACTTTAGGATTAACAGACATTTGAACCGCTTCTAATACGTCAC
GTCCTGCAAGGTCAATTGCAGCAGCACGTTCGAAAGGAAGATCAATGTCAGCACGTTGTGCAGCAATATTAGCGTCAACAACTCTGTCAA
CATTTCCTCCTGCTAGATAATGCGATTCTAATTGGTTTGTTGTTAATGCTAGTCCTGCTTTATGCGCTTTAATTAATGGCGCTATAACTTTTC
TTGGAGATACACGACGTAAACGCATACCAACCAATGTACCTATACCAACATGAACGCCAGCTGCTAACGCTGAAATCCATAAACCAATGG
GTACAAATGAGAATAAAATAAGTAATGCAACTACTATAATAACTGCTATTACGATAAAACTTAAACTAAACAT

Gene: yqeZ (putative membrane bound hydrolase)

Contig: 07_NODE_2, position: 144431 to 145138, length: 708 nt, orientation: REVERSE

Perfect match to: (11819-97-CP003194-[1682809:1683516:r], highly conserved allele)

Sequence:

TTAATCTACTTCCCTCACAACTACTCTTGTTCCTTCAACTTCAAGGATTTTTACCGTTTTATTGCGTAAAATAAAGTTGCCATCTGAAACAGC
ATCAATACGTTCATTTTCACAAAAAATAATCCCTGCAGGTCGAAGATCTGTAACTGTTTGAGCAGTCTTTCCTACGAGGTGCGAGCGGTTA
TCATGAGAATTGTAACCTGACTCAGAATTAGTTGAATCTTTTAAGATAACTTTATCCAAAAACGGAATCTTTCTGTTGAAAATCTTCACTAA
TATCACCCATTCTACAATCGTTAAAATCAAGGCAACGATAACATTCGCAAGCATAAATAGCAAATTATCACCGAGCGTTGTTATGCTTATA
GTTATCAGTATCATGCCAATAATACCAATTACTGCACCAACTACAAATAATTCAATTACAACTAATATAACGCCAATTGAGAATATTAAGA
TAGAATGCATATTGACATTTCCTTGGATTAGAAATCCCAAAAATAAAATAAGTAATGATAATGTGGCGATAATACCAGCTGCATTGATTTT
TTTAGAGTAAAGTTGATATACAAATCCTAAGAATGTCAAACAGGTTAATATTAACGTAAAAATAGGTTGAACAATTATATTACTAACTTGT
TCAACCCAAGTATCTCCAGCCGTTGATTCCAAGATAGTTGTCATTTGTAAAAAATTATTATAACTCAC

Gene: rpsU (30S ribosomal protein S21)

Contig: 07_NODE_2, position: 145358 to 145534, length: 177 nt, orientation: REVERSE

Perfect match to: (RF122-AJ938182-[1563879:1564055:r], highly conserved allele)

Sequence:

TTATTTGAATTTACGTTTACGTGCAGCTTCTGATTTCTTTTTACGTTTTACGCTTGGTTTTTCGTAAAATTCACGTTTACGTACTTCTTGGATT
GTTCCACTTTTAGAAACTGAACGTTTAAATCTACGTAACGCATCTTCAAGTGATTCATTTTTACGTACTACTGTTTTAGACAT

Gene: mtaB (tRNA methylthiotransferase)

Contig: 07_NODE_2, position: 145827 to 147173, length: 1347 nt, orientation: REVERSE

Sequence:

TTAAACTAAAACTTCTCTATCAGATTTATTTGTTGCGAAATCAACAACTTTAATTGCTTGCCCTTCATTTAATGGATAATTTGCTTGCGTAAT
TTTAACTTTTACAATTTGACCTATGAGTGATTCGTCACCTTCAAATTGTACTTTCATATAATTATCTGCATATCCAACTAATGTGCCTTCTGT
GTCACCCTGTTCCTCAGGAATTACTTCAAGCACATCTTGATCAAATTTAGACGCATATAACTTTCCGAGTTGATTACTTAGCGTAATTAACT
TATGCACCCGTTCATTTTTAATTTCTTCATCAATTTGGTCATCCATTCTTGCAGCTGGCGTGCCAATTCTAGGAGAATAAGGGAAAACATGC
AGTTCAGAGAACTTATGCTTTACGATAAAATCATATGTTTCTTGGAACTCTGCTTCAGTTTCACCTGGGAAACCAACAATTACATCACTCGT
AACTGCCAAGTCTGGTAAAGCTTTATGCAATTTTGTTAATCGTTCTGAAAATCTATCCATTGTATACTTACGTCTCATACGTTTTAATACTGT
ATCTGAACCAGATTGTAATGGAATATGCAAATGACGCACAACTTTTGTTGAACGTTCTAAAACGTCAATTACTTCATCTGTAAGTTGACTT
GCTTCAATTGAAGAAATTCGAATTCGTTCTAATCCATTAATCGTTTCAAGATCACGTAATAATTGGGCCAAGTTATAATCTTTTAAATCTTG
ACCATATCCACCTGTATGAATTCCCGTCAATACAATTTCCTTATATCCTGAATTCACTAGTTGCGTCGCTTGTTCAACTACTTTTTCCGGATC
TCTTGAACGCATTAAGCCACGAGCCCATGGAATAATACAGAATGTGCAGAAGTTGTTACAACCTTCTTGAATTTTTAATGACGCACGTGTT
CTATCTGTAAAATATGGGACATCTAATTCTTCATATTTACGATTTTTCATGATATTTCCAACACCATTAATTGGTTGGCGTTCTTTACGGAAT
TCATCAATGTAACCTAATAGTTTATGTCTATCTTGTGTACCAACTACTACATCGACACCAGGAATTTCCATAATTTCAGCTGATGAAGTTTG
CGCATAACAACCTGTTACACAGATTACAGCATCAGGATTTTGTCTTATTGCACGTCTAATTATTTGACGACTTTTTTTATCACCTGTATTCGT
TACTGTACAAGTATTAATAACAAATACATCAGCATTCGCTTCAAAGTCAACGCGCTCATAGTTTGCTTCTTTAAATAATTGCCAGATTGCTT
CAGTTTCATAATGGTTTACTTTACAACCTAATGTGTGAAACGCAACTGTTGACAT

Gene: rsmE (ribosomal RNA small subunit methyltransferase E)

Contig: 07_NODE_2, position: 147180 to 147932, length: 753 nt, orientation: REVERSE

Perfect match to: (MW2-BA000033-[1656136:1656888:r], allele observed in CC1+CC22+CC30)

Sequence:

TCACCCCATTAATTCTTTTTCATAACTTATTGCACTTAACGCATACAATGGCGCAGTTTCTGCCCGTAAAATTCTCGGTCCAAGACCAACAA
CTGTACTAGTATTACTAAATAATGAAATTTCATTTTCTGACAAACCACCCTCAGGACCAAAAATCATCAACACTTTATCCTGAGCCTTGAAT
TGTTGTAAAGTTTGCTTGAAATTGCTTAACTCACCATCTTTTGCTTCCTCTTCATATGCAATAAGAATATAGTCATAATTATCAATAGTATCA
CAAATTAATTTTAAATTCGACTCGAATTGAATAGATGGAATCACTAAACGATAGCTTTGTTCAGCAGCTTCTTTAATTATTTTTTGCCAACG
CTCTATCTTTTTGGCAACTTTTGCCTCGTTTAATTTAACAATTGAACGTTCCATGCTCACAGCTATAAATGATGAAGCACCCAATTCAGTAG
CTTTTTGTAGCAACCACTCATATTTGTCAGCTTTGATTAGTCCACTGCAAATCGTAACATCAACTGGCAATTCTGTATTAATATTTTGTTTTT

CTTTTAAATCAACTGCAATTTTATCACTTGTTATATCAGCAATTTCACATAAATAAACTGTTTGATCATTAAAAGTTAAAATAATTTTACTAC
CAACATCATATCTCATTACATTTGTTATATGATGAATATCTTCTTTTTTTGTAATAAAAAAACGCTGACTTACATCAGCGTTTTGGTCTATGA
AATAACGTTGCAC

Gene: prmA (ribosomal protein L11 methyltransferase)

Contig: 07_NODE_2, position: 147934 to 148872, length: 939 nt, orientation: REVERSE

Perfect match to: (11819-97-CP003194-[1686312:1687250:r], highly conserved allele)

Sequence:

TTATTCACTCACTTTCTGGCCAACAAGACAAACCCAACCGTTGTCATGTTGTTCTGAAATAATTTTAAAACCTACACGCTCCATATGTGACT
GTATACCTTCATACTTCTCTTTTATAATACCAGAAGTAATAAAATAACCGCCTTCATTTAGAGTATTATAAGCATCTTCAATCATTTCATCAA
TAATATGCGCTAAAATATTTGCTATTACAATATCAAATTTTTCTGTTTCGTCTTTCAATAAGTTACCTGGAACAGCTTCAATTAACGTTTCAC
AATGATTTCTTCTGAAGTTTTCTTTAGCTACACTCACTGCCATTTCATCAATATCCAACGCTTTAATACGTTTTACACCGATTAGATGACTTG
CAATACTTAATATACCTGAGCCAGTACCAACATCAATTACTGAATGCTGTGGCAATACATATGTTTCTATTGCCTTCAAACACATACTTGTA
GTCGGATGATCACCTGTTCCAAAAGCCATACCTGGGTCGAGCTCAATGCAAAGCTCTTCATCCGCTTCTTTAGCATATGTTTCCCAACTAG
GAACTATTGTGAACTTCTTCGACGCTCGGAATGGATGGAAATAGTTTTTCCATTCATTTTCCCAATCCGTCTCTGCAATAATTTGCTCACTG
AATTGAACGTTATGTTGATCAAGTTCATCTAAATTTAATAACTCATCTTTAATTTGCTGTCGCAACTTATCATCATAAGTCATTTCATTAAAA
TAGGCTTTCAATCTTACTCCCTTATCTGGATAATCCTCTTTTTTCAAAGCGTAAATTTCACCGTATTTATCTTCTGGTTGGTTAATTAAATCAT
CTGAATCTTCTATCACGACACCATTTGATCCATGATTTTCAAGTATATTGGTAGCCAATTCTACTGCTTCATGATTAATAATAATTGAAAGC
TCTGTCCAGTTCAT

Gene: dnaJ (chaperone)

Contig: 07_NODE_2, position: 148876 to 150015, length: 1140 nt, orientation: REVERSE

Perfect match to: (N315-BA000018-[1612239:1613378:r], highly conserved allele)

Sequence:

TTATTCTCCCTTAAAGAATCTTTTTGCTCTATCTTTAAAATTCGAAGGTTGTTCATTAATTTCTTCACCATTTAATTGGGCAAATTCTTTCATT
AGTTCTTTTTGTCTATCTGTTAATTTAGTAGGCGTTACTACTTTAATATCAACATATAAATCTCCGTATCCATAGCCATGAACATTTTTTATA
CCCTTTTCTTTTAAGCGGAATTGCTTACCTGTTTGTGTACCAGCAGGGATTGTTAACATAACTTCATTATTTAATGTTGGTATTTTTATTTCA
TCGCCTAAAGCTGCTTGTGGGAAGCTAACATTTAATTTGTAATAAATATCATCACCATCACGTTTAAATGTTTCAGATGGTTTAACTCTAAA
TACTACGTATAAATCACCAGCAGGTCCTCCATTCACGCCTGGAGAGCCTTCACCAGCTAATCTAATTTGTTGTTCATTGTCGACACCTTCAG
GTACTTTCACTTCTAATTTAACTGTTTTATTTTCAGTACCTTTTCCGTGACATGTTGGACAAGCTTCTTCAAATTCTTGACCACTTCCATTACA
TTTAGGACAAACTTGTTCAGTACGAACTCTACCTAAAATTGTGTTTTGTTCTACAGCTACATGACCAGCGCCATTACAGTAACTACAAGTCT
TTTTACTTGTTCCAGGCTTTGCACCATCACCATGACATGTTTCGCATGTTACATCTTTACGGATTGAAATTTCTTTTGTTGTACCAAATACCG
CTTCTTCAAATGTTAATGTCATTGTATACTGAAGATCATCACCTTTTTGCGGTGCATTTGGATCTCTTTGTCTGCCGCCACCGAAGAAAGAG
CTAAAGATATCTTCAAAACCGCCGCCACCGAAGCCACTAAAACCGCCAAAGTCAGAGCCATTGAATCCTTGTCCACCAAAACCTTGTGGA
CCATCATGTCCAAATTGATCATAGCTTGCGCGTTTATTATCATCACTTAAAACTTCATAGGCTTCAGAAATTTCTTTAAACTTTTCATCTGCA
CCTTCTTCTTTGTTAATATCTGGATGATATTTTTTCGAAAGCTTTCGATACGCTTTTTTGATTTCATCTTTTGAAGCATCTTTACTAATGCCTA
AAACTTCATAATAGTCTCTTTTGGCCAC

Gene: dnaK (chaperone)

Contig: 07_NODE_2, position: 150151 to 151983, length: 1833 nt, orientation: REVERSE

Perfect match to: (11819-97-CP003194-[1688529:1690361:r], highly conserved allele)

Sequence:

TTATTTTTTGTCGTCGTCTTTTACTTCTTTAAATTCAGCATCTTCTACAGTACTATCGTTGTTTTGACCAGCATTTGCACCTTGTGCTTGTTGT
TGCTGTTGAGCCGCTTGCTCATATACTTTTGCTGATAATTCTTGAATCACTTTTTCAAGTTCTTCTTTTTTAGATTTAATATCTTCTATATCTT
GACCTTCTAAAGCAGTTTTAAGAGCGTCTTTTTTCTCTTCAGCAGATTTTTTATCTTCTTCACCGATATTTTCGCCTAAATCAGTTAAAGTTT
TTTCAACTTGGAATACTAGACTGTCAGCTTCGTTTCTTAAGTCTACTTCTTCACGACGTTTTTTATCTGCTTCAGCGTTAACTTCAGCATCTT
TTACCATACGGTCGATTTCTTCGTCTGATAATGAAGAACTTGATTGAATTGTAATTCTTTGTTCTTTATTTGTACCTAAGTCTTTTGCAGTTA
CATTTACAATACCGTTTTTATCGATATCAAACGTTACTTCAATTTGAGGTTTACCACGTTCAGCTGGTGGAATATCAGTCAATTGGAATCTA

CCAAGTGTTTTATTATCCGCAGCCATTGGACGTTCACCTTGTAATACGTGTACATCTACTGATGGTTGATTATCTACTGCTGTTGAATAGAT
TTGAGATTTAGATGTAGGAATCGTAGTGTTACGTTCAATTAACGTATTCATACGTCCACCTAAAATTTCAATACCTAAAGATAGTGGTGTT
ACGTCTAATAATACTACGTCTTTAACGTCACCTGTGATAACGCCACCTTGGATTGCAGCTCCCATTGCCACTACTTCGTCCGGGTTTACTCC
TTTGTTAGGTTCTTTACCGATTTCTTTTTTGACAGCTTCTTGTACTGCTGGAATACGAGTTGATCCACCAACTAAGATAACTTCATCGATATC
TGAGTTTGTTAAGCCAGCGTCTTTCATTGCTTGGCGTGTAGGTTCCATTGTTCTTCTAATTAATGAATCTGATAATTCTTCAAATTTAGAAC
GAGTTAAGTTTACTTCTAAGTGTAATGGACCGTTTTCACCAGCTGAGATAAATGGTAATGAGATTTGAGTTTGTGATACACCTGATAAGTC
TTTTTTAGCTTTTTCAGCAGCATCTTTCAAACGTTGTAATGCCATTTTATCTTGAGATAAGTCTACGCCATTTTCTTTTTTGAATTCTGCAACT
AGGTAGTCAATAATTACTTGGTCAAAATCATCACCGCCAAGTTTGTTGTCACCGGCTGTTGATAGTACTTCGAATACACCGTCACCTAATT
CTAGGATAGATACGTCAAATGTACCGCCACCTAAGTCAAAAACAAGAACTTTTTCATCTTTATCAGTTTTATCTAAACCATATGCTAATGCT
GCAGCTGTTGGTTCATTAATGATACGCTCAACTTCTAAACCAGCAATTTTACCAGCATCTTTAGTTGCTTGACGTTCAGCATCGTTAAAGTA
TGCAGGTACTGTAATTACAGCTTTGTCAACTTTCTCACCTAAATAGCTTTCAGCTGTATTTTTTAAGTTTTGTAAAATCATAGCTGAGATTTC
TTGTGGTGTGTATGATTTACCTTCAATATCTACTTTATAATCAGTACCCATATGACGTTTAATAGATTGAACAGTGTTTGGGTTTGTAATAG
CTTGACGTTTAGCTACTTCACCAACTTGAGTTTCTCCATTTTTGAAAGCTACAACAGATGGTGTTGTACGTGAACCTTCAGGGTTTTGAATT
ACTTTTGGCTCATCGCCTTCTAATACTGTTACACATGAATTTGTTGTACCTAAGTCTATACCAATAATTTTACTCAT

Gene: grpE (cofactor of DnaK)

Contig: 07_NODE_2, position: 152052 to 152678, length: 627 nt, orientation: REVERSE

Perfect match to: (MW2-BA000033-[1661008:1661634:r], highly conserved allele)

Sequence:

TTATTGGTTTACTTTGACCATTGATGGTCTTAATACTCTATCTTTAAGCTTGTATCCTTTTTGTAGTTCTTGAGTGATTTCGCCAGATTCAAA
ATCAGGGTTATCATCTTGAACTACAGCTTGGTGAATATTTGGATCAAATGCTTCACCTTCAGTTTTAATAACTTCAAGACCATTATCTTTTA
GTGCGTTAATCAAACTTTCATGCACCATTTGTACACCTTTTTGAAGAGATTTAAAAGTCTCATCATCACCTTCAATTTGAAGTGCACGTTCT
ATATTGTCTATTGCTGGTAAAATATCTGTTAACACACGTTGTGCTTGATATGTTTTATTTATTTCATTTTCTTTTTGAATTCTACGCTTATAAT
TTTCAAACTCAGCGTAGAGCCTTAAATATTTCTCTTCGTTTTCATCTGCTAATTGTTGAAGTTCATTAATTTTTTGATCTTTTGGATCTATTTC
TTCAATAACATTCTCGTCAGACGTTTCTTCTATTGCTTCATCTTGTAAATGACCTTTACTTTCTTCAGCTTGTTCAACTGAATCATCAATATTT
TGTTTGACGTTTGTTTCTTCAACTGTTGATTCAGTGTTTTTTTCAACTGATTCGTCTTTATTTGTCAT

Gene: hrcA (heat-inducible transcription repressor)

Contig: 07_NODE_2, position: 152710 to 153687, length: 978 nt, orientation: REVERSE

Perfect match to: (TCH130-ACHD01000016-[1386:2363], allele observed in CC72+CC30+CC72+CC772)

Sequence:

TTACCAAATTCTATTTAATAATTGAATGACATTTTGATAATGCATAGCTGTAGGTCCAATCACAGCGATTTGACCTTTTAACGTTTCATCAA
AATGATATTGACTTGTTACAATTGAAATATCACTTAAGCTGTCATCAATTTCATTACCAATTTTTACATTAATATTTGGTGAAGATATATCTT
GTAATAATTCTGCAATTCTATTTGATTCTATATATTGTAGAATGGGCTGAATTGAAGATACATTACTTTCATTCAATGCATCAATAAGTTTA
ACCTTTCCACCCATATAAATGCTATTACTTTGATTAGAAATATGATTATTCATCGTATTTAACAATTTATTGATAAATATTTCTTCCTGCTCTG
ATTGAACAAAAGAGACAATATCATCTTGTAAATTCTGATTAAACTCAGTTAGTTTGTTTGTAACAAAATTTGATATTGTATTTAGTTTGTCA
TTATTAAACGGTATGTCTGAAGCAAGATGTACATGCTCAACATGACCTGATGAAAATACGATAACCATTATAACTAAATTAGGATTAGCA
CGAATCAAGTGTACATTATTGATAATATCTTGTTTATGATTAGGATGAACAACTAAAGTTGTATATTGAGATATATTTGATAATTCATCTGC
AAAATATGTCAATGCTGATGATACATCATATTGATTCTCAACTAACAATTGATTTAATCGTCTTAATTTATTTGTTTTTTGATGAGATGTTTG
TTCAAGTAAACGATTGACATAATACCTAAAACCTAATTGTGATGGCGAACGCCCTGAAGAACTATGTGTCTTTTCGATATAGTTTAAATCT
TCAAGCTGTTTCATCTCATTTCTAATTGTAGCAGGACTAACATTCAAGTTATGTCGCTCAATTAGTGTTTTAGAACCAACGGGTTGTCCAAA
ATCAACATAATCCTCAACAATTGCGTTTAATATACTCAATTGCCTATCTGTAATCAT

Gene: hemN (oxygen-independent coproporphyrinogen III oxidase)

Contig: 07_NODE_2, position: 153788 to 154945, length: 1158 nt, orientation: REVERSE

Perfect match to: (MW2-BA000033-[1662744:1663901:r], allele observed in CC1+CC5)

Sequence:

TTAATCATTTATTAGGAAAGCTTCAAAAACCTCATTACCTATGACTTTCCCTCGCTTTGTTAGTGCAATCGCATCGTTCTTTTCTACAATTAA
TTCCTTCTCTTTTAAATTATTTATTGTTTGACCAAAGACACTTTCAATAGATTGGTCAAACTTCTTTTTGAACCTACTACTACTCACACCTTCA
TTTAAACGCAAACCAAGAAACATTTCTTCTTCCATTCTCTCAGTCAAAGAAGGTTTATTTGATACTAAAATTGCTTTACTTCTTTATTTATA
GCTTTGATATAATGATTCACTGGATTGATATTCGTATAACGCACACCATCTACATAACCACTTGCACCTGCTCCAAATCCATAATATTCCTC
ATTAAACCAGTAAACCTTATTATGTTCTGATTCATGGCCATCTAATGCAAAATTAGATATTTCGTATTGATGGAAAGGAGATTGTTCTATCT
TAGACATCAGCAACTGATACATGTCAGCACCTAAATCCTCATTAGGAAGTTTAAGCAACCCTTTTCTATACATATTATAAAATTGGGTTTTA
GGTTCAAGTATTAAGCCGTAACTCGAAATATGTTGAATATCCATATCTAAAGCTAGATCTAAACTTTGTTCAAAATCTTCAATCGTCTGTTT
CGGTAAATGATACATTAAATCTAAACTGATTGATTTAATACCTGCGTTTTTAGCATTTAACACCGAAGTGTAAATATCTTCAGTATTGTGCG
TTCTACCTAAAACAGACAATAACTCCGGCTTGAATGTTTGAACGCCCATTGAAATTCTGTTTACTCCATATTTTTCTAATAGTTGGACTTTCT
CTTTAGTTAACTCATCAGGATTTGCTTCAAATGTATACTCGCCTGTGATTGTAAACGTATCACGTATTGCTTTAAGTAATCTTTCCAACTGA
TTAATAGAAAGGGCCGTTGGTGTGCCGCCACCTACATACATGGTCTTTAAGTTCCTATATTTTGCTGTAGACATTTCTGTTATTAGTGCATC
TAAGTACTCATCTACAGGTTGATTCTGTATAAAATATTTATTGAAATCACAATATGTACATATTCTTACACAAAATGGAATATGTATATATG
CACTTTGTACCGTCATTTTAATGCCCCGCTTCTCAGACTGTTTATCCAA

Gene: STAR (Staphylococcus aureus repeat element)

Contig: 07_NODE_2, position: 155055 to 155339, length: 285 nt

Sequence:

GGGAGTGAGATAGAAATAATAAAGAACCACTAATGATTTATTATGTAGTGGCTCTTACACATTAGCCAGATGTAATATGTCCATAAAAAT
AAAATTACATGAGTAAAACTCATGCATAAGGGAGTGGGACAGAAATGATATTTTCGCAAAATTTATTTCGTTGTCCCACCCCAACTTGCAT
TGTCTGTAGAAATTGGCAATCCAATTTCTCTATGTTGGGGCCCCACCCCAACTTGCATTGCCTGTAGAATTTCTTTTCGAAATTCTCTATGT
TGGGGCCCCACC

Gene: SIRU01 (staphylococcal interspersed repeat unit 1)

Contig: 07_NODE_2, position: 155225 to 155392, length: 168 nt

Sequence:

CCAACTTGCATTGTCTGTAGAAATTGGCAATCCAATTTCTCTATGTTGGGGCCCCACCCCAACTTGCATTGCCTGTAGAATTTCTTTTCGAA
ATTCTCTATGTTGGGGCCCCACCCCAACTTGCACATTATTGTAAGCTGACTTTTCGTCAGCTTCTATGTTGGGGCC

Gene: lepA (elongation factor 4)

Contig: 07_NODE_2, position: 155536 to 157359, length: 1824 nt, orientation: REVERSE

Perfect match to: (Strain_21343-AHKV01000043-[88344:90167:r], highly conserved allele)

Sequence:

TTATTCGTCATCCATTTTCAATACAGCCAAGAAAGCATCCTGTGGAATTTCAACATTACCAACTGCTTTCATCTTAGCTTTACCTGCTTTTTG
TTTTTCAAGTAATTTACGTTTACGGCTTATGTCACCGCCATAACATTTAGCTAAAACGTTTTTACCCATTGATTTAATATTTGTACGCGCTAC
AATTTTTTGTCCTATTGCAGCCTGTACAGGTACTTCAAATTGCTGTCTTGGAATTAACGTTTTAAGTTTTTCAACTAATGCTTTACCACGTTC
ATATGCAAAATCTCTATGAACTATGAAGCTTAGCGCATCCACTTTATCACCGTTTAATAAAATATCCATCTTAACTAAATTACTTTCTTTATT
TTCGATGAATTCATAATCAAATGATGCATATCCTTTAGTATTAGATTTAAGTTGATCGAAGAAATCAAATACAACTTCAGCTAAAGGTAAT
TCATAAACAATATTTACACGAATATCATCTAAATAGTCCATATTTATAAATTGTCCACGTTTACGTTGACATAATTCCATTACTGCACCTACA
TAGTCATTTGGAACCATCATAGTTGCACGAACATATGGCTCAAATATTTTATCAATTTTATCACGATCTGGCATTTGTGCTGGGTTATCAAC
CGTCACTTCTGAACCGTCCCTTAAAATACATTGATAAATTACAGATGGTGCTGTTGCAATTAATTCAATGCCAAATTCTCTTTCAATTCTTTC
TTGAATTATTTCCATGTGTAACATACCTAAGAAACCAGTTCTATAACCAAAACCTAATGCTTGTGACGATTCAGGCTCAAATTCTAATGAT
GCATCATTCAATTGTAATTTTTCTAATGCTTCTCTTAAATCATTATAATTTTTGTTATCTATTGGGAACAGTCCGCAATATACCATTGGATTC
ATTTTCTTATAACCTTGCAATGGTTCTGATGCAGGTCTACTAGCTAATGTGATGGTGTCACCAACCCTAGAATCATCAACATTTTTAATACT
TGCAATAATATAACCAACATCACCAACTGTTAATTCATCAACTGGAAGCTGCTTAGGTGTATTAATTCCAACTTCTGTTACTTCGAACTCTT
TACCAGTCGCCATCATTCGAATTTTATCTCCGGCTTTAACAACACCGTCTACAATTCTTATCGATGAAATTACCCCTCTATATGGATCATACT
CAGAATCAAATATTAACGCTTTTAGTGGTGCTTCTGGGTCGCCATCTGGAGCTGGCACAACTTCAACTATTTTCTCTAGTATCTCTTCAATT
CCAATGTTAGATTTAGCACTTGCTAAAACAACATCGTCTTGGTCTAAACCTATCATATCTTCAATTTCTTGTTTCACGCGTTCAGGTTCTGCA
GCAGGTAAATCAATTTTGTTAATAACAGGCAATAACTCTAACTCATTATCTAATGCTAAATAAACATTTGCTAATGTTTGTGCTTCGATACC
TTGAGCCGCATCTACTACTAAAATCGCGCCCTCACAAGCTGCCAAAGAACGTGACACTTCATATGTAAAATCGACGTGTCCAGGCGTATC

GATTAAATGGAATGTATAAGTATTTCCATCTTTAGCTTCGTACTTTAAACGAACTGCGTTTAATTTGATTGTAATACCACGTTCTCTTTCTAA
ATCCATTGAATCTAGTAACTGATCTTGCATATCTCTTGTTTCAACTGATTTGGTATTTTCTAAAATTCTATCAGCCAATGTAGATTTTCCGTG
GTCAATATGTGCTATAATCGAGAAATTCCTTATATTCTCTCTTCTTTTTAAGCGTTGCTCATTATCCAT

Gene: rpsT (30S ribosomal protein S20)

Contig: 07_NODE_2, position: 157705 to 157956, length: 252 nt, orientation: FORWARD

Perfect match to: (RF122-AJ938182-[1576162:1576413], highly conserved allele)

Sequence:

ATGGCAAATATCAAATCTGCAATTAAACGTGTAAAAACAACTGAAAAAGCTGAAGCACGCAACATTTCACAAAAGAGTGCAATGCGTAC
AGCAGTTAAAAACGCTAAAACAGCTGTTTCAAATAACGCTGATAATAAAAATGAATTAGTAAGCTTAGCAGTTAAGTTAGTAGACAAAGC
TGCTCAAAGTAATTTAATACATTCAAACAAAGCTGACCGTATTAAATCACAATTAATGACTGCAAATAAATAA

Gene: holA (DNA polymerase III, delta subunit)

Contig: 07_NODE_2, position: 158001 to 158975, length: 975 nt, orientation: REVERSE

Perfect match to: (MW2-BA000033-[1666957:1667931:r], allele observed in CC1+CC80+CC188+CC361)

Sequence:

TTATAAAGATAGAATGAATAATTCCAGTATTAACTGTTTATCCATATATGATGATTTAAGTTTATAATCAGTTTCCGCACAAGCATCTATAA
TATTCAATAATTCATCAAGTTGATAATGTCTTACTTGTCCTAACGCTAATTTTACTCTGTATGGATGTACGCCTATTGTTTTAGCAATTTGCT
GTCCACTATATCCTTTTTGACTCAAAATCTTACATTGATAAAATAATCGGTAATTACTTGTGATTAGTGCAAGTAATTTAATAGGTTCTTCTT
TCATAGTTATTAAATCTTTTACTAAATGAATTGCTTGTTCTTTCTTTCTTTTCTGAATGTATTCAGTCAGTAAAAATACATTTTGCTCTAAACT
TCTATTAATAATTTGGTTAACATCCTGCTTATTAATTGTTGGTCTATCGCCTAAAAATAAAATCAACTTTTCTATCTCTTGTGAGACAATATT
AAAGTTAATACCTGTCAACTCAATAAATAAATCTAATGCATCTCTTTTGATATCTTTGAAATTCTCATTTAATTTACTTTGAATCCATTTTTTT
ATTTCTTCTTCAGACATTTGCTCTATTTTTTTAAGCCTTGCATGCTTTTTTAGAGTTTTAGTTAACTTTTTTCTTTCATCAAGTTTATTTTGATA
TATCTCAAAGACAATCAAATTTTCGCCATCATATTTTTCAATAAATTCTATTAATTGATTTACATTATGAGCCATATCTTTTGGCGCTTTTTCA
CCTGTAAATATATATGCATTTTTAACCAAAATTGCTTTTTTATCTGAAAAGAAAGGCAATGTTAATGTTTCTTCAACAATTGGTGCAATCTC
TGTTTCGTATAAATTATATTTCACAAAGTTAAAGTCATCTCTATCACTTTTCAAAAATTGTGATATAATTTCTGCACTTTGTTTTTCAACCAAT
TCAGGCACATCTCCATAAATAGCTACAATATTGTCGCTCAT

Gene: comEC (DNA internalization-related competence protein)

Contig: 07_NODE_2, position: 159032 to 161233, length: 2202 nt, orientation: REVERSE

Perfect match to: (MW2-BA000033-[1667988:1670189:r], allele observed in CC1+CC5+CC22+CC25+CC361)

Sequence:

CTATAAACCACTTGCATTTCCATAAGAGTTTGAATCAACTTTTAAATTATCATCTAAGTCAATTGTAACTTGACCGTTTTGTTGACTATTGTA
AATGCGACTGCGAATCCTTTGCAATCGTTTAACAACTTCTATATTAGGAAGATGATACATATTGTTCTTCCCAGAAGAAATCAAACTTATTT
TAGGCTTAATCATCTCTATAAATTCTTTAGAACTACTTGTCTTGCTCCCATGATGACCTACTTTTAAAATATCAATCTCCGGCAAGTTATATT
TTTTTAGTAGTAAAGATTCATTATTTTTACTAGCATCGCCCATTAATAAAACTTTTTTATTTTGATATGTAATCATAGTAATAATCGAATACT
CATTTTTATCTCGGCTATTTGGAATAAAACTATCAAAAAATAGAAAACTACTATCTCCAAGTTTAAAACTACTAACTTGTCTTACATCTATA
AGTTTAATGTTGTACTTATGGCTTAATTTCGATAATAACATCAATGTATTACTACTATATCCCTTATTGTATATCTCTATATGTTTAATTTTAA
TATGACTAATAATATATTCCACTTCACCAATATGGTCATTGTGTGGATGTGTTAAAATTAGATACTCTAATTCATTTATCCCTCTTTCATTTA
GCGTTGGTAAAATATGATATTTAGAAATTGAATAACTAGGTTGTTTAGTATCATCAATCACTTTCCCACCTGTATCAATCAAGACATTTTGG
TTCTTACCACCTTCATATAAAATACTGTCTCCCTGCCCCACATTTAACATTGTAATTTTGTGATGTGAATTTGTTGGAAACGTTATTAATAAT
GTCAGAATAATTATAGTCCAAACCGTAACCAATATATATTTACGTTTAGCCAATAACCAAAATATGTAATAAACAGAAATTATAAATATTA
TAAATATCCAATCATTAAACTTGGTAACAGAAAAATGTGATTGCTTGATTCTTGTGAATAGGTCTAGTAACCAGTCATGAAAATTAAAACT
TAGGTCAACCAAGTAATTTAGCGGCGTTAATCCCACAATAAAATGACTTGTAATAAAGAATAAAATAGATAGCGGAAACAATATAATCGA
ATAGTACGGTACAAAAATCAAATTAGATAAAAATCCCACCCATTGAAGTTGATGAAAGTTTGGAATGGCAACGATAAATGAAGCTAATT
GTGCAATAAACGTAATTATGAATAATGATTGTAACTTTGACAATTGCTGTAAAAAAGGAAAAAGTAGCATAATAAAAAATGAAATGATG
AATGAAAATTGAAATCCAATATCATAAACAACTAGTGGATTTAAAATAAACATAATTATAAATGCAAATGCTAATAGCTGAATACCCTTTA
TTTTAATTTGCTTAGTAATAAGCAGTACAAGAGTTGTCATTATTATAGCTCTTACAGCACTAGGTGCATAATTTGTGTATTGAGCAAATAA

AGCTAATACAATGATTGTAATTCCTTTAATGACAAATAAAGGTAAATTTAATCGTTTTAAAGGTTTGTAAATTAAGAATACAATTGCAGCT
ATATGCGAGCCACTAACTGCCAGCAAATGATATATACCTATCTCTTTAACACGTTCCTTAAATTGCTCATTAATTTCTTTTACGTCACCAGTA
ATCAATGCCATAATACGATCCGGAAACTTAATACCCGAATCATAAATTCGATTCATTATAAACTGTTTATGTTTCTCAATTAAATTAGACCG
GTTCGATTCTAGGCAGCTTTGTACAACTATACTTTGTAATTTAAGAGTTACAAACTTATTGTCATTAACTTTGAATTGTCCTTTAACAATACA
ATTACGACTTTCAATTTTCTTTAAATCAAAATTCTTTTTATTTGTTAAAAAGAAAGGATATATTTCATTTTTTAAACTCAACCTACCTTTATAT
GTGTCACTACCTTGACGTTGAATTTGGATTACTTGAGCACGCTCATTAAACTGAGAATTACGTTCAATATAATTGATATAATTAAATATTGC
TTGTTGTGAATAATGTAAATACCATGAGGAGAAAATGATTAAAAAGAGAGAAATAGGGGCATAAACGATTTTATTTTTACGATAAGTAAT
ATACAAAAGTAAAATGAAAAGAAATGTAGAGAGCACTTTGCTAGAATTCCAAAGCACTCCTACAATCATTGATAACGCGACATACAGCAA

Gene: comEB (competence protein EB)

Contig: 07_NODE_2, position: 161238 to 161669, length: 432 nt, orientation: REVERSE

Perfect match to: (RF122-AJ938182-[1579697:1580128:r], highly conserved allele)

Sequence:

TTAACCTTTAGTCAGATATTTAGCAACATATTCTGGTGAAAATGGAATTTTTTTAAATTCAATACCAGATTGTTTGAGTAATTTAGTTGCAT
ATTCATGGTTATGATAATCTTCTGCATAGTAGATACGCTTTATACCTGCTTGAATAATTGACTTTGTACAATTTAGGCATGGAAAATGAGT
AACATAGATTGTTGCACCTTCAGTAGATACACCTTGTTTTGCACATTGTAATAAAGCATTCATTTCTGCATGTATCGTTCTGATACAATGTC
CATCTTCAATTAAACATCCTTCATCTATACAATGCACCTCGCCAGCTACAGAGCCATTATAACCACCAGCAATAATACGATTATCCTTAACA
ATCGTTGCACCTACAGATAATCTTTGACAAGTTGAACGTAATGCTAGCAAATGACTTTGTGCCAT

Gene: comEA (competence protein EA)

Contig: 07_NODE_2, position: 161791 to 162477, length: 687 nt, orientation: REVERSE

Perfect match to: (MW2-BA000033-[1670747:1671433:r], allele observed in CC1+CC188)

Sequence:

TTATATCGTGAAATAAGATTTCAGTTTATCAAAAGTTTTACTTCCAAAACCTTTTACTTTTTTCAAATCGTCAATTTCTTGAAATGCACCTTG
TTGGTTGCGATATTCAACAATTGCATTAGCTTTAGCTTGCCCTACTCCAGGAACAGACATCAATTCTGATACAGATGCGGTATTTAAATTT
ACTTTAGTATTATTTGTGTTCCCATTTTTTACATGCACACTGTTTACTCCAATTTGTGGTTCAACATTCTTTTGTCCTTTATGAGGTATGAAA
ATCATTTTTTGATCTGTTAATTTTTCAGACAAATTAATTTGACTTACATCTGCATCCTCCAATAATTGTGCTTTATCAAGTAAATCAACTACT
CTATCCTTAGATGTCATTTTATAAACATTAGGATGTTTAACAGCACCTTTTACATCGACATATACAGGACCCTTATTTTTGGAATTATCTCCA
TCTTTGACCTGGACATCTTCTACTTTGGACAAACTACTATTTTCACTAGTGCTTTGTTTCAGAGCATTATCTTTATTTTCAAAATTTCTTGAA
GTATAATCATCTTGTCTCCAGAATATAAAACCAATTAATACCATAATTAAAACAACAGCACTTATAATATATAACTTCCATTGAGTTAAAAA
ATCTTTATAGCGTAATAAAAATTGATACAATAAAACCAC

Gene: yqeM (putative protein)

Contig: 07_NODE_2, position: 162517 to 163233, length: 717 nt, orientation: REVERSE

Perfect match to: (MW2-BA000033-[1671473:1672189:r], highly conserved allele)

Sequence:

TTATTTTTTCGCAATGAAAAACAATCTTTCTGCATCTTCATTATGTTCATCTATATTAAAATCAGTAAATGTTTCAACATGTTTAAAACCAAC
TTGAGCTAACCAAGACAAATATGTCTTTTCATCAAATGTTCTCTGAAAATGAGACTCATCAAATCTTGAATATGTTTCATCTTCATGTCGAA
TGAAAAATGTCATATCATGATAAACACTTAAAGGTAAATCTCCTTGCACAGCATCCCATGCTAAAAAAATGTCCCCTTTATCATCAATATA
ACTTTGATTATTAAACAAAGTCATCATTTTATAAACAGTATGTACATCAAAAATAAATACACCTGAATCAGTCAGATGATGATAAACATTG
ATGAATGTTTCAATCACTGCAGTTTCATCTTGCAAATAATTTAGAGAGTCACAAAAAATAGTGATGATATCAAATTGTTGTTGCAAATCAA
AAGATGTCATATCTCCTTCAAGCCAATTTACATTTGCTGATTTTTGAGCTGCAACAGTCAACATATCAACACTTAAATCCATACCAGTAACA
TTACCTAAAGCTTCTAATTGAACTGTTAAACTACCAGTACCGCATCCAATATCTAAAATATTTGATTCATCTTTGCAGTGATTTTTTACAATT
TCAAACCATTTTTCATATGGTTGATCTTGAGTCAATTGATCGTACACTAGGCTCATTTCTGCATATTGCGACAT

Gene: rsfS (ribosomal silencing factor)

Contig: 07_NODE_2, position: 163236 to 163589, length: 354 nt, orientation: REVERSE

Perfect match to: (MW2-BA000033-[1672192:1672545:r], highly conserved allele)

Sequence:

TTAATACGCAACCTGACTATATGATTCTAATGGTGCATCTTGATATAACTTTTCAATATTATAATAATTTCTTTCGTCTTTATGGAAAACATG
TACCACAACATCAGCTAAGTCAATTAATATCCAACGCGCTTCATTGTATCCTTCCATACGTTTTACTTCTATATTTTGTTCATTGGCTACTTCT
TTCACCGCTCTAGCAATCGCTTGAACTTGTCGTTCATTATTTCCGTGCGTTACAACAAAATAATCTGTCATATCGCTGATACCTTTCATTTCT
AAAGAAATCGTATCTTCGCCTTTTTTATTGTCAATTGCATCCACAGCAATTGCTAATAATTCTTGTGAATTCAT

Gene: yqeK (putative hydrolase)

Contig: 07_NODE_2, position: 163590 to 164174, length: 585 nt, orientation: REVERSE

Perfect match to: (MW2-BA000033-[1672546:1673130:r], allele observed in CC1+CC25+CC188+CC772)

Sequence:

TTAATCATCCTTTATTCTTTCGTCACTATAGTTATAATAATTTAAACAGTCAATCGTCTTATTATATACCGTAATATCTTTCTGTATTAAAAAT
AGTACTGTGCGTTTAGAAATTTCATAAATTGTCTTATCTAAACTACCTTGATTGTATGCCATATCTCGAATATCATCAACTCCTGGGATTGT
TCTTCCAGGTTCGATGTAATCTGCAATAAAAATCAGTTTTTCAGTTTTTGTCATTTGTTGACGTCCAGTAGTATGGTATTTGATAGCCATTA
ATACTTCCTCATCATTGATACCATATTCATGTTCCATGATTGCTGCACACACAGGGCCATGCAATATTTCACTACCATAACTCAGTAGATCA
TTACCTAATTCGTATTGTCGAACAATTTGATACATTTTACCTAAATCATCATATTTACAGAAATCATGTAATACACCTGCTAATTCTACTTTA
CTAGTGTCTCCATCATAAATTTCTGCCAATTTAATAGCTGTTTCTGCAACTCTTAAAGAATGATTATAACGTTTCTCTGGCAGTTTCTCTTTT
GCAAGCCGTTTTGCTTTTTCAATGTTCAT

Gene: nadD (nicotinate-nucleotide adenylyltransferase  (Deamido-NAD (+) pyrophosphorylase)  (Deamido-NAD (+) diphosphorylase))

Contig: 07_NODE_2, position: 164164 to 164733, length: 570 nt, orientation: REVERSE

Perfect match to: (N315-BA000018-[1627469:1628038:r], highly conserved allele)

Sequence:

TCAATGTTCATATAATCCTTCCCCCTTAATATAGTTTTCAACGGATTTAGGAACAAGAACTTGGATAGATTTCCCTTCACTAACTCTTTGTCG
AATCATTGTCGAACTTATATCTACCCTAGGTATCTGAATTGCAATCATAGCATTTTCAACATTTTGACTATTTTTGTCTCGATTTACAACTAC
AAAAGTAACCATTTCTTTTAAGTATTCAATTTGATACCATTTCTCTAGTTGGTTATACTGATCCGTCCCAATAACAAAGTACAACTCACTGT
CTTTGTGTTGCTCCTTGAATGCCTTGATCGTGTCATAGGTATAACTCTGACCACCACGTTTAATTTCATCGTCGCAAATATCTCCAAAACCA
AGCTCGTCGATAATCATCTGTATCATTGTTAATCTGTGCTGAACATCTATAAAATTATTGTGCTTTTTCAATGGAGACATAAAACTAGGTAA
AAAATAAAATTCATCTGGCTGTAATTCATGAAATACTTCGCTAGCTACTATCATATGTGCAGTATGGATAGGGTTAAACTGACCGCCGTAA
AGTACTATCTTTTTCAT

Gene: yhbY (RNA-binding protein)

Contig: 07_NODE_2, position: 164736 to 165026, length: 291 nt, orientation: REVERSE

Perfect match to: (N315-BA000018-[1628041:1628331:r], highly conserved allele)

Sequence:

TTATGGCAATTCAATTTCTTTATTTTCTTTAGATTCTCTATAAATCACTATCATAGATCCAATCACTTGCACTAATTCACTACGCGTAGCTTC
GCTTAATGTTTCAGCTAATTCTTTTTTATCATCAAAGTTATTTTGTAGTACATGTACTTTAATCAATTCTCTGTTTTCTAACGTATCATCTATT
TGTTTAATCATATTTTCGTTGATACCGCCTTTTCCAATTTGAAAAATCGGATCAATATTGTGTGCTAAACTTCTTAAGTATCTTTTTTGTTTG
CCAGTAAGCAT

Gene: aroE (shikimate 5-dehydrogenase)

Contig: 07_NODE_2, position: 165030 to 165836, length: 807 nt, orientation: REVERSE

Perfect match to: (MW2-BA000033-[1673986:1674792:r], highly conserved allele)

Sequence:

TTATTCTCCTTTTAATTGTTGTAAAACTGCTGTTTTCATAGAATTAATATCAGCATCTTTATTAGTCCAAATTTTAAAGCTTTCCGCACCTTG
GTAAACAAACATATCTAAGCCATTATAAATATGGTTTCCCTTGCGCTCTGCTTCCTCTAAAATAGGTGTTTTATACGGTATATAAACAATAT
CACTCATTAAAGTATTGGGAGAAAGATGCTTTAAATTAATAATACTTTCGTTATTTCCAGCCATACCCGCTGGTGTTGTATTAATAACGAT
ATCGAATTCAGCTAAATACTTTTCAGCATCTGCTAATGAAATTTGGTTTATATTTAAATTCCAAGATTCAAAACGAGCCATCGTTCTATTCG
CAACAGTTAATTTGGGCTTTACAAATTTTGCTAATTCATAAGCAATACCTTTACTTGCACCACCTGCGCCCAAAATTAAAATGTATGCATTT
TCTAAATCTGGATAAACGCTGTGCAATCCTTTAACATAACCAATACCATCTGTATTATACCCTATCCACTTGTCATCTTTTATCAAAACAGT
GTTAACTGCACCTGCATTAATCGCTTGTTCATCAACATGATCTAAATACGGTATGATACGTTCTTTATGAGGAATTGTGATATTAAAGCCA
TCTAATTCTTTTTTCGAAATAATTTCTTTAATTAAATGAAAATCTTCAATTGGAATATTTAAAGCTTCATAAGTATCATCTAATCCTAAAGAA
TTAAAATTTGCTCTATGCATAACGGGCGACAAGGAATGTGAAATAGGATTTCCGATAACTGCAAATTTCAT

Gene: yqeH (putative GTP-binding protein)

Contig: 07_NODE_2, position: 165850 to 166950, length: 1101 nt, orientation: REVERSE

Perfect match to: (N315-BA000018-[1629155:1630255:r], allele observed in CC5+CC1+CC8+CC88+CC772)

Sequence:

TTATAAAATAGAATTTCTTAATACAACATCAACATTTTTAGGAACACGAACGATTACTTTAGCTCCTGGTCCTATAGTTATAAAGCCTAGAC
CAGAGATCATAACATCGCGTTTCTCTTTGCCTGTTTCAAGTCTAACAGCCTTTACCTCATTAAGATCAAAATTTTGTGGATTTCCAGGTGGC
GTTAATAAATCGCCAAGTTGATTACGCCATAAATCATTGGCCTTCTCCGTTTTAGTACGATGTATATTCAAGTCATTAGAAAAGAAACAAA
CTAACGGACGTTTACCACCTGATACATAATCTATGCGCGCTAGACCGCCGAAGAATAATGTTTGCGCCTCATTTAATTGATATACGCGTTG
TTTTATTTCTTTCTTAGGCATAATAATTTTCAATTCTTTTTCACTAACTAAATGCGTCATTTGGTGATCTTGAATAATACCTGGTGTATCATAC
ATAAATGATGTTTCATCTAAAGGAATATCTATCATATCTAAAGTTGTTCCAGGGAATCTTGAAGTTGTTACTACATCTTTTTCACCAACACT
AGCTTCAATCAGTTTATTAATCAATGTAGATTTCCCAACATTCGTTGTCCCTACAATATACACATCTTCATTTTCTCGAATATTCGCAATTGA
TGATAATAAGTCGTCTATGCCCCAGCCTTTTTCAGCTGAAATTAATACGACATCGTCAGCTTCCAAACCATATTTTCTTGCTGTTCGTTTTAA
CCATTCTTTAACTCGACGTTTATTAATTTGTTTCGGCAATAAATCCAATTTATTTGCTGCTAAAATGATTTTTTTGTTTCCGACAATACGTTT
AACTGCATTAATAAATGATCCTTCAAAGTCAAATACATCCACGACATTGACGACAATACCCTTTTTATCCGCAAGTCCTGATAATAATTTTA
AAAAGTCTTCACTTTCTAATCCTACATCTTGAACTTCGTTATAATTTTTCAAGCGGAAACAACGTCTGCAAATCACGTCATCACGAAACATA
TTATGCTCTGGTACAAAACCAGGTTTATTTTTATCTTCAGATTGAAGTGGCGCACCACAACCGATACATTTTAAAATGTCAGACAA

Gene: yqeG (HAD-superfamily hydrolase)

Contig: 07_NODE_2, position: 166951 to 167478, length: 528 nt, orientation: REVERSE

Perfect match to: (N315-BA000018-[1630256:1630783:r], highly conserved allele)

Sequence:

TCAATTTTCCTCCCATGTGATATAACCTTTTTTACTGAAATGACGTAATAATCGTCTTTCAATTAATCTATTAAACTTAGTAATAAAGCCATC
AGTTCGTTTAACTGGAACAACCATAATTGTATATAGACCTCGACGATTACCACCAAATACATCAGTAAGCATTTGGTCACCTATAACAACA
GTTTGATCTGGTCTGATATTCATCTTAGTTATTGCTTTATCAAACGCTTTCCCCATTGGCTTTCTCGCTTTAAAAATAAAATCGATGTCTAAA
TGCTGACTAAAACTAGCAACACGAGACTCATTATTATTAGACACGATTGTAATAGTGATTCCTTTTTCATTAGCTTCCTTAAACCATGCTTT
AACACGTTCTGTAGGTTCTTTAACATCCCAACCTACTAGCGTATTATCTAAATCTGTAATAATACCTTTAACGCCTTTGTCCACTAACTTGTC
TAAATCAATTTGAAATATTGATTGAACATATGAATTCGGCATAAAAAACTTGCGAACTAAACCCAT

Gene: mtnN (5'-methylthioadenosine/S-adenosylhomocysteine nucleosidase)

Contig: 07_NODE_2, position: 167498 to 168184, length: 687 nt, orientation: REVERSE

Perfect match to: (MW2-BA000033-[1676454:1677140:r], allele observed in CC1+CC15+CC188)

Sequence:

TTATAATTGAGACACTAATGCTTCAACAGTTTGACTTGATGATACAGCTGCTTTTTCTAAAAATGCTTCGAAGCTCATTTCCGCTTCTCCATT
TGCTAAGTCTGAAACTGCACGAACTACAACAAATGGTACATTAAATTGATAACATGTTTGTGCAATTGCAGTTGCTTCCATTTCAACCGCC

ATCGCATTTGGAAATGCTTTTTTAATTTTTTGACGTTGTTCAACACTACCGATAAAGCTATCACCACTTACAATTAAGCCTACTTTAGCTGTT
AATTGTTGTTGTTGTACAACTTGAGATACTTTTTCTATTAAAGGTTTACTTGATTGAAATGCTACCGGCATCTGTGGTATTTGTCCATATTC
ATAACCAAATGCTGTTGCATCCGCATCATGATATTTTACATCATCACTTATAAGAACGTCACCTACATTTAAACTTTCATCTAAAGCTCCAG
CAGAACCTGTATTAATAATGACGTCCGGTTTAAACTTATTAATTAATAATGTCGTAGAAATTGCAGCATTAACTTTTCCAATGCCGCTTTGG
GTAATCACTACTTCTCTATCTTTTAAAATGCCAGTATAAAATTTAACATGTGCAACTGAAATTTCGCTTAATTGTGTTAATTTATTTTTTAAT
ATTGTTACTTCTTCTTCCATGGCACCAATTATACCAATCAT

Gene: Q5HFG1 (putative protein)

Contig: 07_NODE_2, position: 168498 to 168767, length: 270 nt, orientation: FORWARD

Perfect match to: (MW2-BA000033-[1677454:1677723], highly conserved allele)

Sequence:

ATGGCTGAAAATAATCAAAATAGTCTCGTAACAAAGATAGCTACATACGGCAGTTTTATTGCAATTGCGTCGTTTGTCATTTTATTTATAA
GTATATTTTTAAAGTTTTCATTTAATATCGAGTTTACAGCAACAATTATGAACATATGTCGCTACACTCTAATTCTAGGATTTATACTTATGT
CCTTACCTGATGTTGTTGATAAAAACATCAAGAAAATTATATTCGATGCATTTATTATCATCGTCTTTATCTTCTTTTTACTATAG

Gene: entX (putative enterotoxin homolog)

Contig: 07_NODE_2, position: 168969 to 169721, length: 753 nt, orientation: TRNC-RVRS (no start codon)

Sequence:

TTATGATTTGAATAAATAGATATCTAAATGCAATTTGTCACTATTGATAGTTTTATTATCAGCATACATTTTTAATACCTCATAACCATACTC
ACCATTTAAATTATAAAAATTATATTCTATATTTTGTTCATCATCATTATGATATTTAACATACCCTTTAACAATGTCGCCACCTTGTTCGTAC
AACTTATACTTCTCATTCAAATAATTTCTTAATTGAACATCTAATTCTTGAATGGTAACAATTTTCTTTTTCGTTTTTACTGCTGTTAATTCTA
TTTCTGCTTGATGTCCGTCAATCCATAAATTACAAGGTATATTCTTATAATCATCGTACTTATTATTGTCACTTAAAGTAACACCACCATAAC
TACATTGCGTTTTATTGGTTGCTCCCCCATGACATCCATAACCATAATTTCCAGCAAATATATCGATATCCTTATTTATAAATTTCTTTGAAA
GTGCCTCATTTTCGAATTCAACTTTAAAATCTTTTTTCCAATCATCATTTACGAACATGTCATGAAATAACAAATCATGCTTCAACAGTCTAT
CGTTTGTGTTTTTATTTTCAAGCTGAGTTGGATTGATGAATGACATCTTAGCATTACTTAGACGCTTTGAATCAAACTTACTTTTATGATGT
AAGTCTGAATATTCGATTGCACTTGCTGAATTTGTCAAAATTTCTTCTAACAATAATATTAAAATAAATCCTGTTAAATATTTAACTTCAAAC
TCCCCAAA

Gene: Q5HFF9 (Fido domain protein)

Contig: 07_NODE_2, position: 170428 to 171648, length: 1221 nt, orientation: REVERSE

Perfect match to: (GR1-AJLX01000009-[145194:146414], allele observed in CC361+CC772+CC1290)

Sequence:

TTATTTTATAATCGAATTTAGAAATTCATCACTTATCTCATAAATTATTGGTCTACTTTTAATTTTAACTAAGTAGTTATCATATTTTTTAATT
GTATTATTAATTTTTACTCTTCCTACTCCCAATATGTTTTCTAAATCAATTAATGTTAATCTATTTCTTTTATTTCCAAACAATTTATCCATTGA
AAGGAGATACAAAAATTCATAATCTATCTGTTTATAATGACTAGTTAAATAAAGCGTTAATTTTTCAGTCGCATCCATTTTTGGTATTAACT
CATCAAGTATTCTTTCTTGTCCTGCTATCAATAGCTCAAGCATTGTGTCAATAAATTCCGTCAAATCACCACAATTCAAATGATTTGAAGCA
GTCATAAACGCCTTGTAATATTTCGATTTATTTCTATTTATAACATATGAAAATGTCAAAGCAGTATAATTATCATAATAATCACTCAAGAG
CTTGGCAATTATAAATCTTCCAACTCTACCATTGCCATCATAAAAAGGATGTATATATTCAAACAGATAATGACTAGCCATGATTTTGAAC
GGCTGAGGCGCATCAAAATATTTTAAAAATGTTAGCATTTCACCTATATATTCAACAATTTTGGTTTCAGGTTGTAACCCAACATGTATATA
TTTATTCGTTGACCCATCATGCACACCGACAAAATTTTTACGAAATAGCTCTCCATCTAACTTATCTTGTTCGTTAATTTCATTTGAAACTAA
TTTATCATAAATCGCTCTAATGTCTCTTACATTATCAACTTTAATTTTTTTATTAAGTTCTATCTCTTTATATTGATCCACGAGGCCTCTGAAC
TTAAGAAATTCTGATGCTTGGTTATTTAACGCATGTGCAATCTCTTGTTTAGTACTAAATACATTTTCAATTTCATTAGTACTTTGTAATTCA
TCGATTAATAAATCATTAAAATATTGTTCTCTAGCTGCATATGGTAAAGAATTAAGTGCTCTATCAATTTTTCTACTATTAATTGATATTAAT
TCTTGTTTTTTTGATAGATTTTTAGTCACCATAAAGAATAGAGGATATTCCAAATCATTAACTTTTTTTCCATTTTCCATAGGTATGATATTA
ATATTAGTATTGAAAGAAGCTAAAGAATTAAATCTTTTAGTATACTCTTCCTTCATTTTACTTTCATTGTGTTCATGAAAAATACTTTTTAAA
GTTCTGTAACCCAT

Gene: ycsG (putative branched chain amino acids transporter)

Contig: 07_NODE_2, position: 171935 to 173164, length: 1230 nt, orientation: REVERSE

Perfect match to: (MW2-BA000033-[1680891:1682120:r], allele observed in CC1+CC188)

Sequence:

TTAAAATAACTGAAGTACTTCTTTGAATGAGAATATACCTGTCAATATTGTAACAAGTACTGCAACGATACCAAAGATGAACATCCAATTT
GGGTGTTTATAATCACCAACAATTGATTTCTTTTTACTTGCAATCAAAATTGCACCTAACGTAATAGGTAAAATCCATCCATTTATCGCGCC
TGCTATAATTAAAAGGCTGATTGGTTTTCCAATAAATAAGAAAATCATTGTTGAAATAACGATAAACACAATCACAATTAAATTACTTCTTT
CGTTAAGTGATTTATGAAGTGTTTTTAAAAATGTTGCGCTTGTGTATGCTGAGCCAATTACTGATGACATAGCTGCAGCAAATAACACAAT
ACCAAAAATATTCTTTCCAATTGGTCCAATTGCGTGTTCAAAAACTGACGCTGGTGGATTTTCAGAACTTAGTGTCACACCTGTTACAACA
ACACCTAATACCGCTAGGAATAGTAACGTTCTCATAATACCTGTAGTTAAAATACCAGCAATTGCTGATTGATTTACAAATGGTAAATATT
GCTTACCTTTAATGCCAGAGTCTAATATACGATGTGCACCTGCAAAGGTAATATAACCACCTACAGTTCCACCAACTAACGTAATGATTGG
CAAGACTAATTTCATTGGATGTTCTGGCGCAAATGTATGCACAAAAGCATCACCATAAGGTGGATTAGAAACAAACATCACATATGCCAC
AACTAAAATCATCACAATACCAAGAATCATTGAAACAACGTCCATAATTTTTTGGCCACTTTTACTTACAAAGATTAATATTGCAAAGATTG
CAGTAATAGCTGCGCCCCATTTTACATCTAATCCAAAAATTGCATTTAAACCTAAACCGGCACCAGCAATATTACCAATATTAAAAGCTAG
ACCACCAAATGCAATCAATATTGAGATAACAGTACCAAGCCCAGGAACAACTTTATTTGATATTTCTTGACCTCTTAAACCAGTTACAACT
AATATGCGCCATATATTAATTTGTGCACCAATGTCAATGATGATAGACAGTAATATGGCAAATGCGAAACTTGCAAAAAATTGTGATGTA
AATACTGCTGTTTGCGTTAAAAATGCTGGCCCAATTGCAGAAGTTGCCATCAAAAATACAGAACCTAATAATAACCTTTTATGATTTTTTG
TGAATTCAAAGTCACTTTCTTTTTTAAGCTTTAAATTTCTTCCCAT

Gene: ycsF (uncharacterized protein family UPF0271)

Contig: 07_NODE_2, position: 173176 to 173928, length: 753 nt, orientation: REVERSE

Perfect match to: (N315-BA000018-[1636542:1637294:r], highly conserved allele)

Sequence:

CTATAAGGATTGAATATCAATGCCTTCTTTCATTAAAATTTCTCTAATTTTCGAAACAAATAATAATGCATGTTCTCCATCACCATGCACACA
AATTGTATCTGCTTGTAACGTTACTTCCTTATTGTTTTTTGAAATAACTTTATTTTCCTTCACCATCTTTAAAACCTGCTTAAGTGCTTCGTCA
GTATCAGTAATCACAGCATCACTTTCTTTTCTACTAACGAGCTGCCCATCATCTTCGTATCGTCTATCAGCAAACACTTCAGAAGCTGTAAT
TAATCCGACATTCTTTGCTTCTGAAATTAGATATGAATTTGCTAATCCTACTAACACTAGTGATGGATCAAAGTCATAAACAGCTTGTGCTA
TAACGCTTGCTATTTCTCTGTCTTTTGCACCCATCTGATACAACGCACCATGCGGTTTAACATGATTAATTTTAACTTGATGAATGCGACAA
AACCCTTGTAATGCACCTAATTGATAAATCATCAAATTATAAATCTCGTCGTTAGAGATATCTATATTTCGTCTGCCAAAGCCTTTCAAATC
AGGTAAACCAGGATGTGCACCTACTGCAACATTATGTGCTTTGGCAAGTTTTACCGTTTCATTCATTACATTTTCATCACCAGCGTGAAAA
CCACAAGCAACATTCGCACTTGTAATTAACGGAATAATTTGATGATCACCACCAAAGGAATAATTTCCAAATGCTTCGCCTAAATCACAAT
TCAAATCAACTCGCAT

Gene: accC2 (biotin carboxylase subunit)

Contig: 07_NODE_2, position: 173928 to 175289, length: 1362 nt, orientation: REVERSE

Perfect match to: (MW2-BA000033-[1682884:1684245:r], allele observed in CC1+CC188)

Sequence:

TTATAATTCCACCCCTTTAACAATTTGATGTTTTTCTAAAAATTTAATATCAACATCTTTTGCATCTCCATCACGATATAGTGGATAATTTAA
AACTGCATATAAAAAATCGGCAGTTGTAGAAAATCCATCTATCACCATTTCATCTAAGGTGGCTTTCAACTTATCAATTGCTGAAGCTCTA
TCATGAGATTTTACAATTACTTTAGCTACTAAAGAATCATAATATGGTGAAACTTGATAACCGTGATATAGTAAAGAATCGACTCGCACAT
TAAAGCCTTGAGGTAAATGTAACGCTGTCACTTTACCTGGTGTTGGTTGAAATTTCTTTTCAGGATTTTCGGCATTTATTCTCGCTTCTATC
ACATGACCATTAAATTGAATATCGCTTTGTGAAAAAGGTAAATGATTATGTTCCAATAAATACAATTGTGCTGCAACCAAATCACGTTCTG
CTCGCATCTCTGTAACAGTATGTTCAACTTGTATTCGAGCATTCATTTCAATAAAGTAATGTGCGGTATCAGTTACTAAAAATTCAATCGTA
CCTGCACTTCTATAATTTGCTGCACGTGCAACTTTAACAGCATCGTTACATATTTGTTGTCGTCTTTCTTCAGTTAATGCTGCACAAGGAGA
TTCTTCGATTAATTTTTGATTTTTACGTTGTACAGAACAATCACGTTCCCCTAAATGTACAAAATTATCCTGCCCATCTCCCATAACTTGAAC
TTCAACATGTTTTGCAACAGGTATAAAAGCCTCAACATAAACACGATCATCATCAAAGTATTTTTTTCCTTCACTTTTAGCTTCTTTAAATGC
CTTTTCTAAATCTTCAGCTTTCTTTACAATACGTATACCTTTACCACCACCGCCACTGGCAGCTTTGATAACAACTGGATAACCGATGTCTTT
GGCAAGCTTCTCAATTTCAGACACATGGTTCACAGCACCATTTGATCCTGGAATCACAGGAACACCTGCATGATGAACTGTTTGTCTTGCT
GTTATTTTATCCCCCATCATTTCCATCGTTTTTTTAGTAGGCCCTATAAACGCTATGCCTTGTTCCTCAACGGTTTGAGCAAATTTTGTTGAT

TCTGATAAAAAGCCATATCCTGGGTGAATTGCATTAGCACCAGTGATTTGTGCAGCAGATATGATGCGGTCAATATTTAAATAACTACCT
AAAGCATTAGCTTCCCCAATACATATAGCTTGATCTGCTAAATGTACATGCAAGCTTTGCTCGTCCCCTTTTGCATAAACTGCTACAGTTTC
AATCCCATATTCTCTGCAAGCTCTTATAATCCTTACAGCAATTTCACCTCTATTCGCAATTAAACAACGAAGCAT

Gene: accB2 (biotin carboxyl carrier protein of acetyl-CoA carboxylase)

Contig: 07_NODE_2, position: 175303 to 175752, length: 450 nt, orientation: REVERSE

Perfect match to: (MW2-BA000033-[1684259:1684708:r], allele observed in CC1+CC5)

Sequence:

TTACTTAATACGTACCAAAACTTGGTCGTATTCAACATTTGTTCCATGATCAGCTACTATTTCAGTAATTTCTCCAGCAACATCTGTTGTTAC
CTCGTTTAATACTTTCATCGCTTCAACATATCCTATAATATCTCCCTTGTTAACTTTGTCACCGACATTCACAATTGGTTCAGTTAATTCTTTA
CTATCTTGTAAAAAGAATGTACCTACCATTGGTGATTTAATGTCATGATAATCATTTGTCGAAACATCGGAGTTATCATTCGCTTTTGAAGC
TGTCAAATCATTATTGTTCATACTTTGATTTGATTGATTACTGTGTGCAGCCAAATGATTCGAGTCAGTGAAGTCAATTTCTATTTCATCTTC
AAAATTTTTATATTTAAATTTCTTAACATCATTTTCCTTCACTAATTTGATTATTTGTTCGATTTTTTCAATATTCAT

Gene: kipA (allophanate hydrolase subunit 2)

Contig: 07_NODE_2, position: 175754 to 176764, length: 1011 nt, orientation: REVERSE

Perfect match to: (MW2-BA000033-[1684710:1685720:r], highly conserved allele)

Sequence:

TTACAAATCCCCTTTTAAAATTGTTGCTAATTTTTTCGAAGTATGTCGCAAGCTAGATGTATCAAAAATTGGAGTCTTTTGATGACTCTTAA
GAATTTCATTAAACAGAGACATTTGTTCCCGATTCTTATCTACAGCTTCTTGGAATGATATCCATTTAAATTGAATTGTATCTTGTGGTTTCA
TCTGTGCTAACTTTGGCAGATCAAATTTGCATACAGTTGCAATTTTGGTATAACCACCTATCGTTTGTTTATCATTAAGCAGAATAATAGGT
TGACCATCATTTGGTACTTGAACACTACCAAGAGCAACCGGTTCAGAAATGATATCTGCTTGATTAATTGGTGCAACGCTGTCACCTTCCA
AACGATAGCCCATACGGTCTGATTGTTCAGTAATTAAATATGGATGATTTACAATTTTCGCTCTAGCCTCTTCAGAAAATGCCTCGAATTG
AGGTCCTTGAAGAATGTGTATAATATTATTTTCTGGCAATAAATCGTCCTGTAAATGAATCGTCTTTCCAATGTTTTCTTTAAAGTCATTATT
TATTTTCACTGTTATTACATCATTAGCTAATAACTTTCTACCTTTGAATCCTCCTATACTGCTTCGGGTATGTGTTGCATAACTTTCAGCAAT
AGGAGGTACGTTGATAGAATGACCAAAAGTAAGATAACCGCGTGCACCTTTGGTTATAGCACCTATTTTTAAAATGTCACCTTTCTCAGCT
AATATGACAGAATTCATTGATATAGTTTTATTATTTAGCGAGGCATTAACACTACCACCGGTTATAACAAATGTATTTTGCGTATTAAATTG
AATGGTAGGACCAATCAAAGTATATTCAATCGCTGGACCATCATTGTTAATTAATAACTGCGCAACCTTAAAACTAAATTGATCCATGGCA
CCTGCGCCTGAAAATCCAATATGTTCATAACCTTTTCTTCCTAGATCTTGTACCGTTGAAAAGAGACCTGGTTGTAAAATCTTAATTGACAT

Gene: kipI (allophanate hydrolase subunit 1)

Contig: 07_NODE_2, position: 176754 to 177488, length: 735 nt, orientation: REVERSE

Perfect match to: (MW2-BA000033-[1685710:1686444:r], allele observed in CC1+CC5)

Sequence:

TTAATTGACATTTTCAATCACCACCCAGTCATCAACATTAAAGTTGCCATCTGATATATCTCTTTCGATTTGTATAAATTCTTGTTCATCTATT
GCATAAAATTGTATCCATTCTCCTGCTTCGTACATTGACATTGGTTCACGCTCGCTGCTAAATACTTTTAACGGTGTGCGTCCAATAATTTG
CCATCCGCCAGGAGAATCTGATGGATATAGTCCTGTTTGATTATTCGCAATACCTACAGAACCTGCATGAATTTTTAACCTTGGCTGATTA
CGTCTAGGTGTATGTAGTTGTTCATCAAGTCCGCCTAAGTATGGAAATCCTGGCATAAATCCTAGCATATATATTAAATAAGGTTTACTTG
TATGTTTTTCAATAACTTGCTCAACAGTTATTCGATTATGCTTTGCTACTTCTTCAATATCTGGTCCATATGTACCACCATATTGAACAGGTA
TTTTAATAATACGATTGGTTTGATTCACAGCATGAACATTTTTTTCATTAAATTTGTTAAGTTCTAAATTTTCAATTAATTTAGAAGATGTTA
TAGCTTGTTCATCAAAATATATTAGAACTGCTCGATACGAAGGGACAATATCTTGAATTTCTAATATTTCTTTTTCTCGTATCCACCGTACC
ATTGCTGTGACATTACGATATGTCTCTTCGGATATTTTATTTTCAAAATAAATCATAATTGTCTGCTCGTTAATAAATCTTACATCCAC

Gene: greA (transcription elongation factor)

Contig: 07_NODE_2, position: 177814 to 178290, length: 477 nt, orientation: REVERSE

Perfect match to: (RF122-AJ938182-[1595986:1596462:r], highly conserved allele)

Sequence:

TTATTGGATATTAACAATTTTTACGTTCATTTCGCCACCATTAGGTAGTGGAACACGAACTTCATCATCTAAACCTTTACCAATTAACGCTTT
AGCCATTGGTGATTCATTTGAAATCTTACCATTAAATGCATCTGATTCAGCTGAACCAACGATTTGATAACTTTCCTCTTCATCACCTGGTA
ATTCTACAAACGTTACTGTTTTACCAATTTTAACAACGTTGTTATCTCCAGTATCTTCAATGATTAATGCATTTCTTAACATATGCTCAATTCT
TTGAATATCTTGTTCGATGAATCCTTGTTCATCTTTTGCTGCATCATACTCAGAGTTCTCTGATAAGTCACCAAATGAACGTGCAACTTTAA
TTTTCTCTACAACTTCAGGACGCTTAACTGTTTTTAATTCTTCAAGTTCACGCTCTAATTTTTCAAAACCTTCTTGAGTCATTGGATATTGCTT
TTGATTTTCCAT

Gene: udk (uridine and cytidine kinase)

Contig: 07_NODE_2, position: 178318 to 178941, length: 624 nt, orientation: REVERSE

Perfect match to: (RF122-AJ938182-[1596490:1597113:r], highly conserved allele)

Sequence:

CTATTGCTTGCTAACTAAAGACTGAATTTTTGTTGTCATAATATCTATTGCAACTTTATTGCTCCCACCTTCAGGAATAATTATATCAGCATA
TTTCTTAGTCGGTTCAATAAATTGGTCATGCATAGGTCTAACAACACTTAAATATTGATTGATAACAGAGTCCATTGAACGCCCACGCTCT
TTAGTATCTCGTGTTAAACGGCGTAATATTCTCAAGTCTGCATCTGTATCAACATATATTTTAACATCCATCATATCACGTAATACCTTATTT
TCTAAAGCGAAAATACCTTCTACGATAATAACATCTTTAGGTTTAAAATCAATGGTAATGTCACTTCTTGTATGACTAGCATAATCATATGT
CGGTACTTCTACTGCTTTACCATTTTTCAAGTCTTTAAGATTTTCAATTAATAAATCATTATCGAATGCAAATGGATGGTCATAATTGGTTTC
TAGGCGCTCGTCGAAAGTCAAGTGCTTTTGATCTTTATAATAGTAATCTTGAGCAAGTAAAGCGACACTATGACCTTCTAAGTTTTTCATA
ATTTCGTTAGTTACAGTTGTTTTTCCTGAGCCAGATCCACCAGCTATGCCAATGATTGTAGTAGCTTTCAT

Gene: yrrO (putative hydrolase)

Contig: 07_NODE_2, position: 178941 to 180209, length: 1269 nt, orientation: REVERSE

Perfect match to: (TW20-FN433596-[1733364:1734632:r], highly conserved allele)

Sequence:

TTAGCCAATTTCCTTTCTCATCATGTTGTTTGGATATATCGGGCGATCCACTTTAATTTGAACGATTTGTAATGGATGGCGCGCCGCGTCTA
AGCTGTTACCTTCTTCATCATAAATTGCTTCTACTACTTGTGTAAATGTTTCAATTTCTGGACCAAAGAATTCTATTTCTTGACCTGGTTTAA
AGTTATTTCGTTGTTGAATAGTCGCAATTTTTGTATCTTCATTATAGTCTAATACCAAACCACAAAAATCAAATGGTGATTTTTTAGATTGTT
GTTGACCAAACATCTGTTCTTCATAACCAGGTGTTCCCTCAAAGAATGCTGGTGCAGTGTCTCTATTTGCACATTTATCTAACTCTATTAAC
CATTCCGGATTAATCTTAAAGTTGTCAGGATCTGCCGCATACGCATCAATGACTTTACGATATACTGAGACAACTGTTGCAATATAATGAA
TTGACTTCATACGTCCTTCAATTTTTAATGAGTCCACACCAATATCCATCATTTGAGGAATTGATTCGATTAATTTTAAATCTTTAGGACTCA
TCGCAAACGGTGTAACTTCACCTTGATTATAAAAAACATCAAGTTCACCATTATCATCAACTTCTAATAATTCATAATCCCAACGGCAACTT
TGACAGCAACCGCCTCTGTTGGAATCCCTTGCAGTCATATGATTACTTAATGTACATCTACCTGAATAGGCGATACACATAGCACCATGAA
TAAATGCTTCGATTTCAATATCTACTTTTTCCTTCATTTCACGCATTTCCATCGCGCCGGTCTCACGTGCTAATACAACACGATCCAATCCTT
CTTCTTTCCAATATTCTACAGCTTTGTAATTAGAAAGTGATTGTTGAGTAGATAAATGAATTTCAAGTTTTGGCGCAACTTCTTTACATGTT
TCGATAATTAAAGGATCTGCAACAATGATACCTGTCGCACCAGTCTTTTCCAAATTACGCAAATATGATTCTAGACCTTCAATATTCTCATC
ATGTGCAATAATATTTGTCGTAACATAAATTTTGGCACCGTAACGGTTCGCAAATTCAACACCTTCAGCTATTTCTTCCATCGTGAAATTAT
CAGCATTTGAACGTAATCCATATTCTTGACCACCTAAAAATACGGCATCAGCGCCATAATGTACTGCTATTTTTAACTTTTCTAAGTTTCCA
GCAGGTGCTAATAATTCTGGTTTCTTCATAACTGTTTTAGGAGTTGATTTAATCTCTTCTATTGTCTTCAT

Gene: yrrN (putative hydrolase)

Contig: 07_NODE_2, position: 180221 to 181144, length: 924 nt, orientation: REVERSE

Perfect match to: (MW2-BA000033-[1689177:1690100:r], allele observed in CC1+CC30+CC72)

Sequence:

TTAATATACCGTTTGTTTATATAAGAAACCTTCGTCAAATGGTCGATGATCAGGTTGAATTTCTTCAATTGGGTCCATCAACATAAATTTCT
CATCTTCATAGATTTCAGGATCTTCATTGTACAAATCTATCGCTTGACGATACTGTTCCGTTACCACATTAATATATTCTTCCGTTTGTAGAA

TACCATCGATTTTAAATGAATCTATACCCGCCTCAAAAAATGGTGCTAATTCTTCAATTAAACAAATGTCGTTTGGTGACATAATGTGCGT
ACCATTGTAATCTTCGTAAACTGGGTAATTATTTTGTCTTTCTTCATCATAAAGTAATAAAGATTGTTCATCATTGCGACGTTCAATTTTCAT
TTGGCGATCTTGGAACGTATAATAATTGCCTAGTAGCATACGCTTTGATTGGAACATACAAGTCATTCCTTGAACTTGCACCTCAATTTCC
ACATTTGAATTTTCTTTTATATTAATAATTTCATCCAAATTCAGCTCACGTGCTAAGACAGCTCTTGATGCGCCTCTTTTACCCCAGTAATTA
CATTGAAAATGATTAGTTACTAACGTCTCTGCATTCCAATGAAGTGGTATTGGATTCTCTTGCGCCTTCACATACATTACTACTGCTGGATC
CCCGAAAATAATTCTGTCAACTCGTATTTCATGTAAAAAATTAATATAATCTTCTACAGCATCTAAATGATAATTATGAAATAATCCATTCA
CCGCCGCATATACTTTTTTATCGTTTTTGTGAGCTAATGCGACAGCCTCTGTCATTTGTTGTCTATTGAATTCCCCTGGAAGTCTTAAACCA
AACTTTTGCTCGCCAATTACAAAAGCATCTGCACCTAAATCAATAAGTGTTTCCATATGGCTTAATGACTTGGGTGTGACAAGTAATTCTG
TCAT

Gene: yrrM (putative acyl-CoA O-methyltransferase)

Contig: 07_NODE_2, position: 181147 to 181785, length: 639 nt, orientation: REVERSE

Perfect match to: (MW2-BA000033-[1690103:1690741:r], highly conserved allele)

Sequence:

TCATTCTCCTTTAATTGAAATCGCTAATCCATCGTCTATATTTAAAAAATTCGTTGTATATCCTGGTTGCTTTATTAACCACTCATTATAATCT
TGAACCTTTTTAACCATTTGTCTTACATTTCTCGATCTAACAATCCCAATATCCGATACAAAACCGTGATATAAAACATTATCTGTAATTACG
AGACCTTGGTGCTTTAAAAGTGGTGTATATATTTCAAAAAATTTCTTTGATTGCGCTTTTGCTGCATCAATAAATATCATATCATAAACTTT
GTCATTTACATTTTCAAATTGCTCTAAAGCATTACCTTCAATAATTCGAACCTGGTTTTCAAAATGATAAGTAGCTAAATTTTGTTTAGCAT
ATTGAATCATCGTTTCATTACGCTCTATCGTTGTGACATGAATGTCATCAGATATAGAAGCGAATTGCATAGAACTATAGCCGATTGCTGT
ACCAATTTCTAAAATATTTTTAACATTATTCATACGAATTAATTGCTTAATTAAATCTAATGTTAAACGATCTACAATTGGCACTTCATTTAC
TTCGGCAAATTCACGCAAAACTTCGATTGAACTATTTTGATGTTGATGTAAATCTATTAAATATTTTTTATTTAGGTCATCCAT

Gene: yrzB (putative anti-sigma factor)

Contig: 07_NODE_2, position: 182070 to 182378, length: 309 nt, orientation: REVERSE

Perfect match to: (N315-BA000018-[1645436:1645744:r], highly conserved allele)

Sequence:

TTATTCTTCCATTTCAGTATTTACAACTTCTTCAATCATGTCCCATTCTTCATCAGTTTCGATTGGTACTAACTTACCACCGTCACCTGACTCA
TCTGGTTCATTGATCATTGGTACAAGCTCAATCATATCGTCTTCATCTGATTGAGCACCTTCTTCAGCTAAGATAACATACTCTTTTTTGAAT
TCAGGATGATAAAATTCTAAAACTTTTCGGTATAAAACTTCATTTCCCTCTTCATCGAATAAAGTTAATAATTCTTCTTCGTTATTAATTTCT
AGTTGTGAATCATGATTATGTTCAGTCAT

Gene: yrrK (putative Holliday junction resolvase)

Contig: 07_NODE_2, position: 182393 to 182821, length: 429 nt, orientation: REVERSE

Perfect match to: (RF122-AJ938182-[1600565:1600993:r], highly conserved allele)

Sequence:

TTAATGTAGTGAATCTAAATAGCCTTGTAAAATAAATACCGCTGCCATTTTATCAATCACTTGTTTTCTTTTTTGTCTTGAAACATCTGCTTC
TAATAATGATCGTTCAGCAGCCATTGTGCTTAATCTTTCATCCCACATCACAATCTCAATAGAAGGATAAGCTTCTAATAATTTTTCTTTATA
TGTTAACGAAGCTTCGCCTCGAAATCCTATTGAATTATTCATGTTTTTAGGTAGTCCTATTACGACTGTACCCACATTATGTTTTTTAATAAT
GTCTACTAATTGGTCAATACCTAATTCATTATTTTCTTCATTGATTCGGAGTGTGTCTAATCCTTGTGCCGTCCAACCCATTATATCACTAAT
TGCAATTCCTACCGTTCTACTACCGACATCGAGTCCTAAAATTTTATGTTGTAACAT

Gene: yrzL (putative protein)

Contig: 07_NODE_2, position: 182825 to 183085, length: 261 nt, orientation: REVERSE

Perfect match to: (MW2-BA000033-[1691781:1692041:r], allele observed in CC1)

Sequence:

TTATTTATTTTGCTCTTTTAAATAGTAAGAAACAAGTTCTTCCATAATAACATCTCTATCAATATGACGAATTTGATTTCTTGCTTCATTTTG
GCGTGGAATATACGCAGGGTCACCTGATAATAAATAACCTACAATTTGGTTTACGGCATTATATCCTCGTTCATCTAATGTTCGATAAACA
TTATTTAAAACATCTCTTACATCTTGCGTTGGAAGTTCTTCATAGTCGAATTTCATTGTTTTATCAAAGTTTTCCAT

Gene: alaS (alanyl-tRNA synthase)

Contig: 07_NODE_2, position: 183148 to 185778, length: 2631 nt, orientation: REVERSE

Sequence:

TTATAGATTTTTAATGTAATCTTTAATAAAGCTTAATGATTTTGAGATATTTTCAGGTTGTGTACCGCCACCTTGAGCCATATCTGGACGAC
CGCCACCTTTACCACCAACGATTGGTGCCATTTGTTTGATAAGATCACCGGCTTTAACGTTATTTGTTAAAGATTTAGGGACAGTTGCAAC
CATCGATACTTTATCATCAACATTACTTGCAAGAATGATAATTGTATCTTGTAGTTTAGATTTAAAATCGTCCATTGTCGAGCGAATTGCTT
TCGCATTTGGTACATCCACTTCAGTAACCAATACTTTATAGCCATTGATTTCTTCAACTTGATTTTCAATATTACCCATTTTAAGTGATGTGA
TTTCTTTGTCACGTTGCTCTAATTGTTTTAATAATGCTTTTTCTTCATCTTGTAATTGTGTTAACTTATCGACTACTTGATCATCAGATTTCAC
TTTCAGCTGTGATTTCATCGTATTAAATTTCTCTTGAATATCTTCTAAATATAAGAAAGCTGCTTTACCTGTTAATGCTTCAATACGACGCAC
ACCAGCTCCTGTACCTGACTCACTTACTATTTTGAATAAGCCAATTTCAGAAGTATTGCGGACATGAATACCACCACATAATTCAATTGAA
AATGGTGCCATATTTACTACACGCACAACATCACCATATTTTTCACCGAATAATGCCATTGCGCCCATTTCTTTAGCTGAAGCAATATCCAT
TTCTTGAATGTTAACGTCAATACCTTTCCAAATTTCTTCATTTACTAAGCGTTCAACTTGATCAATTTCATCATTAGTCATTGGACCAAAATG
AGAGAAATCAAAACGTAAACGATCTGCTTCTACTAGTGAGCCAGCTTGGTTAACATGATCACCCAGTACTGATTTCAACGCTGCATGTAA
TAAATGTGTTGCACTATGGTTCTTTTGAATGTCACGTCGATCATTTTGGTTCACTTCAGCAGACACTGTAGCGCCAACATTTACTTGGCCAA
ATTGTACTACTCCTTTATGCAAGTTTTGACCATTTGGTGCTTTGGTTACTTCACTAACAGCAATTTCAAAATTGTCATTATAAACAATACCTG
TATCCGCAACTTGTCCACCACTGACTGCATAAAATGGTGTTTCCGTTAACATGAAGTATACTGTTTCACCCGCTTCAACTTGTGAAACTTCT
TCACCATTATATATCAAGTGTGTTAGTGTTGTTTGAGCTGTCGCAGTATCATAACCAACAAAAGTACTTGCAGATGTAATATTTTTCAATAC
TTCACTTTGAACTTGCATTGATTGAGAATTTTGACGTGCTTGACGTGCACGATCACGTTGTTGTTGCATTTCTGACTCGAATGTTGTCATAT
CAACTTTCAATCCTGCTTGCACTGCTATTTCTTCAGTTAATTCAATTGGGAACCCGTACGTATCATACAATTTAAATGCATCTTTCCCATTAA
TTTCATTTGTTGTCGCTTTAGCTTTTTTAATTAATTCATTTAAAATCGCTAAACCATCTTCTAATGTTTCATGGAATCGTTCTTCTTCAGACTT
TATAACACGCTTAATGAAATCTGCTTTTTCCTTAACATTTGGATAATATGGTTCCATAATGTCTGCAACAATATCAACAAGTTTGTACATAA
ATGGCTCATTGATTCCTAACGTTTGACTAAAACGAACGGCACGACGTAACAATCGACGTAATACATATCCTCTACCTTCATTGGCAGGTAA
TGCACCATCAGAAATTGCAAATGCAATCGTACGAATGTGGTCAGCAATTACTTTAAATGCCACATCTTGTTCGTTGTTTACTAAATATTGTT
TACCTGATACTTTTTCGATTTCATTCATTATAGGCATAAATAAATCTGTTTCATAGTTAGTACGTACATTTTGAGAAACTGAGGCCATACGC
TCAAGCCCCATGCCAGTATCAATGTTTTTATTAGGTAATGGTGTGTAACTATGATCTTTATTATGATTGAATTCACTAAATACTAAGTTCCA
TACTTCAAGATAGCGTTCATTTTCTCCACCTGGATACATTTCTTCTGCCGGATCGTCTTGTCCATATGCTTCTCCGCGATCATAGAAAATCTC
AGTGTTTGGTCCTGAAGGCCCTTCACCAATATCCCAGAAGTTACCTTCAATGCGAATAATACGACTTTCTTCAAGCCCAATATCTTTATGCC
AAATGTTGTATGCTTCCATATCTTCCGGATGAATCGTAACGTACAATTTATCTGGCTCCATCCCCATCCATTTATCACTCGTTAAAAATTCCC
AAGCAAATTCAATCGCTTCTTGTTTAAAATAATCACCAATTGAGAAGTTACCTAACATTTCAAAGAATGTATGGTGACGCGCTGTGAAACC
AACATTTTCAATATCATTTGTACGAATAGCTTTTTGAGAGTTTACAATTCTTGGCTTTTTAGGTGTTTCACGTCCATCAAAATATTTCTTTAA
TGTTGCTACACCTGAATTAATCCATAATAATGTATCATCATCAATTGGCACTAATGGTGCAGAAGGTTCAACCATATGTCCTTTTTCAACAA
AGAAATCTAGATATTTTTGTCTAATTTCACTCGCTTTTAACTTTTTCAT

Gene: tbox07 (T-box leader element)

Contig: 07_NODE_2, position: 185910 to 186087, length: 178 nt

Sequence:

AAAAGGGACGAACGTTATCGCGGTACCACCCTAGTTATAAATGCAATTCAACACATTTATCACTTTAAATCGACTATACAGTTGTGCATAA
AGTAGCGTTCACTAATGTTTGTTGTACTTTTCACCAACCAGTACATCTCTGATAAACAAATCATTAACTACTCATCTTTATACGAAT

Gene: recD (ATP-dependent DNA helicase)

Contig: 07_NODE_2, position: 186121 to 188598, length: 2478 nt, orientation: REVERSE

Perfect match to: (68-397-ACJT01000043-[15077:17554:r], allele observed in CC30+CC1)

Sequence:

TCAACGTTCTATAAAGTCATACGGCGTGATTTCTCCCATATTAATCATTGGGTCAATTTTAAACATTGTAGCTTCCGTTAATACATTTATATC
TGTTTTTGTTGAATCAGACGTAACTTCTCCACTATCATTCGATGACATTGGCGCTTCTACTTGATCATCTATTGTCGTTTGTGAAGCTCCTGT
ATCATTAGTTGCTGTGTTTTCCAGCATTTCTTCATCTTCTGAATTAAAATAATTTTTCAACAATGTACATAATTGTGTTAAACGCGCTTGACC
ATTTGTTTTTAATCCAATATCAAATGCTTCTGGATCACCAAGTAGAACTAAACTCGTTTTCGCTCTAGTTAAACCAGTATATAATATCGGTC
TTTGTAACATTCTAAAATACTGTTTAACAATAGGCATGATAACAATAGGAAATTCTGAACCTTGTGATTTATGGATTGATGTACAATAAGC
ATGTGTTAATTCCATCATATCTTGTTTCGTAAATGTAATTTCATTACCTTCAAAATCCACAACAAGTACATCTTTATTAAGGGCATTTTCTTT
CGCCCAAAAAATACCAACAATAACTCCTATGTCACCATTGAATATGTTATCATTTGGCCTATTAACAAGTTGTAATACTTTGTCACCTTTTCT
AAAGACTACATCACCAAACTCAATTTCTCGTGTGTCTTTCTTTTTAGGGTTTAAAATATCTTGTAAAACTTGATTTAAACGTTTAATACCGG
CATTTCCTTTATACATTGGTGCAAGCACTTGAATATCAGCCATAGTATACCCTTTATTAACAGCACTAGTAACTACCTTCTCTACAACTGTT
GGTATTTGGTTTGCCTGACAGTTAATAAAACTTCTATCATGAAAACGCTGTGTAATATCAATTTTCTGACCCAACTTCATTCGATGTGCTAA
TTCTATAATGCTTGAACCATCTTGTTGACGATATACTTCAGTCAGATTAACTCGTGGTATAGCTTTCGATTCAATTAAATCTTTAAATACTTG
ACCAGGACCTACAGAAGGCAATTGGTCCTCATCACCTACAAATATCAATTGTGCATCTAAAGGAACTGCACTTAAAAATTGGTGGAACAA
CCAAGTATCTACCATAGACATCTCATCAATGATTATGAGTCGTGCGTTTATTTCATTTTCTAATATATCCTCTGGCTTTGTGTCTTGATTCCA
ACCTATTAAACGATGAATCGTCATAGCTTCTAATCCAGTTGACTCTTGTAGTCTCTTGGACGCTCTTCCTGTTGGCGCTGCTAATACAACTG
GATAATCATCATTGACATAATCATCATAATCTAATGATAAGCCATGAATCTCAGCATATAATTCAACAATACCTTTAATTACTGTCGTTTTTC
CTGTTCCCGGTCCACCGGTTAATAGCATCACCTTAGAATTGATAGCCGTTTGCAAAGCTTCTTTTTGTGAAGCTGCATAGTTCACTTGATTC
GCATCTTCTATTTCACCAATATGCATTTGTAAATCTGACTGTTCAATTTCTGTAAGTTTATTTGTATGCGTCTTTATTCTGAATAAGTTTTGA
ACACTTTTGATTTCAGAATAATACAAACTTGGAATTGCAACTTGTTCATTGTCAATAATTAGTCGTTTTTCCTCATTTAAGTATTGCAACATT
TCGTCTAATTTTTCAGGTTCGATGACCTCTTCATCTTGATAATTTAATACATCAACCGTTAAATCTATAACAACATTGATAGGCAAATATGT
ATGTCCCTGTTTAATACATTCTTCTTCTAACGTATAGAGCAACGCAGCTTTTAATCGTTCATTATCGTTATAAGCGATACCAATATTTCTAGC
AAGTTGATCTGCTTTATTAAAACCAATACCTTTAATATCATAAATCAATTGATATGGATTTCGATCTAAAATAGTCAGTGTATCGCCGAGAT
AAAACTGATAAATTGCCATTGAAAGTTTAGGACCAAACCCTAAATCATGTAAACGAATCATTATTTTTTCAGATTCTTGATTTGCTGAAATT
TGTTCTGCAATTTGTTTCTGTTTCTTTTTAGATAATCCCGAAACTTTTTCTAGCACTGAATGGTCATCTAATATATCATTTATCGCATTGTCAC
CTAATGTATTAACAATATTTTGAGCTGTCTTTTTACCTACACCTTTAAACAAATCACTAGATAAATAACTTATAATTGCTTCTTTCGTTTGTG
GCATTTCTTTTTCAAAAGTCTCTGCTTTTAATTGTTTACCATAACGTGGATGATCAACAACTTGCCCTTTAAATGTGTAGACATCGCCTTCAA
CAATATTCGGAAGAAACCCTACAACAGTTGGCATTGTATCAAAGTCTTCATTTGTTTCAATAGTATCTACTTTAAGCACTGTATAAAAATTA
TCACTGTTTTGAAACAATATCGCTTCAACAGTACCTTTGATCATTGAATAATCAAATAGTGTAGGGTCTGACAT

Gene: yrrB (tetratricopeptide repeat family protein)

Contig: 07_NODE_2, position: 188600 to 189268, length: 669 nt, orientation: REVERSE

Perfect match to: (MW2-BA000033-[1697556:1698224:r], allele observed in CC1+CC188+CC361)

Sequence:

TTACTCCTCCTCTTTCATTTTAGTGAATGTTTTCAGCGCATGCTGACTTAATAAGTGTTTAGGGTCGATAGTCACAGCTTCTTTAAAATGAG
TTATTGCTTCATCAATATCTTCATTTTTCATAAATAACGCTAAGCCCAAATTGTATCTTGCATCAACATGATTTTTATCAATCGTTAATACAT
GTTTAAGTTGAGTTATGGCTTCATTAAACATTTCTAATTGACATAATACAAGACCATATTGAAATTGAACTTCTGCATCTTTGTCTTTATCTA
GTTCCGCAGCAGTCATTAAATACGGCAATGCCAGCTTAAATGATTCTAACTGATTAAACGCCATACCGATCATATAATTACAATCAACTTG
TTCAATCTCTGTTTGTAATGCTTGTTGATATAATTTAATAGCTTCTTGATAACGTTGCTGATTATAATATACATTTGCTAGATTATAAAATAC
GACGCCATTCTTCGGATCTATTGTTAAAGCTTTTTGGAAAAAGCGCTCTGCCTTTTCAATCTCATTCGCATCAGCAAGTACGATACCAGCAT
TAATATAATTTTCAATAATTGTAGGATTTTCTTCGATATTTCCGAACAATGCTTGTAACGCTTCTTCTATTTTTCCATTTTGTATGTATTGATA
AATTGTTTGTTGATCTATCAT

Gene: STAR (Staphylococcus aureus repeat element)

Contig: 07_NODE_2, position: 189378 to 189596, length: 219 nt

Sequence:

GGGAATGGAATAGAAATGATTAAGAACCATTAACGGTTTATTATGTAATGGTTCTTCCACATTAGCCACTACTATTATGTACTTAAAAATA
GGAATACATAATTAGACTCATGCATAGGGAGTGGGACAGAAATGATATTTTAACAAAATTAAATTCGTTTATCCCCAACTCGCATTGCCT
GTAGAATTTCTTTACGAAATTCTCTGTGTTGGGGCCCC

Gene: SIRU01 (staphylococcal interspersed repeat unit 1)

Contig: 07_NODE_2, position: 189543 to 189712, length: 170 nt

Sequence:

CCAACTCGCATTGCCTGTAGAATTTCTTTACGAAATTCTCTGTGTTGGGGCCCCATCCCCAACTTGCACATTATTGAAATCTGACTTTTGGT
CAGCTTCTGTGTTGGGGCCCCGTCCCCAACTTCCATTGCCTGTTGAAATTGGGGTCCCAATTTCTCTGTGTTGGGGCC

Gene: mnmA (tRNA-specific 2-thiouridylase)

Contig: 07_NODE_2, position: 189849 to 190967, length: 1119 nt, orientation: REVERSE

Perfect match to: (MW2-BA000033-[1698805:1699923:r], allele observed in CC1+CC30+CC96+CC97)

Sequence:

TTATACAACATAATTTAATTGACCTTCATTTTTGAACACATCGTCAATTGTTGCTCCACCAAGACACACATCACCTTGATAAAAAACAACTG
CTTGTCCAGGTGTGATTGCTCTTACTGGCTCAGCAAAAGTAACACGTAGTGCATGGTCGTTTTCACGTTTCACAAAAACTTTCGTATCTTTT
TGGCGATATCTAAATTTAGCTGTACATTCAAAACCTTGATCTAAGTCATTATCTTCTGGATTTACAAATGAATAGTCTGAAGCAATTAAGT
AATCACTGTATAATGCATCGTGATGGAATCCTTGTTCTACATATAAAACATTATCTTTTAGATTTTTACCGACAACAAACCAAGGATCGCCA
TCTCCACCTATACCTAAACCATGTCTTTGTCCTATTGTGTAATACATCAACCCACTATGTTTACCCATTTTCTTACCATCAAGTGTTATCATAT
CACCCGGTTGCGCAGGTAGATATTGAGATAAAAATGTTTTAAAGTTTTTTTCGCCGATAAAACAAATACCTGTAGAATCTTTTTTCTTAGC
AGTAACTAGTCCTTGTTCTTCAGCAATTCGACGCACTTCAATCTTTTCGATGTCGCCAATTGGGAACATCACTTTTGAAAGTTGTTGTTGGG
ATAATTGATTCAAGAAGTATGTTTGATCTTTATTATTATCTACACCACGTAACATTTCAACATGACCATCTTCATGACGATGTATGCGTGCG
TAATGCCCTGTTGCTACATAATCTGCACCTAAATTCATCGCATGATCTAAAAAGGCTTTAAACTTAATTTCTTTATTACACATAACGTCTGG
GTTTGGAGTACGACCTTTTTTATATTCATCTAAGAAATACGTAAAGACTTTATCCCAATATTCTTTTTCAAAATTAACAGCGTAATATGGAA
TGCCAATTTGATTACACACTTCAATAACATCGTTGTAATCTTCAGTTGCAGTACATACGCCATTTTCGTCAGTGTCATCCCAGTTTTTCATAA
ATATGCCAATGACATCATAACCTTGTTCTTTTAAGACGTGGGCTGTTACAGAACTATCTACACCGCCTGACATACCAACGACAACACGTAT
ATCTTTATTTGACAA

Gene: csd1 (cysteine desulfurase 1)

Contig: 07_NODE_2, position: 190968 to 192110, length: 1143 nt, orientation: REVERSE

Perfect match to: (JH1-CP000736-[1779103:1780245:r], allele observed in CC5+CC97+CC772)

Sequence:

TTATGACTCCTCCTTAAATTTAAAATATATTTTATGAATTTCAGCTACAATTGCATTAATTTCATTTTCAGTAGTCAATTCGTTAAAACTAAA
TCGAATCGAATGATTTGATCGCTCTTCATCTTCGAACATTGCATCTAAAACATGCGACGGTTGTGTAGAGCCTGCTGTACATGCAGATCCA
GACGACACATAGATTTGTGCCATATCCAACAATGTTAACATCGTTTCAACTTCAACAAACGGAAAATATAGATTTACAATATGGCCTGTAG
CATCCGTCATTGAACCATTTAATTCAAATGGAATCGCTCTTTCTTGTAATTTAACTAAAAATTGTTCTTTTAAATTCATTAAATGAATATTGT
TATCGTCTCGATTCTTTTCTGCTAATTGTAATGCTTTAGCCATCCCGACAATTTGTGCAAGATTTTCAGTACCTGCACGGCGTTTCAATTCTT
GTTCACCGCCAAGTTGAGGATAATCTAATGTGACATGGTCTTTAACTAATAATGCACCGACACCTTTTGGTCCTCCAAACTTATGAGCAGT
AATACTCATTGCGTCGATCTCAAATTCGTCAAACTTAACATCAAGATGTCCAATTGCTTGAACCGCATCAACATGGAAATATGCATTTGTC
TCAGCAATAATATCTTGAATATCATAAATTTGTTGCACTGTGCCAACTTCATTATTTACAAACATAATAGATACTAAAATCGTCTTATCTGT
AATTGTTTCTTCAAGTTGGTCTAAATCAATAGCACCTGTATCATCAACATCTAGATATGTTACATCAAAACCTTCTCGCTCTAATTGTTCAA
AAACATGTAACACAGAATGATGTTCAATCTTCGATGTGATAATGTGATTACCCAATTGTTCATTTGCTTTTACTATGCCTTTAATTGCCGTA
TTATTCGATTCTGTTGCACCACTCGTAAATATAATTTCATGTGTATCTGCACCAAGTAATTGTGCAATTTGACGTCTTGACTCATCTAAATAT
TTACGCGCATCTCTTCCCTTAGCATGTATTGATGATGGATTACCATAATGCGAATTGTAAATCATCATCATCGCATCTACTACTTCAGGTTT
TACTGGTGTGGTCGCAGCATAATCTGCATAAATTTCCAT

Gene: limB2 (putative monooxygenase locus 2)

Contig: 07_NODE_2, position: 192422 to 193435, length: 1014 nt, orientation: FORWARD

Perfect match to: (MW2-BA000033-[1701378:1702391], allele observed in CC1+CC97)

Sequence:

ATGGCGATTAAATATTCAGCATTAAACCTTGTCCCTATTCGAGAAGGTGAAGATGAACGAACAGCAATTAATGATATGGTTAAGCTCGCA
CAACATTTAGACGAATTATCATATGAAAGATATTGGATTGCTGAACACCATAACGCTCCCAACCTAGTAAGTTCAGCGACTGCTTTATTGA
TTCAACATACGTTAGAACATACGAAACACATACGTGTAGGTTCTGGAGGAATCATGTTACCTAACCATGCTCCATTAATCGTTGCGGAAC

AATTTGGCACGATGGCAACATTATTTCCAAATCGTGTCGATTTAGGATTAGGACGTGCACCTGGAACAGATATGATGACCGCAAGTGCAT
TAAGACGAGATCAACATGATGGTGTTTATAAATTTCCGGAAGAAGTTTCATTATTACAACAATATTTCGGTCCCGCTCACCAACAAGCATA
TGTTCGTGCTTATCCAGCAGTAGGTAAAAATGTGCCTTTATATATTCTTGGTTCTTCAACAGATTCTGCACATTTAGCTGCTCGTAAAGGGC
TTCCATATGTGTTCGCTGGACATTTTGCACCTCAACAAATGAAAGAAGCTATCGAAATTTACAAAACGTTATTTGAACCTTCTGATGTATTA
GACGAACCTTATGTTATTGTATGTTTAAATACAATCGTTGCTGAAAATGATGACGAAGCACAATATTTAGCTTCATCTATGGCACAAGTAA
TGGTTAGTATCACTCGTGGCAGAATGCAGCCCGTTCAACCGCCAACACATGAACTACAAAATATATTAACGCCGAGAGAATATGCGATG
GCTATGGAAAGACAGAAAATATCATTAATAGGTTCAGAAAATACTGTTCAACAAAAAATTCAAGATTTTATGGAAACTTATGGTGAAGTC
AACGAAATTATGGCAATAAGTTATATTTATGATAAAGATATGCAATTAGACTCTTATCGTCGGTTCAAGAATGTTATAAATCAGATAAATG
AAAAAAACACTTTATAA

Gene: Q5HFD8 (putative small protein)

Contig: 07_NODE_2, position: 193672 to 193818, length: 147 nt, orientation: REVERSE

Perfect match to: (N315-BA000018-[1657152:1657298:r], highly conserved allele)

Sequence:

TTATTTCTCTTTATCTTCATTTTCTTTTTTCTCTTCGTTATTCGATCCTGTATATTCATTTATCTTATCTTTTACATTTTTAACTTGTTCATTATCG
CTATTTTTAAATTTTTCTACTGCGTCTTTAGCTTTATCCATAAAACTCAT

Gene: csbD-L2 (stress response protein, locus 2)

Contig: 07_NODE_2, position: 193858 to 194040, length: 183 nt, orientation: REVERSE

Perfect match to: (N315-BA000018-[1657338:1657520:r], highly conserved allele)

Sequence:

TTATTTTTTAAGTTTATCAATTGCATCAGTTATTTTGTTTTTAGCATTTTCAACAACTTCTTTTGCTTTACCAGTCGCTTTATCTTGCTGACCTT
CTTTTTCTAATTCTTTGTTATCAGTAACGTTACCTACTGTTTCTTTAACATTTCCTTTAAATTGATCGAACTTACTTTCGTCTGCCAT

Gene: cymR (oxidation-sensing transcriptional regulator)

Contig: 07_NODE_2, position: 194140 to 194562, length: 423 nt, orientation: REVERSE

Perfect match to: (MW2-BA000033-[1703096:1703518:r], allele observed in CC1+CC22+CC97+CC188+CC361)

Sequence:

TTAAATATAAAACATGTATCCGTCTAAATCTTCGCTTGTATCTACATATTCCGCTAAATATTTCAATGTTGTATTATCTAAAACATCTCTCAC
TGCATCTCTCATGCGAATCCATAGTTGTTTTTGCGCAGGTGGTTCTGATTCAATACTTTCAACAAATGTAATTGGACCTTCCAACAGTCTTA
TAATATCCCCTGCTGAGATTTCTTCCGCTGGCACTCTTAATTGGTATCCACCTTTAGCACCGCGTACACTTCGAATTAACCCCGCATTTCTTA
AAGGACCTACAAGCTGTTCTAAATATAAATCACTCAAATTATTTTCTTCAGCAATTGACTTTAATGATATACATCCTTGCCCCTCTTTTTTAG
CAAGAGAAATCATCAATGTAAGTCCATATCTCCCTTTAGTAGAAATTTTCAT

Gene: rarA (DNA-dependent ATPase)

Contig: 07_NODE_2, position: 194647 to 195921, length: 1275 nt, orientation: FORWARD

Perfect match to: (MW2-BA000033-[1703603:1704877], allele observed in CC1+CC25+CC97+CC239)

Sequence:

GTGAGTACAGAACCATTAGCATCGAGAATGCGCCCAAAAAATATAGATGAAATCATTTCCCAACAACATTTAGTTGGACCAAGAGGCATT
ATCAGAAGAATGGTTGATACAAAAAAATTAACTTCAATGATTTTTTATGGTCCACCTGGTATAGGCAAAACAAGTATTGCCAAAGCAATTT
CGGGCAGTACGCAATATAAATTCAGACAATTGAATGCTGTAACTAACACTAAAAAAGATATGCAACTTGTTGTTGAAGAAGCTAAAATGT
CTGGTCAAGTTATCTTGTTATTAGATGAAATACATCGACTAGATAAAGCTAAACAAGACTTTTTATTACCTCATTTAGAAAATGGCAAAAT
CGTCTTGATCGGTGCTACAACTTCAAATCCTTATCATGCTATCAATCCAGCGATTCGTTCAAGAGCGCAAATTTTTGAGTTATATCCTTTAA
ATGATGAAGATGTGCGCCAAGCGTTAACTCGTGCAATAGAAGATGAAGAGAATGGTTTGAAAACATATCAACCCAAAATTGATGAAGAT

GCCATGACCTACTTTTCTACACAAAGTCAAGGTGATGTTCGTAGTGCGTTAAATGCATTGGAATTAGCTGTATTAAGCGCAGATAATGAC
AAAGACGGTTATCGACATGTTACATTGCAAGATGCTAAAGACTGTTTACAAAAAGGTGCATTTGTAAGTGATAAGGATGGTGACATGCA
TTACGATGTTATGAGCGCTTTCCAAAAATCTATCCGTGGTAGCGACGTCAATGCCGCTTTACATTATTTAGCACGATTAATTGAAGCTGGA
GATTTACCTACAATAGTTCGACGATTACTTGTAATTAGCTATGAGGATATAGGCTTAGCCTCACCTAATGCTGGTCAGAGAACACTTGCTG
CTATTGAATCAGCAGAACGTCTAGGTTTACCAGAAGCTAGAATTCCACTAAGCCAAGCAGTAATCGAACTATGCTTATCACCTAAGTCAA
ATTCAGCAATGAGTGCCATTGATAGTGCATTGTCCGATATTAGAAACGGTCATGTGGGCCAAATTCCAAACCATTTAAAAGATGGACATT
ATCAAGGTGCTAAAGATCTAGGCCGATCTATTGGTTACAAATATCCACACCAATATGTTAATGGCTATGTTTCACAGCAATATTTACCTGA
TAAACTTAAAAACAAAATTTATTATGAACCAAAAACGACATCTAAAAGTGAACAACAACTCAAAGAAATATATAACAACTTACTTAAACA
AAGGCCGTAA

Gene: yrvM (putative N6-threonylcarbamoyladenosine cyclase)

Contig: 07_NODE_2, position: 196082 to 196855, length: 774 nt, orientation: REVERSE

Perfect match to: (N315-BA000018-[1659562:1660335:r], highly conserved allele)

Sequence:

TTATTGCCCTTTGTCTTTAATGCGACGAACTGGAATATCTTTTAATACGTCATTCACCACATAACTAGCACAAATTAATCCAACAACACTTG
GCACAAAGGCATTTGAAGAAGGTGGCATTTGTCCTTTTCGATTGATAGCATTTTTATCTCCAACTATATCTTTTACATCTTCTCTTATGACA
ATTGGACTTTCATCTGAAAATACAACTGGAATCCCTTTACGAATTCCTAGTTTTTTCAATTTTTGACGAATAATTTTGGCCATTGGATCGGT
ATGTGTTTTAGAGATATCTGAAATTGTAAAACGTGTTGGATCTGTTTTATTTGCAGCACCCATACTGGAAATCACTTTAATCCCTCGGTCAA
GACACTCTTTCATTAAGTGTACTTTGTACATTATTGTATCACTTGCATCTACAAAATAATCTATATCGTAGTTATCGAAAATTTCTTCATATG
TCTCTTCTGTATAAAACATATGTAAGGGCGTGACTTTACAATCTGGATTAATTAATTTAATACGTTCTTCCATCAAAGAAACTTTACTTTGT
CCTACCGTTGTAGTTAAAGCGTGTAATTGTCTGTTTACATTTGTAATATCAACATCATCTTTATCTATTAATATAATATGACCAATATTCGTT
CTTGCTAATGCTTCAGCAGCAAATGAACCAACACCTCCAACGCCAAGTATGACAACAGTTTGTTGCTTCAATAAATCTAAACCTTGTTGTC
CAATCGCTAGTTCATTTCTTGAAAATTGATGTTTCAT

Gene: ssrS (6S RNA)

Contig: 07_NODE_2, position: 197015 to 197246, length: 232 nt

Sequence:

ATAAAAATACGCAAGACAAAGTCTTGCGTATCGATAGAGTCCGTATTGCCGTAGTTATAATAGCTTGATCATTCGGCCTGTTATATACAG
GTGGGTGCCCTGTTTCTTGTTTTGTACGTCCTTCATATAAGGCGTGTACGCTGCAAGAAAACCCATTGGGCTCCCTTGATCAAAGAGTGTT
AGGCCCAAATTAAAAAGCAAACTTACGAACAACTCAGATGACTATCTTATG

Gene: aspS (aspartyl-tRNA synthase)

Contig: 07_NODE_2, position: 197316 to 199082, length: 1767 nt, orientation: REVERSE

Perfect match to: (Strain_21193-AFEG01000006-[67211:68977:r], allele observed in CC25+CC5+CC25+CC361+CC425)

Sequence:

TTAGTGACGAATTCGCAAAGAAAGTTCTTCTAATTGTTTATCAGAAACTTCACCAGGCGCATTCGTTAATAAACATGTAGCAGATGCTGTT
TTAGGGAATGCGATTGTATCTCTCAAGTTTGTTCTATTAGTCAATAACATGACTAATCGGTCTAATCCTAATGCAATACCGCCATGTGGTG
GTGCACCATATTTAAATGCATCTAGTAAGAAGCCGAACTGTTCTTGTGCTTGTTCTTTAGTAAATCCAAGAACTTCGAACATTTTTTCTTGT
AACTCACCATCATGAATTCTGATTGAACCGCCACCTAATTCATAACCATTTAATACTATGTCATAAGCATTTGCCTCAGCTTCTTCTGGCGC
AGTGCCAAGCTTAGCAATATCAGCTTCTTTTGGAGATGTAAATGGATGATGTGCTGCAACGTAACGTTTCGCATCTTCATCATATTCCAAT
AATGGCCAATCTGTCACCCATAAGAAGTTTAATTTTGTTTCATCGATTAAACCTAATTCTTTAGCTAATTTGACACGTAATGCACCTAAACT
TTGTGCAACGACATTTGGTTTGTCTGCAACAAACATTACTAAGTCACCAGCTTCAGCACCAGTTAATGTAAGTAATGTTTCAACATTTTCTG
TTTCAAAGAAACGTCCAATTGGACCTGTCAAACCATCTTCCACAACTTTAACCCACGCTAATCCTTTAGCACCATAGATGTTTACAAATTCT
GTTAAAGCATCCATATCTTTACGAGTATATTGTTCAGCTGCACCTTTAGCGACAATTGCTTTAATTTCACCATCATTTTCAACAGTATCTTTA
AATACTTTAAAGTCCATATCACGTCCTAATTGAGAAACGTCAATTAATTCCATTTCAAAACGCGTATCTGGTTTATCAGAACCATAGCGAC
GCATCGCTTCTTTATATGTCATGCGTGGGAAAGCGCCATTAATTTCAACGCCTTTAACTTCTTTAACAACTTTTTTAAGCATTTCTTCACCCA
TTTGCATCACATCTTCTTGGTCTACAAAACTCATTTCAATATCGACTTGTGTAAATTCAGGTTGACGATCTGCACGTAAATCTTCGTCACGG
AAGCATTTTACGATTTGGTAGTATTTGTCAAATCCACTAATCATCAATAATTGCTTAAATAATTGTGGTGATTGTGGTAATGCATAAAATTC

ACCATCATGAACACGAGATGGTACTAAATAGTCACGTGCACCCTCAGGTGTTGACTTCGTTAGTACTGGTGTTTCGATGTCAAAGAACCC
TTCATCATCCAAATATTGACGAATAGAACGTGTAATTTGATGTCTCATTTTAAATGTTTGCGCTAACTCTTGACGACGTAAATCTAAATAAC
GGTATTTTAATCGAATATTTTCATCAACGTTAACATTTTCTTCATTTATAGAAAATGGTGGTGTCTCAGATTTATTAATCACTTTAATATTTG
TAACTTGTACTTCAACTTGGCCAGTTTTAATTTTAGGATTAACTGTTTCAGGGTCACGCTTCGTAACTGTACCTTGAACTTCTACAACATATT
CAGAACGTACTGTTTCAGCAATTTTCAATGCCTCTTCTGAAAATGCAGGATTAAACACGACTTGTACAATTCCTTCTCTATCTCTTAAATCA
ACGAAAATCAATCCACCTAGGTCACGACGATTGTTAACCCATCCTTTTAATGTAATTTCTTGTCCTAAAAATGCTTCAGTAACTAATCCACA
ATAAGTTGTTCTCTTACTCAT

Gene: hisS (histidyl-tRNA synthetase)

Contig: 07_NODE_2, position: 199098 to 200360, length: 1263 nt, orientation: REVERSE

Perfect match to: (08-02119-CP015645-[463713:464975], allele observed in ST582+CC8+CC15+CC239)

Sequence:

CTACTTCTTAAAATATTCGACTAATGCGTCTAATTCAATTGTTTCAGATTCACCAGTTGTCATATTTTTAACATCGATTTTATTATTTTCTAAT
TCTTGATCACCAATAACGATTGTAAACTTGGCACCTAAACGGTCTGCTTGTTTCATTTGTCCTTTAATTTTACGCTGTAAGTAGTCTTTATCT
GCTTTAATACCATTATGTCTCAAATGATTTAATAACTTCACAGCATATCGATCTGCTTGATCACCCATTGTAACAATGAATAAATCTAAGTT
TTCTTCAATATCTAATTCGATACCTTCTTCTTCAAGTGCAAGCAATAATCGTTCTATACTTAGCGCAAAACCAATACCTGTTTCACTTGGACC
ATCTAGCAATTCTAATAAACCATTATAACGGCCACCACCACAAAGCGTTGTAATGGCACCATCATAGTTAGGGTTATCCATCATTAATTCA
AATGCTGTATGTGTATAATAATCCAATCCACGAACTAAGTTAGGATCTTCAATATATGGAATACCTAAATCATCTAAATAAGCTTTTACTTG
TTCATAATATGCCTTAGATTCCTCATTTAAGAAATCAGTGATTCTAGGTGCAGTCTTAATCGCTTCTTTATCACGGTCAACTTTACAATCCA
AAATTCGCATCGGATTTGTATGCAAACGTGATTGACAATCTGAACAAAATTCATGAATTACTGGTTCAAAGTGTTTCACTAACGCTTCGTT
ATATTCTTTTCGAGACGCCATATCCCCTACACTATTAATAACAAGCTTTAAATGTTTTAATCCAAATGATTGATAAATATGCATAACCATAG
CTAATACTTCTGCATCTACGCTAGGATTTTCAGCACCAATAGCTTCTACACCAAATTGATTAAATTGACGATAGCGCCCTTTTTGCTTACGT
TCATATCTAAACATCGGTCCATTGTAATAAAGTTTAATTGGTTGGTTTGGATTACCTTGCATTTTATGTTCAATATATGAACGCACAACTGC
AGCTGTTCCCTCAGGTCTTAATGTAATACTTCTATCGCCTTTATCTTTAAATGTATACATTTCTTTTTGTACGACATCGGTTGAATCACCAAC
ACCTCTTGCAAAAAGATCTGTACTTTCAAAAATTGGTGTTCTTATTTCTTTATAATTATAAAATGTCATTAATTCATCTAATTGATTTTCAAT
GTAACGCCATTTCTTTGAATCTTCAGGTAAAATATCCTGCGTCCCTCTAGGTATTTTAATCAT

Gene: lytH (N-acetylmuramoyl-L-alanine amidase)

Contig: 07_NODE_2, position: 200821 to 201696, length: 876 nt, orientation: REVERSE

Perfect match to: (08-02119-CP015645-[462377:463252], highly conserved allele)

Sequence:

CTACGCAGAAAAATAAATTTTAAGGCCATCAACAATTGCTTGTTCTAAAATTTGTCTATGTAATTGATCTTTAATCATCGTTTCATCAGTTG
GGTTACTAATATAACCTAATTCTAATAAAACAGCAGGAACTTTTGTTTGTCTTAACACTTGATAATTTTCTTGTCTTGAACCGCGATTAGAA
AGTAGACCTTTCTTCTGAATCGTAGCGTCTAACGTATCTGCTAAAGCTCTTTGATTATCATGATACCAATAAACTGTCATTCCATTTGCATT
AGATGATTCTAATGCATCATTATGTATACTCAAATAGGCATCGCCTTTGATATCACGATTTTCTAGTGAAACATATGTATCGTCTGTTCTTG
TCATCTTAACAGTTGCGCCTTCTTTTTCTAAAGTACGCTGCAATTCTTTTGCTGTTTTCAACGTATAGTCTTTTTCTAAACTTTTATATTTAGT
ATTGCTTGAAGCACCCTGGTCACTACCTCCATGACCAGGATCAAGCACTATTGTTTTACCTTGCAAAGGATTTTTCTCCTTCGTATTATCCG
CGACAATATCTAAATTTGTGTGCCATCCAGCTATCCAACCTTTTTCATTACTGGATGTATCTTCAACTTCAATCCATTTACCTACTTTACCAA
TCTTTTTAAAATGGTCACCTTTCTCAACTTTATATATGACTGGATACGCAGCGTTTGGACCTGTACGTAATTCAGCATTTTCAGTTATCGTG
ATGTTCCCACTATCTTCACTATTGCTATTCAGCAATAAAAATAAAAAGATGATAAATAAGACAAAGGCAATCACTACTATTAGAGTACGTT
TATTTTTAAGACCCTTTTTAGATAACCATGCCTCTATTTTTTTCAT

Gene: dtd (D-tyrosyl-tRNA (Tyr) deacylase)

Contig: 07_NODE_2, position: 201693 to 202145, length: 453 nt, orientation: REVERSE

Perfect match to: (N315-BA000018-[1665173:1665625:r], highly conserved allele)

Sequence:

TCATTGAATTTTGCCGTCCTGACTTTCATAAATAATAGTGACTGGACCATCATTATTTATGCTAACATTCATGTGTGTTCCAAATTCACCTGT
TTTCACAGTAAGACCATACGCTCGTAGCGCATCATTAAAATACTCATAAATTTTTACCGCTTGATCAGGATTTTTAGAATTTGAGAAACCT

GGACGGTTACCTTTTTTTACATCTGCATAGAGAGTAAATTGTGAAACTGATAGTATTTCACCATTCATTTGTTGGATATTAAAGTTTAATTT
ATTATTGTCATCTTCAAATAATCTTGCATTAGCAATTTTCTTTGCAATTACATCTGCATCTTGCTCTGTAGAGTTCTGACCGATACCGACTAA
TAAACAATATCCTTTTTTGATTTGATTATTTAATGTATCATTCGTCACCGATGCTTCTTTAACTCTTTGTACAACTACTTTCAT

Gene: relA (ppGpp synthase I)

Contig: 07_NODE_2, position: 202157 to 204346, length: 2190 nt, orientation: REVERSE

Perfect match to: (N315-BA000018-[1665637:1667826:r], highly conserved allele)

Sequence:

CTAGTTCCAAACTCTTGTTACTGTATAAACATCACCAAGTTGTTTGATCTTTTCTACCACACGATAAACATCATTCACGTTTTTCACCATGAC
ACTAATATTTATTATTGCATTTTTATCAATATCTGAACGTCCTGAAACTTTAATTAAATTGCCGGCTGTCGAGCTAACAGCTTGTAGTACTT
CATTCAACAAGCCATTTCGGTCATACGCAGTTACCTCTAAATCAACCTGATATTTTTGAGTTGCGTCTTTTGATTTTACCCATTCAACATTAA
TTAGTCGTTCAGTTTCGTTCTTAATATTTGGGCAATCAGTGCGATGTACTTTAATACCGTGACCTTTGGTGATATAACCTACAATATCATCA
CCTGGTATAGGATTACAACATTTTGACAACTTGATAAGTACATTTTCTAAACCTTCTACATAGACACCACTATCAGTAATGATGTTGTCTTT
AATAGGCAATGATTTCGTAACTTCTTGTGCTTCATTTAAAGCACGTTGTTTATCTAAAATACGTTGTCTTTCAGTTAATTTATTAACAATCTG
TAAGGATGTCACGCCGCCAAATCCTACAGCTGCGAATAAATCATCTTCATTTGCAAAGTTATATTTTTCATTAACAACCTGAATATTTTTCT
CTGTCAAAATATCTTCGACTCTAAATCCTTGCTCTTTTATTTCAACTTCAACCATCATTCGGCCTTTTTCAATATTAGATGAACGATCTTGTTT
TTTGAAGAAACTTTTAATTTTACCTTTGGCACTAGACGATTTAACAATTTTCAACCAATCACGACTTGGTCCATATGAATGTTTACTAGTAC
GTATTTCAACAATATCGCCTGTTTGTAAAATATAGTCAATTGGTACAATTTTGCCATTCACCTTGGCACCAATCATCTTATTACCTACTTCAC
TGTGAATCGCATAAGCAAAATCAATCGGCACAGCACCATATGGCAACTCAATAACATCACTCGCTGGGGTAAATGCGTATACTTTGTCAC
TCTGTAAGTCATATTTTAAGGTTTCCATAAATTCTTGAGCGTCAGACGATGTATGATCCGCTTCAGCTAATTCTTTTAACCAATTTAACTTAT
TTTGATAAGTTTGATCTTTTTCACTTACTTTTTTACCTTCTTTGTAAGCCCAGTGTGCTGCAACACCATGCTCAGCAATTTCGTGCATATCAA
ACGTTCGTATTTGGATTTCGAGCGGGTCTCCATTTGGACCTACTACTGTAGTATGCAATGACTGATACAAATTTTGTTTAGGCATTGCAAT
ATAATCTTTAAAACGTCCTGGCATCGGTTTCCATAACGTATGCACCAACCCAAGTATCGCATAACAATCATTAATAGAATTGACAATAACA
CGTATCGCCAACAAATCAAAAATTTGATCAAATTGTTTTTTCTGCTTCATCATTTTCCGATAAATACTGTAAATATGTTTAGGTCTACCATTT
ATATCGCCTTCGATATTCATTCGGTCCATTTCAGTACGTATTCTATCAATAGCCGTTTCGATATACGCTTCACGTTCACTACGTTTCTTCTTC
ATTAAATTGACTATTCTAAAATATTGCACATTATCAATATAACGAAGAGCCGTATCTTCTAGTTCCCATTTAATTGTATTAATACCAAGACG
ATGTGCTAAAGGTGCATAAATTTCTAATGTTTCTCGAGAAATTCTAATTTGTTTTTCACGCGGCATGGCTTTCAAGGTACGCATATTATGTA
ATCTGTCTGCTAATTTCACCAAAATTACGCGTACATCTTTGGCAATCGCAATAAATAACTTGCGATGATTTTCAGCTTGTTGTTCTTCTTTTG
AGCGGTATTTTACTTTTTTAAGCTTCGTCACACCATCAACAATTCGAGCAACTTCTTCATTGAACATTTCTTTTACATCTTCAAATGTATACG
GTGTATCTTCAATTACATCATGCAAAAAACCTGCGACAATCGTCGGTCCGTCTAATCGCATTTCTGTTAAAATACCTGCAACTTGTATAGG
ATGCATAATGTATGGTAATCCGTTTTTTCGGAACTGACCTTTATGTGCTTCATAAGCAATATGATAGCTTTTTAAAACATACTCATATTCAT
CTGCTGACAAATATGATTTTGCTTTGTGAAGAACTTCATCTGCACTATATGGATATTCGTTGTTCAT

Gene: apt (adenine phosphoribosyltransferase)

Contig: 07_NODE_2, position: 204774 to 205292, length: 519 nt, orientation: REVERSE

Perfect match to: (N315-BA000018-[1668254:1668772:r], highly conserved allele)

Sequence:

TTATTCGTCGTATGAGATTAAACTCATAACATCGTAATCTTTAATTTTTTCAATACCATTTAAATATTTCAATTCAATTATAAATGCAATACC
TACTACGATACCGCCTAATTTTTCAACTAATTTTATTGCTGCTTCAATCGTACCACCAGTAGCTAATAAATCATCTGTAATTAACACACGTT
GACCTGGTTTAATTGCATCTTTGTGCATTGTTAAAACATTTGTACCATATTCTAGGTCATACTCATAACGAATGACTTCACGAGGTAATTTC
CCTTCTTTTCTAACAGGTGCAAAGCCAATCCCCATTGAATAAGCTACAGGACAGCCAATGATAAAGCCACGCGCTTCAGGTCCTACAACG
ATATCAACATCTCTGTCTTTTGCGTATTCTACAATTTTATCTGTTGCATAGCCATATGCTTCACCATTATCCATAATTGTAGTAATATCCTTG
AAACTAACACCTGGTTTCGGCCAATCTTGAACTTCTGATACGTATTGCTTTAAATCCAT

Gene: recJ (single stranded DNA-specific exonuclease)

Contig: 07_NODE_2, position: 205314 to 207587, length: 2274 nt, orientation: REVERSE

Perfect match to: (MW2-BA000033-[1714270:1716543:r], allele observed in CC1)

Sequence:

TCACGACAATTGTGACTTTATCCAATTTTTTATTTCTGAAAAATCTTGATATAATAATTGCTTTTCAACATCCATACGTTGTTGTCTTAATTG
ATATACTTTGCTGGAATCAATCGATCTTTTATCAGGTTGTTGATTGATTCGAATTAAACCATCTTCTTGTGTTACAAATTTTAAGTCTAAGA
AAACTTTCAACATGAATTTAAGTGTATCTGGTTTCACACTTAAATGTTGACACAATAACATACCCTCTTTCTGGATATTTGTTTCTTGTTTAG
TTATTAATGCTTTATAACACTTTTTAAAAATATCCATATTAGGTATACCATCGAAGTAAATCGAATGATTATGTTGCAAAACTATATAAAGT
TGAGAAAATTGCAGTTGTTGCAAGGAATTAGACAAGTCTTCCATTGACGTTGGTAAATCTCTTAATACTACTTTATCAGTTTGTTGTTTAAT
TTCTTCACCATAATAATATTCATTCGCATTTACTTTATCACTTTTAGGATGAATAAGCACGACAATATTTTCATCATTTTCTGTAAAAGGTAA
ACTTTTTCGCTTACTTCTATAATCTAATATTTGCTGTTCATTCATCGCAATATCTTGAATAATTATTTGCGGTGATTGATTACCATTCCATTCG
TTGATTTGAACAGATCCTAATATATTAATTGGCTGTTCATCTTGTAACTCAGGTTCTAAGTGTCCATTTTGCCAAAATAGCGCGGCGATATT
ACTTTCACCAAGTGTCAATTTTAGATGATTTTTTTGTTGACCTATCGCCTTAACTGAAGAAACTGATAAATCATCCATTTCAAAAATAGGTC
TAGAAAAATCTGTTCCGAAGGGTCTTAAACGATTCATATCACGAATATTTTTAATCGTTATATCATTTTCTGTTAATAATACATCTACTTGCT
TTACGGGATCTAACGAAGTTGTTTTAGATAATTCTTTCATCCATTTATTTAAACCTTCAGCTAACGATTCTATATTTTCAATATCCATCGTCA
TACCTGCAGCCATATGATGGCCGCCAAATTTAGCGATTAACTCTTGATGTGCTGATAGTATTTCAAACATCGACACTTGATCAATTGATCT
GGCGGAACCTTTTGCATGATTTTGCTCCCTATCAATATTTAAAATTAATGTTGGCAAAGCAAATGTTTCGACAATTTTCGAAGCAACAATA
CCTAAGACACCTTCATGCCAATTTTCTTTTGCTAAAAGTAAAAATAAATCTCCCTTTTTAACTTTCGTTTCTGCCATAGCCATTGCTTCTTCTG
TGATAGTTGCTACAATATCTTTTCTTTCACGGTTAAAATGTTCAACTTGTTCTGCTAAAAATGCAGCTTCTTCTTCGTCGTCAGTCATCAACA
ATTCGCAAGCTAATGATGCGTCATCTAAACGACCTACAGCATTAAGTCTAGGTCCAATAATAAAACCAATTGTTTCTTCATCAATATTGTCA
TTGTATCCCGCTTCTTTTAGCAATGCTTTAACAGAGGTCGGACATTGATCATTTAAGACTTTTAATCCTTGTTTCACTAATGATCGATTTTCA
TCAGTTAAGGATACTAAATCCGCAATGGTACCTATCGCAACTATTGCTTTAAAATAATCAGGTACATTTTCAATCAATGCTTGTGCTAATTT
GTATGCAACACCTGCACCACACAATTGTTGGAACGGATAATTAAACGATGGATGCATTGGATGTACGATTGCATATGCTTCTGGTAATGT
ACTACCAATTTCATGATGATCAGTTACAATGACATCAACTCCTAAATCTTGAACCATTTTAATTTCATTATGACCTTGTATGCCATTATCAAC
AGTTATGATTAATGTTATGCCTTCATCATGAGCATTTCTAAATGCTAGTTCGTTTGGTCCATATCCTTCGGTAAAGCGGTTAGGAATATGCC
ATCCTACTTGTGCACCTAAAAGTTGTAATGTTGTCACTAAAATTGTAGTTGAGGTAACACCGTCAGCATCGTAATCACCATAAACTAGGAT
TTTCTCATCATTCGCTATCGCTCTTTTAATTCTTTCAATAGTCTTAGTCATATCGCTCAATTGCAATGCATCATGATTGATATCTGTATCTGA
AATGATGGATTCTATTGCTTGTTCATCAATAATCGATTTACTTTCTAATATTTTTTTTACGATTGGCGTTAACTTTAATTTTGATGTTAATTCA
TCACTTATGTATTCAGCTGGTTTAGTTAATTTCCACTTATACTTCGGTTTAATCAT

Gene: secDF (preprotein translocase fused subunits D and F)

Contig: 07_NODE_2, position: 207790 to 210069, length: 2280 nt, orientation: REVERSE

Perfect match to: (MW2-BA000033-[1716746:1719025:r], allele observed in CC1-ST582)

Sequence:

TTAAACTAAAATCTTTTCATCGTTCGATTTCTTTTCTTTATATACAACTAATTTGTGTTTCGGCGATTTTTTCAGCTGACGTTTTTTCATTATTC
CCCATAGCGGAACGGCAATGAAGATTGAAGAGAATACACCAGAAATCAATCCGATAAATAATGCTAAAGTAAAGTTGAATATCGTAGGA
GCACCGAAGAATAGTATAGCAACTACTACTACAATAACTGTTAATACTGTATTAATTGAACGTGTCATTGTCTGTCTAATTGATCTATTAAC
GATATCATCAATTTGTTCTGTTGTCGTAATCACTTTAACCTTTTGTAAGTTTTCACGTACACGGTCAAACGTTACGATTGTATCATTAATTGA
ATAACCGACAATTGTTAATACAGCGGCGATAAATGTTAAATCTACTTCAATTCTAAATAAACTGAAAATCGCTACTATAATGAATACATCA
TGTAATAATGCCAATACAGATGAAAGACCCATTCGCCATTCAAATCGTAATGATACATAGATGATGATACCTATCGATGCATAGATTAAT
GCAAGCATTGCATTTTTTGCTAATTCCTGTCCAATAATTGGTGATACAGTATTAATTTGAGGTGTGTCACCGAATTTCGATTTAATATTATC
ACTCAATTTATTATCTTGAGCACGCGTTAAATCGTCTTTAAATTGAACAGTTGCTACTTTATTGTCTTTACCATTGATCTGAATTTGATCCGC
TTTAAGTCCACTATCTTTTACAACTTGCTCTACCTTTTGTTGAGTAATTGCTTGTTTAGATTGGAAATCTACGCGTGTACCACTTGAGAAATC
AATTCCTAAGTTTAACTTGAAGATATAAAGAATAACTAAACCGACAACTACAATTAAAATACTTACTCCAATTAATGGCTTAGCTAATTTA
ACAAAATTCCATTTCTCGAATGAAGTTTTAAGGTCATGAACATCTACACCTTCATTAATATCATGTCGTTTATTCTTTTTAACACCAAATAAC
CAAAATTGATTTTTGAATATATTTGATGAAACAAGTAATGATAATAAGAATCTTGATAAGAACACGGCTGTAACAAAGATCATTAGAATA
CCTAATAATAACATTGTCGCGAAACCTTTAACTGAACTTTCACCGAAGAAGAATAATACTGCTGCGGCGATAACTGTTGTTAAGTTAGAAT
CAAAAATTGTTAGGAATGAACTTTTGTTTGCTTTAGAAAAGGCTTGCTTTATCGTTCTACCTATTCGAAGTTCATCCTTAATACGCTCATAC
ATGATAATATTGGCATCTACAGCCATACCTACACCTAATACCAACGCCGCTAATCCTGGTAAAGTTAGAACCCCGGAAATGAAATTAAAT
GCTACTAACGTTAGATAGATATAAGTTGTCAATGCAATAATCGCTACTAAACCAGGTAATCGGTAGAATCCAAGCATGAATAAATAAATT
AATGCTACACCAATAAACGATGCAAACACAGTTTTATCTAATGCATCTTGACCAAATTGGGCACCTACTGAGTTTGAATAAATTTCTTTCA
AGTCAACTGGTAAAGAACCTGCATTTAACAATTCGGCGATTTGTTTTGCTTTTTTAACGCCTTCTTGTCCTTTAAATCCACCCGAGATTTCTA
CGCTATCAGAATTGATTGGTTGATCAACACTTGCTGCAGAAATAAATTTAGGGTTTTTCTTTTGTGCTTCTTTTTTATAGCTGTCACCTTTTT
TGAAATCTAACCAAACAACCATGACATTATCACGTTTCTTAGAGATTTCTTCCGTTACTTTTTTAAATTTGTTTTTGTCTTTTACTTTAAAAGT
AACTGTAGGCTGGTTTGTTTCCTGTTTAAATTCTTGTTTGGCAGATCCCTGTTTAATATCAGAACCGCTTAATTTTACTTTATCTTCTGCATC
GCGAATTGTTAAATTAGCTTGAGAAGATAAAATTTTACGTGCTTCATTCTGGTCTGTTACACCAGCAAGTTGTACTCTAATTCTATTAGGTT
CTTCAACTTGAATTTTAGGTTCCGAAACACCTAAAACGTTAACACGATTTTCTAATGTTTGCGCTGTTGATTGTAAGGCTTTTTTATCTATTT
TGTCGCCTTTATTTAAAGGATCGACTTGATAAAGCACCTCAAATCCACCTTGCAAATCAAGTCCTAAATTGACATTCTTTATAACACTTTTA
TAAGTTGCAGCCATTCCGGCAAACAACAATACGACTAAAAGCAAGAACGCAATTATTCTACTACTTTTCTTCAC

Gene: yajC (preprotein translocase subunit yajC)

Contig: 07_NODE_2, position: 210344 to 210604, length: 261 nt, orientation: REVERSE

Perfect match to: (RF122-AJ938182-[1628361:1628621:r], highly conserved allele)

Sequence:

TTATGAAGGGTCAACTTGTTTAATAGCAGGTTTTTCGAAAGTTAATTCAGTACCATGACCATTAACTGTAATAACAACAGTTGTTTCATCTA
CTGCTTTAACAGTACCTTTAATACCACCAATAGTTGTAATTCTTTGACCAGATTGAATGTTATTAATCAACTCACGATGCTGTTTCGCACGT
TTTTGTTGTGGTCTGATCATCAAGAAATACATAACCGCAAAAATTACGACTATATATATTAGTAATGAAAATTGCAT

Gene: tgt (queuine tRNA-ribosyltransferase)

Contig: 07_NODE_2, position: 210623 to 211762, length: 1140 nt, orientation: REVERSE

Perfect match to: (N315-BA000018-[1674103:1675242:r], highly conserved allele)

Sequence:

TTAAAAGTTTTTTGGGTTCTCAACATTTAATCCATATTGCTCGAAGAATTCTTCTTTGAAATCTAAAAGACGATCTTCTCGAATGGCTTGTC
TTATATCTTCCATTAATTTTAGCAGAAAATGTAAATTATGAATAGTAGTAAGACGAATACCAAAAGTTTCCTCTGCCTTGATTAAATGACGT
ATATACGCTCTTGAATAGTTTTGACATGTATAACAGTCACAATTCTCATCTAACGGTCTTAAATCATCTGCAAATTTTGCATTTTTAATAACT
AAACGACCTTGCGATGTCATACAAGTACCATTTCTGGCAATACGTGTCGGTAAGACACAATCAAACATATCCATGCCGCGAATACTACATT
CGATTAACGCATCTGGAGATCCTACACCCATTAAATATCTTGGTTTATCTTTAGGCATAAACTGCTCTGTATGTTCAACCATTTTATACATA
ACCGGTTTAGGTTCACCAACTGACAAACCGCCGATTGCATAACCAGGAAAATCTAATTCTACTAAATCCTTTGCACTTTGTTCTCTTAAATC
TTCATATTCGCCACCTTGTATAATGCCGAACAATGCTTGATCTTCAGGTCTTTGGTGTGCATCTAGACATCTTTTCGCCCAACGTGTTGTAC
GTTCAATAGATTTTTTTACATAATCATATTCAGCAGGCATCGGTGGACATTCATCAAATGCCATCATAATATCAGATCCTAAATCATTTTGA
ATTTGCATTGATTTCTCAGGACTCAAAAATAATTTAGACCCATTAGTATGATGTCTAAATTCCACGCCTTCTTCTGTAATTTTACGTAAATTA
CTTAAACTAAACACTTGGAAACCGCCTGAATCTGTAAGAATCGGACCATCCCAATTCATGAATTTATGTAATCCCCCAGCGTGTTTGATAA
TATCATTTCCGGGTTGTAACCACAAATGATATGTGTTGCCCAAAATGATTTTTGCTTCAATTTGTCTTAACTCTTCTGGACTCATTGTTTTAA
CGGTTGCTTTAGTACCAACTGGCATAAACATAGGTGTTTCAAATGAACCGTGTGGTGTGTGCACGATACCTAAACGCGCACCTGATTGTT
TACAAGTTTTAATGTGTTCGTATGTTACTGCAGGCAT

Gene: queA (S-adenosylmethionine:tRNA ribosyltransferase-isomerase)

Contig: 07_NODE_2, position: 211785 to 212810, length: 1026 nt, orientation: REVERSE

Perfect match to: (MW2-BA000033-[1720741:1721766:r], highly conserved allele)

Sequence:

TTATATAATTAACATTGCATCGCCAAAACTAAAGAATCTATATTCTAAATTTACTGCTGTTTTATAAGCATTCAGAACATTTTCACGAGTAC
TAAACGCTGATACTAGCATAACTAATGTTGATTTTGGTAAATGAAAATTAGTAATCTGGCCATCAATTGCTTTAAAATCAAATCCTGGATA
AATAAATATATTAGTCCAGCCACTCGTTTCAACAAATTTATCATGATCGCGTCGAATTGTTTCAAGTGTACGTGTTGAAGTTGTACCAACT
GATATAATGCGATGTCCTTTGGACTTAGTATCATTTAATAAATCAGCTGTTTCTTGTGTCATTTGATAATATTCACTATGCATTTCGTGGTC
ATTCACATCATCGACGCTCACCGGTCTAAACGTACCTAACCCAACATGTAATGTAACAAATGCGATATTAACGCCTTTATTTTTAATTTCAG
TTAATAACTCATCAGTAAAATGTAATCCTGCTGTTGGTGCTGCCGCTGAACCACTTTCTTTAGCGTAAACTGTTTGATAACGATCTGGATC
ATCTAAACGTTCTTTGATGTATGGTGGCAGTGGCATTTCCCCTAATTCATCTAATCTTTCTTGTAAAATACCTTCATAATGTAAACGCATGA
TGCGTCCACCTTGATCCATTTCTTTTATGCACTCAGCTATAATTTTGCCATTACCAAAATTCAATTTATTACCAACTTTAATACGCTTAGCTG
GTTTCAGTAAGACTTCCCAATCATTACCTTCAATTTGAGTTAACATTAACATTTCAACTTTTGCACCAGTTTCTTCTTTTAAACCAAAAAGTC
TAGCTGGCATTACTCGCGTATCGTTAAGCACTAATGTATCACCAGGTCTAAAATACTCAATGATATCTTTGAAATGTAAATGTTTCATTTCA
CCAGTTTCTCTATCCATGACTAATAAACGACTATGATCACGATCTTTTAAAGGCGTTTGAGCAATTAATGATTCTGGTAAGTCATAGTCAA
ATTCTTCAATATTCAC

Gene: ruvB (Holliday junction ATP-dependent DNA helicase subunit B)

Contig: 07_NODE_2, position: 212812 to 213816, length: 1005 nt, orientation: REVERSE

Perfect match to: (RF122-AJ938182-[1630829:1631833:r], highly conserved allele)

Sequence:

TTATCCTCTCTCCTCATTCGACTTTGCAAAATGTTCATAAGCTAATGGTGTTGCTTTTCTGCCACGTGGCGTACGTTCTAAAAAGCCTTTCT
GAATAAGAAATGGCTCATAAACGTCCTCAATTGTAATACGTTCTTCACCAATTGTTACGGCAATCGTATCTAAACCAACAGGTCCACCATT
ATACTGCTTAATAATACAGTTCATCATTTTATGATCAATGTAATCTAGTCCGTGTTGATCAACTTGAAGTAAACCTAATGCGTGCTTCGTTG
TTTCAATGTATATTTGTTCATCTTCATTCACTTGCTGGAAGTCTCTTACCCGCTTCAATAGTCGATTTGCTACTCTTGGAGTCCCTCTAGAAC
GTTTAGCAAGTTCAATGGCACTTTCTTCATCAATACCTGTGCCTAAAACCTCAGCTGTTCTAATAATGATTTCTTTTAAATCTGATTCATTAT
AATATTCTAATCTTAAGTGCACACCAAATCGATCCCTTAGTGGACCTGTTAAGCTGCCAGCTCGCGTTGTTGCACCTACCAAAGTGAATGG
AGGTAAGTCGATACGGATACTTCTAGCCTCATCGCCTTTACCAATGATAATATCTAAAAAGAAATCTTCCATTGCAGGGTATAACACTTCT
TCAACAACACTACTCAGTCTGTGTATTTCATCAATAAACAATACATCTCCAGGTTGAAGTCCTGATAAAATTGCAGCCAAATCACCAGGTC
TTTCTAATGAAGGCCCTGATACTGTACGTATATTAACTTCCATTTCATTGGCAATGATATTAGATAATGTTGTCTTACCTAATCCAGGGGG
GCCAAAAAGCAATACATGATCTAATGGTTCATGACGAAGTTTAGCCGCTTTAATAAATACTTCTAAATTACTTTTTATTGAATTTTGACCAA
TATATTGTCGTAATCTCGTAGGTCTAAGCGACAATTCGAAATCAGTTTCTTCACTATGCATTGATTGATCAACCATACGCTCATTCAT

Gene: ruvA (Holliday junction ATP-dependent DNA helicase subunit A)

Contig: 07_NODE_2, position: 213848 to 214450, length: 603 nt, orientation: REVERSE

Perfect match to: (N315-BA000018-[1677328:1677930:r], highly conserved allele)

Sequence:

TTAAGATACAACTAATTGAAGACCTGCCTTAACAGCTTCATCAACTGAGTCATATTTATTTTTATTTAACGTTTTCTCAACTTTTGCAAGCTC
TCGTTTAGAATAACCTAATGCTTCTAACGCTAACATTGCTTCTTGCACGAATTGATCTTGCACCGTCGAAGTAGCGTCTACTTGTAATAATG
AATCGCTATCTTCTTCAGTGATTTTCACTTTACCTTTTAAATCTAAGACAATCTGTCTTGCCGTTTTCTTACCAATTCCTGGGAATTTAGTTA
AATACGTATCATTTTCATTTTCAATGGCACGTTTTACTTCATTAGGCGTACTTGTCGCTAAAATAGCTAAAGCTGATTTCGGACCAATACCA
GTAACTTTAATTAAACTCAAGAACATATCTTTCTCTTCTTCACTACTAAATCCATACAATAATTGTGCATCTTCACGAACAATTAAAGATGTA
CGAATTAAAACTTCATGATCTAGATGCTTTTGAAAACGATAAGAATTTGGTGTTTGAATTTCATAACCAACACCAGCAGTTTCAACAACTA
CGTGTGTAGGATATAAATGTGTTAACTTACCTTTGACATACGCGTACAT

Gene: pheB (ACT domain-containing protein)

Contig: 07_NODE_2, position: 214464 to 214922, length: 459 nt, orientation: REVERSE

Perfect match to: (MRSA252-BX571856-[1788403:1788861:r], highly conserved allele)

Sequence:

TTACATACTCATACTAATTAATTCTACTTTTGATACATAATCTAAATTTCTCAAAGCACCAATAACATCTTCTACTGAAGTTTCTTTAGATTTA
GCATTCAGTGATAATGTTATTGTTGCTTTTTCTTCCATTGGAATACTTTGATGAATCGTTAATACAGATAGTTCTAACTTTGATATAACATCT
AGTACACGTGCCAACATACCCACAATATCAGTTACATATAAAATTAATGTAAATTCTCGATGGTCAAGCATTTTATCGTCTACTGGAAATA
TCGTTTCTCTATATTTATAAAAAGCACTTCTAGATAGATCAAACTGTTTAACGGCATCATAAATGGACAATGTCGGATCACTTTTTAAGGC
ATCTTTAATCTTCAATGTTTTAACCACGGATTCAGGCAAGACATCTTCTCTAATTAAATAAAACTTTTTATAATCTTTATTGTCCATCAT

Gene: obgE (GTPase obg)

Contig: 07_NODE_2, position: 214932 to 216224, length: 1293 nt, orientation: REVERSE

Perfect match to: (N315-BA000018-[1678412:1679704:r], highly conserved allele)

Sequence:

CTATTCAACGAATTCAAATTCTCCGCCAAGAATTCTAACGATATCACCATTTTTACAACCACGTTCTCTAAGTGCATCATCAATACCCATCG
AACGCATTTGACGAGCAAATCGACGTACTGCTGGATCACTGTTAAAGTCAGTCATTTTAAACATTCTTTCAATAGCATTACCACTTACCAC
ATAAGCACCATCATCATCTCTTGAAATTGTAAATTTATCTTGTGACGGTGTATGTTTATATAATACTCGGTTAATGCCAACTGACTCCTCTT
CTTCAACTGTGAAGTCAACATCTTTATATTCTTCTAATTTATCTGCTATTGCATATAATAATTGATCAATATTATCACGCGTTATTGTTGAAA
CTGGAATAACTGGCACATCTTCGCCAATTTCTTCTTTAAACAAGTTTAAATTATCTTGTGATTCAGGTAAATCCATCTTGTTAGCTACTACG
ATTTGAGGTCTATCTTCTAAACGTTGCTCGTACGCAGCTAATTCTTGATTAATGACTTTATAATCTTCAATAGGTTCTCTACCTTCAGAACC

GCTCATATCAATCATGTGAACAATAACTTTTGTTCTCTCTACATGTCTTAAAAATTGATGTCCTAATCCAACGCCATCAGATGCACCTTCAA
TTAAACCTGGTAAATCTGCCATAACAAAACTACGTTGATCAGGCGTTGAAACAACACCTAGATTTGGTTTAATCGTTGTAAAATGATATGC
CCCAATTTTAGGCTTAGCTTTTGAAACGATAGATAATAAAGTCGATTTACCCACACTAGGGAAACCTACTAATCCTACATCAGCTAATAAT
TTCAATTCTAAAGATACATCTAATTCCTCACCTGGTTCACCTTTTTCACTGAAGTCAGGTGCAGGGTTTCTAGGTGTTGCAAAACGTGAATT
ACCTCGGCCACCTCGACCGCCCTTCGCTACTACAGCTCTTTGACCATCTTCAACAAGATCTGCTAACACTTCGTCTGTTTCAACATTTTTAAT
AATTGTACCAGGTGGAACTTTTAATACTAAATCTTCCGCATTTTTACCATGCATATTACTACTTTGGCCATTTTCACCTTTGCTTGCTTTAAA
ATGACGTTGATATCTAAAATCTAATAACGTTCTTAAACCTTCATCCACTTCAAATACGACTGAAGCACCTTTACCACCGTCACCGCCAGCTG
GTCCACCAAATGGTACATATTTTTCTCTTCTATATGCGGTAATACCATTACCACCATCACCGGCTTTAAGAGATATTTTGACTTGATCGACA
AACAT

Gene: SIRU15 (staphylococcal interspersed repeat unit 15)

Contig: 07_NODE_2, position: 216303 to 216586, length: 284 nt

Sequence:

ATTAGGGGCTCTTATGCAGTTGCTTTTAAATTTACTTTACTCAAATTGCTGATATTTCCGAGTACCAAATATGATACTTTTTTTGCTTTTTAT
ACTGCAACTGTATTCTTTTGTCCCCTTATAGTTTAGCGATTAGGGGCTCTTATGCAGTTGCTTTTAAATTTATTTTACTCAAATTGCTGATAT
TTCCGAATACCAAATATGATACTTTTTTTGCTTTTTATACTGCAACTGTATTCTTTTGTCCCCTTATAGTTTAGCGATTAGGGGCTCTTATGC
AGTTG

Gene: rpmA (50S ribosomal protein L27)

Contig: 07_NODE_2, position: 216709 to 216993, length: 285 nt, orientation: REVERSE

Perfect match to: (RF122-AJ938182-[1634464:1634748:r], highly conserved allele)

Sequence:

TTATTCAGCTACTGCATATACAGAAACTTGTTTTTTGTCGCGACCTTTACGTTCGAATTTAACAACGCCGTCGATTTTAGCGAATAATGTAT
CATCGCCACCACGACCTACATTTTCACCAGGGTAAATTTTAGTACCACGTTGGCGATATAAAATTGAACCACCTGTTACGAATTGACCGTC
AGCACGTTTAGCACCTAAGCGTTTTGATTCAGAGTCACGTCCGTTTTTTGTAGAACTTACCCCTTTTTTAGATGCGAAGAATTGTAAGTTTA
ATTTTAACAT

Gene: DUF464 (putative protein)

Contig: 07_NODE_2, position: 217005 to 217325, length: 321 nt, orientation: REVERSE

Perfect match to: (N315-BA000018-[1680354:1680674:r], highly conserved allele)

Sequence:

TCACTTATAATTTAATCTAATATTCTCATTATATTCTTCTTCAATAGTTTGTAAAGACACAAGCATTGTTTGAAGAATTAGTTGCGCTTCATC
GTTATTTGTATCAACGCTTCTTATATGAAAATGACCACCATTGTCGTCATAATTGATATCTGGTCTCTCAGATGTCAATCCTATAATCGCAT
TAACACTACCAAACAATACAGCTGAAGCTCCAGCACAAACGATATCATGACCATATTCACCATGGTCAGCATGGCCATCCATAATAACGT
CTGTTACTTTGCCTTCATCATTAACTGTAATATCAACAGTAATCAT

Gene: rplU (50S ribosomal protein L21)

Contig: 07_NODE_2, position: 217331 to 217639, length: 309 nt, orientation: REVERSE

Perfect match to: (RF122-AJ938182-[1635086:1635394:r], highly conserved allele)

Sequence:

TTACGCGTTGATTTTATCGATTGTTAATTTAGTGTATGGTTGACGATGGCCTTTTTTACGTTTTGAATTTTTACGACGTTTGTATGTGAATAC
AGTGATTTTTTTACCGCGACCTTGTTTATTAACAGTAGCAGTAACTGTTGCACCTTCAACTGTTGGCGCTCCAACTTTAACTGAATCTCCAC
CTACAAATAATACTTTATCAAATGTAAAAGTATCTCCTTCGTTTACGTCTAATTTTTCAACGAAGATTTCTTGACCTTCTTCTACTTTGATTTG
TTTTCCACCTGTTTCAATAATAGCAAACAT

Gene: L21_leader (ribosomal protein L21 leader RNA)

Contig: 07_NODE_2, position: 217652 to 217974, length: 323 nt

Sequence:

CTGTATAATAAGTCACGCCATACATAGGTGACATTTCGTTATTGAGTCAAGCGTTTGCACTCTACGTTGTTTCGGTCGCAACTCGAGTCAT
TTATAAACATAAACTCTTCTCGTTTCTTCCTTCAACGCCTTGATTGACAATCGCTTTCTTTCAATAACTTCTGCAAATGAGTCAAGCGTTTGC
ACTCTACGTTGTTTCGGTCGCAACTCGAGTCATTTATAAACATAAACTCTTCTCATTTCTTCCTTCAACGCCTTGATTGACAATCGCTTTCTT
CATTAAAAATGTTCTTGAACCTATATTTGAGCGGTTGTATGTAGCT

Gene: mreD (cell shape determinant D)

Contig: 07_NODE_2, position: 218001 to 218531, length: 531 nt, orientation: REVERSE

Perfect match to: (N315-BA000018-[1681224:1681754:r], highly conserved allele)

Sequence:

TTACCATTGACGACGTTTCATGTCAATGTCATTGTTTGTTTTTTTAAGGAACTTTATAATCAACGGATAAAGCATAATCAACAGTACAAAAT
TCATAATTAATGTTGGCAATAATCTAAAGACTACAAAATGAATAATATCAAATTGAATGAATCCTAACATACCGTATATTAATGCCACATA
GACTTCTAATAATAAGGTGCTGGCTAATATAATAATGAATAACATCGAATGATCTTTGTAAAAAATTTTAAAGAATCGATCTATAAGTGCT
AAAAACAATATATAGCCAAATAAGTACACTCCATAAATACTACCAAAGTATACATCAGTCATTACGCCTAAAAATATGCTGAGCAATAATG
ATACGCCAAAGCCACGATACACTACCATCATTAAAATATACATAAATGTAAGGTGTGGTACAAATACAAGTTCAAACTTACCTATATGCAT
TGGAATAAGAAGCCCAATTGCAGTATCTATATAAAATAGTAAAATACCTATCAAAAAATAATACAGTGTACGCAT

Gene: mreC (cell shape determinant C)

Contig: 07_NODE_2, position: 218531 to 219373, length: 843 nt, orientation: REVERSE

Sequence:

TTATTTATCCCTGCTTTCATCATCAGGAATTGTTTTAGGATCTCTTTTTGCAACATAAACATGACTCAAATCTGTTAAGTCTGCACCAGTCTT
AACCCTAACTTCTTTAGCTAAGCCGTATTGATCATTTTGAACCTTAGTCACTTCTCCTATATATAAATTACTTGGTAGTTGATCAGCTAATCC
ACTTGTAACGACTTTATCACCTTTTGAGATATTATCTCTATTATTAATGTCACTAATTACAAGTTCTGAGTTCTTTTCATCATAACGATCAAT
TAAACCAAATATATTTTTAGAACCGTGTTGTATATTTACAGATAATTTACCCGCACGTGTATTAGTTGAGATTAAATCAACTTGTGAAGAA
AATTTATTAACTTTAGTAACTCTTCCAACAAAACCTTGTGATGTCATCACAGCCATATTTGAAGTTATACCTGCTTTAGATCCCTTATCAATT
ACAATTGTATTCATCCACTGATCCGGATTTCTTGCTAAAACCGTAGTAGAAATAGGATCAAATTTTGAAATATCTTTTAAATCAAGCTCTTT
TTTTAATTTTTCATTTTCCGCTTCTAATTGTTGGTTCTTAGATTCTAACTGGCTAATCTTATTTTTAGATTCTTTAGAATCTCCTTTTTTAAAAA
AGTCCCCAATCGTACCAGCAACAAAATTAACTGGATAGCTCACAACTCGTTGTCCAAAAGACACAGAATCACCTATATATTGTTCAGGAG
GTGATTGAGATTGTGAACGTATGGACAGCCCAATTAATGCAATAAAAACGATAATTGCACATAAAACAACAATTAATTTGTTATTTTTAAA
AAACTTAAGCAC

Gene: Q5HFB3 (putative protein)

Contig: 07_NODE_2, position: 219765 to 220238, length: 474 nt, orientation: REVERSE

Perfect match to: (CA347-CP006044-[1731943:1732416:r], highly conserved allele)

Sequence:

CTATTGATAAGCATTTTCGGGTTTTAGTTGTAAATTTTGCCCTAATTCATTTAAGTCTTGTTGCATTTCAAATTCAGTATTGTAAACACGCAT
TGACTCATCTCCAAATTTATAAAGAATAAATTCGTCTCCTCTTTGACCTATAATATATTGATCATTATAAGCCATGCGATTCATTCCAGACA
CAGCCATAAACTCTTGTTTATCTATCATTTTAAATACATTTTTAATTTGGCTTAATGGTACATTCTTTATTAAATCATTTTCTTTTAATGAATA
TTTCTCACCACTTACATAAACATGTTGGGCATCATTAGTGGGTTGCGTAACTTGATTCATTTGATCATTATCGTGATTTACAGGATTCCAAT
CTTGTTCTTTTAAAAAAGGTGCGTATTTCAAGGCGATATAAACAATTATCACAATTGCTATTACTGCTATAATGTTTTTAATCATACTAAAT
ATCAATCTCAT

Gene: Q5HFB2 (putative protein)

Contig: 07_NODE_2, position: 220712 to 220996, length: 285 nt, orientation: REVERSE

Perfect match to: (N315-BA000018-[1683935:1684219:r], highly conserved allele)

Sequence:

TTACTTACGCCCAAATGCGTTAGATAAAAATCGTATCAGCGCGTAGCTACCACTTAGTAAAAGGCCCCAAACGATTCCAGTAAAAATTGA
GCCGATTGCTACACTTAATAAAGGAAAAGCTTGTTTCGTAACGATCACACCTGCTAACGCCGTTACTAAAATACCTAACGTAGATAAAGCT
AATGTTATTAATAGATTGCGCTTTGTTACAGGTTCATTTTTAATATTTAACTGCATGGTATAAACCATTGGCACGAGAACCCCGATAATAAT
AAATAGAGTCAC

Gene: radC (DNA repair protein C)

Contig: 07_NODE_2, position: 221064 to 221750, length: 687 nt, orientation: REVERSE

Perfect match to: (MW2-BA000033-[1730020:1730706:r], highly conserved allele)

Sequence:

TCAATCATTTTCATCAAAGTAACCCGCTTCTACAAGACTGGTAAATCTATTATCACCGATTATAATATGATCCAATAAATCTATCCCTAAAA
TCAAACCACACTCCTTCAACCTCATTGTTGTTATGATATCTTCTTGTGAGGGCGTTACATCACCGGATGGATGATTATGAACTGCGATGAT
TGCATTGGCATTTTCTCTCACCGCAATACTAAAAATTTCACGTGGATGTACAATCGAACTATTTAATGTACCTTTAAAAACACAGGTTTCTT
TAATCACTACATTTTTTGAATTTAACAATAAAATGACAAAATGTTCTTGTGTTAAATCTTTCATTGTTGGAATCATATAATCAGCAACATCA
CTTGGTTGCGTTATTTTTATACGATTATTTTCAGCTCTTCTCCCCATCCTTTCCCCTAACTCAAATGCTGCTTTTAAAGTAATTGCTTTTTGTA
ATCCAATCCCTTTAACTTTTATCAAATCGTTAATTGAAGATTTTTTCAATTCATTCAGATTCGAAGCAGATTTAAGCAGTTCATTACTAATGT
CTATGCTCGAGAATCCTTTTCTTCCGGTGTTAATTAATATAGCTAATAATTCTGTATTCGAAAGACTTTTTGCACCATGGCTTAACAAACGT
TCTCTTGGCATTTCTGAAGTTACCATTTCTTTAATTTTCAA

Gene: comC (pseudopilin signal peptidase)

Contig: 07_NODE_2, position: 221747 to 222454, length: 708 nt, orientation: REVERSE

Perfect match to: (MW2-BA000033-[1730703:1731410:r], highly conserved allele)

Sequence:

TCAAAAATATACGCCTCCTAAAAATTGATGGATATCATTATAAAAAAGTGAATTGATAAAAAAGGAAATAAATATAAATGGAACAAGGG
GTAATAGTTTAATCGGCTTAAATATCATGGTAATTAAAGCAACTAAACCAGCAATGACAAATGTAAATAAAATGACATAAATAGTGAATT
GGAGAGGGAAAAACAAAGAAAGTGCAGATATTAGTAAAACGTCACCATAACCAATATATGCCCGAAATAAAAAGTAGAATATATGCGT
GGTCATACTAATAATGATAAAAGCTACTGGATAAATCATACTTAACGAGAGAGAAACGATACAATAAATTATAATTAAGCGACAATCTAA
CATTAAAGAAGTGATATCGGTCATAGTAAAAATAAGCAGAAAAACATATGTAGTTATAAATAGCGTAGCATTTACGTATGTGAAATCATA
CTTAATAAAGACGATAGGTATTAAAGCAAAGGTTTCCCCTAAGAAATGTGTTAGGGAAATACGCTTTCGACAGTTTCGACATCGCCCTTTT
AATAATAAAAAACTAATAATCGGCATTAATTCATACCATTTGAGTGACGAATTACAATAATCACATTTCGATCTTCTATGTAAATAATCAAA
TGACGTTTCTTCTATAGATATAAATTGATATAGAAAACTAAAAATACAACTGCAACTATAAGATAACAATACTACCAA

Gene: folC (folylpolyglutamate synthase/dihydrofolate synthase)

Contig: 07_NODE_2, position: 222724 to 223995, length: 1272 nt, orientation: REVERSE

Perfect match to: (RF122-AJ938182-[1640282:1641553:r], highly conserved allele)

Sequence:

TTATAATGCTTCAAAGTCTAATTTTGATTTAACTTCACTTATGAAATACAGACTACCGGTAATTACTAATGTATCACCTTGATAATTTTTTAT
AAATTCAACGTAGTCATCTACTAATTGTATTTCATCATTTTCAATACTACCTACAATTTCTTCTTTGCGTAACGCTTTCGGAAAATCAAATTC
AGTTGCATAAAACGTATGCGCAATTAAACTTAAATGTTTGACCATCTCGTTAATCGGTTTTCCGTTTATTGCTGAGAACAAAATATCTACTT
TTTCTTTATCATGGTACTGTTTAATTGTATCAATTAGAGCATCTATACTCTCTGAATTATGTGCGCCATCCAAAATGATTAAAGGTTTGTCAT
GCACCTGCTCAATACGTCCAGTCCAACGAACTGATTCAATACCGTCTATCATCTTATTGAAATCTAATTCAATTAATCCTTGTTCATTTAATT
CAATAAGAGCTGTTATGGCTAATGCAGCATTTTGTTTCTGATGTTCACCTAACATGCTTAAAATGATTGTTTCTAATTCATAATCTTTATAA

CGGTAAGTAAATTCATCATTTTGCGATACAACAACAATTTCTCTATCTAATTCAATTGGCTTTGCATGTTGTTCAATTGCGCGTTCACGAAC
ATATTTTAATGCATCTTCATTTTTAACAGCATATATCACTGGAACGTTAGGCTTTATAATCGCGCCTTTATCCCTAGCAATATCTAGATAAG
TACCACCTAAAATATCTGTATGGTCTAGACCGATACTAGTTAAGATTGATAAAACCGGTGTAAAGACATTTGTCGAATCGTTCTTTATACC
CAATCCAGCCTCAACAATGACAAAATCAACAGGATGTATTTCACCAAAATATAAAAACATCATCGCTGTGATTATTTCGAATTCAGTTGCA
ACACCTAAATCTGTTTCACGTTCCATCATTTCACTTACTGGTTTAATACGTGATACTAATTCTACAATAGCGTCATTTGATATTGGCACACCA
TTTAGACTAATTCGTTCATTAAATGTTTCAATAAACGGCGACGTAAATGTACCTACTTCATAACCATTTTCAACTAAAGCTGTTCTAAGGTA
AGCAACTGTAGAGCCTTTACCATTTGTGCCACCTACATGAATACCCTTAATGTTATTTTGAGGATTATTAAATTGTGCTAGCATCCATTCCA
TACGTTTAACACCTGGTTTGATGCCAAATTTAGTTCTTTCGTGTATCCAATACAAGCTCTCTAGGTAATTCAT

Gene: valS (valyl-tRNA synthase)

Contig: 07_NODE_2, position: 224008 to 226638, length: 2631 nt, orientation: REVERSE

Perfect match to: (N315-BA000018-[1693943:1696573:r], highly conserved allele)

Sequence:

CTATGCTTTTAATTGTTCAATTCTTGCCTTCACACCATCATATTTTTCTTGATAATCTTGTTTTTTACGTTTTTCTTCATTTATAACCTTTTCAG
GTGCTTTACTTACAAAGTTTTCATTAGAGAGCTTTTTATCTACTCTATCTAATTCGCTTTGAAGTTTAGCTAATTCTTTTTCCAAACGGCTGA
TTTCCTTATCCATATCAATTAGCCCTTCTAATGGTAATACCACTTTACCTGCAATTACAACTGATGTCATTGCTTTCTCAGGAATTTCCACGT
CAGTGCTAATATTTAAGGTACTAGGATTACAGAATTTGATTAAATAATCTTTGTTTTGTGATAAAGTTGTTTCAATTTCTTTATCTTTAGCTT
GAATTAAAATAGGTATTTCTTTAGACAATGGCGTATTTACTTCTACACGTGATTGTCTTACAGATTTAATGATTTCAACAAGTTGTTGCATT
GTTTGTTTACTTTCTTCAAAAATCAATGATTCACGCACTTCTGGCCATGAAGCTTTAACAATTGTGTCACCTTCATGTGGTAAACTTTGCCA
TATTTTCTCTGTTACAAATGGCATGAATGGATGTAGCATTCTCATAATATTGTCTAAAGTATAACTCAATACTGAACGTGTAACTTGTTTTT
GTTCTTCATCATTACTATTCATTGGAATTTTACTCATTTCAATGTACCAATCACAGAAATCATCCCAAATGAAATTATATAATGCACGTCCAA
CTTCGCCGAATTCATATTTGTCACTTAAATCAGTAACTGTTGCAATCGTTTCATTTAAACGTGTTAGAATCCATTTATCTGCTAATGATAAG
TTACCACTTAAATCGATATCTTCAACTTTAAAGTCTTCACCGATATTCATTAAACTGAAACGTGCCCCATTCCAGATTTTATTGATAAAGTTC
CACACTGACTCAACTTTTTCAGTTGAGTATCTTAAATCATGTCCTGGAGATGAACCTGTTGCTAAGAAGTAACGCAAGCTATCAGCACCGT
ATTCGTCAATAACATCCATTGGATCCACACCATTACCTAATGATTTACTCATCTTACGCCCGTCTTCAGCACGAACTAAACCGTGTAATAAT
ACATCATTAAATGGACGACGATCTGTAAATTCTAAGCCTTGGAATATCATGCGTGCTACCCAGAAAAAGATAATATCGTAACCTGTAACT
AAGGCATTTGTTGGGTAGTATCGTTTAAAGTCTTCACTTTCTAAATCAGGCCAACCTAACGTAGAGAAAGGCCATAAAGCACTTGAGAAC
CACGTATCTAATACATCTTCATCTTGTTGCCAATTTTCAATATCAGTTGGCGCTTCTTCTCCAACATATATTTCGCCTGTTTCTTTATGATACC
AAGCCGGAATTTGATGACCCCACCATAATTGTCTTGAAATCGTCCAATCTCTAATATTTTCCATCCATTGGTTAAATGTATGTTCGAAACGT
TGCGGATAAAAATCAATACGATCATCTGTTTTTTGGTTATCTAATGAACGTTTCGCTAAGTCTTCCATGCGCACAAACCATTGTGTTGATAA
ATATGGTTCAACAACAGCGCCAGATCGTTCTGAATGACCTACAGAATGAACATGATCTTCAATCTTGATAACTAAATCTTGTTCTTTTAAAT
CTTTAACTAGCTGTTTACGACAATCAAAACGGTCCATACCTTCATATTTACCCGCTTTGTCGTTCATTTTACCATTTTCATCCATAACGATAA
TATTTTCTAATTGATGTCTTTGACCAATTTCAAAATCATTAGGGTCATGTGCTGGTGTCACTTTCATAGCACCAGAACCGAAGTCTATATCA
ACATACTCATCTGCTAAAATAGGCAGTTCGCGTCCTACGATTGGTAATATAACAGTTTTACCGATTACATCTTTGTATCGTTCGTCATTAGG
GTTAACAACAATCGCTGTATCACCTAACATCGTTTCTGGTCTTGTTGTTGCAATTTCAATAAAACCTTCACCATCAGCGTAAGGATATTTAA
AATGATAAAACGCACCTTGAACATCTTCATGTATTACTTCAATATCAGATAAAGCTGTACGTGCTTTAGGATCCCAATTTATAATACGTTCG
CCACGATAAATAATTCCTTTATTGTATAAATCAACAAAAACTTTTTTAACTGCTTTACTTAAACCTTCATCTAAAGTAAAACGTTCTCTACTA
TAATCTAAACCTAGACCTAATTTAGCCCATTGCGCACGAATAAATGACGCATACTCTTCTTTCCAATCCCATGCCTGTTCTAAAAACTTTTC
ACGACCAAGATCATATCTAGTTATTCCTTGTTCATTTAATTTAGCTTCTACCTTTGCCTGTGTCGCAATACCAGCATGATCCATACCTGGTA
AGTATAACGTATCGTATCCTTGCATACGTTTCATACGTGTAATGATATCTTGTAAAGTCGTATCCCATGCATGTCCTAAATGTAATTTACCA
GTTACATTTGGTGGCGGGATAACAATTGTATATGTTTCTTTTGATTTATCTTCTGACGGTTTAAAATAACCATTCTTTACCCATTCTTCATAA
CGTCCCGCTTCAACTTCACGAGGATCATATTTTGGTTTCATTTCCAT

Gene: tbox08 (T-box leader element)

Contig: 07_NODE_2, position: 226682 to 226983, length: 302 nt

Sequence:

AAATAGGACGGATATTCCGTGGTACCACCTATATTCAAGAAGGATGTTTAATATCAAATTCACTCTTTTAACATAATTGGAATAATCATAC
CAATACTATCATCGTGAAATTTGAAATGCTTCATCTCTTCAAGCACTCTAGATTATGATTAACGCTCAAACACGTCTTAGCCTACTATTAAT
CACGTTCAGCTAAGATACTCTGTGGGCTACCTTCAGTAAAAATCATTTACATACTCACACCAAATCATATGCTCTCTTTAAAATAATTTGAA
CTTACTCTTCCCAAATCCTATATTAAA

Gene: tag (DNA-3-methyladenine glycosidase)

Contig: 07_NODE_2, position: 227041 to 227601, length: 561 nt, orientation: FORWARD

Perfect match to: (N315-BA000018-[1696976:1697536], allele observed in CC5+CC1+CC9+CC45I+CC72)

Sequence:

ATGAATGAATGCGCATTTGGTACTAAAGATCCAGTCTACTTAAACTATCATGATCATGTATGGGGACAACCGCTCTATGATAGCAAGGCA
TTGTTTAAACTTTTAGCATTAGAATCACAACATGCTGGGCTATCTTGGTTAACTATTTTAAAAAAGAAAGAAGCCTATGAAGAAGCATTTT
ATGATTTCGAACCAGAAAAGGTAGCACAAATGACCGCTCAAGATATCGACCGCTTAATGACTTTTCCAAATATCGTTCATCATCGTAAAAA
ATTAGAAGCAATTGTTAATCAAGCTCAAGGGTATTTAAAAATTGAACAAGCATATGGTAGTTTTAGTAAATTTTTATGGTCTTATGTAAAT
GGTAAGCCTAAAGATTTGCAGTATGAACATGCTTCTGATCGTATCACAGTTGATGATACTGCAACACAACTATCTAAAGATTTAAAACAAT
ACGGGTTTAAATTTTTAGGTCCAGTAACAGTATTTTCGTTTTTAGAAGCAGCTGGTTTATATGATGCACATTTAAAAGATTGTCCATCAAA
GCCTAAACACAATTAA

Gene: abrB (putative ammonia monooxygenase)

Contig: 07_NODE_2, position: 227824 to 228897, length: 1074 nt, orientation: REVERSE

Perfect match to: (MW2-BA000033-[1736780:1737853:r], highly conserved allele)

Sequence:

CTAATCTTCACGCTTATTCGAACGATATTTTAAAAAGTAATTAATTCCTGGTGCAATAATAAATAGGATGAAAAAGATTCGGAAAATATGA
TAACTCGTAATCATAGCAACATCGGCACCAGTAGCTAATGCAACTAAAACTATCTGATTAACCCCTCCTGGTGCTGCACCAAGAAACAATT
CATTAATAGGATTATTATCAAAGAAATGTATGACATAAACCATGATTAGCGCACCAATTATCAACATAATATTTTGAATTGTAATTGCGAT
TGCTAGTCTACCTTTTAAATCTGACAATAAATGCGCAATTTGAACTCCAATTCTAATCATATATATTAGTTGTGCCATGTTCAACAACCAAT
GATCTAGTGTAAATGTTAAACCTGTAGAAAAATTCCAAACAATTAATACAATGAGTGGTGCTAATAATTGAAATGTTGGAAACTTTATTTT
AGACATAATTAGATAAACTATAAAGATAGCTATCGCTAAAATAACTATTTGCCCTATGTTTAATACTTGTGATAAAGGCAAGACTTTTGTT
AACTTTCCATTCGCATGCATGTTACCATCATGAAAAAAATATGAAATGAACGGTACTAAAACAACAACAAATATAATTCGTGATGTTTGCG
TTAAGCTAACAACTAACAAATTAGCACGTTTGTCTTGTTCAGCCATGACCAGCATTTGTGTTAGTGCTCCTGGTATAACACTTAAAATAGC
TGTTTCTGTATTAATACGTGCAATTTTTTTAAAAATAAATGCCATTACTATTGCAATTAATAATATCGAAATAGATACAACAATAATCGAAA
GCCAATTGTCTTTAATATCCATAACGACATTTTTCGTAAACGTTGATCCGATTTGCACACCTAATAGTACAATACCTAATTCACTAAGTAAG
AATGGCCATTTAATATCAAGTTTGAAAACTTTTACACAAATGATTGATGCGATAATAGGACCAAACATAAATGGGAGTAATACGTGCGAT
GAATACAGTAGAATACTAATAAAAAATGATAAAACGAACACAATGAAATTATTTCTATATATCATTGTCAT

Gene: gsaA (glutamate-1-semialdehyde 2,1-aminomutase, locus 1)

Contig: 07_NODE_2, position: 229093 to 230379, length: 1287 nt, orientation: REVERSE

Perfect match to: (MW2-BA000033-[1738049:1739335:r], highly conserved allele)

Sequence:

TTATTTTACAATACGACTTAAAGCCGTATCAAATGCTTGAATCGTTTTTTCAATATCTTCTTTCGTGTGTGCCGTAGATAGGAATGTACCTT
CAAATTGAGATGGTGGTAAAAACACACCTTCTTTTGCCATTTCTCGATACATTTCTGCAAATAATTTCAAATCACTTTTATTCGCTTGTTCAA
AATTAGTTACAGGTCCTTCATTTAAGAAATAACCAATCATTGAACCTGCTCTATTTACAGTTATTGGTACATTGTGTTTAGCAAATACACGC
TTTAAACCGTCTTCAAGTATATCGCCTAACATATTAAAATACTCATATGTCTCTGGCGTTAATTGGCTTAACGTTTCATAACCACTTGTCATT
GCAAGAGGATTTCCTGATAACGTACCCGCTTGATAAATATTTCCTAATGGTGCTATATGATCCATGATTTCTTTTTTACCACCAAAAGCACC
TACAGGTAGTCCTCCACCGATAACTTTTCCTAAGCAAGTTAAATCTGGTGTCACACCAAAGTAACCTTGTGCACAATGATAACCGACTCTG
AAACCAGTCATTACTTCATCGAAAATTAGCAATGCGCCGTATTCAGTCGTAATATTTCTTAATCCCTGTAAAAAACCTTCAATCGGCGGTA
CGACACCCATATTACCAGCAACAGGTTCTACGATTACACCAGCAATATCGTCTCCAAATTTTTCGAAAGCGATTTTAAGTGCATCTAAATC
ATTGTATGGAACTGTAATTGTATTTTTAGCAATACCTTCAGGCACACCAGGAGAATCCGGCAATCCTAATGTTGCCACCCCAGAACCAGCT
TTGATTAATAACGAATCACTATGACCATGATAGCAACCTTCAAATTTCACAATTTTATTTCTTCCAGTATAACCACGTGCTAATCTTAAAGT
ATCCAATGTAGCTTCTGTACCAGATGACACCATACGCACTTTTTCTATTGAAGGTACTCGGTCAATAACGAGCTGCGCCAATTTATTTTCAA
GTAATGTTGATGCACCAAAACTTGTACCTTTATCAATTGCTTCATGTAAATGACTAATAACTTGAGGGTCTCTATGTCCTAAAATAAGTGG
CCCCCAACTTAGTACATAGTCGATATACTCGTTACCATCGATATCATAAATTTTTGAACCTTTACCGTGATCCATAAAAATTGCTGGTGTAT
CTACTGATTTAAATGCGCGTACTGGACTATTTACACCACCAGGCATTAAAGTTTCAGCAACCTTCATTGCTTCTTCTGATTTCGTATATCTC
AT

Gene: hemB (porphobilinogen synthase)

Contig: 07_NODE_2, position: 230427 to 231401, length: 975 nt, orientation: REVERSE

Perfect match to: (NN50-BAEA01000025-[279907:280881:r], allele observed in CC4803+CC8+CC22+CC30+CC80)

Sequence:

TTATTTATCTAAATAGCGACAAATGTCCTTTGCAAAATACGTAATAATCATATCAGCACCTGCACGTTTCATTGAAACCATTTGTTCCATAA
CGACACGTTCTTCATCTATCCAACCATTTTGTGCCGCTGCTTTAGTCATACTATATTCTCCACTCACATTATATGCAACAACTGGAACATTCG
TATGATTTTTAACATCTCGAACTATGTCTAAATAACTTAGAGCAGGTTTAACAATCATCATGTCGCACCCTTCTTTAAGATCACTTTCTAATT
CACGAAGTGCTTCCAAACGGTTAGCAGGGTCCATCTGATACGTTTTTCTATCCCCAAATGATGGCGCTGAATCTGCTGCATCTCTAAAAGG
TCCAAAGAAACTTGATGCATACTTGACACCATAACTCATTATAGGAATATTGTAATAGCCGGCTTCATCTAATCCACGACGAATTTCAGCA
ACAAAACCATCCATCATATTACTTGGCGCAATAATATCAGCACCAGCTTCCACTTGAGAAATTGCTGTTTTAACAAGCAGTGGCAATGATT
TATCATTGTCAACGTCATGTGTATGGTCATCAATCACGCCACAATGACCATGATCAGTATATTCACATAAACAAGTGTCTGCAACAATTAA
TAAGTCATCATACATTTTTTTAGCAATACGTGTTGCCTGTTGAATAACACCATCGTGAATGTATGCACCAGTACCTATATCATCTTTTGAGT
TTGGAACACCGAAAAACATAATGGCACGTATGCCTAAGTCATAAGCTTCTTTTAATTCACTTTCAAGTAAATTCAAACTGATTTGGTATAC
ACCTGGCAATGACTTAATTTCTTTTTTCACATCGTCTTTTTCAACTACAAAAATTGGATATATTAAATCTTCTTTTCTTACATGATTCTCTCTA
ACCATATCTCTCATTGTCGCTGATGATCTCAATCTTCTATGTCTATCAAATTTCAT

Gene: hemD (uroporphyrinogen III synthase)

Contig: 07_NODE_2, position: 231404 to 232072, length: 669 nt, orientation: REVERSE

Perfect match to: (N315-BA000018-[1701339:1702007:r], highly conserved allele)

Sequence:

TTAGCCCCTACTTTCTAAAATCTTTTCAATTAGTGATTCGAGTGTTTGAATTTCTGCAATTGTTACTGGTTGTTGATATGATTTAATGGTCCG
TGCTGTTTGTTCTCCAATAGCAAAATACGACTTGAATTTTGGTACAAATCCTTCATTAAAATAATAACGTACTGCCGACGAACTTGAAAAT
GTTAATGCATCGATTTGTTGATGTTCTATCATTTCTTTAACATCTTGTATATTTTGTTTGTTAGGCACTGAAGTATATAAATCTATTTTAACA
ACTTCATTATCTTTAGATAACGCTGCTAATAACAATGGTCTCGCCAATTCACTCGAAGGCAAAAGTATTTTTTGGTTAGTTTGATTAAATGA
TTTTAAAAATCCTTCTTGAGAAAAGTCGTTTGGCATAAAATCAACTCGAATGCCAAGTGATTCACAATATTGCGCTGTCTTACTTCCTATCA
CAGCAATGTTATCAACATTAATTCCTTTTAGATATTTATAAAAGAATTTCACAGCATTTTTAGATGAAAAAATAAGCCAGTCATAGCGTTG
ATTTAACAAATGAATATCAAAATTTAGTGGCTTTATATCAATAAAGGGTTTGTGAATAATTGATACTAAATCACTTTGCATGTCATTTGTTT
GTGTCATAACTACAACTGGCTTCAT

Gene: hemC (porphobilinogen deaminase)

Contig: 07_NODE_2, position: 232094 to 233020, length: 927 nt, orientation: REVERSE

Perfect match to: (MW2-BA000033-[1741050:1741976:r], allele observed in CC1)

Sequence:

TTAATGTTGTTCATTTAAGCGTTTTATAATTTCATAAGCACCTTGCTCTTTTAATTTGTTACTCACTGTTTTGCCTAACTCAACCGGATCTGTT
CCGTTCATTGTATATTCAAATCGTTCTTTACCATCTGGGGTCATAATTAAACCTGTAAATTCGATTTCTTTTTGATCTGAGATTGTAGCATAT
CCTGCGATTGGCACCTGACAACTACCATCCATTTCTGCTAAAAACGTTCGTTCAGCAGTCACACATTTTGCAACCTCATCATTATGTACTTT
GCTTAATAATGTTAATAGTTCTTCATCGTCACTACGACATTCTATCCCTAAAGCACCTTGTCCGATTGCAGGTAACAATGTATCTCTATCAA
GATAAGATGTTACAATATCATCTGACCAGCCCATTCTTCTTAAACCAGCTGCAGCTAAAATAATCGCATCATAATCTTCAGTTTGTAACTTT
TCTAATCGTGTATCTATATTACCTCTAATCCATTTAATCTCTAAATTAGGATACTTAGATAATATTTGTGCACCACGACGTAATGAACTAGT
ACCAATAATACTGCCTTCTGGCAATTGGGATAGTGGTGTATGTGTTTTAGAAATATACGCATCAAAAGGTAATTCTCTATCAGGGATACA
ACCTAATGTTAAACCTTCCGGAATTACACTTGGTACATCTTTAAGCGAGTGTATTGCCATATCGATATTTTTTTCAAAAAGTTCATGTTGTA
TTTCTTTAACAAATAAGCCTTTGCCTCCGACTTTAGACAATTGCTTATCTACTATACGATCGCCTTTCGTGACAATTTCTTTAATTTCAATTTC
TAGATTTGGCTCGACAGCTTTTAATTTATCAATAAATTGCTGGCTTTGTGTTAAAGCTAATTTACTTCTTCTGGAGCCAACGACTAATTTAC
GCAT

Gene: hemX (membrane uroporphyrinogen III methylase)

Contig: 07_NODE_2, position: 233062 to 233877, length: 816 nt, orientation: REVERSE

Perfect match to: (CIG290-AIES01000017-[446231:447046:r], allele observed in CC45I+CC45IV8)

Sequence:

TCAATTCACAAAATGTGTTGCAAAAAATAAATTAATCATATTTAAGCAAAATAAAATAATGTTATAGTATATTAAATACCTTGAATTCAACC
ATTTGTTGATTCTAAGCAAAATATAACTTCCATATAATACTGTAATAATTGAAGAGAGTATTACCTTCGGGTCAATGAATATACGTTCACC
AACTGAAATTACACCCCACTGTGTACCTAAAATAATACTAAATATGAGAATTATCCACCCACTTAACGTTGAGTAAAACACAATTGATTCA
AGTGTAGCAACGCTACCTATTCTAAAGTATTTTTGATCAAAACGTTTTTCCTTCAAATTACGGTATTGCATGATATACAGTAATGCATTGAC
AAAAGCTAAGGCAAAGAAGACATAACTTAACACAGCTAGACCGATATGGACTAACAGTAACTCGTCTACAACAGCAATTTTCTGAACCTT
ATTAGTATAATGTGTCGGTTGAAATGTATTCATCCCTAAAAGTGTTAGCCCTATTAAATTCAAGAAAAACACAGAGAAATTCAAAACTTTG
ATAAGGTTAAGAATTAAAGAGATTGAAATAATCAGCCAACTTAAAGTATAGAATACATCTGAAATAGACCCTAATGGAATATGTCTAGTT
TGTATAATAAAAATAGATAAAGAGATTGTTTGTAAAACCCAAACAATCCCCAATAAATATATGCCTAAACTTCTAATCTTATGACTTTTTTG
GACAAAATCATAAAAATAGCAAATGATACTGATTAAGTATATTAATAATATAATTTCATTGAATCGAATAAACAGGTTTTCTTGCAT

Gene: hemA1 (glutamyl-tRNA reductase)

Contig: 07_NODE_2, position: 233899 to 235245, length: 1347 nt, orientation: REVERSE

Perfect match to: (ED133-CP001996-[1732798:1734144:r], highly conserved allele)

Sequence:

TTATTCAAAACTAAAGATACGTCGCGCTGAAATTTCCTTGACCTTGCTTTCTTTTTGTTGCTTCGCTTGCTCATGAGGACATTCAGCTTCGA
TATCAAATATATTTTGAAATAGCTCTAATTTTTCATTACTTTTCTTATCACTACTTAATTCTTTGGCCTGTTTAATAGGATCTTTCAACATTTG
ATTGATGATACTTTTTGTATGTTTAGATATAATTTTACGCTCTCTTTCGCTTAACCCTGGCAATTTACGATCAATACTGTCCATCGTTTCTGC
TTGAATTGCCATAGCTTTTTCACGTAAAGCTCTAATCACTGGAACAACACCCAACATACTAATCCACTCATTGTGTGCATGTATTTCTGCAG
GAATTTGTTCCGAAATTGTTGCAGCCGCTAATTGTCGCTCACGTAAGTTTGCATCAACTAAACCTTTTAAGTCATCAACATCATAATTAAAG
ATGTTTGTGATGGCACTAATACCAGGTTCAATATCTCGAGGAACTGCAATATCAATCAATACTAGTGAATCTTGCTTTCTATTTTCTGCAAT
TCTTTCTATCATTTCATTTGTAATGATATAAGATTGTGCACTCGTTGAACTAATCACAATATCTGCACTTTCAAGTAAATTTGGTAATGATG
ATAGTTCATCATATTTCACTTGATGCTTTGCTGCTAATTTCATAGCATTTTCAATTGTTCTATTTACTACTGTAATATCAGTAATTCCAGAAC
CAAGAAGATTTAATAGTGATAATTCACTCATTTCCCCTGCACCAATAATGATAGCTTGCTTACTTTTCAATTTGCCAAATACTTTTTTCGCCA
ACTCGACCGCAGCATAAGACACACTTACAGCATTATCAGCTATATCTGTTTCATTATGTGCTCTTTTTGCAAAAGTAATTGCCTGTTTAAAT
AGATGATTAAAAATTGTTCCTGTCGTACCTGTGCTTTGCGCTAAGAAAAATGCATCTCTTATTTGACCTAAAATTTGAGTTTCTCCAAGTAC
GATTGAATCTAAACCAGAAGTGACACGCAATAAATGTTCTACTGCTTCGTCCCCCACTTTTACTTCTGACATTGCTTTAATATCATCTACTTC
AAATCCAAATGCACGAGCTAGAAATCGTTGAATATAGTAACGACCTGTGTGAATTTGATCAACAACAGCATATACTTCAGTTCGATTACAT
GTTGATAATATGACATTTTCTAAAATAGATTTAGTTTCATATAAATCTTCATGGGCAATTCGTAAGGCATCATCTCTAAAAGCAACTTGCTC
TCTTAGTGCAACATCAGCTGTGCGATGATTTATACTAATTGCAATAAAATGCAT

Gene: engB (GTP-binding protein)

Contig: 07_NODE_2, position: 235462 to 236052, length: 591 nt, orientation: REVERSE

Perfect match to: (TW20-ST239-FN433596-[1796130:1796720:r], highly conserved allele)

Sequence:

CTATGAAATATACGGTTCAATTAAATTCCATATTTGTTGTTGTTTATTATTTTGAATTGATGAATAACTTACAATTGTATCGTCTGGGTCCAT
ATCTAATTGTGTCTTAATATTTTTAATATGCTTTTGAACTTTACCTTTTGGAATTTTGTCTTCTTTAGTGCATATAACTAAAGTAGGAATATC
AAAATGTTTCAAATAATTGTACATTAAGATATCATCTTGTGTTGGATCATGTCTTAAATCAACTAATTGAATAACTAATTGCAAATTCTCTC
TCTTAGTTATATATTCCTCAATCATTTTCCCAAATTTTTCACGTTGTGTTTTACTTACTTTAGCATATCCATACCCTGGAACATCCACAAAAAT
AAGTTGTTCATCTATATTATAAAAATTTAACGTTTGCGTTTTGCCGGGTTGCTGTGATGTACGTGCCATATTTTTTCTGCCAATCATACTATT
GATAAATGTAGACTTACCTACATTAGATCGACCGCTCAGTGCAACTTCAGACAATTCTGTTTCTGGATATTGTTCTTCTTTTACTGCACTAA
TGATTAATTCTATATTATTAGGATTAACTTTCAT

Gene: clpX (ATP-dependent chaperone protease, ATP-binding subunit)

Contig: 07_NODE_2, position: 236206 to 237468, length: 1263 nt, orientation: REVERSE

Perfect match to: (N315-BA000018-[1706141:1707403:r], highly conserved allele)

Sequence:

TTAAGCTGATGTTTTACTATTATTAATTAAATTGCCTTCTGCGTCGTATAGTTCTGGTTCAGTTTCTTCATTAATTGTTTGTGCTGTAATAAC
TACCTTCGTTACATTTTCGTTAGAAGGCACATCAAACATAATATCGATTAACGATTCTTCTATGATTGAACGTAAACCACGCGCACCTGTTT
TTCTTTCAATTGCTTTTTCACTAATTGCTGATAAAGCTTCTTCAGTGAACTCTAAATCCACATCATCTAATTCCAGCATTTTAGTATATTGTTT
CACAAGTGCATTTTTAGGTTGCGTTAAGATGTTTTTCAACGCAGTTACATCTAATGTTTCTAAATTAGCTACAATTGGCACACGTCCGATAA
ATTCAGGAATCAAACCATAGGCTTGCAAATCTTCTGGGCGAATTTGTGCTAATAATGCTTGTTCGTCATATTTATCAGCTTCATTGCTTGAG
AAACCAATAACTTTTTCACCAAGACGGCGCTTAATCACTTCTTCAATACCATCAAAGGCACCACCAAGAATAAATAAGATATTTGTTGTAT
CAATTTGAATCATTTCTTGGTTTGGATGTTTGCGTCCACCTTGTGGCGGAACACTTGCAGTCGTACCTTCTAAGATTTTAAGCAATGCTTGT
TGAACACCTTCACCTGAAACGTCACGTGTTATAGATGTGTTTTCAGATTTACGTGCAATTTTATCAATTTCATCTACATAAATAATACCTTTT
TCGGCTTTATCAATGTCAAAGTCAGCTGCTTGAATTAATCTCAACAAGATATTTTCAACATCATCGCCTACATAACCAGCTTCAGTTAAACT
TGTCGCATCTGCAATTGCAAATGGTACATTCAACGTCTTGGCTAATGTTTGAGCTAATAATGTTTTACCACTACCTGTTGGCCCAATTAATG
CAATGTTACTTTTTTGTAATTCAACATCATCTTCTTTTGGTCCTAATTGTTGAATACGCTTATAGTGGTTATAAACAGCTACAGCTAAAGATT
TTTTAGCTTTTTCTTGACCAATAACATATTCGTTTAAATGATCCATAATTTCTTTAGGAGTAGGTAATTCTGTCATCGCTTCAGAAGTGTTTT
GAGCTAATTCTTCTTCGACGATTTCTGAGCATAATTCAATACACTCATTACAAATATATACACCACTTCCTGCTACAAGTTTTTTTACTTGAT
CTTGGTCTTTTCCGCAGAAAGAGCATTTCAAATTTTCTTCATCTTCATTGAATTTAAACAT

Gene: tig (trigger factor)

Contig: 07_NODE_2, position: 237619 to 238920, length: 1302 nt, orientation: REVERSE

Perfect match to: (MW2-BA000033-[1746575:1747876:r], highly conserved allele)

Sequence:

TTAATCTTCTTTAGTTCCTTCAACGAACTTTGCGTTATCTCTTAATAAATCGATAACTTTTTGGATACGAACATCATTTTTAATGATATCAGT
ATTACCTAAAGTATTTTTGATATCTTCAACTGAGATATTAAATTGTTTACTCATTTTTTCTAATTCTTTATCGATATCTTCATCAGTAGCTTCG
ATTTTTTCAGCTTCAGCGATCGCAGTTAAAGTTAAGTTAGTTTTAACACGTTGTTCTGCATCGTCTTTCATTTGCTCTCTTAATTGAGTTTCA
TCTTGACCTGAGATTTGGAAGTACGTTTGTAAATCTAAACCTTGTTGTTGAATTCTTTGTGCAAATTCAGACACCATACGATCTAATTCAGT
ATTAACCATTGCTTCAGGAATATCGATTGTTGTATTATCAGTAGCTTTTGTAATCGCTTCTTCTTTTTCAACATTTTCAGCATCTGTAGCTTTT
TGTTCAGCTAAACGTTTACGTAAGTTTTCTTTGTACTCGTCTACTGTATTTGCTTCTGCATCTAATTCATTAGCAATTTCATCTGTTAATTCTG
GAACTTCTTTAAATTTAATTTCGTTAACTTTTGTTTTGAAAGTTGCTTCTTTACCAGCTAATTCTTCAGCATGGTATTCTTCTGGGAATGTTA
CGACAACATCTTTTTCTTCGTCAACTTTCATACCTTCTAATTGCTCTTCGAAACCAGGTATGAATGAACCTGAACCGATTTCTAAATCGTAA
CCTTCAGCTTGTCCACCTTCGAATTCTTCTCCGTCAACTGAACCACTAAAGTCAATGTTAACTGTGTCGCCATTTTCAACAACACCATCTTCT
TTAACTACCATTTCAGCTAAATGTCCTAAGCTGTGGTCAATCGCTTCTTGTAACTCATCATCAGATAATTCAGTTTCTTGTTTTTCAATTTCA
AGACCTTTATAGTCTCCTAATTTAACTTCTGGCTCAACTGTAACTGTTGCTTCAAAAATGAAATCTTTACCTTTTTCAATTTGAGTAACACTT
ACTTCTGGTTGTGCAACTGGTTTAATATCAGTTTCGTCAATTGCTTCACCATAAGCATCTGGTAATAAAATGTCGATAGCATCTTGATATAA
TGCTTCTACACCAAAGCGTTGTTCAAAAATTGGACGTGGCACTTTACCTTTACGGAATCCAGGTACGTTAATTTGTTTAACCACTTTTTTGA
ATGCTTGATCTAACGCTTTGTTTACTTTTTCTGCAGGAACAGTAACAGTTAATAAACCTTCGTTACCTTCCTTTTTTTCCCAAGTTGCTGTCA
T

Gene: ysoA (tetratricopeptide repeat domain protein)

Contig: 07_NODE_2, position: 239083 to 240012, length: 930 nt, orientation: REVERSE

Perfect match to: (N315-BA000018-[1709018:1709947:r], highly conserved allele)

Sequence:

TTAACTATTATTATTCAAATCTAATTGCTGAATAAAATTAAATGCATCGATACTTTGTAATTCACATTTAAGTCCTAACATATTTTTGAAATA
ACATTCGTATGCATTAATCCATTTATTCGTTTCAAACAAAGTTTCAATATCAATTGGATAAATCATGATAGAGTGATTATTCATAATATGGT
GTGCTTCTTCAGCGATATGTAATGCACCATCATTTAATGTTTCCATAACGTTAGGTATAACTTTTTCTTTCAGTGTTGTATGTTCTAGCCCAT
TTAAATTAGCTGGTACAAAAGTTACATCCATACCATACTTTTCAATTGTCAGTTCTTGTGTACAATTTGCAAACCTTAAATACTCAATCATTA
AACTAATGAGGTTATAACTGTACGTATTAGATTTTAATATATATAATACCGTTTCTTGAAATTGAAAATGACCATTGTCTATTAACTTCAAA
ATCAAGTGCGTCTGTTCCCTCATTGATAACGTATCAAAATCAGTTAATGACTGAGTCAATCGTTTTTCATCTTCAATTAATTTTGACTTAGC
AAATTCTTTAAGAGGATGTAATGCCATTCTTGTTTTGTGATCTTTGACTTCATCAATAATTTGATGAATTACTTCTACCGCTTCAAAATATTG
TCCCAAACCAATCAAACTTTTCACGTAATAAATCATCAATGCATCATATTGTTGTATGCCAGTTTTCAATAAGACAATTGTTTCTTCTCTTAA
TTCAAGAAATGACCCCGTCTCATATAACATACGGCATTTCATCATTGCTATTTCTTCAGTGAGTTCAAATTGACGTTCATATTGCACAATAT

ATTCATACATCAGGTCATAGTTTTGGTCTTGTTCAGCACGTTTTATATCTTTATATAACTTTTTCGAAGAGTTAGGAAACTGAATAATATCT
GACAT

Gene: ymaB (enzyme involved in deoxyribonucleotide synthesis)

Contig: 07_NODE_2, position: 240031 to 240639, length: 609 nt, orientation: REVERSE

Perfect match to: (COL-CP000046-[1755659:1756267:r], highly conserved allele)

Sequence:

TTATAAATCTTGAAGGATTAATGCGCTCCACGTTTCGAAATCATCATATGACTCTATGTTGCCTTTTTCAACCCATTTTATTCGTAAAGTATC
TGTTTCTTTAGCTTCTACATCATTCGTACTTACAGTGATTTTAAATACAACACCAATATGTACCTTGCCCACTTCATTATTATCGTCATTAATA
AAACCGATATATTCCATATTTTGTGAATCTTGCTCACTTAAACCTACTTCTTCTTCTAATTCTCTCTGTGCATTAACTCTCAATACTTCGTTAA
TAGATTCTGCTCCTGGAACATCATTCATATGACCGCCTACACCTATTGAAGATTGTCCATGCAATCGAGCTTCTCCACCGCCAGATAATCG
TTCATACACTAATATCTCGCCATGCTCATTTTCAAGTAAACAATAAGAAATGAGTTGTTTATATGATGGATCCTCTTCCATATCGCCGCGTC
GCTTAACTTCATATTGACTTAGCGCATCAAAAATATTTTGACCTTCTGGTTTATTCTTATTTAAAAACCCATTGAAAGTATTCTTTTCATTATT
AAAAATAATTTCTCTAGGTACTACAATGATTTGTTCATCAAATTTAGACAT

Gene: rplT (50S ribosomal protein L20)

Contig: 07_NODE_2, position: 240781 to 241137, length: 357 nt, orientation: REVERSE

Perfect match to: (N315-BA000018-[1710716:1711072:r], highly conserved allele)

Sequence:

TTATTTTAAAGCATCTTTAGCTTTAGTTACTAATTGAGCAAATGCTTTTTCGTCAGAAATTGCGATTTCTGATAACATTTTACGGTTAATGTC
GATACCAGCTTTTTTCAAACCGTTCATTAAACGTGAGTAGCTCATTTCATGTTGACGAGCTGCTGCGTTGATACGTGTAATCCATAATTTAC
GGAAGTCACGTTTACGTTGACGACGGTCACGGAAAGCATATTGACCTGATTTCATTACTTGTTGCTTAGCTACTTTGTATAATGTATGTTT
TGAACCGAAGTAACCTTTAGCTAATTTAATCGTTTTTTTACGACGCGCTCTTGTTACTGTTCCACCTTTAACTCGTGGCAT

Gene: rpmI (50S ribosomal protein L35)

Contig: 07_NODE_2, position: 241184 to 241384, length: 201 nt, orientation: REVERSE

Perfect match to: (N315-BA000018-[1711119:1711319:r], highly conserved allele)

Sequence:

TTATTTTTTGTATGCTAATAATTGTTTTACACGTTTCATATCGCTCTTAGACACTAATCTAGCTTTACGTAATTGACGTTTTTGTTTAGTGCTC
TTGTTTGCGAATAAGTGAGATGTGAAAGCTCTTGAACGTTTTAATTGACCTGAAGCAGTTCTTTTAACACGTTTAGCTGCTCCGCGGTGAG
TTTTCATTTTTGGCAT

Gene: infC (translation initiation factor 3)

Contig: 07_NODE_2, position: 241413 to 241940, length: 528 nt, orientation: REVERSE

Perfect match to: (MW2-BA000033-[1750369:1750896:r], highly conserved allele)

Sequence:

TTATTTTTCAGCTGTTGGCGCTAACATGATAAACATTTGACGCCCGTCCATTTTAGGTTTTTGTTCAACTGTTGCTATATCTTTGCATTCATC
TGCATATTTTTCTAGCACACGTTGACCAATTTCCTTATGCGTAATGGCACGCCCTCTGAAACGAATAGATACTTTACATTTATCGCCTTTAG
TTAAGAATTTACGTCCGTTTTTCAACTTAGTTTGGAAATCATGTTCCTCAATTGTTGGACTTAAACGAATTTCTTTAACATTGATAATTTTTT
GTTTCTTTTTCATTTCTTTTTCTTTTTTCTGTTGTTCGAATTTGAATTTACCGTAATCCATAATTCTTGCAACTGGTGGTTTCGCATTCGGTGC
AACGACCACTAAGTCTAAATCTACACGTTCAGCCATTTCTAAAGCTTCACGCTTTGATTTAACACCAATTTGTTCACCATCTTGACCGATTA
AACGTAATTCTTTTGCACGAATTTTGTCATTGATTTGAGTTTGATCTTTTGCTATGGTTGACAC

Gene: lysP1 (putative amino acid permease)

Contig: 07_NODE_2, position: 242169 to 243662, length: 1494 nt, orientation: REVERSE

Perfect match to: (08-02119-CP015645-[420140:421633], highly conserved allele)

Sequence:

TTAACCTTTTATTTCATCCATTGACACGTCTTGACGTAAATCTACTTGTTCTAATGGAATTTTTTTCGTTTTATATCGAAGCTTATGATAAAT
AAAGAATGCTAAAAATACTGGGATTCCCATATACGTAATTAAGAAGCGACTAAAATTAAAATCTCCTGTTTTAATAAAGTCAACATCTTGA
CCAATAATTACTACAATACATAAAAAGCCAGCAAATAATGGTCCGAATGGGAATAATTTAGCAGTATATTTTAATTTAGATTTGTCATAAT
TTTGTTTATCAAATGCTCTTCTAAATCGATAATGACTTACTGCTATACCTACCCAAGCAATAAAACCAGTTAAACCACTTGCTGCAACGATA
TATTCGTATGCACCTTTTGTAAGGCTTTGTAATACGAAAATTATCACTACAATGATTGCTGTAACGAGTAACGACATATATGGCACACCGT
TTTTATTTGTTTTACCAAATGCTTCAAACGCTAATTTATCTTTACTCATTGAATATAGCATTCGAGTTGAAGCATACATACCTGAGTTACCTG
CTGATAACACAGACGTTAAAATGACTGCATTCATAAATGATGCTGCAAACGCAAATCCAGCATTTTTAAACACTAATGTGAATGGAGACG
TTGCTACATTATCACTACCACCCATTAATGCACTACTATCATAAGGAATTAACATACCGATAACAAAAATGGCTAAAATGTAAAATAATAA
AATTCTCCAGAATACTTGTTTAATTGCTTTCGGCACAGCACGTTCAGGATTTTCTGATTCACCAGCCGTAATACCAATTAACTCAGTACCTT
GGAATGAGAAACCAGCGATTAAGAATACACCTAGAATTGATAATAAACTTCCTCCTAAGTTGCCACCAAGAATAGGACCTTCACCTTTATT
AAATATTTCGAATCCTACAACATGACCACCCATGATTCCGACAATCGTTAATAAACCAATTGCAATGAAAACAATAACTGTAACCACTTTT
ATCAATGCCAACCAGTATTCACTTTCACCATAGACGCGAACTGATAACGAATTCAGACTAAAAATTATAACTAAGAACAACGCACTCCATG
CCCAAGCGGGTATGCCTTGCAATGGTGTCCAATATTGAATGACTTGTGCTGCAATCGTAATATCTGCTGCTACAGTTACTACCCAGTTAAA
CCAATAGTTCCAACCAAGCGCAAACCCTAAAGATGGATCAACAAATCTTGTAGCATATGTACTAAATGAACCTGATACTGGCAAATACGT
AGCCATTTCGCCAAGTGACGTCATTAAGAAAAATACCATTATTCCGATAATTGCGTATCCTATTAATGCACCCAAAGCACCTGCATCATGA
ATTGCTCCACCAGAAGTTACAAATAAACCTGTACCAATACAACCCCCAATCGCAATCATAGAAATATGACGATCTTTAAGTCCCCTTTTGA
CAACATTGTTACTTTCATTTTGAACTTTTGACAT

Gene: Lys_riboswitch (lysine riboswitch)

Contig: 07_NODE_2, position: 243807 to 243982, length: 176 nt

Sequence:

ATATAAGGTAGCACATCACATTCTGTGACAGTACAGTTCCTATTCGAAAACTGTCCCAATAGATATAATTTATGGTTTATATCTATTTCGGC
ATCTTTACCTTTCACTTGTTCAACTTATGTACCATAAATACTTCTGACAAGTTACTAATTAAACATGCAACCTCTAACTCAATT

Gene: thrS (threonine--tRNA ligase)

Contig: 07_NODE_2, position: 244088 to 246025, length: 1938 nt, orientation: REVERSE

Sequence:

CTATCTATGTTTTTTTAAACGAATTTCATCAACTAGATTCCAGATAAATTCATCTTTTTCAACTGTTTCTTGGTCTTGCGATCCATATTGACG
CACATTCACTTGATTATTTTCAACTTCCTTATCCCCAACTACGATTTGATAAGGTATTTTTTGCATTTGAGCTTCTCTAATTTTATAACCCATT
TTTTCATTACGGTCATCAATACTTACACGAACGCCTTGAGATTTCAATTCATCTTGTAATTGGCGCGCATAATCATAATGTAAATCAACGTT
AACTGGAATGATTTGAACTTGTTTTGGCGCTAACCAAGTTGGGAATGCACCTTTAGTTTCTTCAGTTAAAAATGCTACAAATCGTTCCATT
GTTGATACAACACCACGATGAATAACAACTGGACGATGATGTTCACCATCTTGACCAATATAAGTTAAATCAAAACGTTCTGGTAATAAG
AAATCAAGTTGTGCTGTTGATAATGTCTCTTCTTTACCCATCGCTGTTTTAACTTGAACATCTAGTTTCGGACCATAGAATGCCGCTTCACC
AATCGCTTCTTCGTACGATAAGCCAAGCTCATCCGCTGCCTCTTTAAGCATATTTTCAGCTTTATTCCACATATCATCATCATCAAAGTACTT
TTCTTTATCTTCAGGGTCTCTATAACTTAATCTAAAGCTATAATCCTCGAAACCAAAGTCTTTATACACATCAATAATCATGTTTACAACGC
GTTTGAATTCTTCTTTAATTTGATCAGGTCGAACAAAGATATGTGAATCATTTAAAGTCATACCACGAACACGTTGTAATCCTGATACAGC
ACCACTTGCTTCATATCTATGCATCGTTCCTAGCTCAGCGATACGGATAGGTAATTCACGATATGAATGTGGTTTATTCGCATAAATCATC
ATATGATGTGGACAGTTCATTGGACGTAATACCATAGATTCAGTTTCATCTAACTGCATTGGTGGGAACATATCTTCTTGATAGTGATCCC
AGTGACCAGATGTTTTGTATAAATCAACATTAGCAAGTACTGGTGTATAAACGTGGTCATACCCCATGCTCACCTCTTTATCAACAATGTA
ACGTTCAATTTCACGTCTAATTGTTGCACCGTTAGGTAACCATAATGGCAAACCAGCACCAACTAATTGGCTATTTGTGAATAATTCTAAC
TCTTTACCAATTTTACGATGATCACGTTCTTTACGCTCTTCTAACATTTGTAAATGTGCTTTTAATTCTTTTTTATCAAAGAAAGCAGTACCG
TATATACGTTGTAACATTTTGTTGTTACTATCTCCACGCCAGTATGCACCTGCTGTAGATAATAGTTTAAACTCTTTAATTTTAGCTGTTGAT
GGAACGTGAACTCCACGACATAAATCAGTAAAATCACCTTGACTATATAATGTTACATTTTCATCTTCAGGAATCGCGTCGATTAATTCTA
ATTTGTATTCATCATTGCTGAATAACTCTTTCGCTTCATCTCGTGAAACCACTTTTCGTTCGATTTTCATATTTTCGTTAACGATTTGTTTCAT
TGTCTTTTCAATTTGTTCAAAGTCATCAGATGAGATGTTTTGGTCAATGTCGAAGTCATAGTAGAATCCACCTTCTATTACAGGACCTACAC
CAAATTTAACATTACCATATAACCTTTTAATCGCGTGTGCCATTAAATGTGCAGCAGAATGACGTAATACCTCTAACGCTTCTTCACTACCT

GGTGTCACAATTTCAATTGATCCATCAGTTTCAAGCGGTTTTGTTAAATCTACAAGTTGCCCGTTAAATTTGCCGGCAACAGCTTTTTTACG
TAATCCAGGACTAATTGATTGTGCTATATCTTCAGTAGTAGTACCTTTATCAAACGCCTTTTTATTACCATCTGGAAATTGAATATTAATTT
GTTCCAT

Gene: tbox09 (T-box leader element)

Contig: 07_NODE_2, position: 246185 to 246396, length: 212 nt

Sequence:

ACAAGGGACGAGATCGTCGTGGTACCACCCTAGTTATTTAATACAATTTATGTATTAAATATCTCTGCTTAAGATAACGGTCTTGATCCGG
GTATTCATTACAAATACCATAAATGAAGTAGTAATCATCTAATTTATTAACCATATTCTCATCAAACAATGGCTTTCTGTGTAATATTGATT
AGATCATCTTGTCTTCAATCATTTAAACG

Gene: dnaI (helicase loader)

Contig: 07_NODE_2, position: 246437 to 247357, length: 921 nt, orientation: REVERSE

Perfect match to: (11819-97-CP003194-[1784801:1785721:r], highly conserved allele)

Sequence:

TCAATTGTTTCTGAAATTTTCTCCTGATAAAAAATATGGTGTTGACAAAGATTTGACACGTTCAATAATACGTGCTGCTTTAGTCTTCTCTT
CACCATCACGAGTCATCGCTAAATGATGTTCCAATTCACTATAGTCAAAATTAGAACTAAAGAATGTTGGTAATTCATGAACCATTCGATA
ATGTAGCAAAGGTCCAATTACCTCATCTCTCACCCATGGAGTCACTTCTTCAGCCCCAATATCATCAAGCATTAAAATGTTTGCTTCTCTTA
CGCGATGTAATTTCTTTTCAAAAGAACCATCTTTAAAGCCACCTTTTAATGTTCTAATAAATTCCGGTAAATAAATAATTGTCGAACGTACC
TTCTTAGATTTGAGCTGATTCGCAATTGCACCTAGAATAAAAGATTTACCTGTCCCAAATGGACCATAAAGGTAAAGGCCTTTCACTTGTT
CCCCATTAGTTATTGCTGTACAAATATCATCTGCTGCCATAGCTACATCAAGACGGTCTCGTTGATTCATATAAATATCTTTCAATTTGGCA
TTTAAAGTATCTCGTTGCATATGATGAGATGTAATTAGCTCAGCTTCAAAGCGTTCTTCGTCGTACTTGATTTTACATGGGCATTGTAAATA
GCGTATTTTAATTCGGTTATTATCAACATATAACTCAGGCACATGCCCCTTTACGAAATTTGGACAATCAGCAAATTTATGACCGTCATAAT
GTTTTTGTTGATCTTTATACTCTTGTAACACATTTAAGTCTTCATCAATCATAGCATTCGTTAATTCAGCTCGATGCGCTTCCAAAAATTGCT
TAACATCTGGGTCATTGATTACTTCTTTTTTTATCTTTTCTATTCTTTTTTCAAAGTCCTGCGACGTGTTAATTATACTTTTAAATTGCTTCAT

Gene: dnaB (chromosomal replication initiation and membrane attachment protein)

Contig: 07_NODE_2, position: 247357 to 248757, length: 1401 nt, orientation: REVERSE

Sequence:

TTATTGACTGTCCTCCTCCCATTTTTTAGATAATTTATCTAGAAATGCTTGTCGATCTTGCTCTAATTGTTGATCATCTACGCTATTATCTTTA
GCCGAATCTTCTTCACTAGGTTTATCTCTATTTTCTAACCATTTAGGTGTTTTTTCTTTTGAAATACGATTACGCTGCCCAAAGTATGAACCA
CGCTTTTGGTAATTTCCGCTAGAACCCTCATTTTTAGGTTGATTAACTTTTTTAGCGTAATTATATGCTTCTTTAGCTGTCTTAATACCTTTTT
TCTTCCAATTTGATGCTATTTCTAAAATATACGCTTTAGGAAGTTTCATATCTTCTTTTAACATGACAAATTGCAACAAAATATTAATGACG
CCAAAAGACATTTTTTCACGTTCAATTAATTCTTCAACCATTGTCTTTTGCGATATAGTTGGTTCTGATTCAGACCAAGAAGCTAACATATC
AATTGGACTCGTTTGTTCAAGTAACTCAAACCATTCATCACTTTGTGGCTTTGGATTCACTTCTGAAGATTTGCCCGTCGAAGATGATGTA
GCAGGAGATTTCACCTGTAATTTAGGCATTTGATTTTCATGTTCCATTAAGTAATACGAGCGTGCTTGTTTACGCATTTCTTCAAAGGATAA
CTGTTGTCCACTTGTAATTGAATTTAAAATAACATGCTTCATGCCATCTGCTGTTAAACCATATAAAGTCGCGAGTTGTGTTATTAAACGCT
TTGCATCTTTGGTAACAATGTCTTGACTAATAAAATGTTTACCTAACATTTGTCTCAACATTTCAAAGTCAAAAGATTCATTTGATAAATCG
ATACCTTGGTACGGTTCATTAATCGGAATATCACTTGTATCGATATCTATTTTTGTAGACGGCACTTTAAAAACATCAGTAAATTGTCTTGT
TACCTGTTTAAATTCACTCAAATCAATTTGTTGATACTCAAAGTATTTCTTCAACTCATGAAATCGACGATGCTCGACTTCACTATATAAAA
AGATTGACAACATTGGATCATTAAAAAACAAATGTGCTGAAGGCGGTTGAATTAATTGGTAAACAAATTGTGTTTCTTGTTCATCATGTTT
GACAAACGCCTTTAACAATCCAATCGCTTCAAGTAAGTCCATTTGTTGTCTAAACTCTAGTAAATTAATTTTAAGTTCATTCATAAAAATAT
AATGAGAAAGAATCAATGTTTCATTATGACTTTCTTTAACGAATTGAGTCATAAAATGGTATAAACCCACTGCTTGTGTTCCAATTAGCGG
TGTATACAGCCGATTCAATACCTCTAAATGATTCGTATTTAAATCAAAGTGTTGCATAACTTTGAATTGATCCTTTGGTCTTAAGCCGAATT
CGAAGGCTTGTCGTCCCAT

Gene: nrdR (transcriptional repressor of nrdD/nrdG operon)

Contig: 07_NODE_2, position: 248758 to 249228, length: 471 nt, orientation: REVERSE

Perfect match to: (08-02119-CP015645-[414572:415042], highly conserved allele)

Sequence:

TTAAGCATCACTCCGTTTGTTTTCGCTTAAAATCCCTTGCATCGATGCTAACAATTGATCAACATCTTTAAATTCTTTATAGACTGATGCAAA
TCTAACATATGAAACTTGATCAACATGCATTAACAAGTTCATAACGTGTTCACCTATATCTCGTGAAGACACTTCCGTATGACCTTCATCTC
GTAATTGCCATTCAACCTTGTTTGTTATGTCTTCAAGTTGTTGATATCTAACTGGTCGTTTCTCACAAGAACGCACAAGTCCATTAAGTATC
TTTTCTCTTGAAAACTGCTCTCTTGTGCCATCTTTTTTCACAACTATAAGCTGACTAACTTCGATATGTTCAAATGTAGTGAAACGTGTTCCA
CAATTTTCACATTCTCTTCGTCTTCGAATGGCATTTAATTCATCGGCATGCCTTGAATCTACAACTTTAGATTGTGTAGAATTACATTTCGG
GCATTTCAT

Gene: gapB (glyceraldehyde 3-phosphate dehydrogenase, locus B)

Contig: 07_NODE_2, position: 249439 to 250464, length: 1026 nt, orientation: REVERSE

Perfect match to: (MW2-BA000033-[1758395:1759420:r], highly conserved allele)

Sequence:

TTAACTTGCACTTACAGTTTCTTTTGATGTCAAAAGTGCTCCAATTTGCTCAGCAACATCTACAACTCTATTTGAATAACCCCATTCATTATC
ATACCAAGCAATAACTTTTACTTTATTCCCTGACATGACCATTGTTGATTTTGCATCAATAATAGCTGAATTTGGATTAGTATTAAAATCAA
CAGACACTAGTGGTTGATGTTCGACTTCTATGATACCTTCTAAACCTGCATTTTCAAAAGCTTGGTTTACTTCTTCTGCAGTTACTTCTTTTT
CTAAATCAACAACTAAATCAACGAGCGATACATTCTTTGTTGGTACACGTAATGCCATGCCGTGTAATTTACCTTCTAATTCTGGTAATACT
TCTTTTAAAGCTTTCGCCGCACCAGTAGAAGTAGGAATAATGCTTTCATTACATGAACGTGCACGTCTTAAATCTTTATGTGGATTATCAAT
ATTTTTTTGGTCATTTGTAATAGCGTGAACAGTAGTCATTAAACCATTAACTATTCCAAACTGATTATTTAAAACTTTTGCAACTGGACCAA
TGCAATTAGTAGTACATGAAGCATTACTAAAAATGTCAAATGCTTCTATATCTAATTGGTTATCATTTACGCCTTTAACTACCATTTGAACA
TGTCCACCTTTTGAAGGACCAGTTAACAAAACTTTTTTGGCACCTGCTTTAATATGTGCGATGGCTTTATCACCATGATTAAATTTACCAGT
TGCATCTATAGCAATATCGATATCTAATTCTTTCCATGGCAAGTTTTCAGGATTGCGATCAGCAACCAATTTAATTTTATGATCTCCAACTT
GCAATCCATTTTCAATCGGTTCAACTTTTAGATTATATTTTCCATGTGTCGTATCGTAATTGATTAAATGTGCAATTGTTTCGGGTGGATAA
CTAGCATTTATCGCTACTACATTTAAATTTTTATTTTGTAATGCAATACGTAATACCATTCTTCCAATTCTACCCATACCATTAATTGCAATAT
TCGTTGACAT

Gene: coaE (dephospho-CoA kinase)

Contig: 07_NODE_2, position: 250634 to 251257, length: 624 nt, orientation: REVERSE

Perfect match to: (MW2-BA000033-[1759590:1760213:r], highly conserved allele)

Sequence:

TTAATCTTCTTCTCCGTAATTCGGCTTTTCAATATAACCTTCTTCTTCTAACAATCTCTCAAGGTTTTGTTTTAATTCAAGTTTATCCCCTAAA
TTATCGATAACATGATCGGCCATTCGGCTTTTTTTATCAATAGAAATTTGGCTATAGACACGTGCTTTCGCATCTTCTAATGACAAATTATT
ACGTTGCATTAAACGATCCATTTGTATACTTTCAGAAGTGTATACAACCCACACTTCGTCTACTGTATTTTCCAATTCATTTTCAAATAATAA
TGGAATATCCATGATTACATTATATCCTTGTTTTAAATATTCTTGCTTTTCTTCTTCCATAATATCTCGCACGATAGGATGTATGATAGCATT
TAATTCTAAGCGTTTTTCTGGATGATTAAACACTAGATCACCCATATAACGACGATTCATCTCACCATTTTCATCAATTGCTTCATCACCAA
AGACTTCTCGTACTTGAGCTAAACCTTTACTCCCCTTTTTAACAGCTTCCCTGGCTGCTTTATCAGCATCTACTACTTTAAAACCGAATACGG
ATAAGAGTTCTGATACTGTTGATTTTCCTGAGGCGATTCCACCTGTTAGACCAATAACTTTCGGCAT

Gene: mutM (DNA glycosylase)

Contig: 07_NODE_2, position: 251273 to 252145, length: 873 nt, orientation: REVERSE

Perfect match to: (N315-BA000018-[1721209:1722081:r], highly conserved allele)

Sequence:

TTATTTTTGACATACTGGACAATAATGACTATTTCTTGTCGCGATGATTTTTGTTTCAATTTGACTTCCACACACTTTGCATACCGGCTGCTT
ATATACATTAAGATGCAATTGCATCTCACCAGTTTTTCCATCAGCATGACGATAATCTGAAATACTTGTACCGCCATATTTAATACCTTCTT
CTAGTACTTCTCTAACATAATAAAAAACCATTTCTTGTTGTTGGTGTGTTAAGTCTTTTACTTTTTTATCTGGTAAAACACCTGCACGAAACA

ACGCTTCACATGCGTAAATATTTCCACAACCTGCGATTACTTTATGATCCAAAATCACTTGTTTGATTGGTTTATTCTTATTAGACTGTTGAT
GAATTCGATTTAAATAATACGTCAATGCTTCATTTGAAAAAGGTTCAGGCGCTATTTCTAAAAATGAAGGATAAGATGCTACAGACGCAA
CATTTCTAATTTCTCCAAAACGACGTATATCTGAATAAATTAACTTTTTGTCATTTGACAACTCAAAAATAACATGCCAATGCTTACGATAA
TTAGGTATCATAATATCTTCAAGTTCATCTACAATGAAAAAACCGCCCGCCATACCTAAATGACTAATTAATGTACGTTGTTCTCGTTTATT
ATCTAGCTGAAAAACGATATATTTACTTCTTCGTTCTACATTTGTAATGGTATAGCCTTCCGATAAAGTTTTAAAAGTATCTAATTCAATTCC
TTTTATAATTGTTTCCTTGCCTTGAGCTTTACCTTCGATTACTTTATCCGAAAATATAACGTGTTCAATTTTTTGATTTATAACGTAGGGTTC
AATTCCTCTTTTTACATGTTCTACTTCTGGTAATTCGGGCAT

Gene: polA (DNA-directed DNA polymerase I)

Contig: 07_NODE_2, position: 252161 to 254791, length: 2631 nt, orientation: REVERSE

Sequence:

TTATTTTGCATCATACCAAGTTGCACCATAACTTGAGTCTACTTTTAATGGAACATCTAATTGCAATGCATTTTCCATTATCTCTTCTACAAA
TTCACTAAATGAATCTACTTCTGACTTAGGTACTTCAAAAATTAATTCATCGTGTACTTGTAATAATAGTTTAGCTTGATATGTTGTCTCTTT
CATTTTTTGAGCAAATTTAACCATTGCCAGTTTAATGATATCTGCAGCACTGCCCTGTATTGGCGTATTCATAGCAGTACGTTCAGCAAAG
CCGCGTAAATTAAAGTTACGACTCGTAATATCAGGAATATAGCGTCGACGATGTAGCAATGTTTCCACGTAACCTAAAGCTTTGGCATCTT
TTACAATATCAGACATATATTGTTTTACACCTGGGAAACTAGCTAAATAATCATCAATGAATGCTTTTGCTTTTTTACGAGTAATACCTAAA
CTTTGACTTAAACCATAATCACTTATCCCATAAACAATTCCAAAGTTAACTGCTTTTGCTTGACGACGCATTAAACTATCGACTTGATCAGC
TTCTACACCAAATACTTTCATAGCAGTTGCCGTATGAATATCATCGCCGTTGATAAATGCTTCTTTCATACTCTCATCTTGTGTAATGTGTGC
TAATACACGCAATTCAATTTGAGAATAATCTGCTGATAATATAACGCTATCTTTTGAAGTTGGTTTAAAGGCTTTTCTTATTTTACGCCCTTC
TTCAAGTCTAACCGGAATATTTTGTAAATTAGGATCTACACTTGATAAACGTCCAGTTTGCGCTAAAGTTTGATTAAAACGTGTATGGATA
CGTTGATCATCACTAATTACCTTTTGCAATCCTTCAACATAAGTAGACTGTAACTTAGATAATTGACGATATTCTAAAATATAATCAATGAT
AGGATGTTCACCTTGCAATTGCTCTAAGACATCTACAGCAGTAGAATATCCTGTTTTCGTCTTTTTAATAACAGGTAATTGTAATGTCTCAA
ACAATACAACACCTAATTGCTTAGGAGAATTTATATTAAAATCTTCACCAGCTGCATCATGGATATTTCGAATCAAGACGTCTAATTTTTCT
TGAATTTCTTTTTCCATTTCTTCTAAATCATGAACATCTGTAAATATACCAATTTCTTCCATTTCACTTAAAATCTTAGCTAGCGGTAGCTCTA
AATCAGCCAAGAGTTCTACCTGATTGTATTCTTCTAATTGTTTATCCATATTTGGTTTCGCAAAGTAAATTGCATCAGTAATAGAAGCAACA
TATGGATTTAAAACATCATCTTCAGGTACCTTAAATTTCTTACCTTTTCCATATATACTCACATCGTCTTTCACAAAACTTTGACCGTACAAT
GAAACAACTGATTGAACATCACTAATCGTACGAGATGGATCAATTATATAACTGGCCAACATAATATCGAAAGAAATATTTTGAATATCA
ATCCCCAATCTATGTGATGCTACATATGTTTTTTTAGCATCATATACGACTTTTTTCGAATTCGGATTTTCTAACCATGAAACTAGTTCGACA
TAATTATTTATGTCATCCGCATTAATTACAATATGTTTCTCACCTGTAAATAAAGAGAATTTTAAAATATTATTTCGCAAATAGTTACCACC
GTCTAATTCGAAATGGATGGCCGCTTCTTTCAATGAAGTAAAATCAATATTATCAAAAGACGTTTCAATTTCAAATGTCTTTTCTATTGCAT
CTTCAACGCTTGCTGATTGATCAATGTCAGCCAACAATTGTTTGAATTCTAACTTCTTAAACAATTCGATTTTTTCTTGTTGTTCATCTTGAT
GAGTCATTAACGTATCTTCAAGTTTTACTTCAATCGGACTATCTACATTAATCGTTGCTAATTCTTTACTCATTAATGCATCTTCTTTGCTATT
TTGAAGTTTTTCTTTTAACTTTTTACCTGAAATTTCATCTAAATGTTCATAGACACCTTCTACTGTGTCAAATTGGTTTAGCAATTTTATTGCT
GTTTTCTCTCCAACACCTGCAACACCTGGTATATTATCAGAAGTATCTCCCATTAATCCTTTCATATCAATAATTTGATTAGGTGTTAACCCG
TTGTATTTTTCCGCAATAAAGTCAGGTGTATAATGATCAACATCAGTAACACCTTTTTTAGTGTAATAAATGGTTACATTATCCGTTGCAAG
TTGTGTTAAATCTCGGTCTCCCGTAATAATAATTGTCTGAAATCCCGCTTTATCTGCTTCTTTACTTAAAGTTCCGATAATATCATCTGCCTC
ATAGTTATCTAATTCATAACGTTTAATATGATAAGCATCTAATAATTGGCGAATATAAGGAAATTGCTCACTTAGTTCAGGCGGCGTTTTC
TGGCGTCCACCTTTATATTCACTATATTTTTCATGTCTGAAAGTCGTTTTACCTGCATCAAACGCTACTAAAAAATGATTTGGCTTTTCTTCT
TTTAAAATCTTCTCTAGTAACATTGCAAAACCATATACTGCATTGGTATGAATGCCTGCTTTGTTTGATAACAAAGGTAATGCATAAAAAG
CTCTAAAACTTAAGCTATTACCATCGATTAATACTAATTTATTCAC

Gene: Q2YTE9 (putative membrane protein)

Contig: 07_NODE_2, position: 255085 to 256572, length: 1488 nt, orientation: REVERSE

Perfect match to: (Strain_21334-AGTW01000036-[33875:35362:r], highly conserved allele)

Sequence:

TTATGAATCGTCTGAACCAAATGGAATATATTTAACTTTGGTCATTTTCGGTAATTCTTCATAATTATTAAACCACTCTTGGTAATTTTTATT
GATTGGTGACGGATGATGCATATAATGTTCAAACGGCAATGATTCCACTGTATAAACCTCTTGTCGTGGATGATCTTTAATTTGTTGTTTT
AATAACTGAACTCTTTTTTCATGTTCATAATGAACATAAATAAATGCACTAAGATATATCACAGCTAAAACTAGTGATGCACCTTTAATAAA
ATTAACATTGATTGACTTATATTTCCGAAATTCTTTTAATAAAATAATTAAAATGATTACATGTATCGTATAAACAATCAAAAAATTACCTG
GTTCTATTGGAGTAACAATGACTAGTGTCGACGCCGAAACACATATTGCAATAAGTAAGGAATATAAAGTGATTTGTGTTTTACGATCAT
TAATAGACAAATAAATACCTACAAATATCGAAAACGCAAAGTAACCACATACAATTACGTTCACAAAACCAACCAATCCAATATCTGTATT
TTTATTTAATAAAAACTGATTGTAAAATAGTAAATAATAAAGCGGTAAAGTGATAAATCCTATCATAATGATACGACGCTTTAATATTGTT

AAATGTACATACCGATCACTTCTAAGCAGTAGATAAATAATAAGCGCTGCAATCATACTTAAAATTATAATCTGACTGAAAATGACACCAT
ATGGAAGTGACGTTGATATCATTTCTGCAAATTTTGAAAAGATACCTTGATTATTTGAAACTTGTTGATATTCTGATCCTTCAAATAAAATT
TTGCGATAATTTGGATTGGAAAACATAATGATTGTACCTATAGTAGCAATCATAAATGAAAACAATAATTTATAATTAAGTGTTCGATTTG
CAGTAAATTCATATAGAAAAGCTAAAATTAAAATGATACAGTGGAATAATGTCACATTCTCCATAAATAATTGACCGAAGAAGCATAAAA
CATAGAATAGTACAGTAACACTTACCGGCTGCTTTTCTTTATATATAATTGCATTAATACAATAATAAATAATAAAGAGTGAAATTAGTGT
TGATGTCGCATAATTATAAAATCCTGCAAACCAGCCATATGTATCTGCATAAATAGCACTTGGTAAAATTAACATTAAAGAAAATGCTAAC
AAATAATAGCTTGTCCACGCTTTACAACGTGTAATGTGCATAATCATCCAAATGATGCCCATACTAATGAGACCATATGAAAGCCAACGTA
ACCAGCTTACATGTACAGCTATAATTTCAAAGATATTTCCGATATAGCGACCATTAAGTGATGCAAATCCTACTTTTAAAATATCAGTATTA
TAATTGCTAAACCATTGTAAATCATCGTGCATGAGTGGTAGTAAGATACCCATAAAAGTATAAAACAATAATATCGCAATTAATATCAAA
GTTGTCTTGTGTAATTGAATTGTTTTCAC

Gene: phoR (two component sensor/regulator of alkaline phosphatase, sensor histidine kinase)

Contig: 07_NODE_2, position: 257071 to 258732, length: 1662 nt, orientation: REVERSE

Perfect match to: (MW2-BA000033-[1766026:1767687:r], allele observed in CC1+CC15+CC101-ST582)

Sequence:

TTATTCTTTATAATCTTTTAGAATAACTTTGAACGTTGAGCCTTTGCCAACTTGACTATTCACTTCAATATTGCCTTGGTGGGCCTCTACTAT
ATGCTTTGTAATTGATAATCCAAGACCTGTCCCACCAGAATCTCTACTTCGTGCTTTATCTACTCTATAAAATCTTTCAAAAATACGTTGTTG
GTCTTCTAATTTAATACCTATACCAAAATCTTGTACTTCGAAAATGACACGAAAGTCATCTCGATACACACGAACGTTAATATCTCCATCTT
CATAAGAATAGTTAATTGCATTCGTTAACAAATTCGTGATAACTTGAGCAATTTTACTTTCTTGTGCTTTAACAATGACATCTTTTTCAATAT
CAGTATGAATGGAAATATTTTTTTGATTGGCTTGAGTCATCATATTATCAATAATACGCCGCGTTAAATCTGATAAATTCATATAGTCTGTA
TCTAACTCTGTATGTTGCTCAATATGCGATAAATCTAACAGATCTGTTACTAAAGATTCTATTCGATTTGATTCTTTTAAAATTATGTTTAAA
AACATGTCTAATGATTCCGCATCATTTTTTGCACCATCAATAAGCGTTTCAGCAAACCCCTTAATTGAAGTAATAGGTGTTTTTAATTCATG
TGAAACATTTGCTACAAACTCACGTCTTAGATTTTCAAGTTGTTTCAGATTTGTTATGTCATGCATCACAACTAAAATCCCTTGCAAACTTTT
TTGAGACCTAGTTAAAATCGGAACGCATGAAATATCAAAGTACTTGGCATGGACTTGGTTTATTGCAACTTCCAATTGTTCATAAATAGGT
TTTTCAACTTTAAAACTTTCTAAAATTAATTGCTCAATTTCAGTATTAACATAACCGTGATAGCCTACTTGTTCAATATTATGCGAGATGTTG
AACTGTTCATAATACGCTTTATTTGCAACAACGATTTTTCCATTTCGATCTATCATTAAAATAGCACTTGGAATATTTTCAATCGTTGTTTTT
AAACGGTTGGATTGAATTTTTTGCTCATTATTAAGCTTTTGAAGGCGTCGTGCTAAATCATTGGTAGACACAAAAAGCGCTTTAGTTTCTA
CAACATTACTTTCAGGTACACGTATGTGATAATAACCATTTGCCAACAATTGTGTTGCATAAGTAACTTCTTGAATGGGACGGATTAATGT
ACGCTTAAAACTACGGCTTGCAAAATACAGACAAATGAGTACAACTAAACATGTCAAAATAAGATATTTCCACAACGTCCAATGCATTTCT
GTAATATCGTTATTGTAACCTTTAATCCATACATGATAACCGTTAACCTTCTTATTAAAAATAAAAACGTCCCTTTTTTGAATATAGCTATCA
CTATTTGGGATAGCTTTCAGTTTGCCACTACTAAACACCTTTTTATCATGCTTAGTTATCAATAAATCTATATTTTGCTGTTTTACAATTTCTT
TAACTTTATCAATCTCATTATCTTGGACTAAATAAATATATGATCTTGCATCTGTTGCTAGAGCTTGTTCGTGTTTTTCTGATAAAACATATG
TGATGGAAGCGTGAATAATAATGCCTAATGTAACAAAACTGATAATTAATATACTGCTTATCAATAACATTAAGCGGTGGTGAAACTTCA
T

Gene: phoP (two component sensor/regulator of alkaline phosphatase, transcriptional regulator)

Contig: 07_NODE_2, position: 258732 to 259436, length: 705 nt, orientation: REVERSE

Perfect match to: (MW2-BA000033-[1767687:1768391:r], allele observed in CC1+CC25+CC239-ST582)

Sequence:

TCATTGTTCTTTAGGTCTTTCCAATTTATAGCCTAAGCCACGCACAGTTTTAATAAGTTGTGGCTTCTTAGGATTATCTTCTAATTTATCTCT
TAAATGACTGATATGTACATCGACAATTCTTGAGTCTCCTGCAAATTCATAATTCCATACCGTATTTAACATATGTTCTCTCGTAATGACTCT
GCCTTGTCTTTCTATCAAATAAAGCAAGAGTTCAAATTCTTTCGGTGTTAGCTCCAATAACTCATTATGCTTATATACTTCAAAATAATCGG
GTCTAATACGTATCGATCCGATGGTAATATCATCATCAACCTCTTCTACTTCTTTTACAAATTGAGAACGTCTTAAAATGGCTTTCACACGG
GCAACAACTTCTCTAGGTGAAAAAGGCTTAGTCATATAGTCATCGGCACCTAATTCTAAACCTAATACCCGATCAAATTCATCATTTTTCGC
CGTTAACATTAATATAGGGACTAAATTTTTATTTGTTCTTACAGTCTTACATACGTCAATGCCATCTTTTTTAGGTAGCATAACATCTAAAAT
AATTAAATCTGGCTGTTCACTTTCTACCTTTTCTAAAGCCTCATCACCATCAAATGCGACAACAACTTCATAACCAGCTGTTTCTAAGTTATA
TTTAAGTAATGTTACGATTGAATGTTCGTCATCTACTACCAACACTTTTTGCGACAT

Gene: citC (NADP+-dependent isocitrate dehydrogenase)

Contig: 07_NODE_2, position: 259826 to 261094, length: 1269 nt, orientation: REVERSE

Perfect match to: (Strain_21193-AFEG01000006-[129916:131184:r], allele observed in CC25+CC5+CC239)

Sequence:

TTATTTTAAATTTTTAATCAATTCATCTGCAAATGCTGATGTAGAAACTTCTTCAGCACCATCCATTAAACGGGCAAAGTCATAAGTAACAA
CTTTTGAAGCAATTGTATCTTCAATTGAATCTGTAATCTTATCTGCCGCTTCTTGCCATCCTAAATGTTCTAACATTAATACAGAACTTAAAA
TTACTGAAGATGGATTCACTTTATTTAAACCTGCATATTTTGGAGCTGTACCATGTGTTGCTTCAAAAATAGCATGACCTGTTTCATAATTA
ATGTTTGCACCTGGCGCAATACCAATACCACCAACTTGTGCAGCTAAAGCATCTGAAATATAGTCACCATTCAAGTTCATAGTTGCTACAA
CATCATGCTCAGCTGGACGAGTTAAAATTTGTTGTAAGAAAATGTCAGCAATAGAATCTTTAATGATAATCTTGCCTTCTTTTTCAGCTTTT
TCTTGAGCAGCATTAGCAGCATCTCTGCCTTCTTTTTCAACAATTTCGTCATATTGTTGCCAAGTGAATACTTGATCACCAAATTCAGATAA
TGCTAAATCGTAACCCCACTGCTTAAATGAGCCTTCTGTAAATTTCATAATATTACCTTTATGAACTAAAGTAACTGATTTACGGTTATTAT
CGATAGCATATTGTATAGCTGCTCTAACTAATCGCTCAGTTCCTTCTTTAGAAACTGGTTTAATACCAATACCTGAAGTTTCTGGGAATCGA
ATATTTGTCGCACCCATTTCGTTTTGTAAGAAGTCAATTACCTTTTTAACTTCTGTTGTACCTTCTTTAAATTCAATACCAGCATAAATGTCTT
CAGTATTTTCACGGAAAATAACCATATCAACATCTTGTGGACGTTTAACAGGTGATGGTACTCCTTTAAACCAACGTACCGGTCTTAAACA
AGTAAATAAATCTAATTCTTGGCGTAAAGCCACATTTAATGATCTAATACCACCACCAATTGGTGTTGTTAAAGGTCCTTTAACAGCAATT
AAATATTCTTTAATTGTATCAAGTGTTTCTTGAGGTAACCATTCACCAGTTGTATCAAATGCTTTTTGGCCAGCTAGCACTTCTTTCCATTCA
ATGCGTTTTTCGCCATTATAGGCTTTCTCAACAGCAGCATCTATAACTCGGCTTGCTGCCTTCCAAATATCCGGTCCAATTCCATCACCGAT
AATAAATGGGATAATTGGTTCATTAGGTACGTTTAATCCTTCAGTTCCTTGAGTAATTTTTTCTGCAGTCAT

Gene: citZ (citrate synthase)

Contig: 07_NODE_2, position: 261143 to 262264, length: 1122 nt, orientation: REVERSE

Perfect match to: (MW2-BA000033-[1770098:1771219:r], highly conserved allele)

Sequence:

TTATTTTCTTTCTTCAAGCGGGATATACTTACGATTCGTTTCGCCAATATATTTCGCTCTAGGACGCATAATTCTATTATCTTTATATTGTTCT
AAAATATGAGCAATCCATCCTGCAGAACGACTTACAGCAAAGATTGGCGTGAATAAGTCATGAGGTATTTCCATACAGTGATAAACACTC
GCACTATAAAAATCAACATTAGGAATTAATCCTTTTTCTTCTGCCATACGTTTTTCCATTTTCACTGACATTTCAAATAATTCTTCACGACCA
GCGTCTTTCGTAATTTGACGGCTCATTTCTCTTAAATATTTCGCTCTAGGATCACCATCTTTATATACACGATGACCGAAGCCCATTACTTTA
TCTTTATTAGCAAATTTTTCATCTAAGTAAGCATCAACATTTTCAATTGACCCAATCTCAGATAACATCGTCATAACTTGTTCGTTTGCACCA
CCATGTAATGGCCCTTTCAGAGAACCTACAGCTGCTACAATACCTGAGTACATATCTGACAATGATGATACCGCACAACGTGCTGTAAAT
GCAGATGCGTTCAACTCATGATCAGCGTGTAAAATAAGTGCTTTATTGAAGGCTTCTACTTCTATATCTGTTGGTAATTCCCCACGTAACA
TATATAGGAAGTTTGCCGCATAACTTAAGTCAGGATTAGGCTTAAGTGGTTCTTTATCTTGTCTTACTCGAGCAAACGCTGTAACTAATGA
TGCTACTTTAGCCTGTATACGCATTGCTCTTTCATAACGATTTTCATCTGATTCATTTTCAGCATCAGGATCGAAATGTGCAATATATGATA
ATGACGTACGTAATGCTGTCATTGGATGCACGTGATCTGTAACATACTCCTCAAAATGTGTATACACACGAGGATTTAATGTCATGTATTG
ATTTAATTTCCCTTTTAGATGAGCAAGCTCTTCTTCGTTTGGCAATCTATAGTTCCATAATAGGAAAATAACTTCTTCAAATTGCGCATTTTC
AGCTAGATCATCAATATCATAGCCGGCATAAGTCAATTGACTTTCAATAATTGAACTTATTTTAGTCTCCGCTGCGATAACCCCTTCTAAAC
CTCTTTGTAATTCTGCCAT

Gene: aapA1 (amino acid permease locus 1)

Contig: 07_NODE_2, position: 262612 to 263973, length: 1362 nt, orientation: FORWARD

Perfect match to: (N315-BA000018-[1732548:1733909], highly conserved allele)

Sequence:

ATGGCTGAAAAATTACAAAGGGAACTGAGCAATCGCCACATACAATTAATTGCAATTGGCGGTGCAATTGGTACAGGCTTATTCTTAGGT
GCTGGTCAAACGATTGCATTAACCGGCCCTTCAATTCTATTAACATACATCATTATAGGATTTATGTTATTTATGTTTATGCGAGGTTTAGG
AGAAATCATTATACAGAATACTGAATTTAAATCTTTCGCAGATGTAACCAATACATATATTGGGCCTTTTGCAGGATTTGTTACCGGATGG
ACATACTGGTTCTGTTGGATTATTACAGGTATGGCTGAAGTAACGGCTGTGGCAAAATATGTTAGCTTTTGGTTCCCAGAAATTCCAAACT
GGATAAGTGCACTATTTTTGTGTACTGTTATTAATGTCATTCAACCTACTTAGCGCAAGACTTTTCGGAGAATTAGAATTTTGGTTCTCTATC
ATTAAAATAGCGACAATTATTGGTTTAATAGTAGTTGGTTTCGTCATGATTCTATTTGCATTTAAAACTCAATTCGGGCATGCCAGTTTCAC
AAATTTATATGAACACGGCATATTCGCTAAAGGTGCTTCTGGATTCTTTATGTCTTTCCAAATGGCACTATTCTCATTTGTAGGAATTGAAA
TGATTGGTGTTACAGCTGGGGAAACAAAAGATCCAGTTAAAACAATTCCAAAAGCAATTAACAGTGTACCCATTAGAATTTTAATATTTTA
CGTTGGGGCGTTAGCGGTTATCATGTCTATTATCCCTTGGCAGCAAGTTGATCCTGATAACAGTCCATTCGTAAAATTATTCGCATTGATC
GGTATTCCGTTTGCTGCGGGCTTGATTAATTTTGTAGTATTAACCGCTGCTGCTTCATCATGTAATAGTGGTATATTCTCAAATAGCCGTAT
GCTTTTCGGTTTATCAAGTCAACAACAAGCACCTCCGAACTTTTCTAAGACGAATAAATATGGCGTTCCACATGTTGCAATCTTTGCTTCAT

CAGCATTATTACTTGTGGCAGCATTACTAAACTATATTTTCCCAGATGCGACAAAAGTATTTACGTATGTGACTACCATCTCTACAGTGTTA
TTTTTAGTTGTATGGGGTCTGATTATCATTGCATATATCAATTATAGTCGTAAAAACCCAGATCTACATAAAAATGCTACGTACAAACTATT
AGGTGGTAAATATATGGGCTACTTAATATTTGTATTCTTCATTTTTGTGTTCGGGTTATTATTTATTAATGTTGATACAAGACGTGCAATTT
ATTTTATTCCGATTTGGTTTATACTTTTAGCATTTATGTACTTAAGATATAAACGTATCGCTGCTAAATCAAATAAATAA

Gene: pykA (pyruvate kinase)

Contig: 07_NODE_2, position: 264321 to 266078, length: 1758 nt, orientation: REVERSE

Perfect match to: (MW2-BA000033-[1773276:1775033:r], allele observed in CC1+CC188)

Sequence:

TTATAGTACGTTTGCATATCCTTCAAAGATTTTACCTTGAGCAGCATCAATCGTAACTAACATGTTATTGCTTATGTTTTTAACAGCTTTTTC
TACACCTACAACTGTTGGAATACCTTTTTCTAAACCAACAATTGCACTTGGTGATGTAATACCATTTTCTTCTGTAATTAAGCCTAAAGCTTT
TTCTACATAAGGTACAAACGTTTCATCGATTGAGTTAGTAACGATAACTTTGTCAGATAAATCTTTACCTTCTAAATCTTTAACAGTTTCAG
CAACTAACGTAGTACCAACAACTGATCCACGTCCAATACCTTGACCATTAGCAATTTCGTCACCAACTAGGTGGATTTTCATCATATTAGTA
GTTCCAGTTTCACCAGTTGGTACACCAGCAGTAATAATGATTAAATCACCATTAGATACTCTACCAGTTTCAACAGCTGTTGCAACTGCAT
TGTTTAACAATGCATCTGTACTCTTACGTCCTTTTTTAACTACAGGTTGAACTCCCCAAACAATTGAACATTGACGTGCAGTTTCTTCACTTG
GAGTCACTGCAATAATGTCTGAATGCGGACGATATTTAGAGATAGTACGTGCCGTTGAACCACTTTCAGTAGCAGCTACAATTGCTTTAA
CATTTAAGTTTAAAGCTGTATGTGCAACCGAAATACCGATAGCATTCACTAATGAAGTTTCAACTAATTTAGTACGATCTGACAATAACTT
TTTGTAATCTTGGGCTGCTTCAGCTGATACAGCAATATTTCTCATTGTTTTAACAGCTTCTTCAGGATATAAACCAGCAGCAGTTTCACCAG
ATAACATTACTGCATCTGTACCATCATAGATTGCGTTGGCAACGTCACTAGCTTCTGCACGTGTAGCACGTGGGTTACGTTGCATAGAATC
TAACATTTGTGTAGCTGTAATAACTGGTTTACCTAATTTGTTACATTGTCTGATTAAATCTTTTTGAACCATTGGTACTTTTTCAGGTGGAAT
TTCAACACCCATGTCACCACGTGCAACCATTAAACCATCAGACACTTCAAGAATTTCCGCAATATTATCAATACCTTCTTGGTTTTCAATTTT
AGGGAATACTGAAATGTTAGCTTTTTGTTCTTCTAAAATTTCACGAATTTCTAAAACATCACTAGGACGACGTACGAAACTTGCTGCAATG
AAGTCAACATTTTCTTTAATACCGAAACGGATATCTTCAGCATCTTTTTCTGTAATACCAGGTAAACTTACTCTTACGCCAGGTAAGTTAAC
ACCTTTTTTGTTTTTAAGCTCACCAGAGTTTAAAATATCACATTTAACTTCTTTTTTAGCATGGTCAATATCTTTAACTTGTAATTCAATTAAG
CCATCATCAAGTAAAATGTATGAACCTACTTGAACATCGTTAATTAAGTTTTCATATGTTACTGAGAACTTTTCAGGTGTTCCTTCAACTTC
ATTCATGCTAACAATAACTTCGTTACCACGTTCAAGTTCAATGATACCGTCTTTCATATTATGCGTACGAATTTCTGGACCTTTTGTATCTAA
TAAAATTGCTACAATTTTGTCTAATCTTTTAGCTACTTTACGAATTGTATCAATTCTACCTTTATGCTCTTCATGACTACCATGTGAAAAGTT
TAATCGTGCAACGTTCATACCAGCATTGATTAATTTCTCAATCATTTCTTCTGATTCTGAAGCTGGTCCAATTGTACATACAATTTTAGTTTT
TCTCAT

Gene: pfkA (6-phosphofructokinase)

Contig: 07_NODE_2, position: 266100 to 267068, length: 969 nt, orientation: REVERSE

Perfect match to: (MW2-BA000033-[1775055:1776023:r], highly conserved allele)

Sequence:

TTATATAGATAACTTGTTAGCAAGTTCATATAGACTATAATCAAATTTATGATCTTTACCATCAAAAATTTCATCAAAAGATGTTGCTACAA
TTTTATTGTTCTTAATTCCAACACCCTTAGCTGTTTCACCTTGCATTAATAAGTCTACCGCATATCCACCTAAACGTGATGCTAAAACTCTAT
CCGCACCTGTTGGGCTACCACCACGTTGAACGTGACCTAACACAGACACTCTATTATCAACATTGATGTATTGTGATAATTCTTTTTGACA
ATCTTGCGCAGTCATACAACCTTCTGCTACAAGAACGATTGAGTGTTTCTTACCACGTTTAATACCTTGTTCAATTTTATCAGCTATTTCTTT
AATATCTGTTTTCACTTCTGGAACTACAATTGTCTCAGCACCAACTGATAATCCAGCCCATAATGCTAGATCTCCACAATCACGGCCCATTG
CTTCAATGATAAATGTTCGTGCGTGACTTGACGCAGTGTCTCTAATTTTGTCGACTAAGCCAATAATCGTATTTAATGCTGTGTCAAATCCA
ATTGTAAAATCAGTACCATTGATATCATTGTCAATCGTACCAGGAATACCGATAGTTTGAATTTCTTTGCATTCCTCACTGATGCGTTGTGC
ACCGCGATAACTACCGTCACCACCAATAACTACAAGGCCCTCAATCCCTCTTTTACGTAAGTTTTCGATTGCAACTTTACGTACTTCTTGCT
CCTTAAACTCTGGACATCTTGCTGAATACAAGAATGTACCTCCACGCTGAATCGTATCCCCAACTGATCCTAATTCAAGTTTATGAATATCA
TCATTTAACAATCCTTGGTAACCATGATACACACCATAAACTTCAATTTCATTGTAAATTGCTGTACGAACAACTGCTCTTACGGCAGCATT
CATTCCAGGTGAATCTCCACCACTAGTTAAAACTGCAATTTTCTTCAT

Gene: accA (acetyl-CoA carboxylase, carboxyl transferase, alpha subunit)

Contig: 07_NODE_2, position: 267337 to 268281, length: 945 nt, orientation: REVERSE

Perfect match to: (08-02119-CP015645-[395519:396463], allele observed in ST582+CC239)

Sequence:

TTATTCTATATAAGAACCGATATTTCTGAATTTTTCAAAGCGATCATTAGCAATTTCATCACGTGATAATGACTCAAGTGAATCTAACTGTG
CAACAAACGCTGATTTAATAGCTAAAGCTTGCTGTTCAATATCTTTATGTGCACCGCCAAGTGGTTCAGAAATGACATCATCTATAATACC
TAATTGCTTAATATCATGGGCAGTAATTTTCATTGTTTCAGCTGCAATTTTAGCCAAATTACTGTCTTTCCATAATAATGCCGCTGCACCTTC
AGGAGATATAACAGAGTAAGTACTATTCTCTAACATCAATACTTTATTGGCAATACCAATACCTAGAGCACCTCCACTGCCACCTTCACCA
ATGACAATCGCAATAACTGGTACTTTTAGTGAAGCCATCTCAATCAAATTTGTTGCGATAGATTCACTTTGTCCACGTTCTTCCGCAGCTTT
ACCAGGATATGCACCTTTTGTATCTATAAATGTAAAGATAGGACGATTGAATTTTTCAGCTTGTTTCATTAAACGTAATGCTTTTCGATAAC
CTTCTGGATGCGCCATACCAAAATTTCGATAAATATTATCTTTTGTATCTTTTCCACGTTGTTGTCCAATAACTGTAACAGCACGACCATTTA
AAAAGCCAATACCACCAATCATTGCTGGATCATCTCTAAAATTACGATCACCATGTAGTTCCATAAACGAATCAAAGATATATGGAATATA
ATCTAGGGTCGTAGGTCTTTCTTGCAAACGCGCAATTTGCACACGATCCCATGGTTTTAGATTTGTATATATTTTTTTAGTTTCTCGTTCCA
ATGACGCTTCAAGCATGTCAATTTCTTCTTGTAAATCCACATCATTTTTATCTTGAGATTCTTTTAAAGATTCAATTTTATTTCGAATTTCAAA
AAGTGGTTTTTCAAAATCTAACAT

Gene: accD (acetyl-CoA carboxylase, carboxyl transferase, beta subunit)

Contig: 07_NODE_2, position: 268281 to 269138, length: 858 nt, orientation: REVERSE

Perfect match to: (Tager_104-CP012409-[2620850:2621707], highly conserved allele)

Sequence:

TTATTTAGTCACCTCTTGATGGATTTTTAGAATTTCAGACAATGTTTGACGCATATTATTACGATGTACAACTTTATCCAATTGTCCATGCTC
TAATAAAAATTCTGCAGTTTGGAAATCATCTGGCAATTTTTCGTTTATTGTCTGTTCAATAACTCGACGACCTGCAAAACCTATCAACGCTT
TTGGCTCACTTAAATTTATATCACCAACTGATGCAAAACTTGCAGATACACCACCAGTAGTTGGATGTGTTAAATATGATATATATAATAG
TCCAGCGTCAGAATGACGTTTTAAAGATACACTGGTTTTACCCATTTGCATCAAGGAAATAATACCTTCTTGCATACGTGCACCACCACTT
GCAGAGAAAAGAATAAATGGTAAACGGTTCTCAGTGCAGTAATCAATGATGCGACATATCTTTTCACCGATAACCGATCCCATACTTCCC
ATTCTAAAACGTGAATCCATGACAGCAACGCCAAATTTCATACCATCTAGTTGCGCTGTACCAGTCACAACTGCTTCTTTAAGACCTGTCTT
TTGTTGGTCCTTTTCAATTTTTTCTAAATAACTTGGAAAATCTAATGGATTCGCAGAGGTCATTCCCTTATCGAATTCTGTAAATGATCCTTC
ATCAGAAATTGCTTCTATACGTTTATACGCAGTTAAAGCAATATGATGATCACAATTAAAGCACACATTTAAATTTTCAGCTAATTCTTTTG
TGTACATAATTTTCTTACACTTTGGACACTTAGTCATAATACCTGCAGGCACATCATTATTTTTAGAGTCTTGTACTGTAAGATATTTCTTTT
TCTTTGTTCGATTAAAAAAATCTTTAAACAT

Gene: maeB (malate dehydrogenase)

Contig: 07_NODE_2, position: 269333 to 270562, length: 1230 nt, orientation: REVERSE

Perfect match to: (N315-BA000018-[1739269:1740498:r], highly conserved allele)

Sequence:

TTATTGTAAATCTGTAAGTTTCATTGTTTTATCATACACATCTTGCGGATCAACTTCAATCCTAGCTACTCCAGATTCCATTGCCGCTTTAGC
AACATTACGAGCAACTGATGGCGCTACACGTTTATCAAACGGTCCTGGGATACAGTAGTCTTCATTTAATTCAGAACTATCGATTAAATCA
GCAATCGCTTCTACAGCTGCCTTTTTCATTTCTTCATTTATATGTGTAGCTTCAACCTCTAATGCACCTCTAAAAATACCAGGGAAAGCTAA
TACATTATTAATTTGGTTAGGATAGTCTGAACGTCCTGTACCAACAACTCGTGCACCTGCCGCTTTGGCATCATCAGGTATTATTTCAGGA
TTTGGATTAGCCATTGCAAATATAATTGGATTATCTGCCATACTCTTAACCATATCTTGTGACAGCGCATTAGCTACAGAAACTCCGATAA
ATACATCTGCGTCTTTTACGACTTCTTCTAAAGACCCTTCAATCTTATCTTTATTTGTCCATTTAGCTACAACATCTTTCGTAGGATTCATACC
ATATGAACGTCCTTCAAAAATTGCGCCTCTTGAGTCACACATAACCATATTTCTTACACCATACGCGTATAGTAATTTAACAATGGCTATTC
CTGCTGCACCAGCACCATTTAGTACAACTTTTATTTTAGCAATATCTTTGTTAACAACTCTCAATGCATTTACCAAACCTGCCATTGTTACAA
TTGCTGTACCATGTTGATCGTCATGGAATACCGGAATATTAGTTTCTTTTTTCAATCGTTCTTCAATTTCAAAACAACGTGGTGCCGAAATA
TCCTCTAAATTAATACCACCATAATTAGGTTCTAACAACTTAACTGTTTTAATGATTTCTTCGGTATCAGTTGTATTTAACGCAATAGGCAC
CCCATTGATACCAGCGAAGCTTTTGAATAATACTGCTTTACCTTCCATTACAGGAATACTTGCTTCAGGTCCAATGTTACCTAAACCTAATA
CCGCTGTTCCATCAGTAATAACTGCAACTGTATTTCCTTTAATTGTGTAATCATATACTTTTCTTTTATCTTCATAAATATCTTTACACGGTTC
AGCAACGCCAGGTGAGTATGCTAAACTTAATTCCTCTTTATTAGTAACTTTTACATTTGGTTTAACTTCTAATTTACCTTGATTACGTTTGTG
CATTTCCAATGCTTCATCTCTTAATGACAT

Gene: dnaE (DNA polymerase III subunit alpha)

Contig: 08_NODE_1, position: 95 to 3292, length: 3198 nt, orientation: REVERSE

Perfect match to: (TW20-FN433596-[1833657:1836854:r], allele observed in CC239+CC20+CC239)

Sequence:

TTATATAAGCCTAATATCACTAGGGTTAAAGGATTGTATAAAATTATTAAACATACTATCTTTTTGATTAATATAGCCTAAAGTAGTCATTT
GTTTAATCGTTTCATCATAAAAGGATAACACAACATCATTAGCATTCTCTTTCGTAGCTTTAATCATCTCTTCAAACATATCTATTTGTGATT
TATTTCTAATTATAATTTGTTTGGCAAATGCTAATTTTTGTTCTTCAAAAGTGGCTAATGTCTGAATCTCATTTATAATTAGTTGACGTTGTT
GCTTTCTATGGTCAAATTTCCCGCTAACTATAAACAAGTCATTATGTGATAACAACTCTTCGTACTTTTTAAACTGATTAGGGAAAATCACA
CCATCTAAAGTTTCAATGCCATCATTTAATGTGACGAATGCCATATTTTGACCATTTTTAGTTCGAATTTGTTTAACTTTATCAAACTGTACT
AATATAGGTTTATTATTCTGCGCGTTACTCAATTTAAATATCGTTAAATATTGTTTGGCAACAAACTTTTTATCTACTGGGTGTTGCGAAAC
ATAAAATCCTAAATATTCTTTTTCGTACTGACTAATAAGTGCATCAGGCAATTCTTCTTTATCTTCATACATCTGTTTTGGCGTTAAAATATC
AAATAAAAAACCATCTTGTTCAATGTTTAAATCGCCATCCAACACTTGATCAATAGCTTGCAACAACGTTGAACGTGTTTTACCAAAAGCA
TCAAACGCTCCCACTAAAATCAGTGCTTCAAGTAACTTTCTCGTTTTGACTCTCTTCGGTATACGTCTAGCAAAATCAAAGAAATCTTTAAA
TTTGCCGTTCTGATAACGTTCATCAACAATCACTTTCACACTTTGATAACCAACACCTTTAATTGTACCAATTGATAAATAAATGCCTTCTTG
GGAAGGTTTATAAAACCAATGACTTTCGTTAATGTTCGGTGGCAATATAGTGATACCTTGTTTTTTTGCTTCTTCTATCATTTGAGCAGTTT
TCTTCTCACTTCCAATAACATTACTTAAAATATTTGCGTAAAAATAATTTGGATAATGGACTTTTAAAAAGCTCATAATGTATGCAATTTTA
GAATAGCTGACAGCATGTGCTCTAGGAAAACCATAATCTGCAAATTTCAGAATCAAATCAAATATTTGCTTACTAATGTCTTCGTGATAAC
CATTTTGCTTTGCACCTTCTATAAAATGTTGACGCTCACTTTCAAGAACAGCTCTATTTTTTTTACTCATTGCTCTTCTTAAAATATCCGCTTC
ACCATAACTGAAGTTTGCAAATGTGCTCGCTATTTGCATAATTTGCTCTTGATAAATAATAACACCGTAAGTATTTTTTAATATCGGTTCTA
AATGCGGATGTAAATATTGAACTTTGCTTGGATCATGTCTTCTTGTAATGTAAGTTGGAATTTCTTCCATTGGACCTGGTCTATACAAAGA
AGTTACAGCCACAATATCTTCAAAGTGTTCCGGCTTTAATTTTTTTAATACACTTCTTACACCGTCAGACTCTAATTGGAATATGCCAGTCG
TATCTCCTTGCGACAACAATTCAAACACTTTTTGATCATCGAACGGAATCTTTTCGATATCAATATTAATACCTAAATCTTTTTTGACTTGTG
TTAAGATTTGATGAATAATCGATAAGTTTCTCAAACCTAGAAAATCTATTTTTAATAACCCAATACGTTCGGCTTCAGTCATTGTCCATTGC
GTTAATAATCCTGTATCCCCTTTCGTTAAAGGGGCATATTCATATAATGGATGGTCATTAATAATAATTCCTGCCGCATGTGTAGATGTAT
GTCTTGGTAAACCTTCTAACTTTTTACAAATACTGAACCAGCGTTCATGTCGATGGTTTCGATGTACAAACTTTTTAAAATCGTCAATTTGA
TATGCTTCATCAAGTGTAATTCCTAATTTATGTGGGATTAAACTTGAAATTTCATTTAATGTAACTTCATCAAACCCCATAATTCTTCCAACA
TCTCTAGCAACTGCTCTTGCAAGCAGATGACCGAAAGTCACAATTCCAGATACATGTAGCTCGCCATATTTTTCTTGGACGTACTGAATGA
CCCTTTCTCGGCGTGTATCTTCAAAGTCAATATCAATATCAGGCATTGTTACACGTTCTGGGTTTAAAAAACGTTCAAATAATAGATTGAA
TTTAATAGGATCAATCGTTGTAATTCCCAATAAATAACTGACCAGTGAGCCAGCTGAAGAACCACGACCAGGACCTACCATCACATCATTC
GTTTTCGCATAATGGATTAAATCACTTACTATTAAGAAATAATCTTCAAAACCCATATTAGTAATAACTTTATACTCATATTTCAATCGCTCT
AAATAGACGTCATAATTAAGTTCTAATTTTTTCAATTGTGTAACTAAGACACGCCACAAATATTTTTTAGCTGATTCATCATTAGGTGTCTC
ATATTGAGGAAGTAGAGATTGATGATATTTTAATTCTGCATCACACTTTTGAGCTATAACATCAACCTGCGTTAAATATTCTTGGTTAATAT
CTAATTGATTAATTTCCTTTTCAGTTAAAAAATGTGCACCAAAATCTTCTTGATCATGAATTAAGTCTAATTTTGTATTGTCTCTAATAGCTG
CTAATGCAGAAATCGTATCGGCATCTTGACGTGTTTGGTAACAAACATTTTGAATCCAAACATGTTTTCTACCTTGAATCGAAATACTAAG
GTGGTCCATATATGTGTCATTATGGGTTTCAAACACTTGTACAATATCACGATGTTGATCACCGACTTTTTTAAAAATGATAATCATATTGT
TAGAAAATCGTTTTAATAATTCAAACGACACATGTTCTAATGCATTCATTTTTATTTCCGATGATAGTTGATACAAATCTTTTAATCCATCAT
TATTTTTAGCTAGAACAACTGTTTCGACTGTATTTAATCCATTTGTCACATATATTGTCATACCAAAAATCGGTTTAATGTTATTTGCTATAC
ATGCATCATAAAATTTAGGAAAACCATACAATACATTGGTGTCAGTTATGGCAAGTGCATCAACATTTTCAGACACAGCAAGTCTTACGG
CATCTTCTATTTTTAAGCTTGAATTTAACAAATCATAAGCCGTATGAATATTTAAATATGCCACCAT

Gene: nrnA (oligoribonuclease)

Contig: 08_NODE_1, position: 3313 to 4254, length: 942 nt, orientation: REVERSE

Perfect match to: (MW2-BA000033-[1783185:1784126:r], highly conserved allele)

Sequence:

TTAGTTAAGTTTTGTGCGTAAAGCTGTAGCAAGTTGCTCAAATTCATCCCAGCTGTCCACTGAAACTCCTGACGCATTCGGATGACCACCG
CCACCAAAATCTTGCGCAATATCATTAATAATCAATTGCCCTTTAGAACGTAATCGACATCTGATTTCATTACCTTCATCGACTGCAAATAC
CCATATTTTCAAGCCTTTGATGTCAGCAATTGTATTAACAAACTGAGATGCTTCATTTGGCTGAATACCGAATTGCTCCAATACATCTTCAG
TTATTTTAACTTGGCAGAATCCATCATCCATAAGTTCGAAATGTTGTAAAACATAACCTTGAAACGGCAACATTTTTGGGTCCTTCTCCATC
ATTTTATTTAAAAGCGCATTATGATCAATATCATGCCCAATTAACTTTCCAGCAATTTCCATAGTATGTTCTGAGGTATTGTTAAAAAGGAA
TCGCCCAGTATCACCGACGATACCAAGATATAAAACGCTCGCGATATCTTTATTAACAATTGCTTCATCATTAAAATGTGAGATTAAATCG
TAAATGATTTCACTTGTAGATGACGCGTTCGTATTAACTAAATTAATATCACCATACTGATCAACTGCAGGATGATGATCTATTTTAATAA
GTTTACGACCTGTACTATAACGTTCATCGTCAATTCGTGGAGCATTGGCAGTATCACATACAATTACAAGCGCATCTTGATATGTTTTATCA
TCAATGTTATCTAACTCTCCAATAAAACTTAATGATGATTCCGCTTCACCCACTGCAAATACTTGCTTTTGCGGAAATTTCTGCTGAATATA
GTATTTTAAACCAAGTTGTGAACCATATGCATCAGGATCTGGTCTAACATGTCTGTGTATAATAATTGTATCGTTGTCTTCGATACATTTCA
TAATTTCATTCAAAGTACTAATCAT

Gene: ytoI (putative protein)

Contig: 08_NODE_1, position: 4708 to 6006, length: 1299 nt, orientation: REVERSE

Perfect match to: (MW2-BA000033-[1784580:1785878:r], allele observed in CC1+CC30)

Sequence:

CTATACTTCGTCAAACATTTGGCATATCACGAGAGCGCTCGCTACTTTGTCGTTTTGACTATGCATGTTCACTTCTATTTTGGCGAAGTTTC
TTCCGACGTCTAGTATGCCAAAGCGCACTGTTATATGTGATTCAATAGGTACTGTTTTAATATACACGATATTTAAGTTCTCTATCATGACA
TTACCTTTTTTAAATTTACGCATTTCATATTGTATTGTTTCTTCTATAATACTTACAAATGCCGCTTTACTTACTGTTCCGTAATGATTGATTA
AAAGTGGTGAAACTTCTACTGTAATTCCATCTTGATTCATTGTTATATATTTGGCGATTTGATCGTTAATTGTTTCACCCATCTGAGGCTGT
CTTCCTAAAAGTTGCATAGACTTTAAAACATCTTGTCTATTAATCACACCCACTGTCTTTTTATTACTCGAAACGACAGGAATCAATTCAAT
ACCTTCCCAAATCATCATATGCGCACAACTTGCTACTGTACTCATAGCATTTACATAAATAGGATTTCGCGTCATCACTTTATCTATTTCGTC
GTCGTCCTTTGTATTAATCATCTCTCGACTTGTTACAATACCTACTAATTTATAAGACTCATTGACTACCGGAAATCTTGTATGGCCAGTTC
GATTCGCCATACGCTTATAATCTGCTATTTTCATCGTATCAAACAGCACAGATAAATCATCTAATGGCGTCATTATATCTTGAACTATTAAG
ATATCTTTTCGTATTTTCTGATTAAAAAGTGCTTTGTTGATAATATTTGCAACTAGAAATGTATCATAACTTGATGATAGAACAGGTAAATC
ATGTTCATTCGCAAAATTAATAACTTTATTAGATGGCTTAAATCCACCAGTAATTAATATAGCCGTACCTCTTTTTAAAGCTTCAATCTGCA
CATCTTCACGATTTCCGACAATCAATAATGTCTTTGGACCAATATATTTTAAAATATCTTTGAGTTCCATTGCTCCAATTGCAAATTTAGATA
CCATCTTAGTGATACCTTTATTGCCACCTAACACTTGGCCATCAATAATATTGACAATTTCATTAAAAGTTAAATGTTCAATTTCATTACGAT
TACGTTTTTCGATTCGAACCGTACCAACACGATCTATCGTTGCGACCATGCCCATTTTATCAGCATCTTTAATTGCACGATATGCTGTCCCT
TCAGATACGTTTAAAAATTTAGCGATTTTACGCACCGAAATTTTAGAGCCTATAGATAACGATTCAATATAATCTAAAATTTGTTCATGTTT
TGTCAT

Gene: uspA (universal stress regulator)

Contig: 08_NODE_1, position: 6211 to 6624, length: 414 nt, orientation: FORWARD

Perfect match to: (N315-BA000018-[1746922:1747335], highly conserved allele)

Sequence:

ATGTATAAAAATATATTACTTGGTGTAGACACTCAGTTAAAAAATGAAAAAGCACTAAAAGAAGTGTCTAAATTAGCTGGCGAAGGTAC
AGTCGTAACAGTTTTAAACGCAATCAGCGAACAAGATGCTCAAGCATCAATTAAAGCAGGTGTTCATTTAAACAAACTTACTGAAGAACG
AAGCAAGCGATTGGAAAAAACACGCAAAGCTTTAGAAGATTATGGTATTGATTATGACCAAATAATTGTTCGTGGTAATGCAAAAGAAG
AACTATTAAAACATGCTAATAGCGGTAAATACGAAATTGTTGTTTTAAGTAACCGTAAAGCAGAAGACAAAAAGAAATTTGTACTTGGAA
GTGTCAGCCACAAAGTAGCAAAACGTGCGACTATCCCTGTATTAATCGTTAAATAA

Gene: ytkL (metal ion-dependent hydrolase)

Contig: 08_NODE_1, position: 6929 to 7618, length: 690 nt, orientation: REVERSE

Perfect match to: (N315-BA000018-[1747640:1748329:r], highly conserved allele)

Sequence:

TTAAAACTGAACAGATTCACCTGGTTTTAAAATTTGCACGTCCCCTACATTAACAGCATCTTTAAATTGTTGTGGATCTTGTTCGATTAATG
GGAATGTATCATAATGAATCGGTACAGAAATTTTTGGTTTAATAAATTCATTAATAGCATAACTTGCATCATCAATACCCATCGTAAAATT
ATCTCCAATTGGTACAAAACATACATCAACTGGATGACGTTTCGCAATAAGTGACATGTCACTAAACAGACCTGTATCACCAGTATGATAA
ATTGTTTTTCCTTCAACTTCAAACACGATACCCATTGGCATACCTAAATAAACTGGAATACCATTTTCATGTGTAAAACTTGAACTATGAAA
TGCTTGAACAAATTTAACGCTTCCGAAATCAAAGTTTGCTTTACCACCAATATTCATACCATGAACATTTTCAACACCGTGATATGAAGAA
AGATAGTCAGCCATTTCTGCACTTCCAATTACTGTTGCTCCTGTTTTCTTTGCTAGTTCCACAACATCACCAAAATGATCAAAATGACCGTG
CGTTAAAACGATATAGTCTACCTGCACTGTTTCAATATTCAAATCACACTTAGGGTTATTTGAAATAAACGGATCTACGATAACCTTTTTGT
TGTTCCCTTCTAAATAAATCGTTGATTGACCATGAAATGATAACTTCAT

Gene: pepQ (Xaa-Pro dipeptidase)

Contig: 08_NODE_1, position: 7793 to 8848, length: 1056 nt, orientation: FORWARD

Perfect match to: (TW20-FN433596-[1841162:1842217], allele observed in CC239)

Sequence:

ATGACAAAAATATCAAAAATAATAGACGAATTGAACAATCAACAAGCTGATGCAGCATGGATTACAACACCGTTGAATGTATATTATTTT
ACTGGATACCGTAGCGAACCCCATGAAAGATTATTTGCATTATTGATTAAGAAAGATGGTAAACAAGTACTATTTTGTCCAAAAATGGAA
GTCGAAGAAGTCAAAGCATCACCTTTCACAGGTGAAATCGTTGGATATTTAGACACTGAAAACCCTTTTTCACTTTATCCTCAAACAATCA
ATAAATTACTAATTGAAAGCGAGCACTTAACAGTAGCACGCCAAAAACAATTAATCTCTGGTTTCAATGTCAATTCATTCGGAGATGTTGA
TTTAACAATCAAACAATTGAGAAATATTAAATCCGAAGATGAAATTAGCAAAATACGTAAAGCTGCTGAGTTAGCAGATAAGTGTATCGA
AATAGGTGTTTCTTATTTAAAAGAAGGTGTGACTGAACGTGAAGTAGTCAACCATATTGAGCAAACTATCAAACAATATGGCGTCAATGA
AATGAGTTTTGATACGATGGTTTTATTTGGAGATCATGCCGCATCACCTCATGGCACACCAGGAGATCGCAGATTAAAAAGCAATGAATA
TGTACTATTTGATTTAGGTGTAATTTATGAGCATTATTGTAGCGATATGACACGTACTATTAAATTTGGTGAACCTAGCAAAGAAGCACAA
GAAATTTATAATATTGTATTAGAAGCAGAAACATCTGCAATCCAAGCAATTAAACCTGGAATACCATTAAAAGATATCGATCATATCGCTA
GAAATATTATTTCAGAAAAAGGTTATGGTGAATATTTCCCTCATCGCTTAGGTCATGGCCTAGGATTACAAGAACATGAATATCAAGATG
TTTCAAGTACTAATTCTAATTTGTTAGAAGCTGGCATGGTTATTACAATCGAACCAGGTATTTATGTACCTGGTGTTGCAGGTGTAAGAAT
TGAAGATGACATACTTGTCACTAATGAAGGATATGAAGTATTAACACATTACGAAAAATAA

Gene: ald2 (alanine dehydrogenase 2)

Contig: 08_NODE_1, position: 9164 to 10282, length: 1119 nt, orientation: REVERSE

Perfect match to: (MW2-BA000033-[1789036:1790154:r], highly conserved allele)

Sequence:

TTATAATTGTAATGCTTCTTCTACAGATTTATATTCCATTTCAAATGCCTCTGCAACGCCTTTATTGGTTACGTGACCTTTGTAAGTATTTAA
ACCTAATGATAATGGTTGATTTGATTTAAATGCTTCTCTATACCCTTTATTAGCTAGCATGAGCGCATAAGGTAGCGTAGCATTATTTAAA
GCTAACGTCGAAGTACGCGGTACTGCACCTGGCATATTTGCAACTGCATAATGAACCACACCATGCTTAATATATGTAGGATCATCATGT
GTCGTAATTTTATCAGTTGTTTCAAAAATACCGCCTTGATCAATAGCAATGTCAATAATAACTGACCCATTTTTCATTTGTTTAATCATGTCT
TCTGTTACAAGTCTTGGCGCTTTAGCACCTGGAATTAAAACTGCACCTATTACTAAATCACTTTGTTTAACATACAACTCAATATTCAACGG
ATTTGACATAATTGTATGTACACGTCCACCGAATAAATCATCTAATTGTTGTAAACGCTTTGGATTAACATCTAAAATCGTAACATCTGCAC
CTAGTCCTAGTGCAATTTTAGCTGCATTTGTTCCTGCTTGACCACCACCGATAATAGTTACTTTACCCTTAGGTACTCCTGGGACACCACCT
AGTAGAATTCCCATACCACCATTAAGTTTTTGTAGGAACTCTGCGCCAACTTGAGCTGACATTCTTCCTGCTACCTCACTCATTGGTGATAA
CAATGGTAAAGATCGGTCTGGTAACTGCACAGTCTCATATGCAATACTAATTACTTTTCTATCTATCAAAGCTTGTGTTAATTTTTCTTCATT
TGCTAAATGAAGATAAGTGAATAATACAAGCCCTTCTTTAAAATATGGATATTCAGATTCAAGTGGTTCTTTAACTTTAATAACCATATCCA
CATCCCAAACTTTTGCTTGTTCAGCAACAATCTCAGCACCTGCTTCTTTGTAATCTACATCTTCAAAGAATGATCCTGAACCCGCATTTGTTT
CCACTAAAACAGTATGCCCACTTTCTACTAAAGCGTGCACACCACTTGGTGATAAACCAACACGATTTTCATTATTTTTAATCTCCCTTGGT
ATACCAATTTTCAT

Gene: yxiE (phosphate starvation protein)

Contig: 08_NODE_1, position: 10423 to 10923, length: 501 nt, orientation: FORWARD

Perfect match to: (MW2-BA000033-[1790295:1790795], highly conserved allele)

Sequence:

ATGATTACTTACAAAAATATTTTAATCGCAGTTGACGGTTCACATGAAGCGGAATGGGCATTTAACAGAGCAGTTGGTGTTGCTAAACGT
AACGATGCGAAGTTAACAATTGTGAATGTAATTGATTCAAGAACGTATTCTTCTTATGAAGTTTATGATGCTCAATTTACTGAAAAATCTA
AGCATTTTGCAGAAGAATTATTAAATGGTTATAAAGAAGTAGCTACTAACGCTGGTGTTAAAGATGTAGAAACGCGTCTAGAGTTTGGCT
CTCCTAAATCTATCATTCCTAAAAAGCTTGCACATGAAATTAATGCAGACTTGATTATGAGTGGTACATCAGGCTTAAATGCCGTGGAAAG
ATTTATTGTTGGTTCTGTATCAGAATCTATCGTTCGTCATGCGCCATGTGACGTGTTAGTTGTTCGTACTGAAGAGTTACCAGCAGACTTC
CAACCACAAGTTGCAACAACTCAATTACGTGAAAAATATCAAAATTAA

Gene: ackA (acetate kinase)

Contig: 08_NODE_1, position: 11169 to 12371, length: 1203 nt, orientation: REVERSE

Perfect match to: (ED133-CP001996-[1781019:1782221:r], highly conserved allele)

Sequence:

TTATTTTAAACCACCGAATGTCATAACATCACGGGCAATCATACTTTCTTCATCTGTTGGAATAACGACAACTTTAACTGGTGAATGAGGA
TAGTTAATAAATCCTTCTTTACCACGTAGTAAGTTTTCATTTTTCTTAGGATCCCAGTAAACACCCATAAATTCTAAGCCTTCAAGAACTTTC
GCACGAATTTCTACTGAGTTTTCACCGATACCTGCTGTAAATACGATAACATCAACACCATGCATTCTCGCAGCATATGATCCAATATATTT
GTGAATTTTAGAAGCAAATACATCTAAAGCCATTTGTGAACGTGCTTTACCTGATTCAGCTTCTTCTGATAAGTCACGTAAATCACTAGAT
GTACCTGATAATCCTAATAAACCTGATTCTTTGTTTAAGATTTCCAATACTTGTTCAGCAGTTTTACCTGTTTTTTCCATAATAAATGGAATT
AAAGCAGGGTCAATATTACCAGAACGAGTACCCATTGTTACACCAGCAAGTGGTGTGAAGCCCATTGATGTATCAATAGATTTACCGCCA
TCGATAGCTGCAATTGATGCTCCATTACCAATGTGACATGAAATAATACGTAAATCTTCAATTGGCTTATCTAACATTTCTGCCGCTCTTTG
TGATACAAATTTATGGCTTGTACCATGGAAACCATACTTACGAATGCCATAATCTTTATAATAATGATATGGCAAGCTATATAGATATGCT
TTTTCAGGCATTGTTTGATGGAATGCTGTATCAAAAATTGCCACATGAGGGATATTTGGTAATAATTTACGGAAAGCACGAATACCCATC
AAGTTAGCTGGGTTGTGAAGCGGTGCTAATTCGCTTAATTCTTCAATTTCCTTTTCAACCTCATCAGTAATAGCTACTGATTCAGGGAATTT
TTCACCACCATGTACAACACGGTGACCTGTTCCATCGATATCGTTAATATCATTAATAATATTGTGCGCTTTAAAAGCATCCAACATGATAT
CAACTGCCTCAACGTGATCCTTGATATCTTGTACTGTTTTAACTTTTTCCCCGTTGACTTCAATTGTAAAAATTGAATCCTTCAATCCGATTC
TTTCTACTAAACCTTTTGTTACTAATTCCTCTTCAGGCATTCTAATTAATTGAAATTTTAATGATGAACTACCAGCATTGATAGCCAAGATTA
ATTTTGACAT

Gene: ytxK (putative nucleic acid methyltransferase)

Contig: 08_NODE_1, position: 12459 to 13406, length: 948 nt, orientation: REVERSE

Perfect match to: (MW2-BA000033-[1792331:1793278:r], highly conserved allele)

Sequence:

TTATTTTTTAGGACGATTTGTGTCCATCCACTGATTTAACTCTGTCATAAATCCTTGAAATTGTGAAGGAATTTTGAAATCAGGAATATTTG
CCAATAATACTTCAACTGGCTTTGTTTCACCCGATTTTTTCTTTTGTAAAATTAATATAGATTTTCGCGCTTTTTCATTTTTAAATAAAGTTGG
TGGTAAATTTAAAAATGCTTGCATCTCTGTCTCTGTTGCAATATATTTTTCAAGCTGTTTTACATGTTCACCTGTAAAAATATTACTTGGTAC
CACTAGAAAGGCATATCCAGCATCTTTTAATGCATTTATTGCTTGTTCTATTAATAAATAATGTGAATAACTATGTCCTTCTTCAAAACCTA
GCTTAAACTCCTTACTTCTTTCATCAATTGGATAATAGCCTACTGGAAAATCACCAATAACGATATCTGCTTCTTCTAATGGTAGTGGCATG
ATGGCATCTTGAGGATACACATCGAAAGGAATTTCTAAGAAGTTTGCTAAATGTACACTAACACGTGATAAAACTGGATCAACTTCAATT
AAATGATGCATAACCGCAATTTCAGGTAACACTTCTTTTACAGTAGCACTTAAATGACCGGCACCACTTGCAATATCAACAATATGTAATT
CTTCTTGGTTGTTCATAAAACGCTCAACTAAAAATCCTAGTATCAATCCAATTGAATCTGGTGTAATTTGATGATTTGCTTGTATCTTTTCTT
CCTGCATTAAACTTAAATATGCAAATTGGAATGCTTTACGTCGATCTTGTAACGTCGATTGTTCTAACAATCCTCTTTCATTGGTATATACTT
GTTCCATTGCTAGCCCAAGATTTTCAATAAAACTTTGGCCATTTTCATTATTTAATGTTTTAGCTTTTTCATCTAATGTATGAAACAAGCGTT
CCATAATTGTTTGTTGTTCTGCCAT

Gene: tpx (thiol peroxidase)

Contig: 08_NODE_1, position: 13529 to 14023, length: 495 nt, orientation: REVERSE

Perfect match to: (MW2-BA000033-[1793401:1793895:r], highly conserved allele)

Sequence:

TTAAATATTTTTGTATGCAGCTAAAGCAGCATCAAAATCTGGGAAATCAGTACCTTCACTAACGATTTCTTTATAAACAACTTTATTATCTG
CATCTAATACAAATACTGCACGAGCTAATAAGCGAAGTTCTTCCATAACAACGCCATAGTTTTCACCAAATGATAAGTCACGGTGGTCACT
TAATGTAATGACATTGTCTAAACCTGCTGAAGCGCACCATCTTTTTTGTGCGAATGGTAAGTCTGCTGAAATTGTAAGCACAATCCCCTCT
TCTTTAGAAGCATCAGAGTTGAATTTGCGAGTCTGCTGATCACAAACACCTGTATCAATTGATGGTACCACACTAATTAATTTCTTTTTACC
AGCATAATCTGCTAATGTTACTTGATTTAAGTCATTATCTAACACTGTAAAATCAGGTGCAAAATCACCTTCATTAATTTGTTGACCTTTTA
AGTGGATTGGTCCACCTTTGAATGTTATTTCAGTCAT

Gene: ytnM (putative transporter)

Contig: 08_NODE_1, position: 14121 to 14891, length: 771 nt, orientation: REVERSE

Perfect match to: (MW2-BA000033-[1793993:1794763:r], highly conserved allele)

Sequence:

TTATACAAATTGTTGAATATAATCAAAAGCATTTTTTAAAATTAAAATAGCAGTAATAATAATAAATAATACTTTTACATAGCCAACACCTT
GTTTGATAGCAAAATGTGCCCCAGTATATGACCCAGCAATCATGCTTGTAGCCATTATTAAACCTATTACATAATCTACTTGTCCTAATACC

ATAAATAATACAAGCGCACCTATATTAGAAGCAAAGTTCAAAACTTTAGCATTTCCTGCTGCACTTAAAAAATCAAAACCAAAGACTAACA
ATACAAAAAGCATAAATGAACCTGTTCCCCCACCTACAAATCCATCATAAAAGCCGATTAATATAAAAAGTGCTGCAAATAGTATGGCTTT
CTTAAATGTAAATTGAGTAAACGTACGTGTATTGCCCCAATCTTTTTTAAGTAATGTGAATATAAACACCGACGAAAGTGCAATAATGATT
AAAGGTTTCAATATTTGTGACGGAACCATCGTTGCAATATATGCGCCACATGCAGATGCCAAAAATACAAAACCAAATAATTTGGCAACA
ACATATAAGTCCACTTTACCGGACCTTATAAACTTTATCGTACTAGTTAAAGAACCAAATGAACTTGCCAATTTATTTGTACCTAAAGCCAC
AGATGGTGGTAGACCGATTGCTAATAATGCTGGCGTAGAAATTAAACCGCCACCCCCTACAACCGAATCTATAAACGCCGCGATAAAACC
AAATAAAATTATGATTATAATCATCGTTAAGTTCAAATCCAT

Gene: thiI (thiamine biosynthesis protein)

Contig: 08_NODE_1, position: 14935 to 16158, length: 1224 nt, orientation: REVERSE

Perfect match to: (TCH959-AASB02000248-[35097:36320:r], allele observed in CC7+CC5+CC15+CC20)

Sequence:

TTATAAAAAGTCATTTATTAATTGGTTTGTTTGCTGTTCTTTAATAGTTTTATAATCACTAGTTATTTCAAGTGTTTCAATATTTTCAACAGC
ACGATTAATCATCTCTTCAAAATCAAAGACGCTTTCATATTGAACTACCTTATCAAAGTTTGGTTCGGTTACTGGATTTTTAGGGGTGAAA
ATTGTACAACAATCTTCAAATGGTTGAATAGATGTTTCAAATGTACCAATTTCTTTCGATTTAATAATAATTTCTTCTTTATCGTAAGTTAAT
AAAGGACGTAATACAGGAGTAGAAGTTACATTATTAATTGCATACATGCTATGAAGTGTTTGACTGGCTACCTGCCCTAGGTTTTCACCAT
TTACAATAGCTAAAGCCCCTATTTGATGTACTAATTTATCAGCAACACGCATCATCATACGTCTCGTTGAAGTCATTGTATATCTTGGATGT
ACAACTTTATTTACCTGTTTTTGCAATTCTGTAAATGGTACAATATGCAATTTAATTGGTCCAACACGTTCAGCTAAAATACGTGTCAATTC
AATAACTTTTTCTTTTGCTTGATCACTTGTAAATGGTGGACTATGGAAATGAATCGCTTCAATTGTTACGCCACGTCTCATCACTTCCATCC
CAGCAACTGGTGAGTCTATACCGCCTGAAAGCATTAGTAACGTCTTACCACCAGTACCAACTGGTAATCCACCTGAACCCGGAACAACTT
CTTCATACATATAAATTGCATCTAATCTAACTTCCACTCGAATTTCATGATCTGGACGTTTGACATTCACTGAAATATTGTCGAAGTGCTTC
AATACTGCACCACCCAATTCACGCTGTAATTCATACGTATCCATTGGGAAATTTTTATCGGCACGCTTCACATCAATTTTAAATGTGCTGTT
TTCTTCAAATTGCTGCGCAAATTTAATTGCCGCTGCACTCATTGCCTCTATTGTTTTTTCTACTTTTAATACTGGACTAATAGATTTAATACC
GAAAATTTTTGATAATCGATATGTTATTTCATTTATATCTGCATGGTCTTCAAGTTCAATATACATACGATCTCGTTTGCCTTTAACGACAAA
CCCATCAAGTCCTTTTAATGACTTATTTACATTATTTCTTAATTGATTTACAAATTTTTTTCTATTTGAACCCTTTAATGTTAACTCCCCGTATC
TAACAAGCAAGTGATCATACTTCAT

Gene: csd2 (cysteine desulfurase 2)

Contig: 08_NODE_1, position: 16158 to 17297, length: 1140 nt, orientation: REVERSE

Perfect match to: (04-02981-CP001844-[1791420:1792559:r], allele observed in CC5+CC72+CC772)

Sequence:

TTATTTTAGCAACTCCTTAATTTCCTCATAAATGATGATAAATATTTCTTTAAACCTTGCTATATCTTCTTTAGTTGTAGTAGCCCCAAATGA
TAATCTTATACTACCTTCAATAGATTTGTCTGATAATCCCATTGCAGCCAATACTTCATTTAATTTATTACGTTTAGATGAACAAGCACTCGT
CGTAGATATCATAATGTCATATTTTGAAAAAGCATTAACTAATACTTCACCTTTTACGCCAGGAAAACTAATATTTAAAACGAATGGTGAA
CCAGAAGTTGAAGAATTAATATAAACTCCATGATATTTATTTAAAAATTGACGGACGTCATTATTTAACACAGTAACAAATGCATTCAATG
CTTCAAAGTTTTCATTAGCTATCTTCATCGCTTTAACCATTGCAATATCATTTGGCAAATTAACTGTTCCACTTCTAACGCCATATTCTTGAC
CACCACCATGGACAGTTGGTTCAACATTTTGAATGTGATTTACAAGTAAGACGCCTTGGCCTTTTAAACCATTAAACTTGTGTCCACTTAA
ACTAATACTATCTATGTTATTGAGATCCATTGAAATTTTGCCGAATGCTTGAACCGCATCTACATGAAAATGTGCCTTAGGATAATTTTTTA
TAACTTTAGCCATTTGTGGAATAGGCTGTATTTGTCCAGTTACATTATTTACATACATACATGTTACTAAACCGACTTTGTCTGACATTAATT
CTTTGAAGTGTTCTAAGTTAATACTGCCATCTTTCTTTACATCAACATATTTAACTTTAAATCCTTCGTGTGCTTCCAAATATCTTACAACCTC
TAATACGGACGGATGCTCTAACACGGATGTAATTATTTCCTTCGCTGTATCAAATTTACGATAGGCAATACCTTTTAAAGCAAGATTATTG
GATTCAGTTGCACCACTAGTGAATACAACATCATAATTTGTTTTTGAATTAATCATTGCATTAATTTGGGCTTTTGCTTGTTGTAGTAATTG
ATTTGCCTGCAAACCAGCTTTATGCGGACTATTCGGATTATAATACATTGATTGATTTACTTTTAAATAAGTATCTAACACTTCTTCAAATG
CTTTCGTCGTTGCCGCATTATCTAGATATATCAA

Gene: ezrA (septation ring formation regulator)

Contig: 08_NODE_1, position: 17669 to 19363, length: 1695 nt, orientation: REVERSE

Perfect match to: (COL-CP000046-[1805997:1807691:r], allele observed in CC8+CC398+CC772)

Sequence:

CTATTGCTTAATAACTTCTTCTTCAATATGTTTAGTAACACCTGGCTCAACACTTTCAAGAGCTTGCTCTGCAATTTCAATCGCACGCTTATA
GCGATTATTTTTAAATAATCGTTCAGCTTCATTTAAGCTCTTATCAACATTGCTATAGTCCTTACGATATCTATTTCCATATTGAATTAATTTC
TCTGCATAAACAGCATTAACAAGAACATCATTTGCTTCATCTTCAAATGTATTCATTTGAATCACAATTTTAGACACTTTATCTTTTAACTGT
TTAACGTGTATTGGACGTTCACTAAATTGTTCGTTAACATCACGAACTTCATGATCAATTTCATTTTTCATGATGATAAACCTTTCAGGAAC
GCTTGTTAAGTTAGAAGCAAGTAATCGACGATACACTTCTTCTTTCTTCGATTGGACTCGTAGCAGATTGTCTTCTGCTTCTGCTTCATCTT
CACGCAATTGAATCAGATGATTTTGTAGCTTTTCTTGTTTGTCATTAATAACTGTGACATGATCTTCTAAATATTGTAAATTATCCTGAACCT
CGCTATATCGCACAGCAGATTTAGACATTTCTTTTAAAATATCATCATATACAGAAATTAAACTTTGAATTTCATTTTCAAATTGACGAACA
CTTTGAGCATCAGATTCATTTATATAGTAGTTTTCACGTACATATTCAATTTCTGTTTGCAATGTATAATTCATGTCTTTAGCTTTGAATAAG
TTATCCGTAATGATATCTTTTGTTTCTTCGACATCATTTTTAGCTTTAACTTCATGTTCAATTAAATCATACATGTCATCTAACTTATCATTGA
TATTAGCTAGTTTATCATTAGCTTCTTCTAATTCTAAGCGGCTAATTAATGGTTCAACGAAACTAAGCTCTGTTTTTAAGCTTTGTAATGTAC
TGTCTACTTTTACGTGATCCAGATCATACCCTTCAACTTTAAGATCACGGCAACCATATTTTAAATCTTGGAATTGACCAGGTAATTCTTTTT
GAGTTTCTCTAATTAATTCTGGTATTTCTTCCATATAAGATCTTAGCTGTTTCATTTGTTCATTCAAGGCAGCTATATGGTTGTGCGCTTGTA
CATAATTACCATCAGCTTTTAGTACTTCATATTGCTCTAACCTTGGCTCAAACTTTTCAATTTCAGTTTCAAGTAGACTTGCTGCCTCACCAA
ATTGATGACGATTTGCTAAAACATCACGTTTCATTTCACGATAATCAACCTTACATTTGTCATATAATTCATCATTATCTTTGTATAACGCAA
TAATTTCATTTACATCTTCTAATTGTTGCTGATAGCTTTGTTCGTAACTATCCATCAACTCATTTGCATCATCAATTTCACTTTGAGATGCGTT
GAAACTAAATTTATCTAATAAAGCCTCAGCATTATGGATTTTTTCTTCCACAGGAGCTAGATACTTATTTGTACTTTCTACGTTGTCCTTTTT
CATTGCATCGTATTTCGTTTTTGTTTCACCTTTTAAATTCAACTTAGATAATTGTGCAAGGTTTTGATCAAAAGGTAACGTCTCAATTTCATT
TTTACGTTCGATTGCTTTTTCAATAATTTGTCGTTTATTTGAACGTAAATAGAATAATACACCTACAGCAATCAATATAATCACAATTATTGC
CAAAATGATATATAACACCAT

Gene: ytsP (GAF domain protein)

Contig: 08_NODE_1, position: 19500 to 19964, length: 465 nt, orientation: FORWARD

Perfect match to: (ATCC51811-ST1-ADVP01000005-[26032:26496:r], allele observed in CC1+CC772)

Sequence:

ATGACAACAATTAACCCAACAAACTACACATTATTAAAGAAACAAGCAGCAAGCCTTATTGAAGATGAACATCATATGATTGCTATTTTAA
GTAACATGTCTGCCTTATTAAATGATAATCTAGATCAAATTAATTGGGTCGGCTTTTACTTATTGGAACAAAATGAACTTATACTTGGACCT
TTCCAAGGACACCCCGCTTGTGTCCACATTCCAATTGGAAAAGGTGTATGTGGTACAGCCGTTTCAGAACGTCGTACACAAGTTGTAGCT
GATGTCCATCAATTCGAAGGACATATCGCTTGTGATGCTAATAGTAAGTCTGAGATTGTCGTTCCAATTTTCAAAGATGATAAAATTATCG
GCGTCTTAGATATCGATGCCCCTATAACTGATCGATTTGATGACAATGACAAAGAACATCTTGAAGCAATTGTTAAAATTATTGAAAAGC
AACTCGCATAA

Gene: rpsD (30S ribosomal protein S4)

Contig: 08_NODE_1, position: 20208 to 20810, length: 603 nt, orientation: FORWARD

Perfect match to: (MW2-BA000033-[1800080:1800682], highly conserved allele)

Sequence:

ATGGCTCGATTCAGAGGTTCAAACTGGAAAAAATCTCGTCGTTTAGGTATCTCTTTAAGCGGTACTGGTAAAGAATTAGAAAAACGTCCT
TACGCACCAGGACAACATGGTCCAAACCAACGTAAAAAATTATCAGAATATGGTTTACAATTACGTGAAAAACAAAAATTACGTTACTTA
TATGGAATGACTGAAAGACAATTCCGTAACACATTTGACATCGCTGGTAAAAAATTCGGTGTACACGGTGAAAACTTCATGATCTTATTA
GCAAGTCGTTTAGACGCTGTTGTTTATTCATTAGGTTTAGCTCGTACTCGTCGTCAAGCACGTCAATTAGTTAACCACGGTCATATCTTAGT
AGATGGTAAACGTGTTGATATTCCATCTTATTCTGTTAAACCTGGTCAAACAATTTCAGTTCGTGAAAAATCTCAAAAATTAAACATCATC
GTTGAATCAGTTGAAATCAACAATTTCGTACCTGAGTACTTAAACTTTGATGCTGACAGCTTAACTGGTACTTTCGTACGTTTACCAGAAC
GTAGCGAATTACCTGCTGAAATTAACGAACAATTAATCGTTGAGTACTACTCAAGATAA

Gene: ugpQ2 (chromosomal glycerophosphodiester phosphodiesterase)

Contig: 08_NODE_1, position: 21026 to 21769, length: 744 nt, orientation: REVERSE

Perfect match to: (MW2-BA000033-[1800898:1801641:r], highly conserved allele)

Sequence:

TCACTTAATAAATAATTTCGGATTATCTGTAATTAATCCATCTACACCCATTTGTCTTAATTTTTCTCCAGTTTTCAATTTATTAACTGTGTAT
GGCATAACCTGTAATTGATGATGATGTGCTTTATCAACAAATTTTCTGGTCACTAATGCATAATTAGGATTAACATAACTAGCAATTTGAG
CAATTCTTGAAAAGTTTGGCTTTTTATACCAATAATTACGTTTACTACAAAGCACACCTAATTCATATATACTGCCCAATGTGTTTAACTTTT
CGATGCATTCGATATCAAAAGATTGTATAACTACTTGTGTCGCATCGACTTTCTTTTCTTCCAAAAATGCCAGCAATTTACATTCTATTTCTG
GATATAAATTCGGACTTTTCAATTCAATCAATAGCTTTTTATCATACTTTAAGCATAATGAAAGTACTTCATCTAACGTGGGTATTCGTTCT
CCCTTAAAAGCAACATCTTTATAACTACCAAAATCAAATGATTTTAATTGCGATAATGTGTAATCAGAAATACGCCCCTTACCATCCGATGT
TCTATCAATTGTTTCATCATGTATCACAACAAAATGTTGGTCTTTGGTCAAATGAACATCTATTTCTAACATAGCAACATTGAGCCCCATTA
CCTCTCGATAACCGACCATTGTATTTTCAGGAAAATCACTCGGCAATCCACGGTGCGAAACAATTTGTAATTCATCTTTCAGTTTATTCAGA
GTCAT

Gene: osmC (peroxiredoxin-like)

Contig: 08_NODE_1, position: 21849 to 22295, length: 447 nt, orientation: FORWARD

Perfect match to: (MW2-BA000033-[1801721:1802167], allele observed in CC1+CC188+CC772)

Sequence:

TTGCATCAACATGACTTTAAAGTCCAAACTTCTTGGCAAGGTGGTCGTAACAATGTCGGAAACGTTCAAGGCGACATACTTTCAGAGAAT
ATTTCTATACCTGCTTCCTTAGGTGGTGTTGGTTTAGGAACAAATCCCGATGAATTGTTAGTATCAGCCGCTTCATCATGTTATATCATCTC
ATTAGCAGCTACTCTTGAACGTGCAAAGTTCACAGATATTTCAATTGAACAACAATCGATTGGAACAGCTTGTTTAAATAACGGAAAATTC
AGTATGTCAAAAATTGTGCACCATCCTCAAATTCAAATTCCAAGTGATCAAATAGCACAATTAGAAAAGCGATTACCAAAATTGATAACA
ATTGCAGATAATAATTGCATGATTTCAAATGCTGTAAGAAATAATGTGGACATAAAAATTTATCCCATCATTCAGGCCAAATAA

Gene: pucG (vitamin B6-dependent  (S)-ureidoglycine glyoxylate aminotransferase)

Contig: 08_NODE_1, position: 22409 to 23569, length: 1161 nt, orientation: FORWARD

Perfect match to: (MW2-BA000033-[1802281:1803441], allele observed in CC1+CC5+CC6+CC8+CC630)

Sequence:

ATGTATTATCATCAACCGTTGTTATTAACACCTGGCCCAACCCCTGTACCTGATGCCATTATGAGAGAAATTCAAGCACCTATGGTTGGTC
ATCGTTCTAAAGATTTTGAAGACATCGCACAACAAGCATTTCAAGGTCTAAAGCCAATATTTGGGAGTCAAAATGATGTACTTATTTTAAC
ATCTAGCGGTACAAGCGTCTTGGAGGCTAGTATGTTGAACATTGTAAACCCTGAAGATCACTTCGTTGTCATTGTTTCAGGTGCCTTTGGT
AACCGATTTAAACAAATTGCACAAACTTATTACAAAAATGTGCATATTTATGACGTAACATGGGGAGAAGCTGTAGATGTCAAAGATTTC
ATCAATTTCCTTTCAACTTTAAATGTTGAAGTGAAAGCAGTATTTAGTCAATATTGCGAAACATCTACGACAGTGCTACACCCTATTCACG
AGTTAGGAAATGCCATCAATCAATTTAATAGTAATATTTATTTTGTAGTTGACGGCGTAAGTTGCATTGGTGCTGTTGATGTTGACATTAA
CAAAGATAAAATTGATGTACTTGTTTCTGGTAGTCAAAAAGCAATTATGTTACCTCCAGGATTAGCTTTTGTAGCTTATAGCCACCGTGCA
AAAGAACGTTTCAAAGAAGTAACTACGCCAAAATTTTATCTAGACTTAAATAAATACATTTCGTCACAAGCTGACAATTCTACACCGTTCA
CACCAAATGTGTCTTTATTTAGAGGTGTAAATGCATACGTTGAAACCGTAAAAGCAGAAGGTTTCAATCACGTAATAGCACGACACTATG
CAATTAGAAATGCATTAAGAAGCGCCTTAAAAGCATTAGATTTAACTTTATTAGTCAATGATAAAGATGCATCTCCAACGGTTACAGCATT
CAAACCTAATACAAATGATGAAGTGAAAATAATCAAAGATGAACTTAAAAATCGGTTTAAAATAACAATTGCTGGTGGTCAAGGCCATCT
TAAAGGTCAAATTTTAAGAATTGGTCATATGGGGAAAATTAGTCCTTTCGATATTTTATCGGTAGTATCTGCTTTAGAAATTATTTTAACTG
AACACCGTAAAGTTAACTATATCGGTAAAGGTATATCAAAATATATGGAGGTTATTCATGAAGCAATTTAA

Gene: serA (D-3-phosphoglycerate dehydrogenase)

Contig: 08_NODE_1, position: 23556 to 25160, length: 1605 nt, orientation: FORWARD

Perfect match to: (MW2-BA000033-[1803428:1805032], allele observed in CC1+CC5)

Sequence:

ATGAAGCAATTTAATGTACTCGTTGCAGATCCCATATCAAAAGATGGTATCAAAGCATTATTAGATCACGAACAATTCAATGTAGATATTC
AAACTGGCTTGTCCGAAGAAGCATTAATCAAAATTATACCTTCATACCATGCTTTAATCGTTCGTAGTCAAACTACGGTTACTGAAAATAT
CATAAATGCTGCTGATTCTTTAAAAGTAATCGCACGCGCCGGTGTTGGTGTAGATAATATTAATATTAATGCTGCAACATTAAAAGGTATT
TTAGTTATTAATGCCCCAGATGGTAATACGATTTCAGCTACTGAACATACACTGGCAATGTTATTATCAATGGCACGAAATATTCCGCAAG
CACACCAATCACTTACAAATAAAGAATGGAATCGAAATGCATTTAAAGGTACTGAGCTTTATCATAAAACATTAGGTGTCATTGGTGCTG
GTAGAATTGGTTTAGGTGTTGCTAAACGTGCGCAAAGTTTCGGAATGAAAATACTAGCTTTTGACCCTTACTTAACGGATGAAAAAGCAA

AATCTTTAAGCATTACGAAGGCAACAGTTGATGAGATTGCCCAACATTCTGATTTCGTTACATTACATACACCACTAACACCTAAAACAAA
AGGCTTAATTAATGCTGACTTTTTTGCCAAAGCAAAACCTAGTTTGCAAATAATCAATGTGGCACGTGGTGGTATTATTGATGAAAAGGC
GCTAATAAAAGCATTAGACGAAGGACAAATTAGTCGGGCAGCTATCGATGTGTTTGAACATGAACCTGCAACTGACTCGCCTCTTGTTGC
ACATGATAAAATTATTGTTACACCTCATTTGGGTGCTTCAACAGTCGAAGCTCAAGAAAAAGTGGCAATTTCTGTTTCAAATGAAATCATC
GAAATTTTAATTGATGGTACTGTAACGCATGCAGTGAATGCACCTAAAATGGACTTAAGCAATATAGATGATACTGTAAAATCATTCATC
AATTTAAGCCAAACAGTTGGTGAATTAGCTATTCAATTAATGTACAATGCACCAAGCTCTATTAAAATTACGTACGGTGGCGACTTAGCCT
CTATTGATAGTAGTTTATTAACACGTACAATTATTACTCATATTTTAAAAGATGATCTTGGTCCTGAAGTCAATATTATCAATGCTCTAATG
TTGTTAAATCAACAACAAGTGACATTAAATATTGAAAATAATAAAGCAGAGACAGGTTTTAGTAACTACTTAGAGGTAGAACTATCAAAC
GATAGCGATTCCGTTAAAGTTGGCGCTTCTGTCTTTACAGGTTTCGGTCCAAGAATTGTTAGAATTAATAATTTTTCTGTAGACTTAAAGC
CAAATCAATATCAAATTGTGTCATATCATAATGATACTCCAGGTATGGTAGGAAAAACTGGCGCATTGTTAGGTAAATACAATATCAACA
TTGCATCTATGACTTTAGGTAGAACTGAAGCGGGCGGAGATGCGCTAATGATTTTATCCGTTGATCAACCTGTTTCAAACAATATAATTGA
TGAACTTAAACAAGTTGGTGAATACAATCAAATTTTCACAACTGAATTGACGGTACAGTCATAA

Gene: Q5HF49 (haloacid dehalogenase-like hydrolase)

Contig: 08_NODE_1, position: 25310 to 26440, length: 1131 nt, orientation: REVERSE

Perfect match to: (MW2-BA000033-[1805182:1806312:r], allele observed in CC1+CC22+CC188+CC361)

Sequence:

TTACAAATTATCTAGTACACCTCTAAGTTCACCTAAATGATTAATAACATAGTCGGCATGATGCGCTTCTAACTCACCTGCAGCATCCTTAC
CTTTTAAACCTGTTAATGTTCCAATAAACGTTGCACCTATTTTTTGAGCACTTAATAAGTCAGCTAACGAATCGCCTACTATAAATACGTCA
TCTTTATTTACAATGTTATCTTGCTTATTGATATAAGATTCATATTTATCGCGATTATTACCATATAAAGCTGCGATATAACTAAAAGGATTC
GGCTTTCCTAATGGTCGTGCTTGCGGATACATATTCTCTGCTTCTAAAACATCACTTGCTGTTGCAATAAAATCAGCTTCAAAATATGGTAA
CAATCCTAAATTTTCAAATGGCACAACAGTCTCAGTATAAGGACGACCTGTTGCAATACCTAATTCGAAACCAGCACCTTTTAAATCATTC
AGAAGTACCTTAACTTCATCTACTGGTCTCAAAATAATTTCTTGATAAATATAACCTGTCTTAAAAGTAGTTCGTGCTATTTTCTTTTCAACA
TCTTCATACAACTTCGATCCTAAATACCATTCTTGATAAACTTCTTGCGCTAACGTCCATAATGCACCCCTTCAAACTAAATAAAGTAGCATC
CGAAACATGTAACTCTGTTGTTGCAAACTCTTCCAAAGCAGCATAAATATTATTTTTACCAACTTTTACATTATCCAAAAATTGTAAAGGTA
GTTGTTCATTTAAATTAAAACAGTCTGCTAAGTTTGTGCTTATATTTTGCAACTTCAATTCGACAGGCTCATCTTGATACATGAATGCCTCA
ATTTCATCATGTGATAACTTTTTCAAAATATCAATTAAATGAATACTAAAAACGATAAATAACATATCCCAATTTGAATTTAGCCCTAGCGA
TTTTAATTTGTTTAAAATCTTATCTTTTTGAAAAAATTCGATTCCTAATGTCTTGTATATCGTTATCAGTCAAAGTTTCCCAATCTATATGTGAA
TGAAGACCTAAATAACACTTATCCATTAATAATTCATATACCGTTAATGCAGAGACATCGAAACAACGTTCTTCACTTAAAAAAACGCCAT
CAACATCAAATAAAATTTTCTTCAC

Gene: nagE (PTS system, N-acetylglucosamine-specific IIBC subunit precursor)

Contig: 08_NODE_1, position: 26547 to 28013, length: 1467 nt, orientation: REVERSE

Sequence:

TTATAAATGTTTTTCAATTTCATCAGCAACCTGCTGTACGTGTGTACCGACAATAACTTGAGTTGAATGTTTACCATTAACAGTAACACCAA
CTGCACCGGCGTTTTTAATCTTCTGTTGATCAATAATAGATGTGTCTTTTAACTCTAGACGCAACCTTGTTGTACAATTAGTTAAATTAACA
ATATTCTCTTGACCACCTAAACCTTCTAATATTTGTATAGCATGTTGATGATATTTACTTTGTTTAATATCATTTTCACCAGGAGCAATATTA
TCTTTTACAACTGTTGGATCAACTAATTCATTTTCACCTCTACCAATCGTATTCAAGTTAAATACTTGGATTACTACACGGAAAATCACATA
GTATAAGATGAAAAATACAACACCTTGAACAAGCAACATCAATGGATGATTTGATACCGGATTAATTAGTGATAACACATAATCTATCAA
ACCTGCACTAAATGAAAATCCAGCTGTCCAATGGAATGTAGCTGCGATAAATAAAGATAATCCTGTTAATAACGCATGAACAACATATAA
GATTGGCGCAACAAACATAAATGCAAACTCAATCGGCTCTGTAACACCAACGAAAAATGCTGCAACTGAACTCGCTAGGAACCAACCGT
AAACTTGTTTTTTCTGAGTCGTTTTAGCTGTATGATACATTGCTAACGCAGCCGCTGGAATACCGAACATCATGATTGGGAAGAATCCCGC
TTGATAGCGTCCTGTAATACCTTTTATAGCATCTTTGCCACTTTGGAATTTACCAATATCATTAATACCAATCGTATCAAACCAGAACACAC
TATTCAGTGCATGATGTAATCCTGTAGGAATTAATAGTCTGTTAGCAACACCATATATGAAAGCACCAAACGATCCTAAACCAACTATAGA
TTCACCAAATTTTACAATCCATGAATAAAGTAGTGGCCATAAGAATAACAATATGACAACTAAAAATGTACAGTAAAATGCAGTCATAAT
TGGAACTAGACGTTTACCACTAAAAAATGATAATGCTAATGGTAATTCTGTTTCACTAAACTTATTGTATGCATAAGCTGCTATTAAACCTA
TTACAATACCAACAAAGACATTGCCATTATTCATCTTTTCAAAAGCTGAATTTATTTCCGAAGCTTTCATTCCTAATAAAGGCGCTAATTTC
ATTGGTGATAATACAACTGTAACTAAAAAATATCCTAACGTAGCTGCAAGCGCGACTGCACCATCATTTTTCTTTGCCATTCCTATAGCTAC
ACCAATTGCAAATAAAATACCTAATTGCTCTAAAATCGTAGTACCTACCGTAGTAAAGAACATTGCGATTTTCGGCGTCGCATGAAGTGC
ATTTAACGTATTACCAATTCCGGCAATAATTGCTGCAGCCGGTAAAATGGCAACTGGTAACATTAACGAACGCCCTAAATTTTGGAAAAA
TTTATACAT

Gene: plsC (1-acylglycerol-phosphate  (1-acyl-G3P))

Contig: 08_NODE_1, position: 28141 to 28758, length: 618 nt, orientation: REVERSE

Perfect match to: (MW2-BA000033-[1808013:1808630:r], highly conserved allele)

Sequence:

TTATAAACTTTTTACAATTTCATGCAATTCTTGTTGTAACTTTGCTGTTCGTGTTTCAATCTCTTTTGTAATATAATCGATACGCTCGTTTCGT
TTTAAATCTTTAGGTAAATCGTTAATATCGATTGGTTTACCAATATTTATGTATGCTTGTCCTGTTAAAAGACCGTGAATCTTAGTAGGACC
AACATAAGCAACAGGTAATATTGGTGACTTACTTAACATTGCAATTGTTGAAGCACCACGTTTCAAAGGTGCACCTTCTTGCGATGTGCG
AGAACCTGTTGGGAAGATACCAACTGTCTTATTATCTTTCAACAAATTGATTGGGCGTTTTAAAGTACTAGGTCCTGGATTTTCACGATCT
ACAGGAAATGCATTTAAAGACGTTAAAAATTTACCAATCCATTTATTTTTGAATAATTCTTTTTTAGCCATATAATGAATTTGATTAGGATA
TAATGCCATACCTAGCATAATGACTTCGTTATAACTTTCATGCGTACAAGTTACGACATATTTACTATCCTTAGGAATATTATCTTTACCGA
TTACGTATAATGATTTTGACATTTTAACTAAAATGAAATTCAAAATCTTACTAATCACTGAATACAT

Gene: htrC (putative serine protease)

Contig: 08_NODE_1, position: 28930 to 30204, length: 1275 nt, orientation: FORWARD

Perfect match to: (MW2-BA000033-[1808802:1810076], allele observed in CC1+CC5)

Sequence:

ATGTCAGATTTTAATCATACAGATCATTCTACAACAAACCATAGCCAAACACCTAGATACAGAAGACCTAAATTTCCATGGTTTAAAACAG
TCATCGTTGCATTGATTGCTGGAATTATTGGTGCACTTCTAGTACTTGGTATAGGCAAAGTATTAAATAGTACAATTTTAAATAAAGATGG
TTCAACTGTTCAGACAACAAATAATAAAGGTGGCAATCAATTAGACGGTCAAAGCAAGAAATTCGGTACCGTTCATGAAATGATAAAATC
TGTCTCCCCTACAATTGTTGGAGTTATTAACATGCAAAAAGCATCAAGTGTAGACGACTTATTAAAAGGCAAATCATCTAAACCATCTGAA
GCTGGAGTAGGTTCAGGTGTTATCTATCAAATAAACAACAATTCAGCTTATATCGTTACAAACAATCATGTTATTGATGGCGCAAATGAA
ATTAGAGTCCAATTACATAATAAAAAACAAGTTAAAGCGAAATTAGTTGGTAAAGATGCAGTAACTGATATTGCTGTACTTAAAATTGAA
AATACAAAAGGTATTAAAGCGATTCAATTCGCCAACTCTTCAAAAGTACAAACTGGCGATAGCGTATTCGCAATGGGTAACCCATTAGGA
TTACAATTTGCTAACTCTGTAACATCTGGTATCATTTCAGCAAGCGAACGTACTATTGACGCTGAGACAACTGGTGGCAATACAAAAGTCA
GCGTTCTTCAAACAGATGCTGCTATTAACCCAGGTAACTCAGGTGGCGCATTAGTAGATATTAATGGTAATTTAGTTGGTATTAACTCAAT
GAAAATTGCTGCGACACAAGTTGAAGGTATCGGGTTTGCTATTCCAAGTAATGAAGTTAAAGTAACAATTGAACAACTTGTAAAACATGG
TAAAATTGACCGCCCTTCGATTGGTATTGGTTTAATTAATTTGAAAGATATTCCTGAAGAAGAGCGCGAGCAACTTCATACTGATAGAGA
AGACGGTATTTATGTCGCCAAAGCTGATAGTGATATTGATCTTAAAAAAGGTGATATTATTACAGAAATTGATGGCAAGAAAATTAAAGA
TGATGTTGATTTAAGAAGCTATTTATATGAAAATAAAAAACCTGGTGAATCAGTCACTGTTACCGTTATCCGTGATGGTAAAACAAAAGA
AGTTAAAGTGAAATTAAAACAACAAAAAGAACAACCAAAACGTCAAAGCCGATCAGAACGTCAATCACCTGGCCAAGGCGATAGAGATT
TCTTTAGATAA

Gene: tyrS (tyrosyl-tRNA synthase)

Contig: 08_NODE_1, position: 30298 to 31560, length: 1263 nt, orientation: REVERSE

Perfect match to: (N315-BA000018-[1772528:1773790:r], highly conserved allele)

Sequence:

TTATTGATAGTTAACCATGAAGTATTTTTTCTTACCGCGACGAATAATCGTAAATTCGCCATCAATTTTATCTTCTGGTGCTAAAGCATAAT
TAACATCTTGTTGTCTCTCACCATTAATATAAATCGCACCATTGTTAACATCTTCACGTGCTTGTCGTTTAGAAGGAGAAATGCCTGTTTCA
ATAAGGACTTCAACGATATTTGTTGTGTCATTTGATAATGTCACTTGAGGCACATCTTTAAATCCATCTTTTAATTCTTTCGCTGATAATGAT
TTTAAATCACCACTAAATAATGCTTGTGAAATACGGATTGCATCATTTAATGCATCTTCACCATGAATAAATTTAGTTACTTCTTCAGCTAA
TGTTTTTTGAGCTTCACGTAAATGCGGTGCTTCATTTTTAGATTGTTCTAAGCGATCAATTTCTTCTTTTCCTAAGAAAGTAAAGTATTTTAA
GAATTTAATTACATCTTCGTCTGATTGATTAATCCAGAATTGATAAAATTCATAAGGACTTGTTTTTTCAGCATCTAACCAAACAGCACCTG
ACTCAGACTTACCAAATTTCTTACCATCTGATTTAGTTACAAGCGGAATAGTTAAACCGTATGCGTCTGTTTGACCATACATACGACGCATT
AATTCAATACCACTTGTGATATTACCCCATTGATCTGATCCACCTACTTGAATCTTACAATTCAATTCTCTATTCAAATGACCGAAATCAATA
GCTTGTAAAATCGTGTATGTGAATTCTGTATATGAAATACCATGTTCTAAACGACTTTGGATTGAATCTTTACCTAACATGTAATTAACGCC
GACGTGTTTACCATAGTCACGTAAAAAGCTAATTAATGAGATTTGTCCTAACCAGTCTCTATTATTAACAAGCACTGCACCATGGTCTGTT
CCAAATTCAAAAATATTGTGCATTTGCTTACTAATACCTTCGATATTTTTATCTACTTGTTCTTCTGTTTGTAGCACACGTTCTTCTGATTTAC
CTGATGGATCACCAATCATACCTGTACCACCGCCAATTAAAACGATAGGACGATGTCCATGTTCTTGAAAACGTCTTAATGTTAAGAATG

GTAGTAAGTGACCAATATGTAAACTATCTGCCGTTGGATCGGCACCGCAGTATAACGTCACTTGTTCTTTATTTAATAAATCTTCAATACCT
TGTTCATCAGTTTGTTGATAAATAAGACCTCTCCATTTTAAATCTTCAATTAATACATTCGTCAT

Gene: tbox10 (T-box leader element)

Contig: 08_NODE_1, position: 31652 to 31853, length: 202 nt

Sequence:

TGTAAGGGCGCGATTGCACGTTACCACCAAACTTAAACATAATCATAAGATAATGTTCACTCTATTAATGATACGTTCATTAATAAACGTA
GGACATGTTAGTTATAAAGGTGTATTCATATTATTAAAAACACTAGTTCACAGCGACCACTAGCTCTCTGATGATTTCAAATAATATTACTT
GTCCTTTTATCCTATTCTT

Gene: sgtA (monofunctional transglycosylase A)

Contig: 08_NODE_1, position: 31942 to 32847, length: 906 nt, orientation: FORWARD

Perfect match to: (MW2-BA000033-[1811814:1812719], allele observed in CC1+CC5+CC8+CC239-ST582)

Sequence:

ATGACGAATCAAGACAACAATCATCAATTGAATCATCGTATATATCATTTTGAAAAGATATATAAAGCTATCAAACATGTCATTGTTTTTAT
ATTTATGATTTTCATTGCCATCGTTGCTATCGCTGTGATTGCGATGTCTTTATATTTTCATCATTTAACTAAAACGTCCGACTCATTATCAGA
TGATGCTTTAATAAAAAAAGTTCGACAAATACCTGGCGATGAATTATTAGATCATAATAACAAAAATTTATTATATGAGTATAACCATTCT
CAAAACTCACTCATTATAGGCCCTAAAACATCAAGTCCAAATGTCATTAAAGCATTAACGTCATCTGAAGACACTTTATTTTATAAACATG
ATGGCATCTTACCAAAGGCGATTTTAAGAGCAATGATACAAGATATTTTTAATACTGATCAAAGTTCAGGTGGTAGCACAATTACACAAC
AACTTGTTAAAAATCAAGTTCTTACCAACGAAAAAACATATAGTAGAAAAGCAAATGAACTTCGCCTAGCAATTAGATTAGAACACCTAC
TCTCAAAAGATGAAATTATATATACATATTTAAATATAGTTCCCTTCGGTAGAGATTATAATGGCGCTAATATTTCCGGAATTGCATCCGCT
TCATATAGTTTGTTTGGTATTCCACCAAAAGATTTATCAATTGCACAATCTGCATACCTTATCGGTTTGTTGCAAAGCCCTTATGGCTATAC
ACCCTACGAAAAAGATGGAACATTAAAATCGGATAAAGATTTGAAATATAGTATCCAAAGACAACATTATGTATTAAAGCGTATGTTAAT
CGAAGATCAAATCACTGAAAAAGAATACAACGACGCATTAAAATATGATATTAAATCACATTTGTTAAATCGAAAAAAGCGTTAA

Gene: isdH (haptoglobin-binding surface anchored protein)

Contig: 08_NODE_1, position: 33050 to 35737, length: 2688 nt, orientation: REVERSE

Perfect match to: (ATCC51811-ST1-ADVP01000005-[10259:12946], allele observed in CC1)

Sequence:

TTATTTAGATTCTTTTCTGAATTTAGGAATGAATAAAGCTAACATACCTAATAACGCATATAAGCCCCACCATGATTGGCTTGAAGTTGTTT
CTCCAGTTTTTGGTAGCATTTTAGATTGACTAAGTTTGTTTTCTTTCGATGGTGTGCCGGCTTTTTCTTTTGTTTTCACTGTTTTATCTACAGT
TTTATGAATATCACTCGGCAGATGTTCAGTTGTTTTTTTGTCAGTAACTTTGTCTGTATTATTATAATTTTGTTTCACTACGTCAAGCTTTGC
TGCTTTTCCAGTATGATTATTTTTATCGGCAATATGATTCAGCTGTATGACTTTGTCTTTATTTTTATTAGAGTCTTTATCAGTATCGACATTC
GATGACATACCAACGCTATTATCGGCACCTTTATCCACATTTCTATCAGTATCTTTGGCAATTTGAGTATCCATTTCTTTTAAATCATACTTA
TCGAAGTGATTATTATCCGACATATCGGATAAATGATCAACATCATGTTGCACATCTTTATCAATATTATTATCAGCATCTTTAACCACGTC
AGAATCTGGTTCTATCACATCTGCTTTATCAGACGCATCTTTAGGATTTGTTGCAGTGCTGCTATTTTCAGCTACATCTGTATCAGCAACCT
TACCTTCTTGTCCTGTTTGTACATTTAGCGGTTCACTCGTGTTATTTTGTGATGTATCATCATCTTTTGTATTGATATCCTGATTTATAATTCT
GACATGATATTGACCTTCATAACCAATGTTTGCCACAACGACTTTAACAATCGCATTGTAAACTGCTTTGTCAGGTATATATGGGAAAATC
AACGTTCTAGAATTATTTTTAGGATCTTTAGAAACAGTAGTGACACGTTTACCTTCTACAATTAAATCTTTCCAGTAACTGTCATCCTTTGTT
TTCATCACTACATATTTTTGACCATTTAAAGTTGCTGTATAGAATGGATGTTCAACAAAGCCGTCCATAACTGACTCACTATTTTCTTCACTT
TCAAAAACAACAAAATGCGCTTCTTGTACATCTGTTAATTGATCATTTGTAGGTGTAACATTTTCAAATTCCGTCACTGCTGATTTAACCTG
ATCAGCTAACTCTACTCTAGTTTGATCTAATTTCTTTTTATATTCCGCCTTATATTTTTCCGGCAATTTCTCTTGTAATTTTTCTAATTCATAAA
CTTGTCTTTCTAACGTTTTAGCTTTGTGATACGGAGCTAATAATTTTTGTAAATTGTATGTTTCCTCATCCACATAGTCGTCTGGGTTATTAG
TAATAGGCTGTGCAAAGACCATTAGCGTATAATCATAGTCTTCATGGATGTTCTCACCATATTCAATAGATGACACAATTTTAACTTCTCTC
GTACCATTAGATACTGGGAAACGAATATAGGCATAATCTTTATCAGAATCATATGATACTAATTCGACTGGTAACTTTTTGTCACCTTCAT
AAACTTCAAATTTCTTCCATGTTGAAGCAGTCTTTAAACCTAATTCAATTATTGGTCCTGTTTTTGTAAAAATGACAGTTGCTGGTTCAACA
GTACTAGCATAATGATAGAACTGTCTTTCACCTTTATCATTTTTCATTTGAAAATCAATTGGTCGCCAATTATCAGCTGTATGTTCTTTATCG
ATGATAGCCGGGTTTTTAATTGCATCTTGTAGTGATTCATCTGCTGGTGGATACTGTTGATTAACATCCGACTGATTACTTGACTCATTCGT

TTTATCGTTAGTATTACCATTAGAATTTGTTTCATGAGCTGGTTGGCTTGACGCTTGATCTGCATTCGCTGACGATTTTGGTTGTGCAGGTT
GACTCATATTTGTCGTTGCCTGCGGTTGATTATTAGCATTGTTGATCGTTGATGTATTTTGATTAGATGTATTCGTGTTTGTTTGATTACTTG
CGTCTGAACTTGATTGATCATTCGTTACTACTGCATCATTTGTATCTGATTTCACAAGTGAAGGATCGTTATAAATAGGTTTAGCAAATACT
AATTTAGTATAATCATAATTTGTTTCTTCTCCATCATCAATTTGAGTCGAAGAAACAATTTTCAATTCTTGTGTGCCATCTGAAACTGGGAA
TCGAATATAGGCATGGTCTTCTGGTACAGGACTATATGATACAAGTCTCACTGGTAATTTTTGATTGTTTTCATAGACTTCAAACTTTTTCC
ATGTTGAAGCAGTATTGATGTCTAATTCAACTTCTGCTTTCTTTTTAGTGTAATACACATCTGCTGGATCTTTGATGCTGAAAAAGTGATAG
TATTGCGTTTCATTGTTTTTATCTAATAATTGGAAATTGACTTGTTCTCTTGGACCTATATCATGTTCTTTATTTTCTAATGCAGGATCTTTAA
TTGCATCTTTAAGTGATTCATCCGCTGCAGGATAGGTTTTCGCAGTGTTTGCTGGTTGCGTAGCAGGTTGTGTTTGATTTGTATCCTTTGGT
GGCTGAGTTGTAGTTGCATTATTATTTTGATTTTCCGAGATTTTATCTGAAGTATTTGTATTTTCTGCTGCTTGTGCTTGATGTTGAGAAGT
AATTAAAAATAGTGTACTGACAATGACCGATGCAACGCCTAGAATTGATTTTCTAATAGAATAGAAAGACCTTAATTTTGGGTGATGTTT
GTTCAT

Gene: fhs (formyl-tetrahydrofolate synthase)

Contig: 08_NODE_1, position: 36071 to 37738, length: 1668 nt, orientation: REVERSE

Perfect match to: (MW2-BA000033-[1815943:1817610:r], allele observed in CC1+CC30)

Sequence:

TTAGAATAACCCAATTGCATGACCATCATCAGTAACATCCATGTTTAATGCTGCTGGTTTTTTAGGTAAACCAGGCATAGTCATGATTGCA
CCTGTCAACGCTACGATAAATCCTGCACCTGTTTTCGCTTCTAATTCACGAATTGTAATTTCAAATCCTGATGGTGCACCTAACAACGTTTG
ATCATCTGAGAATGAATATTGTGTTTTCGCCATACATACTGGGTAATTATCCCAACCATTTTCTTTAAATTGTTTTAATTGTTTTTGCGCTTT
ACTGCTAAACGTTACTTTTGAACCGCCATAGATTTCAGTCACAATCTTTTCAATCTTTTGCTCTAATGGTAATTCTAATTCATATAAAGGTTT
AAATGAATTAGGTTGATCAATGACTTCTAATACTTCATTTGCTAAGTCAACGCCACCTTTACCACCTTTTTCCCAAACTTCAGTTAAGGCAA
TTCGTACGTTATTTTCTTTAGCCCAAGATTTTACATATTCTACTTCTGCATCGGTATCATGTATAAATGCATTAATTGCAACAACCGGTTCTA
CACCGAATTTTTTAATATTATTAACATGACGCTCTAAATTAACAATTCCTGCTTTTACTGCTTCTACATTTTCTTCTTTTAAATTATCTTTCGCT
ACACCACCATGCATTTTTAACGCACGAATTGTCGCAACAACAACGACAGCTGCCGGATCAAATCCTGCTTCACGCGCTTTAATGTCCATGA
ATTTTTCAGCGCCTAAGTCTGAACCAAATCCAGCTTCCGTTACAACGATATCAGCTAAATCACGTGCTGTTTCAGTTGCTAAAATTGAGTT
ACAACCGTGTGCGATATTCGCAAATGGTCCACCATGAACTAATGCTGGTGTCCCTTCAATTGATTGTACTAAGTTTGGTTTAATTGCATCTT
TTAAAATCATTGCAAGTGCACCTTGCACTTTTAAATCTGCAACTGTAACTGGCTTGCGATCTCTAGTATAACCAATAGTAATACGACTAATT
TTATCTTTTAAGTCTTTAATACTTCTACTTAAACATAAAATCGCCATAATTTCAGACGCTACTGTAATATTAAAGCCATCTTCACGTGGTACA
CCATTTGTAGGTCCACCTAACCCAACATTTACATGTCTAAGTGCACGATCATTCATATCTAATACACGTTTCCATTCAATACGTCTTTGATC
GATTCCTAATTCATTACCTTGGTGAATATGATTATCTATAAACGCAGACAATGCATTATTAGCAGTTGTAATCGCATGGAAATCCCCATTA
AAATGTAAGTTGATATCTTCCATAGGTAAGACTTGCGCATAACCACCACCAGTCGCACCACCTTTAATACCAAATGTTGGTCCTAAAGCAG
GCTCTCTTAATGCAACCATAACGTTTTTATTTAACTCATGGAATGCATCAGCTAAACCAACTGTAACCGTTGATTTACCTTCACCAGCTGGT
GTTGGGCTCATCGCAGTTACTAAAACAACTTTCCCTTTGTTTTCTCTTGGCGTAATTTTATTAATGTCGATTTTAGCTTTGTAATGACCATAA
GGTTCTAATGCATCCTCTGAAATACCTACTGATGCAGCAATATCCTTAATTGGTTGTAGTGTTGATTGATTCGCAATATCTAAATCTGATAA
ATGAGTCAA

Gene: acsA1 (acetyl-coenzyme A synthase)

Contig: 08_NODE_1, position: 38178 to 39884, length: 1707 nt, orientation: REVERSE

Perfect match to: (MW2-BA000033-[1818050:1819756:r], allele observed in CC1+CC80)

Sequence:

TTATTCCATTGTACTTAAATCCCCTGCATCTAAATTTAATTCCCAAGCTTTTAATACACGTCTCATAATTTTACCTGAGCGTGTTTTAGGTAA
TTTATCTTTAAATTCGATTTCACGTGGTGCTGCATGTGCCGACAAACCTTCTTTAACAAATAAACGAATTTCTTCTTTTAATTCGTCTGTTGG
TTCATATCCTTTTCTCAGTGCAACAAACGCCTTAATTATTTCACCGCGAACCGGATCAGGTTTACCAATAATTCCTGCTTCGGCAACTGCTT
CGTGTTCAACCAATTTAGACTCAACCTCAAATGGTCCAACTCGTTCACCAGCTGTCATAATTACATCATCAACACGTCCTTGGAACCAGAA
GTAACCATCTTCATCTTTATATGCCGAATCACCAGATACATACCAGTCTCCAATAAAATATGACTTATATTTTTCTGGATTCTTCCAGATAC
GATACATCATTGATGGCCAGCCTTTTTTTATAGCAAGGTTGCCCATTCGATTTGGTGGTAATTCATTACCTGCATCATCGATAATTGCAGCT
TGAATACCAGGTAATGGTTTGCCCATTGAGCCAAGCTTGACGTCCATCGTTGGATAGTTAACAATCATATGTCCACCTGTTTCTGTCATCC
ACCAAGTATCTAACACCGTTAAACCGTATACTTTTTTCGCCCATTTTATAACTTCAGGATTTAAAGGCTCACCTACTGATAGAATCGAACGT
AACGATGACAAGTCATATTTCTCAACAATATCGTCACCAGCACTCATTAACATTCTTAAAGCTGTTGGTGCCGTATACCAAATCGTCACTTT
AAAATCTTCAATCATACTATACCACTGTTCTGGTGAAAAGCGACCACCAGCTATACAATTTGTAGCGCCATTTAACCATGGTGCAAAAATA
CCATAAGATGTTCCTGTAACCCAACCTGGATCTGCTGTACACCAATAAACATCATCTTCTTGTAAATCTAATACATATTTCCAGAAATATA
GTGCACTAACATTGCTTGTTGAACATGCAATACACCTTTAGGTTGCCCAGTAGAACCTGATGTATAATGTAAAATCAAACCATCATCCGAC

TTTAACCATTCAATGTCAAATTCATCGCTAGCAGTTTCCATCAAACTAATGAAGTCTATGTAATTGTCTTCTACATCCTCATCTACGACAAC
AATTTTTTTCAAGTTCGGTAATTTATCTACAGGTACTCGAGGTAACAATGCCTTATTAGTAATTAACACTTTAGCTTCACTGTTCTCTAATCT
ATCCGCAACTGCCTTTTCCATAAATGCTTCAAATAACGGCCCAACAATTGCACCAATTTTTAAAACACCTAACAACGCAAAATATAGTTCA
GGTGTACGCGACATAAATATAAATACTCTGTCACCTTTGTCAACTTCTGCATGTTCAGACAAAACATTCGCTGCTTTATTAGATAACCGTTG
CATATCTTTATAAGTATACGATTCTTTTCTGTACTCATCTTTGTAATTTAACGCTATTTTATCCCCTAATCCTTGATCTACATGGCGATCTATA
CATTCATATGCCATGTTCATTTTTCCAGTTTCACTCCAAGAAAATGCTTGTTCTACGTCTTTCCAATCAAAAGTATTATATATTTCTTCATAAT
CTTTAAGGTTATGTTTACCTTGCGCTCCTTTATAAACTTCGACTTTCAT

Gene: acuA (acetoin utilization protein A)

Contig: 08_NODE_1, position: 40053 to 40685, length: 633 nt, orientation: FORWARD

Perfect match to: (TCH959-AASB02000248-[60210:60842], allele observed in CC7+CC1+CC12+CC80+CC361)

Sequence:

ATGAATCATTTAAAGACGTATCAATCCGAAGATTATTACATTCATGACAAGCAATTTGTTATTGAAGGTCCTTTAACATACGAAGATTTGA
AAGCGCTTACTTTCGATGCGCATTTAACCGCATTTAGAGATGCTGAAGATCAGTATGAAGCTTTGTTAGAAATTACAACATTACCAGAAG
GTAGAATTTATGTTGCTCGCCAAGATCAACTCATTGTGGGTTATGTCACTTTCCACTATCCTGATGAAATTGAGCGCTGGTCTACAGGTAA
CCTTCCATATTTAATCGAATTGGGGGCAATTGAAGTCAGCATCAATTTTAGGCAATTACATCTTGCAGAAAAGCTAATACAACTTAGCCTT
TCTACACCAGAATTCGAGGATTATATCGTTATAACTACTGAATATTACTGGCATTGGGATTTAAAAAATTCAAAGTTAGATGTATTTGACT
ATAAAAAATTAATGCAGCGGTTAATGGCAACTGGTGGACTTGAAATATTCGCTACAGATGATCCAGAAATAACAAGTCATCCAGCTAATT
GTTTAATGGCAAGAATTGGCAAAAATATTACATTAGAACAGCAACAAGCGTTTGATGATATTCGTTATATGAATCGGTTTTTCTTTTAA

Gene: acuC (acetoin utilization protein C)

Contig: 08_NODE_1, position: 40710 to 41879, length: 1170 nt, orientation: FORWARD

Perfect match to: (MW2-BA000033-[1820582:1821751], allele observed in CC1+CC15+CC361+CC772)

Sequence:

ATGCAACAACATTCATCAAAAACTGCATATGTTTATTCAGATAAGTTATTACAATATCGATTTCATGACCAACATCCCTTCAATCAAATGCG
TTTAAAATTAACAACAGAGCTACTTTTGAATGCAAATTTATTGTCTCCAGAACAAATAGTACAACCTAGAATTGCAACAGATGACGAATTA
ATGTTAATTCATAAATATGATTACGTCGAAGCAATTAAGCATGCTTCACATGGCATTATCAGTGAAGATGAGGCTAAGAAATATGGATTA
AATGATGAAGAGAATAGTCAATTTAAGCATATGCACCGCCATAGTGCCACAATTGTTGGAGGCGCTTTAACTTTAGCAGATCTTATTATG
TCAGGCAAAGTATTAAATGGTTGTCACTTAGGTGGTGGATTGCATCACGCTCAACCTGGTCGAGCTAGTGGTTTTTGTATATACAATGAT
ATTGCAATTACCGCACAATACTTAGCTAAAGAATACAATCAACGCGTTTTAATCATAGATACCGATGCACATCATGGAGATGGTACACAA
TGGAGTTTCTATGCCGATAACCATGTTACTACTTATTCTATCCATGAAACCGGAAAATTTCTTTTCCCAGGCTCTGGTCACTATACTGAGCG
CGGTGAAGATATCGGCTATGGACACACTGTAAATGTCCCACTTGAACCGTATACAGAAGATGCATCATTTTTGGAGTGTTTTAAATTAAC
AGTTGAGCCTGTCGTAAAGAGTTTTAAACCTGATATTATTCTAAGCGTAAATGGTGTCGATATACATTATCGTGATCCACTAACTCATCTA
AATTGTACGTTACATTCATTATATGAAATTCCATATTTTGTAAAATATTTAGCTGATTCTTATACGAATGGAAAGGTAATTATGTTTGGTGG
CGGAGGCTACAATATTTGGAGAGTCGTACCACGTGCATGGAGTCATGTATTCTTAAGTTTAATTGATCAACCAATTCAAAGTGGTTATTTA
CCGTTAGAATGGATTAATAAATGGAAACATTATTCATCTGAATTATTACCTAAAAGATGGGAAGATCGTTTAAATGATTATACCTATGTCC
CCCGCACAAAAGAAATTAGTGAAAAAAATAAAAAATTAGCTTTACATATAGCGAGTTGGTACGAATCTACTCGTCAATAA

Gene: ccpA (catabolite control protein A)

Contig: 08_NODE_1, position: 41977 to 42966, length: 990 nt, orientation: REVERSE

Perfect match to: (MW2-BA000033-[1821849:1822838:r], highly conserved allele)

Sequence:

TTATTTTGTAGTTCCTCGGTATTCAATTCTGTGAGGTAAAACTACATTTGGTTCTTCTATCTTTTCATCGTTCATATATTTGTTAATAAGCG
CATCCCTACTGCACCGATATCATATAATGGTTGAATAACACTAGAAAGTTGTGGTCTAACCATCTCAACTAATCGTGTATTATTGAAACTA
ATAATTTGTAATTCCTCTGGAACTTTAATACCAGCATCCATTGCACTATGCATAATACCAATTGCTTCTTCGTCGCTGATACATAAAATGGC
ATCTGGCAAATTGCCTTTCATTTTGGCAAAAGCTTTTACGCCTTCTTTATAACTTTCAGCACCAGAACAATTCAATGTATCACCTAATTGAA
GGCCATTTTTATTTAACACTTCAGTTAAACCTTCTAAAACATCTTCTTGAGCTTTTTTAGAATGTTCTCCACCTACTAAAGCAAATGATTTAG
CGCCTTTTTCAATTAATTCTCCCGTAATTTCTTTCGCAGCTTCAGTAAAATCAATATTAACTGATGCTATATGTGCATCCTTACCATTTGTTCC

TGATACTACTACAGGTACAGATGATTGATTTATCAATTCTTTCATTTCTTCAGTAATTGTACCACCAAGGAAAATAATACCATCAACCTGTT
TACTTAATAAGTTATTAAAAATTTCTTTTTCCTTTTCAGGATCGTTATCTGAATTTGAAATAATTGAGTGATATTTATACATTGTTGCAATAT
CTTCAAGTCCACGAGCAAGTTGTGAATAATAGATATTAGATATATCTGGAATGATCACACCTACTGTTGTTGTCTTTTTACTAGCTAAACCT
CTAGCAACAGCATTTGGACGATAATTCAAACGCTTAATGACTTCGTTAACTTTATTTTTAGTTTCTGCTTTAACATTTTGGTTCCCATTAACA
ACACGCGACACTGTGGCCATAGAGACACGCGCTTCTCTTGCTACATCATATATAGTAACTGTCAT

Gene: aroA2 (bifunctional chorismate mutase/phospho-2-dehydro-3-deoxyheptonate aldolase)

Contig: 08_NODE_1, position: 43506 to 44597, length: 1092 nt, orientation: REVERSE

Perfect match to: (08-02119-CP015645-[348149:349240], highly conserved allele)

Sequence:

TTATTTTAACTTTTTAGCGTTATATAAATCAGCTAAAGGCTTTAATTCATCATAAAATGCTTGGAATTCATCTAAATCCATTTGTTGACCCGC
ATCACTAAGTGCAACAGATGGATCTGGATGCACCTCAGCCATAACTCCATCAGCACCAACTGCTAATGCTGCTTTCGCAGTTGGTAACAT
GATATCTTTACGACCTGTACTATGCGTTACATCTACCATGACTGGTAAGTGTGTACCTTGTTTTAAAATTGGTACTGCTGAAATATCTAAAG
TGTTACGTGTCGCCTTTTCATAAGTTCGGATTCCACGTTCACATAAAATAATGTTTTGATTACCTTGTGAAGCAATGTATTCAGCTGCATAA
ACAAACTCTTCGATTGTAGCAGATAAACCACGTTTTAATAGAATAGGCTTTTTCGTACGGCCAGCTTCTTTTAATAACTCGAAGTTTTGCAT
ATTACGTGCACCAATTTGGAATACGTCTAAATACTCATCAGCCACTTCAAAATCATTTGGATTTACGATTTCGCTGACAACATTTAAATCAT
ATTTATCTTTAATCTGTTTAAGTATTTTAAGTCCTTCAACACCTAGGCCTTGGAAATCATATGGTGATGTACGTGGTTTAAATGCACCGCCA
CGAATAAATTTTTCACCTTTAGCATGTAAGTTTTTAGCAACAGCTTCAACTTGTTCAAATGATTCAACTGAACATGGCCCAAATACAAATGA
TTTATTGCCGTCTCCAATAATGCCCCCATTATCAAATGTTACAATCGTATCTTCAGGTTTCAACTTACGTGATACATATAAATGTTTTTCATT
TTCAGATTTTTGTAAATCTGTAGAGGCTTTGAAAATTTCTTTAAATAATTGCTTAATAGTATTATCGTTGAATGGTCCTTTGTTACTATCGAT
TAAGTCGTTAAGCATTTCTTTTTCACGTTGTGGATCATAGATACGTGTACCTTGTTTTAATTTTTCTTCCCCAATTTTTTGTGCTAGTTCACCA
CGTTTAGATAATAAGTCTAAAATTTGATGATTCAGTGATACAATCTCACTTCTGTATGATTCTAATTTATTACTCAT

Gene: Q99TC6 (putative protein)

Contig: 08_NODE_1, position: 45297 to 46661, length: 1365 nt, orientation: REVERSE

Perfect match to: (MW2-BA000033-[1825169:1826533:r], allele observed in CC1)

Sequence:

TTAGTCATTGAATGTACGTTTCTCTATTTTACTATTAGCTTTTTCAACTTTAGCATTTGGCGTTTTTGATGCATTGGATGATTTAGCATTTGTT
GTTTTTGAATTCTTATTACTAGTTTGATTATTCTTTTGAGTTGACTTACTTGAACTACTTTGTTTCTTTGCACCTTGTGATGATTTCTTATTAT
GTTGCTTTTTCTGACCTGAAGTTTTATTTGTAGATGCTTTTGATGCATTTCGTTTATTTGAAGGTGTTGTTTTTTTAGATTGTTTACCTGATTT
CTTGTCAACAGCTGTATTATTAGTTGTTTTTTCATCAGCTTTACGTGTAATAACACCATTTTCAAATTTAGCTTCTTTTTGTTTAACAGTGTCT
TTATTATCTATATTAACTGTCTCTGACTCTGATACATCATTCTTTTTAGTAACTAATTGAGGAATTTCTTTCAAGTCATTTTTAGCAACTGGTT
TTTCTGCAAATAACGCTTCAGTTAATTGACTCTCTTTTGAACCTGGTGTTAATTTAGCTTGCTTCTGTTTTGCAGCGTTTGCTAATCTTTCAG
CTTGTGACTCTTCTTTCGATACCTTTAATGCCACTGCTTTTGATTCTTCATTTGATACAGCTACACTTTTATCTGTTTCTGCTTGTGCTTCTCTT
TTAGCTTCTTGAATCTCTTGTGCCTCTTGTGATGTATCACTTAAATTATTTGCACTTGCTTCTTCTTTTATTGCTGCTTGTTGTGCTTTTAATG
CCGCTGCTTTTGATTCTTCATTTGATACAGCTGCACTTTTATCTGTTTCTGCTTGTGCTTCTTTTTTAGCTTCTTGAATCTCTTGTGCCTCTTGT
GATGTATCACTCAAATTATTTGCACTTGCTTCTTCTTTTATTGCTGCTTGTTGTGCTTTTAATGCCGCTGCTTTTGATTCTTCATTTGATACAG
CTGCACTTTTATCTGTTTCTGCTTGTGCTTCTTTTTTAGCTTCTTGAATCTCTTGTGCCTCTTGTGATGTATCACTCAAATTATTTGCACTTAC
TTCTTCTTTTATTGCTGCTTGTTGTGCTTTTAATGCTGCTTGCTCATTTTTAGATTTGTTTAAAAATCCTTCAACACGTTCTTTTGTAAAGGCA
ACCGTTTCTTCAAGTTGCGTTTTTCTTTCTTCAAATTTTTGCGACAGTTCTTGTTCTTTGACTTTTAAATCATCTGCTTTTTGATAAACTTTATT
TTTAAAATACAAACCTAAAGCTGAACCAACAAGCGCGCCAGTTATAAAACTAACAACAAAATCTTTACGGTTGGGTAATGGTTCATTTTG
ATAAGTGTGTTTATTTTTTAATGTTCTTTTATTATTTTGTTGTTGCGTCAT

Gene: yoxC (general stress protein)

Contig: 08_NODE_1, position: 46735 to 47226, length: 492 nt, orientation: REVERSE

Perfect match to: (N315-BA000018-[1788940:1789431:r], highly conserved allele)

Sequence:

TTATTTATCTACTCGAGAAGTATAGCTATGATTTGCATCAGTTGCTACATTATTAGCTTTGTAATTTGCACTTCCACGACGGTAGTGTCTAT
TTTGCCATTTGTCTGCAATTTCCATTGCAACATTTGACCATTGAACAACTTGTGAGATTTTATCTTCATTTTGAGAAATATTATGTGTAATTG
AATTTGTTACACGATCTACAGAGCTGTTTAACGTTTGTACTGAGTCACCGATACCTTTAACAGCATCTACAACTGAGTTTAAACGATCTACT
TTACCTTGGATATCCTCAGTTAAACGGTTTACTTTATGAAGTAAATCTGTTGTTTCACGAGTAATACCTTGAACTTGACCTTCTACACCGTC
AAGTGTTTTTGCAACATAATCTAAGTTTTTCTTAACAGAATTTAATACAGCTACGATACCGATACATAAAATTAAGAATGCAATCGCAGCG
ATAATTCCAGCAATTGGTAAAATCCAATCCAT

Gene: murC (UDP-N-acetylmuramate--L-alanine ligase)

Contig: 08_NODE_1, position: 47300 to 48613, length: 1314 nt, orientation: REVERSE

Perfect match to: (MW2-BA000033-[1827172:1828485:r], highly conserved allele)

Sequence:

TTAAAACGCATTTTTCATGCCTAATTTATCTAAATATGCATTTTGTAATTTTTGAATATCACCTGCACCCATAAATAAAACAACAGCATTATC
AAATTGTTCTAATACATTAATAGAATCTTCATTAATTAACGATGCACCTTCAATTTTATCAATTAAATCTTGTATCGTTAATGCGCCAGTATT
TTCTCTAATTGATCCAAAAATTTCACATAAGAATACACGATCTGCTTTACTTAAACTTTCTGCAAATTCATTTAAAAATGCTTGTGTTCTAGA
GAAAGTGTGTGGTTGAAATACTGCAACAACTTCTTTATGTGGATATTTCTTTCGTGCTGTTTCAATTGTAGCACTAATTTCTCTTGGATGGT
GTGCATAATCATCTACAATAACTTGATTTGCAATTGTAGTTTCATTGAAACGACGTTTAACACCACCAAACGTTTCTAATGCTTCTTTAATA
TTTGTAACATCTAGCTTCTCTAAATAACTAATCGCAATTACAGCTAATGCATTTAAAACTGTATGGTCACCATATTGTGGAGACAGGAAGT
GATCATAAAACTCACCATCCACATACACATCAAAAGCAGTACCTTTATCCGTAATTTGAATATTTTGAGCATAAATGTCATCCGAATCTTTA
AATCCATAGTAATAAATTGGAACATCTGCTTCAATTTTACGTAGATGTTCATCATCACCCCAAGCAATAATACCTTTTTTAACATTATGTGC
CATTTCTTGGAATGCATCAAAAACATCATTAATATCTTTAAAATAATCAGGATGATCGAAATCAATATTTGTCATAATTGCGTAATCAGGTT
TATAACTTAAAAAGTGACGTCTATATTCACATGCCTCAAAAGCGAAATAATCACTTTCAGGCAATCCCATACCTGTGCCATCACCAATTAA
AAATGAAGTCTTTTTATCACCATTCATAACATGTGATAATAAACCTGTTGTAGAAGTTTTACCATGTGCACCAGTTACAGCTACTGAAGTA
TATTGATCAATAATCTGTCCTAAAAAATCATTATAACTTACAACATCTAATTTCAATTGATGTGCACGTACTATTTCTTCATGGCTACTCGCG
AATGCATTACCTTGTATAACTACCATATCTTCTTTTATGTTATTAGCATCAAATGGTAATATTTTTATCCCCTTATTTCTAAGAGCAACTTCTG
TAAATACGTAGTTCTCAATATCCGATCCTTGAACTTCATGTCCTAAATCATGCATGATTTGTGCTAATGAACTCATGCCAGAACCTTTAATT
CCGACAAAATGATAGTGTGTCAT

Gene: Q5HF33 (putative cell division protein)

Contig: 08_NODE_1, position: 48637 to 52461, length: 3825 nt, orientation: REVERSE

Sequence:

TTATTCTTTATTTAAATCTGCTTCCGTAACATAAACATCCCTTGGTTTTGAACCATTAGCACTCGAAACATAACCGAGTTGCTCTAATTGATC
GATAATTCTTGCTGCTCTATTATAGCCAATTTGGAAATGTCTTTGGATTAATGATGTTGAAATATGTCCTTCATTAACCATAAATGCACAAA
CATCATCAAATAATTCATCTTGTGATTGTGTTTGTGTTTTTTTCAACAATTCTTTTTCTTCAAATAGATAGTCCGGTTCTCTTTGTTGTTTGAT
AAAATCAACAACATCATCAATTTCGTCATCAGAAACAAATGTACCTTGAACTCTAATCGGTTTATTCATACCGCTACCAAGATATAACATAT
CGCCATATCCTAACAAGCGTTCTGCTCCACCACTGTCTAATATCGTTCTCGAATCTACACTTGATGATACCATAAATGCAATTCTTGTTGGT
ATGTTGGCTTTAATTAAACCTGTAATTACATTGACAGATGGTCTTTGCGTAGCTACTAACATATGAATACCACATGCTCTTGCTTTTTGAGC
AATTCTAGCAATAGACTGCTCAACTTCTTGCGGAGCCATCATCATTAAATCAGCCAACTCATCAATTACAATGACAATTTTTGGCATTCTTT
CATCATATGGTGCTTTTTTGTTAAATGCTGTTATATTGCGTACATGGTAATGTGCAAATAACTTATAACGTCGTTCCATTTCTTCTACGGCC
CATTTTAAACTCTGTGTAGCTGCTTTGACATCTGTAATTACCGGTGCAACTAAATGTGGCAAACCATTATAAGGAGCTAATTCAACCATTTT
TGGATCGATAAGTAATAATCTTAATTCCTCAGGGTGATTTTTATATAGTAAAGACATCAAAATACTATTGATACAAACTGATTTCCCTGATC
CAGTTGCACCTGCAATTAGTGCGTGTGGCGTTTTAGCAATATCCATAAGTAATGGTTCATTATTAATTCTATACCCCATCGCAACTGTTAAT
TTAGATTCAGCATTTTTAAAACTTGGAGATTCAATAATAGAACGTAAGTTGACTGTCGTTGGATTTTGGTTCGGAACTTCAATACCAACAC
GACTAGTTCCTGGAATAGGCGCTTCTATACGAATATCTTTCGCTGCCAATGCCATTTTAATGTCATCTTGTAATGCCGTAATTCTTGAAACT
TTAACACCTTTTTCAACTGATAATTCAAATCTTGTAACACTTGGACCTTCAGTTACATCTTGTACTTCTGCAGGTACATTAAAGTAAAATAA
TGCGTCATTCAGTTCTTTCTTTTTATCTGTAATCCAGTCCTCGTCCGACTCAATAACTTGTGGTTCTTCTAGTAATGAAACACTTGGCAATTT
AATATTTGGGCCTTTACGAATCATCGGCTTAGATGTTTGTACAGCTTGATTTGTTGCAACATCTTGTTGGTTCGTCCGAGGTCTATTTTCAT
TAGCTGTATCTTCATTTTGATTTGAAAATGAAGGCTGTAAATCTTTTTGTTGATCATCATTATCTTGTTGTCCACTAGTATTGTTTGGATGTG
TTGTTTCTTCGCTTTCTTCAGTTATGTCGCTTACTTCTGAAACTGAAGACGAAGTTGATTGATCAACTTGCTCATCTTCCAATTGATTATCAT
CTTGGCTTTCTTCAAATATTTCCGTTTGAGTTGAATCAGCAGAAGGTTTCTGCTCTGAATATTGTTGTGCATTTTGATAATCATTTTCTGTTT
CTGCATGGCCAATAAGTTGATTGTTCTCAACATTGTTTGATGTCATATTATTTGTTTTATATGCATTTGTGTTTGACTCTTGTGAATCAAATC
TTGATGATGGTGCGGCTTGACTCGCAGGCTTGCTTTCACTCACAGCTTGTTTACTTTGTACAGGCTTTAATTCAGGCACATTGACTTTTGAA
TGCTTTTTACGATCCATCATACGCTTTTTATCAGATGGCGTCATGACAACATTAAATGGTCTTTTACTTAAAGTTGAAACCTTTTCAGTCTGT

TTCTTTGGATTTACGTTTTCAATAGTTTTCTCTTCGACATTTTGTTCACTTTCAGCTGCTTCGTTTTGCACATTTTCTGTAATTTCTGCGTTAT
GCTTTTCTATTTCATTCATGCTTGAATCTTCCACAACATGACCATCATTCACATCATTCGTTTTCAATGAGTTGACATTTAAGTTTGTTTGGT
CATCTTTTTTATGACGCGGTGCTAATTCAATTTCATTATCAACCGTTTTATCGATATTATTAACTTGAGATTCAGTTACTGCGTGCTCTTGCG
TTTTAGGAGCGTCTACATGCATTTCAGACGCGTTTCTATTGGTATTATCTTCGATATTTGAAGTAGTATCCTCATCTGCTTCTGTATCATTTA
ATTCATAAAATTCTGCATCATCGATTTGACTTCCTGAATATTCTTCTTTATTCGTTGCTCCATCTTCTTCTGAATCAGCAATTAATCTGGATT
GATTTTTTAGTTCATCTTTATCATTTACTTCAACGTTATGTTGTAGTGCTGATTGATGTTGAGACGTTACATTCGAAACCGTAACTTCATCAT
CTGACAATTGTTTTGTTGTCGATACTTGATTCAAACTAACTTCCTCATAATTGTATGGACTTTCATCTACATGAGTGTCATTAGTGTCTTCAT
TTTGTATTGACGCTTGTTTATTTTCATCATTTGATACAGTTTCATTCTCTATGCCATTTGTATGAAGCTGACTAACATCTGTACTATTATCTGT
TAAGTCACTATCATCATTTAATGAACTATCACCAACATAGCGTTCCGCTTGTTTAGCATACATTTCATCAATTGCACGTTGTATTGCATCTTG
CTCTTCGTTTTTATGTTCTTCACGCTTTTGTTGCAACGCTTTTTTAAATCGACGCTTTTGAAGCACTTTACGTTCTCGTTCACGTCTAATTTCT
TCAACAATTTGTGAAGCATAAATATTTTCAATTTTGATAGTATTATCAACTTTTGAATAATTAGGCATTGATTTTGTTGTTGATGATACATTT
TCATCAGATGATTTAGATGCAGTTCCTTGTTTGACAACACTATCATTTTGTTTTTCTTGTTCTAATTGTTTATTTTGAGACGTTTGCGTCTTA
GCTACATATTTATCATATTTTTGTTTATCTGACTCAACTTTTTCTGAAGGTTTACTTACAGGGATACGACCATTTTCTAACTTTTTAGGTTTCA
TTGTGCCAAAAAATAGCTGACGGTACCTCTGAAGTCTTGAAACTTTCTTTGTGATAATCTGGAGTTGAATCTTTAGCACGATGATTTGTATT
ATTAGAATACATATTATGTGTTTTTGACTTGTGCGTTTCCTTCTCAATACCATTAATTGCAGAAACATATGTACCTGGCTTATTCGTATGGT
AATGTGAATGATCTTTATATTTTATACTTTGCTGTGATATTTTAGAATTCCCACGTTGTTCACTATAATTTTGTTCTTCAGTTGTTTGATTTCT
TCTACGGCGATGTCGTTTTTGTGAACGAGAATCGTGGCTTTGTTTGCGATAGTCTCGATGGTATTGTTCTTTTTCATCTGAAATAGTATCTG
CAGATTGTTCAACATTTTCATTTTCATAAGCTACGCTCATAGGAAAACGGAATTTTCCCCTCGGACGACTATAAATATCATTATTTTGAGGC
AGTAATGAGTCATGATCGTTATCTATATTTTGTGATTCTTGACGTCTTTTTTTCTTTCTATGAATCAAGTCATCATTTGAATCATTATCTTCGC
CGAATAATTTATCAAACCAGCTCAT

Gene: pheT2 (tRNA-binding domain protein)

Contig: 08_NODE_1, position: 52482 to 53078, length: 597 nt, orientation: REVERSE

Perfect match to: (Strain_21193-AFEG01000006-[193409:194005:r], allele observed in CC25+CC5+CC25+CC361)

Sequence:

TTATTCAAAAAAATGCCTGTCCAATTTCATAGCTGTCATTTAATACCATAATACCTTTTTCTTCAGGTGCATTAGGTAAATTCAATTCTTTCAT
TGAACAAATCATACCGCTTGAGGCAACACCACGTAATTCAGCATCTTTAATTACCATACCGCTAGGCATCACTGCACCTACTTTAGCAACA
ACAACTTTCTGTCCAGCTTCAACGTTAGGCGCGCCACATACAATTTGTAATGTGTCATTTCCAACGTTTACATTTAGTACACTTAATTTATCT
GCATCAGGATGTTTGTCTTTAGTTTCAACGTAGCCAACTACAAATTTCGGTGATAGATCAGCATTTAATTTATAATCAAAACCAGCTTCTG
AAATACGCTTTTGGAATACATTTACAAGTTCATCAGTTAATTTAATATGACCTTTTTCTTCAATTGTTATATCTTTTGAAATTTCAAAAATAT
TATAACCTACAACATTACCTTCATTAGTAATTTCAACAACATTACCTTTTTTATTGTAGTTTAATTCACCTTCAACTGGTTCAATTTGTAAAA
ATGCGACATCTCCTACATATTTAGGATTGTAAAATAAATTCAT

Gene: Q5HF31 (putative protein)

Contig: 08_NODE_1, position: 53107 to 53964, length: 858 nt, orientation: REVERSE

Perfect match to: (COL-CP000046-[1841588:1842445:r], highly conserved allele)

Sequence:

CTATTTATCTTTATTAAATTTACGACGATTTGCTTCTAAACGCTGAATCACGTTTGGATCTCTTTTTTGTTTATTATTTTTACCTAAAATAAAT
ATCGGTTCAAGATGACCCTGTTTATATCCAAAGGATAATGATGTAATTGGAACTAGACCTTTAGTGAAAAATTCCATTGTTAAATGTGCCA
TCACATCATATCCTGTTTTATTGCGTATATCTGCAATAATTAACACATCTTGGTGTGGCACTGCTACGAGCATTTCGCCTTGACATTGTGCC
TCAATTTCATTTAAAAATGCAGTATTTAGTATCCTACTTGCATCATACCCGTCATTTGAGTTAATAAAATAAAAAATATTACCTTTTACTTCA
TCAGTCGTATATGAATTTGACAATTTTCTAACATTAAACAGAGACATTTCTCTTATTTGTTGTTCAGTTAACTTCAAATCCTCTAACATGCTT
TCGTCAATTAGACGATATGATTTCCCTAAATCGACTGCATAATAAACTGCTGTTTCTGCAGTATGCTCATCATAGATAAAAGGAACACCTT
GTTTAGTTTTTTTATCAAAGCTAGTCGCTCTAATGACAGGCATAATTTGACTAGATGATATACTCTCAAGGGTTTTATCTGCCATTTGTGCA
ATAGCTTCATCAACGTAATAAACAATTTCATCTACAATTTTTTCTTTTTTATCTTCATATTTTGCGACTATAGCGTTAAGTTTAATCGTGATAC
CTTTGTTATTATCTGTTCGATAAATACGCAAAGTTTCTTCTTCACGATTAAATTTAAAATCAACGTCTAAATGGCTTAAACGTTCCTTTAATT
TATCTCTCATTTGAAAGGTATTCAT

Gene: ytpP (putative thiol-disulfide oxidoreductase)

Contig: 08_NODE_1, position: 54064 to 54375, length: 312 nt, orientation: REVERSE

Perfect match to: (MW2-BA000033-[1833936:1834247:r], highly conserved allele)

Sequence:

TTACACGTATTGAGCTAAAAATGCATCTATCTGTTCAATTGATTTTCGTTCTTTTCCAATATAACTTCCAAGCAGTTCTCCATTTTTATATACT
AGAAAACTTGGAATACCCATAATACCATTTTCAATACAAATATCCATAAATTTATCACGGTCTACTGATACGAAGTCAAACATAGGATATC
TCGCTTCTAATTCCGGTAAATCTGGTTCTATCACTCTACAATCTGGACACCAGCCTGCAGTGAATTCAAATACTGTAGCACCTTGTTTTAAA
GATTCAAATTGTTGTTCTGATTCAAGTTGTTTCAT

Gene: pepA1 (glutamyl aminopeptidase, locus 1)

Contig: 08_NODE_1, position: 54440 to 55516, length: 1077 nt, orientation: REVERSE

Perfect match to: (08-02119-CP015645-[337236:338312], allele observed in ST582+CC15+CC188)

Sequence:

TTATTTATATTGTAATGTTTCTATTTGATTATTATCTAAATTACAAATGGCTTCTGAAAGTAAAGATCTAGCTGCAAAATAGTCTCTTATATC
AAATACTGAGTCTGTACTATGAATATATCGTGCACATACACCAATAACTGCAGTCGGAATACCAATATTAGCTTTATGAATTTCTCCACCAT
CTGTTCCACCTGGTGACATATAGTATTGATGTTCAATGTCATGTGCTTCTACTAACTTTAATAAATAGTCTCTAAATACAGGCTTTAAAATC
ATTGTACCGTCTTTTATGCGAATTAACGTCCCTTTACCAAGTTCACCAGATAATGGTTGGCTTCCTTTAACGTCATTGGCAGGTGAACAATC
GACTACAAATGCAACGTCTGGGTCTATCATCTCTGCAGATGCTTTCGCACCTCGTAATCCAACCTCTTCTTGAACATTTGCGCCAACATACA
AGTCTACATCTAATTCTATATCTTTTAATAATTCTAGTATTTCAATTGCCAAGACACAACCATAACGATTATCCCATGCTTTAGCACTATATC
GATGTTCAGATAACTGTGTGAATGGCGTGTGAGGTACAATTGTATCTCCTATATCTATTCCGCGCTCACGCACCTCATCTTCATTTTGAGC
ACCTATATCTAATGTTAAATCTTTAATTTCCGGTGCGCCTTCACTACCAGTACGAAAATGTTTAGGTATATTAGAAACAACACCGATAATTT
TATCGCCATTTCTATTTTTAATTACTAAGCGTTGTCCTTGCCAAATATCATTTGCAACACCACCTAAATTTGTGAATTGAATCATTCCATTTTT
AGTGATATTTGTAATCATAAATCCGATTTCATCCATATGTGCTGCAATCATTACACGTTTTGCATTTGGATTTTTAGATTTTTTCACACCAAA
AAATCCACCCATACGATTTTCAATAAATTCATCTACGTACGGCGCCATTTGCTGAGTCATATAATTTTTTACTTCTTCTTCAAAACCTGGTGC
CCCATGAAGCTCAGTTAAAGTTTGAATTCGTTGTAATGTTACTTTTTTATTTATGTTCAT

Gene: ytzB (putative small protein)

Contig: 08_NODE_1, position: 55603 to 55914, length: 312 nt, orientation: FORWARD

Perfect match to: (MW2-BA000033-[1835475:1835786], highly conserved allele)

Sequence:

ATGACTAAACTGAAATATATAATTCCAACAATAATTGCAGTAGCCATTGTAATTATTTCTACCATTTCAATCATCCAATATATTAATCGTAA
ACGCTATAATCCCGTTAAAGTACTTAATGAAGTAAAATCATATTTTATGAATGTCAAAGGCTCATATATCGTTTATGAACCATTCGTTCATC
CTGAAACTGATAAATACCGTTTAGTTTATCAAGGTGGAATTACAACTATTAAAAATGGTCAAAATATTCATTATGATTTTTATGCAGATGC
ATATACTGGTGAAGTCATTAACATTGTAGAGCGTTAA

Gene: ytnP (metallo-beta-lactamase superfamily protein)

Contig: 08_NODE_1, position: 56039 to 56881, length: 843 nt, orientation: REVERSE

Perfect match to: (MW2-BA000033-[1835911:1836753:r], allele observed in CC1+CC188)

Sequence:

TTAGTTATTATCAACTAATGTTTCACGTAAAATATATGCATCTATGTTTTCACCATCATCGCTGTATTTTACAGCAAAGTAGTTTTCATCATG
ATAAAACAAGAACCAATATTGTTGCTGAATAAAATATGGTATCATGCGTTCTTTTTCACGAATCGATTGCATAGGATAATCATCATATGCC
GTTACCCATAGAGGATTTTTATGTGCAGTAGTTGGGAATATATCACCCATATGAACTGCTTTATCTCCTTGACTTTCAATCGTAATAATCGT
GTGGCCAAAGCTATGACCTCCACTATGTTGCATCTTGATACCCGGAACCGGTTCAAAATGTTTTTCGAATAAAATCAACTTGTTACTATAA
TCGCCTTTATTCTTATCCCAGTAAGTTGATTTACTTCTTATATTAGGTGCAATAAACTCATGCCACTCATCTTGTTGCACAACATGAATCGCA
TTTTCAAAAATTGCATGTCCCGCTTGATCAGTCAAACCGGCAGCATGATCAAAATGCATATGTGTCATTAGCACATAATCAATATCCTTTG
GCGTTAAATTATAATTTGCCAAATCAGTAATAATATGACTTTCTTCATCTACTCCAAAATTACGTAATTGCTTTTCAGATAATTTACCATTAC
CAATACCCGCATCTATAATCAAATTATATTGAGCCGTTTGAATCAAAATTGGATGTGTCGGTAAATTGATTTGATTTCGTTCATTTGCATTG

TATTGCTTTGACCACAACGGCTTCGGAACAACACCAAACATTGCACCGCCATCCATTTTTGTATTGCCGCCATTTAGATAATGAATAGATA
TATCCCCGATTTTCAC

Gene: trmB (tRNA  (guanine-N (7)-)-methyltransferase)

Contig: 08_NODE_1, position: 57351 to 57995, length: 645 nt, orientation: REVERSE

Perfect match to: (TCH70-ACHH02000010-[63414:64058], allele observed in CC1+CC22)

Sequence:

TTATTTTTGTGAATGGAATTTCGCTTCCATACGATAAATACGTGACCCTTTATTCGAAAATTTCTTTTCATATTCTGTTAGAATATTACTGCC
ATCGTCTTCTTGATGTAAATTTAGATTTATTTTTGTAAAATACATTCCAAATTGAGACATACTTTCTAAACTGTAGGCAAATAGTCCTCTGTT
ATCAGTTTTAAAATGTAAATCTCCTTCATCATTTAAGATTTGTTGATACAACGCTAAAAACGTATGATACGTTAAACGTCGTTTTGCATGAC
GATTTTTTGGCCATGGATCTGAAAAGTTCAAATAAATACGCGAAACTTCGCCGTCTTTAAAATATTCATTTAATTCAATGGCATCATTACAA
ATAATCTTTAAATTTGTTAAACTCATCTCTTTAACTTTATCCAATACTTTATAAACGATACTTTTCTCACGTTCCATTGAAATATAGTTAATAT
GAGGATTTTGAGCAGCTAATGTTGTAATAAACTGCCCCATACCCGAACCAATTTCAATGTGTATCGGTTGCGTTTTATCAAACCATTCAGT
CATTTTCCCTGCATGTTGACCGTCCATGTCAACCAATTCAGGATGATCTTTTAAATAATCTTCAGCCCATGGTTTGTATCGAACTCTCAT

Gene: ytmP (putative phosphotransferase)

Contig: 08_NODE_1, position: 58010 to 58801, length: 792 nt, orientation: REVERSE

Sequence:

TTAAATAAACATGTTACTATTCATAACTTGATTTAGGAATTTAAGCCAAGTGTTCATATCCTTATATCTTTTTTGCTCTTCATACCATTGCAC
AAGACCTATAGATTGAATTACCGTATACCATTTCATACGTTTATTTAAATTCAAGCTCTCTTGAACACCATATGTTTCAAGCCATTCAGACC
ATTGTTGTTGTGGAACATAGTTGTAAAGCAGCATTCCGATATCAATTGCCGGGTCTGCGATCATTGCACCTTCCCAATCGACTAAAAATAG
TTCATCTCGATCAGATAATAACCAATTATTATGATTCACATCACCATGTACAACAGTGAAAAAACGTGAATCTAAACTCGGTATATGCTCTT
CTAAATAGGTTAATGATTTTCTCACAATATGATGTGTTAAAACTTCTCTTGATAAAGAGGCATTAATTTTATTAAGCATAATCTCAGGAGTA
ATAGGTTCCATTTCCATACGCTTTAACATACTTAATAAAGGTCTAGAATTGTGTATCTTCTTTAATAAATGTGCAACTCTTGTTTGCTTCATT
TCGTTTAAAGATAGTTCACGGCCATTTTTCCAATGTTGTGCTGTAACAACCTCGCCTGTTTCTATGCGCTTCGTCCATACTAATTTGGGCAC
AATACCTTCTGCTGATAATGCCGCAATAAATGGATTTGAATTTCGTTTTAAAAACAACTTTTGTCCATCTTGTTCAGCCATATATGCTTCGC
CAGATGCACCACCTGCAGAATCAAGTGTCCACCCTAATTGATAAAACTGCTCCAA

Gene: daaA (D-alanine aminotransferase)

Contig: 08_NODE_1, position: 59352 to 60200, length: 849 nt, orientation: REVERSE

Perfect match to: (08-02119-CP015645-[332543:333391], allele observed in ST582+CC22+CC239)

Sequence:

TTAAATACTGTGTGACTCTATATACTTTTCAAATCCTTCTTGTAGTTGACGTGTAATTGGGCCAACTTTACCATCATTAACTGGTTCACCATC
TAATTTAATAACAGGTGTAACCTCAGCTGAAGTACTTGAAACAATAACTTCATCTGCGTTTCTCAAGAAATCTACAGTAAACGTTTCTTCTT
TAAATGGGATATTATAGTCTTCGGCAATTTTTTTAATTACAATTCGTGTAATACCATTAAGAATATAGTTGTTAATCGGATGTGTATAAATC
ACACCGTCTTTAATTGCATAAGCATTACTTGAAGATCCTTCAGTGACAGTTTCGCCTCGATGTTGGATTGCTTCAACGGCATTGTATTTTAC
AGCATATTCTTTTGCTAATACATTTCCTAATAAATTCAAGCTTTTAATGTCGCAACGTAACCATCGGATATCTTCAACGGTAACACCATTCA
CACCATTTTCTAAATGATCATAAGGACGATCATAACTCTTTGTATAAGCAACAATTGCTGGTTCTACTTCAGGTGTTGGGAAGCTATGATT
TCTTTCAGCTACACCTCGCGTTGCTTGAATATAAATTGCCCCAGTTTCAATTTGATTCATATCAACTAATTTACGAGATAGTTCAATTAATTC
TTCTACAGAATAATTTAAATCTAAACCAATCTCATTGGCACTACGTAAAAATCTTTCATAATGTTCTGTTACTGTAAATAACTTACCATTATA
TACTCGAATGTATTCATAAATACCATCGCCAAATACGTATCCTCTGTCGTTGTATGAAACCTTTGCTTCACTTGGACTTACAAACTCACCAT
TTAAAAAAATTTTTTCCAT

Gene: ytjP (Mn (2+)-dependent dipeptidase)

Contig: 08_NODE_1, position: 60204 to 61613, length: 1410 nt, orientation: REVERSE

Perfect match to: (MW2-BA000033-[1840076:1841485:r], allele observed in CC1+CC30)

Sequence:

TTATTCCTCCACGCATAATGAATAAATTGCTTCTAAGTAAATACTAGTTGCGTTAAATAACTGTTTTTTAGTGATATATTCATTTTTCTGATG
CATTAAATCTTCAGAATCACTAAACATTGCGCCAAATGCTACACCCTTGTCTAAGTTTCTCGCATAAGTACCGCCACCTATAGTATAAGGTT
CAGTCATATCATTTGTTTGATTTCTATATGCAGTAACTAACTTTTGTACAAAAGGATCATTTTTATCAACATAATGTGGTGGTTGGACTTTA
CCTAATTTCACTTCAAAGCCATATTGTTGAATCTCATTTGCAAAACGATCCATAGCTTTTTCAAATTCAAATCCTTCTGGATAGCGCAAGTT
GATACCGAAAAGACCCGCATTTTCATTATCATATGTAATAACACCAATGTTATTTGTCACGTCACCCATGACATCTGTATGGAATTTCATTC
CCATCTTTTCACCAAAATCTGAATCAAATAAGTAGCGATTACTAAATGCTACAAACGCTTGTGCATTATTATCAAGATTTAATGATGCCAA
GAATTTTAGTAAGTAAAGACCCGCATTCACACCGATAGATGGATCCATACCATGAACCGCTTTACCTTCAACTGTTAAAACTAGAATGCCA
CTATCAACAGTACTATCACCTTGTAAATGATTTTGTTCTAAAAAGTACTCAAAGTCTTGAATAACATCTGTCATATTTTCTTTAACAAGCACT
CTTGCTTCTGCATGATCAGGCACCATGTTGTAACGTTCACCAGATTTAAAAGTTATTAATTCATAATCAGGTTCATCTTGATCTTCAGCAAG
CTTATTTTGAACTAAGTCAAATGTTGTAATGCCTTTTTCACCATGAATACACGGAAATTCTGCATCTGGTGCAAAACCTAATGTTGGCATTT
CTTCTGTTTTAAAATAGCGATCCGTACATTTCCAATCAGATTCTTCATCCGTACCAATAATCATATGAATACGTTTCTTCCAATCCACATTCA
TATCTTCTAATATCTTAATTGCATAATAAGCAGCAATTGTTGGACCTTTGTCATCAAGTGTACCTCTAGCTATGATAGCATCTTCTGTTACA
ACCGGCTCGAACGGATTACTATCCCATCCATCACCAGCAGGAACAACGTCAACATGACATAAGATACCTAATACGTCATTTCCTTTACCTG
CCTCAATTCTTCCTGCAATATGATCCACATCATGTGTTGTAAATCCATCTCTATGTGCAATTTCATACATGTAGTCTAATGCCTTACGAGGA
CCTGGACCAACTGGTGCGTCTTCTGATGCTTTTGCATCATCTCTCACACTTTCAATTGCTAATAATCCTTTTAAGTCATTAATGATTTGATCT
TCGTATTGTTGAACTTTTTCTTTCCACAT

Gene: Q5HF22 (putative protein)

Contig: 08_NODE_1, position: 62306 to 62728, length: 423 nt, orientation: REVERSE

Perfect match to: (MW2-BA000033-[1842177:1842599:r], allele observed in CC1+CC88)

Sequence:

TTATTCTTTATCAGATAGTGCATTTTTATTCTTTTTTAAGTCTTCTTCAGTGACGATACGTAAATTATTATTTGGTGCGGCGCCACCTTCATC
ATCAAATTTACCTTTTTCAATACTTTCGTCAGTCTTATTGTCATATTCGGTAAATTTTGATTTTTCTTCTTCGAAAAATGCTTTTGGATTATTT
TTTAATCTATTAGCATATTCTTTCGGATTTGTTTTTACTTCTTTAATTGTTTCATTAGCAATTGTTCCTAATTGCGTCGCTTTATCCTTAGCATT
ATCTTTATAGCTTTGAGGATCTTGTTTATATTTATTATATTCTGCTTTCAGCTTGTCACGACTATCTTTACGTGTAACAAGTACAGCTGCTAC
AGCGCCACCTATACCCAAAATCGCTTTAAATAAATTACCTTTTGCCAT

Gene: rsuA (pseudouridine synthase)

Contig: 08_NODE_1, position: 62745 to 63440, length: 696 nt, orientation: REVERSE

Perfect match to: (TCH959-AASB02000249-[731:1426:r], allele observed in CC7+CC1+CC88)

Sequence:

TTATTTATAATTTAATTTGTCAAAATCATTTTCAGTTAATAAACGATATTCTCCTGAATCTAGATTGCTGTCCAATTCTAAATCAGCAATTTT
GATACGTCTTAAATGTAATACCTCATTTTGAATGCTATGAAACATTCGTTTAACTTGATGATATTTTCCTTCATAAATTGTTACGTGTGACG
TTTGATTATCAATATAAGTTAAAATTGCAGGCTTAACCTTGCCATCAGACAGTGTTACACCCTCTTTAAAAGCTTGAATGTCGTCTTCAGTG
ATAGGATTTGCTGAAATAACTTCATATTTTTTAGAAACATGTTTGTTTGGACTCATTAATTCATGATTAAAATCACCATCATTCGTTATCAAT
AAAAGCCCTTCTGTATCTTTATCAAGACGACCAACCGGAAAAAATATTTAGATGTTGGTATTCAGGTATTAAATCAATAACGGTTTTTGAAT
GATGATCTTCAGTTGCTGATATATAATCTTTTGGCTTATTTAACATAATATAGACATTTTCAATGTATTCTATTAATTCTCCACGAACTGTTA
TCTTATCGTTTTCTGGTTCTATATGTGTTTTTGGTGATTTAATTACTTGTTCGTTGACATTTACAAGGCCTTTTTTAAGTAACTGTTTGACCTC
ATTACGTGTACCGACGCCCATATTTGCTAAAAATTTATCTATTCTCAT

Gene: ytgP (putative membrane protein)

Contig: 08_NODE_1, position: 63437 to 65098, length: 1662 nt, orientation: REVERSE

Perfect match to: (MW2-BA000033-[1843308:1844969:r], allele observed in CC1+CC25+CC49+CC361)

Sequence:

TCATCGTAAAAACCTAACTCTACGTCTTAATTTTTCAGGAATTTCACCTAAGAATTCGTCCGCAAGACGCGTTTTAATTGTGATTGTACCGT
AAATTAGAATACCTACTGTAACACCTAAAATAATAATGATTAAGTAACCAAGTTTAGTAGGTTCTAAGAATAGATTTGCAAGGAAGAATA

CTAATTCTACACCTAGCATCATAATAAATGAATACAAGAATATTTTTGCAAAATGAATCCAACTATAGCTGAATTTAAACTTCGCATATTTT
TTAAGAATATAGAAATTACATCCAATTGCAAATAATAATGCGATACTAGTACTTAAAATTGCACCAGGTGTATGGAATAACATAATTAATG
GATAGTTTAACGCTAACTTGATAACTACAGAAGCTAAAATAACATAAACTGTTAATTTCTGTTTATCTATACCTTGTAACATTGATGCCGTT
ACACTTAATAGTGAAATTAGTATTGCTACAGGCGCATAATAGAATAATAAGCGACTACCATCATGGTTAGGGTCATGACCTAAAACAATT
GGATCGTAACCATAGAAAACTGTGAATAATGGTTGTGCCAAGGCCATAATTCCAATACTAGCTGGAACAGTTATAAACATTAATACACCA
ATAGATGTTCTAATTTGATGATGCATTTCATGTAAGCGACCTTCTGCAAATGTTTTTGTAATAAAAGGAATTAAACTCACTGCAAAACCAG
CACTTAATGATGTCGGAATCATTACAATTTTATTAGTTGACATATTTAGCATATTAAAGAATATATCTTGTAACTGTGAAGGTATACCAACT
AAAGATAAAGCACCGTTATGTGTAAATTGATCTACTAAGTTAAATAATGGATAATTCAAACTTACAATAACGAACGGAATACTATAAGCA
ATAATTTCTTTATACATCTTGCCATATGACACATCTATATCTGTGTAATCAGATTCGACCATACGATCAATATTATGCTTACGCTTTCTCCAG
TAATACCAGAGTGTGAATATACCAATAATCGCACCAACTGCTGCTGCAAAAGTAGCAATACCATTGGCTAATAAAATAGAGCCATCAAAG
ACATTTAGTACTAAATAACTTCCGATTAATATGAAAATCACGCGTGCAATTTGCTCAGTTACTTCTGACACTGCTGTTGGCCCCATAGATTT
ATAACCTTGGAATATACCTCTCCATGTCGCTAATACAGGAATAAAGATAACAACCATACTAATGATTCTTATAATCCAAGTAATATCATCG
ACTGACCAACCGTTTTTATCATGAACGTTTCTAGCTAATGTTAATTCAGAAATATAAGGTGCTAGGAAATACAGTACCAAGAAACCTAAAA
CACCGGTAATACTCATTACAATAAAACTCGATTTATAAAATTTCTGACTGACTTTATATGCCCCAATAGCATTATATTTCGCAACATATTTC
GAAGCTGCTAATGGTACACCTGCTGTCGCAACTGCAATTGCAATATTATATGGTGCATAAGCGTATGTGAACGGCGCCATATTTTCTTGTC
CACCAATTAAATAGTTGAATGGAATGATAAAAAGTACGCCCAATACCTTGGTAATTAATATACTAATGGTAATTAAAAAGGTTCCACGCA
CCATTTCTTTACTTTCACTCAT

Gene: Q1Y9Y5 (NAD (FAD)-utilizing dehydrogenase)

Contig: 08_NODE_1, position: 65505 to 66773, length: 1269 nt, orientation: FORWARD

Sequence:

ATGTATCAAACAATTATTATCGGAGGCGGACCTAGCGGCTTAATGGCGGCAGTAGCTGCAAGCGAACAAAGTAGCAGTGTGTTACTCAT
TGAAAAAAAGAAAGGTCTAGGTCGTAAACTCAAAATATCTGGTGGCGGTAGATGTAACGTAACTAATCGATTACCATATGCTGAAATTAT
TAAGAACATTCCTGGAAATGGGAAATTTTTATATAGTCCCTTTTCAATTTTTGATAATGAATCCATCATAGATTTTTTTGAGTCTAGGGGTG
TTAAATTAAAAGAAGAAGATCACGGGCGTATGTTTCCAGTTTCCAACAAAGCACAAGACGTGGTTGATACATTAGTGACAACTATCGAAC
GCCAACATGTAACGATTAAAGAAGAAGAAGCTGTTAGTAGAATCGAAGTTAATACAGACCAAACTTTCACTGTACATACTCAAAATAATA
GTTATGAAAGCCATTCGCTAGTGATTGCTACAGGTGGTACAAGTGTCCCTCAAACTGGTTCAACTGGTGATGGTTATAAGTTCGCACAAG
ATTTAGGTCATACCATTACTGAGTTATTCCCGACCGAAGTTCCAATTACATCAGCTGAACCTTTCATCAAATCCAATCGTCTAAAAGGTTTA
AGTTTAAAAGATGTTGAATTGTCAGTACTTAAGAAAAATGGTAAAAAACGCATCAGTCATCAAATGGATATGTTATTTACTCATTTTGGTA
TCAGTGGTCCAGCTGCATTAAGATGTAGTCAGTTTGTTTATAAAGAACAAAAAAATCAAAAGACACAGCACATTTCTATGGCAATCGATG
CATTTCCTGAATTAAACCATGAACAATTAAAACAACACATCACATCATTATTATCGGACACACCAGATAAAATCATTAAAAACAGTTTGCA
GGGTCTAATTGAAGAGCGCTACTTACTGTTCATGCTGGAACAAGCAGGAATCGATGAAAATACCACATCACATCACTTATCAAATCAACA
ATTGAACGACTTAGTAAATATGTTTAAAGGGTTTGTATTTAAGGTGAACGGGACATTACCTATAGATAAGGCATTTGTCACAGGTGGTGG
TGTGTCACTTAAAGAAATTCAACCTAAAACAATGATGTCTAAATTAGTTCCGGGATTATTTTTATGTGGTGAAGTATTAGATATACATGGT
TATACTGGTGGTTATAATATTACAAGTGCACTCGTAACAGGACATGTCGCTGGATTATATGCCGGACATTACTCACATGCATCAATGGAA
TAA

Gene: sasC (LPXTG-sorted surface protein C)

Contig: 08_NODE_1, position: 66890 to 73450, length: 6561 nt, orientation: REVERSE

Sequence:

TTATGATTCTTTTTCGTTTTTAGTACGTCTTCTAGCTAACAAAGCCGCACCTGTAATCAGTGCAAATTCTTTCAATGGTAAATCCATTTCTTC
AGAACCTGTATTTGGAAGTTCTTTTTCAACTTTGCGCGATTCATGTGTCTCTTCTTTTTTAATAGGCGTACAAACTTTTGGAGCTGGCTGAA
TTACTTTTGGTGATACTTTCATCGCTTCAGCTGGTAATTTAATTGCTAAAATTTCATCAACAATGAATTGCGTATGTTGTTTGATGTCATTTA
ATGTCGCATCTTCATCAATCATTCTATTGCCATCTGCAACATATTGATCAATTAATACTTTTACTTTAGCTAATTGTTCTGGTGTTGCGATCG
CTTTGAATTTCGCATATGTTTGTTGAGCAATGTTATCAATGCGCAGTAAGCTATTTTCTTTTTCAGTAATTACTGCTTCTATATCGCTTAATG
CAACATTAAATCGTTTTAAAACTGCATCAACATCAGCATTAGTGCGTGCTGTTTTTAATTCTTCATCCATTTGTAATTTTAAAGCAGTTATA
GCTTTTAATGCATCAGCCTTATTACGATCACTTACTTTTCGATAATTTTGCACTAAAGCAGTGACGCGTGCAAGATCATCATTAATCGTTTT
TTCAGCATCTGGCTTTTTAATAGGATGTACATCTAAATCATGTATTGTTTGTAGATTTAATGATGCTGTTTTATCAACTTGTGCATTGCTAC
GATCTTGATCAATTTGTCCAATAGCAGTGTCATAAATATTTTGTAACTGTGCTAATATACTATTTCTTTCTTCTACCGTTGCTTGAATATTCG
CTTCAATTGCTTGTTTTTTATCGTTGAATAATGTTGTCAATTGTTCTCGAGCAGACGCCTTTCTGCTAATAACAGGTTCGATTTCACGAATTT
CGTTTTTCTCATCATGCAATAAATATGCCACATCAGCATTAGTCACTGCACTAGCAATTTGTTGTTTAGCTTTAATTAACTCTTTTTCAACTT
GTGCTATTGCAATATTTTGTTCTTCATCTGTCGCTTCGTTATTTGCTTTAATTAAATTAATTTTATTTGTAGCGATATTTTGAATTTGTTGTAA
TGCTGTTGCTTTAACTGTTGTCGCTGGTTTAATTTTTGAAATAATATTTTGAGCATCTATACTATCTTGATTAACTTGGGCAGTCTTATCTGC

ATGATTGATCTGATCAATAGCCTGATTAAGTGCTTGTTCTACTAAATGTTTAGCAGCTAGTCTTTCTTCTTCAGTTGATAAATCGCTTTGAT
CGATTAGTGCATTTTGAGCTTCGGCTTTTACACCAACAGATTGACGCGCTGCTGGTTTAACTTGAACTTTAGGTAAAATCACTTTGATGTT
GTCGTTGCCATCAGTCTCAGTTCGATCCACTTCTGCATTCGTTTTGTTTTGTGCAATGTCATTTTTAATTGTATTTACAATTTTATTTAAAGTA
TCAATAGCAACATCTCTTTCATCTTGAGTAGTATCCAACGTATTTCGGATTGCATCGAGTTGATTTTTATTATTTTCTTCAATGCTATCAAGC
GCAGCTCGTTTACGTCTTACTTTAGGTTTTATTTGCTCAATTGCCTTGATTGTTTGATTTCTAACATCAGTAACAGCAGCATCTTGATTTGTA
TTTTCTATTTCTTGTTGCGCTTGTTTGAGTGTGTCGCTAATTAATTGATTCGCTTCATCTAATTCATCAACTGTTGCATGTGGTGTATCTTTTA
TTGATTCTACTTGATTTTCTGCACTTGCTTTTATTGCTTGTTGTGCTTCAGGCTTAATTACAATATGAGGTTGTACACCTTTTAGTGTAGCAA
TGCCATTTGTTTCAACACGTTTCACATCATTATTCGTTACTGCTTGATTGATGTTTTGTAATGCAAGTTTTTTATTATTCGCTAATTGATTTAA
AGCAACTTGTTTTTCTTCATCAGTCGCATGTTCAGCTTGCTCTATTTCTTGCTTTTTAGCCTCATATTGTTGCTTTACTGCATCTCTAGCAGCT
GCTCTAACAATATGTTCAGGCGCTACTAAAGCAATGCTATCAAGCGCTTGACTTGTTGTATCATCAACTTGTTGATTTGTTCTATTATTCGT
AATATCTGTCATGGCTTGATTTACAAATTCATTGATTTTATCTAGTGCTACTTGTCTTTCTTCTGCTGTTGCTTCTTTATCCTGATTAATCTTA
GCACGTAATTCATTCGCTTTTTGATTGATTTTTTCACGTGCAGCTGGTTTAACTTTTGTTTCAGGTTGAATAATTTTAATCGCTGATACACCA
TTTGTTGCTGCTTGATTCACCTGACTATTCGTTTGAGCTTGGTCAATAGCTGCAAGTGCTTTTTCTTTTTCTTTAGCTAATGCTTGTGAAGCA
ACTTCTTTCTCATTATCTGTTGAATCAAGACTATTATCAATTTGCTGTTGCTTTTCTTTAACAGCTTTTTCAATATCTGCAATTGCCTTTGGTT
TAATTACTACTTCAGCTTCAACATTATCTATAGCATTTAACGCTTGATTTGTAGTTGTGTCTACCTGATCATTTGTTTGGTTTTGATTAATTT
GATTAAATGCTTGATCTTTAAGTTGATTGATTTGATTAACAGCAGCCTGCTTTTCTTCGTCAGTTGCATTAGGTGTTTGTTTAACCGCTTCA
ATACGCTTCGCCACTTCAGCAGTGATTTTATCTCGCGCTGCTTGTTTTTTTACTACGTCAACTTGAACAGCATCGATATTATTCTCTGCTACT
GTCGCAGCTTGGTCTACTTCTGCATTTGTGTTAGCTTGTTTAATACTTTCAATAGCTTGTTGTCTGTCTTGATTTAAAGTATTGATCGCAGC
ATTTTTCTCATCATTCGTTGCATCTGGTGTAGCATTGATTTCAGCTAATTTAGCATTATAATGCTGATTGATTTGTGCTAATGCTGCAGGTTT
TTTAACAATATTTGGCTGAATCGCATTAATTGCTTTTGTACCTAATTGTTGCGCTTGATCTACTTCCGCATTTGTATCAGCTTGATTTATATT
ATTAATTGCCGTTGCTAACTCTTGATCCACTTGATTTAAATCCACTTGCTTTTCTTCAGTTGTTGCATTTGTGTTTTGATTAATTTCTTGCTTT
TTAGCAGTTGCTAAATCATTTAATACACCTGTAGCAGTTTGTTTCTTCGTTACATGCGGTTGAACTGCGCCGATTTGATTGACTGCATCGTC
TCTAATACTATTGACCATCGCAGTAGTAGATGTCACACCAATATCAGTTAATGCTCTATTTTTAAGTGTATTGACACGATTTATCGCTTCTT
GTTTCTCTTCTCGAGTCGCGCCAGGTGTAGCATTGATATTTGTTATCGCCTCTCGCGCTTTATCATTTACAACATTGCGAGCATCCGTTTTA
ACTTGTGTTGCCGGTTGAATCACTACTATATTTTGTGTGCCCTGATCTTTTGCTTGTGCAACTTCTTGATTCGTATCTGCTGCATTAATATTT
TGCTTCGCTGTGGCTACAGCTTGATCAAGTAATTGTTGTGCTGCTTGTTGTTCTTCGAGCGTCGCATCATTATTATTAAATATCGTATTATG
TTGCGTTTCCGCACTTTTATCGATGGCATTTTTTGCATCTGTTTTTACCTTTGTAGCTGGTGTAATTGCTTGTATTCCTTGTATCGCATTTGTC
TTCACTTGTTCCACTTCAGCATTGGTATTAGCGTTTAAAATGTTTGTGTTTGCTGCAGTTACAGCAGCATTCACTTTGTCAATTGCTGCTTGT
CTTTCTTCTTGAGTCGCATCAGGATTAGCATTGATCTCTGCGATATGTTGTTGTGCATCATGTGATACGGCATCCCTTGCAGCTTGCTTAAC
AACAGTTACAGGAGCAATTGGATTAATGGCATTTAGACCATCTCCTTTGGCGTTATCAACATCAGCATTTGTTGTTGCTTGATTGATTTGTT
CTAAAGCATGGTTGGTTGCTTGAGTTAATTCGTTCAATGCTGCATTTTTCTCTTCCTGAGTTGCTTCTCTATTACTATTTATTTGTTGTCTTTT
CGTTGCTGTTGCTTGGTTAATTGCATCTCTTGCAGCTTGTTTATGTGTCACTTGAGGAACAACTGATCCAATAGTATTGATGCCATTATTTT
TAGCTGTTTCAACGTCAGCATTTGTAGTAGCATTCGTAACATTATCAATAGCATCTGTTTCATCCGTAGCTAATTGATTTATTGCATCTTGA
ATCTCGTCTTCAGTAGCATCTGGTGTTGCATTGATAATTTCCCTTTGTTTCGTTGCTTTATCACGTATTGCTTTTTTAGCATTTGGTTTAACA
ACCGGTGTTGCAGTATCCCCACTTAAGGTCTGTATACCTTGATCTTTGATTCTAGTAACGCCATCATCAGTCGTTTGATCACCAATATCACC
AATAATATTGCCTTTATGTTCCTCGATAACTTGTATTGCTGCTTGTTTTTCTTCATCTGTCAGTTCATCATTTTGATTAACTAAATCTTCCATT
TGATCAGCTTTTTGATTTACTGCATTTTCAGCATCAACACTTCGAATTAAAGTATGTTGCATCTGATTAGTTAATGTATCAATATCTGCTTGA
GATACTCTTTTATTTAATGGTACATTGTTACGATTTTCAGCTAAAATTGTTTGTGCACGTTTTTTAAGATCATTAAAGATATCTAATGATGCA
AATGTATAATCAGCTTGTTGAATACGTCTGTCAACTTCGGCTTGTAATGCATCTTTATTCATGATGATATCGATAGTATATGGATTTGTACT
TACAGTATGACTTTCTGCAGCTGAATTAATGAAATCTTGTGTATATGTTTTATACGTTAATGTATCATTAAATGTTACTGTTCTTGGTGTCG
GTACATTATTAACACGCAACTTATACTTTAAATCCAATATTTTATCAGGCATTAGTCGTGCAGGTGAATTCGTTGTGCCACCACCAGTGCTT
TTAATTGTTATCACTCGATTCGCCGCATCATATGTAACATTCATATCATTAACATCAACGCCTGAATTGTTACTTGGAAAATCTTTAGTCAA
TGAATTGTTCACATATTCTACCCCTTCAGGTAATTGAATTTTATAAACAAATTCATCTGTGTCTAAAGAAGCACCAGAATTACCATTATTTTT
TAGTGATGTTGTAACTGTAAATTCTTTATTATTCGTTGCTGTGGGTTCCATTGTTCGTCTTTCAACATAAACATGTGACCCAGAATGAAGTC
CGATAGAGTCAACAAAGCTATAGTATTTGTAACCATCTTTTAGTTGATAAATGCCACGCGCATCTGTTATTGCGTCATTTTTAGGTACAAA
TTGAATTTTGAGATTTCTCACATTATCAGGTACTTTAAATAAACGCAAAGTTGGACCGCCTTCAACAGTCTTTTCAGCAATCGTATCATTAG
TATCAGCATTTTTGATAATAACATTTGTTGCGCCTTGACCATTTTTAGTTGGCATTGTATTAAATTCAAAGATTAATTCAGAATTTGGATTTA
CTGTTAAAGATTTTTCAATACCATTAAAATCTCCATGATTACTAGCGTCCGTCCCGCTTATACGTCCTAATGCAAGTACATTGCCTTGTGCC
TGATAGGTTCTGTTGCTCCCAGCCTCAAACATACTTGTTCTAACCATCGCATGACTTAGCACACCTACTTTACCACCATTGATAAGTGTAAA
GCCTGGTAAATTATCAACAACTGTTACTGATGGTACAGAACGGTTGGTACTTGGTCTAATACTATTGTCATCAAATGACAGCACTTCGTTA
GGTGCATTTTGTCCTGCATTATTGGCATTAGGATCAGTTGTAGGCGTGTATGGCGCTGTAATTGCAACTGGTGCACCACCGTTTCCTGCCG
CTGCAGCCCCTGGATCTGCTGGAGTTGCATTAGGATCTGCCGGTGCCGCACGTCTACTTCTCTTTTTCGGTCTATTAGATGCTGGTTCAGC
AATTGCAACTAGCTCTGGTTTATCTGAAGAATGACGAACATCTTCTTGAATTTCCTTTAAAGTTAGATGTCCTCCTGAACCATTTTCATTTG
TTTTATTTGGTACATTTAATGTGGTATTATTCGACGCTACATCATTATTATTAGCGTTTGACACTGTCTCAGTTGCTGTTGTATTGCCATCTG
TATGATTATTGTTTTGTGCTGAAGGCGTACTTGATTGCACTGATGTTGGCGTAGCTTGATTTACTATACTACCATTATTATGATTAACCAAT
GCTTGATTCGTTGCTTGATTGGTTGTTGCAGATTGATTAGGTGTATTCTGCGCACTATTTGCTAAACCTCTATTATTAGCAACATCTTTATCT
TGTGAATTTACAGGTGTTGCTTGATTAGTATCGCTTTGTACATTATTATCCGTAGTTAAGGCTTGTGCACCATTTGGGTTTGAAAGTAATA
AAACTGTTCCGATTAAAGTAGAGAATATGCCTACTTTATACTTCCTAATACTATATTTATTTTTCTTTAACAAATTCAT

Gene: ytwF (putative sulfur transferase)

Contig: 08_NODE_1, position: 73776 to 74087, length: 312 nt, orientation: REVERSE

Perfect match to: (MW2-BA000033-[1853647:1853958:r], highly conserved allele)

Sequence:

TTAAATACTTTTTATTTCCAAACCTTCATCGCCCCATGCGTGCATGCCGCCTTCGACATTTACGGCATCAATGCCATTTGCCTCTAAATATTC
TACAACTTTAGCGCTTCGAACTCCACCAGCACATACAATATAATATATTTCATTTTTATTAAATGAATTTAAATTATCCGGAATGGTATCCA
TTGGAATCAACTTTGCATTAGGAATATATCCCATTGCTGTTTCTTCGTCAGTACGAACATCAACAATTTGAACTGGTTTAGATTCTAAAAGT
TTGTTTTTTAATTCATCTGTAGTAATTGACTTCAT

Gene: leuS (leucyl-tRNA synthetase)

Contig: 08_NODE_1, position: 74109 to 76529, length: 2421 nt, orientation: REVERSE

Perfect match to: (MW2-BA000033-[1853980:1856400:r], allele observed in CC1+CC8+CC239+CC772)

Sequence:

TTATTTAGCTACAATATTGACTAATTTTTGAGGAACAGCGATGACTTTCATGATGTCTTTACCTTCAATACTCGCTTTAACATTGTCATTAGA
TAAGGCAATTTCTTGCATTTCTTCTTTTGATGTATCTTTAGCAATTTTAATTTTAGCTCTCAATTTACCATTTACTTGAACAACGATTTCTACT
TCATCATCTACAAGTAGTGCTTCGTCATAAGTTGGCCAAGGTTGGTACGTAATAGACTCTTCATGTCCTAATTTTGACCATAATTCTTCACC
GATATGTGGTGCAATAGGTGCTAACATTTTAACGAAGCCTTCAATGTAAGGTTTATAAACTTCATCAACTTTATAACACTCATTAATAAAT
ACCATTAATTGACTAATAGCAGTATTAAATCCTAATGTTTCAAAGTCTTCTGTTACCTTTTTAACAGTTTGGTTATAAACTTTATCTAAAGAT
TTATTATTTGTAGTTACAATTTTTGAACTCAATGTTCCATCTTCATTTACCATTAAACGCCATACGCGATCTAAGAATCGACGAGACCCATC
TAATCCTTTTTCACTCCATGCAATTGCAGCATCTAAAGGTCCCATAAACATTTCGTAAAGACGTAAAGTATCTGCACCATGAGACTGTACT
ATATCATCAGGATTGATTACATTTCCTTTAGATTTACTCATCTTCTCATTACCTTCTCCTAAAATCATACCTTGGTTAAATAATTTTTGGAAA
GGTTCTTTAGTAGGTACGATACCCAAATCATAAAGGACTTTATGCCAAAATCTTGCATATAATAAGTGAAGAACCGCATGTTCTACTCCAC
CGATATATAAATCAACAGGTAACCAATGTTTTAATTTTTCAGGATCTGCTAACATATTTTCATTTTTAGGATCGATGTAACGTAAATAATAC
CAACAACTACCTGCCCATTGTGGCATTGTATTTGTTTCACGACGTCCTTTCATACCTGTTTTTTCATCTACAACATTTACAAATGAATCAATA
TTAGCTAGTGGAGACTCACCAGTCCCTGATGGCTTGATTTCATCTGTTTCAGGTAACAACAATGGTAGCTCTTCTTCAGGAACAGTTGTCA
TTGTTCCATCTTCCCAATGAATGACAGGAATTGGTTCGCCCCAATAACGCTGACGACTGAATAACCAATCTCTTAATTTGTAATTAACTTTC
TTTTCGCCAGCACCTTTTTGCTCTAATAATTGAATAGCTTTAGTAATTGCCGCTTCATTTTCTAAACCATCAAGTTCACCAGAATTAATATGT
TTACCTTCACCAGTGTATGCTGCTTCTTCAACATTTCCACCTTCGATGACTTCAATGATTGGCAAATCAAACTTTTTAGCAAATTCATAATCT
CTGTCATCATGCGCTGGTACTGCCATAATTGCTCCAGTACCATATGTTGATAATACATAATCAGCAATCCAAATTTGTACTTTTTCACCAGA
TAAAGGATTAATTGCATATGCACCAGTAAACACACCTGATTTATCTTTTGCTAAATCTGTACGTTCTAAATCTGACTTTTTAGAAGCTTCTG
TTTGATAAGCTTTTACTTTTTCTTTATATTCATCAGTTGTAATTGAATTAACTAATGCATGTTCAGGACTTAAGACTAAGAATGATGCACCA
TAGATTGTATCTGGTCTAGTCGTAAATACTTCTACTTTTCCTTCCGCTTCCGTATTATCTACATCAAATGAAACTTTGGCCCCTTCAGAACGT
CCAATCCAATTGCGCTGCATATCTTTTAAAGATTCAGGCCAATCTAAATCATCTAAATCTGCTAATAATTGATCTGCATATTCTGTGATTTT
AAGTACCCATTGTTTCATCGGCTTACGATAAACTGGATGTCCACCACGTTCAGAGACACCATCAATCACTTCTTCGTTAGATAAAACAGTG
CCTAATGCTGGACACCAGTTAACTGCAACTTCATCAACGTATGCTAAACCTTTGTTATATAACTGTATGAAAATCCACTGTGTCCATTTATA
GTATTCTGGATCTGTTGTATTAACTTCACGATCCCAATCATAACTGAACCCTAATTCTTTAATTTGTCGTTTAAAAGTTTGGATATTTTTCTT
TGTAAATTCACGTGGGTCGTTGCCAGTGTCTAAAGCATATTGCTCTGCTGGTAATCCGAATGCATCCCACCCCATCGGATGTAATACATTA
TATCCTTGCATTCTTTTATATCTTGAAATGATATCTGTTGCTGTATAGCCCTCAGGATGTCCAACATGTAAACCAGCACCTGATGGATATGG
AAACATGTCTAAAGCATAAAATTTCTTTTGACCTAAGTTATCATTTGTTTTAAATGTTTTATTTTCGTCCCAATAGTCTTGCCATTTCTTTTCA
ATTTGATTGTGGTTGTAATTCAA

Gene: yttB (major facilitator superfamily transporter)

Contig: 08_NODE_1, position: 76820 to 78001, length: 1182 nt, orientation: REVERSE

Perfect match to: (MW2-BA000033-[1856691:1857872:r], allele observed in CC1+CC30)

Sequence:

TTATGCATCTATTTTTTTAGGTTGCGTATTATTCTCCTTGAAAACCATTAATAATATTAATGCAAATACAAGTAGTAGCATCATACCGATAA
ACATCATGCGCATATTAAACGCATCAACTAATACACCACCAAGAAATGGACCAAATGCTTTTCCTACTGTAGCAGCTGAATTCACAAAACC
TTGGTACTGTCCTTGCTTACCATCTGGCGCTAACTGATTGGCTATAGTTGGAACTGCTGGCCATACAAACATTTCTCCAAAAGTTAAAATA
ATCATACCGACAACAAATATTGTAAAGTTTTCGGCAAAACTCGTGACAAAGAACGACAACATAAAAATGATGATGCCGACAAACATTTGC

TTCTTTAAGTTTCCTTTTAACAGATAGAGAATCGGTTTAATTAATGGTTGTGCTACTAAAATCATTATTCCGTTAATTGTCCATAAAACACT
ATATTGTGCCATTGAAATATTAATAGATTGTGTAAATGAAGCGATTGTAGACTCCCATTGAATATATGCAACCCAACAAATTGCAAACATT
GCACAAATTAGTACTAATGAAATAAATCTTGCTTTATTCTTTTTACCAGTAATATCTAATTGAGTTGGATATTTAACTTTCGCATTAATTTCA
ATATTAAATTGCGTTACCGCGACAAGCGCAAACACAACATACATAATAAGATTGGCTAAAAAGATATAGTTAAAGCTAAATTCTGCGACA
AAGCCGCCCATTGCAGCACCGACAGCCACACCAATATTTTGCGCTAAGTATATCGCATTAAACGTTTGTCTTCCGCCATTTGGCCACACTG
CTCCAGCCATAGCGTATATCGCAGGAATAATCATTCCGCCACCAAACCCTAACATTACAAGCCATACAGCATACCAAGGCCACCCGTGAA
AGAAATTAAGTAGCGTTGTACTACAAAGACAAGTGAAAGTTCCAATTAAAATCGTCTTGTATCCACCTAATTTATCAAATAGTGAACCACC
TAATAAGTTTCCAATAACCATGCCAAATGAATTTATCATTAGCACTAAACCAGCAACAGTTAAACTTTTTCCAAGTTCTTGTTTCATATAAA
TTGTATTTAAAGGCCACAAAAAACTGGAACCAGTAATATTTAACGCCATGCCAATTACTAGCCACCAGACTGATTTAGGTATATTCAT

Gene: ytqA (putative Fe-S oxidoreductase)

Contig: 08_NODE_1, position: 78111 to 79064, length: 954 nt, orientation: FORWARD

Perfect match to: (MW2-BA000033-[1857982:1858935], allele observed in CC1+CC8+CC239)

Sequence:

ATGGGCAATCATTTCCAATACGCTTTTGAAAACAAACGTTATCACACATGGAATTACCATTTAAAAAATAAATTTGGACAAAAAATATTTA
AAGTTGCATTGGATGGCGGGTTTGACTGTCCTAACCGCGATGGCACTGTAGCACATGGTGGATGTACATTTTGTTCTGCTGCAGGTAGCG
GAGACTTTGCAGGTAATCGTGCAGATTCAATCGCAGTACAATTTAAAGAAATTAAAGAAAAGATGCATGAGAAATGGCACGAAGGAAA
ATATATTGCTTATTTTCAGGCATTTACAAATACACATGCGCCAGTTGAAGTATTAAAAGAAAAATTCGAACCTGTACTTAAAGAACCGGGT
GTTGTAGGATTATCTATTGGTACGCGTCCTGACTGTCTACCAGACGATGTTGTCGAATATTTAGCAGATTTGAATCAACGAACATACTTAT
GGGTTGAATTAGGACTACAAACAATCCATCAATCAACATCTGATTTAATCAATCGTGCCCATGATATGAAAACTTATTATGATGGTGTGGC
AAAATTACGTAAGCATAATATCAATGTATGTACACACATCATTAATGGCTTACCTGGCGAAGACTATGACATGATGATGGCTACTGCCAA
AGAAGTTGCACAAATGGATGTACAAGGTATTAAAATTCATTTACTTCATTTGTTAAAAGGTACACCGATGGTAAAACAATACGATAAAGG
TTTATTAACTTTTATGACTCAAGAAGAGTACACAAACCTAGTTGTGGACCAATTAGAAGTGATTCCCCCTGAAATGATCGTTCACCGAATT
ACCGGTGATGGTCCAATAGATATCATGGTCGGTCCAATGTGGAGTGTTAATAAATGGGAAGTATTAAATGGCATCGATGCTGAATTAGC
ACGTAGAAATTCATATCAAGGCTTGCGTTACAAGTCTAAGGTGAAGCAATGA

Gene: ytqB (rRNA methylase)

Contig: 08_NODE_1, position: 79061 to 79624, length: 564 nt, orientation: FORWARD

Perfect match to: (COL-CP000046-[1867762:1868325], highly conserved allele)

Sequence:

ATGAAATTAGAACGTATACTCCCTTTTTCAAAAACACTTATTAAACAACATATAACACCAGAAAGTATTGTTGTAGACGCAACTTGCGGTA
ACGGCAATGACACTTTATTTTTAGCCGAACAAGTACCAGAAGGACATGTTTATGGTTTCGACATTCAAGATTTAGCTTTGGAAAATACAC
GTGATAAAGTTAAGGATTTCAATCATGTTTCTTTAATAAAAGATGGACATGAAAATATTGAACATCATATAAATGATGCACATAAAGGTC
ATATTGATGCAGCCATCTTTAACCTAGGTTATTTGCCTAAAGGTGATAAATCTATCGTGACAAAGCCTGACACGACAATCCAAGCTATTAA
TTCATTGCTATCATTAATGTCAATTGAAGGTATTATTGTACTTGTTATATATCATGGTCATAGCGAAGGACAAATTGAGAAGCATGCATTG
CTTGATTACTTGAGTACTTTAGATCAAAAGCATGCGCAAGTTTTGCAATATCAATTTTTAAACCAACGTAATCATGCTCCATTCATTTGTGC
CATAGAAAAAATTTCTTAA

Gene: rot (repressor of toxins)

Contig: 08_NODE_1, position: 79744 to 80145, length: 402 nt, orientation: REVERSE

Perfect match to: (N315-BA000018-[1821811:1822212:r], allele observed in CC5+CC8)

Sequence:

TTACACAGCAATAATTGCGTTTAAACTATTTTGCATTGCTGTTGCTCTACTTGCAATCGCATCACTGATGAAATTCAACAACTCTACTTTCTC
TTGTTGTAACTTTTCATTAAAATGAATAATAACTGTTCTTTCATCGTCAACAGGACGCTCTTTGTAAATCCATTCTAATTCAACTAAATTATT
ATACGTTCTCGTACGCTTATACGGTTTAACTTCAACAAATCTGTCCATTTCTTTAAGCGTCATAGAACCTTTTTGCCATAAAGTTAGTAAAA
TTAAAATTTCTTCTCTAGACATTTTGTATTCGCTTTCAATCTCGCTGAAAATTGAGTTAATGTCACCCAAAAGTGTTTCTAATTGCAAAATCC
CAAATACAGTGTCGTTATTTACTTTTTTCAT

Gene: pldB1 (putative lysophospholipase, locus 1)

Contig: 08_NODE_1, position: 80719 to 81546, length: 828 nt, orientation: REVERSE

Perfect match to: (08-02119-CP015645-[311092:311919], allele observed in ST582+CC1+CC1290)

Sequence:

TTAAATTTCTACAATTTCGTCATCTTCAACAATAAAGCCCATTGTATTGACGCTGTTATTTAAGAAAGTCAGAATATAACGCATTACTTCAT
CGCGTTCTGGCTCATTGTGAACCTCGTGGTAAAAACCTTGCCAAGCTTTAAAATATAATTCAGGTGTTTGATATTTTTCTTTAAACTCATCA
ATTGCCCTAGTATCAACAATTAAATCCTTCGTTCCATACATTAATAGCGTTGGCATTGGTTGAATGTCATGAATATGAGCCATCGTATCTTT
CATCGTCTCATTAATTGTATTATACCAATGATACGTTGCTTTTTTTAACATTAAACCATCGTTAACTGTTTCTTCAACAATTTCTAAATTACGT
GTTAAATCTTTTGGTTCTACACCAACATTAAAACGTGTGTCTTTTGAAATTTTACCTATATTTGAAACAAGTTTATCTTTACGATTTTTTCCAT
TCTTTTGAAGTTCTAGCATAGGAGAAATTAACATCATCCCCTCGATTGGCAATTCTACTTTTTCAAGTAAATTTAATAAAATCAAACCGCCA
AGTCCTACCCCTAATACATAAGTAGGAATTTTATATTCATTAGCTATCTTTAACCAGTCTAGCAAACTTTCGTGATACGTTTGAAAGTTTTC
AATTTGTCCTTTATTAGCTCTTGAAGTTTGACCTTGACCAGGCAAATCTCCCATAATCACATGATAGCCATTTCTTCTTAACATCGTAATAAC
ATATGCATATCTTCCCGTATGTTCTAATATATTATGAGCAATAACAACGACGCCTTTCGCATCATTTTCAGCTTCCCACTTCCACAT

Gene: putA (proline dehydrogenase)

Contig: 08_NODE_1, position: 81780 to 82781, length: 1002 nt, orientation: FORWARD

Perfect match to: (08-02119-CP015645-[309857:310858:r], allele observed in ST582+CC1+CC15)

Sequence:

ATGGCACTATTAAAGAATTTTTTTATCGGATTATCTAATAATAGTTTTTTAAACAACGCAGCAAAAAAAGTGGGCCCACGTTTGGGCGCCA
ATAAAGTCGTTGCCGGAAATACAATTCCAGAGTTAATTAATACAATCGAATACTTAAATGACAAGAATATCGCTGTTACAGTAGACAATTT
AGGGGAATTTGTCGGTACAGTTGAAGAAAGTAATCATGCTAAAGAACAAATTTTAACAATTATGGACGCGCTTCATCAACATGGCGTAA
AGGCACATATGTCTGTTAAATTGAGTCAGTTAGGTGCAGAATTCGACTTAGAATTAGCTTACCAAAATTTAAGAGAGATTTTACTTAAAG
CAAATACTTACAACAATATGCATATAAATATTGATACTGAAAAATATGCTAGCCTGCAACAAATTGTTCAAGTTTTAGATCGATTAAAAGG
CGAATTTAGAAATGTTGGTACTGTAATTCAAGCATATTTATACGATAGCCATGAATTAGTTGATAAGTACCAAGATTTACGATTACGTTTG
GTTAAAGGTGCATATAAAGAAAACGAATCAATTGCATTTCAATCTAAGGAAGACGTAGATGCAAATTACATCAAAATAATTGAACAACGT
TTGTTAAACGCACGCAATTTCACTTCAATTGCAACACATGACCATCGCATCATTAATCATGTAAAACAATTTATGAAAGAAAATCACATTG
AAAAAGATCGTATGGAATTCCAAATGCTCTATGGTTTTAGATCAGAGTTAGCAGAAGAAATCGCAAATGAAGGCTATAATTTCACTATTT
ATGTACCTTATGGCGATGATTGGTTTGCGTATTTTATGAGAAGATTAGCAGAACGCCCACAAAACCTATCTCTTGCTGTAAAAGAATTTGT
GAAACCTGCTGGCTTAAAACGTGTTGGCATAATTGCAGCTTTAGGAGCTACAGTTATGTTAGGTTTAAGTACAATTAAAAAATTATGCCG
TAAATAG

Gene: ribH (6,7-dimethyl-8-ribityllumazine synthase)

Contig: 08_NODE_1, position: 82903 to 83367, length: 465 nt, orientation: REVERSE

Perfect match to: (MW2-BA000033-[1862774:1863238:r], allele observed in CC1)

Sequence:

CTATGCTTTTATAGATTTTAATAAATTAGCCATTTCAATTGCACTTACTGCTGCTTCGGCACCTTTATTACCAGCTTTCGTACCTGCTCTTTCC
ACAGCTTGTTCAATACTTTCAGTCGTTAAAATACCAAATATGACTGGTACATTAGTTTGATCATTTACTTTAGAAACACCTTTCGCGACTTC
ATTACAAACATAATCATAATGAGACGTAGCACCGCGAATTACGCATCCTAATGTAATTATTGCATCATAATTTCCTGATGAGGCTAATTTT
TTAGCTACTAAAGGAATTTCAAACGCACCTGGCACAAATGCTACATCAATATTGTCTTCATTAACATCATGTCGAATCAAAGTATCTTTTGC
ACCTTCAAGTAATCTTCCAGTGATAAAATCATTAAATCGACTAACTACGATTGCAACTTTCAAATCTTTTCCAATTAATTTACCTTCAAAATT
CAT

Gene: ribA (bifunctional 3,4-dihydroxy-2-butanone-4-phosphate synthase/GTP cyclohydrolase II)

Contig: 08_NODE_1, position: 83380 to 84561, length: 1182 nt, orientation: REVERSE

Perfect match to: (Strain_21193-AFEG01000006-[224356:225536:r], allele observed in CC25)

Sequence:

CTATATTAAATGACCCATTTTTATTTTTTTCGTTTCCATATAATCATGATTATGTACCGTTTCTGGTACGATAACTTCAATTCTTTCTGCAATA
TCAATGCCATATTGTTTTAATCCCTCAAATTTACTTGGATTATTACTTAATAAATTGATATGTTCGATGTTAAAATATTTTAAAATCTGTGCA
GCAATATGATAATCTCGCAAATCTTCATCAAAACCTAATGCTAAATTTGCAGTTACTGTATCATATCCTTGCTCAATTAATTCATATGCGCG
TAATTTGTTTAACAATCCTATGCCACGACCTTCTTGAGGTAGATAAATAATCATGCCACCATGTTCATTGATATACTTCATAGACGATTCAA
GTTGAGCACCACAATCACAACGTTGACTATGGAAAATATCGCCTGTAAGGCACGCAGAATGTAAGCGTACATTTTCATGTTGTCGAATTG
CACCTTTTGTCAGTACAACTATCTCTTCATCTGTGTAAGTCGCTTTAAAACCATACATATCAAATGTTCCGAAATCTGTAGGCATTTTCACTT
TTGCCTTAAATTCAATTTCTGGTTCTAATTTTTTACGATATTCAATTAAATCATCAATCGTAATCATCTTTAATTGATGTTTTTCTTTAAACTT
TTGTAAATCTTGTCCTTTCGCCATCGTGCCGTCATCATTCATAATCTCACAAATGACACCAGCGGGCTTGGCACCAGTAAGTTTAGCTAAA
TCAACAGCCGCTTCTGTGTGTCCATTTCTAGCTAATACGCCTTTATCTTGTGCTACTAATGGAAATAAATGACCAGGACGATTAAAATCTTT
AGCTTCACTACTAGGATCAATGAGCTTTTTGGCAGTCAATGTACGTTCATAAGCACTAATTCCTGTTGTTGTATCTACATGATCAATACTTA
CTGTAAATTGCGTACCAAAGATGTCGGAGTTATCATCAACCATTTGTACCAAATCCAAACGTTGTGCAATATCTTTAGACACTGGTGCGCA
TATTAATCCCCTTGCTTCTTTCGCCATAAAATTAATGGTATTATCGTTCATCCATTCAGTGACCGCTACTAAATCACCTTCATTTTCACGATT
CTCATCATCTACTACAATAATTGTTTCTCCATTTTTTAAAGCCATTAAAGCACTGTCAATATTATCGAATTGCAT

Gene: ribB (riboflavin synthase subunit alpha)

Contig: 08_NODE_1, position: 84572 to 85204, length: 633 nt, orientation: REVERSE

Perfect match to: (RF122-AJ938182-[1773440:1774072:r], highly conserved allele)

Sequence:

CTAAAAACCAAATGCTCTTAATTTATCTACAGATAATTGGTCTTTATCTTTATTTAAAATATTTTCAACATATTTAAACAAAACGTCTGTTTC
TAAATGTACTTTATCTCCTAATTTTTTGGATGATAAAATCGTTGAACGCCTCGTTTCTGGAATAAGATGAATGTCAAAACTGTTATCATGCT
TATCAAATACCGTTAGACTTACACCATCCACAGTAATAGACCCTTGCTTAACTAACTGATTATTAATATGTTGGCTACATTGAATCGTAATA
ATTTTGGCATTGGCTGTTTCATTTATTTTTGAAACTGTTCCTAGTTCATCTACATGACCGAGGACAAAATGTCCACCAAACCTACCGTTACC
ACTCATGGCACGCTCTAAATTTACTTCTGATTGTCGCTTAACATCTGCTAAATAGGTTTTATTTTCAGTGCCTTTAATTACTTGAACAGTAAA
AGATGTCTGATCAAAATCAATCACTGTTAAACATGCACCATTAACACTGATGGAATCACCAATATGCATATCTGCCGTAATCTTATGTGCT
TCGATTTCAATCGTCCTGACTGATTGACGAATTTGAACACTTTTAACGACACCTATTTCTTCAACGATGCCAGTAAACAT

Gene: ribD (riboflavin specific deaminase)

Contig: 08_NODE_1, position: 85211 to 86254, length: 1044 nt, orientation: REVERSE

Perfect match to: (MW2-BA000033-[1865082:1866125:r], allele observed in CC1+CC239)

Sequence:

TCACTTCTTTCGTAAAGTTAATTTAACATTTTGATTTAATAACTCGGAATGAACAATTTCAAATTGGTTCGCATCTGGTATCTCAATCACATC
ATTTGTTTGATAAAATTGATAATTTCCAGATCCGCCAATTAATTTCGGGGCATAATAGAGAATAAATTCATCTATATAATTAGATTGGAGA
AATTCTGAAGTAGTGGTTGGACCTGCCTCGACTAGCAAAGTTCCAACTCCTCTTTTATATAAATTGTGAAGAATTGTTGTTAAATCGCAAG
ACTTCAAGTAAATAATTTCAATATGTGTTTGATTGGTTGTTAAATTTGGATTTTCAGTATATATCCAAATTGGTGTTGATTCATCTTGATAA
ATTTGCTGATTAAAATGAATATTCCCAGACTTAGACAATATTACTTTTATAGGGTTTTTTCCATCTTGAATACGTGTAGTATATTGTGGATC
ATCTAATTCAACTGTACGTCTTCCAGTTAACACTGCGTCGTGTCGATGTCTTAACTTATAGACATCTTGTTTAACCTCTTTGTTAGTAATCCA
TTGACTTTGTCCATTATCATTCGCTTGTTTACCATCTAAACTTGCAGATACTTTCACTGTAATTTGTGGCAGTTGCTTTGCTTTTGCTTTAAA
AAAGTCTTGGTATAATTGTGATGCCCGTTCATCATCAACGCATTCAACCTCAATACCGTGAGCCCGTAACGTCTCATCACCATGTGTGTCT
AACGAATTGTCTTTTGTTGCGTAAACTACTTTAGTTATCTTACAATCAATAATTTTGTTAACACAGGGTGGTGTTGAACCAAAATGACTACA
TGGCTCTAACGTAATATAAATCGTCGCACCTTCAGCATTTTGTTGTGCCATATCAAGTGCTTGAACCTCCGCATGCTTGTCACCTTTTCTCA
AGTGTGCACCAATACCAACAATCCTACCTTCTTTAACTACAACAGCGCCAACGGGTGGATTAACACCTGTTTGACCTTGTACCATATTTGC
AAGTTGAATCGCATAATCCATAAATTGACTCAA

Gene: Q5HF04 (putative pyridine nucleotide-disulfide oxidoreductase)

Contig: 08_NODE_1, position: 86735 to 88237, length: 1503 nt, orientation: REVERSE

Perfect match to: (N315-BA000018-[1828799:1830301:r], allele observed in CC5+CC1)

Sequence:

TTATTGATTGTCACTGCGCTGTTGTTGCTCATTCGATTCTAAAGCATCATATAATTGAGATACTTTATGCGCAACTTGTTCTACAATCATTTT
CACACCATTTCGTAGTTTATTAACACCGTTTGTCATTTGACCTATCGCAATCATATTTGTTAATGTTCCAAACCTTGGACTAATAACTTGATT
GGTTTCCGGAATGATTTGTATGCCTCCCATTGGGTGTGCTTGTACAATTTGTCTATTTTCAAGATTTTTAATTAATTGATCATCTTGATCCAA
TTCATTTAAATGACTTTTTGCACCTGTCGCGTTAATGACAACATTATATATGTCTACTGATTCTTGGTTTTTGTATGAAAAATAATACAACTT
GCCATCATGTTTCACATCTTCTAAATCTTTTTTCAAAATTAAAGACTTATTTTCTATTAATTCAATAATTAGTTCAGCAGTTCTTGGAGGCAT
TGGATTTGAATTTAATTGAATCATCTTTGAGTATTTTTGATTAAATTGATGTTGGTCTTCAATACTTAAGCTATTCCATATCCAATTTAAATT
CTCTTTCAAATGTTCAATCATACTTTGGAAAATGCCCATTTCTGTTGGACGCGCTAAATCATACTTCAAATCTGCAATATGATTTCCTGTAC
GTCTATATACTAATTTTTTAAAATCAATGTCATATTCAGCACATTCTTTTAAAAATAAAGAAACTAAAGTATCAAGCGGTGCATTGCCGAA
ATGATGTTTTTTAATGTCATTTAATTTGTCTTTAGTTAAGTACTTGAATGTCACGTCTATCATTGTACCTCTTACACTTGGTAAATGAGCAGA
ACGACTCGTCATAGTAATTGGTAATTTCGGATGATGAGCAGCAACATAACGGACAACATCTAAACTGGCAAGGCCTGTACCAATAATCGC
AATATCGTCCAGTTCATTTACTTCGTCTAACGTATTATATGTTGGATAAGGCGTAGCGATATATCCTTTTTTACCCTTTAAGTTATATGGATC
ATGGTAGGCAAATGTACCACATGTTAAAAATACATAATCGTACGCTTGCCATGATTGTTCTGAATTTGTAGTACATATGTAATAAGTTAAA
TTCGTTTCATCGATATTAGAATTTGTATAAATCTCTTGAACTTTATTATAATTAGTTGATATATTTGGATATTTTTTCGTGAACATAGATAAA
TAAGATTTCATATAATGTCCGAATACAAATCTCGGTAAATATGCAGGTTCATCAAAATTAAAATCAGTTTGTTGTTTATACCACTTCCAAAA
TTCAGTCTCATCATCTAAATTTAAACTCATCTTTTTCGAAGGCATATTAATTAGCAGCTCAGAACTATCATTTTGAAATGGTACGCCCTGTC
CCATATTTACTTTATCATCGTATAAATCTATATCTAATTGATTAAACTTCGGGTGCTTAACTAACTCTCTCAATACACTTACACCAGCAGTTC
CCATGCCTATTATTGCTACACGCAT

Gene: arsR (repressor of arsenic resistance operon)

Contig: 08_NODE_1, position: 88760 to 89074, length: 315 nt, orientation: FORWARD

Perfect match to: (MW2-BA000033-[1868631:1868945], allele observed in CC1)

Sequence:

ATGACGTATAAAGAACTAGCAACATTTTTAAAAGTTTTATCAGATTCAAGCAGATTAGAAATACTAGATTTACTTTCTTGTGGAGAGTTAT
GCGCTTGTGATTTGTTAGCACATTTTCAATTCTCTCAACCTACACTTAGCTATCATATGAAAGCATTAGTAAAAACCAACTTAGTTACGACA
CGAAAAATCGGAAATAAACATTTATACCAGCTTAATCATAATATTTTTGAGTCCGTAATTAATAACTTGTCAAAGGTTCATACCTCTAACCA
ACGATGTATTTGTCATAACCTTAAGACTGGTGAATGCTAA

Gene: arsB (arsenical pump membrane protein)

Contig: 08_NODE_1, position: 89074 to 90366, length: 1293 nt, orientation: FORWARD

Perfect match to: (ECT-R2-FR714927-[1777347:1778639], allele observed in CC5)

Sequence:

ATGATGACAACTTTAGCGACACTCATTTTTCTAGTAACTTTATTATTTGTATTATGGCAACCTAAAGGCCTAGATATTGGCATTACCGCATT
AACTGGTGCCTTTATTGCTGTTATTACTGGTGTTGTAAGTTTTTCCGATGTTTTCGAAGTAACAGGTATTGTTTGGAATGCTACTTTGACTT
TTGTCTCAGTCATTCTTATTTCATTAATATTAGATAAAGTTGGATTATTCGAATGGTCAGCTATTCACATGCTTCATGCTTCAAAAGGCAAT
GGTTTAAAAATGTTCGTTTATATCATATTATTGGGTGCCATTGTTGCTGCATTTTTCGCAAATGATGGCGCAGCGTTAATCTTAACGCCTAT
TGTATTAGCGATGGTTAAAAATATAGGTTTTAGTAAGCGGGCCATATTCCCTTTTATTATTGCGAGTGGTTTTATAGCTGACACAACTTCTT
TACCTTTGATCGTGAGCAATCTAGTGAATATTATATCTGCTGATTATTTTCATATAGGATTCATTCGATATTTTAGTAGAATGATTATACCT
AATTTATTCTCACTTTTAGCAAGTATTATAGTATTGTGGTTATATTTTAGAAAGGCGATACCTAAAACGTTTGATGATAATAATATAAAGCA
TCCTAAAGATGCCATTAATGATTTAAAGCTATTTAAAATTTCATGGATTGTGCTAGTTATATTACTTTTCGGCTATCTTATCAGTGAATTTAC
TAAAATTCCGGTATCAATTTTCACTGGAATCATTGCTTTTATTTTTCTAATGTTGGCTCGTAAATCAAATGCTTTAAATATTAAGCAAGTCAT
TAAGGGCGCACCTTGGAATATAGTATTATTTTCAATTGGTATGTATATCGTCGTATTCGGCTTAAGAAATGCTGGCATTACTTTAATATTG
GCTAAAATATTAGAATATATTTCCAATTACGGTCTATTTAGCACTATTTTGGGAATGGGCTTCATTTCAGCGTTTTTATCATCAATAATGAA
TAATATGCCTACAGTTTTAATAGATGCGATTGCTATTGGTCAATCAAATGTCCATGGCATGTTAAAAGAAGGCCTAATTTATGCGAATGTT
ATCGGTTCTGATTTAGGTCCAAAAATTACACCGATAGGCTCTCTAGCTACATTACTGTGGTTACACGTCTTAACACAAAAAGATGTTAAGA
TTTCTTGGGGCACATACTTTAAAACCGGTATCATCATTACAATTCCAGTACTATTTATAACCCTCATAGGGTTGTATCTAACACTTATCATAT
TTTAA

Gene: gad-sagB (glucosaminidase B)

Contig: 08_NODE_1, position: 90453 to 91307, length: 855 nt, orientation: REVERSE

Perfect match to: (08-02119-CP015645-[300710:301564], allele observed in ST582+CC1+CC5+CC8+CC30)

Sequence:

TTACTTATTCAAATGTTTACTGTCATCTTTATACACAAAGTATTTGAAGTATTTTCCTTCAGTCTTCATGTTCTTATAAAAGTCAGCGATAAT
TGTTGCATTACTTTCTGCCCACTTAATATCAGTAGCATATTGATGTTCTCCTGGATTTTTTGGATTCCATCTCATACTATACAATGTATTTTG
ATCTGTGCTTGATAAGAAGTGCTTATGAATGAAATCAGCACCGCCTGAAATAGCTTTTTCAGGTGTATCCCAACCATGCTTTTTAGCATAT
TCTGCACCTGTTTTAATTGGGTCTTTATCAAGGGCTCCTACTCCATAGAAATTGTAGTATTTTTTGCCATCAATTTCGACTCCATTAGCTAAT
TCACTTTTAACTGCGCCAGTTTCTAATAATGCATGTGAAATTAAATAAACTTCGTTAACGTGCTTATCTTTAGCAGCTTTTAAGAAATCATC
CGTATGTTTCAATAACGTTGGTCTATCTACTAACATACGTTTAATTCTATTTTTATCAATCCCTTGATACTTTGATAAATCTAAAAATTGATA
TTTTTGCTTTTCATTATCGATAAAAGTACCGCTATCCATTGCACTTTTAATTTCAGTTGCAGATGCATCTCTCCATGCATCATTCTTTTTATTT
GATACCTGTTGACTCGTATAATTATTTATTTGTTTCTTTGCTGCATCGTTTAATGTAACATTTAACTTTTCAATCTTAATGTCGGATTTAACAT
GTTTGAAAAATATCTGATCGGATATCATTGAGAAAAATAAAAATGAGACAACAGCAAATATGACAACAAGTCCTATTATTCCAAAAATAG
AACCTTTCTTGTGTTTATTCAT

Gene: Q5HEZ9 (putative protein)

Contig: 08_NODE_1, position: 91583 to 91807, length: 225 nt, orientation: REVERSE

Perfect match to: (MW2-BA000033-[1871454:1871678:r], highly conserved allele)

Sequence:

TTACTTAGTTTCTTTTTTATAATCCATCATAATAAAACTTGCTGCATTTGATCTATTTTTATAATAGTACTGATTTAAATCAATCGTACCTTCA
ATTTGACCATCACGATACATCATTTCATTAGTGAAGTTATTTATCATCACGAAATCAGCGCGATCTCTTATAACATCTAAATCGTCGTTTCG
TGCAAAAAAATGTTCTAAATTTAAATGTGCGTAATCCAT

Gene: sigS (RNA polymerase sigma factor S)

Contig: 08_NODE_1, position: 92006 to 92476, length: 471 nt, orientation: FORWARD

Perfect match to: (MW2-BA000033-[1871877:1872347], highly conserved allele)

Sequence:

TTGAAATTTAATGACGTATACAACAAACACCACAAAATCATACACCATCTTTTAAAAAAATATAATATTAGCTATAATTATGATGAGTATTA
TCAACTACTCTTGATAAAAATGTGGCAATTGAGTCAGATATATAAACCCTCAAGCAAGCAATCTTTATCCTCTTTTTTATTCACTCGATTAA
ATTTTTACCTTATCGATTTATTCAGACAACAAAATCAATTAAAAGATGTCATTTTATGTGAGAATAATTCACCAACATTAACTGAACAACCA
ACATACTTTAATGAACATGACCTTCGTTTACAAGATATCTTCAAGCTTTTAAATCAAAGAGAAAGACTATGGCTCAAACTATACCTTGAAG
GATACAAGCAATTTGAAATTGCTGAAATCATGTCATTATCGCTTTCAACGATTAAATTAATTAAGATGTCCGTTAAGCGTAAATGCCAACA
TAATTTTAATTAG

Gene: Q5HEZ7 (putative protein)

Contig: 08_NODE_1, position: 92589 to 93032, length: 444 nt, orientation: FORWARD

Perfect match to: (N315-BA000018-[1834653:1835096], highly conserved allele)

Sequence:

TTGCAAGACAATTCTACTAAATATCTACTTTATATCCAAACTGCTACTTCAAACCATCTCGAAACAAATTGTGTCTTTTTACATTGCGATTAC
ATTCTTAAAGTTCCAATTAACAAACTCGTTTCATATTATGCGAAATTGCATTTATCATCACAAAGTGTGCTAATTGAGACTGCAAAAAACAT
ACTAAATATTAATAAACTGGTTCCTATTTACATCAACCCAAAAACTATACTTTTTCCGTTAAAACATAAACGTGCACCGATACAAATTTATA
TCAATGCACATTATATTGTTGGTATGACTGCCATAGAGAATTCAACATTAATACATTTTCAAGAAGGCATTCAGCTAGAAGTTGATGAGCC
ATTTTCTCTAGTTTCAAAAAAAATGTCATGAAAGTTTAGCTTTGAAGCACTTCATTGAAAATACTATTTCGAATTAA

Gene: Q5HEZ6 (putative membrane protein)

Contig: 08_NODE_1, position: 93019 to 93462, length: 444 nt, orientation: REVERSE

Perfect match to: (MW2-BA000033-[1872890:1873333:r], highly conserved allele)

Sequence:

CTATTTCGAATTAAATTTAATTTCTAAAAGTTTCTTAGTAAAGTATGATGTCACAATTTTTATAAAATATATGCTAACAATCATTAATAAAAT
CGCTCCAATAATTGTTAAAATAACAACAGTTGTTGATAAATCTACCTTAGGACTAACGAAAAATTGTACGCCAGATAGACTTGTAATTAGC
CCAAATAAAAACAAGCCTAATAAAGCAACATAAGCAAACATCATCACTACTAATGTAACAAACATACTATAACTTTTTGTTTCTTTATTTCG
AAATACATTTTGTCTTAAGTAAGCATTAGCAAAGGTAACAGGCTTATTTAAGTTAACGCTGTCATCATCAGATATAGAAGACAATCGTTGA
TCTAATGCTTGTTTCTCCTTCTTGTTCAAAAACCATAATTGCTTTTTAACTTTTTTATCAAATGATGCTTTATTCAT

Gene: Q5HEZ5 (putative CAAX protease)

Contig: 08_NODE_1, position: 93758 to 94393, length: 636 nt, orientation: FORWARD

Perfect match to: (11819-97-CP003194-[1902345:1902980], highly conserved allele)

Sequence:

ATGCAAAAATTCAAAGACTTTTTTTACGATGATTTATCGGTTACACGAGGAAATTATTTTTTAACTTTAATGGCAGCATTTTTTATTACTATC
ATTTTATTTATCGGCATAGTTGTCAGTGAAGTACATTTACTTTATAGCATGCTAATTGTATTAGTAGGTTTAATTCTATTGAGGCTATTCAA
AATCAATTTATTCTCTTTTAAAAAATTAACATTGTCTCAAGTTATTTATATTATAGGCGGTGCACTATTAATTTATGGGTTAGATAATCTTTA
TTTATATTTTCATGACGTACCGGCAAATGAACAACAATTAGAGCAAGAAATACGAAATACACCATTCTATATTTCTATTTTCACTGTTACCA
TCATCCCCGCTATTGTGGAAGAAATTGTTTTTCGCGGTATGATAATAAGGGTTATCTTCAGAAAACACTTGTTTTTAGGGTTAATTGTGTCT
AGTTTAGTTTTTGCATCATTACACGAATCTGACACTTGGATTGGTTATTTACCTTACTTATATTCTGGTTTGATTTTTGGTATAATTTATATA
AAAACAAAACGATTAGAAGTGGTAATATTCATGCACTTCTTAAATAACTTGTTAGCTCTGCTCTTTATAATATGGGGATAA

Gene: tal (transaldolase)

Contig: 08_NODE_1, position: 94833 to 95546, length: 714 nt, orientation: REVERSE

Perfect match to: (MW2-BA000033-[1874704:1875417:r], highly conserved allele)

Sequence:

TTATAGAATAGAAAGACCTGAAGATTGAATATCTTTCGCAAAGCCTTTAACTGTATCTACTGATAATTCGTTAATATCGCGACCTAAGTTT
GTATTCACTTTTTTCACAACATCTGCAGGGCATGTAATAATATCTGCACCAATTTCATCAGCTTGAATCACATTGAATAATTCGCGGCAACT
TGCCCATAATAATTTAACGCCGTCTTTACTATGCGTAACTTTTACAGCCTCTTTCATTAATGGTAATGGATCTACGCCTGTATCTGCAATAC
GTCCTGCAAATACTGAAACATATGTTGGCACACCTTCAGTTACTGCTTCAGTTATTTCTTTAACTTGTTCAATTGTGTAAACAGCCGTAACG
TTTAATCTCACATTGTCAGCTGAAAGCTTTTTAATTAAAGGAATCGTTGATTCACCTTTTGTATTTACAATAGGAATTTTAACAAATACATTT
TCGCCATATTGTTTTAGAATTGCTGCTTCTTTTTCCATAGTTTCTAAATCGTCTGCAAATACTTCAAATGAAATTGAAGCATCTGGAATTTCT
TTCACAGCTTCTTCAGCAAAAGCTTTGTAATCTGTTACGCCCGCTTTCGCCATTAAACTAGGATTTGTTGTAAAACCATCCACTTGTTTGTTT
TTATAAGCTGCTTTCATTTCTTCAATATCTGCACCGTCCGCAAATACTTCTACATTTAGTTTAGCCAT

Gene: Q7A500 (putative protein)

Contig: 08_NODE_1, position: 95806 to 96108, length: 303 nt, orientation: FORWARD

Perfect match to: (MW2-BA000033-[1875677:1875979], highly conserved allele)

Sequence:

ATGTCTCGTTCAAAAAAATACTTTTACTTATCTAGCTTAATGATTATTTTAAGCTTTTTCTTTAATACAAATAACGTTTTCCTAAGTGGACTTT
TTAATTCTTTTATTAAATTAATACTTTTCTGCAGTGTTATTAACTCAATTGTACTAATTTTGTCTATAATTTTTGCAGATCGTTCAATTAAATC
ACTAAAGCCTGATGCAGATTGGATTAGAATTGCGAGTAAAAGTTTGCCTTGGATTATTCTAATTGTTATTTTAGTACATATCTTTTCAATTG
TTCGTACATTCGGTTTTATTTAA

Gene: crcB1 (protein CrcB homolog 1)

Contig: 08_NODE_1, position: 96286 to 96729, length: 444 nt, orientation: FORWARD

Perfect match to: (MW2-BA000033-[1876157:1876600], allele observed in CC1+CC8)

Sequence:

ATGCATCGACAATTTTTGTCGTCGCGTTGCCAAAACCTCTTTTTTAAATTCAAACTACTTCTTTTCGAGGTGAACCAAATGCAATATGTATA
TATTTTTATCGGTGGTGCTTTAGGCGCTTTATTACGTTACCTCATTTCTTTTCTGAATACTGACGGAGGTTTTCCAATCGGAACACTGATAG
CCAATTTGACTGGTGCCTTTGTAATGGGATTGCTAACAGCCTTAACAATTGCATTTTTTTCAAACCATCCGACGCTAAAAAAAGCTATTAC
GACTGGTTTTCTTGGTGCTTTAACGACTTTTTCAACATTTCAATTAGAATTAATACATATGTTTGATCATCAACAATTTATAACTTTACTACT
ATATGCTGTAACAAGTTATGTCTTTGGTATTTTGTTATGTTACGTCGGTATAAAACTAGGTGGTGGTTTATCATGA

Gene: crcB2 (protein CrcB homolog 2)

Contig: 08_NODE_1, position: 96726 to 97079, length: 354 nt, orientation: FORWARD

Perfect match to: (MW2-BA000033-[1876597:1876950], allele observed in CC1)

Sequence:

ATGATATCAATCATTTTAGTCATGATTGGCGGCGGTTTCGGTGCAATTACTAGAAGTGCCATTACTGATTATTTTAATCATAAATTTACTTC
AAAGTTACCTATCGCAACATTGATAGTAAATCTAGTTGGTAGTTTTTTAATTGGATTAAATATAGGCTTATCAATTTCAATCTCATGGTTCC
CTGCGTTCTTTGTTACCGGTTTTTTAGGTGGCTTAACAACTTTCTCAACGTTAGCCAAGGAACTTACACTAATGATGACGCCAAAATTTAAT
ATTAACCTTTTTCTCAATTATTCACTTTTACAATTCATCATTGGATTTATAGCTTGTTATATTGGCTATCATATTTAA

Gene: tx_universal2 (rho-independent terminator)

Contig: 08_NODE_1, position: 97321 to 97359, length: 39 nt

Perfect match to: (Strain_21331-AGTV01000040-[104403:104441], allele observed in CC398)

Sequence:

TAGAATTGAAAAAAGCTTGTTACAAGCGCATTTTCGTTC

Gene: ytbE (glyoxal/methylglyoxal reductase)

Contig: 08_NODE_1, position: 97462 to 98295, length: 834 nt, orientation: REVERSE

Perfect match to: (N315-BA000018-[1841140:1841973:r], allele observed in CC5+CC1+CC361)

Sequence:

CTATCCTTCAAAAGTTTTTGGATCAGGTCCAATTCTCTTATCTTGATTTAAACCATCAATTCGCGTCATTTGTTCATCTGATAATTCGAAATC
AAATATTTGGAAGTTTTCAGAGATTCTGTTTGGTGTTACCGATTTAGGGATTGTAACCACACCATGCTGCACATTCCATCTTAAAACAACTT
GGGCAGGTGACTTTCCTAATTCTTGAGCAATGTCTTTAATTGTCTCATCATTTAAAATTTGTGCATTCATCAATGGTGACCAAGATTCCATC
ACGATATGTTGTGCTGCCAAATATAATTTCAATTTATGTTGCGTTAAATATGGATGATATTCAACCTGATTAATTACAGGTTTAATTGACAC
TTGTGCCAACAAAGCTTCCAAATGTTCAGGTTCAAAATTGCTGACACCTATATTTTTAACTTTATTATTTTTATATAAATCTTCCATACCTTTC
CATGTATCAACCATTACGGCTTCGTTCGTACCTGGCCAATGTACTAGATACAAATCTAAGTATTTTAAACCTAATCTAGATAAACTAGCTTC
GTAAGCAGCTGCTACATTTTCACGACCGAAATCCTCAAAATATAATTTTGAAGTAATAAATAAGTCTTCTCTAGCAATACCAGTTGACTCC
AATCCGGCACGAATGCCAGCACCTACTTGTTCTTCATTCCCATAAACTTTTGCGGTATCAATACTACGATATCCTTGTTCAATGGCATACTT
AACACTTTCCATGCAATTTTCATCATTTTCCACACGAAATGTCCCTAAACCAATTTGTGGCATCGTGTTTCCATTATAAAATGTTTTAACCTC
CAT

Gene: nrd (nuclease-related domain  (NERD))

Contig: 08_NODE_1, position: 98507 to 99415, length: 909 nt, orientation: REVERSE

Perfect match to: (MW2-BA000033-[1878378:1879286:r], allele observed in CC1)

Sequence:

TTAATTGAATGATTCAATTTTATCCATCATTTGTTGTAAGTCTTCCACGTTGTATTGAATACGACCATGGAATACAAATTTGTTAAAGAACT
CGTCTAATTGTTCAGCACCGACAAGCACTTTGACAGCACTATTTTGATTATAATTTGAAATCGTTACATCGCCTTCATTTTTAGGATTAAAG
TATAAAATTGAAGTTGGCGTATATTTGGCACCTAATTCTTTTTGTAAGTCTTCAGCCAATTGTTTAATCGCCTCAATTTGATCTGAATAATTT
ACAAAAGATAATGAACGTTTGTCATCATTTTGATCCATCACAATAGTTTGTGGTCTAGATTTATCTAAATCCAATGTATCAAATACTTGTTC
CATTGGTGGTAAATCTTTAAATTGACCGCCACTAATACCATTATAAACATGACCTTTTAACAATTGAGAATCAATAATATAAAGACCAGTT
CTTGTTAATACTAAATGACTAATTCGTTCAATATTATTAAAGCCATCCTTTGGTAAAAAGATATTTGCCATAATGTGCATATCTTCTGGTCG
AATTCGTTTTTCTTTAACTAATCTTTCACGAATACCAATTAATCTCATGTCCGTTACATATTCACTATGATTTTTCGAGAACAATTTTAATGC
GTCAATCTCACGATCTTTTGTACTAACCATGTGATTATAATCTTCTTGTTGTTTTGTAATTGTCTTTTTATTTTGAATACGCTCTTTCTCTAAA
GCTTCTTCATGAGACTTTTTAATGTTTTGTTCTTGTTGTTCATACTTTTCTTCTGTTTGTCGCTTAACTTTTTTCTTACTACCTAAGGTAACTAA
GAAAAGGACAAAAAAGATTAATGCAATGACTACTGCAATAATGAGTCCAATGACTATCGGTGAAGATAAATCCAT

Gene: metK (S-adenosylmethionine synthetase)

Contig: 08_NODE_1, position: 99540 to 100733, length: 1194 nt, orientation: REVERSE

Perfect match to: (N315-BA000018-[1843215:1844411:r], allele observed in CC5+CC1)

Sequence:

TTATTTTACTGCGTCTTTTAATTCTTCCACTTTGTCTAATTTTTCCCATGGGAATAAGACATCTGTACGTCCAAAATGACCATAAGCAGCAGT
TTGTTTGTAAATCGGTTGTTTCAAATCAAGCATTTTAATAATACCTGCAGGTCTTAGGTCAAAGTGTTTTCTAACTGCTTCGACAAGTTGCC
CTTCAGAAACTTTACCTGTTCCAAATGTATCAATTGCAATTGACACTGGTTCTGCAACACCAATCGCATATGCCAATTGTACTTCACATTGA
TCTGCTAAACCTGCTGCAACAATATTTTTAGCCACATAACGTGCAGCGTATGCAGCTGAACGGTCTACTTTTGTAGGATCCTTACCACTGA
AGCATCCGCCACCATGACGTGCATAGCCACCGTACGTATCAACAATGATTTTACGTCCTGTTAATCCTGCATCACCTTGAGGTCCACCGAT
TACAAAGCGTCCTGTAGGATTGATGTAGAATTTAGTTTGTTCATTAATCAAGTTTTCTGGAACAGTTGGATAAATGACATGTGCTTTAATG
TCTTCTTGAATTTGTTCAAGTGTCACATCCTCAGCATGTTGTGTTGATACGACAATCGTATCAATACGTACTGGGTTATCATTTTCATCATAT
TCAACAGTGACCTGAACTTTACCGTCTGGTCGTAAATAATTTAACGTACCATCTTTACGCACATCTGATAAACGTTTTGCCAATTGATGTGA
TAAATAAATTGCTAGAGGCATATACGTCTCTGTTTCATTCGTTGCGTAACCAAACATTAAACCTTGGTCACCTGCACCTGTTGCTTCAATTT
CTTCTTCGCTATCTTTATCACGATACTCTAATGCTTTATCCACGCCTTGTGCAATGTCAGGTGATTGTTCATCAATCGCAGTTAAAATTGCCA
TTGTTTCATAATCATAACCATATTTTGCTCTTGTGTATCCAATTTCTTTAATTGTTTCTCTAACAACTTTCGGAATATCAACATATGTTGTTGT
AGAAATTTCGCCGGCGATCAATGCCATACCTGTTGTAACAGTTGTTTCACAAGCTACACGTGCATTTGGGTCGTCTTTTAAAATAGCATCT
AATATTGCATCTGACACTTGGTCAGCGATTTTATCTGGGTGTCCTTCTGTAACAGACTCTGAAGTAAATAATCGTTTGTTATTTAACAT

Gene: pckA (phosphoenolpyruvate carboxykinase [ATP])

Contig: 08_NODE_1, position: 101105 to 102697, length: 1593 nt, orientation: FORWARD

Perfect match to: (ED98-CP001781-[1867387:1868979], allele observed in CC5)

Sequence:

ATGTCAGTAGACACATACACTGAAACAACTAAAATTGACAAATTACTGAAAAAACCAACGTCACATTTTCAACTTTCGACGACACAACTTT
ATAATAAAATCTTAGACAATAACGAAGGGGTATTAACAGAACTTGGTGCTGTTAATGCAAGTACTGGAAAATATACTGGTCGTTCGCCTA
AAGACAAATTTTTTGTCTCTGAACCTTCATATAGAGATAACATTGATTGGGGAGAAATTAATCAACCTATCGATGAAGAAACTTTCTTGAA
GTTATACCATAAAGTACTAGACTATTTAGATAAAAAAGATGAACTATACGTATTTAAAGGCTACGCTGGTAGCGATAAAGATACAATGTT
AAAACTTACAGTCATCAATGAATTAGCATGGCATAATTTATTTGCTAAAAATATGTTTATTAGACCTGAATCAAAAGAAGAAGCTACAAA
GATTAAACCTAACTTCACTATCGTTTCTGCCCCACATTTTAAAGCAGATCCAGAAGTTGATGGTACTAAATCTGAAACCTTTGTCATTATTT
CATTTAAACACAAAGTCATTTTAATCGGCGGTACTGAATACGCTGGTGAAATGAAAAAAGGTATCTTCTCTGTAATGAATTATCTCTTACC
GATGCAAGATATTATGAGCATGCATTGCTCAGCAAACGTTGGTGAAAAAGGCGATGTTGCATTATTCTTTGGTCTATCTGGCACTGGTAA
AACAACCTTATCGGCTGATCCACACCGAAAACTAATCGGTGATGATGAACACGGCTGGAATAAAAACGGGGTCTTTAATATCGAAGGTG
GCTGCTATGCAAAAGCAATTAATCTTTCCAAAGAAAAAGAACCACAGATTTTTGACGCAATCAAATATGGTGCGATTTTAGAGAACACTG
TAGTTGCAGAAGATGGTTCAGTGGACTTTGAAGACAATCGTTATACAGAAAACACTCGTGCCGCTTATCCAATTAATCACATTGACAATAT
TGTAGTACCATCAAAAGCAGCACATCCAAATACAATTATTTTCTTAACTGCGGATGCATTTGGTGTTATTCCACCGATTTCAAAGTTAAATA
AAGACCAAGCAATGTATCATTTCTTGAGTGGTTTCACTTCTAAATTAGCTGGTACAGAGCGTGGTGTGACAGAACCTGAACCATCATTCTC
AACATGTTTCGGAGCACCGTTCTTCCCGTTACACCCTACTGTTTACGCTGATTTATTAGGTGAACTTATCGATTTACATGATGTTGATGTTT
ATCTTGTTAATACTGGATGGACTGGCGGAAAATATGGTGTAGGACGTAGAATCAGCTTACATTACACACGTCAAATGGTAAACCAAGCG
ATTTCTGGCAAATTGAAAAATGCAGAATATACAAAAGATAGTACGTTTGGTTTAAGCATTCCTGTAAAAATTGAAGATGTACCGAAAACA
ATTTTAAATCCAATTAATGCTTGGAGCGACAAAGAGAAATATAAAGCACAAGCAGAAGATTTAATTCAACGTTTTGAAAAGAACTTCGAA
AAATTTGGTGAAAAAGTTGAACATATTGCTGAAAAAGGTAGCTTCAACAAATAA

Gene: ytmA (putative peptide hydrolase)

Contig: 08_NODE_1, position: 103075 to 103845, length: 771 nt, orientation: REVERSE

Perfect match to: (MW2-BA000033-[1882946:1883716:r], allele observed in CC1+CC9)

Sequence:

CTAGCTATGCAACTCAACTTGGTTCATAAACTCTTTAATATAAGTCAATGTTTCAACCATCGCTGGTGGTCTTGGCACATGTCCTTCTGCCA
TTTGATAAAATGTTTCATGCGTGGCACCTTTTAACTCTAGTTGGTCCGCTAAATAATACGCATGATGAATACCAACTTGCTGGTCTTTCCCT
CCATGTACAATTAATATTGGCGGACTGTTTTCATTAATGTTTGGAATCGCTTGGCGTGCCTCATATGCCGCTCGATCTTTTTTCGGATGACC
AATCATTCTTCGTAGCATGCCTCTTAAATCGACACGTTCTTCATACATTAAATCAATATCTGAGACACCACCCCAGATTGTATAACTTGTTA
CTGGTAAGTCTTGAAATGTCAACAATCCTTGTAAACCACCTCGCGAAAAACCAACCATGTGGATAAATGCATGTGGATATTTATCATGTA
GCAACCTTAATAATTGCGTCACATCATTTAAATCGCCACGGTAAAATTCGTCTTTACCTTCACTCCCATTGTTACCTCGGTAGTATGGCCCA
ATCACTAAAGTTTGACTATCTGAAAATTGCATTAATCTACCTGCGCGCACACGTCCTACTTGACCTTTGCCACCTCGCAAATATACTACAAT
GCGATTTACTTCATGATGTGGTGTCATCATTAAAGCTTTTACTTGTAAGTCATCTGACAAATATGTAATTTCTTCGAATTGATGCGTAAAAG
ATTCAATTGGCATTCGTTTACGTTTGATAAAACCCAA

Gene: rppH (8-oxo-dGTPase  (antimutator))

Contig: 08_NODE_1, position: 103826 to 104305, length: 480 nt, orientation: REVERSE

Perfect match to: (MW2-BA000033-[1883697:1884176:r], allele observed in CC1+CC398)

Sequence:

TTACGTTTGATAAAACCCAAGTGATTGCACCCTCTCTACGCATTTTAAAATGGTACTATCTTGCAGTAAGAAACTCCGTTGTGCGAGTTCA
ATATCATTGATACAGTTAAACAACACTGGCCCTGCTGTTTCTAAATAATCGTTCTTGCTTACCAATGATTCAACTTCGATAAAATATACATC
TTTTACAAAATCAGTTTGATCATGTGTTTCAATGGTATATTGTGCTATGTAATGAATATTTTTAACTTTGGCGCCTGTTTCTTCATATAATTC
ACGGGTAACTGCTTCAGCACTACTTTCCCCGCGTTCCCTTTTACCACCAGGAAATTCAATACCCCGTAAATTATGTTTGGTAAAAAGCAATT
GATTTTTAAACGTTGGAATAGCTAGCACATGATTGCCATCTGCTATCTCATTATCCTTTTTAAATGTCAAATTAACTTGACGATTATCTTTAT
CCCTAAACTTCACGCGCAT

Gene: ytjA (membrane protein insertion efficiency factor)

Contig: 08_NODE_1, position: 104365 to 104622, length: 258 nt, orientation: FORWARD

Perfect match to: (MW2-BA000033-[1884236:1884493], allele observed in CC1+CC10)

Sequence:

ATGAAAAAGATATTCTTGGCGATGATTCATTTTTATCAACGTTTCATTTCGCCACTCACTCCACCAACTTGTCGTTTTTATCCAACATGTTCA
GAGTACACTAGAGAAGCGATTCAATACCACGGTGCTTTCAAAGGCCTTTATTTAGGTATCCGTCGTATTTTAAAATGTCATCCGCTTCATA
AAGGTGGCTTTGACCCTGTTCCGTTAAAAAAAGACAAGTCAGCAAGCAAGCATTCACATAAACATAACCATTAA

Gene: menC (O-succinylbenzoic acid synthetase)

Contig: 08_NODE_1, position: 104619 to 105620, length: 1002 nt, orientation: REVERSE

Perfect match to: (MW2-BA000033-[1884490:1885491:r], allele observed in CC1+CC80)

Sequence:

TTAATATGGTTGTAATTGAGTTATATCCACTAAAGGGGGGCGAAATTCGAGTCGCCCCTCTTTTAATATGCCTGAATGCGCCACCACATCT
TGTTCAAAATAATAACCTGCTGGTGTAACATCTCCTGGATAATCACCTTTACGAGCAAGCATCGCTGTAAAATAACGGCTTAAACCATATT
CGTACATGCCGCCAATAACCACTTTTACACCATGACTTTTCAAAGTATCAATTGCCGTTTGCACTTTATCAATGCCACCTAGACGAAATGGT
TTTAATACAACAACTTTCACATTGTATAATTCTATCAAATTAATTATGTCCAACAACGATGTTGCCTTTTCATCAAGGGCTATTGGAGGTAT
TGTTCCATCCACTACTTCATCAAGCATGGAGATATCTTTAAATGGCTCTTCGATATAAAGAACCTGTTCACGCGCTAATAACTGTAACTGT
GTGAAATCTTGACGATCCAAGGACTCATTTGCATCTATAACCAATTGAAAGTGAAACTCTAATTCCCGTAACACTCTAATTTGATGCATGA

TTTGAGGTGTCCATTTTAATTTAATTCTGGCCGGCTTAGTTGCTTTTAATGACTCTAGTTGTTTATTTGATAAGCCGCTCACTGTCGCTCCAT
ATGCTACTGAAAATGAAGGCAGTACATGAAACATTTGATACAATGCCATGACAATAGTTGCCCTTGCAGCAGGCGTATTTTCCAATGAAT
CTACTAATTTTAGTGCTGCTTCATACGTTTCAAATGATTTATTTCTATTATCTTCGAACCATTGCTCAATTACATGTTTCACTGAAGCAATTG
TTTCATGATCATACCAATCTGTTTGAAAAGCGTTACATTCCCCGAAATATGCATTTCCTTTATCATCAATCAATTCGATAAACAAACAATCA
CGATGTGTTAAAGTGACTTTCGGTGTTACAATTTGTGACTTAAATGGCTCACTATATTTATAAAAATGCAAAGCTGTCAACTTCAT

Gene: menE (O-succinylbenzoic acid CoA ligase)

Contig: 08_NODE_1, position: 105625 to 107103, length: 1479 nt, orientation: REVERSE

Perfect match to: (TCH70-ACHH02000010-[14307:15785], allele observed in CC1+CC80)

Sequence:

TCATCCTCTATACAACTTATTTCTTTGTAATTTACCTGTTGATGTATAAGGTAAAGTATCAACCTTTTCAAAGTGTTTCGGTACTTTATATTT
CGCTAAATGTTGTGATAAATATGCAATCAATTGTGCCTTTGAAATGTCACTTTCACTGACAAAATATAATTTAGGCACTTGTCCCCAAGTAT
CATCAGGATGCCCTACACATACTGCGTCACTGATACCTGGAAATTGCTTCGCTACCGTTTCAATTTGATATGGATAAATATTTTCACCGCCA
CTAATAATTAAATCTTTACGTCGGTCATAAATCATGACATAACCTTCATGATCTATTTCAGCAATGTCACCCGTATTAAAATAACCATTTTCA
AACGTACCCGTTAAATCTGTTGGATACAAATATCCATTCATCACATTGGCACCTTTAATCATTAATTCTCCATGACCTTCTTTATTAGGATTT
TTAATTTTAACGTCTACATTGGCACTTGGCATCCCTACAGTGTCAGGACGTGCATGCAACATTTCCGGTGTTGCTGTTAAAAATTGTGAAC
ATGTCTCAGTCATACCAAATGAATTATAAATTGGCAGGTTATATTGTAATGCCGTCTCTATCAAAGTGGCAGATAATTTAGCACCGCCGAG
TAATATTTTTTGCAAATTATAAGGTTCATGTAAACCTTGTTGCATAAGCCAATTTAAAGTTTGTGGCACAAGCGAAATGTGCGTGATGCGT
TCATTTTTAATTATCGTTAAAATTTGTTCGGCATTGAATTTATCAACAATGCGCACAGTAAACCCTTCAATAACAGCTCTTAAAAGTACACT
GAGACCCGAAATATGATAAATCGGCAAGACAGATAGCCAATTAGTATCACGATCAAATCCCAAGCTCTCTTTACATCCGATTGCACTGGC
ATAATGATTACGAAACGTTTGCGGCACCGCTTTTTGAGGGCCAGTTGTCCCTGATGTAAACATAATCGATGCAATGTCATCTAAATTAAAT
GAAGTATTTAATATGTTGGACGGCGACTCTTTCGGCACCACAGTTTCATTCGATGTATCATATTGGATATCCATTGTGTTGTCCAACAAAC
CGTTCATTGTAATATCCGTTCCAGCGAATTCAATATCATCCAGCGATACAATTTGAAACCCTCGCAATTCCAGTGGCAAGGTACAAAAAAT
CAATTGTACATCGATTGACCTCATCTGATTCTTCATCTCATTAGGTGTCAACCTTGTATTAATCATCGCAATTTCAATATTTGCCAACCAACA
AGCATGTATTAAAATGATCGATTGAATCGAATTATCTATGTATAGCCCGACACGAGATTGTTGATAAGCCTTGAGTCTTTTAGCCAATAGA
CTCGCTTCACAGTATAAATTTTGATAAGTATAAGATTCTTGACCGTCTGTTATCGCAATATGATGTCCATTTTGTTGTGCTTGTTTATATAAC
CAAAAGTCCAT

Gene: Q5HEY1 (lipoprotein)

Contig: 08_NODE_1, position: 107262 to 107744, length: 483 nt, orientation: REVERSE

Perfect match to: (MW2-BA000033-[1887133:1887615:r], allele observed in CC1+CC9+CC80)

Sequence:

TCAGTTATTTTTATTTAATTTTAGTGTCATTCTGTCATTTTGATGTGGTGATTTACCCATTGTTGCCACATCATCTGCAATGTCAATTGGTAT
ACGATTCATGTCTTGTAATACACTTAAATGGAATACTTCATCATCTAAATTTTCAATGAGATATACATAATATGTTATCTTATCCTTTTTATA
TTTTAACGTTTTCCAAAAGTCCGACTTGCAATTCAATACATTATCCGGAATATATTCAATAAATAAGTAACGTTTGCTGCCTACTTTGTCTA
CGAAATATTTTACAGTGCCTTTTTCTATACCTCTTATATGTGCATAGTCTGCTGAAAAGTAAATACTACCTATTGTTTCATTATGTTGTTGTA
TTTCAAATCGTTGGCCTACTATTTTATTATTTGTGCCACAACTACTTAAAAAAATCAGTAGATATAGCATTAAACATATTTTCATCCCCTTGA
ATTTTAAAGACTTTTTCAA

Gene: Q5HEY0 (putative calcium binding protein)

Contig: 08_NODE_1, position: 108020 to 108670, length: 651 nt, orientation: FORWARD

Perfect match to: (MW2-BA000033-[1887891:1888541], allele observed in CC1+CC9+CC80)

Sequence:

ATGAAAATAACATATAAATATAGAGGAGATTTACCTTTGAATACAGAGAACAACAAGAATCAAAACCGATCTGTTAAAAATTCTGAAAGA
CGTGGCATGTTAAAAGGATGCGGCGGTTGCCTTATTTCTTTCATTTTATTAATAATCTTATTATCAGCCTGTTCAATGATGTTTAGTAATAA
TGACAATTCCACTAGTAATCAATCATCAAAAACGCAATTAACTCAAAAAGATGAAAATAAAAATGAAGATAAGCCTGAGGAAAAATCAG
AAACAGCAACAGATGAGGATTTACAATCAACCGAAGAAGTACCCGCAAATGAAAATACTGAAAATAATCAACATGAAATTGATGAAATA
ACAACAAAAGATCAATCAGATGATGAAATTAACACACCAAACGTTGCAGAAGATAAATCACAAGACGACTTGAAAGATGATTTAAAAGA

AAAGCAACAATCAAGTAACCATCATCAATCCACGCAACCTAAGACCTCACCATCAACTGAAACAAACACGCAACAATCATTTGCTAATTGT
AAGCAACTTAGACAAGTATATCCGAATGGTGTCACTGCCGATCATCCAGCATATCGACCACATTTAGATAGAGATAAAGATAAACGTGCA
TGTGAACCTGATAAATATTAA

Gene: DUF4352 (immunoprotective extracellular protein)

Contig: 08_NODE_1, position: 108751 to 109746, length: 996 nt, orientation: FORWARD

Perfect match to: (MW2-BA000033-[1888622:1889617], allele observed in CC1+CC6+CC9+CC80)

Sequence:

ATGAGCAATCAATTCAAAAGCGAAGAAGAGCGAAGACAATGGGAACAATTCCAAGCTTTCCAAAATCAACAAAACCAACAGAACCAGCA
ATACGGACAAAAGAAATCTAAAAAAGGATGGTTCTGGGGCTGTGGTGGTTGTCTAGTATTATTTATTTTAATTATCATCGGTATTTCAGCT
TGTACAGCTGGTATTACAGGTAACCTTGGCGGAAATAGTTCTAAAGAAACGAACAAAACCCATAAAATTGGAGAGACTGTAAAAAATGG
TGATCTTGAAGTCACAGTTAATTCAGTTGAAACTATGAAATCAGTTGGTCCATCTATCGCGCCAACAAATGCTAAAGGTACATTTGTCGTT
GCTGATGTGACGATTAAAAACAAAGGTAAAGAAGCGTTAACAATTGATAGTTCAATGTTTAAACTGAAATCTGGTGATAAAACATTTGAA
GCAGATAATACAGGCTCAATGTCTGCTAATCAAAATGACAACGGTAGTATAGAAAATTCATTTTTCTTACAGCGTATAAATCCTGATAGCA
CTGCTCAAGGTAAAATTGTTTTCGATGTGTCAGAAAACATAGCCAACGCAAAAGATAAAAAATTAGAAGTTATTTCTAGTTTATTTAGCGT
CAAGAAGATTACATTTGATTTATCCGATGCTAAAAAAACATCAAAAGCTAAAAAAGACAAGCAAGATACAGAAGTAGCTGCTGCGAGTT
CAAATAGCGATAATGTAAGTTATGAAGCTTCGGCTACTACACCTGCTACAACTTCTAGTGCAAATAATGATTCTGAAGAAAATGAACAGT
CTAGTAAAGATGAAGATAAGCAGAATGCGTCTAAAAGTGATAAATCTAGTGTAGAAAAAAGTGAATCTAATGAAGAAACTGCTACTGTA
GAATCAGCACCTCAAAGCAAACCTGTTACAAGTGAAGCACCACCTAACCAAAATAATCACAACGAAGATAGCATGTACGACGCTTCAACA
GAATAA

Gene: Q5HEX8 (putative lipoprotein)

Contig: 08_NODE_1, position: 109820 to 110446, length: 627 nt, orientation: FORWARD

Perfect match to: (MW2-BA000033-[1889691:1890317], allele observed in CC1+CC80)

Sequence:

ATGAAATTCAAAGCTATCGTTGCCATTGCATTATCATTGTCACTATTAACTGCTTGCGGTGCTAATCAACATAAAGAAAATAATAGTAAAT
CAAATGACACTAATAAAAAGACGCAACAAACTGACAACACTACACAGTCAAATACAGACAAGCAAATGACACCACAAGAAGCCGAAGAT
ATCGTTCGAAACGATTACAAAGCAAGAGGTGCTAACGAAAATCAAACATTAAATTATAAAACAAATCTTGAACGAAGTAATGAACATGA
ATATTATGTTGAACATCTAGTCCGCGATGCAGTTGGCACACCTTTAAAACGTTGCGCTATTGTTAATCGACACAATGGTACGATTATTAAT
ATTTTTGATGATATGTCAGAAAAAGATAAAGAAGAATTTGAAGCATTTAAAAAGAGAAGCCCTAAATACAACCCAGGTATGAGTGATCA
TGATGAAACAGATGGTGAGTCAGAAGACATTCAACATCATGACAATGATAATAACAAAGCAATTCAAAATGACATACCAGATCAAAAAG
TCGATGATAAAAGTGATAAAAATGCTGTTAATAAAGAAGAAAAACACGATAACGGTACAAATAATTCTGAAGAAACTAAAGTTAAATAA

Gene: Q5HEX7 (putative protein)

Contig: 08_NODE_1, position: 110487 to 110831, length: 345 nt, orientation: FORWARD

Perfect match to: (MW2-BA000033-[1890358:1890702], allele observed in CC1+CC80)

Sequence:

ATGATAACATTTGAAAATATACAACAACTTGAAAAATACACCTTAATGACTATGCATGGTCTTTTTAATCAACTTAAACTCGGTATTATTTC
AATCGACAATGCAGAGCATACGCTCTTTACACCTTATATGATGGAAACACTCTCTTCCCTAGGCATGAAAGACAGCATTGTCGATTTAATT
CATAAAGGGACTGAATTAGAAGACTTTGCAGCATTTAATTTATCAATTGAAGATACAGTTACAGTCTGTTTACAAAGAACTGAAGAACTA
TTAAATCAATACAAAAATGTGGAATTCAATGACAAAATATTAATCAATTGGCGTATTATGCAAAAGTAATAG

Gene: A5ITW8 (putative protein)

Contig: 08_NODE_1, position: 110929 to 111501, length: 573 nt, orientation: FORWARD

Perfect match to: (MW2-BA000033-[1890800:1891372], allele observed in CC1)

Sequence:

GTGGAGAAAAATGAATATATAGCTAAATATAATGAATATAGTCAATTATTAGACGCTACATACTCGCAAGCCGTAGCATACCTTTTAAAT
AAATATGGCGCTGTAACCGATGACTATTATAAGGAAAAATCATACACGCGATTTTTAAATGGAGAAATCAAAAGTATTACAAAAGGAAA
ATACACTAGAGCTGGTGAAGGATTATATTGTCATCACATAAGCGAAGACAAATTCCAAAACTTATCTGACCTAAGATTCATTTCCGAATTT
AAGTACTCATACAACTATCAAAAGAAAGAAAACTTAGTTTACTGCGATTTAATCGAGCATTTAATTTTACATGCAATTATTACAAAAGAAT
CCAATGGCCAATTTGGTGTGGCTGGATTATGTCAAATGATCAAACCAACAGTCATTGATTGGTACATTAGCGAATATAATCCAAAACCAG
CATGGATGCAAGCCACCAAAGCACGTGCCTATTTGCCTAGAATATTAGTAGAGAAACTACTCATTAAAATTGACGATATGTTAAAAGAAA
TAGAAATATATGATTTCCTTGAGTCTAGATAA

Gene: Q6GFQ3 (putative protein)

Contig: 08_NODE_1, position: 111650 to 111768, length: 119 nt, orientation: TRNC-RVRS (no start codon)

Perfect match to: (MW2-BA000033-[1891521:1891559:r], highly conserved allele)

Sequence:

TTAGTTTTCTTTTGGTTTTAATAAATCGACTAGATTTTCACAATATTTATCAAATATGTATTCCTAAATTATACAGCCTTAATCCAGCAGCTA
CTTTCGAAACTTCCAACTTAGTTGAT

Gene: DUF955-L1 (putative bacteriophagal protein)

Contig: 08_NODE_1, position: 111699 to 112256, length: 558 nt, orientation: REVERSE

Perfect match to: (11819-97-CP003194-[1920162:1920719:r], allele observed in CC80+CC1+CC6+CC80)

Sequence:

TCAAATATGTATTCCTAAATTATACAGCCTTAATCCAGCAGCTACTTTCGAAACTTCCAACTTAGTTGATATAAGGTTCAATAGTTTGTTTC
ATTCTTTTTCAGATAAACCAGAACTTAAATTGATATTATTGACTTCATAAAAATTATAGACTAATGCCTCTATTTGCTTTTTAGGCATAAGTA
AGTCGACTGAAAACTGATTTACGTCGCTTTCATAAATCATTTCATGTAAATTCTTTAGACTATTATCGTTGTTATCTTTAATTTTGTATAAAT
AACGGCCGAGTTCACGAGCTATTGCAAATCTTGTATTATTAATCGAGTGATTATTATTGATATAAATCGTTCTTCCACTTAAATAACCCGAA
GTATTACCCTCCATTTTAATATATTTAACTTTTAAATTAAGTTGAAATAATAGCTTGTCTATGTCAATAGCAAAGTGTTCAGAAGTAATAAA
AAGTTGATCCATTTTGTCCTTAACTAATGGCTGAATTAATTGAACTATTTCTGGTTCTAAAATATCTTCATAATGAACTTTCTCAATAACTTT
CAA

Gene: Q5HEX3 (putative protein)

Contig: 08_NODE_1, position: 112816 to 113199, length: 384 nt, orientation: REVERSE

Perfect match to: (MW2-BA000033-[1892688:1893071:r], allele observed in CC1)

Sequence:

TTACTGATATCCCCATTTTTCTCCTGTCAGAACGTATTCCCCGGCTGCATTCTTTTTAGTTTTTAATTTTATATATATTCCCTTACTAGTATCA
ATATTCGATGCAGCTATAGATCCAAGGAAACCACCAACACCTGCGCCAACTAAACCACCTGTTAGTGCAGAAATAACGCTAATAGCCCCC
GCACCTAAAGCAGCTATTGTTTTTGTATATGCAGAAGAAAGATATAATGTTGCAGTATCTTTACCTGTTTCTACATATTGAGTTTTACCCGC
TCTTAATTGGTCTTCAGCTTTATATTTTTTTATTTCTTCTTTTGTAAATATATCTTCCAGTTTATAACCTTTTTTCTCAAGTTCATCAAATAAAT
TTTGGTTACTCAA

Gene: tnpIS3 (transposase IS3 family fragment)

Contig: 08_NODE_1, position: 113605 to 113970, length: 366 nt, orientation: TRUNCATED

Sequence:

GATACTGAATATTGATGTGCATTCTTTTGAATGACTTCTATTTTTGCCCCATAATCAGCGCTACTTGCTTTAAAATATCGTTCTCCATTTTTA
AATGTTGAACTTCTTTGCGTAATTTAATCAGCTCTTTTTCTTCATCCGATAAGTTATCTTGGTGATTGAATGTACCCGTGTTTTGATGTTGCT

TTATCCATTTTCCTACATTTTATAACCGCCATTTACAAACGTCGAAGGTGTGAAATCATACTCGCGTATAATTTCATTCCTAGGCTTACCATT
TTTATATAATCTAACCATTTGTAACTTAAACTCTGAACTAAATGATCTTCTTTCTCTTGTCATAATAAAATCGCCTACTTTCTTAAA

Gene: tnpIS3-var2 (transposase IS3 family truncated)

Contig: 08_NODE_1, position: 113669 to 113946, length: 278 nt, orientation: TRNC-FRWD (no stop codon)

Perfect match to: (COL-CP000046-[1906458:1906735:r])

Sequence:

TTGCTTTAAAATATCGTTCTCCATTTTTAAATGTTGAACTTCTTTGCGTAATTTAATCAGCTCTTTTTCTTCATCCGATAAGTTATCTTGGTGA
TTGAATGTACCCGTGTTTTGATGTTGCTTTATCCATTTTCCTACATTTTATAACCGCCATTTACAAACGTCGAAGGTGTGAAATCATACTCG
CGTATAATTTCATTCCTAGGCTTACCATTTTTATATAATCTAACCATTTGTAACTTAAACTCTGAACTAAATGATCTTCTTTCTCTTGTCAT

Gene: sprA-L1 (small pathogenicity island RNA A, Locus 1)

Contig: 08_NODE_1, position: 114098 to 114316, length: 219 nt

Sequence:

AGTCGCCTATCTCTCAGGCGTCAATTTAGACGCAGAGAGGAGGTGTATAAGGTGATGCTTATTTTCGTTCACATCATAGCACCAGTCATC
AGTGGCTGTGCCATTGCGTTTTTTTCTTATTGGCTAAGTAGACGCAATACAAAATAGGTGACATATAGCCGCACCAATAAAAATCCCCTCA
CTACCGCAAATAGTGAGGGGATTGGTGTATAAGTAAAT

Gene: sprA-L8 (small pathogenicity island RNA A, Locus 8)

Contig: 08_NODE_1, position: 114098 to 114308, length: 211 nt

Sequence:

AGTCGCCTATCTCTCAGGCGTCAATTTAGACGCAGAGAGGAGGTGTATAAGGTGATGCTTATTTTCGTTCACATCATAGCACCAGTCATC
AGTGGCTGTGCCATTGCGTTTTTTTCTTATTGGCTAAGTAGACGCAATACAAAATAGGTGACATATAGCCGCACCAATAAAAATCCCCTCA
CTACCGCAAATAGTGAGGGGATTGGTGTAT

Gene: ldr_fst-L1 (hemolytic peptide)

Contig: 08_NODE_1, position: 114149 to 114244, length: 96 nt, orientation: FORWARD

Perfect match to: (N315-BA000018-[1856535:1856630], highly conserved allele)

Sequence:

GTGATGCTTATTTTCGTTCACATCATAGCACCAGTCATCAGTGGCTGTGCCATTGCGTTTTTTTCTTATTGGCTAAGTAGACGCAATACAAA
ATAG

Gene: DUF1433-var9 (DUF1433 family protein)

Contig: 08_NODE_1, position: 114816 to 115193, length: 378 nt, orientation: REVERSE

Perfect match to: (MW2-BA000033-[1894688:1895065:r], highly conserved allele)

Sequence:

TTATTTATTGGACGTATTTTGCTCTTTTTTTATTTCAGAAACTGACTTAGGATTTTTATTAAATTTTCTACCCAATTCATCTGTATAAGAAATA
TCGGTATCAAATTGAAAATCATCAACAGATCTTATACCAGCTGTAAATGATAACTTTTTATCATTATTTATATAACCACTAATAGAATAACC
ATCCATTGGGTTTTCTTTAAAATTAGCGAAGCTTATATTTTTATAACCTTTCACATTGTACTTCATATAAATCGTTATTCTTTCTTTTTGTTCA
TCATAATATTTCTTTTCGTCATATTTCATCTTCAAAAAGATTCCGCCTAGAATCAATATTAAAATTATAATTACAATCATAAGATATTTTTTAT
TCAA

Gene: Q2FXD2 (putative NTPase domain protein)

Contig: 09_NODE_6, position: 160 to 631, length: 472 nt, orientation: TRNC-RVRS (no stop codon)

Sequence:

CGTGATCTTGCTTATTTATGTTTAAATTCAAAAACTCTTCGCATAAGTCTTGAGTTAAAATAATATCTTTTTTACTATCGAATAATATATTAT
GTGGATTTGAAAAATTCTTATTTTTAATATTATTATTTTGATTTCCAATAAATATAAACTTTAATCGATAACCTTCTTCCATTTTATTTGTTAA
ATACTCTTTTTTTAGAGTGTCCTCTATTTTTTGCTTTTTACACGTTGCGGTAACTTGAATAATAATTTTATTCGTATTATCTATAAGATCAAAT
GCTGGTGCATTTTTTTGTTTTTTATTATGATTCTCAAGTGAATACCCATATACAATATTTAACAGGTCTCTAAAAAAATATTCGGAATATAT
ATGATTATTTAAAATATTTATCTTTCCTAGTTCTTCAATCTTAGTATTAAGTCTAATTAGTGTATCAAGAAATTTTTCTTGATTCGTTGATCGT
TTCAA

Gene: hsdS-2 (type I restriction-modification system site-specificity determinate)

Contig: 09_NODE_6, position: 1481 to 2680, length: 1200 nt, orientation: REVERSE

Perfect match to: (ATCC51811-ADVP01000004-[795:1994], allele observed in CC1)

Sequence:

TTAAATAAACATTTTTTGTAATAGTCCTTTTTTACGTTCTTTTAATAACTCAATTCTATTCATTTGATTATTCATTTTATTGTCTATAGATTTTA
ACAATGCACTTACTTTATCTTGTTCAGTGAGACAAGGTATTTTAAGATTTATATTGCATAAGTTCGCATGAGTTAATTTAGCTGGTGCATTA
CCTGTGACAAATGCTCGTAGTTCTTTAAAATTTAAATAATAATTCATAAAAAACAAATTATGATCATTACTTTTAACTACATGCGCATGATT
ATTTACCCAGTATTGCCCATTAGCAATAAAGCTACTCGTCTCAAACTGCCCCCATTTTGCACCATCTTCTCCTATTAGTAATCGTTCTTCATT
ATTGAATAAATAATCTTTTACGTAATCAATAATTCCAGTTGCACCATAGTAAGGGTATAACCCCTTTTCTCTTAATGAAGAAGTAATTGGTT
TTCTTCTATTATTTTCAAAGATAAAGATGTCTTTTATGAATTTGTTTTCCCACTCTGGATATTCTTCACCATTCTCATCTTTGAATCGCAATTC
TTGTGTGAAGATTTTCTGCATATAGCCTTTTTTCTGTTGTTGAAGCAATTCAAGTTTTTGTTCTTCTAATTCAATTTGTCGGTCGAGTTTGCT
GAAGAACTTGCCTATTTTTTGCTGTTCTTCAAATATAGTTGGGGTGAAGATTTTTAAATTAGCAATTTCTTTGAAGTTTAGACCTTCTCGAC
TACCTCCACTTTGTGCAAGGAAAATTTTCCTTTTACCTTTTCTTGATAATAGATACTGTCCAAAAAAATTATAATAATACTCTTTTTTCAATCT
AATAATACATACATGTTGATTTAAATTAGCATGCGTTTCAACTATCGAATTAATGGCTGTTCTACCTATTGATGCTCCTGTAATATTTAAAA
GAACATCACCATAGTACGTTCTACTATTTTTCATCTCATCATCTATATCTTTACTAATATAAACTAAGTCATTAAGATTTAATTTACCATTTCT
AATATTTTGACTCCTTAAAAATGGTATGCCTTTGTTTGTATAGTTTTCACTTCCACCTTTGGGAGTCTTTCCACTACCTATTTTGGTAGTAAG
ATTCCCTAACTTCTTCTCTTCCCATTCGCCCTCAAATCCTGGGAATCTCAACTCTGGCACATTTTTCGTTTGTGTATTACTCAT

Gene: hsdM (type I restriction-modification system DNA methylase)

Contig: 09_NODE_6, position: 2673 to 4229, length: 1557 nt, orientation: REVERSE

Perfect match to: (N315-BA000018-[1859152:1860708:r], highly conserved allele)

Sequence:

TTACTCATCTTTCAACACCCCAAGTTCTTTCAGGTATGCATTGATTTCTTGTTCAATTTCTGCGATTTCTTTGTCGATATTTTTCAAATCTTGT
TGGACTTGATCTAAATCAATTGGTGCTTCTTCTTCGAATGTATCAACATATCGCGGTATGTTTAAGTTGTAATCGTTATCGGCGATCTCTTG
TAATGTCGCGCTGTAGCTATATTTATCAATCGTTGCTTTACGCTTATATGTGTCTATAATACGTTCGACTTGGGCATCGCTTAAATGGTTTT
GATTTTTTCCTTTTTCAAAATCATTGGATGCATCGATAAATAGTACGTTGTCGTCTTGTTGGCGACATTTTTTAAATACTAAAATACATGTT
GGAATACTTGTCCCATAGAAAATATTGGCTGGTAACCCAATCACGGCTTCTAAGTAGTTCTTTTCTTCTATTAAATAGCGACGAATCACAC
CTTCTGCGGCACCACGGAATAATACACCATGTGGGAGTACGACTGCCATGGTACCTTCATCGTCTAGGTAATGTACCATGTGTTGAATAA
AGGCAAAGTCTGCTTTGGATTTTGGCGCAAGCTTGCCGTAACCACTGAATCGTTCATCATTTTCAAATTTTGAATCTGCTGTCCATTTCGCA
CTGTATGGTGGGTTCGCAATAACCGCATCAAATGTATTGCCTAAAAAGGCTGGATTTTCCAATGTGTCATCATTACGGATCTCGAAGTTCT
CATAACGCACATCATGTAATAACATATTCATGCGTGCTAAGTTGTATGTAGTATTGTTACGTTCTTGTCCGAAATAACGATACACTTGCGTT
TCTTTACCAACACGTAACAACAATGAACCGGAACCACATGTTGGGTCGTACACGTGACGTAATTTATCTTTACCGTCTGTGACAATCTTCG
CCAGTATCTTAGATACTTGTTGTGGTGTATAGAACTCGCCTGCTTTTTTACCCGCTGTCGCCGCAAAGCGCCCGATTAGGAATTCATATGC
ATCACCTAACATATCAATTTCCATGTCACTGTGAACGAATGGTAAGTCGTCAAGATTAACCATGACTTTAGAGATTAAAGCAGTACGTTCT
TTGACATTGTTACCTAGTCGCGTTGAACTCAAATCCATATCGCTGAACAGCCCGATAAAGTCATTTTCACTTTCTTCACCTAATGTAGATGT
TTCAACTTTGCGAATCGCCGTCGCTAGATGTTCTATATCGAAATCTTGCGTTTCAATTTCACGAATCATCGCACTGAATAAATCTTGTGGCT
CAATGAAGTAACCGACTTGATCAATTAATTCTGCTTTTAAGTCTTCACGATATTCTTCATCTGCCCATGCTTCTTGATACGTGATGTCTTCAC

CTGACAAGGCATCTGCATATTCTTGTTCGGCTTTTTCAGATAAGAAGCGATAGAAAATCAAGCCTAAAATGTAATTACGGAATTCACTCGC
ATCCATGTTCCCTCTTAAATCATTCGCAATCGACCATAATTTTTTATGTAATTCAGCTTGTTGCTGACGTTGTTTTTCAGTAATAGACAT

Gene: splF (serine protease F)

Contig: 09_NODE_6, position: 4592 to 5311, length: 720 nt, orientation: REVERSE

Perfect match to: (COL-CP000046-[1916577:1917296:r], highly conserved allele)

Sequence:

TTATTTATCTAAATTATCTGCAATGAATTTCTTAATTTCAGGAGAGAAATAAACAGCAAATCCTCTTGTGCTTTCACCTGATGGCTTATTAC
CGGCATAGATTACACCAATAGCTTCGTGTTTACTATTTAATATAGGTGAACCAGAGCTACCAGGCTGAATAATTGCATCCGATGACACTAT
ATTCCCATTCACTGATAATACTTTACCAGTTGATTCATACATTTGTAGTTTATTTCCATTAGGATTTGGATAACCAATGACTGATATAGGTTC
ATTTTCTTTAGCTTCTGATGCTATATTAAATTTACTAGTGAAATCTTTGAATTTTCTACCTTTTGGTTGTGTTGATTTTTCTTCAACTTGTACA
ACCGCAATATCTTCTTTACCAGGATAATCTACAATCTTAGTAACTTTATAAAGTCCACCACCGTTATTATAAAAACCATTAGGATGTGCTTT
GATTTCATCACCGACTTTCATGTGATAGGTAACATGTTTATTGGTAATGATTGTATGATTTCCAACTACAAATCCTGTTCCAGCGCCCATCC
ATGTAACACCACTGTATGGTGCAACATTTGTATTTGTAATTTGTTTAACAGTATTTTCGGCTTTGGCTGTTTGTTGAATACCTTCAACCATT
GTTGTGCCGACACCAGTTATTGATGTTAAAATCGTCAATGCTGCAATACTTTTGATGATTATATTTTTATTCAT

Gene: splD1 (serine protease D)

Contig: 09_NODE_6, position: 6336 to 7055, length: 720 nt, orientation: REVERSE

Sequence:

TTATTTATCTAAATTATCTGCAATAAATTTCTTAATTTCAGGAGAGAAATAAACAGCAAATGACCTTGTACTTTCACCTGTTGGTTTATCAC
TAGCATACATAACACCAATTGCTTCTCGCTTACTATTTAATATAGGTGAACCAGAGCTGCCAGGTTGGACAACCGCATCAGATGTCACTAT
ATTTCCATTCACTGATAGTACTTTACCAGTTGATTCATACATTTGTAGTTTATTTCCATTAGGATTTGGATAACCAATGACTGATATAGGTTC
ATTTTCTTTAGCTTCTGATGCTATATTAAATTTGCTAGTGAAATCTTTGAATTTTCTACCTTTTGGTTGCGTTGATTTTTCTTCAACTTGTACG
ACCGCAATATCTTCTTTACCAGGATAATCTACAATCTTAGTAACTTTATAAAGTCCACCACCGTTATTATAAAAACCATTAGGATGTGCTTT
GATTTCATCACCGACTTCCATGTGATAAGTAACATGTTTATTGGTAATGATTGTATGATTCCCAACTACAAATCCTGTTCCAGCGCCCATCC
ATGTAACACCACTGTATGGTGCAACATTCGTGTTGGTAATTAATTTCACACTATTTTCTGCTTTGGCTGTTTGTTGAATACCATCAACCACT
GTTGTGCCGACACCAGTTATTGATGTTAAAATCGTCAATGCCGCAATACTTTTGATGATTATATTTTTATTCAT

Gene: splC (serine protease C)

Contig: 09_NODE_6, position: 7176 to 7895, length: 720 nt, orientation: REVERSE

Perfect match to: (Strain_21259-AFTS01000022-[179823:180542:r], allele observed in CC72)

Sequence:

TTATTGTTCAATGTGCTTTTGAATAAAATCTTTGATTTGAGGCGTAAAGTATACGGCACCATTATATTCAGAACCAATTTTTCCAATACCGC
CATACACCACACCTATGACCTCATTGTTAGAATTTAGAACTGGTGATCCTGAATTCCCGGGTTCAATGTATGCATCAAAATTTAAAATATT
GTCTTTGATTCTTTTTATAGTTCCTGTAGATTCAAACTGTTTAAAACTATTTTGAGCAGGTAATGGGTAACCAATAACTTTAATTTTGTCATC
AACTTTAGCATCTTTCGCAAAATTTAATGCTTGGACATTTTCATTAAAATTAAAGCCTTTTGGTCCACGTTCAACTGCTTGTTCTTCAATATT
CATGACAGAGATGTCTTCATCACCCGGATAATCAGAAATGCTTTTAATTTTATATATACCACCATTTCCTTTGTCACCGTCTGGATGGGCAG
TAATTCTATCGCCAACTTTATAATCTTTTGATACATGTTTATTGGTGATAATTGTATTTTTTCCAATTACAAAACCTGTCGCATCTTTAAATG
AAACGACGCCATTATATGGAAAAATATTTGTATCTTTAACTTGCGTAACATTCTTCTCTGCATTTGCTATTTGTTGTGTCTCTTCAACGACTG
CAGCATTTATTCCAGTTACTGAGGTTAGAATGGCTAATGCTGCCATGCTTTTAATGACTATATTTTTATTCAT

Gene: splB (serine protease B)

Contig: 09_NODE_6, position: 7953 to 8675, length: 723 nt, orientation: REVERSE

Perfect match to: (MW2-BA000033-[1905276:1905998:r], allele observed in CC1+CC25+CC72+CC97+CC834)

Sequence:

TTATTTATCTATGTTTTCTGCAATGAATTTTTTAATTTCTGGTGTAAAGTAGACGCCATATGCATTTCTGTTATCATCATTTTTTACATCAGA
AGCAAAATGAATACCAACTAATTCGTTGTTGCTGTTTAATACAGGTGATCCAGAGTTTCCGCTTTCAGTATGCGCTGAATATACAATACTG
CTACCTTCTACTGACATCACAGGGCCAGTTGACTCATATAAAACATATTTATTTTTGTATGGGTGTGGATAACCAATCACTTTAATTCGCTC
ACCAGCTTTAGCCCCTGCCGCATATTTGAATGGCGTTACATTATCATTAAAATTAAAGCCTTTTGGTCCACGTTCTATTGCACGCTCTTCAA
CTTGAATGACTGATACATCTTCTTTACCTGGATAATTAATAATCTTTTTAATCGAATAAATACCACCATTACCTTTATCACTATTTGGATGTG
CAGTAATTCTATCGCCCACTTTGTAATTTTTCGACACATGTTTATTTGTTAAAATAGTATTCTTTCCAACTACAAATCCAGTTGCACTTTTAA
AAGCAACTACACCAGTATATGGAAAAATATTTGTATCTTTAACTTTTGTGACATTATTTTCTGCTTTGGCAGTTTGTTGTACTTCCTCAACCA
ATGTTATTCCAATACCTGTTACAGATGTTAAAATTGTTAATGCTGCTAAACTCTTGATGACTACGTTTTTGTTCAT

Gene: splA (serine protease A)

Contig: 09_NODE_6, position: 8800 to 9507, length: 708 nt, orientation: REVERSE

Perfect match to: (TW20-FN433596-[1968024:1968731:r], allele observed in CC239+CC1+CC8+CC239+CC4803)

Sequence:

TTATTTTTCAATATTATTTTGAATAAATTCTTTTAATTGTGGTGTGAAATAAACACCGAAATTCTTTTCAGATTCATCTTTTCCACTACCTGCA
TATAAAATACCAATCAGTTCATGTTTAGAATTCAATACAGGAGATCCTGAATTACCTGGTTGTGCATACGCATCAAATTCCATAAACGTTC
CACTGATATGGTTAATCGTTCCTGTCGATTCAAACATTTTATATTTTGTTTGTGCACCCTTTGGATAACCAATAACAGAAATTCTATCTTTCA
CTTTTGCTCCGTCTGCAAATTTTGTATAACTAACGTTCTTATTAAAATTCAAACCTTCTGTACTTGTTTCATGAACATGAACTATCGCAAGGT
CTTCTTTTCCGGGATATTCTACAATGTCTTTAACGTCGTAGTTTCCTCCGCCTTTACCTTTACTCGAATGATGTGCTGATACTCTATTTTTAA
AAATATCATTACTTTTAGCGATATGTTTGTTAGTTACGATTGTATTTTTACCAACAACTACACCAGTACCACCCACAAATGCTACCACTGAA
TTGTATGGTTCCTTAGTTGCATCGGTAATTTCTTTGACATTCTTTTCTGCTTTGGCAATTGAATGAGGCTGATTAGAAATATTTTCAGCAAA
ACCAAGAGATGTTAAAATAGTTAAAGCAGTTAAACCTTTAACCATTACATTTTTATTCAT

Gene: A6QHZ7 (putative protein)

Contig: 09_NODE_6, position: 9967 to 10158, length: 192 nt, orientation: FORWARD

Perfect match to: (MW2-BA000033-[1907290:1907481], allele observed in CC1+CC8+CC239+CC4803)

Sequence:

ATGGAATTTTTTTCGAGATATGTTATTTCGGGGATCAGATTTTGCGATTTTGCCTTGGATAGAGTAGTTGCGATTTGTAAATACGTTTTTCA
GGTTCGATTCAATTTCGTTGTTAAGATAATATGGATTAGTTTCACTGACATGTTGATGCATGTCTTTTTTGTTCTGATAAAGTATAAAATGT
TTACTTAA

Gene: Q2FXC0-ear2 (putative protein)

Contig: 09_NODE_6, position: 10471 to 11037, length: 567 nt, orientation: FORWARD

Perfect match to: (MW2-BA000033-[1907794:1908360], allele observed in CC1+CC8+CC239)

Sequence:

ATGAATACAAAATTTTTAGGTAAAACATTAGTAGCAAGTGCTTTAGTATTAACAACATTAGGAACAGGCTTACATTCTTCATACTTAGGAT
TAGATACAAATAAAGTTGTTAAAACGGCTAAAGCAGAAGAAAAAATGACAGATGGTCAATTATGGAAAAAAGTTAAAGATTCATTACAT
GATTCTGATATTATTTTAAGTAACGAGTACGAAACAATCAACGTTACTTATTTATTAAGCAATGGATATTCTAGCAGCGTTTCTGCTCCTGG
AAATGATGATGGAGGCCATCTAACACAATCTATAGACTTTAAAGGATTAAAACAAATTGATTTAACTAAGGAAAATGTCTACGATGACTT
TAACAAAAAGCTAGATGCTAAAAATACATGGAATTCTCTAACCGAGAAGTTAAAAGGTCTTGGTTTACTACAAAATGGCCAAAAAGTATC
TATTTATTCTTCAGATAGCTCTTCTCCAGTCTCGGGTAAAGTGGGAGAAGGAGTAACTAGTGGTGGCGAAAATACTCTAACTAAACGCTT
CATCAACAAAATCACAATTGATTAA

Gene: sprB (small pathogenicity island RNA B)

Contig: 09_NODE_6, position: 11039 to 11509, length: 471 nt

Sequence:

CCTTAAACTCAAATTTCCTTAATTCATAAAACATTAATAACTAACCCTTTCTTACAAACCACCTAGCCCATAATATATTAAAACGTCATTTTT
ATTTCAATTAGCGGAAAATAAAAAAACCATTCCGCATTAACTTTGGCAAGTTGGAATGGTTAAAAAAATATTTTAGAAGCCAGCGTTTGG
CTTGCTTAGACGACATGTTCGCGCATGTCGTCTTATTTTTACATTTATATAATAACACGCAATTCAATAAAATGTCTAATCAATAAACCTCA
ATTACATTCAAATTCAAAAACTTTTCACCTAATATTCATCGCTTAAATAAACATTACGTTTAATTAAACAGACATTGTCAATCGCGTCTAATA
CCCCTGCTGTCCGAAAACAAAATTATTTTGTATTCATATTTAACTGTTCTCTTTCCTCCCTTTTCAACGCAACTAAATTTCTTTTACCGTTAGA
GCCCCAATC

Gene: epiG (lantibiotic ABC transporter, transmembrane permease)

Contig: 09_NODE_6, position: 11511 to 12209, length: 699 nt, orientation: REVERSE

Perfect match to: (MW2-BA000033-[1908834:1909532:r], highly conserved allele)

Sequence:

TTAATCTTTAATTGTTCTACCGCTCCATTTATTAAATCCTTTAAAGAGTAAAACTGCTAATAGCAACGTGATAATAATATAGATTGCCAATG
TTAATGTAACTGGTATACTCCCTTCGATAAACATATAAACGTAACGTGTAGCATATGTGATTGGTAAATAGAACCACGAATGATCTCCAAG
CACTTCTAATCCAAAATAAACGTTAAAAATAAACATTAAAACTCCGACAACAATAGCCATTACATCTTTAATGAAAATACTAAAAATAAAA
AGTAGCAGTAATATAATTACATTGAAAAACAATGATACGCCTATAAACATAAGTGTTATTTTCATATCATGTGAATGCCACAATAAATTAA
TTGATGCTAATAGAATACATATGGCTGTAATAGTATAAGTAAAAATCATTGATGCATTTAACCAATTTAGCCTATTAGCTTTTCCTAAAATA
TGATTAAAGTGACCAATATTTTCTTCAAAATTGATAACTTGATAGACGTTTATAGAAATTAATAGCGATGTAATTGCATTAAAACTCGCTG
TAAACAAACTTATTTGTCGACCATTCCATAAATTTACGTTTAAATACCAATTTATAAATAATATAAACAATATGGTTACAATAATGGGTACA
AATGTGAGCGCTTGCTTAGAAAATTTCAACTTACATGACTTTAATTCGTTAATTATCAT

Gene: epiE (lantibiotic ABC transporter, transmembrane permease)

Contig: 09_NODE_6, position: 12206 to 12967, length: 762 nt, orientation: REVERSE

Perfect match to: (MW2-BA000033-[1909529:1910290:r], highly conserved allele)

Sequence:

TCATTTTAATCGCCAACTTTTCTTATTATTTAAAAATGTTACTATCGCGAAAATAATGATGCTAACAATAATCGAAATATATAAGGCATTGA
GATCATTAAAGTATTTTGAATTATGGTTTACAACTAAACCATTAGGTAGTATACCAAGCGTAATAAGCGGGATTCTCTGCATTATCCCCCA
CGGCAACACCCAAAATAAACTCTTCAATGTTAAAAACAAGACACAAATGACTGATAATACTAAGTTTATTAATATTGATACGAACACACCA
AAGTATCGAGTTAATAAAAAGTTGAGCGGTATCAATGGTAGAGATACTACATACATCAACAATATTGTCACCAATAACAACATAGCATTA
ACCGGATGTGGATTAATAATTAGGTCACCTATATAAGCAATAATAAATACTAAAAAGCAATGTACCAAAAATGCTATTGATAAAATGAAA
ATCTTTGCTCTTATTTCTTTTGTAATCGACCAATTATTACTTAAGTAATAATTAAATGATTTATTTCTCATTTCAATTTTAAATAACGAATTAC
AAGCCATACATAATACAATCGGGATGAAAGCAATTGGCCAAATATTAAATAGTAAAGTTATATATGGTGACACACTATTCGCTGTTCCCG
TATTACTTTTGGCGAATAAGACTGTGAAAATAGCAAAACAAAGAAATACCAGCGGACTTATTAATAAAACATACATACTGAAAGAATTTT
TAAATTTGATACCTTCTATCTTTAAATGTATCAT

Gene: epiF (lantibiotic ABC transporter, ATP-binding protein)

Contig: 09_NODE_6, position: 12964 to 13656, length: 693 nt, orientation: REVERSE

Perfect match to: (MW2-BA000033-[1910287:1910979:r], allele observed in CC1)

Sequence:

TCATTTGTAATCACCTTTCGTTATTTTGAAGAATATCTCTTCTAAGTTTTCATCTTTGTTATTTCTTTGCTGATATTTTAGCTCACCCTCATGA
ATAATACCGATATGATCTGCTAAAACTTGGATTTCAGATAAAATGTGACTTGAAATAATAATACTAGTACCTTGTTCTGTTAATAATTTTAG
AAGTTCTCTAAGTTCTTGGATTCCATATGGGTCTAAACCATTAGATGGTTCGTCTAATACTAAAATTTCTGGCTTTTTAATTAATGCCATTG
CAATTCCAAGTCTTTGTTTCATACCTAAAGAAAAGTCCTTAACCTTTTTCTTTTTATCGACATTTAAATTGACTAAGCTTAAAACACTATTAA
TTTCGCTGAAATCAACGCTTTCATTTAAACACACAATTTTCAAATTATCTTGTGCACTTAAATGATTATATGTCGCAGGTGTTTCAATAAGC
GAACCAACTTTGTGCAAATCATTTCGTGTCATTGGTCTATTATCTAATTTAATTTCCCCTGAATCTTGTTGAAGTATGCCACATATAATTTTC
ATAAGTGTCGTTTTACCGGCTCCATTAATACCAAGTAATCCATAAACTTCGTTATTTTCTAAAGTGATATTAATATGCTTTAATACATCTTGA
TTTTTAAACCGTTTCGAAATGTTTTCAGTAACTAATTTTGCCAT

Gene: epiP (autocleaving serine protease)

Contig: 09_NODE_6, position: 13679 to 15052, length: 1374 nt, orientation: REVERSE

Sequence:

TTAACTTGCTTTTTGATTTGCTACATTTAATGCTTTATACACATCAAGCTCACCATGCCCATATCTACTAAATGGTTTATTATTCTTAGATGTC
CCATGCTGATATAACAATTCAATCGCTTTATCTGGATGTTTTTCAAGATGATATTTATCAATGATTAAAGCTAGTGCACCCGAAACTTTAGG
TGTGGCTAATGAAGTTCCAGCTTGATAAATATATCTTCCGTTATTGGCAGTAGTTAAAATGTTCTCCTTATGCATATACCCTTCATTCATCC
ATTTATCCACACCGAATTGATTTAAATAAGCAAATGATCCTCCGGGCGCAGCAATATCTGTATAATTCATACCAAAATTGGAAAACTCAGA
TAGATTACTCTTTTGATCTGTAGATCCTACTGTAACGACATTGTCCATAGATGCAGGAACATCTTTCACTTCGCCATTACCTTGATATTCAC
GCTGTAATTTTAGTTTCTGTTTGTCATTGACATCAATACCATCATTACCAGCTGCAGCAACAACGATAGATTTTTTCTTCTTGGCGTAATTG
ATTGCTTTCTGTAACGCATCGTATTCTACTTTTTCATCTTTTCTAAATGTTTGATGGTCATTTTTGTCCAAAATAATATAACTACCAACACTAA
TATTAATGACTTGATTTCCATCATTTGCAGCTTGAACAATCGCTTTTGATACCCAAAGCAGTTCTGTTTTTTTACTACCAAACACGCGATAC
ATTGTAAATTTGTTATTCGGTGCAACACCTATTAACTTACCATTAGCACTCGTTTGACCCGACACCATCGTGCCATGTCCTTTCCTATCATTG
ACATCGTGAACATCACCTGTTTCCTCCGGTTCAGTACCTCTAAAACCGTTTAAAGGTACTAAATTTTTAGAATCAGTCGAGAAATTATTTTT
CAAATCGTCATGGTTTTTCATCACACCTGTATCTATGATTGCTATTTTGGTGTTAGCATGTTTTGGCAAATCATCATACGATGCACCATTATT
GGTTATTTTATTCATATCCCATTGTCTTGAAAATAATGACTCATTTGATGTTCTGTCTATTGTTTTCTCGCTAGTAATACAAGTTGAACATGT
GGCATTGATATATTTAATATCATTTTTATAGTTTGCTAAAGCATTAGCATGCATTTTCGTCATTTTAATCTGTGCCACATGTAATTCCGGTAT
ATTATAGACAACGTTTAAGGACTTCTTTTTAACTAACTTGTTAAATGTTGCTGTATTTTTGTATTCAACACTGTAATATAGTTCTTCTGACGC
TGATGCATTACTCATTGTAATAGATATTAAAAGACTCAATATTATTAAACTAATAATAGCGCGCTTTATGATTTTCAT

Gene: epiD (lantibiotic epidermin biosynthesis protein EpiD)

Contig: 09_NODE_6, position: 15062 to 15580, length: 519 nt, orientation: REVERSE

Perfect match to: (MW2-BA000033-[1912385:1912903:r], highly conserved allele)

Sequence:

TCAAATGAATTCCAGAACTTTATATGGTTCAGGTGCGACAACATTCTTTTTAAATGTTTTTGACGCTAATTCATAACTTTCTGAAATATTTG
CTGGATATATTGATACACCATAATCTTTTAATAATCGAATATTATTTTGAGTAACTGGATTTTCCCACATTCGTAAATTCATATTTGGAAAT
ATAGAAAGTTTTTCAAAAGCTGTATGACAAATAGTTAATAATAAATTGTCACATATACCATTTGCAATTTTATTAATCGTATTAGAAGTCGC
AGGTAAAATAATAATCTTGTCATGTTTATTTGCTATATCTACATGATTTAAAAAAGGGTCTTCAAATTCGTCATAATAGTTATCGCAAAATT
GCTTTAATATTTCACCATTAATAAACTTTCTACCGTTTGTTGACGCGATTACATTTACTTCATCAAATTTTGATTTTAATTCAATAATATAGT
GGCTAATATTGATACTATTAACTGAGCCACACAAACAAATCAATACATTTTCTCCCAT

Gene: epiC (lantibiotic epidermin biosynthesis protein C)

Contig: 09_NODE_6, position: 15596 to 16840, length: 1245 nt, orientation: REVERSE

Perfect match to: (MW2-BA000033-[1912919:1914163:r], highly conserved allele)

Sequence:

TCAGCTAATTAAAAACGCATTGGTCCAATTTTTTTCATTGAGAATTTTTGTATTTTTCAGTGTCATCAATGTTAGATAGACCCCTAATTCACC
TTCTAAAAGGCCAACTTTCCTACTTTTAAATACATCTTGCTTATTTTCATTAATGTCTATAAAATTCACCAGATAATCTTCCTTATAATGAGA
AATCAGTTTATTTATTAATACAGTGATATAATCAGACACTTCATTTAATTCAAAATTCAAATTCATAATTGTTAACATTAATATTTGTGATGA
CAATCCATGACAAATAGTTGGACTTATTAAATTTTCATCTTTATCATTTACTACTTGTAGCATCACTTTTTTAGACATTTTAATTATTTCATCA
TCTTGTAATGCTTGGCCGATTAAAAACAACGTCGTCATTACACCCGTATTGCCATAACACCAACCATTCCGAATAAAATTGAAATGATTAC
GTTCAGAAATTAAATCGTAACGCTGCAACCATCTTTCGTGGTTACAAAATTTTTCGTCCATGATAAATTTGTACATGTCTTTTAATATGTGC
TGATGATTTTCAATCGTAATTCCTTTAATCACGCATAATGCAAATAAAGACATCGGTCCTAGCACTCCATGTGCAAGGCCAAGATTGATAT
TACCTTCAGTAAAATAATTTTTATCAGACTCTAAAAATTGACTTTCTTGTGGGACTACCCAGCTATTTTTAGAATATTGAATATCTTTAAAGT
ATACTAATATGCTTTTTAATGCTTTAACATTAAATTCATTCTCATCTACTCTATTTAGCAAATATCTCCCTATCCCAGCTAATCCACTGACAGT
ATCATAATTTAATGGATTTAATGGTTCTTGTAATATTTGATCAAATTTATTTTCTATTTCATTTACAATGTGACCATCAATATTATTTAAAATA
TTTTGATAATTTTTGCCATTTTGCGATGCTAAATCCATTGAAAATGCAATACCTGAAAGTCCACCAAACAAAGAATTGTTATATTGACCACT
CTGTAAAATATGGTGCTAATTTCATGATGTACTTATGCACAATTTGCTCAGTATTAATATCGAAAACTTTTTGATAAGCATCTAAAAACAAAA
TGATACCTGGTATACCATGTGATAAAGTTGAAGGTTCAAAATAATCGGTTTCAGTAGATGCTTTTGAAATGAAATCATCTACTTCTGAAAT
CTCTTGAAACTTCTTCTTCAATATTAGATTCATATCAATCAT

Gene: epiB (lantibiotic epidermin biosynthesis protein EpiB)

Contig: 09_NODE_6, position: 16833 to 19826, length: 2994 nt, orientation: REVERSE

Perfect match to: (MW2-BA000033-[1914156:1917149:r], allele observed in CC1+CC8+CC80)

Sequence:

TCAATCATTTTTGTCACCACACCAATGCTTTTGAGTTTTAACAATTTCTTTTACAATAGATAAAACAAATGTTTCTTGATCCTTATCAATGCC
AAATATTCGATTGCAGCGCATATGAATTAAGCTGCCAATAATCCTTGACCTTTTAGTAGTTAAAGTTTTTTTAAGCCCTTCATTTAATTTGCT
CAAAATTTGGTTGCCATCTTTTAAAATTCGATAAATACCACTATAGTGTTTAGCCATATATTCAAAATTATTAGCAGGATTACATATAGTTA
CAAGTTCATTTTTAAAAGGTCTAATATCTTTACTTTTAAATGATGTAGGTACATAGTTATTAACAATTTCCATACGCTCTTCATTAGAAATTG
AAAACATCTCAAATAAATACATTATTGAAATAGCAACAATATATGGTCGATCAAACTTAAAATCATTTTCAATAATATTGACAGCTAAATT
CGTGTCATATTCAAAGAAGGCTTCTATTTCATTAATCGTGTTCTTTCCACCATATCTAAAAAATTCTGGCTCGTATGATACAAATTCATAAT
CTGAAACTTCTGTCGTTTGTCGAACATGTGGTAACCAATTCTTCAAGATGCTGTATATTTCTGCGTAATTTTCGTCATTCCTGAATAACCTC
AACTTTAAAATGTCACCTTGTTTGATATAACGCATTAAAAAATATTGATCTATATCTCCCTTATCTTTAAGATGTTTTACAAATGGATACAA
ATTGTCGATGATAAATGTATCTTGCGATGGCTTATCGATATGAATATGTAATGCAAACCAGTCTTTGTCATATTCAATATCTGTATTTTTAT
ATTTTGGAATTTCGATTTCGGGTCCTCGATAAGATGATTTTTTATAAATTGGTGTAACAATTTCATACACATGATCATTCTTTGATTGAGGT
AAAAATGATTCTACTAAACGTACACGTTTATGCTTCTTATATTCTTTCATCAATAAATATCTATGATTAGCTAATGATAAATTTAGTAGAAG
CTTGTTATCTCCATAAACTAAATTCACGATATTAGGTATATTGAATTGTTCTTGATATTTCAAAAATTGTTGATCCCATTCTTCATTTTTAGG
TAAAGGTAATACCATCTCGTTAATTTTCCAATATGCTGGTTTAAGAATGACATTTTTATATCTGATTCGTGGTGAATAACTAAATGAATCAA
TACTCACATCATTCATAGGTTCAATGCATTTCACAGATTCCATTGAAATTTCTCGTAACAATCGATATAAATTACATTCTTTTAAAAAGTTAT
[truncated: 899,441 more chars]
