# Supplementary material for: Molecular investigations on a chimeric strain of Staphylococcus aureus sequence type 80
Source: PLoS One. 2020 Oct 14;15(10):e0232071. doi: 10.1371/journal.pone.0232071 (PMC7556507; doi:10.1371/journal.pone.0232071)
Supplement: S5 File — S5 Table/3, Genes in Deviating Region 2 in comparison to canonical CC80, to Oerebro-086360 and to canonical CC1. (PDF) [file pone.0232071.s005.pdf]

Supplemental file 5, Table 1: Genes in Deviating Region 1 in comparison to canonical CC80, to Oerebro-086360 and to canonical CC1.

	**Comparison of Dresden-275757 to CC80 reference sequence 11819-97 (CP003194)**	**Comparison of Dresden-275757 to Oerebro-086360**	**Comparison of Oerebro-086360 to MW2**	**Provenance in Dresden-275757**	**Provenance in Oerebro-086360**
walR	0 mismatches/702 bases (0%)	2 mismatches/702 bases (0.28%)	4 mismatches/702 bases (0.57%)	CC80	unknown
walK	5 mismatches/1827 bases (0.27%)	12 mismatches/1827 bases (0.66%)	14 mismatches/1827 bases (0.77%)	CC80	unknown
walH	18 mismatches/1335 bases (1.35%)	36 mismatches/1335 bases (2.7%)	36 mismatches/1335 bases (2.7%)	CC80	unknown
walI	0 mismatches/789 bases (0%)	19 mismatches/789 bases (2.41%)	19 mismatches/789 bases (2.41%)	CC80	unknown
walJ	17 mismatches/801 bases (2.12%)	0 mismatches/801 bases (0%)	18 mismatches/801 bases (2.25%)	unknown	unknown
sasH	95 mismatches/2319 bases (4.1%)	0 mismatches/2319 bases (0%)	65 mismatches/2319 bases (2.8%)	unknown	unknown
orfX	16 mismatches/480 bases (3.33%)	0 mismatches/480 bases (0%)	14 mismatches/480 bases (2.92%)	unknown	unknown
**ACME-III (see Table 2 and Suppl. file 6)**	*Not present in 11819-97 (which has SCCmec IVc instead)*	Identical element (see text and Supplemental file 7)	*Not present in MW2 (which has SCCmec IVa instead)*	ACME-III	ACME-III
**Q6GKL1**	*Not present in 11819-97/in canonical CC80*	0 mismatches/576 bases (0%)	*Not present in MW2/in canonical CC1*	unknown	unknown
**Q6GKL6**	*Not present in 11819-97/in canonical CC80*	0 mismatches/159 bases (0%)	*Not present in MW2/in canonical CC1*	unknown	unknown
**ORF-CM14**	*Not present in 11819-97/in canonical CC80*	0 mismatches/780 bases (0%)	*Not present in MW2/in canonical CC1*	unknown	unknown
dusC	29 mismatches/987 bases (2.94%)	0 mismatches/987 bases (0%)	26 mismatches/987 bases (2.63%)	unknown	unknown
**A6TXM6**	23 mismatches/204 bases (11.27%)	0 mismatches/204 bases (0%)	25 mismatches/204 bases (12.25%)	unknown	unknown
**A6QD71**	31 mismatches/297 bases (10.44%)	0 mismatches/297 bases (0%)	32 mismatches/297 bases (10.77%)	unknown	unknown
**Q5HJT2**	*Absent from Dresden-275757 and canonical CC80*	*Absent from Dresden-275757 and Oerebro-086360*	*Absent from Oerebro-086360 but present in canonical CC1*	unknown	unknown
**Q6GD34**	*Absent from Dresden-275757 and canonical CC80*	*Absent from Dresden-275757 and Oerebro-086360*	*Absent from Oerebro-086360 but present in canonical CC1*	unknown	unknown
**A6QD75**	*Absent from Dresden-275757 and canonical CC80*	*Absent from Dresden-275757 and Oerebro-086360*	*Absent from Oerebro-086360 but present in canonical CC1*	unknown	unknown
**A6QD76**	*Absent from Dresden-275757 and canonical CC80*	*Absent from Dresden-275757 and Oerebro-086360*	*Absent from Oerebro-086360 but present in canonical CC1*	unknown	unknown
**A8YZ18**	*Absent from Dresden-275757 and canonical CC80*	*Absent from Dresden-275757 and Oerebro-086360*	*Absent from Oerebro-086360 but present in canonical CC1*	unknown	unknown
**Q6GKK6**	22 mismatches/612 bases (3.6%)	1 mismatches/612 bases (0.16%)	17 mismatches/612 bases (2.78%)	unknown	unknown
**Q7A890**	*Not present in 11819-97*	1 mismatches/3153 bases (0.03%)	104 mismatches/3153 bases (3.3%)	unknown	unknown
**Q2YUT2**	1 mismatches/483 bases (0.21%)	0 mismatches/483 bases (0%)	2 mismatches/483 bases (0.41%)	CC1 or CC80	CC1
plc	26 mismatches/987 bases (2.63%)	0 mismatches/987 bases (0%)	1 mismatches/987 bases (0.1%)	CC1	CC1
***lpl*-SAOUHSC_00052**	17 mismatches/771 bases (2.2%)	0 mismatches/771 bases (0%)	1 mismatches/771 bases (0.13%)	CC1	CC1
***lpl*-SAOUHSC_00053**	97 mismatches/771 bases (12.58%)	0 mismatches/771 bases (0%)	1 mismatches/771 bases (0.13%)	CC1	CC1
***lpl*-MW0073**	100 mismatches/693 bases (14.43%)	0 mismatches/693 bases (0%)	0 mismatches/693 bases (0%)	CC1	CC1

	**Comparison of Dresden-275757 to CC80 reference sequence 11819-97 (CP003194)**	**Comparison of Dresden-275757 to Oerebro-086360**	**Comparison of Oerebro-086360 to MW2**	**Provenance in Dresden-275757**	**Provenance in Oerebro-086360**
***lipC3*-MW0074**	42 mismatches/1377 bases (3.05%)	0 mismatches/1377 bases (0%)	0 mismatches/1377 bases (0%)	CC1	CC1
**Q8NYT6**	17 mismatches/2238 bases (0.76%)	1 mismatches/2238 bases (0.04%)	20 mismatches/2238 bases (0.89%)	CC1	CC1 ?
**Teg15as**	0 mismatches/244 bases (0%)	0 mismatches/244 bases (0%)	0 mismatches/244 bases (0%)	*Related in all lineages in question*	*Related in all lineages in question*
**Q8NYT5**	23 mismatches/1179 bases (1.95%)	0 mismatches/1179 bases (0%)	1 mismatches/1179 bases (0.08%)	CC1	CC1
norC	27 mismatches/1389 bases (1.94%)	0 mismatches/1389 bases (0%)	0 mismatches/1389 bases (0%)	CC1	CC1
nptA	14 mismatches/1662 bases (0.84%)	0 mismatches/1662 bases (0%)	0 mismatches/1389 bases (0%)	CC1	CC1
**Q2YUS5**	17 mismatches/1776 bases (0.96%)	0 mismatches/1776 bases (0%)	1 mismatches/1776 bases (0.06%)	CC1	CC1
**DUF1648**	0 mismatches/474 bases (0%)	0 mismatches/474 bases (0%)	0 mismatches/474 bases (0%)	*Related in all lineages in question*	*Related in all lineages in question*
***lctP*-locus1**	20 mismatches/1593 bases (1.26%)	0 mismatches/1593 bases (0%)	0 mismatches/1593 bases (0%)	CC1	CC1
txbi_lctP	1 mismatches/72 bases (1.39%)	0 mismatches/72 bases (0%)	0 mismatches/72 bases (0%)	CC1	CC1
txbi_proteinA	1 mismatches/62 bases (1.61%)	0 mismatches/62 bases (0%)	0 mismatches/62 bases (0%)	CC1	CC1
spa	Dresden-275757: *spa* t 1849<br>**07-23-34-33-13**<br>11819-97: *spa* t044<br>**07-23-12-34-34-33-34**	Dresden-275757: *spa* t 1849<br>**07-23-34-33-13**<br>Oerebro-086360: *spa* t1242<br>**07-23-12-34-34-16-34-33-13**	Oerebro-086360: *spa* t1242<br>**07-23-12-34-34-16-34-33-13**<br>MW2: *spa* t128<br>**07-23-23-21-16-34-33-13**	*Related in all lineages in question*	*Related in all lineages in question*
tx_sarS	0 mismatches/63 bases (0%)	0 mismatches/63 bases (0%)	0 mismatches/63 bases (0%)	*Related in all lineages in question*	*Related in all lineages in question*
sarS	5 mismatches/753 bases (0.66%)	0 mismatches/753 bases (0%)	0 mismatches/753 bases (0%)	CC1	CC1
sirC	7 mismatches/999 bases (0.7%)	0 mismatches/999 bases (0%)	0 mismatches/999 bases (0%)	CC1	CC1
sirB	4 mismatches/996 bases (0.4%)	5 mismatches/996 bases (0.5%)	0 mismatches/996 bases (0%)	CC1 or CC80	CC1
sirA	0 mismatches/993 bases (0%)	6 mismatches/993 bases (0.6%)	9 mismatches/993 bases (0.91%)	CC80	CC1 ?
sbnA	0 mismatches/981 bases (0%)	1 mismatches/981 bases (0.1%)	0 mismatches/981 bases (0%)	*Related in all lineages in question*	*Related in all lineages in question*
sbnB	1 mismatches/1011 bases (0.1%)	2 mismatches/1011 bases (0.2%)	1 mismatches/1011 bases (0.1%)	CC1 or CC80	CC1
sbnC	4 mismatches/1755 bases (0.23%)	9 mismatches/1755 bases (0.51%)	0 mismatches/1755 bases (0%)	CC80	CC1
sbnD	3 mismatches/1257 bases (0.24%)	0 mismatches/1257 bases (0%)	3 mismatches/1257 bases (0.24%)	CC1 or CC80	CC1
sbnE	16 mismatches/1737 bases (0.92%)	17 mismatches/1737 bases (0.98%)	8 mismatches/1737 bases (0.46%)	CC1 or CC80	CC1
sbnF	0 mismatches/1740 bases (0%)	8 mismatches/1740 bases (0.46%)	13 mismatches/1740 bases (0.75%)	CC80	CC1 ?
sbnG	4 mismatches/777 bases (0.51%)	4 mismatches/777 bases (0.51%)	1 mismatches/777 bases (0.13%)	CC1 or CC80	CC1
sbnH	5 mismatches/1203 bases (0.42%)	3 mismatches/1203 bases (0.25%)	2 mismatches/1203 bases (0.17%)	CC1 or CC80	CC1
sbnI	3 mismatches/765 bases (0.39%)	4 mismatches/765 bases (0.52%)	2 mismatches/765 bases (0.26%)	CC80	CC1
**Q5HJP4**	*Absent from Dresden-275757 and 11819-97*	*Absent from Dresden-275757 but present in Oerebro-086360*	1 mismatches/408 bases (0.25%)	CC80	CC1

Supplemental file 5, Table 2: The ACME-III element in Dresden-275757 and Oerebro-086360.

Gene	Description/gene product and comments	Orientation	Start position in SCC	End position in SCC	Start position in genome	End position in genome	Comparison of Dresden-275757 to Oerebro-086360
orfX	23S rRNA methyltransferase with the SCC integration site being located at the 3' end of *orfX*.	Forward	1	480	33682	34162	0 mismatches/480 bases (0%)
**sRNA6**	Antisense RNA associated with *orfX*.		181	464	33862	34146	0 mismatches/284 bases (0%)
**DR_SCC**	Direct repeat of SCC, 19 nt of the 3' end of the coding sequence of *orfX*.		462	480	34143	34162	0 mismatches/19 bases (0%)
dam5	Type II restriction-modification system, endonuclease and methyltransferase. A reference sequence for this gene is from strain K12S0375, GenBank JYGF01000026.1 [127750:130506].	Forward	775	3522	34456	37204	0 mismatches/2748 bases (0%)
**helicase**	DNA helicase, associated with *dam*, putative restriction system. A reference sequence for this gene is from strain K12S0375, GenBank JYGF01000026.1 [130502:132466].	Forward	3512	5477	37193	39159	0 mismatches/1966 bases (0%)
**"YeeC"**	YeeC-like protein. 1381/1442(96%) identities and 4/1442 gaps compared to strain FORC_090, GenBank CP029198.1:39824-41262 (FORC090_0030)	Reverse	5480	6920	39161	40602	0 mismatches/1441 bases (0%)
**A9UFT0**	LPXTG protein homologue. A reference sequence for this gene is from strain C427-ST42, GenBank ACSQ01000048.1 [74:295].	Reverse	6685	6906	40366	40588	0 mismatches/222 bases (0%)
**Q9KX75**	Putative protein, encoded on SCC elements. A reference sequence for this gene is from strain C427-ST42, GenBank ACSQ01000048.1 [310:813].	Reverse	6921	7423	40602	41105	0 mismatches/503 bases (0%)
**Q7A207**	Putative protein, encoded on SCC elements. A reference sequence for this gene is from strain C427-ST42, GenBank ACSQ01000048.1 [829:1143].	Reverse	7439	7750	41120	41432	0 mismatches/312 bases (0%)
**Q7A206**	Putative protein, encoded on SCC elements. A reference sequence for this gene is from strain C427-ST42, GenBank ACSQ01000048.1 [1230:1580].		7752	7838	41433	41520	0 mismatches/87 bases (0%)
ccrB-1	Cassette chromosome recombinase B, type 1. A reference sequence for this gene is from strain JCSC6690, GenBank AB705452.1 [7432:9057:r].	Reverse	8654	10282	42335	43964	0 mismatches/1629 bases (0%)
ccrA-1	Cassette chromosome recombinase A, type 1. 1268/1352 (94%) identities and 4/1352 gaps compared to strain JCSC6690 SCCmec type IX, GenBank AB705452.1 [10431-10962].	Reverse	10303	11652	43984	45334	0 mismatches/1350 bases (0%)
ORF No KK12	513/532 (96%) identities and 1/532 gaps compared to strain JCSC6690 SCCmec type IX, GenBank AB705452.1 [10431-10962].		11655	12185	45336	45867	0 mismatches/531 bases (0%)
cch	Cassette chromosome helicase.	Reverse	12188	13974	45868	47656	0 mismatches/1788 bases (0%)
**orf7795**	Putative protein from ACME element of strain C427. A reference sequence for this gene is from strain C427-ST42, GenBank ACSQ01000048.1 [7795:9354].	Reverse	14398	15957	48079	49639	0 mismatches/1560 bases (0%)
**IR_IS431**	Inverted repeat of IS431.		16224	16239	49905	49921	0 mismatches/16 bases (0%)
**tnpIS431-06**	Transposase for IS431.	Reverse	16284	16958	49965	50640	0 mismatches/675 bases (0%)

Gene	Description/gene product and comments	Orientation	Start position in SCC	End position in SCC	Start position in genome	End position in genome	Comparison of Dresden-275757 to Oerebro-086360
**“DLJ55_14705”**	Hypothetical protein. 97% identity compared to, GenBank CP029627.1:2809799-2816653, strain MOK042; 98% identity compared to CP029650.1:1401-8447, a plasmid from strain AR_0471, GenBank.	Forward	17243	24273	50924	57955	Cannot be assessed because of discrepancies between Nanopore and Illumina sequences.
**IR_IS431**	Inverted repeat of IS431.		24603	24618	58284	58300	0 mismatches/16 bases (0%)
**F8WKF9**	Putative membrane protein. A reference sequence for this gene is from strain C427-ST42, GenBank ACSQ01000050 [548:900].	Forward	24821	25173	58502	58855	0 mismatches/353 bases (0%)
**EHQ67276**	Putative protein, branched-chain amino acid transport domain protein. A reference sequence for this gene is from strain C427-ST42, GenBank ACSQ01000050 [1158:1583].	Forward	25431	25856	59112	59538	0 mismatches/426 bases (0%)
***opp3A*/A8YYZ6**	Putative S-adenosyl-L-methionine-dependent methyltransferase from SCC elements. A reference sequence for this gene is from strain FPR3757, GenBank CP000255.1 [77130:77945].	Forward	26265	26903	59946	60585	0 mismatches/639 bases (0%)
opp3B	Nickel/peptide ABC superfamily ATP binding cassette transporter, membrane protein	Forward	28456	29412	62137	63094	0 mismatches/957 bases (0%)
opp3C	Oligopeptide permease, channel-forming protein	Forward	29412	30179	63093	63861	0 mismatches/768 bases (0%)
***opp3D*/A8YZ00**	Nickel/peptide ABC superfamily ATP binding cassette transporter, ABC protein, known from ACME elements.	Forward	30146	30913	63827	64595	0 mismatches/768 bases (0%)
***opp3E*/A8YZ01**	Nickel/peptide ABC superfamily ATP binding cassette transporter, ABC protein, known from ACME elements.	Forward	30907	31540	64587	65222	0 mismatches/635 bases (0%)
**tnp_A8YYY6**	Transposase.		31627	32293	65308	65975	0 mismatches/667 bases (0%)
**DR_SCC**	Direct repeat of SCC.		33683	33701	67364	67383	0 mismatches/19 bases (0%)

Supplemental file 5, Table 3: Genes in Deviating Region 2 in comparison to canonical CC80, to Oerebro-086360 and to canonical CC1.

	**Comparison of Dresden-275757 to CC80 reference sequence 11819-97 (CP003194)**	**Comparison of Dresden-275757 to CC1 reference sequence MW2 (BA000033)**	**Comparison of Dresden-275757 to Oerebro-086360**	**Provenance in Dresden-275757**
salA	0 mismatches/1065 bases (0%)	0 mismatches/1065 bases (0%)	0 mismatches/1065 bases (0%)	CC1 or CC80
ycnB	1 mismatches/1443 bases (0.07%)	0 mismatches/1443 bases (0%)	0 mismatches/1443 bases (0%)	CC1 or CC80
sepA	1 mismatches/468 bases (0.21%)	1 mismatches/468 bases (0.21%)	0 mismatches/468 bases (0%)	CC1 or CC80
sdrM	4 mismatches/1344 bases (0.3%)	0 mismatches/1344 bases (0%)	0 mismatches/1344 bases (0%)	CC1 or CC80
hlIII	3 mismatches/684 bases (0.44%)	3 mismatches/684 bases (0.44%)	0 mismatches/684 bases (0%)	CC1 or CC80
urtF	3 mismatches/1188 bases (0.25%)	3 mismatches/1188 bases (0.25%)	0 mismatches/1188 bases (0%)	CC1 or CC80
yvsG	0 mismatches/516 bases (0%)	0 mismatches/516 bases (0%)	0 mismatches/516 bases (0%)	CC1 or CC80
ynzG	0 mismatches/261 bases (0%)	0 mismatches/261 bases (0%)	0 mismatches/261 bases (0%)	CC1 or CC80
**Q5HE31**	3 mismatches/1371 bases (0.22%)	0 mismatches/1371 bases (0%)	0 mismatches/1371 bases (0%)	CC1 or CC80
htsC	0 mismatches/969 bases (0%)	0 mismatches/969 bases (0%)	0 mismatches/969 bases (0%)	CC1 or CC80
htsB	10 mismatches/1032 bases (0.97%)	0 mismatches/1032 bases (0%)	0 mismatches/1032 bases (0%)	CC1
htsA	0 mismatches/984 bases (0%)	0 mismatches/984 bases (0%)	0 mismatches/984 bases (0%)	CC1 or CC80
**tnpIS1**	*Absent from CP003194*	2 deletions in MW2/34 bases (5.88%)	0 mismatches/34 bases (0%)	CC1
**Q5HE27**	4 mismatches/1071 bases (0.37%)	0 mismatches/1071 bases (0%)	0 mismatches/1071 bases (0%)	CC1
rhbC1	18 mismatches/1758 bases (1.02%)	0 mismatches/1758 bases (0%)	1 mismatches/1757 bases (0.06%)	CC1
**Q5HE25**	13 mismatches/1194 bases (1.09%)	2 mismatches/1194 bases (0.17%)	1 mismatches/1194 bases (0.08%)	CC1
rhbC2	16 mismatches/1977 bases (0.81%)	0 mismatches/1977 bases (0%)	0 mismatches/1977 bases (0%)	CC1
asp23	12 mismatches/510 bases (2.35%)	1 mismatches/510 bases (0.2%)	0 mismatches/510 bases (0%)	CC1
**DUF2273**	0 mismatches/240 bases (0%)	0 mismatches/240 bases (0%)	0 mismatches/240 bases (0%)	CC1 or CC80
**Q5HE21**	1 mismatches/549 bases (0.18%)	2 mismatches/549 bases (0.36%)	0 mismatches/549 bases (0%)	CC1 or CC80
opuD2	20 mismatches/1563 bases (1.28%)	0 mismatches/1563 bases (0%)	0 mismatches/1563 bases (0%)	CC1
**Q5HE19**	0 mismatches/1008 bases (0%)	0 mismatches/1008 bases (0%)	0 mismatches/1008 bases (0%)	CC1 or CC80
qorA	6 mismatches/1002 bases (0.6%)	0 mismatches/1002 bases (0%)	0 mismatches/1002 bases (0%)	CC1
**DUF915**	1 mismatches/870 bases (0.11%)	1 mismatches/870 bases (0.11%)	0 mismatches/870 bases (0%)	CC1 or CC80
lacG	2 mismatches/1413 bases (0.14%)	0 mismatches/1413 bases (0%)	0 mismatches/1413 bases (0%)	CC1
lacE	0 mismatches/1719 bases (0%)	10 mismatches/1719 bases (0.58%)	0 mismatches/1719 bases (0%)	CC1 or CC80
lacF	6 mismatches/312 bases (1.92%)	0 mismatches/312 bases (0%)	0 mismatches/312 bases (0%)	CC1
lacD	20 mismatches/981 bases (2.04%)	1 mismatches/981 bases (0.1%)	0 mismatches/981 bases (0%)	CC1
lacC	4 mismatches/933 bases (0.43%)	4 mismatches/933 bases (0.43%)	0 mismatches/933 bases (0%)	CC1 or CC80
lacB	4 mismatches/516 bases (0.78%)	3 mismatches/516 bases (0.58%)	0 mismatches/516 bases (0%)	CC1
lacA	6 mismatches/429 bases (1.4%)	0 mismatches/429 bases (0%)	0 mismatches/429 bases (0%)	CC1
**tx_lacR**	5 mismatches/68 bases (7.35%)	0 mismatches/68 bases (0%)	0 mismatches/68 bases (0%)	CC1
lacR	6 mismatches/756 bases (0.79%)	0 mismatches/756 bases (0%)	0 mismatches/756 bases (0%)	CC1

	Comparison of Dresden-275757 to CC80 reference sequence 11819-97 (CP003194)	Comparison of Dresden-275757 to CC1 reference sequence MW2 (BA000033)	Comparison of Dresden-275757 to Oerebro-086360	Provenance in Dresden-275757
cobB	5 mismatches/732 bases (0.68%)	5 mismatches/732 bases (0.68%)	0 mismatches/732 bases (0%)	CC1 or CC80
**D9RC03**	*Absent from CP003194 (100%)*	0 mismatches/98 bases (0%)	0 mismatches/98 bases (0%)	CC1
**DUF3885**	*Absent from CP003194 (100%)*	14 mismatches/609 bases (2.3%)	14 mismatches/609 bases (2.3%)	Unknown, but other than CC80
**D9RC05**	*Absent from CP003194 (100%)*	0 mismatches/249 bases (0%)	0 mismatches/249 bases (0%)	CC1
**Q5HE05**	*Absent from CP003194 (100%)*	0 mismatches/126 bases (0%)	0 mismatches/126 bases (0%)	CC1
yvgN2	6 mismatches/849 bases (0.71%)	0 mismatches/849 bases (0%)	0 mismatches/849 bases (0%)	CC1
adhR	2 mismatches/417 bases (0.48%)	0 mismatches/417 bases (0%)	0 mismatches/417 bases (0%)	CC1
hysA	201 mismatches/2434 bases (8.19%)	0 mismatches/2448 bases (0%)	1 mismatches/2448 bases (0.04%)	CC1
att_nyEtd	*Absent from Dresden-275757*	*Absent from Dresden-275757 and MW2*	*Absent from Dresden-275757 and Oerebro-086360*	Absent (as in CC1, unlike CC80)
hsdS-etd	*Absent from Dresden-275757*	*Absent from Dresden-275757 and MW2*	*Absent from Dresden-275757 and Oerebro-086360*	Absent (as in CC1, unlike CC80)
hsdM	*Absent from Dresden-275757*	*Absent from Dresden-275757 and MW2*	*Absent from Dresden-275757 and Oerebro-086360*	Absent (as in CC1, unlike CC80)
etD	*Absent from Dresden-275757*	*Absent from Dresden-275757 and MW2*	*Absent from Dresden-275757 and Oerebro-086360*	Absent (as in CC1, unlike CC80)
**F3TKB7-var1**	*Absent from Dresden-275757*	*Absent from Dresden-275757 and MW2*	*Absent from Dresden-275757 and Oerebro-086360*	Absent (as in CC1, unlike CC80)
edinB	*Absent from Dresden-275757*	*Absent from Dresden-275757 and MW2*	*Absent from Dresden-275757 and Oerebro-086360*	Absent (as in CC1, unlike CC80)
**F5W4X2**	*Absent from Dresden-275757*	*Absent from Dresden-275757 and MW2*	*Absent from Dresden-275757 and Oerebro-086360*	Absent (as in CC1, unlike CC80)
**att_nyEtd**	*Absent from Dresden-275757*	*Absent from Dresden-275757 and MW2*	*Absent from Dresden-275757 and Oerebro-086360*	Absent (as in CC1, unlike CC80)
**Q5HE00**	*Absent from Dresden-275757*	*Absent from Dresden-275757 and MW2*	*Absent from Dresden-275757 and Oerebro-086360*	Absent (as in CC1, unlike CC80)
eapH-1	6 mismatches/426 bases (1.41%)	0 mismatches/426 bases (0%)	0 mismatches/426 bases (0%)	CC1
alsD-L1	12 mismatches/705 bases (1.7%)	0 mismatches/705 bases (0%)	0 mismatches/705 bases (0%)	CC1
alsS	32 mismatches/1665 bases (1.92%)	0 mismatches/1665 bases (0%)	0 mismatches/1665 bases (0%)	CC1
**Q8NVB9**	4 mismatches/183 bases (2.19%)	0 mismatches/183 bases (0%)	0 mismatches/183 bases (0%)	CC1
**rpsI**	0 mismatches/399 bases (0%)	0 mismatches/399 bases (0%)	0 mismatches/399 bases (0%)	CC1 or CC80
**rplM**	1 mismatches/438 bases (0.23%)	1 mismatches/438 bases (0.23%)	1 mismatches/438 bases (0.23%)	CC1 or CC80
**L13_leader**	0 mismatches/70 bases (0%)	0 mismatches/70 bases (0%)	0 mismatches/70 bases (0%)	CC1 or CC80
truA	14 mismatches/804 bases (1.74%)	0 mismatches/804 bases (0%)	0 mismatches/804 bases (0%)	CC1
ecfT	6 mismatches/807 bases (0.74%)	0 mismatches/807 bases (0%)	0 mismatches/807 bases (0%)	CC1
ecfA1	6 mismatches/861 bases (0.7%)	0 mismatches/861 bases (0%)	0 mismatches/861 bases (0%)	CC1
ecfA2	8 mismatches/810 bases (0.99%)	0 mismatches/810 bases (0%)	0 mismatches/810 bases (0%)	CC1
rplQ	0 mismatches/369 bases (0%)	0 mismatches/369 bases (0%)	0 mismatches/369 bases (0%)	CC1 or CC80

	Comparison of Dresden-275757 to CC80 reference sequence 11819-97 (CP003194)	Comparison of Dresden-275757 to CC1 reference sequence MW2 (BA000033)	Comparison of Dresden-275757 to Oerebro-086360	Provenance in Dresden-275757
rpoA	0 mismatches/945 bases (0%)	0 mismatches/945 bases (0%)	0 mismatches/945 bases (0%)	CC1 or CC80
rpsK	1 mismatches/390 bases (0.26%)	0 mismatches/390 bases (0%)	0 mismatches/390 bases (0%)	CC1 or CC80
rpsM	0 mismatches/366 bases (0%)	0 mismatches/366 bases (0%)	0 mismatches/366 bases (0%)	CC1 or CC80
rpmJ	0 mismatches/114 bases (0%)	0 mismatches/114 bases (0%)	0 mismatches/114 bases (0%)	CC1 or CC80
infA	0 mismatches/219 bases (0%)	0 mismatches/219 bases (0%)	0 mismatches/219 bases (0%)	CC1 or CC80
adk	0 mismatches/648 bases (0%)	2 mismatches/648 bases (0.31%)	0 mismatches/648 bases (0%)	CC1 or CC80
secY1	1 mismatches/1293 bases (0.08%)	1 mismatches/1293 bases (0.08%)	0 mismatches/1293 bases (0%)	CC1 or CC80
rplO	1 mismatches/441 bases (0.23%)	0 mismatches/441 bases (0%)	0 mismatches/441 bases (0%)	CC1 or CC80
rpmD	0 mismatches/180 bases (0%)	0 mismatches/180 bases (0%)	0 mismatches/180 bases (0%)	CC1 or CC80
rpsE	0 mismatches/501 bases (0%)	0 mismatches/501 bases (0%)	0 mismatches/501 bases (0%)	CC1 or CC80
rplR	0 mismatches/360 bases (0%)	0 mismatches/360 bases (0%)	0 mismatches/360 bases (0%)	CC1 or CC80
rplF	0 mismatches/537 bases (0%)	0 mismatches/537 bases (0%)	0 mismatches/537 bases (0%)	CC1 or CC80
rpsH	0 mismatches/399 bases (0%)	1 mismatches/399 bases (0.25%)	0 mismatches/399 bases (0%)	CC1 or CC80
rpsZ	0 mismatches/186 bases (0%)	0 mismatches/186 bases (0%)	0 mismatches/186 bases (0%)	CC1 or CC80
rplE	0 mismatches/540 bases (0%)	0 mismatches/540 bases (0%)	1 mismatches/540 bases (0.19%)	CC1 or CC80
rplX	0 mismatches/318 bases (0%)	0 mismatches/318 bases (0%)	0 mismatches/318 bases (0%)	CC1 or CC80
rplN	0 mismatches/369 bases (0%)	0 mismatches/369 bases (0%)	0 mismatches/369 bases (0%)	CC1 or CC80
rpsQ	0 mismatches/264 bases (0%)	0 mismatches/264 bases (0%)	0 mismatches/264 bases (0%)	CC1 or CC80
rpmC	0 mismatches/210 bases (0%)	0 mismatches/210 bases (0%)	0 mismatches/210 bases (0%)	CC1 or CC80
